# Plasma membrane lipid–protein interactions affect signaling processes in sterol-biosynthesis mutants in *Arabidopsis thaliana*

**DOI:** 10.3389/fpls.2014.00078

**Published:** 2014-03-18

**Authors:** Henrik Zauber, Asdrubal Burgos, Prashanth Garapati, Waltraud X. Schulze

**Affiliations:** ^1^Max Planck Institute of Molecular Plant PhysiologyGolm, Germany; ^2^Max-Delbrück-Centrum für Molekulare MedizinBerlin-Buch, Germany; ^3^Plant Systems Biology, University of HohenheimStuttgart, Germany

**Keywords:** microdomains, sterols, sphingolipids, signaling

## Abstract

The plasma membrane is an important organelle providing structure, signaling and transport as major biological functions. Being composed of lipids and proteins with different physicochemical properties, the biological functions of membranes depend on specific protein–protein and protein–lipid interactions. Interactions of proteins with their specific sterol and lipid environment were shown to be important factors for protein recruitment into sub-compartmental structures of the plasma membrane. System-wide implications of altered endogenous sterol levels for membrane functions in living cells were not studied in higher plant cells. In particular, little is known how alterations in membrane sterol composition affect protein and lipid organization and interaction within membranes. Here, we conducted a comparative analysis of the plasma membrane protein and lipid composition in Arabidopsis sterol-biosynthesis mutants *smt1* and *ugt80A2;B1*. *smt1* shows general alterations in sterol composition while *ugt80A2;B1* is significantly impaired in sterol glycosylation. By systematically analyzing different cellular fractions and combining proteomic with lipidomic data we were able to reveal contrasting alterations in lipid–protein interactions in both mutants, with resulting differential changes in plasma membrane signaling status.

## Introduction

The plasma membrane contains numerous lipid species with different physicochemical properties. The three major classes are glycerolipids, sphingolipids and sterols. Spontaneous phase separation was shown to occur in artificial membranes involving mainly lipids of higher hydrophobicity such as sphingolipids, sterols or long-chain phospholipids (Karnovsky et al., 1982; Thompson and Tillack, 1985). These experiments extended the fluid mosaic model of Singer and Nicolson (Singer and Nicolson, 1972) of protein–lipid organization by adding sub-compartmental occurrences of specific protein and lipid compositions in the plasma membrane in so-called microdomains. Since then, biological evidence for such membrane domains came from many organisms and different biological contexts (Simons and Toomre, 2000; Lucero and Robbins, 2004; Bhat and Panstruga, 2005; Rajendran and Simons, 2005; Lingwood et al., 2009; Cacas et al., 2012). With a reported size of microdomains ranging from nanoscale up to microscale (Edidin, 2001; Cacas et al., 2012), direct visualization of such domains in living cells remains challenging. Sterol-dependent protein localization was reported in various plant systems (Borner et al., 2005; Roche et al., 2008; Kierszniowska et al., 2009; Minami et al., 2009; Carmona-Salazar et al., 2011; Navarro-Lérida et al., 2012) and selected proteins with sterol-dependent membrane location were shown to exhibit a patchy organization within the plasma membrane (Bariola et al., 2004; Parton and Hancock, 2004). The biological function of these microdomains was especially linked to signaling and transport processes in several independent studies (Simons and Toomre, 2000; Borner et al., 2005; Langhorst et al., 2005; Kierszniowska et al., 2009; Staubach and Hanisch, 2011; Stuermer, 2011). The current working model of sterol-rich microdomains involves controlled dynamic association/dissociations of particular proteins with the distinct local sterol-lipid environment. Support for this model in plants came from stimulus-dependent membrane microdomain localization of the *Arabidopsis* flagellin receptor and the ion channel SLAH3 using a combination of proteomic and cell biology approaches (Keinath et al., 2010; Demir et al., 2013).

Large-scale proteomic analysis of microdomain-associated proteins in plants has so far been mainly based on the treatment of purified plasma membranes with non-ionic detergents. Fractionation of the plasma membrane after detergent treatment results in a high density detergent soluble fraction (DSF) that contains membranes and proteins solubilized by detergent treatment, and a sterol-enriched detergent-resistant membrane (DRM) fraction with associated proteins (Lingwood and Simons, 2007). Proteomic analysis of such DRMs elucidated a specific set of DRM-resident proteins especially involved in signaling or transport (Shahollari and Berghöfer, 2004; Kierszniowska et al., 2009). Furthermore, the sterol-dependence of these proteins was validated by using sterol-depleting agents such as methyl-β-cyclodextrin (mβcd) (Ilangumaran and Hoessli, 1998; Zidovetzki and Levitan, 2007; Kierszniowska et al., 2009). However, these approaches were criticized as being prone to artifacts due to *in vitro* modification of plasma membranes after disruption of the cells (Tanner et al., 2011).

System-wide studies of membrane composition in living systems with endogenously altered sterol levels are rare and have so far only focused on single-protein examples (Lauwers and André, 2006). Endogenous alteration of sterol levels can be achieved by nutritional manipulations in sterol auxotrophic species (Entchev and Kurzchalia, 2005). In sterol autotrophic species such as plants, sterol diets obviously have no major effects. Alternatively sterol synthesis can be manipulated applying sterol-depleting drugs (He et al., 2003; Benveniste, 2004; Schrick et al., 2004a) or sterol synthesis inhibitors (He et al., 2003; Benveniste, 2004) on living cells. Nevertheless, secondary side-effects of applying drug treatments to living cells are hard to control, especially when these drugs are poisonous to the cell. In contrast, mutants inhibited in various steps of sterol biosynthesis (Schrick et al., 2000, 2002, 2004a, 2012a; Souter et al., 2002) display altered sterol profiles without the need of drug treatment. Indeed, reported sterol profiles of sterol-biosynthesis mutants showed significant shifts in total sterol composition (Schrick et al., 2002, 2004a; Boutte et al., 2008). All of these sterol-biosynthesis mutants exhibit a strong dwarf phenotype and are sterile (Schrick et al., 2000, 2002, 2004a, 2012a; Clouse, 2002). Although this pleiotropic phenotype could partially result from alterations in sterol- (He et al., 2003) and brassinosteroid-signaling (Clouse and Sasse, 1998), strong perturbations of general plasma membrane structures and microdomain functions are also likely. Therefore, these sterol-biosynthesis mutants are ideal systems for an in-depth characterization of microdomain protein composition and plasma membrane signaling status in the context of an endogenously-altered membrane sterol-composition.

We used the mutant *smt1* (Schrick et al., 2002, 2004a; Willemsen et al., 2003; Fujioka, 2010) for its reported qualitative and quantitative alterations in sterol levels. *smt1* encodes one of three sterol-methyl-transferases in *Arabidopsis* (Fujioka, 2010). Even though *smt1* mutants exhibit a typical dwarf like phenotype at the whole plant level, its phenotype is less visible on non-differentiated callus systems of *smt1* (Supplemental Figure 2 and Schrick et al., 2002), which makes it an ideal system for the propagation of cell material on a large scale. In contrast, the *ugt80A2;B1* (DeBolt et al., 2009) double knockout mutant lacks the function of the only two sterol-UDP-glycosyltransferases in *Arabidopsis*. This mutant is fertile, but was shown to lack sterolglycosides (DeBolt et al., 2009; Schrick et al., 2012b) with only small effects on embryo development, root length or general sterol composition (DeBolt et al., 2009). Therefore, *ugt80A2;B1* is an optimal further candidate for studying loss of sterol-related function (glycosylation) on sterol–protein interactions in the plasma membrane. We performed a systematic comparative analysis between both mutants with a particular focus on how altered sterol composition affects cellular protein–lipid interactions. Thereby, the DRM/DSF abundance-ratio of proteins and lipids (Zauber et al., 2013) was shown to be a useful proxy of sterol–protein interactions in *smt1*. We used a systems biology-based approach involving label-free proteomics as well as lipidomics to identify disturbed protein–sterol interactions with implications on stress signaling in these mutants.

## Results

The following work aims at a thorough assessment of the role of sterols for membrane protein and lipid composition. Based on mass spectrometric analysis of proteins and lipids in the sterol-biosynthesis mutants *smt1* and *ugt80A2;B1* we suggest significant roles of sterols in membrane-based signaling processes.

### Detection of sterol-dependent protein candidates by DRM/DSF distribution analysis

**Figure 1:**
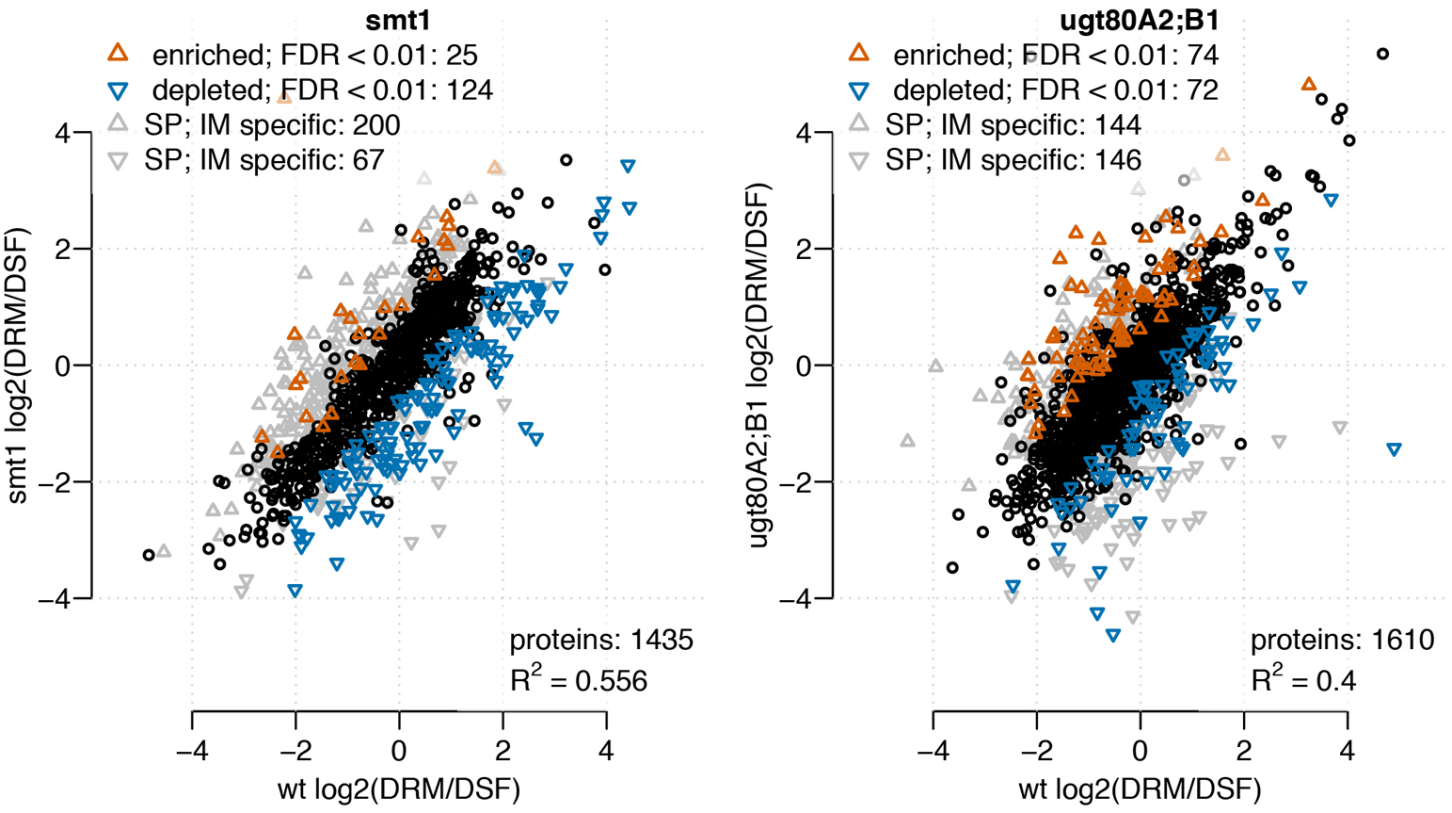
**Scatterplot of log_2_ transformed DRM/DSF protein abundance ratios from *smt1* (*left*) and *ugt80A2;B1* (*right*), each in comparison to DRM/DSF distributions in wildtype**. Proteins with Unicorn scores above the threshold defining a false discovery rate lower than 1% were considered to be significantly altered in their distribution between wildtype and the respective mutant. In *smt1* more proteins exhibited a decreased DRM/DSF ratio compared to wildtype, while among enriched proteins the number of co-purifying proteins from intracellular membrane (IM) and soluble protein (SP) fractions was much higher. In *ugt80A2;B1*, similar numbers of proteins were found to be enriched (red) or decreased (blue) in their DRM/DSF abundance ratios.

To characterize the role of sterol composition for the localization of proteins within membrane microdomains, we studied the distribution of proteins between sterol-rich (detergent resistant membranes, DRM) and sterol depleted (detergent sensitive fraction, DSF) membrane fractions. From 3716 identified proteins, 1435 and 1610 could be analyzed based on the abundance ratio distribution between DRM/DSF in *smt1* or *ugt80A2;B1*, respectively (Figure 1). In *smt1* most proteins showed decreased abundance in DRM while a similar number of proteins were enriched or depleted relative to DRM fractions for *ugt80A2;B1*. In total, 149 and 146 proteins (classified as responsive proteins) were found with a differential DRM/DSF distribution ratio in *smt1* and *ugt80A2;B1*, respectively, as compared to the wildtype (Figure 1). Since alterations in sterol composition or glycosylation is the primary result of the mutations, we particularly expected to observe altered sterol–protein interactions. Therefore, in the following sections all distribution changes between DRM/DSF ratios will be expressed relative to the DRM fraction, which in the wildtype is the sterol-enriched membrane fraction. By comparing abundances of proteins identified in DRM and DSF against abundances in fractions of soluble proteins (SP) and intracellular membranes (IM), co-purifying proteins were defined if their highest abundance was either in SP or IM. These co-purifying proteins were found to be specifically enriched in DRM fractions of *smt1* as reported previously (Zauber et al., 2013). In contrast, occurrence of co-purifying proteins was equally distributed between DRM and DSF fractions in *ugt80A2;B1*. Most of the co-purifying proteins were of cytosolic location, but mitochondrial, vacuolar, plastidial, nuclear and endoplasmic reticulum proteins were also present, according to SUBA3 (Tanz et al., 2013) (Supplemental Figure 2). Among the co-purifying proteins, we identified also a number of plasma membrane located proteins which showed significantly higher abundance in IM and SP. These proteins were not included in later analysis of abundance DRM/DSF ratios in order to minimize effects related to altered membrane trafficking in the mutants (Zauber et al., 2013).

**Figure 2:**
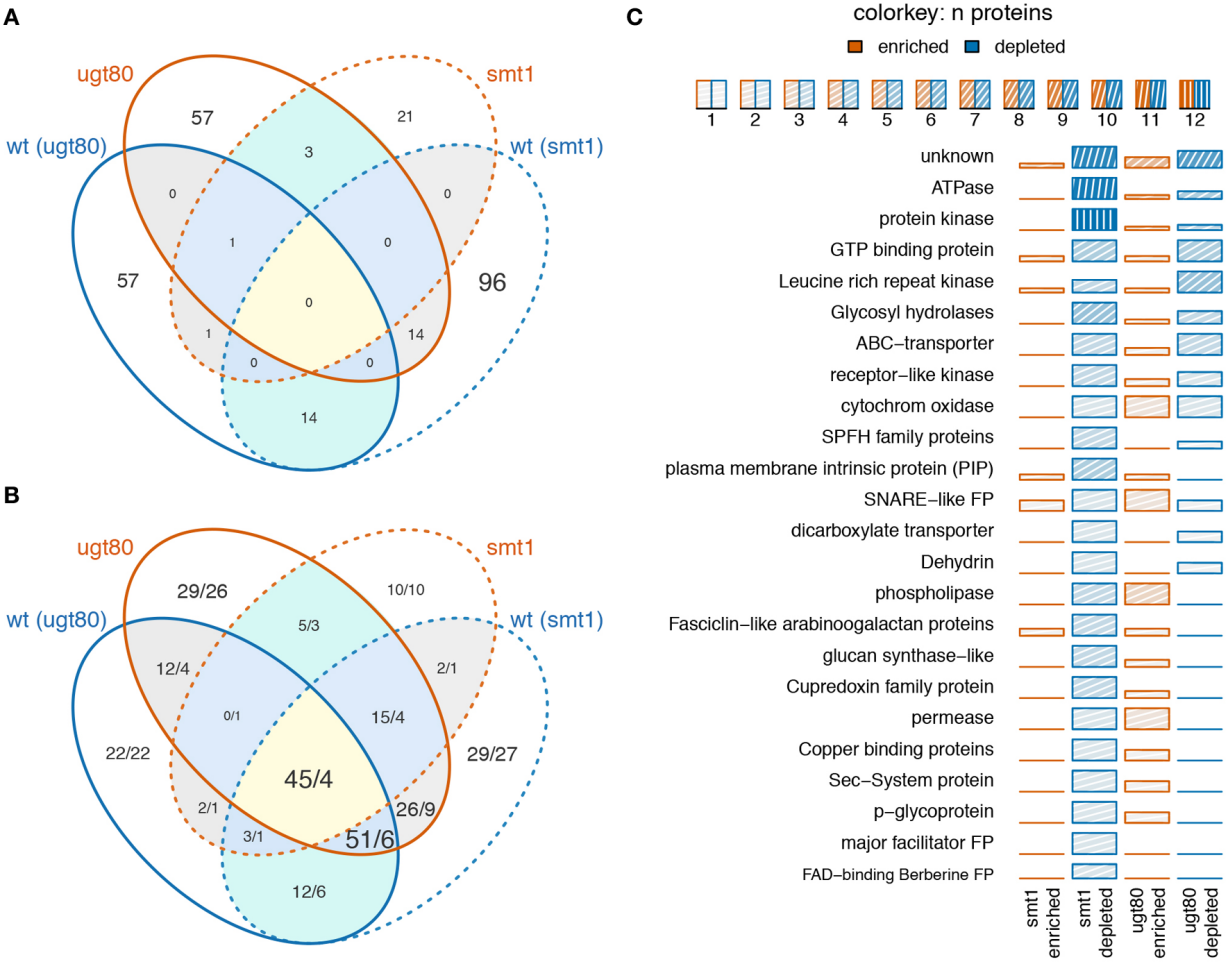
**Venn diagram displaying the number of overlapping proteins **(A)** or protein groups **(B)** being enriched (red) or depleted (blue) in *smt1* or *ugt80A2;B1* (ugt80) DRM fractions**. Numbers in **(B)** indicate the number of unique proteins represented in the overlapping protein groups, separated by a slash. Low overlap was observed between proteins **(A)** but analysis based on protein groups showed a higher degree of overlap **(B)**. A quantitative analysis of significantly-altered protein abundances based on manually-curated protein groups is displayed in **(C)**. Only protein groups represented by at least two proteins are shown. The number of proteins in protein groups is indicated by color intensity. wt, wildtype; ugt80, *utg80A2;B1*; FP, family protein.

In summary, 14 proteins showed decreased abundance in DRM in both mutants and 14 proteins were depleted in *smt1* but enriched in *ugt80A2;B1* (Figure 2A). Only a small fraction of proteins seems to be responsive in both mutants while the overlap based on hand-curated protein groups is much higher (Figure 2B). In this study, the largest two classes of overlapping proteins cover already 96 of all responding proteins and were represented in 9 protein groups and proteins of unknown functions. Within these overlapping protein groups, most proteins were strongly decreased in *smt1* and enriched or depleted in *ugt80A2;B1*. Protein groups that were enriched in *smt1* were only represented by one protein across all comparisons (Supplemental Table 1). However, the majority of all identified sterol-dependent proteins showed reduced abundance in DRM fractions in both *smt1* and *ugt80A2;B1* (Figure 2C). This included ATPases, protein kinases, leucine-rich repeat kinases (LRR kinases), GTP binding proteins, glycosyl-hydrolases, SNARE like family proteins, ABC-transporters, receptor-like kinases, SPFH protein family and “early responsive to dehydration” proteins (ERD). Cytochrome oxidases (Asard et al., 2001) with two proteins responding in both mutants were depleted and enriched in DRM of *ugt80A2;B1*. Additionally some co-purifying proteins were also found in this category, such as dicarboxylate transporter, glucose-6-phosphate/phosphate-translocator and an ADP-ribosylation-factor (Supplemental Table 1). However, these co-purifying groups were generally represented by one or two proteins only.

The second big class of protein groups was generally depleted from DRM in *smt1* but enriched in DRM in *ugt80A2;B1*. In total, 13 protein groups represented by 41 individual proteins showed this pattern. Examples with the highest number of unique proteins were the group of phospholipases, fasciclin like arabinogalactan proteins (FLA), plasma membrane intrinsic proteins (PIP) and cupredoxins (SKU; Figure 2C). Interestingly, the accepted plant microdomain marker remorin was depleted from the DRM fraction in *smt1*, but not in *ugt80A2;B1* (Supplemental Table 1). In both mutants 12 protein groups showed a similar trend in their abundance distributions relative to in the DRM fraction. From these only one was enriched and 11 were strongly reduced in abundance (Table 1).

**Table 1:** **Selected protein groups showing significant alterations in DRM/DSF protein abundance ratios in *smt1* and *ugt80A2;B1***.

**Protein group**	**Abbreviation**	***smt1***						***ugt80A2;B1***					
		**Proteins**	**Trend**	**PTM**				**Proteins**	**Trend**	**PTM**			
				**Pal**	**Myr**	**GPI**	**P**			**Pal**	**Myr**	**GPI**	**P**
**SAME TREND**													
Nitrite reductase	Nitrit.Red	at2g15620.1↑	↑	0	0	0	1	at2g15620.1↑	↑	0	0	0	1
ABC-transporter	ABC	at2g47000.1↓; at2g47800.1↓	↓	1	0	0	2	at1g30400.1↓; at1g59870.1↓; at3g53480.1↓; at2g47800.1↑	↓	1	0	0	3
Aspartyl protease	Asp.Pro	at3g02740.1↓	↓	1	0	1	0	at3g02740.1↓	↓	1	0	1	0
ATPase	ATPase	at1g78900.1↓; at2g21410.1↓; at3g42050.1↓; at4g29900.1↓; at4g39080.1↓; at5g57110.1↓	↓	2	0	0	5	at1g63440.1↓; at3g21180.1↓; at5g57110.1↓; at1g78900.1↑; at4g39080.1↑	↓	1	0	0	4
Cytochrom oxidase	CYT	atmg00160.1↓; at2g07727.1↓	↓	1	0	0	1	at3g14610.1↓; at4g22690.1↓; at2g47380.1↑	↓	0	0	0	2
Dehydrin	ERD	at1g30360.1↓; at1g32090.1↓	↓	0	0	0	2	at1g76180.1↓	↓	0	0	0	1
Dicarboxylate transporter	DCT	at5g64290.1↓; at5g12860.1↓	↓	0	1	0	1	at5g64290.1↓	↓	0	0	0	0
G6P/P translocator	G6p.P	at5g54800.1↓	↓	0	0	1	1	at5g54800.1↓	↓	0	0	1	1
GTP binding protein	GTP.b.Prot	at1g06400.1↓; at3g46060.1↓	↓	2	0	0	1	at3g11730.1↓; at4g34460.1↓; at5g27540.1↓; at5g47200.1↓; at4g39520.1↑	↓	4	0	0	1
Receptor-like kinase	RLK	at1g14390.1↓; at3g14840.2↓; at3g46290.1↓; at3g17840.1↓; at3g21630.1↓	↓	0	0	0	5	at3g46290.1↓; at3g51550.1↓; at3g24550.1↑	↓	4	0	0	5
SPFH family proteins	SPFH	at1g69840.1↓; at3g01290.1↓; at5g51570.1↓	↓	2	2	0	3	at3g01290.1↓	↓	1	1	0	1
			∑:	9	3	2	22		∑:	12	1	2	19
**MUTANT SPECIFIC TREND**													
Sec-system protein	Sec	at1g72160.1↓	↓	0	0	0	1	at1g29310.1↑	↑	1	0	0	0
Copper binding proteins	Copper.B	at5g20650.1↓	↓	1	0	0	1	at4g12290.1↑	↑	0	0	0	0
Cupredoxin family protein	SKU	at4g12420.1↓; at4g25240.1↓; at5g51480.1↓	↓	2	0	2	2	at1g76160.1↑	↑	0	0	0	1
Fasciclin-like arabinoogalactan proteins	FLA	at4g12730.1↓; at5g44130.1↓	↓	1	0	2	0	at5g55730.1↑	↑	1	0	1	1
Glucan synthase-like	GSL	at2g36850.1↓; at3g07160.1↓; at4g03550.1↓	↓	0	0	0	3	at4g30270.1↑	↑	0	0	0	0
Inorganic pyrophosphatase	PPase	at1g15690.1↓	↓	0	0	0	1	at1g15690.1↑	↑	0	0	0	1
p-glycoprotein	PGP	at1g02520.1↓; at2g39480.1↓	↓	1	0	0	2	at1g02530.1↑	↑	1	0	0	1
Permease	permease	at5g09400.1↓; at5g62890.1↓	↓	0	0	0	2	at5g62890.1↑; at1g19770.1↑	↑	0	0	0	1
Phospholipase	P.lipase	at3g08510.1↓; at4g36945.1↓	↓	2	0	1	1	at3g08510.1↑; at5g67130.1↑	↑	2	0	1	1
Respiratory burst oxidase	Resp.burst.ox	at5g47910.1↓	↓	0	0	0	1	at5g47910.1↑	↑	0	0	0	1
Glycosyl hydrolases	Glu.Hyd	at3g13560.1↓; at5g58090.1↓; at1g11820.2↓; at3g04010.1↓	↓	1	0	2	0	at3g04010.1↓; at5g05460.1↑	↑↓	1	0	1	0
Protein kinase	P.kin	at3g54030.1↓; at4g23250.1↓; at1g11330.2↓; at1g48210.1↓; at3g17410.1↓; at5g12480.1↓	↓	6	3	0	4	at1g11330.2↓; at5g46570.1↓; at1g06700.1↑; at2g30740.1↑	↑↓	4	1	0	2
Unknown	Unknown	at1g53625.1↓; at1g29980.1↓; at1g31130.1↓; at1g73650.3↓; at2g30930.1↓	↓	1	0	1	1	at5g63190.1↓; at3g08950.1↓; at3g47200.1↓; at3g54290.1↓; at5g11680.1↓; at3g49720.1↑; at1g05150.1↑; at2g19080.1↑; at3g60600.1↑; at5g52420.1↑	↑↓	4	1	1	5
ATP/ADP carrier	ATP.ADP	at5g01500.1↑	↑	0	0	0	0	at1g15500.1↓	↓	0	0	0	1
CaLB domain FP	Ca-Lipid-Binding.FP	at3g61050.1↓; at5g07300.1↑	↑↓	0	0	0	1	at5g37740.2↓	↓	0	0	0	0
			∑:	15	3	8	20		∑:	14	2	4	15

*Proteins were selected for significant lipid–protein dependencies based on network analysis using a false discovery threshold <1%. Arrows indicate increased or decreased DRM/DSF abundance ratios. Postulated post-translational modifications (PTM) were cumulated for each protein group considering palmitoylation (Pal), myristoylation (Myr), glycosyl-phosphatidyl-inositol anchor (GPI), and Phosphorylation (P)*.

The last fraction included mainly protein groups that were previously shown to be sterol-dependent, such as ATPases, ABC-transporters, leucine-rich repeat kinases, dehydrin, receptor-like kinases, and SPFH proteins (Borner et al., 2005; Browman et al., 2007; Kierszniowska et al., 2009; Minami et al., 2009). Core protein groups being enriched in *ugt80A2;B1* DRM fraction but depleted in *smt1* included cupredoxins, fasciclin-like arabinogalactan proteins, plasma membrane intrinsic proteins, and lipid signaling relevant proteins with phospholipase C activity. In addition, proteins involved in membrane trafficking were differentially affected such as Sec and SNARE-like proteins.

The group “kinases” included a large number of signaling active proteins likely to alter the signaling status in both mutants. Association of the proteins with DRMs was not in general dependent on the presence of post-translational modification sites (Table 1). However, modification status could affect degree of lipid protein associations, as was suggested for SLAH3 (Demir et al., 2013).

### Alteration in lipid profiles in both mutants

**Figure 3:**
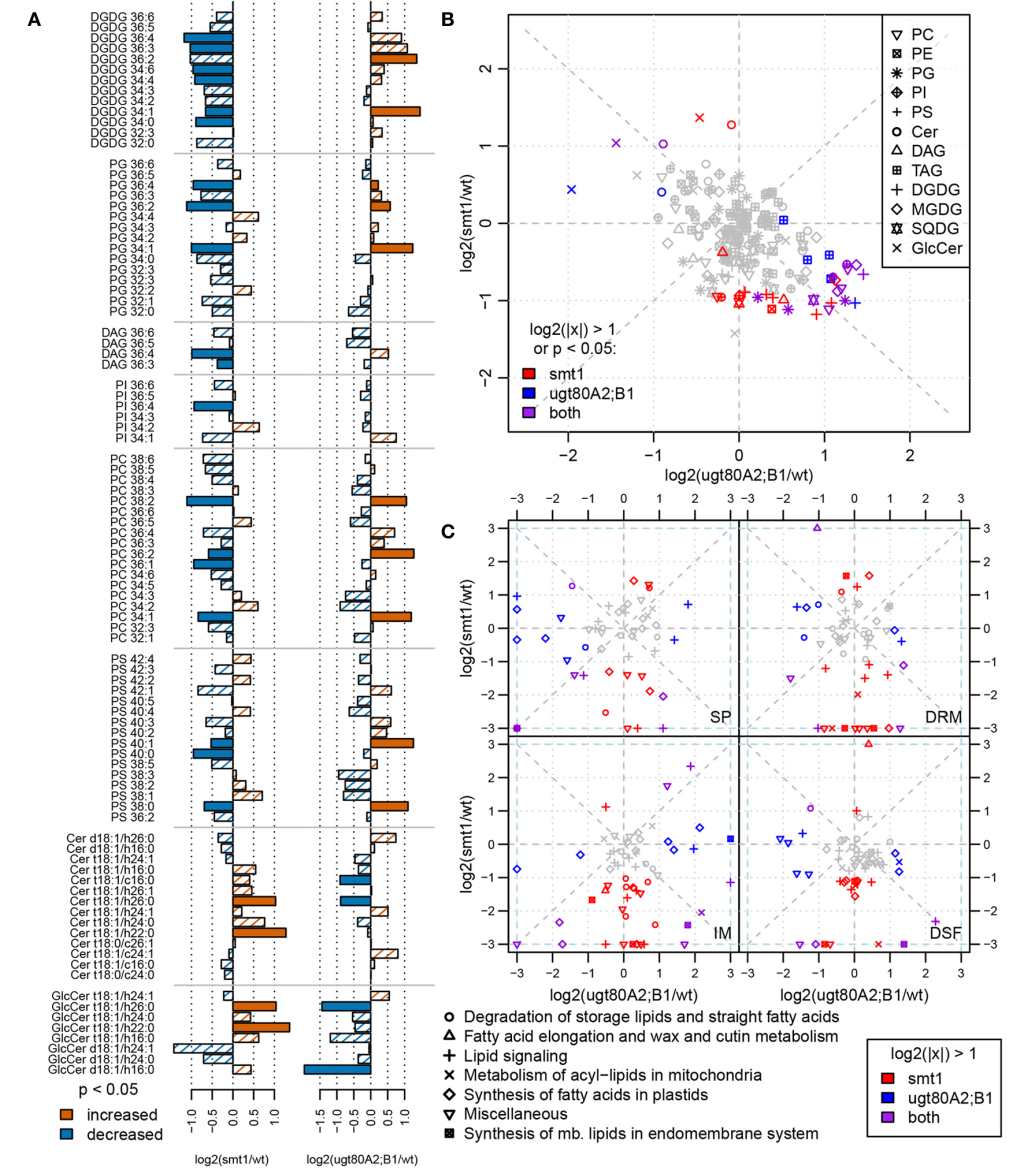
**Comparison of log_2_ transformed lipid abundances from *smt1* and *ugt80A2;B1* related to wildtype **(A)****. Lipids with a higher-than two-fold change or significant difference in abundance (*t*-test; α < 0.05) are colored as indicated in the legend. Lipid abundance changes of *smt1* and *ugt80A2;B1* relative to wildtype display a generally inverse pattern **(B)**. Specific changes in proteins involved in lipid metabolism correlate with lipid distributions in respective fractions (IM, SP, DRM, DSF) **(C)**. Isomer forms of detected ceramides and glycosylceramides are shown in Supplemental Table 2. PC, phosphatidylcholine; PE, phosphatidylethanolamine; PG, phosphatidylglycerol; PI, phosphatidylinositol; PS, phosphatidylserine; Cer, ceramide; GlcCer, glycosylceramide; DAG, diacylglycerol; MGDG/DGDG, mono-/digalactosyldiacylglycerol; SQDG, sulfoquinovosyldiacylglycerol; TAG: triacylglycerol; SP, soluble proteins; IM, intracellular membranes; DRM, detergent resistant membranes; DSF, detergent soluble fractions.

Changes in sterol composition are the primary effect of the mutations (Schrick et al., 2002, 2004a; DeBolt et al., 2009). Since we were interested in how this altered sterol composition and glycosylation status affected other type of lipids in the membranes we complemented the proteomic investigation by a lipidomic analysis. Both mutants exhibited an altered membrane lipid profile compared to wildtype (*t*-test, α = 0.05). Although there were only a few changes observed after multiple testing correction for single compounds, analysis at the class level show a contrasting lipid composition between both mutants (Figure 3B). This observation could be confirmed through a separate analysis of the sterol biosynthesis mutant *cpi*. *cpi* exhibits stronger alterations in its whole-plant sterol composition (Boutte et al., 2008) than *smt1*. Further, this mutant shows a similar but stronger dwarf-like phenotype as *smt1* (Schrick et al., 2004a; Boutte et al., 2008). Concordantly the lipid profile of *cpi* shows a similar but even stronger global shift of lipid abundances as *smt1* (Supplemental Figure 3). Affected plasma membrane lipid classes in *smt1* and *ugt80A2;B1* were ceramides (Cer), glycosylceramides (GlcCer), phosphatidylglycerol (PG; 36 acylcarbons), phosphatidylserines (PS; 40 acylcarbons), and phosphatidylcholines (PC; 36 acylcarbons) (Figure 3A; Supplemental Table 2). The largest relative change was evident for glycosylceramides (GlcCer), which showed a general increased abundance in *smt1* and decreased abundance in *ugt80A2;B1*. Two GlcCer species with lower hydroxylation status showed interestingly a decreased abundance also in *smt1*.

### Alterations in lipid synthesis-related proteins correlate with lipid abundance profiles

The observed differential lipid composition in *smt1* and *ugt80A2;B1* could be related to alterations in the abundance of proteins involved in lipid synthesis (Figure 3C). All four fractions SP, IM, DRM, and DSF were analyzed using two-fold changes as a significance-threshold. Large differences in the abundance of proteins involved in lipid synthesis were especially visible in the IM fraction. In this study, proteins involved in lipid degradation were strongly decreased in the *smt1* IM fraction, while they showed a slight increase in *ugt80A2;B1*. Proteins involved in fatty acid synthesis in the plastid (Supplemental Table 3) were strongly decreased in *smt1* and increased in the *ugt80A2;B1* IM fraction. Interestingly, a decreased abundance was observed for acyl carrier proteins (ACP2, ACP3) in the IM fraction in both mutants. Similarly, proteins involved in the synthesis of lipids at the endoplasmic reticulum (ER) were affected, which could possibly explain the observed changes in membrane lipids. These proteins were in general decreased in the smt1 mutant, but increased in ugt80, such as putative cholinephosphate cytidylyltransferase and ethanolaminephosphate cytidylyltransferase, involved in head group synthesis; glycerol-3-phosphate dehydrogenase, involved in glycerol synthesis and involved in the synthesis of long-chain fatty acids (Beaudoin et al., 2009).

The majority of proteins involved in lipid signaling (hand curated categories, see Supplemental Table 3) shows a plasma membrane localization according to SUBA3 (Tanz et al., 2013). Therefore, alterations in abundance of these proteins should be viewed with regards to altered distributions between DRM and DSF fractions. A general decrease in the abundance of proteins involved in lipid signaling in the DRM as well as DSF could be observed for *smt1*. Based on DRM/DSM ratios, the phospholipases C2 and D γ 1 were depleted from *smt1* DRM fractions but significantly enriched in *ugt80A2;B1* DRM fractions. Interestingly, cell-wide DAG abundance in *smt1* was significantly decreased indicating a reduced phospholipase C activity in *smt1*. Furthermore, SMT2 was found with an increased abundance in IM and DSF fractions of *ugt80A2;B1* while it was not detected in *smt1*. SMT2 acts at the branching step of sterol synthesis which leads to sitosterol synthesis through isofucosterol (Fujioka, 2010). Isofucosterol levels were slightly but not significantly elevated in *ugt80A2;B1* (DeBolt et al., 2009) which might be linked to the observed increased abundance of SMT2.

### The link between sterol-dependent proteins and ceramides

**Figure 4:**
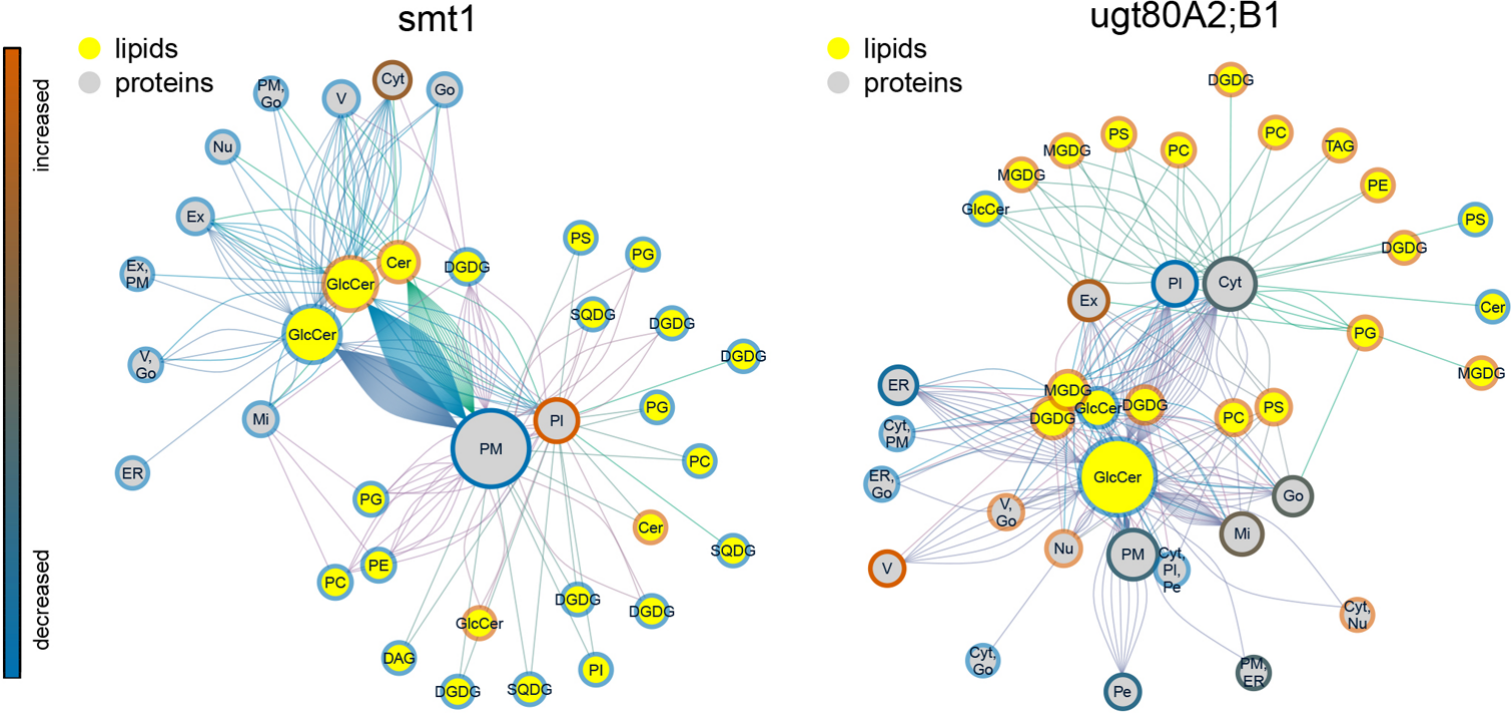
**Lipid–protein correlation network displaying proteins according to subcellular localization in SUBA3 (Tanz et al., 2013)**. Edges were selected for significance using a false discovery threshold lower 1%. For both mutants glycosylceramide shows the highest degree indicated by node diameter. In particular an inverse relationship of increased (blue node frame) ceramides and plasma membrane proteins is visible for *smt1*, while in *ugt80A2;B1* more sterol-dependent proteins correlated with decreased (red node frame) levels of glycosylceramides. In general, lipid–protein connections were grouped by their subcellular localization. Frame node color indicates decrease (blue) or increase (blue) in sterol dependency of proteins or abundance of lipids (yellow node color). Edge colors are linked to the different lipid nodes. Lipids: PC, Phosphatidylcholine; PG, Phosphatidylglycerol; PS, Phosphatidylserine; Cer, Ceramide; GlcCer, Glycosylceramide; MGDG/DGDG, Mono-/Digalactosyldiacylglycerol; SQDG, Sulfoquinovosyldiacylglycerol. Subcellular localizations: PM, plasma membrane; Pl, plastid; ER, endoplasmatic reticulum; V, vacuole; Go, Golgi apparatus; Ex, extracellular; Mi, mitochondrion; Pe, peroxysome; Nu, nucleus; Cyt, cytosol.

**Figure 5:**
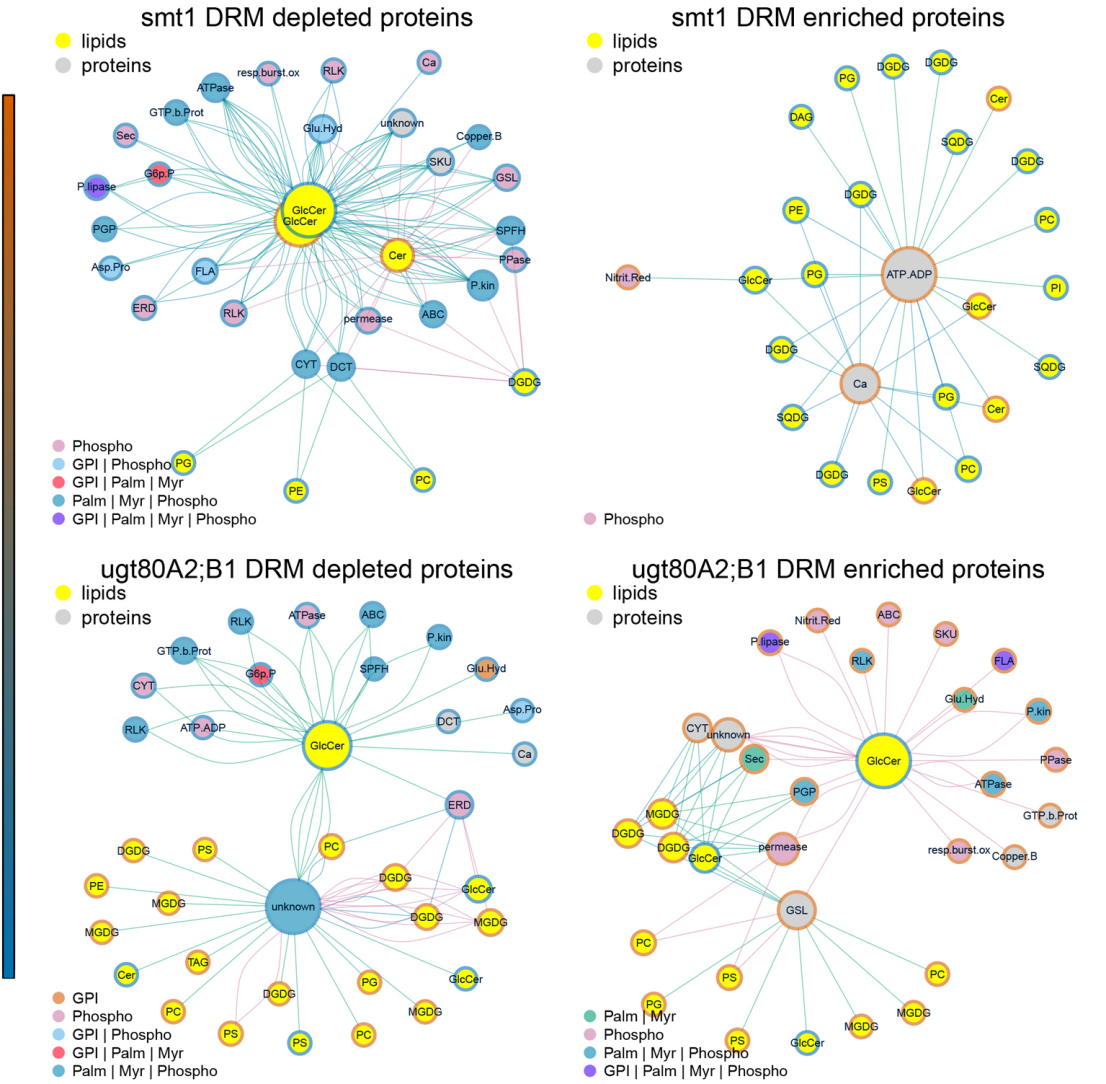
**Subnetworks of lipid–protein interactions (Figure 4) filtered for edges between commonly identified protein groups of *ugt80A2;B1* and *smt1***. Networks were further separated into proteins exhibiting an increased (red) or decreased (blue) DRM/DSF ratio. Abbreviations of protein groups are explained in Table 1 and Lipid species in Figure 4. Proteins could be divided into ceramide-dependent or non-dependent classes which show a high degree in edges with galactolipids or glycerolipids. From these networks a list of proteins with biologically-relevant alterations in protein abundances was obtained and is shown in Figure 1. Node color of proteins is according to postulated posttranslational modifications (PTM) relevant for protein sterol/lipid interactions. Abbreviations and color code are explained in Figure 4. GPI, Glycosyl-Phosphatidyl-Inositol; Palm, Palmitoylation; Myr, Myristoylation; Phospho, Phosphorylation.

The statistical significance of a co-occurrence between sterol-dependent proteins and specific lipid abundances was analyzed using a network based approach (Figures 4, 5). Pairwise scores between lipid abundances and sterol-dependence of proteins were calculated as a measure for lipid–protein correlation. These scores were based on log_2_ fold changes of lipid abundances in mutant compared to wildtype multiplied by the Unicorn scores (Zauber et al., 2013) of the identified sterol-dependent proteins. The significance of protein–lipid correlations displayed as network edges, was determined by applying an FDR threshold of 0.01%. This FDR-threshold was determined from score populations derived from randomized Unicorn-scores and randomized lipid abundance ratios. Based on this correlative relationship we propose lipid–protein interactions. Networks based on protein localizations (SUBA3; Tanz et al., 2013) showed that most protein abundances correlated significantly with abundances of glycosylceramides, which showed the highest degree among the lipid nodes in both networks (Figure 4). In *smt1*, proteins localized to the plasma membrane had a degree of 167 and were exclusively depleted (blue node frame) from DRM fractions. Similarly, in *ugt80A2;B1* plasma membrane protein were mainly connected to glycosylceramides (GlcCer). Phospholipids and galactolipids showed a generally lower degree of connectivity and were with a few exceptions connected to plastid or plasma membrane proteins. In *ugt80A2;B1*, proteins showed positive as well as negative correlations to depletion of GlcCer at equal frequencies. In general, proteins assigned to vacuolar, extracellular or nuclear localizations showed an enrichment in *ugt80A2;B1* while only plastidial and cytosolic proteins were enriched in *smt1*.

**Figure 6:**
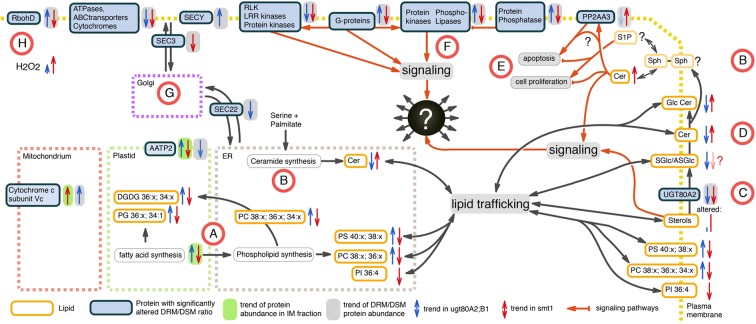
**Working model of altered proteins and lipids in *smt1* (blue) and *ugt80A2;B1* (red) based on the set of identified core proteins from network analysis**. **(A)** Fatty acid and glycerolipid synthesis is decreased in *smt1* and increased in *ugt80A2;B1*, with significant alterations in abundances of distinct glycerolipids. **(B)** Alteration in ceramide (Cer) *de novo* synthesis was not observable. Additionally, alterations in activity of salvage synthesis pathways (Van Brocklyn and Williams, 2012) could explain changes in abundances of ceramides in the mutants. **(C)** Glycosylation of sterols (SGlc/ASGlc) might additionally happen in plasma membrane catalyzed by UGT80A2, which was depleted from DRM in both mutants. **(D)** GlcCer levels are inversely altered in both mutants implying an additional sterol-dependent pathway for ceramide glycosylation in the plasma membrane. **(E)** Alterations in Cer activated phosphatase PP2 (Nickels and Broach, 1996) observed in a DRM-dependent response of PP2 subunit PP2AA3 indicate an unbalance in activation of apoptosis and cell proliferation in both mutants. **(F)** Phospholipase signaling was sterol-dependent in a discordant manner in both mutants. **(G)** Vesicle trafficking is likely affected in both mutants. **(H)** RbohD shows discordant sterol affinity, but lead to increased ROS production in both mutants. Lipids: PC, phosphatidylcholine; PG, phosphatidylglycerol; PS, phosphatidylserine; PI, phosphatidylinositol; Cer, ceramide; GlcCer, glycosylceramide; DGDG, digalactosyldiacylglycerol; SGlc, sterolglycosides; ASGlc, acylated sterolglycosides; Sph, sphingosine.

Subnetworks were constructed from edges of commonly-identified protein groups in *smt1* and *ugt80A2;B1* (Figure 5). This analysis resulted in a core set of 27 protein groups represented by 63 proteins in *smt1* and 59 different proteins in *ugt80A2;B1*. In *smt1*, 60 DRM-depleted proteins remained in the subnetwork and were mainly associated with the decrease of GlcCer and Cer and interestingly to one decreased GlcCer with a lower hydroxylation status. In contrast, only 3 DRM-enriched proteins remained in the *smt1* network (Figure 5 left graph) including two co-purifying proteins (plastidial ATP/ADP translocator and nitrite reductase) and one calcium dependent copine like protein. Proteins in this network were mainly linked to phospho- and galactolipids in contrast to proteins depleted in *smt1* DRM, which were almost exclusively linked to GlcCer and Cer. In *ugt80A2;B1*, proteins were almost evenly distributed between the subnetworks, with 31 depleted and 27 enriched proteins. These proteins could be divided into two classes: a larger class connected to one of the most depleted glycosylceramides and a smaller group mainly linked to enriched galacto- and phospholipids. Based on this network analysis and the observed lipid profiles, a working model for biologically relevant alterations in lipid and signaling pathways was constructed (Figure 6).

In our study, fatty acid and glycerolipid synthesis appeared to be decreased in *smt1* and increased in *ugt80A2;B1* (Figure 6A). This is supported by the significant alterations in detected phospho- and galactolipids (Figure 3). We are not certain whether the *de novo* synthesis pathway of ceramide biosynthesis was affected as we could not identify most of the proteins involved in this process by the proteomic analysis. However, the alkaline phytoceramidase (CES1) showed a decreased DRM/DSM protein abundance ratio in *ugt80A2;B1* which could indicate alterations in salvage synthesis pathways of ceramides (Van Brocklyn and Williams, 2012). In contrast, CES1 was not detectable in *smt1* DSM fraction, so no conclusions regarding the CES1 DRM/DSM distribution in *smt1* could be drawn (Figure 6B).

Glycosylation of sterols (SGlc/ASGlc) is likely to also happen in the plasma membrane and this pathway could be catalyzed by UGT80A2. This Glycosyl-transferase was depleted from DRM in both mutants (Figure 6C) and is listed in the PhosPhat database as a phosphorylated protein (Durek et al., 2010; Zulawski et al., 2013) and might be involved in dynamic microdomain remodeling processes. Because several GlcCer levels are inversely altered in both mutants anaylzed here, it implies an additional sterol-dependent pathway for ceramide glycosylation in the plasma membrane (Figure 6E). Therefore, a dynamic glycosylation of ceramides may also be involved in microdomain organization and remodeling. Furthermore, ceramide signaling pathways seem to be altered in the sterol-mutants and the observed elevated levels of Cer in *smt1* might activate phosphatase PP2A (Nickels and Broach, 1996). A ceramide dependent activation of PP2A was previously observed in yeast triggering apoptosis (Nickels and Broach, 1996). Here we observed a sterol-dependent response of the PP2A subunit PP2AA3 (Figure 6D) that could additionally also be a result of ceramide signaling events. PP2AA3 was previously shown to be involved in various signaling processes in plants (Zhou et al., 2004; Blakeslee et al., 2008; Dai et al., 2012a,b). Therefore, misbalances in signaling events might contribute to the strong phenotype of *smt1* with cells remaining in a continuous apoptotic status.

In addition, phospholipases showed opposing responses in their degree of DRM residence in both mutants (Figure 6F) and regulation of protein–protein interactions with G-proteins, protein kinases and phosphatases might involve different degrees of lipid phase separation. Vesicular transport might be affected in both mutants as indicated by differential DRM residences of vesicle trafficking proteins SEC3 and SEC22 (Figure 6G). The plant NADPH oxidase RbohD showed discordant changes in DRM/DSM ratio distributions between *smt1* and *ugt80* (Figure 6H). Based on the prediction of perturbed H_2_O_2_ levels, as indicated by responses of RbohD (Liu et al., 2009) (Table 1), as well as based on reported linkages of ceramide signaling to reactive oxygen signaling (summarized in Berkey et al., 2012; Van Brocklyn and Williams, 2012), we measured intrinsic H_2_O_2_ levels in both mutants studied (Supplemental Figure 4). Consistent with our prediction we observed disturbed oxidative response levels in both mutants with threefold higher H_2_O_2_ concentrations in *smt1* and only one fold higher levels in *ugt80A2;B1* relative to wildtype (Figure 6G).

## Discussion

The proteome- and lipidome-wide alterations observed in *smt1* and *ugt80A2;B1* can largely be brought down to specific alterations in membrane structure and signaling properties. Membrane structures of the mutants are mainly affected by the intrinsically altered sterol composition and sterol glycosylation status (Schrick et al., 2002, 2004b; DeBolt et al., 2009). By combining information from systematic proteomics with information from lipidomics, a number of biologically-relevant processes could be identified and linked to new aspects in interpretation of the phenotypes of both mutants.

### Structural effects of altered membrane lipid environments on DRM protein composition

We could show that besides changed sterol composition, particularly alterations in ceramide (Cer) and glycosylceramide (GlcCer) abundances were related with changes in protein DRM/DSF distribution in the mutants. Cer abundance as well as overall glycosylation status of microdomain lipids, in addition to sterols, could potentially affect recruitment and residence times of proteins in membrane microdomains.

Recently we could show, that *smt1* exhibits a protein DRM/DSF distribution that resembles methyl-β-cyclodextrin (mβcd) treated DRM compositions (Zauber et al., 2013). This suggests a structure-related impact of altered sterol–protein interactions in the plasma membrane. In contrast to *smt1*, the whole-plant phenotype of *ugt80A2;B1* is less severe and adult plants are remarkably healthy. Consistently, the sterol profile of *ugt80A2;B1* was shown not to be strongly altered, apart from a depletion of sterolglycosides. However, low abundant sterols were slightly decreased (cycloarthenol and cholesterol) or increased (isofucosterol) (DeBolt et al., 2009). Nevertheless, strong changes in the glycero- and sphingolipids and DRM/DSF protein distributions were visible for this mutant. These changes were in many aspects discordant to the changes in *smt1*. While the differential proteomic profile suggests plasma membrane structure can be affected in different ways by both mutant backgrounds, a general depletion of known sterol-dependent proteins was observed for both mutants. This suggests that structural perturbations in the plasma membrane could reduce sterol–protein interactions in both mutants with global effects on protein and lipid residence and recruitment to microdomains. Furthermore, the glycosylation status of microdomain lipids seems to play a crucial role for protein recruitment, as was shown in *ugt80A2;B1* mutant. Glycosylresidues in microdomains might be one of the triggers for reinforcement or prevention of protein recruitment into these microdomains or, alternatively, general microdomain formation (Table 1). Glycosylhydrolases previously identified as resident to membrane microdomains (Kierszniowska et al., 2009; Gupta and Surolia, 2010) might be involved in the dynamic control of the glycosylation in microdomain lipids.

A small number of DRM-dependent microdomain proteins was identified as co-purifying in DRM and DSF fractions and could not be automatically filtered by pairwise comparisons with intracellular membrane and soluble protein fractions. The purity of plasma membrane preparations over two-phase systems was reported to reach 95% based on activity measurements of known plasma membrane proteins (Bérczi and Asard, 2003; Mika et al., 2004; Schindler and Nothwang, 2006; Lüthje, 2008). Nevertheless, major proteins of endomembrane systems can introduce biases into plasma membrane analysis (Bérczi and Asard, 2003, Griesen et al., 2004; Kjell et al., 2004; Preger et al., 2005; Lüthje, 2008). The differential distribution of these proteins in both mutants may indicate biologically relevant structural changes in membranes leading to an altered protein abundance and/or distribution. Within the core set of proteins selected from the network analysis a glucose-6-phosphate/phosphate translocator and a dicarboxilic acid transporter were significantly altered in their DRM/DSF ratios (Table 1). Analysis of copy numbers revealed, that these proteins even had higher copy numbers in DSF than in IM fraction, where they would have been expected (Supplemental Figure 5). While the DRM/DSF ratios rather reflects alterations in sterol–protein interactions particularly of the plasma membrane, sterol dependence of plastidial or mitochondrial membrane proteins is debatable due to very low amounts of sterols in these organelle membranes (Block et al., 2007). However, if protein abundances are drastically altered it is possible that these changes affect abundances across all analyzed fractions. Even though the exact structural basis for the apparent changes in sterol–protein affinities remains unclear for these co-purifying proteins, their observed alterations in *smt1* may also be linked to disturbed membrane trafficking, as explained later.

### Alterations in signaling pathways localized at the plasma membrane

The strong changes in membrane lipid composition in the mutants may have direct implications on signaling pathways that are initiated at the plasma membrane. Due to the strong changes seen in cellular lipid composition, observed alterations in plasma membrane signaling can for the most part be attributed to structural alterations in the plasma membrane. Since microdomain-forming lipids, such as ceramides, are particularly affected, specific alterations in microdomain formation and dynamic protein recruitment are likely. As microdomains were shown to be related to signaling, it is likely that *smt1* and *ugt80A2;B1* have altered sterol-sphingolipid-protein interactions. Therefore, signaling involving the plasma membrane may not function properly in response to environmental or cellular stimuli. In the mutants, different signaling pathways seem to be affected, involving not only signaling proteins but also microdomain specific lipids. As signaling properties have been shown in particular for sterols (He et al., 2003) and ceramides (Cer) (Nickels and Broach, 1996; Chen et al., 2008; Alden et al., 2011; Van Brocklyn and Williams, 2012), a diverse set of signaling pathways may be constitutively altered in *smt1* and *ugt80A2;B1*. The impact of these mutations on plant phenotypes is challenging to interpret as it is most likely a combination of structural effects and altered signaling properties of the membrane. Nevertheless, the systems biology-based approach applied in this study proposes two signaling pathways affecting the phenotype of both mutants.

Cer and sphingosine-1-phosphate (S1P) are both involved in signaling but regulate contrary processes (Breslow and Weissman, 2010; Van Brocklyn and Williams, 2012). In the currently accepted model, both lipids are directly related to sphingosine and the balance of the involved reactions is crucial for the activation of either apoptosis or cell proliferation processes when Cer or S1P is high, respectively. This model was shown to be ubiquitous among different species and seems to play a role in plants as well (Stunff et al., 2004). As one target of this signaling pathway, the phosphatase PP2A in yeast was shown to be activated by ceramides (Nickels and Broach, 1996). In support of this, the positive regulatory subunit PP2AA3 (Marmagne et al., 2007; Dai et al., 2012a,b) of PP2A phosphatase showed a significant increase in DRM/DSF ratios in *smt1*, but was slightly decreased in *ugt80A2;B1*, although this was not significant (Supplemental Figure 5). As PP2A is involved in cell growth limiting processes, the activity of PP2A could therefore contribute to the extreme pleiotropic phenotype of *smt1*. Alterations in PP2A activity can also be considered to be a direct effect of observed elevated Cer levels in *smt1*. If levels of S1P were raised in *ugt80A2;B1* and decreased in *smt1*, alterations in S1P and Cer levels might explain the inverse lipid profiles observed for both mutants in comparison to wildtype. Following this hypothesis, both mutants could be oppositely unbalanced in signaling processes that regulate apoptosis and cell proliferation (Van Brocklyn and Williams, 2012) (Figure 6E). To investigate this further, sphingosine kinase, ceramide synthase, ceramidase as well as S1P-phosphatase could serve as targets of future studies.

Furthermore we could also identify an inverse abundance profile for phospholipases between both mutants. The phospholipase signaling cascade was shown to involve particularly G-proteins, protein kinases as well as phosphatases (Nickels and Broach, 1996). All these protein groups showed an inverse DRM abundance in the two mutants indicated by inverse DRM/DSF distribution ratios between *ugt80A2;B1* and *smt1*. Phospholipase activity was shown to be linked to microdomains as recently summarized (Gardiner and Marc, 2013). Therefore, we postulate a regulation of the detected phospholipases that involves protein segregation in membrane microdomains. In this model, recruitment of phospholipases and their regulating proteins into microdomains brings them into close proximity and could trigger phospholipase activity. Thereby, phosphorylation of the identified phospholipases could be a potential mechanism for regulating the recruitment to microdomains. Accordingly, the identified phospholipase 2C was reported to be phosphorylated in sucrose and nitrate starvation/resupply experiments (PhosPhat; Durek et al., 2010) and phosphorylation-dependent recruitment of proteins to membrane microdomains has now been reported in the context of ABA signaling (Demir et al., 2013). Interestingly, sterol–protein interactions among these proteins were increased in *ugt80A2;B1*. This exemplifies once more that glycosylation of microdomain lipids could directly affect the recruitment of sterol-dependent proteins.

### Evidence for sterols as donors of glycosylresidues in a ceramide glycosylation pathway

In *ugt80A2;B1*, the observed significant decrease of glycosylated ceramide (GlcCer) species correlates with the depletion of glycosylated sterols (DeBolt et al., 2009). GlcCer in beans were shown to be mainly present in the outer plasma membrane leaflet (Lynch et al., 1997). It was therefore suggested that glycosylresidues from sterolglycosides might serve as precursors also for ceramide glycosylation (Lynch and Phinney, 1995). Depletion of GlcCer in *ugt80A2;B1* strongly supports this finding in *Arabidopsis*, but it does not prove location of this process at the plasma membrane. Interestingly, UGT80A2 was depleted from DRM fractions in the *smt1* mutant and was shown to also be depleted in an independent mβcd treatment of plasma membranes (Zauber et al., 2013). Furthermore, according to TAIR (Poole, 2007), UGT80A2 is localized to the plasma membrane and was exclusively identified in DRM and DSF fractions in our analysis. This raises the possibility that sterol glycosylation indeed occurs at the plasma membrane in addition to sterol glycosylation events happening in the endomembrane system. Therefore, sterol glycosylation events at the plasma membrane might be particularly involved in controlling lipid remodeling processes and lipid–protein interactions induced by dynamic lipid glycosylation processes. An additional level of control could thus be imposed by phosphorylation, as UGT80A2 exhibits a treatment-specific phosphorylation status according to PhosPhat database (Durek et al., 2010; Zulawski et al., 2013). Our current working model suggests therefore, that a dynamic glycosylation status of microdomains may be specifically adjusted to environmental and cellular conditions. In case sterol glycosylation occurs at the plasma membrane, the available pools of glucose-derivates for sterol glycosylation might accumulate in *ugt80A2;B1* which increases the probability for glucose derivates being incorporated into other extracellular structures, such as cell wall components. Consistent with this hypothesis, *ugt80A2* and *ugt80B1* mutants were shown to incorporate more glucose residues into their cell walls (DeBolt et al., 2009). This effect was also observed by an analysis of the mucilage of *ugt80A2;B1* seeds (Supplemental Figure 6). Interestingly, proteins with a putative function in glucan synthesis showed altered sterol–protein interactions in *smt1* as well as *ugt80A2;B1* (Table 1) and *smt1* was reported to have lowered cellulose levels (Schrick et al., 2004a). Thus, our data strongly supports the existence of plasma membrane-bound lipid glycosylation.

### Inverse effects on lipid synthesis pathways in both mutants

Both mutants exhibited inverse abundance profiles in most of the altered lipids. Interestingly, the changes in lipid abundances correlated with protein abundances involved in lipid synthesis. Synthesis of acyl chains takes place in the plastid, while esterification of fatty acids occurs in both the plastid and the endoplasmic reticulum to give rise to membrane lipids (Benning, 2008). The analysis of proteins involved in the lipid synthesis pathways suggested that fatty acid synthesis in general could be decreased in *smt1* and elevated in *ugt80A2;B1*. The overall trend of increased lipid synthesis in *ugt80A2;B1* was even supported by the abundances of proteins involved in lipid degradation (Figure 3C). In general, no significant alterations could be observed in triacylglyceride abundance profiles (Supplemental Figure 3). For this reason, global starvation effects on fatty acid synthesis can presumably be excluded in the mutants. While the used callus system was suppressed in photosynthesis activity, alterations in lipid synthesis require adjustment of energy supply in form of ATP to the plastid in the mutants. Such a scenario could explain the observed altered protein abundances of the plastidial ATP/ADP transporter AATP2 in the IM fraction of both mutants (Figure 6 and Supplemental Figure 5).

### General implications of lipid–protein interactions in plasma membrane functions

A major function of the plasma membrane is the build-up and maintenance of a transmembrane electric potential through a number of ATPases (Flickinger et al., 2010). Further, a constitutive redox system involving cytochromes (Asard et al., 2001; Lüthje et al., 2005) and NAD(P)H oxidoreductases (Lüthje et al., 1997; Sparla et al., 1997; Bérczi and Møller, 1998a,b; Trost et al., 2000; Lüthje, 2008) was identified and linked to regulation of redox processes for inorganic nutrient uptake (Böttger and Lüthen, 1986; Döring and Lüthje, 1996; Robinson et al., 1997, 1999; Waters et al., 2002). Cytochromes and ATPases were both significantly decreased in their DRM/DSF ratio in *ugt80A2;B1* and *smt1*, indicating the relevance of sterols and sterol glycosylation also for redox processes and transport. The detailed implications of alterations in these membrane functions are beyond the scope of our study. However, it needs to be pointed out, that phase separation in membranes was shown to also be dependent on pH gradients in giant vesicles (Staneva et al., 2012) underlining the biological significance of the observed alterations in cytochromes and ATPases identified in this study. Nevertheless, only the co-purifying cytochrome c oxidase showed an increased DRM/DSF ratio in *ugt80A2;B1* and is involved in mitochondrial respiration for generating ATP. Elevated levels of cytochrome c oxidase were also observed in IM and SP fraction in both mutants, indicating a possible increase in cell respiration. Thus, elevated respiration in both mutants could be linked to perturbed cell homeostasis as a result of alterations in cell signaling status.

Membrane trafficking in cells is an obligatory process and interconnects most cellular membrane structures in the cell. In this study, we identified proteins being significantly and discordantly altered in their DRM/DSF ratio abundance between the two mutants, which indicates distinct alterations in membrane trafficking pathways. For instance, in *ugt80A2;B1* membrane trafficking from ER to Golgi apparatus seems to be altered indicated by decreased DRM/DSF abundance ratio of SEC22. In contrast, vesicle movement from Golgi apparatus to the plasma membrane shows alterations particularly in *smt1*, with SEC3 displaying a decreased DRM/DSF ratio. Further SEC14 was shown to be a major factor for localizing recruitment of microdomain proteins to the plasma membrane (Curwin et al., 2013). Even though this protein was not significantly altered in the DRM/DSF distribution, when analyzing log_2_ fold-change or DRM/DSF protein abundance ratios, it showed depletion from DRM and DSF fractions in *smt1* but was increased in *ugt80A2;B1* (Supplemental Figure 5).

The very high amounts of co-purifying proteins in the DRM fraction of *smt1* might be even linked to disturbed membrane trafficking. However, our data at this point does not allow conclusions on whether structural changes or affected signaling pathways in involved membranes are triggering these effects. Since sterols were shown to be rapidly transported by MT to the plasma membrane (Hartmann and Benveniste, 1987; Moreau et al., 1998; Grebe et al., 2003), disturbed membrane trafficking would likely cause alterations in the sterol gradient from ER over Golgi apparatus to the plasma membrane. Therefore, the high amount of co-purifying proteins in *smt1* DRM fraction suggests an accumulation of sterols in non-plasma-membrane structures. This could then explain high amounts of co-purifying “non-plasma membrane” proteins by sterol-enriched patches in other membrane types in *smt1*. A similar effect was described for maize roots treated with fenpropimorph (Grandmougin et al., 1989). There, it was shown, that unusual sterols accumulated, but were incorporated into membranes with drastic effects on the cellular distribution of sterols leading to enrichment of free sterols in the ER (Grandmougin et al., 1989). Such an effect was also observed in *smt1*, in which functional categories of DRM co-purifying proteins linked to ER and related membrane structures (Supplemental Figure 7) are dominant among the co-purifying proteins with typical sterol-dependent abundance distributions. Therefore, although total sterol amounts are likely depleted in *smt1* plasma membrane, subcellular sterol distributions may additionally be affected.

## Conclusion

Sterol-biosynthesis mutants have strong pleiotropic phenotypes, which are obviously the result of several overlaying effects related to perturbations in membrane structure and signaling properties. In order to understand the underlying biological processes in more detail, systems biology-based approaches as exemplified in this study, are helpful in dissecting the pathways involved. In particular, the changes that were observed in lipid abundances were of a general systemic nature and have not been considered so far when studying sterol–protein relationships. Within this study we could connect alterations in the mutants' lipidomes and proteomes resulting in a consistent working model of specifically altered biological processes in *ugt80A2;B1* and *smt1*. With respect to effects on sterol–protein interactions, we could show that (1) particularly an interplay between sterols, sphingolipids and proteins is necessary for formation and maintenance of membrane subcompartmentation in form of microdomains. We could (2) confirm disturbed H_2_O_2_ signaling in *smt1* and *ugt80A2;B1* connecting this signaling pathway to lipid/sterol environments. In addition, (3) modifications, such as glycosylation of lipid species (sterols, ceramides, or sphingolipids) may also serve as additional regulating factors for microdomain formation and dynamic glycosylation status of microdomains might control and alter specific recruitment of proteins to membrane subcompartments. Our work herewith provides a thorough assessment and working model of the roles of lipid–protein interactions for cellular signaling processes.

## Methods

### Cell suspension culturing

Heterozygous seeds from *smt1* mutant named *cphT357* (Schrick et al., 2002) with a point mutation at T357 in the *smt1* (AT5G13710) locus were germinated and homozygous seedlings showing the *cph* typical dwarf like phenotype were selected (Supplemental Figure 8). Seedlings from homozygous *ugt80A2;B1* (A2: AT3G07020; B1: AT1G43620) double mutant (DeBolt et al., 2009; Schrick et al., 2012b) (Supplemental Figure 8) were immediately used for callus initiation. Callus cultures were initialized from leafs on 6.8% Agar in full mineral Murashige-Skoog-Medium (Murashige and Skoog, 1962) with the freshly added components 3% sucrose, 200 mg/l myoinositol, 1 mg/l 2,4-dichlorophenoxyacetic acid and 0.25 mg/l kinetin for *smt1*, *ugt80A2;B1* and corresponding wildtype Ler-0 or Col-0, respectively (*A. thaliana*). Established callus cultures (Supplementary Figure 1) were chopped and directed to solution culture using the same agar free medium. *Arabidopsis* cell suspension cultures were subcultured to fresh medium every week. For all analyses up to three or four biological replicates were collected for *smt1* or *ugt80A2;B1*, respectively and their corresponding wildtypes.

### Sample preparation and protein extraction

The protocol for sample preparation was recently described (Zauber et al., 2013) and will only be briefly summarized. Frozen cell powder was mixed with 2 volumes of cold extraction buffer (100 mM Hepes-KOH pH 7.5, 250 mM sucrose, 3 mM KCL, 0.1 mM EDTA, 1 mM DTT and addition of μl/l protease inhibitor cocktail (Thermo Scientific)). Supernatant after ultra centrifugation was used for extracting soluble proteins (SP). Microsomal pellet fraction was resuspended (5 mM KH_2_PO_4_, 0.33 M sucrose, 3 mM KCL, 0.1 mM EDTA, 1 mM DTT, protein inhibitor cocktail) and plasma membrane (PM) and intracellular membranes (IM) containing organelle membranes were separated using aqueous two phase system (Schindler and Nothwang, 2006). IM pellet and PM pellet were resuspended in 25 mM Tris buffer (pH 7.5; 150 mM NaCl; 5 mM EDTA; 1 mM DTT). Samples were adjusted to equal protein amounts of 100 μg using Bradford (Bradford, 1976). All PM fractions were mixed with TritonX 100 and incubated for 30 min at 4°C using a protein detergent ratio of 1:15 at a total TritonX 100 concentration of 1%. A detergent resistant membrane fraction (DRM) and a detergent soluble fraction (DSF) was separated on a sucrose gradient (1.8 mM; 1.6 mM; 1.4 mM and 0.15 mM) by ultra centrifugation. From the four fractions DRM, DSF, SP, and IM, proteins were extracted using methanol/chloroform extraction.

### Lipid extraction

From frozen callus, aliquots of 25 mg were prepared in 1.5 mL Eppendorf tubes, maintaining the sample constantly frozen. The aliquots were suspended in 1 mL of a pre-cooled 1:3 methanol/methyl tert-butyl ether (MTBE) solution. After 10 min ultrasonication and shaking for 30 min at 4°C, 500 μl of a 3:1 water/methanol solution were added to the samples. After mixing, the tubes were spun down for 5 min in a table top centrifuge. The organic phase was dried down in a SpeedVac and stored at −20°C before being analyzed. Four to five biological replicates were analyzed per mutant or wildtype line.

### LC-MS/MS analysis and protein identification

Up to three replicate injections, containing 25 μg of protein, were analyzed by LC-MS/MS using nano-flow HPLC (Proxeon Biosystems) and an Orbitrap hybrid mass spectrometer (LTQ-Orbitrap XL, Thermo Scientific) as mass analyzer. Peptides were eluted from a 75 μm analytical column (Reprosil C18, Dr. Maisch GmbH) on a linear gradient, running from 5% to 80% acetonitrile in 90 min at a flow rate of 250 nL/min. Up to five data-dependent MS/MS spectra were acquired in the linear ion trap for each FTMS full-scan spectrum acquired at 60,000 full-width half-maximum resolution settings with an overall cycle time of approximately 1 s. Raw file peak extraction, protein identification and quantitation of peptides was done by MaxQuant (version 1.2.2.5) using a protein sequence database of *Arabidopsis thaliana* (TAIR10, 35386 entries, www.arabidopsis.org). For protein identification by Andromeda search engine implemented in MaxQuant (Cox and Mann, 2008), carbamidomethylation and N-terminal protein acetylation were used as fixed modifications and methionine oxidation as a variable modification. Trypsin was selected as a digestive enzyme and for database search, up to two missed cleavages were allowed. Precursor ion tolerance was set to 6 ppm, mass tolerance for fragment ion matching was 0.5 Da. Standard settings in MaxQuant involving peptide false-discovery rate of 0.01, minimum peptide length of 6 amino acids and enabled retention time correlation, over a time window of 2 min, were used.

### Peak identification and quantification of lipids

GeneData software was used to pre-process the chromatogram raw files, that is, baseline correction, chemical noise subtraction, chromatogram alignment and peak detection. Pre-processing parameters were set to the same values as described (Giavalisco et al., 2011). After pre-processing, a list of detected peaks (a retention time and m/z pair) and a matrix with their respective intensities for each sample were obtained. A targeted search for the glycerolipid species of interest was carried out using the in-house developed R package “grms,” and based on the library compiled by Giavalisco et al. (2011). The software first performs a retention time correction of the output matrix based on previously identified markers with known m/z values and retention times. Then, the compounds were searched by comparing their specific m/z, expected adduct and retention times within user-given tolerance thresholds. A mass tolerance of 5 ppm and retention time deviation of 0.5 min were used to identify the lipid species. Further confirmation was achieved by manually inspecting the chromatograms.

### Statistical analysis

Statistical analysis of all datasets was done using open source scripting language R, version 2.15.2 (Team, 2009). Analyses involved functions from the following packages: “gplots” (Warnes et al., 2013a), “igraph” (Han et al., 2010, 2011), “gdata” (Warnes et al., 2013b), “seqinr” (Charif et al., 2007), and “grImport” (Murrell et al., 2012). Lipids detected in positive and negative mode were combined before normalization. For normalization only lipids with a variation lower than the median variation of all lipids across all analyzed samples were used. Triacylglycerides (TAG) were excluded for calculating total ion-intensity sums per sample. Finally, all lipids were expressed as fraction of total ion-intensity sums. For further analysis, data was z-scaled and linearly transformed to positive values for log_2_ transformation. Differential abundances were tested using *t*-test (α = 0.05). Protein intensities were calculated from peptide ion-intensity based on the table evidence.txt derived from MaxQuant. This table was processed further with the cRacker platform (Zauber and Schulze, 2012) for automated analysis of peptide intensities. Parameters were set as described (Zauber et al., 2013). Principle steps involved peptides exclusion if measured in less than 70% of one of the fractions. Further peptide intensities within each sample were normalized to fraction of total ion-intensity sums. Normalized peptide intensities were median scaled and median averaged. cRacker exports were directly used for analyzing DRM/DSF protein abundance ratios using Unicorn. The applied algorithm was recently described (Zauber et al., 2013). Unicorn is based on bootstrapping intensities from ratios for statistical analysis of protein distribution between two fractions. False positives were controlled by calculating a data specific threshold based on randomized data. False discovery rates (FDR) were set to <1%. Significant protein candidates were further filtered out against higher abundance proteins in SP or IM fraction, applying a pairwise comparison using *t*-test (α = 0.01). At this step the applied statistics was used to filter out co-purifying proteins. Thats why a multiple testing correction was not applied to be more stringent in the filtering.

### Network analysis

A combined score for definition of lipid–protein correlations was calculated by multiplying lipid log_2_ fold changes in mutant versus wildtype with scores from comparative DRM/DSF protein abundance analysis (Unicorn scores). Thereby, only significantly altered proteins were included. Significance of lipid–protein dependence was defined by a false discovery threshold <1%. This threshold was calculated from randomized sampling of lipid ratios and protein Unicorn scores. Networks were visualized using the “igraph” (Han et al., 2010, 2011) R-package. Protein subcellular localizations were obtained from SUBA3 (Tanz et al., 2013). Phosphorylation data was obtained from PhosPhat database (Durek et al., 2010; Zulawski et al., 2013). Myristoylation data was taken from TAIR and was based on work from Thierry Meinnel's group. Protein palmitoylation was predicted using CSS-Palm software 3.0 (Ren et al., 2008). GPI-anchoring of proteins was predicted using *Arabidopsis* specific predictions obtained from GPI-DB (Poisson et al., 2007). Palmitoylation and GPI-anchoring predictions were FDR (FDR <0.05%) controlled, running both algorithms with randomized sequences from analyzed proteins in parallel.

### Conflict of interest statement

The authors declare that the research was conducted in the absence of any commercial or financial relationships that could be construed as a potential conflict of interest.

## Supplementary material

Supplemental Figure 1 **Photographs from callus cultures**.

Supplemental Figure 2 **Subcellular localizations of responding proteins according to SUBA3 (Tanz et al., 2013) in DRM/DSF for *smt1* (left) and *ugt80A2;B1* mutant (right)**.

Supplemental Figure 3 **Barplots of log_2_ transformed intensity ratios of all detected lipids**.

Supplemental Figure 4 **H_2_O_2_ contents in callus from smt1, ugt80A2;B1, and corresponding wildtypes**.

Supplemental Figure 5 **Barplots of calculated protein abundances from all analyzed proteins across all experiments**.

Supplemental Figure 6 **Barplots of amounts from mucilage side chain residues using HPLC system for detection after hydrolysis of purified mucilage from seeds (Vasilevski et al., 2012)**.

Supplemental Figure 7 **Mapman functional categories of co-purifiying proteins showing a significantly increased DRM/DSM protein abundance ratio in *smt1***.

Supplemental Figure 8 **Homozygous *ugt80A2;B1* was confirmed using a PCR screen as described in DeBolt et al. (2009)**.

Supplemental Table 1 **List of proteins with significantly different DRM/DSF distributions in mutants versus wildtype**.

Supplemental Table 2 **List of analyzed lipids**.

Supplemental Table 3 **List of responding proteins involved in lipid synthesis**.
