# Supplementary material for: Plasma membrane lipid–protein interactions affect signaling processes in sterol-biosynthesis mutants in Arabidopsis thaliana
Source: Front Plant Sci. 2014 Mar 18;5:78. doi: 10.3389/fpls.2014.00078 (PMC3957024; doi:10.3389/fpls.2014.00078)
Supplement: Supplemental Figure 1 — Photographs from callus cultures. [file DataSheet4.ZIP › supplemental figure 2.pdf]

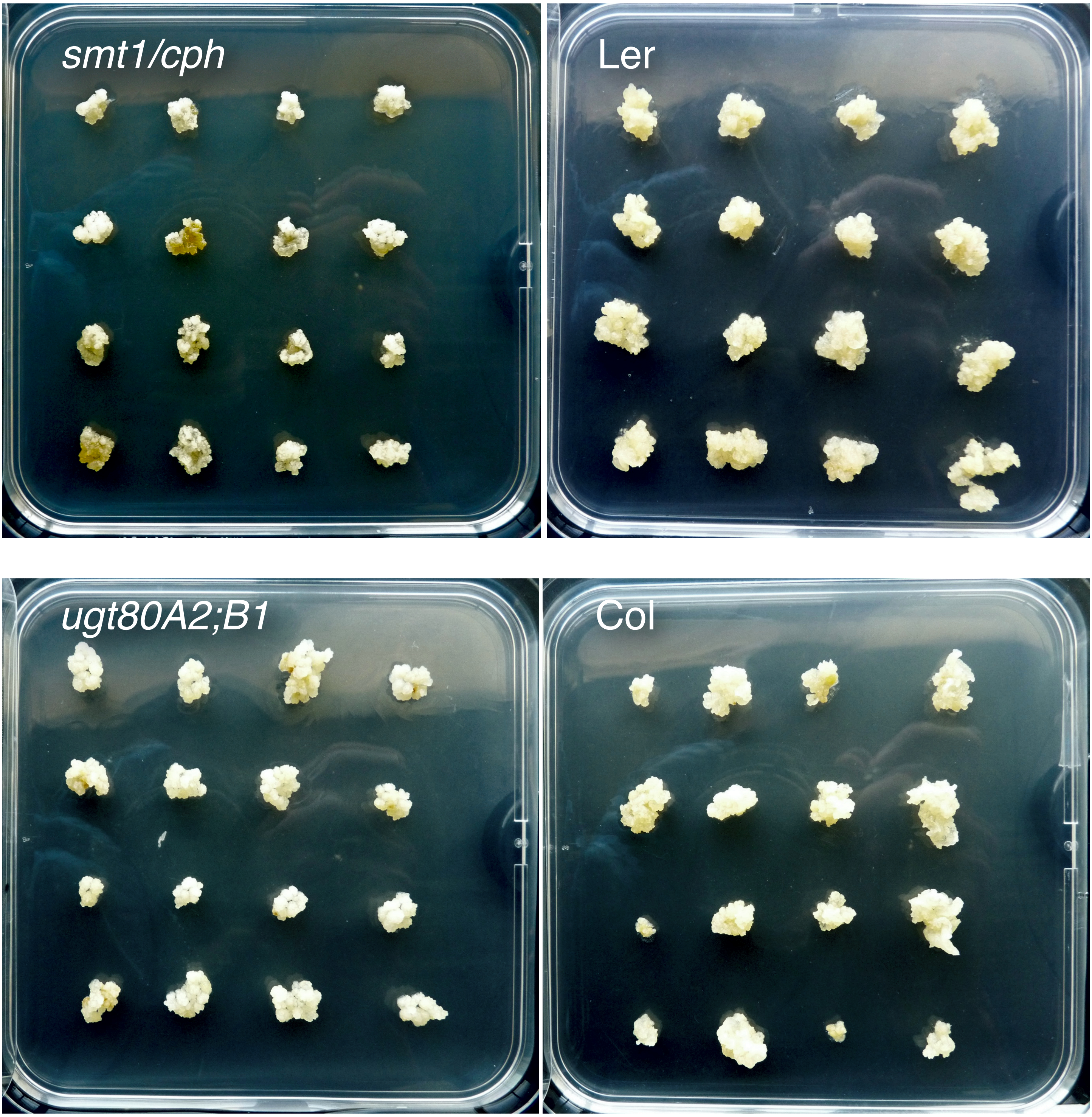

Supplemental Figure 2:
Callus cultures of *smt1* and *ugt80A2;B1* mutant on the left and corresponding wild type on the right. Callus was plated with equal sizes on MS agar (3% sucrose, 1mg/l 2,4D, 0.25 mg/l kinetin, 200mg/l myo-Inositol). After 3 weeks *ugt80A2;B1* shows no difference in size to wild-type (Col). In contrast, *smt1* is smaller than wild type (Ler).
