# Supplementary figures and images for: Plasma membrane lipid–protein interactions affect signaling processes in sterol-biosynthesis mutants in Arabidopsis thaliana
Source: Front Plant Sci. 2014 Mar 18;5:78. doi: 10.3389/fpls.2014.00078 (PMC3957024; doi:10.3389/fpls.2014.00078)

MapMan functional categories  
of excluded proteins from smt1 DRM/DSM ratio analysis

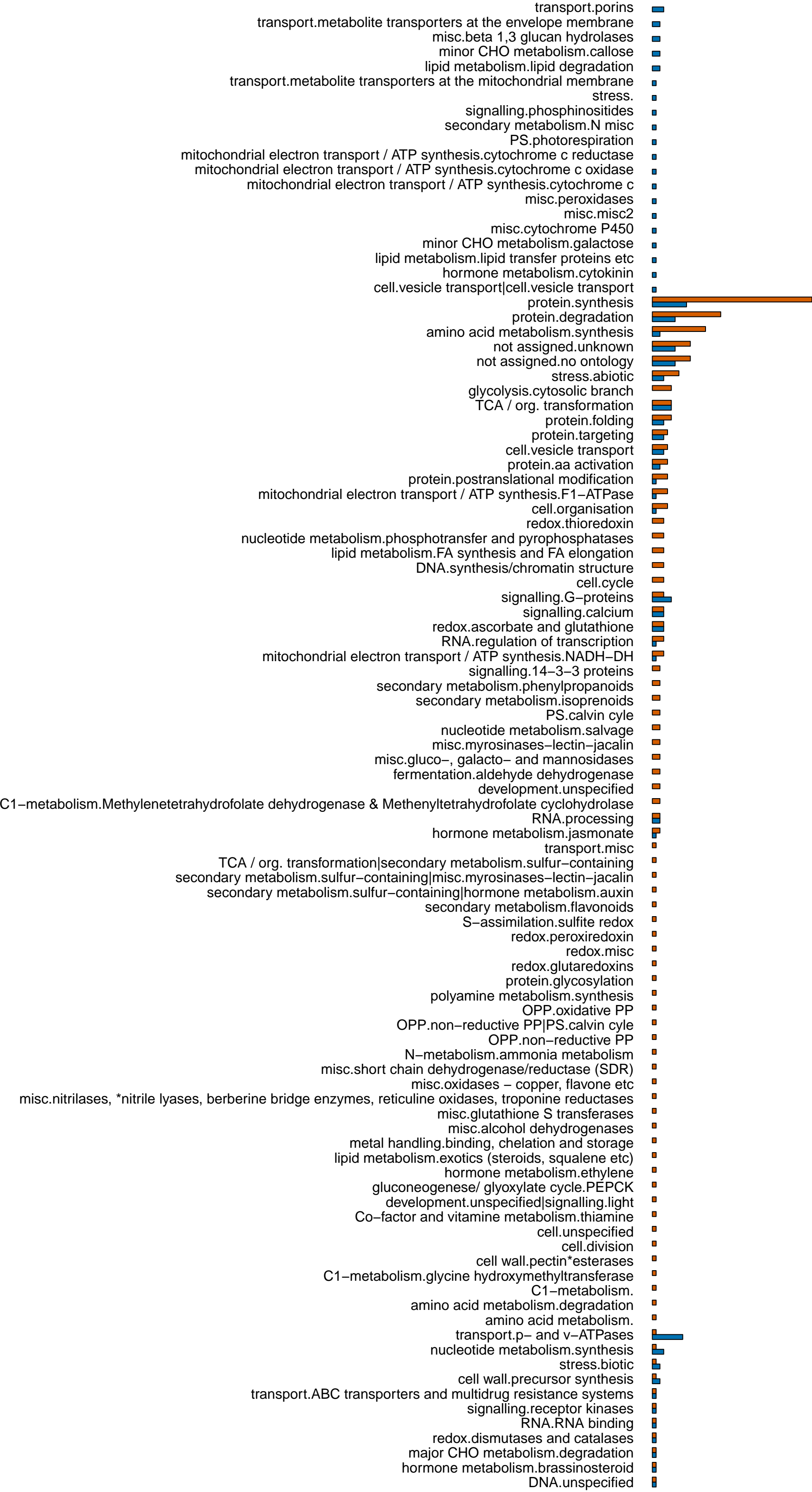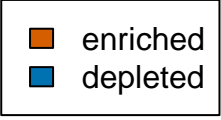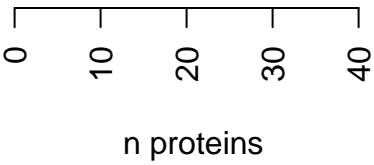

Supplement: Supplemental Figure 1 — Photographs from callus cultures. [file DataSheet4.ZIP › supplemental figure 6.pdf]

A)

*cph-T357/smt1*

wt

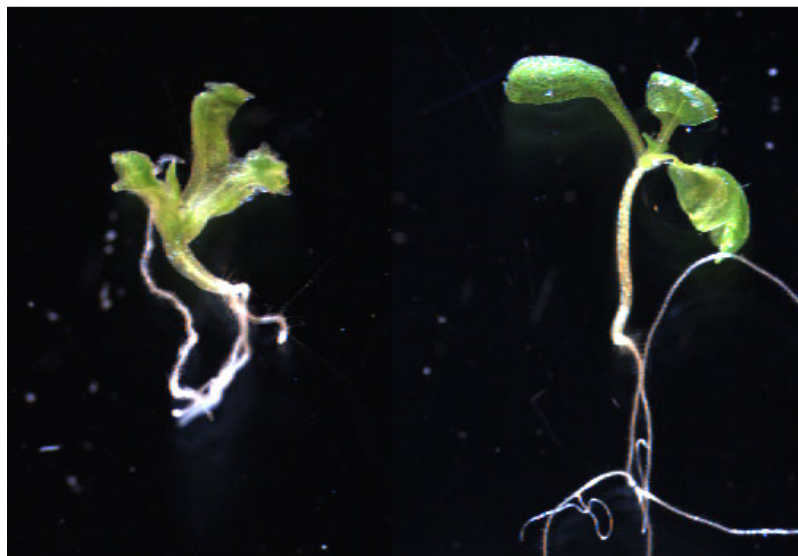

B)

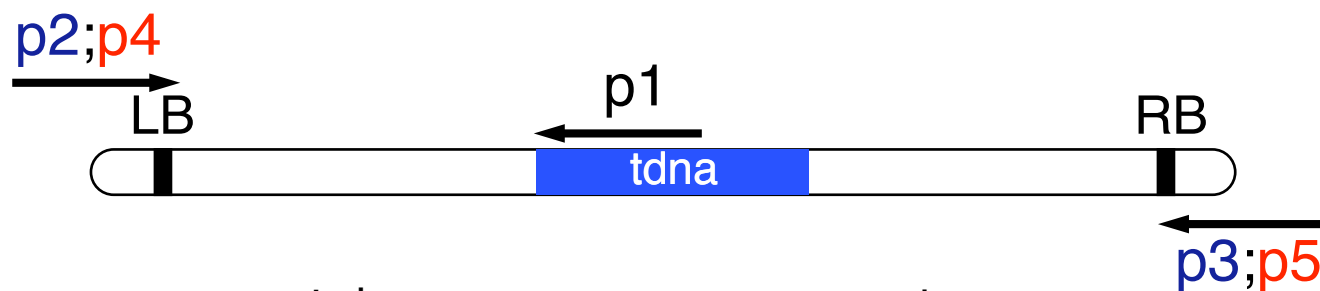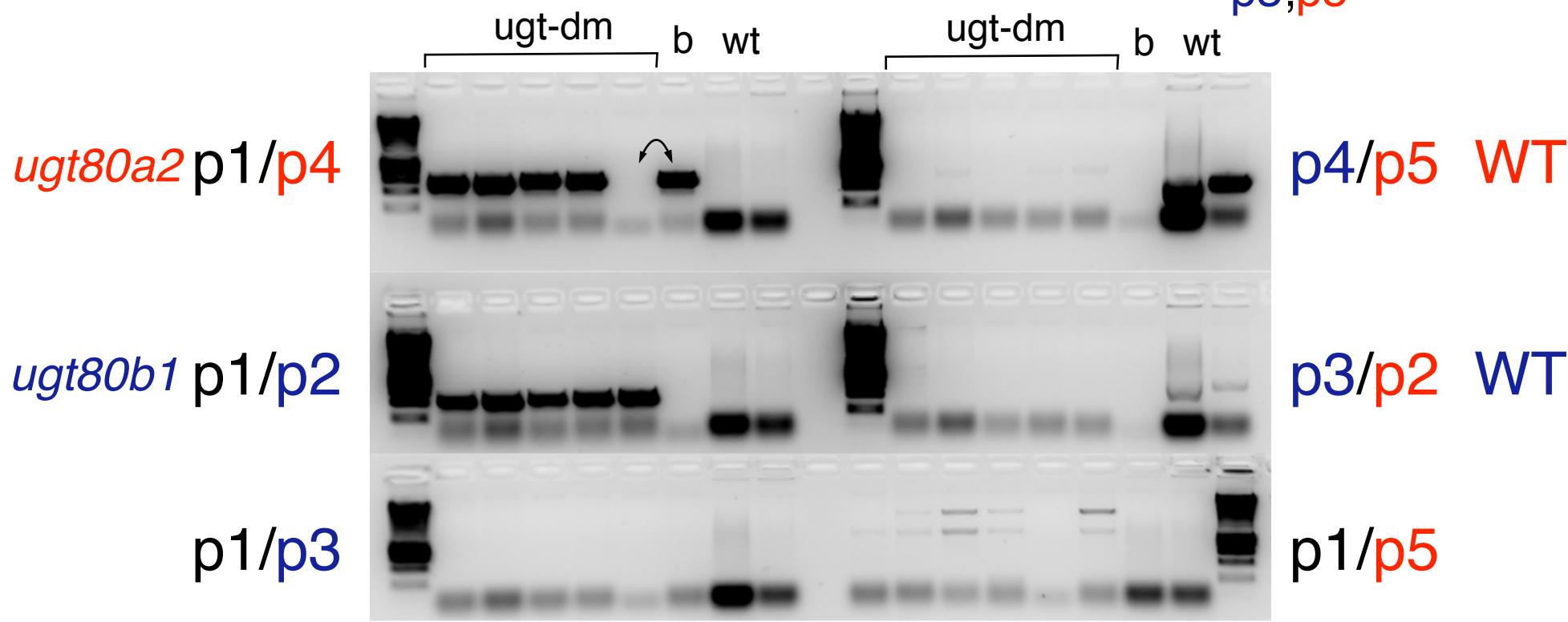

Supplement: Supplemental Figure 1 — Photographs from callus cultures. [file DataSheet4.ZIP › supplemental figure 7.pdf]

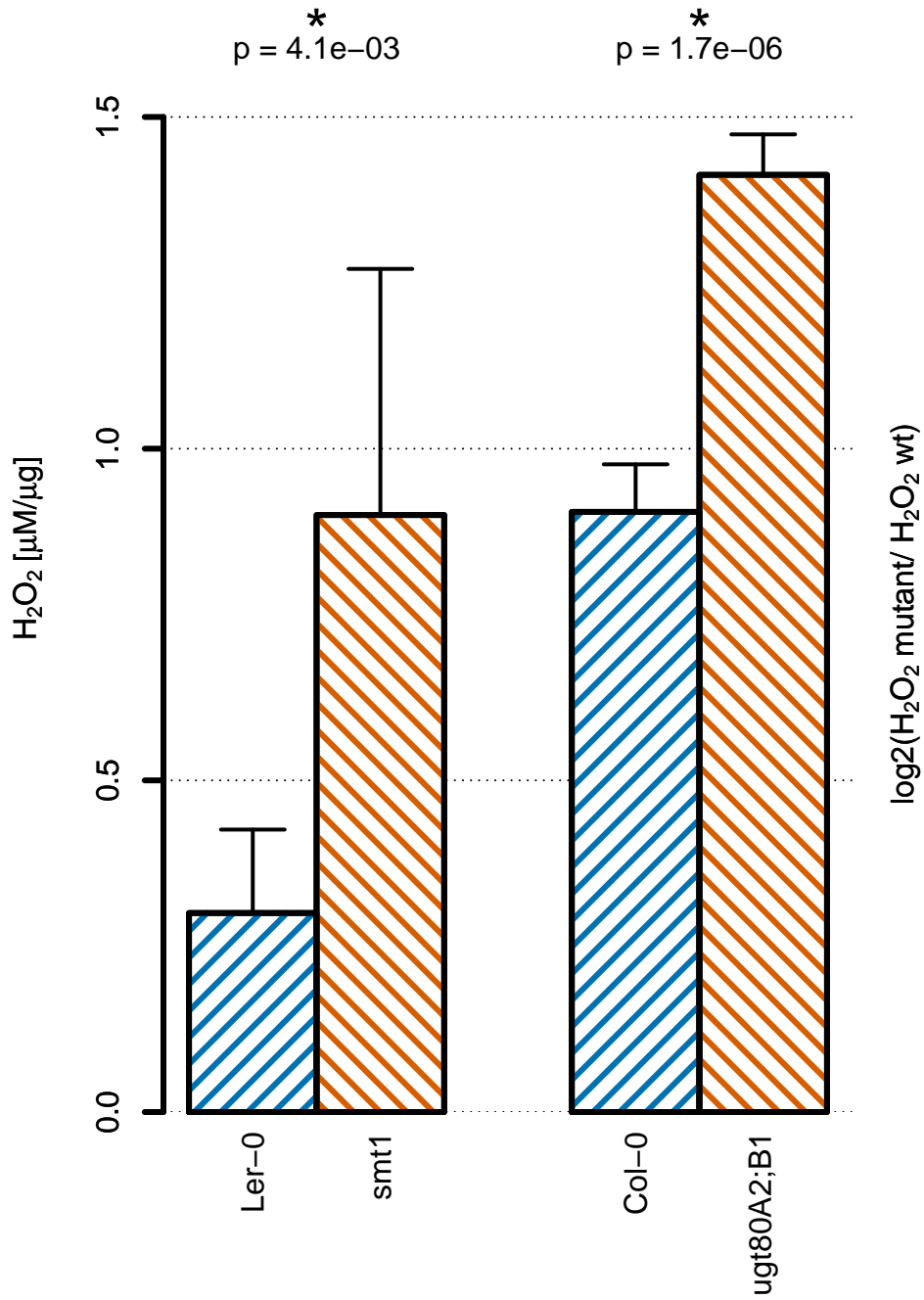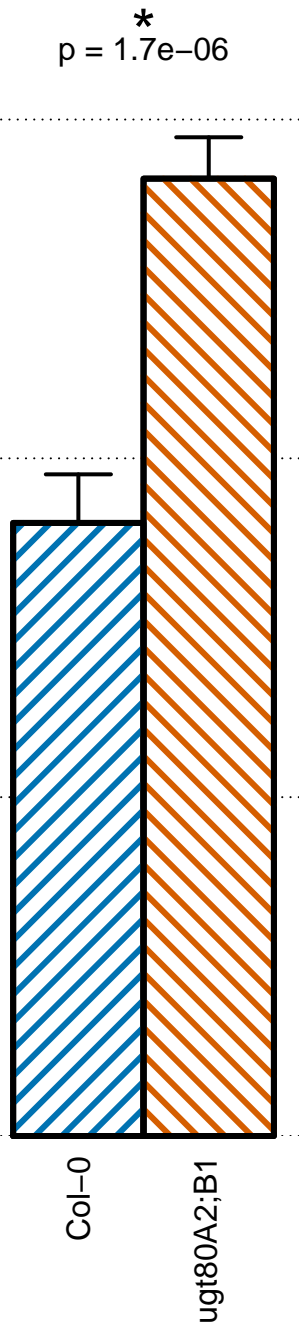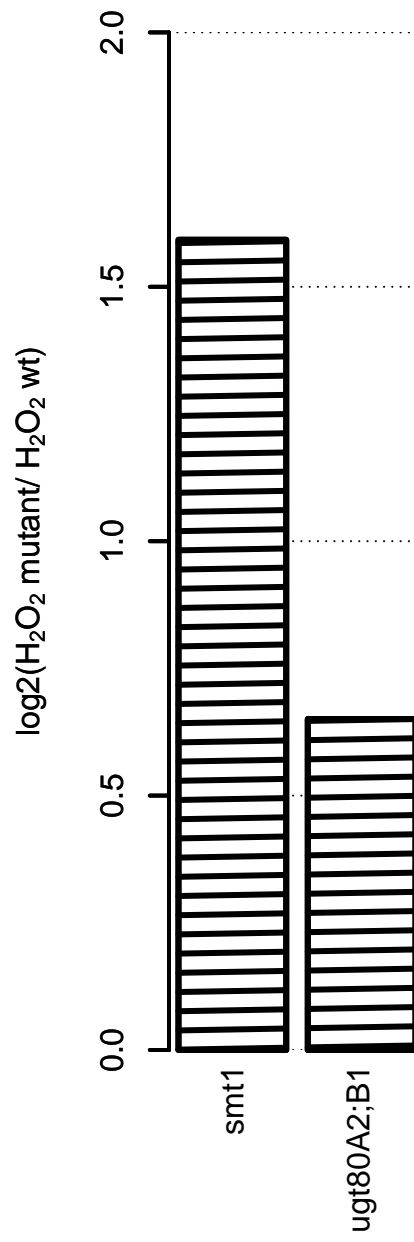

Supplement: Supplemental Figure 1 — Photographs from callus cultures. [file DataSheet4.ZIP › supplemental figure 8.pdf]

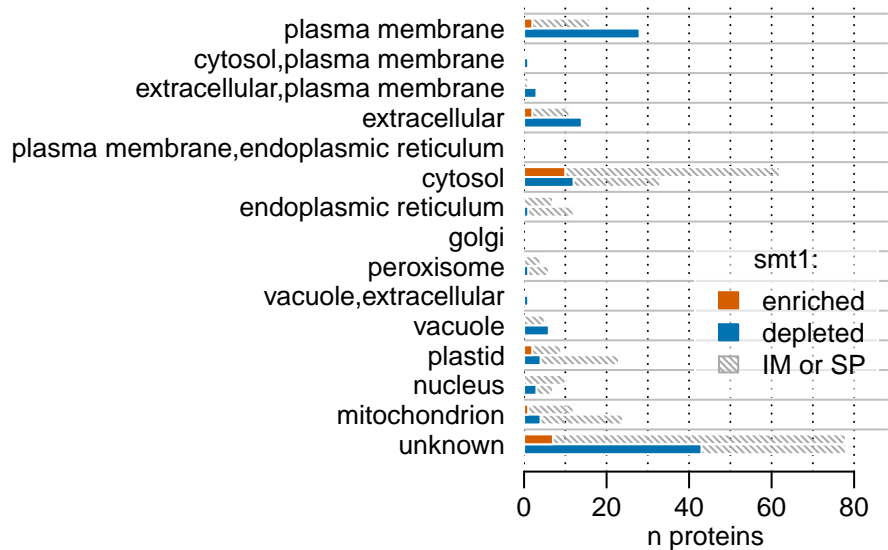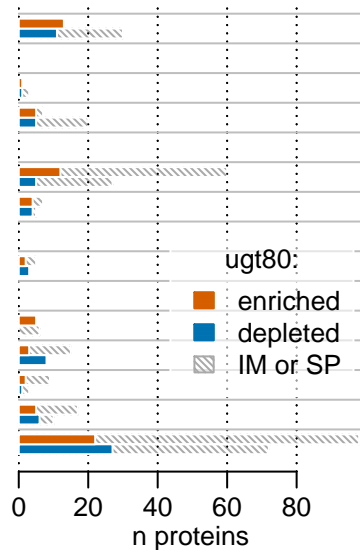

Supplement: Supplemental Figure 1 — Photographs from callus cultures. [file DataSheet4.ZIP › supplemental figure 1.pdf]

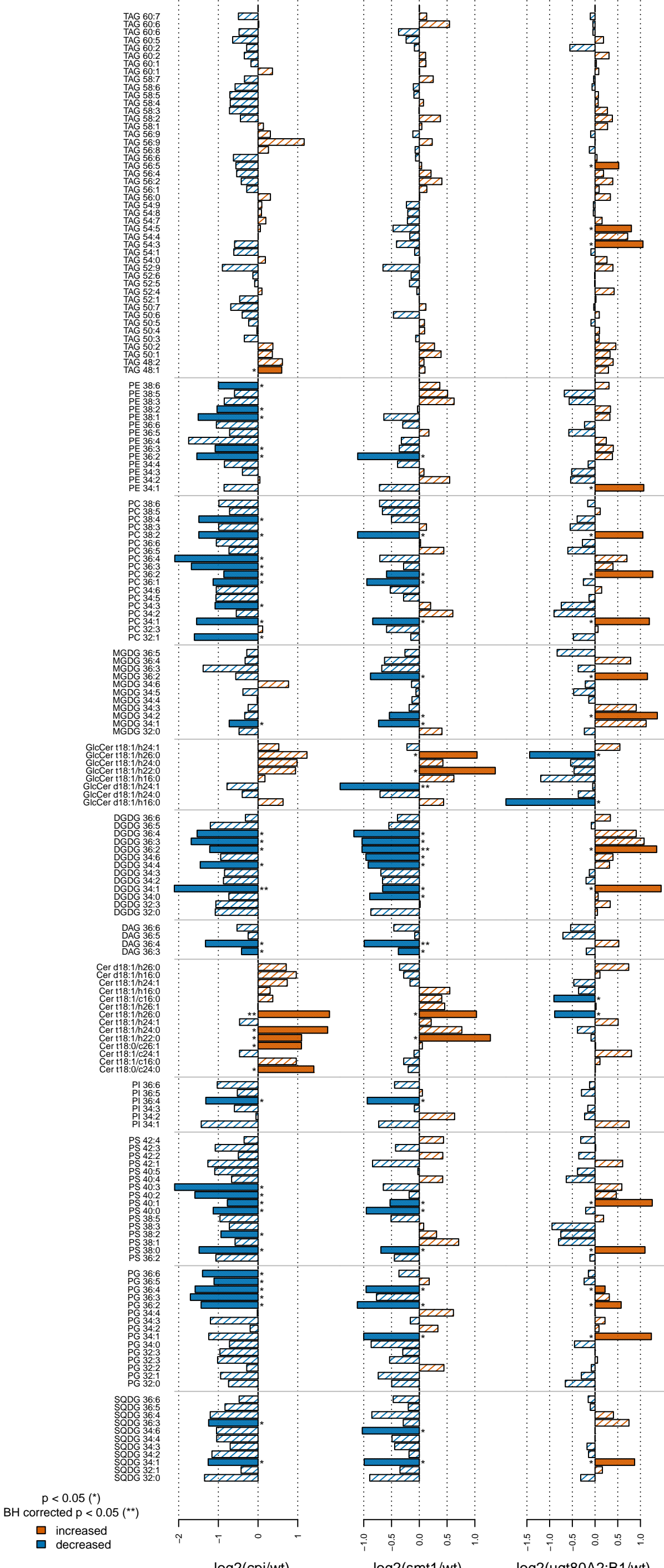

Supplement: Supplemental Figure 1 — Photographs from callus cultures. [file DataSheet4.ZIP › supplemental figure 3new.pdf]

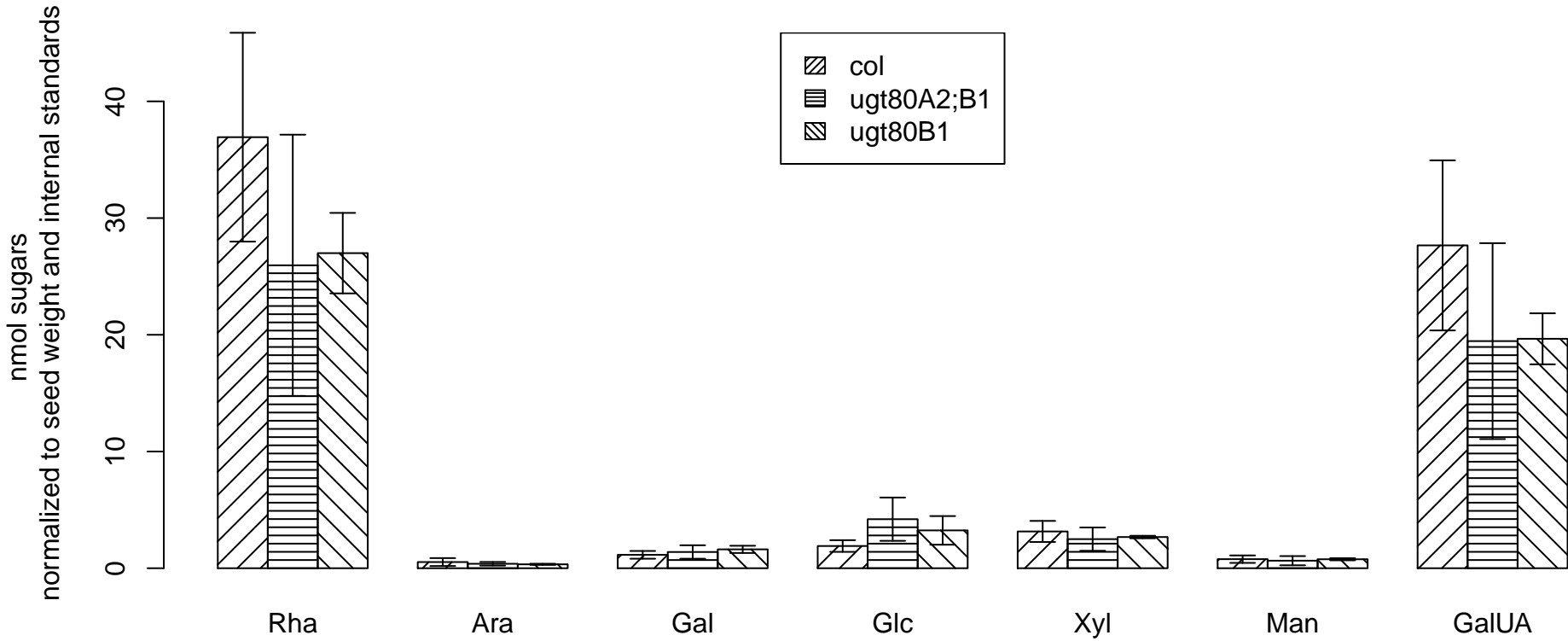

Supplement: Supplemental Figure 1 — Photographs from callus cultures. [file DataSheet4.ZIP › supplemental figure 5.pdf]
