# Supplementary material for: Plasma membrane lipid–protein interactions affect signaling processes in sterol-biosynthesis mutants in Arabidopsis thaliana
Source: Front Plant Sci. 2014 Mar 18;5:78. doi: 10.3389/fpls.2014.00078 (PMC3957024; doi:10.3389/fpls.2014.00078)
Supplement: Supplemental Figure 1 — Photographs from callus cultures. [file supplemental figure 4.pdf › supplemental figure 4.pdf]

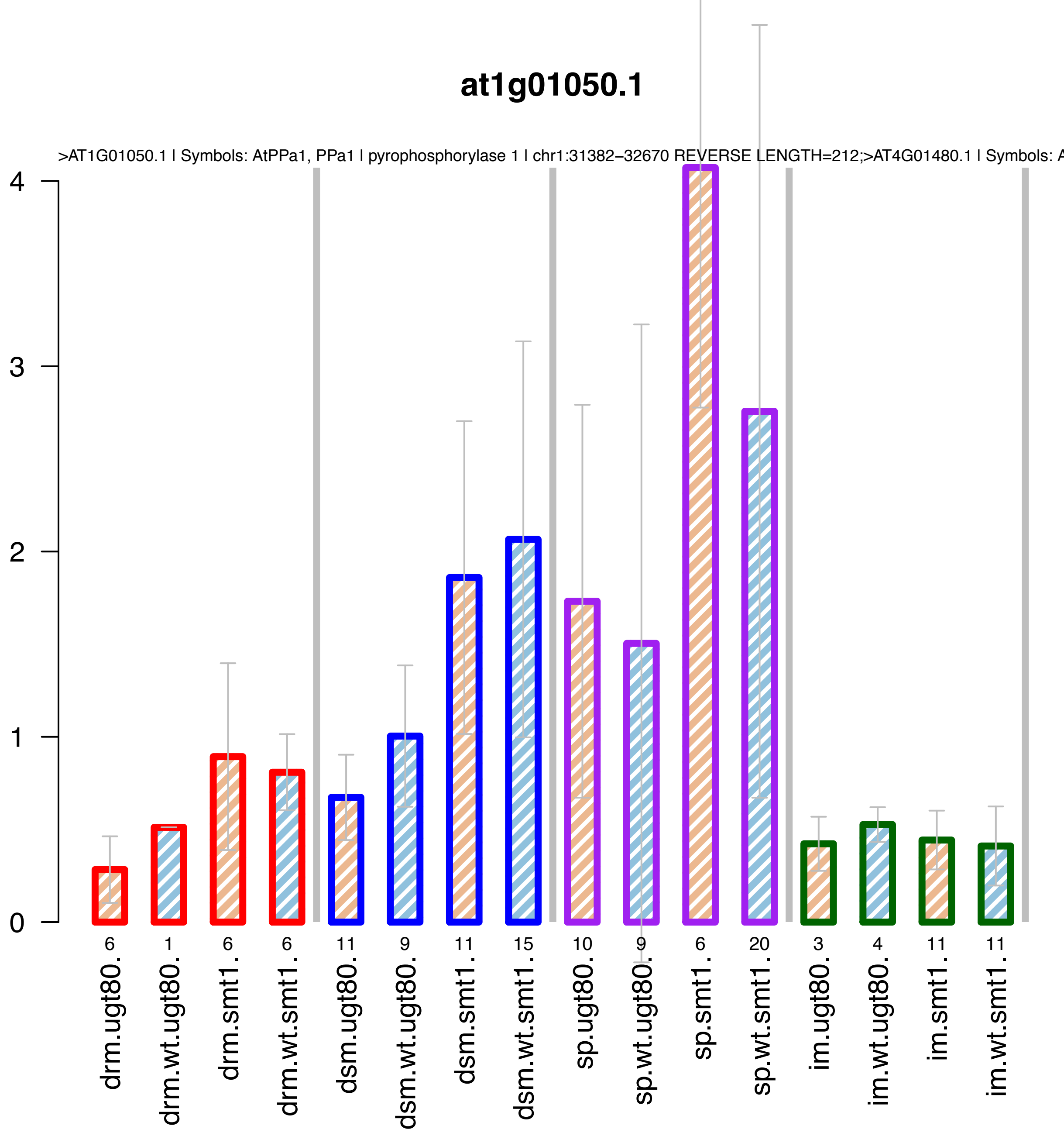
at1g01050.1
>AT1G01050.1 | Symbols: AtPPa1, PPa1 | pyrophosphorylase 1 | chr1:31382–32670 REVERSE LENGTH=212;>AT4G01480.1 | Symbols: A
4
3
2
1
0
6
1
6
6
11
9
11
15
10
9
6
20
3
4
11
11
drm.ugt80.
drm.wt.ugt80.
drm.smt1.
drm.wt.smt1.
dsm.ugt80.
dsm.wt.ugt80.
dsm.smt1.
dsm.wt.smt1.
sp.ugt80.
sp.wt.ugt80.
sp.smt1.
sp.wt.smt1.
im.ugt80.
im.wt.ugt80.
im.smt1.
im.wt.smt1.

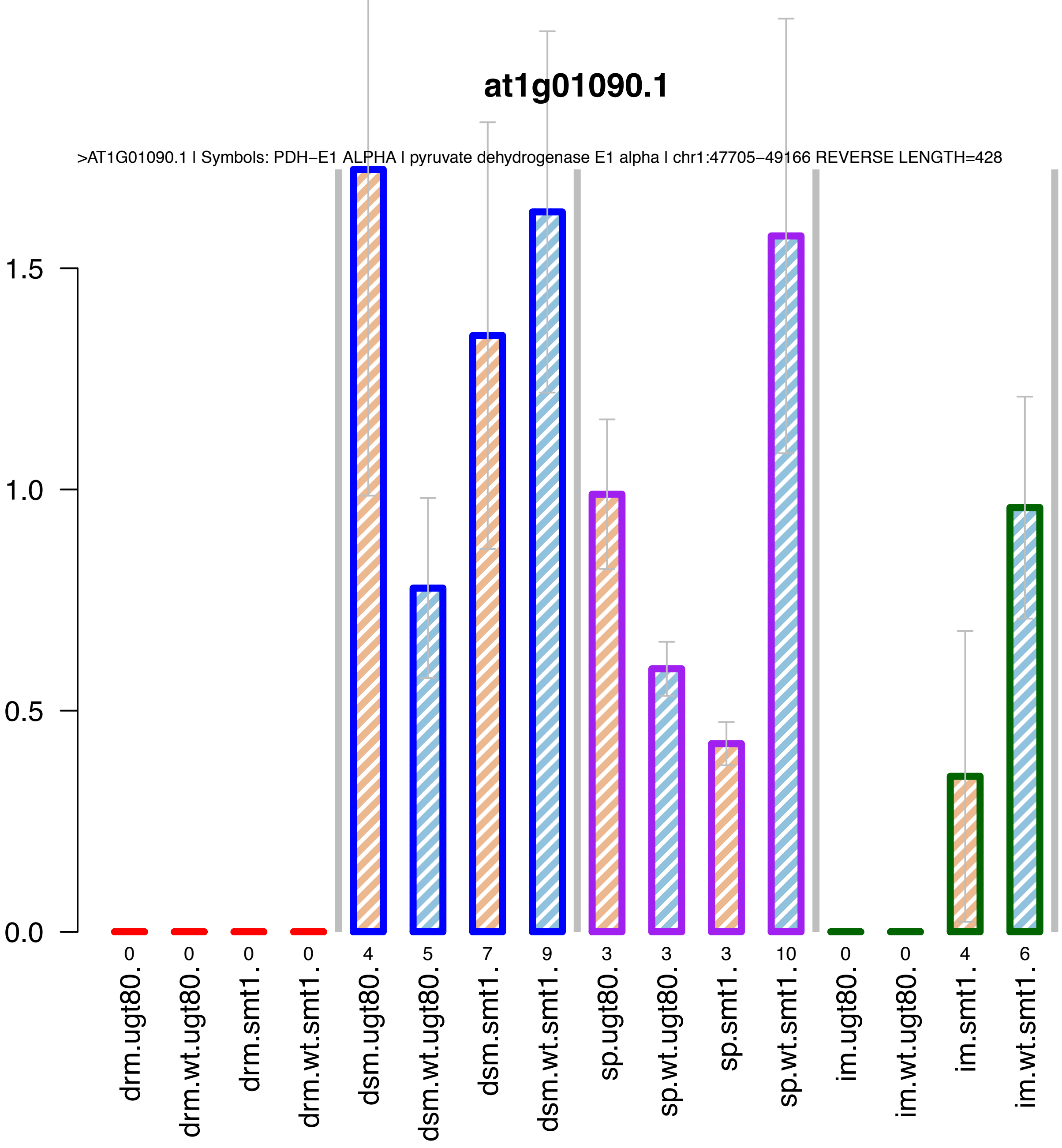
at1g01090.1
>AT1G01090.1 | Symbols: PDH−E1 ALPHA | pyruvate dehydrogenase E1 alpha | chr1:47705−49166 REVERSE LENGTH=428
0.0
0.5
1.0
1.5
0 0 0 0 4 5 7 9 3 3 3 10 0 0 4 6
drm.ugt80.
drm.wt.ugt80.
drm.smt1.
drm.wt.smt1.
dsm.ugt80.
dsm.wt.ugt80.
dsm.smt1.
dsm.wt.smt1.
sp.ugt80.
sp.wt.ugt80.
sp.smt1.
sp.wt.smt1.
im.ugt80.
im.wt.ugt80.
im.smt1.
im.wt.smt1.

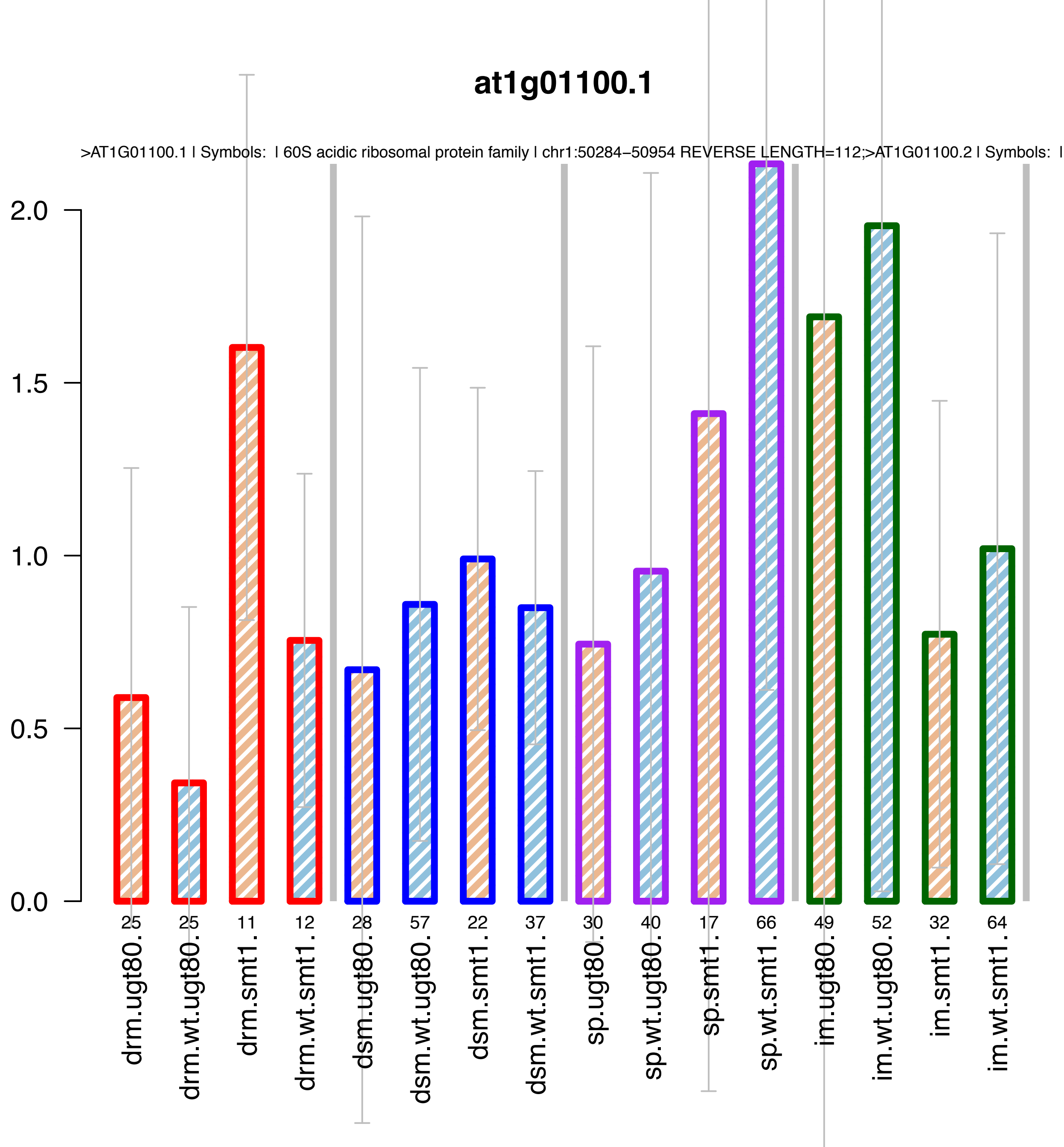
at1g01100.1
>AT1G01100.1 | Symbols: | 60S acidic ribosomal protein family | chr1:50284-50954 REVERSE LENGTH=112;>AT1G01100.2 | Symbols: |
2.0
1.5
1.0
0.5
0.0
25
25
11
12
28
57
22
37
30
40
17
66
49
52
32
64
drm.ugt80.
drm.wt.ugt80.
drm.smt1.
drm.wt.smt1.
dsm.ugt80.
dsm.wt.ugt80.
dsm.smt1.
dsm.wt.smt1.
sp.ugt80.
sp.wt.ugt80.
sp.smt1.
sp.wt.smt1.
im.ugt80.
im.wt.ugt80.
im.smt1.
im.wt.smt1.

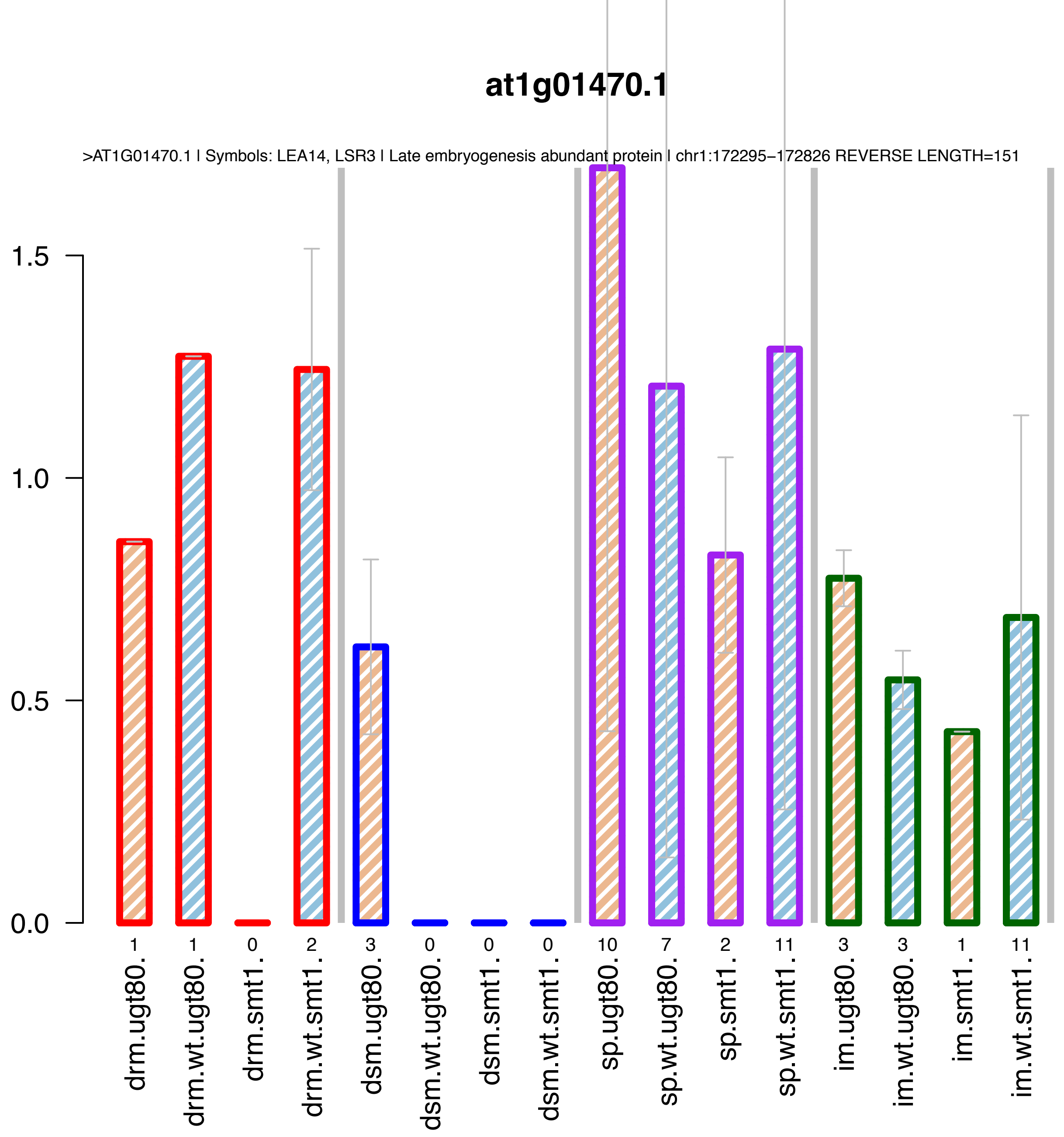
at1g01470.1
>AT1G01470.1 | Symbols: LEA14, LSR3 | Late embryogenesis abundant protein | chr1:172295-172826 REVERSE LENGTH=151
0.0
0.5
1.0
1.5
1 1 0 2 3 0 0 0 10 7 2 11 3 3 1 11
drm.ugt80..
drm.wt.ugt80..
drm.smt1..
drm.wt.smt1..
dsm.ugt80..
dsm.wt.ugt80..
dsm.smt1..
dsm.wt.smt1..
sp.ugt80..
sp.wt.ugt80..
sp.smt1..
sp.wt.smt1..
im.ugt80..
im.wt.ugt80..
im.smt1..
im.wt.smt1..

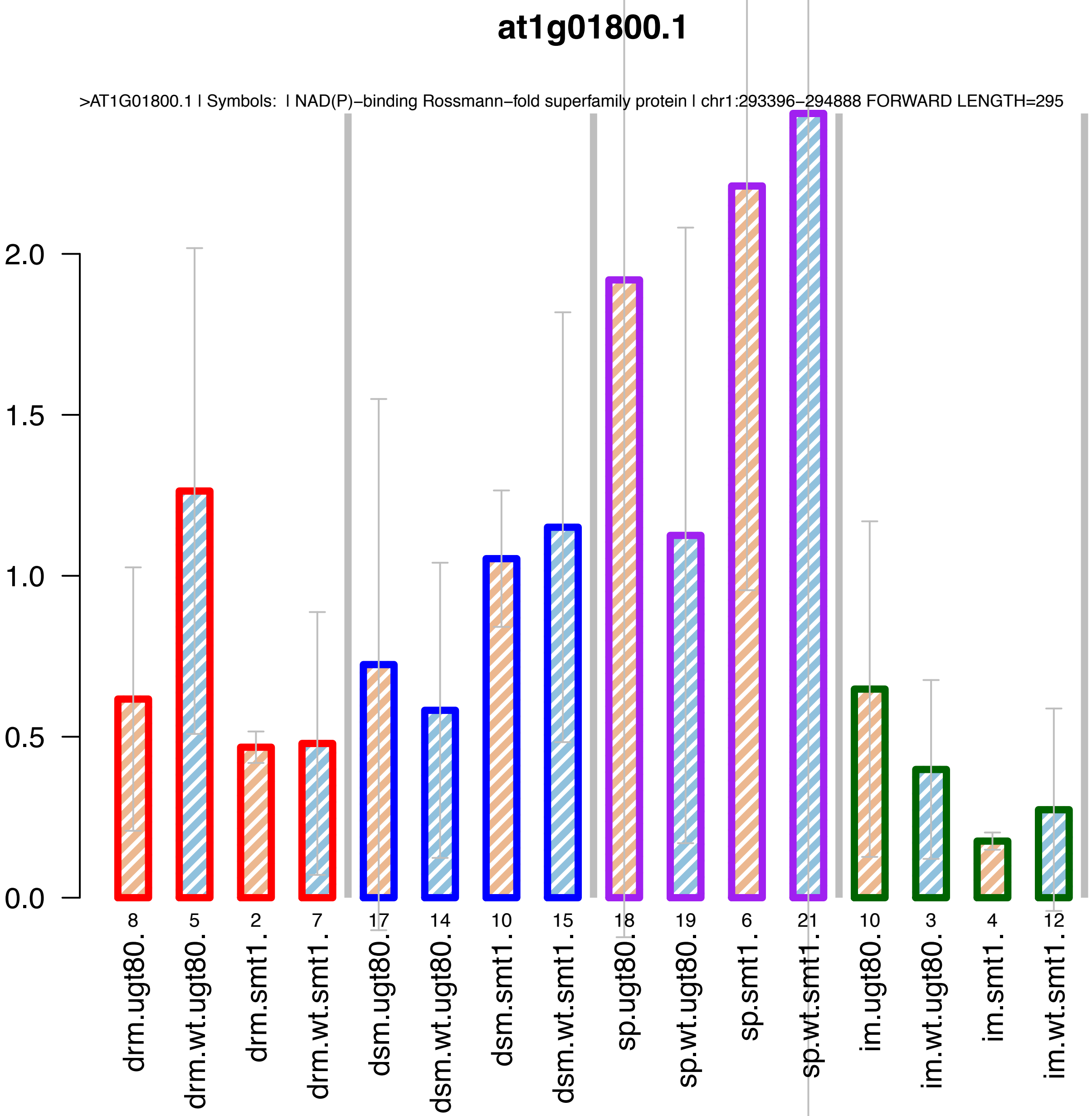

at1g01800.1
>AT1G01800.1 | Symbols: | NAD(P)−binding Rossmann−fold superfamily protein | chr1:293396−294888 FORWARD LENGTH=295
0.0
0.5
1.0
1.5
2.0
8
5
2
7
17
14
10
15
18
19
6
21
10
3
4
12
drm.ugt80..
drm.wt.ugt80..
drm.smt1..
drm.wt.smt1..
dsm.ugt80..
dsm.wt.ugt80..
dsm.smt1..
dsm.wt.smt1..
sp.ugt80..
sp.wt.ugt80..
sp.smt1..
sp.wt.smt1..
im.ugt80..
im.wt.ugt80..
im.smt1..
im.wt.smt1..

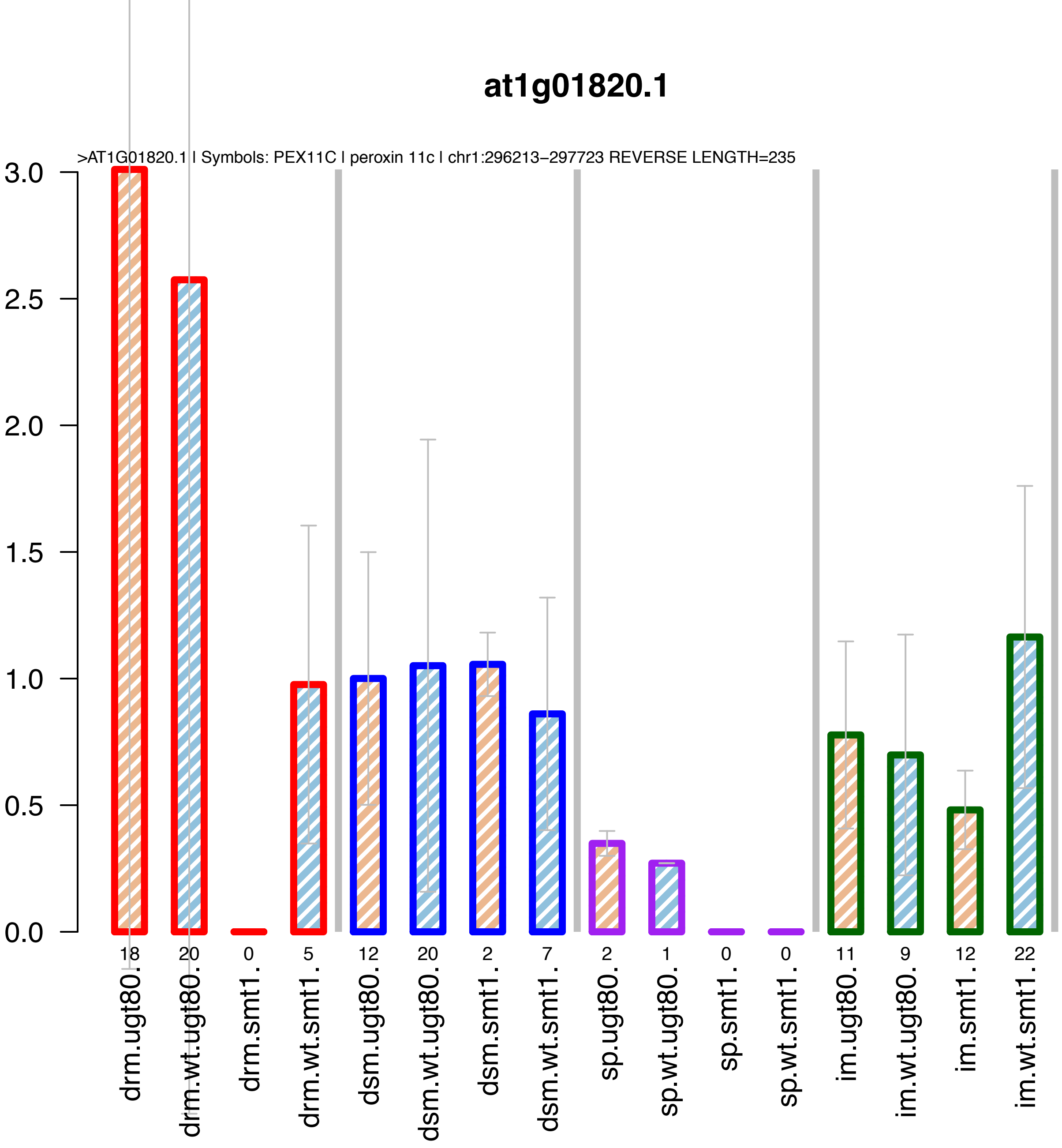

at1g01820.1
>AT1G01820.1 | Symbols: PEX11C | peroxin 11c | chr1:296213-297723 REVERSE LENGTH=235
3.0
2.5
2.0
1.5
1.0
0.5
0.0
18
20
0
5
12
20
2
7
2
1
0
0
11
9
12
22
drm.ugt80.
drm.wt.ugt80.
drm.smt1.
drm.wt.smt1.
dsm.ugt80.
dsm.wt.ugt80.
dsm.smt1.
dsm.wt.smt1.
sp.ugt80.
sp.wt.ugt80.
sp.smt1.
sp.wt.smt1.
im.ugt80.
im.wt.ugt80.
im.smt1.
im.wt.smt1.

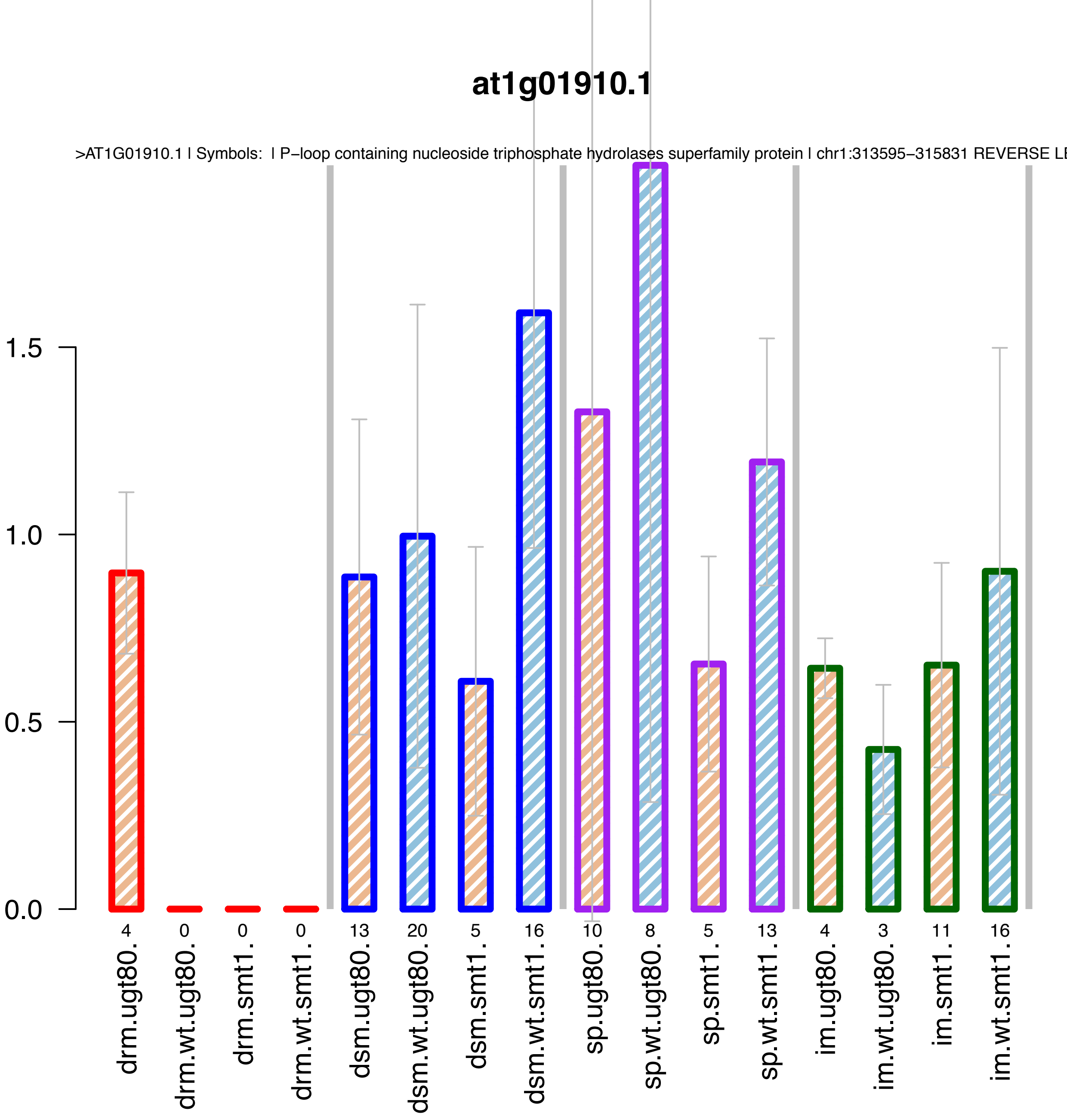
at1g01910.1
>AT1G01910.1 | Symbols: | P−loop containing nucleoside triphosphate hydrolases superfamily protein | chr1:313595−315831 REVERSE LE
0.0
0.5
1.0
1.5
4
0
0
0
13
20
5
16
10
8
5
13
4
3
11
16
drm.ugt80.
drm.wt.ugt80.
drm.smt1.
drm.wt.smt1.
dsm.ugt80.
dsm.wt.ugt80.
dsm.smt1.
dsm.wt.smt1.
sp.ugt80.
sp.wt.ugt80.
sp.smt1.
sp.wt.smt1.
im.ugt80.
im.wt.ugt80.
im.smt1.
im.wt.smt1.

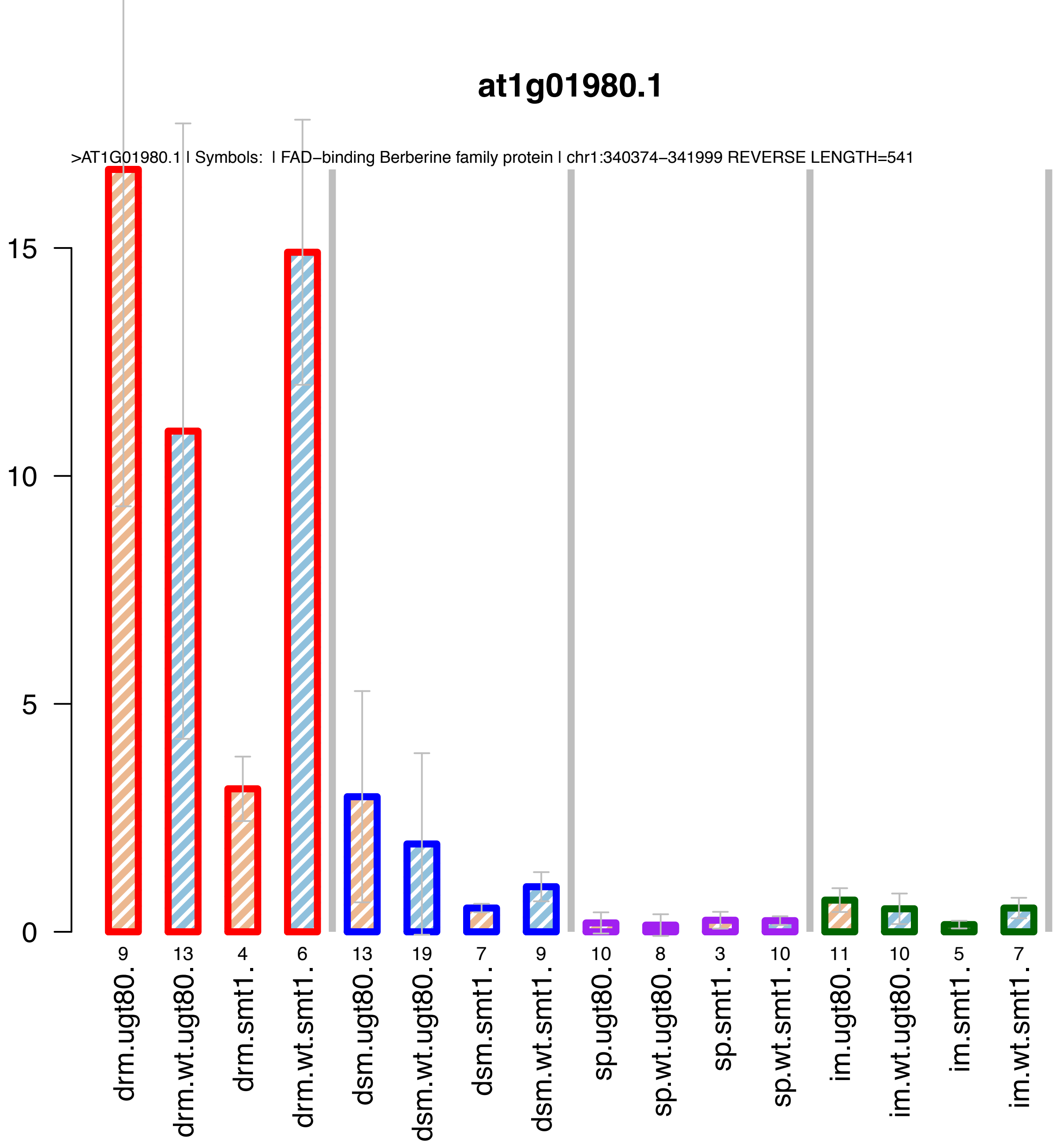

at1g01980.1
>AT1G01980.1 | Symbols: | FAD−binding Berberine family protein | chr1:340374−341999 REVERSE LENGTH=541
15
10
5
0
9
13
4
6
13
19
7
9
10
8
3
10
11
10
5
7
drm.ugt80.
drm.wt.ugt80.
drm.smt1.
drm.wt.smt1.
dsm.ugt80.
dsm.wt.ugt80.
dsm.smt1.
dsm.wt.smt1.
sp.ugt80.
sp.wt.ugt80.
sp.smt1.
sp.wt.smt1.
im.ugt80.
im.wt.ugt80.
im.smt1.
im.wt.smt1.

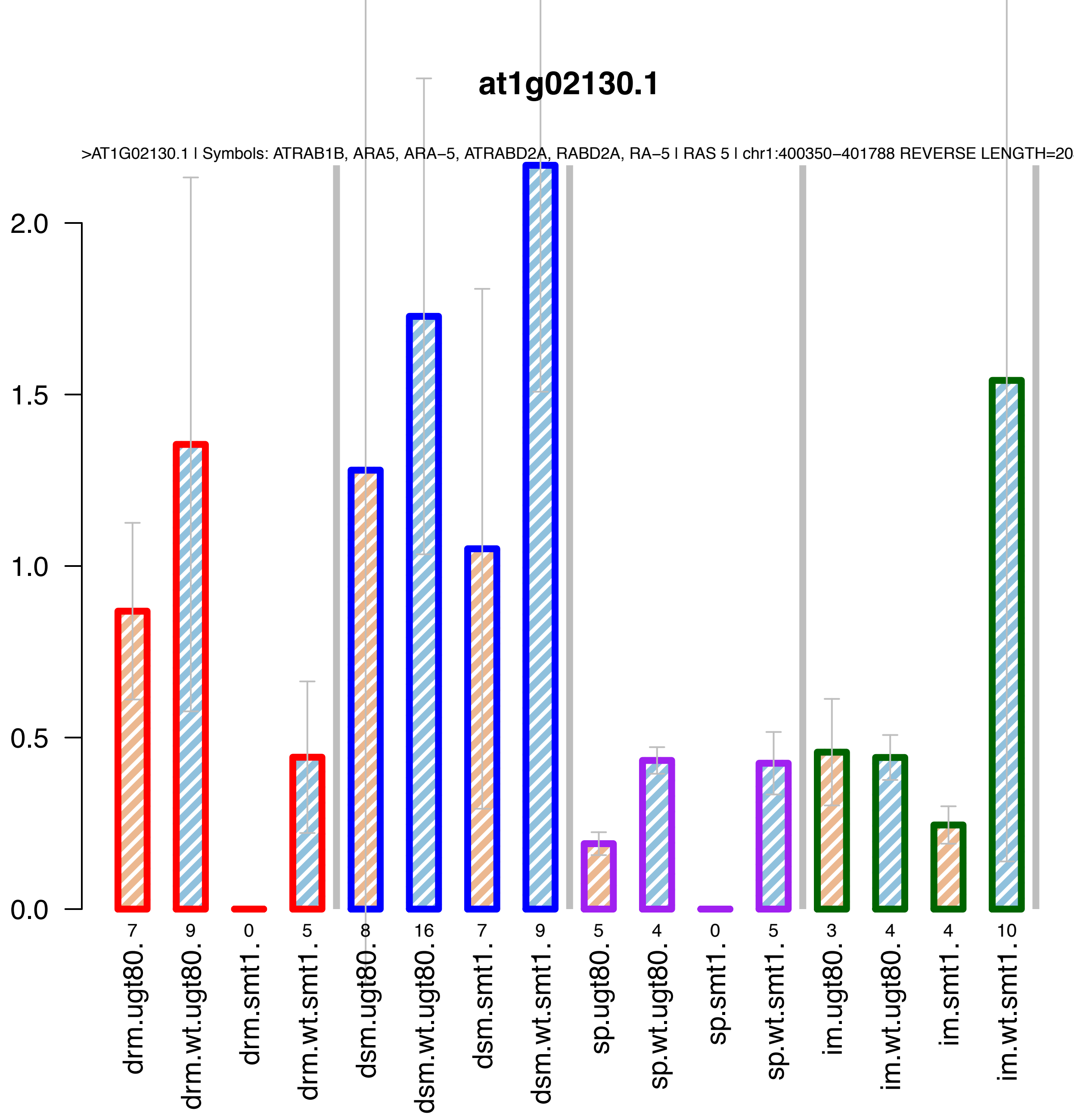

at1g02130.1
>AT1G02130.1 | Symbols: ATRAB1B, ARA5, ARA-5, ATRABD2A, RABD2A, RA-5 | RAS 5 | chr1:400350-401788 REVERSE LENGTH=20
0.0
0.5
1.0
1.5
2.0
7
9
0
5
8
16
7
9
5
4
0
5
3
4
4
10
drm.ugt80.
drm.wt.ugt80.
drm.smt1.
drm.wt.smt1.
dsm.ugt80.
dsm.wt.ugt80.
dsm.smt1.
dsm.wt.smt1.
sp.ugt80.
sp.wt.ugt80.
sp.smt1.
sp.wt.smt1.
im.ugt80.
im.wt.ugt80.
im.smt1.
im.wt.smt1.

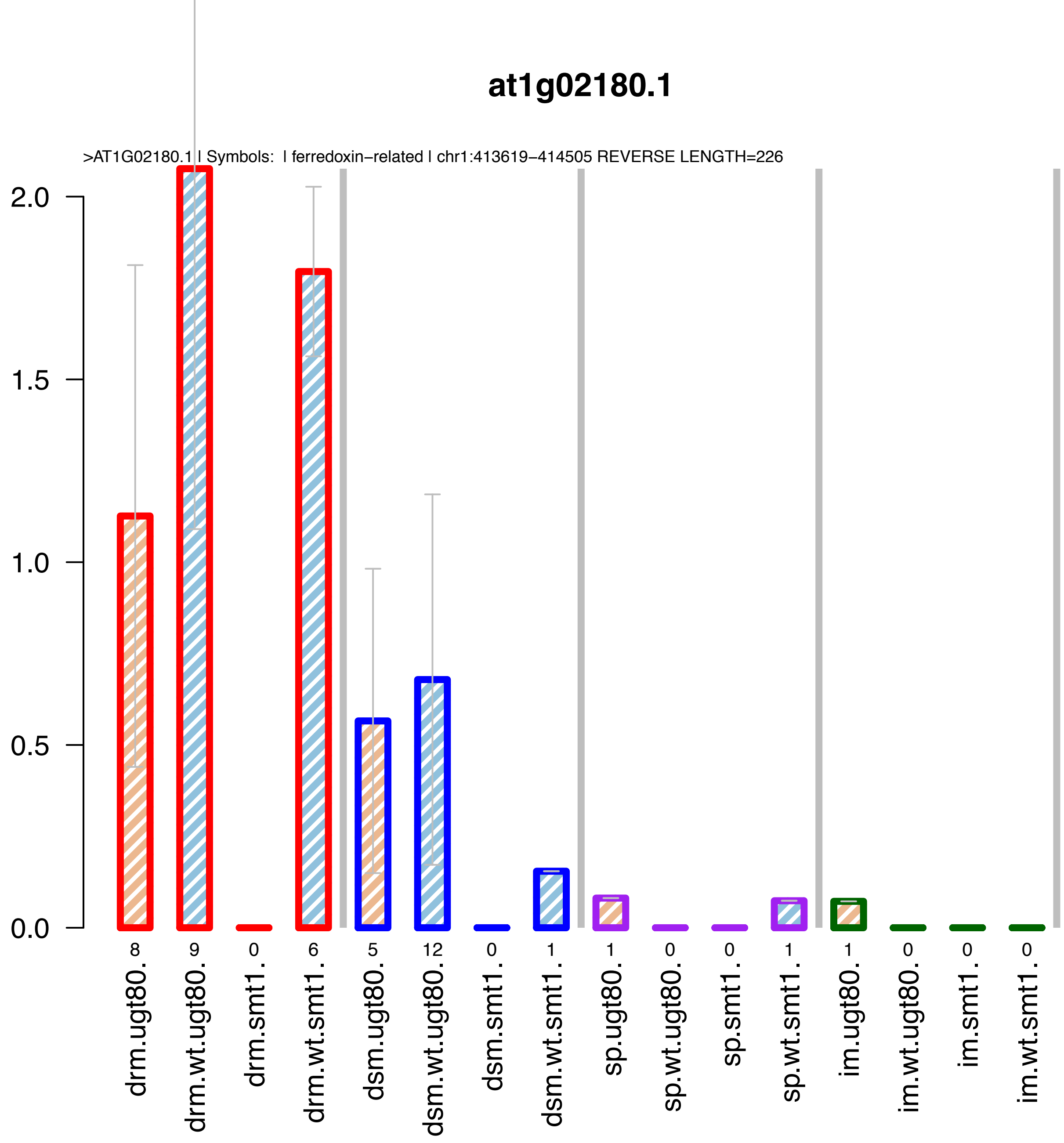
at1g02180.1
>AT1G02180.1 | Symbols: | ferredoxin−related | chr1:413619−414505 REVERSE LENGTH=226
2.0
1.5
1.0
0.5
0.0
8
9
0
6
5
12
0
1
1
0
0
1
1
0
0
0
drm.ugt80.
drm.wt.ugt80.
drm.smt1.
drm.wt.smt1.
dsm.ugt80.
dsm.wt.ugt80.
dsm.smt1.
dsm.wt.smt1.
sp.ugt80.
sp.wt.ugt80.
sp.smt1.
sp.wt.smt1.
im.ugt80.
im.wt.ugt80.
im.smt1.
im.wt.smt1.

# at1g02305.1

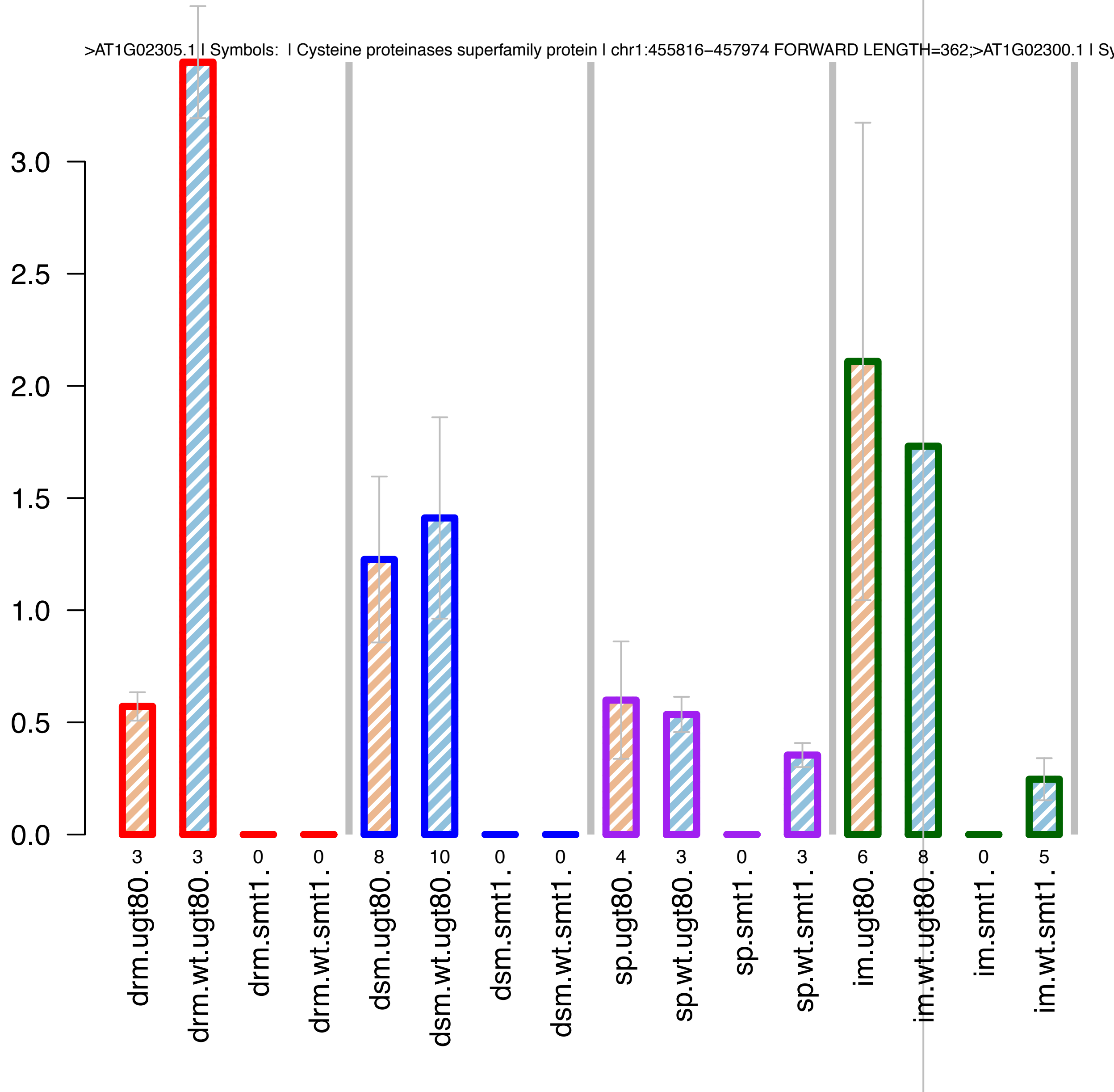
>AT1G02305.1 | Symbols: | Cysteine proteinases superfamily protein | chr1:455816-457974 FORWARD LENGTH=362;>AT1G02300.1 | Sy
3.0
2.5
2.0
1.5
1.0
0.5
0.0
3
3
0
0
8
10
0
0
4
3
0
3
6
8
0
5
drm.ugt80.
drm.wt.ugt80.
drm.smt1.
drm.wt.smt1.
dsm.ugt80.
dsm.wt.ugt80.
dsm.smt1.
dsm.wt.smt1.
sp.ugt80.
sp.wt.ugt80.
sp.smt1.
sp.wt.smt1.
im.ugt80.
im.wt.ugt80.
im.smt1.
im.wt.smt1.

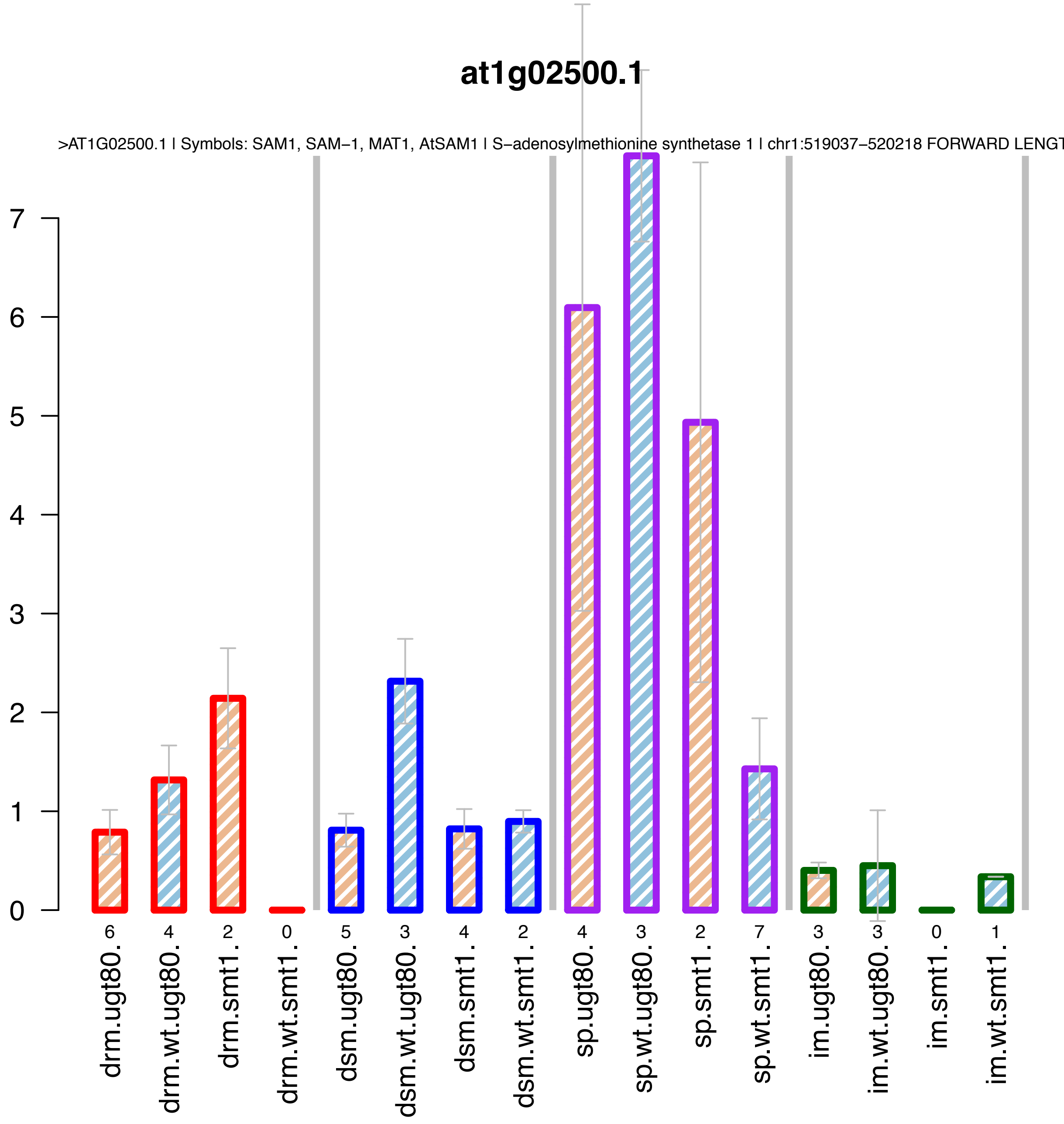
at1g02500.1
>AT1G02500.1 | Symbols: SAM1, SAM−1, MAT1, AtSAM1 | S−adenosylmethionine synthetase 1 | chr1:519037−520218 FORWARD LENGT
0
1
2
3
4
5
6
7
6
4
2
0
5
3
4
2
4
3
2
7
3
3
0
1
drm.ugt80.
drm.wt.ugt80.
drm.smt1.
drm.wt.smt1.
dsm.ugt80.
dsm.wt.ugt80.
dsm.smt1.
dsm.wt.smt1.
sp.ugt80.
sp.wt.ugt80.
sp.smt1.
sp.wt.smt1.
im.ugt80.
im.wt.ugt80.
im.smt1.
im.wt.smt1.

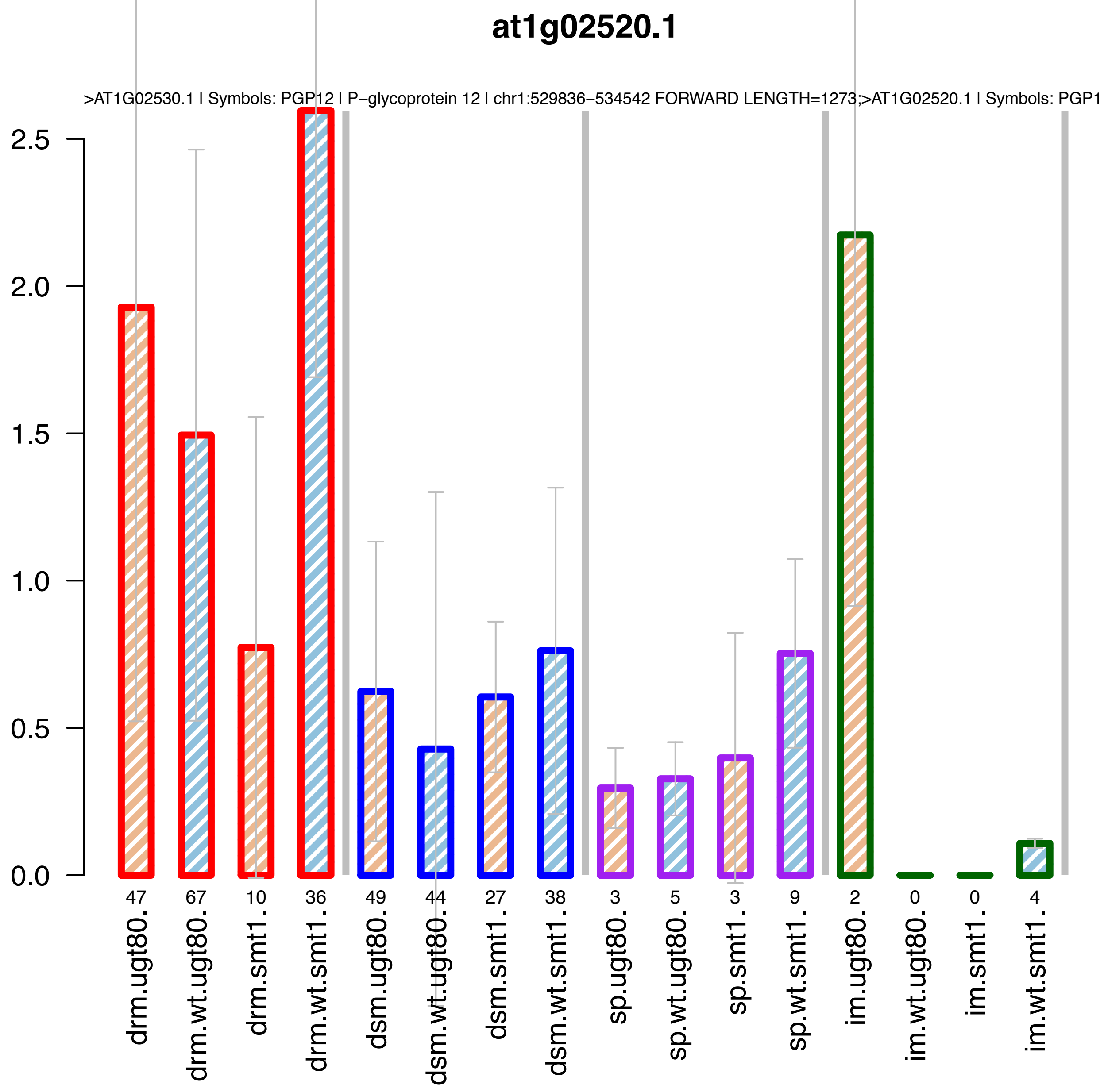

at1g02520.1
>AT1G02530.1 | Symbols: PGP12 | P−glycoprotein 12 | chr1:529836−534542 FORWARD LENGTH=1273;>AT1G02520.1 | Symbols: PGP1
0.0
0.5
1.0
1.5
2.0
2.5
47
67
10
36
49
44
27
38
3
5
3
9
2
0
0
4
drm.ugt80.
drm.wt.ugt80.
drm.smt1.
drm.wt.smt1.
dsm.ugt80.
dsm.wt.ugt80.
dsm.smt1.
dsm.wt.smt1.
sp.ugt80.
sp.wt.ugt80.
sp.smt1.
sp.wt.smt1.
im.ugt80.
im.wt.ugt80.
im.smt1.
im.wt.smt1.

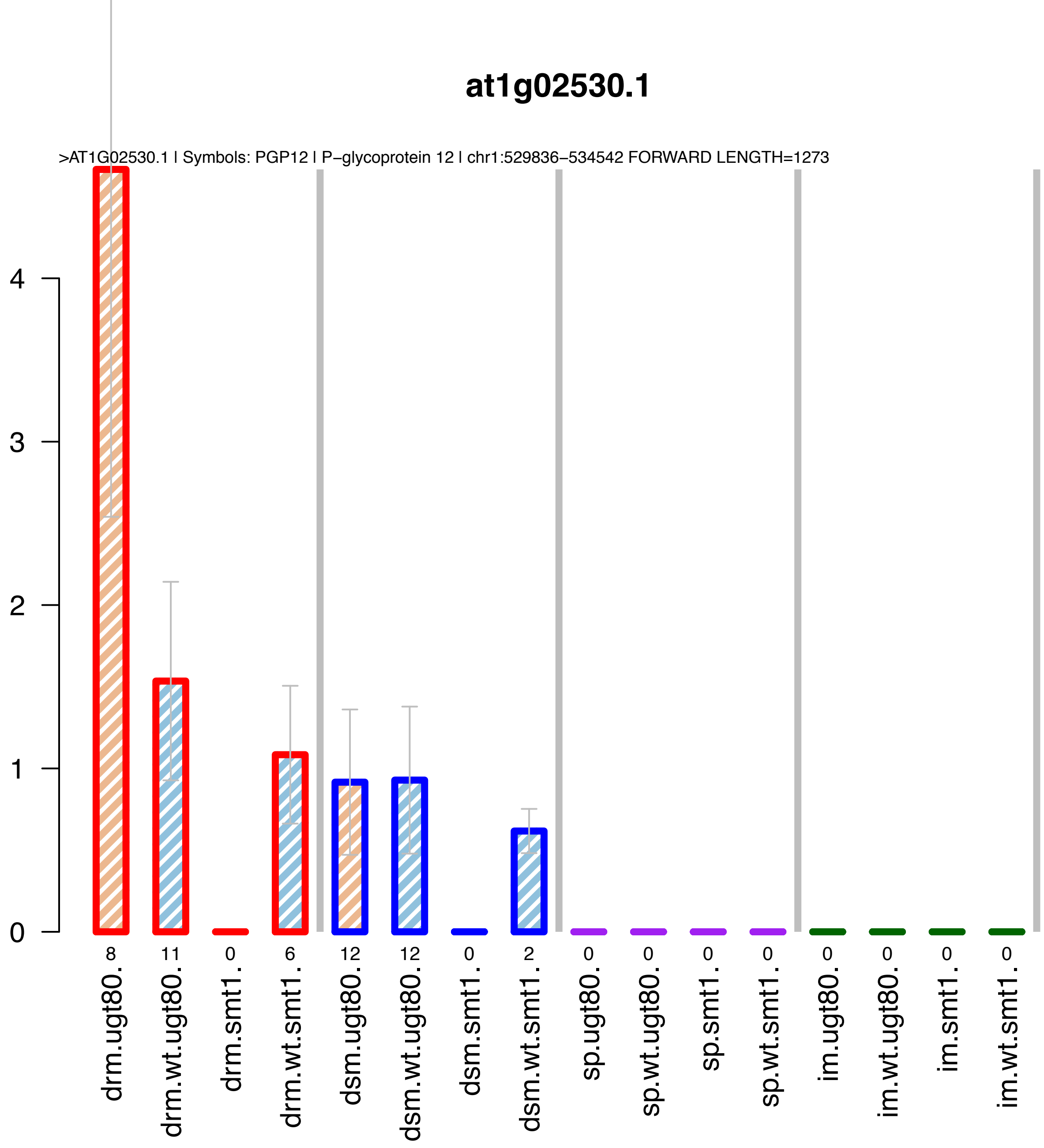

at1g02530.1
>AT1G02530.1 | Symbols: PGP12 | P−glycoprotein 12 | chr1:529836−534542 FORWARD LENGTH=1273
0
1
2
3
4
8
11
0
6
12
12
0
2
0
0
0
0
0
0
0
0
drm.ugt80.
drm.wt.ugt80.
drm.smt1.
drm.wt.smt1.
dsm.ugt80.
dsm.wt.ugt80.
dsm.smt1.
dsm.wt.smt1.
sp.ugt80.
sp.wt.ugt80.
sp.smt1.
sp.wt.smt1.
im.ugt80.
im.wt.ugt80.
im.smt1.
im.wt.smt1.

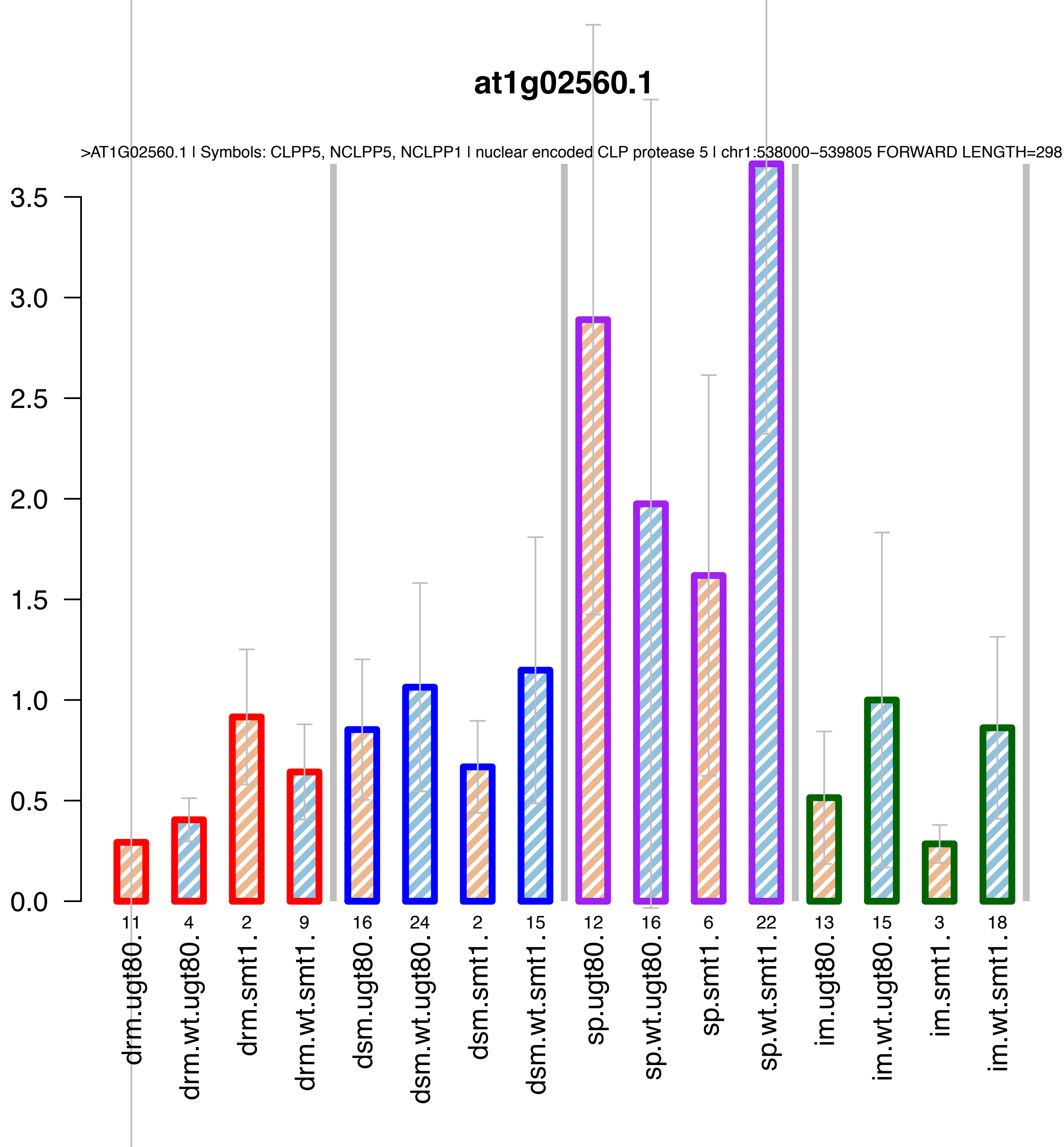
at1g02560.1
>AT1G02560.1 | Symbols: CLPP5, NCLPP5, NCLPP1 | nuclear encoded CLP protease 5 | chr1:538000–539805 FORWARD LENGTH=298
3.5
3.0
2.5
2.0
1.5
1.0
0.5
0.0
11
4
2
9
16
24
2
15
12
16
6
22
13
15
3
18
drm.ugt80..
drm.wt.ugt80..
drm.smt1..
drm.wt.smt1..
dsm.ugt80..
dsm.wt.ugt80..
dsm.smt1..
dsm.wt.smt1..
sp.ugt80..
sp.wt.ugt80..
sp.smt1..
sp.wt.smt1..
im.ugt80..
im.wt.ugt80..
im.smt1..
im.wt.smt1..

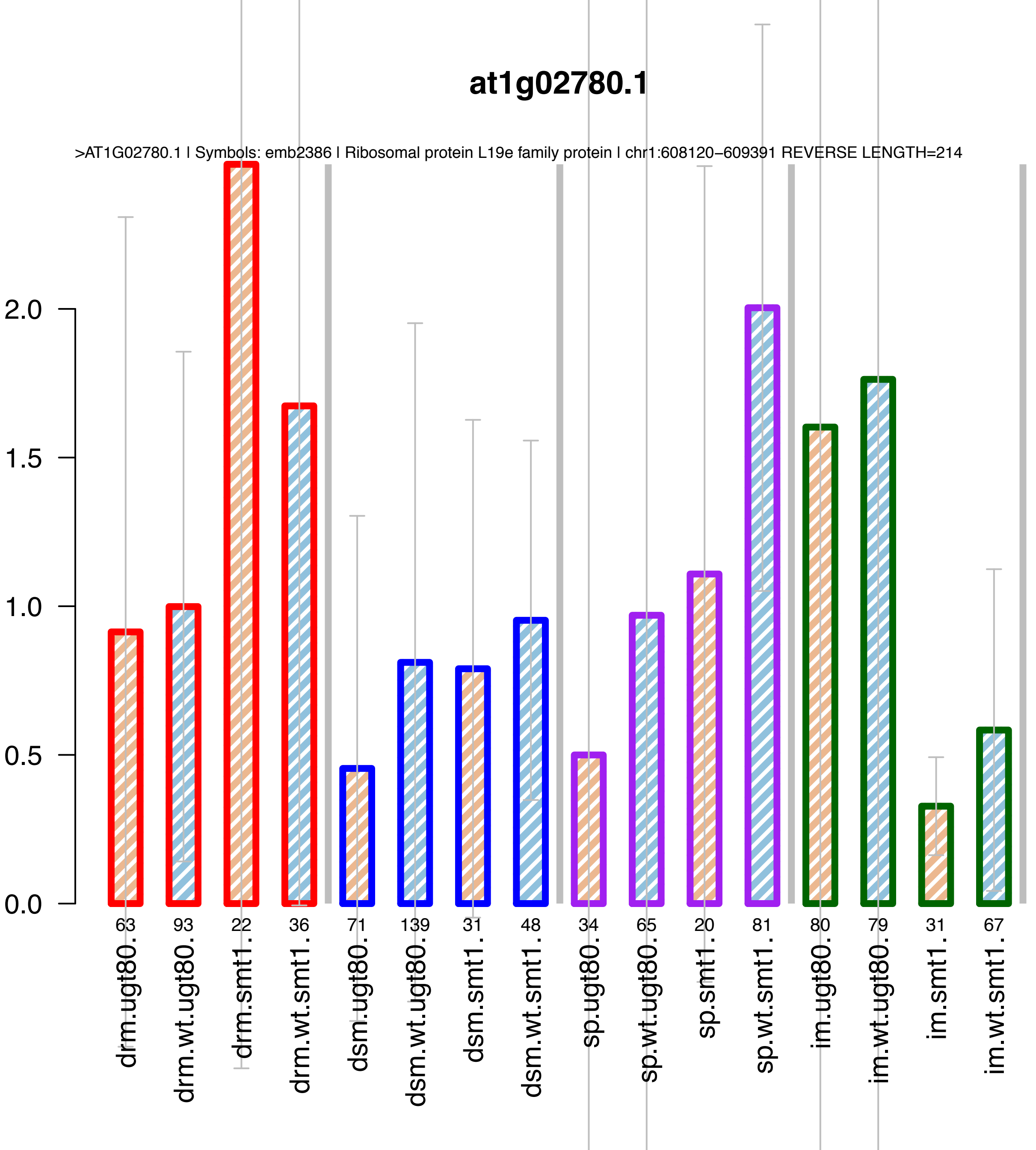
at1g02780.1
>AT1G02780.1 | Symbols: emb2386 | Ribosomal protein L19e family protein | chr1:608120-609391 REVERSE LENGTH=214
2.0
1.5
1.0
0.5
0.0
63
93
22
36
71
139
31
48
34
65
20
81
80
79
31
67
drm.ugt80.
drm.wt.ugt80.
drm.smt1.
drm.wt.smt1.
dsm.ugt80.
dsm.wt.ugt80.
dsm.smt1.
dsm.wt.smt1.
sp.ugt80.
sp.wt.ugt80.
sp.smt1.
sp.wt.smt1.
im.ugt80.
im.wt.ugt80.
im.smt1.
im.wt.smt1.

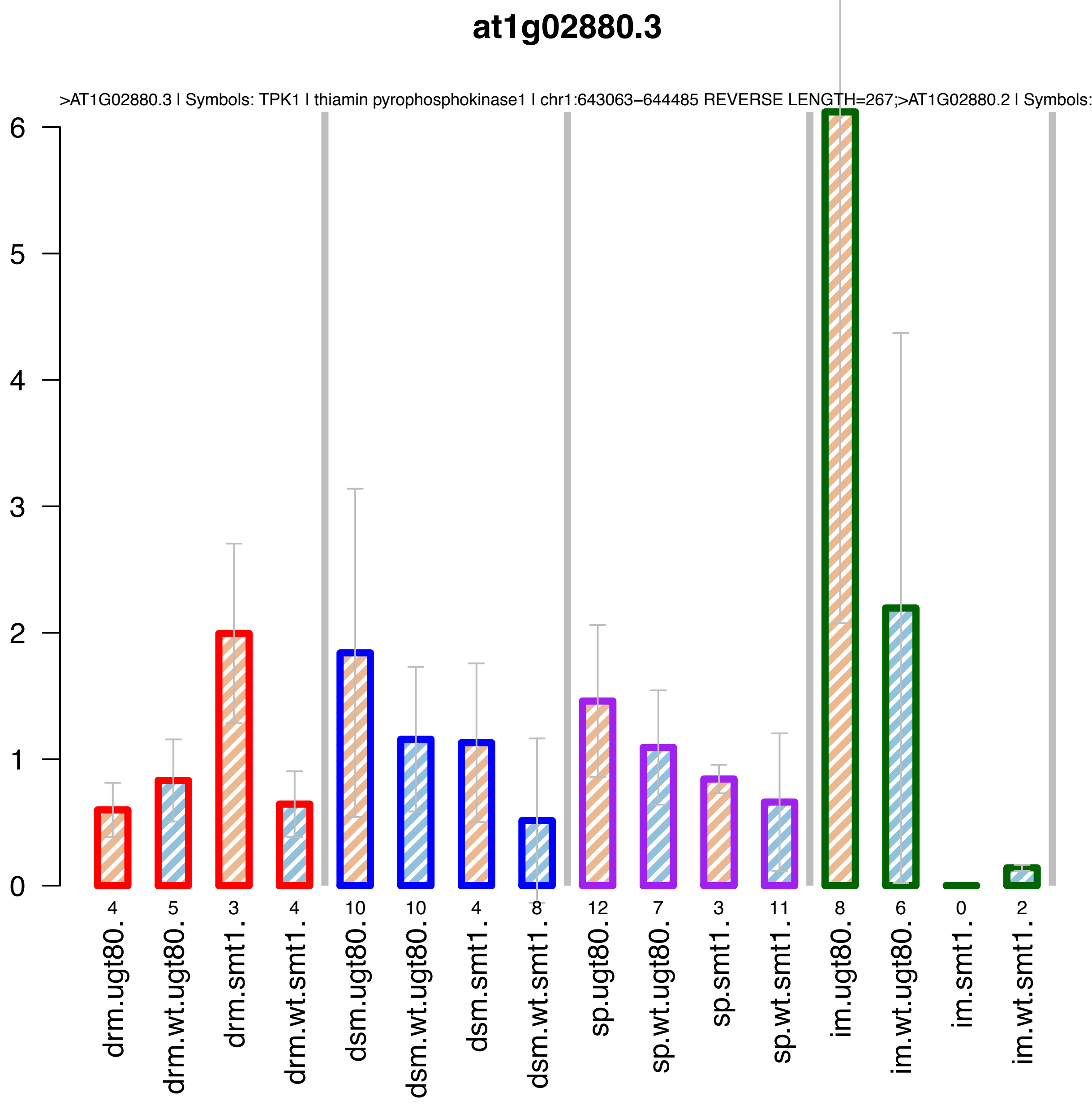

at1g02880.3
>AT1G02880.3 | Symbols: TPK1 | thiamin pyrophosphokinase1 | chr1:643063-644485 REVERSE LENGTH=267;>AT1G02880.2 | Symbols:
0
1
2
3
4
5
6
4
5
3
4
10
10
4
8
12
7
3
11
8
6
0
2
drm.ugt80.
drm.wt.ugt80.
drm.smt1.
drm.wt.smt1.
dsm.ugt80.
dsm.wt.ugt80.
dsm.smt1.
dsm.wt.smt1.
sp.ugt80.
sp.wt.ugt80.
sp.smt1.
sp.wt.smt1.
im.ugt80.
im.wt.ugt80.
im.smt1.
im.wt.smt1.

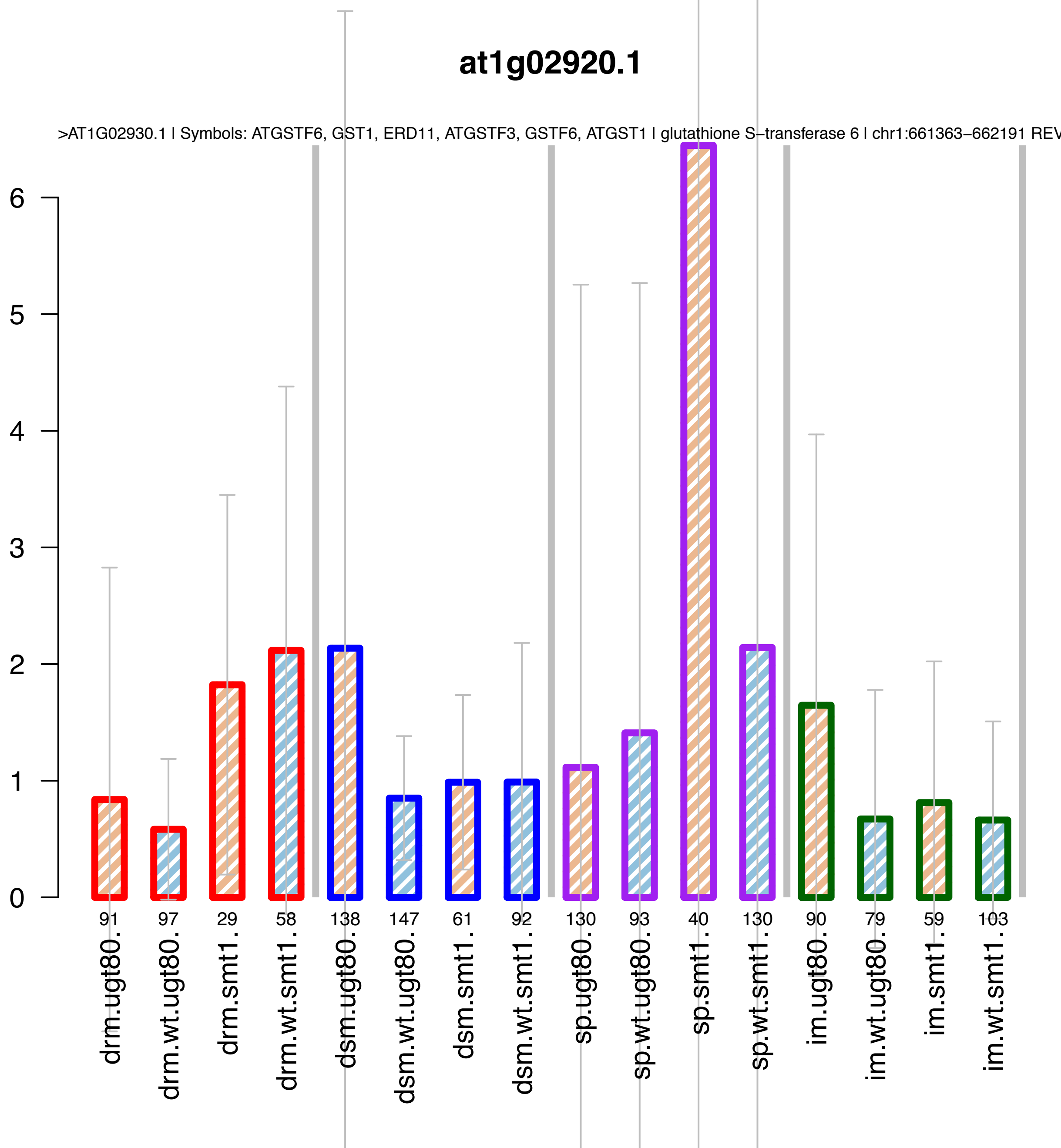
at1g02920.1
>AT1G02930.1 | Symbols: ATGSTF6, GST1, ERD11, ATGSTF3, GSTF6, ATGST1 | glutathione S−transferase 6 | chr1:661363−662191 REV
0
1
2
3
4
5
6
91
97
29
58
138
147
61
92
130
93
40
130
90
79
59
103
drm.ugt80.
drm.wt.ugt80.
drm.smt1.
drm.wt.smt1.
dsm.ugt80.
dsm.wt.ugt80.
dsm.smt1.
dsm.wt.smt1.
sp.ugt80.
sp.wt.ugt80.
sp.smt1.
sp.wt.smt1.
im.ugt80.
im.wt.ugt80.
im.smt1.
im.wt.smt1.

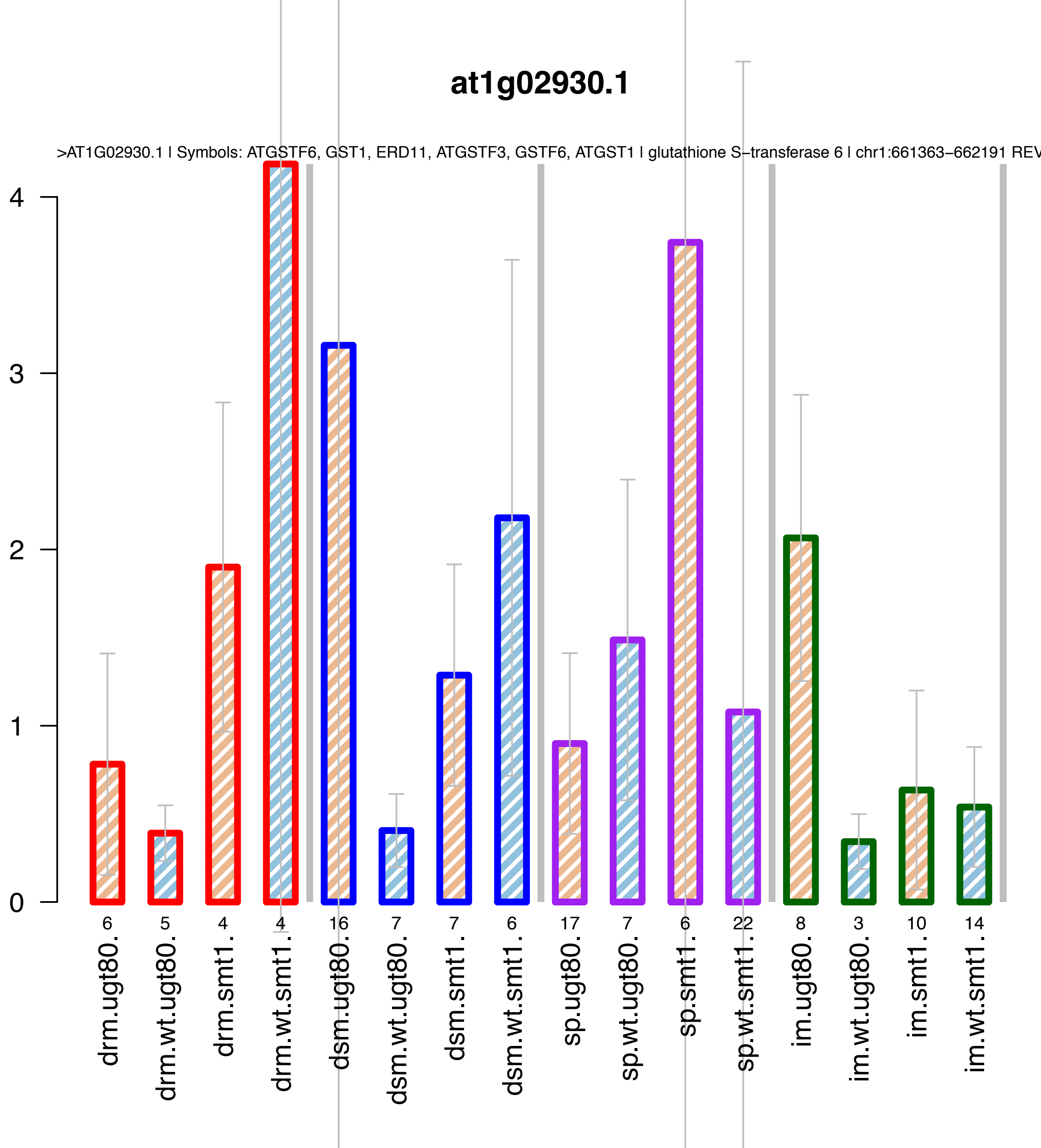
at1g02930.1
>AT1G02930.1 | Symbols: ATGSTF6, GST1, ERD11, ATGSTF3, GSTF6, ATGST1 | glutathione S–transferase 6 | chr1:661363–662191 REV
0
1
2
3
4
6
5
4
4
16
7
7
6
17
7
6
22
8
3
10
14
drm.ugt80.
drm.wt.ugt80.
drm.smt1.
drm.wt.smt1.
dsm.ugt80.
dsm.wt.ugt80.
dsm.smt1.
dsm.wt.smt1.
sp.ugt80.
sp.wt.ugt80.
sp.smt1.
sp.wt.smt1.
im.ugt80.
im.wt.ugt80.
im.smt1.
im.wt.smt1.

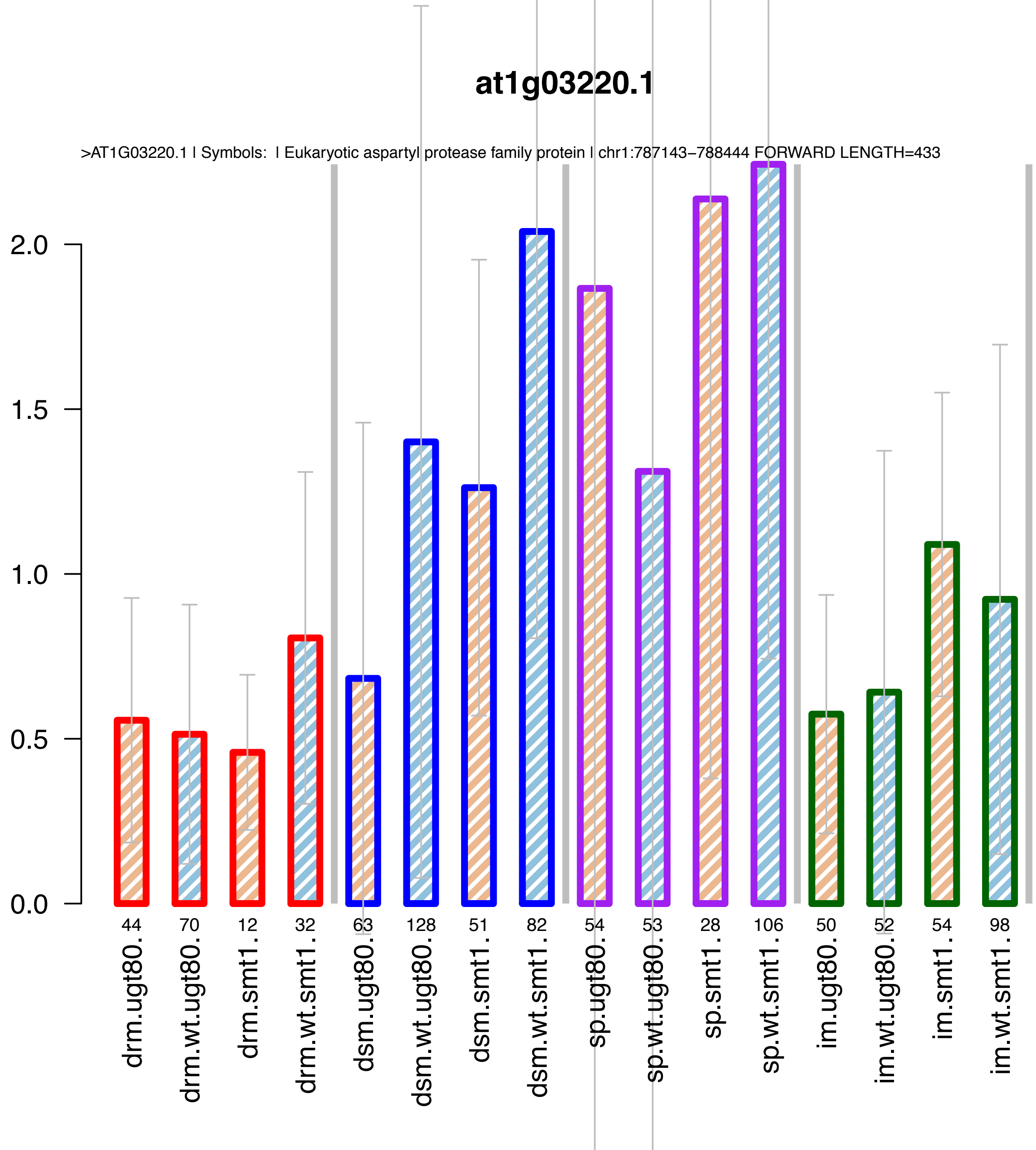

at1g03220.1
>AT1G03220.1 | Symbols: | Eukaryotic aspartyl protease family protein | chr1:787143-788444 FORWARD LENGTH=433
2.0
1.5
1.0
0.5
0.0
44
70
12
32
63
128
51
82
54
53
28
106
50
52
54
98
drm.ugt80..
drm.wt.ugt80..
drm.smt1..
drm.wt.smt1..
dsm.ugt80..
dsm.wt.ugt80..
dsm.smt1..
dsm.wt.smt1..
sp.ugt80..
sp.wt.ugt80..
sp.smt1..
sp.wt.smt1..
im.ugt80..
im.wt.ugt80..
im.smt1..
im.wt.smt1..

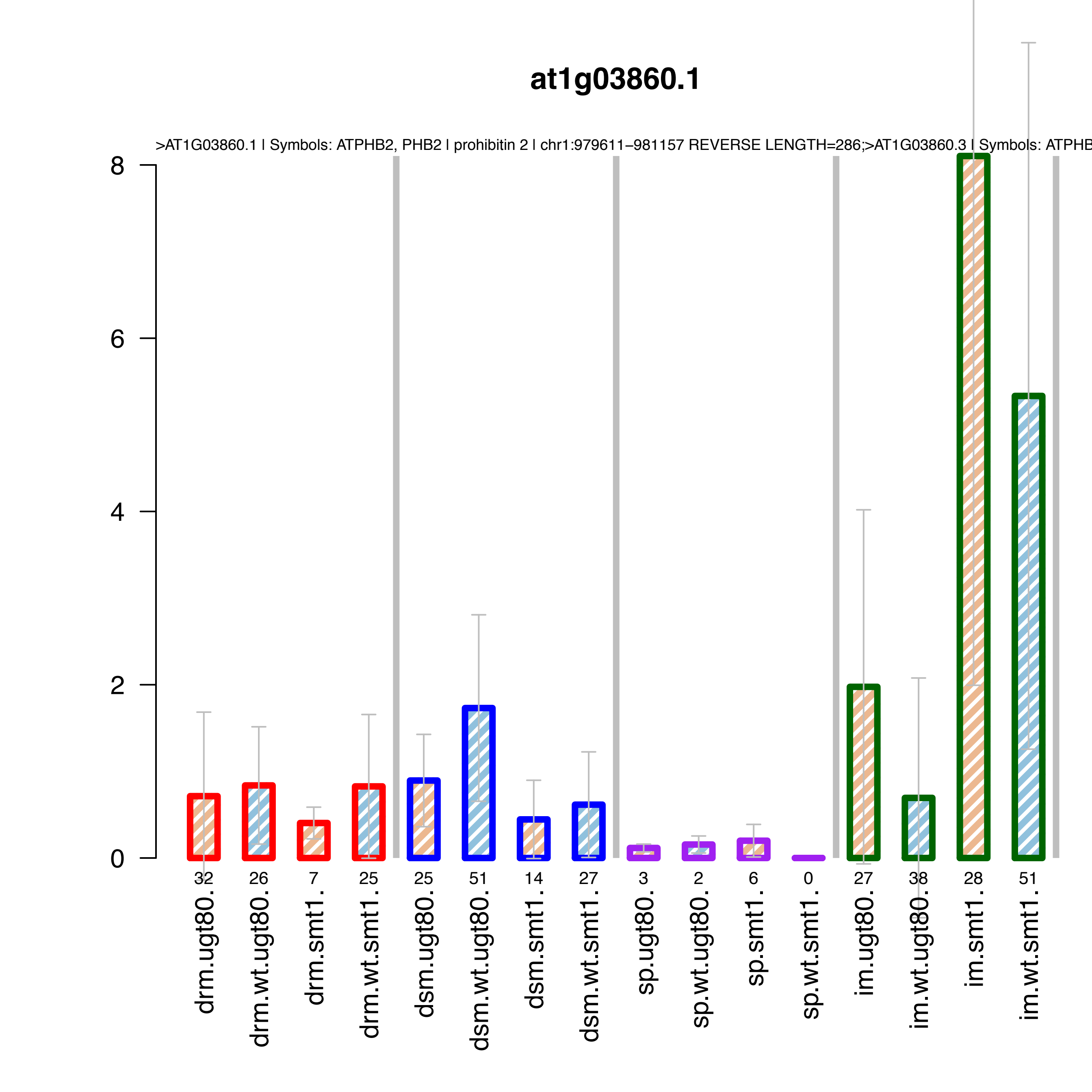

at1g03860.1
>AT1G03860.1 | Symbols: ATPHB2, PHB2 | prohibitin 2 | chr1:979611-981157 REVERSE LENGTH=286;>AT1G03860.3 | Symbols: ATPHB
8
6
4
2
0
32
26
7
25
25
51
14
27
3
2
6
0
27
38
28
51
drm.ugt80.
drm.wt.ugt80.
drm.smt1.
drm.wt.smt1.
dsm.ugt80.
dsm.wt.ugt80.
dsm.smt1.
dsm.wt.smt1.
sp.ugt80.
sp.wt.ugt80.
sp.smt1.
sp.wt.smt1.
im.ugt80.
im.wt.ugt80.
im.smt1.
im.wt.smt1.

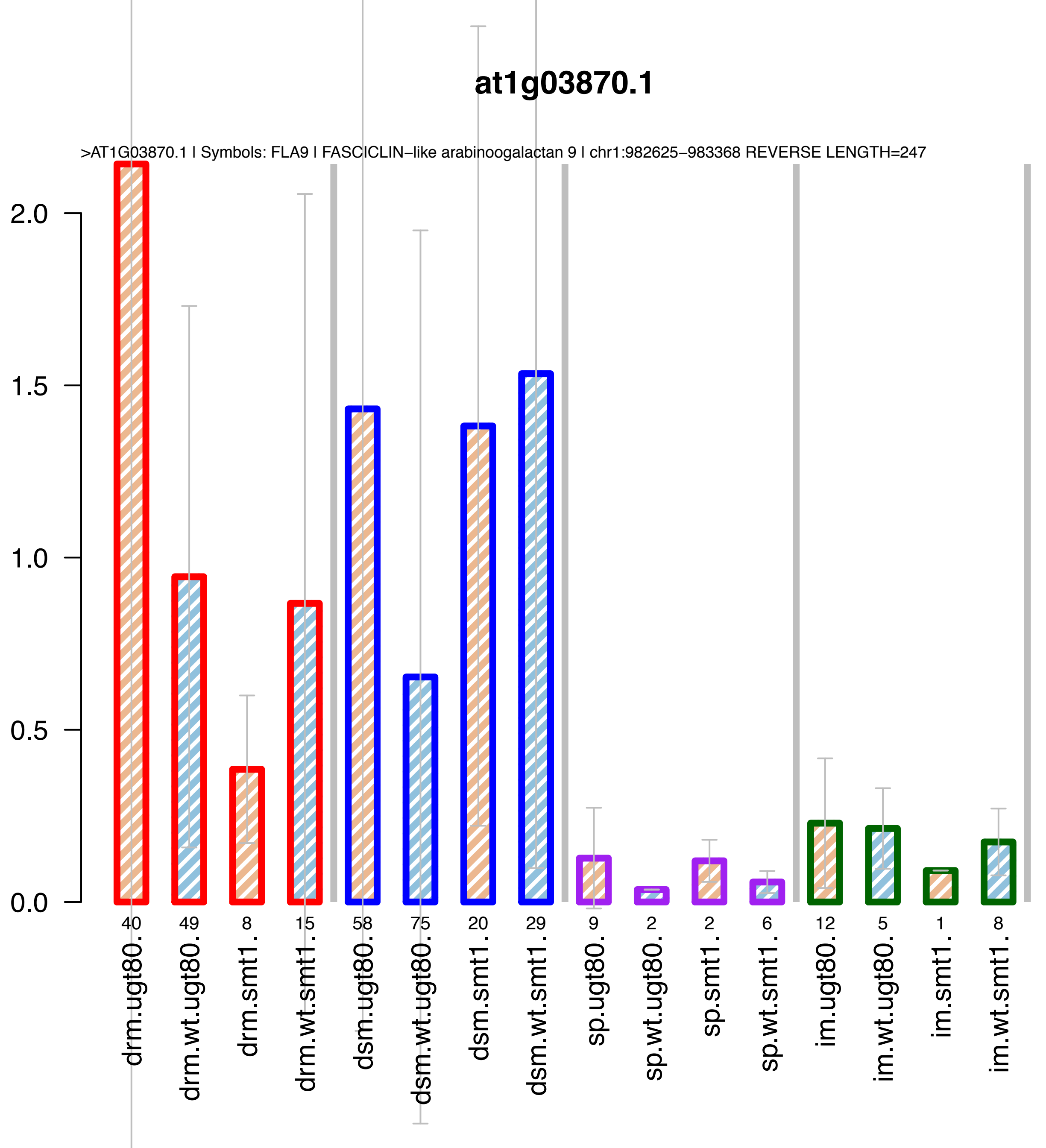

at1g03870.1
>AT1G03870.1 | Symbols: FLA9 | FASCICLIN−like arabinoogalactan 9 | chr1:982625−983368 REVERSE LENGTH=247
0.0
0.5
1.0
1.5
2.0
40
49
8
15
58
75
20
29
9
2
2
6
12
5
1
8
drm.ugt80.
drm.wt.ugt80.
drm.smt1.
drm.wt.smt1.
dsm.ugt80.
dsm.wt.ugt80.
dsm.smt1.
dsm.wt.smt1.
sp.ugt80.
sp.wt.ugt80.
sp.smt1.
sp.wt.smt1.
im.ugt80.
im.wt.ugt80.
im.smt1.
im.wt.smt1.

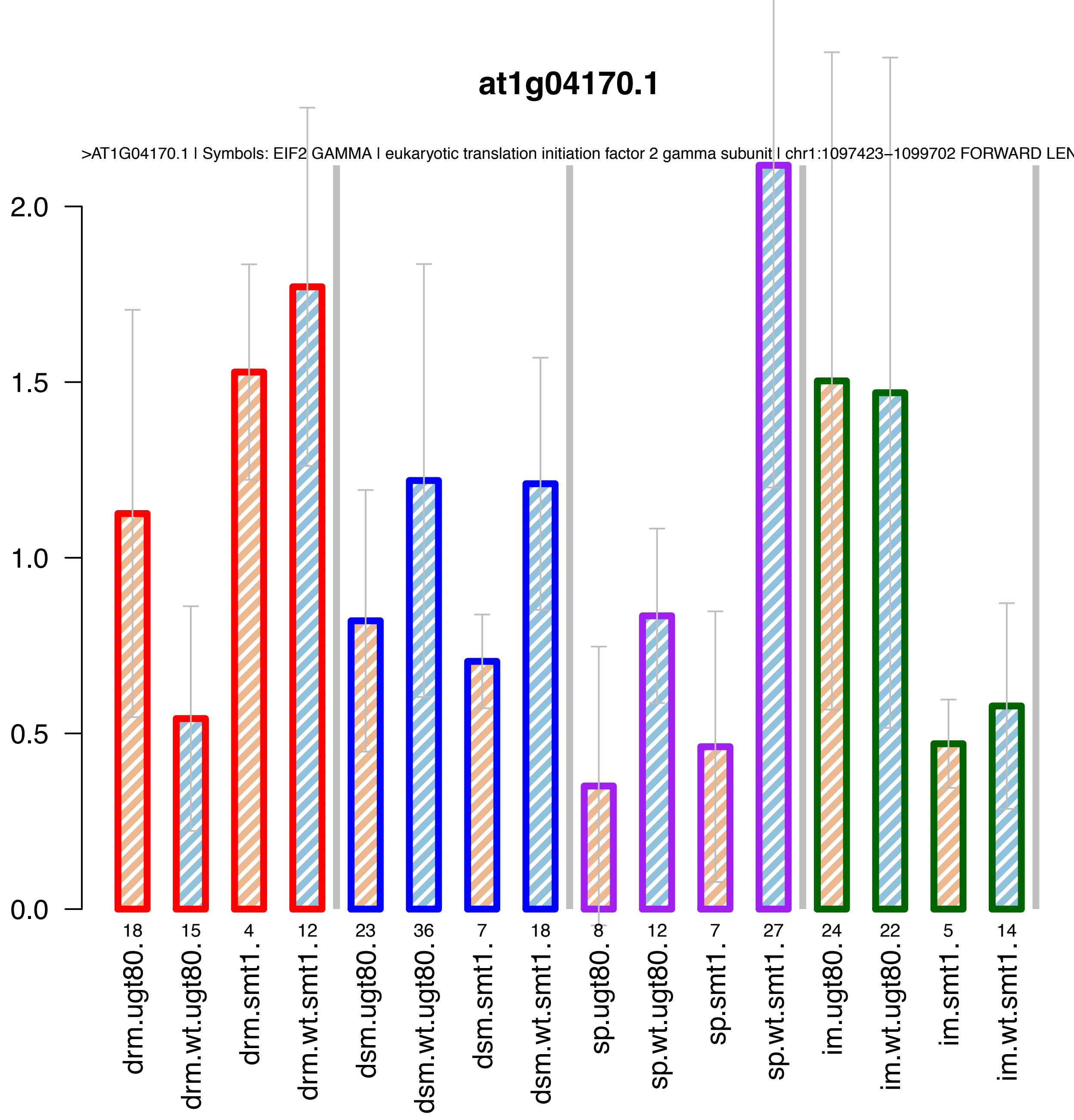
at1g04170.1
>AT1G04170.1 | Symbols: EIF2 GAMMA | eukaryotic translation initiation factor 2 gamma subunit | chr1:1097423-1099702 FORWARD LEN
2.0
1.5
1.0
0.5
0.0
18
15
4
12
23
36
7
18
8
12
7
27
24
22
5
14
drm.ugt80..
drm.wt.ugt80..
drm.smt1..
drm.wt.smt1..
dsm.ugt80..
dsm.wt.ugt80..
dsm.smt1..
dsm.wt.smt1..
sp.ugt80..
sp.wt.ugt80..
sp.smt1..
sp.wt.smt1..
im.ugt80..
im.wt.ugt80..
im.smt1..
im.wt.smt1..

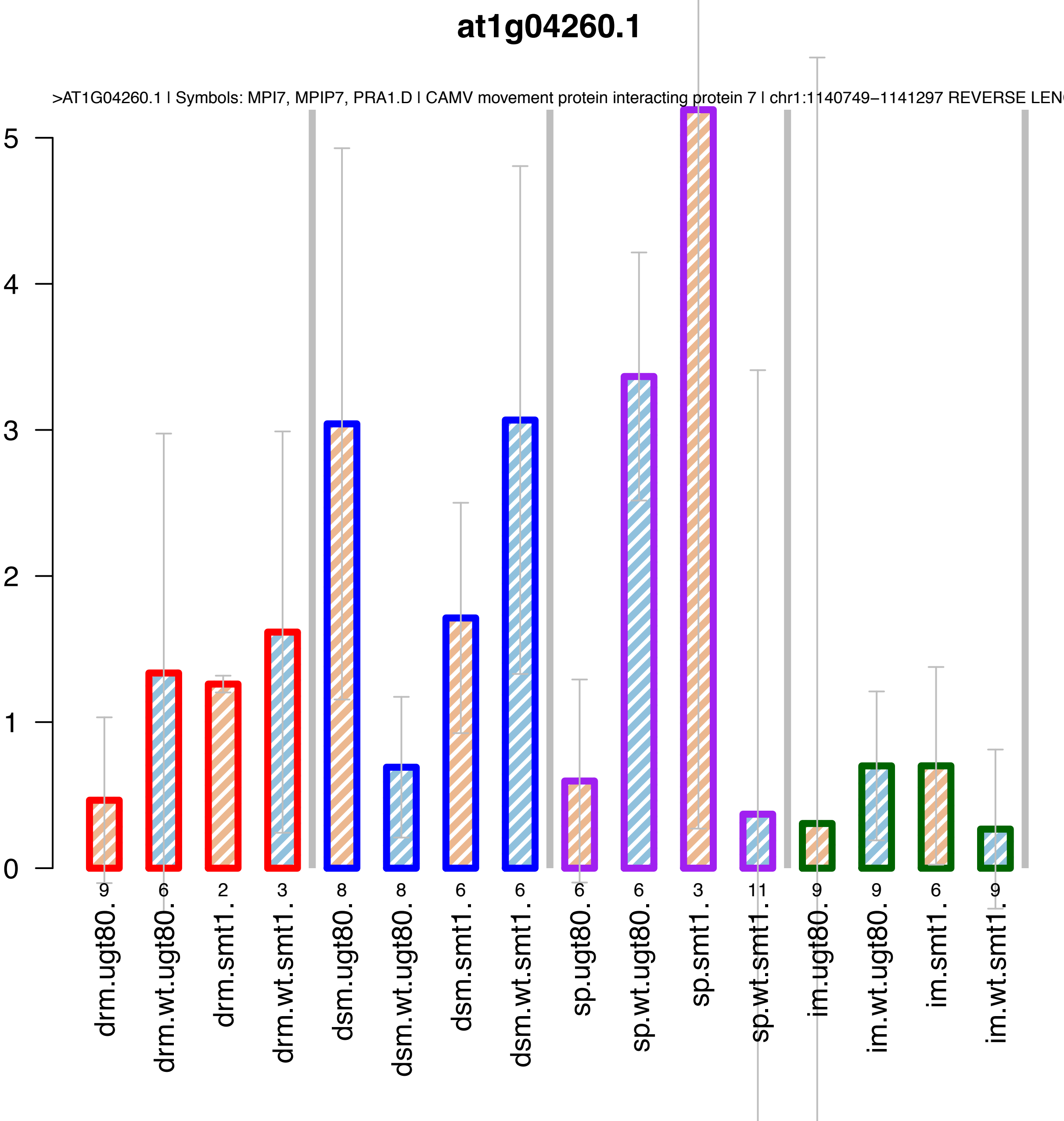
at1g04260.1
>AT1G04260.1 | Symbols: MPI7, MPIP7, PRA1.D | CAMV movement protein interacting protein 7 | chr1:1140749-1141297 REVERSE LEN
0
1
2
3
4
5
9
6
2
3
8
8
6
6
6
6
3
11
9
9
6
9
drm.ugt80.
drm.wt.ugt80.
drm.smt1.
drm.wt.smt1.
dsm.ugt80.
dsm.wt.ugt80.
dsm.smt1.
dsm.wt.smt1.
sp.ugt80.
sp.wt.ugt80.
sp.smt1.
sp.wt.smt1.
im.ugt80.
im.wt.ugt80.
im.smt1.
im.wt.smt1.

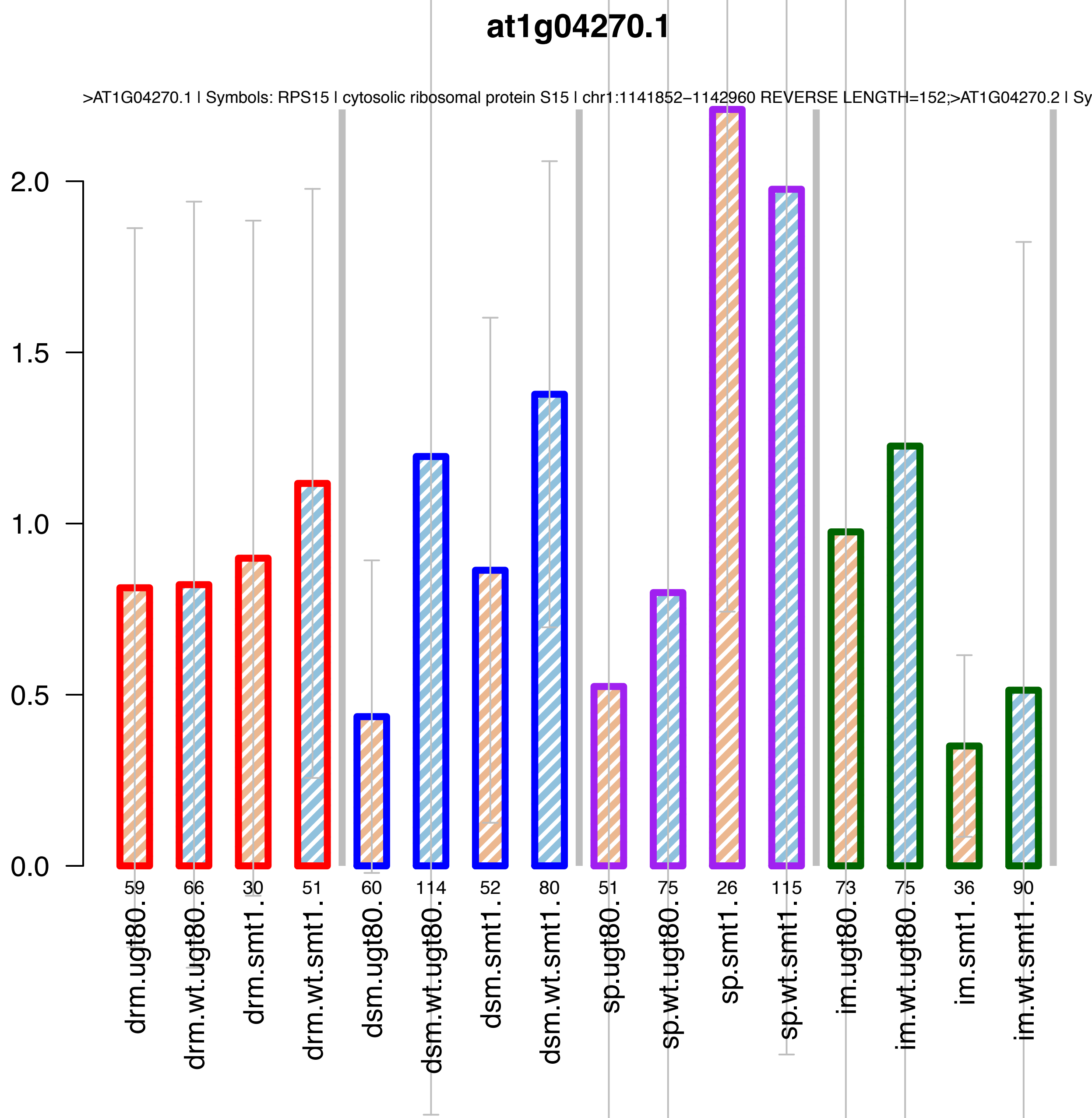
at1g04270.1
>AT1G04270.1 | Symbols: RPS15 | cytosolic ribosomal protein S15 | chr1:1141852–1142960 REVERSE LENGTH=152;>AT1G04270.2 | Sy
2.0
1.5
1.0
0.5
0.0
59
66
30
51
60
114
52
80
51
75
26
115
73
75
36
90
drm.ugt80..
drm.wt.ugt80..
drm.smt1..
drm.wt.smt1..
dsm.ugt80..
dsm.wt.ugt80..
dsm.smt1..
dsm.wt.smt1..
sp.ugt80..
sp.wt.ugt80..
sp.smt1..
sp.wt.smt1..
im.ugt80..
im.wt.ugt80..
im.smt1..
im.wt.smt1..

# at1g04410.1

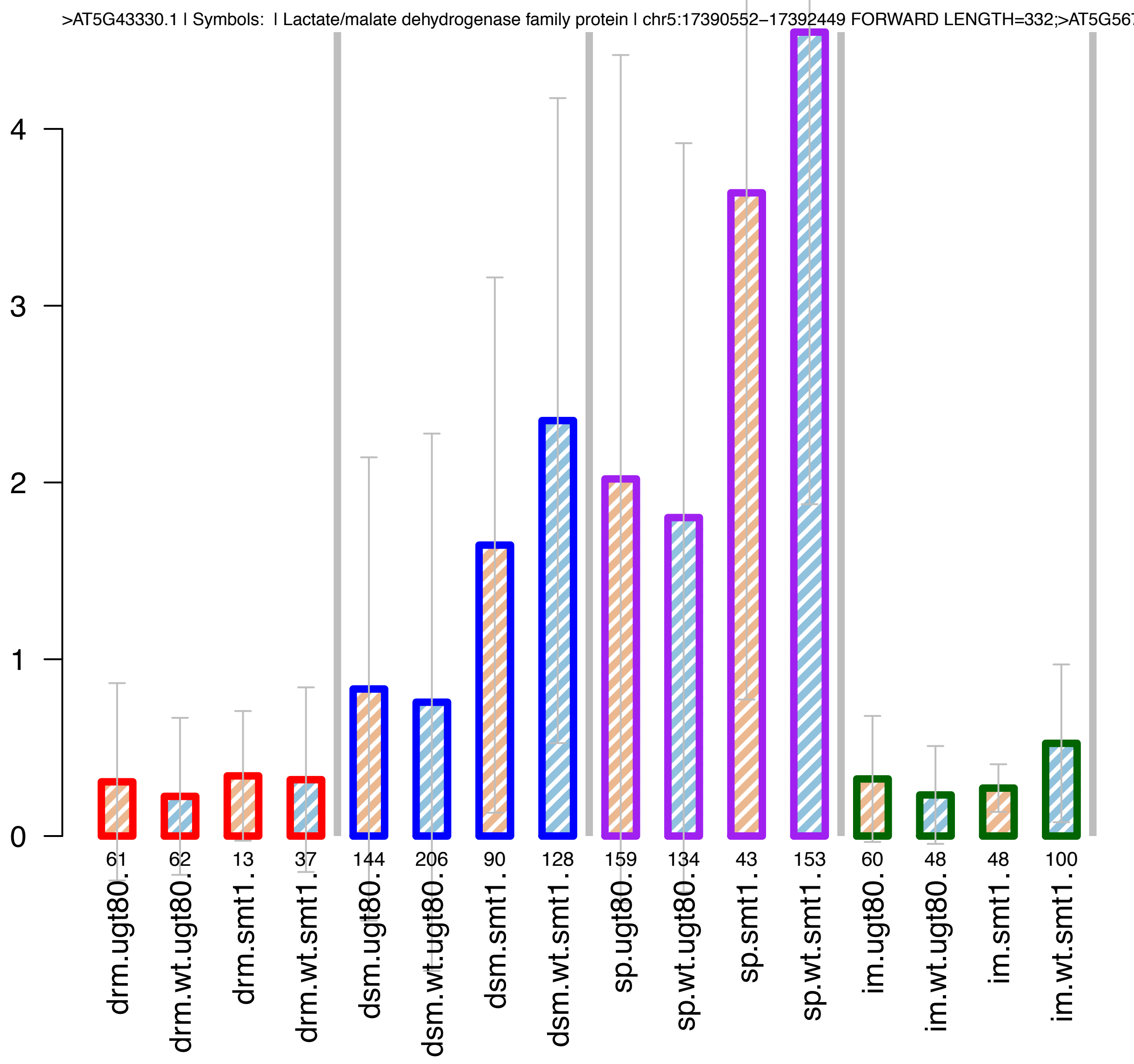

>AT5G43330.1 | Symbols: | Lactate/malate dehydrogenase family protein | chr5:17390552-17392449 FORWARD LENGTH=332;>AT5G56
0
1
2
3
4
61
62
13
37
144
206
90
128
159
134
43
153
60
48
48
100
drm.ugt80..
drm.wt.ugt80..
drm.smt1..
drm.wt.smt1..
dsm.ugt80..
dsm.wt.ugt80..
dsm.smt1..
dsm.wt.smt1..
sp.ugt80..
sp.wt.ugt80..
sp.smt1..
sp.wt.smt1..
im.ugt80..
im.wt.ugt80..
im.smt1..
im.wt.smt1..

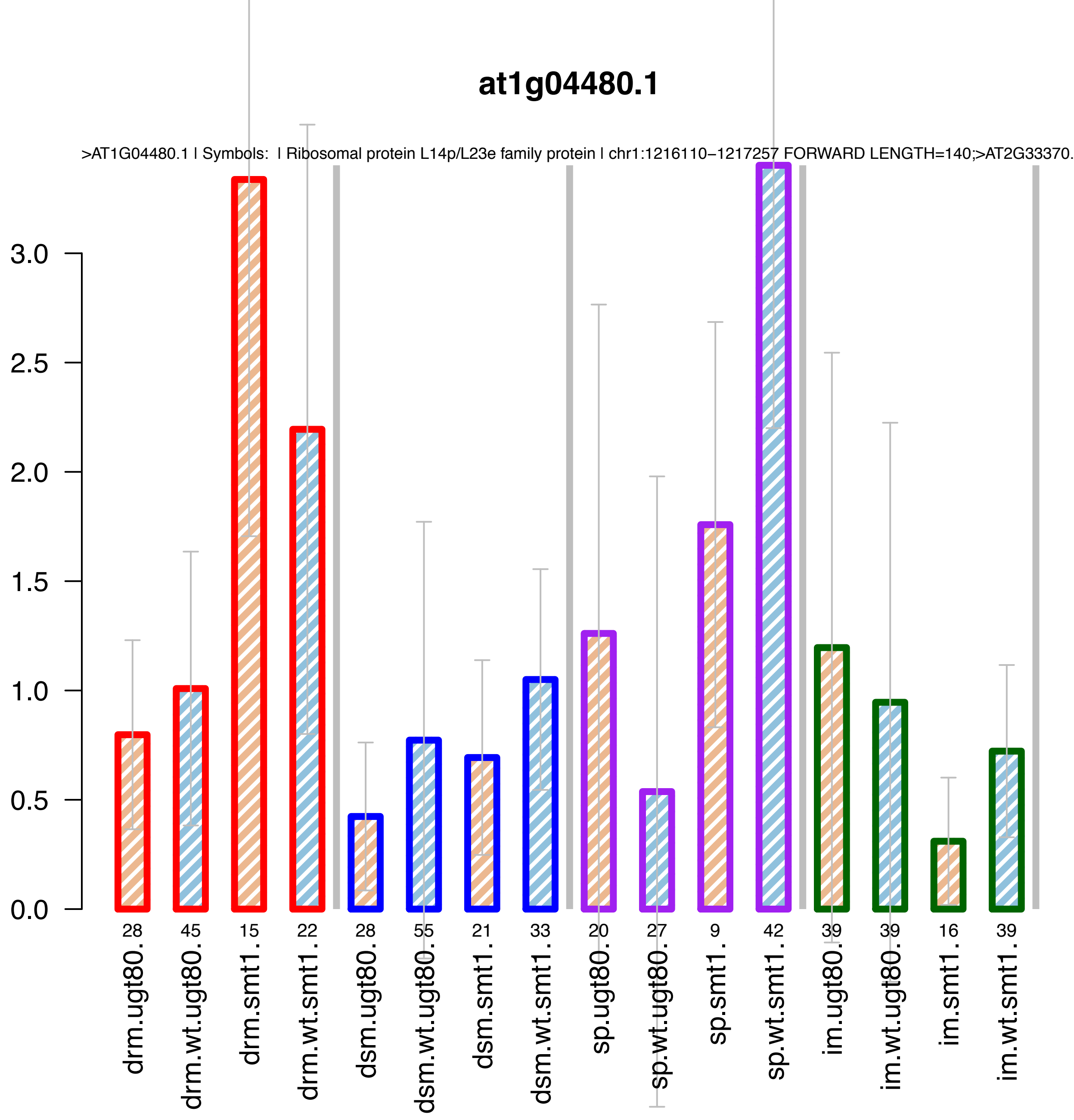

at1g04480.1
>AT1G04480.1 | Symbols: | Ribosomal protein L14p/L23e family protein | chr1:1216110-1217257 FORWARD LENGTH=140;>AT2G33370.
3.0
2.5
2.0
1.5
1.0
0.5
0.0
28
45
15
22
28
55
21
33
20
27
9
42
39
39
16
39
drm.ugt80..
drm.wt.ugt80..
drm.smt1..
drm.wt.smt1..
dsm.ugt80..
dsm.wt.ugt80..
dsm.smt1..
dsm.wt.smt1..
sp.ugt80..
sp.wt.ugt80..
sp.smt1..
sp.wt.smt1..
im.ugt80..
im.wt.ugt80..
im.smt1..
im.wt.smt1..

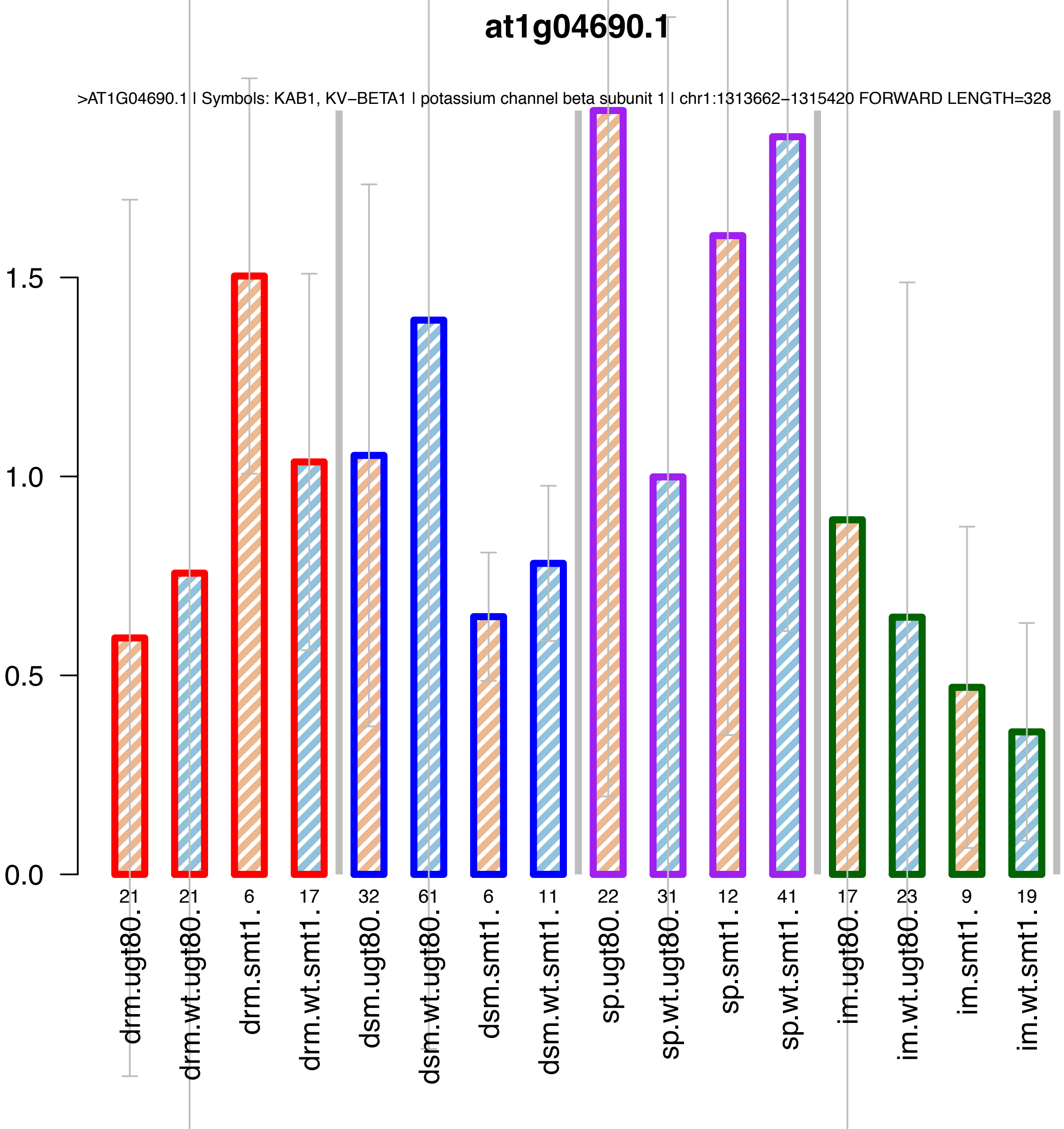
at1g04690.1
>AT1G04690.1 | Symbols: KAB1, KV−BETA1 | potassium channel beta subunit 1 | chr1:1313662−1315420 FORWARD LENGTH=328
0.0
0.5
1.0
1.5
21
21
6
17
32
61
6
11
22
31
12
41
17
23
9
19
drm.ugt80..
drm.wt.ugt80..
drm.smt1..
drm.wt.smt1..
dsm.ugt80..
dsm.wt.ugt80..
dsm.smt1..
dsm.wt.smt1..
sp.ugt80..
sp.wt.ugt80..
sp.smt1..
sp.wt.smt1..
im.ugt80..
im.wt.ugt80..
im.smt1..
im.wt.smt1..

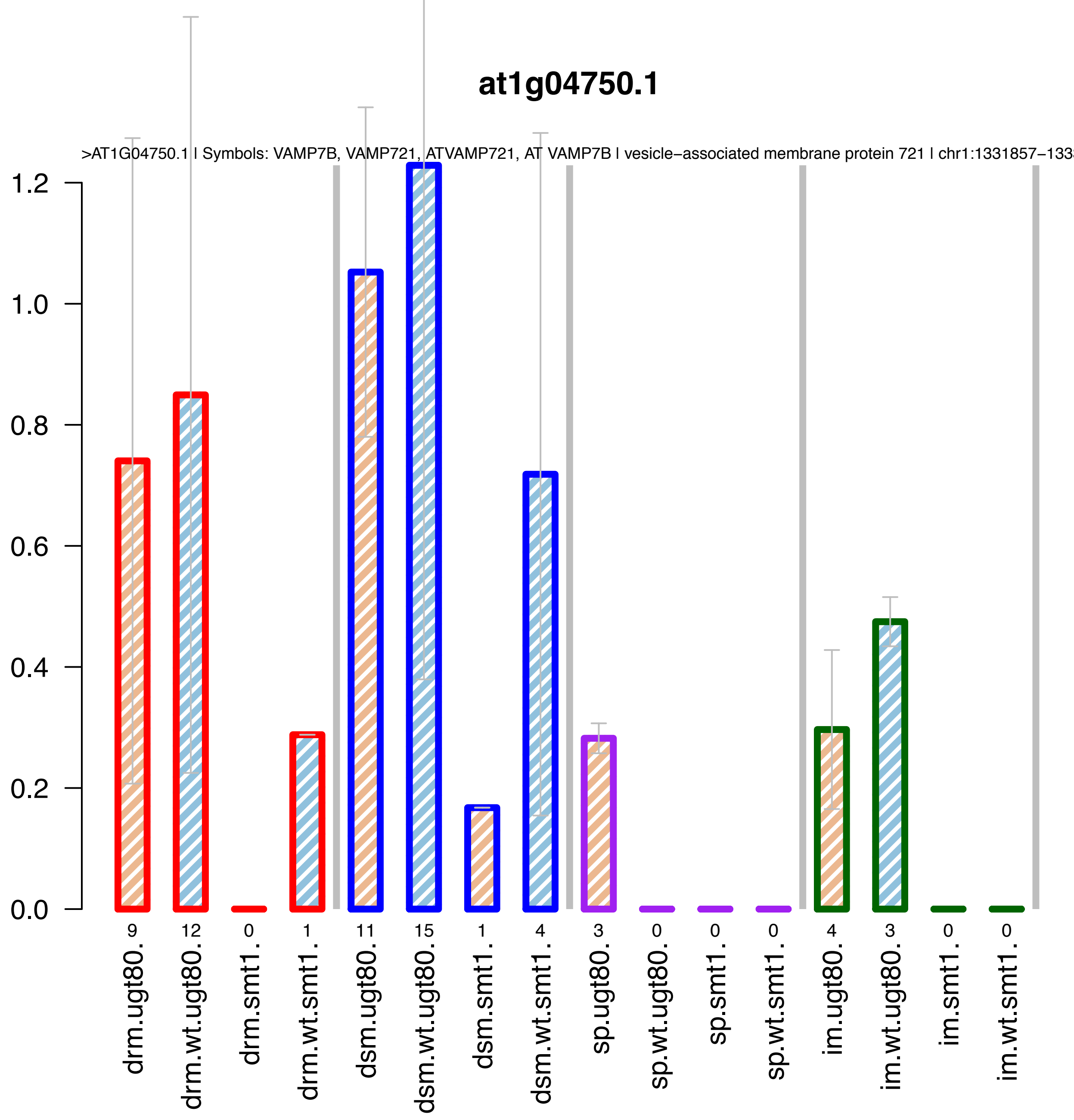

at1g04750.1
>AT1G04750.1 | Symbols: VAMP7B, VAMP721, ATVAMP721, AT VAMP7B | vesicle-associated membrane protein 721 | chr1:1331857-133
1.2
1.0
0.8
0.6
0.4
0.2
0.0
9
12
0
1
11
15
1
4
3
0
0
0
4
3
0
0
drm.ugt80.
drm.wt.ugt80.
drm.smt1.
drm.wt.smt1.
dsm.ugt80.
dsm.wt.ugt80.
dsm.smt1.
dsm.wt.smt1.
sp.ugt80.
sp.wt.ugt80.
sp.smt1.
sp.wt.smt1.
im.ugt80.
im.wt.ugt80.
im.smt1.
im.wt.smt1.

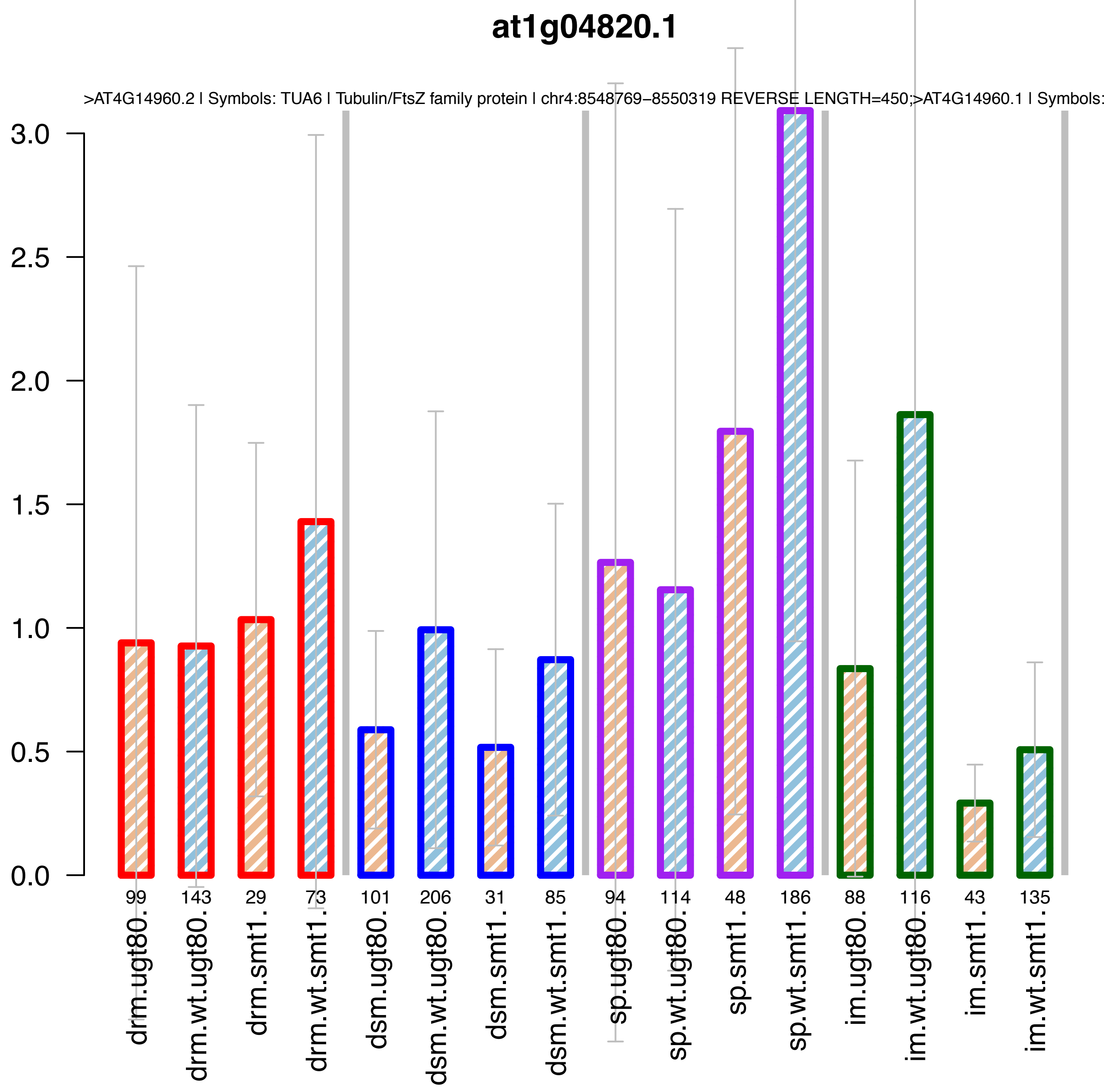
at1g04820.1
>AT4G14960.2 | Symbols: TUA6 | Tubulin/FtsZ family protein | chr4:8548769–8550319 REVERSE LENGTH=450;>AT4G14960.1 | Symbols:
3.0
2.5
2.0
1.5
1.0
0.5
0.0
99
143
29
73
101
206
31
85
94
114
48
186
88
116
43
135
drm.ugt80..
drm.wt.ugt80..
drm.smt1..
drm.wt.smt1..
dsm.ugt80..
dsm.wt.ugt80..
dsm.smt1..
dsm.wt.smt1..
sp.ugt80..
sp.wt.ugt80..
sp.smt1..
sp.wt.smt1..
im.ugt80..
im.wt.ugt80..
im.smt1..
im.wt.smt1..

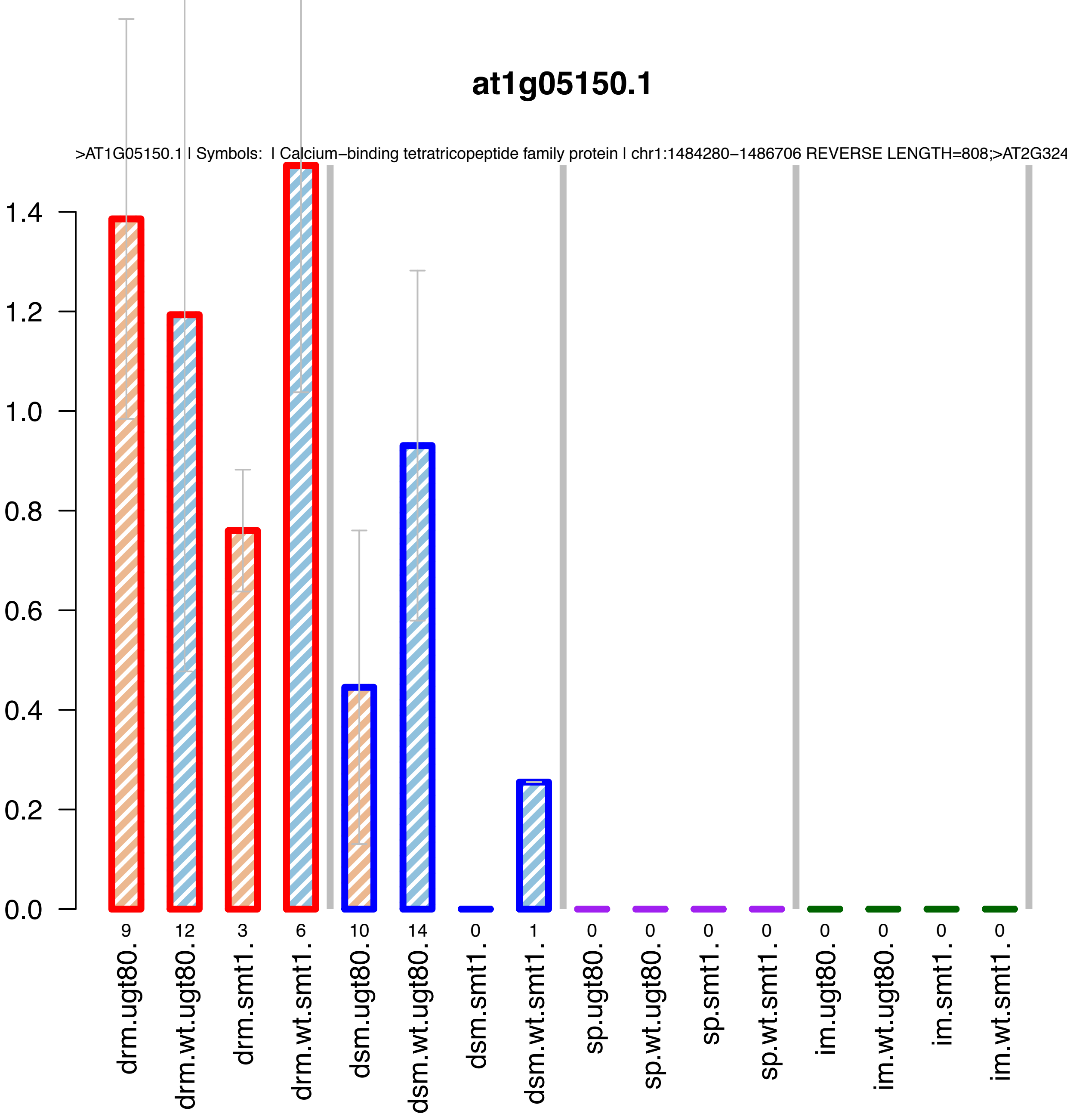

at1g05150.1
>AT1G05150.1 | Symbols: | Calcium–binding tetratricopeptide family protein | chr1:1484280–1486706 REVERSE LENGTH=808;>AT2G324
1.4
1.2
1.0
0.8
0.6
0.4
0.2
0.0
9
12
3
6
10
14
0
1
0
0
0
0
0
0
0
0
drm.ugt80.
drm.wt.ugt80.
drm.smt1.
drm.wt.smt1.
dsm.ugt80.
dsm.wt.ugt80.
dsm.smt1.
dsm.wt.smt1.
sp.ugt80.
sp.wt.ugt80.
sp.smt1.
sp.wt.smt1.
im.ugt80.
im.wt.ugt80.
im.smt1.
im.wt.smt1.

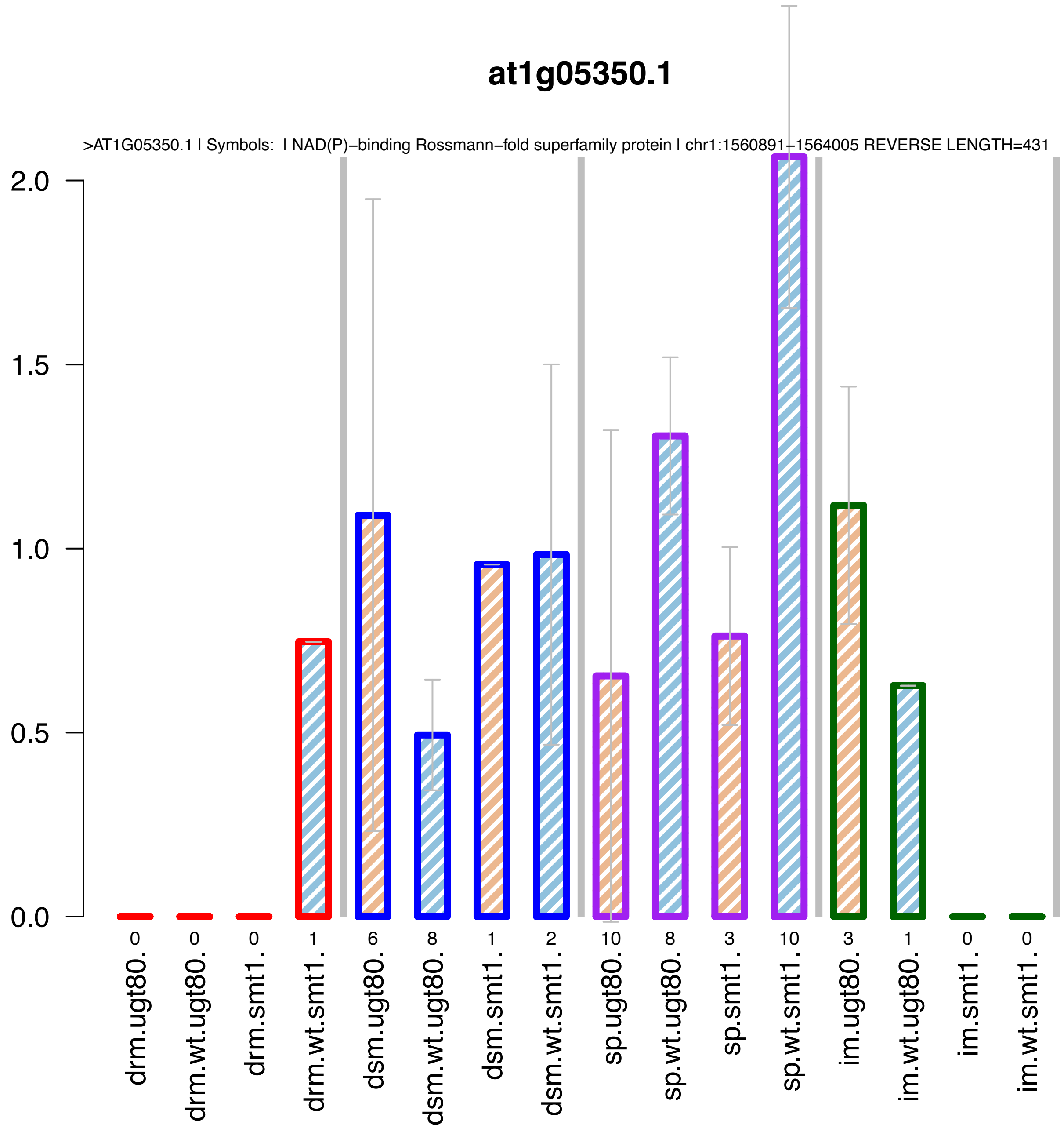
at1g05350.1
>AT1G05350.1 | Symbols: | NAD(P)−binding Rossmann−fold superfamily protein | chr1:1560891−1564005 REVERSE LENGTH=431
2.0
1.5
1.0
0.5
0.0
0
0
0
1
6
8
1
2
10
8
3
10
3
1
0
0
drm.ugt80.
drm.wt.ugt80.
drm.smt1.
drm.wt.smt1.
dsm.ugt80.
dsm.wt.ugt80.
dsm.smt1.
dsm.wt.smt1.
sp.ugt80.
sp.wt.ugt80.
sp.smt1.
sp.wt.smt1.
im.ugt80.
im.wt.ugt80.
im.smt1.
im.wt.smt1.

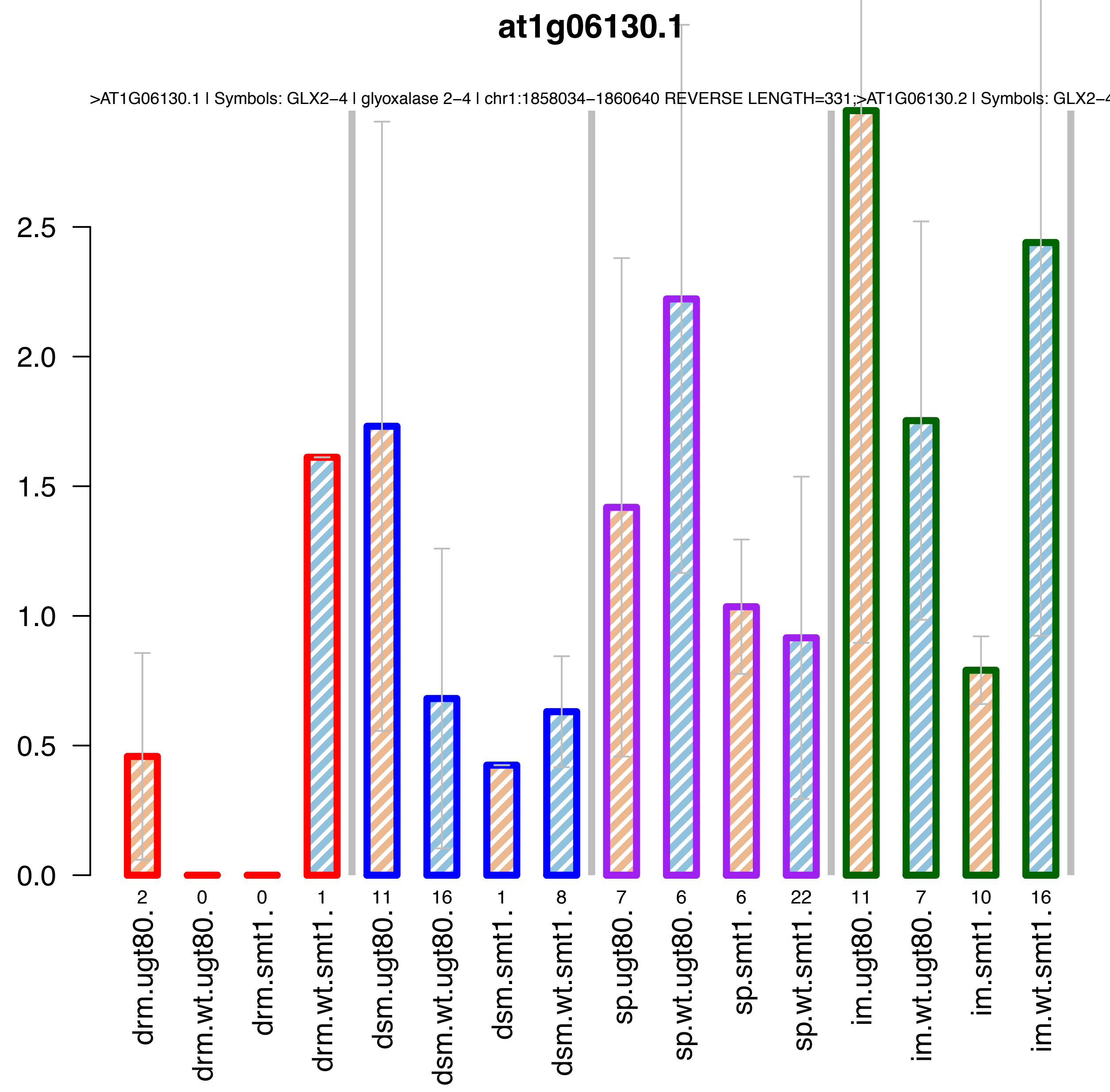
at1g06130.1
>AT1G06130.1 | Symbols: GLX2−4 | glyoxalase 2−4 | chr1:1858034−1860640 REVERSE LENGTH=331;>AT1G06130.2 | Symbols: GLX2−4
0.0
0.5
1.0
1.5
2.0
2.5
2
0
0
1
11
16
1
8
7
6
6
22
11
7
10
16
drm.ugt80..
drm.wt.ugt80..
drm.smt1..
drm.wt.smt1..
dsm.ugt80..
dsm.wt.ugt80..
dsm.smt1..
dsm.wt.smt1..
sp.ugt80..
sp.wt.ugt80..
sp.smt1..
sp.wt.smt1..
im.ugt80..
im.wt.ugt80..
im.smt1..
im.wt.smt1..

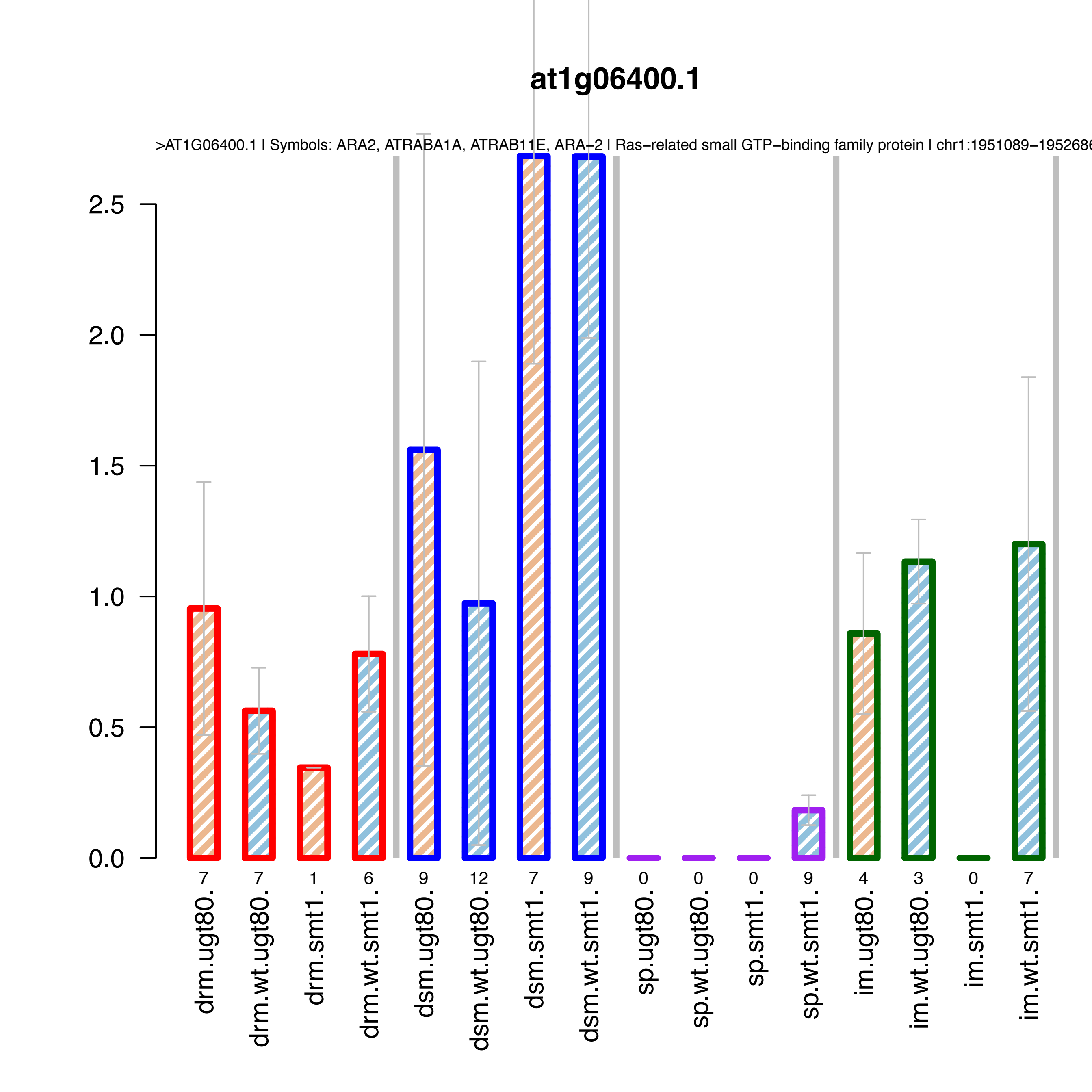

at1g06400.1
>AT1G06400.1 | Symbols: ARA2, ATRABA1A, ATRAB11E, ARA−2 | Ras−related small GTP−binding family protein | chr1:1951089−1952686
2.5
2.0
1.5
1.0
0.5
0.0
7
7
1
6
9
12
7
9
0
0
0
9
4
3
0
7
drm.ugt80.
drm.wt.ugt80.
drm.smt1.
drm.wt.smt1.
dsm.ugt80.
dsm.wt.ugt80.
dsm.smt1.
dsm.wt.smt1.
sp.ugt80.
sp.wt.ugt80.
sp.smt1.
sp.wt.smt1.
im.ugt80.
im.wt.ugt80.
im.smt1.
im.wt.smt1.

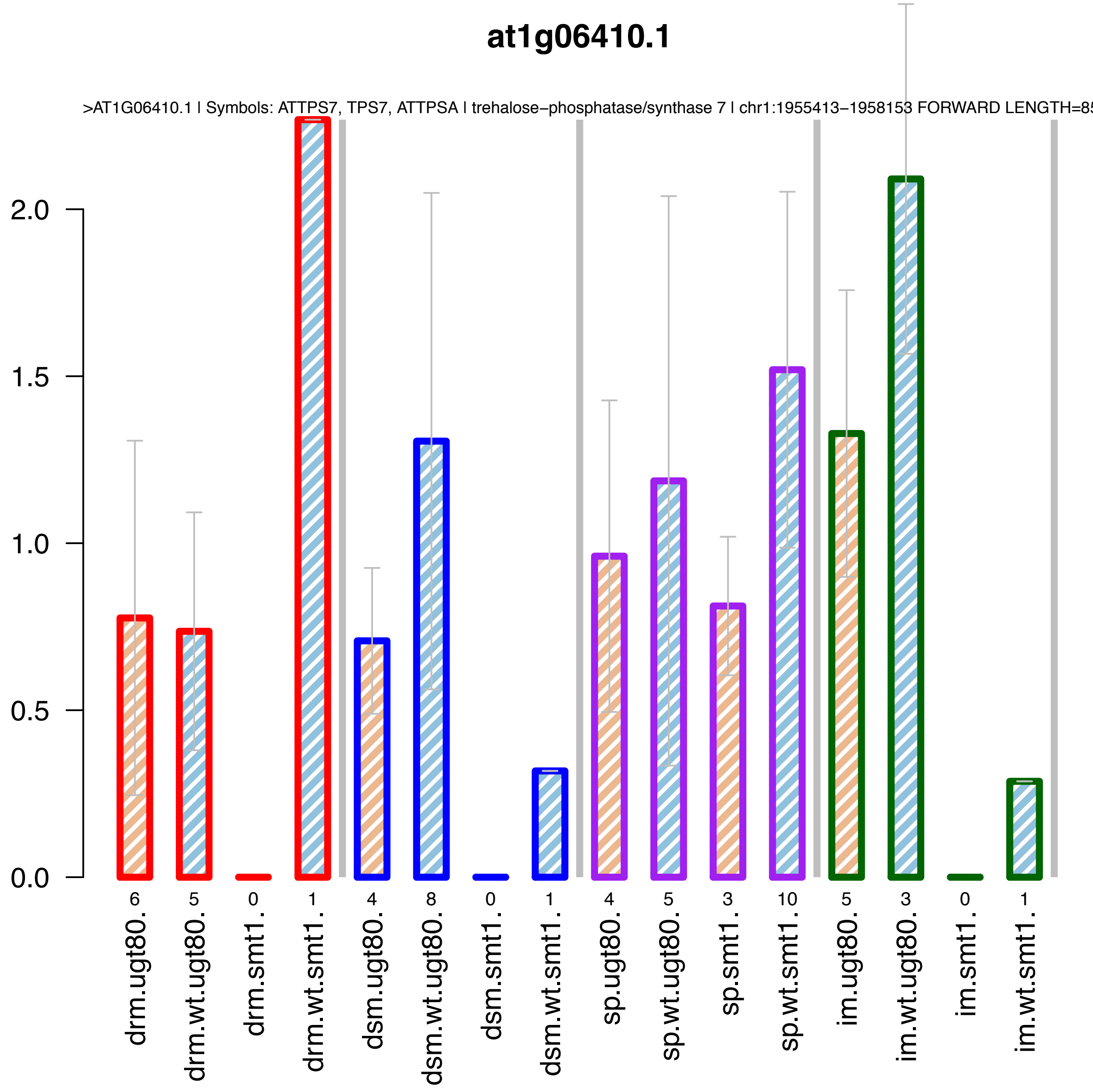
at1g06410.1
>AT1G06410.1 | Symbols: ATTPS7, TPS7, ATTPSA | trehalose−phosphatase/synthase 7 | chr1:1955413−1958153 FORWARD LENGTH=85
2.0
1.5
1.0
0.5
0.0
6
5
0
1
4
8
0
1
4
5
3
10
5
3
0
1
drm.ugt80.
drm.wt.ugt80.
drm.smt1.
drm.wt.smt1.
dsm.ugt80.
dsm.wt.ugt80.
dsm.smt1.
dsm.wt.smt1.
sp.ugt80.
sp.wt.ugt80.
sp.smt1.
sp.wt.smt1.
im.ugt80.
im.wt.ugt80.
im.smt1.
im.wt.smt1.

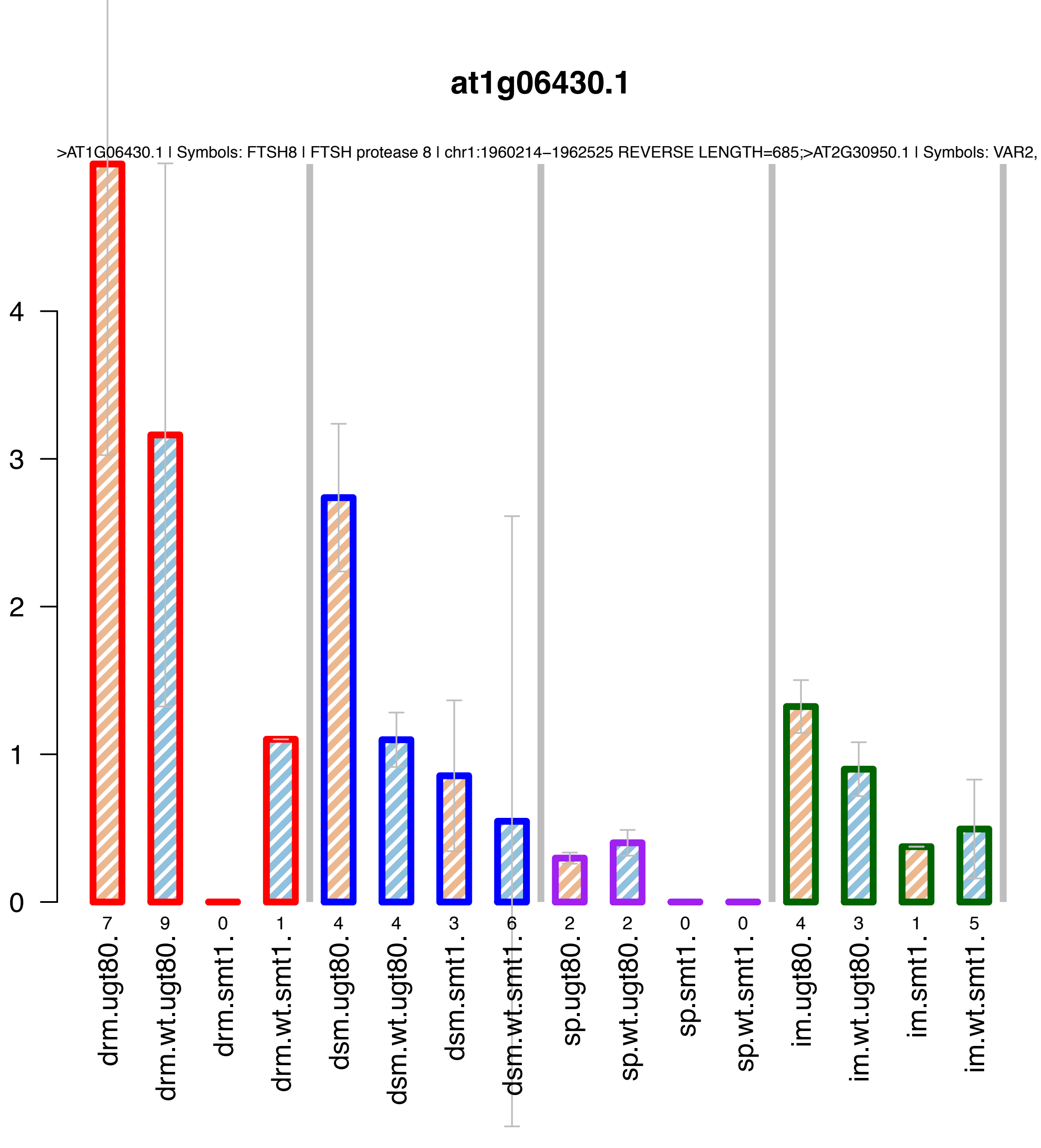

at1g06430.1
>AT1G06430.1 | Symbols: FTSH8 | FTSH protease 8 | chr1:1960214−1962525 REVERSE LENGTH=685;>AT2G30950.1 | Symbols: VAR2,
0
1
2
3
4
7
9
0
1
4
4
3
6
2
2
0
0
4
3
1
5
drm.ugt80.
drm.wt.ugt80.
drm.smt1.
drm.wt.smt1.
dsm.ugt80.
dsm.wt.ugt80.
dsm.smt1.
dsm.wt.smt1.
sp.ugt80.
sp.wt.ugt80.
sp.smt1.
sp.wt.smt1.
im.ugt80.
im.wt.ugt80.
im.smt1.
im.wt.smt1.

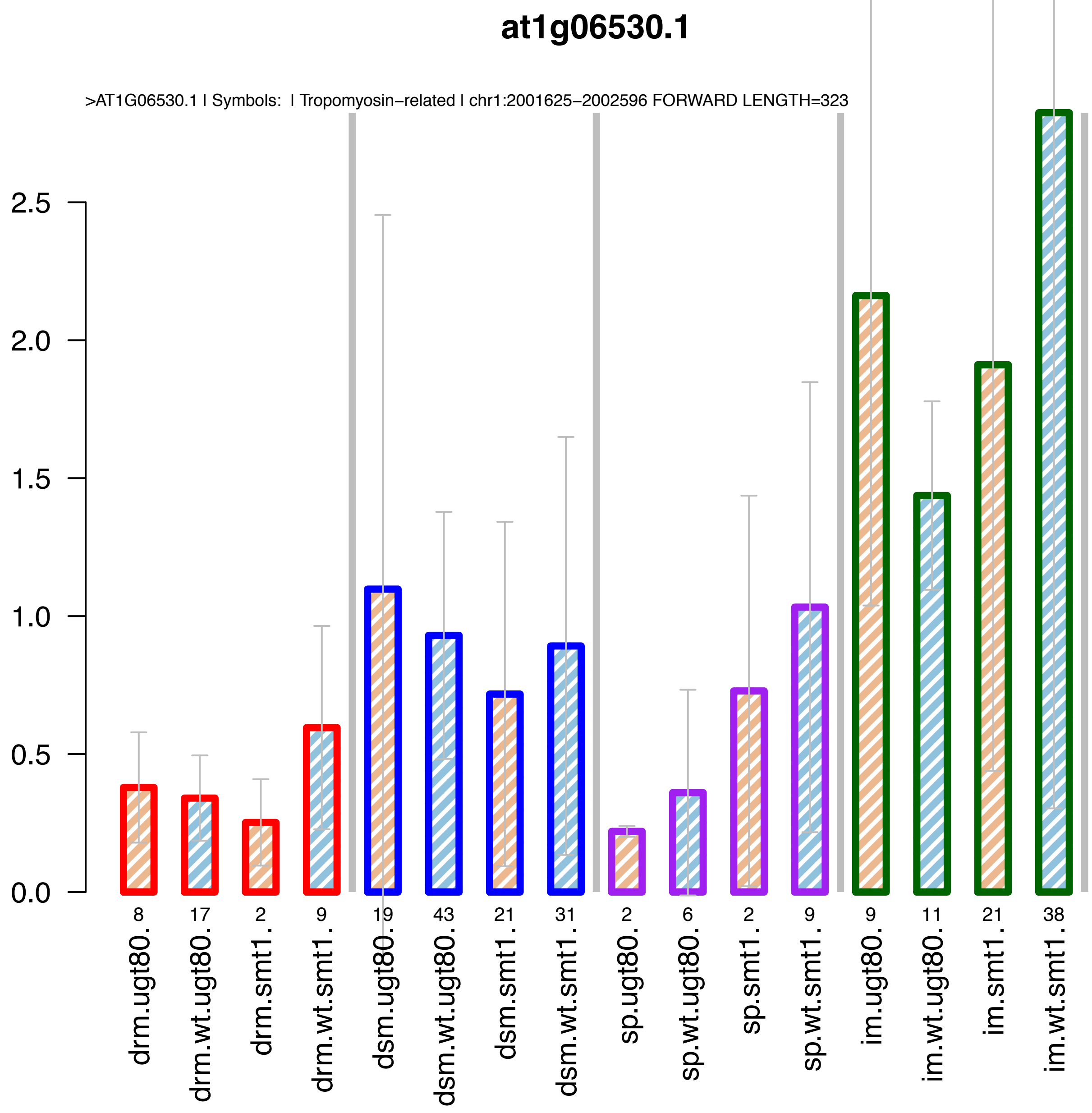

at1g06530.1
>AT1G06530.1 | Symbols: | Tropomyosin−related | chr1:2001625−2002596 FORWARD LENGTH=323
0.0
0.5
1.0
1.5
2.0
2.5
8
17
2
9
19
43
21
31
2
6
2
9
9
11
21
38
drm.ugt80.
drm.wt.ugt80.
drm.smt1.
drm.wt.smt1.
dsm.ugt80.
dsm.wt.ugt80.
dsm.smt1.
dsm.wt.smt1.
sp.ugt80.
sp.wt.ugt80.
sp.smt1.
sp.wt.smt1.
im.ugt80.
im.wt.ugt80.
im.smt1.
im.wt.smt1.

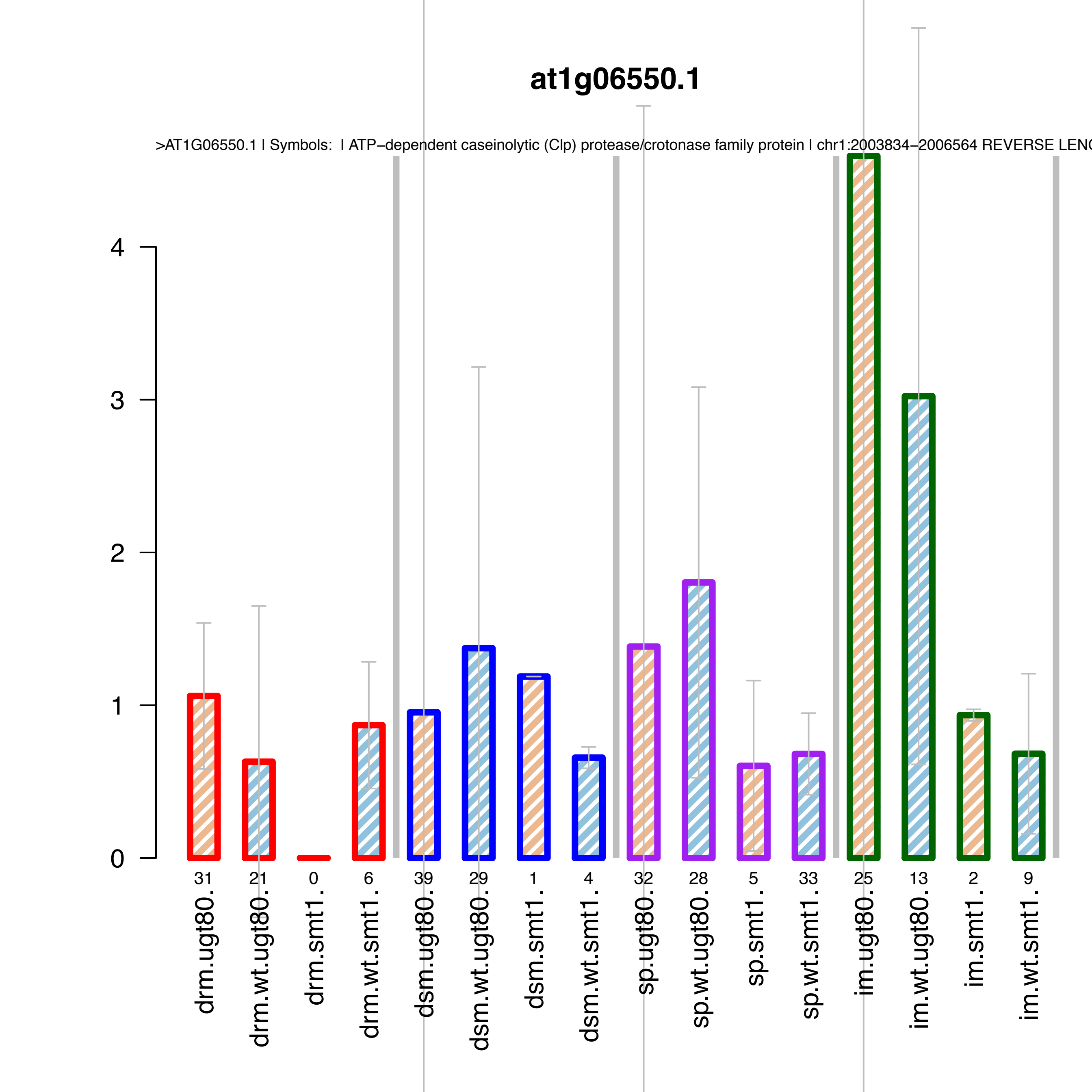

at1g06550.1
>AT1G06550.1 | Symbols: | ATP−dependent caseinolytic (Clp) protease/crotonase family protein | chr1:2003834−2006564 REVERSE LENG
4
3
2
1
0
31
21
0
6
39
29
1
4
32
28
5
33
25
13
2
9
drm.ugt80..
drm.wt.ugt80..
drm.smt1..
drm.wt.smt1..
dsm.ugt80..
dsm.wt.ugt80..
dsm.smt1..
dsm.wt.smt1..
sp.ugt80..
sp.wt.ugt80..
sp.smt1..
sp.wt.smt1..
im.ugt80..
im.wt.ugt80..
im.smt1..
im.wt.smt1..

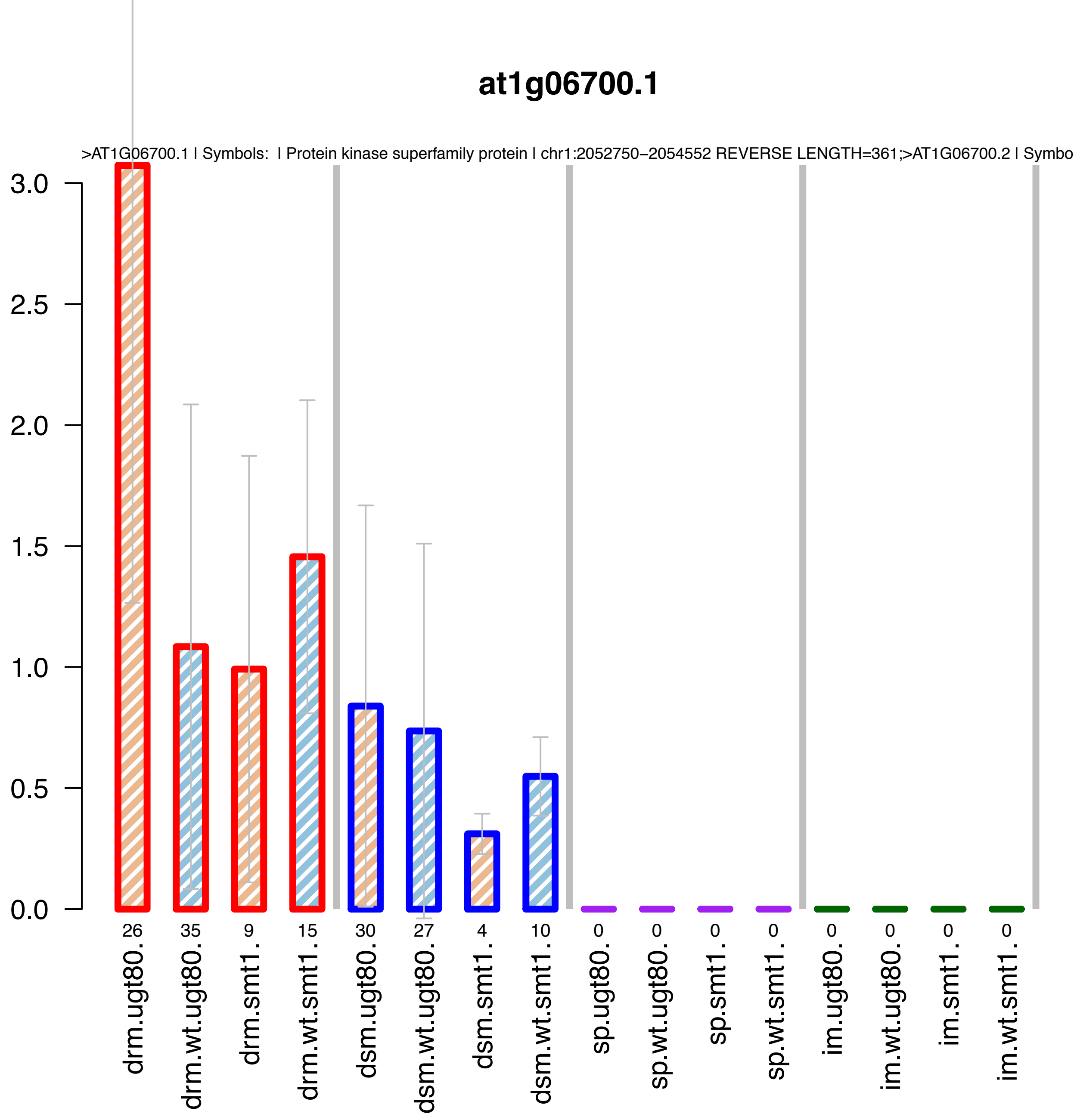
at1g06700.1
>AT1G06700.1 | Symbols: | Protein kinase superfamily protein | chr1:2052750-2054552 REVERSE LENGTH=361;>AT1G06700.2 | Symbo
3.0
2.5
2.0
1.5
1.0
0.5
0.0
26
35
9
15
30
27
4
10
0
0
0
0
0
0
0
0
drm.ugt80.
drm.wt.ugt80.
drm.smt1.
drm.wt.smt1.
dsm.ugt80.
dsm.wt.ugt80.
dsm.smt1.
dsm.wt.smt1.
sp.ugt80.
sp.wt.ugt80.
sp.smt1.
sp.wt.smt1.
im.ugt80.
im.wt.ugt80.
im.smt1.
im.wt.smt1.

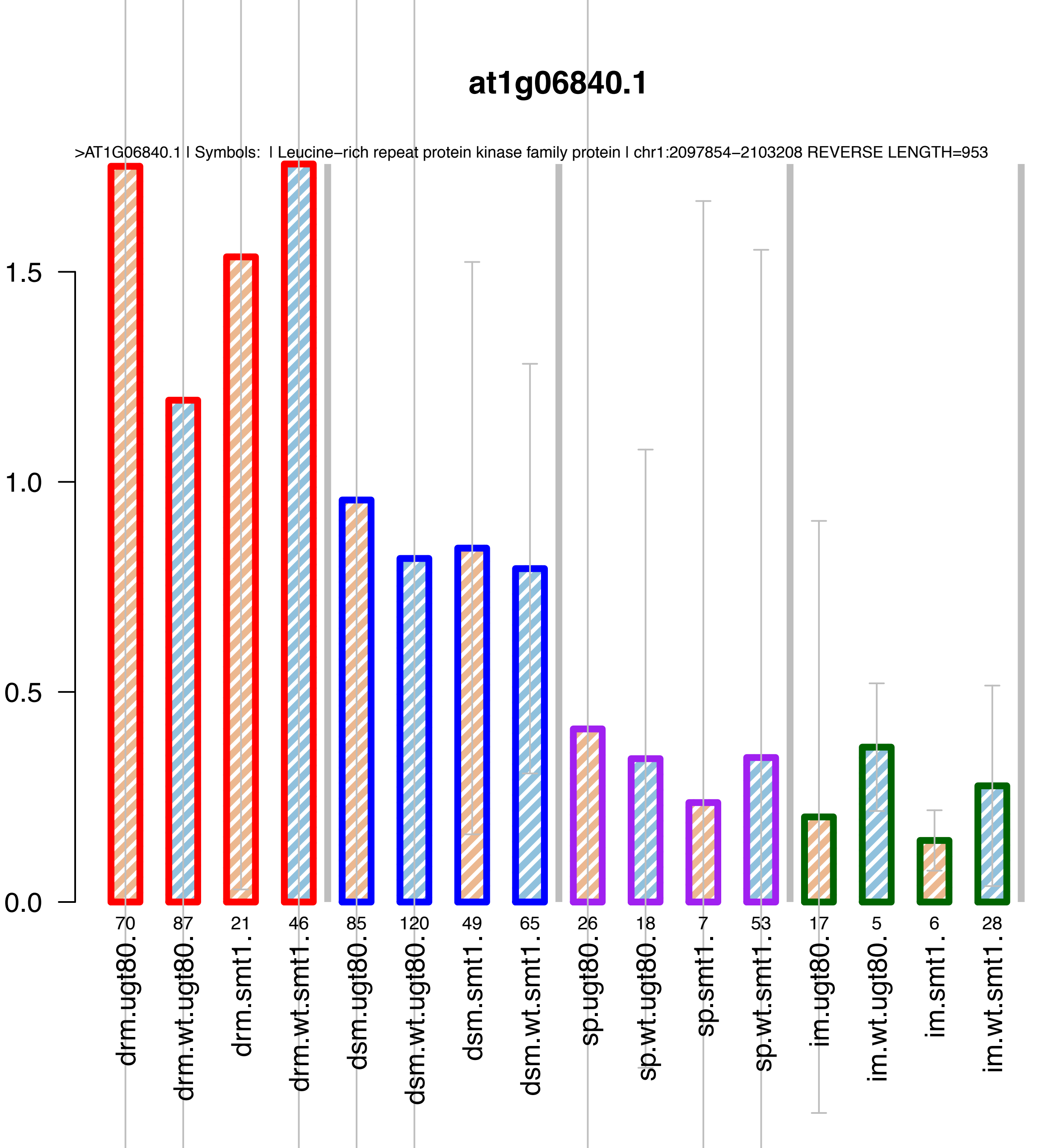
at1g06840.1
>AT1G06840.1 | Symbols: | Leucine−rich repeat protein kinase family protein | chr1:2097854−2103208 REVERSE LENGTH=953
0.0
0.5
1.0
1.5
70
87
21
46
85
120
49
65
26
18
7
53
17
5
6
28
drm.ugt80..
drm.wt.ugt80..
drm.smt1..
drm.wt.smt1..
dsm.ugt80..
dsm.wt.ugt80..
dsm.smt1..
dsm.wt.smt1..
sp.ugt80..
sp.wt.ugt80..
sp.smt1..
sp.wt.smt1..
im.ugt80..
im.wt.ugt80..
im.smt1..
im.wt.smt1..

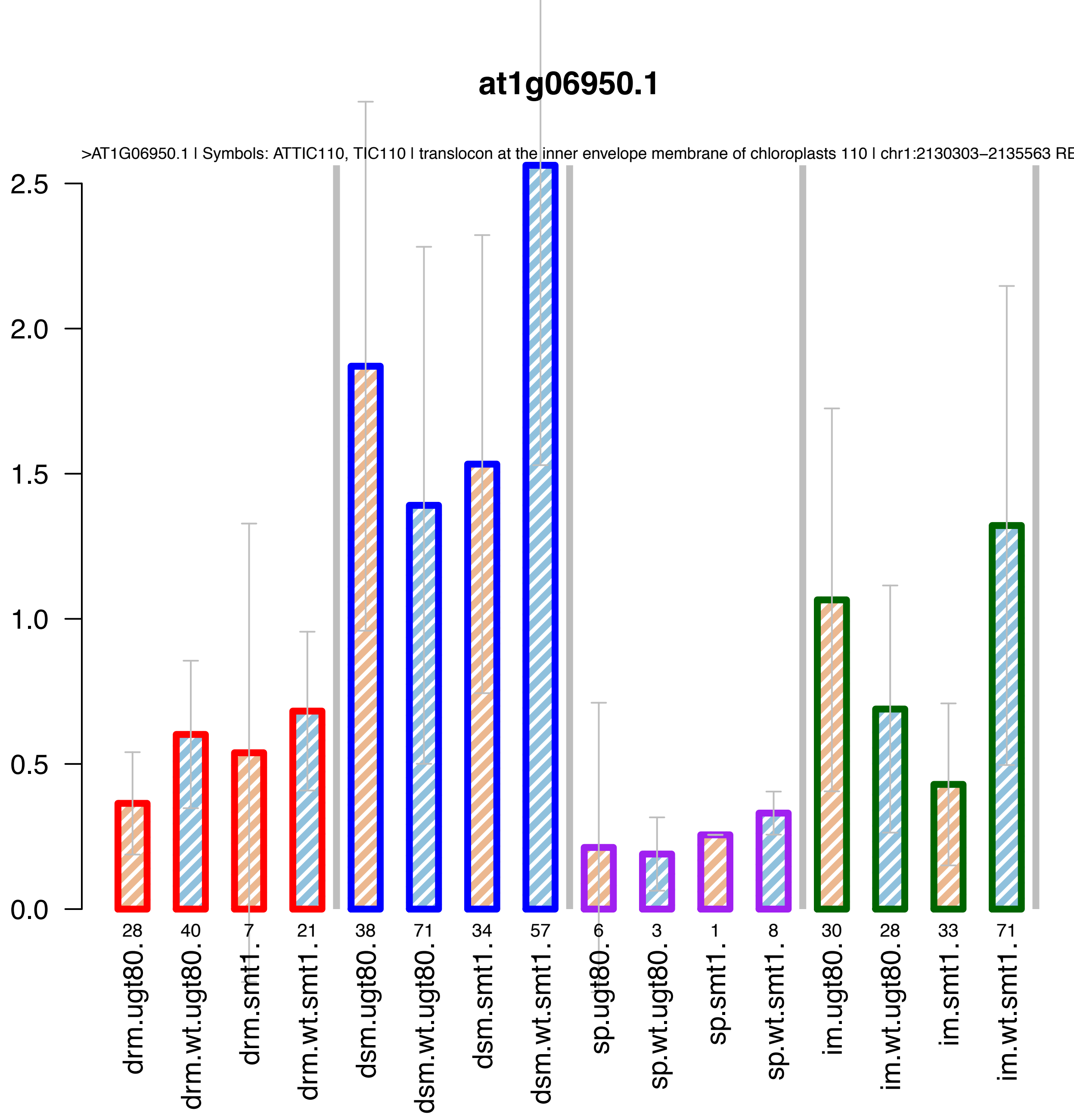

at1g06950.1
>AT1G06950.1 | Symbols: ATTIC110, TIC110 | translocon at the inner envelope membrane of chloroplasts 110 | chr1:2130303-2135563 RE
2.5
2.0
1.5
1.0
0.5
0.0
28
40
7
21
38
71
34
57
6
3
1
8
30
28
33
71
drm.ugt80.
drm.wt.ugt80.
drm.smt1.
drm.wt.smt1.
dsm.ugt80.
dsm.wt.ugt80.
dsm.smt1.
dsm.wt.smt1.
sp.ugt80.
sp.wt.ugt80.
sp.smt1.
sp.wt.smt1.
im.ugt80.
im.wt.ugt80.
im.smt1.
im.wt.smt1.

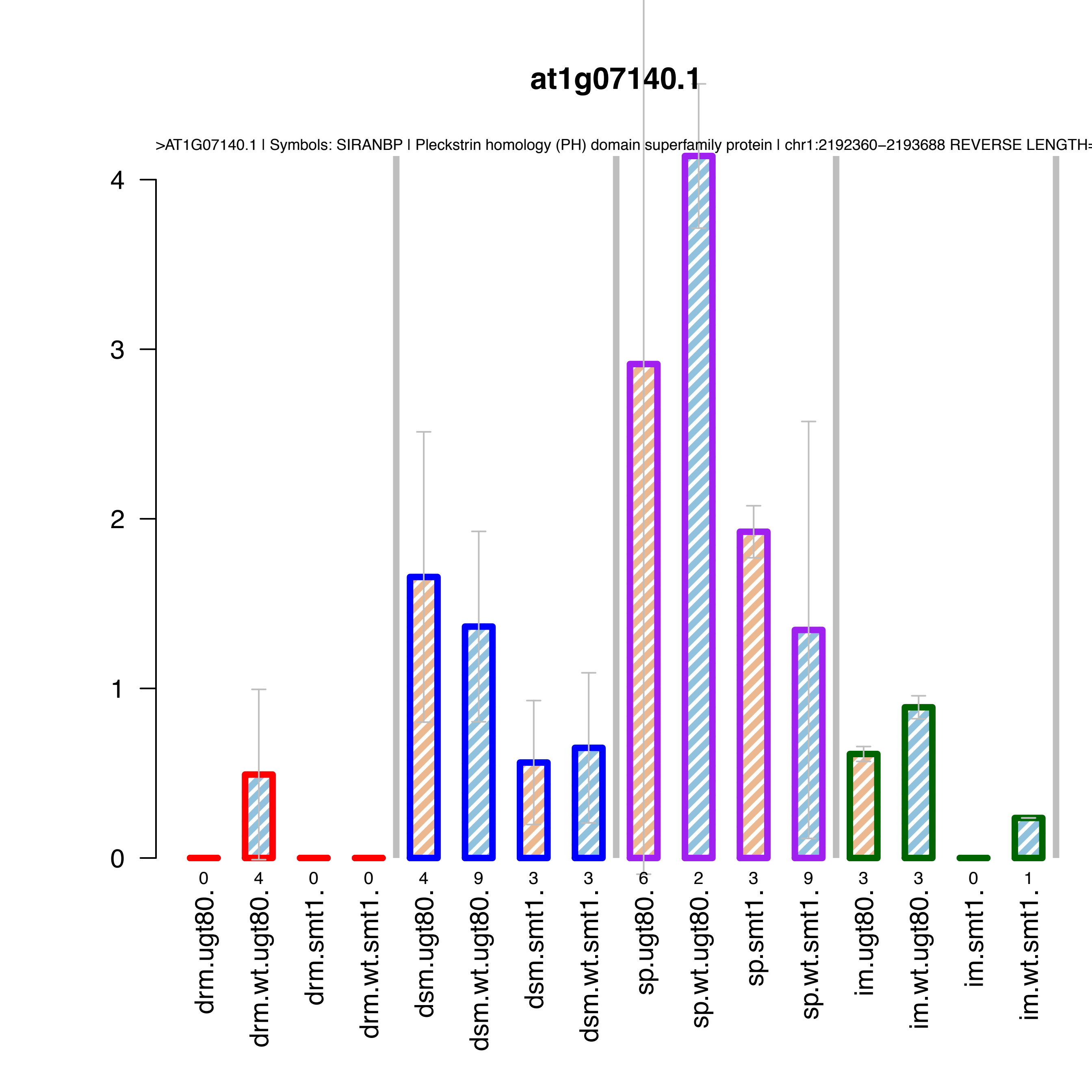

at1g07140.1
>AT1G07140.1 | Symbols: SIRANBP | Pleckstrin homology (PH) domain superfamily protein | chr1:2192360-2193688 REVERSE LENGTH=
0
1
2
3
4
0 4 0 0 4 9 3 3 6 2 3 9 3 3 0 1
drm.ugt80.
drm.wt.ugt80.
drm.smt1.
drm.wt.smt1.
dsm.ugt80.
dsm.wt.ugt80.
dsm.smt1.
dsm.wt.smt1.
sp.ugt80.
sp.wt.ugt80.
sp.smt1.
sp.wt.smt1.
im.ugt80.
im.wt.ugt80.
im.smt1.
im.wt.smt1.

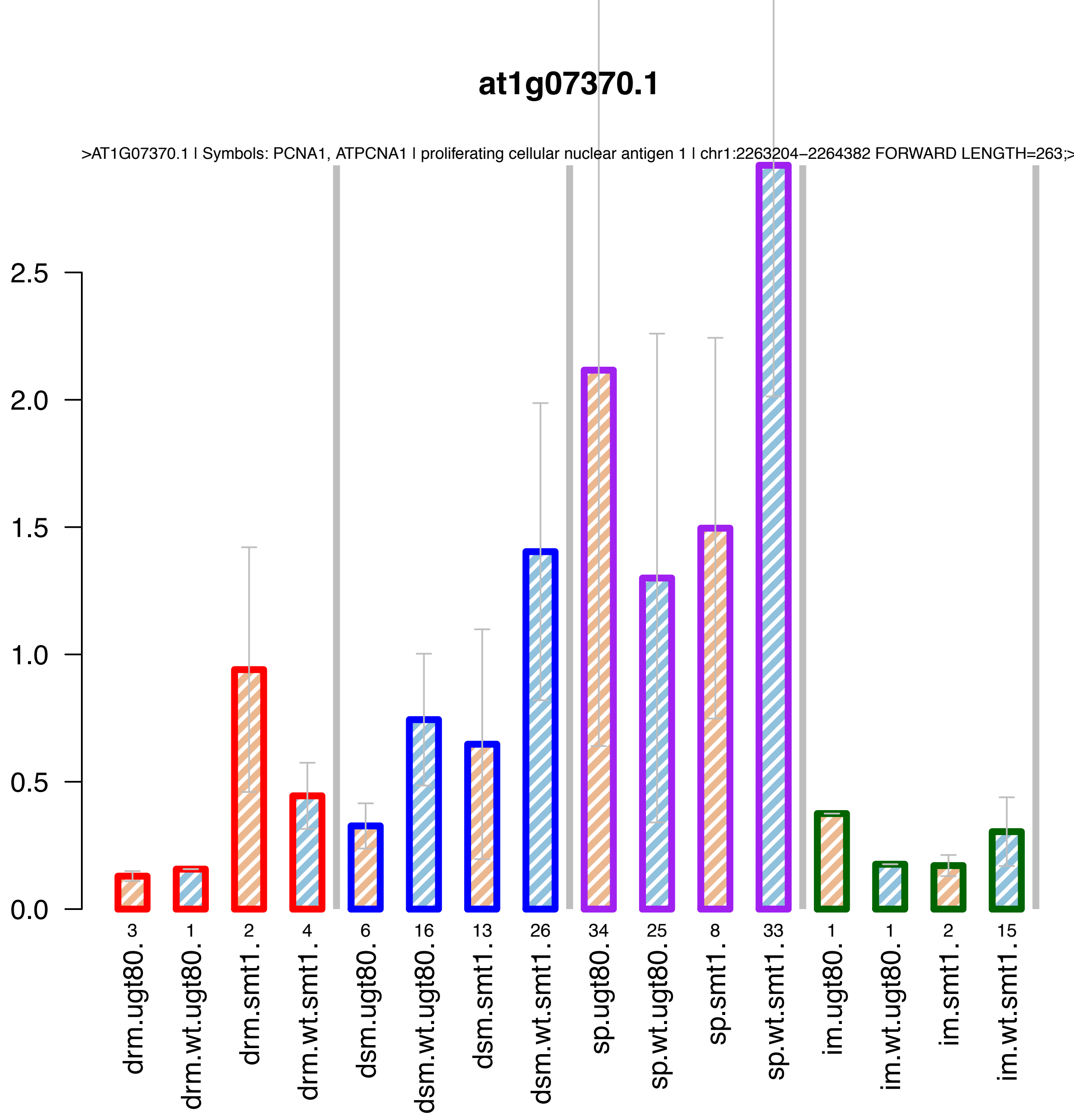

at1g07370.1
>AT1G07370.1 | Symbols: PCNA1, ATPCNA1 | proliferating cellular nuclear antigen 1 | chr1:2263204-2264382 FORWARD LENGTH=263;>
0.0
0.5
1.0
1.5
2.0
2.5
3
1
2
4
6
16
13
26
34
25
8
33
1
1
2
15
drm.ugt80.
drm.wt.ugt80.
drm.smt1.
drm.wt.smt1.
dsm.ugt80.
dsm.wt.ugt80.
dsm.smt1.
dsm.wt.smt1.
sp.ugt80.
sp.wt.ugt80.
sp.smt1.
sp.wt.smt1.
im.ugt80.
im.wt.ugt80.
im.smt1.
im.wt.smt1.

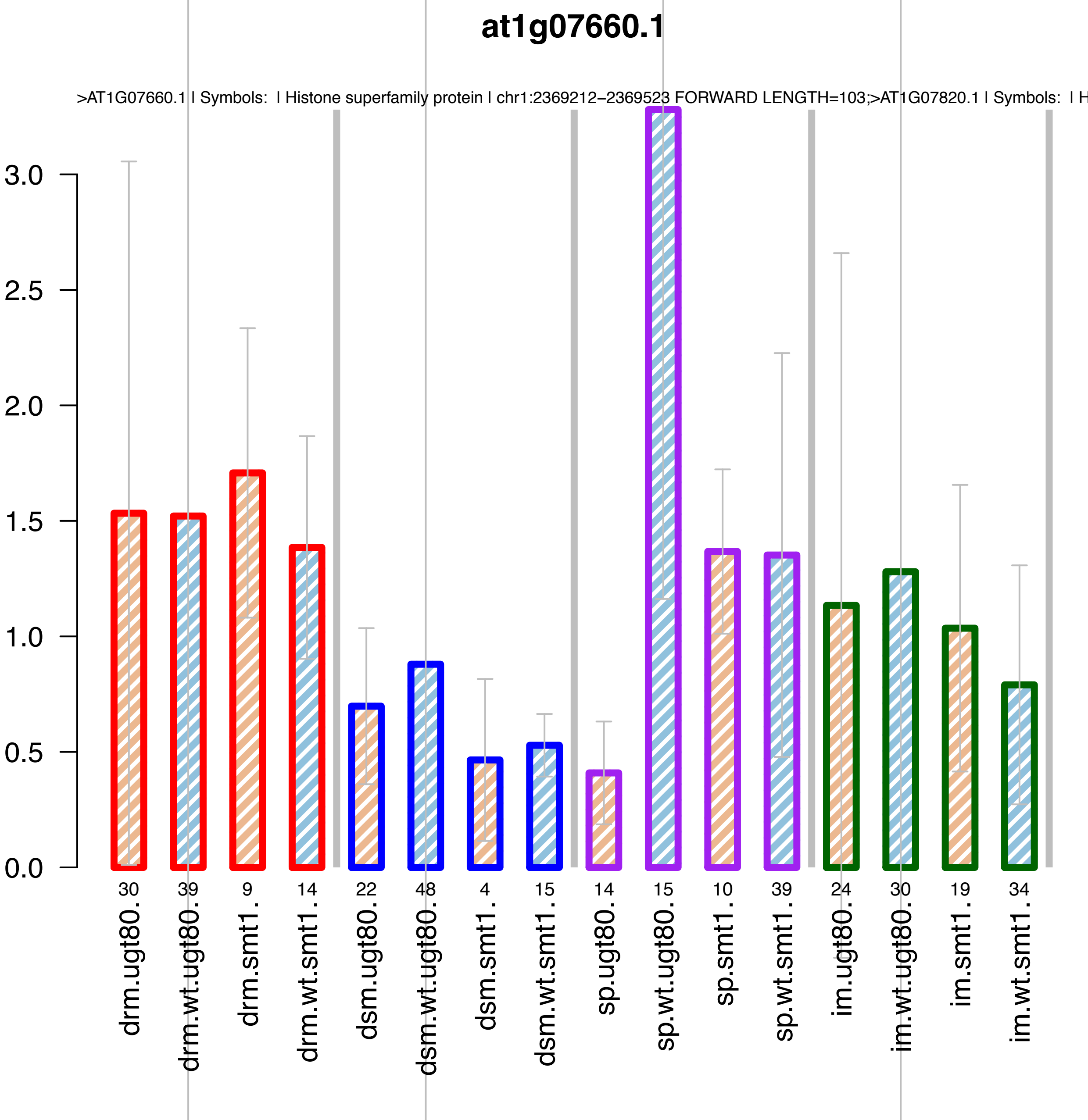

at1g07660.1
>AT1G07660.1 | Symbols: | Histone superfamily protein | chr1:2369212-2369523 FORWARD LENGTH=103;>AT1G07820.1 | Symbols: | H
3.0
2.5
2.0
1.5
1.0
0.5
0.0
30
39
9
14
22
48
4
15
14
15
10
39
24
30
19
34
drm.ugt80..
drm.wt.ugt80..
drm.smt1..
drm.wt.smt1..
dsm.ugt80..
dsm.wt.ugt80..
dsm.smt1..
dsm.wt.smt1..
sp.ugt80..
sp.wt.ugt80..
sp.smt1..
sp.wt.smt1..
im.ugt80..
im.wt.ugt80..
im.smt1..
im.wt.smt1..

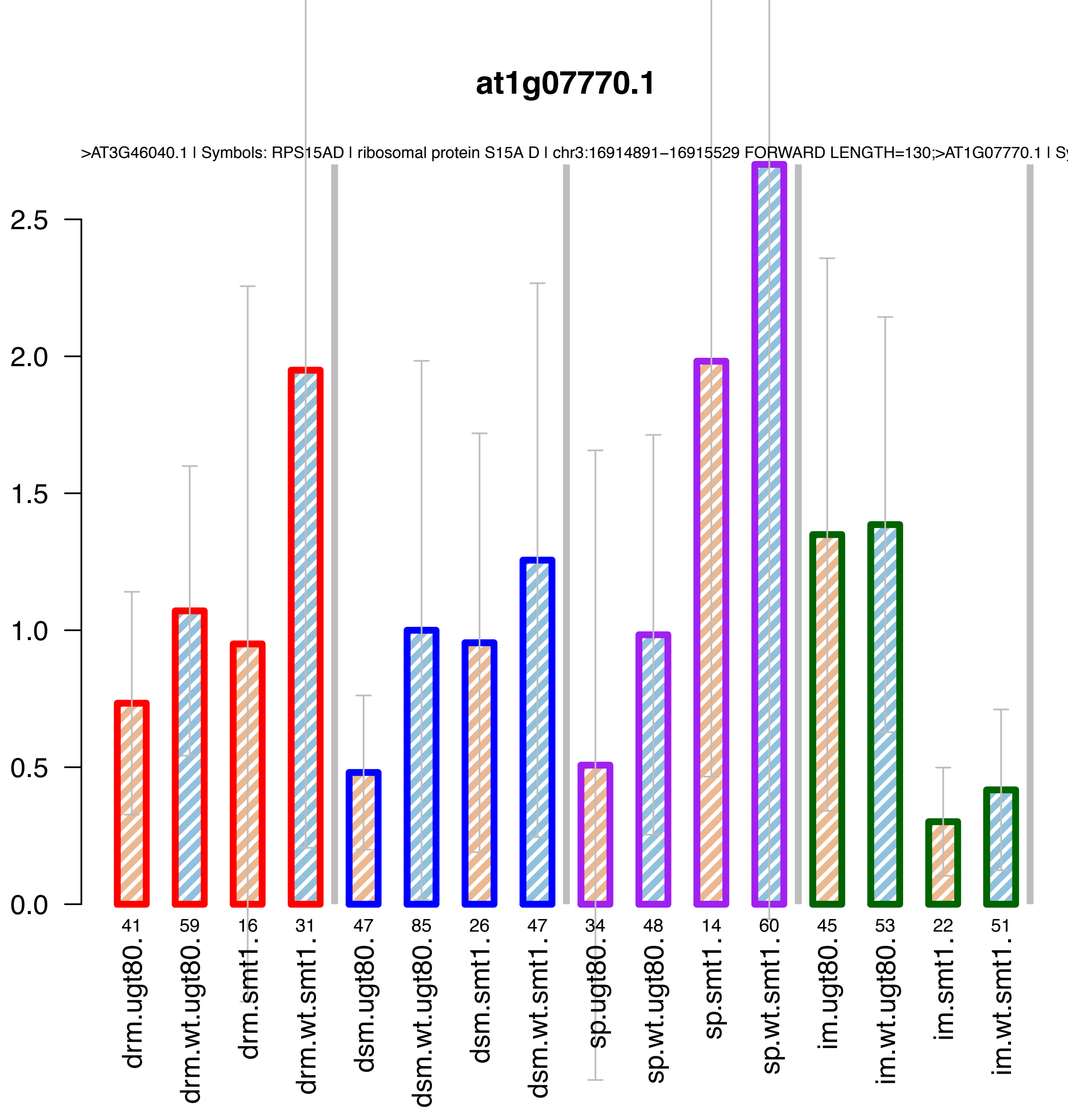
at1g07770.1
>AT3G46040.1 | Symbols: RPS15AD | ribosomal protein S15A D | chr3:16914891−16915529 FORWARD LENGTH=130;>AT1G07770.1 | Sy
2.5
2.0
1.5
1.0
0.5
0.0
41
59
16
31
47
85
26
47
34
48
14
60
45
53
22
51
drm.ugt80.
drm.wt.ugt80.
drm.smt1.
drm.wt.smt1.
dsm.ugt80.
dsm.wt.ugt80.
dsm.smt1.
dsm.wt.smt1.
sp.ugt80.
sp.wt.ugt80.
sp.smt1.
sp.wt.smt1.
im.ugt80.
im.wt.ugt80.
im.smt1.
im.wt.smt1.

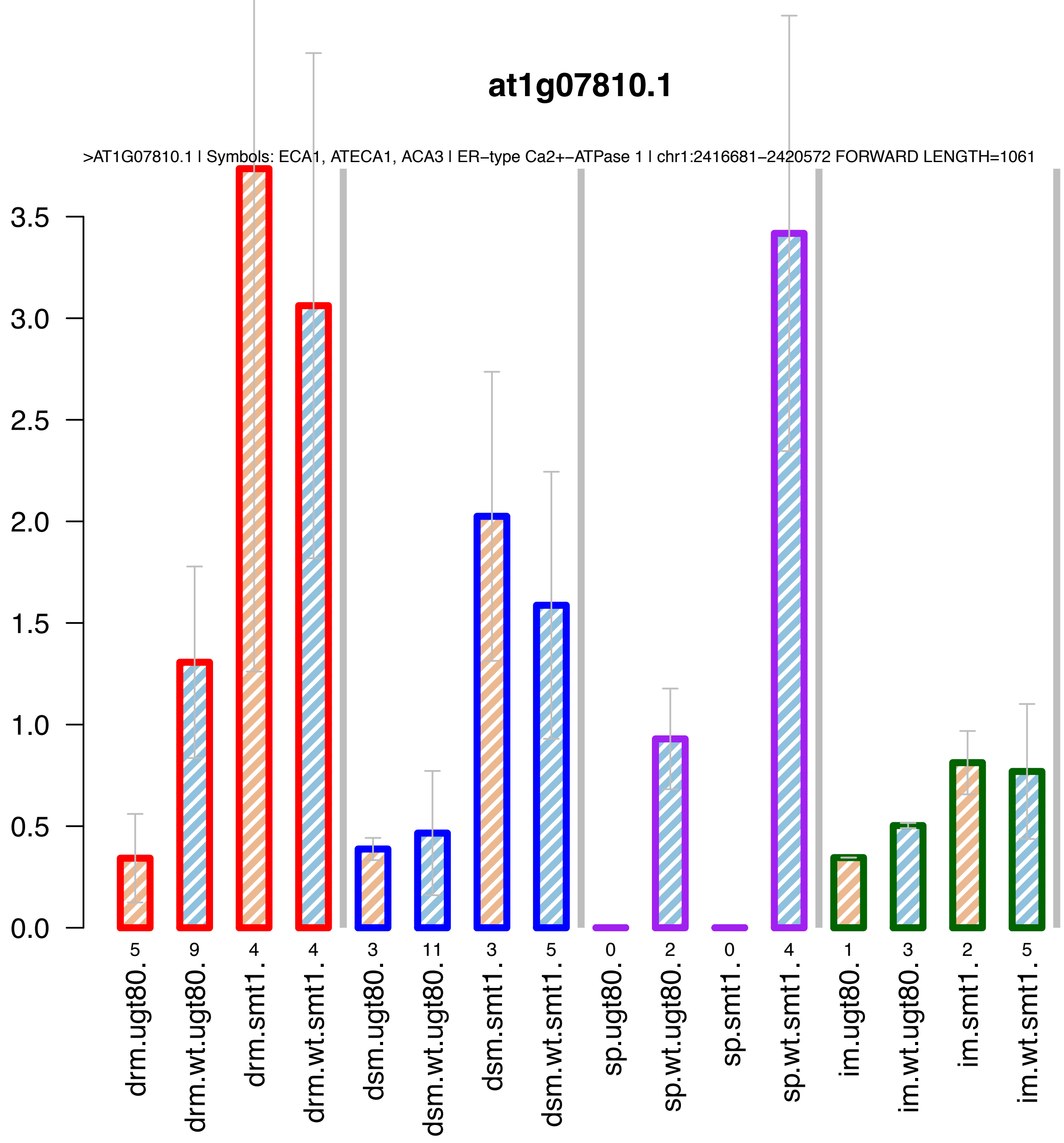

at1g07810.1
>AT1G07810.1 | Symbols: ECA1, ATECA1, ACA3 | ER−type Ca2+−ATPase 1 | chr1:2416681−2420572 FORWARD LENGTH=1061
0.0
0.5
1.0
1.5
2.0
2.5
3.0
3.5
5
9
4
4
3
11
3
5
0
2
0
4
1
3
2
5
drm.ugt80.
drm.wt.ugt80.
drm.smt1.
drm.wt.smt1.
dsm.ugt80.
dsm.wt.ugt80.
dsm.smt1.
dsm.wt.smt1.
sp.ugt80.
sp.wt.ugt80.
sp.smt1.
sp.wt.smt1.
im.ugt80.
im.wt.ugt80.
im.smt1.
im.wt.smt1.

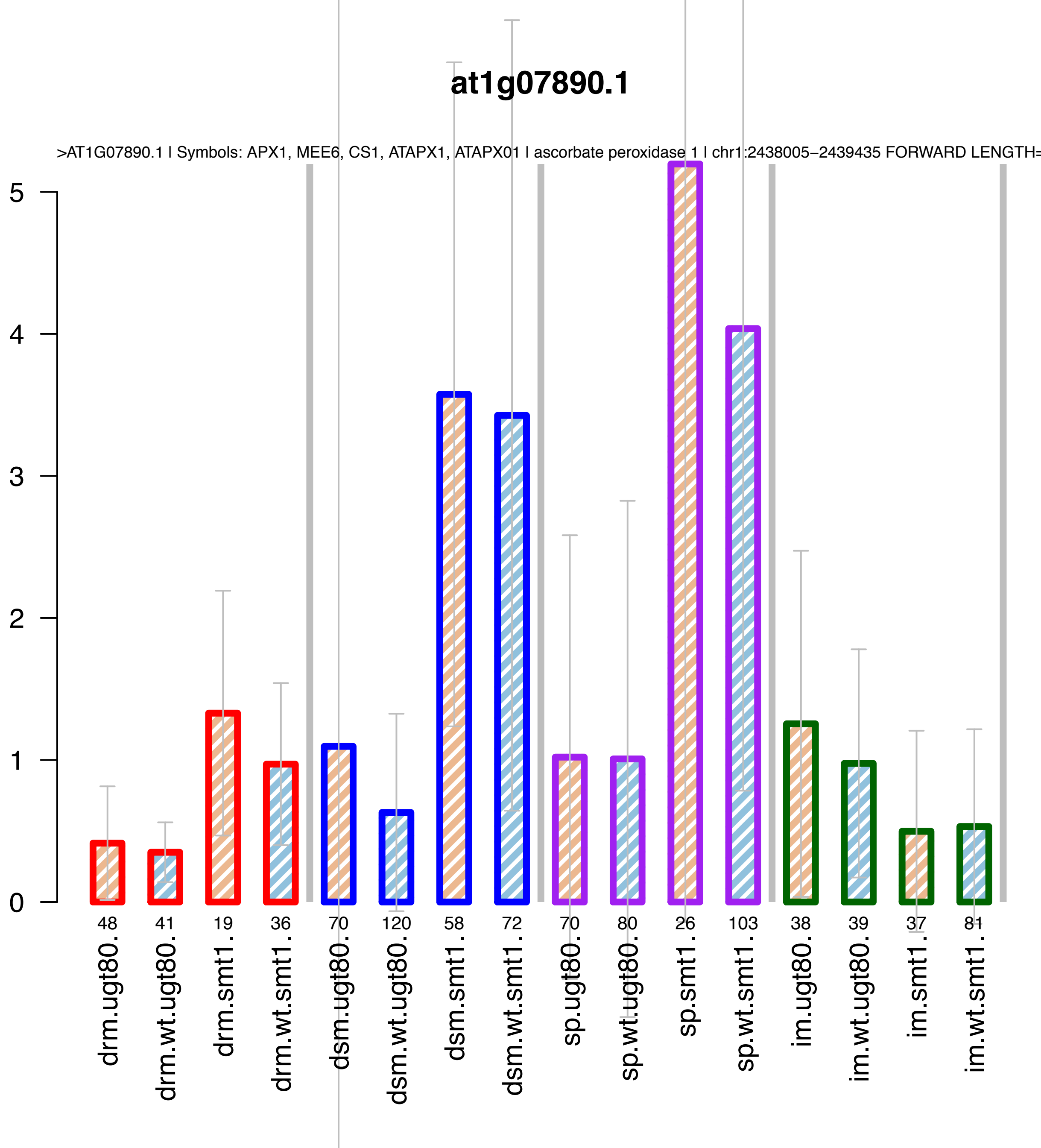

at1g07890.1
>AT1G07890.1 | Symbols: APX1, MEE6, CS1, ATAPX1, ATAPX01 | ascorbate peroxidase 1 | chr1:2438005-2439435 FORWARD LENGTH=
0
1
2
3
4
5
48
41
19
36
70
120
58
72
70
80
26
103
38
39
37
81
drm.ugt80.
drm.wt.ugt80.
drm.smt1.
drm.wt.smt1.
dsm.ugt80.
dsm.wt.ugt80.
dsm.smt1.
dsm.wt.smt1.
sp.ugt80.
sp.wt.ugt80.
sp.smt1.
sp.wt.smt1.
im.ugt80.
im.wt.ugt80.
im.smt1.
im.wt.smt1.

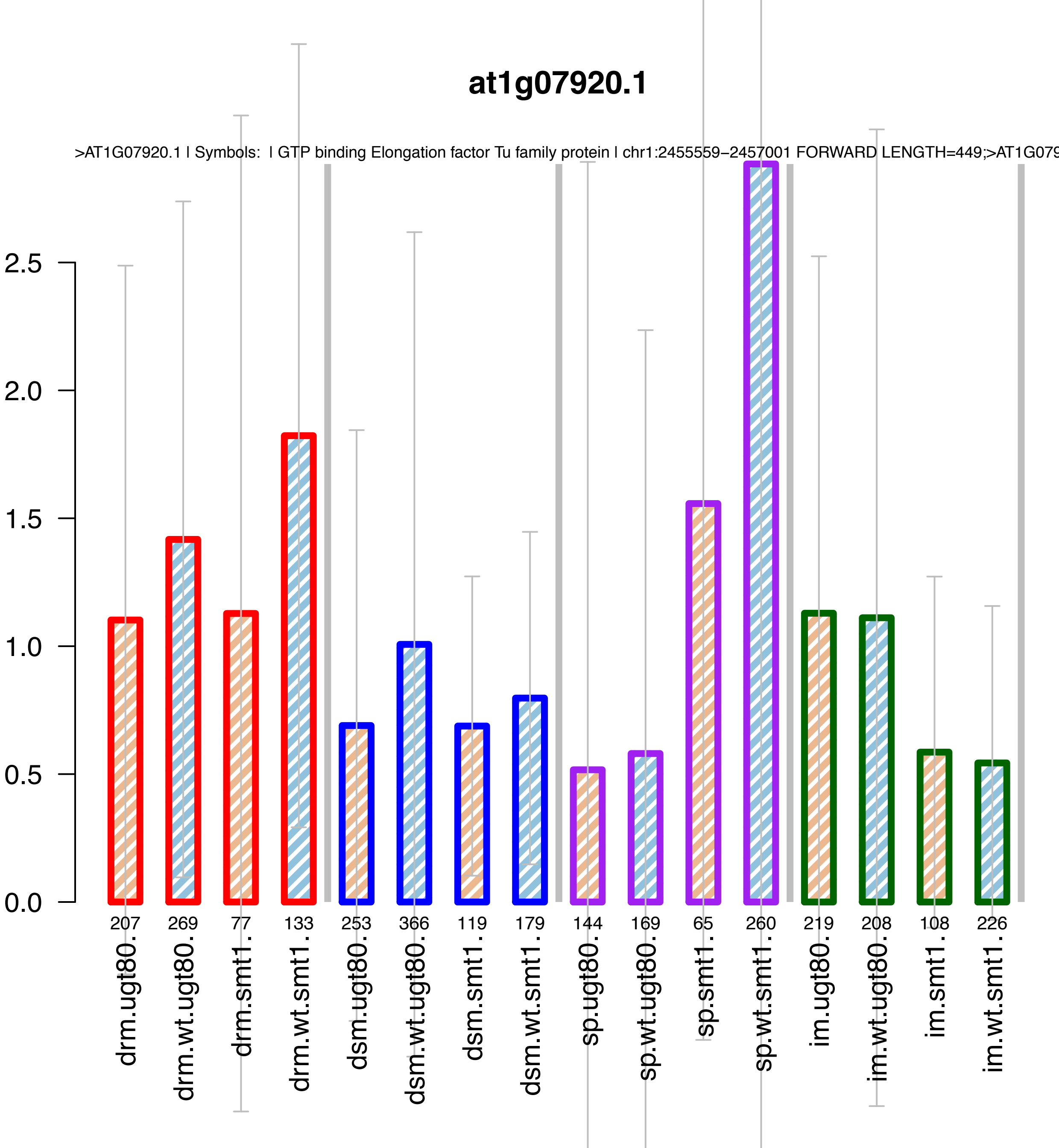

at1g07920.1
>AT1G07920.1 | Symbols: | GTP binding Elongation factor Tu family protein | chr1:2455559-2457001 FORWARD LENGTH=449;>AT1G079
0.0
0.5
1.0
1.5
2.0
2.5
207
269
77
133
253
366
119
179
144
169
65
260
219
208
108
226
drm.ugt80..
drm.wt.ugt80..
drm.smt1..
drm.wt.smt1..
dsm.ugt80..
dsm.wt.ugt80..
dsm.smt1..
dsm.wt.smt1..
sp.ugt80..
sp.wt.ugt80..
sp.smt1..
sp.wt.smt1..
im.ugt80..
im.wt.ugt80..
im.smt1..
im.wt.smt1..

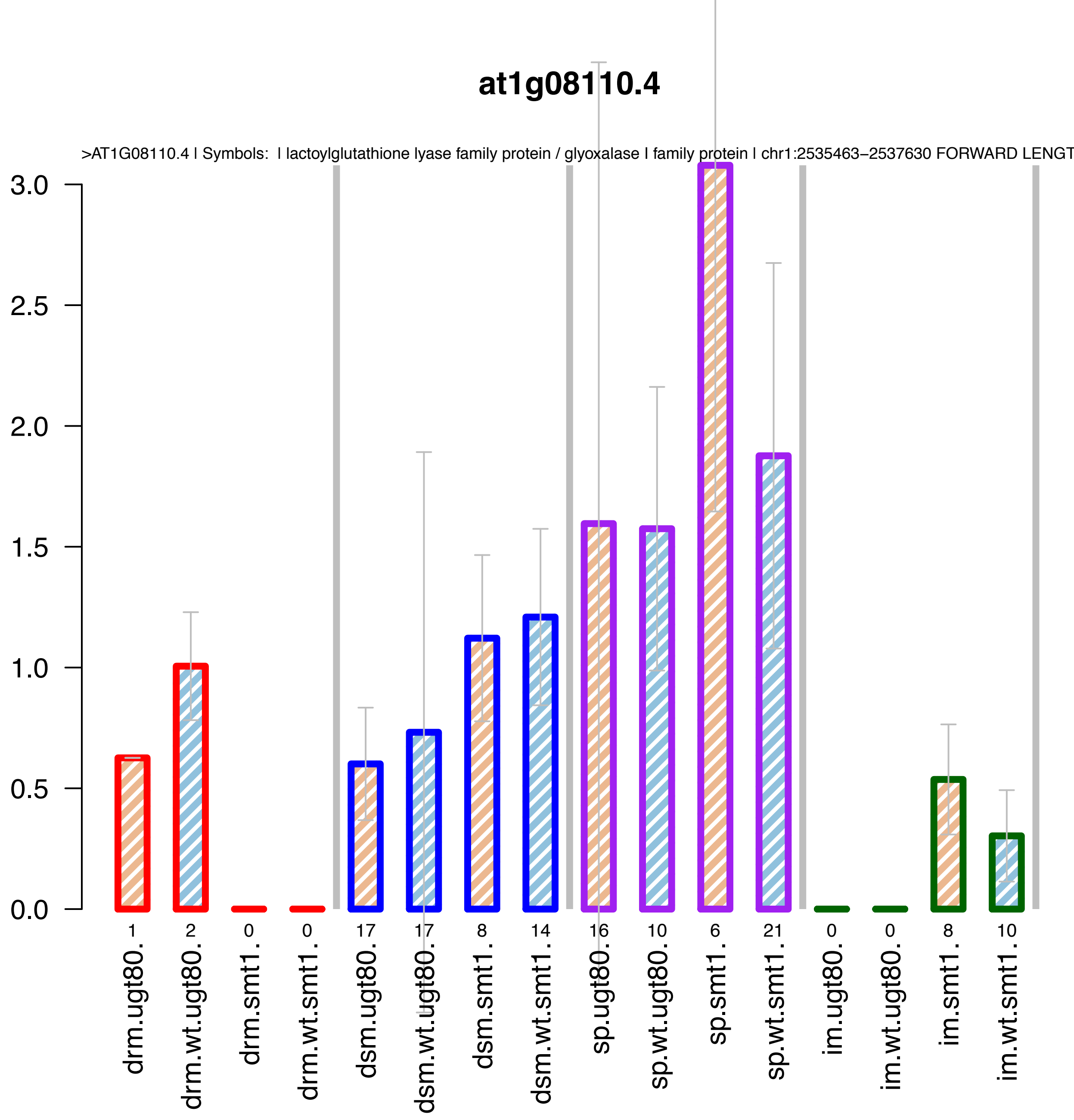

at1g08110.4
>AT1G08110.4 | Symbols: | lactoylglutathione lyase family protein / glyoxalase I family protein | chr1:2535463-2537630 FORWARD LENGT
3.0
2.5
2.0
1.5
1.0
0.5
0.0
1
2
0
0
17
17
8
14
16
10
6
21
0
0
8
10
drm.ugt80.
drm.wt.ugt80.
drm.smt1.
drm.wt.smt1.
dsm.ugt80.
dsm.wt.ugt80.
dsm.smt1.
dsm.wt.smt1.
sp.ugt80.
sp.wt.ugt80.
sp.smt1.
sp.wt.smt1.
im.ugt80.
im.wt.ugt80.
im.smt1.
im.wt.smt1.

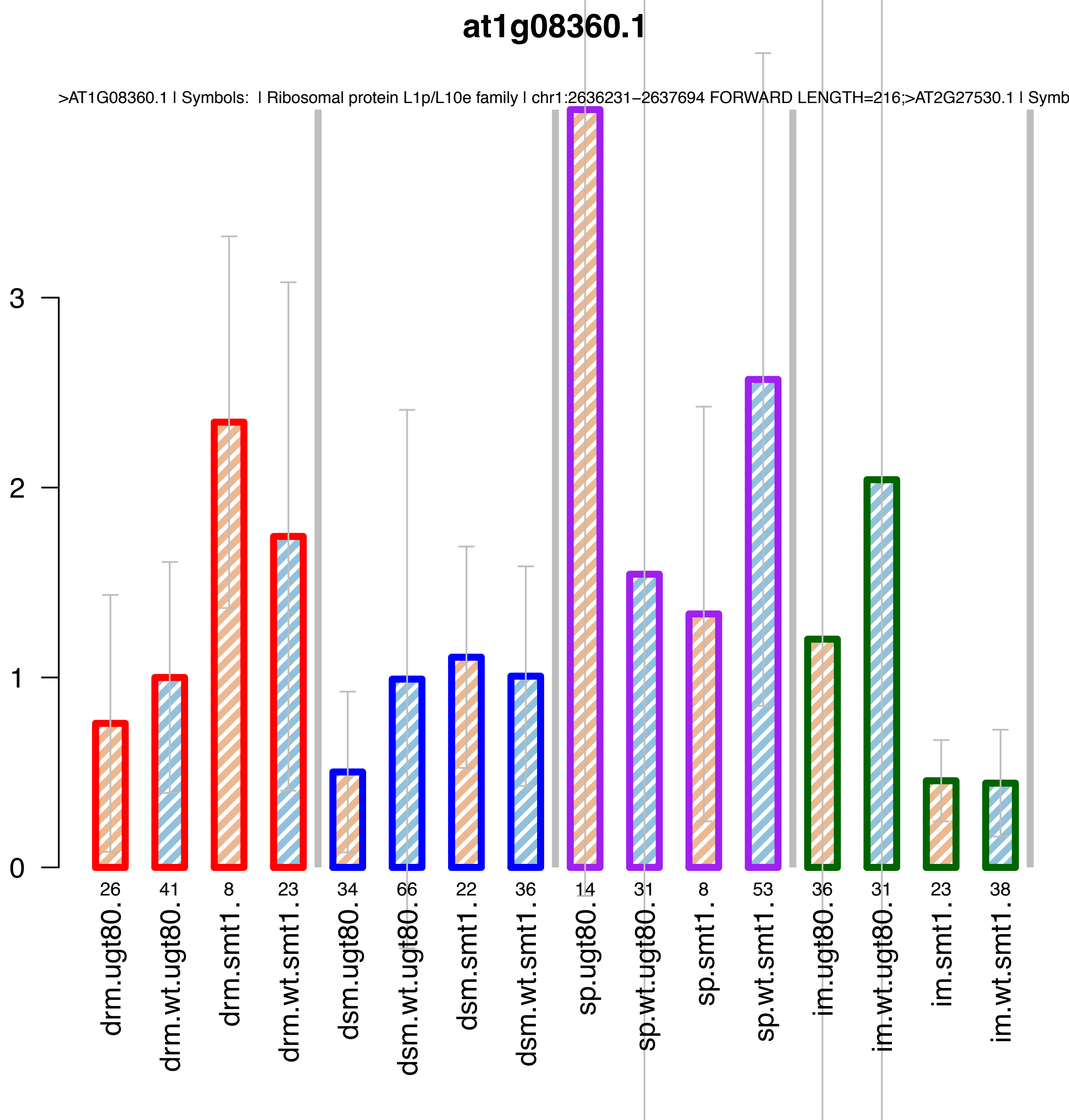
at1g08360.1
>AT1G08360.1 | Symbols: | Ribosomal protein L1p/L10e family | chr1:2636231-2637694 FORWARD LENGTH=216;>AT2G27530.1 | Symb
0
1
2
3
26
41
8
23
34
66
22
36
14
31
8
53
36
31
23
38
drm.ugt80.
drm.wt.ugt80.
drm.smt1.
drm.wt.smt1.
dsm.ugt80.
dsm.wt.ugt80.
dsm.smt1.
dsm.wt.smt1.
sp.ugt80.
sp.wt.ugt80.
sp.smt1.
sp.wt.smt1.
im.ugt80.
im.wt.ugt80.
im.smt1.
im.wt.smt1.

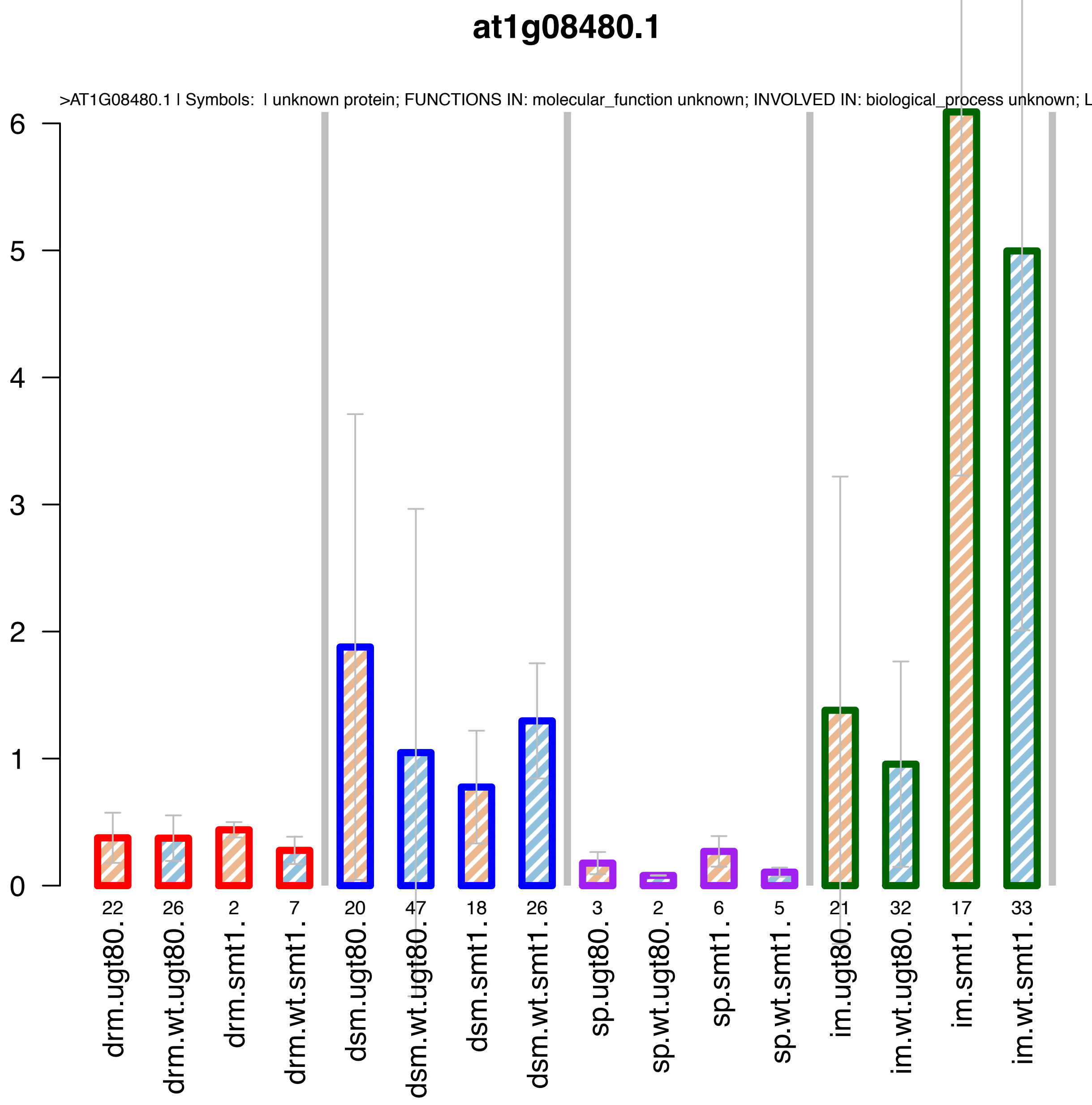

at1g08480.1
>AT1G08480.1 | Symbols: | unknown protein; FUNCTIONS IN: molecular_function unknown; INVOLVED IN: biological_process unknown; L
0
1
2
3
4
5
6
22
26
2
7
20
47
18
26
3
2
6
5
21
32
17
33
drm.ugt80..
drm.wt.ugt80..
drm.smt1..
drm.wt.smt1..
dsm.ugt80..
dsm.wt.ugt80..
dsm.smt1..
dsm.wt.smt1..
sp.ugt80..
sp.wt.ugt80..
sp.smt1..
sp.wt.smt1..
im.ugt80..
im.wt.ugt80..
im.smt1..
im.wt.smt1..

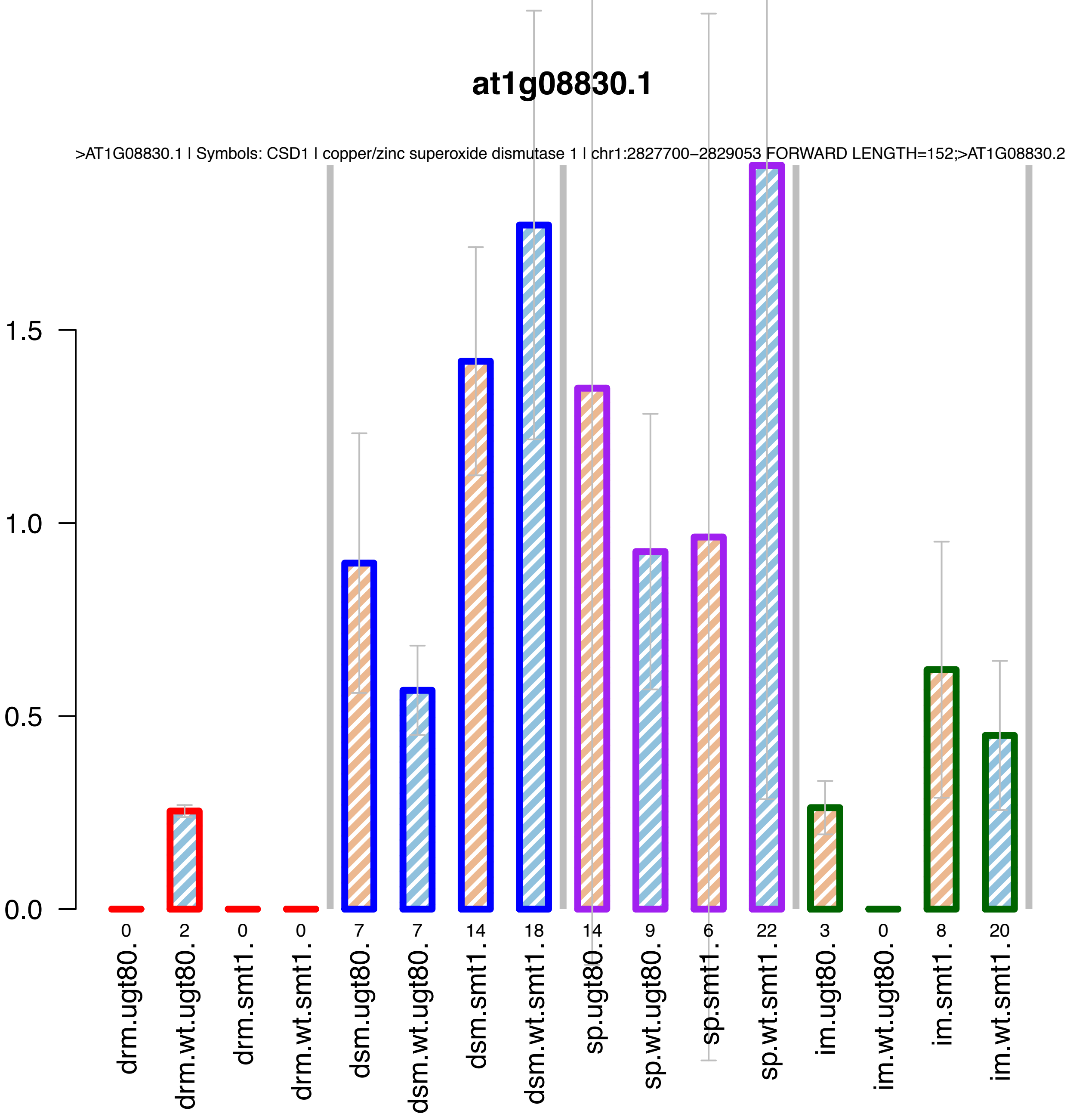
at1g08830.1
>AT1G08830.1 | Symbols: CSD1 | copper/zinc superoxide dismutase 1 | chr1:2827700-2829053 FORWARD LENGTH=152;>AT1G08830.2
0.0
0.5
1.0
1.5
0
2
0
0
7
7
14
18
14
9
6
22
3
0
8
20
drm.ugt80.
drm.wt.ugt80.
drm.smt1.
drm.wt.smt1.
dsm.ugt80.
dsm.wt.ugt80.
dsm.smt1.
dsm.wt.smt1.
sp.ugt80.
sp.wt.ugt80.
sp.smt1.
sp.wt.smt1.
im.ugt80.
im.wt.ugt80.
im.smt1.
im.wt.smt1.

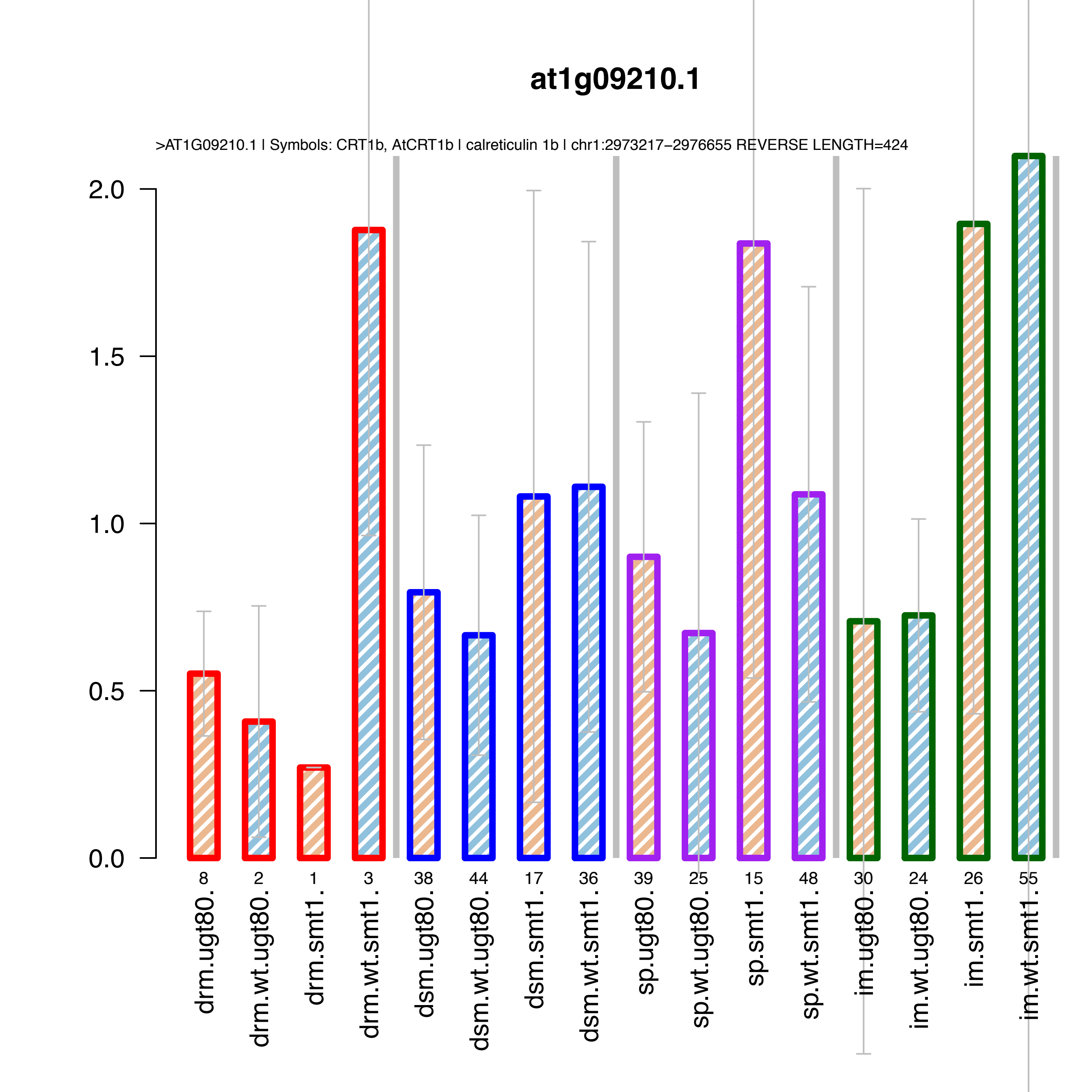

at1g09210.1
>AT1G09210.1 | Symbols: CRT1b, AtCRT1b | calreticulin 1b | chr1:2973217-2976655 REVERSE LENGTH=424
2.0
1.5
1.0
0.5
0.0
8
2
1
3
38
44
17
36
39
25
15
48
30
24
26
55
drm.ugt80..
drm.wt.ugt80..
drm.smt1..
drm.wt.smt1..
dsm.ugt80..
dsm.wt.ugt80..
dsm.smt1..
dsm.wt.smt1..
sp.ugt80..
sp.wt.ugt80..
sp.smt1..
sp.wt.smt1..
im.ugt80..
im.wt.ugt80..
im.smt1..
im.wt.smt1..

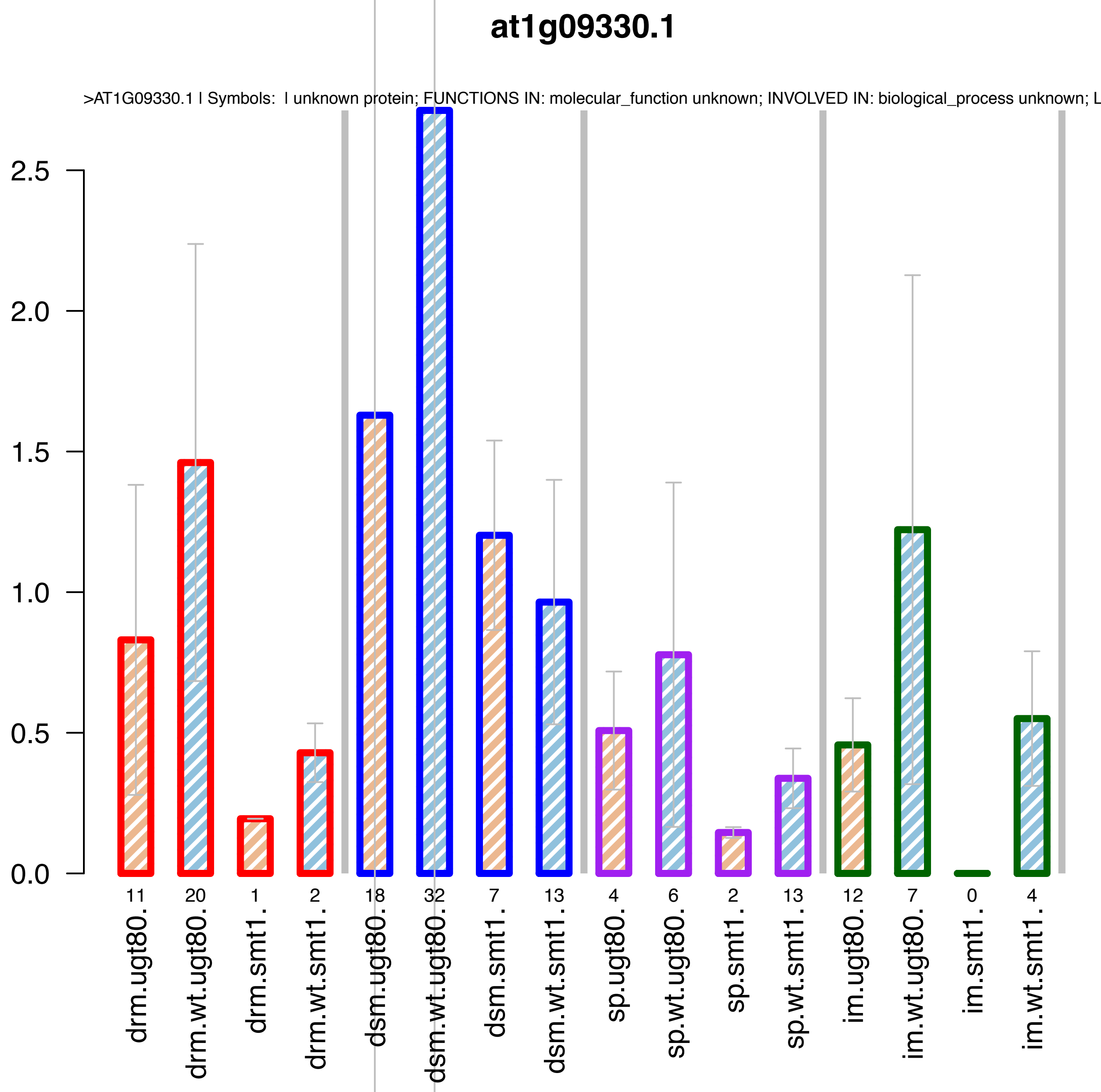

at1g09330.1
>AT1G09330.1 | Symbols: | unknown protein; FUNCTIONS IN: molecular_function unknown; INVOLVED IN: biological_process unknown; L
2.5
2.0
1.5
1.0
0.5
0.0
11
20
1
2
18
32
7
13
4
6
2
13
12
7
0
4
drm.ugt80.
drm.wt.ugt80.
drm.smt1.
drm.wt.smt1.
dsm.ugt80.
dsm.wt.ugt80.
dsm.smt1.
dsm.wt.smt1.
sp.ugt80.
sp.wt.ugt80.
sp.smt1.
sp.wt.smt1.
im.ugt80.
im.wt.ugt80.
im.smt1.
im.wt.smt1.

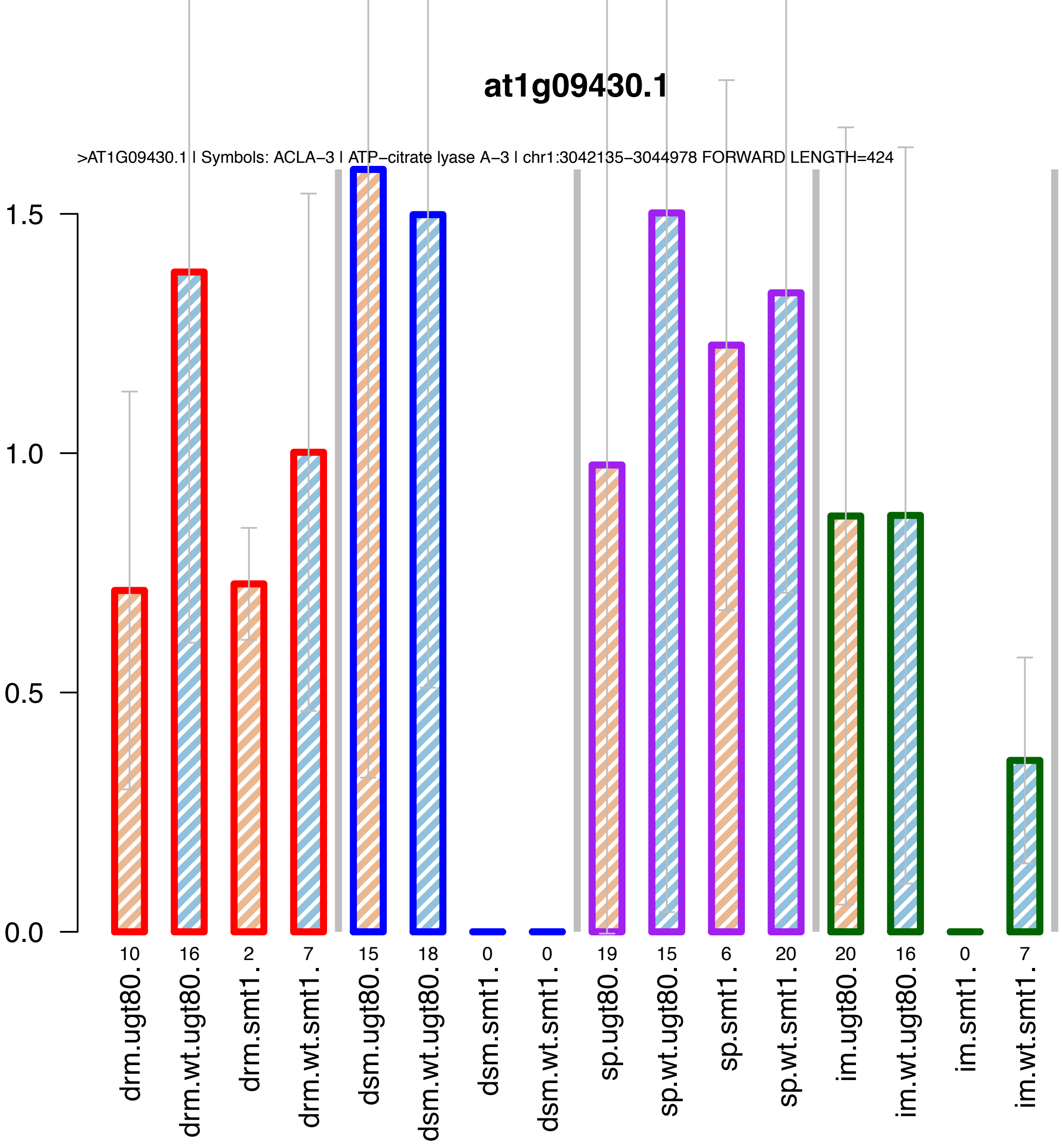

at1g09430.1
>AT1G09430.1 | Symbols: ACLA-3 | ATP-citrate lyase A-3 | chr1:3042135-3044978 FORWARD LENGTH=424
0.0
0.5
1.0
1.5
10
16
2
7
15
18
0
0
19
15
6
20
20
16
0
7
drm.ugt80.
drm.wt.ugt80.
drm.smt1.
drm.wt.smt1.
dsm.ugt80.
dsm.wt.ugt80.
dsm.smt1.
dsm.wt.smt1.
sp.ugt80.
sp.wt.ugt80.
sp.smt1.
sp.wt.smt1.
im.ugt80.
im.wt.ugt80.
im.smt1.
im.wt.smt1.

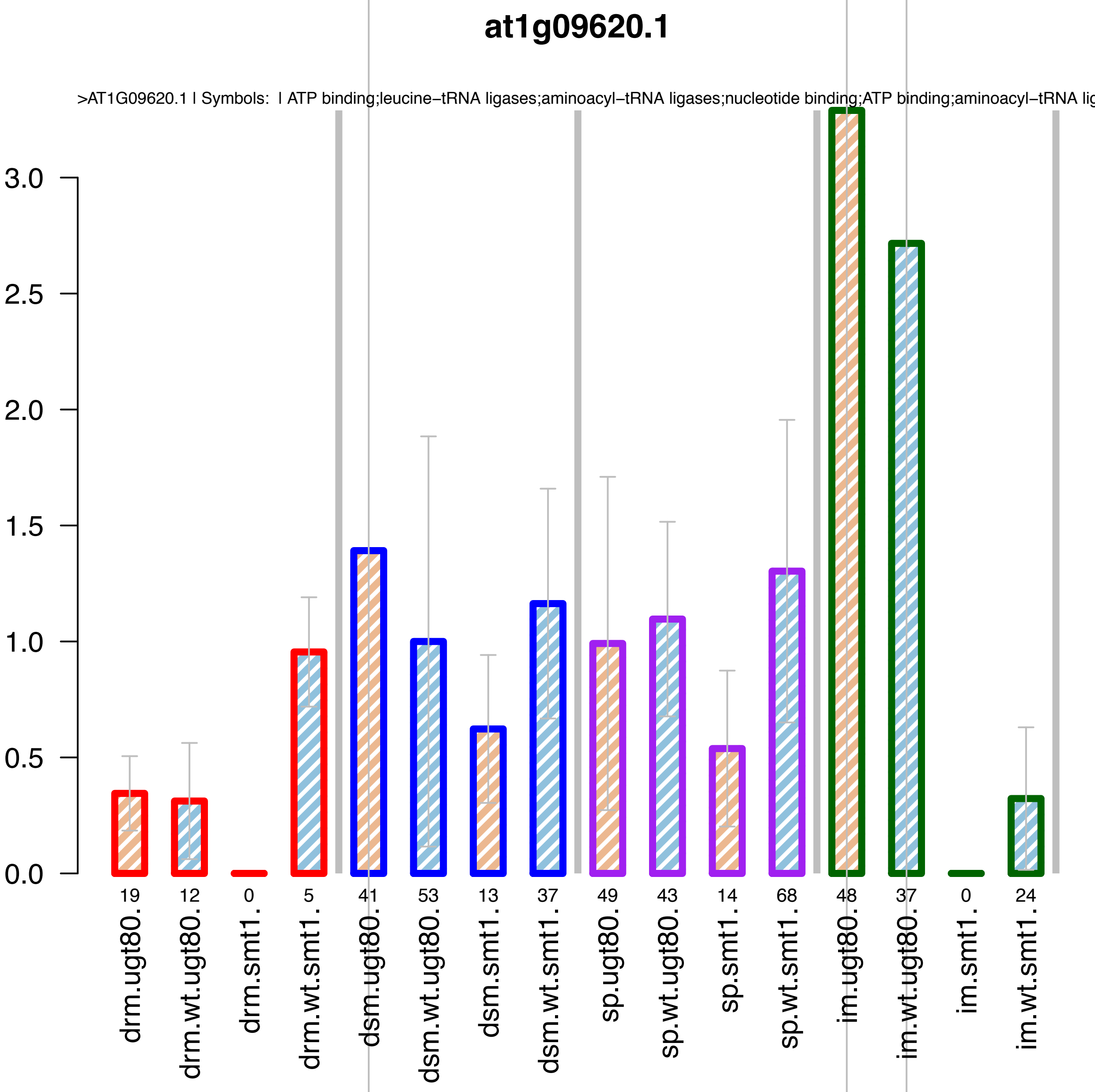

at1g09620.1
>AT1G09620.1 | Symbols: | ATP binding;leucine−tRNA ligases;aminoacyl−tRNA ligases;nucleotide binding;ATP binding;aminoacyl−tRNA lig
3.0
2.5
2.0
1.5
1.0
0.5
0.0
19
12
0
5
41
53
13
37
49
43
14
68
48
37
0
24
drm.ugt80.
drm.wt.ugt80.
drm.smt1.
drm.wt.smt1.
dsm.ugt80.
dsm.wt.ugt80.
dsm.smt1.
dsm.wt.smt1.
sp.ugt80.
sp.wt.ugt80.
sp.smt1.
sp.wt.smt1.
im.ugt80.
im.wt.ugt80.
im.smt1.
im.wt.smt1.

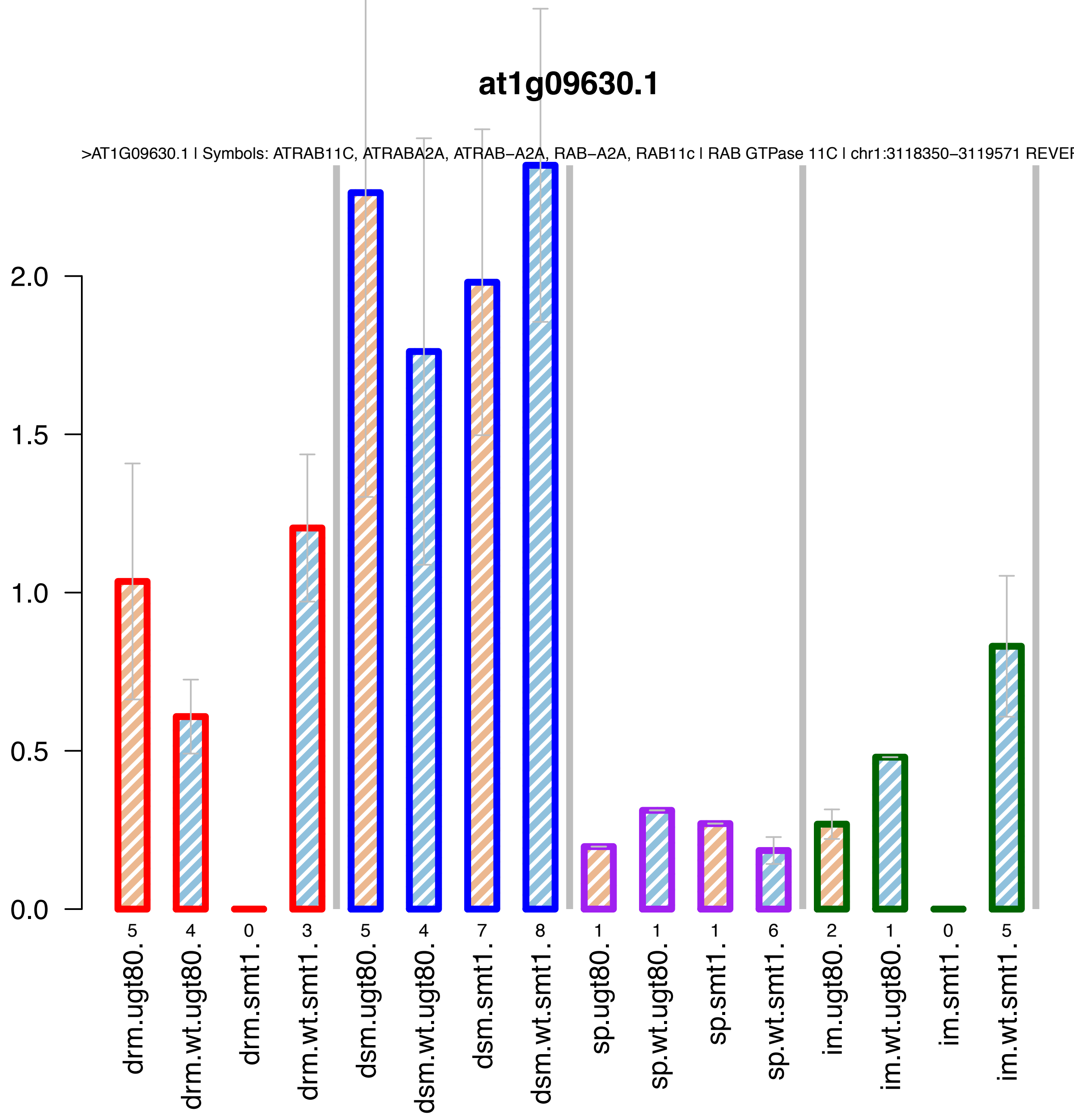

at1g09630.1
>AT1G09630.1 | Symbols: ATRAB11C, ATRABA2A, ATRAB−A2A, RAB−A2A, RAB11c | RAB GTPase 11C | chr1:3118350−3119571 REVER
2.0
1.5
1.0
0.5
0.0
5
4
0
3
5
4
7
8
1
1
1
6
2
1
0
5
drm.ugt80..
drm.wt.ugt80..
drm.smt1..
drm.wt.smt1..
dsm.ugt80..
dsm.wt.ugt80..
dsm.smt1..
dsm.wt.smt1..
sp.ugt80..
sp.wt.ugt80..
sp.smt1..
sp.wt.smt1..
im.ugt80..
im.wt.ugt80..
im.smt1..
im.wt.smt1..

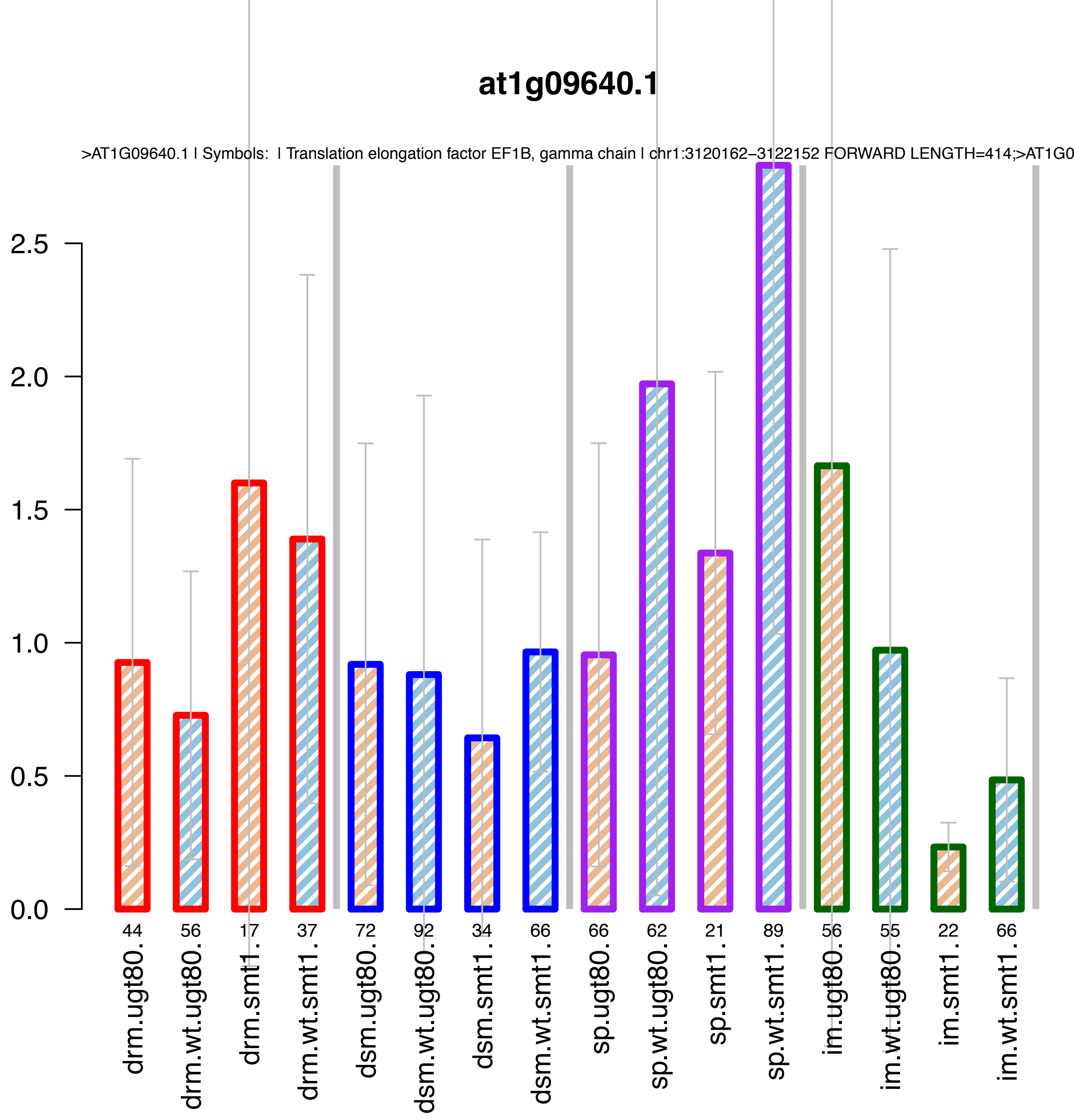

at1g09640.1
>AT1G09640.1 | Symbols: | Translation elongation factor EF1B, gamma chain | chr1:3120162–3122152 FORWARD LENGTH=414;>AT1G0
0.0
0.5
1.0
1.5
2.0
2.5
44
56
17
37
72
92
34
66
66
62
21
89
56
55
22
66
drm.ugt80..
drm.wt.ugt80..
drm.smt1..
drm.wt.smt1..
dsm.ugt80..
dsm.wt.ugt80..
dsm.smt1..
dsm.wt.smt1..
sp.ugt80..
sp.wt.ugt80..
sp.smt1..
sp.wt.smt1..
im.ugt80..
im.wt.ugt80..
im.smt1..
im.wt.smt1..

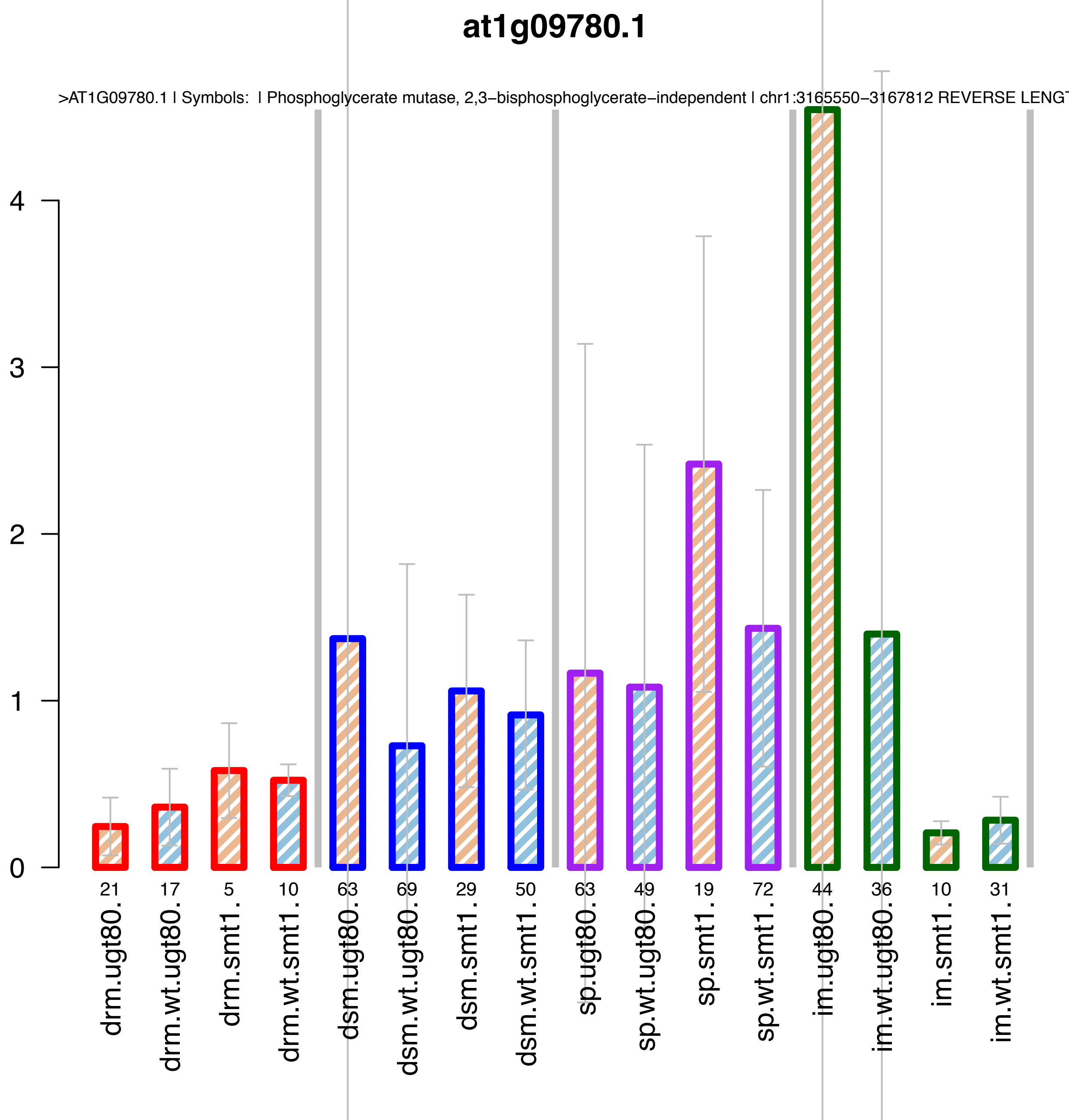

at1g09780.1
>AT1G09780.1 | Symbols: | Phosphoglycerate mutase, 2,3-bisphosphoglycerate-independent | chr1:3165550-3167812 REVERSE LENGT
0
1
2
3
4
21
17
5
10
63
69
29
50
63
49
19
72
44
36
10
31
drm.ugt80.
drm.wt.ugt80.
drm.smt1.
drm.wt.smt1.
dsm.ugt80.
dsm.wt.ugt80.
dsm.smt1.
dsm.wt.smt1.
sp.ugt80.
sp.wt.ugt80.
sp.smt1.
sp.wt.smt1.
im.ugt80.
im.wt.ugt80.
im.smt1.
im.wt.smt1.

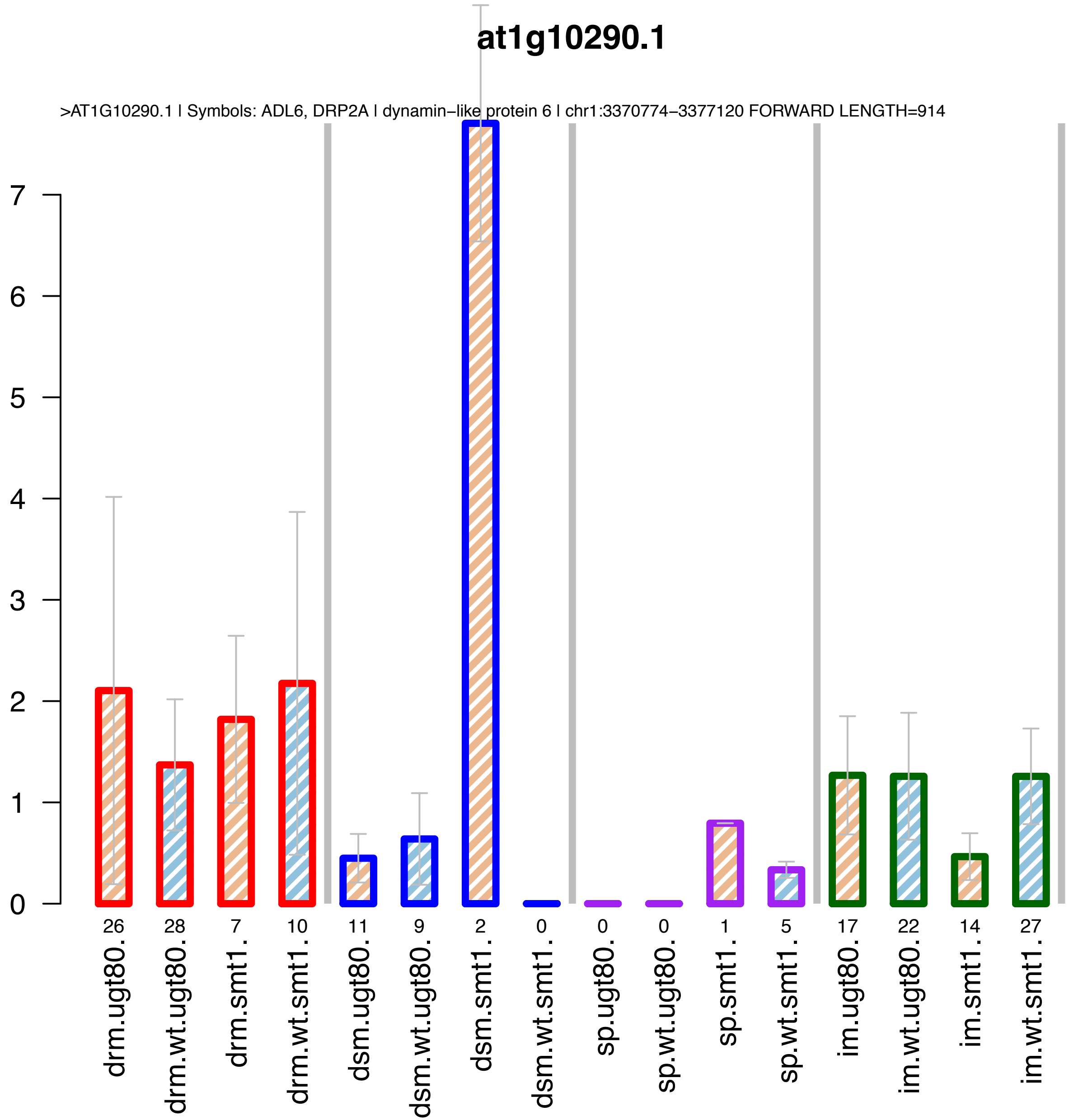

at1g10290.1
>AT1G10290.1 | Symbols: ADL6, DRP2A | dynamin−like protein 6 | chr1:3370774−3377120 FORWARD LENGTH=914
0
1
2
3
4
5
6
7
26
28
7
10
11
9
2
0
0
0
1
5
17
22
14
27
drm.ugt80.
drm.wt.ugt80.
drm.smt1.
drm.wt.smt1.
dsm.ugt80.
dsm.wt.ugt80.
dsm.smt1.
dsm.wt.smt1.
sp.ugt80.
sp.wt.ugt80.
sp.smt1.
sp.wt.smt1.
im.ugt80.
im.wt.ugt80.
im.smt1.
im.wt.smt1.

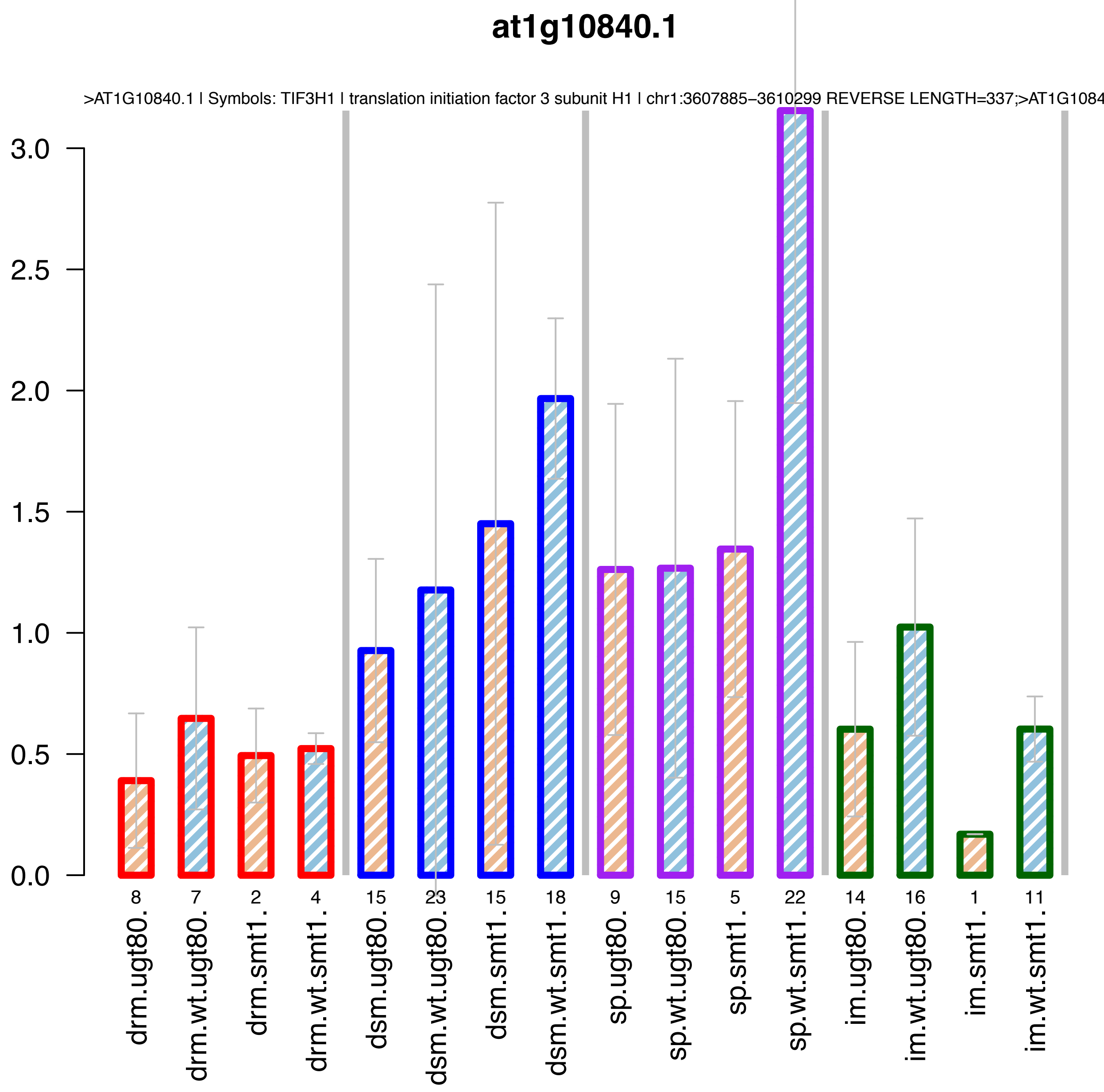
at1g10840.1
>AT1G10840.1 | Symbols: TIF3H1 | translation initiation factor 3 subunit H1 | chr1:3607885–3610299 REVERSE LENGTH=337;>AT1G1084
3.0
2.5
2.0
1.5
1.0
0.5
0.0
8
7
2
4
15
23
15
18
9
15
5
22
14
16
1
11
drm.ugt80.
drm.wt.ugt80.
drm.smt1.
drm.wt.smt1.
dsm.ugt80.
dsm.wt.ugt80.
dsm.smt1.
dsm.wt.smt1.
sp.ugt80.
sp.wt.ugt80.
sp.smt1.
sp.wt.smt1.
im.ugt80.
im.wt.ugt80.
im.smt1.
im.wt.smt1.

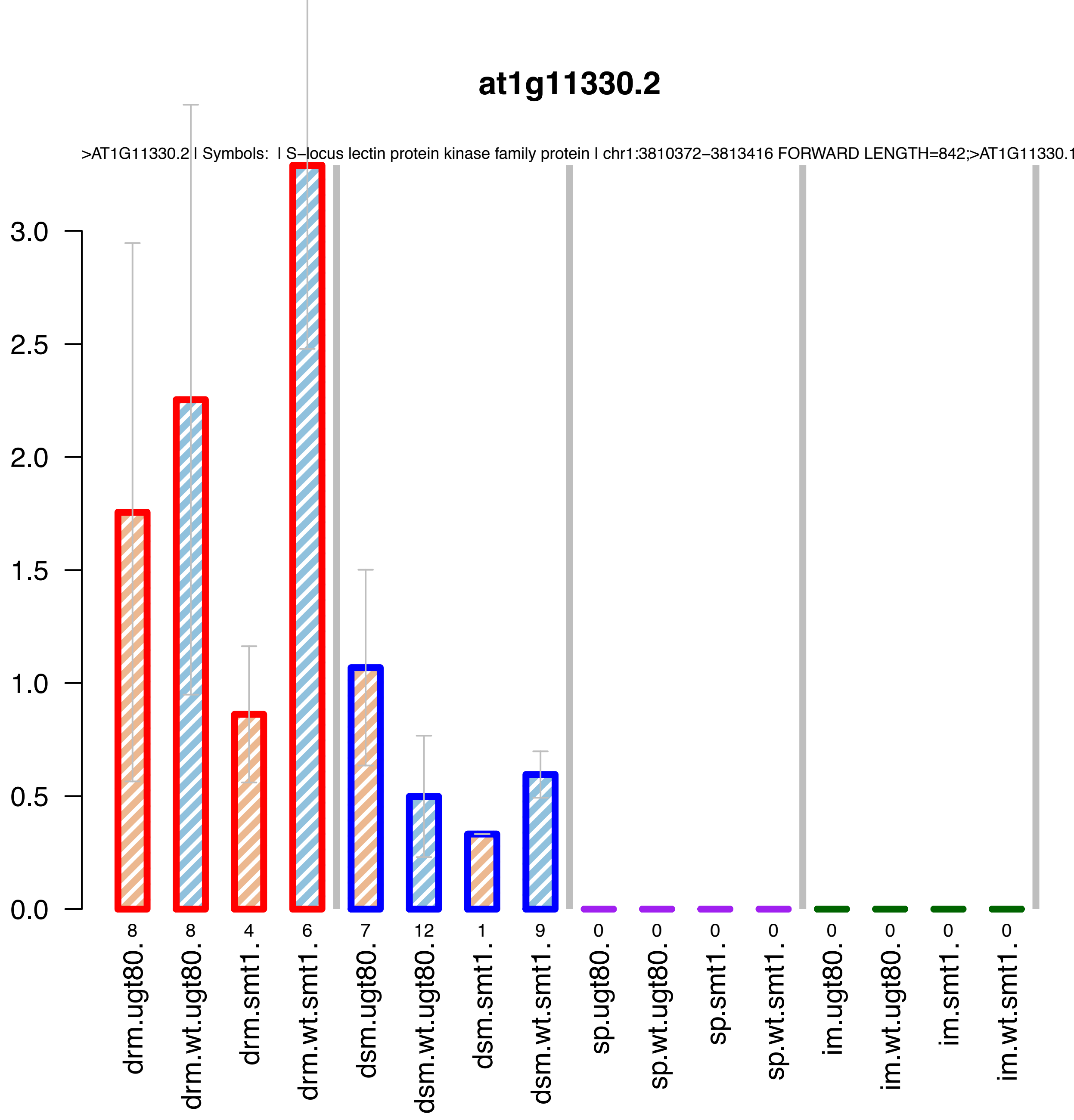

at1g11330.2
>AT1G11330.2 | Symbols: | S−locus lectin protein kinase family protein | chr1:3810372−3813416 FORWARD LENGTH=842;>AT1G11330.1
3.0
2.5
2.0
1.5
1.0
0.5
0.0
8
8
4
6
7
12
1
9
0
0
0
0
0
0
0
0
drm.ugt80.
drm.wt.ugt80.
drm.smt1.
drm.wt.smt1.
dsm.ugt80.
dsm.wt.ugt80.
dsm.smt1.
dsm.wt.smt1.
sp.ugt80.
sp.wt.ugt80.
sp.smt1.
sp.wt.smt1.
im.ugt80.
im.wt.ugt80.
im.smt1.
im.wt.smt1.

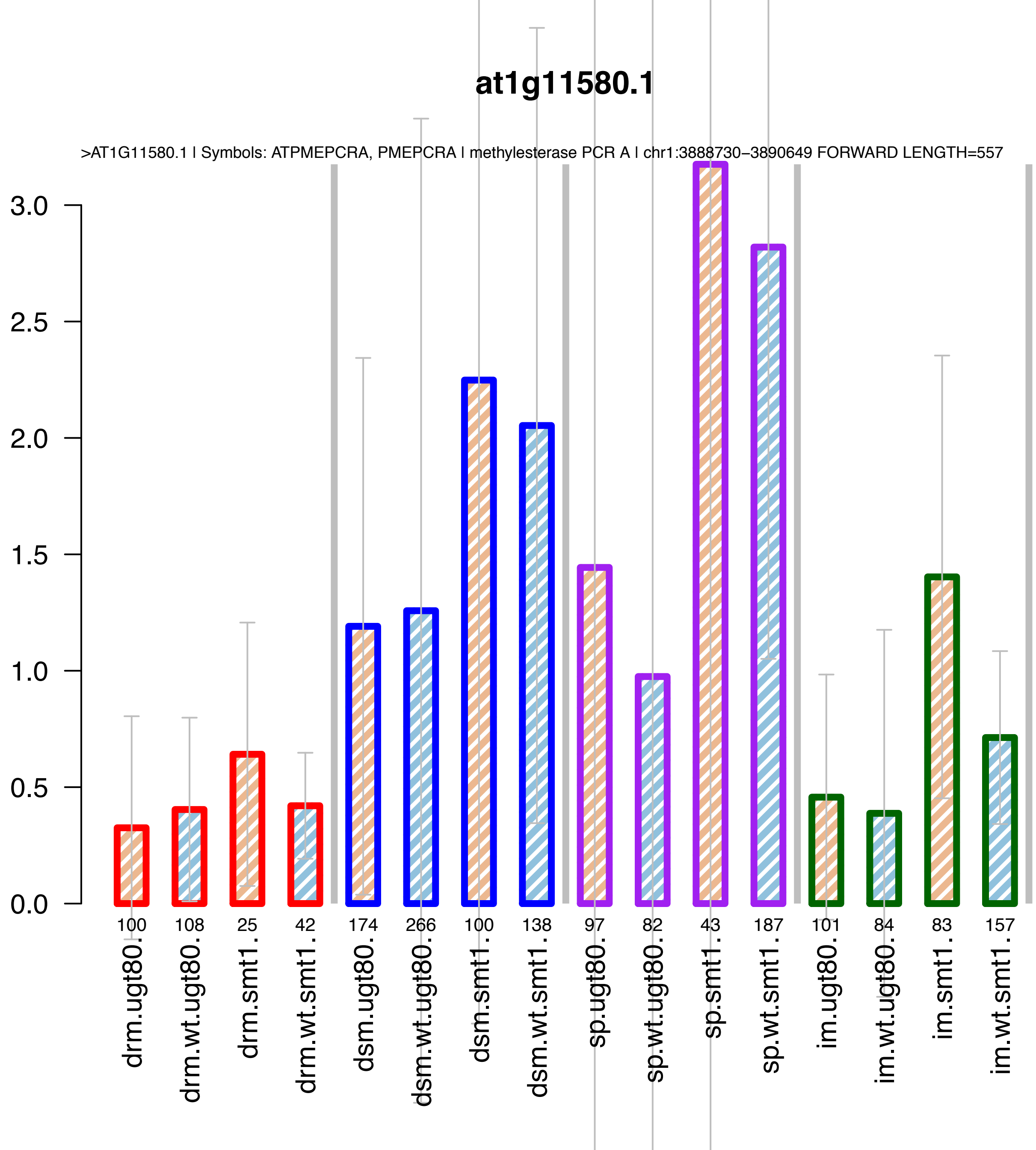
at1g11580.1
>AT1G11580.1 | Symbols: ATPMEPCRA, PMEPCRA | methylesterase PCR A | chr1:3888730-3890649 FORWARD LENGTH=557
3.0
2.5
2.0
1.5
1.0
0.5
0.0
100
108
25
42
174
266
100
138
97
82
43
187
101
84
83
157
drm.ugt80.
drm.wt.ugt80.
drm.smt1.
drm.wt.smt1.
dsm.ugt80.
dsm.wt.ugt80.
dsm.smt1.
dsm.wt.smt1.
sp.ugt80.
sp.wt.ugt80.
sp.smt1.
sp.wt.smt1.
im.ugt80.
im.wt.ugt80.
im.smt1.
im.wt.smt1.

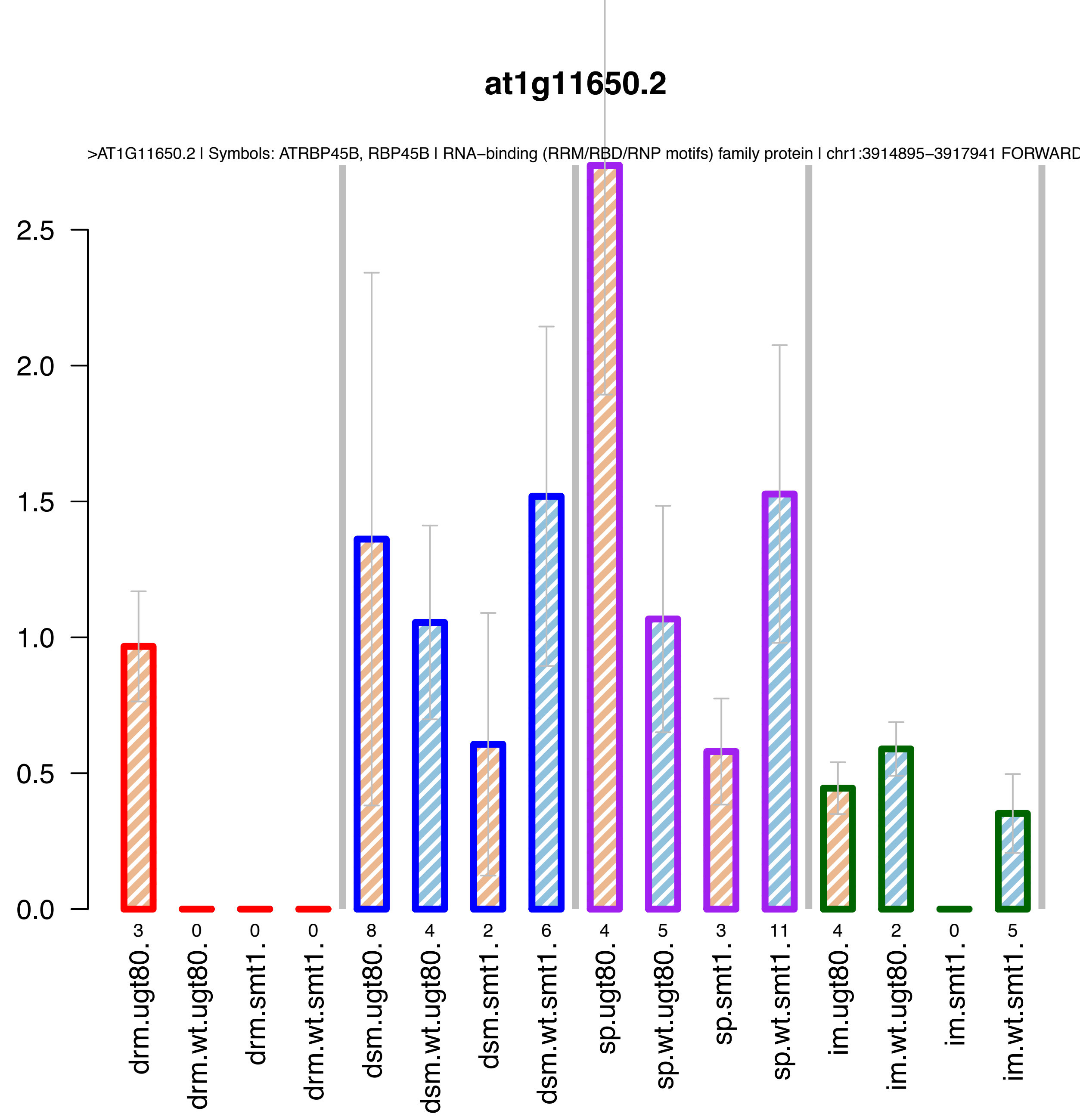
at1g11650.2
>AT1G11650.2 | Symbols: ATRBP45B, RBP45B | RNA−binding (RRM/RBD/RNP motifs) family protein | chr1:3914895−3917941 FORWARD
0.0
0.5
1.0
1.5
2.0
2.5
3
0
0
0
8
4
2
6
4
5
3
11
4
2
0
5
drm.ugt80.
drm.wt.ugt80.
drm.smt1.
drm.wt.smt1.
dsm.ugt80.
dsm.wt.ugt80.
dsm.smt1.
dsm.wt.smt1.
sp.ugt80.
sp.wt.ugt80.
sp.smt1.
sp.wt.smt1.
im.ugt80.
im.wt.ugt80.
im.smt1.
im.wt.smt1.

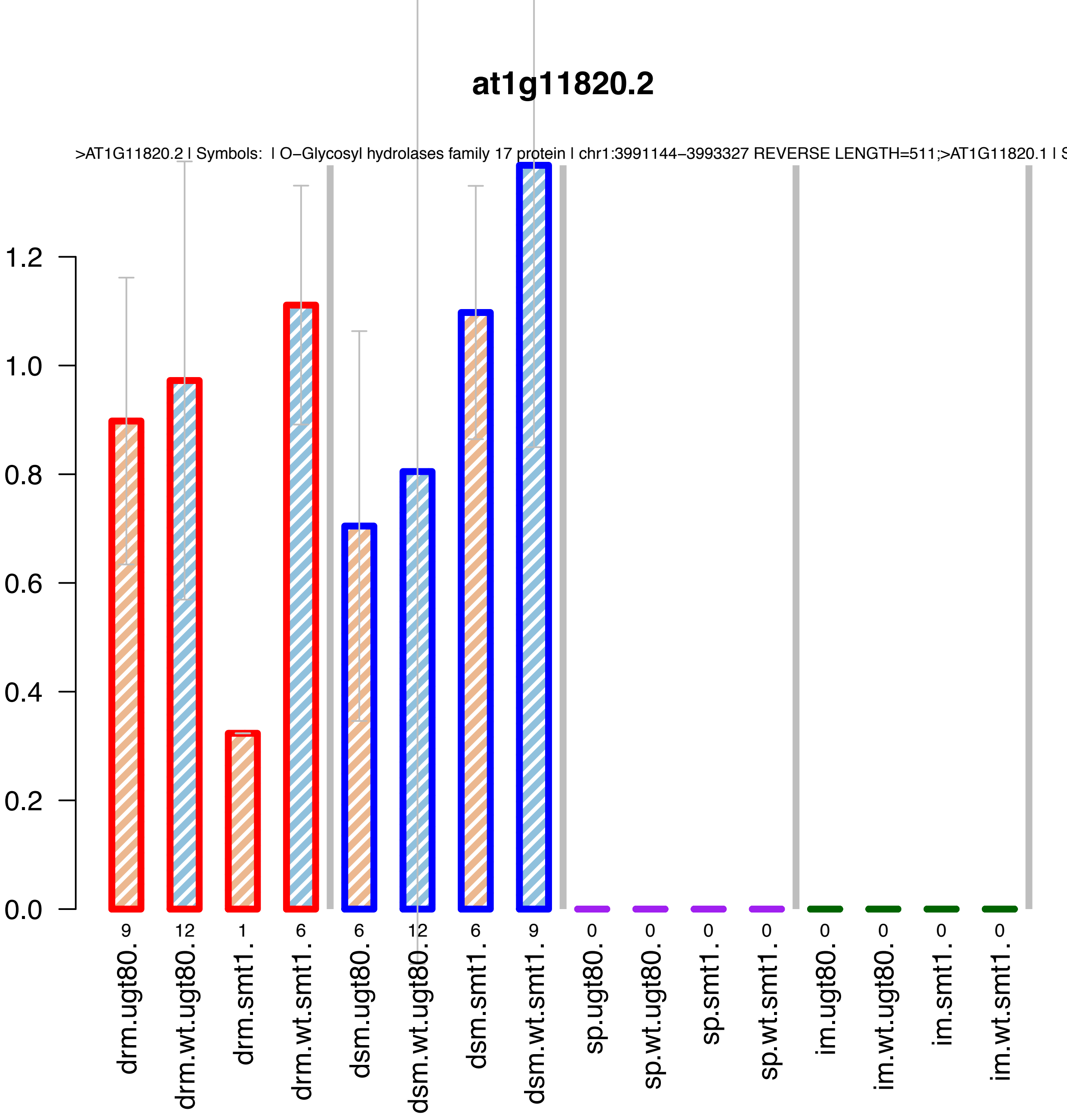

at1g11820.2
>AT1G11820.2 | Symbols: | O−Glycosyl hydrolases family 17 protein | chr1:3991144−3993327 REVERSE LENGTH=511;>AT1G11820.1 | S
0.0
0.2
0.4
0.6
0.8
1.0
1.2
9
12
1
6
6
12
6
9
0
0
0
0
0
0
0
0
drm.ugt80.
drm.wt.ugt80.
drm.smt1.
drm.wt.smt1.
dsm.ugt80.
dsm.wt.ugt80.
dsm.smt1.
dsm.wt.smt1.
sp.ugt80.
sp.wt.ugt80.
sp.smt1.
sp.wt.smt1.
im.ugt80.
im.wt.ugt80.
im.smt1.
im.wt.smt1.

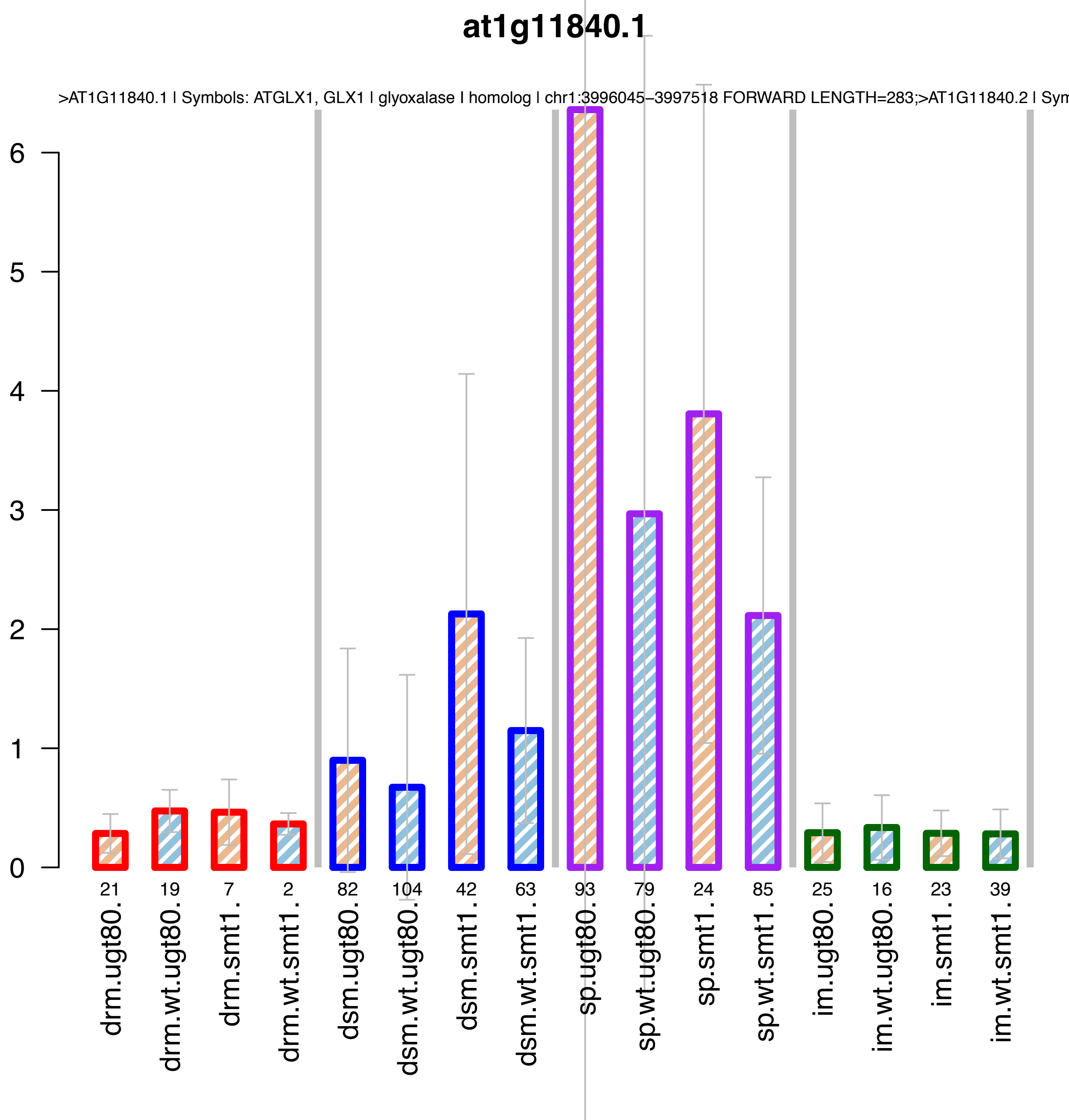
at1g11840.1
>AT1G11840.1 | Symbols: ATGLX1, GLX1 | glyoxalase I homolog | chr1:3996045-3997518 FORWARD LENGTH=283;>AT1G11840.2 | Sym
0
1
2
3
4
5
6
21
19
7
2
82
104
42
63
93
79
24
85
25
16
23
39
drm.ugt80.
drm.wt.ugt80.
drm.smt1.
drm.wt.smt1.
dsm.ugt80.
dsm.wt.ugt80.
dsm.smt1.
dsm.wt.smt1.
sp.ugt80.
sp.wt.ugt80.
sp.smt1.
sp.wt.smt1.
im.ugt80.
im.wt.ugt80.
im.smt1.
im.wt.smt1.

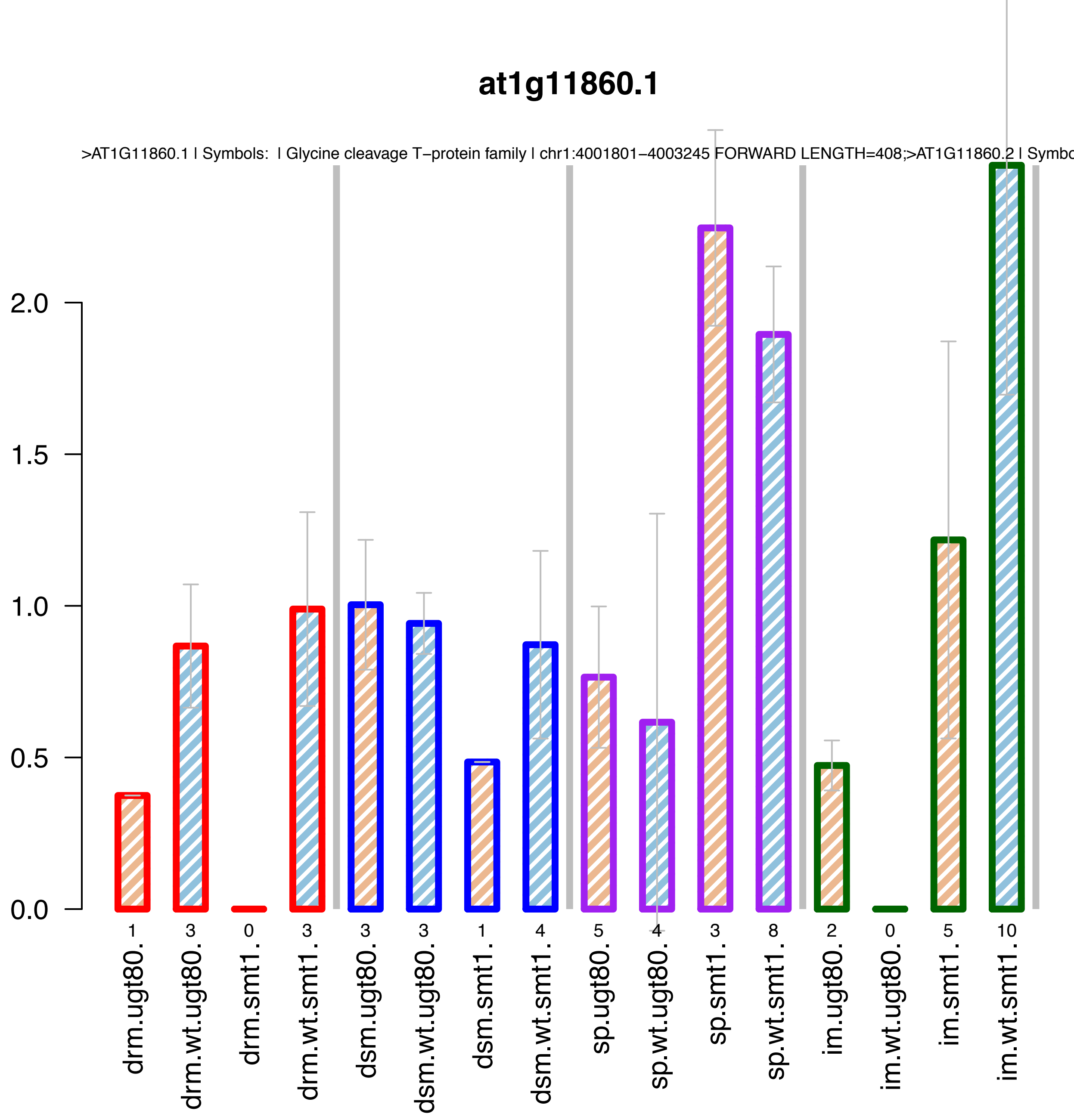
at1g11860.1
>AT1G11860.1 | Symbols: | Glycine cleavage T−protein family | chr1:4001801−4003245 FORWARD LENGTH=408;>AT1G11860.2 | Symbo
0.0
0.5
1.0
1.5
2.0
1
3
0
3
3
3
1
4
5
4
3
8
2
0
5
10
drm.ugt80.
drm.wt.ugt80.
drm.smt1.
drm.wt.smt1.
dsm.ugt80.
dsm.wt.ugt80.
dsm.smt1.
dsm.wt.smt1.
sp.ugt80.
sp.wt.ugt80.
sp.smt1.
sp.wt.smt1.
im.ugt80.
im.wt.ugt80.
im.smt1.
im.wt.smt1.

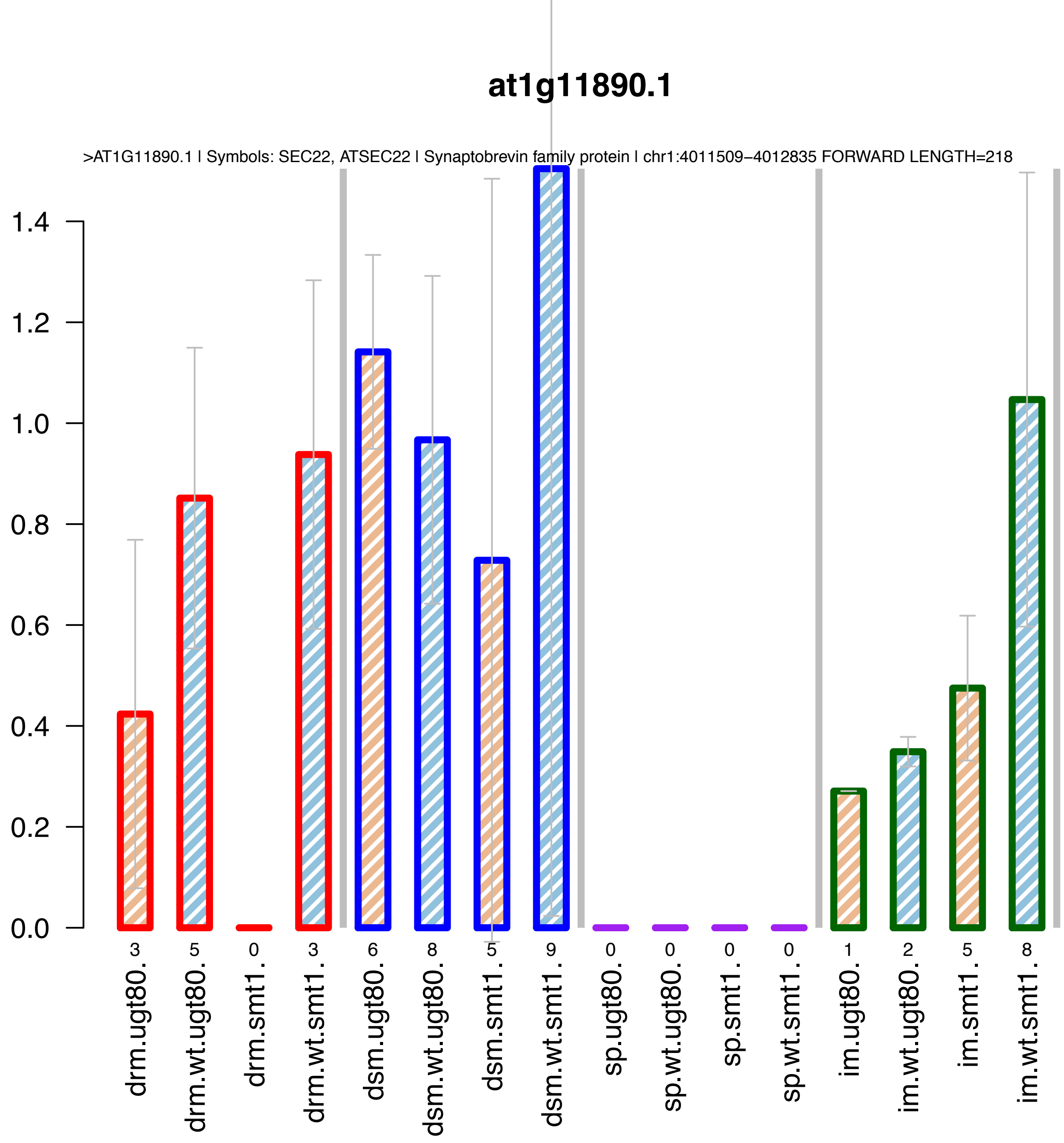

at1g11890.1
>AT1G11890.1 | Symbols: SEC22, ATSEC22 | Synaptobrevin family protein | chr1:4011509-4012835 FORWARD LENGTH=218
0.0
0.2
0.4
0.6
0.8
1.0
1.2
1.4
3
5
0
3
6
8
5
9
0
0
0
0
1
2
5
8
drm.ugt80.
drm.wt.ugt80.
drm.smt1.
drm.wt.smt1.
dsm.ugt80.
dsm.wt.ugt80.
dsm.smt1.
dsm.wt.smt1.
sp.ugt80.
sp.wt.ugt80.
sp.smt1.
sp.wt.smt1.
im.ugt80.
im.wt.ugt80.
im.smt1.
im.wt.smt1.

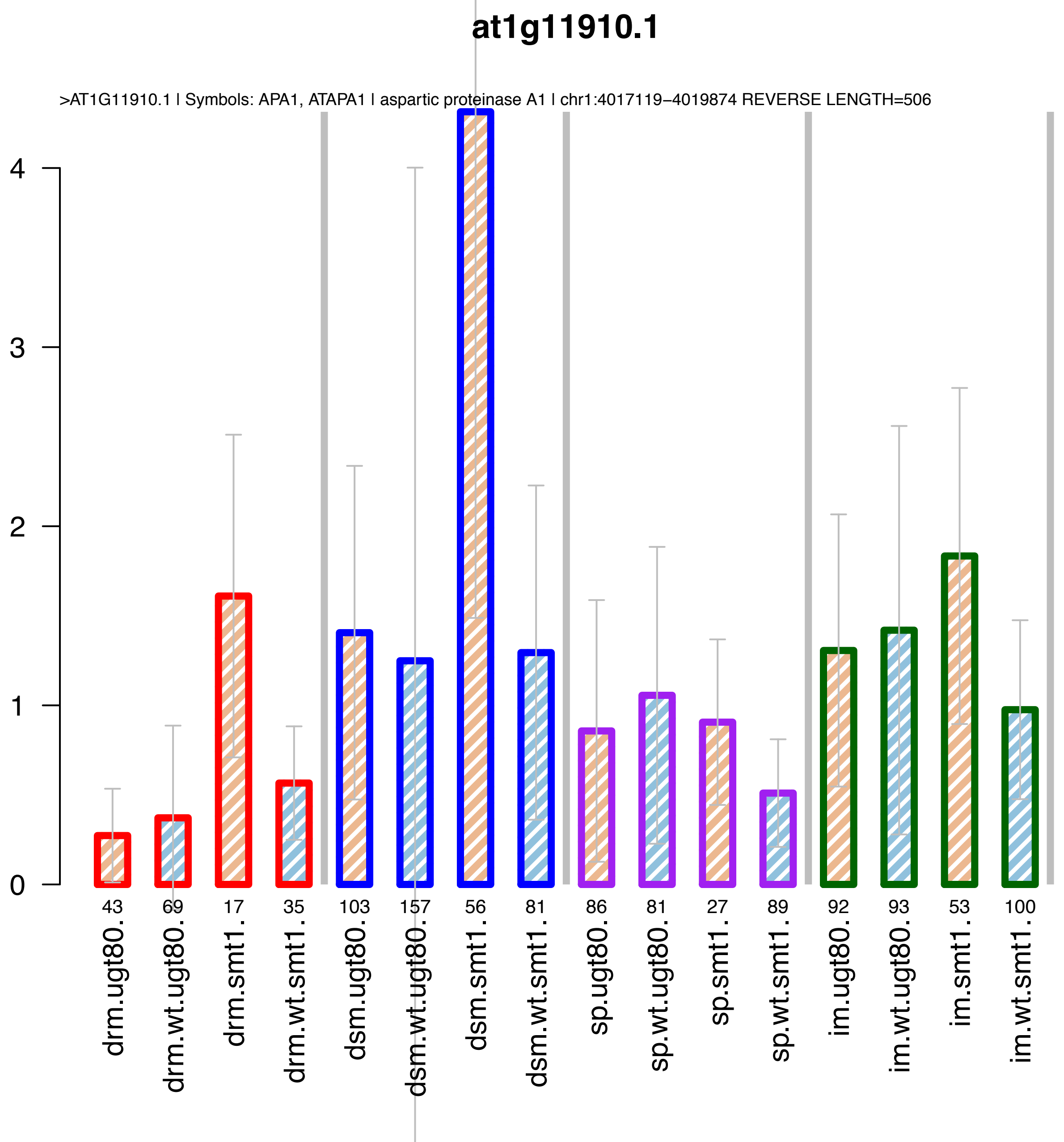

at1g11910.1
>AT1G11910.1 | Symbols: APA1, ATAPA1 | aspartic proteinase A1 | chr1:4017119-4019874 REVERSE LENGTH=506
0
1
2
3
4
43
69
17
35
103
157
56
81
86
81
27
89
92
93
53
100
drm.ugt80.
drm.wt.ugt80.
drm.smt1.
drm.wt.smt1.
dsm.ugt80.
dsm.wt.ugt80.
dsm.smt1.
dsm.wt.smt1.
sp.ugt80.
sp.wt.ugt80.
sp.smt1.
sp.wt.smt1.
im.ugt80.
im.wt.ugt80.
im.smt1.
im.wt.smt1.

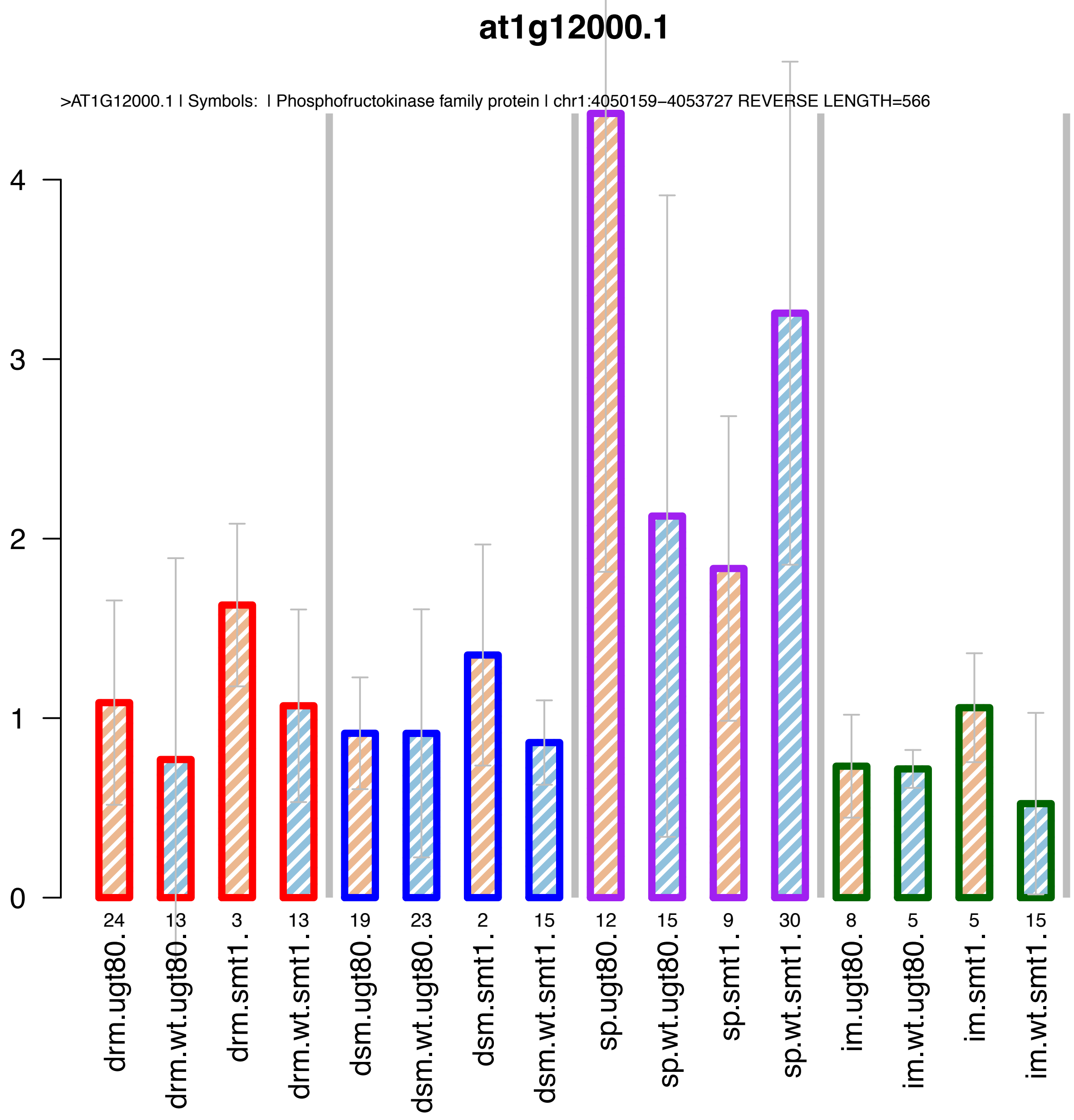

at1g12000.1
>AT1G12000.1 | Symbols: | Phosphofructokinase family protein | chr1:4050159-4053727 REVERSE LENGTH=566
0
1
2
3
4
24
13
3
13
19
23
2
15
12
15
9
30
8
5
5
15
drm.ugt80.
drm.wt.ugt80.
drm.smt1.
drm.wt.smt1.
dsm.ugt80.
dsm.wt.ugt80.
dsm.smt1.
dsm.wt.smt1.
sp.ugt80.
sp.wt.ugt80.
sp.smt1.
sp.wt.smt1.
im.ugt80.
im.wt.ugt80.
im.smt1.
im.wt.smt1.

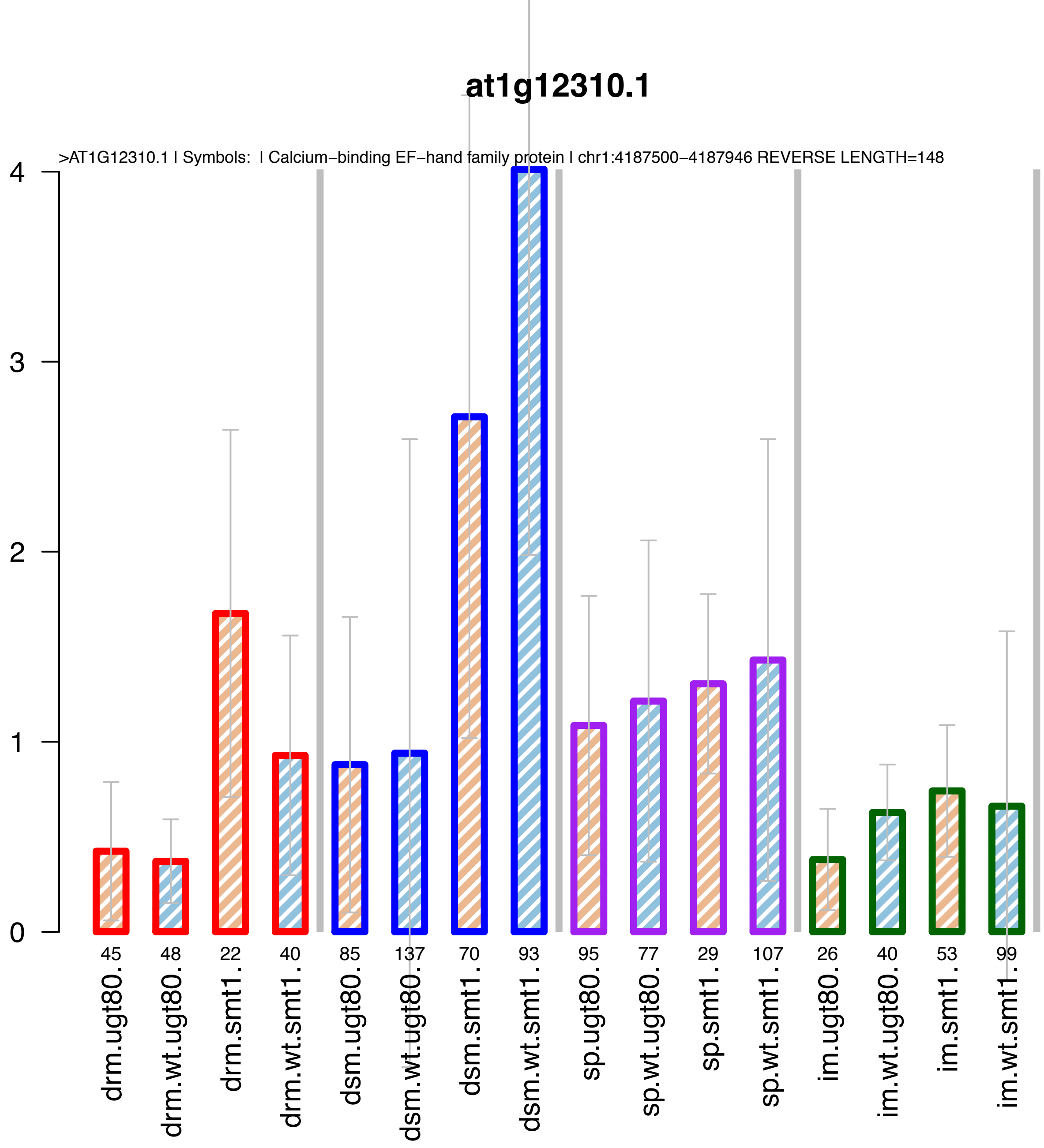

at1g12310.1
>AT1G12310.1 | Symbols: | Calcium−binding EF−hand family protein | chr1:4187500−4187946 REVERSE LENGTH=148
0
1
2
3
4
45
48
22
40
85
137
70
93
95
77
29
107
26
40
53
99
drm.ugt80.
drm.wt.ugt80.
drm.smt1.
drm.wt.smt1.
dsm.ugt80.
dsm.wt.ugt80.
dsm.smt1.
dsm.wt.smt1.
sp.ugt80.
sp.wt.ugt80.
sp.smt1.
sp.wt.smt1.
im.ugt80.
im.wt.ugt80.
im.smt1.
im.wt.smt1.

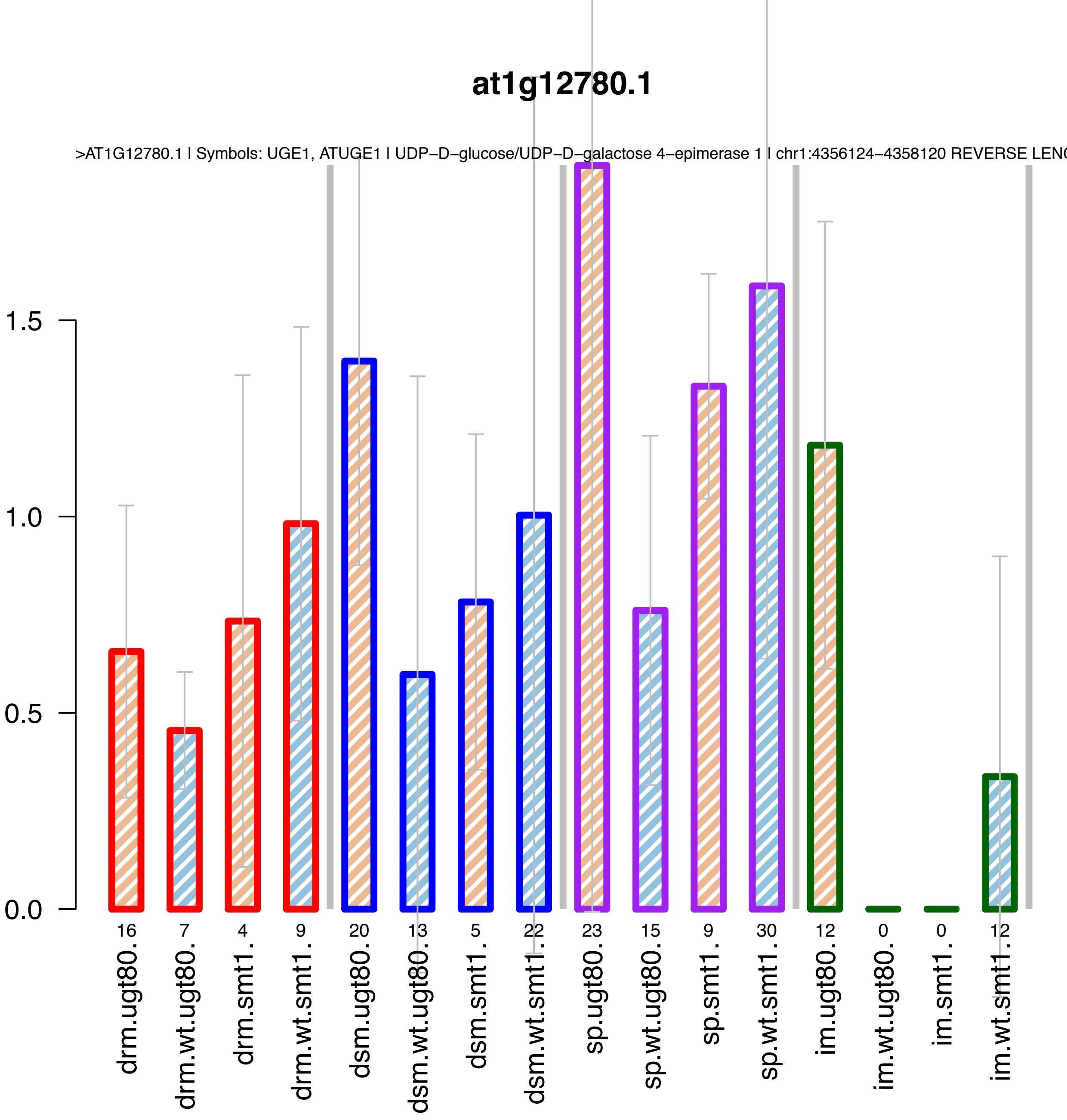

at1g12780.1
>AT1G12780.1 | Symbols: UGE1, ATUGE1 | UDP-D-glucose/UDP-D-galactose 4-epimerase 1 | chr1:4356124-4358120 REVERSE LENG
1.5
1.0
0.5
0.0
16
7
4
9
20
13
5
22
23
15
9
30
12
0
0
12
drm.ugt80.
drm.wt.ugt80.
drm.smt1.
drm.wt.smt1.
dsm.ugt80.
dsm.wt.ugt80.
dsm.smt1.
dsm.wt.smt1.
sp.ugt80.
sp.wt.ugt80.
sp.smt1.
sp.wt.smt1.
im.ugt80.
im.wt.ugt80.
im.smt1.
im.wt.smt1.

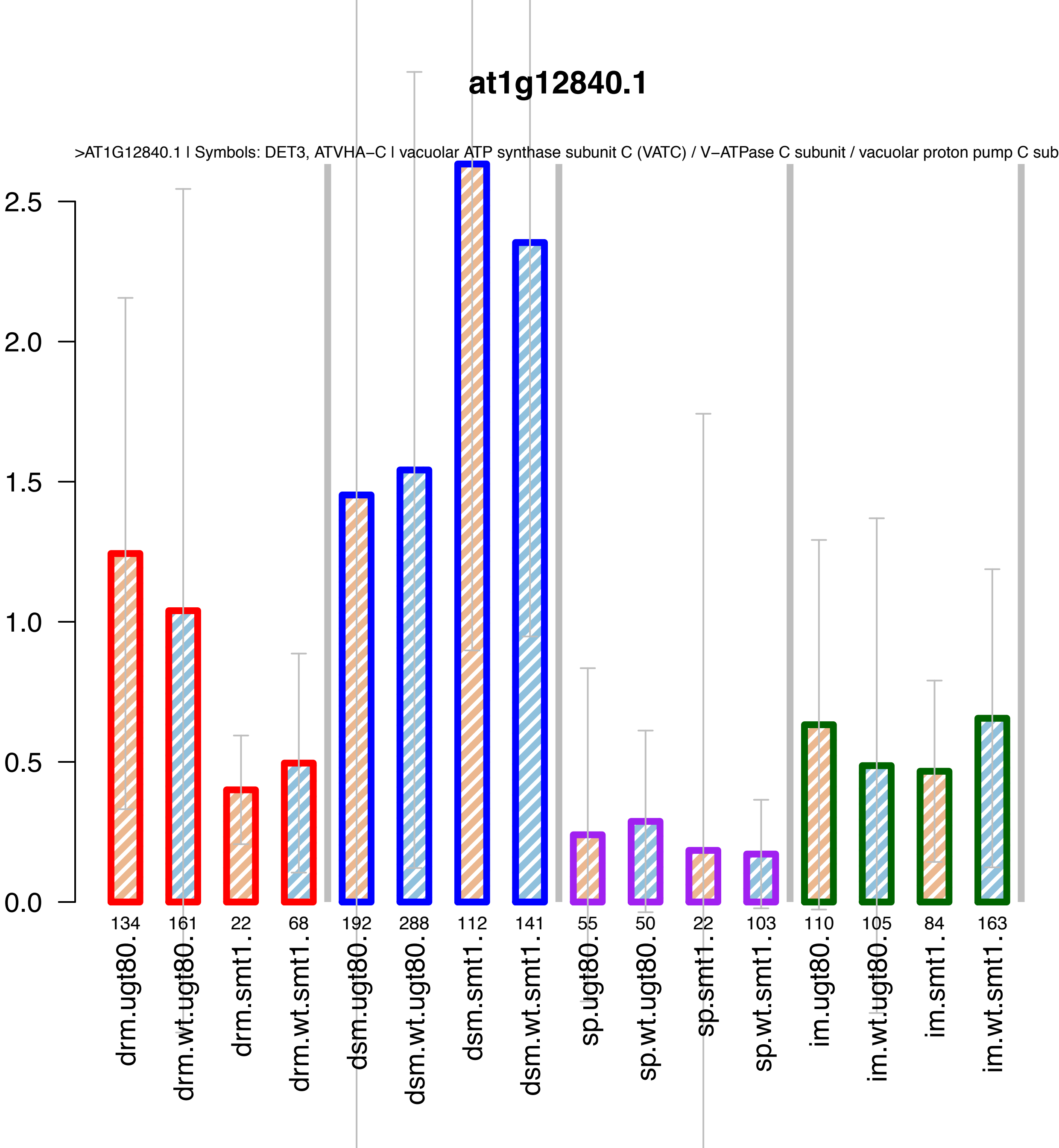
at1g12840.1
>AT1G12840.1 | Symbols: DET3, ATVHA-C | vacuolar ATP synthase subunit C (VATC) / V-ATPase C subunit / vacuolar proton pump C sub
2.5
2.0
1.5
1.0
0.5
0.0
134
161
22
68
192
288
112
141
55
50
22
103
110
105
84
163
drm.ugt80..
drm.wt.ugt80..
drm.smt1..
drm.wt.smt1..
dsm.ugt80..
dsm.wt.ugt80..
dsm.smt1..
dsm.wt.smt1..
sp.ugt80..
sp.wt.ugt80..
sp.smt1..
sp.wt.smt1..
im.ugt80..
im.wt.ugt80..
im.smt1..
im.wt.smt1..

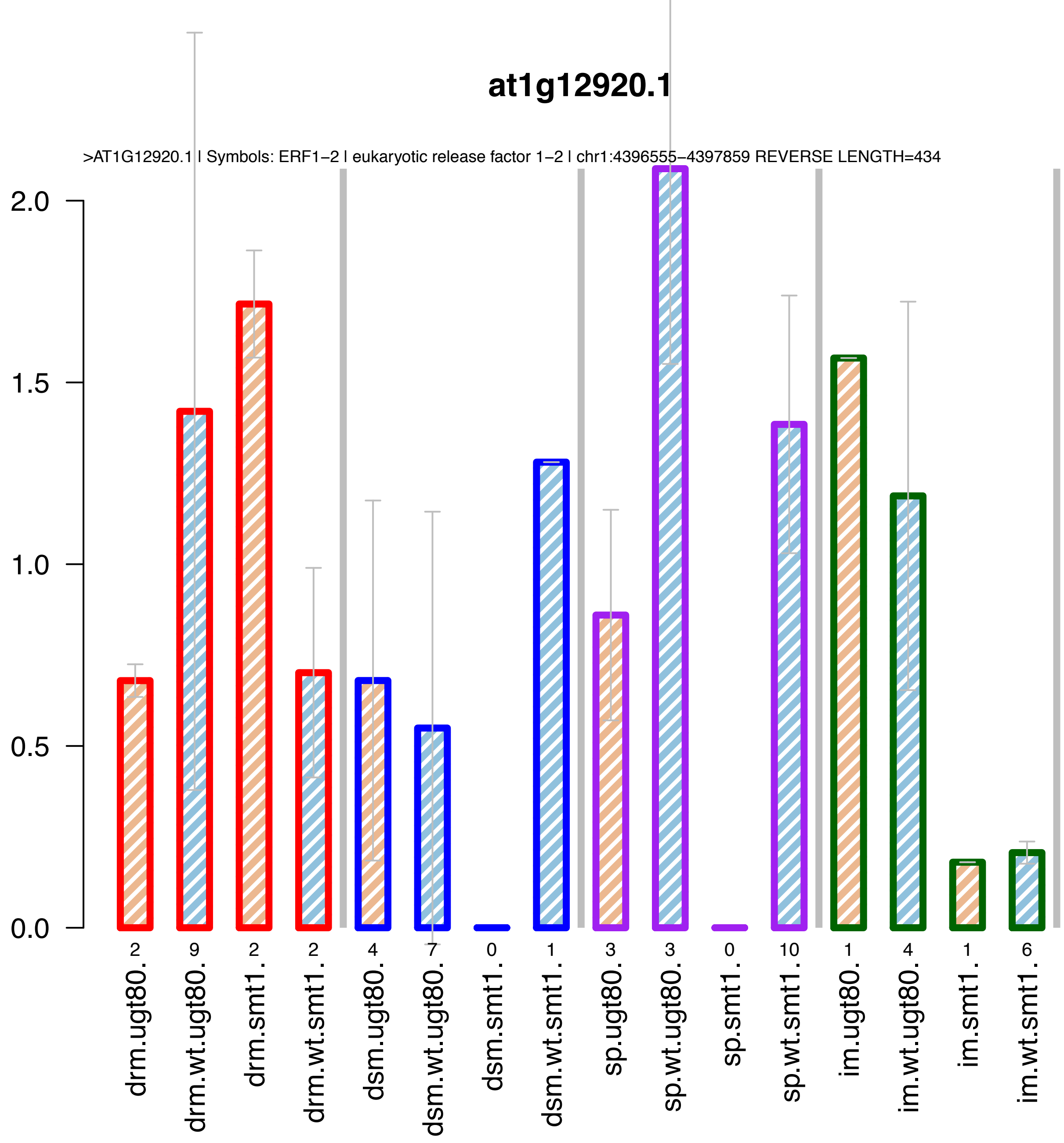

at1g12920.1
>AT1G12920.1 | Symbols: ERF1−2 | eukaryotic release factor 1−2 | chr1:4396555−4397859 REVERSE LENGTH=434
2.0
1.5
1.0
0.5
0.0
2
9
2
2
4
7
0
1
3
3
0
10
1
4
1
6
drm.ugt80..
drm.wt.ugt80..
drm.smt1..
drm.wt.smt1..
dsm.ugt80..
dsm.wt.ugt80..
dsm.smt1..
dsm.wt.smt1..
sp.ugt80..
sp.wt.ugt80..
sp.smt1..
sp.wt.smt1..
im.ugt80..
im.wt.ugt80..
im.smt1..
im.wt.smt1..

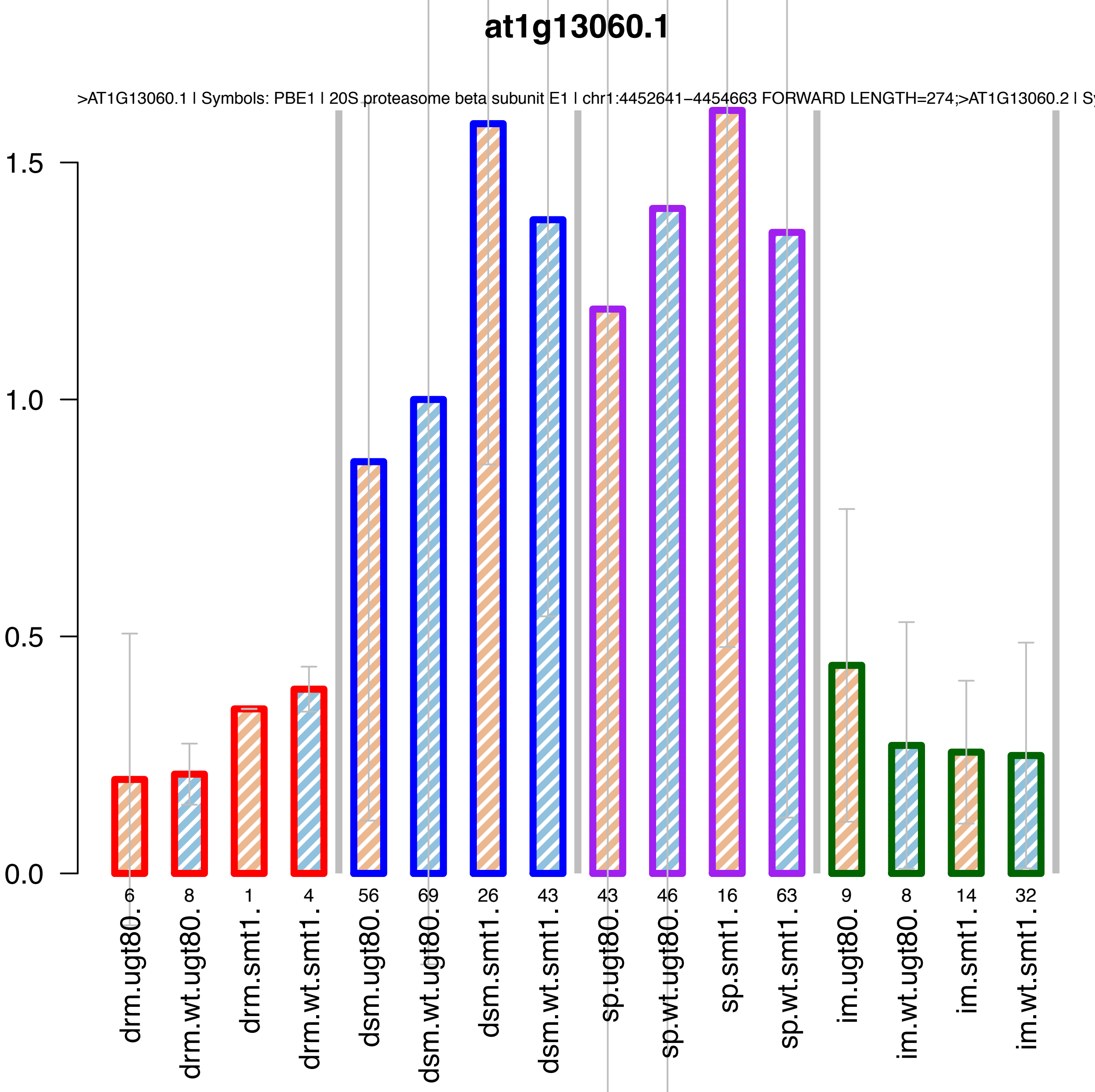

at1g13060.1
>AT1G13060.1 | Symbols: PBE1 | 20S proteasome beta subunit E1 | chr1:4452641−4454663 FORWARD LENGTH=274;>AT1G13060.2 | Sy
1.5
1.0
0.5
0.0
6
8
1
4
56
69
26
43
43
46
16
63
9
8
14
32
drm.ugt80..
drm.wt.ugt80..
drm.smt1..
drm.wt.smt1..
dsm.ugt80..
dsm.wt.ugt80..
dsm.smt1..
dsm.wt.smt1..
sp.ugt80..
sp.wt.ugt80..
sp.smt1..
sp.wt.smt1..
im.ugt80..
im.wt.ugt80..
im.smt1..
im.wt.smt1..

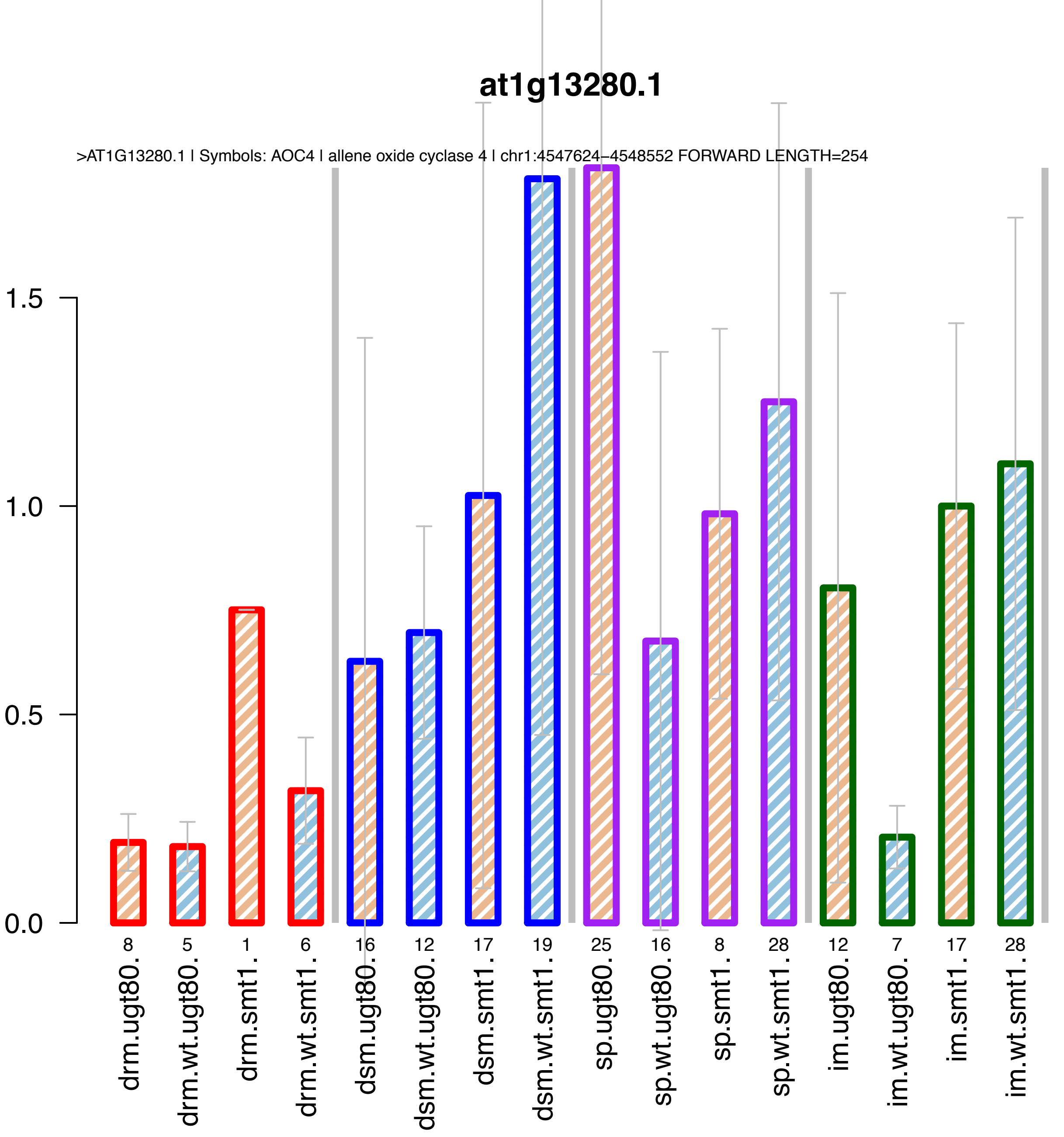

at1g13280.1
>AT1G13280.1 | Symbols: AOC4 | allene oxide cyclase 4 | chr1:4547624–4548552 FORWARD LENGTH=254
0.0
0.5
1.0
1.5
8
5
1
6
16
12
17
19
25
16
8
28
12
7
17
28
drm.ugt80..
drm.wt.ugt80..
drm.smt1..
drm.wt.smt1..
dsm.ugt80..
dsm.wt.ugt80..
dsm.smt1..
dsm.wt.smt1..
sp.ugt80..
sp.wt.ugt80..
sp.smt1..
sp.wt.smt1..
im.ugt80..
im.wt.ugt80..
im.smt1..
im.wt.smt1..

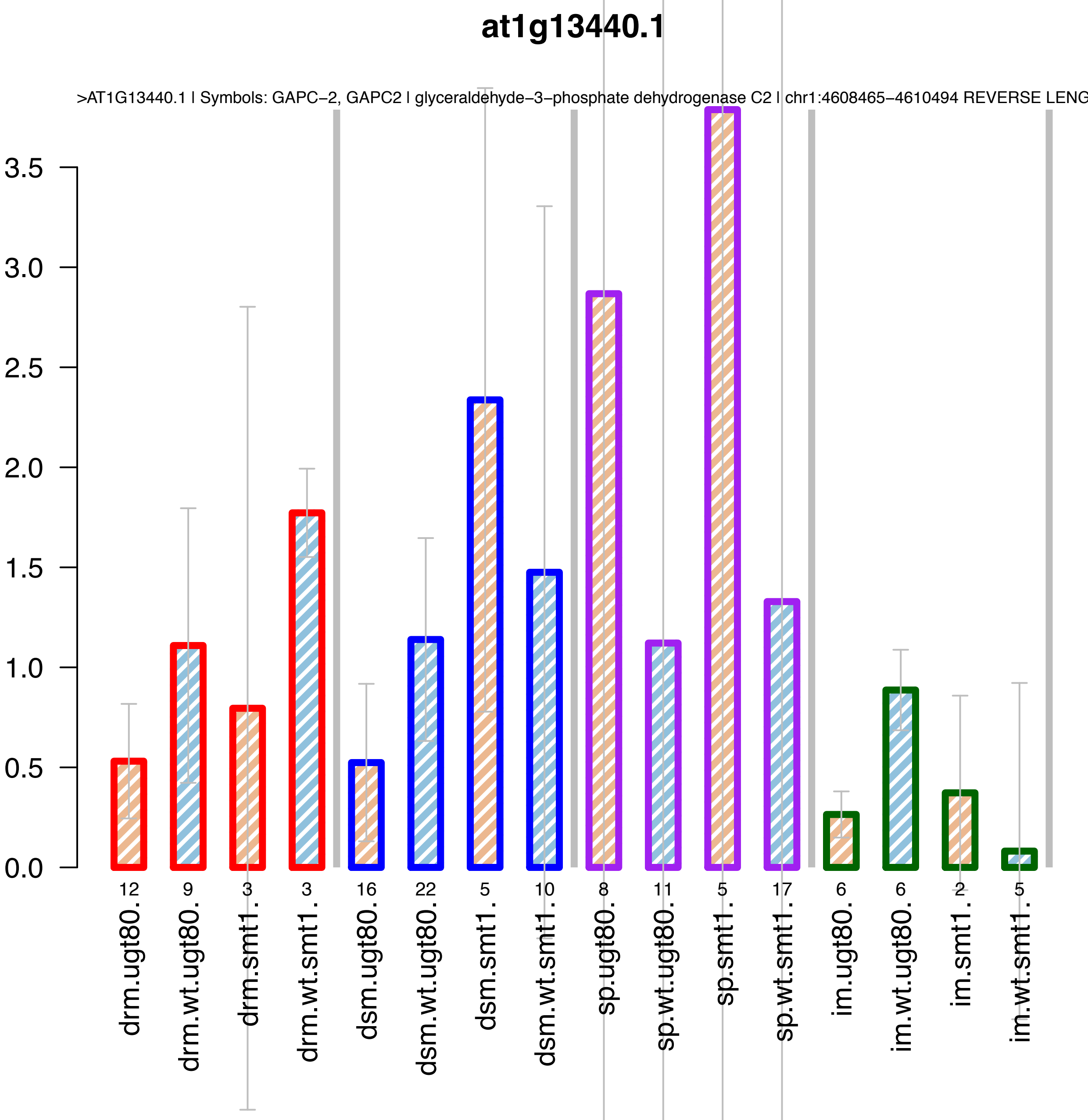

at1g13440.1
>AT1G13440.1 | Symbols: GAPC-2, GAPC2 | glyceraldehyde-3-phosphate dehydrogenase C2 | chr1:4608465-4610494 REVERSE LENG
3.5
3.0
2.5
2.0
1.5
1.0
0.5
0.0
12
9
3
3
16
22
5
10
8
11
5
17
6
6
2
5
drm.ugt80.
drm.wt.ugt80.
drm.smt1.
drm.wt.smt1.
dsm.ugt80.
dsm.wt.ugt80.
dsm.smt1.
dsm.wt.smt1.
sp.ugt80.
sp.wt.ugt80.
sp.smt1.
sp.wt.smt1.
im.ugt80.
im.wt.ugt80.
im.smt1.
im.wt.smt1.

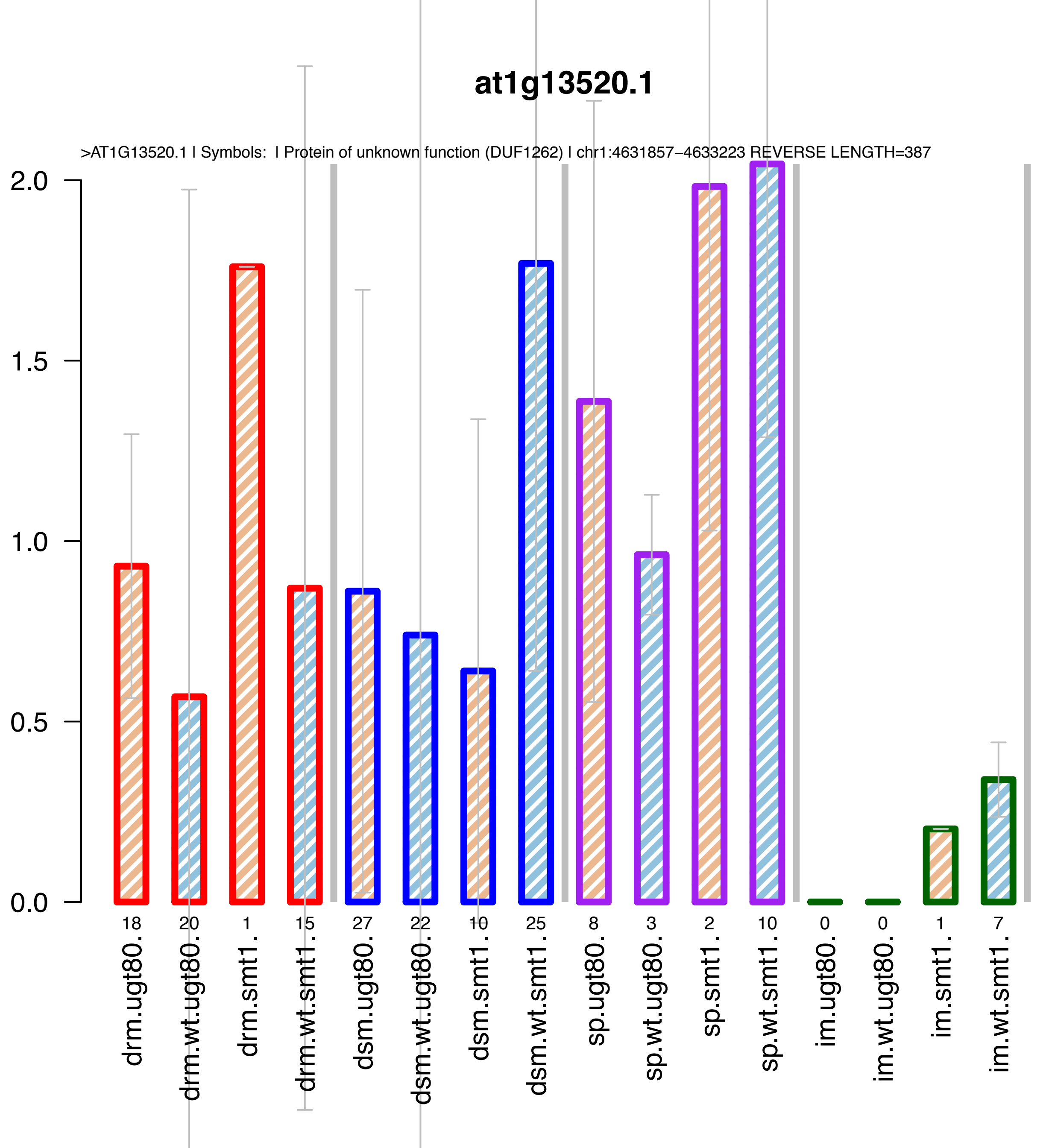
at1g13520.1
>AT1G13520.1 | Symbols: | Protein of unknown function (DUF1262) | chr1:4631857-4633223 REVERSE LENGTH=387
2.0
1.5
1.0
0.5
0.0
18
20
1
15
27
22
10
25
8
3
2
10
0
0
1
7
drm.ugt80..
drm.wt.ugt80..
drm.smt1..
drm.wt.smt1..
dsm.ugt80..
dsm.wt.ugt80..
dsm.smt1..
dsm.wt.smt1..
sp.ugt80..
sp.wt.ugt80..
sp.smt1..
sp.wt.smt1..
im.ugt80..
im.wt.ugt80..
im.smt1..
im.wt.smt1..

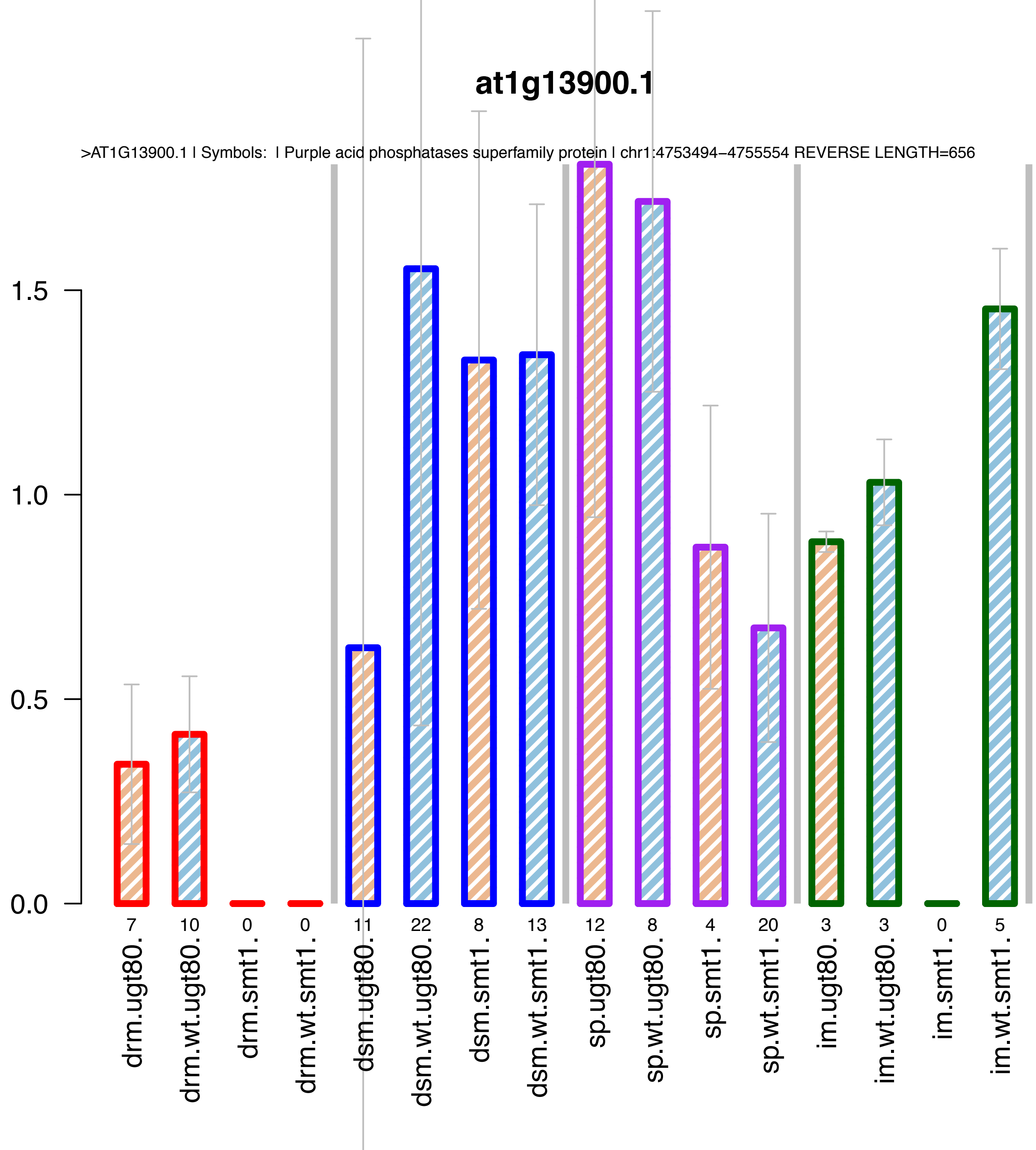
at1g13900.1
>AT1G13900.1 | Symbols: | Purple acid phosphatases superfamily protein | chr1:4753494-4755554 REVERSE LENGTH=656
0.0
0.5
1.0
1.5
7
10
0
0
11
22
8
13
12
8
4
20
3
3
0
5
drm.ugt80.
drm.wt.ugt80.
drm.smt1.
drm.wt.smt1.
dsm.ugt80.
dsm.wt.ugt80.
dsm.smt1.
dsm.wt.smt1.
sp.ugt80.
sp.wt.ugt80.
sp.smt1.
sp.wt.smt1.
im.ugt80.
im.wt.ugt80.
im.smt1.
im.wt.smt1.

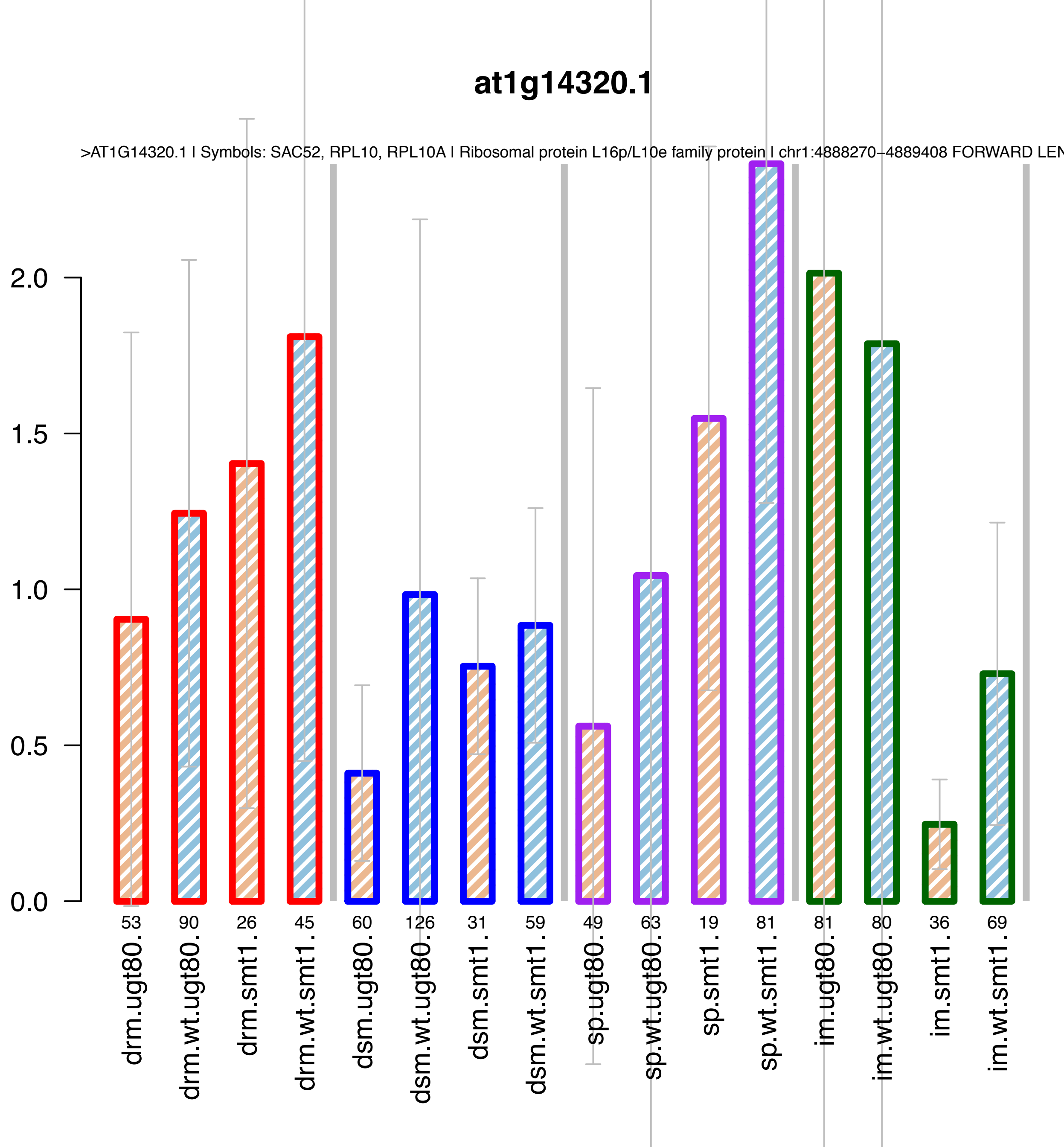

at1g14320.1
>AT1G14320.1 | Symbols: SAC52, RPL10, RPL10A | Ribosomal protein L16p/L10e family protein | chr1:4888270-4889408 FORWARD LEN
2.0
1.5
1.0
0.5
0.0
53
90
26
45
60
126
31
59
49
63
19
81
81
80
36
69
drm.ugt80.
drm.wt.ugt80.
drm.smt1.
drm.wt.smt1.
dsm.ugt80.
dsm.wt.ugt80.
dsm.smt1.
dsm.wt.smt1.
sp.ugt80.
sp.wt.ugt80.
sp.smt1.
sp.wt.smt1.
im.ugt80.
im.wt.ugt80.
im.smt1.
im.wt.smt1.

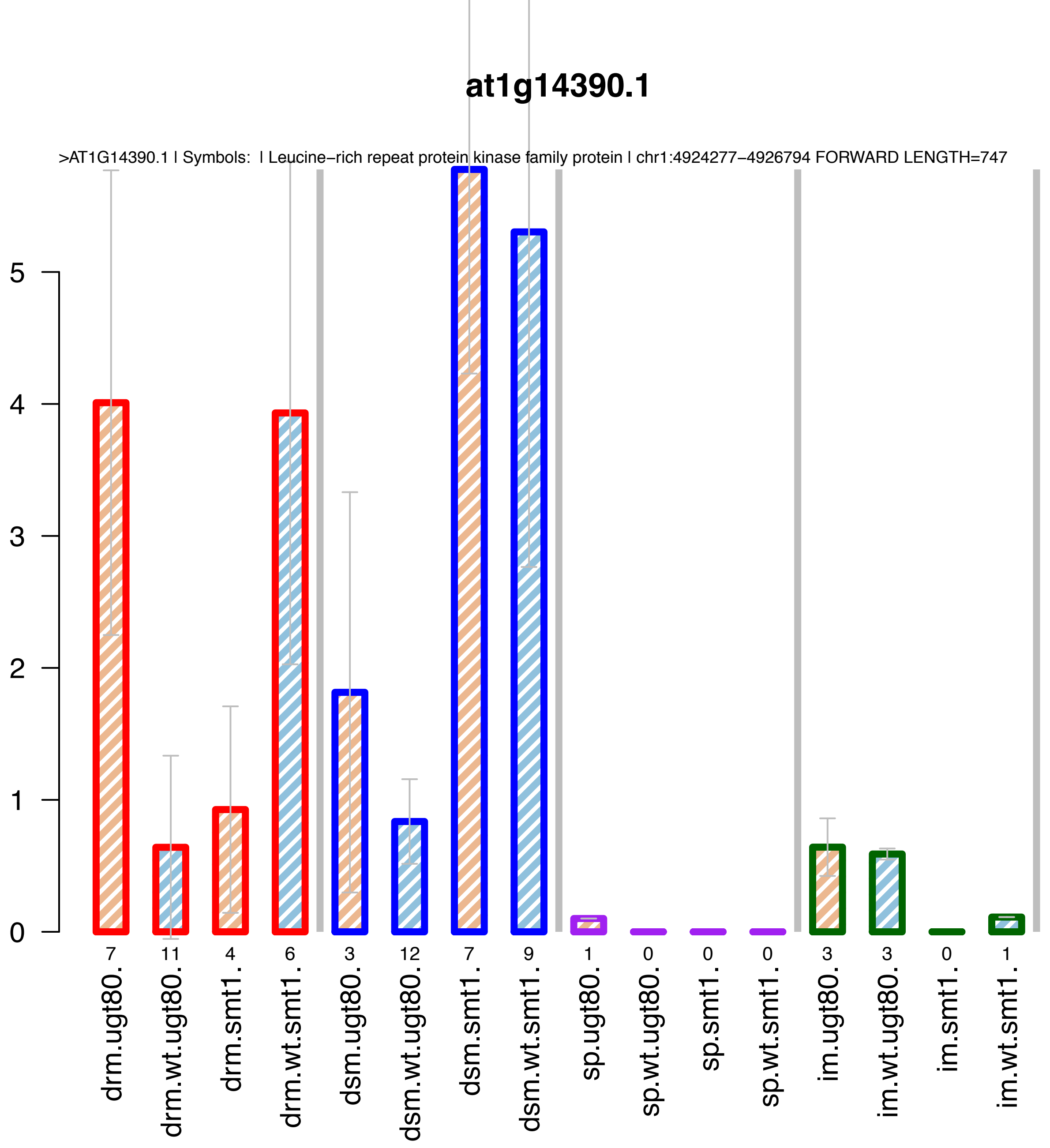

at1g14390.1
>AT1G14390.1 | Symbols: | Leucine−rich repeat protein kinase family protein | chr1:4924277−4926794 FORWARD LENGTH=747
0
1
2
3
4
5
7
11
4
6
3
12
7
9
1
0
0
0
3
3
0
1
drm.ugt80.
drm.wt.ugt80.
drm.smt1.
drm.wt.smt1.
dsm.ugt80.
dsm.wt.ugt80.
dsm.smt1.
dsm.wt.smt1.
sp.ugt80.
sp.wt.ugt80.
sp.smt1.
sp.wt.smt1.
im.ugt80.
im.wt.ugt80.
im.smt1.
im.wt.smt1.

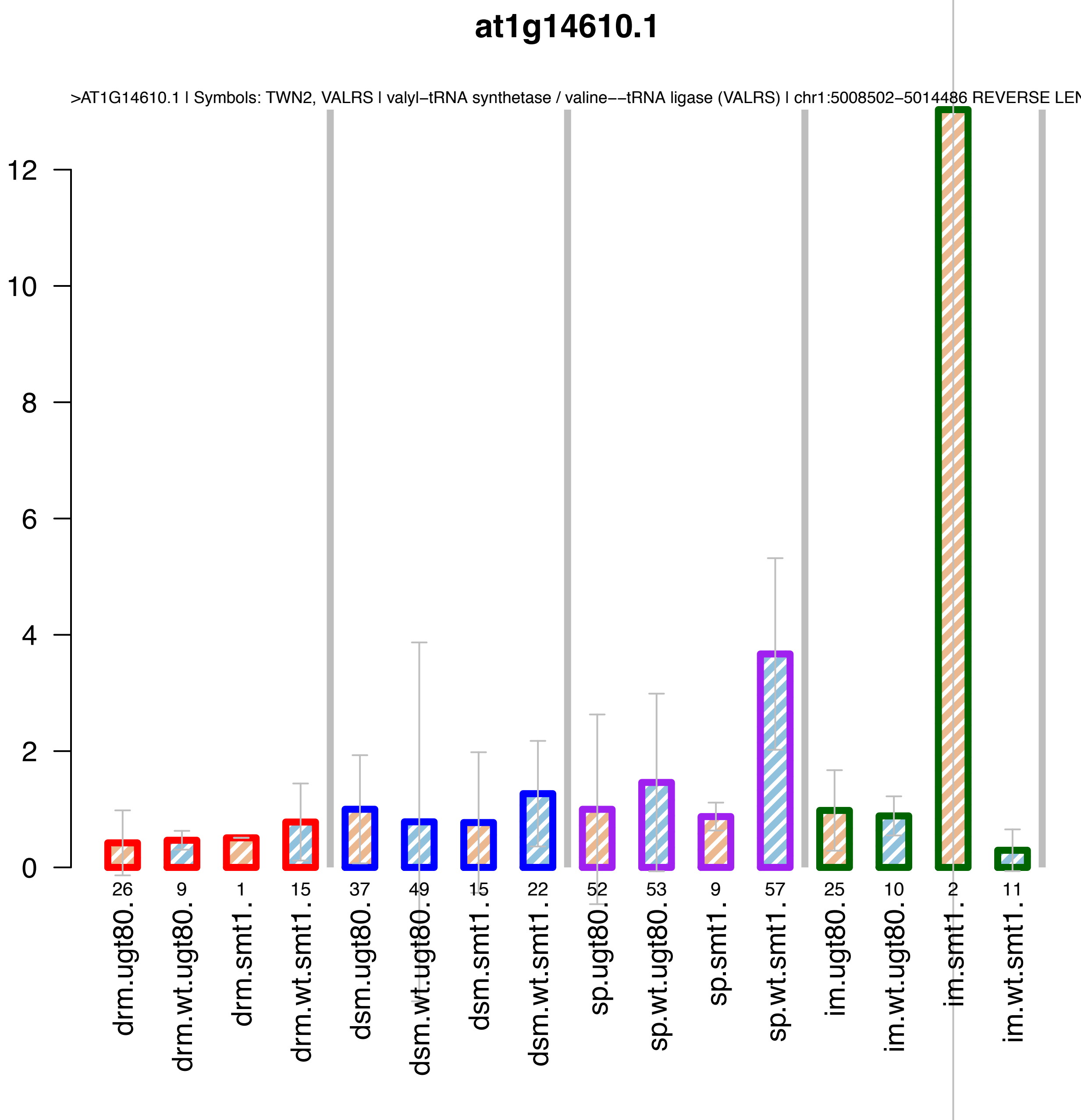

at1g14610.1
>AT1G14610.1 | Symbols: TWN2, VALRS | valyl–tRNA synthetase / valine––tRNA ligase (VALRS) | chr1:5008502–5014486 REVERSE LEN
0
2
4
6
8
10
12
26
9
1
15
37
49
15
22
52
53
9
57
25
10
2
11
drm.ugt80.
drm.wt.ugt80.
drm.smt1.
drm.wt.smt1.
dsm.ugt80.
dsm.wt.ugt80.
dsm.smt1.
dsm.wt.smt1.
sp.ugt80.
sp.wt.ugt80.
sp.smt1.
sp.wt.smt1.
im.ugt80.
im.wt.ugt80.
im.smt1.
im.wt.smt1.

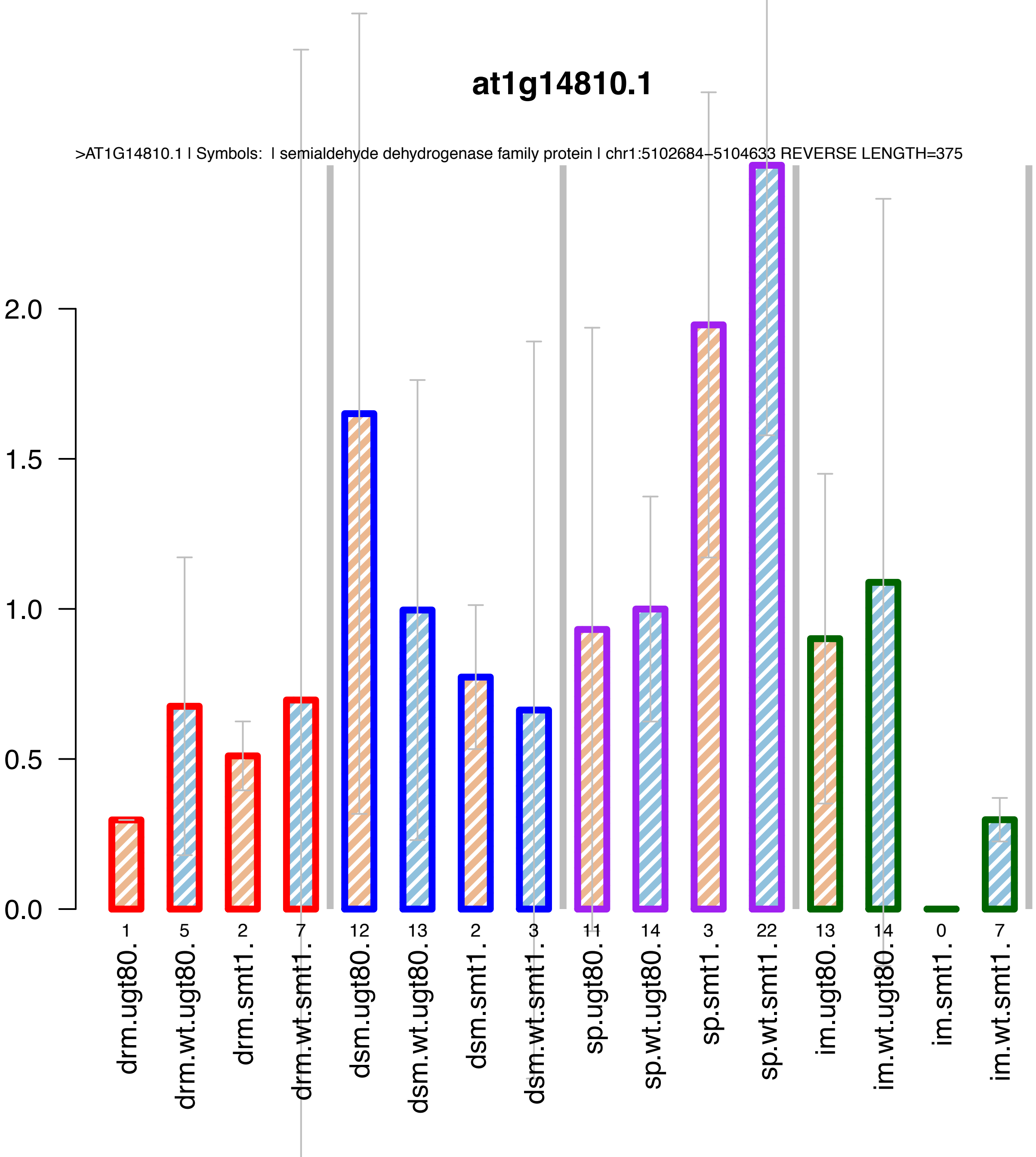
at1g14810.1
>AT1G14810.1 | Symbols: | semialdehyde dehydrogenase family protein | chr1:5102684-5104633 REVERSE LENGTH=375
0.0
0.5
1.0
1.5
2.0
1
5
2
7
12
13
2
3
11
14
3
22
13
14
0
7
drm.ugt80.
drm.wt.ugt80.
drm.smt1.
drm.wt.smt1.
dsm.ugt80.
dsm.wt.ugt80.
dsm.smt1.
dsm.wt.smt1.
sp.ugt80.
sp.wt.ugt80.
sp.smt1.
sp.wt.smt1.
im.ugt80.
im.wt.ugt80.
im.smt1.
im.wt.smt1.

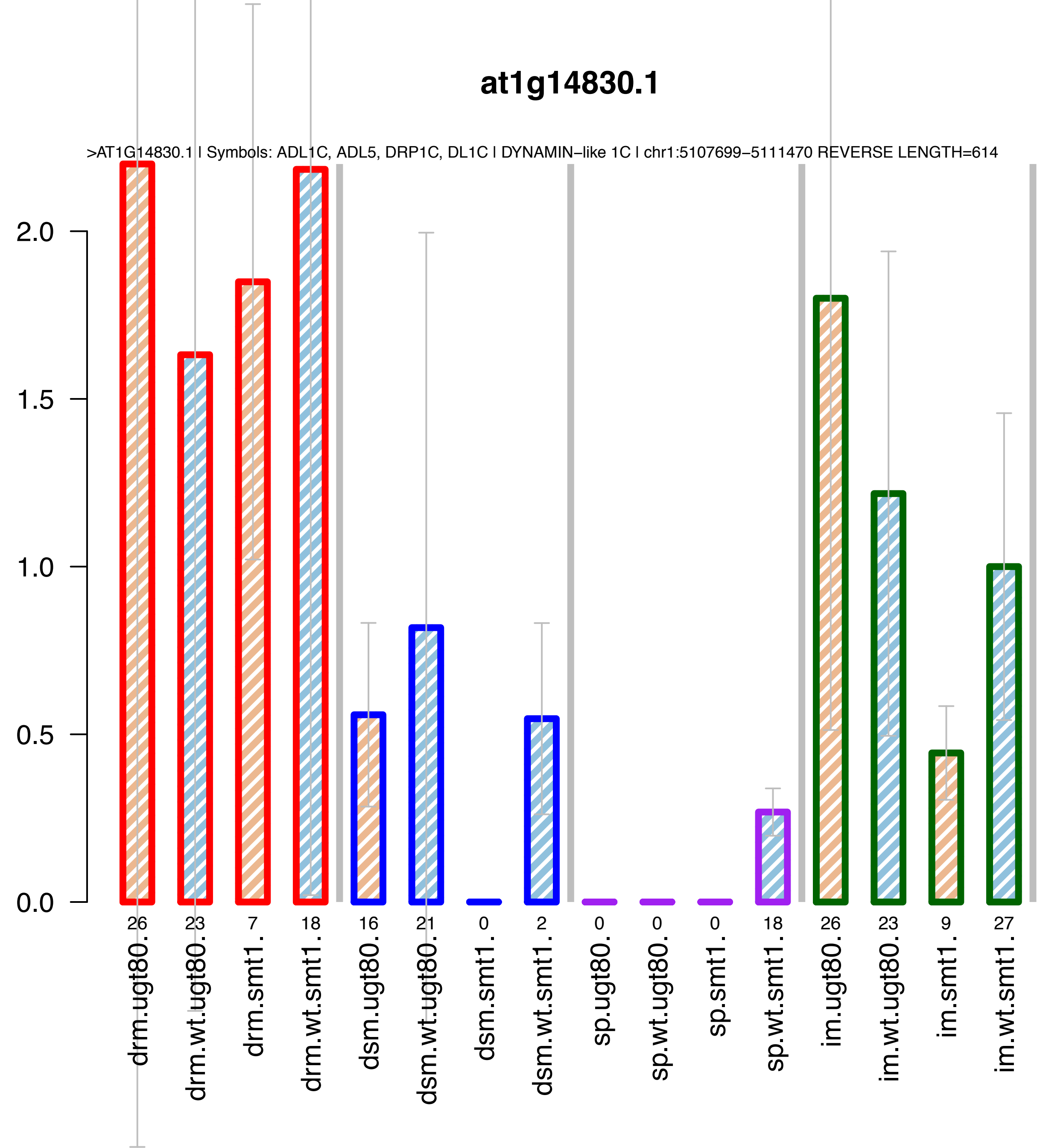

at1g14830.1
>AT1G14830.1 | Symbols: ADL1C, ADL5, DRP1C, DL1C | DYNAMIN-like 1C | chr1:5107699-5111470 REVERSE LENGTH=614
2.0
1.5
1.0
0.5
0.0
26
23
7
18
16
21
0
2
0
0
0
18
26
23
9
27
drm.ugt80.
drm.wt.ugt80.
drm.smt1.
drm.wt.smt1.
dsm.ugt80.
dsm.wt.ugt80.
dsm.smt1.
dsm.wt.smt1.
sp.ugt80.
sp.wt.ugt80.
sp.smt1.
sp.wt.smt1.
im.ugt80.
im.wt.ugt80.
im.smt1.
im.wt.smt1.

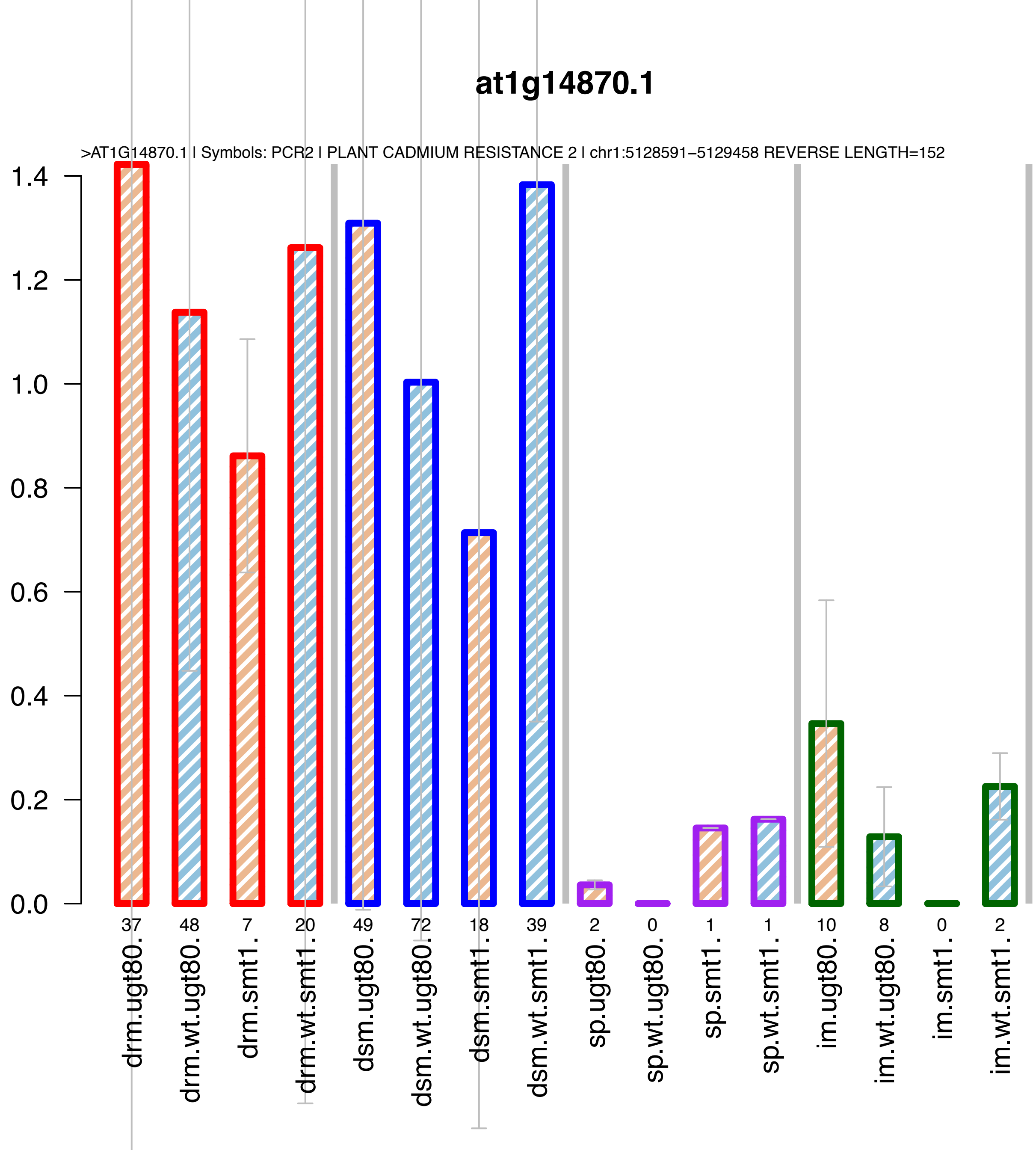

at1g14870.1
>AT1G14870.1 | Symbols: PCR2 | PLANT CADMIUM RESISTANCE 2 | chr1:5128591–5129458 REVERSE LENGTH=152
0.0
0.2
0.4
0.6
0.8
1.0
1.2
1.4
37
48
7
20
49
72
18
39
2
0
1
1
10
8
0
2
drm.ugt80..
drm.wt.ugt80..
drm.smt1..
drm.wt.smt1..
dsm.ugt80..
dsm.wt.ugt80..
dsm.smt1..
dsm.wt.smt1..
sp.ugt80..
sp.wt.ugt80..
sp.smt1..
sp.wt.smt1..
im.ugt80..
im.wt.ugt80..
im.smt1..
im.wt.smt1..

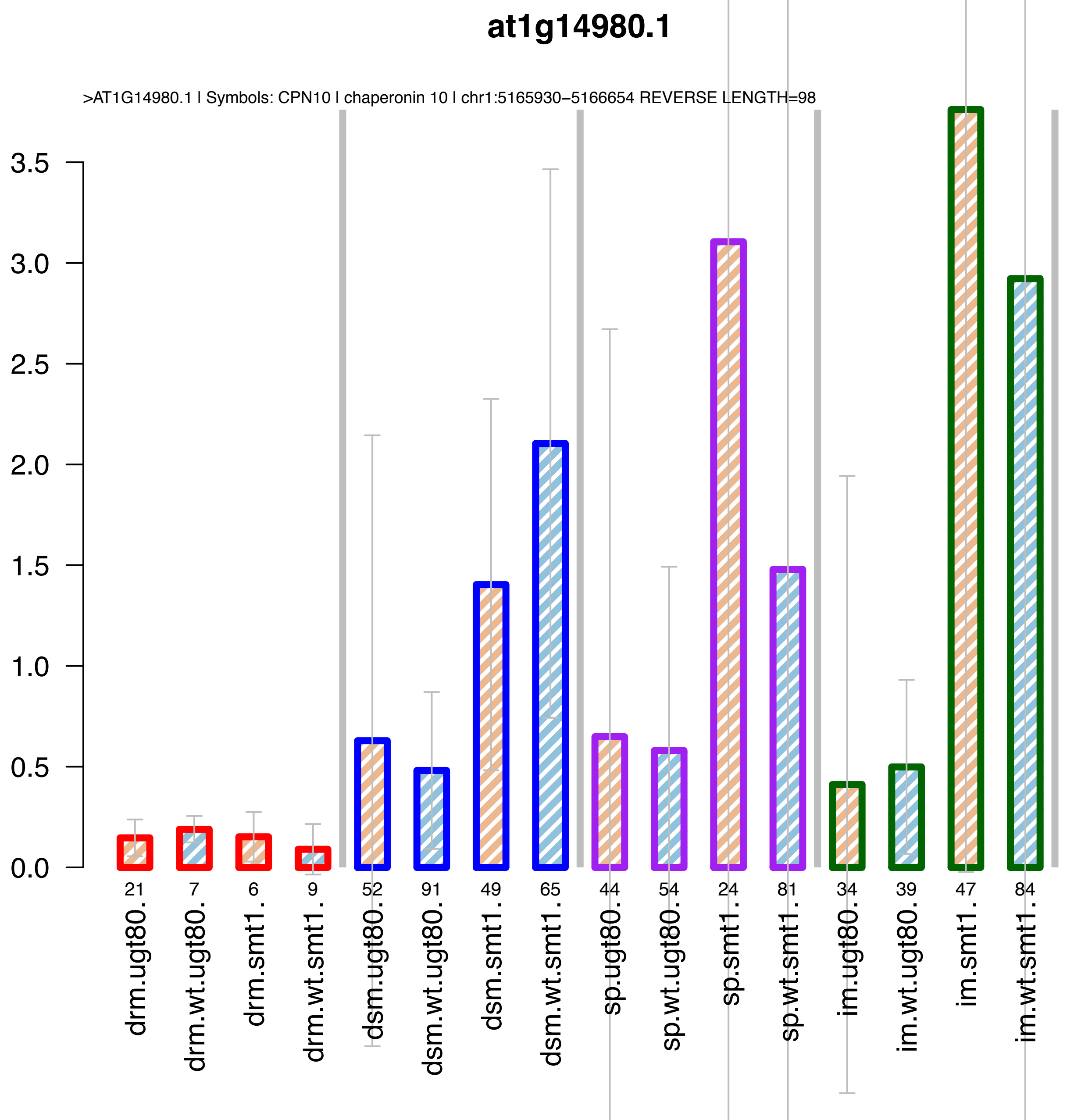

at1g14980.1
>AT1G14980.1 | Symbols: CPN10 | chaperonin 10 | chr1:5165930-5166654 REVERSE LENGTH=98
0.0
0.5
1.0
1.5
2.0
2.5
3.0
3.5
21
7
6
9
52
91
49
65
44
54
24
81
34
39
47
84
drm.ugt80..
drm.wt.ugt80..
drm.smt1..
drm.wt.smt1..
dsm.ugt80..
dsm.wt.ugt80..
dsm.smt1..
dsm.wt.smt1..
sp.ugt80..
sp.wt.ugt80..
sp.smt1..
sp.wt.smt1..
im.ugt80..
im.wt.ugt80..
im.smt1..
im.wt.smt1..

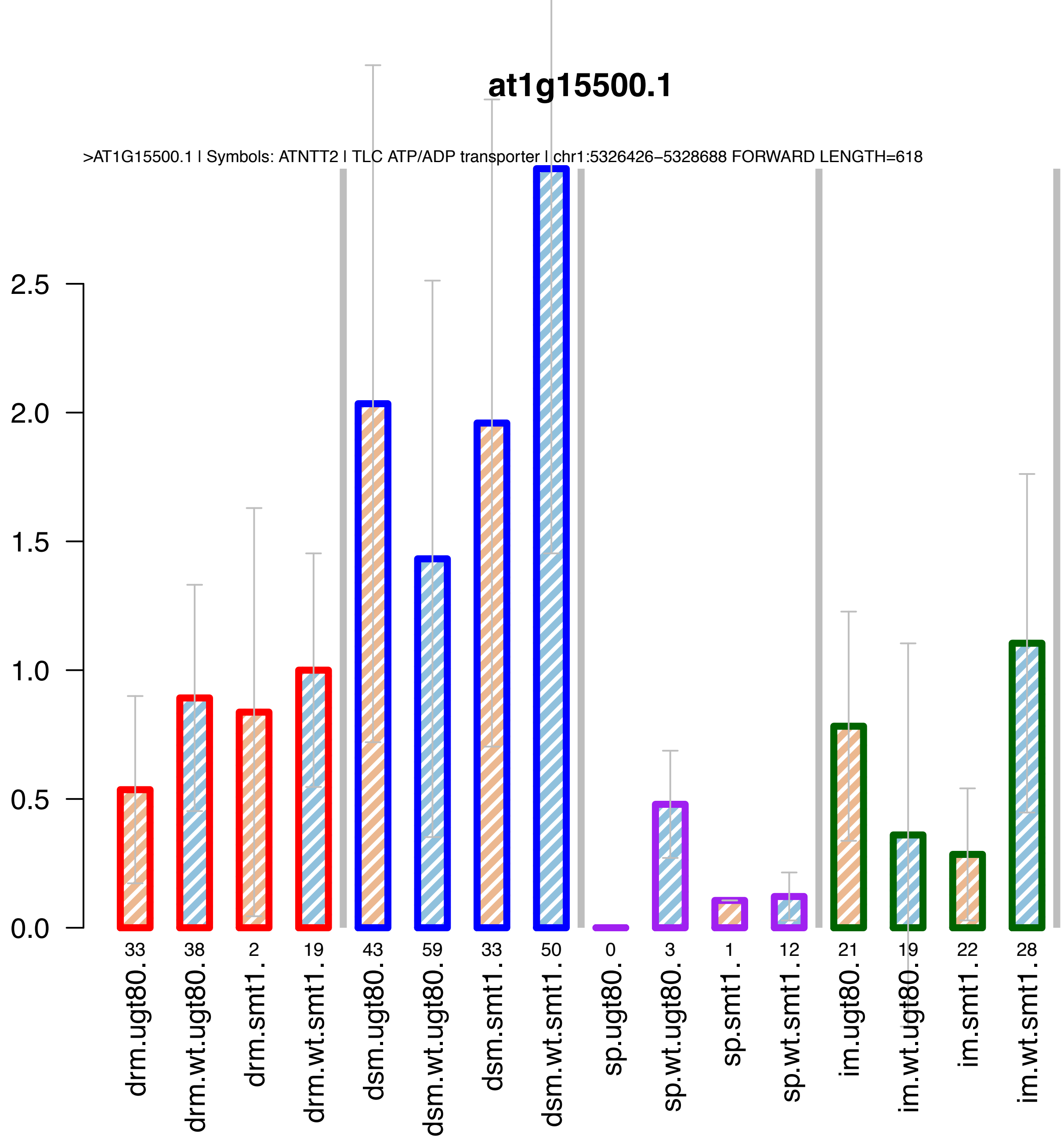

at1g15500.1
>AT1G15500.1 | Symbols: ATNTT2 | TLC ATP/ADP transporter | chr1:5326426-5328688 FORWARD LENGTH=618
0.0
0.5
1.0
1.5
2.0
2.5
33
38
2
19
43
59
33
50
0
3
1
12
21
19
22
28
drm.ugt80..
drm.wt.ugt80..
drm.smt1..
drm.wt.smt1..
dsm.ugt80..
dsm.wt.ugt80..
dsm.smt1..
dsm.wt.smt1..
sp.ugt80..
sp.wt.ugt80..
sp.smt1..
sp.wt.smt1..
im.ugt80..
im.wt.ugt80..
im.smt1..
im.wt.smt1..

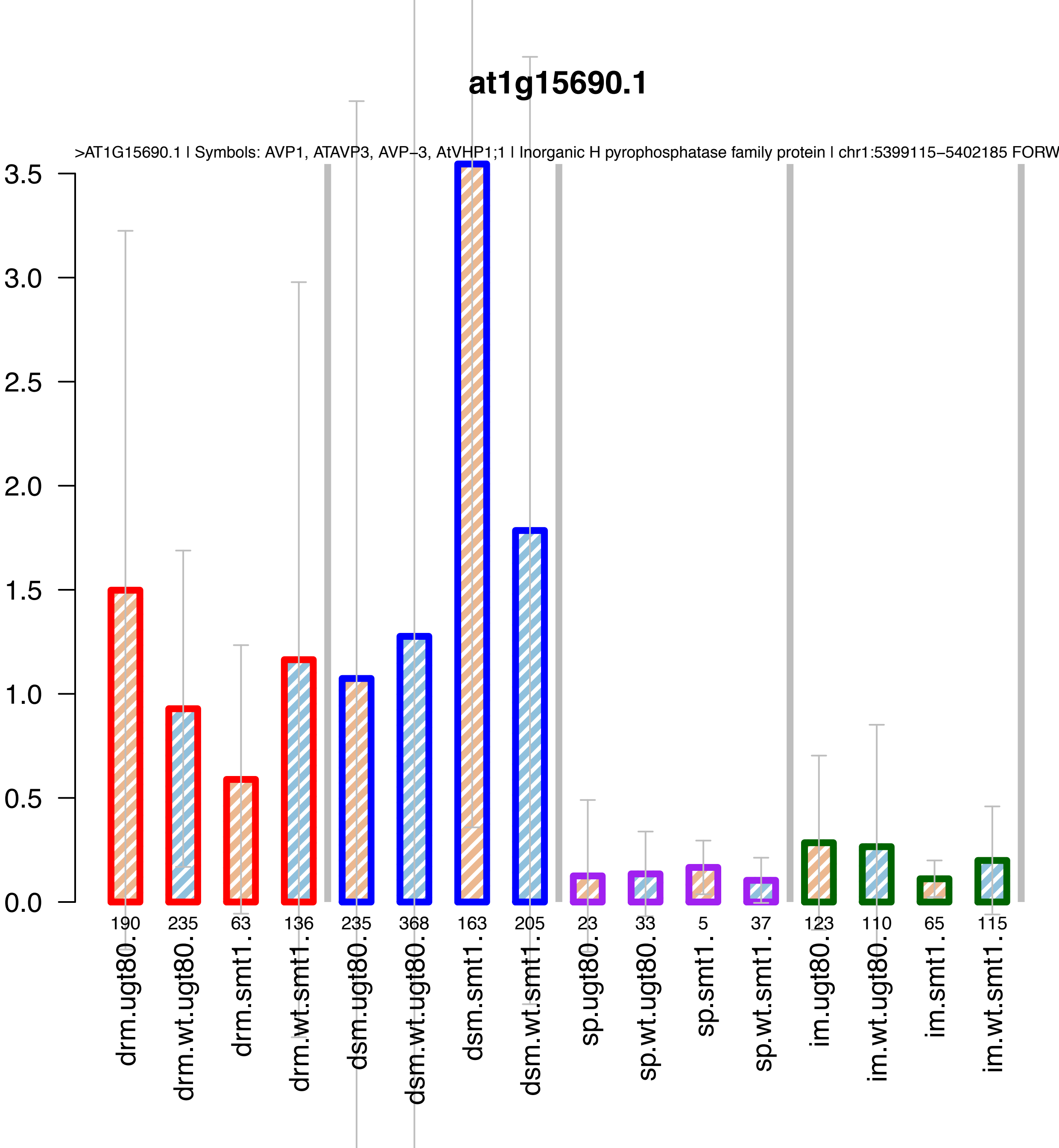
at1g15690.1
>AT1G15690.1 | Symbols: AVP1, ATAVP3, AVP−3, AtVHP1;1 | Inorganic H pyrophosphatase family protein | chr1:5399115−5402185 FORW
3.5
3.0
2.5
2.0
1.5
1.0
0.5
0.0
190
235
63
136
235
368
163
205
23
33
5
37
123
110
65
115
drm.ugt80..
drm.wt.ugt80..
drm.smt1..
drm.wt.smt1..
dsm.ugt80..
dsm.wt.ugt80..
dsm.smt1..
dsm.wt.smt1..
sp.ugt80..
sp.wt.ugt80..
sp.smt1..
sp.wt.smt1..
im.ugt80..
im.wt.ugt80..
im.smt1..
im.wt.smt1..

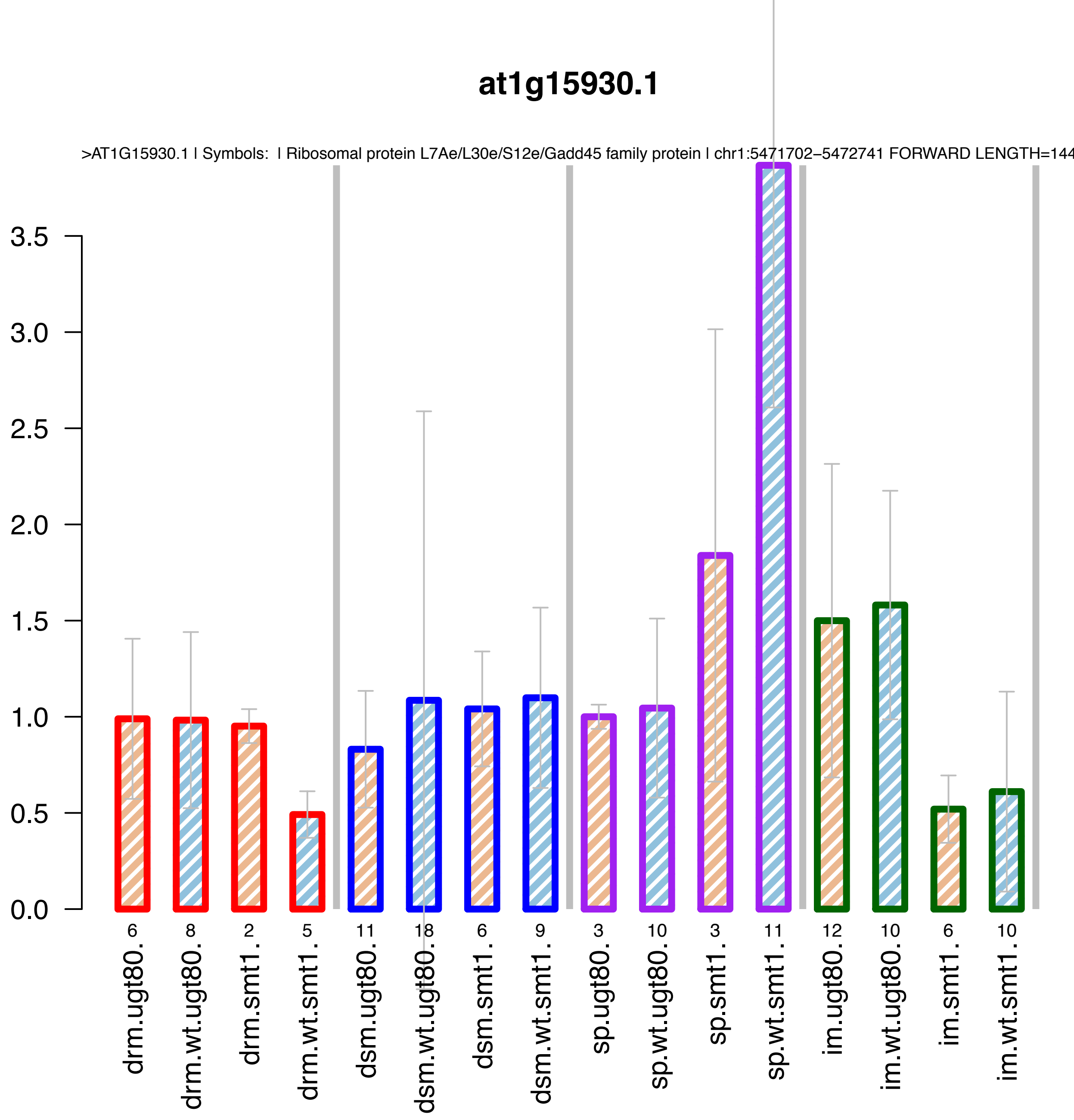
at1g15930.1
>AT1G15930.1 | Symbols: | Ribosomal protein L7Ae/L30e/S12e/Gadd45 family protein | chr1:5471702-5472741 FORWARD LENGTH=144
3.5
3.0
2.5
2.0
1.5
1.0
0.5
0.0
6
8
2
5
11
18
6
9
3
10
3
11
12
10
6
10
drm.ugt80..
drm.wt.ugt80..
drm.smt1..
drm.wt.smt1..
dsm.ugt80..
dsm.wt.ugt80..
dsm.smt1..
dsm.wt.smt1..
sp.ugt80..
sp.wt.ugt80..
sp.smt1..
sp.wt.smt1..
im.ugt80..
im.wt.ugt80..
im.smt1..
im.wt.smt1..

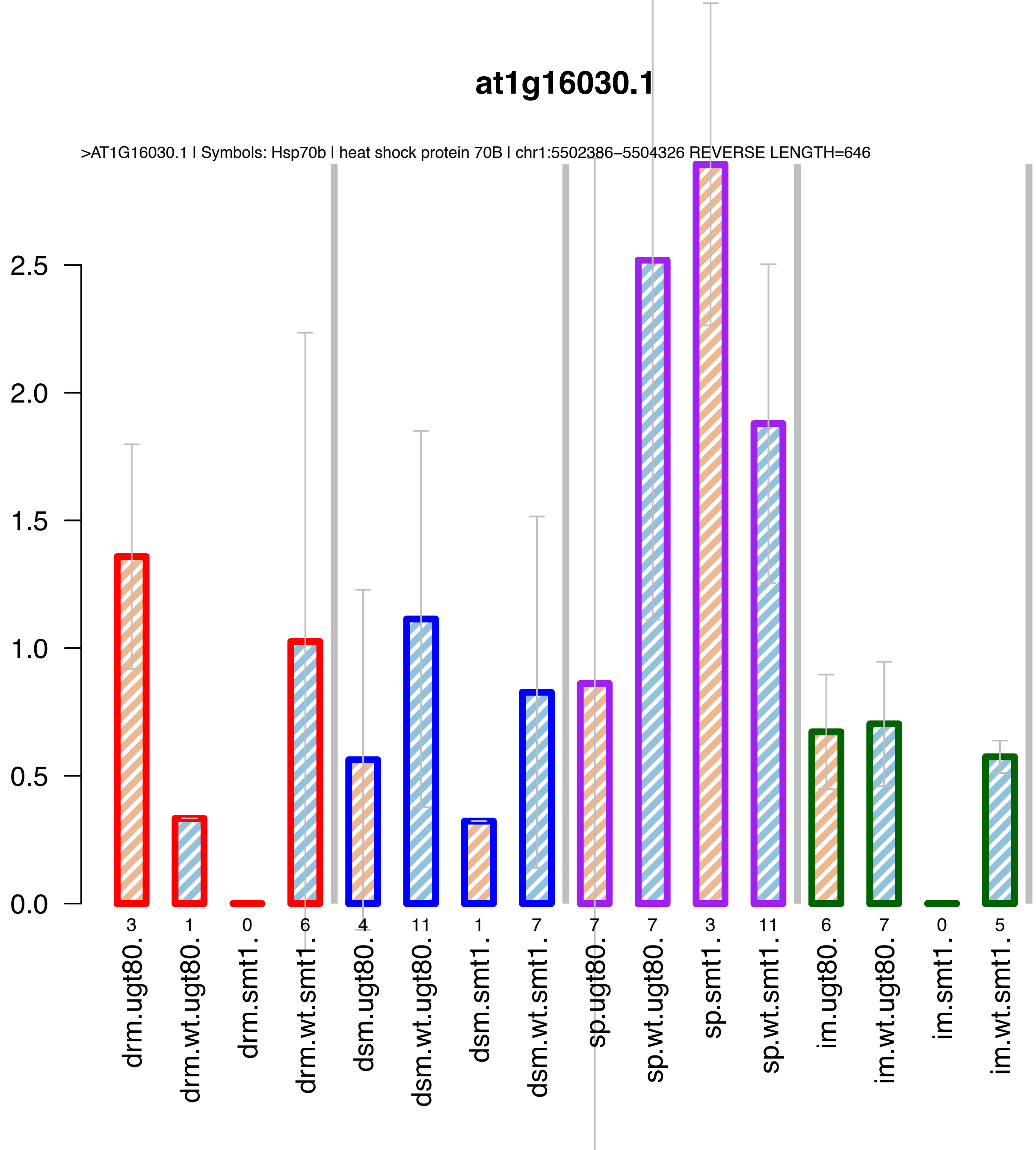

at1g16030.1
>AT1G16030.1 | Symbols: Hsp70b | heat shock protein 70B | chr1:5502386-5504326 REVERSE LENGTH=646
0.0
0.5
1.0
1.5
2.0
2.5
3
1
0
6
4
11
1
7
7
7
3
11
6
7
0
5
drm.ugt80..
drm.wt.ugt80..
drm.smt1..
drm.wt.smt1..
dsm.ugt80..
dsm.wt.ugt80..
dsm.smt1..
dsm.wt.smt1..
sp.ugt80..
sp.wt.ugt80..
sp.smt1..
sp.wt.smt1..
im.ugt80..
im.wt.ugt80..
im.smt1..
im.wt.smt1..

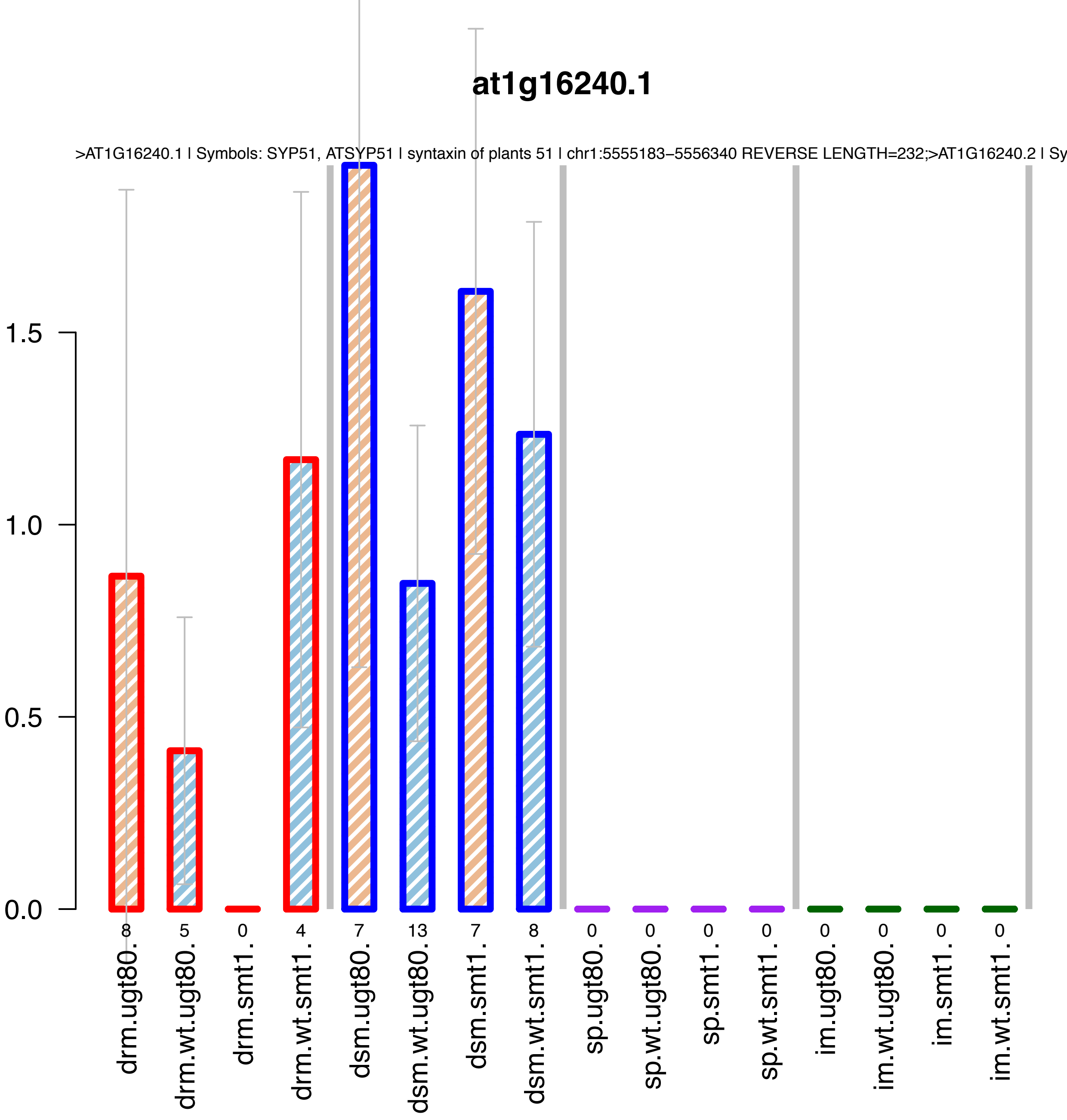
at1g16240.1
>AT1G16240.1 | Symbols: SYP51, ATSYP51 | syntaxin of plants 51 | chr1:5555183–5556340 REVERSE LENGTH=232;>AT1G16240.2 | Sy
1.5
1.0
0.5
0.0
8
5
0
4
7
13
7
8
0
0
0
0
0
0
0
0
drm.ugt80.
drm.wt.ugt80.
drm.smt1.
drm.wt.smt1.
dsm.ugt80.
dsm.wt.ugt80.
dsm.smt1.
dsm.wt.smt1.
sp.ugt80.
sp.wt.ugt80.
sp.smt1.
sp.wt.smt1.
im.ugt80.
im.wt.ugt80.
im.smt1.
im.wt.smt1.

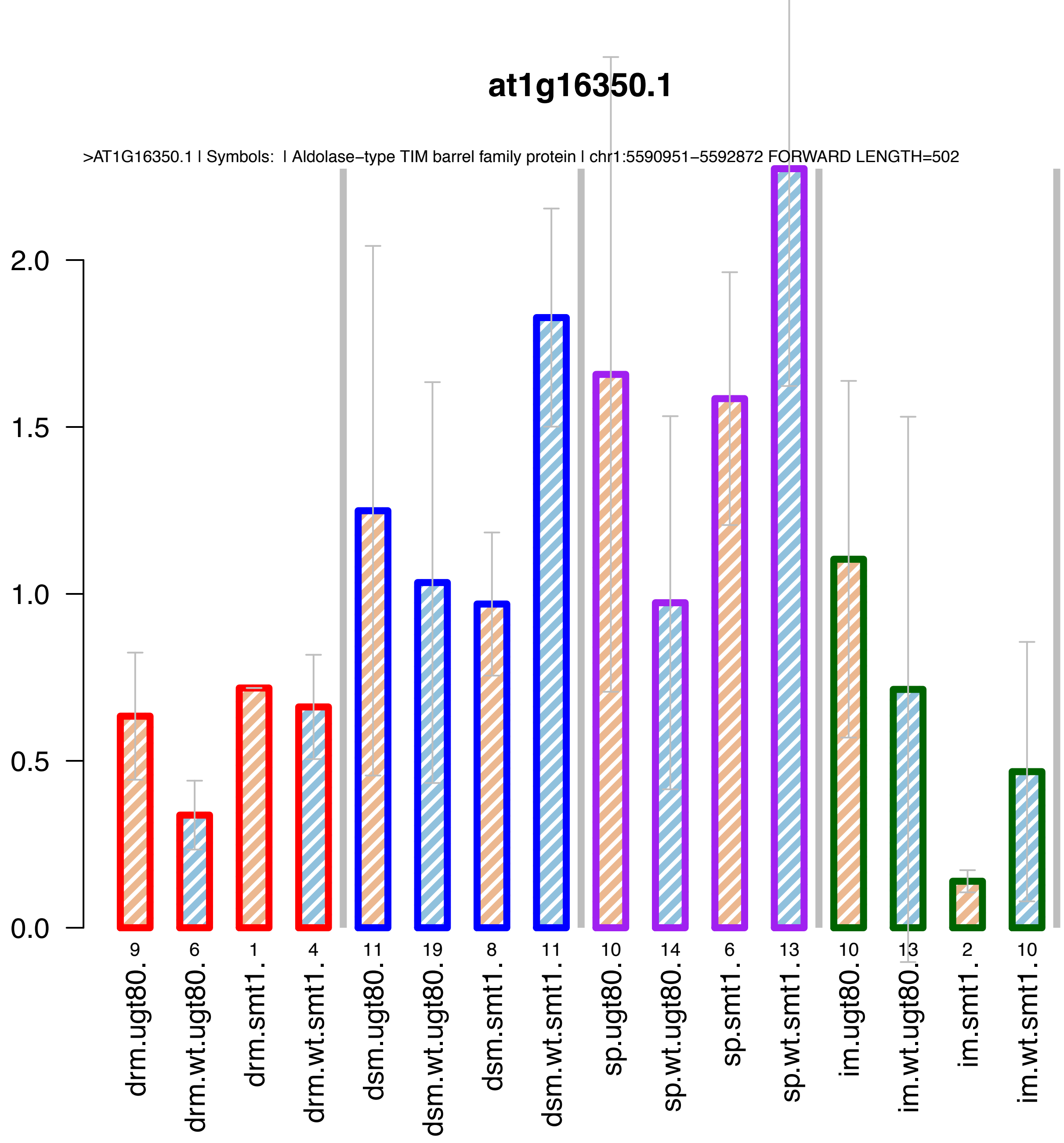

at1g16350.1
>AT1G16350.1 | Symbols: | Aldolase−type TIM barrel family protein | chr1:5590951−5592872 FORWARD LENGTH=502
2.0
1.5
1.0
0.5
0.0
9
6
1
4
11
19
8
11
10
14
6
13
10
13
2
10
drm.ugt80..
drm.wt.ugt80..
drm.smt1..
drm.wt.smt1..
dsm.ugt80..
dsm.wt.ugt80..
dsm.smt1..
dsm.wt.smt1..
sp.ugt80..
sp.wt.ugt80..
sp.smt1..
sp.wt.smt1..
im.ugt80..
im.wt.ugt80..
im.smt1..
im.wt.smt1..

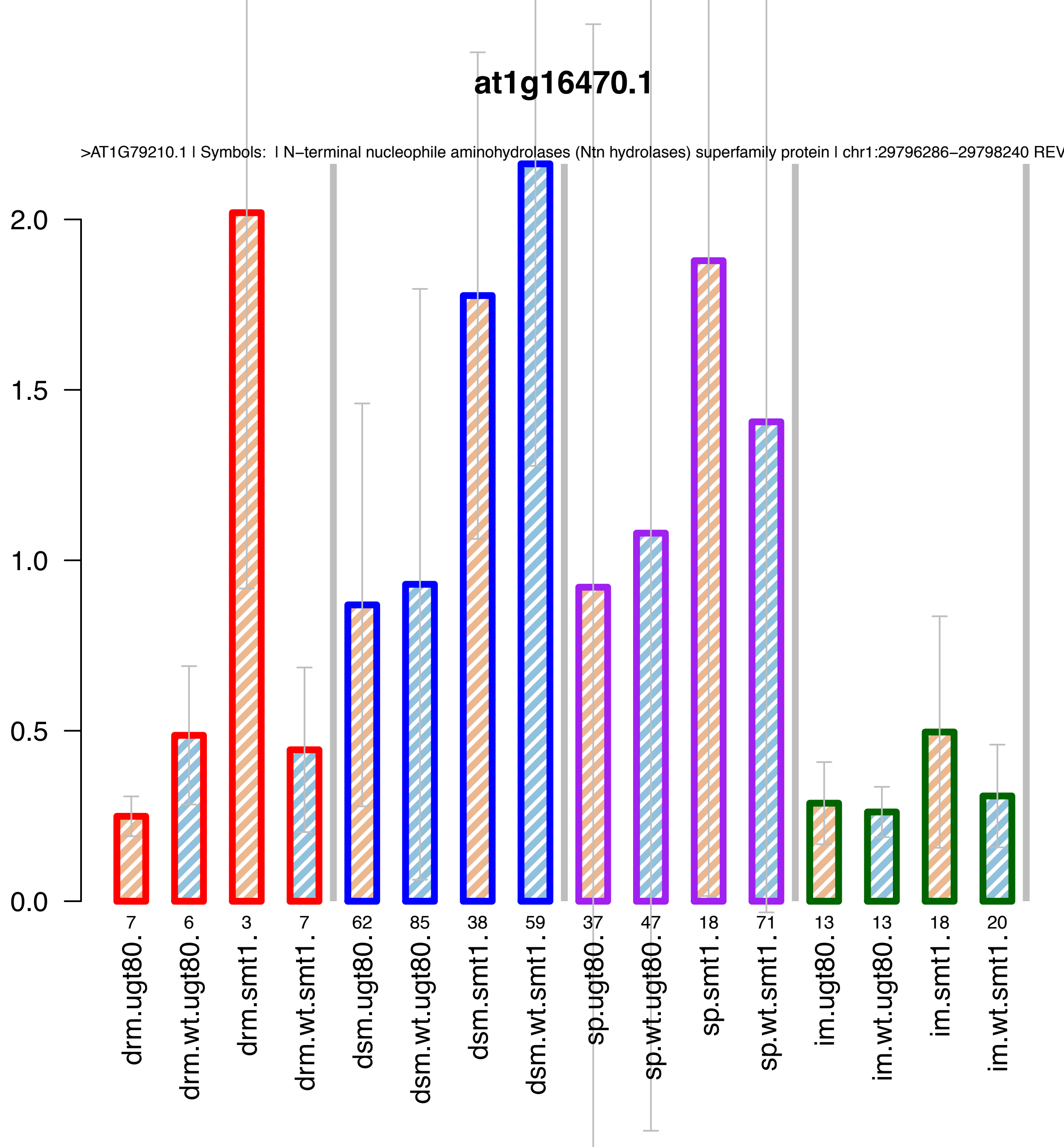
at1g16470.1
>AT1G79210.1 | Symbols: | N−terminal nucleophile aminohydrolases (Ntn hydrolases) superfamily protein | chr1:29796286−29798240 REV
2.0
1.5
1.0
0.5
0.0
7
6
3
7
62
85
38
59
37
47
18
71
13
13
18
20
drm.ugt80.
drm.wt.ugt80.
drm.smt1.
drm.wt.smt1.
dsm.ugt80.
dsm.wt.ugt80.
dsm.smt1.
dsm.wt.smt1.
sp.ugt80.
sp.wt.ugt80.
sp.smt1.
sp.wt.smt1.
im.ugt80.
im.wt.ugt80.
im.smt1.
im.wt.smt1.

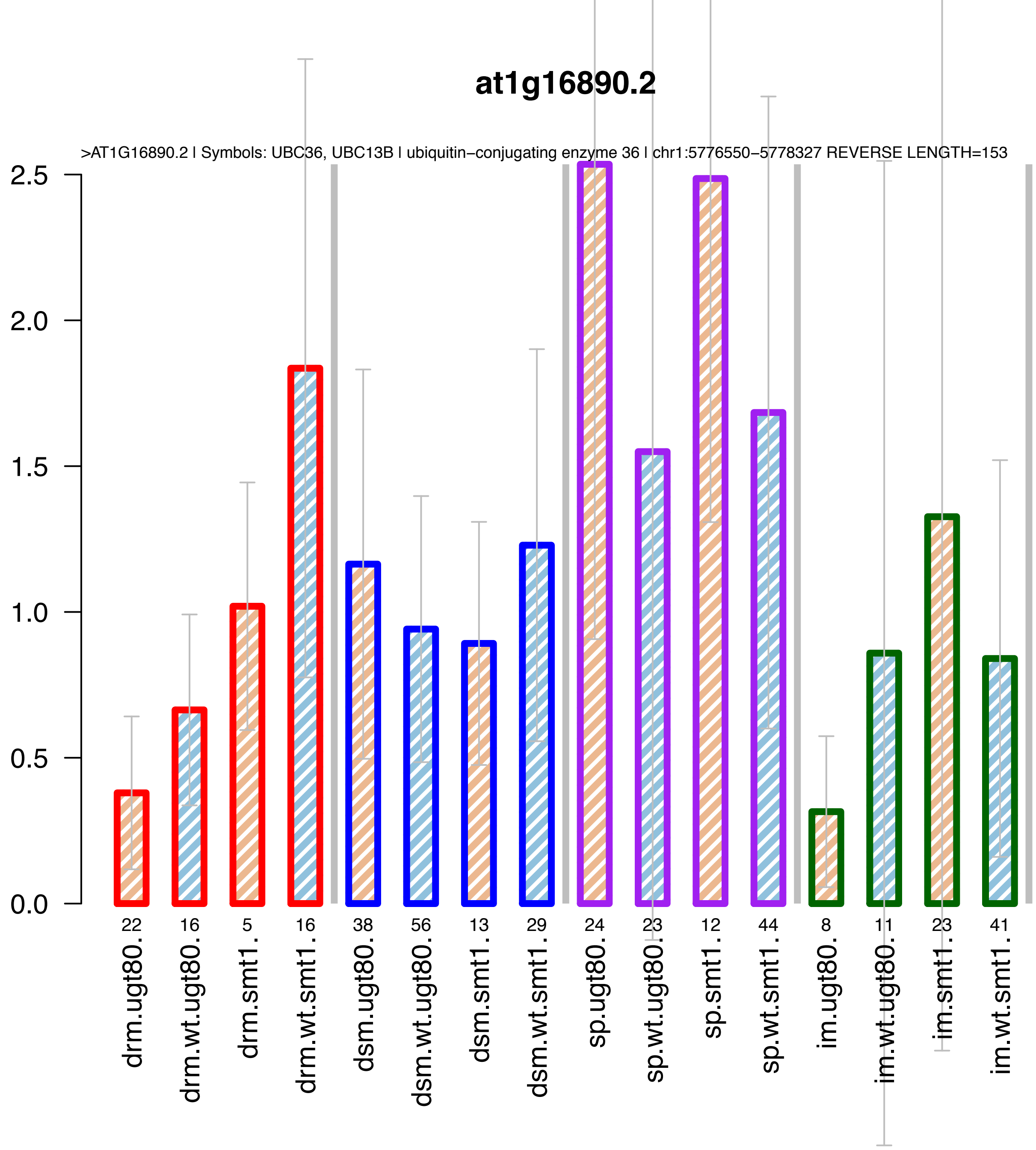
at1g16890.2
>AT1G16890.2 | Symbols: UBC36, UBC13B | ubiquitin−conjugating enzyme 36 | chr1:5776550−5778327 REVERSE LENGTH=153
2.5
2.0
1.5
1.0
0.5
0.0
22
16
5
16
38
56
13
29
24
23
12
44
8
11
23
41
drm.ugt80..
drm.wt.ugt80..
drm.smt1..
drm.wt.smt1..
dsm.ugt80..
dsm.wt.ugt80..
dsm.smt1..
dsm.wt.smt1..
sp.ugt80..
sp.wt.ugt80..
sp.smt1..
sp.wt.smt1..
im.ugt80..
im.wt.ugt80..
im.smt1..
im.wt.smt1..

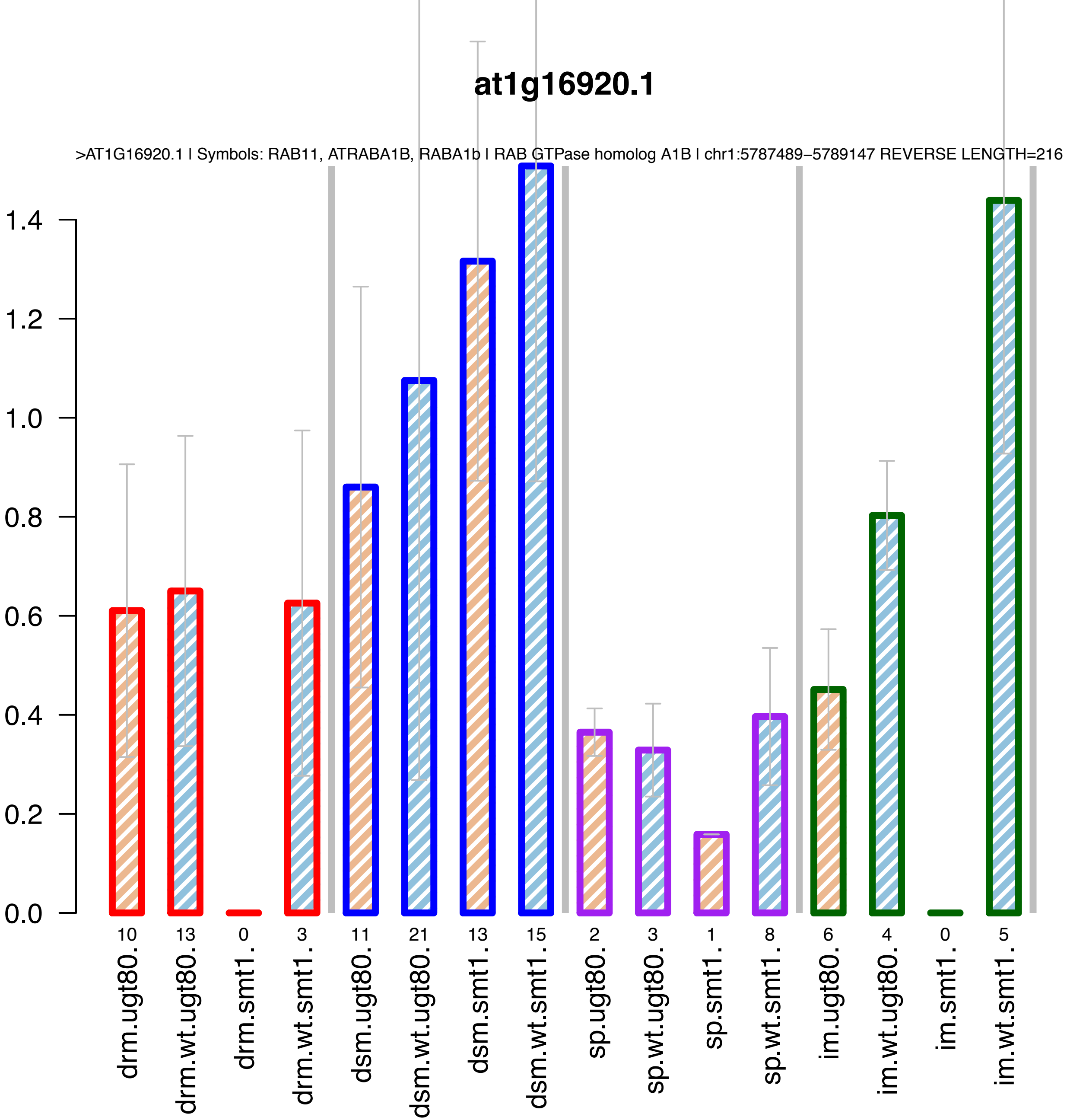
at1g16920.1
>AT1G16920.1 | Symbols: RAB11, ATRABA1B, RABA1b | RAB GTPase homolog A1B | chr1:5787489−5789147 REVERSE LENGTH=216
1.4
1.2
1.0
0.8
0.6
0.4
0.2
0.0
10
13
0
3
11
21
13
15
2
3
1
8
6
4
0
5
drm.ugt80.
drm.wt.ugt80.
drm.smt1.
drm.wt.smt1.
dsm.ugt80.
dsm.wt.ugt80.
dsm.smt1.
dsm.wt.smt1.
sp.ugt80.
sp.wt.ugt80.
sp.smt1.
sp.wt.smt1.
im.ugt80.
im.wt.ugt80.
im.smt1.
im.wt.smt1.

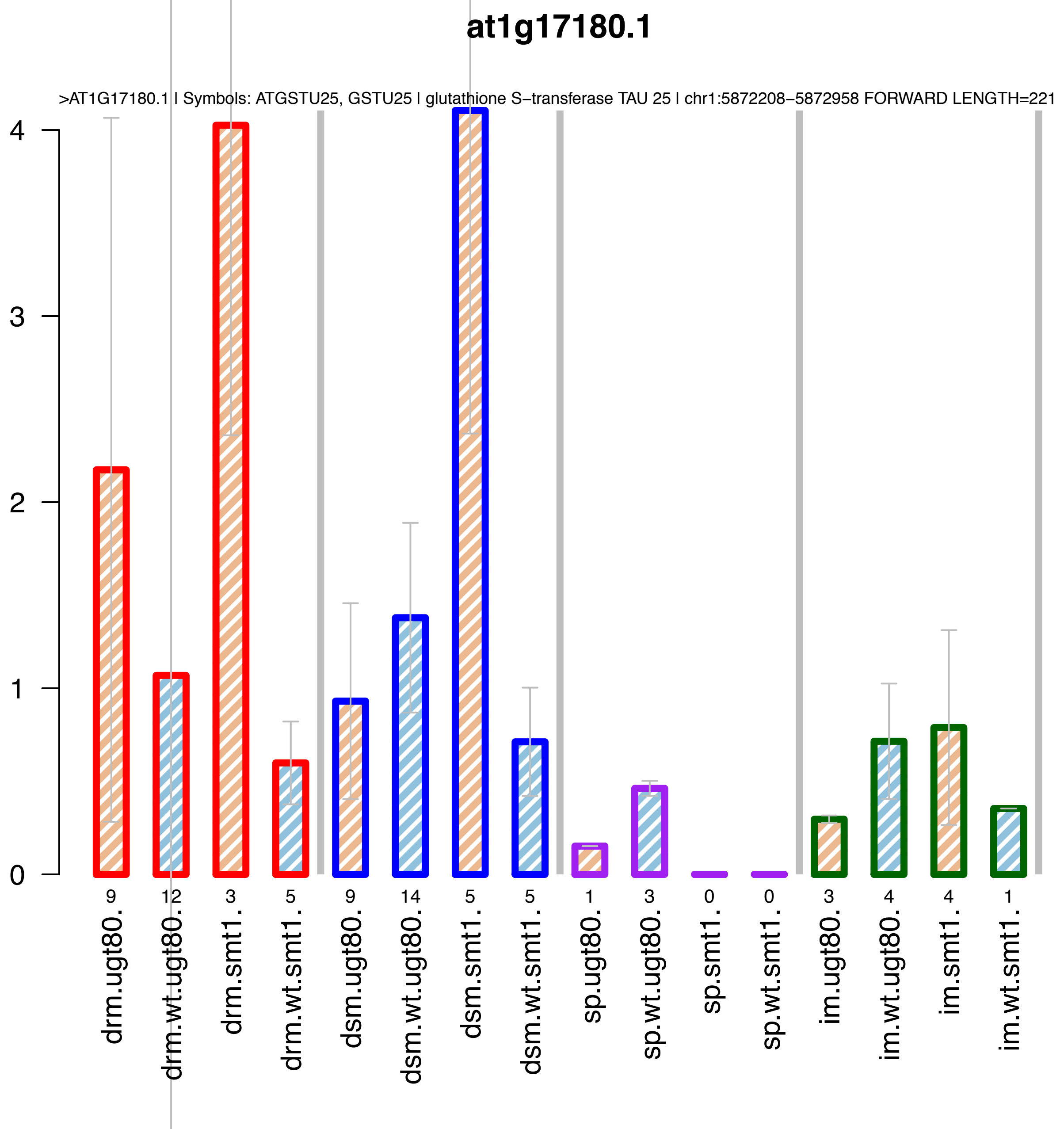
at1g17180.1
>AT1G17180.1 | Symbols: ATGSTU25, GSTU25 | glutathione S−transferase TAU 25 | chr1:5872208−5872958 FORWARD LENGTH=221
0
1
2
3
4
9
12
3
5
9
14
5
5
1
3
0
0
3
4
4
1
drm.ugt80.
drm.wt.ugt80.
drm.smt1.
drm.wt.smt1.
dsm.ugt80.
dsm.wt.ugt80.
dsm.smt1.
dsm.wt.smt1.
sp.ugt80.
sp.wt.ugt80.
sp.smt1.
sp.wt.smt1.
im.ugt80.
im.wt.ugt80.
im.smt1.
im.wt.smt1.

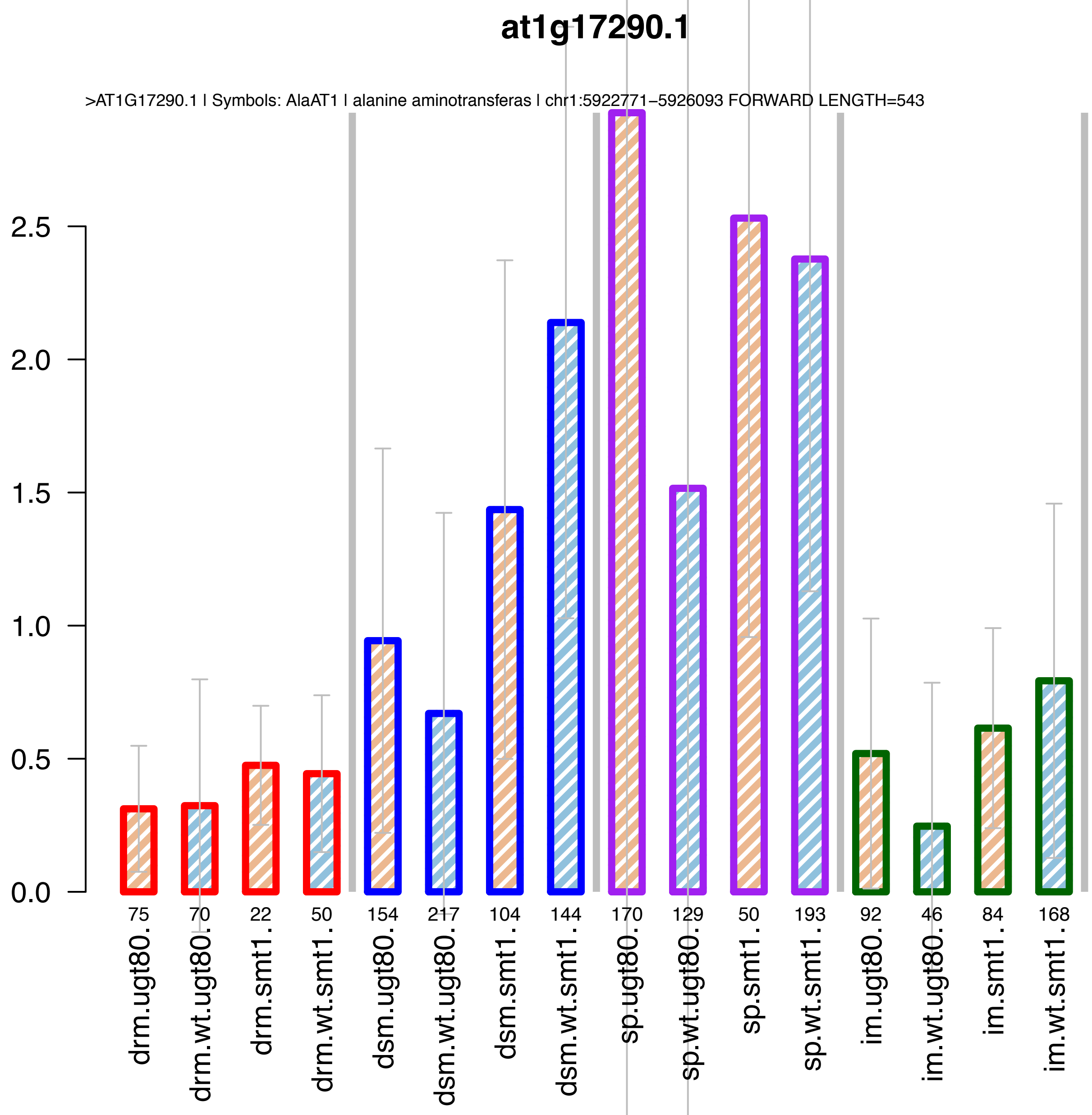
at1g17290.1
>AT1G17290.1 | Symbols: AlaAT1 | alanine aminotransferas | chr1:5922771−5926093 FORWARD LENGTH=543
0.0
0.5
1.0
1.5
2.0
2.5
75
70
22
50
154
217
104
144
170
129
50
193
92
46
84
168
drm.ugt80..
drm.wt.ugt80..
drm.smt1..
drm.wt.smt1..
dsm.ugt80..
dsm.wt.ugt80..
dsm.smt1..
dsm.wt.smt1..
sp.ugt80..
sp.wt.ugt80..
sp.smt1..
sp.wt.smt1..
im.ugt80..
im.wt.ugt80..
im.smt1..
im.wt.smt1..

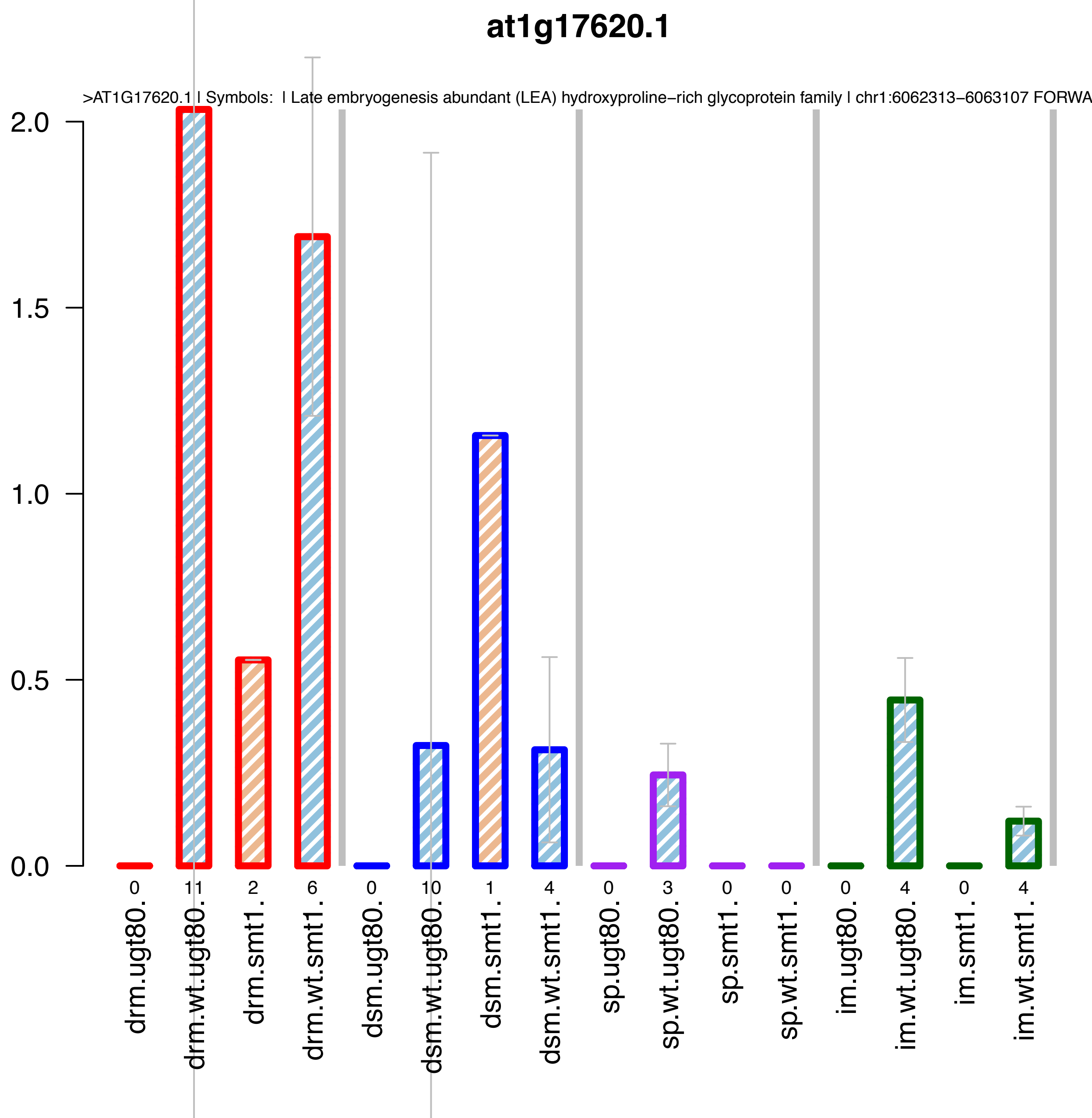
at1g17620.1
>AT1G17620.1 | Symbols: | Late embryogenesis abundant (LEA) hydroxyproline-rich glycoprotein family | chr1:6062313-6063107 FORWA
2.0
1.5
1.0
0.5
0.0
0
11
2
6
0
10
1
4
0
3
0
0
0
4
0
4
drm.ugt80.
drm.wt.ugt80.
drm.smt1.
drm.wt.smt1.
dsm.ugt80.
dsm.wt.ugt80.
dsm.smt1.
dsm.wt.smt1.
sp.ugt80.
sp.wt.ugt80.
sp.smt1.
sp.wt.smt1.
im.ugt80.
im.wt.ugt80.
im.smt1.
im.wt.smt1.

# at1g17880.1

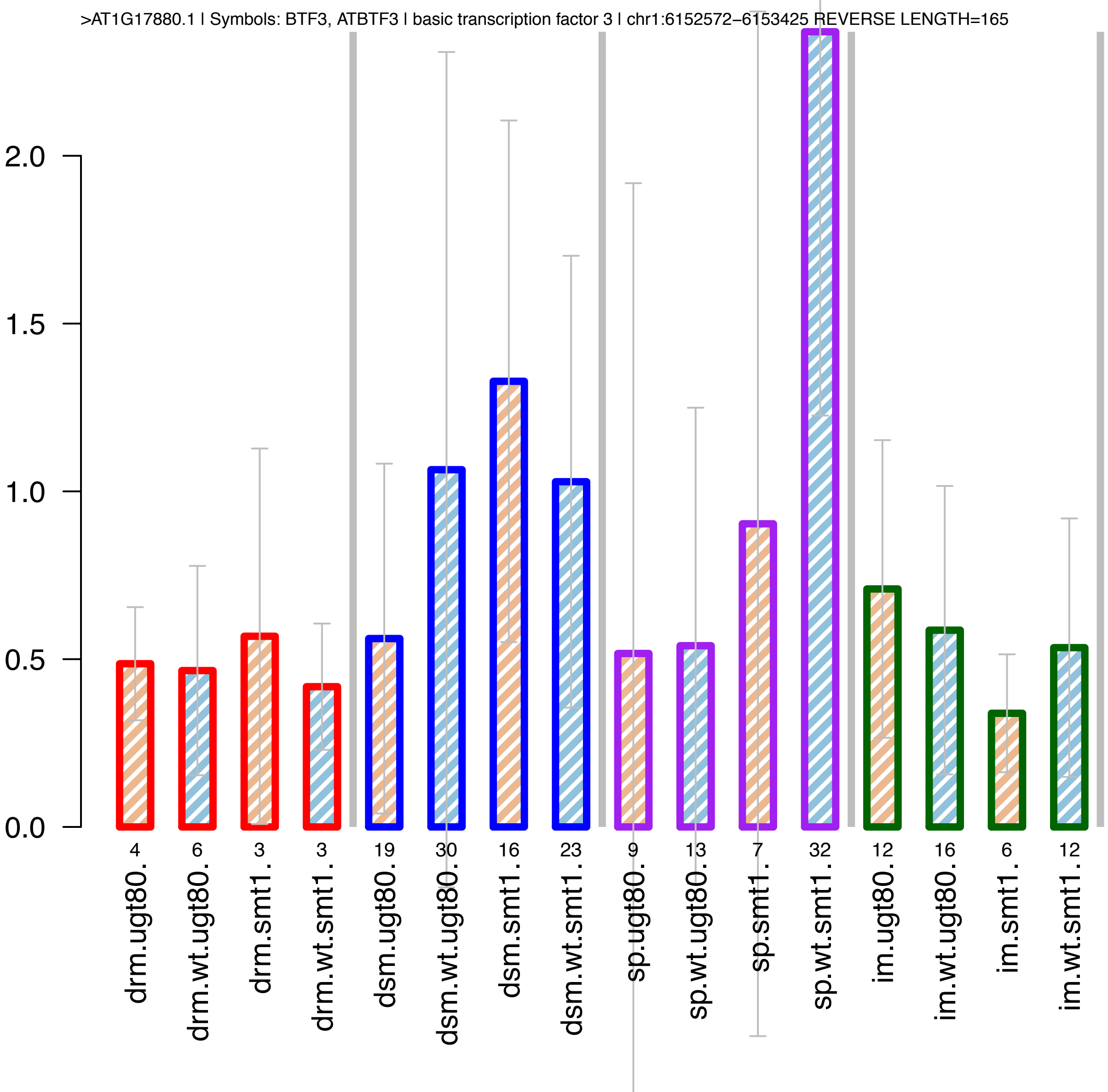

>AT1G17880.1 | Symbols: BTF3, ATBTF3 | basic transcription factor 3 | chr1:6152572–6153425 REVERSE LENGTH=165
0.0
0.5
1.0
1.5
2.0
4
6
3
3
19
30
16
23
9
13
7
32
12
16
6
12
drm.ugt80..
drm.wt.ugt80..
drm.smt1..
drm.wt.smt1..
dsm.ugt80..
dsm.wt.ugt80..
dsm.smt1..
dsm.wt.smt1..
sp.ugt80..
sp.wt.ugt80..
sp.smt1..
sp.wt.smt1..
im.ugt80..
im.wt.ugt80..
im.smt1..
im.wt.smt1..

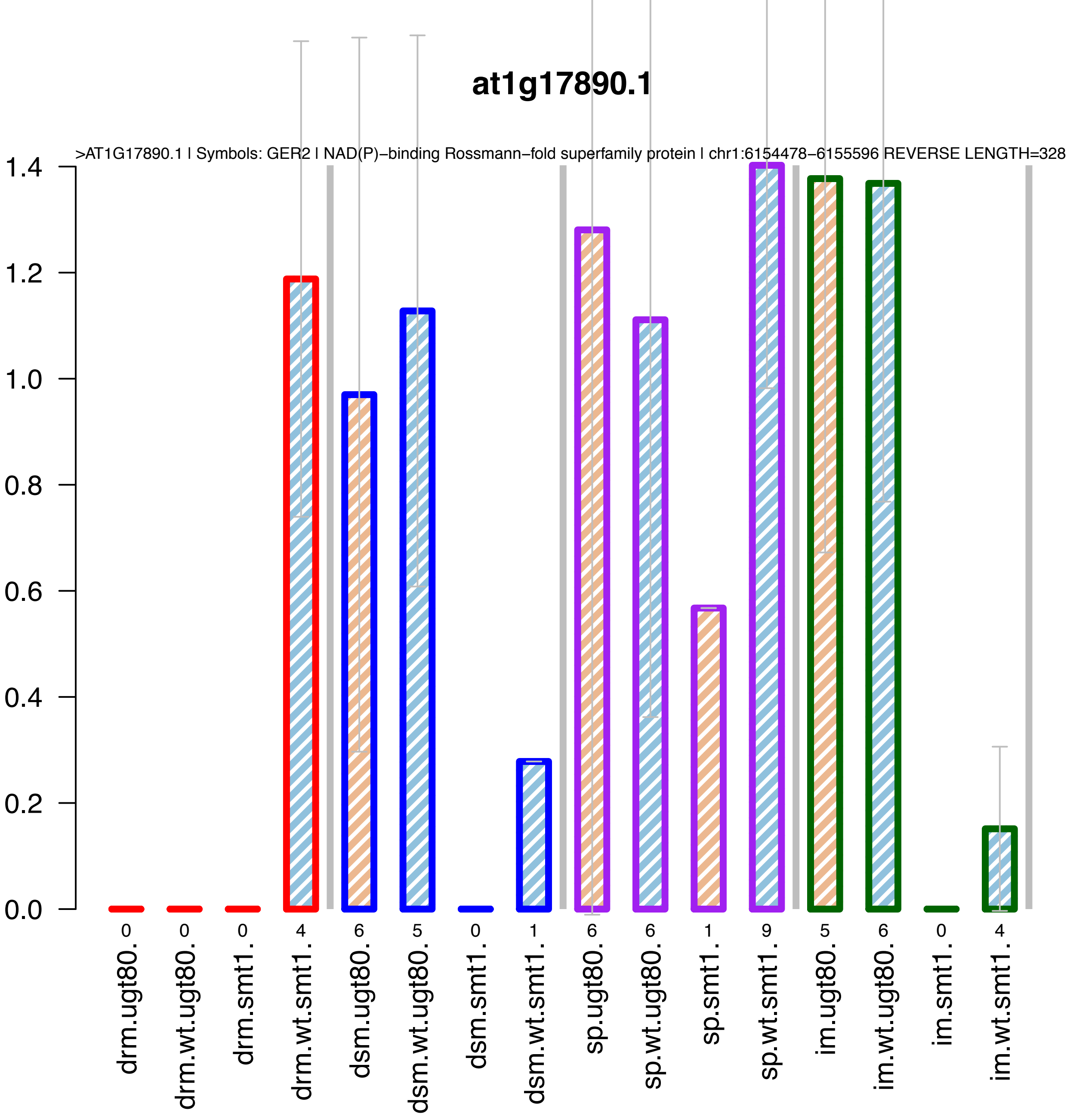

at1g17890.1
>AT1G17890.1 | Symbols: GER2 | NAD(P)-binding Rossmann-fold superfamily protein | chr1:6154478-6155596 REVERSE LENGTH=328
1.4
1.2
1.0
0.8
0.6
0.4
0.2
0.0
0 0 0 4 6 5 0 1 6 6 1 9 5 6 0 4
drm.ugt80.
drm.wt.ugt80.
drm.smt1.
drm.wt.smt1.
dsm.ugt80.
dsm.wt.ugt80.
dsm.smt1.
dsm.wt.smt1.
sp.ugt80.
sp.wt.ugt80.
sp.smt1.
sp.wt.smt1.
im.ugt80.
im.wt.ugt80.
im.smt1.
im.wt.smt1.

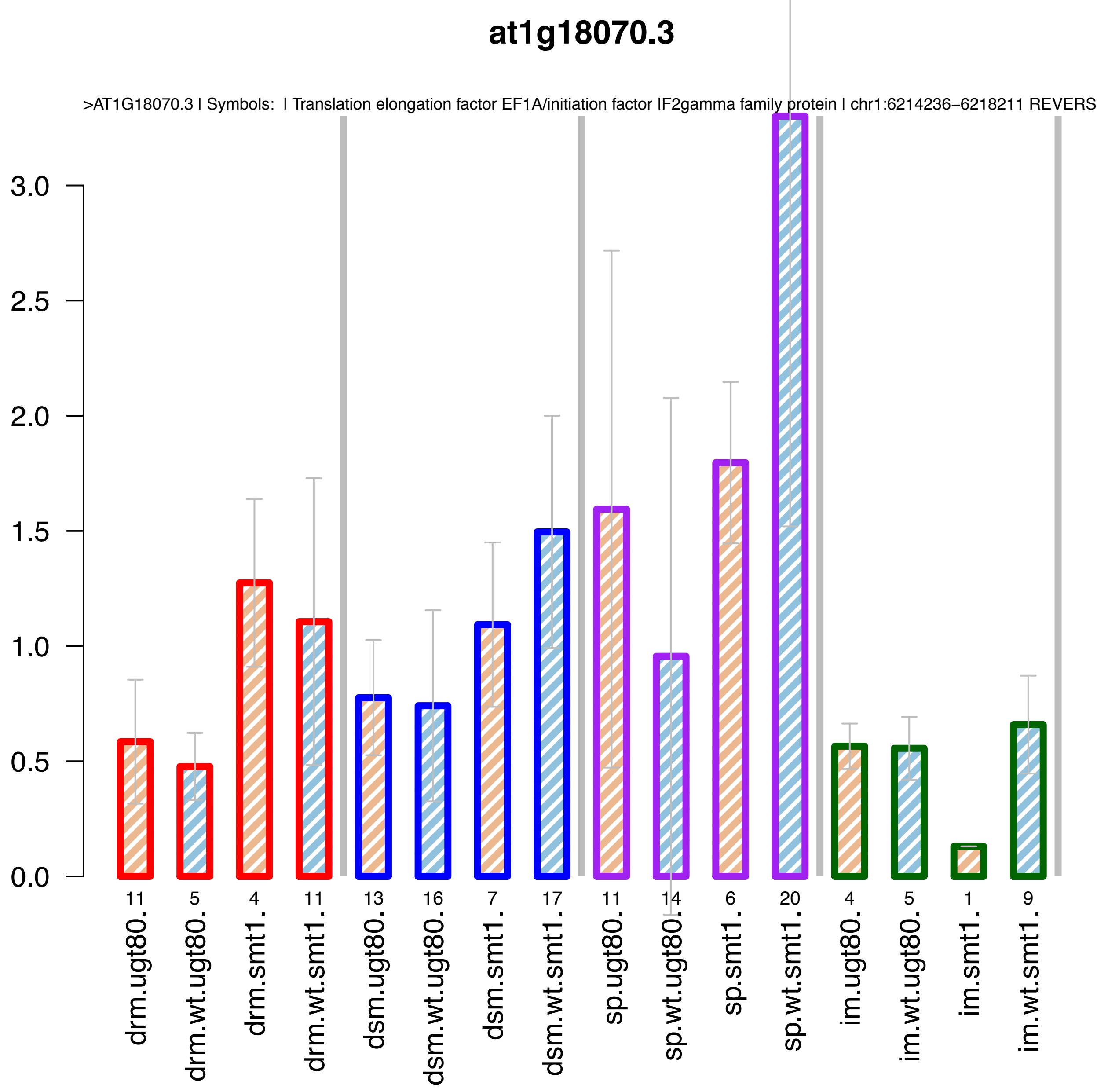

at1g18070.3
>AT1G18070.3 | Symbols: | Translation elongation factor EF1A/initiation factor IF2gamma family protein | chr1:6214236-6218211 REVERS
3.0
2.5
2.0
1.5
1.0
0.5
0.0
11
5
4
11
13
16
7
17
11
14
6
20
4
5
1
9
drm.ugt80..
drm.wt.ugt80..
drm.smt1..
drm.wt.smt1..
dsm.ugt80..
dsm.wt.ugt80..
dsm.smt1..
dsm.wt.smt1..
sp.ugt80..
sp.wt.ugt80..
sp.smt1..
sp.wt.smt1..
im.ugt80..
im.wt.ugt80..
im.smt1..
im.wt.smt1..

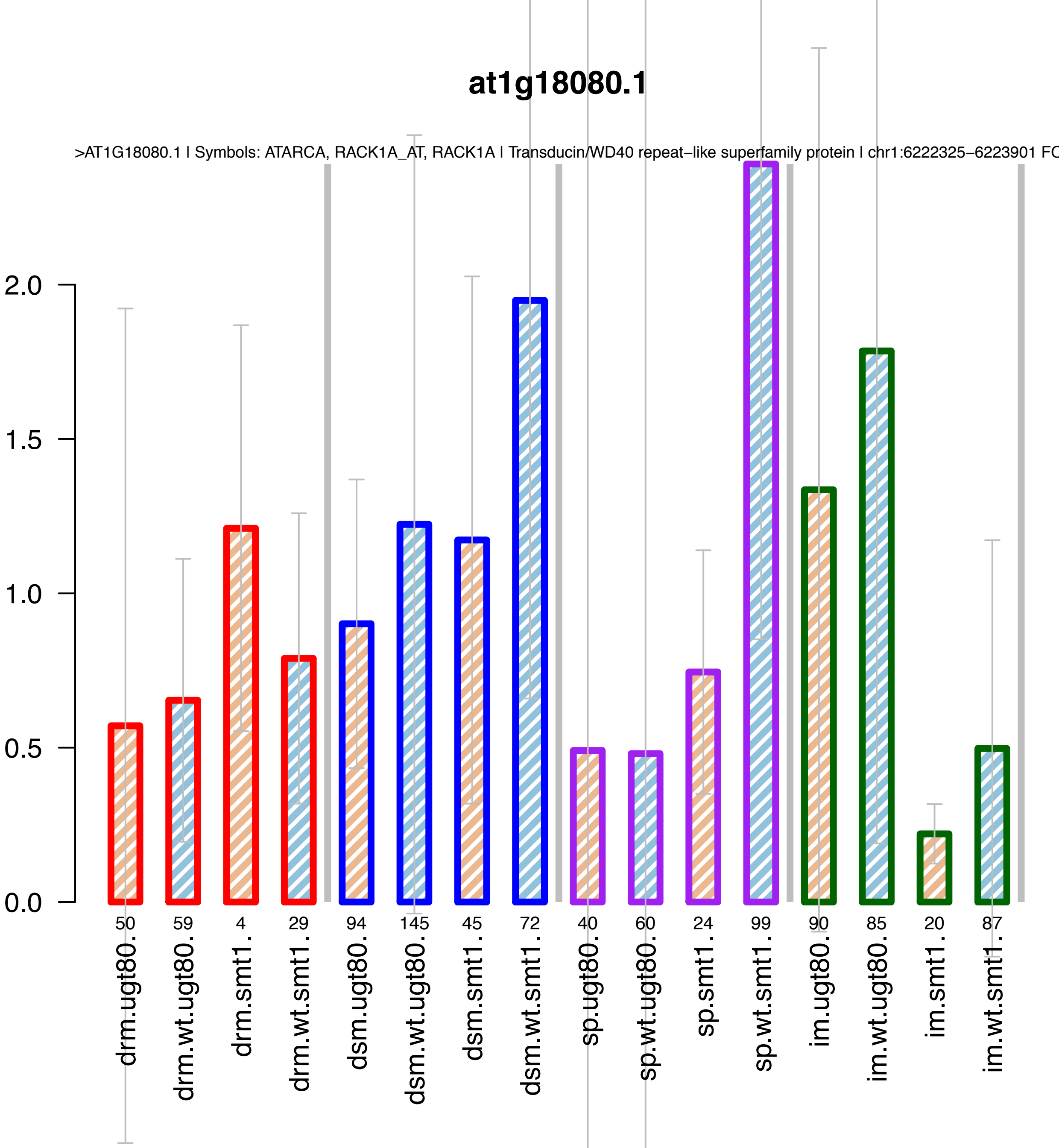

at1g18080.1
>AT1G18080.1 | Symbols: ATARCA, RACK1A_AT, RACK1A | Transducin/WD40 repeat-like superfamily protein | chr1:6222325-6223901 FC
2.0
1.5
1.0
0.5
0.0
50
59
4
29
94
145
45
72
40
60
24
99
90
85
20
87
drm.ugt80..
drm.wt.ugt80..
drm.smt1..
drm.wt.smt1..
dsm.ugt80..
dsm.wt.ugt80..
dsm.smt1..
dsm.wt.smt1..
sp.ugt80..
sp.wt.ugt80..
sp.smt1..
sp.wt.smt1..
im.ugt80..
im.wt.ugt80..
im.smt1..
im.wt.smt1..

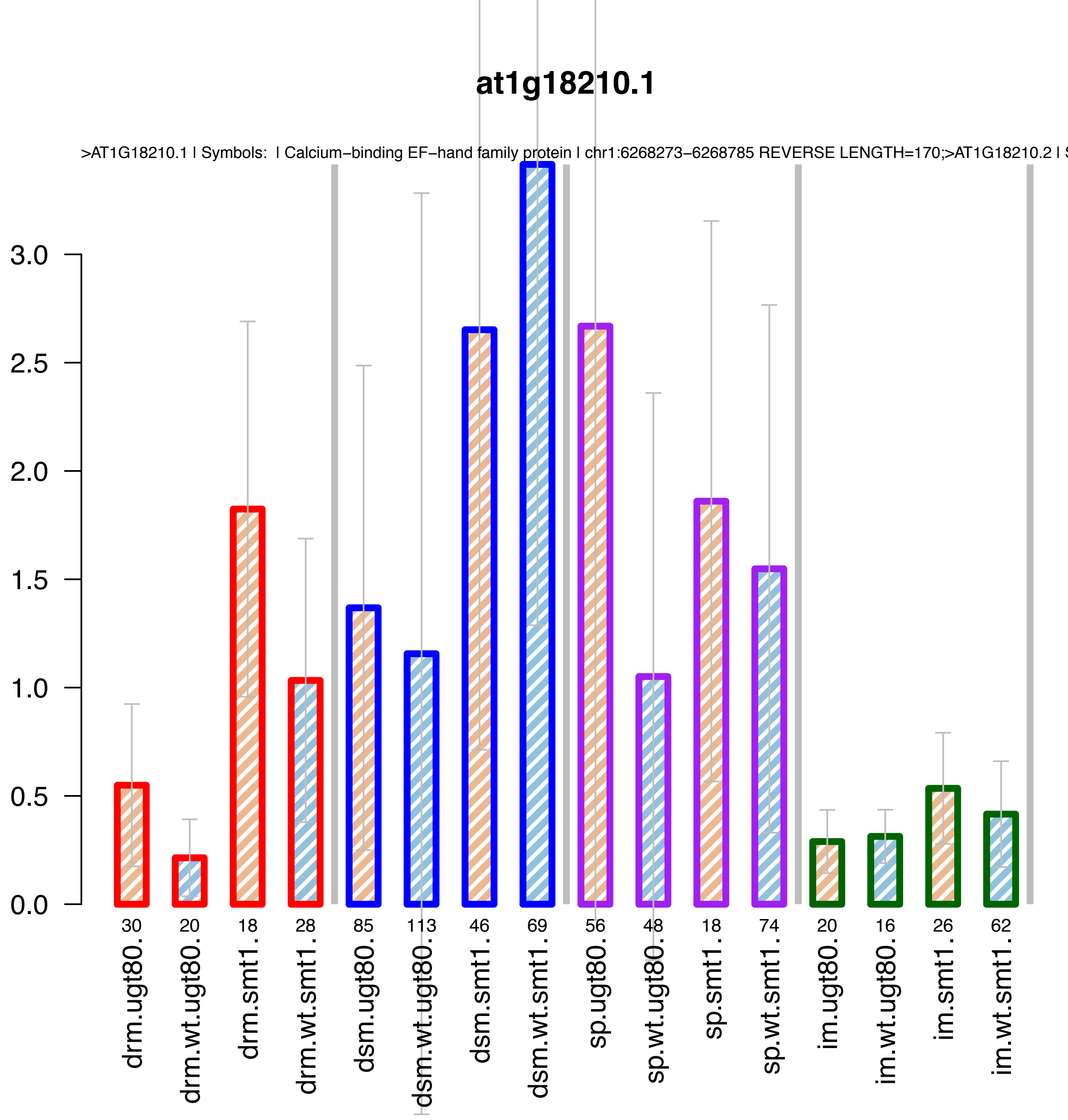

at1g18210.1
>AT1G18210.1 | Symbols: | Calcium−binding EF−hand family protein | chr1:6268273−6268785 REVERSE LENGTH=170;>AT1G18210.2 | S
3.0
2.5
2.0
1.5
1.0
0.5
0.0
30
20
18
28
85
113
46
69
56
48
18
74
20
16
26
62
drm.ugt80.
drm.wt.ugt80.
drm.smt1.
drm.wt.smt1.
dsm.ugt80.
dsm.wt.ugt80.
dsm.smt1.
dsm.wt.smt1.
sp.ugt80.
sp.wt.ugt80.
sp.smt1.
sp.wt.smt1.
im.ugt80.
im.wt.ugt80.
im.smt1.
im.wt.smt1.

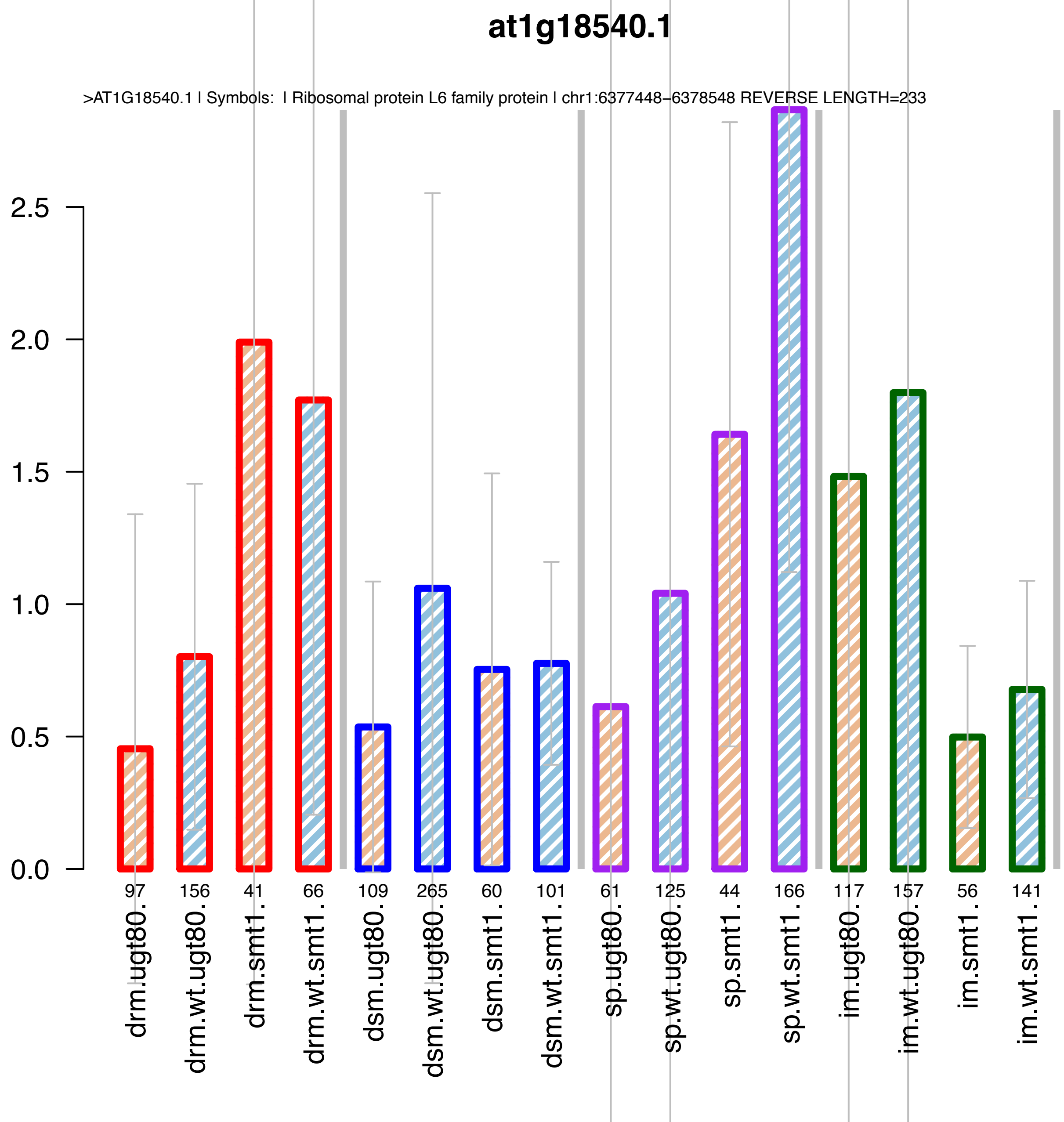
at1g18540.1
>AT1G18540.1 | Symbols: | Ribosomal protein L6 family protein | chr1:6377448-6378548 REVERSE LENGTH=233
0.0
0.5
1.0
1.5
2.0
2.5
97
156
41
66
109
265
60
101
61
125
44
166
117
157
56
141
drm.ugt80..
drm.wt.ugt80..
drm.smt1..
drm.wt.smt1..
dsm.ugt80..
dsm.wt.ugt80..
dsm.smt1..
dsm.wt.smt1..
sp.ugt80..
sp.wt.ugt80..
sp.smt1..
sp.wt.smt1..
im.ugt80..
im.wt.ugt80..
im.smt1..
im.wt.smt1..

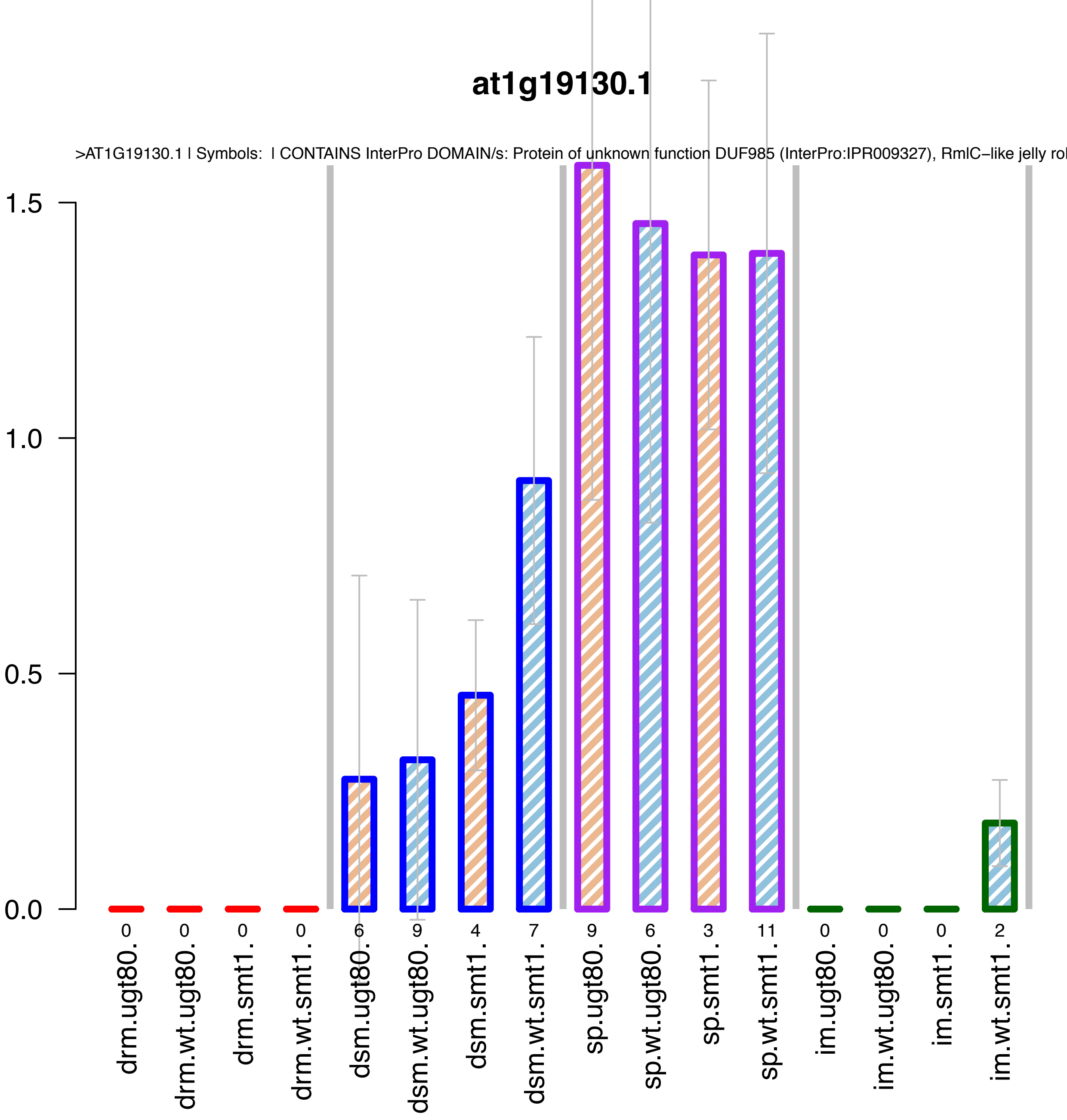

at1g19130.1
>AT1G19130.1 | Symbols: | CONTAINS InterPro DOMAIN/s: Protein of unknown function DUF985 (InterPro:IPR009327), RmlC−like jelly rol
1.5
1.0
0.5
0.0
0
0
0
0
6
9
4
7
9
6
3
11
0
0
0
2
drm.ugt80.
drm.wt.ugt80.
drm.smt1.
drm.wt.smt1.
dsm.ugt80.
dsm.wt.ugt80.
dsm.smt1.
dsm.wt.smt1.
sp.ugt80.
sp.wt.ugt80.
sp.smt1.
sp.wt.smt1.
im.ugt80.
im.wt.ugt80.
im.smt1.
im.wt.smt1.

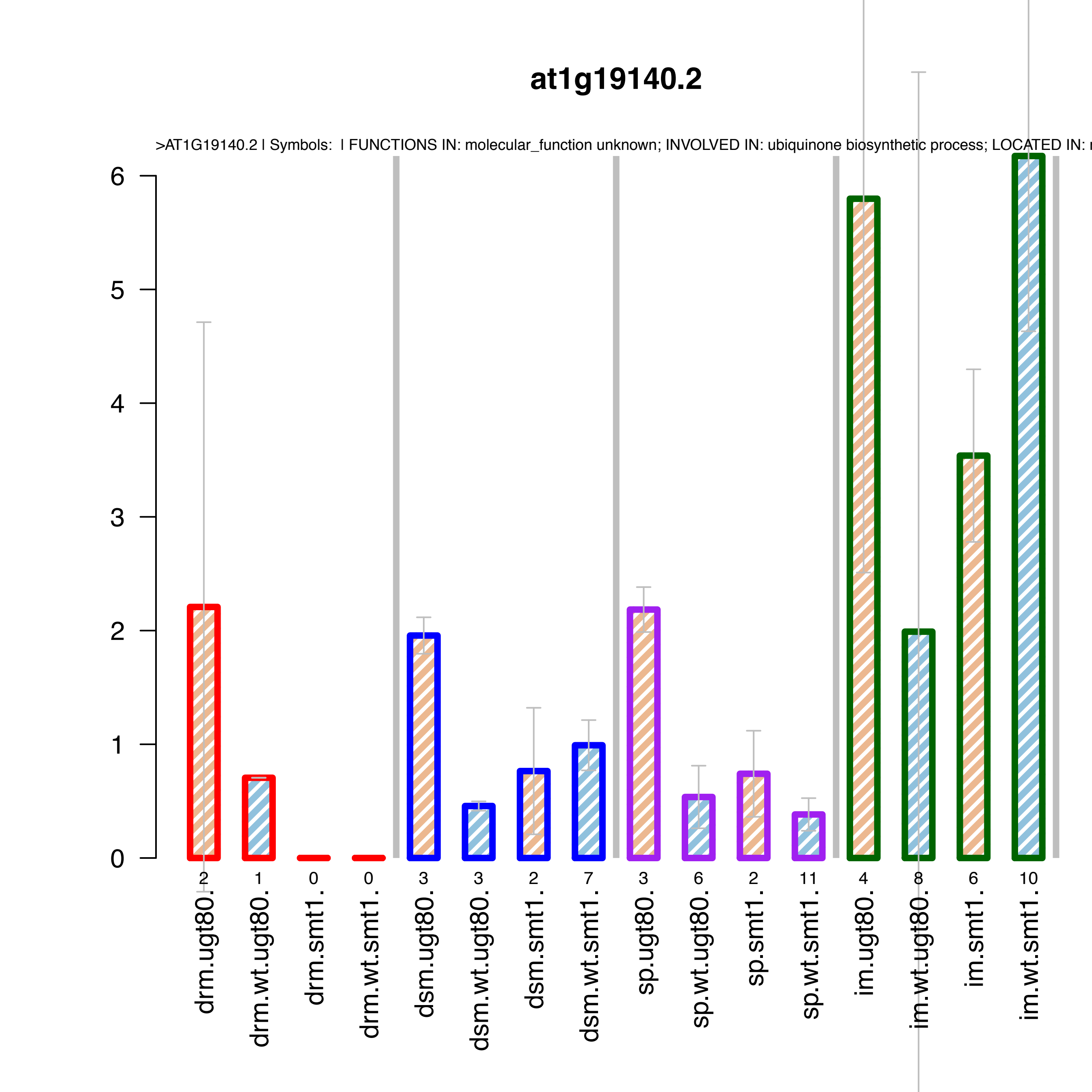

at1g19140.2
>AT1G19140.2 | Symbols: | FUNCTIONS IN: molecular_function unknown; INVOLVED IN: ubiquinone biosynthetic process; LOCATED IN: r
0
1
2
3
4
5
6
2
1
0
0
3
3
2
7
3
6
2
11
4
8
6
10
drm.ugt80.
drm.wt.ugt80.
drm.smt1.
drm.wt.smt1.
dsm.ugt80.
dsm.wt.ugt80.
dsm.smt1.
dsm.wt.smt1.
sp.ugt80.
sp.wt.ugt80.
sp.smt1.
sp.wt.smt1.
im.ugt80.
im.wt.ugt80.
im.smt1.
im.wt.smt1.

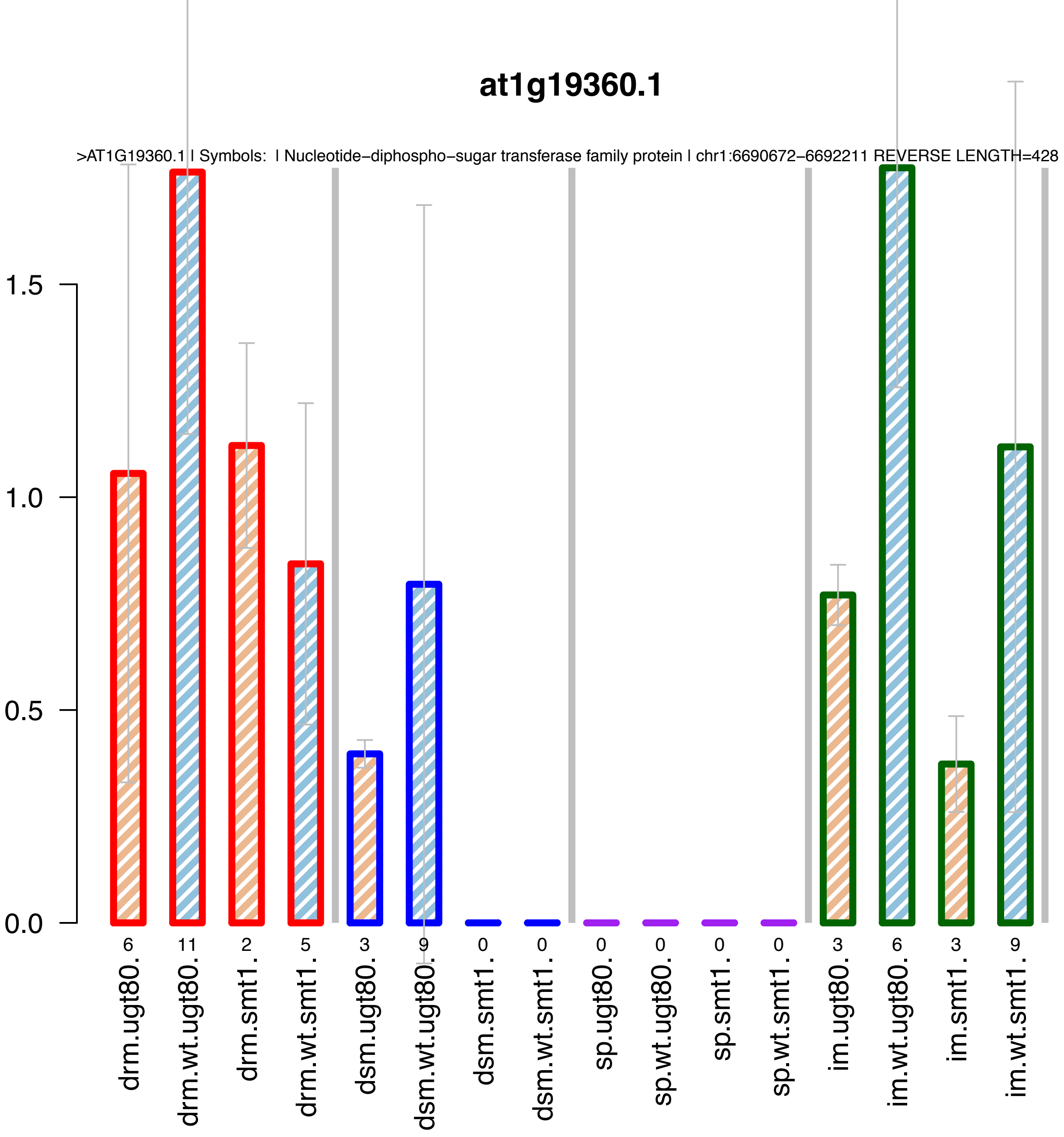
at1g19360.1
>AT1G19360.1 | Symbols: | Nucleotide−diphospho−sugar transferase family protein | chr1:6690672−6692211 REVERSE LENGTH=428
1.5
1.0
0.5
0.0
6
11
2
5
3
9
0
0
0
0
0
0
3
6
3
9
drm.ugt80.
drm.wt.ugt80.
drm.smt1.
drm.wt.smt1.
dsm.ugt80.
dsm.wt.ugt80.
dsm.smt1.
dsm.wt.smt1.
sp.ugt80.
sp.wt.ugt80.
sp.smt1.
sp.wt.smt1.
im.ugt80.
im.wt.ugt80.
im.smt1.
im.wt.smt1.

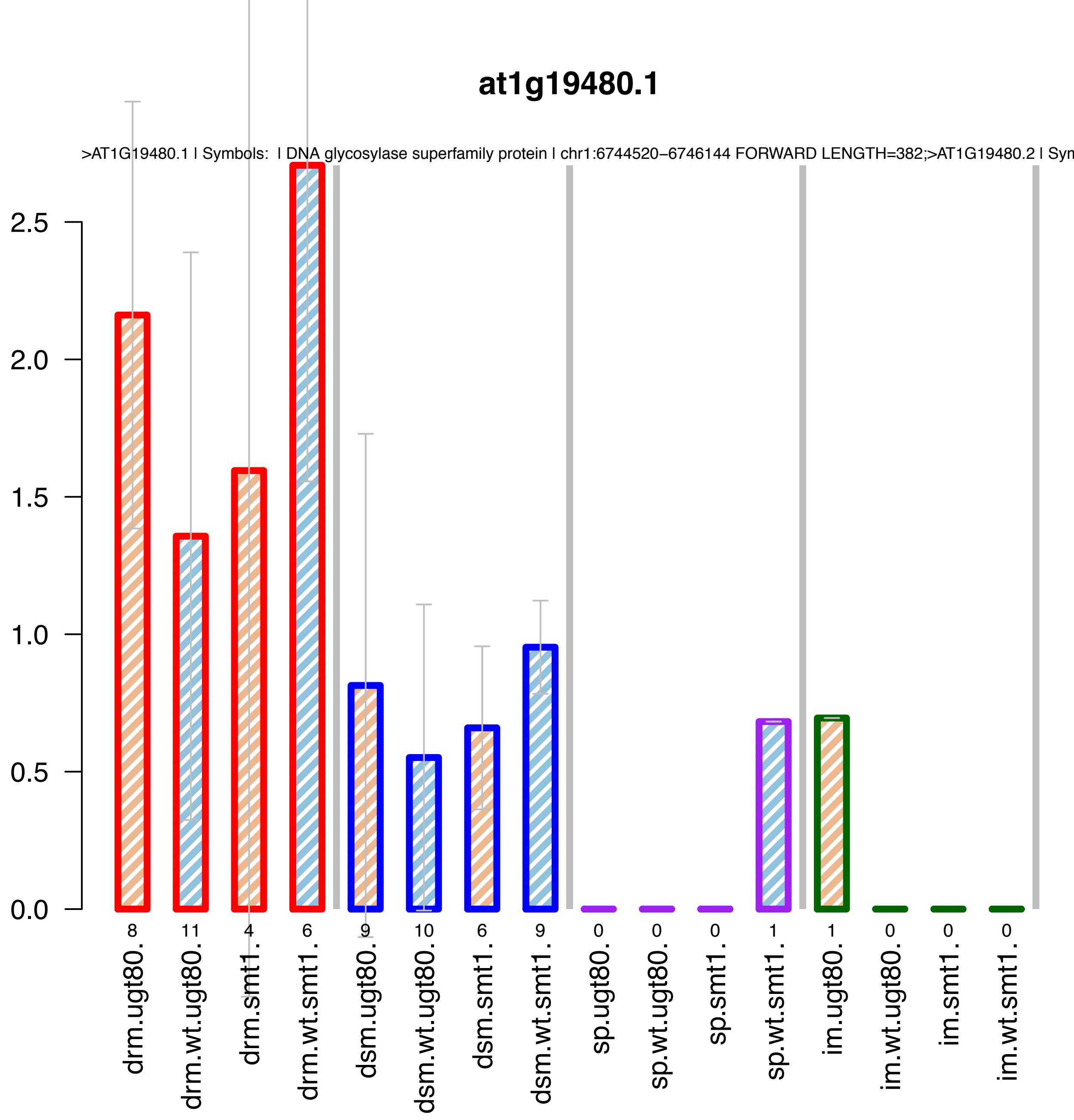
at1g19480.1
>AT1G19480.1 | Symbols: | DNA glycosylase superfamily protein | chr1:6744520–6746144 FORWARD LENGTH=382;>AT1G19480.2 | Sym
0.0
0.5
1.0
1.5
2.0
2.5
8
11
4
6
9
10
6
9
0
0
0
1
1
0
0
0
drm.ugt80.
drm.wt.ugt80.
drm.smt1.
drm.wt.smt1.
dsm.ugt80.
dsm.wt.ugt80.
dsm.smt1.
dsm.wt.smt1.
sp.ugt80.
sp.wt.ugt80.
sp.smt1.
sp.wt.smt1.
im.ugt80.
im.wt.ugt80.
im.smt1.
im.wt.smt1.

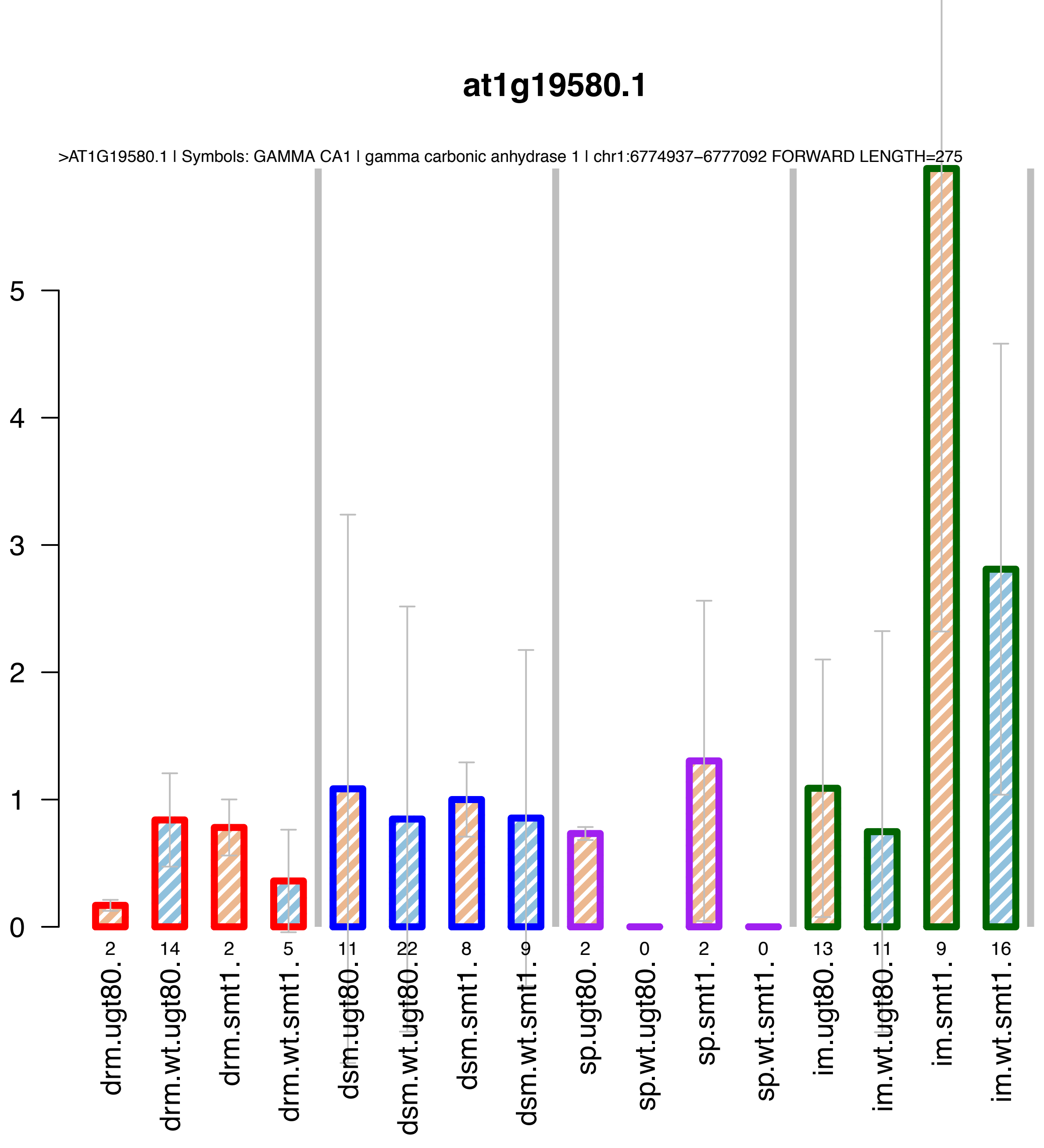

at1g19580.1
>AT1G19580.1 | Symbols: GAMMA CA1 | gamma carbonic anhydrase 1 | chr1:6774937-6777092 FORWARD LENGTH=275
0
1
2
3
4
5
2
14
2
5
11
22
8
9
2
0
2
0
13
11
9
16
drm.ugt80..
drm.wt.ugt80..
drm.smt1..
drm.wt.smt1..
dsm.ugt80..
dsm.wt.ugt80..
dsm.smt1..
dsm.wt.smt1..
sp.ugt80..
sp.wt.ugt80..
sp.smt1..
sp.wt.smt1..
im.ugt80..
im.wt.ugt80..
im.smt1..
im.wt.smt1..

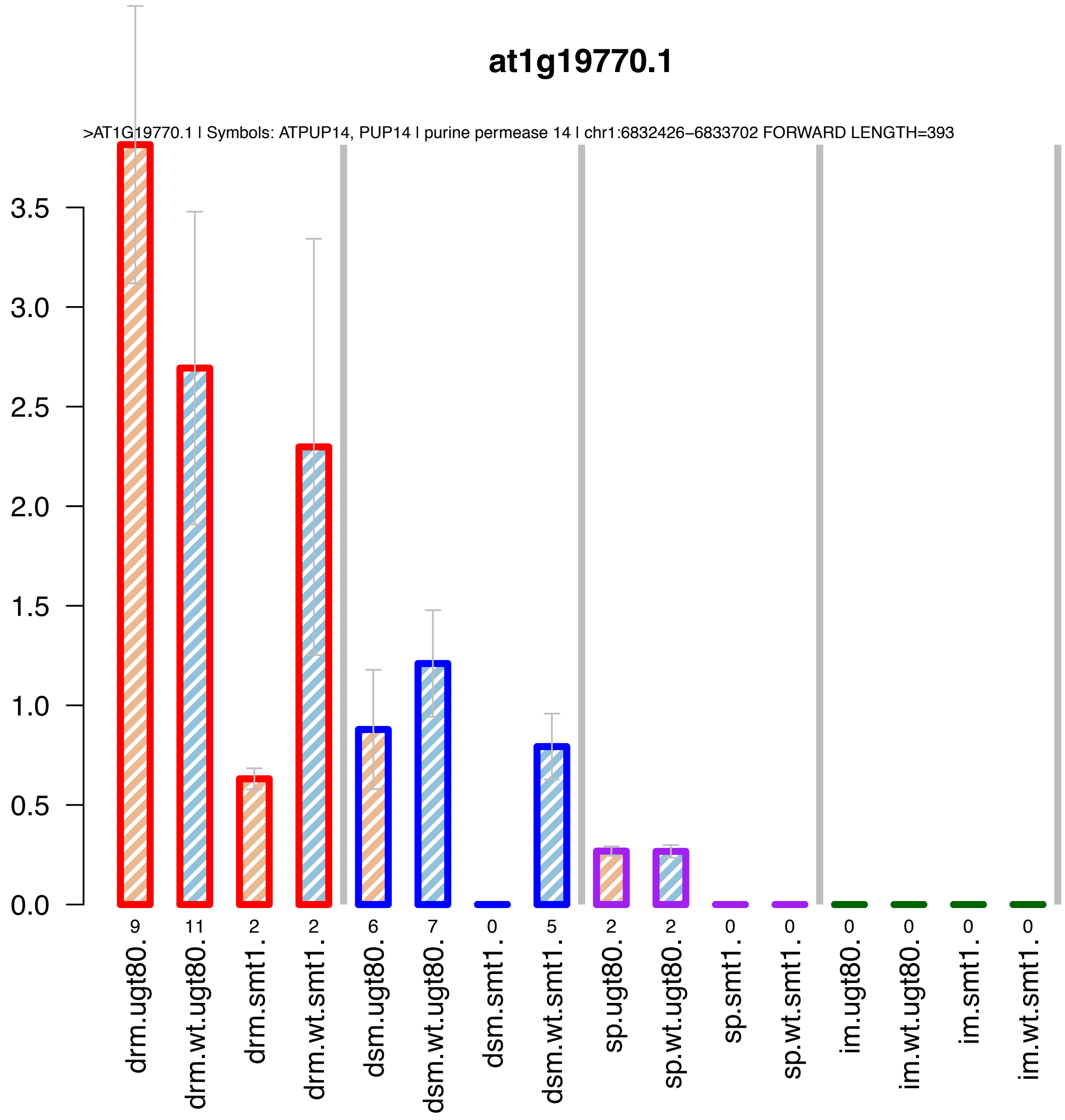

at1g19770.1
>AT1G19770.1 | Symbols: ATPUP14, PUP14 | purine permease 14 | chr1:6832426–6833702 FORWARD LENGTH=393
3.5
3.0
2.5
2.0
1.5
1.0
0.5
0.0
9
11
2
2
6
7
0
5
2
2
0
0
0
0
0
0
drm.ugt80.
drm.wt.ugt80.
drm.smt1.
drm.wt.smt1.
dsm.ugt80.
dsm.wt.ugt80.
dsm.smt1.
dsm.wt.smt1.
sp.ugt80.
sp.wt.ugt80.
sp.smt1.
sp.wt.smt1.
im.ugt80.
im.wt.ugt80.
im.smt1.
im.wt.smt1.

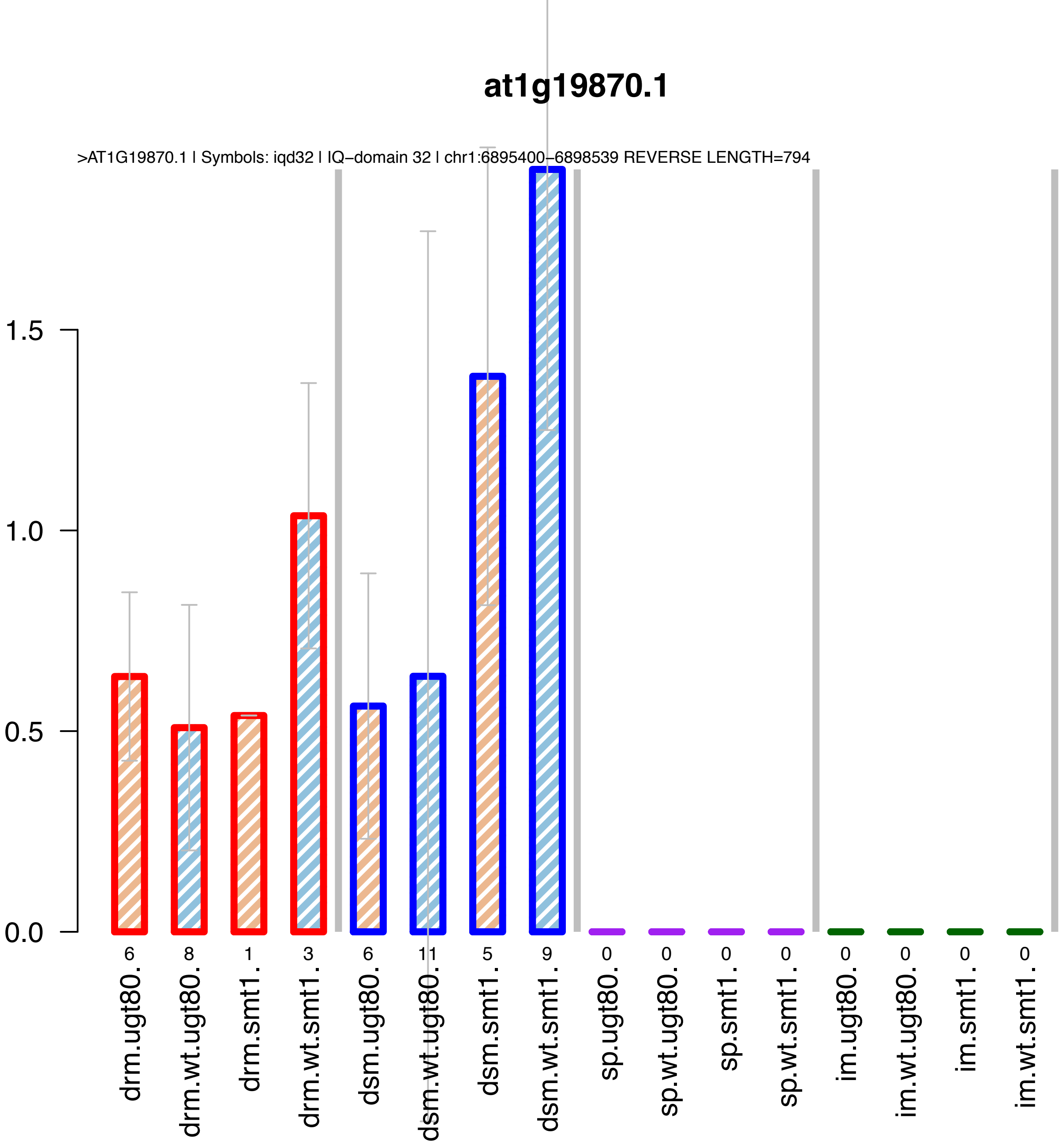
at1g19870.1
>AT1G19870.1 | Symbols: iqd32 | IQ−domain 32 | chr1:6895400−6898539 REVERSE LENGTH=794
1.5
1.0
0.5
0.0
6
8
1
3
6
11
5
9
0
0
0
0
0
0
0
0
drm.ugt80.
drm.wt.ugt80.
drm.smt1.
drm.wt.smt1.
dsm.ugt80.
dsm.wt.ugt80.
dsm.smt1.
dsm.wt.smt1.
sp.ugt80.
sp.wt.ugt80.
sp.smt1.
sp.wt.smt1.
im.ugt80.
im.wt.ugt80.
im.smt1.
im.wt.smt1.

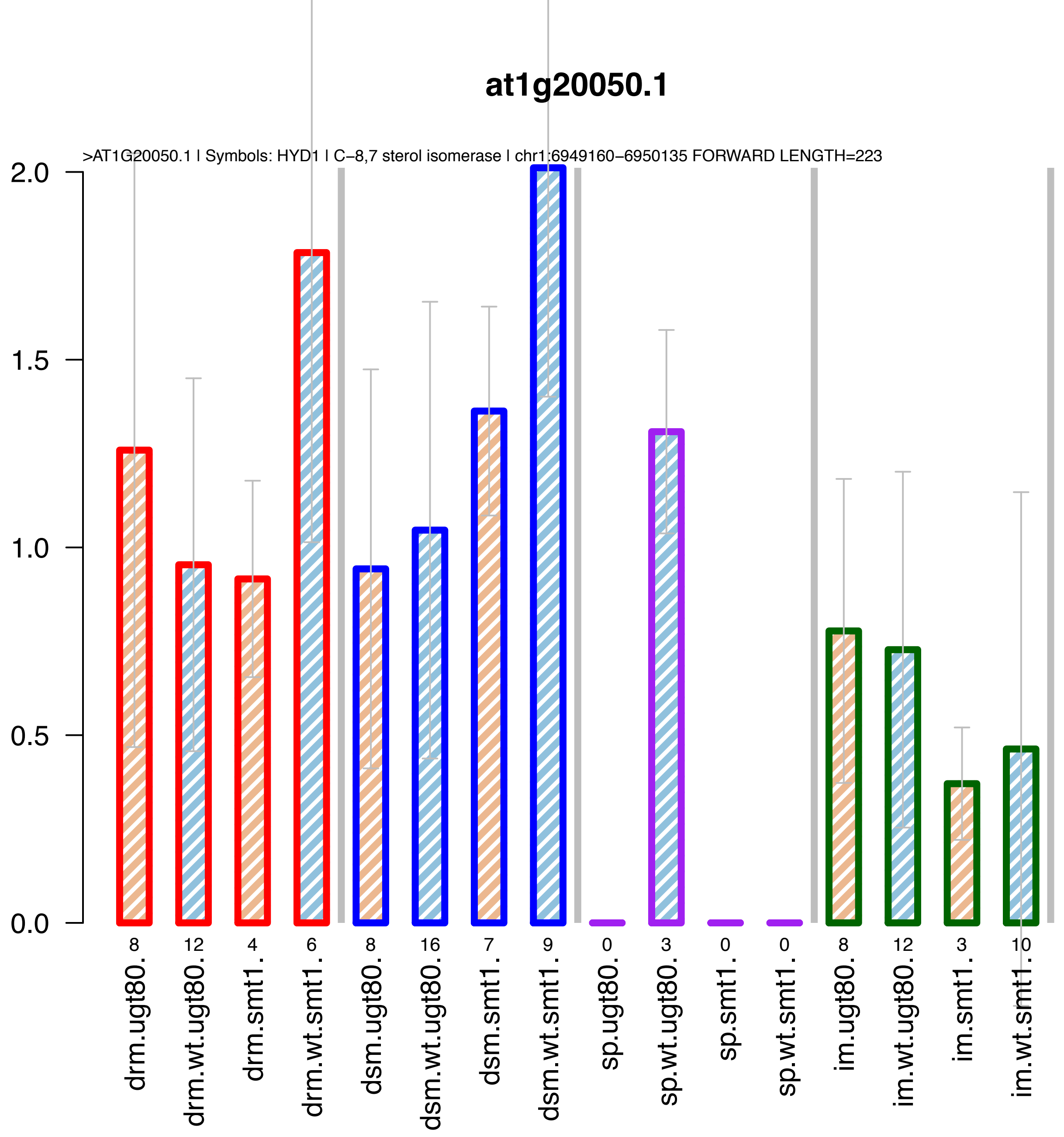

at1g20050.1
>AT1G20050.1 | Symbols: HYD1 | C−8,7 sterol isomerase | chr1:6949160−6950135 FORWARD LENGTH=223
2.0
1.5
1.0
0.5
0.0
8
12
4
6
8
16
7
9
0
3
0
0
8
12
3
10
drm.ugt80..
drm.wt.ugt80..
drm.smt1..
drm.wt.smt1..
dsm.ugt80..
dsm.wt.ugt80..
dsm.smt1..
dsm.wt.smt1..
sp.ugt80..
sp.wt.ugt80..
sp.smt1..
sp.wt.smt1..
im.ugt80..
im.wt.ugt80..
im.smt1..
im.wt.smt1..

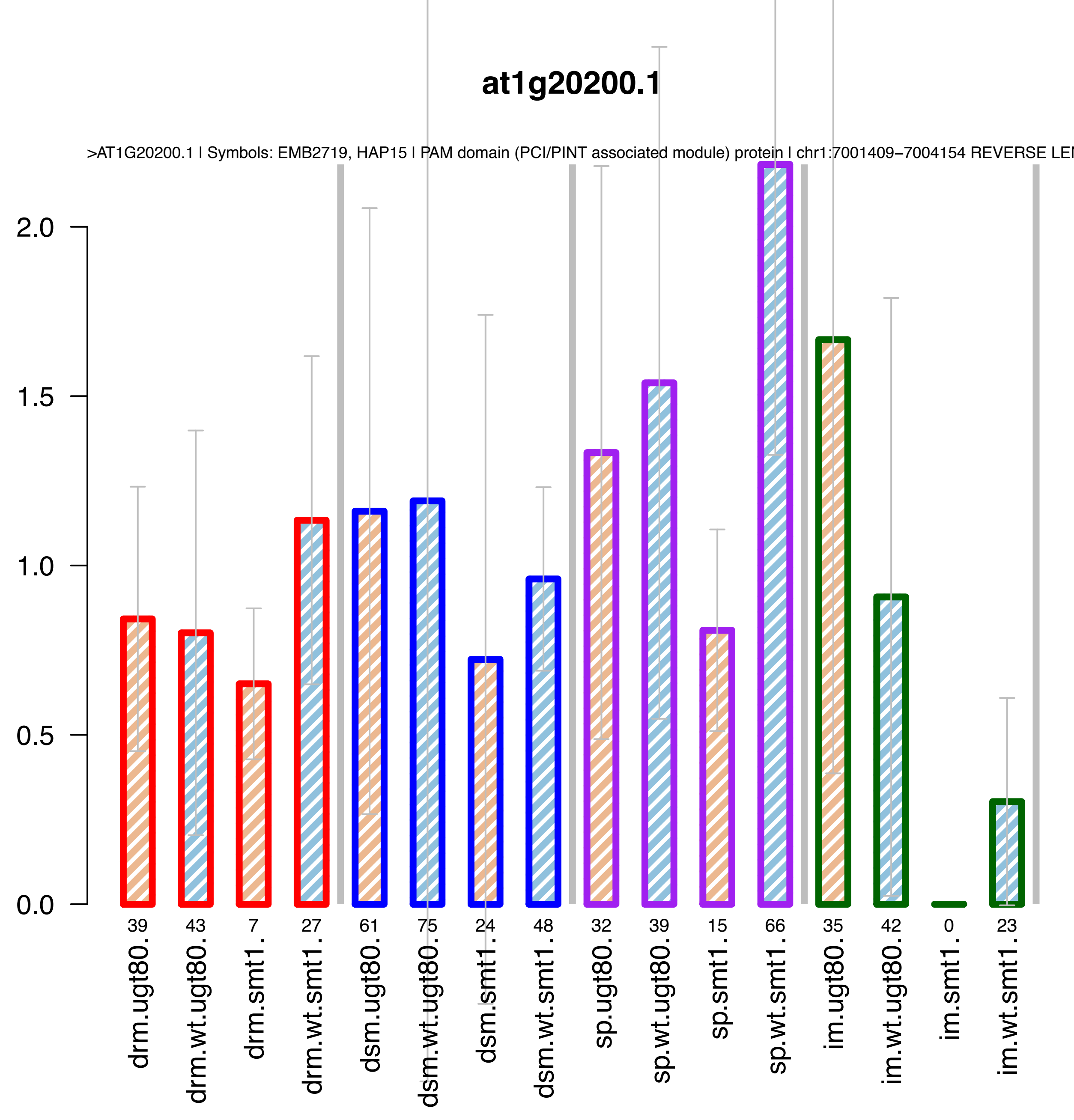

at1g20200.1
>AT1G20200.1 | Symbols: EMB2719, HAP15 | PAM domain (PCI/PINT associated module) protein | chr1:7001409-7004154 REVERSE LEI
2.0
1.5
1.0
0.5
0.0
39
43
7
27
61
75
24
48
32
39
15
66
35
42
0
23
drm.ugt80.
drm.wt.ugt80.
drm.smt1.
drm.wt.smt1.
dsm.ugt80.
dsm.wt.ugt80.
dsm.smt1.
dsm.wt.smt1.
sp.ugt80.
sp.wt.ugt80.
sp.smt1.
sp.wt.smt1.
im.ugt80.
im.wt.ugt80.
im.smt1.
im.wt.smt1.

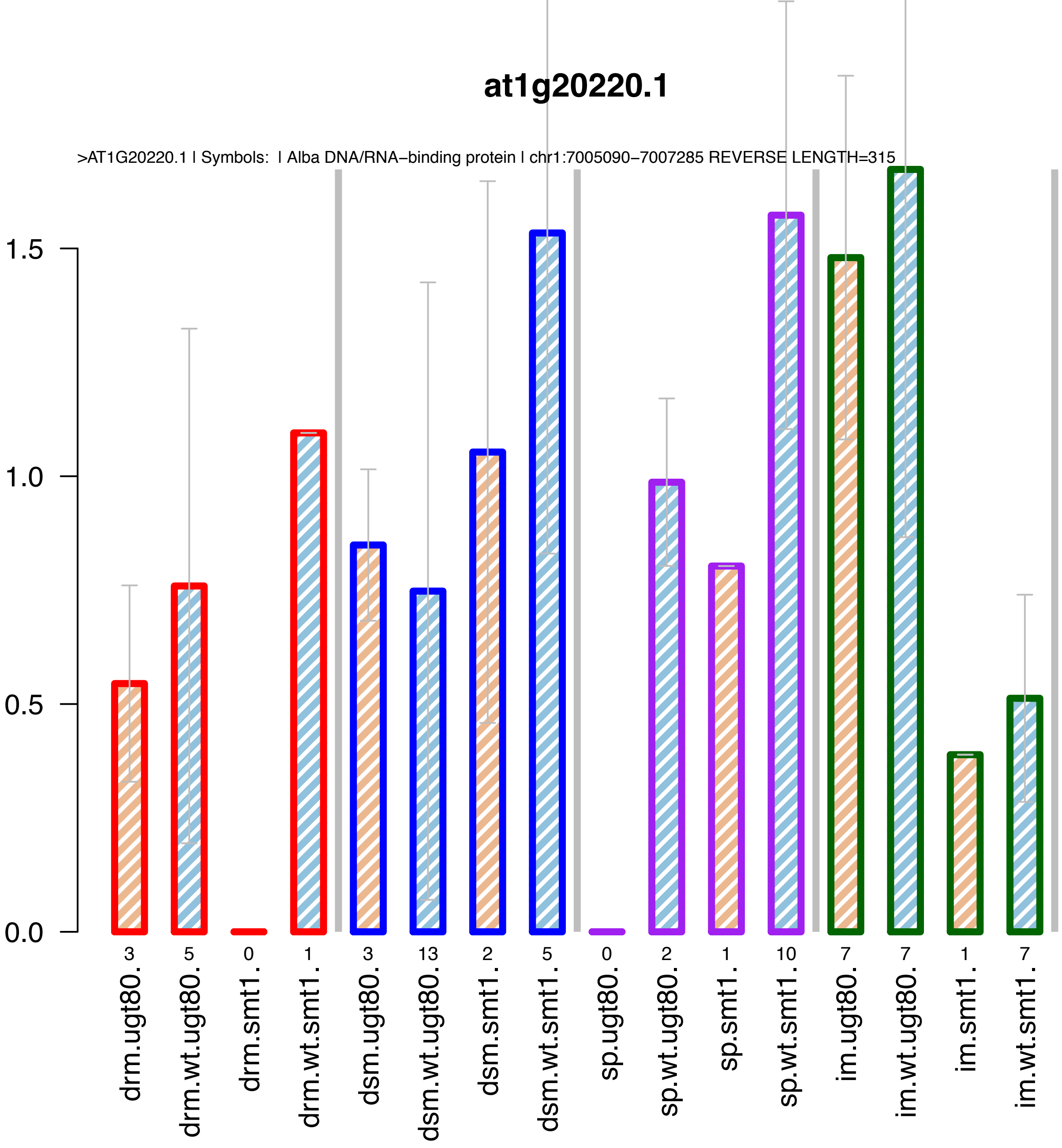

at1g20220.1
>AT1G20220.1 | Symbols: | Alba DNA/RNA-binding protein | chr1:7005090-7007285 REVERSE LENGTH=315
1.5
1.0
0.5
0.0
3
5
0
1
3
13
2
5
0
2
1
10
7
7
1
7
drm.ugt80..
drm.wt.ugt80..
drm.smt1..
drm.wt.smt1..
dsm.ugt80..
dsm.wt.ugt80..
dsm.smt1..
dsm.wt.smt1..
sp.ugt80..
sp.wt.ugt80..
sp.smt1..
sp.wt.smt1..
im.ugt80..
im.wt.ugt80..
im.smt1..
im.wt.smt1..

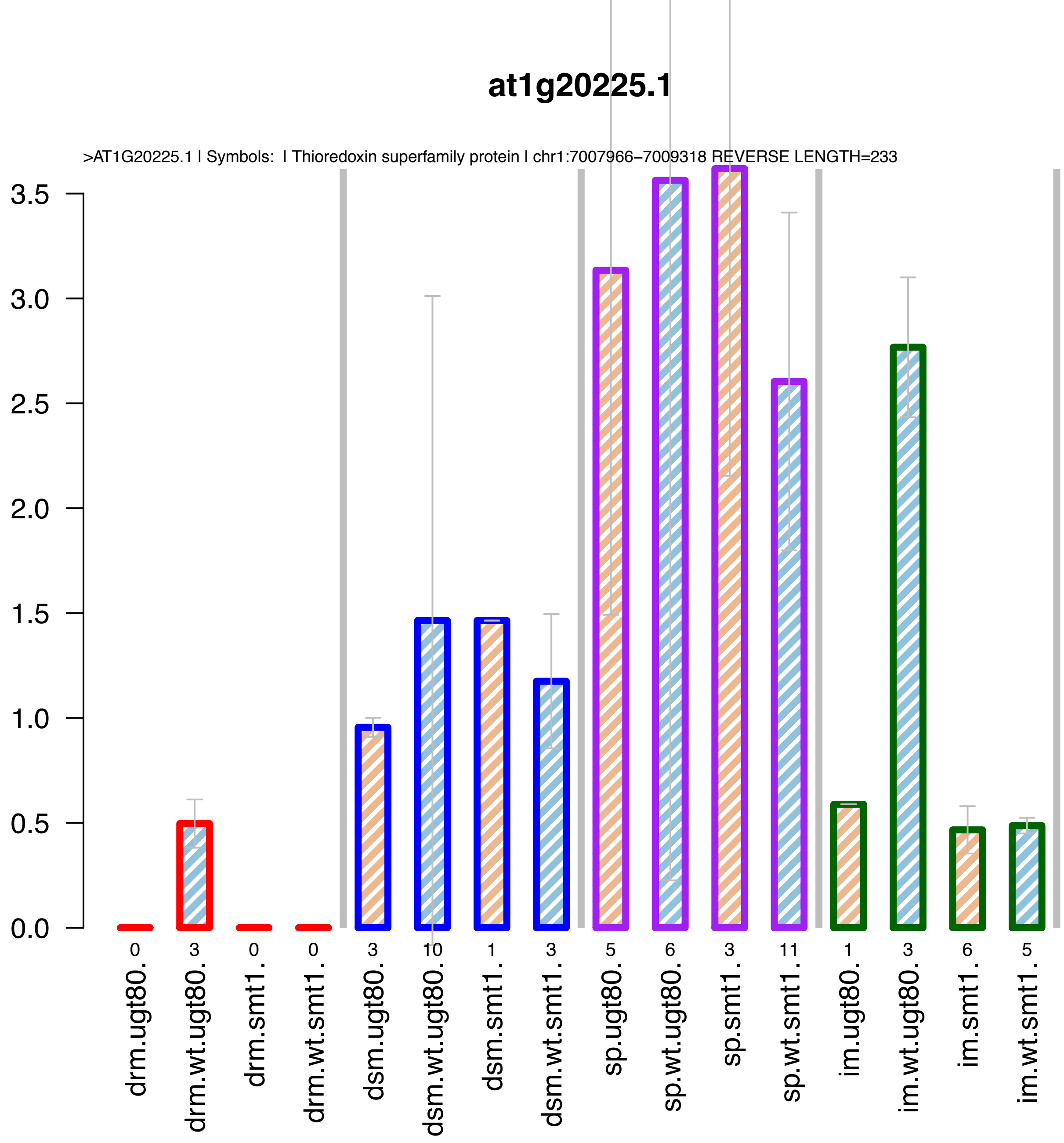
at1g20225.1
>AT1G20225.1 | Symbols: | Thioredoxin superfamily protein | chr1:7007966−7009318 REVERSE LENGTH=233
3.5
3.0
2.5
2.0
1.5
1.0
0.5
0.0
0
3
0
0
3
10
1
3
5
6
3
11
1
3
6
5
drm.ugt80.
drm.wt.ugt80.
drm.smt1.
drm.wt.smt1.
dsm.ugt80.
dsm.wt.ugt80.
dsm.smt1.
dsm.wt.smt1.
sp.ugt80.
sp.wt.ugt80.
sp.smt1.
sp.wt.smt1.
im.ugt80.
im.wt.ugt80.
im.smt1.
im.wt.smt1.

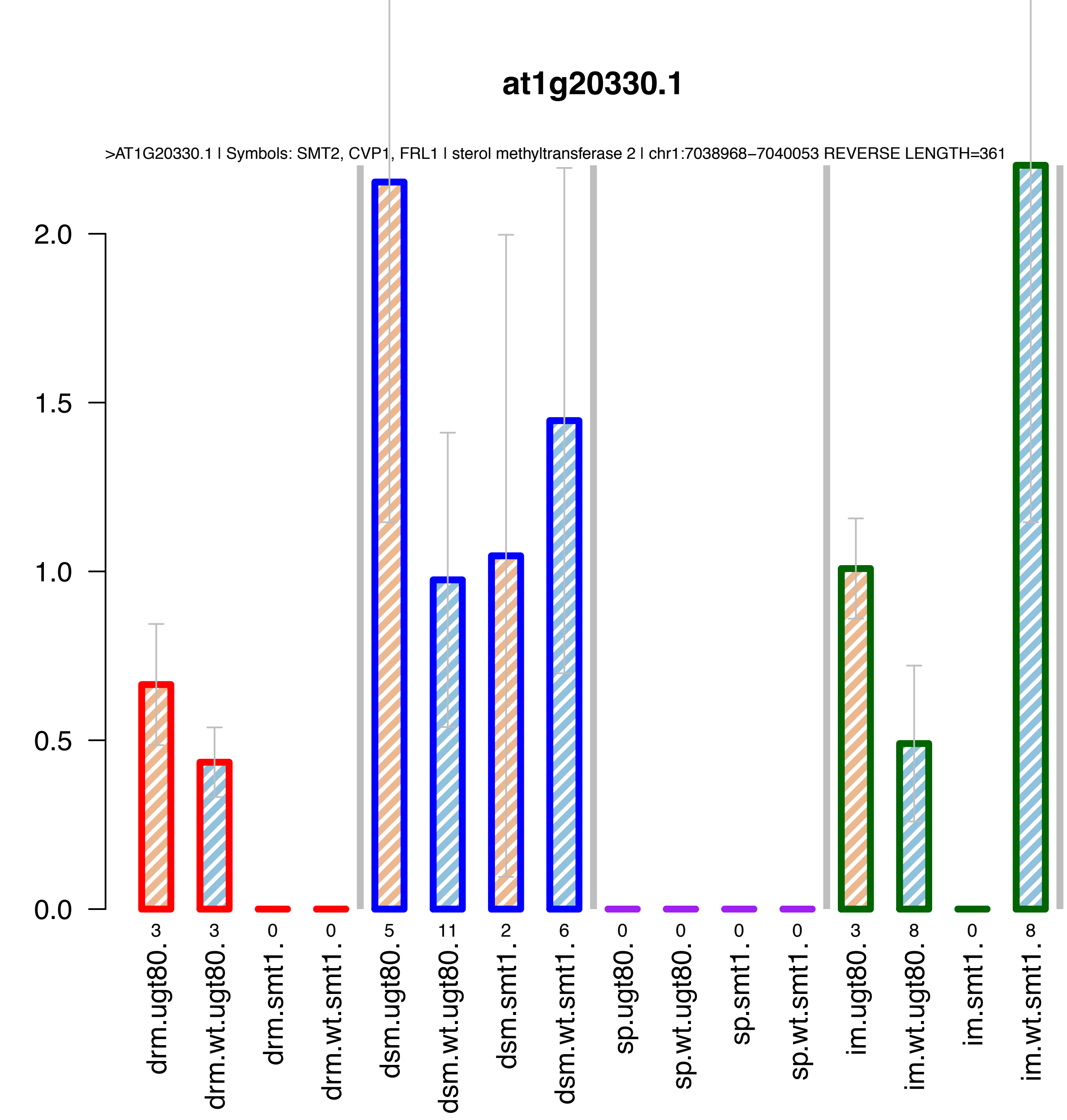
at1g20330.1
>AT1G20330.1 | Symbols: SMT2, CVP1, FRL1 | sterol methyltransferase 2 | chr1:7038968-7040053 REVERSE LENGTH=361
2.0
1.5
1.0
0.5
0.0
3
3
0
0
5
11
2
6
0
0
0
0
3
8
0
8
drm.ugt80..
drm.wt.ugt80..
drm.smt1..
drm.wt.smt1..
dsm.ugt80..
dsm.wt.ugt80..
dsm.smt1..
dsm.wt.smt1..
sp.ugt80..
sp.wt.ugt80..
sp.smt1..
sp.wt.smt1..
im.ugt80..
im.wt.ugt80..
im.smt1..
im.wt.smt1..

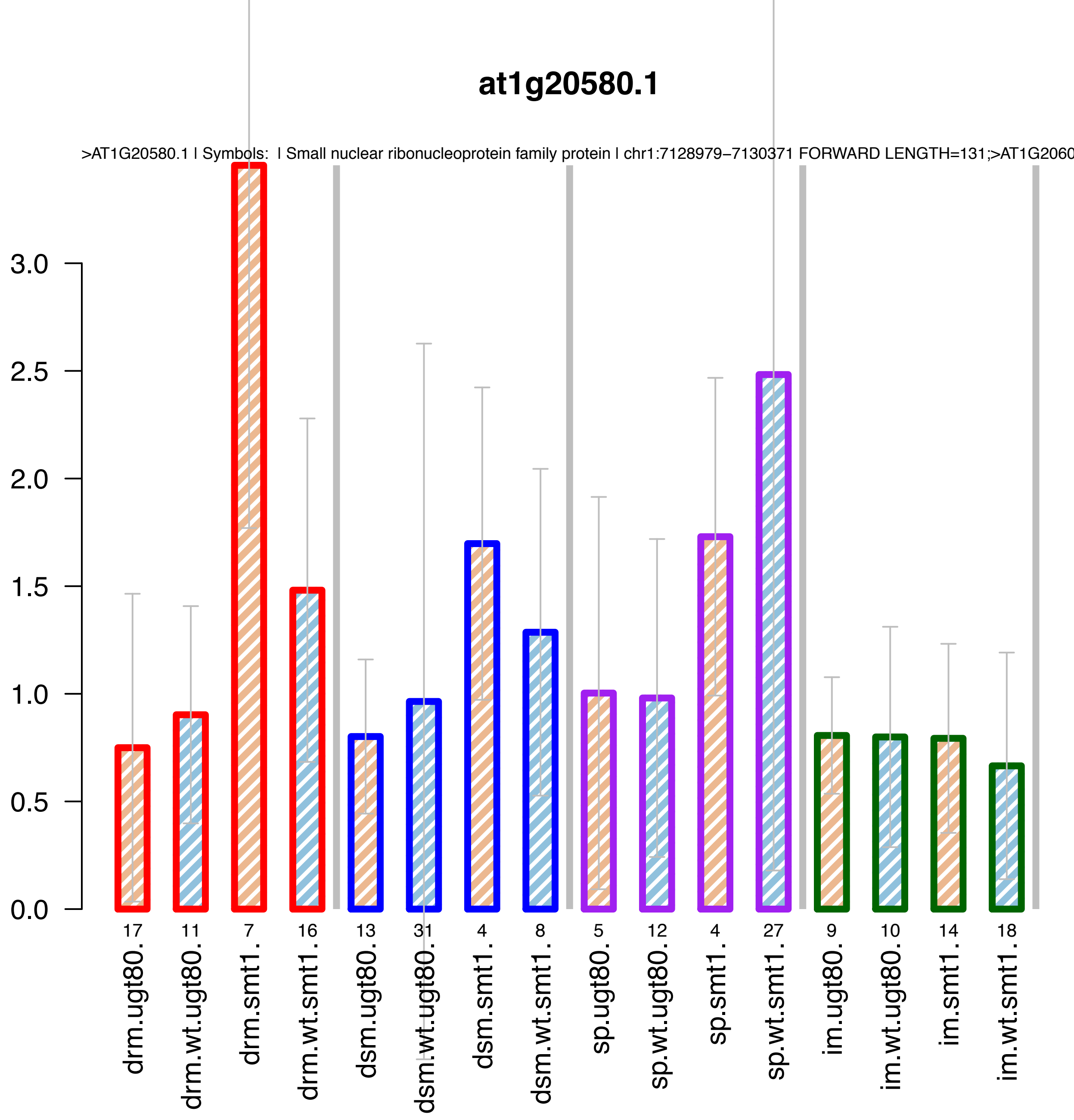
at1g20580.1
>AT1G20580.1 | Symbols: | Small nuclear ribonucleoprotein family protein | chr1:7128979-7130371 FORWARD LENGTH=131;>AT1G2060
3.0
2.5
2.0
1.5
1.0
0.5
0.0
17
11
7
16
13
31
4
8
5
12
4
27
9
10
14
18
drm.ugt80.
drm.wt.ugt80.
drm.smt1.
drm.wt.smt1.
dsm.ugt80.
dsm.wt.ugt80.
dsm.smt1.
dsm.wt.smt1.
sp.ugt80.
sp.wt.ugt80.
sp.smt1.
sp.wt.smt1.
im.ugt80.
im.wt.ugt80.
im.smt1.
im.wt.smt1.

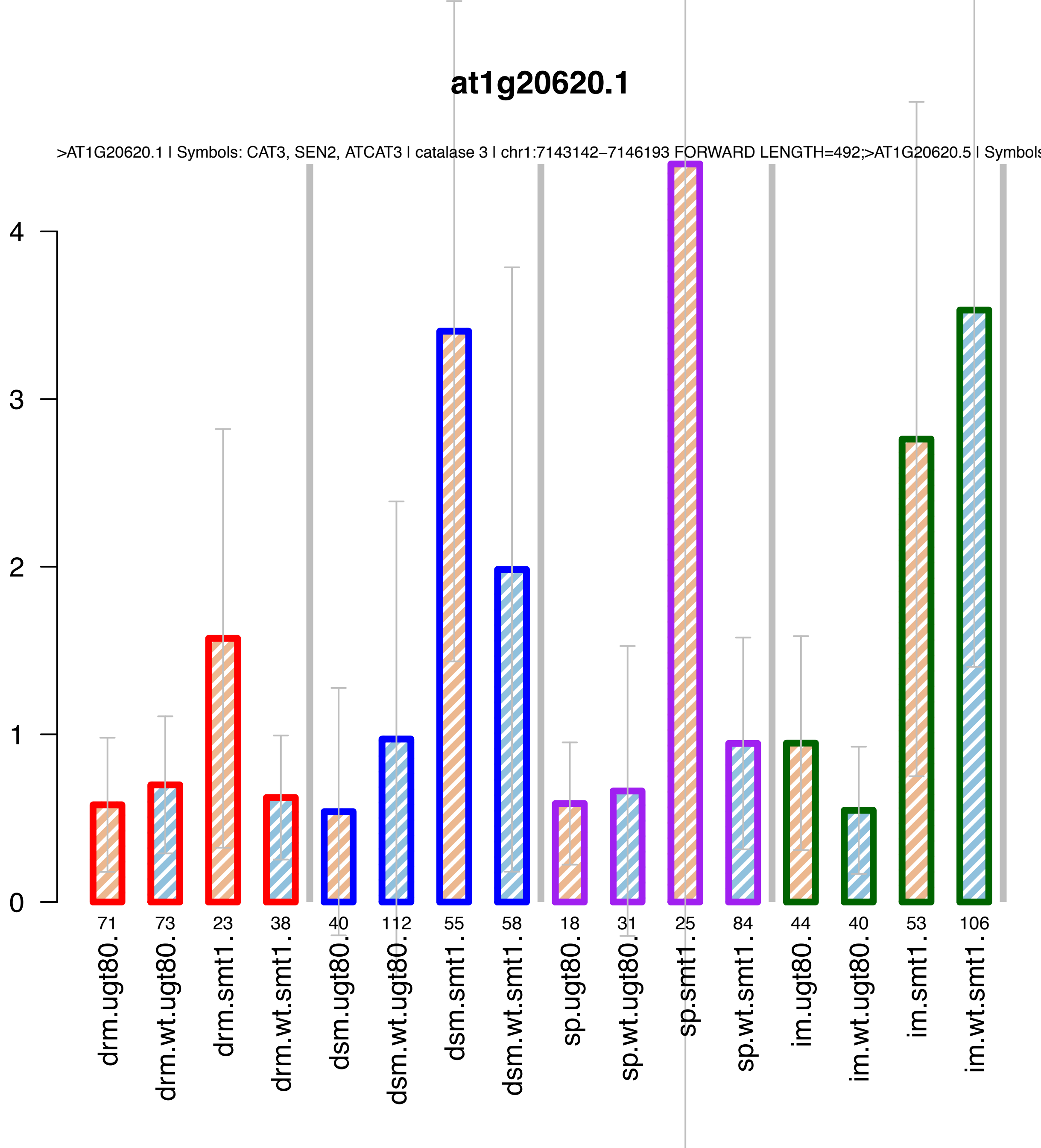
at1g20620.1
>AT1G20620.1 | Symbols: CAT3, SEN2, ATCAT3 | catalase 3 | chr1:7143142–7146193 FORWARD LENGTH=492;>AT1G20620.5 | Symbols
0
1
2
3
4
71
73
23
38
40
112
55
58
18
31
25
84
44
40
53
106
drm.ugt80..
drm.wt.ugt80..
drm.smt1..
drm.wt.smt1..
dsm.ugt80..
dsm.wt.ugt80..
dsm.smt1..
dsm.wt.smt1..
sp.ugt80..
sp.wt.ugt80..
sp.smt1..
sp.wt.smt1..
im.ugt80..
im.wt.ugt80..
im.smt1..
im.wt.smt1..

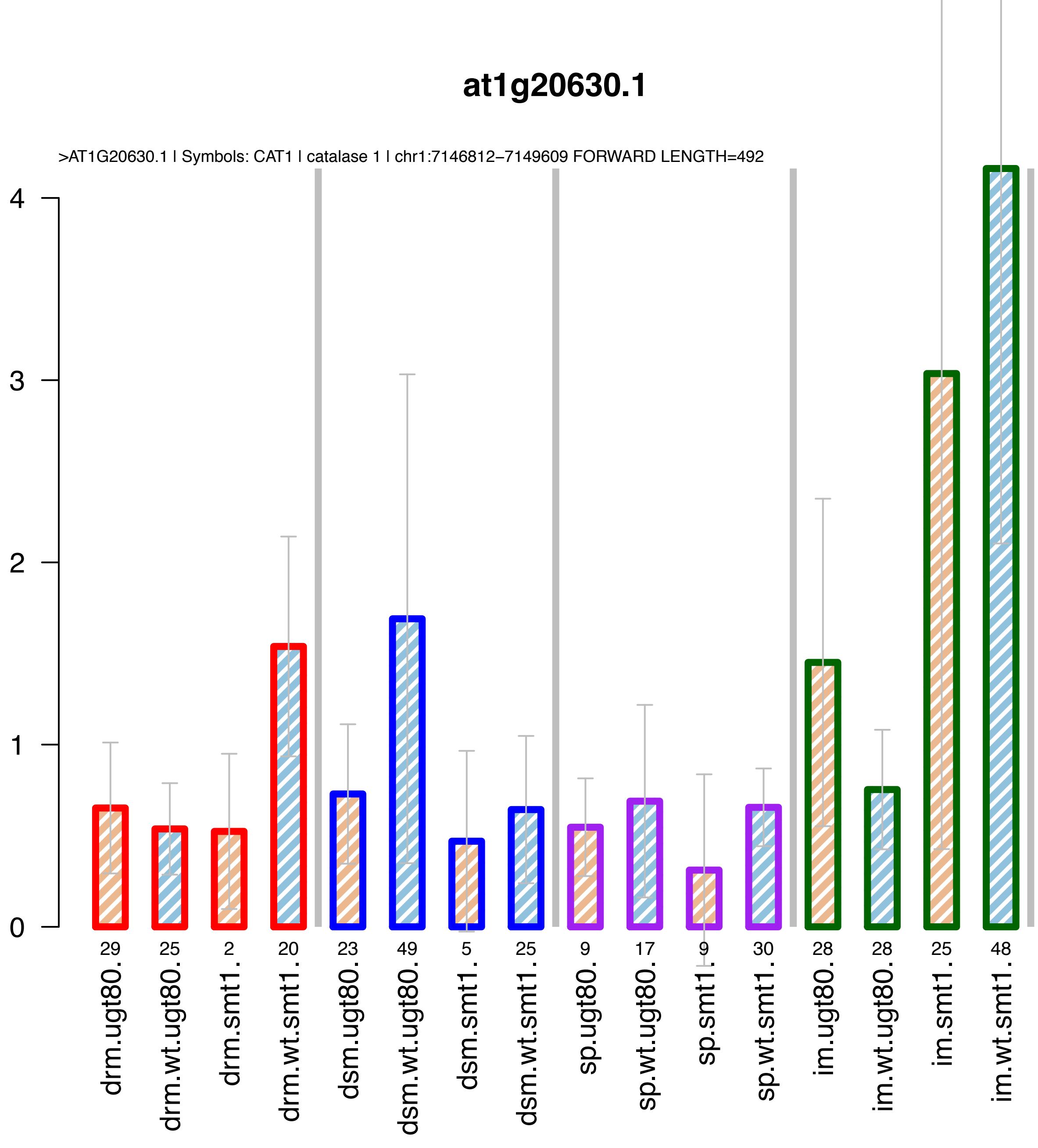
at1g20630.1
>AT1G20630.1 | Symbols: CAT1 | catalase 1 | chr1:7146812–7149609 FORWARD LENGTH=492
0
1
2
3
4
29
25
2
20
23
49
5
25
9
17
9
30
28
28
25
48
drm.ugt80.
drm.wt.ugt80.
drm.smt1.
drm.wt.smt1.
dsm.ugt80.
dsm.wt.ugt80.
dsm.smt1.
dsm.wt.smt1.
sp.ugt80.
sp.wt.ugt80.
sp.smt1.
sp.wt.smt1.
im.ugt80.
im.wt.ugt80.
im.smt1.
im.wt.smt1.

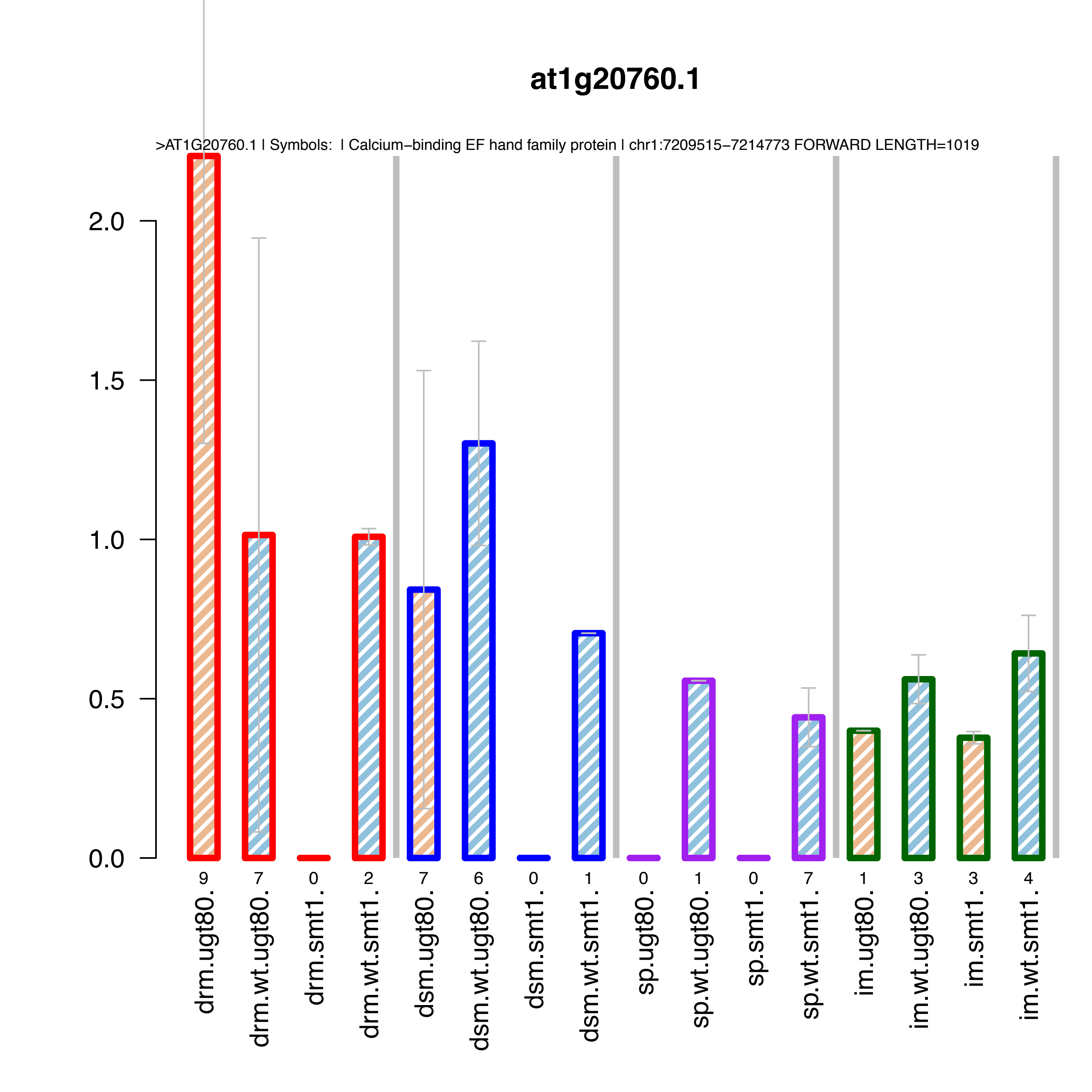
at1g20760.1
>AT1G20760.1 | Symbols: | Calcium−binding EF hand family protein | chr1:7209515−7214773 FORWARD LENGTH=1019
2.0
1.5
1.0
0.5
0.0
9
7
0
2
7
6
0
1
0
1
0
7
1
3
3
4
drm.ugt80.
drm.wt.ugt80.
drm.smt1.
drm.wt.smt1.
dsm.ugt80.
dsm.wt.ugt80.
dsm.smt1.
dsm.wt.smt1.
sp.ugt80.
sp.wt.ugt80.
sp.smt1.
sp.wt.smt1.
im.ugt80.
im.wt.ugt80.
im.smt1.
im.wt.smt1.

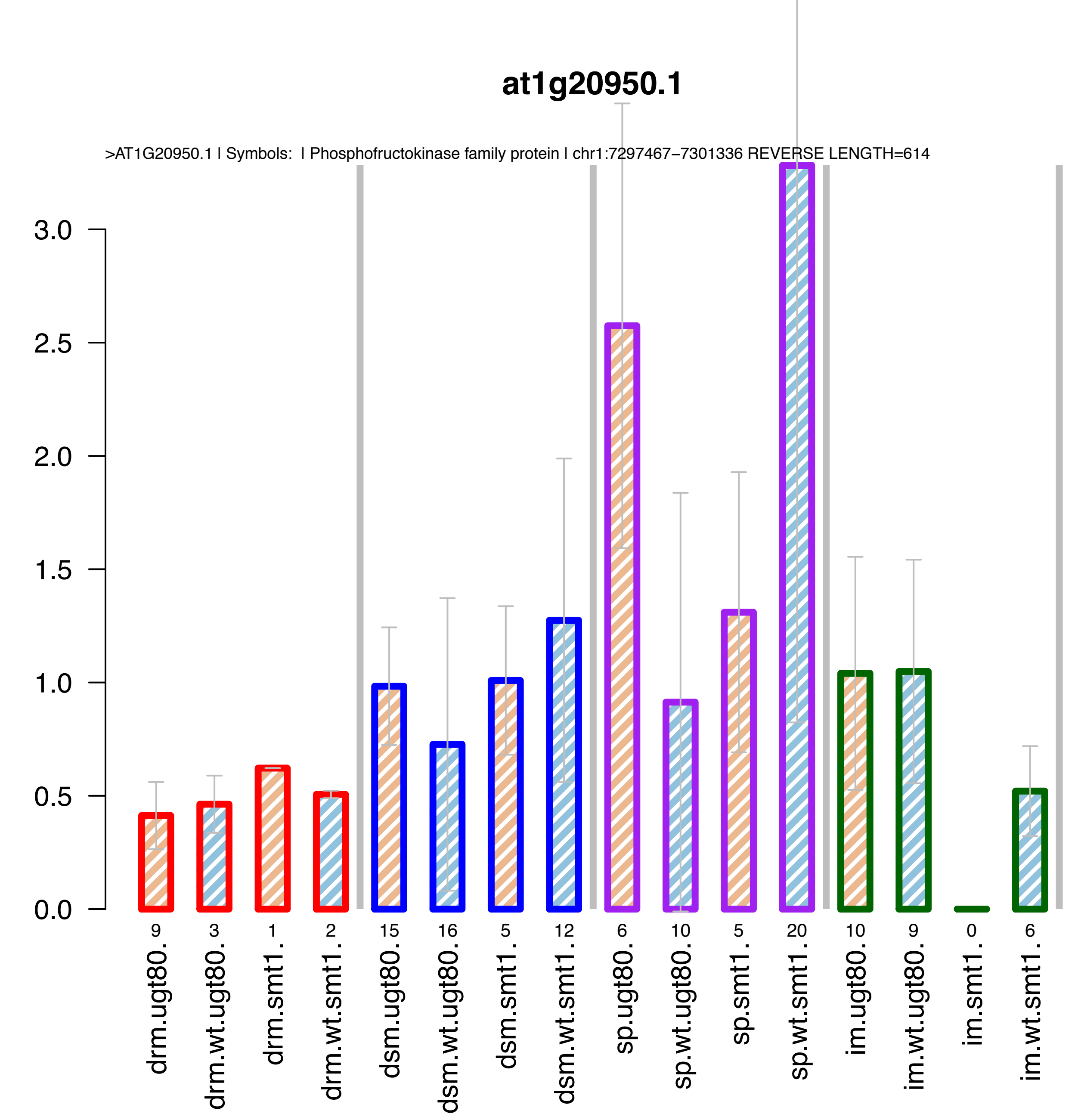

at1g20950.1
>AT1G20950.1 | Symbols: | Phosphofructokinase family protein | chr1:7297467−7301336 REVERSE LENGTH=614
0.0
0.5
1.0
1.5
2.0
2.5
3.0
9
3
1
2
15
16
5
12
6
10
5
20
10
9
0
6
drm.ugt80.
drm.wt.ugt80.
drm.smt1.
drm.wt.smt1.
dsm.ugt80.
dsm.wt.ugt80.
dsm.smt1.
dsm.wt.smt1.
sp.ugt80.
sp.wt.ugt80.
sp.smt1.
sp.wt.smt1.
im.ugt80.
im.wt.ugt80.
im.smt1.
im.wt.smt1.

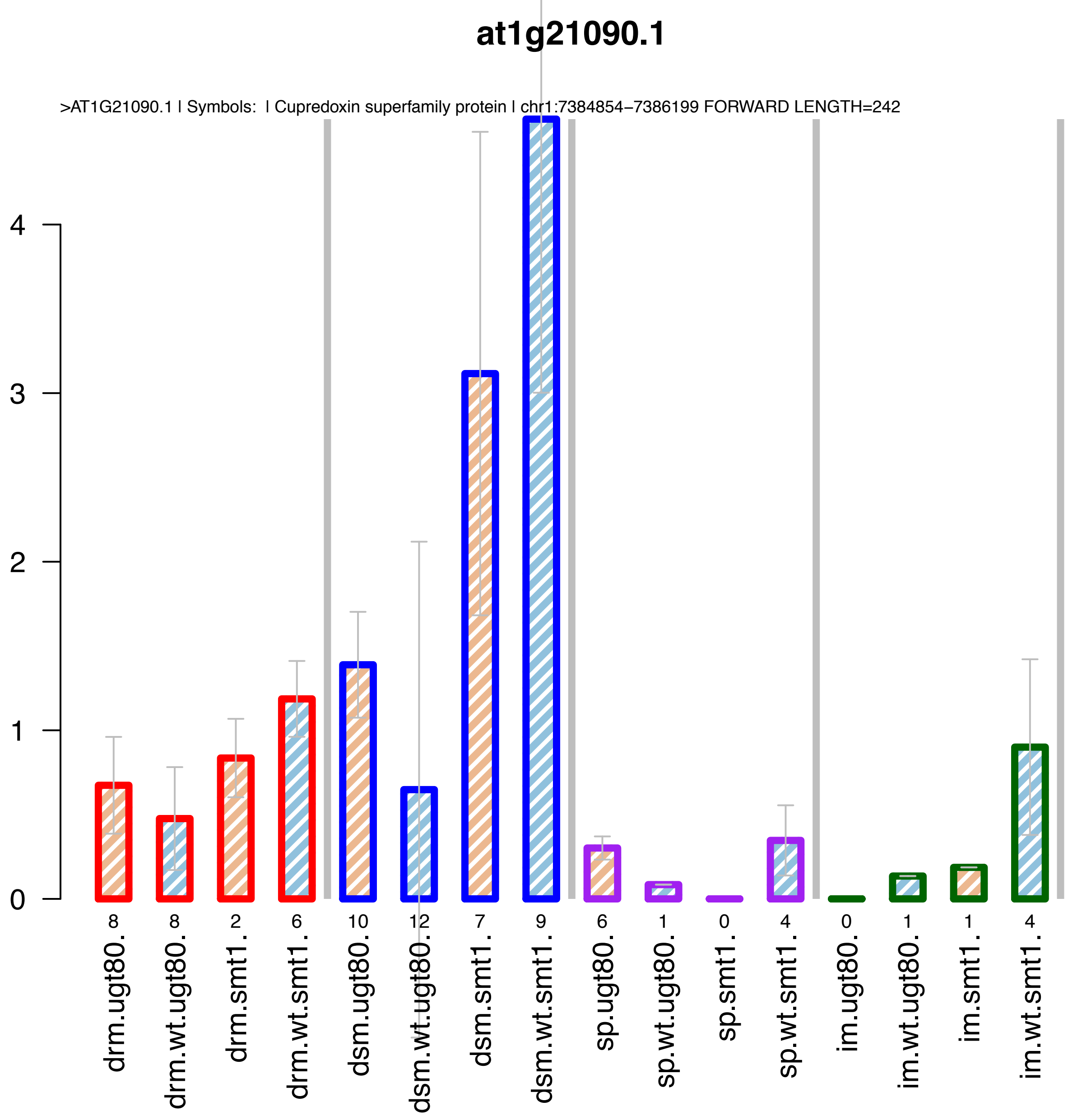

at1g21090.1
>AT1G21090.1 | Symbols: | Cupredoxin superfamily protein | chr1:7384854-7386199 FORWARD LENGTH=242
0
1
2
3
4
8
8
2
6
10
12
7
9
6
1
0
4
0
1
1
4
drm.ugt80..
drm.wt.ugt80..
drm.smt1..
drm.wt.smt1..
dsm.ugt80..
dsm.wt.ugt80..
dsm.smt1..
dsm.wt.smt1..
sp.ugt80..
sp.wt.ugt80..
sp.smt1..
sp.wt.smt1..
im.ugt80..
im.wt.ugt80..
im.smt1..
im.wt.smt1..

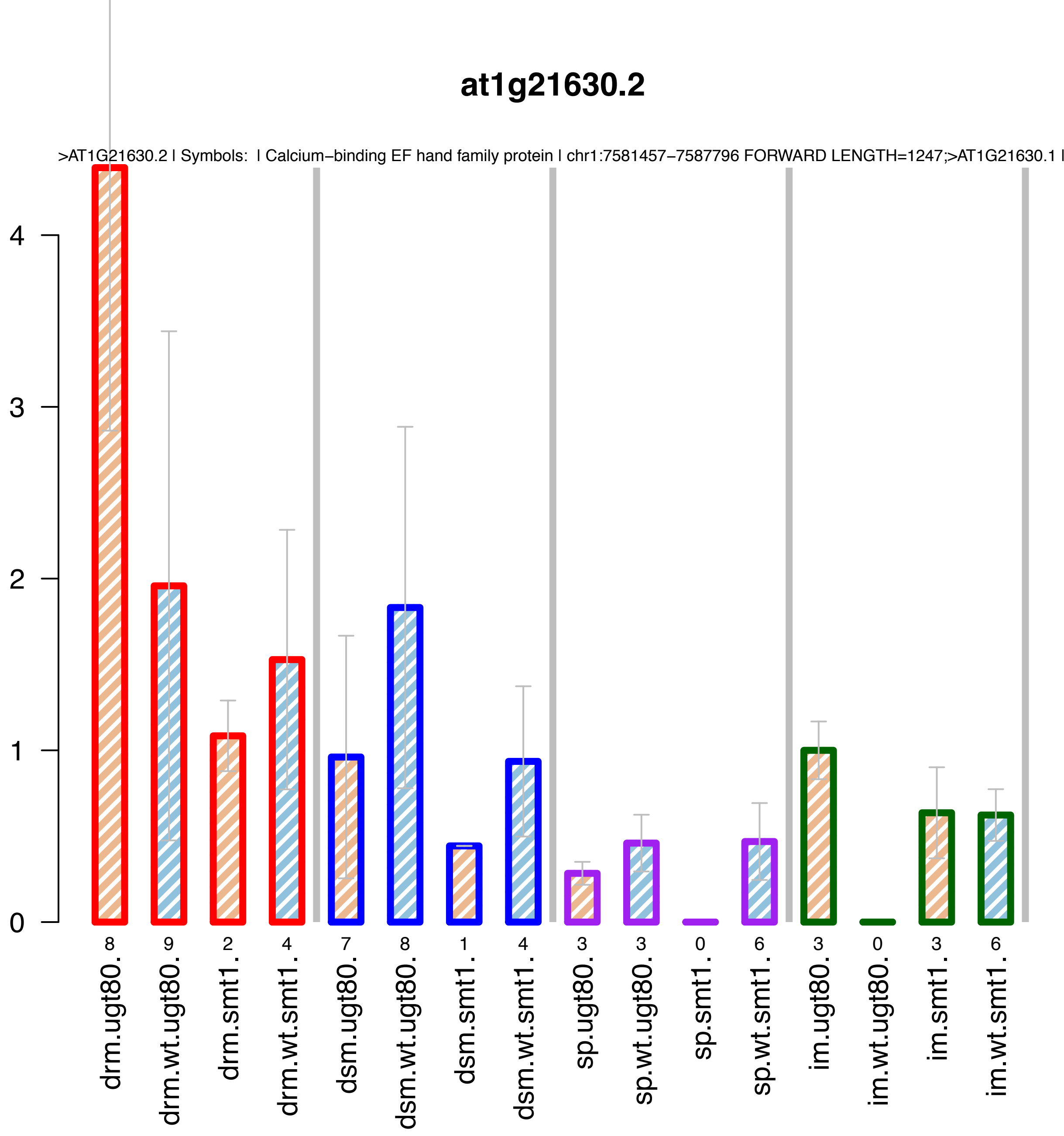

at1g21630.2
>AT1G21630.2 | Symbols: | Calcium–binding EF hand family protein | chr1:7581457–7587796 FORWARD LENGTH=1247;>AT1G21630.1 |
0
1
2
3
4
8
9
2
4
7
8
1
4
3
3
0
6
3
0
3
6
drm.ugt80.
drm.wt.ugt80.
drm.smt1.
drm.wt.smt1.
dsm.ugt80.
dsm.wt.ugt80.
dsm.smt1.
dsm.wt.smt1.
sp.ugt80.
sp.wt.ugt80.
sp.smt1.
sp.wt.smt1.
im.ugt80.
im.wt.ugt80.
im.smt1.
im.wt.smt1.

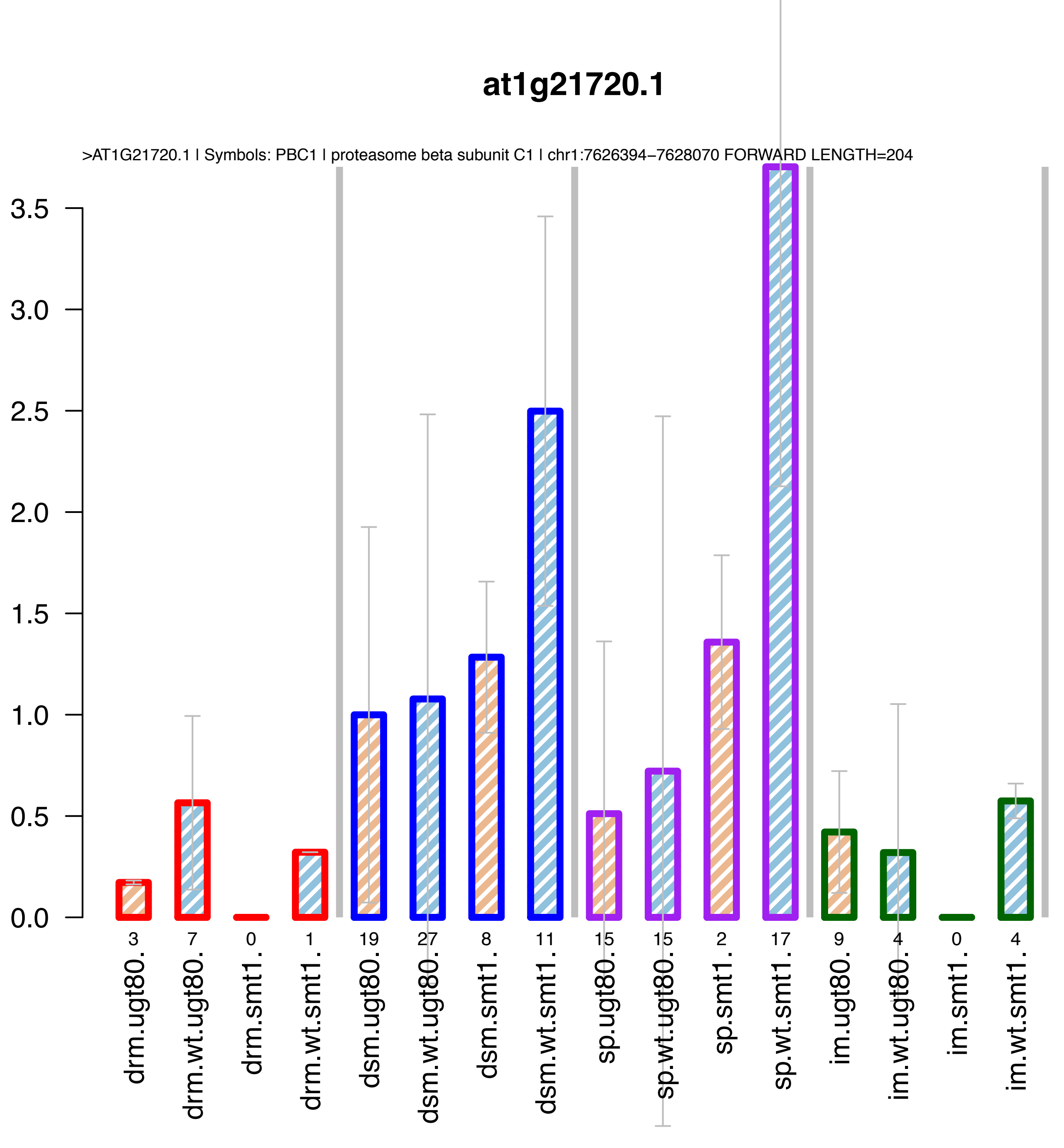

at1g21720.1
>AT1G21720.1 | Symbols: PBC1 | proteasome beta subunit C1 | chr1:7626394-7628070 FORWARD LENGTH=204
3.5
3.0
2.5
2.0
1.5
1.0
0.5
0.0
3
7
0
1
19
27
8
11
15
15
2
17
9
4
0
4
drm.ugt80.
drm.wt.ugt80.
drm.smt1.
drm.wt.smt1.
dsm.ugt80.
dsm.wt.ugt80.
dsm.smt1.
dsm.wt.smt1.
sp.ugt80.
sp.wt.ugt80.
sp.smt1.
sp.wt.smt1.
im.ugt80.
im.wt.ugt80.
im.smt1.
im.wt.smt1.

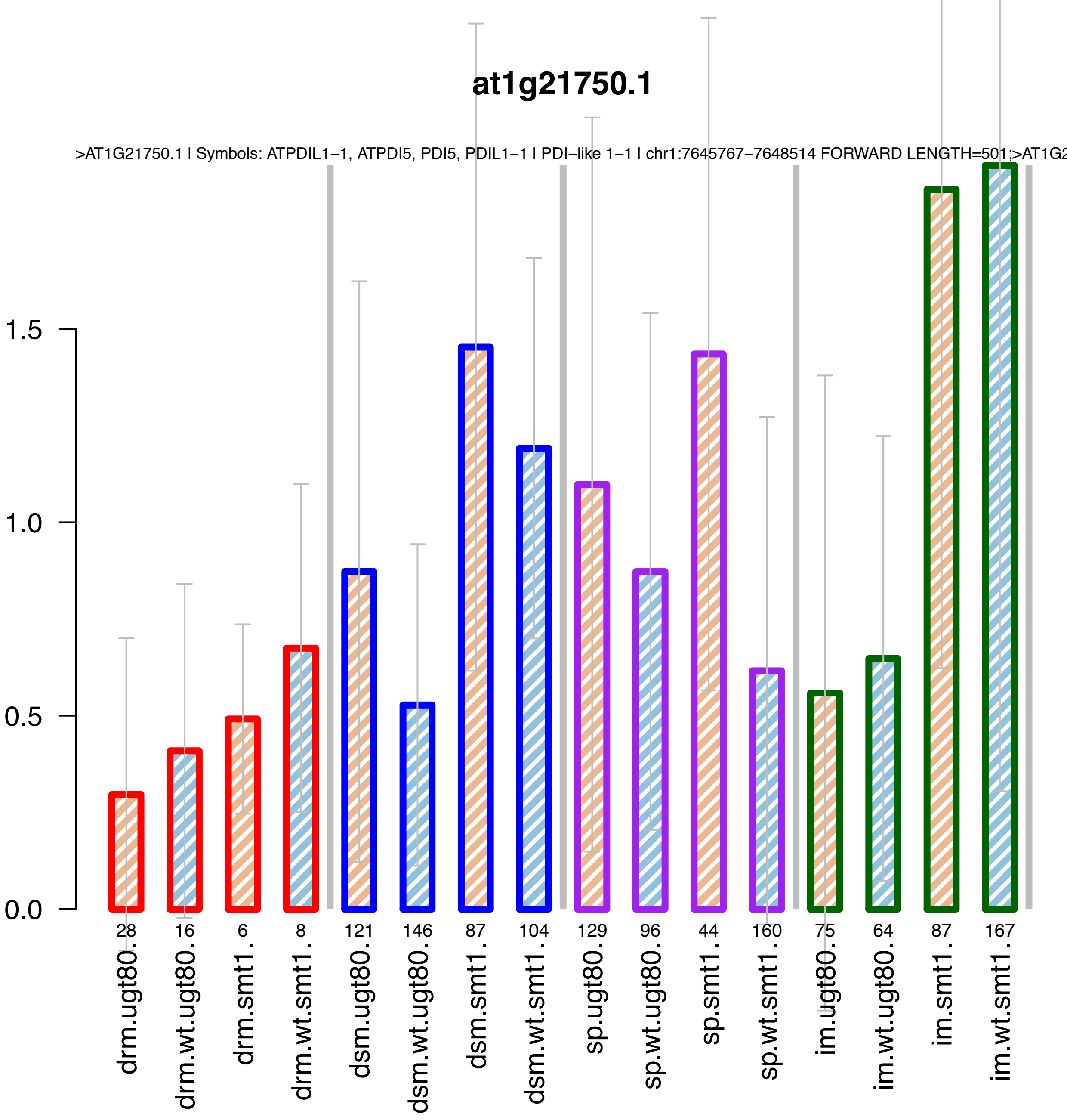

at1g21750.1
>AT1G21750.1 | Symbols: ATPDIL1−1, ATPDI5, PDI5, PDIL1−1 | PDI−like 1−1 | chr1:7645767−7648514 FORWARD LENGTH=501;>AT1G2
1.5
1.0
0.5
0.0
28
16
6
8
121
146
87
104
129
96
44
160
75
64
87
167
drm.ugt80..
drm.wt.ugt80..
drm.smt1..
drm.wt.smt1..
dsm.ugt80..
dsm.wt.ugt80..
dsm.smt1..
dsm.wt.smt1..
sp.ugt80..
sp.wt.ugt80..
sp.smt1..
sp.wt.smt1..
im.ugt80..
im.wt.ugt80..
im.smt1..
im.wt.smt1..

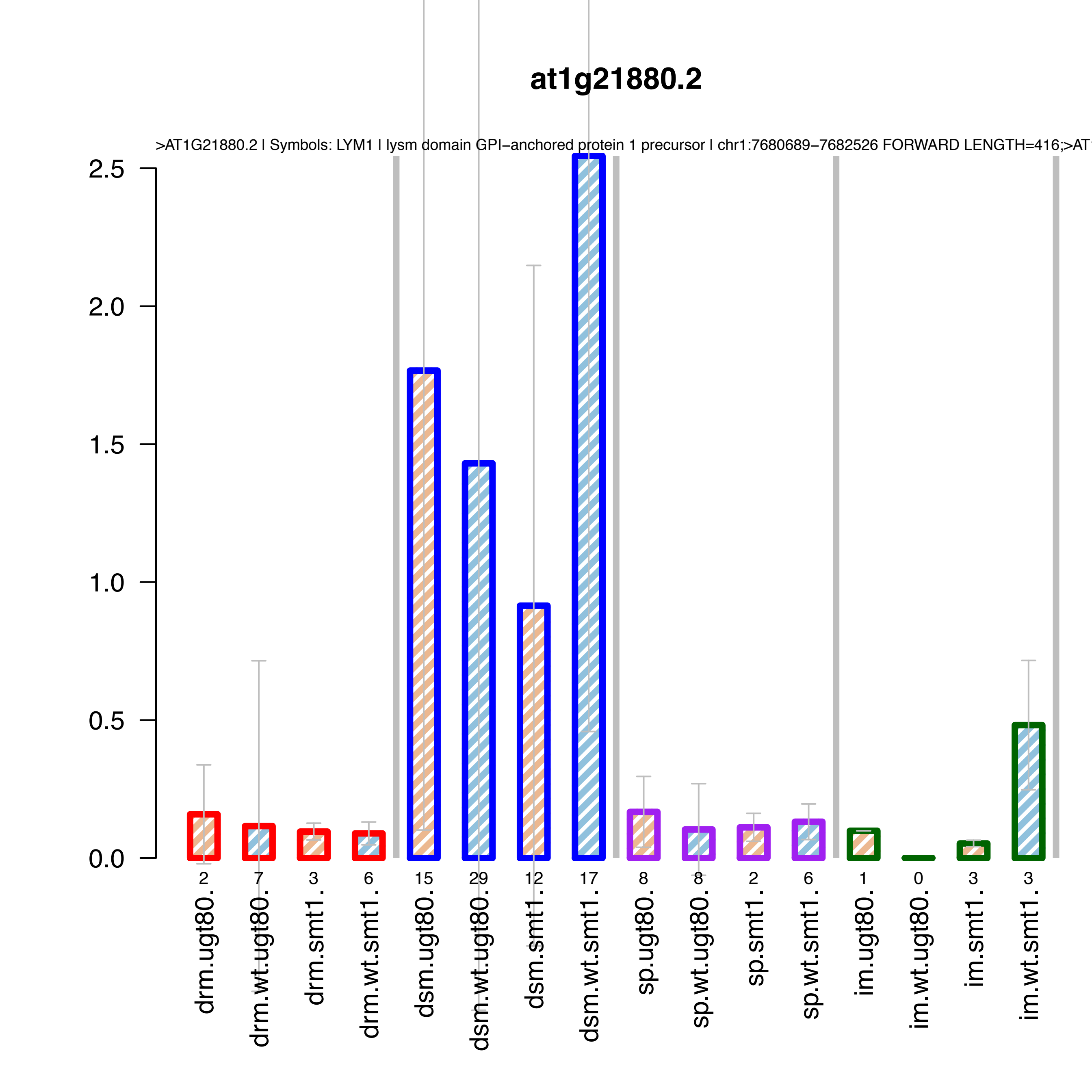
at1g21880.2
>AT1G21880.2 | Symbols: LYM1 | lysm domain GPI−anchored protein 1 precursor | chr1:7680689−7682526 FORWARD LENGTH=416;>AT
0.0
0.5
1.0
1.5
2.0
2.5
2
7
3
6
15
29
12
17
8
8
2
6
1
0
3
3
drm.ugt80.
drm.wt.ugt80.
drm.smt1.
drm.wt.smt1.
dsm.ugt80.
dsm.wt.ugt80.
dsm.smt1.
dsm.wt.smt1.
sp.ugt80.
sp.wt.ugt80.
sp.smt1.
sp.wt.smt1.
im.ugt80.
im.wt.ugt80.
im.smt1.
im.wt.smt1.

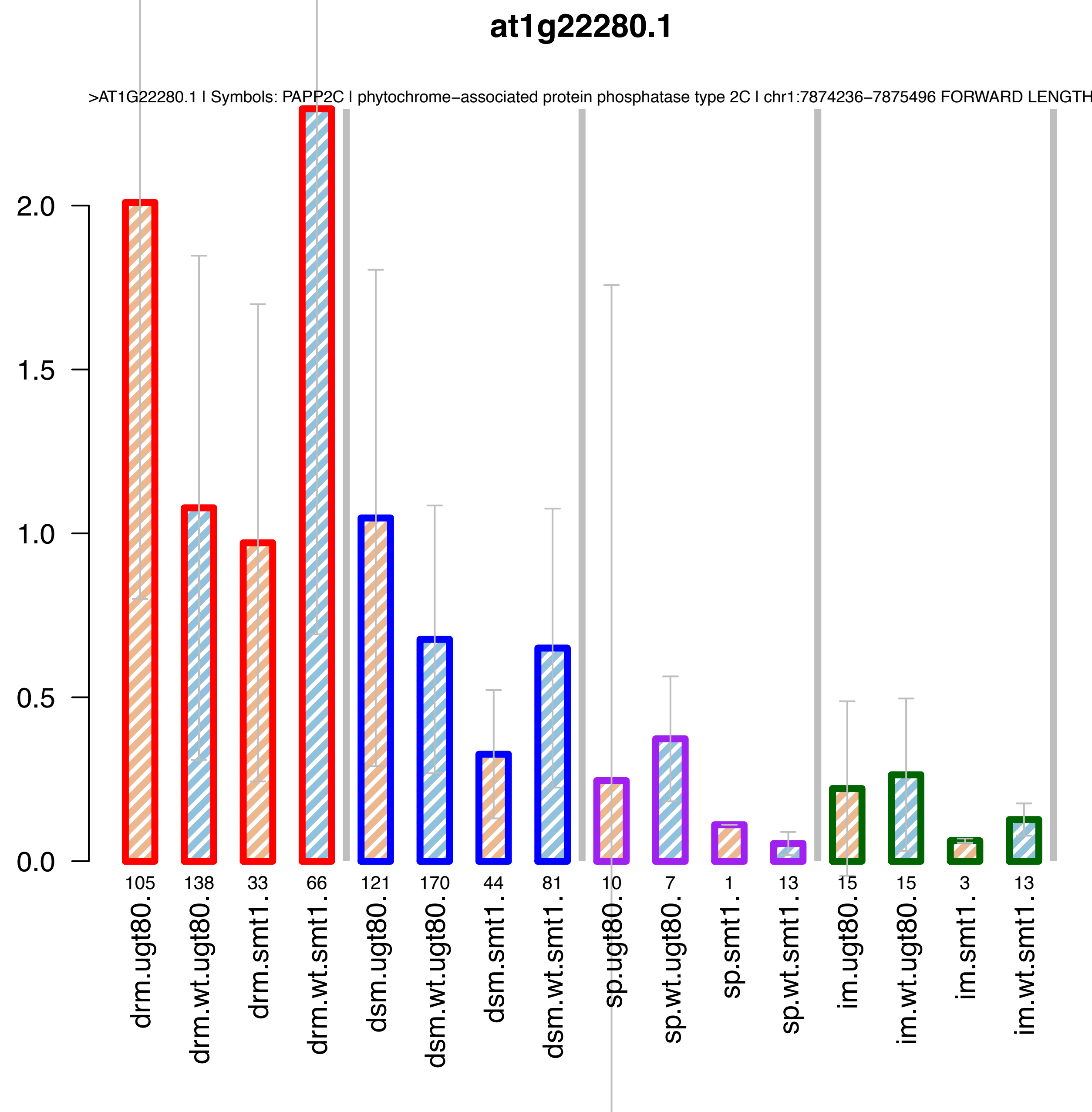
at1g22280.1
>AT1G22280.1 | Symbols: PAPP2C | phytochrome−associated protein phosphatase type 2C | chr1:7874236−7875496 FORWARD LENGTH
2.0
1.5
1.0
0.5
0.0
105
138
33
66
121
170
44
81
10
7
1
13
15
15
3
13
drm.ugt80..
drm.wt.ugt80..
drm.smt1..
drm.wt.smt1..
dsm.ugt80..
dsm.wt.ugt80..
dsm.smt1..
dsm.wt.smt1..
sp.ugt80..
sp.wt.ugt80..
sp.smt1..
sp.wt.smt1..
im.ugt80..
im.wt.ugt80..
im.smt1..
im.wt.smt1..

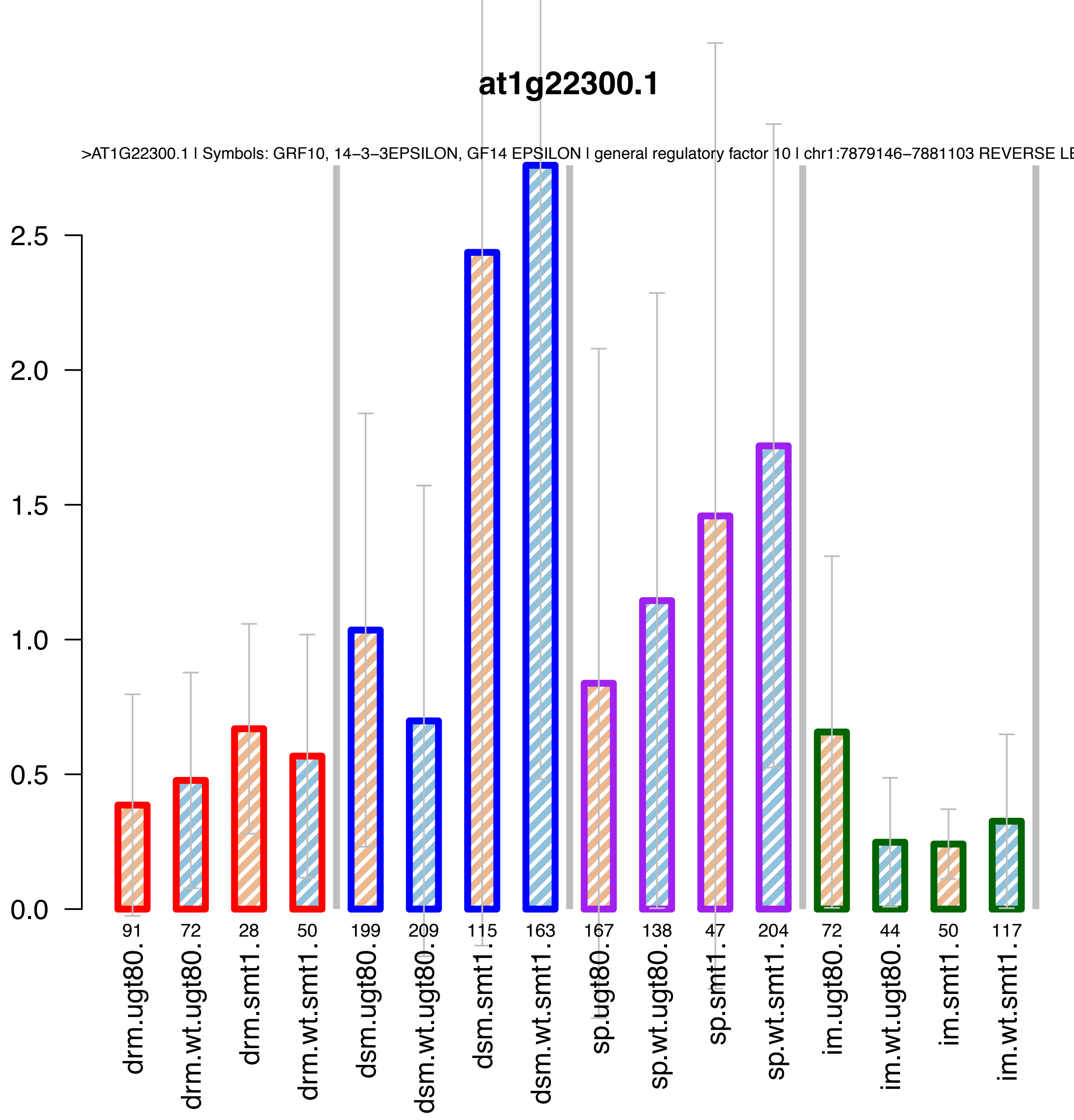

at1g22300.1
>AT1G22300.1 | Symbols: GRF10, 14-3-3EPSILON, GF14 EPSILON | general regulatory factor 10 | chr1:7879146-7881103 REVERSE LE
2.5
2.0
1.5
1.0
0.5
0.0
91
72
28
50
199
209
115
163
167
138
47
204
72
44
50
117
drm.ugt80..
drm.wt.ugt80..
drm.smt1..
drm.wt.smt1..
dsm.ugt80..
dsm.wt.ugt80..
dsm.smt1..
dsm.wt.smt1..
sp.ugt80..
sp.wt.ugt80..
sp.smt1..
sp.wt.smt1..
im.ugt80..
im.wt.ugt80..
im.smt1..
im.wt.smt1..

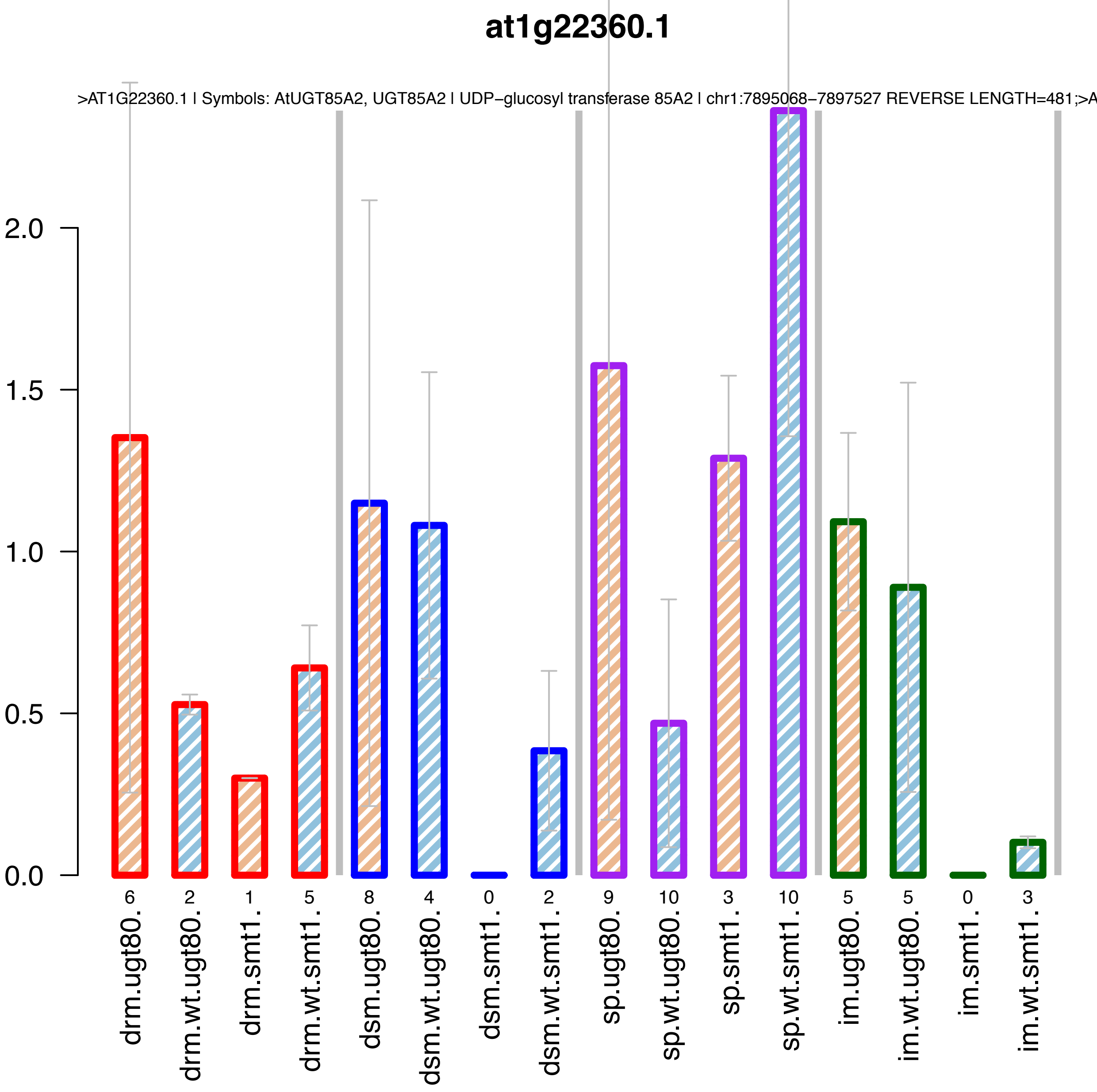

at1g22360.1
>AT1G22360.1 | Symbols: AtUGT85A2, UGT85A2 | UDP−glucosyl transferase 85A2 | chr1:7895068−7897527 REVERSE LENGTH=481;>A
2.0
1.5
1.0
0.5
0.0
6
2
1
5
8
4
0
2
9
10
3
10
5
5
0
3
drm.ugt80..
drm.wt.ugt80..
drm.smt1..
drm.wt.smt1..
dsm.ugt80..
dsm.wt.ugt80..
dsm.smt1..
dsm.wt.smt1..
sp.ugt80..
sp.wt.ugt80..
sp.smt1..
sp.wt.smt1..
im.ugt80..
im.wt.ugt80..
im.smt1..
im.wt.smt1..

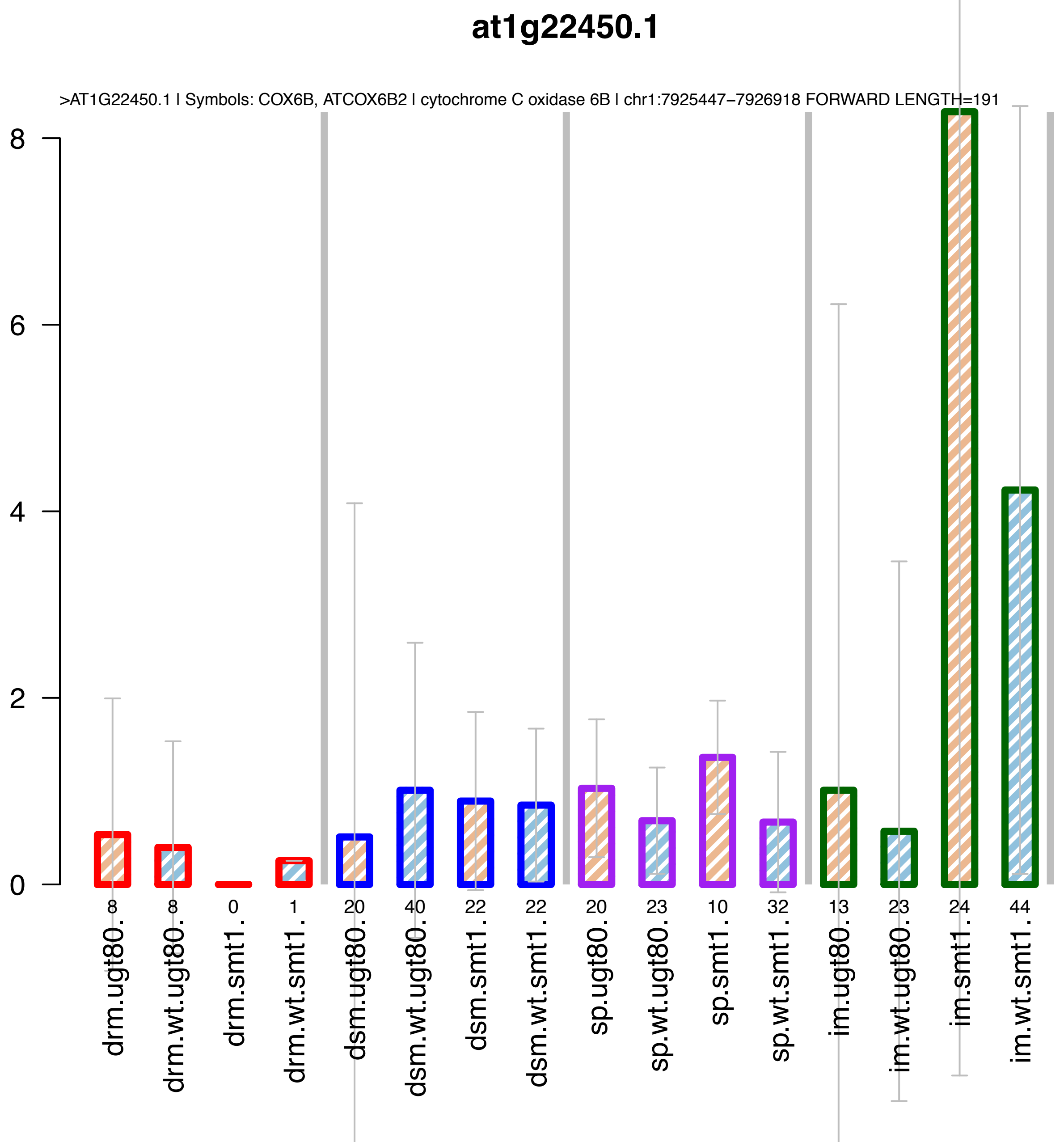

at1g22450.1
>AT1G22450.1 | Symbols: COX6B, ATCOX6B2 | cytochrome C oxidase 6B | chr1:7925447−7926918 FORWARD LENGTH=191
0
2
4
6
8
8
8
0
1
20
40
22
22
20
23
10
32
13
23
24
44
drm.ugt80..
drm.wt.ugt80..
drm.smt1..
drm.wt.smt1..
dsm.ugt80..
dsm.wt.ugt80..
dsm.smt1..
dsm.wt.smt1..
sp.ugt80..
sp.wt.ugt80..
sp.smt1..
sp.wt.smt1..
im.ugt80..
im.wt.ugt80..
im.smt1..
im.wt.smt1..

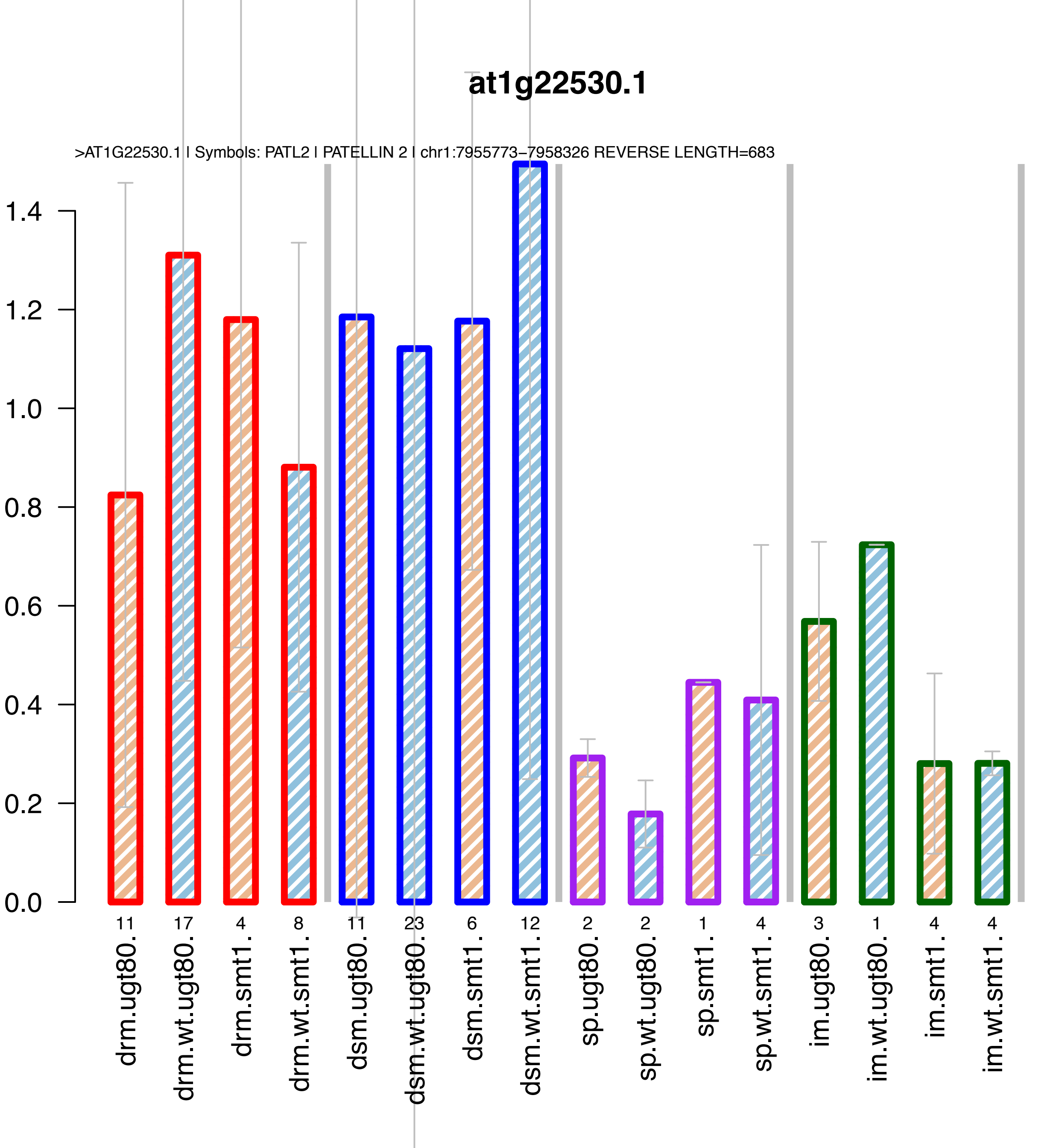

at1g22530.1
>AT1G22530.1 | Symbols: PATL2 | PATELLIN 2 | chr1:7955773-7958326 REVERSE LENGTH=683
0.0
0.2
0.4
0.6
0.8
1.0
1.2
1.4
11
17
4
8
11
23
6
12
2
2
1
4
3
1
4
4
drm.ugt80..
drm.wt.ugt80..
drm.smt1..
drm.wt.smt1..
dsm.ugt80..
dsm.wt.ugt80..
dsm.smt1..
dsm.wt.smt1..
sp.ugt80..
sp.wt.ugt80..
sp.smt1..
sp.wt.smt1..
im.ugt80..
im.wt.ugt80..
im.smt1..
im.wt.smt1..

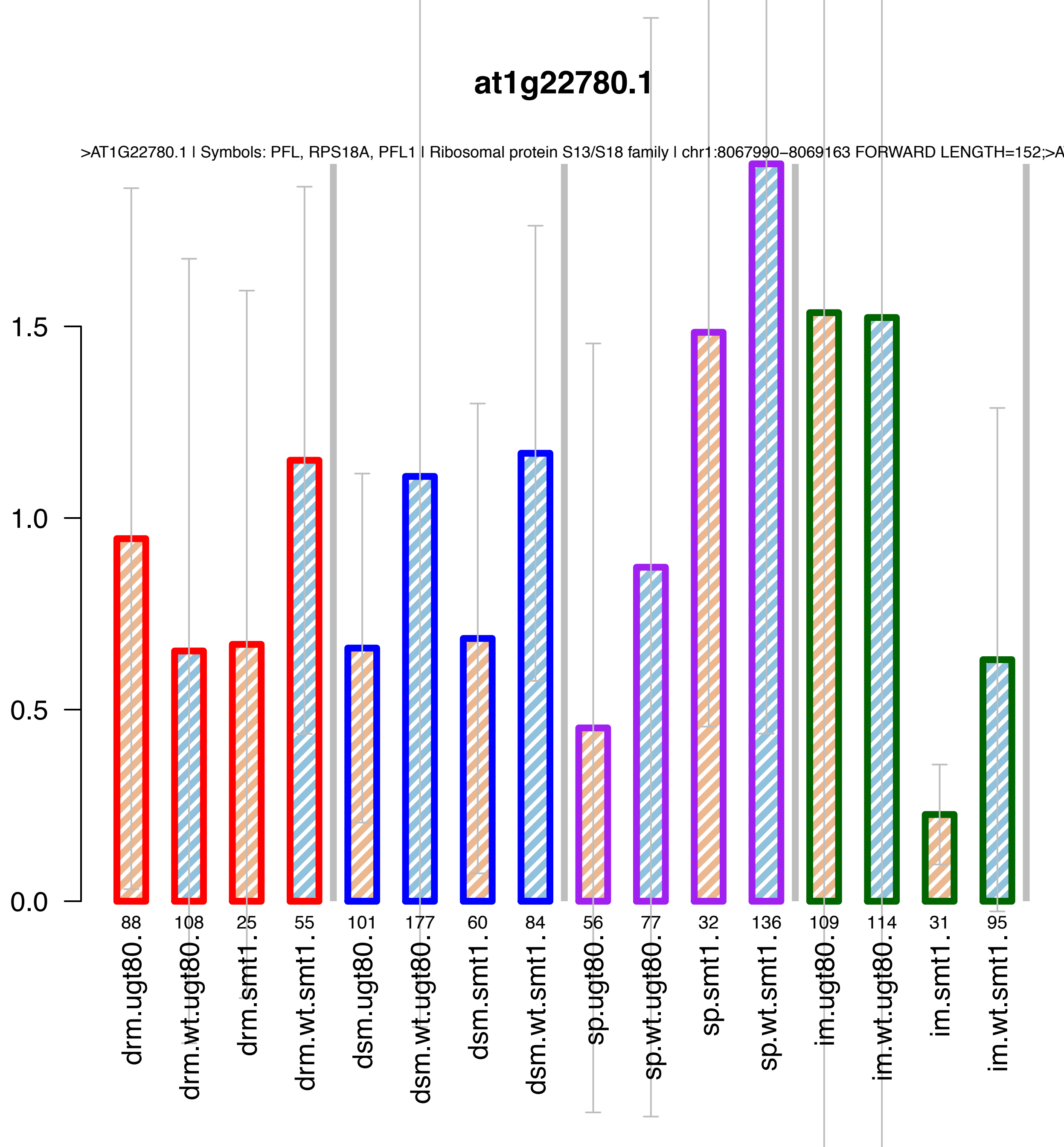

at1g22780.1
>AT1G22780.1 | Symbols: PFL, RPS18A, PFL1 | Ribosomal protein S13/S18 family | chr1:8067990-8069163 FORWARD LENGTH=152;>A
0.0
0.5
1.0
1.5
88
108
25
55
101
177
60
84
56
77
32
136
109
114
31
95
drm.ugt80..
drm.wt.ugt80..
drm.smt1..
drm.wt.smt1..
dsm.ugt80..
dsm.wt.ugt80..
dsm.smt1..
dsm.wt.smt1..
sp.ugt80..
sp.wt.ugt80..
sp.smt1..
sp.wt.smt1..
im.ugt80..
im.wt.ugt80..
im.smt1..
im.wt.smt1..

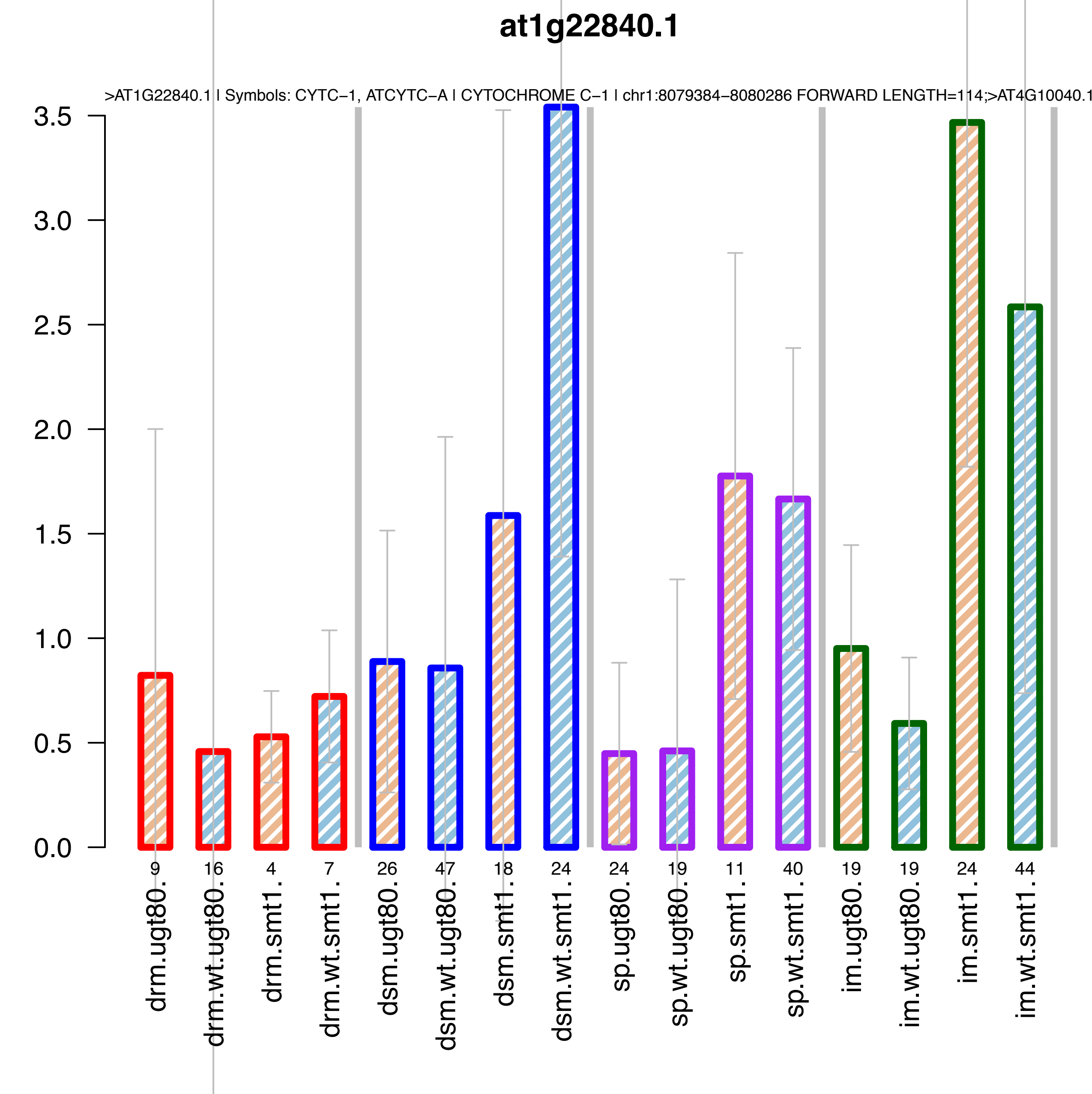
at1g22840.1
>AT1G22840.1 | Symbols: CYTC−1, ATCYTC−A | CYTOCHROME C−1 | chr1:8079384−8080286 FORWARD LENGTH=114;>AT4G10040.1
3.5
3.0
2.5
2.0
1.5
1.0
0.5
0.0
9
16
4
7
26
47
18
24
24
19
11
40
19
19
24
44
drm.ugt80.
drm.wt.ugt80.
drm.smt1.
drm.wt.smt1.
dsm.ugt80.
dsm.wt.ugt80.
dsm.smt1.
dsm.wt.smt1.
sp.ugt80.
sp.wt.ugt80.
sp.smt1.
sp.wt.smt1.
im.ugt80.
im.wt.ugt80.
im.smt1.
im.wt.smt1.

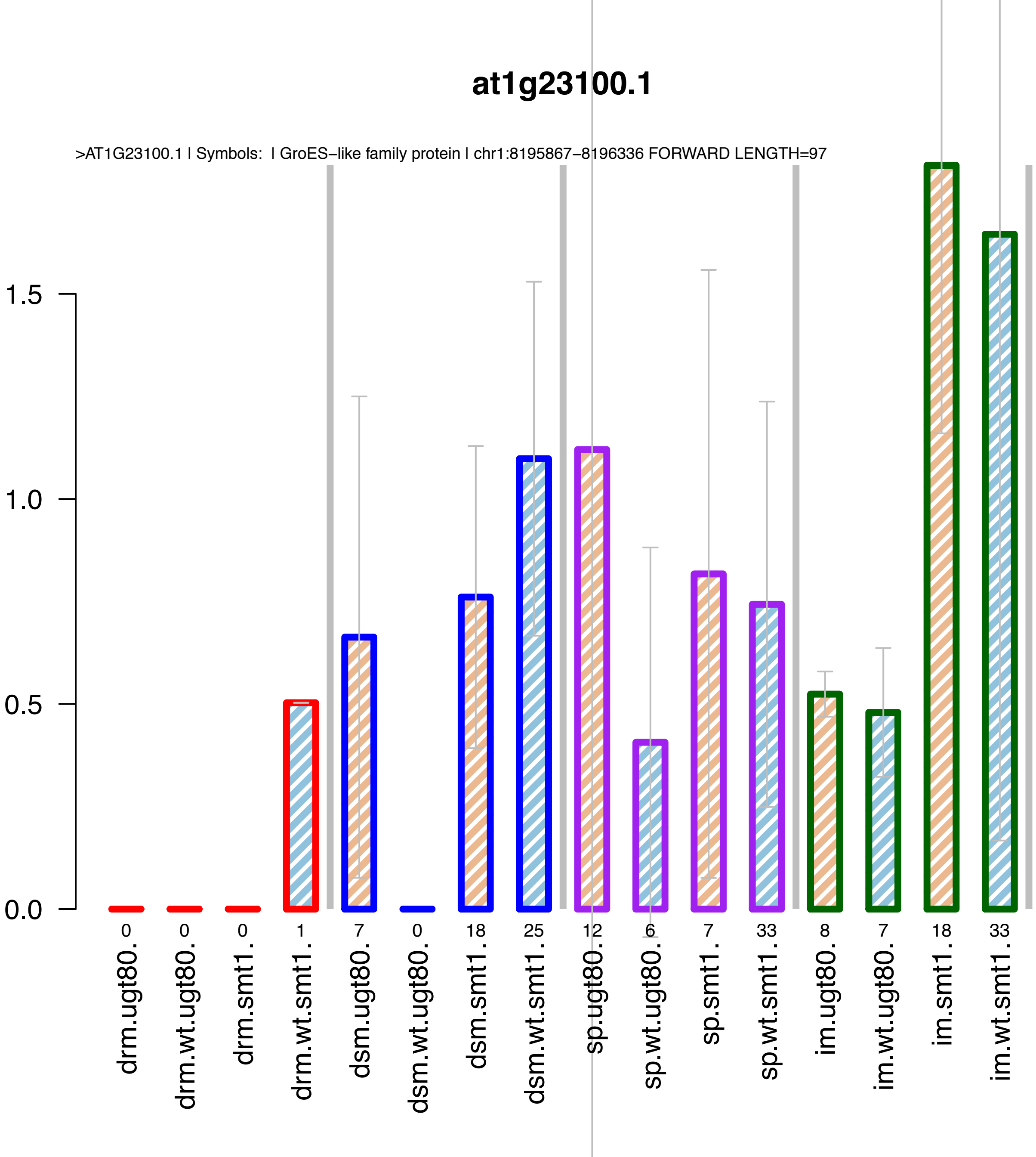

at1g23100.1
>AT1G23100.1 | Symbols: | GroES−like family protein | chr1:8195867−8196336 FORWARD LENGTH=97
0.0
0.5
1.0
1.5
0
0
0
1
7
0
18
25
12
6
7
33
8
7
18
33
drm.ugt80.
drm.wt.ugt80.
drm.smt1.
drm.wt.smt1.
dsm.ugt80.
dsm.wt.ugt80.
dsm.smt1.
dsm.wt.smt1.
sp.ugt80.
sp.wt.ugt80.
sp.smt1.
sp.wt.smt1.
im.ugt80.
im.wt.ugt80.
im.smt1.
im.wt.smt1.

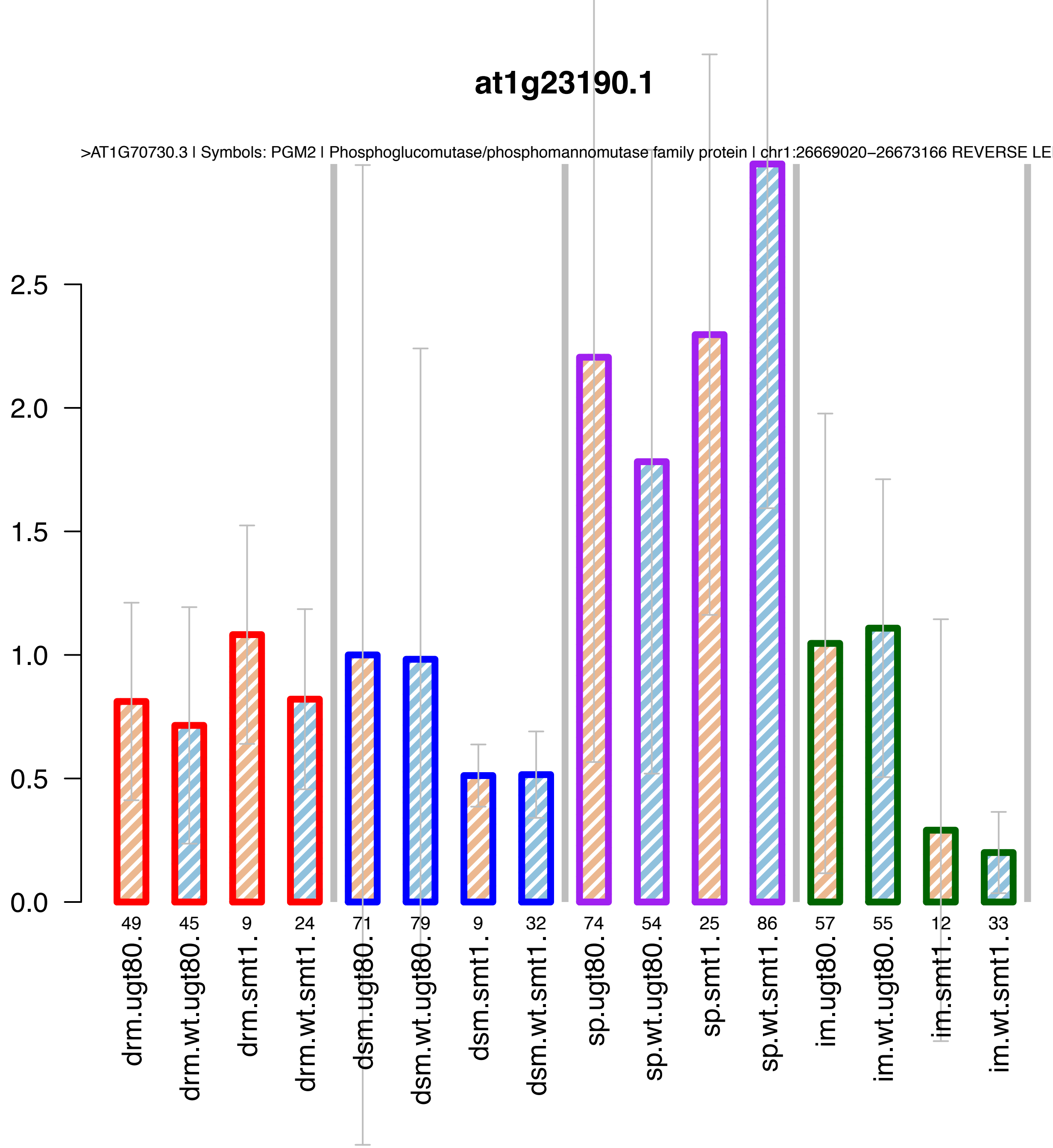
at1g23190.1
>AT1G70730.3 | Symbols: PGM2 | Phosphoglucomutase/phosphomannomutase family protein | chr1:26669020-26673166 REVERSE LEN
2.5
2.0
1.5
1.0
0.5
0.0
49
45
9
24
71
79
9
32
74
54
25
86
57
55
12
33
drm.ugt80..
drm.wt.ugt80..
drm.smt1..
drm.wt.smt1..
dsm.ugt80..
dsm.wt.ugt80..
dsm.smt1..
dsm.wt.smt1..
sp.ugt80..
sp.wt.ugt80..
sp.smt1..
sp.wt.smt1..
im.ugt80..
im.wt.ugt80..
im.smt1..
im.wt.smt1..

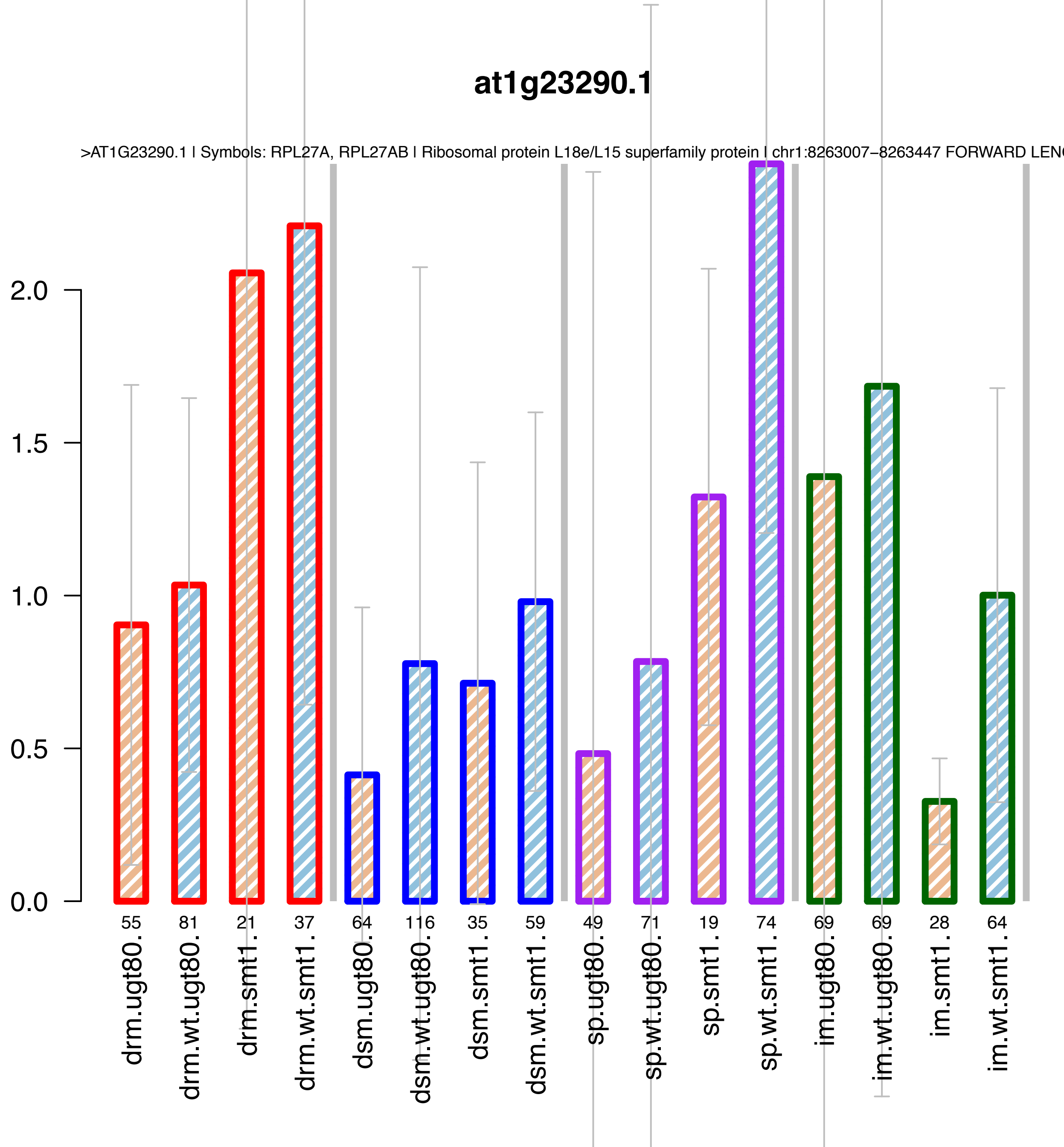

at1g23290.1
>AT1G23290.1 | Symbols: RPL27A, RPL27AB | Ribosomal protein L18e/L15 superfamily protein | chr1:8263007-8263447 FORWARD LEN
2.0
1.5
1.0
0.5
0.0
55
81
21
37
64
116
35
59
49
71
19
74
69
69
28
64
drm.ugt80.
drm.wt.ugt80.
drm.smt1.
drm.wt.smt1.
dsm.ugt80.
dsm.wt.ugt80.
dsm.smt1.
dsm.wt.smt1.
sp.ugt80.
sp.wt.ugt80.
sp.smt1.
sp.wt.smt1.
im.ugt80.
im.wt.ugt80.
im.smt1.
im.wt.smt1.

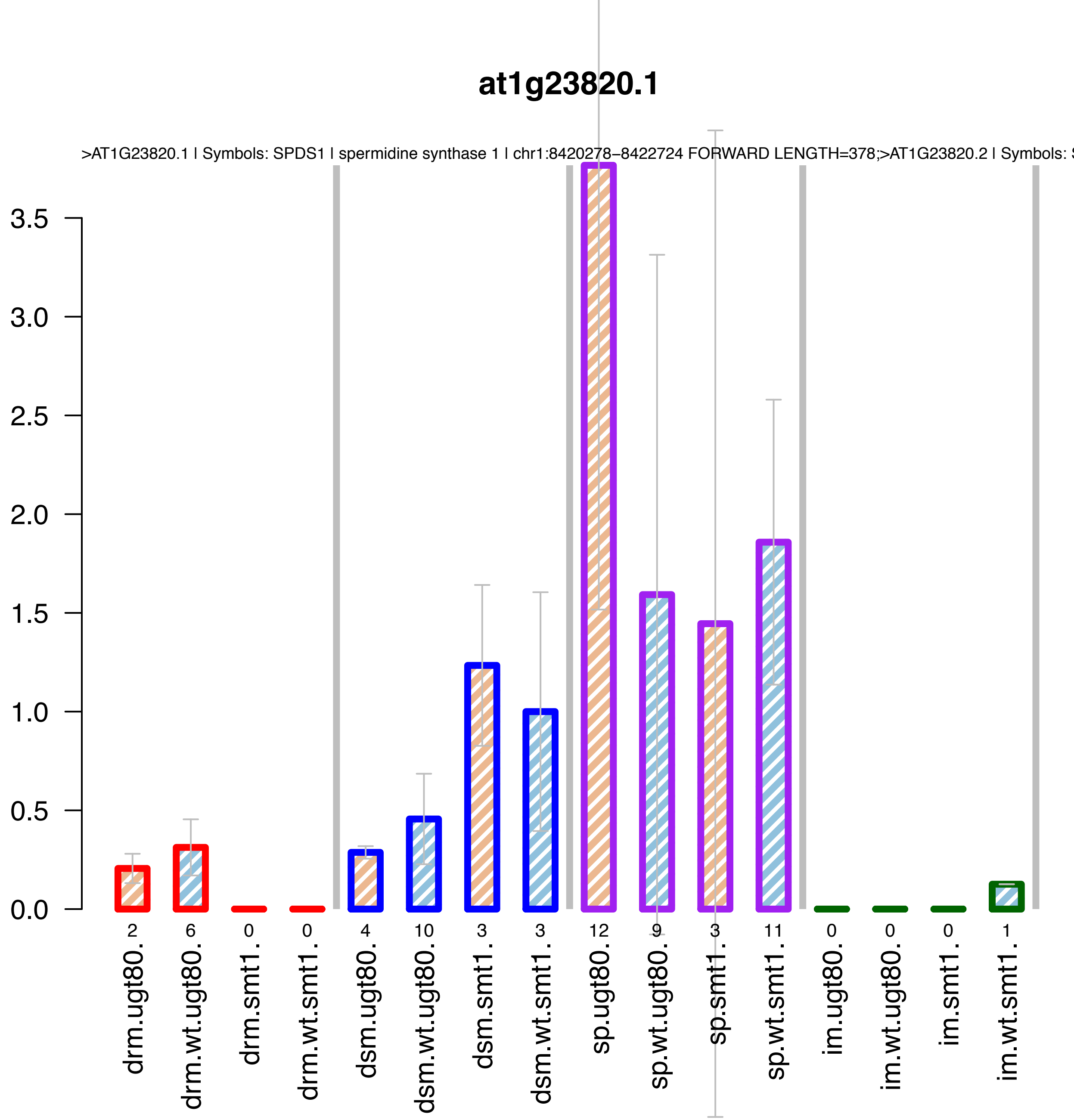

at1g23820.1
>AT1G23820.1 | Symbols: SPDS1 | spermidine synthase 1 | chr1:8420278-8422724 FORWARD LENGTH=378;>AT1G23820.2 | Symbols:
3.5
3.0
2.5
2.0
1.5
1.0
0.5
0.0
2
6
0
0
4
10
3
3
12
9
3
11
0
0
0
1
drm.ugt80.
drm.wt.ugt80.
drm.smt1.
drm.wt.smt1.
dsm.ugt80.
dsm.wt.ugt80.
dsm.smt1.
dsm.wt.smt1.
sp.ugt80.
sp.wt.ugt80.
sp.smt1.
sp.wt.smt1.
im.ugt80.
im.wt.ugt80.
im.smt1.
im.wt.smt1.

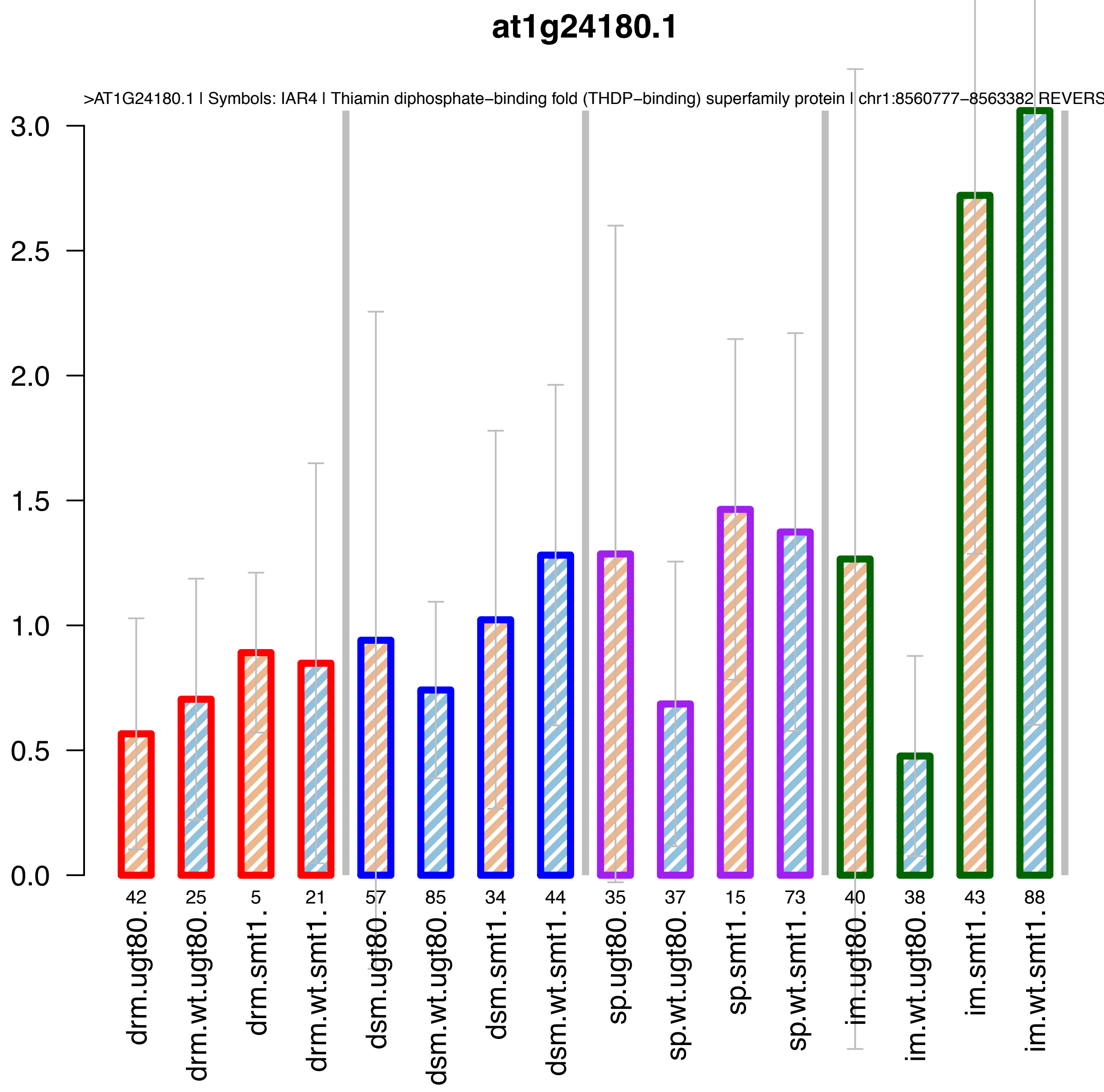
at1g24180.1
>AT1G24180.1 | Symbols: IAR4 | Thiamin diphosphate-binding fold (THDP-binding) superfamily protein | chr1:8560777-8563382 REVERS
3.0
2.5
2.0
1.5
1.0
0.5
0.0
42
25
5
21
57
85
34
44
35
37
15
73
40
38
43
88
drm.ugt80.
drm.wt.ugt80.
drm.smt1.
drm.wt.smt1.
dsm.ugt80.
dsm.wt.ugt80.
dsm.smt1.
dsm.wt.smt1.
sp.ugt80.
sp.wt.ugt80.
sp.smt1.
sp.wt.smt1.
im.ugt80.
im.wt.ugt80.
im.smt1.
im.wt.smt1.

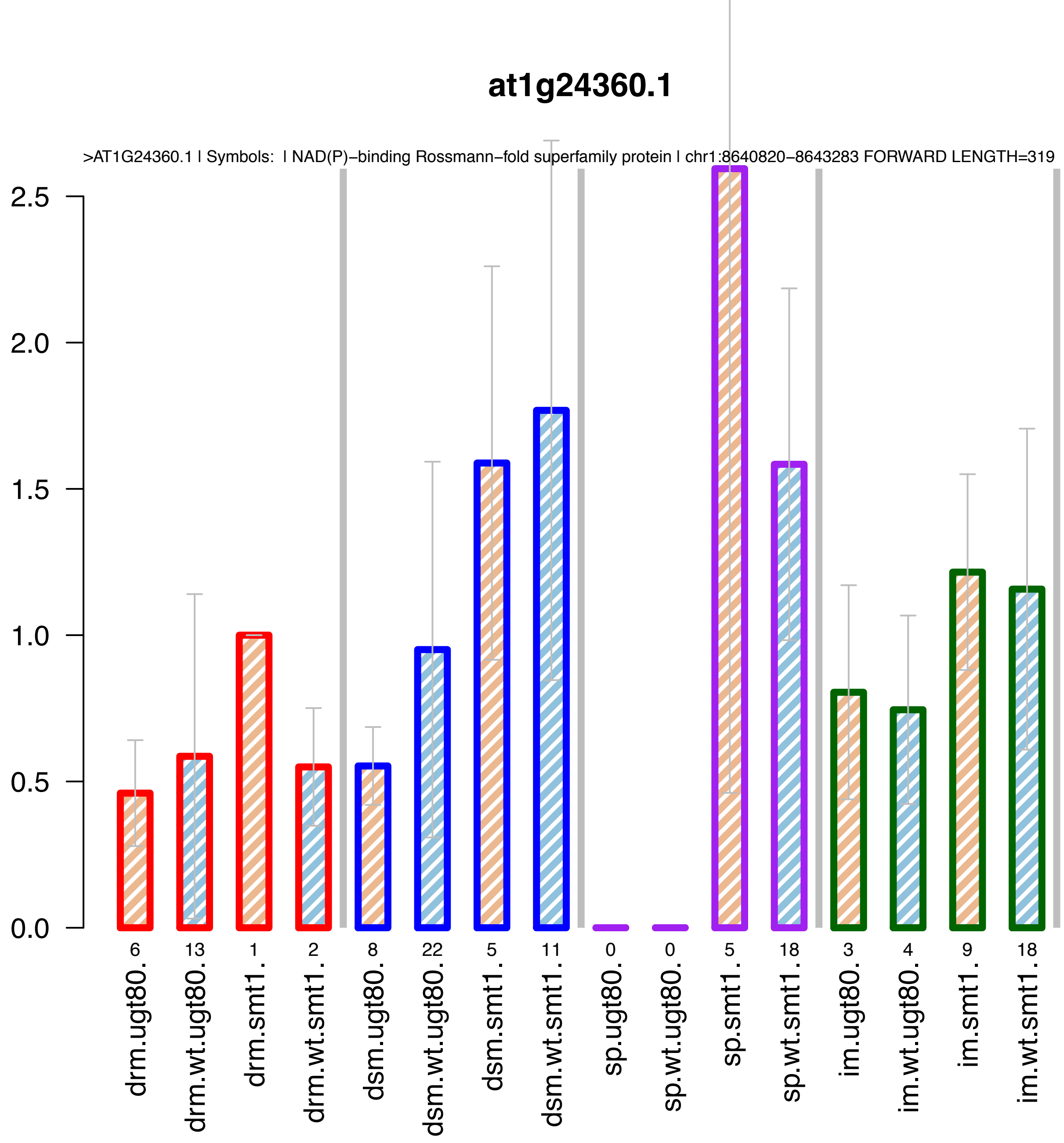

at1g24360.1
>AT1G24360.1 | Symbols: | NAD(P)−binding Rossmann−fold superfamily protein | chr1:8640820−8643283 FORWARD LENGTH=319
0.0
0.5
1.0
1.5
2.0
2.5
6
13
1
2
8
22
5
11
0
0
5
18
3
4
9
18
drm.ugt80.
drm.wt.ugt80.
drm.smt1.
drm.wt.smt1.
dsm.ugt80.
dsm.wt.ugt80.
dsm.smt1.
dsm.wt.smt1.
sp.ugt80.
sp.wt.ugt80.
sp.smt1.
sp.wt.smt1.
im.ugt80.
im.wt.ugt80.
im.smt1.
im.wt.smt1.

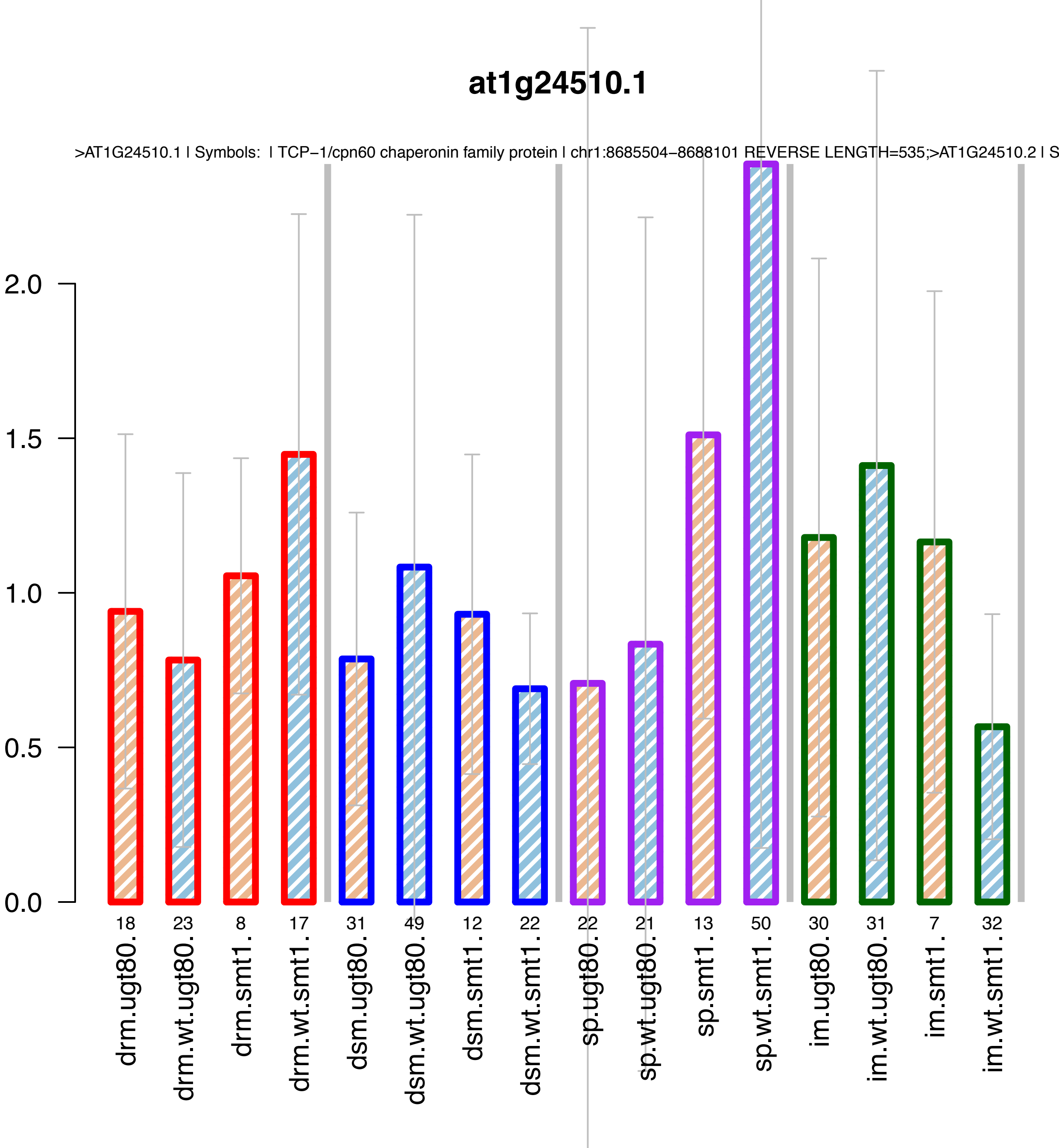

at1g24510.1
>AT1G24510.1 | Symbols: | TCP−1/cpn60 chaperonin family protein | chr1:8685504−8688101 REVERSE LENGTH=535;>AT1G24510.2 | S
0.0
0.5
1.0
1.5
2.0
18
23
8
17
31
49
12
22
22
21
13
50
30
31
7
32
drm.ugt80..
drm.wt.ugt80..
drm.smt1..
drm.wt.smt1..
dsm.ugt80..
dsm.wt.ugt80..
dsm.smt1..
dsm.wt.smt1..
sp.ugt80..
sp.wt.ugt80..
sp.smt1..
sp.wt.smt1..
im.ugt80..
im.wt.ugt80..
im.smt1..
im.wt.smt1..

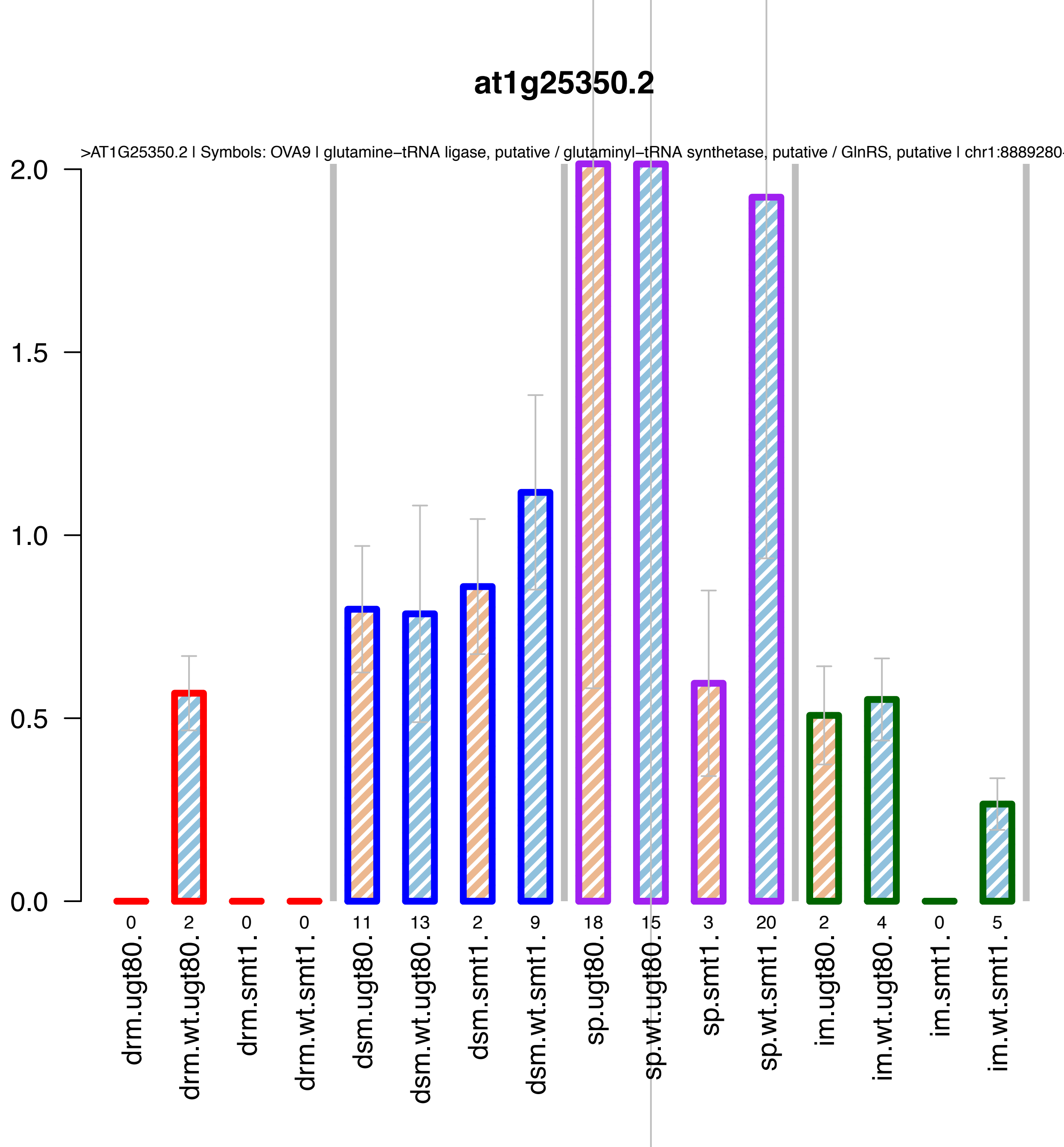
at1g25350.2
>AT1G25350.2 | Symbols: OVA9 | glutamine−tRNA ligase, putative / glutaminyl−tRNA synthetase, putative / GlnRS, putative | chr1:8889280-
2.0
1.5
1.0
0.5
0.0
0
2
0
0
11
13
2
9
18
15
3
20
2
4
0
5
drm.ugt80.
drm.wt.ugt80.
drm.smt1.
drm.wt.smt1.
dsm.ugt80.
dsm.wt.ugt80.
dsm.smt1.
dsm.wt.smt1.
sp.ugt80.
sp.wt.ugt80.
sp.smt1.
sp.wt.smt1.
im.ugt80.
im.wt.ugt80.
im.smt1.
im.wt.smt1.

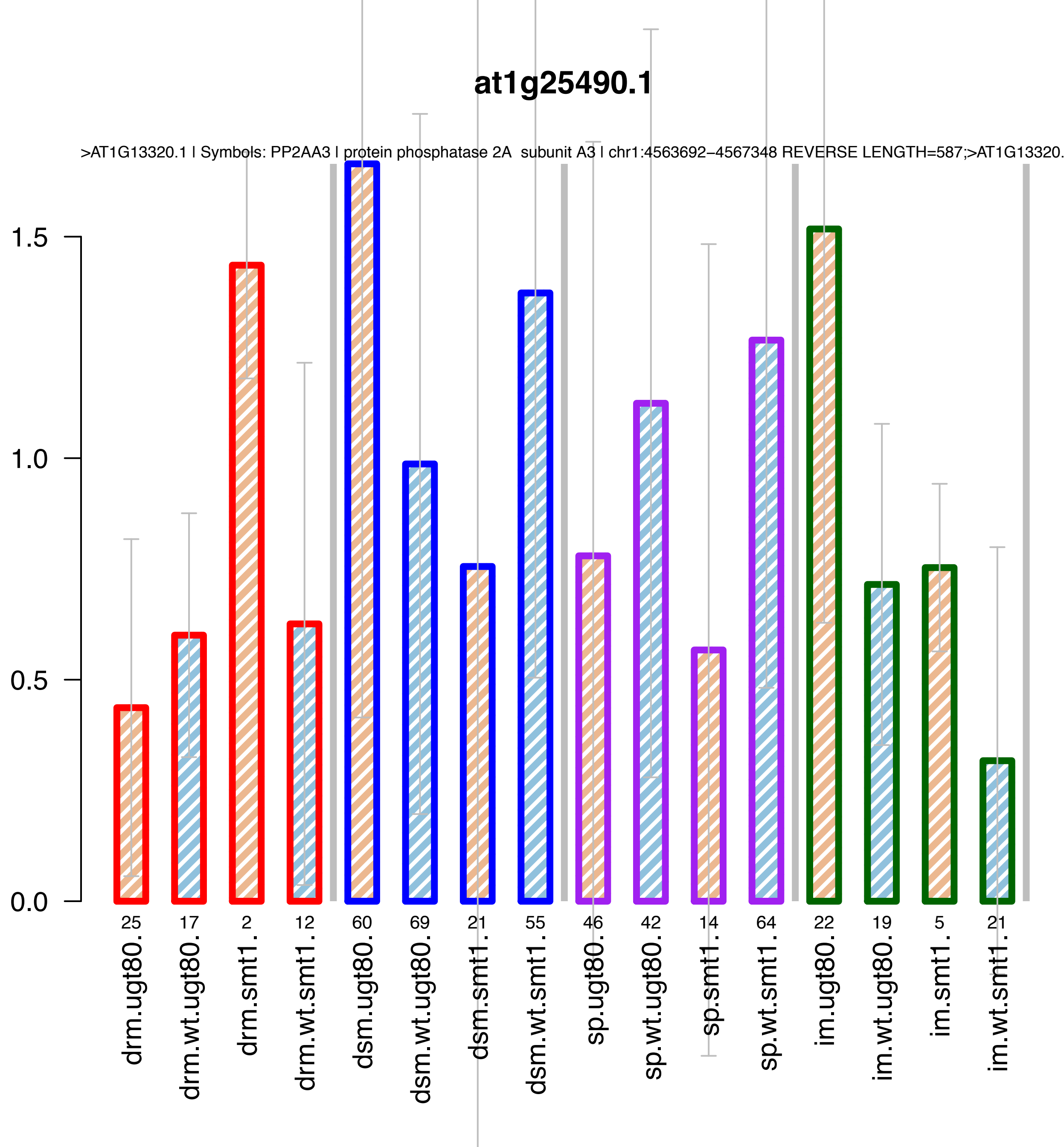

at1g25490.1
>AT1G13320.1 | Symbols: PP2AA3 | protein phosphatase 2A subunit A3 | chr1:4563692-4567348 REVERSE LENGTH=587;>AT1G13320.
1.5
1.0
0.5
0.0
25
17
2
12
60
69
21
55
46
42
14
64
22
19
5
21
drm.ugt80..
drm.wt.ugt80..
drm.smt1..
drm.wt.smt1..
dsm.ugt80..
dsm.wt.ugt80..
dsm.smt1..
dsm.wt.smt1..
sp.ugt80..
sp.wt.ugt80..
sp.smt1..
sp.wt.smt1..
im.ugt80..
im.wt.ugt80..
im.smt1..
im.wt.smt1..

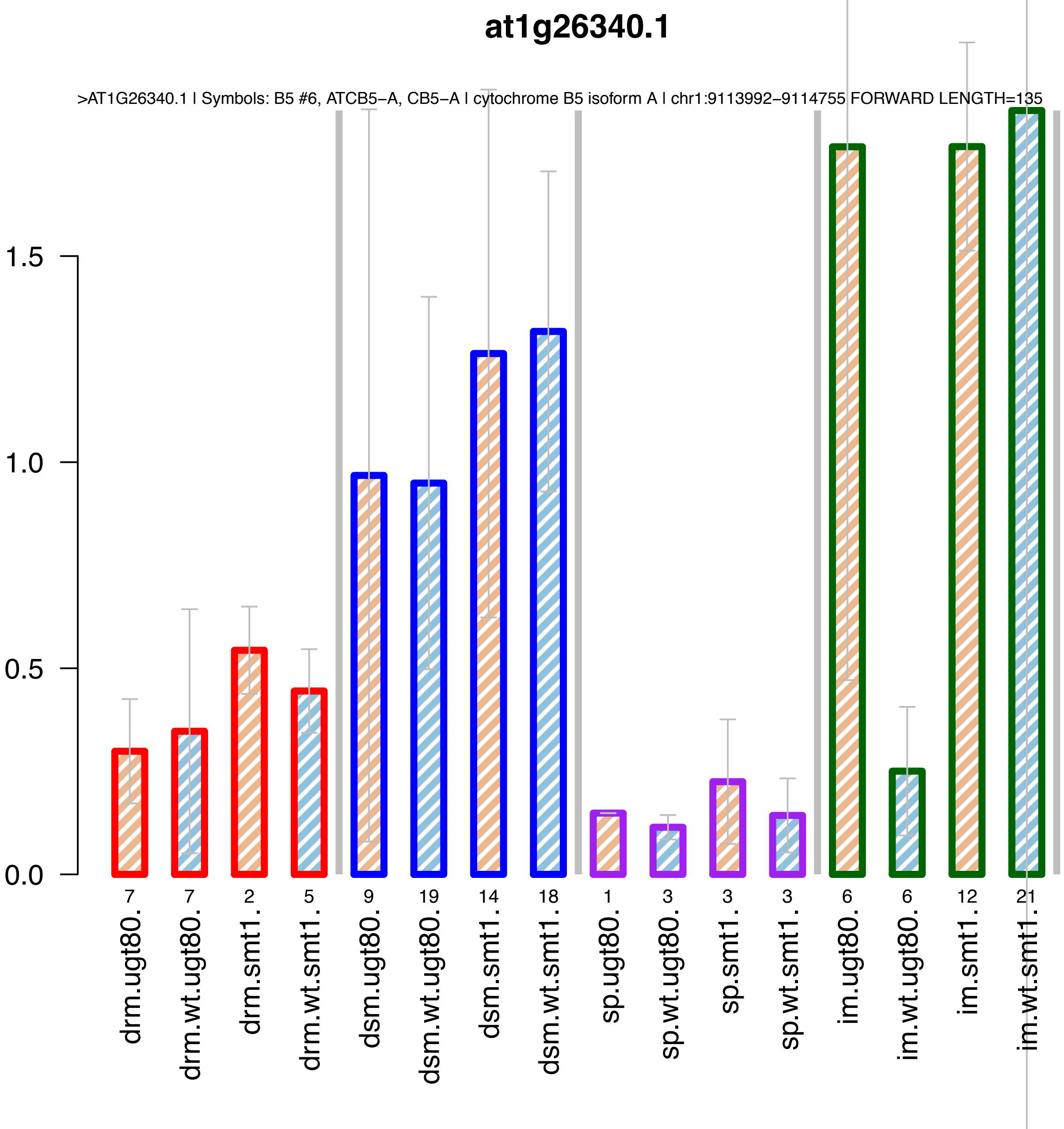
at1g26340.1
>AT1G26340.1 | Symbols: B5 #6, ATCB5−A, CB5−A | cytochrome B5 isoform A | chr1:9113992−9114755 FORWARD LENGTH=135
0.0
0.5
1.0
1.5
7
7
2
5
9
19
14
18
1
3
3
3
6
6
12
21
drm.ugt80.
drm.wt.ugt80.
drm.smt1.
drm.wt.smt1.
dsm.ugt80.
dsm.wt.ugt80.
dsm.smt1.
dsm.wt.smt1.
sp.ugt80.
sp.wt.ugt80.
sp.smt1.
sp.wt.smt1.
im.ugt80.
im.wt.ugt80.
im.smt1.
im.wt.smt1.

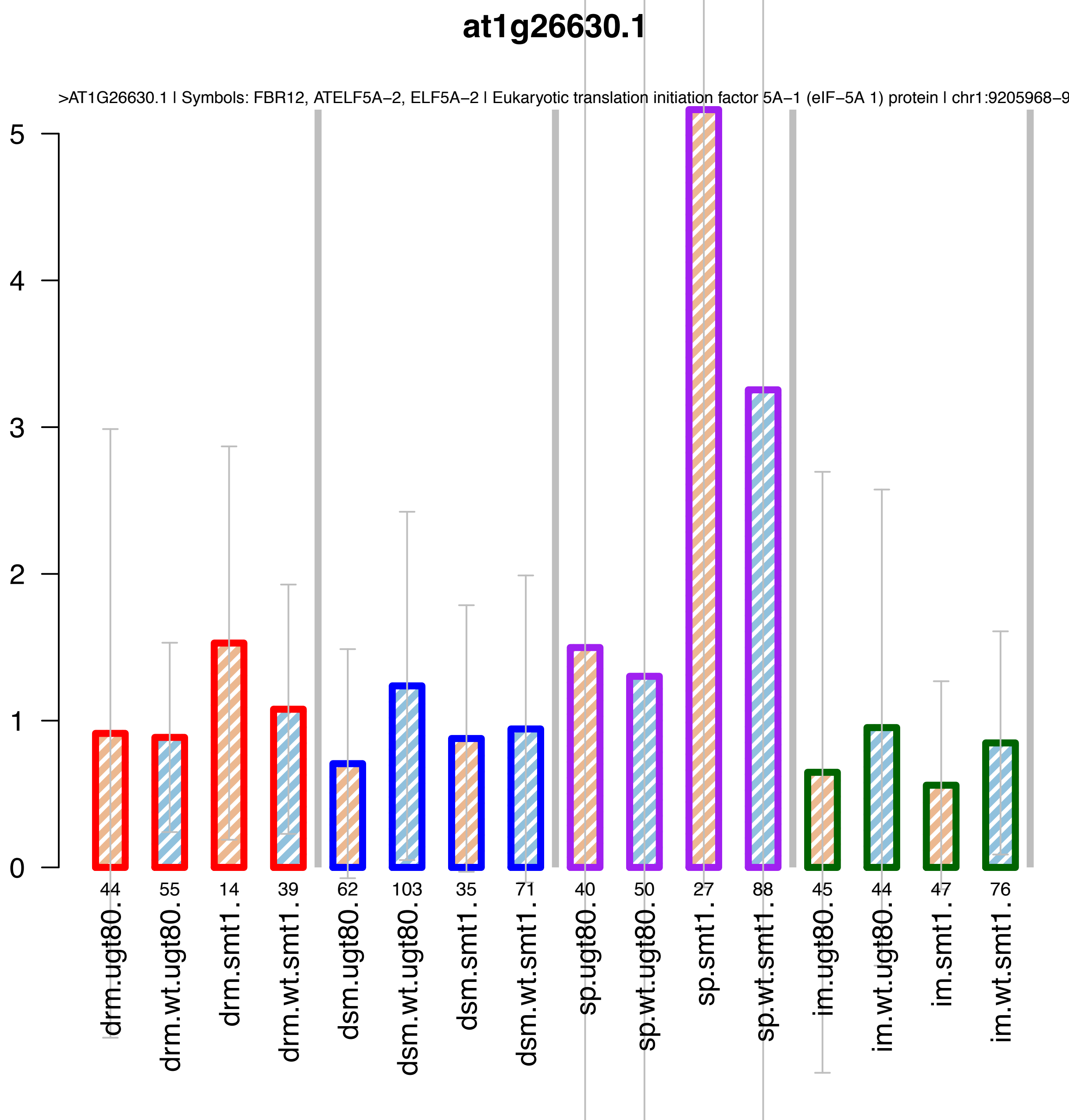
at1g26630.1
>AT1G26630.1 | Symbols: FBR12, ATELF5A−2, ELF5A−2 | Eukaryotic translation initiation factor 5A−1 (eIF−5A 1) protein | chr1:9205968−9
0
1
2
3
4
5
44
55
14
39
62
103
35
71
40
50
27
88
45
44
47
76
drm.ugt80..
drm.wt.ugt80..
drm.smt1..
drm.wt.smt1..
dsm.ugt80..
dsm.wt.ugt80..
dsm.smt1..
dsm.wt.smt1..
sp.ugt80..
sp.wt.ugt80..
sp.smt1..
sp.wt.smt1..
im.ugt80..
im.wt.ugt80..
im.smt1..
im.wt.smt1..

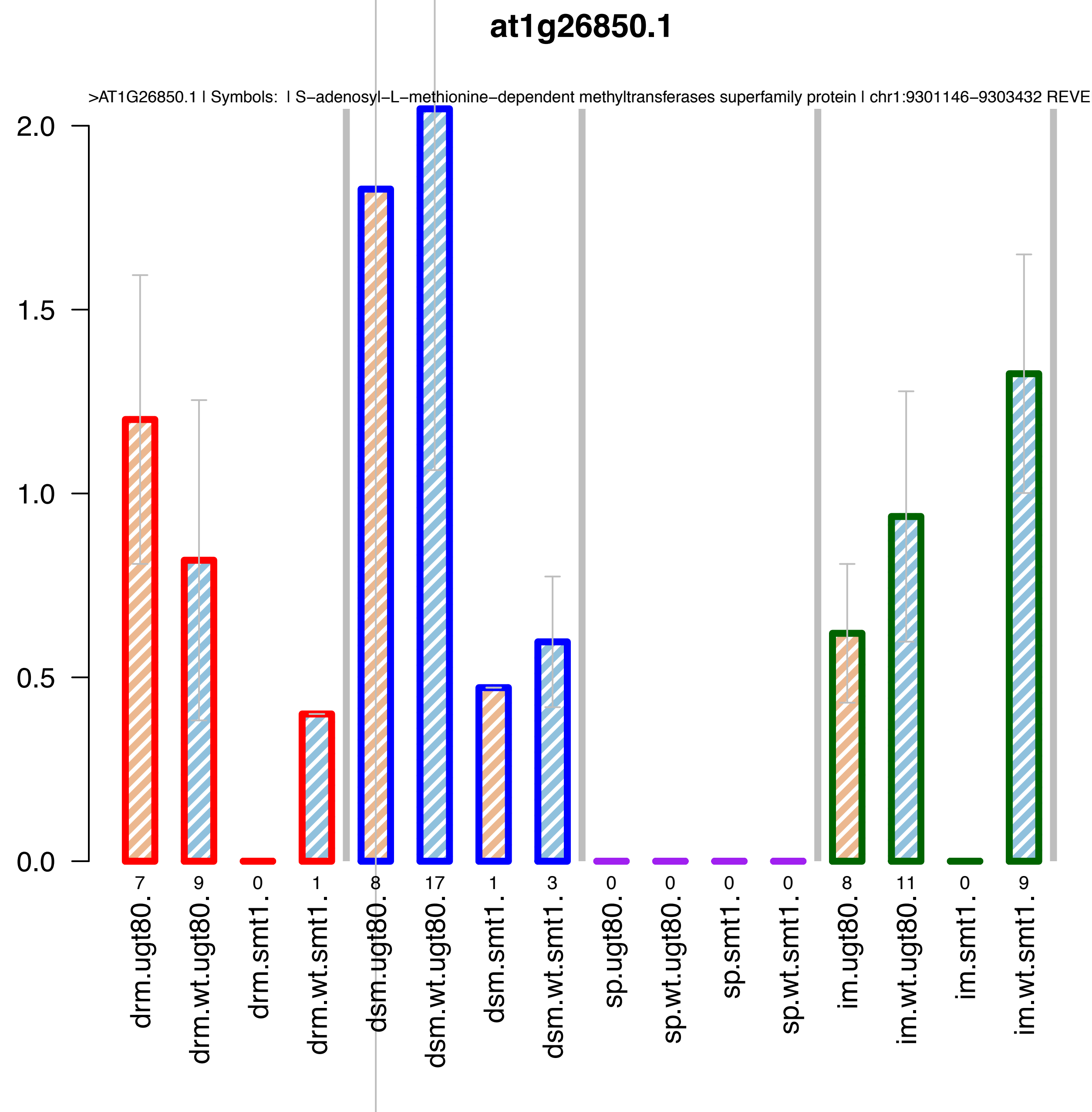

at1g26850.1
>AT1G26850.1 | Symbols: | S–adenosyl–L–methionine–dependent methyltransferases superfamily protein | chr1:9301146–9303432 REVE
2.0
1.5
1.0
0.5
0.0
7
9
0
1
8
17
1
3
0
0
0
0
8
11
0
9
drm.ugt80.
drm.wt.ugt80.
drm.smt1.
drm.wt.smt1.
dsm.ugt80.
dsm.wt.ugt80.
dsm.smt1.
dsm.wt.smt1.
sp.ugt80.
sp.wt.ugt80.
sp.smt1.
sp.wt.smt1.
im.ugt80.
im.wt.ugt80.
im.smt1.
im.wt.smt1.

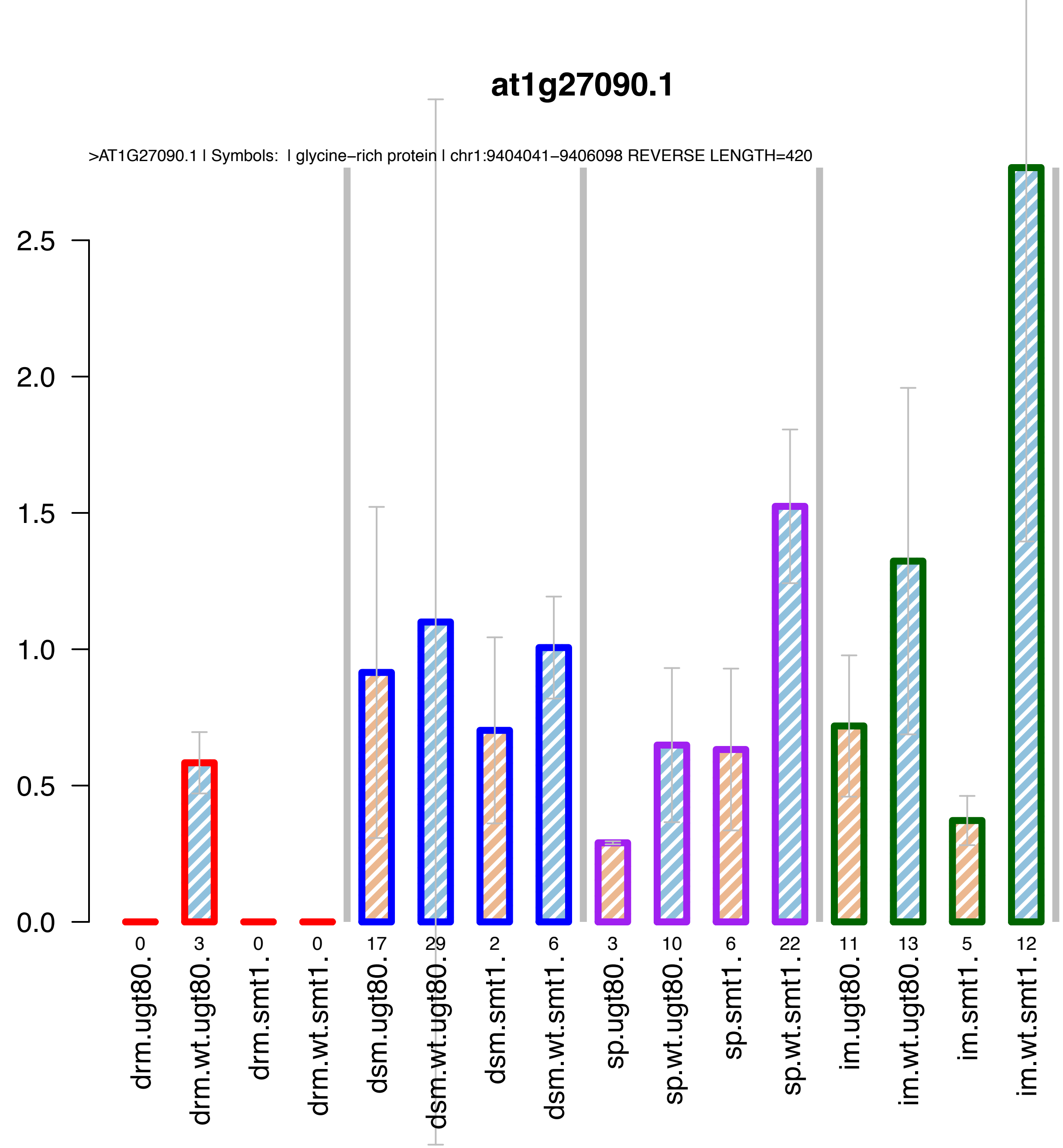

at1g27090.1
>AT1G27090.1 | Symbols: | glycine−rich protein | chr1:9404041−9406098 REVERSE LENGTH=420
0.0
0.5
1.0
1.5
2.0
2.5
0 3 0 0 17 29 2 6 3 10 6 22 11 13 5 12
drm.ugt80.
drm.wt.ugt80.
drm.smt1.
drm.wt.smt1.
dsm.ugt80.
dsm.wt.ugt80.
dsm.smt1.
dsm.wt.smt1.
sp.ugt80.
sp.wt.ugt80.
sp.smt1.
sp.wt.smt1.
im.ugt80.
im.wt.ugt80.
im.smt1.
im.wt.smt1.

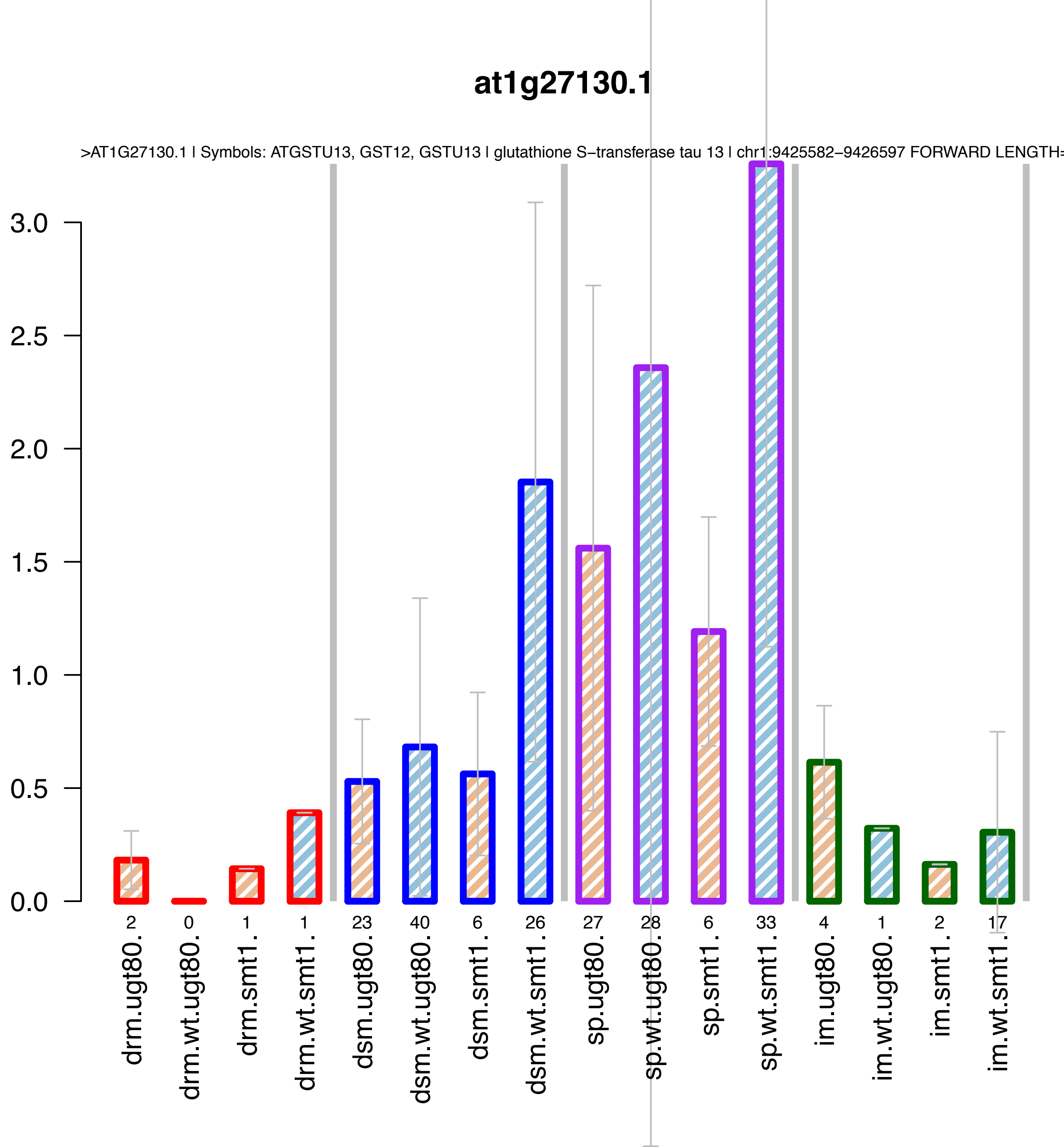

at1g27130.1
>AT1G27130.1 | Symbols: ATGSTU13, GST12, GSTU13 | glutathione S−transferase tau 13 | chr1:9425582−9426597 FORWARD LENGTH=
3.0
2.5
2.0
1.5
1.0
0.5
0.0
2
0
1
1
23
40
6
26
27
28
6
33
4
1
2
17
drm.ugt80.
drm.wt.ugt80.
drm.smt1.
drm.wt.smt1.
dsm.ugt80.
dsm.wt.ugt80.
dsm.smt1.
dsm.wt.smt1.
sp.ugt80.
sp.wt.ugt80.
sp.smt1.
sp.wt.smt1.
im.ugt80.
im.wt.ugt80.
im.smt1.
im.wt.smt1.

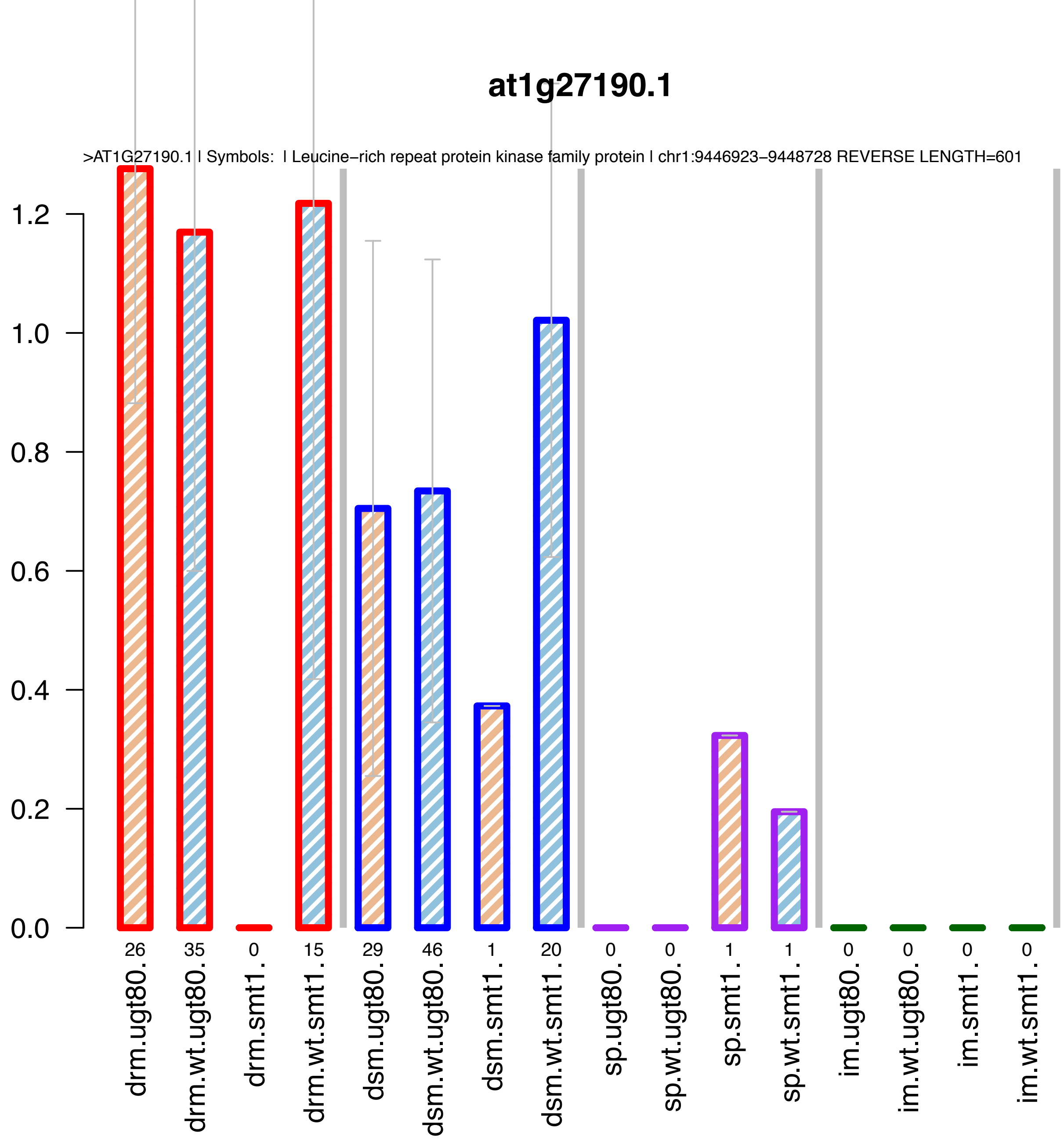
at1g27190.1
>AT1G27190.1 | Symbols: | Leucine−rich repeat protein kinase family protein | chr1:9446923−9448728 REVERSE LENGTH=601
0.0
0.2
0.4
0.6
0.8
1.0
1.2
26
35
0
15
29
46
1
20
0
0
1
1
0
0
0
0
drm.ugt80.
drm.wt.ugt80.
drm.smt1.
drm.wt.smt1.
dsm.ugt80.
dsm.wt.ugt80.
dsm.smt1.
dsm.wt.smt1.
sp.ugt80.
sp.wt.ugt80.
sp.smt1.
sp.wt.smt1.
im.ugt80.
im.wt.ugt80.
im.smt1.
im.wt.smt1.

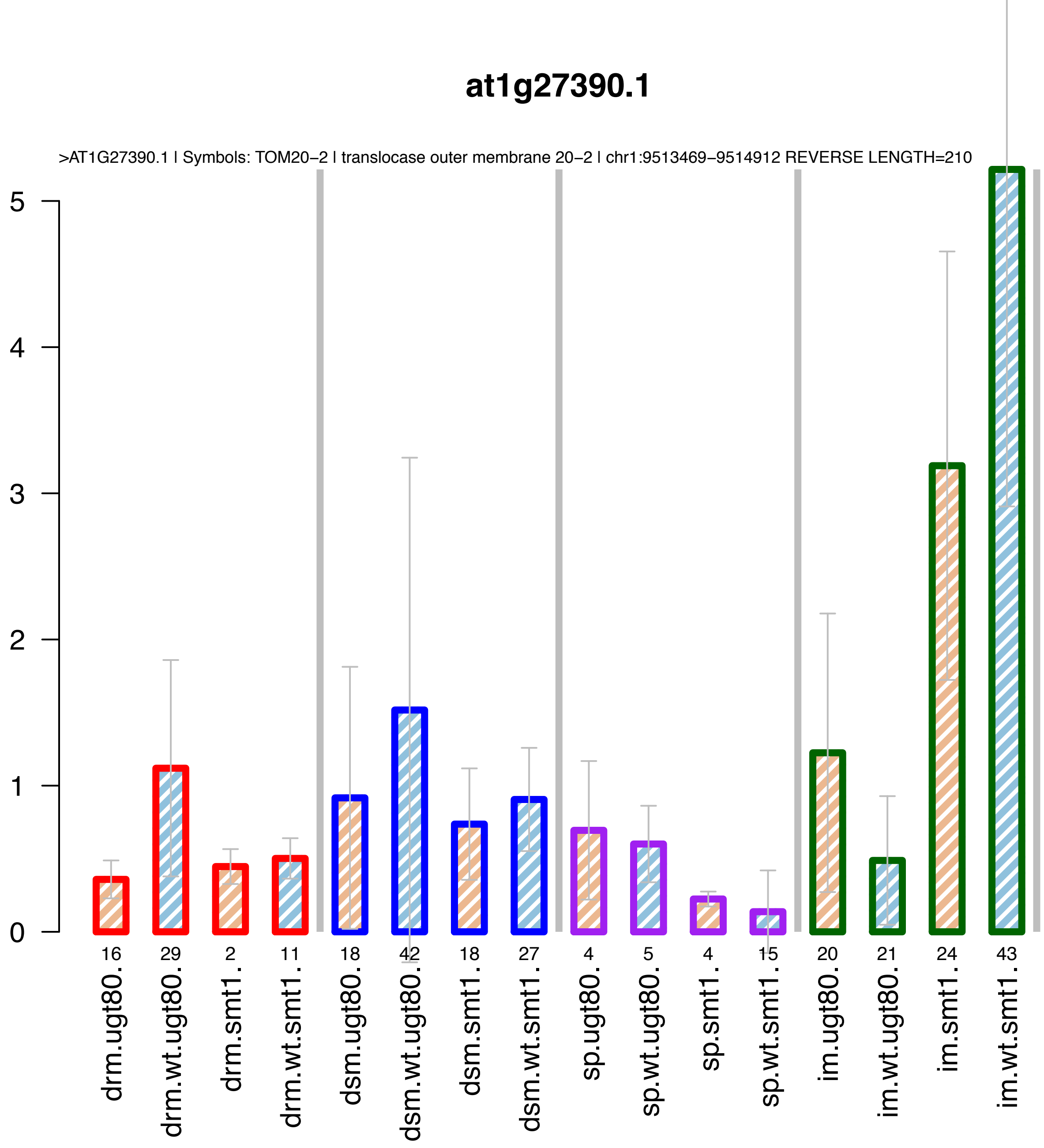

at1g27390.1
>AT1G27390.1 | Symbols: TOM20−2 | translocase outer membrane 20−2 | chr1:9513469−9514912 REVERSE LENGTH=210
0
1
2
3
4
5
16
29
2
11
18
42
18
27
4
5
4
15
20
21
24
43
drm.ugt80.
drm.wt.ugt80.
drm.smt1.
drm.wt.smt1.
dsm.ugt80.
dsm.wt.ugt80.
dsm.smt1.
dsm.wt.smt1.
sp.ugt80.
sp.wt.ugt80.
sp.smt1.
sp.wt.smt1.
im.ugt80.
im.wt.ugt80.
im.smt1.
im.wt.smt1.

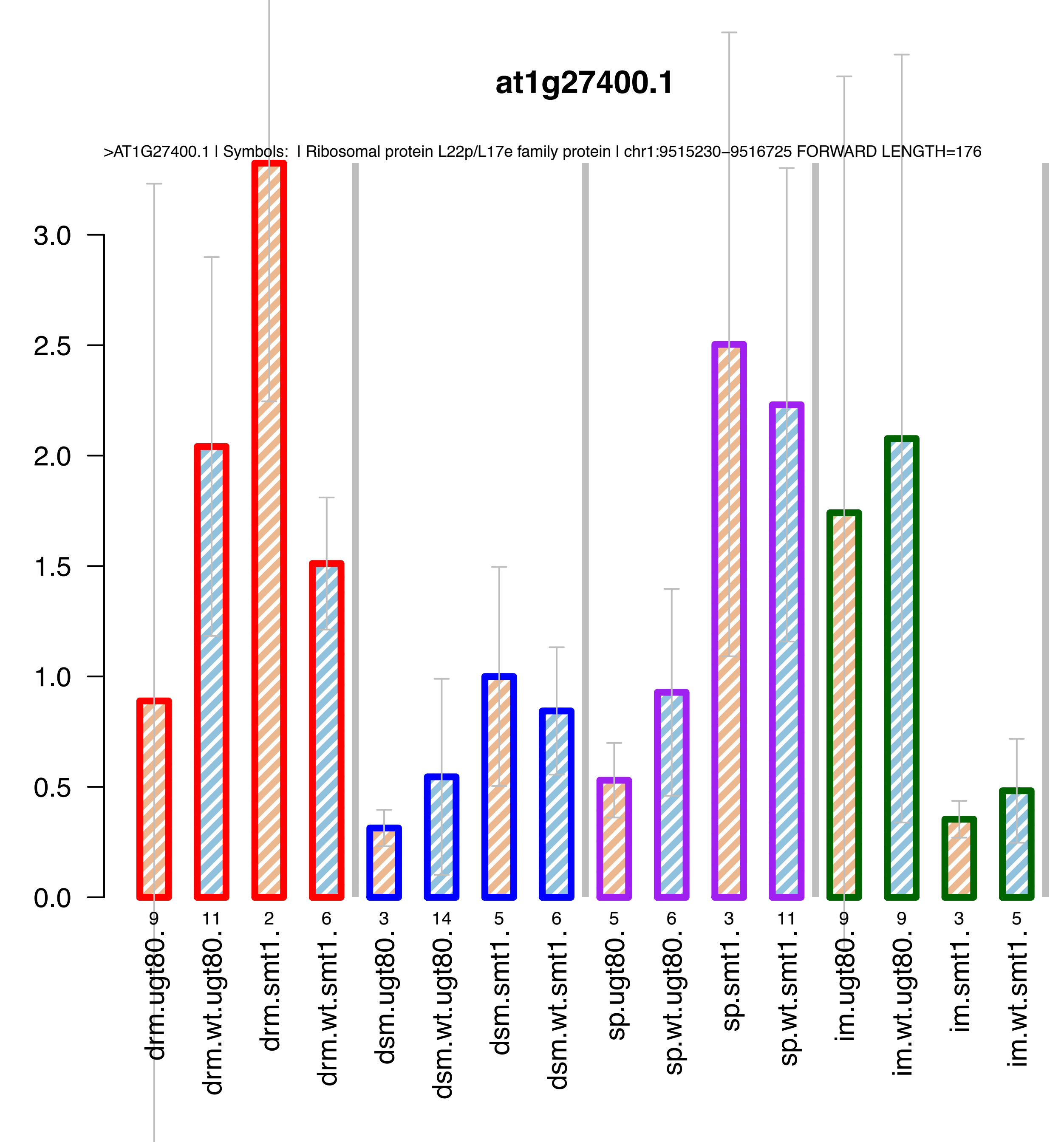
at1g27400.1
>AT1G27400.1 | Symbols: | Ribosomal protein L22p/L17e family protein | chr1:9515230-9516725 FORWARD LENGTH=176
0.0
0.5
1.0
1.5
2.0
2.5
3.0
9
11
2
6
3
14
5
6
5
6
3
11
9
9
3
5
drm.ugt80.
drm.wt.ugt80.
drm.smt1.
drm.wt.smt1.
dsm.ugt80.
dsm.wt.ugt80.
dsm.smt1.
dsm.wt.smt1.
sp.ugt80.
sp.wt.ugt80.
sp.smt1.
sp.wt.smt1.
im.ugt80.
im.wt.ugt80.
im.smt1.
im.wt.smt1.

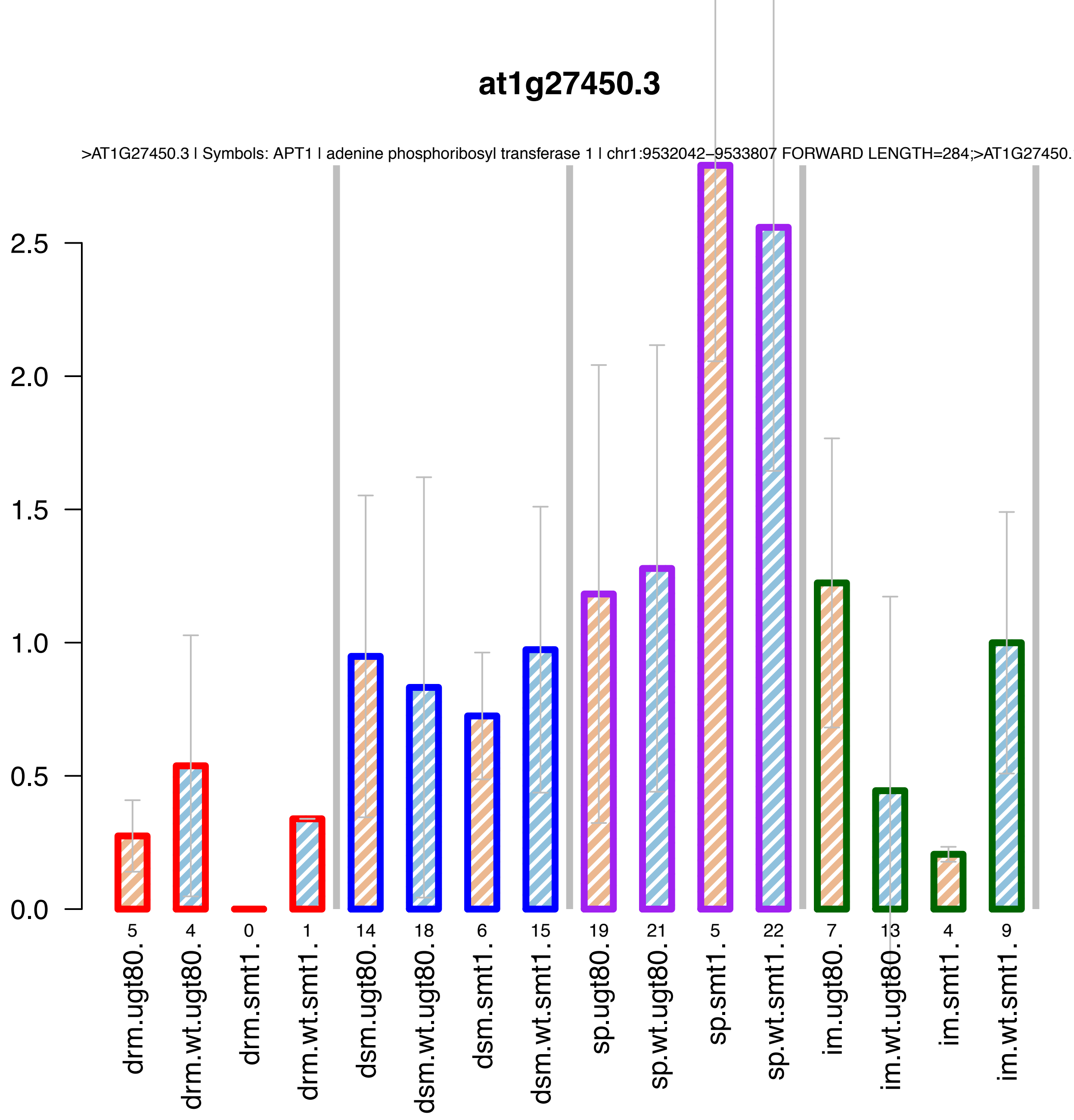

at1g27450.3
>AT1G27450.3 | Symbols: APT1 | adenine phosphoribosyl transferase 1 | chr1:9532042−9533807 FORWARD LENGTH=284;>AT1G27450.
2.5
2.0
1.5
1.0
0.5
0.0
5
4
0
1
14
18
6
15
19
21
5
22
7
13
4
9
drm.ugt80.
drm.wt.ugt80.
drm.smt1.
drm.wt.smt1.
dsm.ugt80.
dsm.wt.ugt80.
dsm.smt1.
dsm.wt.smt1.
sp.ugt80.
sp.wt.ugt80.
sp.smt1.
sp.wt.smt1.
im.ugt80.
im.wt.ugt80.
im.smt1.
im.wt.smt1.

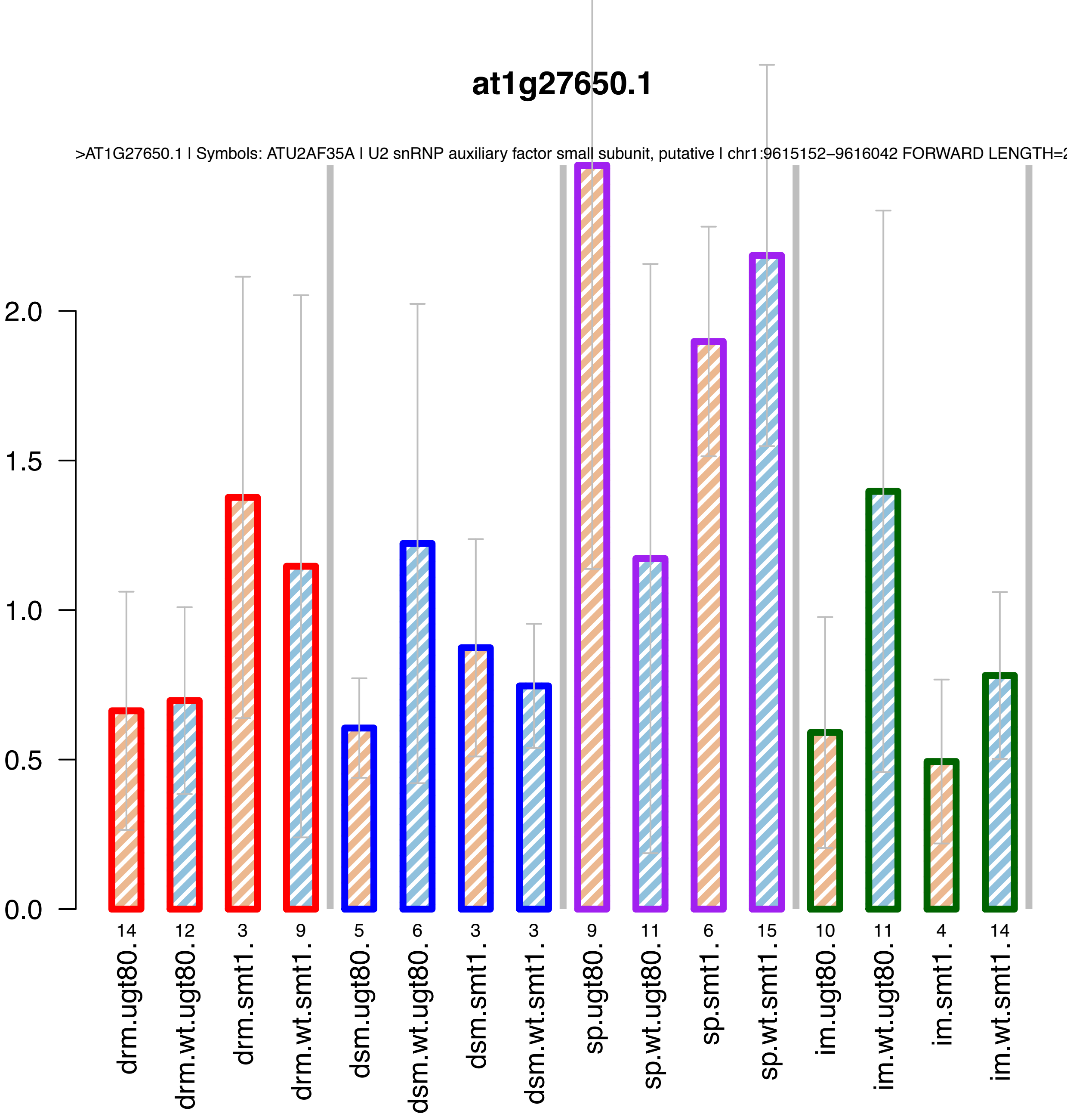

at1g27650.1
>AT1G27650.1 | Symbols: ATU2AF35A | U2 snRNP auxiliary factor small subunit, putative | chr1:9615152–9616042 FORWARD LENGTH=2
2.0
1.5
1.0
0.5
0.0
14
12
3
9
5
6
3
3
9
11
6
15
10
11
4
14
drm.ugt80..
drm.wt.ugt80..
drm.smt1..
drm.wt.smt1..
dsm.ugt80..
dsm.wt.ugt80..
dsm.smt1..
dsm.wt.smt1..
sp.ugt80..
sp.wt.ugt80..
sp.smt1..
sp.wt.smt1..
im.ugt80..
im.wt.ugt80..
im.smt1..
im.wt.smt1..

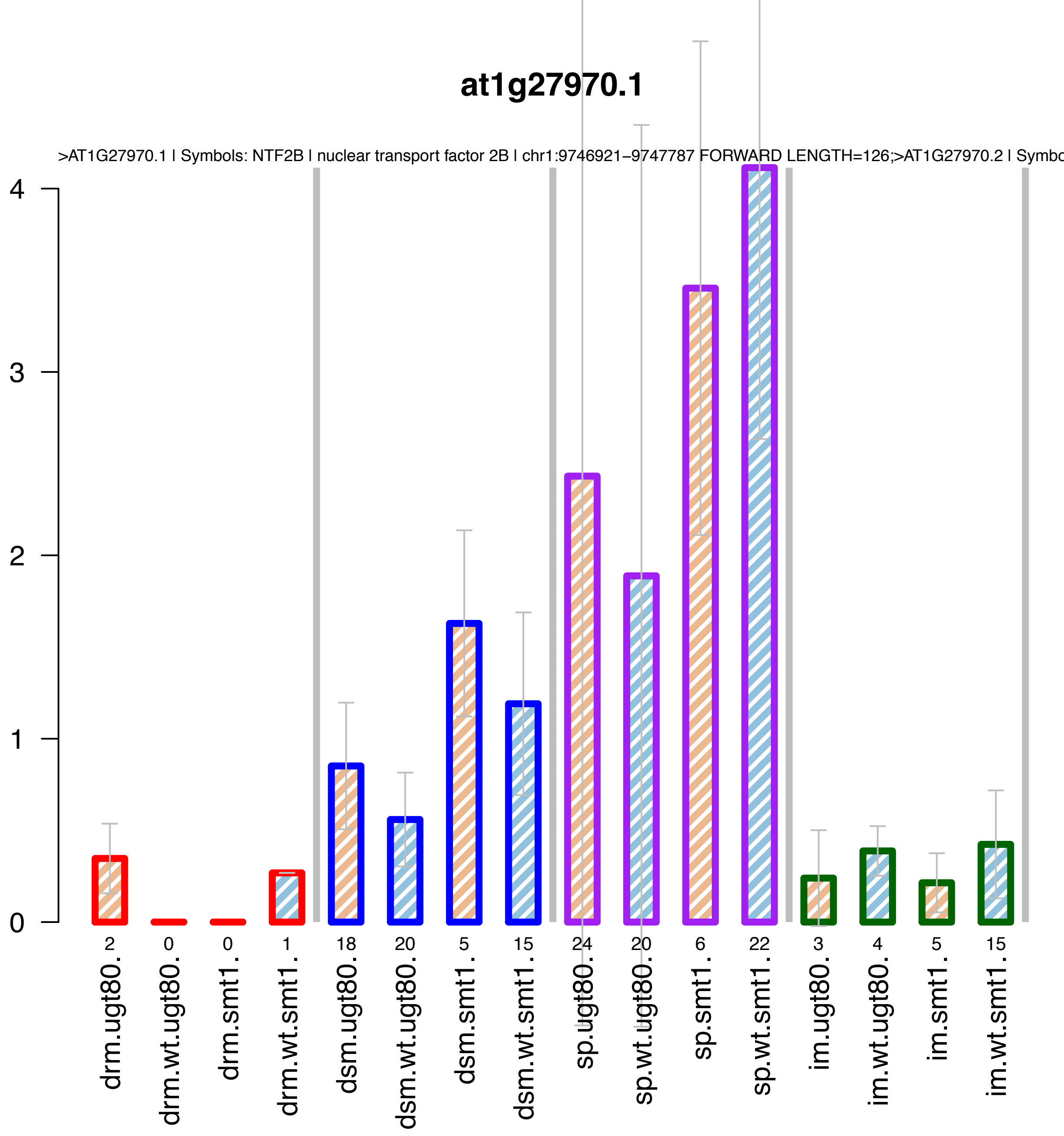
at1g27970.1
>AT1G27970.1 | Symbols: NTF2B | nuclear transport factor 2B | chr1:9746921-9747787 FORWARD LENGTH=126;>AT1G27970.2 | Symbo
0
1
2
3
4
2
0
0
1
18
20
5
15
24
20
6
22
3
4
5
15
drm.ugt80.
drm.wt.ugt80.
drm.smt1.
drm.wt.smt1.
dsm.ugt80.
dsm.wt.ugt80.
dsm.smt1.
dsm.wt.smt1.
sp.ugt80.
sp.wt.ugt80.
sp.smt1.
sp.wt.smt1.
im.ugt80.
im.wt.ugt80.
im.smt1.
im.wt.smt1.

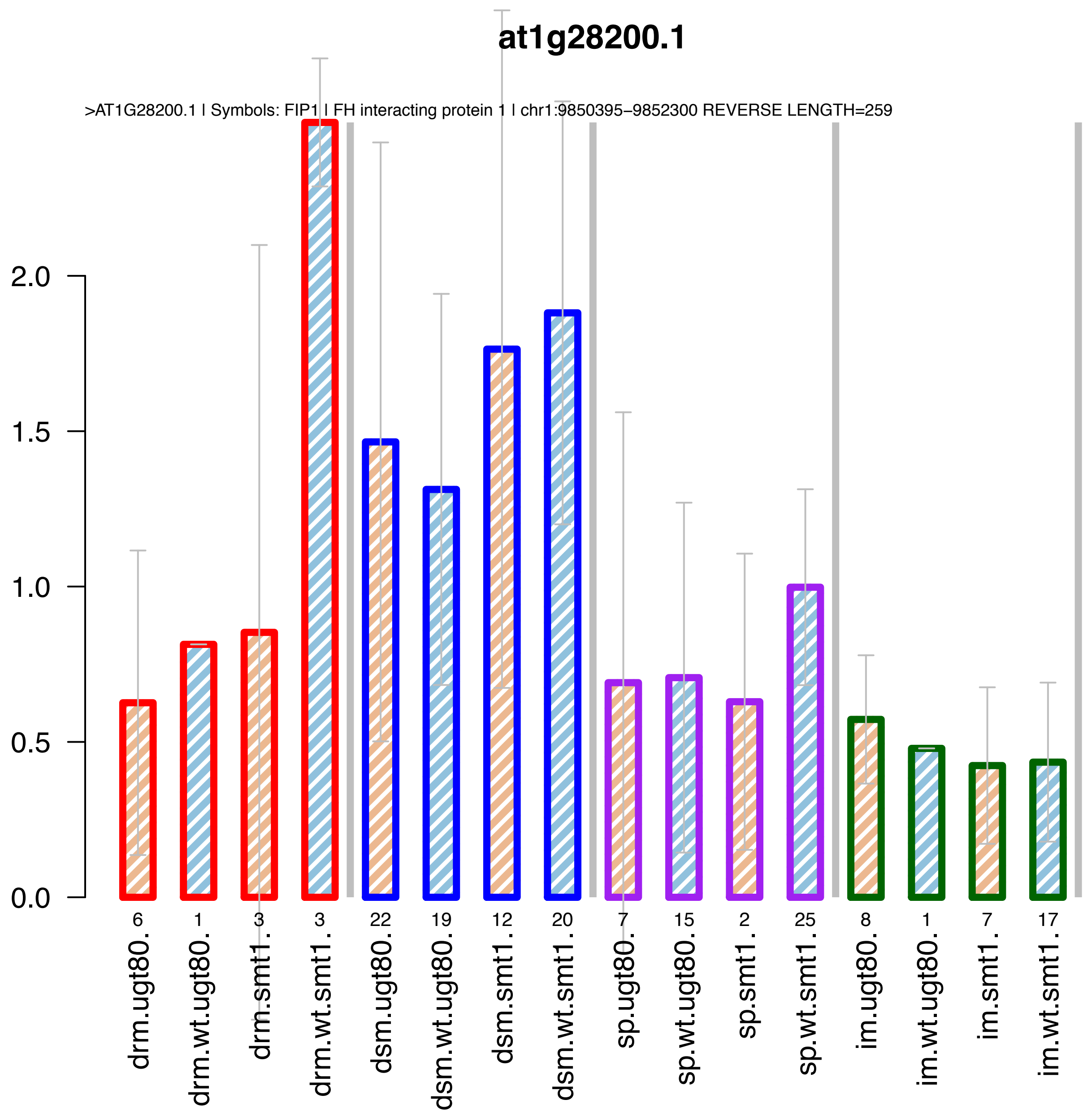

at1g28200.1
>AT1G28200.1 | Symbols: FIP1 | FH interacting protein 1 | chr1:9850395-9852300 REVERSE LENGTH=259
0.0
0.5
1.0
1.5
2.0
6
1
3
3
22
19
12
20
7
15
2
25
8
1
7
17
drm.ugt80.
drm.wt.ugt80.
drm.smt1.
drm.wt.smt1.
dsm.ugt80.
dsm.wt.ugt80.
dsm.smt1.
dsm.wt.smt1.
sp.ugt80.
sp.wt.ugt80.
sp.smt1.
sp.wt.smt1.
im.ugt80.
im.wt.ugt80.
im.smt1.
im.wt.smt1.

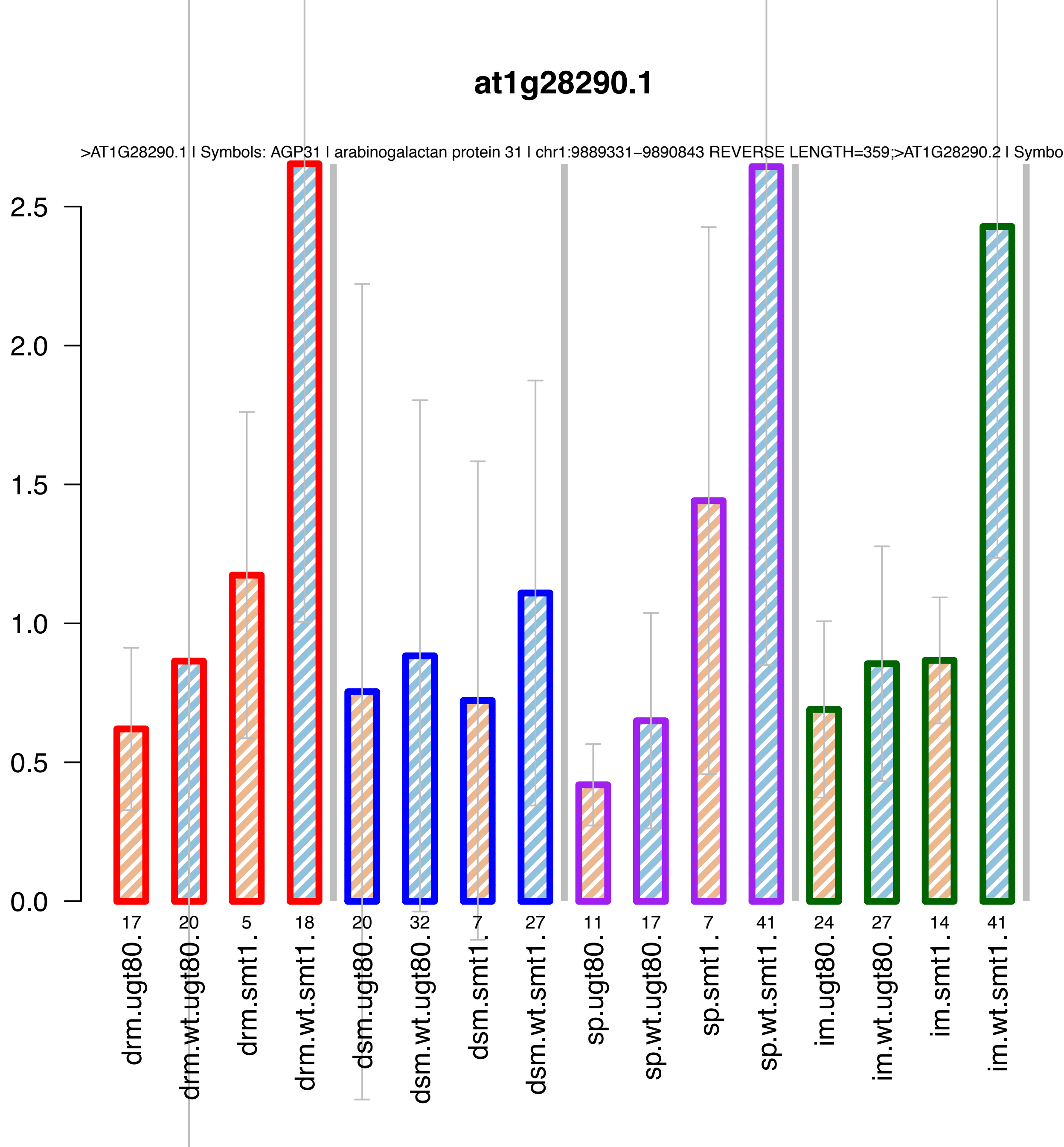
at1g28290.1
>AT1G28290.1 | Symbols: AGP31 | arabinogalactan protein 31 | chr1:9889331−9890843 REVERSE LENGTH=359;>AT1G28290.2 | Symbo
0.0
0.5
1.0
1.5
2.0
2.5
17
20
5
18
20
32
7
27
11
17
7
41
24
27
14
41
drm.ugt80..
drm.wt.ugt80..
drm.smt1..
drm.wt.smt1..
dsm.ugt80..
dsm.wt.ugt80..
dsm.smt1..
dsm.wt.smt1..
sp.ugt80..
sp.wt.ugt80..
sp.smt1..
sp.wt.smt1..
im.ugt80..
im.wt.ugt80..
im.smt1..
im.wt.smt1..

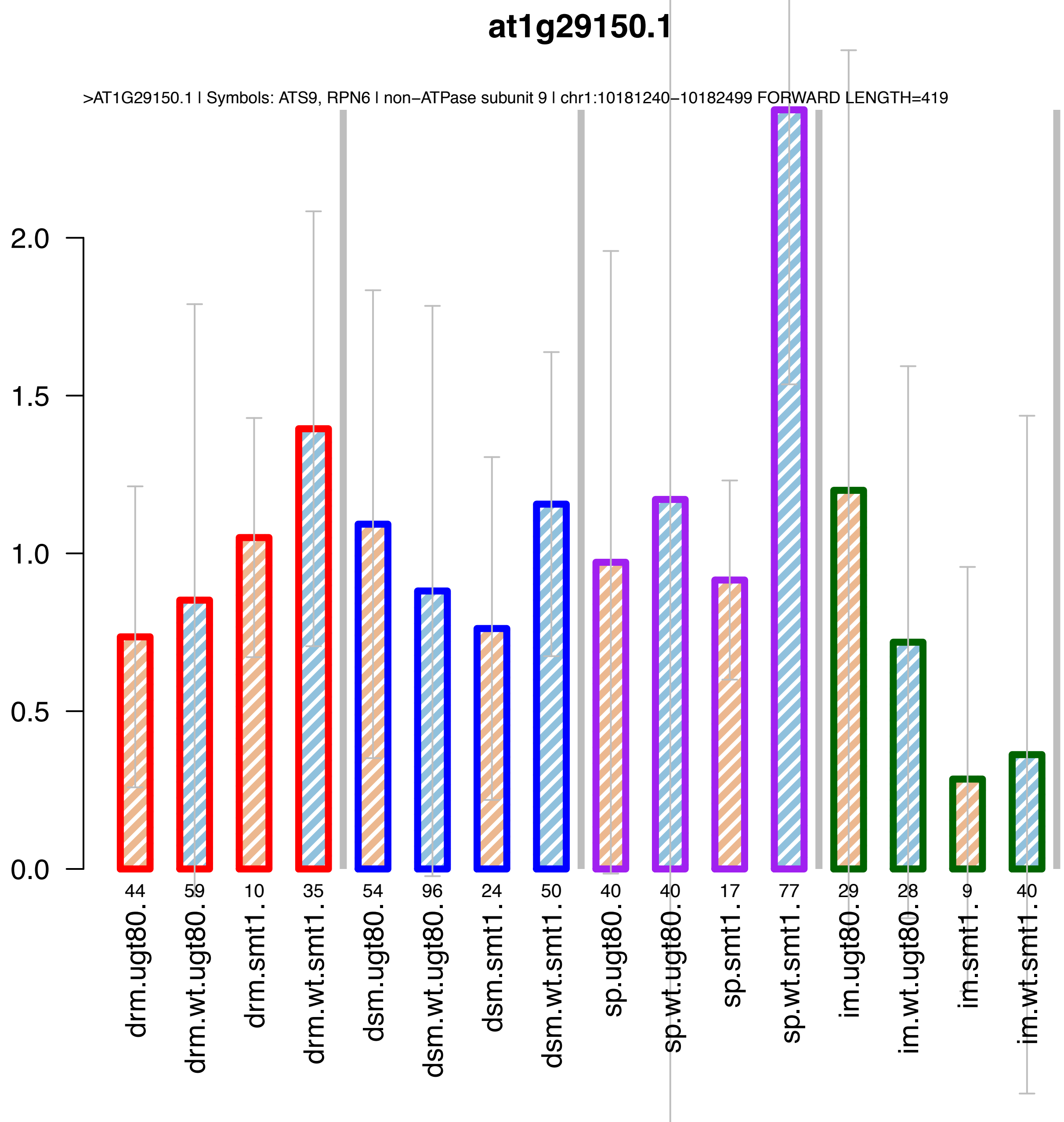

at1g29150.1
>AT1G29150.1 | Symbols: ATS9, RPN6 | non−ATPase subunit 9 | chr1:10181240−10182499 FORWARD LENGTH=419
2.0
1.5
1.0
0.5
0.0
44
59
10
35
54
96
24
50
40
40
17
77
29
28
9
40
drm.ugt80..
drm.wt.ugt80..
drm.smt1..
drm.wt.smt1..
dsm.ugt80..
dsm.wt.ugt80..
dsm.smt1..
dsm.wt.smt1..
sp.ugt80..
sp.wt.ugt80..
sp.smt1..
sp.wt.smt1..
im.ugt80..
im.wt.ugt80..
im.smt1..
im.wt.smt1..

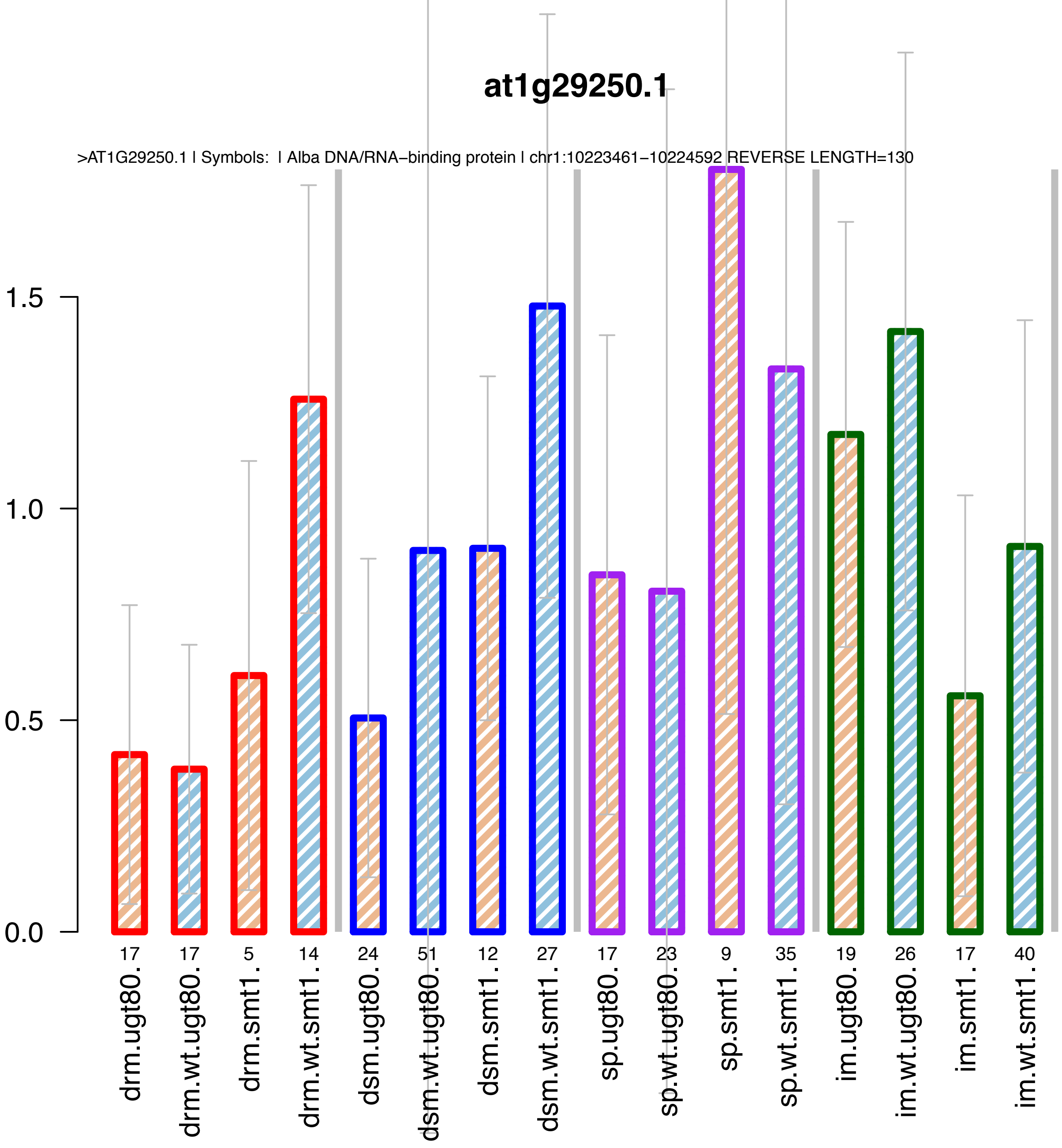

at1g29250.1
>AT1G29250.1 | Symbols: | Alba DNA/RNA-binding protein | chr1:10223461-10224592 REVERSE LENGTH=130
0.0
0.5
1.0
1.5
17
17
5
14
24
51
12
27
17
23
9
35
19
26
17
40
drm.ugt80..
drm.wt.ugt80..
drm.smt1..
drm.wt.smt1..
dsm.ugt80..
dsm.wt.ugt80..
dsm.smt1..
dsm.wt.smt1..
sp.ugt80..
sp.wt.ugt80..
sp.smt1..
sp.wt.smt1..
im.ugt80..
im.wt.ugt80..
im.smt1..
im.wt.smt1..

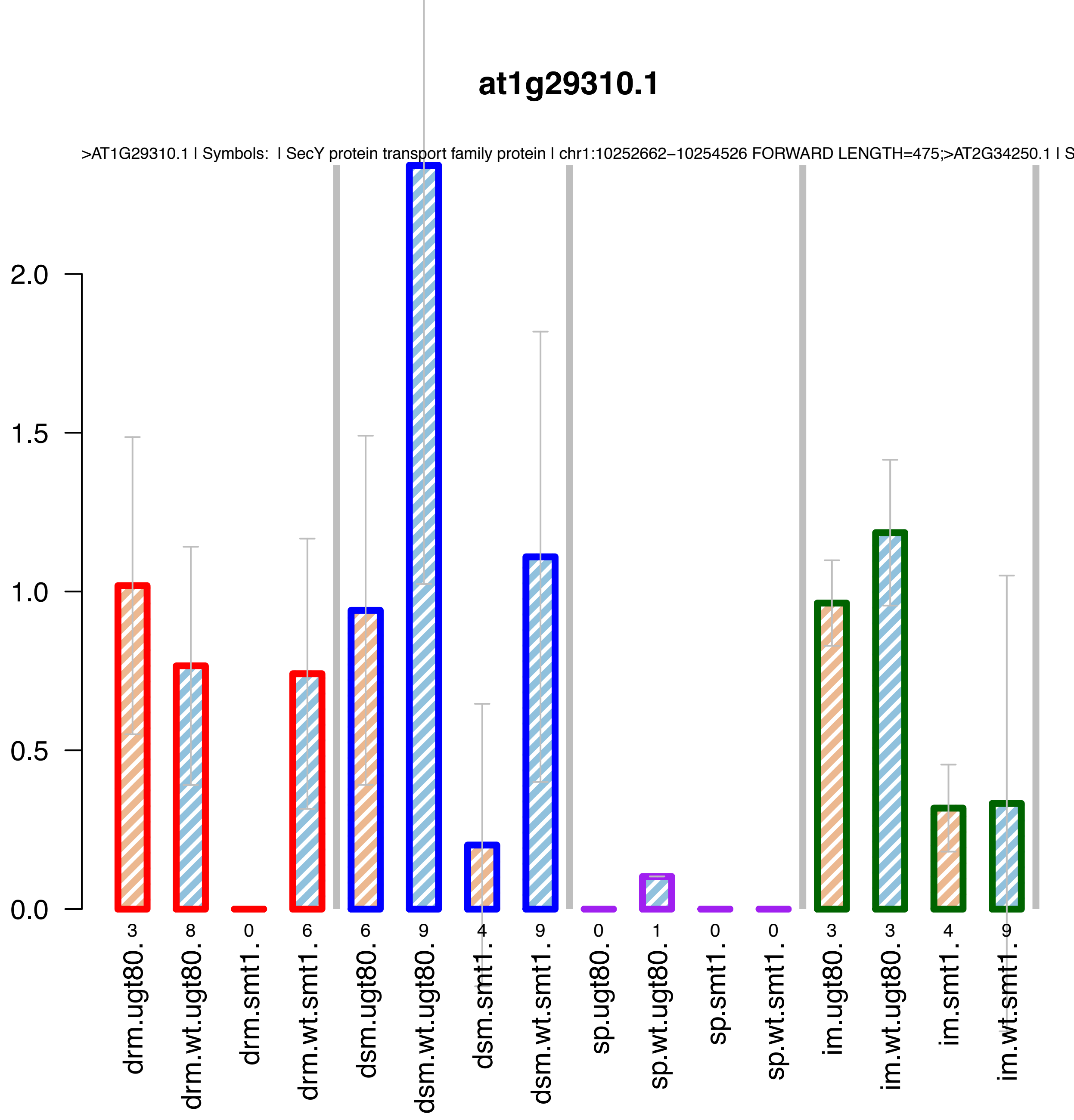

at1g29310.1
>AT1G29310.1 | Symbols: | SecY protein transport family protein | chr1:10252662-10254526 FORWARD LENGTH=475;>AT2G34250.1 | S
2.0
1.5
1.0
0.5
0.0
3
8
0
6
6
9
4
9
0
1
0
0
3
3
4
9
drm.ugt80..
drm.wt.ugt80..
drm.smt1..
drm.wt.smt1..
dsm.ugt80..
dsm.wt.ugt80..
dsm.smt1..
dsm.wt.smt1..
sp.ugt80..
sp.wt.ugt80..
sp.smt1..
sp.wt.smt1..
im.ugt80..
im.wt.ugt80..
im.smt1..
im.wt.smt1..

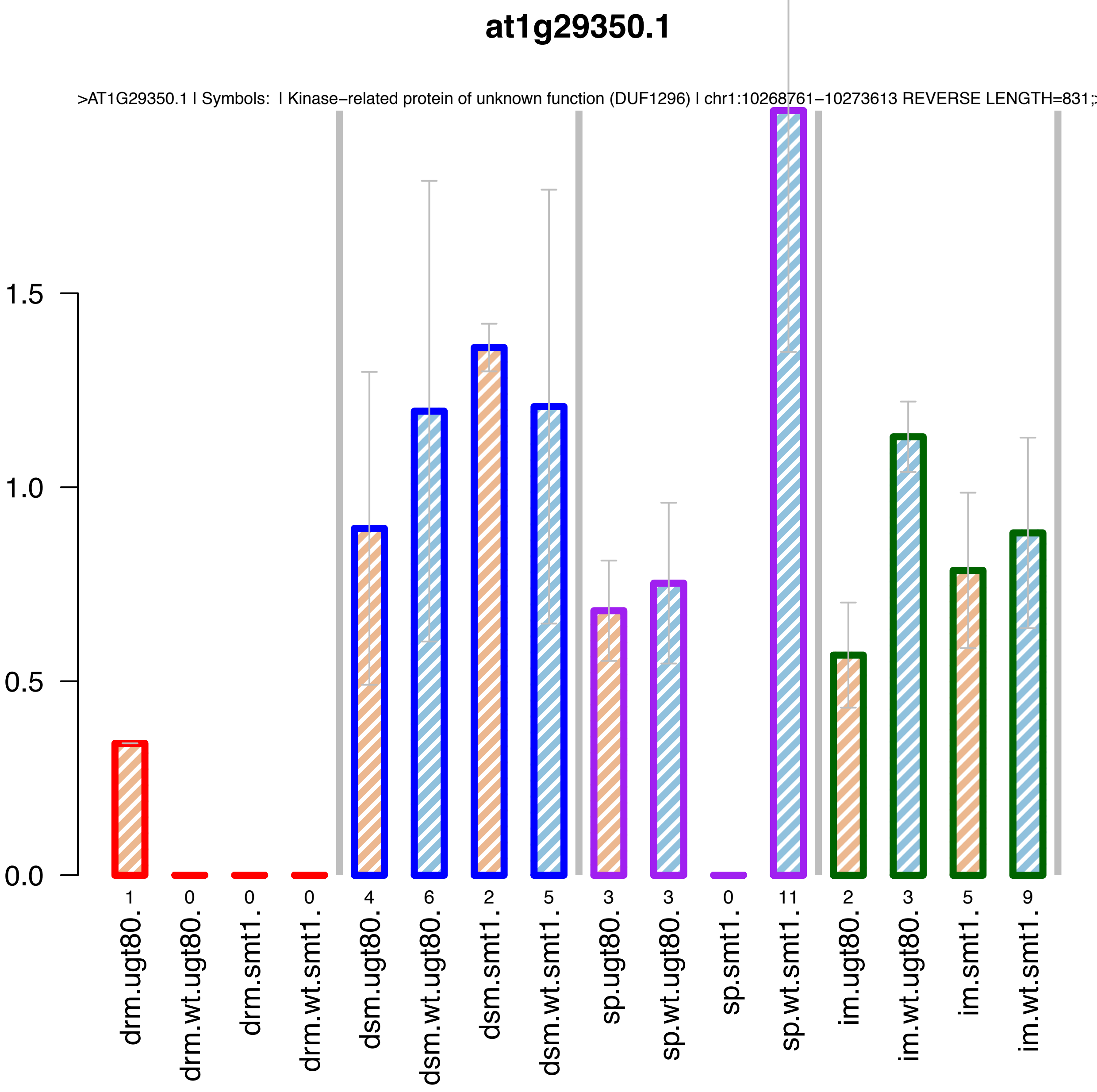

at1g29350.1
>AT1G29350.1 | Symbols: | Kinase−related protein of unknown function (DUF1296) | chr1:10268761−10273613 REVERSE LENGTH=831;
0.0
0.5
1.0
1.5
1
0
0
0
4
6
2
5
3
3
0
11
2
3
5
9
drm.ugt80.
drm.wt.ugt80.
drm.smt1.
drm.wt.smt1.
dsm.ugt80.
dsm.wt.ugt80.
dsm.smt1.
dsm.wt.smt1.
sp.ugt80.
sp.wt.ugt80.
sp.smt1.
sp.wt.smt1.
im.ugt80.
im.wt.ugt80.
im.smt1.
im.wt.smt1.

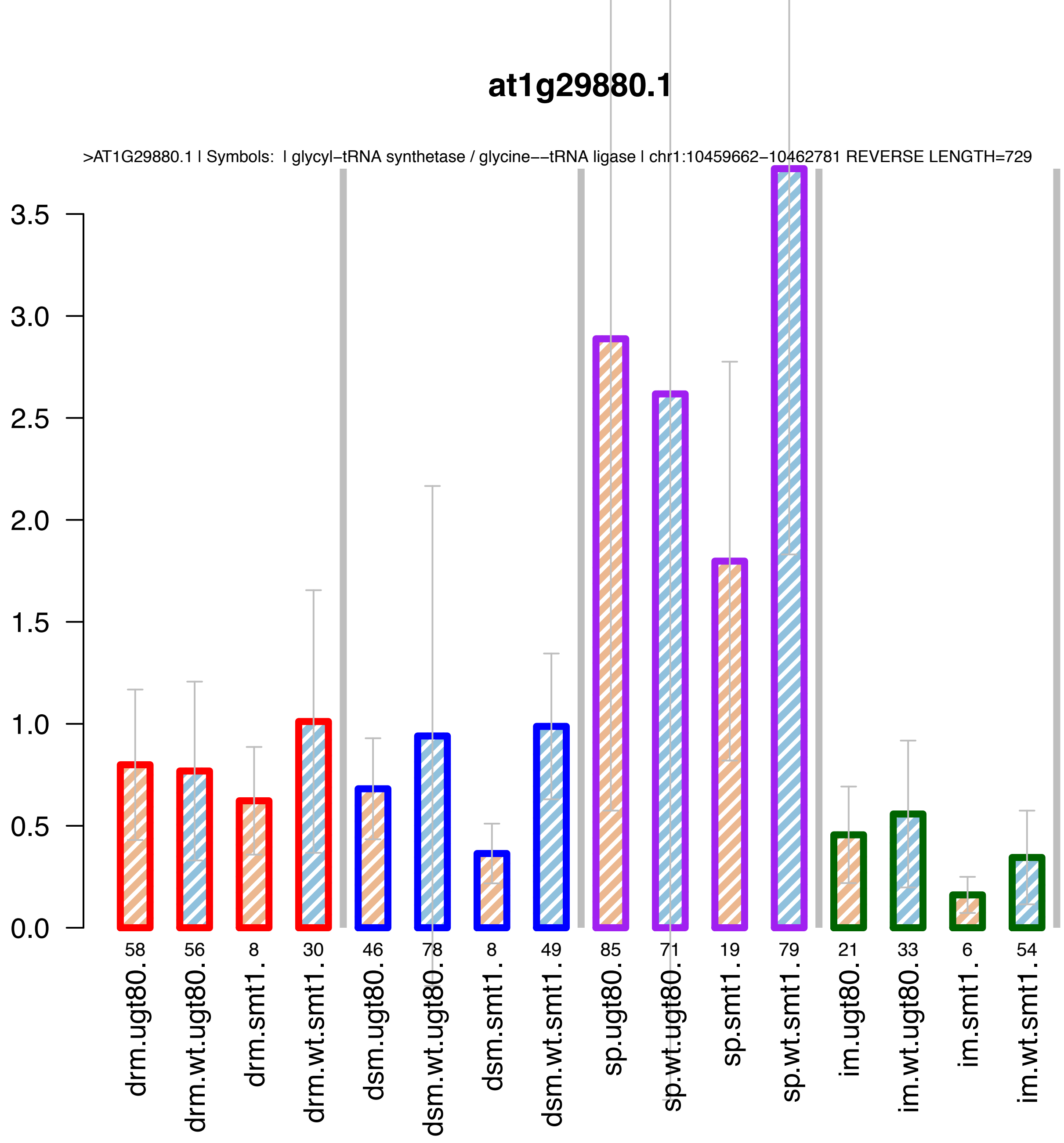
at1g29880.1
>AT1G29880.1 | Symbols: | glycyl−tRNA synthetase / glycine−−tRNA ligase | chr1:10459662−10462781 REVERSE LENGTH=729
0.0
0.5
1.0
1.5
2.0
2.5
3.0
3.5
58
56
8
30
46
78
8
49
85
71
19
79
21
33
6
54
drm.ugt80.
drm.wt.ugt80.
drm.smt1.
drm.wt.smt1.
dsm.ugt80.
dsm.wt.ugt80.
dsm.smt1.
dsm.wt.smt1.
sp.ugt80.
sp.wt.ugt80.
sp.smt1.
sp.wt.smt1.
im.ugt80.
im.wt.ugt80.
im.smt1.
im.wt.smt1.

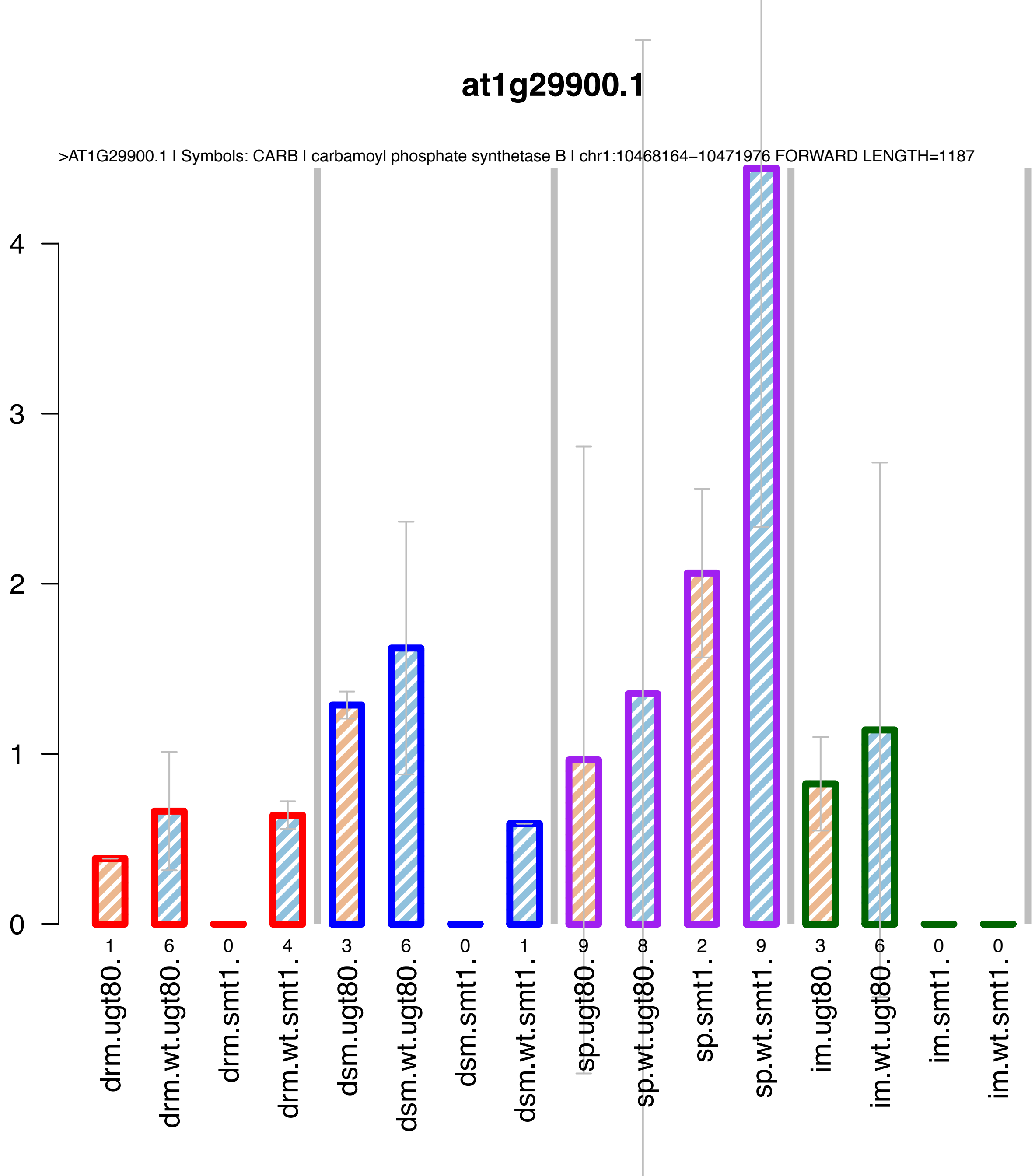

at1g29900.1
>AT1G29900.1 | Symbols: CARB | carbamoyl phosphate synthetase B | chr1:10468164-10471976 FORWARD LENGTH=1187
0
1
2
3
4
1
6
0
4
3
6
0
1
9
8
2
9
3
6
0
0
drm.ugt80..
drm.wt.ugt80..
drm.smt1..
drm.wt.smt1..
dsm.ugt80..
dsm.wt.ugt80..
dsm.smt1..
dsm.wt.smt1..
sp.ugt80..
sp.wt.ugt80..
sp.smt1..
sp.wt.smt1..
im.ugt80..
im.wt.ugt80..
im.smt1..
im.wt.smt1..

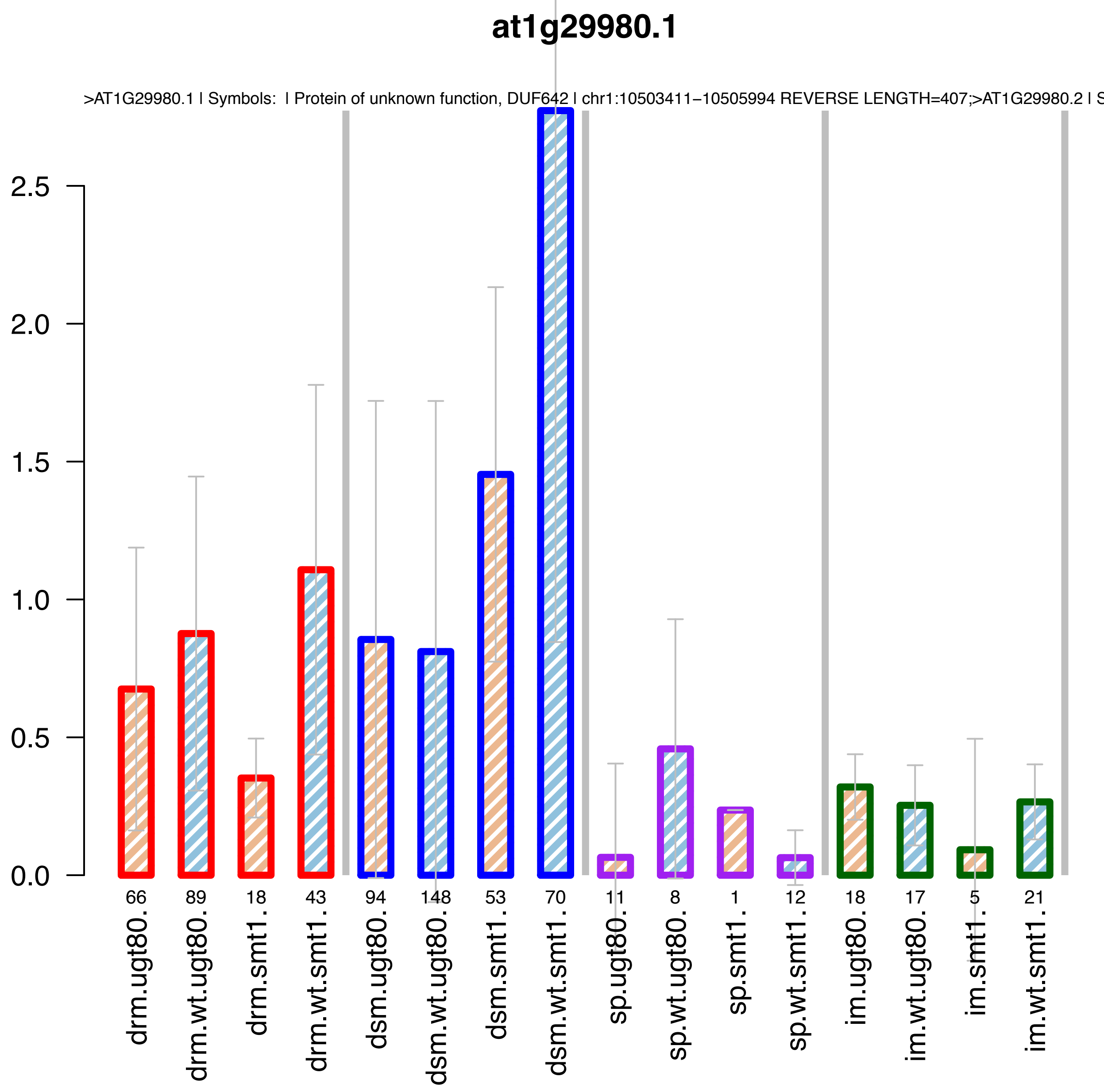

at1g29980.1
>AT1G29980.1 | Symbols: | Protein of unknown function, DUF642 | chr1:10503411-10505994 REVERSE LENGTH=407;>AT1G29980.2 | S
0.0
0.5
1.0
1.5
2.0
2.5
66
89
18
43
94
148
53
70
11
8
1
12
18
17
5
21
drm.ugt80.
drm.wt.ugt80.
drm.smt1.
drm.wt.smt1.
dsm.ugt80.
dsm.wt.ugt80.
dsm.smt1.
dsm.wt.smt1.
sp.ugt80.
sp.wt.ugt80.
sp.smt1.
sp.wt.smt1.
im.ugt80.
im.wt.ugt80.
im.smt1.
im.wt.smt1.

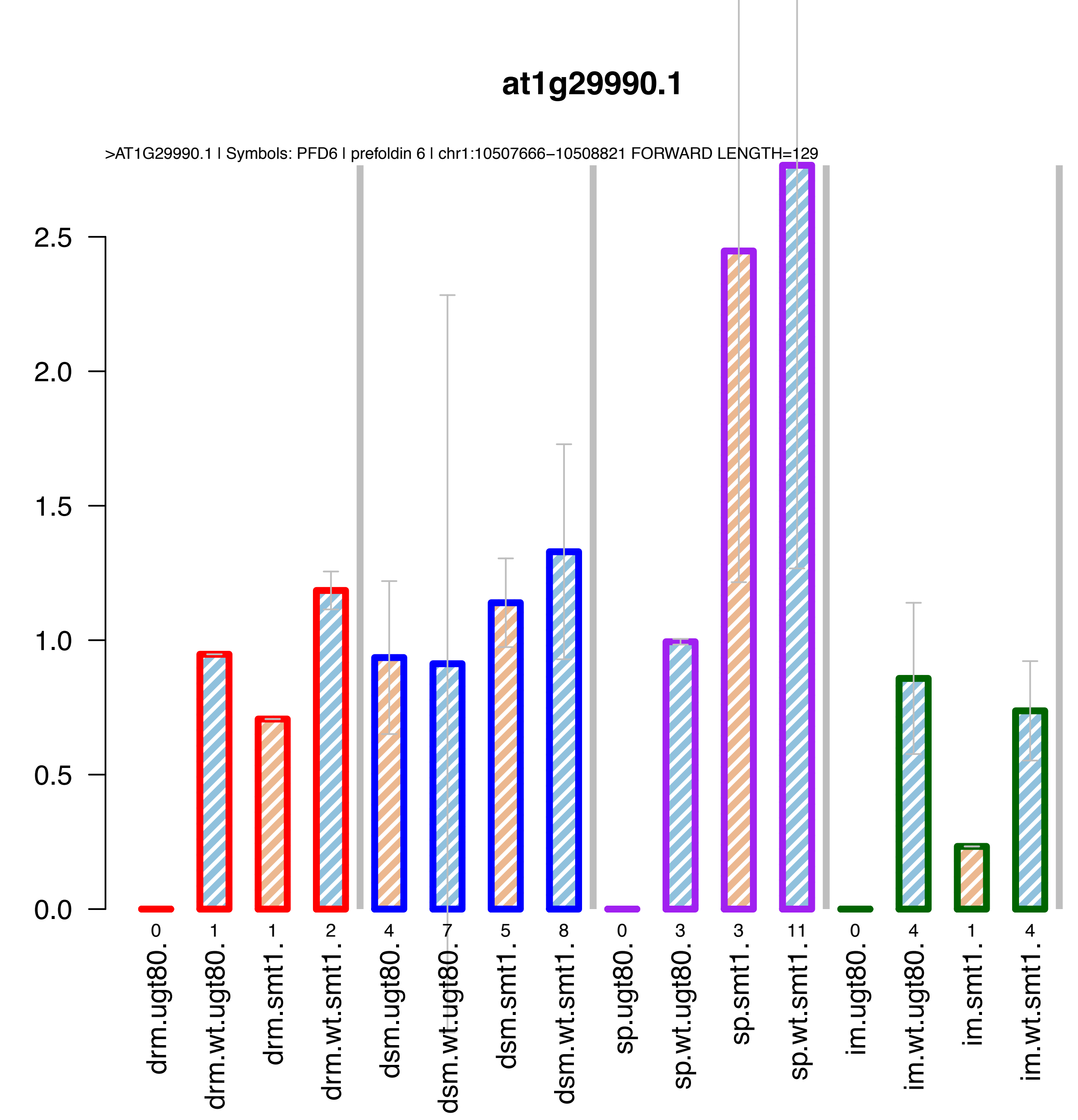
at1g29990.1
>AT1G29990.1 | Symbols: PFD6 | prefoldin 6 | chr1:10507666-10508821 FORWARD LENGTH=129
0.0
0.5
1.0
1.5
2.0
2.5
0
1
1
2
4
7
5
8
0
3
3
11
0
4
1
4
drm.ugt80.
drm.wt.ugt80.
drm.smt1.
drm.wt.smt1.
dsm.ugt80.
dsm.wt.ugt80.
dsm.smt1.
dsm.wt.smt1.
sp.ugt80.
sp.wt.ugt80.
sp.smt1.
sp.wt.smt1.
im.ugt80.
im.wt.ugt80.
im.smt1.
im.wt.smt1.

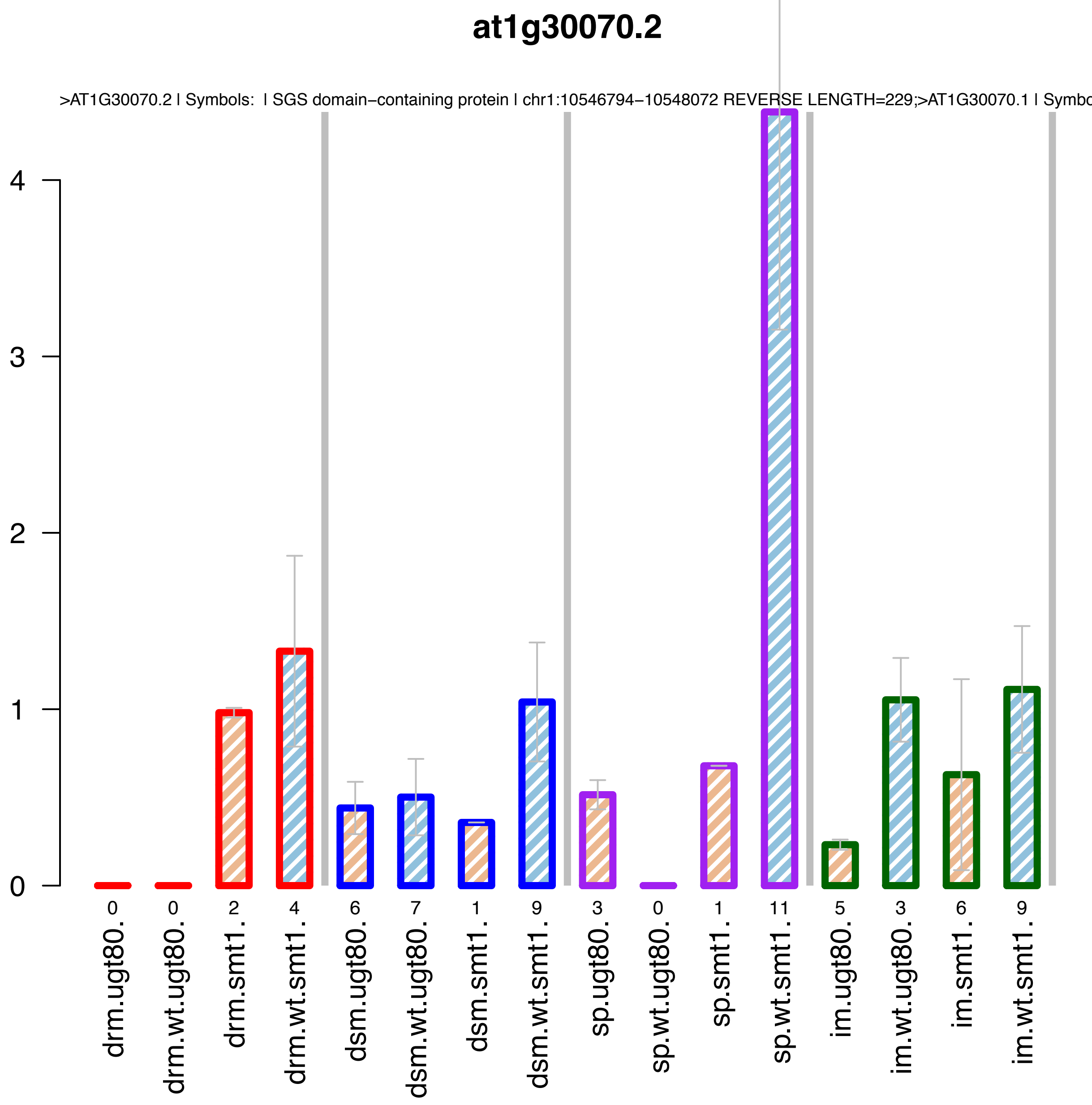

at1g30070.2
>AT1G30070.2 | Symbols: | SGS domain−containing protein | chr1:10546794−10548072 REVERSE LENGTH=229;>AT1G30070.1 | Symbo
0
1
2
3
4
0
0
2
4
6
7
1
9
3
0
1
11
5
3
6
9
drm.ugt80.
drm.wt.ugt80.
drm.smt1.
drm.wt.smt1.
dsm.ugt80.
dsm.wt.ugt80.
dsm.smt1.
dsm.wt.smt1.
sp.ugt80.
sp.wt.ugt80.
sp.smt1.
sp.wt.smt1.
im.ugt80.
im.wt.ugt80.
im.smt1.
im.wt.smt1.

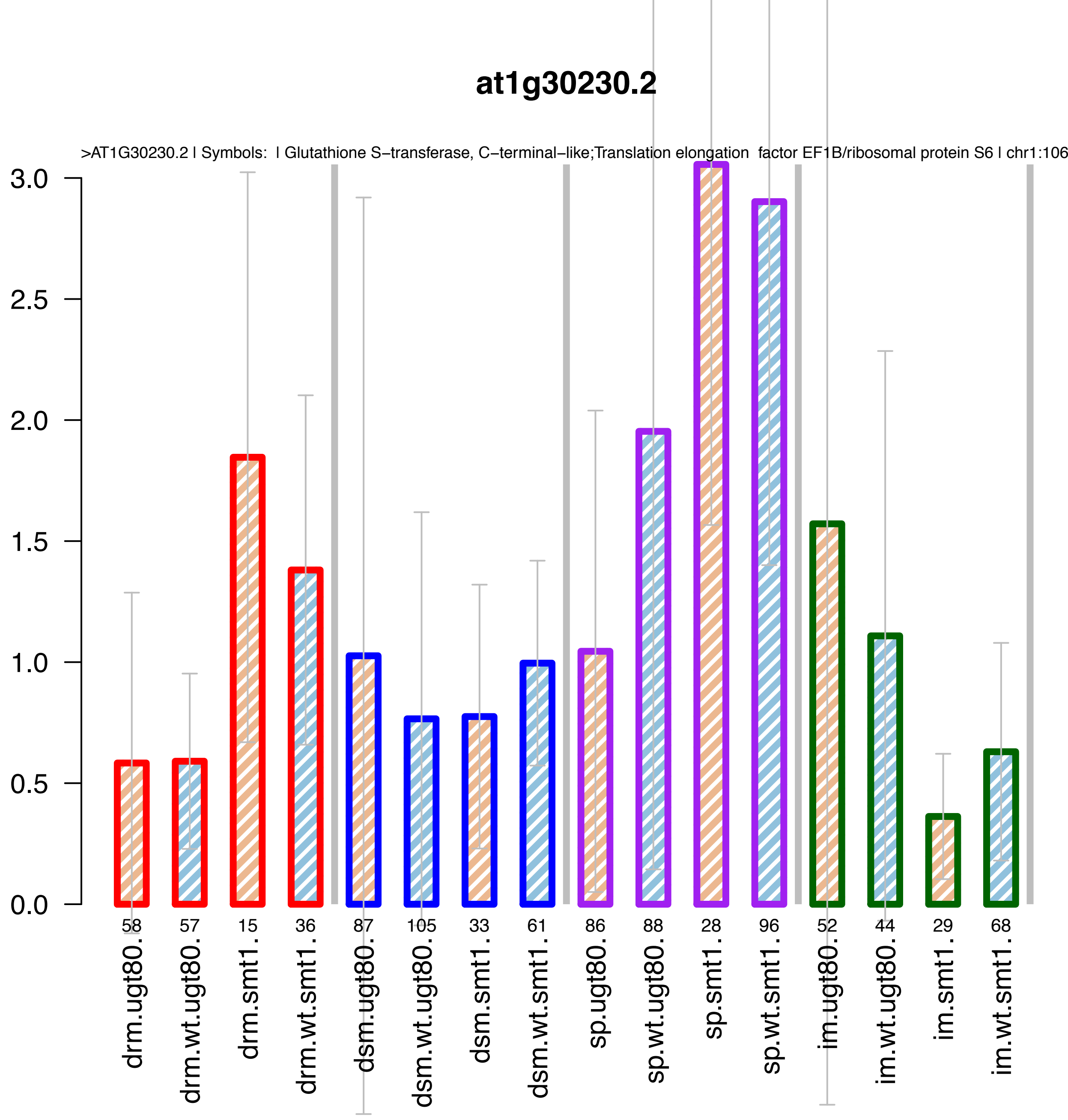

at1g30230.2
>AT1G30230.2 | Symbols: | Glutathione S-transferase, C-terminal-like;Translation elongation factor EF1B/ribosomal protein S6 | chr1:106
0.0
0.5
1.0
1.5
2.0
2.5
3.0
58
57
15
36
87
105
33
61
86
88
28
96
52
44
29
68
drm.ugt80.
drm.wt.ugt80.
drm.smt1.
drm.wt.smt1.
dsm.ugt80.
dsm.wt.ugt80.
dsm.smt1.
dsm.wt.smt1.
sp.ugt80.
sp.wt.ugt80.
sp.smt1.
sp.wt.smt1.
im.ugt80.
im.wt.ugt80.
im.smt1.
im.wt.smt1.

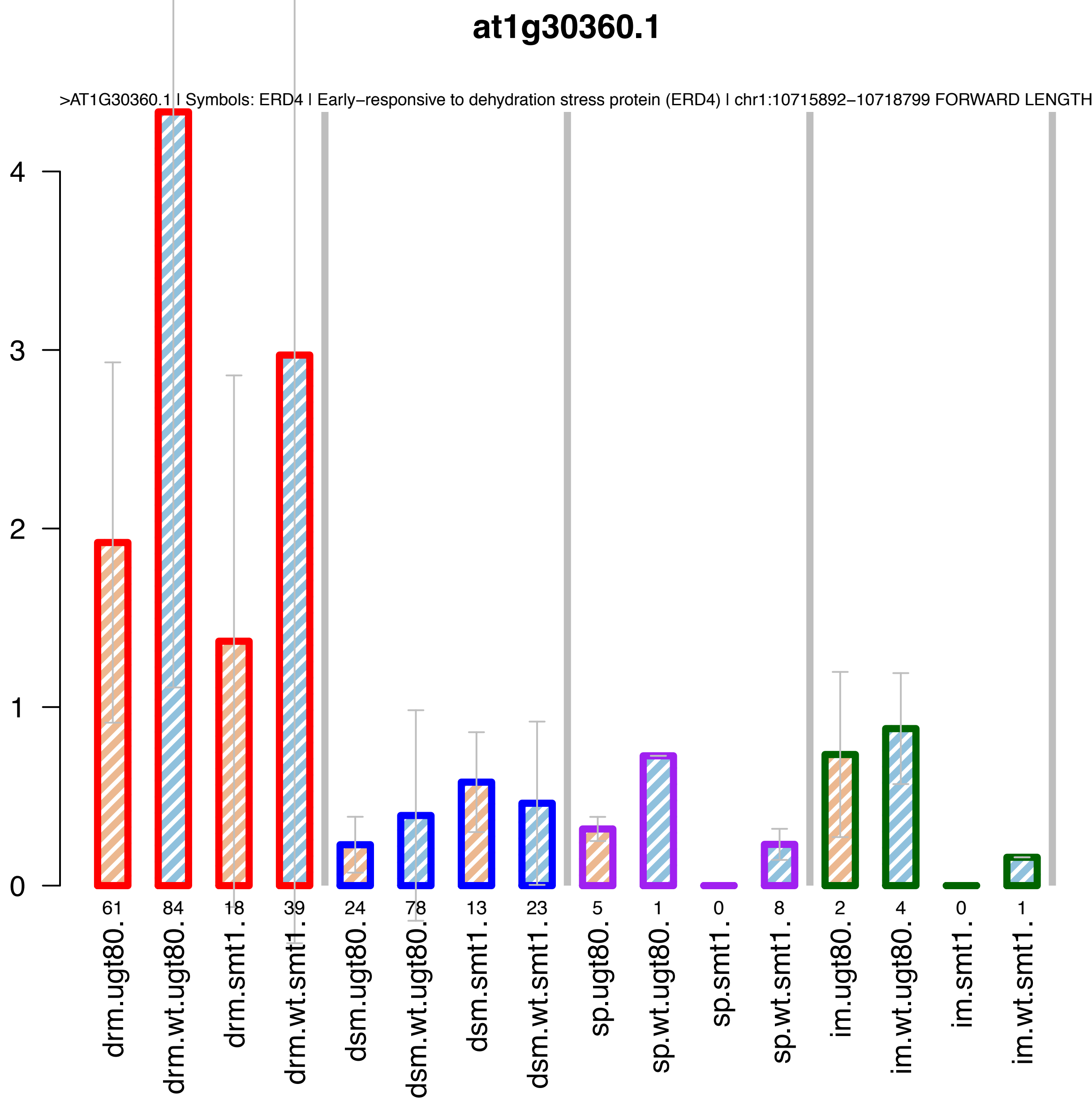
at1g30360.1
>AT1G30360.1 | Symbols: ERD4 | Early−responsive to dehydration stress protein (ERD4) | chr1:10715892−10718799 FORWARD LENGTH
0
1
2
3
4
61
84
18
39
24
78
13
23
5
1
0
8
2
4
0
1
drm.ugt80.
drm.wt.ugt80.
drm.smt1.
drm.wt.smt1.
dsm.ugt80.
dsm.wt.ugt80.
dsm.smt1.
dsm.wt.smt1.
sp.ugt80.
sp.wt.ugt80.
sp.smt1.
sp.wt.smt1.
im.ugt80.
im.wt.ugt80.
im.smt1.
im.wt.smt1.

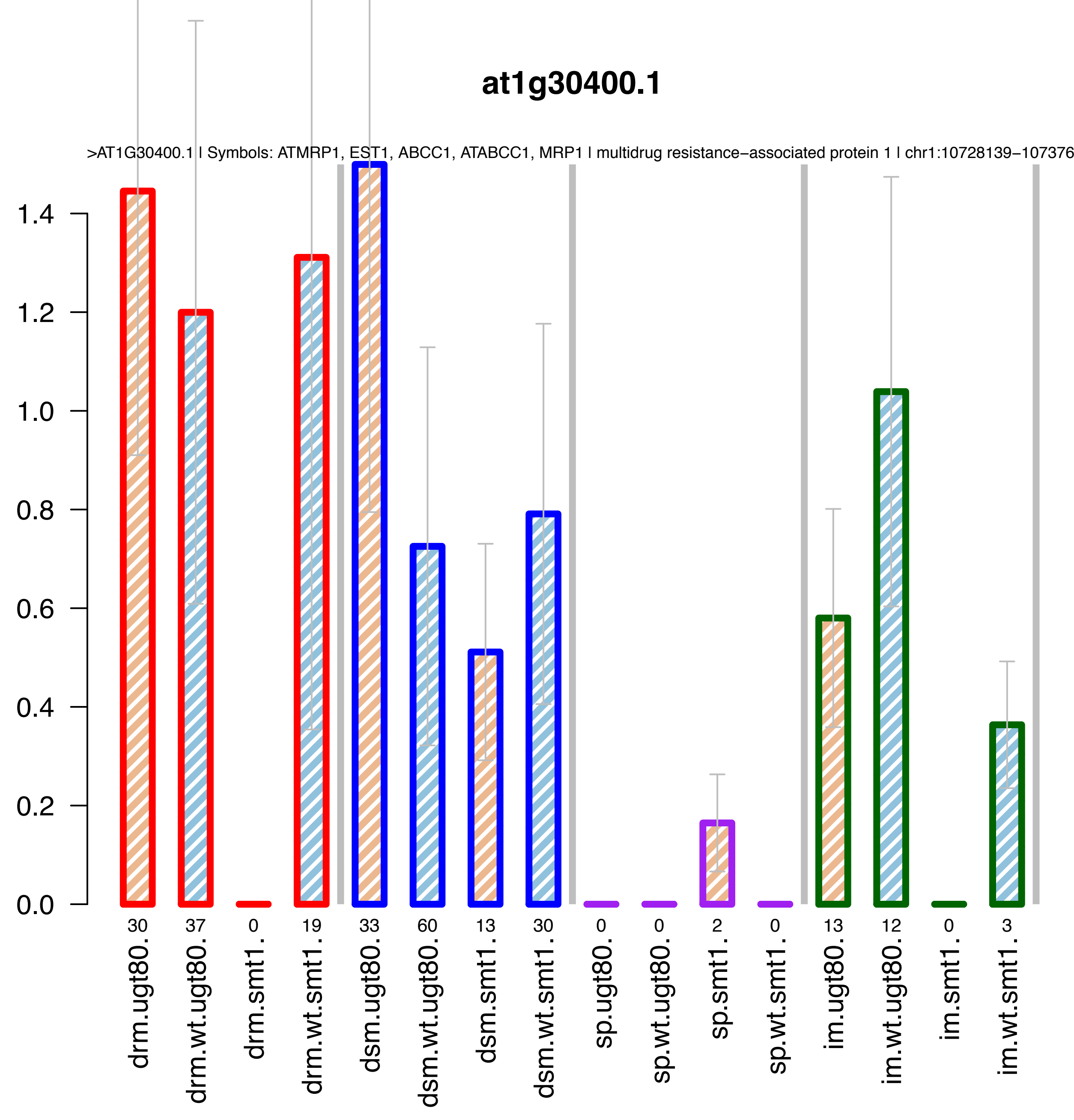
at1g30400.1
>AT1G30400.1 | Symbols: ATMRP1, EST1, ABCC1, ATABCC1, MRP1 | multidrug resistance−associated protein 1 | chr1:10728139−107376
1.4
1.2
1.0
0.8
0.6
0.4
0.2
0.0
30
37
0
19
33
60
13
30
0
0
2
0
13
12
0
3
drm.ugt80..
drm.wt.ugt80..
drm.smt1..
drm.wt.smt1..
dsm.ugt80..
dsm.wt.ugt80..
dsm.smt1..
dsm.wt.smt1..
sp.ugt80..
sp.wt.ugt80..
sp.smt1..
sp.wt.smt1..
im.ugt80..
im.wt.ugt80..
im.smt1..
im.wt.smt1..

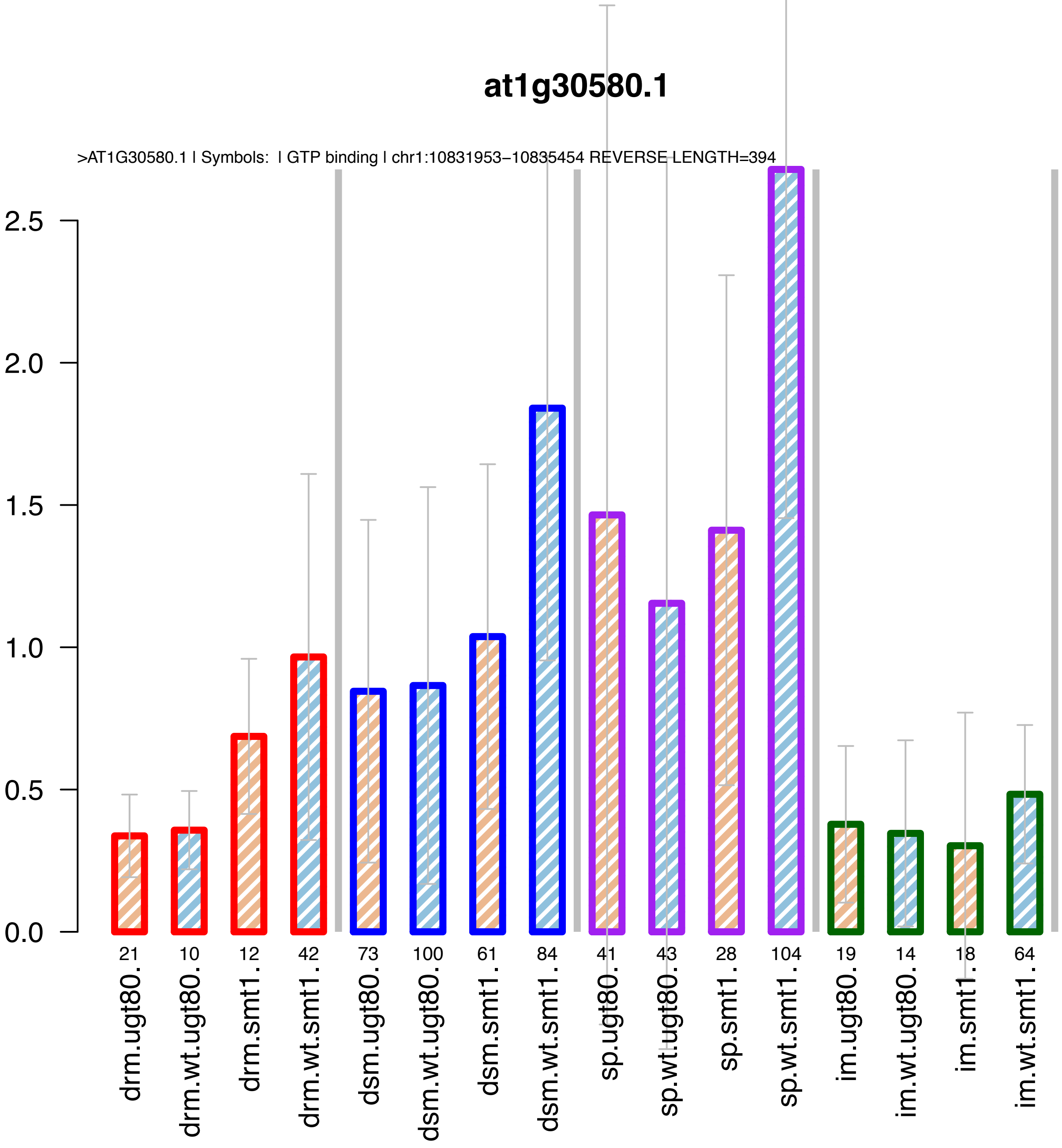

at1g30580.1
>AT1G30580.1 | Symbols: | GTP binding | chr1:10831953-10835454 REVERSE LENGTH=394
2.5
2.0
1.5
1.0
0.5
0.0
21
10
12
42
73
100
61
84
41
43
28
104
19
14
18
64
drm.ugt80..
drm.wt.ugt80..
drm.smt1..
drm.wt.smt1..
dsm.ugt80..
dsm.wt.ugt80..
dsm.smt1..
dsm.wt.smt1..
sp.ugt80..
sp.wt.ugt80..
sp.smt1..
sp.wt.smt1..
im.ugt80..
im.wt.ugt80..
im.smt1..
im.wt.smt1..

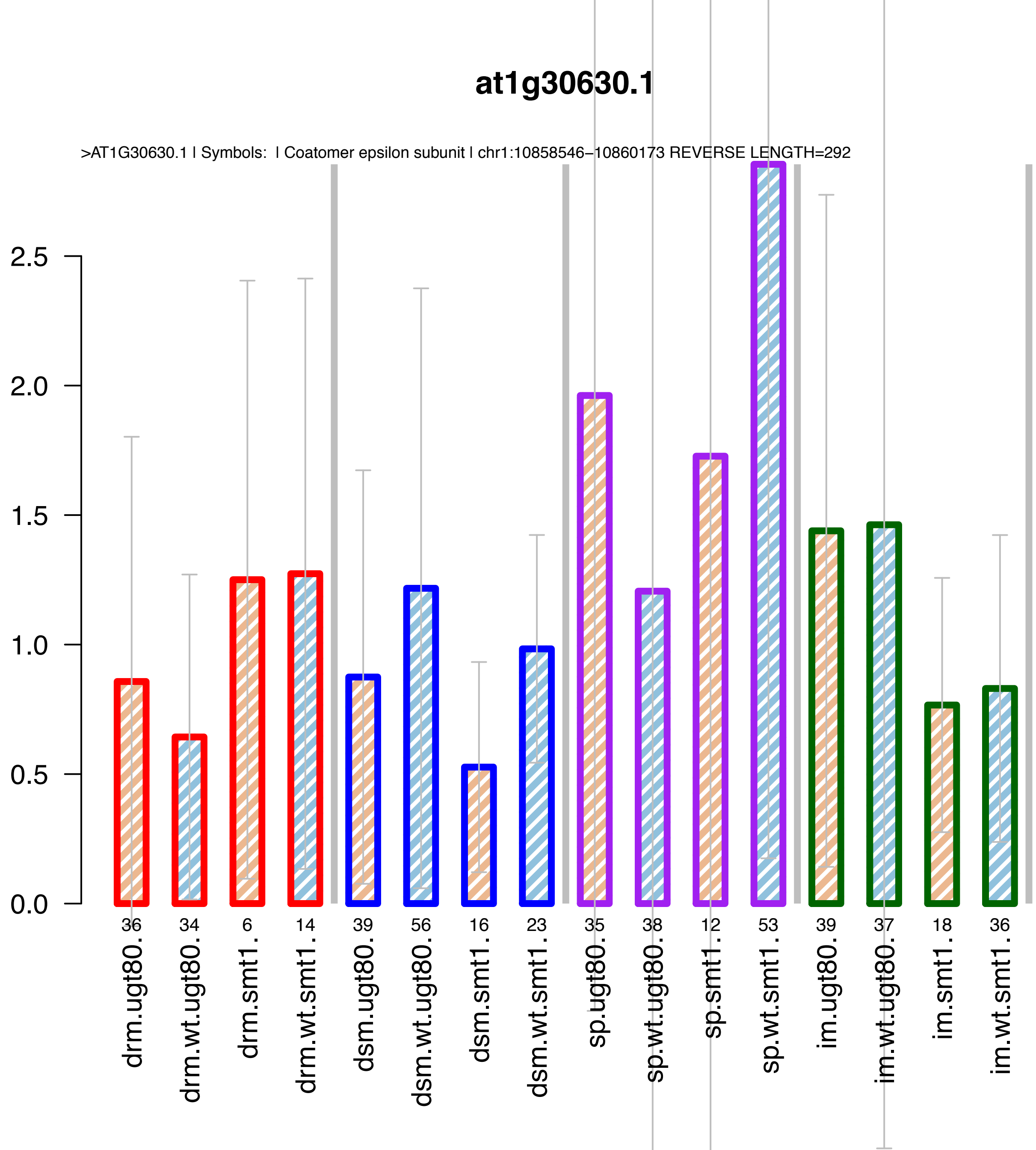

at1g30630.1
>AT1G30630.1 | Symbols: | Coatomer epsilon subunit | chr1:10858546-10860173 REVERSE LENGTH=292
0.0
0.5
1.0
1.5
2.0
2.5
36
34
6
14
39
56
16
23
35
38
12
53
39
37
18
36
drm.ugt80..
drm.wt.ugt80..
drm.smt1..
drm.wt.smt1..
dsm.ugt80..
dsm.wt.ugt80..
dsm.smt1..
dsm.wt.smt1..
sp.ugt80..
sp.wt.ugt80..
sp.smt1..
sp.wt.smt1..
im.ugt80..
im.wt.ugt80..
im.smt1..
im.wt.smt1..

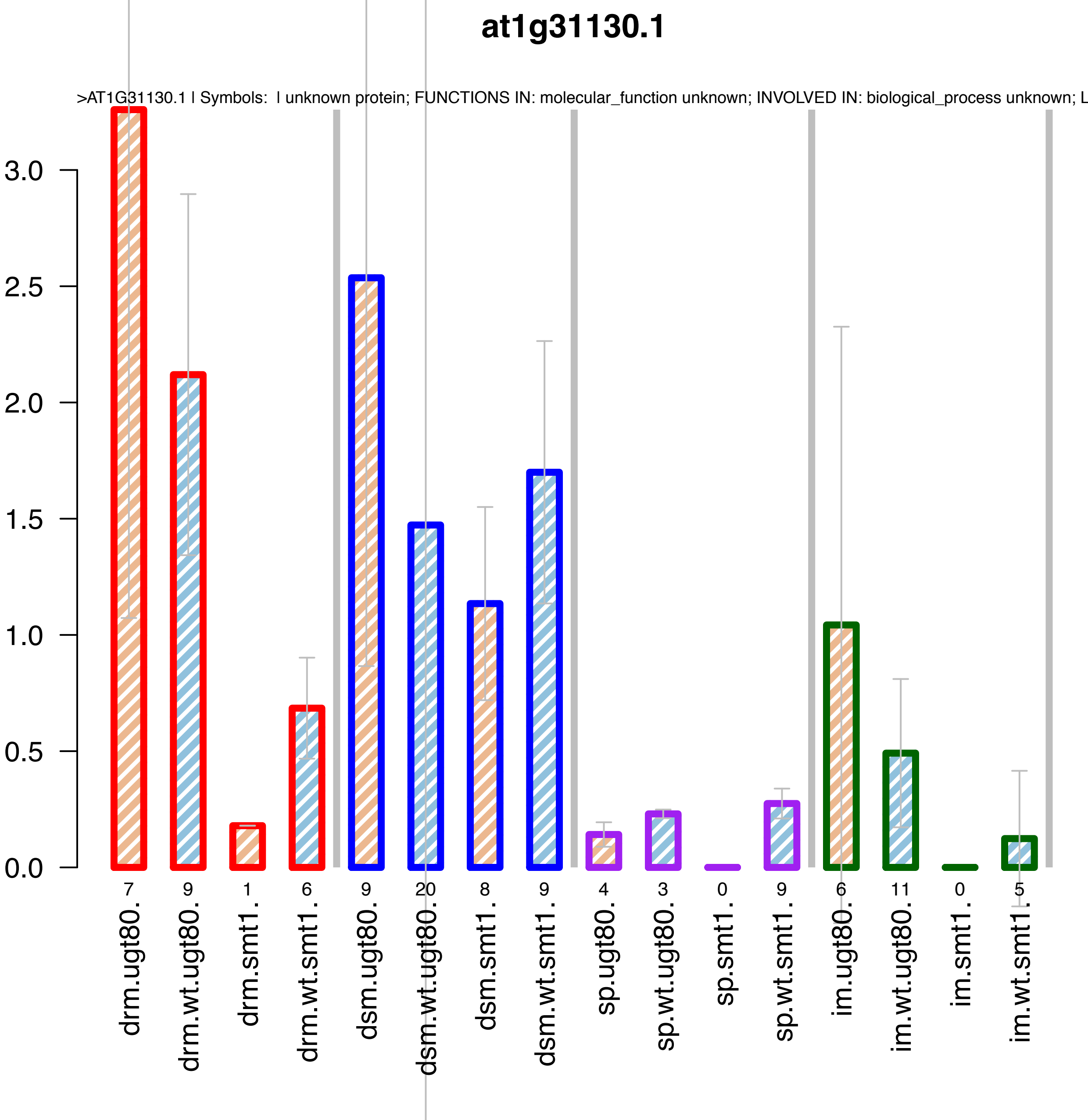
at1g31130.1
>AT1G31130.1 | Symbols: | unknown protein; FUNCTIONS IN: molecular_function unknown; INVOLVED IN: biological_process unknown; L
3.0
2.5
2.0
1.5
1.0
0.5
0.0
7
9
1
6
9
20
8
9
4
3
0
9
6
11
0
5
drm.ugt80.
drm.wt.ugt80.
drm.smt1.
drm.wt.smt1.
dsm.ugt80.
dsm.wt.ugt80.
dsm.smt1.
dsm.wt.smt1.
sp.ugt80.
sp.wt.ugt80.
sp.smt1.
sp.wt.smt1.
im.ugt80.
im.wt.ugt80.
im.smt1.
im.wt.smt1.

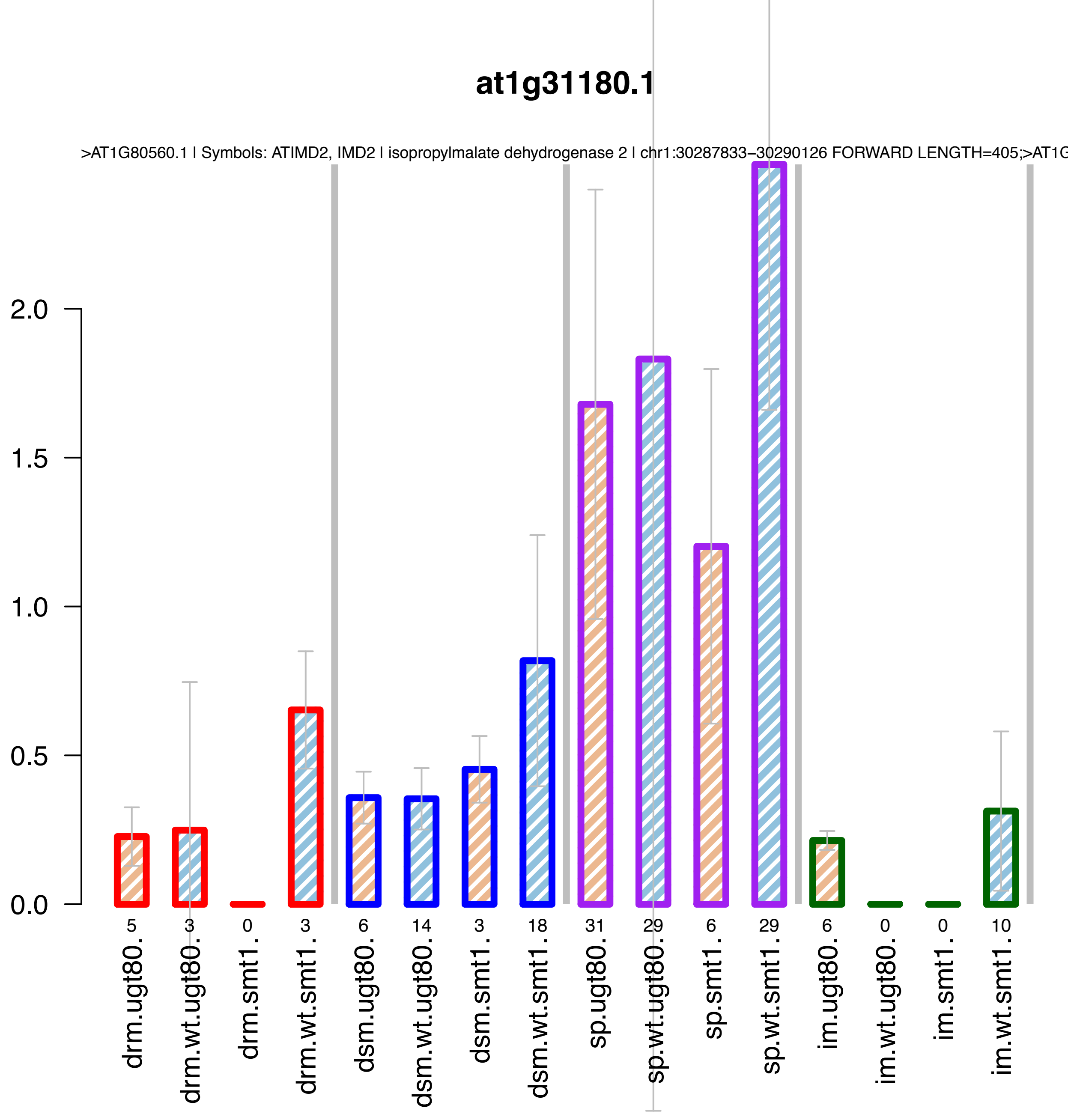
at1g31180.1
>AT1G80560.1 | Symbols: ATIMD2, IMD2 | isopropylmalate dehydrogenase 2 | chr1:30287833–30290126 FORWARD LENGTH=405;>AT1G
2.0
1.5
1.0
0.5
0.0
5
3
0
3
6
14
3
18
31
29
6
29
6
0
0
10
drm.ugt80.
drm.wt.ugt80.
drm.smt1.
drm.wt.smt1.
dsm.ugt80.
dsm.wt.ugt80.
dsm.smt1.
dsm.wt.smt1.
sp.ugt80.
sp.wt.ugt80.
sp.smt1.
sp.wt.smt1.
im.ugt80.
im.wt.ugt80.
im.smt1.
im.wt.smt1.

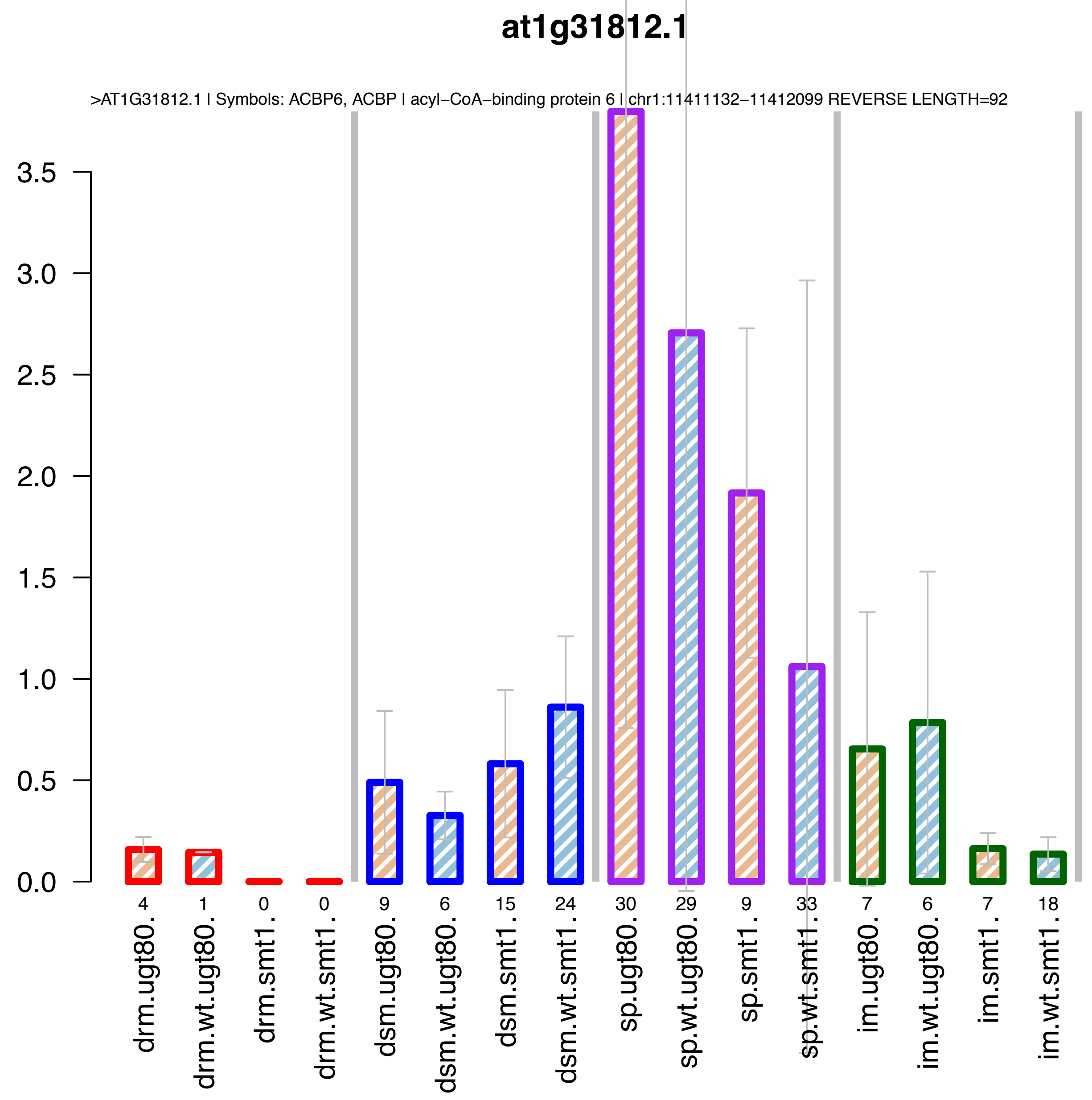

at1g31812.1
>AT1G31812.1 | Symbols: ACBP6, ACBP | acyl−CoA−binding protein 6 | chr1:11411132−11412099 REVERSE LENGTH=92
0.0
0.5
1.0
1.5
2.0
2.5
3.0
3.5
4
1
0
0
9
6
15
24
30
29
9
33
7
6
7
18
drm.ugt80.
drm.wt.ugt80.
drm.smt1.
drm.wt.smt1.
dsm.ugt80.
dsm.wt.ugt80.
dsm.smt1.
dsm.wt.smt1.
sp.ugt80.
sp.wt.ugt80.
sp.smt1.
sp.wt.smt1.
im.ugt80.
im.wt.ugt80.
im.smt1.
im.wt.smt1.

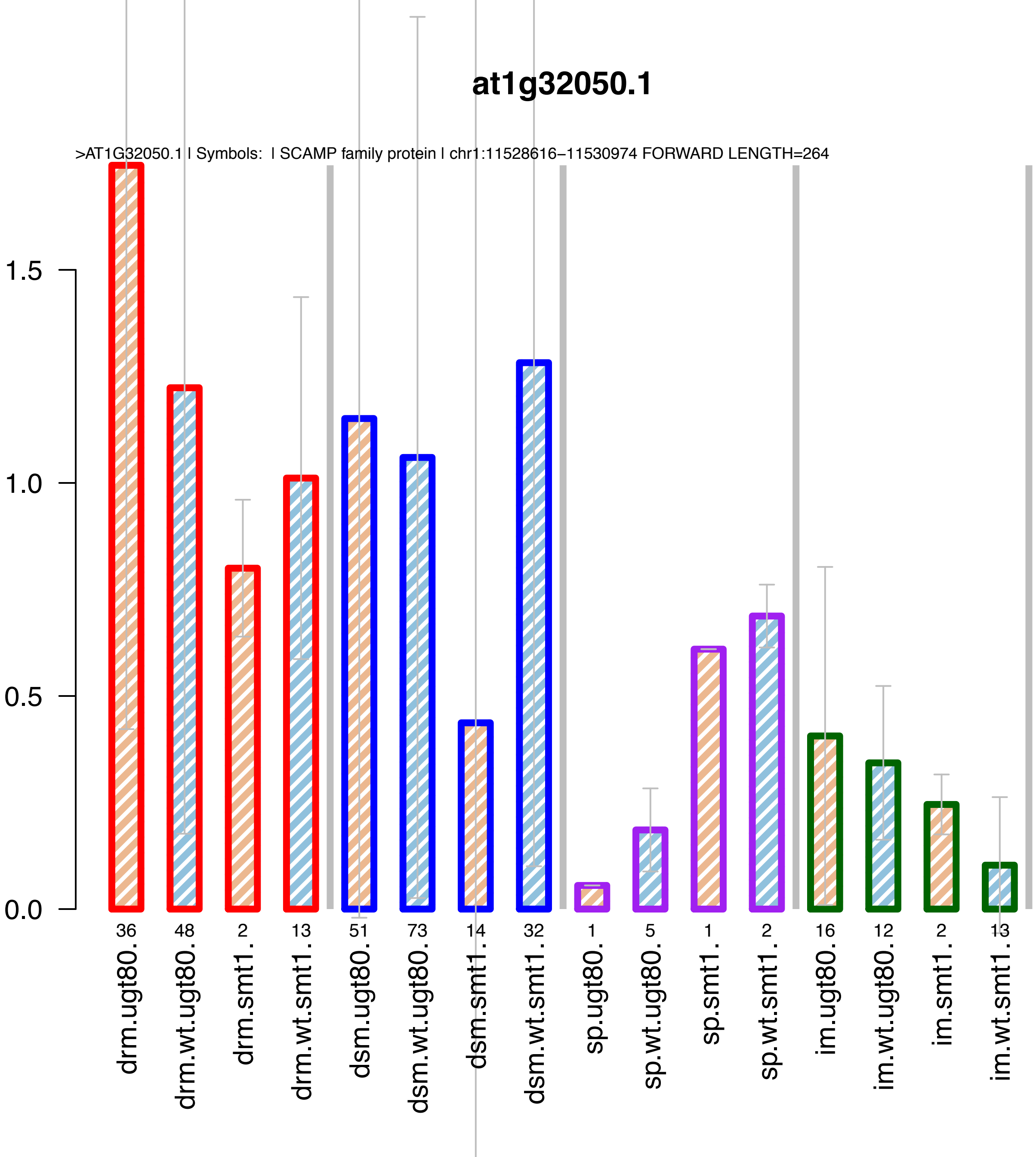

at1g32050.1
>AT1G32050.1 | Symbols: | SCAMP family protein | chr1:11528616-11530974 FORWARD LENGTH=264
1.5
1.0
0.5
0.0
36
48
2
13
51
73
14
32
1
5
1
2
16
12
2
13
drm.ugt80..
drm.wt.ugt80..
drm.smt1..
drm.wt.smt1..
dsm.ugt80..
dsm.wt.ugt80..
dsm.smt1..
dsm.wt.smt1..
sp.ugt80..
sp.wt.ugt80..
sp.smt1..
sp.wt.smt1..
im.ugt80..
im.wt.ugt80..
im.smt1..
im.wt.smt1..

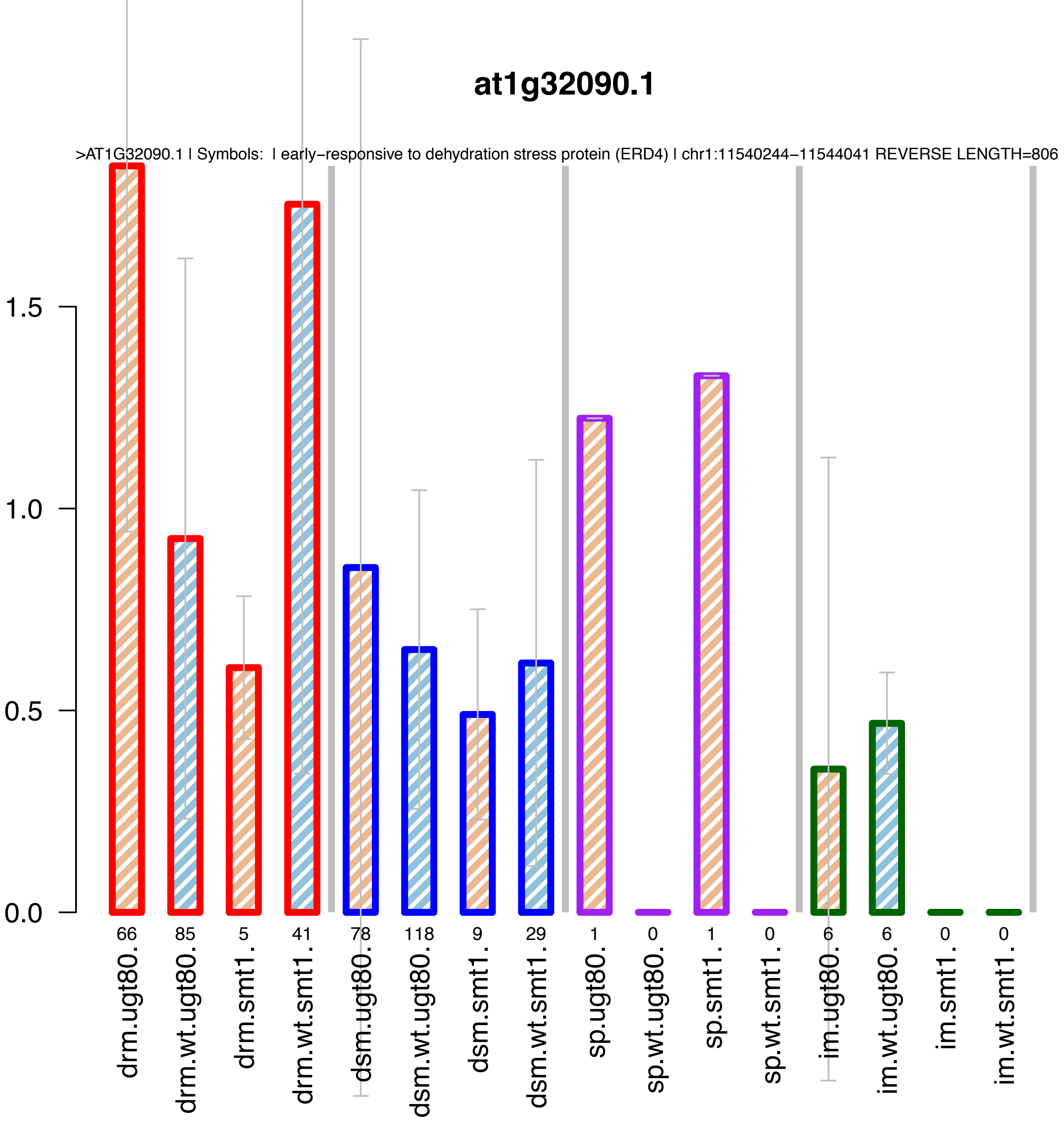

at1g32090.1
>AT1G32090.1 | Symbols: | early−responsive to dehydration stress protein (ERD4) | chr1:11540244−11544041 REVERSE LENGTH=806
0.0
0.5
1.0
1.5
66
85
5
41
78
118
9
29
1
0
1
0
6
6
0
0
drm.ugt80.
drm.wt.ugt80.
drm.smt1.
drm.wt.smt1.
dsm.ugt80.
dsm.wt.ugt80.
dsm.smt1.
dsm.wt.smt1.
sp.ugt80.
sp.wt.ugt80.
sp.smt1.
sp.wt.smt1.
im.ugt80.
im.wt.ugt80.
im.smt1.
im.wt.smt1.

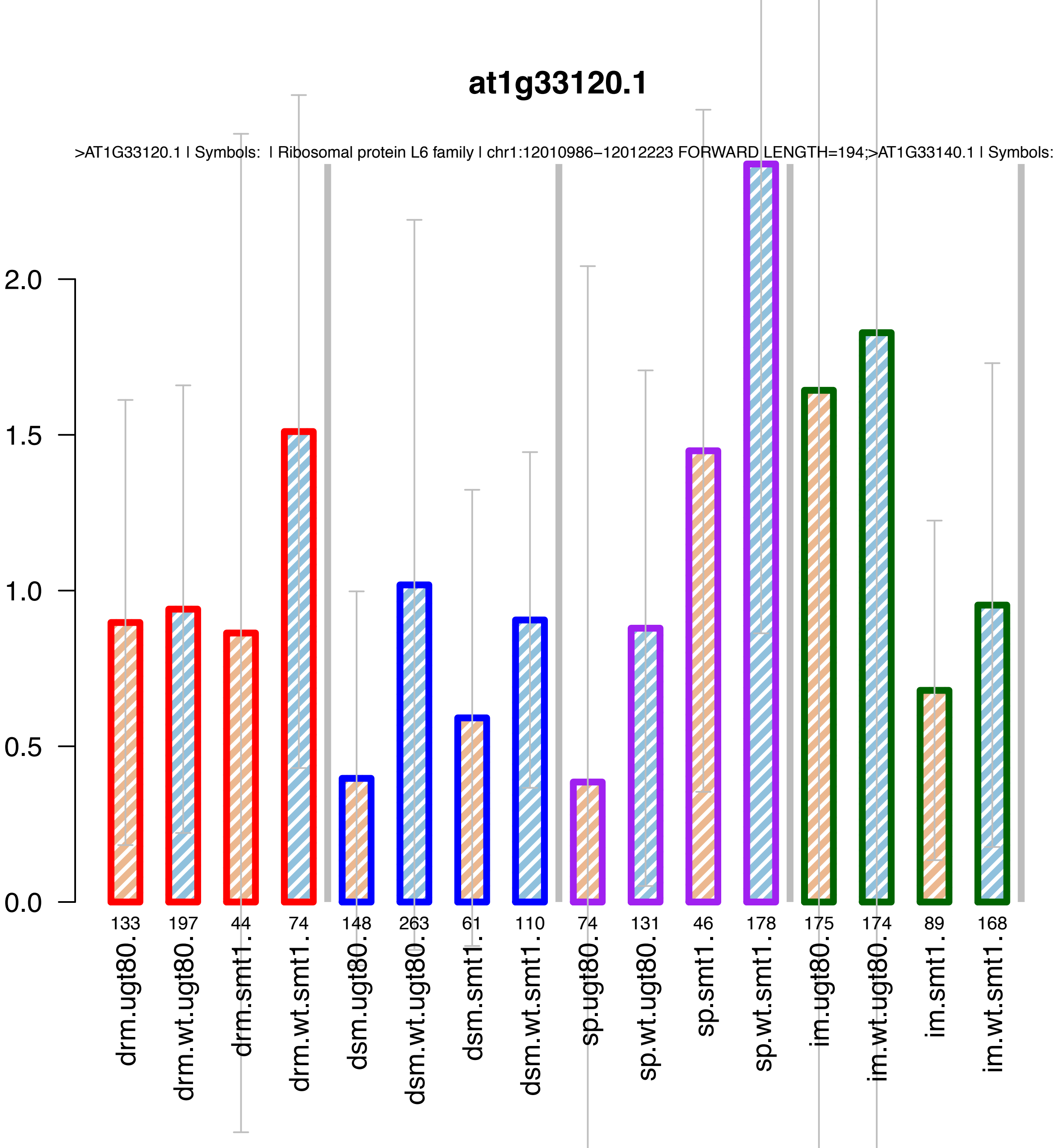

at1g33120.1
>AT1G33120.1 | Symbols: | Ribosomal protein L6 family | chr1:12010986-12012223 FORWARD LENGTH=194;>AT1G33140.1 | Symbols:
0.0
0.5
1.0
1.5
2.0
133
197
44
74
148
263
61
110
74
131
46
178
175
174
89
168
drm.ugt80..
drm.wt.ugt80..
drm.smt1..
drm.wt.smt1..
dsm.ugt80..
dsm.wt.ugt80..
dsm.smt1..
dsm.wt.smt1..
sp.ugt80..
sp.wt.ugt80..
sp.smt1..
sp.wt.smt1..
im.ugt80..
im.wt.ugt80..
im.smt1..
im.wt.smt1..

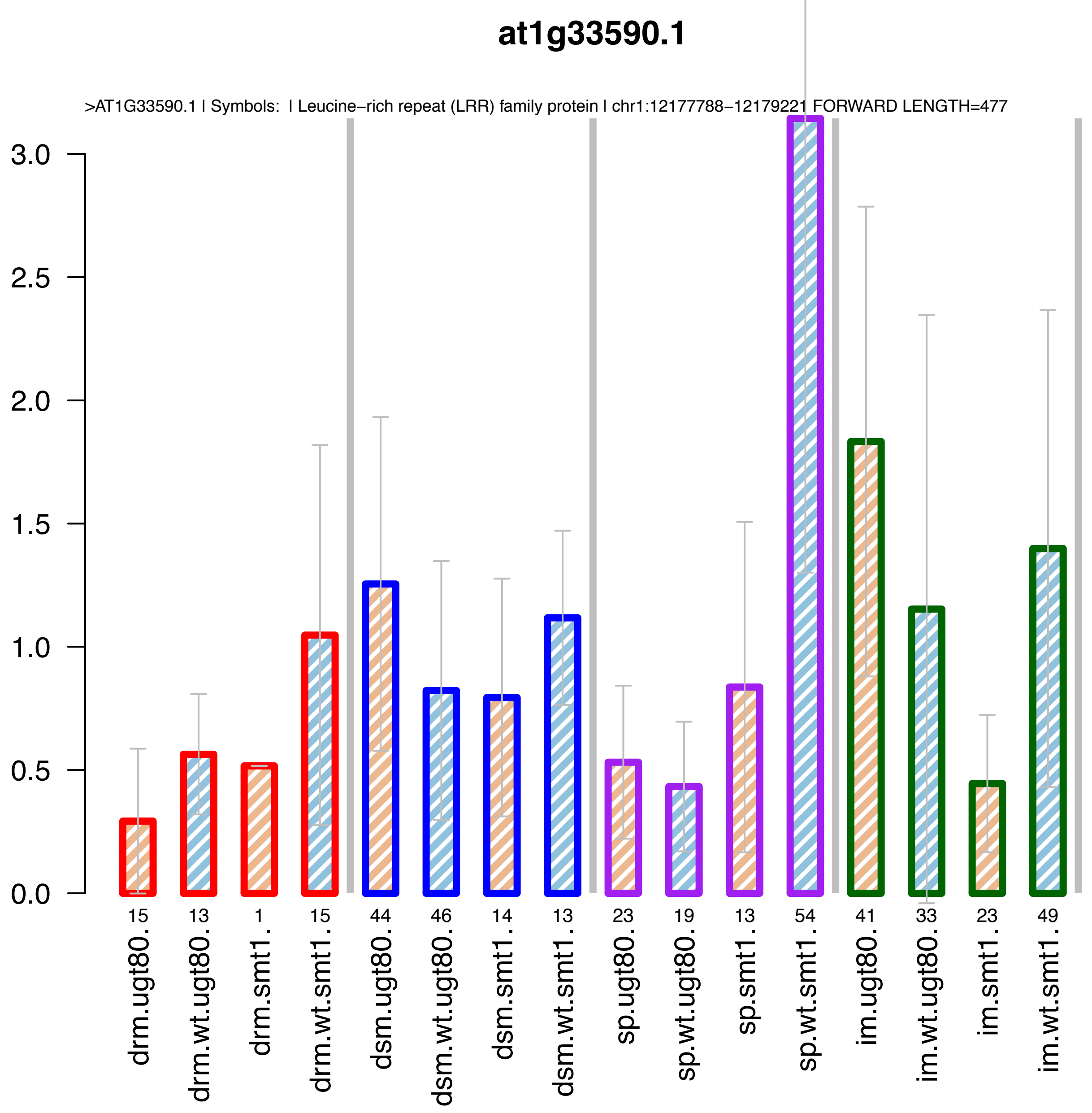
at1g33590.1
>AT1G33590.1 | Symbols: | Leucine−rich repeat (LRR) family protein | chr1:12177788−12179221 FORWARD LENGTH=477
0.0
0.5
1.0
1.5
2.0
2.5
3.0
15
13
1
15
44
46
14
13
23
19
13
54
41
33
23
49
drm.ugt80.
drm.wt.ugt80.
drm.smt1.
drm.wt.smt1.
dsm.ugt80.
dsm.wt.ugt80.
dsm.smt1.
dsm.wt.smt1.
sp.ugt80.
sp.wt.ugt80.
sp.smt1.
sp.wt.smt1.
im.ugt80.
im.wt.ugt80.
im.smt1.
im.wt.smt1.

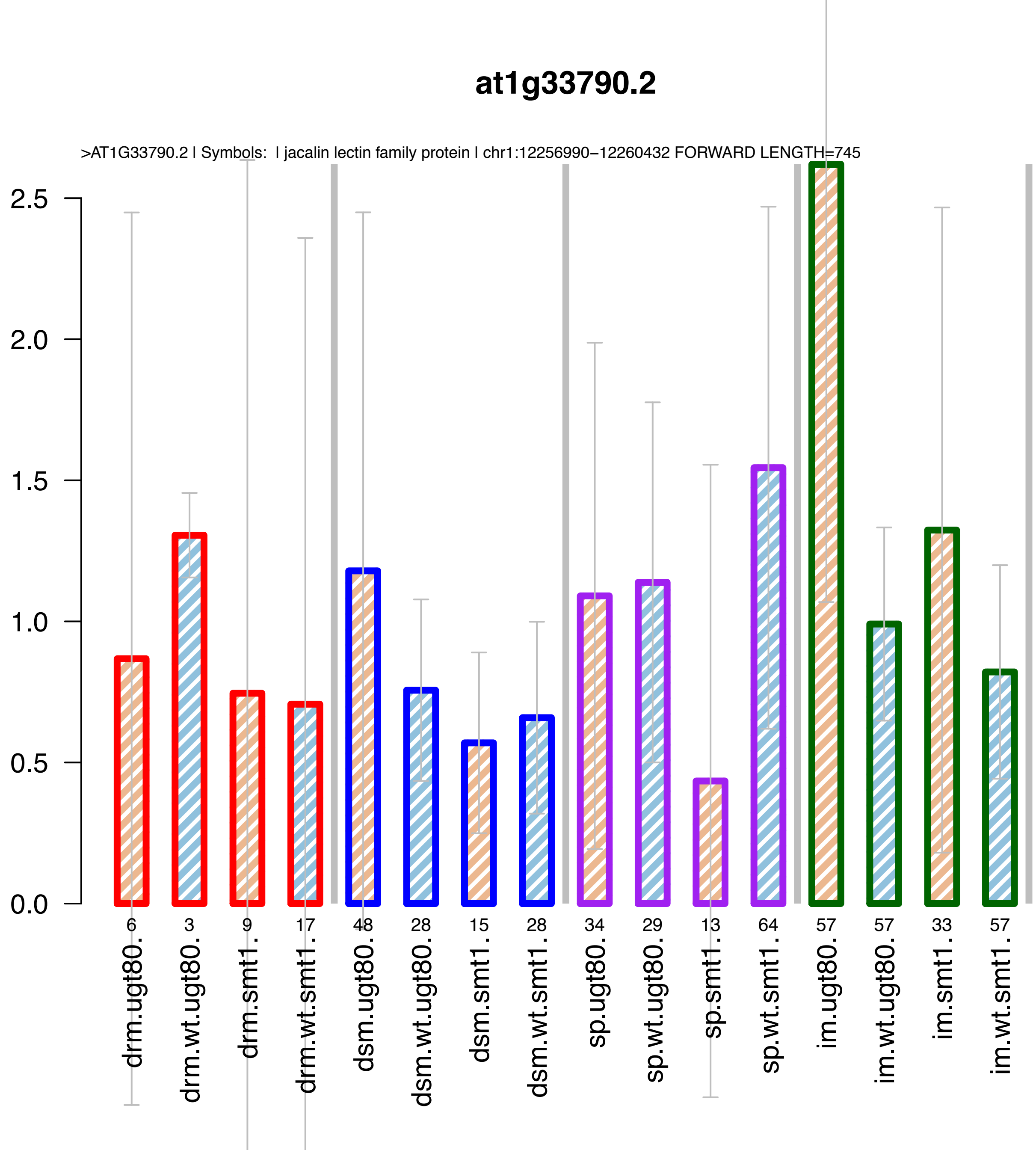
at1g33790.2
>AT1G33790.2 | Symbols: | jacalin lectin family protein | chr1:12256990-12260432 FORWARD LENGTH=745
0.0
0.5
1.0
1.5
2.0
2.5
6
3
9
17
48
28
15
28
34
29
13
64
57
57
33
57
drm.ugt80.
drm.wt.ugt80.
drm.smt1.
drm.wt.smt1.
dsm.ugt80.
dsm.wt.ugt80.
dsm.smt1.
dsm.wt.smt1.
sp.ugt80.
sp.wt.ugt80.
sp.smt1.
sp.wt.smt1.
im.ugt80.
im.wt.ugt80.
im.smt1.
im.wt.smt1.

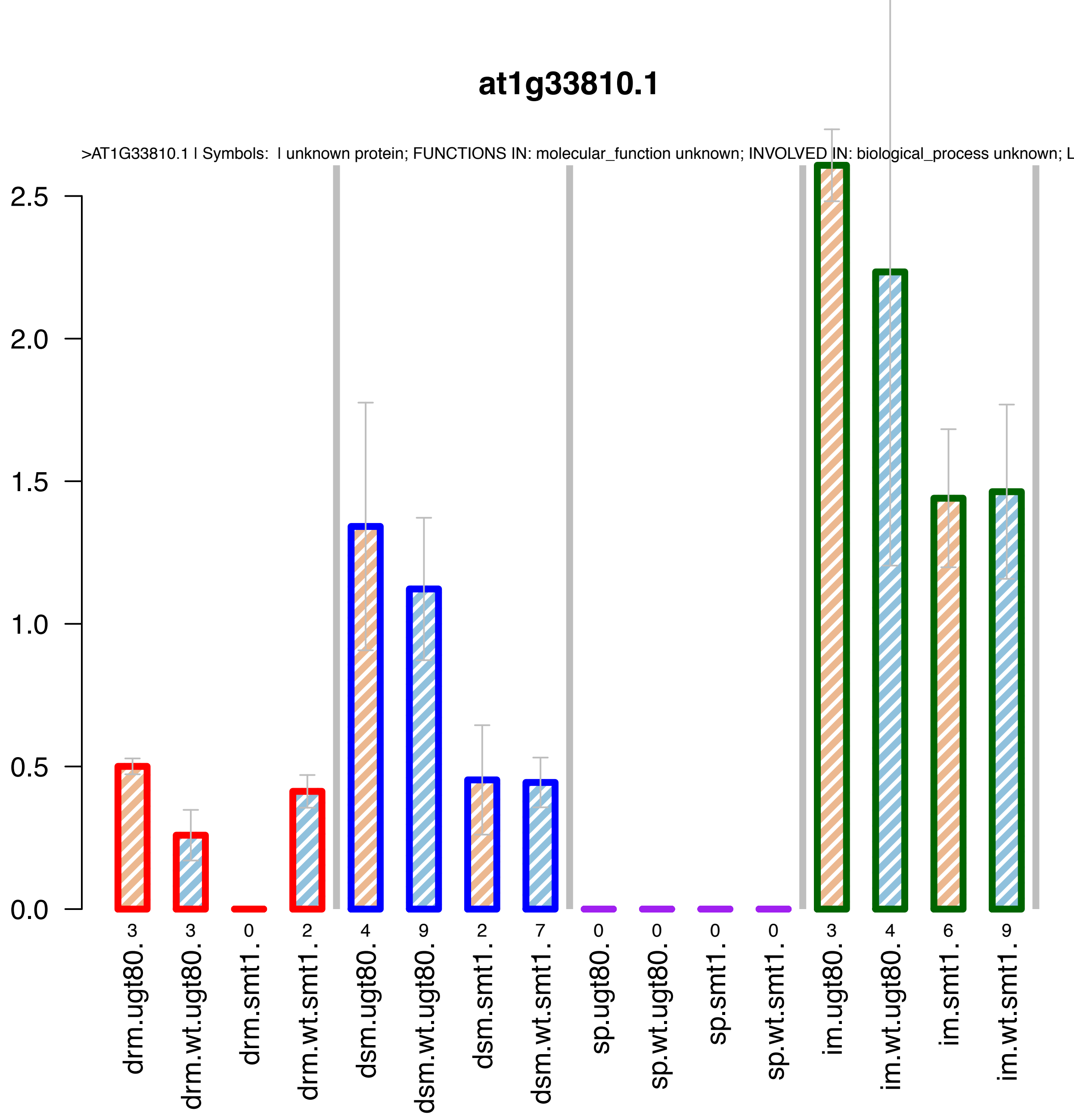

at1g33810.1
>AT1G33810.1 | Symbols: | unknown protein; FUNCTIONS IN: molecular_function unknown; INVOLVED IN: biological_process unknown; L
0.0
0.5
1.0
1.5
2.0
2.5
3
3
0
2
4
9
2
7
0
0
0
0
3
4
6
9
drm.ugt80.
drm.wt.ugt80.
drm.smt1.
drm.wt.smt1.
dsm.ugt80.
dsm.wt.ugt80.
dsm.smt1.
dsm.wt.smt1.
sp.ugt80.
sp.wt.ugt80.
sp.smt1.
sp.wt.smt1.
im.ugt80.
im.wt.ugt80.
im.smt1.
im.wt.smt1.

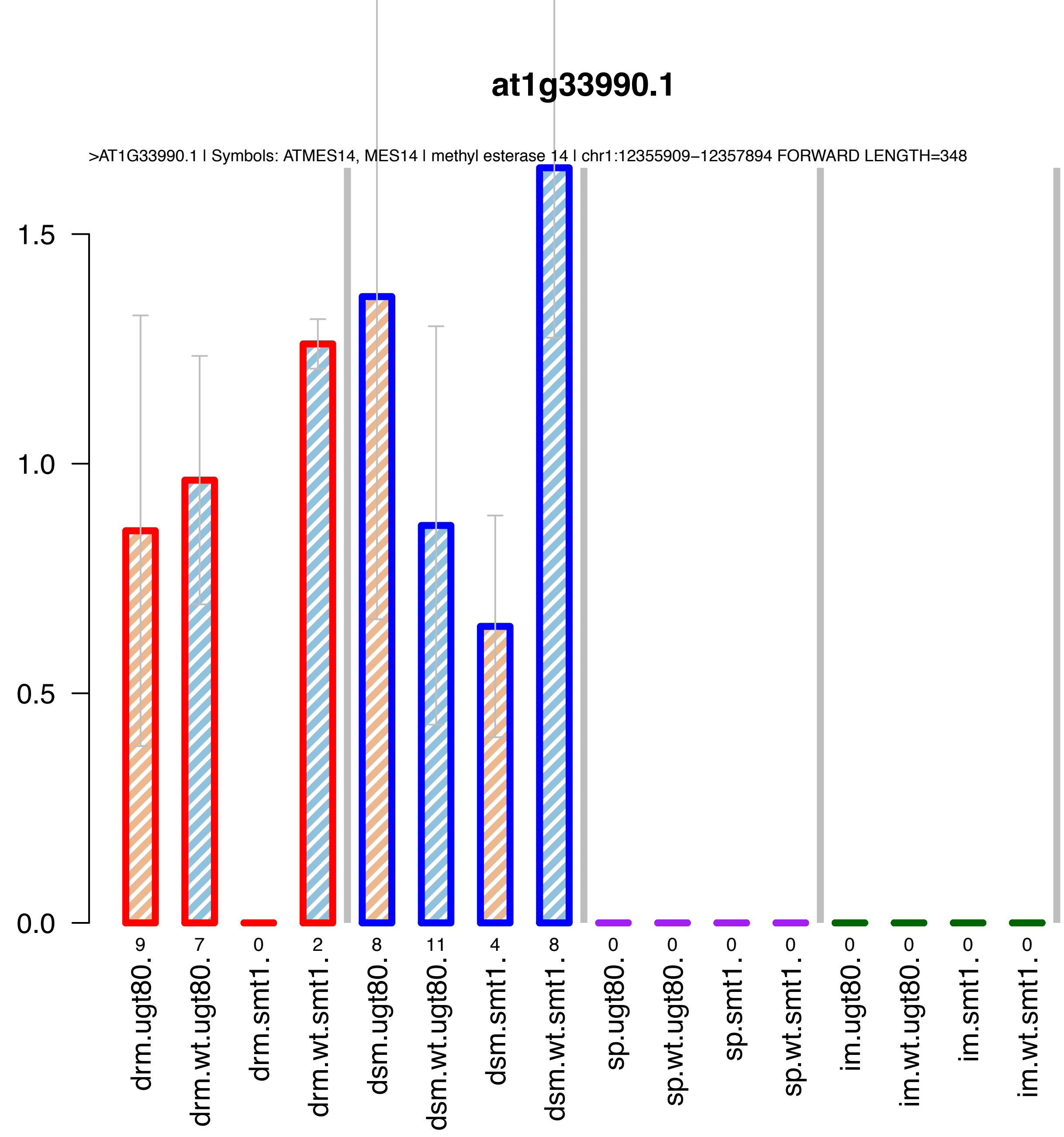

at1g33990.1
>AT1G33990.1 | Symbols: ATMES14, MES14 | methyl esterase 14 | chr1:12355909-12357894 FORWARD LENGTH=348
0.0
0.5
1.0
1.5
9
7
0
2
8
11
4
8
0
0
0
0
0
0
0
0
drm.ugt80.
drm.wt.ugt80.
drm.smt1.
drm.wt.smt1.
dsm.ugt80.
dsm.wt.ugt80.
dsm.smt1.
dsm.wt.smt1.
sp.ugt80.
sp.wt.ugt80.
sp.smt1.
sp.wt.smt1.
im.ugt80.
im.wt.ugt80.
im.smt1.
im.wt.smt1.

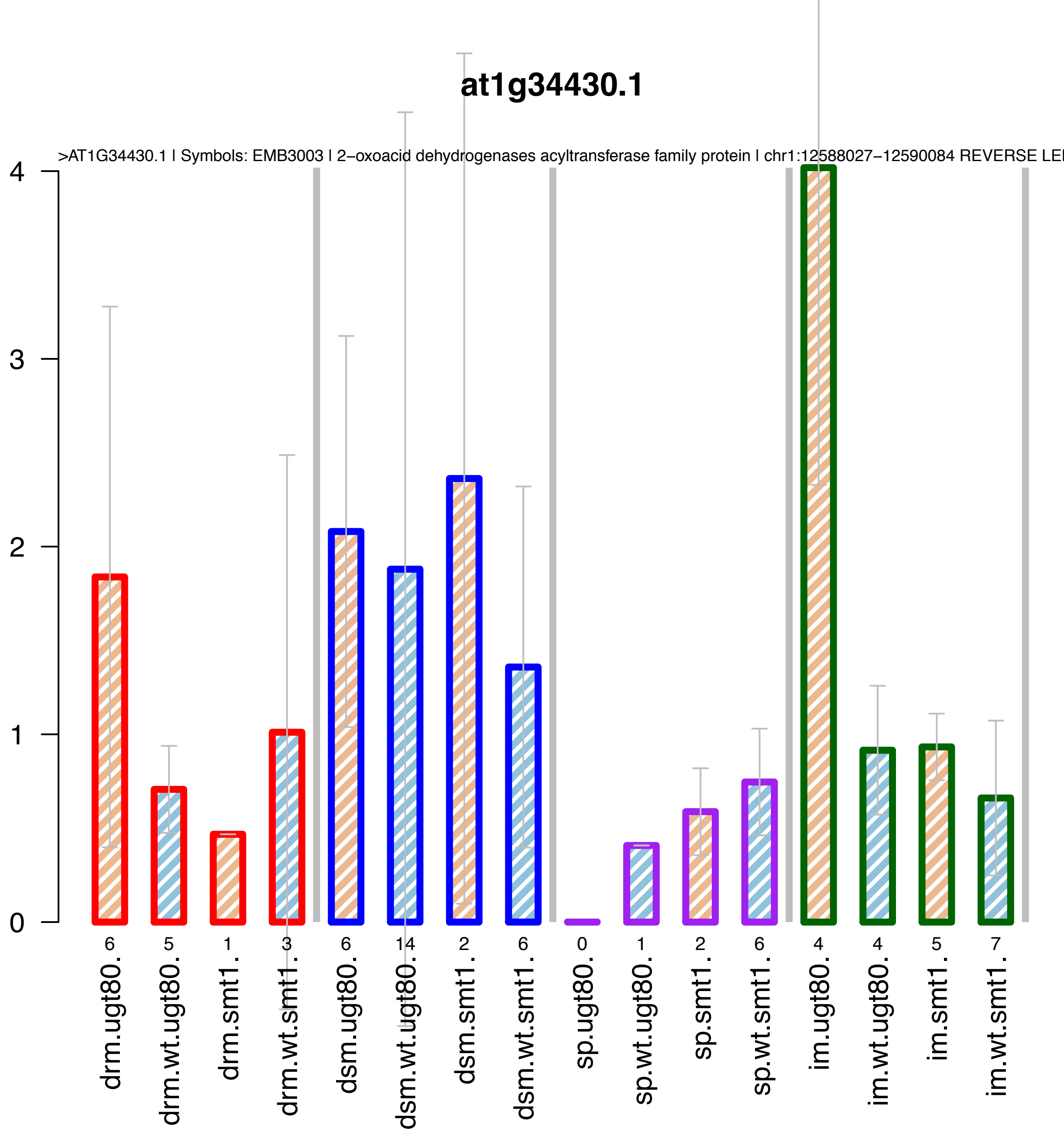

at1g34430.1
>AT1G34430.1 | Symbols: EMB3003 | 2−oxoacid dehydrogenases acyltransferase family protein | chr1:12588027−12590084 REVERSE LE
0
1
2
3
4
6
5
1
3
6
14
2
6
0
1
2
6
4
4
5
7
drm.ugt80..
drm.wt.ugt80..
drm.smt1..
drm.wt.smt1..
dsm.ugt80..
dsm.wt.ugt80..
dsm.smt1..
dsm.wt.smt1..
sp.ugt80..
sp.wt.ugt80..
sp.smt1..
sp.wt.smt1..
im.ugt80..
im.wt.ugt80..
im.smt1..
im.wt.smt1..

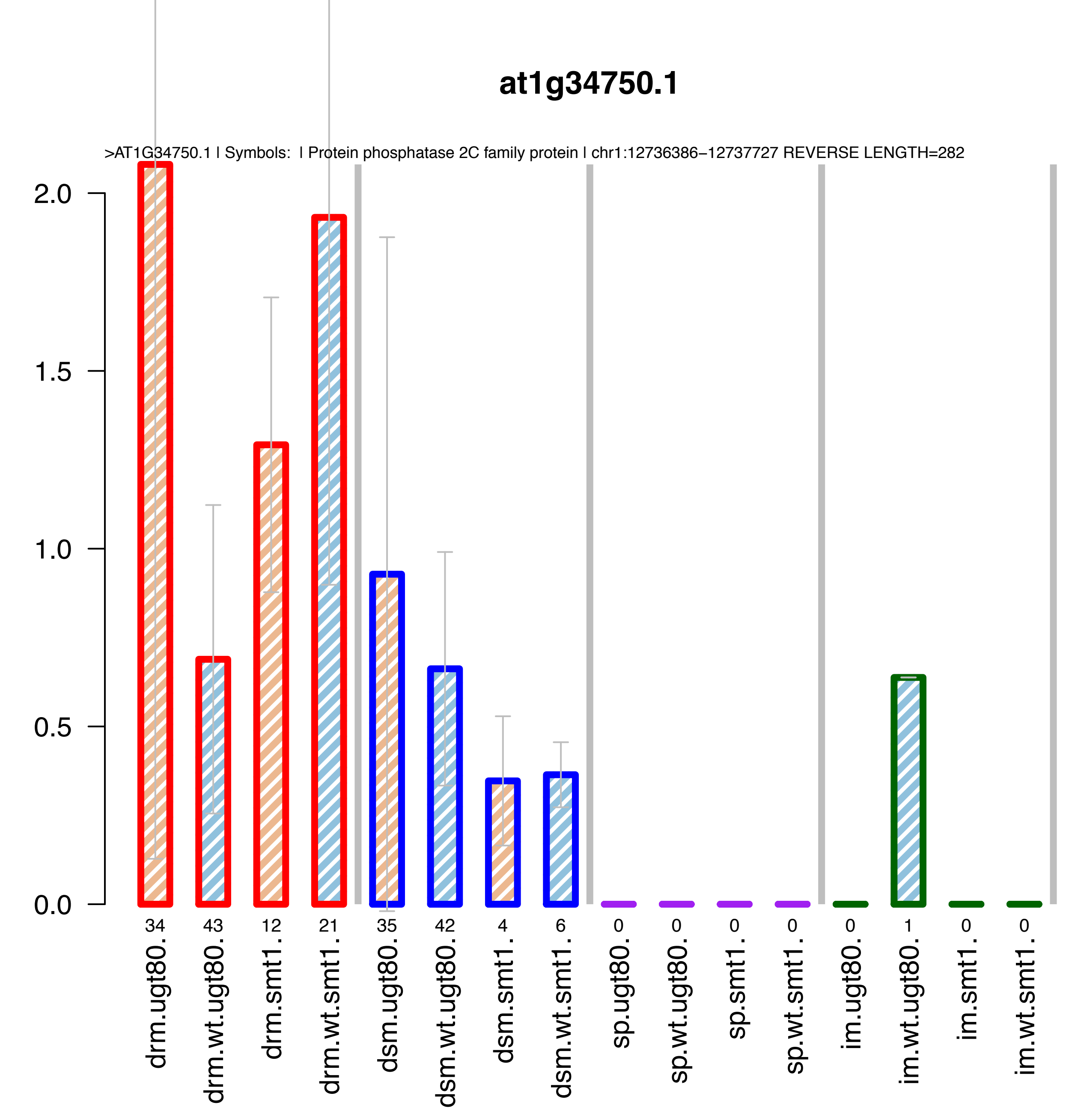

at1g34750.1
>AT1G34750.1 | Symbols: | Protein phosphatase 2C family protein | chr1:12736386-12737727 REVERSE LENGTH=282
2.0
1.5
1.0
0.5
0.0
34
43
12
21
35
42
4
6
0
0
0
0
0
1
0
0
drm.ugt80..
drm.wt.ugt80..
drm.smt1..
drm.wt.smt1..
dsm.ugt80..
dsm.wt.ugt80..
dsm.smt1..
dsm.wt.smt1..
sp.ugt80..
sp.wt.ugt80..
sp.smt1..
sp.wt.smt1..
im.ugt80..
im.wt.ugt80..
im.smt1..
im.wt.smt1..

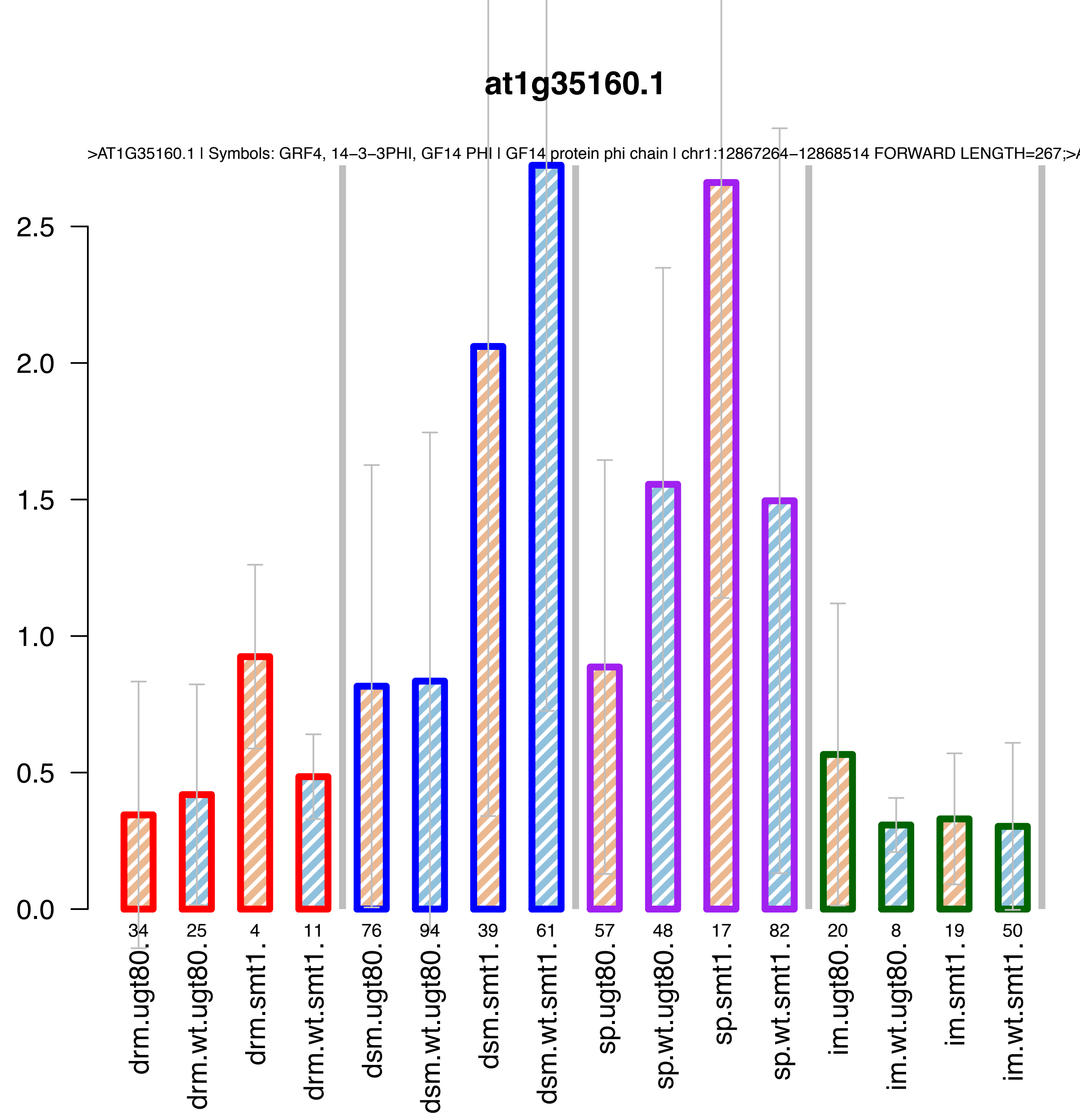

at1g35160.1
>AT1G35160.1 | Symbols: GRF4, 14−3−3PHI, GF14 PHI | GF14 protein phi chain | chr1:12867264−12868514 FORWARD LENGTH=267;>A
0.0
0.5
1.0
1.5
2.0
2.5
34
25
4
11
76
94
39
61
57
48
17
82
20
8
19
50
drm.ugt80.
drm.wt.ugt80.
drm.smt1.
drm.wt.smt1.
dsm.ugt80.
dsm.wt.ugt80.
dsm.smt1.
dsm.wt.smt1.
sp.ugt80.
sp.wt.ugt80.
sp.smt1.
sp.wt.smt1.
im.ugt80.
im.wt.ugt80.
im.smt1.
im.wt.smt1.

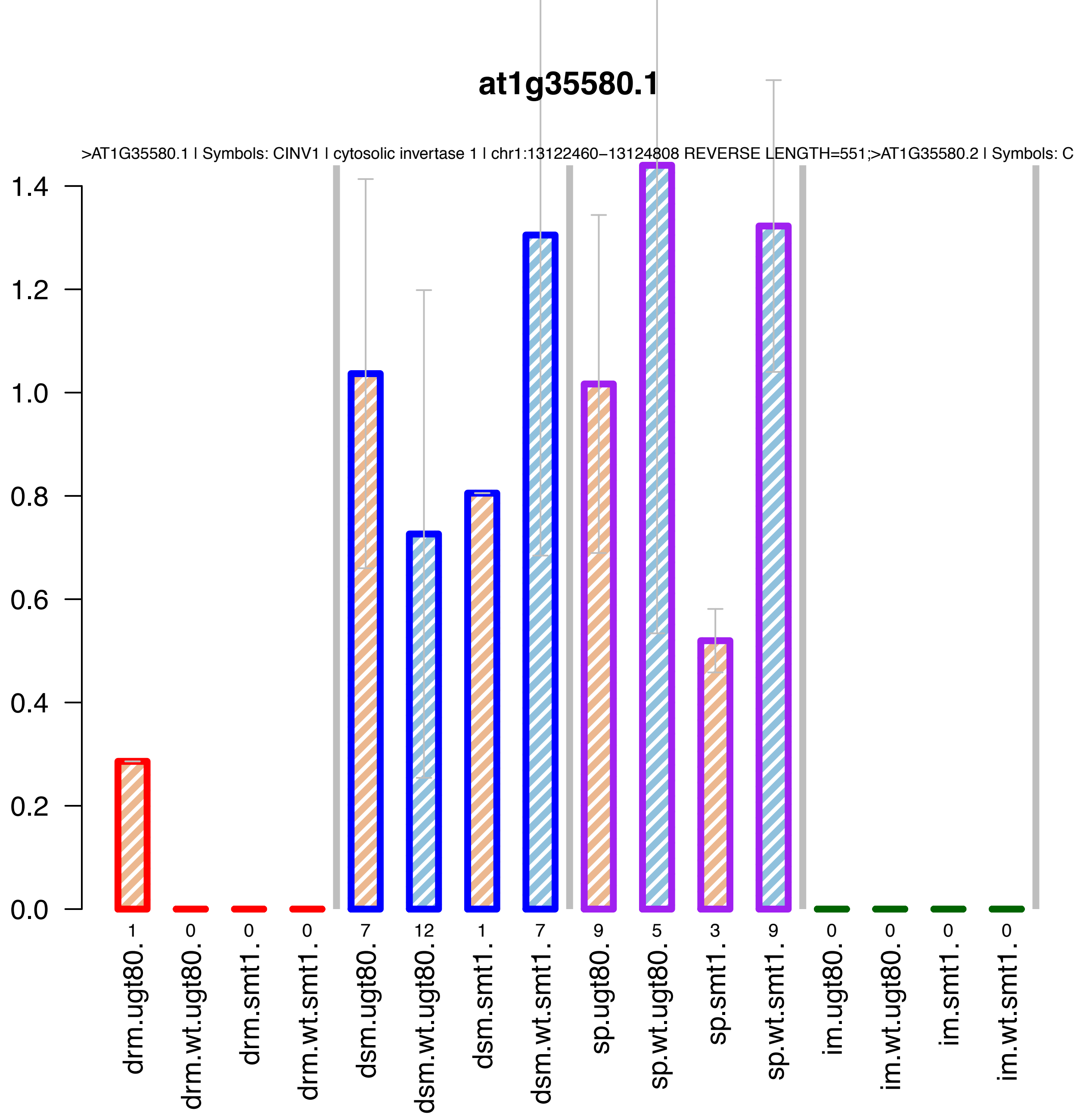
at1g35580.1
>AT1G35580.1 | Symbols: CINV1 | cytosolic invertase 1 | chr1:13122460–13124808 REVERSE LENGTH=551;>AT1G35580.2 | Symbols: C
1.4
1.2
1.0
0.8
0.6
0.4
0.2
0.0
1
0
0
0
7
12
1
7
9
5
3
9
0
0
0
0
drm.ugt80.
drm.wt.ugt80.
drm.smt1.
drm.wt.smt1.
dsm.ugt80.
dsm.wt.ugt80.
dsm.smt1.
dsm.wt.smt1.
sp.ugt80.
sp.wt.ugt80.
sp.smt1.
sp.wt.smt1.
im.ugt80.
im.wt.ugt80.
im.smt1.
im.wt.smt1.

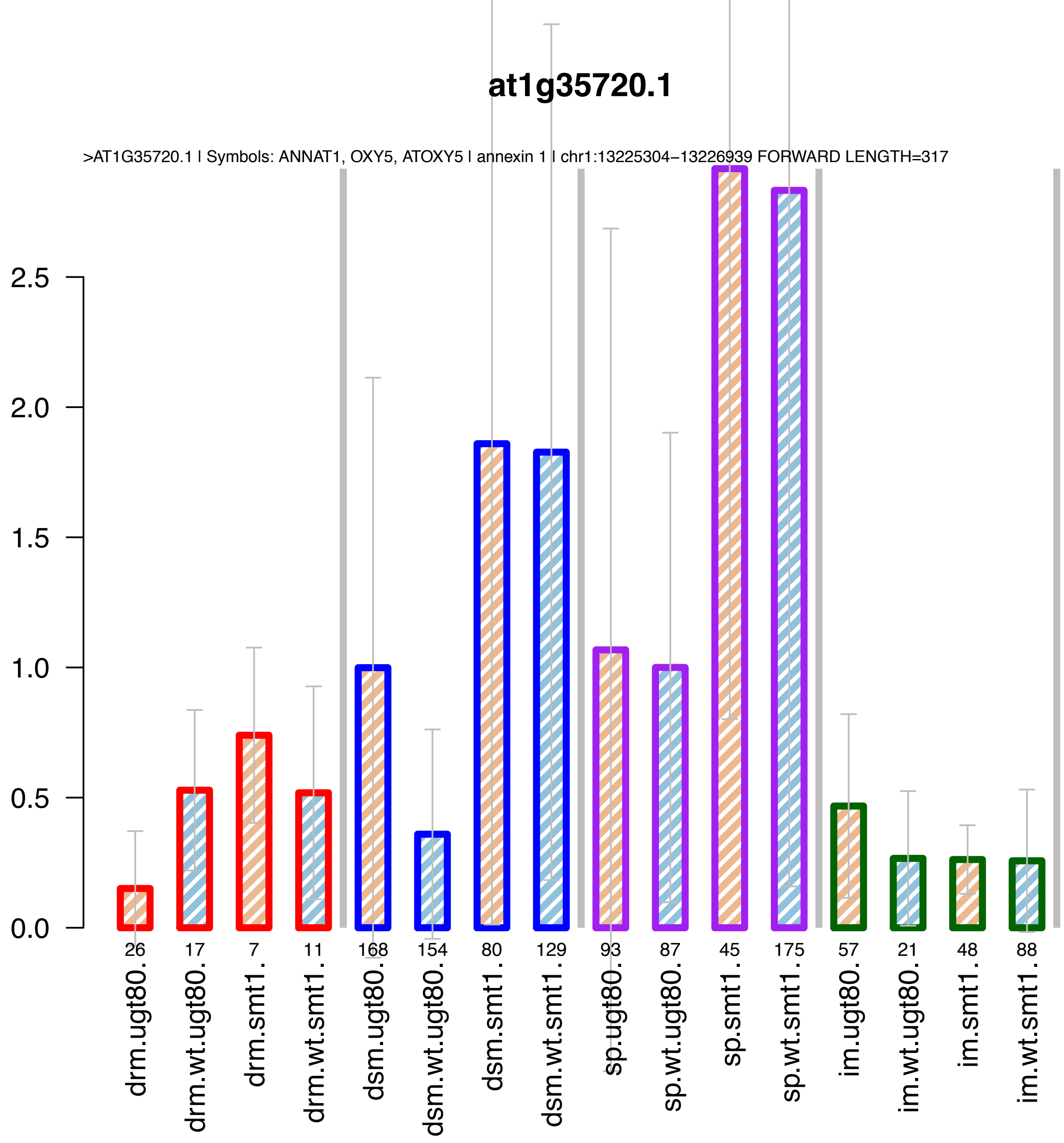
at1g35720.1
>AT1G35720.1 | Symbols: ANNAT1, OXY5, ATOXY5 | annexin 1 | chr1:13225304−13226939 FORWARD LENGTH=317
0.0
0.5
1.0
1.5
2.0
2.5
26
17
7
11
168
154
80
129
93
87
45
175
57
21
48
88
drm.ugt80..
drm.wt.ugt80..
drm.smt1..
drm.wt.smt1..
dsm.ugt80..
dsm.wt.ugt80..
dsm.smt1..
dsm.wt.smt1..
sp.ugt80..
sp.wt.ugt80..
sp.smt1..
sp.wt.smt1..
im.ugt80..
im.wt.ugt80..
im.smt1..
im.wt.smt1..

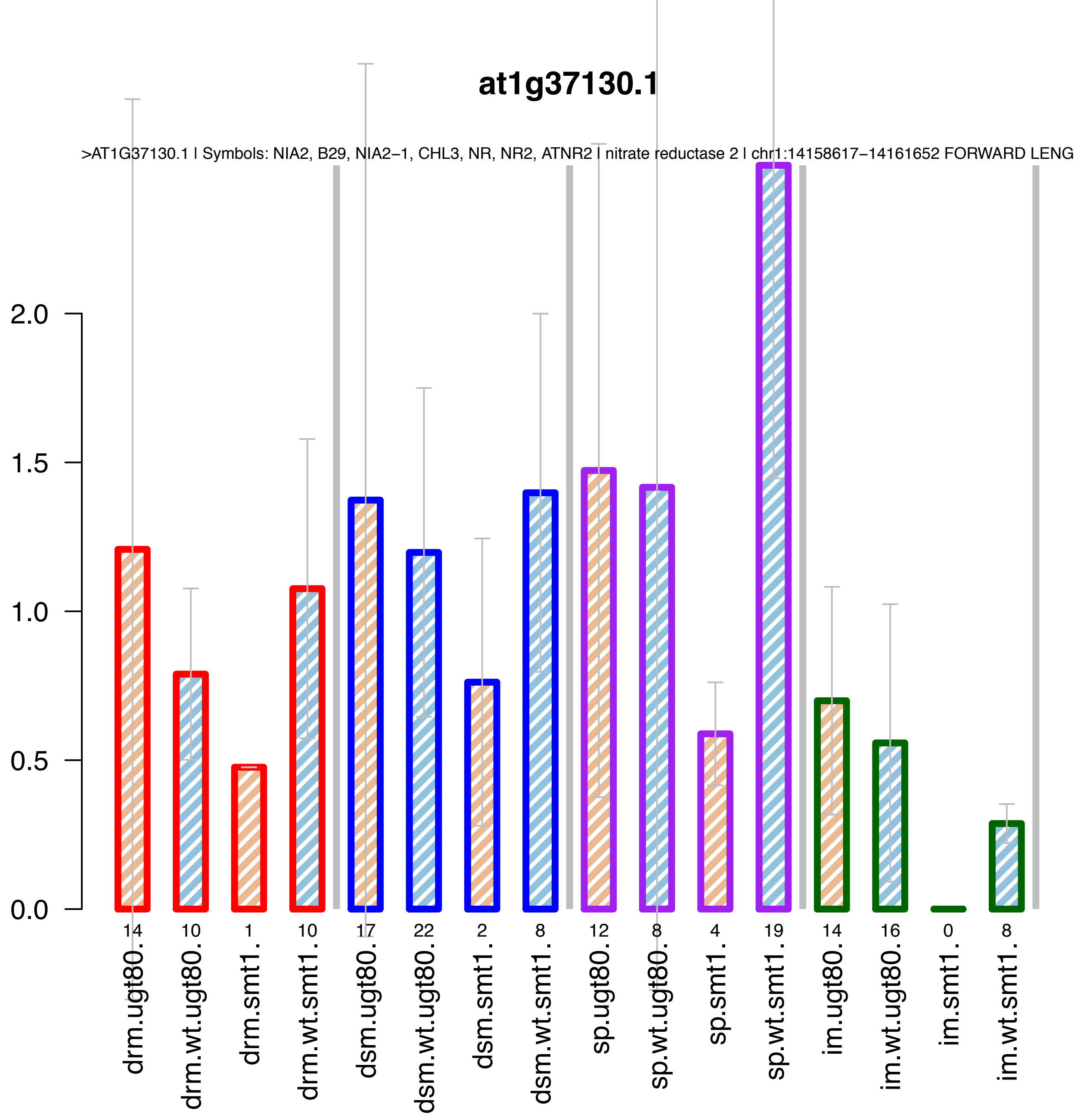
at1g37130.1
>AT1G37130.1 | Symbols: NIA2, B29, NIA2−1, CHL3, NR, NR2, ATNR2 | nitrate reductase 2 | chr1:14158617−14161652 FORWARD LENG
2.0
1.5
1.0
0.5
0.0
14
10
1
10
17
22
2
8
12
8
4
19
14
16
0
8
drm.ugt80..
drm.wt.ugt80..
drm.smt1..
drm.wt.smt1..
dsm.ugt80..
dsm.wt.ugt80..
dsm.smt1..
dsm.wt.smt1..
sp.ugt80..
sp.wt.ugt80..
sp.smt1..
sp.wt.smt1..
im.ugt80..
im.wt.ugt80..
im.smt1..
im.wt.smt1..

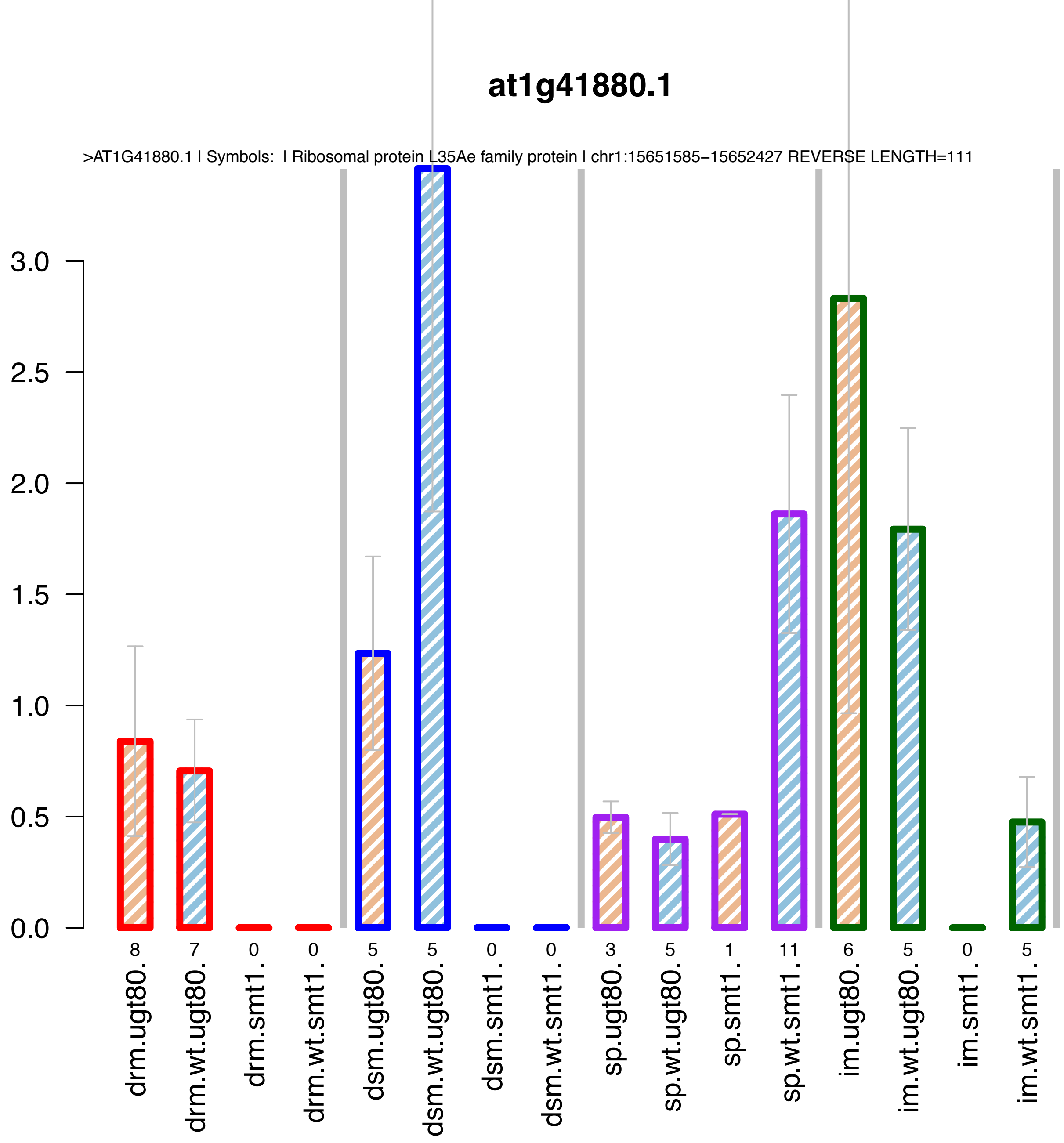

at1g41880.1
>AT1G41880.1 | Symbols: | Ribosomal protein L35Ae family protein | chr1:15651585-15652427 REVERSE LENGTH=111
3.0
2.5
2.0
1.5
1.0
0.5
0.0
8
7
0
0
5
5
0
0
3
5
1
11
6
5
0
5
drm.ugt80.
drm.wt.ugt80.
drm.smt1.
drm.wt.smt1.
dsm.ugt80.
dsm.wt.ugt80.
dsm.smt1.
dsm.wt.smt1.
sp.ugt80.
sp.wt.ugt80.
sp.smt1.
sp.wt.smt1.
im.ugt80.
im.wt.ugt80.
im.smt1.
im.wt.smt1.

# at1g42960.1

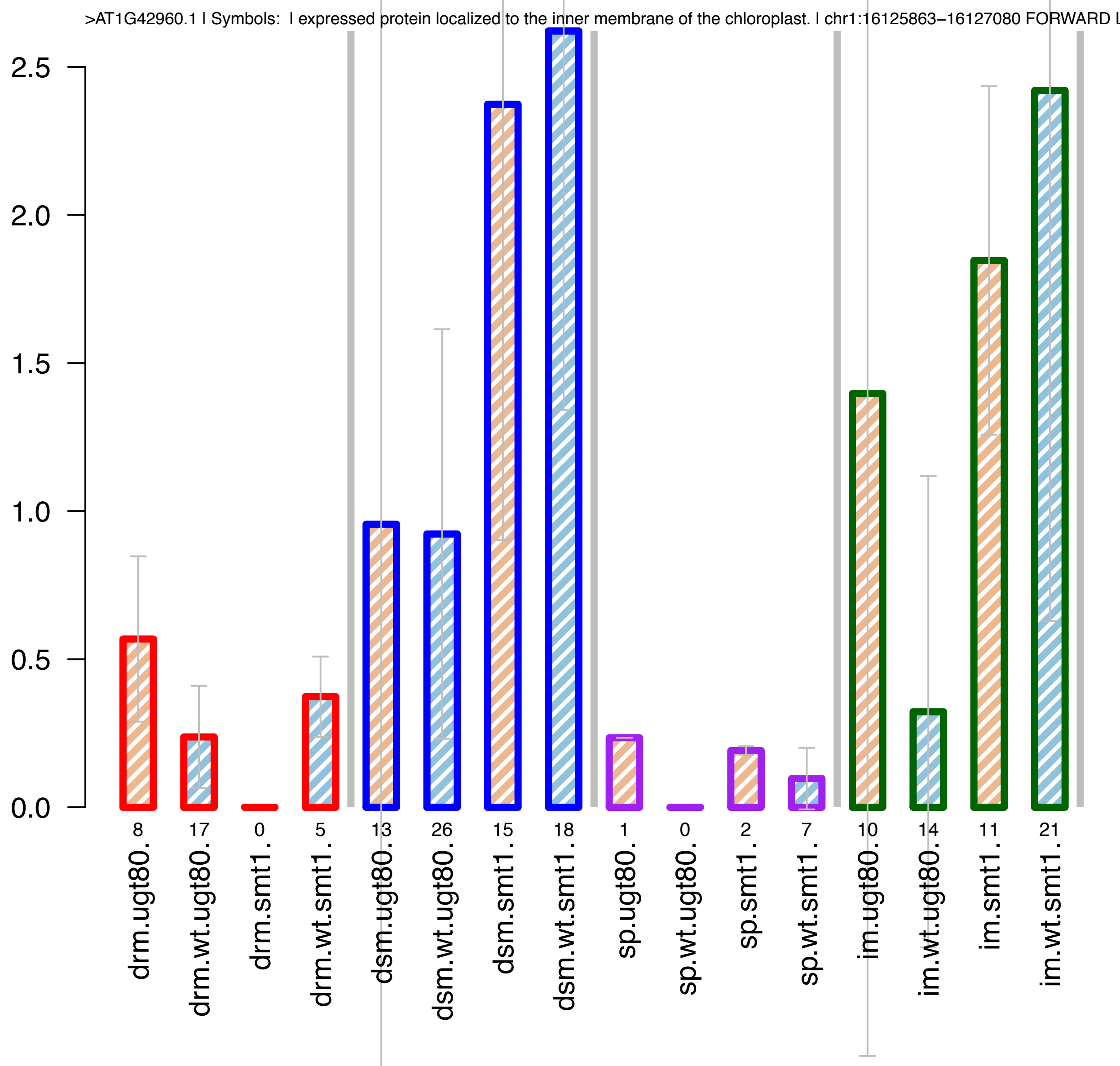

>AT1G42960.1 | Symbols: | expressed protein localized to the inner membrane of the chloroplast. | chr1:16125863−16127080 FORWARD L
0.0
0.5
1.0
1.5
2.0
2.5
8
17
0
5
13
26
15
18
1
0
2
7
10
14
11
21
drm.ugt80..
drm.wt.ugt80..
drm.smt1..
drm.wt.smt1..
dsm.ugt80..
dsm.wt.ugt80..
dsm.smt1..
dsm.wt.smt1..
sp.ugt80..
sp.wt.ugt80..
sp.smt1..
sp.wt.smt1..
im.ugt80..
im.wt.ugt80..
im.smt1..
im.wt.smt1..

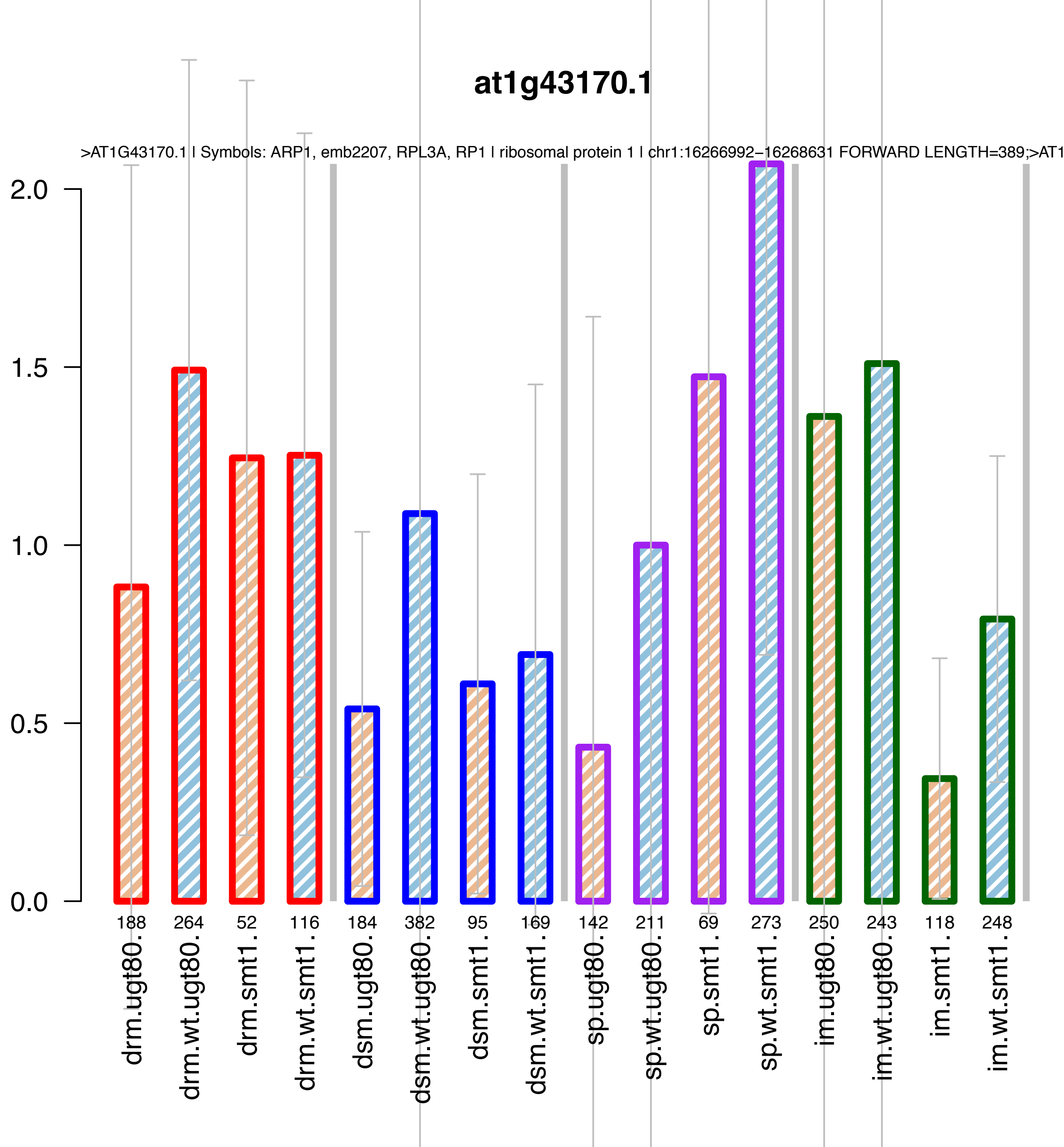

at1g43170.1
>AT1G43170.1 | Symbols: ARP1, emb2207, RPL3A, RP1 | ribosomal protein 1 | chr1:16266992–16268631 FORWARD LENGTH=389;>AT1
2.0
1.5
1.0
0.5
0.0
188
264
52
116
184
382
95
169
142
211
69
273
250
243
118
248
drm.ugt80..
drm.wt.ugt80..
drm.smt1..
drm.wt.smt1..
dsm.ugt80..
dsm.wt.ugt80..
dsm.smt1..
dsm.wt.smt1..
sp.ugt80..
sp.wt.ugt80..
sp.smt1..
sp.wt.smt1..
im.ugt80..
im.wt.ugt80..
im.smt1..
im.wt.smt1..

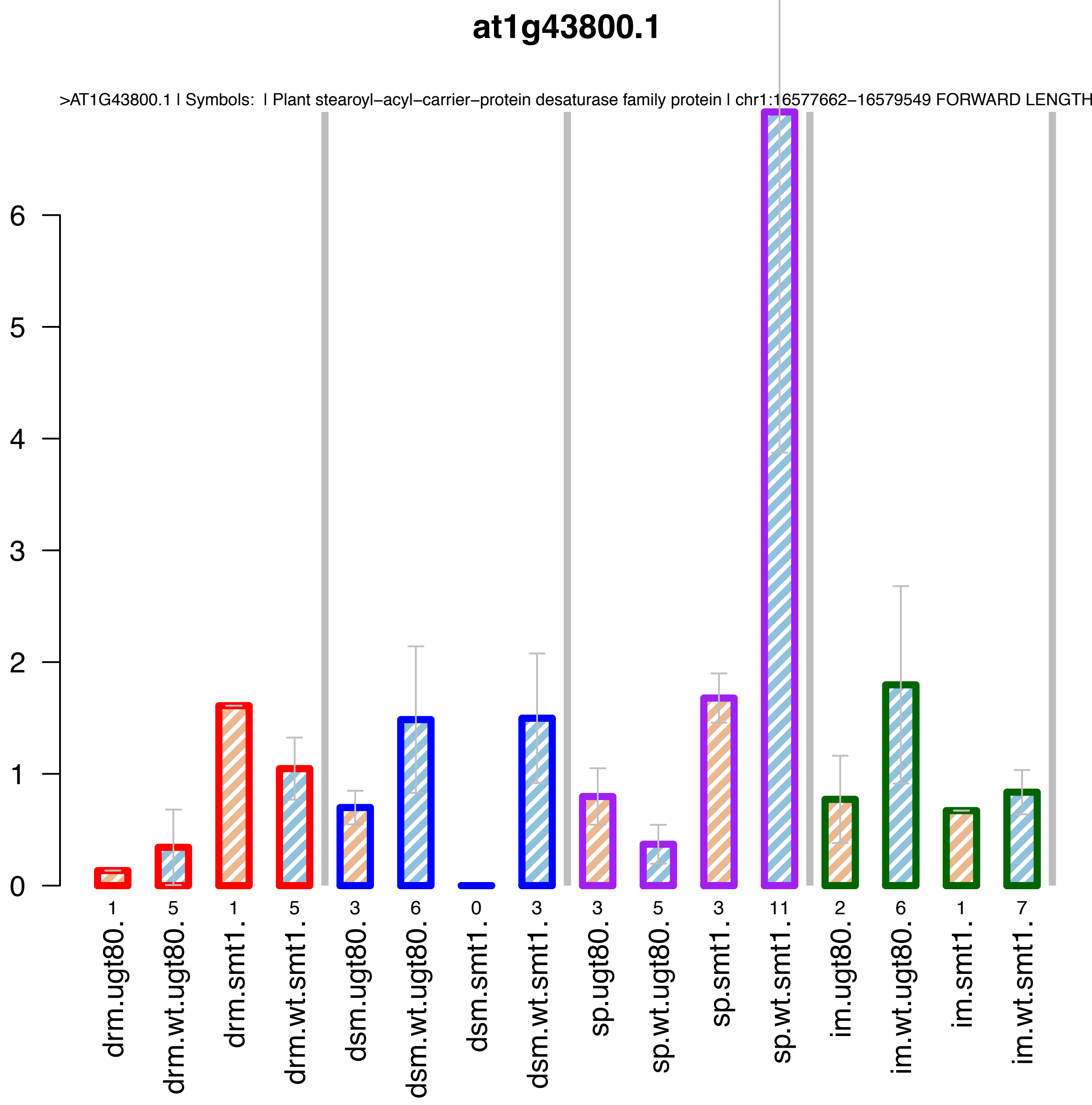

at1g43800.1
>AT1G43800.1 | Symbols: | Plant stearoyl−acyl−carrier−protein desaturase family protein | chr1:16577662−16579549 FORWARD LENGTH
0
1
2
3
4
5
6
1
5
1
5
3
6
0
3
3
5
3
11
2
6
1
7
drm.ugt80.
drm.wt.ugt80.
drm.smt1.
drm.wt.smt1.
dsm.ugt80.
dsm.wt.ugt80.
dsm.smt1.
dsm.wt.smt1.
sp.ugt80.
sp.wt.ugt80.
sp.smt1.
sp.wt.smt1.
im.ugt80.
im.wt.ugt80.
im.smt1.
im.wt.smt1.

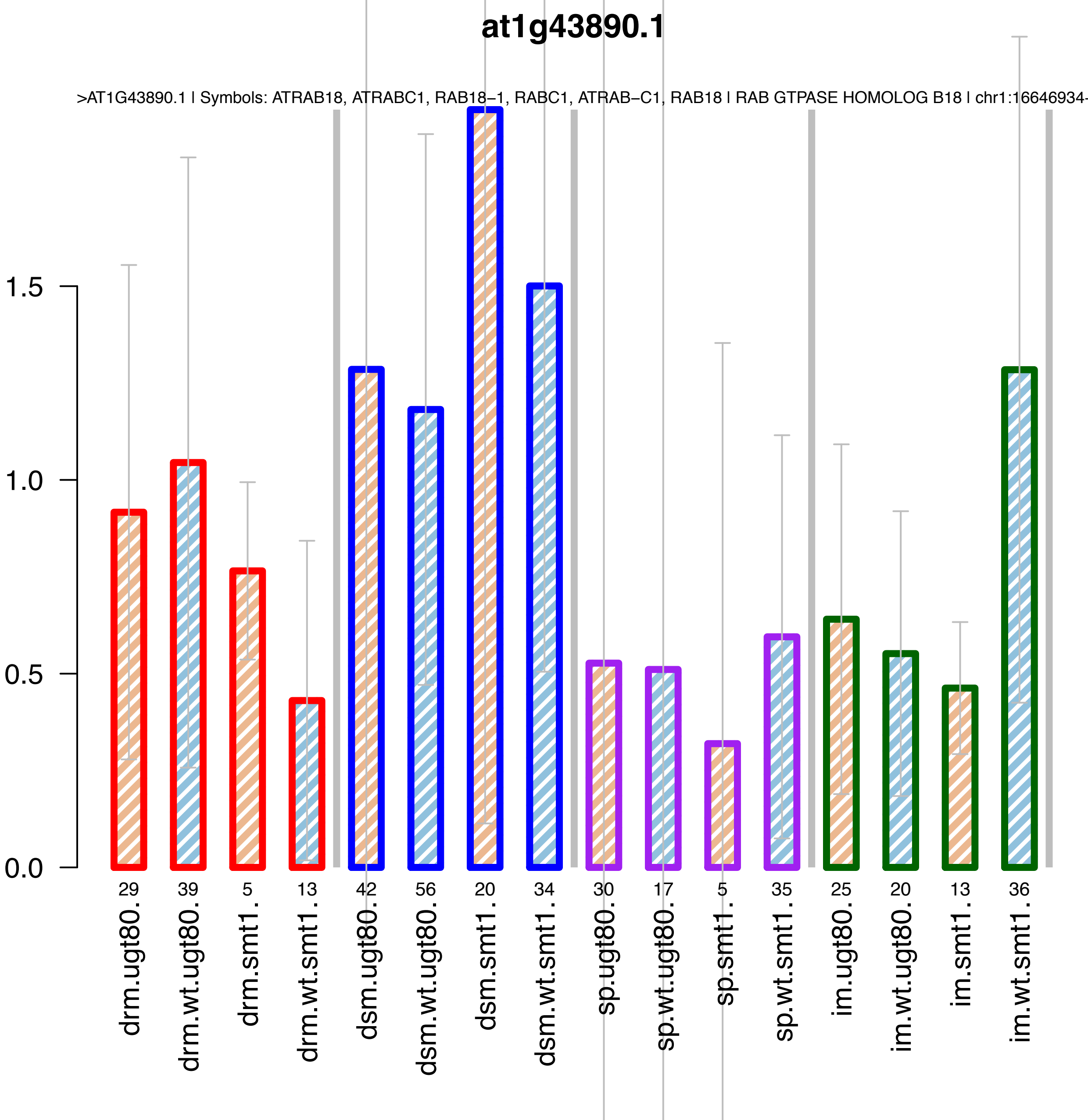
at1g43890.1
>AT1G43890.1 | Symbols: ATRAB18, ATRABC1, RAB18−1, RABC1, ATRAB−C1, RAB18 | RAB GTPASE HOMOLOG B18 | chr1:16646934-
0.0
0.5
1.0
1.5
29
39
5
13
42
56
20
34
30
17
5
35
25
20
13
36
drm.ugt80..
drm.wt.ugt80..
drm.smt1..
drm.wt.smt1..
dsm.ugt80..
dsm.wt.ugt80..
dsm.smt1..
dsm.wt.smt1..
sp.ugt80..
sp.wt.ugt80..
sp.smt1..
sp.wt.smt1..
im.ugt80..
im.wt.ugt80..
im.smt1..
im.wt.smt1..

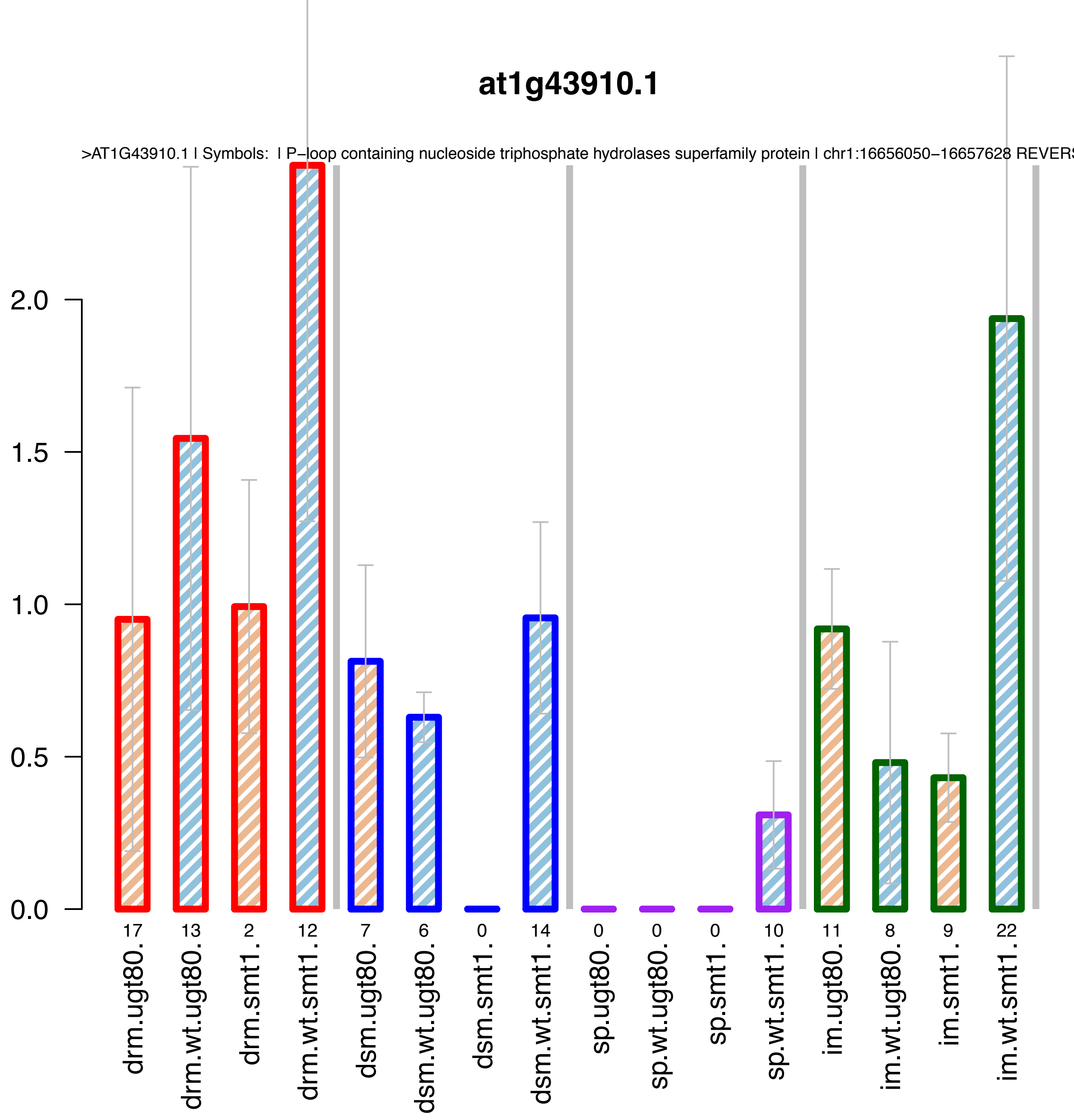
at1g43910.1
>AT1G43910.1 | Symbols: | P–loop containing nucleoside triphosphate hydrolases superfamily protein | chr1:16656050–16657628 REVERS
2.0
1.5
1.0
0.5
0.0
17
13
2
12
7
6
0
14
0
0
0
10
11
8
9
22
drm.ugt80..
drm.wt.ugt80..
drm.smt1..
drm.wt.smt1..
dsm.ugt80..
dsm.wt.ugt80..
dsm.smt1..
dsm.wt.smt1..
sp.ugt80..
sp.wt.ugt80..
sp.smt1..
sp.wt.smt1..
im.ugt80..
im.wt.ugt80..
im.smt1..
im.wt.smt1..

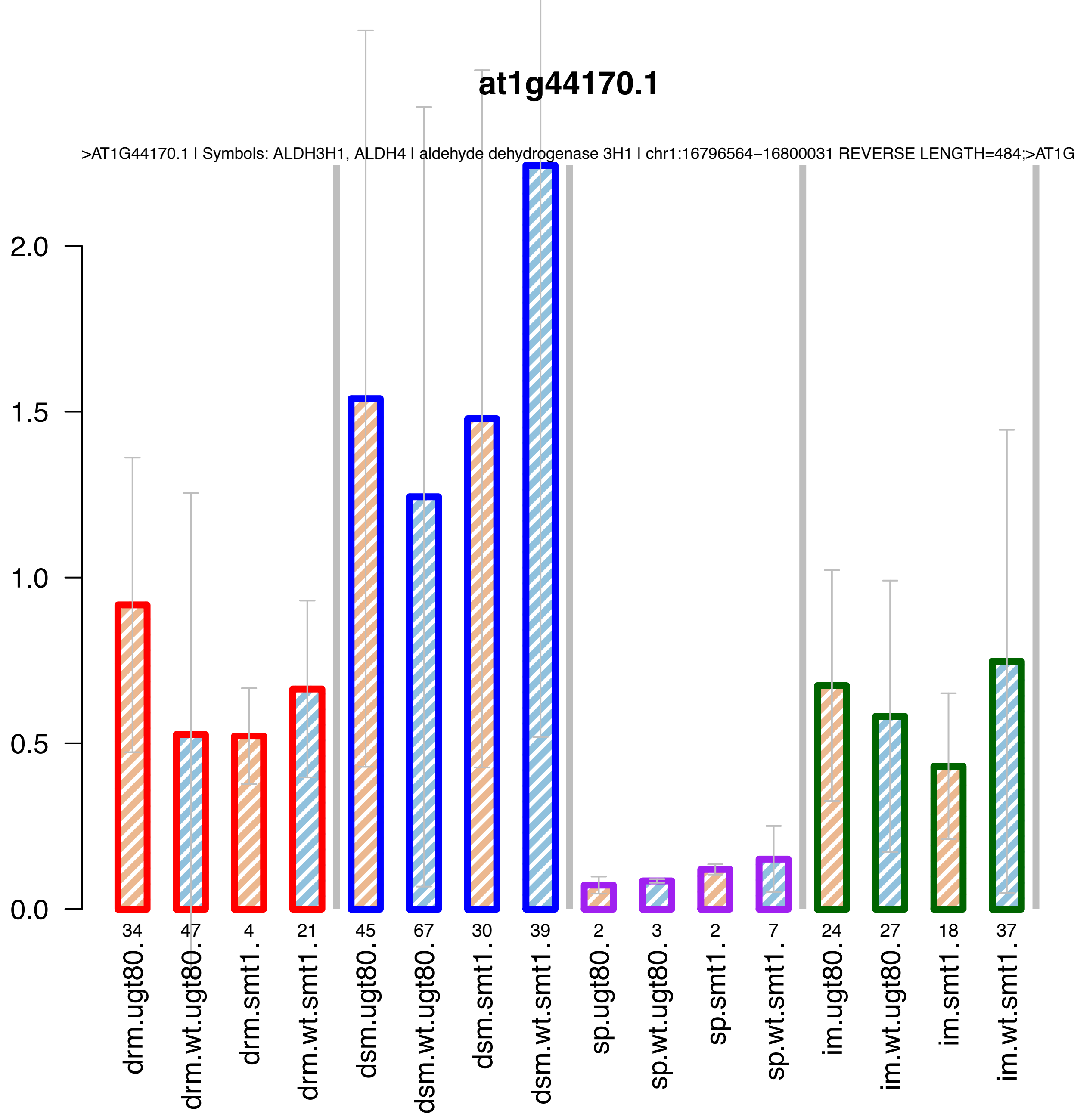

at1g44170.1
>AT1G44170.1 | Symbols: ALDH3H1, ALDH4 | aldehyde dehydrogenase 3H1 | chr1:16796564-16800031 REVERSE LENGTH=484;>AT1G
2.0
1.5
1.0
0.5
0.0
34
47
4
21
45
67
30
39
2
3
2
7
24
27
18
37
drm.ugt80..
drm.wt.ugt80..
drm.smt1..
drm.wt.smt1..
dsm.ugt80..
dsm.wt.ugt80..
dsm.smt1..
dsm.wt.smt1..
sp.ugt80..
sp.wt.ugt80..
sp.smt1..
sp.wt.smt1..
im.ugt80..
im.wt.ugt80..
im.smt1..
im.wt.smt1..

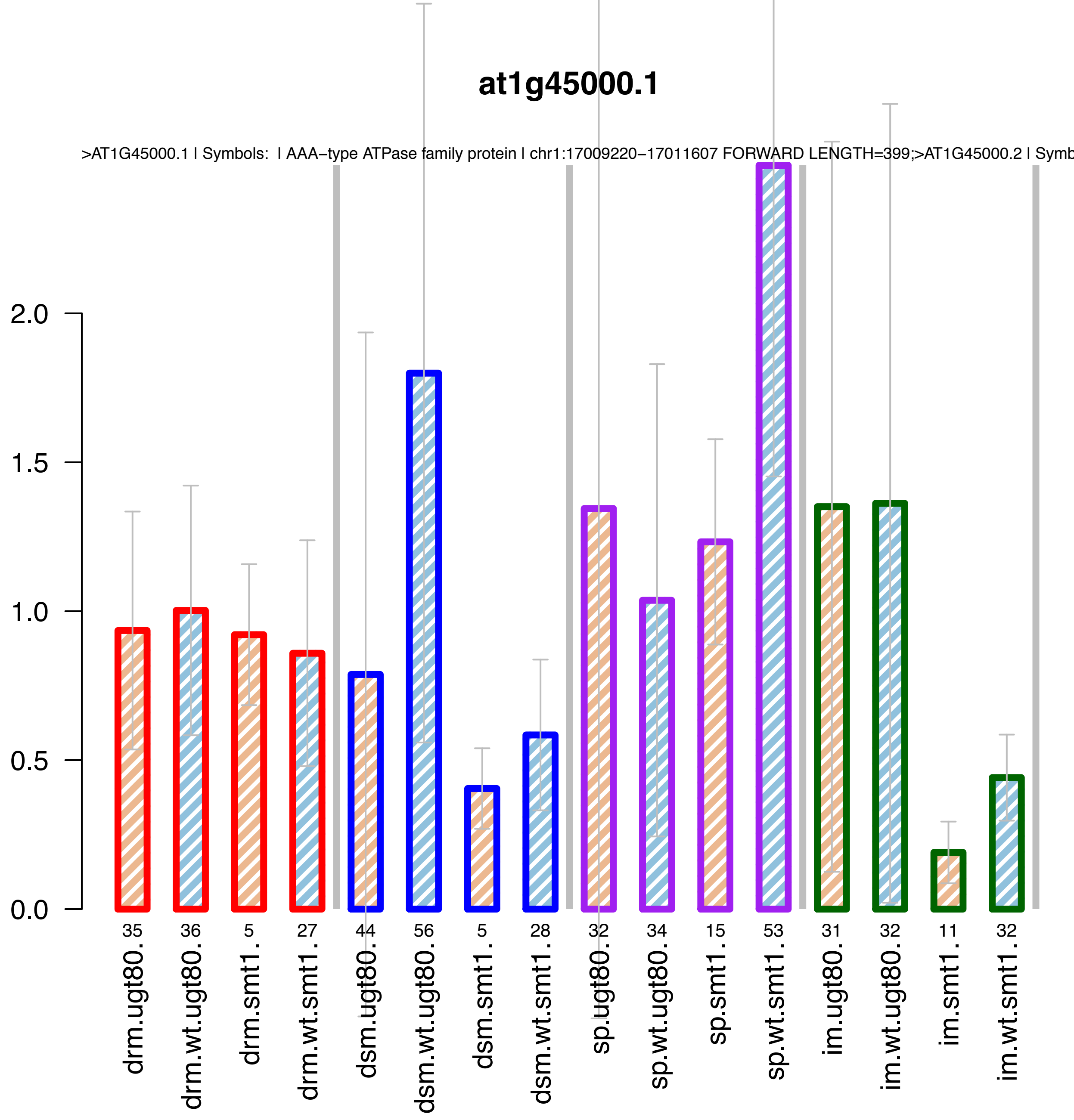
at1g45000.1
>AT1G45000.1 | Symbols: | AAA-type ATPase family protein | chr1:17009220-17011607 FORWARD LENGTH=399;>AT1G45000.2 | Symb
2.0
1.5
1.0
0.5
0.0
35
36
5
27
44
56
5
28
32
34
15
53
31
32
11
32
drm.ugt80.
drm.wt.ugt80.
drm.smt1.
drm.wt.smt1.
dsm.ugt80.
dsm.wt.ugt80.
dsm.smt1.
dsm.wt.smt1.
sp.ugt80.
sp.wt.ugt80.
sp.smt1.
sp.wt.smt1.
im.ugt80.
im.wt.ugt80.
im.smt1.
im.wt.smt1.

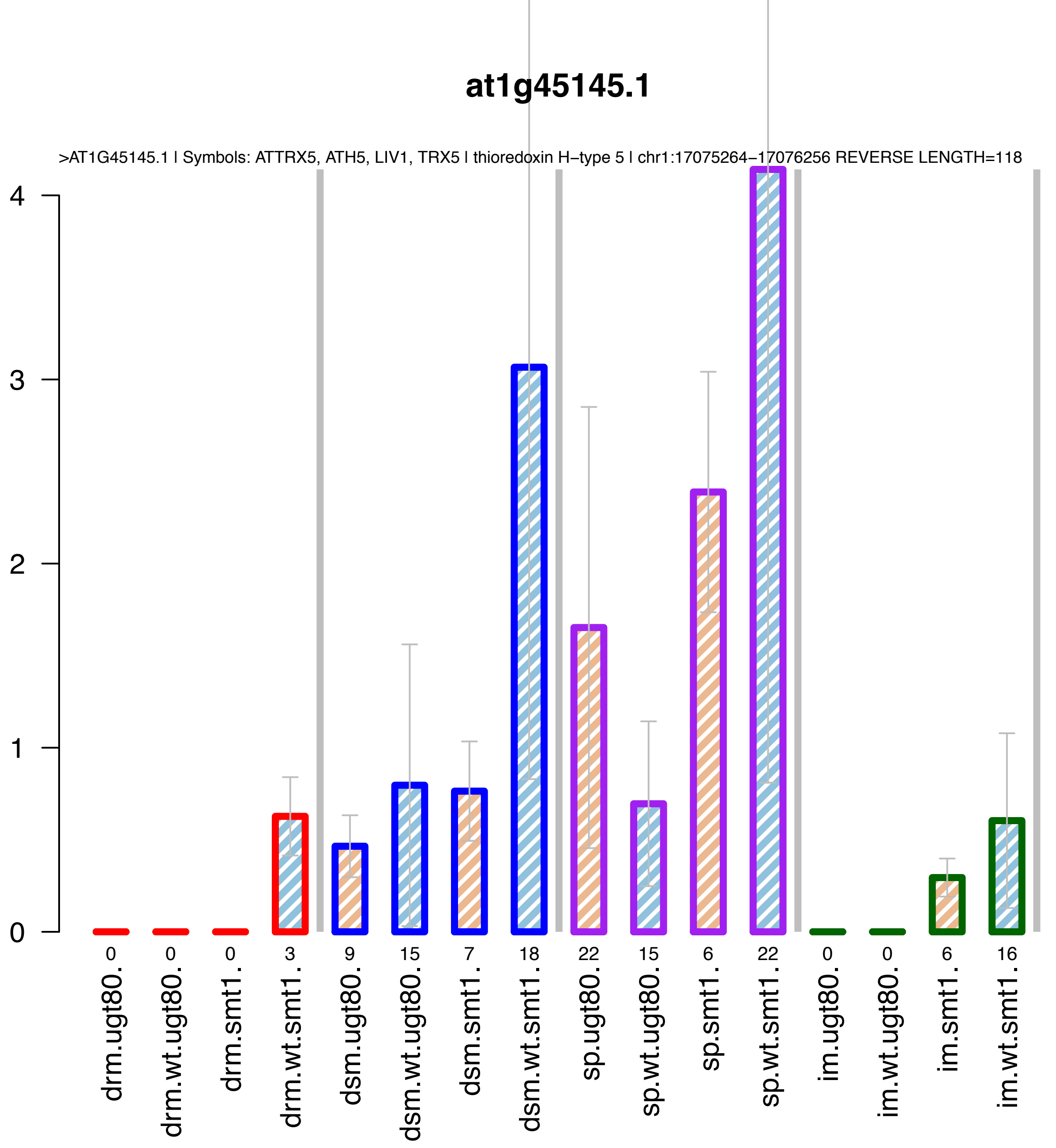
at1g45145.1
>AT1G45145.1 | Symbols: ATTRX5, ATH5, LIV1, TRX5 | thioredoxin H-type 5 | chr1:17075264-17076256 REVERSE LENGTH=118
0
1
2
3
4
0
0
0
3
9
15
7
18
22
15
6
22
0
0
6
16
drm.ugt80.
drm.wt.ugt80.
drm.smt1.
drm.wt.smt1.
dsm.ugt80.
dsm.wt.ugt80.
dsm.smt1.
dsm.wt.smt1.
sp.ugt80.
sp.wt.ugt80.
sp.smt1.
sp.wt.smt1.
im.ugt80.
im.wt.ugt80.
im.smt1.
im.wt.smt1.

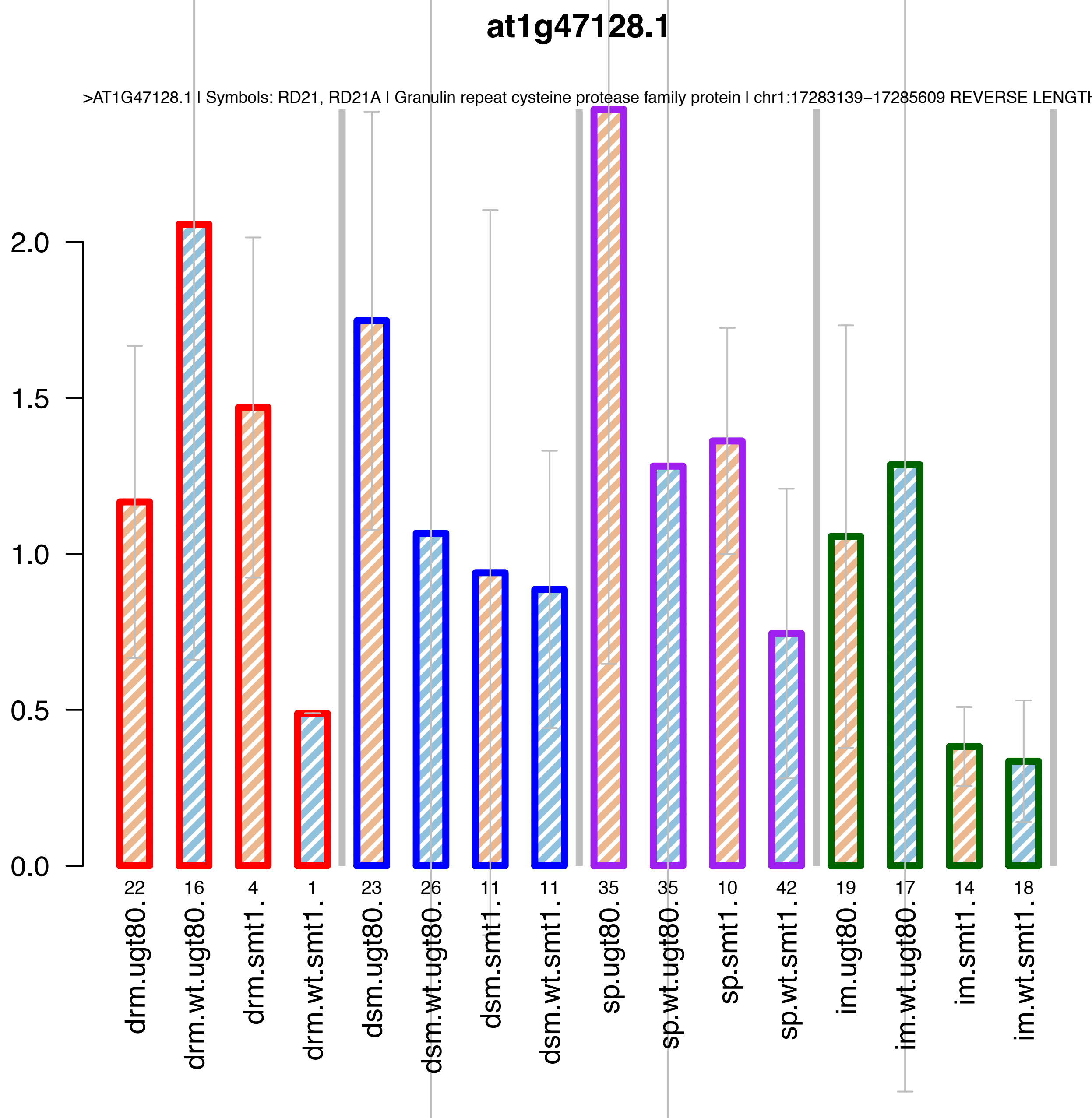
at1g47128.1
>AT1G47128.1 | Symbols: RD21, RD21A | Granulin repeat cysteine protease family protein | chr1:17283139−17285609 REVERSE LENGTH
2.0
1.5
1.0
0.5
0.0
22
16
4
1
23
26
11
11
35
35
10
42
19
17
14
18
drm.ugt80..
drm.wt.ugt80..
drm.smt1..
drm.wt.smt1..
dsm.ugt80..
dsm.wt.ugt80..
dsm.smt1..
dsm.wt.smt1..
sp.ugt80..
sp.wt.ugt80..
sp.smt1..
sp.wt.smt1..
im.ugt80..
im.wt.ugt80..
im.smt1..
im.wt.smt1..

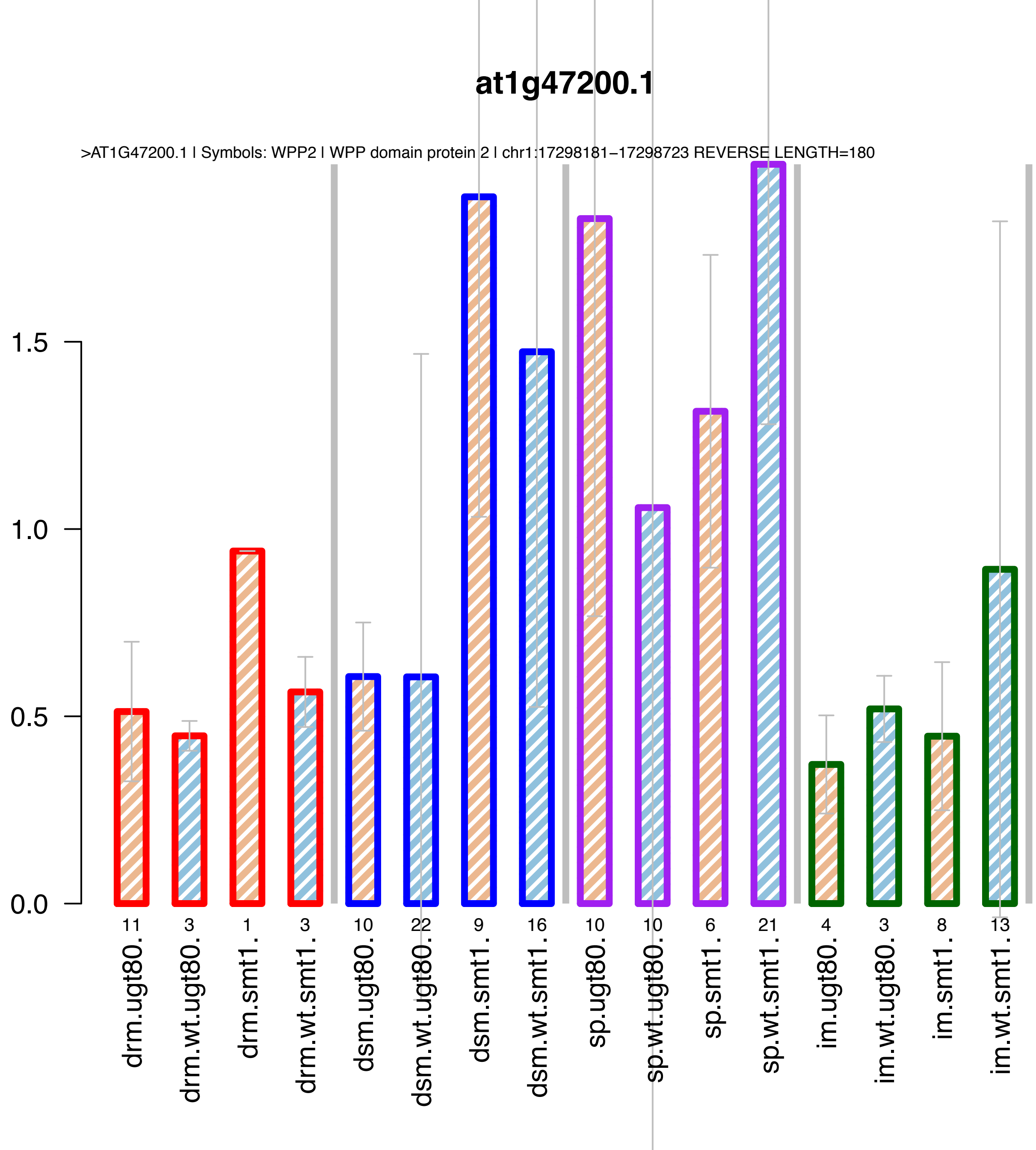

at1g47200.1
>AT1G47200.1 | Symbols: WPP2 | WPP domain protein 2 | chr1:17298181-17298723 REVERSE LENGTH=180
0.0
0.5
1.0
1.5
11
3
1
3
10
22
9
16
10
10
6
21
4
3
8
13
drm.ugt80..
drm.wt.ugt80..
drm.smt1..
drm.wt.smt1..
dsm.ugt80..
dsm.wt.ugt80..
dsm.smt1..
dsm.wt.smt1..
sp.ugt80..
sp.wt.ugt80..
sp.smt1..
sp.wt.smt1..
im.ugt80..
im.wt.ugt80..
im.smt1..
im.wt.smt1..

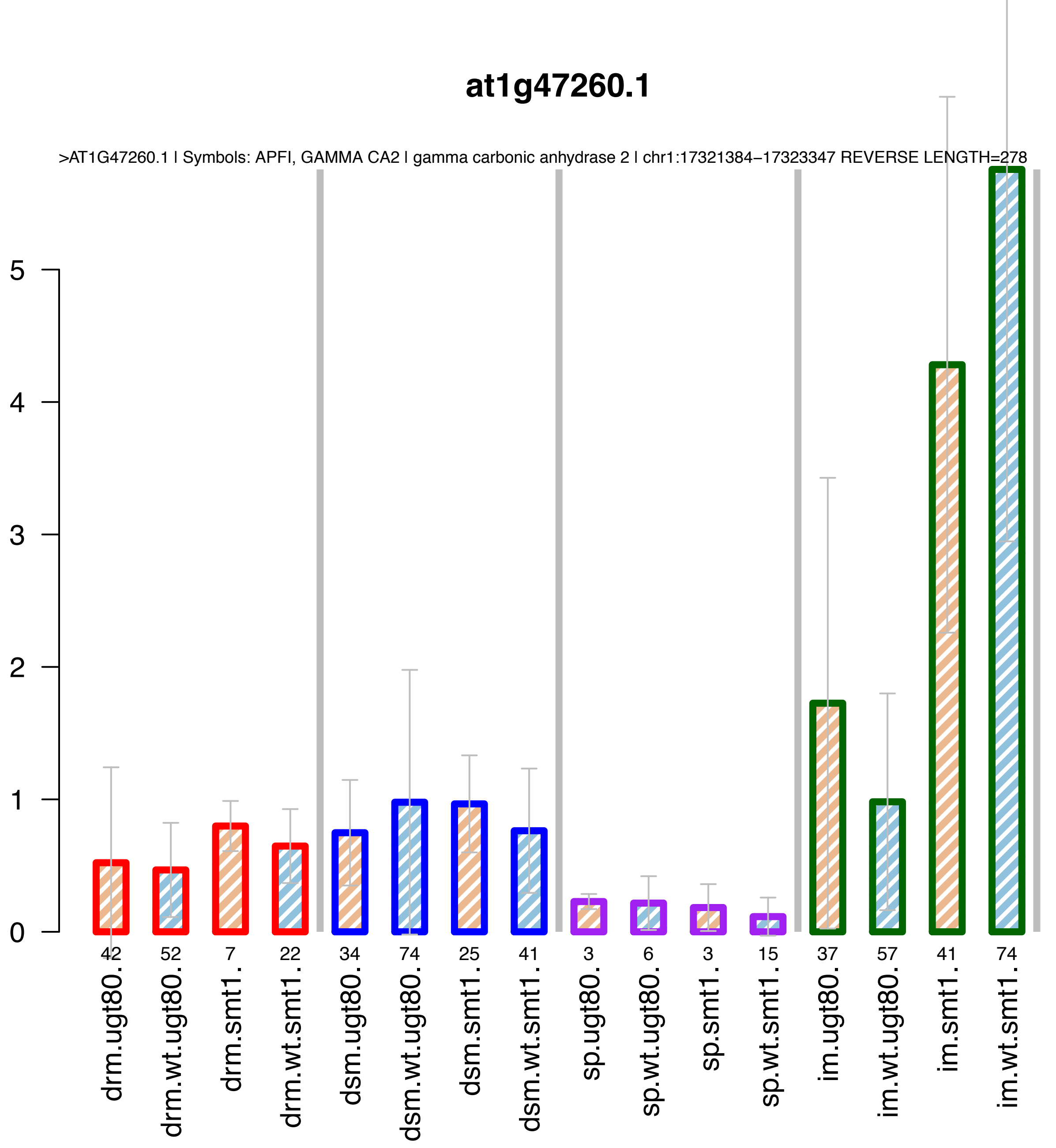
at1g47260.1
>AT1G47260.1 | Symbols: APFI, GAMMA CA2 | gamma carbonic anhydrase 2 | chr1:17321384−17323347 REVERSE LENGTH=278
0
1
2
3
4
5
42
52
7
22
34
74
25
41
3
6
3
15
37
57
41
74
drm.ugt80..
drm.wt.ugt80..
drm.smt1..
drm.wt.smt1..
dsm.ugt80..
dsm.wt.ugt80..
dsm.smt1..
dsm.wt.smt1..
sp.ugt80..
sp.wt.ugt80..
sp.smt1..
sp.wt.smt1..
im.ugt80..
im.wt.ugt80..
im.smt1..
im.wt.smt1..

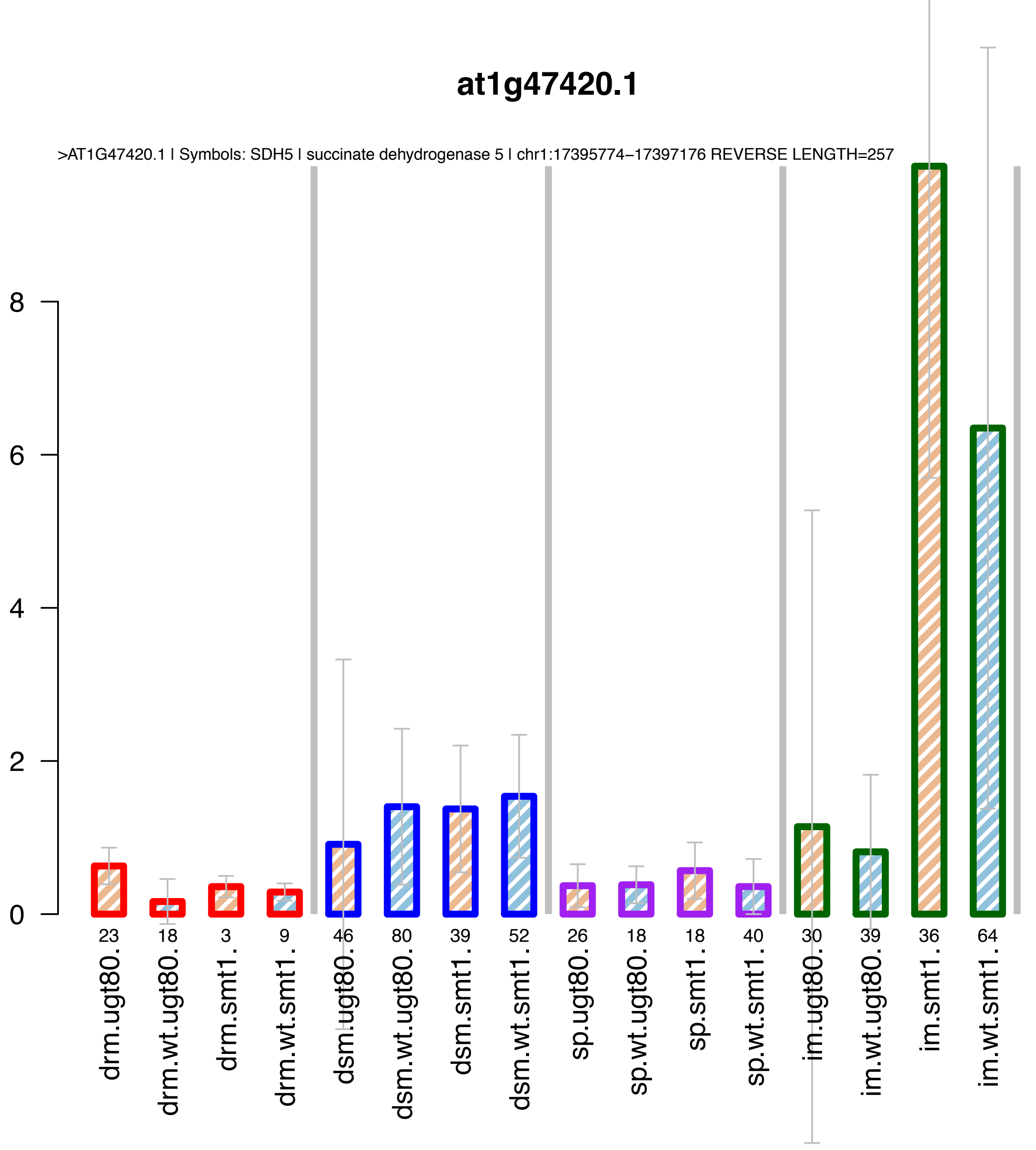

at1g47420.1
>AT1G47420.1 | Symbols: SDH5 | succinate dehydrogenase 5 | chr1:17395774–17397176 REVERSE LENGTH=257
0
2
4
6
8
23
18
3
9
46
80
39
52
26
18
18
40
30
39
36
64
drm.ugt80.
drm.wt.ugt80.
drm.smt1.
drm.wt.smt1.
dsm.ugt80.
dsm.wt.ugt80.
dsm.smt1.
dsm.wt.smt1.
sp.ugt80.
sp.wt.ugt80.
sp.smt1.
sp.wt.smt1.
im.ugt80.
im.wt.ugt80.
im.smt1.
im.wt.smt1.

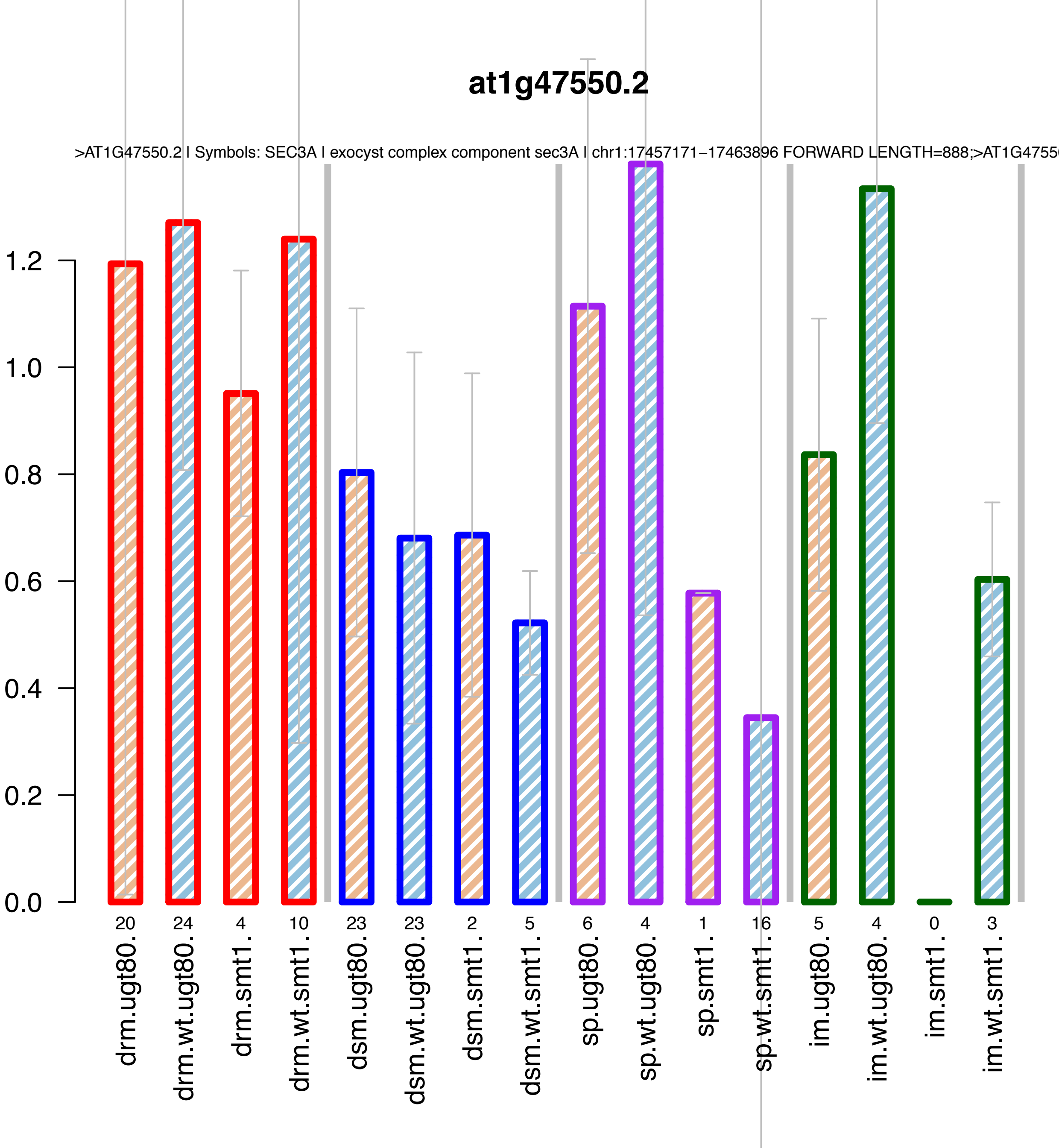
at1g47550.2
>AT1G47550.2 | Symbols: SEC3A | exocyst complex component sec3A | chr1:17457171-17463896 FORWARD LENGTH=888;>AT1G4755
0.0
0.2
0.4
0.6
0.8
1.0
1.2
20
24
4
10
23
23
2
5
6
4
1
16
5
4
0
3
drm.ugt80..
drm.wt.ugt80..
drm.smt1..
drm.wt.smt1..
dsm.ugt80..
dsm.wt.ugt80..
dsm.smt1..
dsm.wt.smt1..
sp.ugt80..
sp.wt.ugt80..
sp.smt1..
sp.wt.smt1..
im.ugt80..
im.wt.ugt80..
im.smt1..
im.wt.smt1..

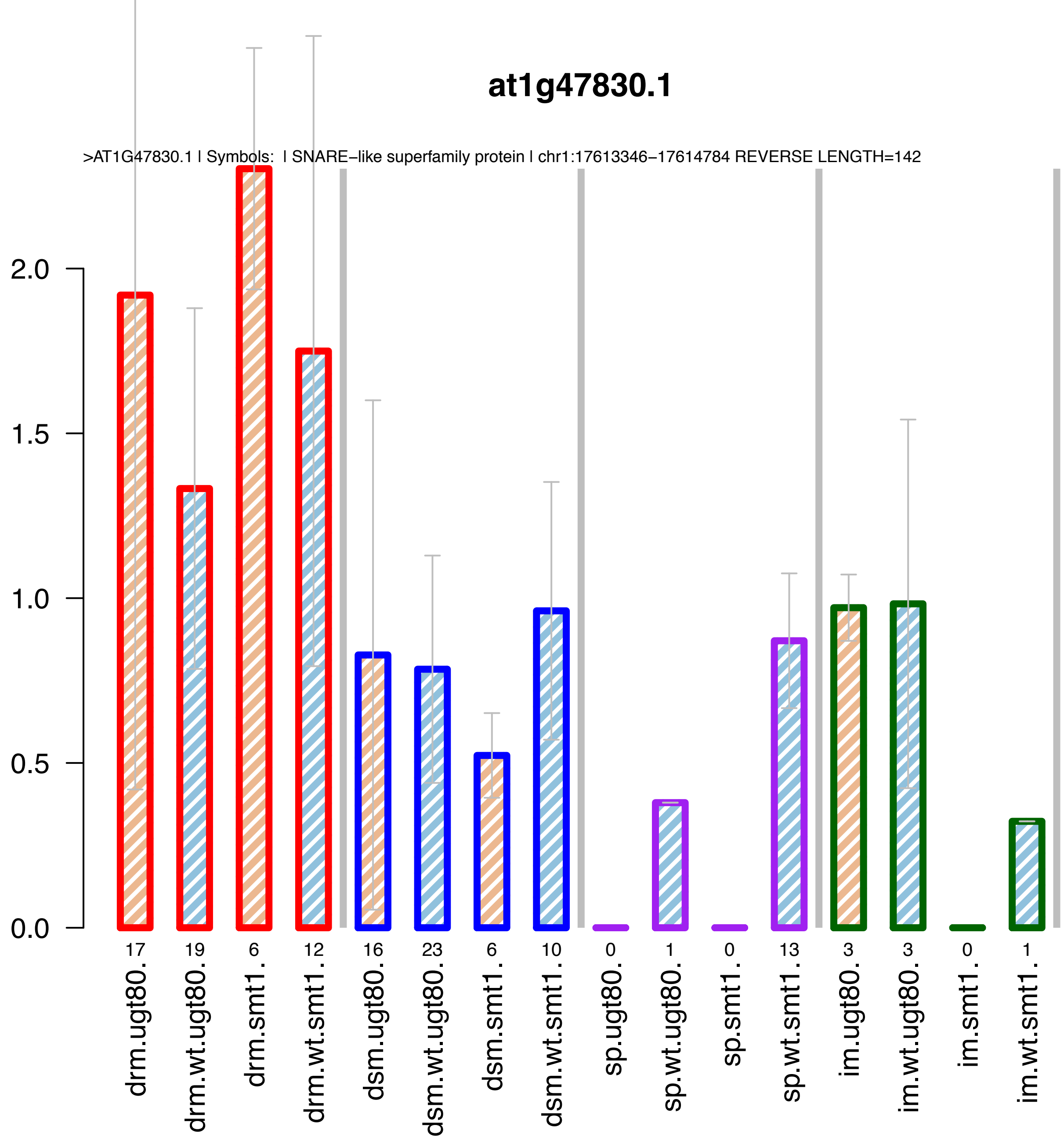

at1g47830.1
>AT1G47830.1 | Symbols: | SNARE−like superfamily protein | chr1:17613346−17614784 REVERSE LENGTH=142
0.0
0.5
1.0
1.5
2.0
17
19
6
12
16
23
6
10
0
1
0
13
3
3
0
1
drm.ugt80.
drm.wt.ugt80.
drm.smt1.
drm.wt.smt1.
dsm.ugt80.
dsm.wt.ugt80.
dsm.smt1.
dsm.wt.smt1.
sp.ugt80.
sp.wt.ugt80.
sp.smt1.
sp.wt.smt1.
im.ugt80.
im.wt.ugt80.
im.smt1.
im.wt.smt1.

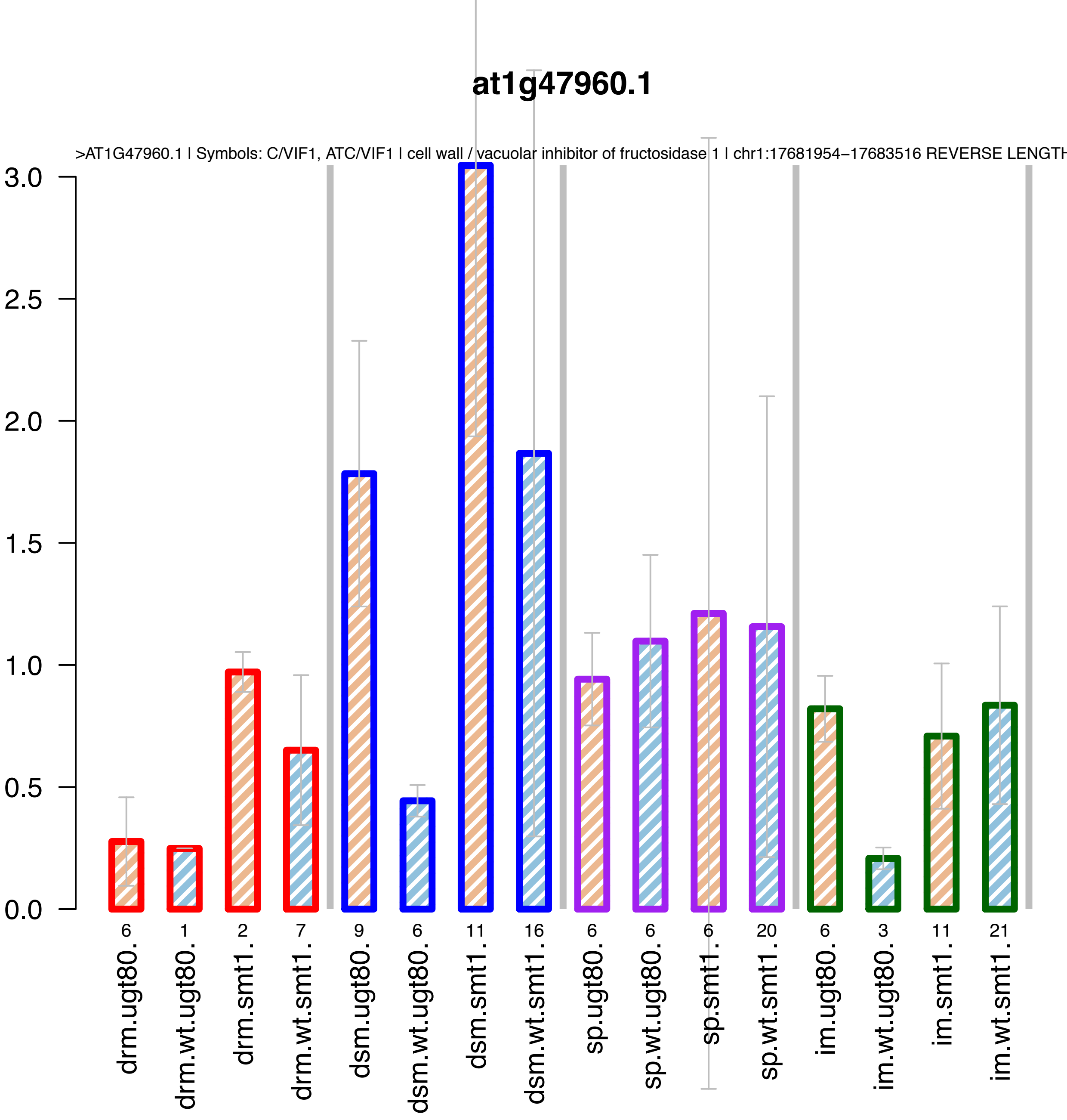
at1g47960.1
>AT1G47960.1 | Symbols: C/VIF1, ATC/VIF1 | cell wall / vacuolar inhibitor of fructosidase 1 | chr1:17681954-17683516 REVERSE LENGTH
3.0
2.5
2.0
1.5
1.0
0.5
0.0
6
1
2
7
9
6
11
16
6
6
6
20
6
3
11
21
drm.ugt80.
drm.wt.ugt80.
drm.smt1.
drm.wt.smt1.
dsm.ugt80.
dsm.wt.ugt80.
dsm.smt1.
dsm.wt.smt1.
sp.ugt80.
sp.wt.ugt80.
sp.smt1.
sp.wt.smt1.
im.ugt80.
im.wt.ugt80.
im.smt1.
im.wt.smt1.

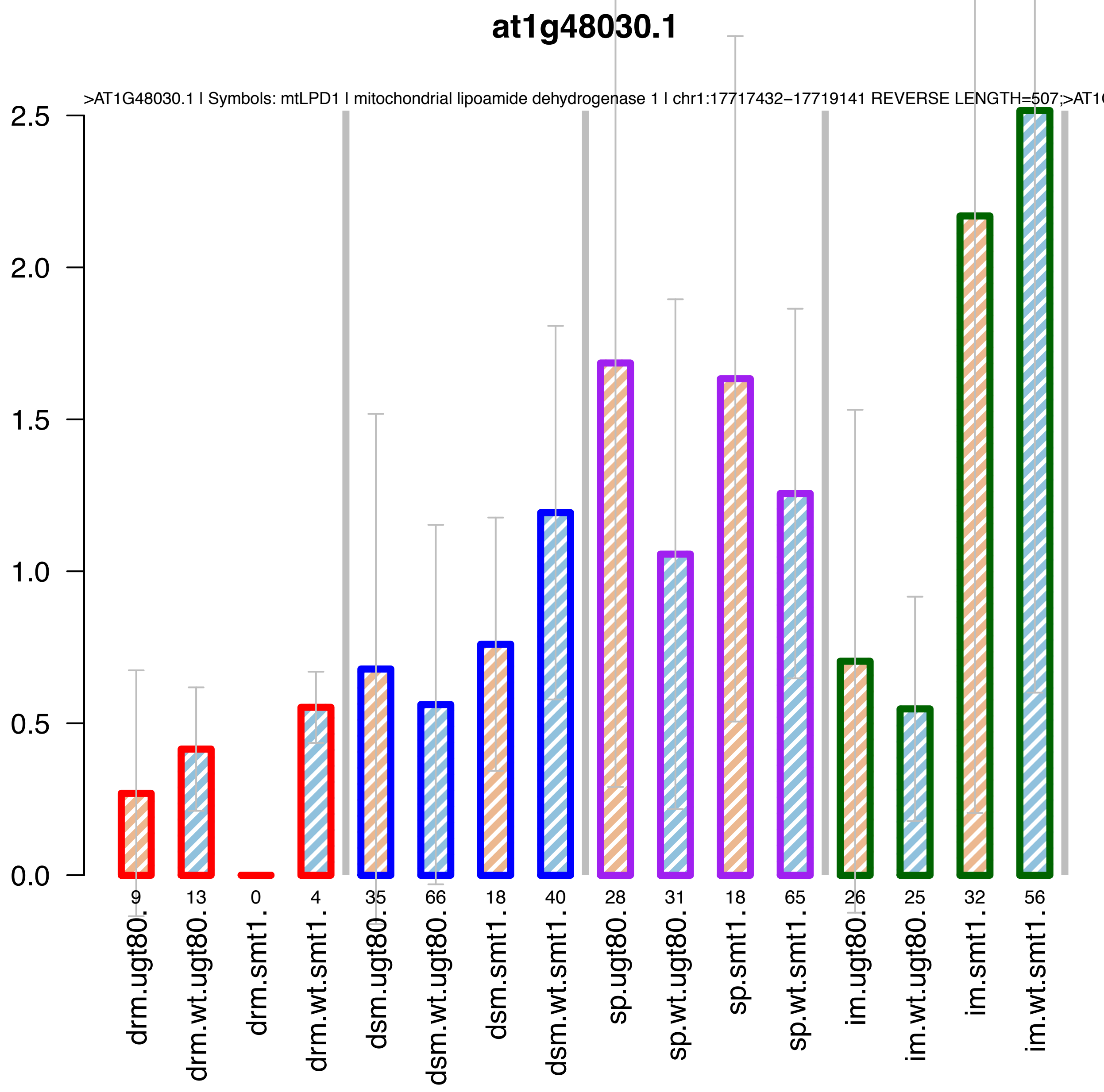

at1g48030.1
>AT1G48030.1 | Symbols: mtLPD1 | mitochondrial lipoamide dehydrogenase 1 | chr1:17717432–17719141 REVERSE LENGTH=507;>AT1
2.5
2.0
1.5
1.0
0.5
0.0
9
13
0
4
35
66
18
40
28
31
18
65
26
25
32
56
drm.ugt80.
drm.wt.ugt80.
drm.smt1.
drm.wt.smt1.
dsm.ugt80.
dsm.wt.ugt80.
dsm.smt1.
dsm.wt.smt1.
sp.ugt80.
sp.wt.ugt80.
sp.smt1.
sp.wt.smt1.
im.ugt80.
im.wt.ugt80.
im.smt1.
im.wt.smt1.

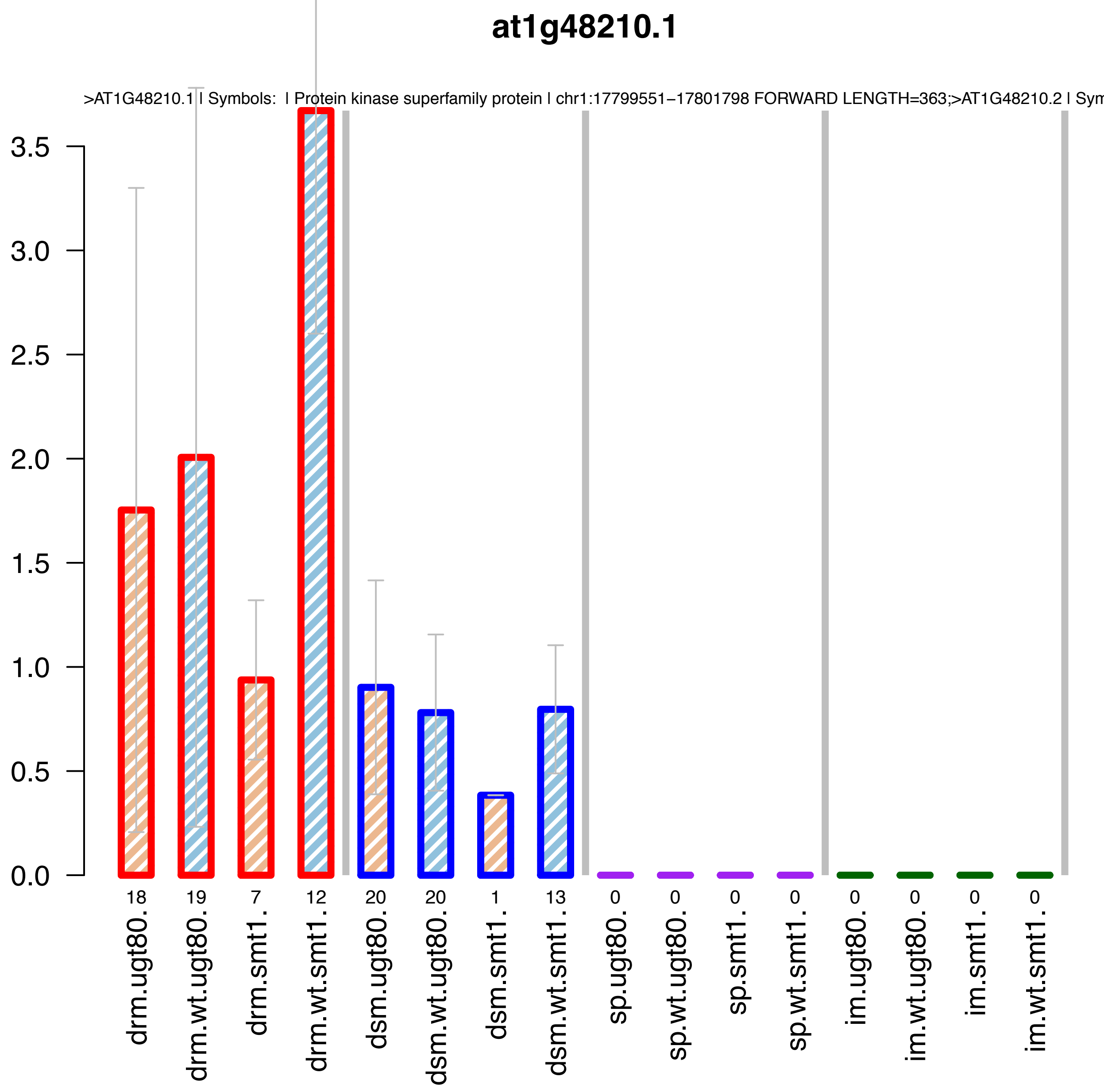
at1g48210.1
>AT1G48210.1 | Symbols: | Protein kinase superfamily protein | chr1:17799551-17801798 FORWARD LENGTH=363;>AT1G48210.2 | Sym
0.0
0.5
1.0
1.5
2.0
2.5
3.0
3.5
18
19
7
12
20
20
1
13
0
0
0
0
0
0
0
0
drm.ugt80.
drm.wt.ugt80.
drm.smt1.
drm.wt.smt1.
dsm.ugt80.
dsm.wt.ugt80.
dsm.smt1.
dsm.wt.smt1.
sp.ugt80.
sp.wt.ugt80.
sp.smt1.
sp.wt.smt1.
im.ugt80.
im.wt.ugt80.
im.smt1.
im.wt.smt1.

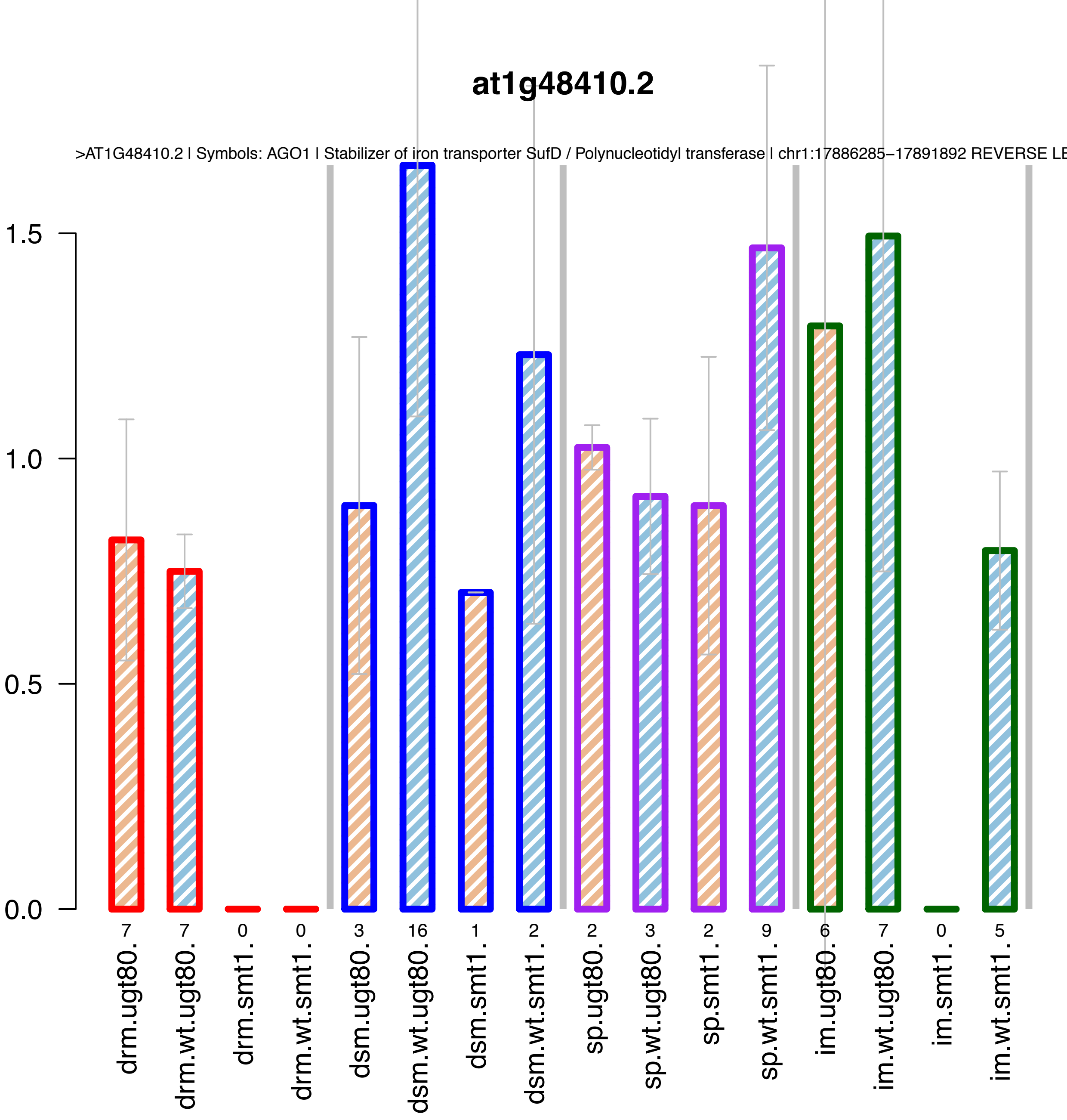
at1g48410.2
>AT1G48410.2 | Symbols: AGO1 | Stabilizer of iron transporter SufD / Polynucleotidyl transferase | chr1:17886285-17891892 REVERSE LE
1.5
1.0
0.5
0.0
7
7
0
0
3
16
1
2
2
3
2
9
6
7
0
5
drm.ugt80.
drm.wt.ugt80.
drm.smt1.
drm.wt.smt1.
dsm.ugt80.
dsm.wt.ugt80.
dsm.smt1.
dsm.wt.smt1.
sp.ugt80.
sp.wt.ugt80.
sp.smt1.
sp.wt.smt1.
im.ugt80.
im.wt.ugt80.
im.smt1.
im.wt.smt1.

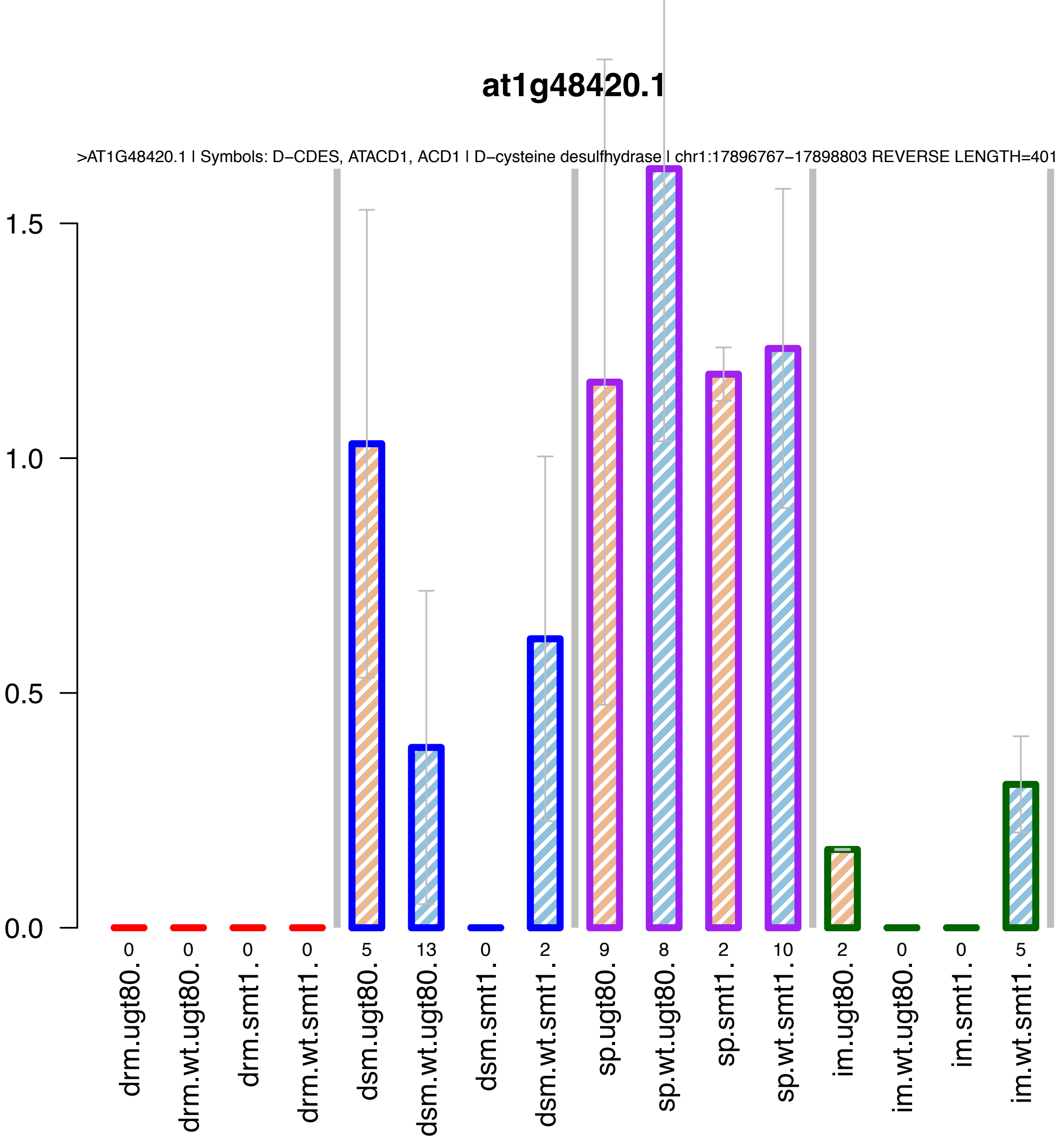

at1g48420.1
>AT1G48420.1 | Symbols: D−CDES, ATACD1, ACD1 | D−cysteine desulfhydrase | chr1:17896767−17898803 REVERSE LENGTH=401
0.0
0.5
1.0
1.5
0 0 0 0 5 13 0 2 9 8 2 10 2 0 0 5
drm.ugt80.
drm.wt.ugt80.
drm.smt1.
drm.wt.smt1.
dsm.ugt80.
dsm.wt.ugt80.
dsm.smt1.
dsm.wt.smt1.
sp.ugt80.
sp.wt.ugt80.
sp.smt1.
sp.wt.smt1.
im.ugt80.
im.wt.ugt80.
im.smt1.
im.wt.smt1.

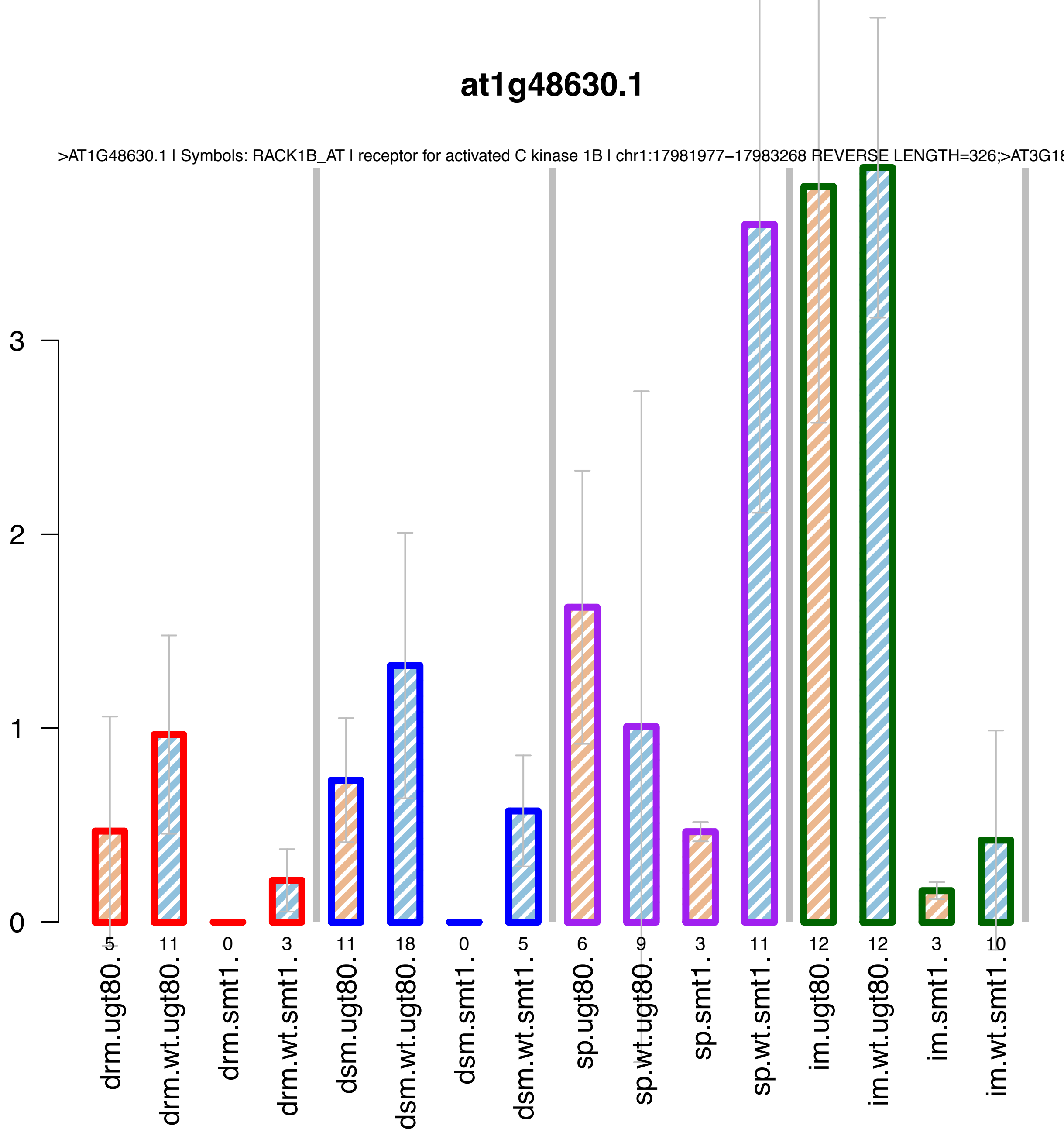

at1g48630.1
>AT1G48630.1 | Symbols: RACK1B_AT | receptor for activated C kinase 1B | chr1:17981977-17983268 REVERSE LENGTH=326;>AT3G1
3
2
1
0
5
11
0
3
11
18
0
5
6
9
3
11
12
12
3
10
drm.ugt80.
drm.wt.ugt80.
drm.smt1.
drm.wt.smt1.
dsm.ugt80.
dsm.wt.ugt80.
dsm.smt1.
dsm.wt.smt1.
sp.ugt80.
sp.wt.ugt80.
sp.smt1.
sp.wt.smt1.
im.ugt80.
im.wt.ugt80.
im.smt1.
im.wt.smt1.

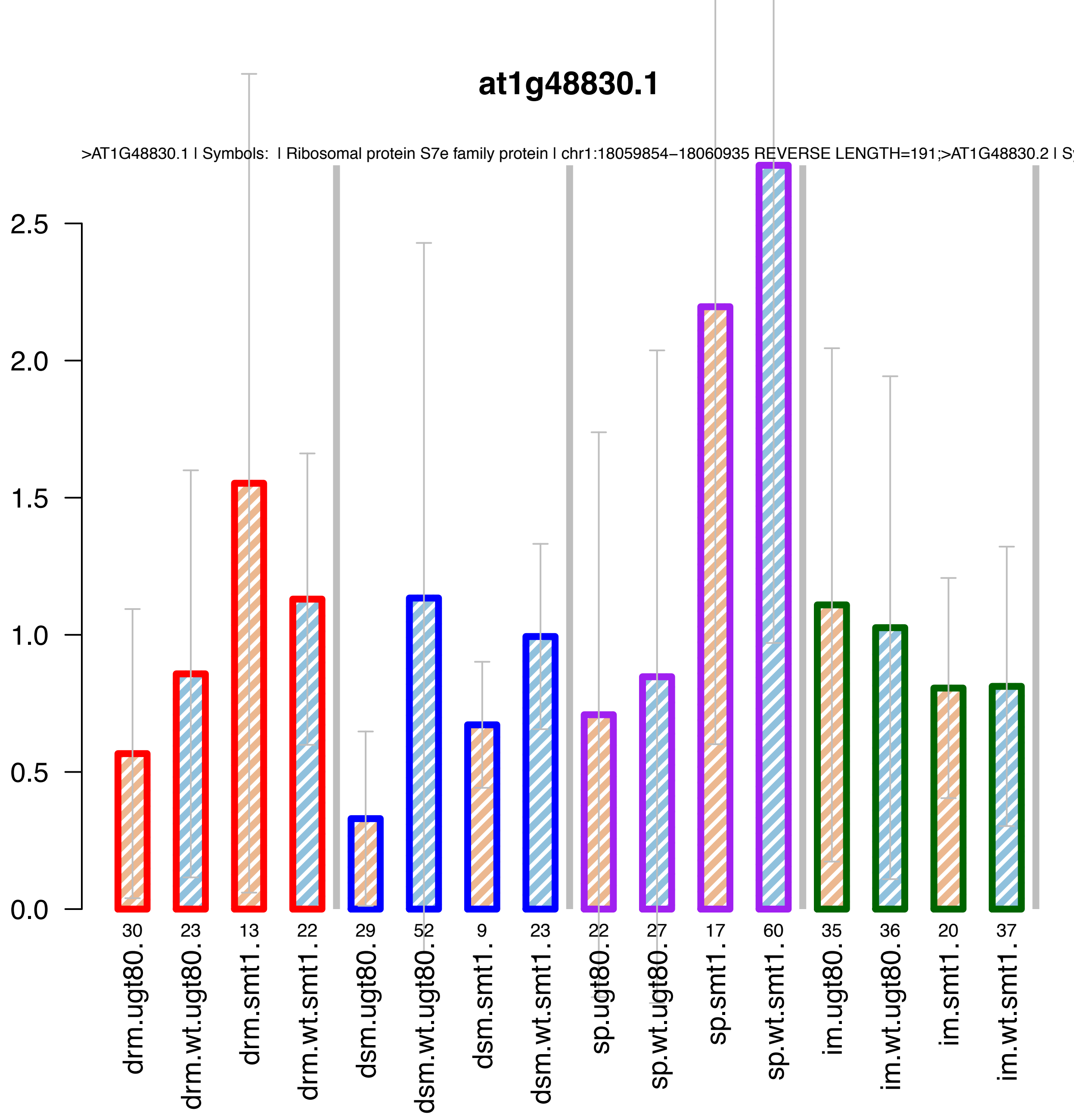
at1g48830.1
>AT1G48830.1 | Symbols: | Ribosomal protein S7e family protein | chr1:18059854-18060935 REVERSE LENGTH=191;>AT1G48830.2 | S
2.5
2.0
1.5
1.0
0.5
0.0
30
23
13
22
29
52
9
23
22
27
17
60
35
36
20
37
drm.ugt80.
drm.wt.ugt80.
drm.smt1.
drm.wt.smt1.
dsm.ugt80.
dsm.wt.ugt80.
dsm.smt1.
dsm.wt.smt1.
sp.ugt80.
sp.wt.ugt80.
sp.smt1.
sp.wt.smt1.
im.ugt80.
im.wt.ugt80.
im.smt1.
im.wt.smt1.

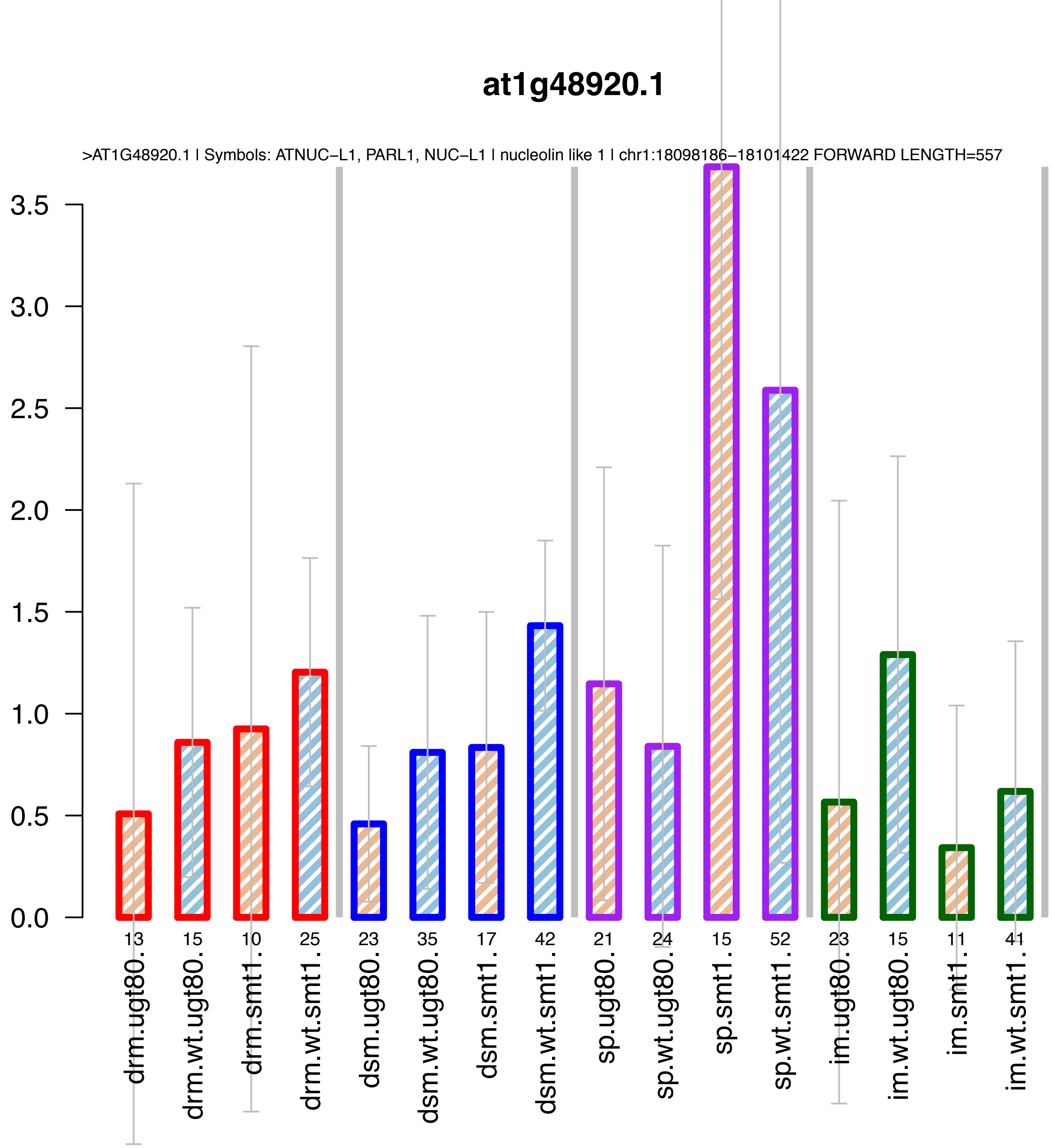

at1g48920.1
>AT1G48920.1 | Symbols: ATNUC−L1, PARL1, NUC−L1 | nucleolin like 1 | chr1:18098186−18101422 FORWARD LENGTH=557
3.5
3.0
2.5
2.0
1.5
1.0
0.5
0.0
13 15 10 25 23 35 17 42 21 24 15 52 23 15 11 41
drm.ugt80..
drm.wt.ugt80..
drm.smt1..
drm.wt.smt1..
dsm.ugt80..
dsm.wt.ugt80..
dsm.smt1..
dsm.wt.smt1..
sp.ugt80..
sp.wt.ugt80..
sp.smt1..
sp.wt.smt1..
im.ugt80..
im.wt.ugt80..
im.smt1..
im.wt.smt1..

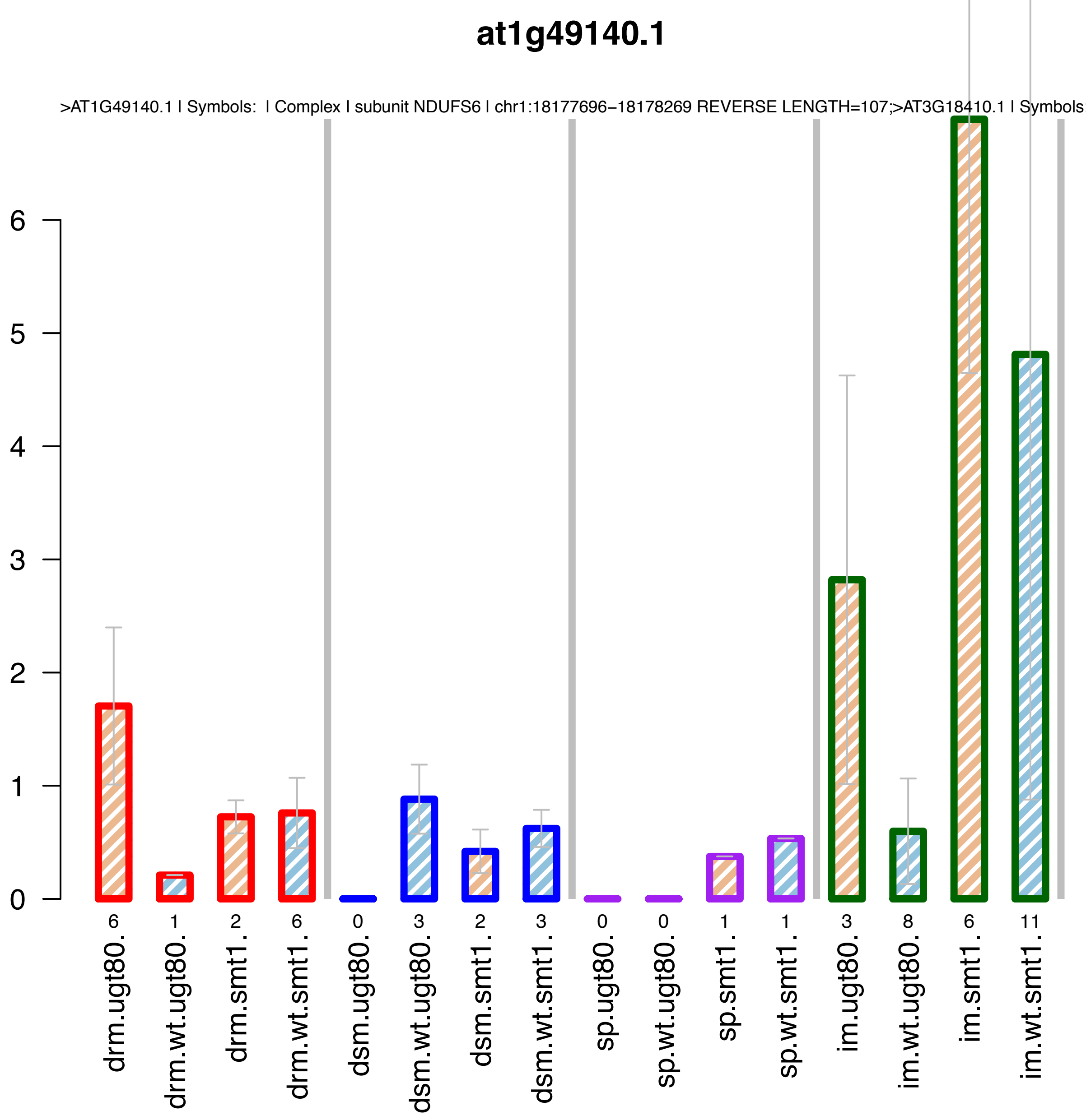

at1g49140.1
>AT1G49140.1 | Symbols: | Complex I subunit NDUFS6 | chr1:18177696-18178269 REVERSE LENGTH=107;>AT3G18410.1 | Symbols: |
0
1
2
3
4
5
6
6
1
2
6
0
3
2
3
0
0
1
1
3
8
6
11
drm.ugt80.
drm.wt.ugt80.
drm.smt1.
drm.wt.smt1.
dsm.ugt80.
dsm.wt.ugt80.
dsm.smt1.
dsm.wt.smt1.
sp.ugt80.
sp.wt.ugt80.
sp.smt1.
sp.wt.smt1.
im.ugt80.
im.wt.ugt80.
im.smt1.
im.wt.smt1.

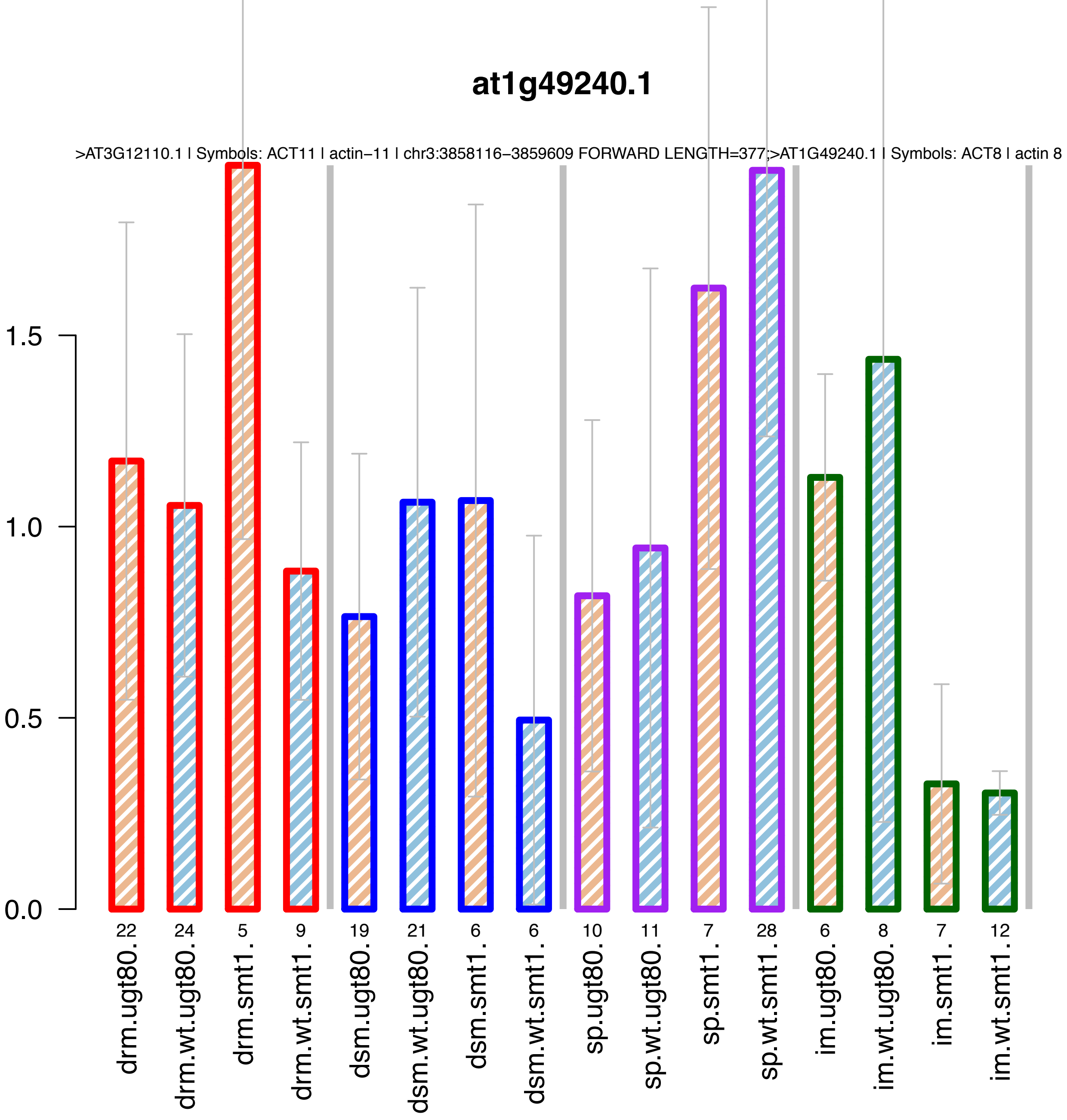

at1g49240.1
>AT3G12110.1 | Symbols: ACT11 | actin−11 | chr3:3858116−3859609 FORWARD LENGTH=377;>AT1G49240.1 | Symbols: ACT8 | actin 8
0.0
0.5
1.0
1.5
22
24
5
9
19
21
6
6
10
11
7
28
6
8
7
12
drm.ugt80..
drm.wt.ugt80..
drm.smt1..
drm.wt.smt1..
dsm.ugt80..
dsm.wt.ugt80..
dsm.smt1..
dsm.wt.smt1..
sp.ugt80..
sp.wt.ugt80..
sp.smt1..
sp.wt.smt1..
im.ugt80..
im.wt.ugt80..
im.smt1..
im.wt.smt1..

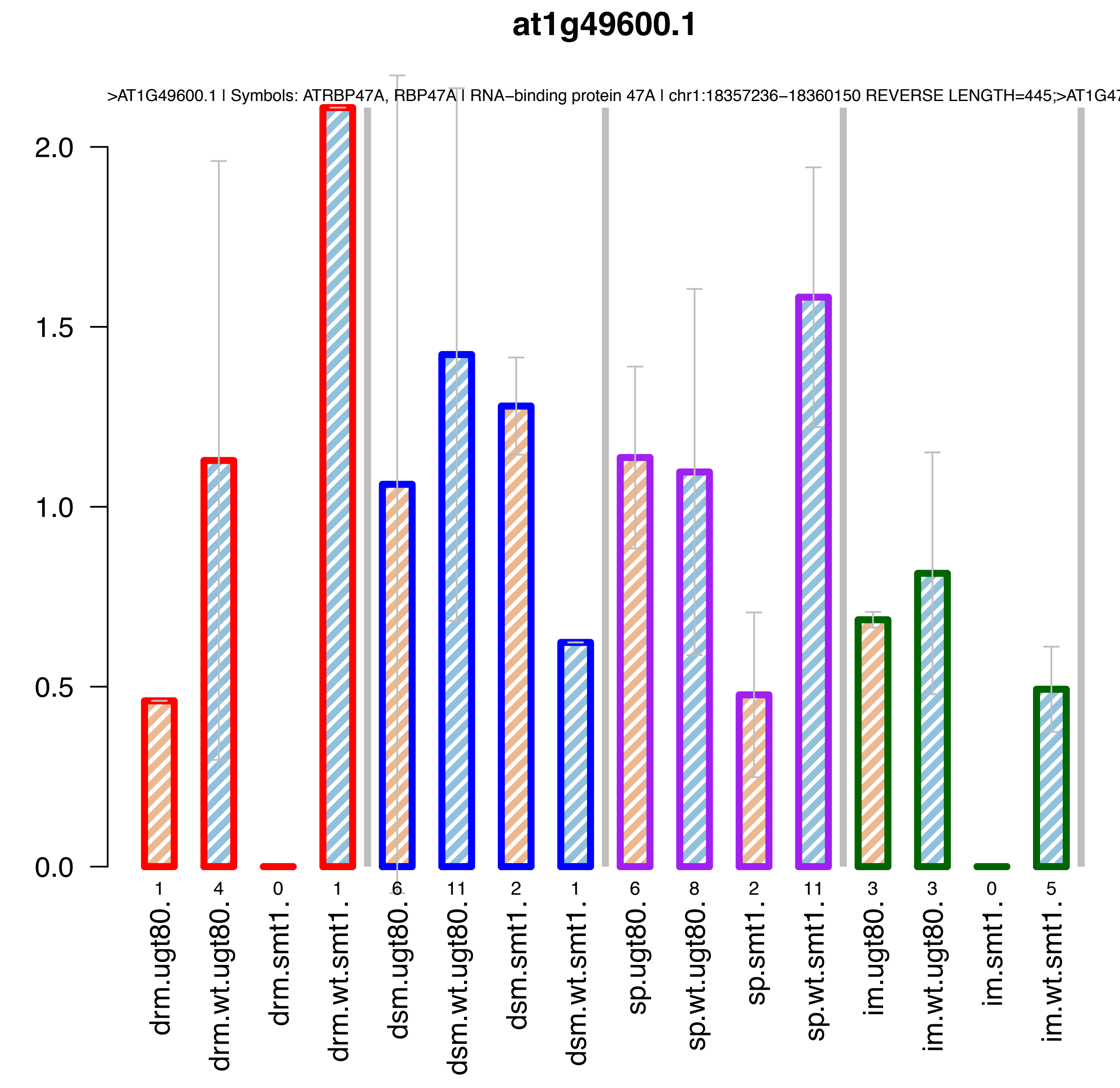
at1g49600.1
>AT1G49600.1 | Symbols: ATRBP47A, RBP47A | RNA–binding protein 47A | chr1:18357236–18360150 REVERSE LENGTH=445;>AT1G47
2.0
1.5
1.0
0.5
0.0
1 4 0 1 6 11 2 1 6 8 2 11 3 3 0 5
drm.ugt80.
drm.wt.ugt80.
drm.smt1.
drm.wt.smt1.
dsm.ugt80.
dsm.wt.ugt80.
dsm.smt1.
dsm.wt.smt1.
sp.ugt80.
sp.wt.ugt80.
sp.smt1.
sp.wt.smt1.
im.ugt80.
im.wt.ugt80.
im.smt1.
im.wt.smt1.

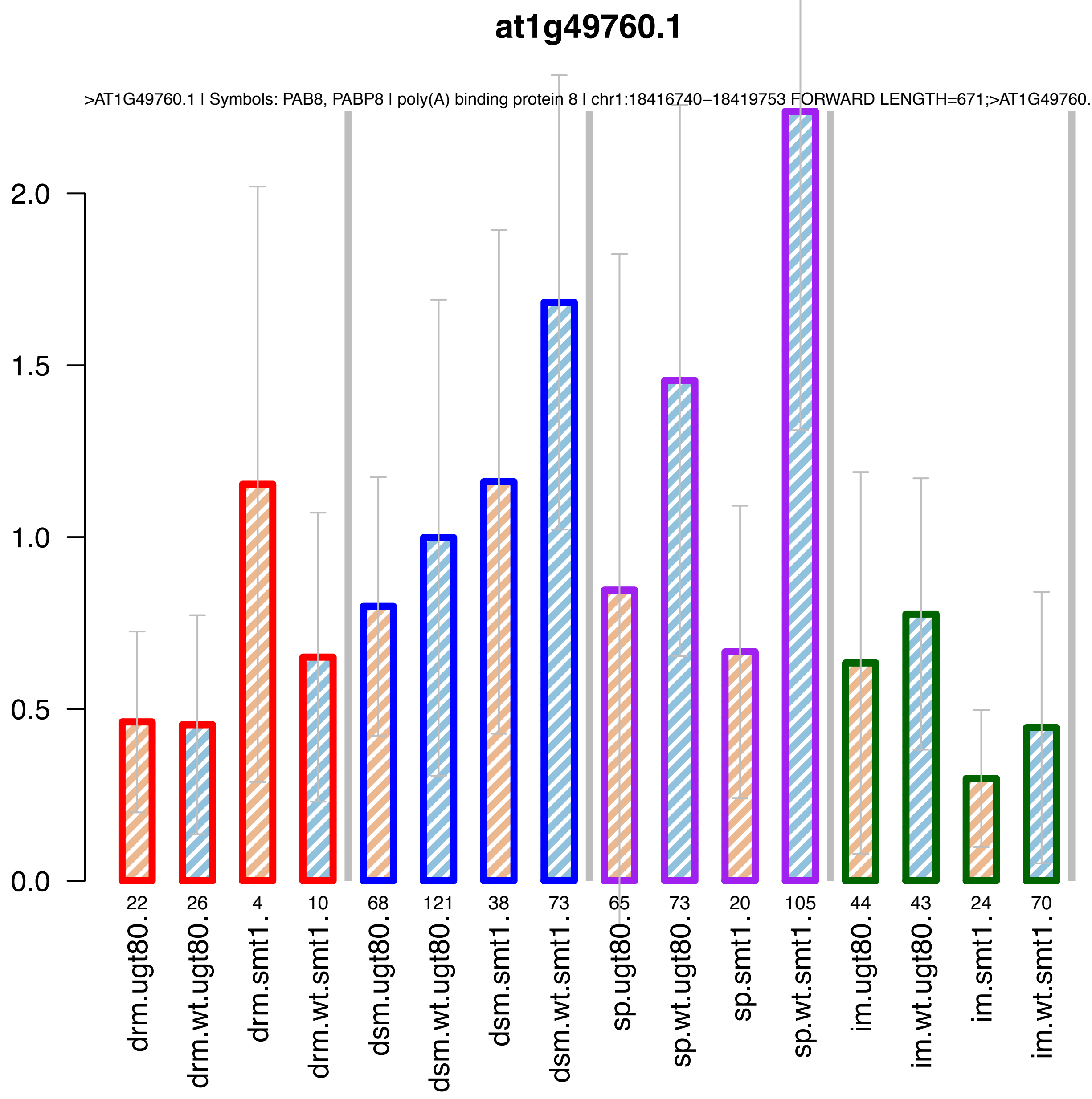

at1g49760.1
>AT1G49760.1 | Symbols: PAB8, PABP8 | poly(A) binding protein 8 | chr1:18416740–18419753 FORWARD LENGTH=671;>AT1G49760.2 |
2.0
1.5
1.0
0.5
0.0
22
26
4
10
68
121
38
73
65
73
20
105
44
43
24
70
drm.ugt80..
drm.wt.ugt80..
drm.smt1..
drm.wt.smt1..
dsm.ugt80..
dsm.wt.ugt80..
dsm.smt1..
dsm.wt.smt1..
sp.ugt80..
sp.wt.ugt80..
sp.smt1..
sp.wt.smt1..
im.ugt80..
im.wt.ugt80..
im.smt1..
im.wt.smt1..

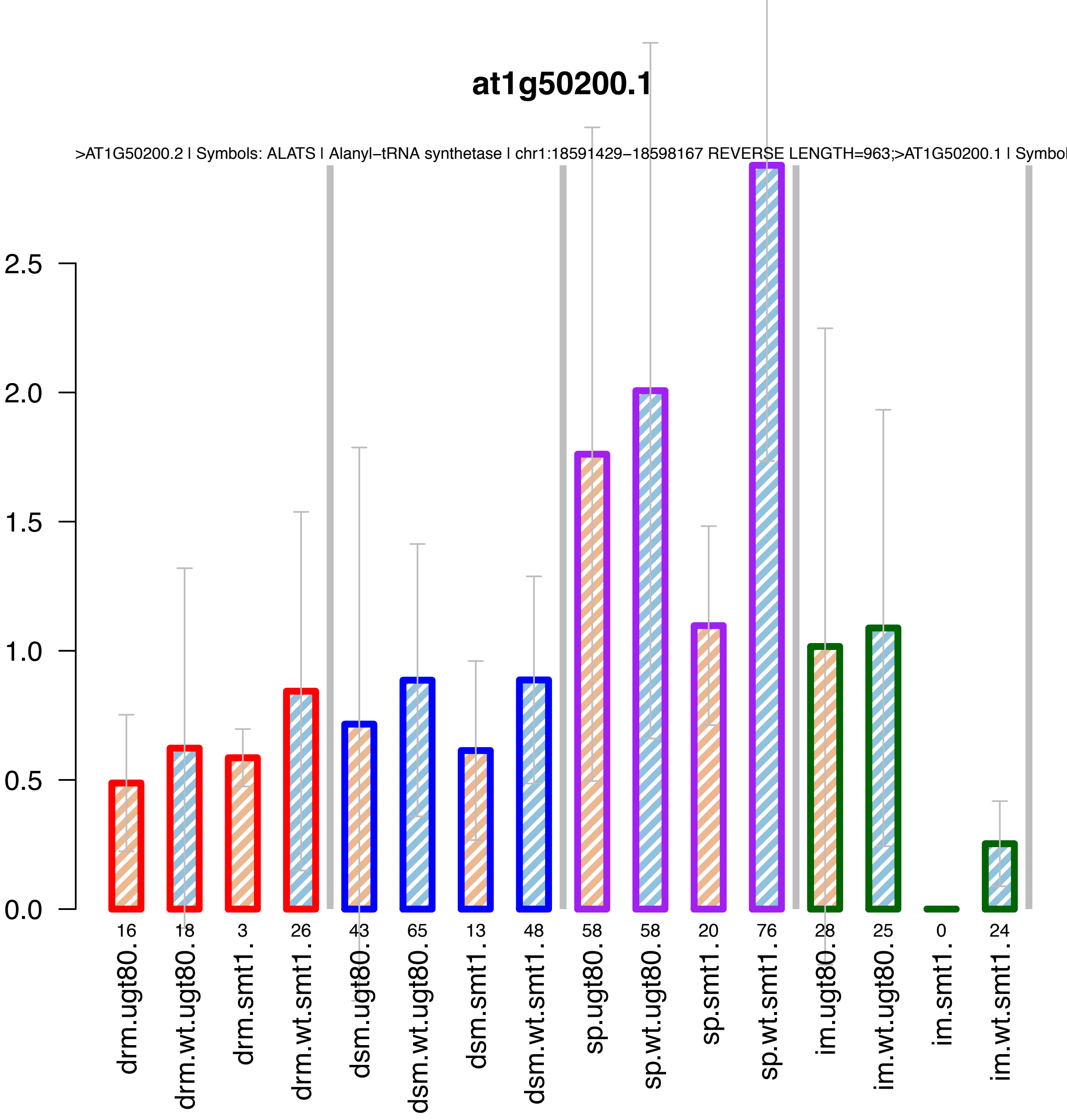

at1g50200.1
>AT1G50200.2 | Symbols: ALATS | Alanyl−tRNA synthetase | chr1:18591429−18598167 REVERSE LENGTH=963;>AT1G50200.1 | Symbol
2.5
2.0
1.5
1.0
0.5
0.0
16
18
3
26
43
65
13
48
58
58
20
76
28
25
0
24
drm.ugt80.
drm.wt.ugt80.
drm.smt1.
drm.wt.smt1.
dsm.ugt80.
dsm.wt.ugt80.
dsm.smt1.
dsm.wt.smt1.
sp.ugt80.
sp.wt.ugt80.
sp.smt1.
sp.wt.smt1.
im.ugt80.
im.wt.ugt80.
im.smt1.
im.wt.smt1.

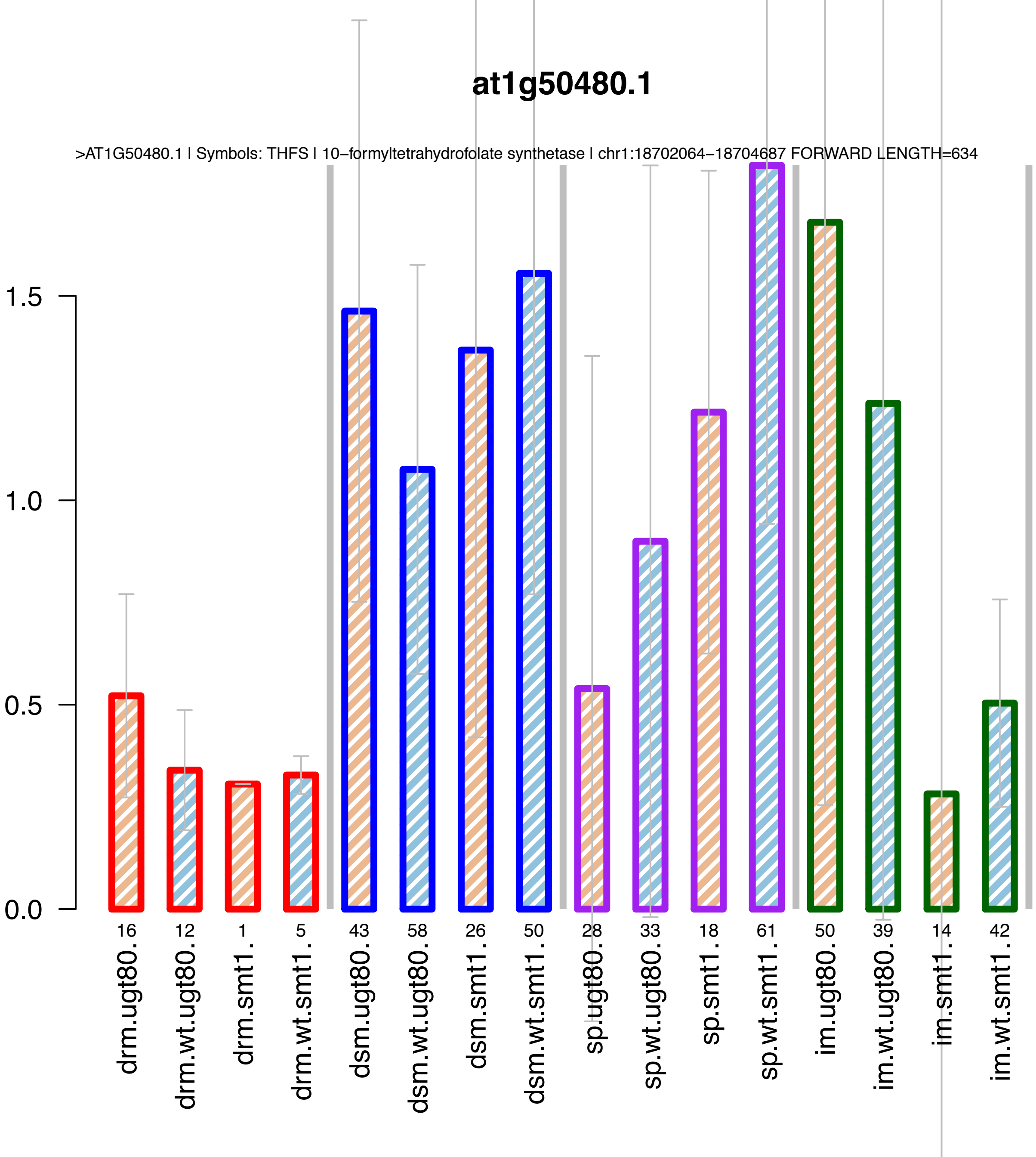
at1g50480.1
>AT1G50480.1 | Symbols: THFS | 10−formyltetrahydrofolate synthetase | chr1:18702064−18704687 FORWARD LENGTH=634
0.0
0.5
1.0
1.5
16
12
1
5
43
58
26
50
28
33
18
61
50
39
14
42
drm.ugt80..
drm.wt.ugt80..
drm.smt1..
drm.wt.smt1..
dsm.ugt80..
dsm.wt.ugt80..
dsm.smt1..
dsm.wt.smt1..
sp.ugt80..
sp.wt.ugt80..
sp.smt1..
sp.wt.smt1..
im.ugt80..
im.wt.ugt80..
im.smt1..
im.wt.smt1..

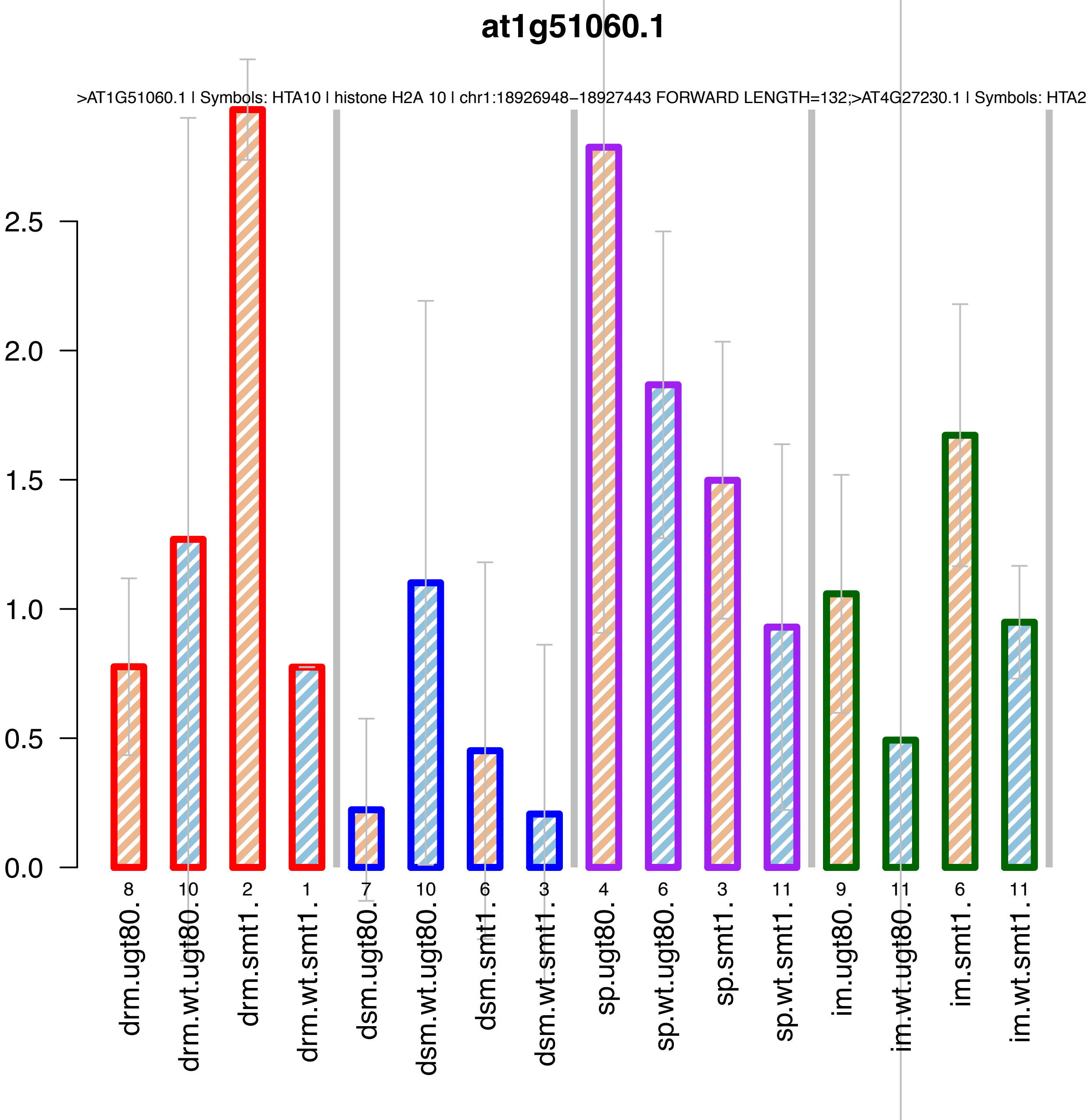

at1g51060.1
>AT1G51060.1 | Symbols: HTA10 | histone H2A 10 | chr1:18926948-18927443 FORWARD LENGTH=132;>AT4G27230.1 | Symbols: HTA2
2.5
2.0
1.5
1.0
0.5
0.0
8
10
2
1
7
10
6
3
4
6
3
11
9
11
6
11
drm.ugt80..
drm.wt.ugt80..
drm.smt1..
drm.wt.smt1..
dsm.ugt80..
dsm.wt.ugt80..
dsm.smt1..
dsm.wt.smt1..
sp.ugt80..
sp.wt.ugt80..
sp.smt1..
sp.wt.smt1..
im.ugt80..
im.wt.ugt80..
im.smt1..
im.wt.smt1..

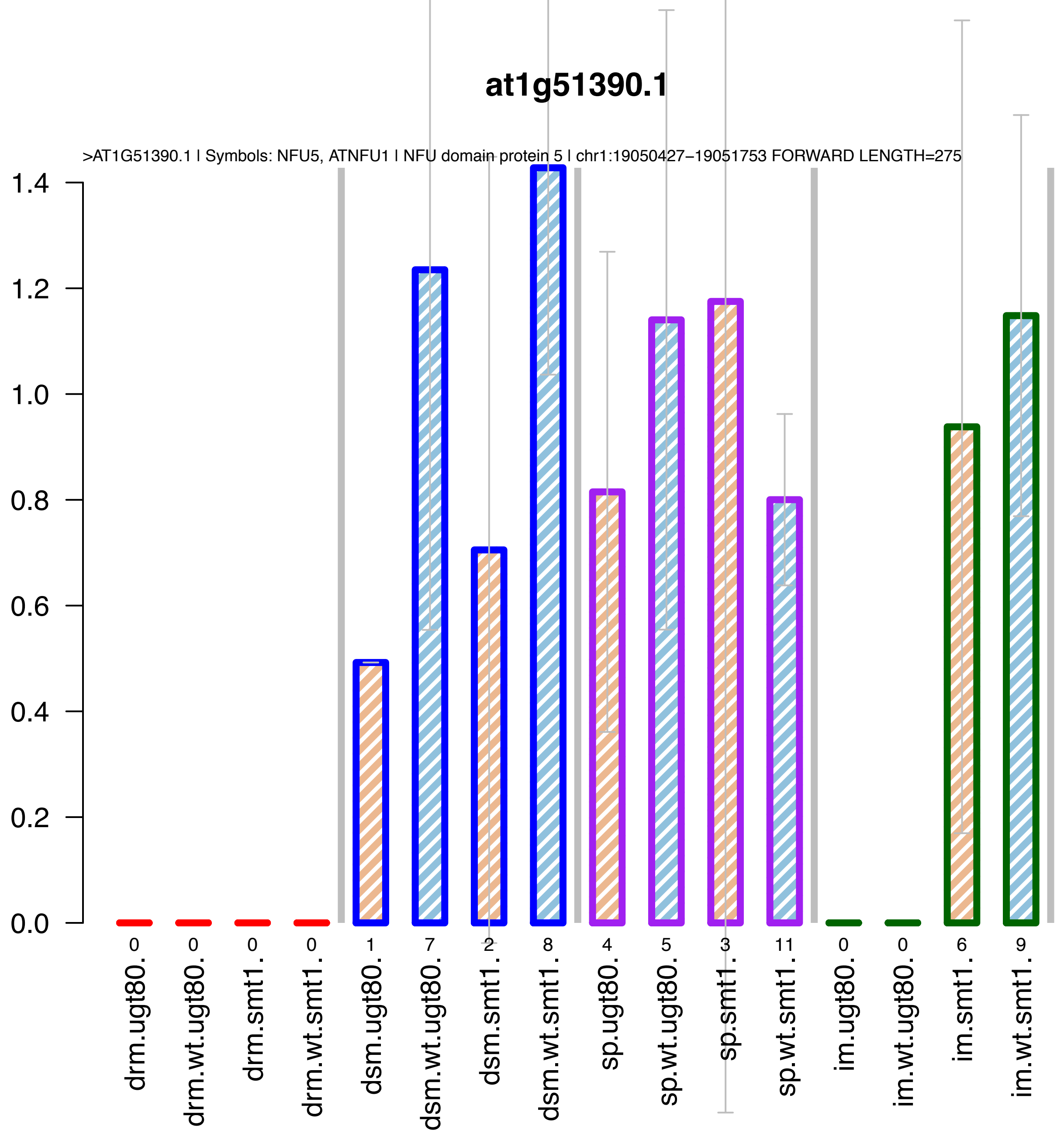

at1g51390.1
>AT1G51390.1 | Symbols: NFU5, ATNFU1 | NFU domain protein 5 | chr1:19050427–19051753 FORWARD LENGTH=275
0.0
0.2
0.4
0.6
0.8
1.0
1.2
1.4
0 0 0 0 1 7 2 8 4 5 3 11 0 0 6 9
drm.ugt80.
drm.wt.ugt80.
drm.smt1.
drm.wt.smt1.
dsm.ugt80.
dsm.wt.ugt80.
dsm.smt1.
dsm.wt.smt1.
sp.ugt80.
sp.wt.ugt80.
sp.smt1.
sp.wt.smt1.
im.ugt80.
im.wt.ugt80.
im.smt1.
im.wt.smt1.

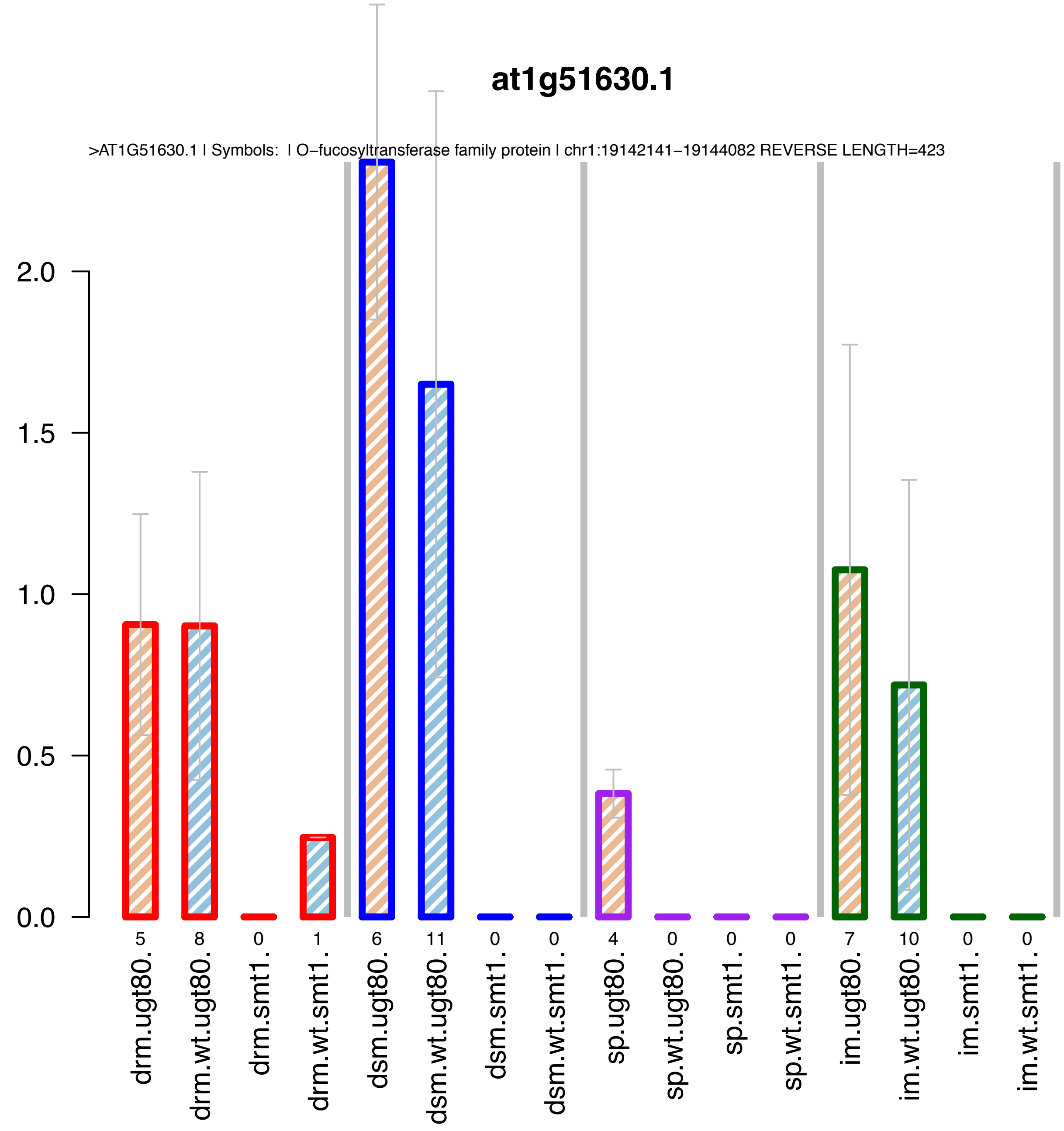

at1g51630.1
>AT1G51630.1 | Symbols: | O−fucosyltransferase family protein | chr1:19142141−19144082 REVERSE LENGTH=423
0.0
0.5
1.0
1.5
2.0
5
8
0
1
6
11
0
0
4
0
0
0
7
10
0
0
drm.ugt80.
drm.wt.ugt80.
drm.smt1.
drm.wt.smt1.
dsm.ugt80.
dsm.wt.ugt80.
dsm.smt1.
dsm.wt.smt1.
sp.ugt80.
sp.wt.ugt80.
sp.smt1.
sp.wt.smt1.
im.ugt80.
im.wt.ugt80.
im.smt1.
im.wt.smt1.

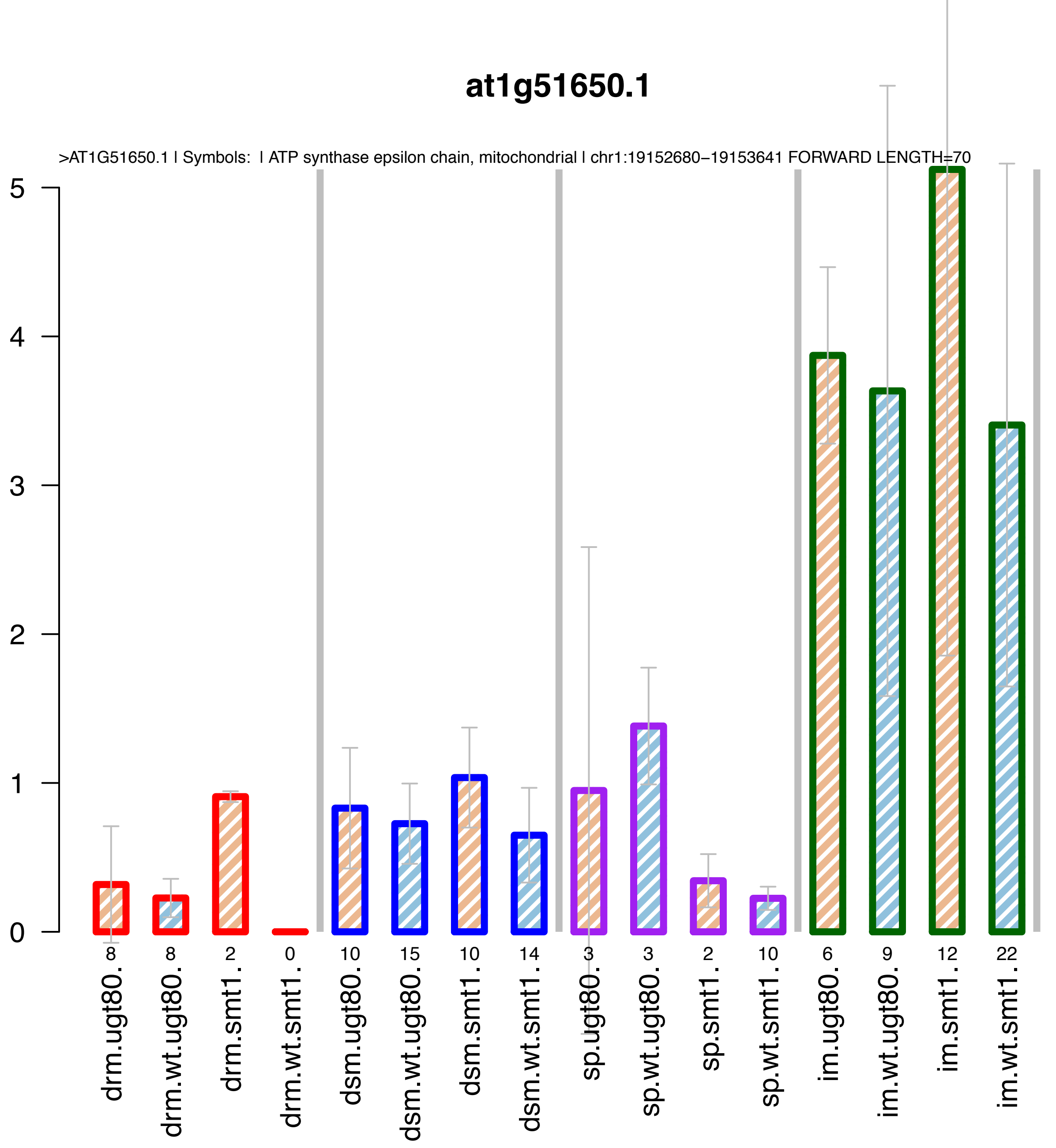

at1g51650.1
>AT1G51650.1 | Symbols: | ATP synthase epsilon chain, mitochondrial | chr1:19152680-19153641 FORWARD LENGTH=70
0
1
2
3
4
5
8
8
2
0
10
15
10
14
3
3
2
10
6
9
12
22
drm.ugt80..
drm.wt.ugt80..
drm.smt1..
drm.wt.smt1..
dsm.ugt80..
dsm.wt.ugt80..
dsm.smt1..
dsm.wt.smt1..
sp.ugt80..
sp.wt.ugt80..
sp.smt1..
sp.wt.smt1..
im.ugt80..
im.wt.ugt80..
im.smt1..
im.wt.smt1..

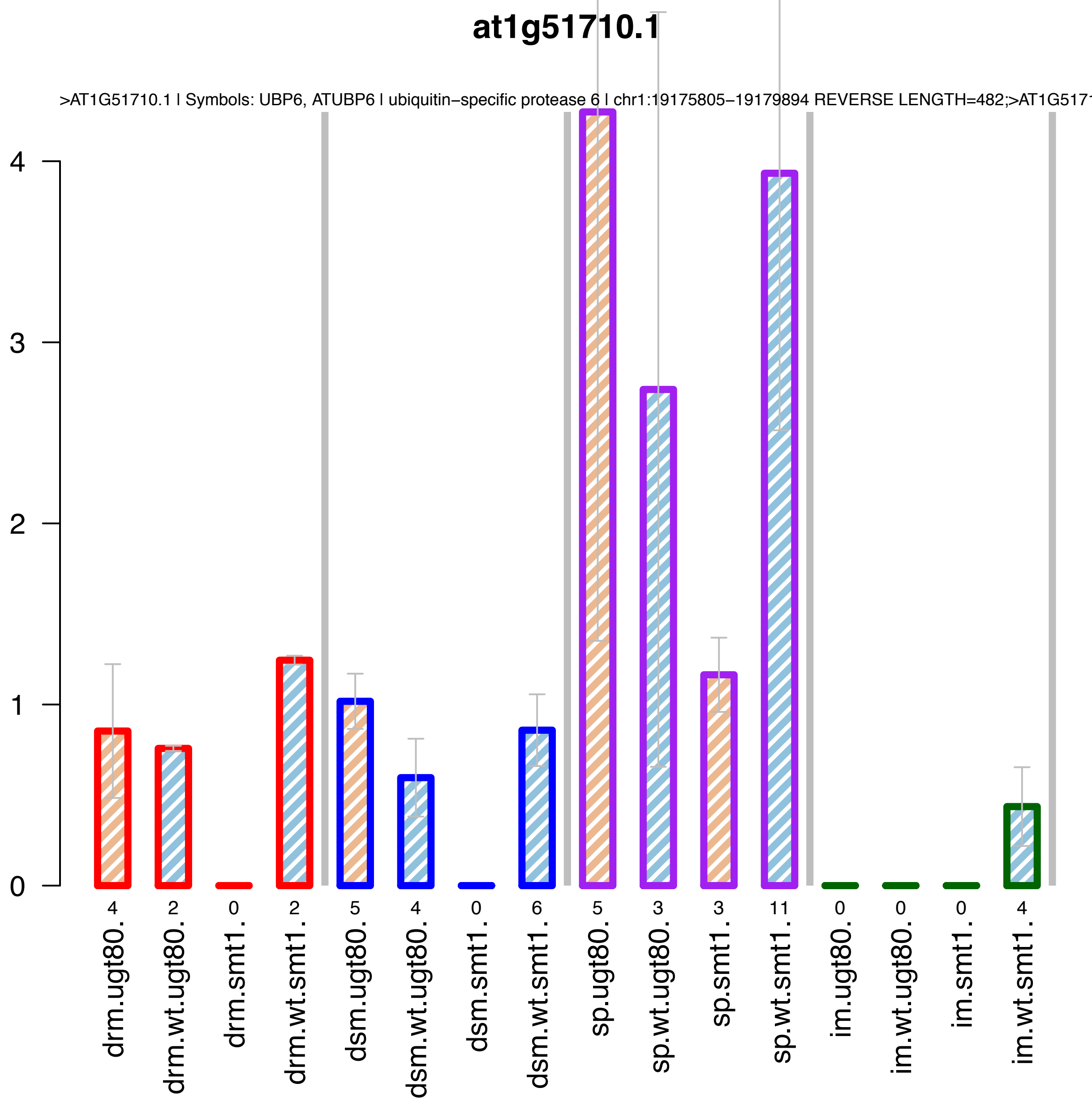

at1g51710.1
>AT1G51710.1 | Symbols: UBP6, ATUBP6 | ubiquitin−specific protease 6 | chr1:19175805−19179894 REVERSE LENGTH=482;>AT1G517
4
3
2
1
0
4 2 0 2 5 4 0 6 5 3 3 11 0 0 0 4
drm.ugt80.
drm.wt.ugt80.
drm.smt1.
drm.wt.smt1.
dsm.ugt80.
dsm.wt.ugt80.
dsm.smt1.
dsm.wt.smt1.
sp.ugt80.
sp.wt.ugt80.
sp.smt1.
sp.wt.smt1.
im.ugt80.
im.wt.ugt80.
im.smt1.
im.wt.smt1.

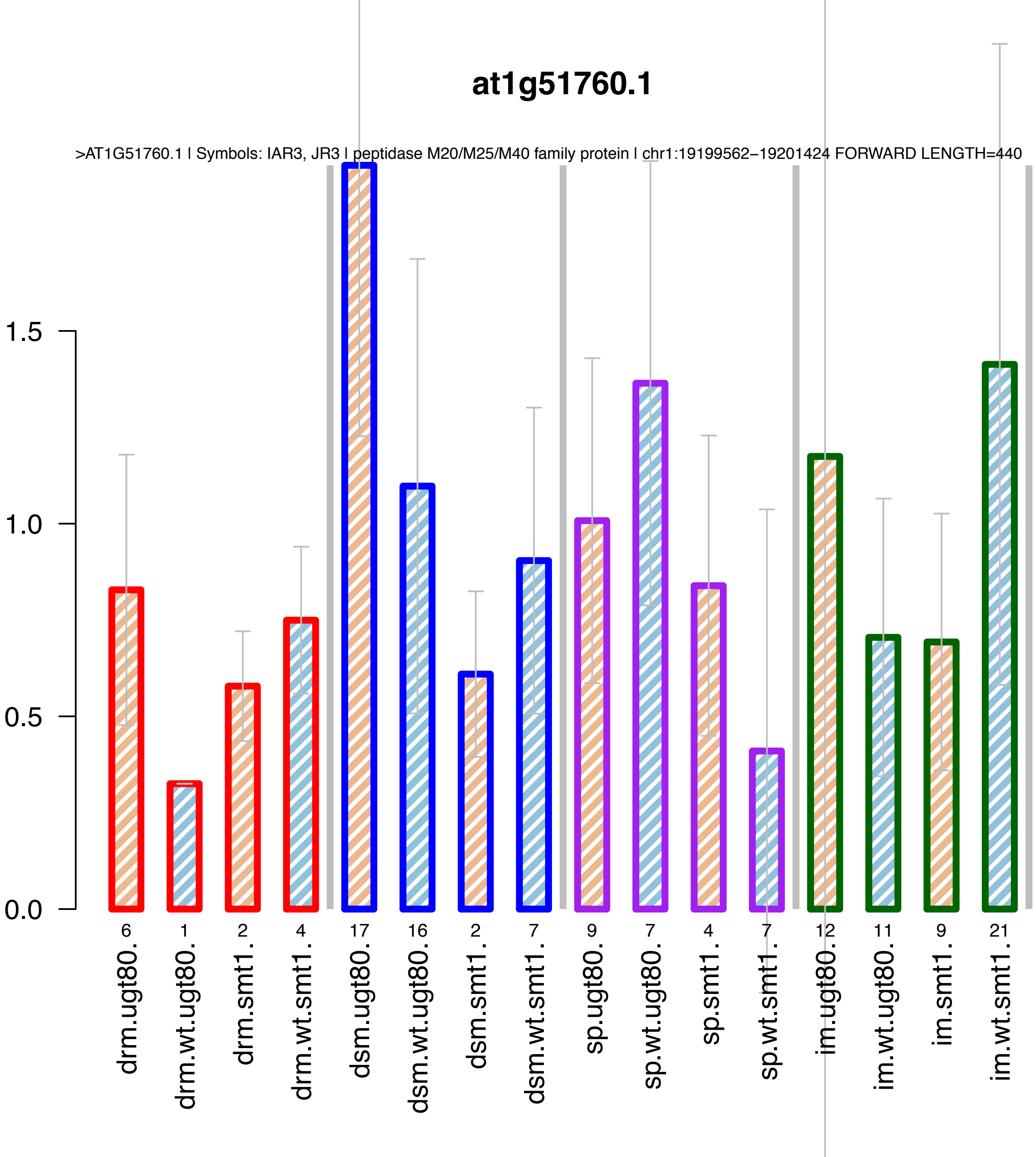

at1g51760.1
>AT1G51760.1 | Symbols: IAR3, JR3 | peptidase M20/M25/M40 family protein | chr1:19199562-19201424 FORWARD LENGTH=440
0.0
0.5
1.0
1.5
6
1
2
4
17
16
2
7
9
7
4
7
12
11
9
21
drm.ugt80..
drm.wt.ugt80..
drm.smt1..
drm.wt.smt1..
dsm.ugt80..
dsm.wt.ugt80..
dsm.smt1..
dsm.wt.smt1..
sp.ugt80..
sp.wt.ugt80..
sp.smt1..
sp.wt.smt1..
im.ugt80..
im.wt.ugt80..
im.smt1..
im.wt.smt1..

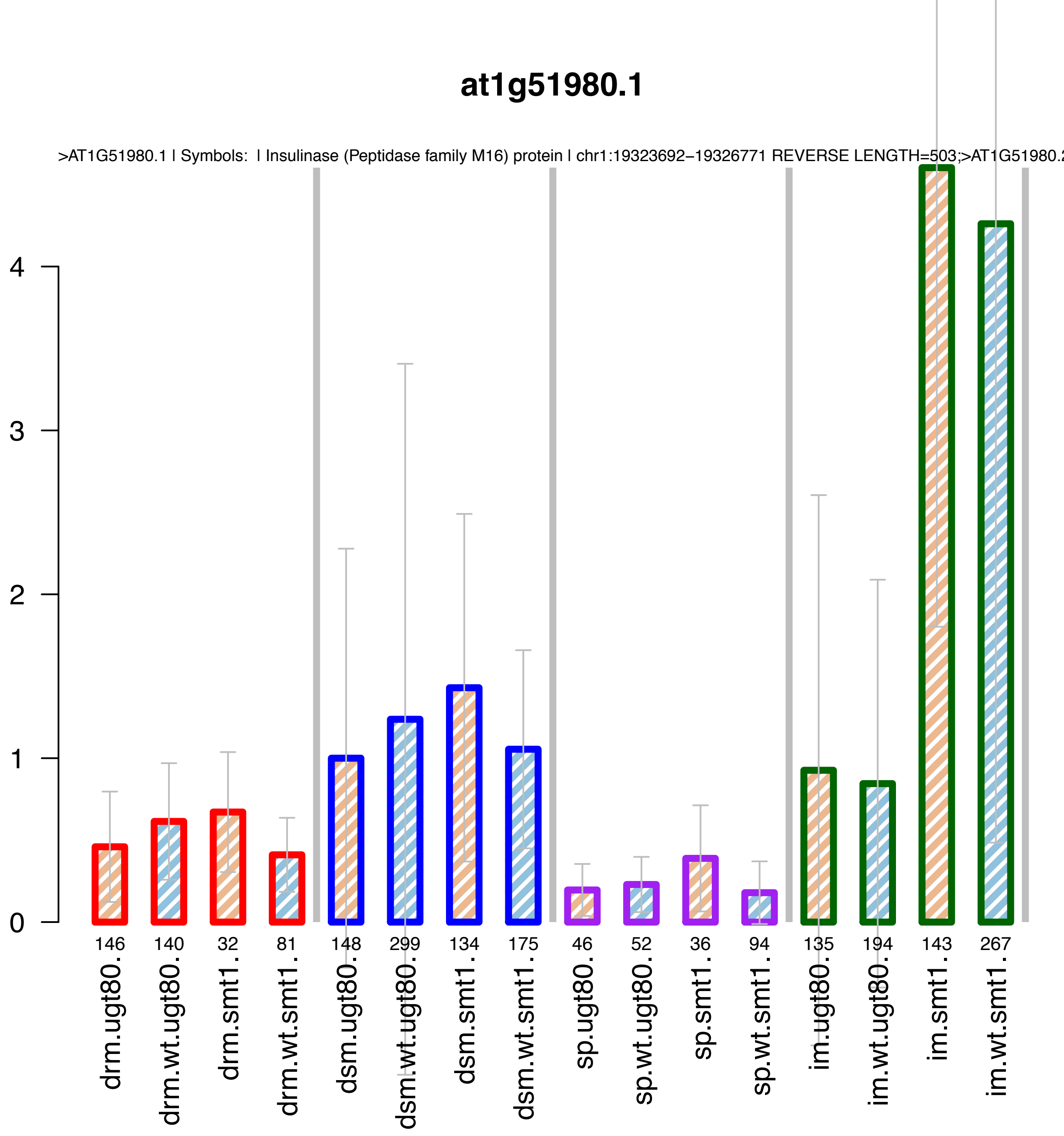

at1g51980.1
>AT1G51980.1 | Symbols: | Insulinase (Peptidase family M16) protein | chr1:19323692-19326771 REVERSE LENGTH=503;>AT1G51980.2
0
1
2
3
4
146
140
32
81
148
299
134
175
46
52
36
94
135
194
143
267
drm.ugt80.
drm.wt.ugt80.
drm.smt1.
drm.wt.smt1.
dsm.ugt80.
dsm.wt.ugt80.
dsm.smt1.
dsm.wt.smt1.
sp.ugt80.
sp.wt.ugt80.
sp.smt1.
sp.wt.smt1.
im.ugt80.
im.wt.ugt80.
im.smt1.
im.wt.smt1.

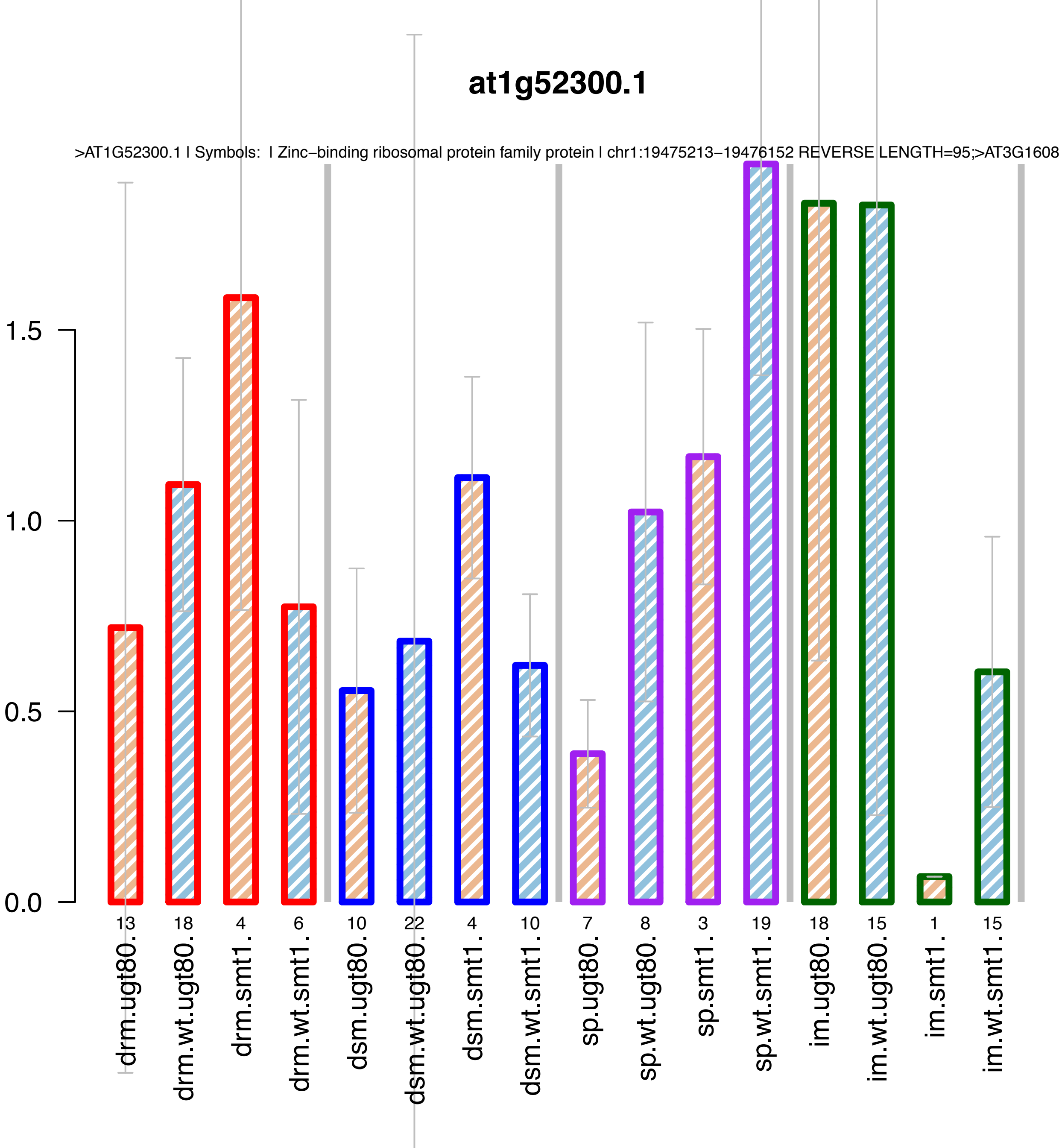
at1g52300.1
>AT1G52300.1 | Symbols: | Zinc−binding ribosomal protein family protein | chr1:19475213−19476152 REVERSE LENGTH=95;>AT3G1608
0.0
0.5
1.0
1.5
13
18
4
6
10
22
4
10
7
8
3
19
18
15
1
15
drm.ugt80..
drm.wt.ugt80..
drm.smt1..
drm.wt.smt1..
dsm.ugt80..
dsm.wt.ugt80..
dsm.smt1..
dsm.wt.smt1..
sp.ugt80..
sp.wt.ugt80..
sp.smt1..
sp.wt.smt1..
im.ugt80..
im.wt.ugt80..
im.smt1..
im.wt.smt1..

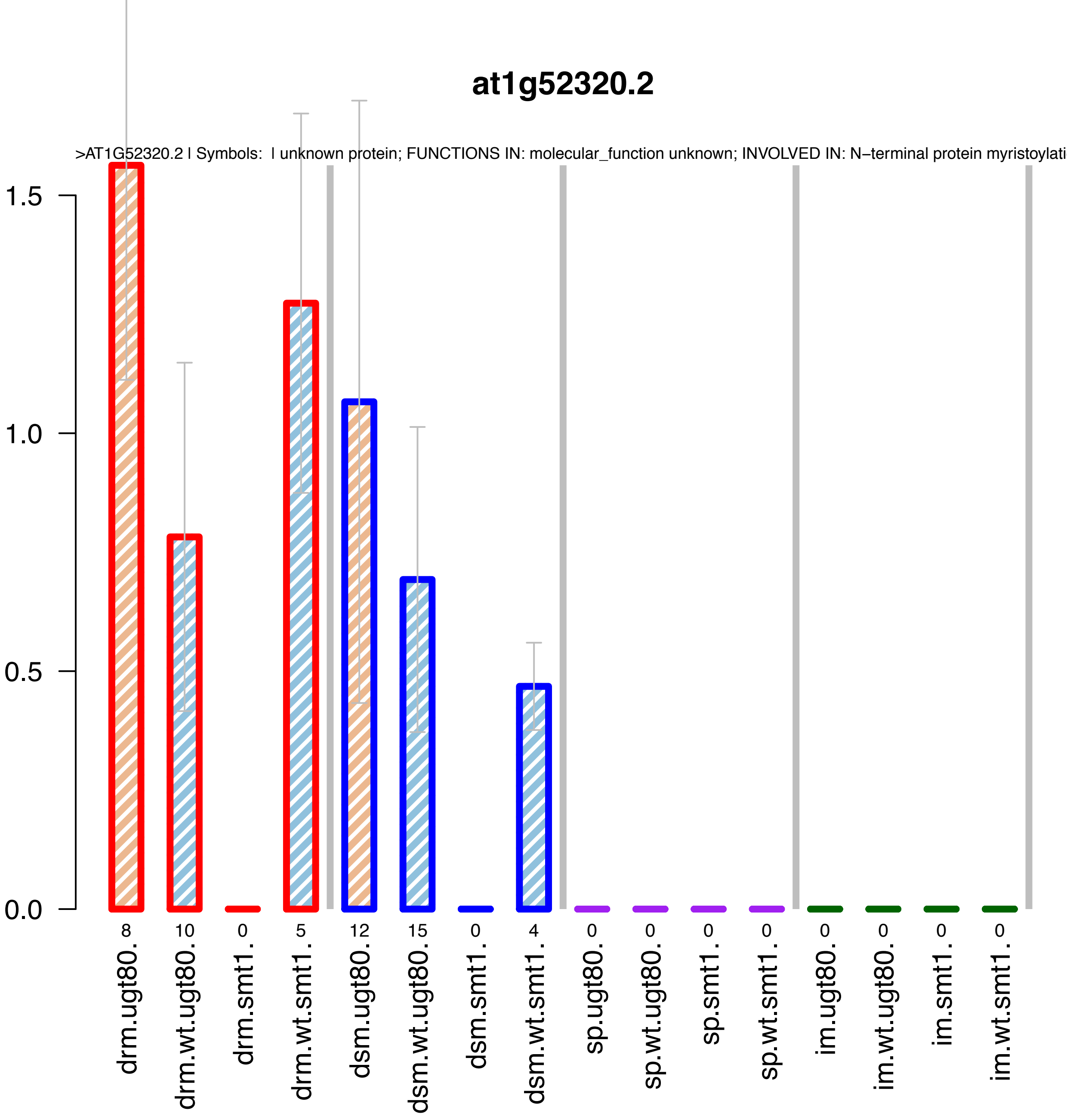

at1g52320.2
>AT1G52320.2 | Symbols: | unknown protein; FUNCTIONS IN: molecular_function unknown; INVOLVED IN: N−terminal protein myristoylati
1.5
1.0
0.5
0.0
8
10
0
5
12
15
0
4
0
0
0
0
0
0
0
0
drm.ugt80.
drm.wt.ugt80.
drm.smt1.
drm.wt.smt1.
dsm.ugt80.
dsm.wt.ugt80.
dsm.smt1.
dsm.wt.smt1.
sp.ugt80.
sp.wt.ugt80.
sp.smt1.
sp.wt.smt1.
im.ugt80.
im.wt.ugt80.
im.smt1.
im.wt.smt1.

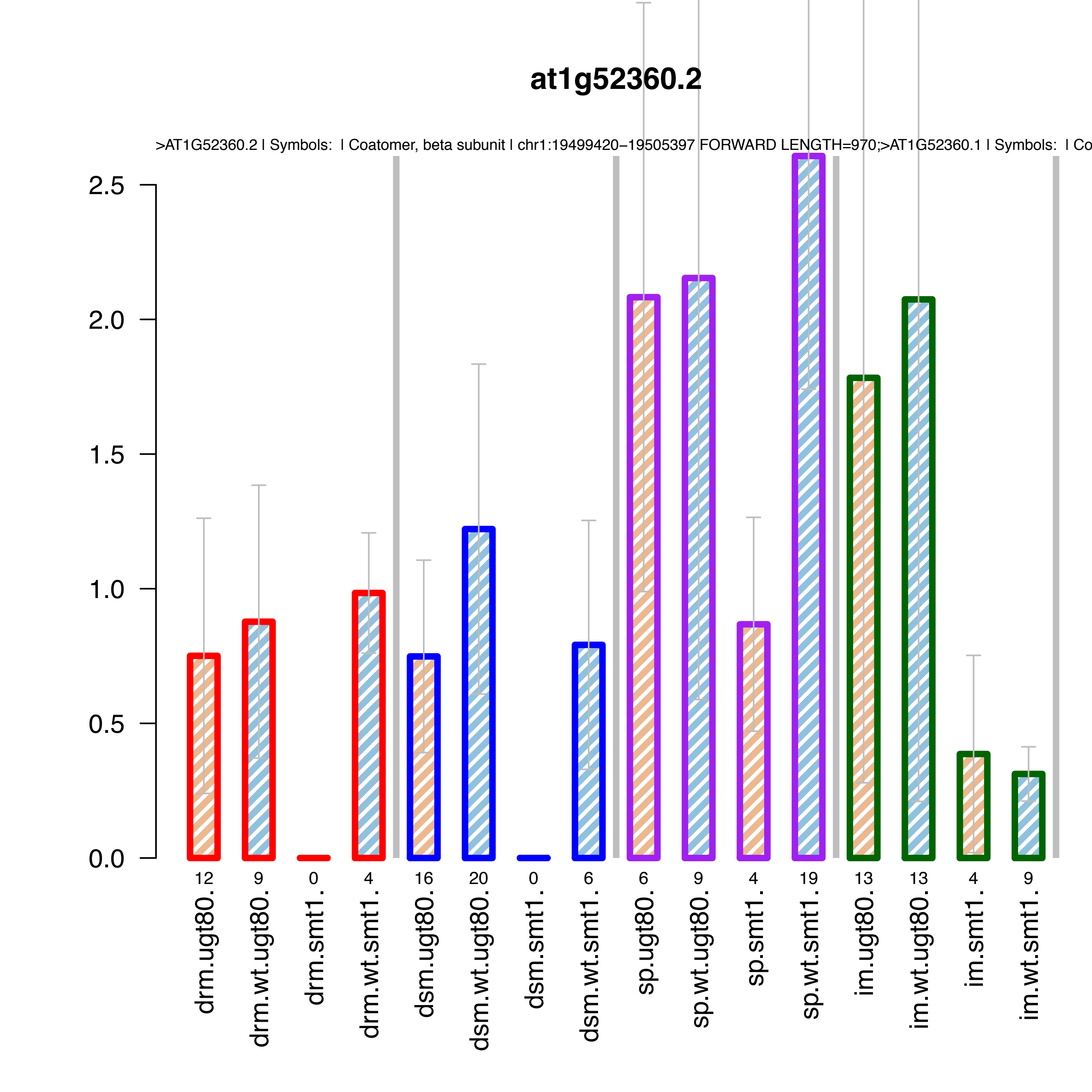
at1g52360.2
>AT1G52360.2 | Symbols: | Coatomer, beta subunit | chr1:19499420−19505397 FORWARD LENGTH=970;>AT1G52360.1 | Symbols: | Co
0.0
0.5
1.0
1.5
2.0
2.5
12
9
0
4
16
20
0
6
6
9
4
19
13
13
4
9
drm.ugt80.
drm.wt.ugt80.
drm.smt1.
drm.wt.smt1.
dsm.ugt80.
dsm.wt.ugt80.
dsm.smt1.
dsm.wt.smt1.
sp.ugt80.
sp.wt.ugt80.
sp.smt1.
sp.wt.smt1.
im.ugt80.
im.wt.ugt80.
im.smt1.
im.wt.smt1.

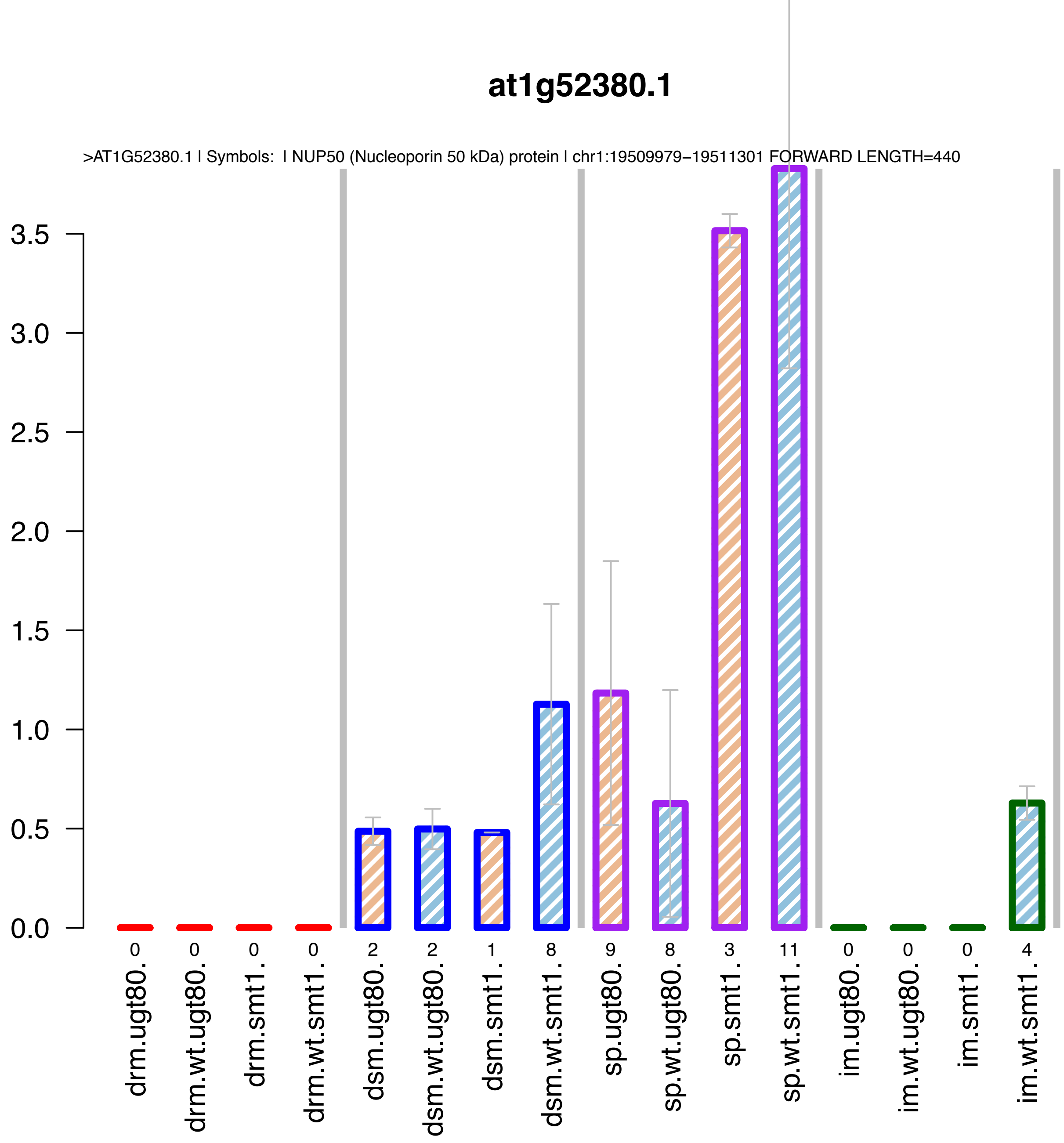

at1g52380.1
>AT1G52380.1 | Symbols: | NUP50 (Nucleoporin 50 kDa) protein | chr1:19509979-19511301 FORWARD LENGTH=440
0.0
0.5
1.0
1.5
2.0
2.5
3.0
3.5
0
0
0
0
2
2
1
8
9
8
3
11
0
0
0
4
drm.ugt80.
drm.wt.ugt80.
drm.smt1.
drm.wt.smt1.
dsm.ugt80.
dsm.wt.ugt80.
dsm.smt1.
dsm.wt.smt1.
sp.ugt80.
sp.wt.ugt80.
sp.smt1.
sp.wt.smt1.
im.ugt80.
im.wt.ugt80.
im.smt1.
im.wt.smt1.

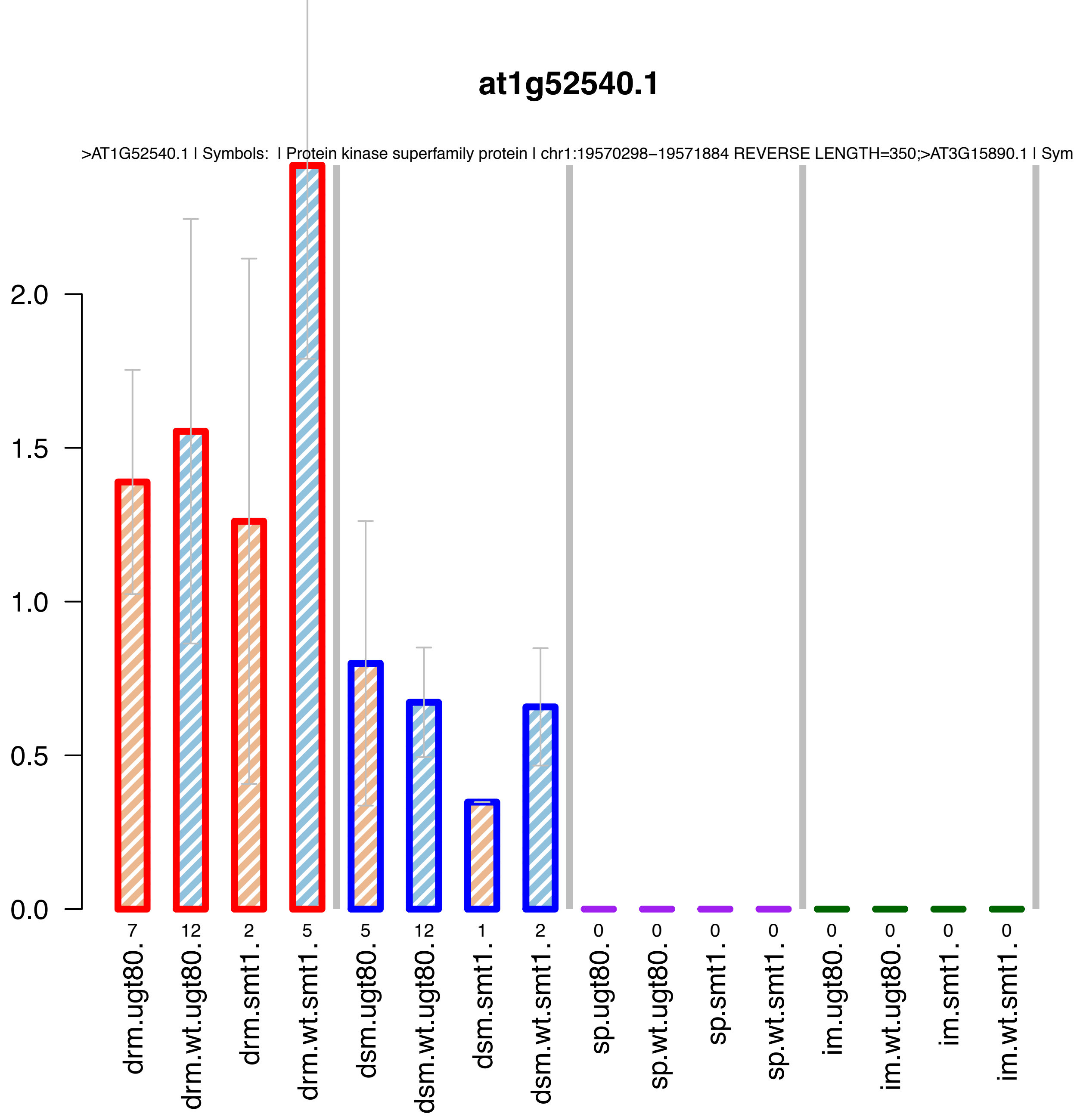

at1g52540.1
>AT1G52540.1 | Symbols: | Protein kinase superfamily protein | chr1:19570298−19571884 REVERSE LENGTH=350;>AT3G15890.1 | Sym
2.0
1.5
1.0
0.5
0.0
7
12
2
5
5
12
1
2
0
0
0
0
0
0
0
0
drm.ugt80.
drm.wt.ugt80.
drm.smt1.
drm.wt.smt1.
dsm.ugt80.
dsm.wt.ugt80.
dsm.smt1.
dsm.wt.smt1.
sp.ugt80.
sp.wt.ugt80.
sp.smt1.
sp.wt.smt1.
im.ugt80.
im.wt.ugt80.
im.smt1.
im.wt.smt1.

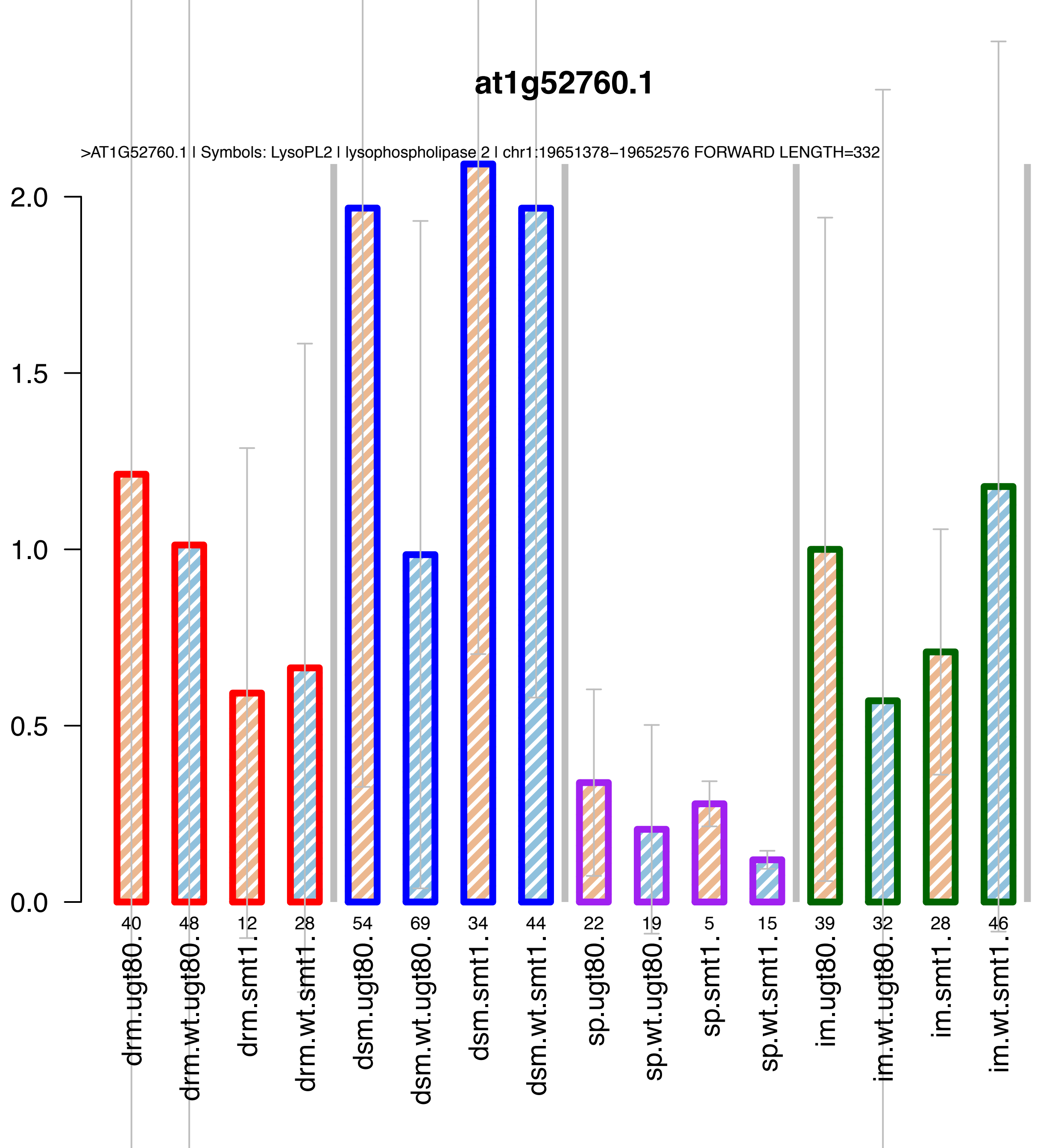

at1g52760.1
>AT1G52760.1 | Symbols: LysoPL2 | lysophospholipase 2 | chr1:19651378-19652576 FORWARD LENGTH=332
2.0
1.5
1.0
0.5
0.0
40
48
12
28
54
69
34
44
22
19
5
15
39
32
28
46
drm.ugt80..
drm.wt.ugt80..
drm.smt1..
drm.wt.smt1..
dsm.ugt80..
dsm.wt.ugt80..
dsm.smt1..
dsm.wt.smt1..
sp.ugt80..
sp.wt.ugt80..
sp.smt1..
sp.wt.smt1..
im.ugt80..
im.wt.ugt80..
im.smt1..
im.wt.smt1..

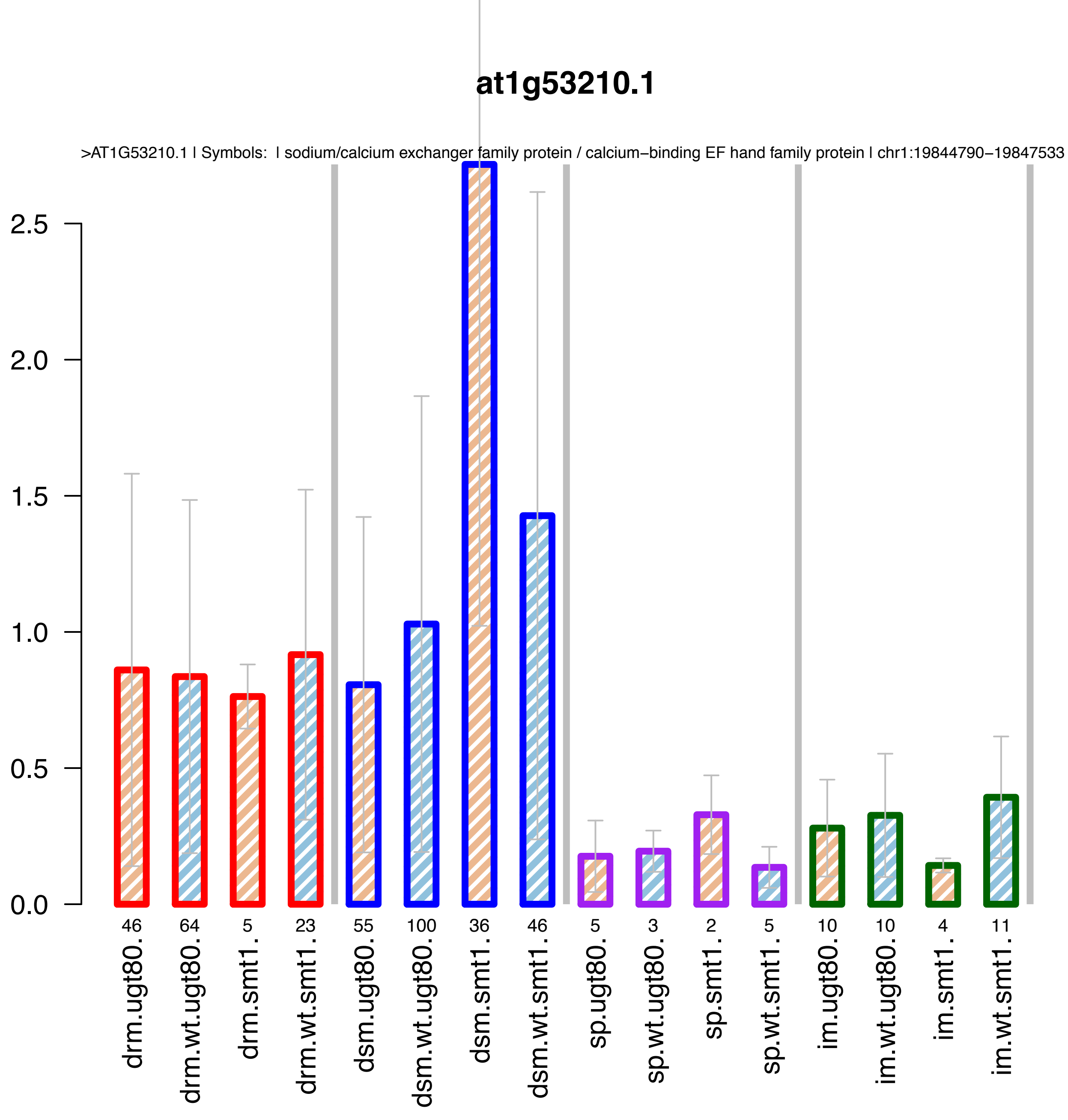
at1g53210.1
>AT1G53210.1 | Symbols: | sodium/calcium exchanger family protein / calcium-binding EF hand family protein | chr1:19844790-19847533
2.5
2.0
1.5
1.0
0.5
0.0
46
64
5
23
55
100
36
46
5
3
2
5
10
10
4
11
drm.ugt80.
drm.wt.ugt80.
drm.smt1.
drm.wt.smt1.
dsm.ugt80.
dsm.wt.ugt80.
dsm.smt1.
dsm.wt.smt1.
sp.ugt80.
sp.wt.ugt80.
sp.smt1.
sp.wt.smt1.
im.ugt80.
im.wt.ugt80.
im.smt1.
im.wt.smt1.

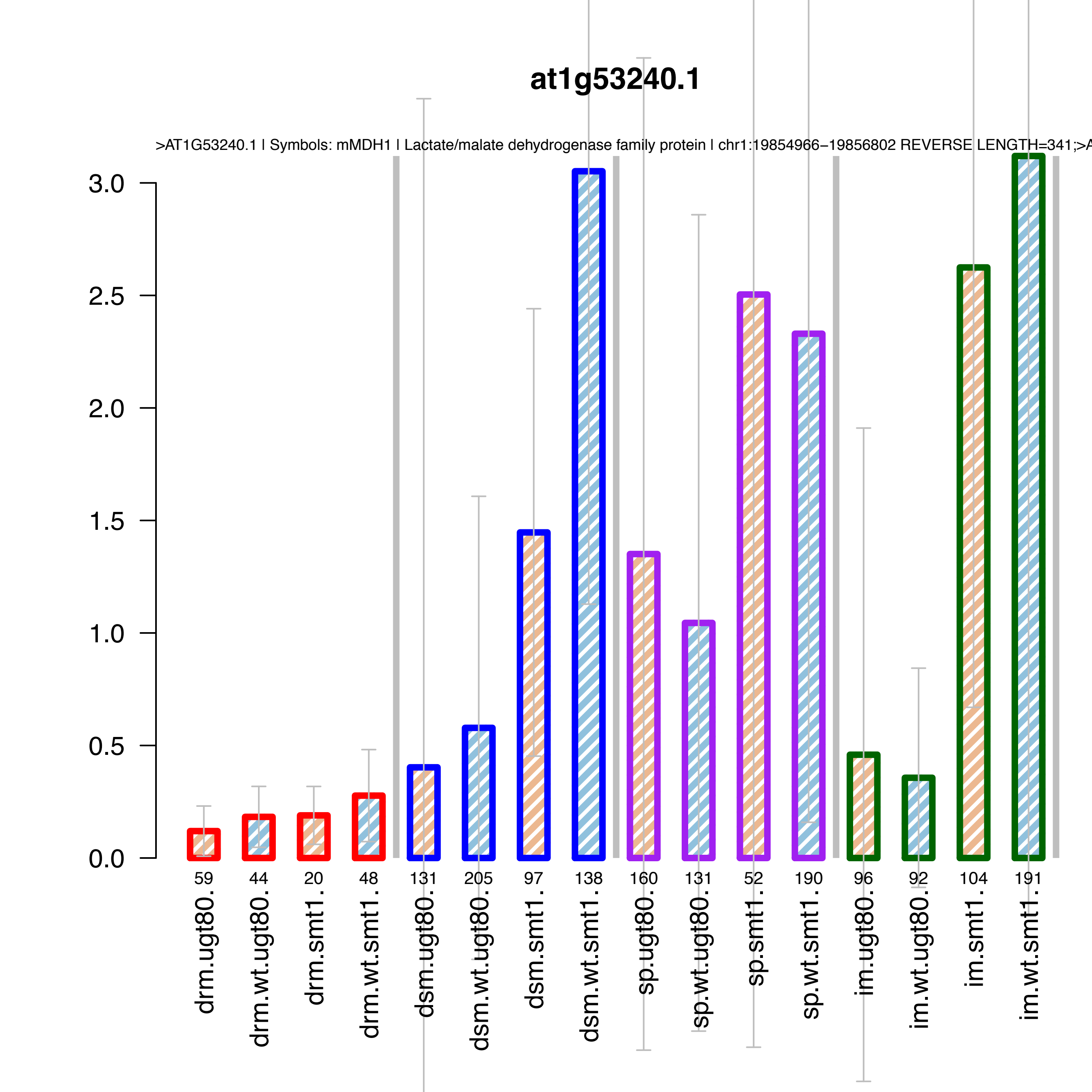

at1g53240.1
>AT1G53240.1 | Symbols: mMDH1 | Lactate/malate dehydrogenase family protein | chr1:19854966-19856802 REVERSE LENGTH=341;>A
3.0
2.5
2.0
1.5
1.0
0.5
0.0
59
44
20
48
131
205
97
138
160
131
52
190
96
92
104
191
drm.ugt80..
drm.wt.ugt80..
drm.smt1..
drm.wt.smt1..
dsm.ugt80..
dsm.wt.ugt80..
dsm.smt1..
dsm.wt.smt1..
sp.ugt80..
sp.wt.ugt80..
sp.smt1..
sp.wt.smt1..
im.ugt80..
im.wt.ugt80..
im.smt1..
im.wt.smt1..

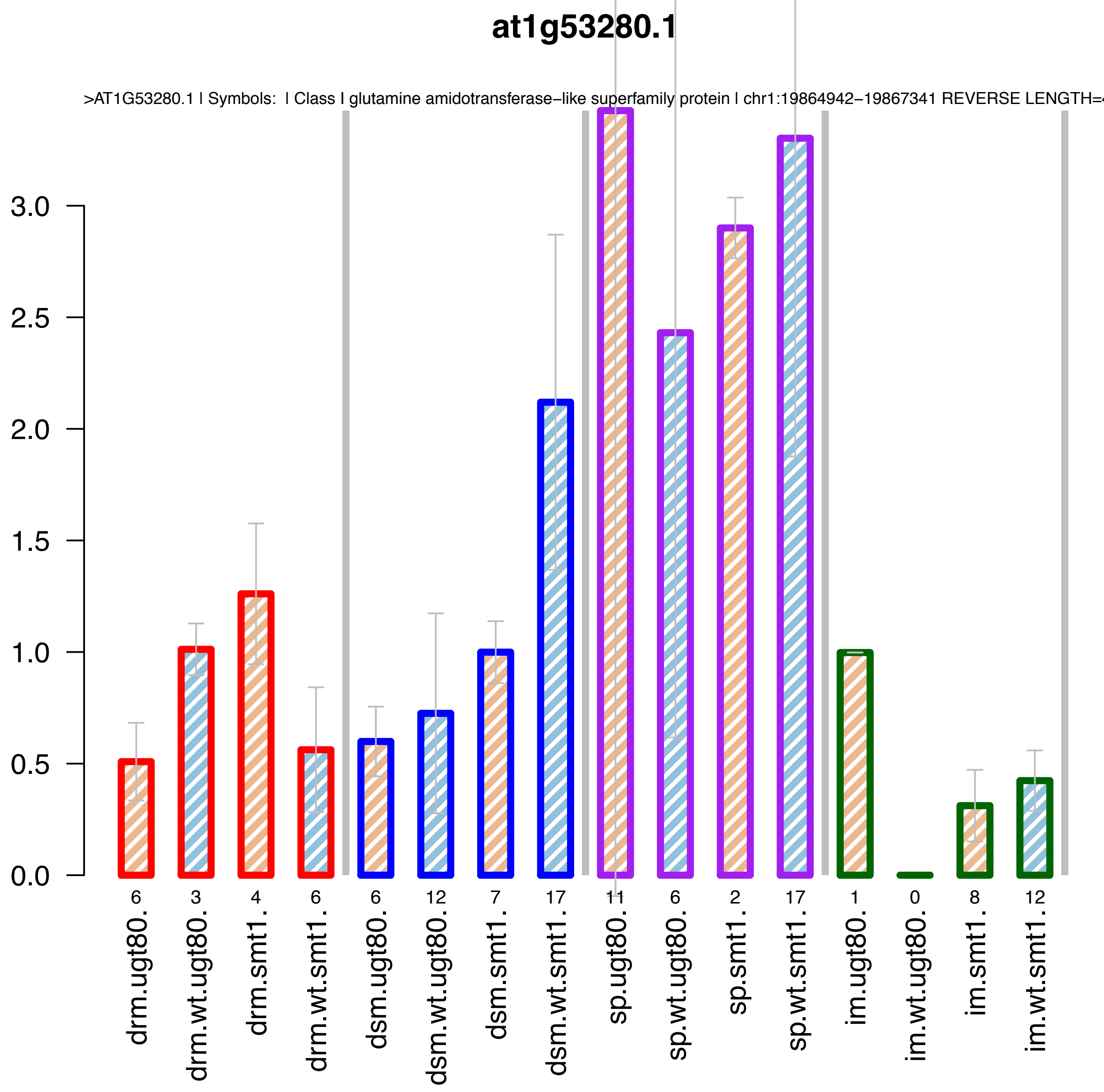

at1g53280.1
>AT1G53280.1 | Symbols: | Class | glutamine amidotransferase−like superfamily protein | chr1:19864942−19867341 REVERSE LENGTH=
0.0
0.5
1.0
1.5
2.0
2.5
3.0
6
3
4
6
6
12
7
17
11
6
2
17
1
0
8
12
drm.ugt80.
drm.wt.ugt80.
drm.smt1.
drm.wt.smt1.
dsm.ugt80.
dsm.wt.ugt80.
dsm.smt1.
dsm.wt.smt1.
sp.ugt80.
sp.wt.ugt80.
sp.smt1.
sp.wt.smt1.
im.ugt80.
im.wt.ugt80.
im.smt1.
im.wt.smt1.

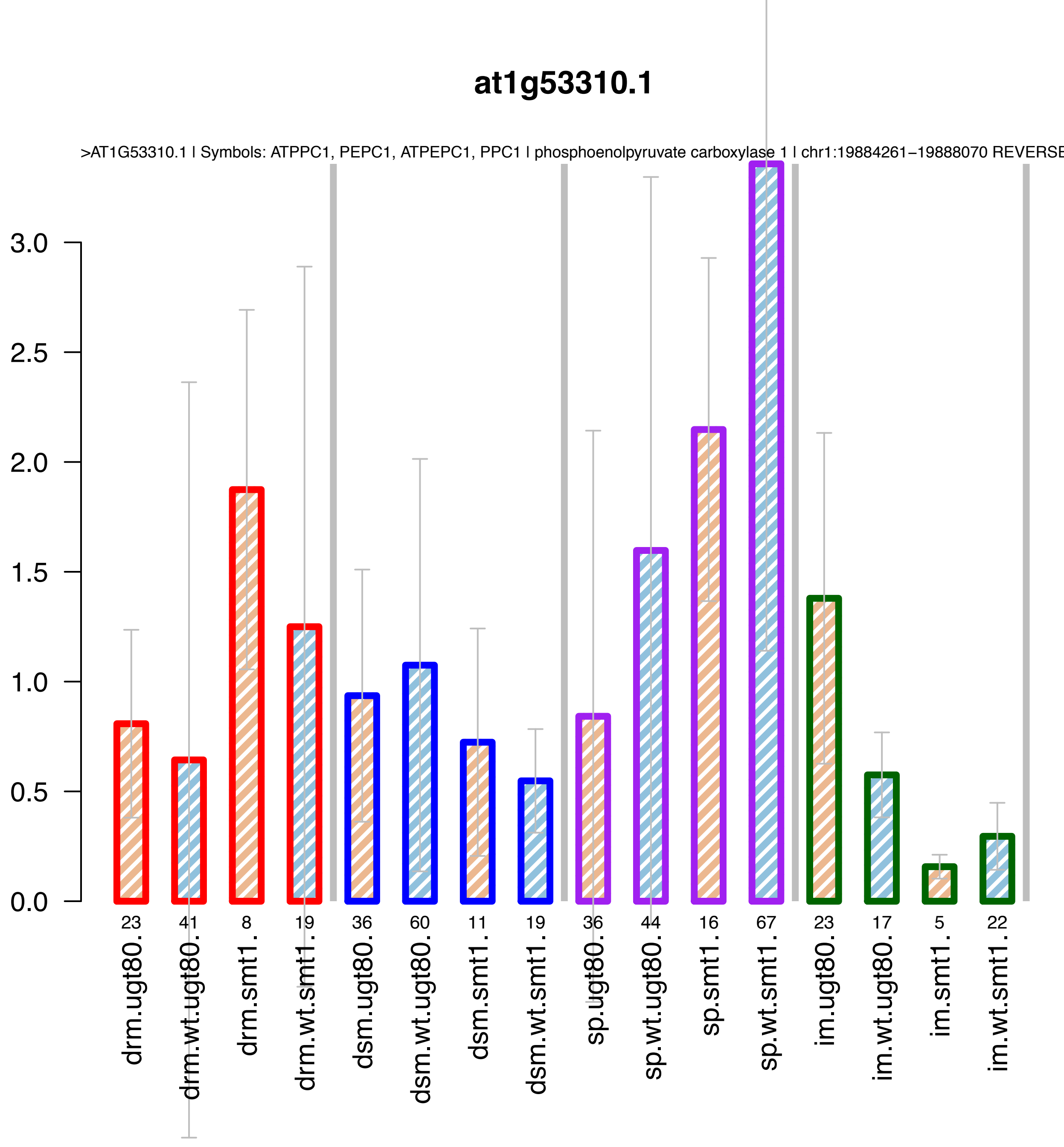

at1g53310.1
>AT1G53310.1 | Symbols: ATPPC1, PEPC1, ATPEPC1, PPC1 | phosphoenolpyruvate carboxylase 1 | chr1:19884261–19888070 REVERSE
3.0
2.5
2.0
1.5
1.0
0.5
0.0
23
41
8
19
36
60
11
19
36
44
16
67
23
17
5
22
drm.ugt80.
drm.wt.ugt80.
drm.smt1.
drm.wt.smt1.
dsm.ugt80.
dsm.wt.ugt80.
dsm.smt1.
dsm.wt.smt1.
sp.ugt80.
sp.wt.ugt80.
sp.smt1.
sp.wt.smt1.
im.ugt80.
im.wt.ugt80.
im.smt1.
im.wt.smt1.

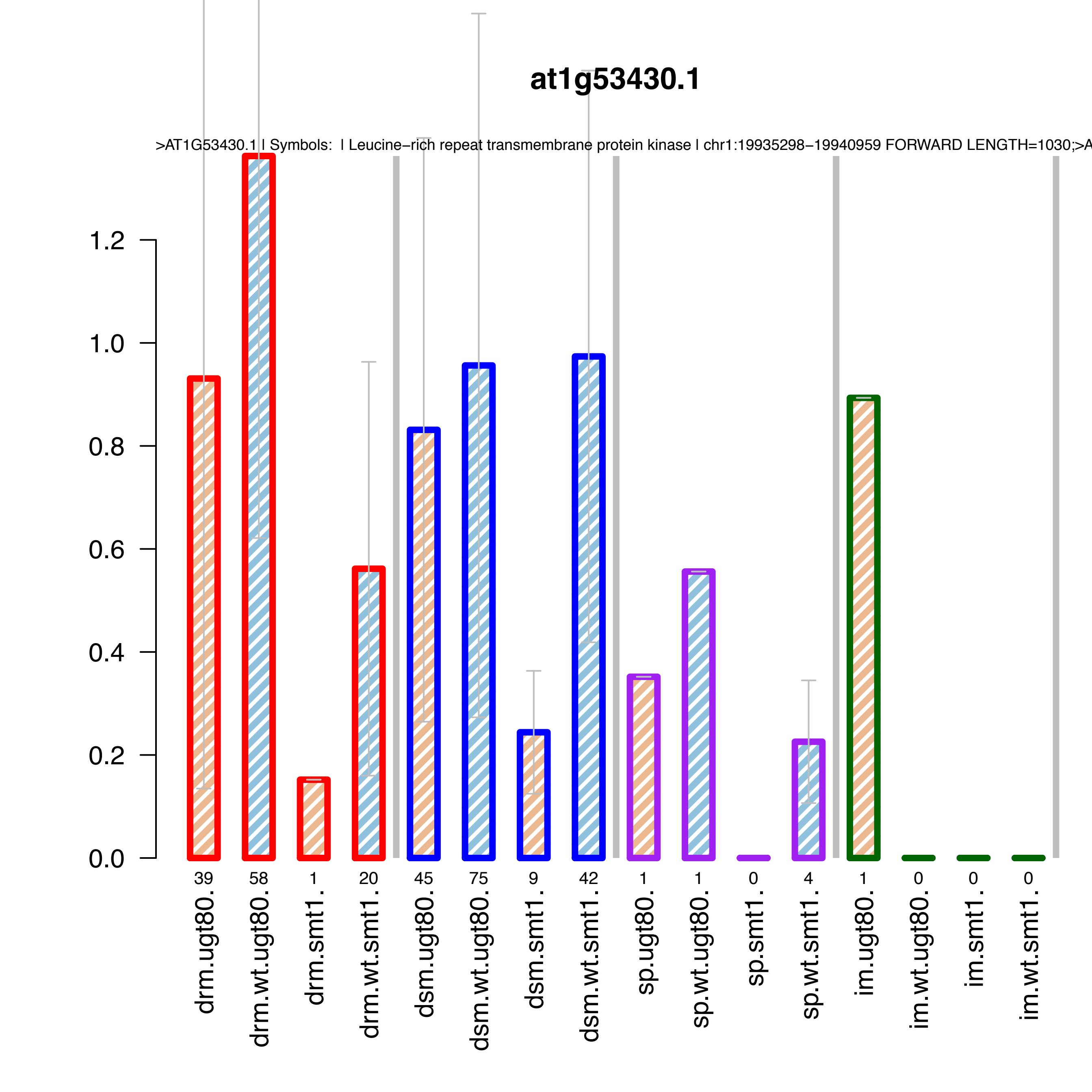
at1g53430.1
>AT1G53430.1 | Symbols: | Leucine−rich repeat transmembrane protein kinase | chr1:19935298−19940959 FORWARD LENGTH=1030;>A
0.0
0.2
0.4
0.6
0.8
1.0
1.2
39
58
1
20
45
75
9
42
1
1
0
4
1
0
0
0
drm.ugt80.
drm.wt.ugt80.
drm.smt1.
drm.wt.smt1.
dsm.ugt80.
dsm.wt.ugt80.
dsm.smt1.
dsm.wt.smt1.
sp.ugt80.
sp.wt.ugt80.
sp.smt1.
sp.wt.smt1.
im.ugt80.
im.wt.ugt80.
im.smt1.
im.wt.smt1.

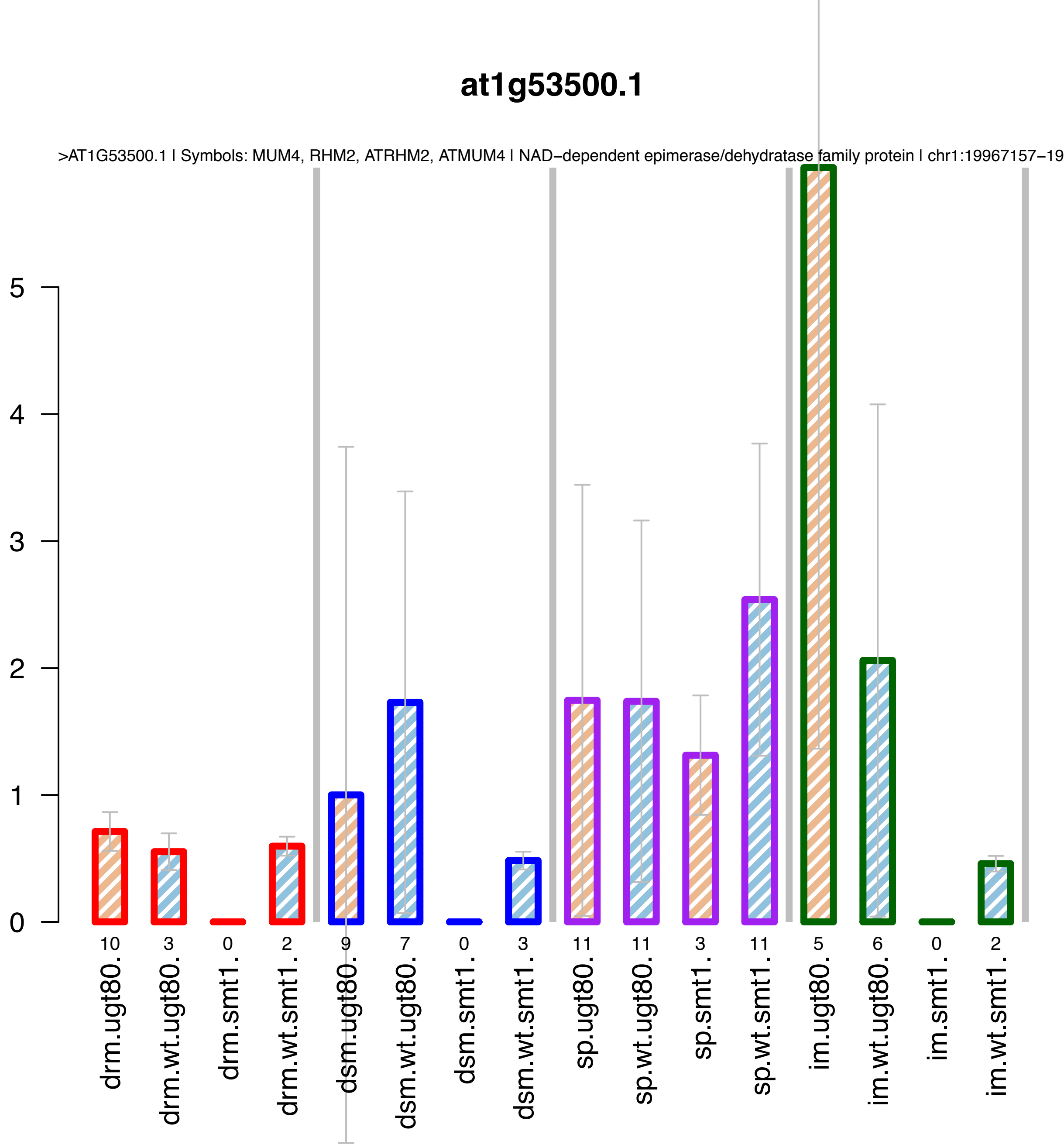

at1g53500.1
>AT1G53500.1 | Symbols: MUM4, RHM2, ATRHM2, ATMUM4 | NAD−dependent epimerase/dehydratase family protein | chr1:19967157−19
0
1
2
3
4
5
10
3
0
2
9
7
0
3
11
11
3
11
5
6
0
2
drm.ugt80.
drm.wt.ugt80.
drm.smt1.
drm.wt.smt1.
dsm.ugt80.
dsm.wt.ugt80.
dsm.smt1.
dsm.wt.smt1.
sp.ugt80.
sp.wt.ugt80.
sp.smt1.
sp.wt.smt1.
im.ugt80.
im.wt.ugt80.
im.smt1.
im.wt.smt1.

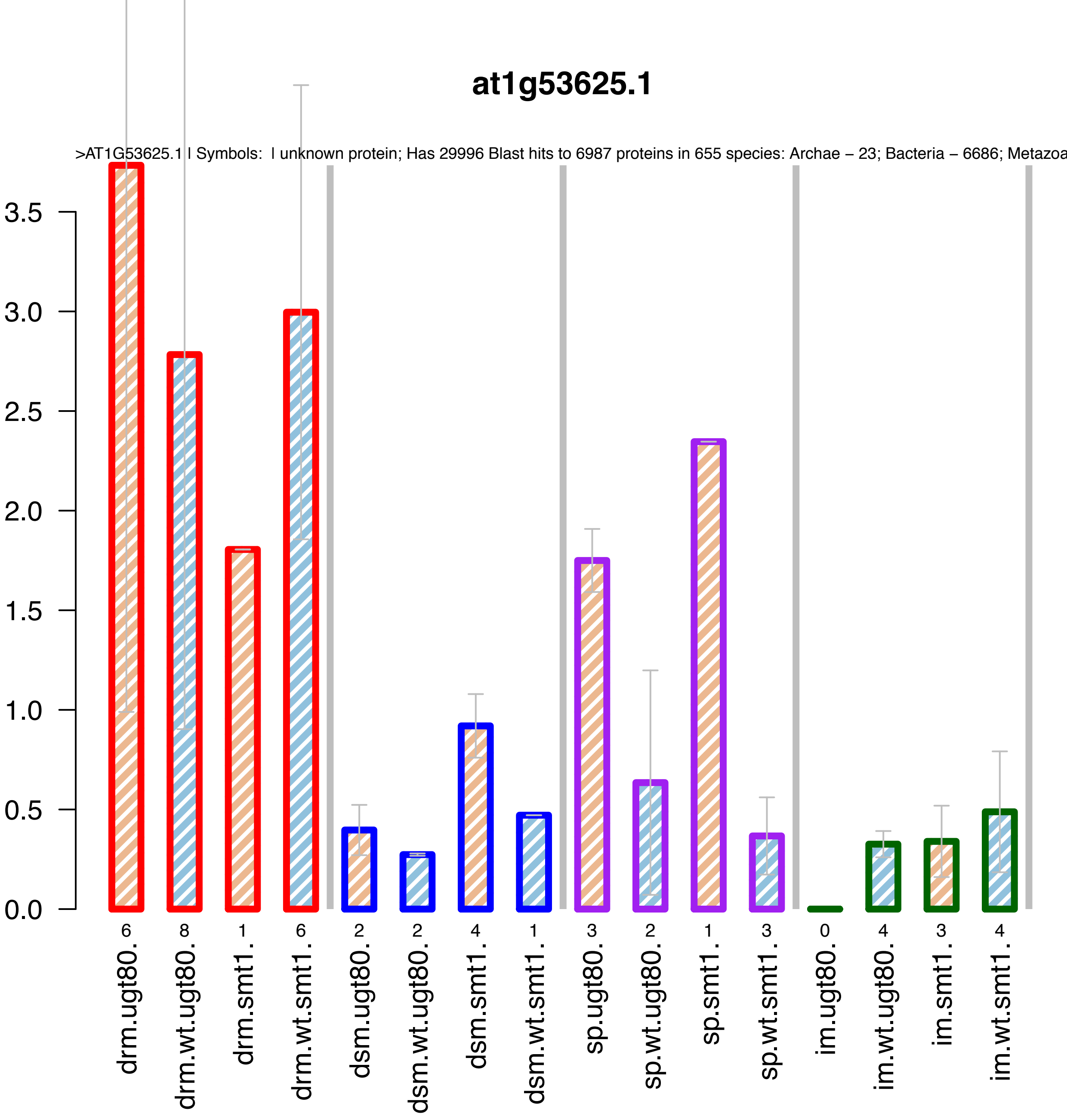

at1g53625.1
>AT1G53625.1 | Symbols: | unknown protein; Has 29996 Blast hits to 6987 proteins in 655 species: Archae – 23; Bacteria – 6686; Metazoa
3.5
3.0
2.5
2.0
1.5
1.0
0.5
0.0
6
8
1
6
2
2
4
1
3
2
1
3
0
4
3
4
drm.ugt80.
drm.wt.ugt80.
drm.smt1.
drm.wt.smt1.
dsm.ugt80.
dsm.wt.ugt80.
dsm.smt1.
dsm.wt.smt1.
sp.ugt80.
sp.wt.ugt80.
sp.smt1.
sp.wt.smt1.
im.ugt80.
im.wt.ugt80.
im.smt1.
im.wt.smt1.

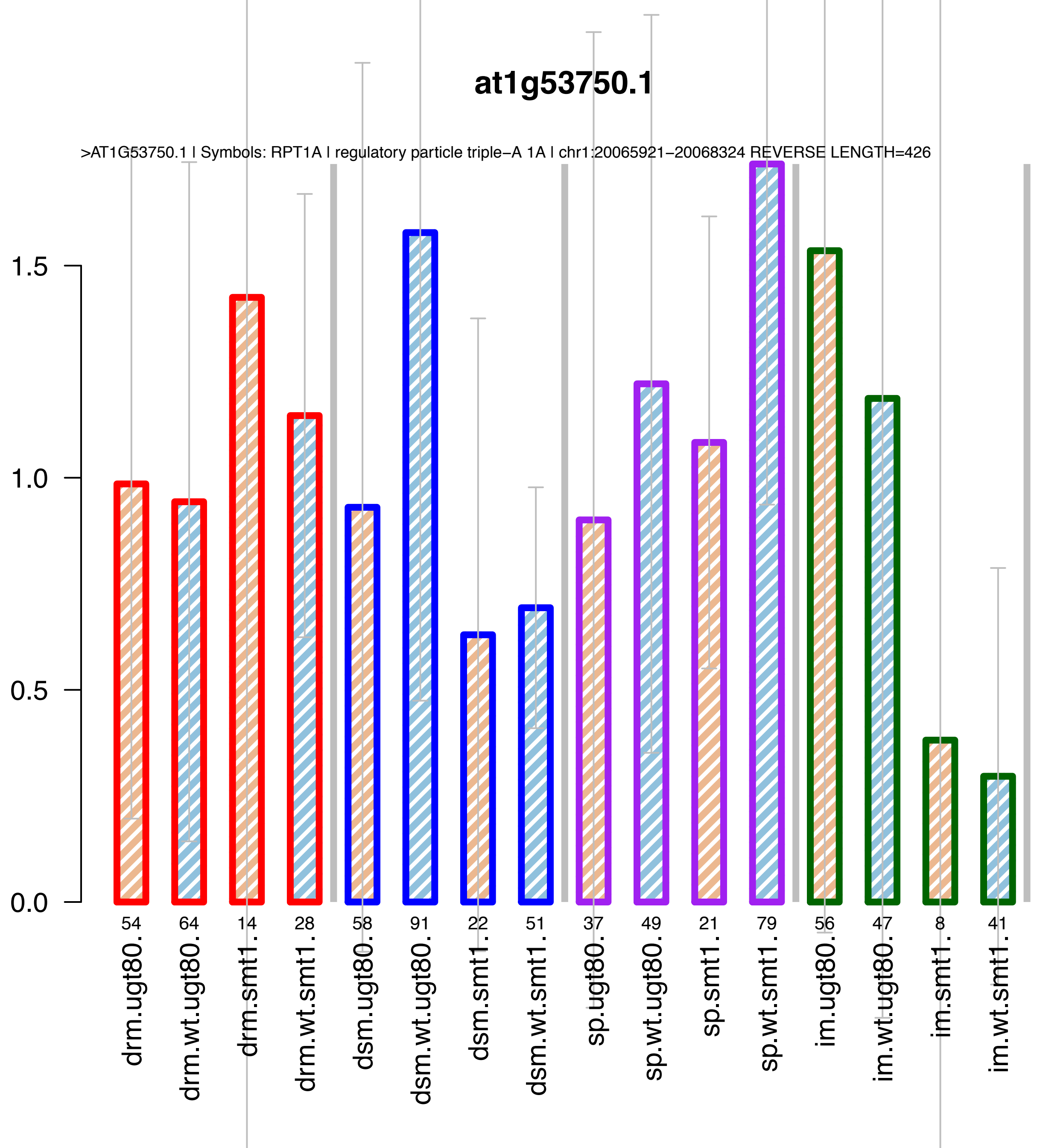

at1g53750.1
>AT1G53750.1 | Symbols: RPT1A | regulatory particle triple−A 1A | chr1:20065921−20068324 REVERSE LENGTH=426
1.5
1.0
0.5
0.0
54
64
14
28
58
91
22
51
37
49
21
79
56
47
8
41
drm.ugt80..
drm.wt.ugt80..
drm.smt1..
drm.wt.smt1..
dsm.ugt80..
dsm.wt.ugt80..
dsm.smt1..
dsm.wt.smt1..
sp.ugt80..
sp.wt.ugt80..
sp.smt1..
sp.wt.smt1..
im.ugt80..
im.wt.ugt80..
im.smt1..
im.wt.smt1..

# at1g53760.1

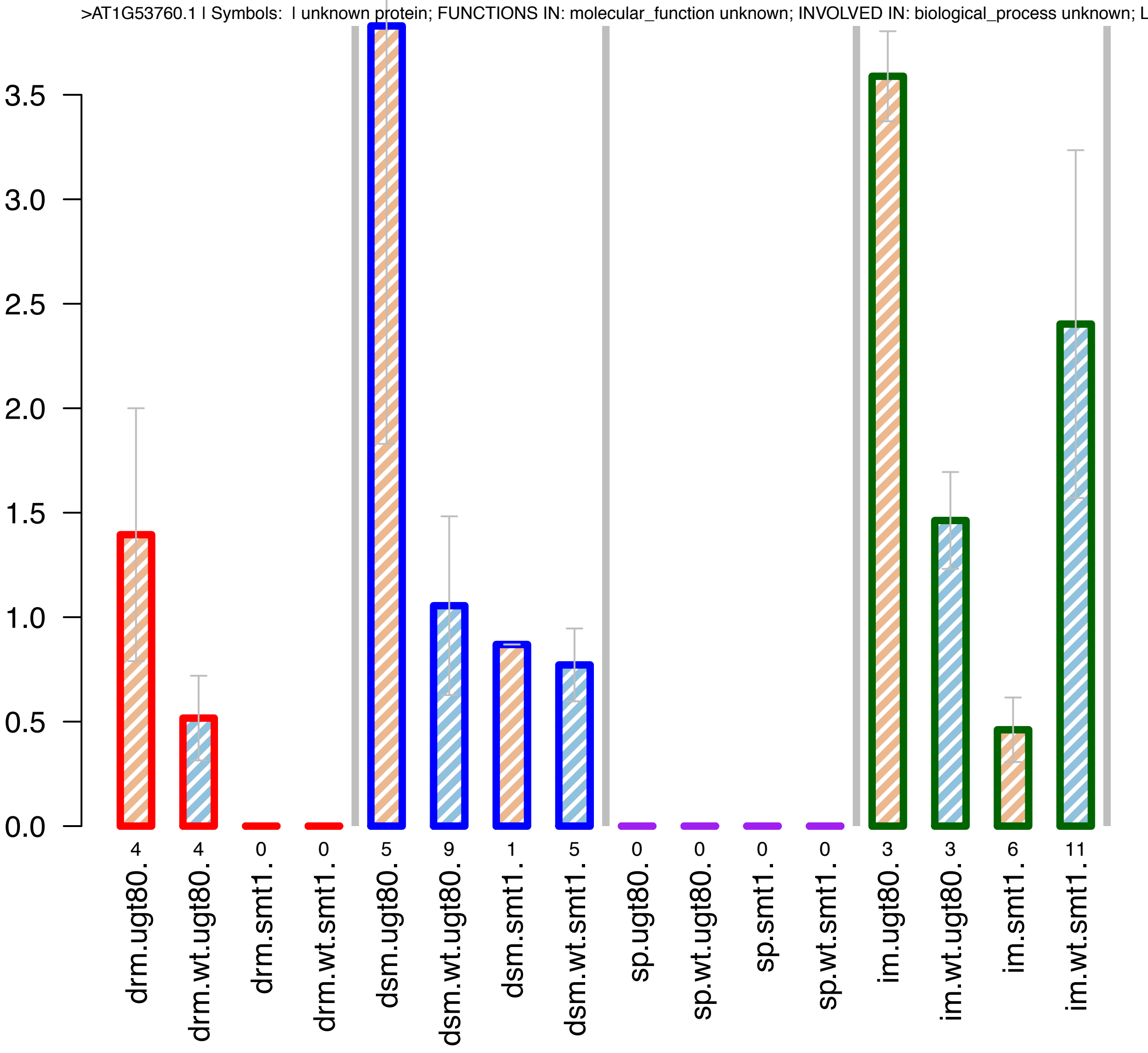

>AT1G53760.1 | Symbols: | unknown protein; FUNCTIONS IN: molecular_function unknown; INVOLVED IN: biological_process unknown; L
0.0
0.5
1.0
1.5
2.0
2.5
3.0
3.5
4
4
0
0
5
9
1
5
0
0
0
0
3
3
6
11
drm.ugt80..
drm.wt.ugt80..
drm.smt1..
drm.wt.smt1..
dsm.ugt80..
dsm.wt.ugt80..
dsm.smt1..
dsm.wt.smt1..
sp.ugt80..
sp.wt.ugt80..
sp.smt1..
sp.wt.smt1..
im.ugt80..
im.wt.ugt80..
im.smt1..
im.wt.smt1..

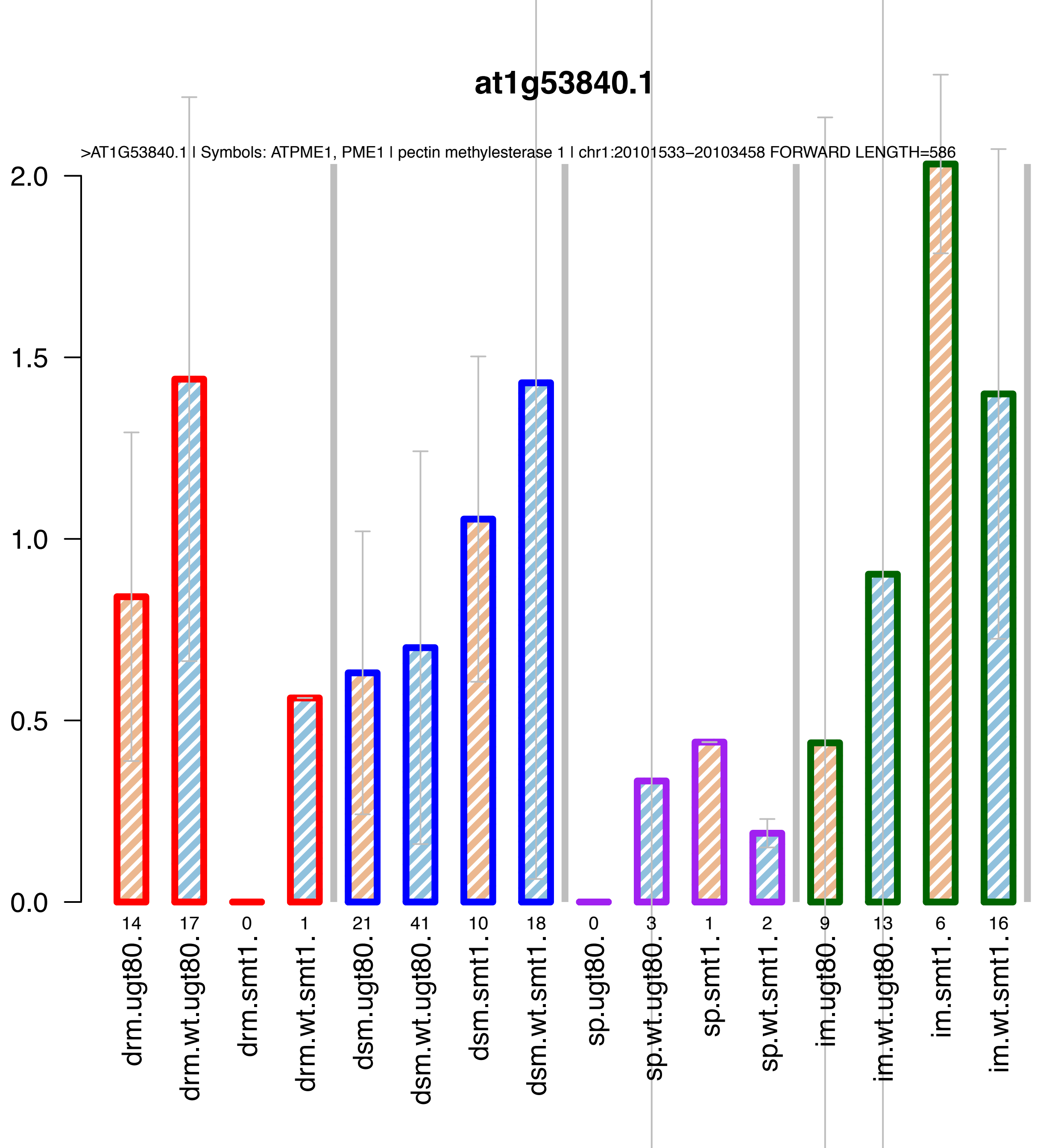

at1g53840.1
>AT1G53840.1 | Symbols: ATPME1, PME1 | pectin methylesterase 1 | chr1:20101533–20103458 FORWARD LENGTH=586
2.0
1.5
1.0
0.5
0.0
14
17
0
1
21
41
10
18
0
3
1
2
9
13
6
16
drm.ugt80..
drm.wt.ugt80..
drm.smt1..
drm.wt.smt1..
dsm.ugt80..
dsm.wt.ugt80..
dsm.smt1..
dsm.wt.smt1..
sp.ugt80..
sp.wt.ugt80..
sp.smt1..
sp.wt.smt1..
im.ugt80..
im.wt.ugt80..
im.smt1..
im.wt.smt1..

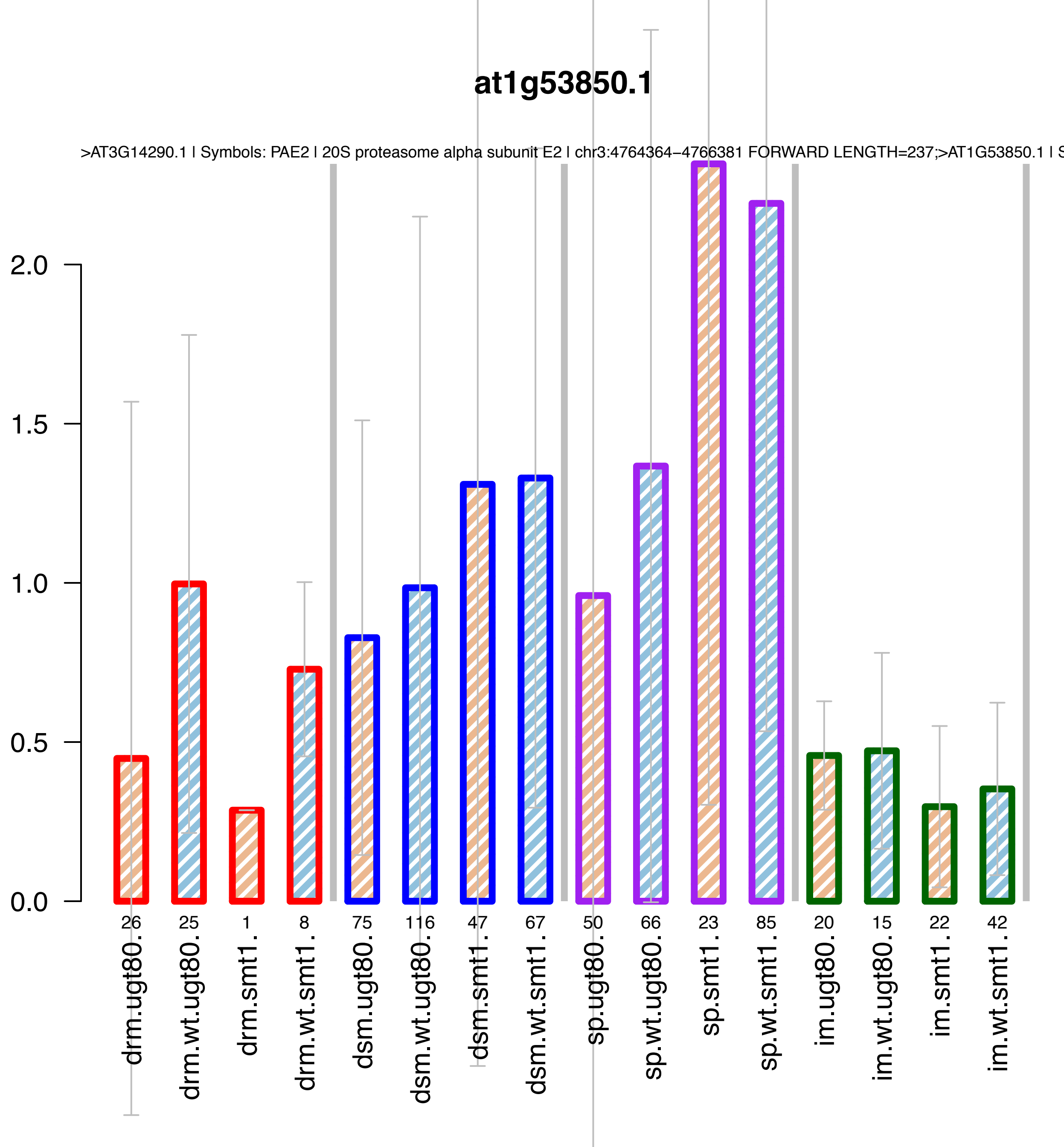

at1g53850.1
>AT3G14290.1 | Symbols: PAE2 | 20S proteasome alpha subunit E2 | chr3:4764364−4766381 FORWARD LENGTH=237;>AT1G53850.1 | S
2.0
1.5
1.0
0.5
0.0
26
25
1
8
75
116
47
67
50
66
23
85
20
15
22
42
drm.ugt80..
drm.wt.ugt80..
drm.smt1..
drm.wt.smt1..
dsm.ugt80..
dsm.wt.ugt80..
dsm.smt1..
dsm.wt.smt1..
sp.ugt80..
sp.wt.ugt80..
sp.smt1..
sp.wt.smt1..
im.ugt80..
im.wt.ugt80..
im.smt1..
im.wt.smt1..

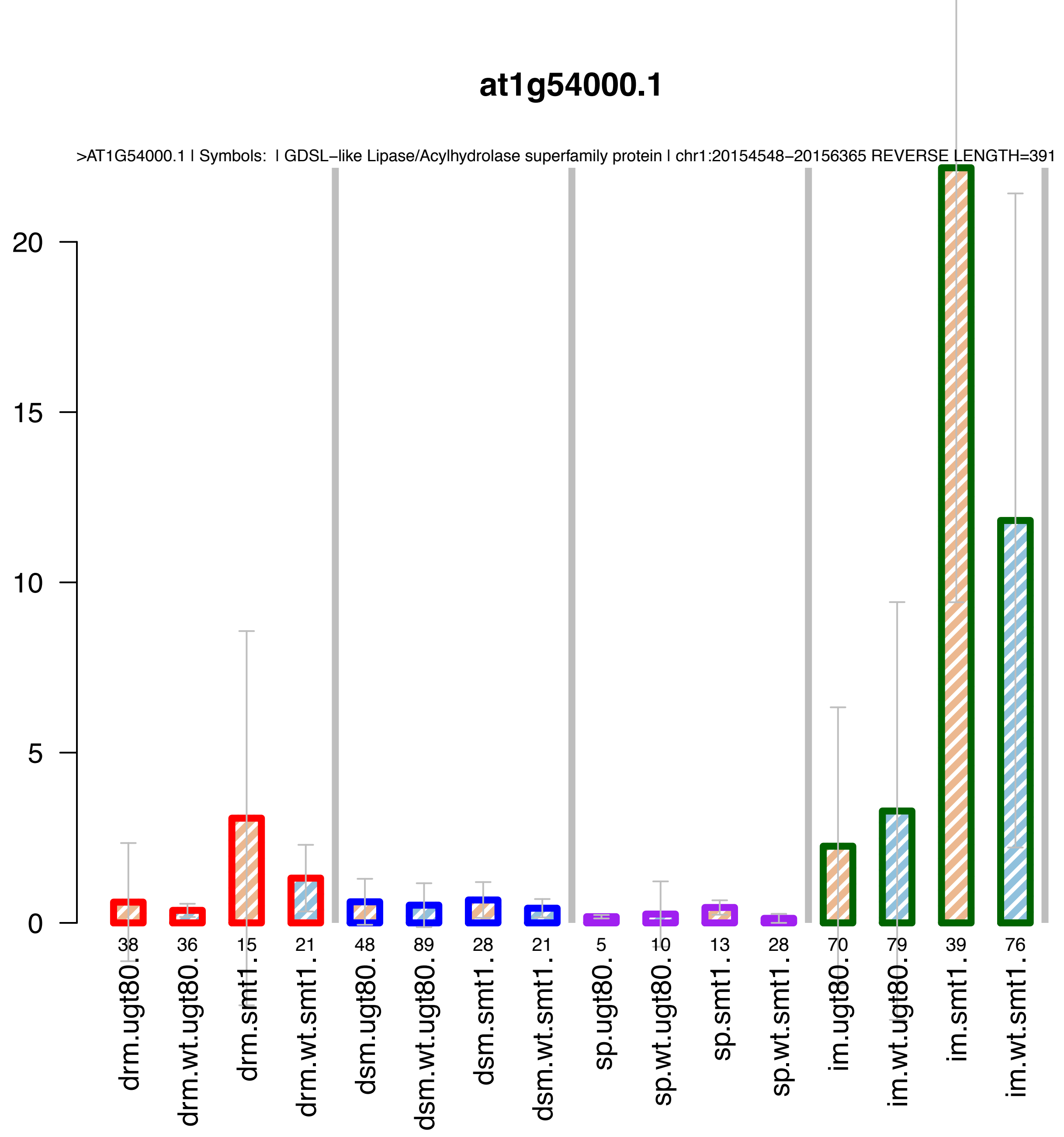

at1g54000.1
>AT1G54000.1 | Symbols: | GDSL−like Lipase/Acylhydrolase superfamily protein | chr1:20154548−20156365 REVERSE LENGTH=391
0
5
10
15
20
38
36
15
21
48
89
28
21
5
10
13
28
70
79
39
76
drm.ugt80.
drm.wt.ugt80.
drm.smt1.
drm.wt.smt1.
dsm.ugt80.
dsm.wt.ugt80.
dsm.smt1.
dsm.wt.smt1.
sp.ugt80.
sp.wt.ugt80.
sp.smt1.
sp.wt.smt1.
im.ugt80.
im.wt.ugt80.
im.smt1.
im.wt.smt1.

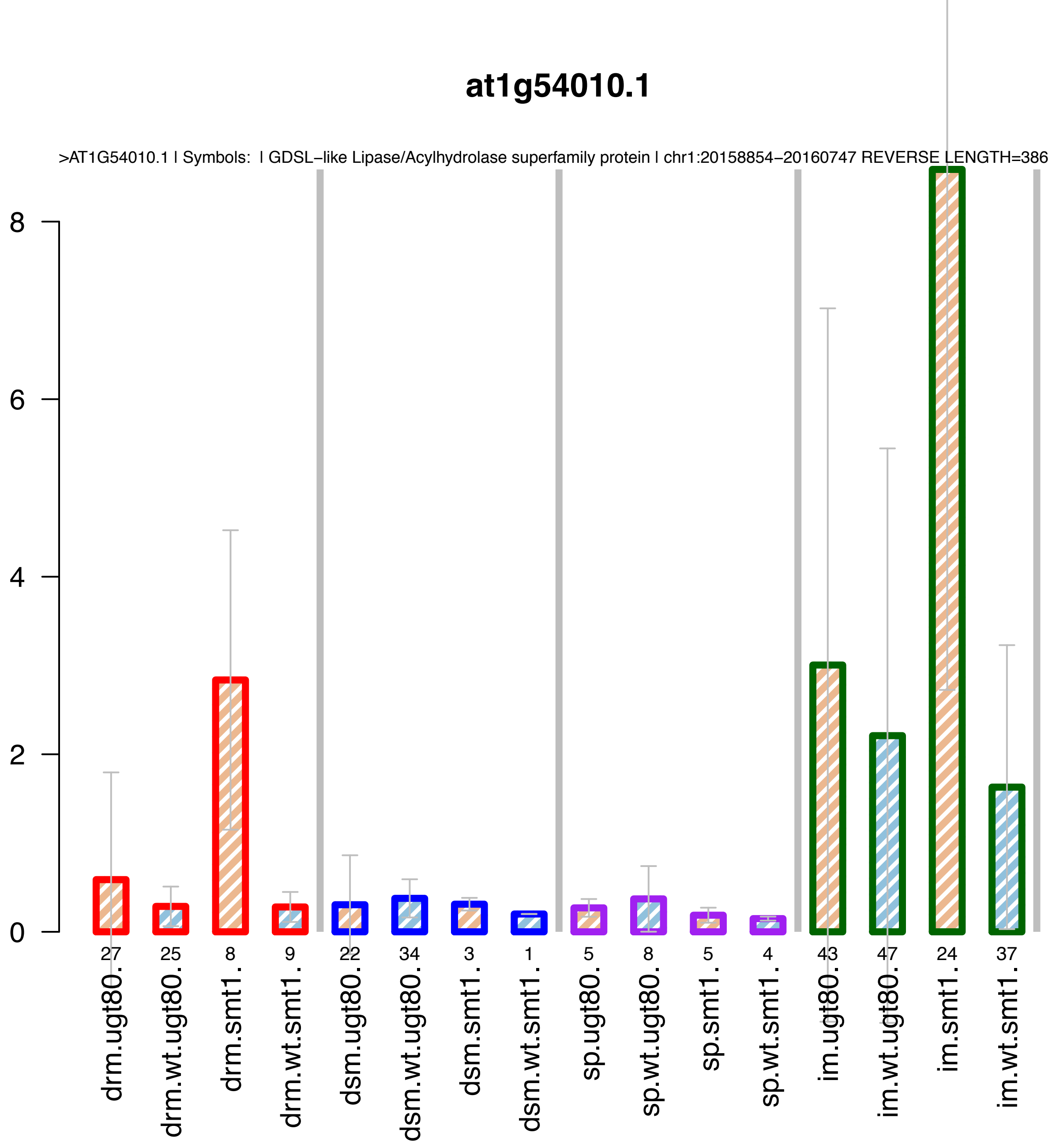
at1g54010.1
>AT1G54010.1 | Symbols: | GDSL−like Lipase/Acylhydrolase superfamily protein | chr1:20158854−20160747 REVERSE LENGTH=386
0
2
4
6
8
27
25
8
9
22
34
3
1
5
8
5
4
43
47
24
37
drm.ugt80.
drm.wt.ugt80.
drm.smt1.
drm.wt.smt1.
dsm.ugt80.
dsm.wt.ugt80.
dsm.smt1.
dsm.wt.smt1.
sp.ugt80.
sp.wt.ugt80.
sp.smt1.
sp.wt.smt1.
im.ugt80.
im.wt.ugt80.
im.smt1.
im.wt.smt1.

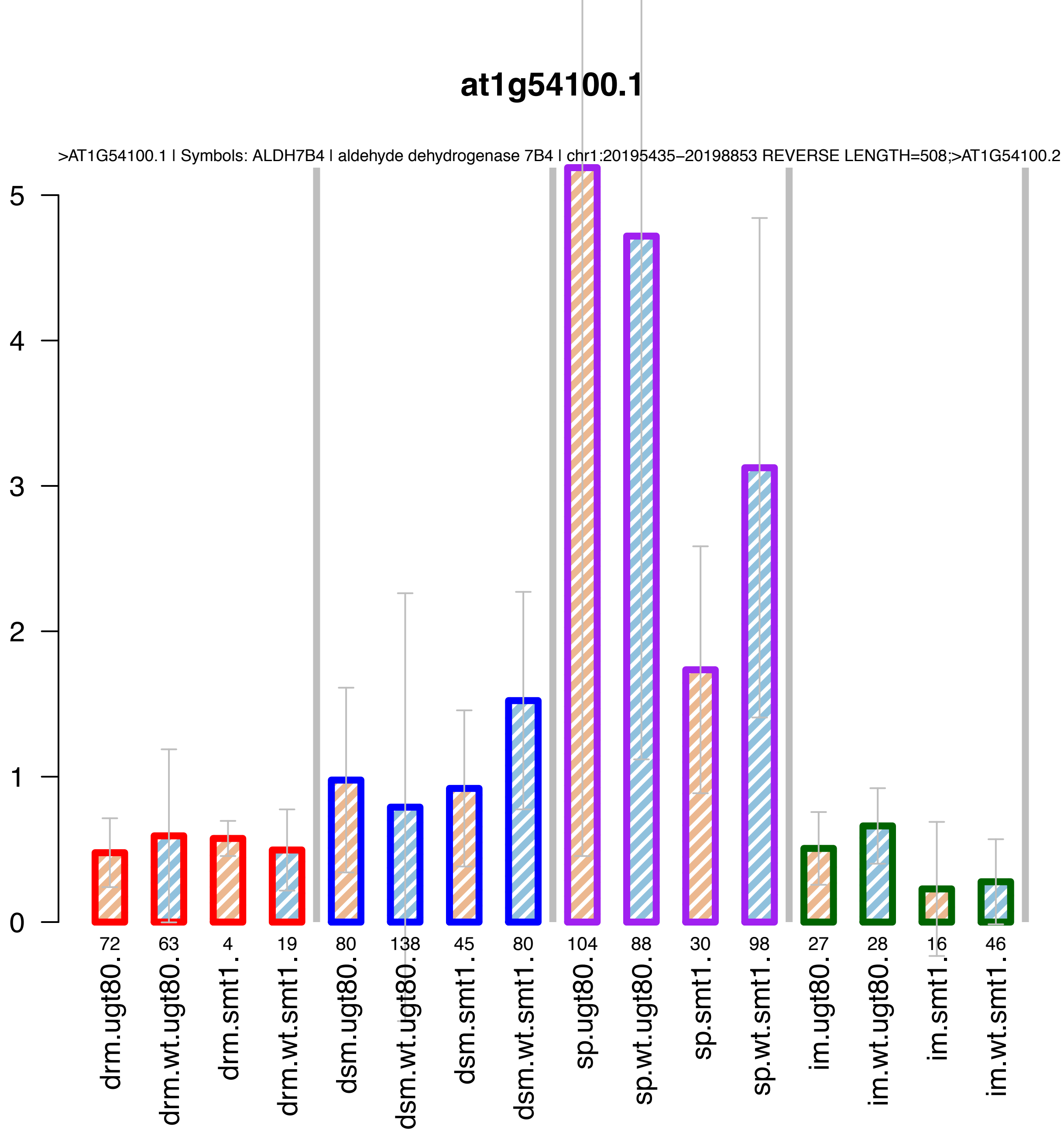

at1g54100.1
>AT1G54100.1 | Symbols: ALDH7B4 | aldehyde dehydrogenase 7B4 | chr1:20195435−20198853 REVERSE LENGTH=508;>AT1G54100.2
0
1
2
3
4
5
72
63
4
19
80
138
45
80
104
88
30
98
27
28
16
46
drm.ugt80.
drm.wt.ugt80.
drm.smt1.
drm.wt.smt1.
dsm.ugt80.
dsm.wt.ugt80.
dsm.smt1.
dsm.wt.smt1.
sp.ugt80.
sp.wt.ugt80.
sp.smt1.
sp.wt.smt1.
im.ugt80.
im.wt.ugt80.
im.smt1.
im.wt.smt1.

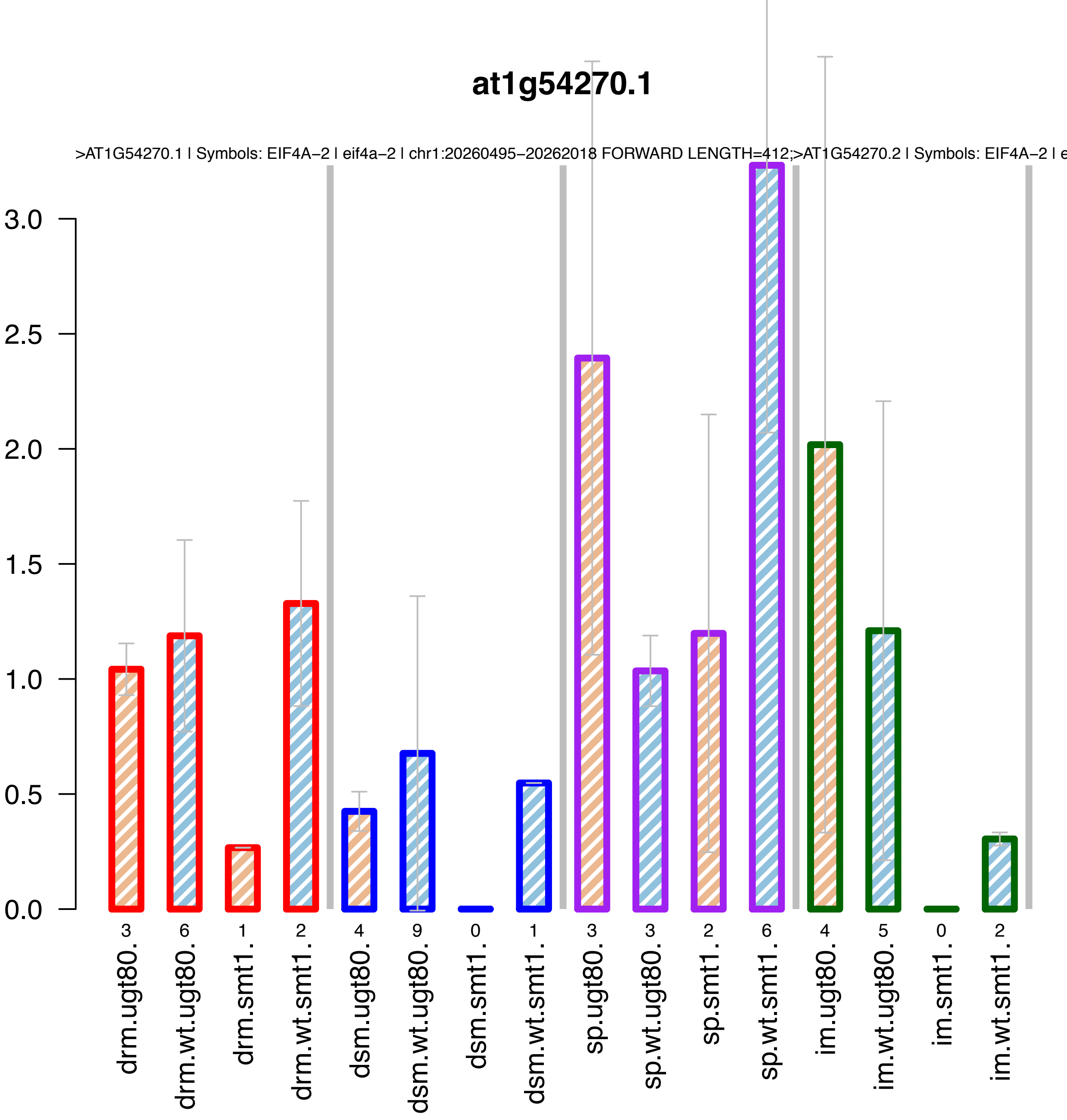
at1g54270.1
>AT1G54270.1 | Symbols: EIF4A−2 | eif4a−2 | chr1:20260495−20262018 FORWARD LENGTH=412;>AT1G54270.2 | Symbols: EIF4A−2 | e
3.0
2.5
2.0
1.5
1.0
0.5
0.0
3 6 1 2 4 9 0 1 3 3 2 6 4 5 0 2
drm.ugt80..
drm.wt.ugt80..
drm.smt1..
drm.wt.smt1..
dsm.ugt80..
dsm.wt.ugt80..
dsm.smt1..
dsm.wt.smt1..
sp.ugt80..
sp.wt.ugt80..
sp.smt1..
sp.wt.smt1..
im.ugt80..
im.wt.ugt80..
im.smt1..
im.wt.smt1..

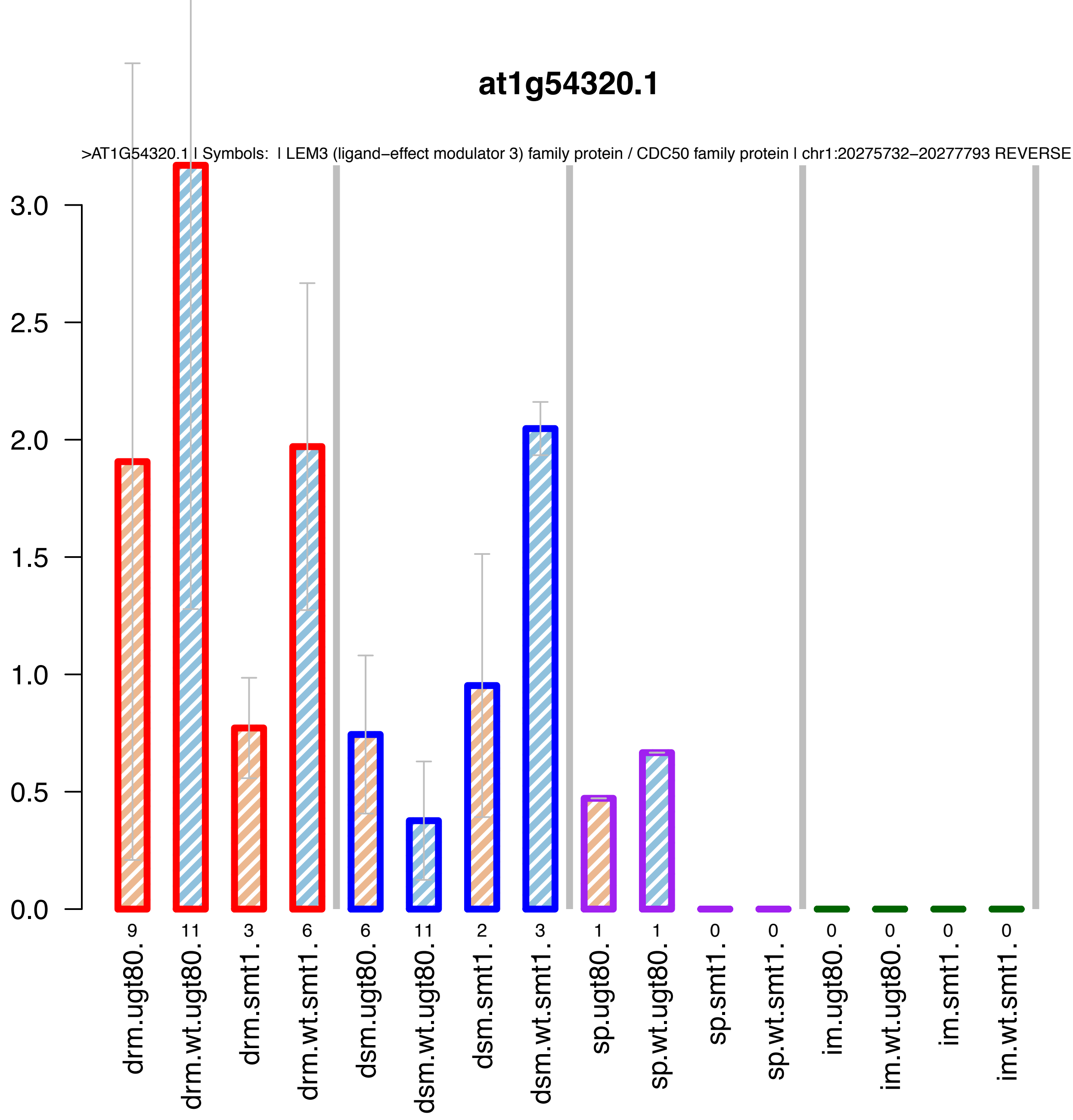

at1g54320.1
>AT1G54320.1 | Symbols: | LEM3 (ligand–effect modulator 3) family protein / CDC50 family protein | chr1:20275732–20277793 REVERSE
0.0
0.5
1.0
1.5
2.0
2.5
3.0
9
11
3
6
6
11
2
3
1
1
0
0
0
0
0
0
drm.ugt80.
drm.wt.ugt80.
drm.smt1.
drm.wt.smt1.
dsm.ugt80.
dsm.wt.ugt80.
dsm.smt1.
dsm.wt.smt1.
sp.ugt80.
sp.wt.ugt80.
sp.smt1.
sp.wt.smt1.
im.ugt80.
im.wt.ugt80.
im.smt1.
im.wt.smt1.

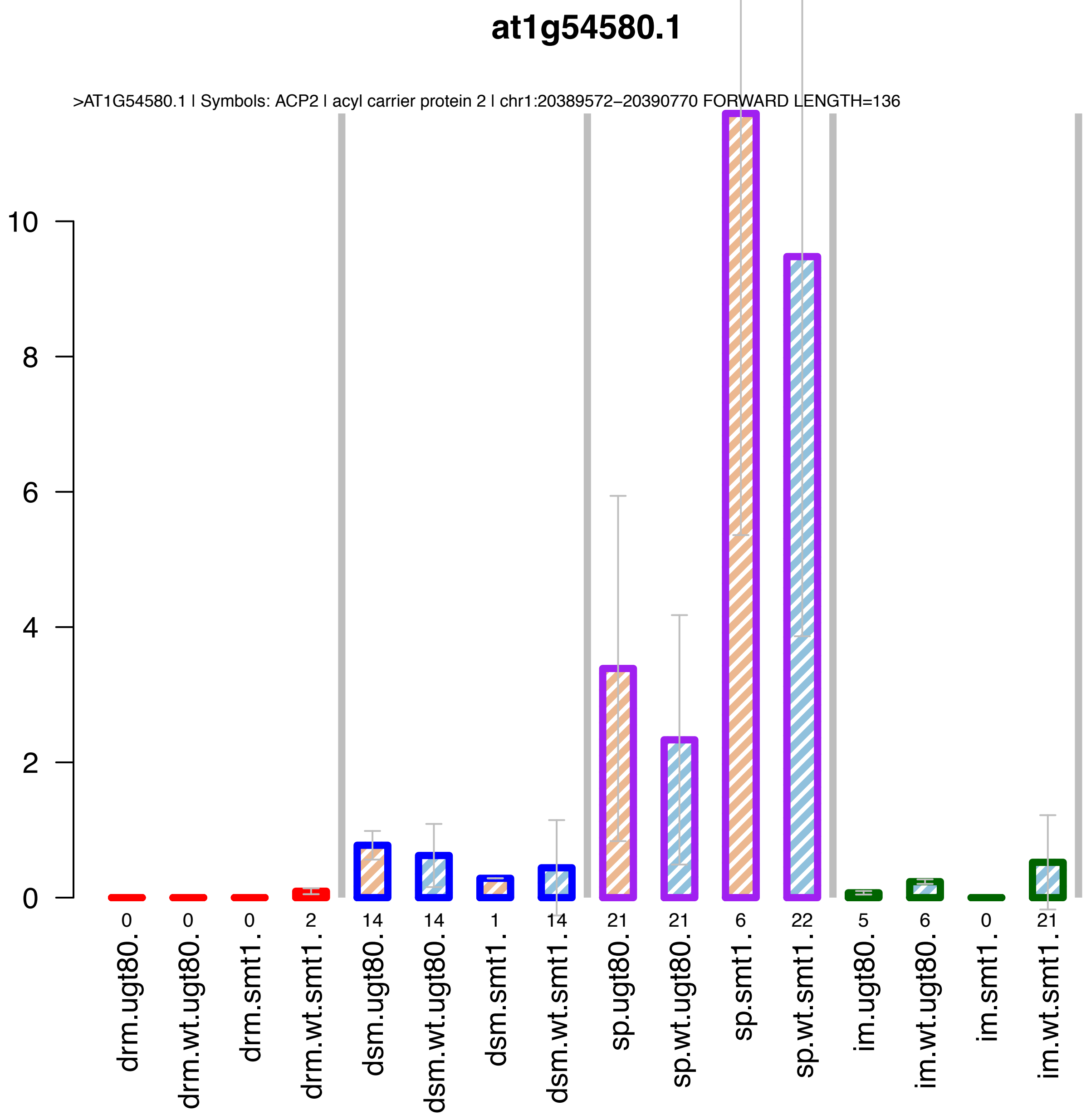

at1g54580.1
>AT1G54580.1 | Symbols: ACP2 | acyl carrier protein 2 | chr1:20389572-20390770 FORWARD LENGTH=136
0
2
4
6
8
10
0 0 0 2 14 14 1 14 21 21 6 22 5 6 0 21
drm.ugt80.
drm.wt.ugt80.
drm.smt1.
drm.wt.smt1.
dsm.ugt80.
dsm.wt.ugt80.
dsm.smt1.
dsm.wt.smt1.
sp.ugt80.
sp.wt.ugt80.
sp.smt1.
sp.wt.smt1.
im.ugt80.
im.wt.ugt80.
im.smt1.
im.wt.smt1.

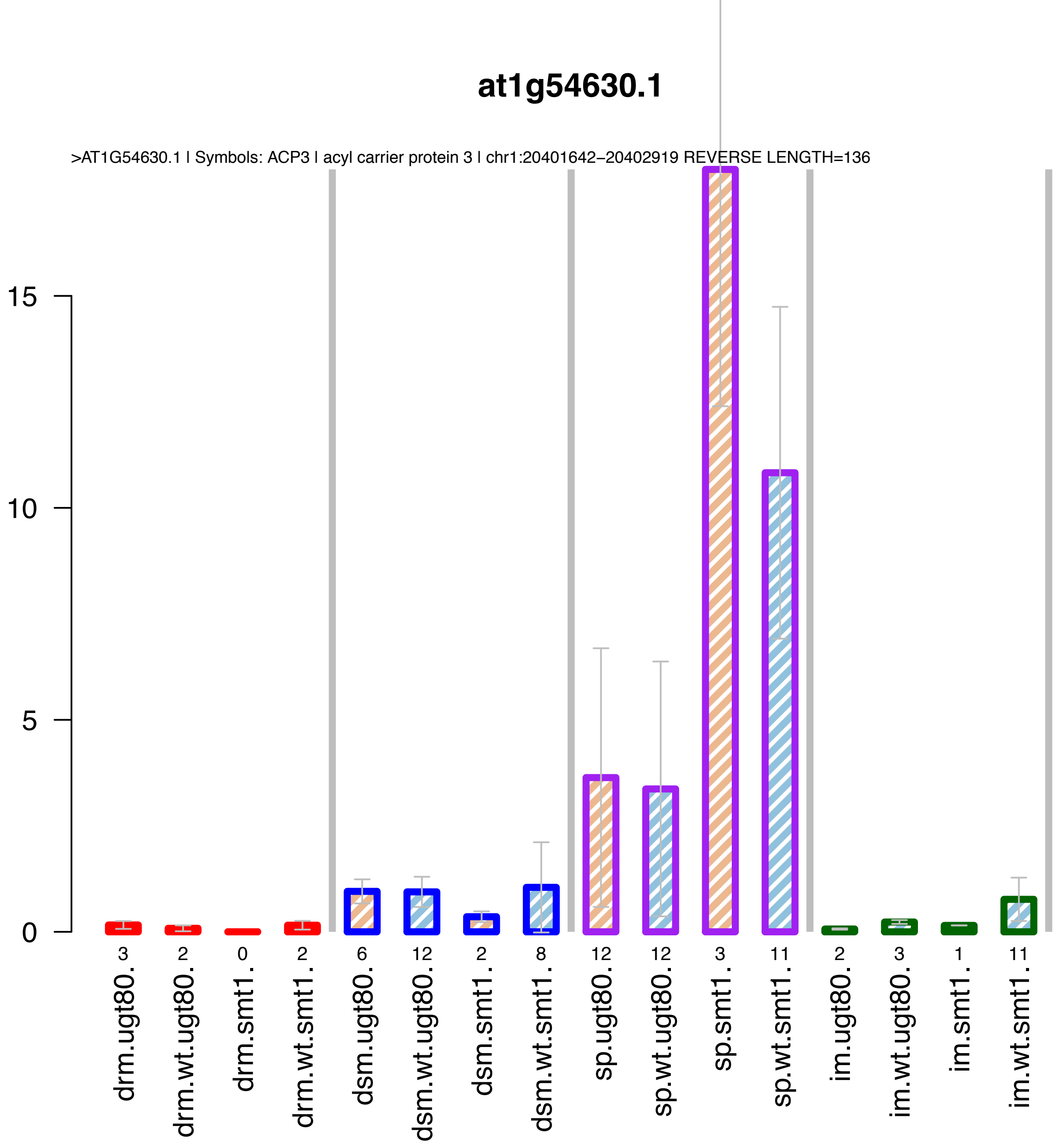

at1g54630.1
>AT1G54630.1 | Symbols: ACP3 | acyl carrier protein 3 | chr1:20401642−20402919 REVERSE LENGTH=136
15
10
5
0
3 2 0 2 6 12 2 8 12 12 3 11 2 3 1 11
drm.ugt80..
drm.wt.ugt80..
drm.smt1..
drm.wt.smt1..
dsm.ugt80..
dsm.wt.ugt80..
dsm.smt1..
dsm.wt.smt1..
sp.ugt80..
sp.wt.ugt80..
sp.smt1..
sp.wt.smt1..
im.ugt80..
im.wt.ugt80..
im.smt1..
im.wt.smt1..

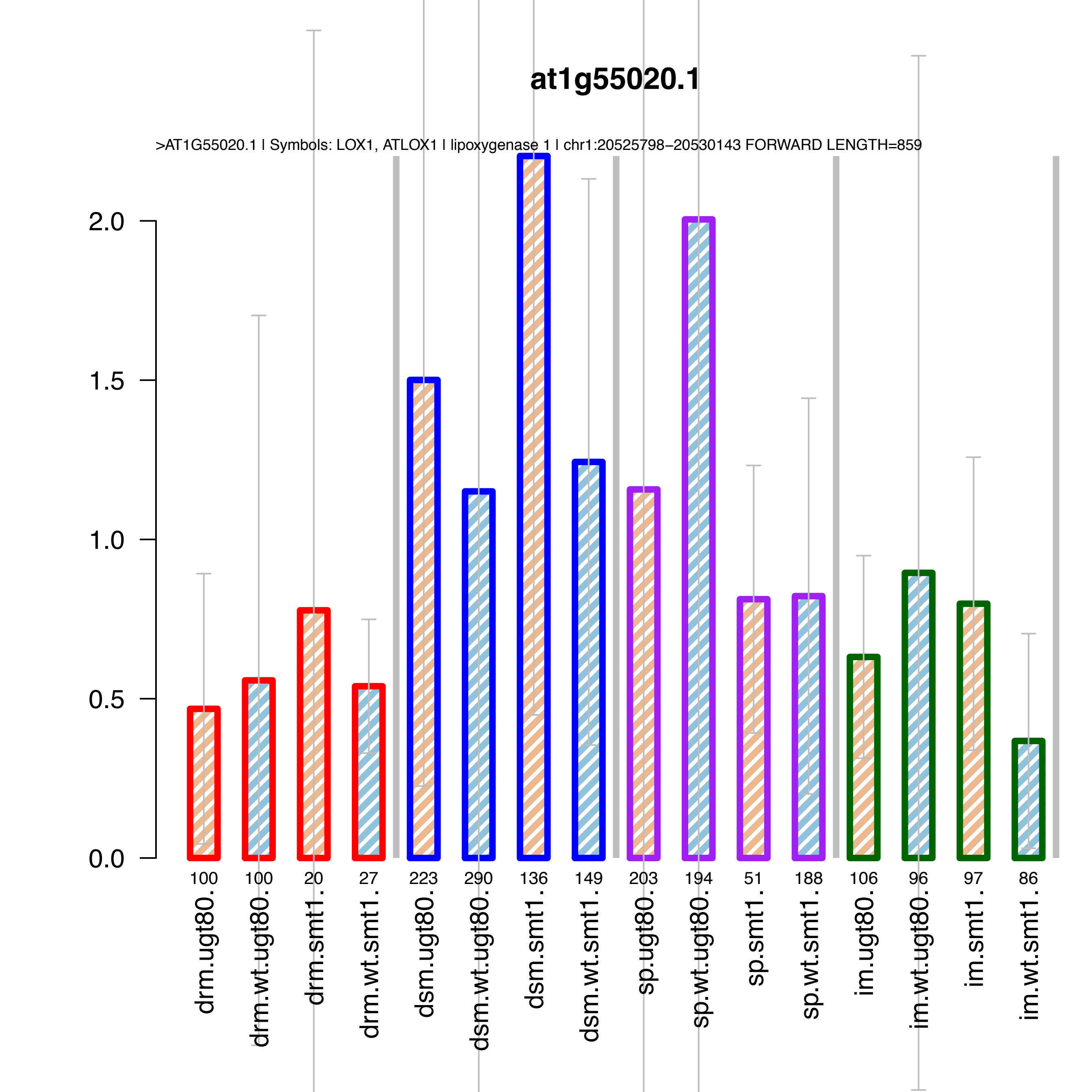
at1g55020.1
>AT1G55020.1 | Symbols: LOX1, ATLOX1 | lipoxygenase 1 | chr1:20525798-20530143 FORWARD LENGTH=859
0.0
0.5
1.0
1.5
2.0
100
100
20
27
223
290
136
149
203
194
51
188
106
96
97
86
drm.ugt80.
drm.wt.ugt80.
drm.smt1.
drm.wt.smt1.
dsm.ugt80.
dsm.wt.ugt80.
dsm.smt1.
dsm.wt.smt1.
sp.ugt80.
sp.wt.ugt80.
sp.smt1.
sp.wt.smt1.
im.ugt80.
im.wt.ugt80.
im.smt1.
im.wt.smt1.

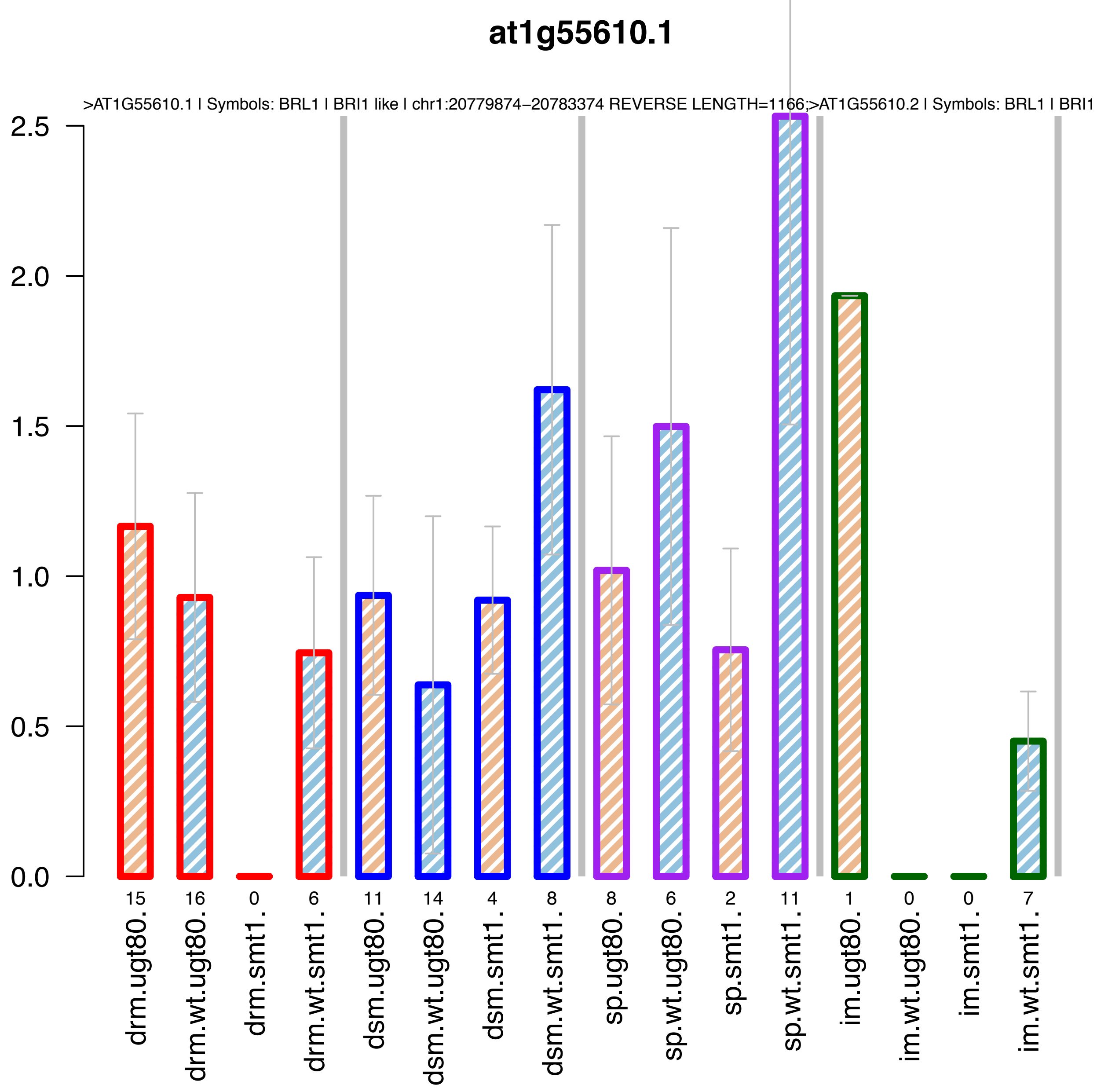

at1g55610.1
>AT1G55610.1 | Symbols: BRL1 | BRI1 like | chr1:20779874-20783374 REVERSE LENGTH=1166;>AT1G55610.2 | Symbols: BRL1 | BRI1
2.5
2.0
1.5
1.0
0.5
0.0
15
16
0
6
11
14
4
8
8
6
2
11
1
0
0
7
drm.ugt80.
drm.wt.ugt80.
drm.smt1.
drm.wt.smt1.
dsm.ugt80.
dsm.wt.ugt80.
dsm.smt1.
dsm.wt.smt1.
sp.ugt80.
sp.wt.ugt80.
sp.smt1.
sp.wt.smt1.
im.ugt80.
im.wt.ugt80.
im.smt1.
im.wt.smt1.

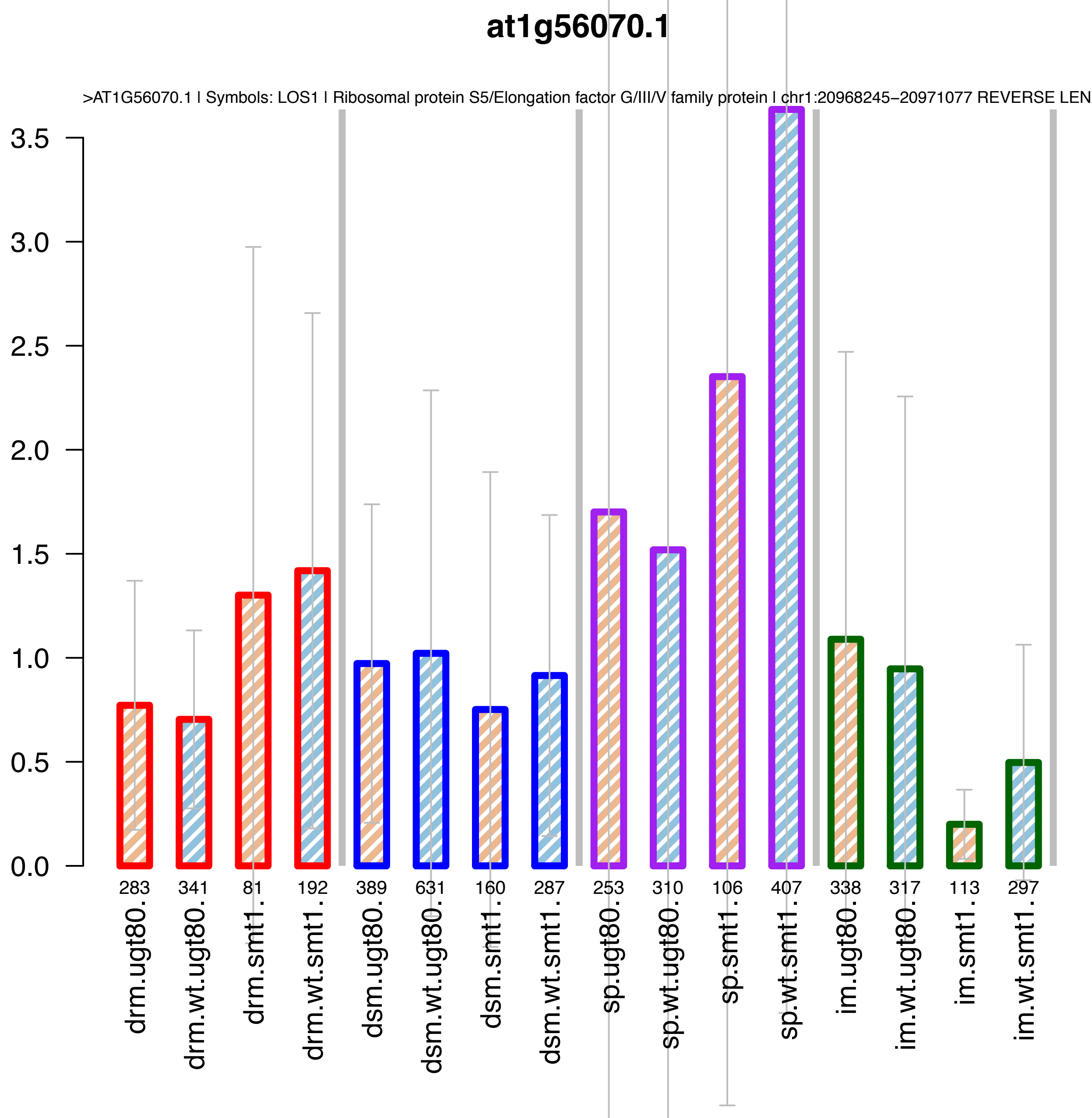

at1g56070.1
>AT1G56070.1 | Symbols: LOS1 | Ribosomal protein S5/Elongation factor G/III/V family protein | chr1:20968245-20971077 REVERSE LEN
3.5
3.0
2.5
2.0
1.5
1.0
0.5
0.0
283
341
81
192
389
631
160
287
253
310
106
407
338
317
113
297
drm.ugt80..
drm.wt.ugt80..
drm.smt1..
drm.wt.smt1..
dsm.ugt80..
dsm.wt.ugt80..
dsm.smt1..
dsm.wt.smt1..
sp.ugt80..
sp.wt.ugt80..
sp.smt1..
sp.wt.smt1..
im.ugt80..
im.wt.ugt80..
im.smt1..
im.wt.smt1..

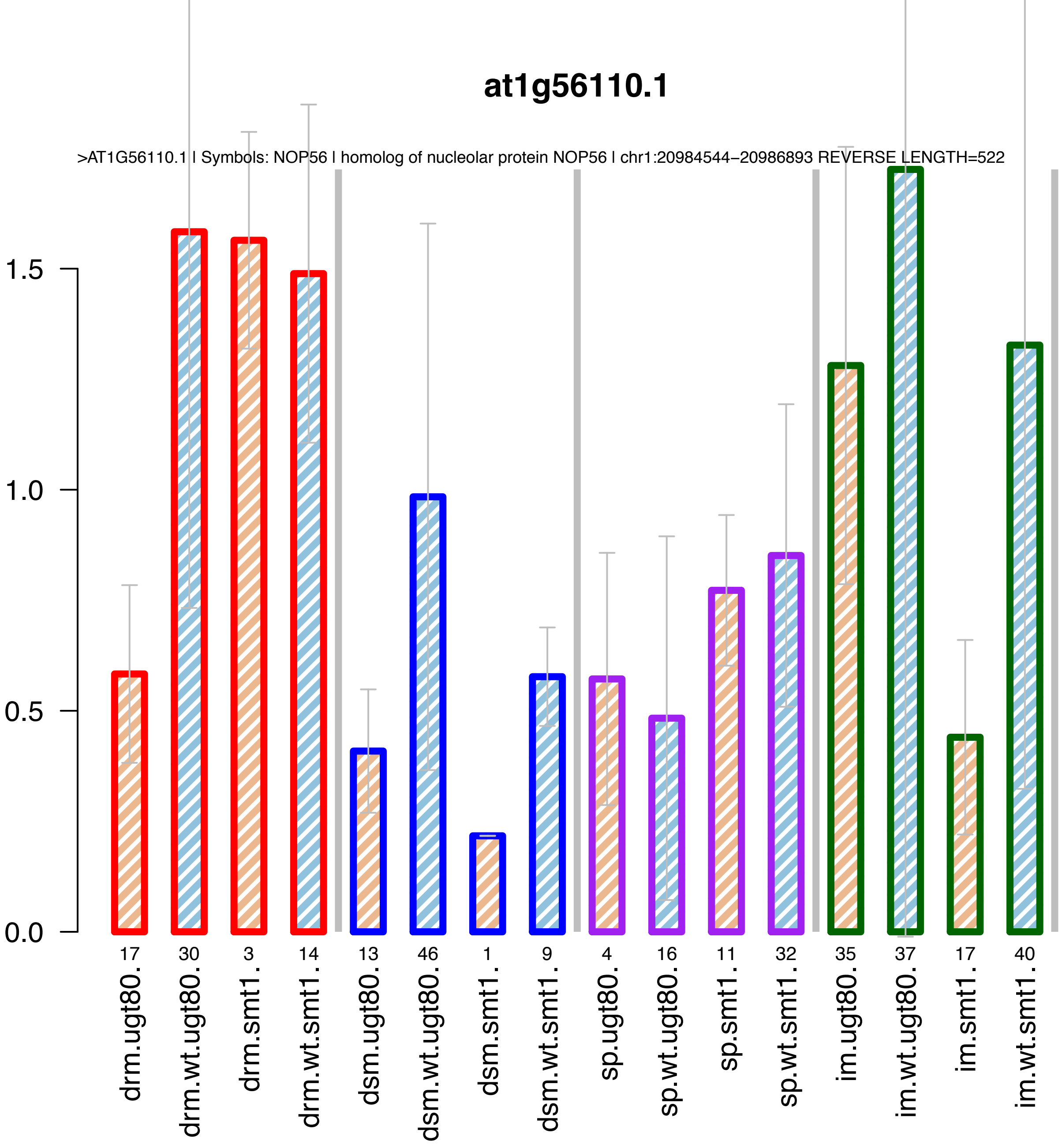

at1g56110.1
>AT1G56110.1 | Symbols: NOP56 | homolog of nucleolar protein NOP56 | chr1:20984544-20986893 REVERSE LENGTH=522
0.0
0.5
1.0
1.5
17
30
3
14
13
46
1
9
4
16
11
32
35
37
17
40
drm.ugt80.
drm.wt.ugt80.
drm.smt1.
drm.wt.smt1.
dsm.ugt80.
dsm.wt.ugt80.
dsm.smt1.
dsm.wt.smt1.
sp.ugt80.
sp.wt.ugt80.
sp.smt1.
sp.wt.smt1.
im.ugt80.
im.wt.ugt80.
im.smt1.
im.wt.smt1.

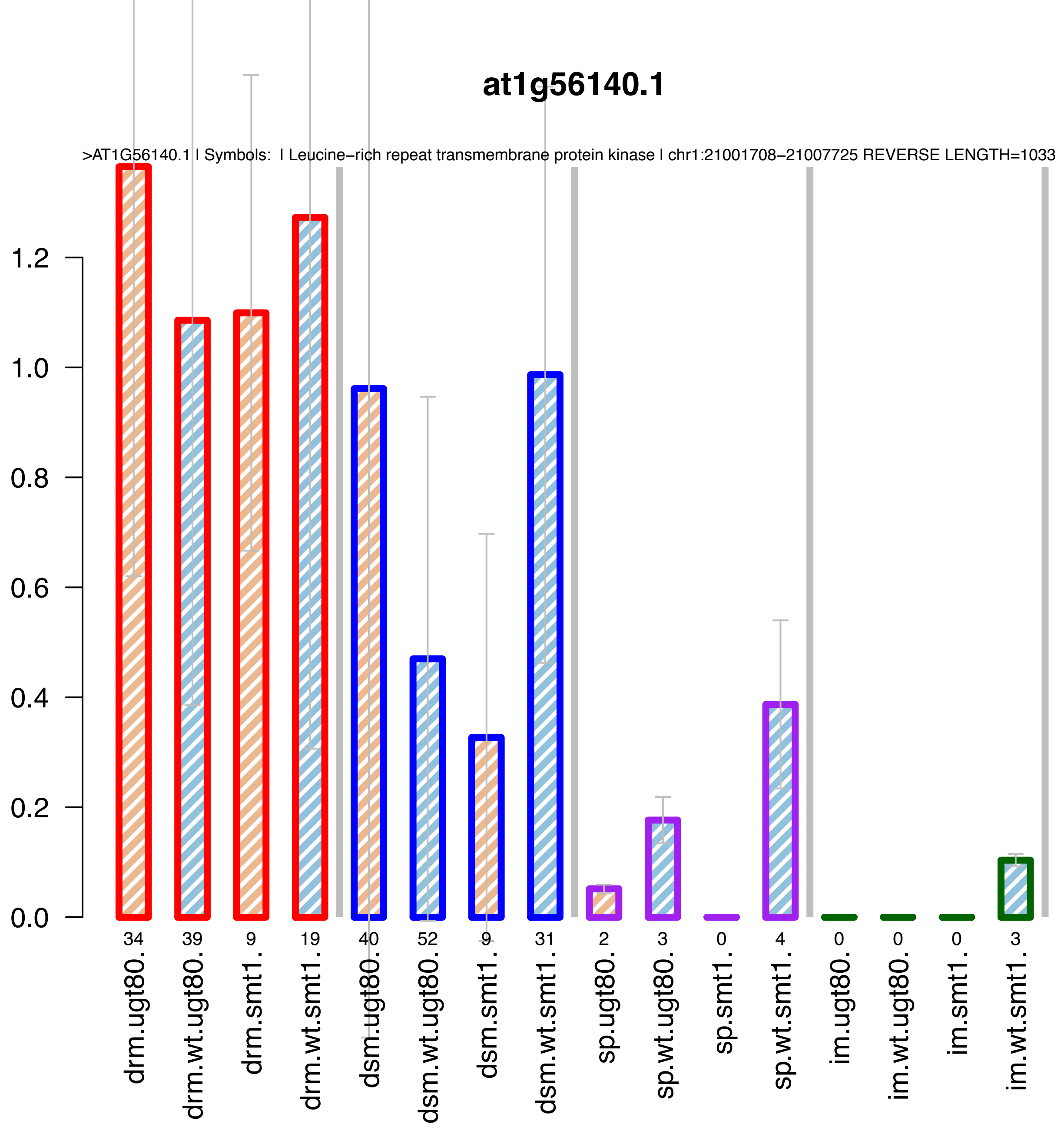

at1g56140.1
>AT1G56140.1 | Symbols: | Leucine−rich repeat transmembrane protein kinase | chr1:21001708−21007725 REVERSE LENGTH=1033
0.0
0.2
0.4
0.6
0.8
1.0
1.2
34
39
9
19
40
52
9
31
2
3
0
4
0
0
0
3
drm.ugt80..
drm.wt.ugt80..
drm.smt1..
drm.wt.smt1..
dsm.ugt80..
dsm.wt.ugt80..
dsm.smt1..
dsm.wt.smt1..
sp.ugt80..
sp.wt.ugt80..
sp.smt1..
sp.wt.smt1..
im.ugt80..
im.wt.ugt80..
im.smt1..
im.wt.smt1..

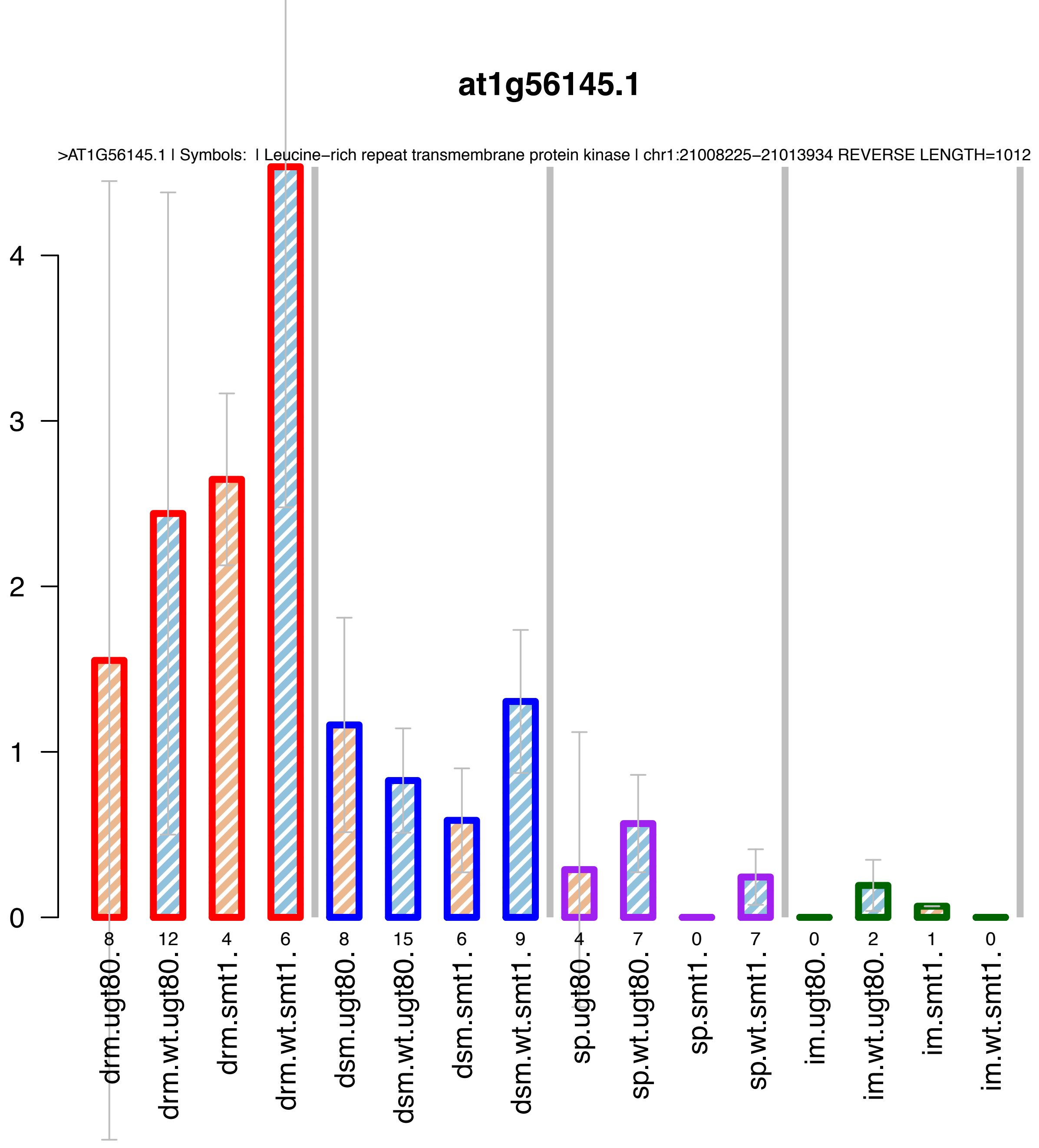

at1g56145.1
>AT1G56145.1 | Symbols: | Leucine−rich repeat transmembrane protein kinase | chr1:21008225−21013934 REVERSE LENGTH=1012
0
1
2
3
4
8
12
4
6
8
15
6
9
4
7
0
7
0
2
1
0
drm.ugt80.
drm.wt.ugt80.
drm.smt1.
drm.wt.smt1.
dsm.ugt80.
dsm.wt.ugt80.
dsm.smt1.
dsm.wt.smt1.
sp.ugt80.
sp.wt.ugt80.
sp.smt1.
sp.wt.smt1.
im.ugt80.
im.wt.ugt80.
im.smt1.
im.wt.smt1.

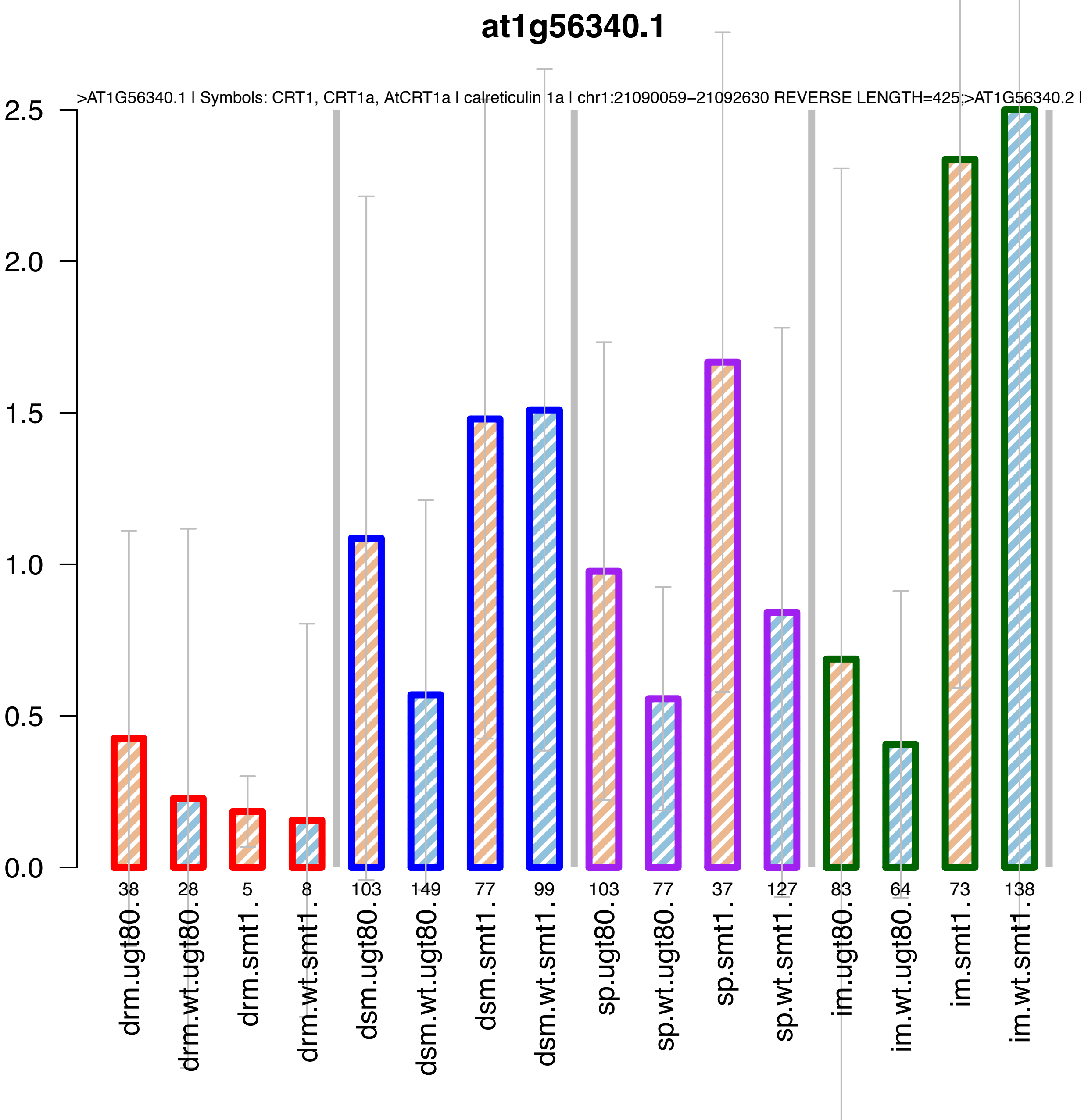
at1g56340.1
>AT1G56340.1 | Symbols: CRT1, CRT1a, AtCRT1a | calreticulin 1a | chr1:21090059-21092630 REVERSE LENGTH=425;>AT1G56340.2 |
0.0
0.5
1.0
1.5
2.0
2.5
38
28
5
8
103
149
77
99
103
77
37
127
83
64
73
138
drm.ugt80..
drm.wt.ugt80..
drm.smt1..
drm.wt.smt1..
dsm.ugt80..
dsm.wt.ugt80..
dsm.smt1..
dsm.wt.smt1..
sp.ugt80..
sp.wt.ugt80..
sp.smt1..
sp.wt.smt1..
im.ugt80..
im.wt.ugt80..
im.smt1..
im.wt.smt1..

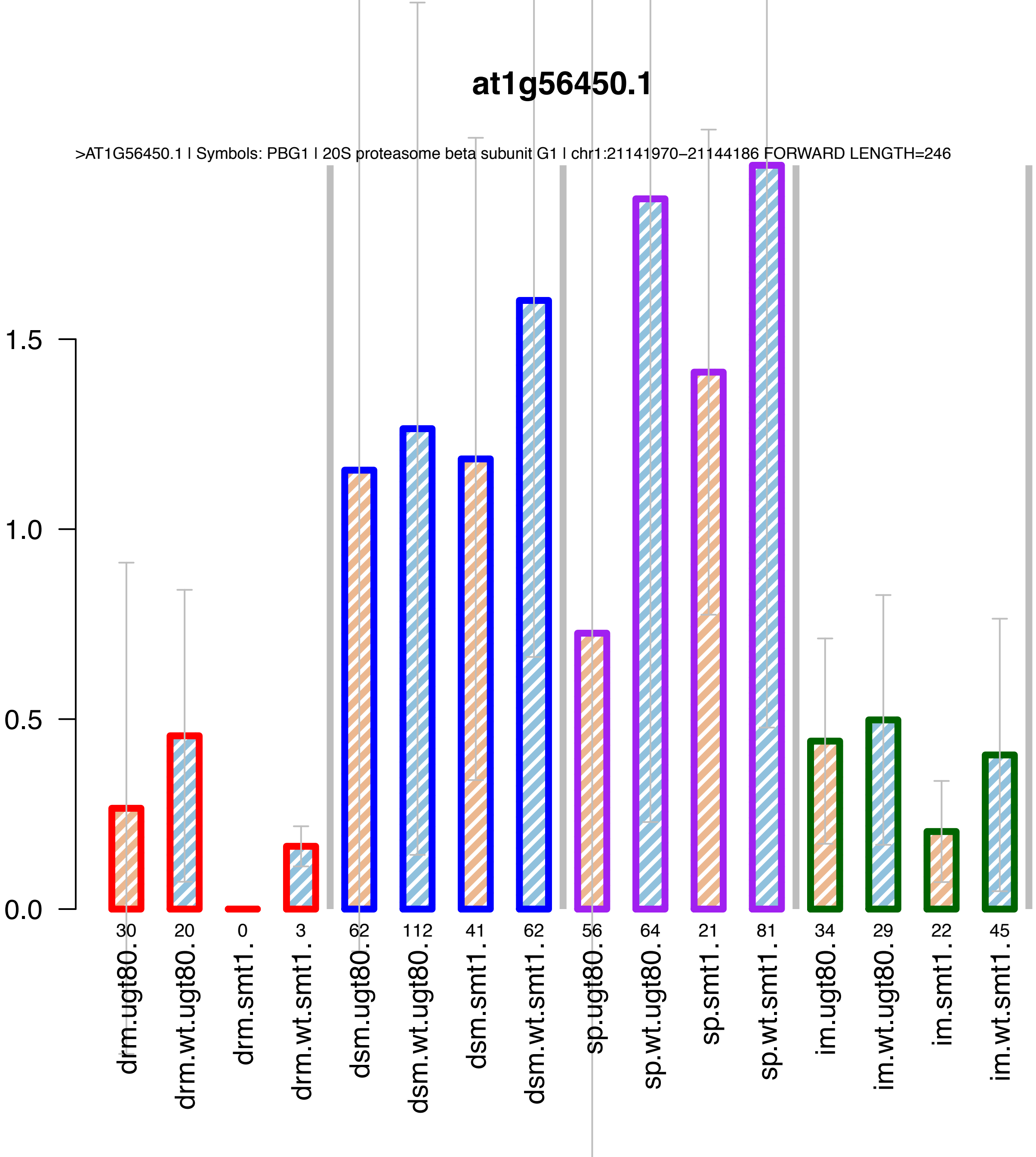
at1g56450.1
>AT1G56450.1 | Symbols: PBG1 | 20S proteasome beta subunit G1 | chr1:21141970-21144186 FORWARD LENGTH=246
0.0
0.5
1.0
1.5
30
20
0
3
62
112
41
62
56
64
21
81
34
29
22
45
drm.ugt80..
drm.wt.ugt80..
drm.smt1..
drm.wt.smt1..
dsm.ugt80..
dsm.wt.ugt80..
dsm.smt1..
dsm.wt.smt1..
sp.ugt80..
sp.wt.ugt80..
sp.smt1..
sp.wt.smt1..
im.ugt80..
im.wt.ugt80..
im.smt1..
im.wt.smt1..

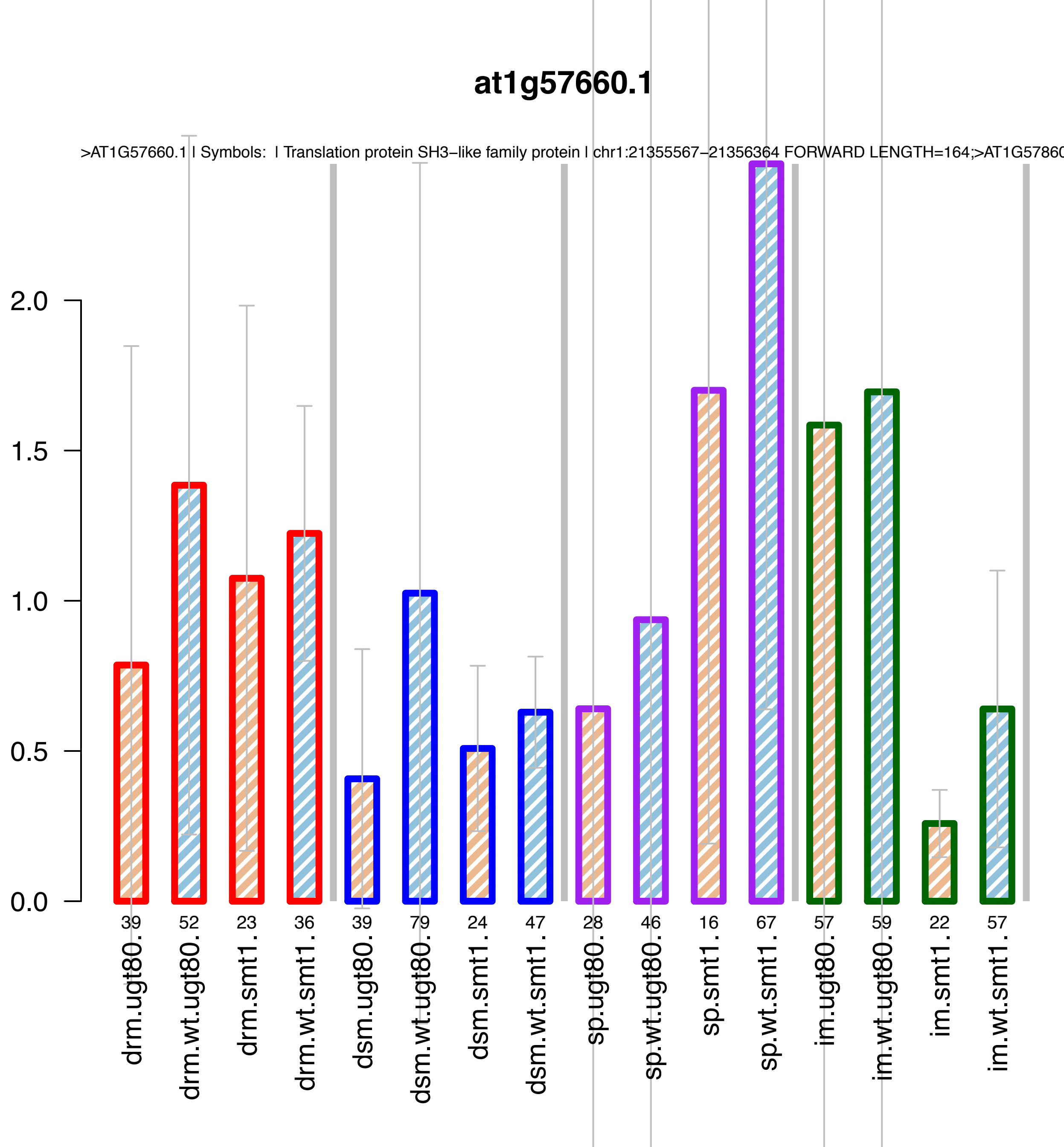
at1g57660.1
>AT1G57660.1 | Symbols: | Translation protein SH3-like family protein | chr1:21355567-21356364 FORWARD LENGTH=164;>AT1G5786
2.0
1.5
1.0
0.5
0.0
39
52
23
36
39
79
24
47
28
46
16
67
57
59
22
57
drm.ugt80..
drm.wt.ugt80..
drm.smt1..
drm.wt.smt1..
dsm.ugt80..
dsm.wt.ugt80..
dsm.smt1..
dsm.wt.smt1..
sp.ugt80..
sp.wt.ugt80..
sp.smt1..
sp.wt.smt1..
im.ugt80..
im.wt.ugt80..
im.smt1..
im.wt.smt1..

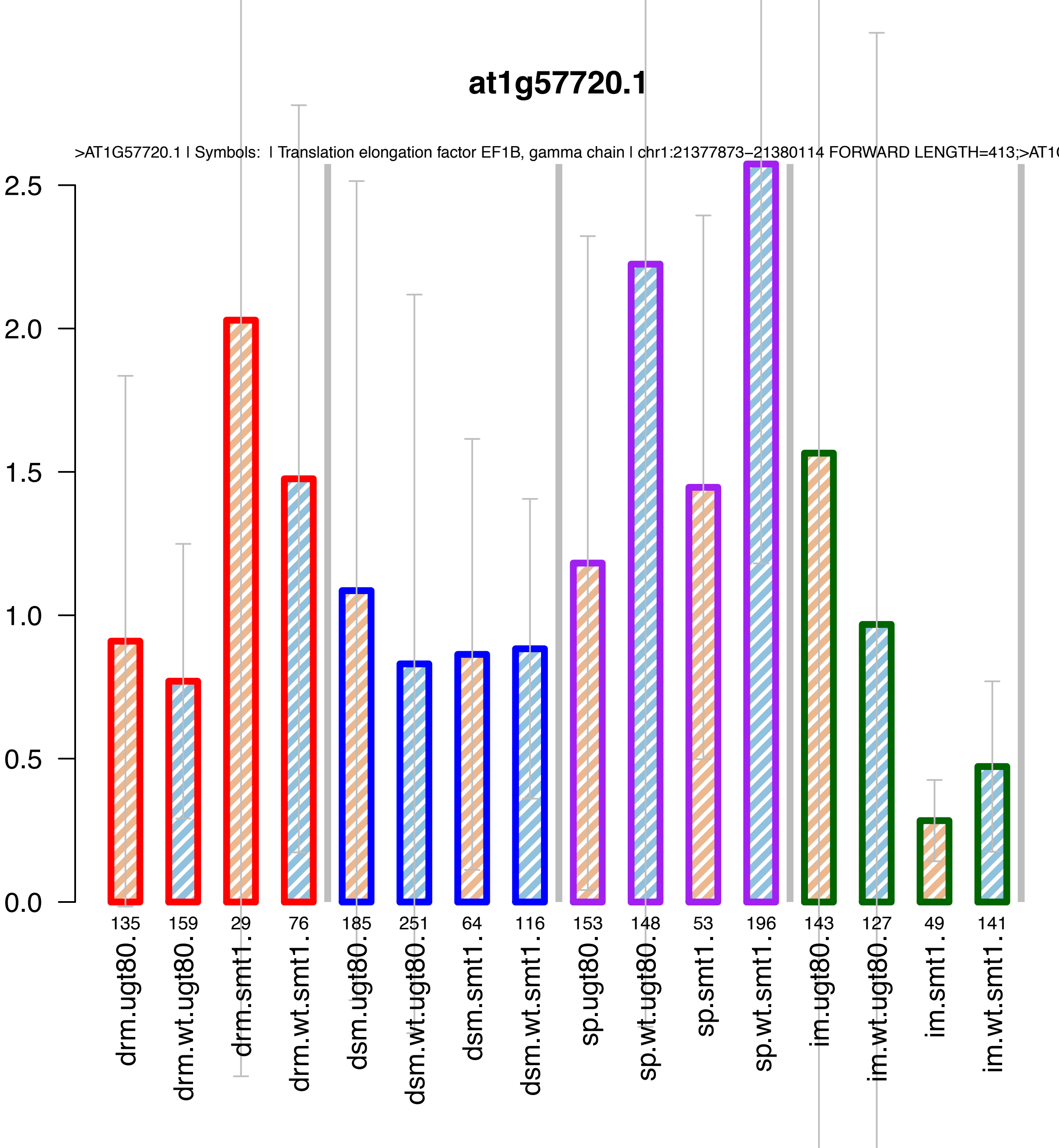

at1g57720.1
>AT1G57720.1 | Symbols: | Translation elongation factor EF1B, gamma chain | chr1:21377873-21380114 FORWARD LENGTH=413;>AT1
2.5
2.0
1.5
1.0
0.5
0.0
135
159
29
76
185
251
64
116
153
148
53
196
143
127
49
141
drm.ugt80..
drm.wt.ugt80..
drm.smt1..
drm.wt.smt1..
dsm.ugt80..
dsm.wt.ugt80..
dsm.smt1..
dsm.wt.smt1..
sp.ugt80..
sp.wt.ugt80..
sp.smt1..
sp.wt.smt1..
im.ugt80..
im.wt.ugt80..
im.smt1..
im.wt.smt1..

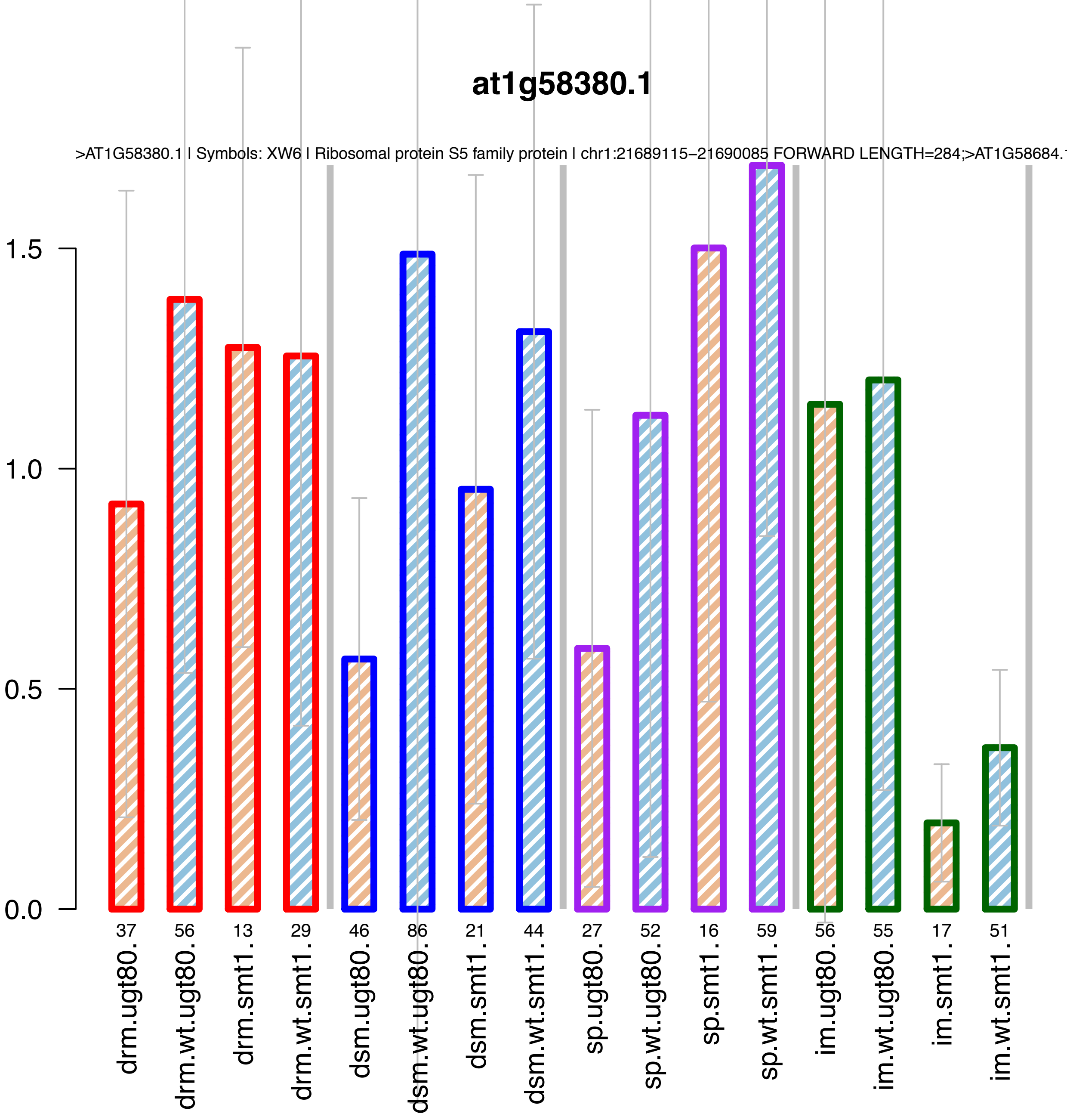
at1g58380.1
>AT1G58380.1 | Symbols: XW6 | Ribosomal protein S5 family protein | chr1:21689115-21690085 FORWARD LENGTH=284;>AT1G58684.1
0.0
0.5
1.0
1.5
37
56
13
29
46
86
21
44
27
52
16
59
56
55
17
51
drm.ugt80..
drm.wt.ugt80..
drm.smt1..
drm.wt.smt1..
dsm.ugt80..
dsm.wt.ugt80..
dsm.smt1..
dsm.wt.smt1..
sp.ugt80..
sp.wt.ugt80..
sp.smt1..
sp.wt.smt1..
im.ugt80..
im.wt.ugt80..
im.smt1..
im.wt.smt1..

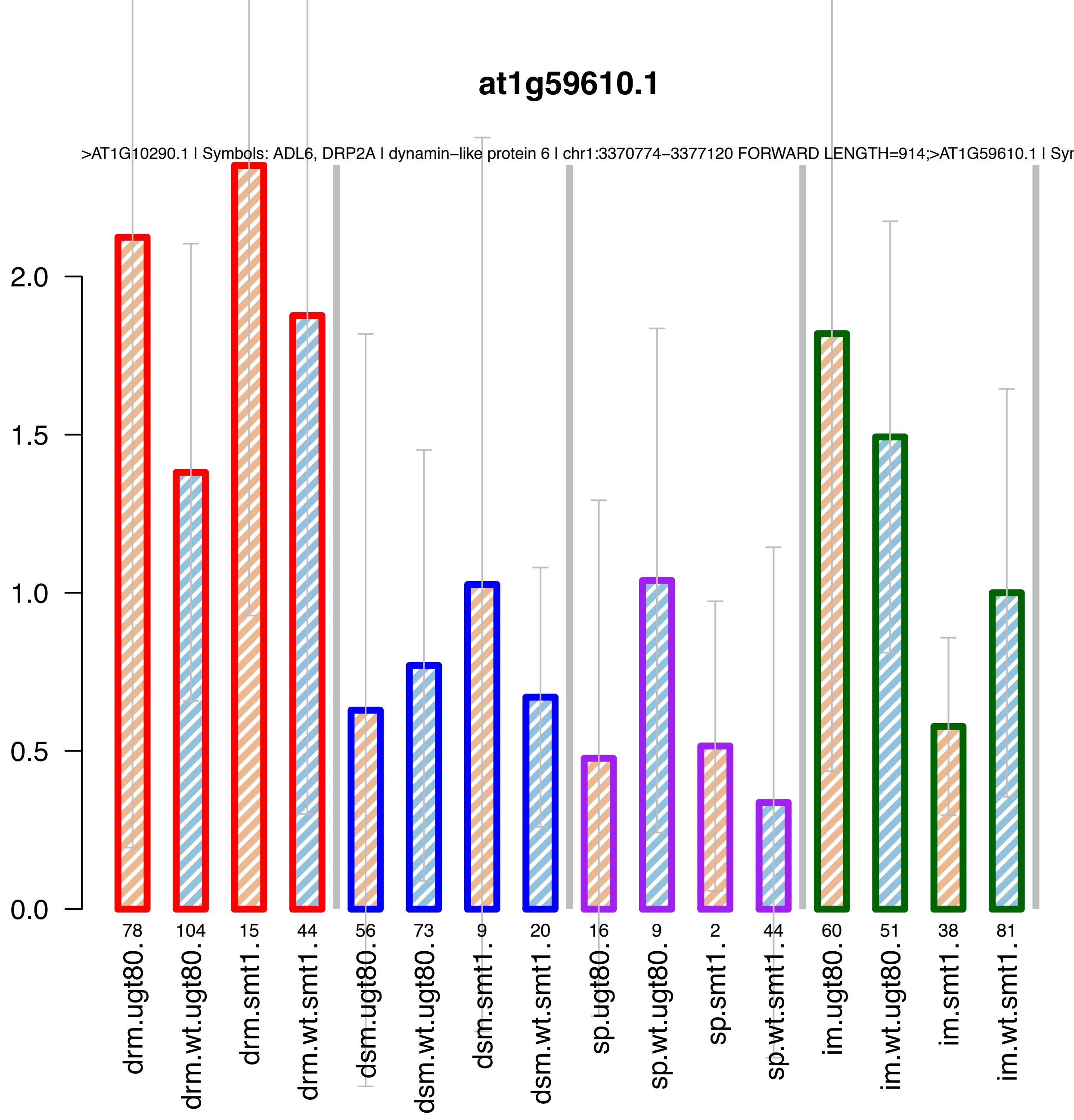
at1g59610.1
>AT1G10290.1 | Symbols: ADL6, DRP2A | dynamin−like protein 6 | chr1:3370774−3377120 FORWARD LENGTH=914;>AT1G59610.1 | Sym
0.0
0.5
1.0
1.5
2.0
78
104
15
44
56
73
9
20
16
9
2
44
60
51
38
81
drm.ugt80..
drm.wt.ugt80..
drm.smt1..
drm.wt.smt1..
dsm.ugt80..
dsm.wt.ugt80..
dsm.smt1..
dsm.wt.smt1..
sp.ugt80..
sp.wt.ugt80..
sp.smt1..
sp.wt.smt1..
im.ugt80..
im.wt.ugt80..
im.smt1..
im.wt.smt1..

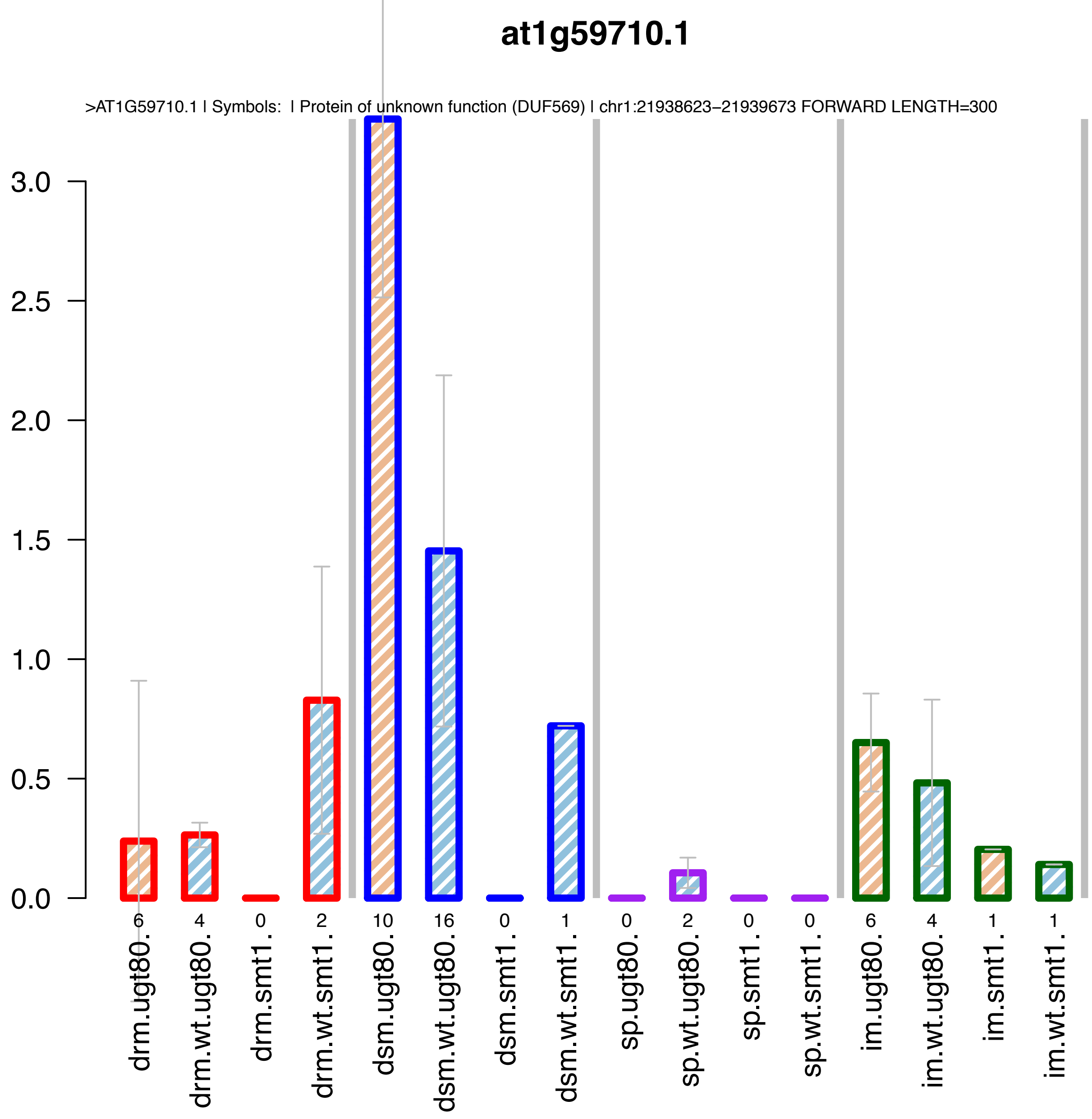

at1g59710.1
>AT1G59710.1 | Symbols: | Protein of unknown function (DUF569) | chr1:21938623-21939673 FORWARD LENGTH=300
0.0
0.5
1.0
1.5
2.0
2.5
3.0
6
4
0
2
10
16
0
1
0
2
0
0
6
4
1
1
drm.ugt80.
drm.wt.ugt80.
drm.smt1.
drm.wt.smt1.
dsm.ugt80.
dsm.wt.ugt80.
dsm.smt1.
dsm.wt.smt1.
sp.ugt80.
sp.wt.ugt80.
sp.smt1.
sp.wt.smt1.
im.ugt80.
im.wt.ugt80.
im.smt1.
im.wt.smt1.

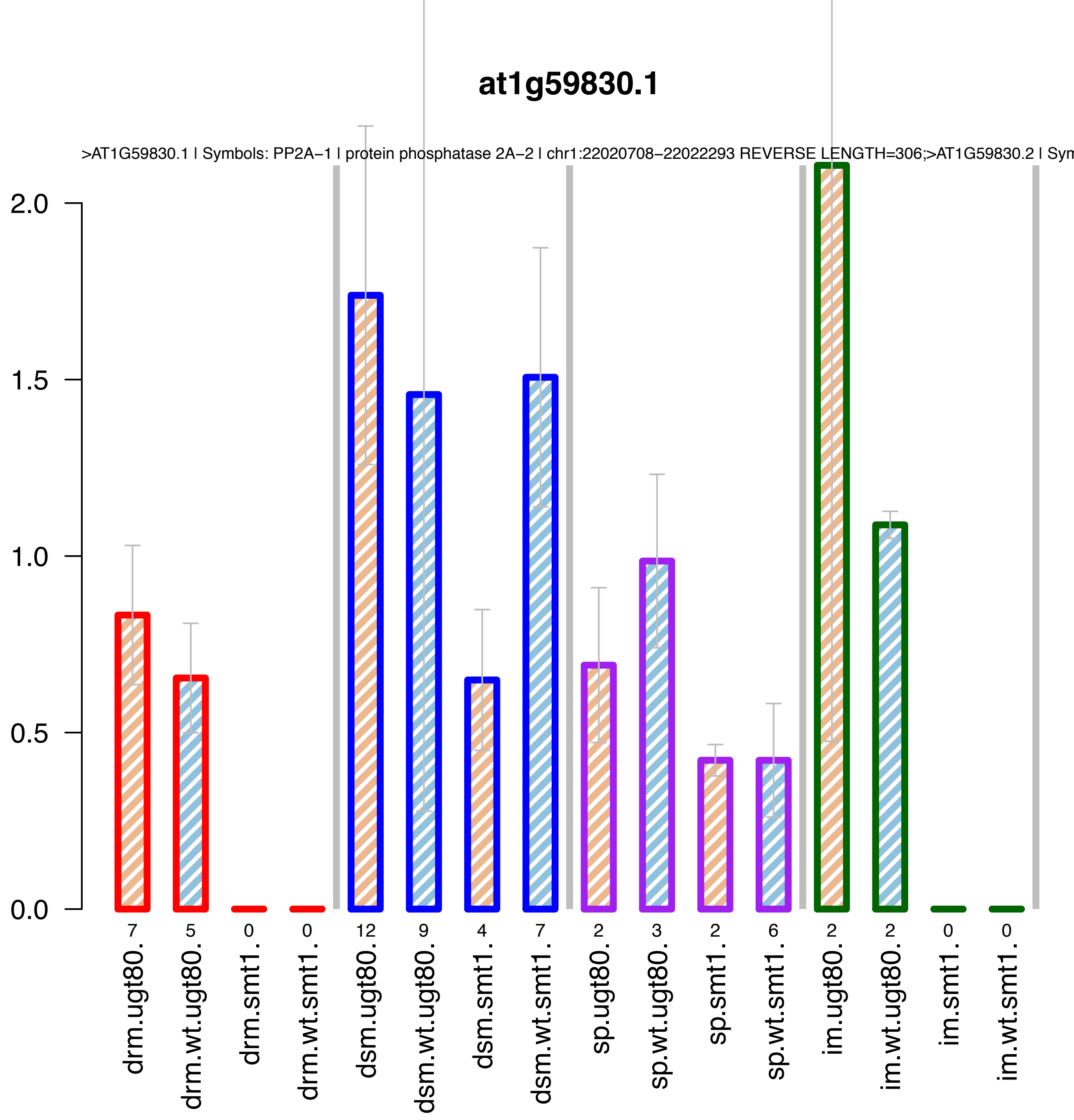
at1g59830.1
>AT1G59830.1 | Symbols: PP2A−1 | protein phosphatase 2A−2 | chr1:22020708−22022293 REVERSE LENGTH=306;>AT1G59830.2 | Sym
2.0
1.5
1.0
0.5
0.0
7
5
0
0
12
9
4
7
2
3
2
6
2
2
0
0
drm.ugt80.
drm.wt.ugt80.
drm.smt1.
drm.wt.smt1.
dsm.ugt80.
dsm.wt.ugt80.
dsm.smt1.
dsm.wt.smt1.
sp.ugt80.
sp.wt.ugt80.
sp.smt1.
sp.wt.smt1.
im.ugt80.
im.wt.ugt80.
im.smt1.
im.wt.smt1.

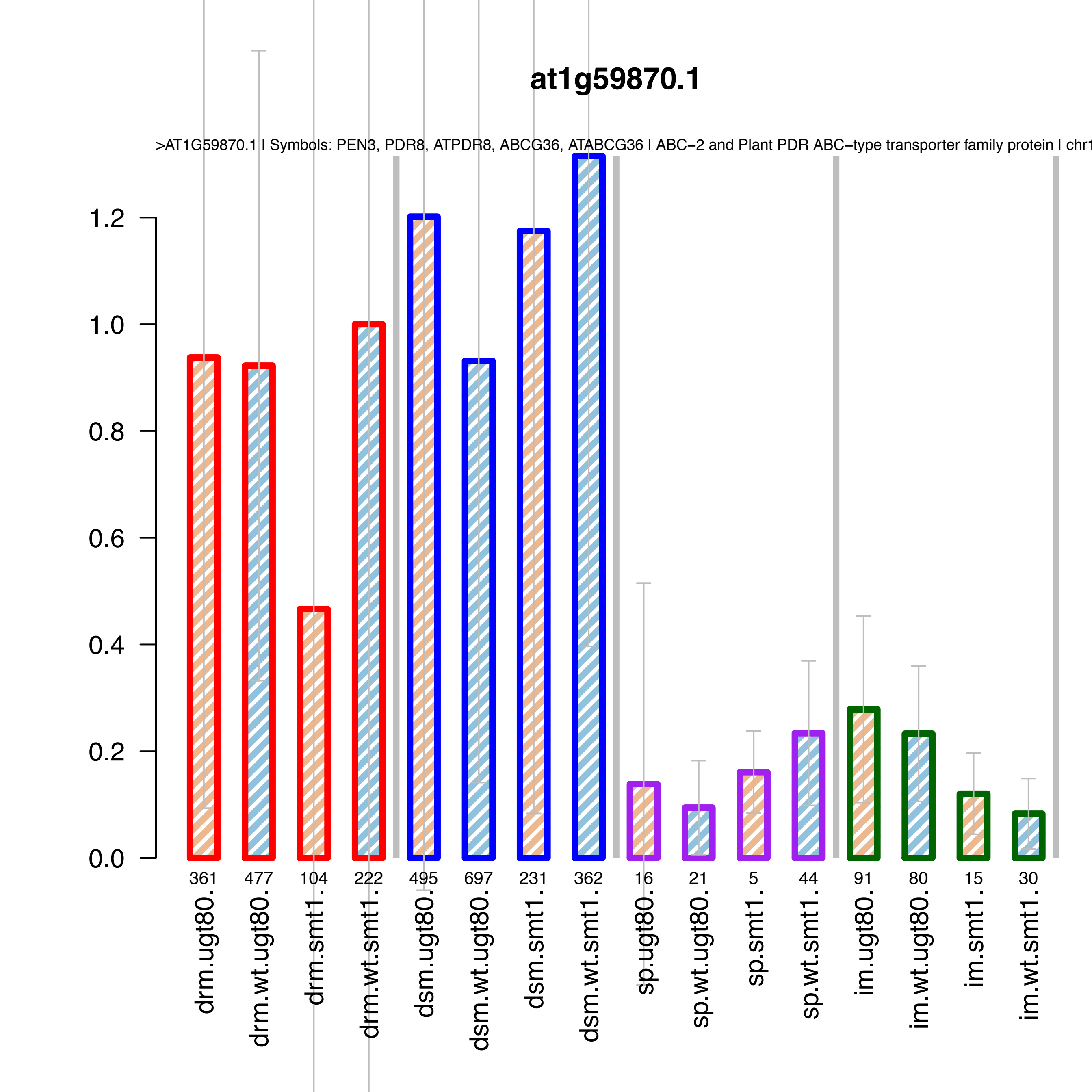

at1g59870.1
>AT1G59870.1 | Symbols: PEN3, PDR8, ATPDR8, ABCG36, ATABCG36 | ABC−2 and Plant PDR ABC−type transporter family protein | chr1
1.2
1.0
0.8
0.6
0.4
0.2
0.0
361
477
104
222
495
697
231
362
16
21
5
44
91
80
15
30
drm.ugt80..
drm.wt.ugt80..
drm.smt1..
drm.wt.smt1..
dsm.ugt80..
dsm.wt.ugt80..
dsm.smt1..
dsm.wt.smt1..
sp.ugt80..
sp.wt.ugt80..
sp.smt1..
sp.wt.smt1..
im.ugt80..
im.wt.ugt80..
im.smt1..
im.wt.smt1..

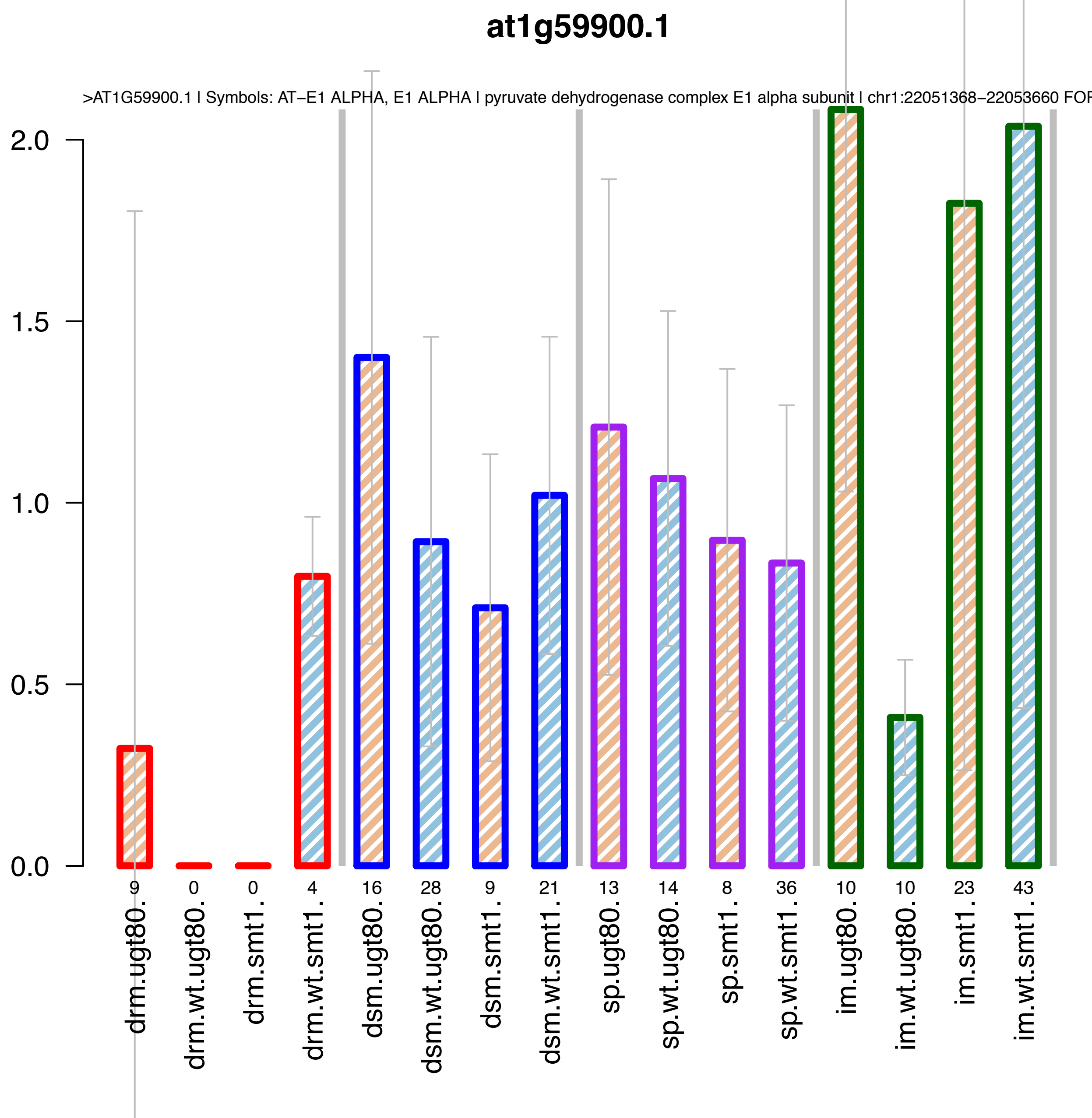

at1g59900.1
>AT1G59900.1 | Symbols: AT−E1 ALPHA, E1 ALPHA | pyruvate dehydrogenase complex E1 alpha subunit | chr1:22051368−22053660 FOR
2.0
1.5
1.0
0.5
0.0
9
0
0
4
16
28
9
21
13
14
8
36
10
10
23
43
drm.ugt80.
drm.wt.ugt80.
drm.smt1.
drm.wt.smt1.
dsm.ugt80.
dsm.wt.ugt80.
dsm.smt1.
dsm.wt.smt1.
sp.ugt80.
sp.wt.ugt80.
sp.smt1.
sp.wt.smt1.
im.ugt80.
im.wt.ugt80.
im.smt1.
im.wt.smt1.

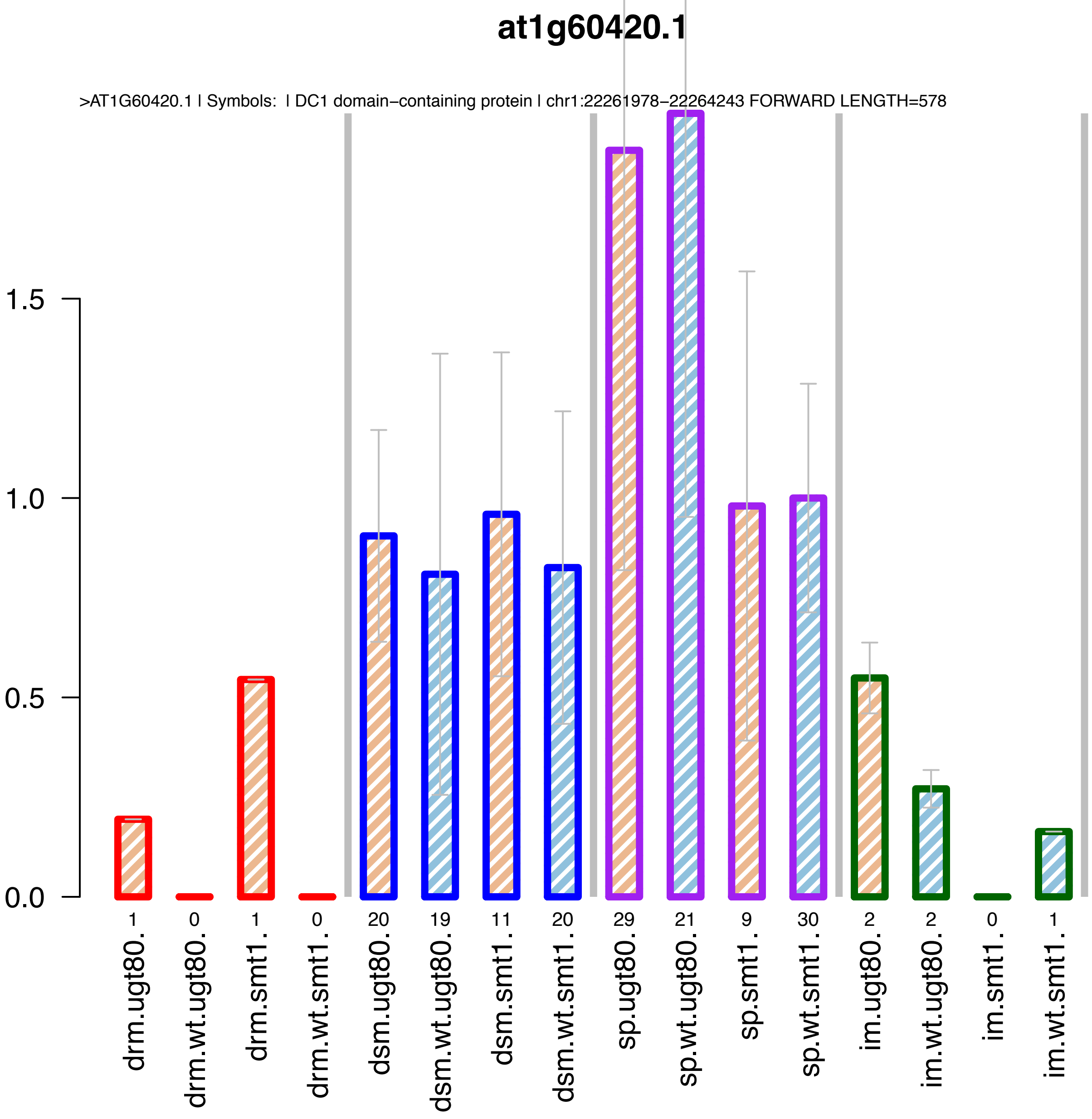

at1g60420.1
>AT1G60420.1 | Symbols: | DC1 domain−containing protein | chr1:22261978−22264243 FORWARD LENGTH=578
0.0
0.5
1.0
1.5
1 0 1 0 20 19 11 20 29 21 9 30 2 2 0 1
drm.ugt80.
drm.wt.ugt80.
drm.smt1.
drm.wt.smt1.
dsm.ugt80.
dsm.wt.ugt80.
dsm.smt1.
dsm.wt.smt1.
sp.ugt80.
sp.wt.ugt80.
sp.smt1.
sp.wt.smt1.
im.ugt80.
im.wt.ugt80.
im.smt1.
im.wt.smt1.

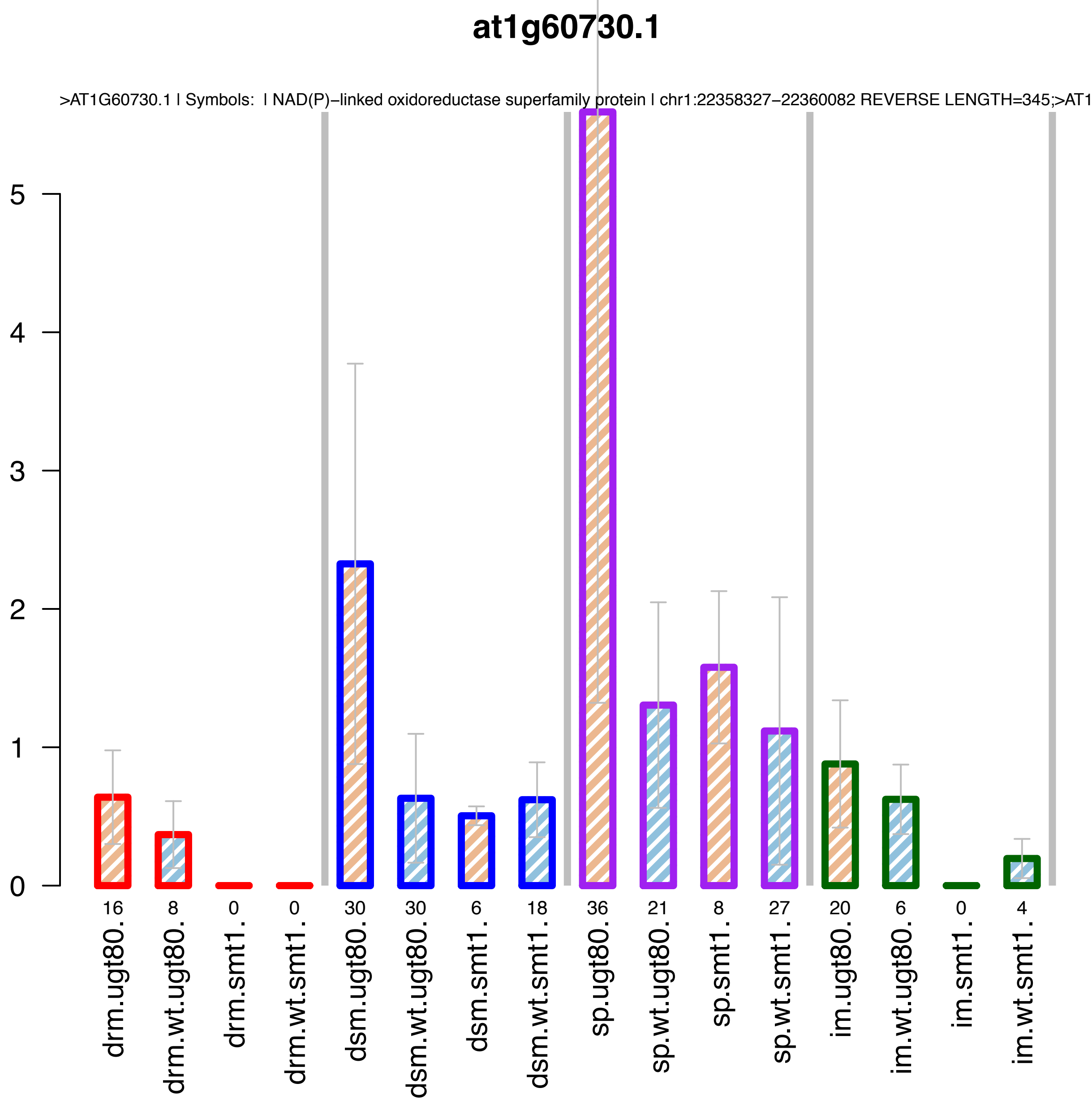

at1g60730.1
>AT1G60730.1 | Symbols: | NAD(P)-linked oxidoreductase superfamily protein | chr1:22358327-22360082 REVERSE LENGTH=345;>AT1
0
1
2
3
4
5
16
8
0
0
30
30
6
18
36
21
8
27
20
6
0
4
drm.ugt80.
drm.wt.ugt80.
drm.smt1.
drm.wt.smt1.
dsm.ugt80.
dsm.wt.ugt80.
dsm.smt1.
dsm.wt.smt1.
sp.ugt80.
sp.wt.ugt80.
sp.smt1.
sp.wt.smt1.
im.ugt80.
im.wt.ugt80.
im.smt1.
im.wt.smt1.

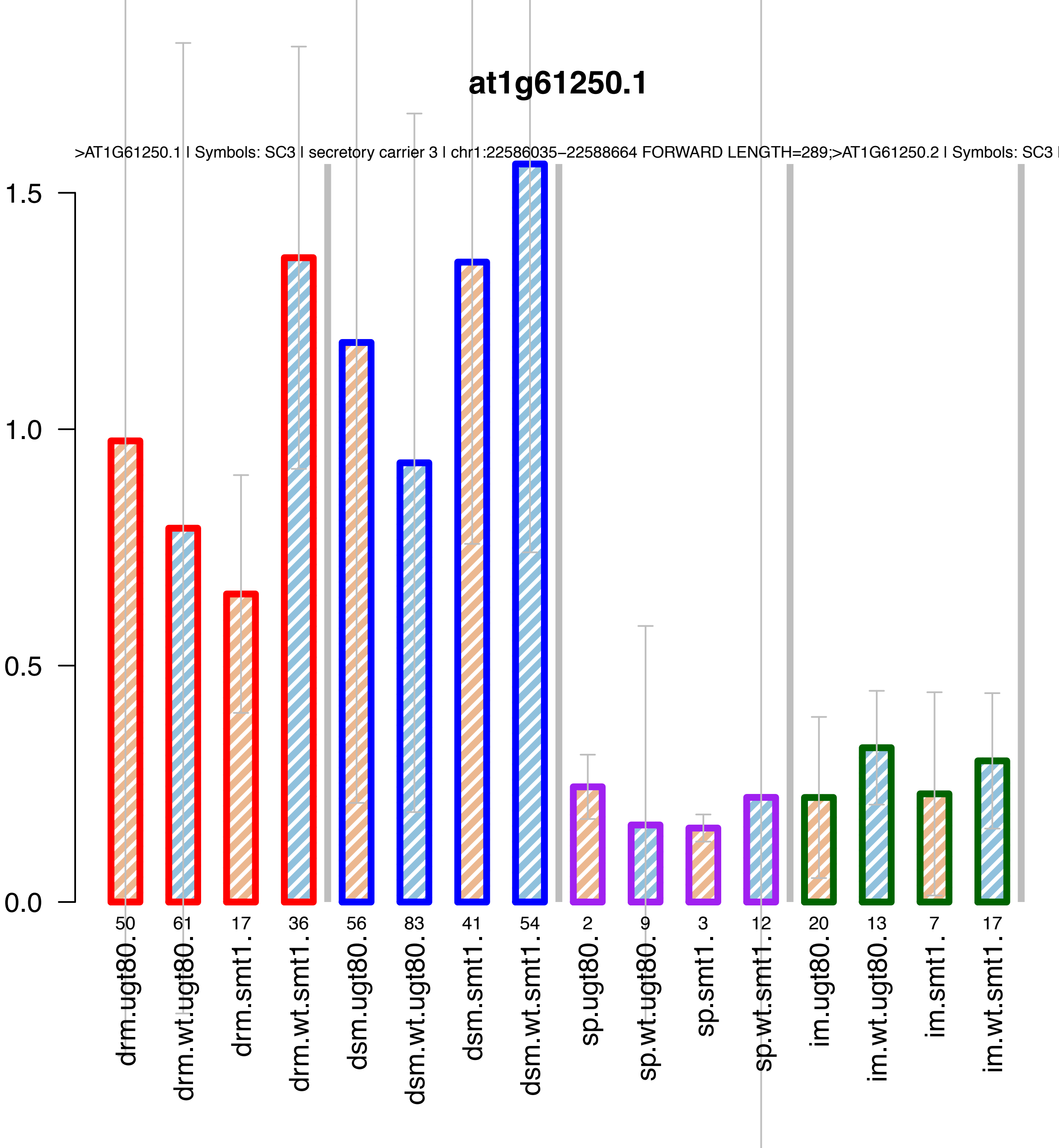

at1g61250.1
>AT1G61250.1 | Symbols: SC3 | secretory carrier 3 | chr1:22586035-22588664 FORWARD LENGTH=289;>AT1G61250.2 | Symbols: SC3 |
1.5
1.0
0.5
0.0
50
61
17
36
56
83
41
54
2
9
3
12
20
13
7
17
drm.ugt80..
drm.wt.ugt80..
drm.smt1..
drm.wt.smt1..
dsm.ugt80..
dsm.wt.ugt80..
dsm.smt1..
dsm.wt.smt1..
sp.ugt80..
sp.wt.ugt80..
sp.smt1..
sp.wt.smt1..
im.ugt80..
im.wt.ugt80..
im.smt1..
im.wt.smt1..

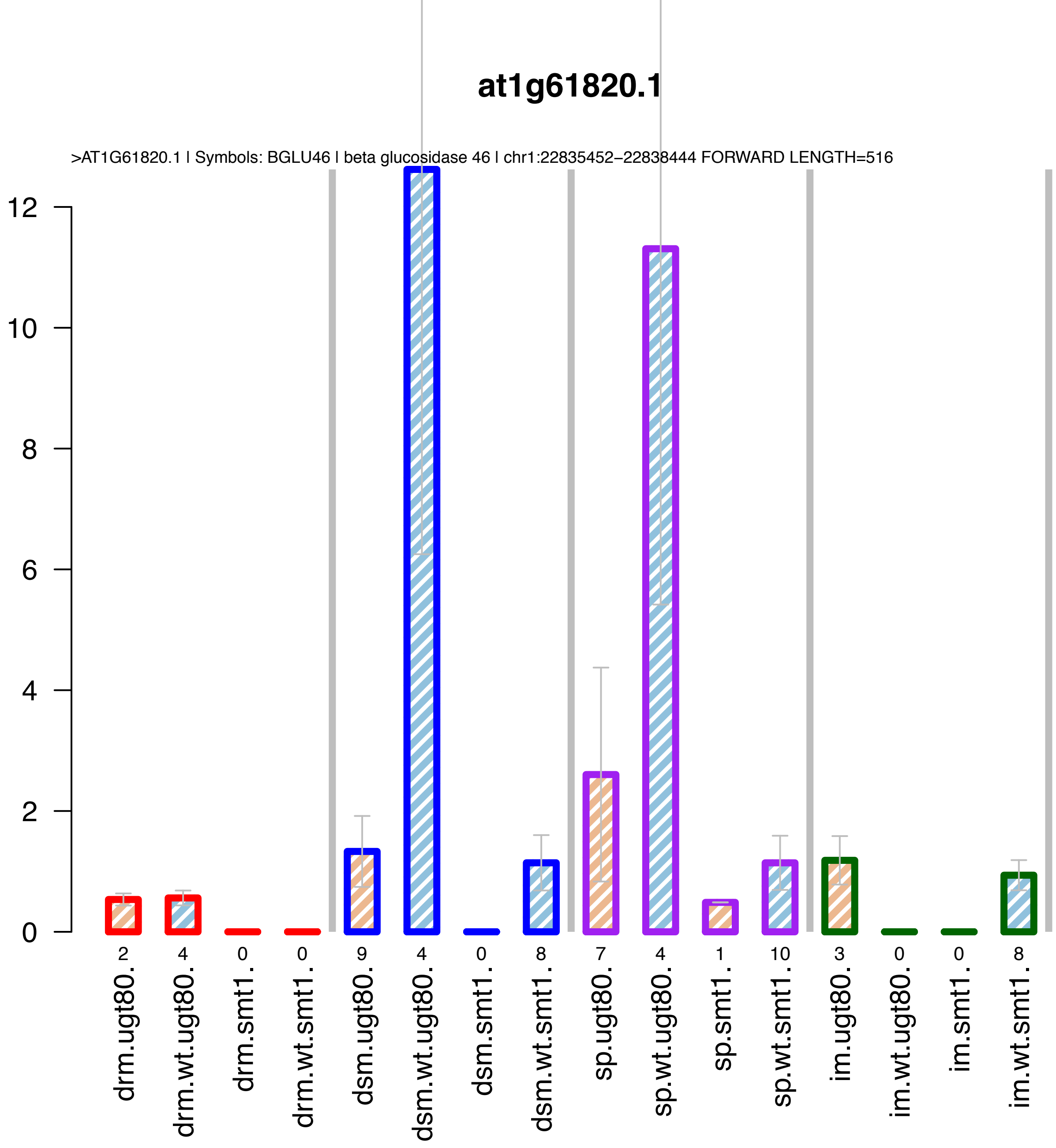

at1g61820.1
>AT1G61820.1 | Symbols: BGLU46 | beta glucosidase 46 | chr1:22835452–22838444 FORWARD LENGTH=516
0
2
4
6
8
10
12
2
4
0
0
9
4
0
8
7
4
1
10
3
0
0
8
drm.ugt80.
drm.wt.ugt80.
drm.smt1.
drm.wt.smt1.
dsm.ugt80.
dsm.wt.ugt80.
dsm.smt1.
dsm.wt.smt1.
sp.ugt80.
sp.wt.ugt80.
sp.smt1.
sp.wt.smt1.
im.ugt80.
im.wt.ugt80.
im.smt1.
im.wt.smt1.

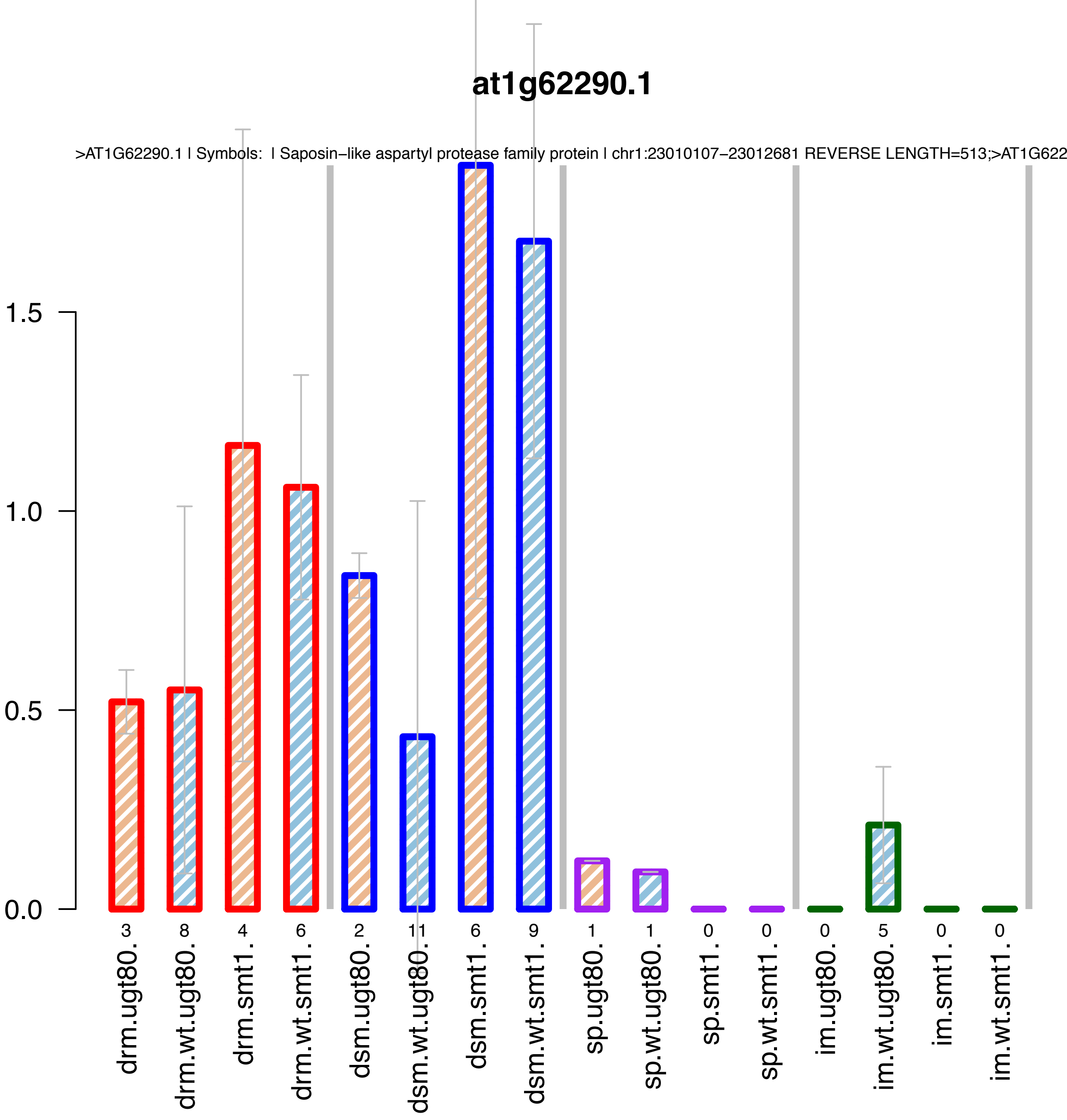

at1g62290.1
>AT1G62290.1 | Symbols: | Saposin−like aspartyl protease family protein | chr1:23010107−23012681 REVERSE LENGTH=513;>AT1G622
0.0
0.5
1.0
1.5
3
8
4
6
2
11
6
9
1
1
0
0
0
5
0
0
drm.ugt80.
drm.wt.ugt80.
drm.smt1.
drm.wt.smt1.
dsm.ugt80.
dsm.wt.ugt80.
dsm.smt1.
dsm.wt.smt1.
sp.ugt80.
sp.wt.ugt80.
sp.smt1.
sp.wt.smt1.
im.ugt80.
im.wt.ugt80.
im.smt1.
im.wt.smt1.

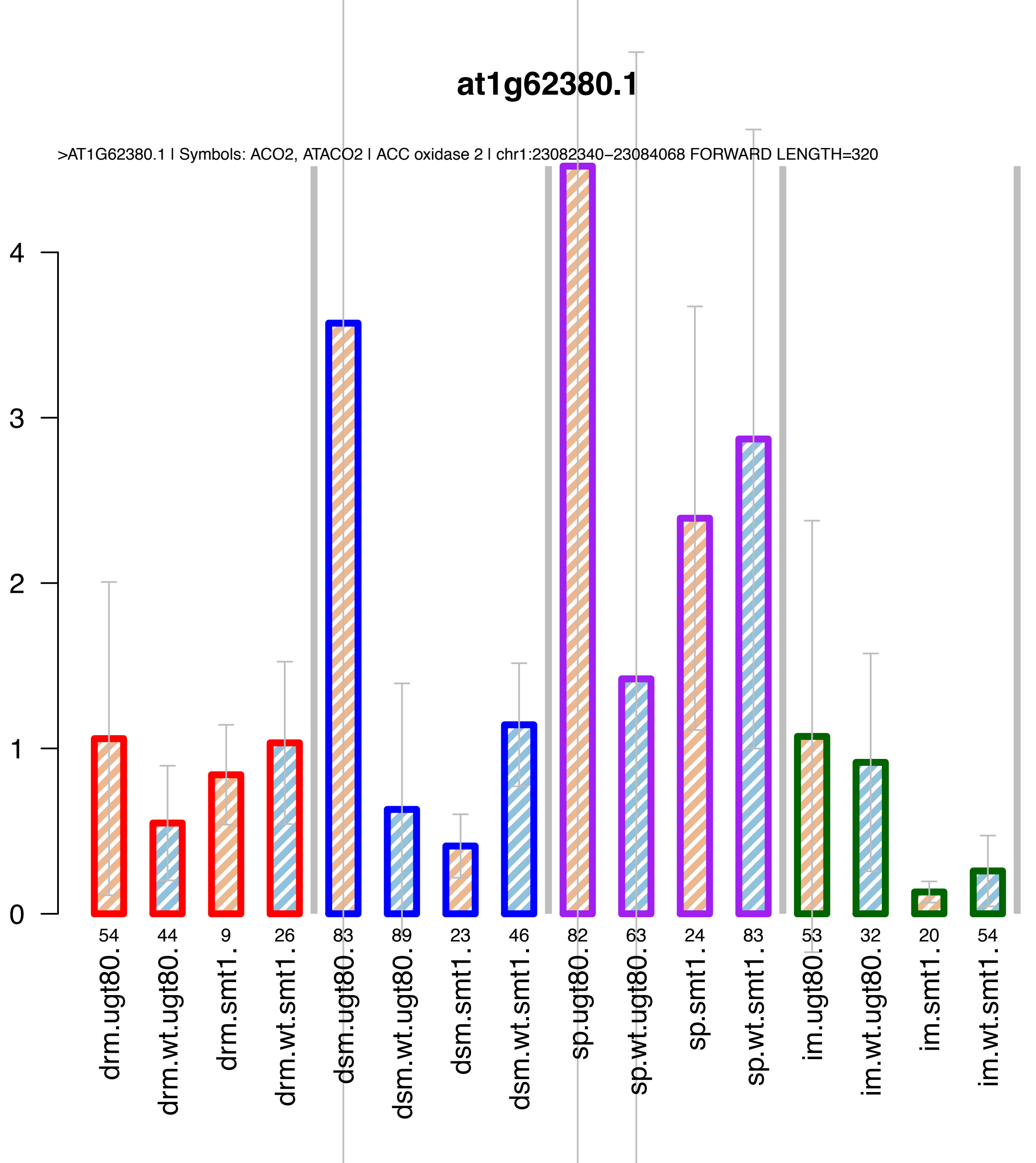
at1g62380.1
>AT1G62380.1 | Symbols: ACO2, ATACO2 | ACC oxidase 2 | chr1:23082340−23084068 FORWARD LENGTH=320
0
1
2
3
4
54
44
9
26
83
89
23
46
82
63
24
83
53
32
20
54
drm.ugt80.
drm.wt.ugt80.
drm.smt1.
drm.wt.smt1.
dsm.ugt80.
dsm.wt.ugt80.
dsm.smt1.
dsm.wt.smt1.
sp.ugt80.
sp.wt.ugt80.
sp.smt1.
sp.wt.smt1.
im.ugt80.
im.wt.ugt80.
im.smt1.
im.wt.smt1.

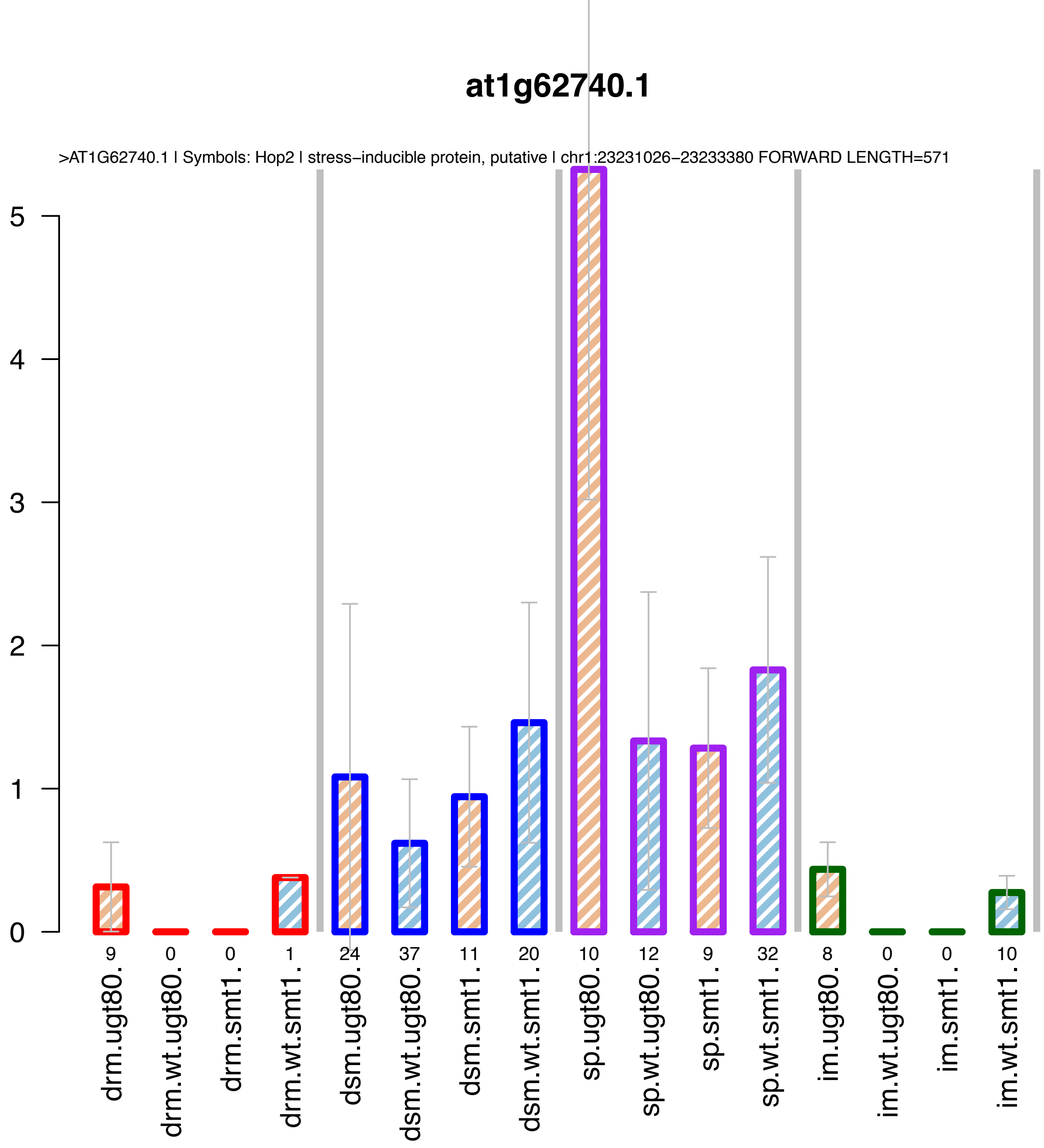
at1g62740.1
>AT1G62740.1 | Symbols: Hop2 | stress−inducible protein, putative | chr1:23231026−23233380 FORWARD LENGTH=571
0
1
2
3
4
5
9
0
0
1
24
37
11
20
10
12
9
32
8
0
0
10
drm.ugt80.
drm.wt.ugt80.
drm.smt1.
drm.wt.smt1.
dsm.ugt80.
dsm.wt.ugt80.
dsm.smt1.
dsm.wt.smt1.
sp.ugt80.
sp.wt.ugt80.
sp.smt1.
sp.wt.smt1.
im.ugt80.
im.wt.ugt80.
im.smt1.
im.wt.smt1.

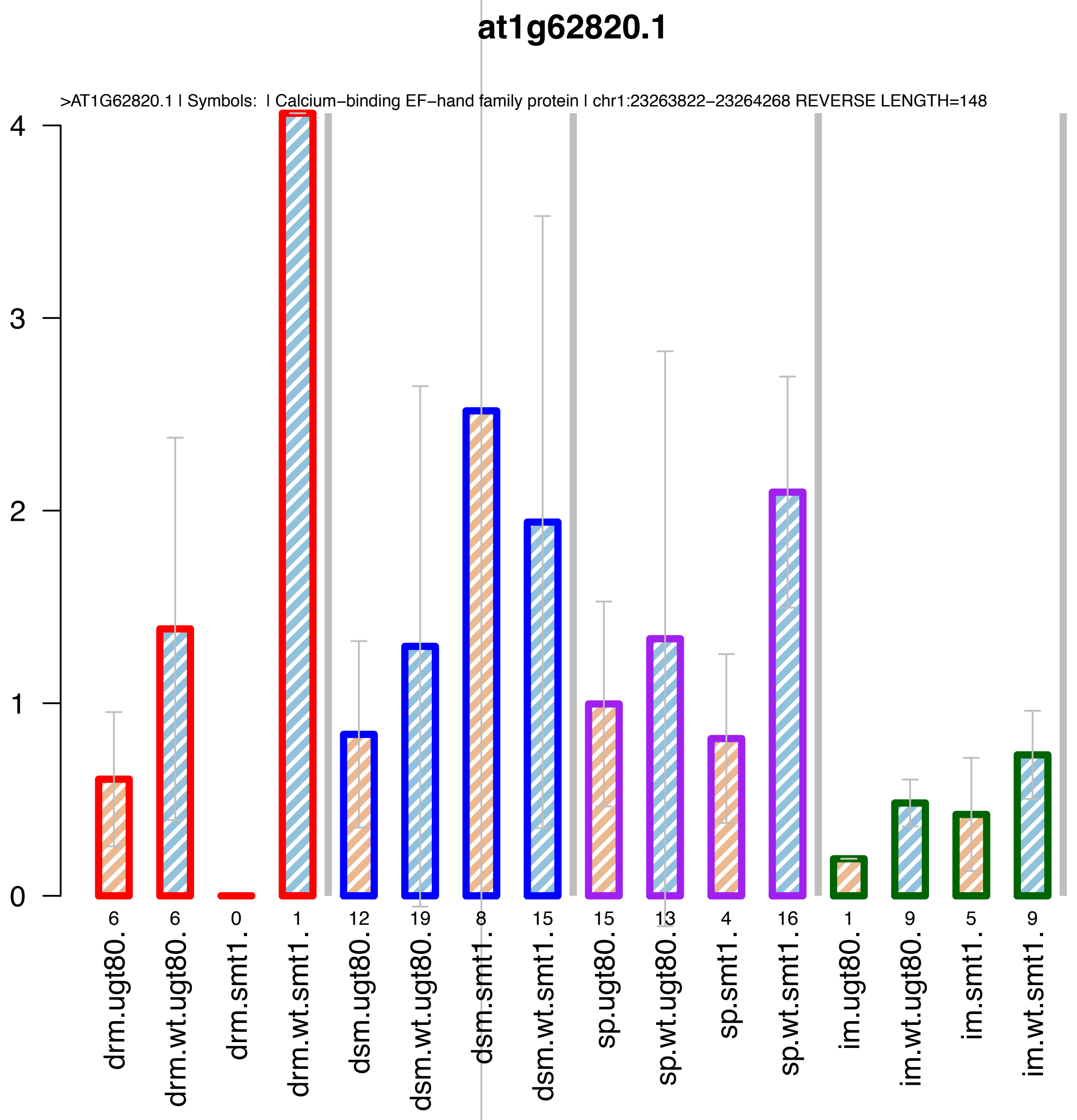
at1g62820.1
>AT1G62820.1 | Symbols: | Calcium-binding EF-hand family protein | chr1:23263822-23264268 REVERSE LENGTH=148
0
1
2
3
4
6
6
0
1
12
19
8
15
15
13
4
16
1
9
5
9
drm.ugt80.
drm.wt.ugt80.
drm.smt1.
drm.wt.smt1.
dsm.ugt80.
dsm.wt.ugt80.
dsm.smt1.
dsm.wt.smt1.
sp.ugt80.
sp.wt.ugt80.
sp.smt1.
sp.wt.smt1.
im.ugt80.
im.wt.ugt80.
im.smt1.
im.wt.smt1.

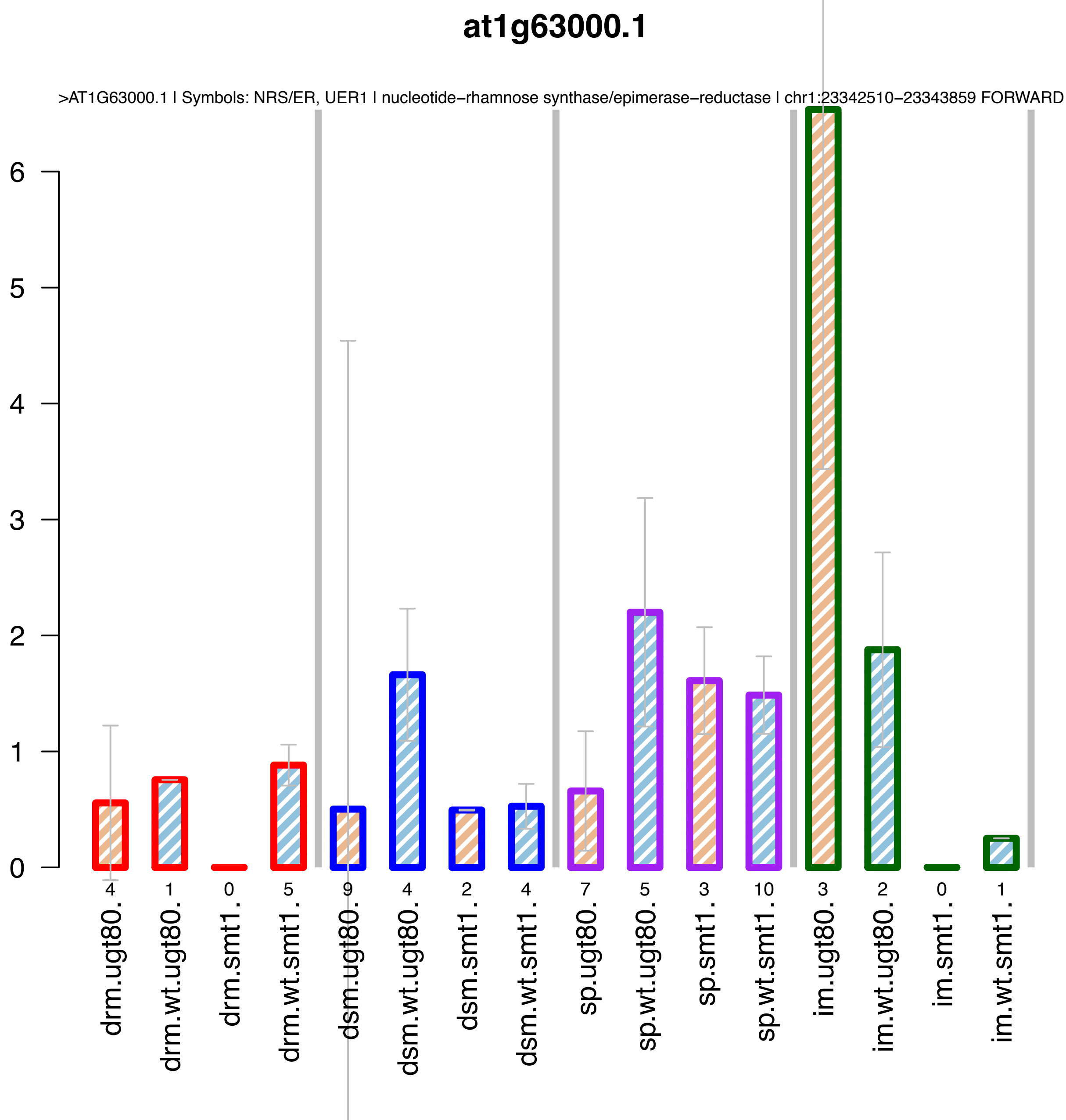

at1g63000.1
>AT1G63000.1 | Symbols: NRS/ER, UER1 | nucleotide−rhamnose synthase/epimerase−reductase | chr1:23342510−23343859 FORWARD
0
1
2
3
4
5
6
4
1
0
5
9
4
2
4
7
5
3
10
3
2
0
1
drm.ugt80.
drm.wt.ugt80.
drm.smt1.
drm.wt.smt1.
dsm.ugt80.
dsm.wt.ugt80.
dsm.smt1.
dsm.wt.smt1.
sp.ugt80.
sp.wt.ugt80.
sp.smt1.
sp.wt.smt1.
im.ugt80.
im.wt.ugt80.
im.smt1.
im.wt.smt1.

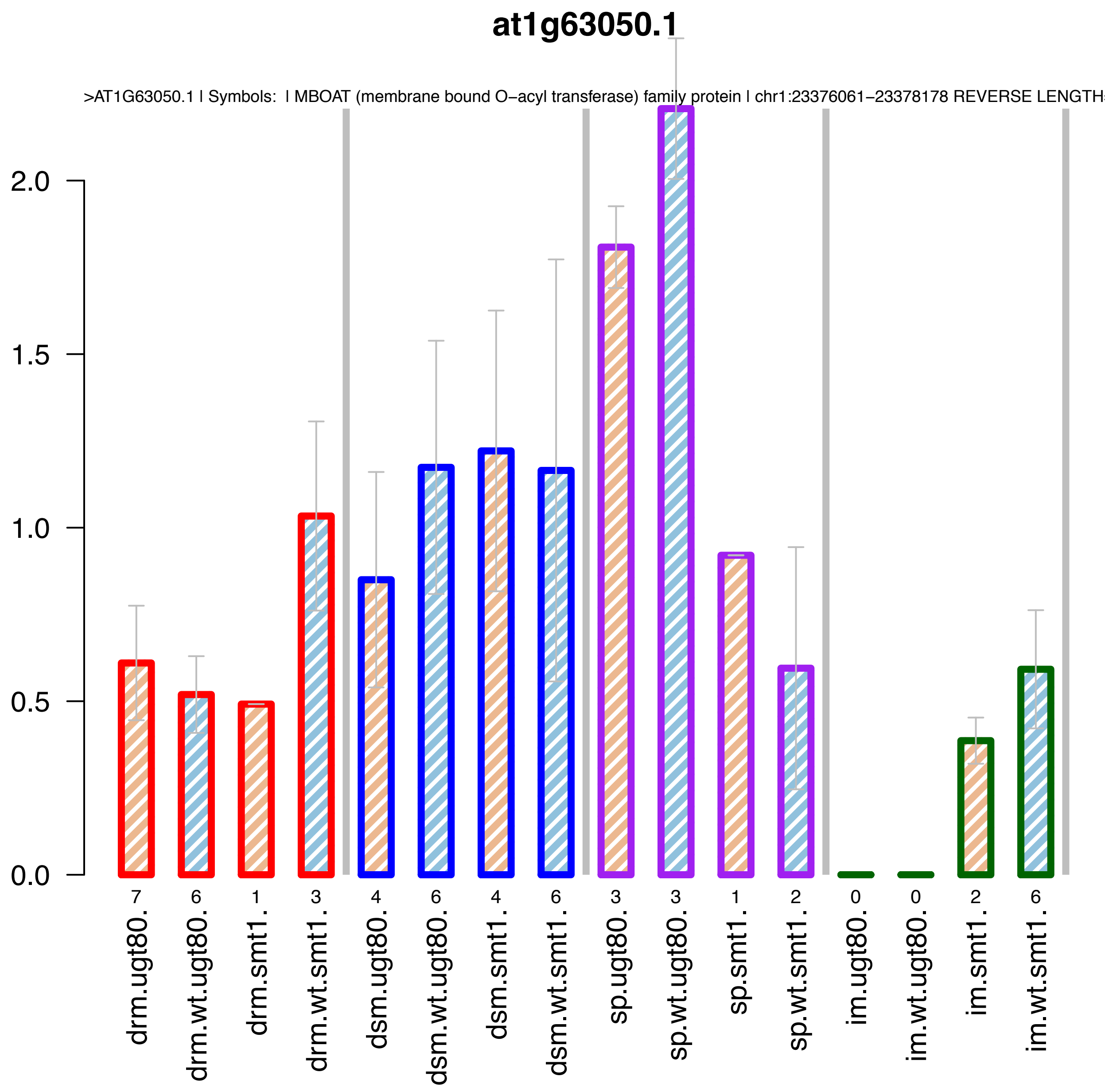

at1g63050.1
>AT1G63050.1 | Symbols: | MBOAT (membrane bound O-acyl transferase) family protein | chr1:23376061-23378178 REVERSE LENGTH
2.0
1.5
1.0
0.5
0.0
7
6
1
3
4
6
4
6
3
3
1
2
0
0
2
6
drm.ugt80.
drm.wt.ugt80.
drm.smt1.
drm.wt.smt1.
dsm.ugt80.
dsm.wt.ugt80.
dsm.smt1.
dsm.wt.smt1.
sp.ugt80.
sp.wt.ugt80.
sp.smt1.
sp.wt.smt1.
im.ugt80.
im.wt.ugt80.
im.smt1.
im.wt.smt1.

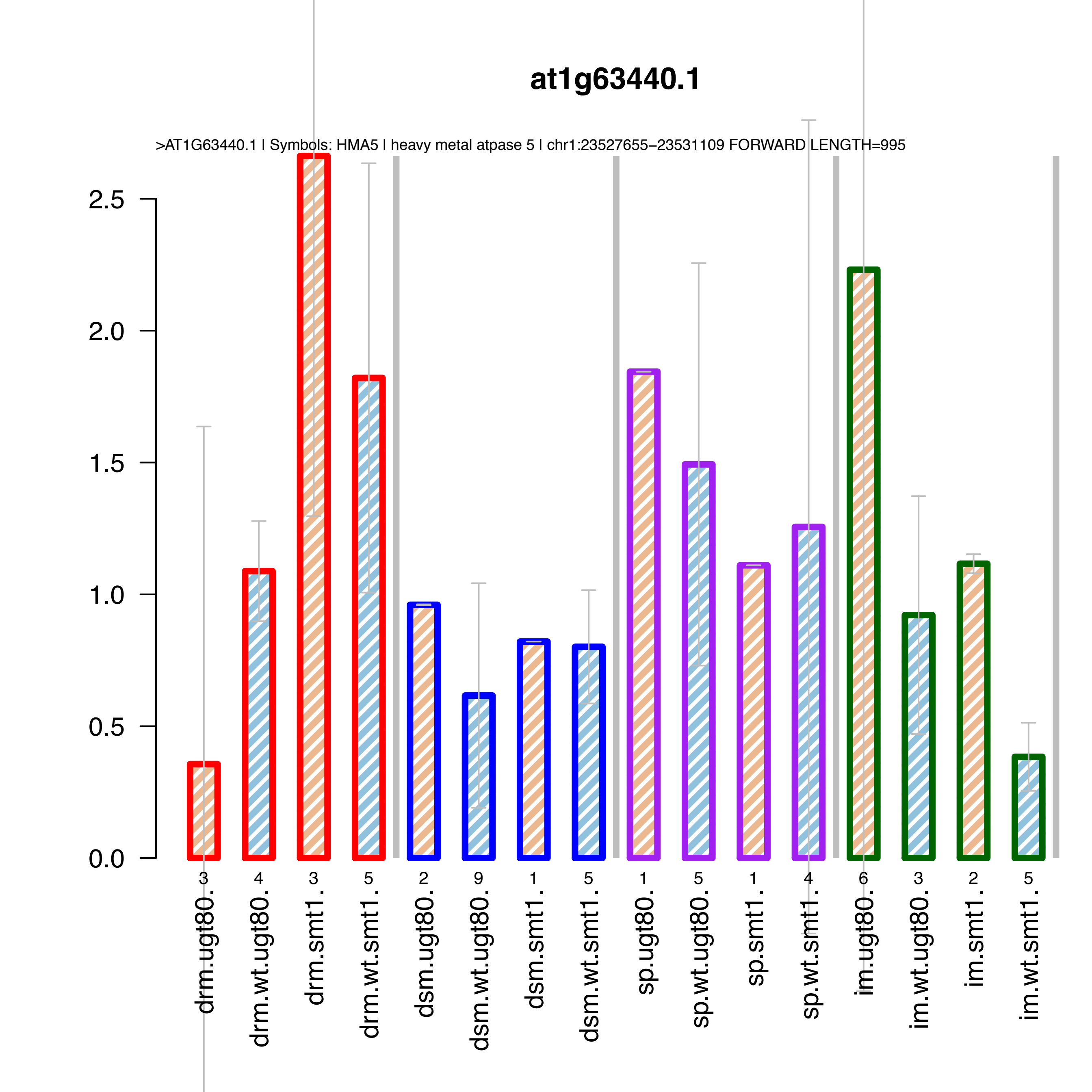
at1g63440.1
>AT1G63440.1 | Symbols: HMA5 | heavy metal atpase 5 | chr1:23527655-23531109 FORWARD LENGTH=995
0.0
0.5
1.0
1.5
2.0
2.5
3
4
3
5
2
9
1
5
1
5
1
4
6
3
2
5
drm.ugt80..
drm.wt.ugt80..
drm.smt1..
drm.wt.smt1..
dsm.ugt80..
dsm.wt.ugt80..
dsm.smt1..
dsm.wt.smt1..
sp.ugt80..
sp.wt.ugt80..
sp.smt1..
sp.wt.smt1..
im.ugt80..
im.wt.ugt80..
im.smt1..
im.wt.smt1..

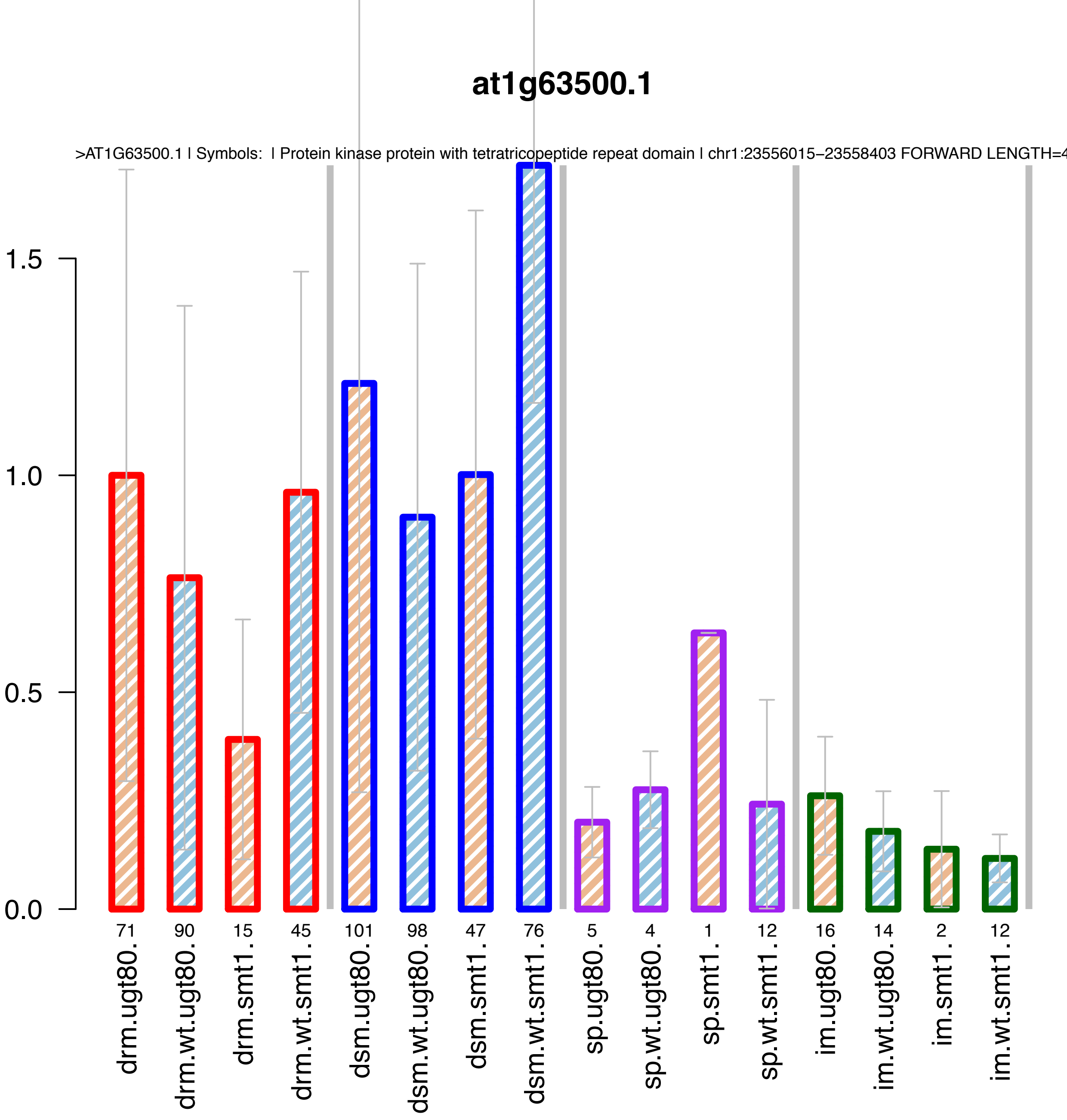

at1g63500.1
>AT1G63500.1 | Symbols: | Protein kinase protein with tetratricopeptide repeat domain | chr1:23556015-23558403 FORWARD LENGTH=4
0.0
0.5
1.0
1.5
71
90
15
45
101
98
47
76
5
4
1
12
16
14
2
12
drm.ugt80.
drm.wt.ugt80.
drm.smt1.
drm.wt.smt1.
dsm.ugt80.
dsm.wt.ugt80.
dsm.smt1.
dsm.wt.smt1.
sp.ugt80.
sp.wt.ugt80.
sp.smt1.
sp.wt.smt1.
im.ugt80.
im.wt.ugt80.
im.smt1.
im.wt.smt1.

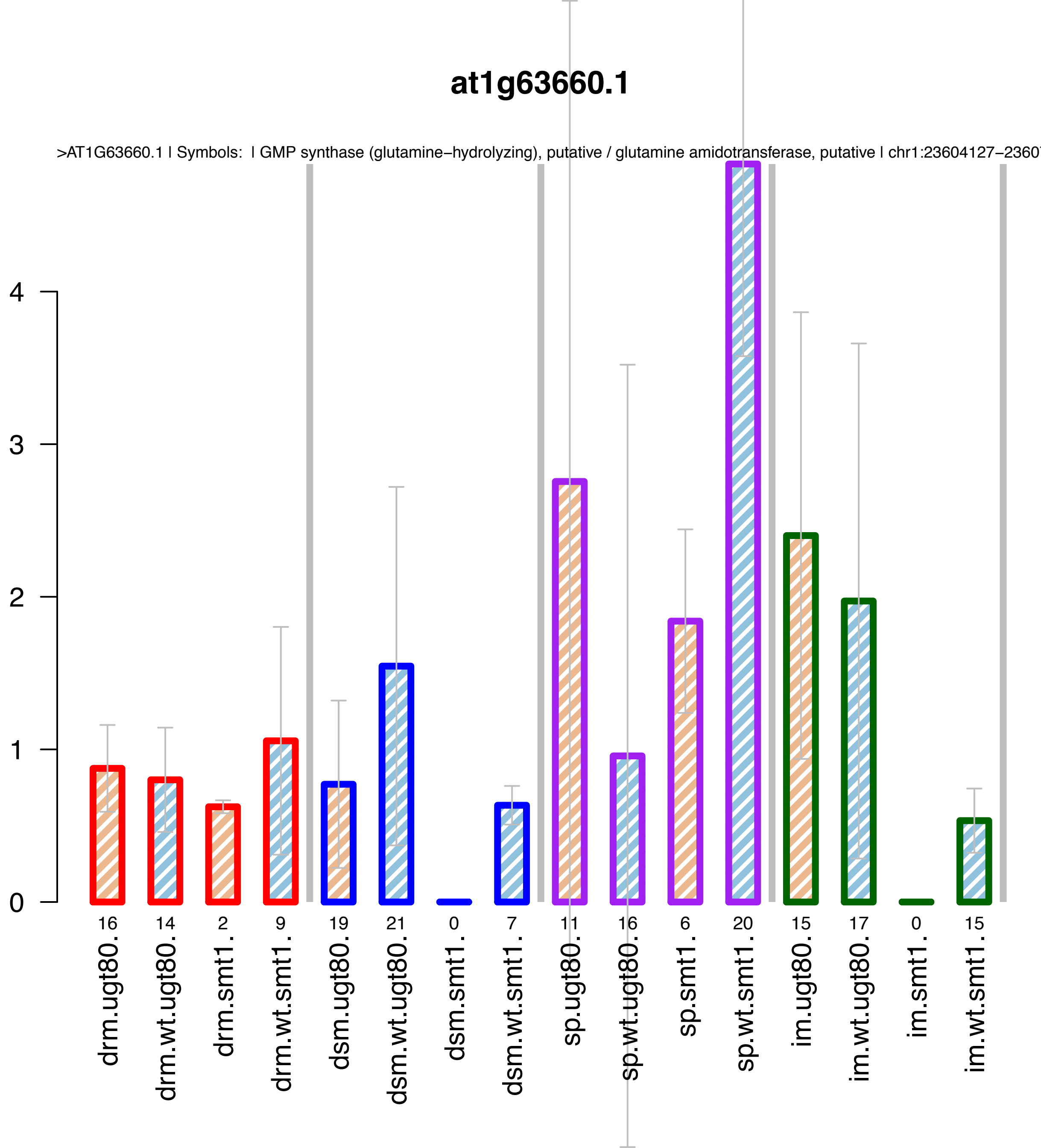

at1g63660.1
>AT1G63660.1 | Symbols: | GMP synthase (glutamine−hydrolyzing), putative / glutamine amidotransferase, putative | chr1:23604127−2360
0
1
2
3
4
16
14
2
9
19
21
0
7
11
16
6
20
15
17
0
15
drm.ugt80.
drm.wt.ugt80.
drm.smt1.
drm.wt.smt1.
dsm.ugt80.
dsm.wt.ugt80.
dsm.smt1.
dsm.wt.smt1.
sp.ugt80.
sp.wt.ugt80.
sp.smt1.
sp.wt.smt1.
im.ugt80.
im.wt.ugt80.
im.smt1.
im.wt.smt1.

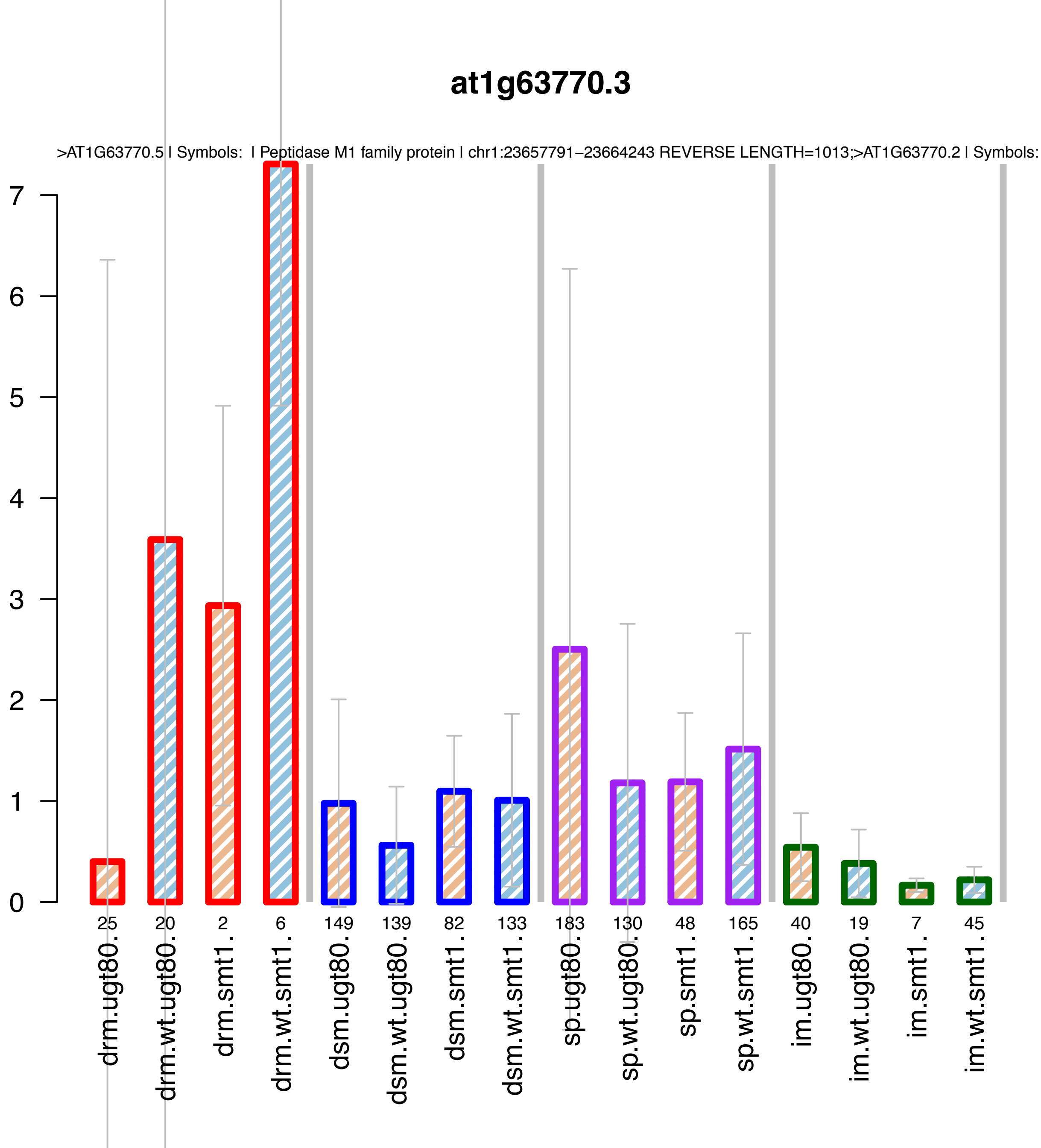

at1g63770.3
>AT1G63770.5 | Symbols: | Peptidase M1 family protein | chr1:23657791−23664243 REVERSE LENGTH=1013;>AT1G63770.2 | Symbols:
0
1
2
3
4
5
6
7
25
20
2
6
149
139
82
133
183
130
48
165
40
19
7
45
drm.ugt80..
drm.wt.ugt80..
drm.smt1..
drm.wt.smt1..
dsm.ugt80..
dsm.wt.ugt80..
dsm.smt1..
dsm.wt.smt1..
sp.ugt80..
sp.wt.ugt80..
sp.smt1..
sp.wt.smt1..
im.ugt80..
im.wt.ugt80..
im.smt1..
im.wt.smt1..

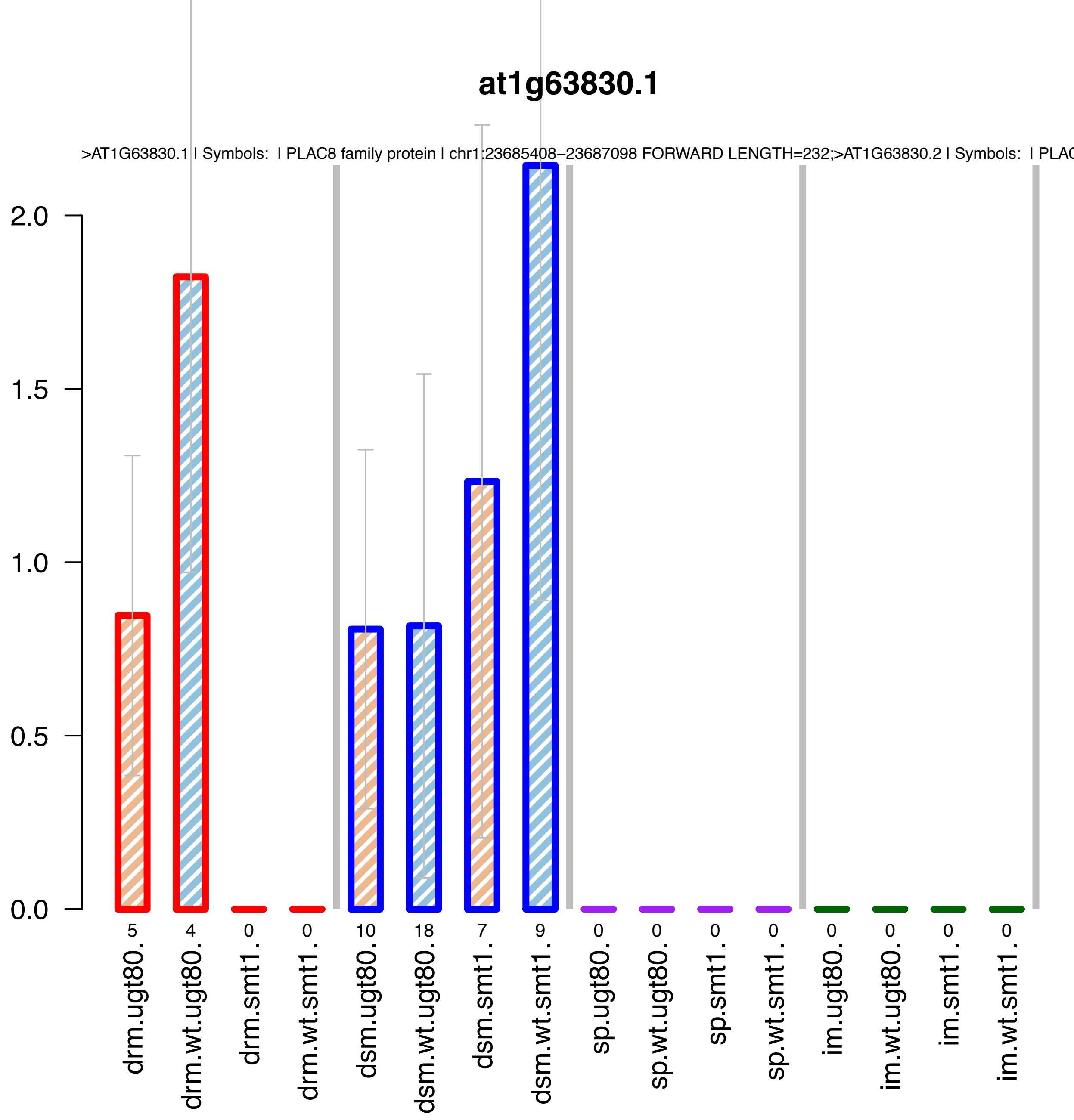
at1g63830.1
>AT1G63830.1 | Symbols: | PLAC8 family protein | chr1:23685408–23687098 FORWARD LENGTH=232;>AT1G63830.2 | Symbols: | PLAC
2.0
1.5
1.0
0.5
0.0
5
4
0
0
10
18
7
9
0
0
0
0
0
0
0
0
drm.ugt80.
drm.wt.ugt80.
drm.smt1.
drm.wt.smt1.
dsm.ugt80.
dsm.wt.ugt80.
dsm.smt1.
dsm.wt.smt1.
sp.ugt80.
sp.wt.ugt80.
sp.smt1.
sp.wt.smt1.
im.ugt80.
im.wt.ugt80.
im.smt1.
im.wt.smt1.

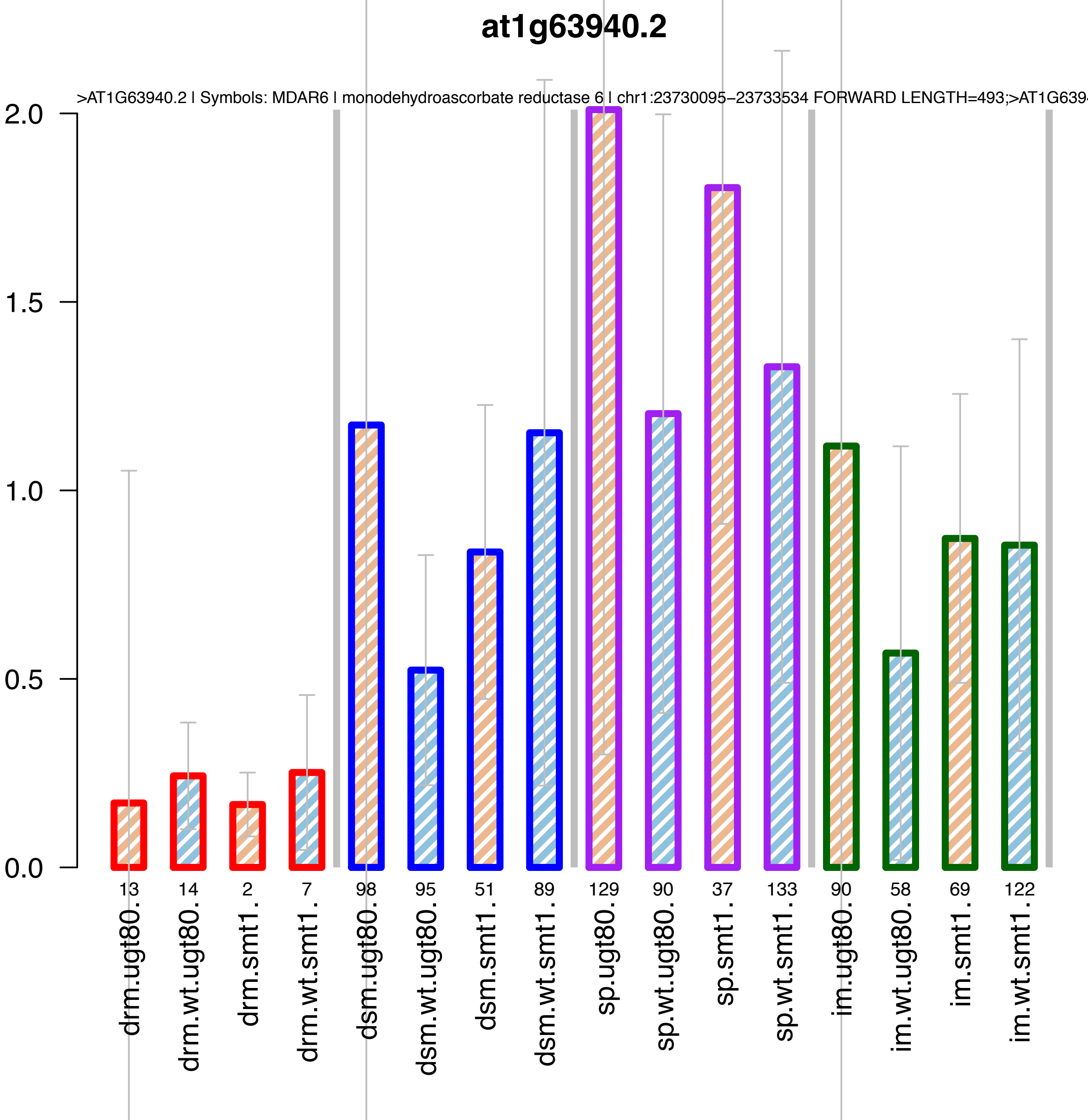
at1g63940.2
>AT1G63940.2 | Symbols: MDAR6 | monodehydroascorbate reductase 6 | chr1:23730095-23733534 FORWARD LENGTH=493;>AT1G639
2.0
1.5
1.0
0.5
0.0
13
14
2
7
98
95
51
89
129
90
37
133
90
58
69
122
drm.ugt80..
drm.wt.ugt80..
drm.smt1..
drm.wt.smt1..
dsm.ugt80..
dsm.wt.ugt80..
dsm.smt1..
dsm.wt.smt1..
sp.ugt80..
sp.wt.ugt80..
sp.smt1..
sp.wt.smt1..
im.ugt80..
im.wt.ugt80..
im.smt1..
im.wt.smt1..

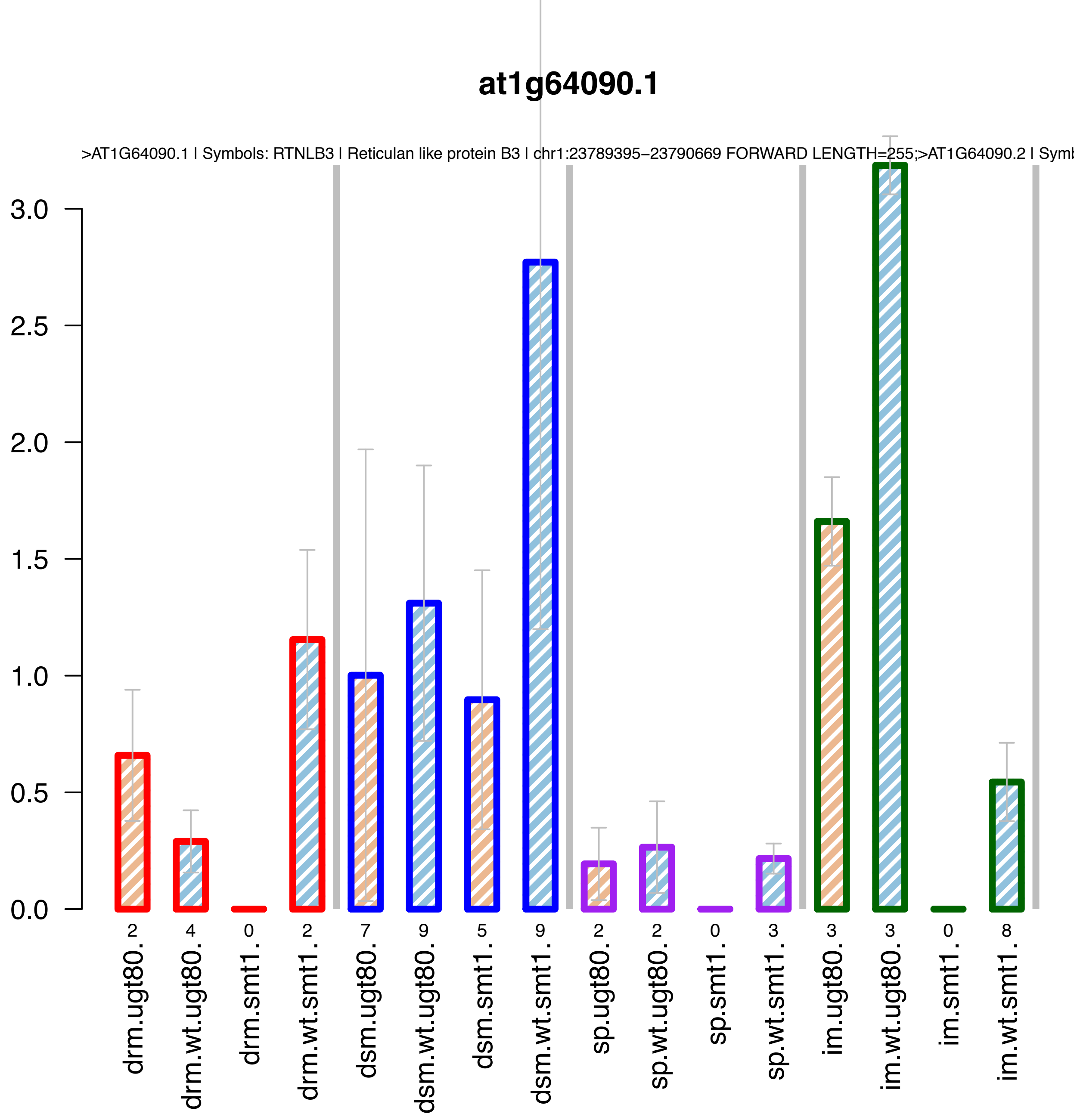

at1g64090.1
>AT1G64090.1 | Symbols: RTNLB3 | Reticulan like protein B3 | chr1:23789395−23790669 FORWARD LENGTH=255;>AT1G64090.2 | Symb
3.0
2.5
2.0
1.5
1.0
0.5
0.0
2
4
0
2
7
9
5
9
2
2
0
3
3
3
0
8
drm.ugt80.
drm.wt.ugt80.
drm.smt1.
drm.wt.smt1.
dsm.ugt80.
dsm.wt.ugt80.
dsm.smt1.
dsm.wt.smt1.
sp.ugt80.
sp.wt.ugt80.
sp.smt1.
sp.wt.smt1.
im.ugt80.
im.wt.ugt80.
im.smt1.
im.wt.smt1.

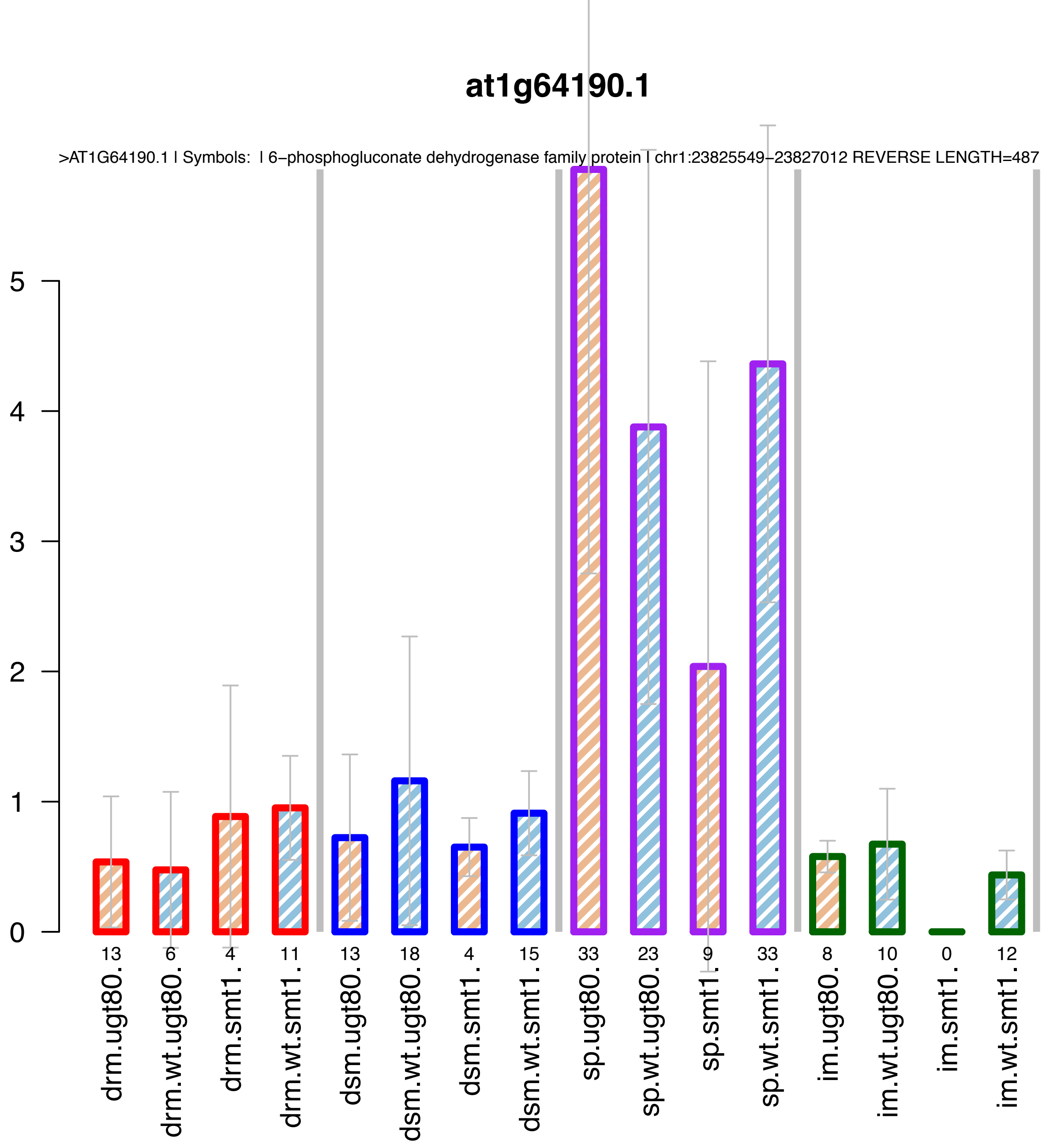
at1g64190.1
>AT1G64190.1 | Symbols: | 6−phosphogluconate dehydrogenase family protein | chr1:23825549−23827012 REVERSE LENGTH=487
0
1
2
3
4
5
13
6
4
11
13
18
4
15
33
23
9
33
8
10
0
12
drm.ugt80..
drm.wt.ugt80..
drm.smt1..
drm.wt.smt1..
dsm.ugt80..
dsm.wt.ugt80..
dsm.smt1..
dsm.wt.smt1..
sp.ugt80..
sp.wt.ugt80..
sp.smt1..
sp.wt.smt1..
im.ugt80..
im.wt.ugt80..
im.smt1..
im.wt.smt1..

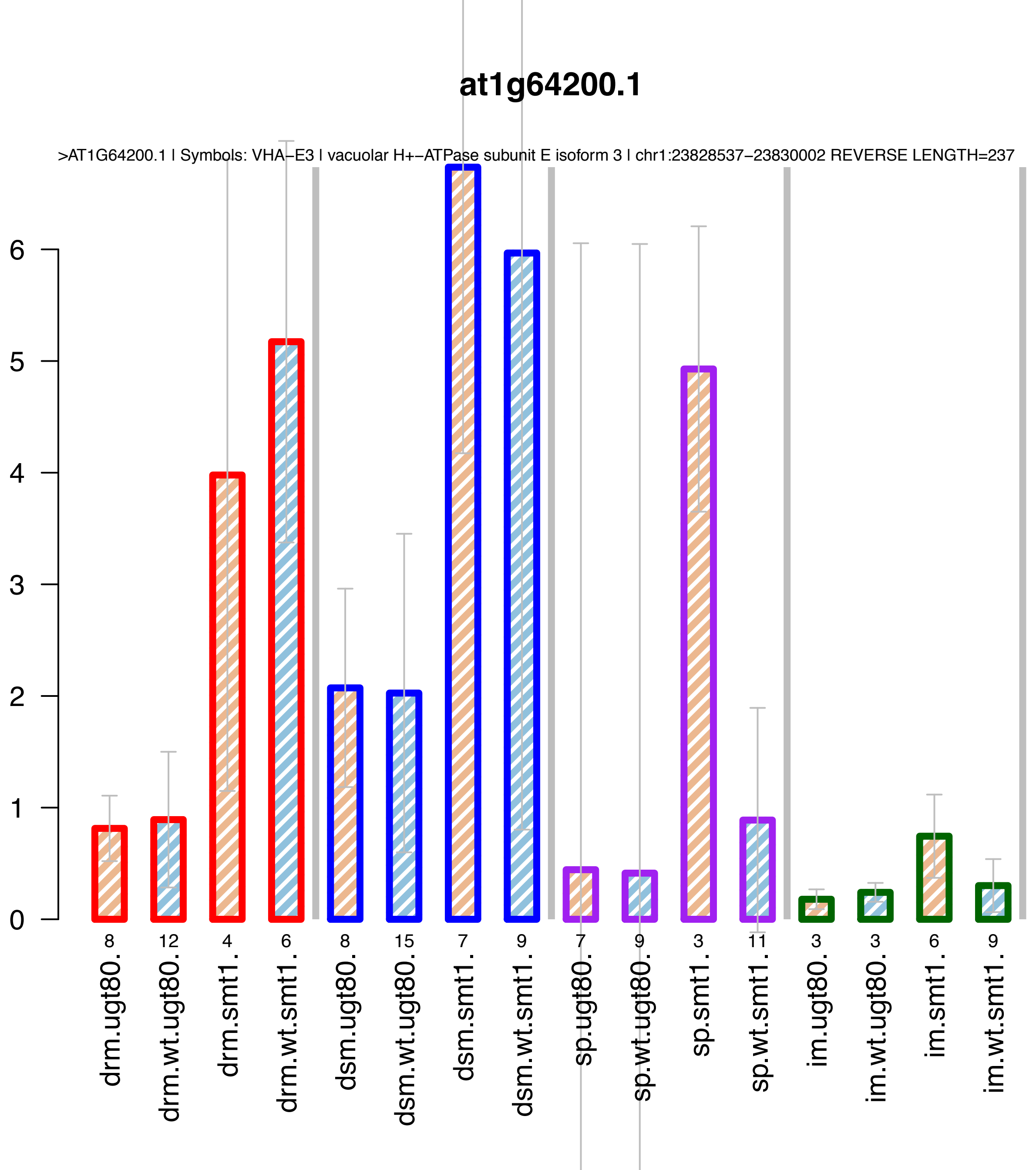

at1g64200.1
>AT1G64200.1 | Symbols: VHA−E3 | vacuolar H+−ATPase subunit E isoform 3 | chr1:23828537−23830002 REVERSE LENGTH=237
0
1
2
3
4
5
6
8
12
4
6
8
15
7
9
7
9
3
11
3
3
6
9
drm.ugt80.
drm.wt.ugt80.
drm.smt1.
drm.wt.smt1.
dsm.ugt80.
dsm.wt.ugt80.
dsm.smt1.
dsm.wt.smt1.
sp.ugt80.
sp.wt.ugt80.
sp.smt1.
sp.wt.smt1.
im.ugt80.
im.wt.ugt80.
im.smt1.
im.wt.smt1.

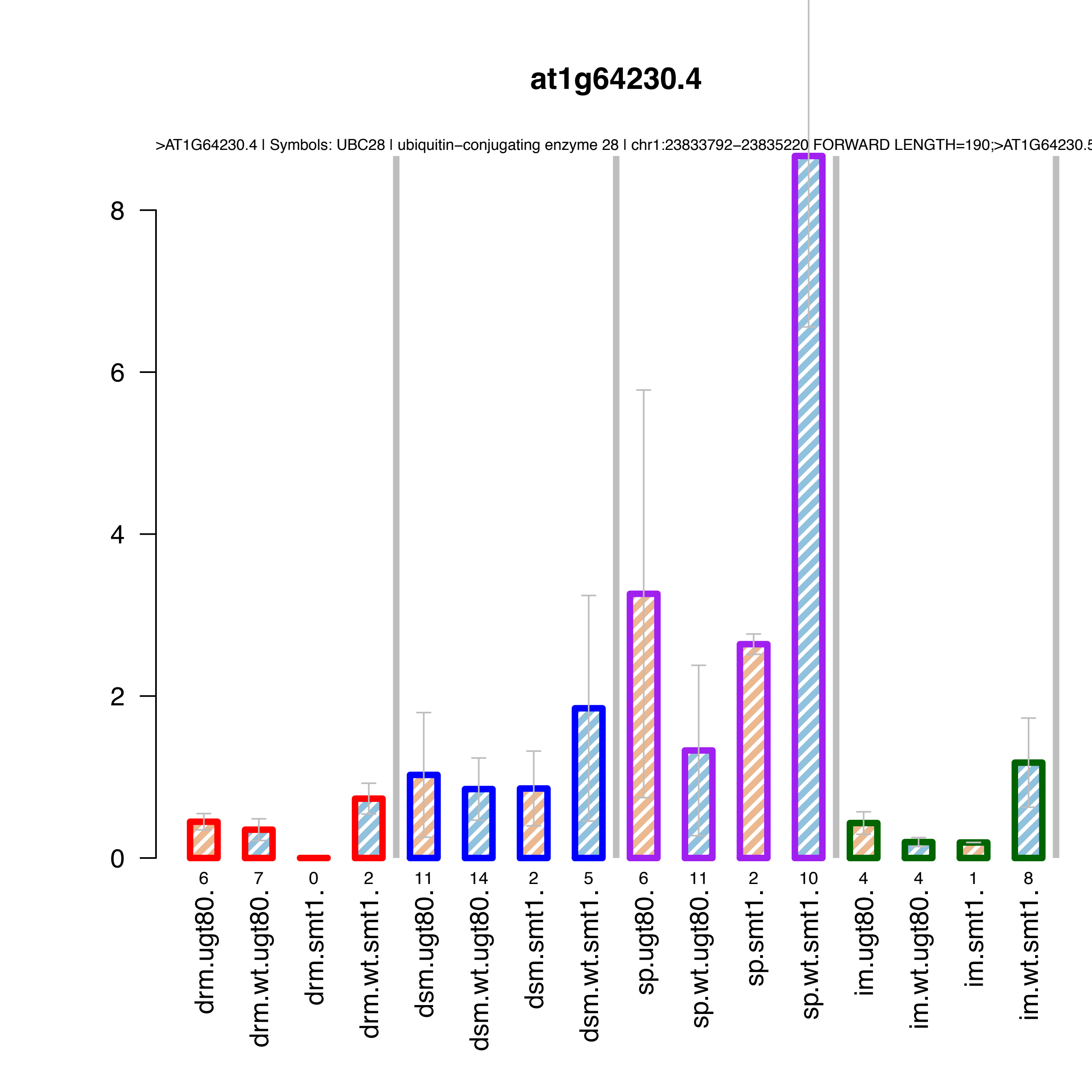

at1g64230.4
>AT1G64230.4 | Symbols: UBC28 | ubiquitin−conjugating enzyme 28 | chr1:23833792−23835220 FORWARD LENGTH=190;>AT1G64230.5
0
2
4
6
8
6
7
0
2
11
14
2
5
6
11
2
10
4
4
1
8
drm.ugt80.
drm.wt.ugt80.
drm.smt1.
drm.wt.smt1.
dsm.ugt80.
dsm.wt.ugt80.
dsm.smt1.
dsm.wt.smt1.
sp.ugt80.
sp.wt.ugt80.
sp.smt1.
sp.wt.smt1.
im.ugt80.
im.wt.ugt80.
im.smt1.
im.wt.smt1.

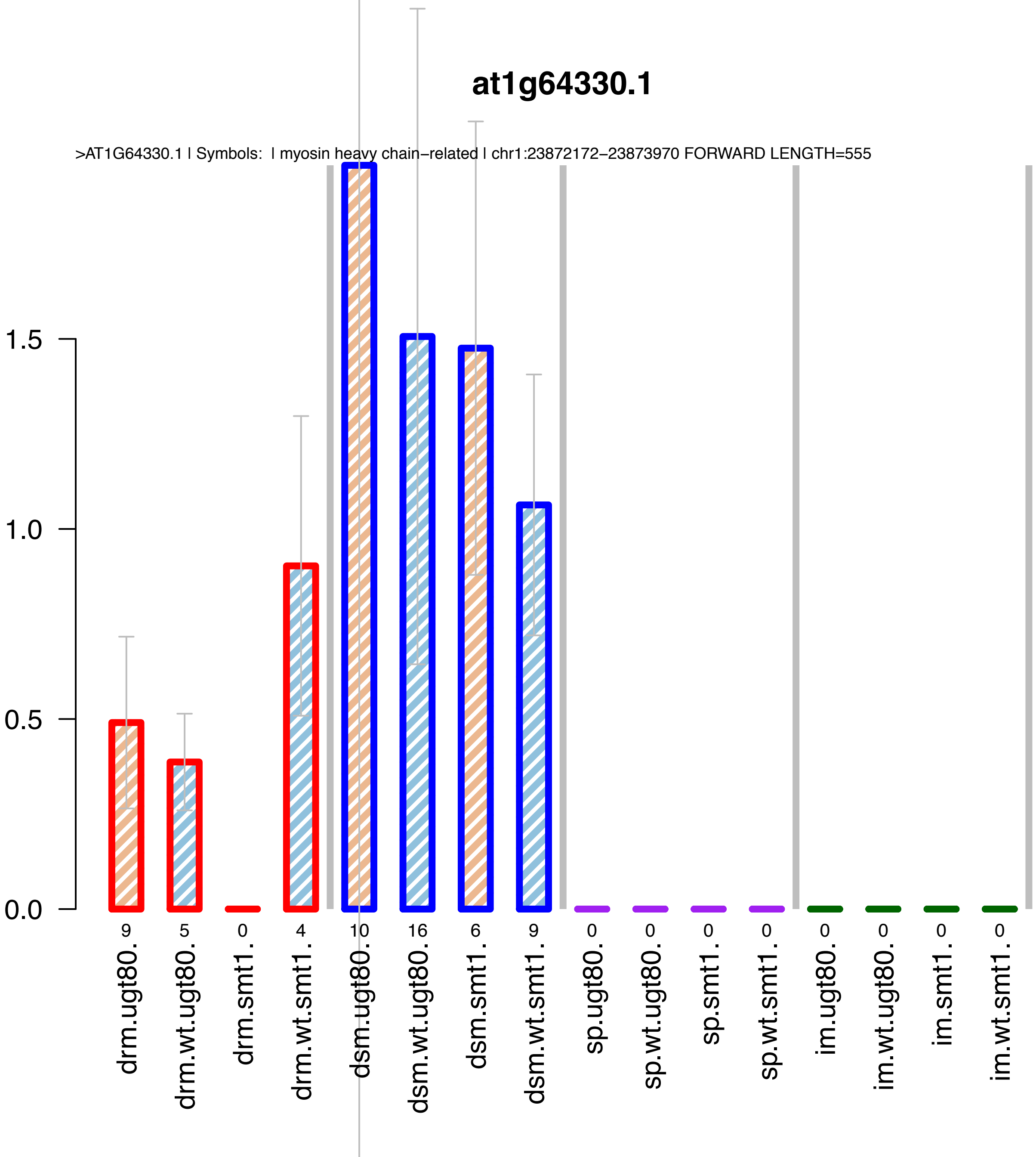

at1g64330.1
>AT1G64330.1 | Symbols: | myosin heavy chain−related | chr1:23872172−23873970 FORWARD LENGTH=555
0.0
0.5
1.0
1.5
9
5
0
4
10
16
6
9
0
0
0
0
0
0
0
0
drm.ugt80.
drm.wt.ugt80.
drm.smt1.
drm.wt.smt1.
dsm.ugt80.
dsm.wt.ugt80.
dsm.smt1.
dsm.wt.smt1.
sp.ugt80.
sp.wt.ugt80.
sp.smt1.
sp.wt.smt1.
im.ugt80.
im.wt.ugt80.
im.smt1.
im.wt.smt1.

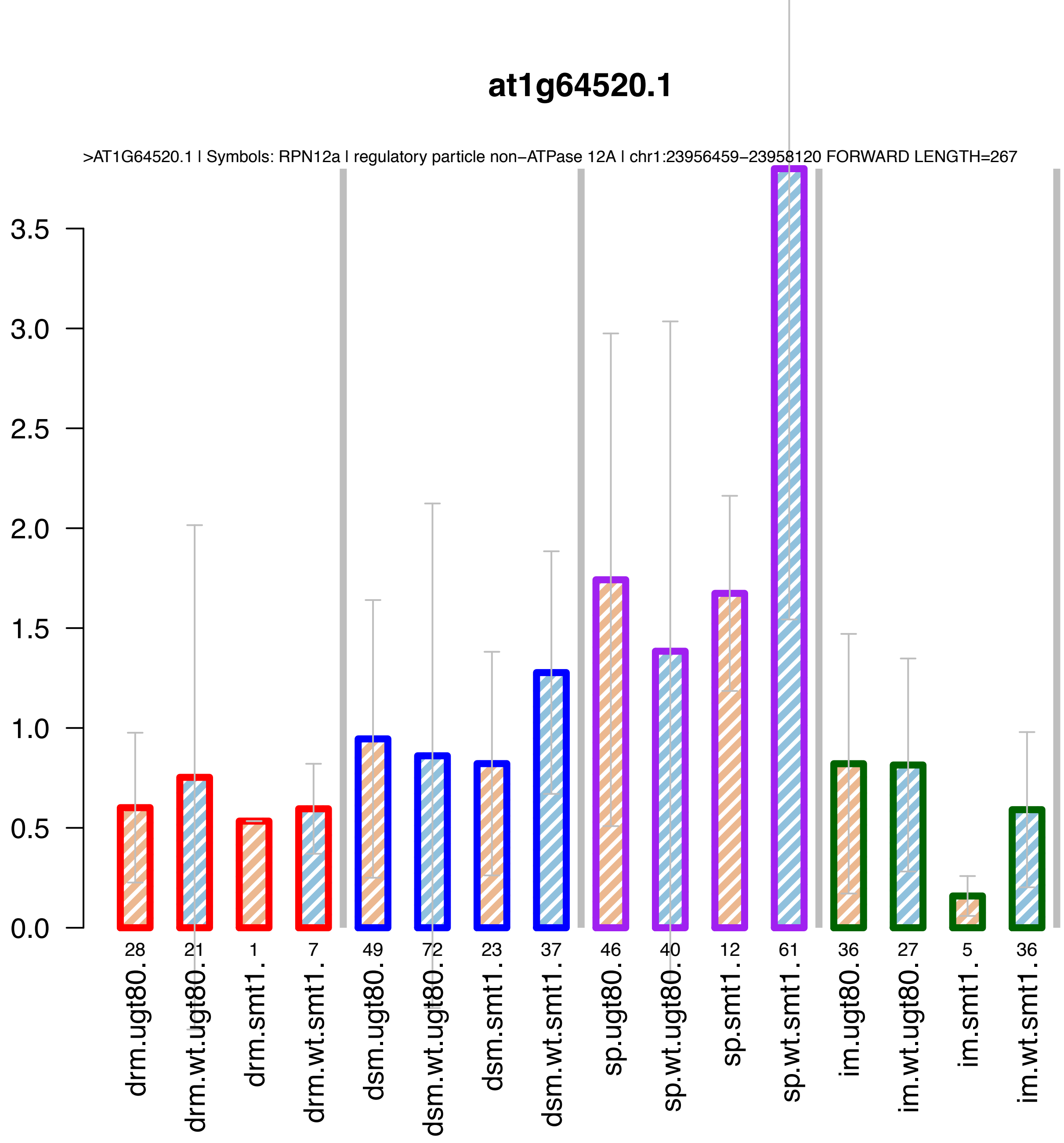

at1g64520.1
>AT1G64520.1 | Symbols: RPN12a | regulatory particle non−ATPase 12A | chr1:23956459−23958120 FORWARD LENGTH=267
3.5
3.0
2.5
2.0
1.5
1.0
0.5
0.0
28
21
1
7
49
72
23
37
46
40
12
61
36
27
5
36
drm.ugt80.
drm.wt.ugt80.
drm.smt1.
drm.wt.smt1.
dsm.ugt80.
dsm.wt.ugt80.
dsm.smt1.
dsm.wt.smt1.
sp.ugt80.
sp.wt.ugt80.
sp.smt1.
sp.wt.smt1.
im.ugt80.
im.wt.ugt80.
im.smt1.
im.wt.smt1.

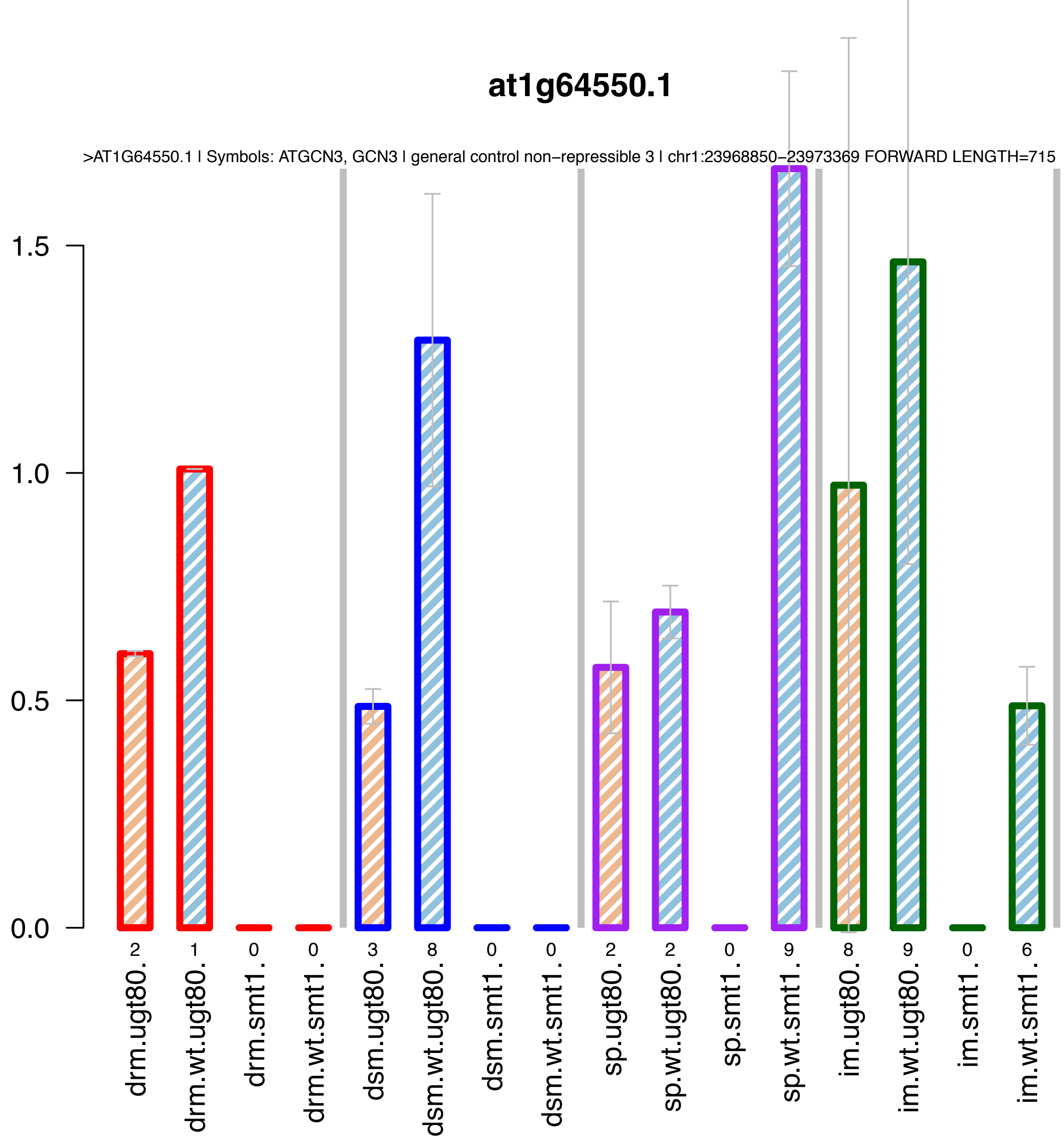

at1g64550.1
>AT1G64550.1 | Symbols: ATGCN3, GCN3 | general control non−repressible 3 | chr1:23968850−23973369 FORWARD LENGTH=715
0.0
0.5
1.0
1.5
2
1
0
0
3
8
0
0
2
2
0
9
8
9
0
6
drm.ugt80.
drm.wt.ugt80.
drm.smt1.
drm.wt.smt1.
dsm.ugt80.
dsm.wt.ugt80.
dsm.smt1.
dsm.wt.smt1.
sp.ugt80.
sp.wt.ugt80.
sp.smt1.
sp.wt.smt1.
im.ugt80.
im.wt.ugt80.
im.smt1.
im.wt.smt1.

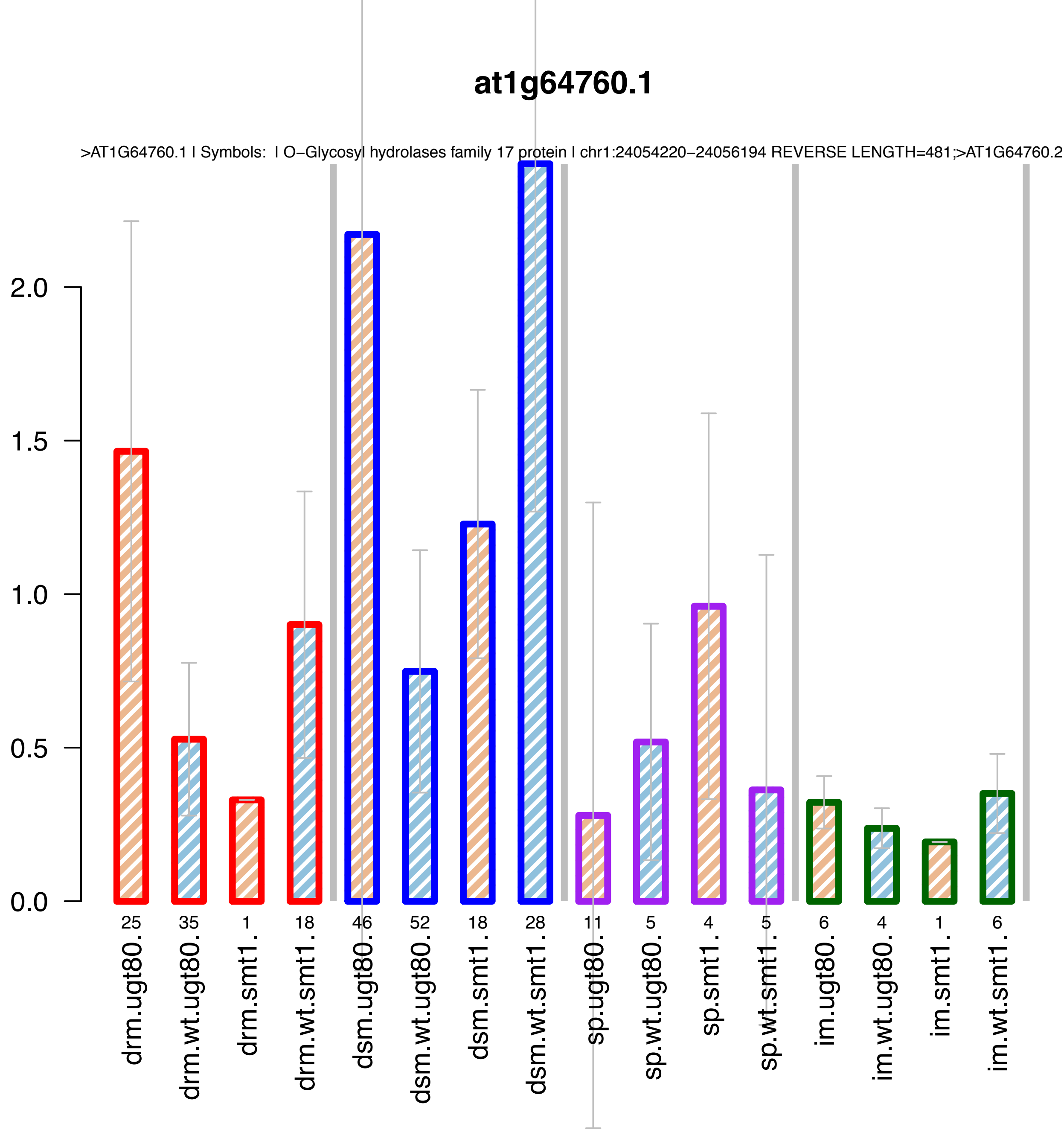

at1g64760.1
>AT1G64760.1 | Symbols: | O−Glycosyl hydrolases family 17 protein | chr1:24054220−24056194 REVERSE LENGTH=481;>AT1G64760.2
2.0
1.5
1.0
0.5
0.0
25
35
1
18
46
52
18
28
11
5
4
5
6
4
1
6
drm.ugt80.
drm.wt.ugt80.
drm.smt1.
drm.wt.smt1.
dsm.ugt80.
dsm.wt.ugt80.
dsm.smt1.
dsm.wt.smt1.
sp.ugt80.
sp.wt.ugt80.
sp.smt1.
sp.wt.smt1.
im.ugt80.
im.wt.ugt80.
im.smt1.
im.wt.smt1.

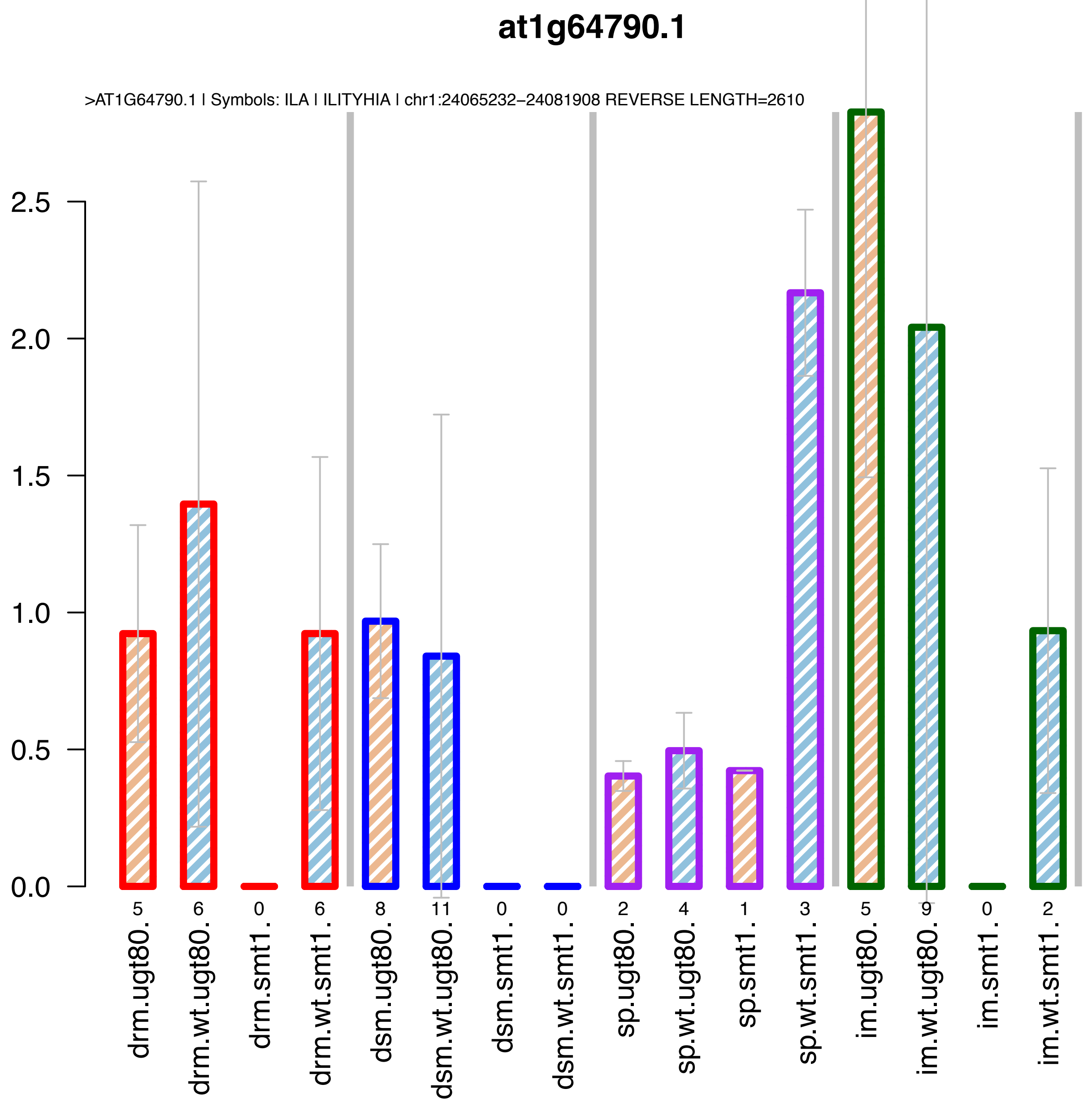

at1g64790.1
>AT1G64790.1 | Symbols: ILA | ILITYHIA | chr1:24065232-24081908 REVERSE LENGTH=2610
0.0
0.5
1.0
1.5
2.0
2.5
5
6
0
6
8
11
0
0
2
4
1
3
5
9
0
2
drm.ugt80.
drm.wt.ugt80.
drm.smt1.
drm.wt.smt1.
dsm.ugt80.
dsm.wt.ugt80.
dsm.smt1.
dsm.wt.smt1.
sp.ugt80.
sp.wt.ugt80.
sp.smt1.
sp.wt.smt1.
im.ugt80.
im.wt.ugt80.
im.smt1.
im.wt.smt1.

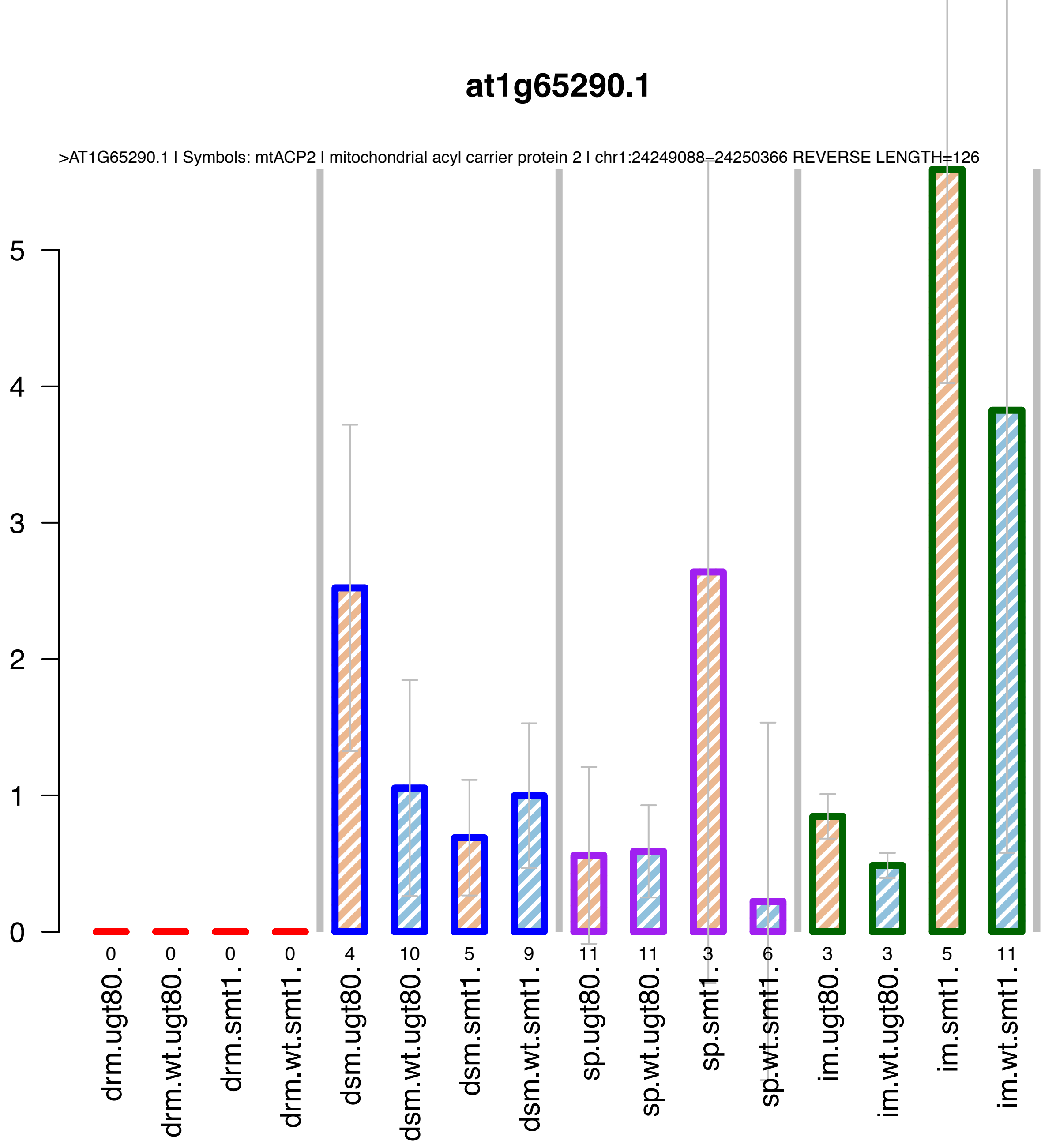
at1g65290.1
>AT1G65290.1 | Symbols: mtACP2 | mitochondrial acyl carrier protein 2 | chr1:24249088-24250366 REVERSE LENGTH=126
0
1
2
3
4
5
0 0 0 0 4 10 5 9 11 11 3 6 3 3 5 11
drm.ugt80.
drm.wt.ugt80.
drm.smt1.
drm.wt.smt1.
dsm.ugt80.
dsm.wt.ugt80.
dsm.smt1.
dsm.wt.smt1.
sp.ugt80.
sp.wt.ugt80.
sp.smt1.
sp.wt.smt1.
im.ugt80.
im.wt.ugt80.
im.smt1.
im.wt.smt1.

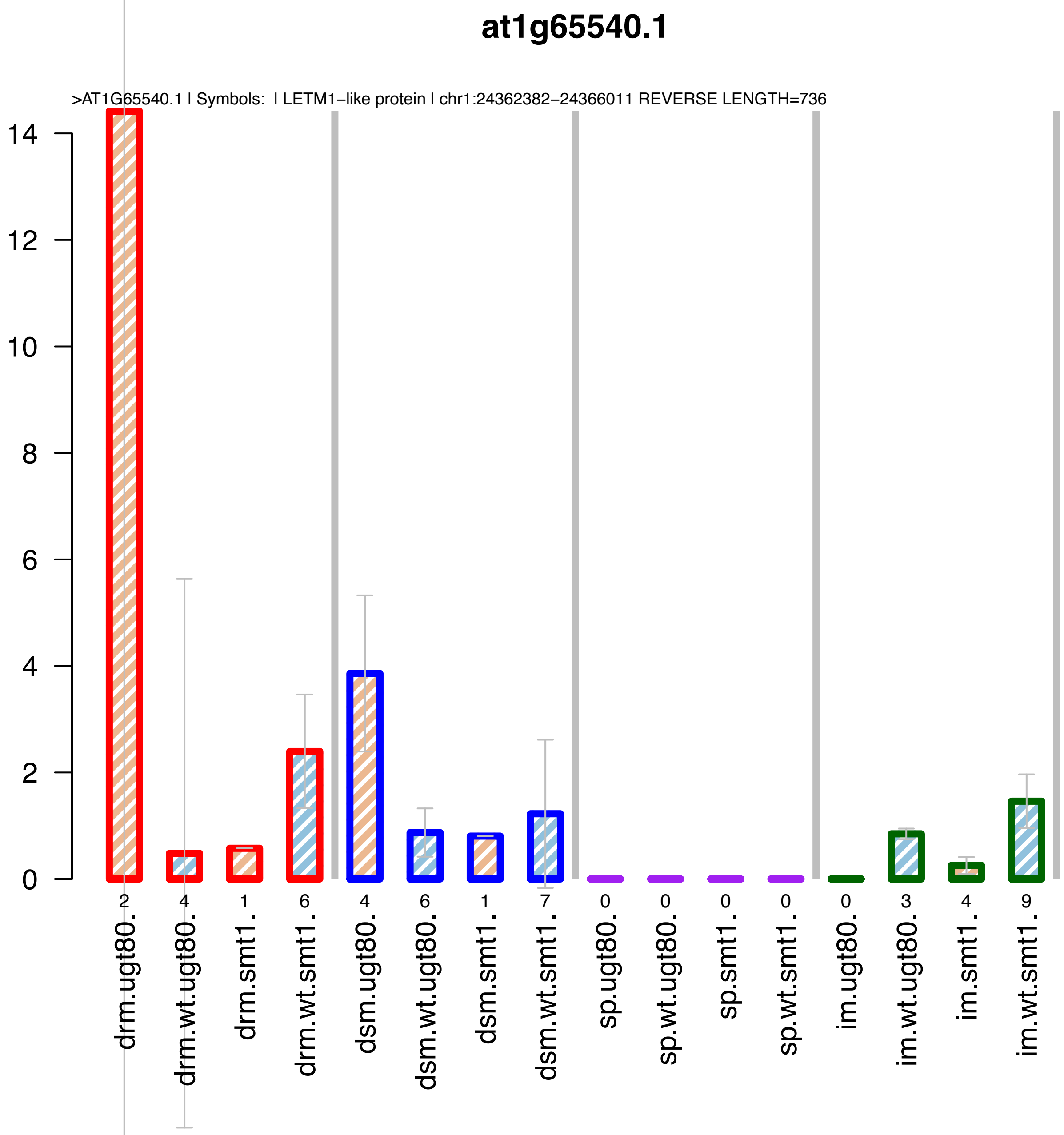

at1g65540.1
>AT1G65540.1 | Symbols: | LETM1−like protein | chr1:24362382−24366011 REVERSE LENGTH=736
0
2
4
6
8
10
12
14
2 4 1 6 4 6 1 7 0 0 0 0 0 3 4 9
drm.ugt80.
drm.wt.ugt80.
drm.smt1.
drm.wt.smt1.
dsm.ugt80.
dsm.wt.ugt80.
dsm.smt1.
dsm.wt.smt1.
sp.ugt80.
sp.wt.ugt80.
sp.smt1.
sp.wt.smt1.
im.ugt80.
im.wt.ugt80.
im.smt1.
im.wt.smt1.

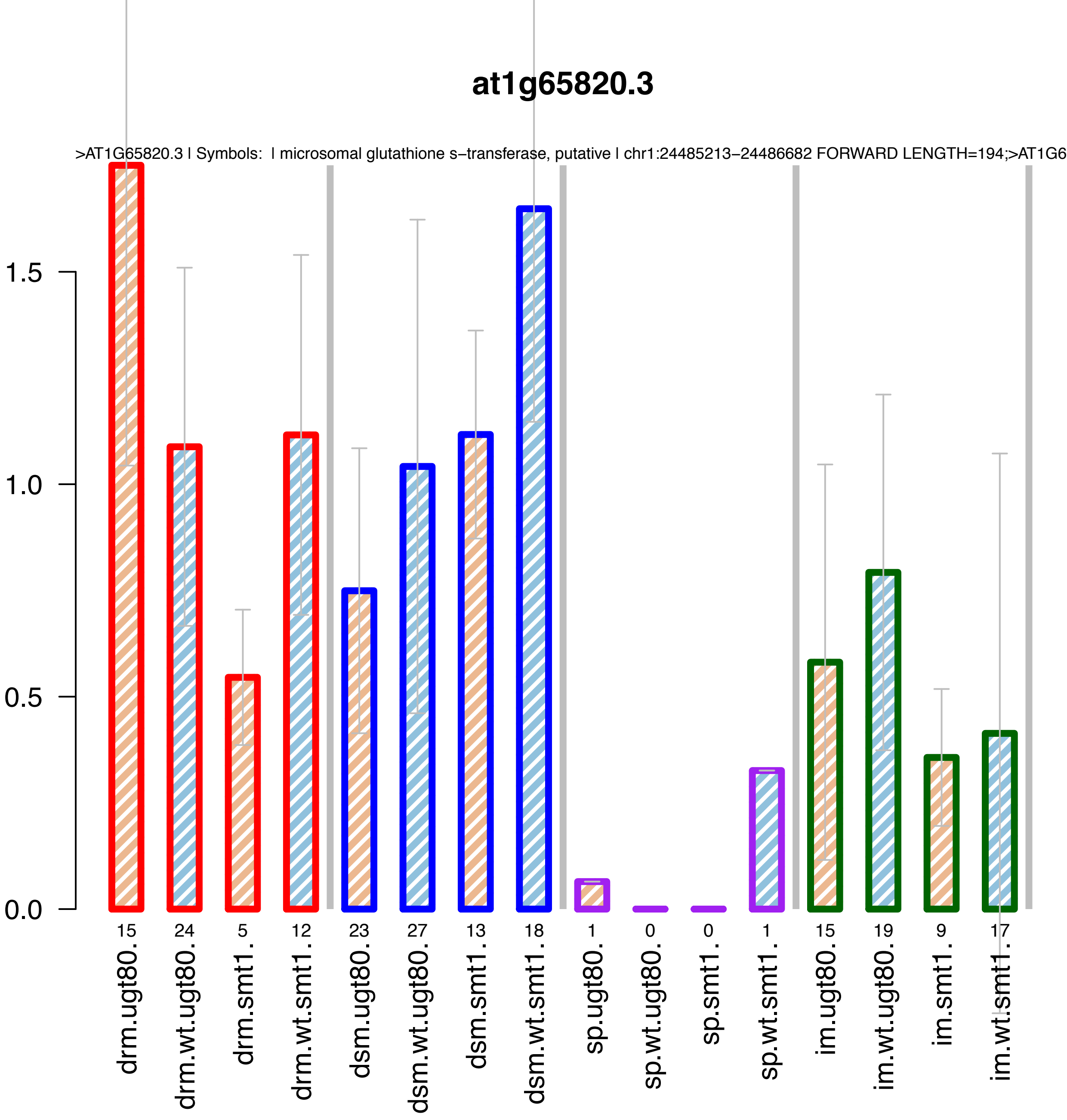
at1g65820.3
>AT1G65820.3 | Symbols: | microsomal glutathione s-transferase, putative | chr1:24485213-24486682 FORWARD LENGTH=194;>AT1G6
1.5
1.0
0.5
0.0
15
24
5
12
23
27
13
18
1
0
0
1
15
19
9
17
drm.ugt80..
drm.wt.ugt80..
drm.smt1..
drm.wt.smt1..
dsm.ugt80..
dsm.wt.ugt80..
dsm.smt1..
dsm.wt.smt1..
sp.ugt80..
sp.wt.ugt80..
sp.smt1..
sp.wt.smt1..
im.ugt80..
im.wt.ugt80..
im.smt1..
im.wt.smt1..

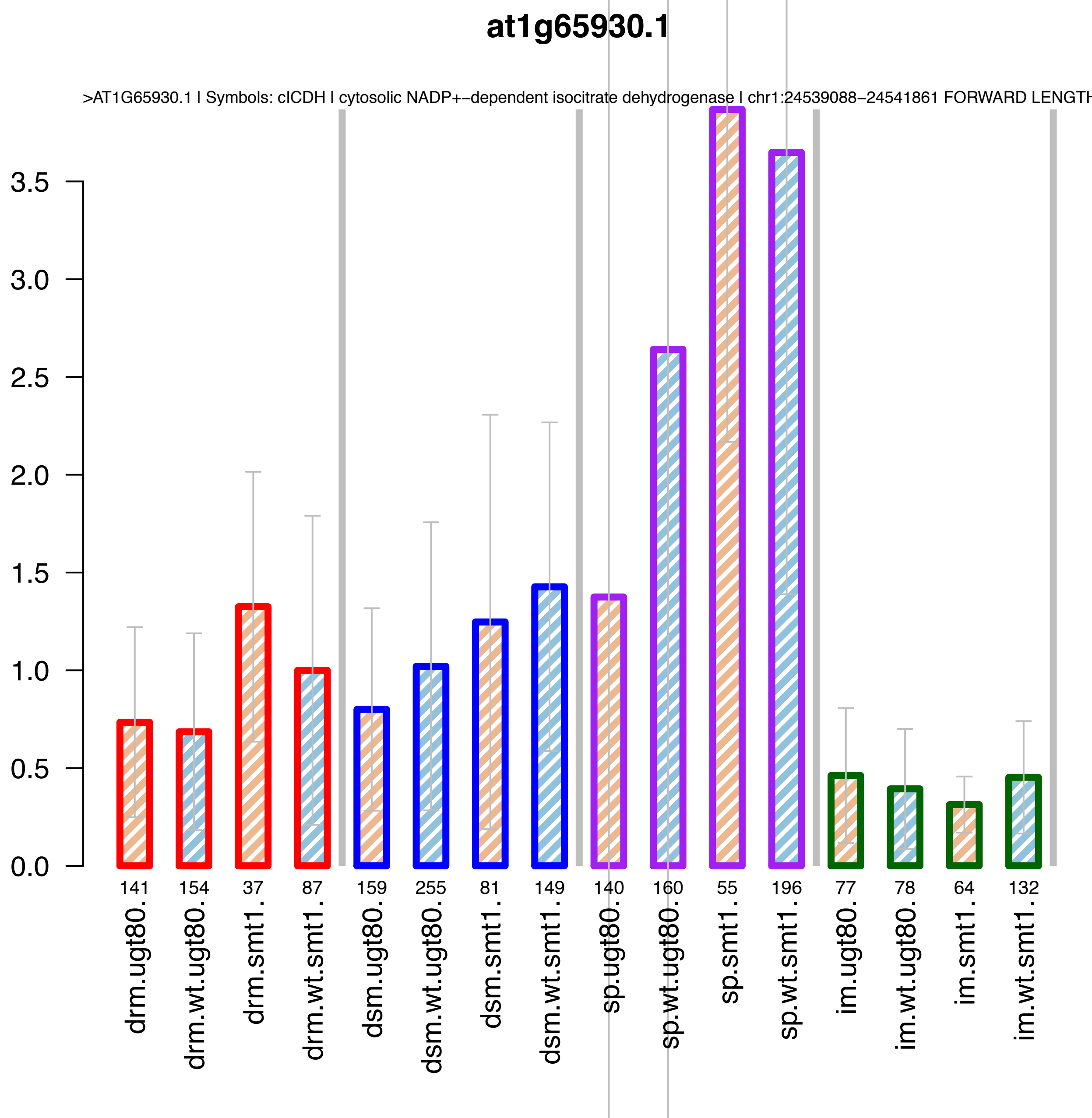

at1g65930.1
>AT1G65930.1 | Symbols: cICDH | cytosolic NADP+-dependent isocitrate dehydrogenase | chr1:24539088-24541861 FORWARD LENGTH
3.5
3.0
2.5
2.0
1.5
1.0
0.5
0.0
141
154
37
87
159
255
81
149
140
160
55
196
77
78
64
132
drm.ugt80..
drm.wt.ugt80..
drm.smt1..
drm.wt.smt1..
dsm.ugt80..
dsm.wt.ugt80..
dsm.smt1..
dsm.wt.smt1..
sp.ugt80..
sp.wt.ugt80..
sp.smt1..
sp.wt.smt1..
im.ugt80..
im.wt.ugt80..
im.smt1..
im.wt.smt1..

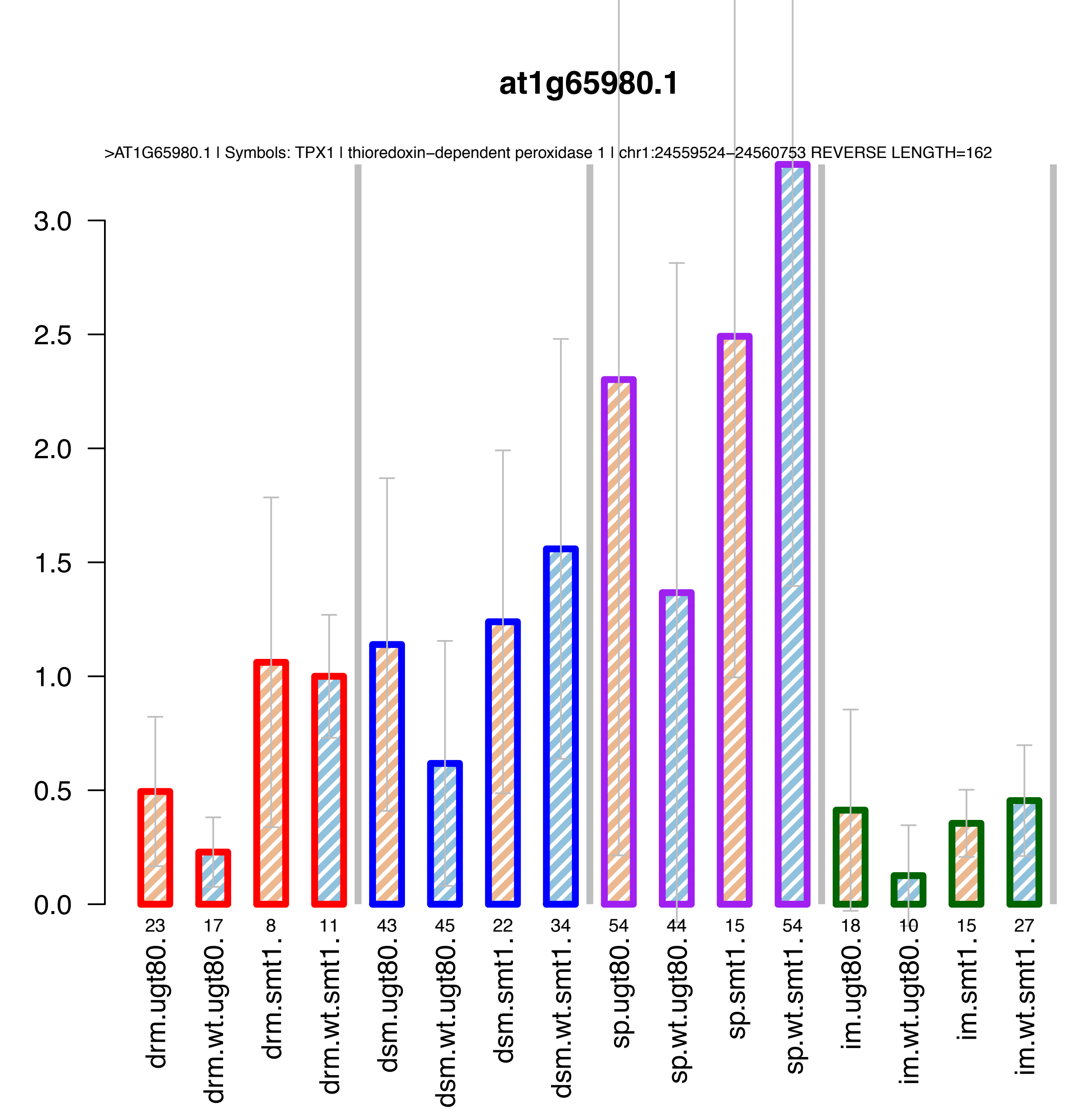

at1g65980.1
>AT1G65980.1 | Symbols: TPX1 | thioredoxin−dependent peroxidase 1 | chr1:24559524−24560753 REVERSE LENGTH=162
0.0
0.5
1.0
1.5
2.0
2.5
3.0
23
17
8
11
43
45
22
34
54
44
15
54
18
10
15
27
drm.ugt80..
drm.wt.ugt80..
drm.smt1..
drm.wt.smt1..
dsm.ugt80..
dsm.wt.ugt80..
dsm.smt1..
dsm.wt.smt1..
sp.ugt80..
sp.wt.ugt80..
sp.smt1..
sp.wt.smt1..
im.ugt80..
im.wt.ugt80..
im.smt1..
im.wt.smt1..

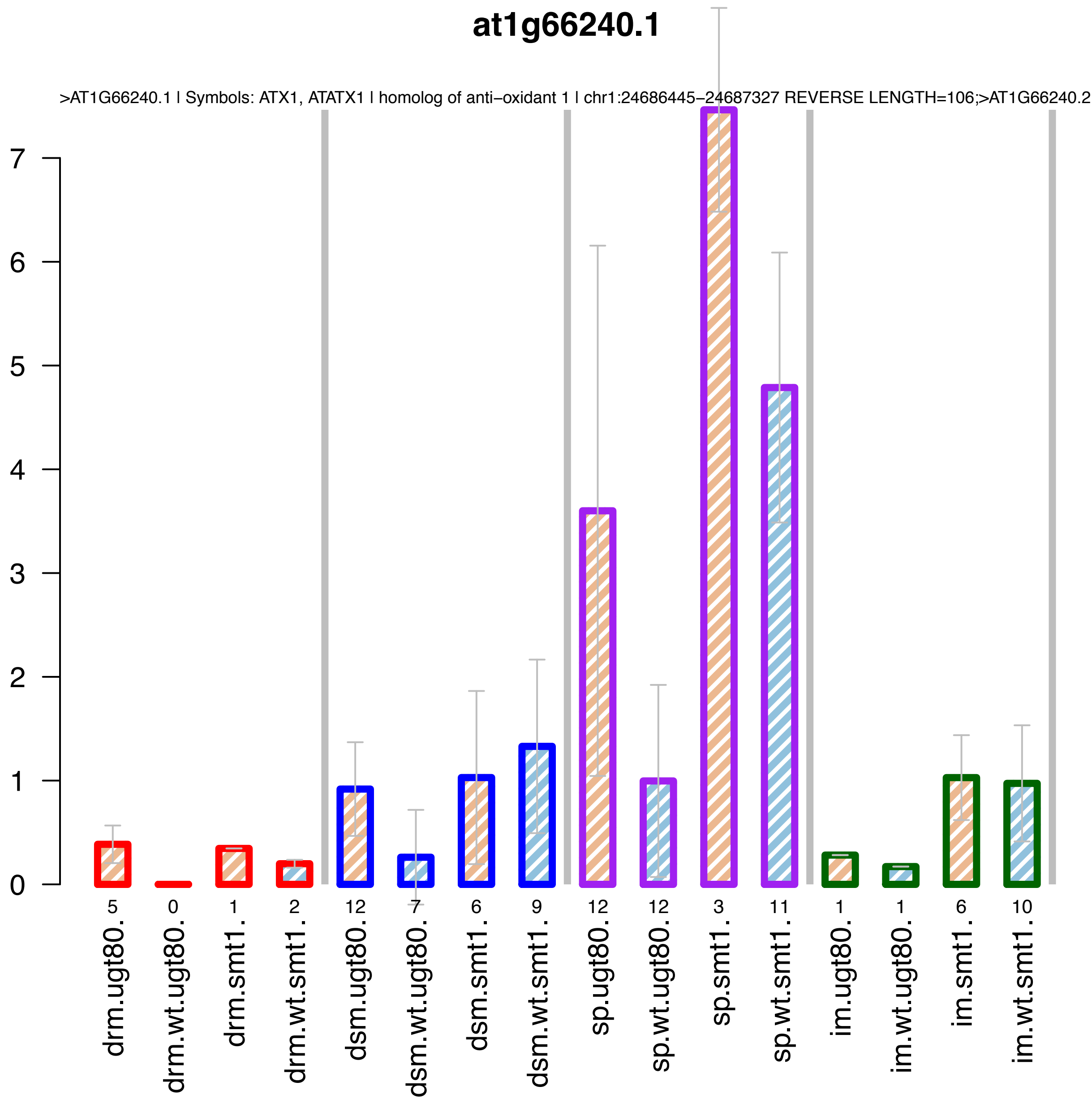
at1g66240.1
>AT1G66240.1 | Symbols: ATX1, ATATX1 | homolog of anti−oxidant 1 | chr1:24686445−24687327 REVERSE LENGTH=106;>AT1G66240.2
0
1
2
3
4
5
6
7
5
0
1
2
12
7
6
9
12
12
3
11
1
1
6
10
drm.ugt80.
drm.wt.ugt80.
drm.smt1.
drm.wt.smt1.
dsm.ugt80.
dsm.wt.ugt80.
dsm.smt1.
dsm.wt.smt1.
sp.ugt80.
sp.wt.ugt80.
sp.smt1.
sp.wt.smt1.
im.ugt80.
im.wt.ugt80.
im.smt1.
im.wt.smt1.

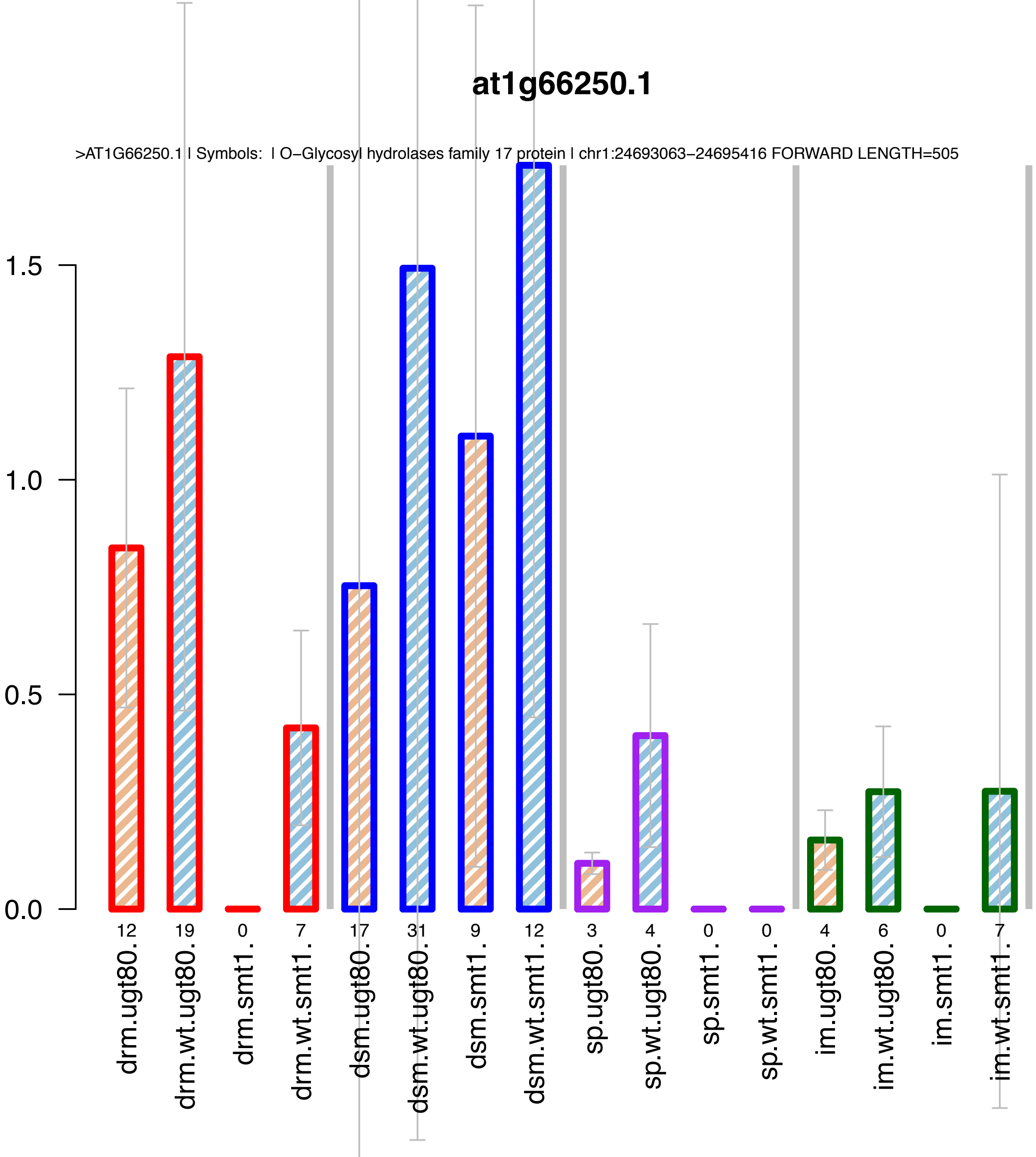

at1g66250.1
>AT1G66250.1 | Symbols: | O−Glycosyl hydrolases family 17 protein | chr1:24693063−24695416 FORWARD LENGTH=505
1.5
1.0
0.5
0.0
12
19
0
7
17
31
9
12
3
4
0
0
4
6
0
7
drm.ugt80.
drm.wt.ugt80.
drm.smt1.
drm.wt.smt1.
dsm.ugt80.
dsm.wt.ugt80.
dsm.smt1.
dsm.wt.smt1.
sp.ugt80.
sp.wt.ugt80.
sp.smt1.
sp.wt.smt1.
im.ugt80.
im.wt.ugt80.
im.smt1.
im.wt.smt1.

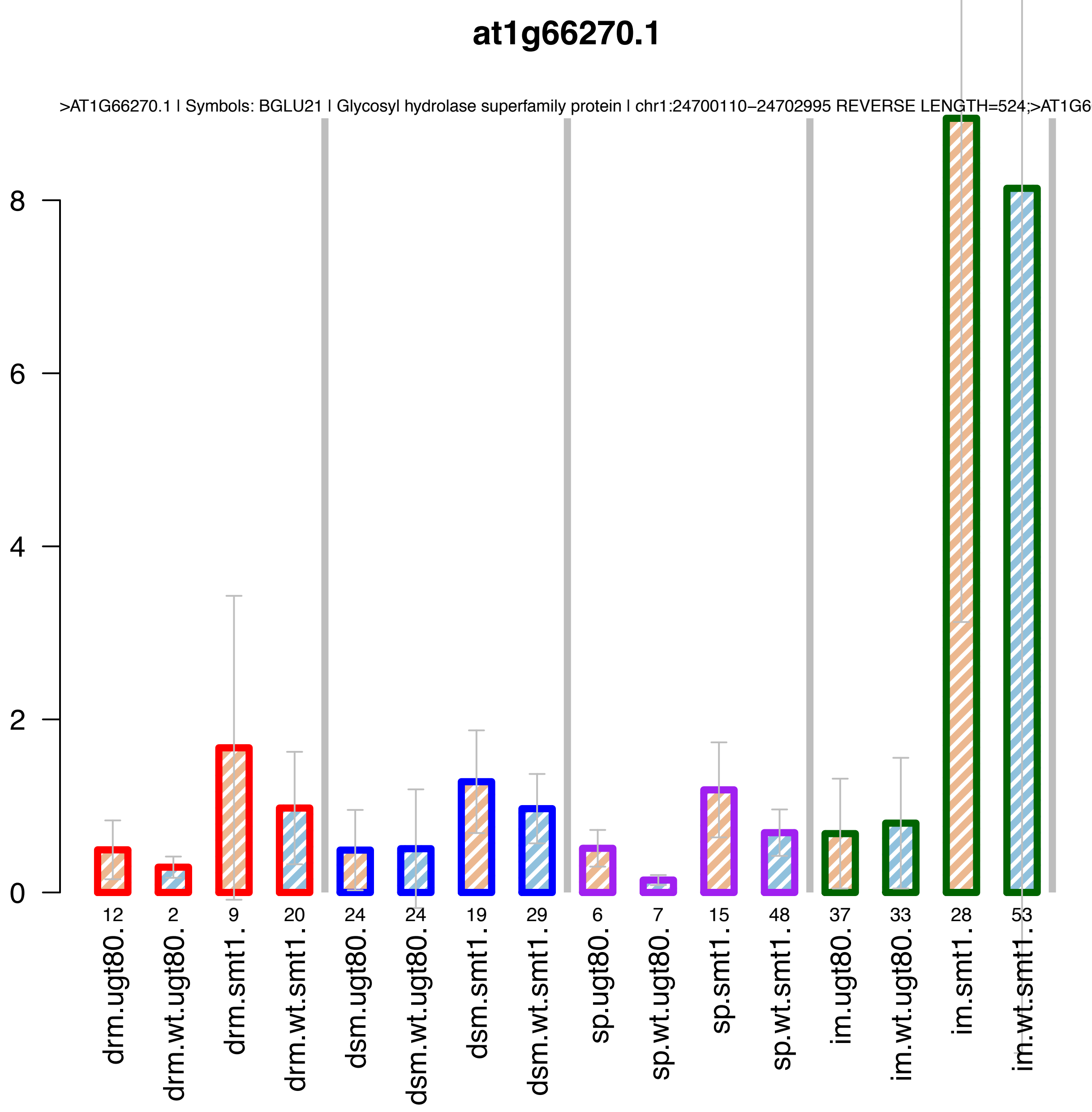
at1g66270.1
>AT1G66270.1 | Symbols: BGLU21 | Glycosyl hydrolase superfamily protein | chr1:24700110-24702995 REVERSE LENGTH=524;>AT1G6
0
2
4
6
8
12
2
9
20
24
24
19
29
6
7
15
48
37
33
28
53
drm.ugt80.
drm.wt.ugt80.
drm.smt1.
drm.wt.smt1.
dsm.ugt80.
dsm.wt.ugt80.
dsm.smt1.
dsm.wt.smt1.
sp.ugt80.
sp.wt.ugt80.
sp.smt1.
sp.wt.smt1.
im.ugt80.
im.wt.ugt80.
im.smt1.
im.wt.smt1.

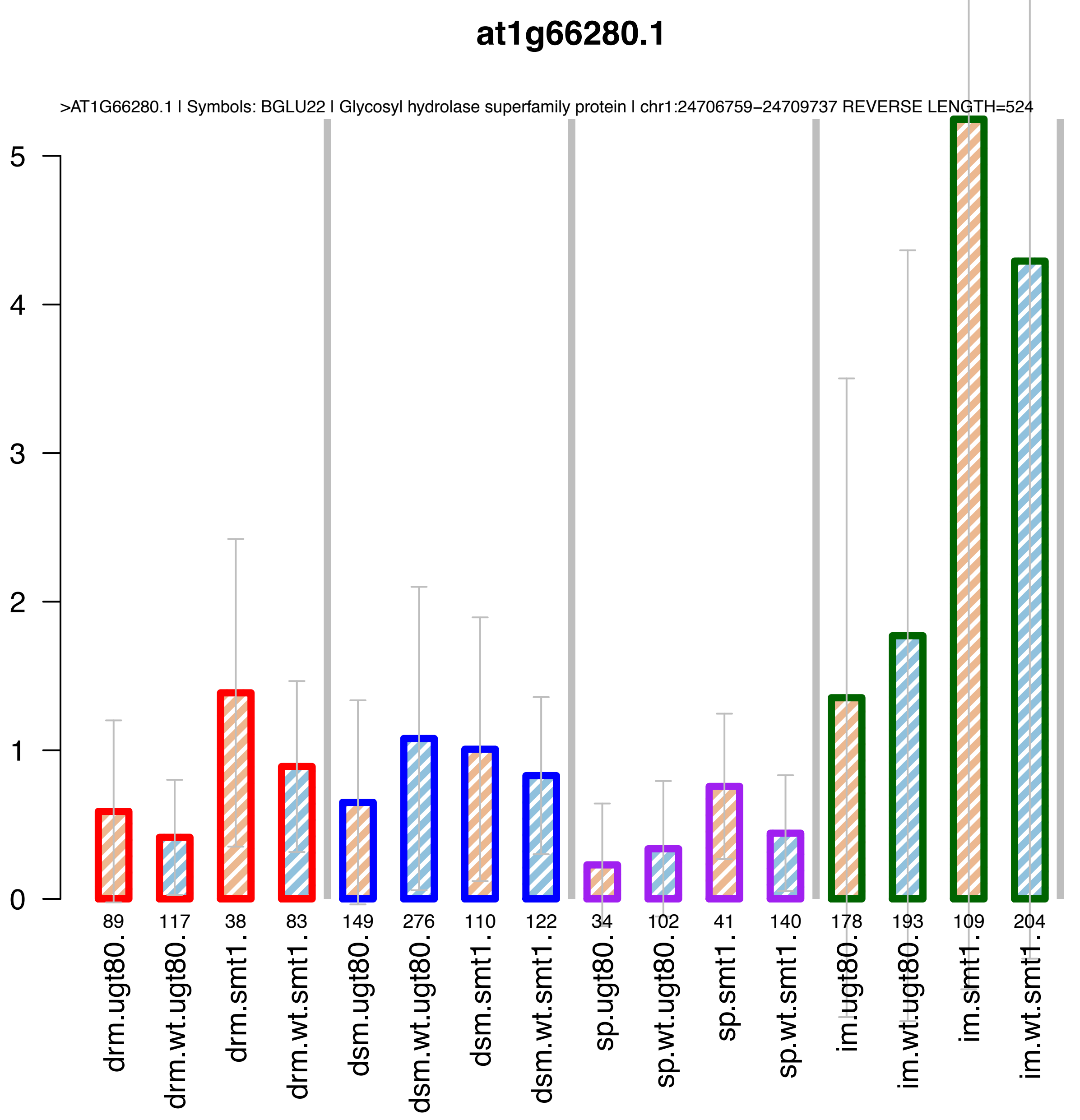

at1g66280.1
>AT1G66280.1 | Symbols: BGLU22 | Glycosyl hydrolase superfamily protein | chr1:24706759-24709737 REVERSE LENGTH=524
0
1
2
3
4
5
89
117
38
83
149
276
110
122
34
102
41
140
178
193
109
204
drm.ugt80.
drm.wt.ugt80.
drm.smt1.
drm.wt.smt1.
dsm.ugt80.
dsm.wt.ugt80.
dsm.smt1.
dsm.wt.smt1.
sp.ugt80.
sp.wt.ugt80.
sp.smt1.
sp.wt.smt1.
im.ugt80.
im.wt.ugt80.
im.smt1.
im.wt.smt1.

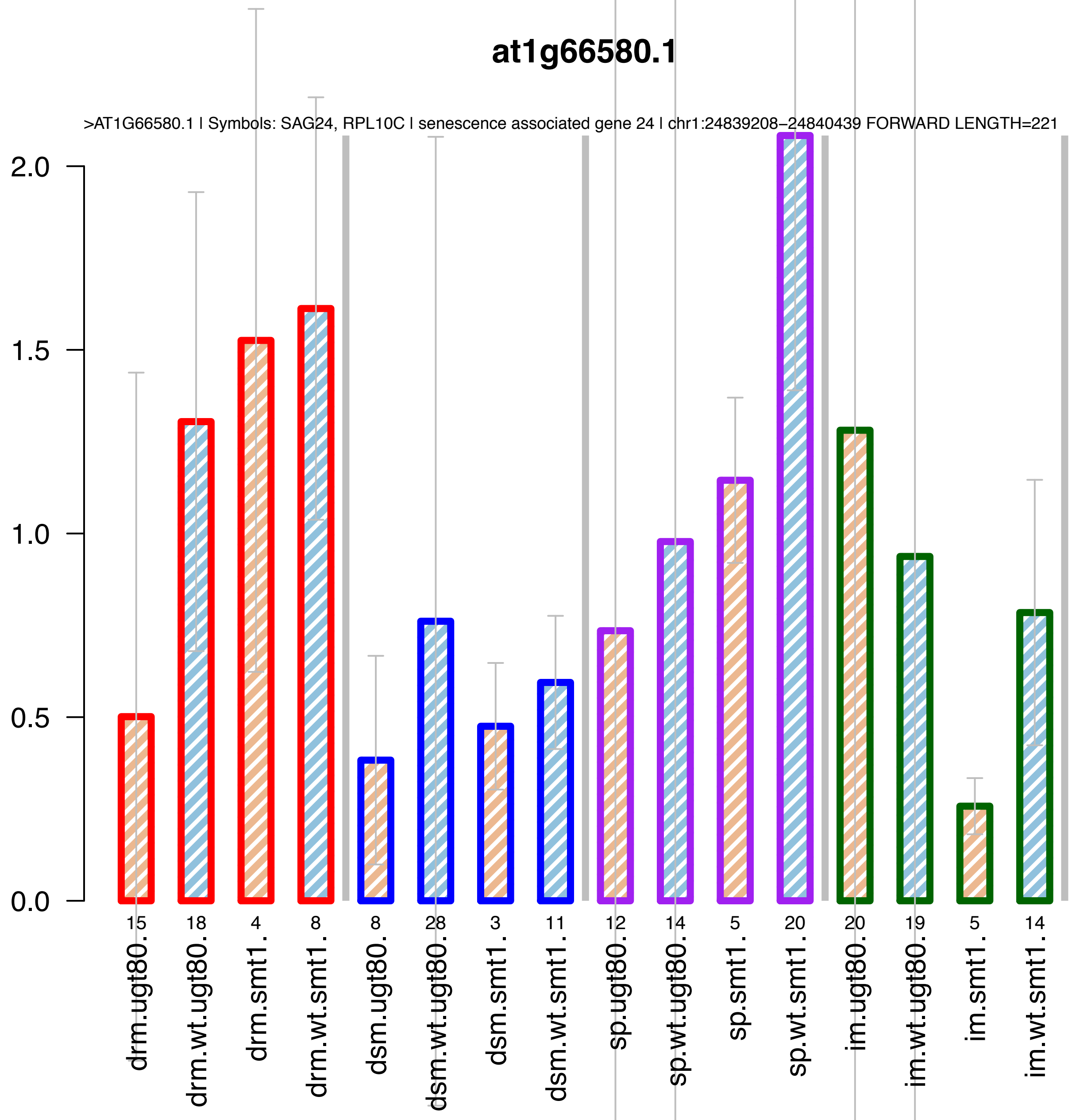

at1g66580.1
>AT1G66580.1 | Symbols: SAG24, RPL10C | senescence associated gene 24 | chr1:24839208–24840439 FORWARD LENGTH=221
2.0
1.5
1.0
0.5
0.0
15
18
4
8
8
28
3
11
12
14
5
20
20
19
5
14
drm.ugt80.
drm.wt.ugt80.
drm.smt1.
drm.wt.smt1.
dsm.ugt80.
dsm.wt.ugt80.
dsm.smt1.
dsm.wt.smt1.
sp.ugt80.
sp.wt.ugt80.
sp.smt1.
sp.wt.smt1.
im.ugt80.
im.wt.ugt80.
im.smt1.
im.wt.smt1.

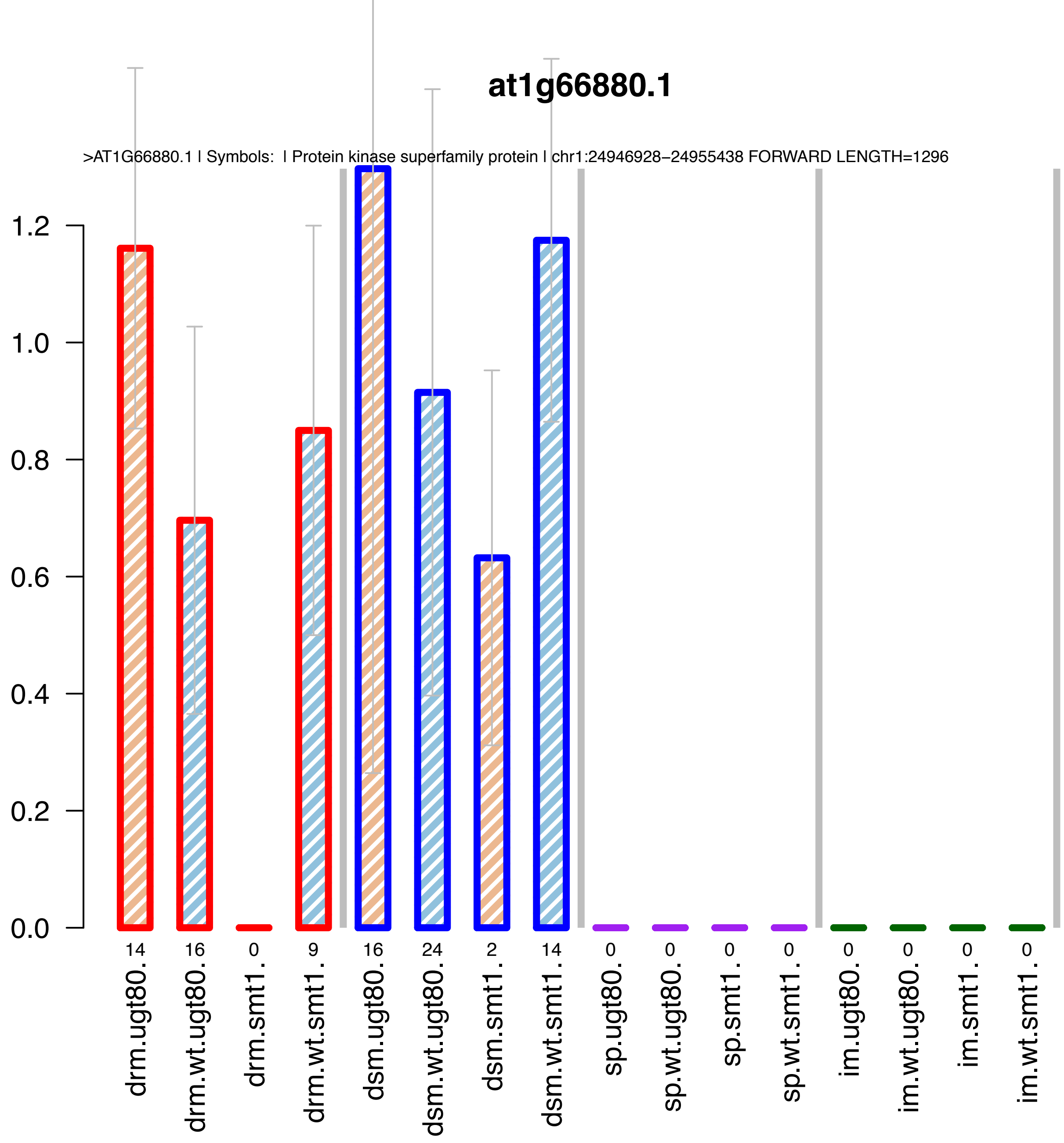

at1g66880.1
>AT1G66880.1 | Symbols: | Protein kinase superfamily protein | chr1:24946928–24955438 FORWARD LENGTH=1296
1.2
1.0
0.8
0.6
0.4
0.2
0.0
14
16
0
9
16
24
2
14
0
0
0
0
0
0
0
0
drm.ugt80.
drm.wt.ugt80.
drm.smt1.
drm.wt.smt1.
dsm.ugt80.
dsm.wt.ugt80.
dsm.smt1.
dsm.wt.smt1.
sp.ugt80.
sp.wt.ugt80.
sp.smt1.
sp.wt.smt1.
im.ugt80.
im.wt.ugt80.
im.smt1.
im.wt.smt1.

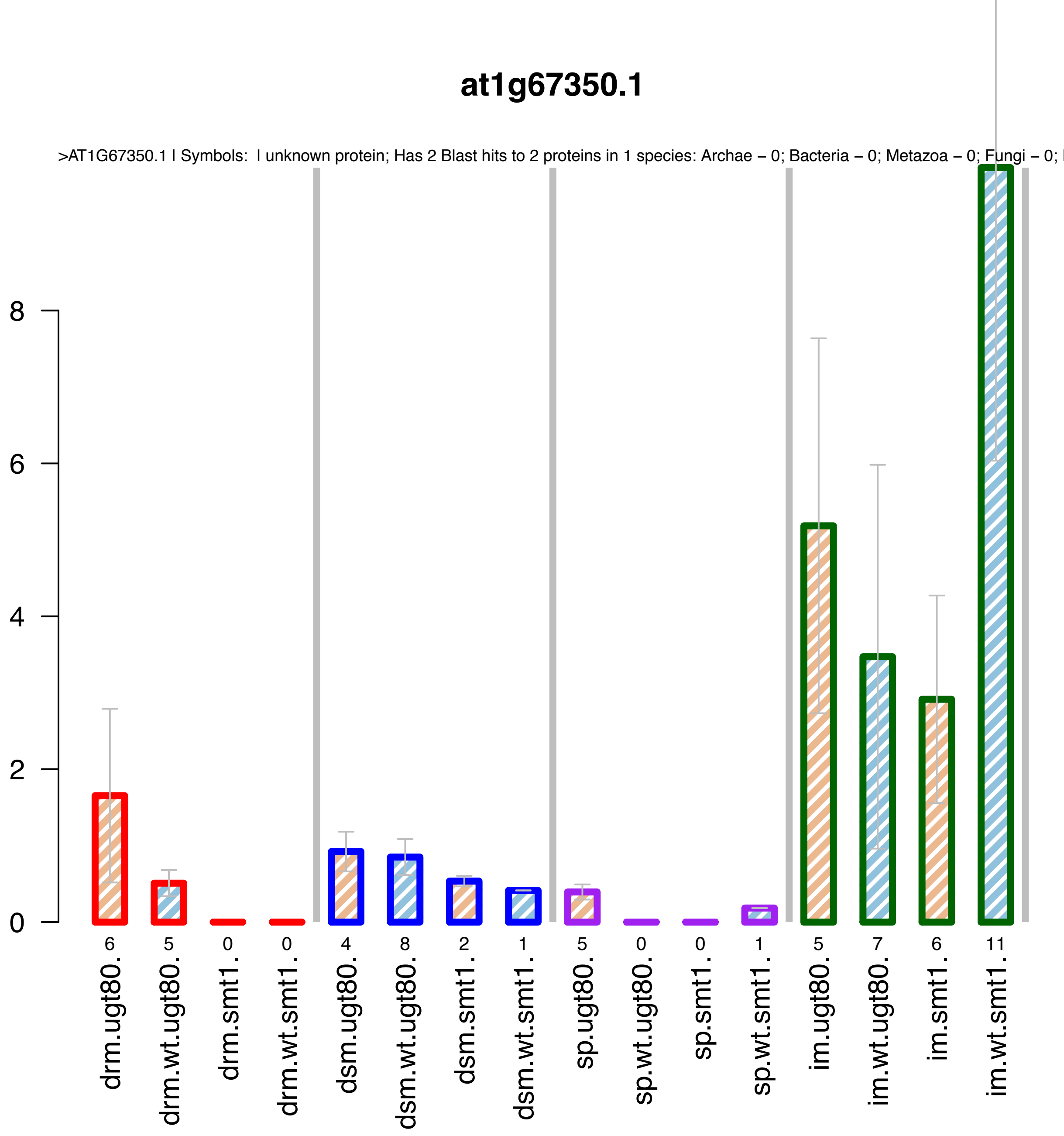

at1g67350.1
>AT1G67350.1 | Symbols: | unknown protein; Has 2 Blast hits to 2 proteins in 1 species: Archae – 0; Bacteria – 0; Metazoa – 0; Fungi – 0;
0
2
4
6
8
6
5
0
0
4
8
2
1
5
0
0
1
5
7
6
11
drm.ugt80.
drm.wt.ugt80.
drm.smt1.
drm.wt.smt1.
dsm.ugt80.
dsm.wt.ugt80.
dsm.smt1.
dsm.wt.smt1.
sp.ugt80.
sp.wt.ugt80.
sp.smt1.
sp.wt.smt1.
im.ugt80.
im.wt.ugt80.
im.smt1.
im.wt.smt1.

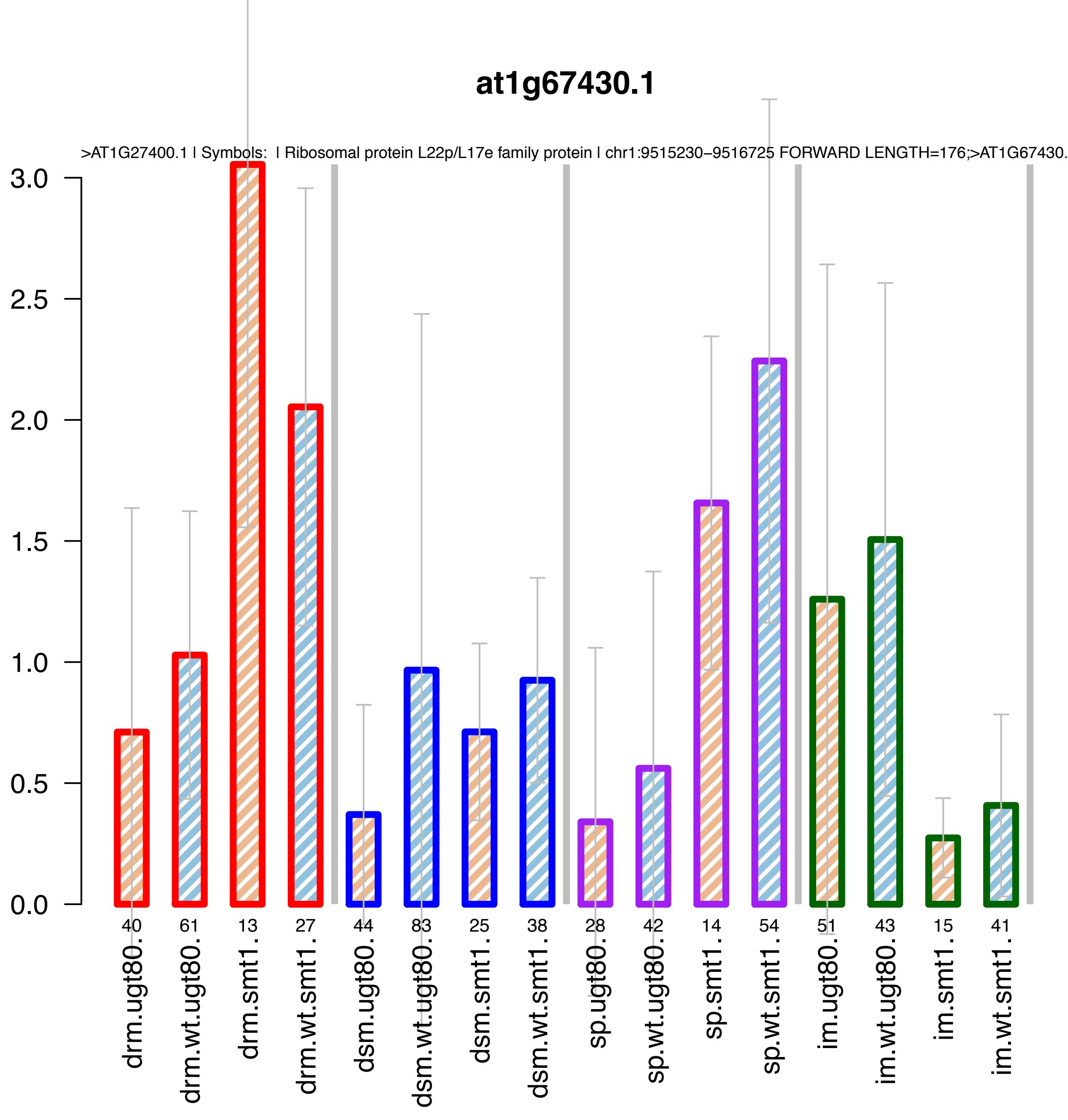
at1g67430.1
>AT1G27400.1 | Symbols: | Ribosomal protein L22p/L17e family protein | chr1:9515230-9516725 FORWARD LENGTH=176;>AT1G67430.
3.0
2.5
2.0
1.5
1.0
0.5
0.0
40
61
13
27
44
83
25
38
28
42
14
54
51
43
15
41
drm.ugt80..
drm.wt.ugt80..
drm.smt1..
drm.wt.smt1..
dsm.ugt80..
dsm.wt.ugt80..
dsm.smt1..
dsm.wt.smt1..
sp.ugt80..
sp.wt.ugt80..
sp.smt1..
sp.wt.smt1..
im.ugt80..
im.wt.ugt80..
im.smt1..
im.wt.smt1..

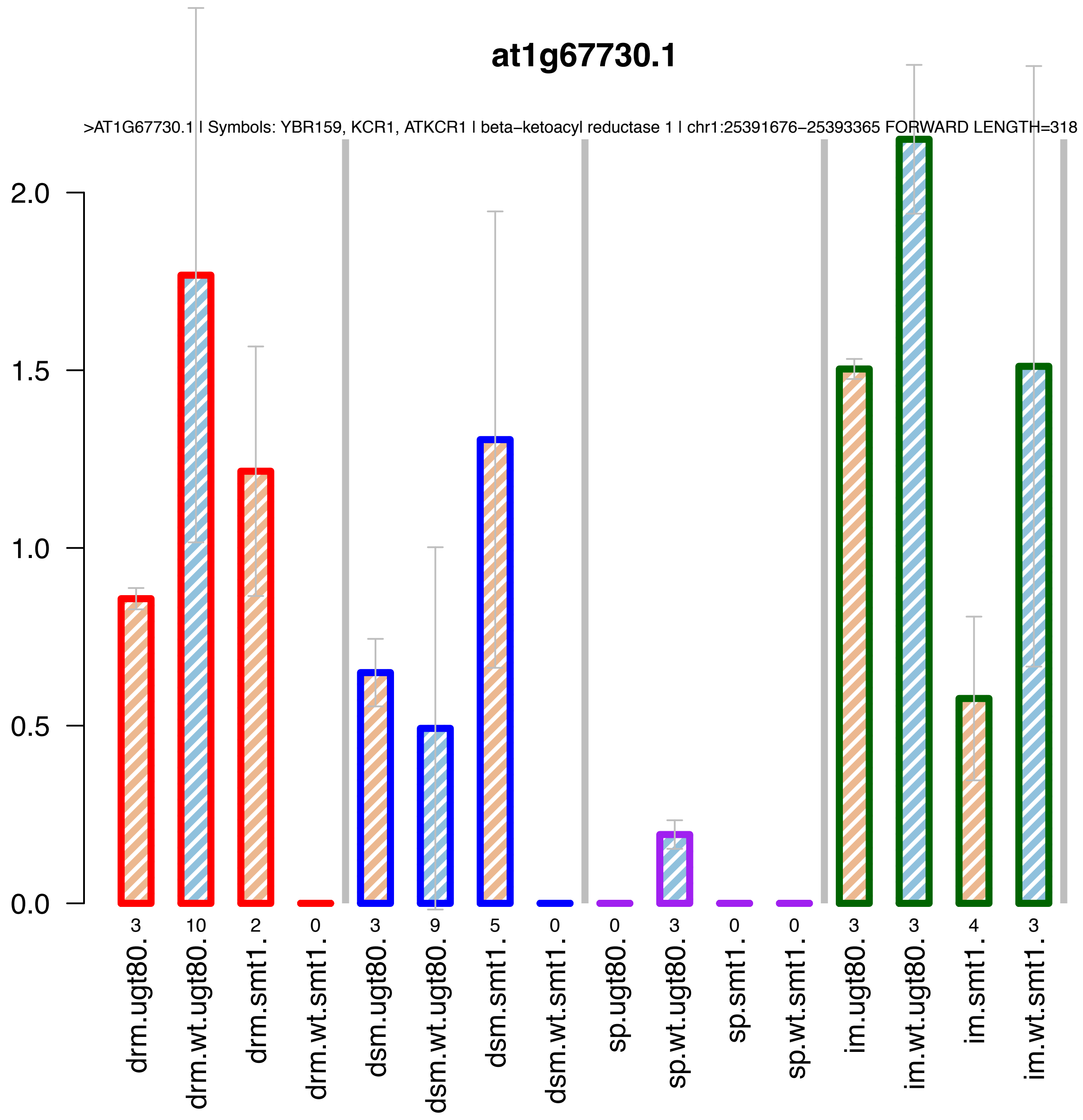

at1g67730.1
>AT1G67730.1 | Symbols: YBR159, KCR1, ATKCR1 | beta-ketoacyl reductase 1 | chr1:25391676-25393365 FORWARD LENGTH=318
0.0
0.5
1.0
1.5
2.0
3
10
2
0
3
9
5
0
0
3
0
0
3
3
4
3
drm.ugt80..
drm.wt.ugt80..
drm.smt1..
drm.wt.smt1..
dsm.ugt80..
dsm.wt.ugt80..
dsm.smt1..
dsm.wt.smt1..
sp.ugt80..
sp.wt.ugt80..
sp.smt1..
sp.wt.smt1..
im.ugt80..
im.wt.ugt80..
im.smt1..
im.wt.smt1..

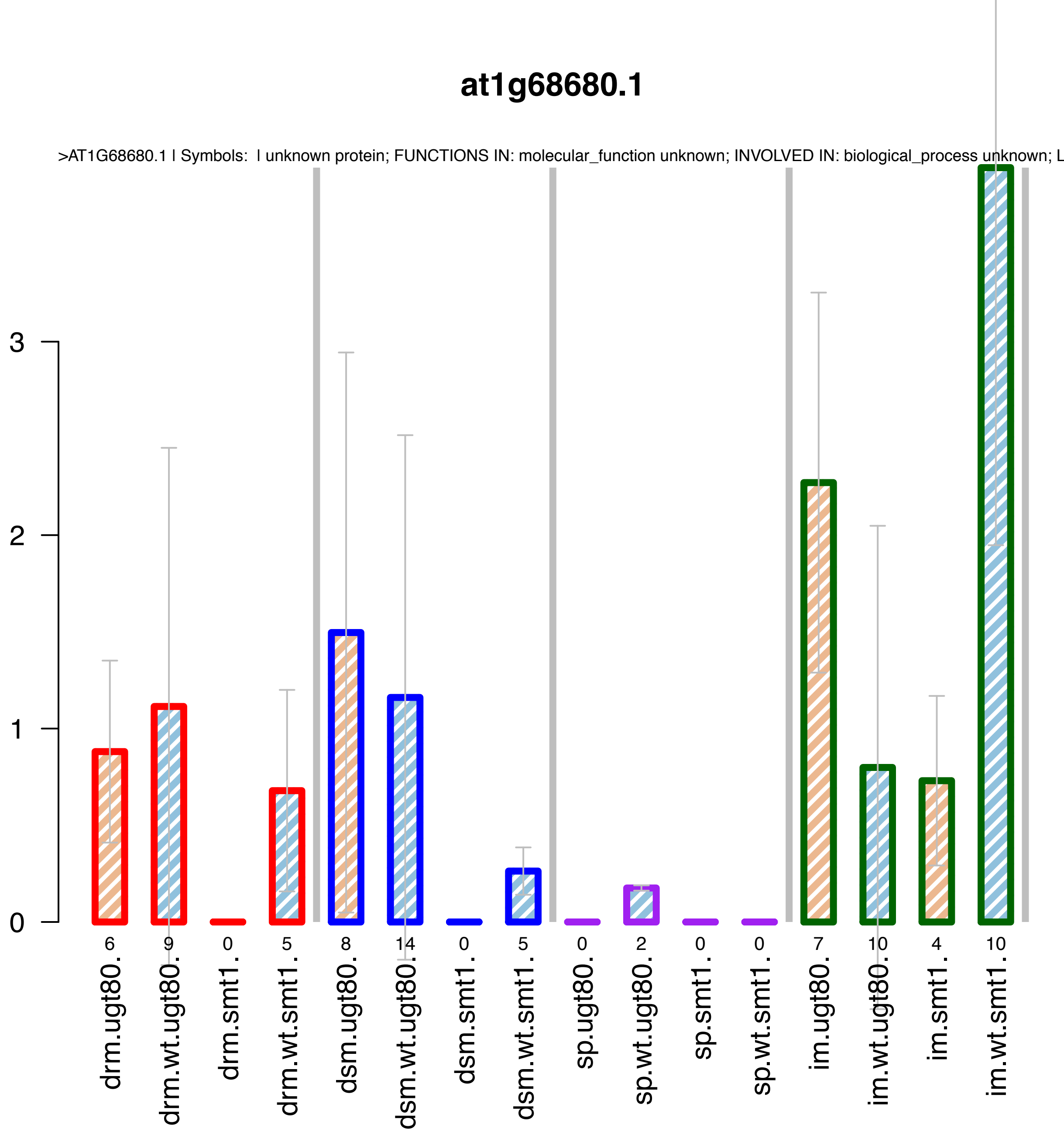
at1g68680.1
>AT1G68680.1 | Symbols: | unknown protein; FUNCTIONS IN: molecular_function unknown; INVOLVED IN: biological_process unknown; L
0
1
2
3
6
9
0
5
8
14
0
5
0
2
0
0
7
10
4
10
drm.ugt80.
drm.wt.ugt80.
drm.smt1.
drm.wt.smt1.
dsm.ugt80.
dsm.wt.ugt80.
dsm.smt1.
dsm.wt.smt1.
sp.ugt80.
sp.wt.ugt80.
sp.smt1.
sp.wt.smt1.
im.ugt80.
im.wt.ugt80.
im.smt1.
im.wt.smt1.

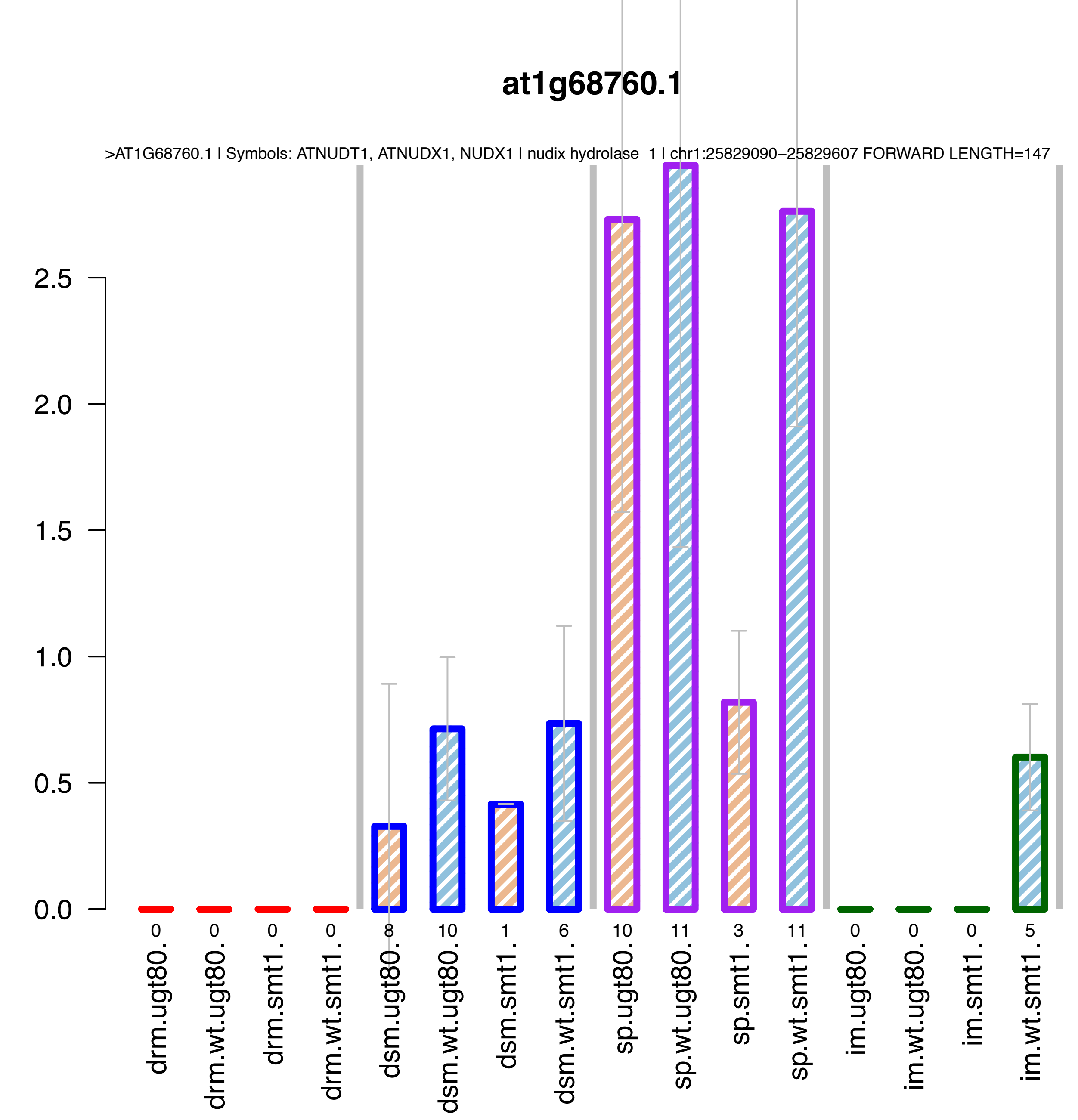

at1g68760.1
>AT1G68760.1 | Symbols: ATNUDT1, ATNUDX1, NUDX1 | nudix hydrolase 1 | chr1:25829090-25829607 FORWARD LENGTH=147
0.0
0.5
1.0
1.5
2.0
2.5
0 0 0 0 8 10 1 6 10 11 3 11 0 0 0 5
drm.ugt80.
drm.wt.ugt80.
drm.smt1.
drm.wt.smt1.
dsm.ugt80.
dsm.wt.ugt80.
dsm.smt1.
dsm.wt.smt1.
sp.ugt80.
sp.wt.ugt80.
sp.smt1.
sp.wt.smt1.
im.ugt80.
im.wt.ugt80.
im.smt1.
im.wt.smt1.

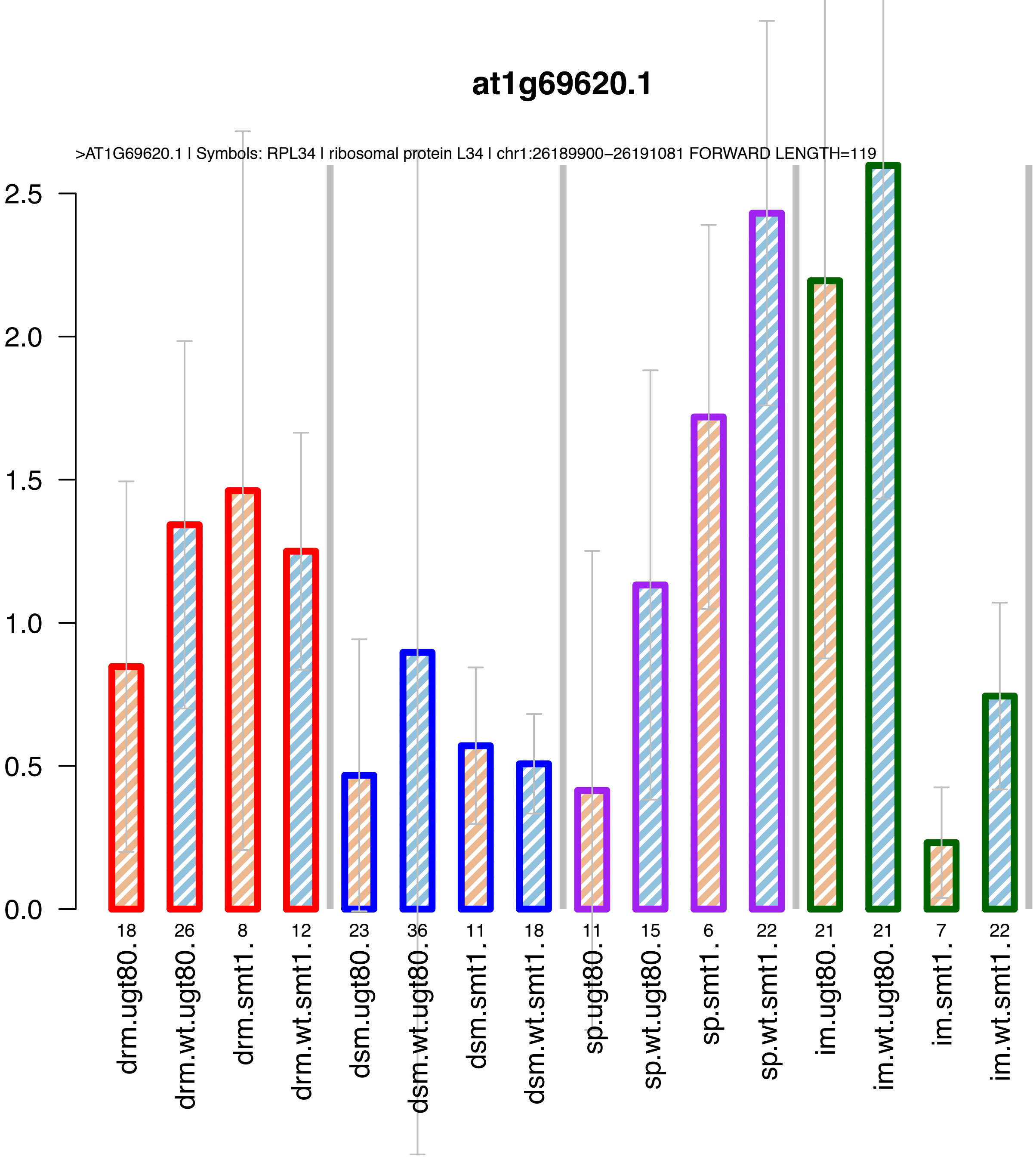

at1g69620.1
>AT1G69620.1 | Symbols: RPL34 | ribosomal protein L34 | chr1:26189900-26191081 FORWARD LENGTH=119
2.5
2.0
1.5
1.0
0.5
0.0
18
26
8
12
23
36
11
18
11
15
6
22
21
21
7
22
drm.ugt80..
drm.wt.ugt80..
drm.smt1..
drm.wt.smt1..
dsm.ugt80..
dsm.wt.ugt80..
dsm.smt1..
dsm.wt.smt1..
sp.ugt80..
sp.wt.ugt80..
sp.smt1..
sp.wt.smt1..
im.ugt80..
im.wt.ugt80..
im.smt1..
im.wt.smt1..

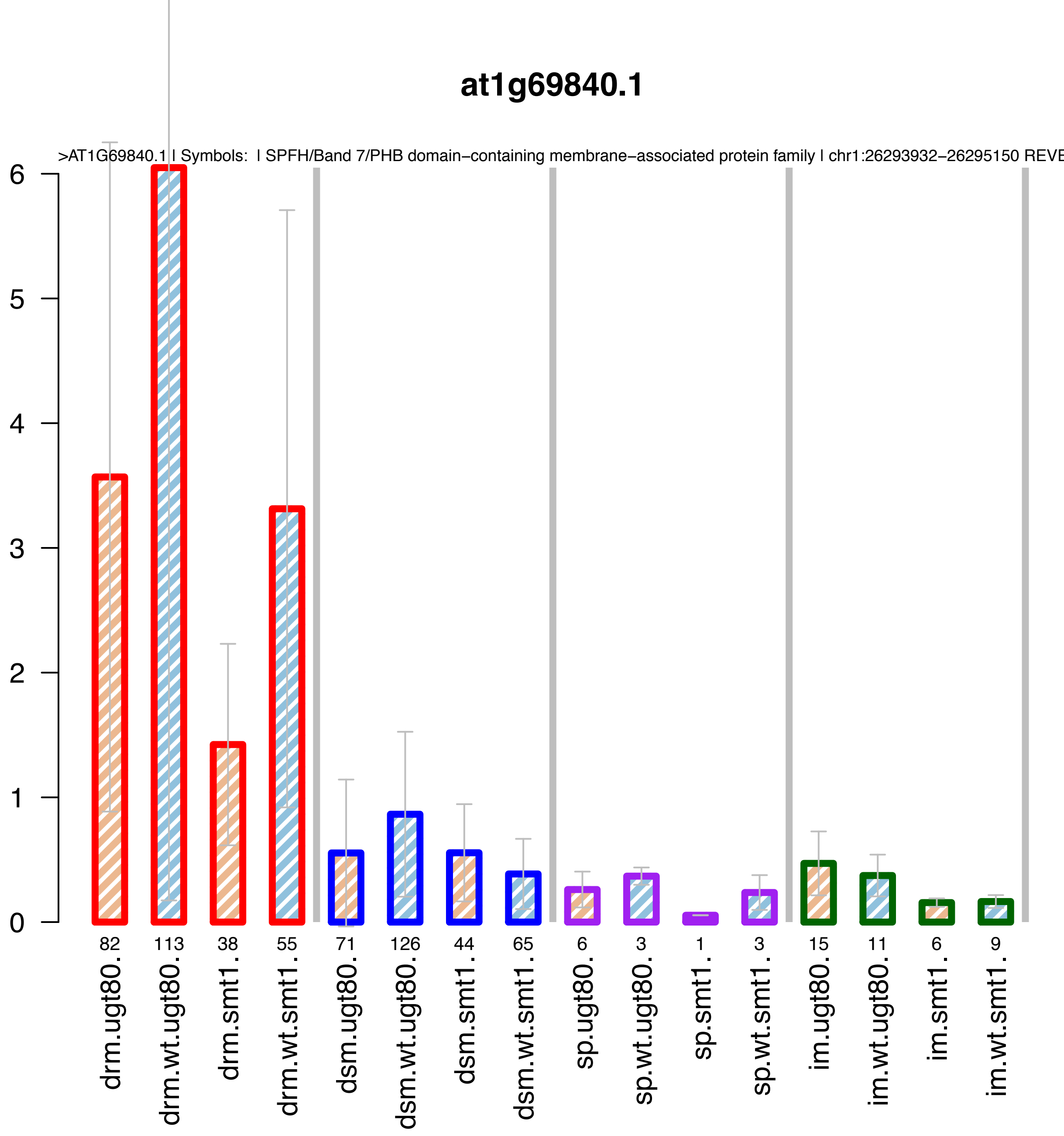
at1g69840.1
>AT1G69840.1 | Symbols: | SPFH/Band 7/PHB domain-containing membrane-associated protein family | chr1:26293932-26295150 REVE
0
1
2
3
4
5
6
82
113
38
55
71
126
44
65
6
3
1
3
15
11
6
9
drm.ugt80.
drm.wt.ugt80.
drm.smt1.
drm.wt.smt1.
dsm.ugt80.
dsm.wt.ugt80.
dsm.smt1.
dsm.wt.smt1.
sp.ugt80.
sp.wt.ugt80.
sp.smt1.
sp.wt.smt1.
im.ugt80.
im.wt.ugt80.
im.smt1.
im.wt.smt1.

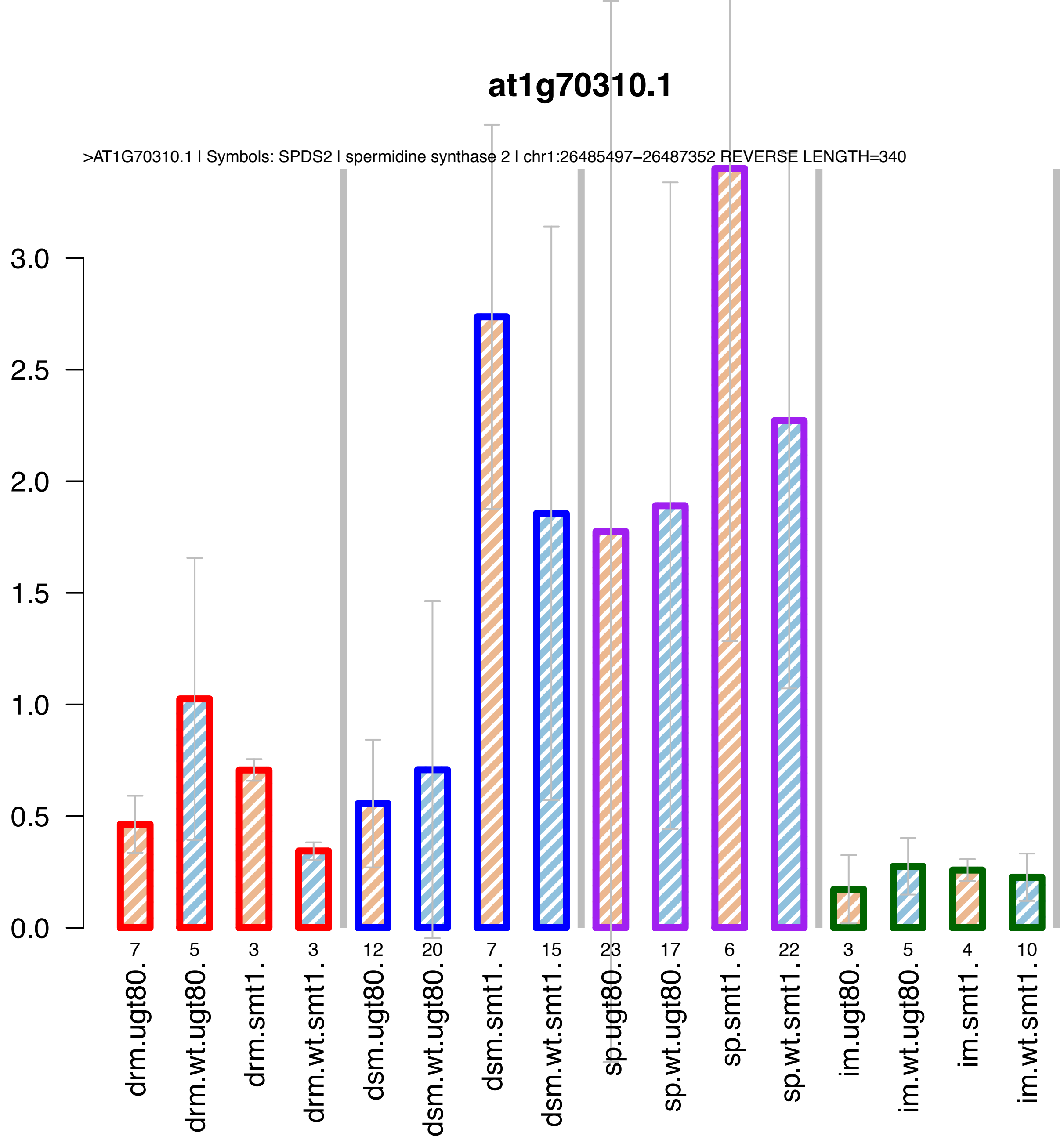

at1g70310.1
>AT1G70310.1 | Symbols: SPDS2 | spermidine synthase 2 | chr1:26485497−26487352 REVERSE LENGTH=340
0.0
0.5
1.0
1.5
2.0
2.5
3.0
7
5
3
3
12
20
7
15
23
17
6
22
3
5
4
10
drm.ugt80.
drm.wt.ugt80.
drm.smt1.
drm.wt.smt1.
dsm.ugt80.
dsm.wt.ugt80.
dsm.smt1.
dsm.wt.smt1.
sp.ugt80.
sp.wt.ugt80.
sp.smt1.
sp.wt.smt1.
im.ugt80.
im.wt.ugt80.
im.smt1.
im.wt.smt1.

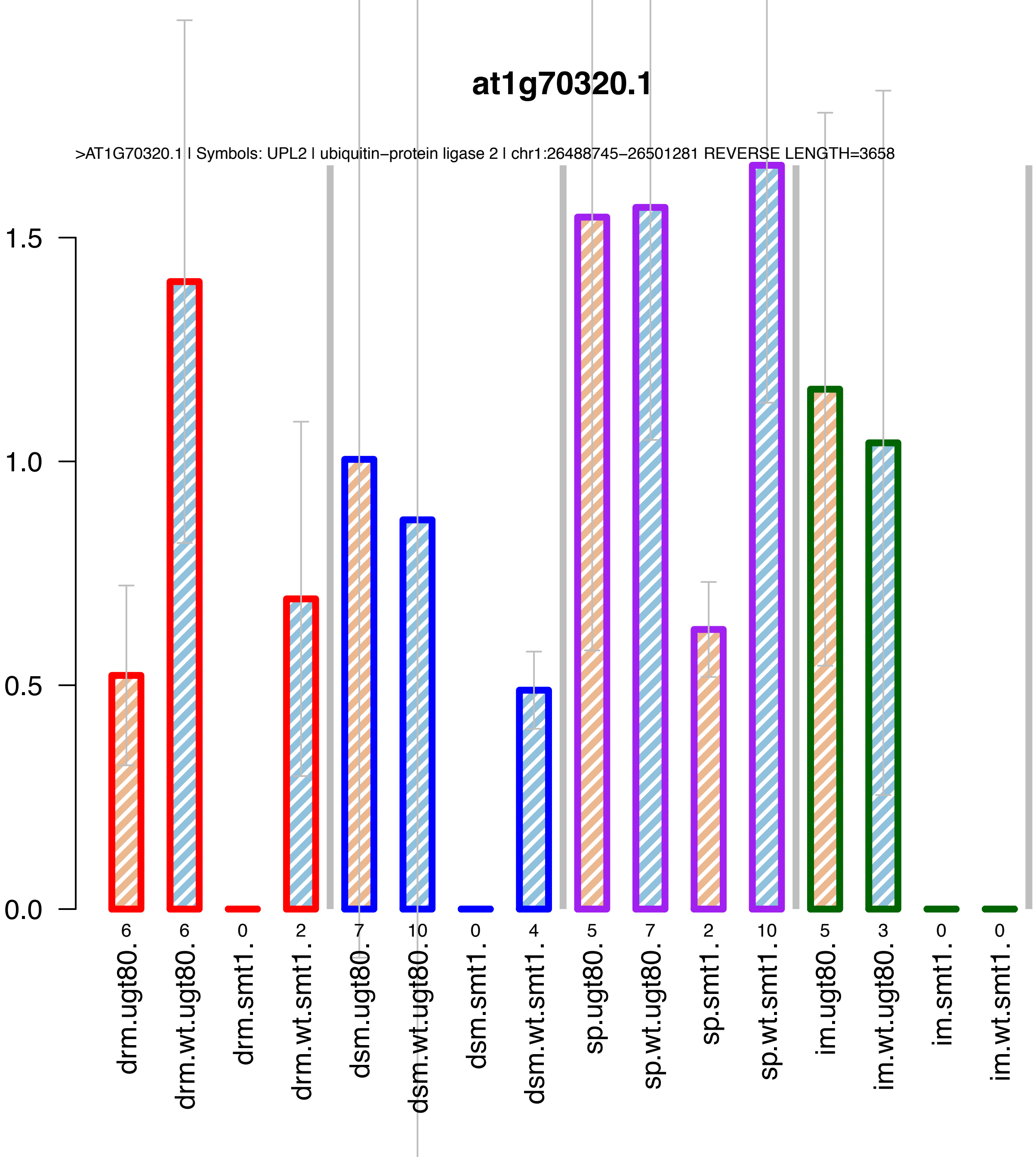

at1g70320.1
>AT1G70320.1 | Symbols: UPL2 | ubiquitin−protein ligase 2 | chr1:26488745−26501281 REVERSE LENGTH=3658
1.5
1.0
0.5
0.0
6
6
0
2
7
10
0
4
5
7
2
10
5
3
0
0
drm.ugt80.
drm.wt.ugt80.
drm.smt1.
drm.wt.smt1.
dsm.ugt80.
dsm.wt.ugt80.
dsm.smt1.
dsm.wt.smt1.
sp.ugt80.
sp.wt.ugt80.
sp.smt1.
sp.wt.smt1.
im.ugt80.
im.wt.ugt80.
im.smt1.
im.wt.smt1.

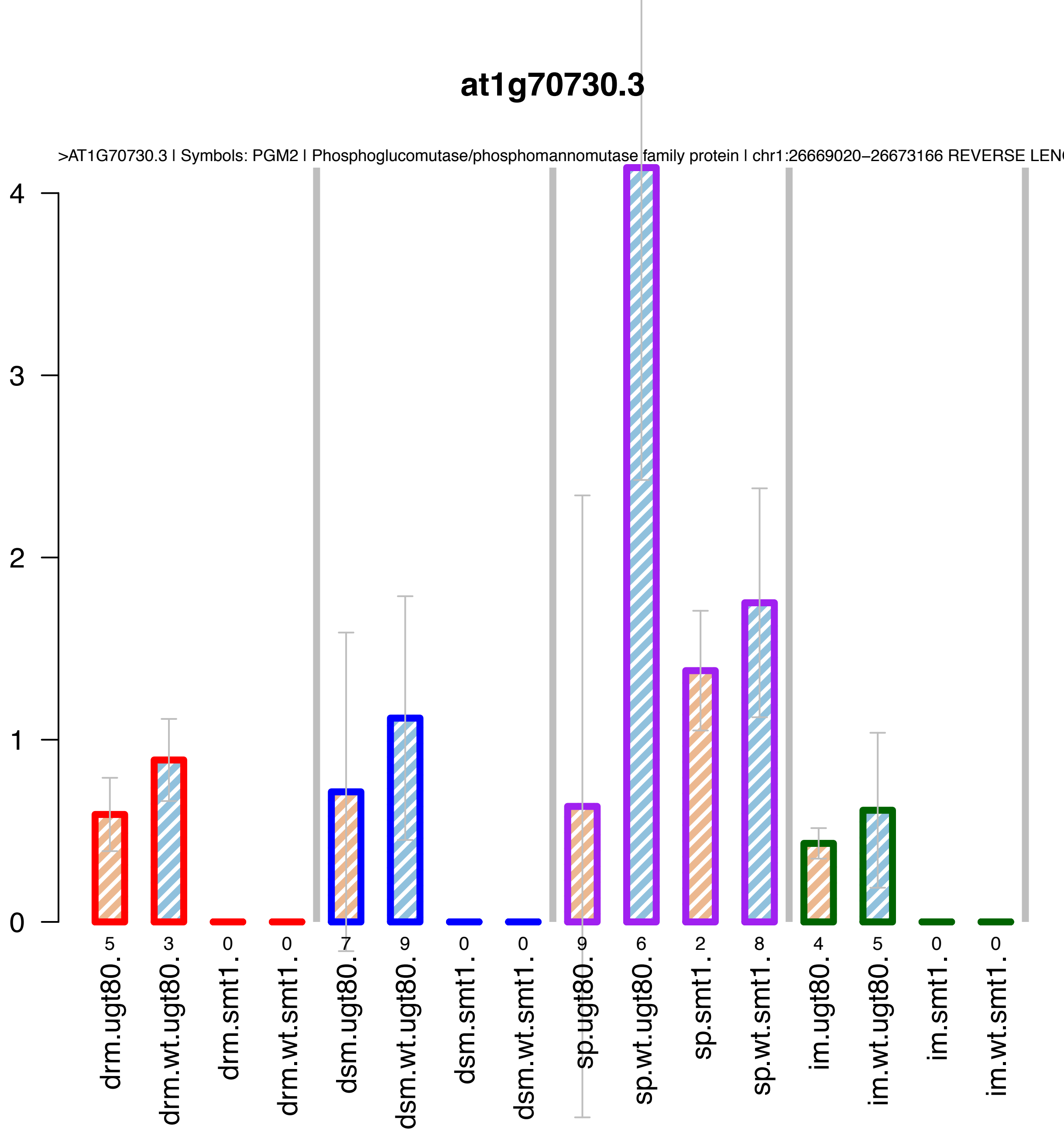

at1g70730.3
>AT1G70730.3 | Symbols: PGM2 | Phosphoglucomutase/phosphomannomutase family protein | chr1:26669020-26673166 REVERSE LENG
4
3
2
1
0
5
3
0
0
7
9
0
0
9
6
2
8
4
5
0
0
drm.ugt80.
drm.wt.ugt80.
drm.smt1.
drm.wt.smt1.
dsm.ugt80.
dsm.wt.ugt80.
dsm.smt1.
dsm.wt.smt1.
sp.ugt80.
sp.wt.ugt80.
sp.smt1.
sp.wt.smt1.
im.ugt80.
im.wt.ugt80.
im.smt1.
im.wt.smt1.

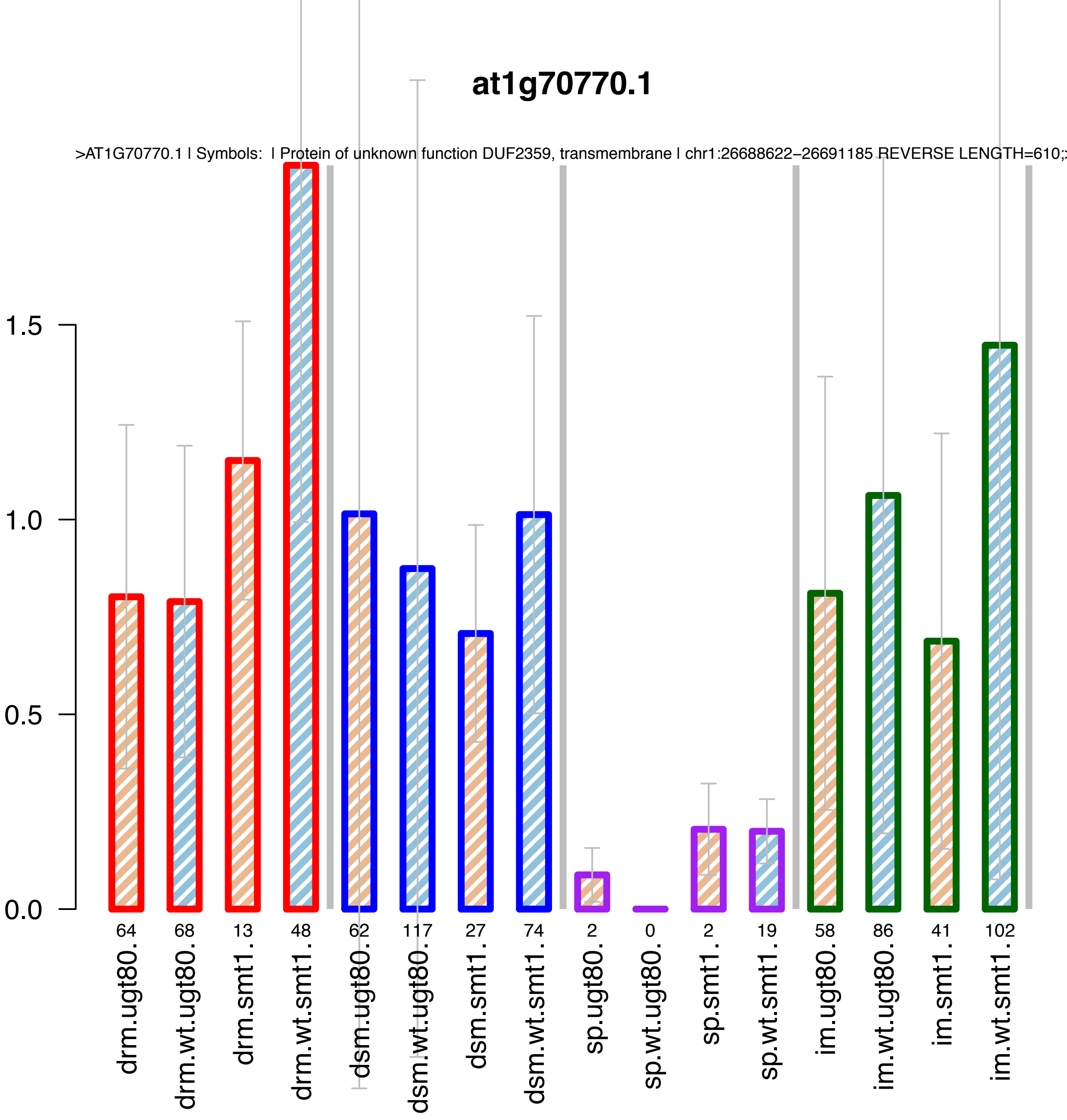

at1g70770.1
>AT1G70770.1 | Symbols: | Protein of unknown function DUF2359, transmembrane | chr1:26688622-26691185 REVERSE LENGTH=610;
0.0
0.5
1.0
1.5
64
68
13
48
62
117
27
74
2
0
2
19
58
86
41
102
drm.ugt80..
drm.wt.ugt80..
drm.smt1..
drm.wt.smt1..
dsm.ugt80..
dsm.wt.ugt80..
dsm.smt1..
dsm.wt.smt1..
sp.ugt80..
sp.wt.ugt80..
sp.smt1..
sp.wt.smt1..
im.ugt80..
im.wt.ugt80..
im.smt1..
im.wt.smt1..

# at1g70810.1

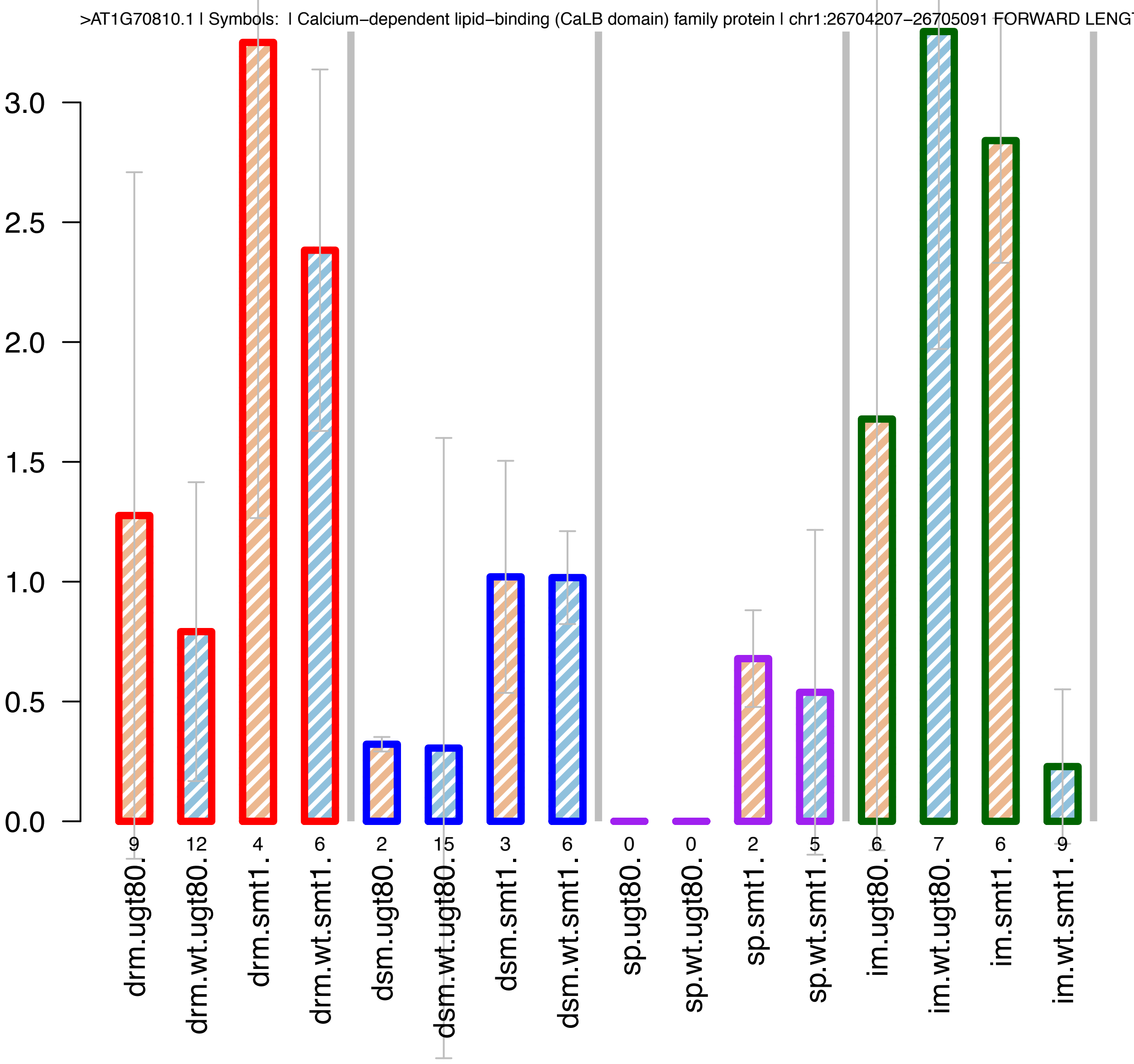

>AT1G70810.1 | Symbols: | Calcium−dependent lipid−binding (CaLB domain) family protein | chr1:26704207−26705091 FORWARD LENG
0.0
0.5
1.0
1.5
2.0
2.5
3.0
9
12
4
6
2
15
3
6
0
0
2
5
6
7
6
9
drm.ugt80.
drm.wt.ugt80.
drm.smt1.
drm.wt.smt1.
dsm.ugt80.
dsm.wt.ugt80.
dsm.smt1.
dsm.wt.smt1.
sp.ugt80.
sp.wt.ugt80.
sp.smt1.
sp.wt.smt1.
im.ugt80.
im.wt.ugt80.
im.smt1.
im.wt.smt1.

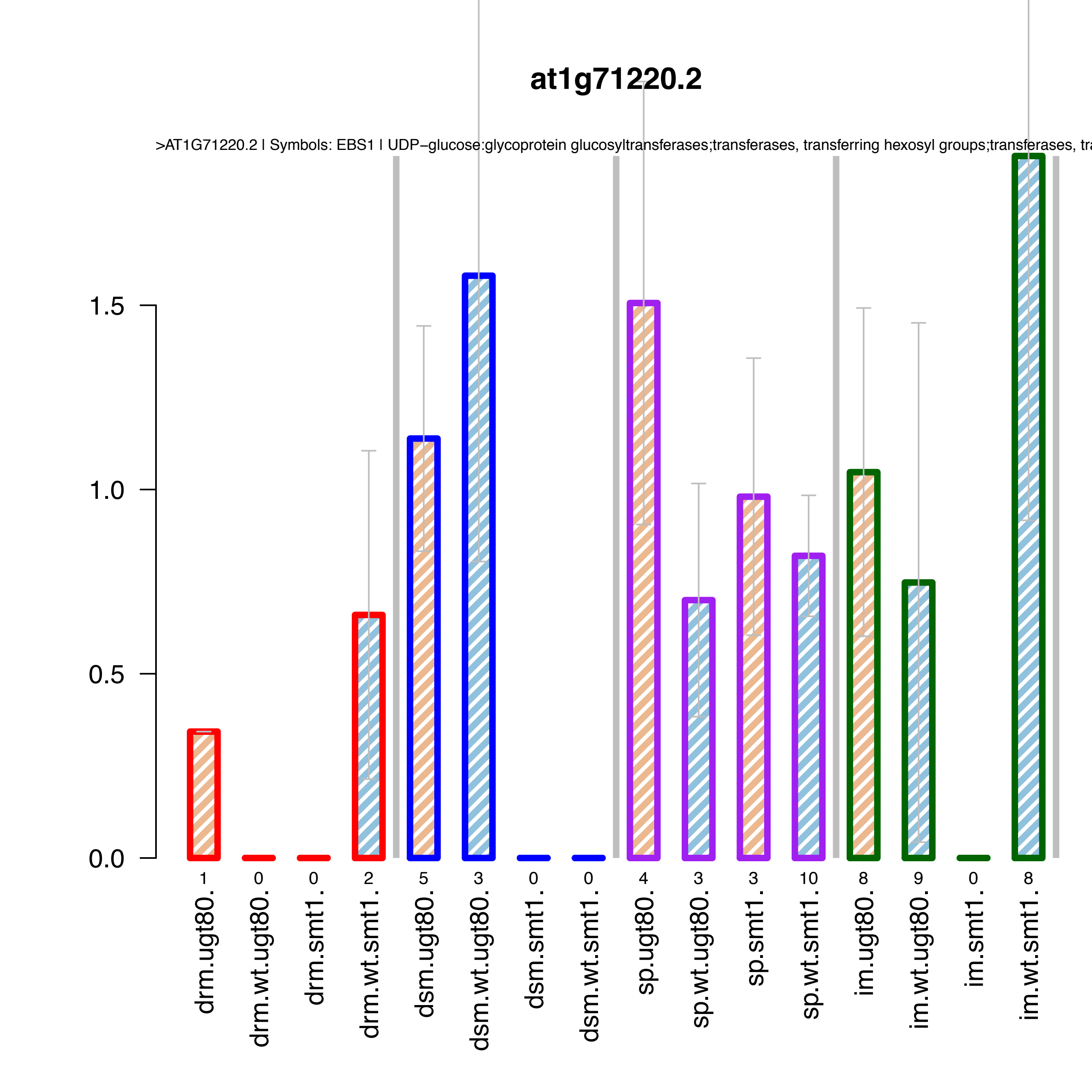

at1g71220.2
>AT1G71220.2 | Symbols: EBS1 | UDP−glucose:glycoprotein glucosyltransferases;transferases, transferring hexosyl groups;transferases, tra
0.0
0.5
1.0
1.5
1
0
0
2
5
3
0
0
4
3
3
10
8
9
0
8
drm.ugt80..
drm.wt.ugt80..
drm.smt1..
drm.wt.smt1..
dsm.ugt80..
dsm.wt.ugt80..
dsm.smt1..
dsm.wt.smt1..
sp.ugt80..
sp.wt.ugt80..
sp.smt1..
sp.wt.smt1..
im.ugt80..
im.wt.ugt80..
im.smt1..
im.wt.smt1..

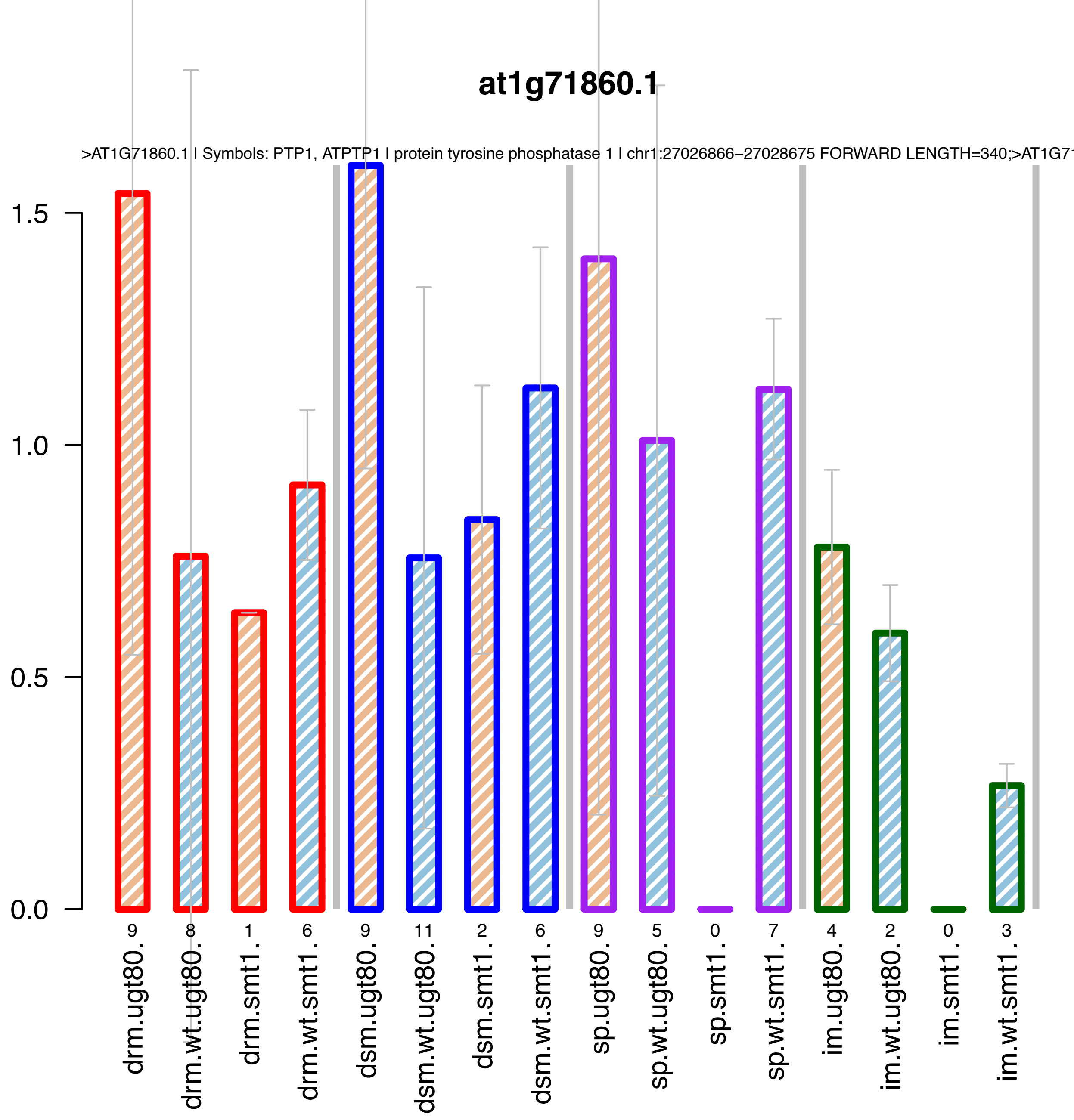

at1g71860.1
>AT1G71860.1 | Symbols: PTP1, ATPTP1 | protein tyrosine phosphatase 1 | chr1:27026866-27028675 FORWARD LENGTH=340;>AT1G7
1.5
1.0
0.5
0.0
9
8
1
6
9
11
2
6
9
5
0
7
4
2
0
3
drm.ugt80.
drm.wt.ugt80.
drm.smt1.
drm.wt.smt1.
dsm.ugt80.
dsm.wt.ugt80.
dsm.smt1.
dsm.wt.smt1.
sp.ugt80.
sp.wt.ugt80.
sp.smt1.
sp.wt.smt1.
im.ugt80.
im.wt.ugt80.
im.smt1.
im.wt.smt1.

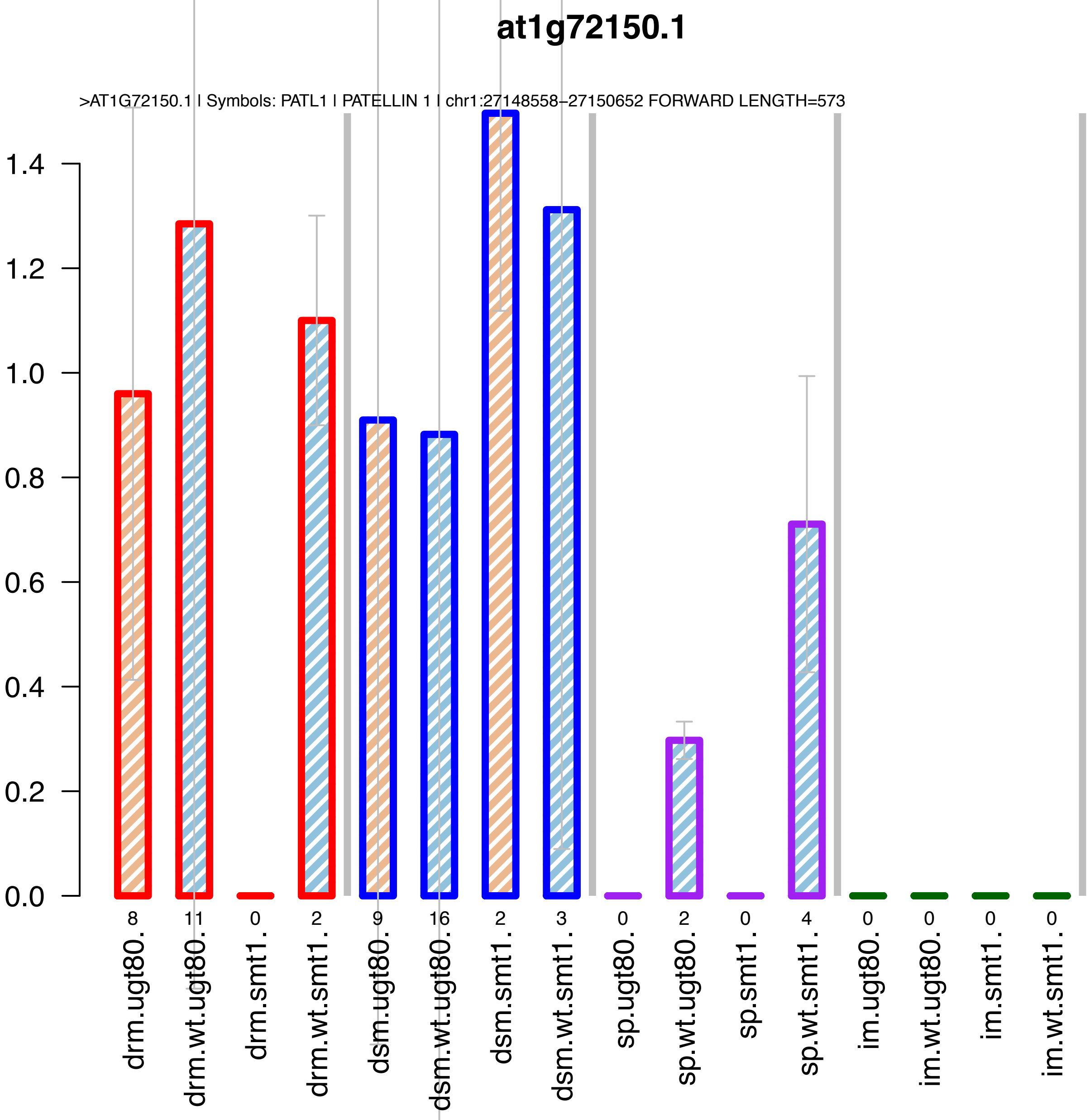

at1g72150.1
>AT1G72150.1 | Symbols: PATL1 | PATELLIN 1 | chr1:27148558–27150652 FORWARD LENGTH=573
1.4
1.2
1.0
0.8
0.6
0.4
0.2
0.0
8
11
0
2
9
16
2
3
0
2
0
4
0
0
0
0
drm.ugt80.
drm.wt.ugt80.
drm.smt1.
drm.wt.smt1.
dsm.ugt80.
dsm.wt.ugt80.
dsm.smt1.
dsm.wt.smt1.
sp.ugt80.
sp.wt.ugt80.
sp.smt1.
sp.wt.smt1.
im.ugt80.
im.wt.ugt80.
im.smt1.
im.wt.smt1.

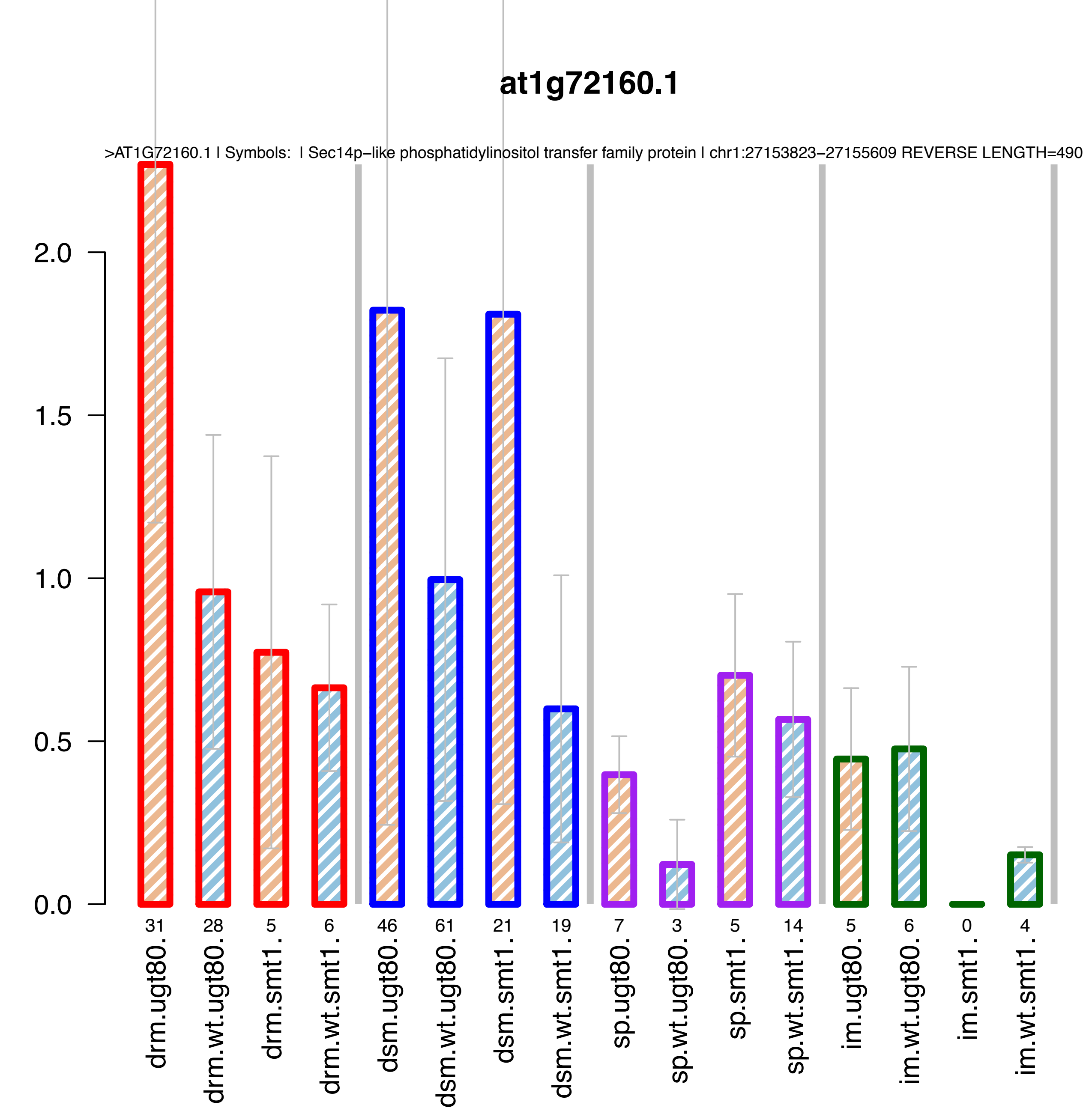

at1g72160.1
>AT1G72160.1 | Symbols: | Sec14p−like phosphatidylinositol transfer family protein | chr1:27153823−27155609 REVERSE LENGTH=490
2.0
1.5
1.0
0.5
0.0
31
28
5
6
46
61
21
19
7
3
5
14
5
6
0
4
drm.ugt80.
drm.wt.ugt80.
drm.smt1.
drm.wt.smt1.
dsm.ugt80.
dsm.wt.ugt80.
dsm.smt1.
dsm.wt.smt1.
sp.ugt80.
sp.wt.ugt80.
sp.smt1.
sp.wt.smt1.
im.ugt80.
im.wt.ugt80.
im.smt1.
im.wt.smt1.

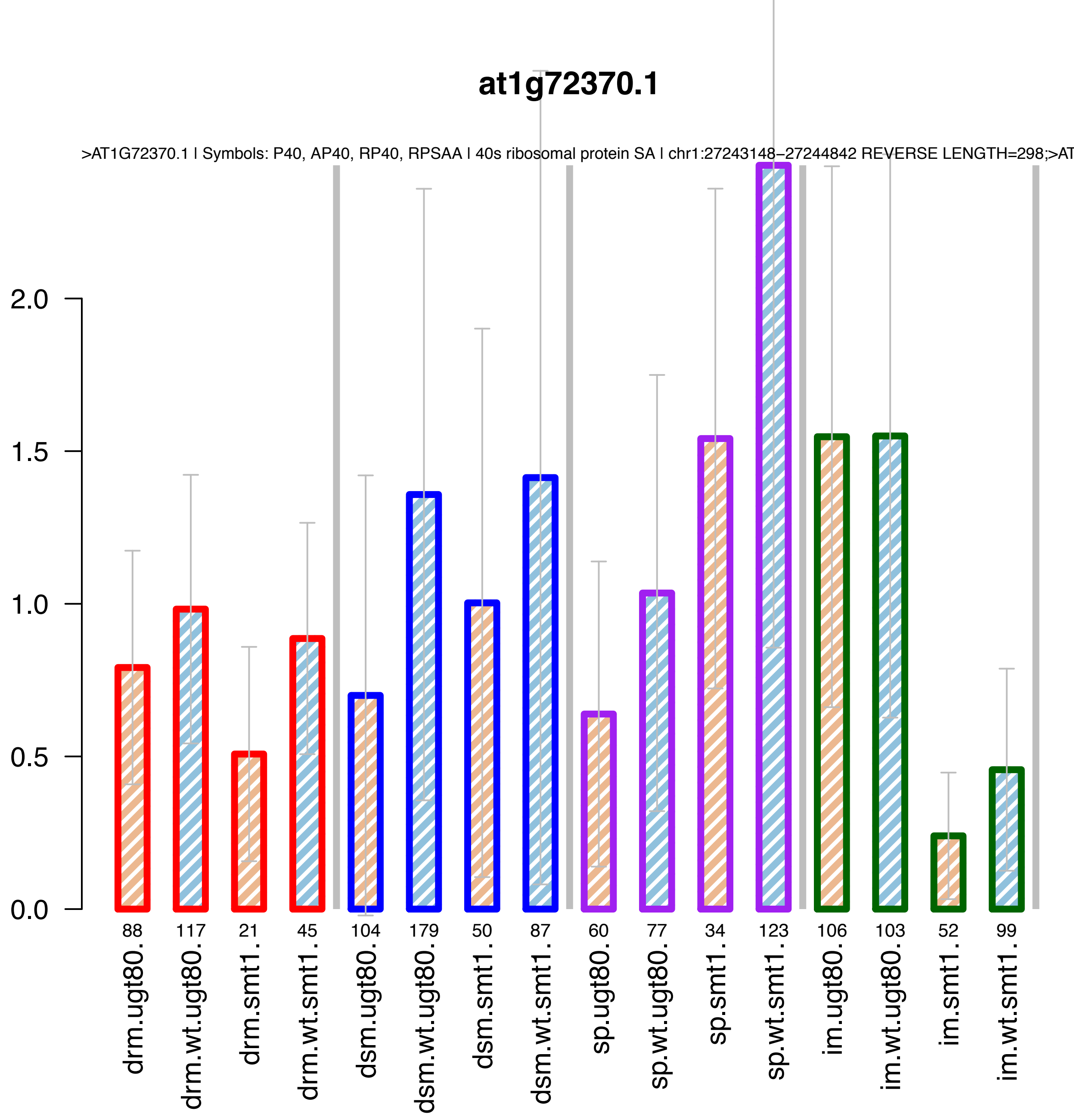

at1g72370.1
>AT1G72370.1 | Symbols: P40, AP40, RP40, RPSAA | 40s ribosomal protein SA | chr1:27243148–27244842 REVERSE LENGTH=298;>AT
0.0
0.5
1.0
1.5
2.0
88
117
21
45
104
179
50
87
60
77
34
123
106
103
52
99
drm.ugt80.
drm.wt.ugt80.
drm.smt1.
drm.wt.smt1.
dsm.ugt80.
dsm.wt.ugt80.
dsm.smt1.
dsm.wt.smt1.
sp.ugt80.
sp.wt.ugt80.
sp.smt1.
sp.wt.smt1.
im.ugt80.
im.wt.ugt80.
im.smt1.
im.wt.smt1.

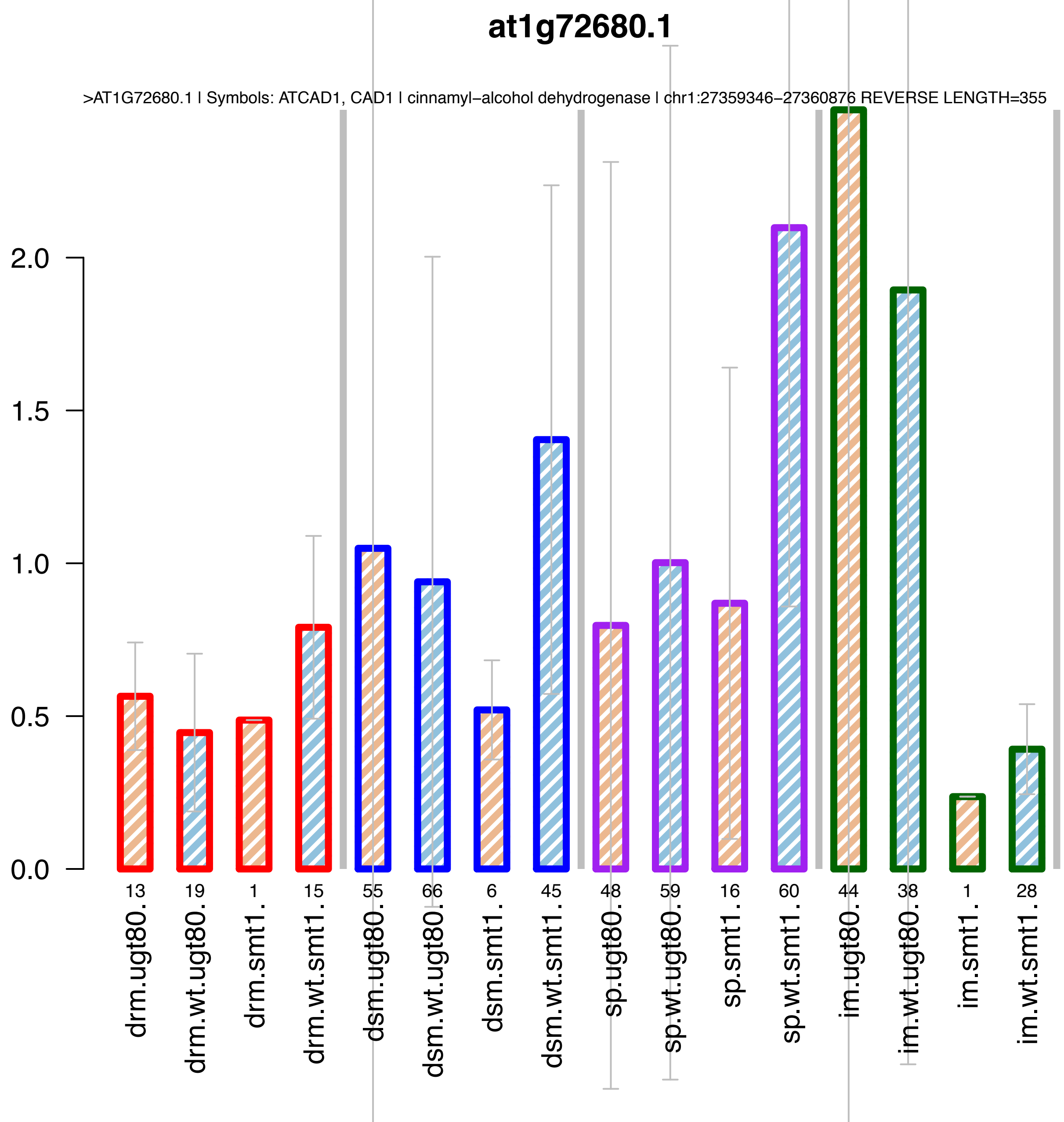

at1g72680.1
>AT1G72680.1 | Symbols: ATCAD1, CAD1 | cinnamyl−alcohol dehydrogenase | chr1:27359346−27360876 REVERSE LENGTH=355
0.0
0.5
1.0
1.5
2.0
13
19
1
15
55
66
6
45
48
59
16
60
44
38
1
28
drm.ugt80..
drm.wt.ugt80..
drm.smt1..
drm.wt.smt1..
dsm.ugt80..
dsm.wt.ugt80..
dsm.smt1..
dsm.wt.smt1..
sp.ugt80..
sp.wt.ugt80..
sp.smt1..
sp.wt.smt1..
im.ugt80..
im.wt.ugt80..
im.smt1..
im.wt.smt1..

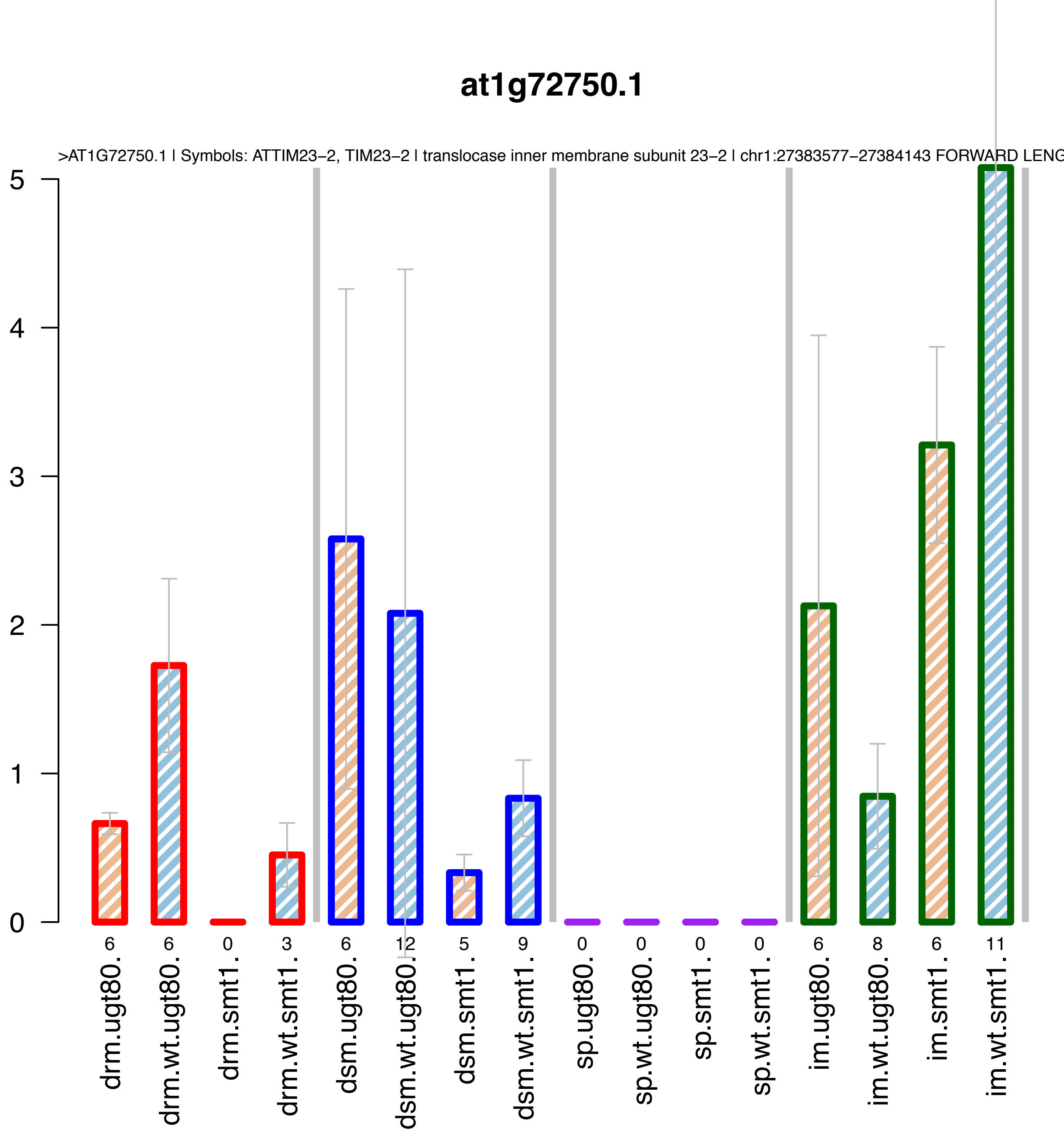

at1g72750.1
>AT1G72750.1 | Symbols: ATTIM23−2, TIM23−2 | translocase inner membrane subunit 23−2 | chr1:27383577−27384143 FORWARD LENG
0
1
2
3
4
5
6
6
0
3
6
12
5
9
0
0
0
0
6
8
6
11
drm.ugt80.
drm.wt.ugt80.
drm.smt1.
drm.wt.smt1.
dsm.ugt80.
dsm.wt.ugt80.
dsm.smt1.
dsm.wt.smt1.
sp.ugt80.
sp.wt.ugt80.
sp.smt1.
sp.wt.smt1.
im.ugt80.
im.wt.ugt80.
im.smt1.
im.wt.smt1.

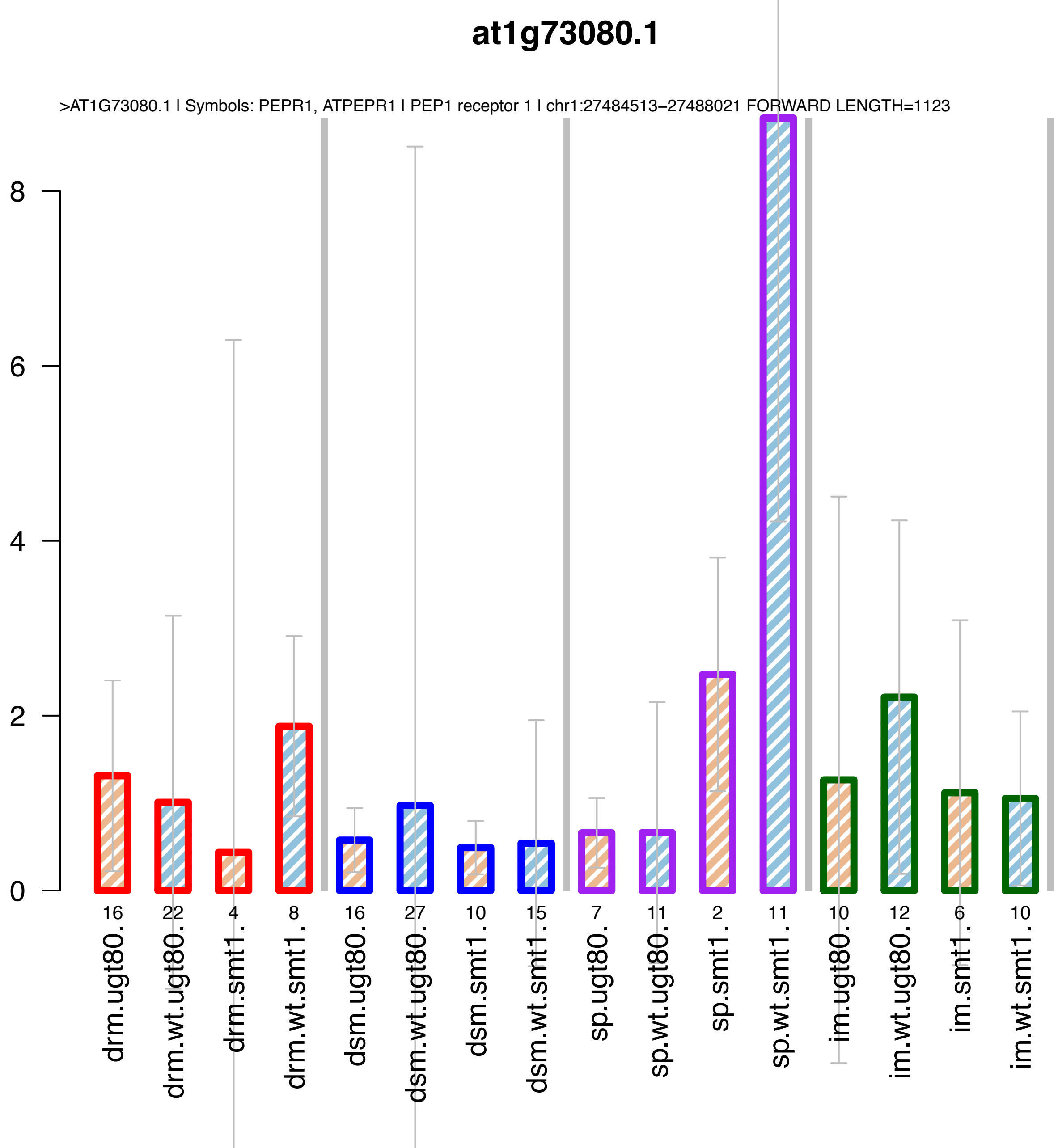

at1g73080.1
>AT1G73080.1 | Symbols: PEPR1, ATPEPR1 | PEP1 receptor 1 | chr1:27484513-27488021 FORWARD LENGTH=1123
0
2
4
6
8
16
22
4
8
16
27
10
15
7
11
2
11
10
12
6
10
drm.ugt80.
drm.wt.ugt80.
drm.smt1.
drm.wt.smt1.
dsm.ugt80.
dsm.wt.ugt80.
dsm.smt1.
dsm.wt.smt1.
sp.ugt80.
sp.wt.ugt80.
sp.smt1.
sp.wt.smt1.
im.ugt80.
im.wt.ugt80.
im.smt1.
im.wt.smt1.

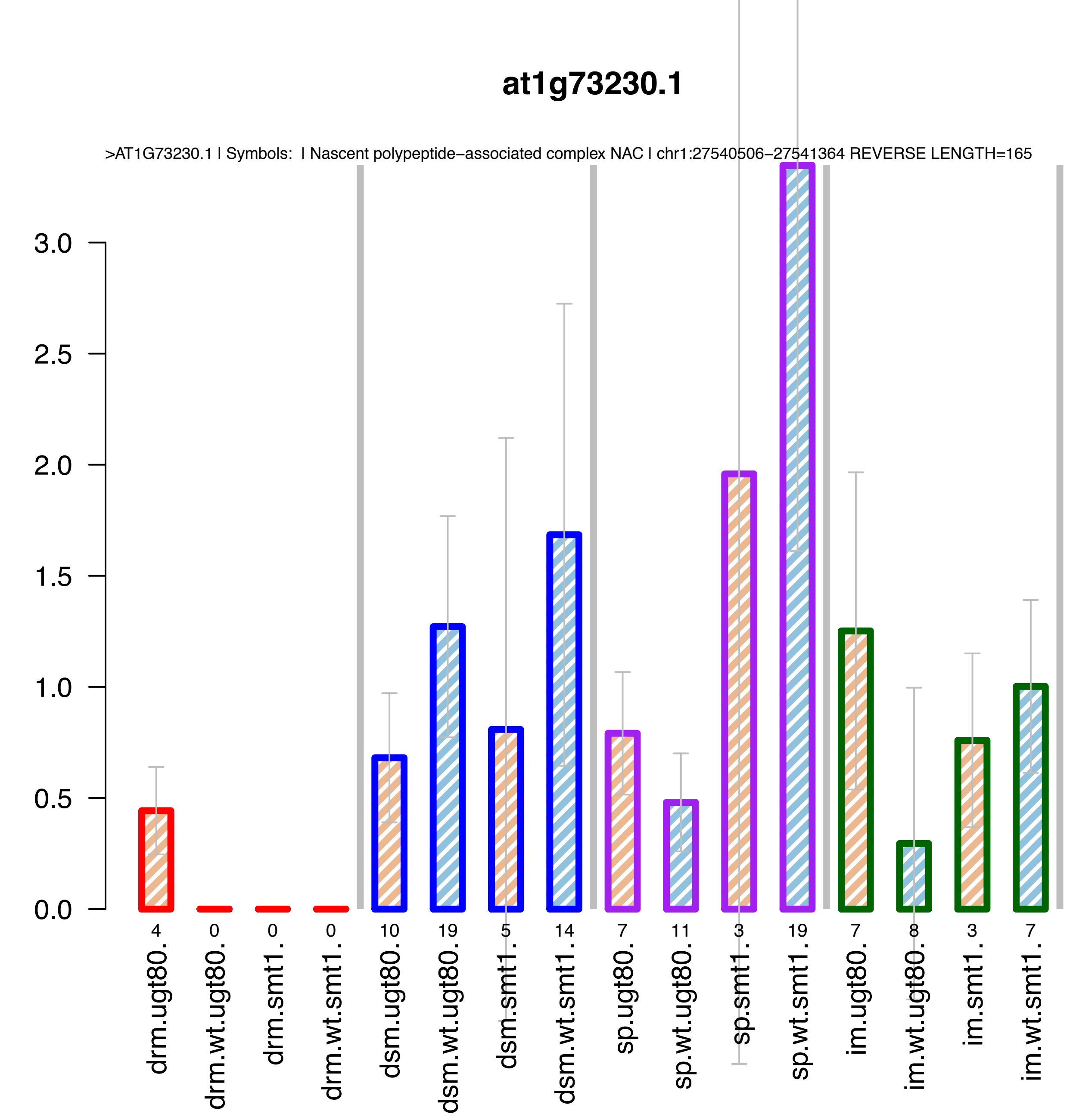

at1g73230.1
>AT1G73230.1 | Symbols: | Nascent polypeptide−associated complex NAC | chr1:27540506−27541364 REVERSE LENGTH=165
3.0
2.5
2.0
1.5
1.0
0.5
0.0
4
0
0
0
10
19
5
14
7
11
3
19
7
8
3
7
drm.ugt80.
drm.wt.ugt80.
drm.smt1.
drm.wt.smt1.
dsm.ugt80.
dsm.wt.ugt80.
dsm.smt1.
dsm.wt.smt1.
sp.ugt80.
sp.wt.ugt80.
sp.smt1.
sp.wt.smt1.
im.ugt80.
im.wt.ugt80.
im.smt1.
im.wt.smt1.

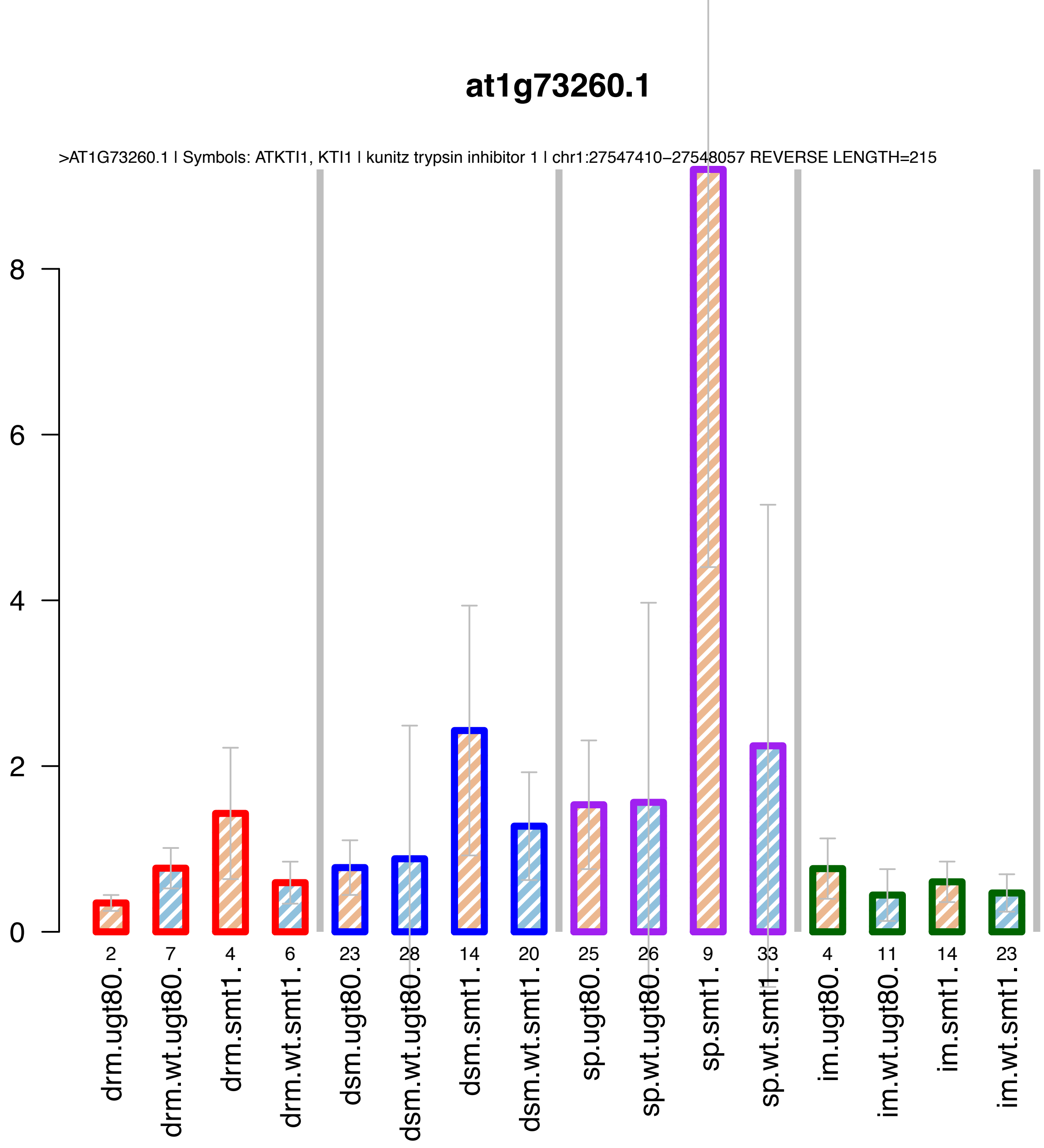

at1g73260.1
>AT1G73260.1 | Symbols: ATKTI1, KTI1 | kunitz trypsin inhibitor 1 | chr1:27547410-27548057 REVERSE LENGTH=215
0
2
4
6
8
2
7
4
6
23
28
14
20
25
26
9
33
4
11
14
23
drm.ugt80.
drm.wt.ugt80.
drm.smt1.
drm.wt.smt1.
dsm.ugt80.
dsm.wt.ugt80.
dsm.smt1.
dsm.wt.smt1.
sp.ugt80.
sp.wt.ugt80.
sp.smt1.
sp.wt.smt1.
im.ugt80.
im.wt.ugt80.
im.smt1.
im.wt.smt1.

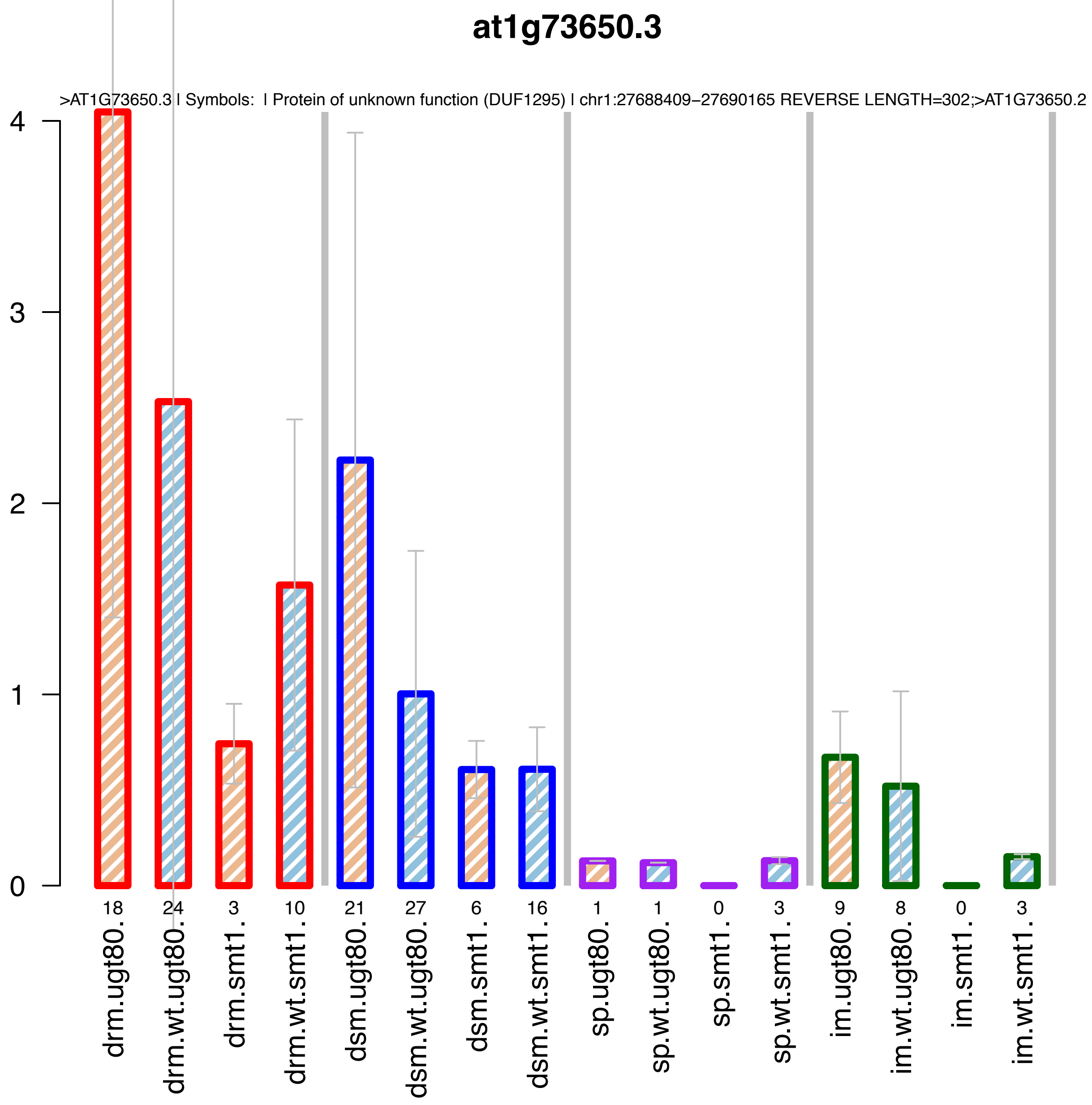

at1g73650.3
>AT1G73650.3 | Symbols: | Protein of unknown function (DUF1295) | chr1:27688409-27690165 REVERSE LENGTH=302;>AT1G73650.2
0
1
2
3
4
18
24
3
10
21
27
6
16
1
1
0
3
9
8
0
3
drm.ugt80..
drm.wt.ugt80..
drm.smt1..
drm.wt.smt1..
dsm.ugt80..
dsm.wt.ugt80..
dsm.smt1..
dsm.wt.smt1..
sp.ugt80..
sp.wt.ugt80..
sp.smt1..
sp.wt.smt1..
im.ugt80..
im.wt.ugt80..
im.smt1..
im.wt.smt1..

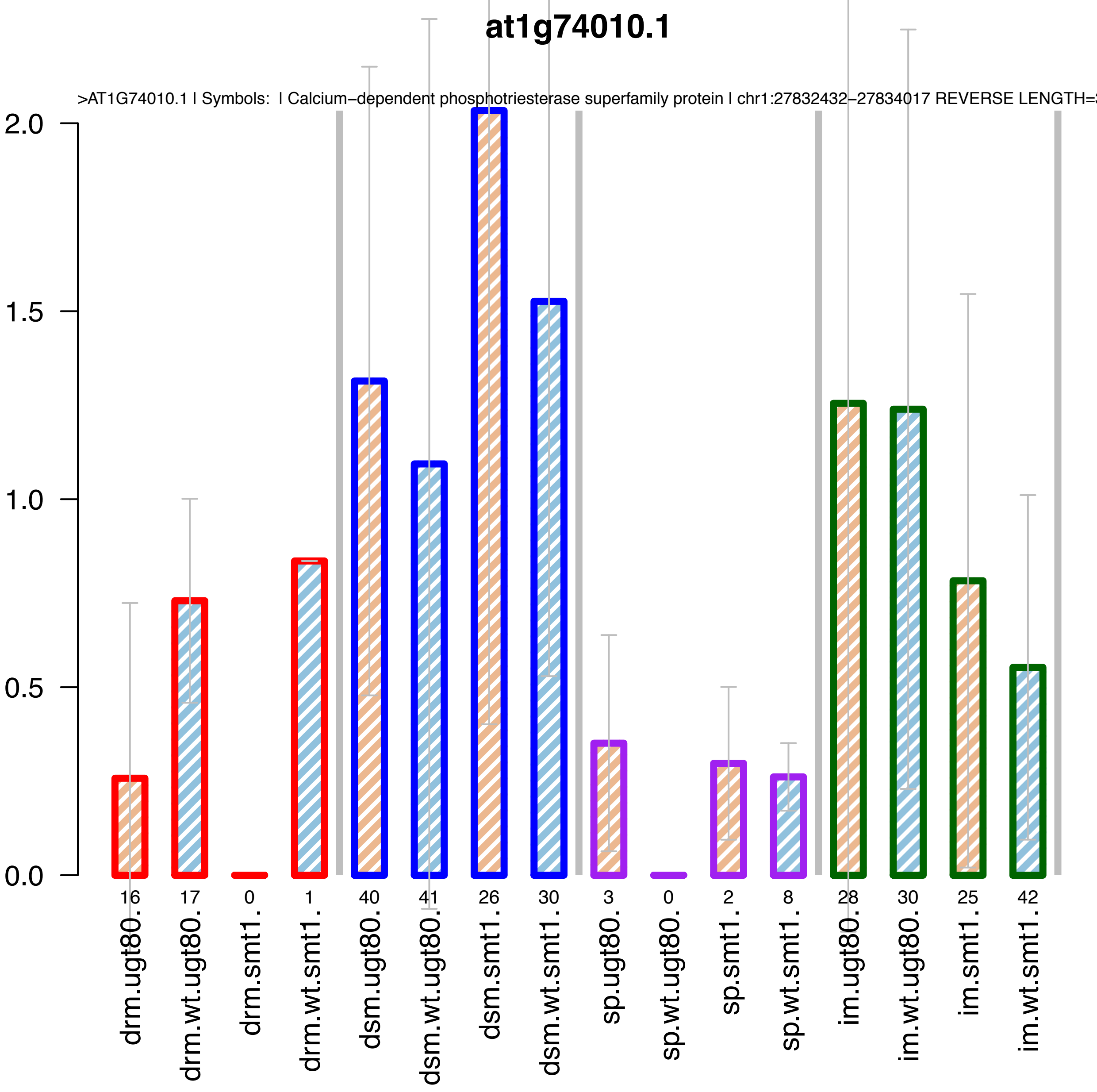
at1g74010.1
>AT1G74010.1 | Symbols: | Calcium−dependent phosphotriesterase superfamily protein | chr1:27832432−27834017 REVERSE LENGTH=
2.0
1.5
1.0
0.5
0.0
16
17
0
1
40
41
26
30
3
0
2
8
28
30
25
42
drm.ugt80.
drm.wt.ugt80.
drm.smt1.
drm.wt.smt1.
dsm.ugt80.
dsm.wt.ugt80.
dsm.smt1.
dsm.wt.smt1.
sp.ugt80.
sp.wt.ugt80.
sp.smt1.
sp.wt.smt1.
im.ugt80.
im.wt.ugt80.
im.smt1.
im.wt.smt1.

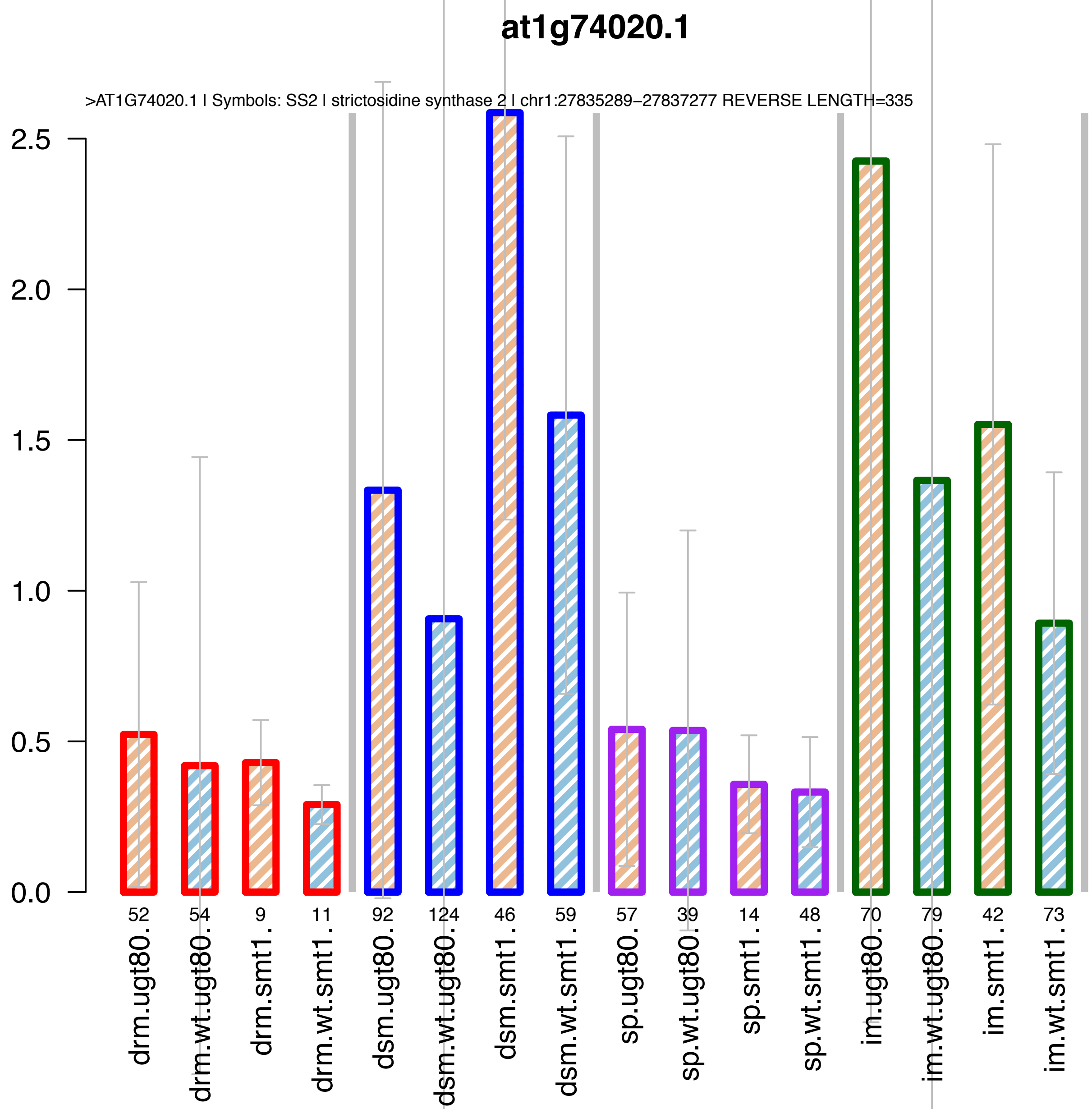

at1g74020.1
>AT1G74020.1 | Symbols: SS2 | strictosidine synthase 2 | chr1:27835289-27837277 REVERSE LENGTH=335
0.0
0.5
1.0
1.5
2.0
2.5
52
54
9
11
92
124
46
59
57
39
14
48
70
79
42
73
drm.ugt80.
drm.wt.ugt80.
drm.smt1.
drm.wt.smt1.
dsm.ugt80.
dsm.wt.ugt80.
dsm.smt1.
dsm.wt.smt1.
sp.ugt80.
sp.wt.ugt80.
sp.smt1.
sp.wt.smt1.
im.ugt80.
im.wt.ugt80.
im.smt1.
im.wt.smt1.

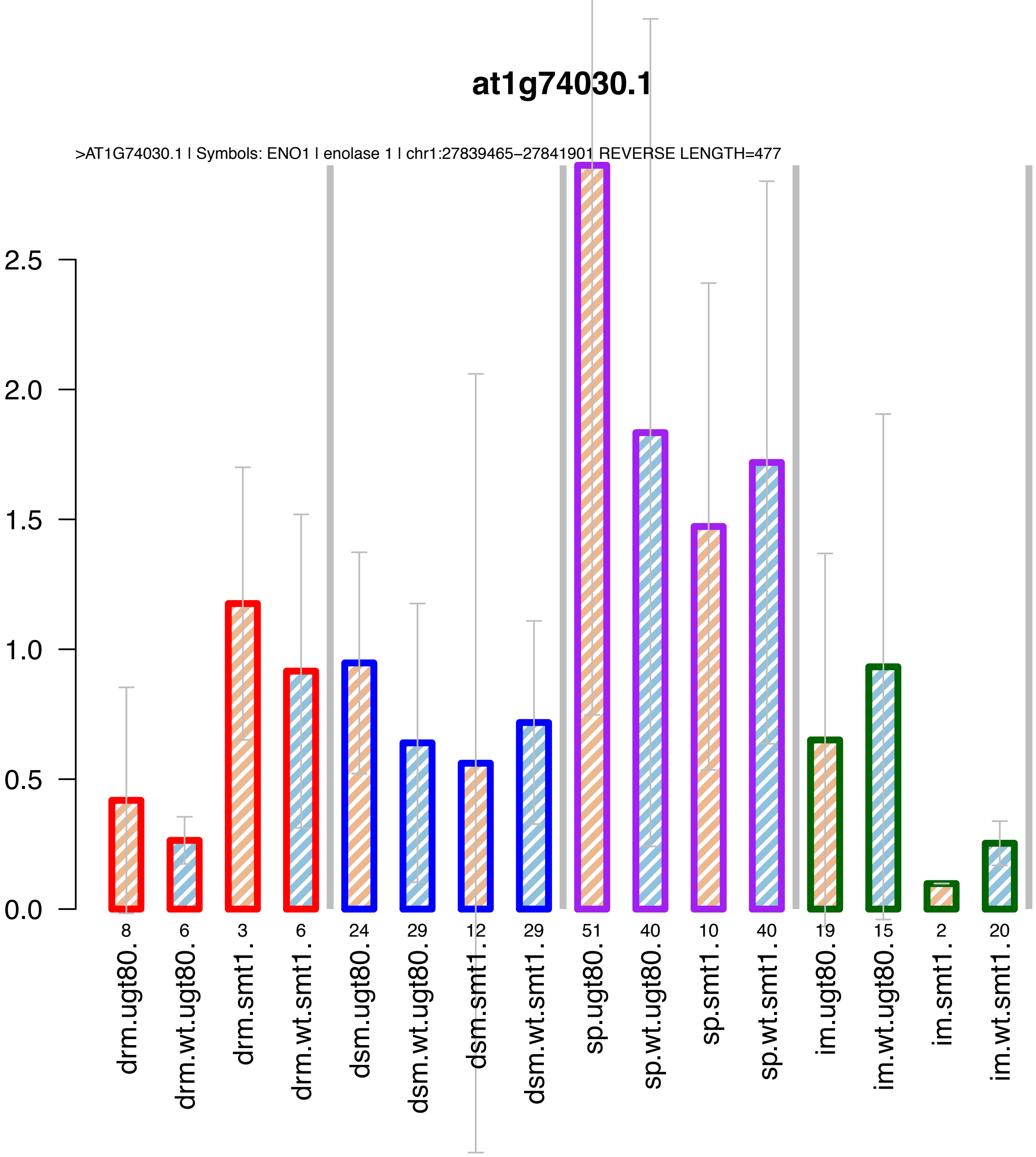

at1g74030.1
>AT1G74030.1 | Symbols: ENO1 | enolase 1 | chr1:27839465-27841901 REVERSE LENGTH=477
2.5
2.0
1.5
1.0
0.5
0.0
8
6
3
6
24
29
12
29
51
40
10
40
19
15
2
20
drm.ugt80..
drm.wt.ugt80..
drm.smt1..
drm.wt.smt1..
dsm.ugt80..
dsm.wt.ugt80..
dsm.smt1..
dsm.wt.smt1..
sp.ugt80..
sp.wt.ugt80..
sp.smt1..
sp.wt.smt1..
im.ugt80..
im.wt.ugt80..
im.smt1..
im.wt.smt1..

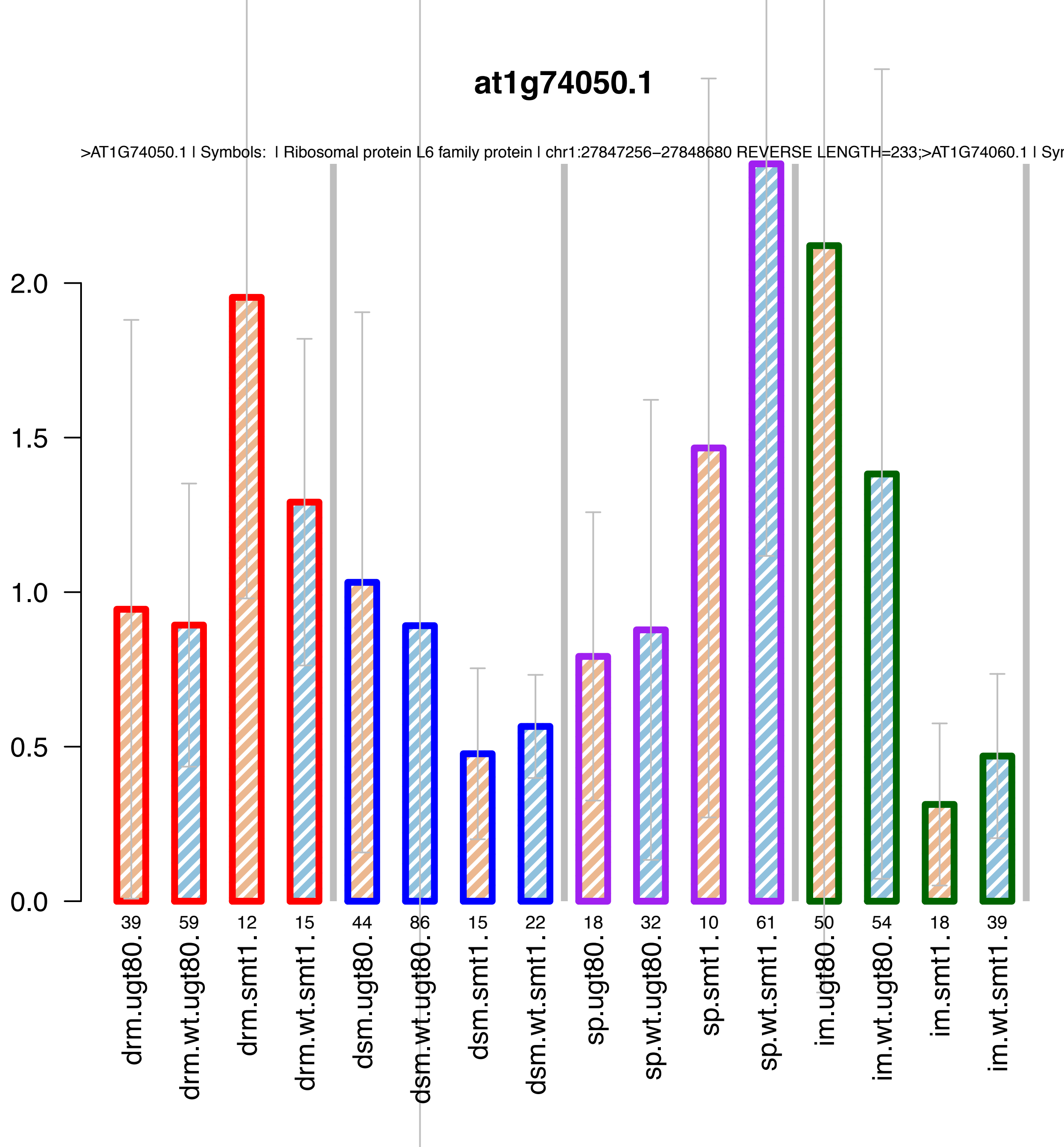

at1g74050.1
>AT1G74050.1 | Symbols: | Ribosomal protein L6 family protein | chr1:27847256-27848680 REVERSE LENGTH=233;>AT1G74060.1 | Syn
2.0
1.5
1.0
0.5
0.0
39
59
12
15
44
86
15
22
18
32
10
61
50
54
18
39
drm.ugt80..
drm.wt.ugt80..
drm.smt1..
drm.wt.smt1..
dsm.ugt80..
dsm.wt.ugt80..
dsm.smt1..
dsm.wt.smt1..
sp.ugt80..
sp.wt.ugt80..
sp.smt1..
sp.wt.smt1..
im.ugt80..
im.wt.ugt80..
im.smt1..
im.wt.smt1..

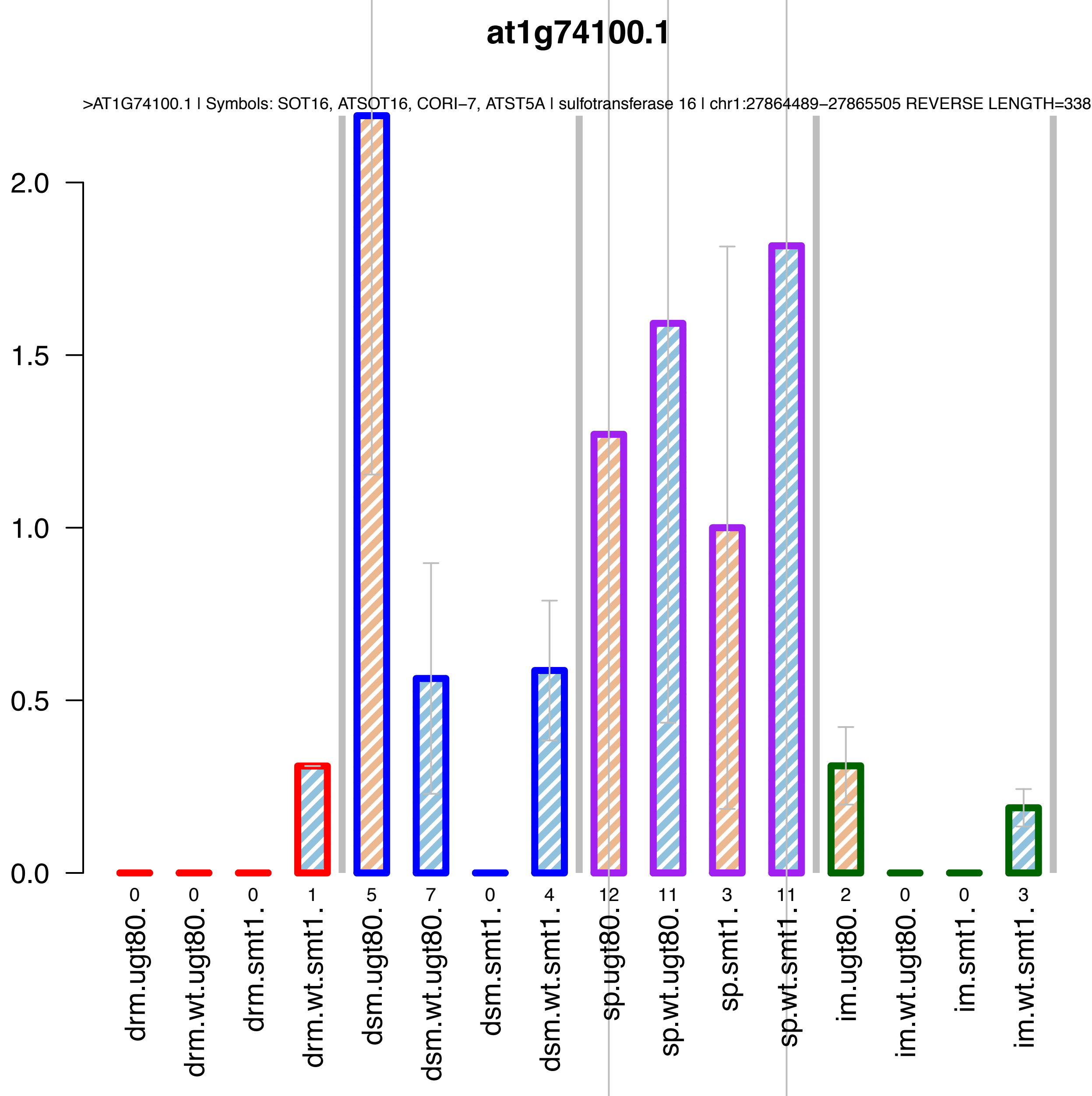

at1g74100.1
>AT1G74100.1 | Symbols: SOT16, ATSOT16, CORI−7, ATST5A | sulfotransferase 16 | chr1:27864489−27865505 REVERSE LENGTH=338
2.0
1.5
1.0
0.5
0.0
0
0
0
1
5
7
0
4
12
11
3
11
2
0
0
3
drm.ugt80.
drm.wt.ugt80.
drm.smt1.
drm.wt.smt1.
dsm.ugt80.
dsm.wt.ugt80.
dsm.smt1.
dsm.wt.smt1.
sp.ugt80.
sp.wt.ugt80.
sp.smt1.
sp.wt.smt1.
im.ugt80.
im.wt.ugt80.
im.smt1.
im.wt.smt1.

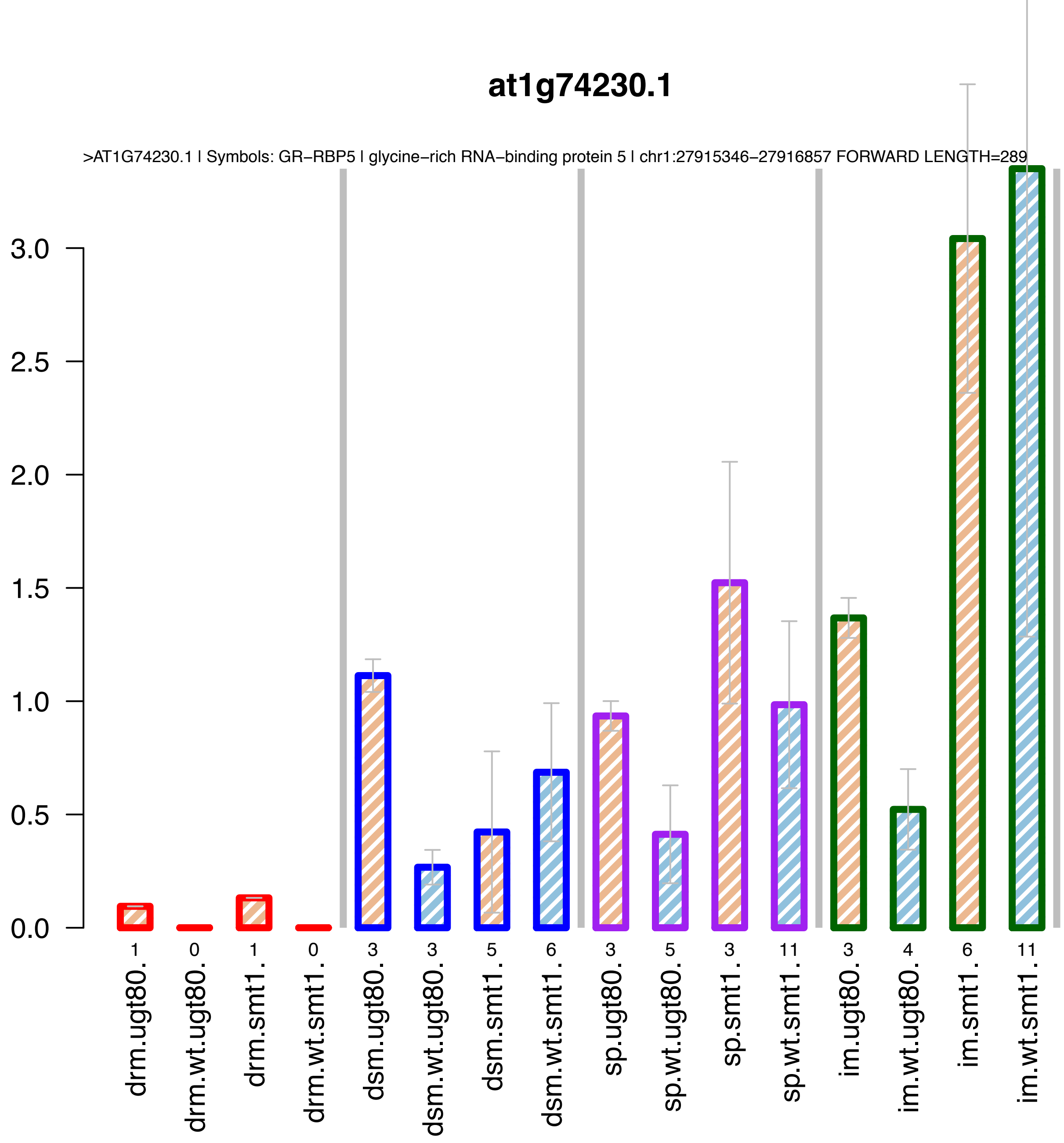

at1g74230.1
>AT1G74230.1 | Symbols: GR−RBP5 | glycine−rich RNA−binding protein 5 | chr1:27915346−27916857 FORWARD LENGTH=289
0.0
0.5
1.0
1.5
2.0
2.5
3.0
1
0
1
0
3
3
5
6
3
5
3
11
3
4
6
11
drm.ugt80.
drm.wt.ugt80.
drm.smt1.
drm.wt.smt1.
dsm.ugt80.
dsm.wt.ugt80.
dsm.smt1.
dsm.wt.smt1.
sp.ugt80.
sp.wt.ugt80.
sp.smt1.
sp.wt.smt1.
im.ugt80.
im.wt.ugt80.
im.smt1.
im.wt.smt1.

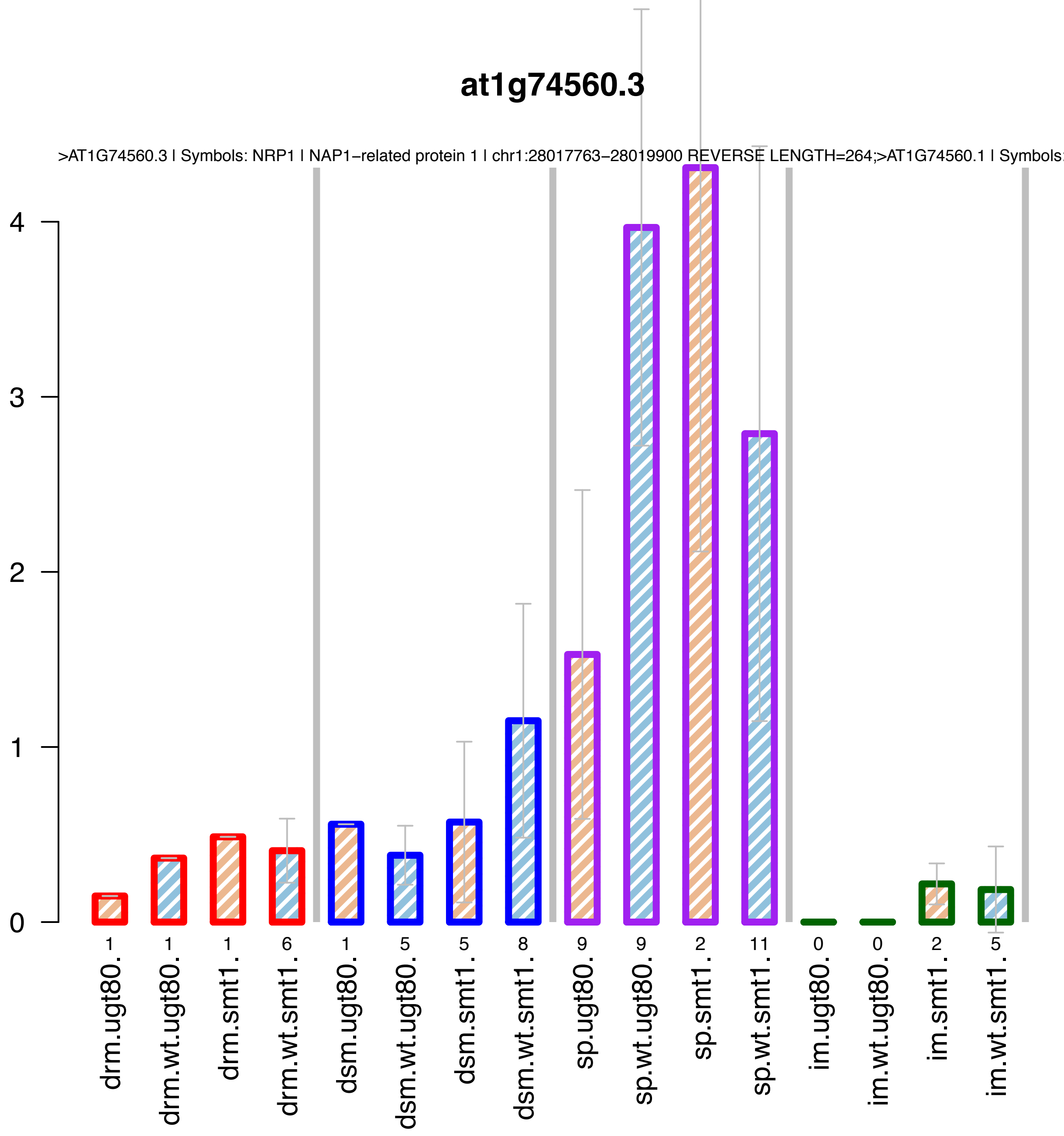
at1g74560.3
>AT1G74560.3 | Symbols: NRP1 | NAP1−related protein 1 | chr1:28017763−28019900 REVERSE LENGTH=264;>AT1G74560.1 | Symbols:
4
3
2
1
0
1
1
1
6
1
5
5
8
9
9
2
11
0
0
2
5
drm.ugt80.
drm.wt.ugt80.
drm.smt1.
drm.wt.smt1.
dsm.ugt80.
dsm.wt.ugt80.
dsm.smt1.
dsm.wt.smt1.
sp.ugt80.
sp.wt.ugt80.
sp.smt1.
sp.wt.smt1.
im.ugt80.
im.wt.ugt80.
im.smt1.
im.wt.smt1.

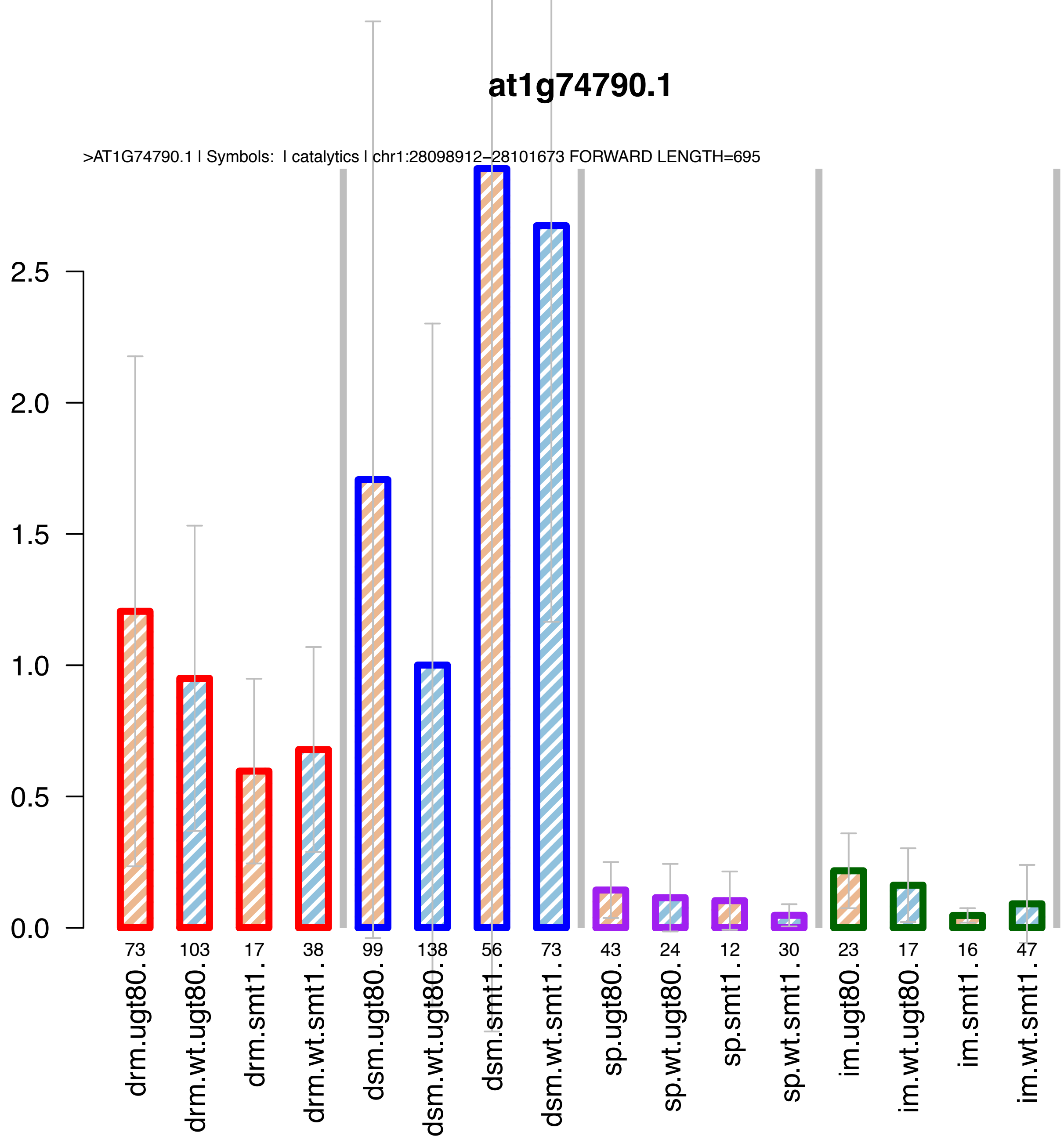

at1g74790.1
>AT1G74790.1 | Symbols: | catalytics | chr1:28098912–28101673 FORWARD LENGTH=695
0.0
0.5
1.0
1.5
2.0
2.5
73
103
17
38
99
138
56
73
43
24
12
30
23
17
16
47
drm.ugt80..
drm.wt.ugt80..
drm.smt1..
drm.wt.smt1..
dsm.ugt80..
dsm.wt.ugt80..
dsm.smt1..
dsm.wt.smt1..
sp.ugt80..
sp.wt.ugt80..
sp.smt1..
sp.wt.smt1..
im.ugt80..
im.wt.ugt80..
im.smt1..
im.wt.smt1..

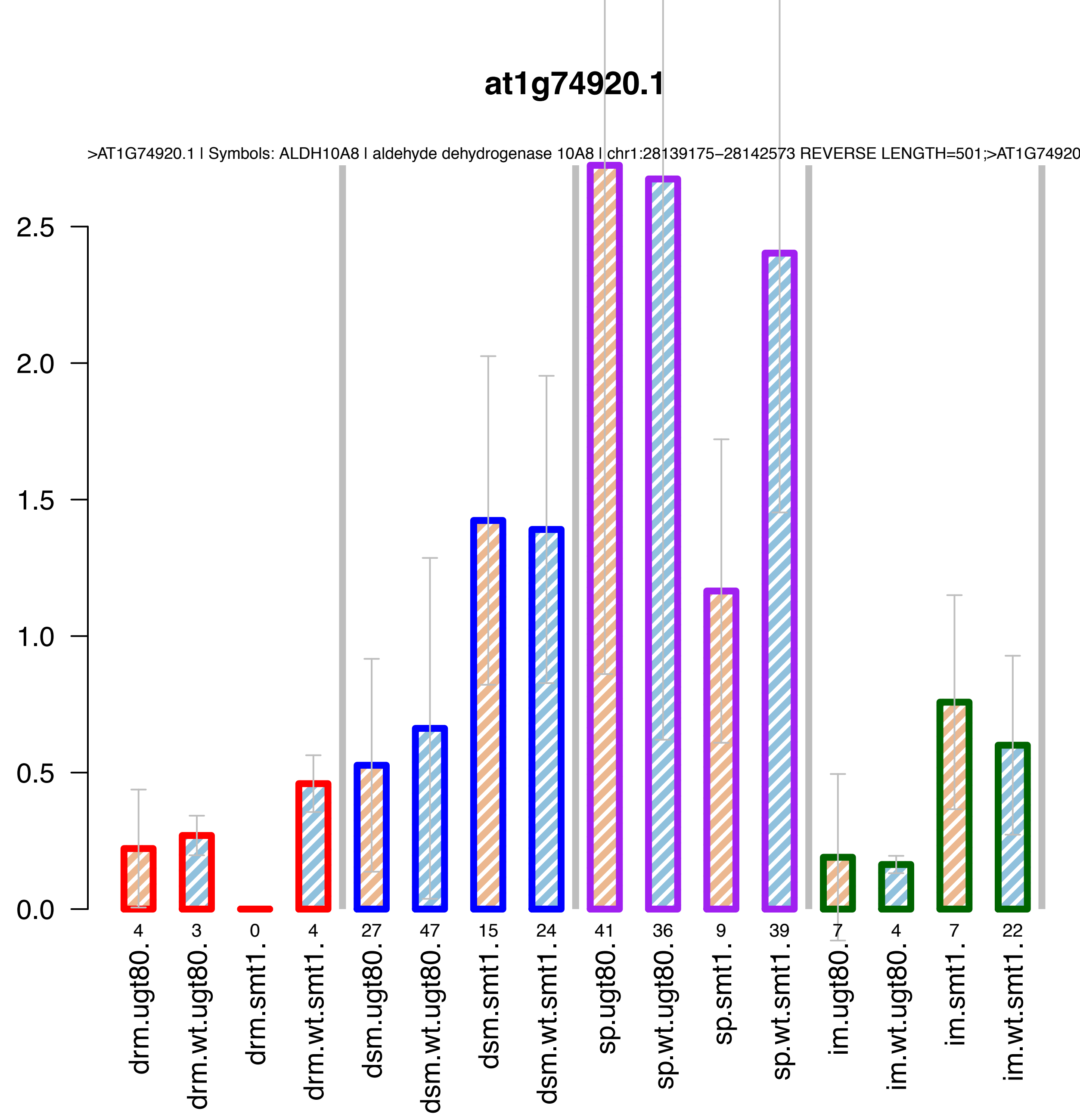
at1g74920.1
>AT1G74920.1 | Symbols: ALDH10A8 | aldehyde dehydrogenase 10A8 | chr1:28139175-28142573 REVERSE LENGTH=501;>AT1G74920
2.5
2.0
1.5
1.0
0.5
0.0
4
3
0
4
27
47
15
24
41
36
9
39
7
4
7
22
drm.ugt80.
drm.wt.ugt80.
drm.smt1.
drm.wt.smt1.
dsm.ugt80.
dsm.wt.ugt80.
dsm.smt1.
dsm.wt.smt1.
sp.ugt80.
sp.wt.ugt80.
sp.smt1.
sp.wt.smt1.
im.ugt80.
im.wt.ugt80.
im.smt1.
im.wt.smt1.

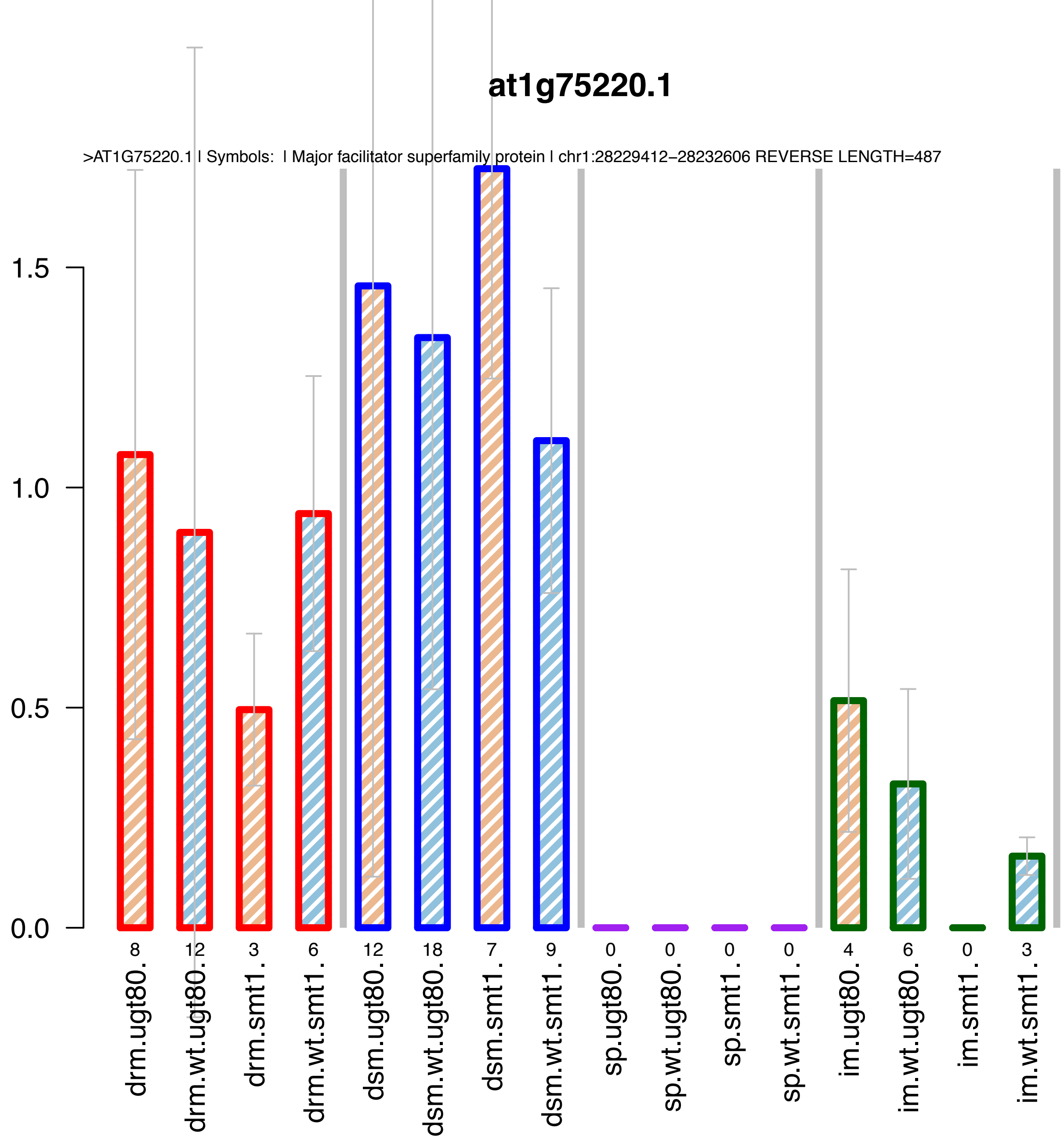

at1g75220.1
>AT1G75220.1 | Symbols: | Major facilitator superfamily protein | chr1:28229412-28232606 REVERSE LENGTH=487
0.0
0.5
1.0
1.5
8
12
3
6
12
18
7
9
0
0
0
0
4
6
0
3
drm.ugt80.
drm.wt.ugt80.
drm.smt1.
drm.wt.smt1.
dsm.ugt80.
dsm.wt.ugt80.
dsm.smt1.
dsm.wt.smt1.
sp.ugt80.
sp.wt.ugt80.
sp.smt1.
sp.wt.smt1.
im.ugt80.
im.wt.ugt80.
im.smt1.
im.wt.smt1.

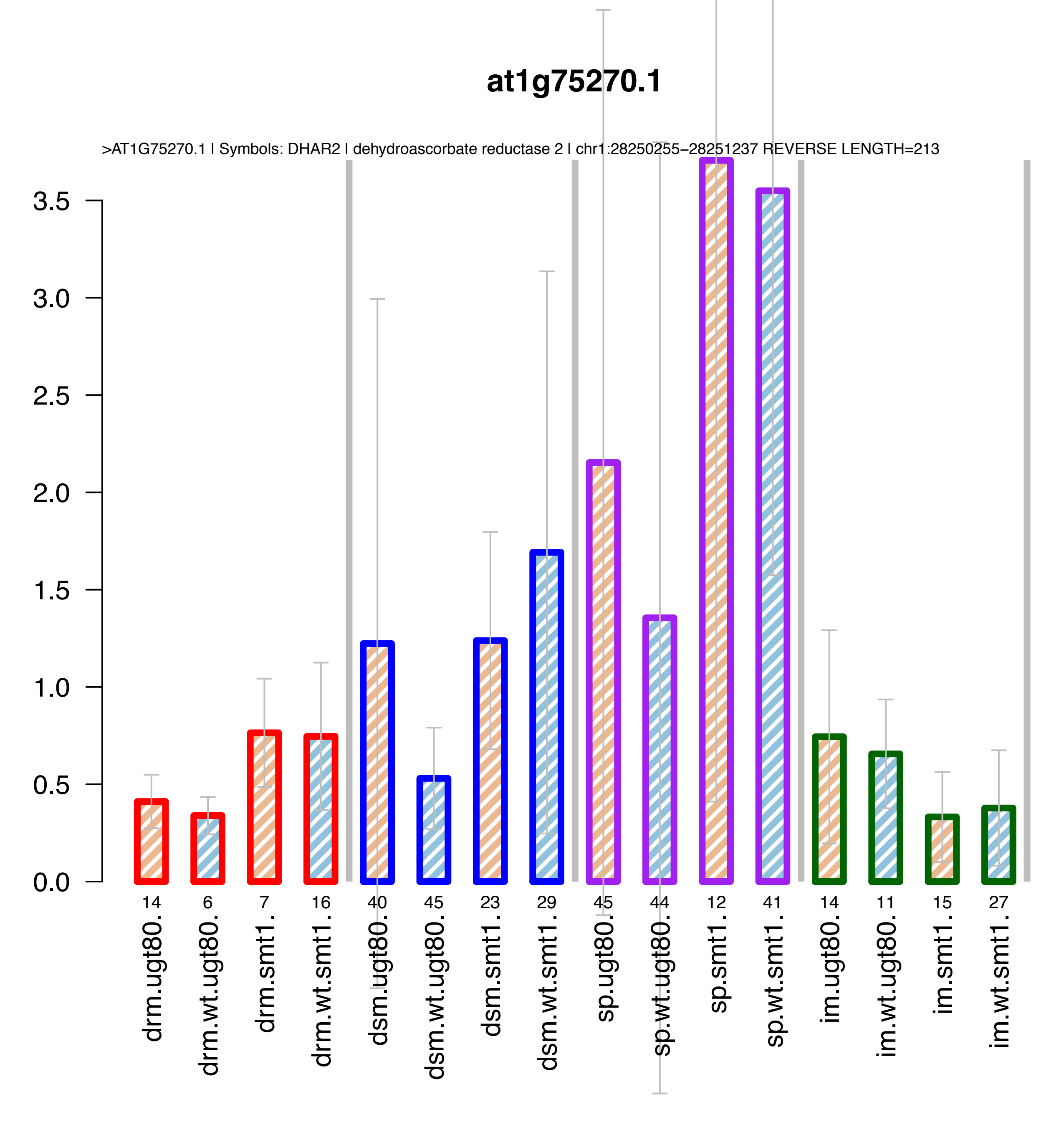

at1g75270.1
>AT1G75270.1 | Symbols: DHAR2 | dehydroascorbate reductase 2 | chr1:28250255−28251237 REVERSE LENGTH=213
0.0
0.5
1.0
1.5
2.0
2.5
3.0
3.5
14
6
7
16
40
45
23
29
45
44
12
41
14
11
15
27
drm.ugt80.
drm.wt.ugt80.
drm.smt1.
drm.wt.smt1.
dsm.ugt80.
dsm.wt.ugt80.
dsm.smt1.
dsm.wt.smt1.
sp.ugt80.
sp.wt.ugt80.
sp.smt1.
sp.wt.smt1.
im.ugt80.
im.wt.ugt80.
im.smt1.
im.wt.smt1.

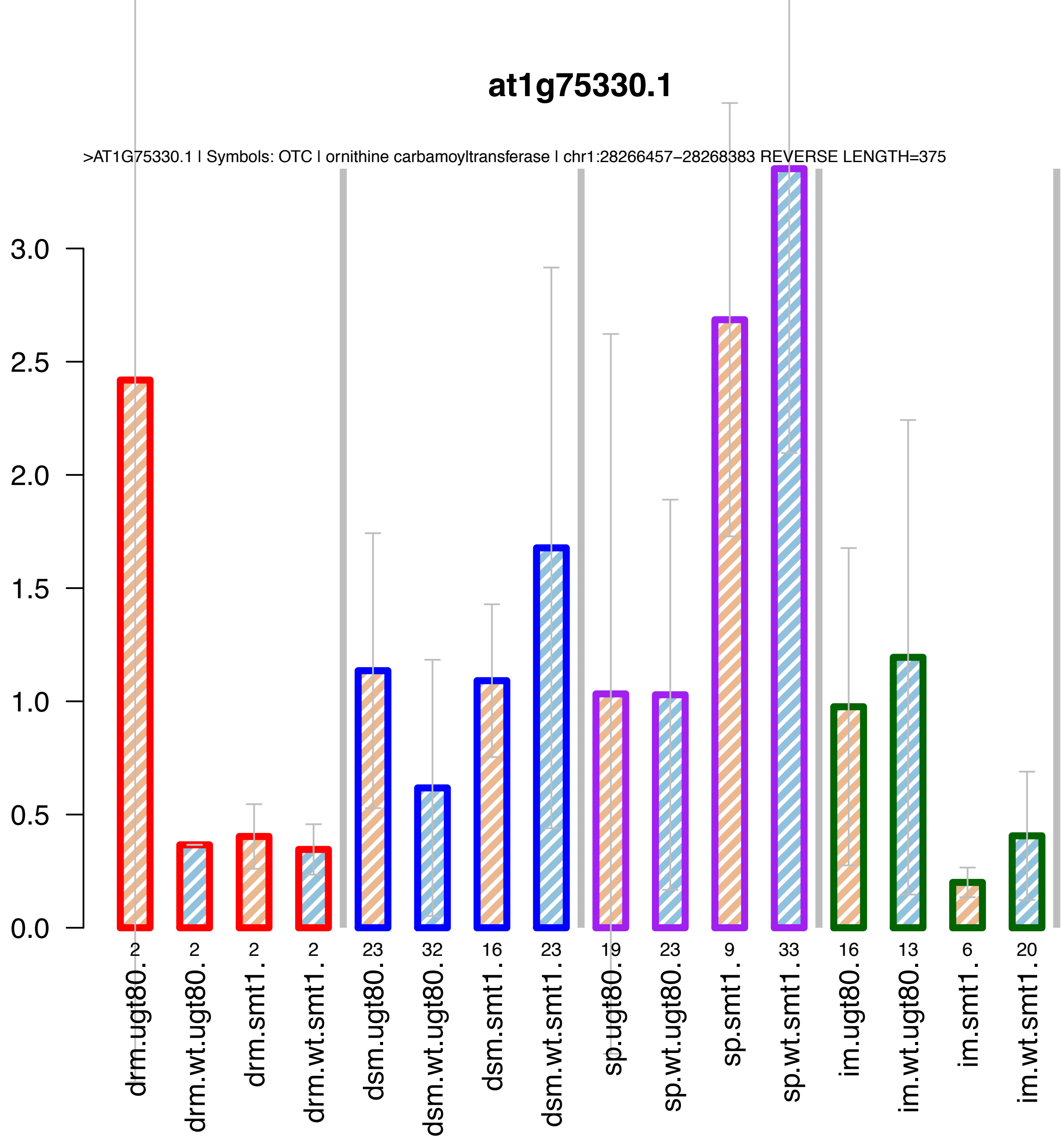

at1g75330.1
>AT1G75330.1 | Symbols: OTC | ornithine carbamoyltransferase | chr1:28266457−28268383 REVERSE LENGTH=375
3.0
2.5
2.0
1.5
1.0
0.5
0.0
2
2
2
2
23
32
16
23
19
23
9
33
16
13
6
20
drm.ugt80..
drm.wt.ugt80..
drm.smt1..
drm.wt.smt1..
dsm.ugt80..
dsm.wt.ugt80..
dsm.smt1..
dsm.wt.smt1..
sp.ugt80..
sp.wt.ugt80..
sp.smt1..
sp.wt.smt1..
im.ugt80..
im.wt.ugt80..
im.smt1..
im.wt.smt1..

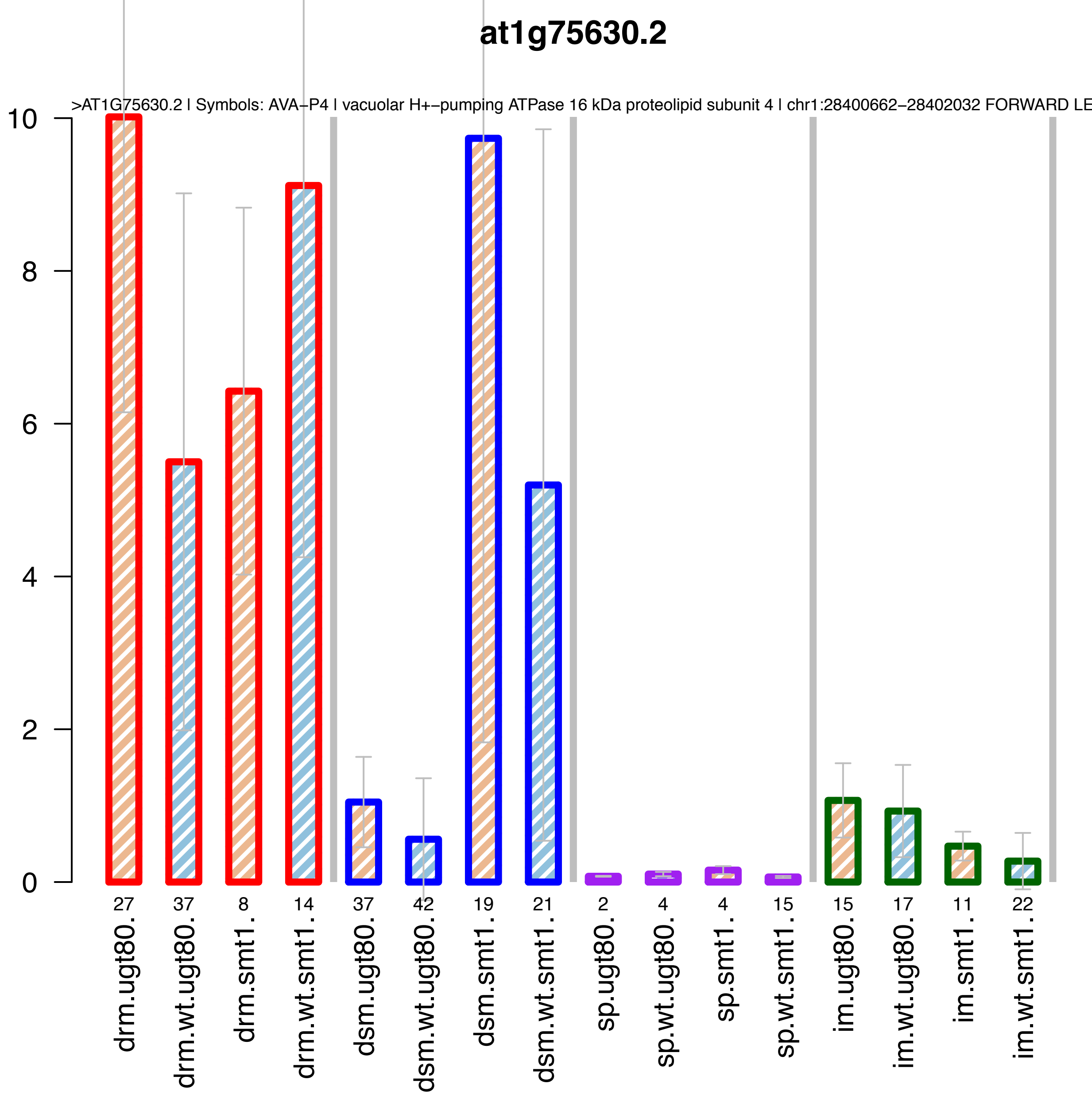

at1g75630.2
>AT1G75630.2 | Symbols: AVA−P4 | vacuolar H+−pumping ATPase 16 kDa proteolipid subunit 4 | chr1:28400662−28402032 FORWARD LE
10
8
6
4
2
0
27
37
8
14
37
42
19
21
2
4
4
15
15
17
11
22
drm.ugt80.
drm.wt.ugt80.
drm.smt1.
drm.wt.smt1.
dsm.ugt80.
dsm.wt.ugt80.
dsm.smt1.
dsm.wt.smt1.
sp.ugt80.
sp.wt.ugt80.
sp.smt1.
sp.wt.smt1.
im.ugt80.
im.wt.ugt80.
im.smt1.
im.wt.smt1.

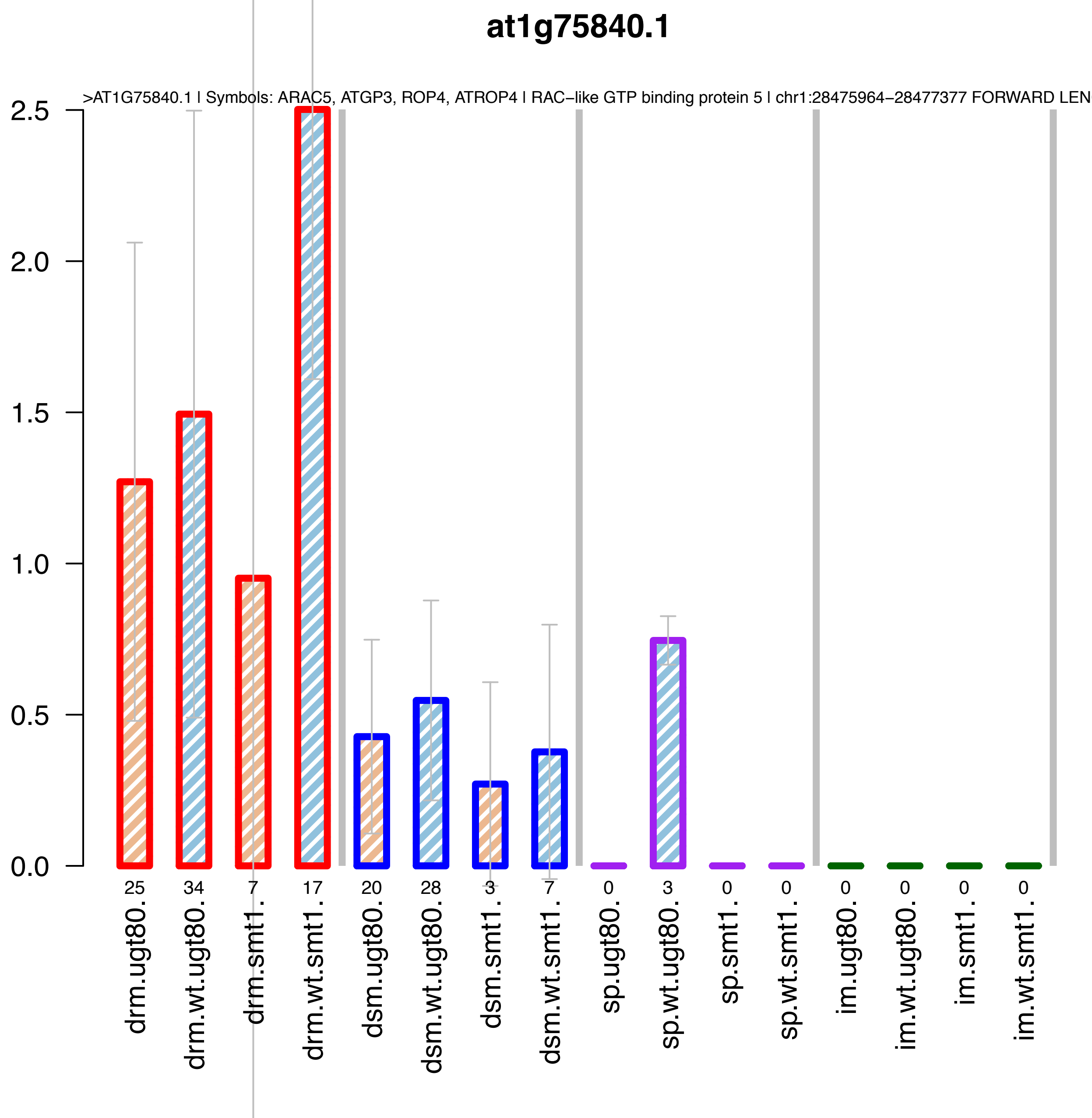
at1g75840.1
>AT1G75840.1 | Symbols: ARAC5, ATGP3, ROP4, ATROP4 | RAC−like GTP binding protein 5 | chr1:28475964−28477377 FORWARD LEN
0.0
0.5
1.0
1.5
2.0
2.5
25
34
7
17
20
28
3
7
0
3
0
0
0
0
0
0
drm.ugt80.
drm.wt.ugt80.
drm.smt1.
drm.wt.smt1.
dsm.ugt80.
dsm.wt.ugt80.
dsm.smt1.
dsm.wt.smt1.
sp.ugt80.
sp.wt.ugt80.
sp.smt1.
sp.wt.smt1.
im.ugt80.
im.wt.ugt80.
im.smt1.
im.wt.smt1.

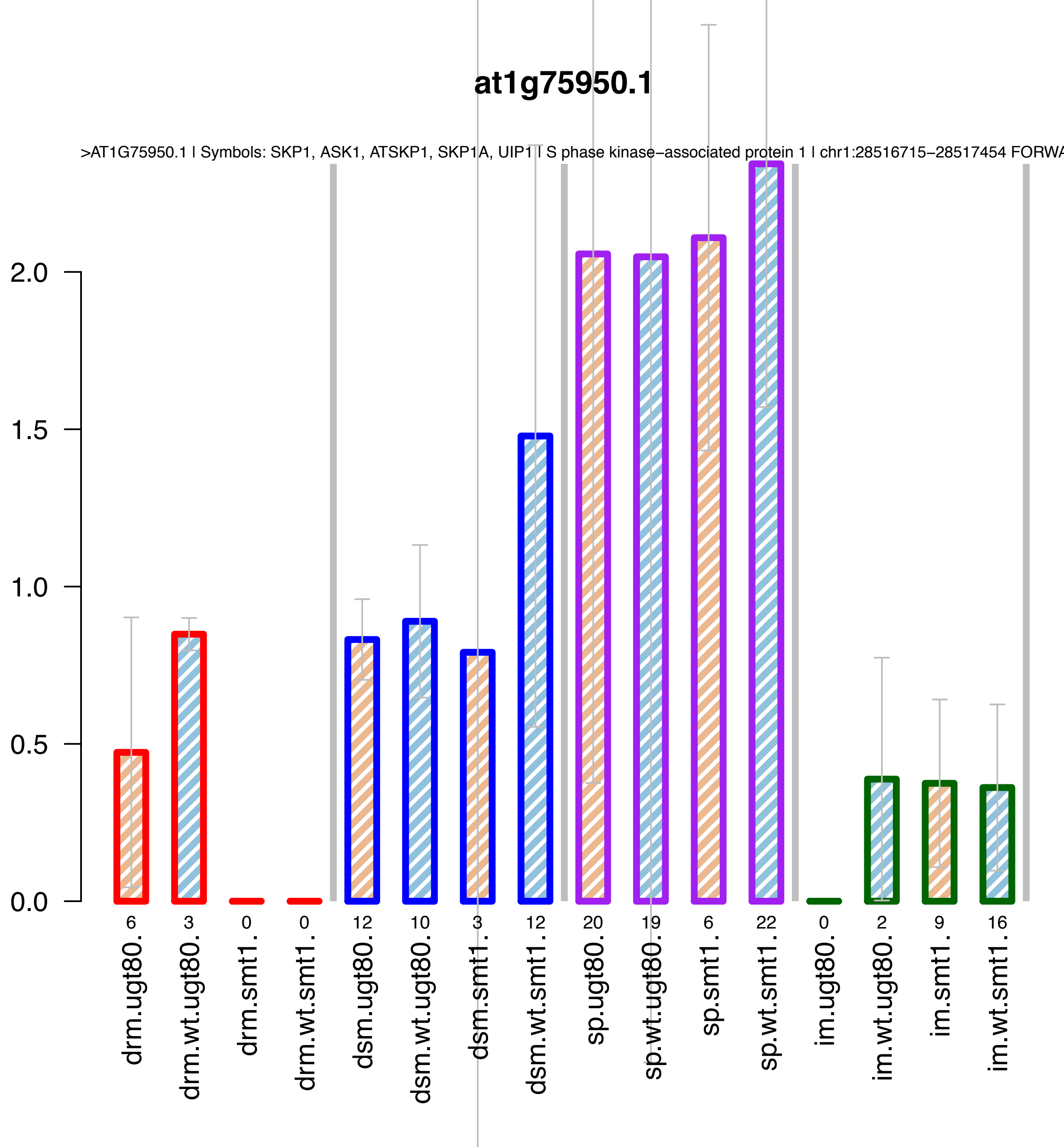

at1g75950.1
>AT1G75950.1 | Symbols: SKP1, ASK1, ATSKP1, SKP1A, UIP1 | S phase kinase-associated protein 1 | chr1:28516715-28517454 FORWA
0.0
0.5
1.0
1.5
2.0
6
3
0
0
12
10
3
12
20
19
6
22
0
2
9
16
drm.ugt80.
drm.wt.ugt80.
drm.smt1.
drm.wt.smt1.
dsm.ugt80.
dsm.wt.ugt80.
dsm.smt1.
dsm.wt.smt1.
sp.ugt80.
sp.wt.ugt80.
sp.smt1.
sp.wt.smt1.
im.ugt80.
im.wt.ugt80.
im.smt1.
im.wt.smt1.

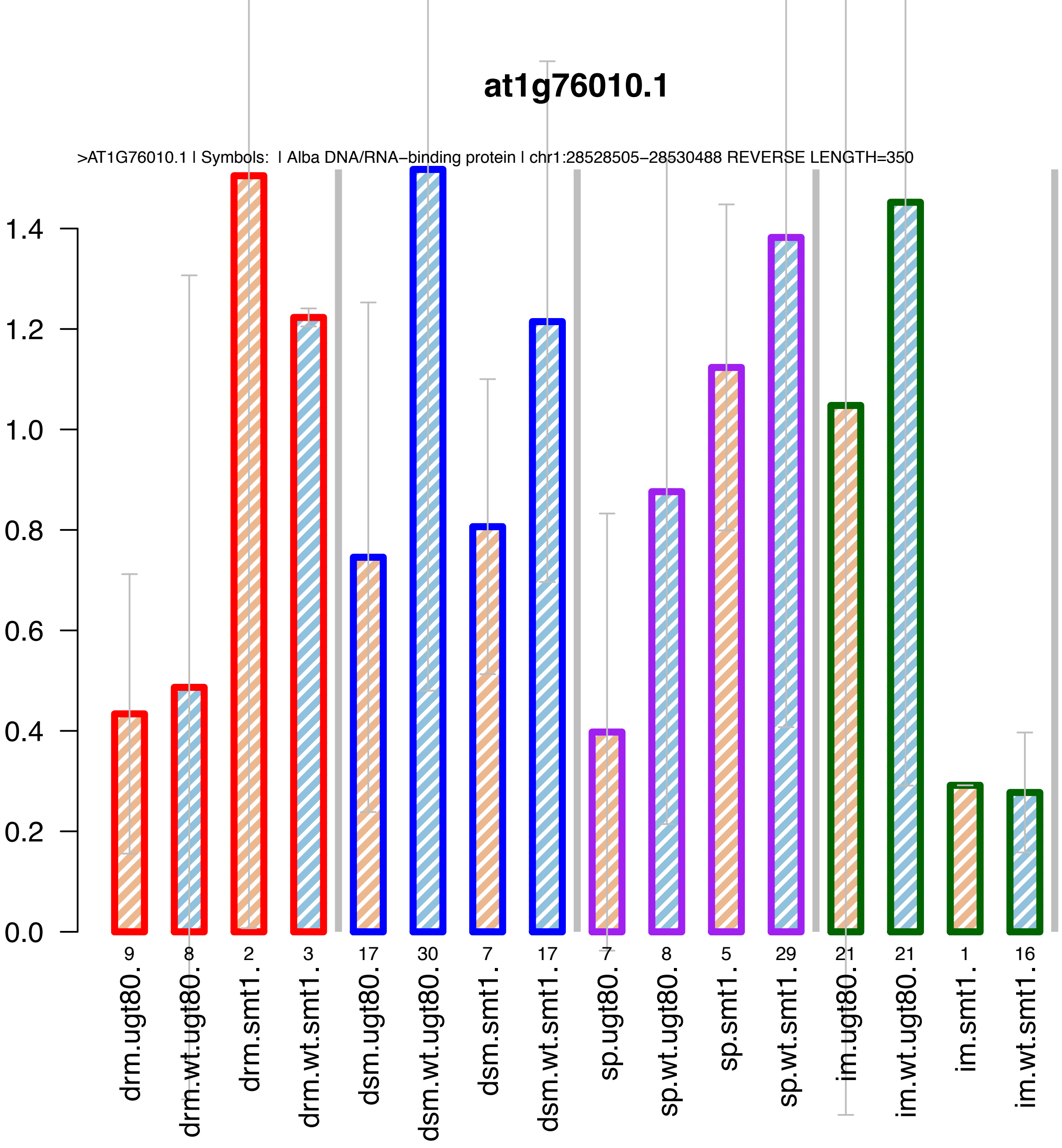

at1g76010.1
>AT1G76010.1 | Symbols: | Alba DNA/RNA−binding protein | chr1:28528505−28530488 REVERSE LENGTH=350
0.0
0.2
0.4
0.6
0.8
1.0
1.2
1.4
9
8
2
3
17
30
7
17
7
8
5
29
21
21
1
16
drm.ugt80.
drm.wt.ugt80.
drm.smt1.
drm.wt.smt1.
dsm.ugt80.
dsm.wt.ugt80.
dsm.smt1.
dsm.wt.smt1.
sp.ugt80.
sp.wt.ugt80.
sp.smt1.
sp.wt.smt1.
im.ugt80.
im.wt.ugt80.
im.smt1.
im.wt.smt1.

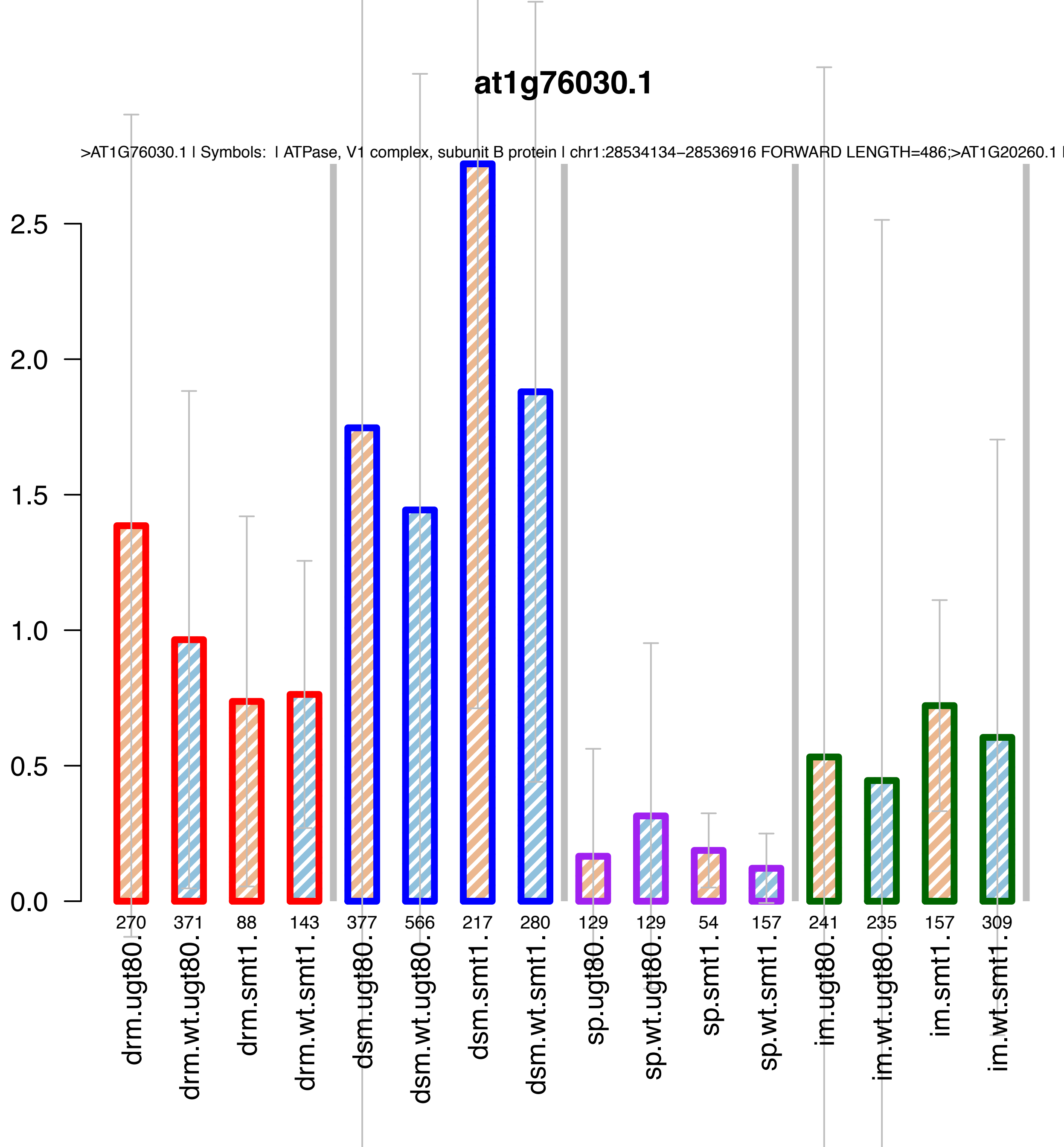

at1g76030.1
>AT1G76030.1 | Symbols: | ATPase, V1 complex, subunit B protein | chr1:28534134-28536916 FORWARD LENGTH=486;>AT1G20260.1 |
0.0
0.5
1.0
1.5
2.0
2.5
270
371
88
143
377
566
217
280
129
129
54
157
241
235
157
309
drm.ugt80..
drm.wt.ugt80..
drm.smt1..
drm.wt.smt1..
dsm.ugt80..
dsm.wt.ugt80..
dsm.smt1..
dsm.wt.smt1..
sp.ugt80..
sp.wt.ugt80..
sp.smt1..
sp.wt.smt1..
im.ugt80..
im.wt.ugt80..
im.smt1..
im.wt.smt1..

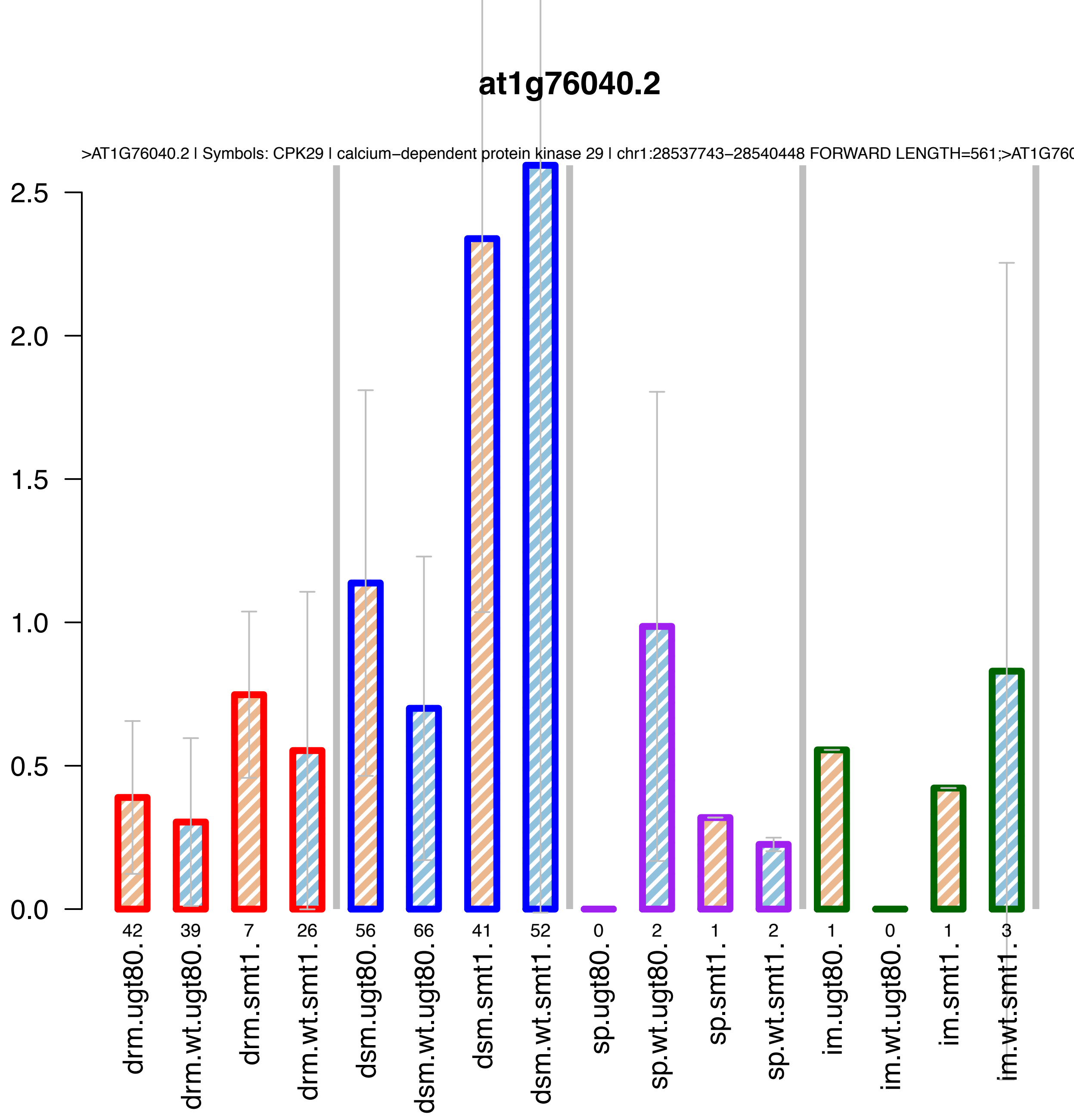

at1g76040.2
>AT1G76040.2 | Symbols: CPK29 | calcium−dependent protein kinase 29 | chr1:28537743−28540448 FORWARD LENGTH=561;>AT1G760
2.5
2.0
1.5
1.0
0.5
0.0
42
39
7
26
56
66
41
52
0
2
1
2
1
0
1
3
drm.ugt80..
drm.wt.ugt80..
drm.smt1..
drm.wt.smt1..
dsm.ugt80..
dsm.wt.ugt80..
dsm.smt1..
dsm.wt.smt1..
sp.ugt80..
sp.wt.ugt80..
sp.smt1..
sp.wt.smt1..
im.ugt80..
im.wt.ugt80..
im.smt1..
im.wt.smt1..

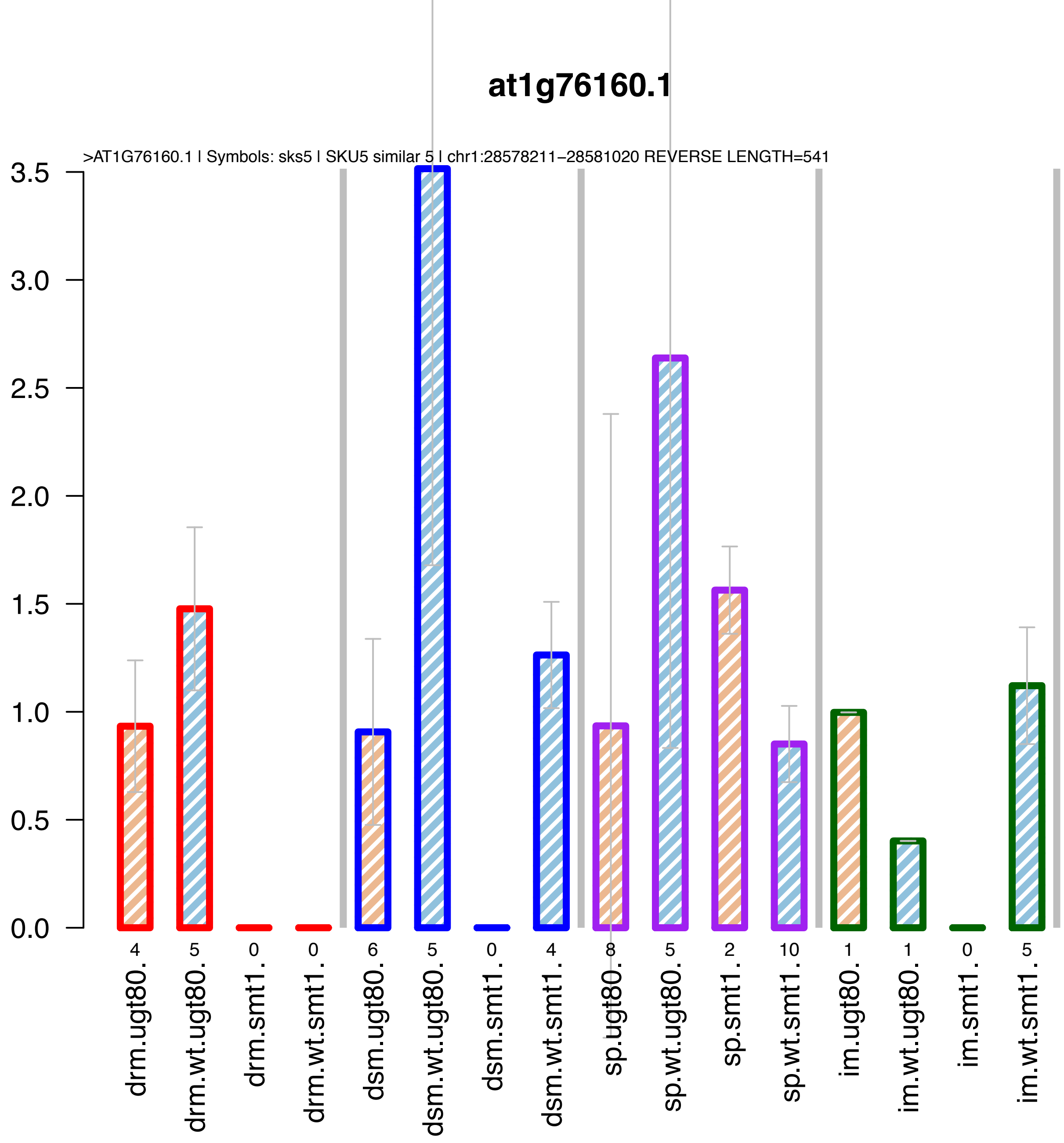

at1g76160.1
>AT1G76160.1 | Symbols: sks5 | SKU5 similar 5 | chr1:28578211−28581020 REVERSE LENGTH=541
3.5
3.0
2.5
2.0
1.5
1.0
0.5
0.0
4
5
0
0
6
5
0
4
8
5
2
10
1
1
0
5
drm.ugt80.
drm.wt.ugt80.
drm.smt1.
drm.wt.smt1.
dsm.ugt80.
dsm.wt.ugt80.
dsm.smt1.
dsm.wt.smt1.
sp.ugt80.
sp.wt.ugt80.
sp.smt1.
sp.wt.smt1.
im.ugt80.
im.wt.ugt80.
im.smt1.
im.wt.smt1.

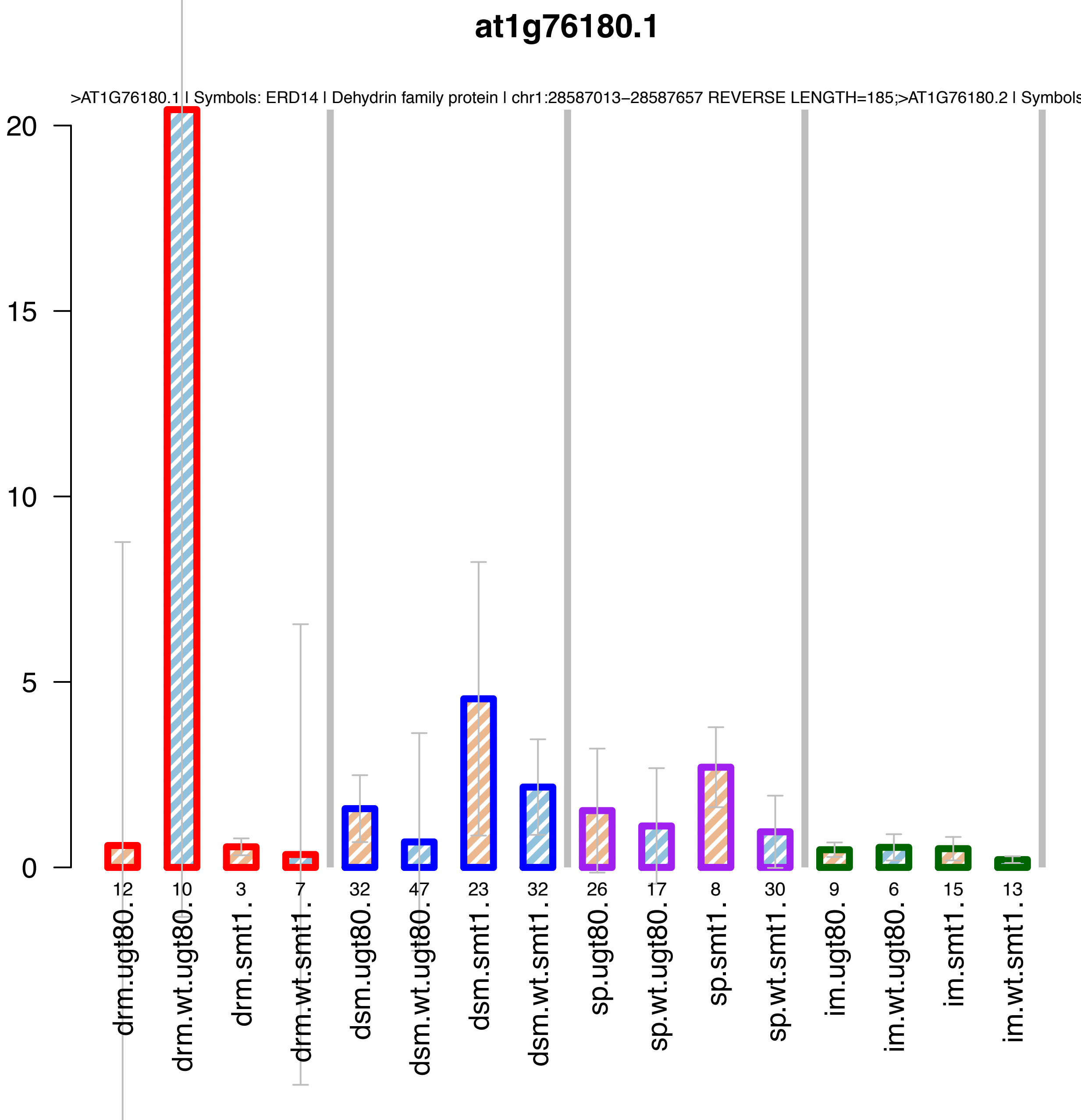
at1g76180.1
>AT1G76180.1 | Symbols: ERD14 | Dehydrin family protein | chr1:28587013–28587657 REVERSE LENGTH=185;>AT1G76180.2 | Symbols
0
5
10
15
20
12
10
3
7
32
47
23
32
26
17
8
30
9
6
15
13
drm.ugt80.
drm.wt.ugt80.
drm.smt1.
drm.wt.smt1.
dsm.ugt80.
dsm.wt.ugt80.
dsm.smt1.
dsm.wt.smt1.
sp.ugt80.
sp.wt.ugt80.
sp.smt1.
sp.wt.smt1.
im.ugt80.
im.wt.ugt80.
im.smt1.
im.wt.smt1.

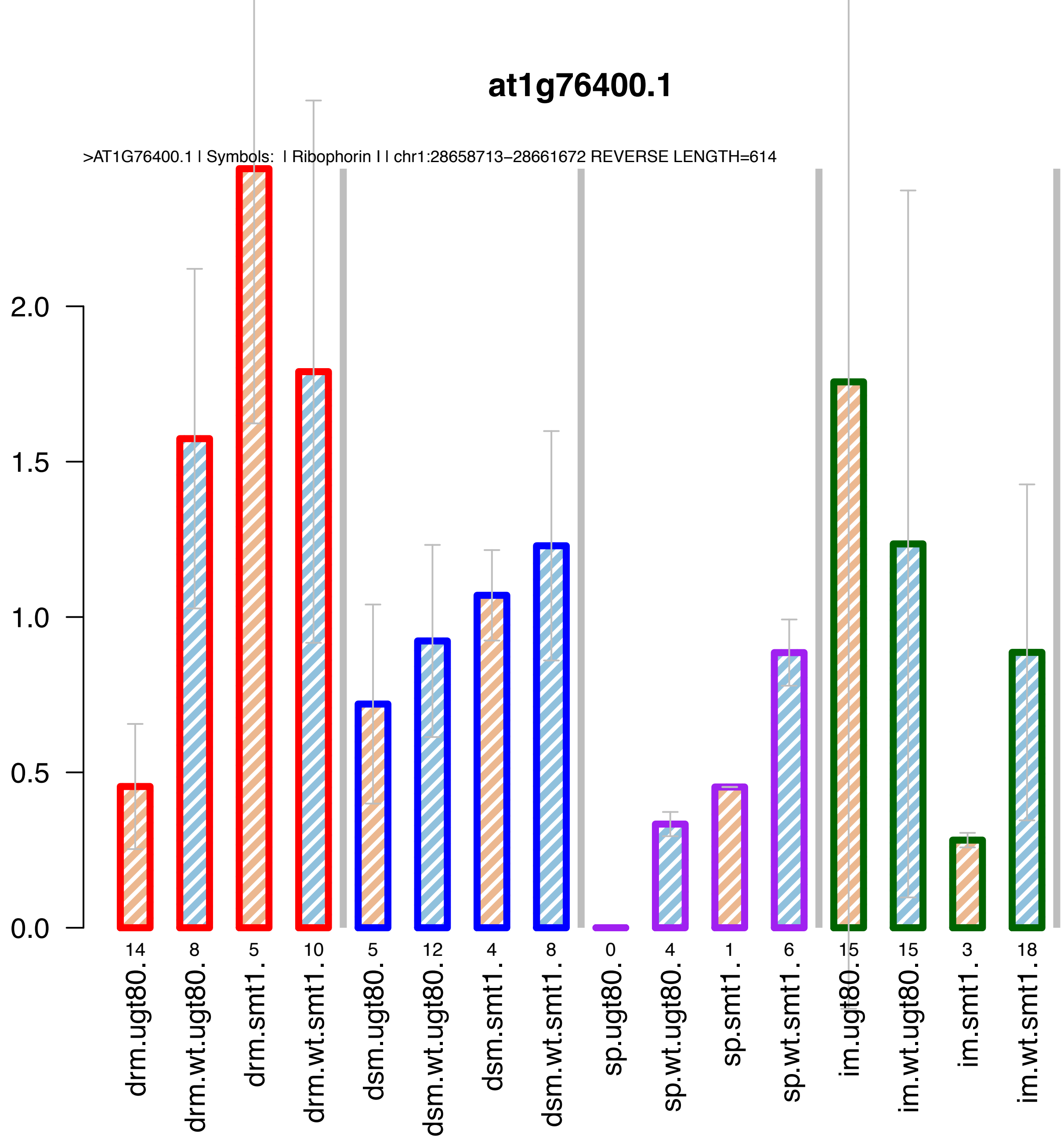

at1g76400.1
>AT1G76400.1 | Symbols: | Ribophorin I | chr1:28658713-28661672 REVERSE LENGTH=614
0.0
0.5
1.0
1.5
2.0
14
8
5
10
5
12
4
8
0
4
1
6
15
15
3
18
drm.ugt80..
drm.wt.ugt80..
drm.smt1..
drm.wt.smt1..
dsm.ugt80..
dsm.wt.ugt80..
dsm.smt1..
dsm.wt.smt1..
sp.ugt80..
sp.wt.ugt80..
sp.smt1..
sp.wt.smt1..
im.ugt80..
im.wt.ugt80..
im.smt1..
im.wt.smt1..

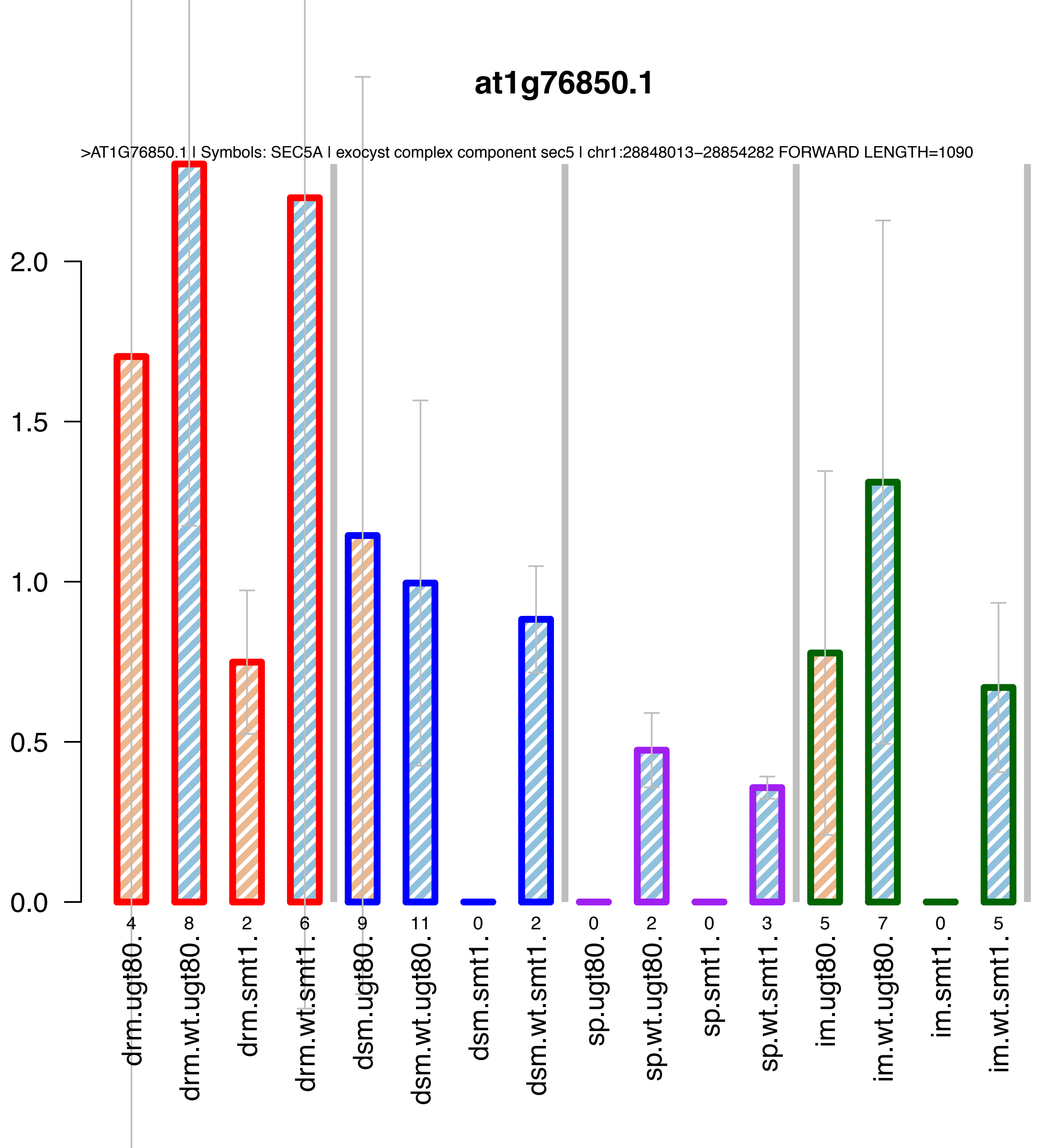

at1g76850.1
>AT1G76850.1 | Symbols: SEC5A | exocyst complex component sec5 | chr1:28848013-28854282 FORWARD LENGTH=1090
2.0
1.5
1.0
0.5
0.0
4
8
2
6
9
11
0
2
0
2
0
3
5
7
0
5
drm.ugt80.
drm.wt.ugt80.
drm.smt1.
drm.wt.smt1.
dsm.ugt80.
dsm.wt.ugt80.
dsm.smt1.
dsm.wt.smt1.
sp.ugt80.
sp.wt.ugt80.
sp.smt1.
sp.wt.smt1.
im.ugt80.
im.wt.ugt80.
im.smt1.
im.wt.smt1.

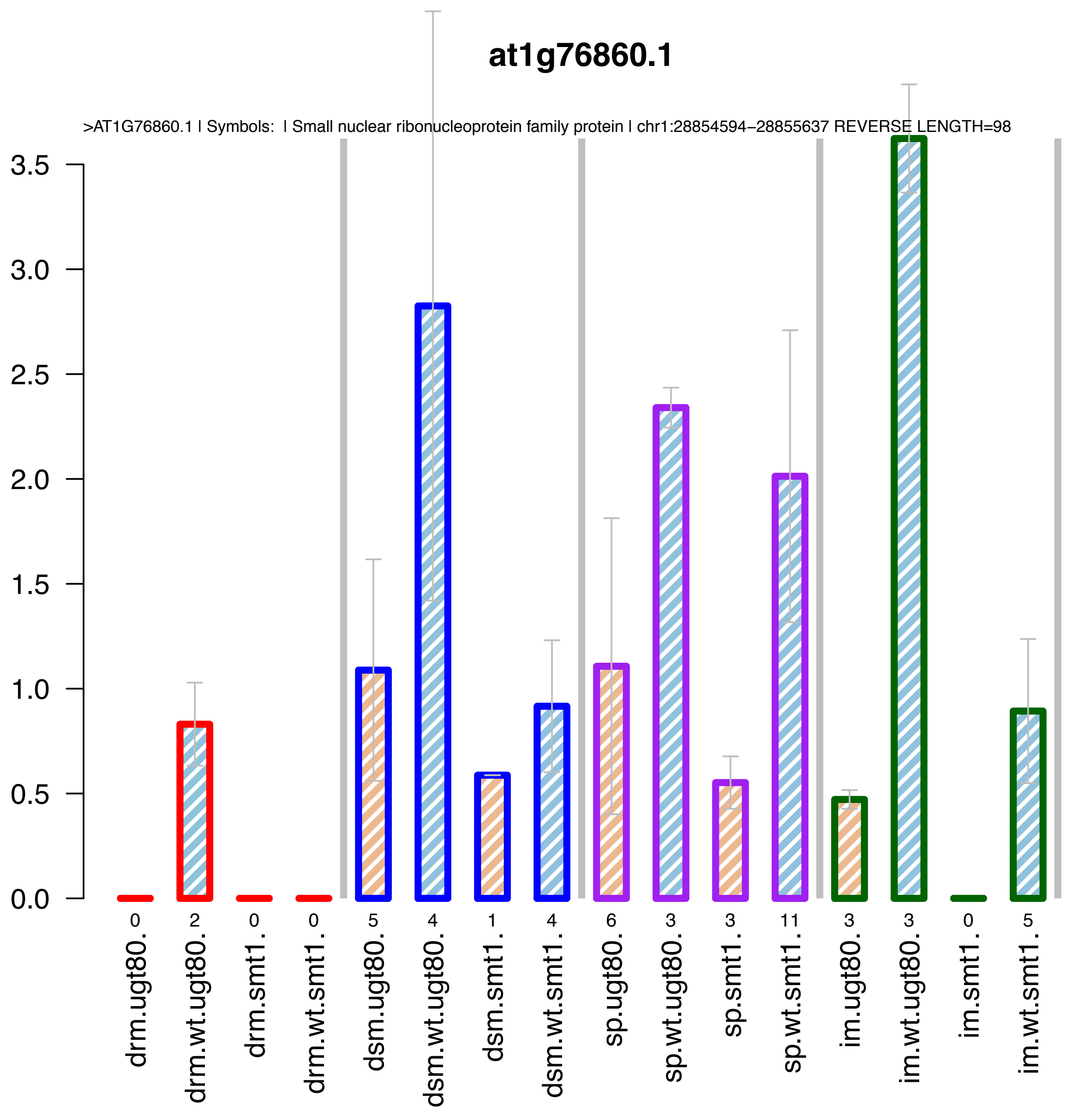
at1g76860.1
>AT1G76860.1 | Symbols: | Small nuclear ribonucleoprotein family protein | chr1:28854594-28855637 REVERSE LENGTH=98
0.0
0.5
1.0
1.5
2.0
2.5
3.0
3.5
0
2
0
0
5
4
1
4
6
3
3
11
3
3
0
5
drm.ugt80.
drm.wt.ugt80.
drm.smt1.
drm.wt.smt1.
dsm.ugt80.
dsm.wt.ugt80.
dsm.smt1.
dsm.wt.smt1.
sp.ugt80.
sp.wt.ugt80.
sp.smt1.
sp.wt.smt1.
im.ugt80.
im.wt.ugt80.
im.smt1.
im.wt.smt1.

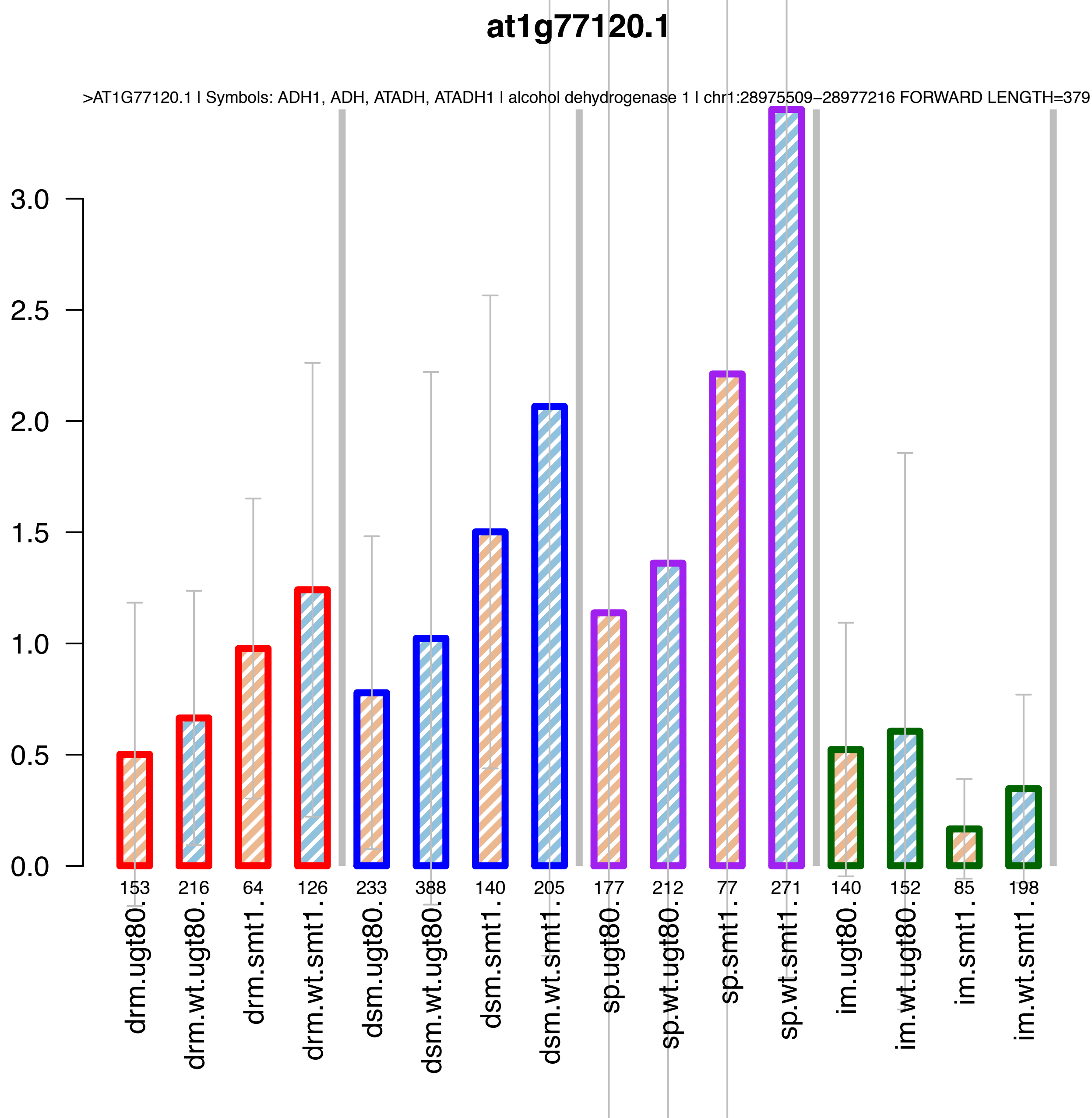

at1g77120.1
>AT1G77120.1 | Symbols: ADH1, ADH, ATADH, ATADH1 | alcohol dehydrogenase 1 | chr1:28975509-28977216 FORWARD LENGTH=379
3.0
2.5
2.0
1.5
1.0
0.5
0.0
153
216
64
126
233
388
140
205
177
212
77
271
140
152
85
198
drm.ugt80..
drm.wt.ugt80..
drm.smt1..
drm.wt.smt1..
dsm.ugt80..
dsm.wt.ugt80..
dsm.smt1..
dsm.wt.smt1..
sp.ugt80..
sp.wt.ugt80..
sp.smt1..
sp.wt.smt1..
im.ugt80..
im.wt.ugt80..
im.smt1..
im.wt.smt1..

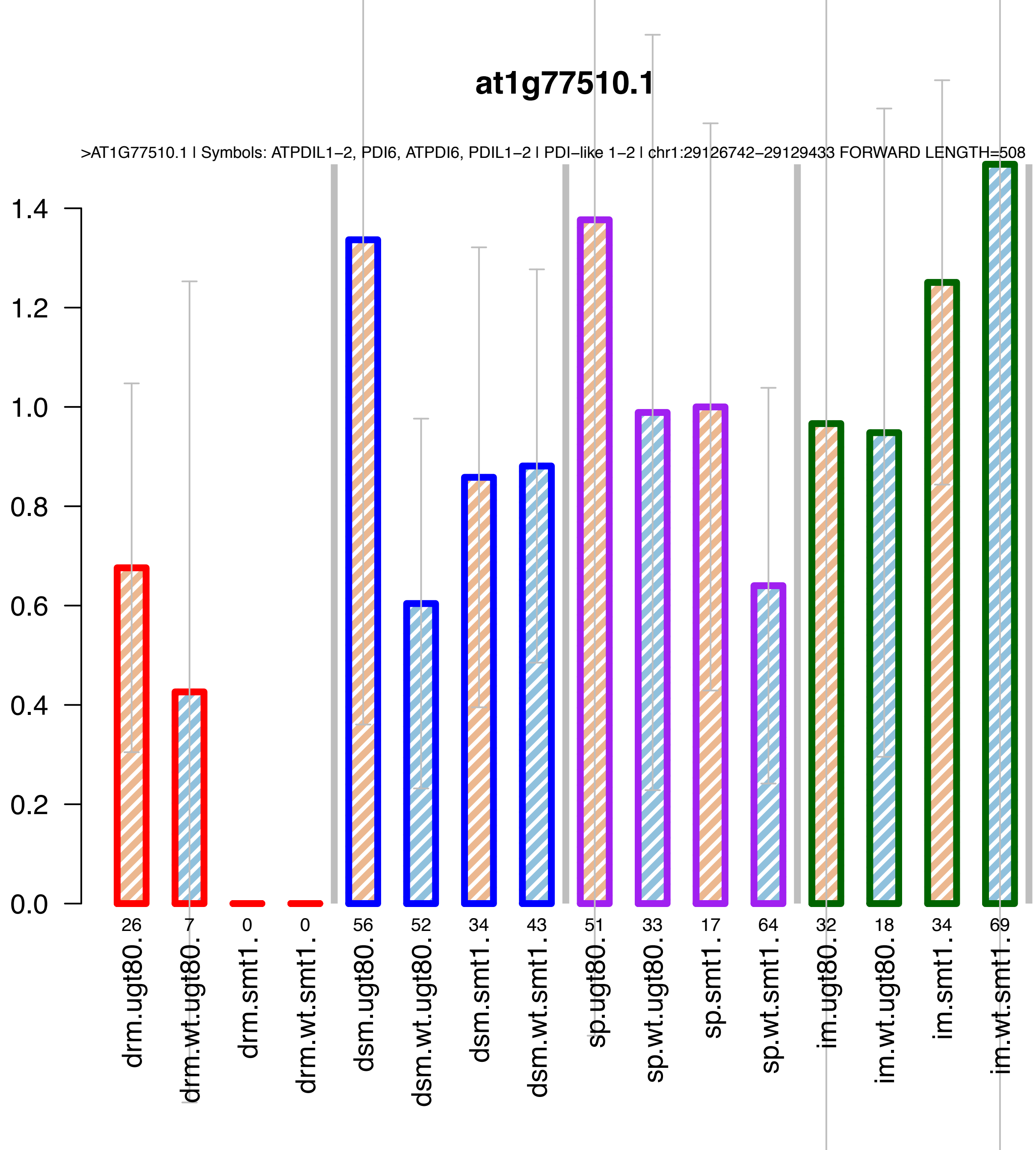

at1g77510.1
>AT1G77510.1 | Symbols: ATPDIL1−2, PDI6, ATPDI6, PDIL1−2 | PDI−like 1−2 | chr1:29126742−29129433 FORWARD LENGTH=508
0.0
0.2
0.4
0.6
0.8
1.0
1.2
1.4
26
7
0
0
56
52
34
43
51
33
17
64
32
18
34
69
drm.ugt80..
drm.wt.ugt80..
drm.smt1..
drm.wt.smt1..
dsm.ugt80..
dsm.wt.ugt80..
dsm.smt1..
dsm.wt.smt1..
sp.ugt80..
sp.wt.ugt80..
sp.smt1..
sp.wt.smt1..
im.ugt80..
im.wt.ugt80..
im.smt1..
im.wt.smt1..

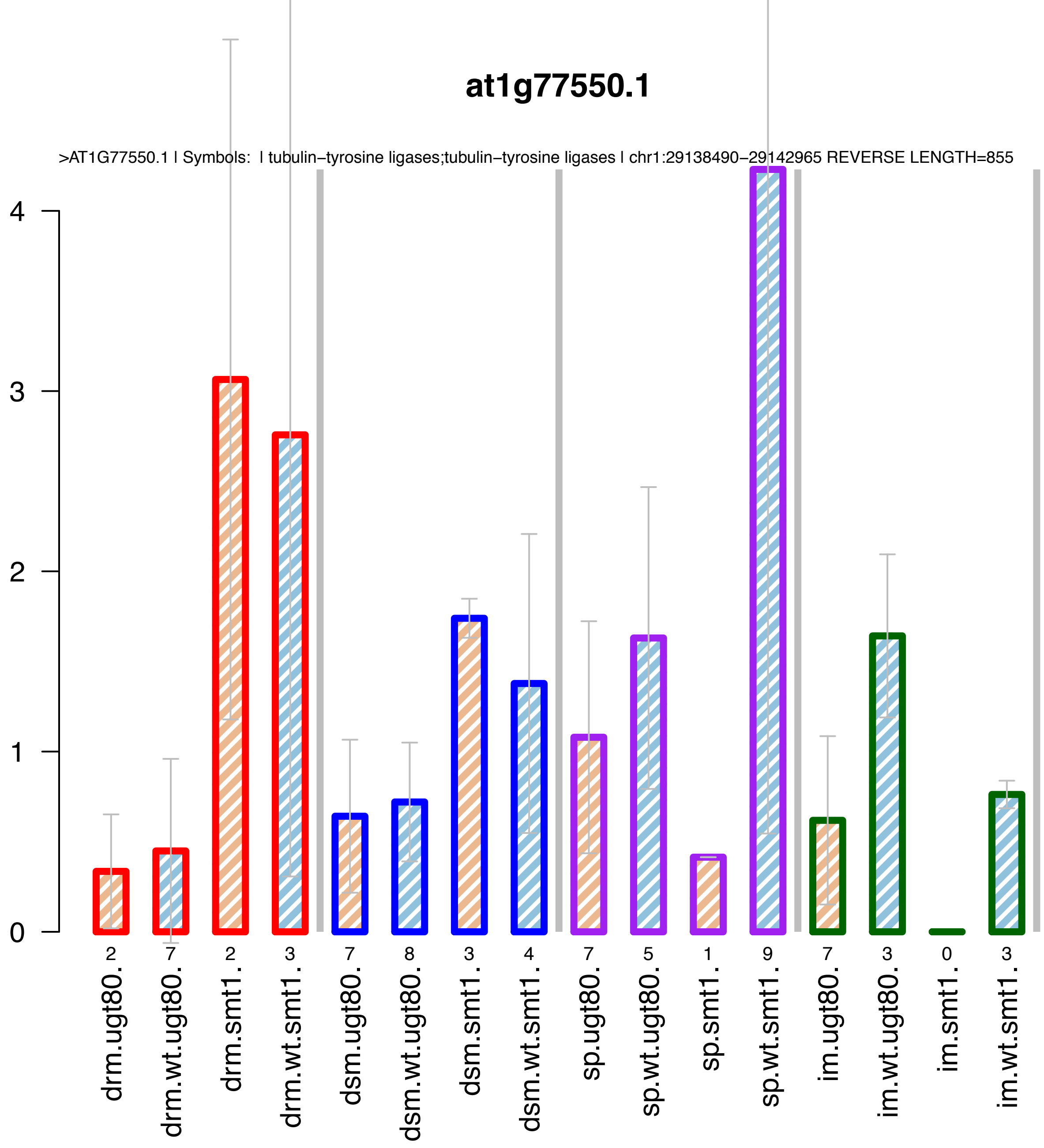
at1g77550.1
>AT1G77550.1 | Symbols: | tubulin−tyrosine ligases;tubulin−tyrosine ligases | chr1:29138490−29142965 REVERSE LENGTH=855
0
1
2
3
4
2
7
2
3
7
8
3
4
7
5
1
9
7
3
0
3
drm.ugt80.
drm.wt.ugt80.
drm.smt1.
drm.wt.smt1.
dsm.ugt80.
dsm.wt.ugt80.
dsm.smt1.
dsm.wt.smt1.
sp.ugt80.
sp.wt.ugt80.
sp.smt1.
sp.wt.smt1.
im.ugt80.
im.wt.ugt80.
im.smt1.
im.wt.smt1.

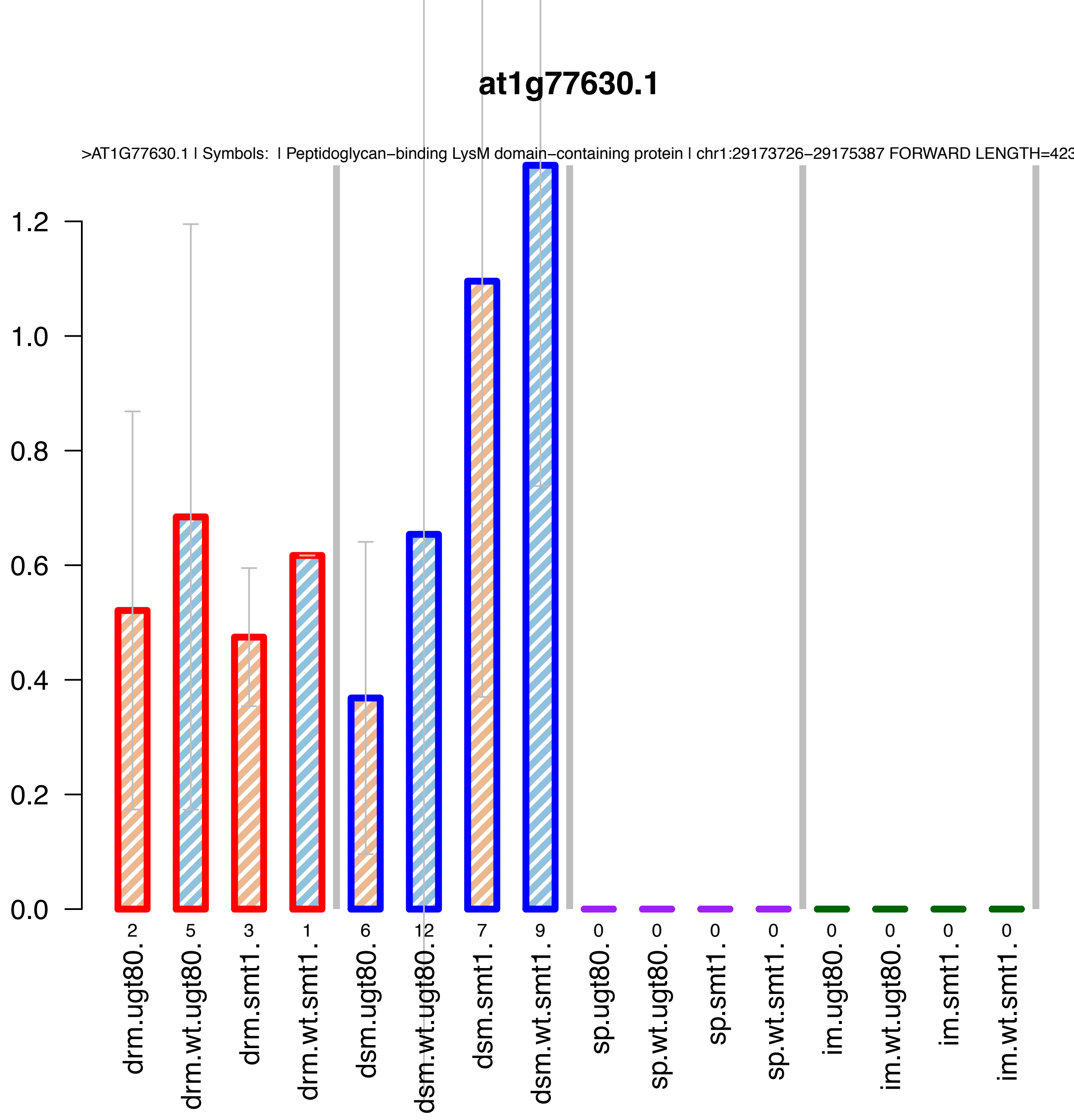

at1g77630.1
>AT1G77630.1 | Symbols: | Peptidoglycan−binding LysM domain−containing protein | chr1:29173726−29175387 FORWARD LENGTH=423
1.2
1.0
0.8
0.6
0.4
0.2
0.0
2
5
3
1
6
12
7
9
0
0
0
0
0
0
0
0
drm.ugt80.
drm.wt.ugt80.
drm.smt1.
drm.wt.smt1.
dsm.ugt80.
dsm.wt.ugt80.
dsm.smt1.
dsm.wt.smt1.
sp.ugt80.
sp.wt.ugt80.
sp.smt1.
sp.wt.smt1.
im.ugt80.
im.wt.ugt80.
im.smt1.
im.wt.smt1.

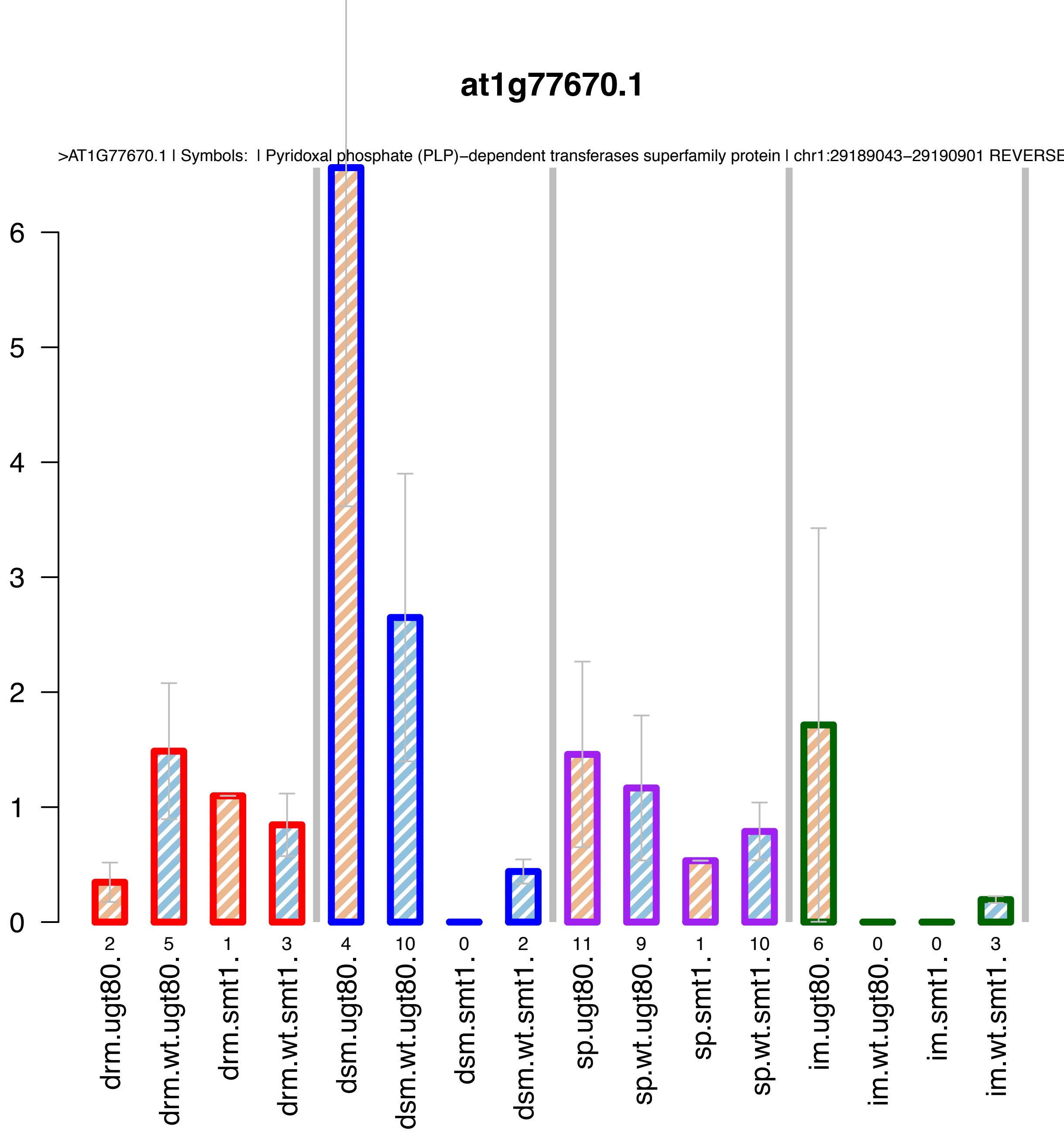

at1g77670.1
>AT1G77670.1 | Symbols: | Pyridoxal phosphate (PLP)–dependent transferases superfamily protein | chr1:29189043–29190901 REVERSE
0
1
2
3
4
5
6
2
5
1
3
4
10
0
2
11
9
1
10
6
0
0
3
drm.ugt80.
drm.wt.ugt80.
drm.smt1.
drm.wt.smt1.
dsm.ugt80.
dsm.wt.ugt80.
dsm.smt1.
dsm.wt.smt1.
sp.ugt80.
sp.wt.ugt80.
sp.smt1.
sp.wt.smt1.
im.ugt80.
im.wt.ugt80.
im.smt1.
im.wt.smt1.

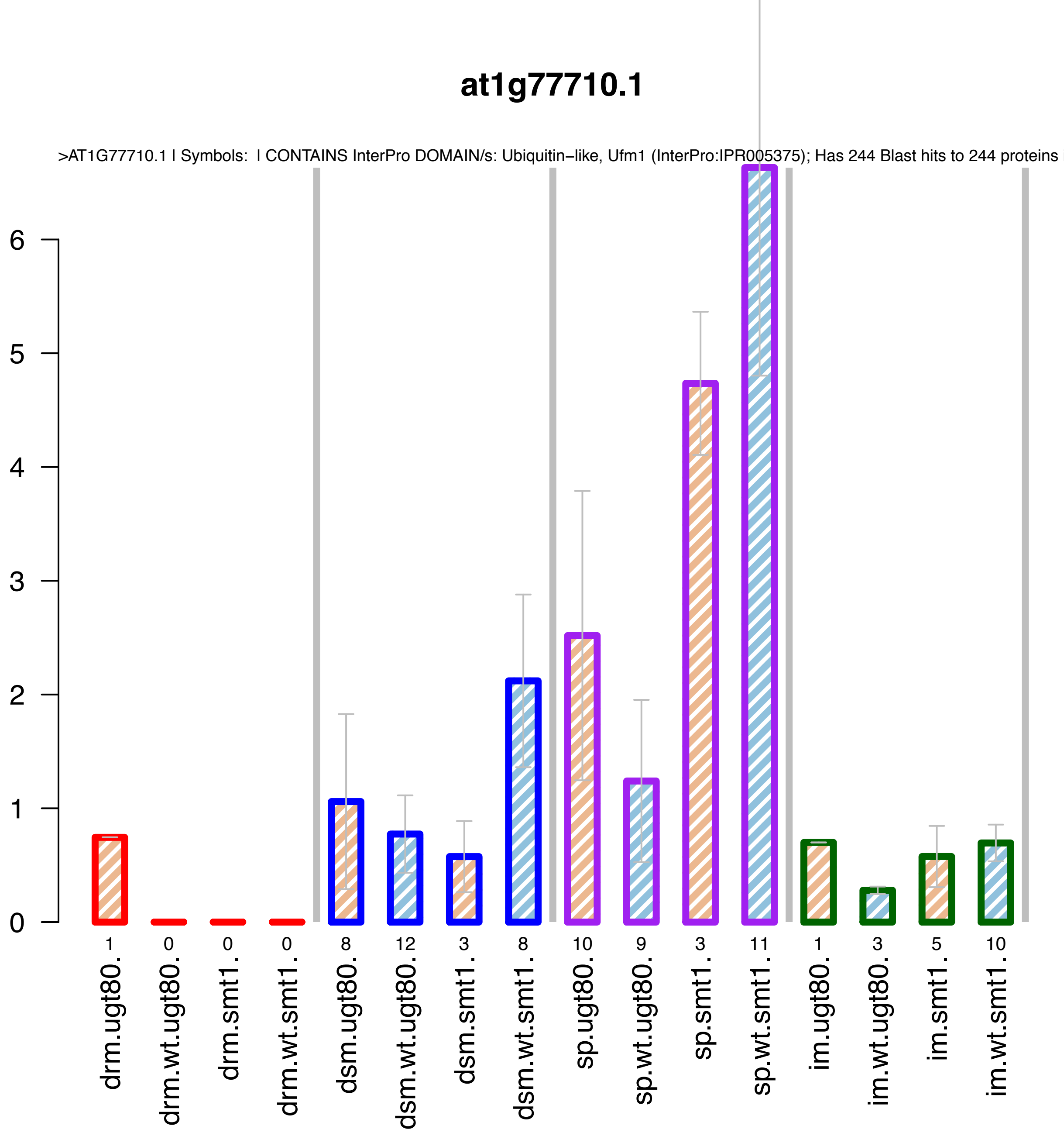

at1g77710.1
>AT1G77710.1 | Symbols: | CONTAINS InterPro DOMAIN/s: Ubiquitin−like, Ufm1 (InterPro:IPR005375); Has 244 Blast hits to 244 proteins
0
1
2
3
4
5
6
1 0 0 0 8 12 3 8 10 9 3 11 1 3 5 10
drm.ugt80.
drm.wt.ugt80.
drm.smt1.
drm.wt.smt1.
dsm.ugt80.
dsm.wt.ugt80.
dsm.smt1.
dsm.wt.smt1.
sp.ugt80.
sp.wt.ugt80.
sp.smt1.
sp.wt.smt1.
im.ugt80.
im.wt.ugt80.
im.smt1.
im.wt.smt1.

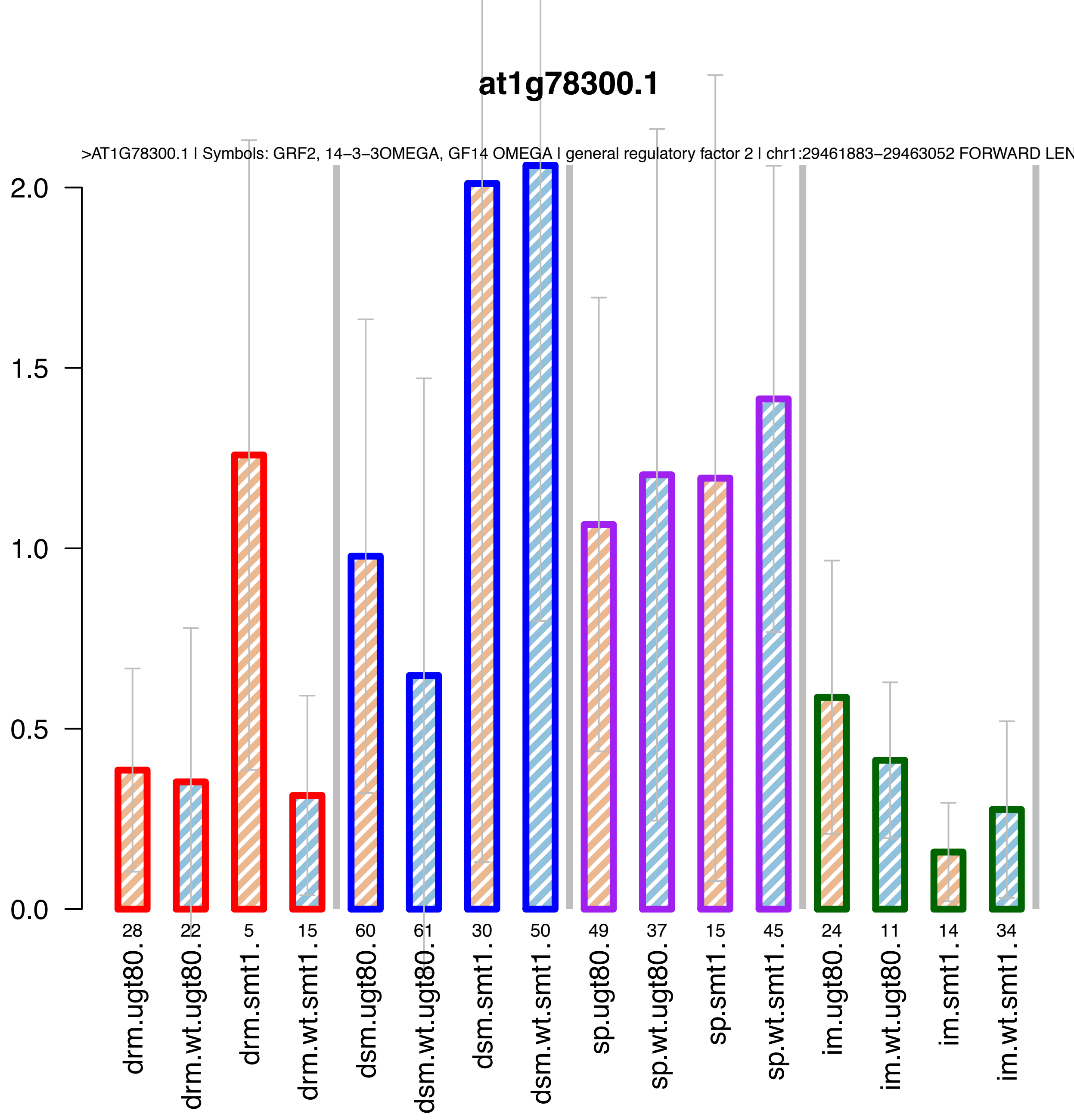
at1g78300.1
>AT1G78300.1 | Symbols: GRF2, 14-3-3OMEGA, GF14 OMEGA | general regulatory factor 2 | chr1:29461883-29463052 FORWARD LEN
2.0
1.5
1.0
0.5
0.0
28
22
5
15
60
61
30
50
49
37
15
45
24
11
14
34
drm.ugt80..
drm.wt.ugt80..
drm.smt1..
drm.wt.smt1..
dsm.ugt80..
dsm.wt.ugt80..
dsm.smt1..
dsm.wt.smt1..
sp.ugt80..
sp.wt.ugt80..
sp.smt1..
sp.wt.smt1..
im.ugt80..
im.wt.ugt80..
im.smt1..
im.wt.smt1..

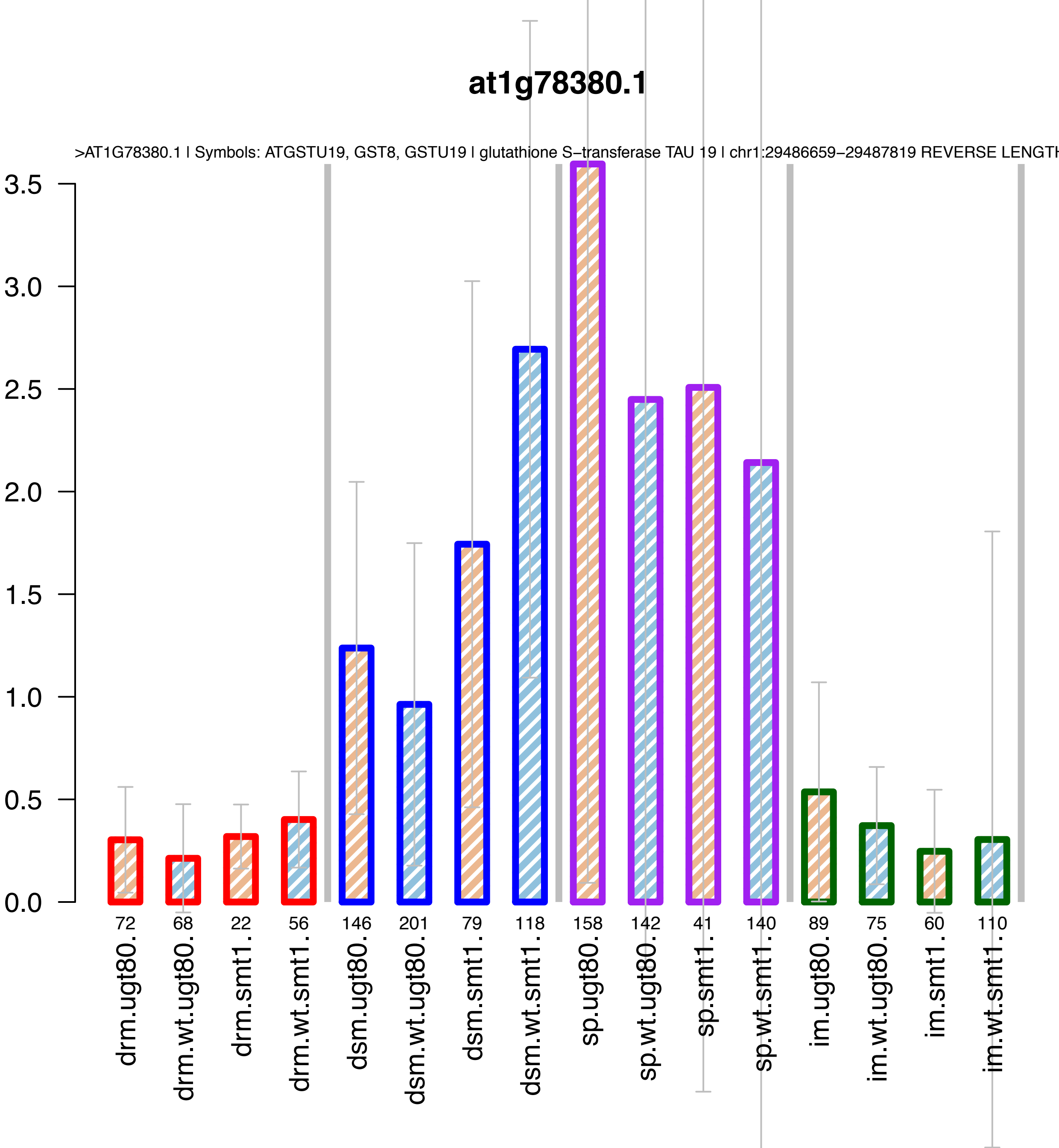

at1g78380.1
>AT1G78380.1 | Symbols: ATGSTU19, GST8, GSTU19 | glutathione S−transferase TAU 19 | chr1:29486659−29487819 REVERSE LENGTH
3.5
3.0
2.5
2.0
1.5
1.0
0.5
0.0
72
68
22
56
146
201
79
118
158
142
41
140
89
75
60
110
drm.ugt80..
drm.wt.ugt80..
drm.smt1..
drm.wt.smt1..
dsm.ugt80..
dsm.wt.ugt80..
dsm.smt1..
dsm.wt.smt1..
sp.ugt80..
sp.wt.ugt80..
sp.smt1..
sp.wt.smt1..
im.ugt80..
im.wt.ugt80..
im.smt1..
im.wt.smt1..

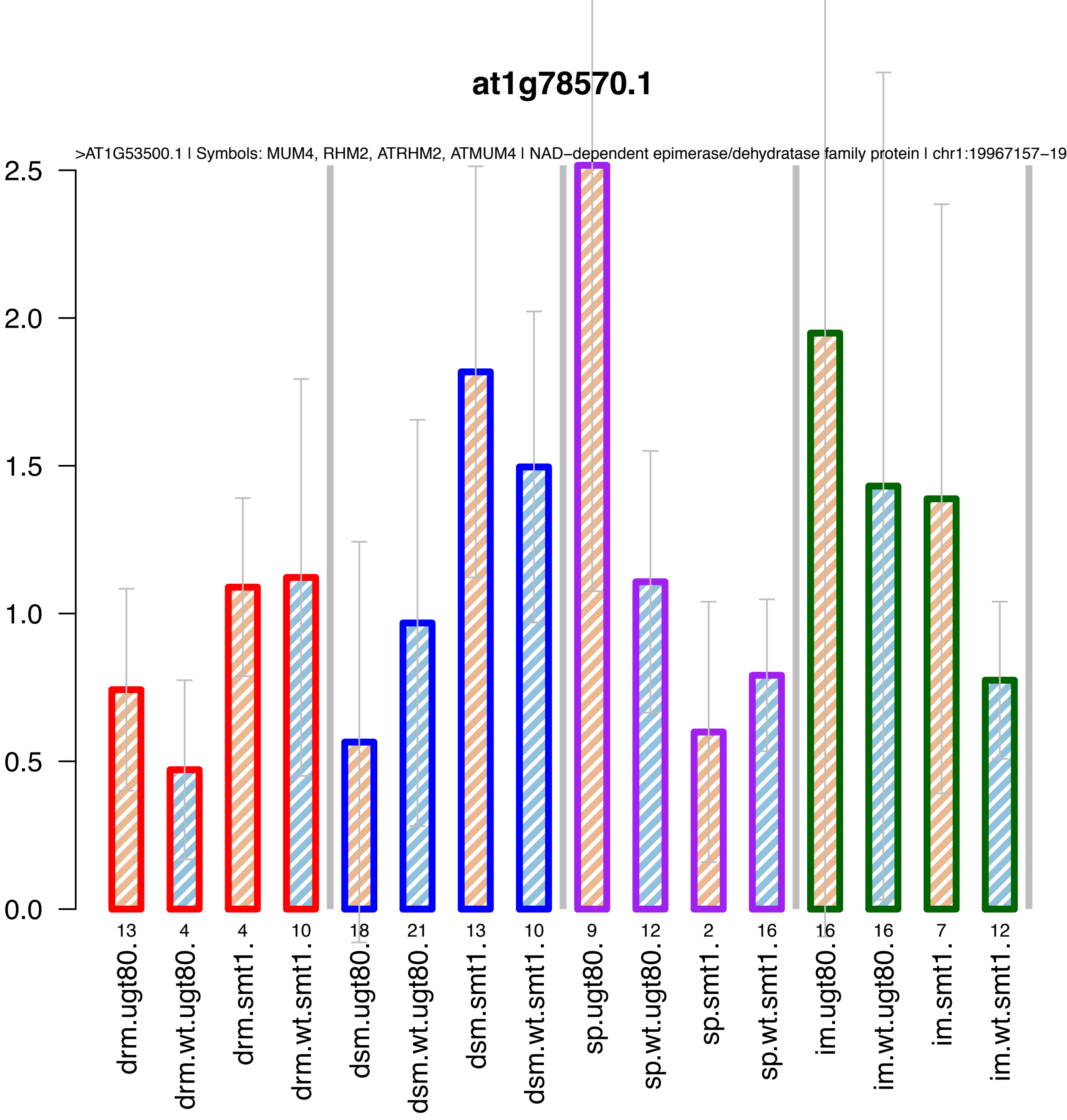
at1g78570.1
>AT1G53500.1 | Symbols: MUM4, RHM2, ATRHM2, ATMUM4 | NAD−dependent epimerase/dehydratase family protein | chr1:19967157−19
2.5
2.0
1.5
1.0
0.5
0.0
13
4
4
10
18
21
13
10
9
12
2
16
16
16
7
12
drm.ugt80..
drm.wt.ugt80..
drm.smt1..
drm.wt.smt1..
dsm.ugt80..
dsm.wt.ugt80..
dsm.smt1..
dsm.wt.smt1..
sp.ugt80..
sp.wt.ugt80..
sp.smt1..
sp.wt.smt1..
im.ugt80..
im.wt.ugt80..
im.smt1..
im.wt.smt1..

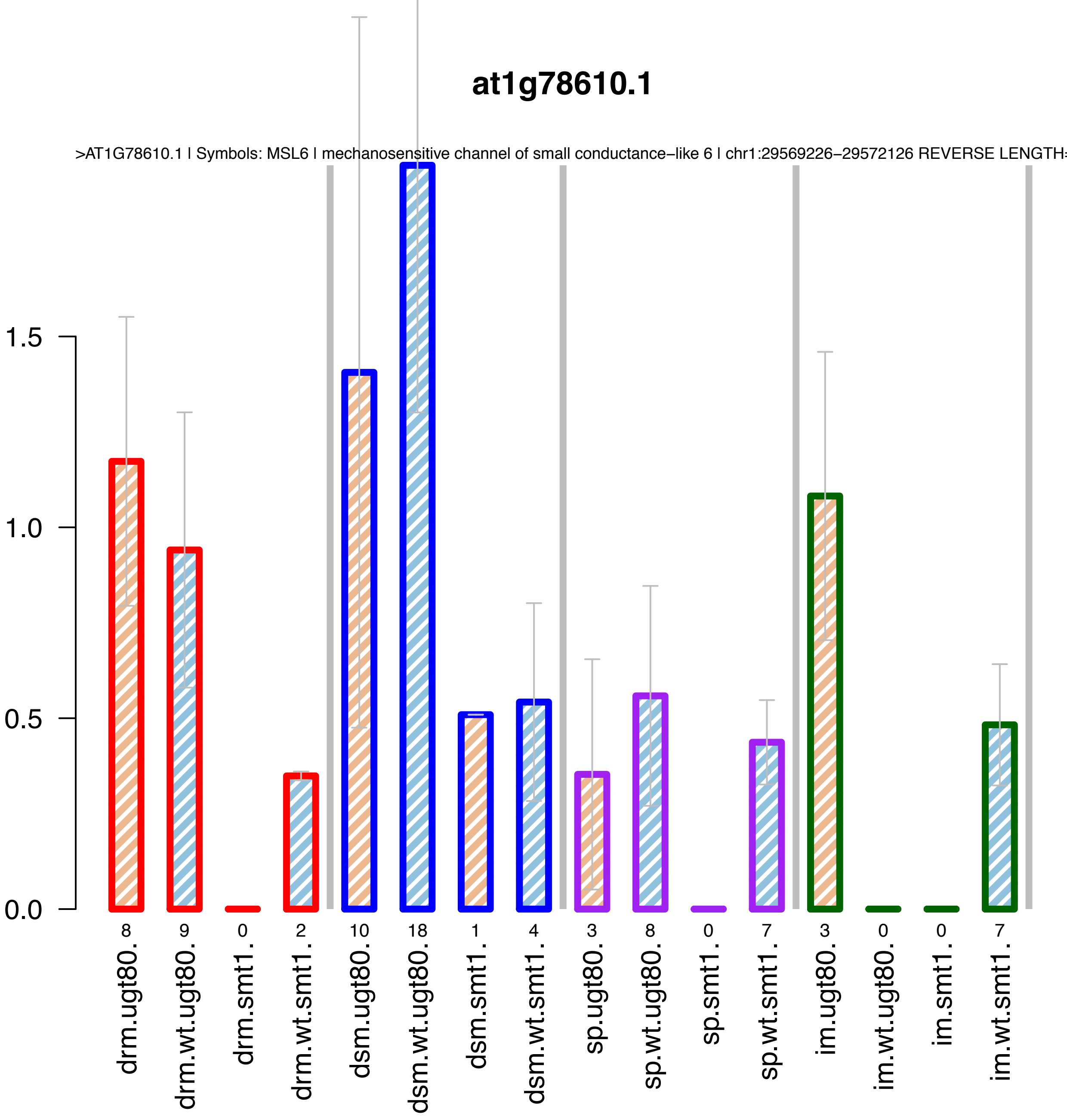
at1g78610.1
>AT1G78610.1 | Symbols: MSL6 | mechanosensitive channel of small conductance-like 6 | chr1:29569226-29572126 REVERSE LENGTH=
0.0
0.5
1.0
1.5
8
9
0
2
10
18
1
4
3
8
0
7
3
0
0
7
drm.ugt80..
drm.wt.ugt80..
drm.smt1..
drm.wt.smt1..
dsm.ugt80..
dsm.wt.ugt80..
dsm.smt1..
dsm.wt.smt1..
sp.ugt80..
sp.wt.ugt80..
sp.smt1..
sp.wt.smt1..
im.ugt80..
im.wt.ugt80..
im.smt1..
im.wt.smt1..

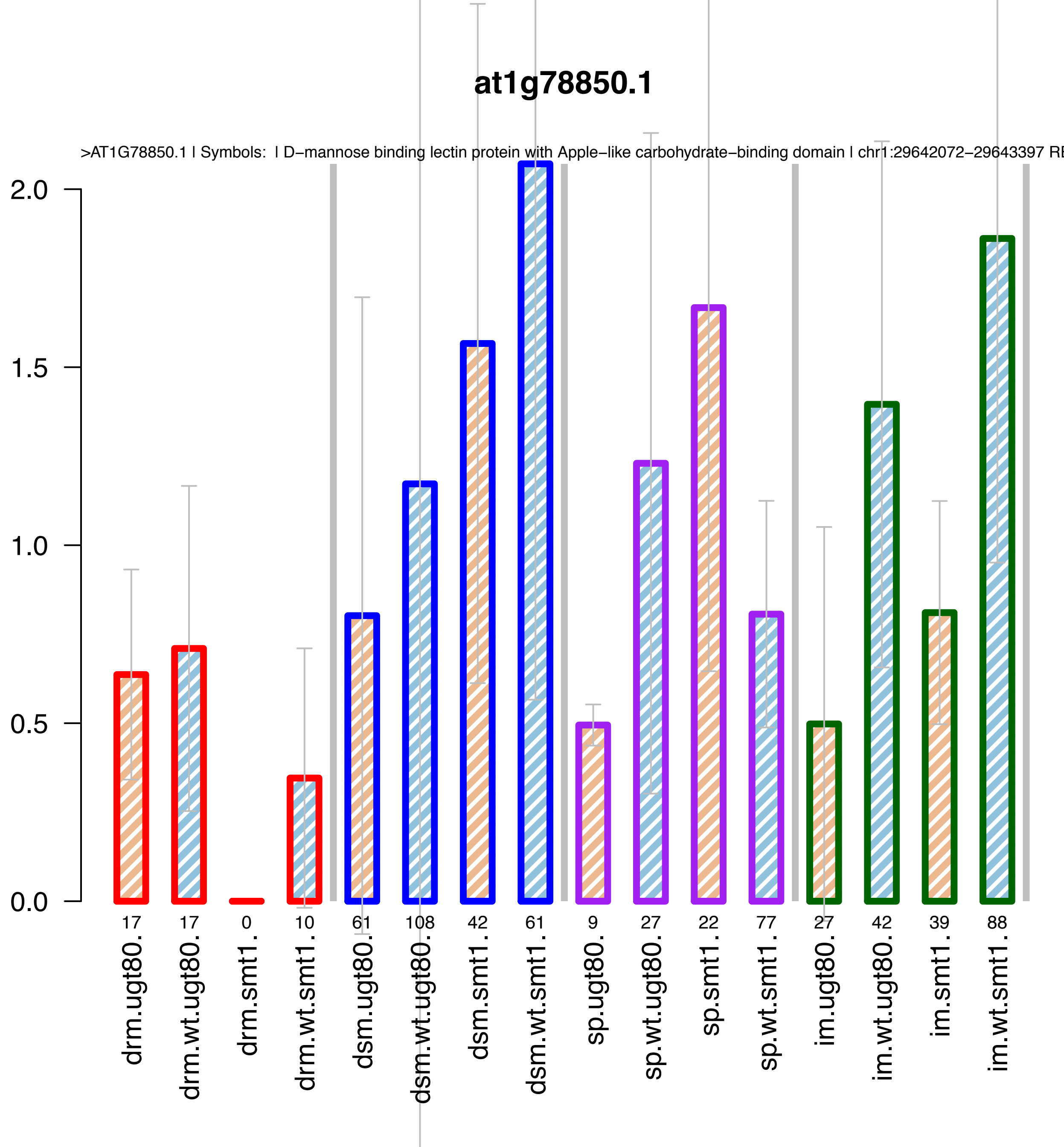

at1g78850.1
>AT1G78850.1 | Symbols: | D−mannose binding lectin protein with Apple−like carbohydrate−binding domain | chr1:29642072−29643397 RE
2.0
1.5
1.0
0.5
0.0
17
17
0
10
61
108
42
61
9
27
22
77
27
42
39
88
drm.ugt80.
drm.wt.ugt80.
drm.smt1.
drm.wt.smt1.
dsm.ugt80.
dsm.wt.ugt80.
dsm.smt1.
dsm.wt.smt1.
sp.ugt80.
sp.wt.ugt80.
sp.smt1.
sp.wt.smt1.
im.ugt80.
im.wt.ugt80.
im.smt1.
im.wt.smt1.

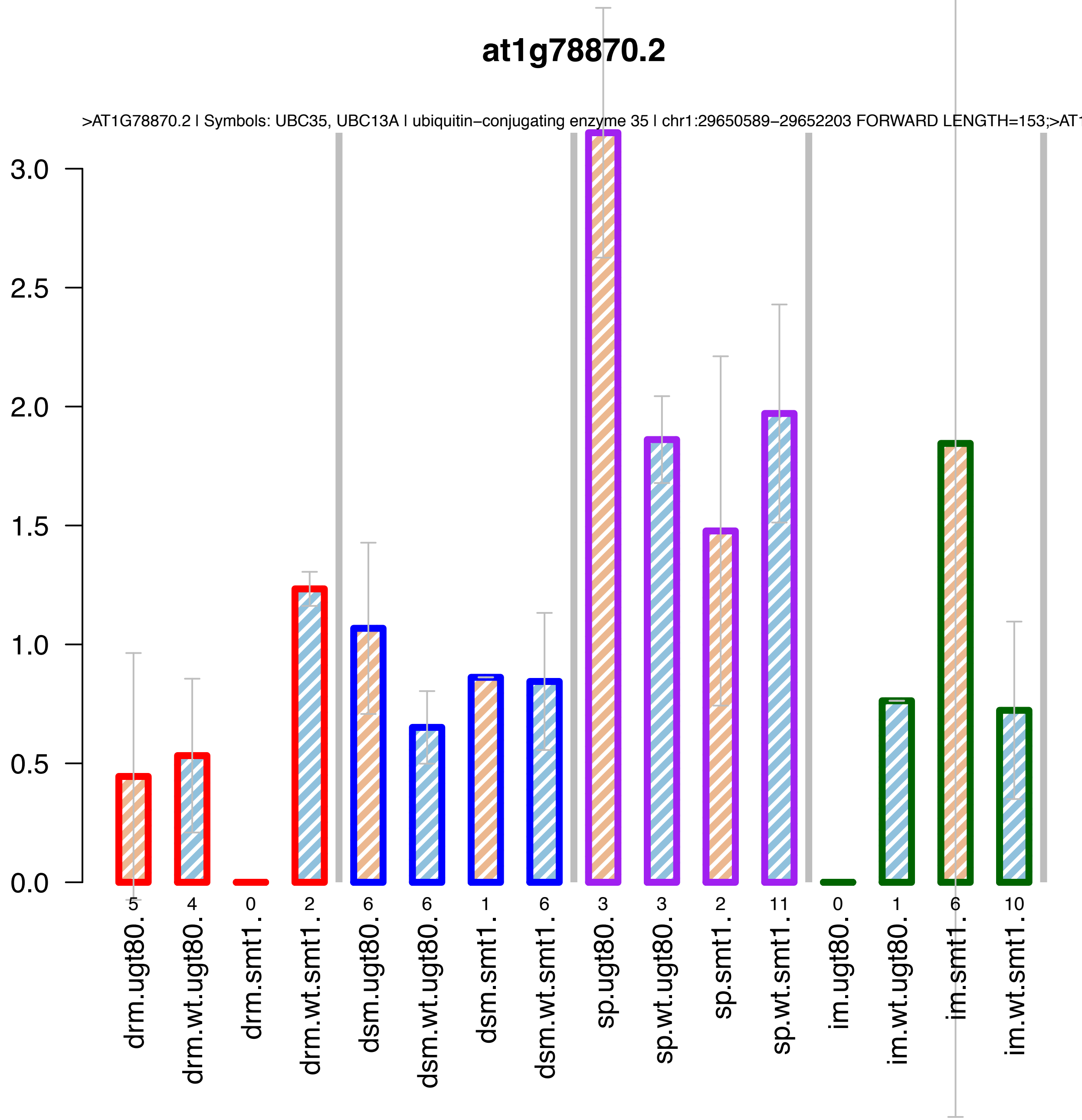

at1g78870.2
>AT1G78870.2 | Symbols: UBC35, UBC13A | ubiquitin−conjugating enzyme 35 | chr1:29650589−29652203 FORWARD LENGTH=153;>AT1
3.0
2.5
2.0
1.5
1.0
0.5
0.0
5
4
0
2
6
6
1
6
3
3
2
11
0
1
6
10
drm.ugt80.
drm.wt.ugt80.
drm.smt1.
drm.wt.smt1.
dsm.ugt80.
dsm.wt.ugt80.
dsm.smt1.
dsm.wt.smt1.
sp.ugt80.
sp.wt.ugt80.
sp.smt1.
sp.wt.smt1.
im.ugt80.
im.wt.ugt80.
im.smt1.
im.wt.smt1.

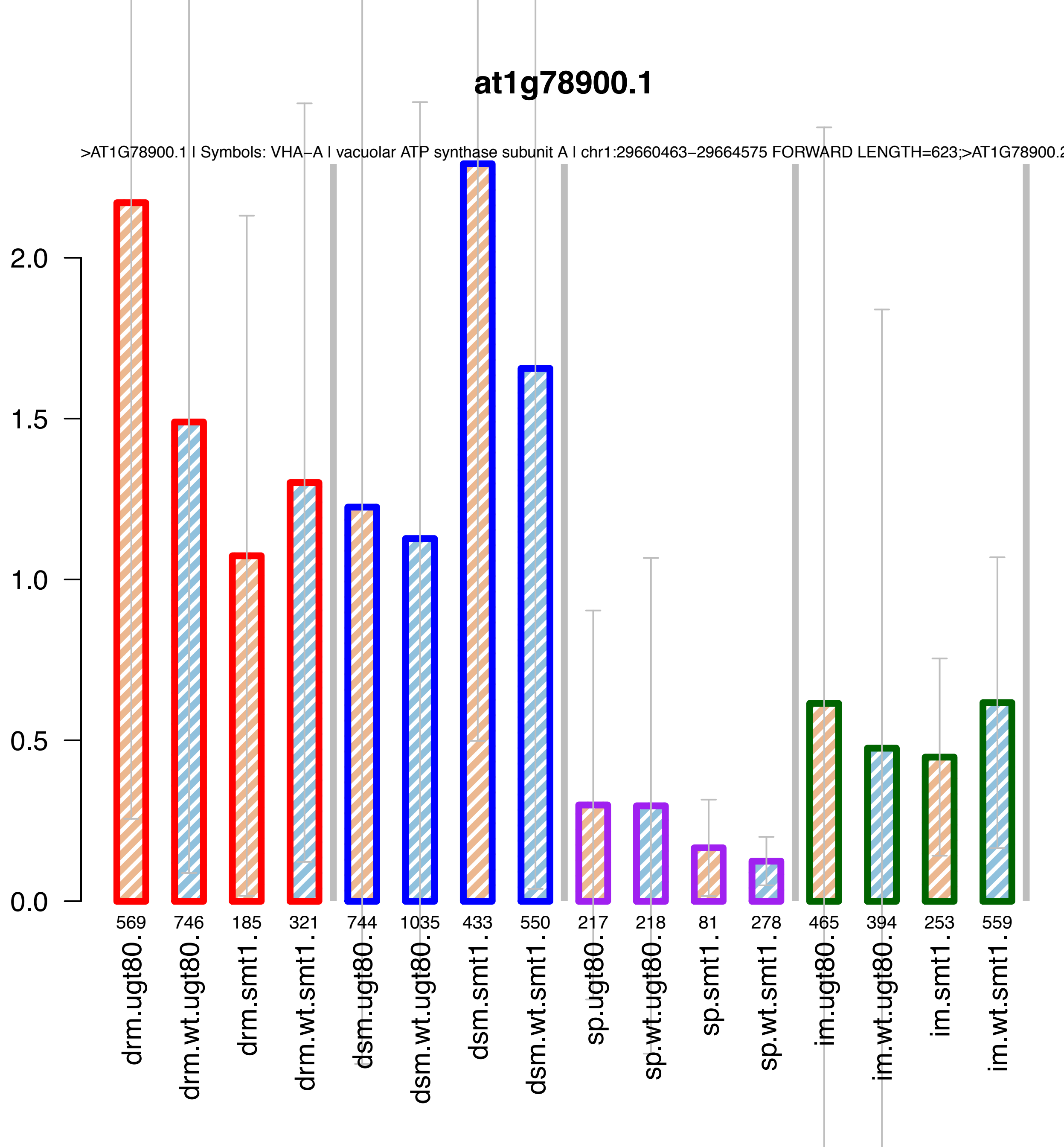

at1g78900.1
>AT1G78900.1 | Symbols: VHA−A | vacuolar ATP synthase subunit A | chr1:29660463−29664575 FORWARD LENGTH=623;>AT1G78900.2
0.0
0.5
1.0
1.5
2.0
569
746
185
321
744
1035
433
550
217
218
81
278
465
394
253
559
drm.ugt80..
drm.wt.ugt80..
drm.smt1..
drm.wt.smt1..
dsm.ugt80..
dsm.wt.ugt80..
dsm.smt1..
dsm.wt.smt1..
sp.ugt80..
sp.wt.ugt80..
sp.smt1..
sp.wt.smt1..
im.ugt80..
im.wt.ugt80..
im.smt1..
im.wt.smt1..

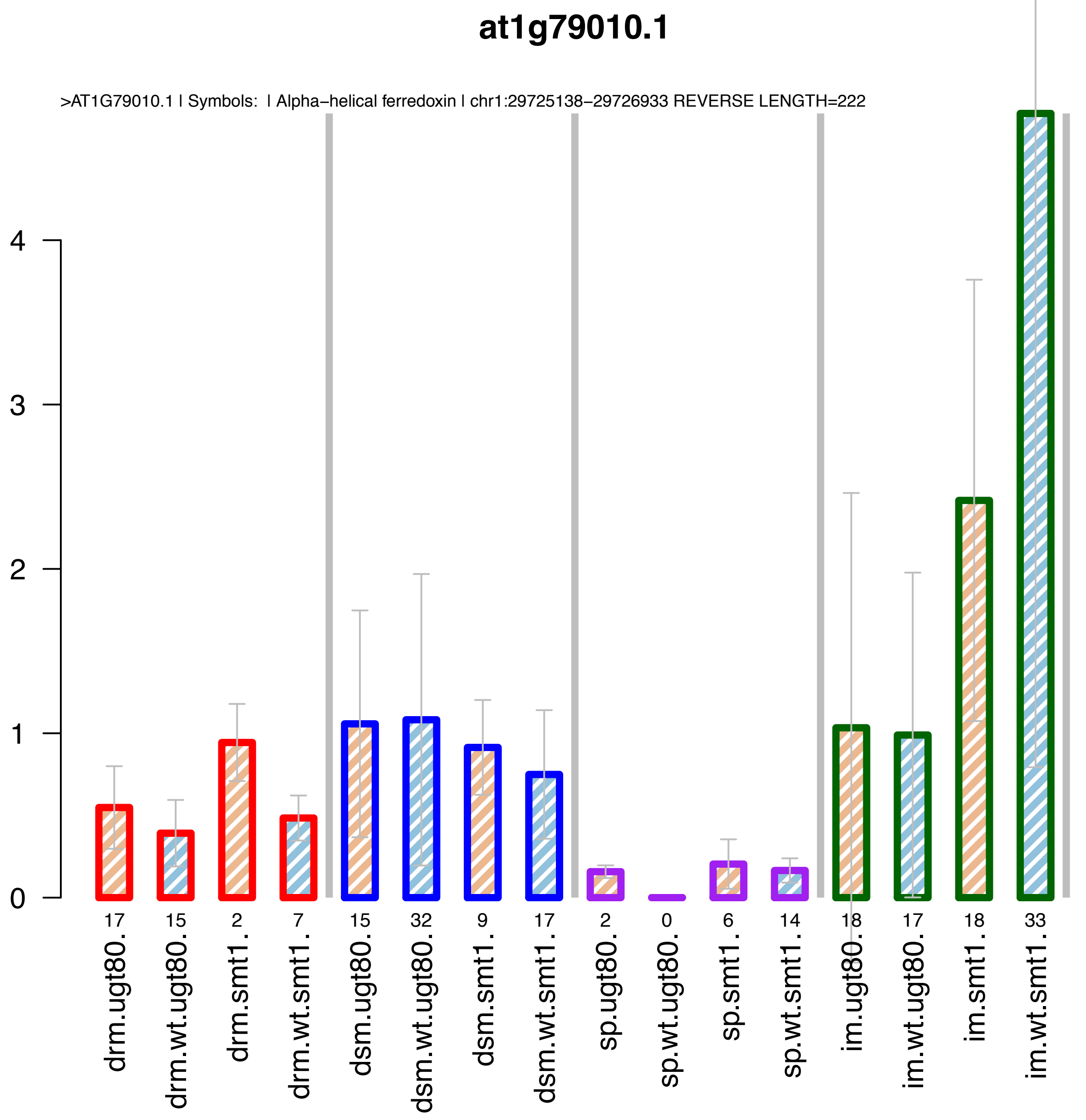

at1g79010.1
>AT1G79010.1 | Symbols: | Alpha−helical ferredoxin | chr1:29725138−29726933 REVERSE LENGTH=222
0
1
2
3
4
17
15
2
7
15
32
9
17
2
0
6
14
18
17
18
33
drm.ugt80..
drm.wt.ugt80..
drm.smt1..
drm.wt.smt1..
dsm.ugt80..
dsm.wt.ugt80..
dsm.smt1..
dsm.wt.smt1..
sp.ugt80..
sp.wt.ugt80..
sp.smt1..
sp.wt.smt1..
im.ugt80..
im.wt.ugt80..
im.smt1..
im.wt.smt1..

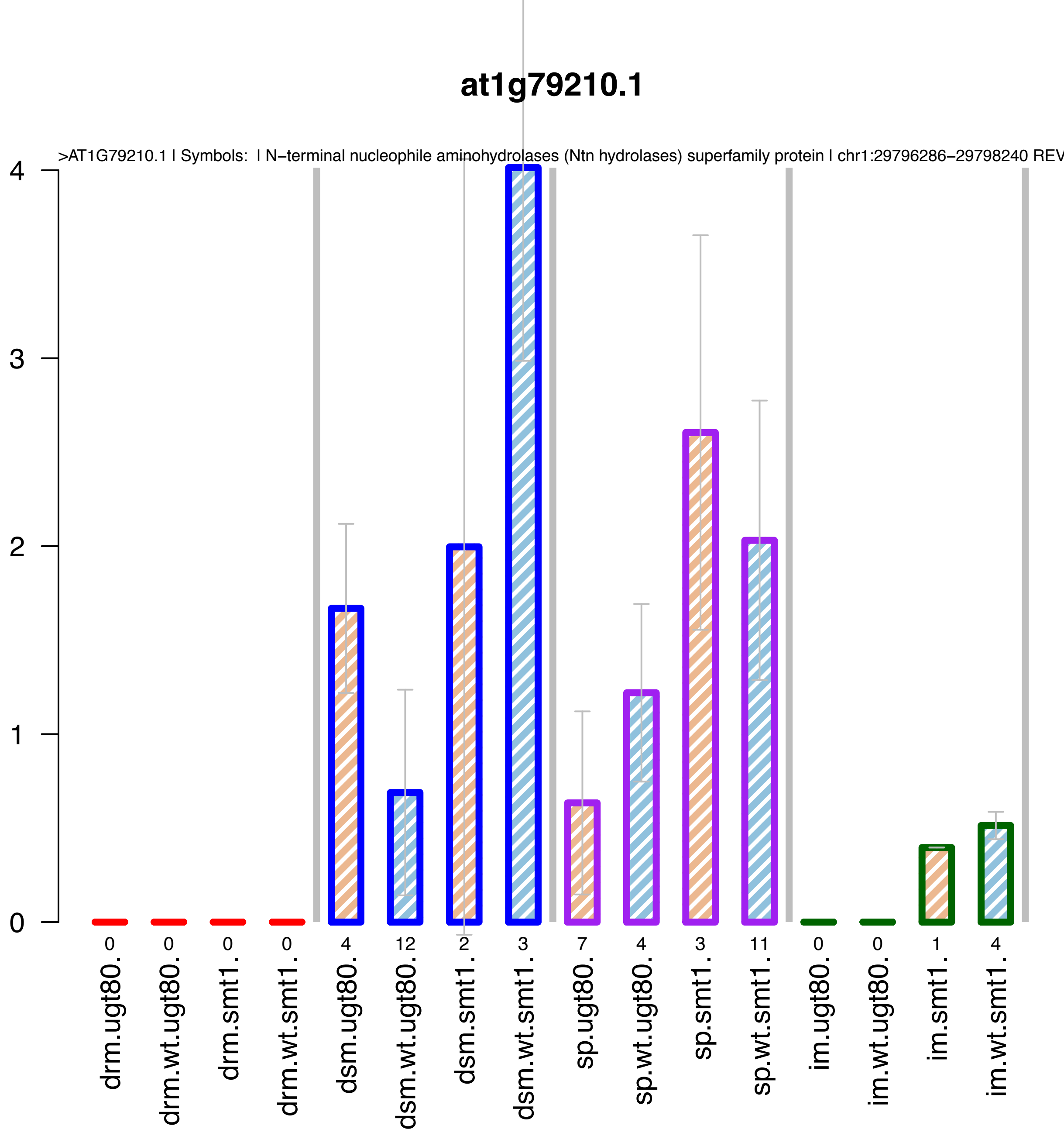

at1g79210.1
>AT1G79210.1 | Symbols: | N−terminal nucleophile aminohydrolases (Ntn hydrolases) superfamily protein | chr1:29796286−29798240 REV
0
1
2
3
4
0 0 0 0 4 12 2 3 7 4 3 11 0 0 1 4
drm.ugt80.
drm.wt.ugt80.
drm.smt1.
drm.wt.smt1.
dsm.ugt80.
dsm.wt.ugt80.
dsm.smt1.
dsm.wt.smt1.
sp.ugt80.
sp.wt.ugt80.
sp.smt1.
sp.wt.smt1.
im.ugt80.
im.wt.ugt80.
im.smt1.
im.wt.smt1.

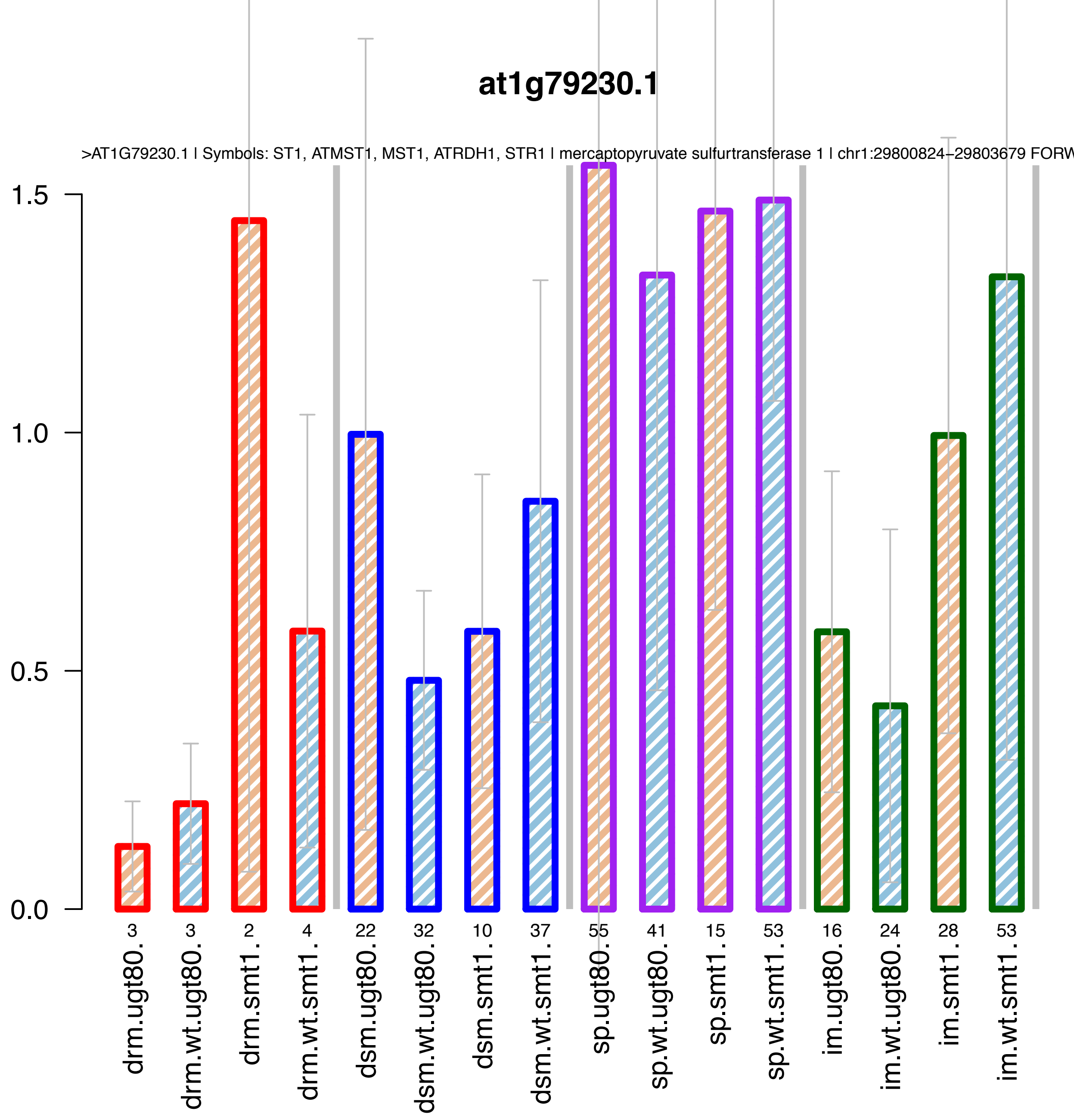

at1g79230.1
>AT1G79230.1 | Symbols: ST1, ATMST1, MST1, ATRDH1, STR1 | mercaptopyruvate sulfurtransferase 1 | chr1:29800824-29803679 FORW
1.5
1.0
0.5
0.0
3
3
2
4
22
32
10
37
55
41
15
53
16
24
28
53
drm.ugt80..
drm.wt.ugt80..
drm.smt1..
drm.wt.smt1..
dsm.ugt80..
dsm.wt.ugt80..
dsm.smt1..
dsm.wt.smt1..
sp.ugt80..
sp.wt.ugt80..
sp.smt1..
sp.wt.smt1..
im.ugt80..
im.wt.ugt80..
im.smt1..
im.wt.smt1..

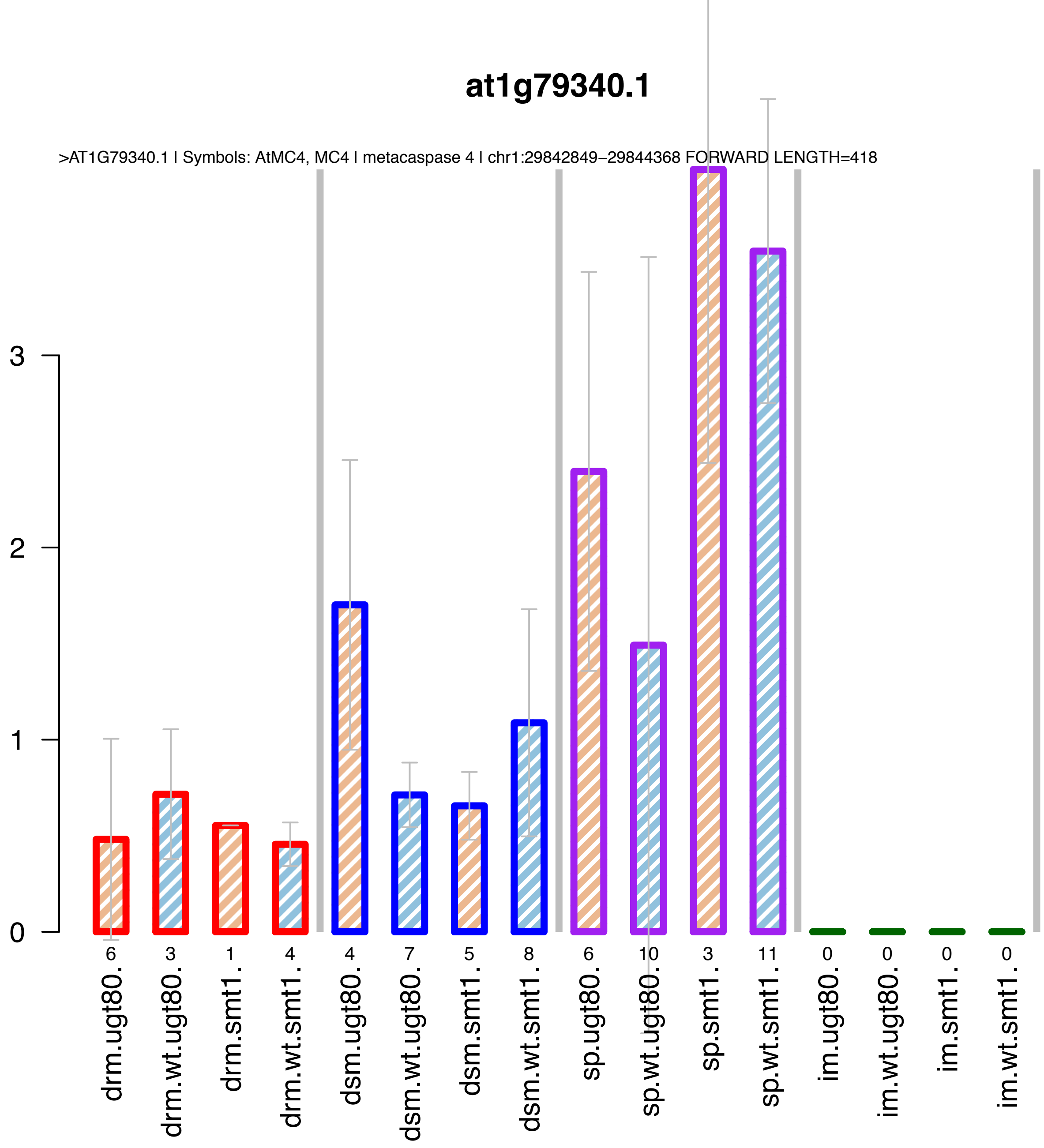

at1g79340.1
>AT1G79340.1 | Symbols: AtMC4, MC4 | metacaspase 4 | chr1:29842849-29844368 FORWARD LENGTH=418
0
1
2
3
6
3
1
4
4
7
5
8
6
10
3
11
0
0
0
0
drm.ugt80..
drm.wt.ugt80..
drm.smt1..
drm.wt.smt1..
dsm.ugt80..
dsm.wt.ugt80..
dsm.smt1..
dsm.wt.smt1..
sp.ugt80..
sp.wt.ugt80..
sp.smt1..
sp.wt.smt1..
im.ugt80..
im.wt.ugt80..
im.smt1..
im.wt.smt1..

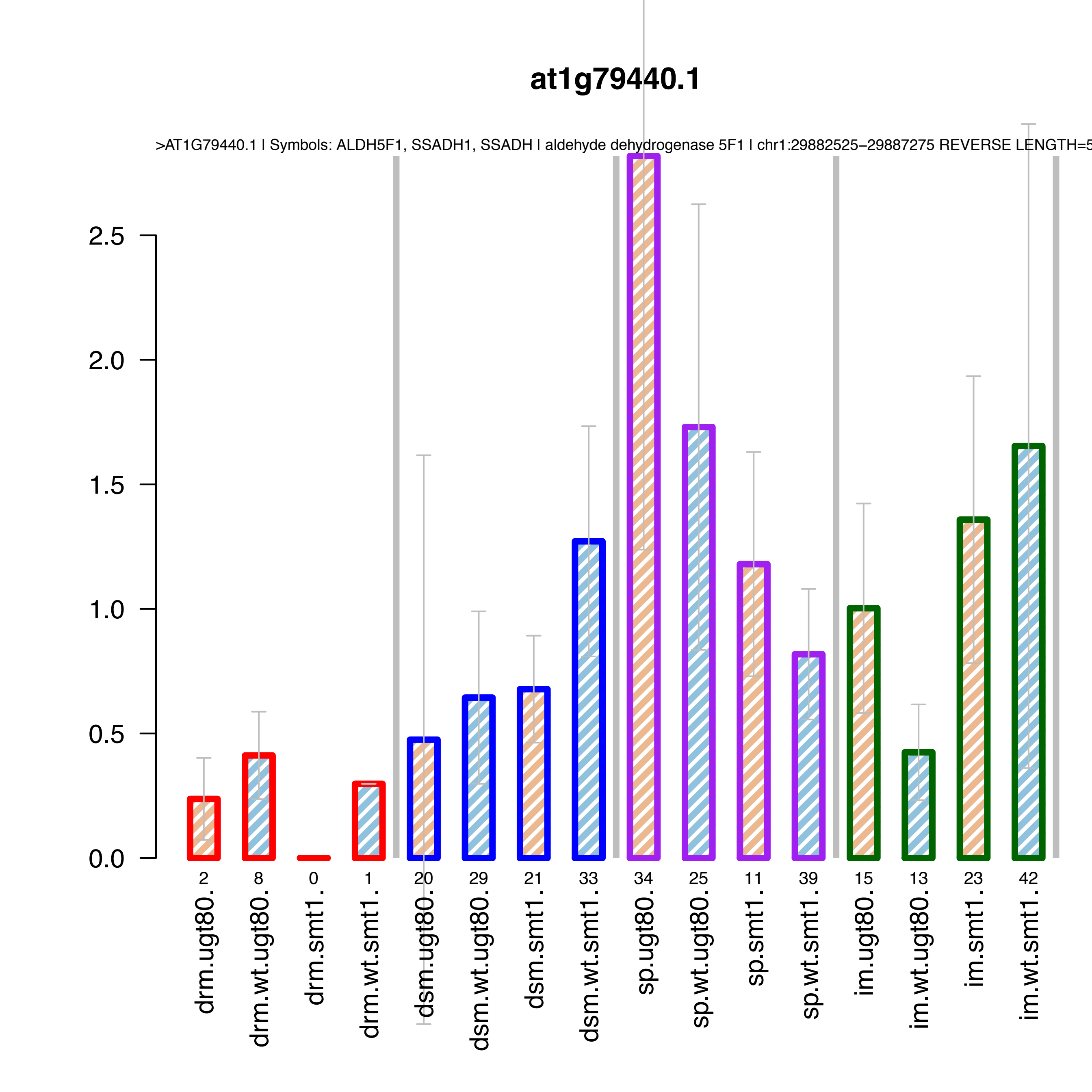
at1g79440.1
>AT1G79440.1 | Symbols: ALDH5F1, SSADH1, SSADH | aldehyde dehydrogenase 5F1 | chr1:29882525-29887275 REVERSE LENGTH=5
0.0
0.5
1.0
1.5
2.0
2.5
2
8
0
1
20
29
21
33
34
25
11
39
15
13
23
42
drm.ugt80..
drm.wt.ugt80..
drm.smt1..
drm.wt.smt1..
dsm.ugt80..
dsm.wt.ugt80..
dsm.smt1..
dsm.wt.smt1..
sp.ugt80..
sp.wt.ugt80..
sp.smt1..
sp.wt.smt1..
im.ugt80..
im.wt.ugt80..
im.smt1..
im.wt.smt1..

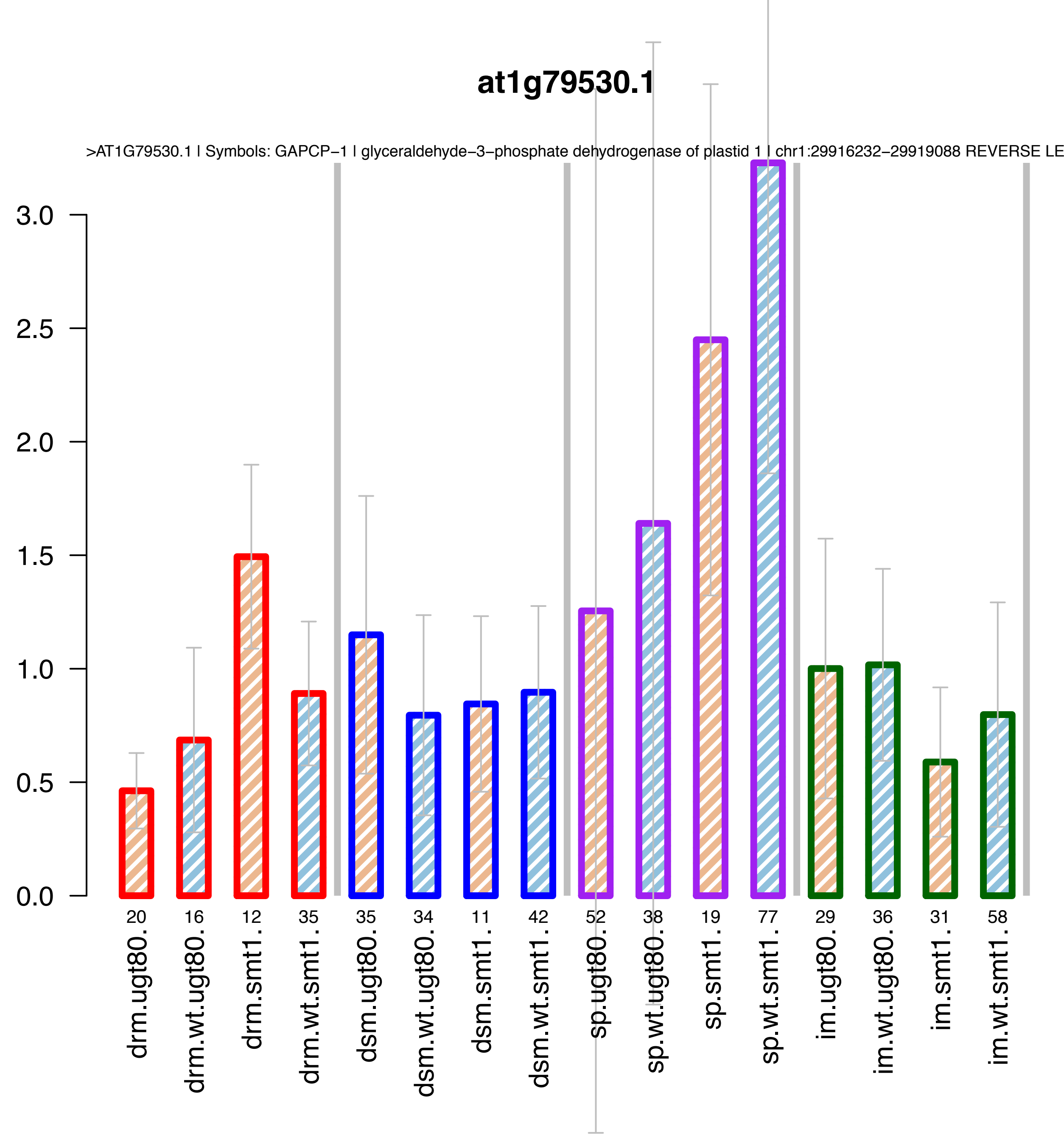

at1g79530.1
>AT1G79530.1 | Symbols: GAPCP−1 | glyceraldehyde−3−phosphate dehydrogenase of plastid 1 | chr1:29916232−29919088 REVERSE LE
0.0
0.5
1.0
1.5
2.0
2.5
3.0
20
16
12
35
35
34
11
42
52
38
19
77
29
36
31
58
drm.ugt80..
drm.wt.ugt80..
drm.smt1..
drm.wt.smt1..
dsm.ugt80..
dsm.wt.ugt80..
dsm.smt1..
dsm.wt.smt1..
sp.ugt80..
sp.wt.ugt80..
sp.smt1..
sp.wt.smt1..
im.ugt80..
im.wt.ugt80..
im.smt1..
im.wt.smt1..

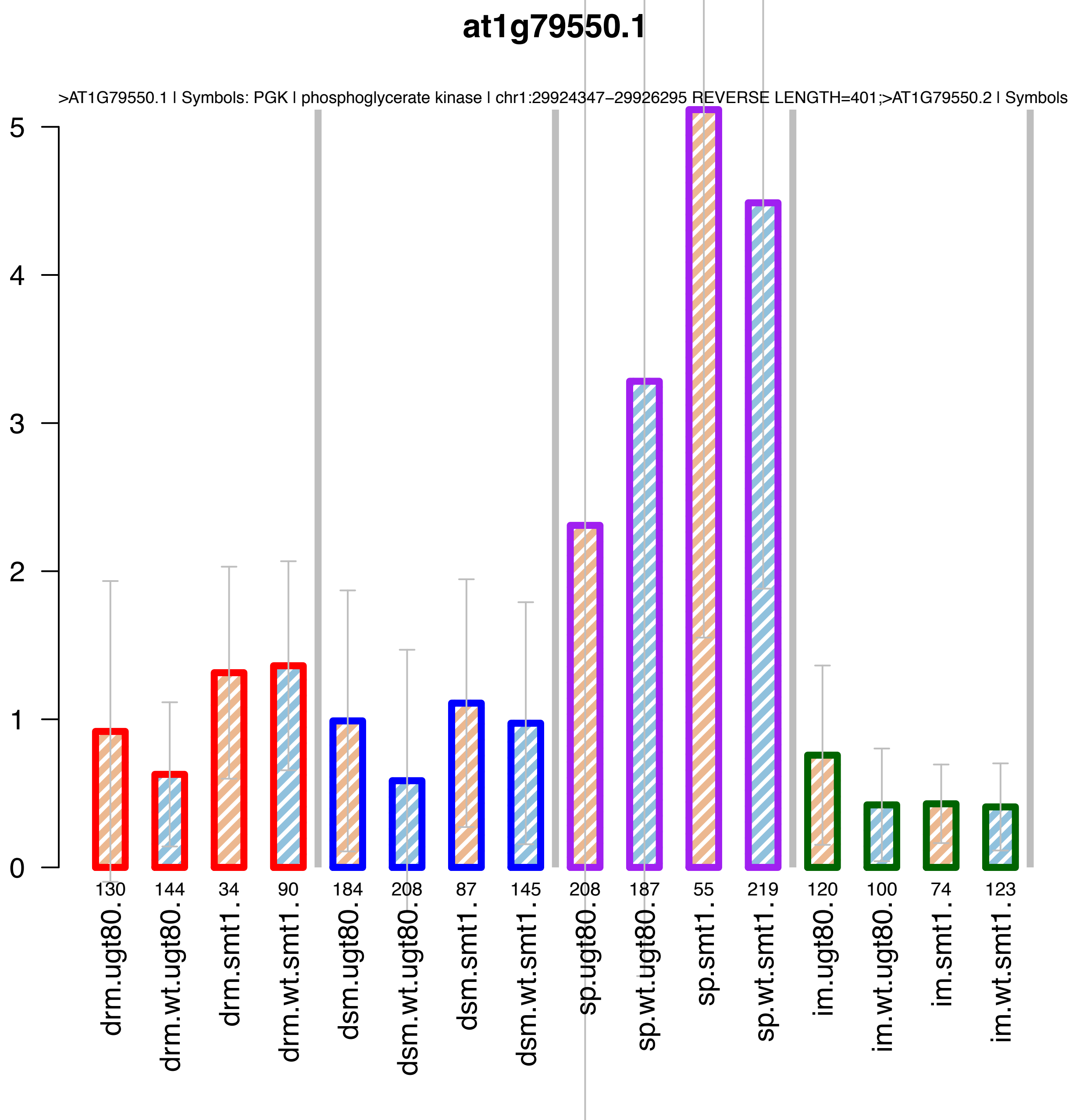

at1g79550.1
>AT1G79550.1 | Symbols: PGK | phosphoglycerate kinase | chr1:29924347-29926295 REVERSE LENGTH=401;>AT1G79550.2 | Symbols
0
1
2
3
4
5
130
144
34
90
184
208
87
145
208
187
55
219
120
100
74
123
drm.ugt80..
drm.wt.ugt80..
drm.smt1..
drm.wt.smt1..
dsm.ugt80..
dsm.wt.ugt80..
dsm.smt1..
dsm.wt.smt1..
sp.ugt80..
sp.wt.ugt80..
sp.smt1..
sp.wt.smt1..
im.ugt80..
im.wt.ugt80..
im.smt1..
im.wt.smt1..

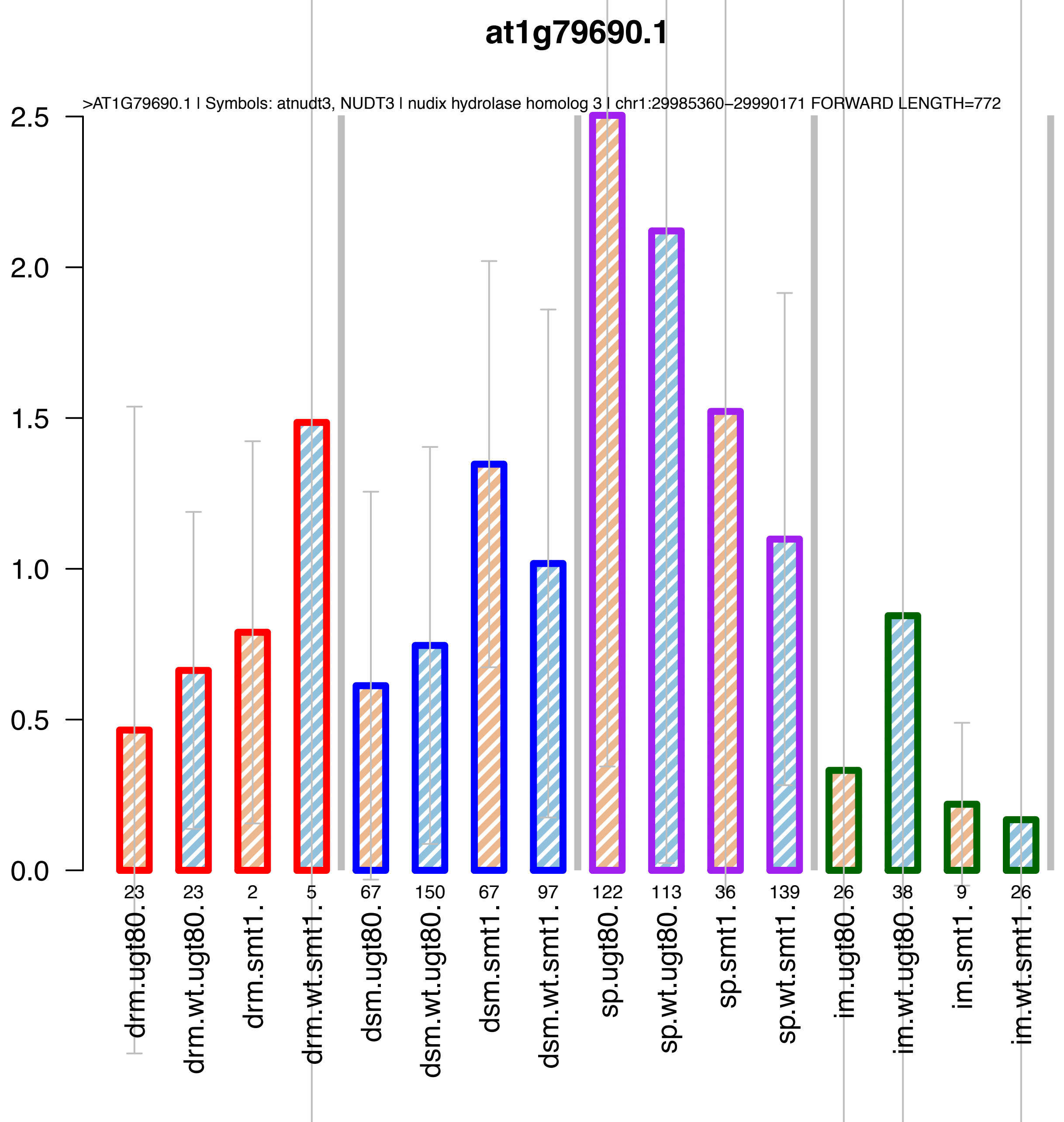
at1g79690.1
>AT1G79690.1 | Symbols: atnudt3, NUDT3 | nudix hydrolase homolog 3 | chr1:29985360–29990171 FORWARD LENGTH=772
2.5
2.0
1.5
1.0
0.5
0.0
23
23
2
5
67
150
67
97
122
113
36
139
26
38
9
26
drm.ugt80..
drm.wt.ugt80..
drm.smt1..
drm.wt.smt1..
dsm.ugt80..
dsm.wt.ugt80..
dsm.smt1..
dsm.wt.smt1..
sp.ugt80..
sp.wt.ugt80..
sp.smt1..
sp.wt.smt1..
im.ugt80..
im.wt.ugt80..
im.smt1..
im.wt.smt1..

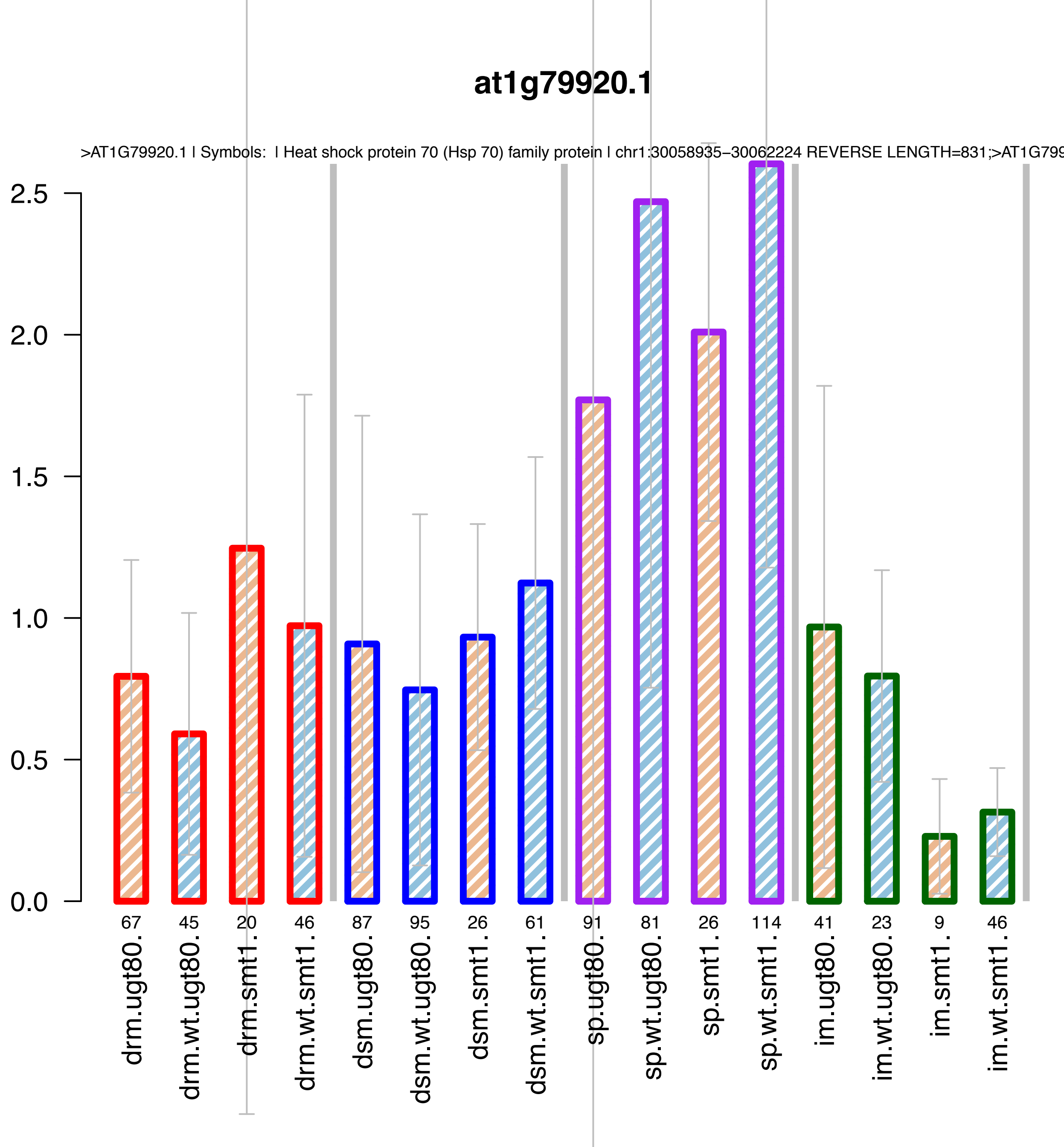

at1g79920.1
>AT1G79920.1 | Symbols: | Heat shock protein 70 (Hsp 70) family protein | chr1:30058935-30062224 REVERSE LENGTH=831;>AT1G799
2.5
2.0
1.5
1.0
0.5
0.0
67
45
20
46
87
95
26
61
91
81
26
114
41
23
9
46
drm.ugt80.
drm.wt.ugt80.
drm.smt1.
drm.wt.smt1.
dsm.ugt80.
dsm.wt.ugt80.
dsm.smt1.
dsm.wt.smt1.
sp.ugt80.
sp.wt.ugt80.
sp.smt1.
sp.wt.smt1.
im.ugt80.
im.wt.ugt80.
im.smt1.
im.wt.smt1.

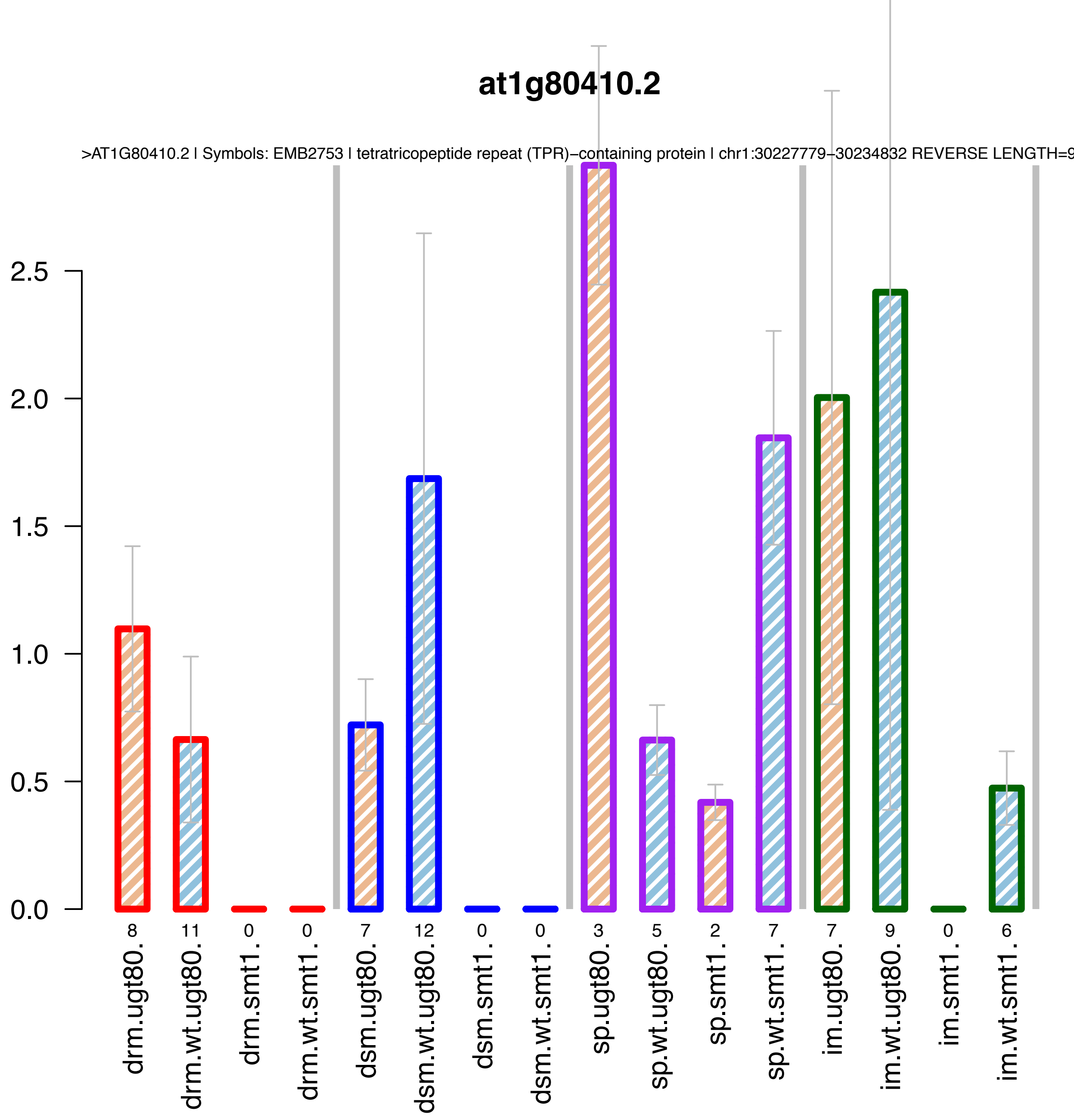

at1g80410.2
>AT1G80410.2 | Symbols: EMB2753 | tetratricopeptide repeat (TPR)−containing protein | chr1:30227779−30234832 REVERSE LENGTH=9
0.0
0.5
1.0
1.5
2.0
2.5
8
11
0
0
7
12
0
0
3
5
2
7
7
9
0
6
drm.ugt80.
drm.wt.ugt80.
drm.smt1.
drm.wt.smt1.
dsm.ugt80.
dsm.wt.ugt80.
dsm.smt1.
dsm.wt.smt1.
sp.ugt80.
sp.wt.ugt80.
sp.smt1.
sp.wt.smt1.
im.ugt80.
im.wt.ugt80.
im.smt1.
im.wt.smt1.

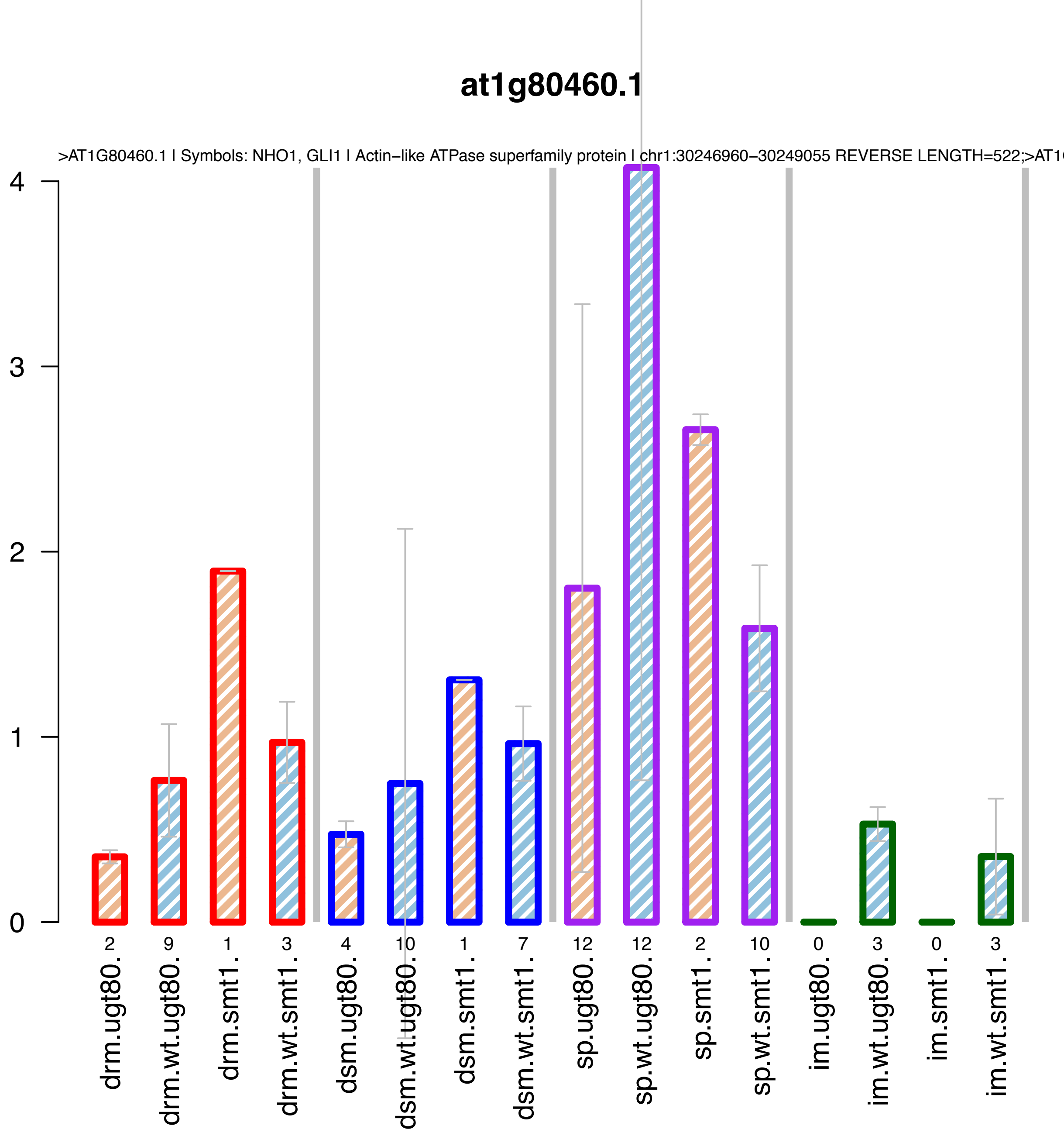

at1g80460.1
>AT1G80460.1 | Symbols: NHO1, GLI1 | Actin-like ATPase superfamily protein | chr1:30246960-30249055 REVERSE LENGTH=522;>AT1
4
3
2
1
0
2
9
1
3
4
10
1
7
12
12
2
10
0
3
0
3
drm.ugt80..
drm.wt.ugt80..
drm.smt1..
drm.wt.smt1..
dsm.ugt80..
dsm.wt.ugt80..
dsm.smt1..
dsm.wt.smt1..
sp.ugt80..
sp.wt.ugt80..
sp.smt1..
sp.wt.smt1..
im.ugt80..
im.wt.ugt80..
im.smt1..
im.wt.smt1..

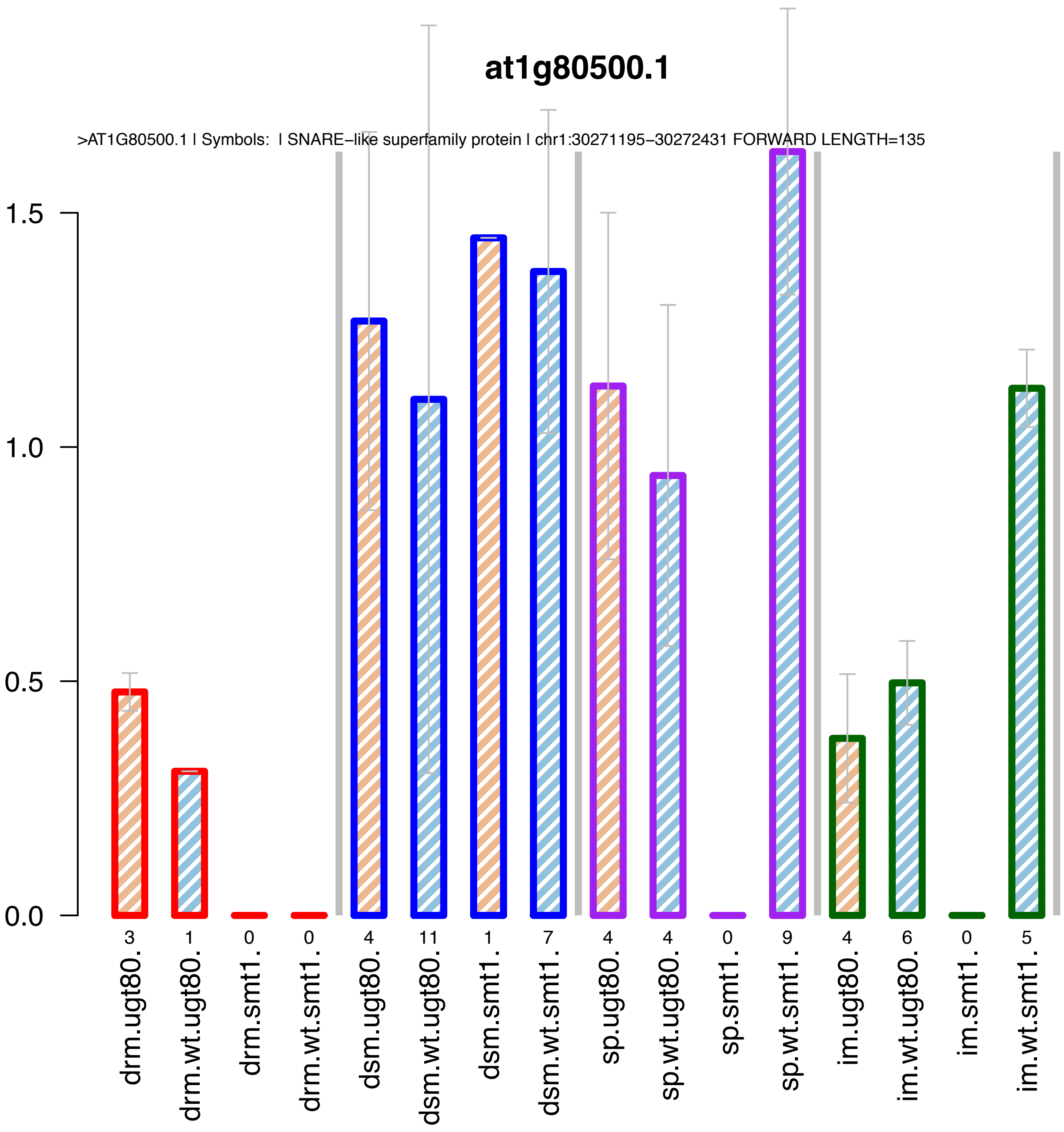

at1g80500.1
>AT1G80500.1 | Symbols: | SNARE−like superfamily protein | chr1:30271195−30272431 FORWARD LENGTH=135
0.0
0.5
1.0
1.5
3
1
0
0
4
11
1
7
4
4
0
9
4
6
0
5
drm.ugt80.
drm.wt.ugt80.
drm.smt1.
drm.wt.smt1.
dsm.ugt80.
dsm.wt.ugt80.
dsm.smt1.
dsm.wt.smt1.
sp.ugt80.
sp.wt.ugt80.
sp.smt1.
sp.wt.smt1.
im.ugt80.
im.wt.ugt80.
im.smt1.
im.wt.smt1.

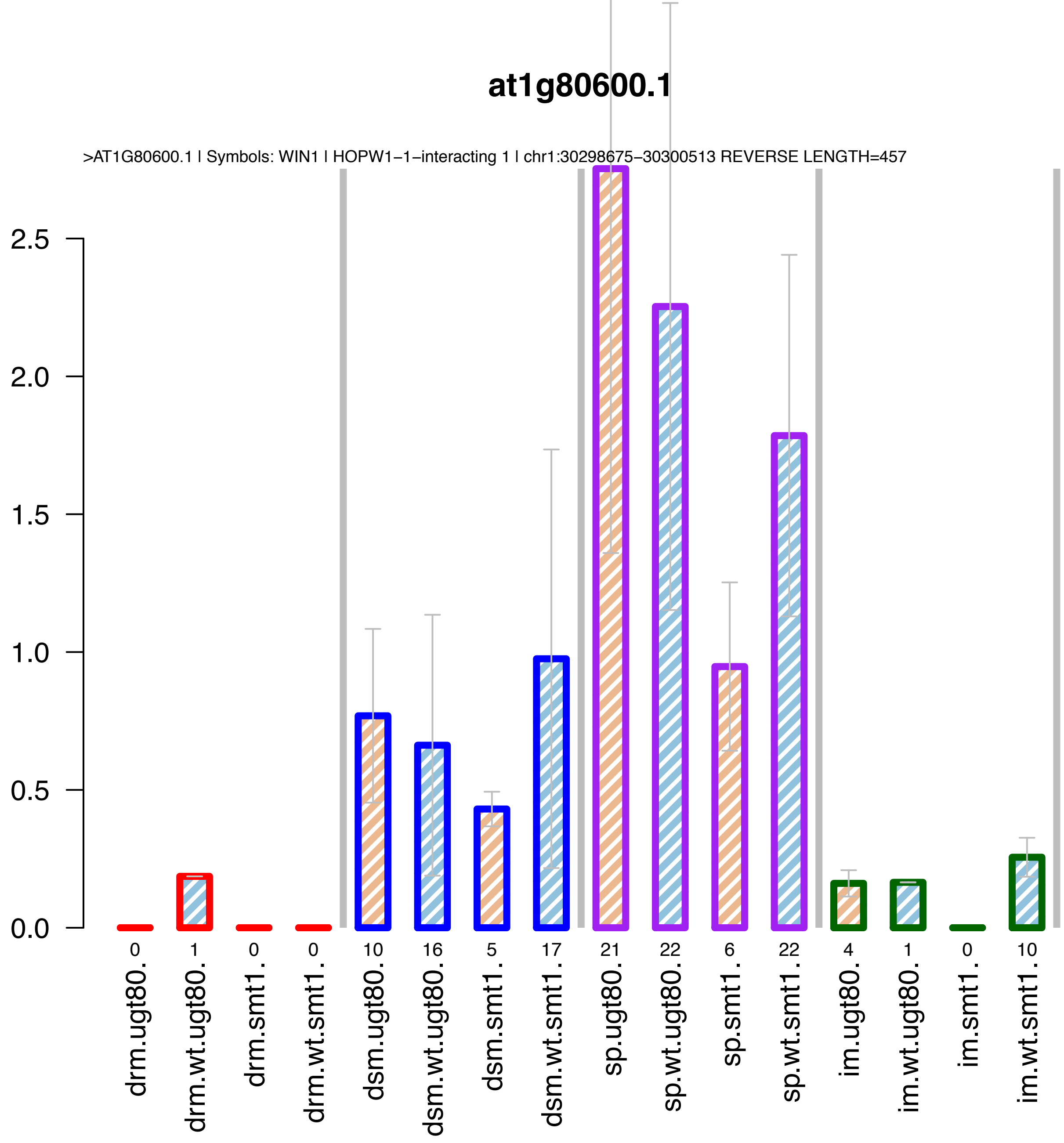

at1g80600.1
>AT1G80600.1 | Symbols: WIN1 | HOPW1−1−interacting 1 | chr1:30298675−30300513 REVERSE LENGTH=457
0.0
0.5
1.0
1.5
2.0
2.5
0 1 0 0 10 16 5 17 21 22 6 22 4 1 0 10
drm.ugt80.
drm.wt.ugt80.
drm.smt1.
drm.wt.smt1.
dsm.ugt80.
dsm.wt.ugt80.
dsm.smt1.
dsm.wt.smt1.
sp.ugt80.
sp.wt.ugt80.
sp.smt1.
sp.wt.smt1.
im.ugt80.
im.wt.ugt80.
im.smt1.
im.wt.smt1.

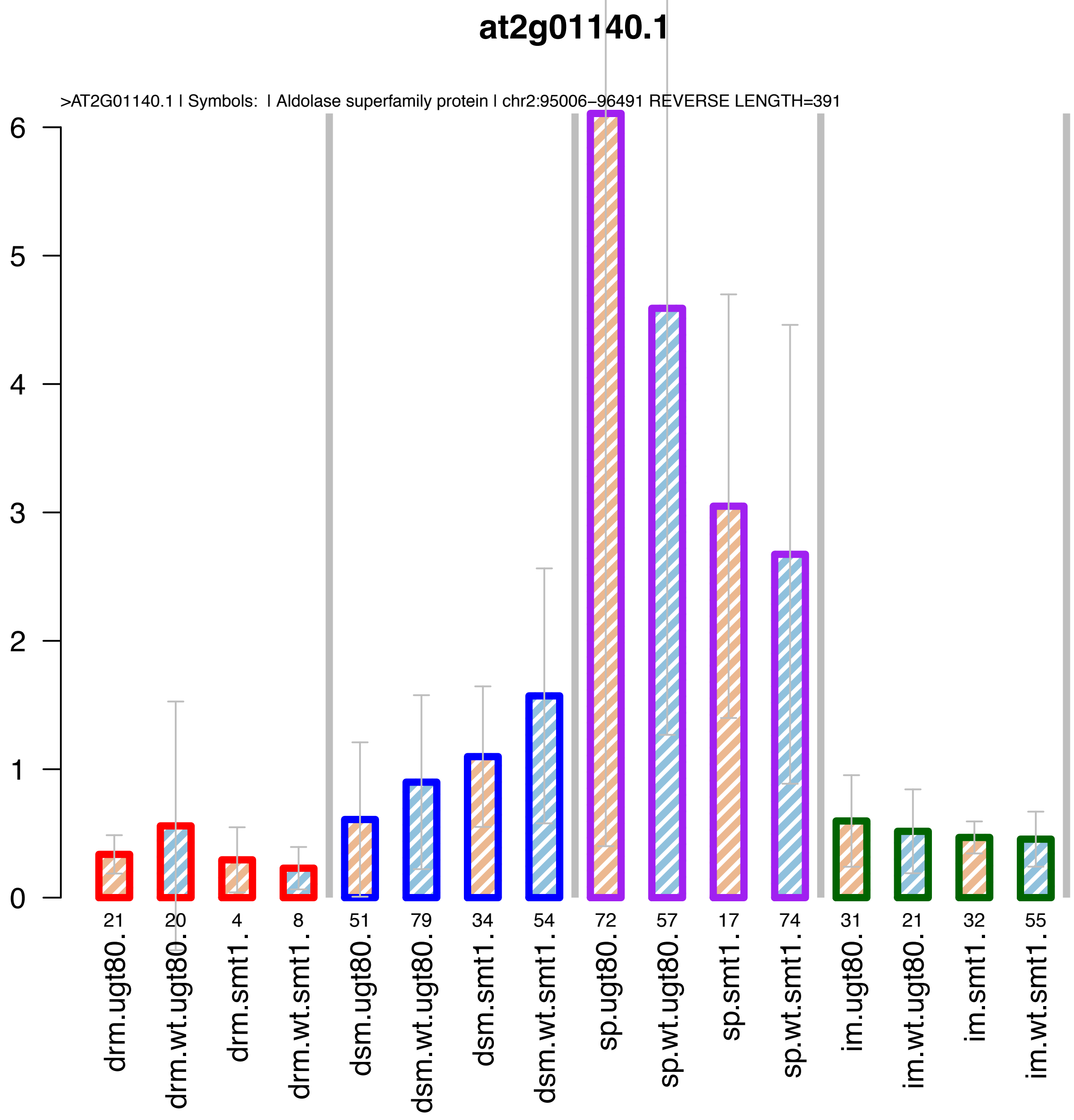
at2g01140.1
>AT2G01140.1 | Symbols: | Aldolase superfamily protein | chr2:95006–96491 REVERSE LENGTH=391
0
1
2
3
4
5
6
21
20
4
8
51
79
34
54
72
57
17
74
31
21
32
55
drm.ugt80..
drm.wt.ugt80..
drm.smt1..
drm.wt.smt1..
dsm.ugt80..
dsm.wt.ugt80..
dsm.smt1..
dsm.wt.smt1..
sp.ugt80..
sp.wt.ugt80..
sp.smt1..
sp.wt.smt1..
im.ugt80..
im.wt.ugt80..
im.smt1..
im.wt.smt1..

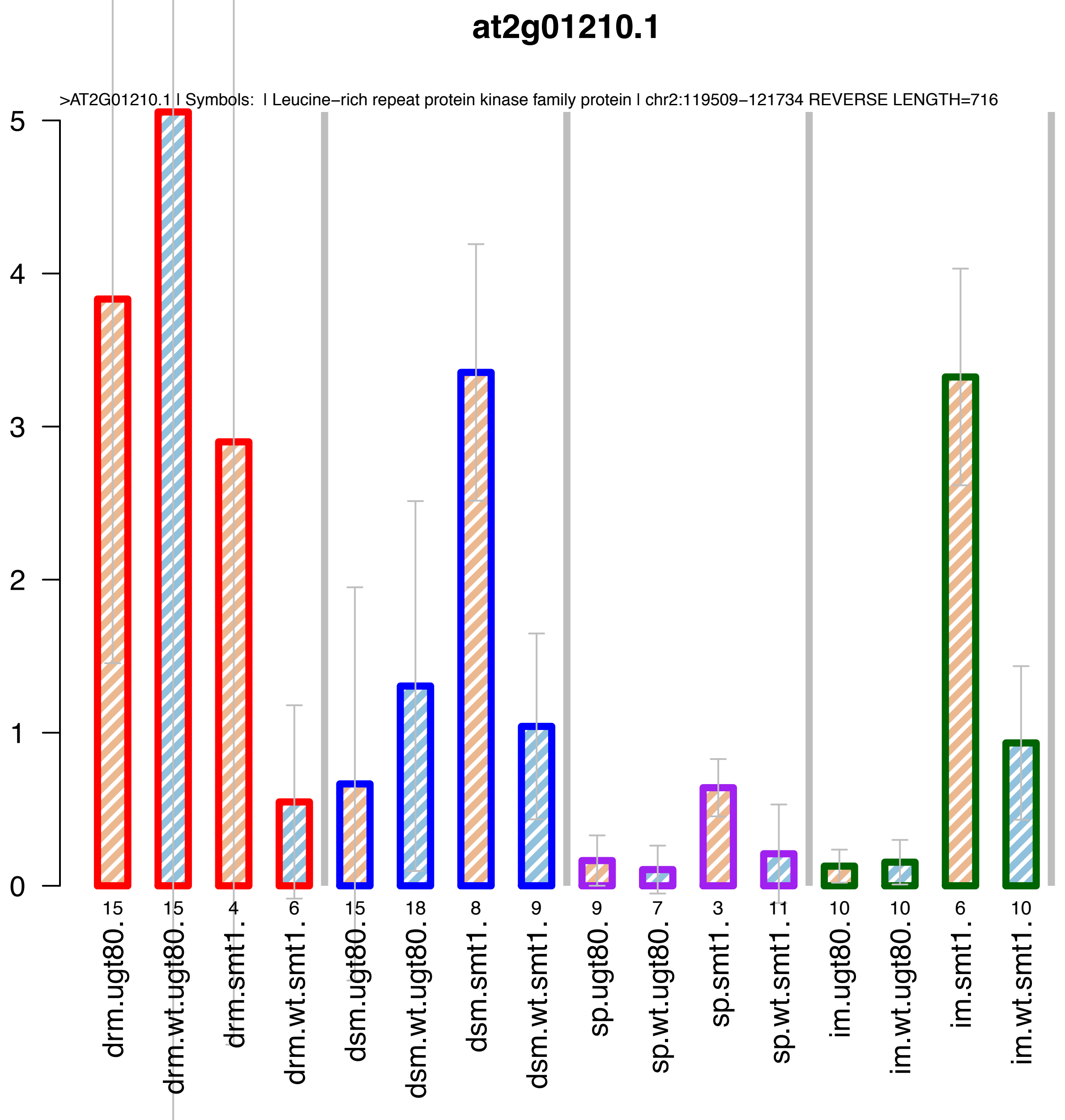

at2g01210.1
>AT2G01210.1 | Symbols: | Leucine−rich repeat protein kinase family protein | chr2:119509-121734 REVERSE LENGTH=716
0
1
2
3
4
5
15
15
4
6
15
18
8
9
9
7
3
11
10
10
6
10
drm.ugt80.
drm.wt.ugt80.
drm.smt1.
drm.wt.smt1.
dsm.ugt80.
dsm.wt.ugt80.
dsm.smt1.
dsm.wt.smt1.
sp.ugt80.
sp.wt.ugt80.
sp.smt1.
sp.wt.smt1.
im.ugt80.
im.wt.ugt80.
im.smt1.
im.wt.smt1.

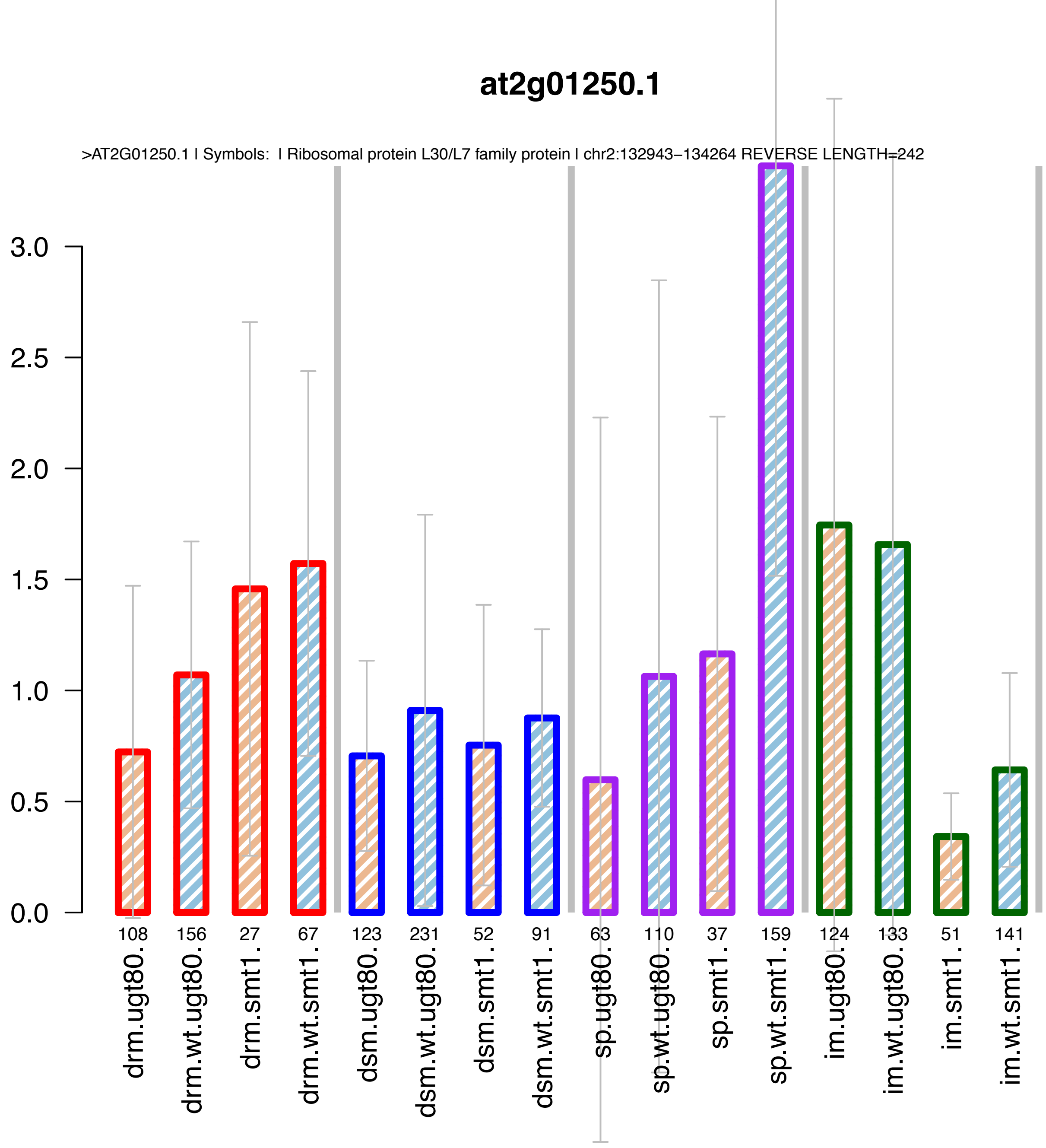

at2g01250.1
>AT2G01250.1 | Symbols: | Ribosomal protein L30/L7 family protein | chr2:132943-134264 REVERSE LENGTH=242
3.0
2.5
2.0
1.5
1.0
0.5
0.0
108
156
27
67
123
231
52
91
63
110
37
159
124
133
51
141
drm.ugt80..
drm.wt.ugt80..
drm.smt1..
drm.wt.smt1..
dsm.ugt80..
dsm.wt.ugt80..
dsm.smt1..
dsm.wt.smt1..
sp.ugt80..
sp.wt.ugt80..
sp.smt1..
sp.wt.smt1..
im.ugt80..
im.wt.ugt80..
im.smt1..
im.wt.smt1..

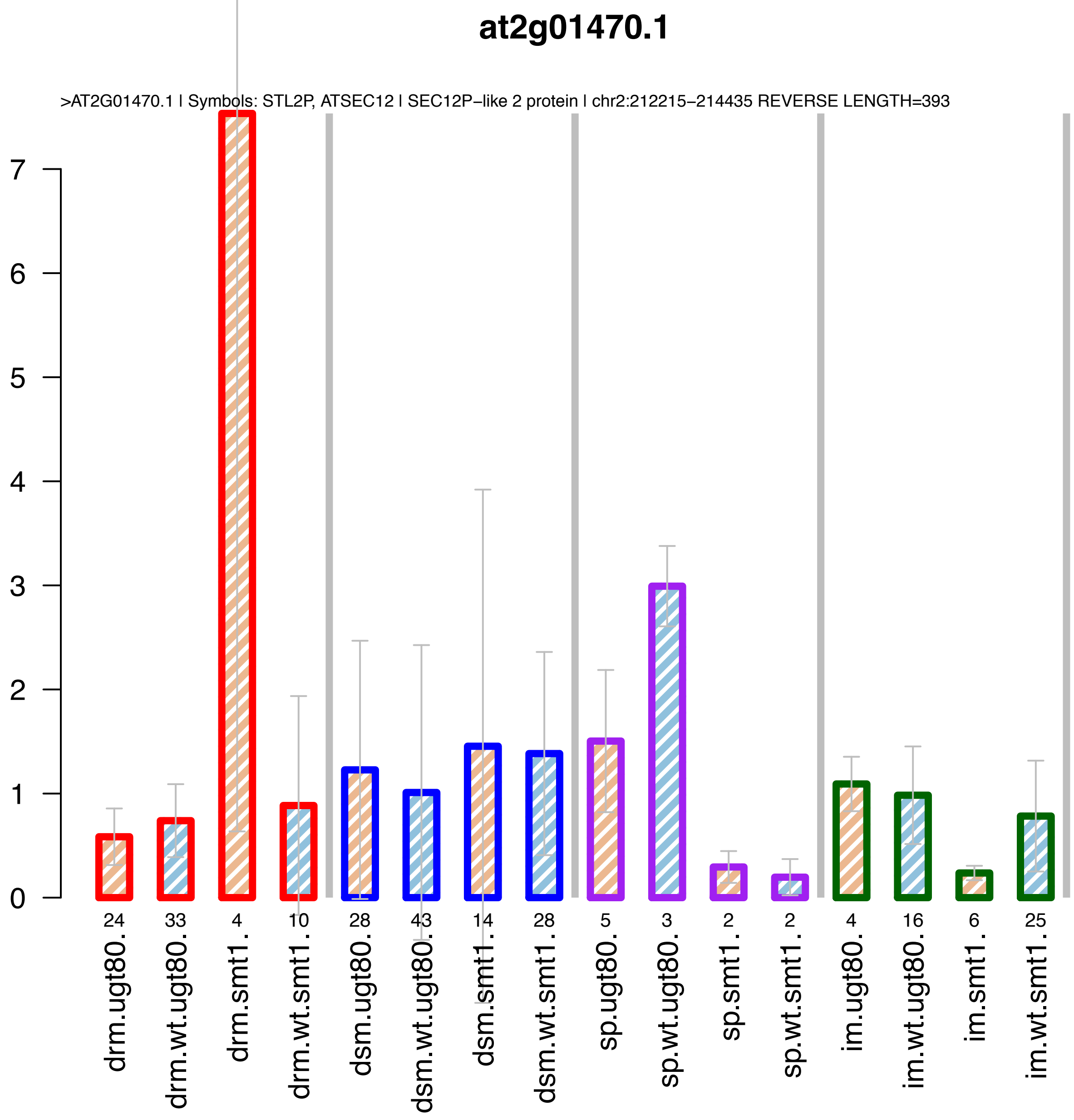

at2g01470.1
>AT2G01470.1 | Symbols: STL2P, ATSEC12 | SEC12P−like 2 protein | chr2:212215−214435 REVERSE LENGTH=393
0
1
2
3
4
5
6
7
24
33
4
10
28
43
14
28
5
3
2
2
4
16
6
25
drm.ugt80..
drm.wt.ugt80..
drm.smt1..
drm.wt.smt1..
dsm.ugt80..
dsm.wt.ugt80..
dsm.smt1..
dsm.wt.smt1..
sp.ugt80..
sp.wt.ugt80..
sp.smt1..
sp.wt.smt1..
im.ugt80..
im.wt.ugt80..
im.smt1..
im.wt.smt1..

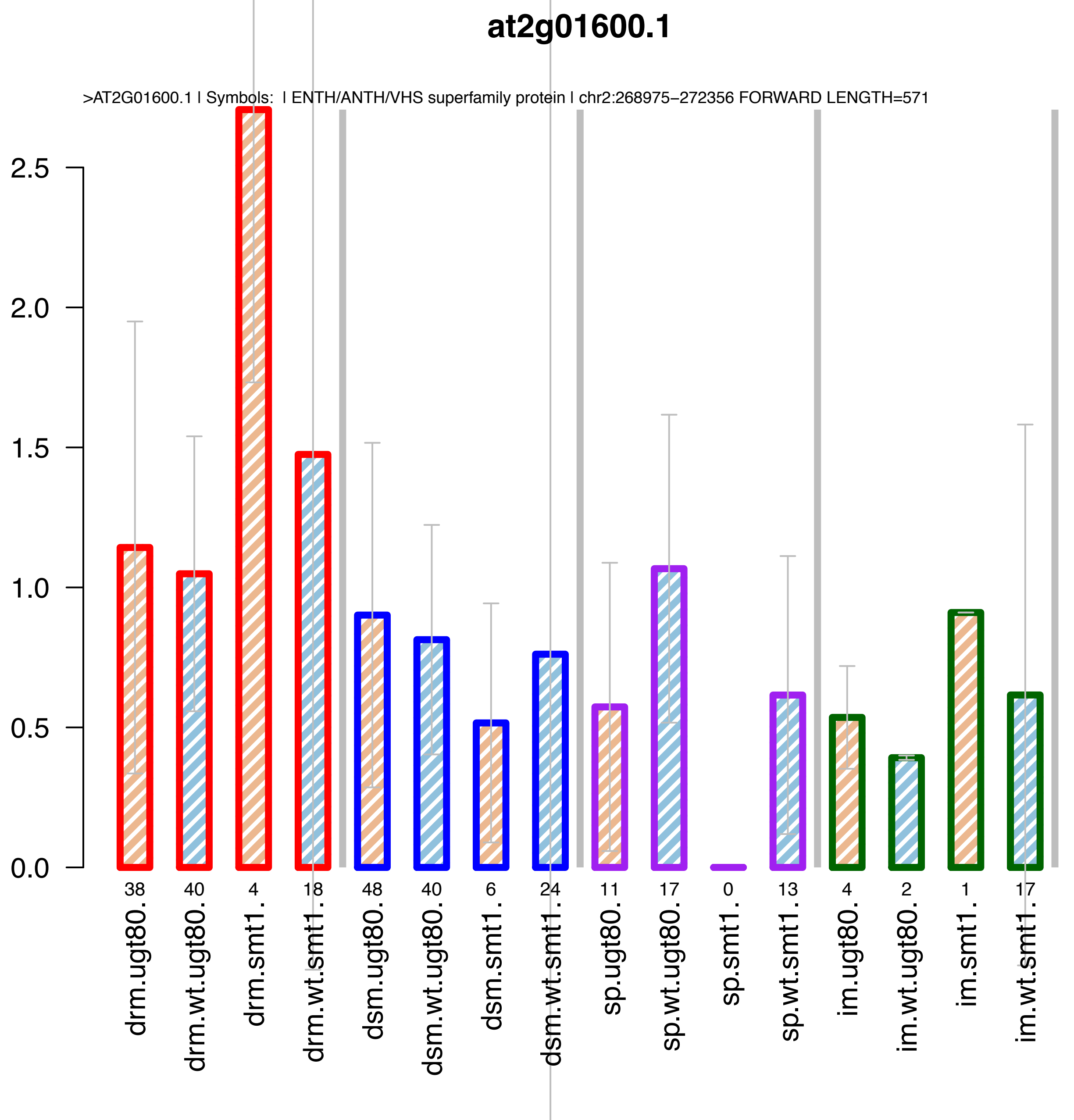

at2g01600.1
>AT2G01600.1 | Symbols: | ENTH/ANTH/VHS superfamily protein | chr2:268975-272356 FORWARD LENGTH=571
2.5
2.0
1.5
1.0
0.5
0.0
38
40
4
18
48
40
6
24
11
17
0
13
4
2
1
17
drm.ugt80..
drm.wt.ugt80..
drm.smt1..
drm.wt.smt1..
dsm.ugt80..
dsm.wt.ugt80..
dsm.smt1..
dsm.wt.smt1..
sp.ugt80..
sp.wt.ugt80..
sp.smt1..
sp.wt.smt1..
im.ugt80..
im.wt.ugt80..
im.smt1..
im.wt.smt1..

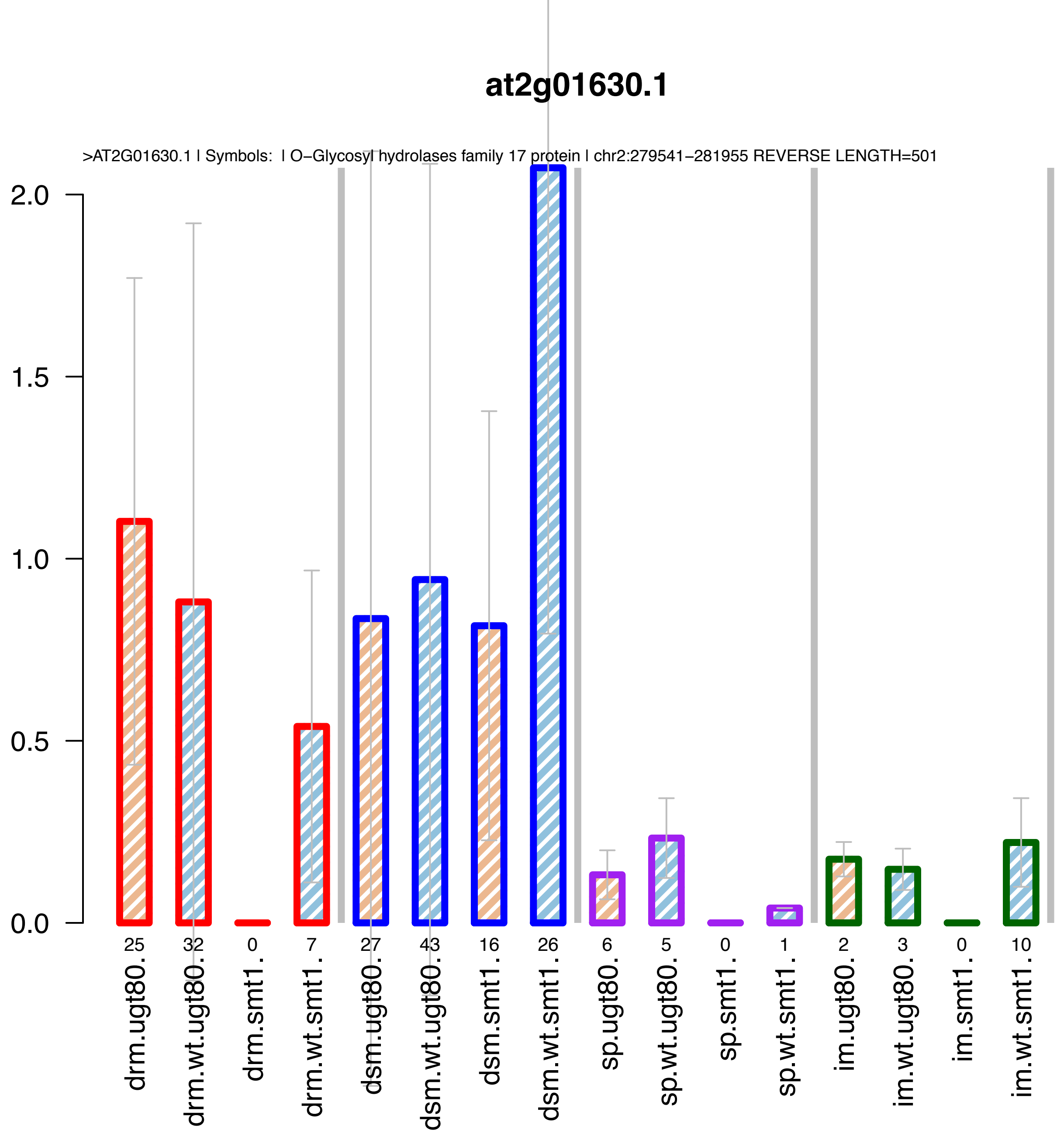

at2g01630.1
>AT2G01630.1 | Symbols: | O−Glycosyl hydrolases family 17 protein | chr2:279541−281955 REVERSE LENGTH=501
2.0
1.5
1.0
0.5
0.0
25
32
0
7
27
43
16
26
6
5
0
1
2
3
0
10
drm.ugt80.
drm.wt.ugt80.
drm.smt1.
drm.wt.smt1.
dsm.ugt80.
dsm.wt.ugt80.
dsm.smt1.
dsm.wt.smt1.
sp.ugt80.
sp.wt.ugt80.
sp.smt1.
sp.wt.smt1.
im.ugt80.
im.wt.ugt80.
im.smt1.
im.wt.smt1.

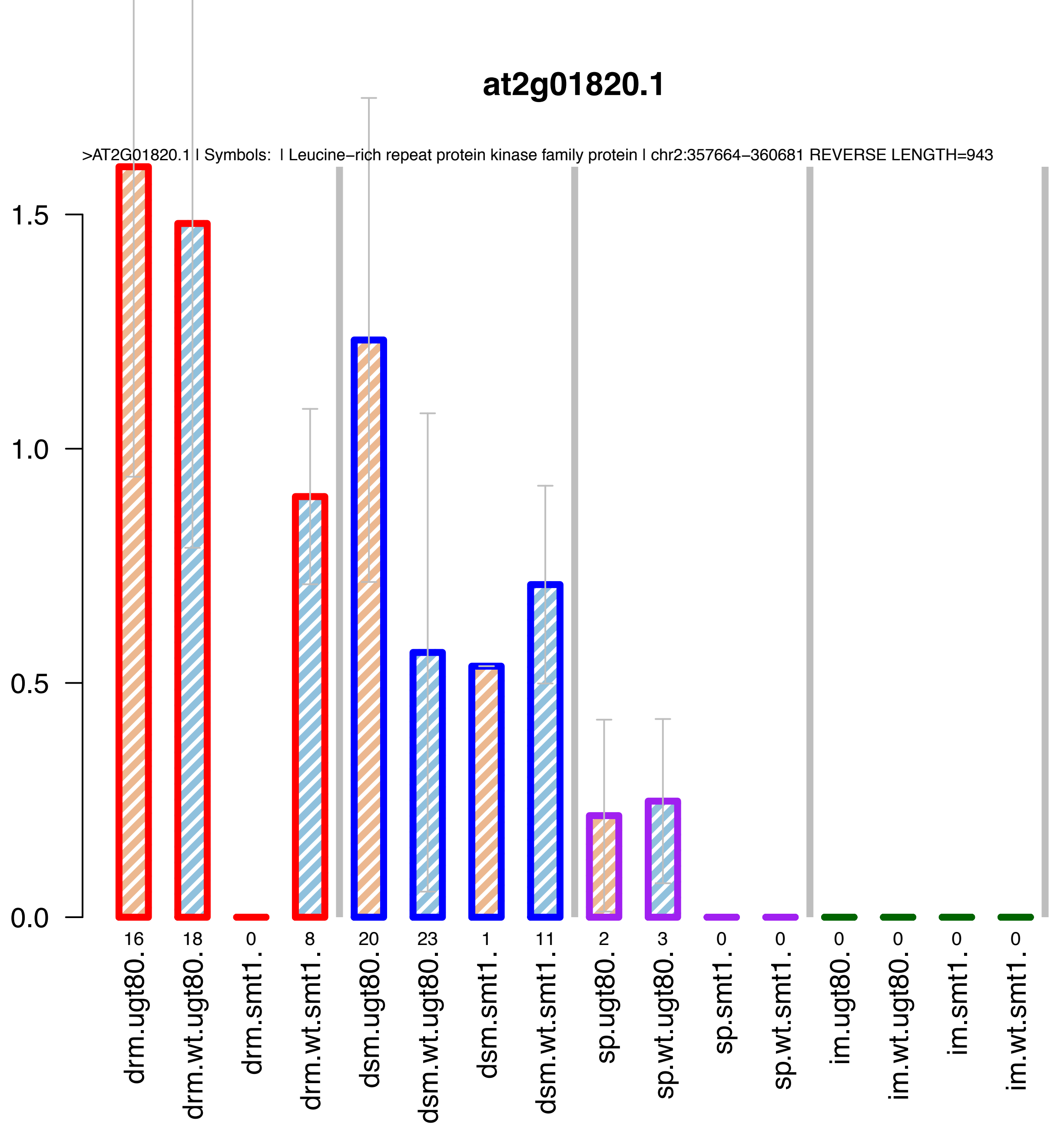

at2g01820.1
>AT2G01820.1 | Symbols: | Leucine−rich repeat protein kinase family protein | chr2:357664−360681 REVERSE LENGTH=943
1.5
1.0
0.5
0.0
16
18
0
8
20
23
1
11
2
3
0
0
0
0
0
0
drm.ugt80.
drm.wt.ugt80.
drm.smt1.
drm.wt.smt1.
dsm.ugt80.
dsm.wt.ugt80.
dsm.smt1.
dsm.wt.smt1.
sp.ugt80.
sp.wt.ugt80.
sp.smt1.
sp.wt.smt1.
im.ugt80.
im.wt.ugt80.
im.smt1.
im.wt.smt1.

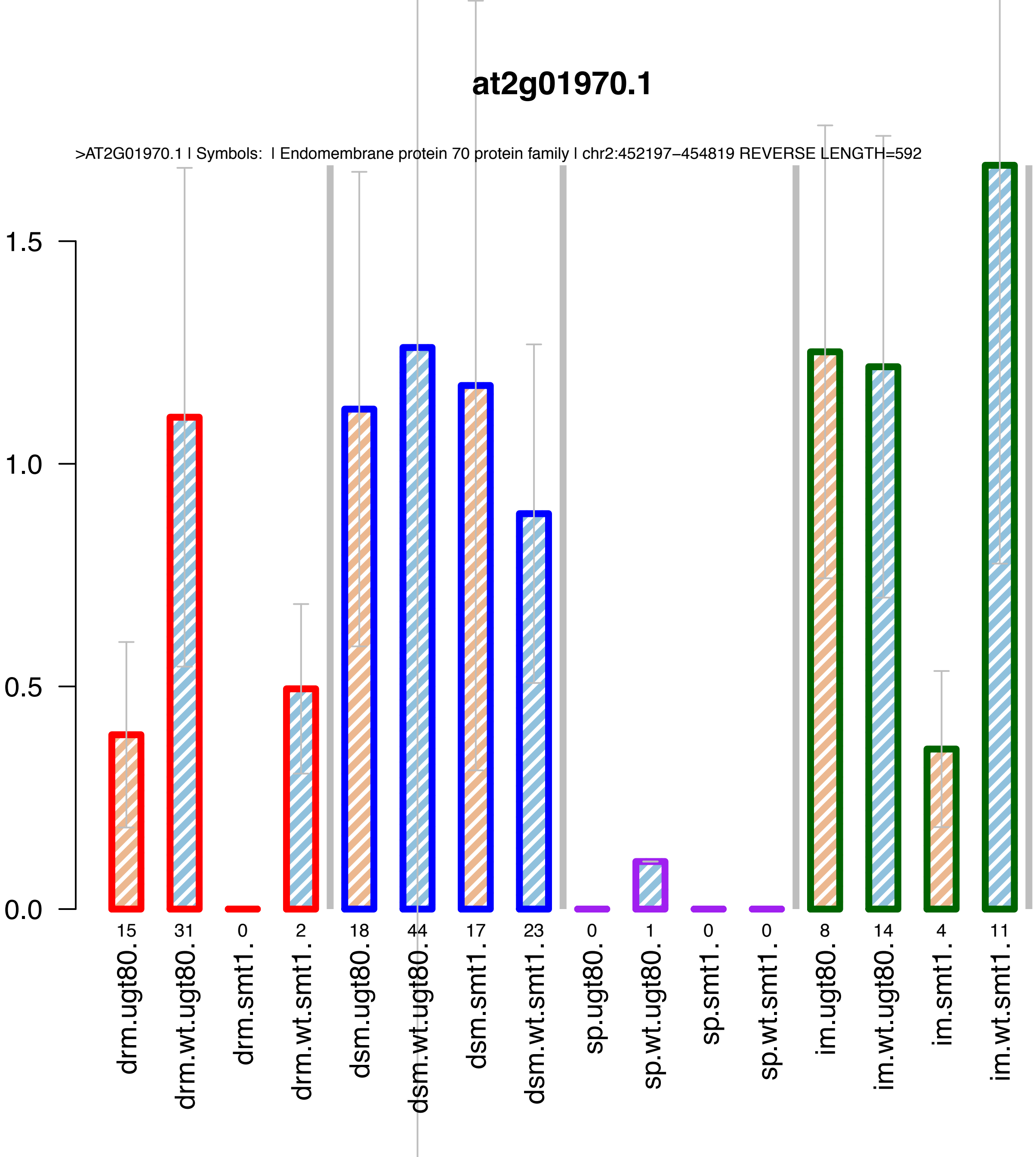

at2g01970.1
>AT2G01970.1 | Symbols: | Endomembrane protein 70 protein family | chr2:452197-454819 REVERSE LENGTH=592
0.0
0.5
1.0
1.5
15
31
0
2
18
44
17
23
0
1
0
0
8
14
4
11
drm.ugt80.
drm.wt.ugt80.
drm.smt1.
drm.wt.smt1.
dsm.ugt80.
dsm.wt.ugt80.
dsm.smt1.
dsm.wt.smt1.
sp.ugt80.
sp.wt.ugt80.
sp.smt1.
sp.wt.smt1.
im.ugt80.
im.wt.ugt80.
im.smt1.
im.wt.smt1.

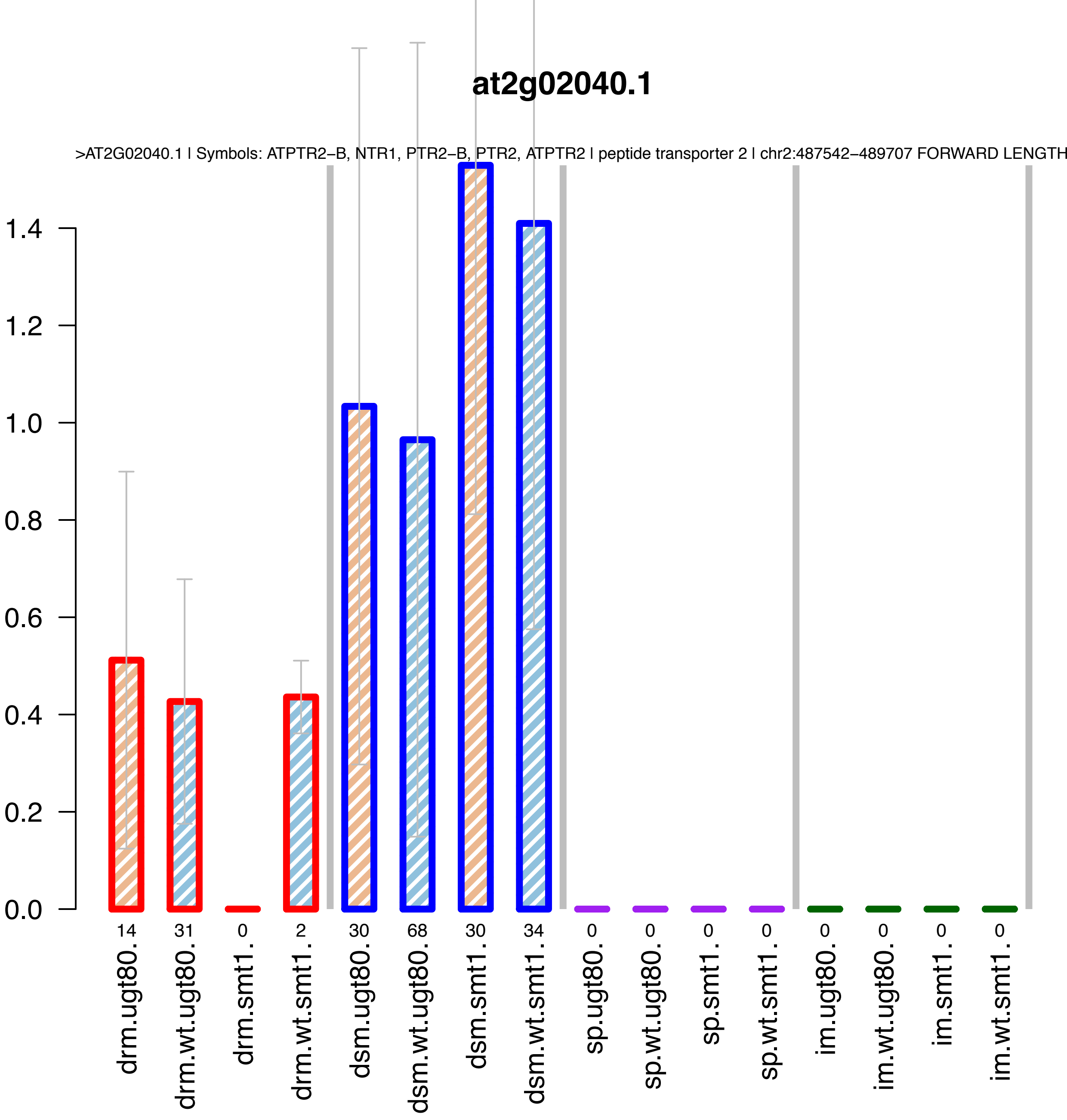
at2g02040.1
>AT2G02040.1 | Symbols: ATPTR2−B, NTR1, PTR2−B, PTR2, ATPTR2 | peptide transporter 2 | chr2:487542−489707 FORWARD LENGTH
0.0
0.2
0.4
0.6
0.8
1.0
1.2
1.4
14
31
0
2
30
68
30
34
0
0
0
0
0
0
0
0
drm.ugt80.
drm.wt.ugt80.
drm.smt1.
drm.wt.smt1.
dsm.ugt80.
dsm.wt.ugt80.
dsm.smt1.
dsm.wt.smt1.
sp.ugt80.
sp.wt.ugt80.
sp.smt1.
sp.wt.smt1.
im.ugt80.
im.wt.ugt80.
im.smt1.
im.wt.smt1.

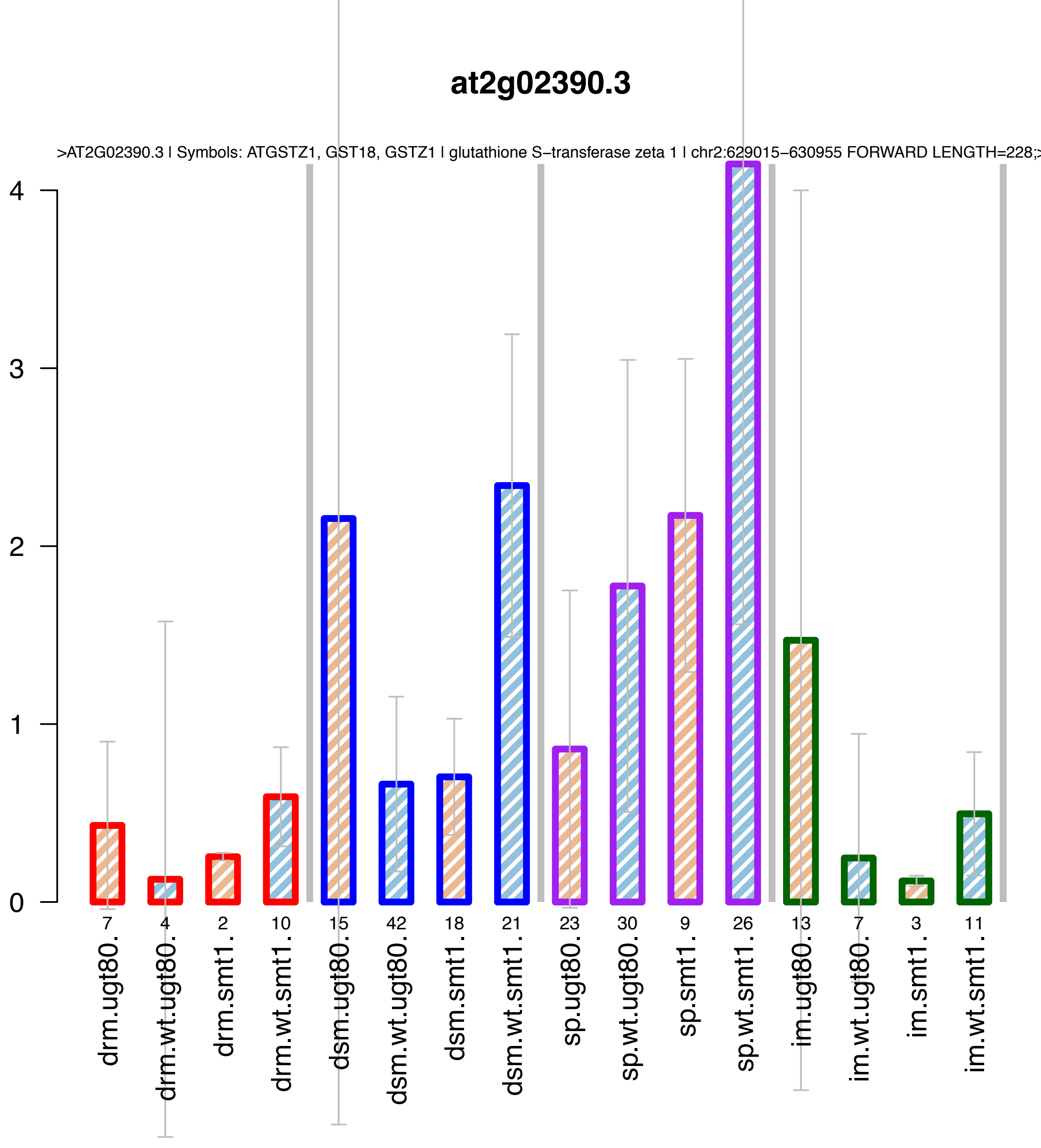
at2g02390.3
>AT2G02390.3 | Symbols: ATGSTZ1, GST18, GSTZ1 | glutathione S-transferase zeta 1 | chr2:629015-630955 FORWARD LENGTH=228;>
0
1
2
3
4
7
4
2
10
15
42
18
21
23
30
9
26
13
7
3
11
drm.ugt80.
drm.wt.ugt80.
drm.smt1.
drm.wt.smt1.
dsm.ugt80.
dsm.wt.ugt80.
dsm.smt1.
dsm.wt.smt1.
sp.ugt80.
sp.wt.ugt80.
sp.smt1.
sp.wt.smt1.
im.ugt80.
im.wt.ugt80.
im.smt1.
im.wt.smt1.

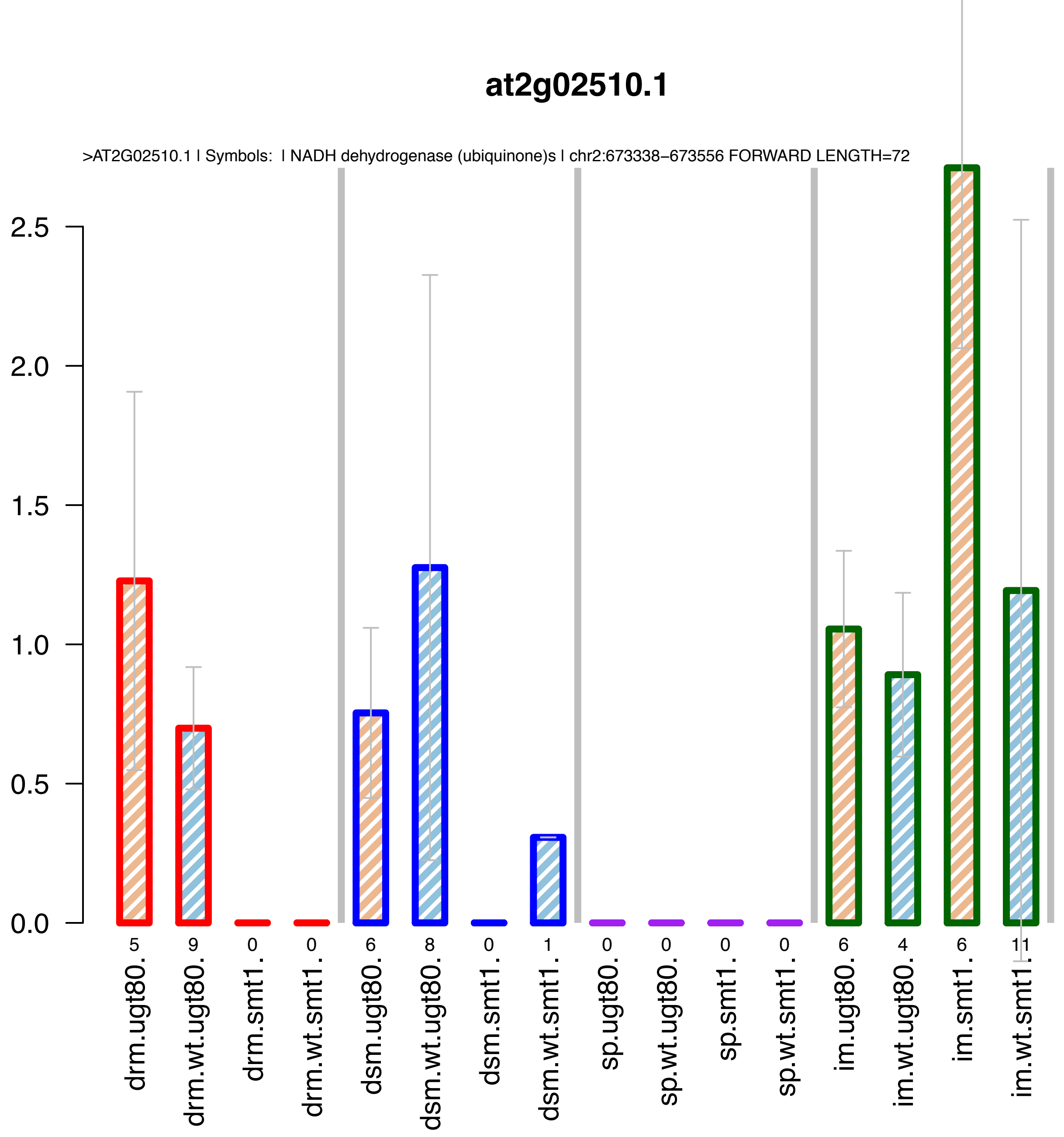
at2g02510.1
>AT2G02510.1 | Symbols: | NADH dehydrogenase (ubiquinone)s | chr2:673338-673556 FORWARD LENGTH=72
0.0
0.5
1.0
1.5
2.0
2.5
5
9
0
0
6
8
0
1
0
0
0
0
6
4
6
11
drm.ugt80.
drm.wt.ugt80.
drm.smt1.
drm.wt.smt1.
dsm.ugt80.
dsm.wt.ugt80.
dsm.smt1.
dsm.wt.smt1.
sp.ugt80.
sp.wt.ugt80.
sp.smt1.
sp.wt.smt1.
im.ugt80.
im.wt.ugt80.
im.smt1.
im.wt.smt1.

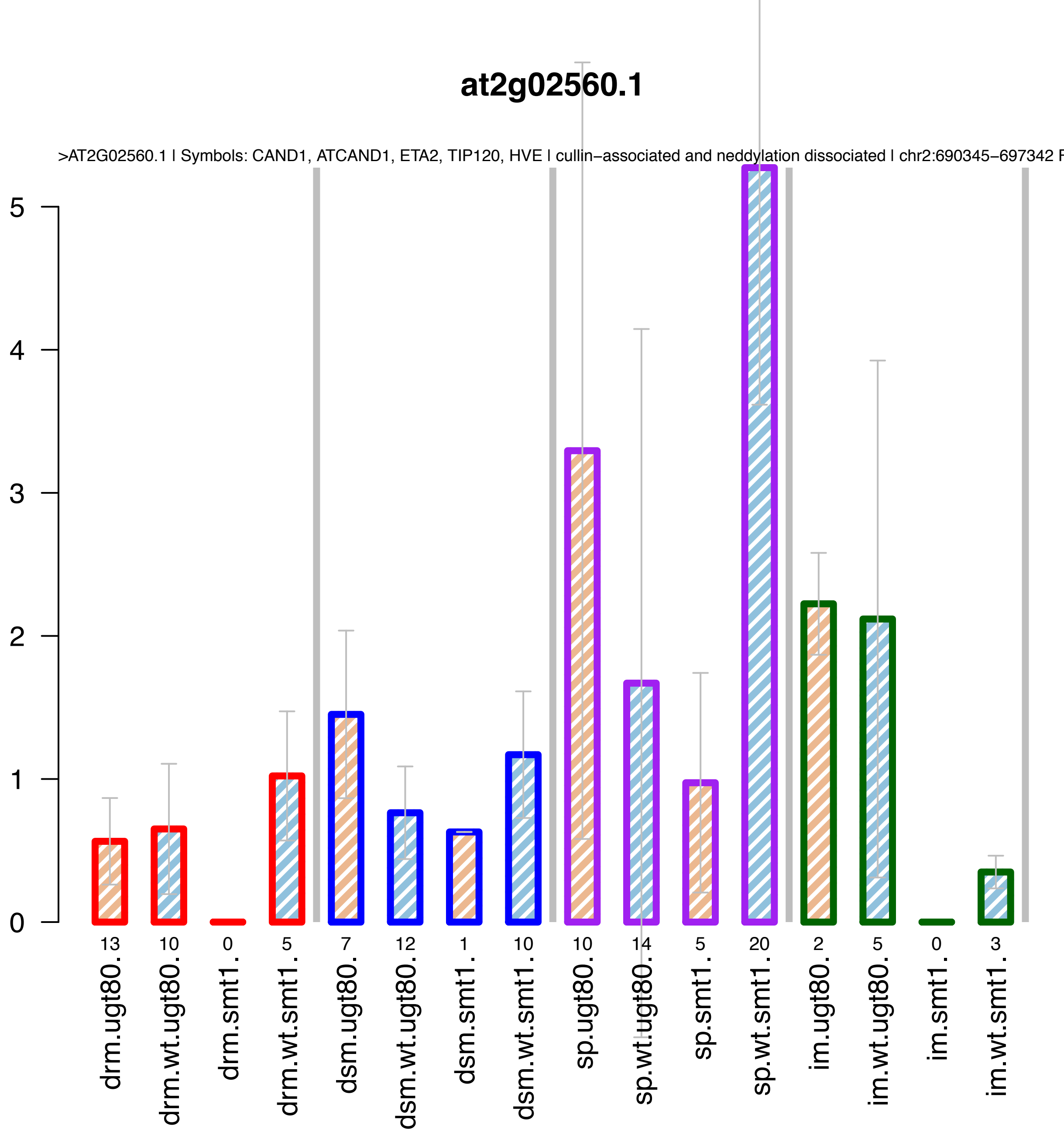

at2g02560.1
>AT2G02560.1 | Symbols: CAND1, ATCAND1, ETA2, TIP120, HVE | cullin−associated and neddylation dissociated | chr2:690345−697342 F
0
1
2
3
4
5
13
10
0
5
7
12
1
10
10
14
5
20
2
5
0
3
drm.ugt80.
drm.wt.ugt80.
drm.smt1.
drm.wt.smt1.
dsm.ugt80.
dsm.wt.ugt80.
dsm.smt1.
dsm.wt.smt1.
sp.ugt80.
sp.wt.ugt80.
sp.smt1.
sp.wt.smt1.
im.ugt80.
im.wt.ugt80.
im.smt1.
im.wt.smt1.

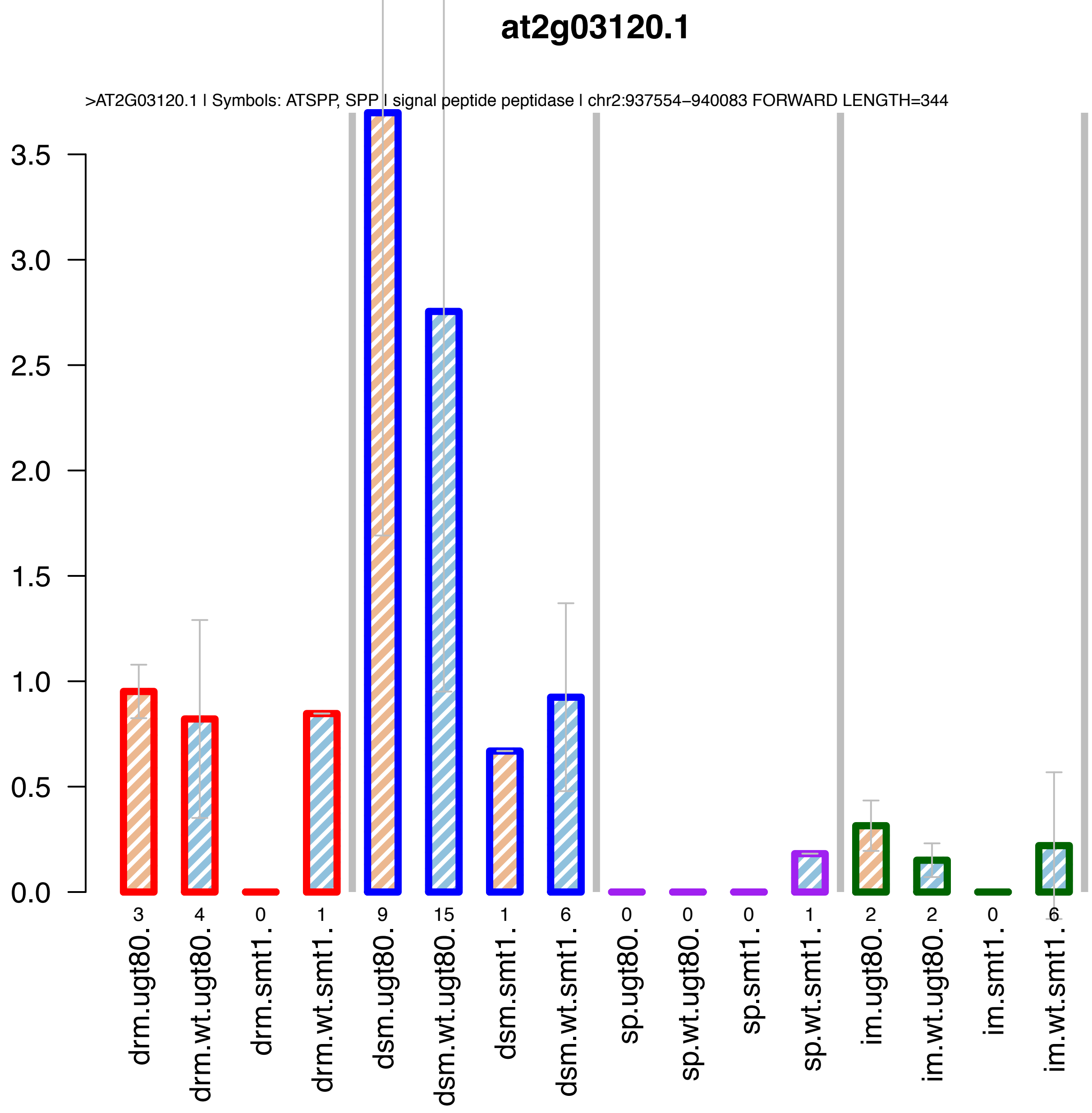

at2g03120.1
>AT2G03120.1 | Symbols: ATSPP, SPP | signal peptide peptidase | chr2:937554-940083 FORWARD LENGTH=344
0.0
0.5
1.0
1.5
2.0
2.5
3.0
3.5
3
4
0
1
9
15
1
6
0
0
0
1
2
2
0
6
drm.ugt80.
drm.wt.ugt80.
drm.smt1.
drm.wt.smt1.
dsm.ugt80.
dsm.wt.ugt80.
dsm.smt1.
dsm.wt.smt1.
sp.ugt80.
sp.wt.ugt80.
sp.smt1.
sp.wt.smt1.
im.ugt80.
im.wt.ugt80.
im.smt1.
im.wt.smt1.

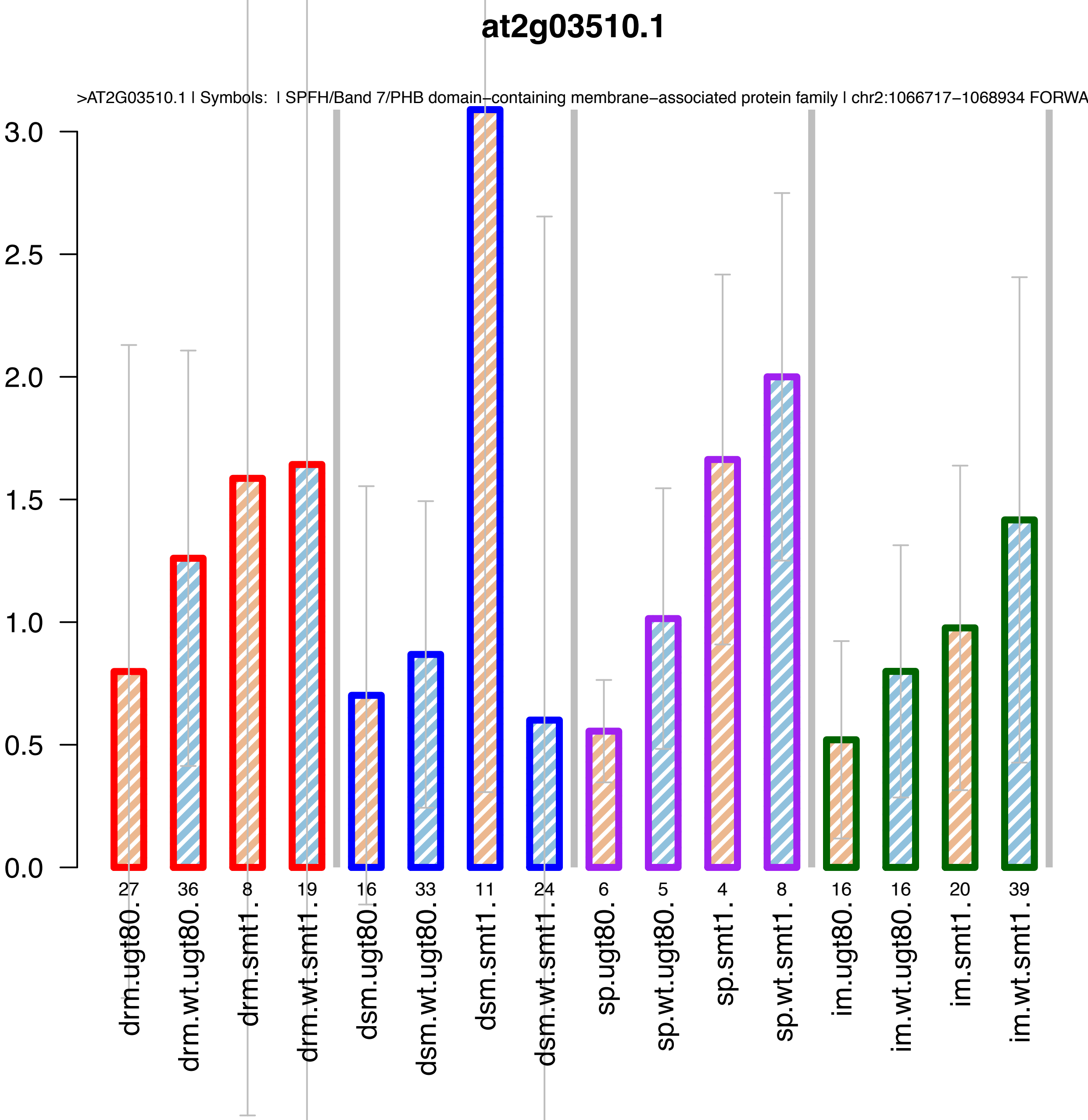

at2g03510.1
>AT2G03510.1 | Symbols: | SPFH/Band 7/PHB domain−containing membrane−associated protein family | chr2:1066717−1068934 FORWA
3.0
2.5
2.0
1.5
1.0
0.5
0.0
27
36
8
19
16
33
11
24
6
5
4
8
16
16
20
39
drm.ugt80.
drm.wt.ugt80.
drm.smt1.
drm.wt.smt1.
dsm.ugt80.
dsm.wt.ugt80.
dsm.smt1.
dsm.wt.smt1.
sp.ugt80.
sp.wt.ugt80.
sp.smt1.
sp.wt.smt1.
im.ugt80.
im.wt.ugt80.
im.smt1.
im.wt.smt1.

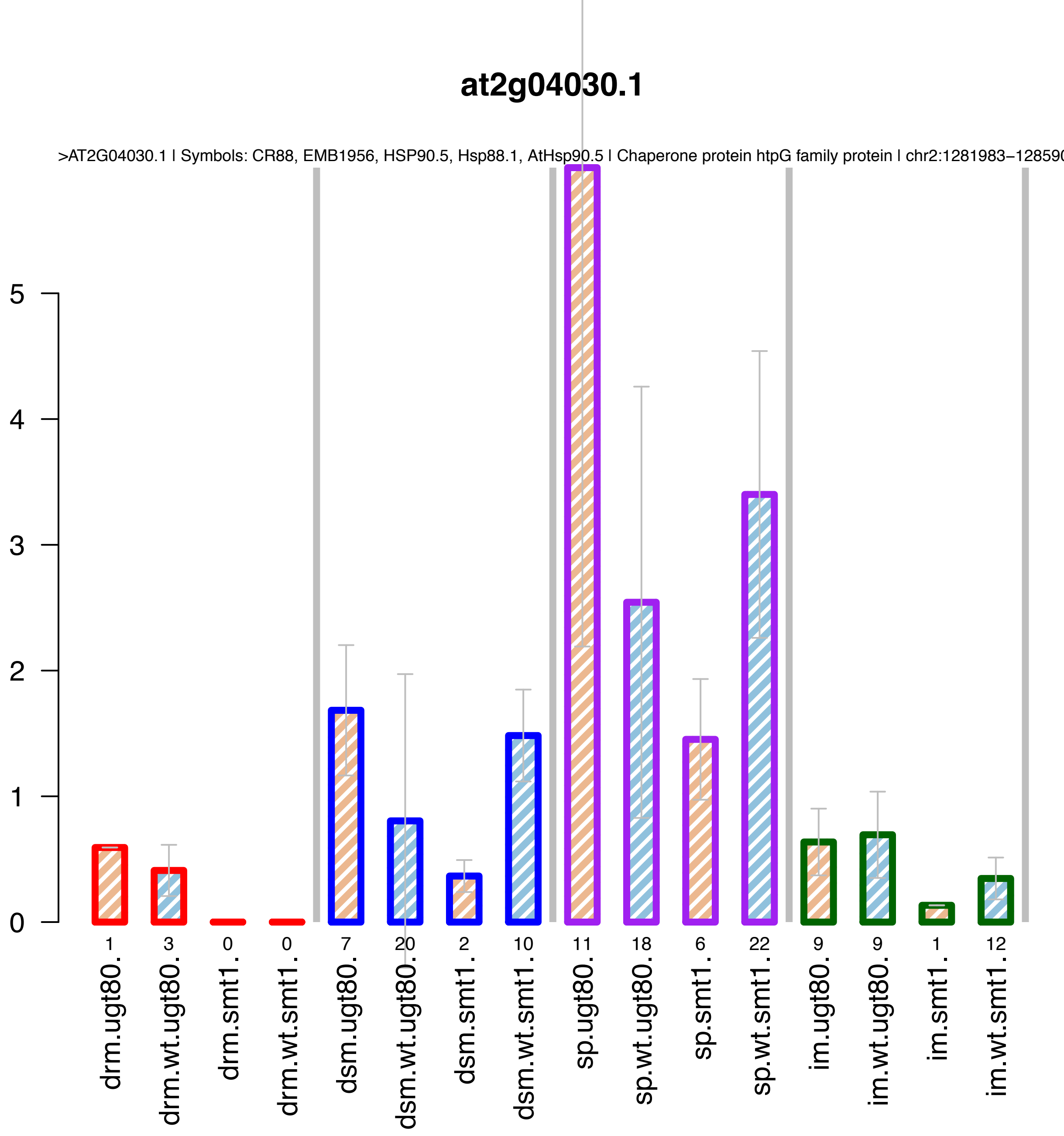
at2g04030.1
>AT2G04030.1 | Symbols: CR88, EMB1956, HSP90.5, Hsp88.1, AtHsp90.5 | Chaperone protein htpG family protein | chr2:1281983–128590
0
1
2
3
4
5
1
3
0
0
7
20
2
10
11
18
6
22
9
9
1
12
drm.ugt80.
drm.wt.ugt80.
drm.smt1.
drm.wt.smt1.
dsm.ugt80.
dsm.wt.ugt80.
dsm.smt1.
dsm.wt.smt1.
sp.ugt80.
sp.wt.ugt80.
sp.smt1.
sp.wt.smt1.
im.ugt80.
im.wt.ugt80.
im.smt1.
im.wt.smt1.

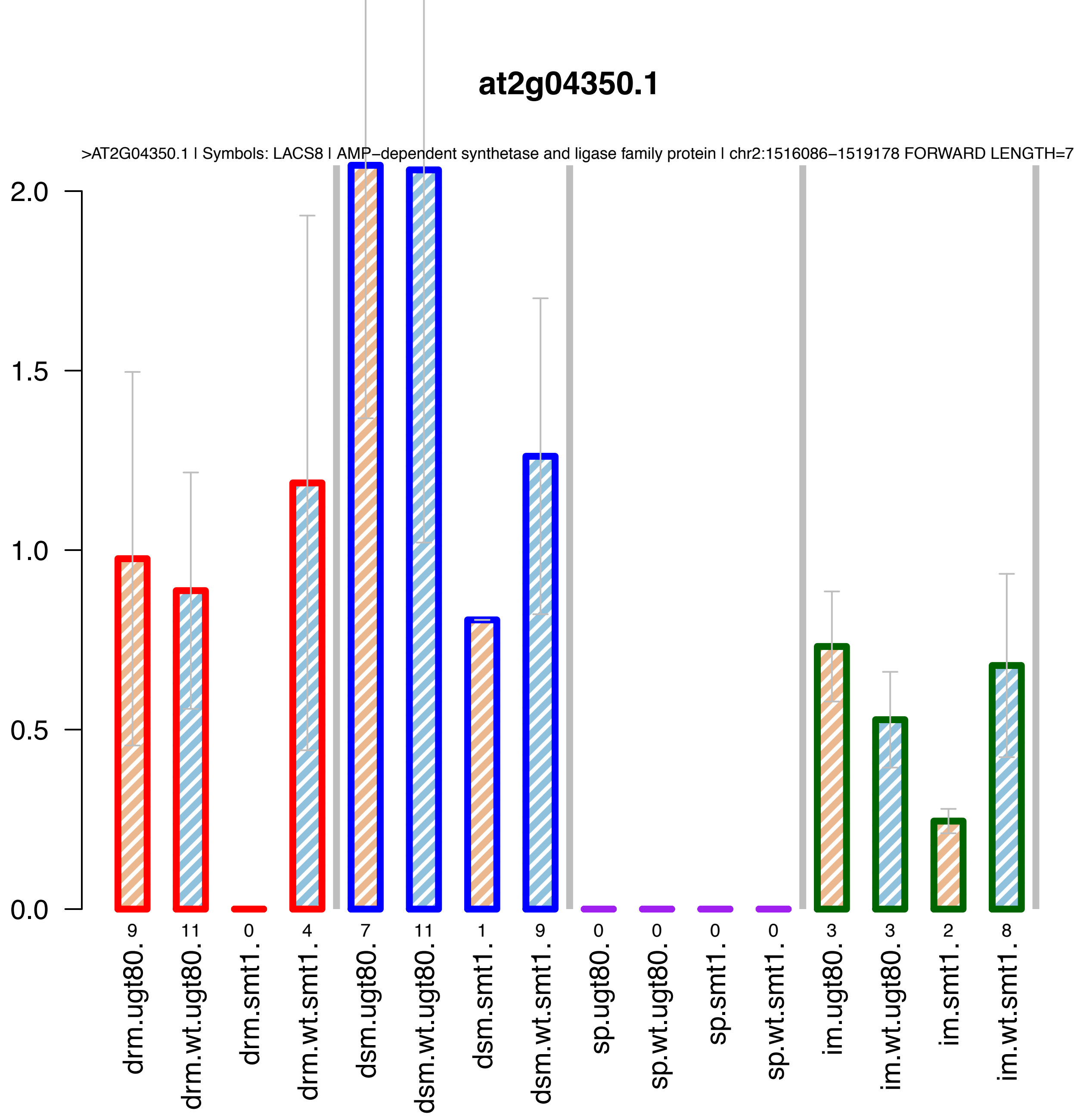

at2g04350.1
>AT2G04350.1 | Symbols: LACS8 | AMP−dependent synthetase and ligase family protein | chr2:1516086−1519178 FORWARD LENGTH=7
2.0
1.5
1.0
0.5
0.0
9
11
0
4
7
11
1
9
0
0
0
0
3
3
2
8
drm.ugt80.
drm.wt.ugt80.
drm.smt1.
drm.wt.smt1.
dsm.ugt80.
dsm.wt.ugt80.
dsm.smt1.
dsm.wt.smt1.
sp.ugt80.
sp.wt.ugt80.
sp.smt1.
sp.wt.smt1.
im.ugt80.
im.wt.ugt80.
im.smt1.
im.wt.smt1.

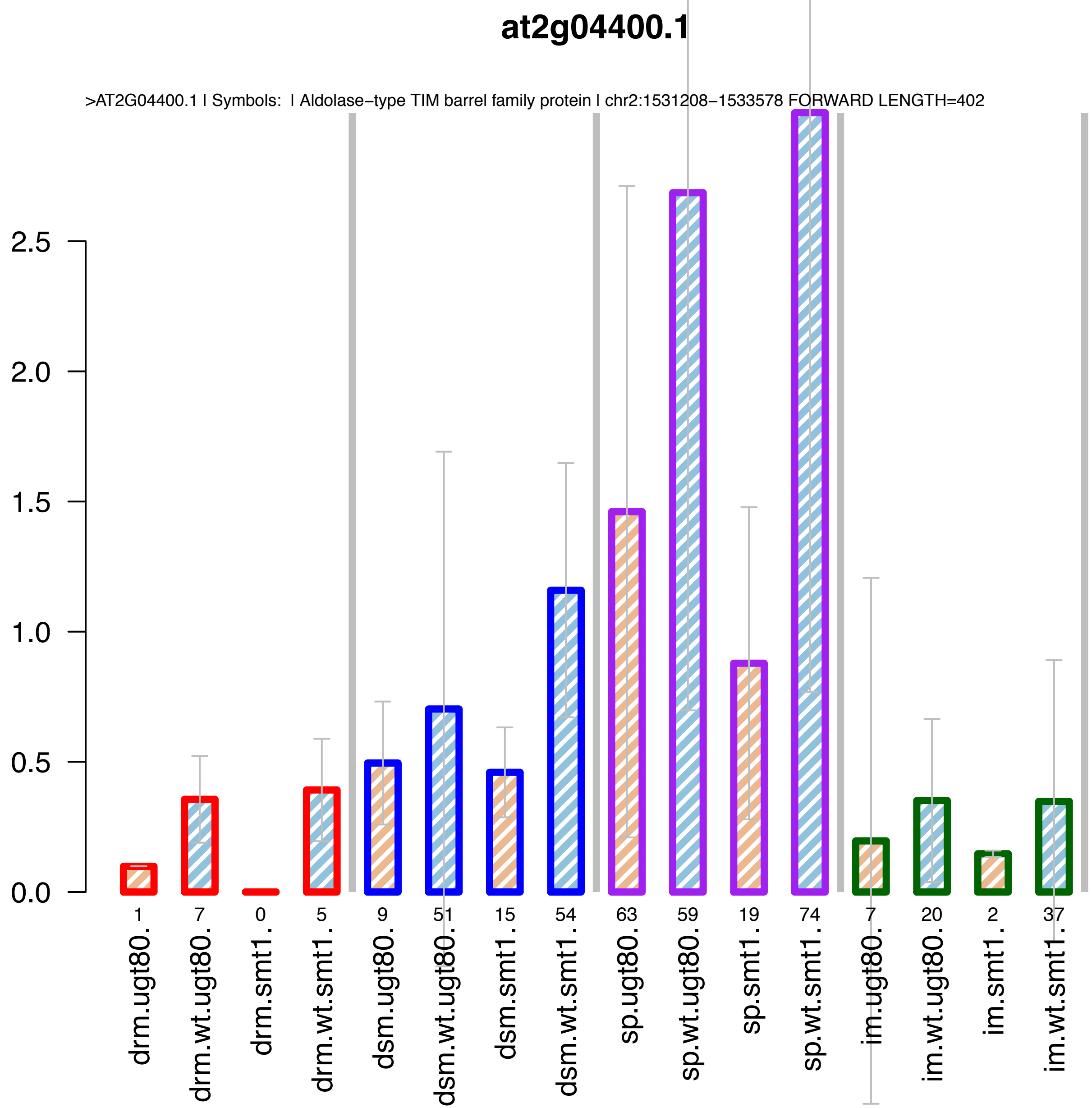

at2g04400.1
>AT2G04400.1 | Symbols: | Aldolase−type TIM barrel family protein | chr2:1531208−1533578 FORWARD LENGTH=402
0.0
0.5
1.0
1.5
2.0
2.5
1
7
0
5
9
51
15
54
63
59
19
74
7
20
2
37
drm.ugt80..
drm.wt.ugt80..
drm.smt1..
drm.wt.smt1..
dsm.ugt80..
dsm.wt.ugt80..
dsm.smt1..
dsm.wt.smt1..
sp.ugt80..
sp.wt.ugt80..
sp.smt1..
sp.wt.smt1..
im.ugt80..
im.wt.ugt80..
im.smt1..
im.wt.smt1..

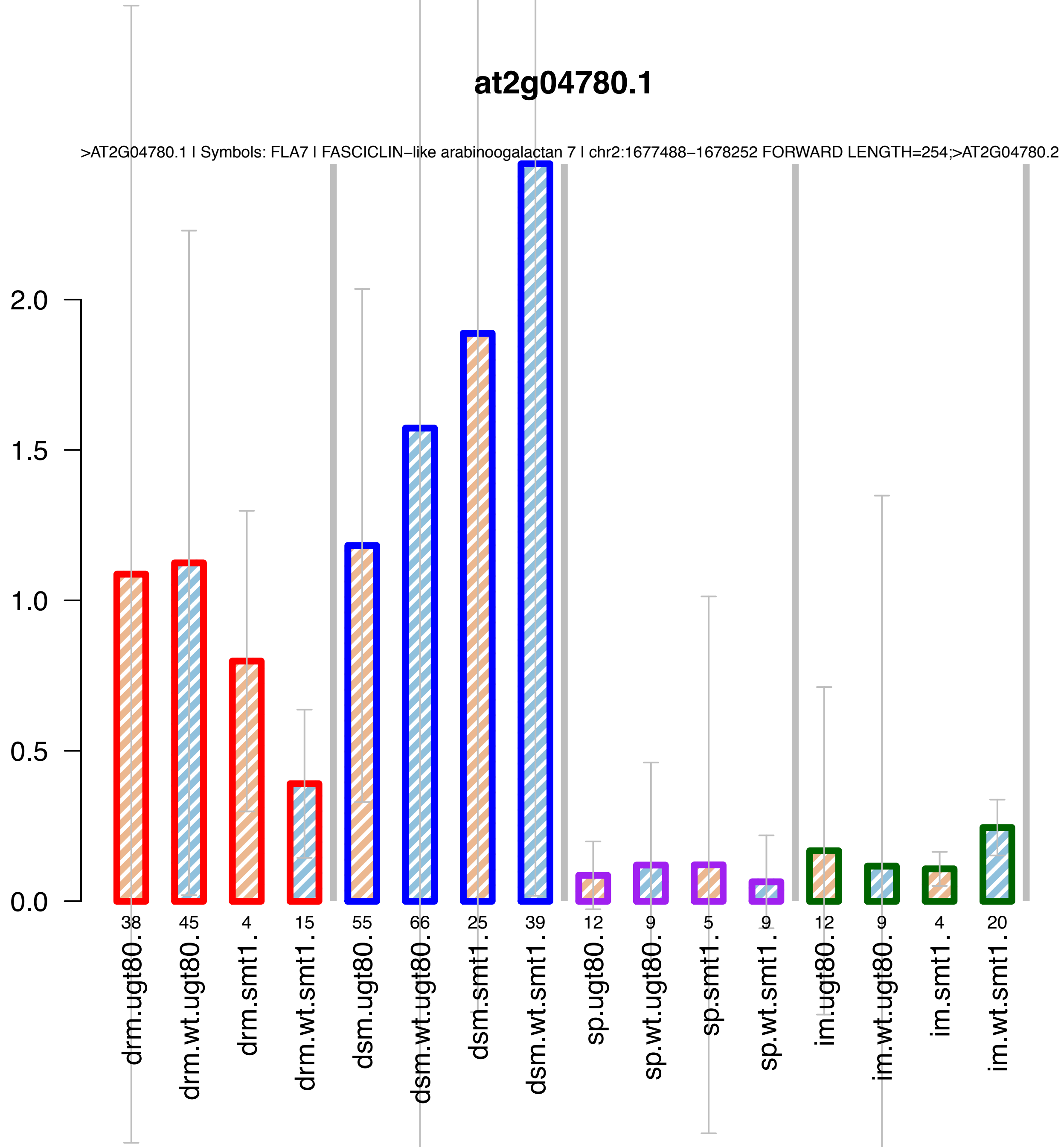

at2g04780.1
>AT2G04780.1 | Symbols: FLA7 | FASCICLIN−like arabinoogalactan 7 | chr2:1677488−1678252 FORWARD LENGTH=254;>AT2G04780.2
0.0
0.5
1.0
1.5
2.0
38
45
4
15
55
66
25
39
12
9
5
9
12
9
4
20
drm.ugt80..
drm.wt.ugt80..
drm.smt1..
drm.wt.smt1..
dsm.ugt80..
dsm.wt.ugt80..
dsm.smt1..
dsm.wt.smt1..
sp.ugt80..
sp.wt.ugt80..
sp.smt1..
sp.wt.smt1..
im.ugt80..
im.wt.ugt80..
im.smt1..
im.wt.smt1..

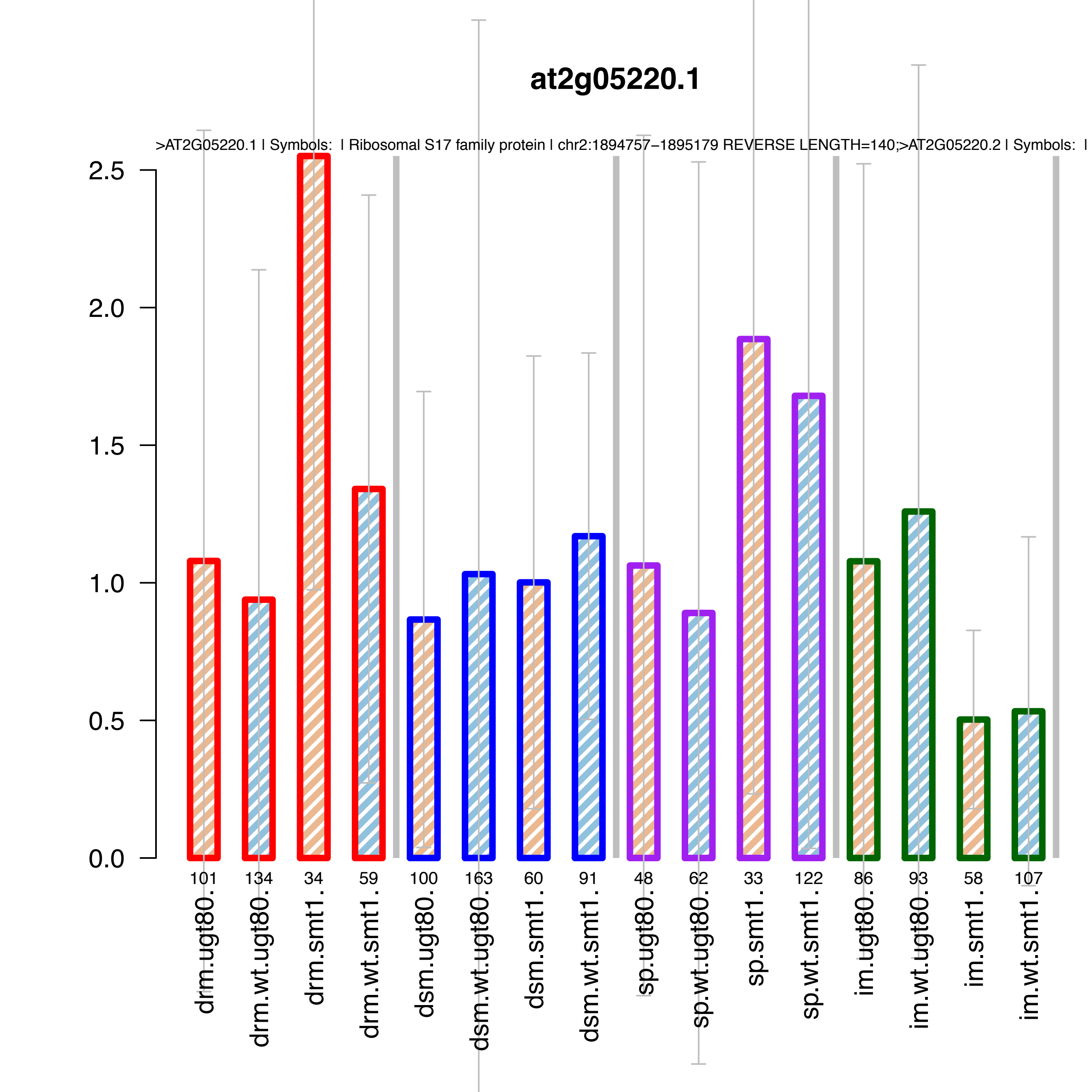

at2g05220.1
>AT2G05220.1 | Symbols: | Ribosomal S17 family protein | chr2:1894757-1895179 REVERSE LENGTH=140;>AT2G05220.2 | Symbols: |
2.5
2.0
1.5
1.0
0.5
0.0
101
134
34
59
100
163
60
91
48
62
33
122
86
93
58
107
drm.ugt80..
drm.wt.ugt80..
drm.smt1..
drm.wt.smt1..
dsm.ugt80..
dsm.wt.ugt80..
dsm.smt1..
dsm.wt.smt1..
sp.ugt80..
sp.wt.ugt80..
sp.smt1..
sp.wt.smt1..
im.ugt80..
im.wt.ugt80..
im.smt1..
im.wt.smt1..

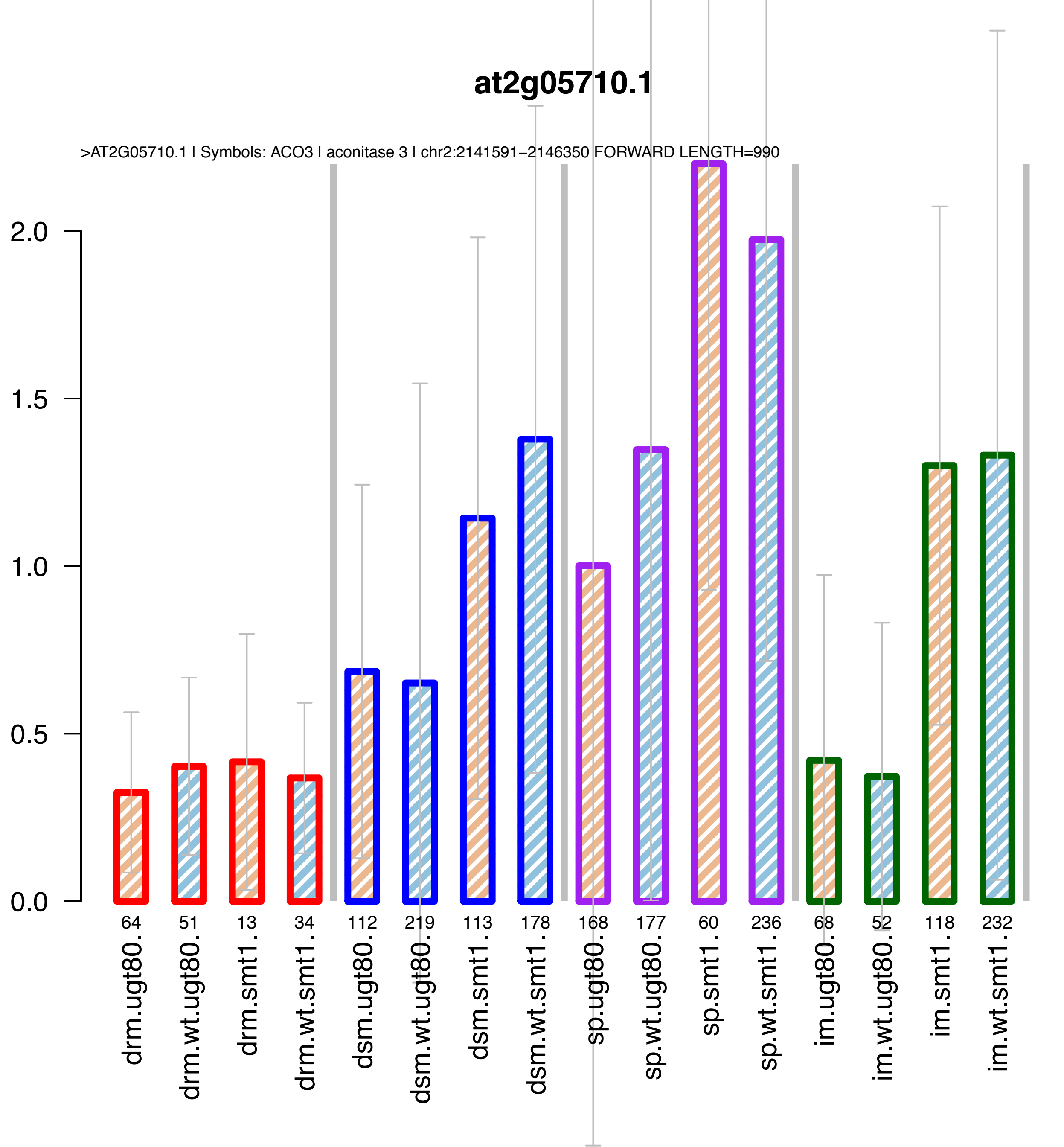

at2g05710.1
>AT2G05710.1 | Symbols: ACO3 | aconitase 3 | chr2:2141591−2146350 FORWARD LENGTH=990
2.0
1.5
1.0
0.5
0.0
64
51
13
34
112
219
113
178
168
177
60
236
68
52
118
232
drm.ugt80..
drm.wt.ugt80..
drm.smt1..
drm.wt.smt1..
dsm.ugt80..
dsm.wt.ugt80..
dsm.smt1..
dsm.wt.smt1..
sp.ugt80..
sp.wt.ugt80..
sp.smt1..
sp.wt.smt1..
im.ugt80..
im.wt.ugt80..
im.smt1..
im.wt.smt1..

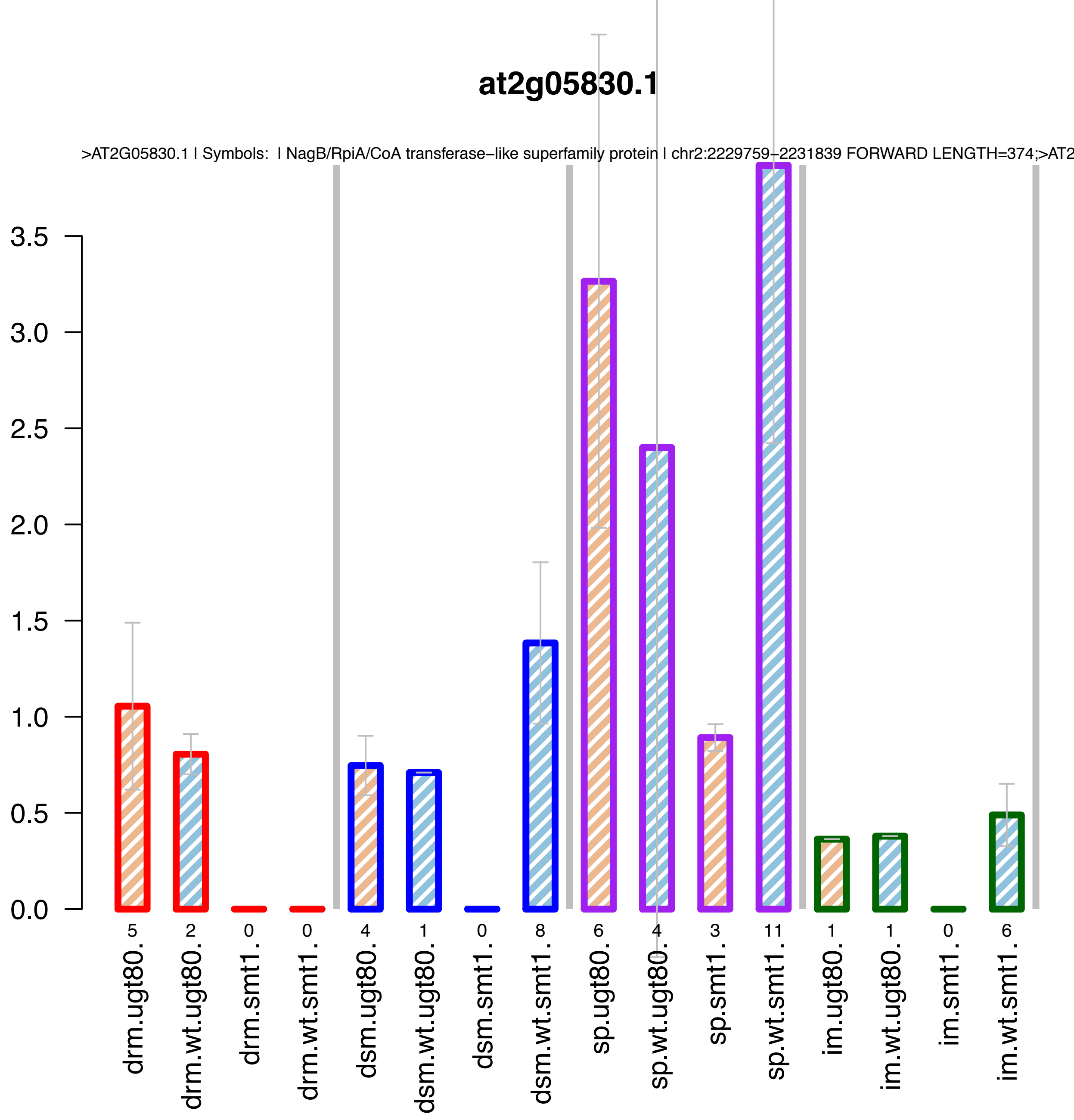

at2g05830.1
>AT2G05830.1 | Symbols: | NagB/RpiA/CoA transferase−like superfamily protein | chr2:2229759−2231839 FORWARD LENGTH=374;>AT2
0.0
0.5
1.0
1.5
2.0
2.5
3.0
3.5
5
2
0
0
4
1
0
8
6
4
3
11
1
1
0
6
drm.ugt80.
drm.wt.ugt80.
drm.smt1.
drm.wt.smt1.
dsm.ugt80.
dsm.wt.ugt80.
dsm.smt1.
dsm.wt.smt1.
sp.ugt80.
sp.wt.ugt80.
sp.smt1.
sp.wt.smt1.
im.ugt80.
im.wt.ugt80.
im.smt1.
im.wt.smt1.

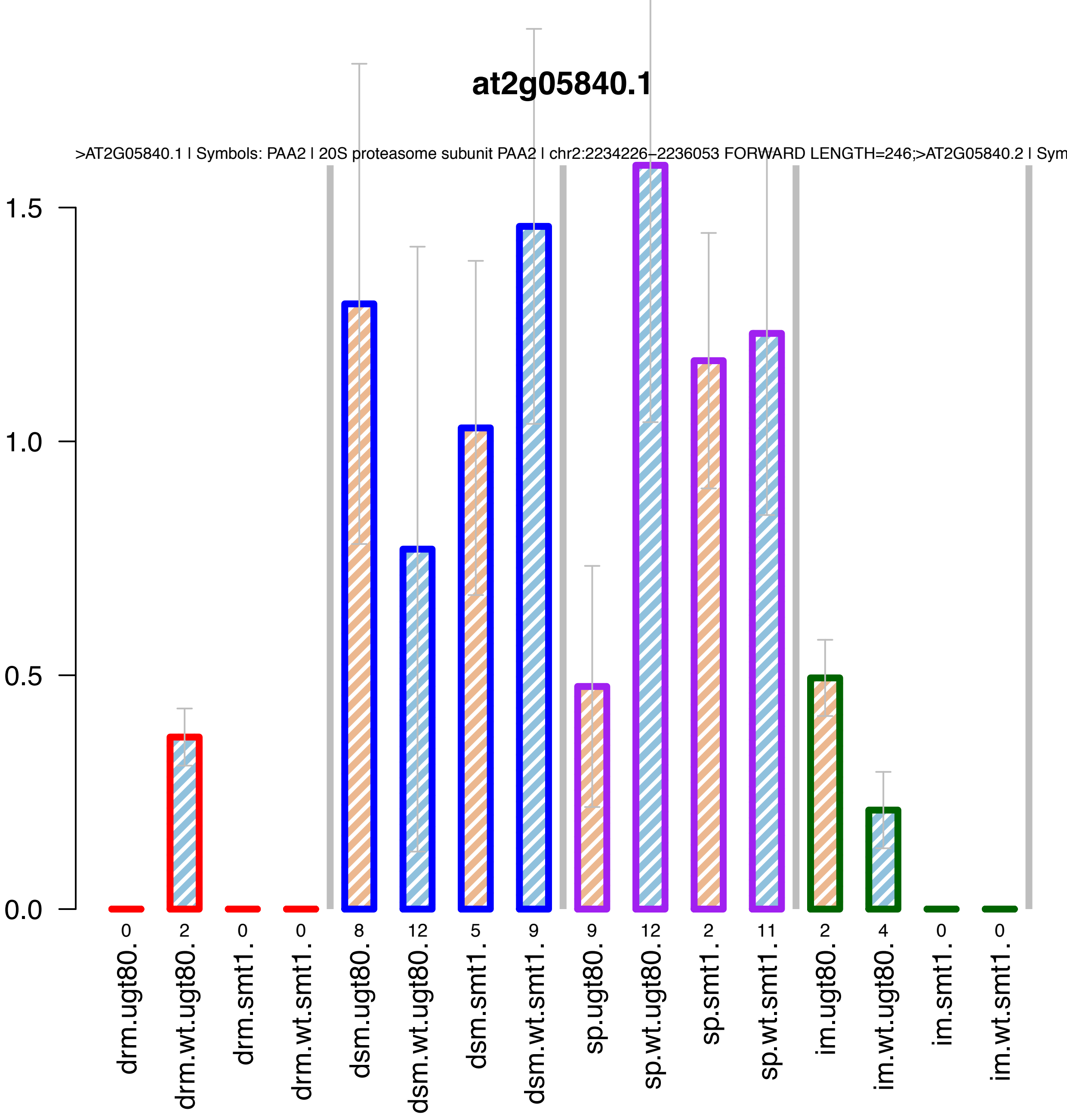
at2g05840.1
>AT2G05840.1 | Symbols: PAA2 | 20S proteasome subunit PAA2 | chr2:2234226-2236053 FORWARD LENGTH=246;>AT2G05840.2 | Sym
1.5
1.0
0.5
0.0
0
2
0
0
8
12
5
9
9
12
2
11
2
4
0
0
drm.ugt80.
drm.wt.ugt80.
drm.smt1.
drm.wt.smt1.
dsm.ugt80.
dsm.wt.ugt80.
dsm.smt1.
dsm.wt.smt1.
sp.ugt80.
sp.wt.ugt80.
sp.smt1.
sp.wt.smt1.
im.ugt80.
im.wt.ugt80.
im.smt1.
im.wt.smt1.

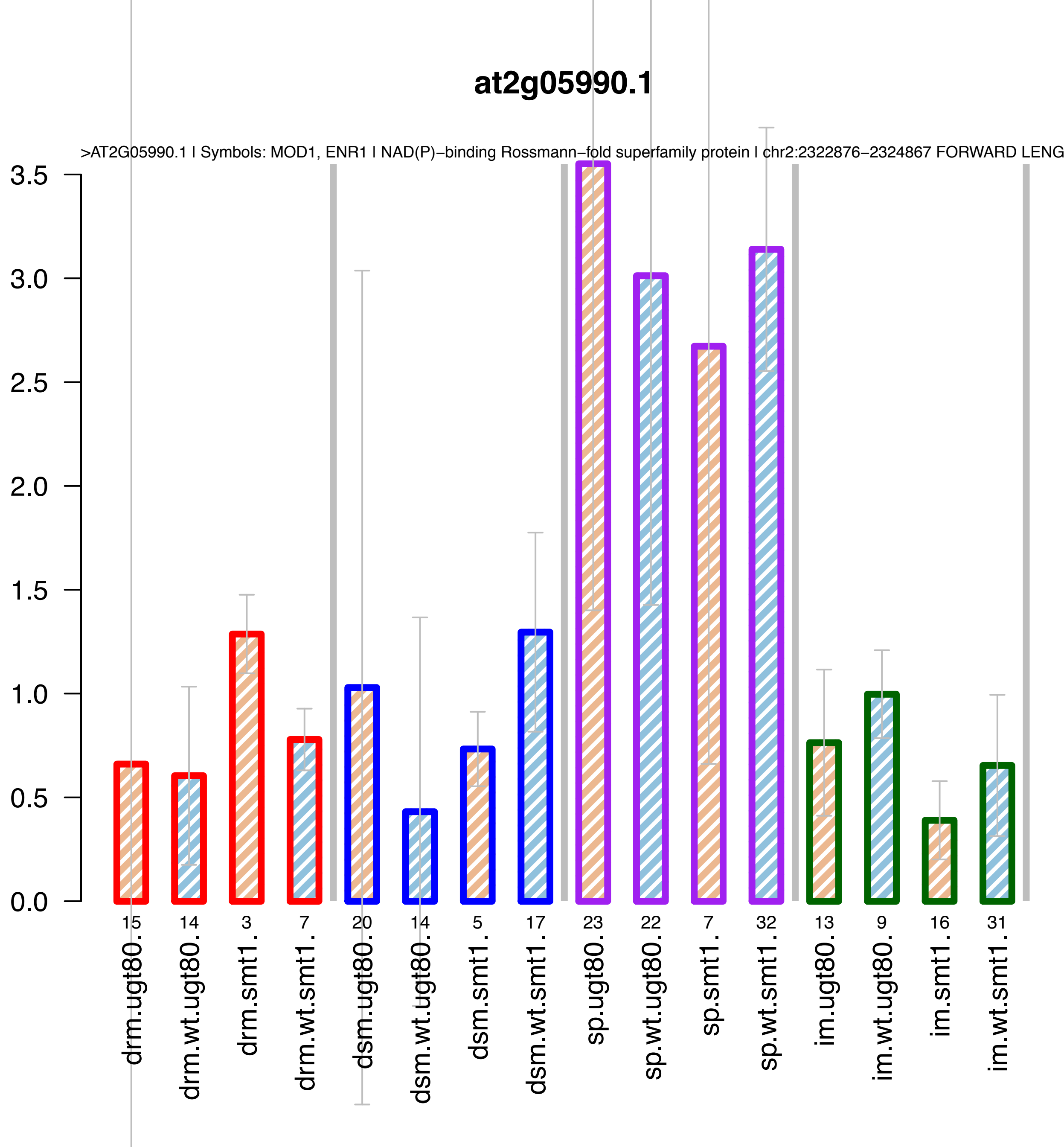
at2g05990.1
>AT2G05990.1 | Symbols: MOD1, ENR1 | NAD(P)–binding Rossmann–fold superfamily protein | chr2:2322876–2324867 FORWARD LENG
3.5
3.0
2.5
2.0
1.5
1.0
0.5
0.0
15
14
3
7
20
14
5
17
23
22
7
32
13
9
16
31
drm.ugt80..
drm.wt.ugt80..
drm.smt1..
drm.wt.smt1..
dsm.ugt80..
dsm.wt.ugt80..
dsm.smt1..
dsm.wt.smt1..
sp.ugt80..
sp.wt.ugt80..
sp.smt1..
sp.wt.smt1..
im.ugt80..
im.wt.ugt80..
im.smt1..
im.wt.smt1..

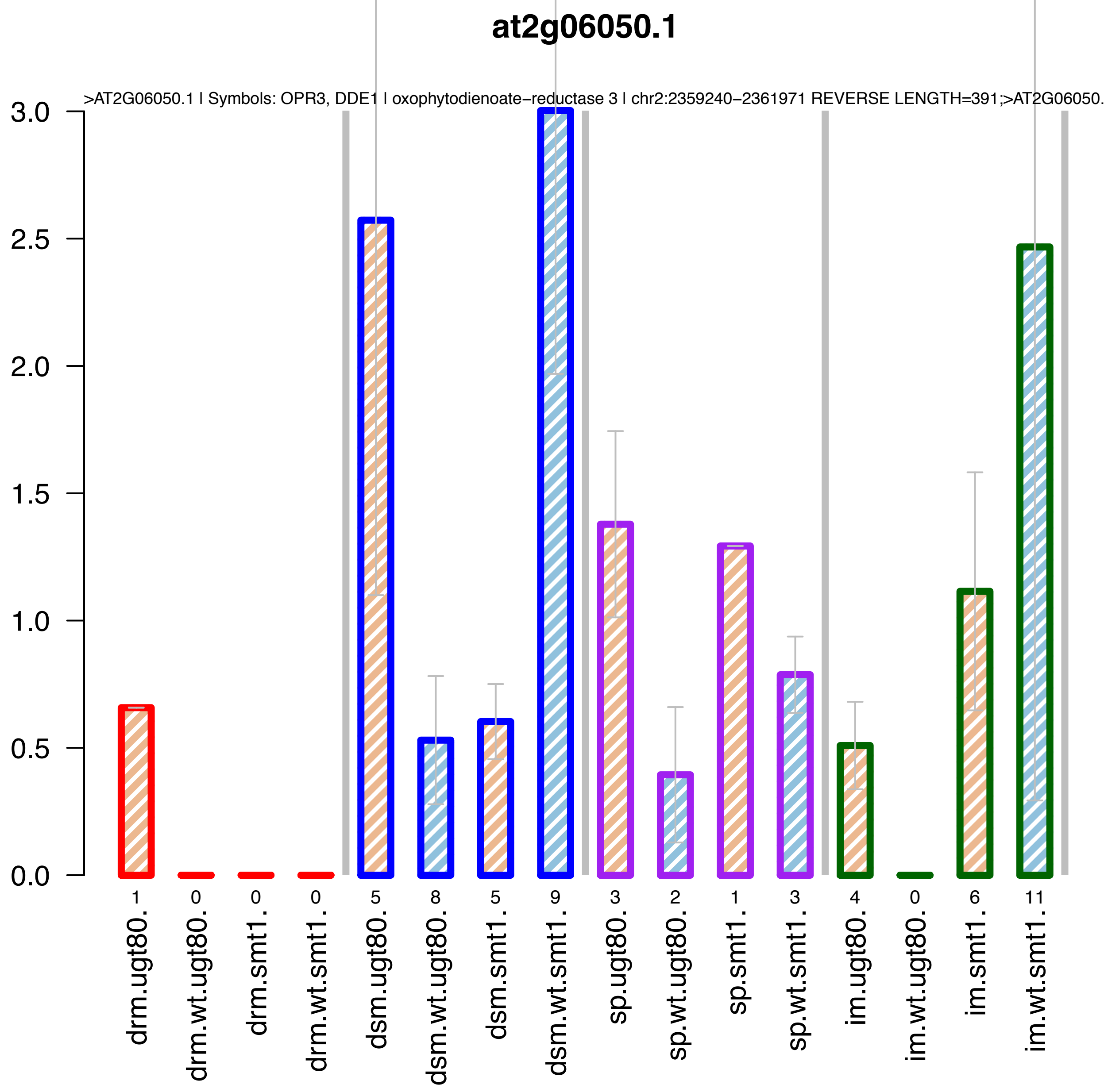
at2g06050.1
>AT2G06050.1 | Symbols: OPR3, DDE1 | oxophytodienoate−reductase 3 | chr2:2359240−2361971 REVERSE LENGTH=391;>AT2G06050.
3.0
2.5
2.0
1.5
1.0
0.5
0.0
1
0
0
0
5
8
5
9
3
2
1
3
4
0
6
11
drm.ugt80.
drm.wt.ugt80.
drm.smt1.
drm.wt.smt1.
dsm.ugt80.
dsm.wt.ugt80.
dsm.smt1.
dsm.wt.smt1.
sp.ugt80.
sp.wt.ugt80.
sp.smt1.
sp.wt.smt1.
im.ugt80.
im.wt.ugt80.
im.smt1.
im.wt.smt1.

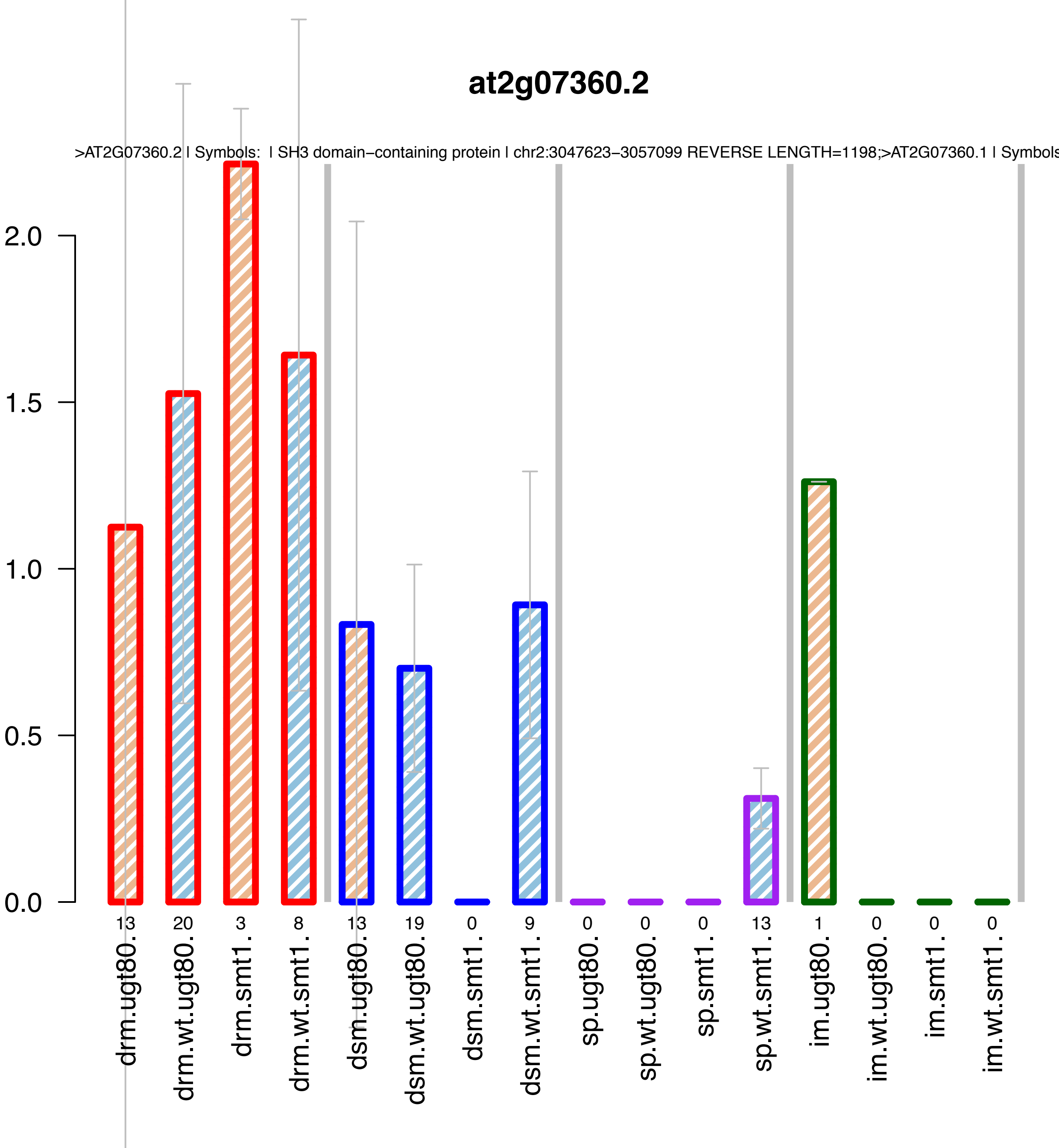

at2g07360.2
>AT2G07360.2 | Symbols: | SH3 domain−containing protein | chr2:3047623−3057099 REVERSE LENGTH=1198;>AT2G07360.1 | Symbols
2.0
1.5
1.0
0.5
0.0
13
20
3
8
13
19
0
9
0
0
0
13
1
0
0
0
drm.ugt80..
drm.wt.ugt80..
drm.smt1..
drm.wt.smt1..
dsm.ugt80..
dsm.wt.ugt80..
dsm.smt1..
dsm.wt.smt1..
sp.ugt80..
sp.wt.ugt80..
sp.smt1..
sp.wt.smt1..
im.ugt80..
im.wt.ugt80..
im.smt1..
im.wt.smt1..

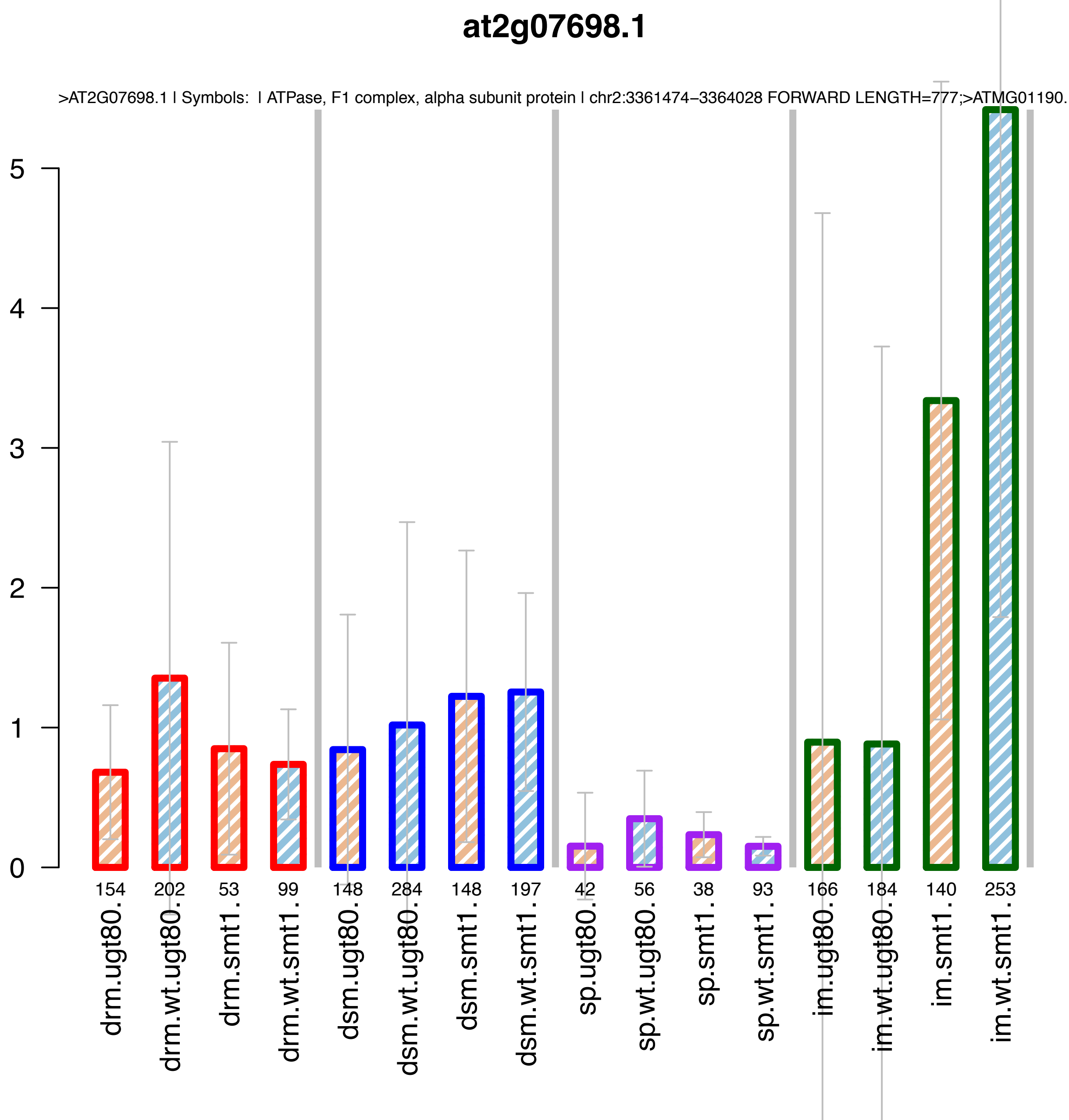

at2g07698.1
>AT2G07698.1 | Symbols: | ATPase, F1 complex, alpha subunit protein | chr2:3361474-3364028 FORWARD LENGTH=777;>ATMG01190.
0
1
2
3
4
5
154
202
53
99
148
284
148
197
42
56
38
93
166
184
140
253
drm.ugt80..
drm.wt.ugt80..
drm.smt1..
drm.wt.smt1..
dsm.ugt80..
dsm.wt.ugt80..
dsm.smt1..
dsm.wt.smt1..
sp.ugt80..
sp.wt.ugt80..
sp.smt1..
sp.wt.smt1..
im.ugt80..
im.wt.ugt80..
im.smt1..
im.wt.smt1..

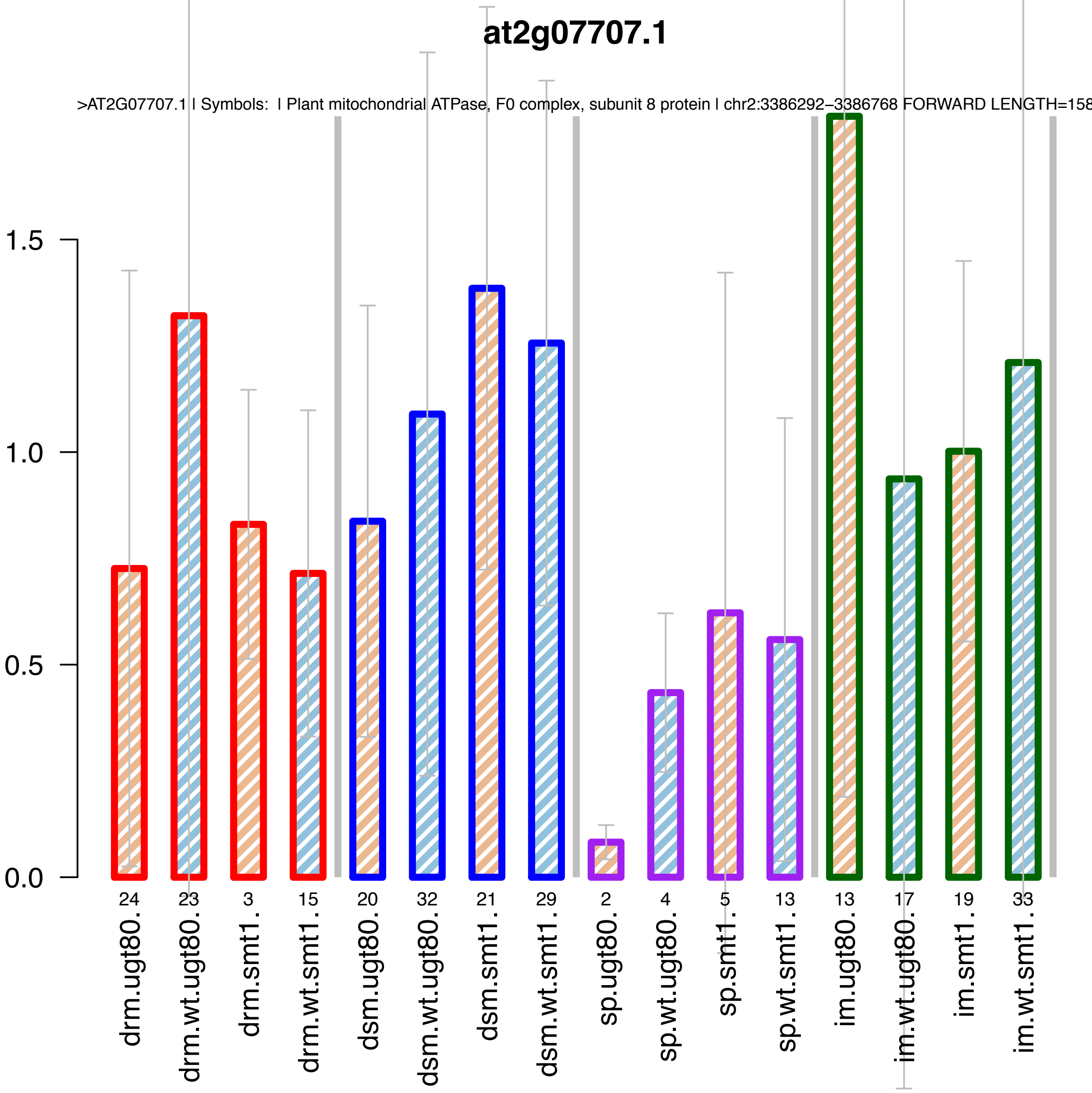

at2g07707.1
>AT2G07707.1 | Symbols: | Plant mitochondrial ATPase, F0 complex, subunit 8 protein | chr2:3386292–3386768 FORWARD LENGTH=158
1.5
1.0
0.5
0.0
24
23
3
15
20
32
21
29
2
4
5
13
13
17
19
33
drm.ugt80.
drm.wt.ugt80.
drm.smt1.
drm.wt.smt1.
dsm.ugt80.
dsm.wt.ugt80.
dsm.smt1.
dsm.wt.smt1.
sp.ugt80.
sp.wt.ugt80.
sp.smt1.
sp.wt.smt1.
im.ugt80.
im.wt.ugt80.
im.smt1.
im.wt.smt1.

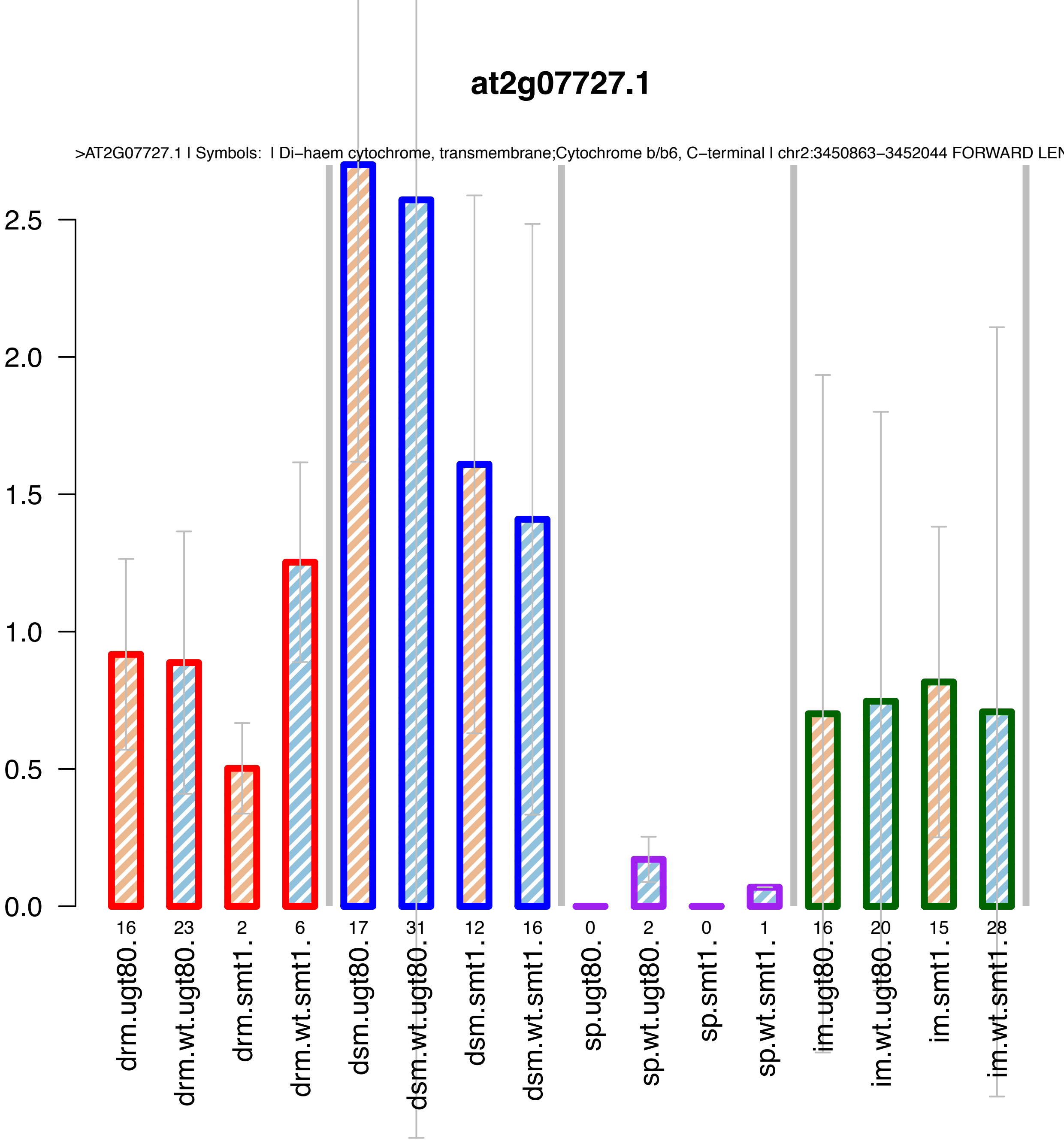

at2g07727.1
>AT2G07727.1 | Symbols: | Di–haem cytochrome, transmembrane;Cytochrome b/b6, C–terminal | chr2:3450863–3452044 FORWARD LEN
0.0
0.5
1.0
1.5
2.0
2.5
16
23
2
6
17
31
12
16
0
2
0
1
16
20
15
28
drm.ugt80..
drm.wt.ugt80..
drm.smt1..
drm.wt.smt1..
dsm.ugt80..
dsm.wt.ugt80..
dsm.smt1..
dsm.wt.smt1..
sp.ugt80..
sp.wt.ugt80..
sp.smt1..
sp.wt.smt1..
im.ugt80..
im.wt.ugt80..
im.smt1..
im.wt.smt1..

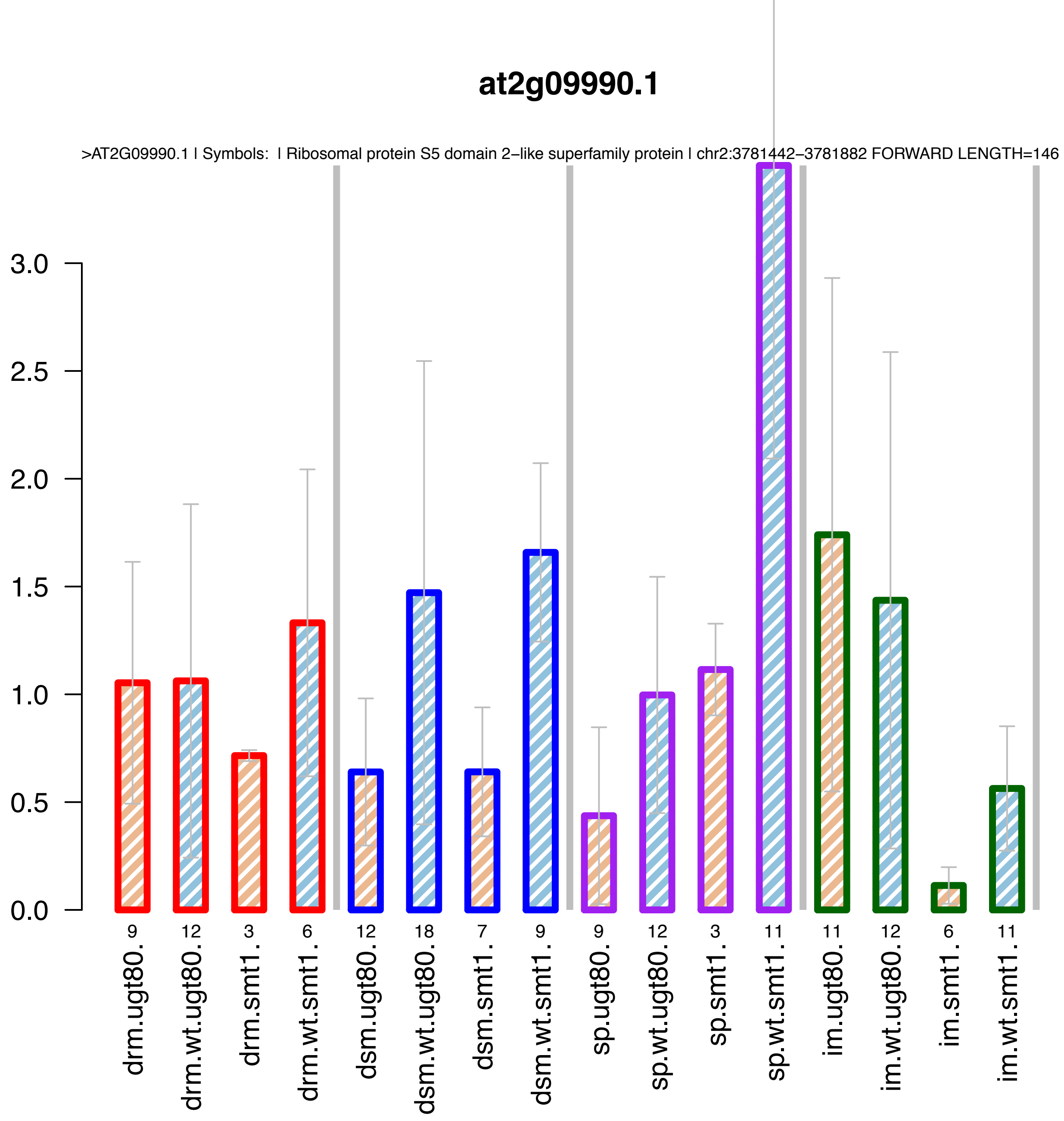

at2g09990.1
>AT2G09990.1 | Symbols: | Ribosomal protein S5 domain 2-like superfamily protein | chr2:3781442-3781882 FORWARD LENGTH=146
0.0
0.5
1.0
1.5
2.0
2.5
3.0
9
12
3
6
12
18
7
9
9
12
3
11
11
12
6
11
drm.ugt80.
drm.wt.ugt80.
drm.smt1.
drm.wt.smt1.
dsm.ugt80.
dsm.wt.ugt80.
dsm.smt1.
dsm.wt.smt1.
sp.ugt80.
sp.wt.ugt80.
sp.smt1.
sp.wt.smt1.
im.ugt80.
im.wt.ugt80.
im.smt1.
im.wt.smt1.

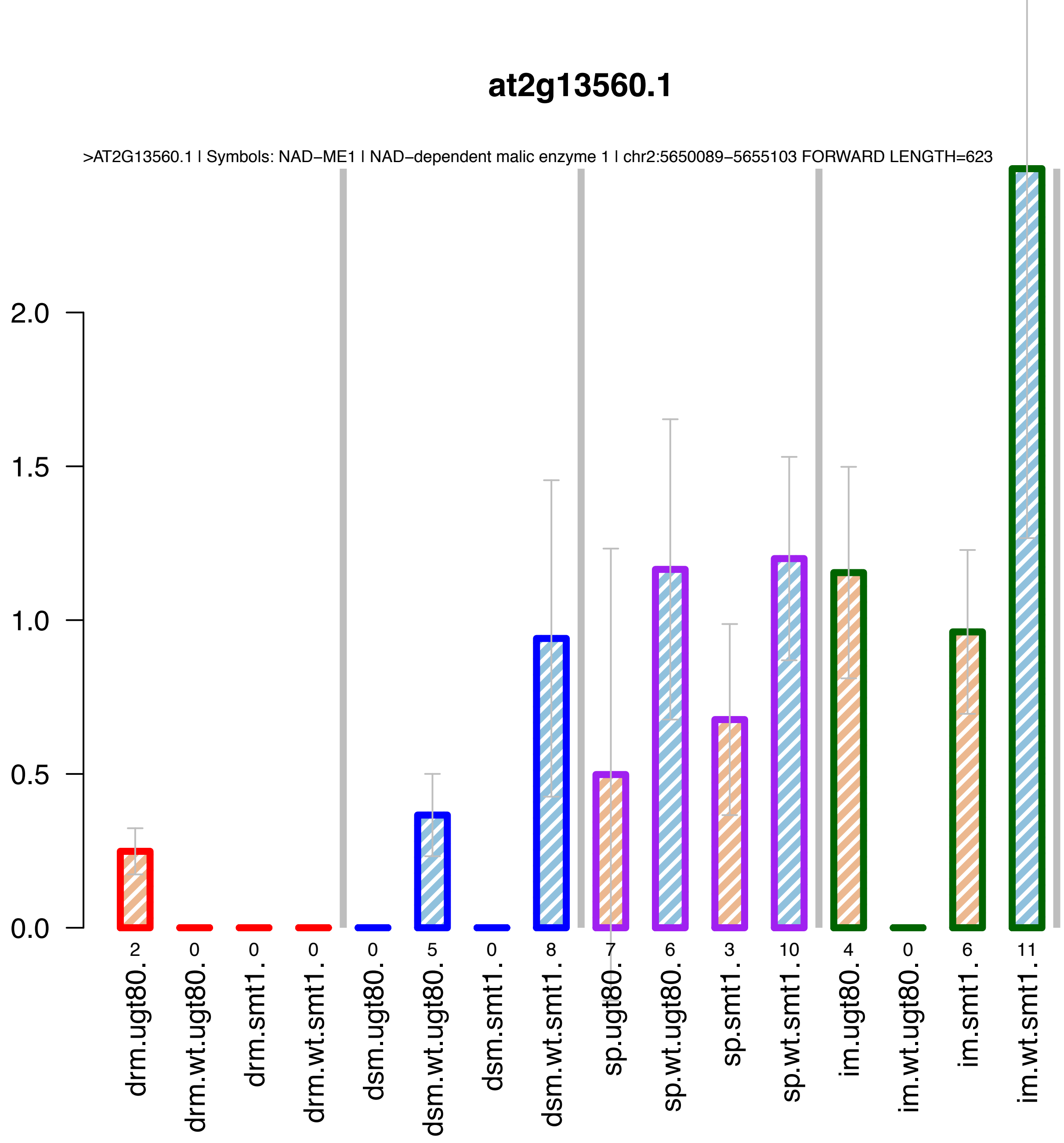

at2g13560.1
>AT2G13560.1 | Symbols: NAD−ME1 | NAD−dependent malic enzyme 1 | chr2:5650089−5655103 FORWARD LENGTH=623
0.0
0.5
1.0
1.5
2.0
2
0
0
0
0
5
0
8
7
6
3
10
4
0
6
11
drm.ugt80.
drm.wt.ugt80.
drm.smt1.
drm.wt.smt1.
dsm.ugt80.
dsm.wt.ugt80.
dsm.smt1.
dsm.wt.smt1.
sp.ugt80.
sp.wt.ugt80.
sp.smt1.
sp.wt.smt1.
im.ugt80.
im.wt.ugt80.
im.smt1.
im.wt.smt1.

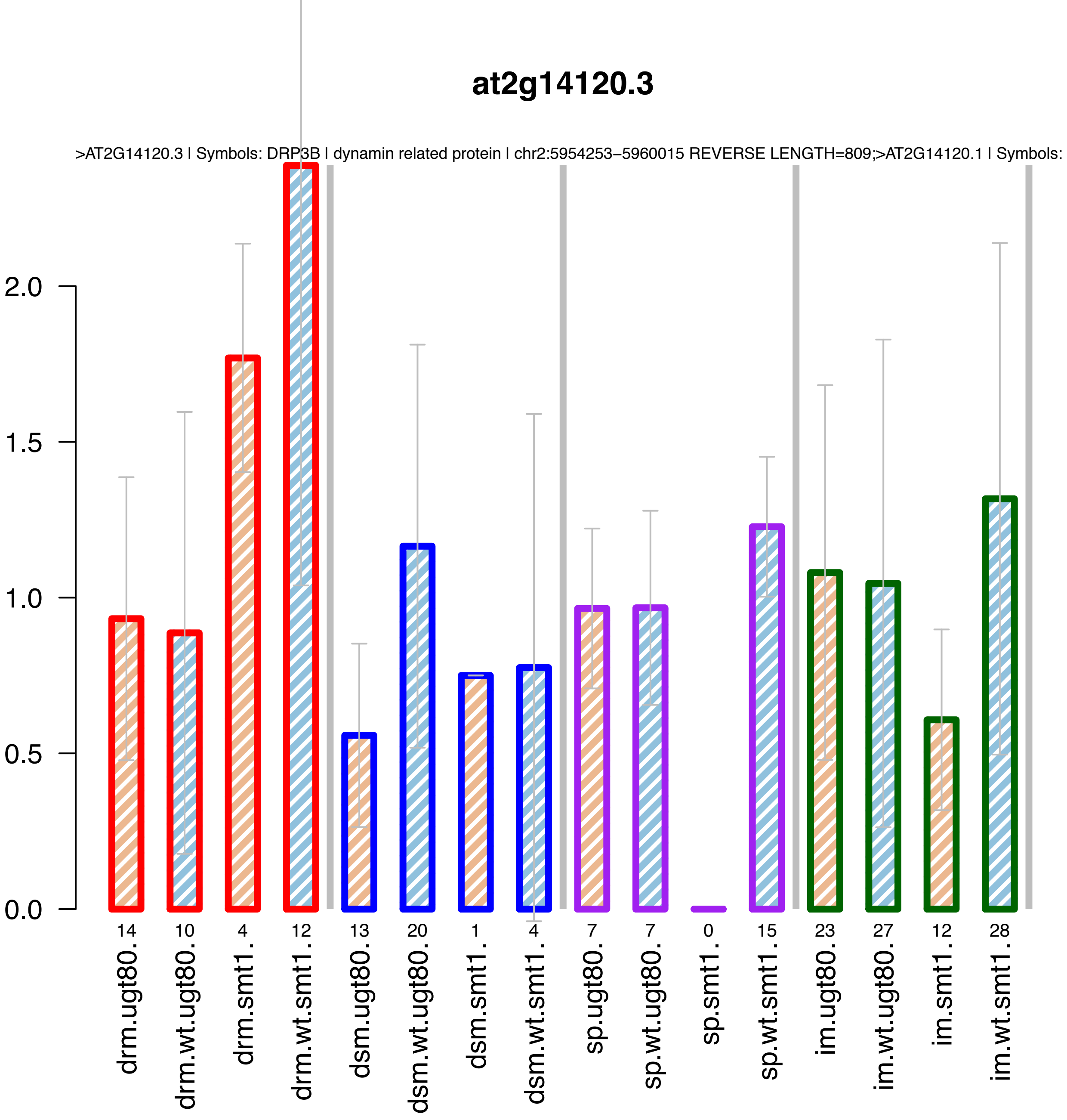

at2g14120.3
>AT2G14120.3 | Symbols: DRP3B | dynamin related protein | chr2:5954253-5960015 REVERSE LENGTH=809;>AT2G14120.1 | Symbols:
0.0
0.5
1.0
1.5
2.0
14
10
4
12
13
20
1
4
7
7
0
15
23
27
12
28
drm.ugt80..
drm.wt.ugt80..
drm.smt1..
drm.wt.smt1..
dsm.ugt80..
dsm.wt.ugt80..
dsm.smt1..
dsm.wt.smt1..
sp.ugt80..
sp.wt.ugt80..
sp.smt1..
sp.wt.smt1..
im.ugt80..
im.wt.ugt80..
im.smt1..
im.wt.smt1..

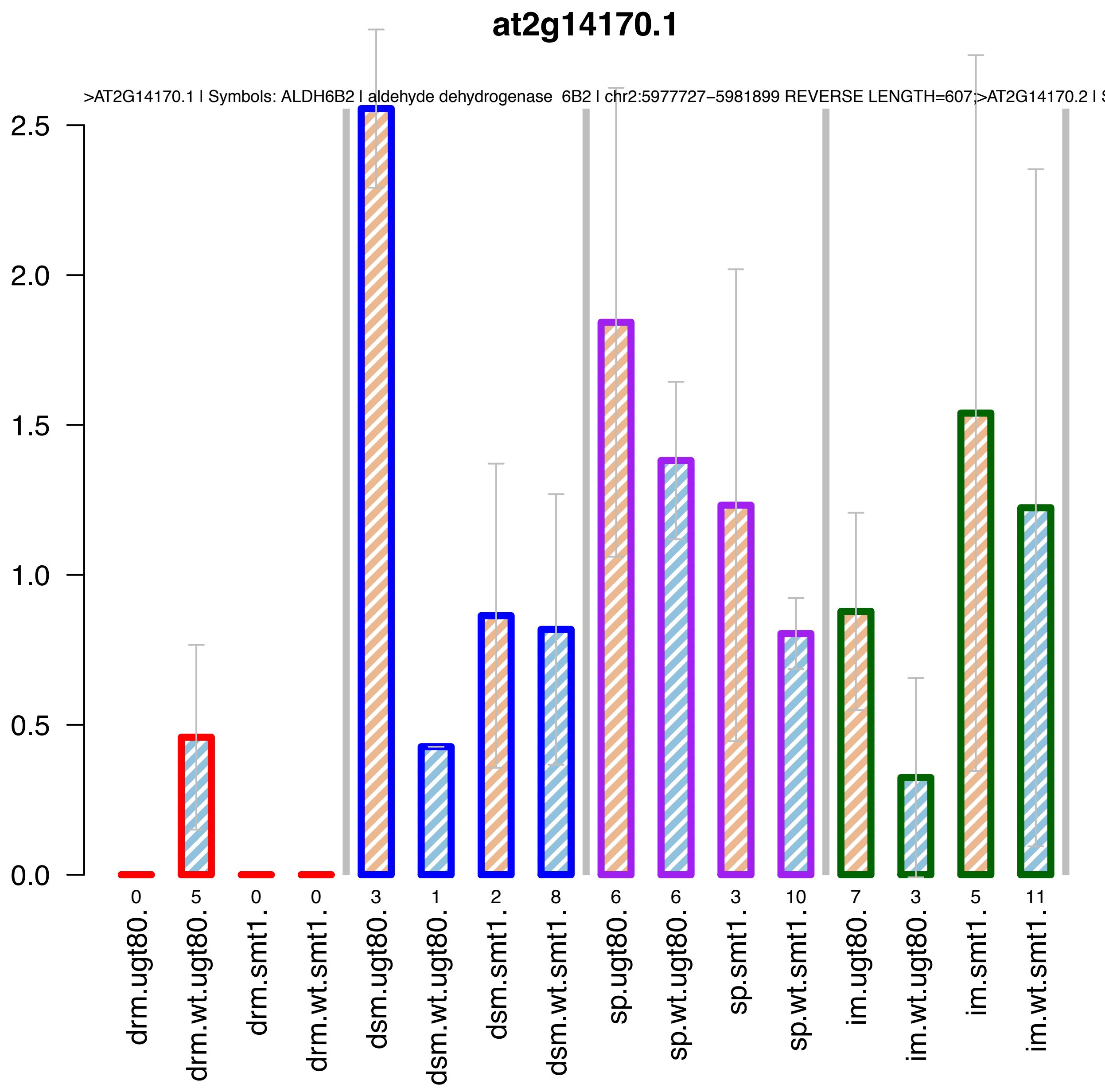
at2g14170.1
>AT2G14170.1 | Symbols: ALDH6B2 | aldehyde dehydrogenase 6B2 | chr2:5977727-5981899 REVERSE LENGTH=607;>AT2G14170.2 | S
0.0
0.5
1.0
1.5
2.0
2.5
0 5 0 0 3 1 2 8 6 6 3 10 7 3 5 11
drm.ugt80.
drm.wt.ugt80.
drm.smt1.
drm.wt.smt1.
dsm.ugt80.
dsm.wt.ugt80.
dsm.smt1.
dsm.wt.smt1.
sp.ugt80.
sp.wt.ugt80.
sp.smt1.
sp.wt.smt1.
im.ugt80.
im.wt.ugt80.
im.smt1.
im.wt.smt1.

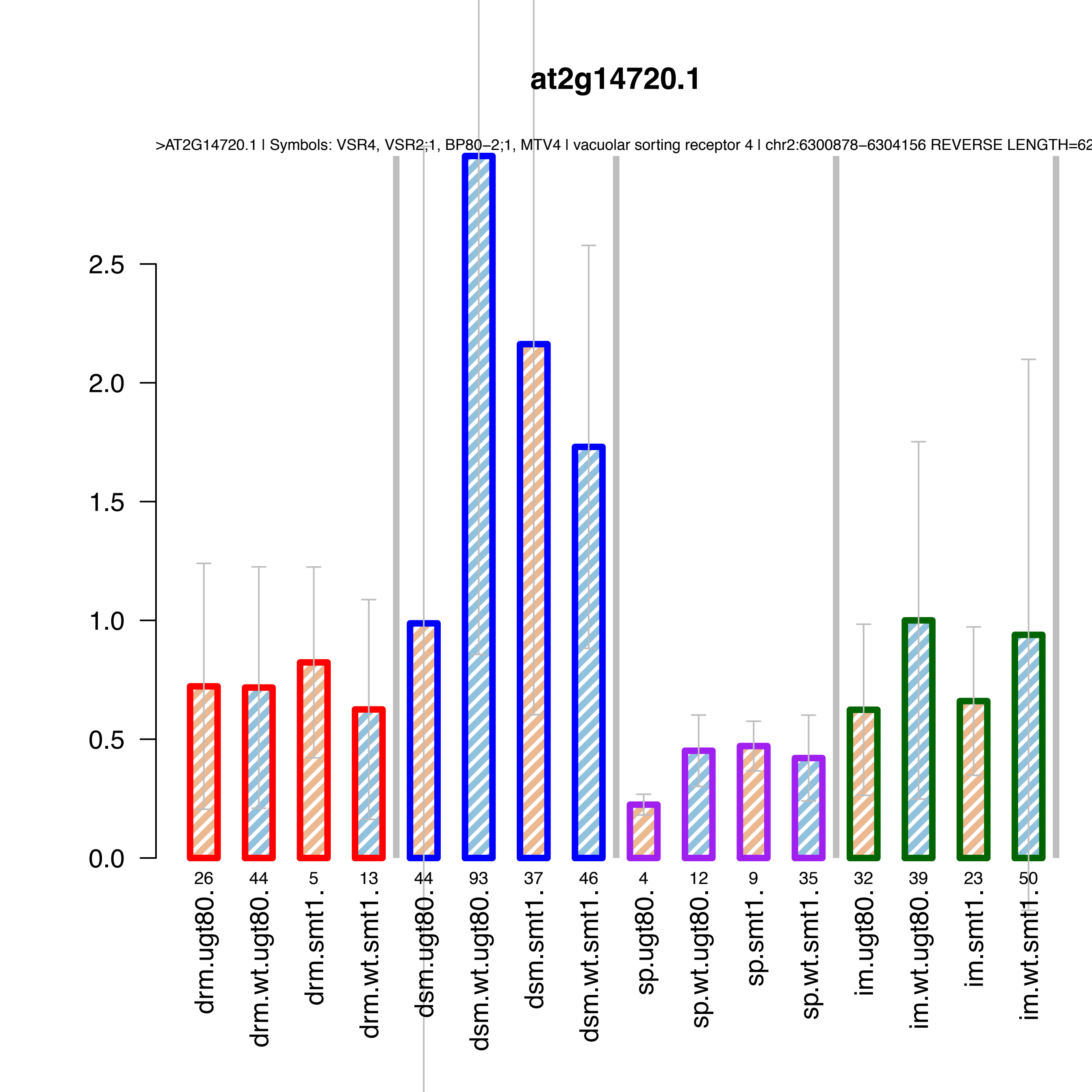

at2g14720.1
>AT2G14720.1 | Symbols: VSR4, VSR2;1, BP80−2;1, MTV4 | vacuolar sorting receptor 4 | chr2:6300878−6304156 REVERSE LENGTH=62
0.0
0.5
1.0
1.5
2.0
2.5
26
44
5
13
44
93
37
46
4
12
9
35
32
39
23
50
drm.ugt80..
drm.wt.ugt80..
drm.smt1..
drm.wt.smt1..
dsm.ugt80..
dsm.wt.ugt80..
dsm.smt1..
dsm.wt.smt1..
sp.ugt80..
sp.wt.ugt80..
sp.smt1..
sp.wt.smt1..
im.ugt80..
im.wt.ugt80..
im.smt1..
im.wt.smt1..

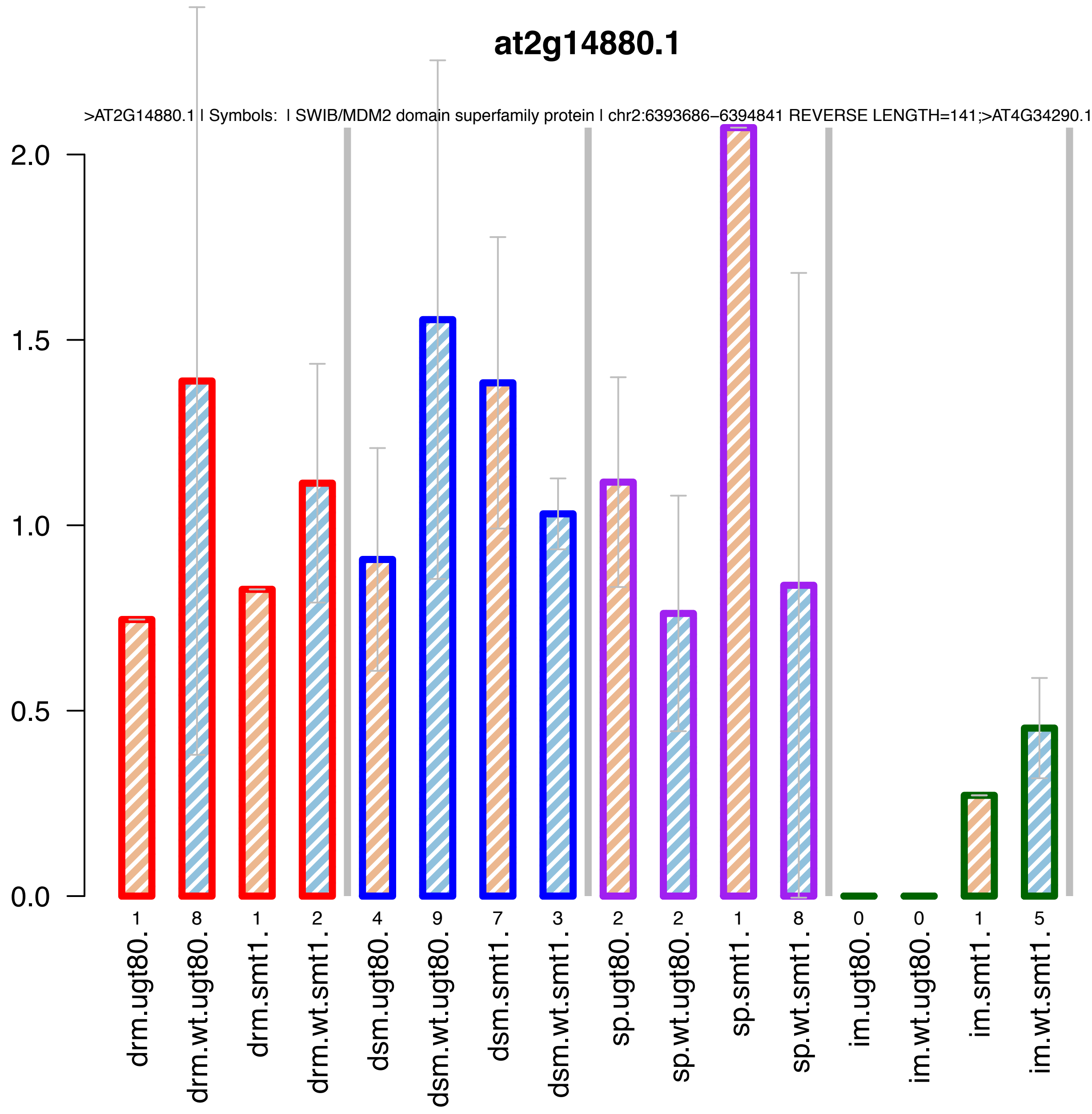

at2g14880.1
>AT2G14880.1 | Symbols: | SWIB/MDM2 domain superfamily protein | chr2:6393686-6394841 REVERSE LENGTH=141;>AT4G34290.1 |
2.0
1.5
1.0
0.5
0.0
1
8
1
2
4
9
7
3
2
2
1
8
0
0
1
5
drm.ugt80..
drm.wt.ugt80..
drm.smt1..
drm.wt.smt1..
dsm.ugt80..
dsm.wt.ugt80..
dsm.smt1..
dsm.wt.smt1..
sp.ugt80..
sp.wt.ugt80..
sp.smt1..
sp.wt.smt1..
im.ugt80..
im.wt.ugt80..
im.smt1..
im.wt.smt1..

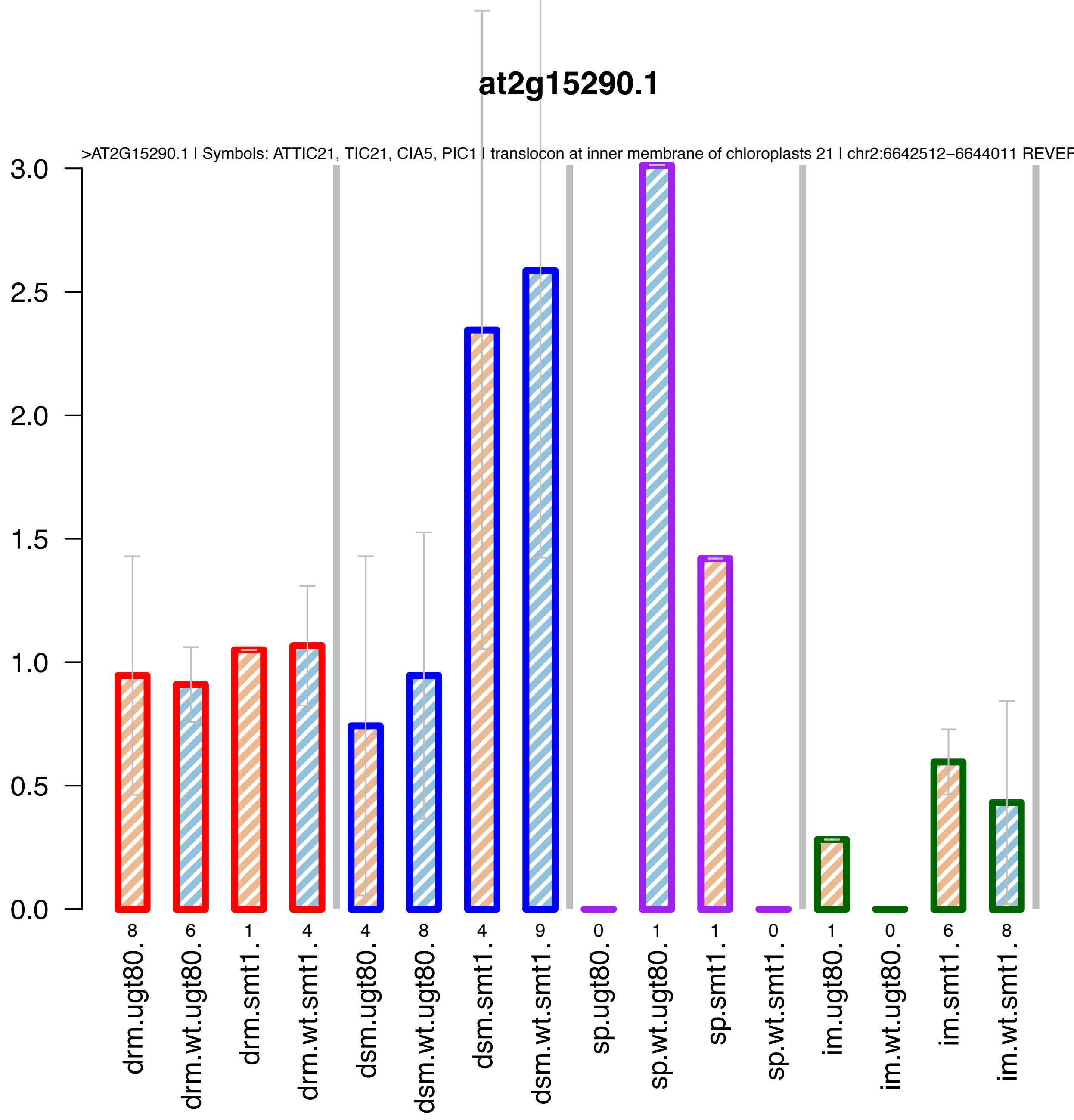
at2g15290.1
>AT2G15290.1 | Symbols: ATTIC21, TIC21, CIA5, PIC1 | translocon at inner membrane of chloroplasts 21 | chr2:6642512-6644011 REVER
3.0
2.5
2.0
1.5
1.0
0.5
0.0
8
6
1
4
4
8
4
9
0
1
1
0
1
0
6
8
drm.ugt80.
drm.wt.ugt80.
drm.smt1.
drm.wt.smt1.
dsm.ugt80.
dsm.wt.ugt80.
dsm.smt1.
dsm.wt.smt1.
sp.ugt80.
sp.wt.ugt80.
sp.smt1.
sp.wt.smt1.
im.ugt80.
im.wt.ugt80.
im.smt1.
im.wt.smt1.

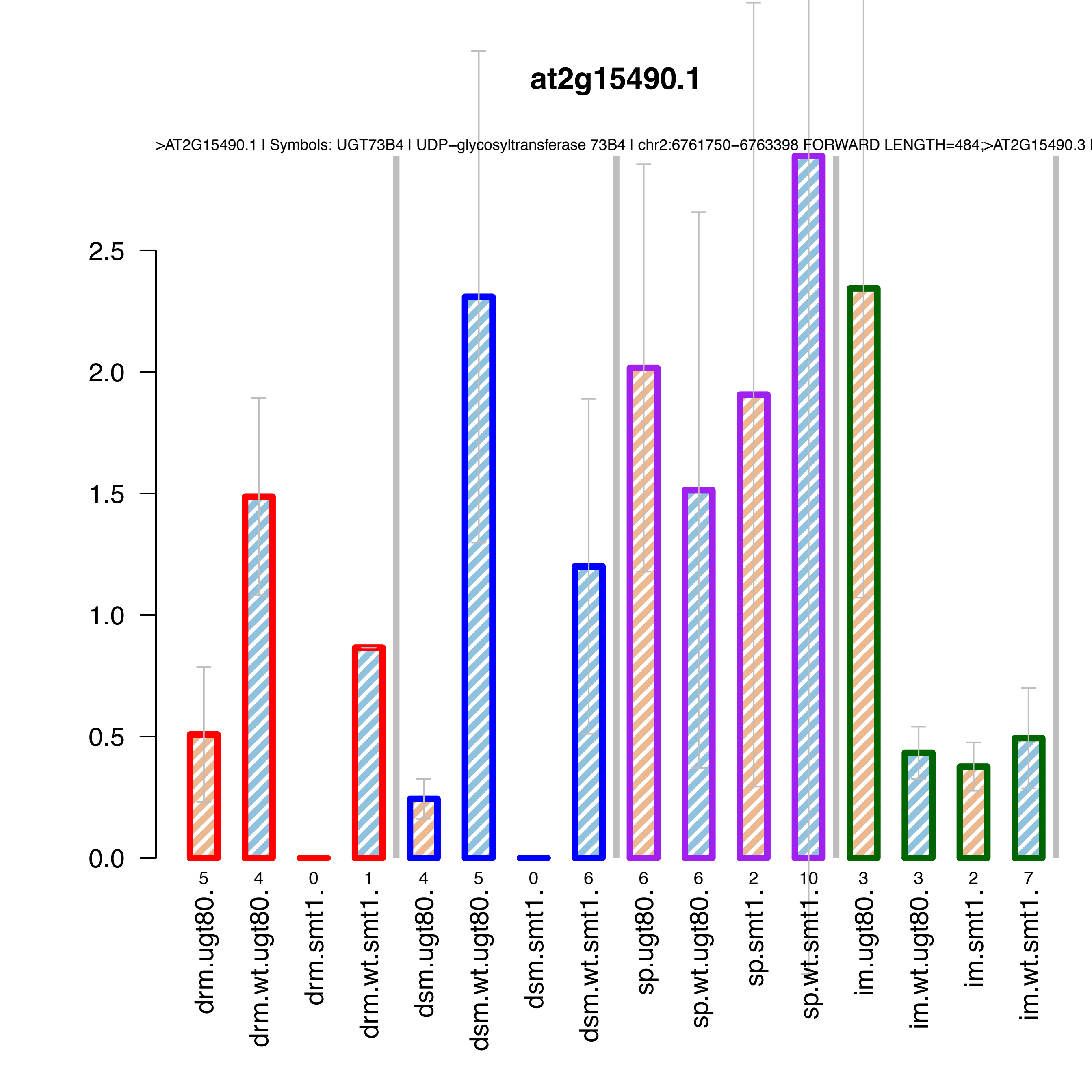
at2g15490.1
>AT2G15490.1 | Symbols: UGT73B4 | UDP−glycosyltransferase 73B4 | chr2:6761750−6763398 FORWARD LENGTH=484;>AT2G15490.3 |
0.0
0.5
1.0
1.5
2.0
2.5
5
4
0
1
4
5
0
6
6
6
2
10
3
3
2
7
drm.ugt80..
drm.wt.ugt80..
drm.smt1..
drm.wt.smt1..
dsm.ugt80..
dsm.wt.ugt80..
dsm.smt1..
dsm.wt.smt1..
sp.ugt80..
sp.wt.ugt80..
sp.smt1..
sp.wt.smt1..
im.ugt80..
im.wt.ugt80..
im.smt1..
im.wt.smt1..

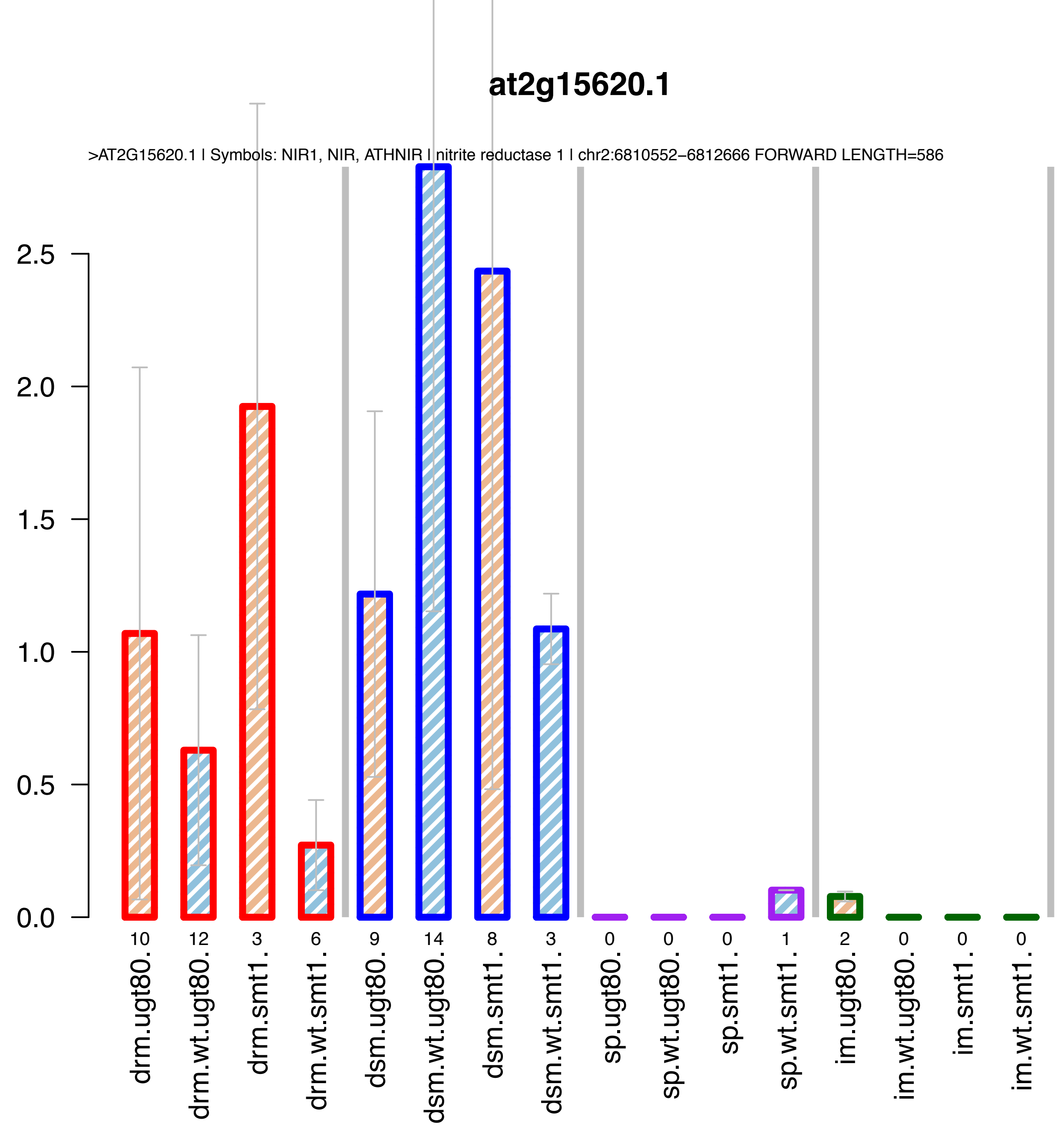
at2g15620.1
>AT2G15620.1 | Symbols: NIR1, NIR, ATHNIR | nitrite reductase 1 | chr2:6810552–6812666 FORWARD LENGTH=586
0.0
0.5
1.0
1.5
2.0
2.5
10
12
3
6
9
14
8
3
0
0
0
1
2
0
0
0
drm.ugt80..
drm.wt.ugt80..
drm.smt1..
drm.wt.smt1..
dsm.ugt80..
dsm.wt.ugt80..
dsm.smt1..
dsm.wt.smt1..
sp.ugt80..
sp.wt.ugt80..
sp.smt1..
sp.wt.smt1..
im.ugt80..
im.wt.ugt80..
im.smt1..
im.wt.smt1..

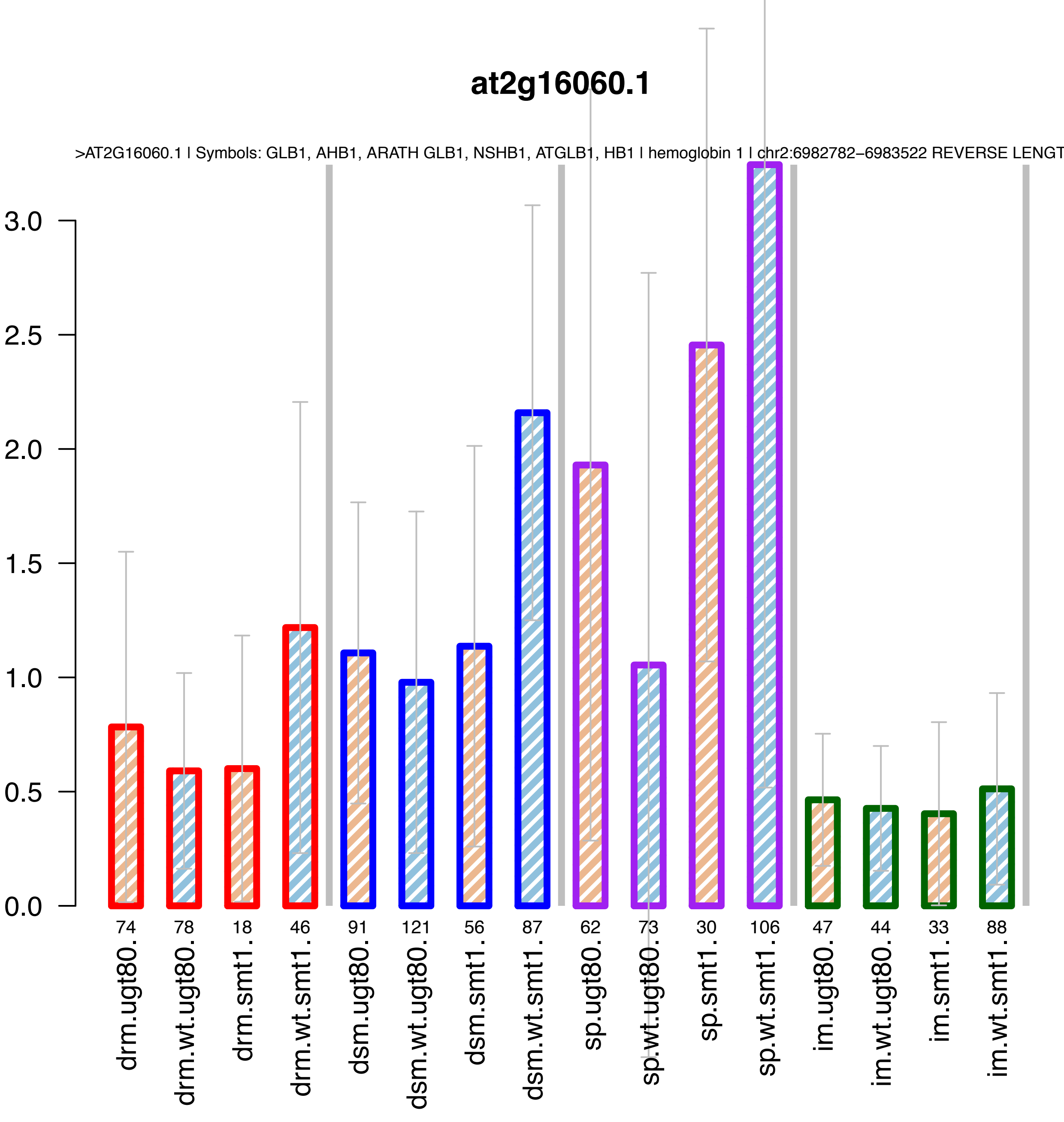
at2g16060.1
>AT2G16060.1 | Symbols: GLB1, AHB1, ARATH GLB1, NSHB1, ATGLB1, HB1 | hemoglobin 1 | chr2:6982782–6983522 REVERSE LENGT
3.0
2.5
2.0
1.5
1.0
0.5
0.0
74
78
18
46
91
121
56
87
62
73
30
106
47
44
33
88
drm.ugt80..
drm.wt.ugt80..
drm.smt1..
drm.wt.smt1..
dsm.ugt80..
dsm.wt.ugt80..
dsm.smt1..
dsm.wt.smt1..
sp.ugt80..
sp.wt.ugt80..
sp.smt1..
sp.wt.smt1..
im.ugt80..
im.wt.ugt80..
im.smt1..
im.wt.smt1..

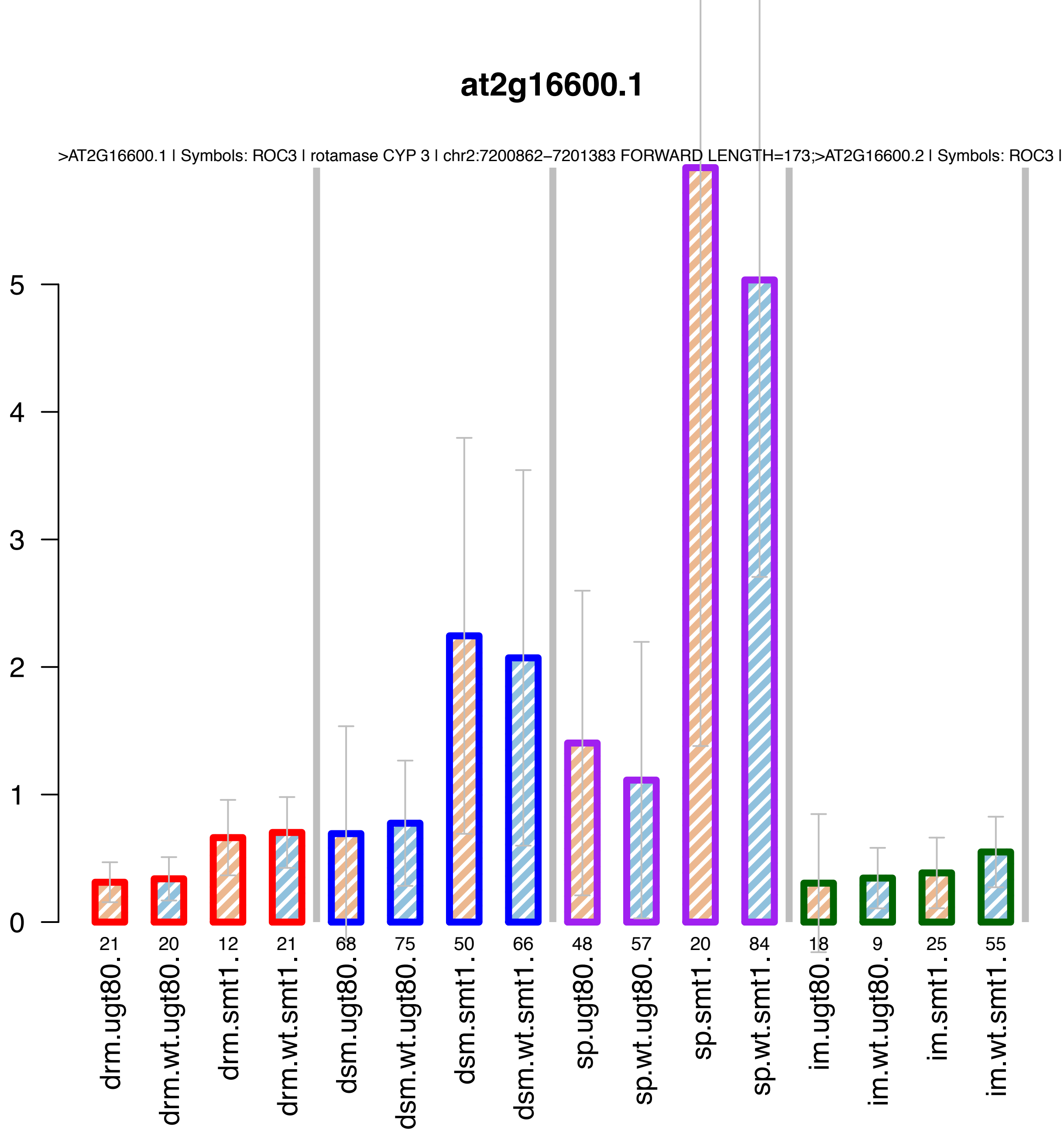

at2g16600.1
>AT2G16600.1 | Symbols: ROC3 | rotamase CYP 3 | chr2:7200862–7201383 FORWARD LENGTH=173;>AT2G16600.2 | Symbols: ROC3 |
0
1
2
3
4
5
21
20
12
21
68
75
50
66
48
57
20
84
18
9
25
55
drm.ugt80.
drm.wt.ugt80.
drm.smt1.
drm.wt.smt1.
dsm.ugt80.
dsm.wt.ugt80.
dsm.smt1.
dsm.wt.smt1.
sp.ugt80.
sp.wt.ugt80.
sp.smt1.
sp.wt.smt1.
im.ugt80.
im.wt.ugt80.
im.smt1.
im.wt.smt1.

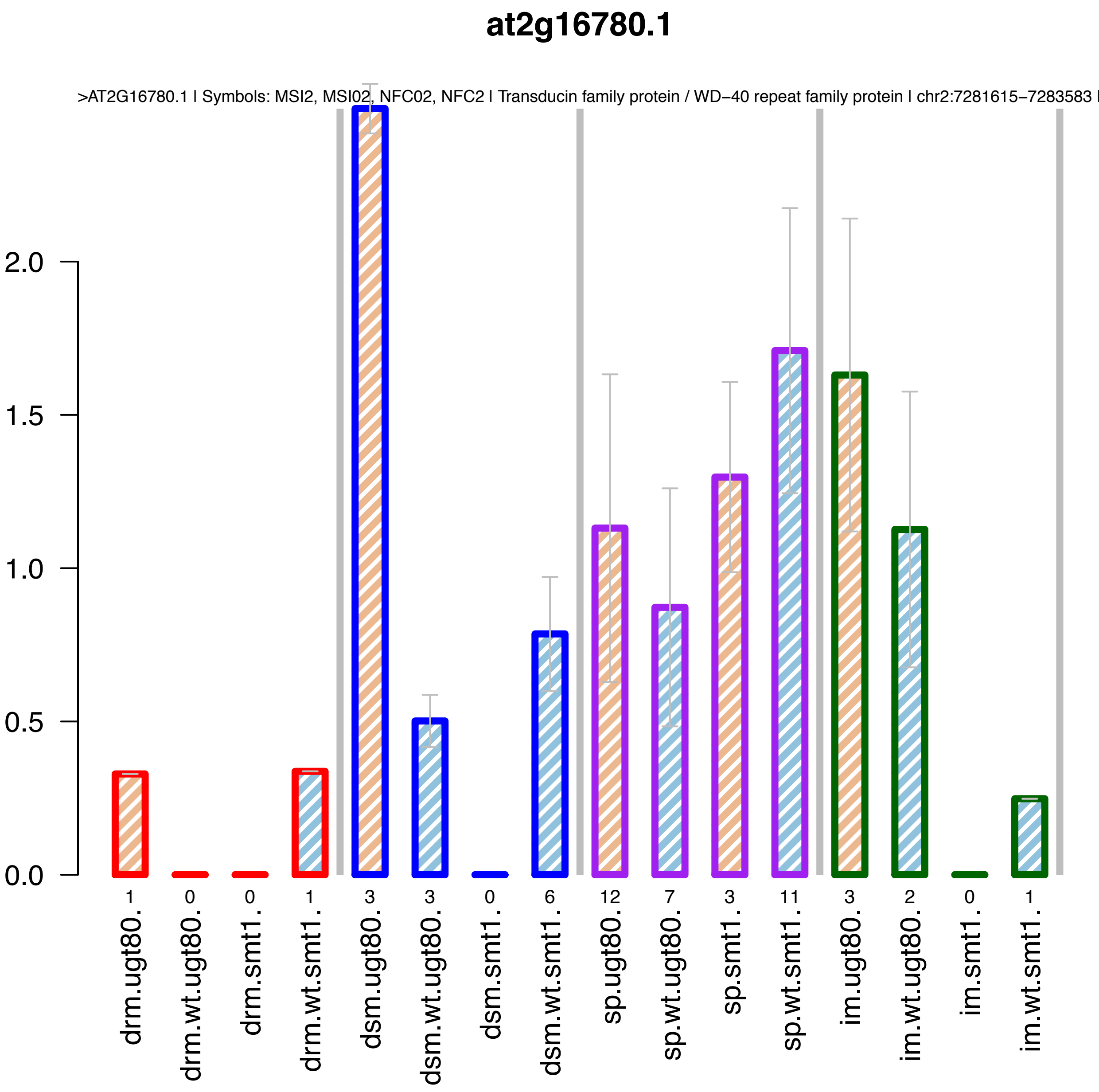

at2g16780.1
>AT2G16780.1 | Symbols: MSI2, MSI02, NFC02, NFC2 | Transducin family protein / WD−40 repeat family protein | chr2:7281615−7283583 |
2.0
1.5
1.0
0.5
0.0
1
0
0
1
3
3
0
6
12
7
3
11
3
2
0
1
drm.ugt80..
drm.wt.ugt80..
drm.smt1..
drm.wt.smt1..
dsm.ugt80..
dsm.wt.ugt80..
dsm.smt1..
dsm.wt.smt1..
sp.ugt80..
sp.wt.ugt80..
sp.smt1..
sp.wt.smt1..
im.ugt80..
im.wt.ugt80..
im.smt1..
im.wt.smt1..

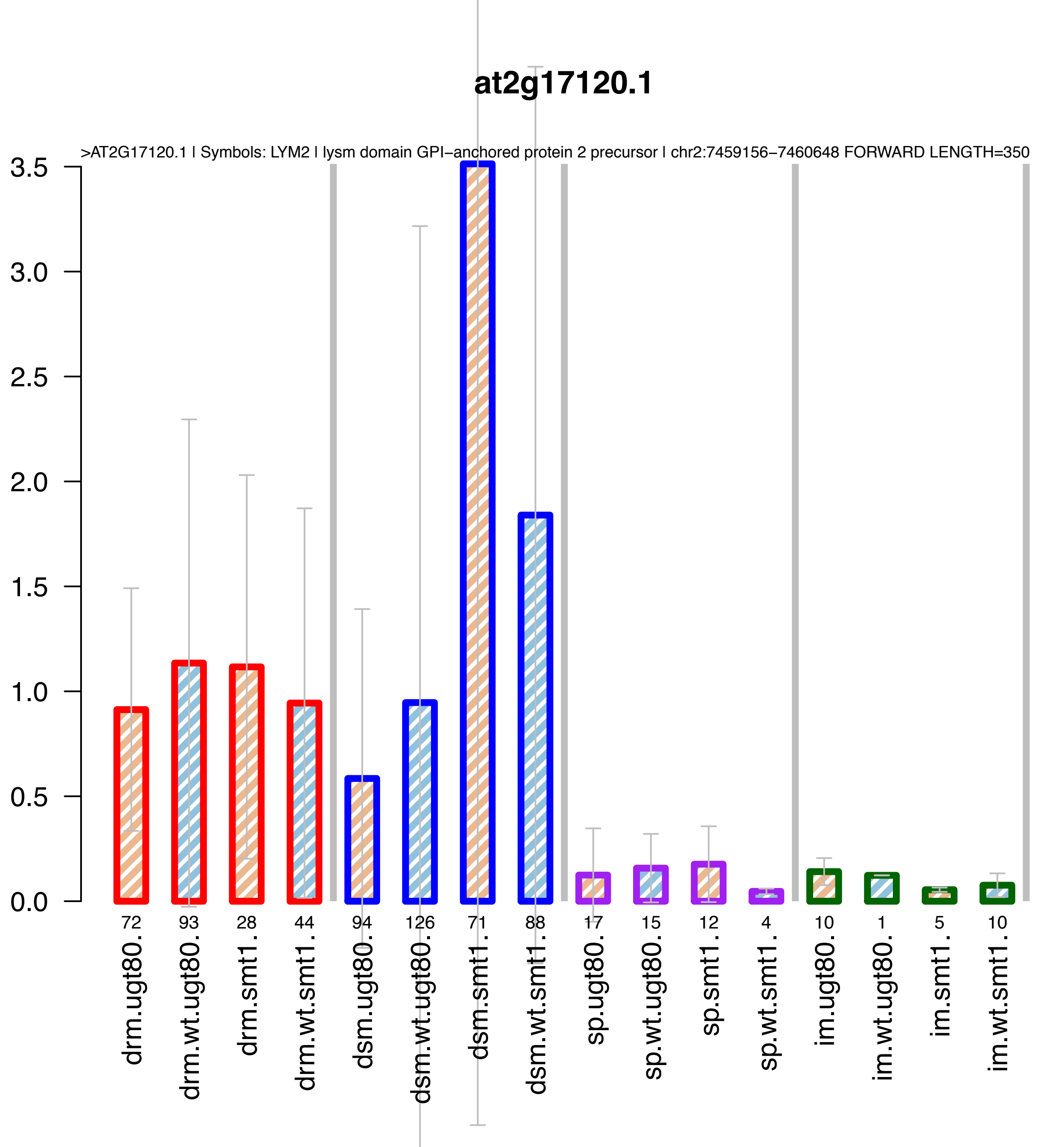
at2g17120.1
>AT2G17120.1 | Symbols: LYM2 | lysm domain GPI−anchored protein 2 precursor | chr2:7459156−7460648 FORWARD LENGTH=350
3.5
3.0
2.5
2.0
1.5
1.0
0.5
0.0
72
93
28
44
94
126
71
88
17
15
12
4
10
1
5
10
drm.ugt80..
drm.wt.ugt80..
drm.smt1..
drm.wt.smt1..
dsm.ugt80..
dsm.wt.ugt80..
dsm.smt1..
dsm.wt.smt1..
sp.ugt80..
sp.wt.ugt80..
sp.smt1..
sp.wt.smt1..
im.ugt80..
im.wt.ugt80..
im.smt1..
im.wt.smt1..

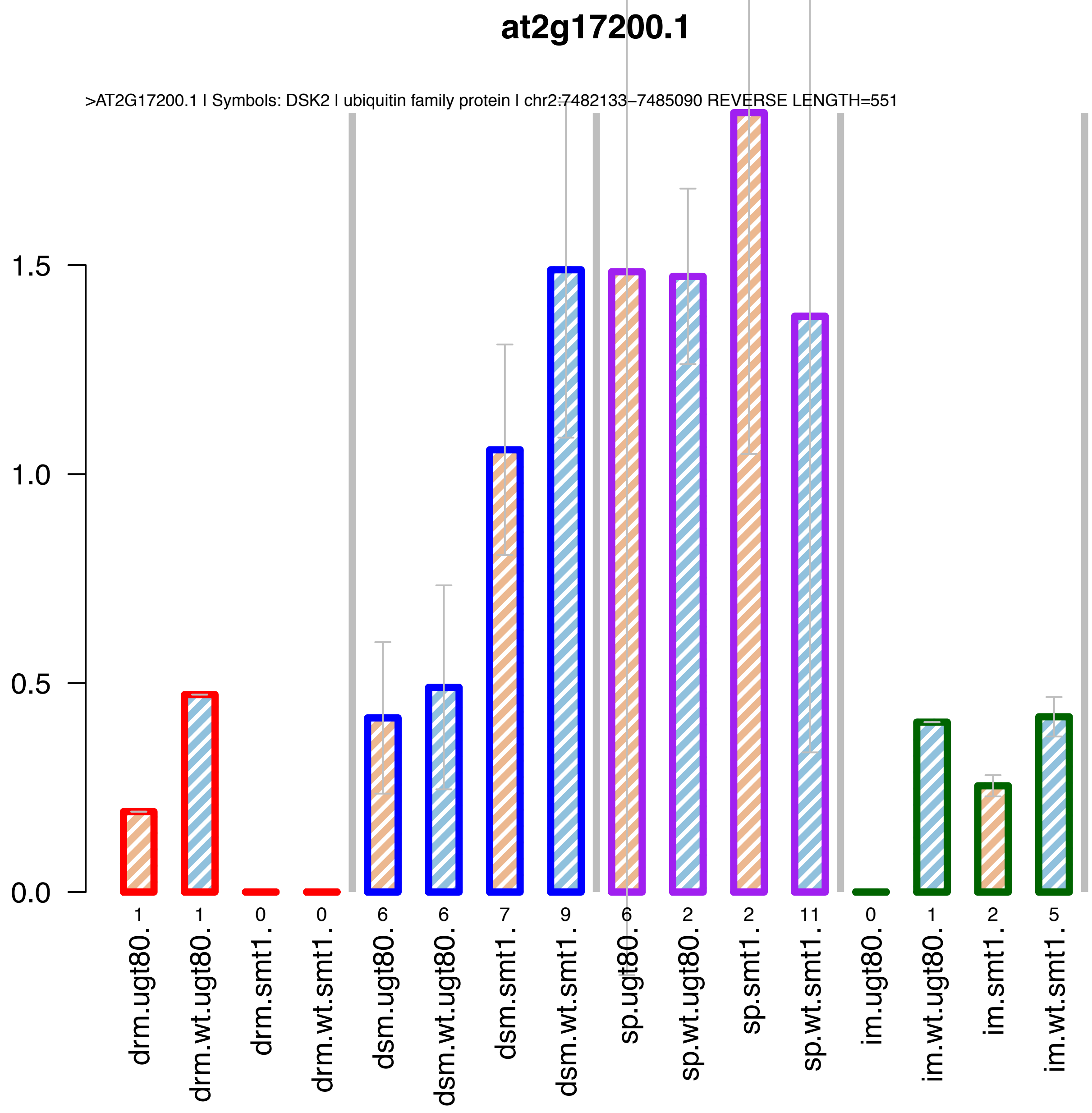

at2g17200.1
>AT2G17200.1 | Symbols: DSK2 | ubiquitin family protein | chr2:7482133-7485090 REVERSE LENGTH=551
0.0
0.5
1.0
1.5
1
1
0
0
6
6
7
9
6
2
2
11
0
1
2
5
drm.ugt80.
drm.wt.ugt80.
drm.smt1.
drm.wt.smt1.
dsm.ugt80.
dsm.wt.ugt80.
dsm.smt1.
dsm.wt.smt1.
sp.ugt80.
sp.wt.ugt80.
sp.smt1.
sp.wt.smt1.
im.ugt80.
im.wt.ugt80.
im.smt1.
im.wt.smt1.

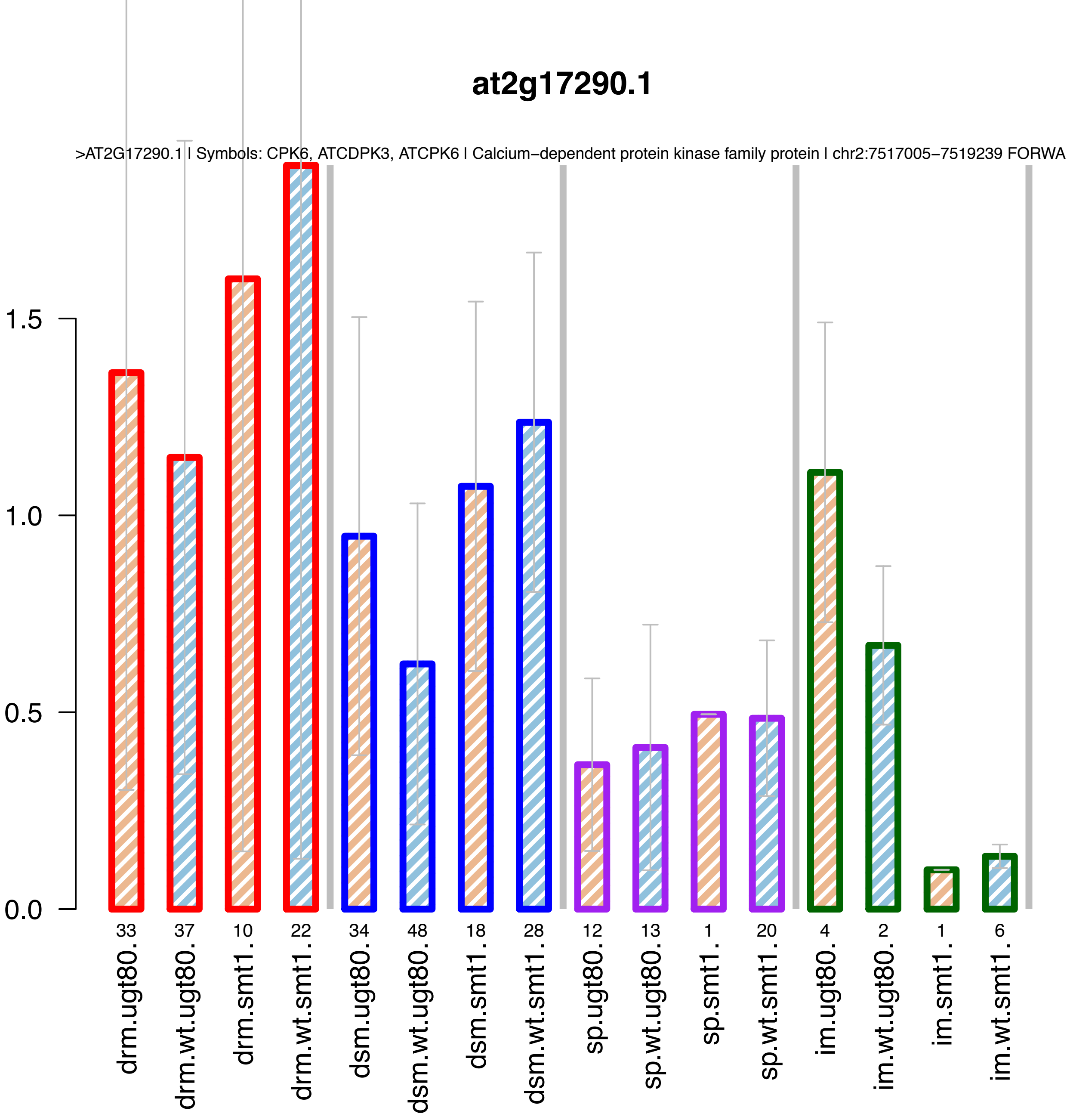

at2g17290.1
>AT2G17290.1 | Symbols: CPK6, ATCDPK3, ATCPK6 | Calcium−dependent protein kinase family protein | chr2:7517005−7519239 FORWA
1.5
1.0
0.5
0.0
33
37
10
22
34
48
18
28
12
13
1
20
4
2
1
6
drm.ugt80..
drm.wt.ugt80..
drm.smt1..
drm.wt.smt1..
dsm.ugt80..
dsm.wt.ugt80..
dsm.smt1..
dsm.wt.smt1..
sp.ugt80..
sp.wt.ugt80..
sp.smt1..
sp.wt.smt1..
im.ugt80..
im.wt.ugt80..
im.smt1..
im.wt.smt1..

# at2g17720.1

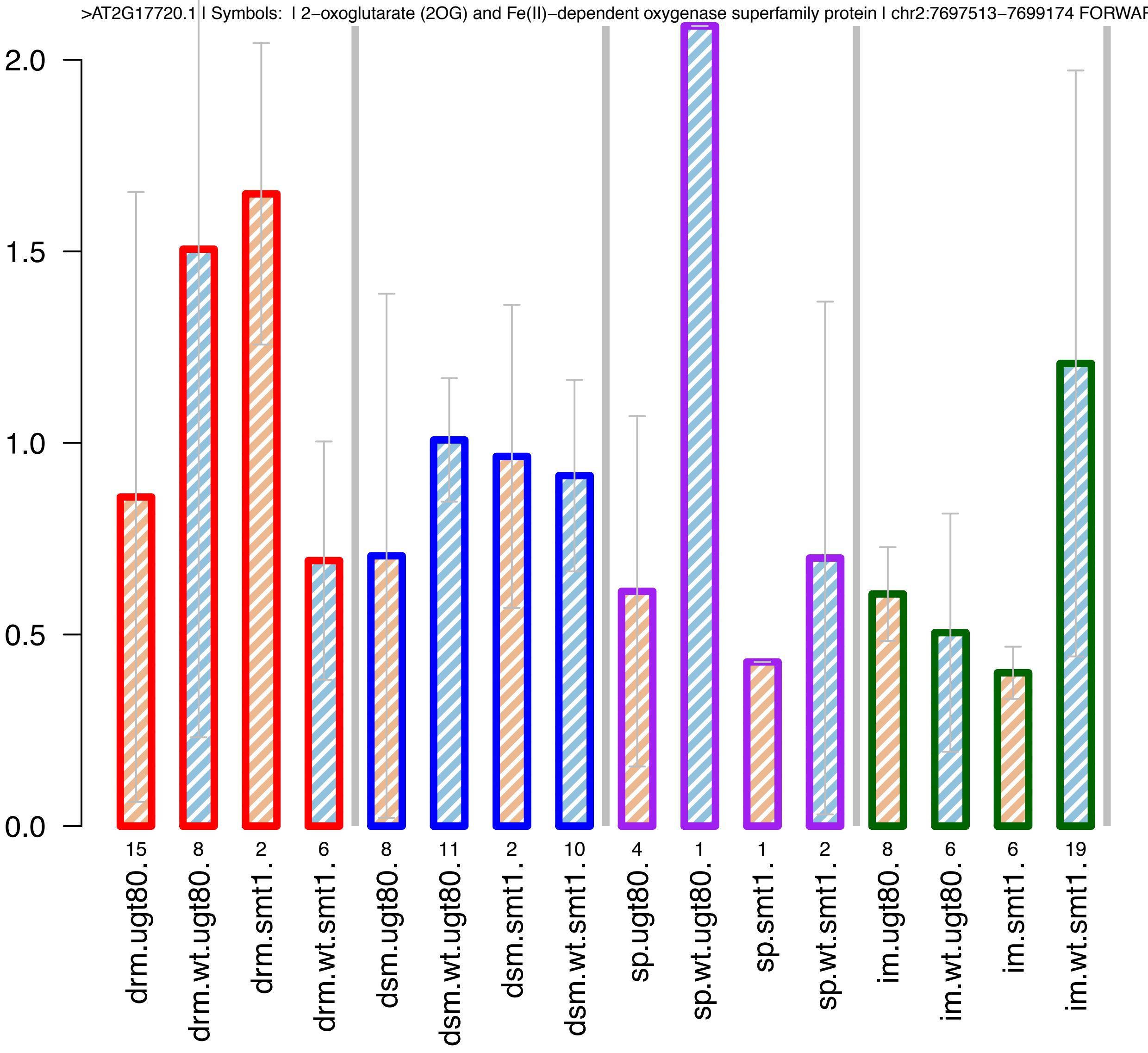
>AT2G17720.1 | Symbols: | 2-oxoglutarate (2OG) and Fe(II)-dependent oxygenase superfamily protein | chr2:7697513-7699174 FORWAR
2.0
1.5
1.0
0.5
0.0
15
8
2
6
8
11
2
10
4
1
1
2
8
6
6
19
drm.ugt80..
drm.wt.ugt80..
drm.smt1..
drm.wt.smt1..
dsm.ugt80..
dsm.wt.ugt80..
dsm.smt1..
dsm.wt.smt1..
sp.ugt80..
sp.wt.ugt80..
sp.smt1..
sp.wt.smt1..
im.ugt80..
im.wt.ugt80..
im.smt1..
im.wt.smt1..

# at2g17850.1

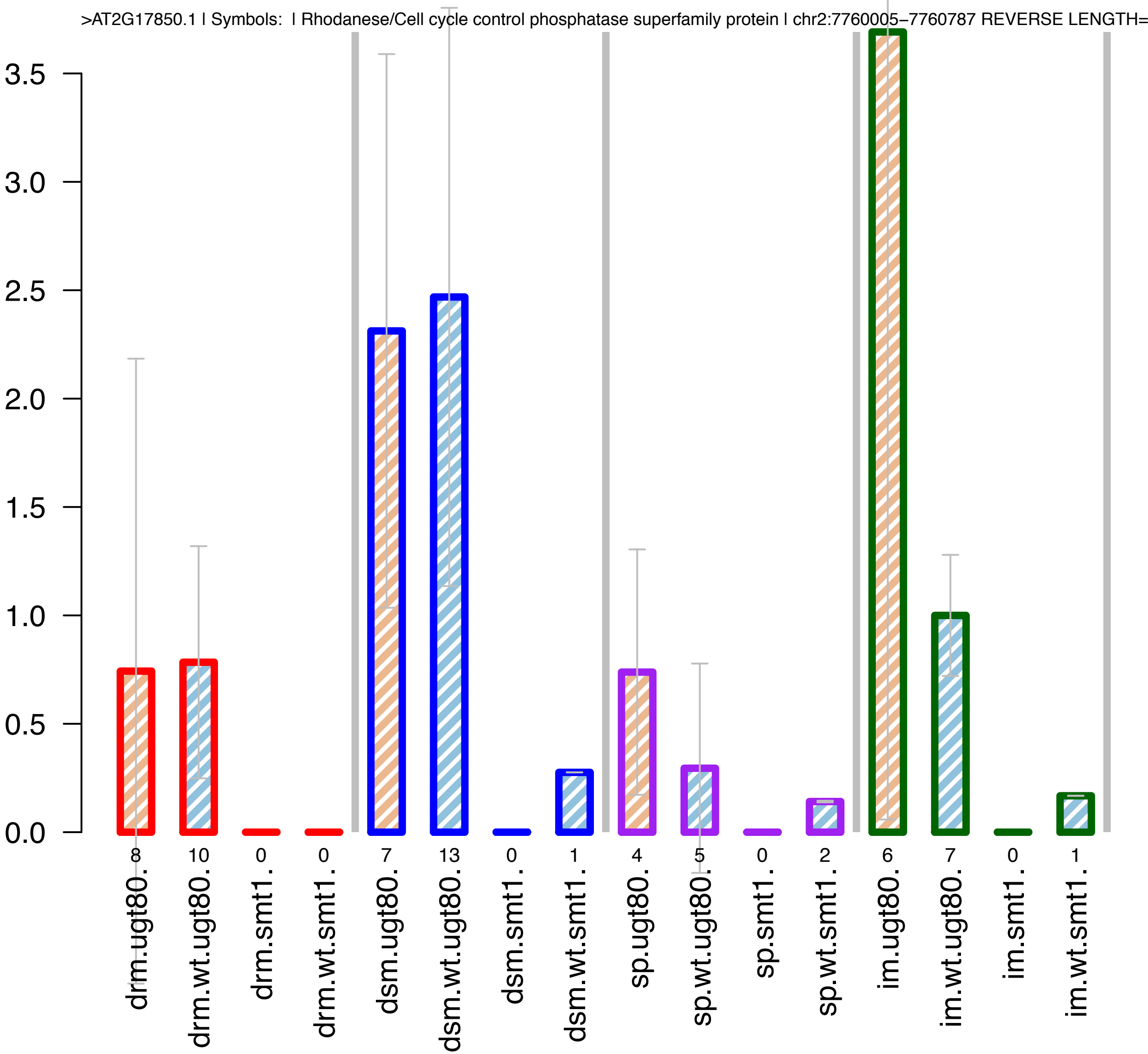

>AT2G17850.1 | Symbols: | Rhodanese/Cell cycle control phosphatase superfamily protein | chr2:7760005–7760787 REVERSE LENGTH=
3.5
3.0
2.5
2.0
1.5
1.0
0.5
0.0
8
10
0
0
7
13
0
1
4
5
0
2
6
7
0
1
drm.ugt80.
drm.wt.ugt80.
drm.smt1.
drm.wt.smt1.
dsm.ugt80.
dsm.wt.ugt80.
dsm.smt1.
dsm.wt.smt1.
sp.ugt80.
sp.wt.ugt80.
sp.smt1.
sp.wt.smt1.
im.ugt80.
im.wt.ugt80.
im.smt1.
im.wt.smt1.

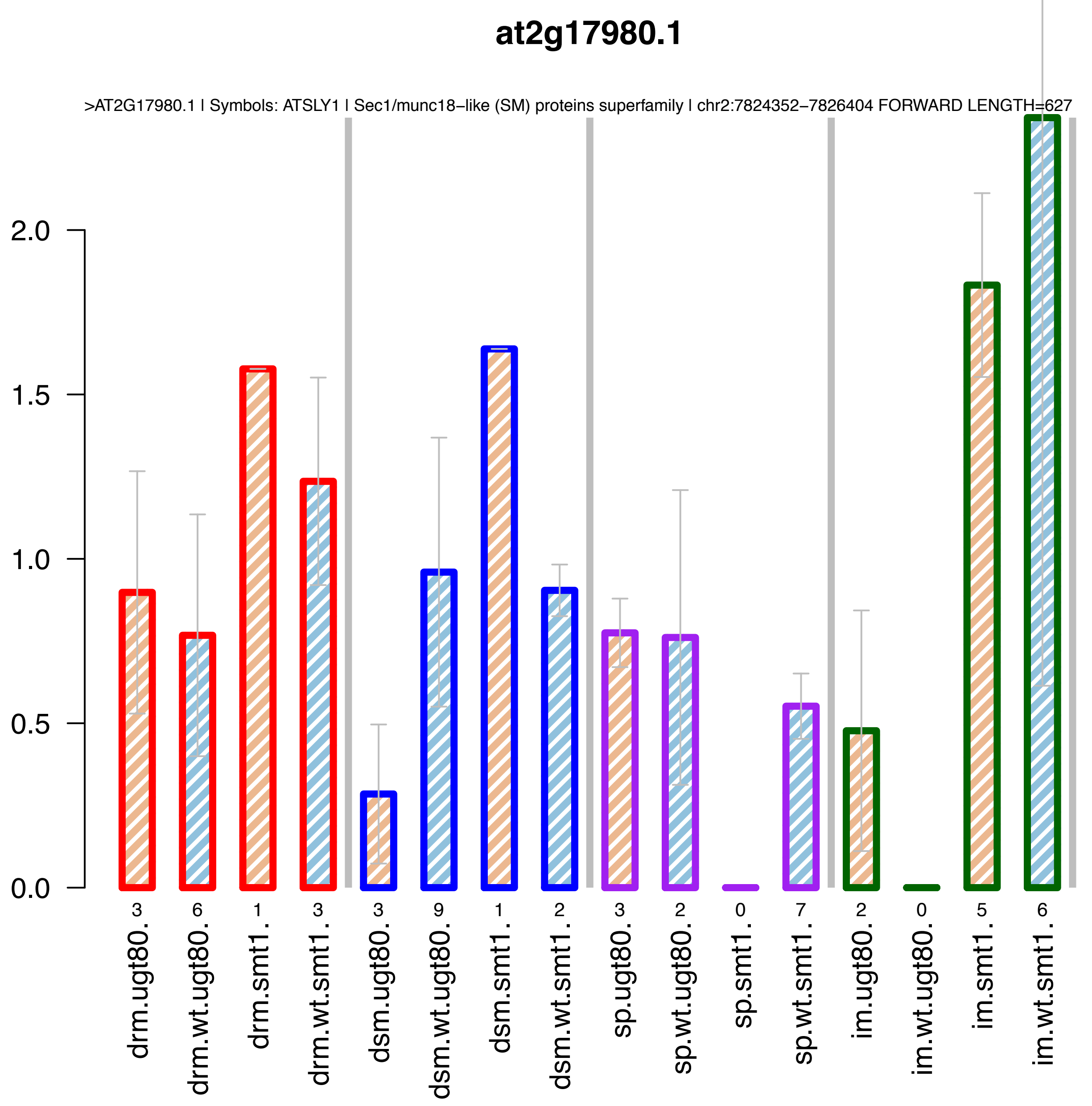

at2g17980.1
>AT2G17980.1 | Symbols: ATSLY1 | Sec1/munc18-like (SM) proteins superfamily | chr2:7824352-7826404 FORWARD LENGTH=627
0.0
0.5
1.0
1.5
2.0
3
6
1
3
3
9
1
2
3
2
0
7
2
0
5
6
drm.ugt80.
drm.wt.ugt80.
drm.smt1.
drm.wt.smt1.
dsm.ugt80.
dsm.wt.ugt80.
dsm.smt1.
dsm.wt.smt1.
sp.ugt80.
sp.wt.ugt80.
sp.smt1.
sp.wt.smt1.
im.ugt80.
im.wt.ugt80.
im.smt1.
im.wt.smt1.

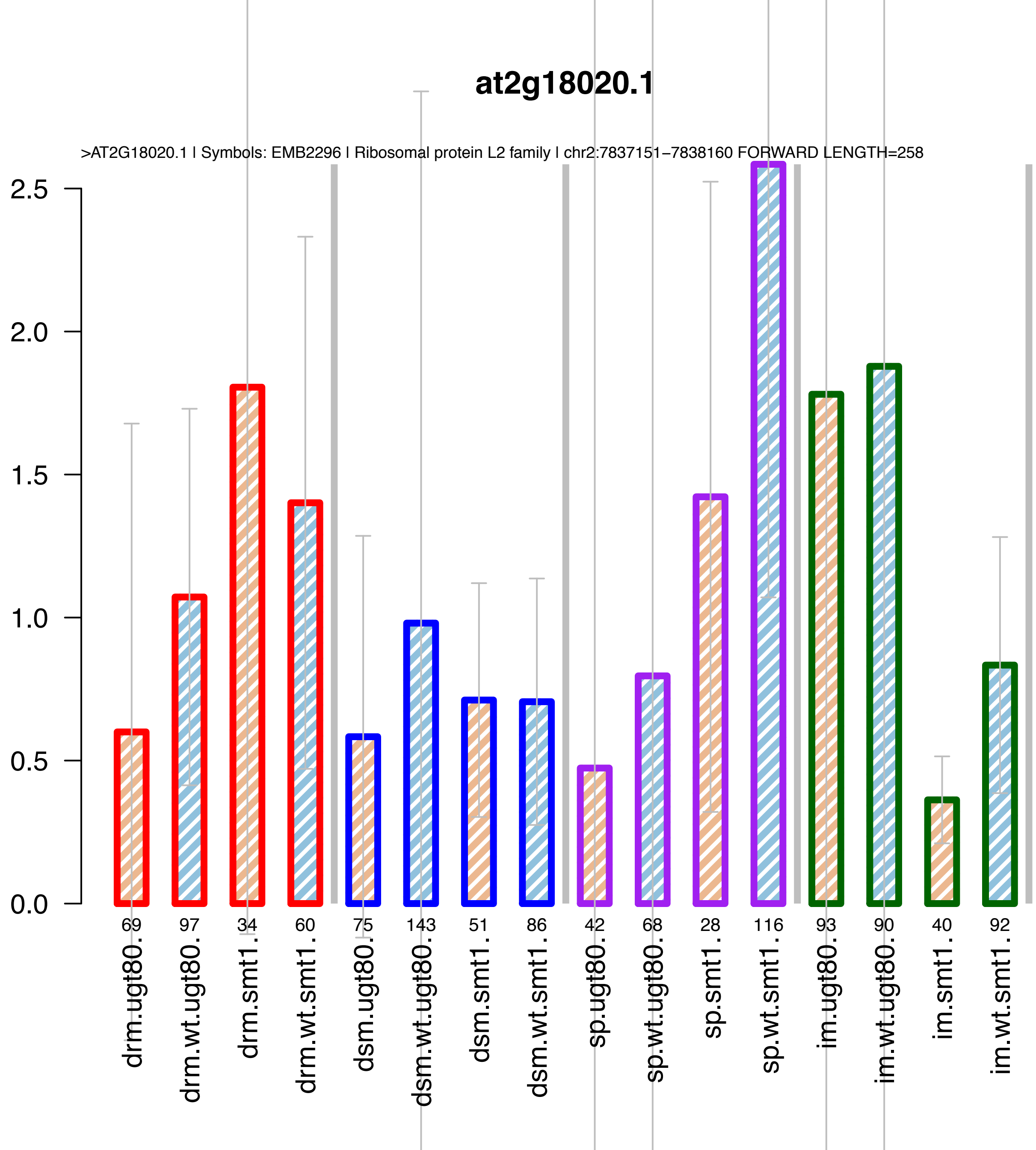
at2g18020.1
>AT2G18020.1 | Symbols: EMB2296 | Ribosomal protein L2 family | chr2:7837151-7838160 FORWARD LENGTH=258
2.5
2.0
1.5
1.0
0.5
0.0
69
97
34
60
75
143
51
86
42
68
28
116
93
90
40
92
drm.ugt80..
drm.wt.ugt80..
drm.smt1..
drm.wt.smt1..
dsm.ugt80..
dsm.wt.ugt80..
dsm.smt1..
dsm.wt.smt1..
sp.ugt80..
sp.wt.ugt80..
sp.smt1..
sp.wt.smt1..
im.ugt80..
im.wt.ugt80..
im.smt1..
im.wt.smt1..

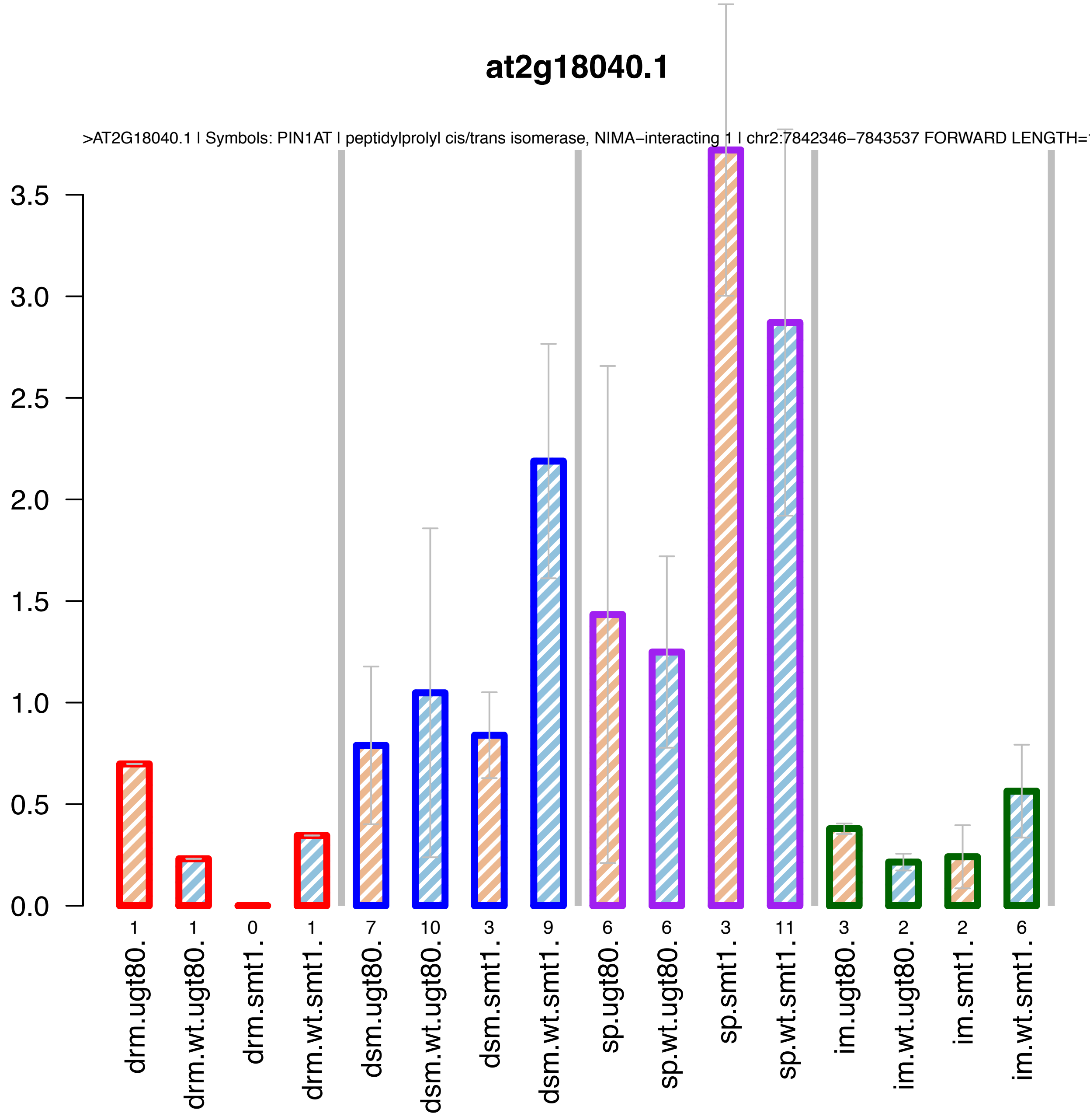
at2g18040.1
>AT2G18040.1 | Symbols: PIN1AT | peptidylprolyl cis/trans isomerase, NIMA-interacting 1 | chr2:7842346-7843537 FORWARD LENGTH=
3.5
3.0
2.5
2.0
1.5
1.0
0.5
0.0
1
1
0
1
7
10
3
9
6
6
3
11
3
2
2
6
drm.ugt80.
drm.wt.ugt80.
drm.smt1.
drm.wt.smt1.
dsm.ugt80.
dsm.wt.ugt80.
dsm.smt1.
dsm.wt.smt1.
sp.ugt80.
sp.wt.ugt80.
sp.smt1.
sp.wt.smt1.
im.ugt80.
im.wt.ugt80.
im.smt1.
im.wt.smt1.

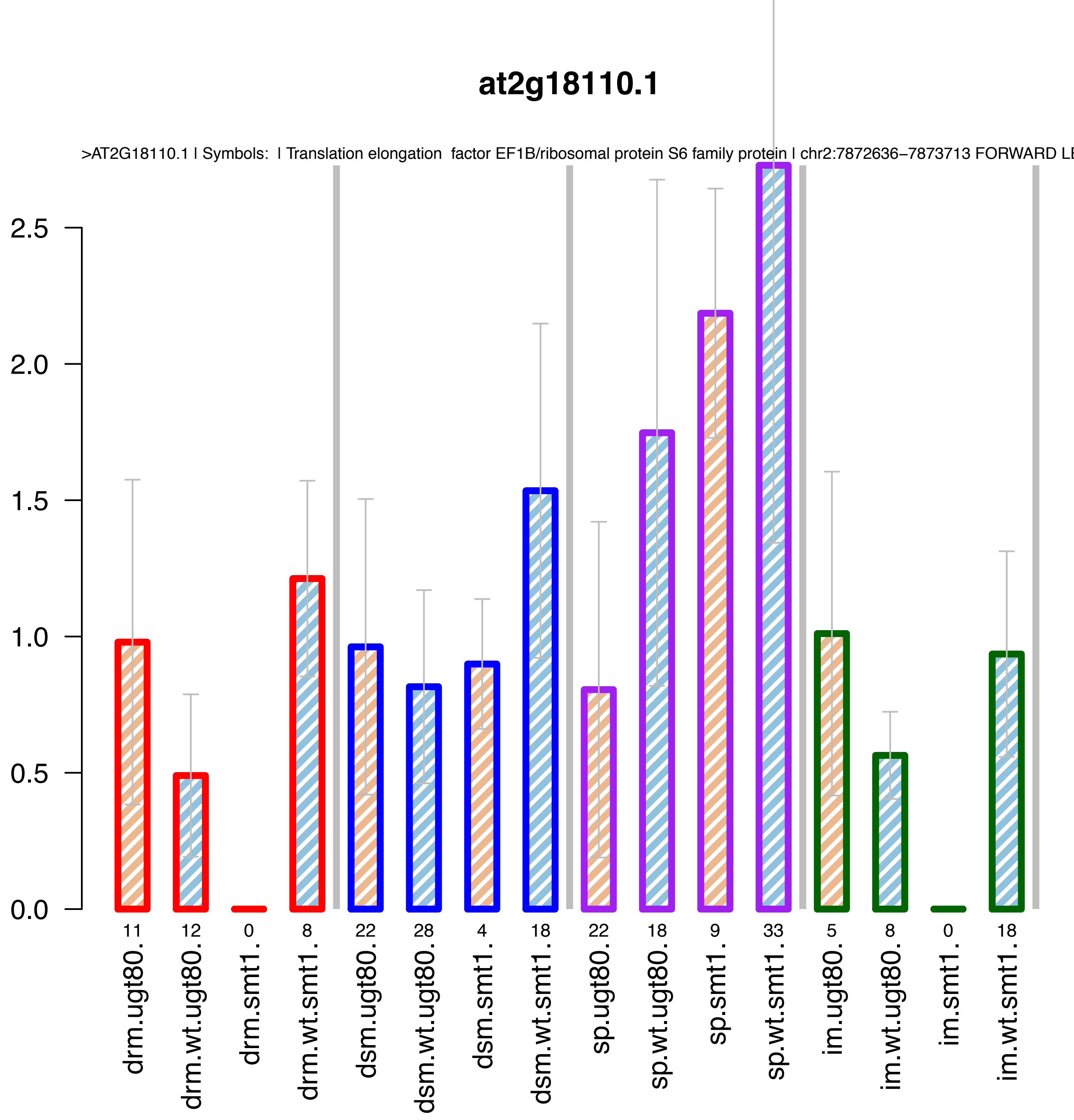

at2g18110.1
>AT2G18110.1 | Symbols: | Translation elongation factor EF1B/ribosomal protein S6 family protein | chr2:7872636-7873713 FORWARD LE
0.0
0.5
1.0
1.5
2.0
2.5
11
12
0
8
22
28
4
18
22
18
9
33
5
8
0
18
drm.ugt80..
drm.wt.ugt80..
drm.smt1..
drm.wt.smt1..
dsm.ugt80..
dsm.wt.ugt80..
dsm.smt1..
dsm.wt.smt1..
sp.ugt80..
sp.wt.ugt80..
sp.smt1..
sp.wt.smt1..
im.ugt80..
im.wt.ugt80..
im.smt1..
im.wt.smt1..

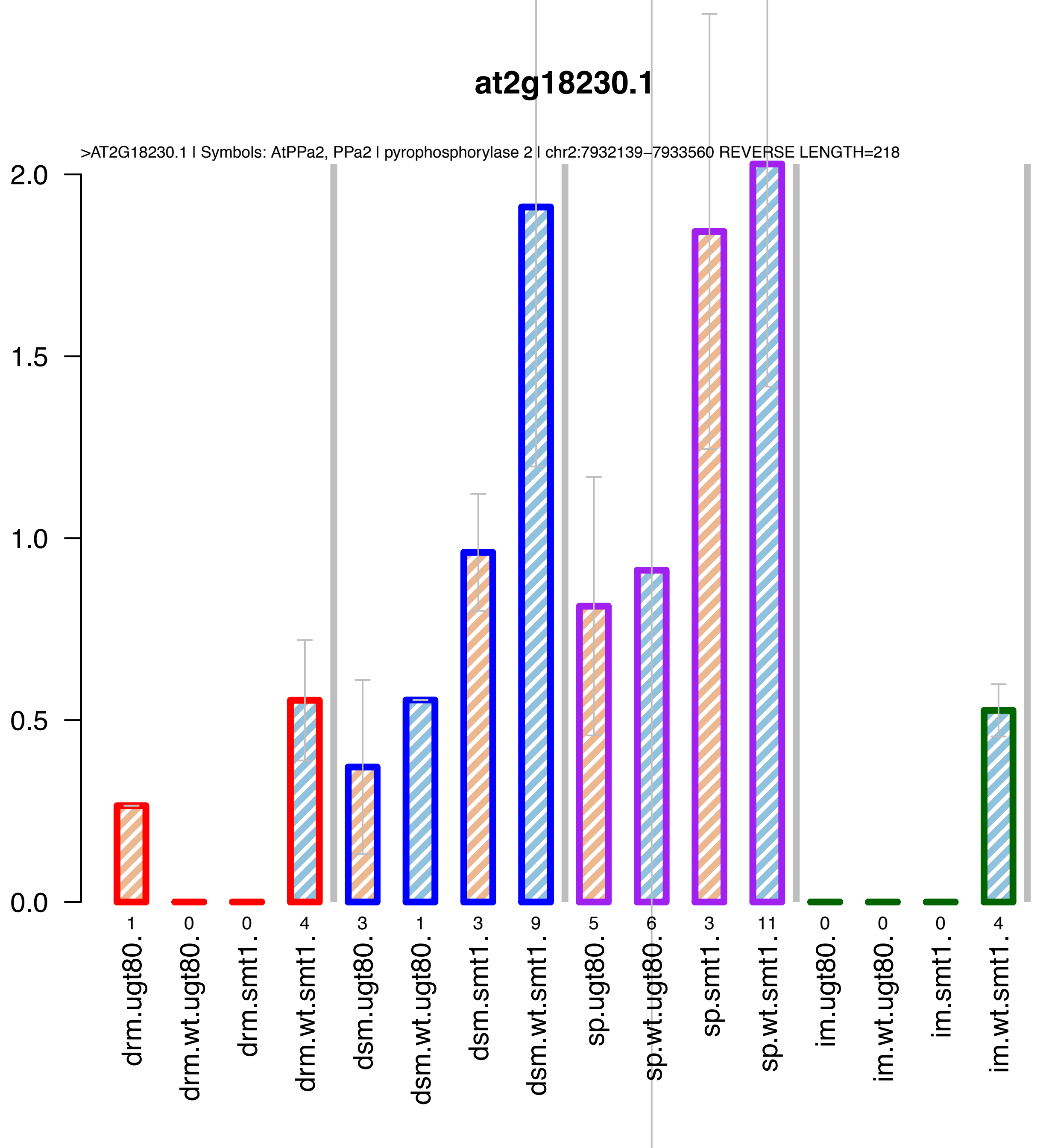

at2g18230.1
>AT2G18230.1 | Symbols: AtPPa2, PPa2 | pyrophosphorylase 2 | chr2:7932139−7933560 REVERSE LENGTH=218
2.0
1.5
1.0
0.5
0.0
1
0
0
4
3
1
3
9
5
6
3
11
0
0
0
4
drm.ugt80..
drm.wt.ugt80..
drm.smt1..
drm.wt.smt1..
dsm.ugt80..
dsm.wt.ugt80..
dsm.smt1..
dsm.wt.smt1..
sp.ugt80..
sp.wt.ugt80..
sp.smt1..
sp.wt.smt1..
im.ugt80..
im.wt.ugt80..
im.smt1..
im.wt.smt1..

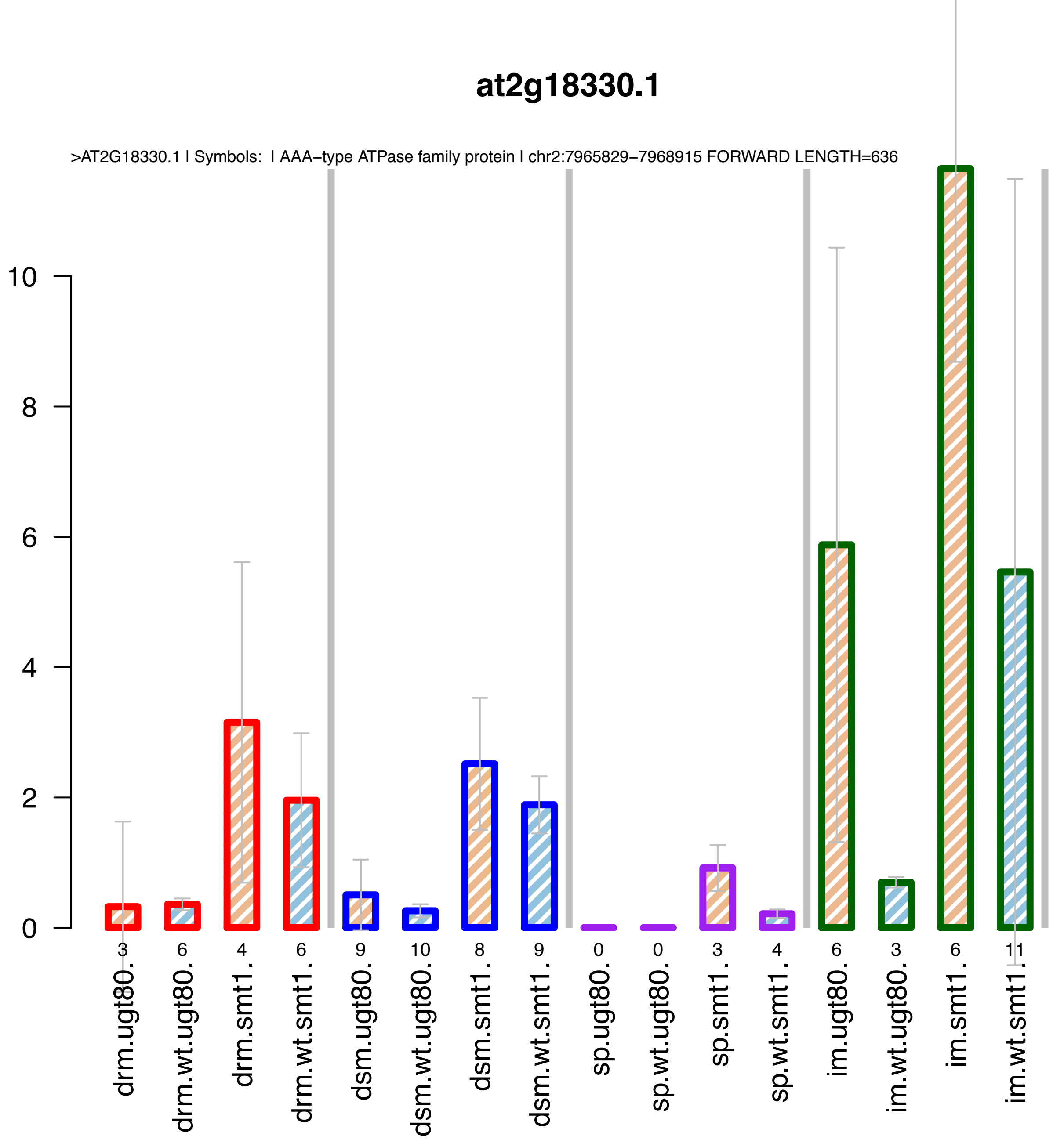
at2g18330.1
>AT2G18330.1 | Symbols: | AAA−type ATPase family protein | chr2:7965829−7968915 FORWARD LENGTH=636
0
2
4
6
8
10
3
6
4
6
9
10
8
9
0
0
3
4
6
3
6
11
drm.ugt80.
drm.wt.ugt80.
drm.smt1.
drm.wt.smt1.
dsm.ugt80.
dsm.wt.ugt80.
dsm.smt1.
dsm.wt.smt1.
sp.ugt80.
sp.wt.ugt80.
sp.smt1.
sp.wt.smt1.
im.ugt80.
im.wt.ugt80.
im.smt1.
im.wt.smt1.

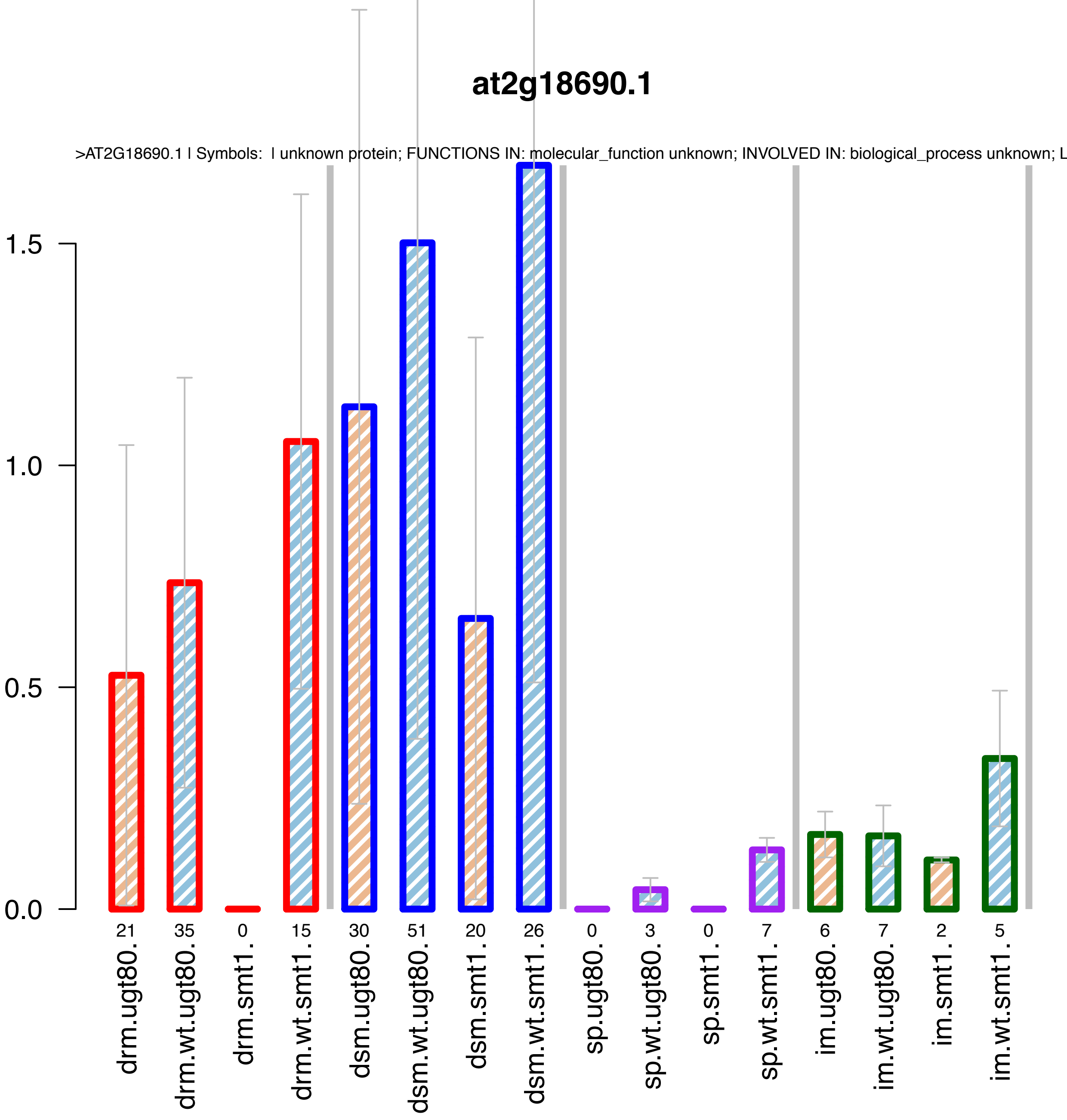

at2g18690.1
>AT2G18690.1 | Symbols: | unknown protein; FUNCTIONS IN: molecular_function unknown; INVOLVED IN: biological_process unknown; L
1.5
1.0
0.5
0.0
21
35
0
15
30
51
20
26
0
3
0
7
6
7
2
5
drm.ugt80.
drm.wt.ugt80.
drm.smt1.
drm.wt.smt1.
dsm.ugt80.
dsm.wt.ugt80.
dsm.smt1.
dsm.wt.smt1.
sp.ugt80.
sp.wt.ugt80.
sp.smt1.
sp.wt.smt1.
im.ugt80.
im.wt.ugt80.
im.smt1.
im.wt.smt1.

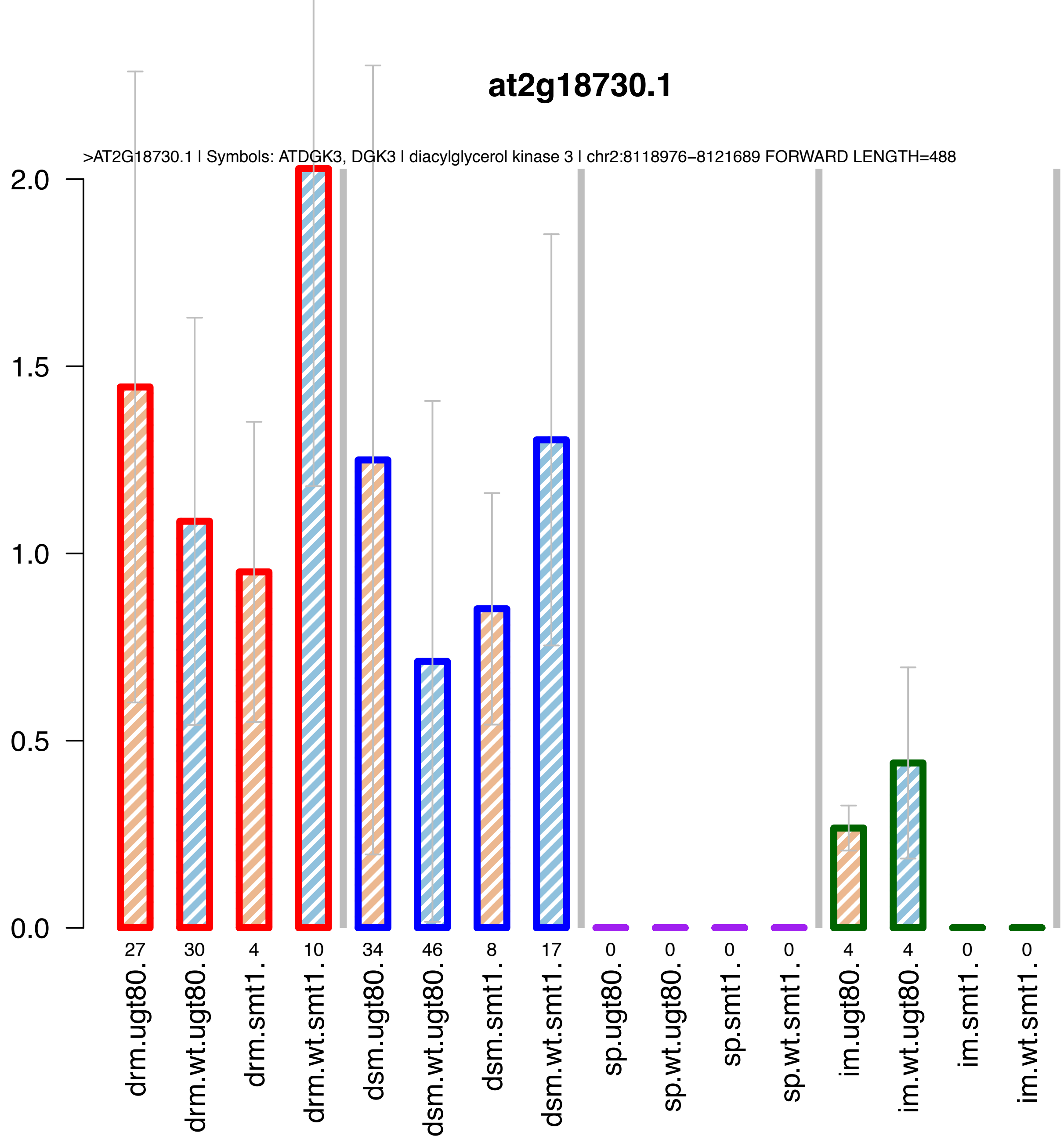

at2g18730.1
>AT2G18730.1 | Symbols: ATDGK3, DGK3 | diacylglycerol kinase 3 | chr2:8118976-8121689 FORWARD LENGTH=488
2.0
1.5
1.0
0.5
0.0
27
30
4
10
34
46
8
17
0
0
0
0
4
4
0
0
drm.ugt80.
drm.wt.ugt80.
drm.smt1.
drm.wt.smt1.
dsm.ugt80.
dsm.wt.ugt80.
dsm.smt1.
dsm.wt.smt1.
sp.ugt80.
sp.wt.ugt80.
sp.smt1.
sp.wt.smt1.
im.ugt80.
im.wt.ugt80.
im.smt1.
im.wt.smt1.

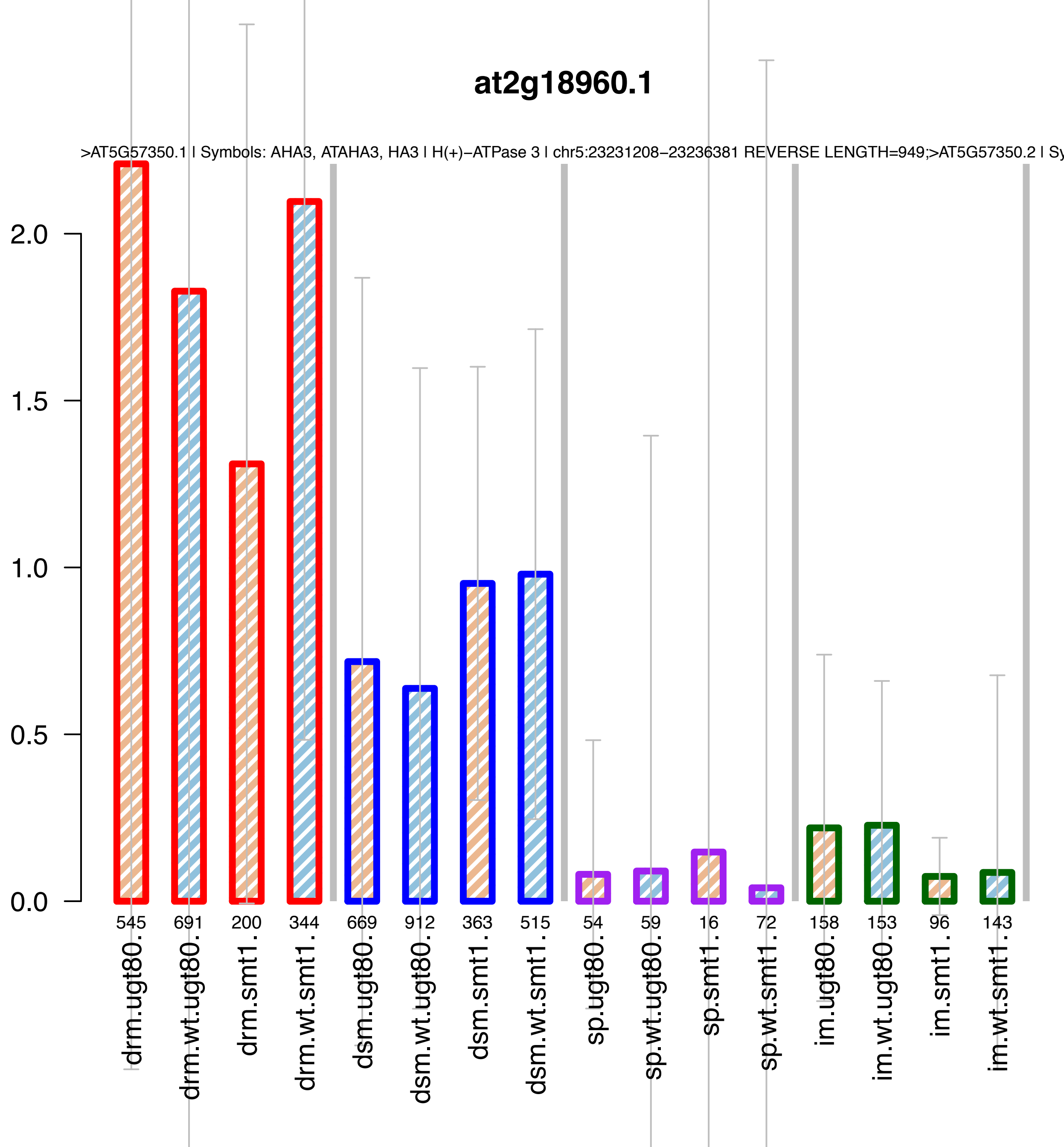

at2g18960.1
>AT5G57350.1 | Symbols: AHA3, ATAHA3, HA3 | H(+)−ATPase 3 | chr5:23231208−23236381 REVERSE LENGTH=949;>AT5G57350.2 | Sy
2.0
1.5
1.0
0.5
0.0
545
691
200
344
669
912
363
515
54
59
16
72
158
153
96
143
drm.ugt80..
drm.wt.ugt80..
drm.smt1..
drm.wt.smt1..
dsm.ugt80..
dsm.wt.ugt80..
dsm.smt1..
dsm.wt.smt1..
sp.ugt80..
sp.wt.ugt80..
sp.smt1..
sp.wt.smt1..
im.ugt80..
im.wt.ugt80..
im.smt1..
im.wt.smt1..

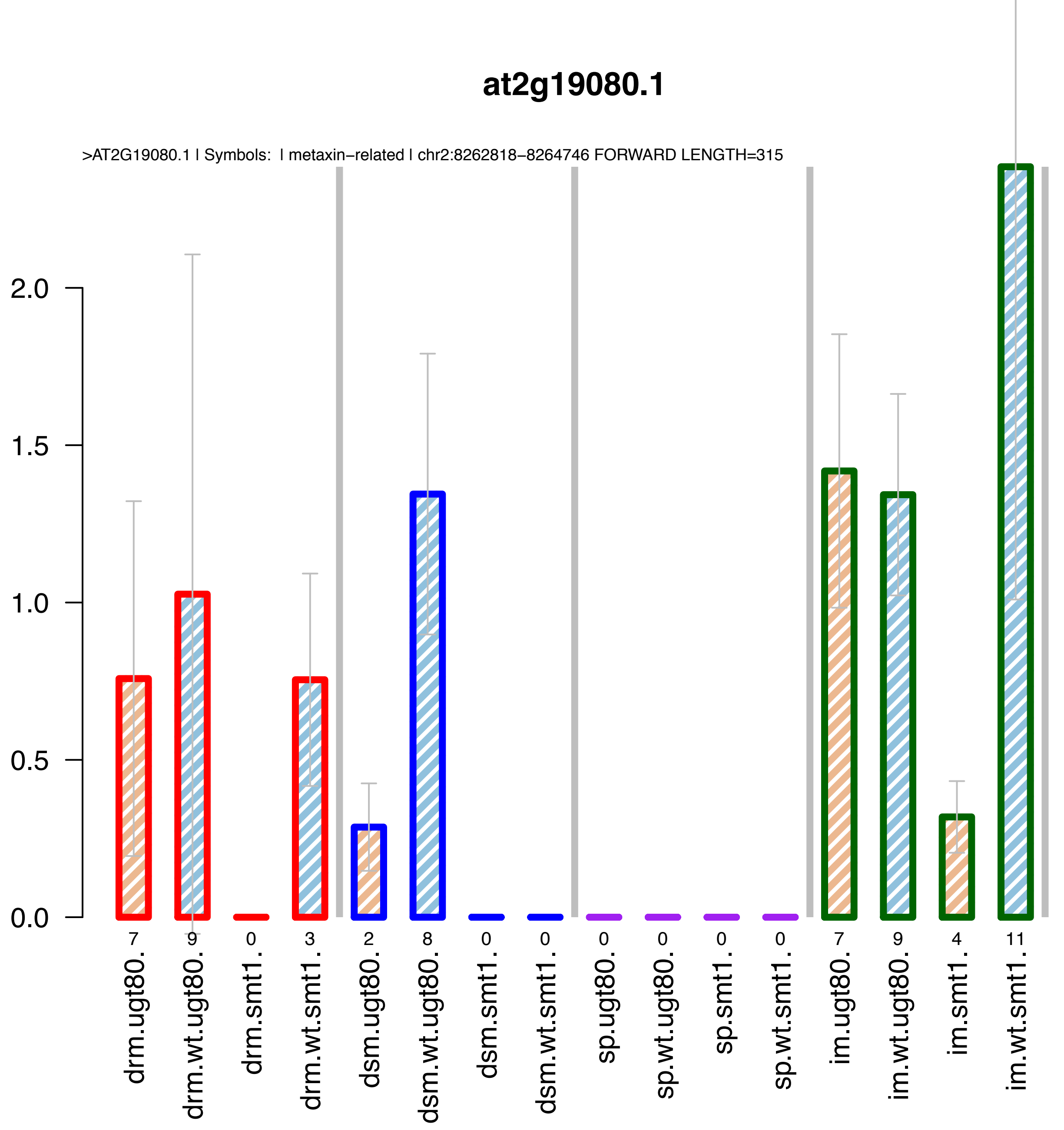

at2g19080.1
>AT2G19080.1 | Symbols: | metaxin-related | chr2:8262818-8264746 FORWARD LENGTH=315
2.0
1.5
1.0
0.5
0.0
7
9
0
3
2
8
0
0
0
0
0
0
7
9
4
11
drm.ugt80.
drm.wt.ugt80.
drm.smt1.
drm.wt.smt1.
dsm.ugt80.
dsm.wt.ugt80.
dsm.smt1.
dsm.wt.smt1.
sp.ugt80.
sp.wt.ugt80.
sp.smt1.
sp.wt.smt1.
im.ugt80.
im.wt.ugt80.
im.smt1.
im.wt.smt1.

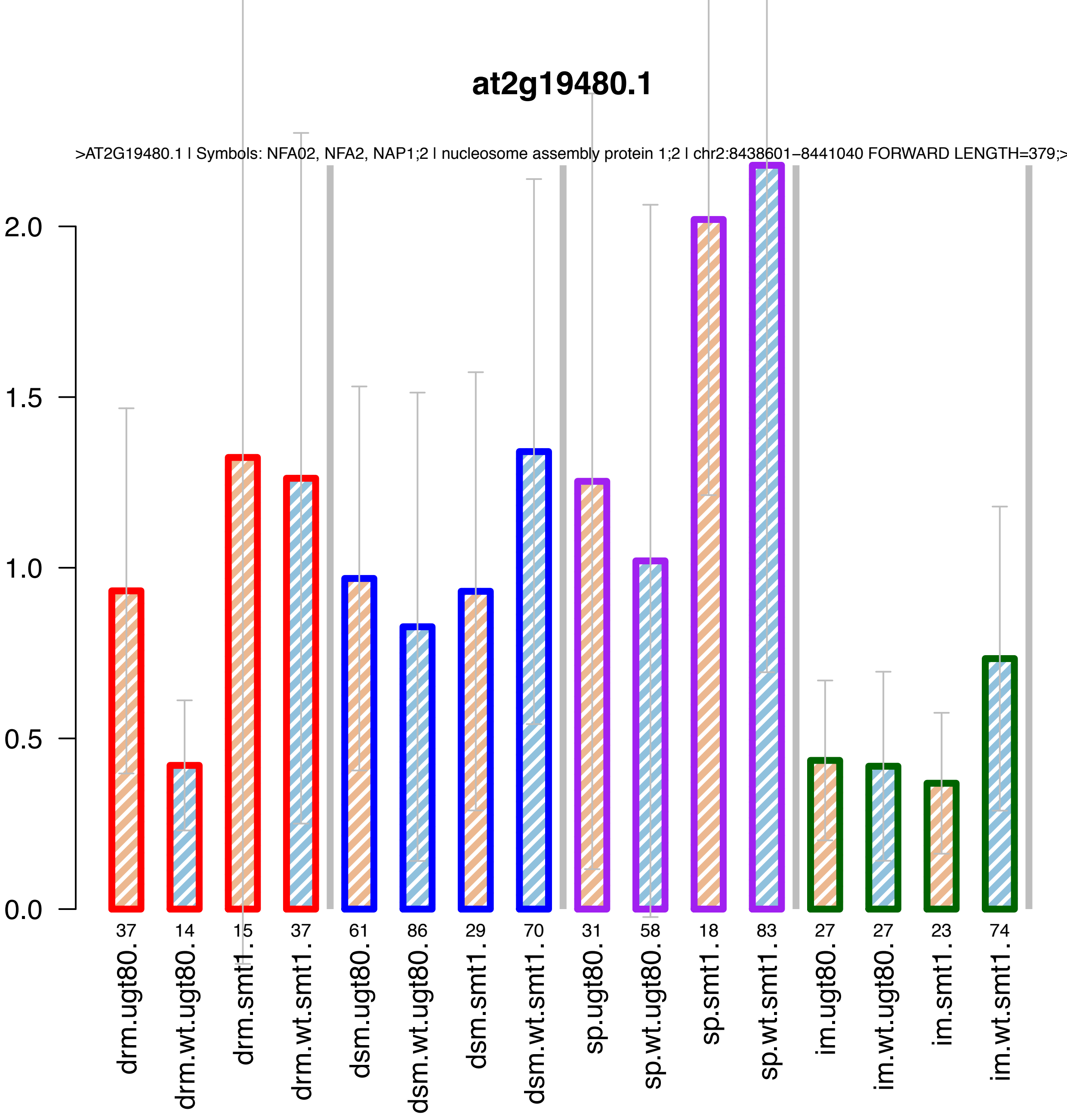

at2g19480.1
>AT2G19480.1 | Symbols: NFA02, NFA2, NAP1;2 | nucleosome assembly protein 1;2 | chr2:8438601-8441040 FORWARD LENGTH=379;>
2.0
1.5
1.0
0.5
0.0
37
14
15
37
61
86
29
70
31
58
18
83
27
27
23
74
drm.ugt80.
drm.wt.ugt80.
drm.smt1.
drm.wt.smt1.
dsm.ugt80.
dsm.wt.ugt80.
dsm.smt1.
dsm.wt.smt1.
sp.ugt80.
sp.wt.ugt80.
sp.smt1.
sp.wt.smt1.
im.ugt80.
im.wt.ugt80.
im.smt1.
im.wt.smt1.

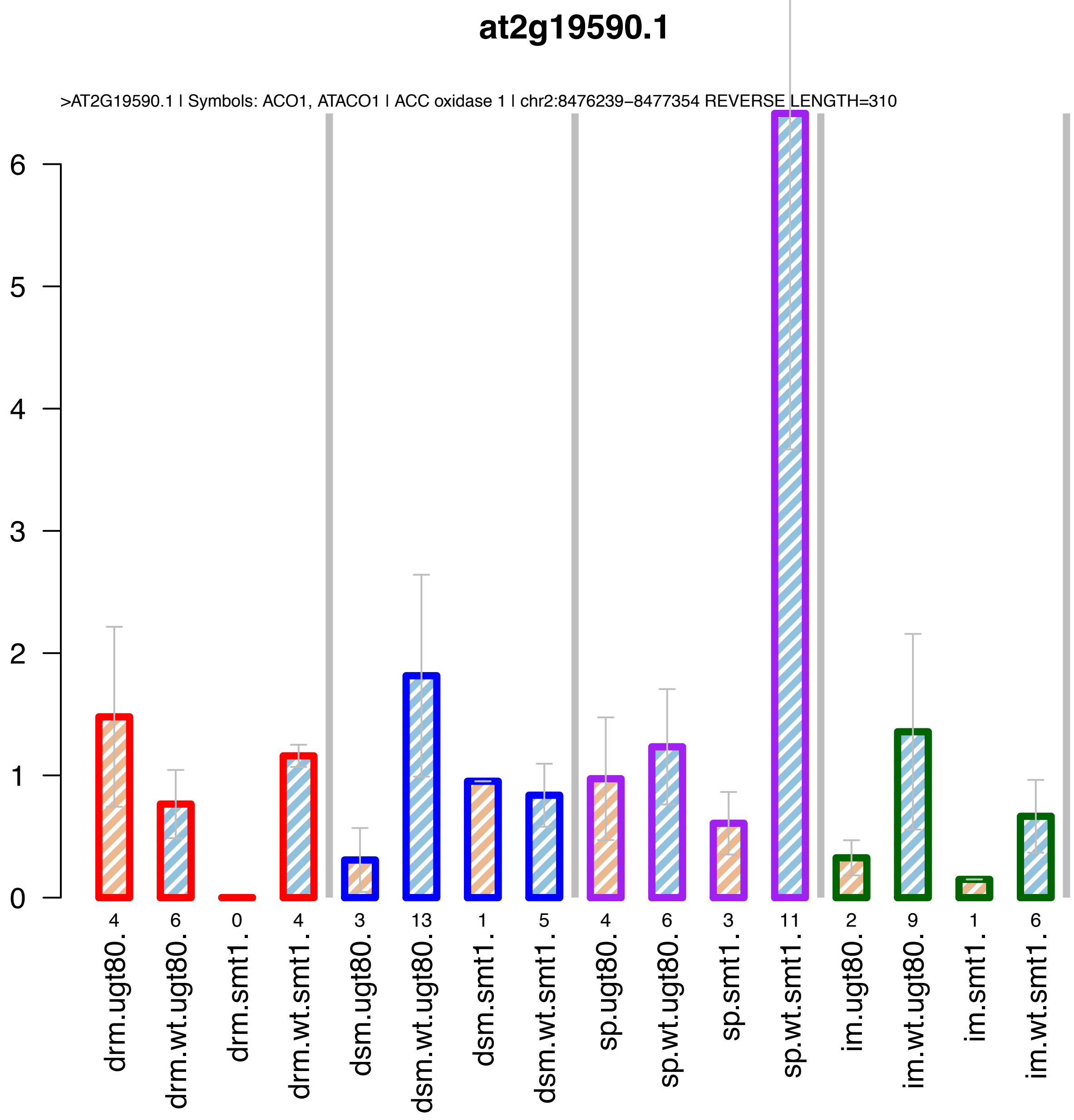
at2g19590.1
>AT2G19590.1 | Symbols: ACO1, ATACO1 | ACC oxidase 1 | chr2:8476239–8477354 REVERSE LENGTH=310
0
1
2
3
4
5
6
4
6
0
4
3
13
1
5
4
6
3
11
2
9
1
6
drm.ugt80.
drm.wt.ugt80.
drm.smt1.
drm.wt.smt1.
dsm.ugt80.
dsm.wt.ugt80.
dsm.smt1.
dsm.wt.smt1.
sp.ugt80.
sp.wt.ugt80.
sp.smt1.
sp.wt.smt1.
im.ugt80.
im.wt.ugt80.
im.smt1.
im.wt.smt1.

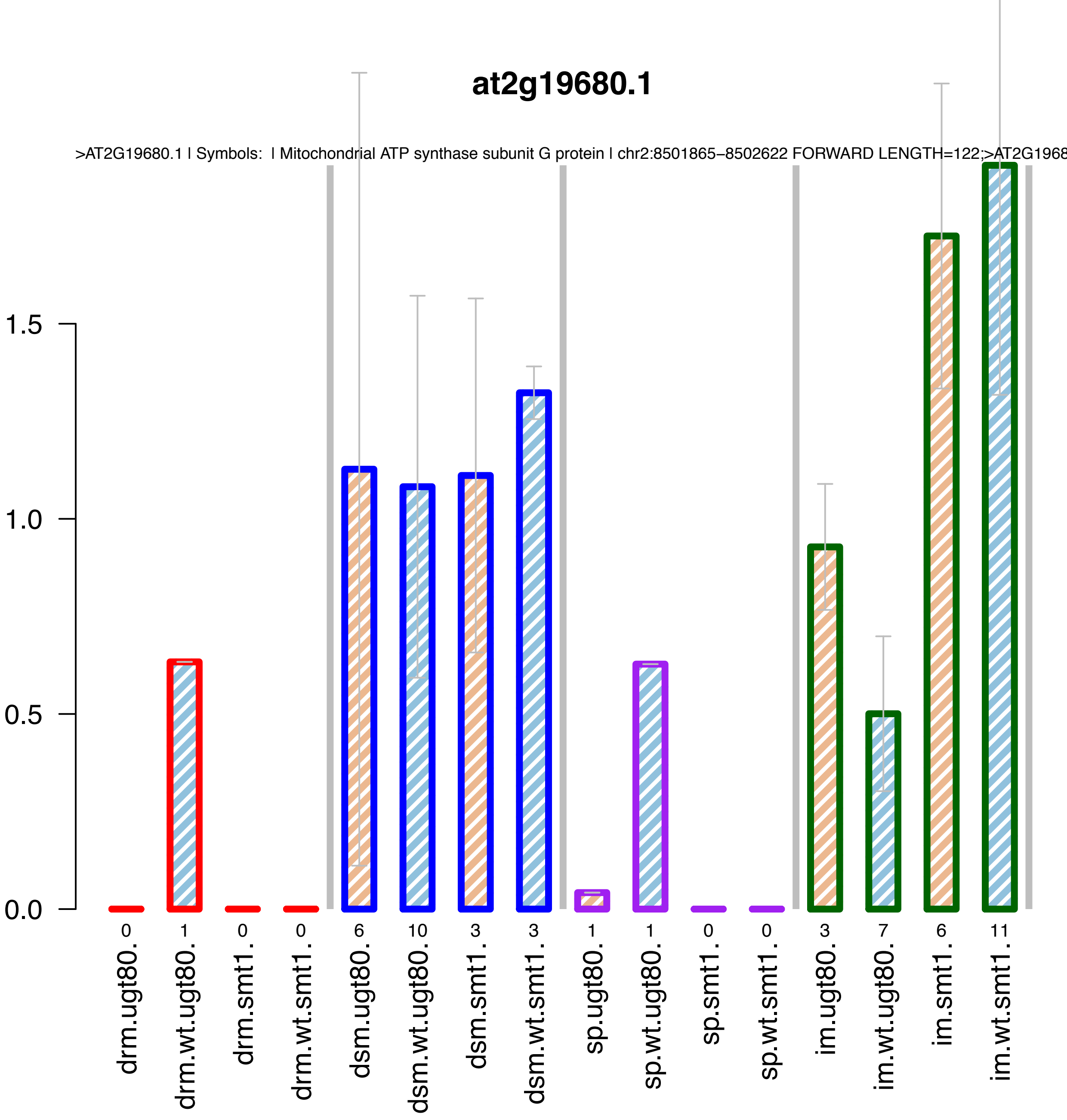
at2g19680.1
>AT2G19680.1 | Symbols: | Mitochondrial ATP synthase subunit G protein | chr2:8501865-8502622 FORWARD LENGTH=122;>AT2G1968
1.5
1.0
0.5
0.0
0
1
0
0
6
10
3
3
1
1
0
0
3
7
6
11
drm.ugt80.
drm.wt.ugt80.
drm.smt1.
drm.wt.smt1.
dsm.ugt80.
dsm.wt.ugt80.
dsm.smt1.
dsm.wt.smt1.
sp.ugt80.
sp.wt.ugt80.
sp.smt1.
sp.wt.smt1.
im.ugt80.
im.wt.ugt80.
im.smt1.
im.wt.smt1.

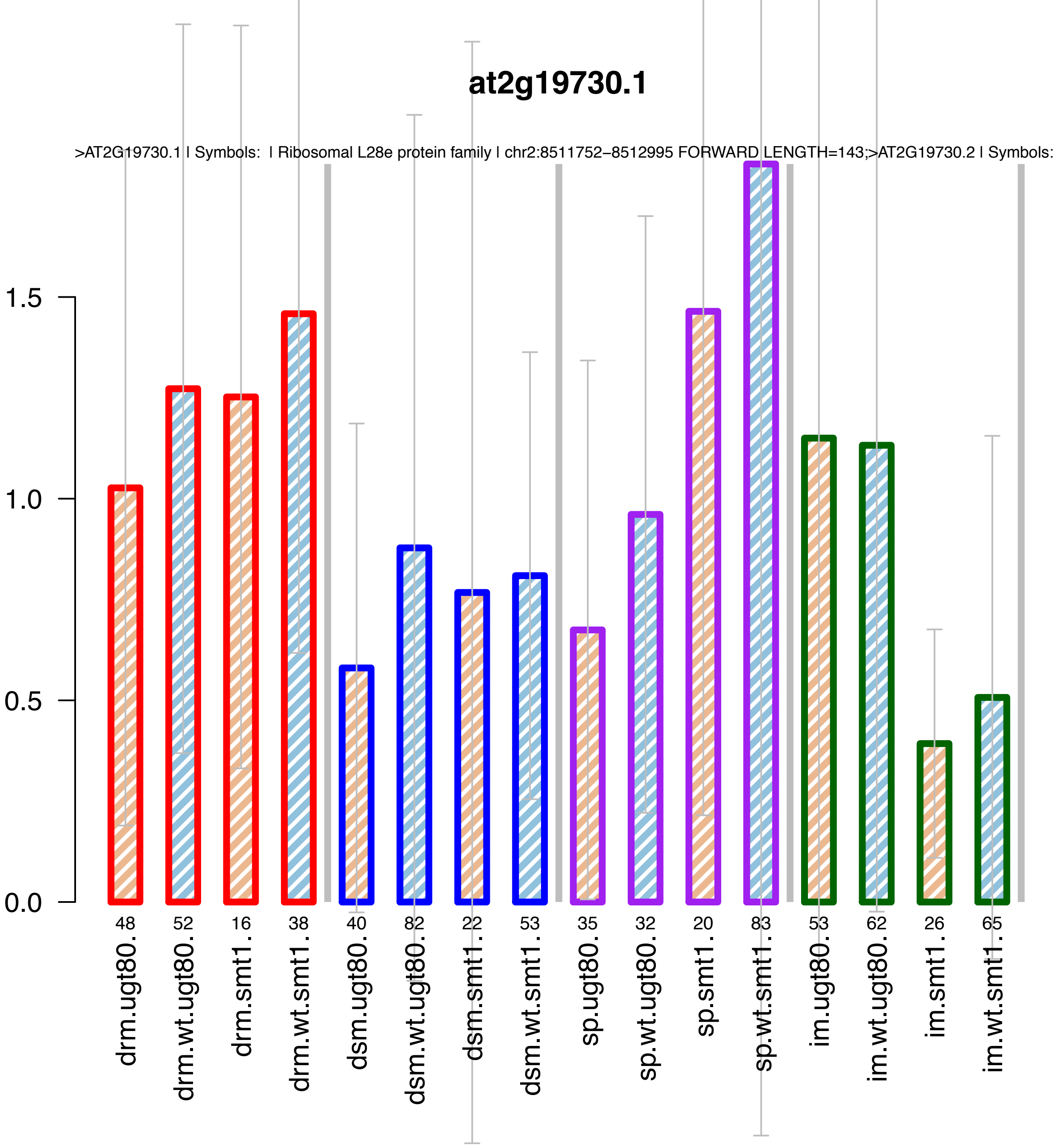

at2g19730.1
>AT2G19730.1 | Symbols: | Ribosomal L28e protein family | chr2:8511752-8512995 FORWARD LENGTH=143;>AT2G19730.2 | Symbols:
0.0
0.5
1.0
1.5
48
52
16
38
40
82
22
53
35
32
20
83
53
62
26
65
drm.ugt80..
drm.wt.ugt80..
drm.smt1..
drm.wt.smt1..
dsm.ugt80..
dsm.wt.ugt80..
dsm.smt1..
dsm.wt.smt1..
sp.ugt80..
sp.wt.ugt80..
sp.smt1..
sp.wt.smt1..
im.ugt80..
im.wt.ugt80..
im.smt1..
im.wt.smt1..

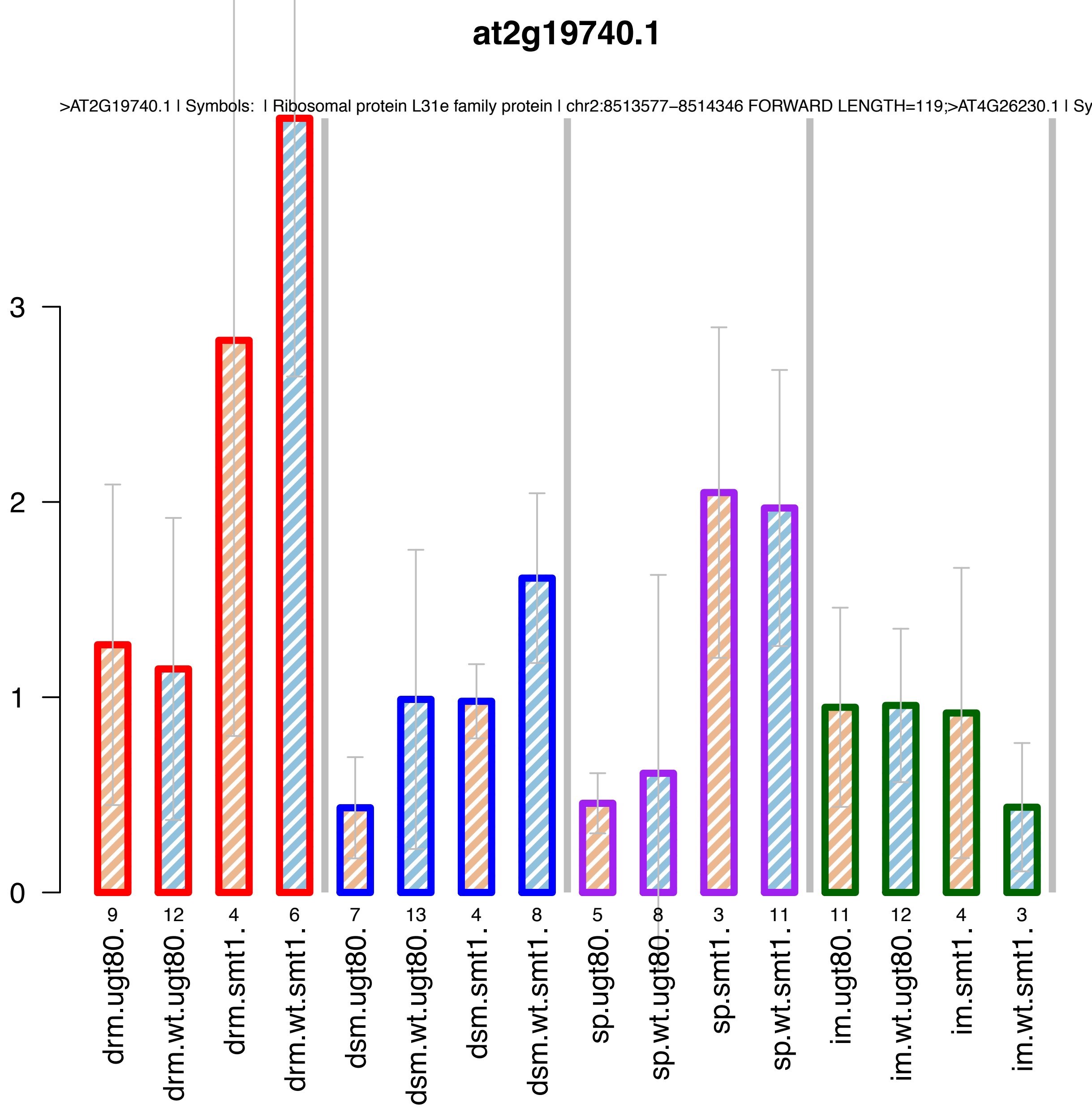

at2g19740.1
>AT2G19740.1 | Symbols: | Ribosomal protein L31e family protein | chr2:8513577–8514346 FORWARD LENGTH=119;>AT4G26230.1 | Sy
0
1
2
3
9
12
4
6
7
13
4
8
5
8
3
11
11
12
4
3
drm.ugt80..
drm.wt.ugt80..
drm.smt1..
drm.wt.smt1..
dsm.ugt80..
dsm.wt.ugt80..
dsm.smt1..
dsm.wt.smt1..
sp.ugt80..
sp.wt.ugt80..
sp.smt1..
sp.wt.smt1..
im.ugt80..
im.wt.ugt80..
im.smt1..
im.wt.smt1..

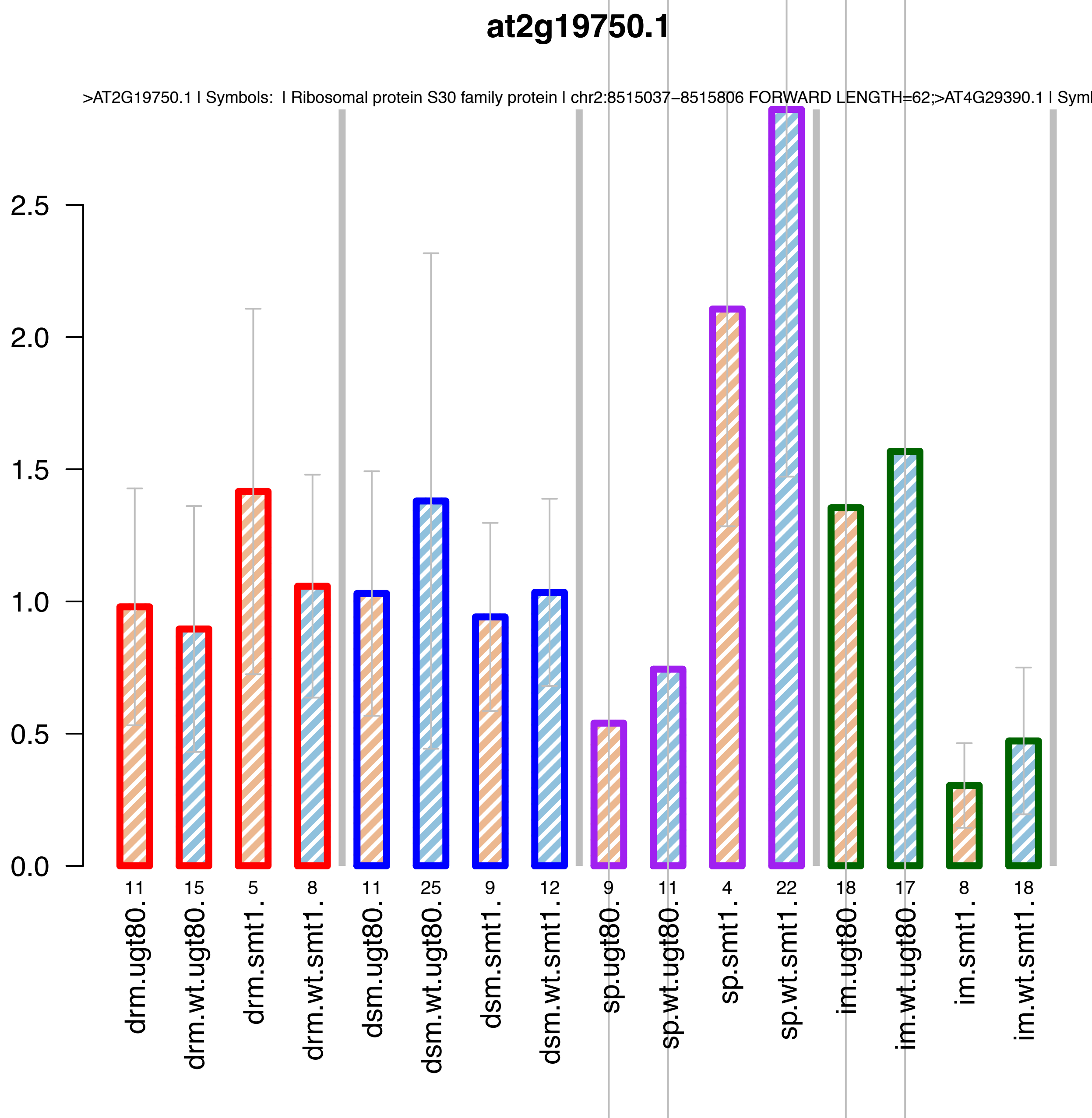

at2g19750.1
>AT2G19750.1 | Symbols: | Ribosomal protein S30 family protein | chr2:8515037-8515806 FORWARD LENGTH=62;>AT4G29390.1 | Symb
2.5
2.0
1.5
1.0
0.5
0.0
11
15
5
8
11
25
9
12
9
11
4
22
18
17
8
18
drm.ugt80..
drm.wt.ugt80..
drm.smt1..
drm.wt.smt1..
dsm.ugt80..
dsm.wt.ugt80..
dsm.smt1..
dsm.wt.smt1..
sp.ugt80..
sp.wt.ugt80..
sp.smt1..
sp.wt.smt1..
im.ugt80..
im.wt.ugt80..
im.smt1..
im.wt.smt1..

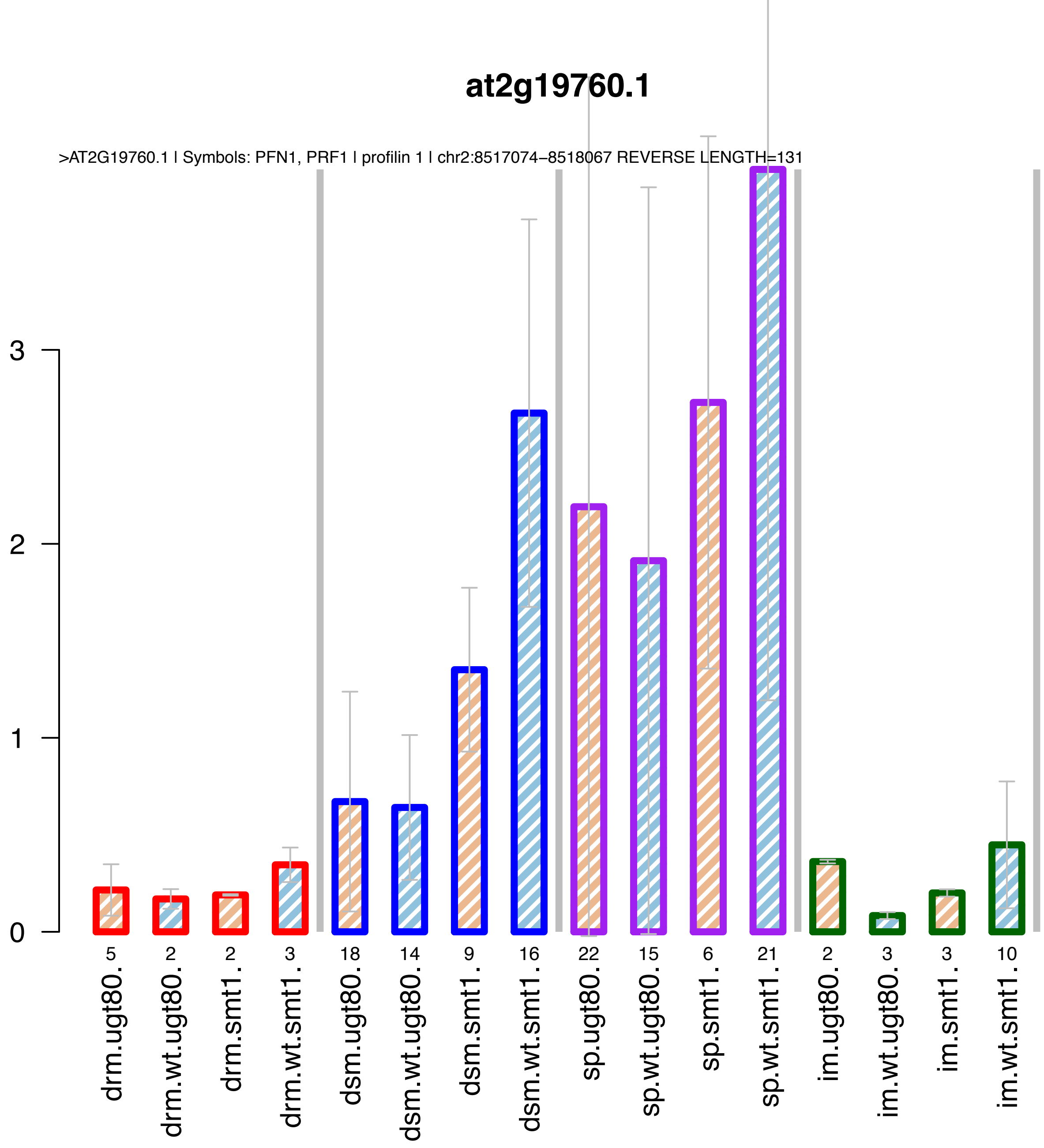

at2g19760.1
>AT2G19760.1 | Symbols: PFN1, PRF1 | profilin 1 | chr2:8517074–8518067 REVERSE LENGTH=131
0
1
2
3
5
2
2
3
18
14
9
16
22
15
6
21
2
3
3
10
drm.ugt80.
drm.wt.ugt80.
drm.smt1.
drm.wt.smt1.
dsm.ugt80.
dsm.wt.ugt80.
dsm.smt1.
dsm.wt.smt1.
sp.ugt80.
sp.wt.ugt80.
sp.smt1.
sp.wt.smt1.
im.ugt80.
im.wt.ugt80.
im.smt1.
im.wt.smt1.

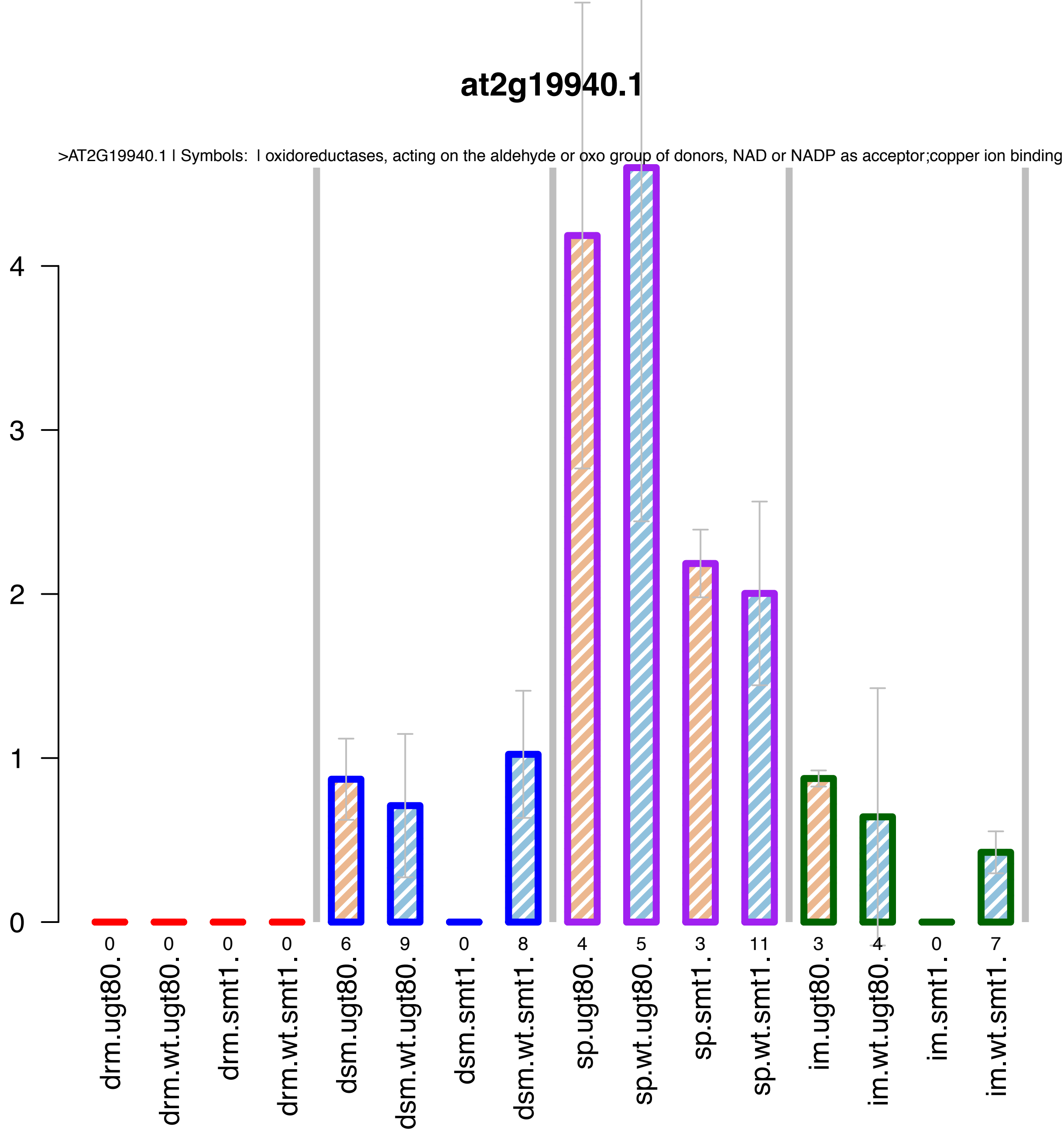

at2g19940.1
>AT2G19940.1 | Symbols: | oxidoreductases, acting on the aldehyde or oxo group of donors, NAD or NADP as acceptor;copper ion binding
4
3
2
1
0
0
0
0
0
6
9
0
8
4
5
3
11
3
4
0
7
drm.ugt80.
drm.wt.ugt80.
drm.smt1.
drm.wt.smt1.
dsm.ugt80.
dsm.wt.ugt80.
dsm.smt1.
dsm.wt.smt1.
sp.ugt80.
sp.wt.ugt80.
sp.smt1.
sp.wt.smt1.
im.ugt80.
im.wt.ugt80.
im.smt1.
im.wt.smt1.

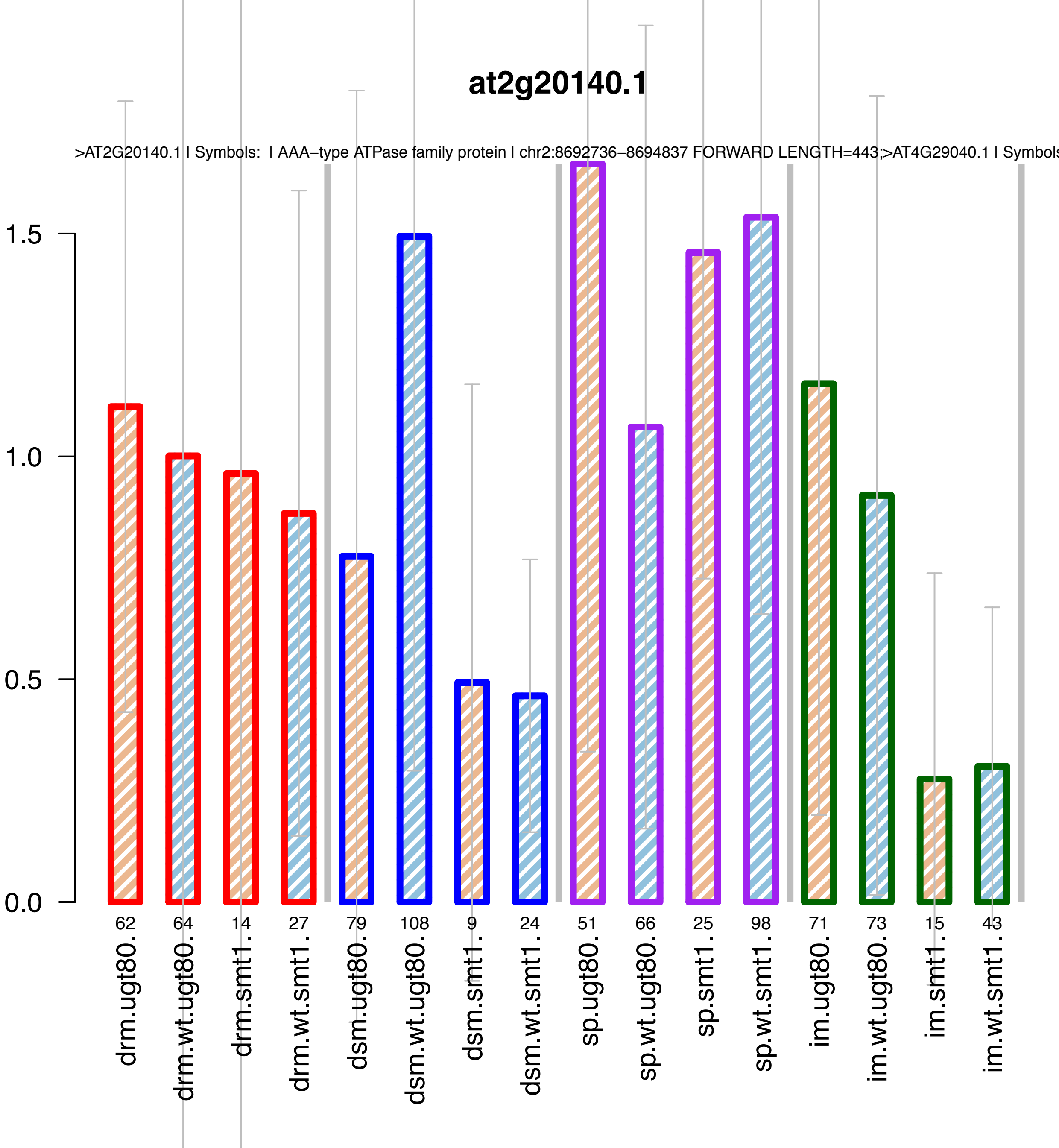
at2g20140.1
>AT2G20140.1 | Symbols: | AAA−type ATPase family protein | chr2:8692736−8694837 FORWARD LENGTH=443;>AT4G29040.1 | Symbols
1.5
1.0
0.5
0.0
62
64
14
27
79
108
9
24
51
66
25
98
71
73
15
43
drm.ugt80..
drm.wt.ugt80..
drm.smt1..
drm.wt.smt1..
dsm.ugt80..
dsm.wt.ugt80..
dsm.smt1..
dsm.wt.smt1..
sp.ugt80..
sp.wt.ugt80..
sp.smt1..
sp.wt.smt1..
im.ugt80..
im.wt.ugt80..
im.smt1..
im.wt.smt1..

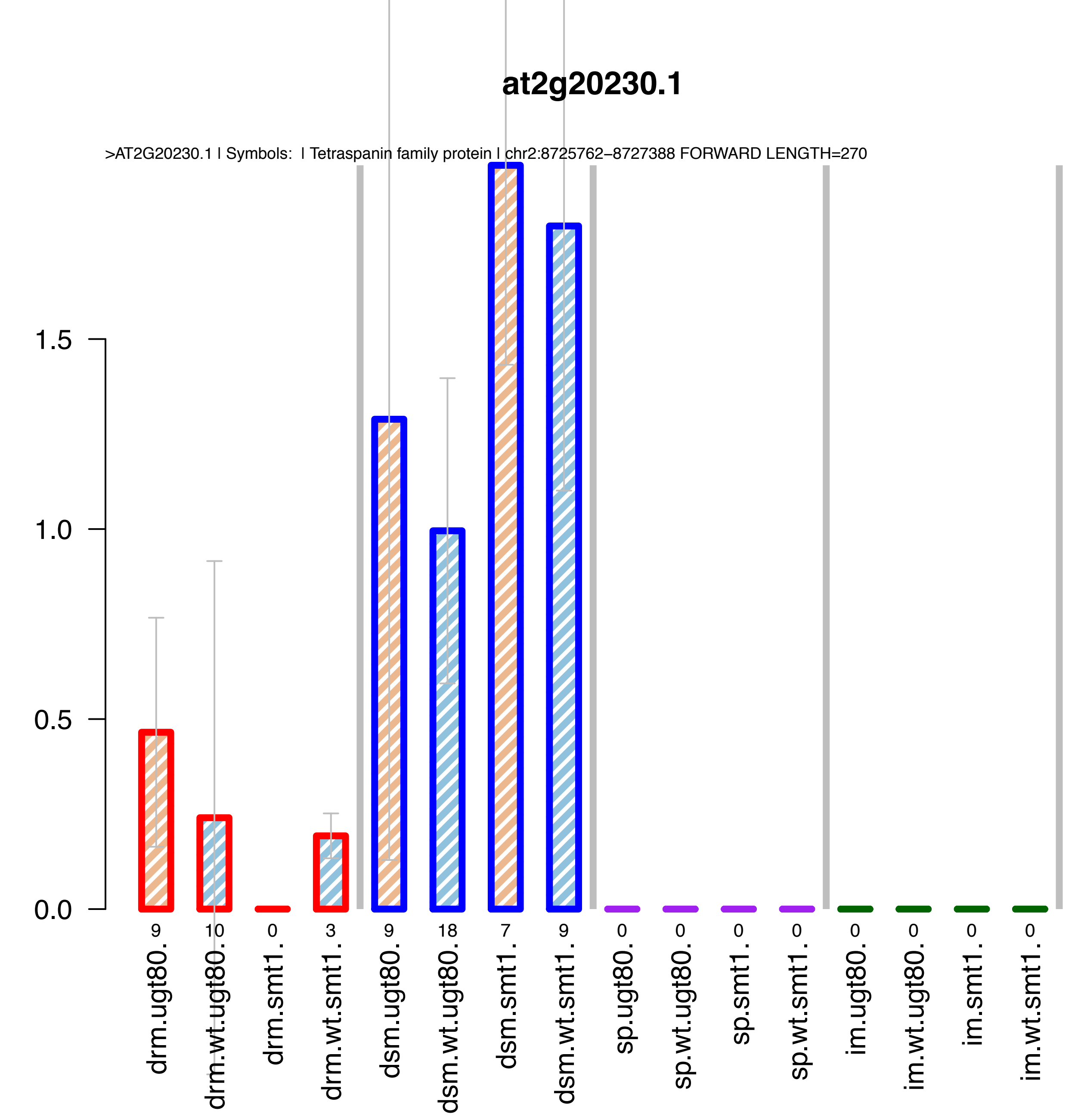

at2g20230.1
>AT2G20230.1 | Symbols: | Tetraspanin family protein | chr2:8725762-8727388 FORWARD LENGTH=270
0.0
0.5
1.0
1.5
9
10
0
3
9
18
7
9
0
0
0
0
0
0
0
0
drm.ugt80.
drm.wt.ugt80.
drm.smt1.
drm.wt.smt1.
dsm.ugt80.
dsm.wt.ugt80.
dsm.smt1.
dsm.wt.smt1.
sp.ugt80.
sp.wt.ugt80.
sp.smt1.
sp.wt.smt1.
im.ugt80.
im.wt.ugt80.
im.smt1.
im.wt.smt1.

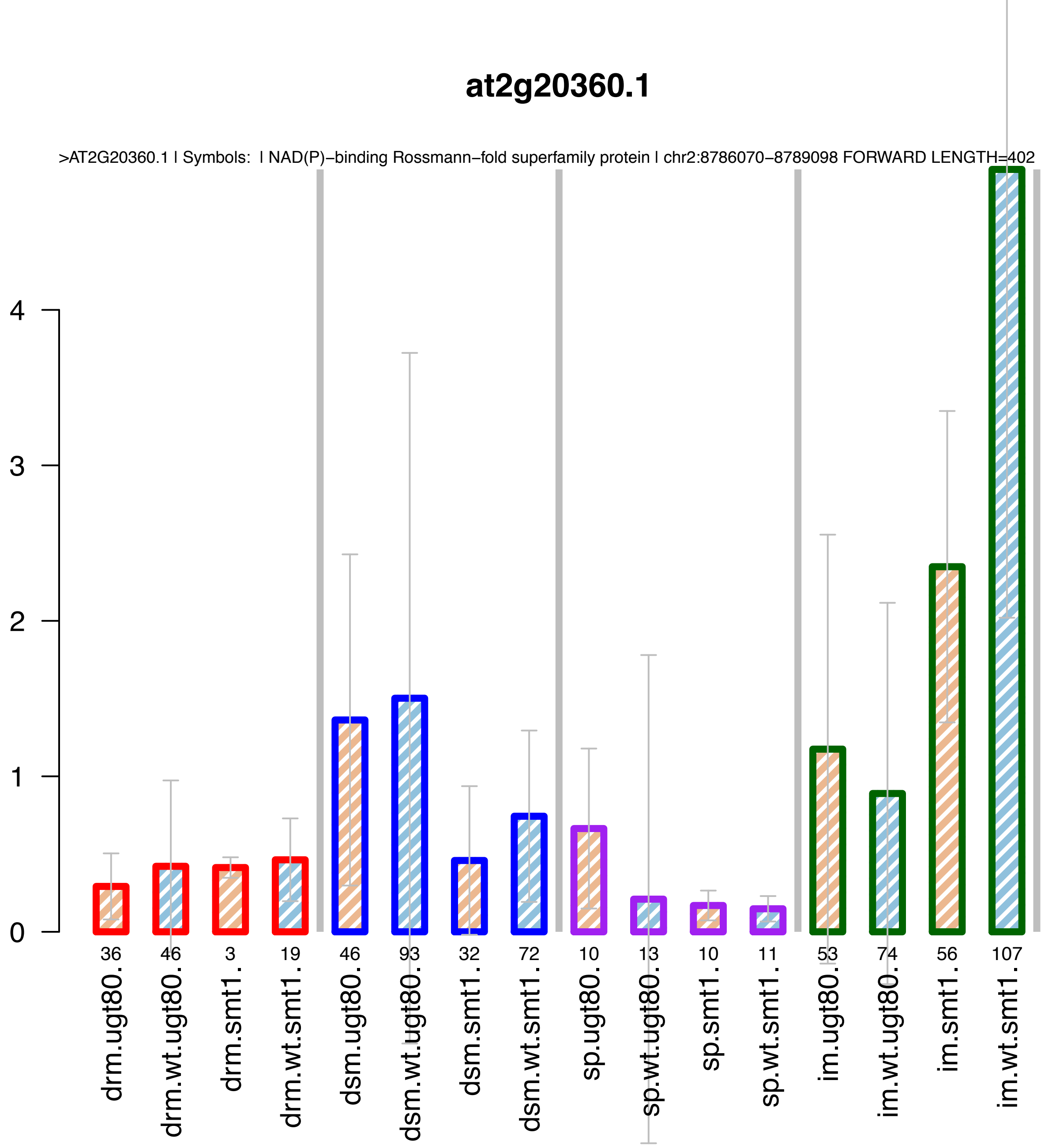

at2g20360.1
>AT2G20360.1 | Symbols: | NAD(P)−binding Rossmann−fold superfamily protein | chr2:8786070−8789098 FORWARD LENGTH=402
0
1
2
3
4
36
46
3
19
46
93
32
72
10
13
10
11
53
74
56
107
drm.ugt80..
drm.wt.ugt80..
drm.smt1..
drm.wt.smt1..
dsm.ugt80..
dsm.wt.ugt80..
dsm.smt1..
dsm.wt.smt1..
sp.ugt80..
sp.wt.ugt80..
sp.smt1..
sp.wt.smt1..
im.ugt80..
im.wt.ugt80..
im.smt1..
im.wt.smt1..

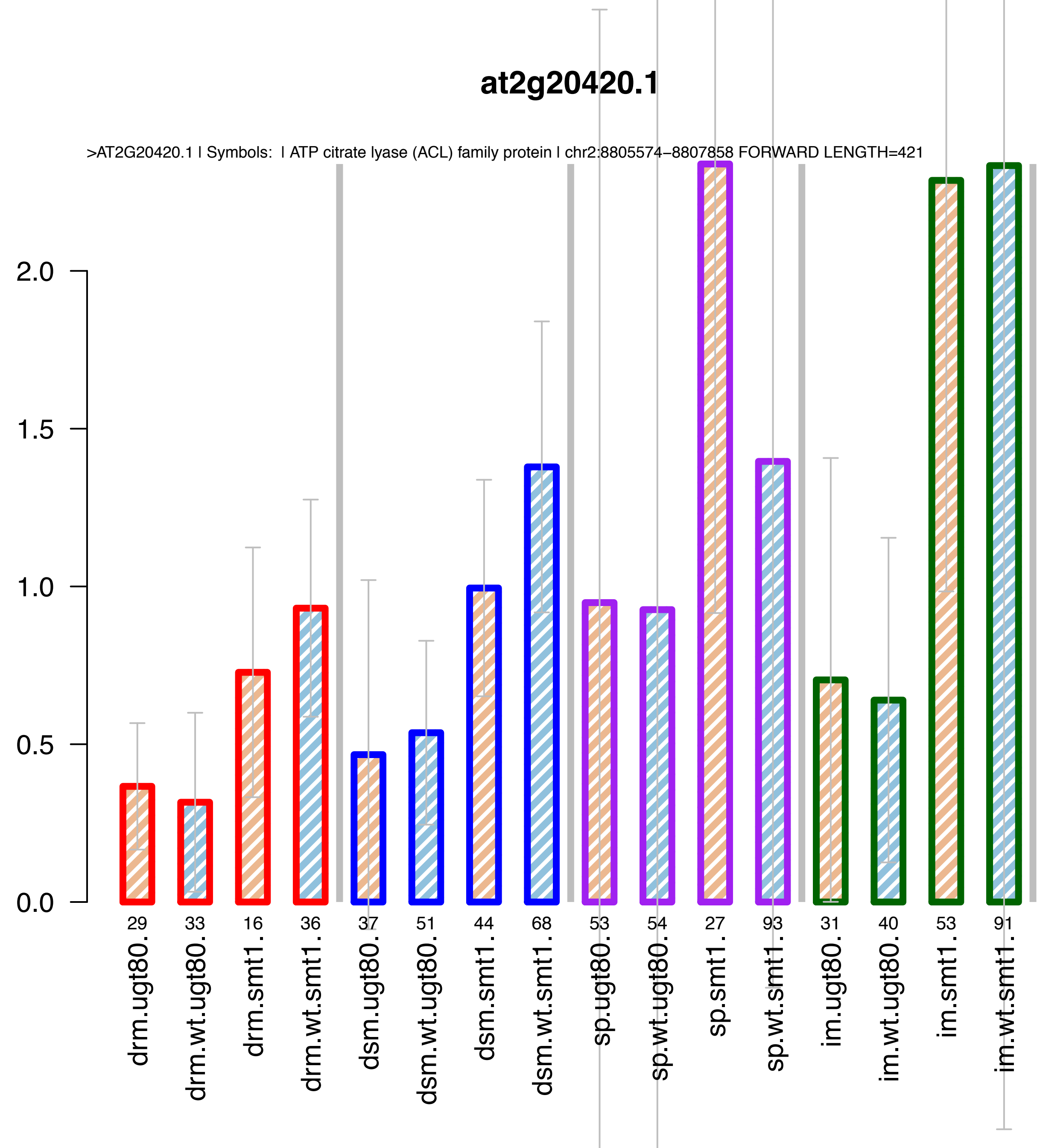

at2g20420.1
>AT2G20420.1 | Symbols: | ATP citrate lyase (ACL) family protein | chr2:8805574-8807858 FORWARD LENGTH=421
2.0
1.5
1.0
0.5
0.0
29
33
16
36
37
51
44
68
53
54
27
93
31
40
53
91
drm.ugt80.
drm.wt.ugt80.
drm.smt1.
drm.wt.smt1.
dsm.ugt80.
dsm.wt.ugt80.
dsm.smt1.
dsm.wt.smt1.
sp.ugt80.
sp.wt.ugt80.
sp.smt1.
sp.wt.smt1.
im.ugt80.
im.wt.ugt80.
im.smt1.
im.wt.smt1.

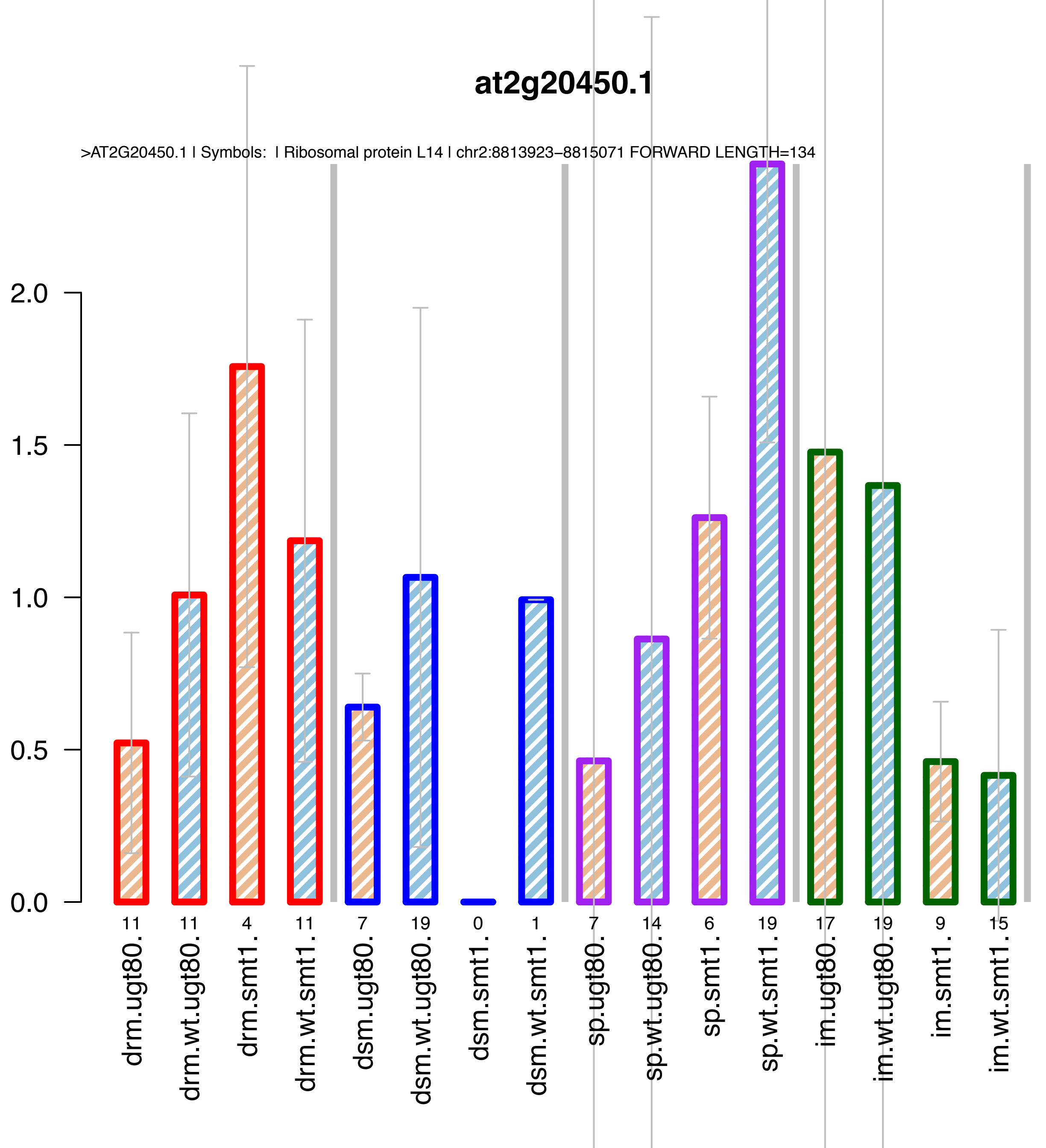
at2g20450.1
>AT2G20450.1 | Symbols: | Ribosomal protein L14 | chr2:8813923–8815071 FORWARD LENGTH=134
2.0
1.5
1.0
0.5
0.0
11
11
4
11
7
19
0
1
7
14
6
19
17
19
9
15
drm.ugt80..
drm.wt.ugt80..
drm.smt1..
drm.wt.smt1..
dsm.ugt80..
dsm.wt.ugt80..
dsm.smt1..
dsm.wt.smt1..
sp.ugt80..
sp.wt.ugt80..
sp.smt1..
sp.wt.smt1..
im.ugt80..
im.wt.ugt80..
im.smt1..
im.wt.smt1..

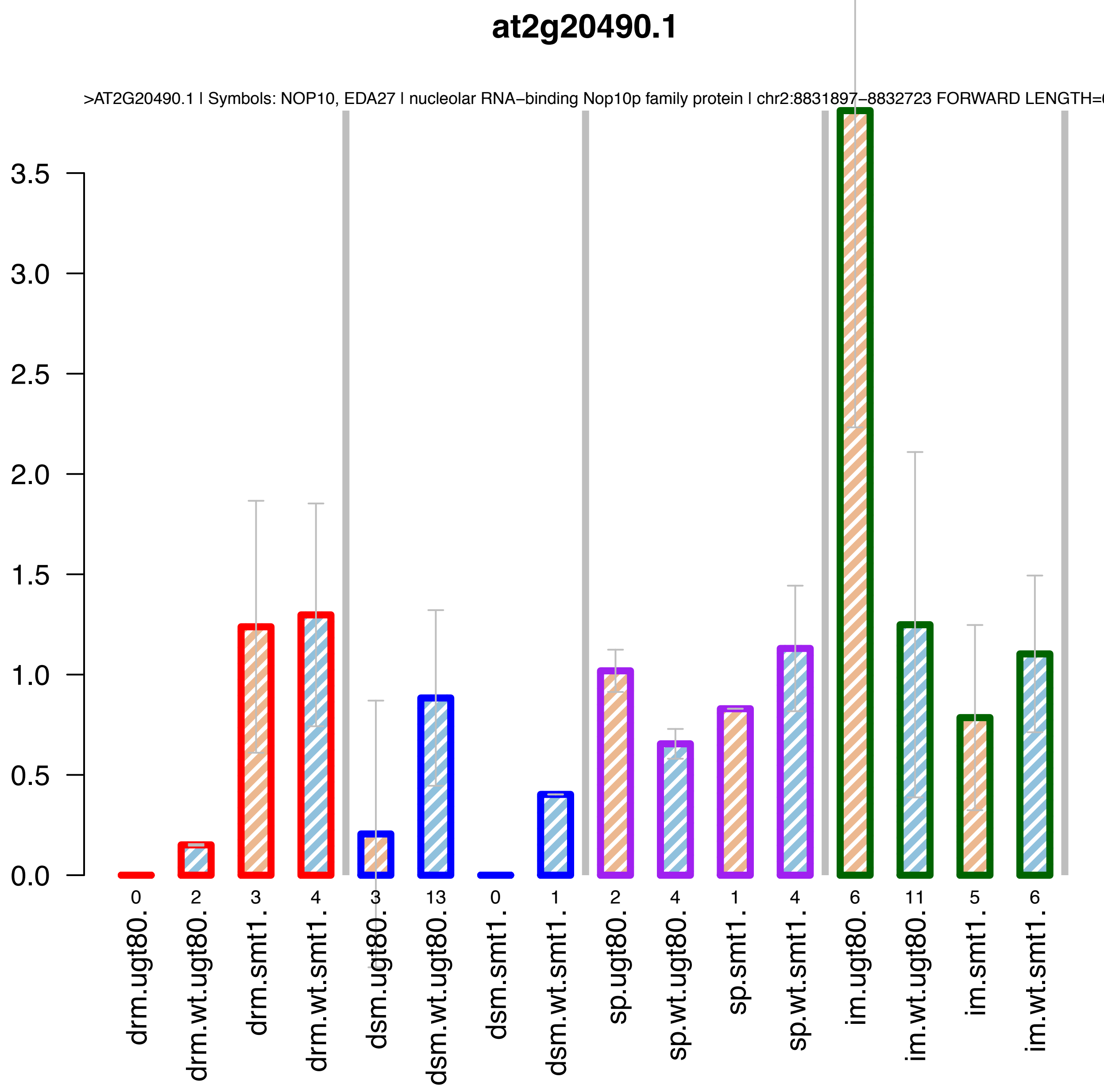

at2g20490.1
>AT2G20490.1 | Symbols: NOP10, EDA27 | nucleolar RNA−binding Nop10p family protein | chr2:8831897−8832723 FORWARD LENGTH=
0.0
0.5
1.0
1.5
2.0
2.5
3.0
3.5
0
2
3
4
3
13
0
1
2
4
1
4
6
11
5
6
drm.ugt80.
drm.wt.ugt80.
drm.smt1.
drm.wt.smt1.
dsm.ugt80.
dsm.wt.ugt80.
dsm.smt1.
dsm.wt.smt1.
sp.ugt80.
sp.wt.ugt80.
sp.smt1.
sp.wt.smt1.
im.ugt80.
im.wt.ugt80.
im.smt1.
im.wt.smt1.

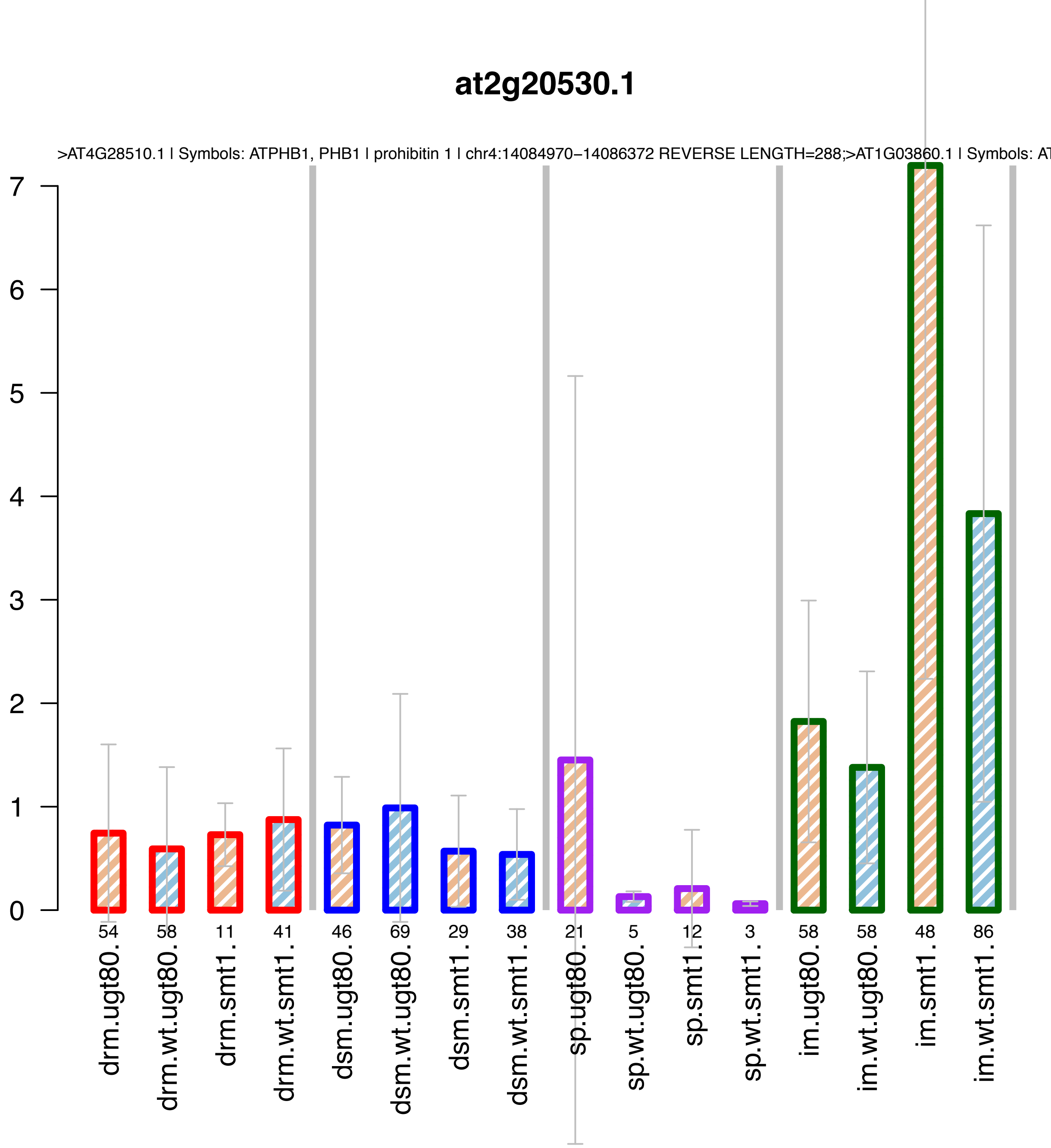
at2g20530.1
>AT4G28510.1 | Symbols: ATPHB1, PHB1 | prohibitin 1 | chr4:14084970–14086372 REVERSE LENGTH=288;>AT1G03860.1 | Symbols: AT
0
1
2
3
4
5
6
7
54
58
11
41
46
69
29
38
21
5
12
3
58
58
48
86
drm.ugt80.
drm.wt.ugt80.
drm.smt1.
drm.wt.smt1.
dsm.ugt80.
dsm.wt.ugt80.
dsm.smt1.
dsm.wt.smt1.
sp.ugt80.
sp.wt.ugt80.
sp.smt1.
sp.wt.smt1.
im.ugt80.
im.wt.ugt80.
im.smt1.
im.wt.smt1.

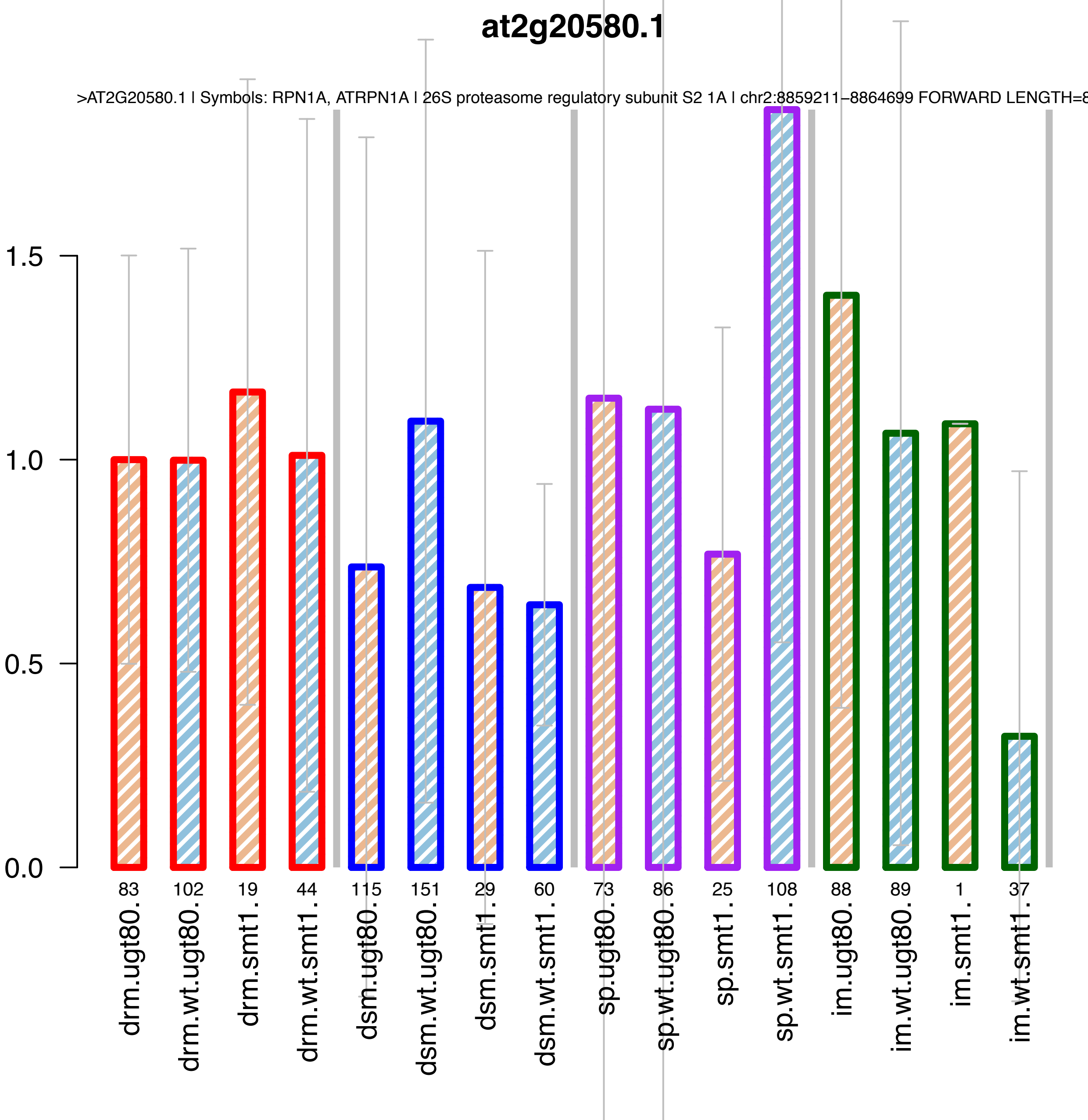

at2g20580.1
>AT2G20580.1 | Symbols: RPN1A, ATRPN1A | 26S proteasome regulatory subunit S2 1A | chr2:8859211-8864699 FORWARD LENGTH=8
1.5
1.0
0.5
0.0
83
102
19
44
115
151
29
60
73
86
25
108
88
89
1
37
drm.ugt80..
drm.wt.ugt80..
drm.smt1..
drm.wt.smt1..
dsm.ugt80..
dsm.wt.ugt80..
dsm.smt1..
dsm.wt.smt1..
sp.ugt80..
sp.wt.ugt80..
sp.smt1..
sp.wt.smt1..
im.ugt80..
im.wt.ugt80..
im.smt1..
im.wt.smt1..

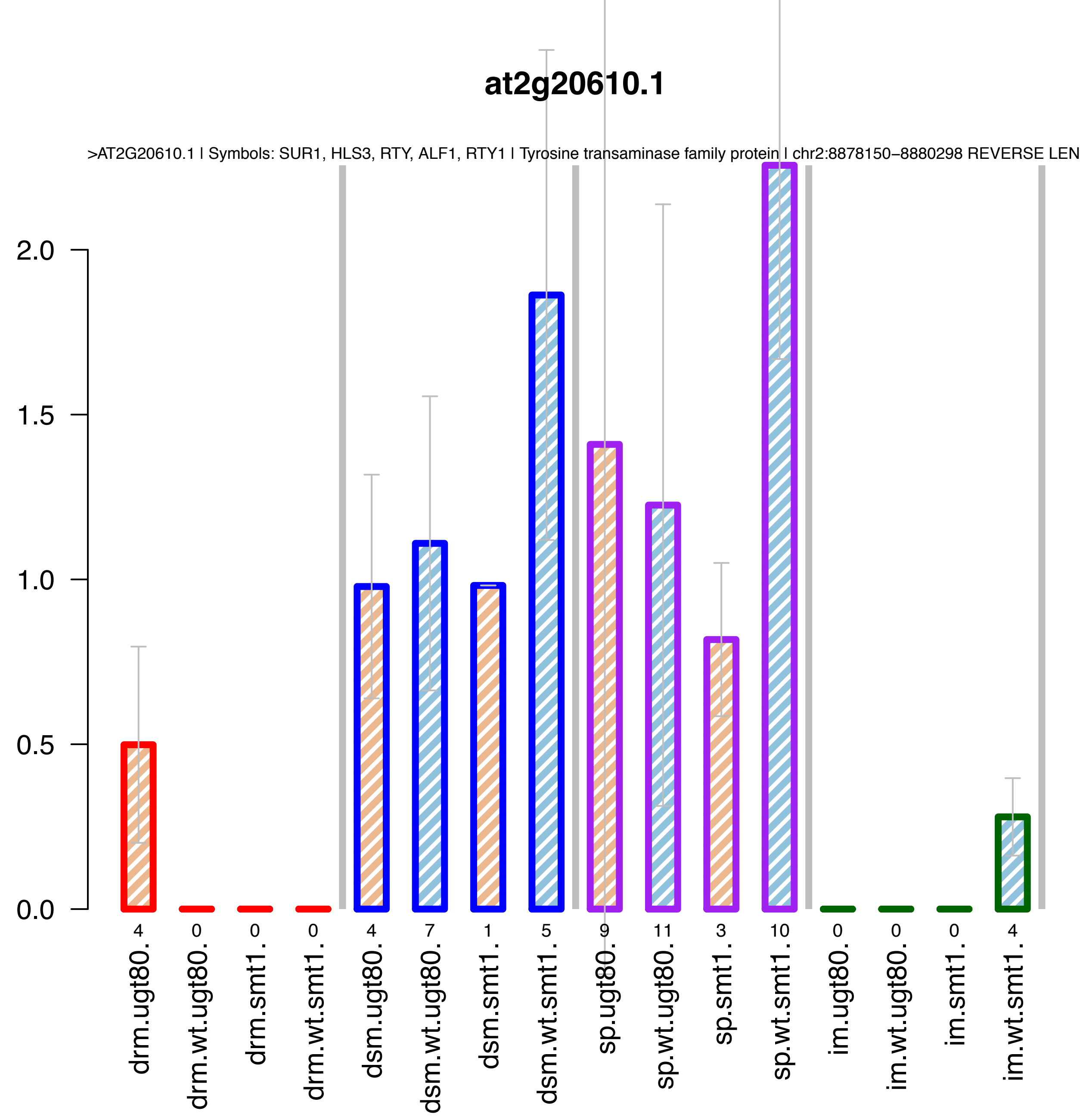

at2g20610.1
>AT2G20610.1 | Symbols: SUR1, HLS3, RTY, ALF1, RTY1 | Tyrosine transaminase family protein | chr2:8878150–8880298 REVERSE LEN
2.0
1.5
1.0
0.5
0.0
4
0
0
0
4
7
1
5
9
11
3
10
0
0
0
4
drm.ugt80.
drm.wt.ugt80.
drm.smt1.
drm.wt.smt1.
dsm.ugt80.
dsm.wt.ugt80.
dsm.smt1.
dsm.wt.smt1.
sp.ugt80.
sp.wt.ugt80.
sp.smt1.
sp.wt.smt1.
im.ugt80.
im.wt.ugt80.
im.smt1.
im.wt.smt1.

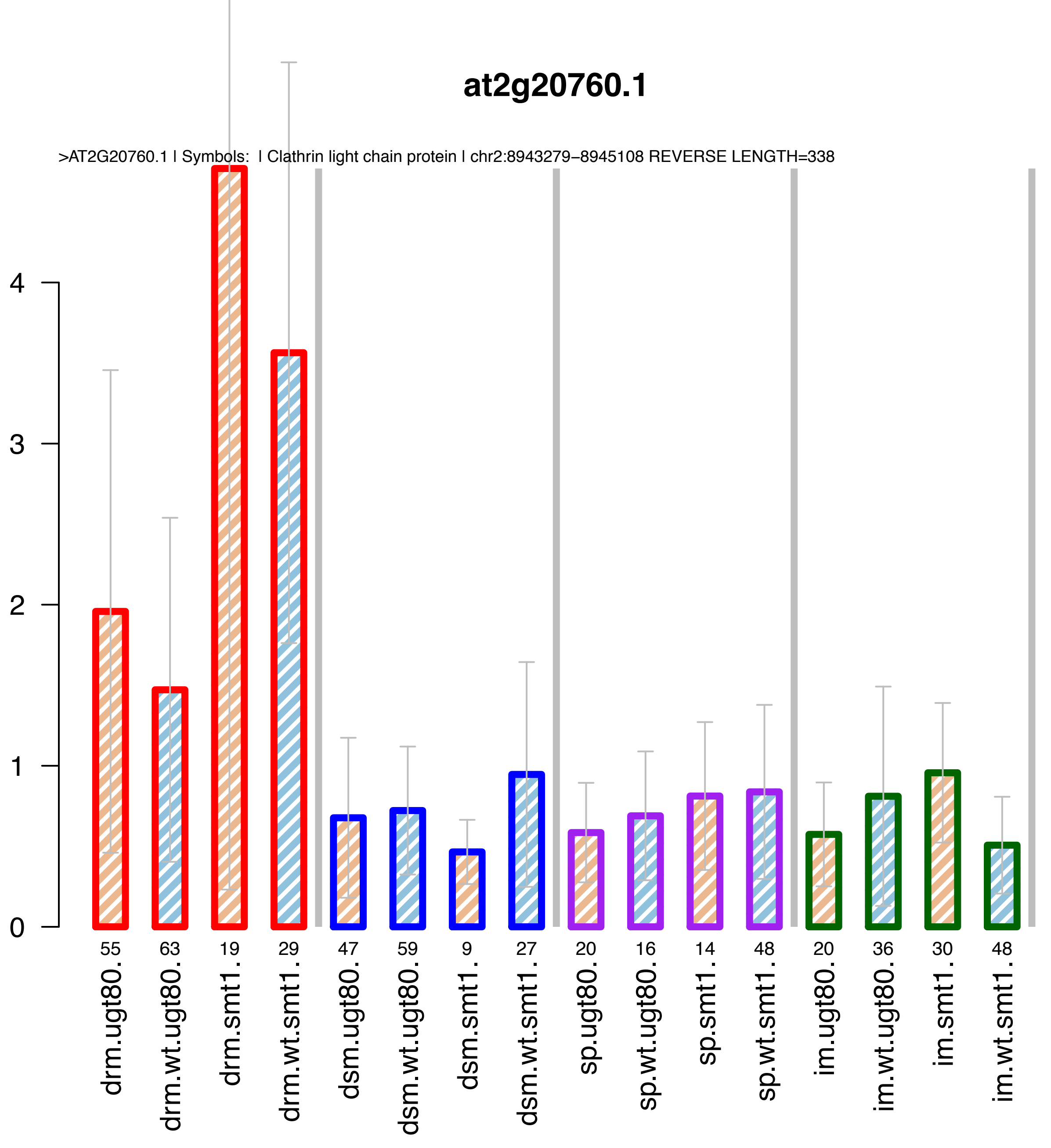

at2g20760.1
>AT2G20760.1 | Symbols: | Clathrin light chain protein | chr2:8943279-8945108 REVERSE LENGTH=338
0
1
2
3
4
55
63
19
29
47
59
9
27
20
16
14
48
20
36
30
48
drm.ugt80.
drm.wt.ugt80.
drm.smt1.
drm.wt.smt1.
dsm.ugt80.
dsm.wt.ugt80.
dsm.smt1.
dsm.wt.smt1.
sp.ugt80.
sp.wt.ugt80.
sp.smt1.
sp.wt.smt1.
im.ugt80.
im.wt.ugt80.
im.smt1.
im.wt.smt1.

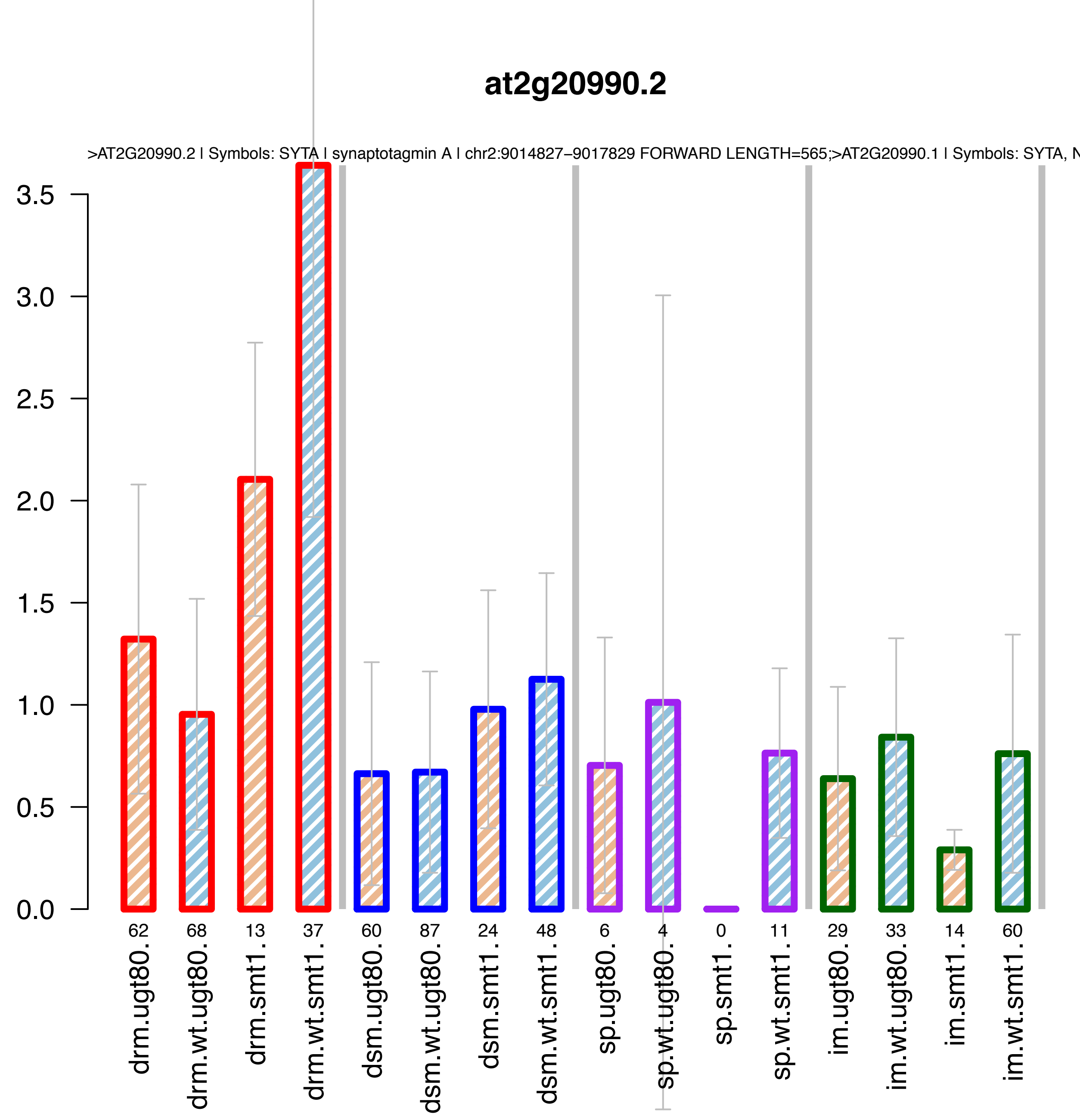
at2g20990.2
>AT2G20990.2 | Symbols: SYTA | synaptotagmin A | chr2:9014827−9017829 FORWARD LENGTH=565;>AT2G20990.1 | Symbols: SYTA, N
3.5
3.0
2.5
2.0
1.5
1.0
0.5
0.0
62
68
13
37
60
87
24
48
6
4
0
11
29
33
14
60
drm.ugt80.
drm.wt.ugt80.
drm.smt1.
drm.wt.smt1.
dsm.ugt80.
dsm.wt.ugt80.
dsm.smt1.
dsm.wt.smt1.
sp.ugt80.
sp.wt.ugt80.
sp.smt1.
sp.wt.smt1.
im.ugt80.
im.wt.ugt80.
im.smt1.
im.wt.smt1.

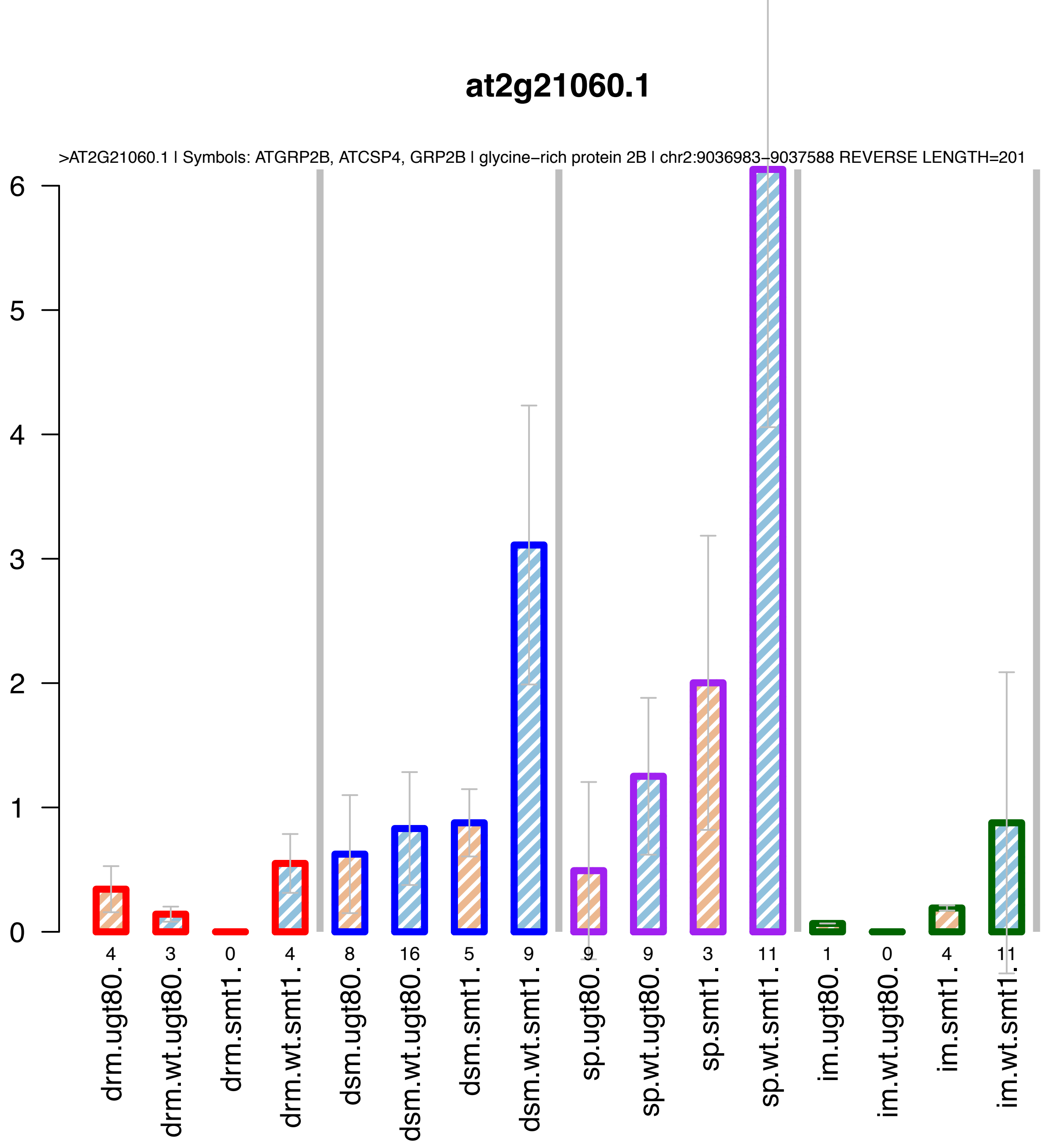
at2g21060.1
>AT2G21060.1 | Symbols: ATGRP2B, ATCSP4, GRP2B | glycine−rich protein 2B | chr2:9036983−9037588 REVERSE LENGTH=201
0
1
2
3
4
5
6
4
3
0
4
8
16
5
9
9
9
3
11
1
0
4
11
drm.ugt80.
drm.wt.ugt80.
drm.smt1.
drm.wt.smt1.
dsm.ugt80.
dsm.wt.ugt80.
dsm.smt1.
dsm.wt.smt1.
sp.ugt80.
sp.wt.ugt80.
sp.smt1.
sp.wt.smt1.
im.ugt80.
im.wt.ugt80.
im.smt1.
im.wt.smt1.

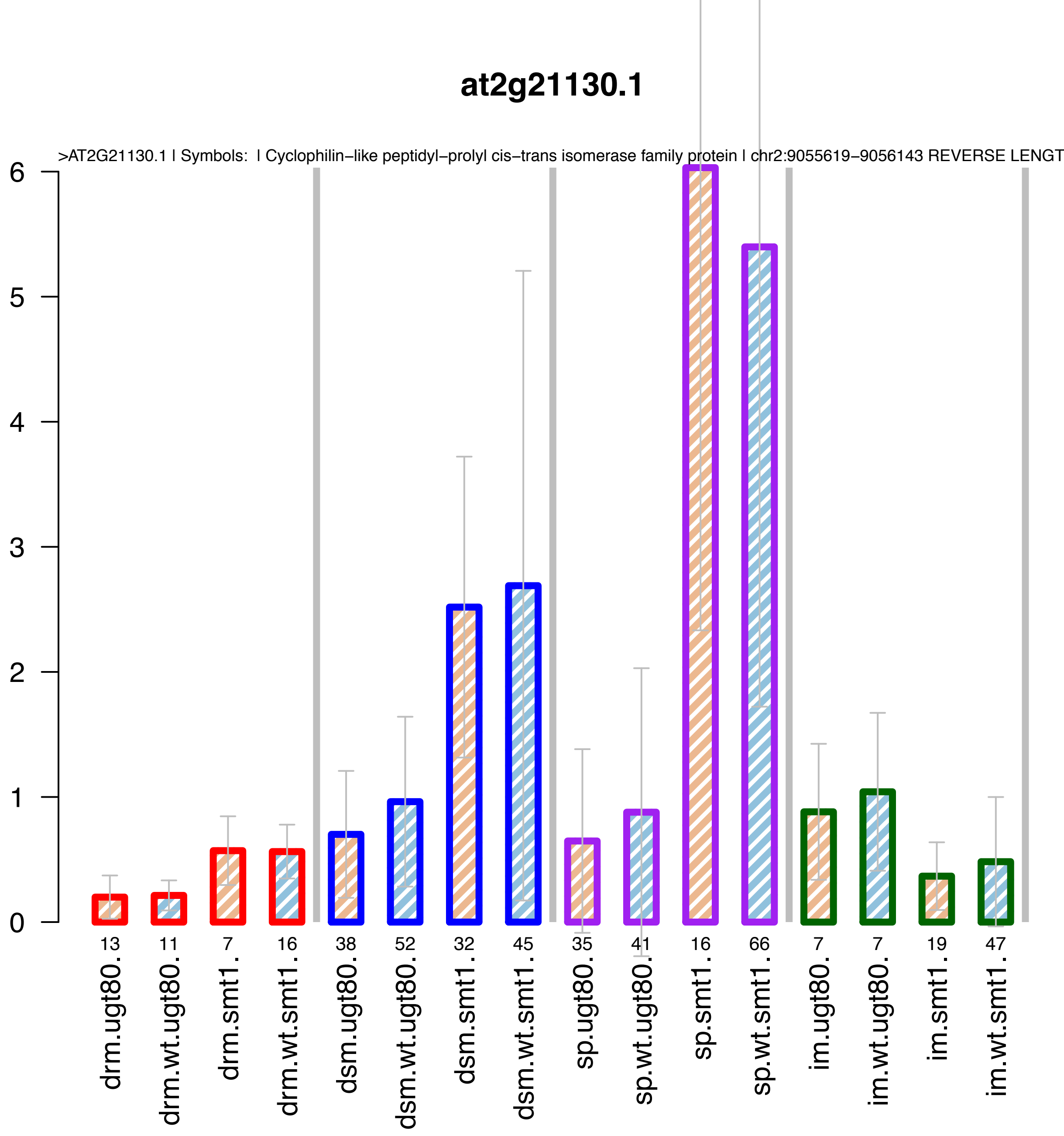

at2g21130.1
>AT2G21130.1 | Symbols: | Cyclophilin−like peptidyl−prolyl cis−trans isomerase family protein | chr2:9055619−9056143 REVERSE LENGT
0
1
2
3
4
5
6
13
11
7
16
38
52
32
45
35
41
16
66
7
7
19
47
drm.ugt80.
drm.wt.ugt80.
drm.smt1.
drm.wt.smt1.
dsm.ugt80.
dsm.wt.ugt80.
dsm.smt1.
dsm.wt.smt1.
sp.ugt80.
sp.wt.ugt80.
sp.smt1.
sp.wt.smt1.
im.ugt80.
im.wt.ugt80.
im.smt1.
im.wt.smt1.

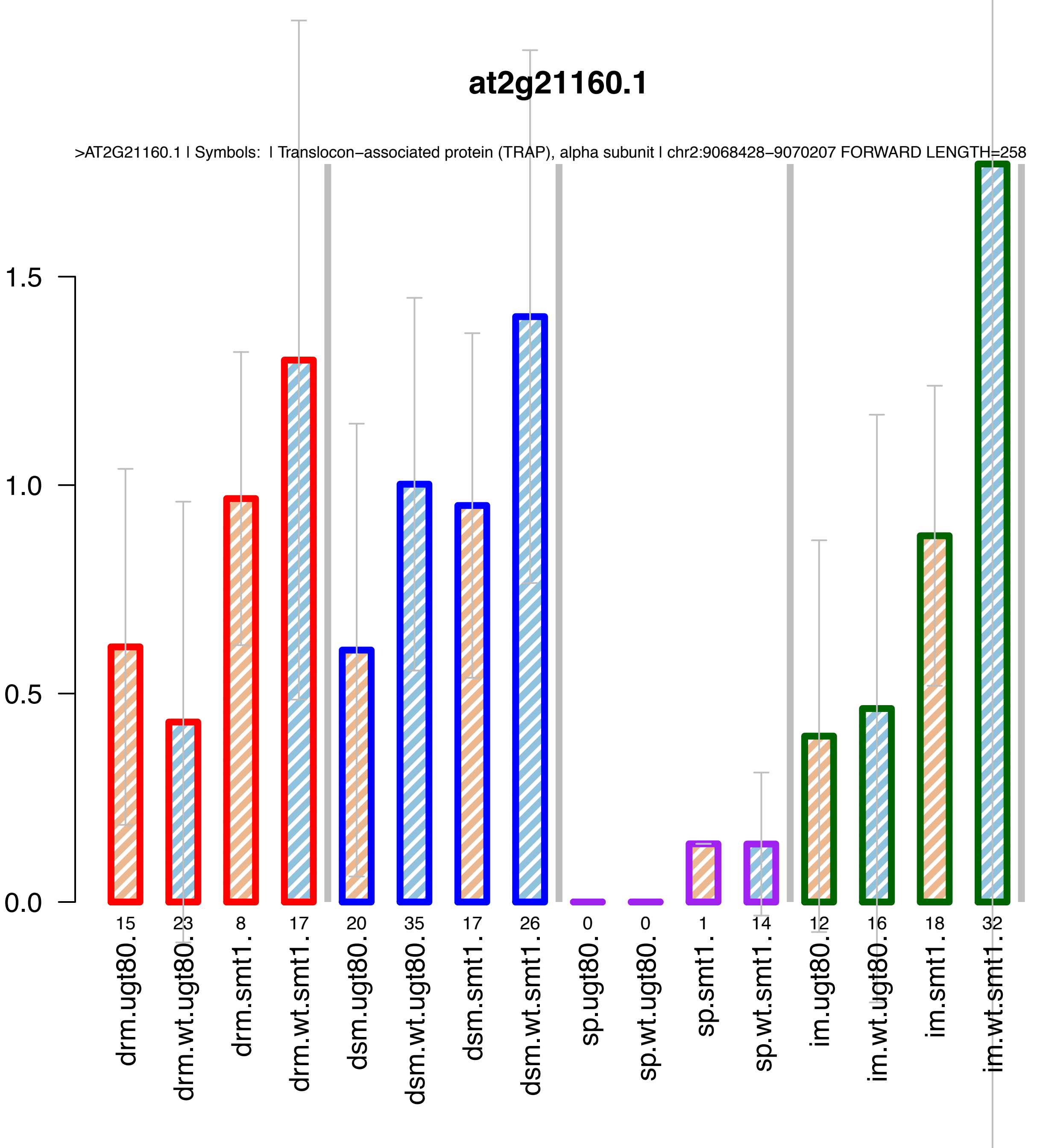

at2g21160.1
>AT2G21160.1 | Symbols: | Translocon-associated protein (TRAP), alpha subunit | chr2:9068428-9070207 FORWARD LENGTH=258
0.0
0.5
1.0
1.5
15
23
8
17
20
35
17
26
0
0
1
14
12
16
18
32
drm.ugt80.
drm.wt.ugt80.
drm.smt1.
drm.wt.smt1.
dsm.ugt80.
dsm.wt.ugt80.
dsm.smt1.
dsm.wt.smt1.
sp.ugt80.
sp.wt.ugt80.
sp.smt1.
sp.wt.smt1.
im.ugt80.
im.wt.ugt80.
im.smt1.
im.wt.smt1.

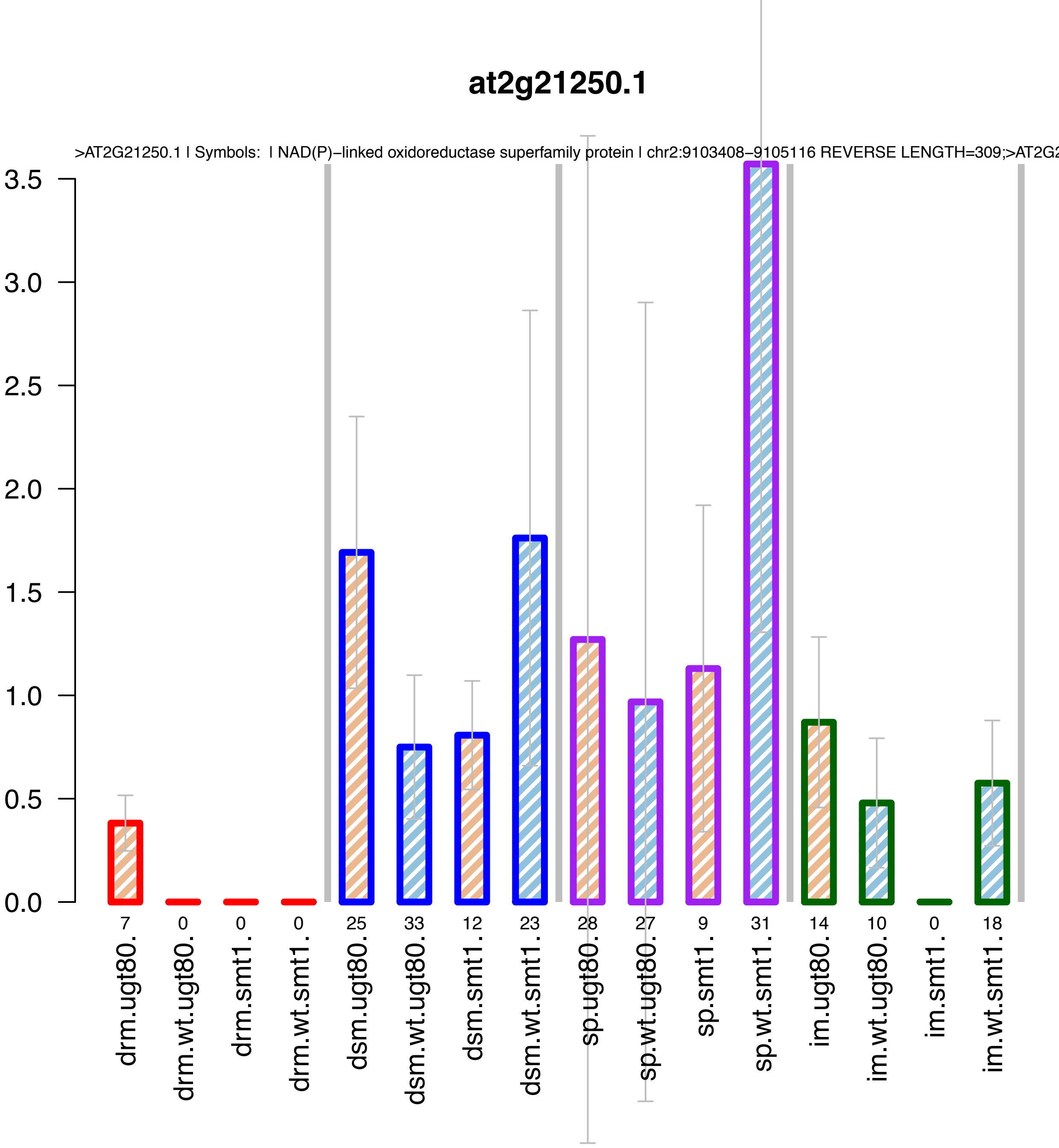

at2g21250.1
>AT2G21250.1 | Symbols: | NAD(P)–linked oxidoreductase superfamily protein | chr2:9103408–9105116 REVERSE LENGTH=309;>AT2G2
3.5
3.0
2.5
2.0
1.5
1.0
0.5
0.0
7
0
0
0
25
33
12
23
28
27
9
31
14
10
0
18
drm.ugt80.
drm.wt.ugt80.
drm.smt1.
drm.wt.smt1.
dsm.ugt80.
dsm.wt.ugt80.
dsm.smt1.
dsm.wt.smt1.
sp.ugt80.
sp.wt.ugt80.
sp.smt1.
sp.wt.smt1.
im.ugt80.
im.wt.ugt80.
im.smt1.
im.wt.smt1.

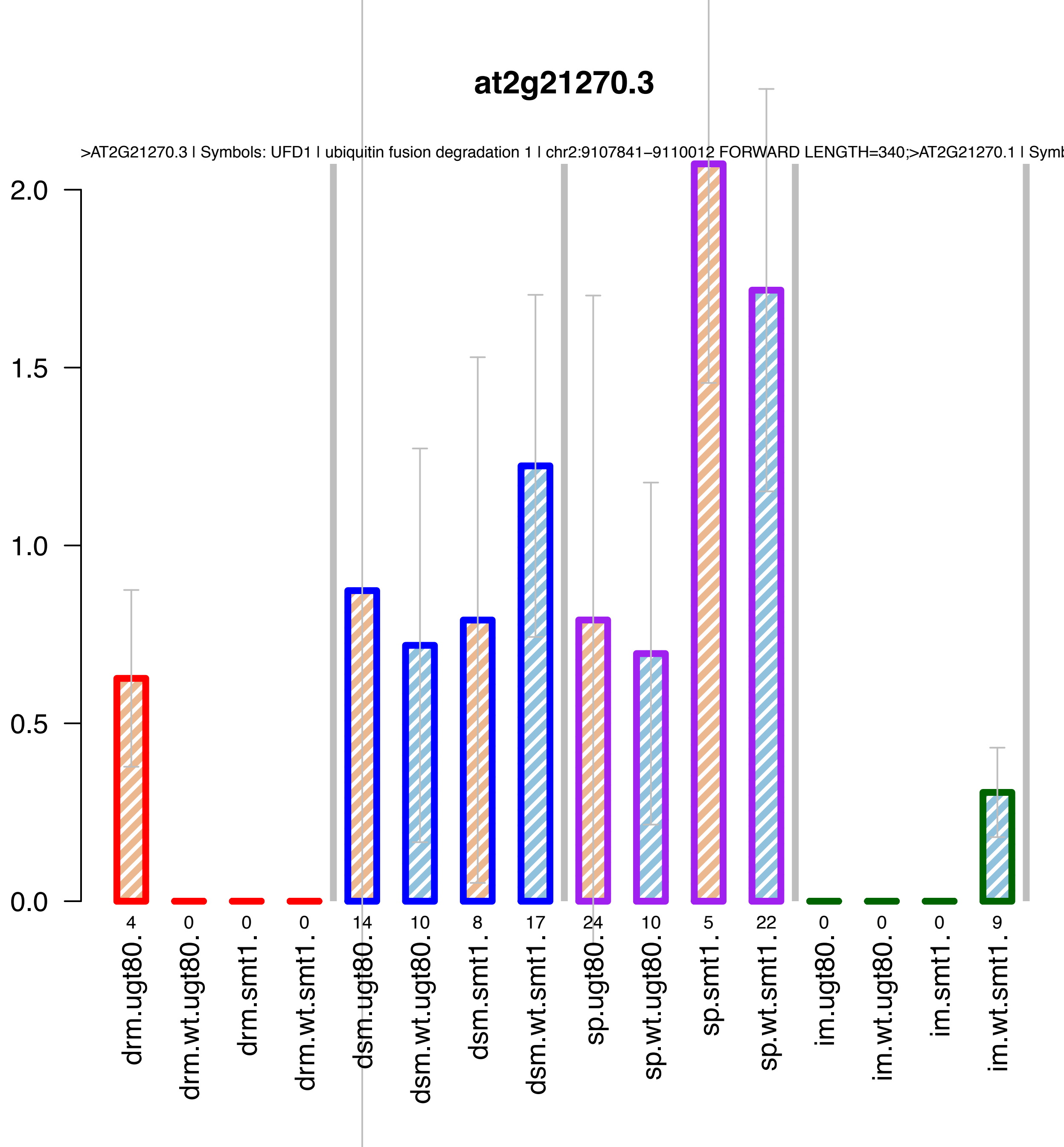

at2g21270.3
>AT2G21270.3 | Symbols: UFD1 | ubiquitin fusion degradation 1 | chr2:9107841–9110012 FORWARD LENGTH=340;>AT2G21270.1 | Symb
2.0
1.5
1.0
0.5
0.0
4
0
0
0
14
10
8
17
24
10
5
22
0
0
0
9
drm.ugt80.
drm.wt.ugt80.
drm.smt1.
drm.wt.smt1.
dsm.ugt80.
dsm.wt.ugt80.
dsm.smt1.
dsm.wt.smt1.
sp.ugt80.
sp.wt.ugt80.
sp.smt1.
sp.wt.smt1.
im.ugt80.
im.wt.ugt80.
im.smt1.
im.wt.smt1.

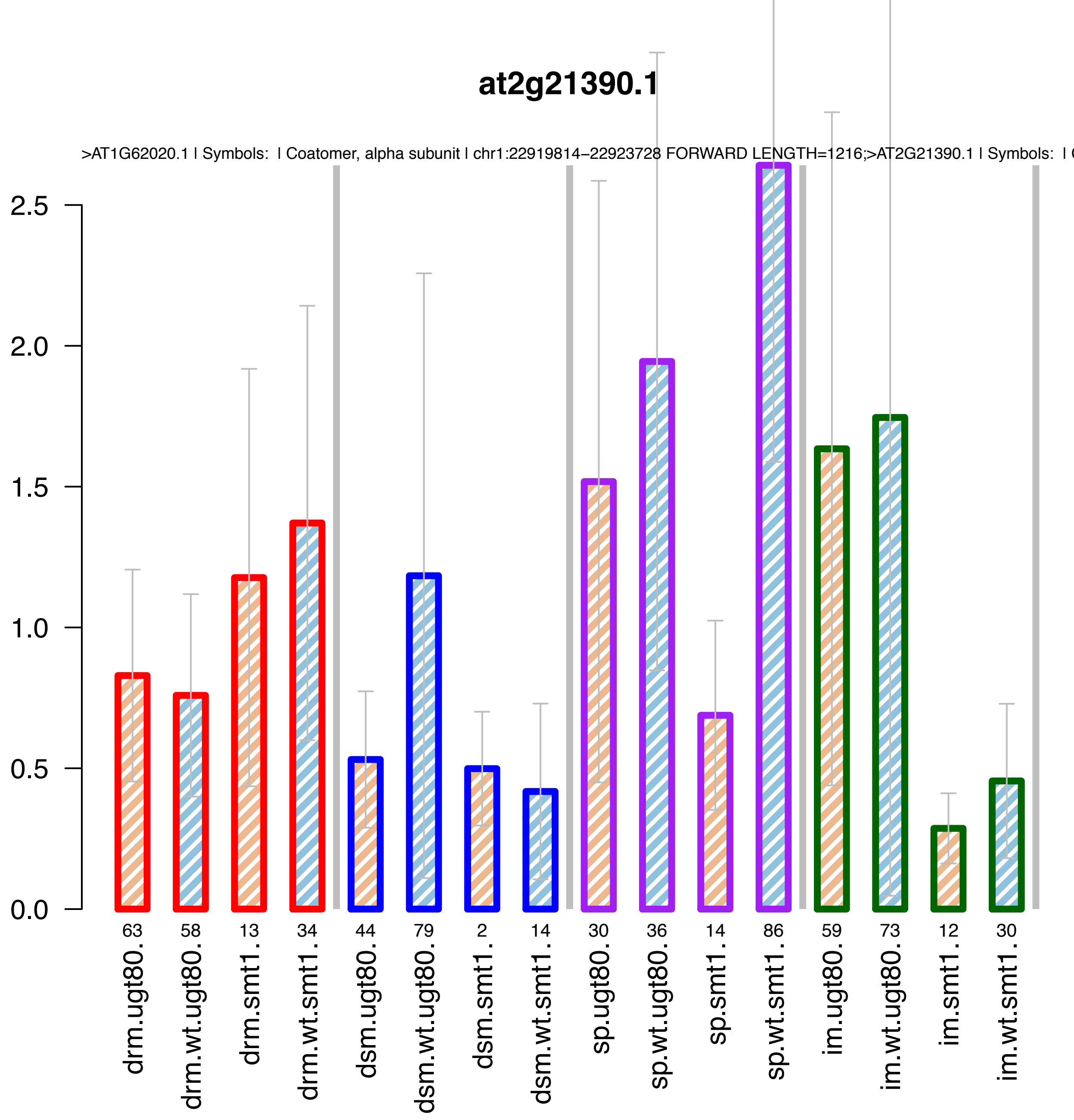
at2g21390.1
>AT1G62020.1 | Symbols: | Coatomer, alpha subunit | chr1:22919814–22923728 FORWARD LENGTH=1216;>AT2G21390.1 | Symbols: | C
0.0
0.5
1.0
1.5
2.0
2.5
63
58
13
34
44
79
2
14
30
36
14
86
59
73
12
30
drm.ugt80.
drm.wt.ugt80.
drm.smt1.
drm.wt.smt1.
dsm.ugt80.
dsm.wt.ugt80.
dsm.smt1.
dsm.wt.smt1.
sp.ugt80.
sp.wt.ugt80.
sp.smt1.
sp.wt.smt1.
im.ugt80.
im.wt.ugt80.
im.smt1.
im.wt.smt1.

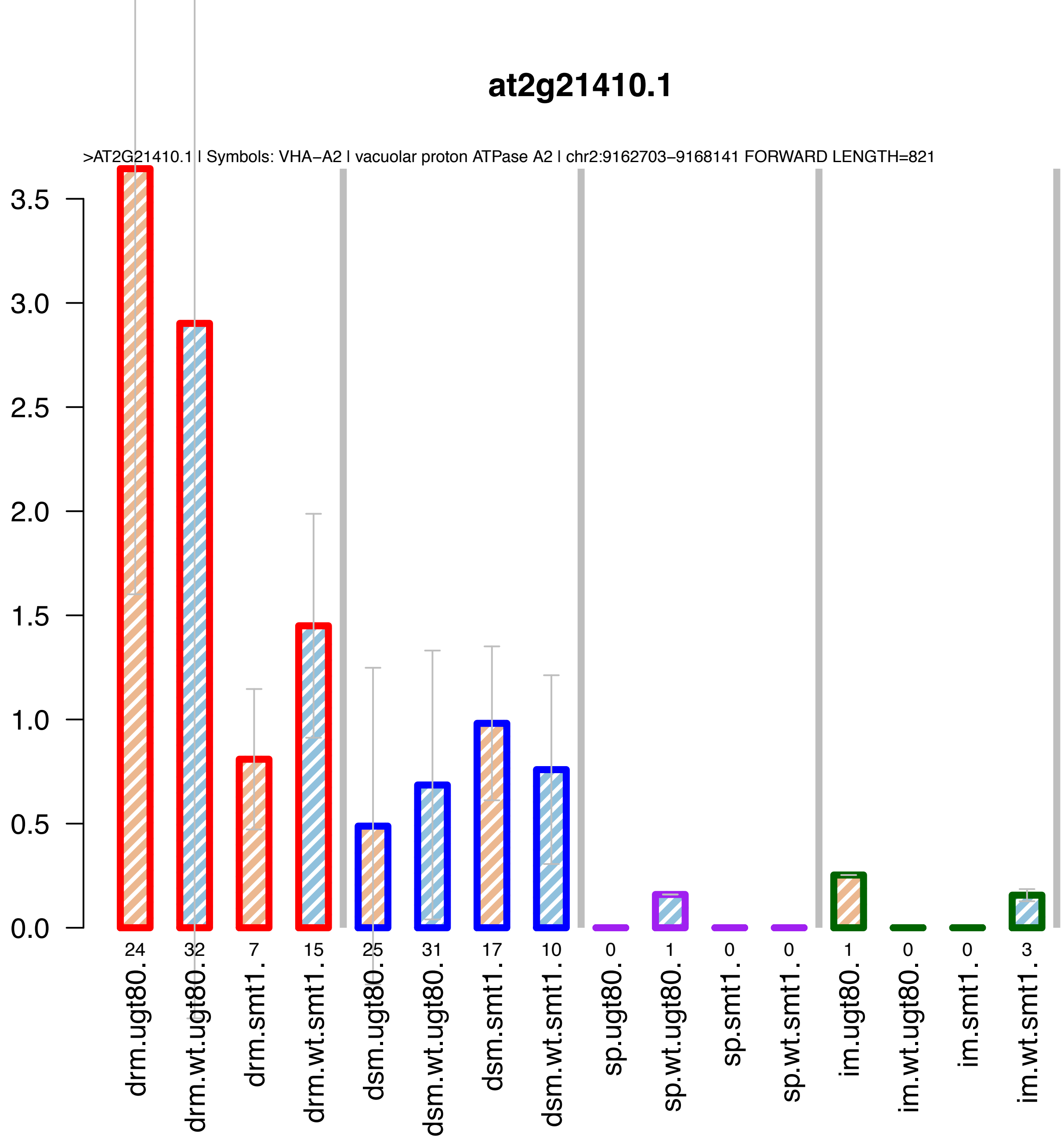

at2g21410.1
>AT2G21410.1 | Symbols: VHA−A2 | vacuolar proton ATPase A2 | chr2:9162703−9168141 FORWARD LENGTH=821
3.5
3.0
2.5
2.0
1.5
1.0
0.5
0.0
24
32
7
15
25
31
17
10
0
1
0
0
1
0
0
3
drm.ugt80..
drm.wt.ugt80..
drm.smt1..
drm.wt.smt1..
dsm.ugt80..
dsm.wt.ugt80..
dsm.smt1..
dsm.wt.smt1..
sp.ugt80..
sp.wt.ugt80..
sp.smt1..
sp.wt.smt1..
im.ugt80..
im.wt.ugt80..
im.smt1..
im.wt.smt1..

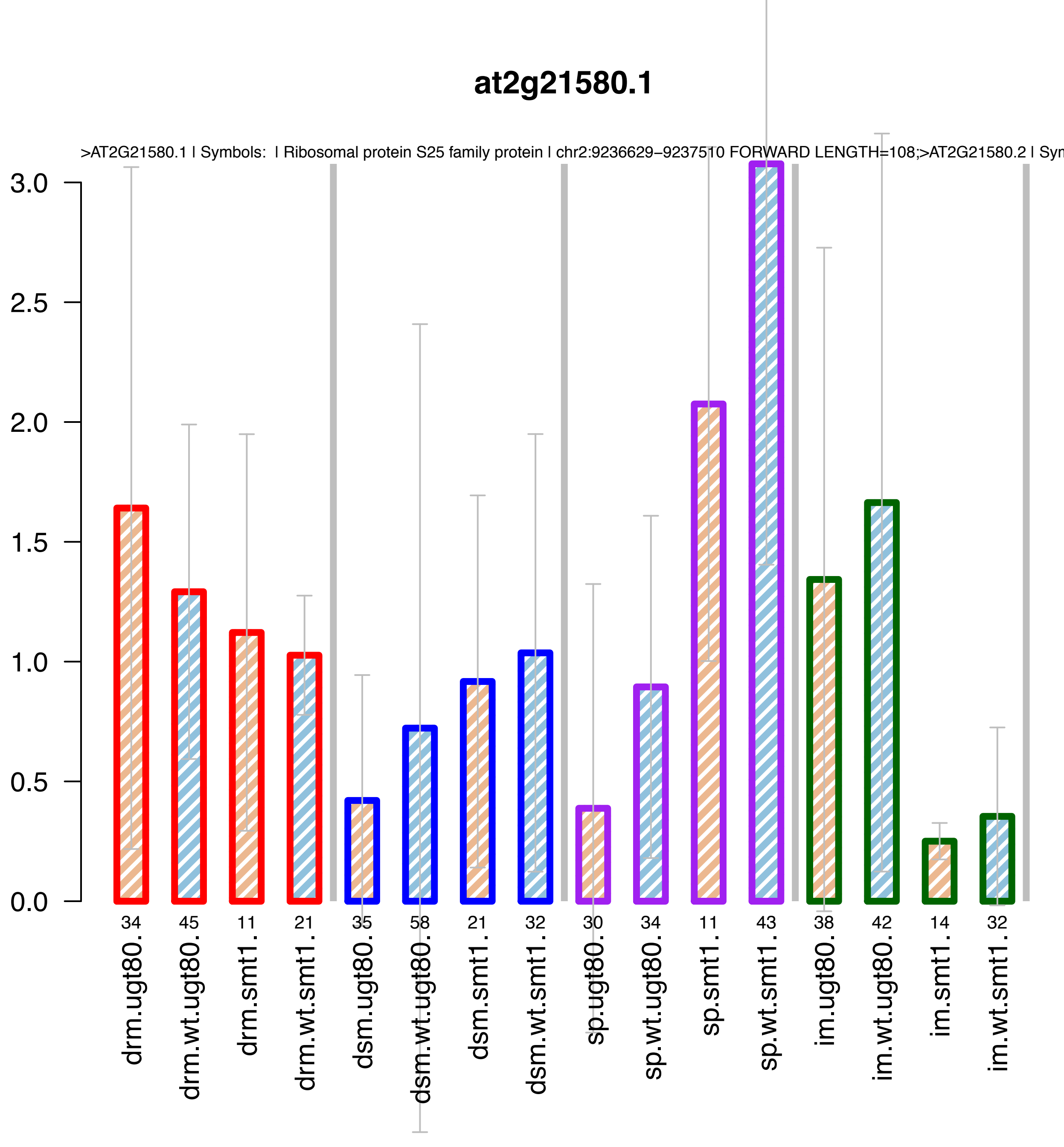

at2g21580.1
>AT2G21580.1 | Symbols: | Ribosomal protein S25 family protein | chr2:9236629-9237510 FORWARD LENGTH=108;>AT2G21580.2 | Syn
3.0
2.5
2.0
1.5
1.0
0.5
0.0
34
45
11
21
35
58
21
32
30
34
11
43
38
42
14
32
drm.ugt80..
drm.wt.ugt80..
drm.smt1..
drm.wt.smt1..
dsm.ugt80..
dsm.wt.ugt80..
dsm.smt1..
dsm.wt.smt1..
sp.ugt80..
sp.wt.ugt80..
sp.smt1..
sp.wt.smt1..
im.ugt80..
im.wt.ugt80..
im.smt1..
im.wt.smt1..

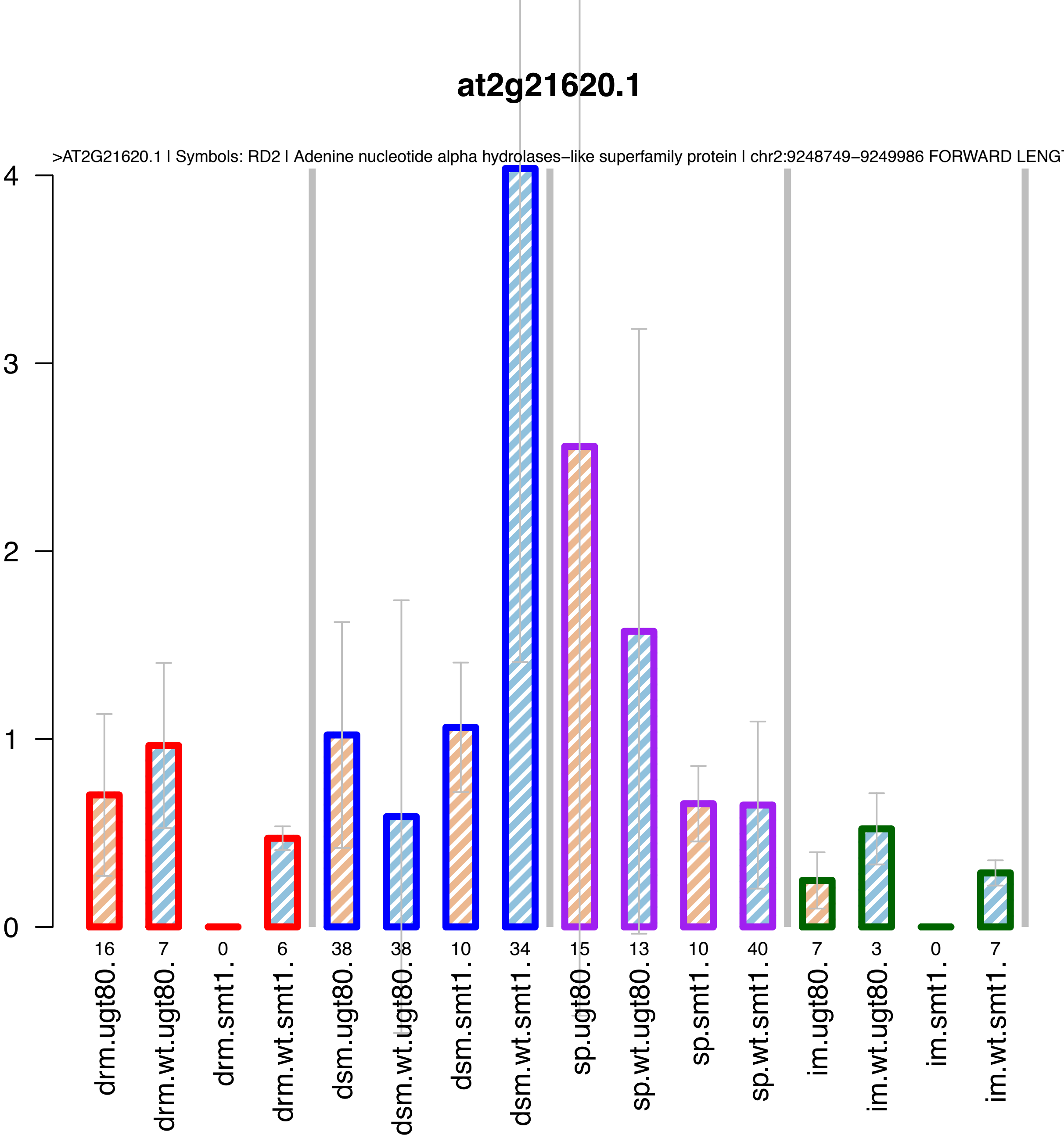

at2g21620.1
>AT2G21620.1 | Symbols: RD2 | Adenine nucleotide alpha hydrolases–like superfamily protein | chr2:9248749–9249986 FORWARD LENGT
0
1
2
3
4
16
7
0
6
38
38
10
34
15
13
10
40
7
3
0
7
drm.ugt80.
drm.wt.ugt80.
drm.smt1.
drm.wt.smt1.
dsm.ugt80.
dsm.wt.ugt80.
dsm.smt1.
dsm.wt.smt1.
sp.ugt80.
sp.wt.ugt80.
sp.smt1.
sp.wt.smt1.
im.ugt80.
im.wt.ugt80.
im.smt1.
im.wt.smt1.

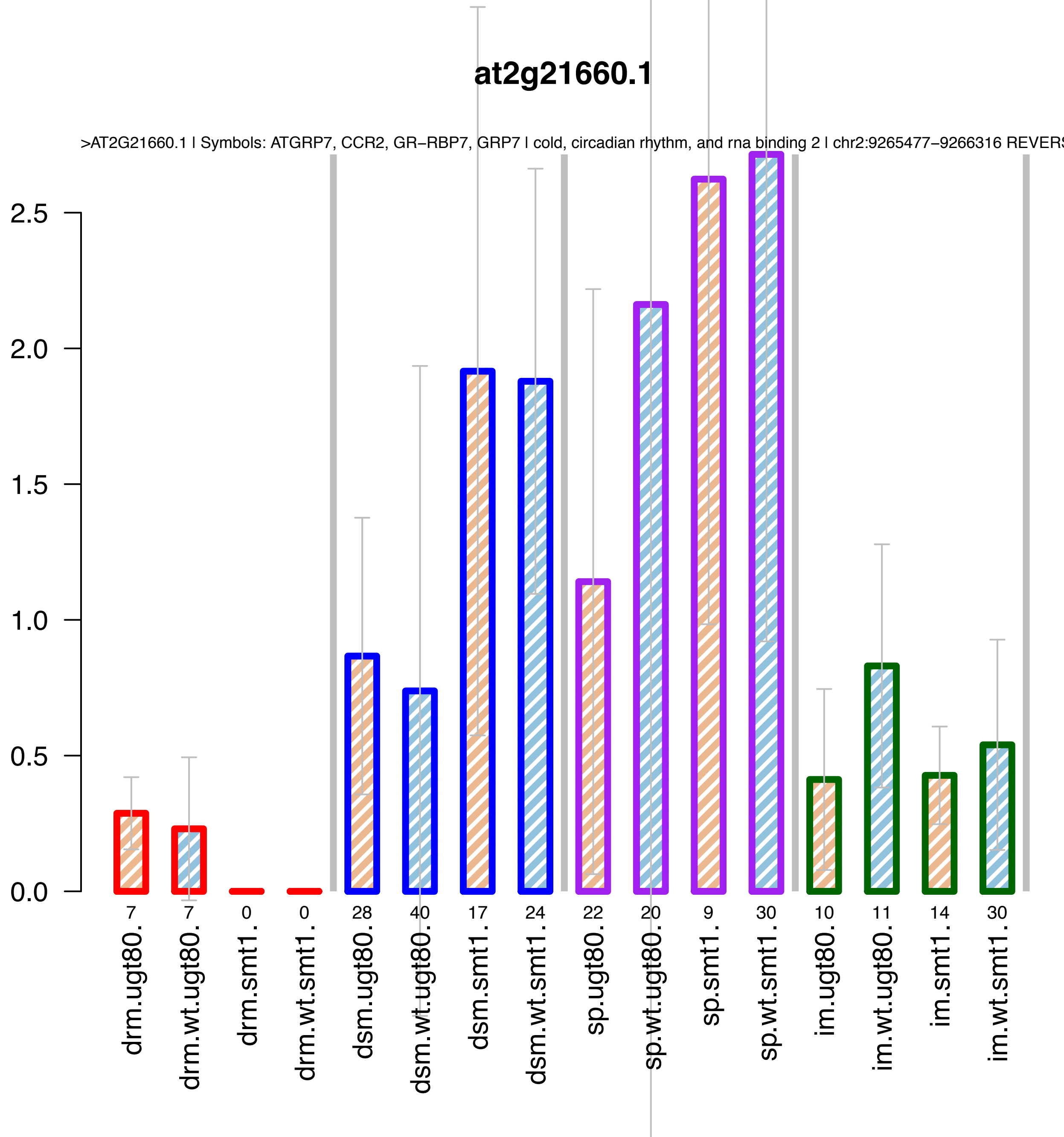

at2g21660.1
>AT2G21660.1 | Symbols: ATGRP7, CCR2, GR−RBP7, GRP7 | cold, circadian rhythm, and rna binding 2 | chr2:9265477−9266316 REVERS
0.0
0.5
1.0
1.5
2.0
2.5
7
7
0
0
28
40
17
24
22
20
9
30
10
11
14
30
drm.ugt80.
drm.wt.ugt80.
drm.smt1.
drm.wt.smt1.
dsm.ugt80.
dsm.wt.ugt80.
dsm.smt1.
dsm.wt.smt1.
sp.ugt80.
sp.wt.ugt80.
sp.smt1.
sp.wt.smt1.
im.ugt80.
im.wt.ugt80.
im.smt1.
im.wt.smt1.

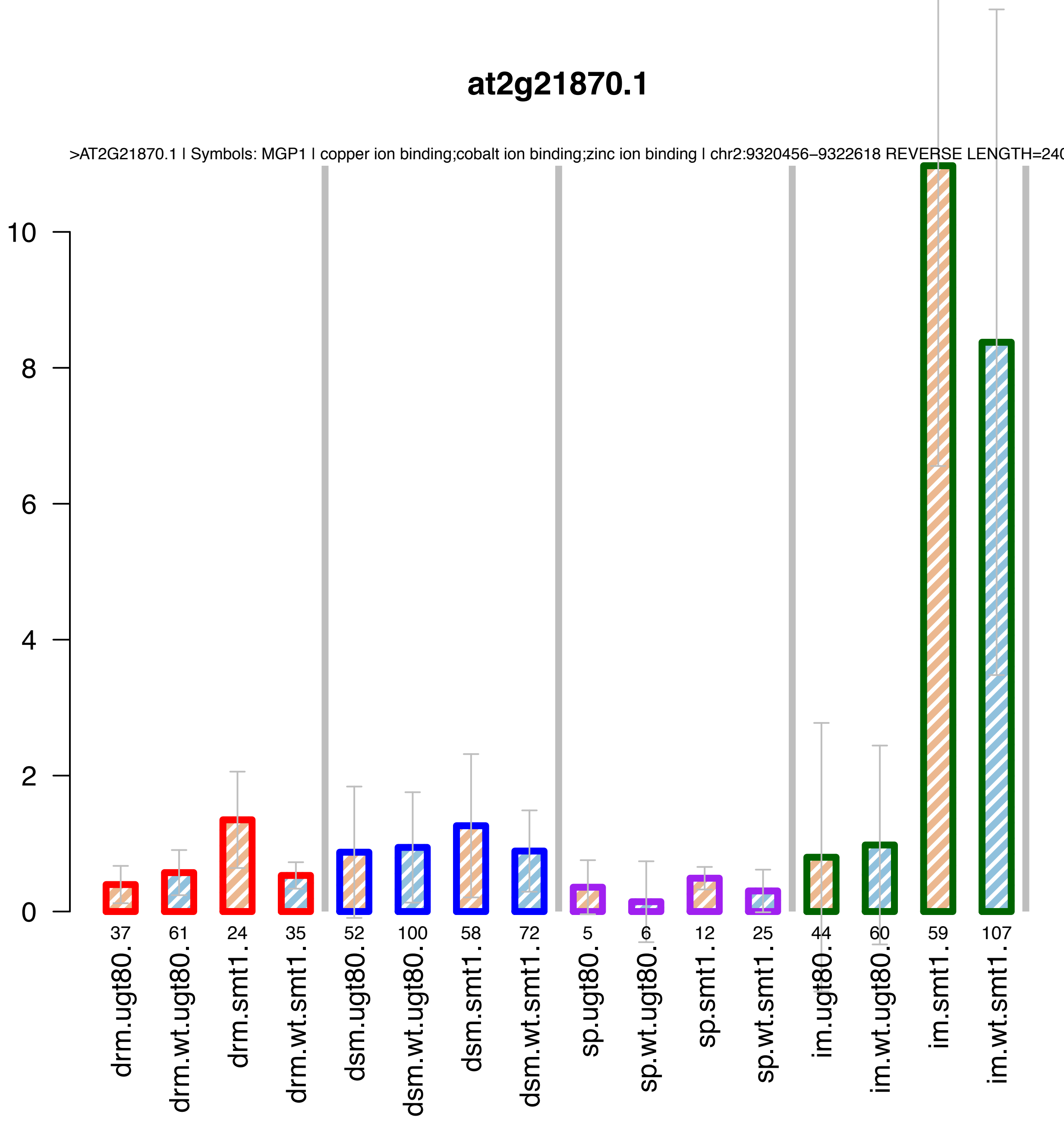

at2g21870.1
>AT2G21870.1 | Symbols: MGP1 | copper ion binding;cobalt ion binding;zinc ion binding | chr2:9320456-9322618 REVERSE LENGTH=240
0
2
4
6
8
10
37
61
24
35
52
100
58
72
5
6
12
25
44
60
59
107
drm.ugt80.
drm.wt.ugt80.
drm.smt1.
drm.wt.smt1.
dsm.ugt80.
dsm.wt.ugt80.
dsm.smt1.
dsm.wt.smt1.
sp.ugt80.
sp.wt.ugt80.
sp.smt1.
sp.wt.smt1.
im.ugt80.
im.wt.ugt80.
im.smt1.
im.wt.smt1.

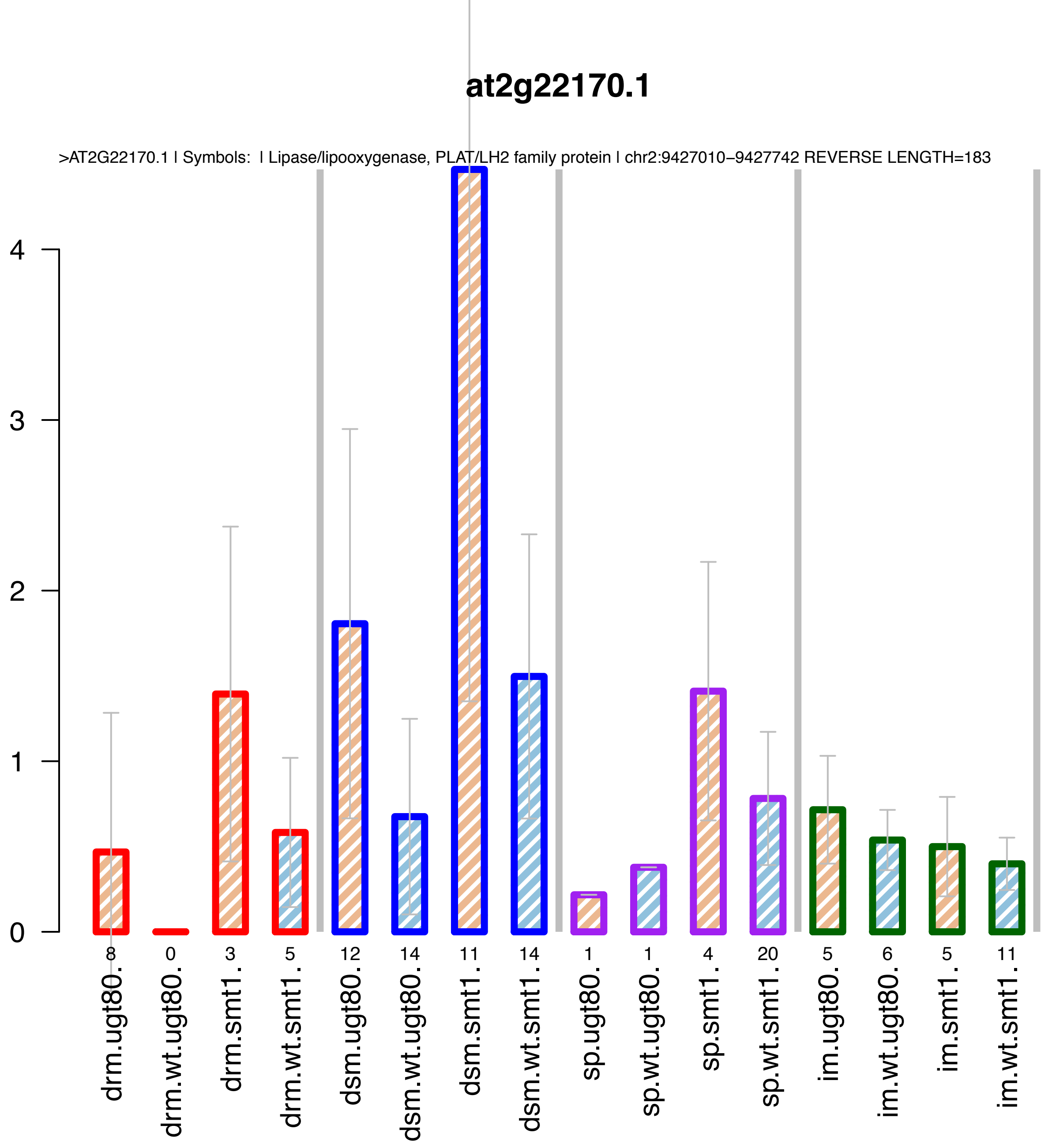

at2g22170.1
>AT2G22170.1 | Symbols: | Lipase/lipooxygenase, PLAT/LH2 family protein | chr2:9427010-9427742 REVERSE LENGTH=183
0
1
2
3
4
8
0
3
5
12
14
11
14
1
1
4
20
5
6
5
11
drm.ugt80.
drm.wt.ugt80.
drm.smt1.
drm.wt.smt1.
dsm.ugt80.
dsm.wt.ugt80.
dsm.smt1.
dsm.wt.smt1.
sp.ugt80.
sp.wt.ugt80.
sp.smt1.
sp.wt.smt1.
im.ugt80.
im.wt.ugt80.
im.smt1.
im.wt.smt1.

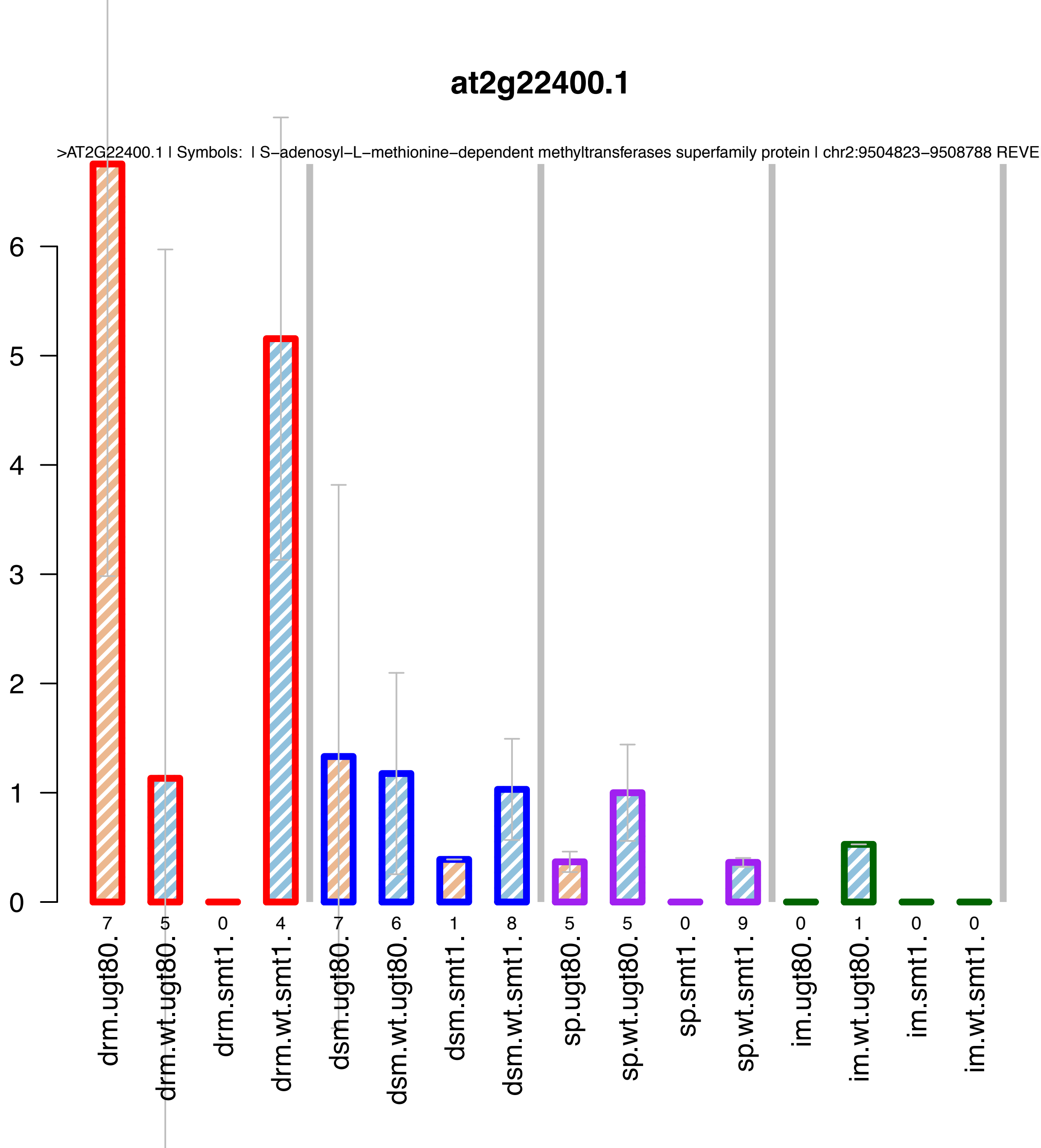
at2g22400.1
>AT2G22400.1 | Symbols: | S–adenosyl–L–methionine–dependent methyltransferases superfamily protein | chr2:9504823–9508788 REVE
0
1
2
3
4
5
6
7
5
0
4
7
6
1
8
5
5
0
9
0
1
0
0
drm.ugt80.
drm.wt.ugt80.
drm.smt1.
drm.wt.smt1.
dsm.ugt80.
dsm.wt.ugt80.
dsm.smt1.
dsm.wt.smt1.
sp.ugt80.
sp.wt.ugt80.
sp.smt1.
sp.wt.smt1.
im.ugt80.
im.wt.ugt80.
im.smt1.
im.wt.smt1.

# at2g22420.1

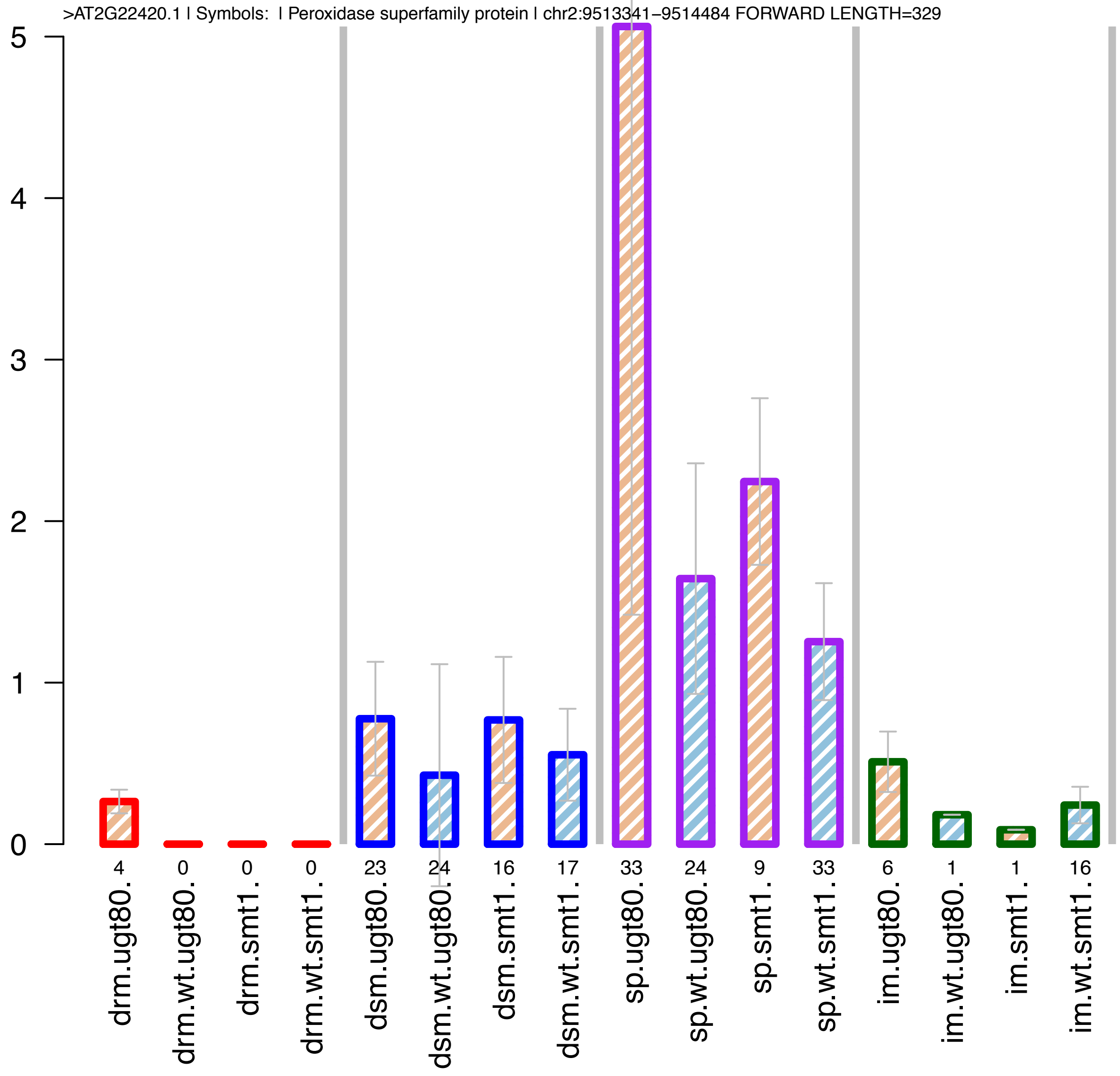

>AT2G22420.1 | Symbols: | Peroxidase superfamily protein | chr2:9513341-9514484 FORWARD LENGTH=329
0
1
2
3
4
5
4
0
0
0
23
24
16
17
33
24
9
33
6
1
1
16
drm.ugt80..
drm.wt.ugt80..
drm.smt1..
drm.wt.smt1..
dsm.ugt80..
dsm.wt.ugt80..
dsm.smt1..
dsm.wt.smt1..
sp.ugt80..
sp.wt.ugt80..
sp.smt1..
sp.wt.smt1..
im.ugt80..
im.wt.ugt80..
im.smt1..
im.wt.smt1..

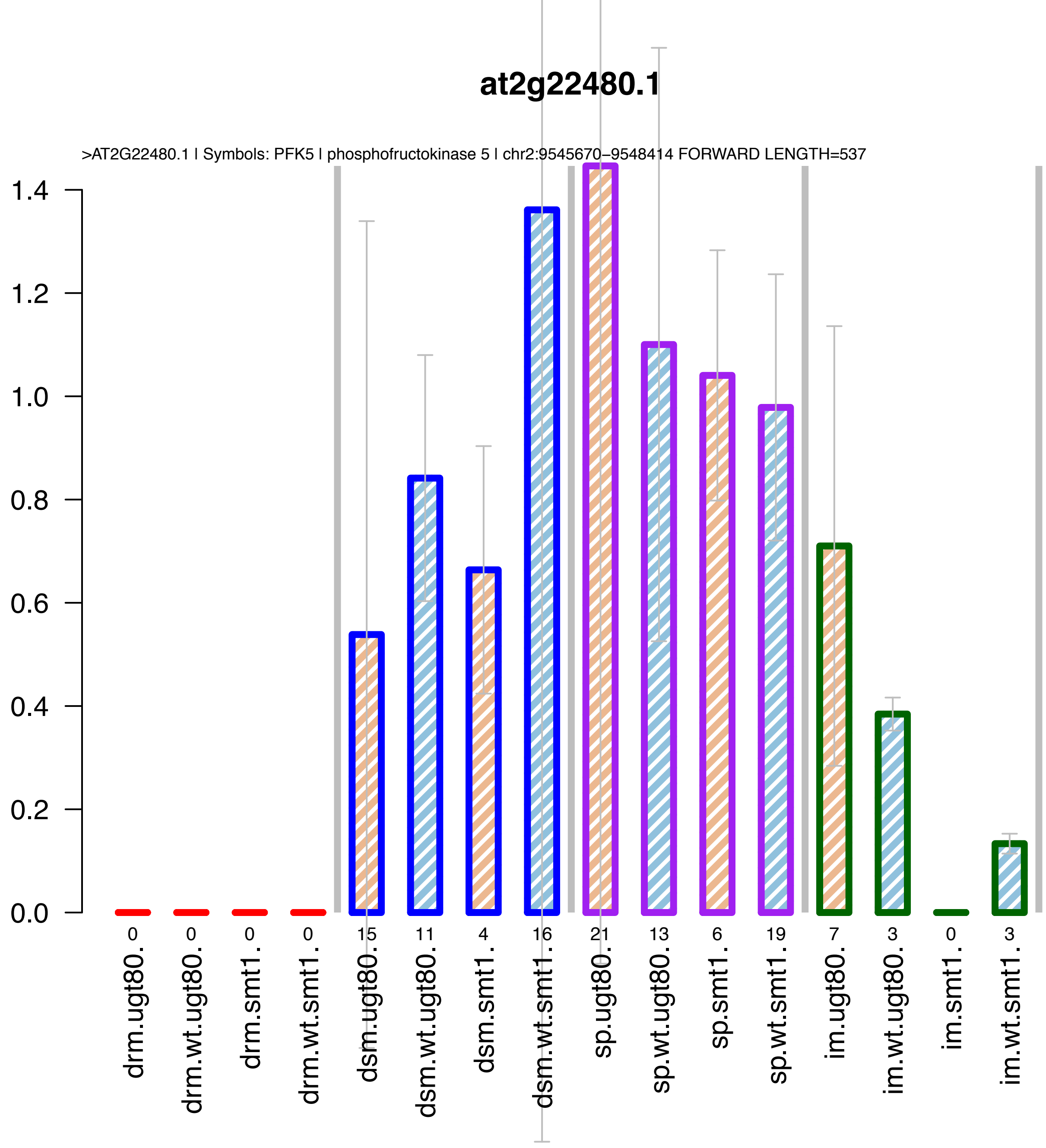
at2g22480.1
>AT2G22480.1 | Symbols: PFK5 | phosphofructokinase 5 | chr2:9545670–9548414 FORWARD LENGTH=537
1.4
1.2
1.0
0.8
0.6
0.4
0.2
0.0
0 0 0 0 15 11 4 16 21 13 6 19 7 3 0 3
drm.ugt80.
drm.wt.ugt80.
drm.smt1.
drm.wt.smt1.
dsm.ugt80.
dsm.wt.ugt80.
dsm.smt1.
dsm.wt.smt1.
sp.ugt80.
sp.wt.ugt80.
sp.smt1.
sp.wt.smt1.
im.ugt80.
im.wt.ugt80.
im.smt1.
im.wt.smt1.

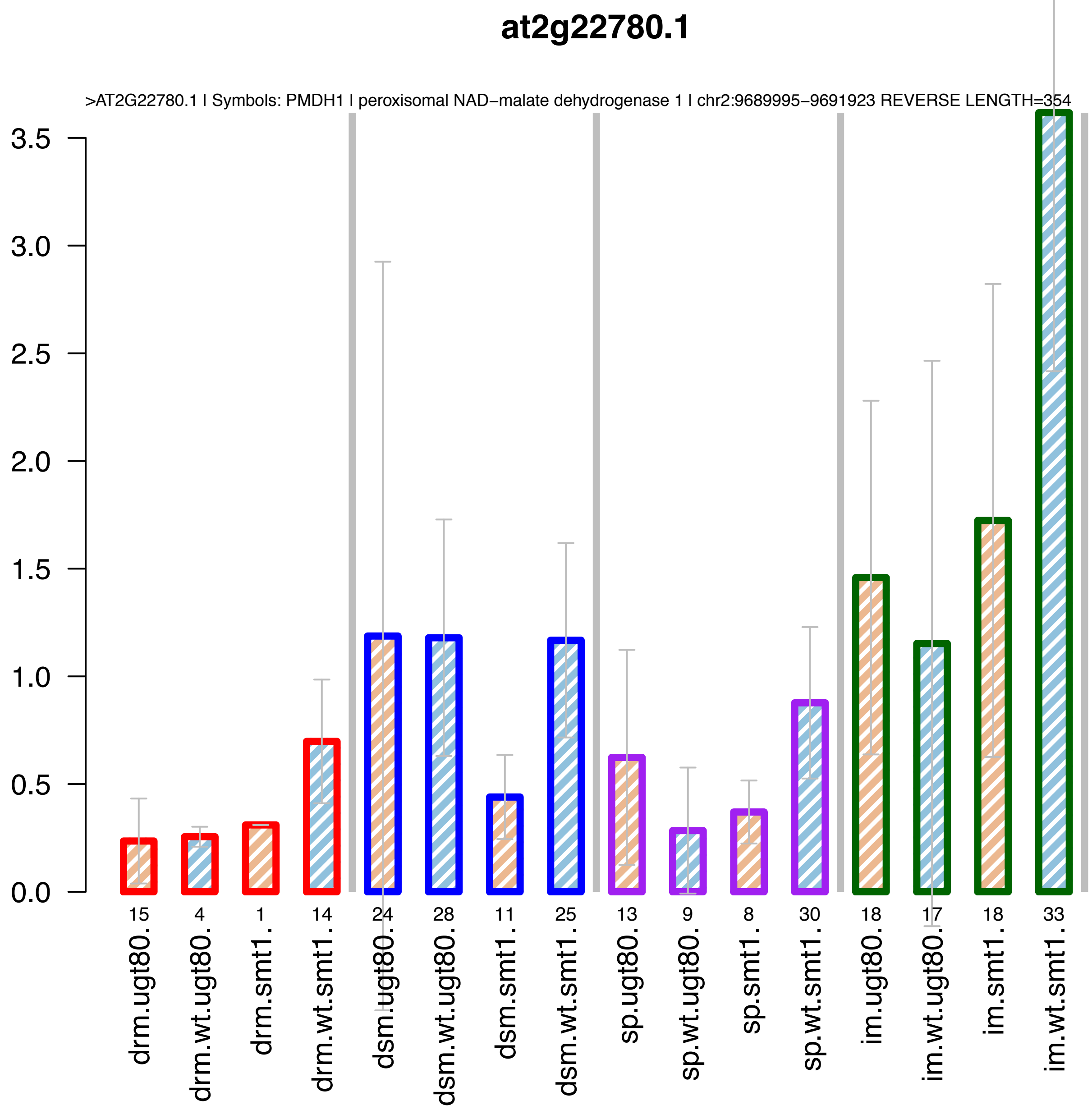

at2g22780.1
>AT2G22780.1 | Symbols: PMDH1 | peroxisomal NAD−malate dehydrogenase 1 | chr2:9689995−9691923 REVERSE LENGTH=354
0.0
0.5
1.0
1.5
2.0
2.5
3.0
3.5
15
4
1
14
24
28
11
25
13
9
8
30
18
17
18
33
drm.ugt80.
drm.wt.ugt80.
drm.smt1.
drm.wt.smt1.
dsm.ugt80.
dsm.wt.ugt80.
dsm.smt1.
dsm.wt.smt1.
sp.ugt80.
sp.wt.ugt80.
sp.smt1.
sp.wt.smt1.
im.ugt80.
im.wt.ugt80.
im.smt1.
im.wt.smt1.

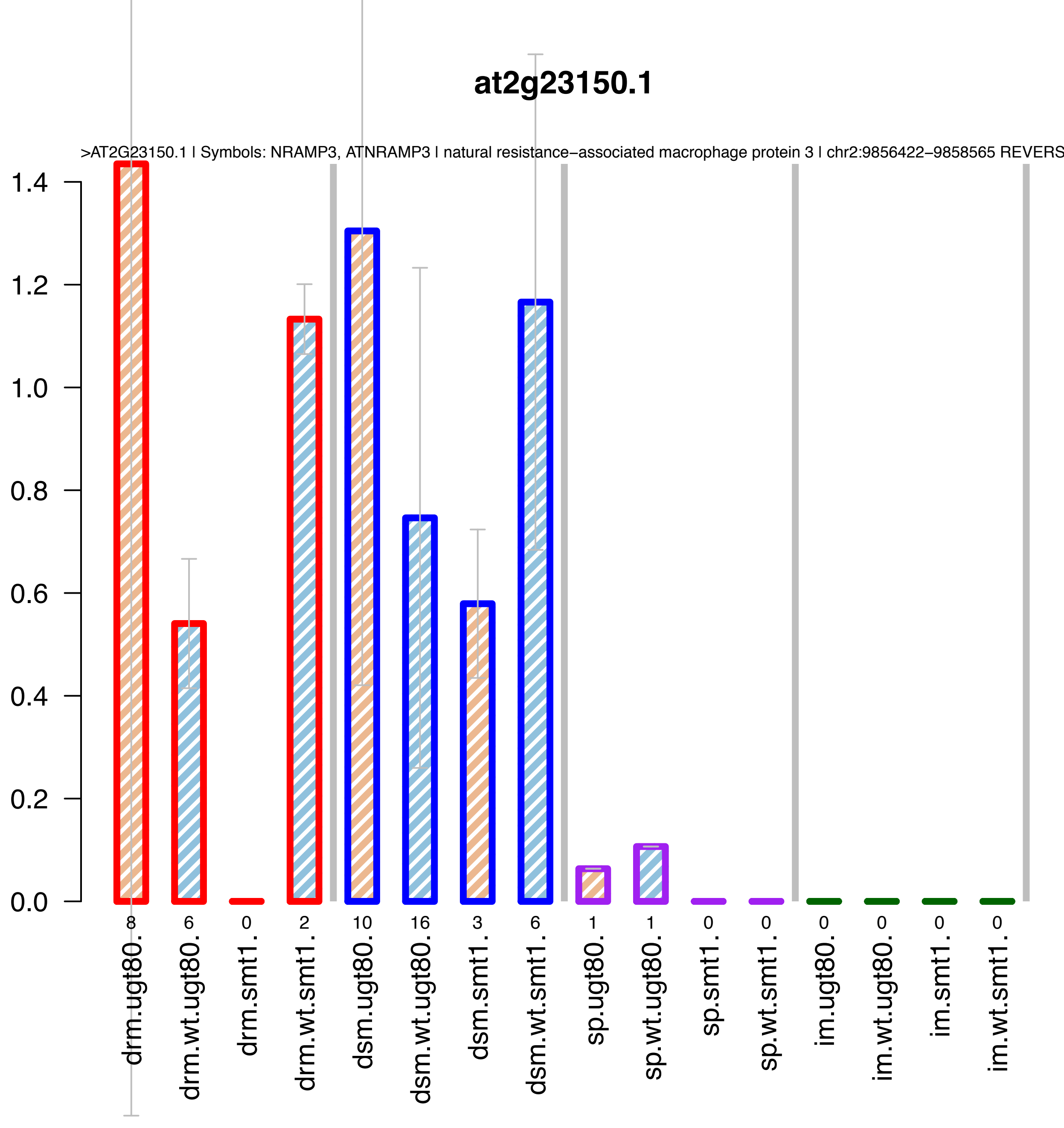

at2g23150.1
>AT2G23150.1 | Symbols: NRAMP3, ATNRAMP3 | natural resistance−associated macrophage protein 3 | chr2:9856422−9858565 REVERS
1.4
1.2
1.0
0.8
0.6
0.4
0.2
0.0
8
6
0
2
10
16
3
6
1
1
0
0
0
0
0
0
drm.ugt80.
drm.wt.ugt80.
drm.smt1.
drm.wt.smt1.
dsm.ugt80.
dsm.wt.ugt80.
dsm.smt1.
dsm.wt.smt1.
sp.ugt80.
sp.wt.ugt80.
sp.smt1.
sp.wt.smt1.
im.ugt80.
im.wt.ugt80.
im.smt1.
im.wt.smt1.

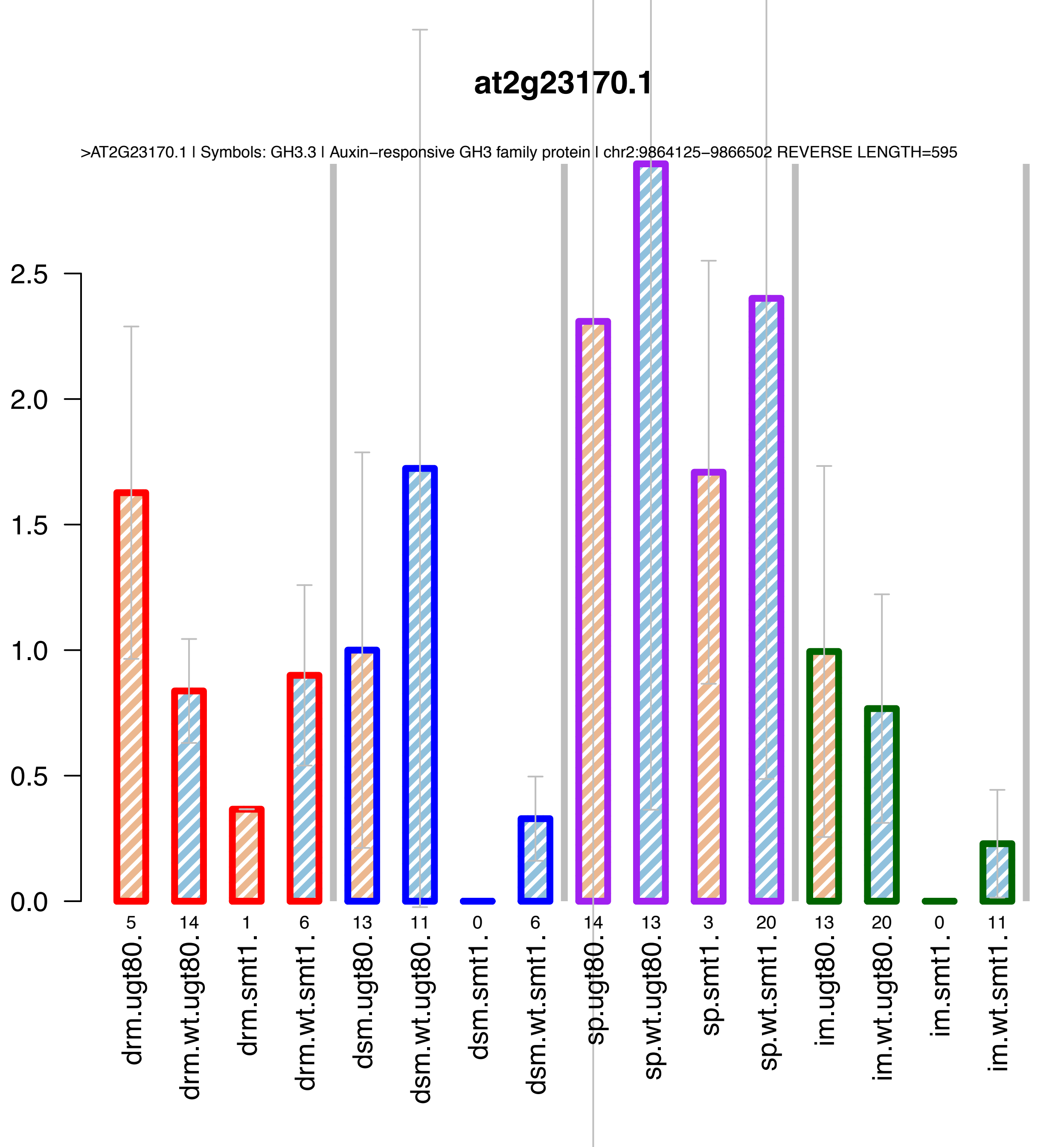

at2g23170.1
>AT2G23170.1 | Symbols: GH3.3 | Auxin−responsive GH3 family protein | chr2:9864125−9866502 REVERSE LENGTH=595
0.0
0.5
1.0
1.5
2.0
2.5
5
14
1
6
13
11
0
6
14
13
3
20
13
20
0
11
drm.ugt80.
drm.wt.ugt80.
drm.smt1.
drm.wt.smt1.
dsm.ugt80.
dsm.wt.ugt80.
dsm.smt1.
dsm.wt.smt1.
sp.ugt80.
sp.wt.ugt80.
sp.smt1.
sp.wt.smt1.
im.ugt80.
im.wt.ugt80.
im.smt1.
im.wt.smt1.

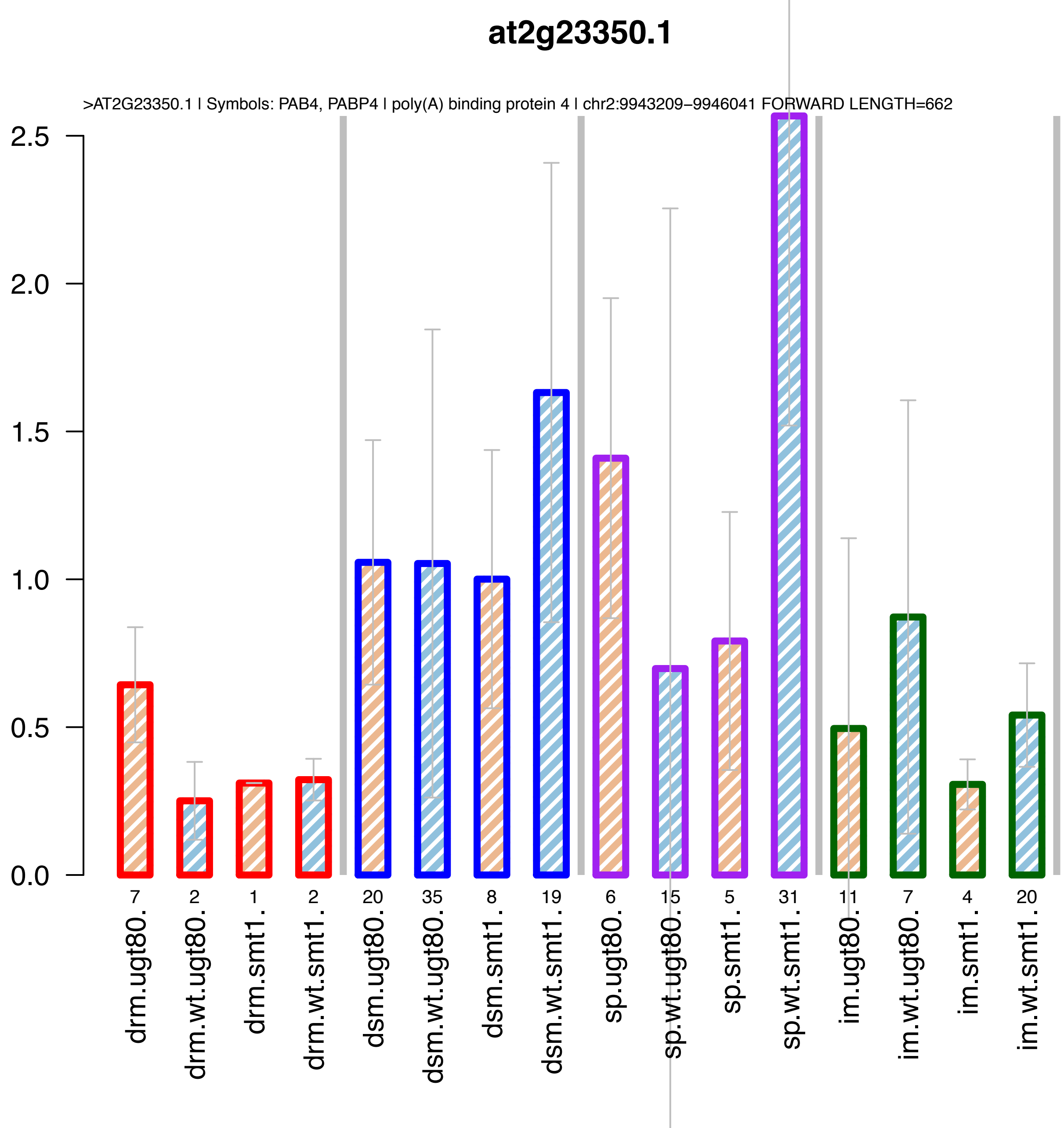

at2g23350.1
>AT2G23350.1 | Symbols: PAB4, PABP4 | poly(A) binding protein 4 | chr2:9943209−9946041 FORWARD LENGTH=662
0.0
0.5
1.0
1.5
2.0
2.5
7
2
1
2
20
35
8
19
6
15
5
31
11
7
4
20
drm.ugt80.
drm.wt.ugt80.
drm.smt1.
drm.wt.smt1.
dsm.ugt80.
dsm.wt.ugt80.
dsm.smt1.
dsm.wt.smt1.
sp.ugt80.
sp.wt.ugt80.
sp.smt1.
sp.wt.smt1.
im.ugt80.
im.wt.ugt80.
im.smt1.
im.wt.smt1.

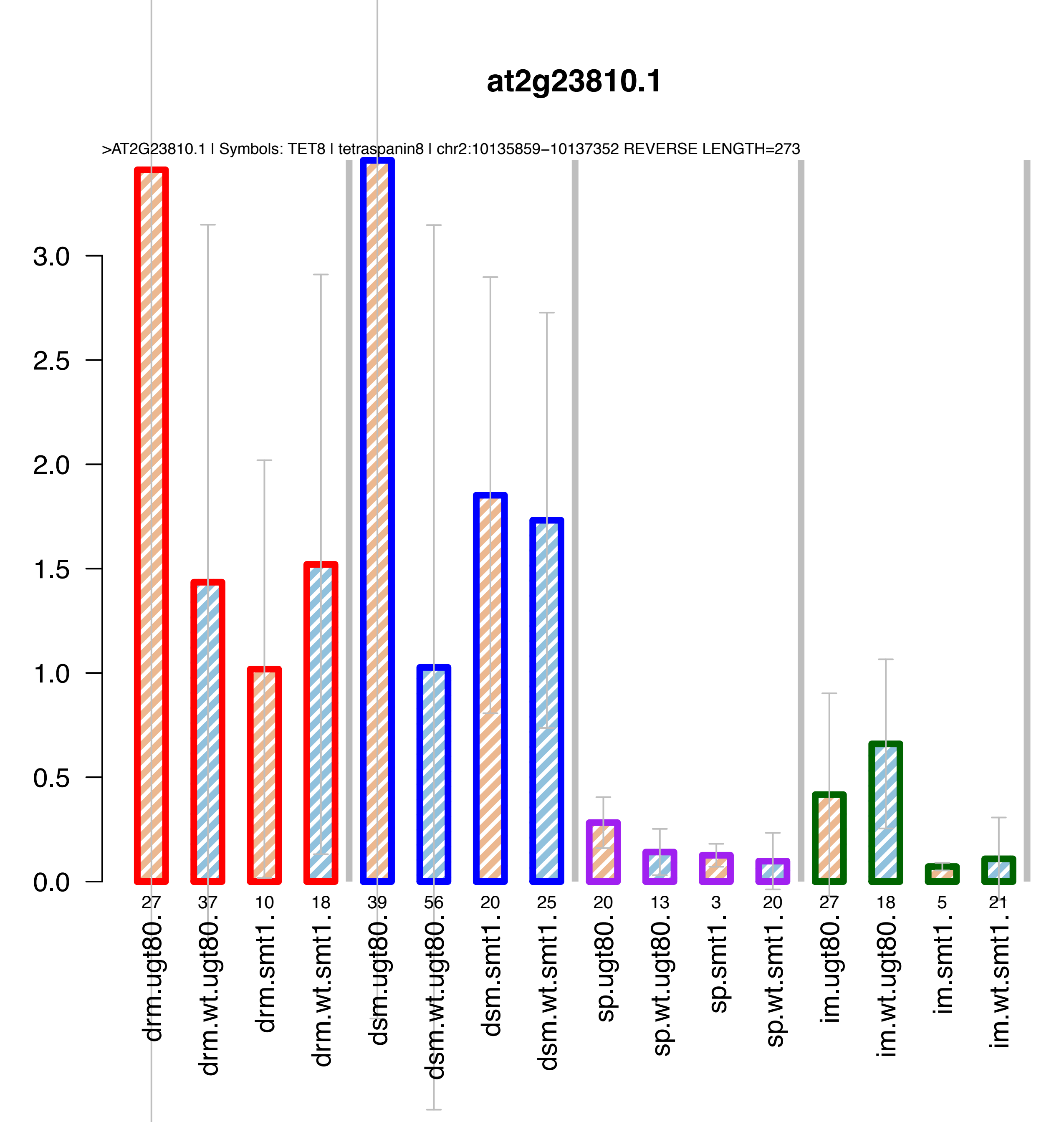

at2g23810.1
>AT2G23810.1 | Symbols: TET8 | tetraspanin8 | chr2:10135859−10137352 REVERSE LENGTH=273
3.0
2.5
2.0
1.5
1.0
0.5
0.0
27
37
10
18
39
56
20
25
20
13
3
20
27
18
5
21
drm.ugt80..
drm.wt.ugt80..
drm.smt1..
drm.wt.smt1..
dsm.ugt80..
dsm.wt.ugt80..
dsm.smt1..
dsm.wt.smt1..
sp.ugt80..
sp.wt.ugt80..
sp.smt1..
sp.wt.smt1..
im.ugt80..
im.wt.ugt80..
im.smt1..
im.wt.smt1..

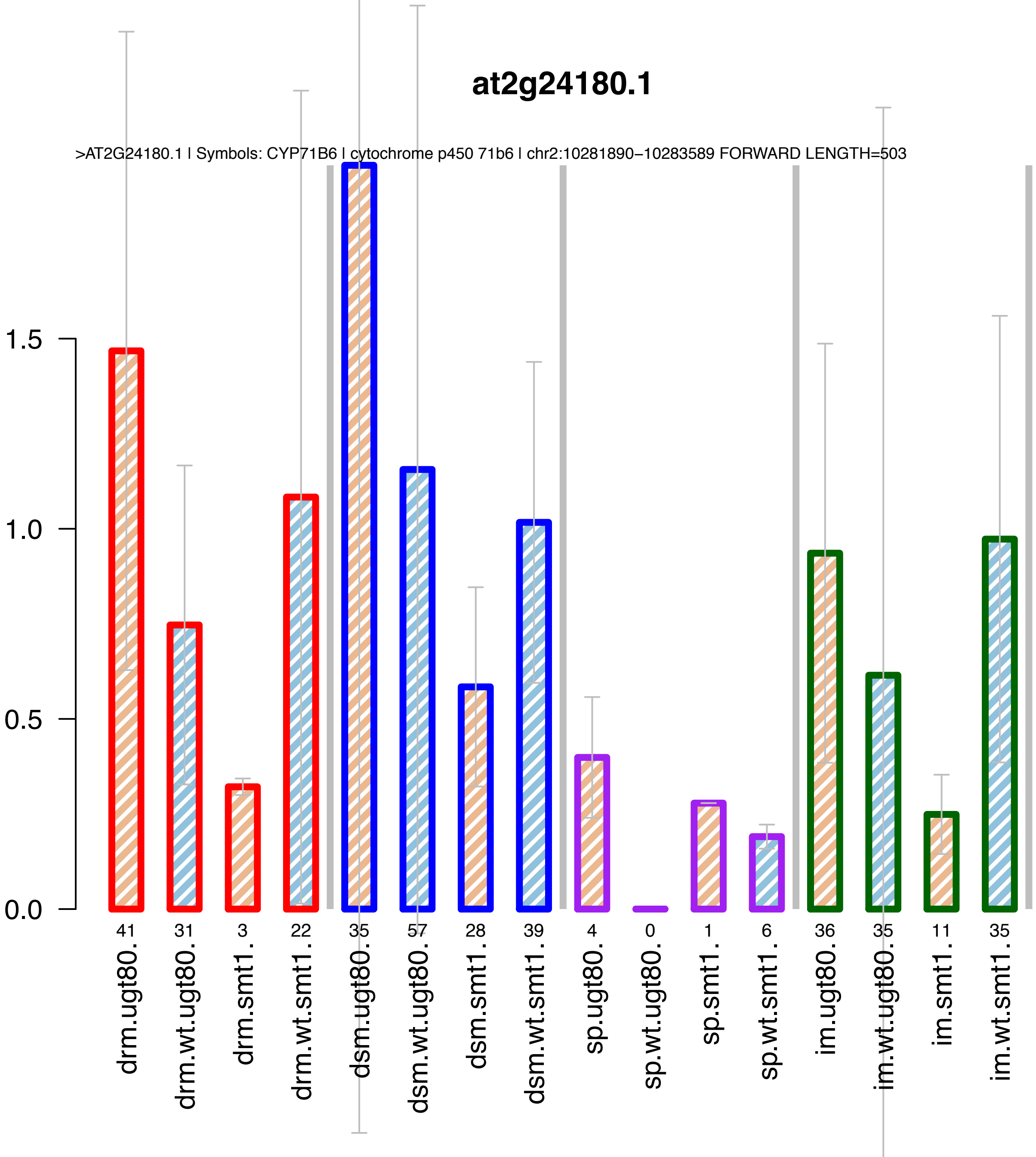
at2g24180.1
>AT2G24180.1 | Symbols: CYP71B6 | cytochrome p450 71b6 | chr2:10281890−10283589 FORWARD LENGTH=503
0.0
0.5
1.0
1.5
41
31
3
22
35
57
28
39
4
0
1
6
36
35
11
35
drm.ugt80.
drm.wt.ugt80.
drm.smt1.
drm.wt.smt1.
dsm.ugt80.
dsm.wt.ugt80.
dsm.smt1.
dsm.wt.smt1.
sp.ugt80.
sp.wt.ugt80.
sp.smt1.
sp.wt.smt1.
im.ugt80.
im.wt.ugt80.
im.smt1.
im.wt.smt1.

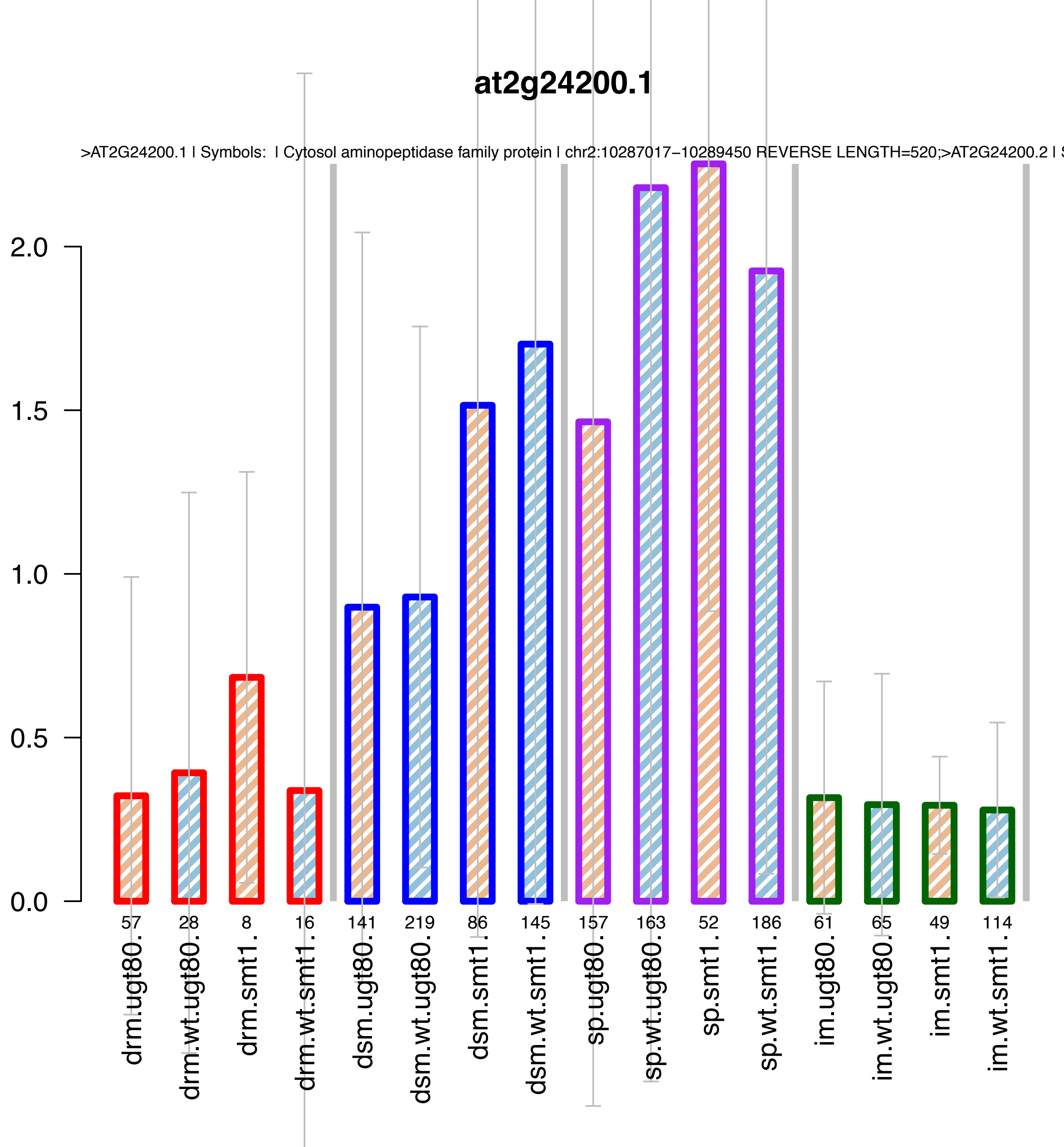

at2g24200.1
>AT2G24200.1 | Symbols: | Cytosol aminopeptidase family protein | chr2:10287017-10289450 REVERSE LENGTH=520;>AT2G24200.2 | S
2.0
1.5
1.0
0.5
0.0
57
28
8
16
141
219
86
145
157
163
52
186
61
65
49
114
drm.ugt80..
drm.wt.ugt80..
drm.smt1..
drm.wt.smt1..
dsm.ugt80..
dsm.wt.ugt80..
dsm.smt1..
dsm.wt.smt1..
sp.ugt80..
sp.wt.ugt80..
sp.smt1..
sp.wt.smt1..
im.ugt80..
im.wt.ugt80..
im.smt1..
im.wt.smt1..

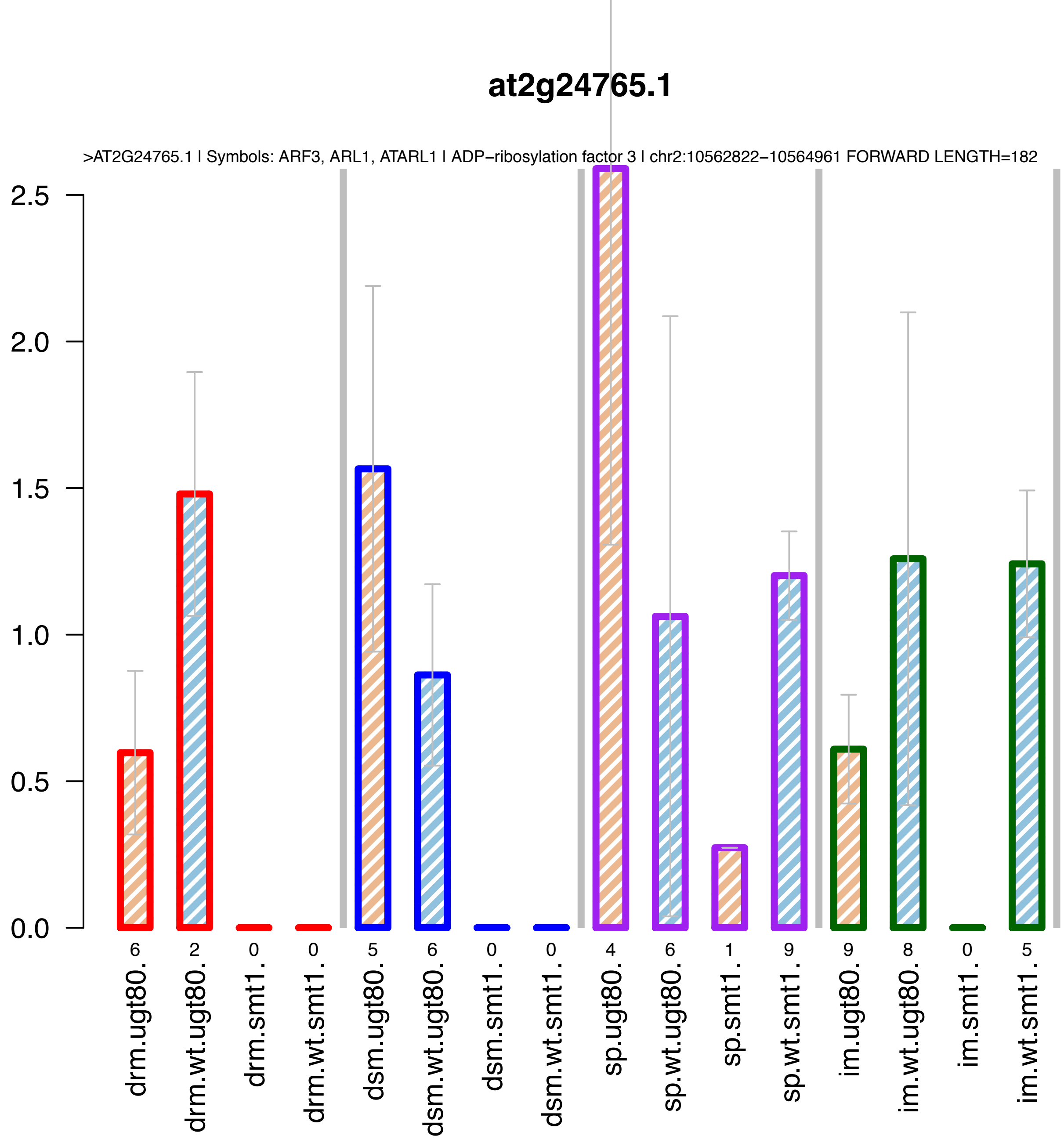

at2g24765.1
>AT2G24765.1 | Symbols: ARF3, ARL1, ATARL1 | ADP−ribosylation factor 3 | chr2:10562822−10564961 FORWARD LENGTH=182
0.0
0.5
1.0
1.5
2.0
2.5
6
2
0
0
5
6
0
0
4
6
1
9
9
8
0
5
drm.ugt80.
drm.wt.ugt80.
drm.smt1.
drm.wt.smt1.
dsm.ugt80.
dsm.wt.ugt80.
dsm.smt1.
dsm.wt.smt1.
sp.ugt80.
sp.wt.ugt80.
sp.smt1.
sp.wt.smt1.
im.ugt80.
im.wt.ugt80.
im.smt1.
im.wt.smt1.

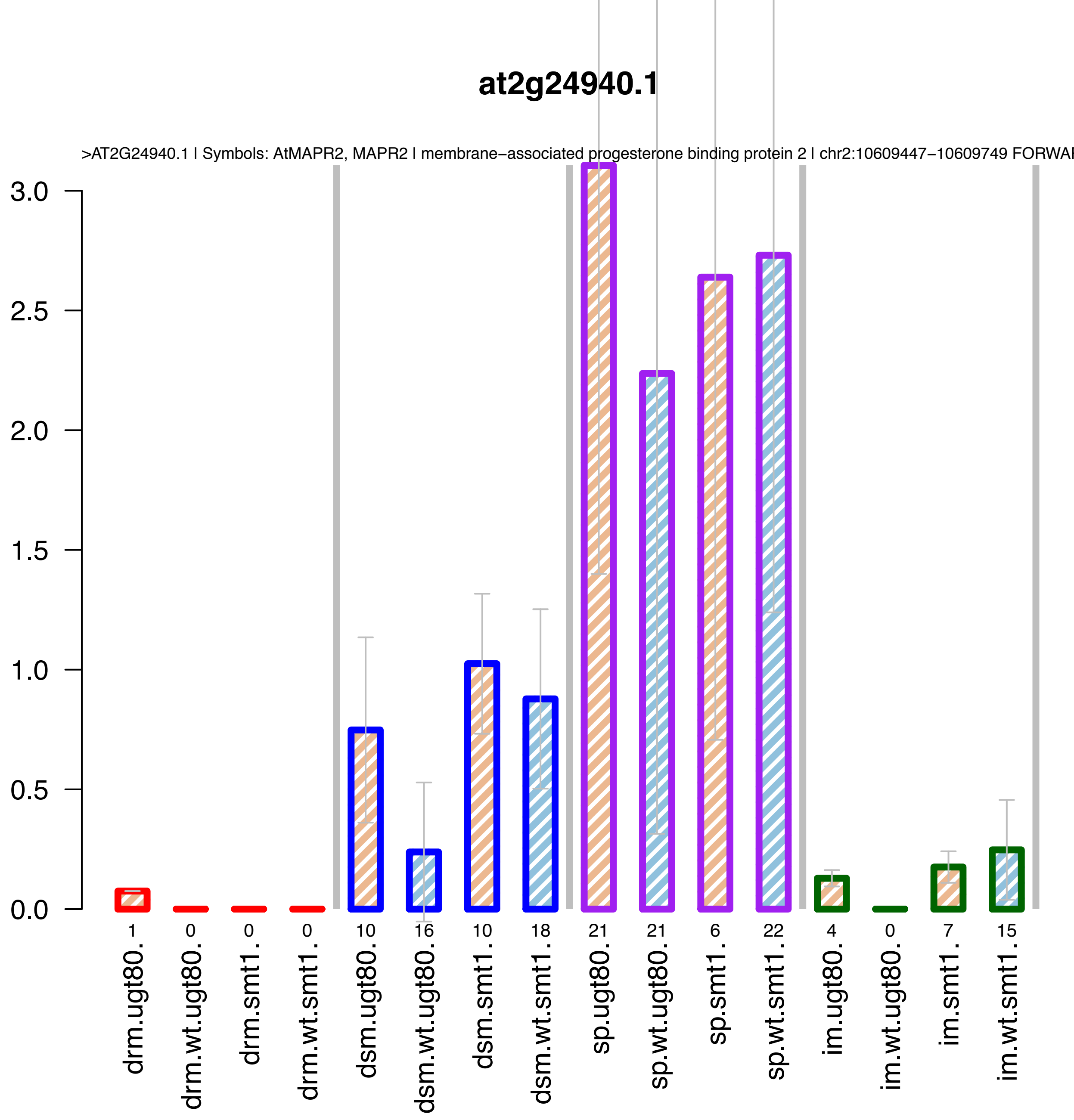

at2g24940.1
>AT2G24940.1 | Symbols: AtMAPR2, MAPR2 | membrane-associated progesterone binding protein 2 | chr2:10609447-10609749 FORWAI
3.0
2.5
2.0
1.5
1.0
0.5
0.0
1
0
0
0
10
16
10
18
21
21
6
22
4
0
7
15
drm.ugt80.
drm.wt.ugt80.
drm.smt1.
drm.wt.smt1.
dsm.ugt80.
dsm.wt.ugt80.
dsm.smt1.
dsm.wt.smt1.
sp.ugt80.
sp.wt.ugt80.
sp.smt1.
sp.wt.smt1.
im.ugt80.
im.wt.ugt80.
im.smt1.
im.wt.smt1.

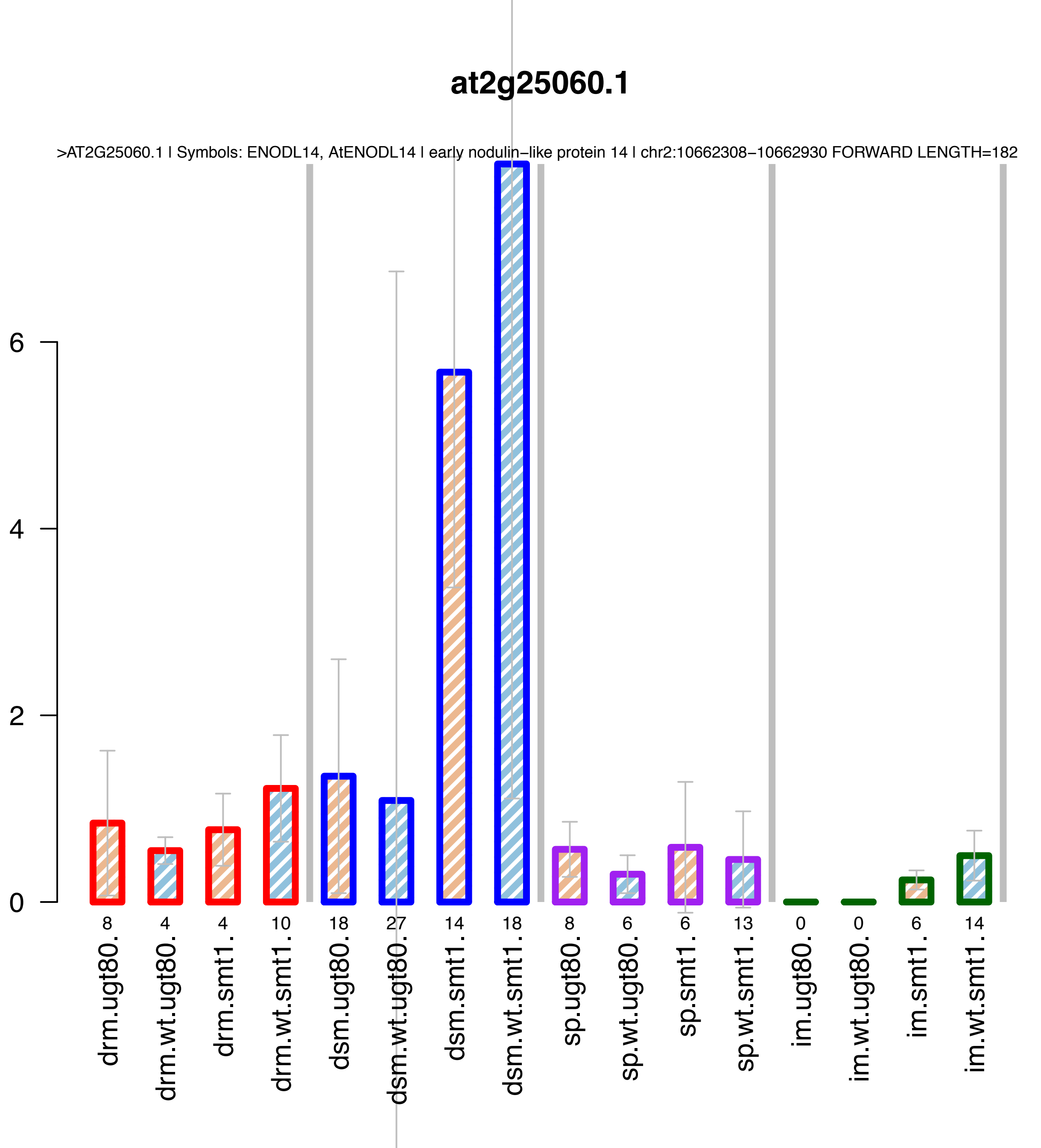
at2g25060.1
>AT2G25060.1 | Symbols: ENODL14, AtENODL14 | early nodulin−like protein 14 | chr2:10662308−10662930 FORWARD LENGTH=182
0
2
4
6
8
4
4
10
18
27
14
18
8
6
6
13
0
0
6
14
drm.ugt80.
drm.wt.ugt80.
drm.smt1.
drm.wt.smt1.
dsm.ugt80.
dsm.wt.ugt80.
dsm.smt1.
dsm.wt.smt1.
sp.ugt80.
sp.wt.ugt80.
sp.smt1.
sp.wt.smt1.
im.ugt80.
im.wt.ugt80.
im.smt1.
im.wt.smt1.

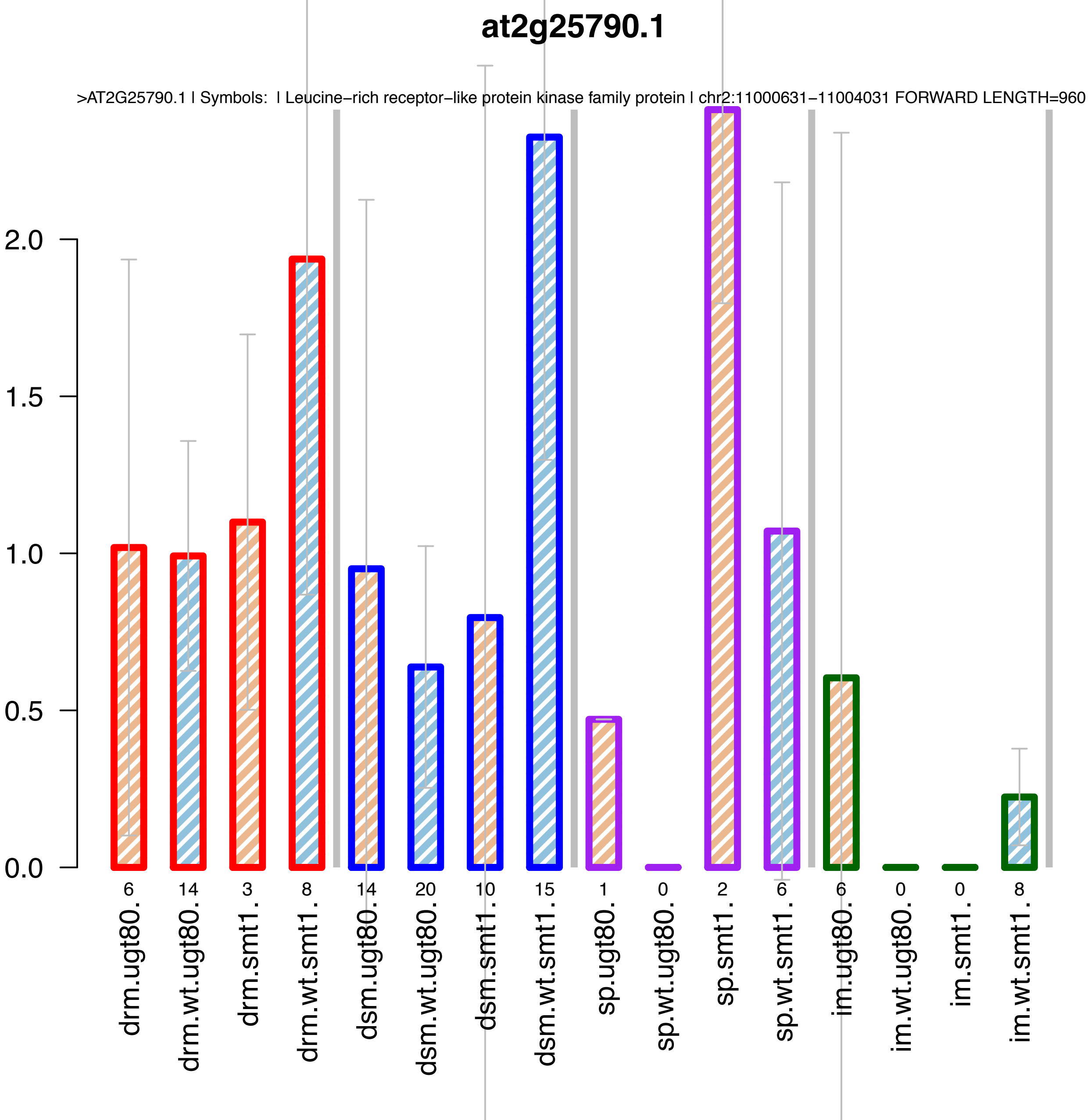

at2g25790.1
>AT2G25790.1 | Symbols: | Leucine−rich receptor−like protein kinase family protein | chr2:11000631−11004031 FORWARD LENGTH=960
0.0
0.5
1.0
1.5
2.0
6
14
3
8
14
20
10
15
1
0
2
6
6
0
0
8
drm.ugt80..
drm.wt.ugt80..
drm.smt1..
drm.wt.smt1..
dsm.ugt80..
dsm.wt.ugt80..
dsm.smt1..
dsm.wt.smt1..
sp.ugt80..
sp.wt.ugt80..
sp.smt1..
sp.wt.smt1..
im.ugt80..
im.wt.ugt80..
im.smt1..
im.wt.smt1..

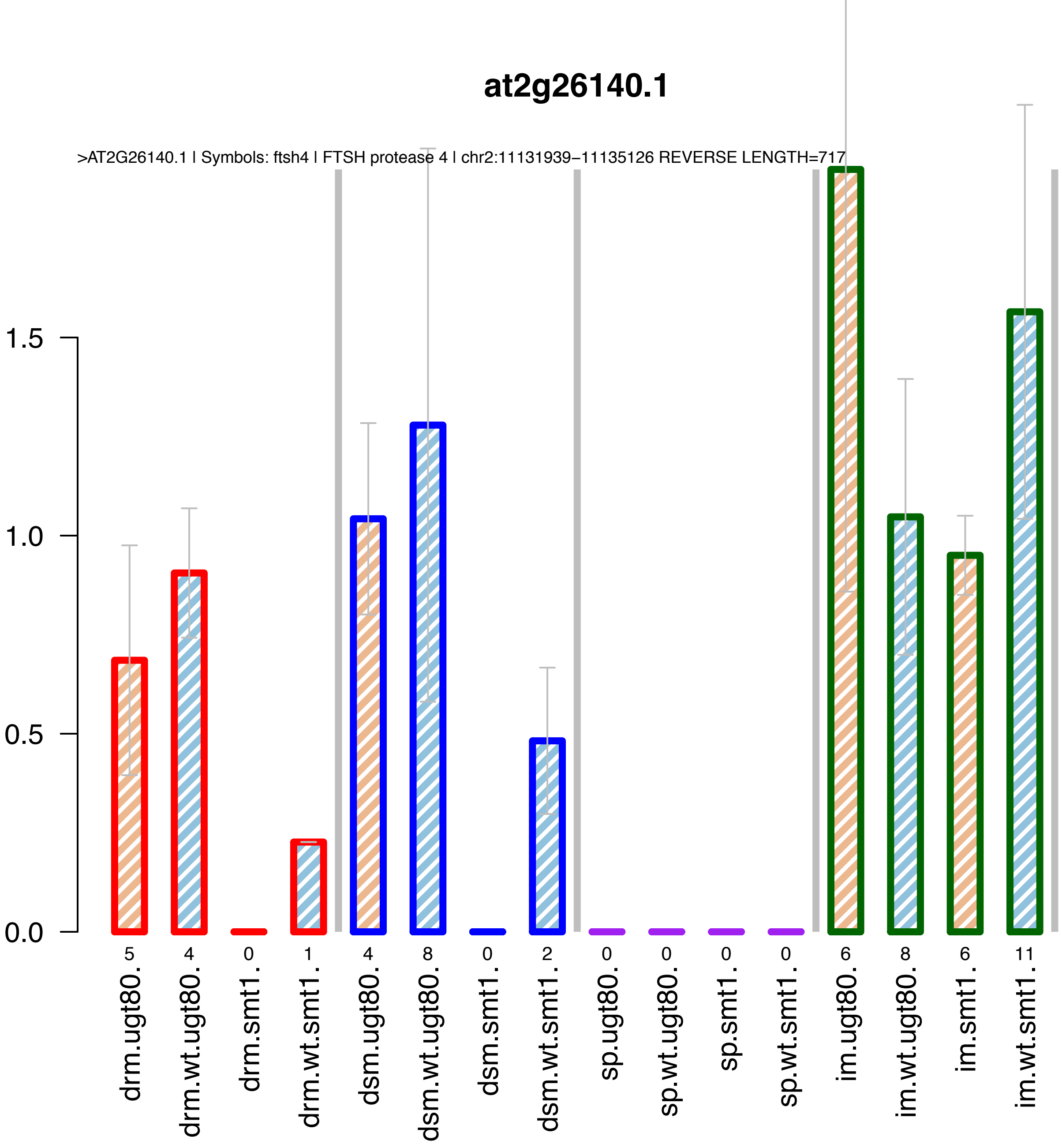

at2g26140.1
>AT2G26140.1 | Symbols: ftsh4 | FTSH protease 4 | chr2:11131939-11135126 REVERSE LENGTH=717
0.0
0.5
1.0
1.5
5
4
0
1
4
8
0
2
0
0
0
0
6
8
6
11
drm.ugt80.
drm.wt.ugt80.
drm.smt1.
drm.wt.smt1.
dsm.ugt80.
dsm.wt.ugt80.
dsm.smt1.
dsm.wt.smt1.
sp.ugt80.
sp.wt.ugt80.
sp.smt1.
sp.wt.smt1.
im.ugt80.
im.wt.ugt80.
im.smt1.
im.wt.smt1.

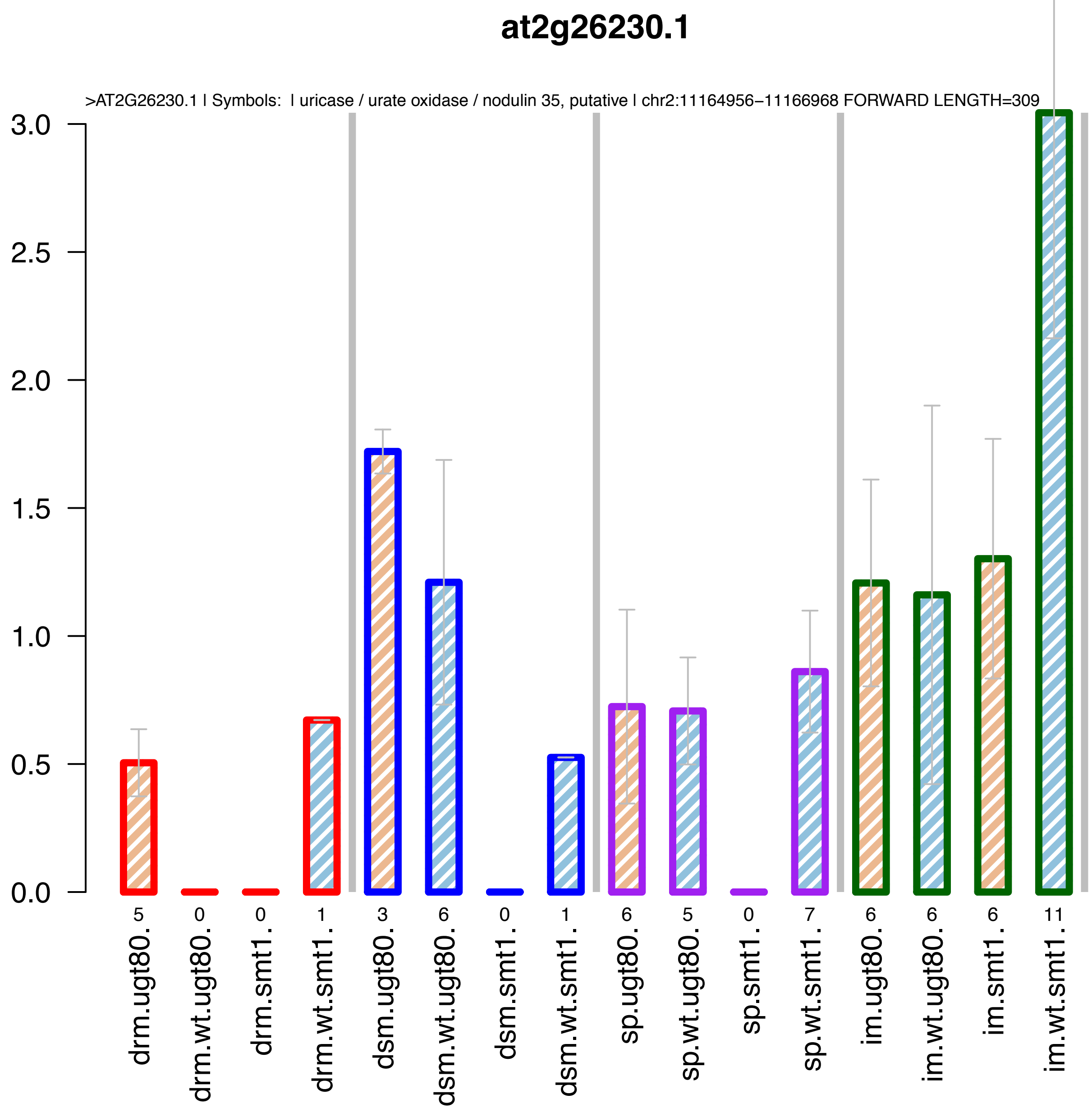

at2g26230.1
>AT2G26230.1 | Symbols: | uricase / urate oxidase / nodulin 35, putative | chr2:11164956-11166968 FORWARD LENGTH=309
3.0
2.5
2.0
1.5
1.0
0.5
0.0
5
0
0
1
3
6
0
1
6
5
0
7
6
6
6
11
drm.ugt80.
drm.wt.ugt80.
drm.smt1.
drm.wt.smt1.
dsm.ugt80.
dsm.wt.ugt80.
dsm.smt1.
dsm.wt.smt1.
sp.ugt80.
sp.wt.ugt80.
sp.smt1.
sp.wt.smt1.
im.ugt80.
im.wt.ugt80.
im.smt1.
im.wt.smt1.

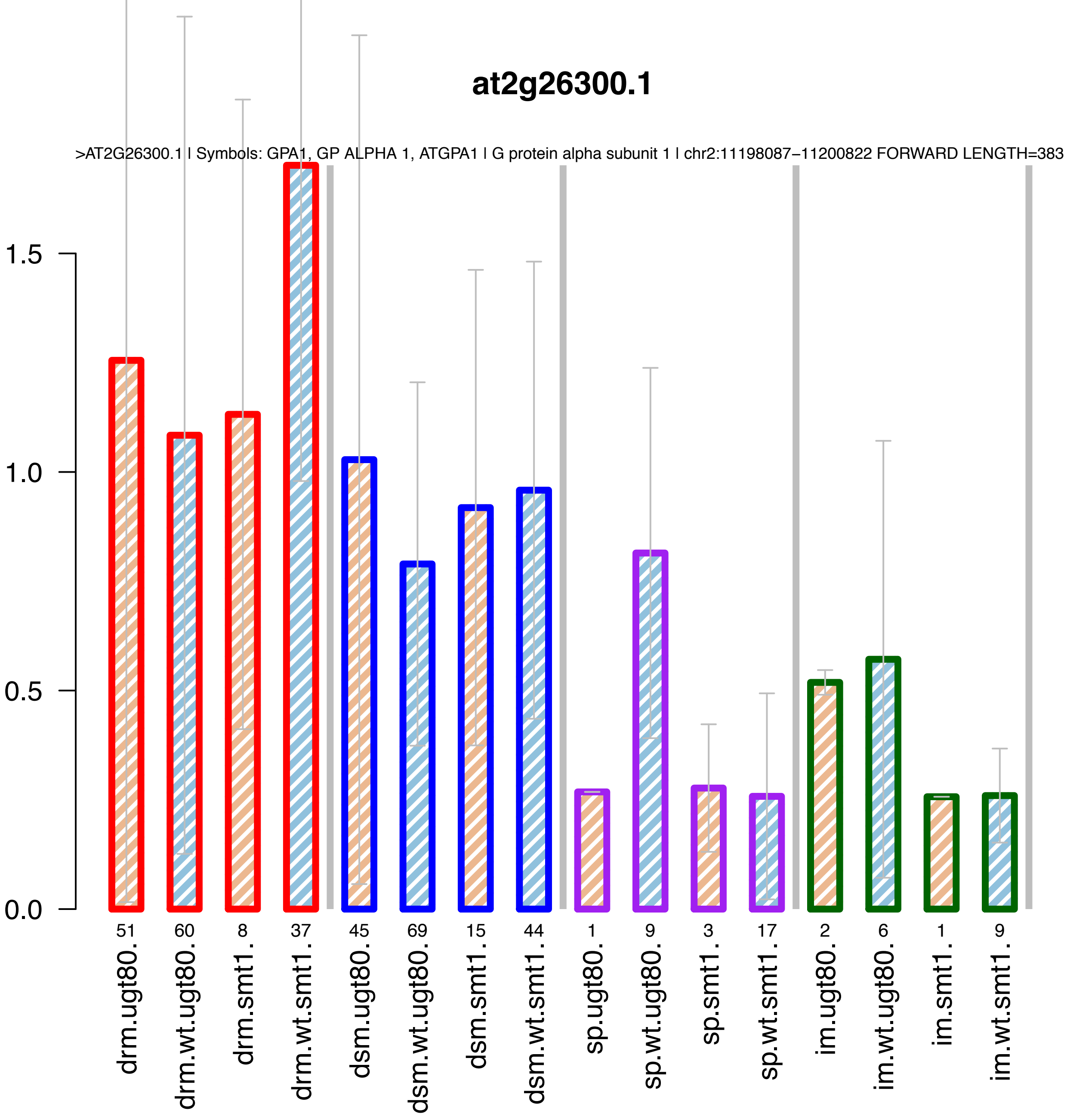
at2g26300.1
>AT2G26300.1 | Symbols: GPA1, GP ALPHA 1, ATGPA1 | G protein alpha subunit 1 | chr2:11198087-11200822 FORWARD LENGTH=383
0.0
0.5
1.0
1.5
51
60
8
37
45
69
15
44
1
9
3
17
2
6
1
9
drm.ugt80.
drm.wt.ugt80.
drm.smt1.
drm.wt.smt1.
dsm.ugt80.
dsm.wt.ugt80.
dsm.smt1.
dsm.wt.smt1.
sp.ugt80.
sp.wt.ugt80.
sp.smt1.
sp.wt.smt1.
im.ugt80.
im.wt.ugt80.
im.smt1.
im.wt.smt1.

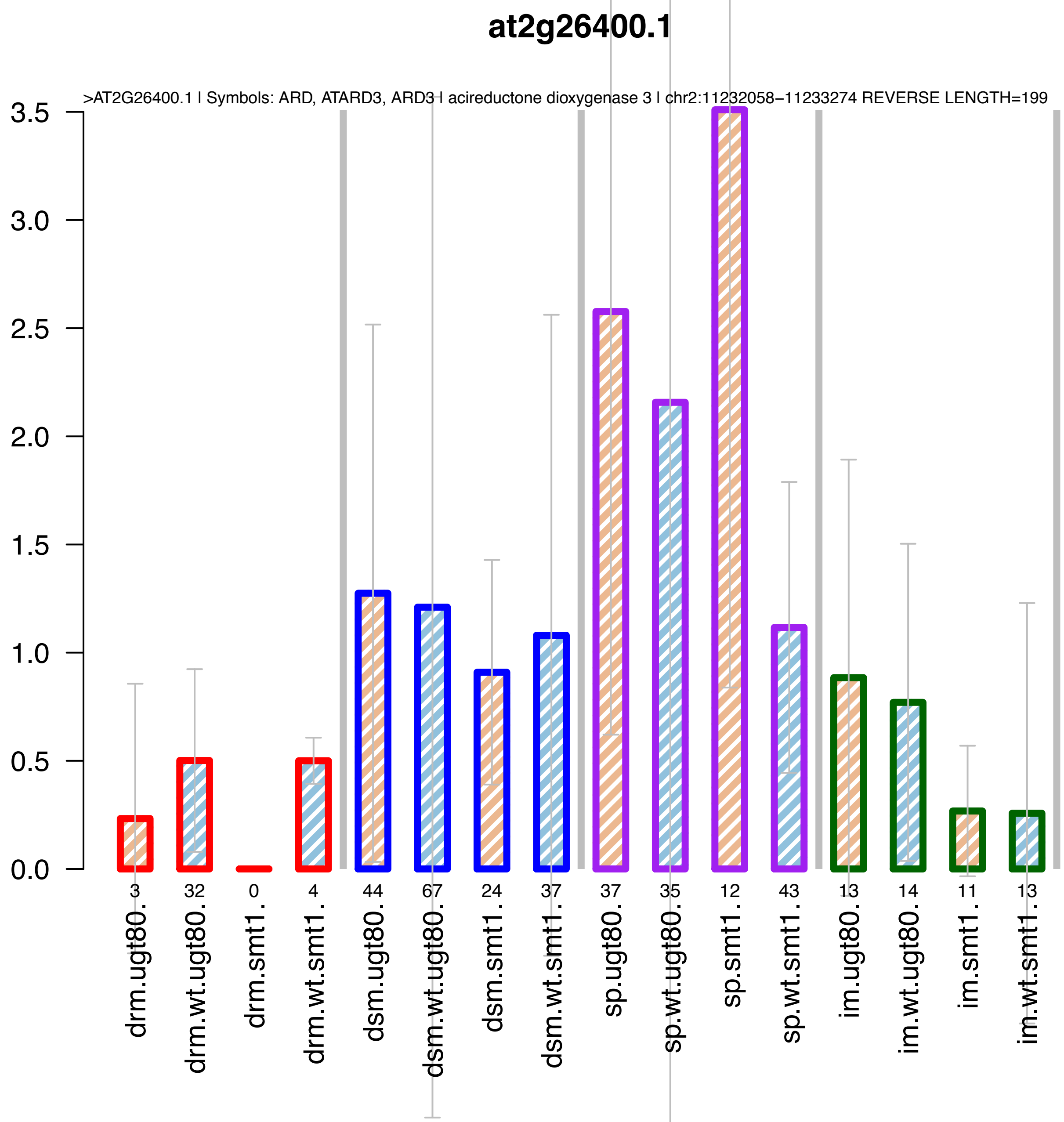
at2g26400.1
>AT2G26400.1 | Symbols: ARD, ATARD3, ARD3 | acireductone dioxygenase 3 | chr2:11232058-11233274 REVERSE LENGTH=199
3.5
3.0
2.5
2.0
1.5
1.0
0.5
0.0
3
32
0
4
44
67
24
37
37
35
12
43
13
14
11
13
drm.ugt80..
drm.wt.ugt80..
drm.smt1..
drm.wt.smt1..
dsm.ugt80..
dsm.wt.ugt80..
dsm.smt1..
dsm.wt.smt1..
sp.ugt80..
sp.wt.ugt80..
sp.smt1..
sp.wt.smt1..
im.ugt80..
im.wt.ugt80..
im.smt1..
im.wt.smt1..

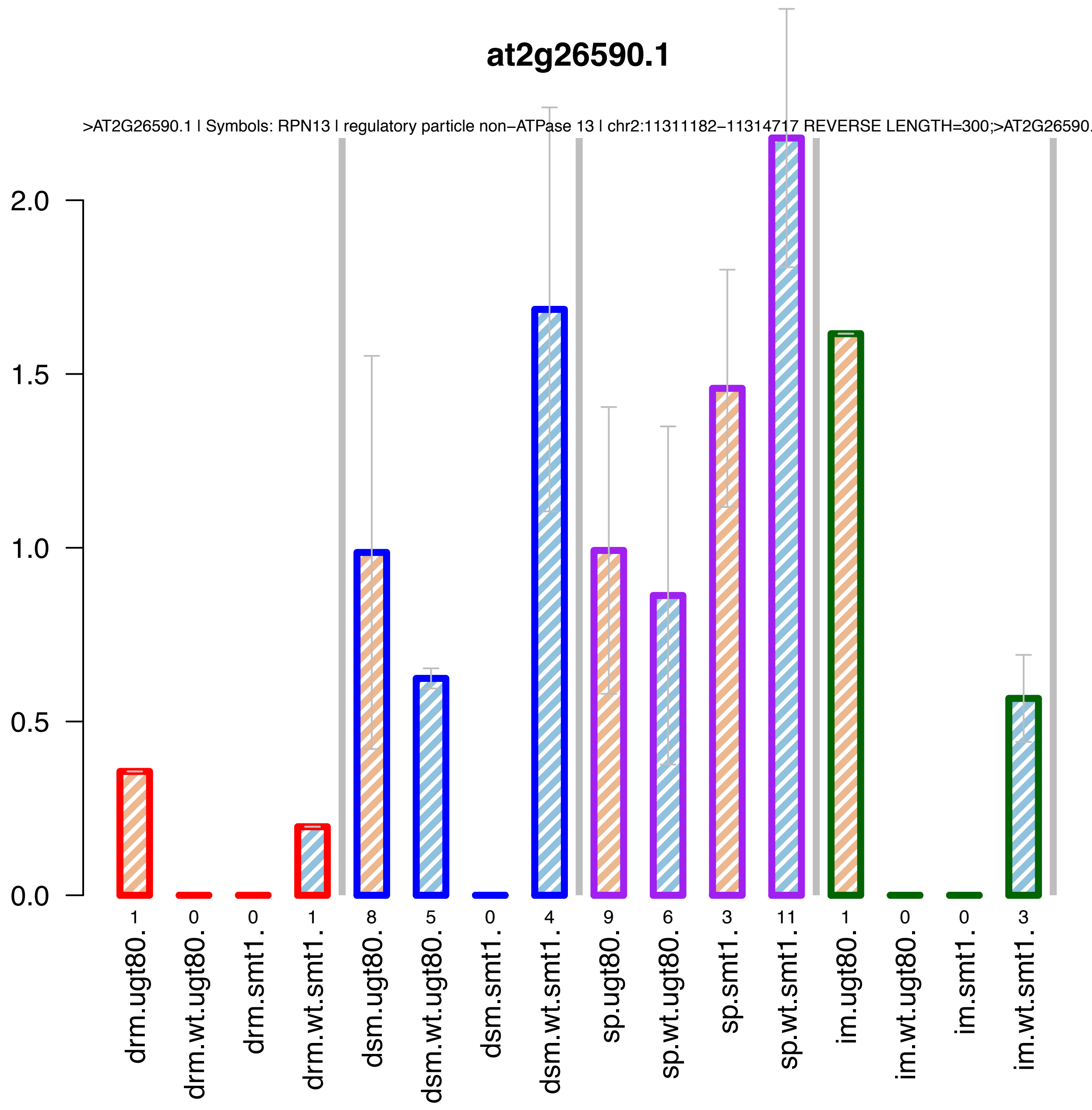

at2g26590.1
>AT2G26590.1 | Symbols: RPN13 | regulatory particle non−ATPase 13 | chr2:11311182−11314717 REVERSE LENGTH=300;>AT2G26590.
2.0
1.5
1.0
0.5
0.0
1
0
0
1
8
5
0
4
9
6
3
11
1
0
0
3
drm.ugt80.
drm.wt.ugt80.
drm.smt1.
drm.wt.smt1.
dsm.ugt80.
dsm.wt.ugt80.
dsm.smt1.
dsm.wt.smt1.
sp.ugt80.
sp.wt.ugt80.
sp.smt1.
sp.wt.smt1.
im.ugt80.
im.wt.ugt80.
im.smt1.
im.wt.smt1.

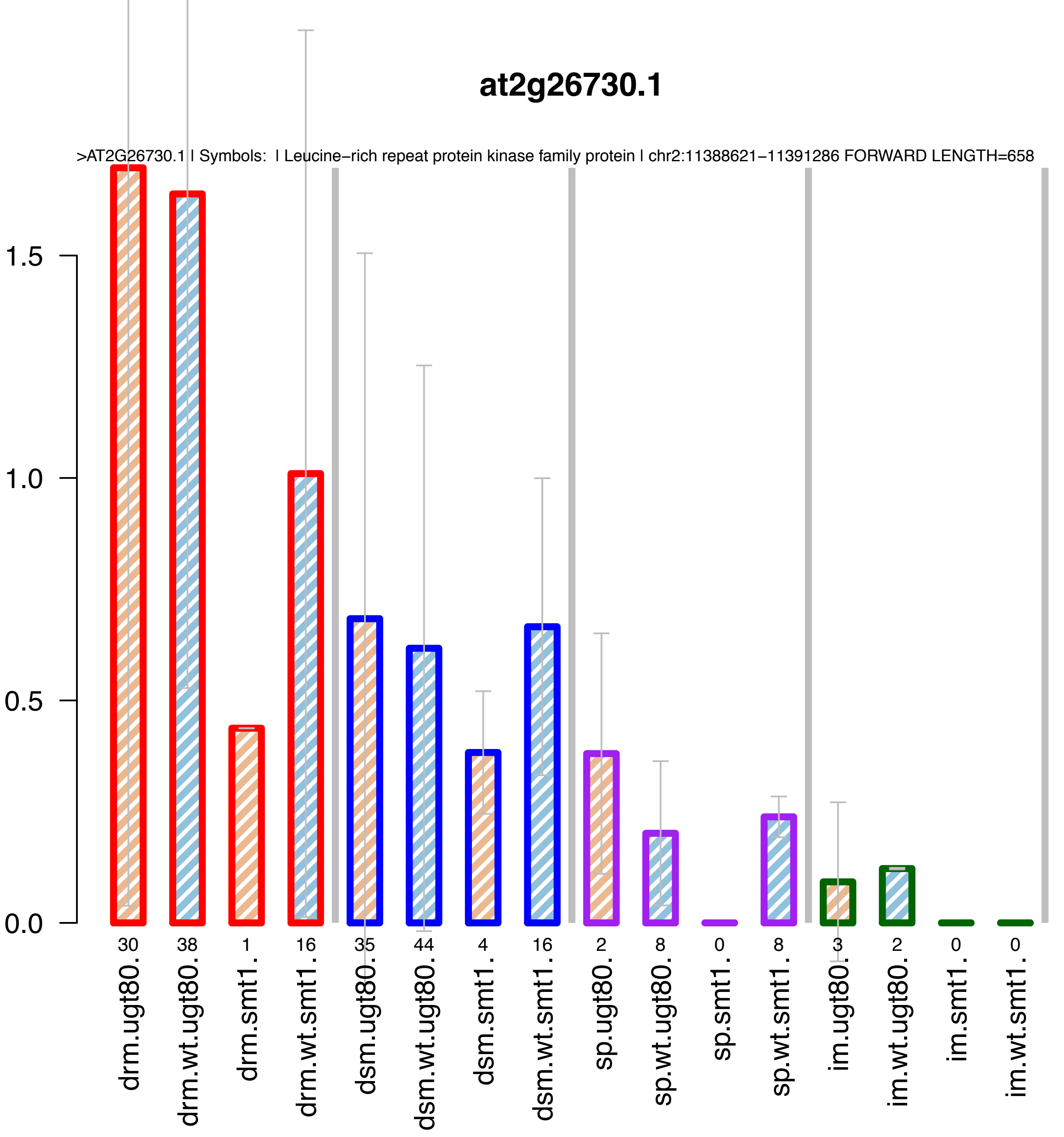

at2g26730.1
>AT2G26730.1 | Symbols: | Leucine−rich repeat protein kinase family protein | chr2:11388621−11391286 FORWARD LENGTH=658
1.5
1.0
0.5
0.0
30
38
1
16
35
44
4
16
2
8
0
8
3
2
0
0
drm.ugt80..
drm.wt.ugt80..
drm.smt1..
drm.wt.smt1..
dsm.ugt80..
dsm.wt.ugt80..
dsm.smt1..
dsm.wt.smt1..
sp.ugt80..
sp.wt.ugt80..
sp.smt1..
sp.wt.smt1..
im.ugt80..
im.wt.ugt80..
im.smt1..
im.wt.smt1..

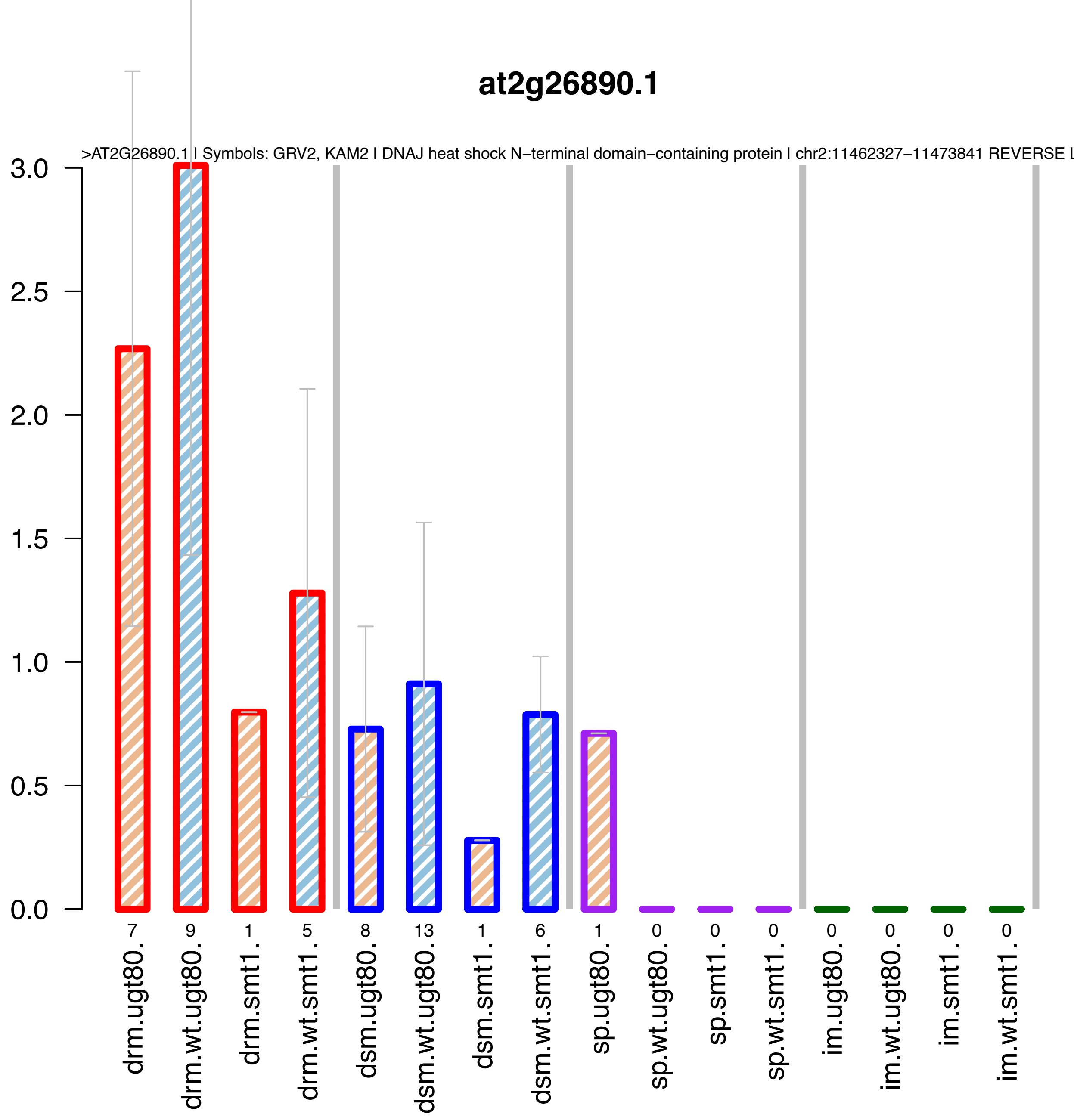

at2g26890.1
>AT2G26890.1 | Symbols: GRV2, KAM2 | DNAJ heat shock N-terminal domain-containing protein | chr2:11462327-11473841 REVERSE L
3.0
2.5
2.0
1.5
1.0
0.5
0.0
7
9
1
5
8
13
1
6
1
0
0
0
0
0
0
0
drm.ugt80.
drm.wt.ugt80.
drm.smt1.
drm.wt.smt1.
dsm.ugt80.
dsm.wt.ugt80.
dsm.smt1.
dsm.wt.smt1.
sp.ugt80.
sp.wt.ugt80.
sp.smt1.
sp.wt.smt1.
im.ugt80.
im.wt.ugt80.
im.smt1.
im.wt.smt1.

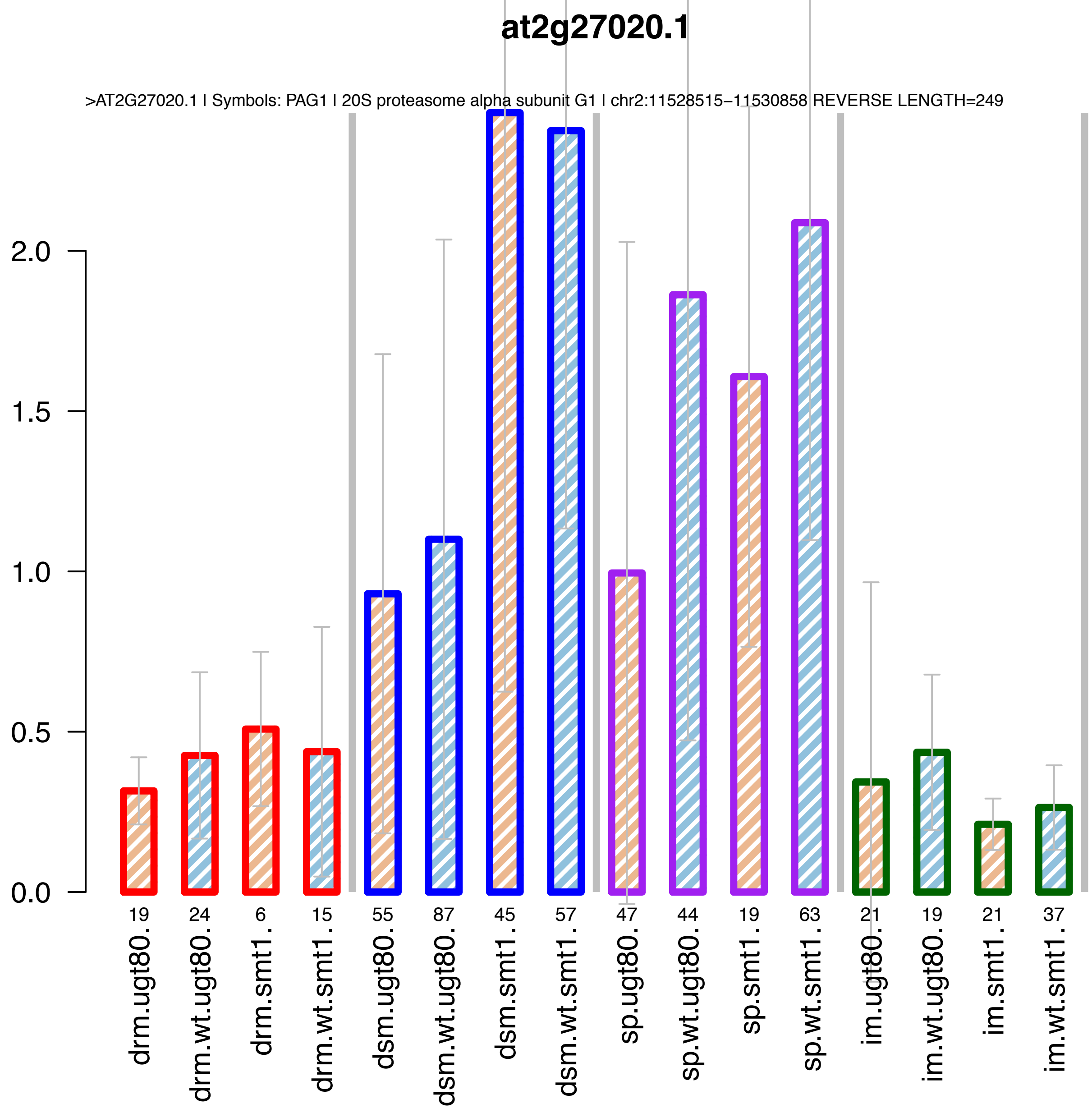

at2g27020.1
>AT2G27020.1 | Symbols: PAG1 | 20S proteasome alpha subunit G1 | chr2:11528515-11530858 REVERSE LENGTH=249
2.0
1.5
1.0
0.5
0.0
19
24
6
15
55
87
45
57
47
44
19
63
21
19
21
37
drm.ugt80.
drm.wt.ugt80.
drm.smt1.
drm.wt.smt1.
dsm.ugt80.
dsm.wt.ugt80.
dsm.smt1.
dsm.wt.smt1.
sp.ugt80.
sp.wt.ugt80.
sp.smt1.
sp.wt.smt1.
im.ugt80.
im.wt.ugt80.
im.smt1.
im.wt.smt1.

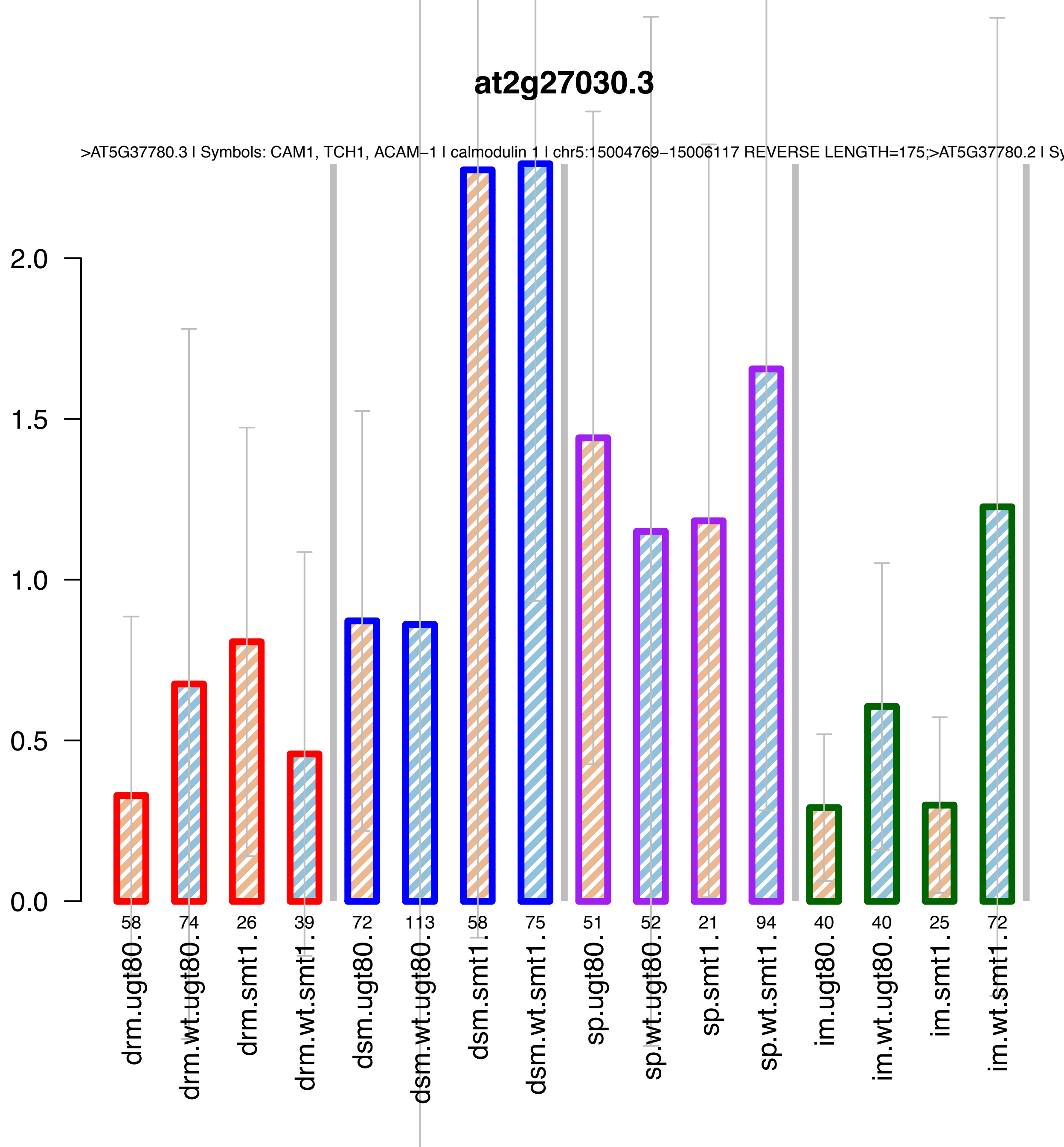

at2g27030.3
>AT5G37780.3 | Symbols: CAM1, TCH1, ACAM-1 | calmodulin 1 | chr5:15004769-15006117 REVERSE LENGTH=175;>AT5G37780.2 | Sy
0.0
0.5
1.0
1.5
2.0
58
74
26
39
72
113
58
75
51
52
21
94
40
40
25
72
drm.ugt80..
drm.wt.ugt80..
drm.smt1..
drm.wt.smt1..
dsm.ugt80..
dsm.wt.ugt80..
dsm.smt1..
dsm.wt.smt1..
sp.ugt80..
sp.wt.ugt80..
sp.smt1..
sp.wt.smt1..
im.ugt80..
im.wt.ugt80..
im.smt1..
im.wt.smt1..

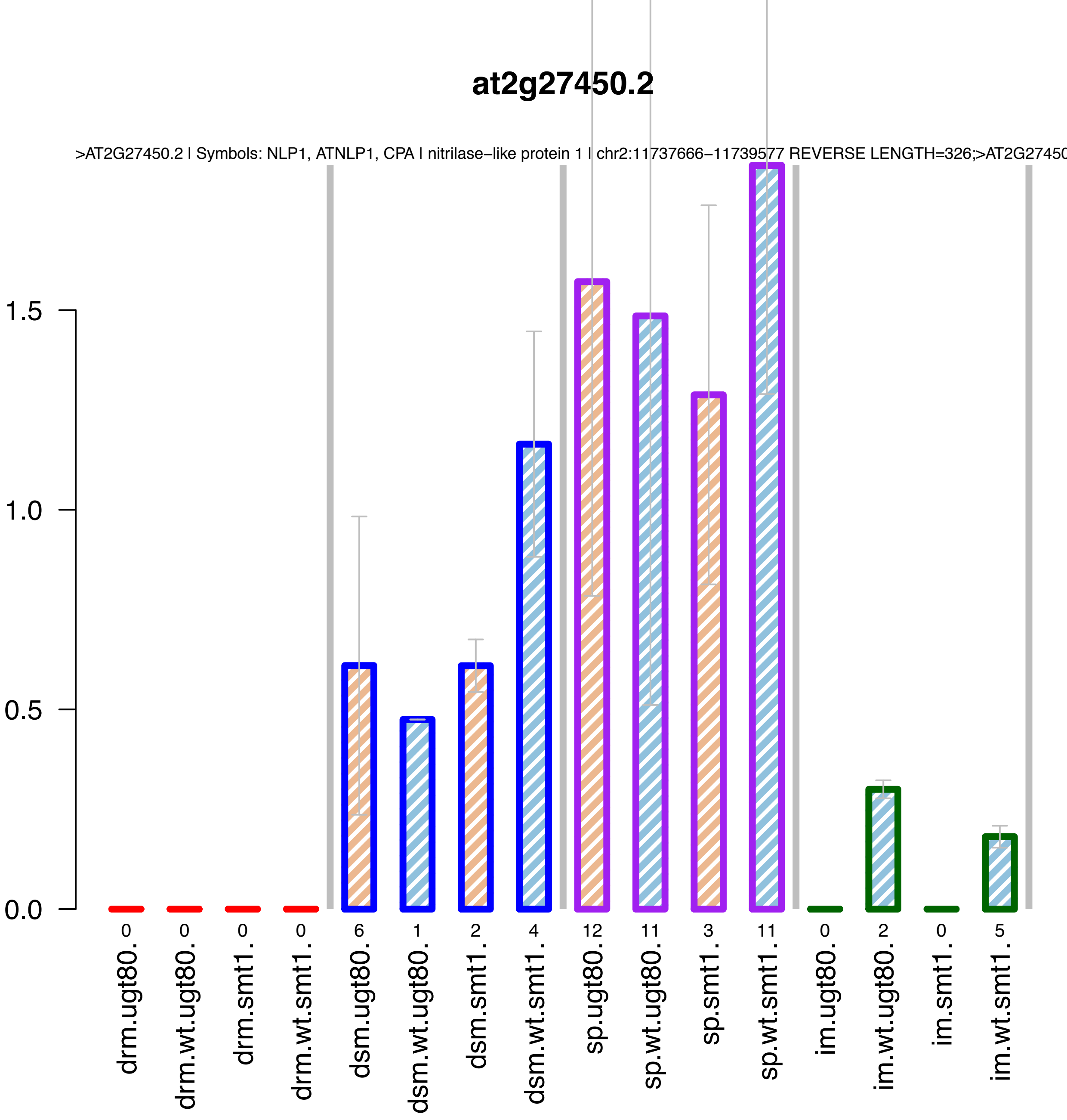

at2g27450.2
>AT2G27450.2 | Symbols: NLP1, ATNLP1, CPA | nitrilase-like protein 1 | chr2:11737666-11739577 REVERSE LENGTH=326;>AT2G27450
1.5
1.0
0.5
0.0
0
0
0
0
6
1
2
4
12
11
3
11
0
2
0
5
drm.ugt80.
drm.wt.ugt80.
drm.smt1.
drm.wt.smt1.
dsm.ugt80.
dsm.wt.ugt80.
dsm.smt1.
dsm.wt.smt1.
sp.ugt80.
sp.wt.ugt80.
sp.smt1.
sp.wt.smt1.
im.ugt80.
im.wt.ugt80.
im.smt1.
im.wt.smt1.

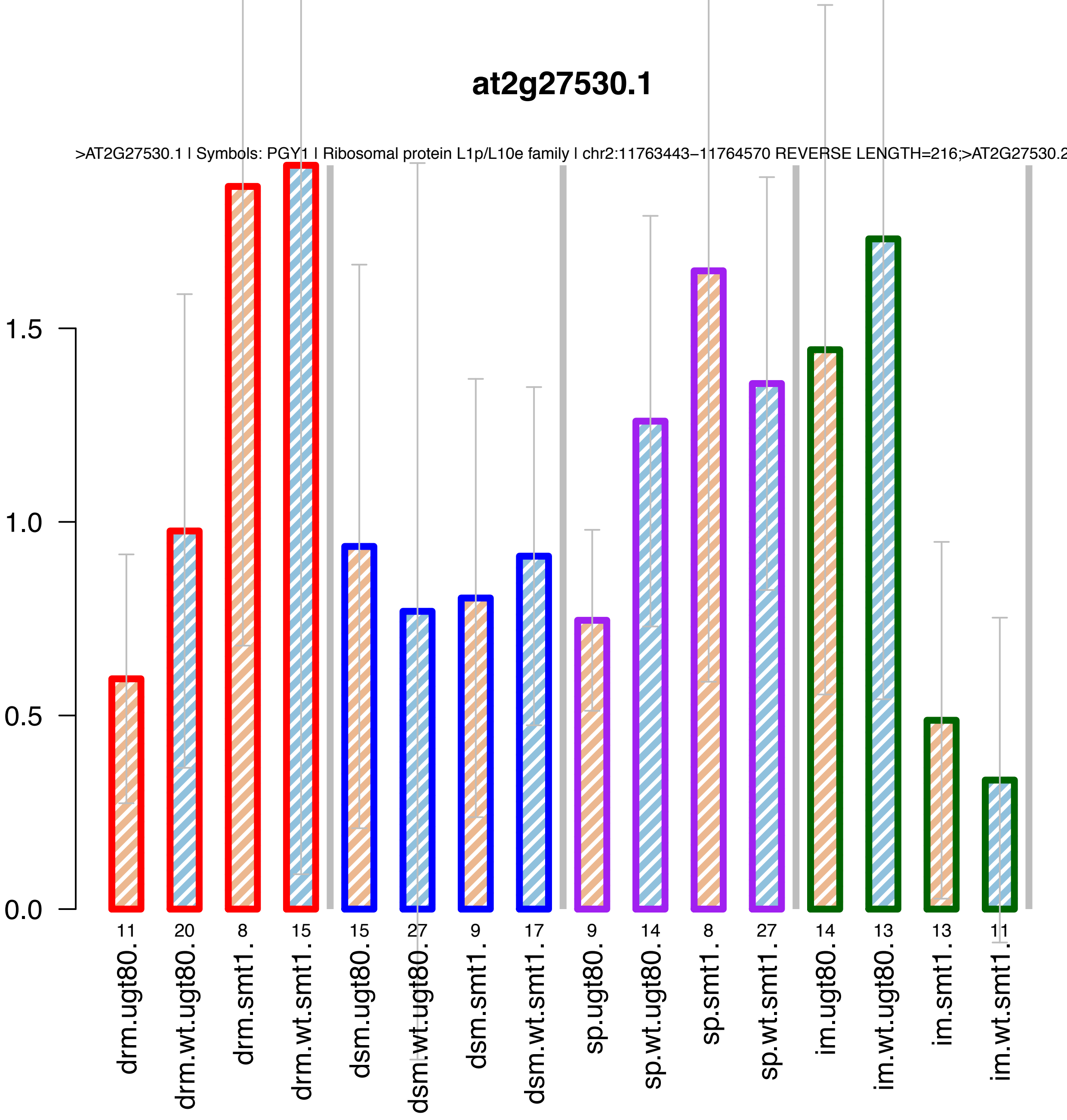

at2g27530.1
>AT2G27530.1 | Symbols: PGY1 | Ribosomal protein L1p/L10e family | chr2:11763443-11764570 REVERSE LENGTH=216;>AT2G27530.2
0.0
0.5
1.0
1.5
11
20
8
15
15
27
9
17
9
14
8
27
14
13
13
11
drm.ugt80..
drm.wt.ugt80..
drm.smt1..
drm.wt.smt1..
dsm.ugt80..
dsm.wt.ugt80..
dsm.smt1..
dsm.wt.smt1..
sp.ugt80..
sp.wt.ugt80..
sp.smt1..
sp.wt.smt1..
im.ugt80..
im.wt.ugt80..
im.smt1..
im.wt.smt1..

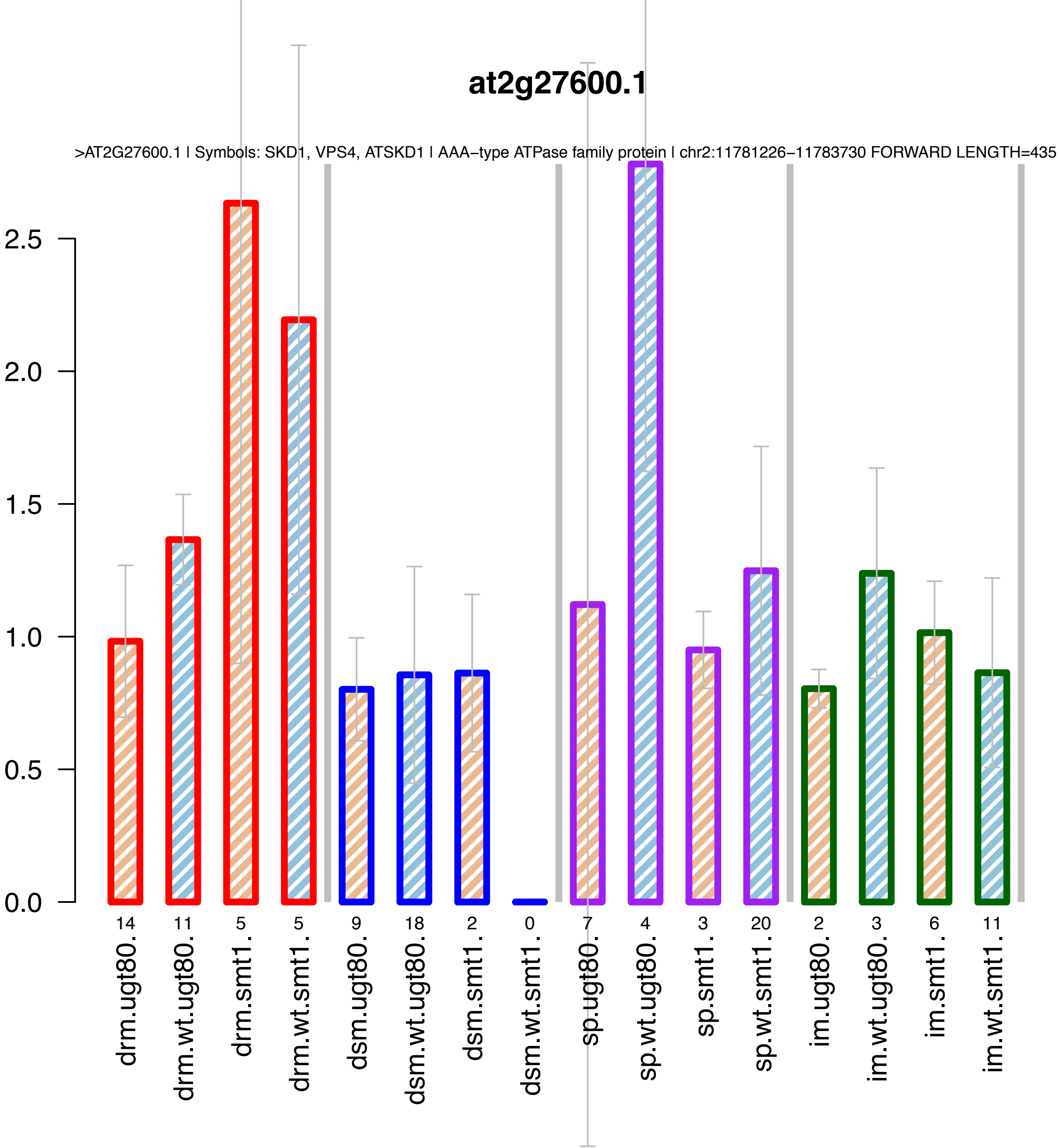
at2g27600.1
>AT2G27600.1 | Symbols: SKD1, VPS4, ATSKD1 | AAA−type ATPase family protein | chr2:11781226−11783730 FORWARD LENGTH=435
0.0
0.5
1.0
1.5
2.0
2.5
14
11
5
5
9
18
2
0
7
4
3
20
2
3
6
11
drm.ugt80..
drm.wt.ugt80..
drm.smt1..
drm.wt.smt1..
dsm.ugt80..
dsm.wt.ugt80..
dsm.smt1..
dsm.wt.smt1..
sp.ugt80..
sp.wt.ugt80..
sp.smt1..
sp.wt.smt1..
im.ugt80..
im.wt.ugt80..
im.smt1..
im.wt.smt1..

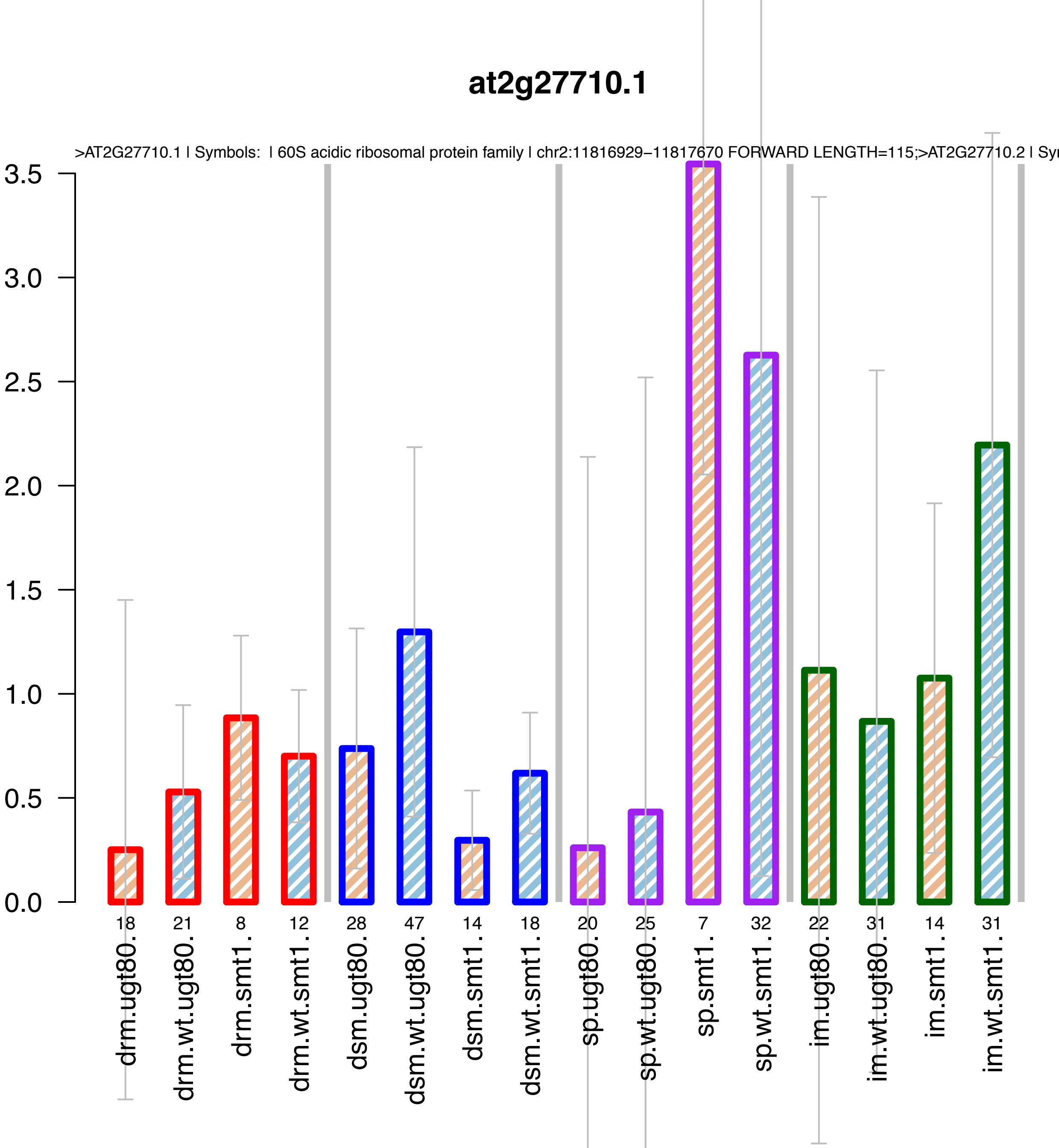

at2g27710.1
>AT2G27710.1 | Symbols: | 60S acidic ribosomal protein family | chr2:11816929-11817670 FORWARD LENGTH=115;>AT2G27710.2 | Sy
3.5
3.0
2.5
2.0
1.5
1.0
0.5
0.0
18
21
8
12
28
47
14
18
20
25
7
32
22
31
14
31
drm.ugt80.
drm.wt.ugt80.
drm.smt1.
drm.wt.smt1.
dsm.ugt80.
dsm.wt.ugt80.
dsm.smt1.
dsm.wt.smt1.
sp.ugt80.
sp.wt.ugt80.
sp.smt1.
sp.wt.smt1.
im.ugt80.
im.wt.ugt80.
im.smt1.
im.wt.smt1.

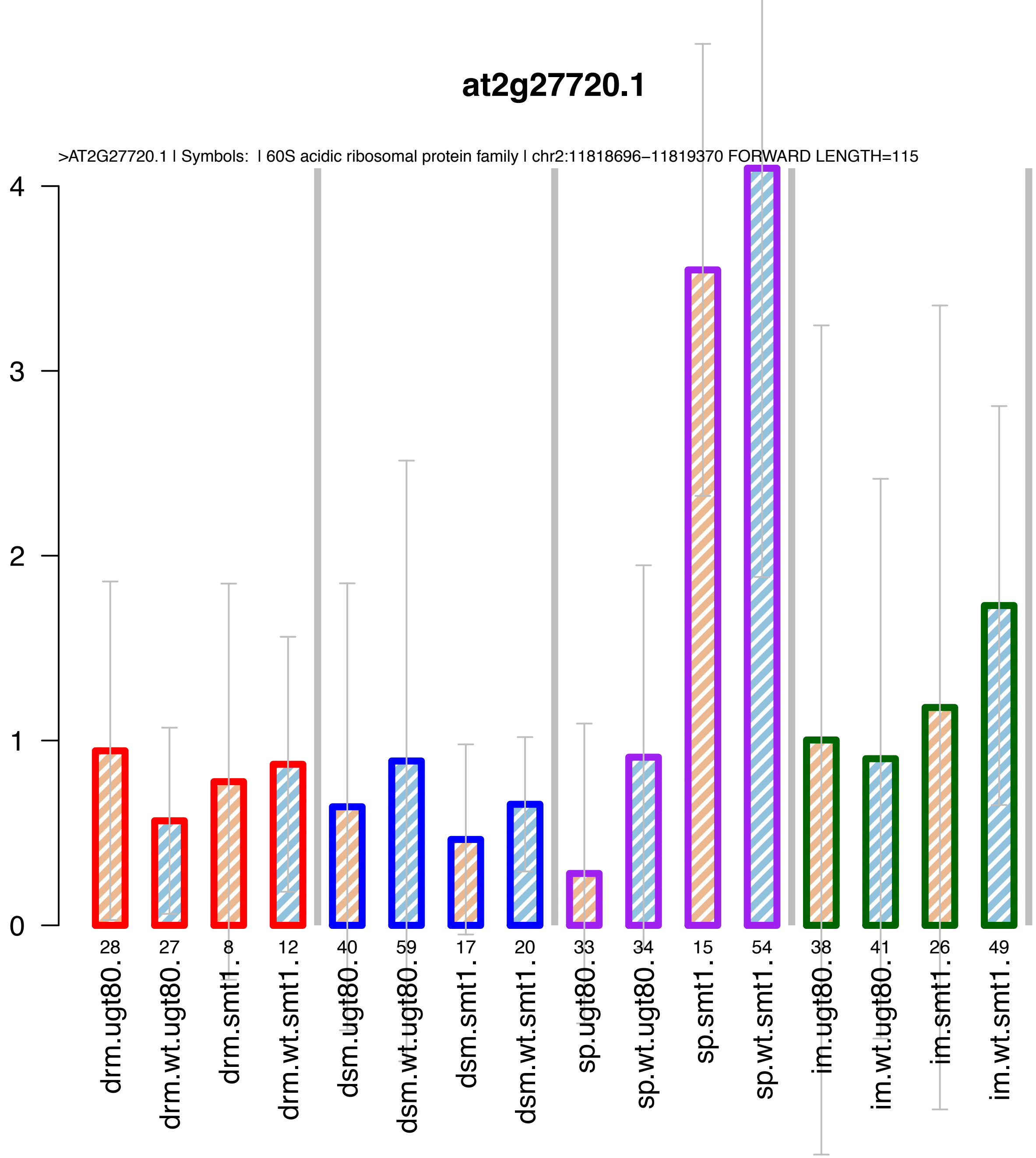
at2g27720.1
>AT2G27720.1 | Symbols: | 60S acidic ribosomal protein family | chr2:11818696-11819370 FORWARD LENGTH=115
0
1
2
3
4
28
27
8
12
40
59
17
20
33
34
15
54
38
41
26
49
drm.ugt80..
drm.wt.ugt80..
drm.smt1..
drm.wt.smt1..
dsm.ugt80..
dsm.wt.ugt80..
dsm.smt1..
dsm.wt.smt1..
sp.ugt80..
sp.wt.ugt80..
sp.smt1..
sp.wt.smt1..
im.ugt80..
im.wt.ugt80..
im.smt1..
im.wt.smt1..

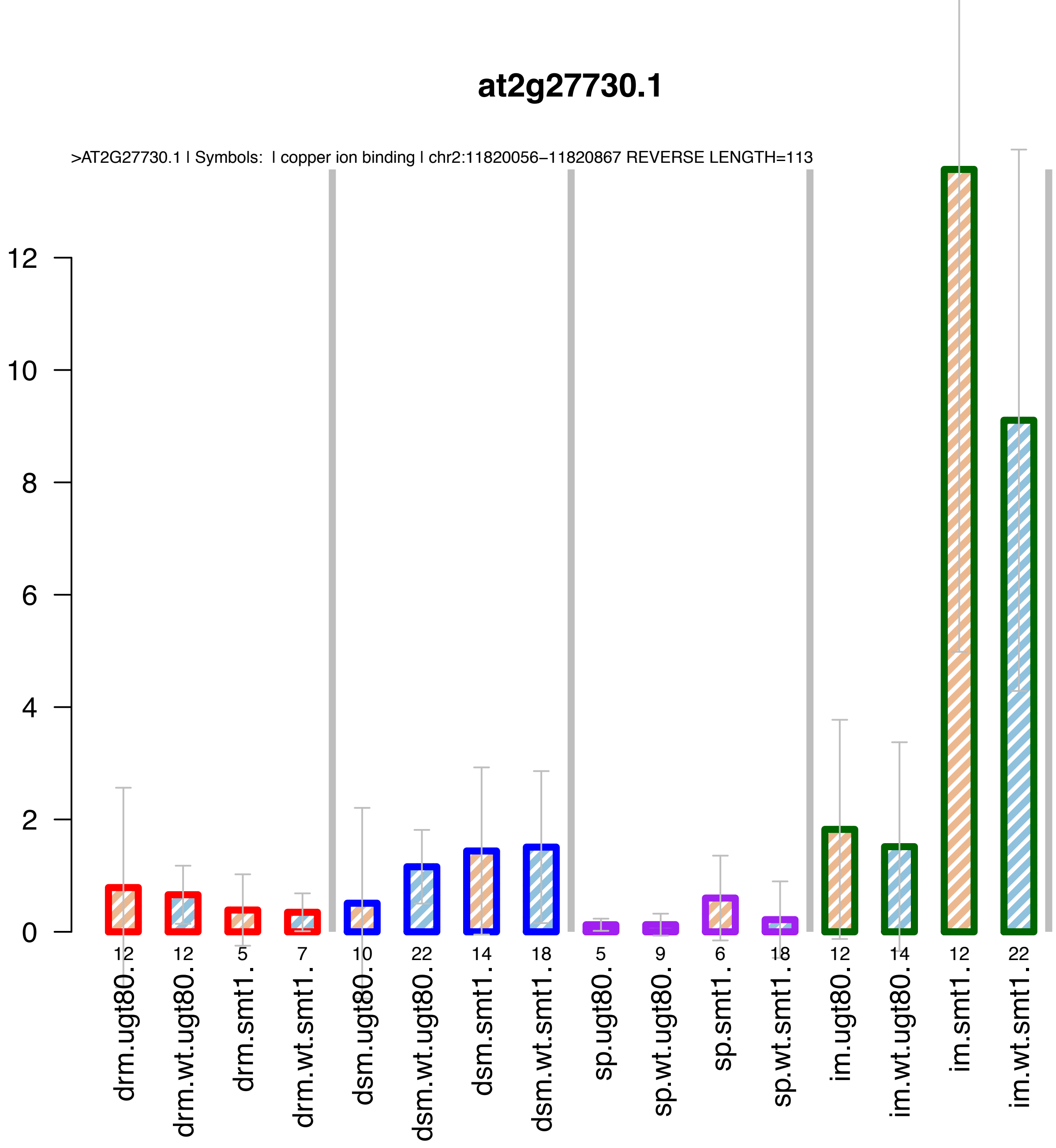

at2g27730.1
>AT2G27730.1 | Symbols: | copper ion binding | chr2:11820056-11820867 REVERSE LENGTH=113
0
2
4
6
8
10
12
12
12
5
7
10
22
14
18
5
9
6
18
12
14
12
22
drm.ugt80.
drm.wt.ugt80.
drm.smt1.
drm.wt.smt1.
dsm.ugt80.
dsm.wt.ugt80.
dsm.smt1.
dsm.wt.smt1.
sp.ugt80.
sp.wt.ugt80.
sp.smt1.
sp.wt.smt1.
im.ugt80.
im.wt.ugt80.
im.smt1.
im.wt.smt1.

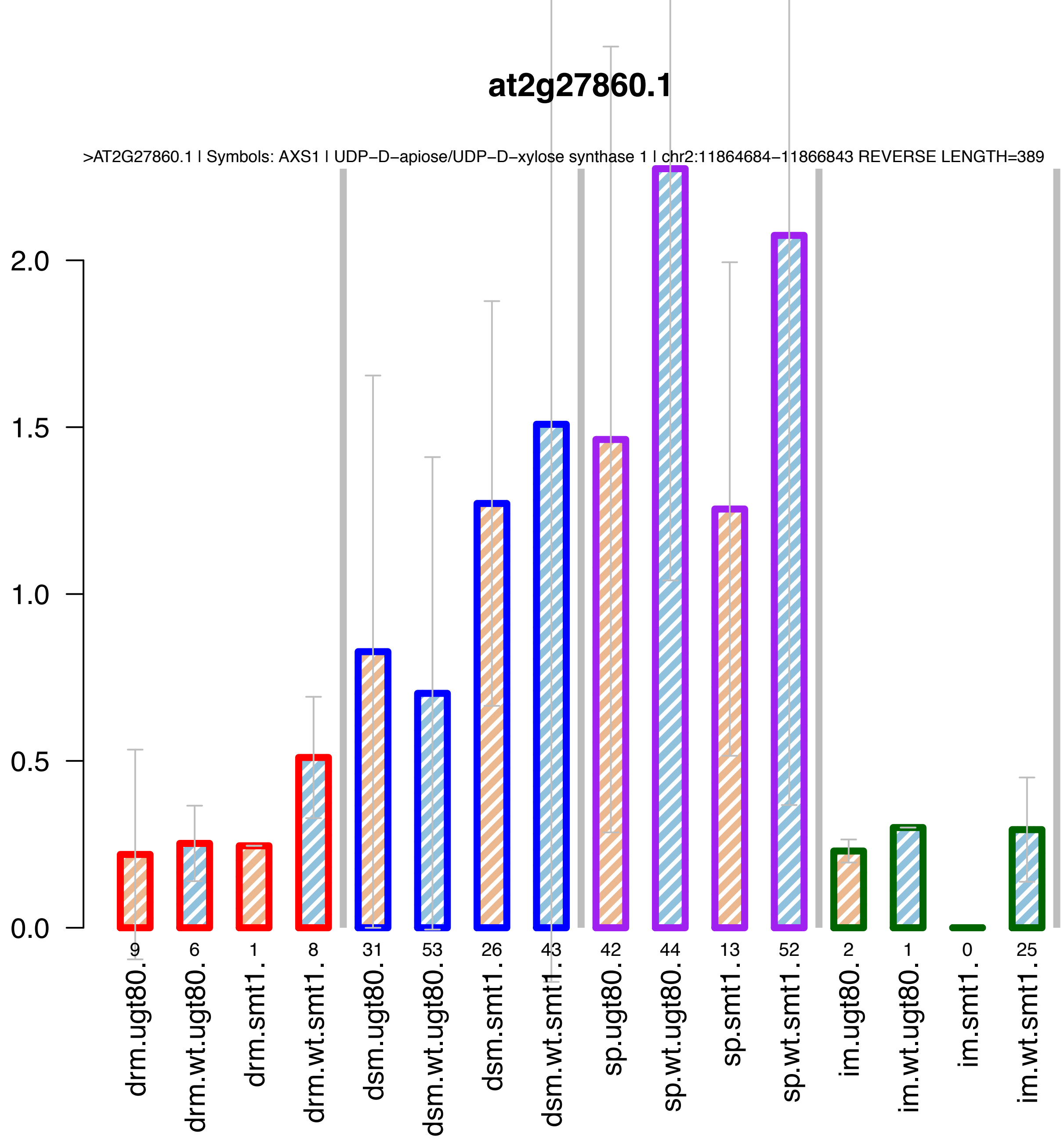

at2g27860.1
>AT2G27860.1 | Symbols: AXS1 | UDP−D−apiose/UDP−D−xylose synthase 1 | chr2:11864684−11866843 REVERSE LENGTH=389
2.0
1.5
1.0
0.5
0.0
9
6
1
8
31
53
26
43
42
44
13
52
2
1
0
25
drm.ugt80.
drm.wt.ugt80.
drm.smt1.
drm.wt.smt1.
dsm.ugt80.
dsm.wt.ugt80.
dsm.smt1.
dsm.wt.smt1.
sp.ugt80.
sp.wt.ugt80.
sp.smt1.
sp.wt.smt1.
im.ugt80.
im.wt.ugt80.
im.smt1.
im.wt.smt1.

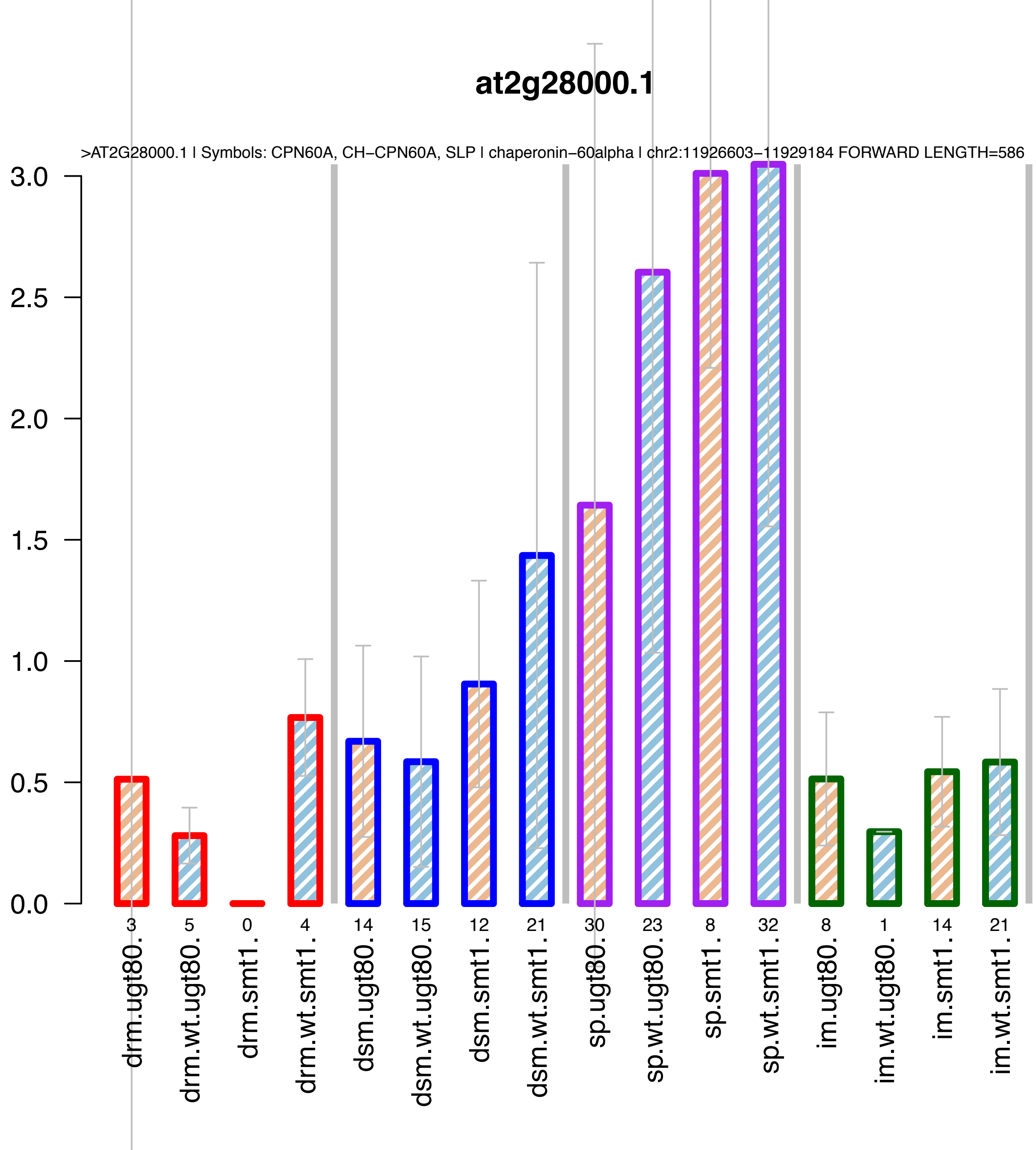

at2g28000.1
>AT2G28000.1 | Symbols: CPN60A, CH-CPN60A, SLP | chaperonin-60alpha | chr2:11926603-11929184 FORWARD LENGTH=586
3.0
2.5
2.0
1.5
1.0
0.5
0.0
3
5
0
4
14
15
12
21
30
23
8
32
8
1
14
21
drm.ugt80..
drm.wt.ugt80..
drm.smt1..
drm.wt.smt1..
dsm.ugt80..
dsm.wt.ugt80..
dsm.smt1..
dsm.wt.smt1..
sp.ugt80..
sp.wt.ugt80..
sp.smt1..
sp.wt.smt1..
im.ugt80..
im.wt.ugt80..
im.smt1..
im.wt.smt1..

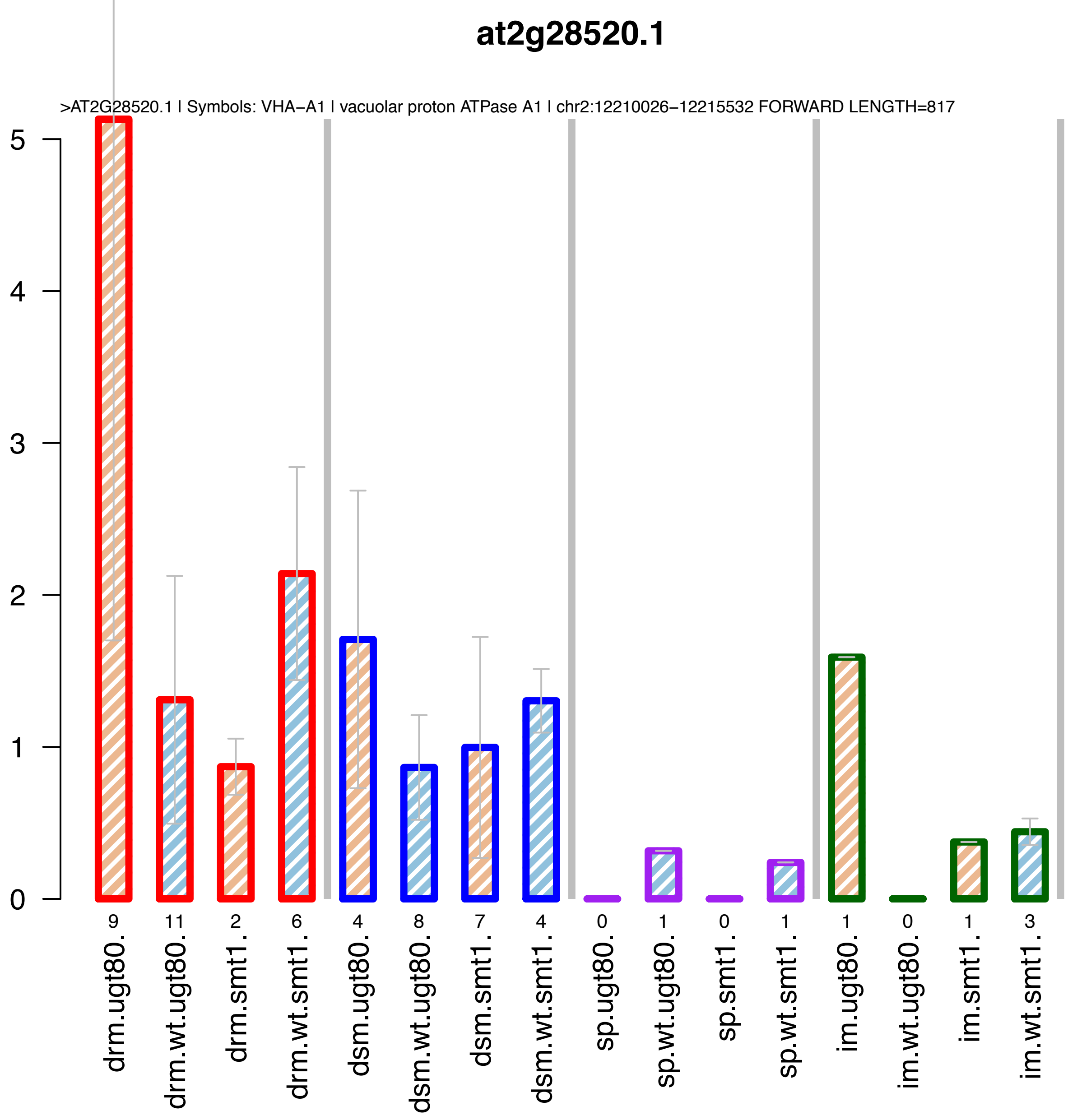

at2g28520.1
>AT2G28520.1 | Symbols: VHA−A1 | vacuolar proton ATPase A1 | chr2:12210026−12215532 FORWARD LENGTH=817
0
1
2
3
4
5
9
11
2
6
4
8
7
4
0
1
0
1
1
0
1
3
drm.ugt80.
drm.wt.ugt80.
drm.smt1.
drm.wt.smt1.
dsm.ugt80.
dsm.wt.ugt80.
dsm.smt1.
dsm.wt.smt1.
sp.ugt80.
sp.wt.ugt80.
sp.smt1.
sp.wt.smt1.
im.ugt80.
im.wt.ugt80.
im.smt1.
im.wt.smt1.

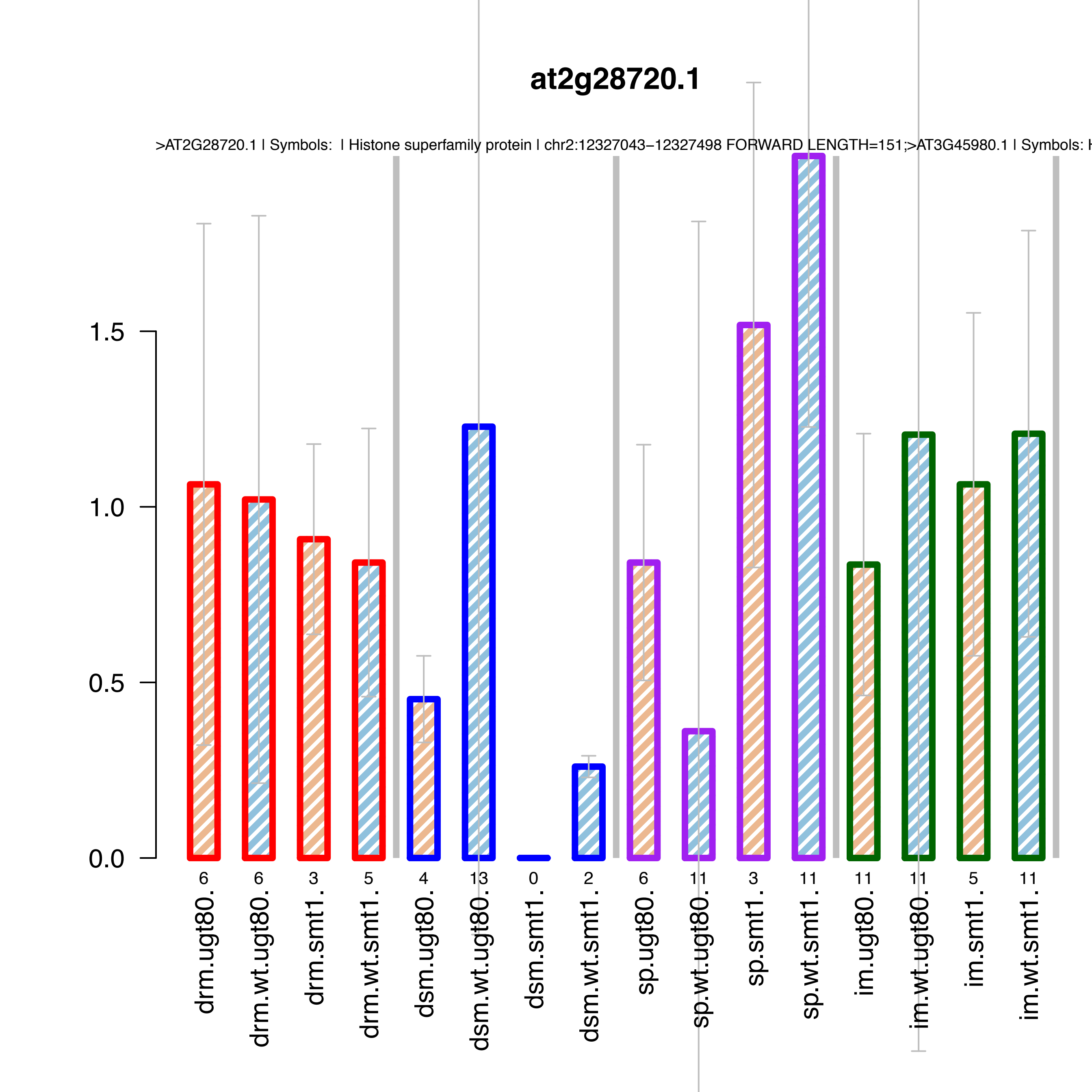

at2g28720.1
>AT2G28720.1 | Symbols: | Histone superfamily protein | chr2:12327043-12327498 FORWARD LENGTH=151;>AT3G45980.1 | Symbols: H
0.0
0.5
1.0
1.5
6
6
3
5
4
13
0
2
6
11
3
11
11
11
5
11
drm.ugt80.
drm.wt.ugt80.
drm.smt1.
drm.wt.smt1.
dsm.ugt80.
dsm.wt.ugt80.
dsm.smt1.
dsm.wt.smt1.
sp.ugt80.
sp.wt.ugt80.
sp.smt1.
sp.wt.smt1.
im.ugt80.
im.wt.ugt80.
im.smt1.
im.wt.smt1.

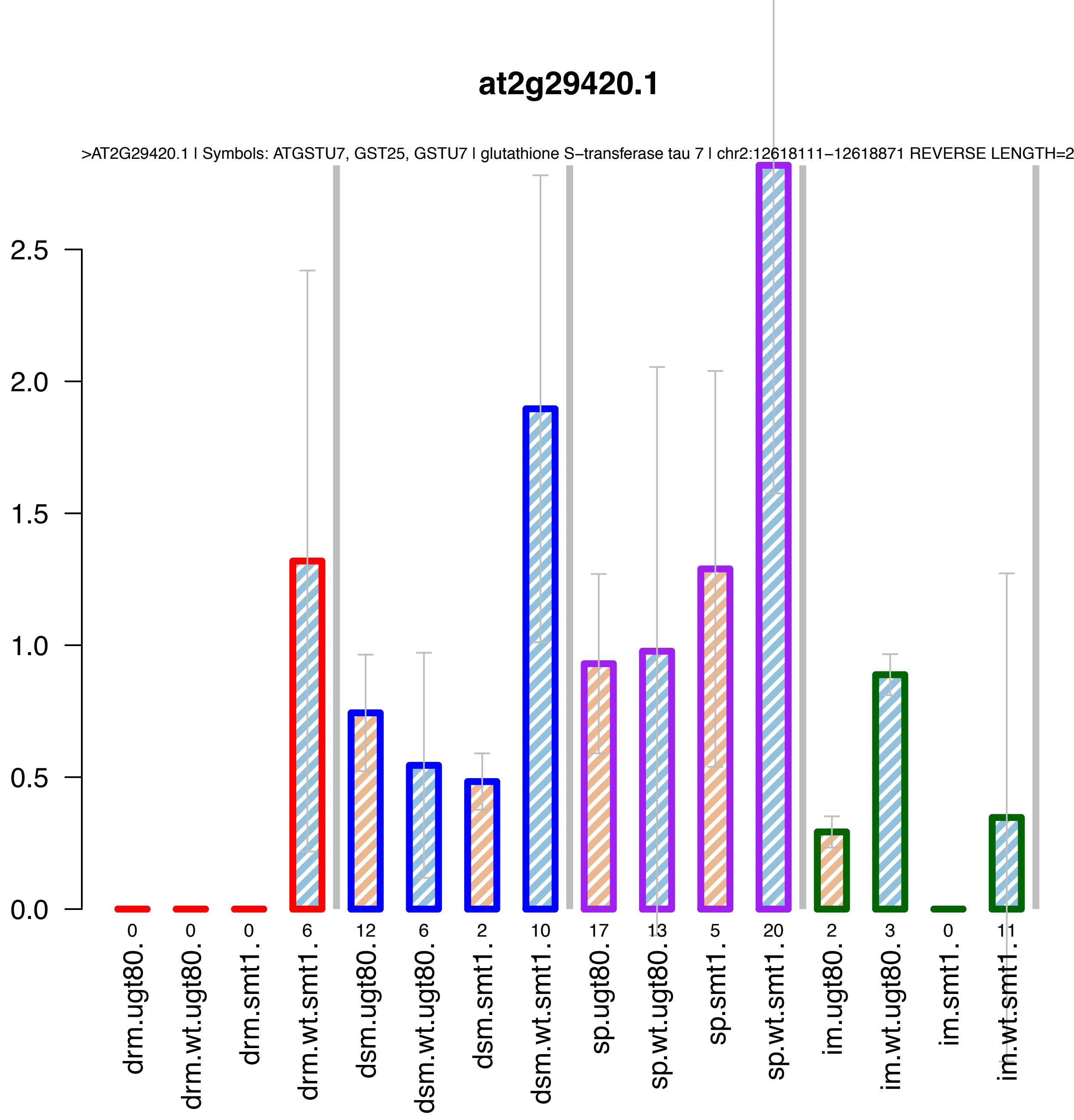

at2g29420.1
>AT2G29420.1 | Symbols: ATGSTU7, GST25, GSTU7 | glutathione S−transferase tau 7 | chr2:12618111−12618871 REVERSE LENGTH=2
0.0
0.5
1.0
1.5
2.0
2.5
0
0
0
6
12
6
2
10
17
13
5
20
2
3
0
11
drm.ugt80.
drm.wt.ugt80.
drm.smt1.
drm.wt.smt1.
dsm.ugt80.
dsm.wt.ugt80.
dsm.smt1.
dsm.wt.smt1.
sp.ugt80.
sp.wt.ugt80.
sp.smt1.
sp.wt.smt1.
im.ugt80.
im.wt.ugt80.
im.smt1.
im.wt.smt1.

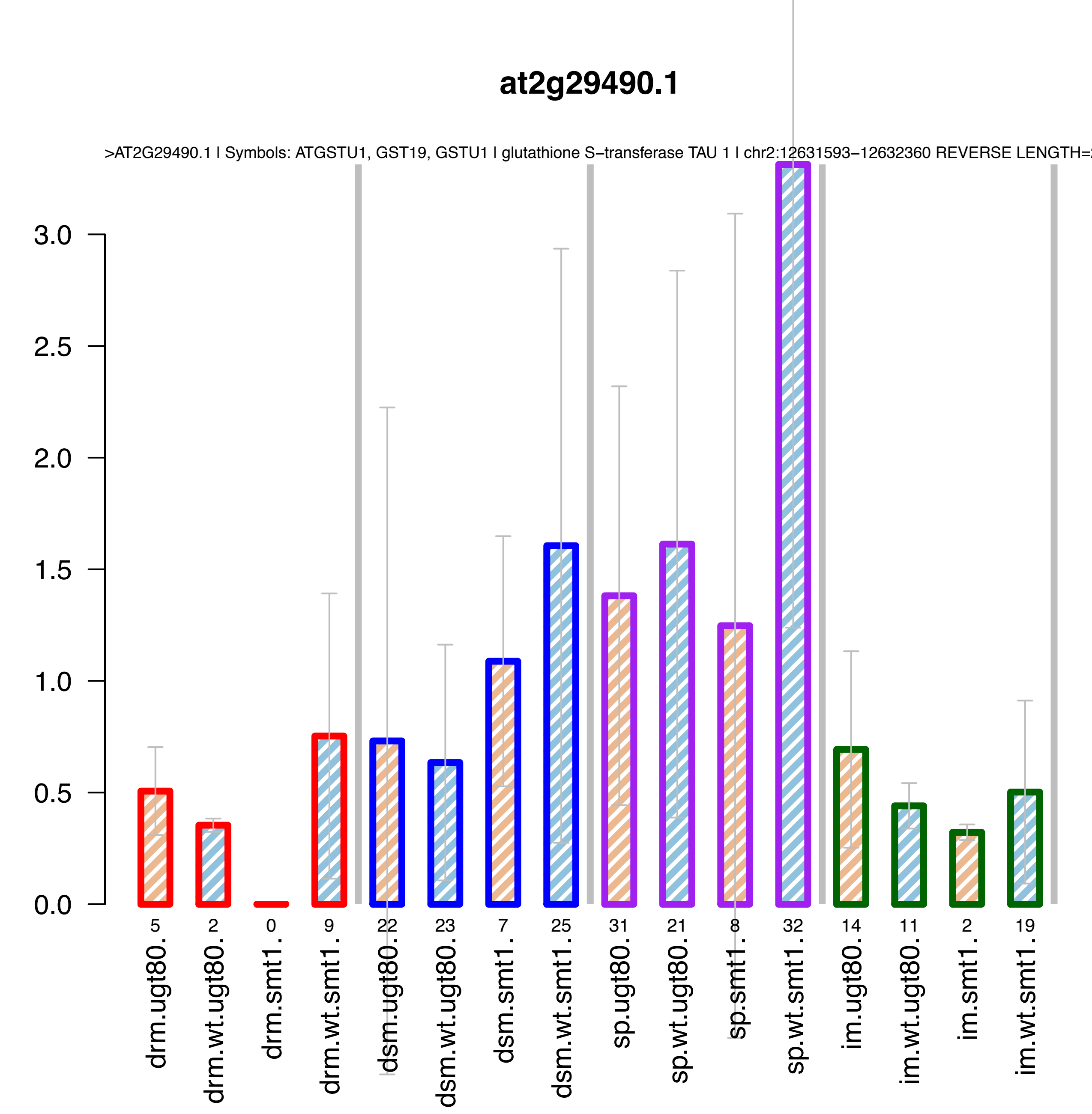

at2g29490.1
>AT2G29490.1 | Symbols: ATGSTU1, GST19, GSTU1 | glutathione S−transferase TAU 1 | chr2:12631593−12632360 REVERSE LENGTH=
0.0
0.5
1.0
1.5
2.0
2.5
3.0
5
2
0
9
22
23
7
25
31
21
8
32
14
11
2
19
drm.ugt80.
drm.wt.ugt80.
drm.smt1.
drm.wt.smt1.
dsm.ugt80.
dsm.wt.ugt80.
dsm.smt1.
dsm.wt.smt1.
sp.ugt80.
sp.wt.ugt80.
sp.smt1.
sp.wt.smt1.
im.ugt80.
im.wt.ugt80.
im.smt1.
im.wt.smt1.

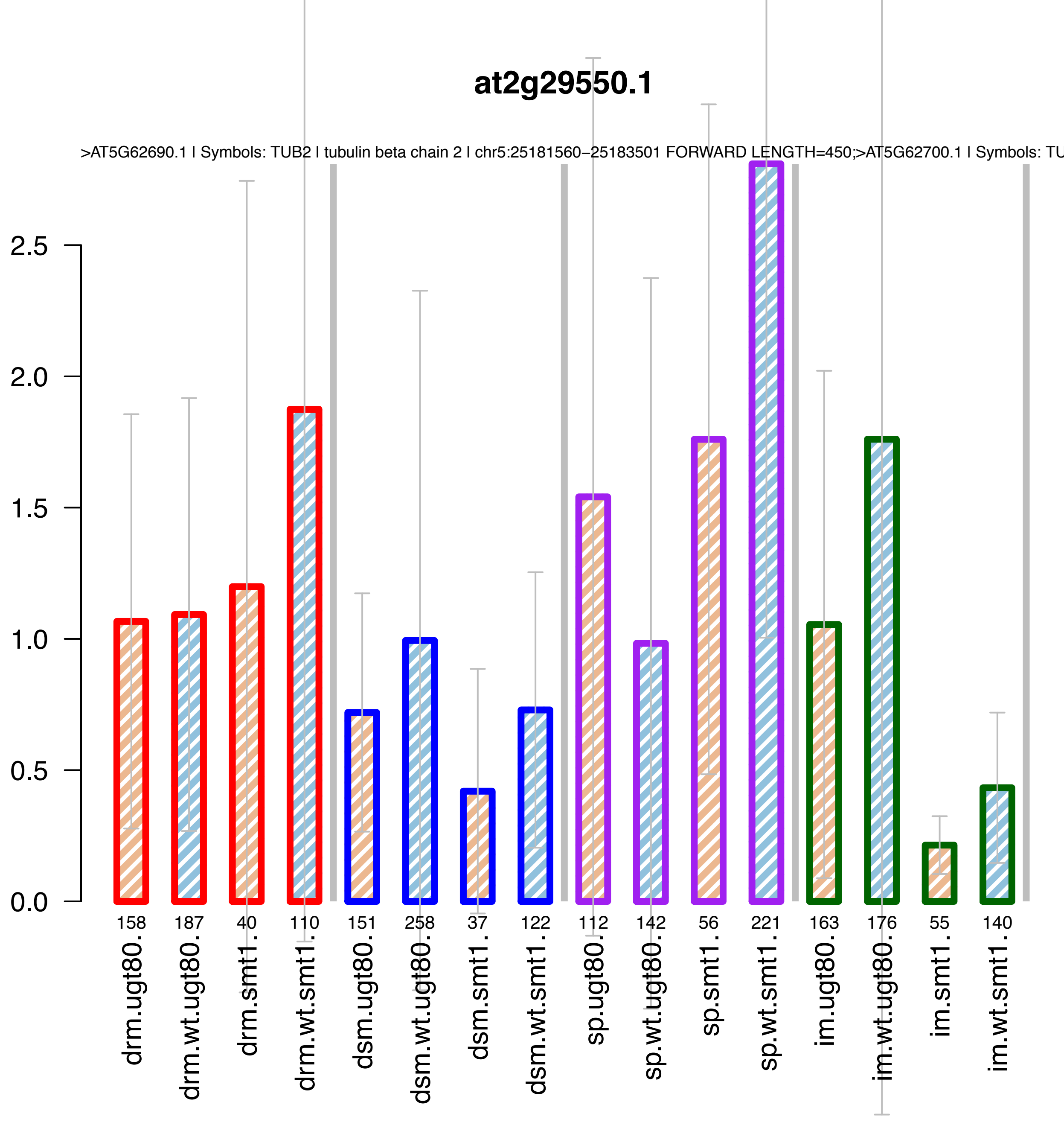

at2g29550.1
>AT5G62690.1 | Symbols: TUB2 | tubulin beta chain 2 | chr5:25181560-25183501 FORWARD LENGTH=450;>AT5G62700.1 | Symbols: TU
2.5
2.0
1.5
1.0
0.5
0.0
158
187
40
110
151
258
37
122
112
142
56
221
163
176
55
140
drm.ugt80..
drm.wt.ugt80..
drm.smt1..
drm.wt.smt1..
dsm.ugt80..
dsm.wt.ugt80..
dsm.smt1..
dsm.wt.smt1..
sp.ugt80..
sp.wt.ugt80..
sp.smt1..
sp.wt.smt1..
im.ugt80..
im.wt.ugt80..
im.smt1..
im.wt.smt1..

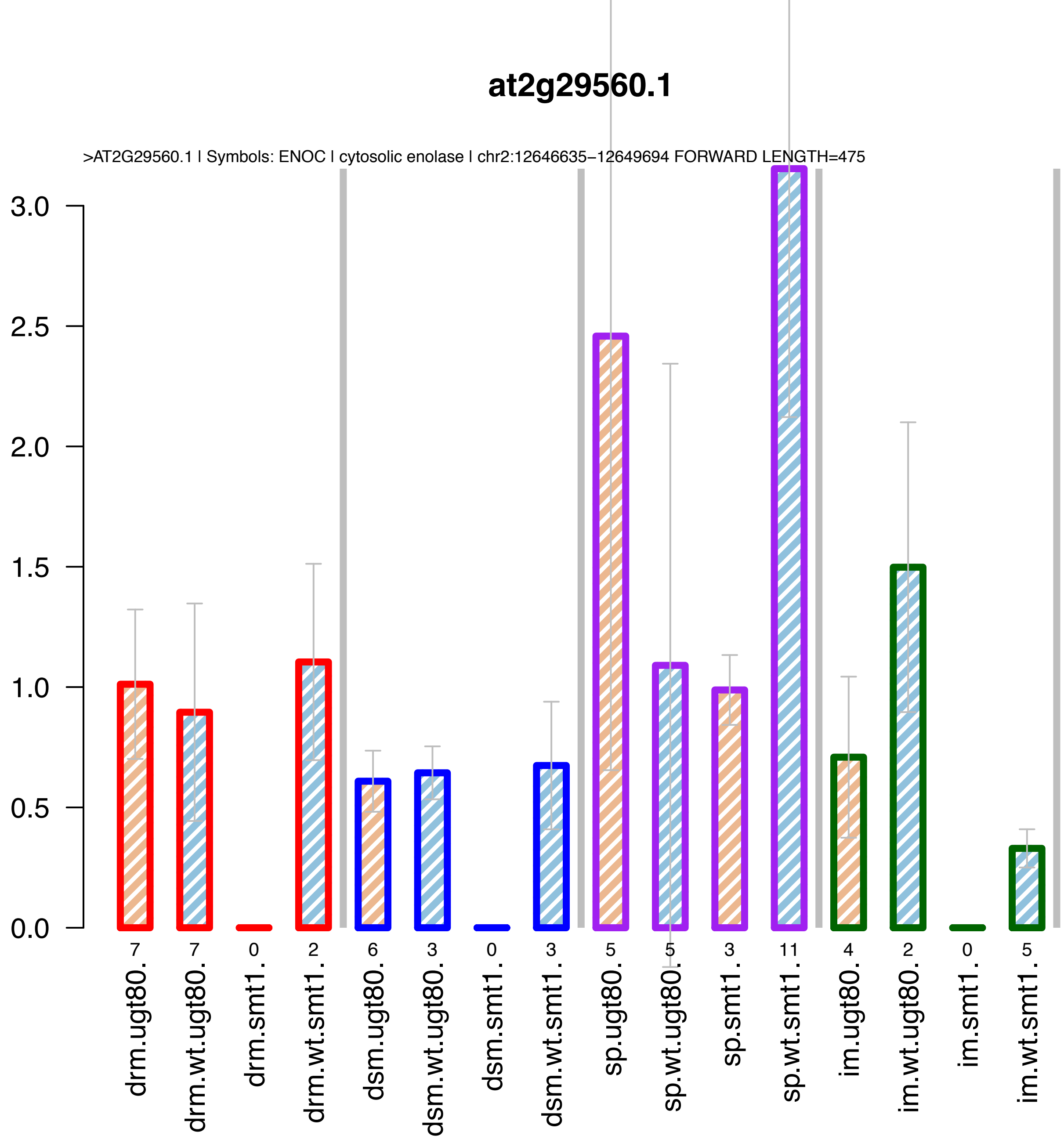
at2g29560.1
>AT2G29560.1 | Symbols: ENOC | cytosolic enolase | chr2:12646635-12649694 FORWARD LENGTH=475
3.0
2.5
2.0
1.5
1.0
0.5
0.0
7
7
0
2
6
3
0
3
5
5
3
11
4
2
0
5
drm.ugt80.
drm.wt.ugt80.
drm.smt1.
drm.wt.smt1.
dsm.ugt80.
dsm.wt.ugt80.
dsm.smt1.
dsm.wt.smt1.
sp.ugt80.
sp.wt.ugt80.
sp.smt1.
sp.wt.smt1.
im.ugt80.
im.wt.ugt80.
im.smt1.
im.wt.smt1.

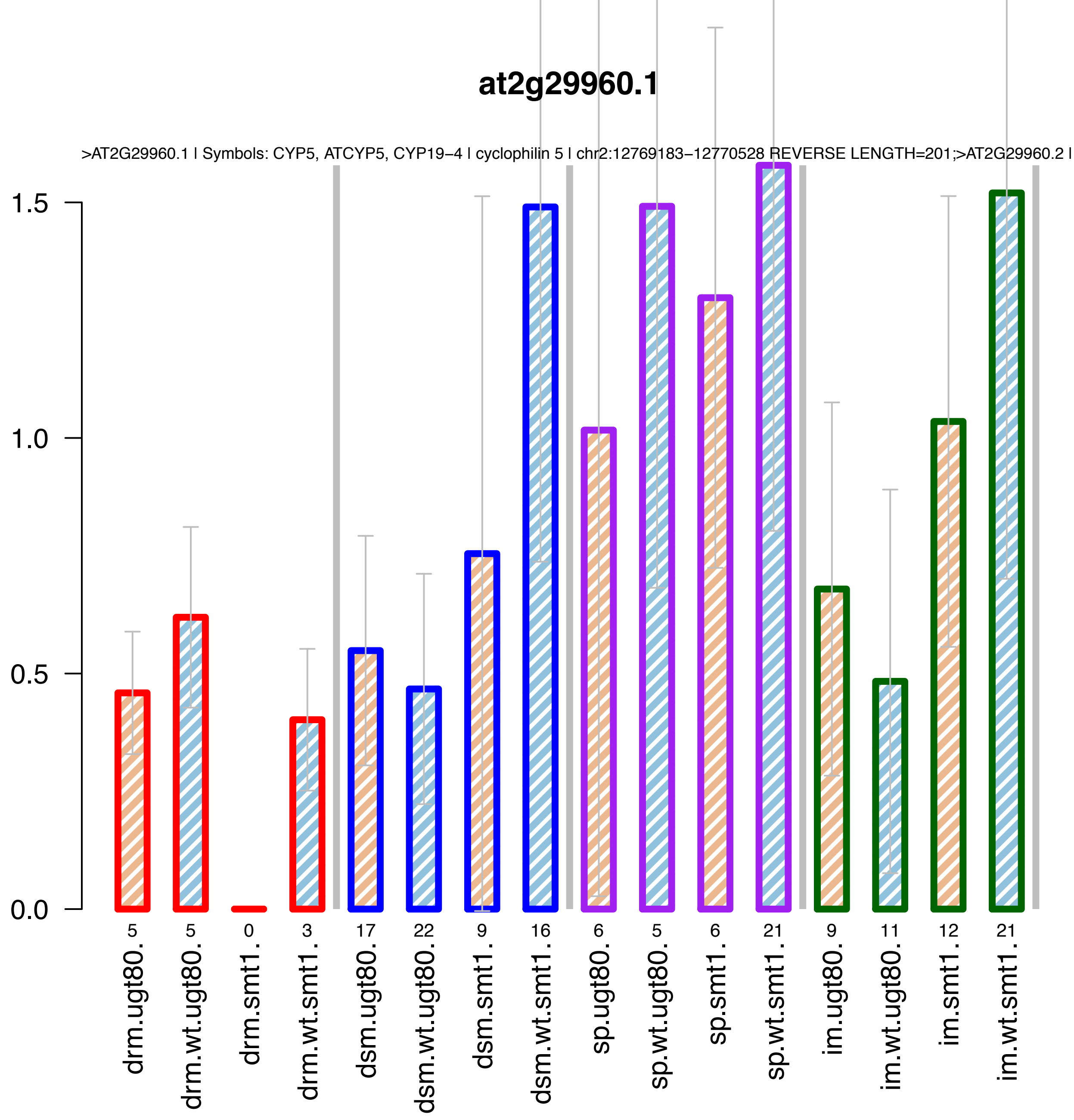

at2g29960.1
>AT2G29960.1 | Symbols: CYP5, ATCYP5, CYP19−4 | cyclophilin 5 | chr2:12769183−12770528 REVERSE LENGTH=201;>AT2G29960.2 |
1.5
1.0
0.5
0.0
5
5
0
3
17
22
9
16
6
5
6
21
9
11
12
21
drm.ugt80.
drm.wt.ugt80.
drm.smt1.
drm.wt.smt1.
dsm.ugt80.
dsm.wt.ugt80.
dsm.smt1.
dsm.wt.smt1.
sp.ugt80.
sp.wt.ugt80.
sp.smt1.
sp.wt.smt1.
im.ugt80.
im.wt.ugt80.
im.smt1.
im.wt.smt1.

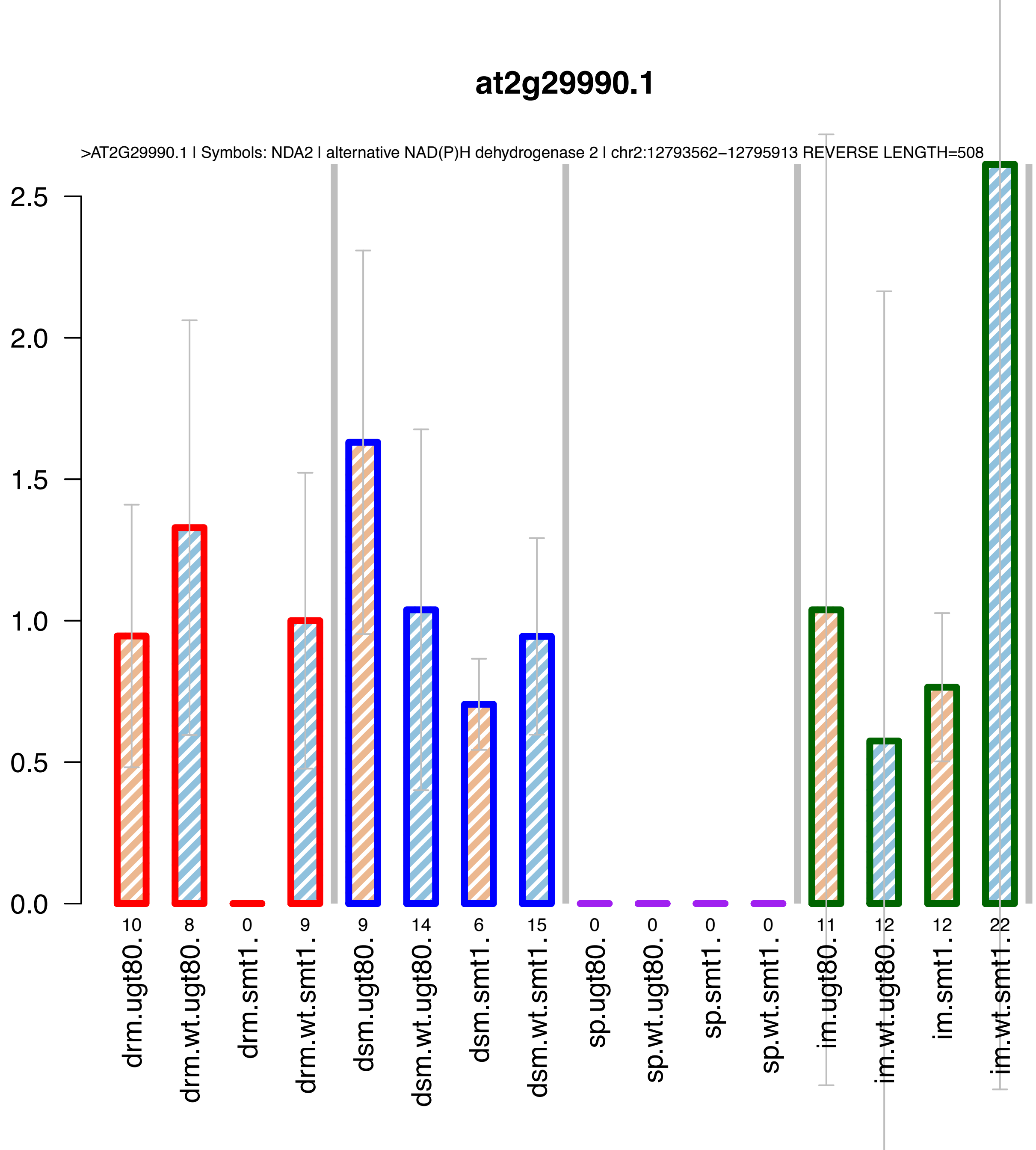
at2g29990.1
>AT2G29990.1 | Symbols: NDA2 | alternative NAD(P)H dehydrogenase 2 | chr2:12793562–12795913 REVERSE LENGTH=508
0.0
0.5
1.0
1.5
2.0
2.5
10
8
0
9
9
14
6
15
0
0
0
0
11
12
12
22
drm.ugt80..
drm.wt.ugt80..
drm.smt1..
drm.wt.smt1..
dsm.ugt80..
dsm.wt.ugt80..
dsm.smt1..
dsm.wt.smt1..
sp.ugt80..
sp.wt.ugt80..
sp.smt1..
sp.wt.smt1..
im.ugt80..
im.wt.ugt80..
im.smt1..
im.wt.smt1..

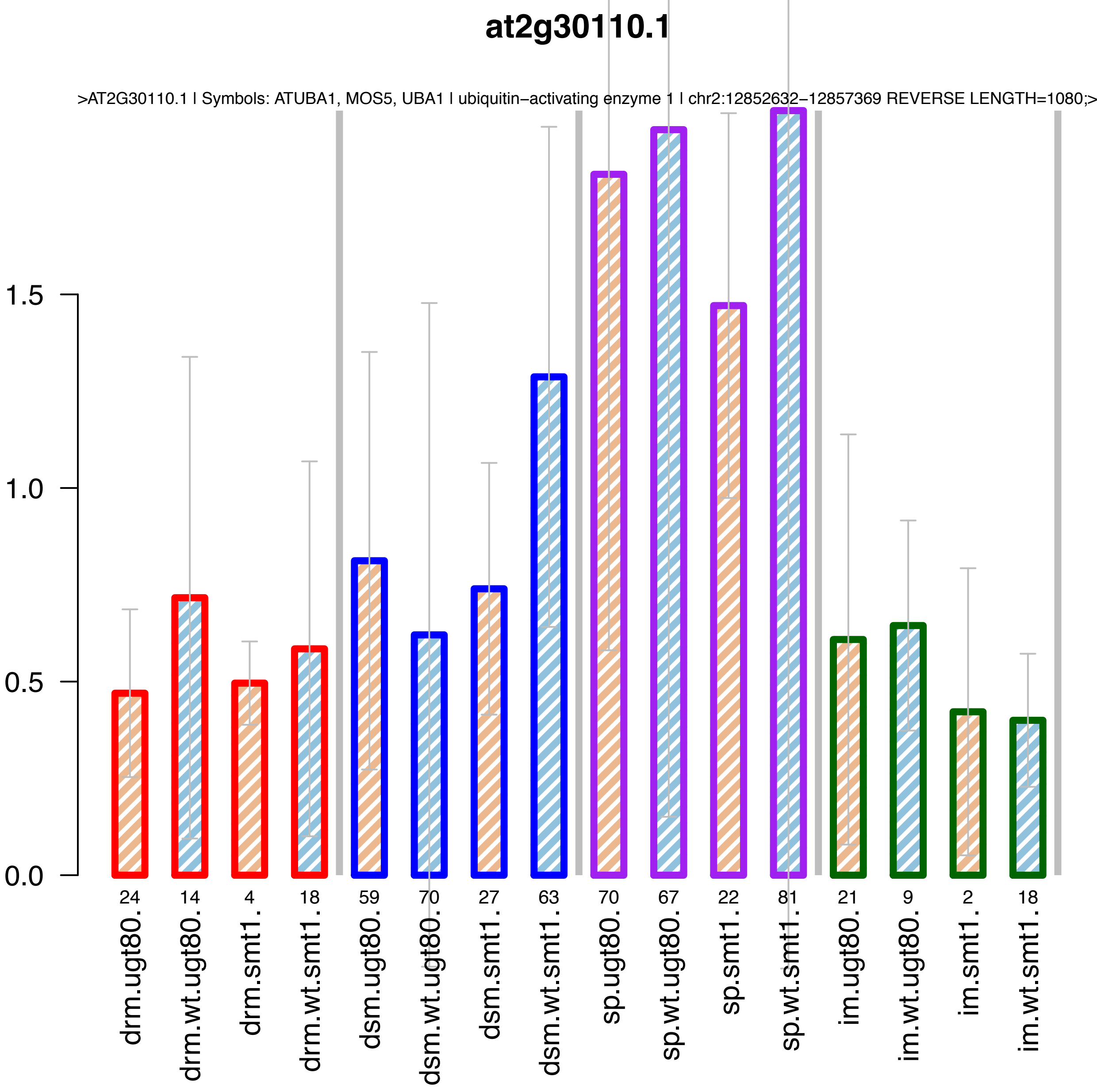

at2g30110.1
>AT2G30110.1 | Symbols: ATUBA1, MOS5, UBA1 | ubiquitin−activating enzyme 1 | chr2:12852632−12857369 REVERSE LENGTH=1080;>
0.0
0.5
1.0
1.5
24
14
4
18
59
70
27
63
70
67
22
81
21
9
2
18
drm.ugt80..
drm.wt.ugt80..
drm.smt1..
drm.wt.smt1..
dsm.ugt80..
dsm.wt.ugt80..
dsm.smt1..
dsm.wt.smt1..
sp.ugt80..
sp.wt.ugt80..
sp.smt1..
sp.wt.smt1..
im.ugt80..
im.wt.ugt80..
im.smt1..
im.wt.smt1..

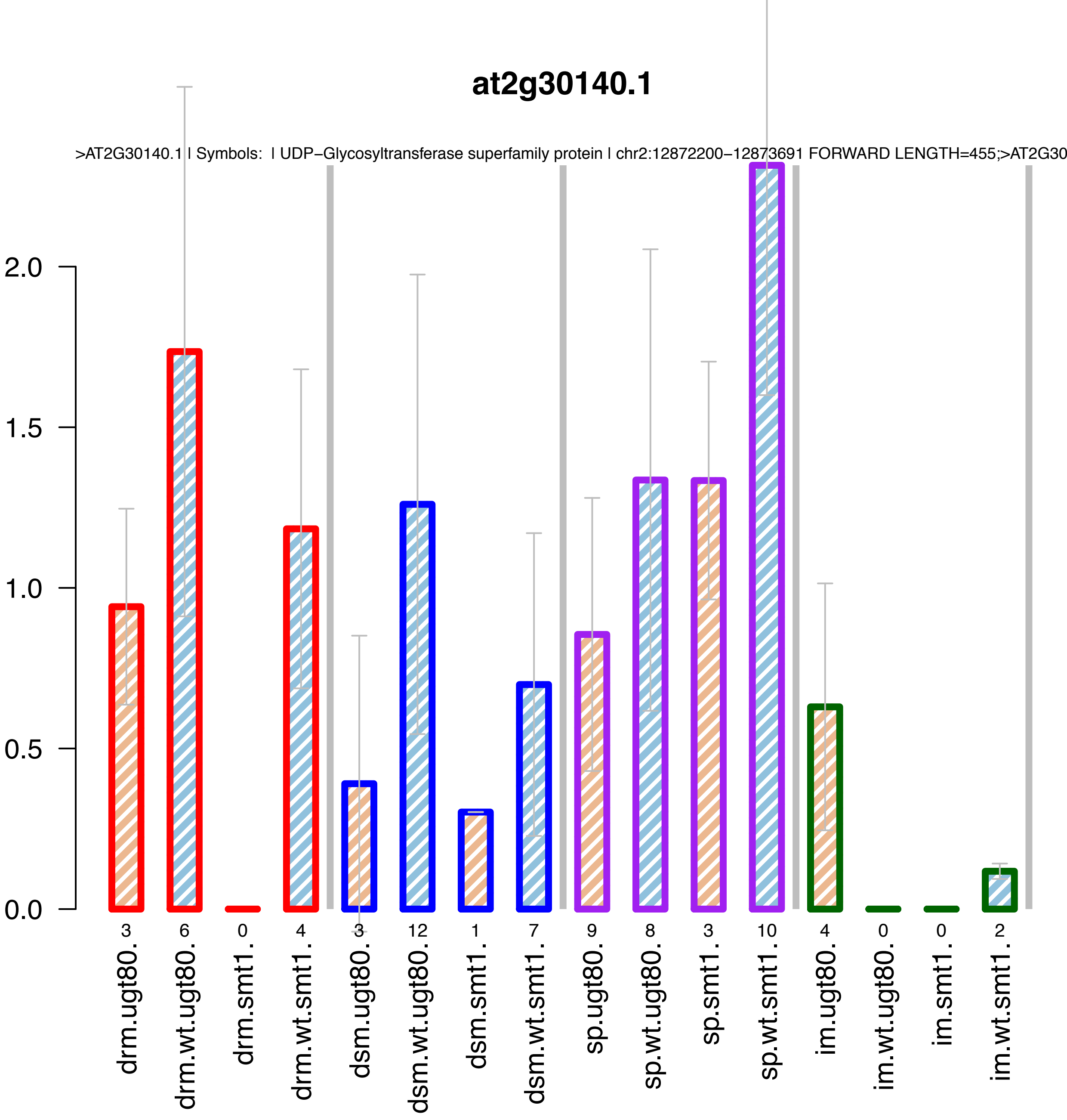

at2g30140.1
>AT2G30140.1 | Symbols: | UDP−Glycosyltransferase superfamily protein | chr2:12872200−12873691 FORWARD LENGTH=455;>AT2G30
2.0
1.5
1.0
0.5
0.0
3
6
0
4
3
12
1
7
9
8
3
10
4
0
0
2
drm.ugt80.
drm.wt.ugt80.
drm.smt1.
drm.wt.smt1.
dsm.ugt80.
dsm.wt.ugt80.
dsm.smt1.
dsm.wt.smt1.
sp.ugt80.
sp.wt.ugt80.
sp.smt1.
sp.wt.smt1.
im.ugt80.
im.wt.ugt80.
im.smt1.
im.wt.smt1.

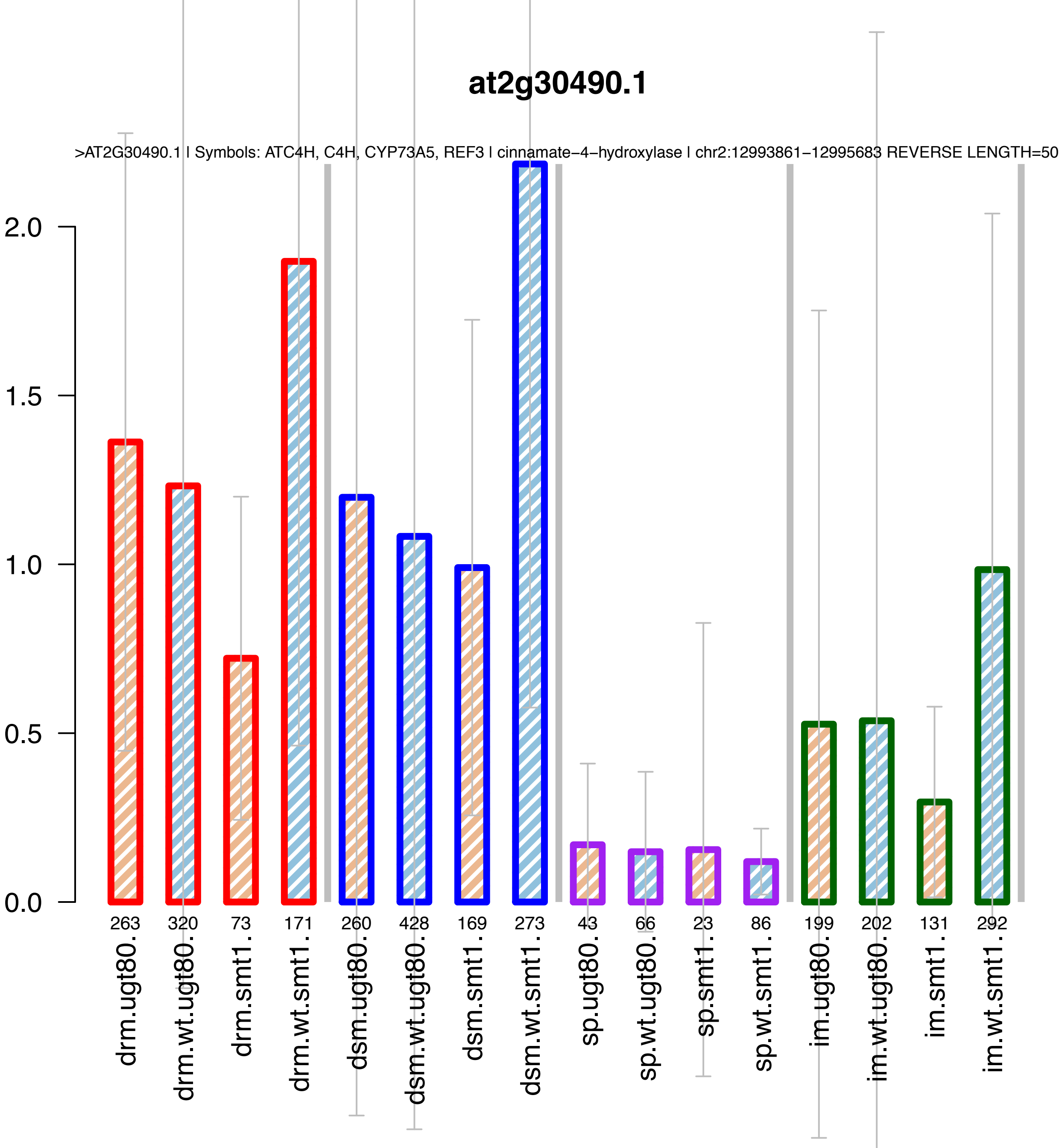
at2g30490.1
>AT2G30490.1 | Symbols: ATC4H, C4H, CYP73A5, REF3 | cinnamate−4−hydroxylase | chr2:12993861−12995683 REVERSE LENGTH=50
2.0
1.5
1.0
0.5
0.0
263
320
73
171
260
428
169
273
43
66
23
86
199
202
131
292
drm.ugt80..
drm.wt.ugt80..
drm.smt1..
drm.wt.smt1..
dsm.ugt80..
dsm.wt.ugt80..
dsm.smt1..
dsm.wt.smt1..
sp.ugt80..
sp.wt.ugt80..
sp.smt1..
sp.wt.smt1..
im.ugt80..
im.wt.ugt80..
im.smt1..
im.wt.smt1..

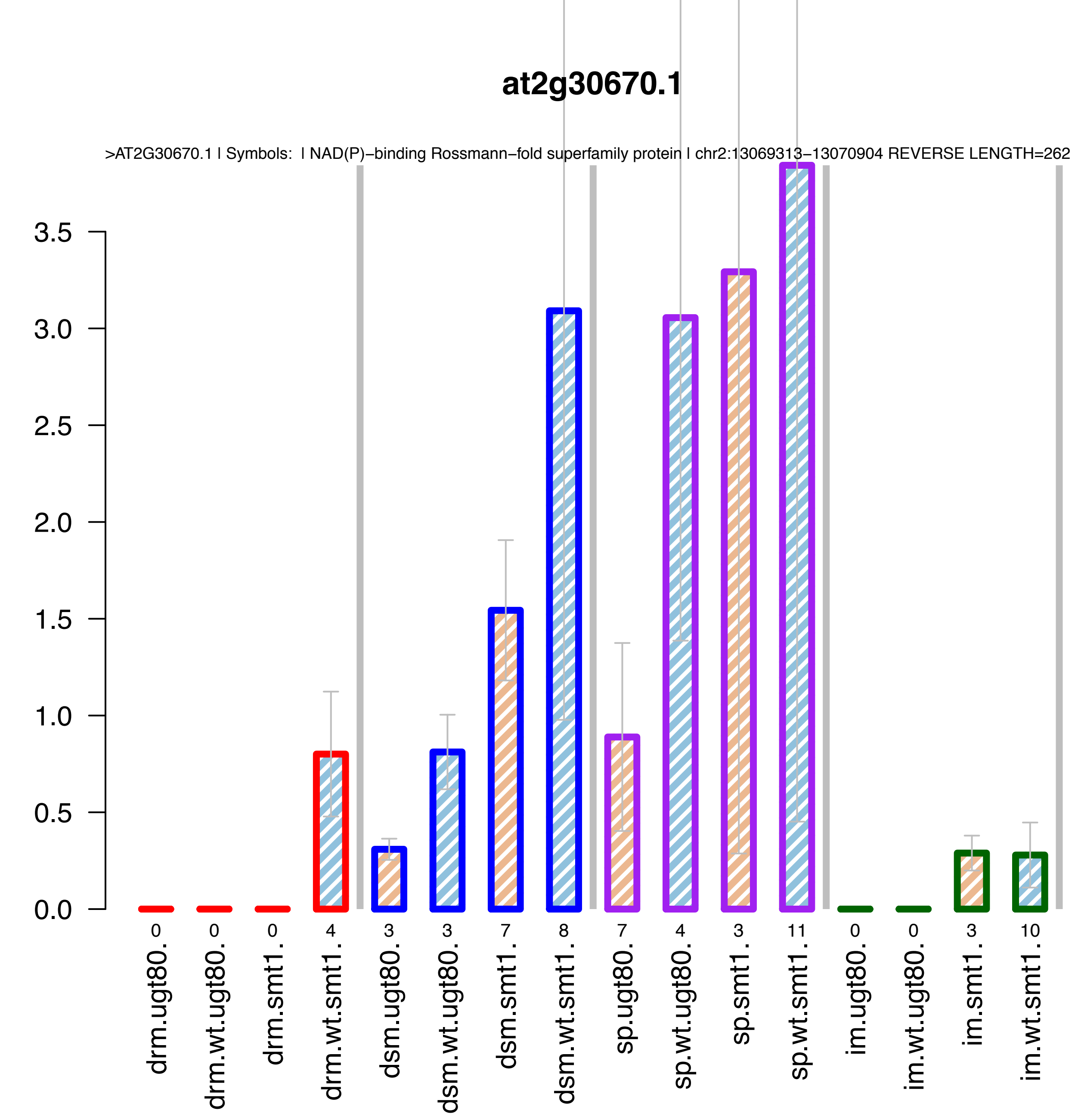
at2g30670.1
>AT2G30670.1 | Symbols: | NAD(P)−binding Rossmann−fold superfamily protein | chr2:13069313−13070904 REVERSE LENGTH=262
0.0
0.5
1.0
1.5
2.0
2.5
3.0
3.5
0 0 0 4 3 3 7 8 7 4 3 11 0 0 3 10
drm.ugt80.
drm.wt.ugt80.
drm.smt1.
drm.wt.smt1.
dsm.ugt80.
dsm.wt.ugt80.
dsm.smt1.
dsm.wt.smt1.
sp.ugt80.
sp.wt.ugt80.
sp.smt1.
sp.wt.smt1.
im.ugt80.
im.wt.ugt80.
im.smt1.
im.wt.smt1.

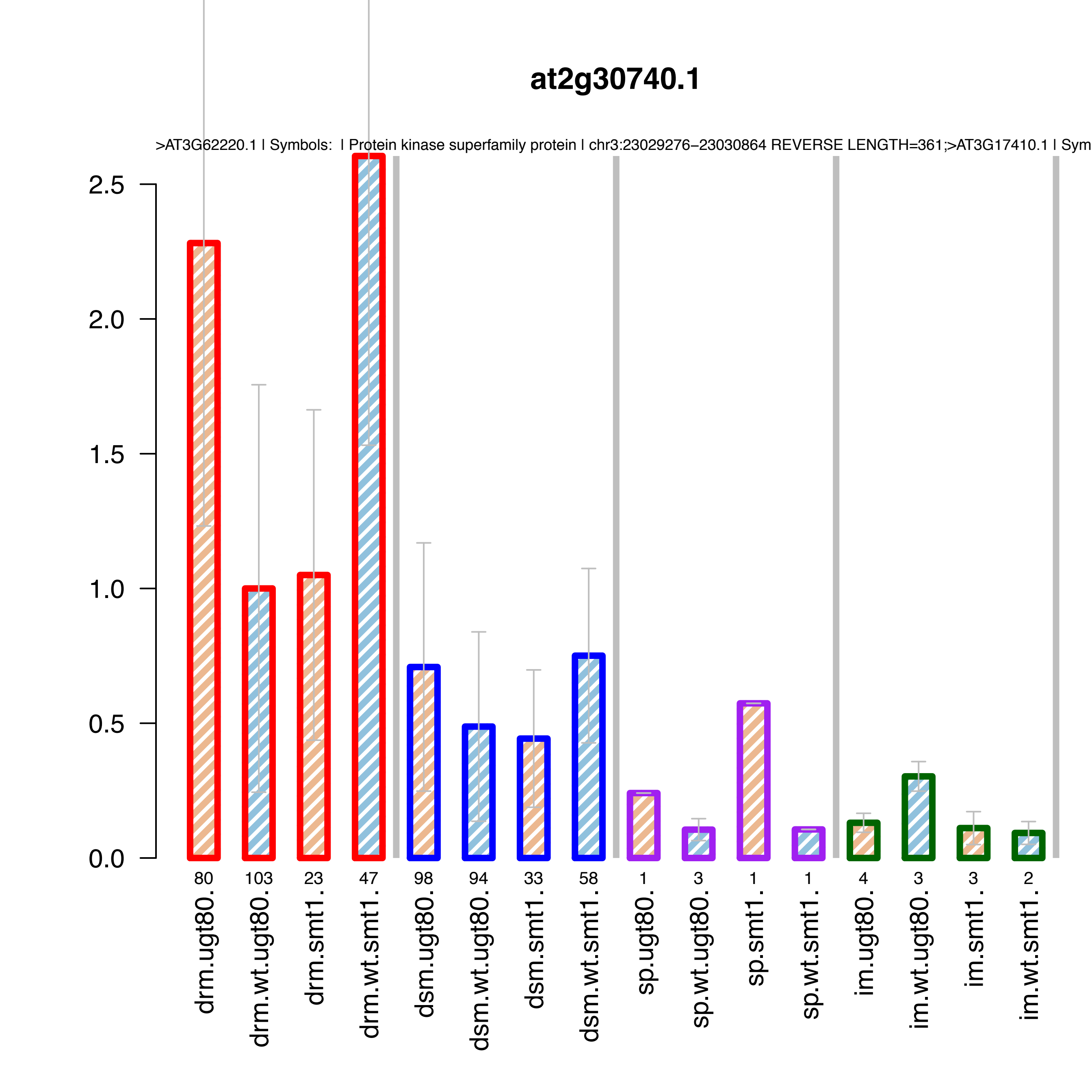

at2g30740.1
>AT3G62220.1 | Symbols: | Protein kinase superfamily protein | chr3:23029276-23030864 REVERSE LENGTH=361;>AT3G17410.1 | Sym
2.5
2.0
1.5
1.0
0.5
0.0
80
103
23
47
98
94
33
58
1
3
1
1
4
3
3
2
drm.ugt80..
drm.wt.ugt80..
drm.smt1..
drm.wt.smt1..
dsm.ugt80..
dsm.wt.ugt80..
dsm.smt1..
dsm.wt.smt1..
sp.ugt80..
sp.wt.ugt80..
sp.smt1..
sp.wt.smt1..
im.ugt80..
im.wt.ugt80..
im.smt1..
im.wt.smt1..

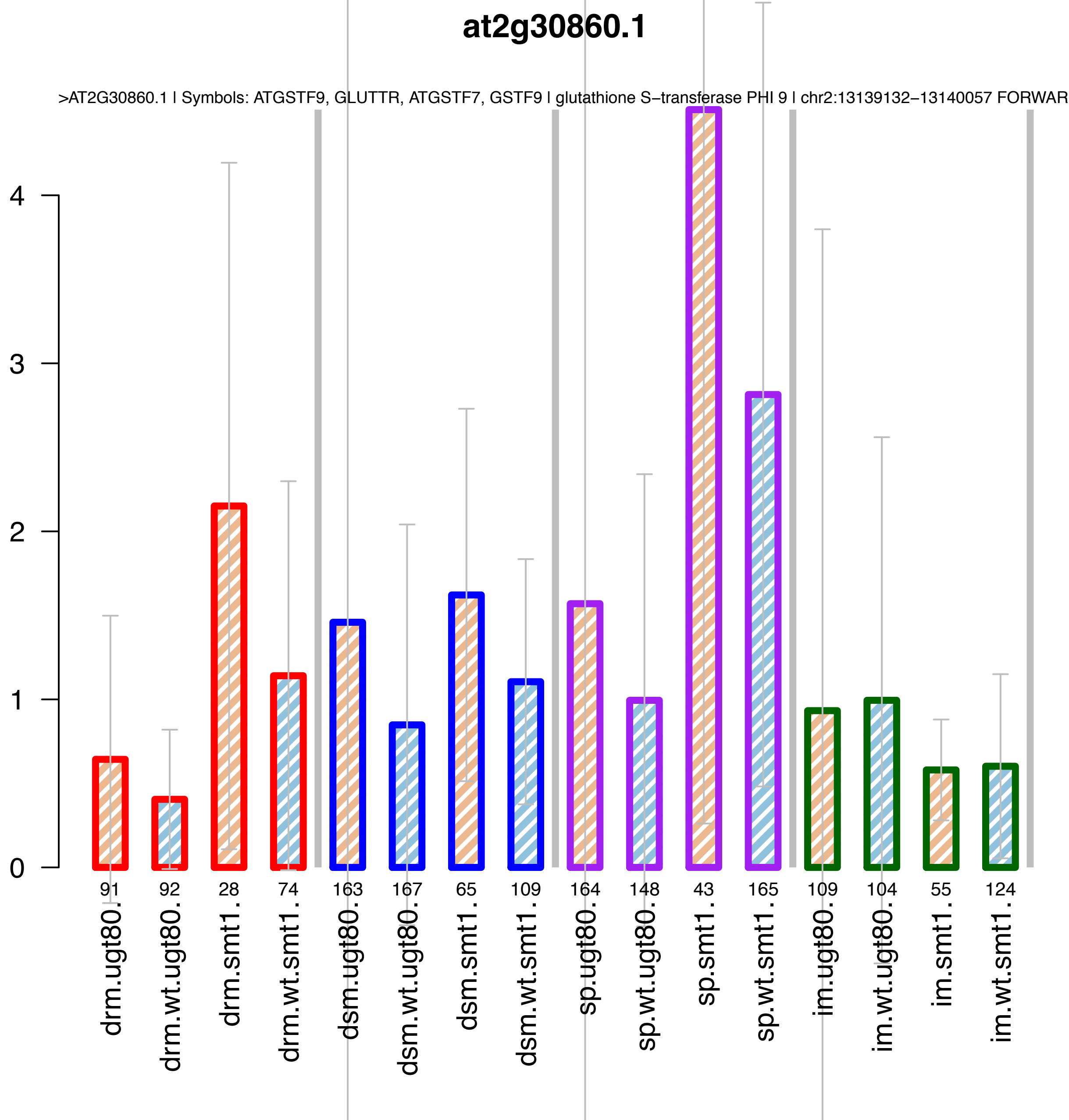

at2g30860.1
>AT2G30860.1 | Symbols: ATGSTF9, GLUTTR, ATGSTF7, GSTF9 | glutathione S−transferase PHI 9 | chr2:13139132−13140057 FORWAR
0
1
2
3
4
91
92
28
74
163
167
65
109
164
148
43
165
109
104
55
124
drm.ugt80.
drm.wt.ugt80.
drm.smt1.
drm.wt.smt1.
dsm.ugt80.
dsm.wt.ugt80.
dsm.smt1.
dsm.wt.smt1.
sp.ugt80.
sp.wt.ugt80.
sp.smt1.
sp.wt.smt1.
im.ugt80.
im.wt.ugt80.
im.smt1.
im.wt.smt1.

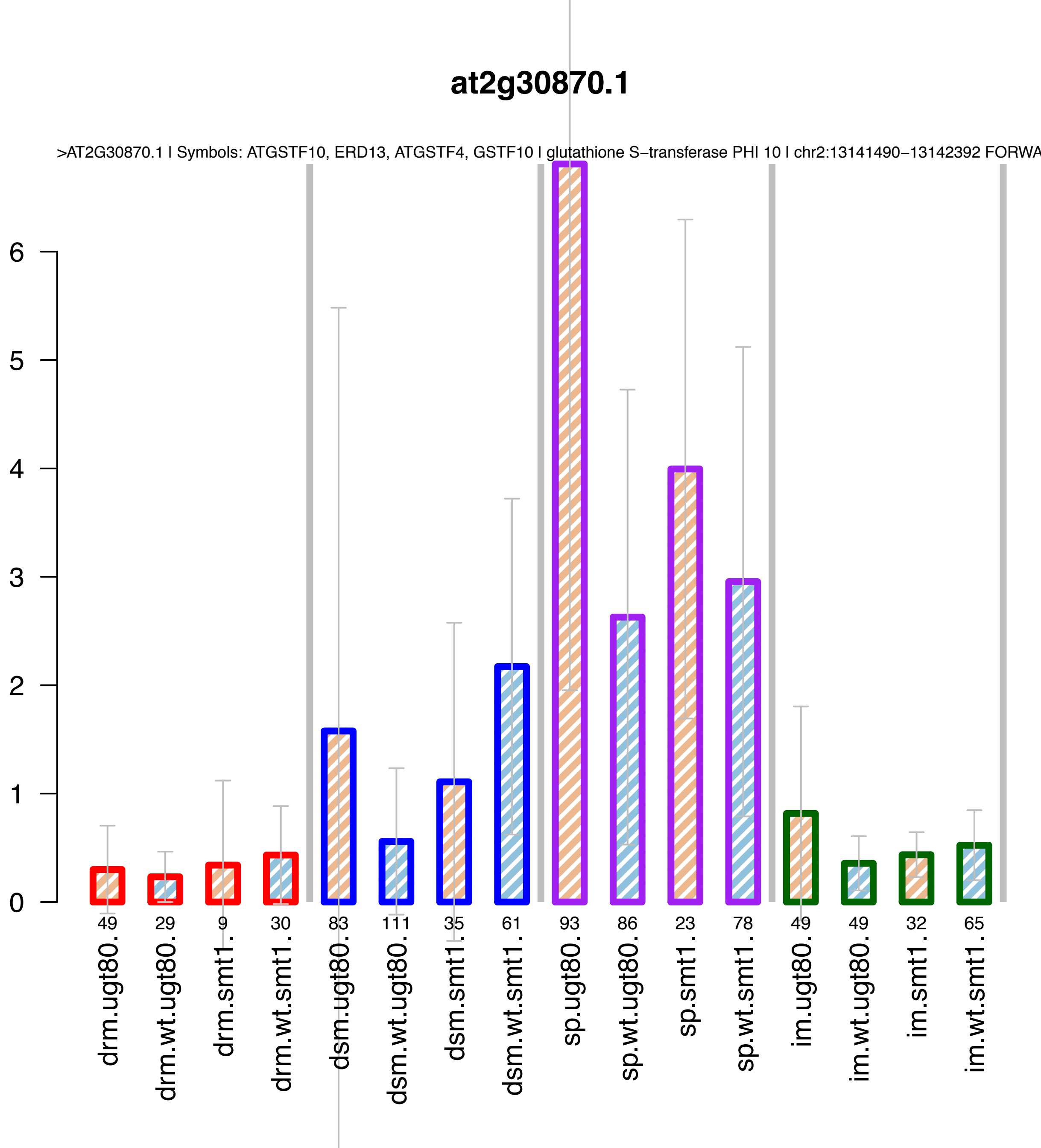

at2g30870.1
>AT2G30870.1 | Symbols: ATGSTF10, ERD13, ATGSTF4, GSTF10 | glutathione S-transferase PHI 10 | chr2:13141490-13142392 FORWA
0
1
2
3
4
5
6
49
29
9
30
83
111
35
61
93
86
23
78
49
49
32
65
drm.ugt80.
drm.wt.ugt80.
drm.smt1.
drm.wt.smt1.
dsm.ugt80.
dsm.wt.ugt80.
dsm.smt1.
dsm.wt.smt1.
sp.ugt80.
sp.wt.ugt80.
sp.smt1.
sp.wt.smt1.
im.ugt80.
im.wt.ugt80.
im.smt1.
im.wt.smt1.

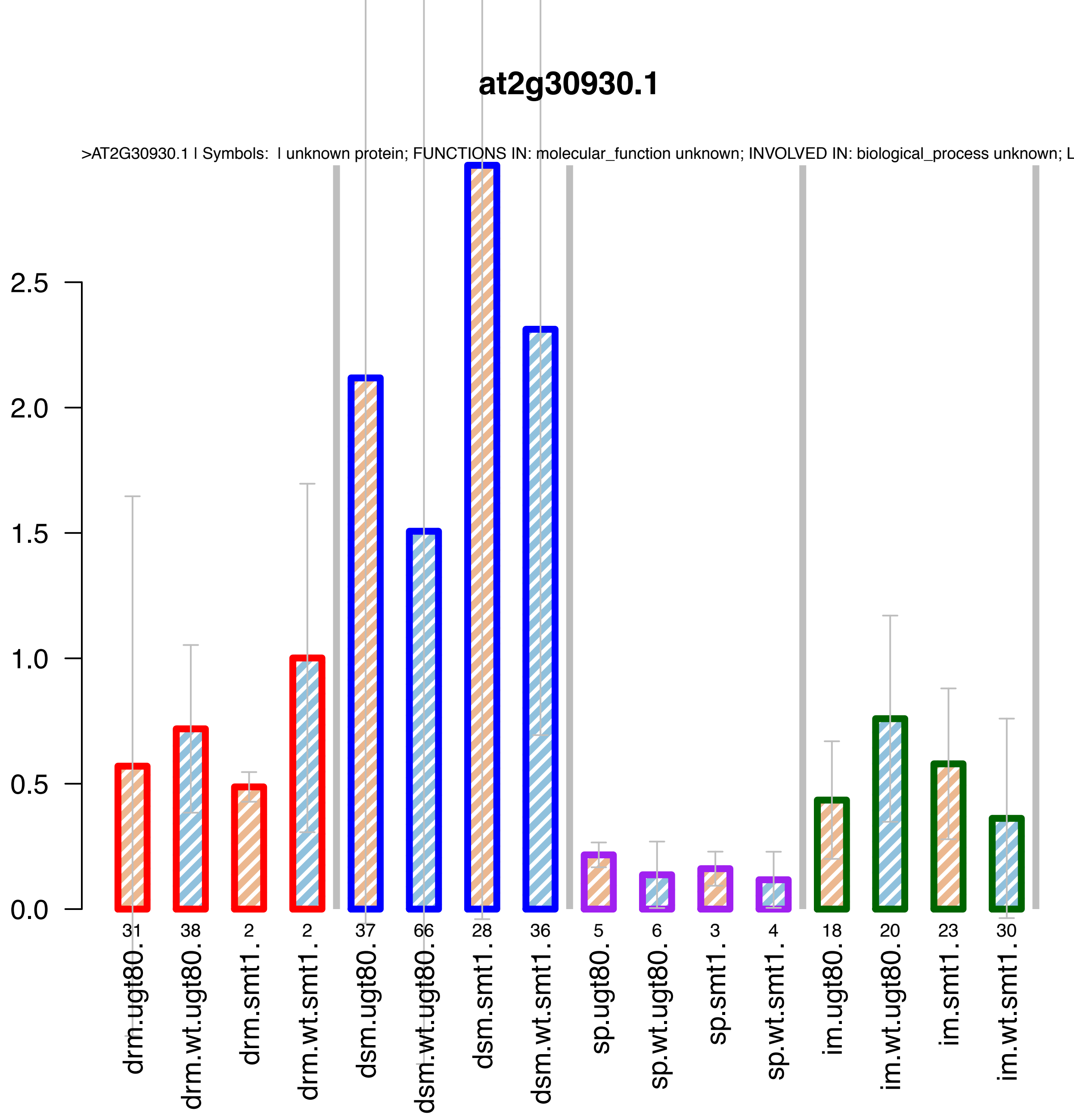
at2g30930.1
>AT2G30930.1 | Symbols: | unknown protein; FUNCTIONS IN: molecular_function unknown; INVOLVED IN: biological_process unknown; L
2.5
2.0
1.5
1.0
0.5
0.0
31
38
2
2
37
66
28
36
5
6
3
4
18
20
23
30
drm.ugt80.
drm.wt.ugt80.
drm.smt1.
drm.wt.smt1.
dsm.ugt80.
dsm.wt.ugt80.
dsm.smt1.
dsm.wt.smt1.
sp.ugt80.
sp.wt.ugt80.
sp.smt1.
sp.wt.smt1.
im.ugt80.
im.wt.ugt80.
im.smt1.
im.wt.smt1.

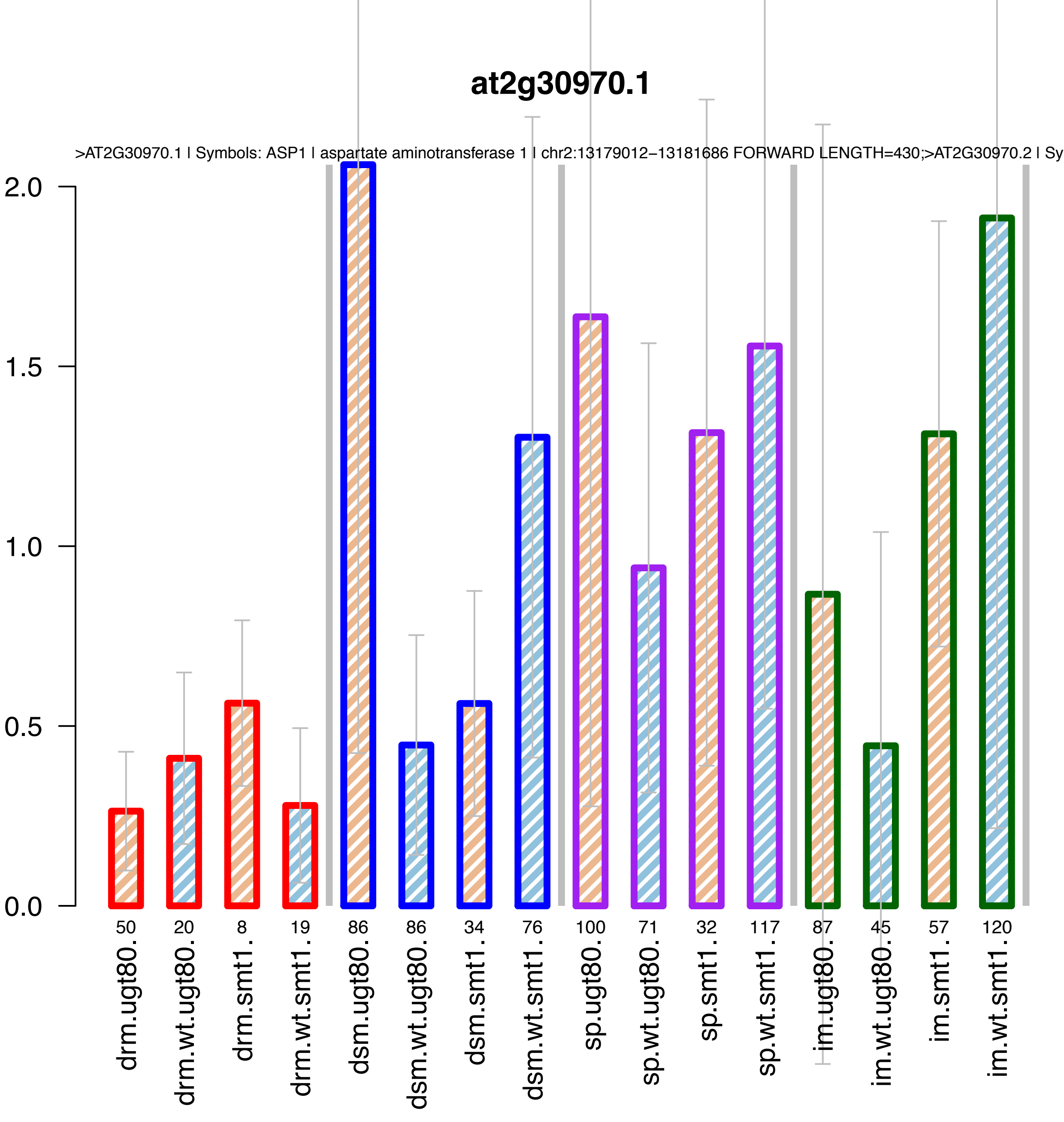

at2g30970.1
>AT2G30970.1 | Symbols: ASP1 | aspartate aminotransferase 1 | chr2:13179012−13181686 FORWARD LENGTH=430;>AT2G30970.2 | Sy
2.0
1.5
1.0
0.5
0.0
50
20
8
19
86
86
34
76
100
71
32
117
87
45
57
120
drm.ugt80..
drm.wt.ugt80..
drm.smt1..
drm.wt.smt1..
dsm.ugt80..
dsm.wt.ugt80..
dsm.smt1..
dsm.wt.smt1..
sp.ugt80..
sp.wt.ugt80..
sp.smt1..
sp.wt.smt1..
im.ugt80..
im.wt.ugt80..
im.smt1..
im.wt.smt1..

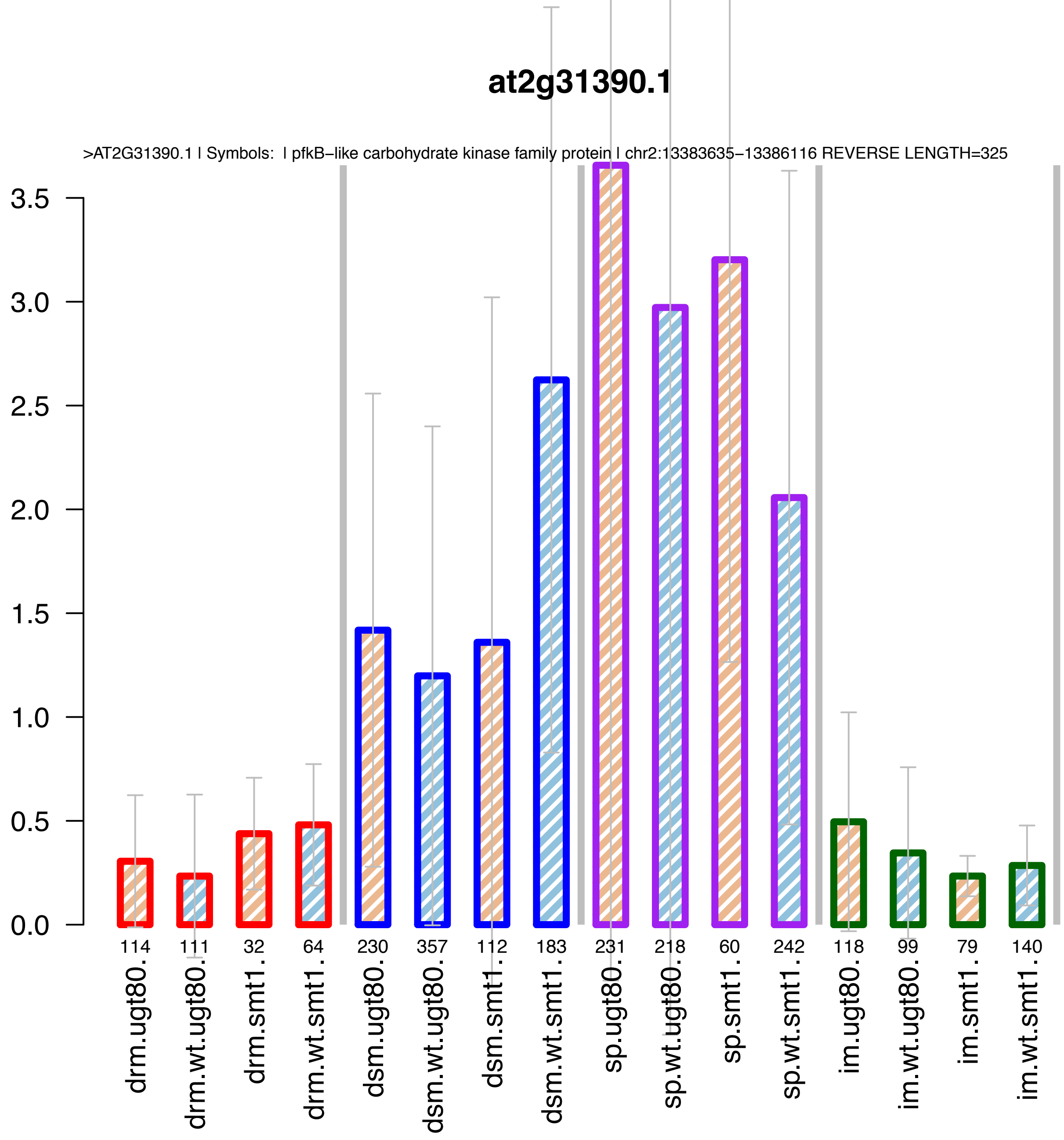

at2g31390.1
>AT2G31390.1 | Symbols: | pfkB-like carbohydrate kinase family protein | chr2:13383635-13386116 REVERSE LENGTH=325
3.5
3.0
2.5
2.0
1.5
1.0
0.5
0.0
114
111
32
64
230
357
112
183
231
218
60
242
118
99
79
140
drm.ugt80..
drm.wt.ugt80..
drm.smt1..
drm.wt.smt1..
dsm.ugt80..
dsm.wt.ugt80..
dsm.smt1..
dsm.wt.smt1..
sp.ugt80..
sp.wt.ugt80..
sp.smt1..
sp.wt.smt1..
im.ugt80..
im.wt.ugt80..
im.smt1..
im.wt.smt1..

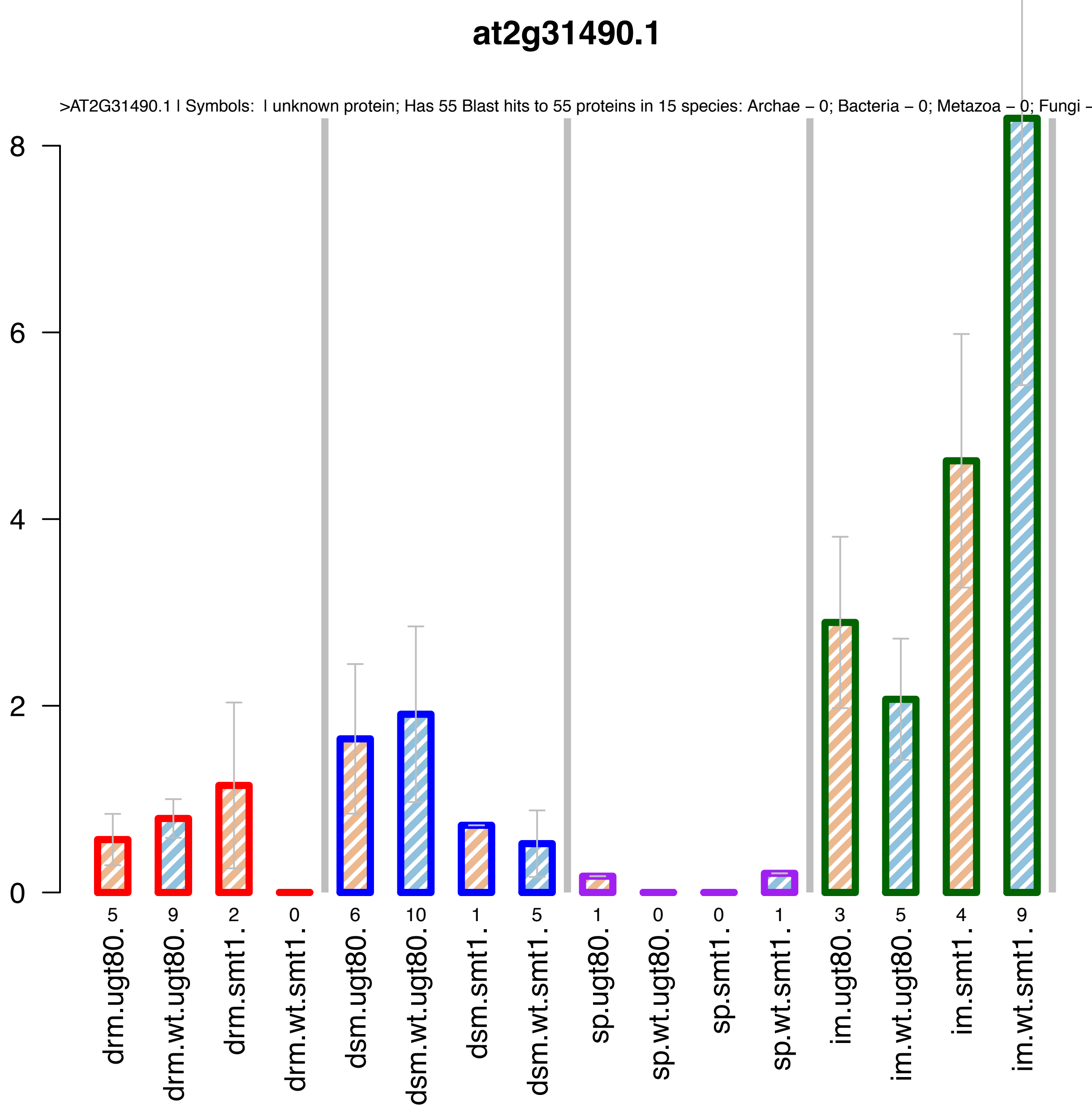

at2g31490.1
>AT2G31490.1 | Symbols: | unknown protein; Has 55 Blast hits to 55 proteins in 15 species: Archae – 0; Bacteria – 0; Metazoa – 0; Fungi –
8
6
4
2
0
5
9
2
0
6
10
1
5
1
0
0
1
3
5
4
9
drm.ugt80.
drm.wt.ugt80.
drm.smt1.
drm.wt.smt1.
dsm.ugt80.
dsm.wt.ugt80.
dsm.smt1.
dsm.wt.smt1.
sp.ugt80.
sp.wt.ugt80.
sp.smt1.
sp.wt.smt1.
im.ugt80.
im.wt.ugt80.
im.smt1.
im.wt.smt1.

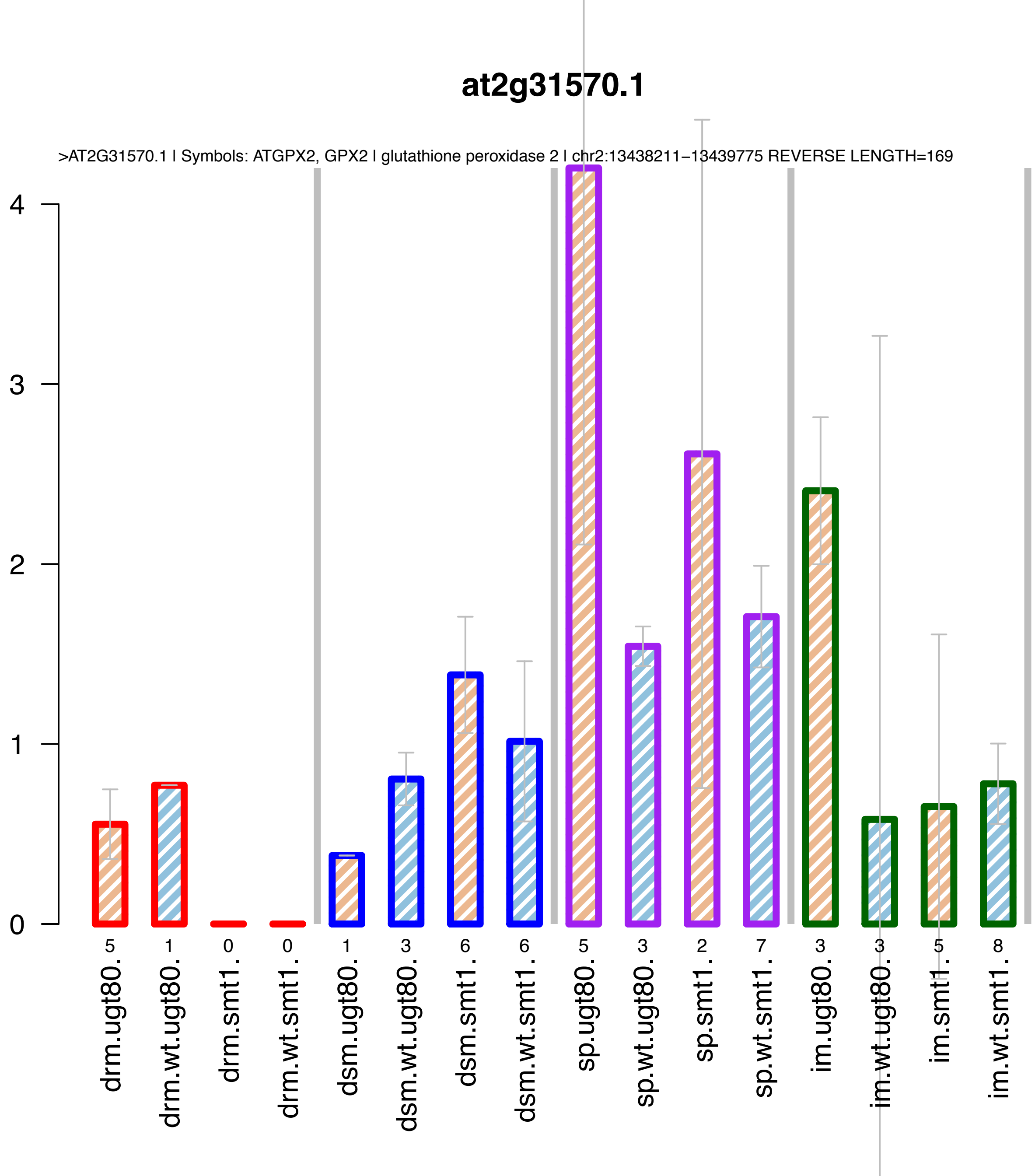
at2g31570.1
>AT2G31570.1 | Symbols: ATGPX2, GPX2 | glutathione peroxidase 2 | chr2:13438211-13439775 REVERSE LENGTH=169
0
1
2
3
4
5
1
0
0
1
3
6
6
5
3
2
7
3
3
5
8
drm.ugt80.
drm.wt.ugt80.
drm.smt1.
drm.wt.smt1.
dsm.ugt80.
dsm.wt.ugt80.
dsm.smt1.
dsm.wt.smt1.
sp.ugt80.
sp.wt.ugt80.
sp.smt1.
sp.wt.smt1.
im.ugt80.
im.wt.ugt80.
im.smt1.
im.wt.smt1.

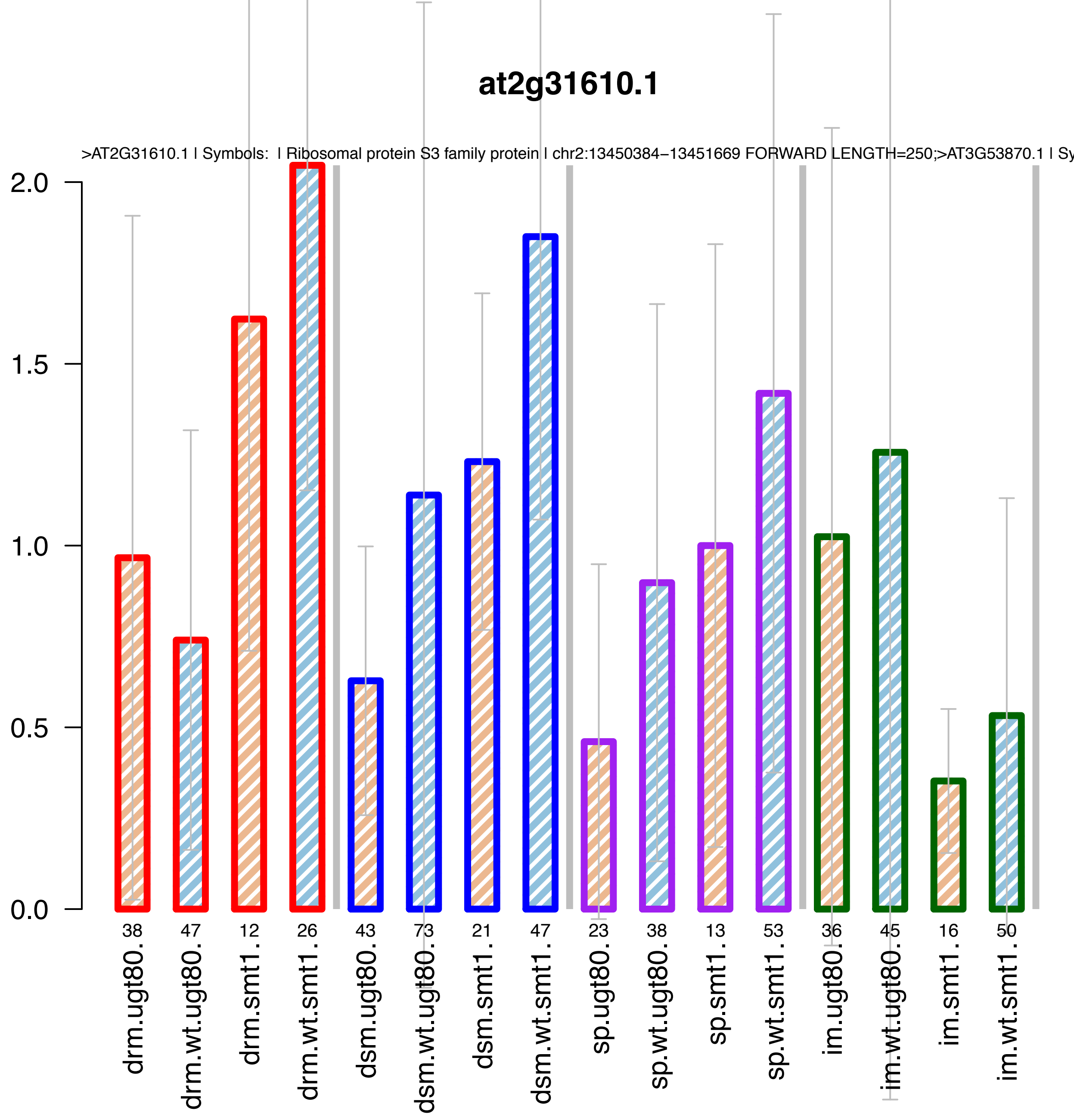

at2g31610.1
>AT2G31610.1 | Symbols: | Ribosomal protein S3 family protein | chr2:13450384-13451669 FORWARD LENGTH=250;>AT3G53870.1 | Sy
2.0
1.5
1.0
0.5
0.0
38
47
12
26
43
73
21
47
23
38
13
53
36
45
16
50
drm.ugt80..
drm.wt.ugt80..
drm.smt1..
drm.wt.smt1..
dsm.ugt80..
dsm.wt.ugt80..
dsm.smt1..
dsm.wt.smt1..
sp.ugt80..
sp.wt.ugt80..
sp.smt1..
sp.wt.smt1..
im.ugt80..
im.wt.ugt80..
im.smt1..
im.wt.smt1..

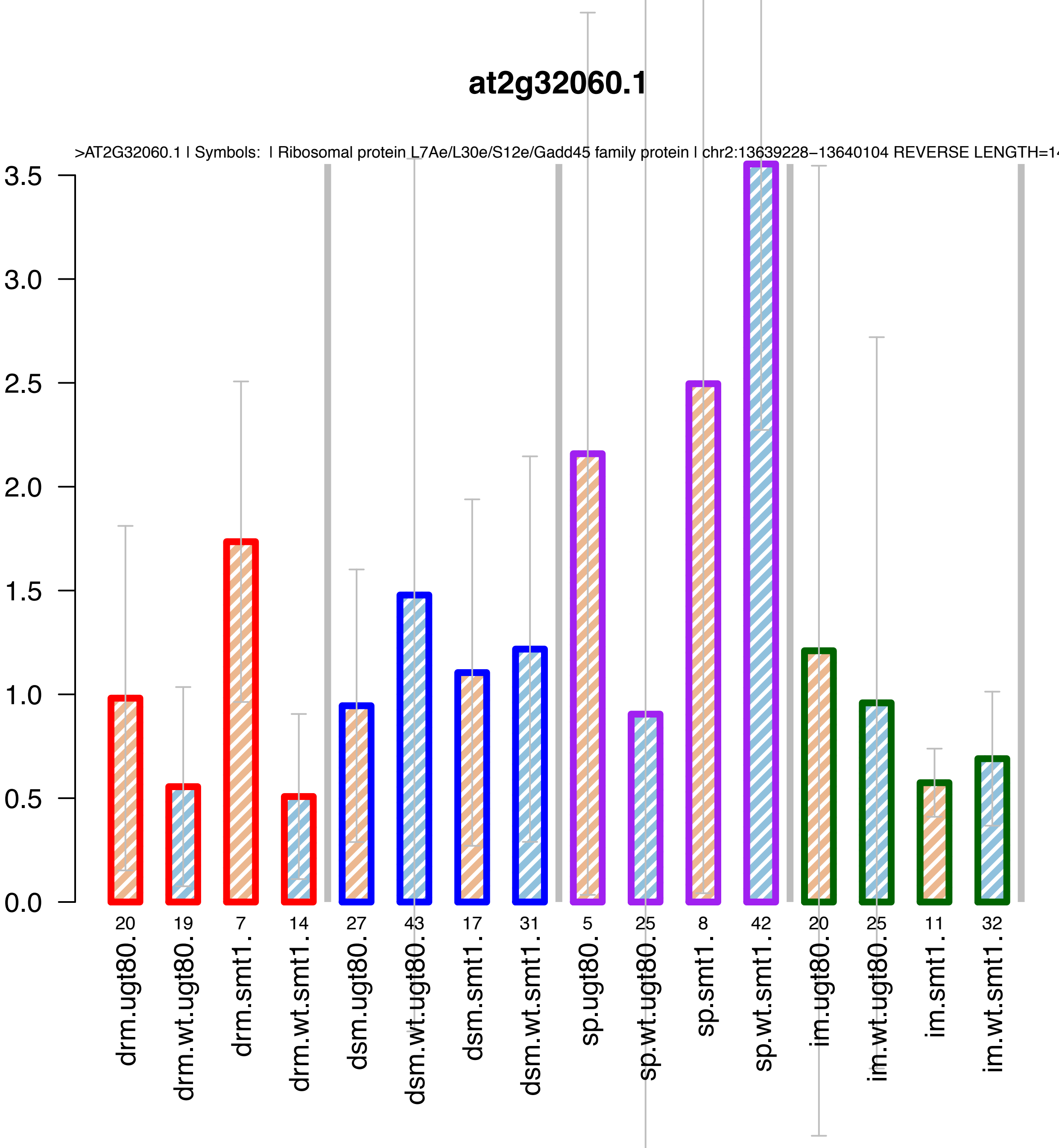

at2g32060.1
>AT2G32060.1 | Symbols: | Ribosomal protein L7Ae/L30e/S12e/Gadd45 family protein | chr2:13639228-13640104 REVERSE LENGTH=1
3.5
3.0
2.5
2.0
1.5
1.0
0.5
0.0
20
19
7
14
27
43
17
31
5
25
8
42
20
25
11
32
drm.ugt80.
drm.wt.ugt80.
drm.smt1.
drm.wt.smt1.
dsm.ugt80.
dsm.wt.ugt80.
dsm.smt1.
dsm.wt.smt1.
sp.ugt80.
sp.wt.ugt80.
sp.smt1.
sp.wt.smt1.
im.ugt80.
im.wt.ugt80.
im.smt1.
im.wt.smt1.

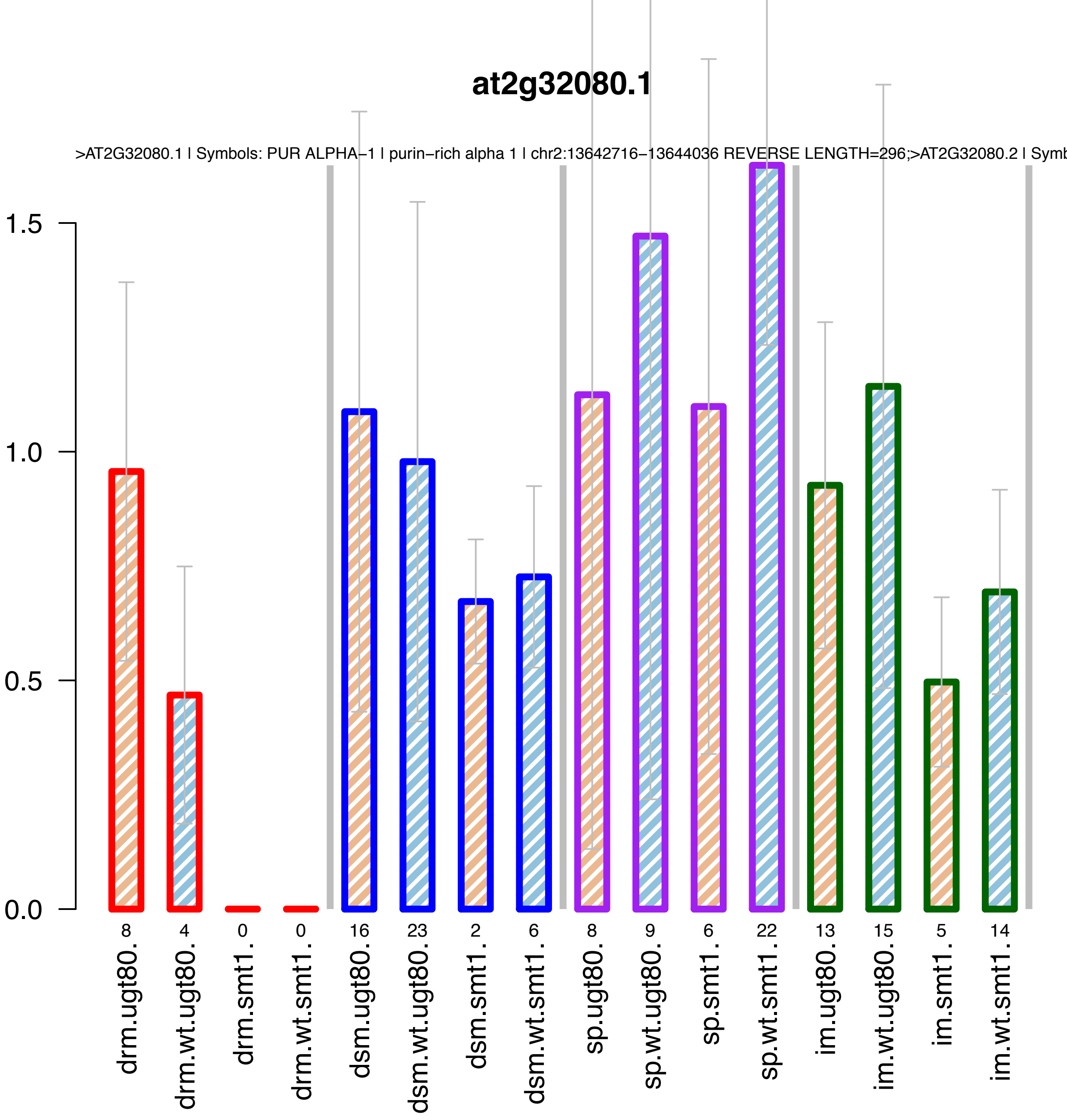
at2g32080.1
>AT2G32080.1 | Symbols: PUR ALPHA-1 | purin-rich alpha 1 | chr2:13642716-13644036 REVERSE LENGTH=296;>AT2G32080.2 | Symb
1.5
1.0
0.5
0.0
8
4
0
0
16
23
2
6
8
9
6
22
13
15
5
14
drm.ugt80.
drm.wt.ugt80.
drm.smt1.
drm.wt.smt1.
dsm.ugt80.
dsm.wt.ugt80.
dsm.smt1.
dsm.wt.smt1.
sp.ugt80.
sp.wt.ugt80.
sp.smt1.
sp.wt.smt1.
im.ugt80.
im.wt.ugt80.
im.smt1.
im.wt.smt1.

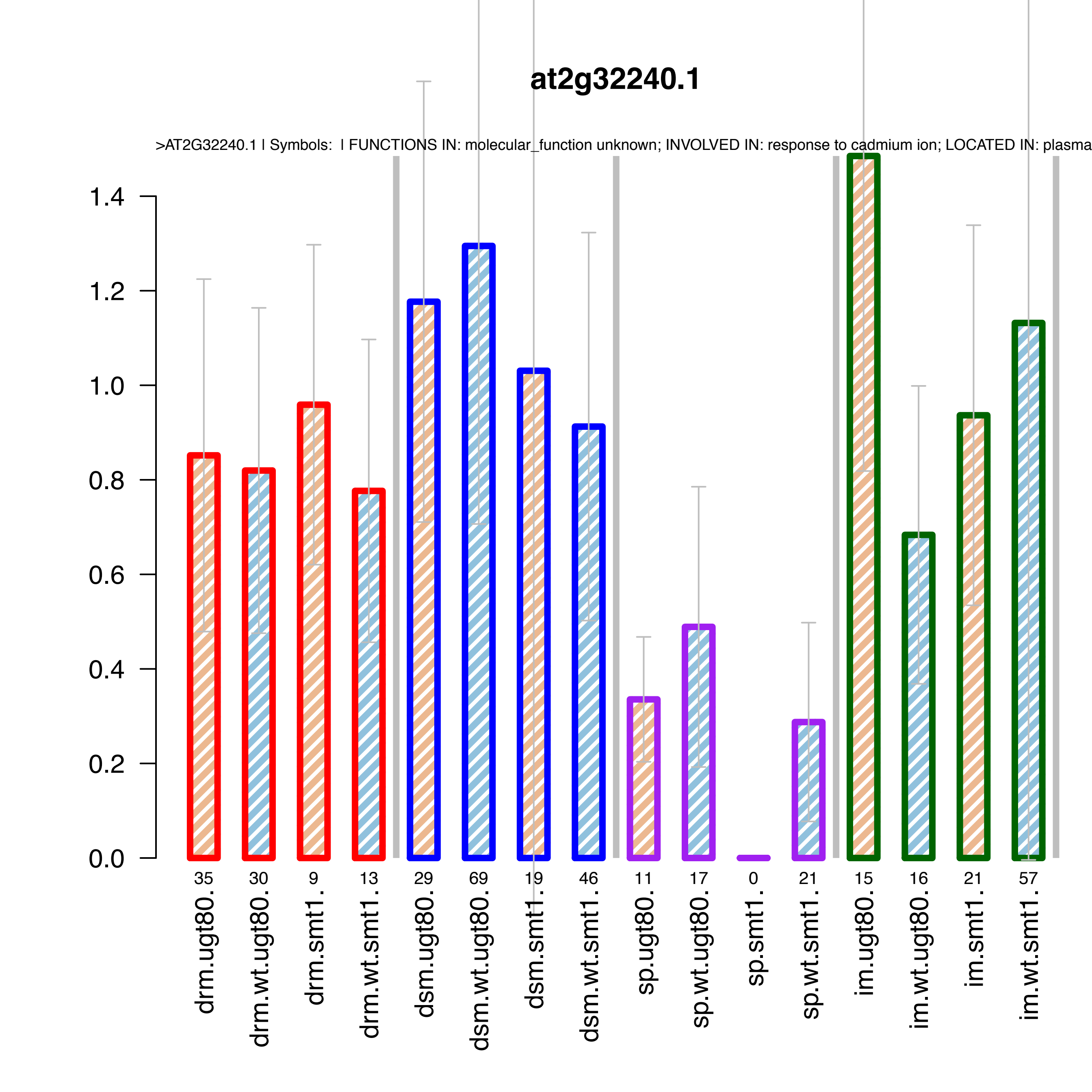

at2g32240.1
>AT2G32240.1 | Symbols: | FUNCTIONS IN: molecular_function unknown; INVOLVED IN: response to cadmium ion; LOCATED IN: plasma
0.0
0.2
0.4
0.6
0.8
1.0
1.2
1.4
35
30
9
13
29
69
19
46
11
17
0
21
15
16
21
57
drm.ugt80.
drm.wt.ugt80.
drm.smt1.
drm.wt.smt1.
dsm.ugt80.
dsm.wt.ugt80.
dsm.smt1.
dsm.wt.smt1.
sp.ugt80.
sp.wt.ugt80.
sp.smt1.
sp.wt.smt1.
im.ugt80.
im.wt.ugt80.
im.smt1.
im.wt.smt1.

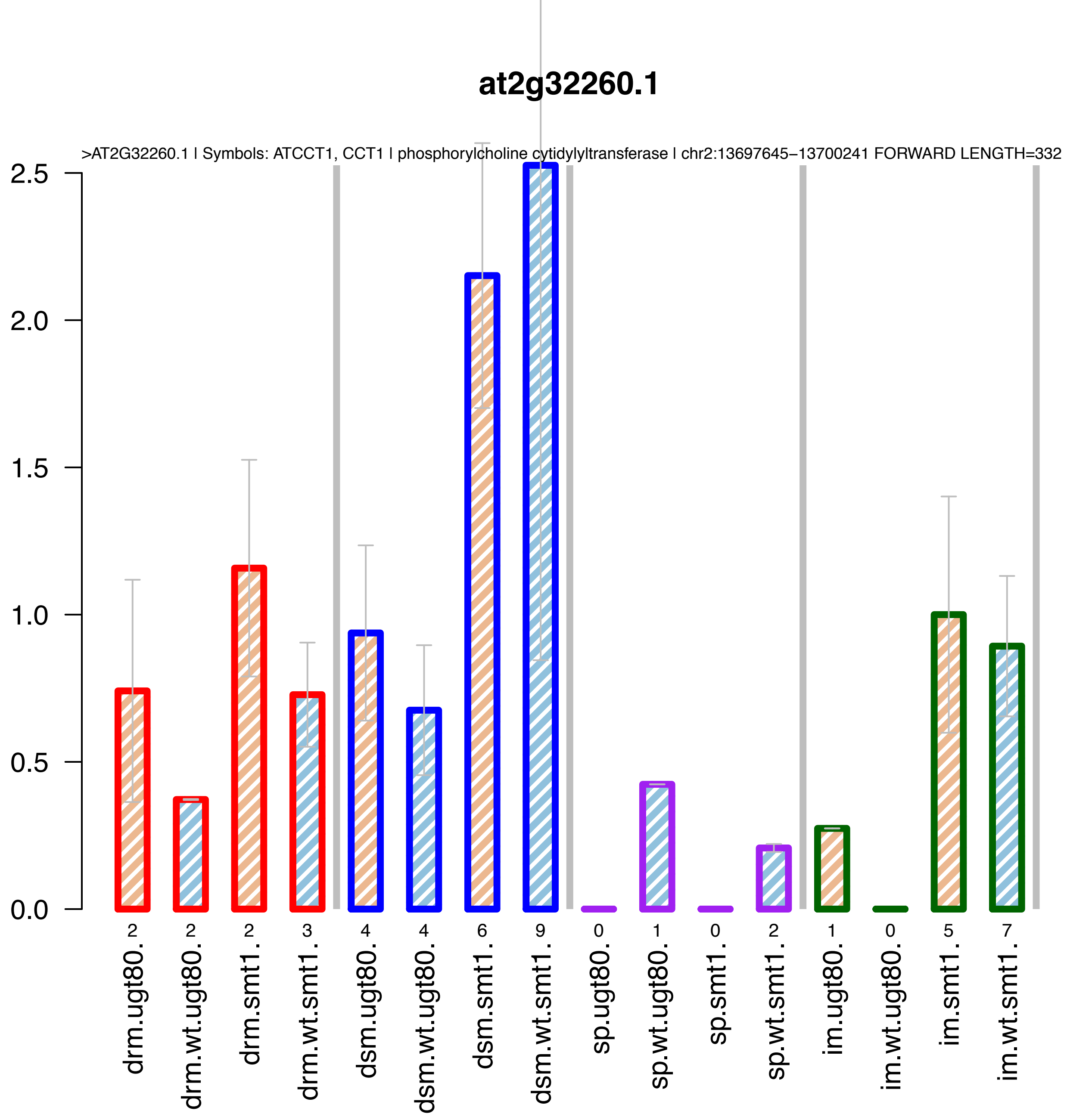

at2g32260.1
>AT2G32260.1 | Symbols: ATCCT1, CCT1 | phosphorylcholine cytidylyltransferase | chr2:13697645-13700241 FORWARD LENGTH=332
0.0
0.5
1.0
1.5
2.0
2.5
2
2
2
3
4
4
6
9
0
1
0
2
1
0
5
7
drm.ugt80..
drm.wt.ugt80..
drm.smt1..
drm.wt.smt1..
dsm.ugt80..
dsm.wt.ugt80..
dsm.smt1..
dsm.wt.smt1..
sp.ugt80..
sp.wt.ugt80..
sp.smt1..
sp.wt.smt1..
im.ugt80..
im.wt.ugt80..
im.smt1..
im.wt.smt1..

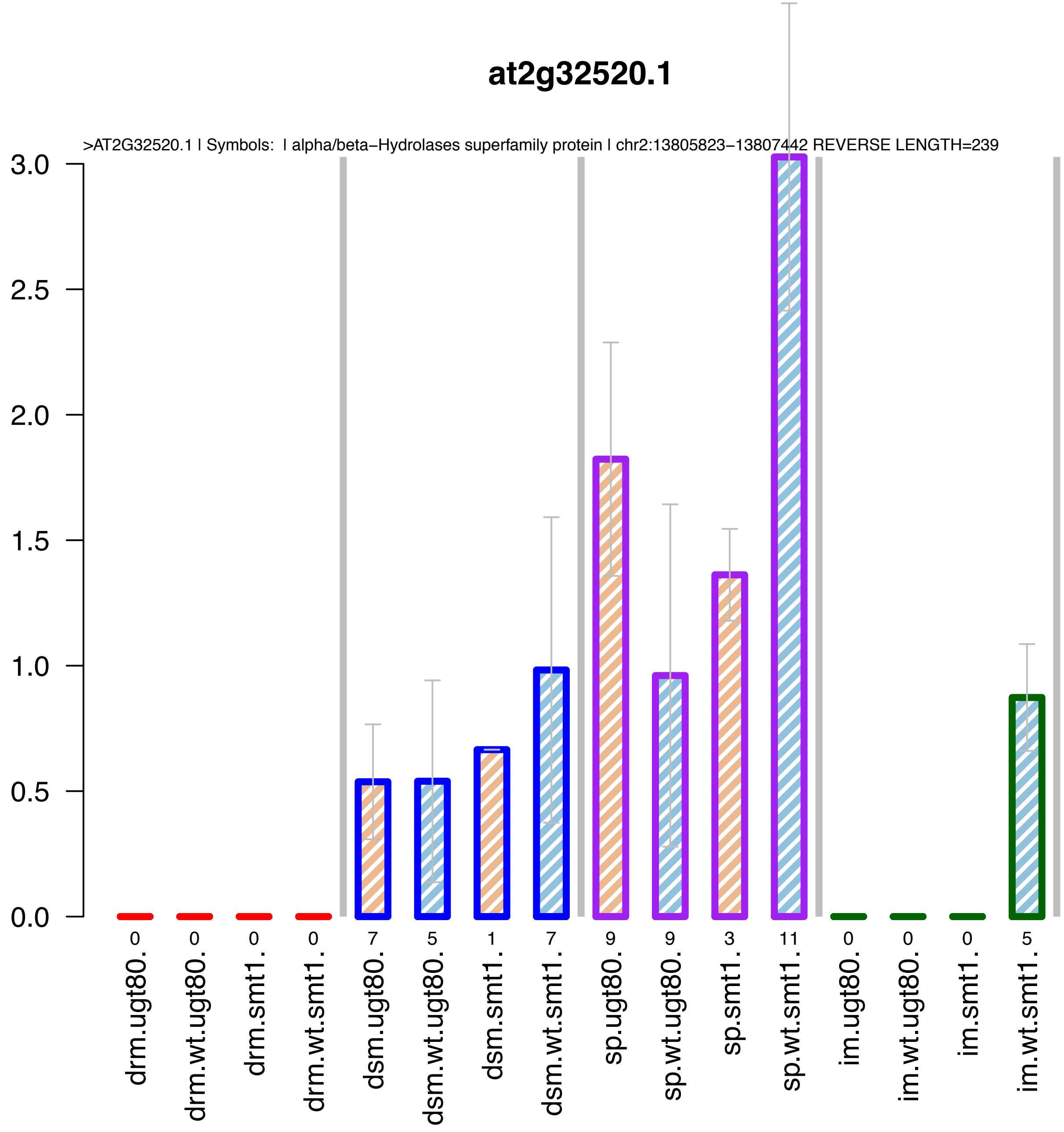

at2g32520.1
>AT2G32520.1 | Symbols: | alpha/beta−Hydrolases superfamily protein | chr2:13805823−13807442 REVERSE LENGTH=239
3.0
2.5
2.0
1.5
1.0
0.5
0.0
0
0
0
0
7
5
1
7
9
9
3
11
0
0
0
5
drm.ugt80.
drm.wt.ugt80.
drm.smt1.
drm.wt.smt1.
dsm.ugt80.
dsm.wt.ugt80.
dsm.smt1.
dsm.wt.smt1.
sp.ugt80.
sp.wt.ugt80.
sp.smt1.
sp.wt.smt1.
im.ugt80.
im.wt.ugt80.
im.smt1.
im.wt.smt1.

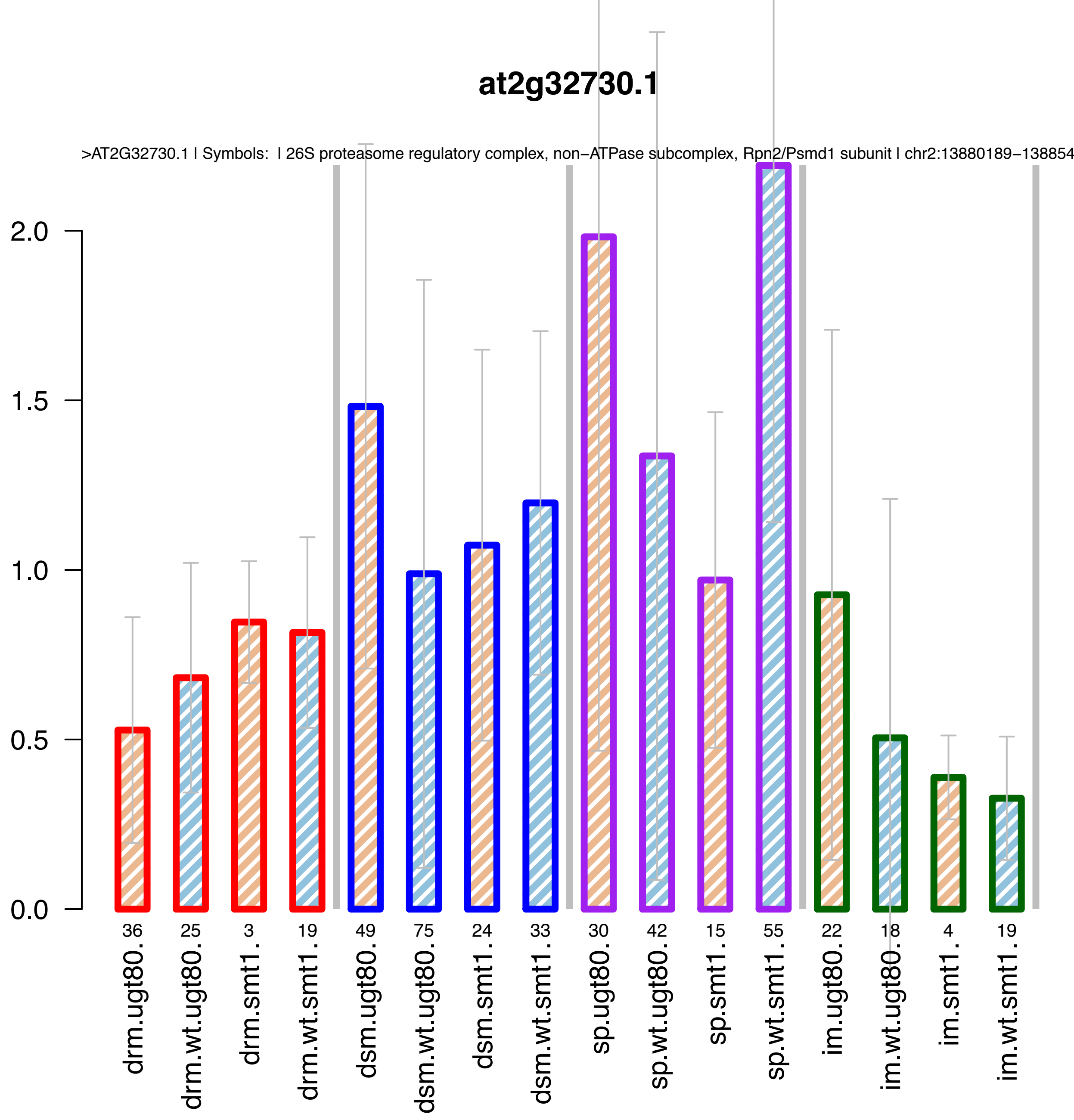
at2g32730.1
>AT2G32730.1 | Symbols: | 26S proteasome regulatory complex, non-ATPase subcomplex, Rpn2/Psmd1 subunit | chr2:13880189-138854
0.0
0.5
1.0
1.5
2.0
36
25
3
19
49
75
24
33
30
42
15
55
22
18
4
19
drm.ugt80.
drm.wt.ugt80.
drm.smt1.
drm.wt.smt1.
dsm.ugt80.
dsm.wt.ugt80.
dsm.smt1.
dsm.wt.smt1.
sp.ugt80.
sp.wt.ugt80.
sp.smt1.
sp.wt.smt1.
im.ugt80.
im.wt.ugt80.
im.smt1.
im.wt.smt1.

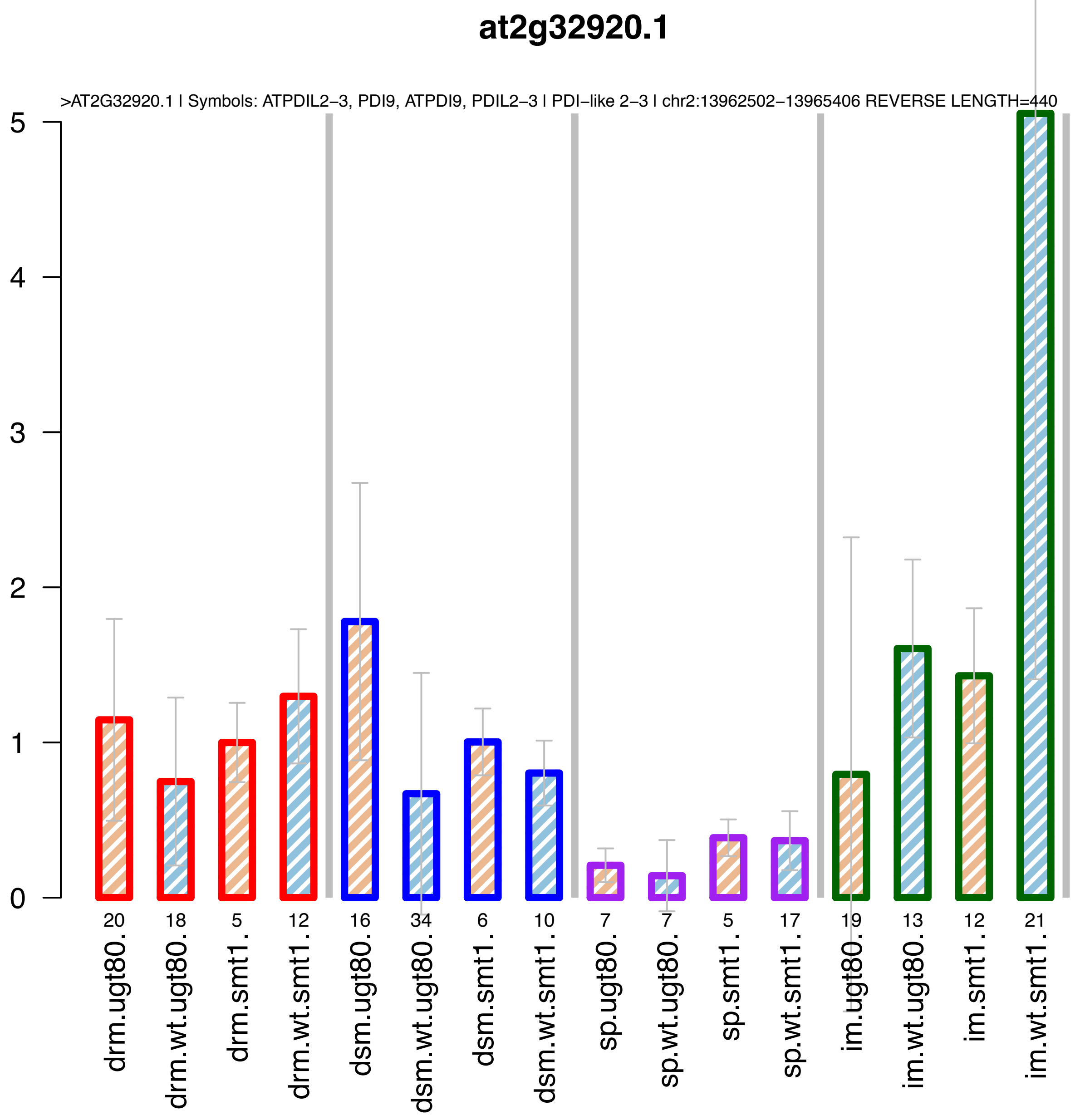

at2g32920.1
>AT2G32920.1 | Symbols: ATPDIL2−3, PDI9, ATPDI9, PDIL2−3 | PDI−like 2−3 | chr2:13962502−13965406 REVERSE LENGTH=440
0
1
2
3
4
5
20
18
5
12
16
34
6
10
7
7
5
17
19
13
12
21
drm.ugt80..
drm.wt.ugt80..
drm.smt1..
drm.wt.smt1..
dsm.ugt80..
dsm.wt.ugt80..
dsm.smt1..
dsm.wt.smt1..
sp.ugt80..
sp.wt.ugt80..
sp.smt1..
sp.wt.smt1..
im.ugt80..
im.wt.ugt80..
im.smt1..
im.wt.smt1..

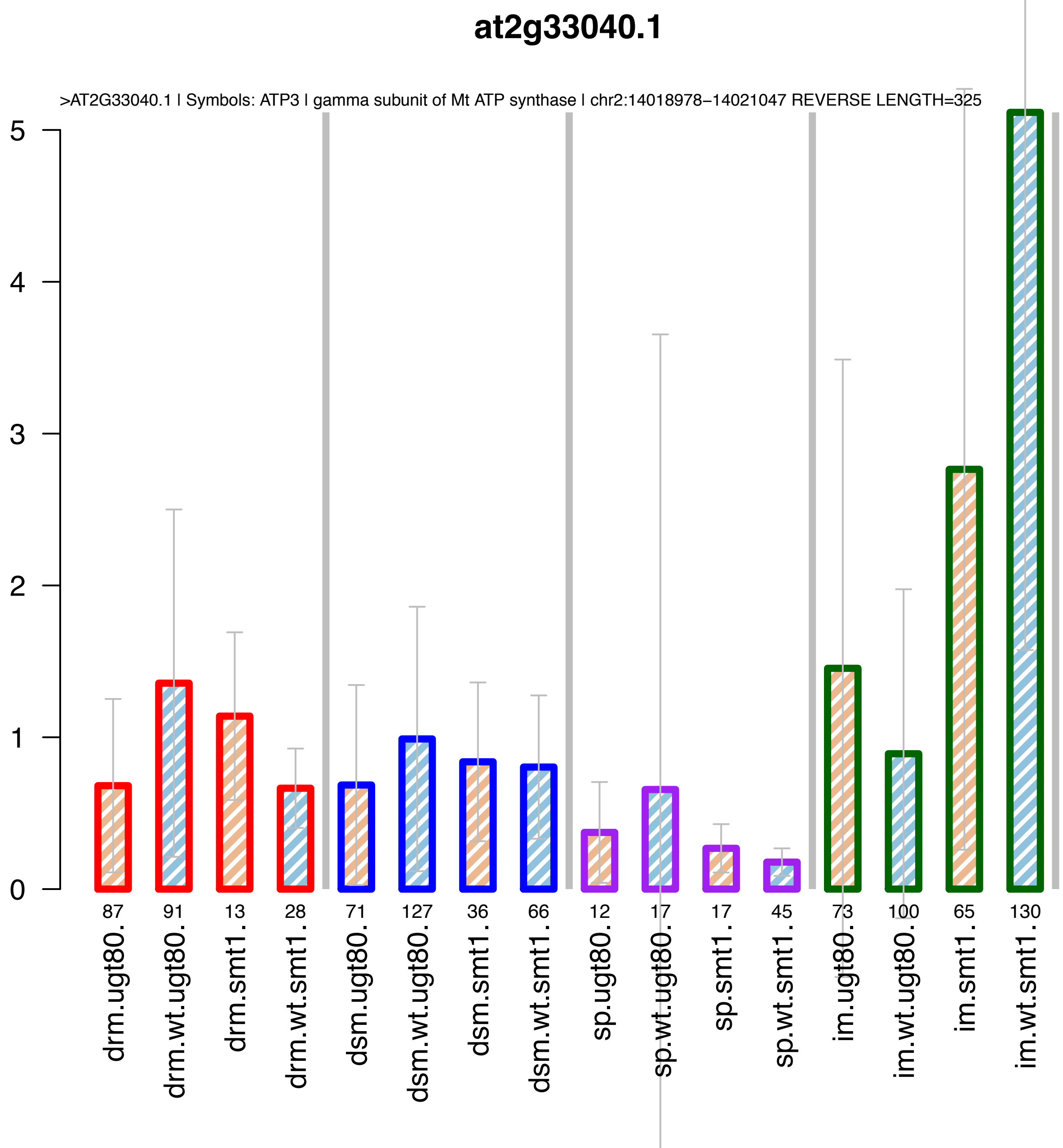

at2g33040.1
>AT2G33040.1 | Symbols: ATP3 | gamma subunit of Mt ATP synthase | chr2:14018978-14021047 REVERSE LENGTH=325
0
1
2
3
4
5
87
91
13
28
71
127
36
66
12
17
17
45
73
100
65
130
drm.ugt80..
drm.wt.ugt80..
drm.smt1..
drm.wt.smt1..
dsm.ugt80..
dsm.wt.ugt80..
dsm.smt1..
dsm.wt.smt1..
sp.ugt80..
sp.wt.ugt80..
sp.smt1..
sp.wt.smt1..
im.ugt80..
im.wt.ugt80..
im.smt1..
im.wt.smt1..

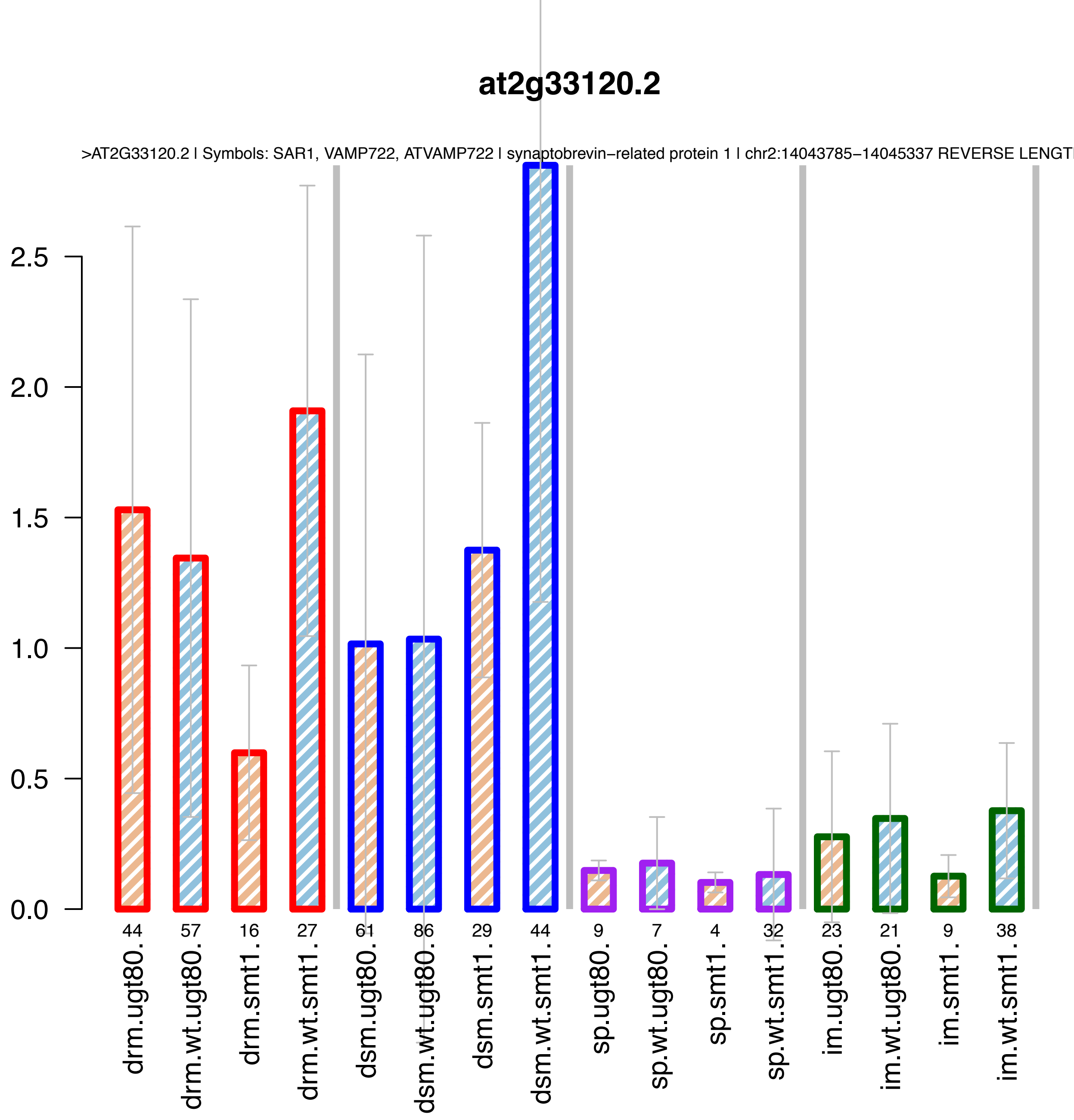

at2g33120.2
>AT2G33120.2 | Symbols: SAR1, VAMP722, ATVAMP722 | synaptobrevin−related protein 1 | chr2:14043785−14045337 REVERSE LENGT
0.0
0.5
1.0
1.5
2.0
2.5
44
57
16
27
61
86
29
44
9
7
4
32
23
21
9
38
drm.ugt80..
drm.wt.ugt80..
drm.smt1..
drm.wt.smt1..
dsm.ugt80..
dsm.wt.ugt80..
dsm.smt1..
dsm.wt.smt1..
sp.ugt80..
sp.wt.ugt80..
sp.smt1..
sp.wt.smt1..
im.ugt80..
im.wt.ugt80..
im.smt1..
im.wt.smt1..

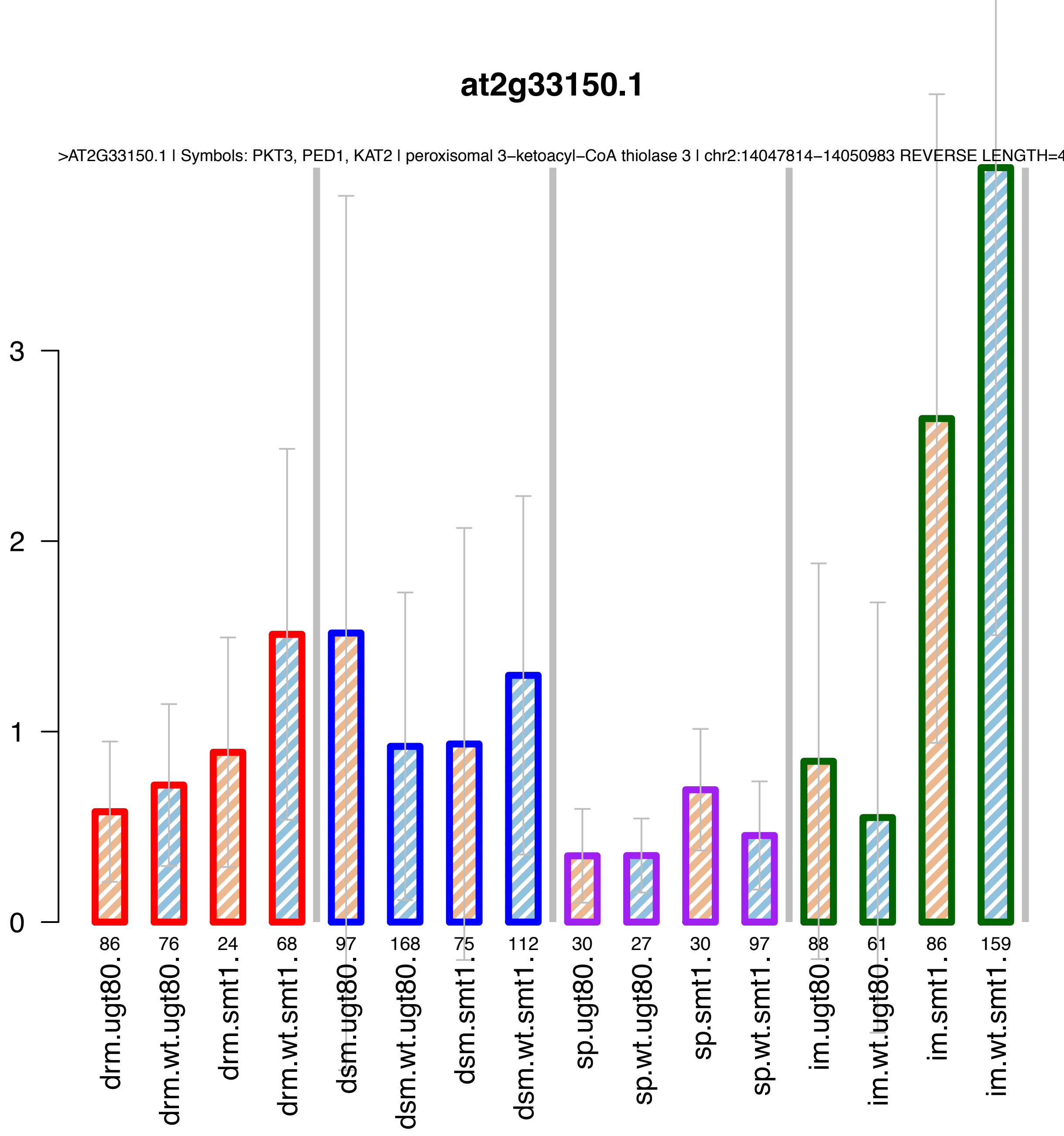

at2g33150.1
>AT2G33150.1 | Symbols: PKT3, PED1, KAT2 | peroxisomal 3−ketoacyl−CoA thiolase 3 | chr2:14047814−14050983 REVERSE LENGTH=4
0
1
2
3
86
76
24
68
97
168
75
112
30
27
30
97
88
61
86
159
drm.ugt80.
drm.wt.ugt80.
drm.smt1.
drm.wt.smt1.
dsm.ugt80.
dsm.wt.ugt80.
dsm.smt1.
dsm.wt.smt1.
sp.ugt80.
sp.wt.ugt80.
sp.smt1.
sp.wt.smt1.
im.ugt80.
im.wt.ugt80.
im.smt1.
im.wt.smt1.

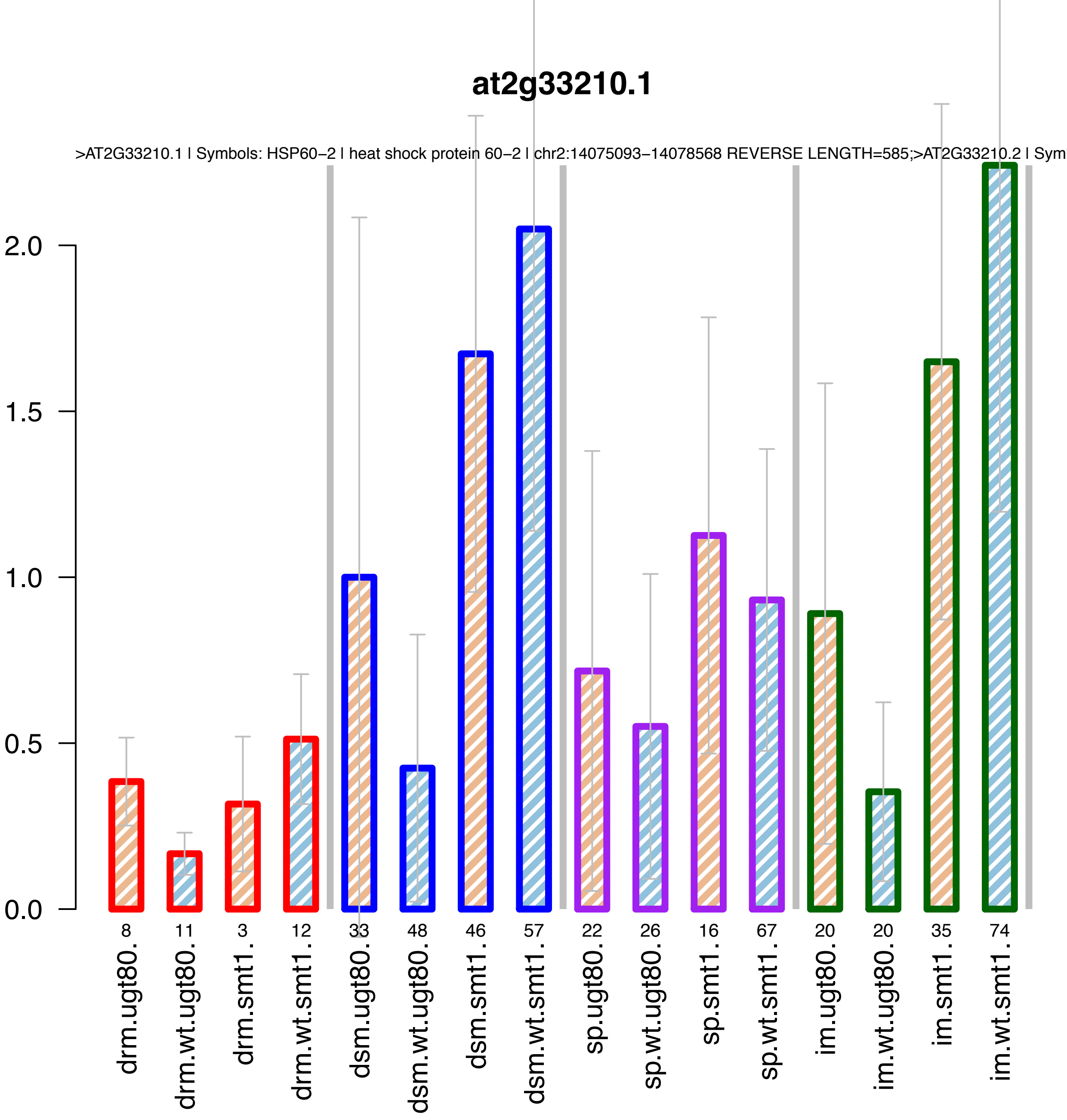

at2g33210.1
>AT2G33210.1 | Symbols: HSP60−2 | heat shock protein 60−2 | chr2:14075093−14078568 REVERSE LENGTH=585;>AT2G33210.2 | Sym
2.0
1.5
1.0
0.5
0.0
8
11
3
12
33
48
46
57
22
26
16
67
20
20
35
74
drm.ugt80.
drm.wt.ugt80.
drm.smt1.
drm.wt.smt1.
dsm.ugt80.
dsm.wt.ugt80.
dsm.smt1.
dsm.wt.smt1.
sp.ugt80.
sp.wt.ugt80.
sp.smt1.
sp.wt.smt1.
im.ugt80.
im.wt.ugt80.
im.smt1.
im.wt.smt1.

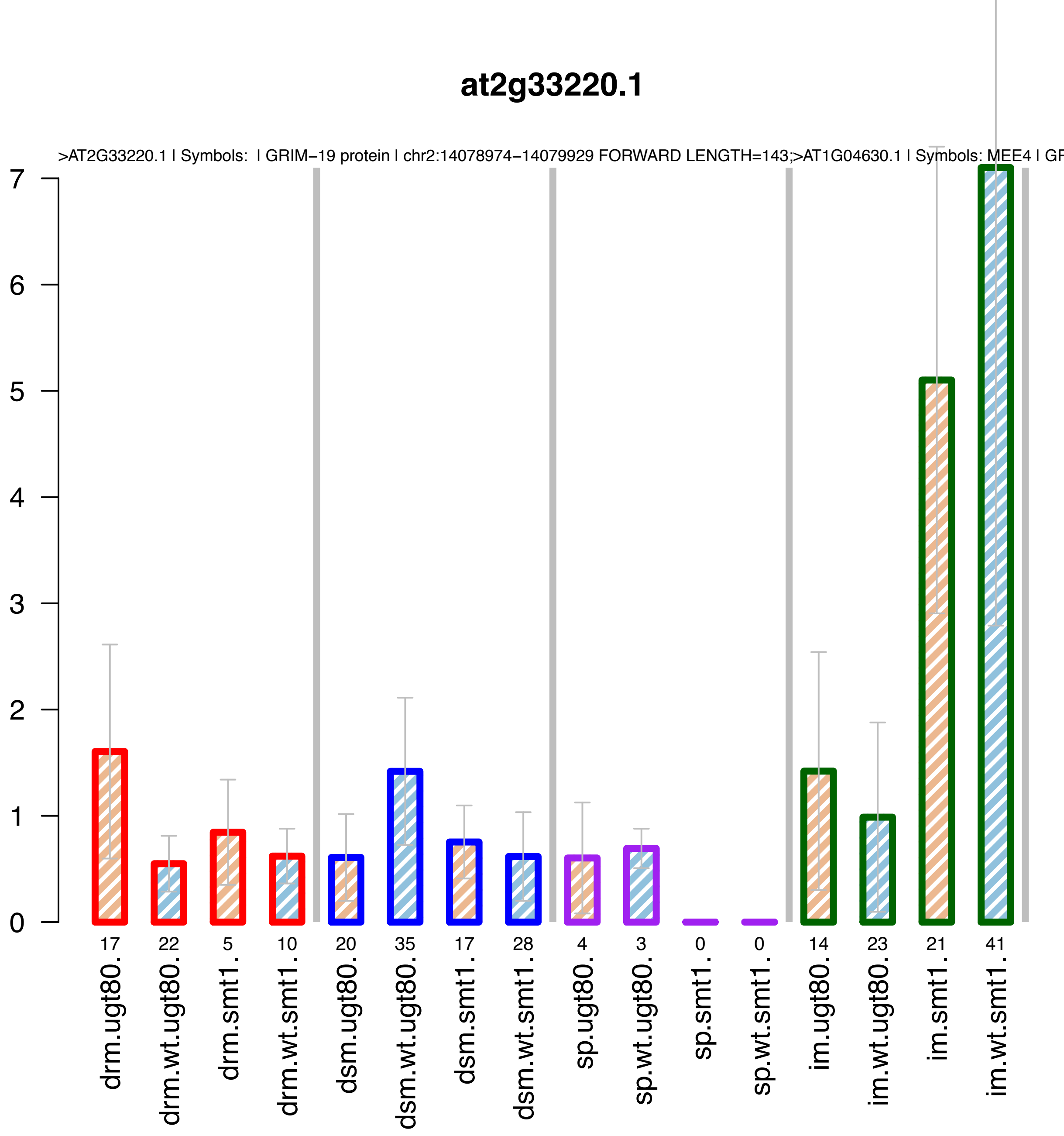

at2g33220.1
>AT2G33220.1 | Symbols: | GRIM−19 protein | chr2:14078974−14079929 FORWARD LENGTH=143;>AT1G04630.1 | Symbols: MEE4 | GF
0
1
2
3
4
5
6
7
17
22
5
10
20
35
17
28
4
3
0
0
14
23
21
41
drm.ugt80.
drm.wt.ugt80.
drm.smt1.
drm.wt.smt1.
dsm.ugt80.
dsm.wt.ugt80.
dsm.smt1.
dsm.wt.smt1.
sp.ugt80.
sp.wt.ugt80.
sp.smt1.
sp.wt.smt1.
im.ugt80.
im.wt.ugt80.
im.smt1.
im.wt.smt1.

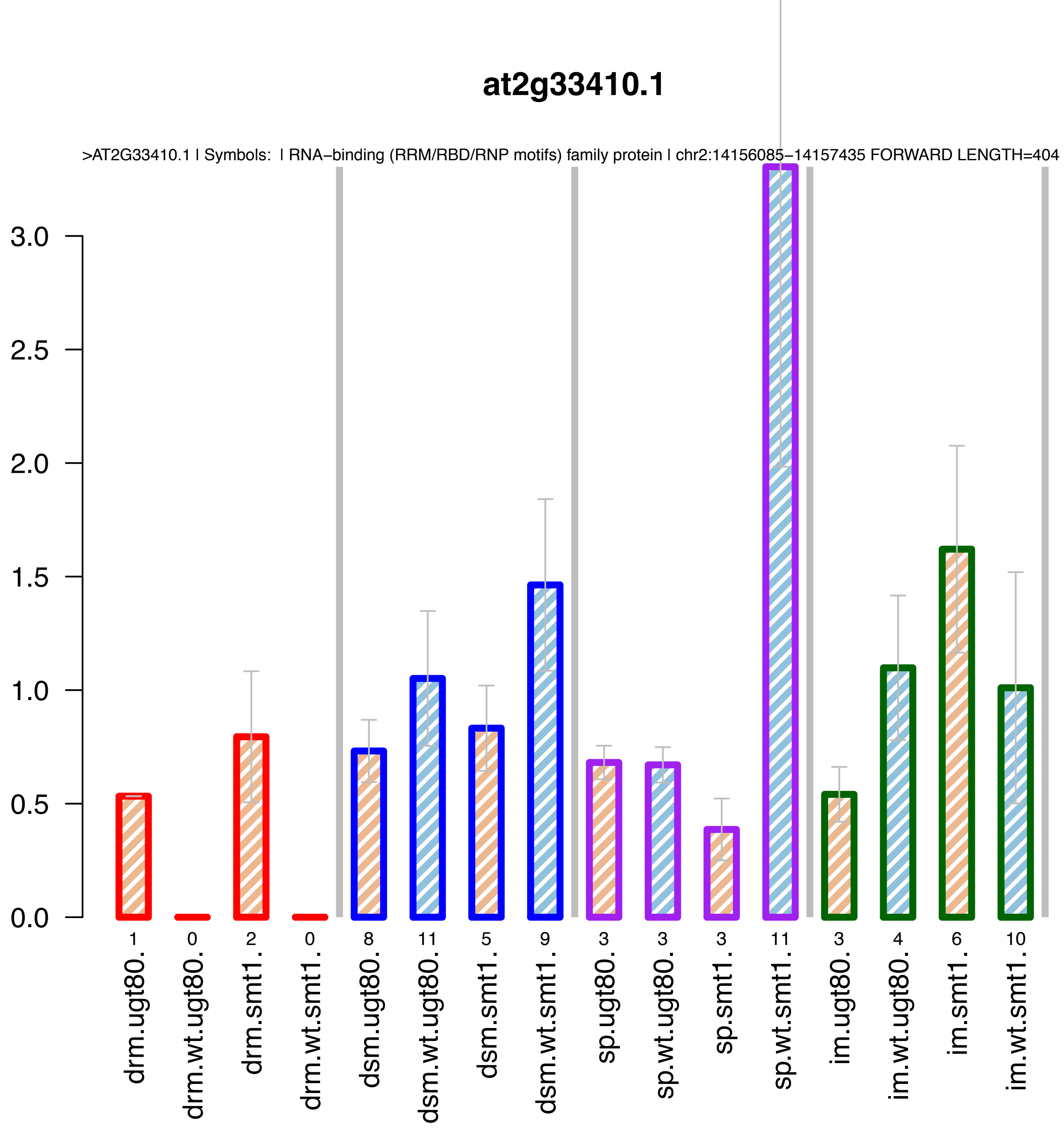
at2g33410.1
>AT2G33410.1 | Symbols: | RNA−binding (RRM/RBD/RNP motifs) family protein | chr2:14156085−14157435 FORWARD LENGTH=404
3.0
2.5
2.0
1.5
1.0
0.5
0.0
1
0
2
0
8
11
5
9
3
3
3
11
3
4
6
10
drm.ugt80.
drm.wt.ugt80.
drm.smt1.
drm.wt.smt1.
dsm.ugt80.
dsm.wt.ugt80.
dsm.smt1.
dsm.wt.smt1.
sp.ugt80.
sp.wt.ugt80.
sp.smt1.
sp.wt.smt1.
im.ugt80.
im.wt.ugt80.
im.smt1.
im.wt.smt1.

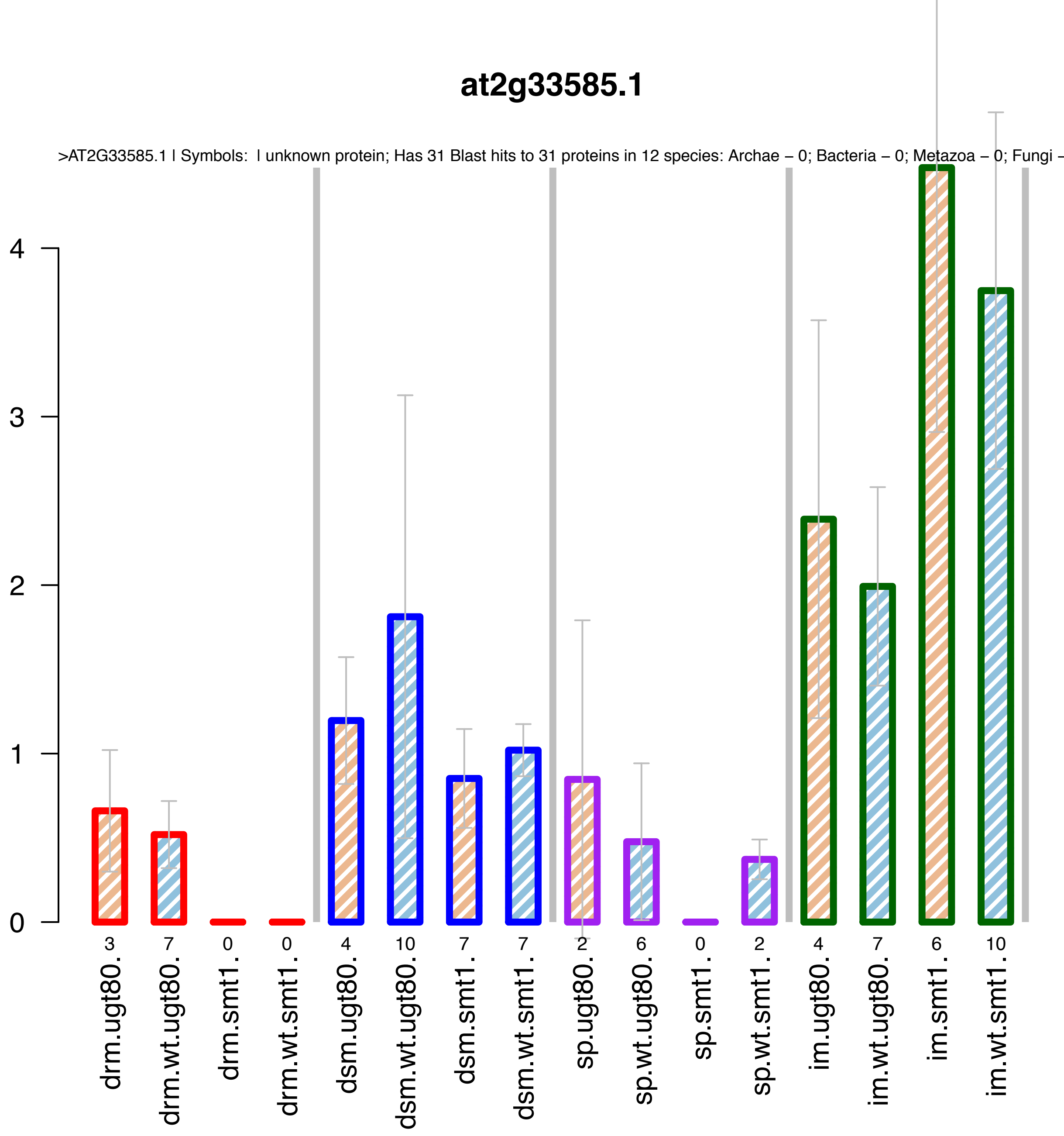

at2g33585.1
>AT2G33585.1 | Symbols: | unknown protein; Has 31 Blast hits to 31 proteins in 12 species: Archae – 0; Bacteria – 0; Metazoa – 0; Fungi –
0
1
2
3
4
3
7
0
0
4
10
7
7
2
6
0
2
4
7
6
10
drm.ugt80.
drm.wt.ugt80.
drm.smt1.
drm.wt.smt1.
dsm.ugt80.
dsm.wt.ugt80.
dsm.smt1.
dsm.wt.smt1.
sp.ugt80.
sp.wt.ugt80.
sp.smt1.
sp.wt.smt1.
im.ugt80.
im.wt.ugt80.
im.smt1.
im.wt.smt1.

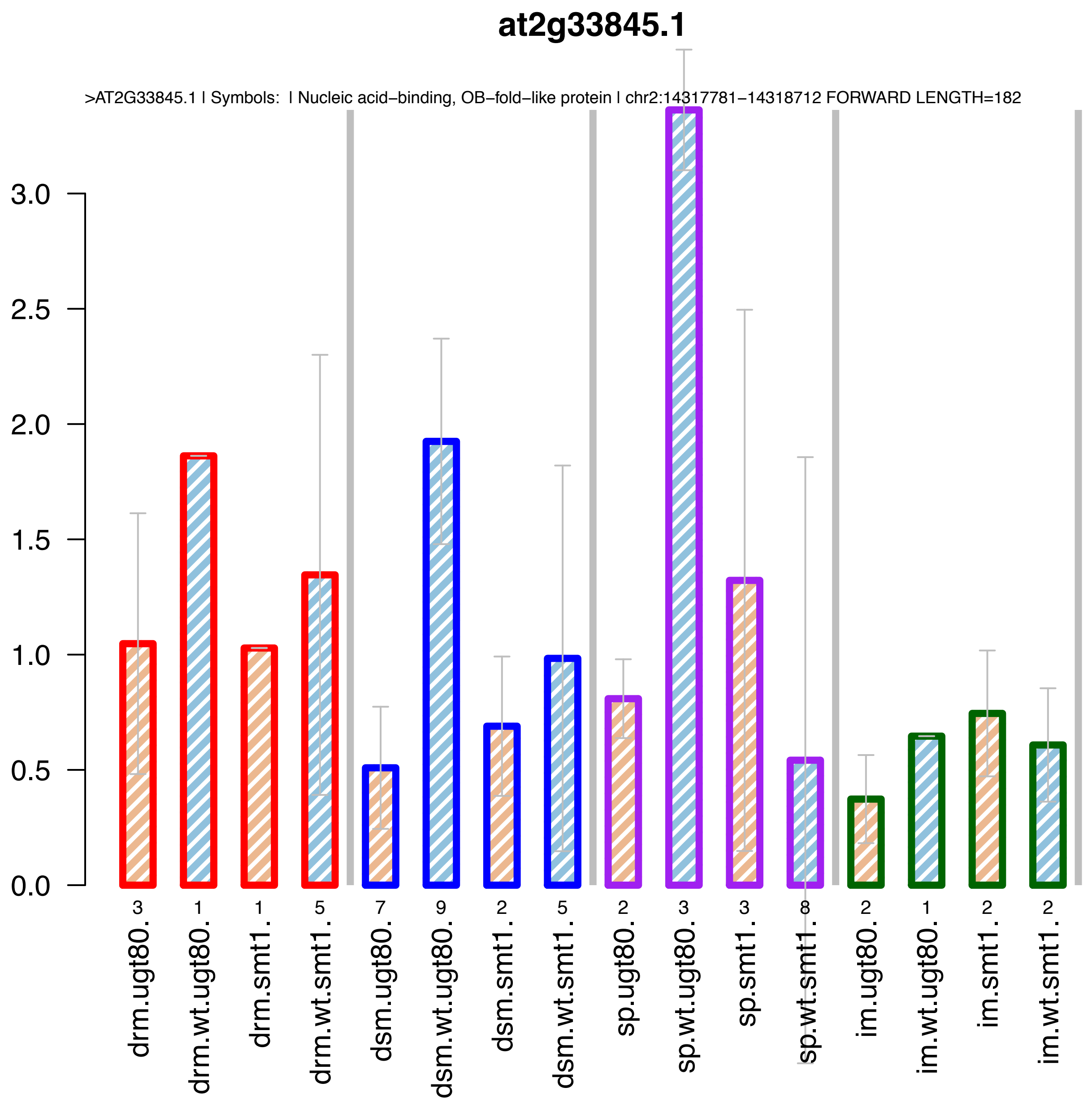

at2g33845.1
>AT2G33845.1 | Symbols: | Nucleic acid−binding, OB−fold−like protein | chr2:14317781−14318712 FORWARD LENGTH=182
3.0
2.5
2.0
1.5
1.0
0.5
0.0
3
1
1
5
7
9
2
5
2
3
3
8
2
1
2
2
drm.ugt80..
drm.wt.ugt80..
drm.smt1..
drm.wt.smt1..
dsm.ugt80..
dsm.wt.ugt80..
dsm.smt1..
dsm.wt.smt1..
sp.ugt80..
sp.wt.ugt80..
sp.smt1..
sp.wt.smt1..
im.ugt80..
im.wt.ugt80..
im.smt1..
im.wt.smt1..

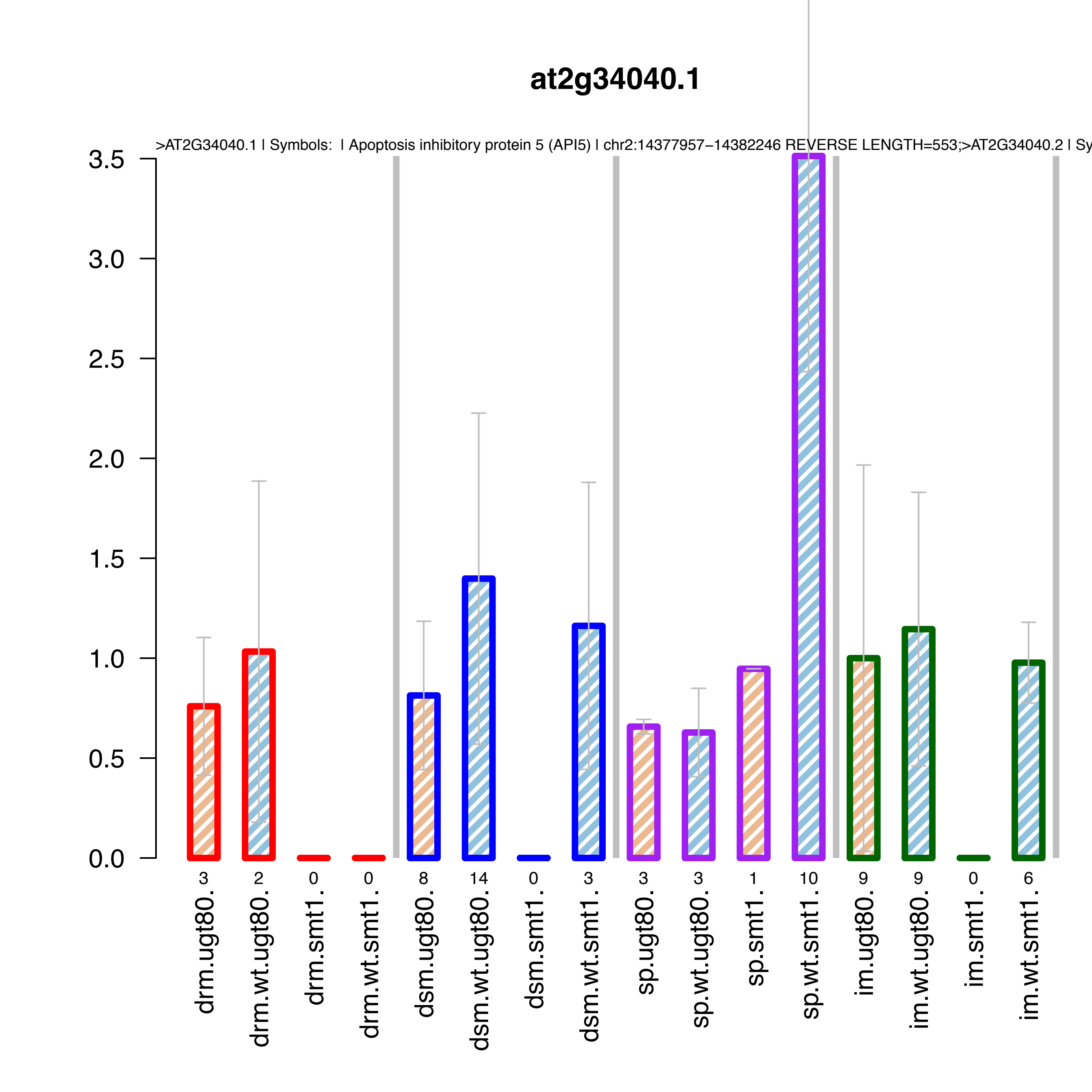

at2g34040.1
>AT2G34040.1 | Symbols: | Apoptosis inhibitory protein 5 (API5) | chr2:14377957-14382246 REVERSE LENGTH=553;>AT2G34040.2 | Sy
3.5
3.0
2.5
2.0
1.5
1.0
0.5
0.0
3
2
0
0
8
14
0
3
3
3
1
10
9
9
0
6
drm.ugt80.
drm.wt.ugt80.
drm.smt1.
drm.wt.smt1.
dsm.ugt80.
dsm.wt.ugt80.
dsm.smt1.
dsm.wt.smt1.
sp.ugt80.
sp.wt.ugt80.
sp.smt1.
sp.wt.smt1.
im.ugt80.
im.wt.ugt80.
im.smt1.
im.wt.smt1.

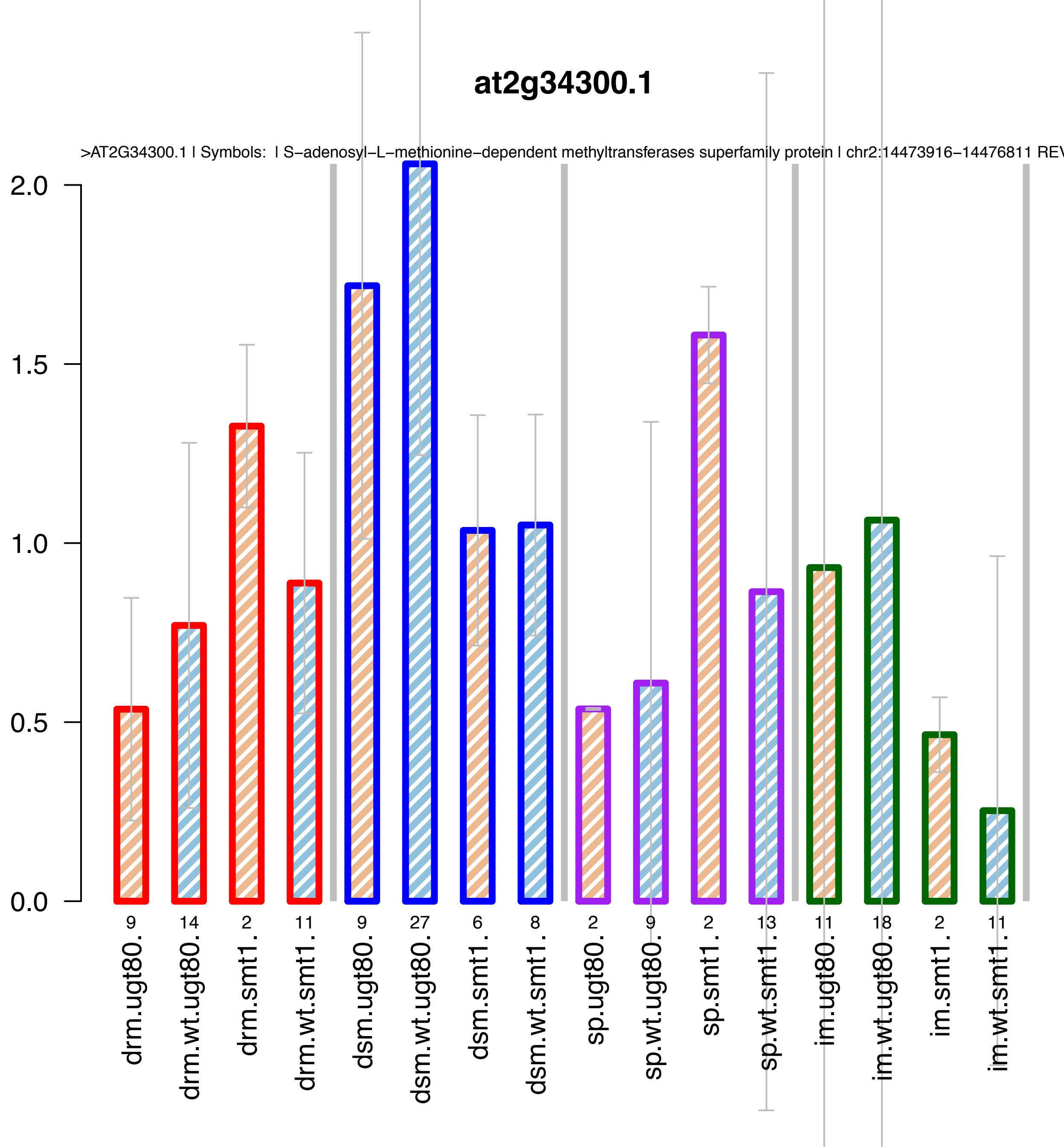

at2g34300.1
>AT2G34300.1 | Symbols: | S−adenosyl−L−methionine−dependent methyltransferases superfamily protein | chr2:14473916−14476811 REV
2.0
1.5
1.0
0.5
0.0
9
14
2
11
9
27
6
8
2
9
2
13
11
18
2
11
drm.ugt80.
drm.wt.ugt80.
drm.smt1.
drm.wt.smt1.
dsm.ugt80.
dsm.wt.ugt80.
dsm.smt1.
dsm.wt.smt1.
sp.ugt80.
sp.wt.ugt80.
sp.smt1.
sp.wt.smt1.
im.ugt80.
im.wt.ugt80.
im.smt1.
im.wt.smt1.

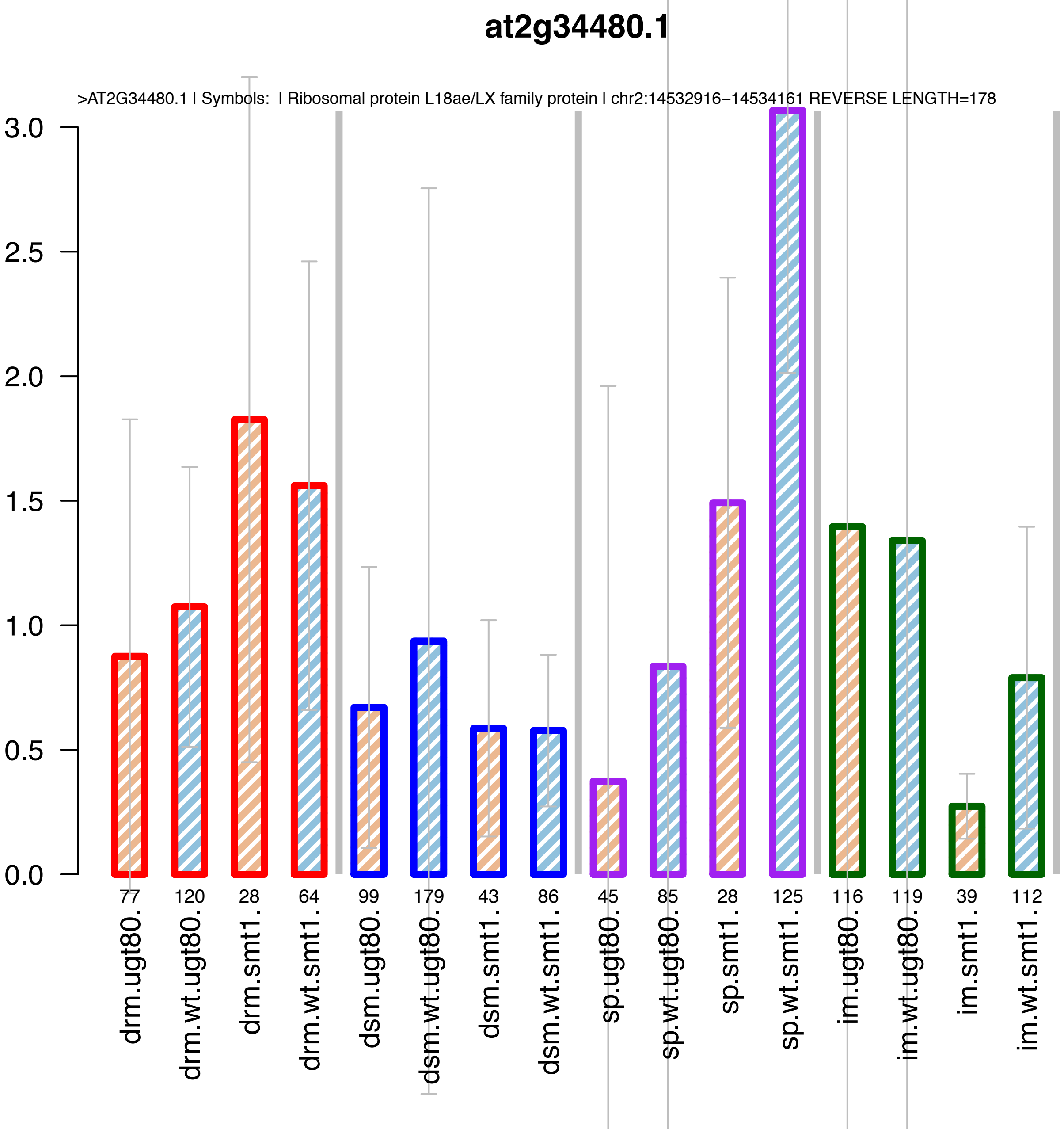

at2g34480.1
>AT2G34480.1 | Symbols: | Ribosomal protein L18ae/LX family protein | chr2:14532916−14534161 REVERSE LENGTH=178
3.0
2.5
2.0
1.5
1.0
0.5
0.0
77
120
28
64
99
179
43
86
45
85
28
125
116
119
39
112
drm.ugt80..
drm.wt.ugt80..
drm.smt1..
drm.wt.smt1..
dsm.ugt80..
dsm.wt.ugt80..
dsm.smt1..
dsm.wt.smt1..
sp.ugt80..
sp.wt.ugt80..
sp.smt1..
sp.wt.smt1..
im.ugt80..
im.wt.ugt80..
im.smt1..
im.wt.smt1..

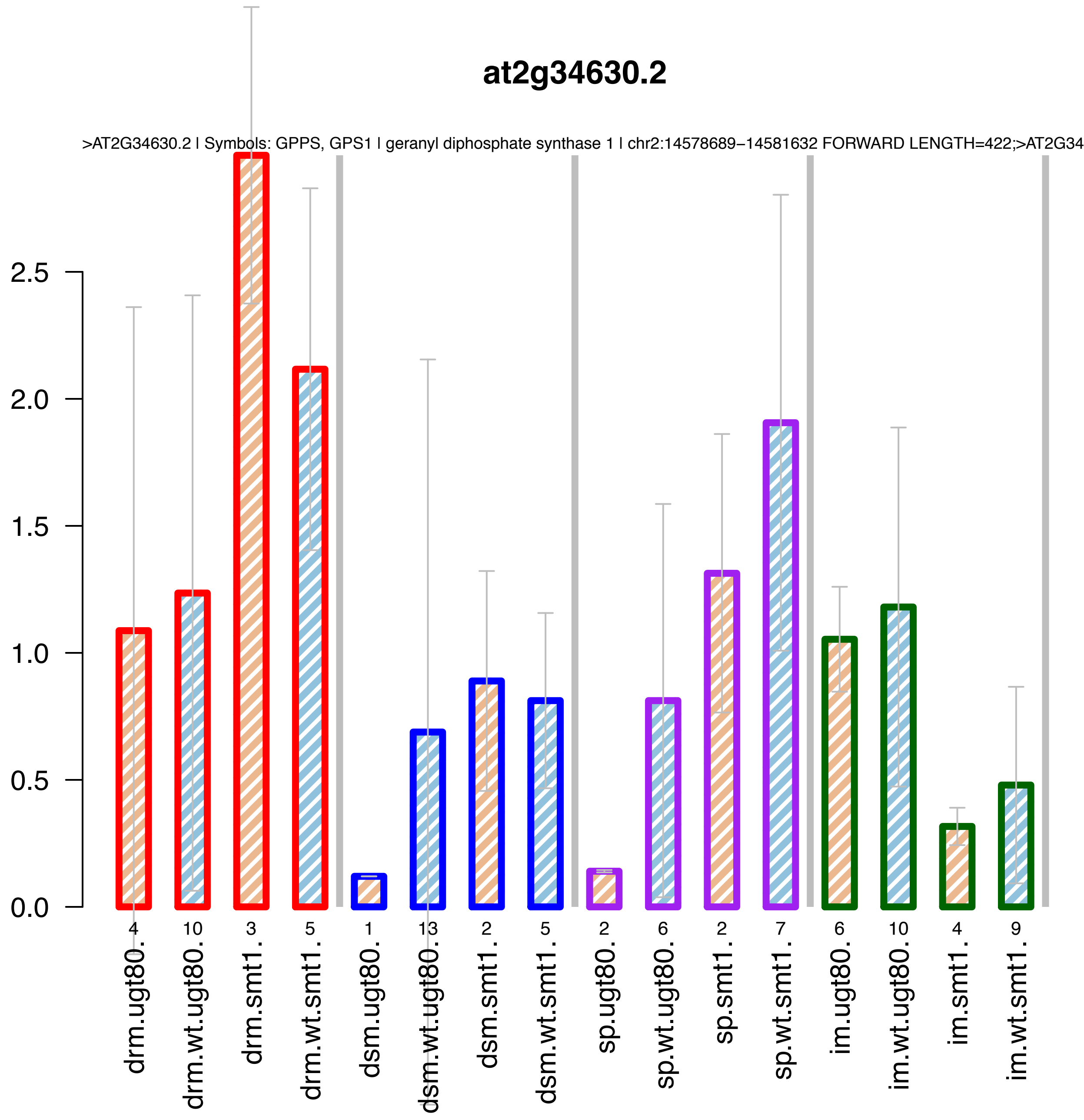
at2g34630.2
>AT2G34630.2 | Symbols: GPPS, GPS1 | geranyl diphosphate synthase 1 | chr2:14578689-14581632 FORWARD LENGTH=422;>AT2G34
0.0
0.5
1.0
1.5
2.0
2.5
4
10
3
5
1
13
2
5
2
6
2
7
6
10
4
9
drm.ugt80..
drm.wt.ugt80..
drm.smt1..
drm.wt.smt1..
dsm.ugt80..
dsm.wt.ugt80..
dsm.smt1..
dsm.wt.smt1..
sp.ugt80..
sp.wt.ugt80..
sp.smt1..
sp.wt.smt1..
im.ugt80..
im.wt.ugt80..
im.smt1..
im.wt.smt1..

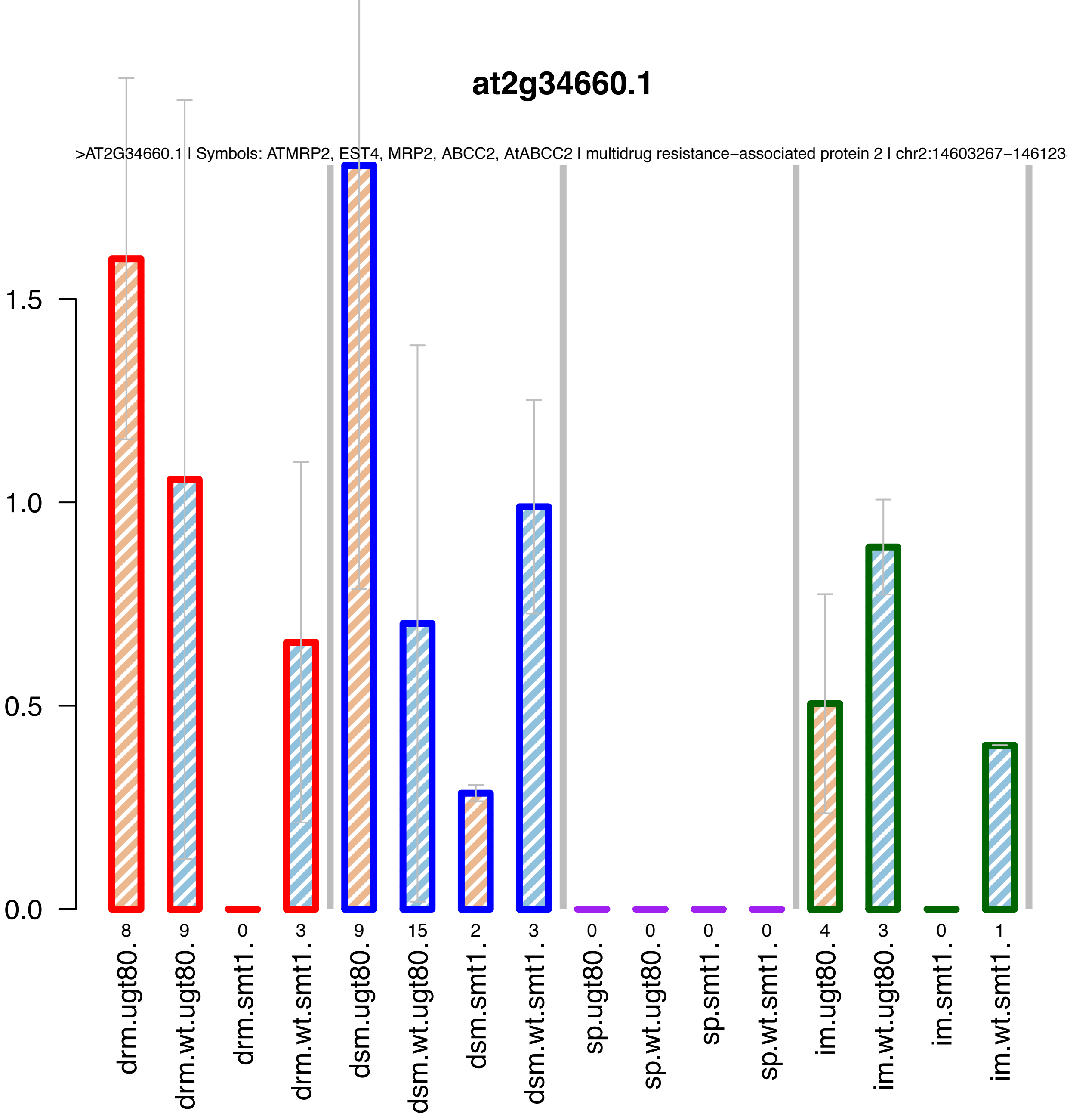
at2g34660.1
>AT2G34660.1 | Symbols: ATMRP2, EST4, MRP2, ABCC2, AtABCC2 | multidrug resistance-associated protein 2 | chr2:14603267-146123
0.0
0.5
1.0
1.5
8
9
0
3
9
15
2
3
0
0
0
0
4
3
0
1
drm.ugt80.
drm.wt.ugt80.
drm.smt1.
drm.wt.smt1.
dsm.ugt80.
dsm.wt.ugt80.
dsm.smt1.
dsm.wt.smt1.
sp.ugt80.
sp.wt.ugt80.
sp.smt1.
sp.wt.smt1.
im.ugt80.
im.wt.ugt80.
im.smt1.
im.wt.smt1.

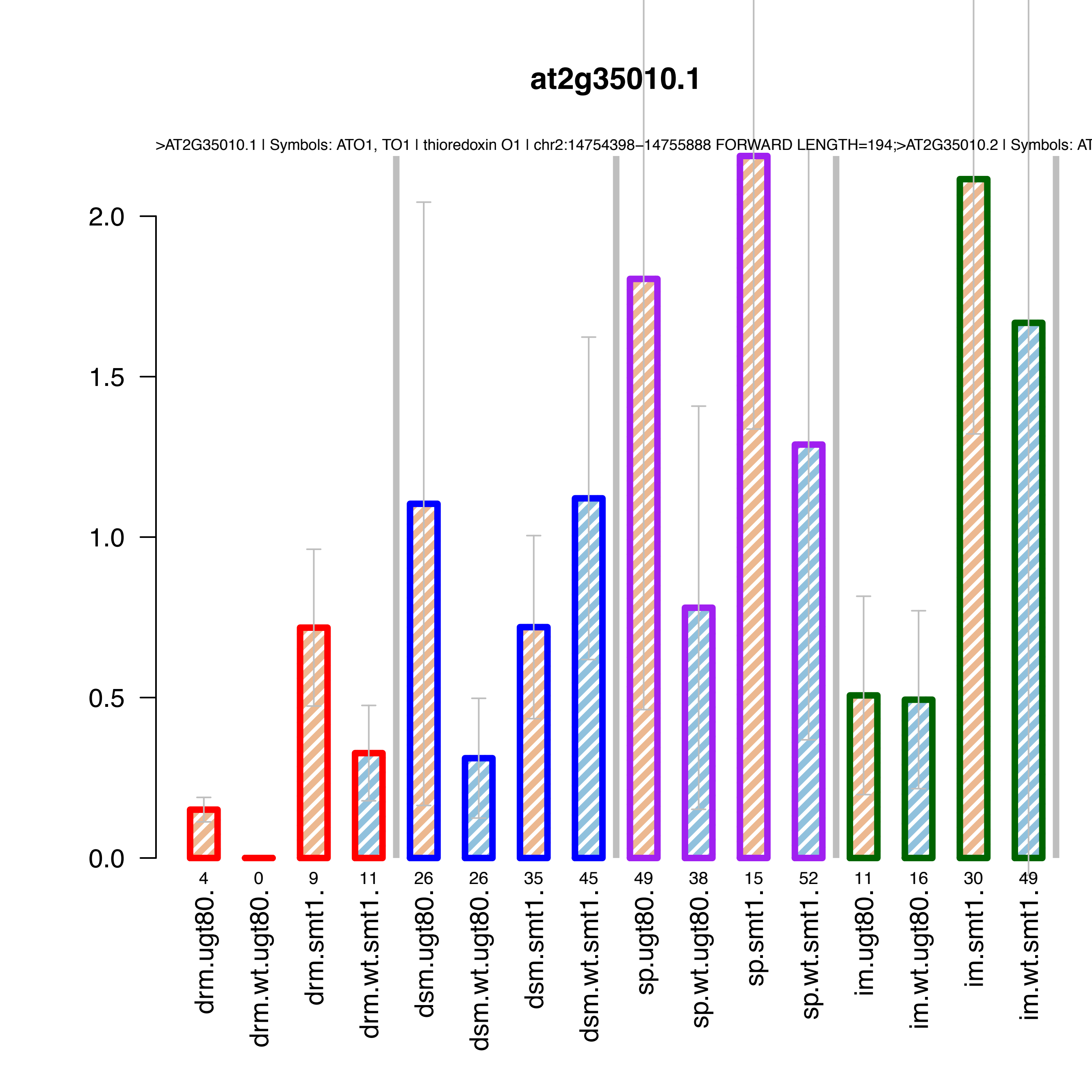

at2g35010.1
>AT2G35010.1 | Symbols: ATO1, TO1 | thioredoxin O1 | chr2:14754398-14755888 FORWARD LENGTH=194;>AT2G35010.2 | Symbols: AT
2.0
1.5
1.0
0.5
0.0
4
0
9
11
26
26
35
45
49
38
15
52
11
16
30
49
drm.ugt80.
drm.wt.ugt80.
drm.smt1.
drm.wt.smt1.
dsm.ugt80.
dsm.wt.ugt80.
dsm.smt1.
dsm.wt.smt1.
sp.ugt80.
sp.wt.ugt80.
sp.smt1.
sp.wt.smt1.
im.ugt80.
im.wt.ugt80.
im.smt1.
im.wt.smt1.

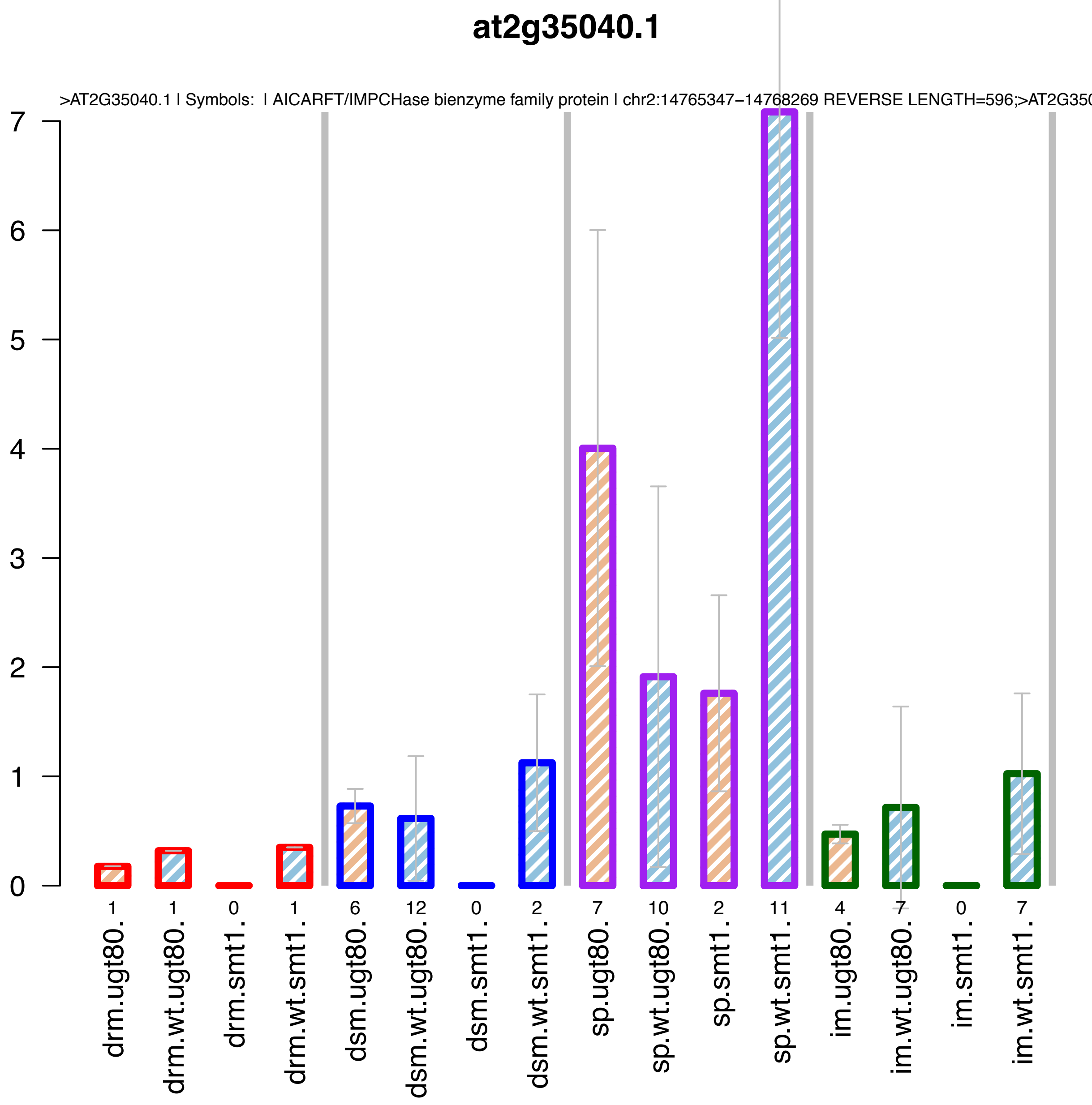

at2g35040.1
>AT2G35040.1 | Symbols: | AICARFT/IMPCHase bienzyme family protein | chr2:14765347-14768269 REVERSE LENGTH=596;>AT2G350
0
1
2
3
4
5
6
7
1
1
0
1
6
12
0
2
7
10
2
11
4
7
0
7
drm.ugt80.
drm.wt.ugt80.
drm.smt1.
drm.wt.smt1.
dsm.ugt80.
dsm.wt.ugt80.
dsm.smt1.
dsm.wt.smt1.
sp.ugt80.
sp.wt.ugt80.
sp.smt1.
sp.wt.smt1.
im.ugt80.
im.wt.ugt80.
im.smt1.
im.wt.smt1.

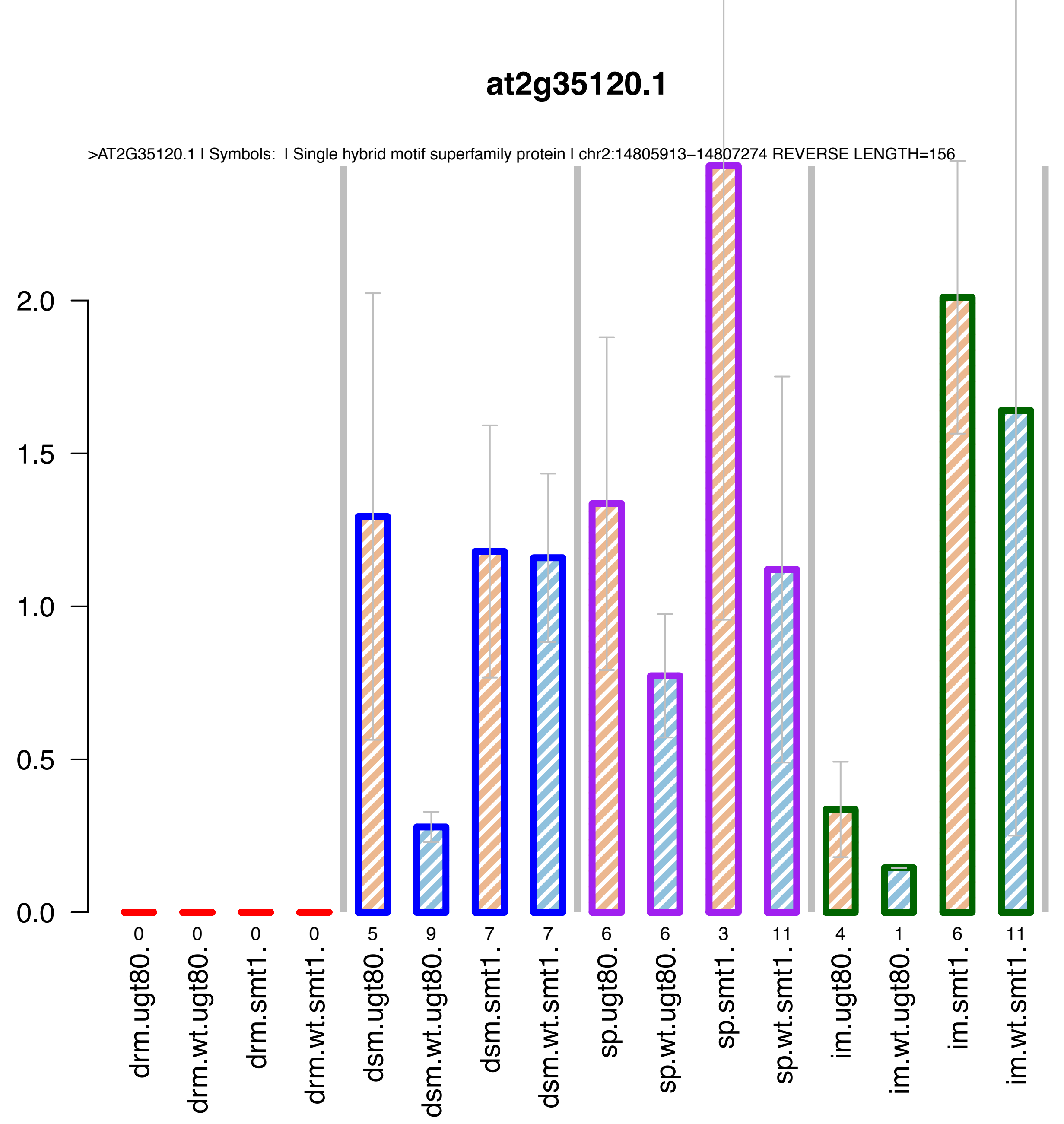
at2g35120.1
>AT2G35120.1 | Symbols: | Single hybrid motif superfamily protein | chr2:14805913−14807274 REVERSE LENGTH=156
0.0
0.5
1.0
1.5
2.0
0 0 0 0 5 9 7 7 6 6 3 11 4 1 6 11
drm.ugt80.
drm.wt.ugt80.
drm.smt1.
drm.wt.smt1.
dsm.ugt80.
dsm.wt.ugt80.
dsm.smt1.
dsm.wt.smt1.
sp.ugt80.
sp.wt.ugt80.
sp.smt1.
sp.wt.smt1.
im.ugt80.
im.wt.ugt80.
im.smt1.
im.wt.smt1.

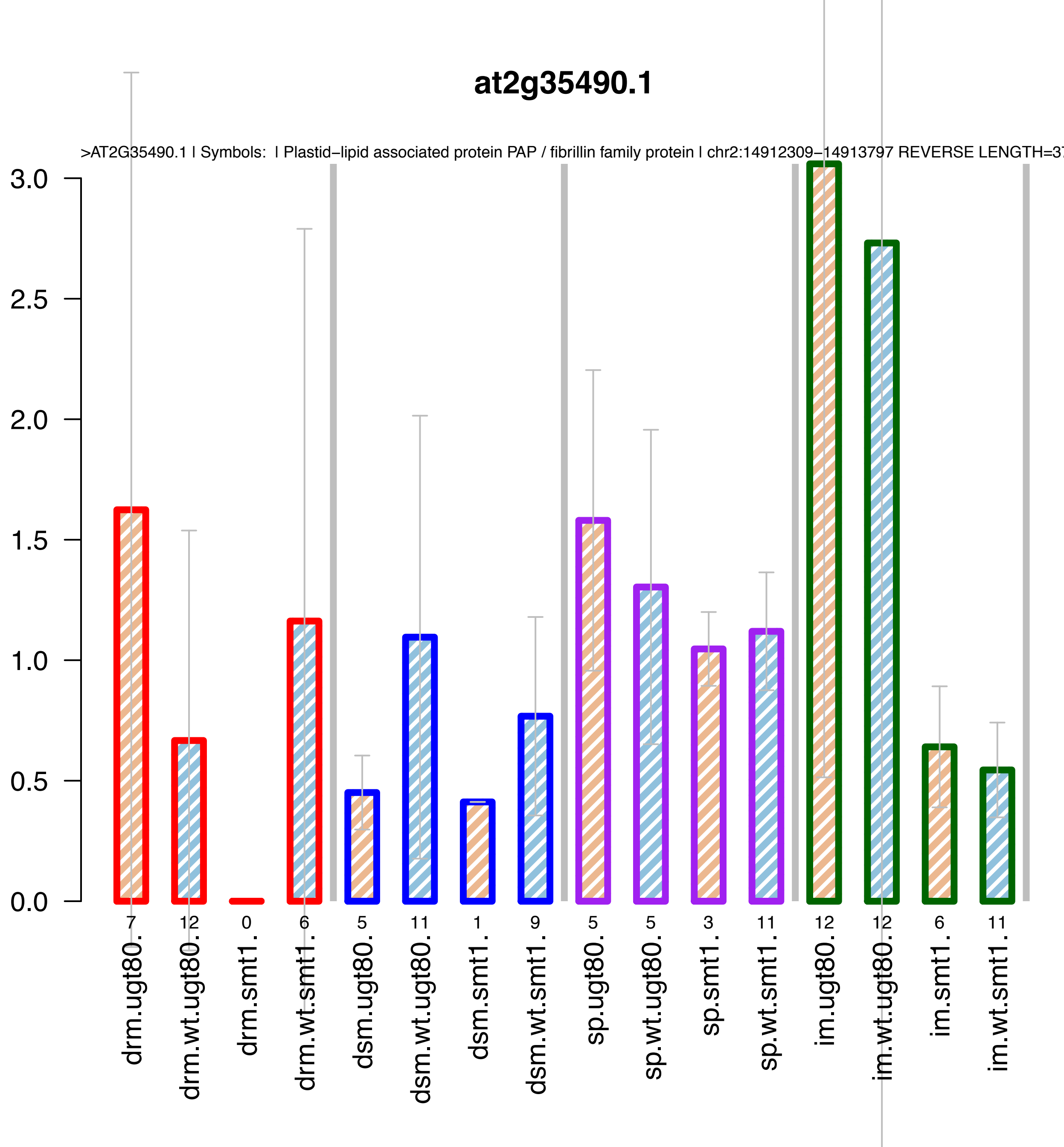

at2g35490.1
>AT2G35490.1 | Symbols: | Plastid−lipid associated protein PAP / fibrillin family protein | chr2:14912309−14913797 REVERSE LENGTH=3
3.0
2.5
2.0
1.5
1.0
0.5
0.0
7
12
0
6
5
11
1
9
5
5
3
11
12
12
6
11
drm.ugt80..
drm.wt.ugt80..
drm.smt1..
drm.wt.smt1..
dsm.ugt80..
dsm.wt.ugt80..
dsm.smt1..
dsm.wt.smt1..
sp.ugt80..
sp.wt.ugt80..
sp.smt1..
sp.wt.smt1..
im.ugt80..
im.wt.ugt80..
im.smt1..
im.wt.smt1..

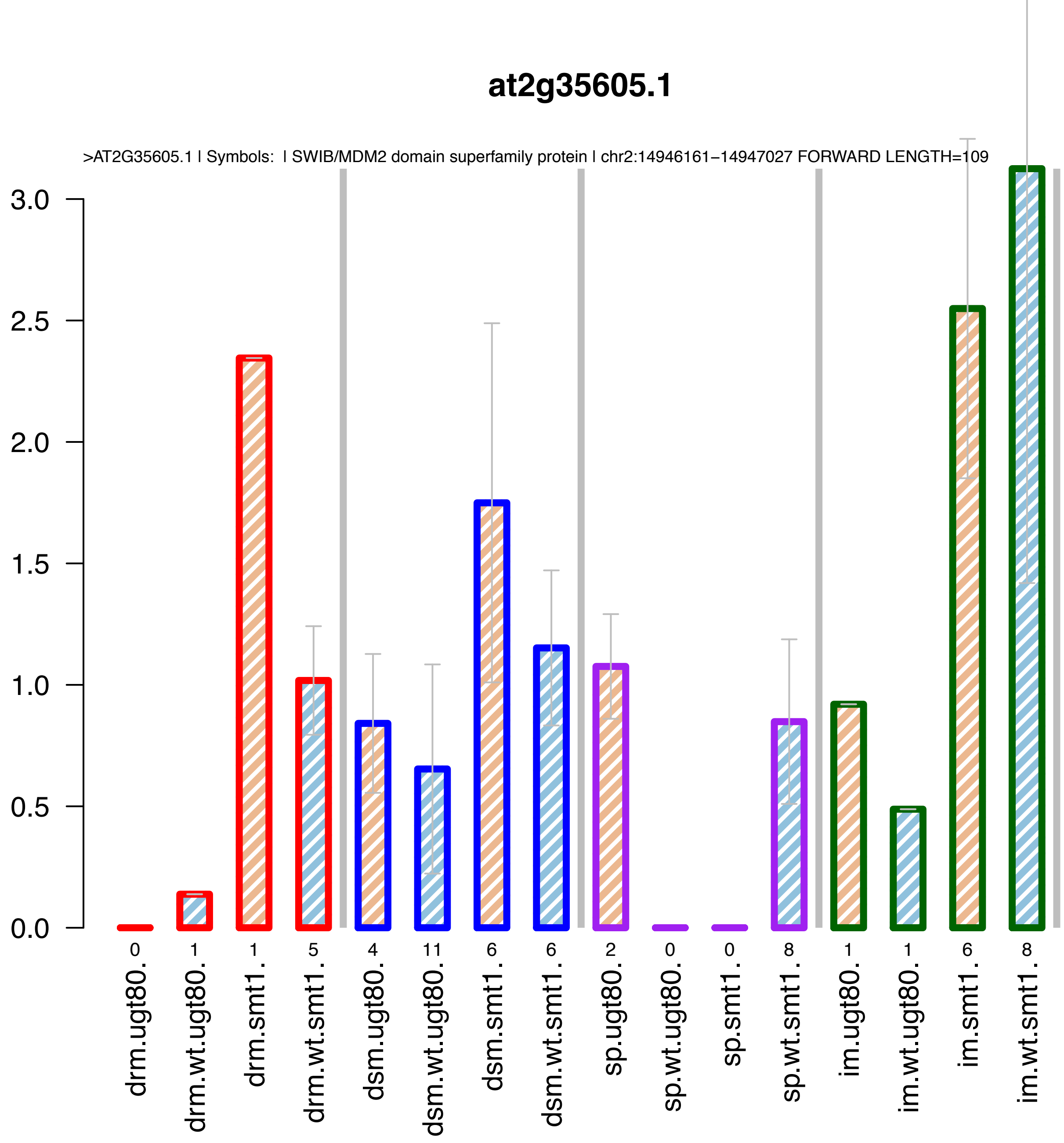

at2g35605.1
>AT2G35605.1 | Symbols: | SWIB/MDM2 domain superfamily protein | chr2:14946161−14947027 FORWARD LENGTH=109
3.0
2.5
2.0
1.5
1.0
0.5
0.0
0
1
1
5
4
11
6
6
2
0
0
8
1
1
6
8
drm.ugt80.
drm.wt.ugt80.
drm.smt1.
drm.wt.smt1.
dsm.ugt80.
dsm.wt.ugt80.
dsm.smt1.
dsm.wt.smt1.
sp.ugt80.
sp.wt.ugt80.
sp.smt1.
sp.wt.smt1.
im.ugt80.
im.wt.ugt80.
im.smt1.
im.wt.smt1.

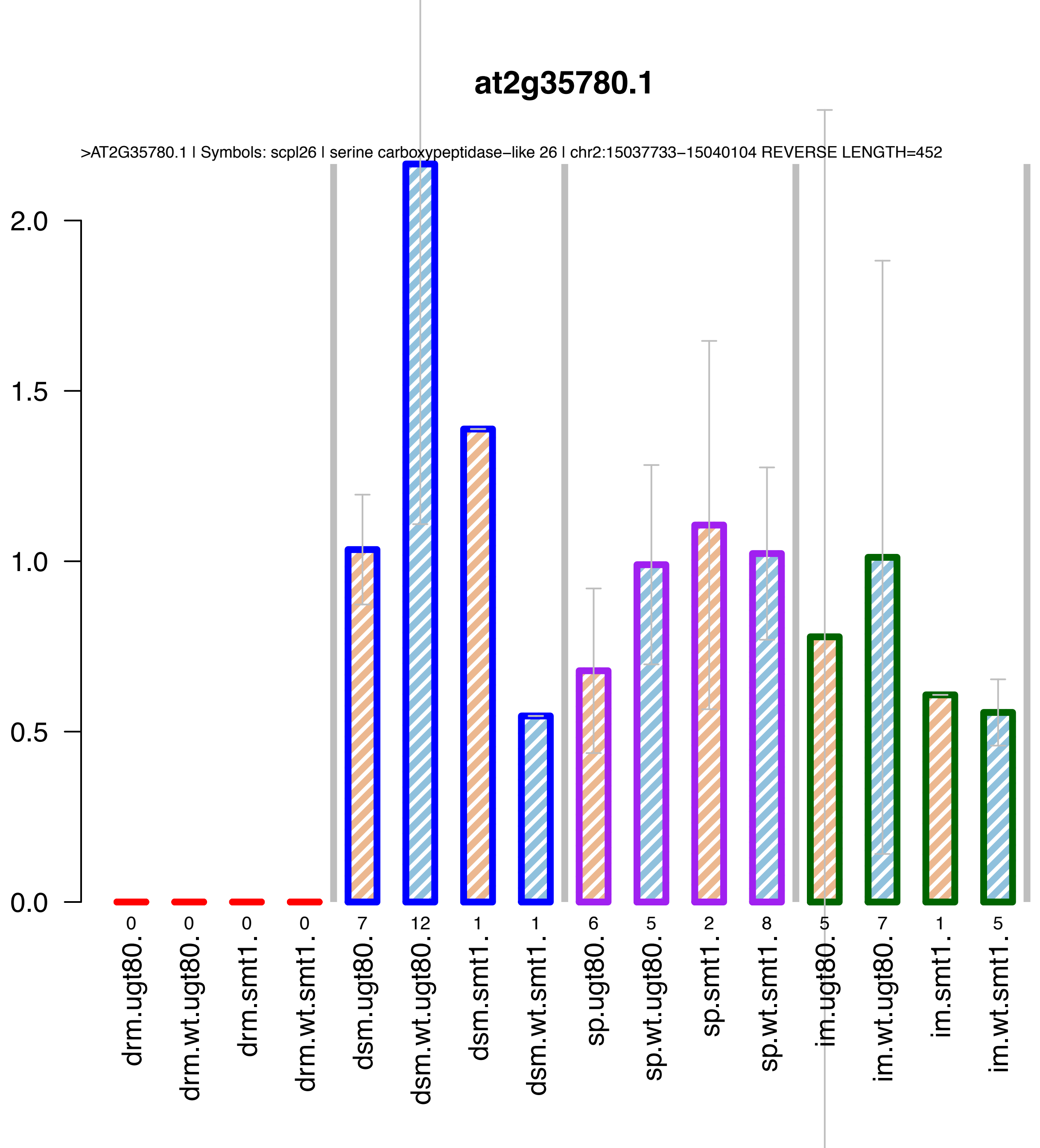

at2g35780.1
>AT2G35780.1 | Symbols: scpl26 | serine carboxypeptidase−like 26 | chr2:15037733−15040104 REVERSE LENGTH=452
0.0
0.5
1.0
1.5
2.0
0 0 0 0 7 12 1 1 6 5 2 8 5 7 1 5
drm.ugt80.
drm.wt.ugt80.
drm.smt1.
drm.wt.smt1.
dsm.ugt80.
dsm.wt.ugt80.
dsm.smt1.
dsm.wt.smt1.
sp.ugt80.
sp.wt.ugt80.
sp.smt1.
sp.wt.smt1.
im.ugt80.
im.wt.ugt80.
im.smt1.
im.wt.smt1.

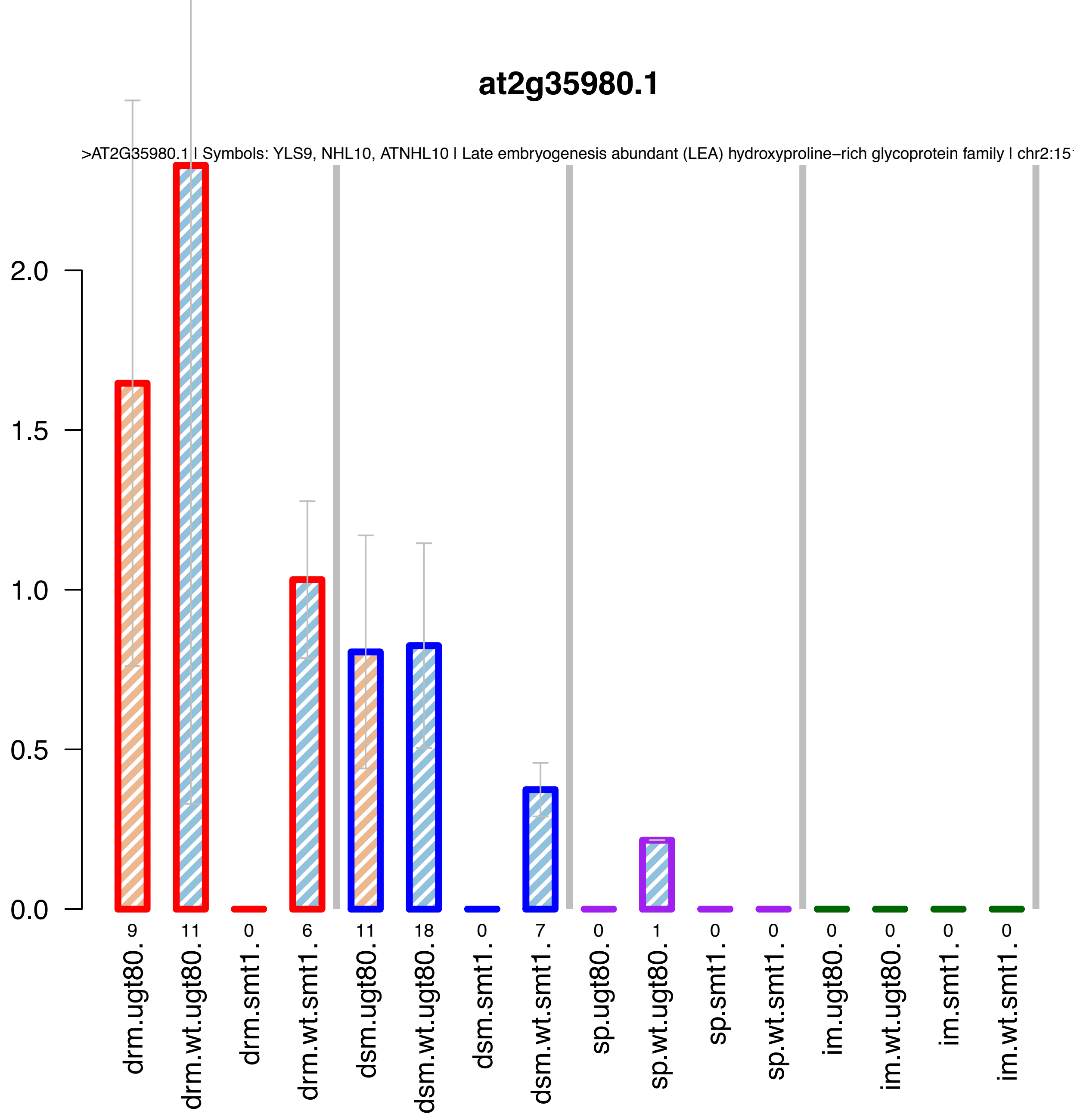

at2g35980.1
>AT2G35980.1 | Symbols: YLS9, NHL10, ATNHL10 | Late embryogenesis abundant (LEA) hydroxyproline–rich glycoprotein family | chr2:15
2.0
1.5
1.0
0.5
0.0
9
11
0
6
11
18
0
7
0
1
0
0
0
0
0
0
drm.ugt80.
drm.wt.ugt80.
drm.smt1.
drm.wt.smt1.
dsm.ugt80.
dsm.wt.ugt80.
dsm.smt1.
dsm.wt.smt1.
sp.ugt80.
sp.wt.ugt80.
sp.smt1.
sp.wt.smt1.
im.ugt80.
im.wt.ugt80.
im.smt1.
im.wt.smt1.

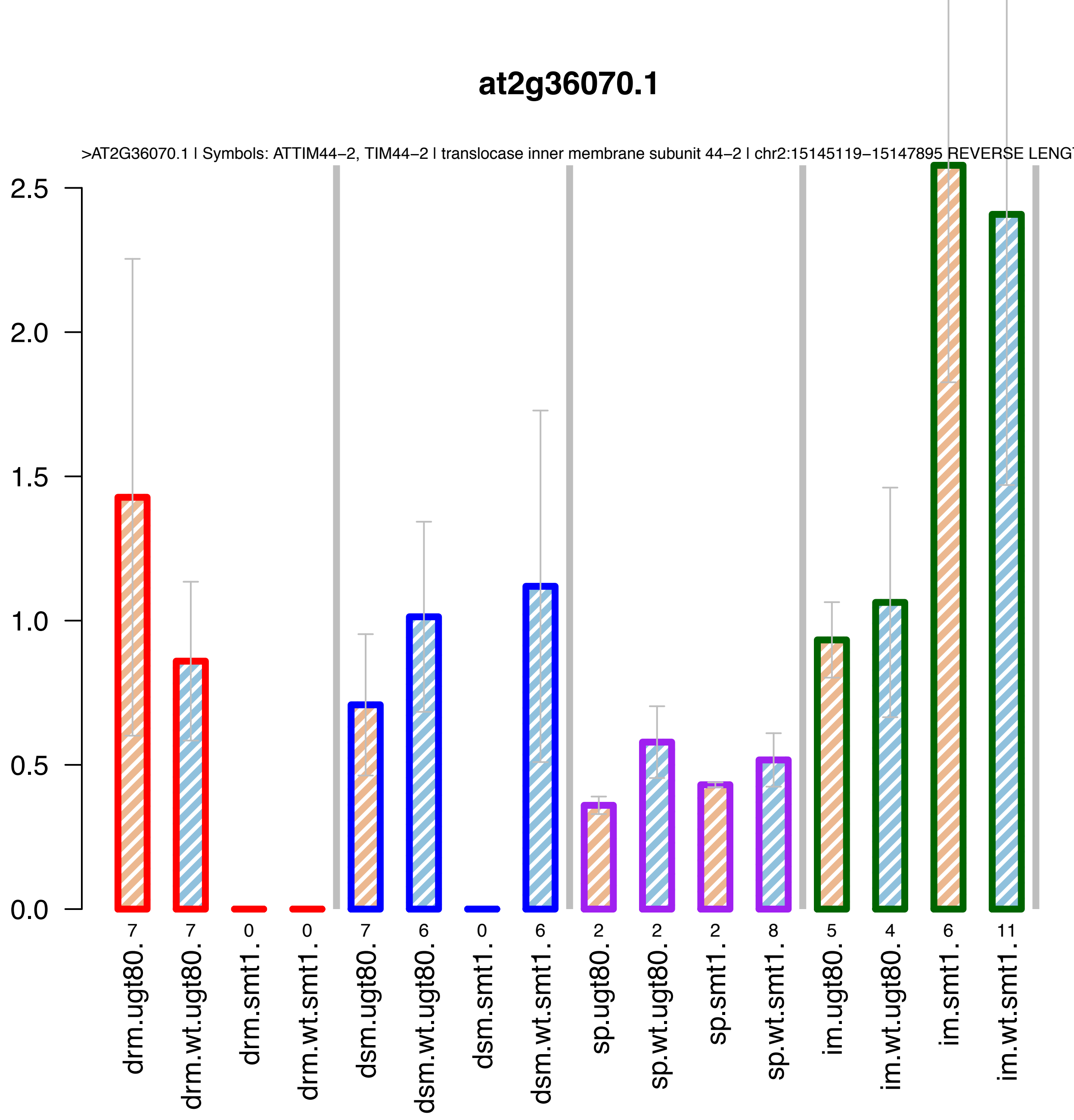

at2g36070.1
>AT2G36070.1 | Symbols: ATTIM44−2, TIM44−2 | translocase inner membrane subunit 44−2 | chr2:15145119−15147895 REVERSE LENG
2.5
2.0
1.5
1.0
0.5
0.0
7
7
0
0
7
6
0
6
2
2
2
8
5
4
6
11
drm.ugt80.
drm.wt.ugt80.
drm.smt1.
drm.wt.smt1.
dsm.ugt80.
dsm.wt.ugt80.
dsm.smt1.
dsm.wt.smt1.
sp.ugt80.
sp.wt.ugt80.
sp.smt1.
sp.wt.smt1.
im.ugt80.
im.wt.ugt80.
im.smt1.
im.wt.smt1.

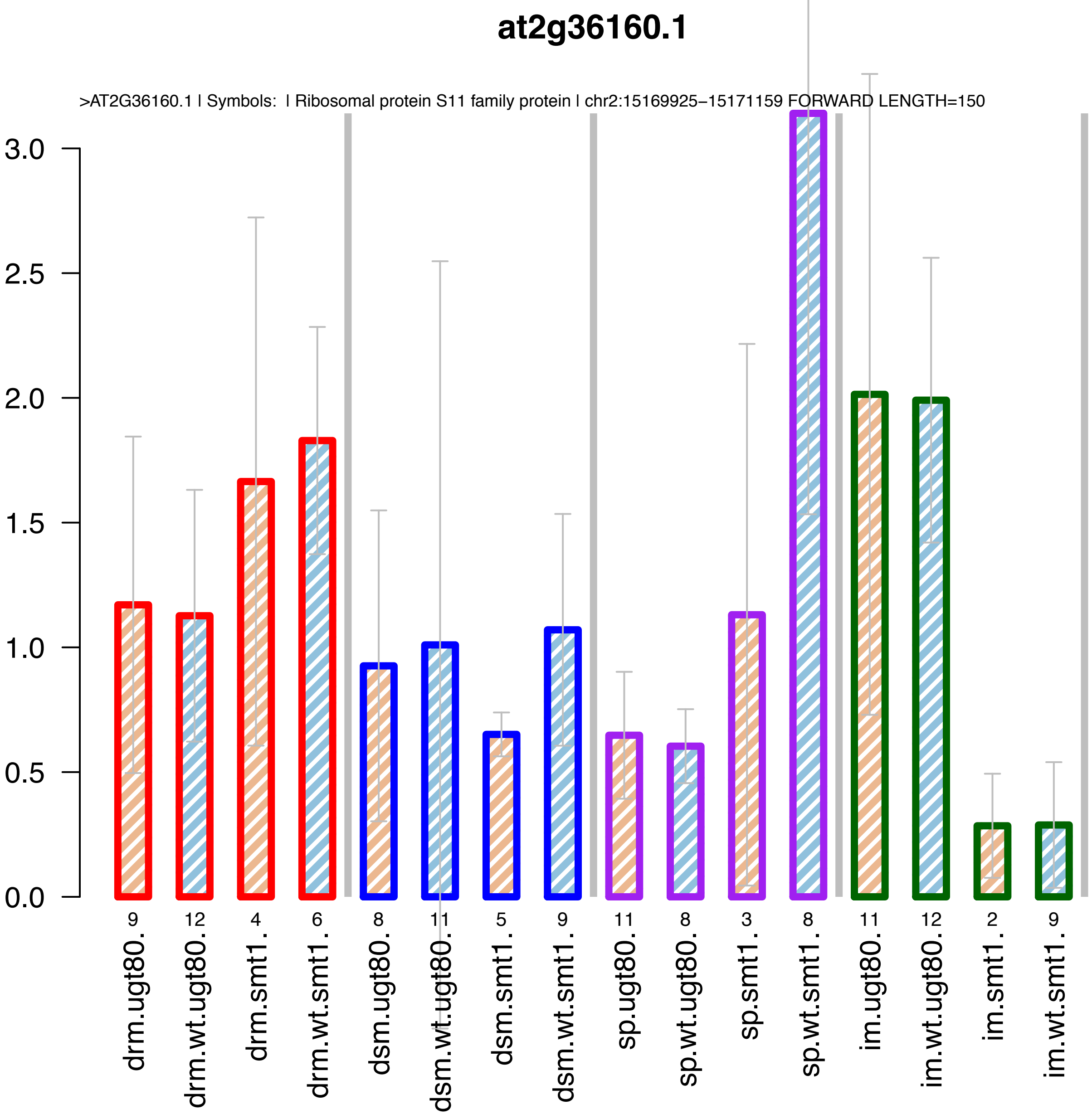

at2g36160.1
>AT2G36160.1 | Symbols: | Ribosomal protein S11 family protein | chr2:15169925-15171159 FORWARD LENGTH=150
3.0
2.5
2.0
1.5
1.0
0.5
0.0
9
12
4
6
8
11
5
9
11
8
3
8
11
12
2
9
drm.ugt80.
drm.wt.ugt80.
drm.smt1.
drm.wt.smt1.
dsm.ugt80.
dsm.wt.ugt80.
dsm.smt1.
dsm.wt.smt1.
sp.ugt80.
sp.wt.ugt80.
sp.smt1.
sp.wt.smt1.
im.ugt80.
im.wt.ugt80.
im.smt1.
im.wt.smt1.

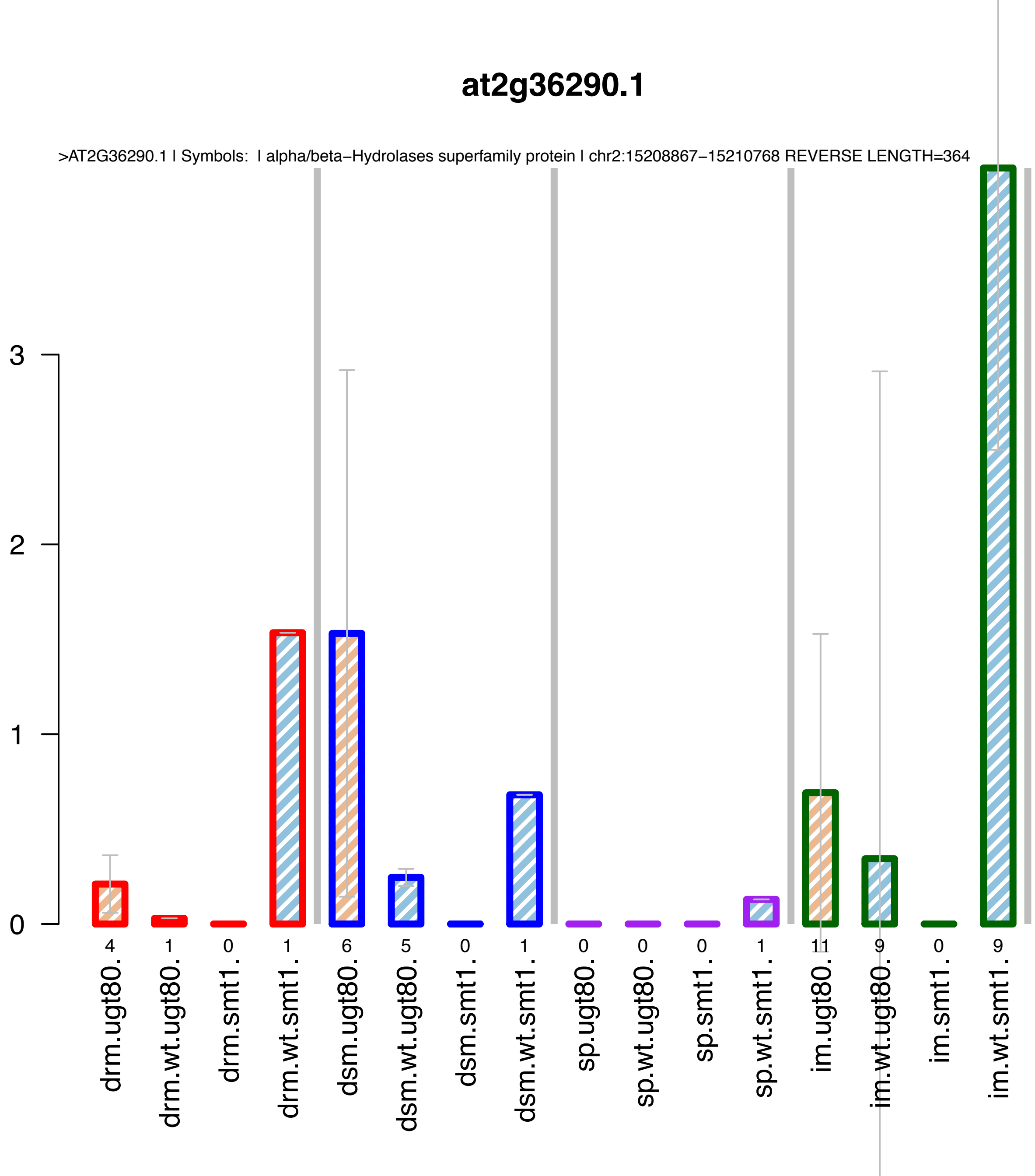

at2g36290.1
>AT2G36290.1 | Symbols: | alpha/beta−Hydrolases superfamily protein | chr2:15208867−15210768 REVERSE LENGTH=364
0
1
2
3
4
1
0
1
6
5
0
1
0
0
0
1
11
9
0
9
drm.ugt80.
drm.wt.ugt80.
drm.smt1.
drm.wt.smt1.
dsm.ugt80.
dsm.wt.ugt80.
dsm.smt1.
dsm.wt.smt1.
sp.ugt80.
sp.wt.ugt80.
sp.smt1.
sp.wt.smt1.
im.ugt80.
im.wt.ugt80.
im.smt1.
im.wt.smt1.

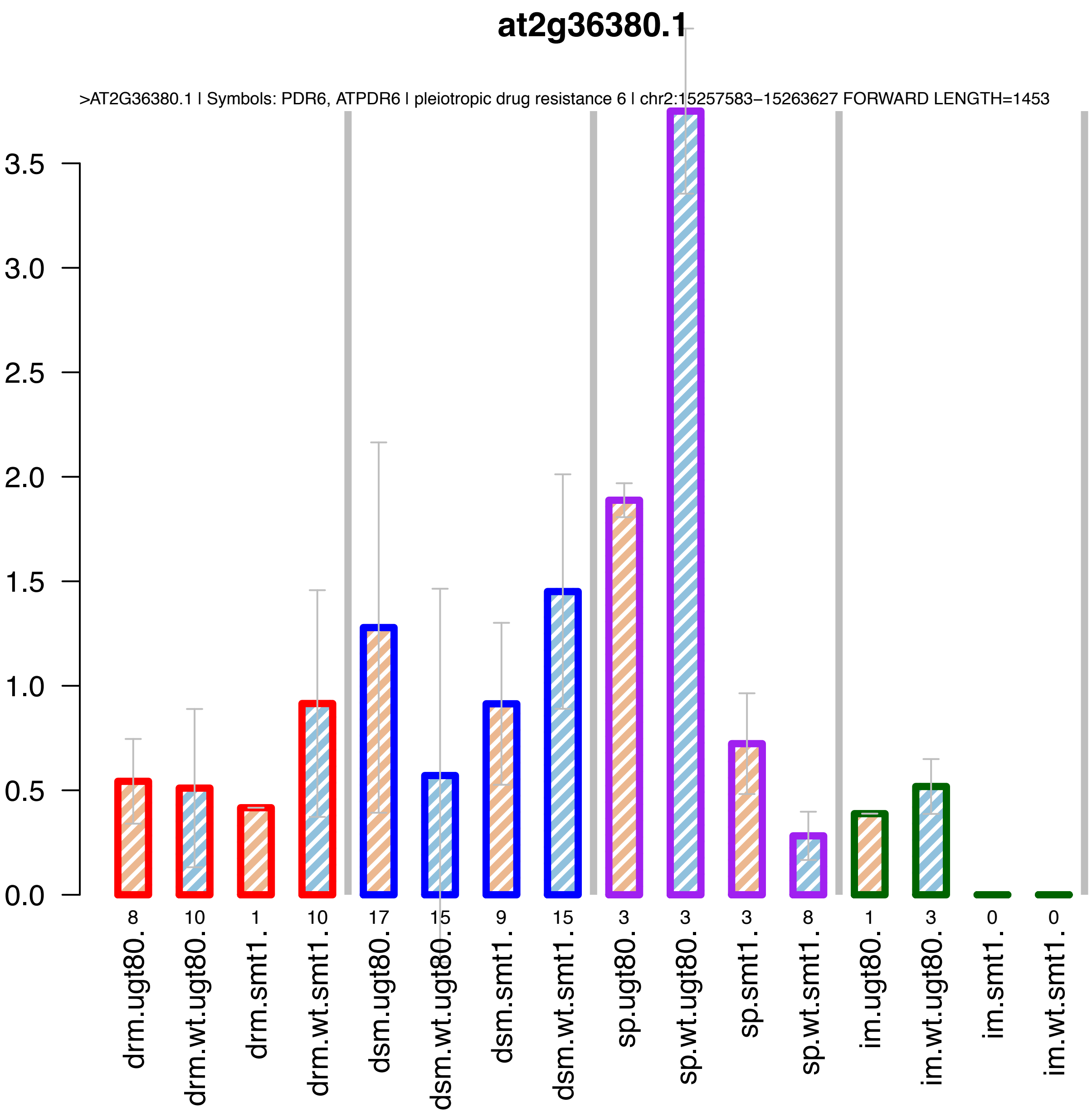
at2g36380.1
>AT2G36380.1 | Symbols: PDR6, ATPDR6 | pleiotropic drug resistance 6 | chr2:15257583−15263627 FORWARD LENGTH=1453
3.5
3.0
2.5
2.0
1.5
1.0
0.5
0.0
8
10
1
10
17
15
9
15
3
3
3
8
1
3
0
0
drm.ugt80..
drm.wt.ugt80..
drm.smt1..
drm.wt.smt1..
dsm.ugt80..
dsm.wt.ugt80..
dsm.smt1..
dsm.wt.smt1..
sp.ugt80..
sp.wt.ugt80..
sp.smt1..
sp.wt.smt1..
im.ugt80..
im.wt.ugt80..
im.smt1..
im.wt.smt1..

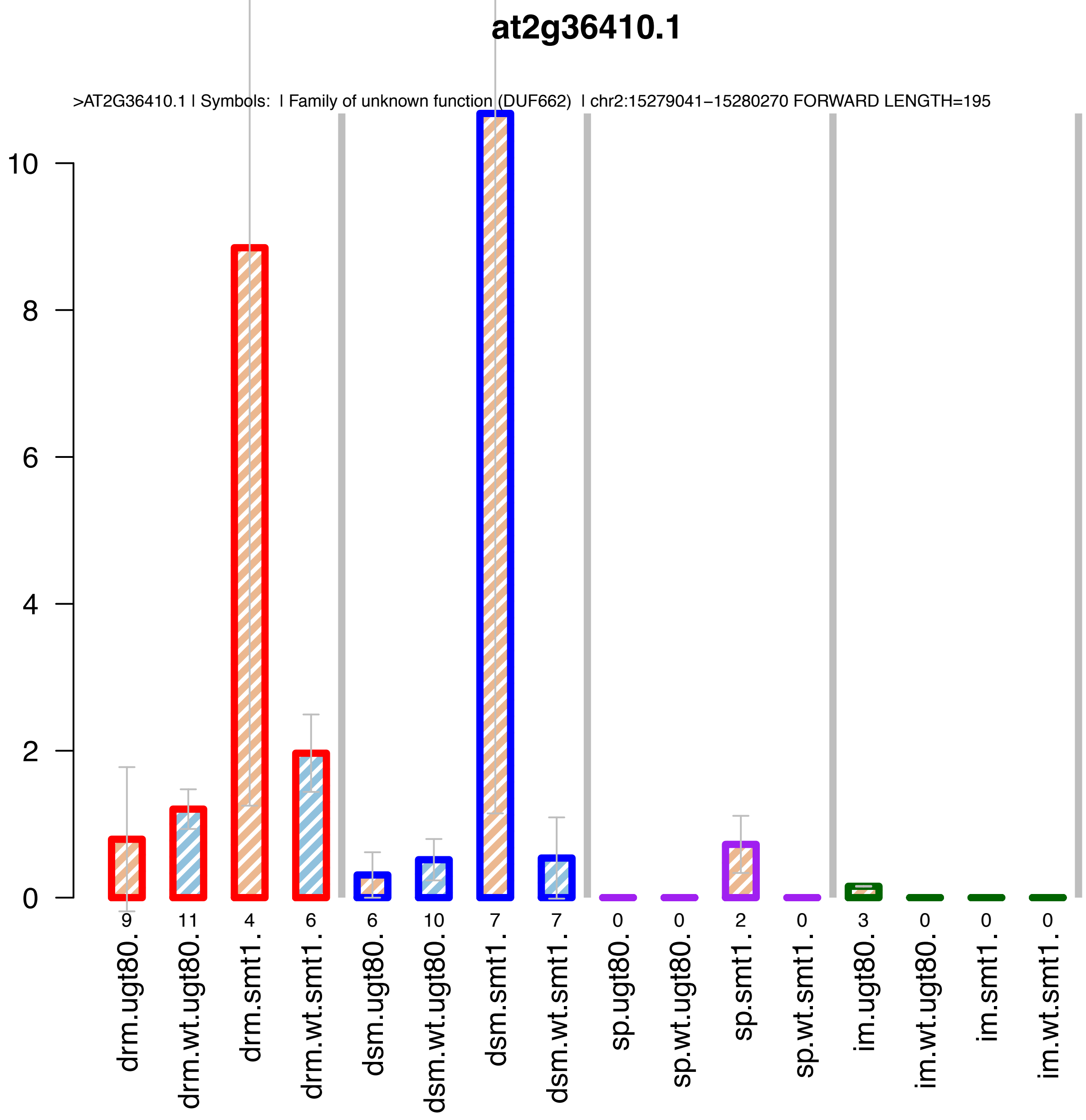

at2g36410.1
>AT2G36410.1 | Symbols: | Family of unknown function (DUF662) | chr2:15279041-15280270 FORWARD LENGTH=195
0
2
4
6
8
10
9
11
4
6
6
10
7
7
0
0
2
0
3
0
0
0
drm.ugt80.
drm.wt.ugt80.
drm.smt1.
drm.wt.smt1.
dsm.ugt80.
dsm.wt.ugt80.
dsm.smt1.
dsm.wt.smt1.
sp.ugt80.
sp.wt.ugt80.
sp.smt1.
sp.wt.smt1.
im.ugt80.
im.wt.ugt80.
im.smt1.
im.wt.smt1.

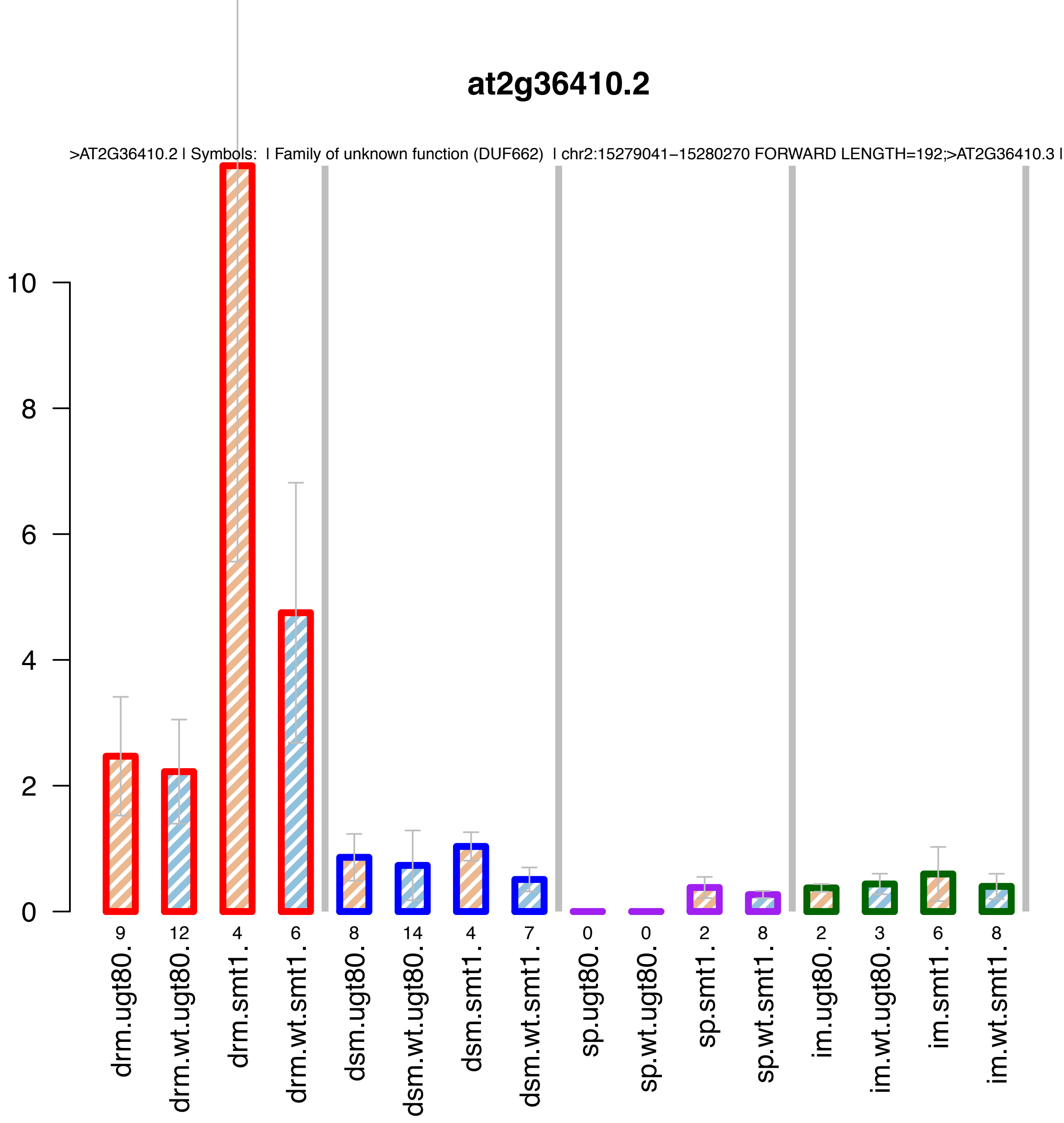

at2g36410.2
>AT2G36410.2 | Symbols: | Family of unknown function (DUF662) | chr2:15279041−15280270 FORWARD LENGTH=192;>AT2G36410.3 |
0
2
4
6
8
10
9
12
4
6
8
14
4
7
0
0
2
8
2
3
6
8
drm.ugt80.
drm.wt.ugt80.
drm.smt1.
drm.wt.smt1.
dsm.ugt80.
dsm.wt.ugt80.
dsm.smt1.
dsm.wt.smt1.
sp.ugt80.
sp.wt.ugt80.
sp.smt1.
sp.wt.smt1.
im.ugt80.
im.wt.ugt80.
im.smt1.
im.wt.smt1.

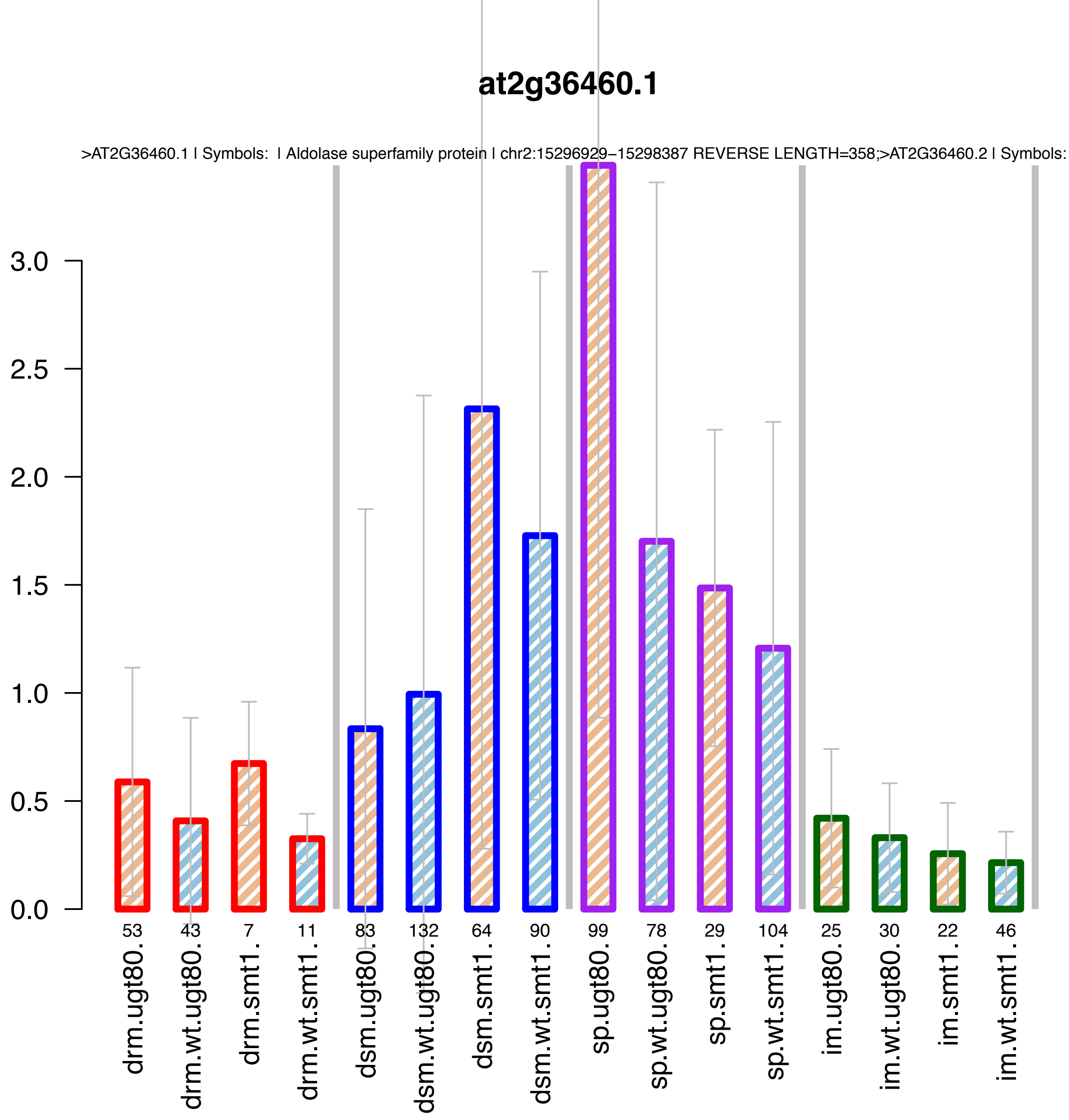
at2g36460.1
>AT2G36460.1 | Symbols: | Aldolase superfamily protein | chr2:15296929-15298387 REVERSE LENGTH=358;>AT2G36460.2 | Symbols:
3.0
2.5
2.0
1.5
1.0
0.5
0.0
53
43
7
11
83
132
64
90
99
78
29
104
25
30
22
46
drm.ugt80..
drm.wt.ugt80..
drm.smt1..
drm.wt.smt1..
dsm.ugt80..
dsm.wt.ugt80..
dsm.smt1..
dsm.wt.smt1..
sp.ugt80..
sp.wt.ugt80..
sp.smt1..
sp.wt.smt1..
im.ugt80..
im.wt.ugt80..
im.smt1..
im.wt.smt1..

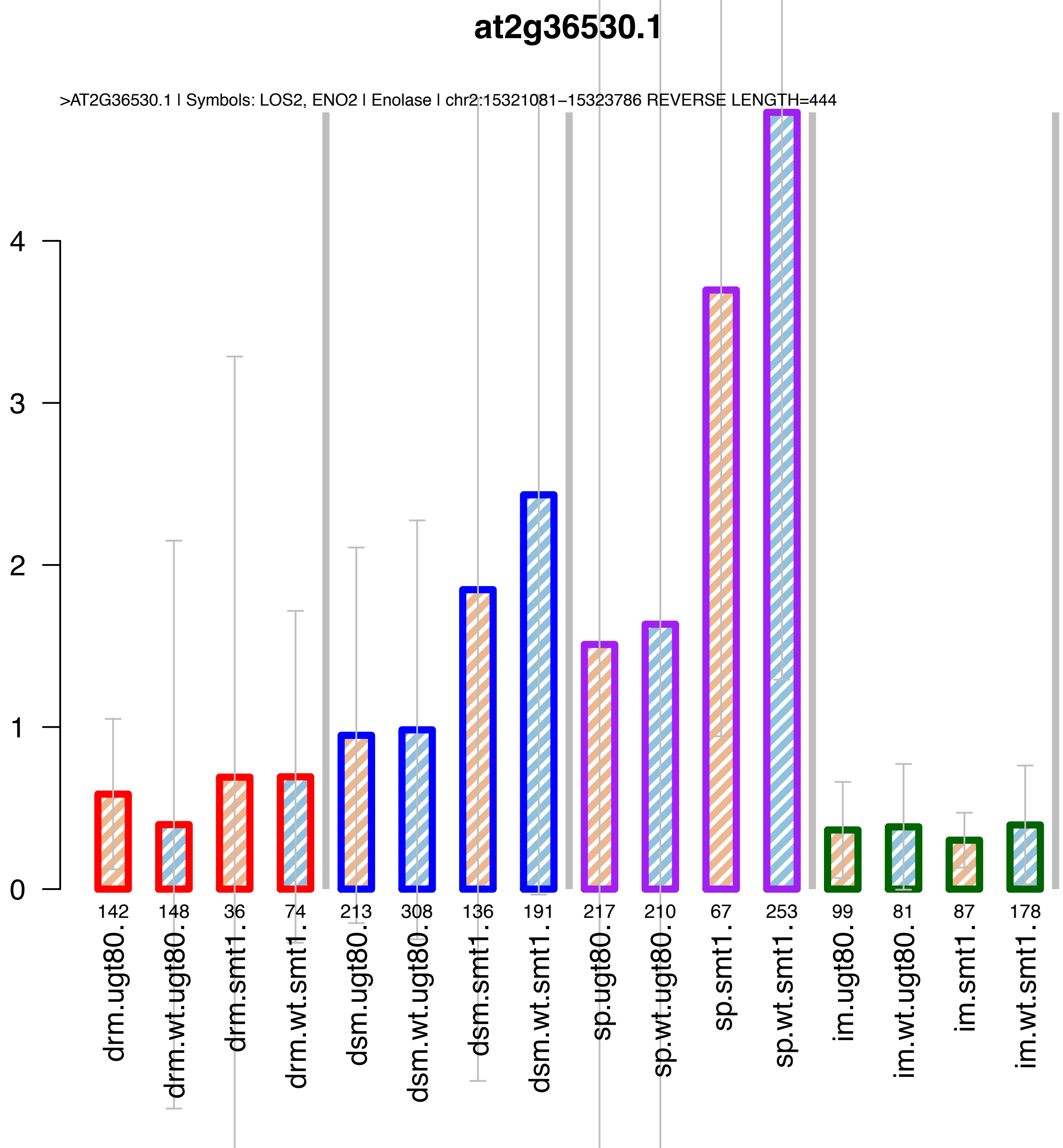

at2g36530.1
>AT2G36530.1 | Symbols: LOS2, ENO2 | Enolase | chr2:15321081−15323786 REVERSE LENGTH=444
0
1
2
3
4
142
148
36
74
213
308
136
191
217
210
67
253
99
81
87
178
drm.ugt80..
drm.wt.ugt80..
drm.smt1..
drm.wt.smt1..
dsm.ugt80..
dsm.wt.ugt80..
dsm.smt1..
dsm.wt.smt1..
sp.ugt80..
sp.wt.ugt80..
sp.smt1..
sp.wt.smt1..
im.ugt80..
im.wt.ugt80..
im.smt1..
im.wt.smt1..

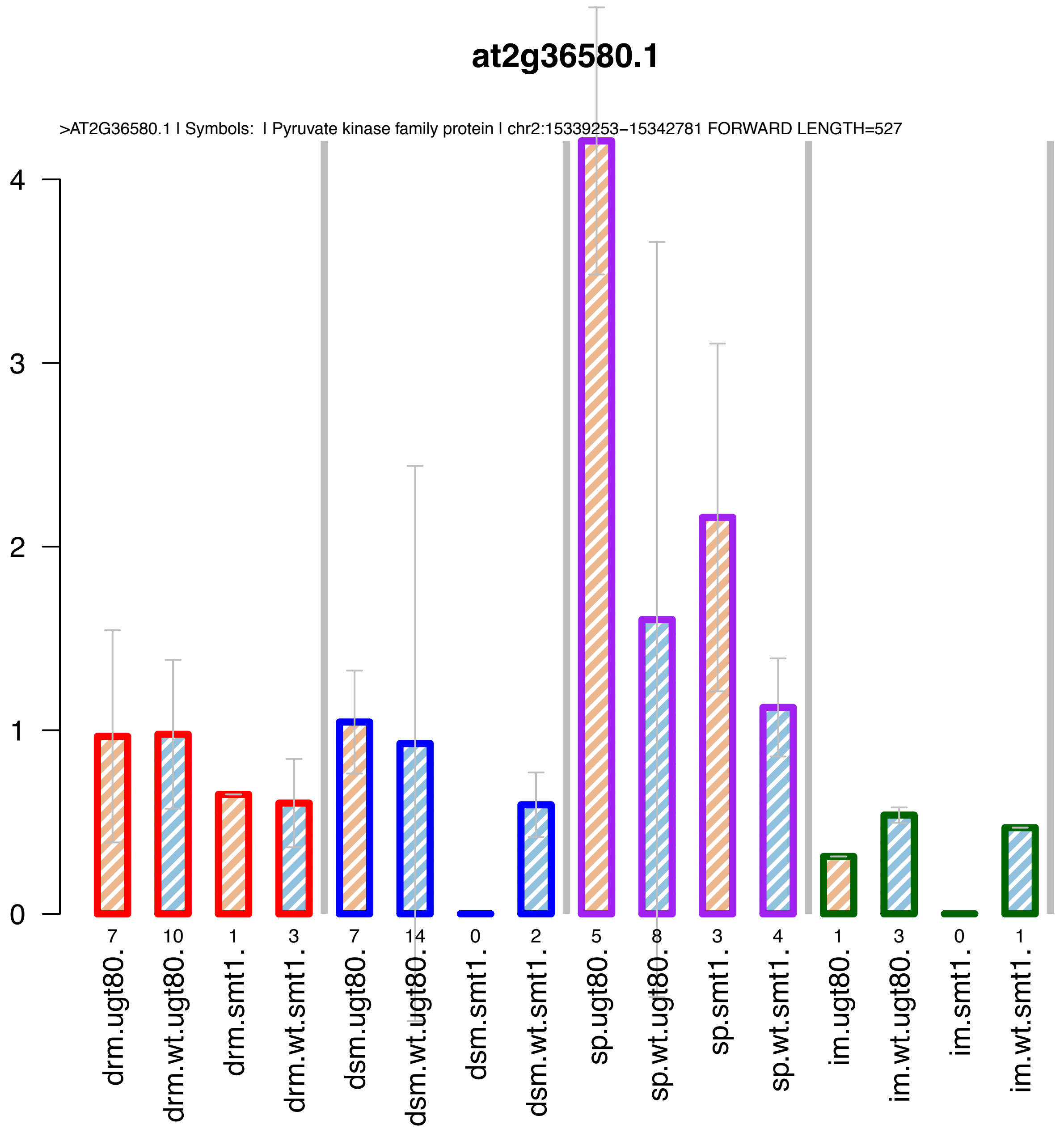
at2g36580.1
>AT2G36580.1 | Symbols: | Pyruvate kinase family protein | chr2:15339253-15342781 FORWARD LENGTH=527
0
1
2
3
4
7
10
1
3
7
14
0
2
5
8
3
4
1
3
0
1
drm.ugt80.
drm.wt.ugt80.
drm.smt1.
drm.wt.smt1.
dsm.ugt80.
dsm.wt.ugt80.
dsm.smt1.
dsm.wt.smt1.
sp.ugt80.
sp.wt.ugt80.
sp.smt1.
sp.wt.smt1.
im.ugt80.
im.wt.ugt80.
im.smt1.
im.wt.smt1.

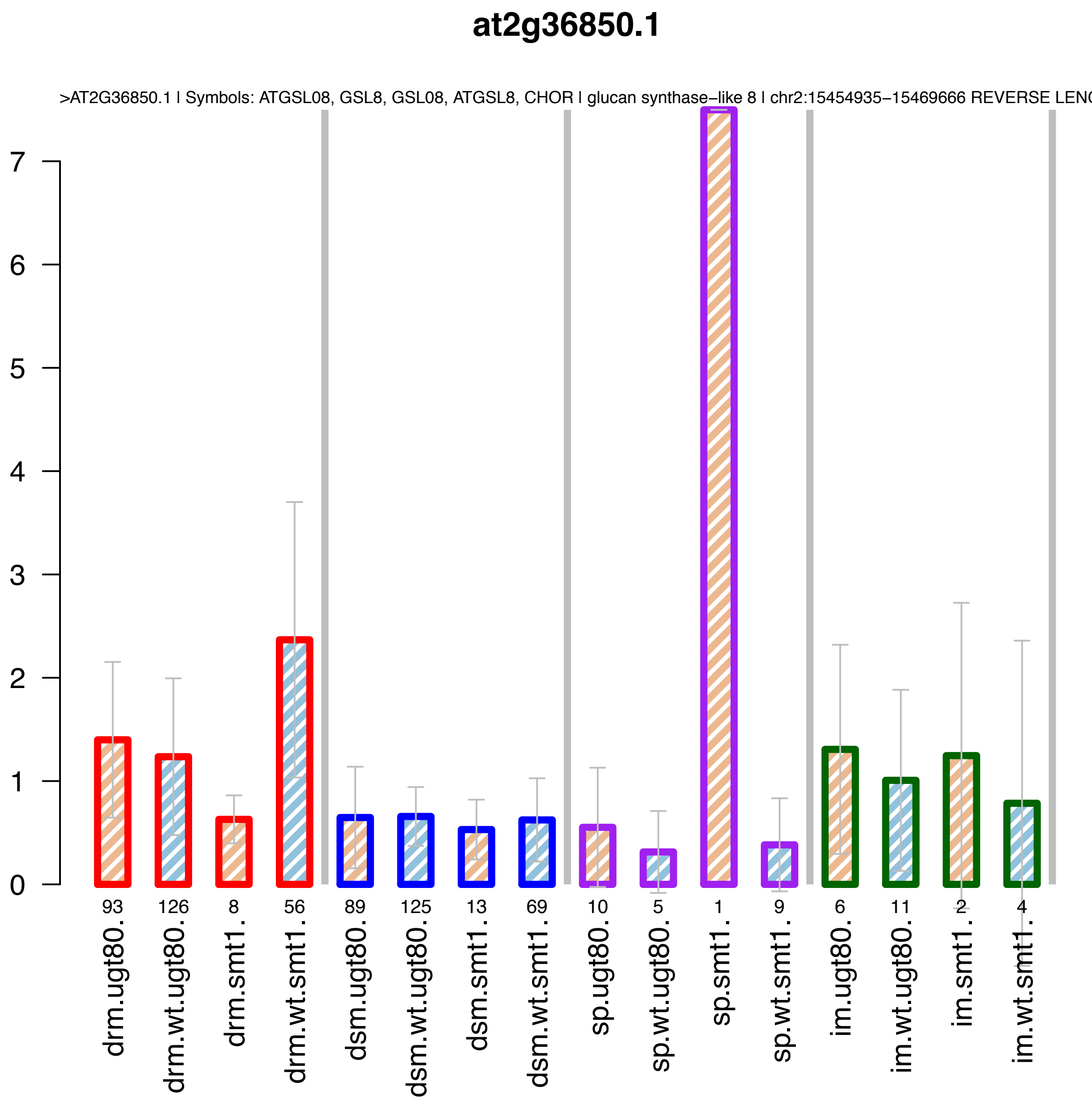

at2g36850.1
>AT2G36850.1 | Symbols: ATGSL08, GSL8, GSL08, ATGSL8, CHOR | glucan synthase−like 8 | chr2:15454935−15469666 REVERSE LENG
0
1
2
3
4
5
6
7
93
126
8
56
89
125
13
69
10
5
1
9
6
11
2
4
drm.ugt80.
drm.wt.ugt80.
drm.smt1.
drm.wt.smt1.
dsm.ugt80.
dsm.wt.ugt80.
dsm.smt1.
dsm.wt.smt1.
sp.ugt80.
sp.wt.ugt80.
sp.smt1.
sp.wt.smt1.
im.ugt80.
im.wt.ugt80.
im.smt1.
im.wt.smt1.

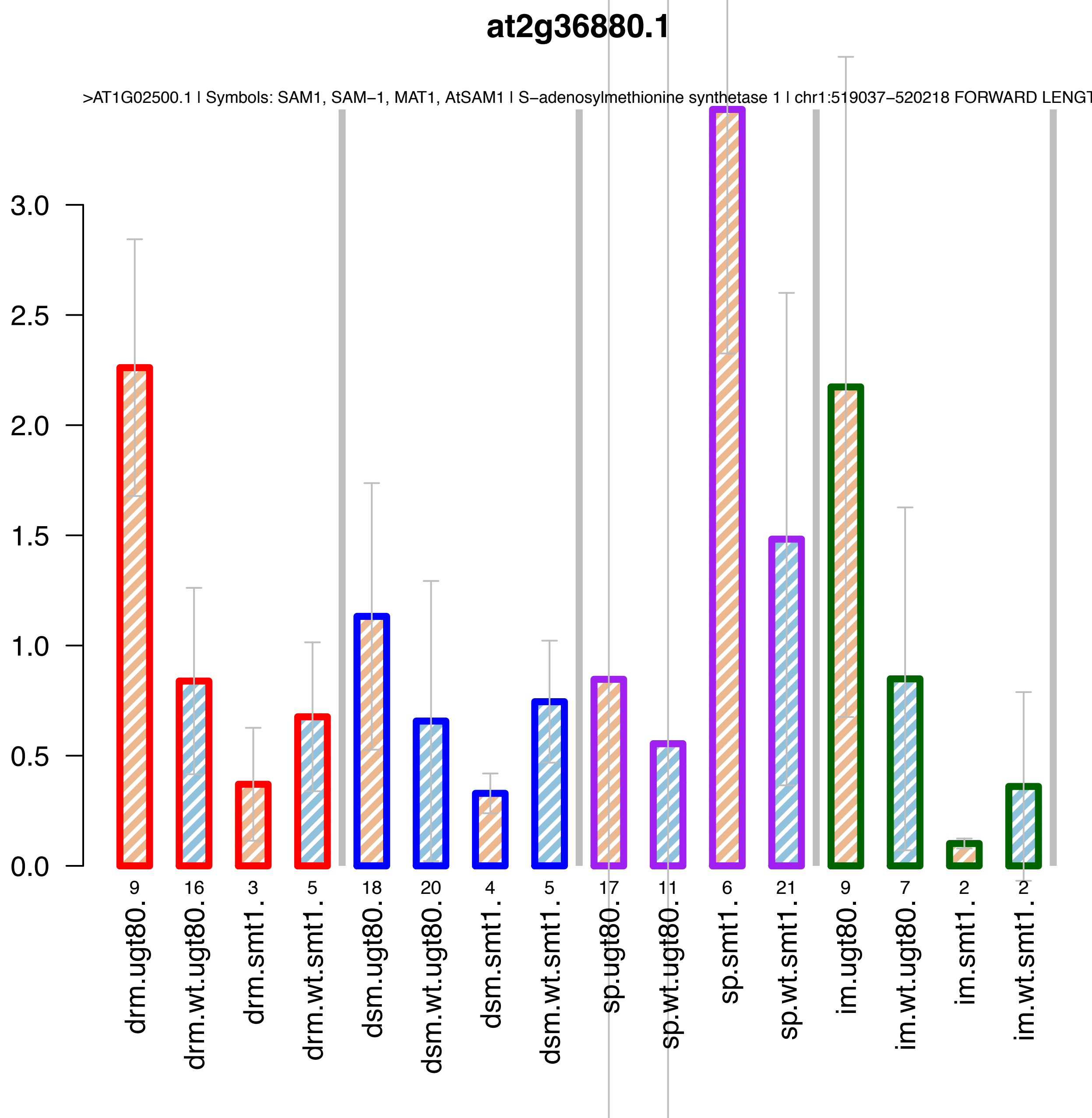

at2g36880.1
>AT1G02500.1 | Symbols: SAM1, SAM−1, MAT1, AtSAM1 | S−adenosylmethionine synthetase 1 | chr1:519037−520218 FORWARD LENGT
0.0
0.5
1.0
1.5
2.0
2.5
3.0
9
16
3
5
18
20
4
5
17
11
6
21
9
7
2
2
drm.ugt80.
drm.wt.ugt80.
drm.smt1.
drm.wt.smt1.
dsm.ugt80.
dsm.wt.ugt80.
dsm.smt1.
dsm.wt.smt1.
sp.ugt80.
sp.wt.ugt80.
sp.smt1.
sp.wt.smt1.
im.ugt80.
im.wt.ugt80.
im.smt1.
im.wt.smt1.

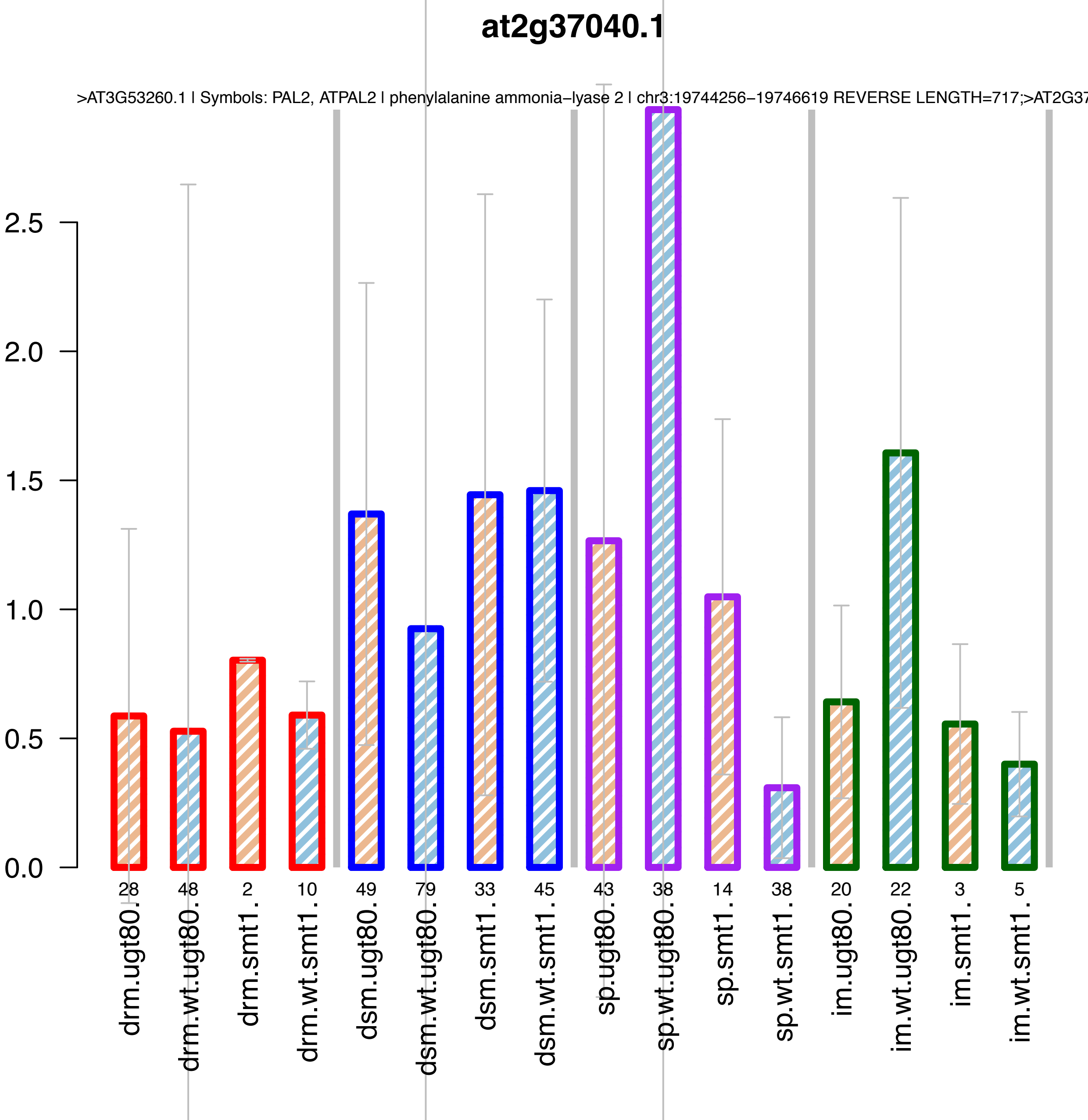
at2g37040.1
>AT3G53260.1 | Symbols: PAL2, ATPAL2 | phenylalanine ammonia−lyase 2 | chr3:19744256−19746619 REVERSE LENGTH=717;>AT2G37
2.5
2.0
1.5
1.0
0.5
0.0
28
48
2
10
49
79
33
45
43
38
14
38
20
22
3
5
drm.ugt80..
drm.wt.ugt80..
drm.smt1..
drm.wt.smt1..
dsm.ugt80..
dsm.wt.ugt80..
dsm.smt1..
dsm.wt.smt1..
sp.ugt80..
sp.wt.ugt80..
sp.smt1..
sp.wt.smt1..
im.ugt80..
im.wt.ugt80..
im.smt1..
im.wt.smt1..

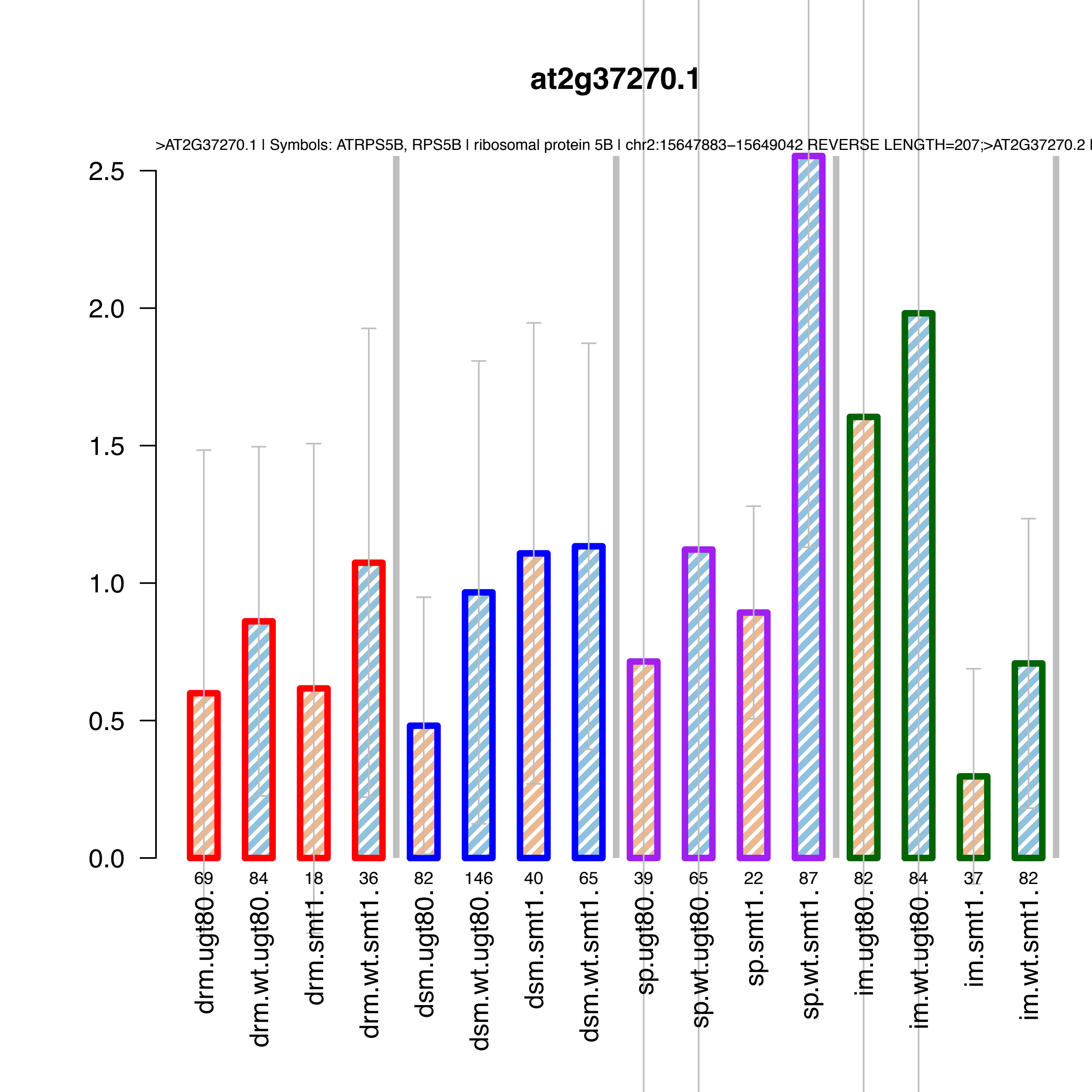

at2g37270.1
>AT2G37270.1 | Symbols: ATRPS5B, RPS5B | ribosomal protein 5B | chr2:15647883-15649042 REVERSE LENGTH=207;>AT2G37270.2 |
2.5
2.0
1.5
1.0
0.5
0.0
69
84
18
36
82
146
40
65
39
65
22
87
82
84
37
82
drm.ugt80..
drm.wt.ugt80..
drm.smt1..
drm.wt.smt1..
dsm.ugt80..
dsm.wt.ugt80..
dsm.smt1..
dsm.wt.smt1..
sp.ugt80..
sp.wt.ugt80..
sp.smt1..
sp.wt.smt1..
im.ugt80..
im.wt.ugt80..
im.smt1..
im.wt.smt1..

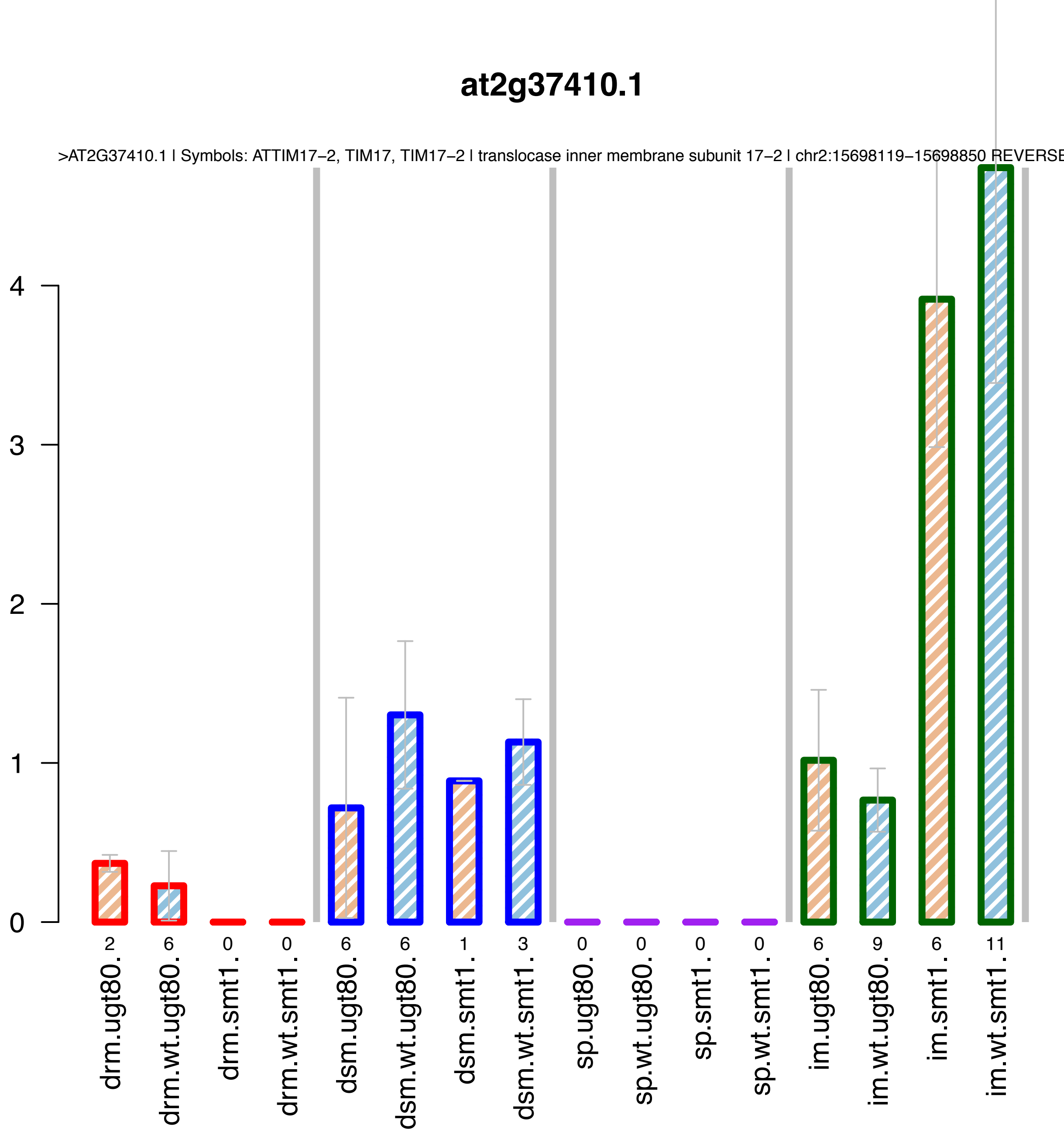

at2g37410.1
>AT2G37410.1 | Symbols: ATTIM17-2, TIM17, TIM17-2 | translocase inner membrane subunit 17-2 | chr2:15698119-15698850 REVERSE
0
1
2
3
4
2
6
0
0
6
6
1
3
0
0
0
0
6
9
6
11
drm.ugt80.
drm.wt.ugt80.
drm.smt1.
drm.wt.smt1.
dsm.ugt80.
dsm.wt.ugt80.
dsm.smt1.
dsm.wt.smt1.
sp.ugt80.
sp.wt.ugt80.
sp.smt1.
sp.wt.smt1.
im.ugt80.
im.wt.ugt80.
im.smt1.
im.wt.smt1.

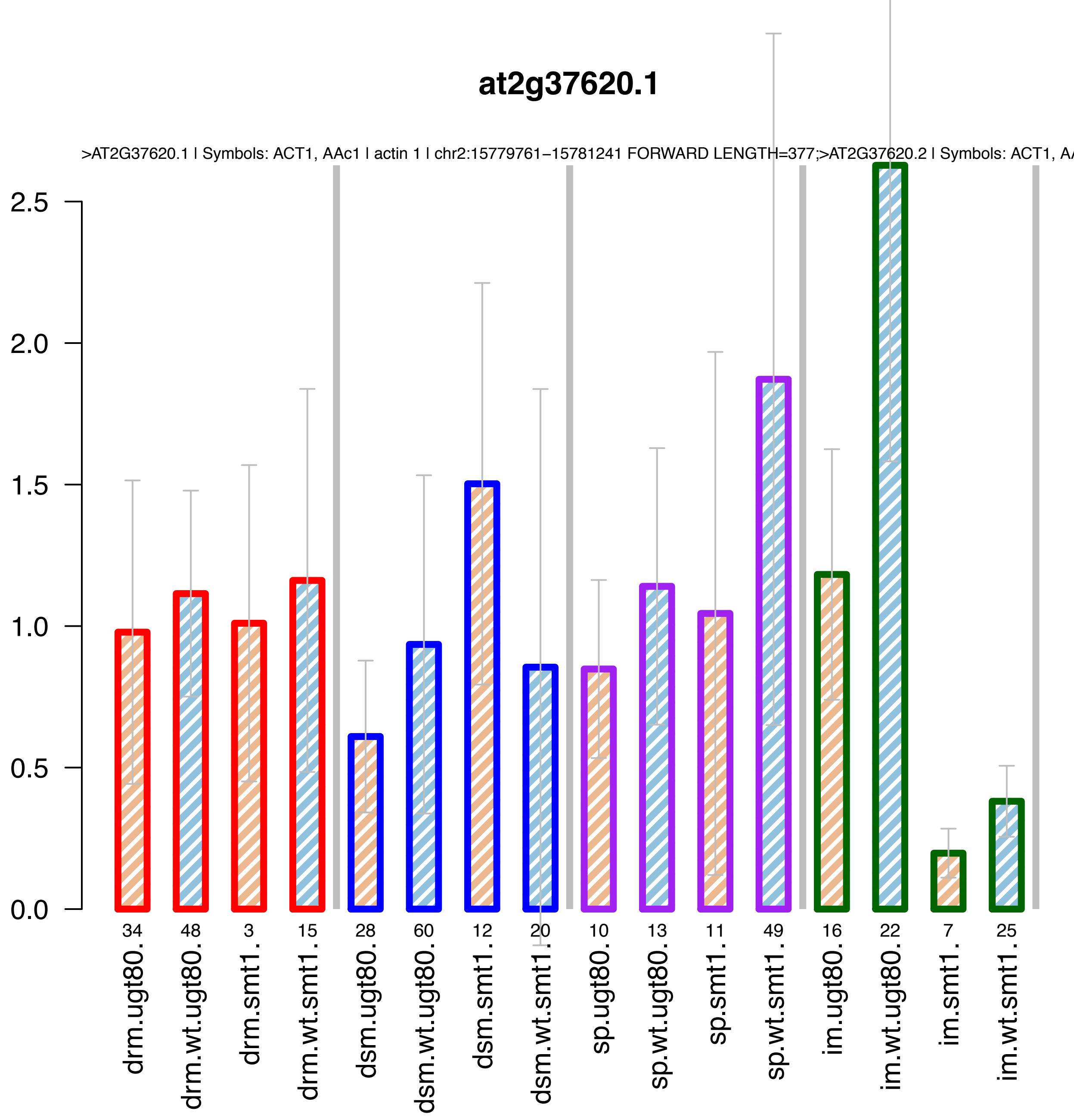

at2g37620.1
>AT2G37620.1 | Symbols: ACT1, AAc1 | actin 1 | chr2:15779761−15781241 FORWARD LENGTH=377;>AT2G37620.2 | Symbols: ACT1, A
2.5
2.0
1.5
1.0
0.5
0.0
34
48
3
15
28
60
12
20
10
13
11
49
16
22
7
25
drm.ugt80..
drm.wt.ugt80..
drm.smt1..
drm.wt.smt1..
dsm.ugt80..
dsm.wt.ugt80..
dsm.smt1..
dsm.wt.smt1..
sp.ugt80..
sp.wt.ugt80..
sp.smt1..
sp.wt.smt1..
im.ugt80..
im.wt.ugt80..
im.smt1..
im.wt.smt1..

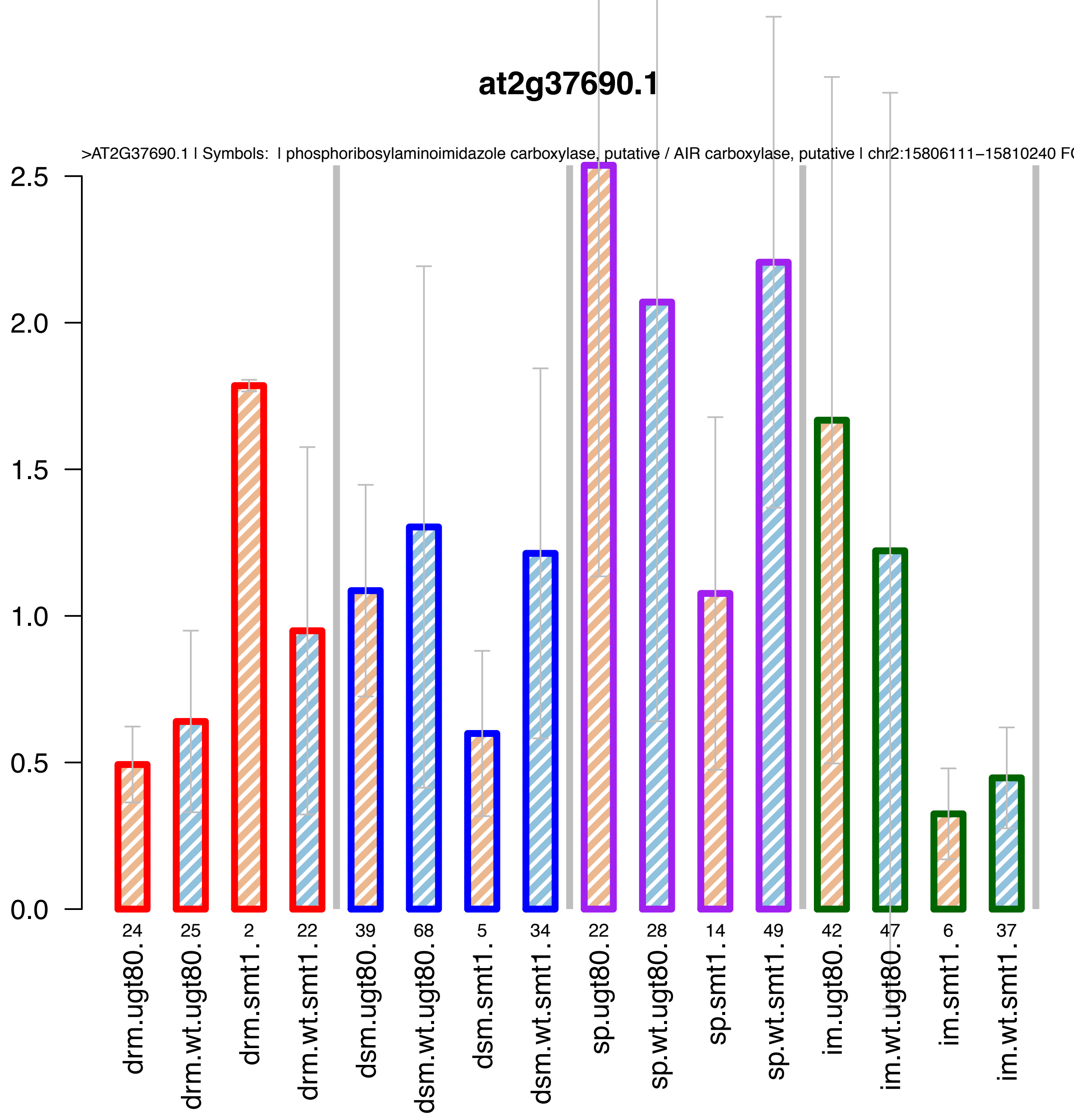

at2g37690.1
>AT2G37690.1 | Symbols: | phosphoribosylaminoimidazole carboxylase, putative / AIR carboxylase, putative | chr2:15806111-15810240 F
2.5
2.0
1.5
1.0
0.5
0.0
24
25
2
22
39
68
5
34
22
28
14
49
42
47
6
37
drm.ugt80..
drm.wt.ugt80..
drm.smt1..
drm.wt.smt1..
dsm.ugt80..
dsm.wt.ugt80..
dsm.smt1..
dsm.wt.smt1..
sp.ugt80..
sp.wt.ugt80..
sp.smt1..
sp.wt.smt1..
im.ugt80..
im.wt.ugt80..
im.smt1..
im.wt.smt1..

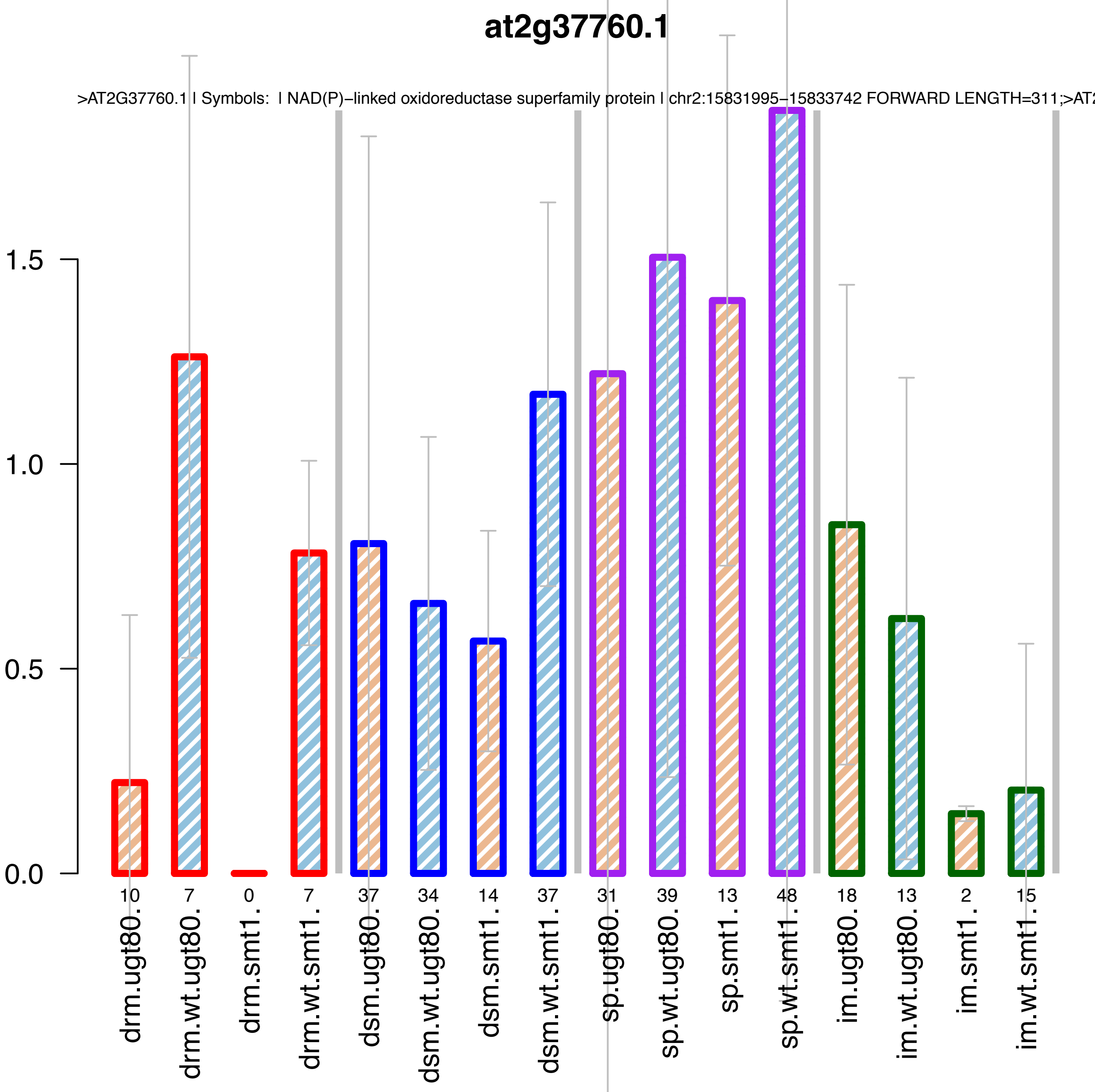

at2g37760.1
>AT2G37760.1 | Symbols: | NAD(P)−linked oxidoreductase superfamily protein | chr2:15831995−15833742 FORWARD LENGTH=311;>AT2
0.0
0.5
1.0
1.5
10
7
0
7
37
34
14
37
31
39
13
48
18
13
2
15
drm.ugt80.
drm.wt.ugt80.
drm.smt1.
drm.wt.smt1.
dsm.ugt80.
dsm.wt.ugt80.
dsm.smt1.
dsm.wt.smt1.
sp.ugt80.
sp.wt.ugt80.
sp.smt1.
sp.wt.smt1.
im.ugt80.
im.wt.ugt80.
im.smt1.
im.wt.smt1.

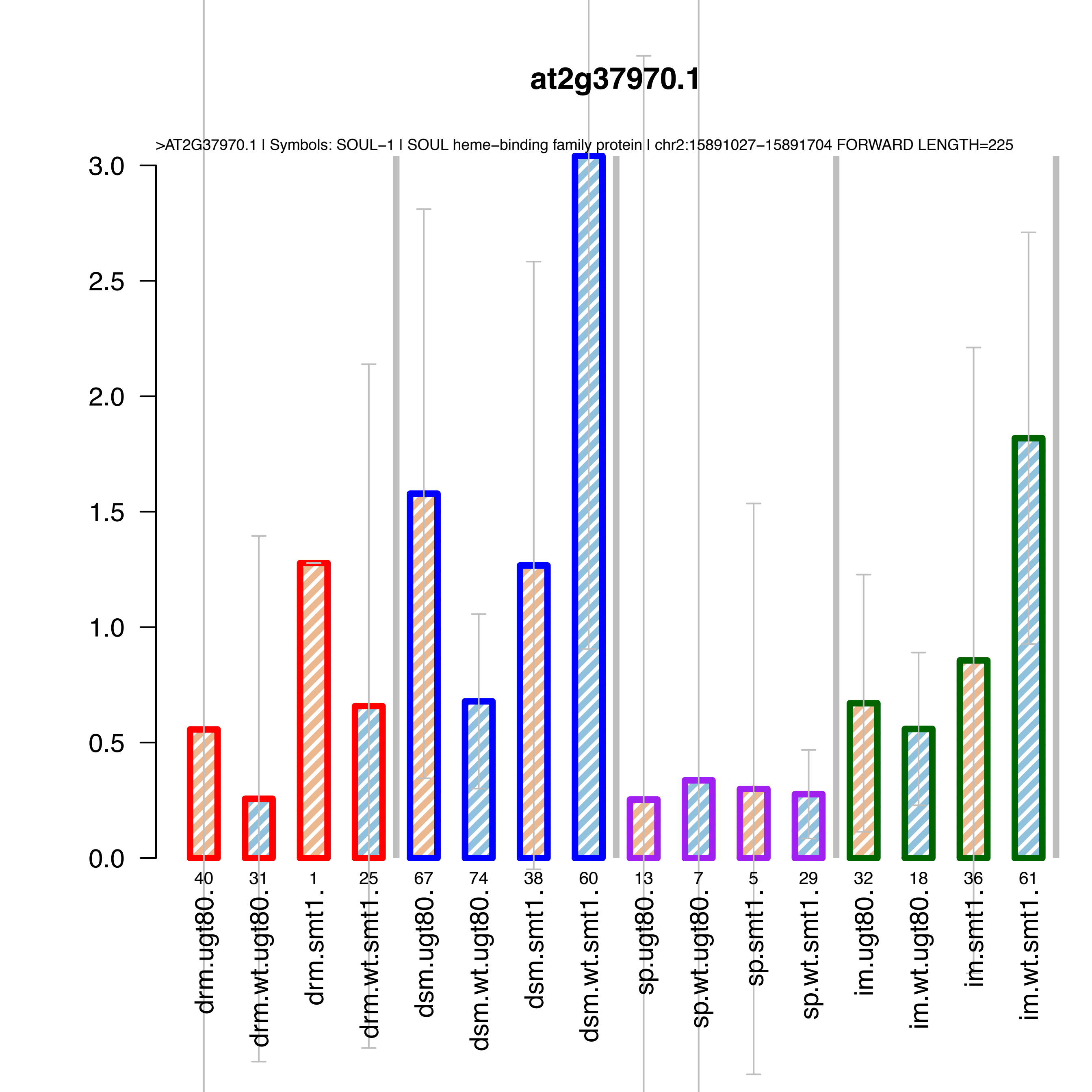
at2g37970.1
>AT2G37970.1 | Symbols: SOUL−1 | SOUL heme−binding family protein | chr2:15891027−15891704 FORWARD LENGTH=225
0.0
0.5
1.0
1.5
2.0
2.5
3.0
40
31
1
25
67
74
38
60
13
7
5
29
32
18
36
61
drm.ugt80..
drm.wt.ugt80..
drm.smt1..
drm.wt.smt1..
dsm.ugt80..
dsm.wt.ugt80..
dsm.smt1..
dsm.wt.smt1..
sp.ugt80..
sp.wt.ugt80..
sp.smt1..
sp.wt.smt1..
im.ugt80..
im.wt.ugt80..
im.smt1..
im.wt.smt1..

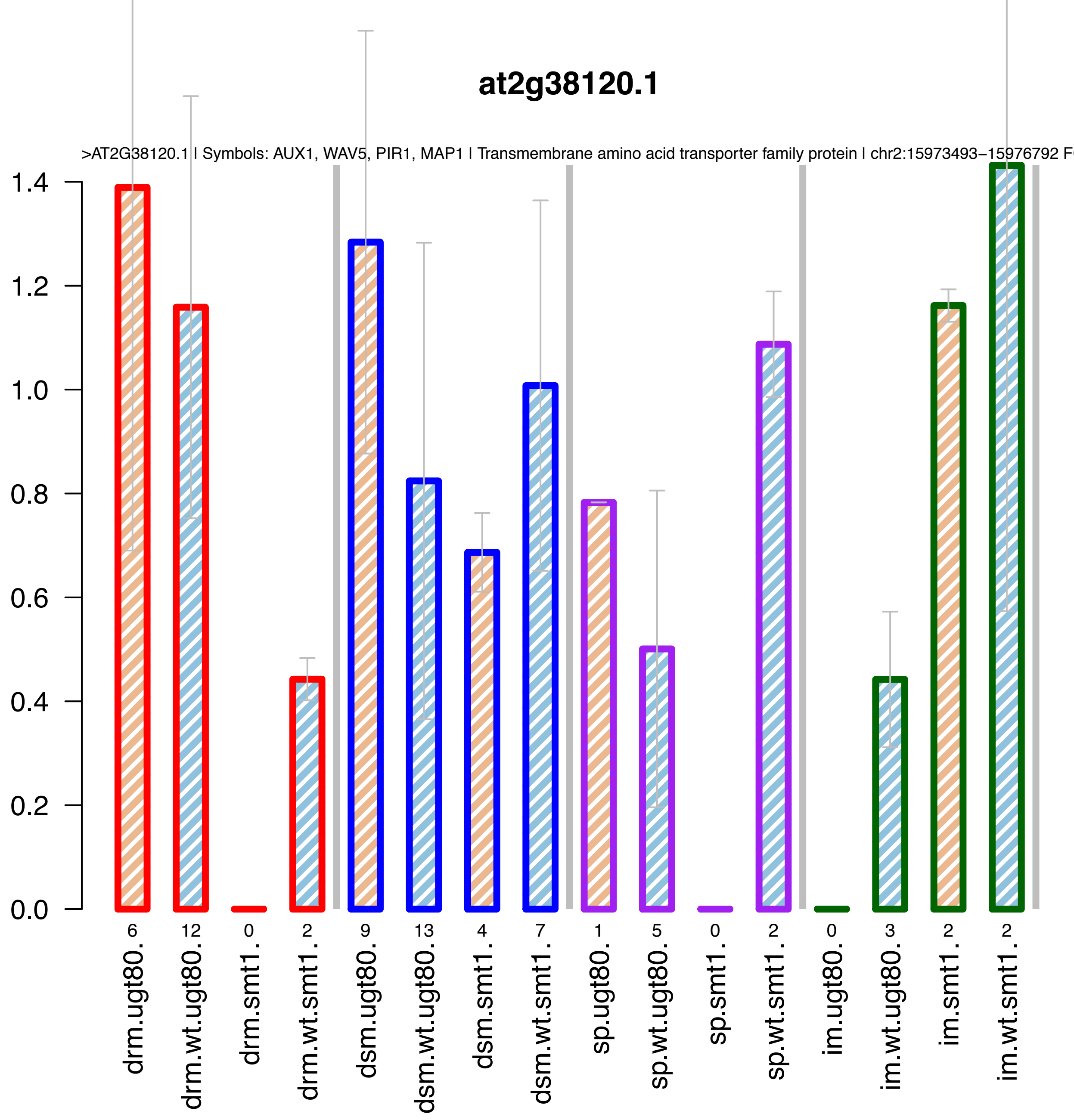
at2g38120.1
>AT2G38120.1 | Symbols: AUX1, WAV5, PIR1, MAP1 | Transmembrane amino acid transporter family protein | chr2:15973493−15976792 F
1.4
1.2
1.0
0.8
0.6
0.4
0.2
0.0
6
12
0
2
9
13
4
7
1
5
0
2
0
3
2
2
drm.ugt80.
drm.wt.ugt80.
drm.smt1.
drm.wt.smt1.
dsm.ugt80.
dsm.wt.ugt80.
dsm.smt1.
dsm.wt.smt1.
sp.ugt80.
sp.wt.ugt80.
sp.smt1.
sp.wt.smt1.
im.ugt80.
im.wt.ugt80.
im.smt1.
im.wt.smt1.

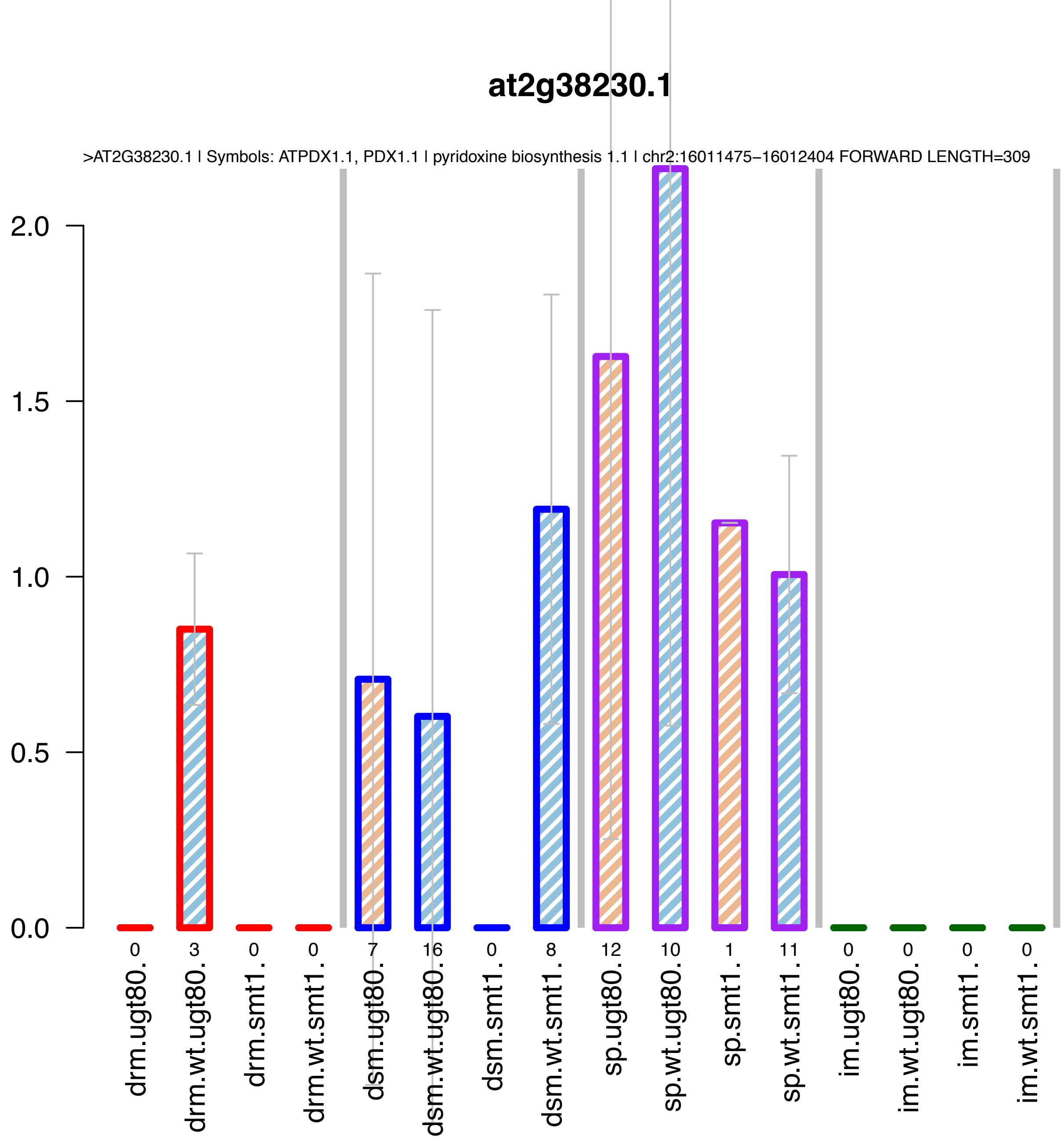

at2g38230.1
>AT2G38230.1 | Symbols: ATPDX1.1, PDX1.1 | pyridoxine biosynthesis 1.1 | chr2:16011475−16012404 FORWARD LENGTH=309
2.0
1.5
1.0
0.5
0.0
0
3
0
0
7
16
0
8
12
10
1
11
0
0
0
0
drm.ugt80.
drm.wt.ugt80.
drm.smt1.
drm.wt.smt1.
dsm.ugt80.
dsm.wt.ugt80.
dsm.smt1.
dsm.wt.smt1.
sp.ugt80.
sp.wt.ugt80.
sp.smt1.
sp.wt.smt1.
im.ugt80.
im.wt.ugt80.
im.smt1.
im.wt.smt1.

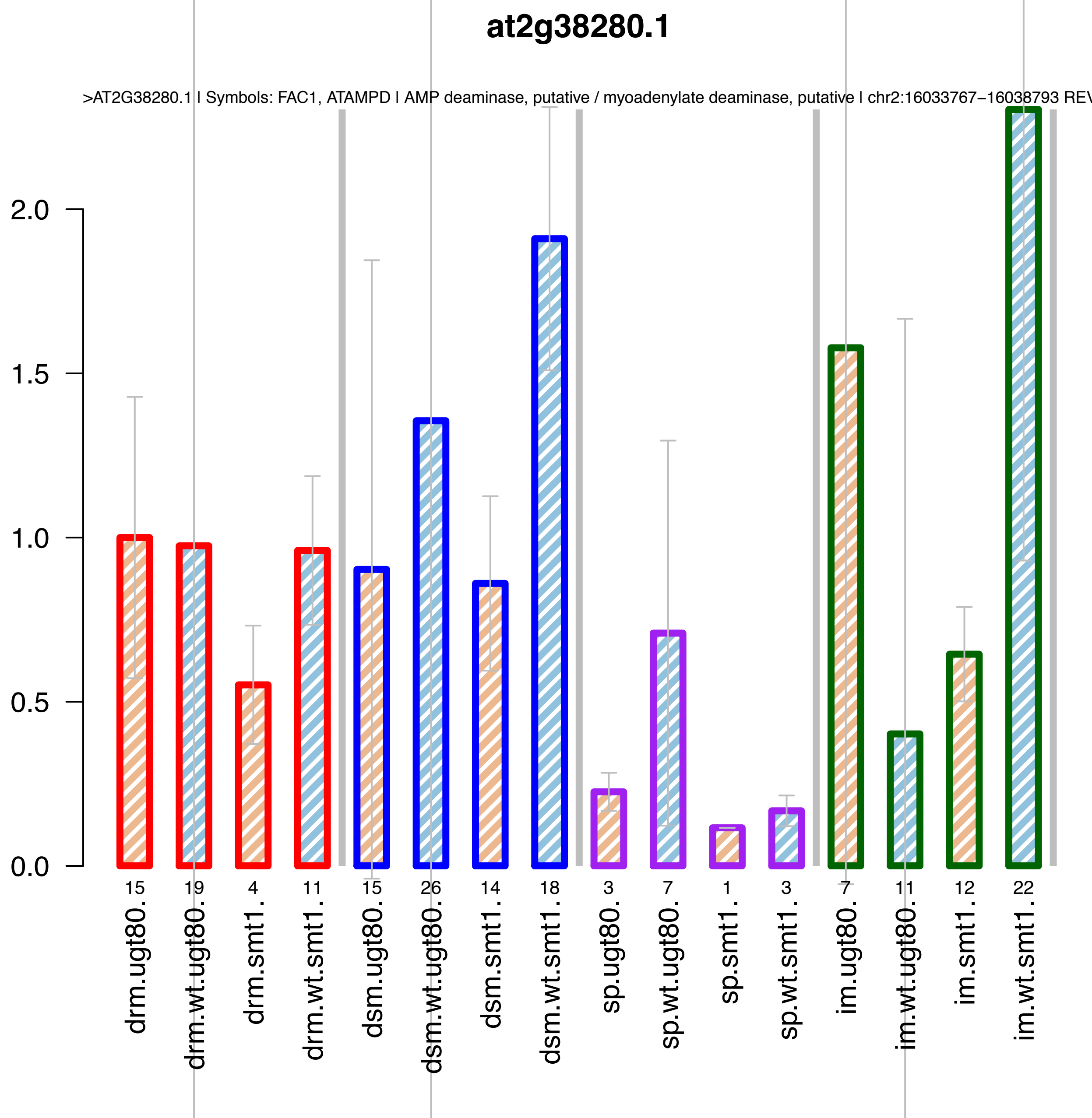

at2g38280.1
>AT2G38280.1 | Symbols: FAC1, ATAMPD | AMP deaminase, putative / myoadenylate deaminase, putative | chr2:16033767-16038793 REV
2.0
1.5
1.0
0.5
0.0
15
19
4
11
15
26
14
18
3
7
1
3
7
11
12
22
drm.ugt80..
drm.wt.ugt80..
drm.smt1..
drm.wt.smt1..
dsm.ugt80..
dsm.wt.ugt80..
dsm.smt1..
dsm.wt.smt1..
sp.ugt80..
sp.wt.ugt80..
sp.smt1..
sp.wt.smt1..
im.ugt80..
im.wt.ugt80..
im.smt1..
im.wt.smt1..

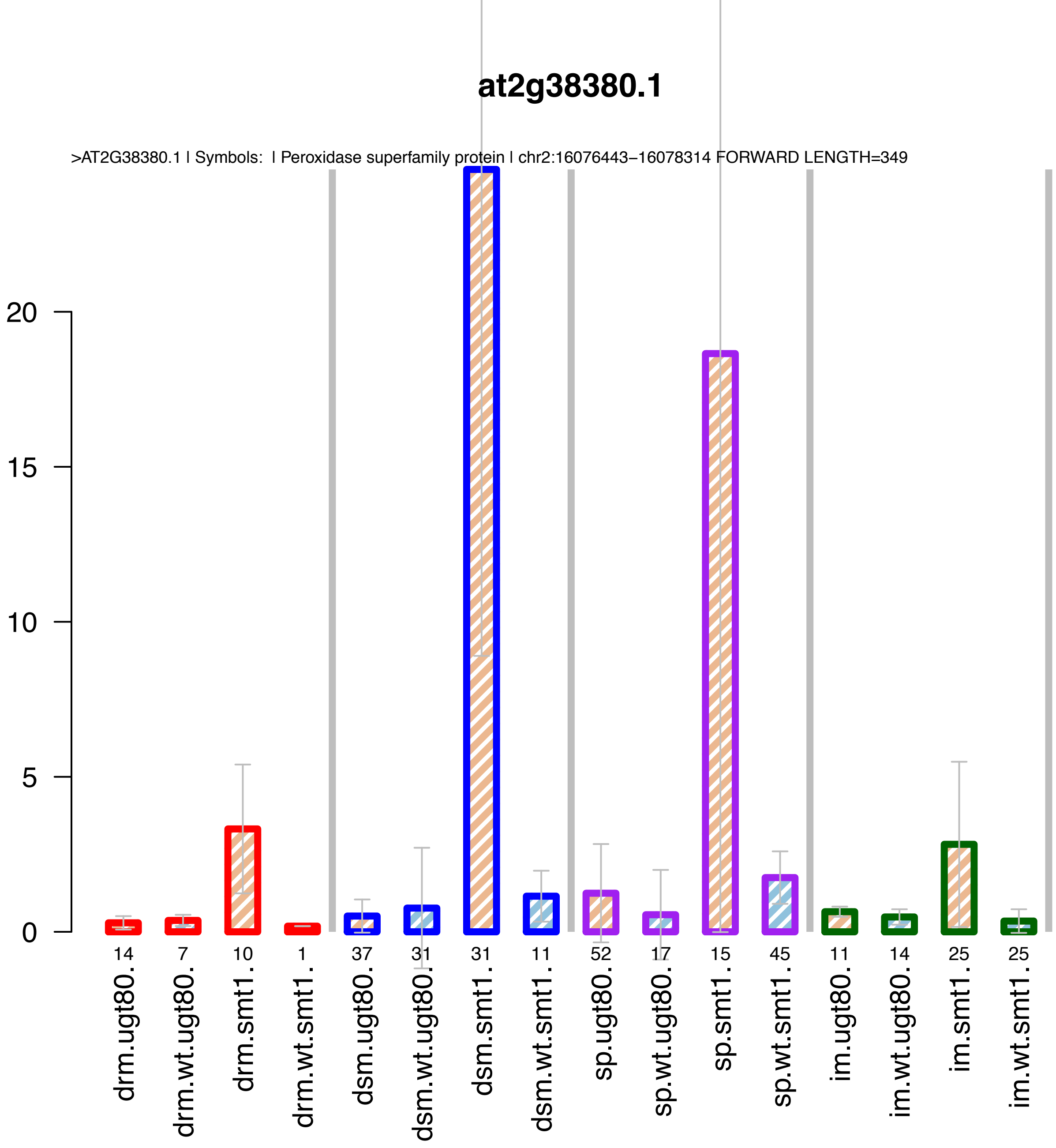
at2g38380.1
>AT2G38380.1 | Symbols: | Peroxidase superfamily protein | chr2:16076443-16078314 FORWARD LENGTH=349
20
15
10
5
0
14
7
10
1
37
31
31
11
52
17
15
45
11
14
25
25
drm.ugt80.
drm.wt.ugt80.
drm.smt1.
drm.wt.smt1.
dsm.ugt80.
dsm.wt.ugt80.
dsm.smt1.
dsm.wt.smt1.
sp.ugt80.
sp.wt.ugt80.
sp.smt1.
sp.wt.smt1.
im.ugt80.
im.wt.ugt80.
im.smt1.
im.wt.smt1.

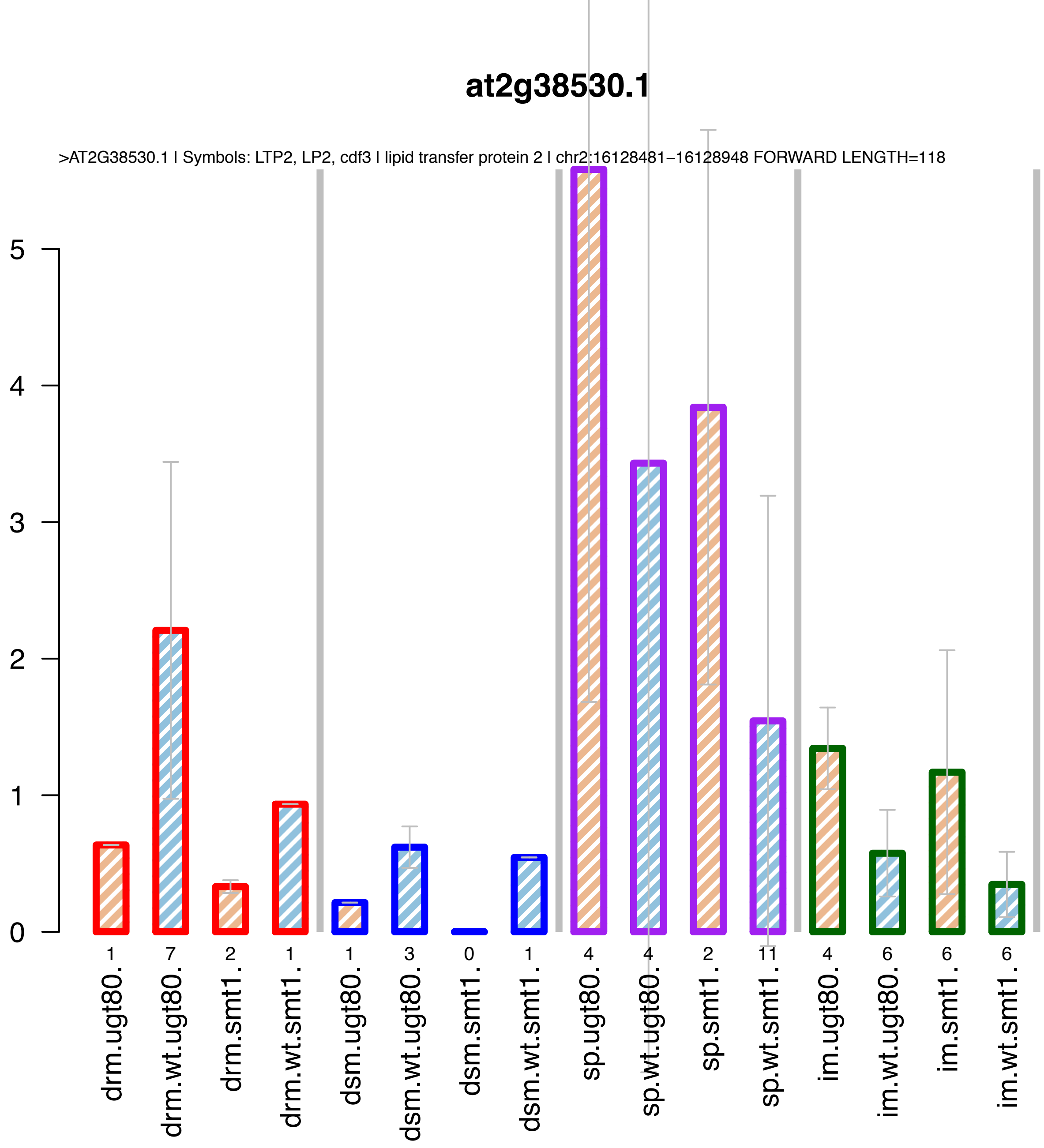

at2g38530.1
>AT2G38530.1 | Symbols: LTP2, LP2, cdf3 | lipid transfer protein 2 | chr2:16128481-16128948 FORWARD LENGTH=118
0
1
2
3
4
5
1
7
2
1
1
3
0
1
4
4
2
11
4
6
6
6
drm.ugt80.
drm.wt.ugt80.
drm.smt1.
drm.wt.smt1.
dsm.ugt80.
dsm.wt.ugt80.
dsm.smt1.
dsm.wt.smt1.
sp.ugt80.
sp.wt.ugt80.
sp.smt1.
sp.wt.smt1.
im.ugt80.
im.wt.ugt80.
im.smt1.
im.wt.smt1.

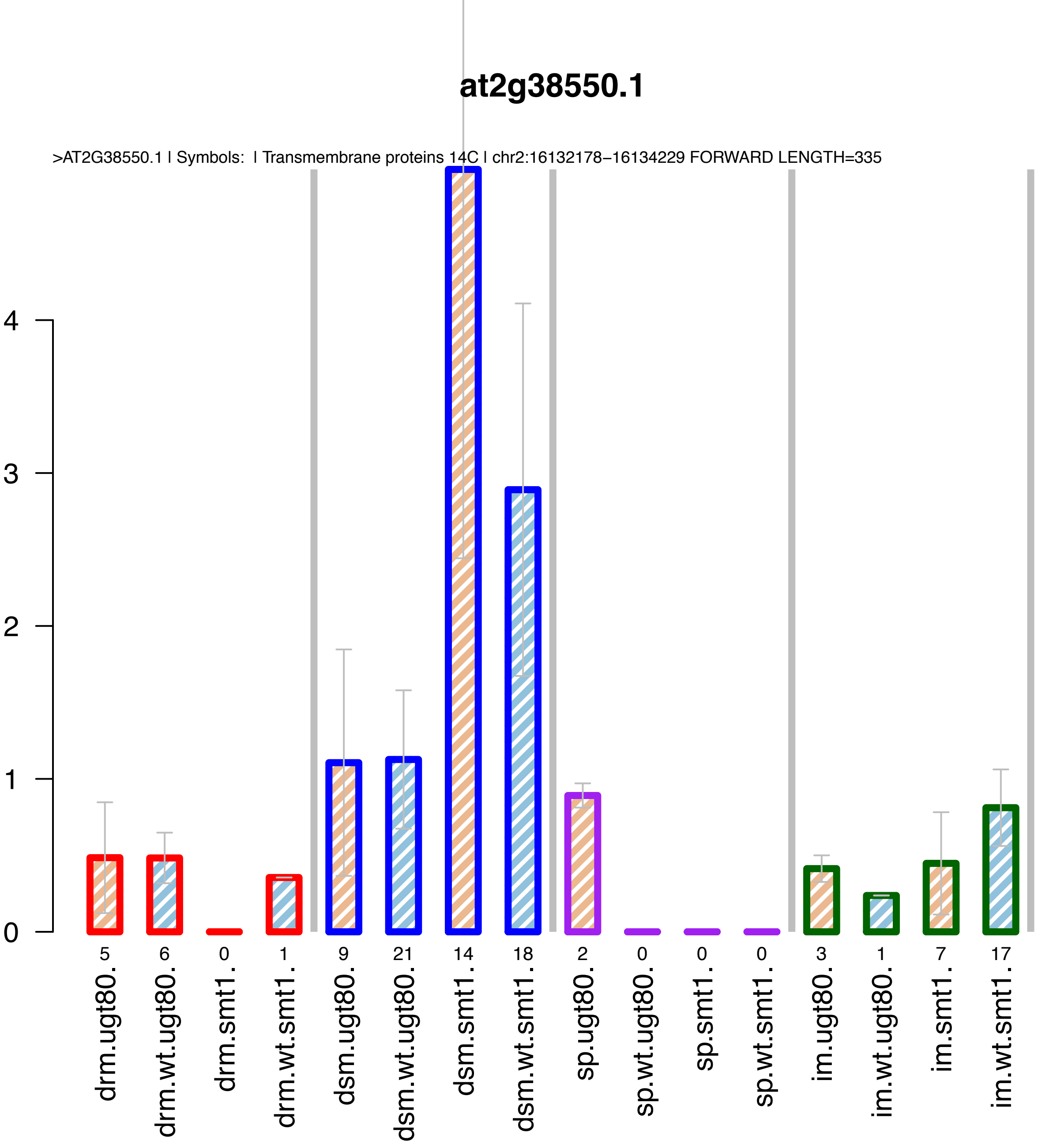

at2g38550.1
>AT2G38550.1 | Symbols: | Transmembrane proteins 14C | chr2:16132178-16134229 FORWARD LENGTH=335
0
1
2
3
4
5
6
0
1
9
21
14
18
2
0
0
0
3
1
7
17
drm.ugt80.
drm.wt.ugt80.
drm.smt1.
drm.wt.smt1.
dsm.ugt80.
dsm.wt.ugt80.
dsm.smt1.
dsm.wt.smt1.
sp.ugt80.
sp.wt.ugt80.
sp.smt1.
sp.wt.smt1.
im.ugt80.
im.wt.ugt80.
im.smt1.
im.wt.smt1.

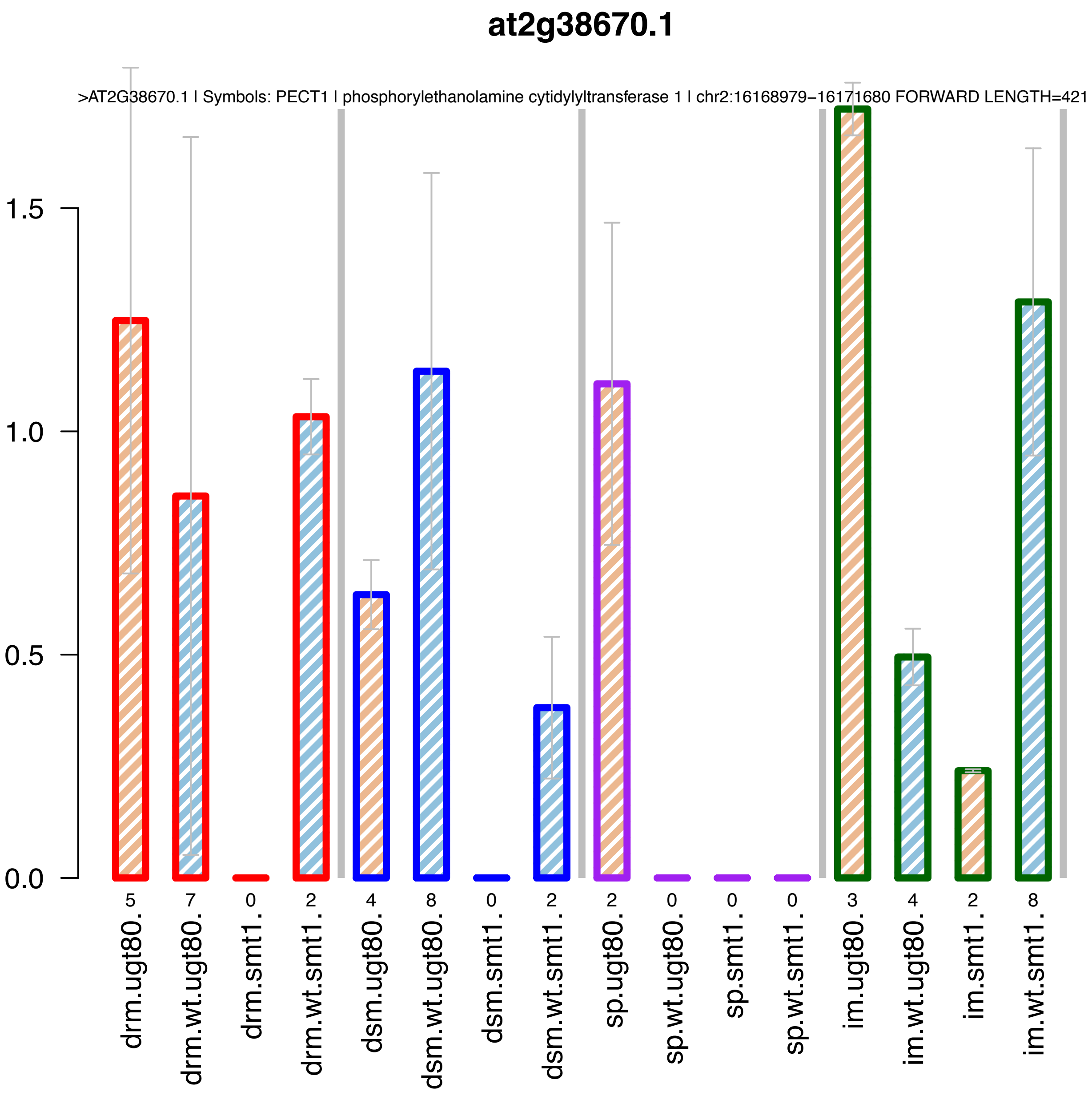

at2g38670.1
>AT2G38670.1 | Symbols: PECT1 | phosphorylethanolamine cytidylyltransferase 1 | chr2:16168979-16171680 FORWARD LENGTH=421
0.0
0.5
1.0
1.5
5
7
0
2
4
8
0
2
2
0
0
0
3
4
2
8
drm.ugt80.
drm.wt.ugt80.
drm.smt1.
drm.wt.smt1.
dsm.ugt80.
dsm.wt.ugt80.
dsm.smt1.
dsm.wt.smt1.
sp.ugt80.
sp.wt.ugt80.
sp.smt1.
sp.wt.smt1.
im.ugt80.
im.wt.ugt80.
im.smt1.
im.wt.smt1.

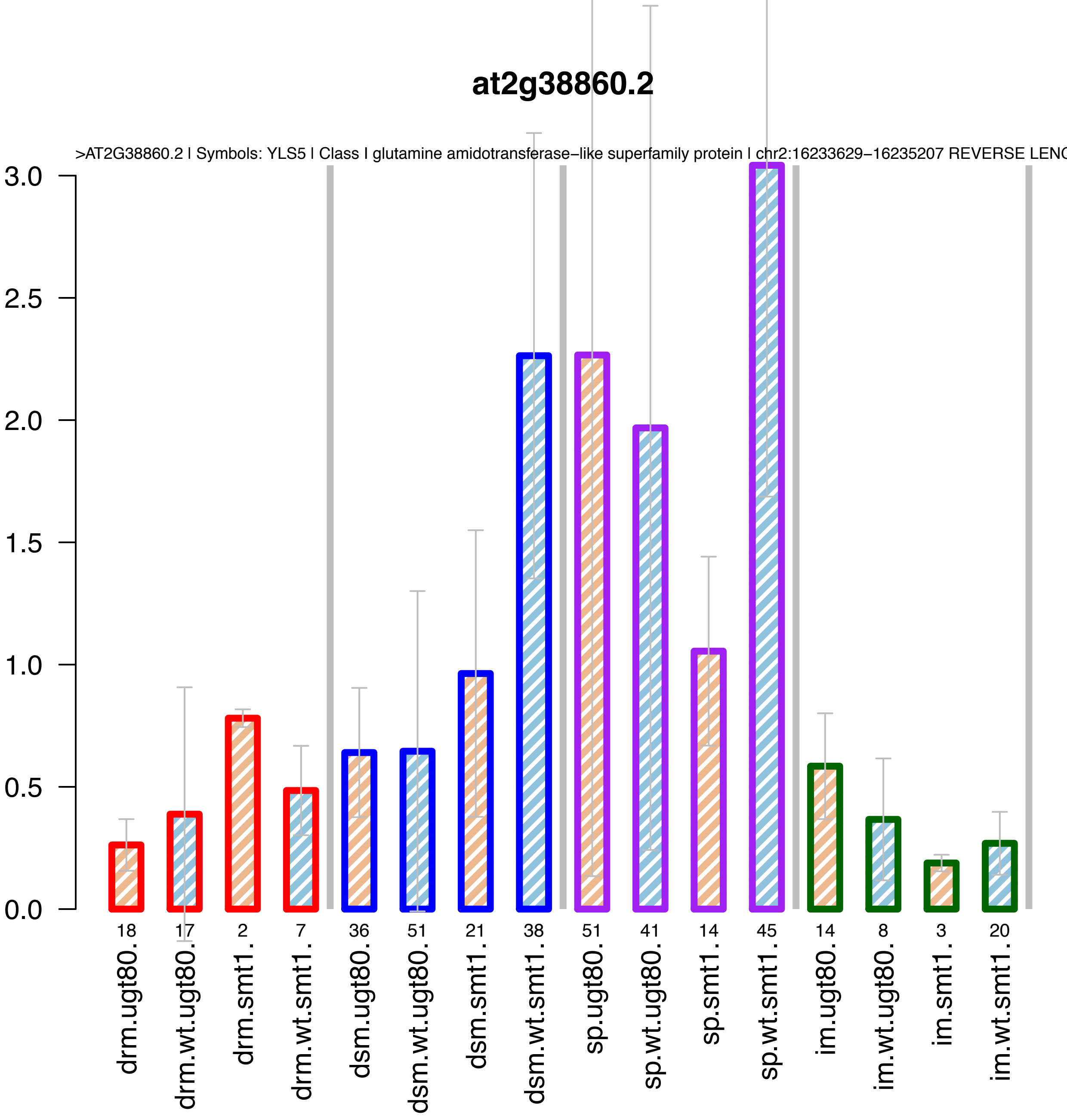

at2g38860.2
>AT2G38860.2 | Symbols: YLS5 | Class I glutamine amidotransferase-like superfamily protein | chr2:16233629-16235207 REVERSE LENG
3.0
2.5
2.0
1.5
1.0
0.5
0.0
18
17
2
7
36
51
21
38
51
41
14
45
14
8
3
20
drm.ugt80..
drm.wt.ugt80..
drm.smt1..
drm.wt.smt1..
dsm.ugt80..
dsm.wt.ugt80..
dsm.smt1..
dsm.wt.smt1..
sp.ugt80..
sp.wt.ugt80..
sp.smt1..
sp.wt.smt1..
im.ugt80..
im.wt.ugt80..
im.smt1..
im.wt.smt1..

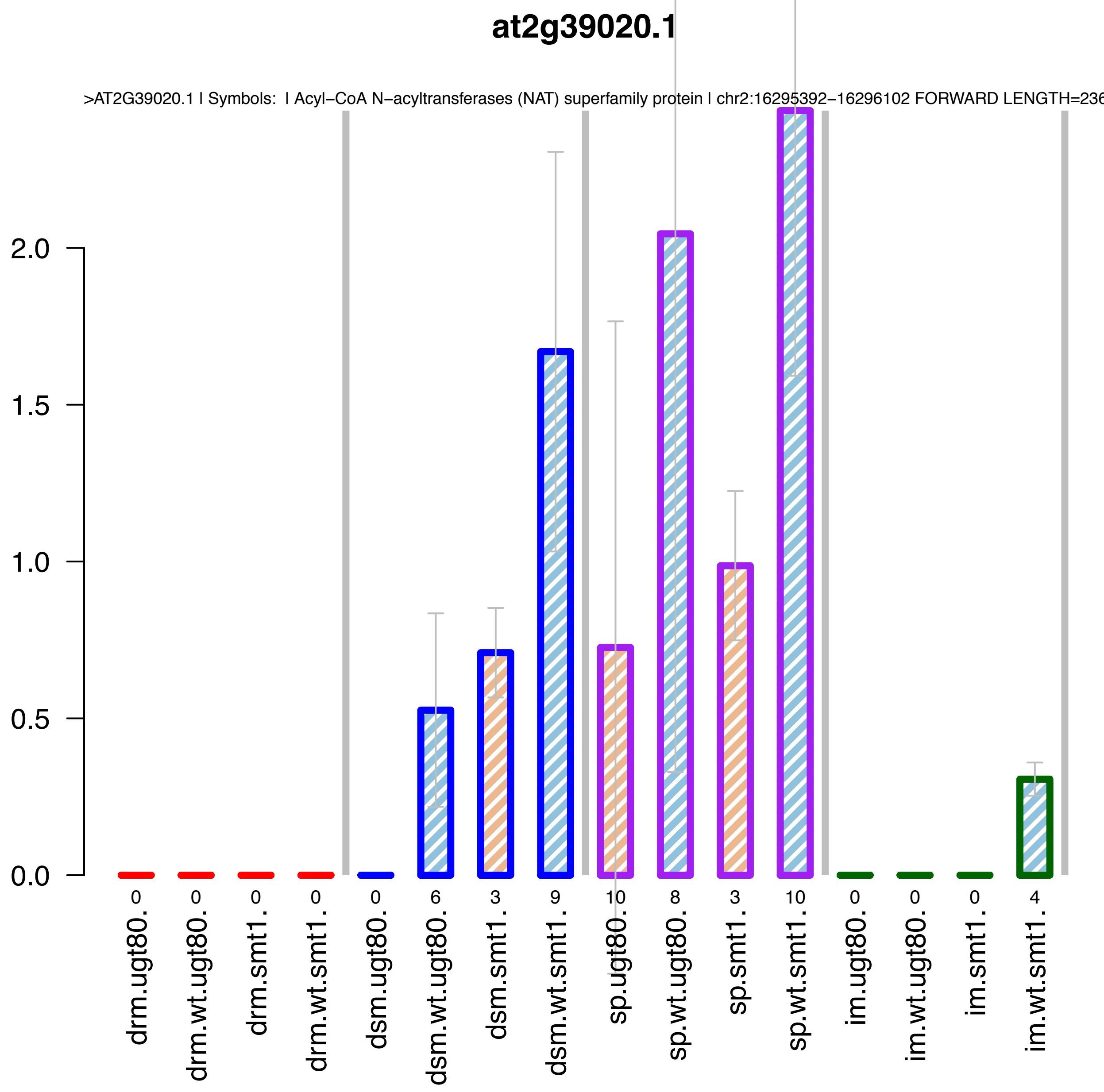

at2g39020.1
>AT2G39020.1 | Symbols: | Acyl−CoA N−acyltransferases (NAT) superfamily protein | chr2:16295392−16296102 FORWARD LENGTH=236
2.0
1.5
1.0
0.5
0.0
0
0
0
0
0
6
3
9
10
8
3
10
0
0
0
4
drm.ugt80.
drm.wt.ugt80.
drm.smt1.
drm.wt.smt1.
dsm.ugt80.
dsm.wt.ugt80.
dsm.smt1.
dsm.wt.smt1.
sp.ugt80.
sp.wt.ugt80.
sp.smt1.
sp.wt.smt1.
im.ugt80.
im.wt.ugt80.
im.smt1.
im.wt.smt1.

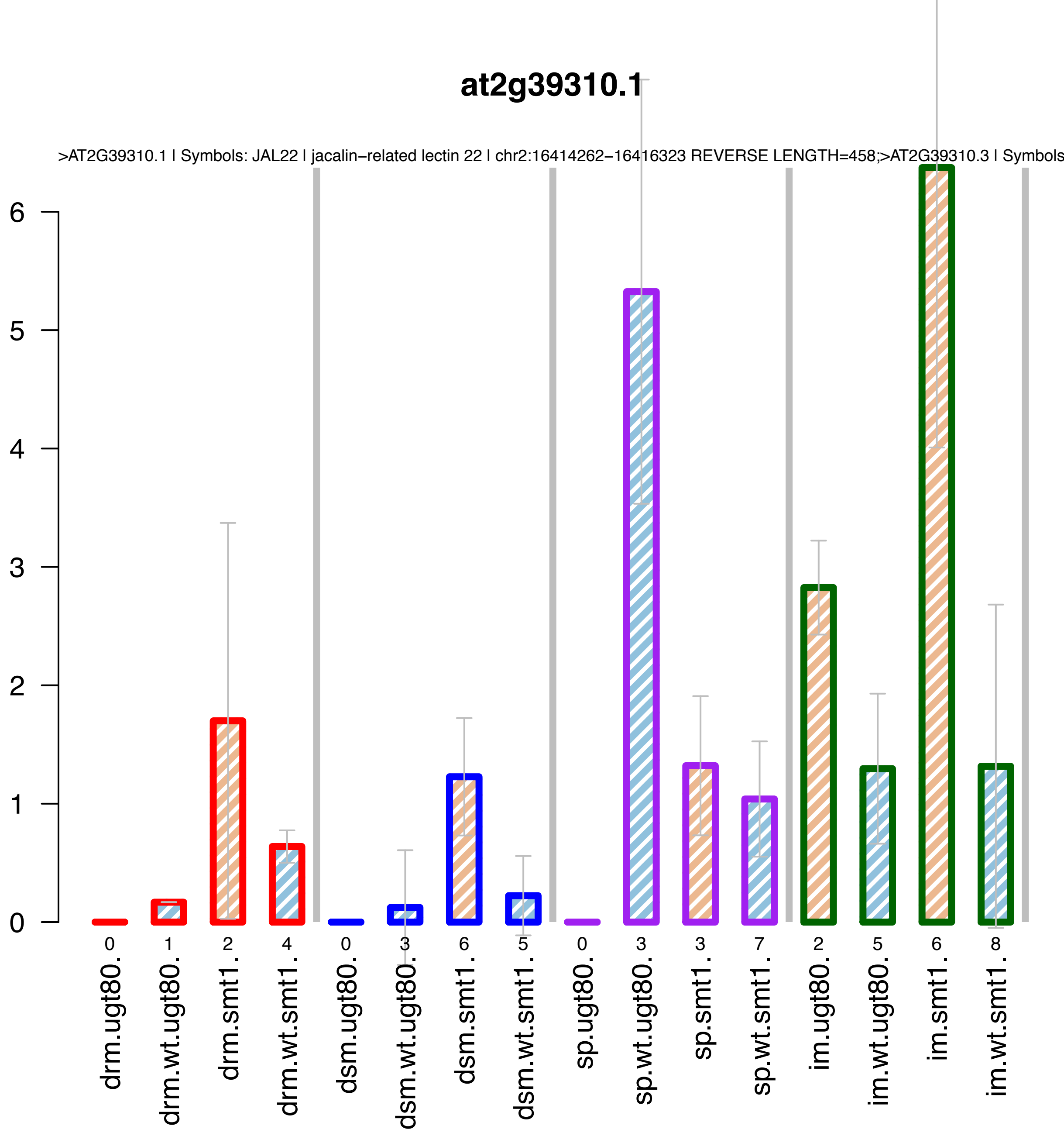

at2g39310.1
>AT2G39310.1 | Symbols: JAL22 | jacalin−related lectin 22 | chr2:16414262−16416323 REVERSE LENGTH=458;>AT2G39310.3 | Symbols
0
1
2
3
4
5
6
0
1
2
4
0
3
6
5
0
3
3
7
2
5
6
8
drm.ugt80.
drm.wt.ugt80.
drm.smt1.
drm.wt.smt1.
dsm.ugt80.
dsm.wt.ugt80.
dsm.smt1.
dsm.wt.smt1.
sp.ugt80.
sp.wt.ugt80.
sp.smt1.
sp.wt.smt1.
im.ugt80.
im.wt.ugt80.
im.smt1.
im.wt.smt1.

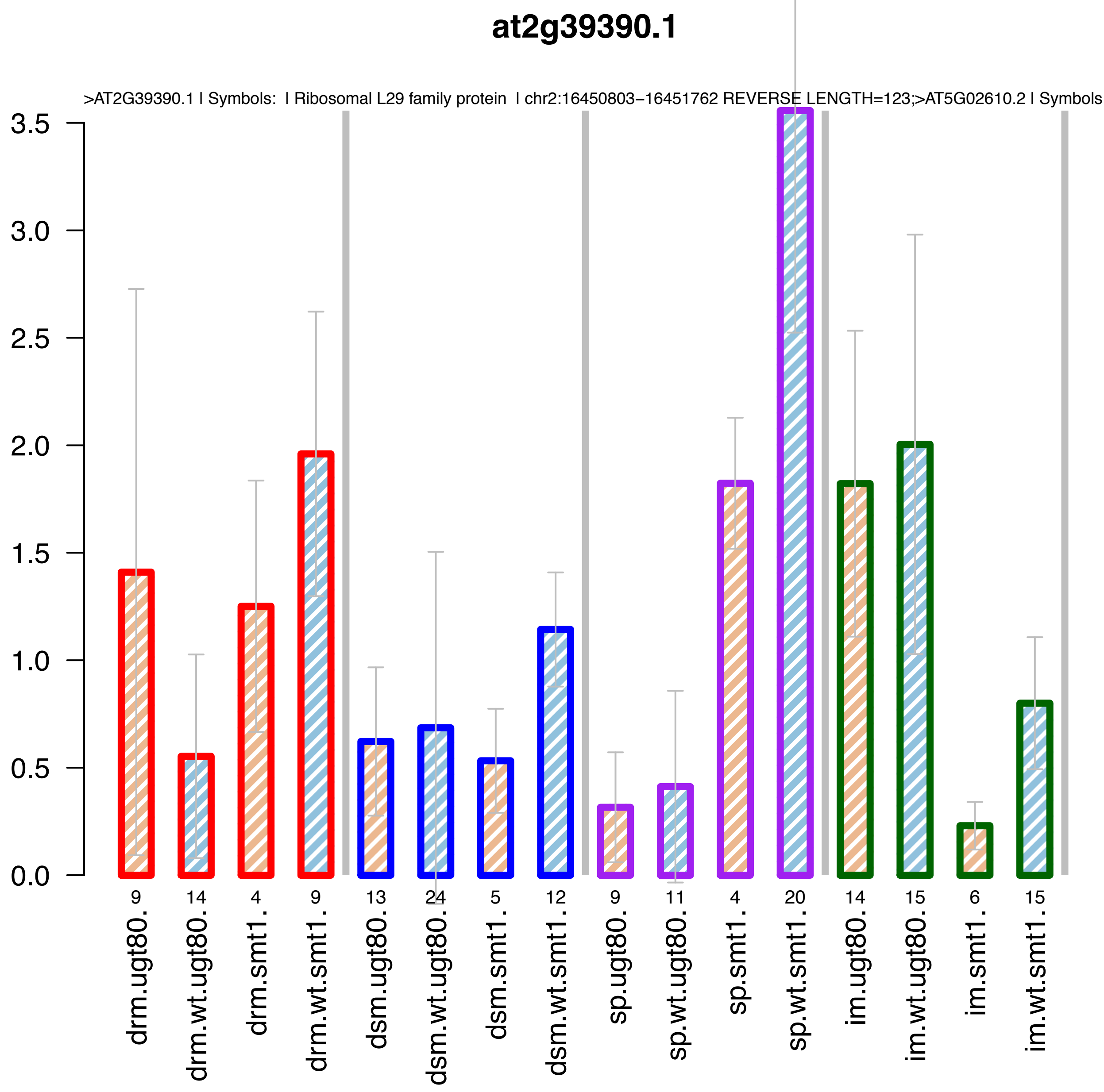

at2g39390.1
>AT2G39390.1 | Symbols: | Ribosomal L29 family protein | chr2:16450803-16451762 REVERSE LENGTH=123;>AT5G02610.2 | Symbols
3.5
3.0
2.5
2.0
1.5
1.0
0.5
0.0
9
14
4
9
13
24
5
12
9
11
4
20
14
15
6
15
drm.ugt80..
drm.wt.ugt80..
drm.smt1..
drm.wt.smt1..
dsm.ugt80..
dsm.wt.ugt80..
dsm.smt1..
dsm.wt.smt1..
sp.ugt80..
sp.wt.ugt80..
sp.smt1..
sp.wt.smt1..
im.ugt80..
im.wt.ugt80..
im.smt1..
im.wt.smt1..

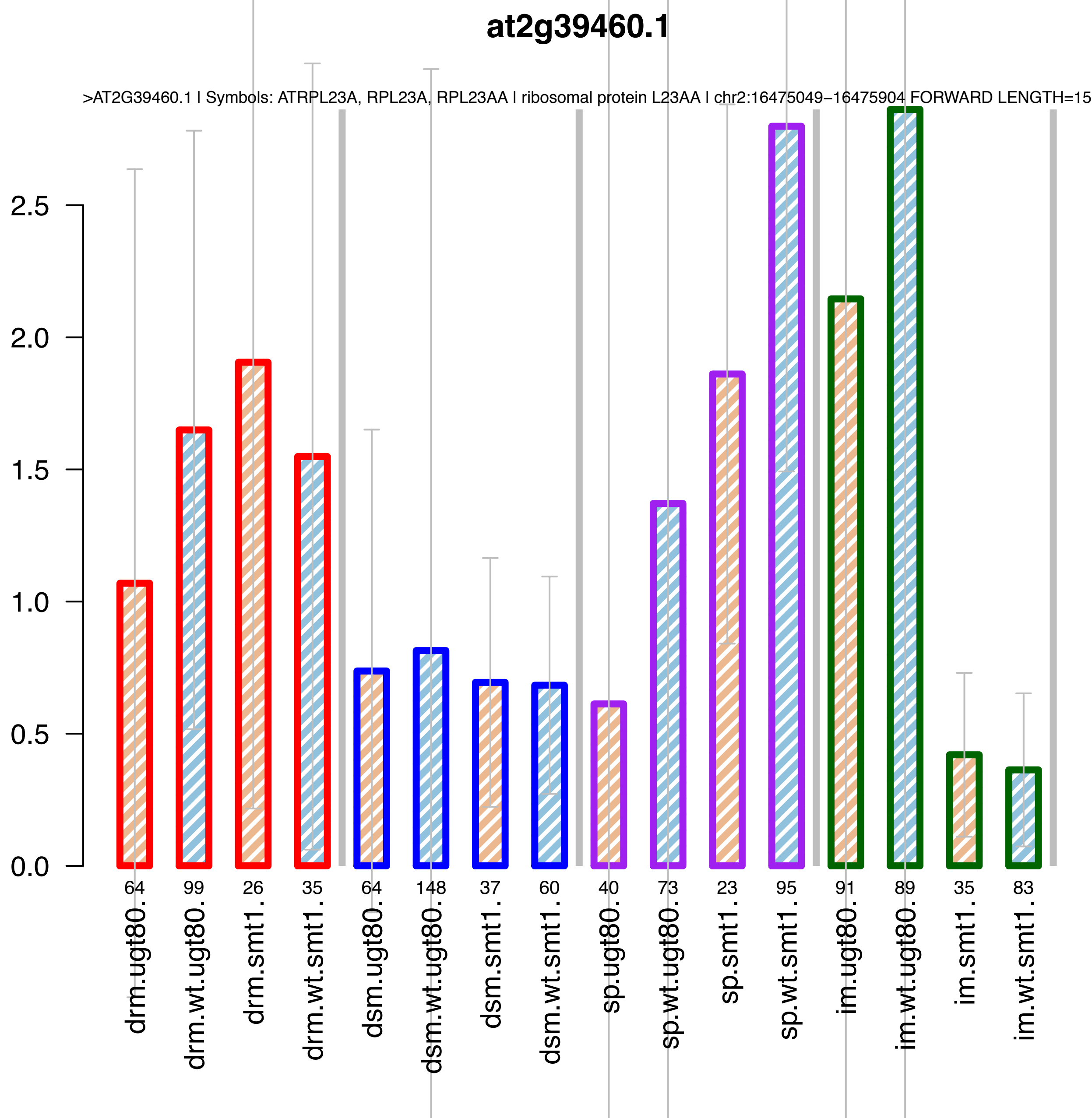

at2g39460.1
>AT2G39460.1 | Symbols: ATRPL23A, RPL23A, RPL23AA | ribosomal protein L23AA | chr2:16475049-16475904 FORWARD LENGTH=15
0.0
0.5
1.0
1.5
2.0
2.5
64
99
26
35
64
148
37
60
40
73
23
95
91
89
35
83
drm.ugt80..
drm.wt.ugt80..
drm.smt1..
drm.wt.smt1..
dsm.ugt80..
dsm.wt.ugt80..
dsm.smt1..
dsm.wt.smt1..
sp.ugt80..
sp.wt.ugt80..
sp.smt1..
sp.wt.smt1..
im.ugt80..
im.wt.ugt80..
im.smt1..
im.wt.smt1..

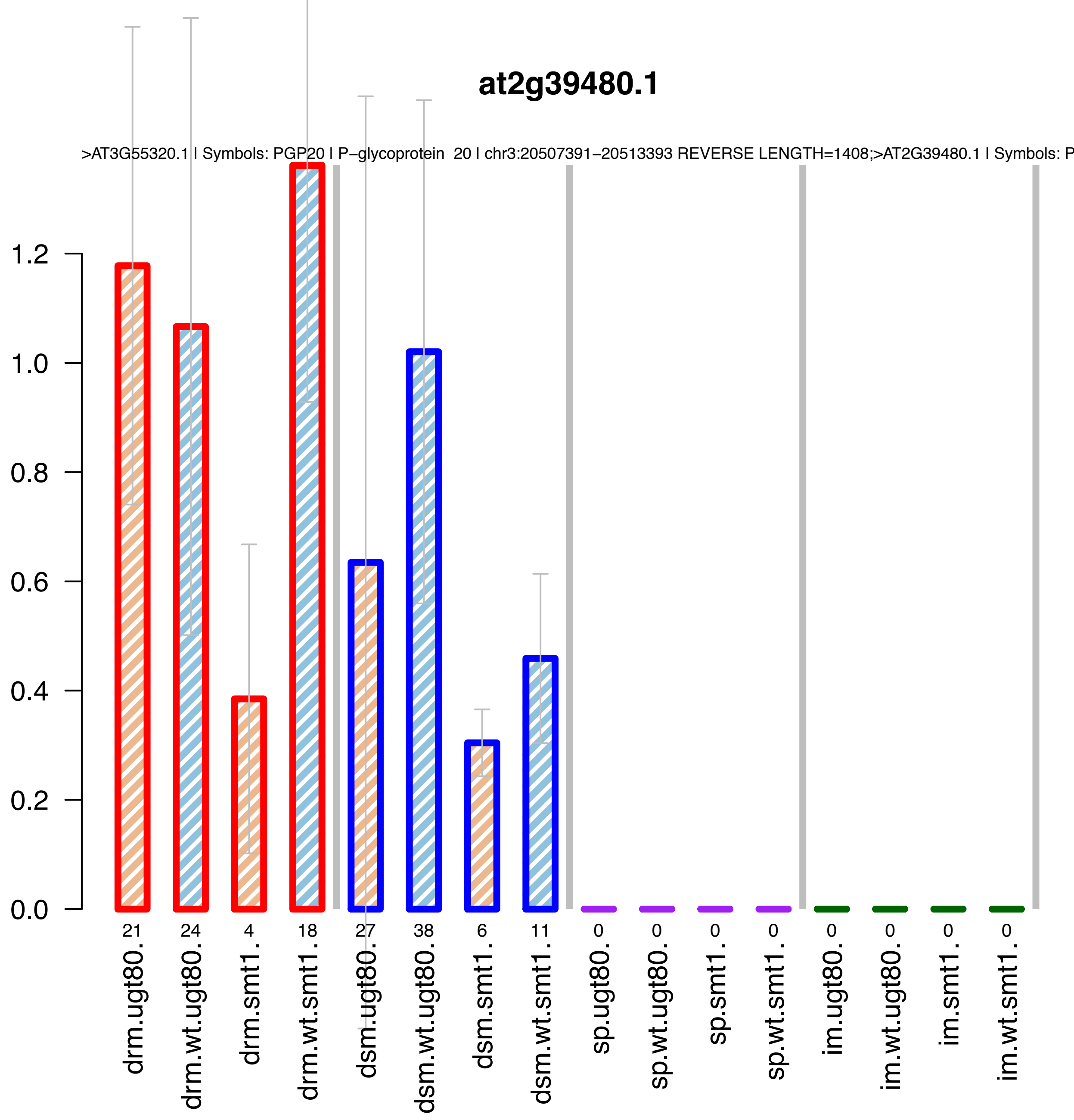

at2g39480.1
>AT3G55320.1 | Symbols: PGP20 | P−glycoprotein 20 | chr3:20507391−20513393 REVERSE LENGTH=1408;>AT2G39480.1 | Symbols: P
0.0
0.2
0.4
0.6
0.8
1.0
1.2
21
24
4
18
27
38
6
11
0
0
0
0
0
0
0
0
drm.ugt80.
drm.wt.ugt80.
drm.smt1.
drm.wt.smt1.
dsm.ugt80.
dsm.wt.ugt80.
dsm.smt1.
dsm.wt.smt1.
sp.ugt80.
sp.wt.ugt80.
sp.smt1.
sp.wt.smt1.
im.ugt80.
im.wt.ugt80.
im.smt1.
im.wt.smt1.

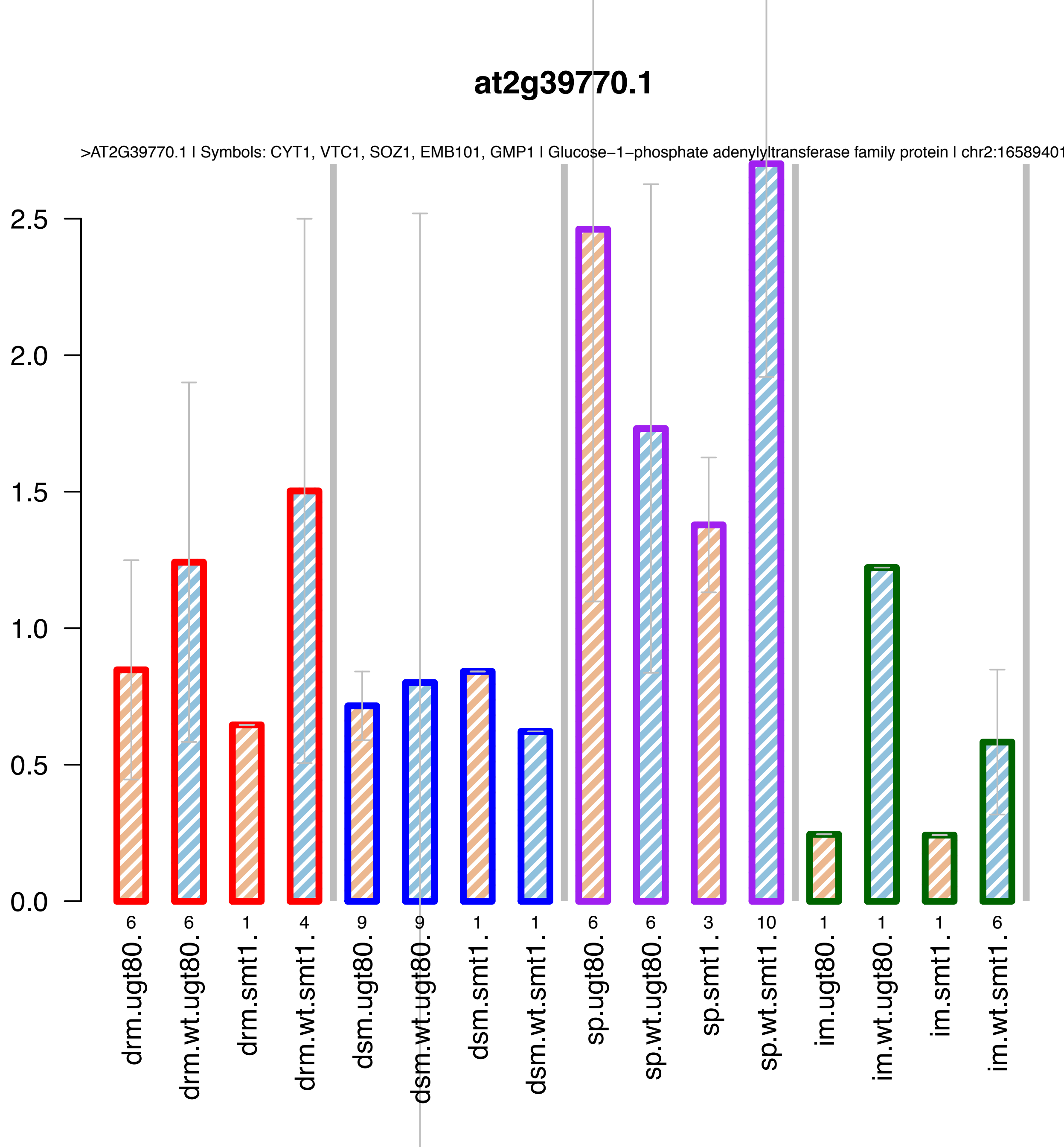

at2g39770.1
>AT2G39770.1 | Symbols: CYT1, VTC1, SOZ1, EMB101, GMP1 | Glucose−1−phosphate adenylyltransferase family protein | chr2:16589401
0.0
0.5
1.0
1.5
2.0
2.5
6
6
1
4
9
9
1
1
6
6
3
10
1
1
1
6
drm.ugt80.
drm.wt.ugt80.
drm.smt1.
drm.wt.smt1.
dsm.ugt80.
dsm.wt.ugt80.
dsm.smt1.
dsm.wt.smt1.
sp.ugt80.
sp.wt.ugt80.
sp.smt1.
sp.wt.smt1.
im.ugt80.
im.wt.ugt80.
im.smt1.
im.wt.smt1.

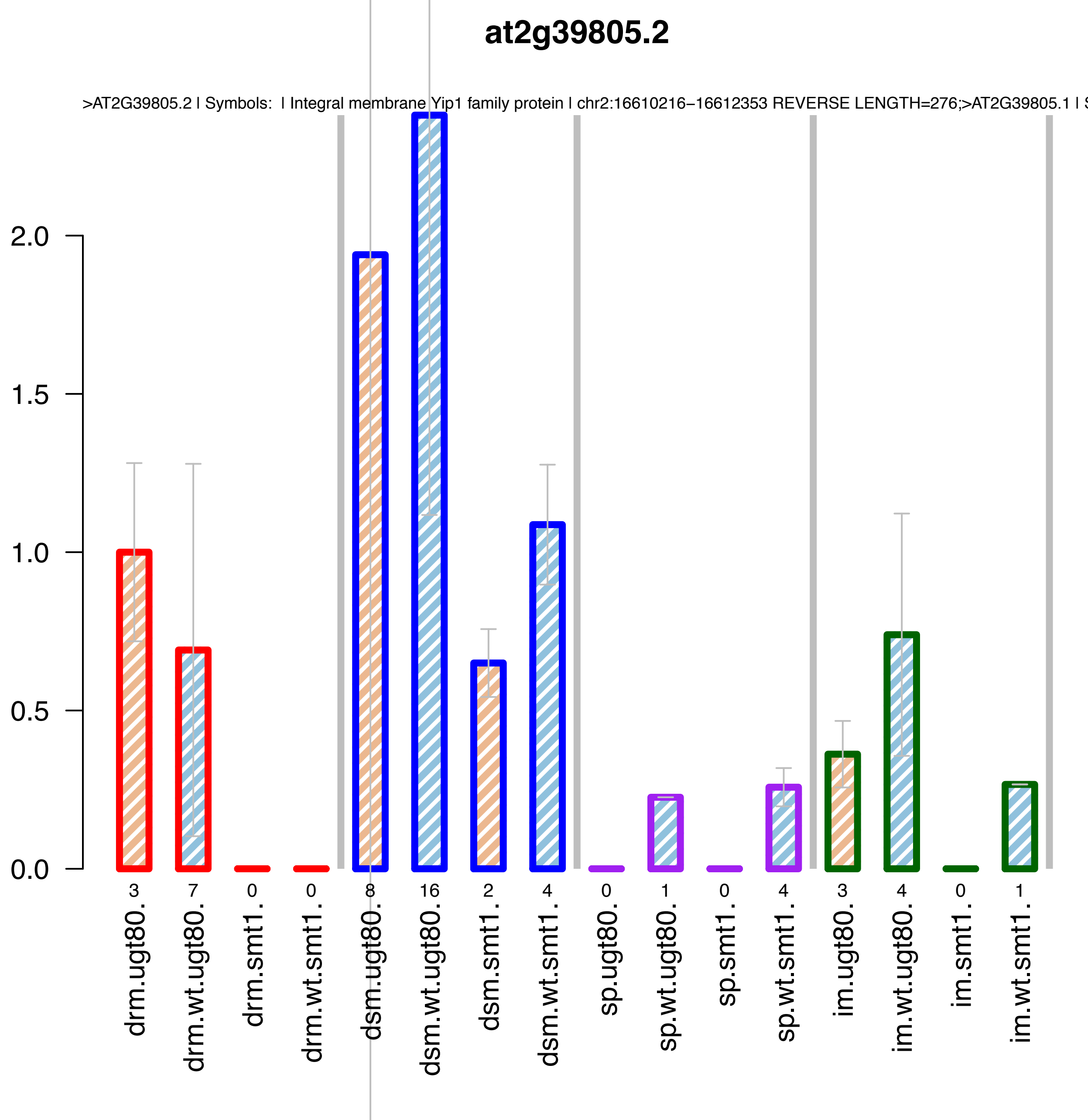

at2g39805.2
>AT2G39805.2 | Symbols: | Integral membrane Yip1 family protein | chr2:16610216–16612353 REVERSE LENGTH=276;>AT2G39805.1 | S
2.0
1.5
1.0
0.5
0.0
3
7
0
0
8
16
2
4
0
1
0
4
3
4
0
1
drm.ugt80..
drm.wt.ugt80..
drm.smt1..
drm.wt.smt1..
dsm.ugt80..
dsm.wt.ugt80..
dsm.smt1..
dsm.wt.smt1..
sp.ugt80..
sp.wt.ugt80..
sp.smt1..
sp.wt.smt1..
im.ugt80..
im.wt.ugt80..
im.smt1..
im.wt.smt1..

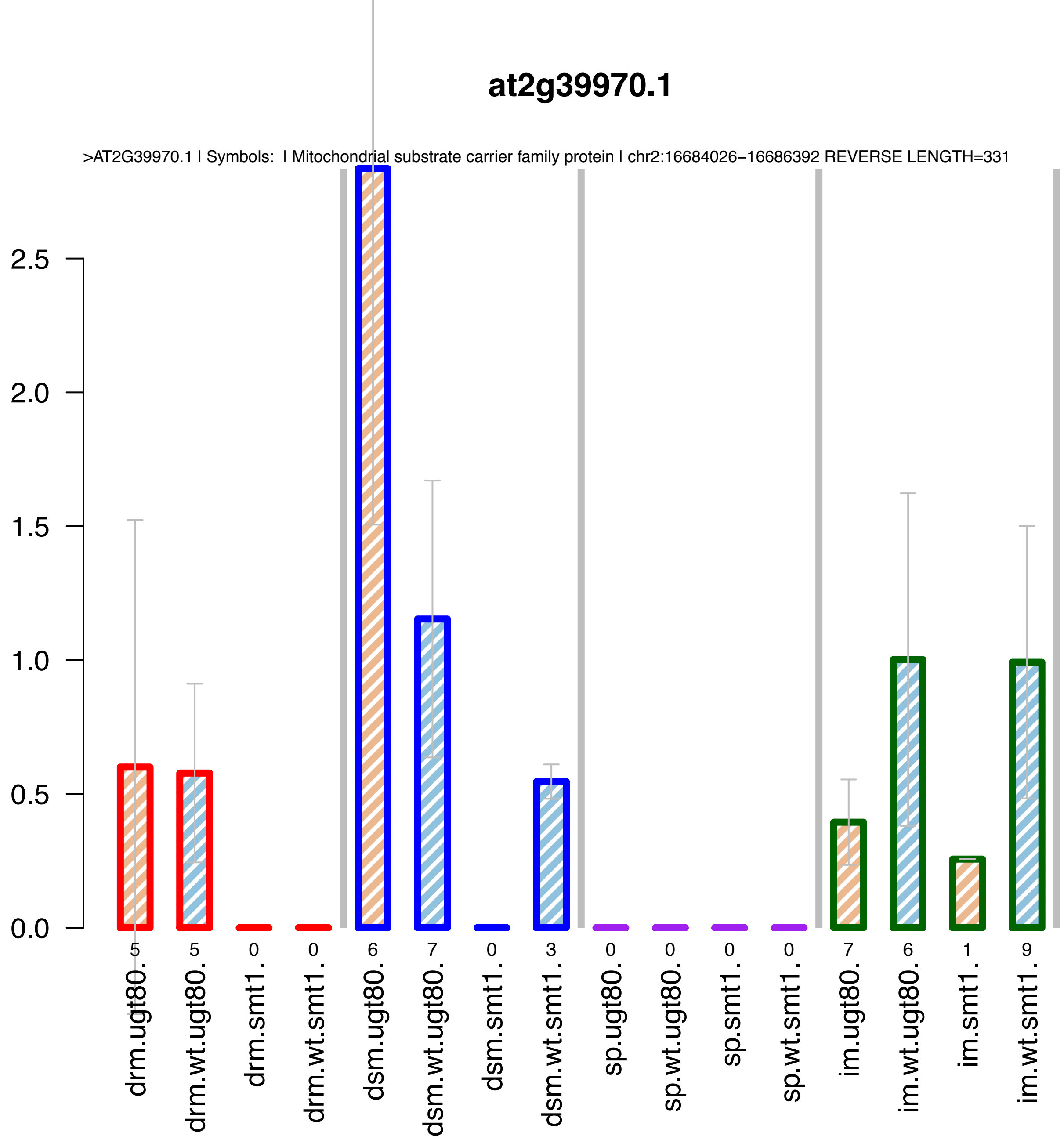

at2g39970.1
>AT2G39970.1 | Symbols: | Mitochondrial substrate carrier family protein | chr2:16684026−16686392 REVERSE LENGTH=331
0.0
0.5
1.0
1.5
2.0
2.5
5
5
0
0
6
7
0
3
0
0
0
0
7
6
1
9
drm.ugt80..
drm.wt.ugt80..
drm.smt1..
drm.wt.smt1..
dsm.ugt80..
dsm.wt.ugt80..
dsm.smt1..
dsm.wt.smt1..
sp.ugt80..
sp.wt.ugt80..
sp.smt1..
sp.wt.smt1..
im.ugt80..
im.wt.ugt80..
im.smt1..
im.wt.smt1..

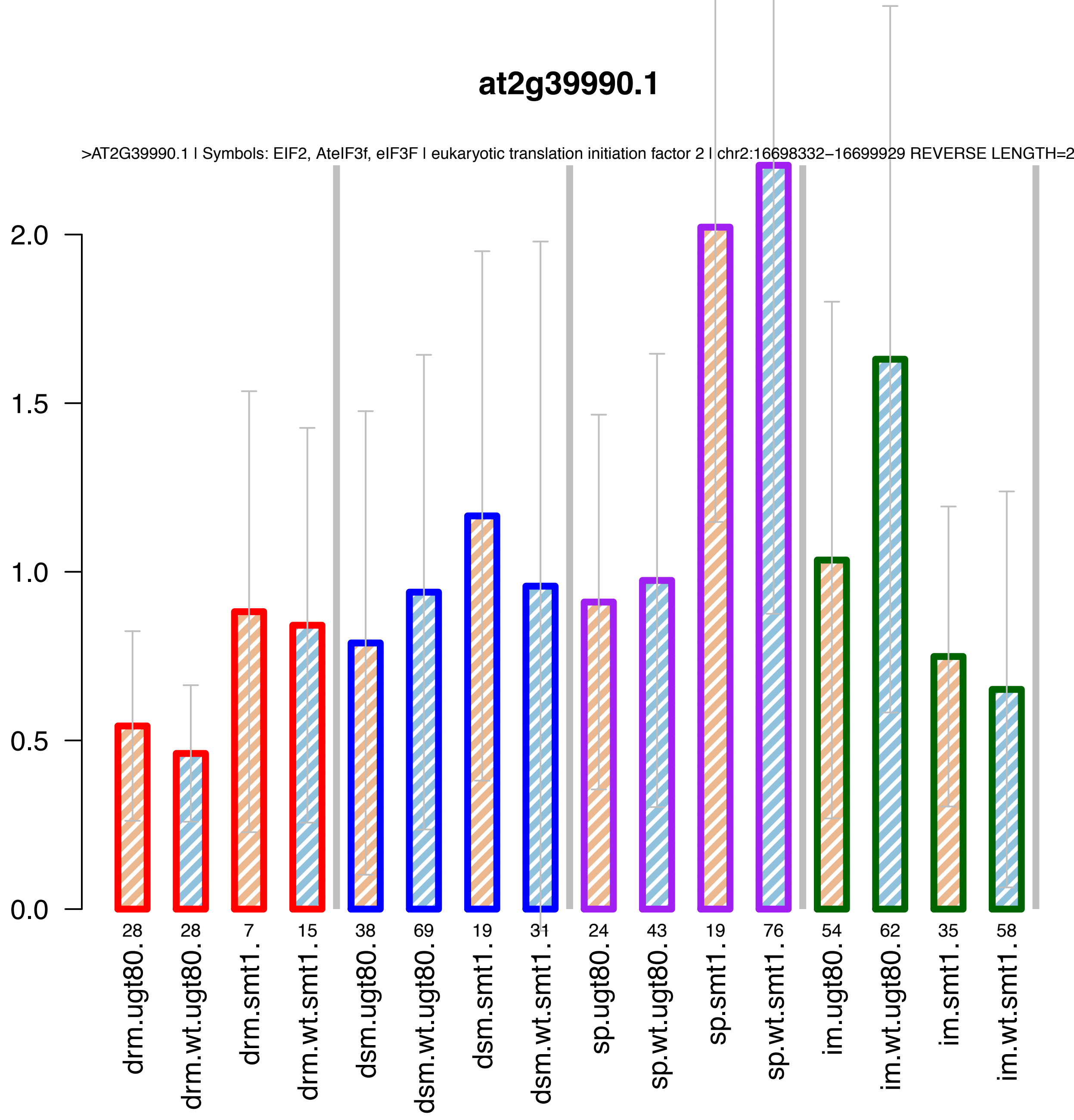

at2g39990.1
>AT2G39990.1 | Symbols: EIF2, AteIF3f, eIF3F | eukaryotic translation initiation factor 2 | chr2:16698332-16699929 REVERSE LENGTH=2
0.0
0.5
1.0
1.5
2.0
28
28
7
15
38
69
19
31
24
43
19
76
54
62
35
58
drm.ugt80.
drm.wt.ugt80.
drm.smt1.
drm.wt.smt1.
dsm.ugt80.
dsm.wt.ugt80.
dsm.smt1.
dsm.wt.smt1.
sp.ugt80.
sp.wt.ugt80.
sp.smt1.
sp.wt.smt1.
im.ugt80.
im.wt.ugt80.
im.smt1.
im.wt.smt1.

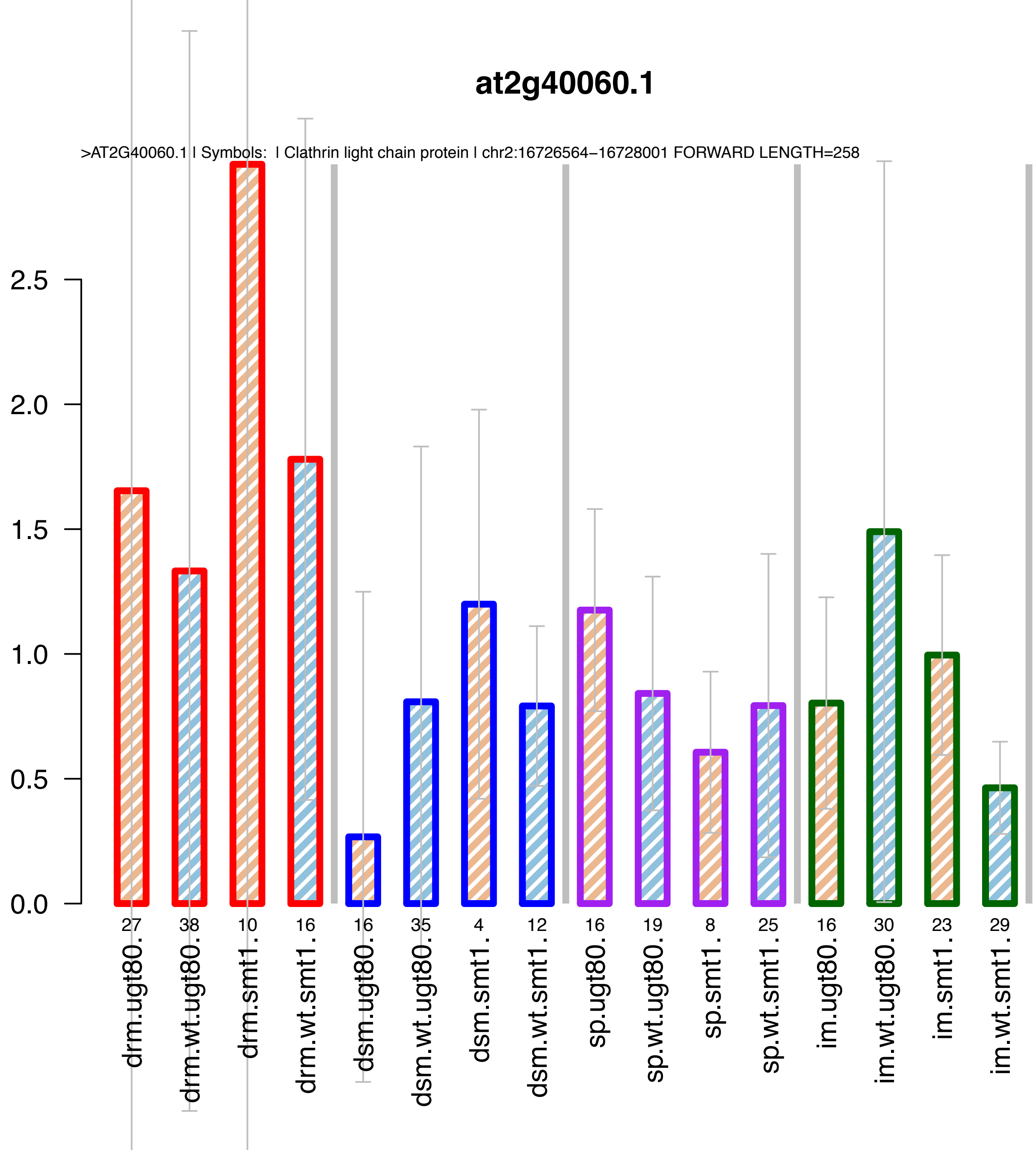

at2g40060.1
>AT2G40060.1 | Symbols: | Clathrin light chain protein | chr2:16726564-16728001 FORWARD LENGTH=258
0.0
0.5
1.0
1.5
2.0
2.5
27
38
10
16
16
35
4
12
16
19
8
25
16
30
23
29
drm.ugt80..
drm.wt.ugt80..
drm.smt1..
drm.wt.smt1..
dsm.ugt80..
dsm.wt.ugt80..
dsm.smt1..
dsm.wt.smt1..
sp.ugt80..
sp.wt.ugt80..
sp.smt1..
sp.wt.smt1..
im.ugt80..
im.wt.ugt80..
im.smt1..
im.wt.smt1..

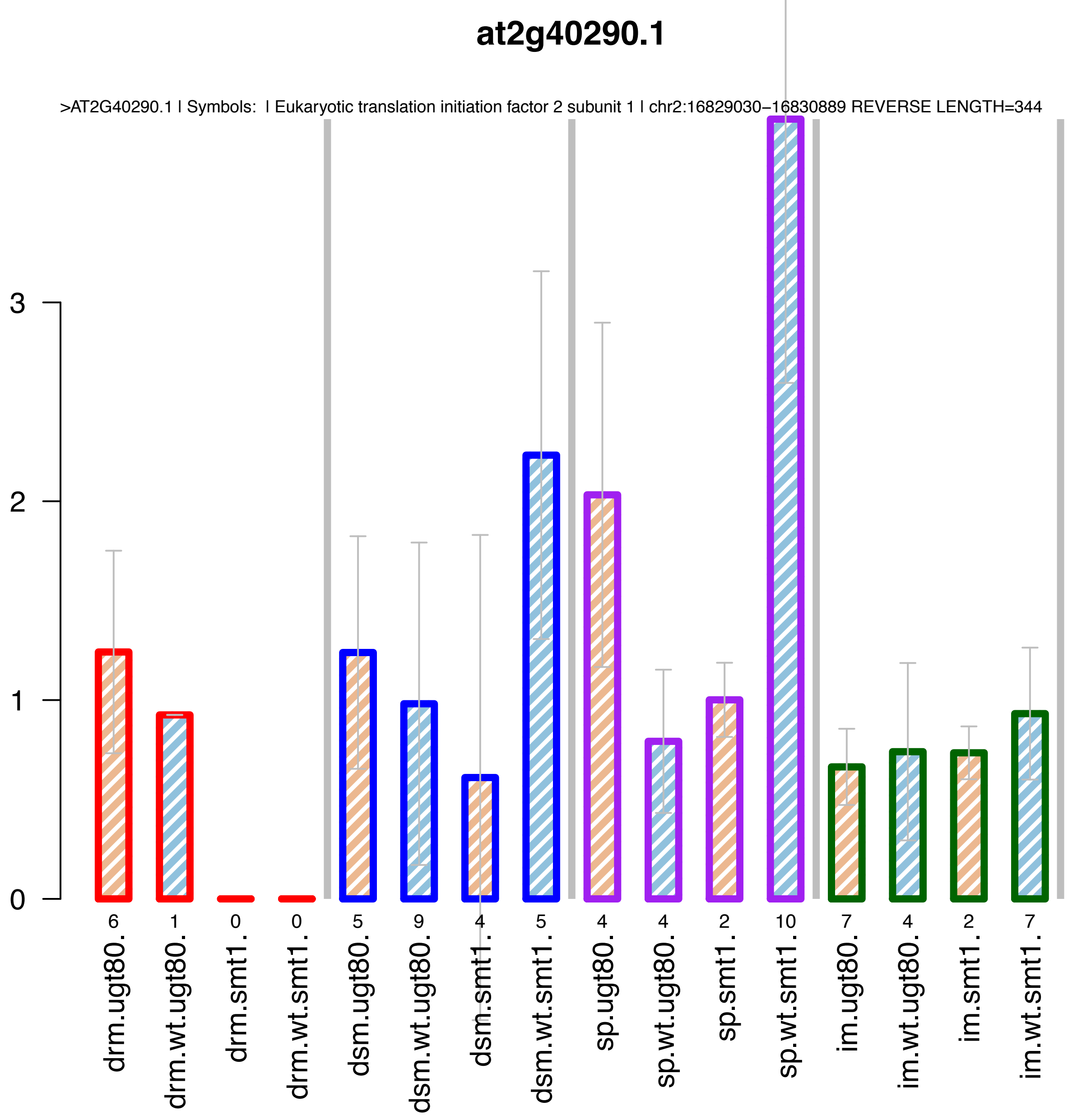

at2g40290.1
>AT2G40290.1 | Symbols: | Eukaryotic translation initiation factor 2 subunit 1 | chr2:16829030−16830889 REVERSE LENGTH=344
0
1
2
3
6
1
0
0
5
9
4
5
4
4
2
10
7
4
2
7
drm.ugt80..
drm.wt.ugt80..
drm.smt1..
drm.wt.smt1..
dsm.ugt80..
dsm.wt.ugt80..
dsm.smt1..
dsm.wt.smt1..
sp.ugt80..
sp.wt.ugt80..
sp.smt1..
sp.wt.smt1..
im.ugt80..
im.wt.ugt80..
im.smt1..
im.wt.smt1..

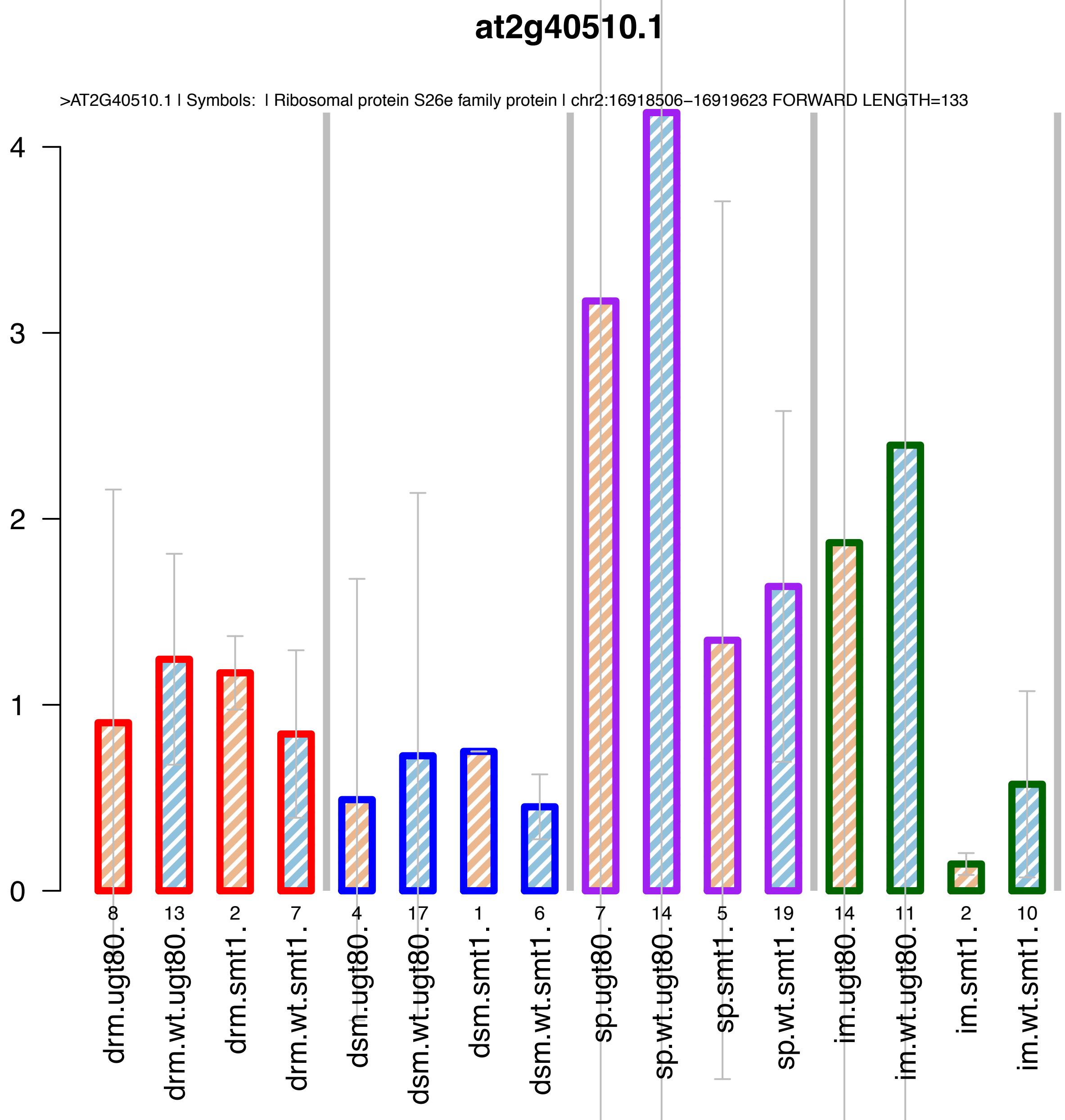

at2g40510.1
>AT2G40510.1 | Symbols: | Ribosomal protein S26e family protein | chr2:16918506−16919623 FORWARD LENGTH=133
0
1
2
3
4
8
13
2
7
4
17
1
6
7
14
5
19
14
11
2
10
drm.ugt80..
drm.wt.ugt80..
drm.smt1..
drm.wt.smt1..
dsm.ugt80..
dsm.wt.ugt80..
dsm.smt1..
dsm.wt.smt1..
sp.ugt80..
sp.wt.ugt80..
sp.smt1..
sp.wt.smt1..
im.ugt80..
im.wt.ugt80..
im.smt1..
im.wt.smt1..

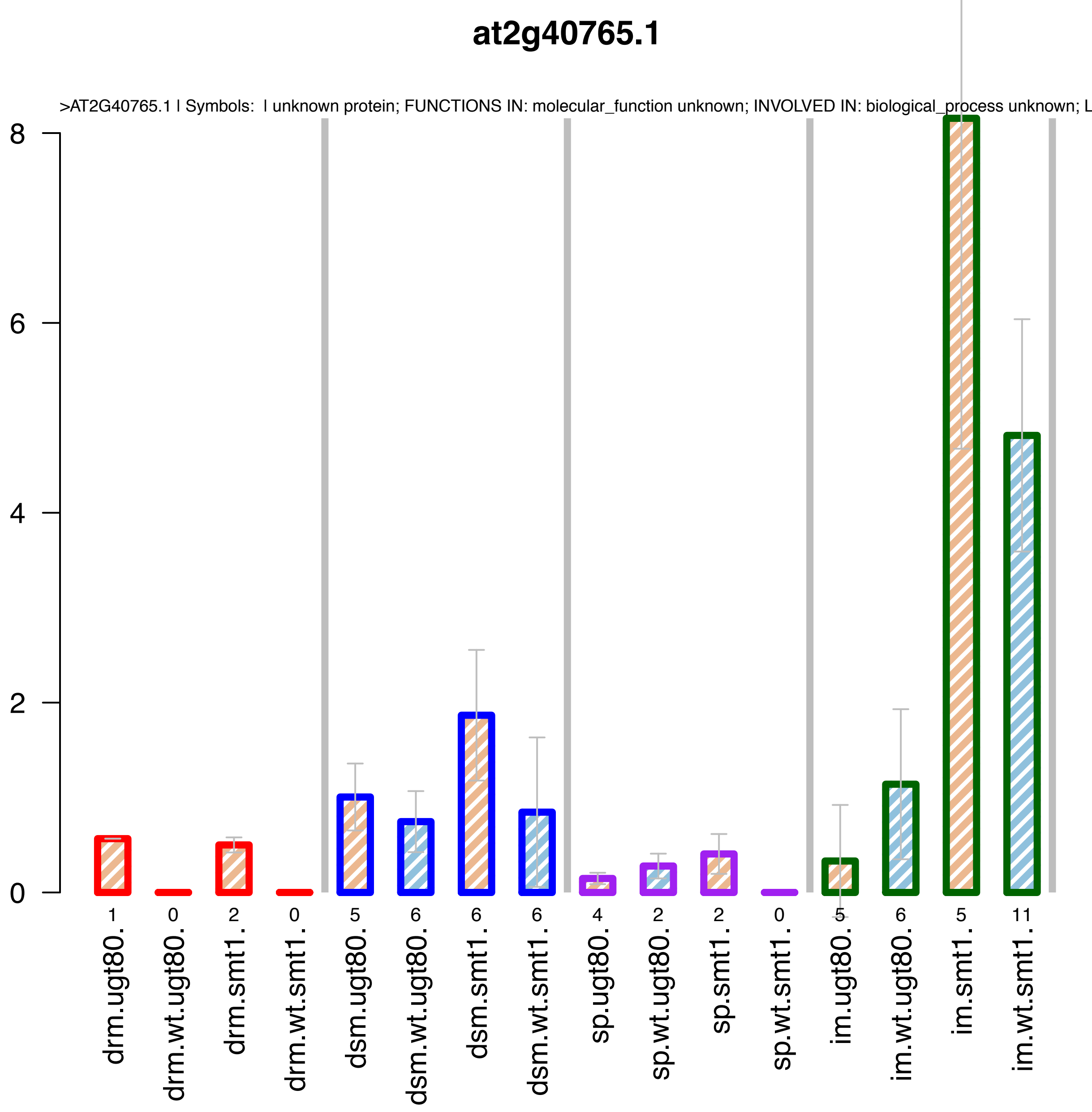

at2g40765.1
>AT2G40765.1 | Symbols: | unknown protein; FUNCTIONS IN: molecular_function unknown; INVOLVED IN: biological_process unknown; L
0
2
4
6
8
1
0
2
0
5
6
6
6
4
2
2
0
5
6
5
11
drm.ugt80.
drm.wt.ugt80.
drm.smt1.
drm.wt.smt1.
dsm.ugt80.
dsm.wt.ugt80.
dsm.smt1.
dsm.wt.smt1.
sp.ugt80.
sp.wt.ugt80.
sp.smt1.
sp.wt.smt1.
im.ugt80.
im.wt.ugt80.
im.smt1.
im.wt.smt1.

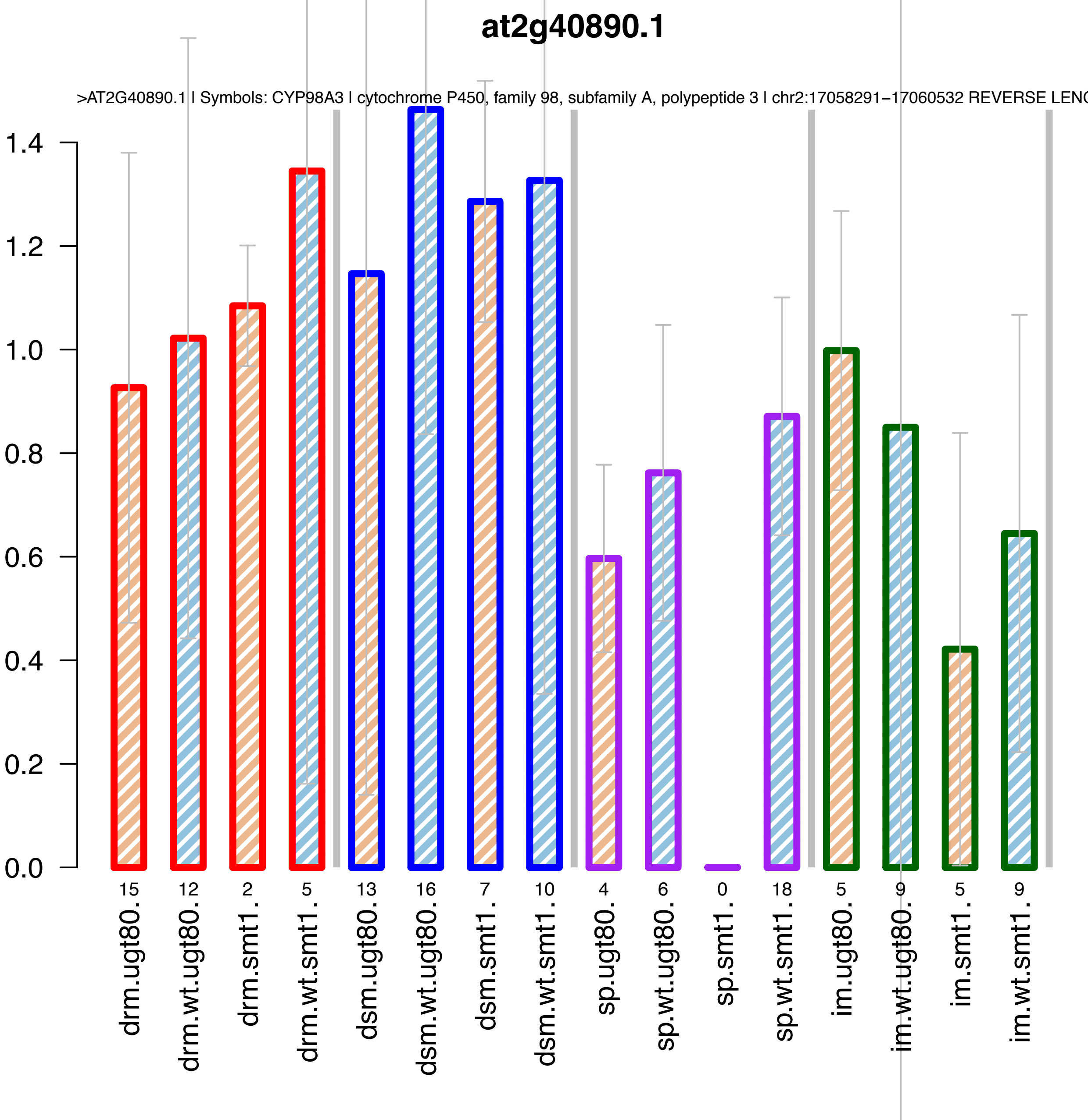

at2g40890.1
>AT2G40890.1 | Symbols: CYP98A3 | cytochrome P450, family 98, subfamily A, polypeptide 3 | chr2:17058291-17060532 REVERSE LENG
1.4
1.2
1.0
0.8
0.6
0.4
0.2
0.0
15
12
2
5
13
16
7
10
4
6
0
18
5
9
5
9
drm.ugt80..
drm.wt.ugt80..
drm.smt1..
drm.wt.smt1..
dsm.ugt80..
dsm.wt.ugt80..
dsm.smt1..
dsm.wt.smt1..
sp.ugt80..
sp.wt.ugt80..
sp.smt1..
sp.wt.smt1..
im.ugt80..
im.wt.ugt80..
im.smt1..
im.wt.smt1..

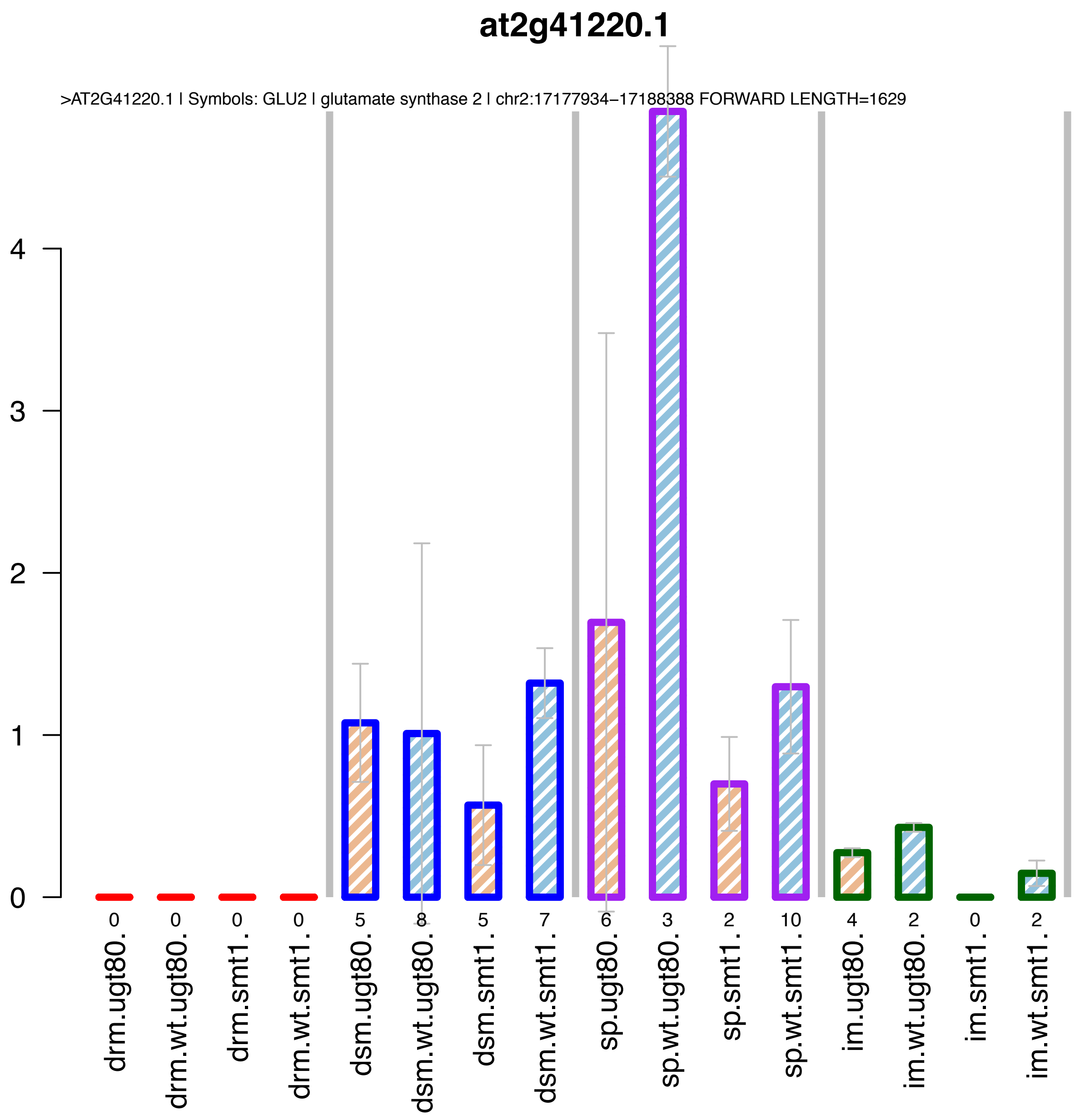

at2g41220.1
>AT2G41220.1 | Symbols: GLU2 | glutamate synthase 2 | chr2:17177934-17188388 FORWARD LENGTH=1629
0
1
2
3
4
0 0 0 0 5 8 5 7 6 3 2 10 4 2 0 2
drm.ugt80.
drm.wt.ugt80.
drm.smt1.
drm.wt.smt1.
dsm.ugt80.
dsm.wt.ugt80.
dsm.smt1.
dsm.wt.smt1.
sp.ugt80.
sp.wt.ugt80.
sp.smt1.
sp.wt.smt1.
im.ugt80.
im.wt.ugt80.
im.smt1.
im.wt.smt1.

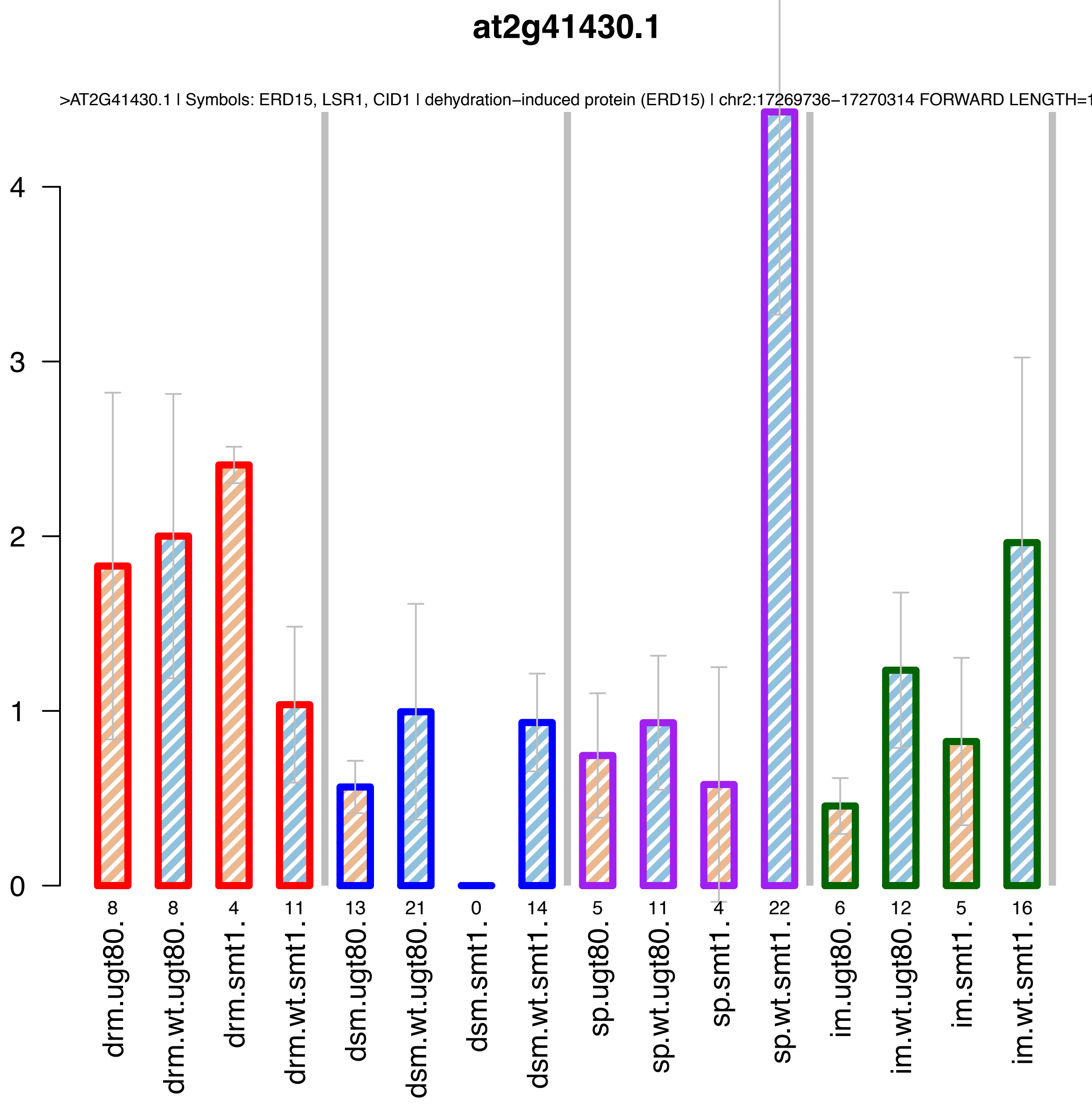

at2g41430.1
>AT2G41430.1 | Symbols: ERD15, LSR1, CID1 | dehydration−induced protein (ERD15) | chr2:17269736−17270314 FORWARD LENGTH=1
0
1
2
3
4
8
8
4
11
13
21
0
14
5
11
4
22
6
12
5
16
drm.ugt80..
drm.wt.ugt80..
drm.smt1..
drm.wt.smt1..
dsm.ugt80..
dsm.wt.ugt80..
dsm.smt1..
dsm.wt.smt1..
sp.ugt80..
sp.wt.ugt80..
sp.smt1..
sp.wt.smt1..
im.ugt80..
im.wt.ugt80..
im.smt1..
im.wt.smt1..

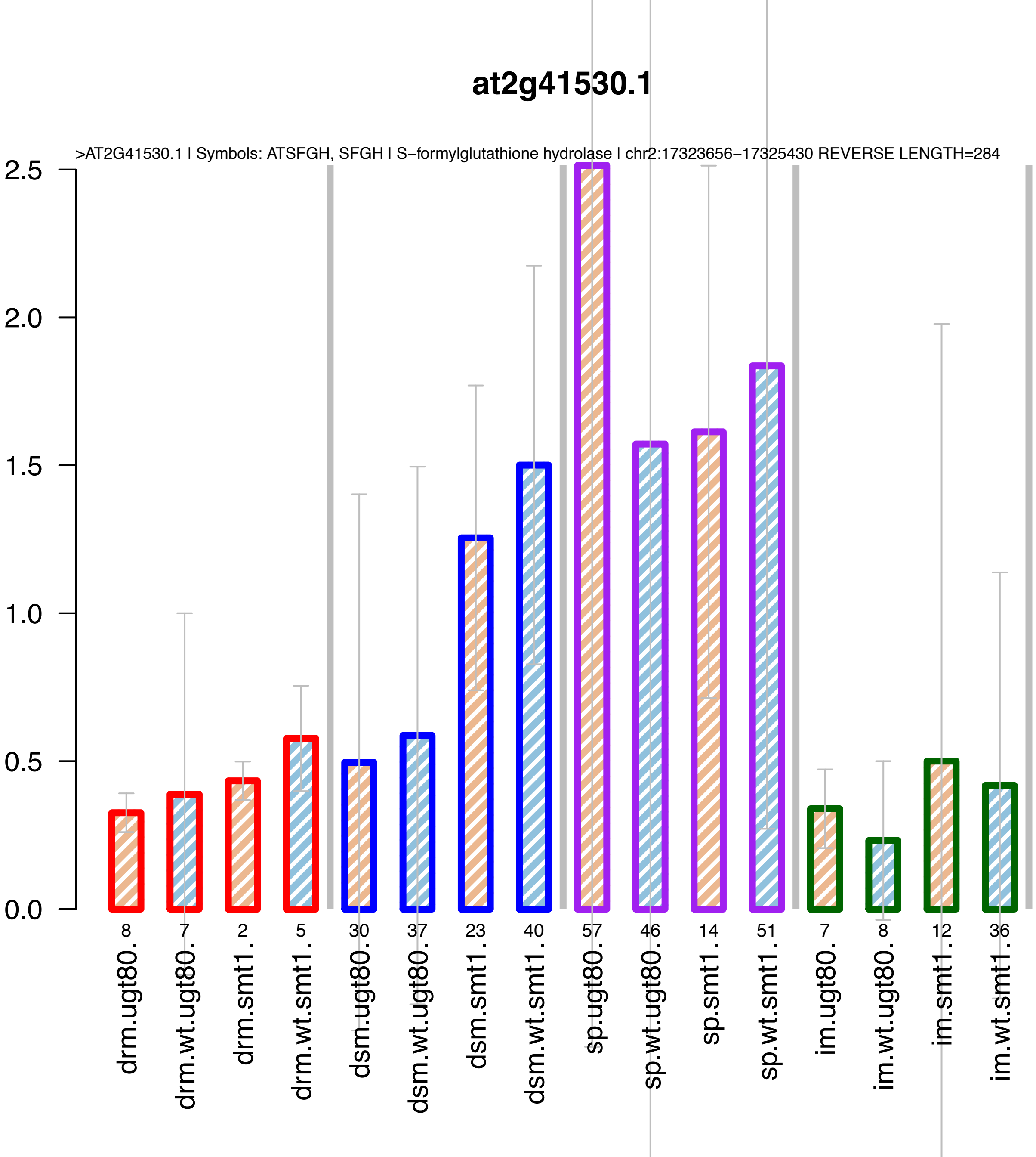

at2g41530.1
>AT2G41530.1 | Symbols: ATSFGH, SFGH | S−formylglutathione hydrolase | chr2:17323656−17325430 REVERSE LENGTH=284
2.5
2.0
1.5
1.0
0.5
0.0
8
7
2
5
30
37
23
40
57
46
14
51
7
8
12
36
drm.ugt80.
drm.wt.ugt80.
drm.smt1.
drm.wt.smt1.
dsm.ugt80.
dsm.wt.ugt80.
dsm.smt1.
dsm.wt.smt1.
sp.ugt80.
sp.wt.ugt80.
sp.smt1.
sp.wt.smt1.
im.ugt80.
im.wt.ugt80.
im.smt1.
im.wt.smt1.

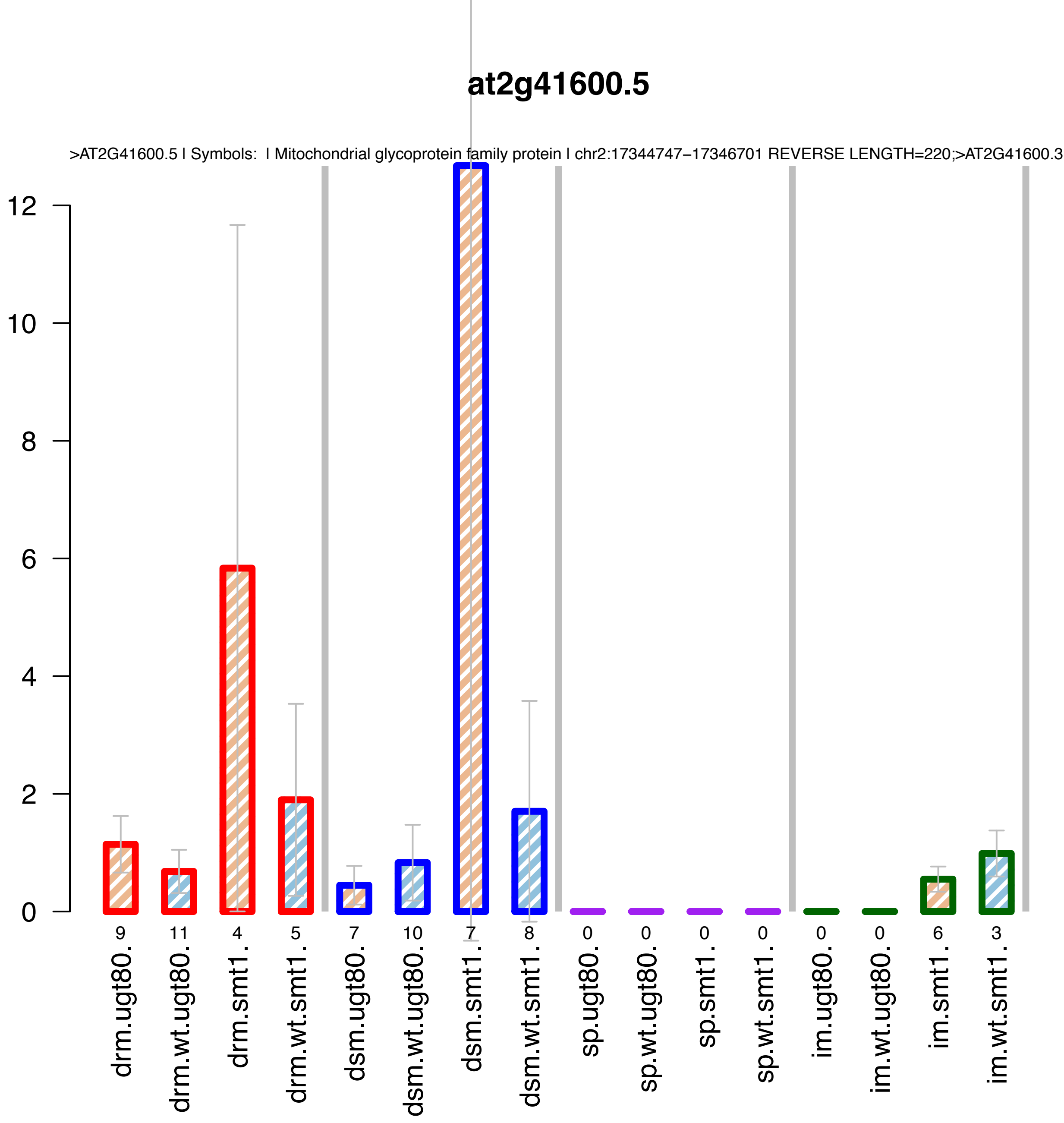

at2g41600.5
>AT2G41600.5 | Symbols: | Mitochondrial glycoprotein family protein | chr2:17344747−17346701 REVERSE LENGTH=220;>AT2G41600.3
0
2
4
6
8
10
12
9
11
4
5
7
10
7
8
0
0
0
0
0
0
6
3
drm.ugt80.
drm.wt.ugt80.
drm.smt1.
drm.wt.smt1.
dsm.ugt80.
dsm.wt.ugt80.
dsm.smt1.
dsm.wt.smt1.
sp.ugt80.
sp.wt.ugt80.
sp.smt1.
sp.wt.smt1.
im.ugt80.
im.wt.ugt80.
im.smt1.
im.wt.smt1.

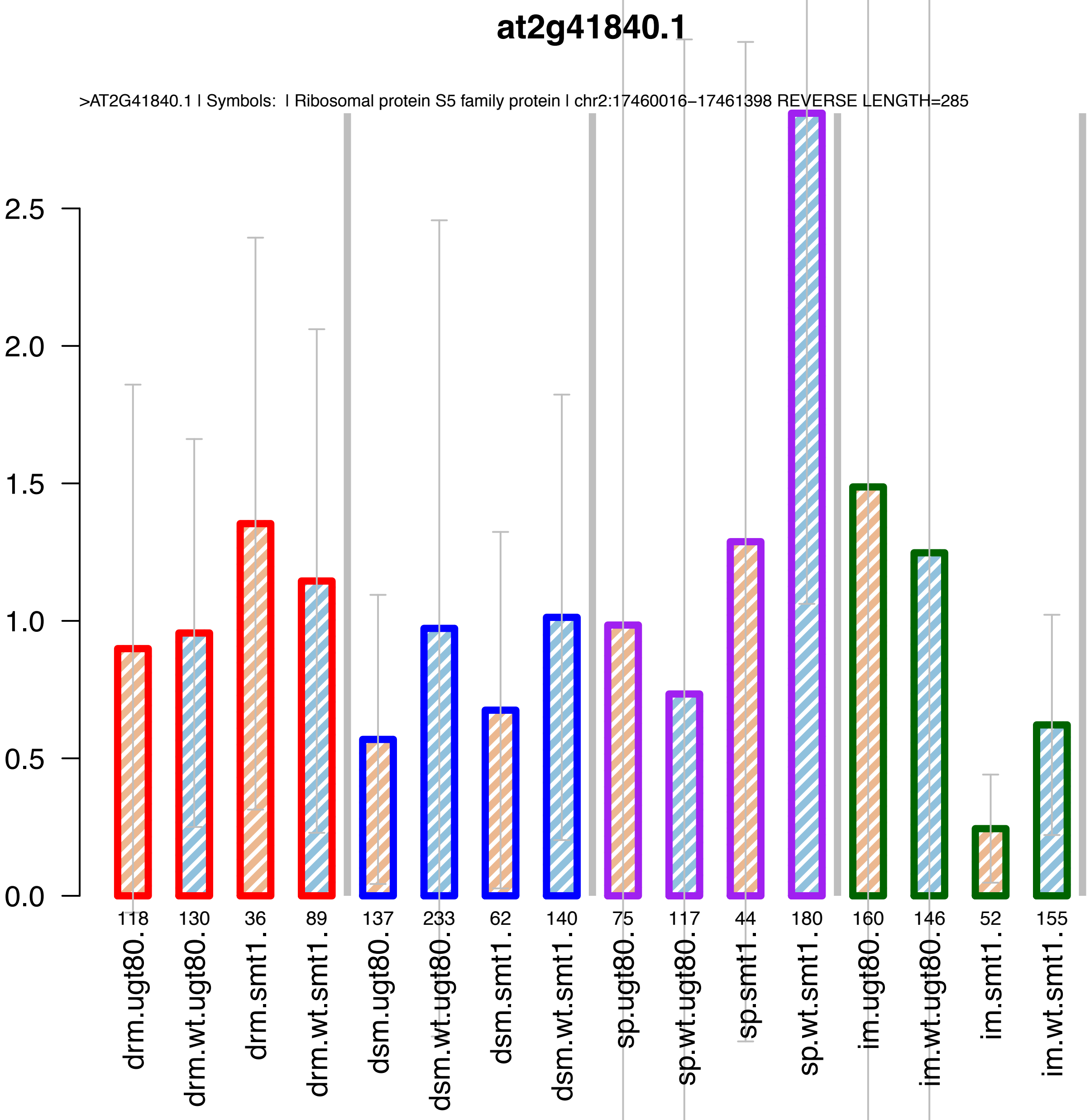

at2g41840.1
>AT2G41840.1 | Symbols: | Ribosomal protein S5 family protein | chr2:17460016-17461398 REVERSE LENGTH=285
0.0
0.5
1.0
1.5
2.0
2.5
118
130
36
89
137
233
62
140
75
117
44
180
160
146
52
155
drm.ugt80..
drm.wt.ugt80..
drm.smt1..
drm.wt.smt1..
dsm.ugt80..
dsm.wt.ugt80..
dsm.smt1..
dsm.wt.smt1..
sp.ugt80..
sp.wt.ugt80..
sp.smt1..
sp.wt.smt1..
im.ugt80..
im.wt.ugt80..
im.smt1..
im.wt.smt1..

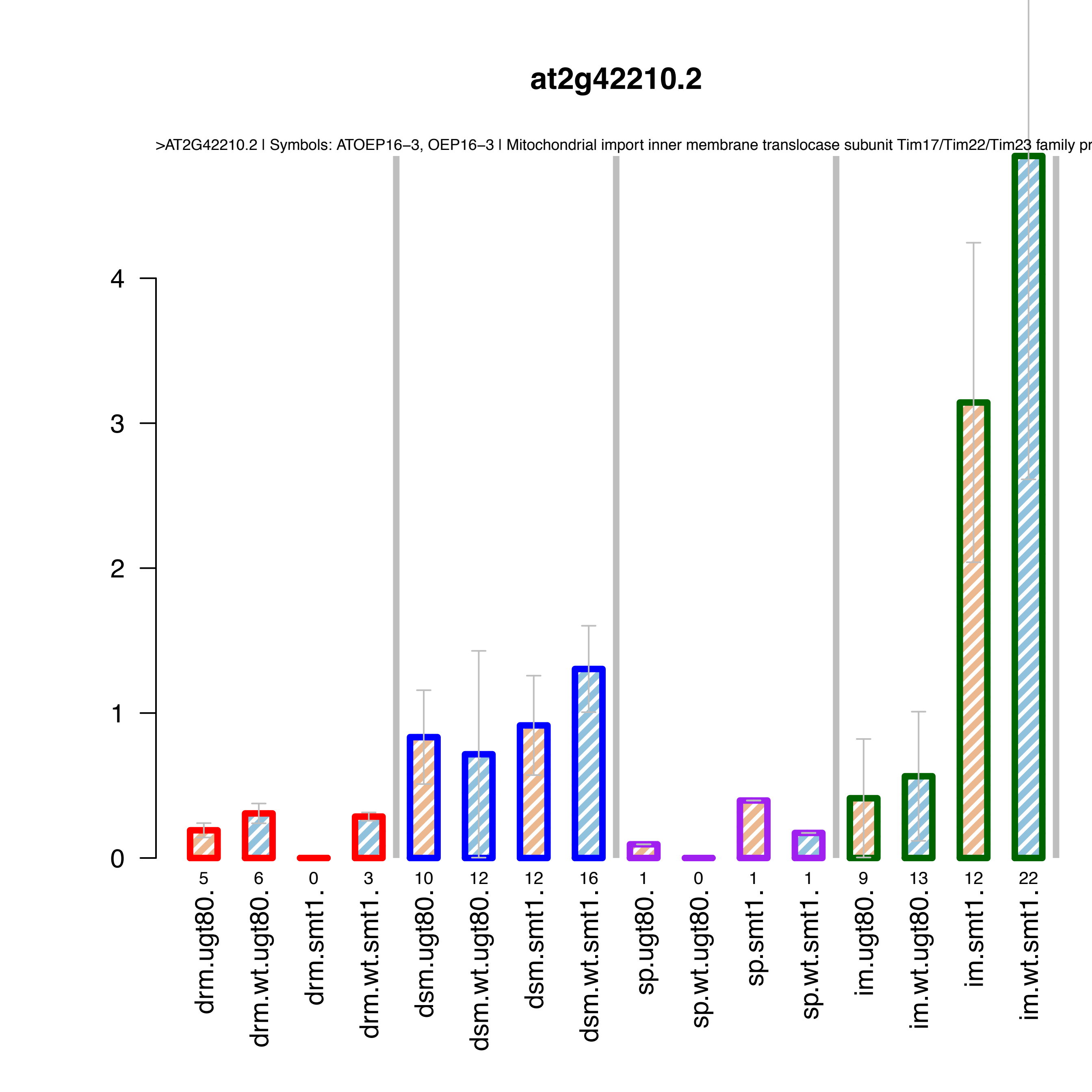

at2g42210.2
>AT2G42210.2 | Symbols: ATOEP16−3, OEP16−3 | Mitochondrial import inner membrane translocase subunit Tim17/Tim22/Tim23 family pr
0
1
2
3
4
5
6
0
3
10
12
12
16
1
0
1
1
9
13
12
22
drm.ugt80.
drm.wt.ugt80.
drm.smt1.
drm.wt.smt1.
dsm.ugt80.
dsm.wt.ugt80.
dsm.smt1.
dsm.wt.smt1.
sp.ugt80.
sp.wt.ugt80.
sp.smt1.
sp.wt.smt1.
im.ugt80.
im.wt.ugt80.
im.smt1.
im.wt.smt1.

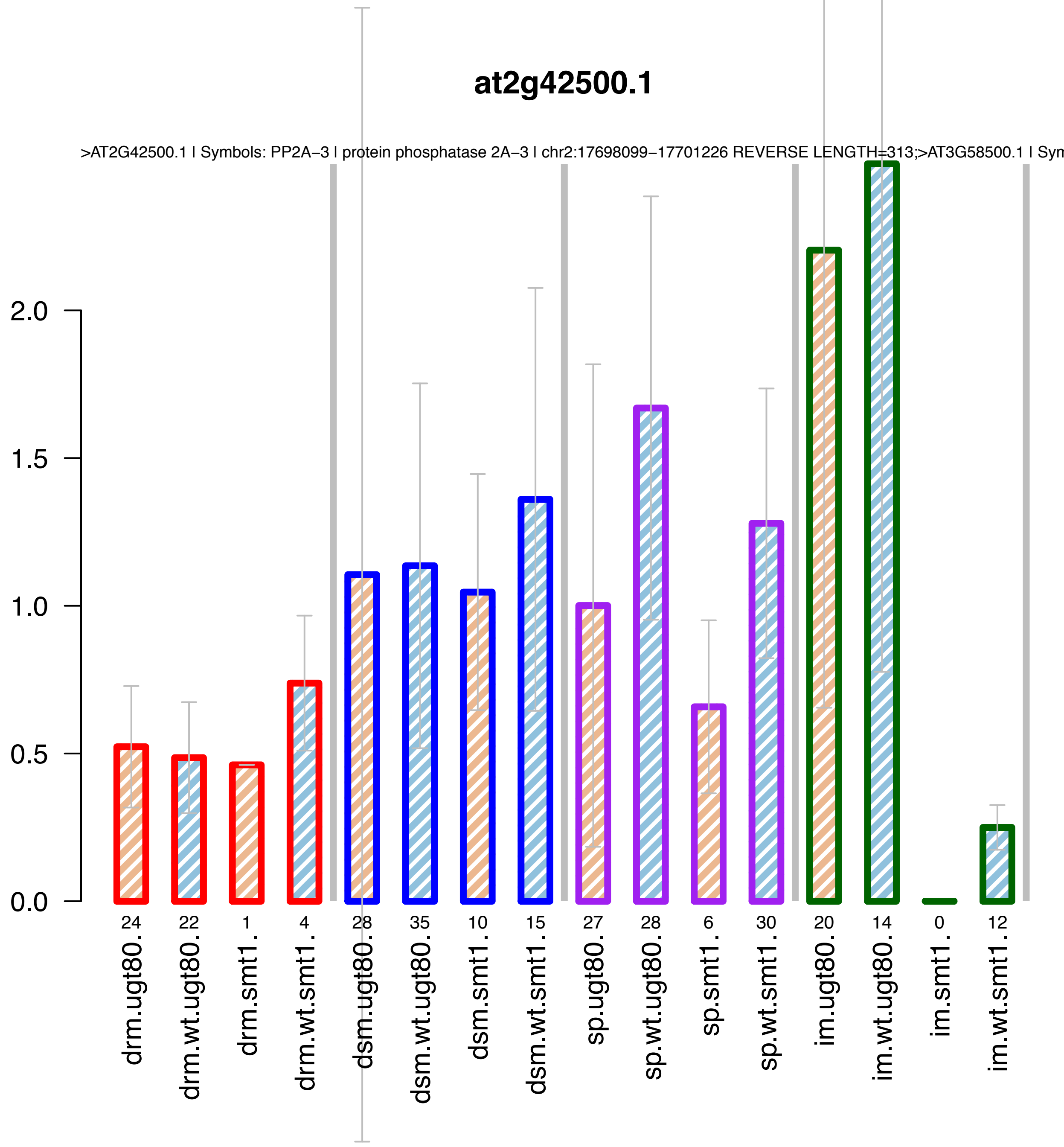

at2g42500.1
>AT2G42500.1 | Symbols: PP2A−3 | protein phosphatase 2A−3 | chr2:17698099−17701226 REVERSE LENGTH=313;>AT3G58500.1 | Sym
0.0
0.5
1.0
1.5
2.0
24
22
1
4
28
35
10
15
27
28
6
30
20
14
0
12
drm.ugt80..
drm.wt.ugt80..
drm.smt1..
drm.wt.smt1..
dsm.ugt80..
dsm.wt.ugt80..
dsm.smt1..
dsm.wt.smt1..
sp.ugt80..
sp.wt.ugt80..
sp.smt1..
sp.wt.smt1..
im.ugt80..
im.wt.ugt80..
im.smt1..
im.wt.smt1..

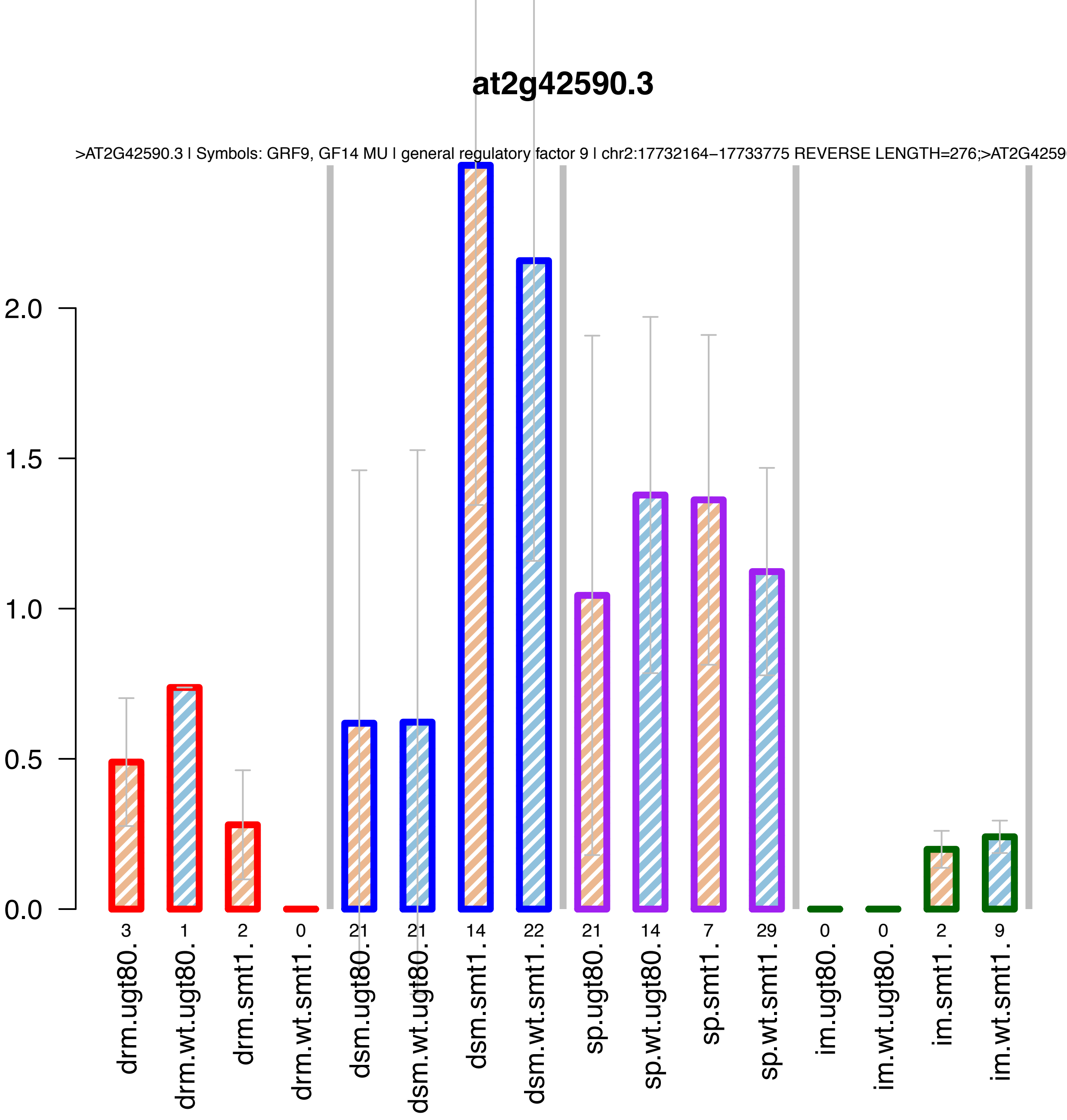

at2g42590.3
>AT2G42590.3 | Symbols: GRF9, GF14 MU | general regulatory factor 9 | chr2:17732164−17733775 REVERSE LENGTH=276;>AT2G4259
0.0
0.5
1.0
1.5
2.0
3
1
2
0
21
21
14
22
21
14
7
29
0
0
2
9
drm.ugt80..
drm.wt.ugt80..
drm.smt1..
drm.wt.smt1..
dsm.ugt80..
dsm.wt.ugt80..
dsm.smt1..
dsm.wt.smt1..
sp.ugt80..
sp.wt.ugt80..
sp.smt1..
sp.wt.smt1..
im.ugt80..
im.wt.ugt80..
im.smt1..
im.wt.smt1..

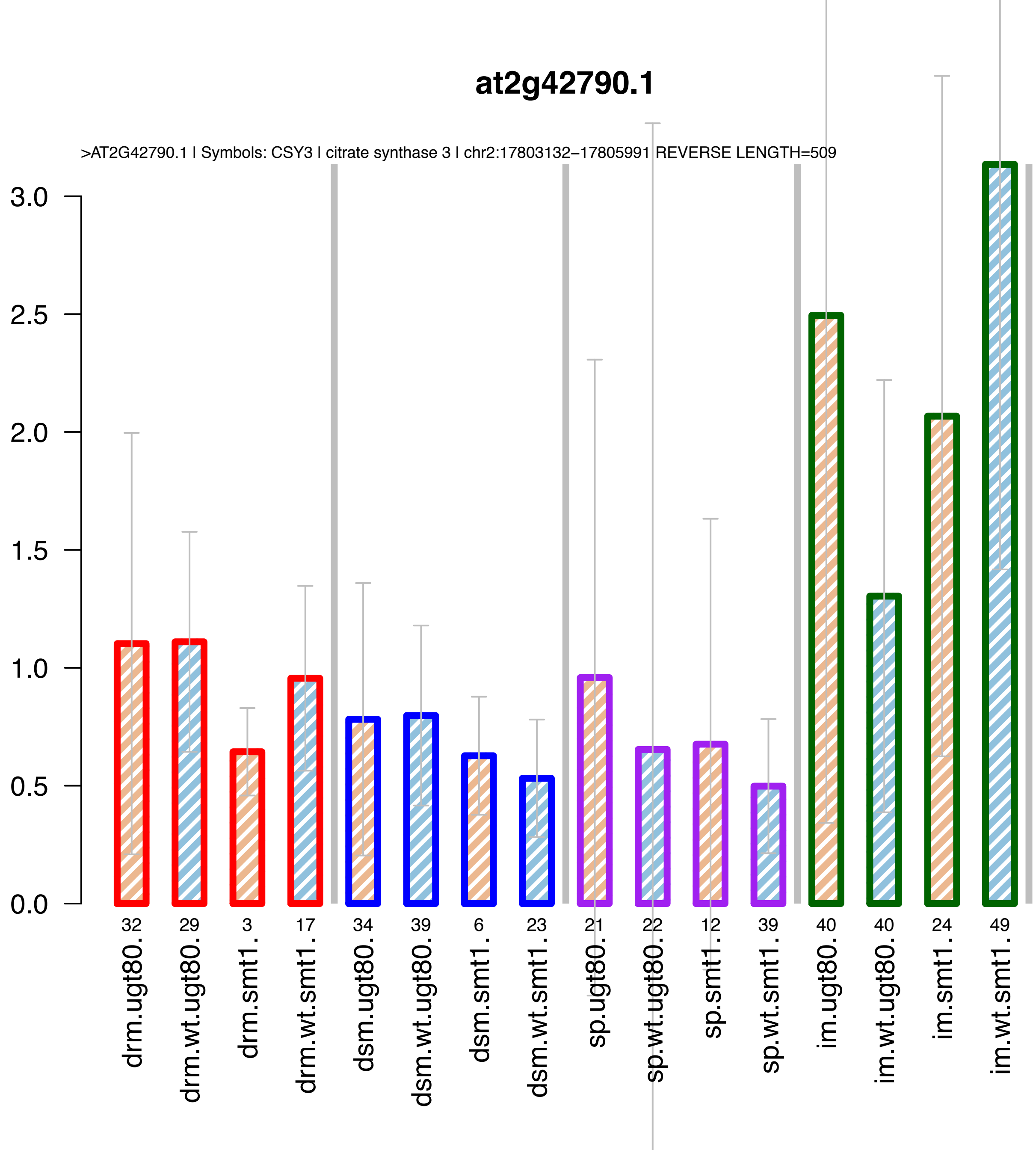

at2g42790.1
>AT2G42790.1 | Symbols: CSY3 | citrate synthase 3 | chr2:17803132–17805991 REVERSE LENGTH=509
3.0
2.5
2.0
1.5
1.0
0.5
0.0
32
29
3
17
34
39
6
23
21
22
12
39
40
40
24
49
drm.ugt80.
drm.wt.ugt80.
drm.smt1.
drm.wt.smt1.
dsm.ugt80.
dsm.wt.ugt80.
dsm.smt1.
dsm.wt.smt1.
sp.ugt80.
sp.wt.ugt80.
sp.smt1.
sp.wt.smt1.
im.ugt80.
im.wt.ugt80.
im.smt1.
im.wt.smt1.

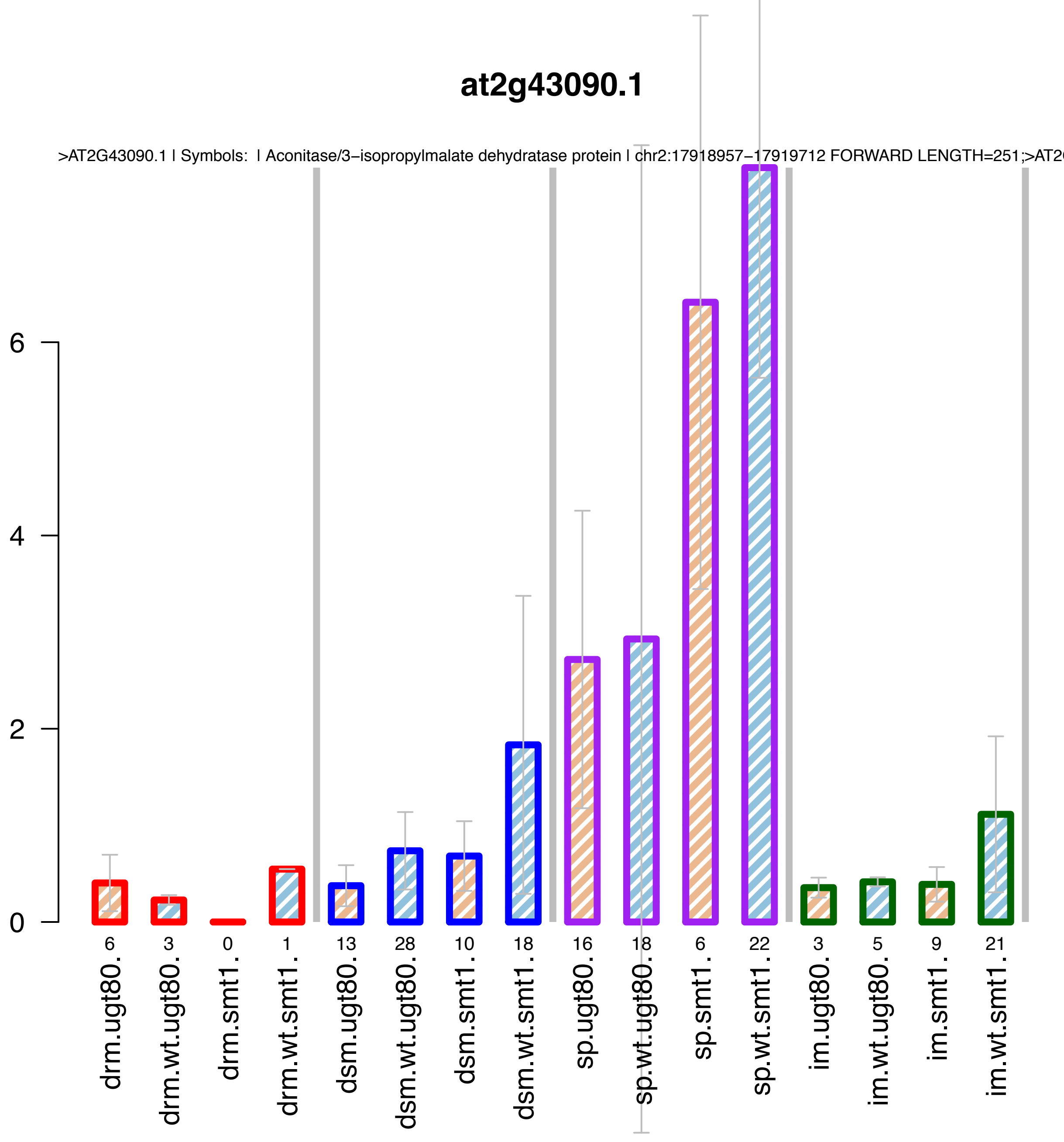

at2g43090.1
>AT2G43090.1 | Symbols: | Aconitase/3-isopropylmalate dehydratase protein | chr2:17918957-17919712 FORWARD LENGTH=251;>AT2
0
2
4
6
6
3
0
1
13
28
10
18
16
18
6
22
3
5
9
21
drm.ugt80..
drm.wt.ugt80..
drm.smt1..
drm.wt.smt1..
dsm.ugt80..
dsm.wt.ugt80..
dsm.smt1..
dsm.wt.smt1..
sp.ugt80..
sp.wt.ugt80..
sp.smt1..
sp.wt.smt1..
im.ugt80..
im.wt.ugt80..
im.smt1..
im.wt.smt1..

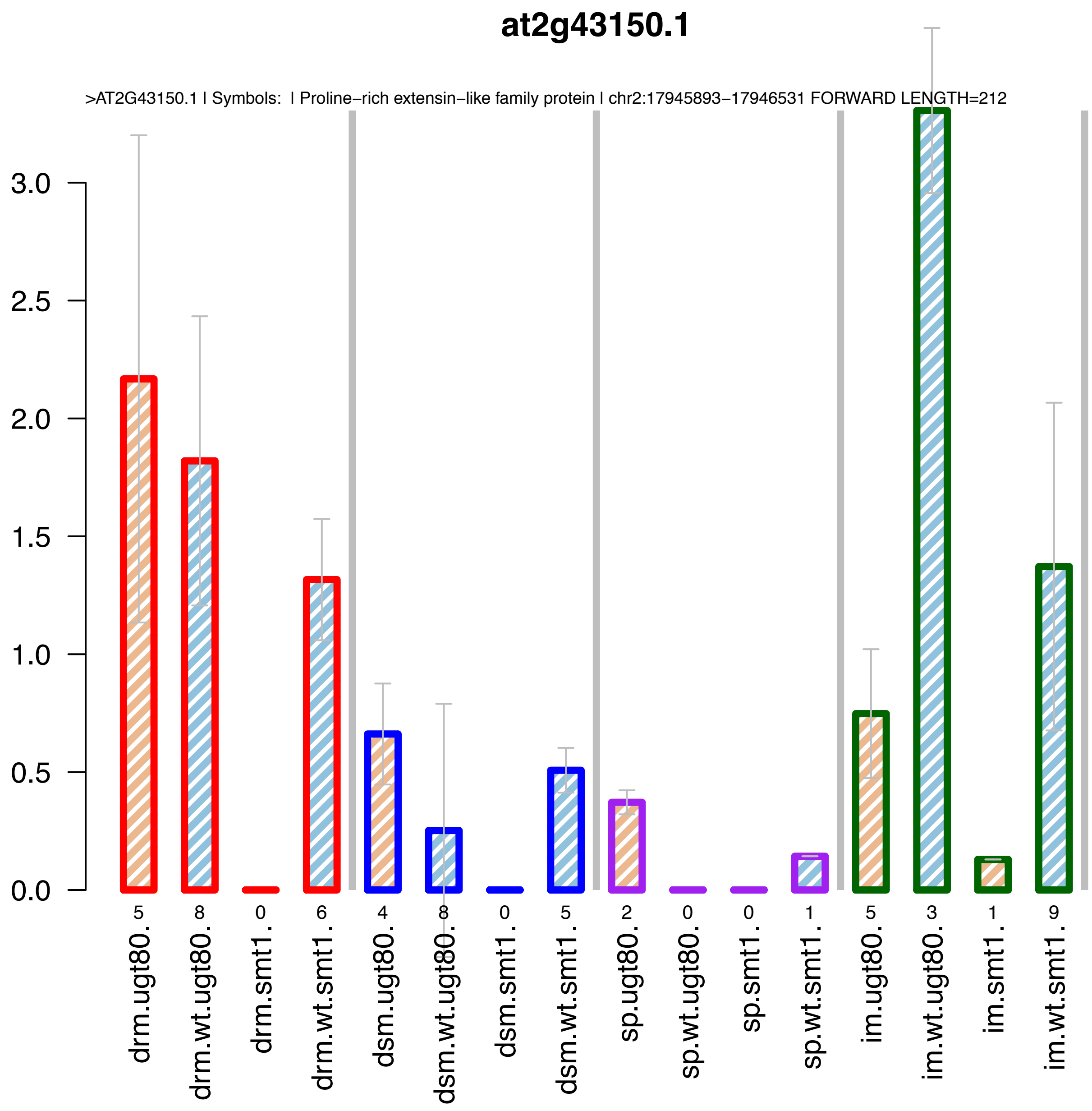

at2g43150.1
>AT2G43150.1 | Symbols: | Proline−rich extensin−like family protein | chr2:17945893−17946531 FORWARD LENGTH=212
0.0
0.5
1.0
1.5
2.0
2.5
3.0
5
8
0
6
4
8
0
5
2
0
0
1
5
3
1
9
drm.ugt80..
drm.wt.ugt80..
drm.smt1..
drm.wt.smt1..
dsm.ugt80..
dsm.wt.ugt80..
dsm.smt1..
dsm.wt.smt1..
sp.ugt80..
sp.wt.ugt80..
sp.smt1..
sp.wt.smt1..
im.ugt80..
im.wt.ugt80..
im.smt1..
im.wt.smt1..

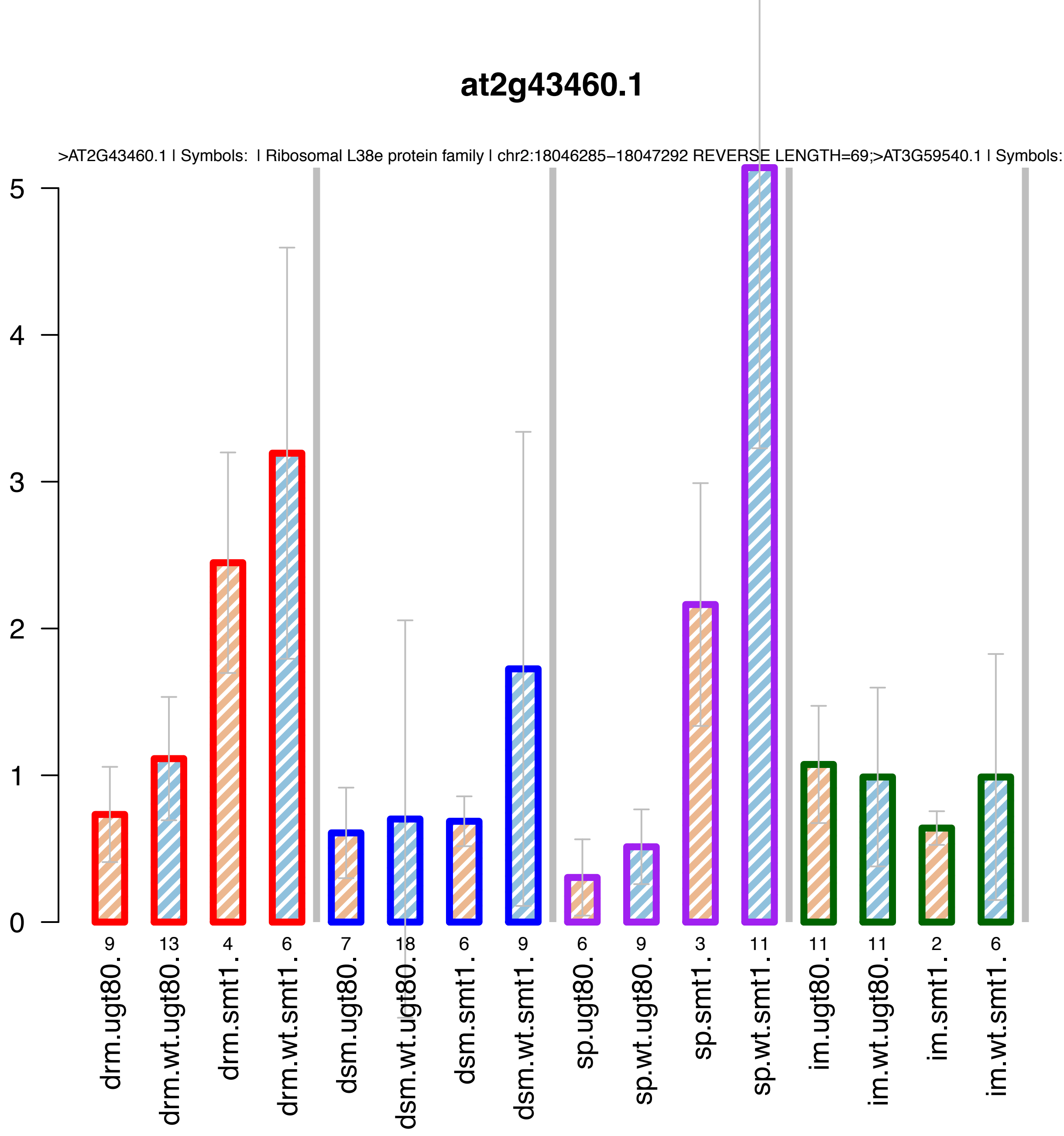

at2g43460.1
>AT2G43460.1 | Symbols: | Ribosomal L38e protein family | chr2:18046285-18047292 REVERSE LENGTH=69;>AT3G59540.1 | Symbols:
0
1
2
3
4
5
9
13
4
6
7
18
6
9
6
9
3
11
11
11
2
6
drm.ugt80.
drm.wt.ugt80.
drm.smt1.
drm.wt.smt1.
dsm.ugt80.
dsm.wt.ugt80.
dsm.smt1.
dsm.wt.smt1.
sp.ugt80.
sp.wt.ugt80.
sp.smt1.
sp.wt.smt1.
im.ugt80.
im.wt.ugt80.
im.smt1.
im.wt.smt1.

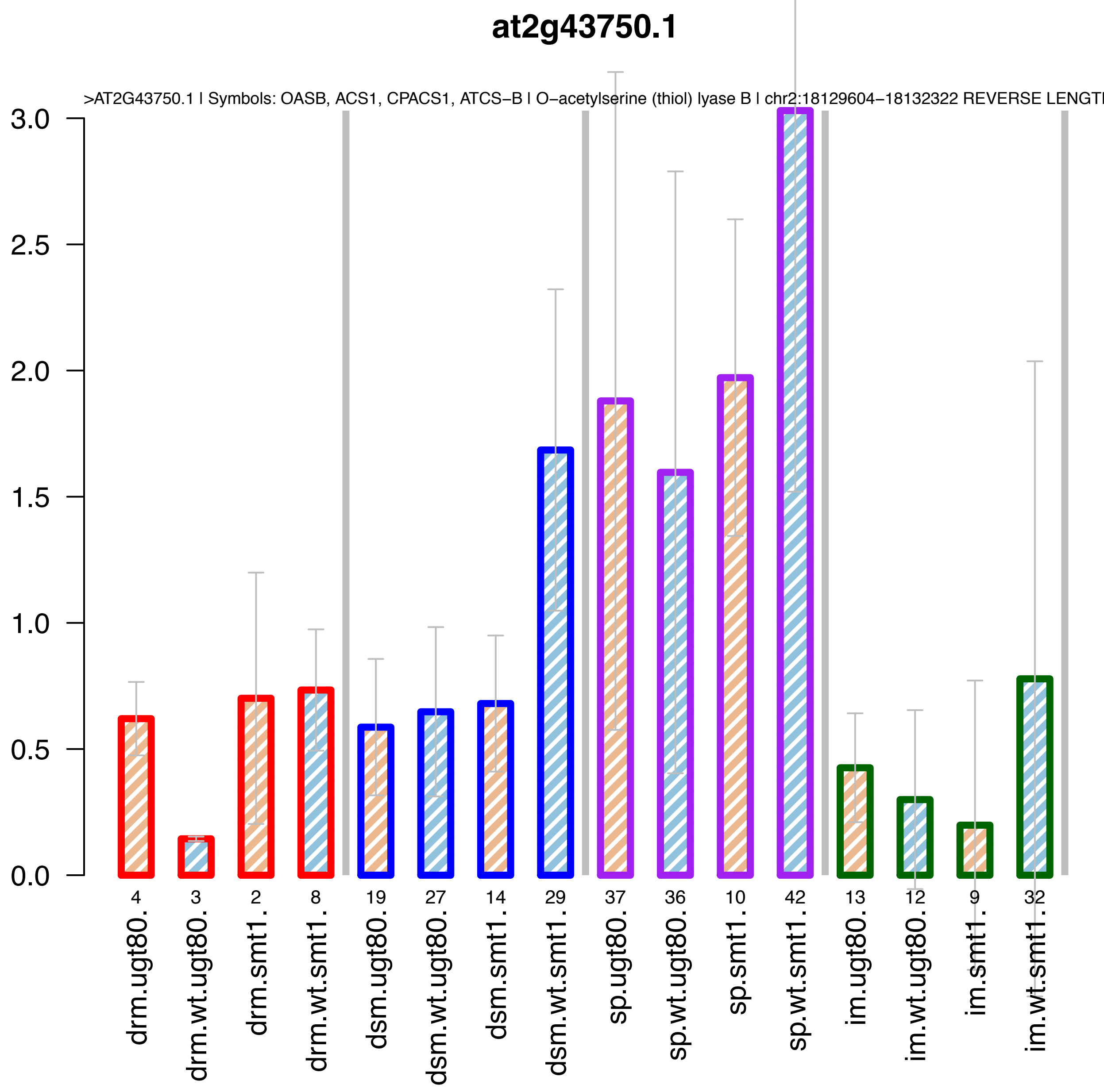

at2g43750.1
>AT2G43750.1 | Symbols: OASB, ACS1, CPACS1, ATCS-B | O-acetylserine (thiol) lyase B | chr2:18129604-18132322 REVERSE LENGTH
3.0
2.5
2.0
1.5
1.0
0.5
0.0
4
3
2
8
19
27
14
29
37
36
10
42
13
12
9
32
drm.ugt80..
drm.wt.ugt80..
drm.smt1..
drm.wt.smt1..
dsm.ugt80..
dsm.wt.ugt80..
dsm.smt1..
dsm.wt.smt1..
sp.ugt80..
sp.wt.ugt80..
sp.smt1..
sp.wt.smt1..
im.ugt80..
im.wt.ugt80..
im.smt1..
im.wt.smt1..

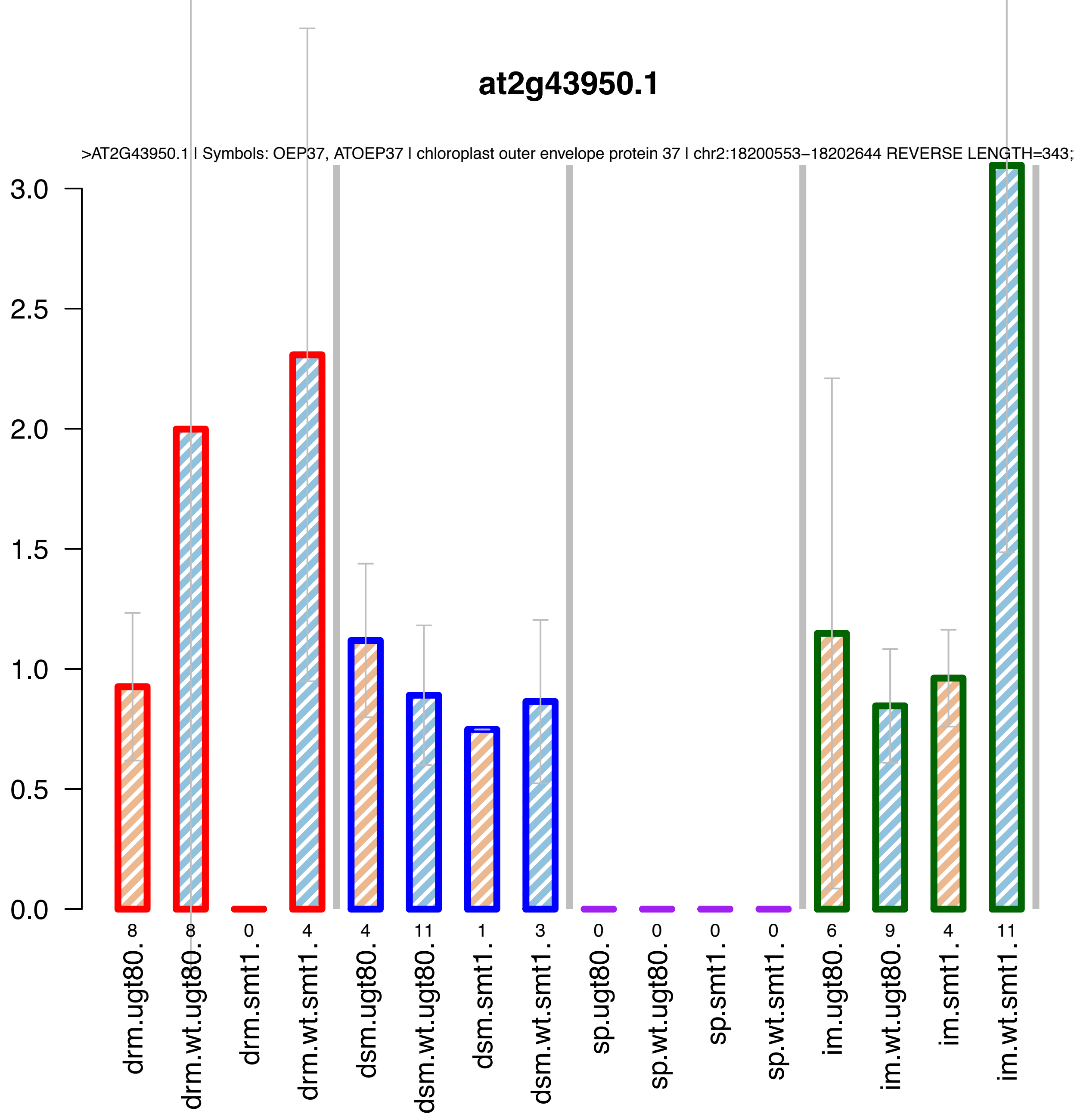
at2g43950.1
>AT2G43950.1 | Symbols: OEP37, ATOEP37 | chloroplast outer envelope protein 37 | chr2:18200553−18202644 REVERSE LENGTH=343;
3.0
2.5
2.0
1.5
1.0
0.5
0.0
8
8
0
4
4
11
1
3
0
0
0
0
6
9
4
11
drm.ugt80..
drm.wt.ugt80..
drm.smt1..
drm.wt.smt1..
dsm.ugt80..
dsm.wt.ugt80..
dsm.smt1..
dsm.wt.smt1..
sp.ugt80..
sp.wt.ugt80..
sp.smt1..
sp.wt.smt1..
im.ugt80..
im.wt.ugt80..
im.smt1..
im.wt.smt1..

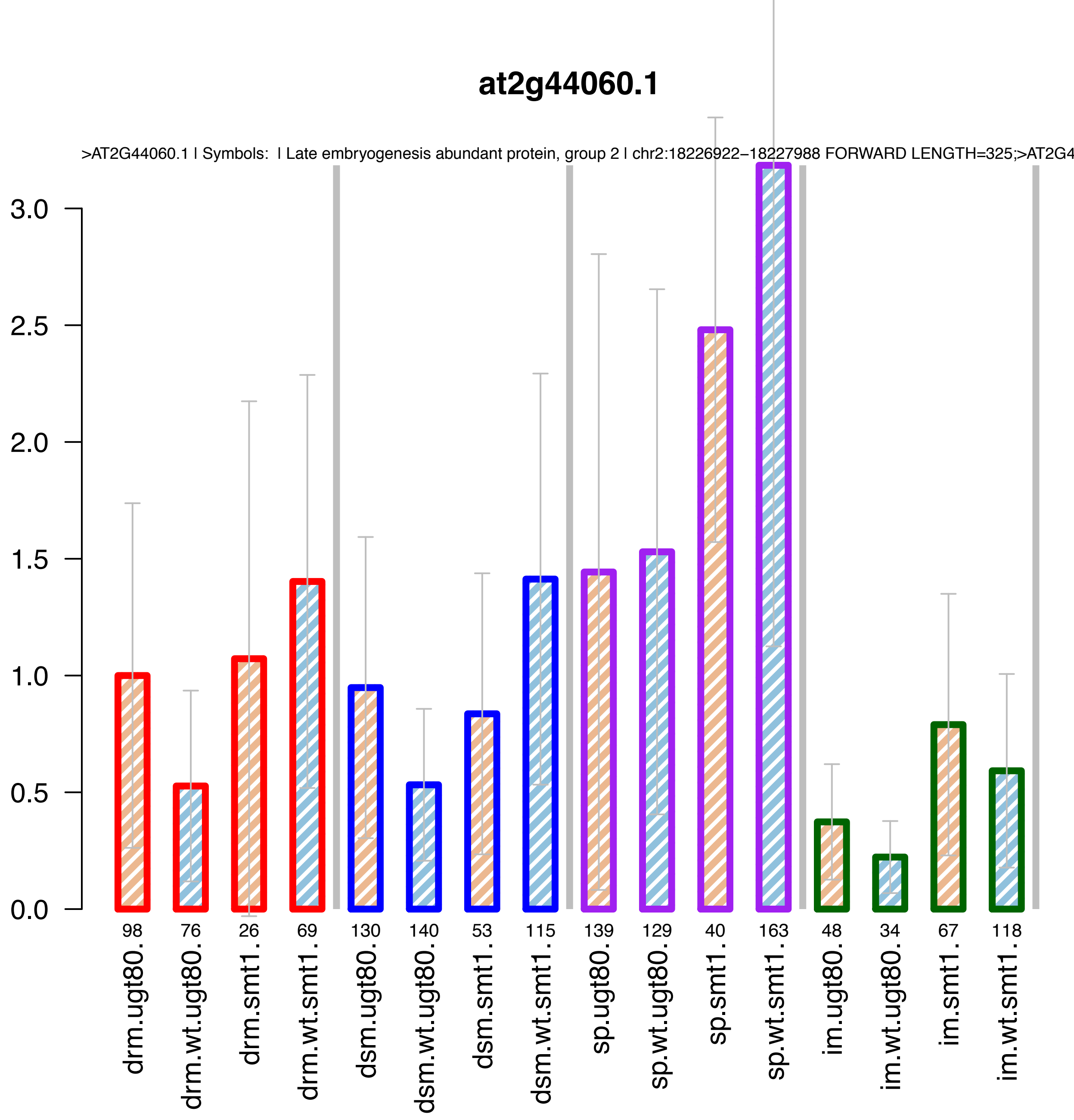

at2g44060.1
>AT2G44060.1 | Symbols: | Late embryogenesis abundant protein, group 2 | chr2:18226922-18227988 FORWARD LENGTH=325;>AT2G4
0.0
0.5
1.0
1.5
2.0
2.5
3.0
98
76
26
69
130
140
53
115
139
129
40
163
48
34
67
118
drm.ugt80..
drm.wt.ugt80..
drm.smt1..
drm.wt.smt1..
dsm.ugt80..
dsm.wt.ugt80..
dsm.smt1..
dsm.wt.smt1..
sp.ugt80..
sp.wt.ugt80..
sp.smt1..
sp.wt.smt1..
im.ugt80..
im.wt.ugt80..
im.smt1..
im.wt.smt1..

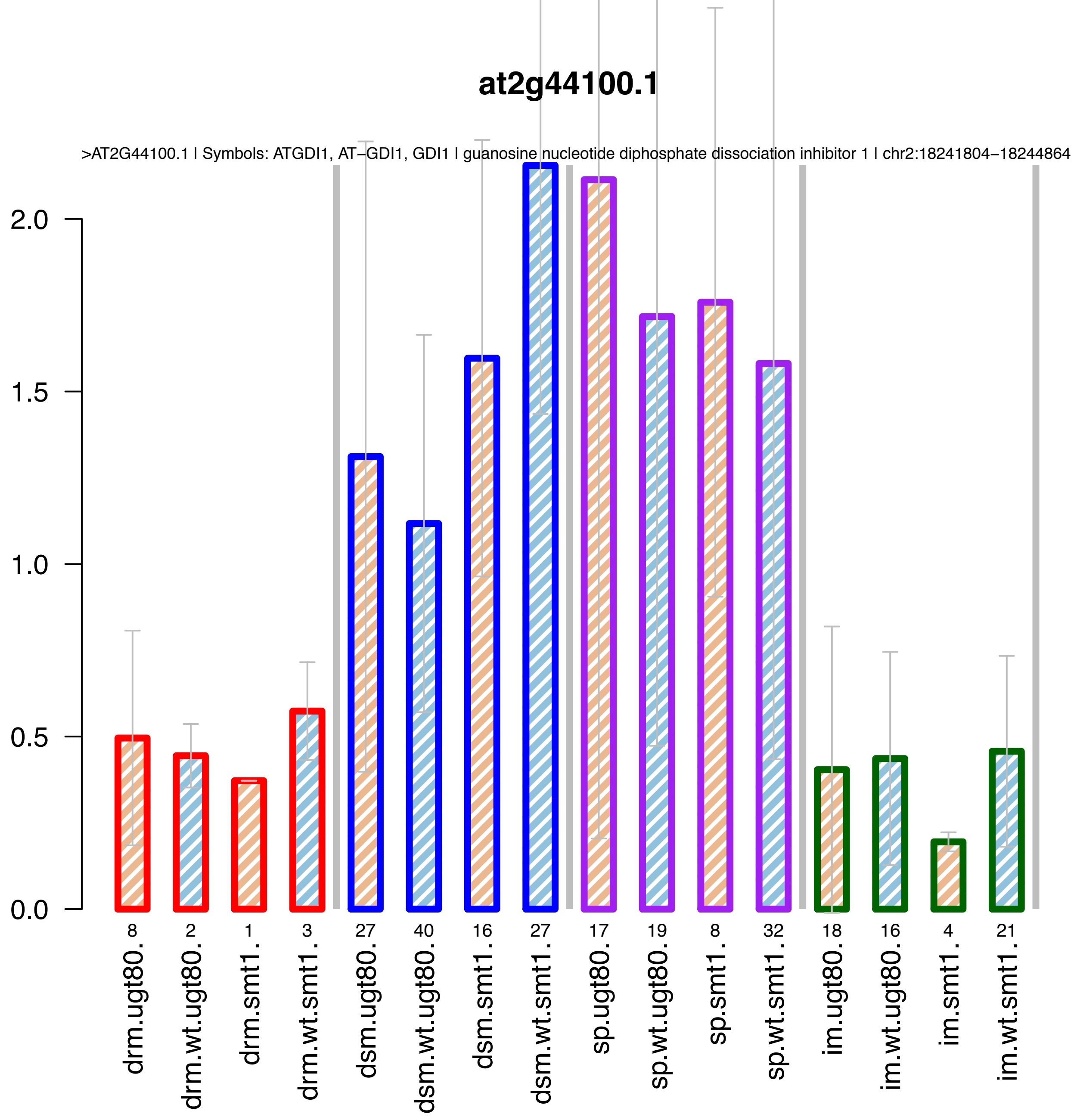
at2g44100.1
>AT2G44100.1 | Symbols: ATGDI1, AT−GDI1, GDI1 | guanosine nucleotide diphosphate dissociation inhibitor 1 | chr2:18241804−18244864
2.0
1.5
1.0
0.5
0.0
8
2
1
3
27
40
16
27
17
19
8
32
18
16
4
21
drm.ugt80..
drm.wt.ugt80..
drm.smt1..
drm.wt.smt1..
dsm.ugt80..
dsm.wt.ugt80..
dsm.smt1..
dsm.wt.smt1..
sp.ugt80..
sp.wt.ugt80..
sp.smt1..
sp.wt.smt1..
im.ugt80..
im.wt.ugt80..
im.smt1..
im.wt.smt1..

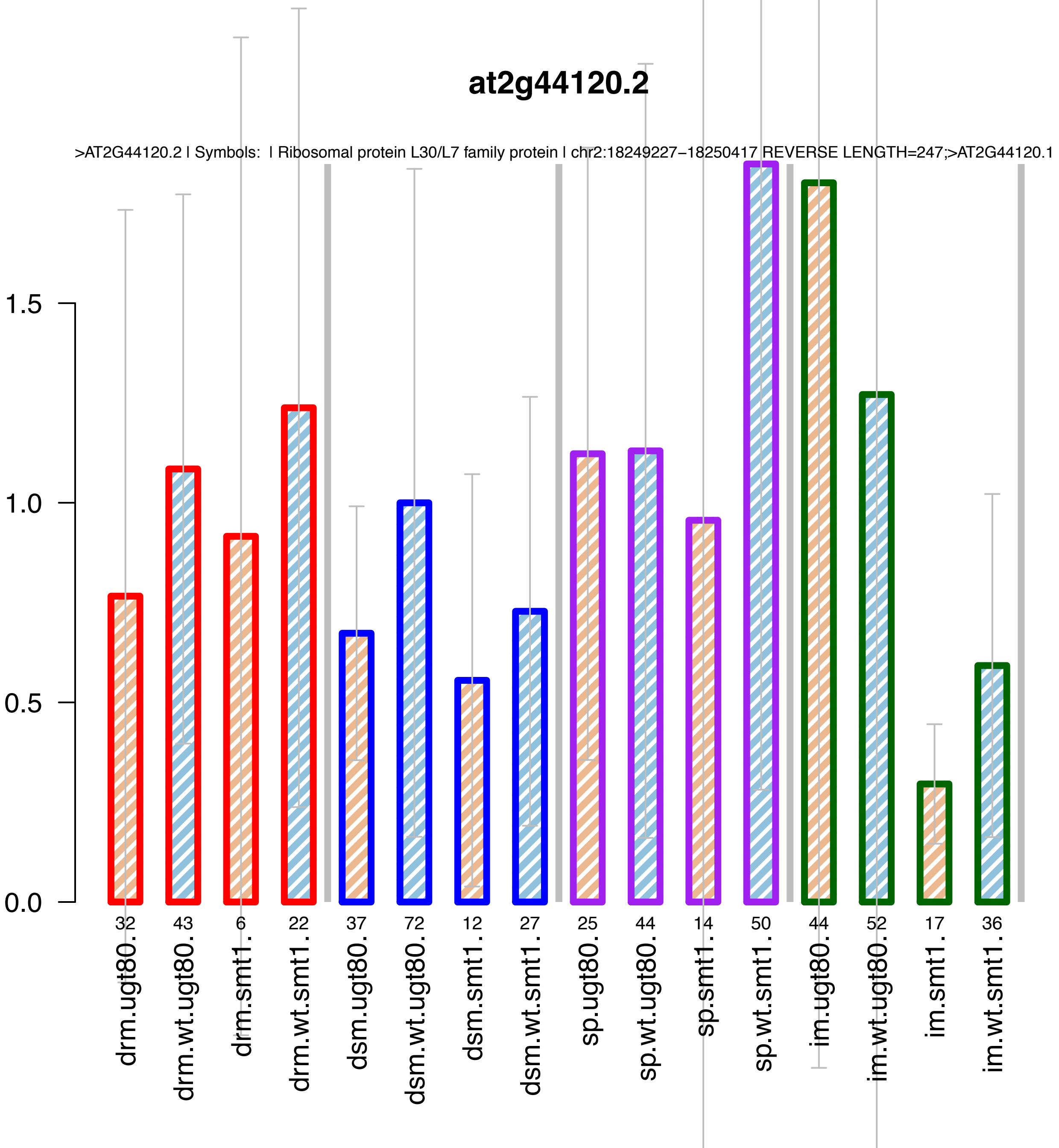
at2g44120.2
>AT2G44120.2 | Symbols: | Ribosomal protein L30/L7 family protein | chr2:18249227−18250417 REVERSE LENGTH=247;>AT2G44120.1
0.0
0.5
1.0
1.5
32
43
6
22
37
72
12
27
25
44
14
50
44
52
17
36
drm.ugt80..
drm.wt.ugt80..
drm.smt1..
drm.wt.smt1..
dsm.ugt80..
dsm.wt.ugt80..
dsm.smt1..
dsm.wt.smt1..
sp.ugt80..
sp.wt.ugt80..
sp.smt1..
sp.wt.smt1..
im.ugt80..
im.wt.ugt80..
im.smt1..
im.wt.smt1..

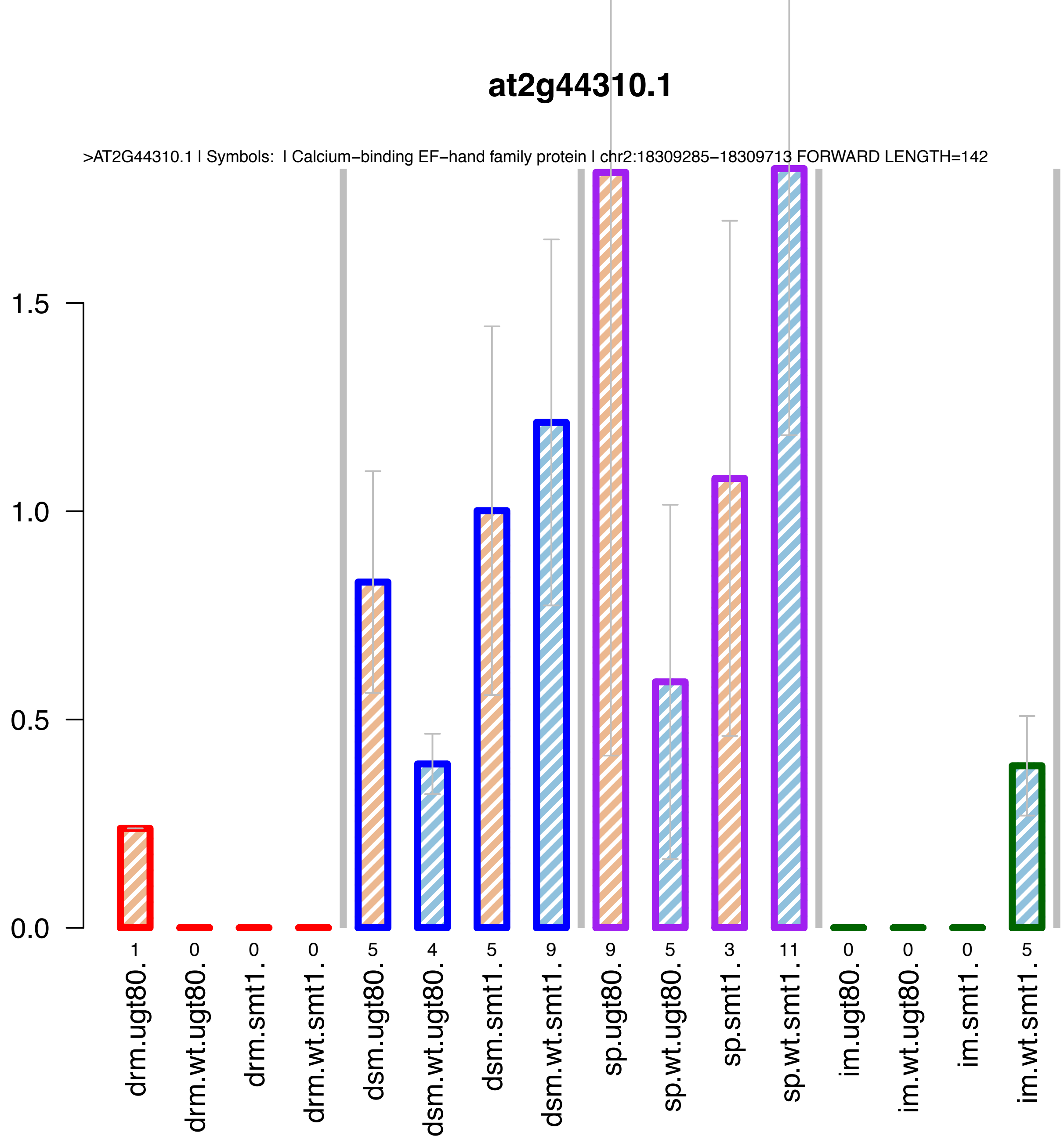

at2g44310.1
>AT2G44310.1 | Symbols: | Calcium−binding EF−hand family protein | chr2:18309285−18309713 FORWARD LENGTH=142
0.0
0.5
1.0
1.5
1
0
0
0
5
4
5
9
9
5
3
11
0
0
0
5
drm.ugt80.
drm.wt.ugt80.
drm.smt1.
drm.wt.smt1.
dsm.ugt80.
dsm.wt.ugt80.
dsm.smt1.
dsm.wt.smt1.
sp.ugt80.
sp.wt.ugt80.
sp.smt1.
sp.wt.smt1.
im.ugt80.
im.wt.ugt80.
im.smt1.
im.wt.smt1.

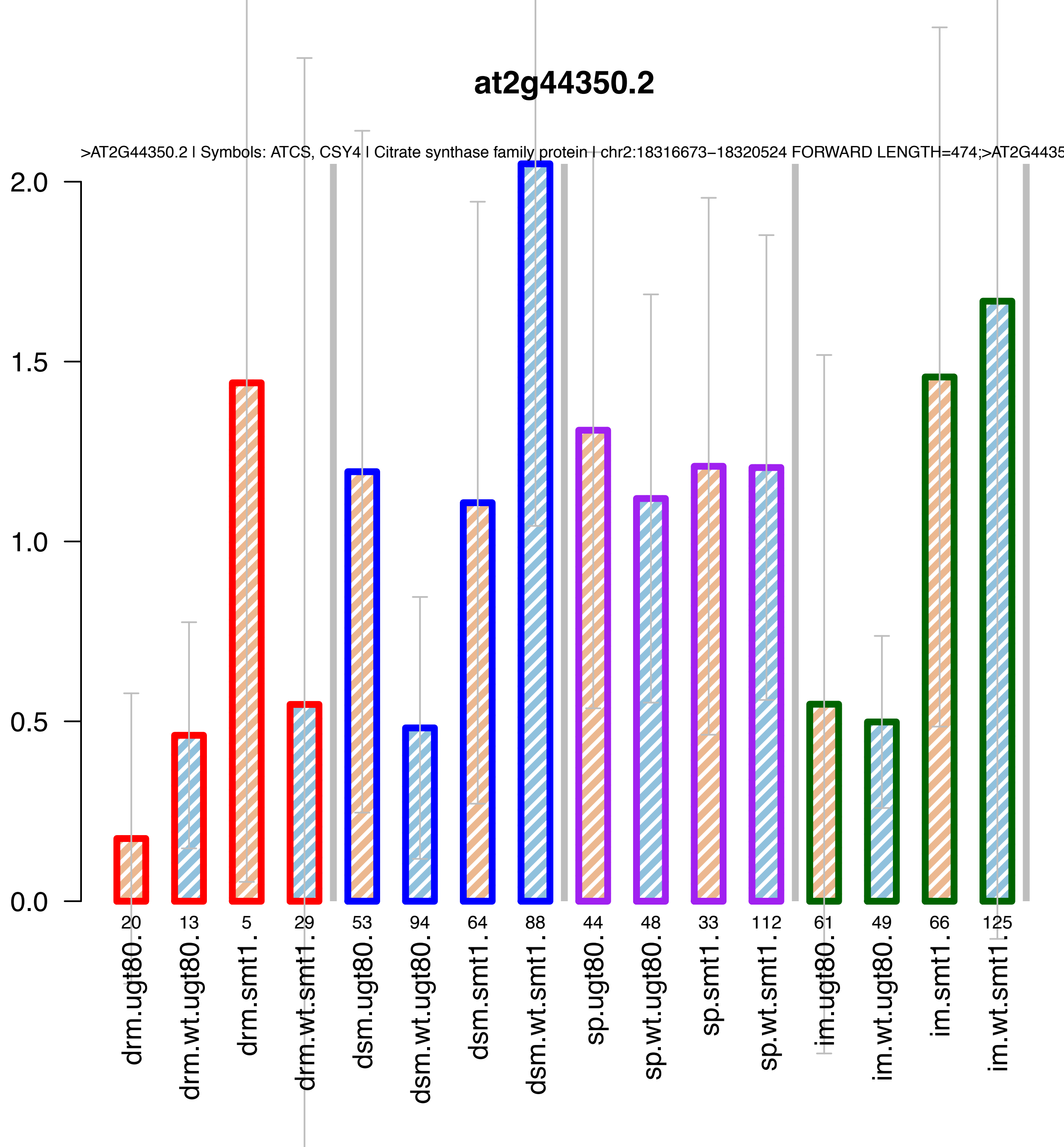
at2g44350.2
>AT2G44350.2 | Symbols: ATCS, CSY4 | Citrate synthase family protein | chr2:18316673-18320524 FORWARD LENGTH=474;>AT2G4435
2.0
1.5
1.0
0.5
0.0
20
13
5
29
53
94
64
88
44
48
33
112
61
49
66
125
drm.ugt80..
drm.wt.ugt80..
drm.smt1..
drm.wt.smt1..
dsm.ugt80..
dsm.wt.ugt80..
dsm.smt1..
dsm.wt.smt1..
sp.ugt80..
sp.wt.ugt80..
sp.smt1..
sp.wt.smt1..
im.ugt80..
im.wt.ugt80..
im.smt1..
im.wt.smt1..

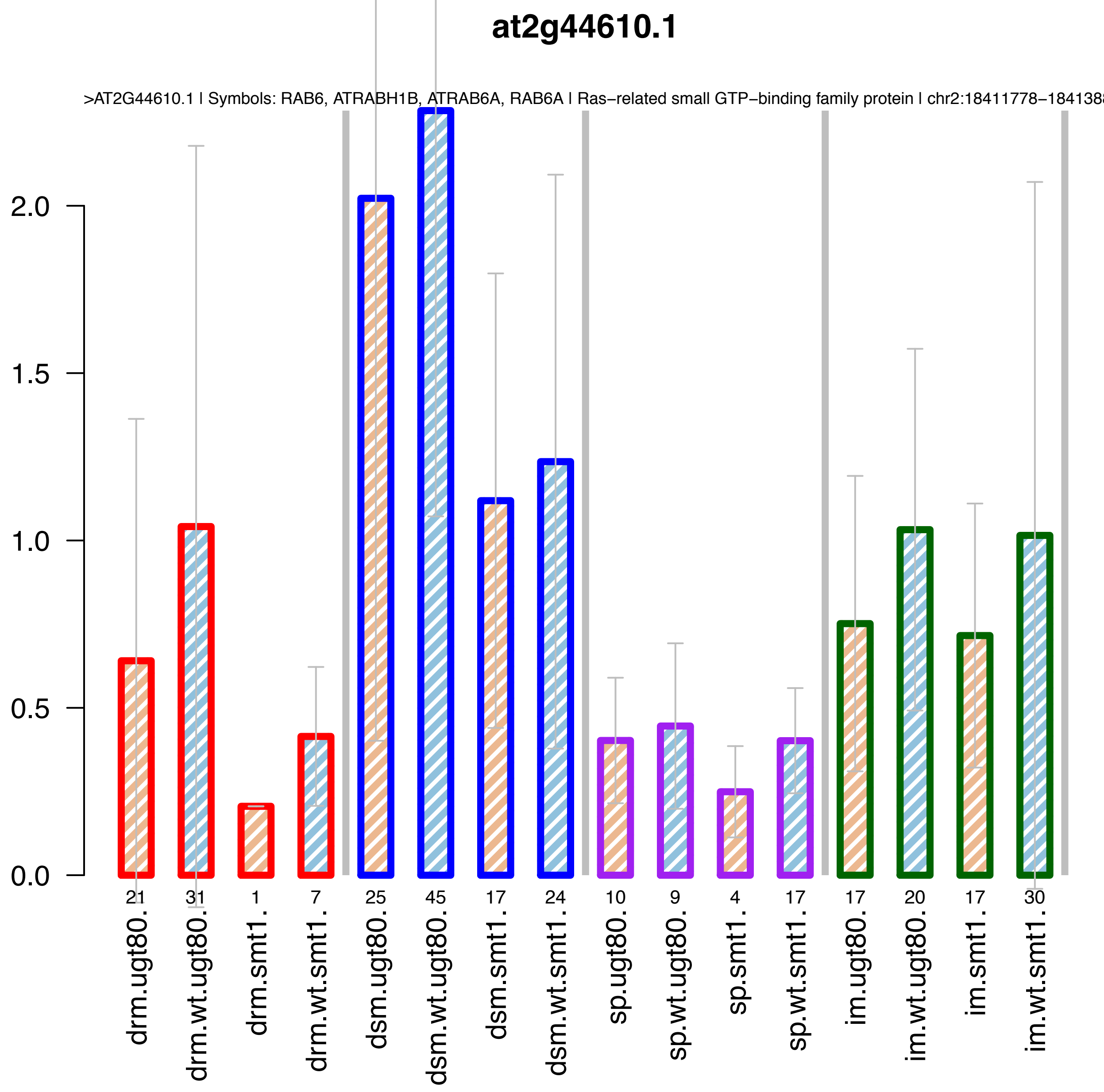

at2g44610.1
>AT2G44610.1 | Symbols: RAB6, ATRABH1B, ATRAB6A, RAB6A | Ras−related small GTP−binding family protein | chr2:18411778−1841138
2.0
1.5
1.0
0.5
0.0
21
31
1
7
25
45
17
24
10
9
4
17
17
20
17
30
drm.ugt80.
drm.wt.ugt80.
drm.smt1.
drm.wt.smt1.
dsm.ugt80.
dsm.wt.ugt80.
dsm.smt1.
dsm.wt.smt1.
sp.ugt80.
sp.wt.ugt80.
sp.smt1.
sp.wt.smt1.
im.ugt80.
im.wt.ugt80.
im.smt1.
im.wt.smt1.

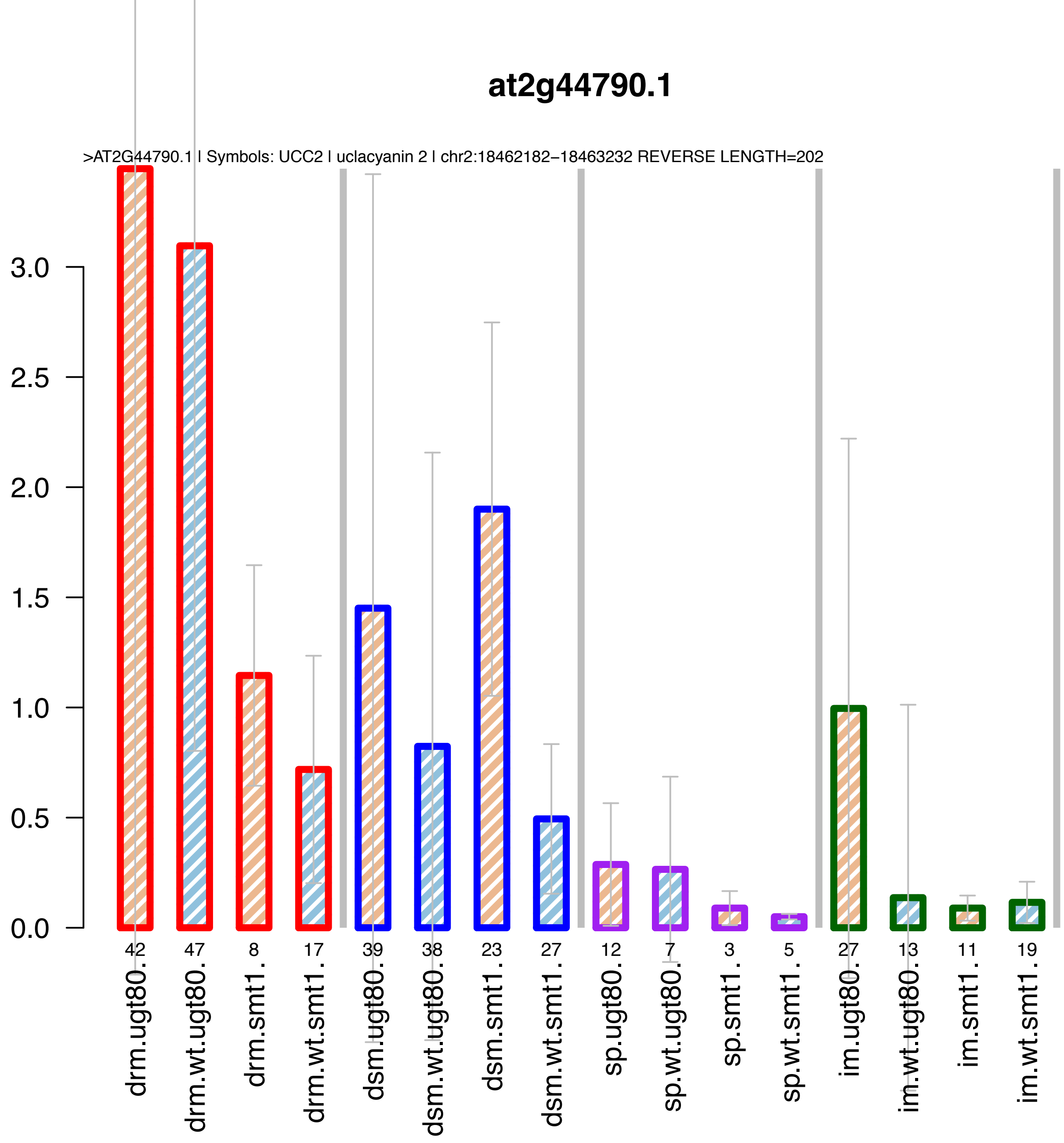

at2g44790.1
>AT2G44790.1 | Symbols: UCC2 | uclacyanin 2 | chr2:18462182-18463232 REVERSE LENGTH=202
0.0
0.5
1.0
1.5
2.0
2.5
3.0
42
47
8
17
39
38
23
27
12
7
3
5
27
13
11
19
drm.ugt80..
drm.wt.ugt80..
drm.smt1..
drm.wt.smt1..
dsm.ugt80..
dsm.wt.ugt80..
dsm.smt1..
dsm.wt.smt1..
sp.ugt80..
sp.wt.ugt80..
sp.smt1..
sp.wt.smt1..
im.ugt80..
im.wt.ugt80..
im.smt1..
im.wt.smt1..

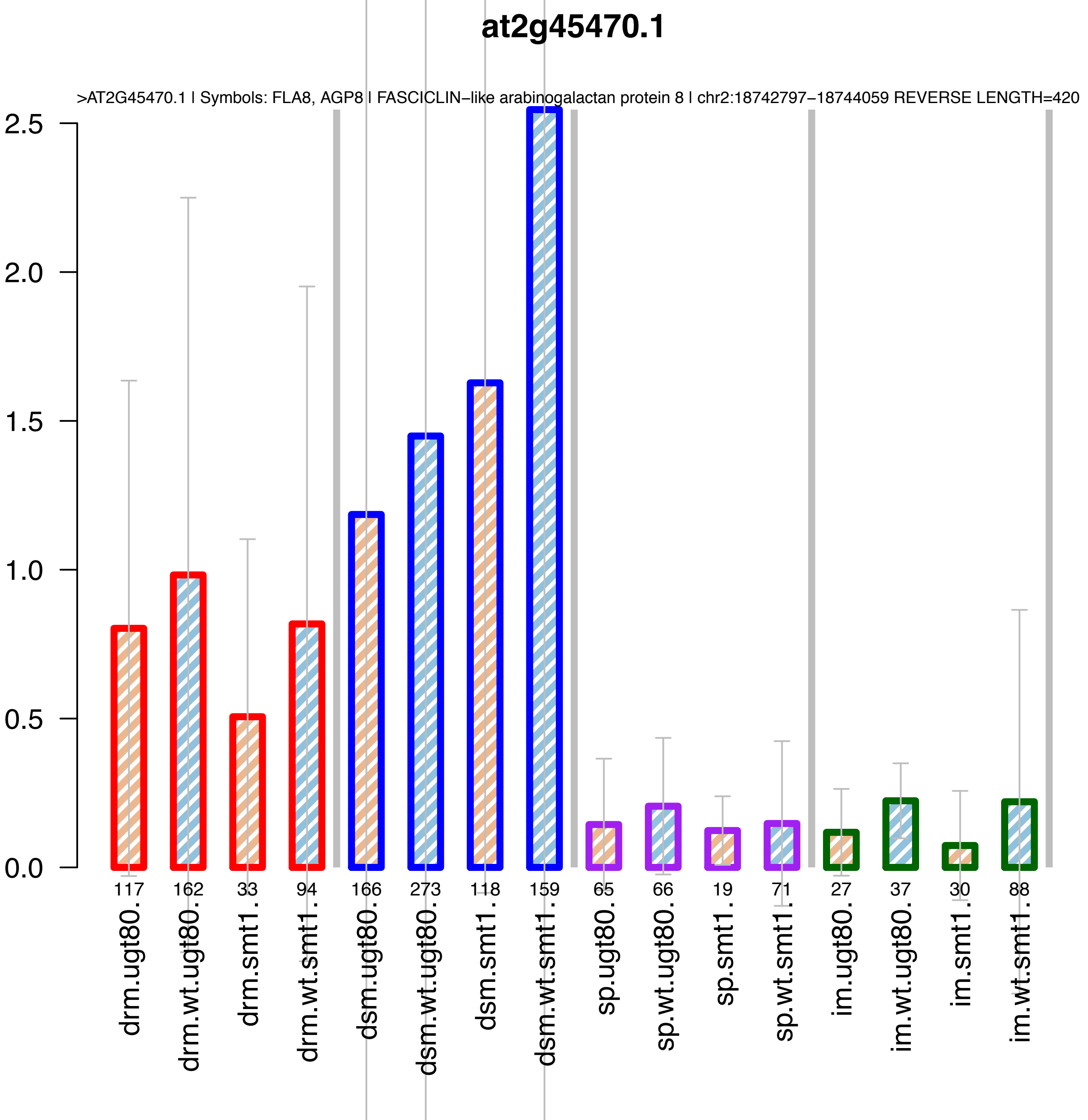
at2g45470.1
>AT2G45470.1 | Symbols: FLA8, AGP8 | FASCICLIN-like arabinogalactan protein 8 | chr2:18742797-18744059 REVERSE LENGTH=420
2.5
2.0
1.5
1.0
0.5
0.0
117
162
33
94
166
273
118
159
65
66
19
71
27
37
30
88
drm.ugt80..
drm.wt.ugt80..
drm.smt1..
drm.wt.smt1..
dsm.ugt80..
dsm.wt.ugt80..
dsm.smt1..
dsm.wt.smt1..
sp.ugt80..
sp.wt.ugt80..
sp.smt1..
sp.wt.smt1..
im.ugt80..
im.wt.ugt80..
im.smt1..
im.wt.smt1..

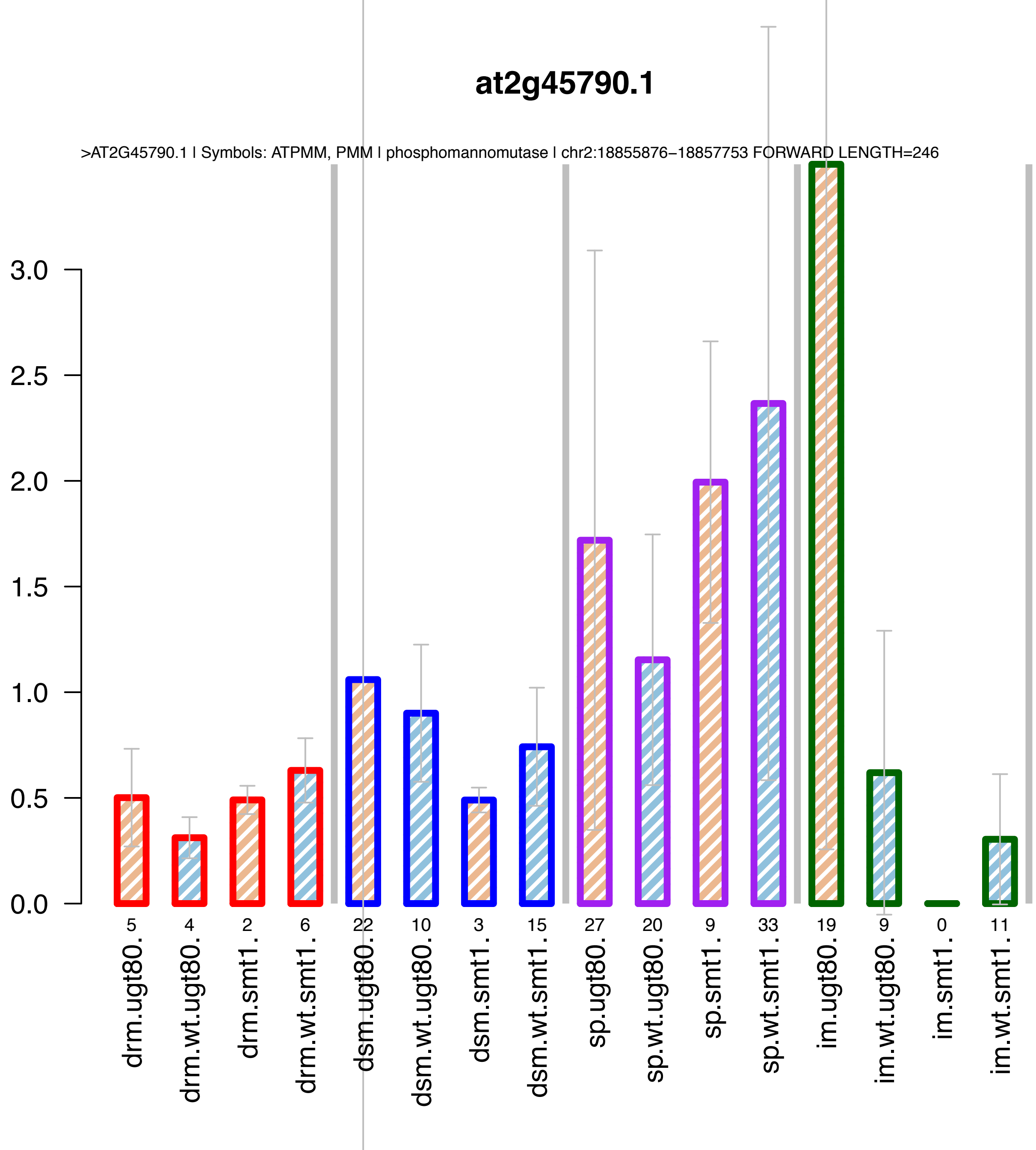

at2g45790.1
>AT2G45790.1 | Symbols: ATPMM, PMM | phosphomannomutase | chr2:18855876-18857753 FORWARD LENGTH=246
0.0
0.5
1.0
1.5
2.0
2.5
3.0
5
4
2
6
22
10
3
15
27
20
9
33
19
9
0
11
drm.ugt80.
drm.wt.ugt80.
drm.smt1.
drm.wt.smt1.
dsm.ugt80.
dsm.wt.ugt80.
dsm.smt1.
dsm.wt.smt1.
sp.ugt80.
sp.wt.ugt80.
sp.smt1.
sp.wt.smt1.
im.ugt80.
im.wt.ugt80.
im.smt1.
im.wt.smt1.

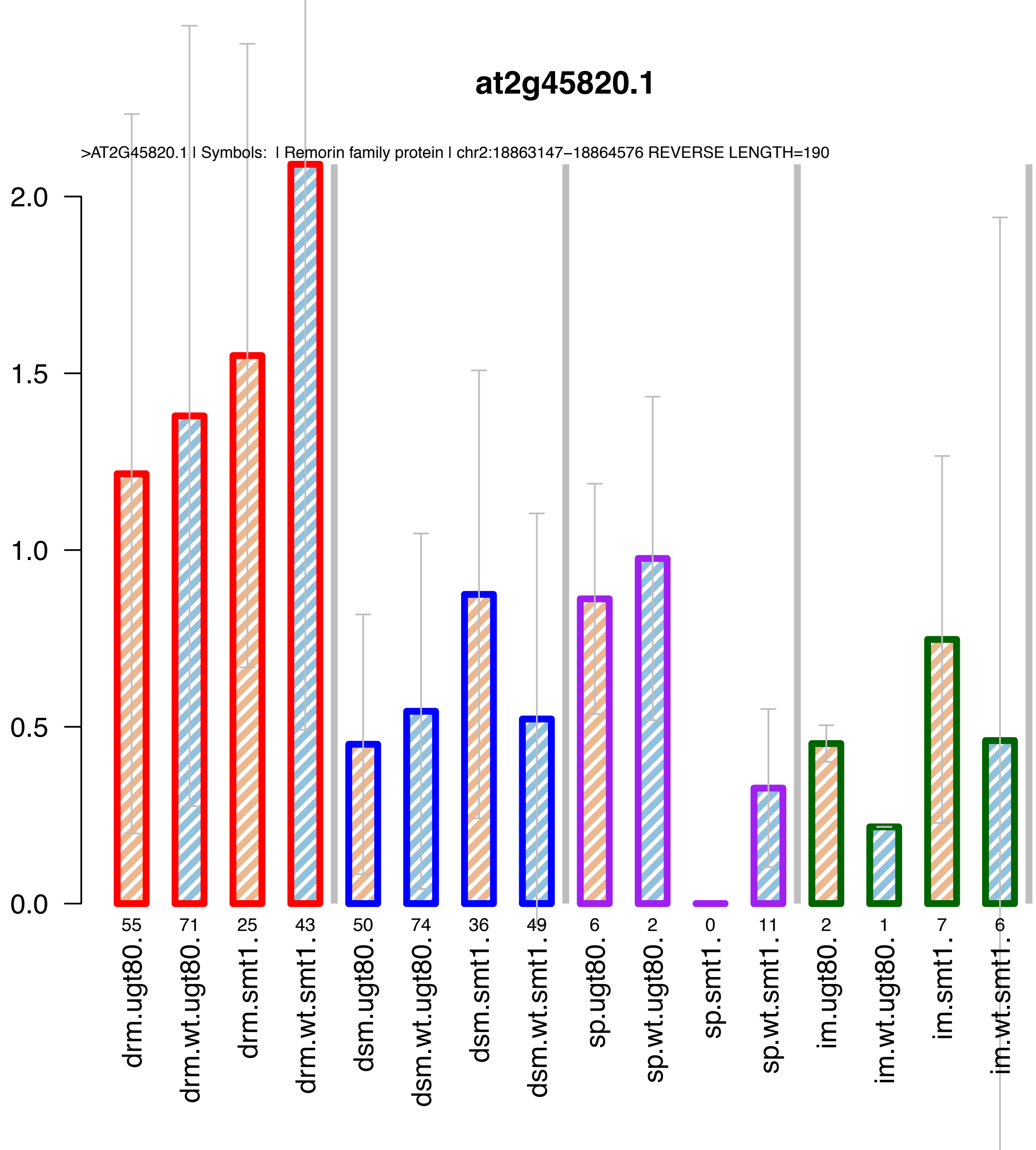
at2g45820.1
>AT2G45820.1 | Symbols: | Remorin family protein | chr2:18863147-18864576 REVERSE LENGTH=190
2.0
1.5
1.0
0.5
0.0
55
71
25
43
50
74
36
49
6
2
0
11
2
1
7
6
drm.ugt80..
drm.wt.ugt80..
drm.smt1..
drm.wt.smt1..
dsm.ugt80..
dsm.wt.ugt80..
dsm.smt1..
dsm.wt.smt1..
sp.ugt80..
sp.wt.ugt80..
sp.smt1..
sp.wt.smt1..
im.ugt80..
im.wt.ugt80..
im.smt1..
im.wt.smt1..

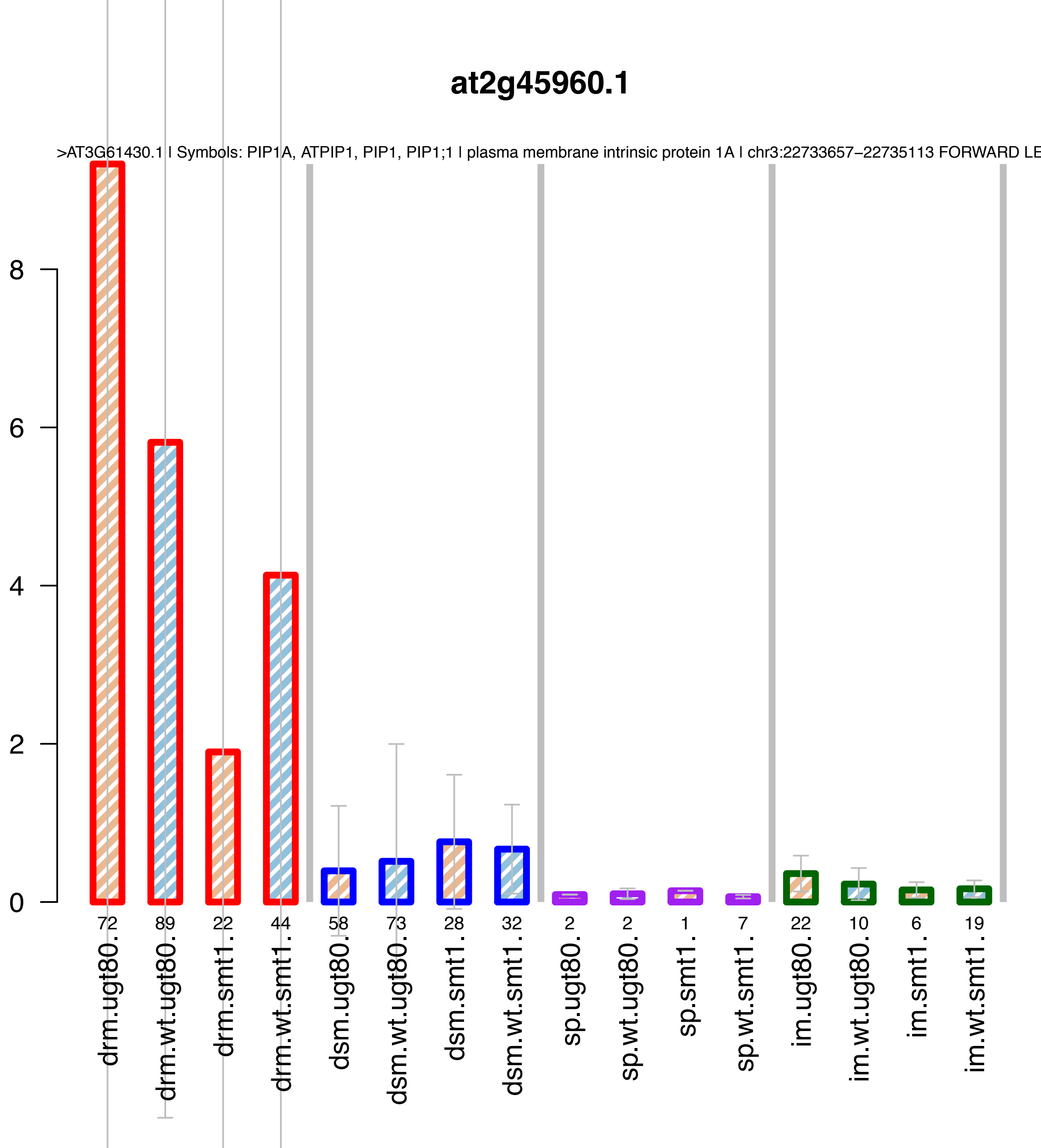

at2g45960.1
>AT3G61430.1 | Symbols: PIP1A, ATPIP1, PIP1, PIP1;1 | plasma membrane intrinsic protein 1A | chr3:22733657–22735113 FORWARD LE
0
2
4
6
8
72
89
22
44
58
73
28
32
2
2
1
7
22
10
6
19
drm.ugt80..
drm.wt.ugt80..
drm.smt1..
drm.wt.smt1..
dsm.ugt80..
dsm.wt.ugt80..
dsm.smt1..
dsm.wt.smt1..
sp.ugt80..
sp.wt.ugt80..
sp.smt1..
sp.wt.smt1..
im.ugt80..
im.wt.ugt80..
im.smt1..
im.wt.smt1..

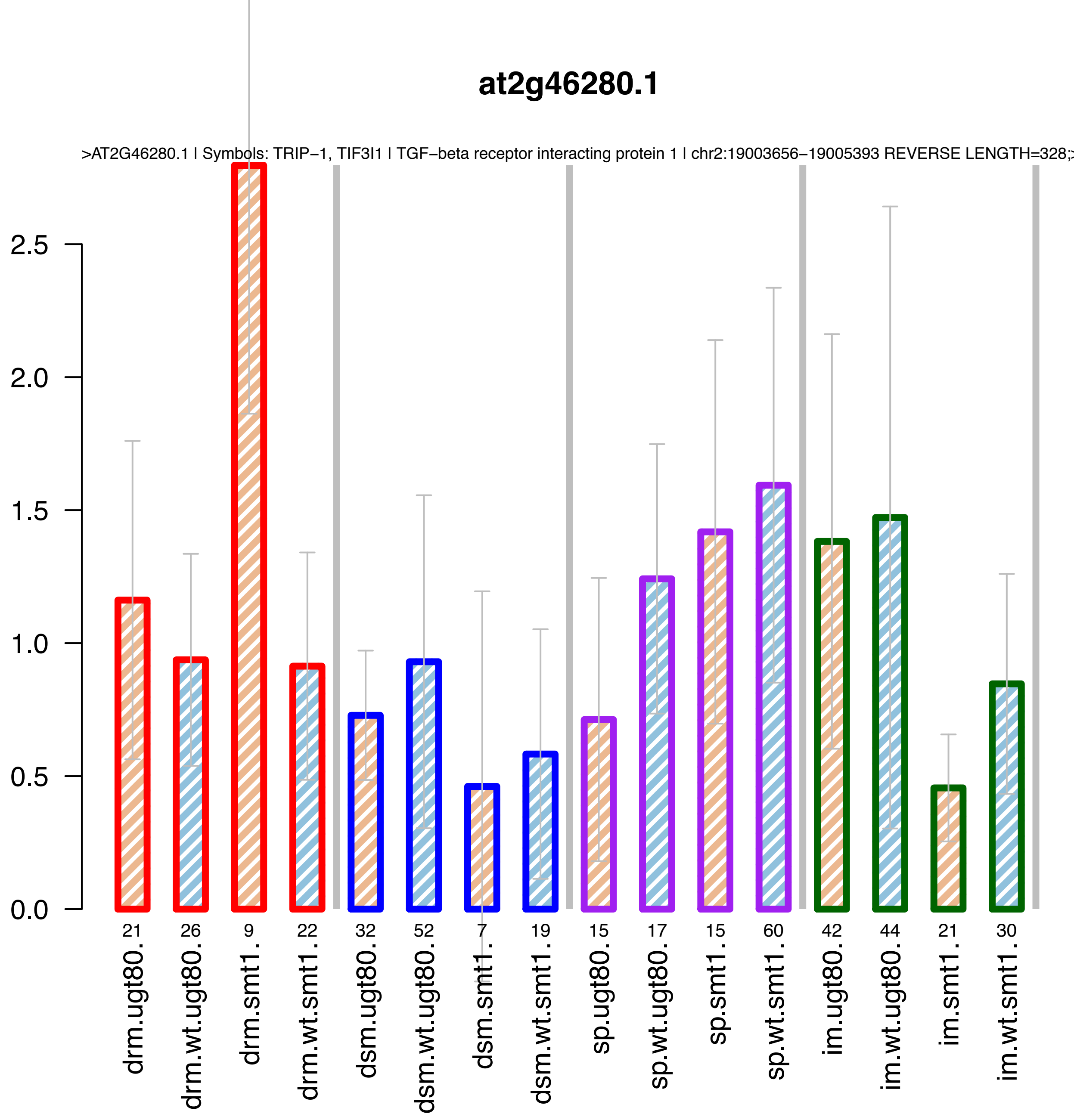

at2g46280.1
>AT2G46280.1 | Symbols: TRIP−1, TIF3I1 | TGF−beta receptor interacting protein 1 | chr2:19003656−19005393 REVERSE LENGTH=328;
0.0
0.5
1.0
1.5
2.0
2.5
21
26
9
22
32
52
7
19
15
17
15
60
42
44
21
30
drm.ugt80..
drm.wt.ugt80..
drm.smt1..
drm.wt.smt1..
dsm.ugt80..
dsm.wt.ugt80..
dsm.smt1..
dsm.wt.smt1..
sp.ugt80..
sp.wt.ugt80..
sp.smt1..
sp.wt.smt1..
im.ugt80..
im.wt.ugt80..
im.smt1..
im.wt.smt1..

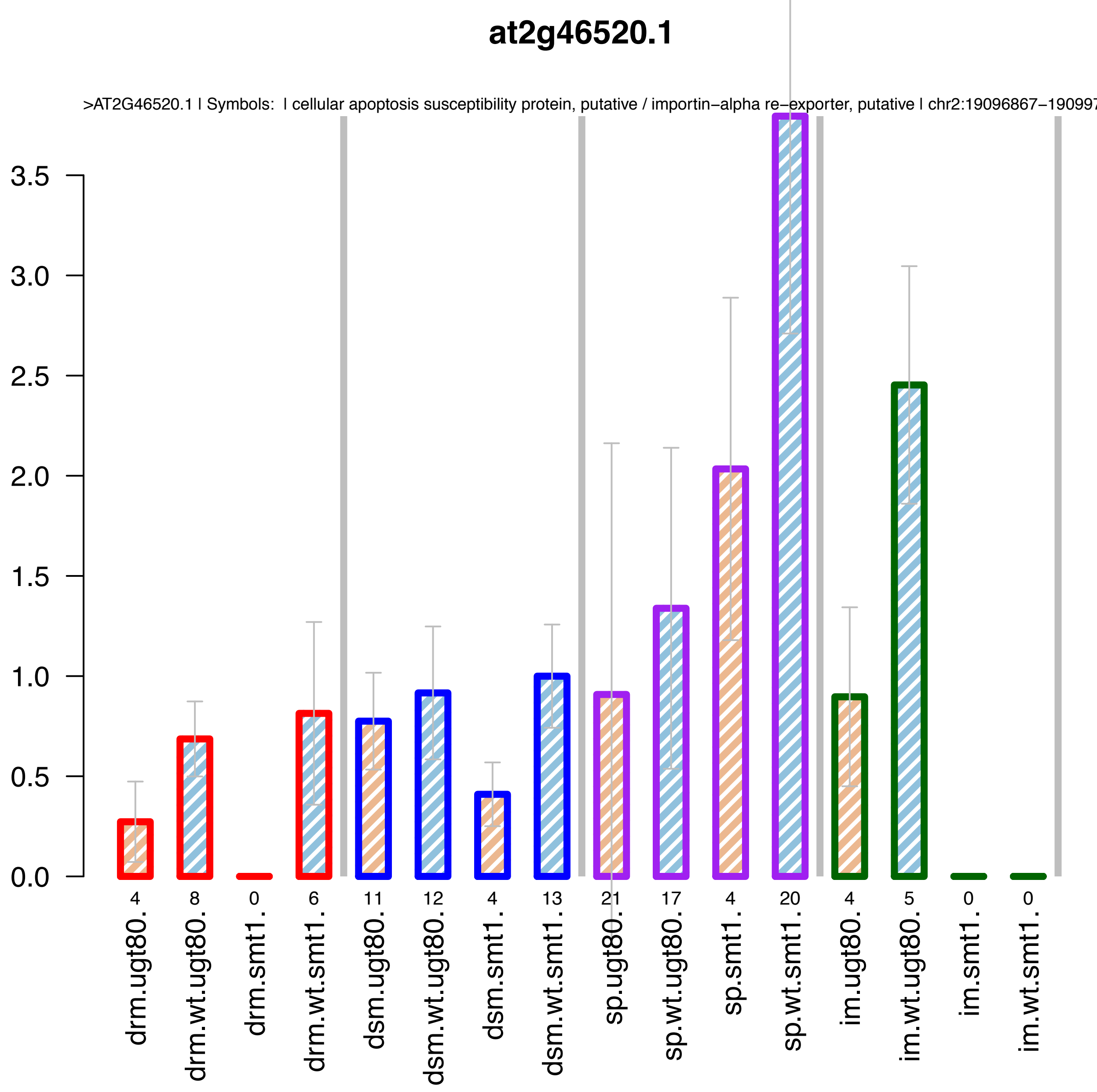

at2g46520.1
>AT2G46520.1 | Symbols: | cellular apoptosis susceptibility protein, putative / importin−alpha re−exporter, putative | chr2:19096867−190997
3.5
3.0
2.5
2.0
1.5
1.0
0.5
0.0
4
8
0
6
11
12
4
13
21
17
4
20
4
5
0
0
drm.ugt80.
drm.wt.ugt80.
drm.smt1.
drm.wt.smt1.
dsm.ugt80.
dsm.wt.ugt80.
dsm.smt1.
dsm.wt.smt1.
sp.ugt80.
sp.wt.ugt80.
sp.smt1.
sp.wt.smt1.
im.ugt80.
im.wt.ugt80.
im.smt1.
im.wt.smt1.

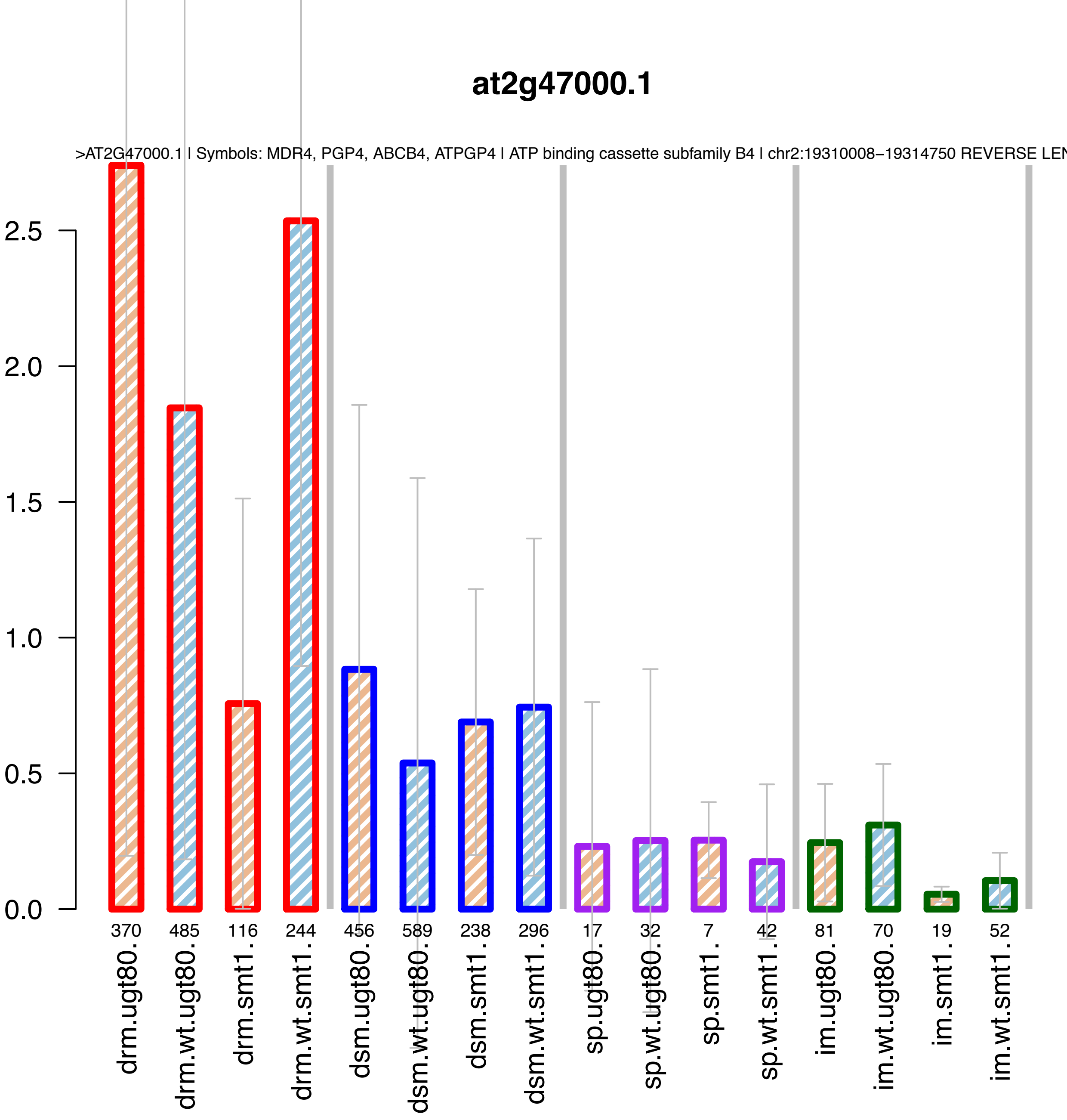
at2g47000.1
>AT2G47000.1 | Symbols: MDR4, PGP4, ABCB4, ATPGP4 | ATP binding cassette subfamily B4 | chr2:19310008–19314750 REVERSE LEN
2.5
2.0
1.5
1.0
0.5
0.0
370
485
116
244
456
589
238
296
17
32
7
42
81
70
19
52
drm.ugt80..
drm.wt.ugt80..
drm.smt1..
drm.wt.smt1..
dsm.ugt80..
dsm.wt.ugt80..
dsm.smt1..
dsm.wt.smt1..
sp.ugt80..
sp.wt.ugt80..
sp.smt1..
sp.wt.smt1..
im.ugt80..
im.wt.ugt80..
im.smt1..
im.wt.smt1..

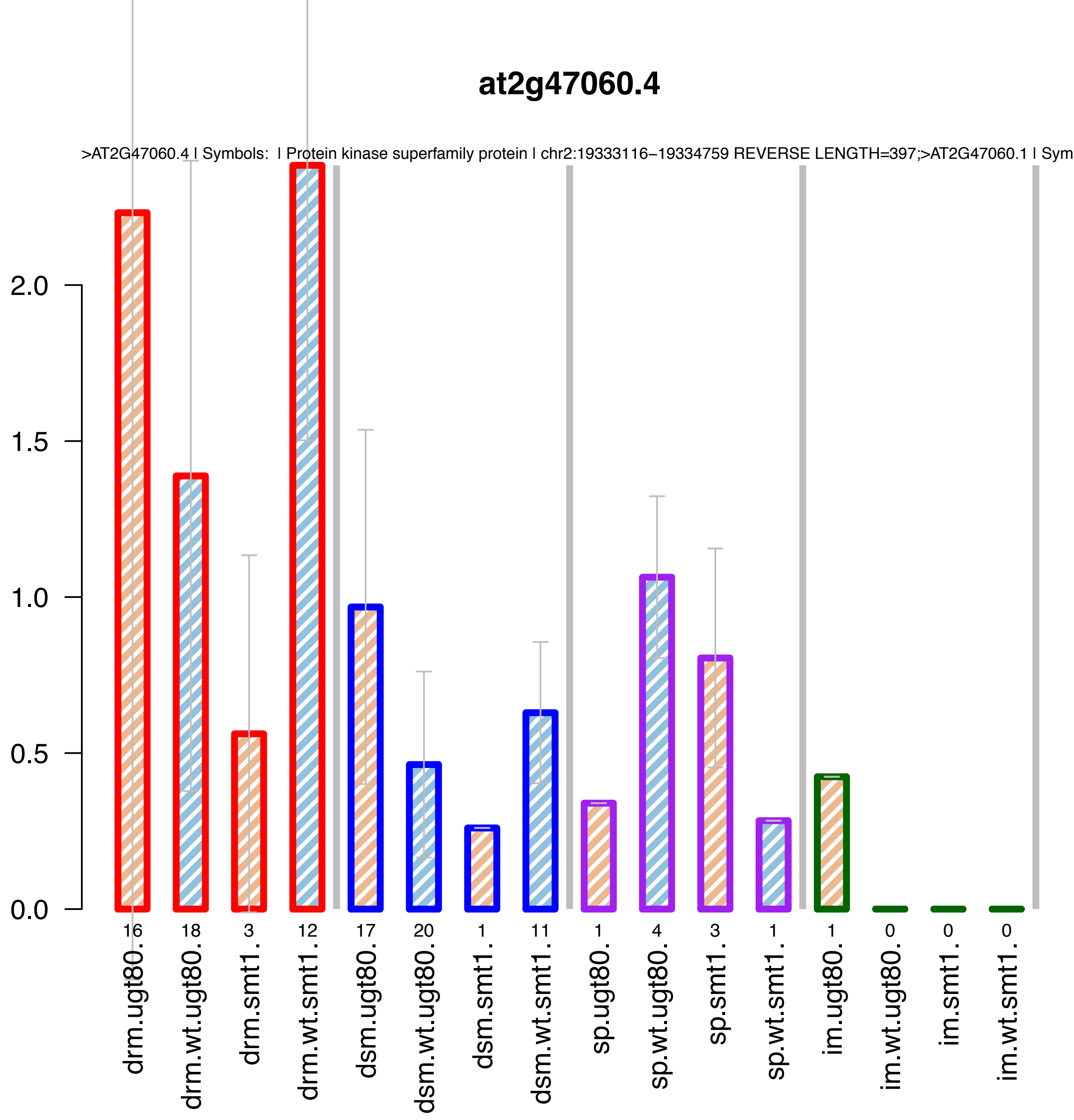

at2g47060.4
>AT2G47060.4 | Symbols: | Protein kinase superfamily protein | chr2:19333116-19334759 REVERSE LENGTH=397;>AT2G47060.1 | Sym
2.0
1.5
1.0
0.5
0.0
16
18
3
12
17
20
1
11
1
4
3
1
1
0
0
0
drm.ugt80.
drm.wt.ugt80.
drm.smt1.
drm.wt.smt1.
dsm.ugt80.
dsm.wt.ugt80.
dsm.smt1.
dsm.wt.smt1.
sp.ugt80.
sp.wt.ugt80.
sp.smt1.
sp.wt.smt1.
im.ugt80.
im.wt.ugt80.
im.smt1.
im.wt.smt1.

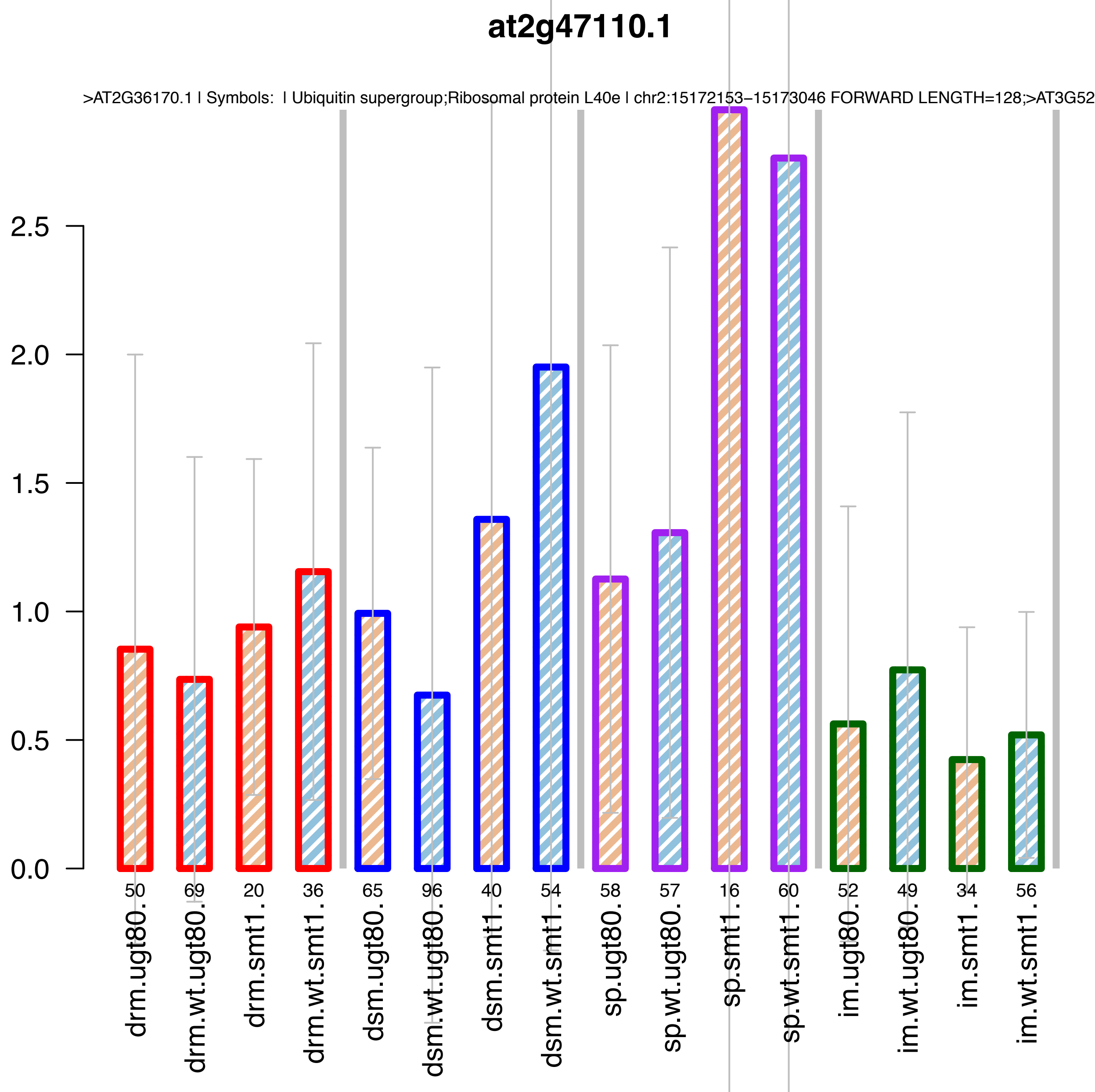

at2g47110.1
>AT2G36170.1 | Symbols: | Ubiquitin supergroup;Ribosomal protein L40e | chr2:15172153–15173046 FORWARD LENGTH=128;>AT3G52
0.0
0.5
1.0
1.5
2.0
2.5
50
69
20
36
65
96
40
54
58
57
16
60
52
49
34
56
drm.ugt80..
drm.wt.ugt80..
drm.smt1..
drm.wt.smt1..
dsm.ugt80..
dsm.wt.ugt80..
dsm.smt1..
dsm.wt.smt1..
sp.ugt80..
sp.wt.ugt80..
sp.smt1..
sp.wt.smt1..
im.ugt80..
im.wt.ugt80..
im.smt1..
im.wt.smt1..

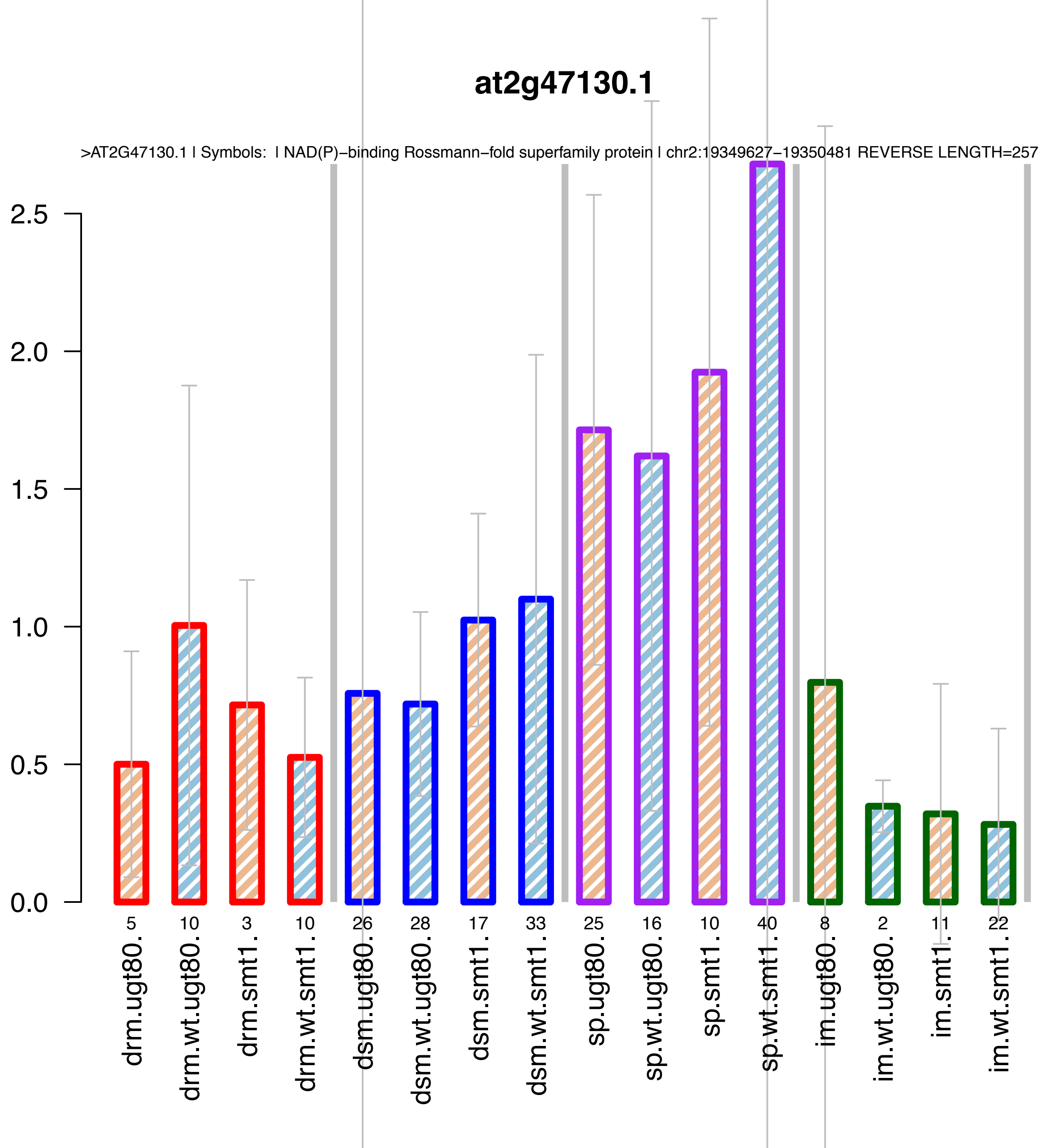
at2g47130.1
>AT2G47130.1 | Symbols: | NAD(P)−binding Rossmann−fold superfamily protein | chr2:19349627−19350481 REVERSE LENGTH=257
0.0
0.5
1.0
1.5
2.0
2.5
5
10
3
10
26
28
17
33
25
16
10
40
8
2
11
22
drm.ugt80.
drm.wt.ugt80.
drm.smt1.
drm.wt.smt1.
dsm.ugt80.
dsm.wt.ugt80.
dsm.smt1.
dsm.wt.smt1.
sp.ugt80.
sp.wt.ugt80.
sp.smt1.
sp.wt.smt1.
im.ugt80.
im.wt.ugt80.
im.smt1.
im.wt.smt1.

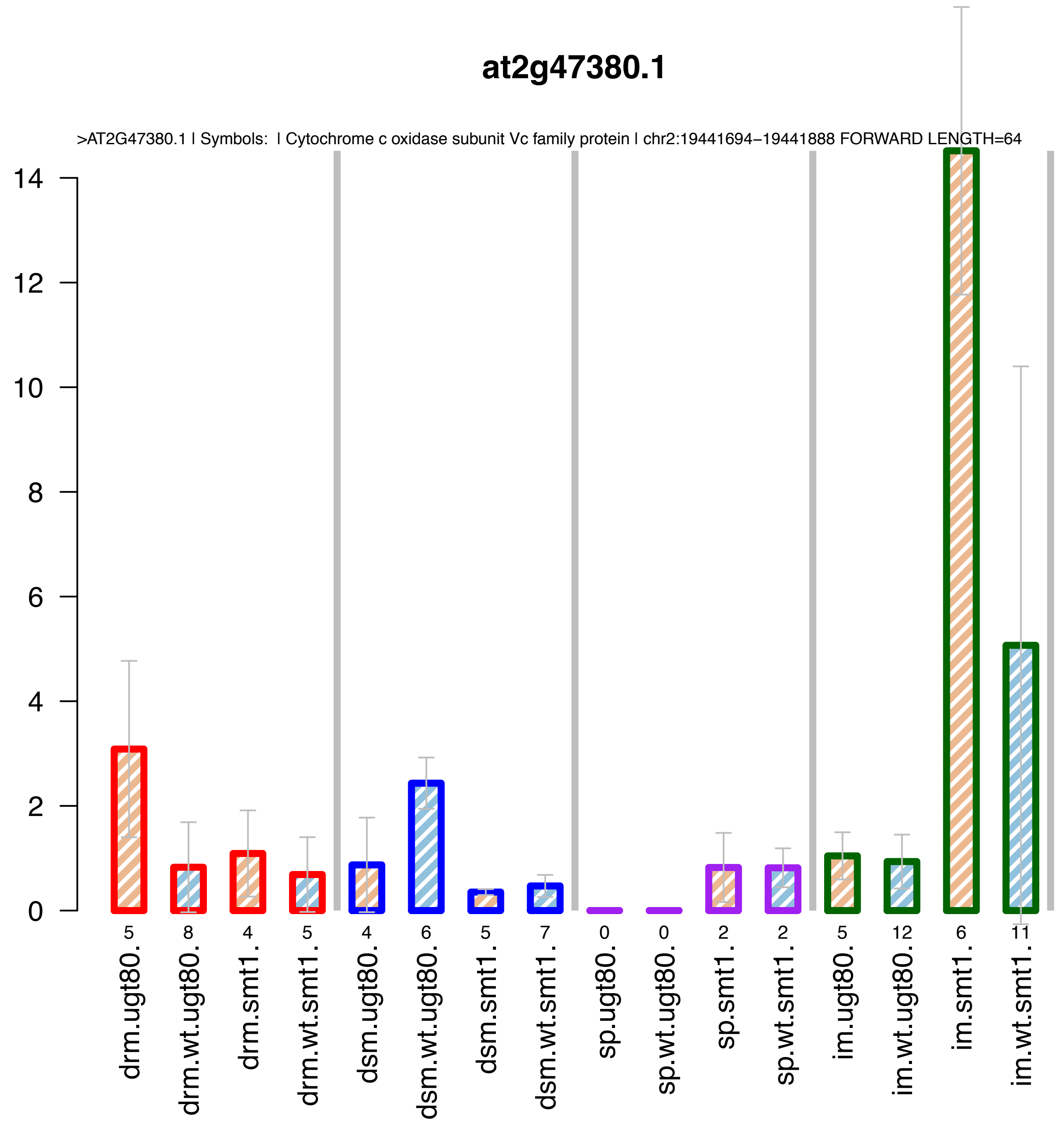

at2g47380.1
>AT2G47380.1 | Symbols: | Cytochrome c oxidase subunit Vc family protein | chr2:19441694-19441888 FORWARD LENGTH=64
0
2
4
6
8
10
12
14
5
8
4
5
4
6
5
7
0
0
2
2
5
12
6
11
drm.ugt80.
drm.wt.ugt80.
drm.smt1.
drm.wt.smt1.
dsm.ugt80.
dsm.wt.ugt80.
dsm.smt1.
dsm.wt.smt1.
sp.ugt80.
sp.wt.ugt80.
sp.smt1.
sp.wt.smt1.
im.ugt80.
im.wt.ugt80.
im.smt1.
im.wt.smt1.

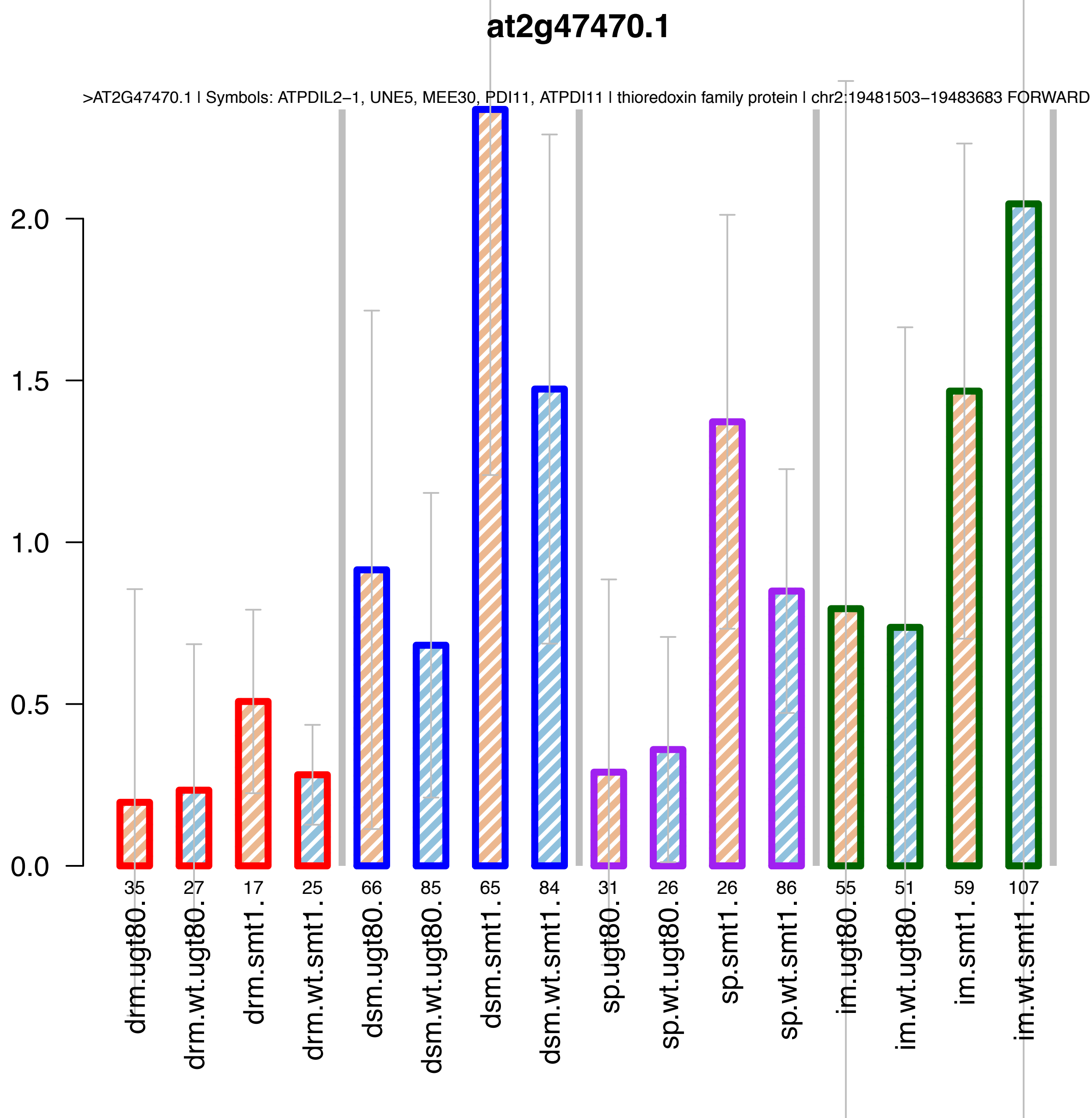
at2g47470.1
>AT2G47470.1 | Symbols: ATPDIL2−1, UNE5, MEE30, PDI11, ATPDI11 | thioredoxin family protein | chr2:19481503−19483683 FORWARD
0.0
0.5
1.0
1.5
2.0
35
27
17
25
66
85
65
84
31
26
26
86
55
51
59
107
drm.ugt80..
drm.wt.ugt80..
drm.smt1..
drm.wt.smt1..
dsm.ugt80..
dsm.wt.ugt80..
dsm.smt1..
dsm.wt.smt1..
sp.ugt80..
sp.wt.ugt80..
sp.smt1..
sp.wt.smt1..
im.ugt80..
im.wt.ugt80..
im.smt1..
im.wt.smt1..

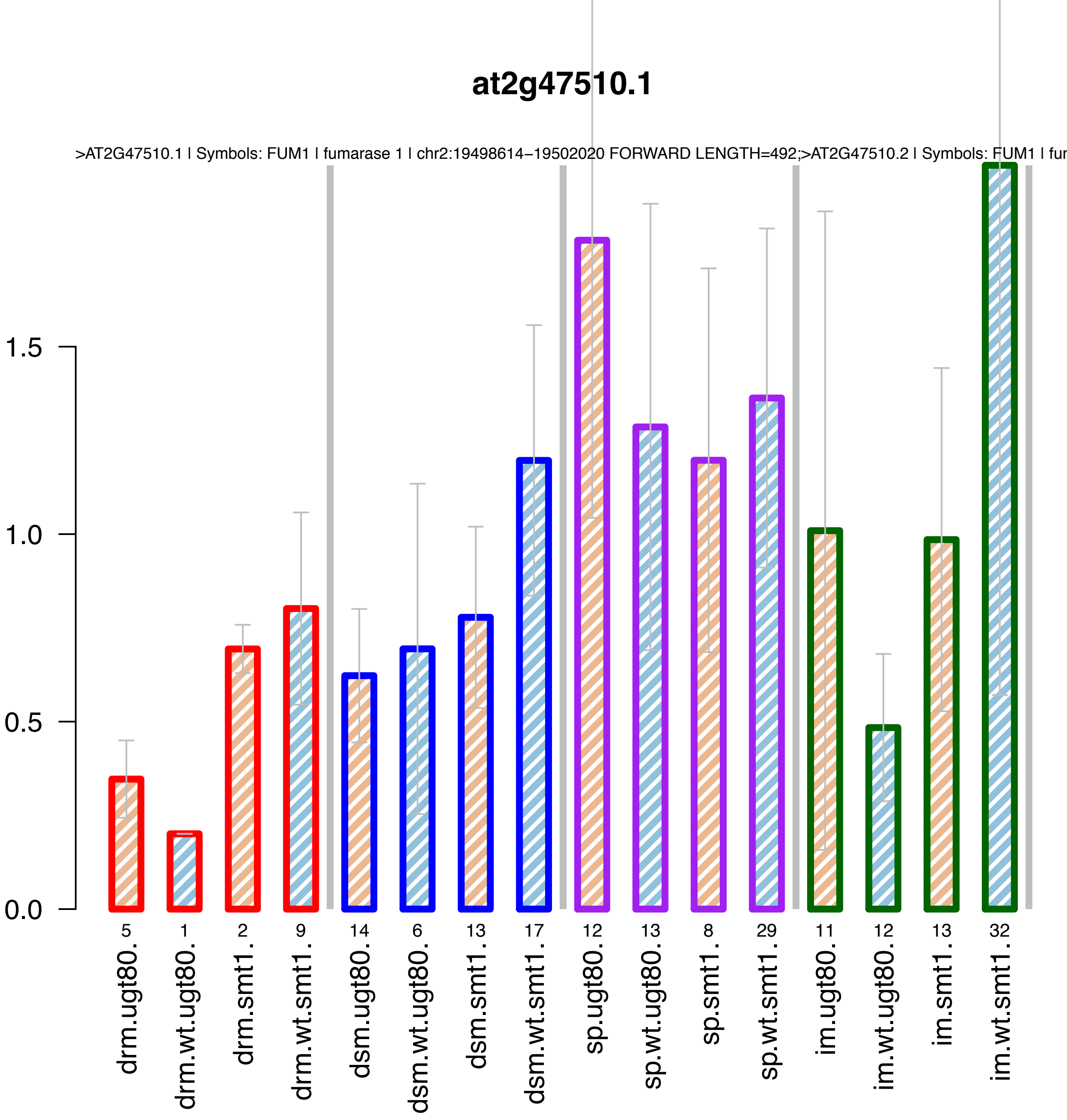

at2g47510.1
>AT2G47510.1 | Symbols: FUM1 | fumarase 1 | chr2:19498614-19502020 FORWARD LENGTH=492;>AT2G47510.2 | Symbols: FUM1 | fur
1.5
1.0
0.5
0.0
5
1
2
9
14
6
13
17
12
13
8
29
11
12
13
32
drm.ugt80..
drm.wt.ugt80..
drm.smt1..
drm.wt.smt1..
dsm.ugt80..
dsm.wt.ugt80..
dsm.smt1..
dsm.wt.smt1..
sp.ugt80..
sp.wt.ugt80..
sp.smt1..
sp.wt.smt1..
im.ugt80..
im.wt.ugt80..
im.smt1..
im.wt.smt1..

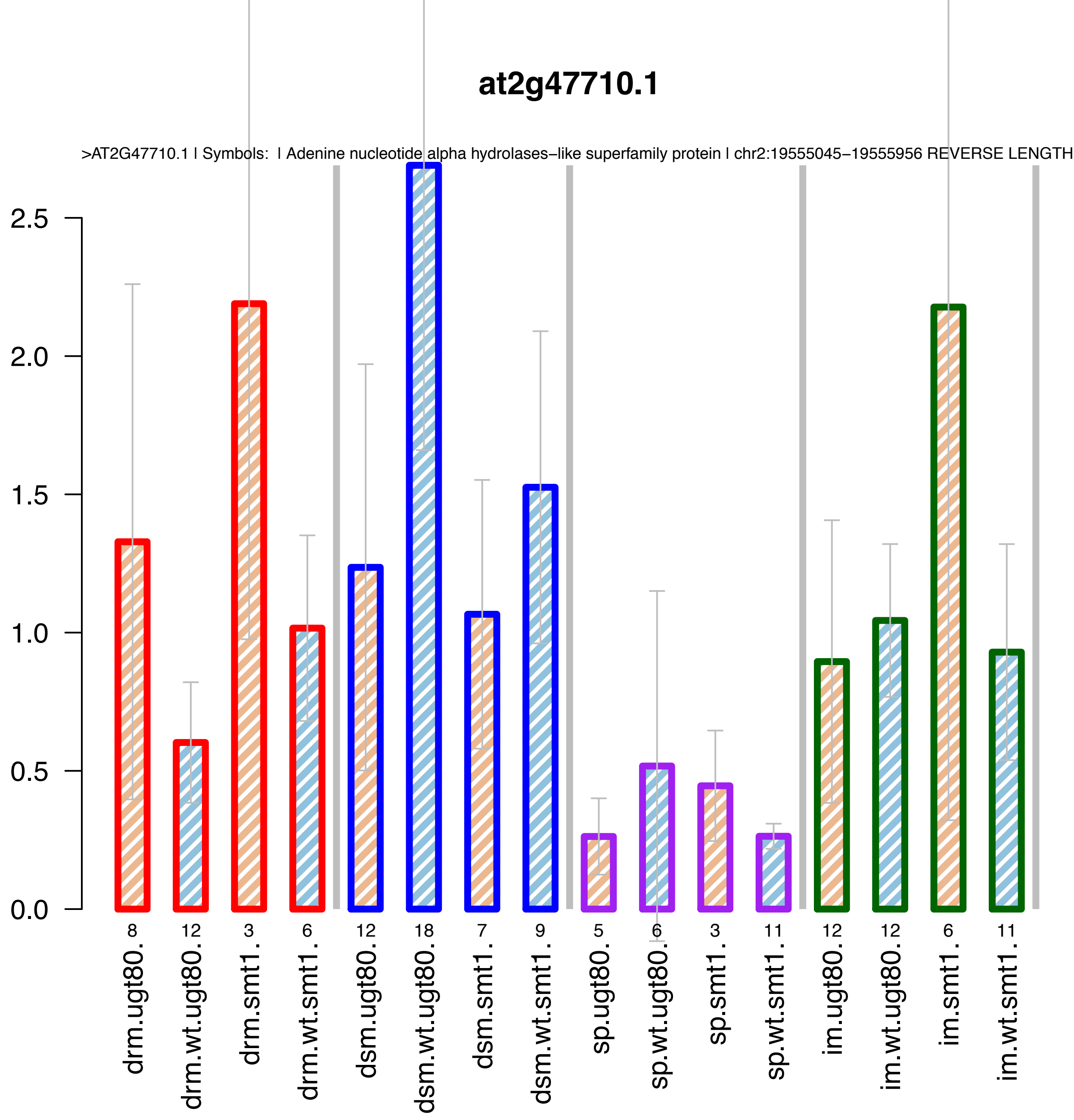
at2g47710.1
>AT2G47710.1 | Symbols: | Adenine nucleotide alpha hydrolases-like superfamily protein | chr2:19555045-19555956 REVERSE LENGTH
0.0
0.5
1.0
1.5
2.0
2.5
8
12
3
6
12
18
7
9
5
6
3
11
12
12
6
11
drm.ugt80..
drm.wt.ugt80..
drm.smt1..
drm.wt.smt1..
dsm.ugt80..
dsm.wt.ugt80..
dsm.smt1..
dsm.wt.smt1..
sp.ugt80..
sp.wt.ugt80..
sp.smt1..
sp.wt.smt1..
im.ugt80..
im.wt.ugt80..
im.smt1..
im.wt.smt1..

# at2g47730.1

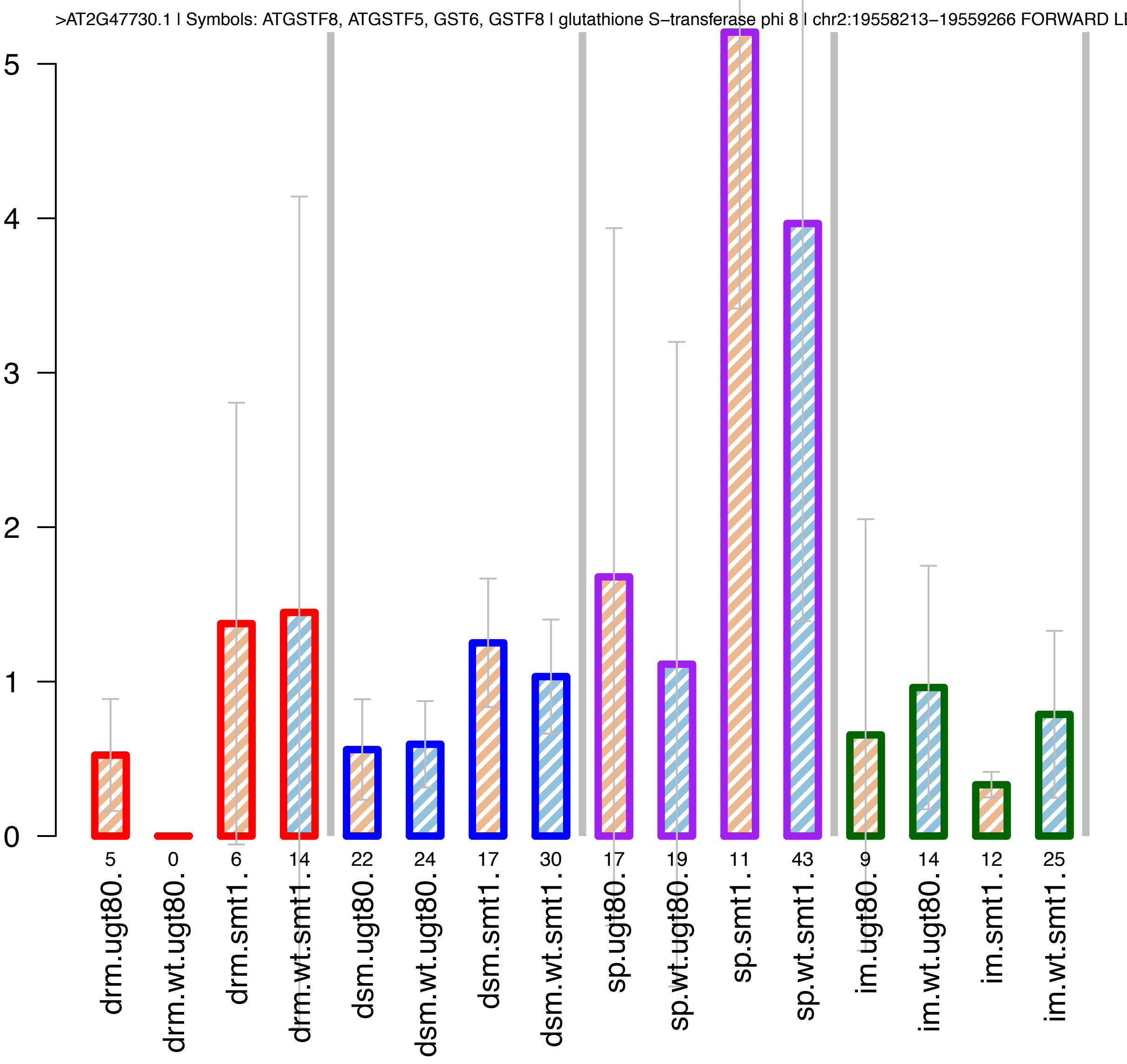

>AT2G47730.1 | Symbols: ATGSTF8, ATGSTF5, GST6, GSTF8 | glutathione S−transferase phi 8 | chr2:19558213−19559266 FORWARD LE
0
1
2
3
4
5
5
0
6
14
22
24
17
30
17
19
11
43
9
14
12
25
drm.ugt80.
drm.wt.ugt80.
drm.smt1.
drm.wt.smt1.
dsm.ugt80.
dsm.wt.ugt80.
dsm.smt1.
dsm.wt.smt1.
sp.ugt80.
sp.wt.ugt80.
sp.smt1.
sp.wt.smt1.
im.ugt80.
im.wt.ugt80.
im.smt1.
im.wt.smt1.

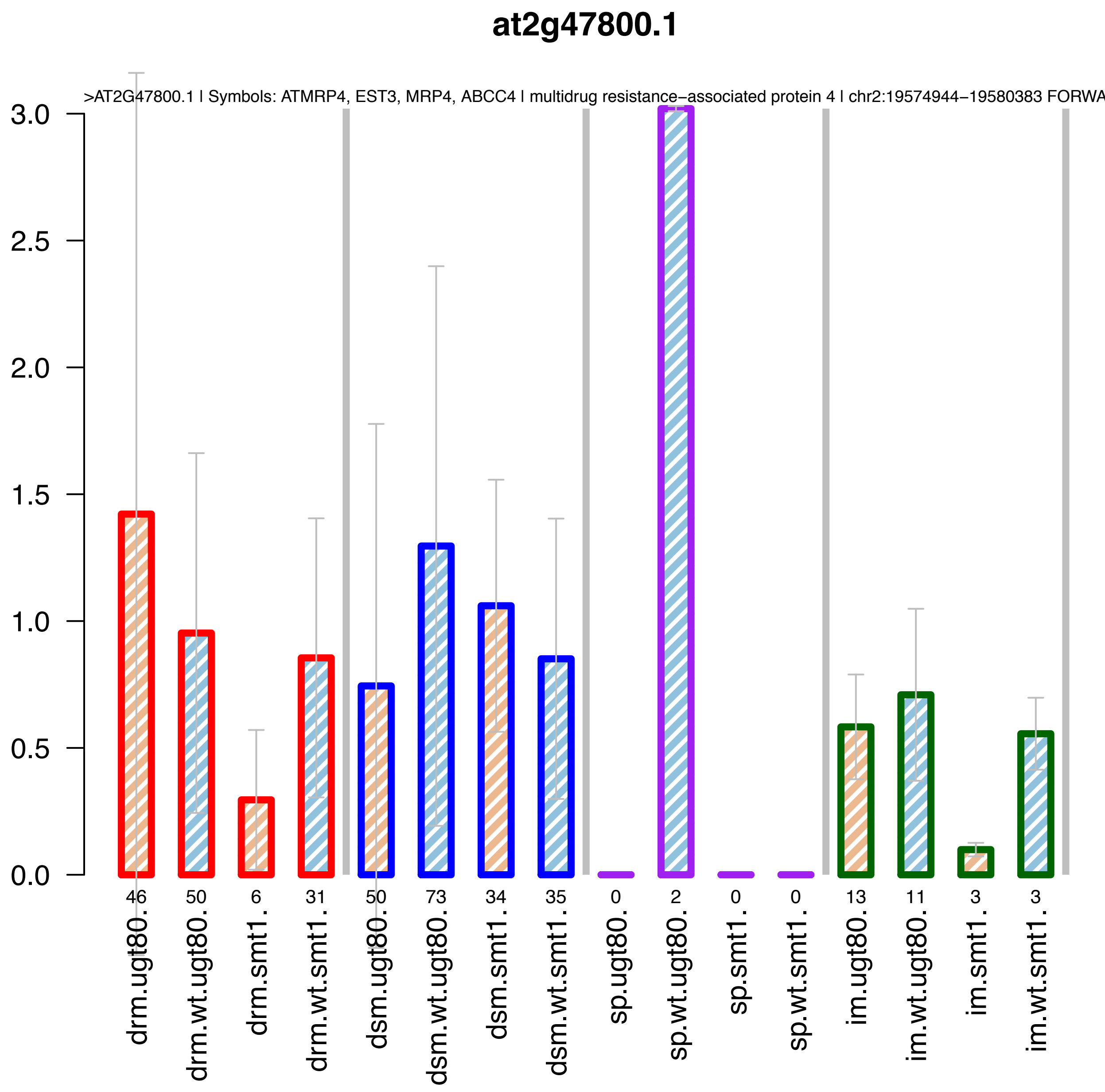

at2g47800.1
>AT2G47800.1 | Symbols: ATMRP4, EST3, MRP4, ABCC4 | multidrug resistance−associated protein 4 | chr2:19574944−19580383 FORWA
3.0
2.5
2.0
1.5
1.0
0.5
0.0
46
50
6
31
50
73
34
35
0
2
0
0
13
11
3
3
drm.ugt80..
drm.wt.ugt80..
drm.smt1..
drm.wt.smt1..
dsm.ugt80..
dsm.wt.ugt80..
dsm.smt1..
dsm.wt.smt1..
sp.ugt80..
sp.wt.ugt80..
sp.smt1..
sp.wt.smt1..
im.ugt80..
im.wt.ugt80..
im.smt1..
im.wt.smt1..

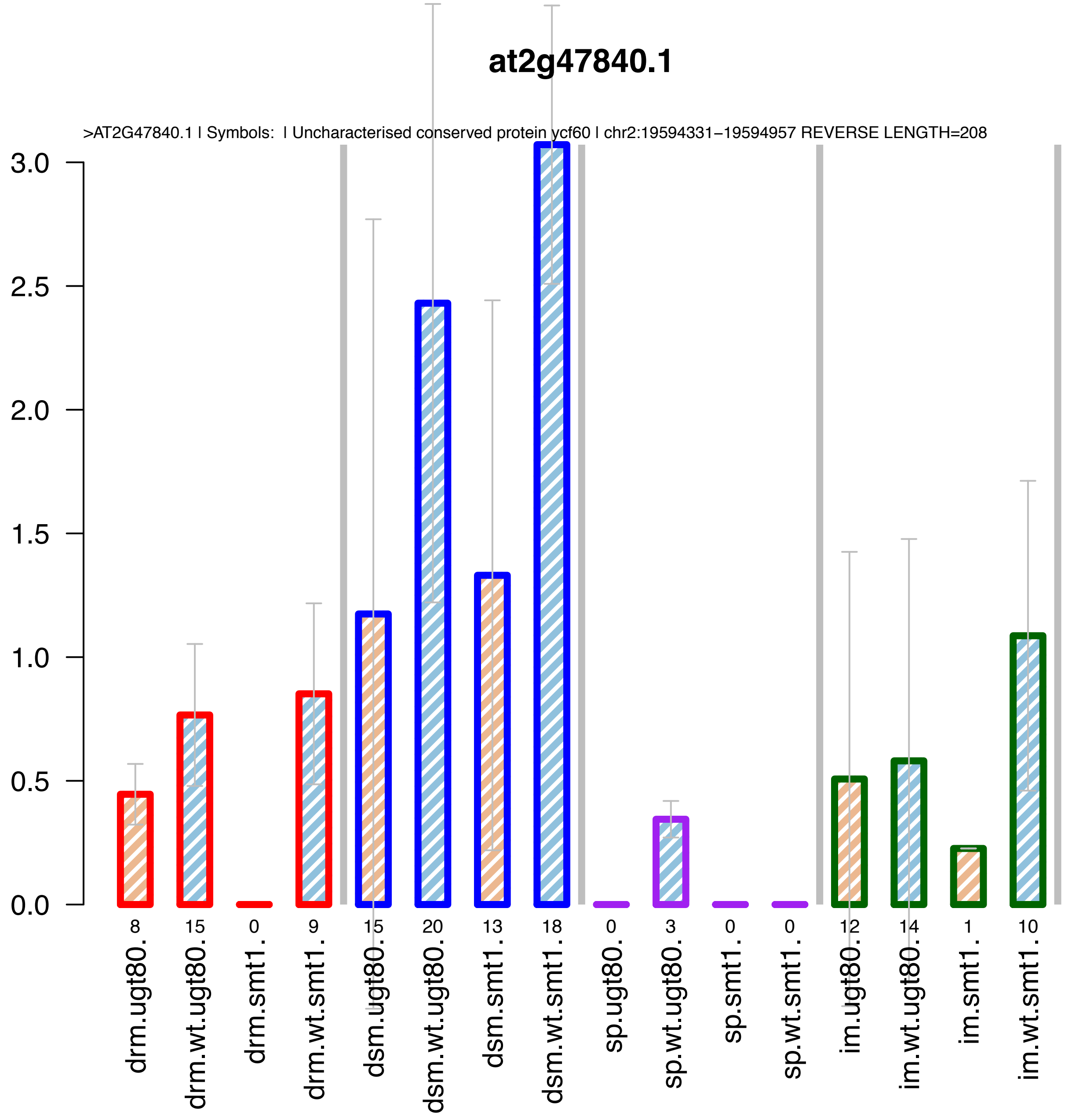

at2g47840.1
>AT2G47840.1 | Symbols: | Uncharacterised conserved protein ycf60 | chr2:19594331−19594957 REVERSE LENGTH=208
3.0
2.5
2.0
1.5
1.0
0.5
0.0
8
15
0
9
15
20
13
18
0
3
0
0
12
14
1
10
drm.ugt80.
drm.wt.ugt80.
drm.smt1.
drm.wt.smt1.
dsm.ugt80.
dsm.wt.ugt80.
dsm.smt1.
dsm.wt.smt1.
sp.ugt80.
sp.wt.ugt80.
sp.smt1.
sp.wt.smt1.
im.ugt80.
im.wt.ugt80.
im.smt1.
im.wt.smt1.

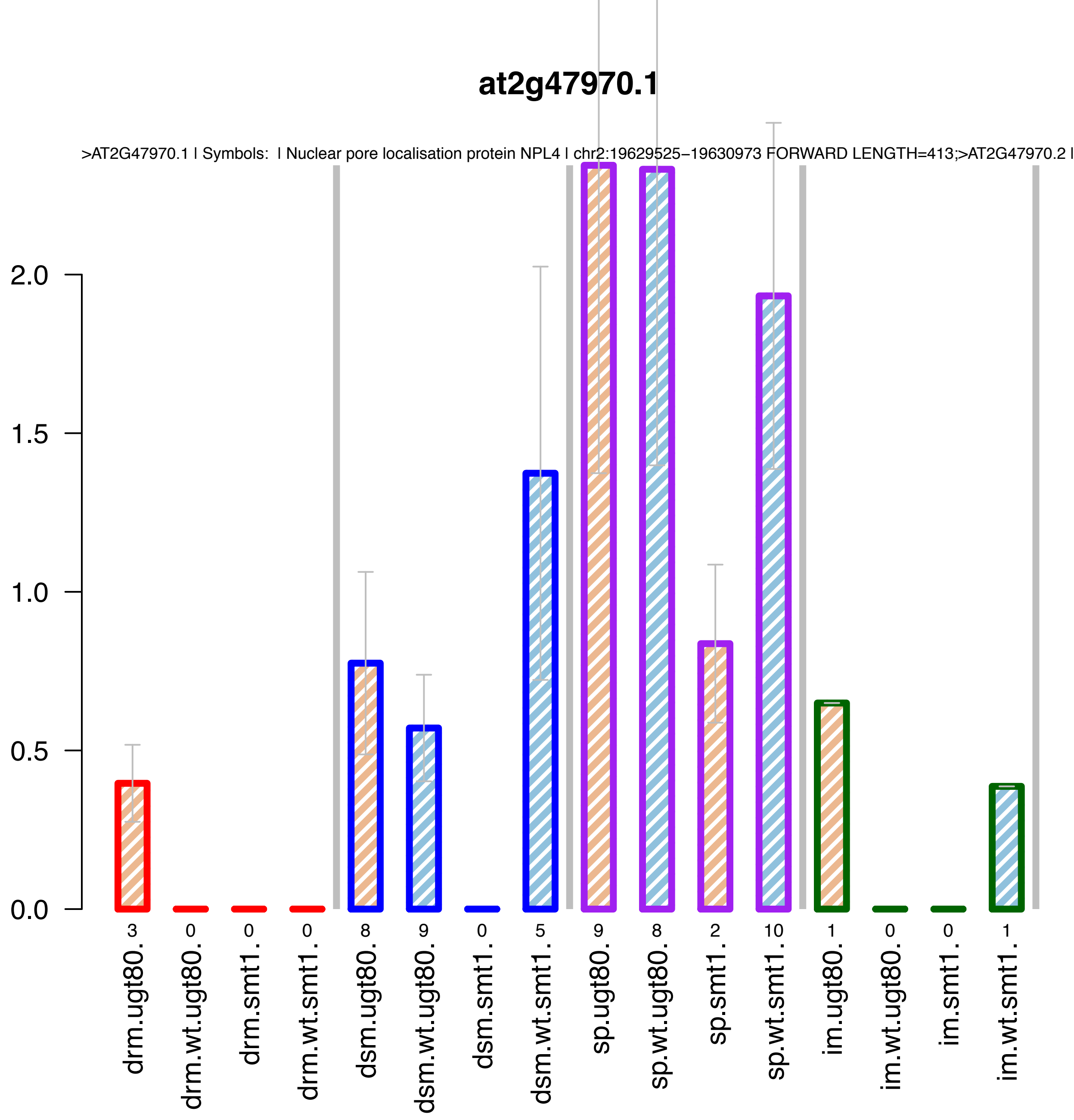

at2g47970.1
>AT2G47970.1 | Symbols: | Nuclear pore localisation protein NPL4 | chr2:19629525-19630973 FORWARD LENGTH=413;>AT2G47970.2 |
2.0
1.5
1.0
0.5
0.0
3
0
0
0
8
9
0
5
9
8
2
10
1
0
0
1
drm.ugt80.
drm.wt.ugt80.
drm.smt1.
drm.wt.smt1.
dsm.ugt80.
dsm.wt.ugt80.
dsm.smt1.
dsm.wt.smt1.
sp.ugt80.
sp.wt.ugt80.
sp.smt1.
sp.wt.smt1.
im.ugt80.
im.wt.ugt80.
im.smt1.
im.wt.smt1.

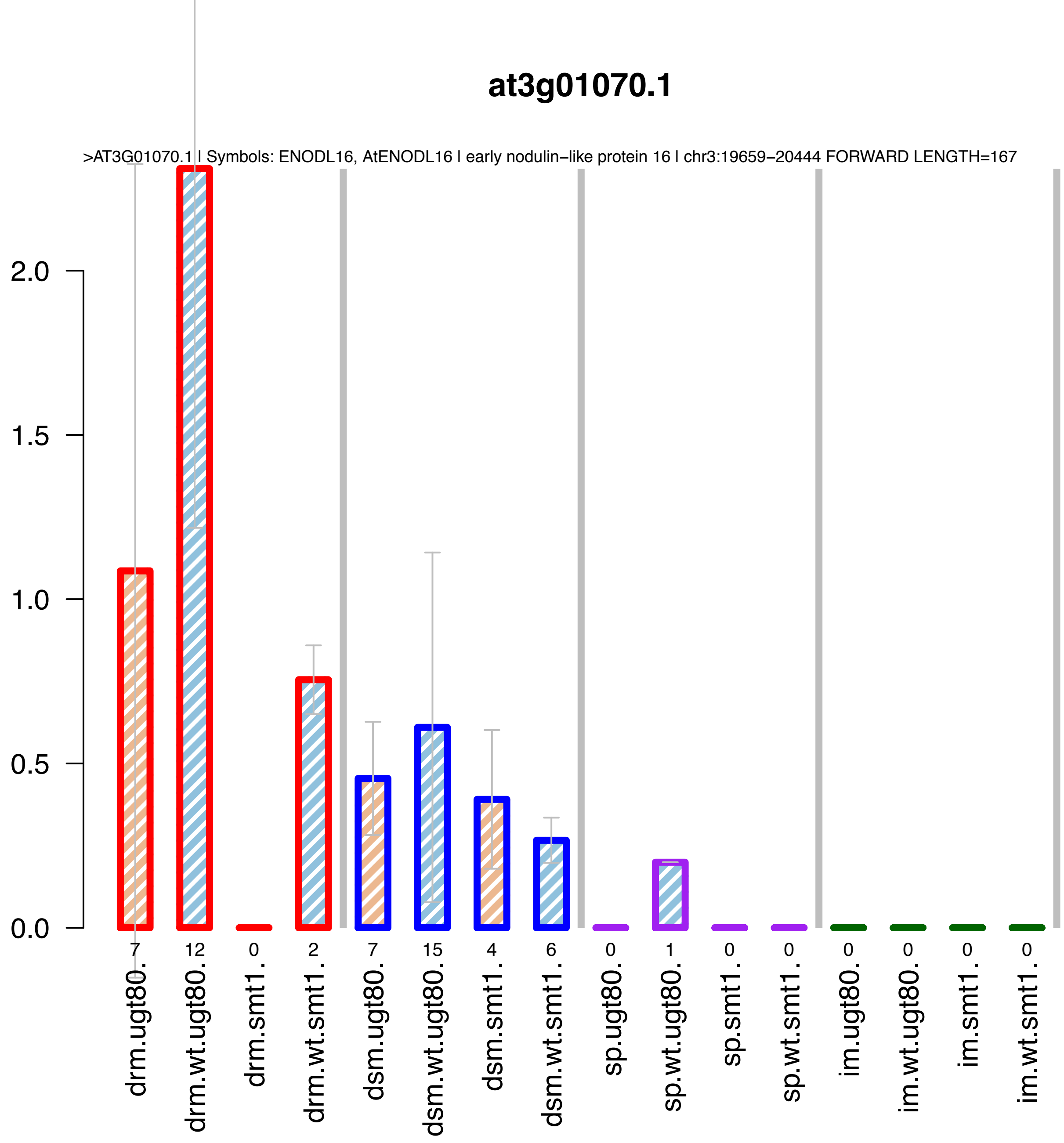

at3g01070.1
>AT3G01070.1 | Symbols: ENODL16, AtENODL16 | early nodulin−like protein 16 | chr3:19659−20444 FORWARD LENGTH=167
2.0
1.5
1.0
0.5
0.0
7
12
0
2
7
15
4
6
0
1
0
0
0
0
0
0
drm.ugt80.
drm.wt.ugt80.
drm.smt1.
drm.wt.smt1.
dsm.ugt80.
dsm.wt.ugt80.
dsm.smt1.
dsm.wt.smt1.
sp.ugt80.
sp.wt.ugt80.
sp.smt1.
sp.wt.smt1.
im.ugt80.
im.wt.ugt80.
im.smt1.
im.wt.smt1.

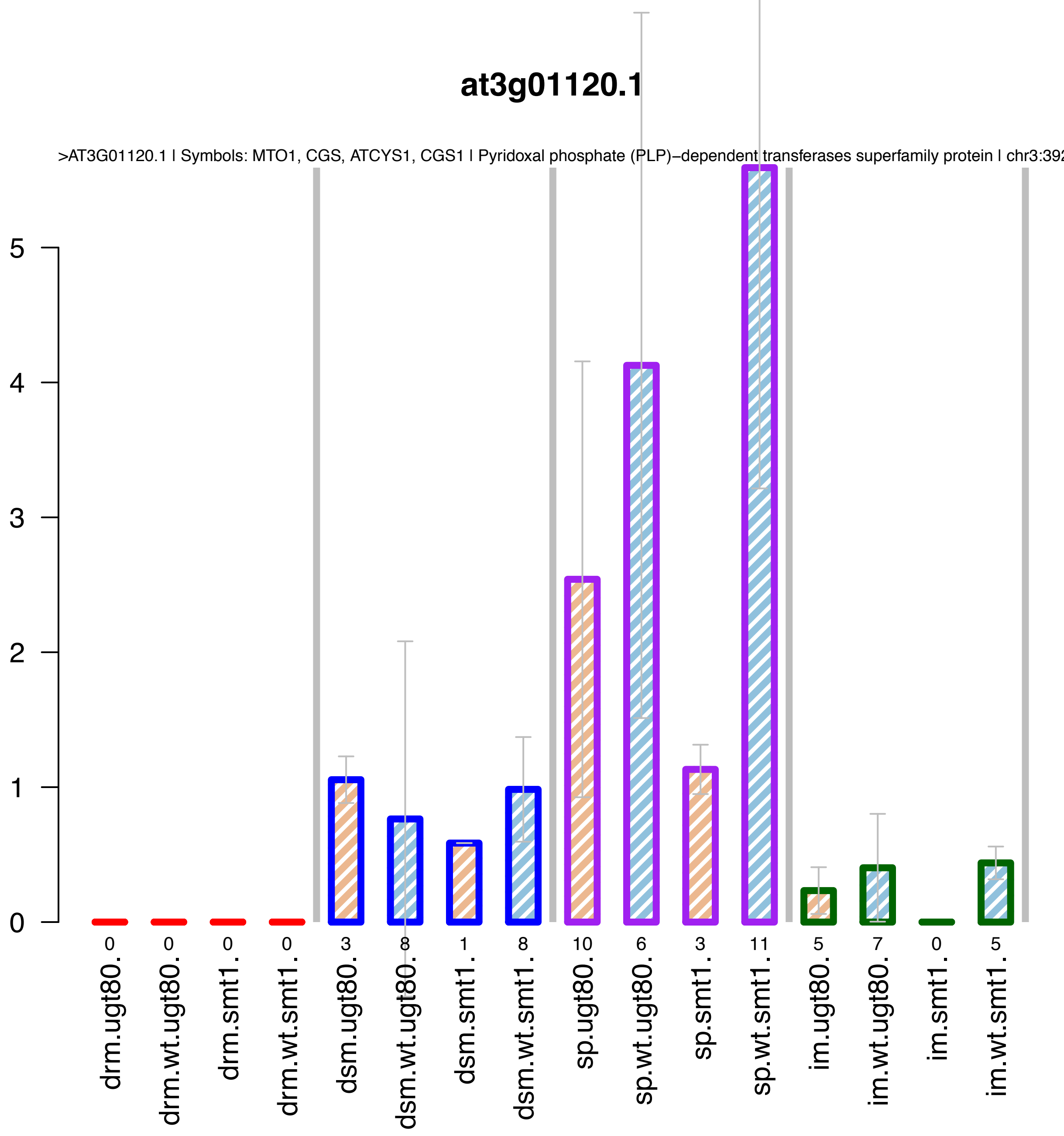

at3g01120.1
>AT3G01120.1 | Symbols: MTO1, CGS, ATCYS1, CGS1 | Pyridoxal phosphate (PLP)−dependent transferases superfamily protein | chr3:392
5
4
3
2
1
0
0
0
0
0
3
8
1
8
10
6
3
11
5
7
0
5
drm.ugt80.
drm.wt.ugt80.
drm.smt1.
drm.wt.smt1.
dsm.ugt80.
dsm.wt.ugt80.
dsm.smt1.
dsm.wt.smt1.
sp.ugt80.
sp.wt.ugt80.
sp.smt1.
sp.wt.smt1.
im.ugt80.
im.wt.ugt80.
im.smt1.
im.wt.smt1.

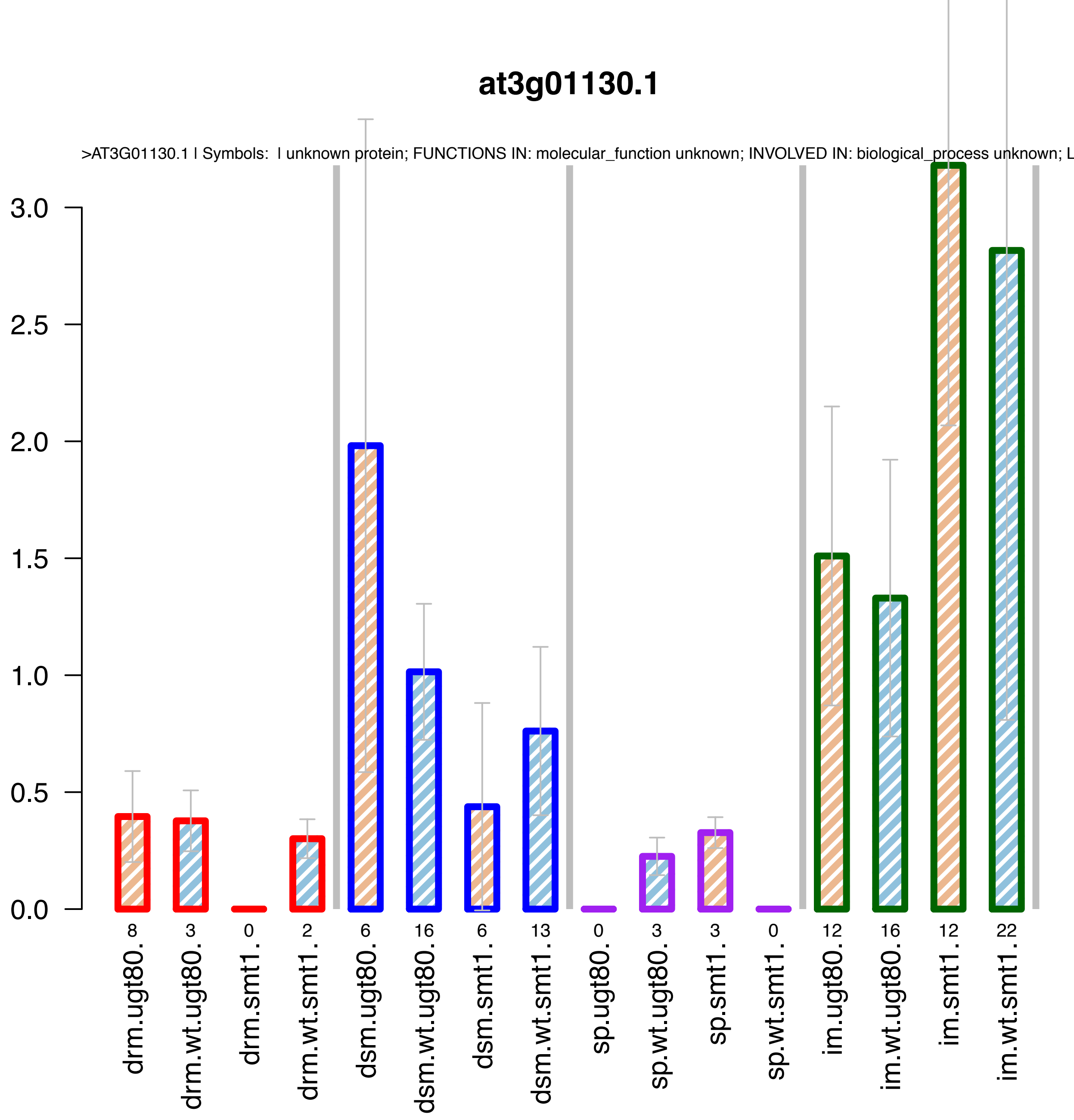

at3g01130.1
>AT3G01130.1 | Symbols: | unknown protein; FUNCTIONS IN: molecular_function unknown; INVOLVED IN: biological_process unknown; L
0.0
0.5
1.0
1.5
2.0
2.5
3.0
8
3
0
2
6
16
6
13
0
3
3
0
12
16
12
22
drm.ugt80..
drm.wt.ugt80..
drm.smt1..
drm.wt.smt1..
dsm.ugt80..
dsm.wt.ugt80..
dsm.smt1..
dsm.wt.smt1..
sp.ugt80..
sp.wt.ugt80..
sp.smt1..
sp.wt.smt1..
im.ugt80..
im.wt.ugt80..
im.smt1..
im.wt.smt1..

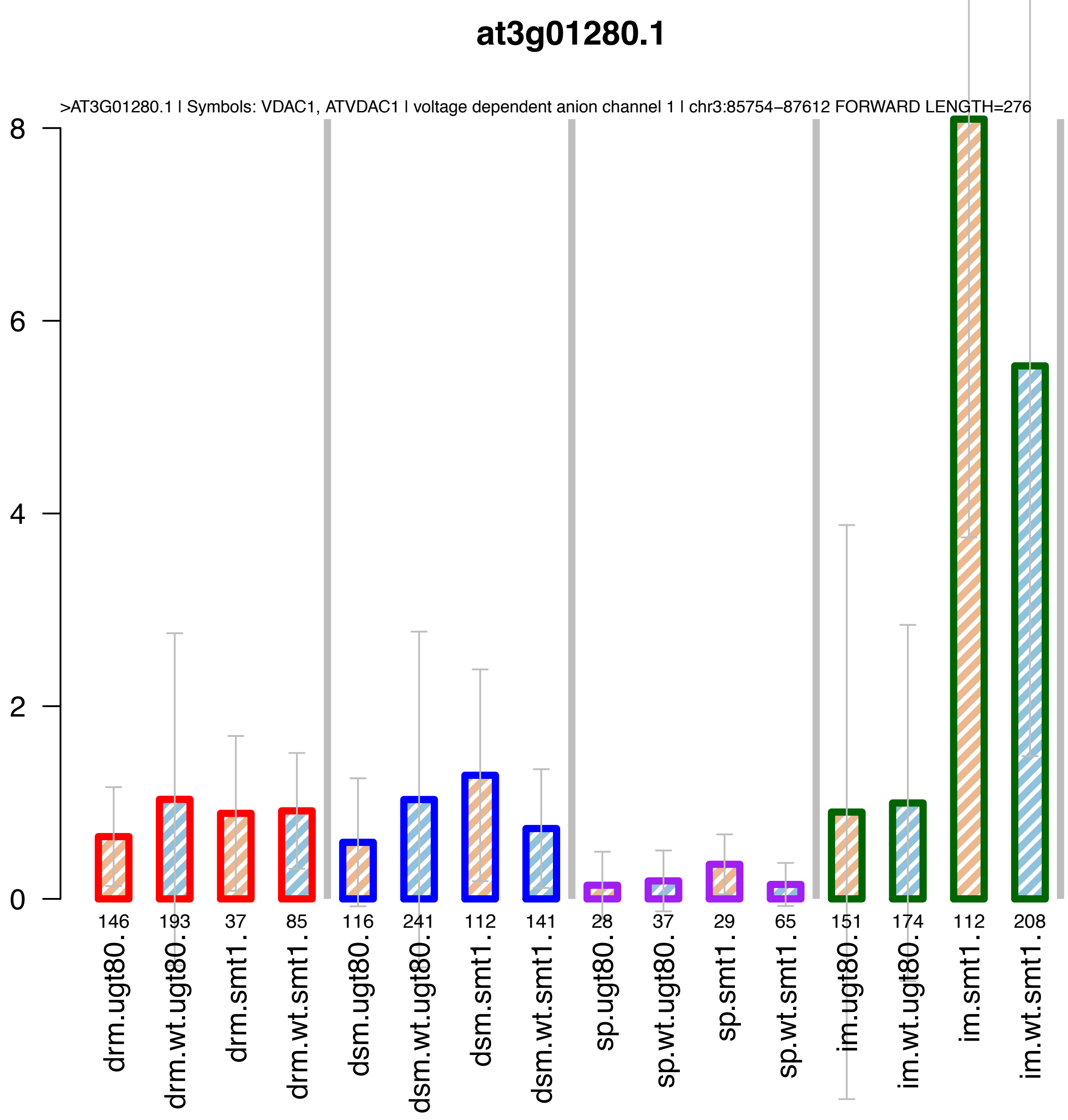

at3g01280.1
>AT3G01280.1 | Symbols: VDAC1, ATVDAC1 | voltage dependent anion channel 1 | chr3:85754-87612 FORWARD LENGTH=276
0
2
4
6
8
146
193
37
85
116
241
112
141
28
37
29
65
151
174
112
208
drm.ugt80..
drm.wt.ugt80..
drm.smt1..
drm.wt.smt1..
dsm.ugt80..
dsm.wt.ugt80..
dsm.smt1..
dsm.wt.smt1..
sp.ugt80..
sp.wt.ugt80..
sp.smt1..
sp.wt.smt1..
im.ugt80..
im.wt.ugt80..
im.smt1..
im.wt.smt1..

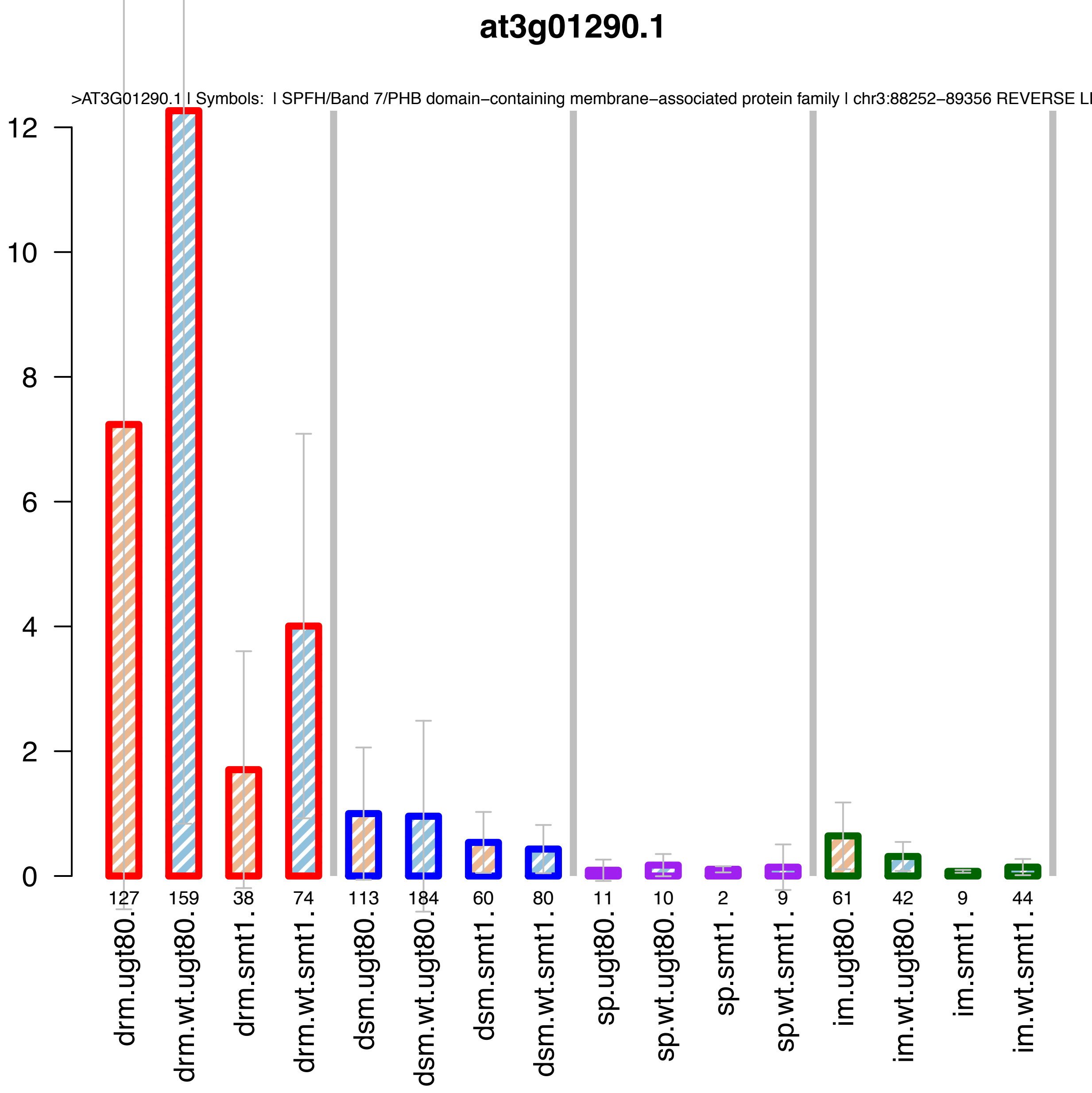

at3g01290.1
>AT3G01290.1 | Symbols: | SPFH/Band 7/PHB domain-containing membrane-associated protein family | chr3:88252-89356 REVERSE LI
0
2
4
6
8
10
12
127
159
38
74
113
184
60
80
11
10
2
9
61
42
9
44
drm.ugt80.
drm.wt.ugt80.
drm.smt1.
drm.wt.smt1.
dsm.ugt80.
dsm.wt.ugt80.
dsm.smt1.
dsm.wt.smt1.
sp.ugt80.
sp.wt.ugt80.
sp.smt1.
sp.wt.smt1.
im.ugt80.
im.wt.ugt80.
im.smt1.
im.wt.smt1.

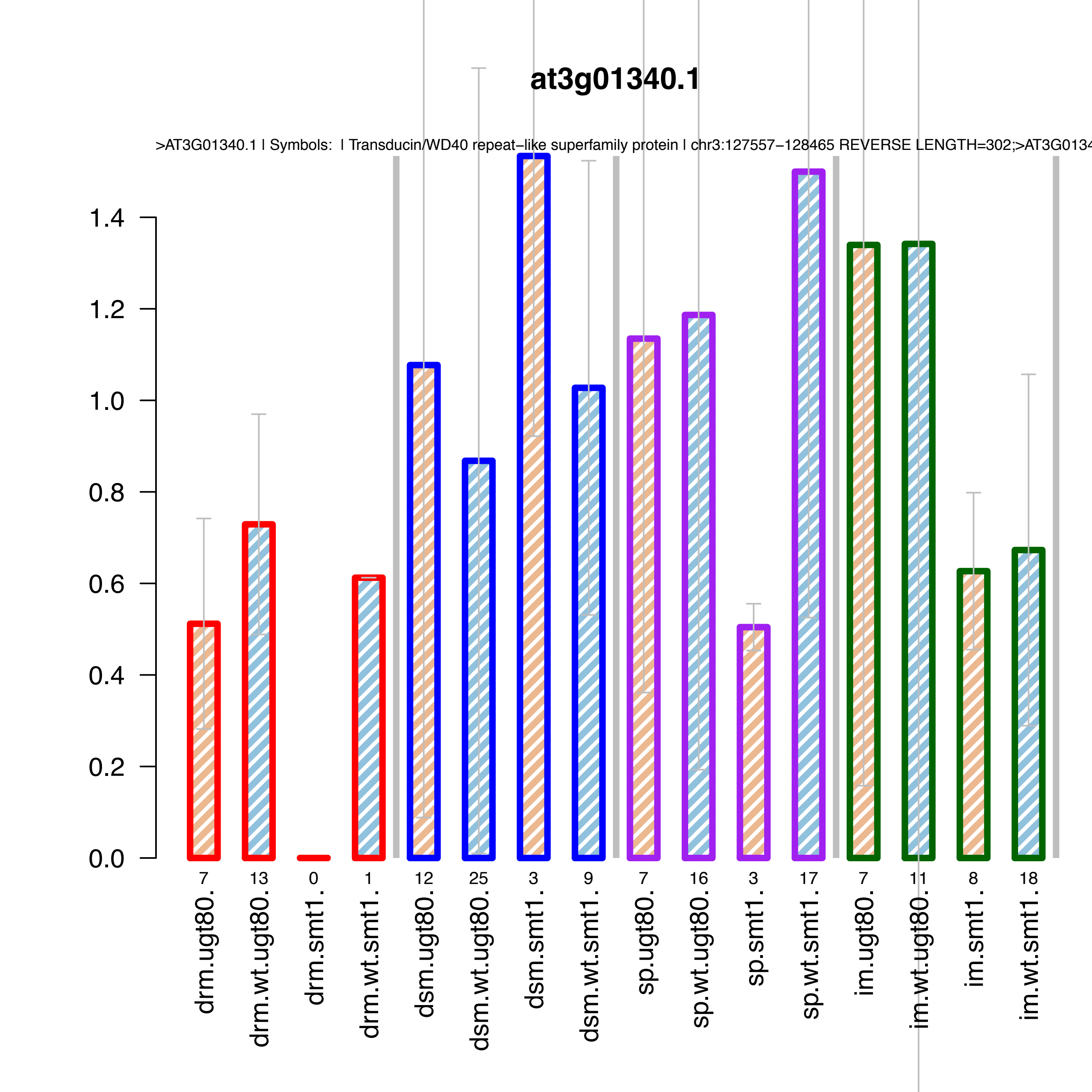

at3g01340.1
>AT3G01340.1 | Symbols: | Transducin/WD40 repeat-like superfamily protein | chr3:127557-128465 REVERSE LENGTH=302;>AT3G0134
1.4
1.2
1.0
0.8
0.6
0.4
0.2
0.0
7
13
0
1
12
25
3
9
7
16
3
17
7
11
8
18
drm.ugt80.
drm.wt.ugt80.
drm.smt1.
drm.wt.smt1.
dsm.ugt80.
dsm.wt.ugt80.
dsm.smt1.
dsm.wt.smt1.
sp.ugt80.
sp.wt.ugt80.
sp.smt1.
sp.wt.smt1.
im.ugt80.
im.wt.ugt80.
im.smt1.
im.wt.smt1.

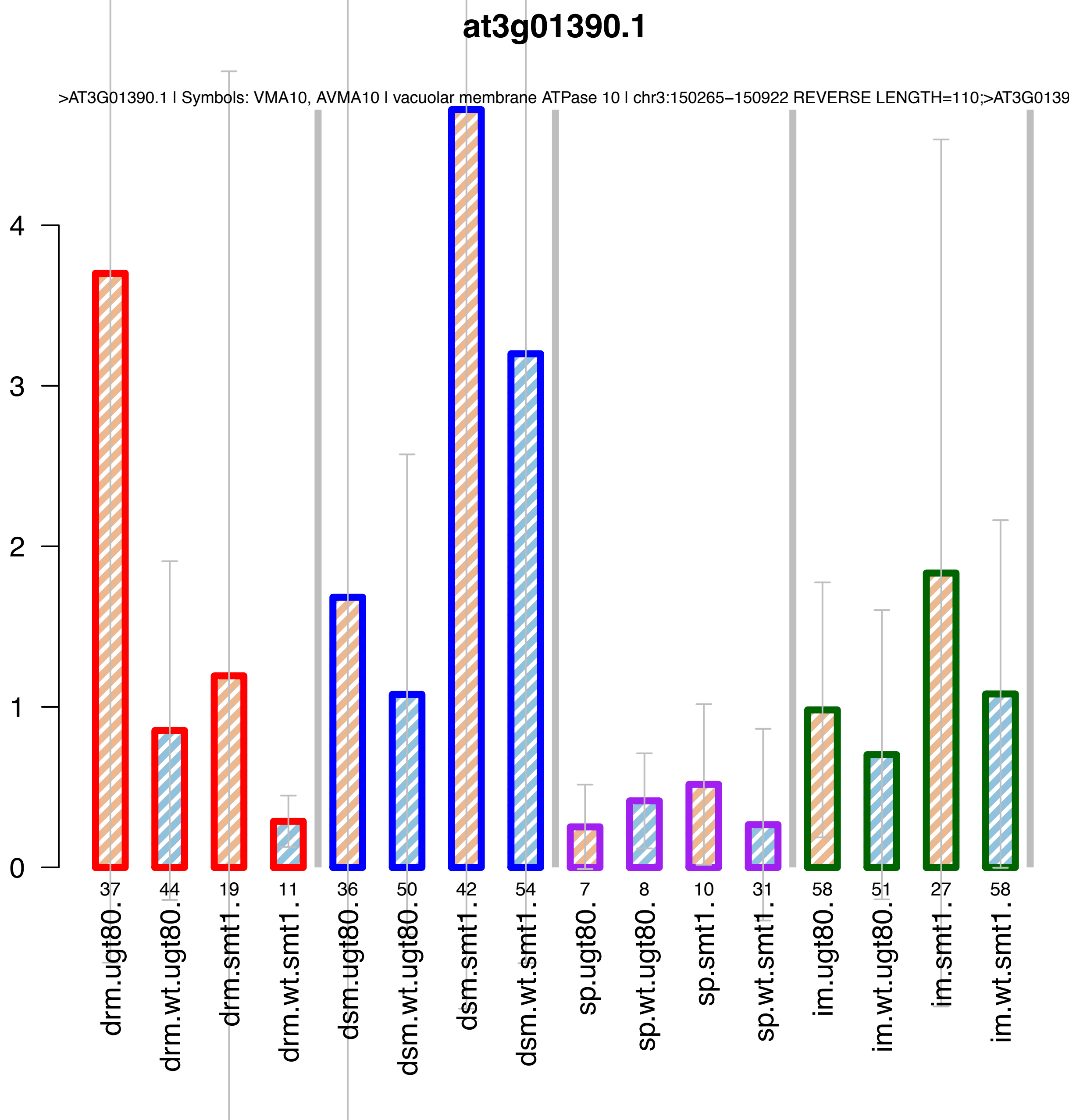

at3g01390.1
>AT3G01390.1 | Symbols: VMA10, AVMA10 | vacuolar membrane ATPase 10 | chr3:150265-150922 REVERSE LENGTH=110;>AT3G0139
0
1
2
3
4
37
44
19
11
36
50
42
54
7
8
10
31
58
51
27
58
drm.ugt80.
drm.wt.ugt80.
drm.smt1.
drm.wt.smt1.
dsm.ugt80.
dsm.wt.ugt80.
dsm.smt1.
dsm.wt.smt1.
sp.ugt80.
sp.wt.ugt80.
sp.smt1.
sp.wt.smt1.
im.ugt80.
im.wt.ugt80.
im.smt1.
im.wt.smt1.

# at3g01640.1

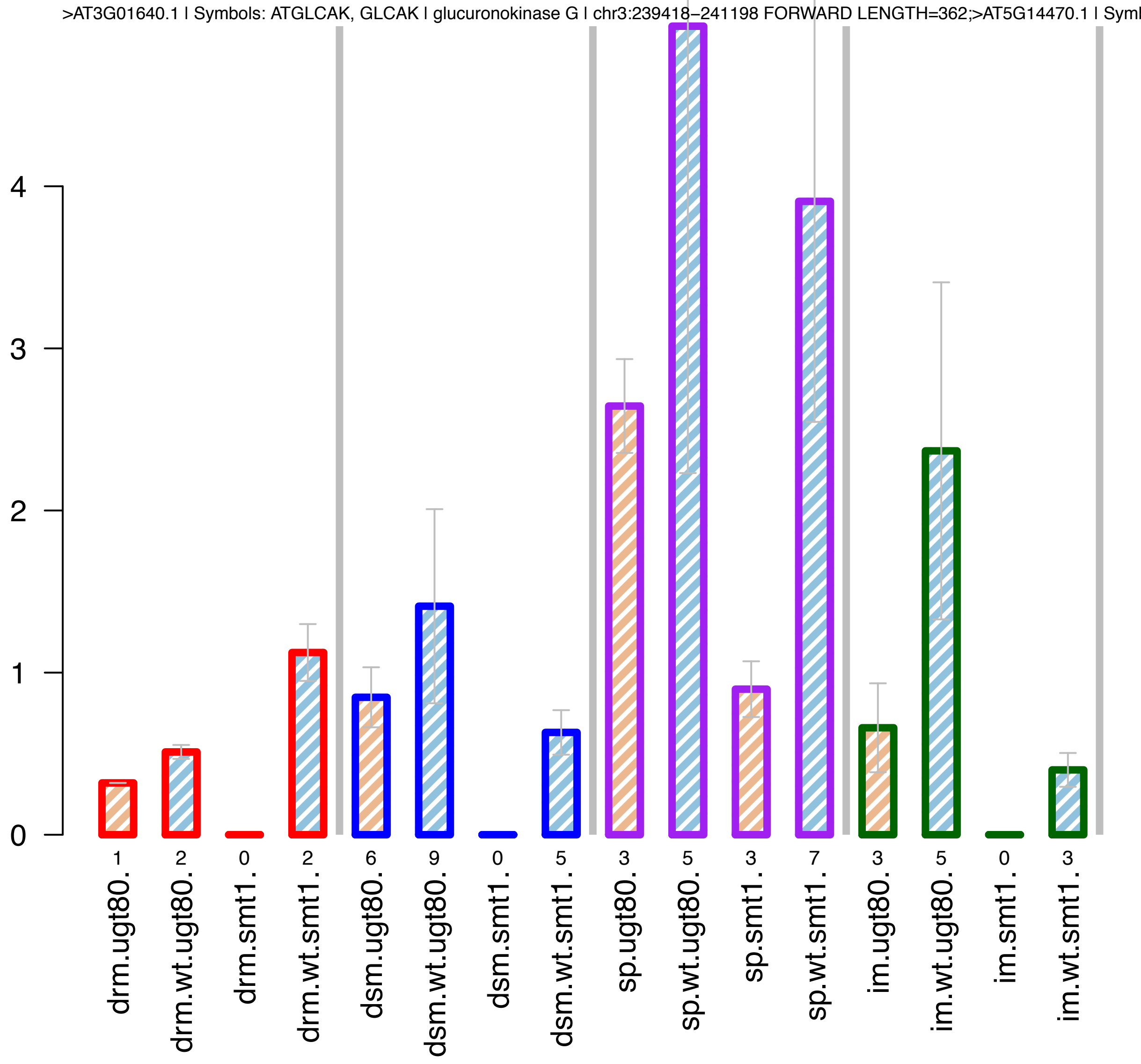

>AT3G01640.1 | Symbols: ATGLCAK, GLCAK | glucuronokinase G | chr3:239418–241198 FORWARD LENGTH=362;>AT5G14470.1 | Symb
0
1
2
3
4
1
2
0
2
6
9
0
5
3
5
3
7
3
5
0
3
drm.ugt80.
drm.wt.ugt80.
drm.smt1.
drm.wt.smt1.
dsm.ugt80.
dsm.wt.ugt80.
dsm.smt1.
dsm.wt.smt1.
sp.ugt80.
sp.wt.ugt80.
sp.smt1.
sp.wt.smt1.
im.ugt80.
im.wt.ugt80.
im.smt1.
im.wt.smt1.

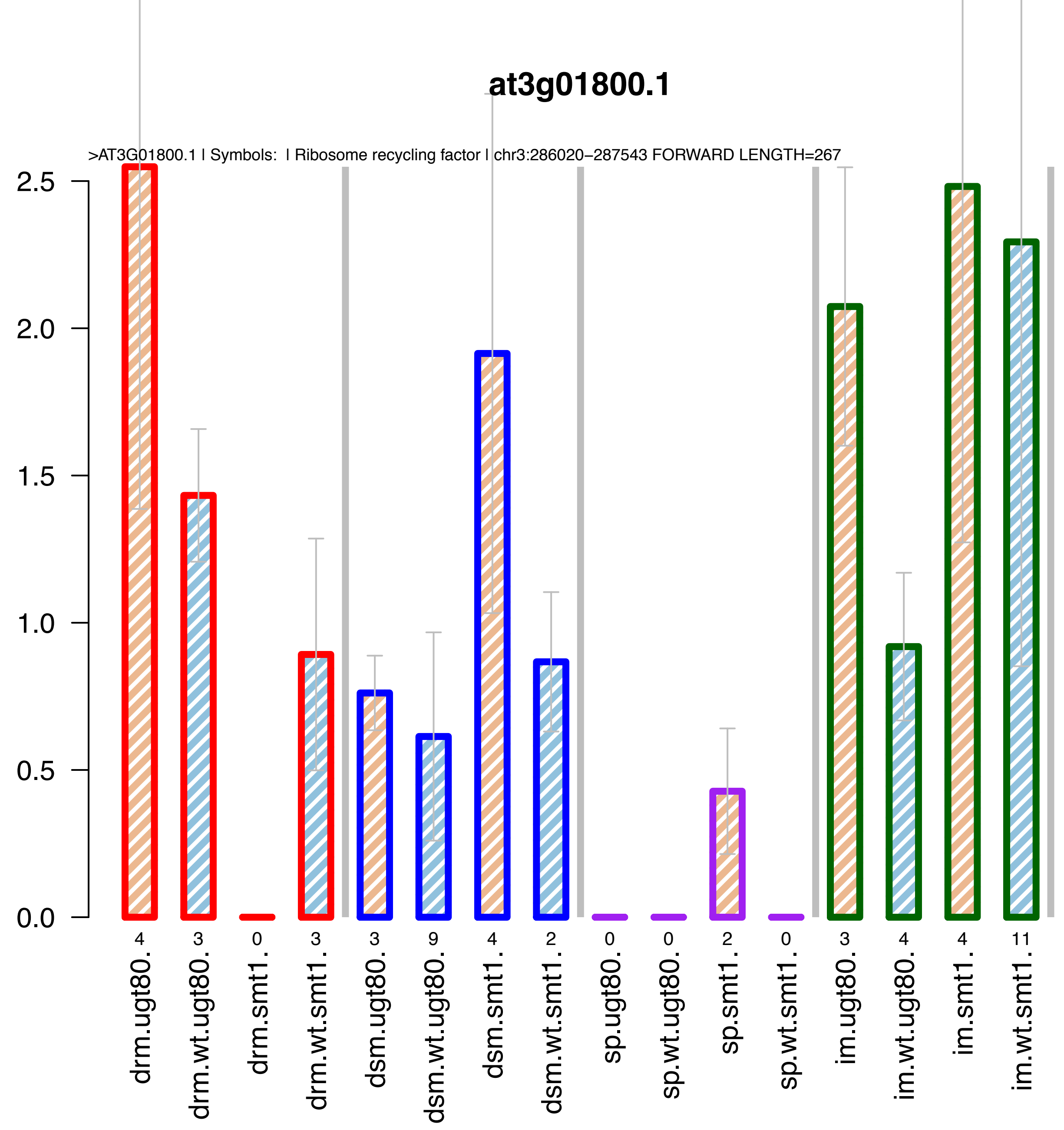
at3g01800.1
>AT3G01800.1 | Symbols: | Ribosome recycling factor | chr3:286020−287543 FORWARD LENGTH=267
2.5
2.0
1.5
1.0
0.5
0.0
4
3
0
3
3
9
4
2
0
0
2
0
3
4
4
11
drm.ugt80.
drm.wt.ugt80.
drm.smt1.
drm.wt.smt1.
dsm.ugt80.
dsm.wt.ugt80.
dsm.smt1.
dsm.wt.smt1.
sp.ugt80.
sp.wt.ugt80.
sp.smt1.
sp.wt.smt1.
im.ugt80.
im.wt.ugt80.
im.smt1.
im.wt.smt1.

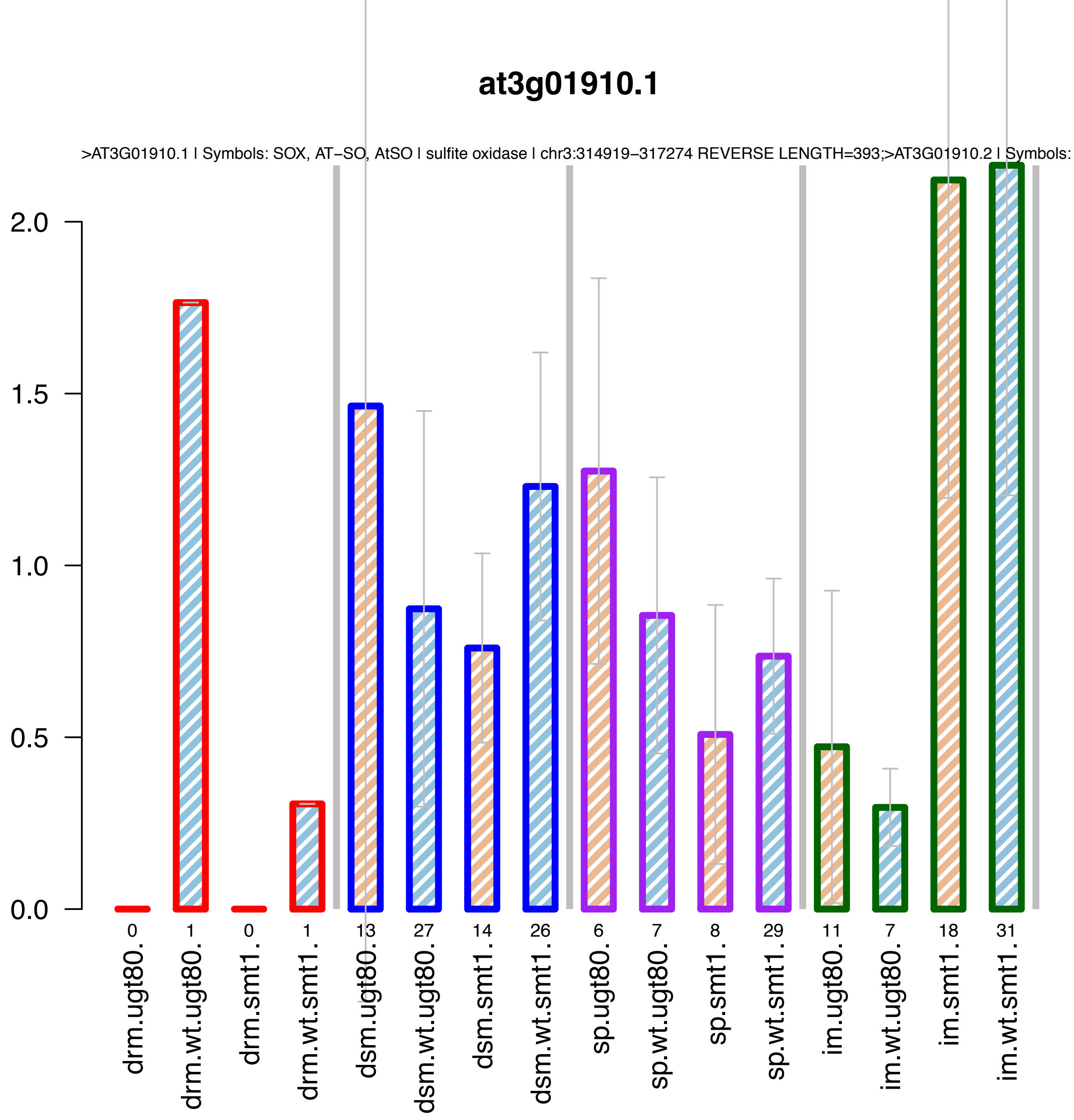

at3g01910.1
>AT3G01910.1 | Symbols: SOX, AT−SO, AtSO | sulfite oxidase | chr3:314919−317274 REVERSE LENGTH=393;>AT3G01910.2 | Symbols:
2.0
1.5
1.0
0.5
0.0
0
1
0
1
13
27
14
26
6
7
8
29
11
7
18
31
drm.ugt80.
drm.wt.ugt80.
drm.smt1.
drm.wt.smt1.
dsm.ugt80.
dsm.wt.ugt80.
dsm.smt1.
dsm.wt.smt1.
sp.ugt80.
sp.wt.ugt80.
sp.smt1.
sp.wt.smt1.
im.ugt80.
im.wt.ugt80.
im.smt1.
im.wt.smt1.

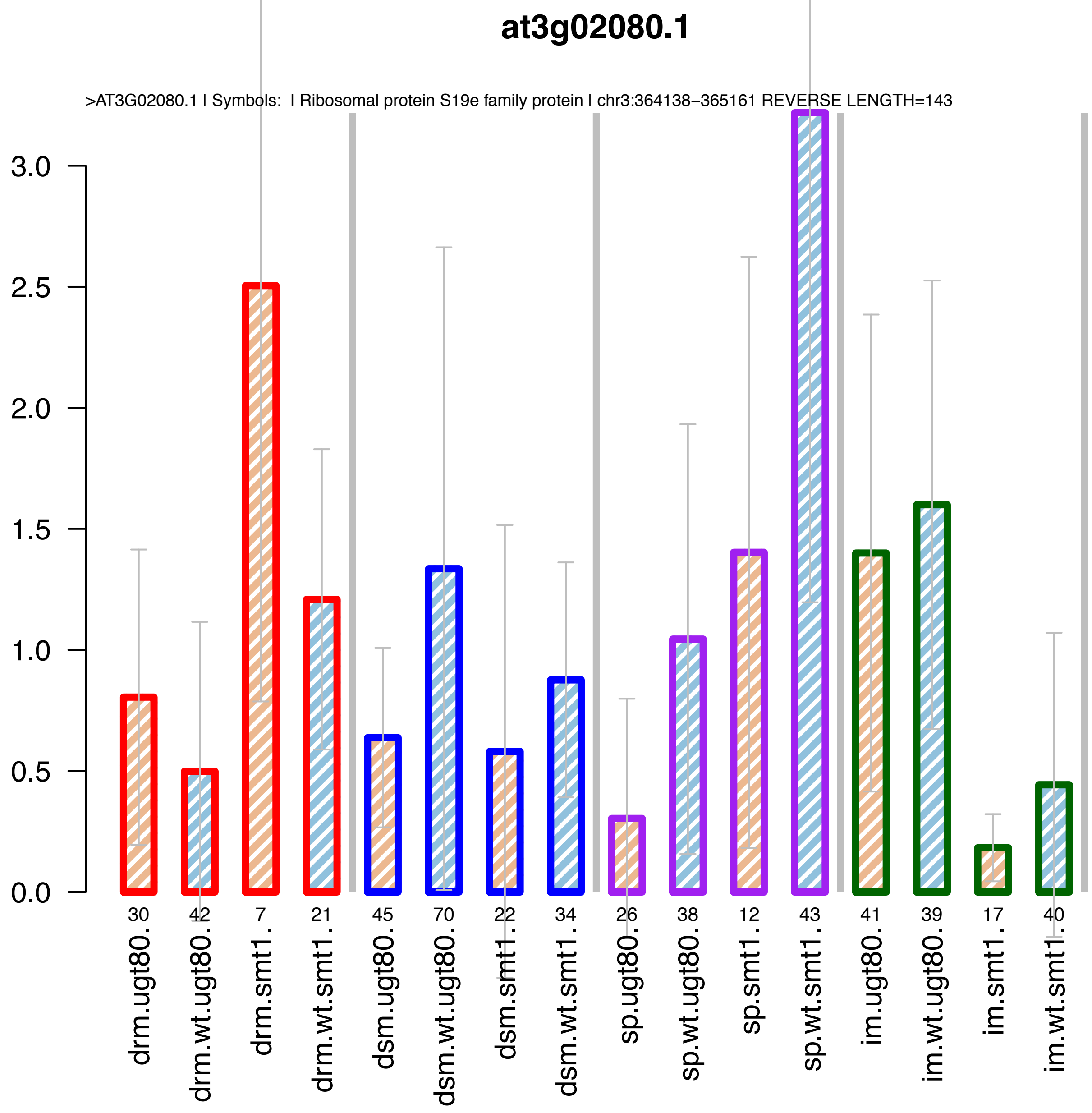
at3g02080.1
>AT3G02080.1 | Symbols: | Ribosomal protein S19e family protein | chr3:364138-365161 REVERSE LENGTH=143
3.0
2.5
2.0
1.5
1.0
0.5
0.0
30
42
7
21
45
70
22
34
26
38
12
43
41
39
17
40
drm.ugt80..
drm.wt.ugt80..
drm.smt1..
drm.wt.smt1..
dsm.ugt80..
dsm.wt.ugt80..
dsm.smt1..
dsm.wt.smt1..
sp.ugt80..
sp.wt.ugt80..
sp.smt1..
sp.wt.smt1..
im.ugt80..
im.wt.ugt80..
im.smt1..
im.wt.smt1..

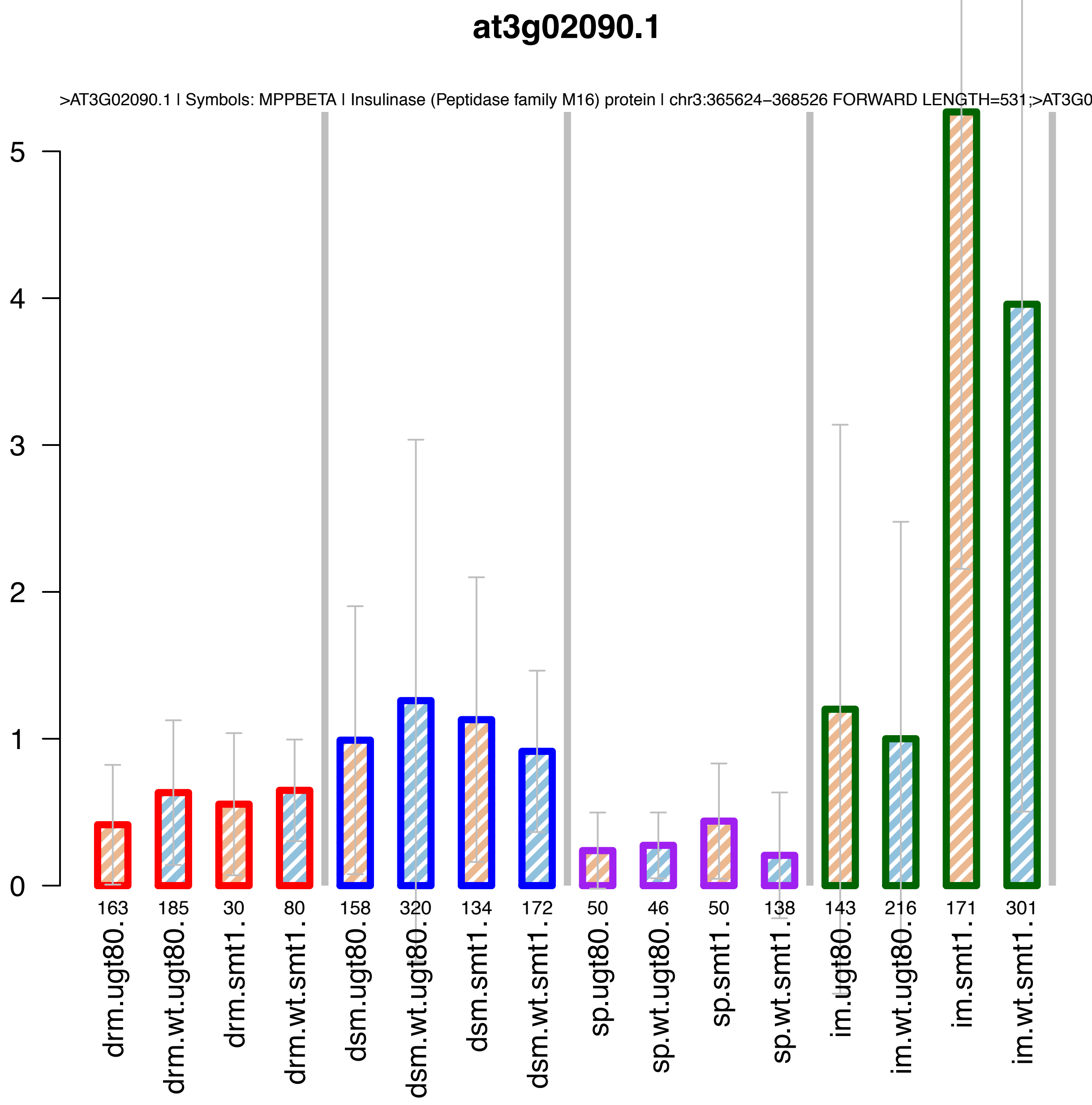

at3g02090.1
>AT3G02090.1 | Symbols: MPPBETA | Insulinase (Peptidase family M16) protein | chr3:365624–368526 FORWARD LENGTH=531;>AT3G0
0
1
2
3
4
5
163
185
30
80
158
320
134
172
50
46
50
138
143
216
171
301
drm.ugt80..
drm.wt.ugt80..
drm.smt1..
drm.wt.smt1..
dsm.ugt80..
dsm.wt.ugt80..
dsm.smt1..
dsm.wt.smt1..
sp.ugt80..
sp.wt.ugt80..
sp.smt1..
sp.wt.smt1..
im.ugt80..
im.wt.ugt80..
im.smt1..
im.wt.smt1..

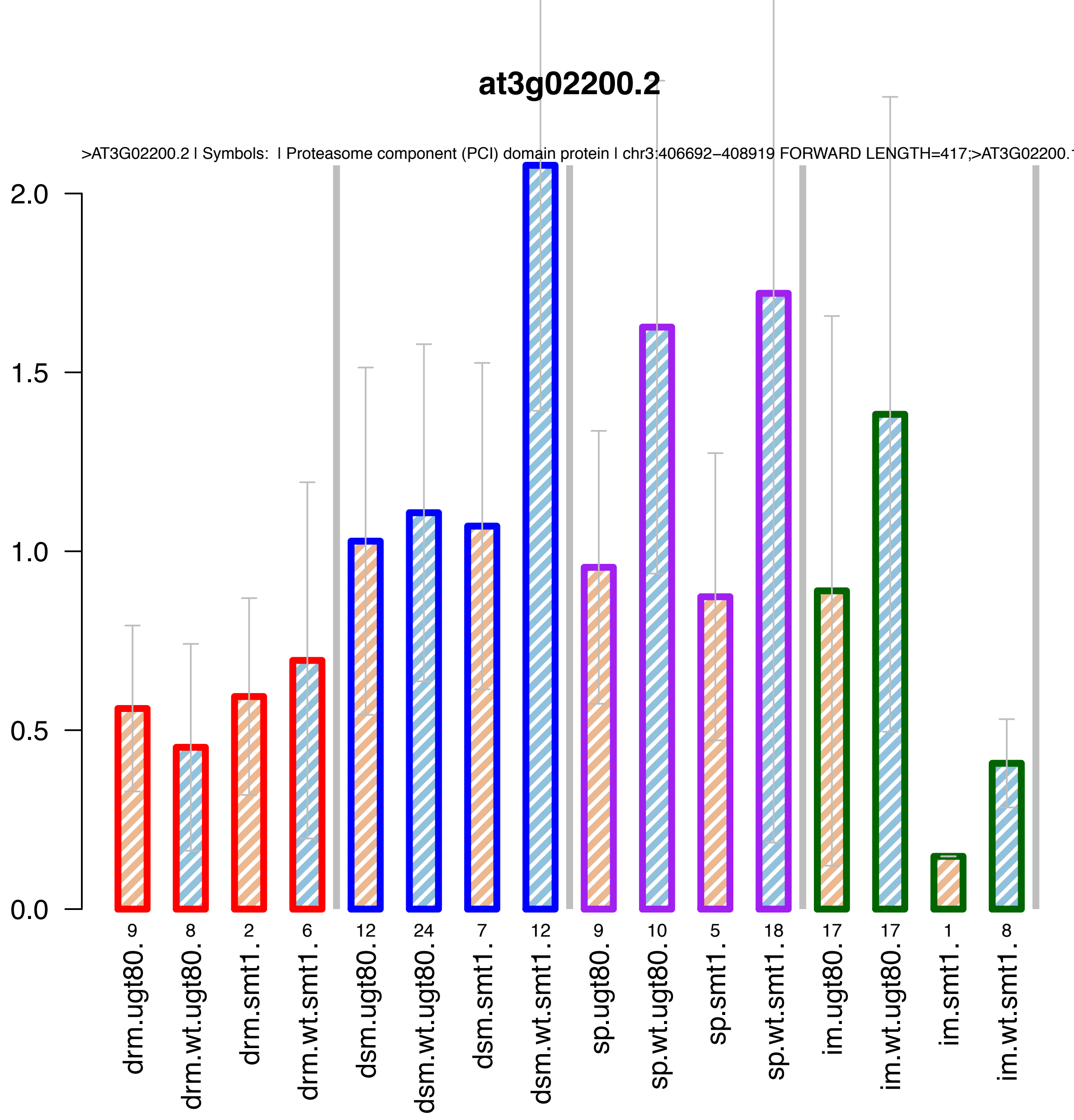

at3g02200.2
>AT3G02200.2 | Symbols: | Proteasome component (PCI) domain protein | chr3:406692-408919 FORWARD LENGTH=417;>AT3G02200.1
2.0
1.5
1.0
0.5
0.0
9
8
2
6
12
24
7
12
9
10
5
18
17
17
1
8
drm.ugt80.
drm.wt.ugt80.
drm.smt1.
drm.wt.smt1.
dsm.ugt80.
dsm.wt.ugt80.
dsm.smt1.
dsm.wt.smt1.
sp.ugt80.
sp.wt.ugt80.
sp.smt1.
sp.wt.smt1.
im.ugt80.
im.wt.ugt80.
im.smt1.
im.wt.smt1.

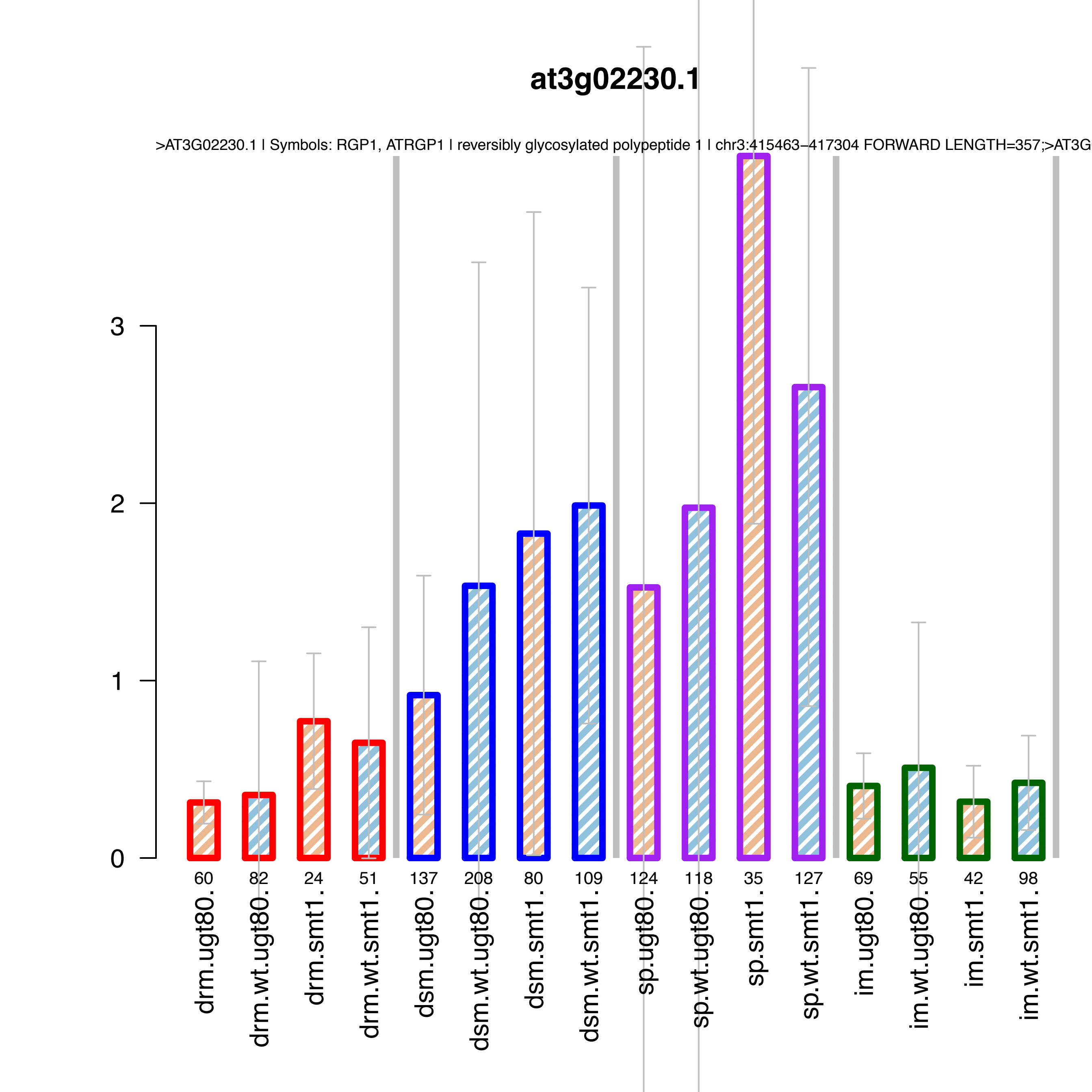

at3g02230.1
>AT3G02230.1 | Symbols: RGP1, ATRGP1 | reversibly glycosylated polypeptide 1 | chr3:415463–417304 FORWARD LENGTH=357;>AT3G
3
2
1
0
60
82
24
51
137
208
80
109
124
118
35
127
69
55
42
98
drm.ugt80.
drm.wt.ugt80.
drm.smt1.
drm.wt.smt1.
dsm.ugt80.
dsm.wt.ugt80.
dsm.smt1.
dsm.wt.smt1.
sp.ugt80.
sp.wt.ugt80.
sp.smt1.
sp.wt.smt1.
im.ugt80.
im.wt.ugt80.
im.smt1.
im.wt.smt1.

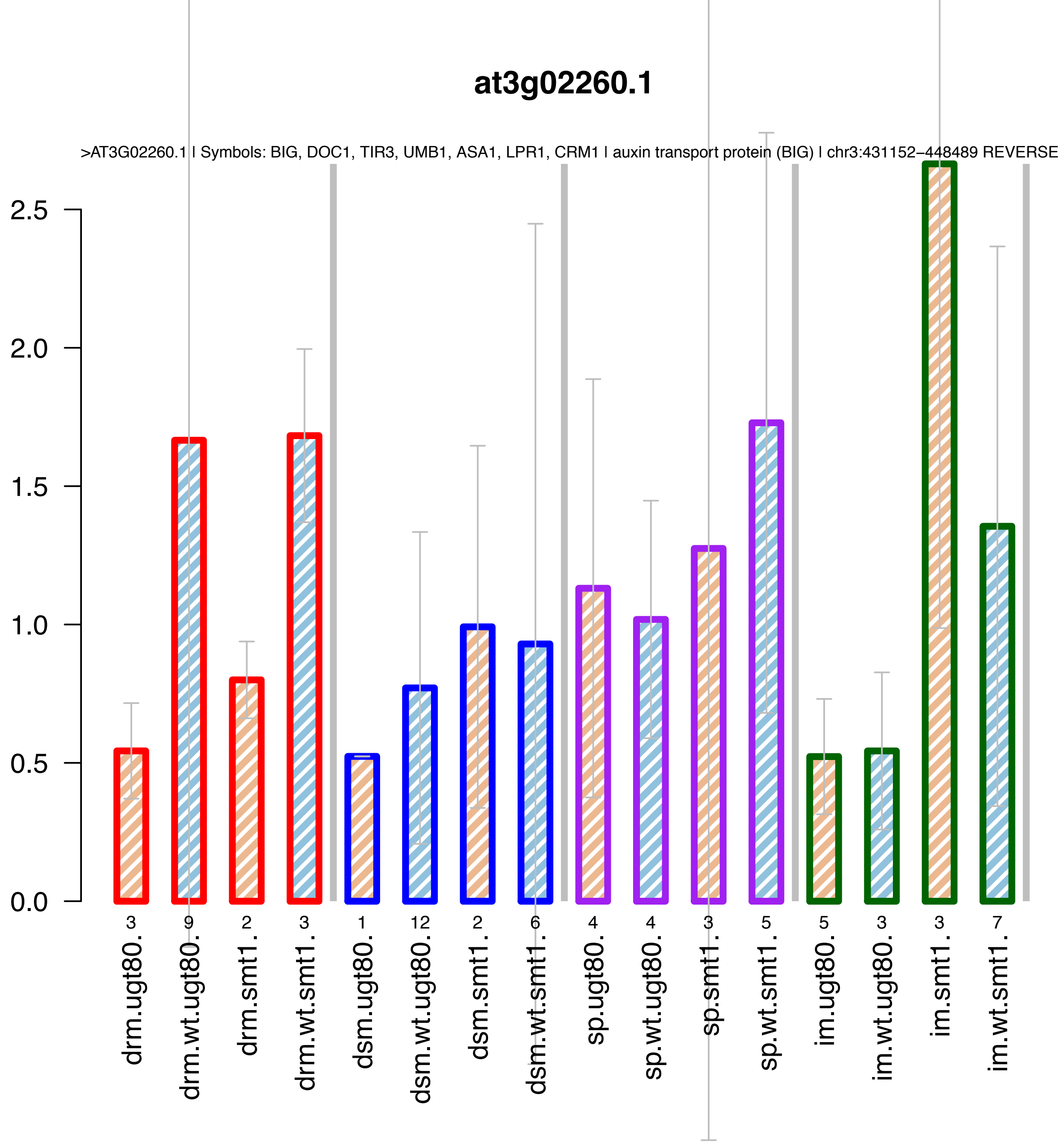
at3g02260.1
>AT3G02260.1 | Symbols: BIG, DOC1, TIR3, UMB1, ASA1, LPR1, CRM1 | auxin transport protein (BIG) | chr3:431152–448489 REVERSE
0.0
0.5
1.0
1.5
2.0
2.5
3
9
2
3
1
12
2
6
4
4
3
5
5
3
3
7
drm.ugt80.
drm.wt.ugt80.
drm.smt1.
drm.wt.smt1.
dsm.ugt80.
dsm.wt.ugt80.
dsm.smt1.
dsm.wt.smt1.
sp.ugt80.
sp.wt.ugt80.
sp.smt1.
sp.wt.smt1.
im.ugt80.
im.wt.ugt80.
im.smt1.
im.wt.smt1.

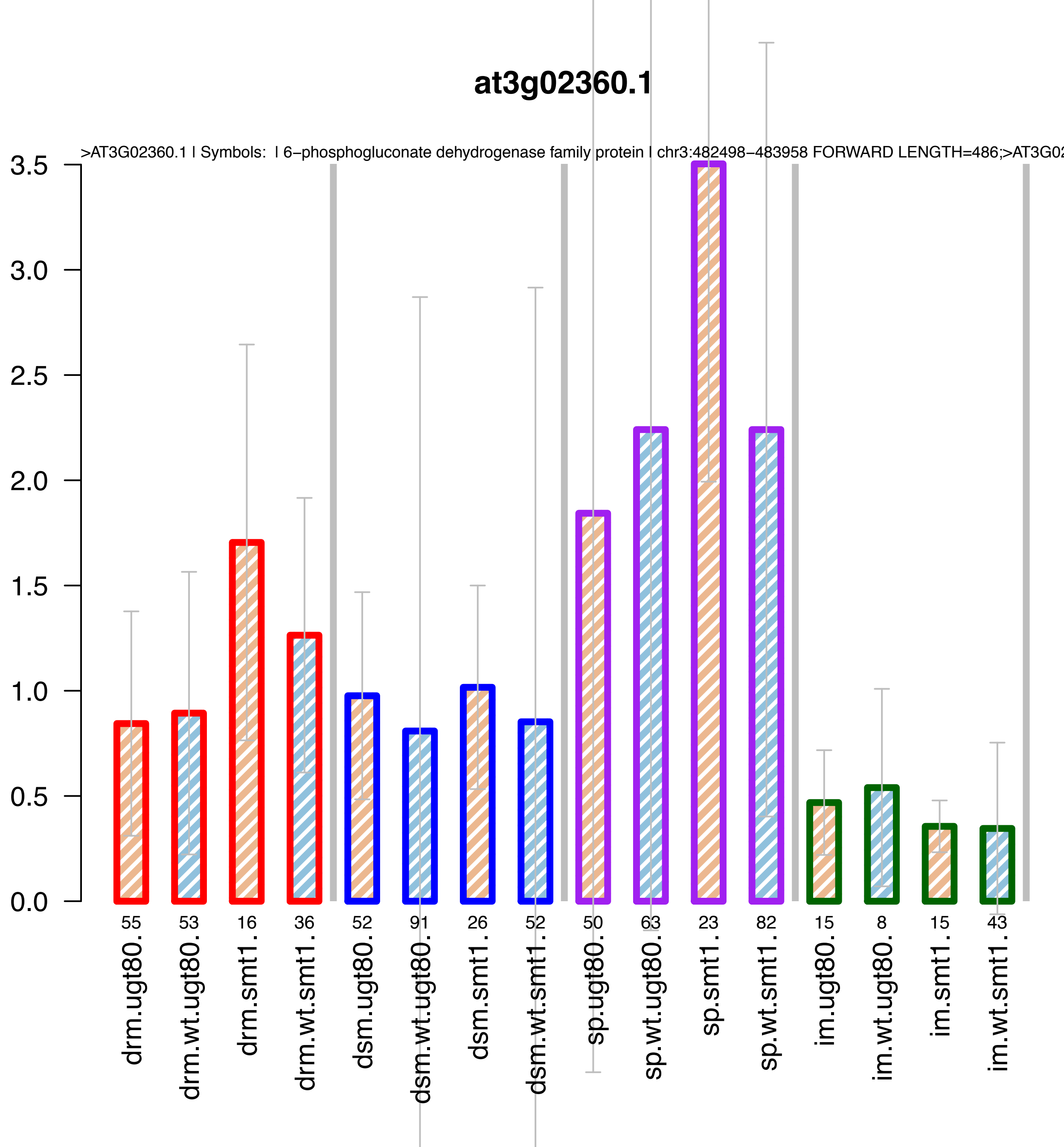

at3g02360.1
>AT3G02360.1 | Symbols: | 6−phosphogluconate dehydrogenase family protein | chr3:482498−483958 FORWARD LENGTH=486;>AT3G02
3.5
3.0
2.5
2.0
1.5
1.0
0.5
0.0
55
53
16
36
52
91
26
52
50
63
23
82
15
8
15
43
drm.ugt80..
drm.wt.ugt80..
drm.smt1..
drm.wt.smt1..
dsm.ugt80..
dsm.wt.ugt80..
dsm.smt1..
dsm.wt.smt1..
sp.ugt80..
sp.wt.ugt80..
sp.smt1..
sp.wt.smt1..
im.ugt80..
im.wt.ugt80..
im.smt1..
im.wt.smt1..

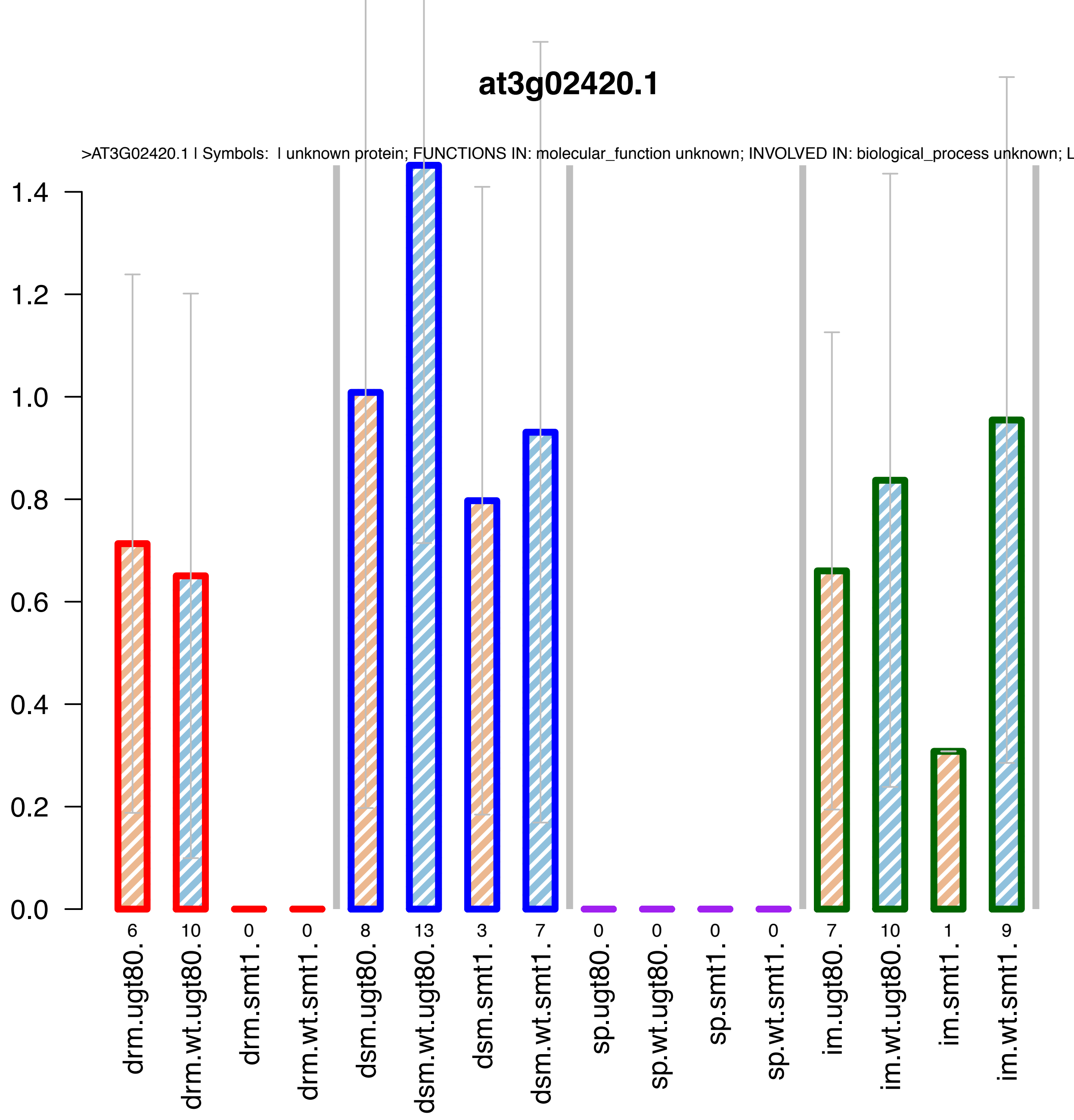

at3g02420.1
>AT3G02420.1 | Symbols: | unknown protein; FUNCTIONS IN: molecular_function unknown; INVOLVED IN: biological_process unknown; L
1.4
1.2
1.0
0.8
0.6
0.4
0.2
0.0
6
10
0
0
8
13
3
7
0
0
0
0
7
10
1
9
drm.ugt80.
drm.wt.ugt80.
drm.smt1.
drm.wt.smt1.
dsm.ugt80.
dsm.wt.ugt80.
dsm.smt1.
dsm.wt.smt1.
sp.ugt80.
sp.wt.ugt80.
sp.smt1.
sp.wt.smt1.
im.ugt80.
im.wt.ugt80.
im.smt1.
im.wt.smt1.

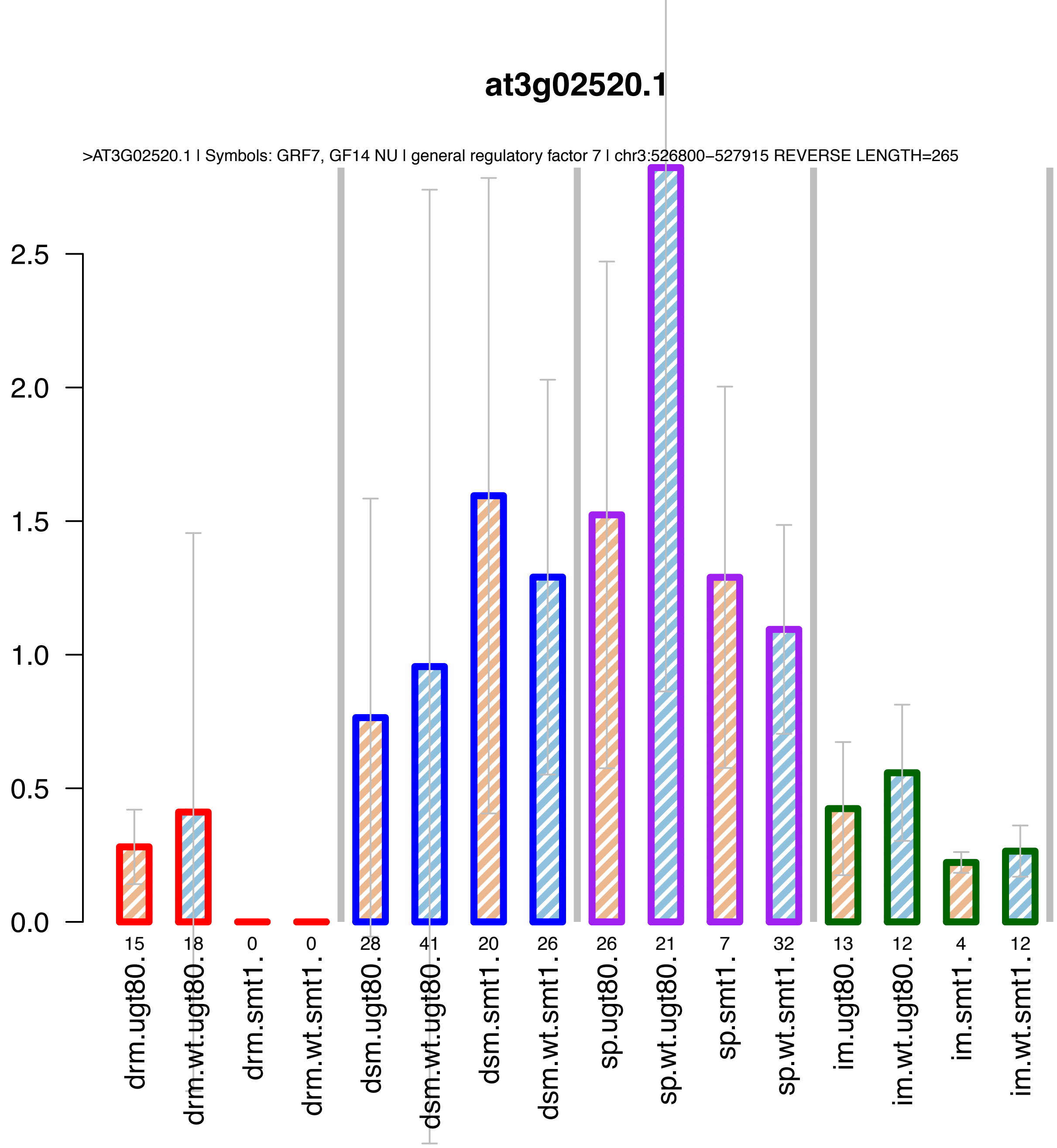

at3g02520.1
>AT3G02520.1 | Symbols: GRF7, GF14 NU | general regulatory factor 7 | chr3:526800-527915 REVERSE LENGTH=265
2.5
2.0
1.5
1.0
0.5
0.0
15
18
0
0
28
41
20
26
26
21
7
32
13
12
4
12
drm.ugt80.
drm.wt.ugt80.
drm.smt1.
drm.wt.smt1.
dsm.ugt80.
dsm.wt.ugt80.
dsm.smt1.
dsm.wt.smt1.
sp.ugt80.
sp.wt.ugt80.
sp.smt1.
sp.wt.smt1.
im.ugt80.
im.wt.ugt80.
im.smt1.
im.wt.smt1.

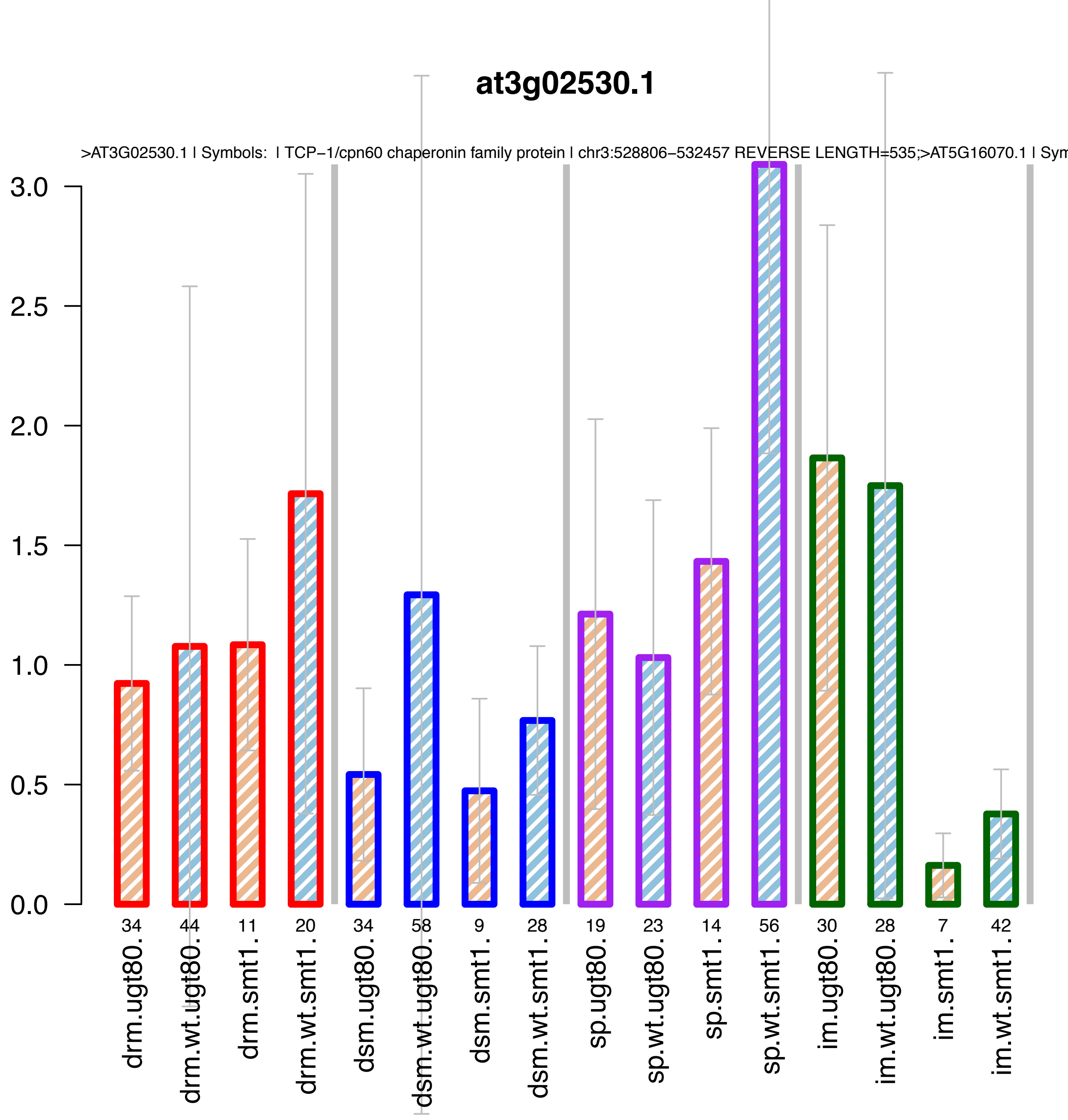
at3g02530.1
>AT3G02530.1 | Symbols: | TCP−1/cpn60 chaperonin family protein | chr3:528806−532457 REVERSE LENGTH=535;>AT5G16070.1 | Sym
0.0
0.5
1.0
1.5
2.0
2.5
3.0
34
44
11
20
34
58
9
28
19
23
14
56
30
28
7
42
drm.ugt80..
drm.wt.ugt80..
drm.smt1..
drm.wt.smt1..
dsm.ugt80..
dsm.wt.ugt80..
dsm.smt1..
dsm.wt.smt1..
sp.ugt80..
sp.wt.ugt80..
sp.smt1..
sp.wt.smt1..
im.ugt80..
im.wt.ugt80..
im.smt1..
im.wt.smt1..

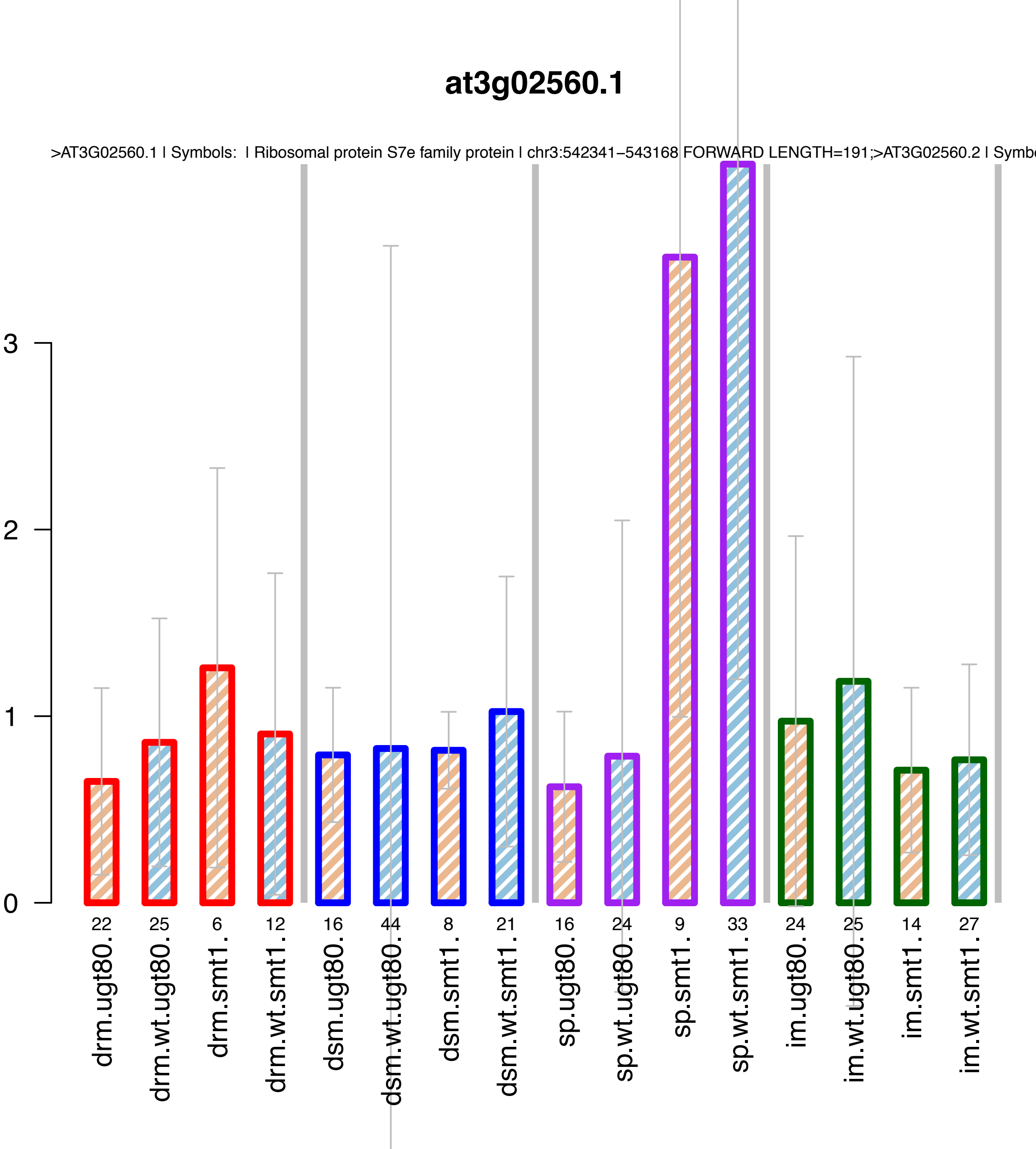

at3g02560.1
>AT3G02560.1 | Symbols: | Ribosomal protein S7e family protein | chr3:542341−543168 FORWARD LENGTH=191;>AT3G02560.2 | Symbo
0
1
2
3
22
25
6
12
16
44
8
21
16
24
9
33
24
25
14
27
drm.ugt80..
drm.wt.ugt80..
drm.smt1..
drm.wt.smt1..
dsm.ugt80..
dsm.wt.ugt80..
dsm.smt1..
dsm.wt.smt1..
sp.ugt80..
sp.wt.ugt80..
sp.smt1..
sp.wt.smt1..
im.ugt80..
im.wt.ugt80..
im.smt1..
im.wt.smt1..

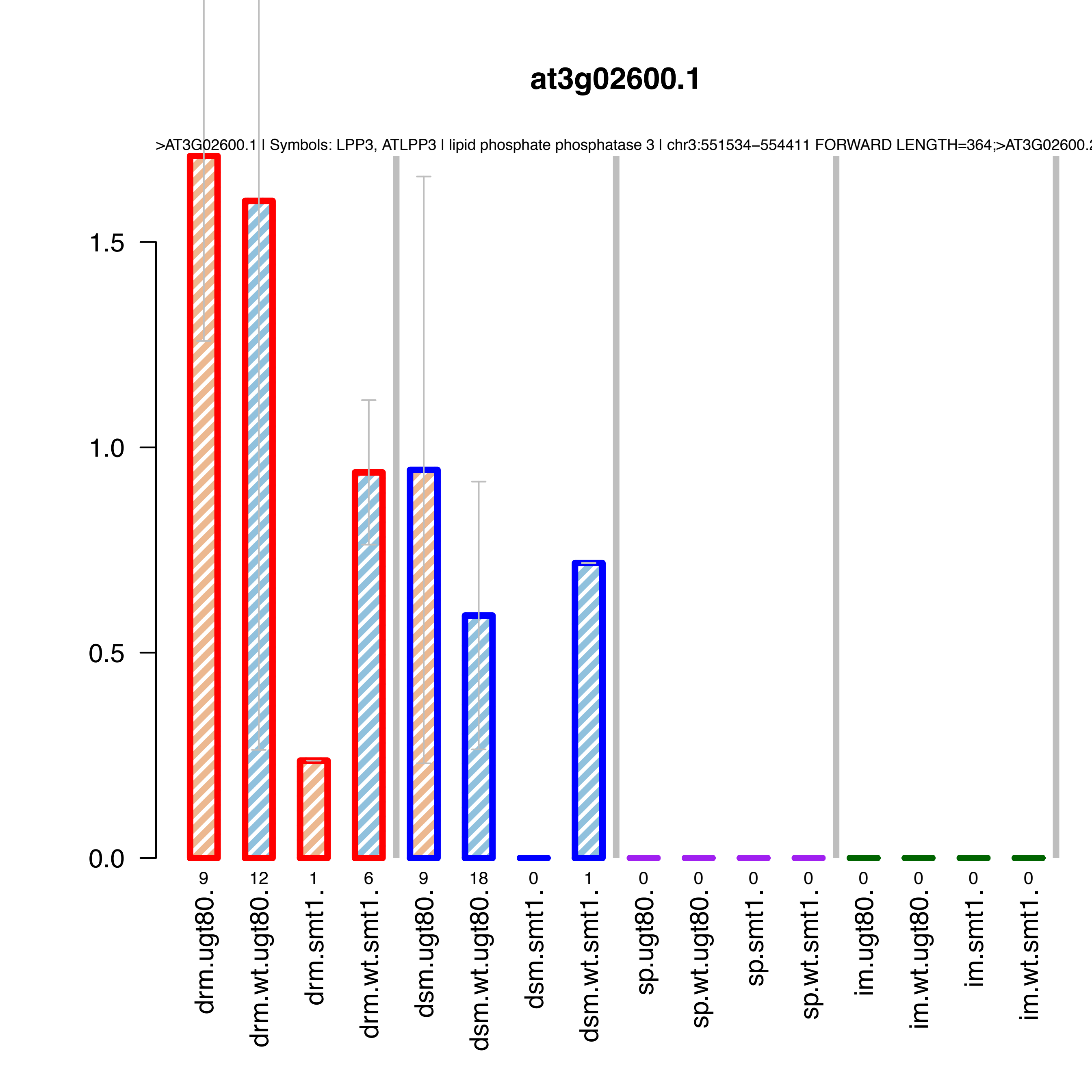

at3g02600.1
>AT3G02600.1 | Symbols: LPP3, ATLPP3 | lipid phosphate phosphatase 3 | chr3:551534-554411 FORWARD LENGTH=364;>AT3G02600.2
1.5
1.0
0.5
0.0
9
12
1
6
9
18
0
1
0
0
0
0
0
0
0
0
drm.ugt80.
drm.wt.ugt80.
drm.smt1.
drm.wt.smt1.
dsm.ugt80.
dsm.wt.ugt80.
dsm.smt1.
dsm.wt.smt1.
sp.ugt80.
sp.wt.ugt80.
sp.smt1.
sp.wt.smt1.
im.ugt80.
im.wt.ugt80.
im.smt1.
im.wt.smt1.

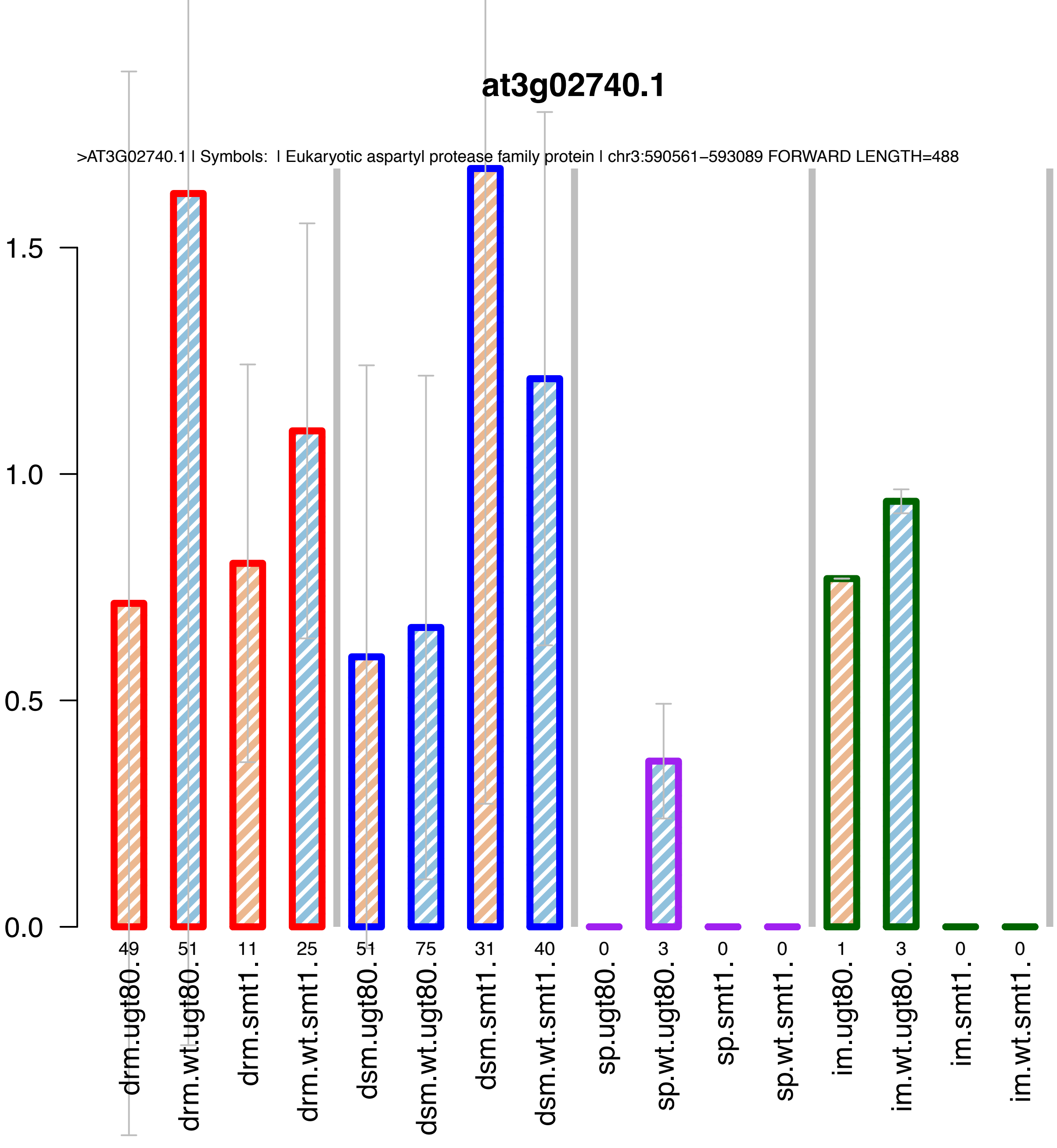

at3g02740.1
>AT3G02740.1 | Symbols: | Eukaryotic aspartyl protease family protein | chr3:590561-593089 FORWARD LENGTH=488
0.0
0.5
1.0
1.5
49
51
11
25
51
75
31
40
0
3
0
0
1
3
0
0
drm.ugt80.
drm.wt.ugt80.
drm.smt1.
drm.wt.smt1.
dsm.ugt80.
dsm.wt.ugt80.
dsm.smt1.
dsm.wt.smt1.
sp.ugt80.
sp.wt.ugt80.
sp.smt1.
sp.wt.smt1.
im.ugt80.
im.wt.ugt80.
im.smt1.
im.wt.smt1.

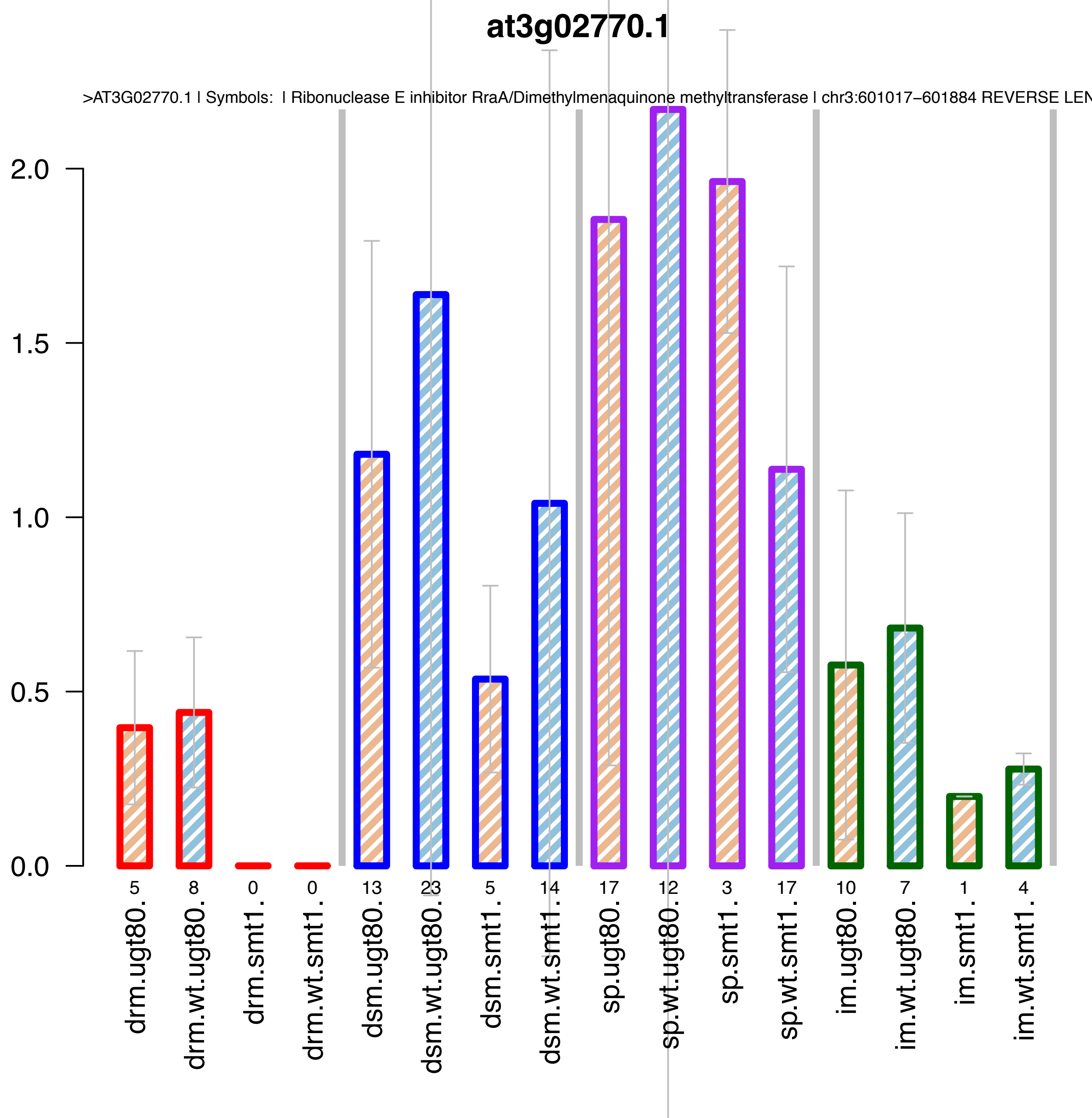
at3g02770.1
>AT3G02770.1 | Symbols: | Ribonuclease E inhibitor RraA/Dimethylmenaquinone methyltransferase | chr3:601017-601884 REVERSE LEN
2.0
1.5
1.0
0.5
0.0
5
8
0
0
13
23
5
14
17
12
3
17
10
7
1
4
drm.ugt80..
drm.wt.ugt80..
drm.smt1..
drm.wt.smt1..
dsm.ugt80..
dsm.wt.ugt80..
dsm.smt1..
dsm.wt.smt1..
sp.ugt80..
sp.wt.ugt80..
sp.smt1..
sp.wt.smt1..
im.ugt80..
im.wt.ugt80..
im.smt1..
im.wt.smt1..

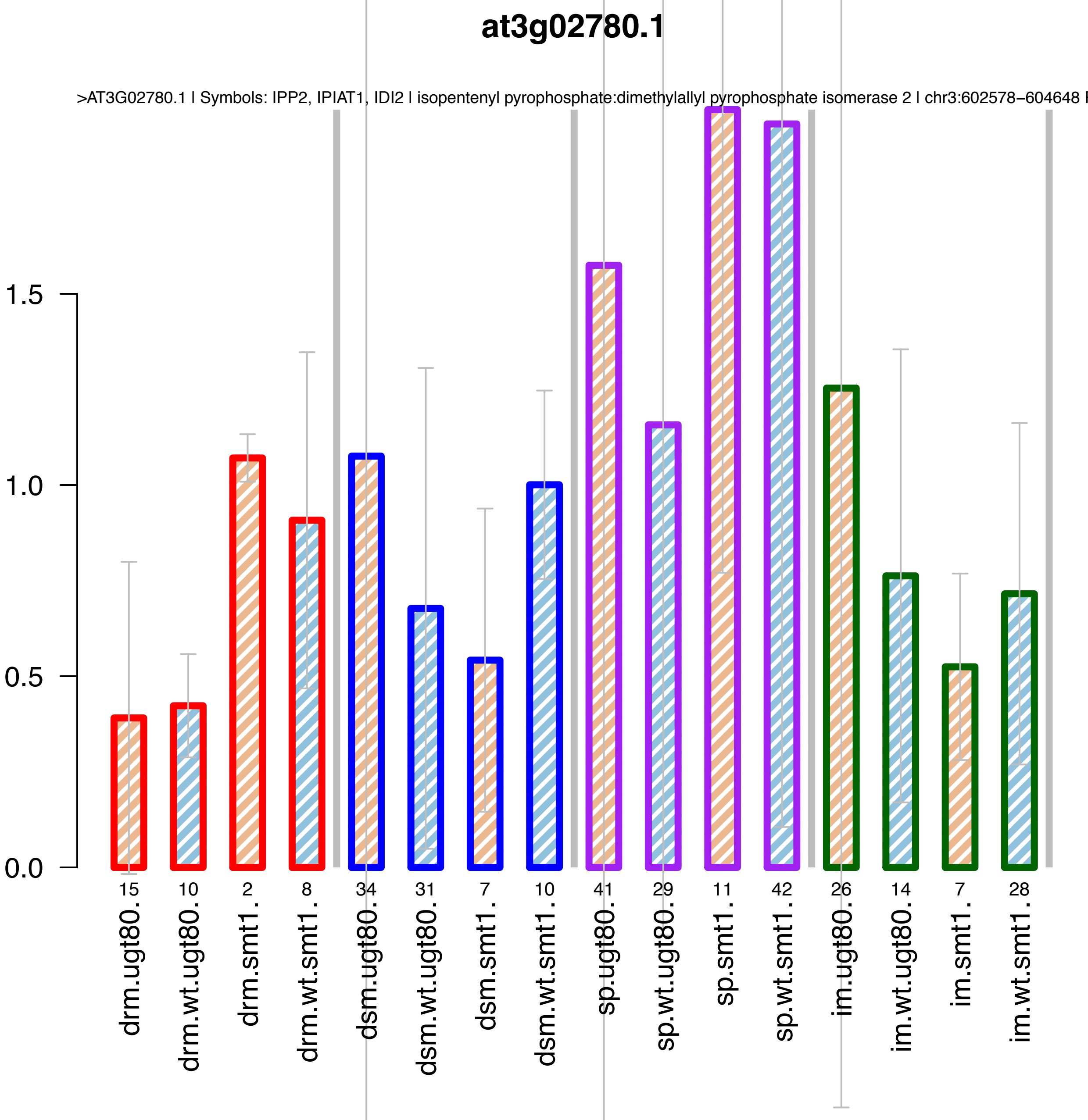
at3g02780.1
>AT3G02780.1 | Symbols: IPP2, IPIAT1, IDI2 | isopentenyl pyrophosphate:dimethylallyl pyrophosphate isomerase 2 | chr3:602578-604648
1.5
1.0
0.5
0.0
15
10
2
8
34
31
7
10
41
29
11
42
26
14
7
28
drm.ugt80..
drm.wt.ugt80..
drm.smt1..
drm.wt.smt1..
dsm.ugt80..
dsm.wt.ugt80..
dsm.smt1..
dsm.wt.smt1..
sp.ugt80..
sp.wt.ugt80..
sp.smt1..
sp.wt.smt1..
im.ugt80..
im.wt.ugt80..
im.smt1..
im.wt.smt1..

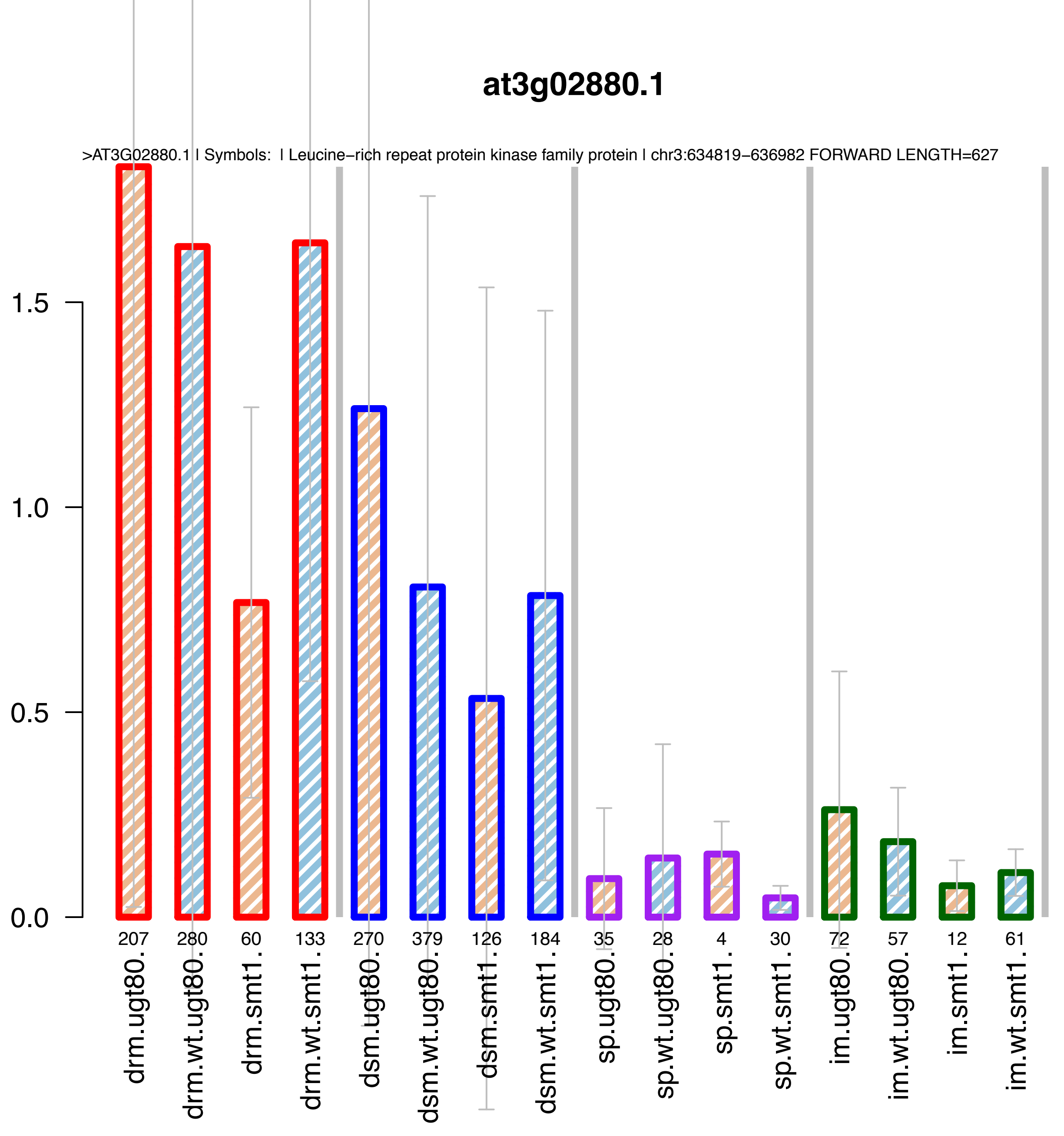

at3g02880.1
>AT3G02880.1 | Symbols: | Leucine-rich repeat protein kinase family protein | chr3:634819-636982 FORWARD LENGTH=627
1.5
1.0
0.5
0.0
207
280
60
133
270
379
126
184
35
28
4
30
72
57
12
61
drm.ugt80..
drm.wt.ugt80..
drm.smt1..
drm.wt.smt1..
dsm.ugt80..
dsm.wt.ugt80..
dsm.smt1..
dsm.wt.smt1..
sp.ugt80..
sp.wt.ugt80..
sp.smt1..
sp.wt.smt1..
im.ugt80..
im.wt.ugt80..
im.smt1..
im.wt.smt1..

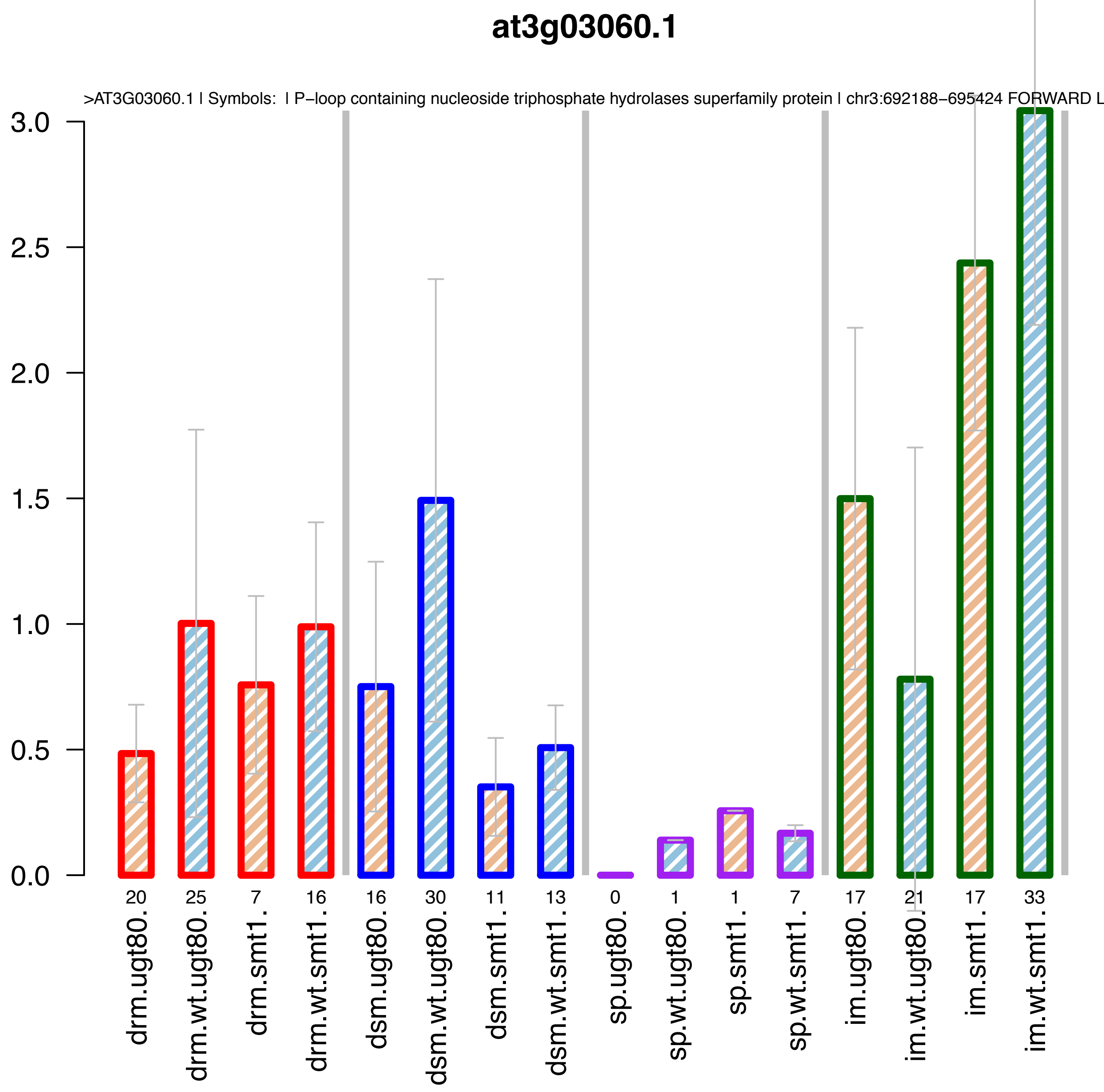

at3g03060.1
>AT3G03060.1 | Symbols: | P−loop containing nucleoside triphosphate hydrolases superfamily protein | chr3:692188−695424 FORWARD L
3.0
2.5
2.0
1.5
1.0
0.5
0.0
20
25
7
16
16
30
11
13
0
1
1
7
17
21
17
33
drm.ugt80.
drm.wt.ugt80.
drm.smt1.
drm.wt.smt1.
dsm.ugt80.
dsm.wt.ugt80.
dsm.smt1.
dsm.wt.smt1.
sp.ugt80.
sp.wt.ugt80.
sp.smt1.
sp.wt.smt1.
im.ugt80.
im.wt.ugt80.
im.smt1.
im.wt.smt1.

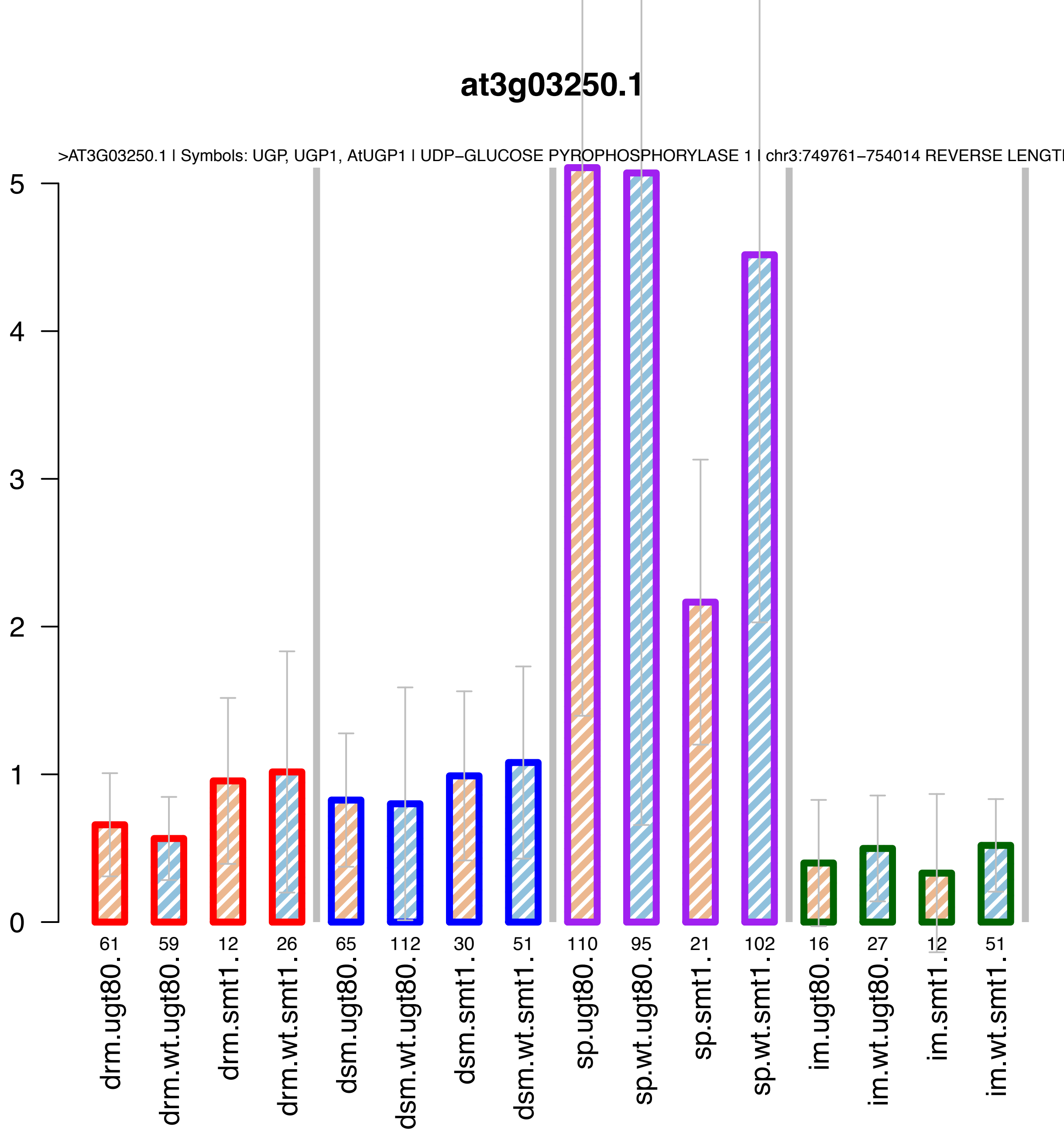

at3g03250.1
>AT3G03250.1 | Symbols: UGP, UGP1, AtUGP1 | UDP−GLUCOSE PYROPHOSPHORYLASE 1 | chr3:749761−754014 REVERSE LENGT
0
1
2
3
4
5
61
59
12
26
65
112
30
51
110
95
21
102
16
27
12
51
drm.ugt80.
drm.wt.ugt80.
drm.smt1.
drm.wt.smt1.
dsm.ugt80.
dsm.wt.ugt80.
dsm.smt1.
dsm.wt.smt1.
sp.ugt80.
sp.wt.ugt80.
sp.smt1.
sp.wt.smt1.
im.ugt80.
im.wt.ugt80.
im.smt1.
im.wt.smt1.

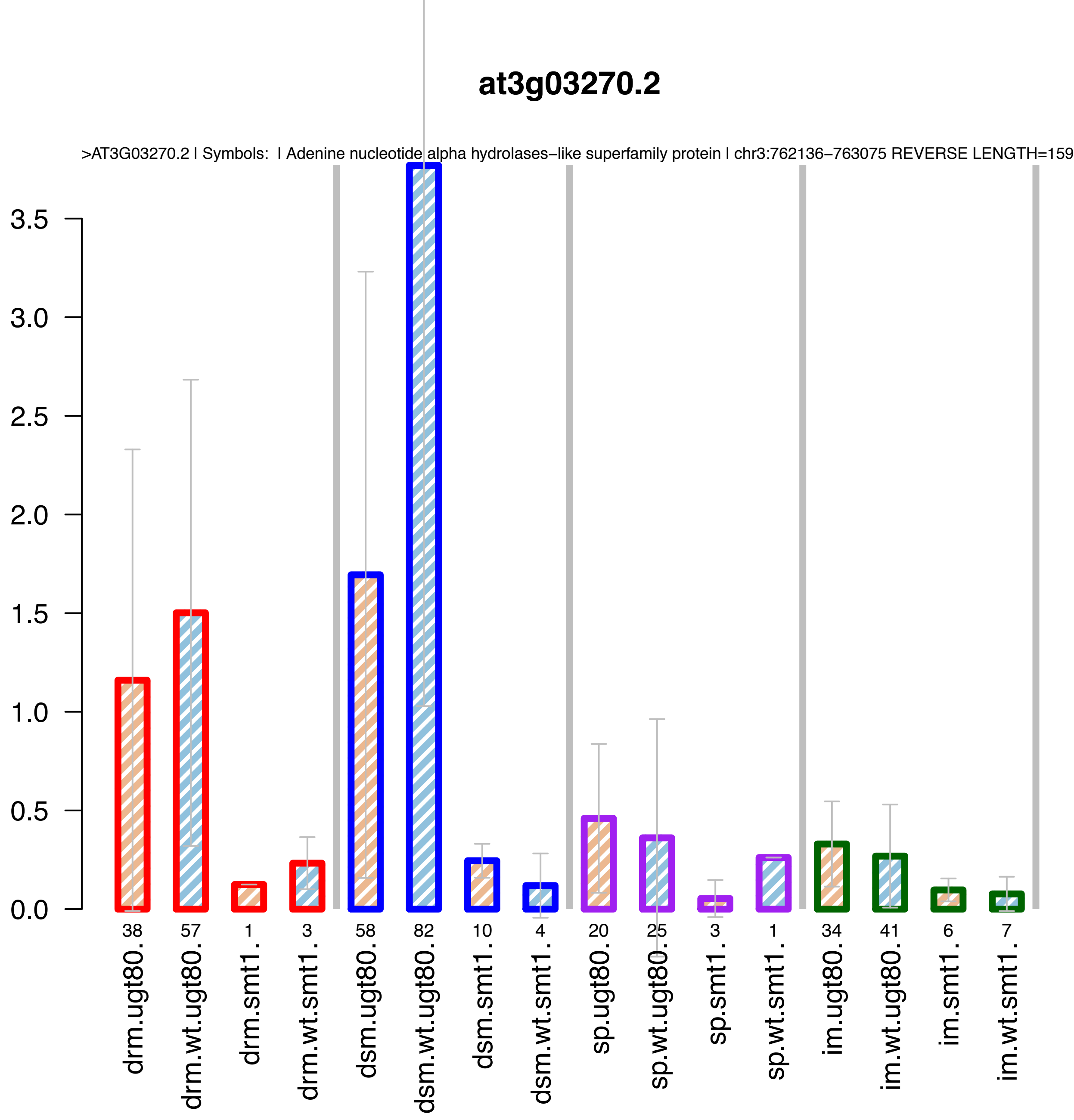

at3g03270.2
>AT3G03270.2 | Symbols: | Adenine nucleotide alpha hydrolases−like superfamily protein | chr3:762136−763075 REVERSE LENGTH=159
0.0
0.5
1.0
1.5
2.0
2.5
3.0
3.5
38
57
1
3
58
82
10
4
20
25
3
1
34
41
6
7
drm.ugt80..
drm.wt.ugt80..
drm.smt1..
drm.wt.smt1..
dsm.ugt80..
dsm.wt.ugt80..
dsm.smt1..
dsm.wt.smt1..
sp.ugt80..
sp.wt.ugt80..
sp.smt1..
sp.wt.smt1..
im.ugt80..
im.wt.ugt80..
im.smt1..
im.wt.smt1..

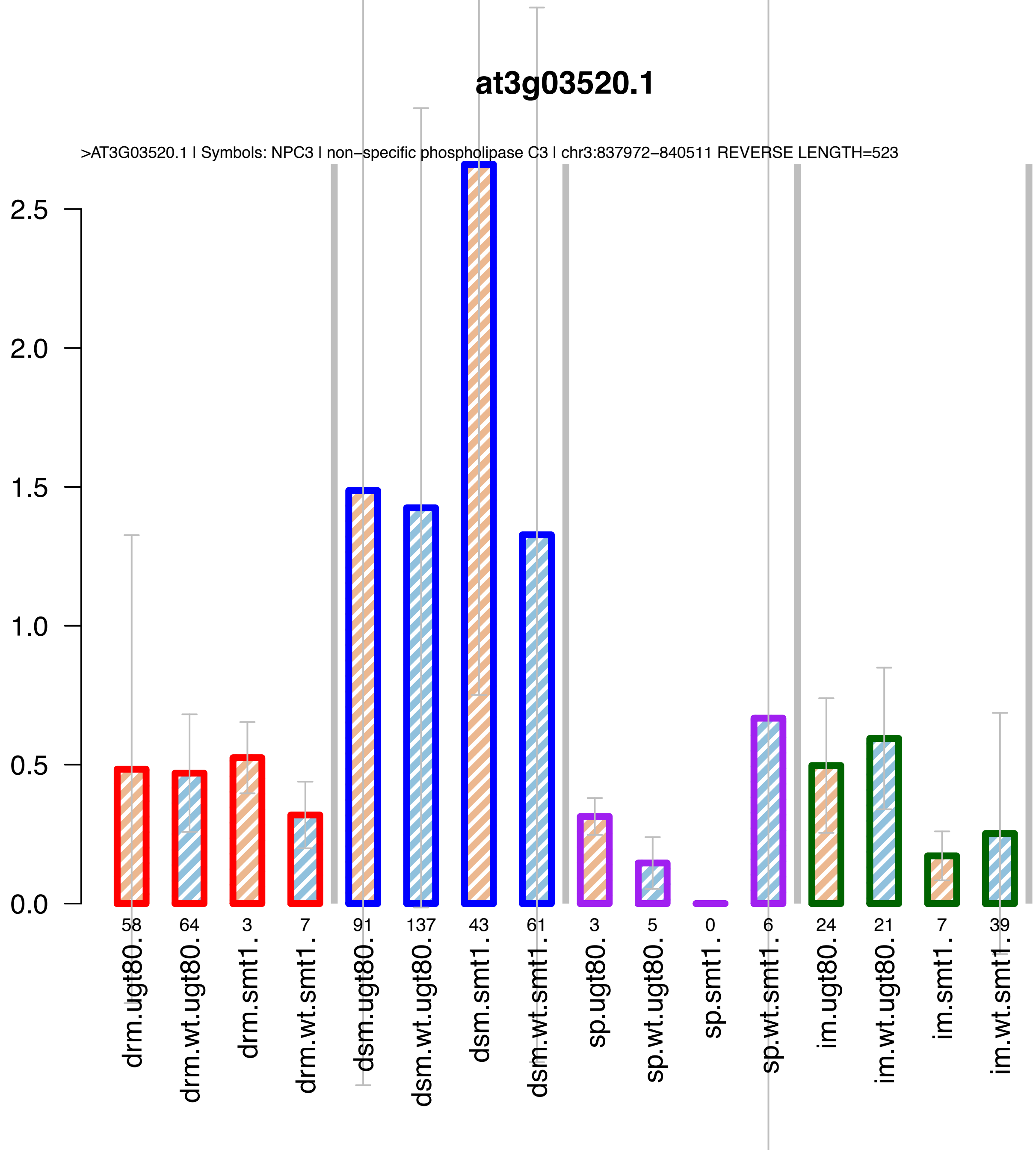
at3g03520.1
>AT3G03520.1 | Symbols: NPC3 | non−specific phospholipase C3 | chr3:837972−840511 REVERSE LENGTH=523
2.5
2.0
1.5
1.0
0.5
0.0
58
64
3
7
91
137
43
61
3
5
0
6
24
21
7
39
drm.ugt80.
drm.wt.ugt80.
drm.smt1.
drm.wt.smt1.
dsm.ugt80.
dsm.wt.ugt80.
dsm.smt1.
dsm.wt.smt1.
sp.ugt80.
sp.wt.ugt80.
sp.smt1.
sp.wt.smt1.
im.ugt80.
im.wt.ugt80.
im.smt1.
im.wt.smt1.

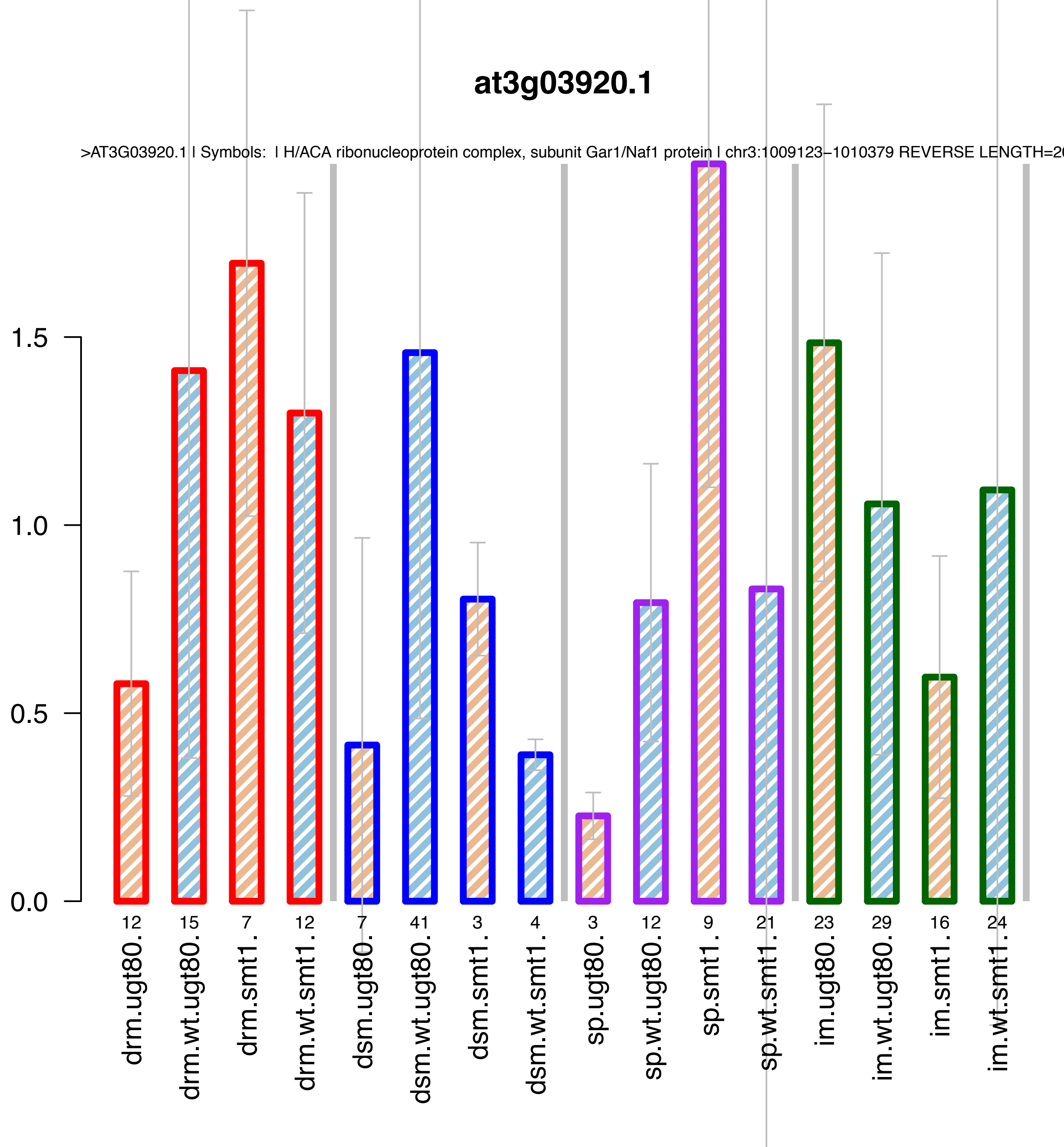

at3g03920.1
>AT3G03920.1 | Symbols: | H/ACA ribonucleoprotein complex, subunit Gar1/Naf1 protein | chr3:1009123-1010379 REVERSE LENGTH=2
0.0
0.5
1.0
1.5
12
15
7
12
7
41
3
4
3
12
9
21
23
29
16
24
drm.ugt80..
drm.wt.ugt80..
drm.smt1..
drm.wt.smt1..
dsm.ugt80..
dsm.wt.ugt80..
dsm.smt1..
dsm.wt.smt1..
sp.ugt80..
sp.wt.ugt80..
sp.smt1..
sp.wt.smt1..
im.ugt80..
im.wt.ugt80..
im.smt1..
im.wt.smt1..

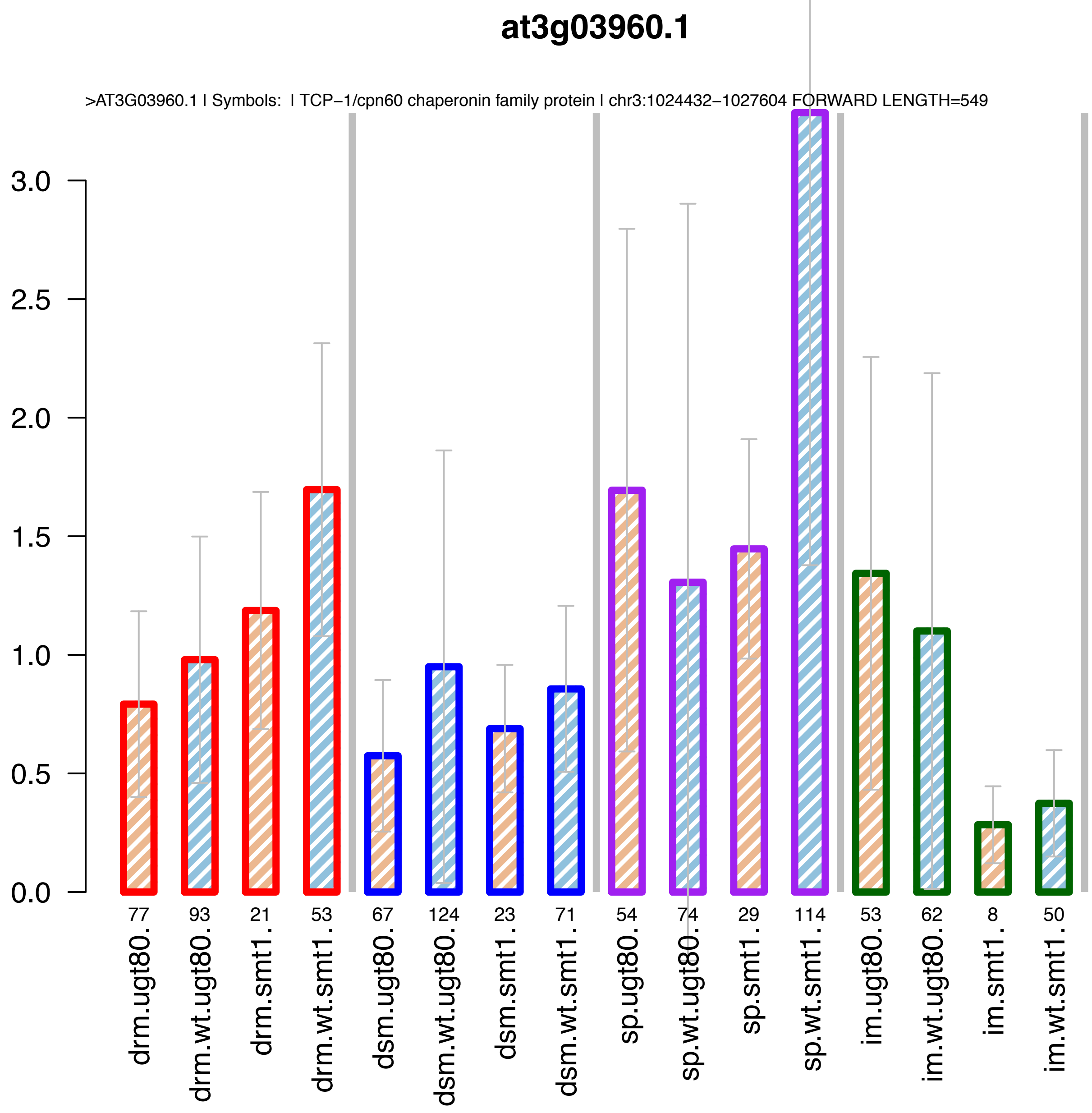
at3g03960.1
>AT3G03960.1 | Symbols: | TCP-1/cpn60 chaperonin family protein | chr3:1024432-1027604 FORWARD LENGTH=549
3.0
2.5
2.0
1.5
1.0
0.5
0.0
77
93
21
53
67
124
23
71
54
74
29
114
53
62
8
50
drm.ugt80..
drm.wt.ugt80..
drm.smt1..
drm.wt.smt1..
dsm.ugt80..
dsm.wt.ugt80..
dsm.smt1..
dsm.wt.smt1..
sp.ugt80..
sp.wt.ugt80..
sp.smt1..
sp.wt.smt1..
im.ugt80..
im.wt.ugt80..
im.smt1..
im.wt.smt1..

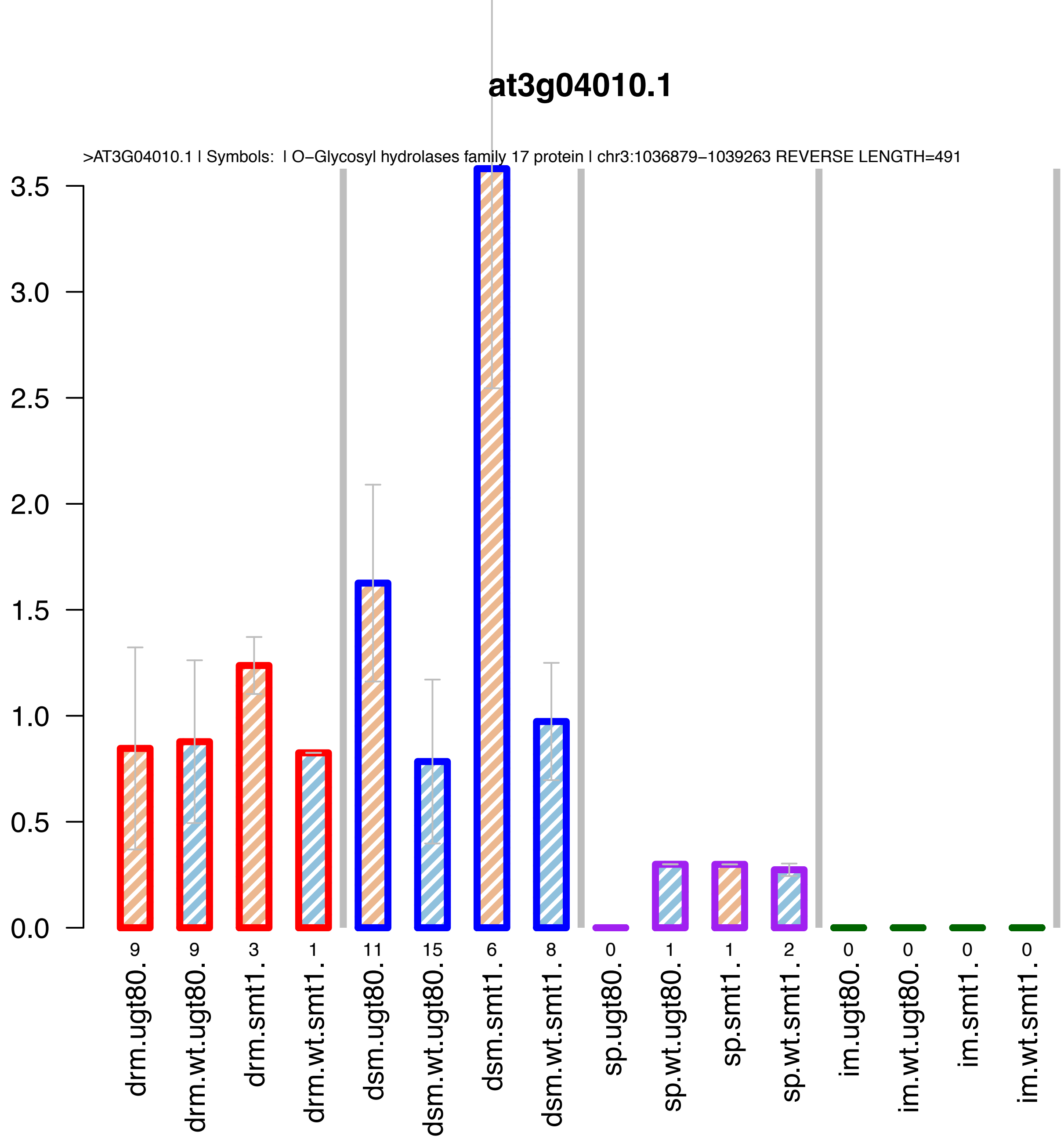

at3g04010.1
>AT3G04010.1 | Symbols: | O−Glycosyl hydrolases family 17 protein | chr3:1036879−1039263 REVERSE LENGTH=491
3.5
3.0
2.5
2.0
1.5
1.0
0.5
0.0
9
9
3
1
11
15
6
8
0
1
1
2
0
0
0
0
drm.ugt80.
drm.wt.ugt80.
drm.smt1.
drm.wt.smt1.
dsm.ugt80.
dsm.wt.ugt80.
dsm.smt1.
dsm.wt.smt1.
sp.ugt80.
sp.wt.ugt80.
sp.smt1.
sp.wt.smt1.
im.ugt80.
im.wt.ugt80.
im.smt1.
im.wt.smt1.

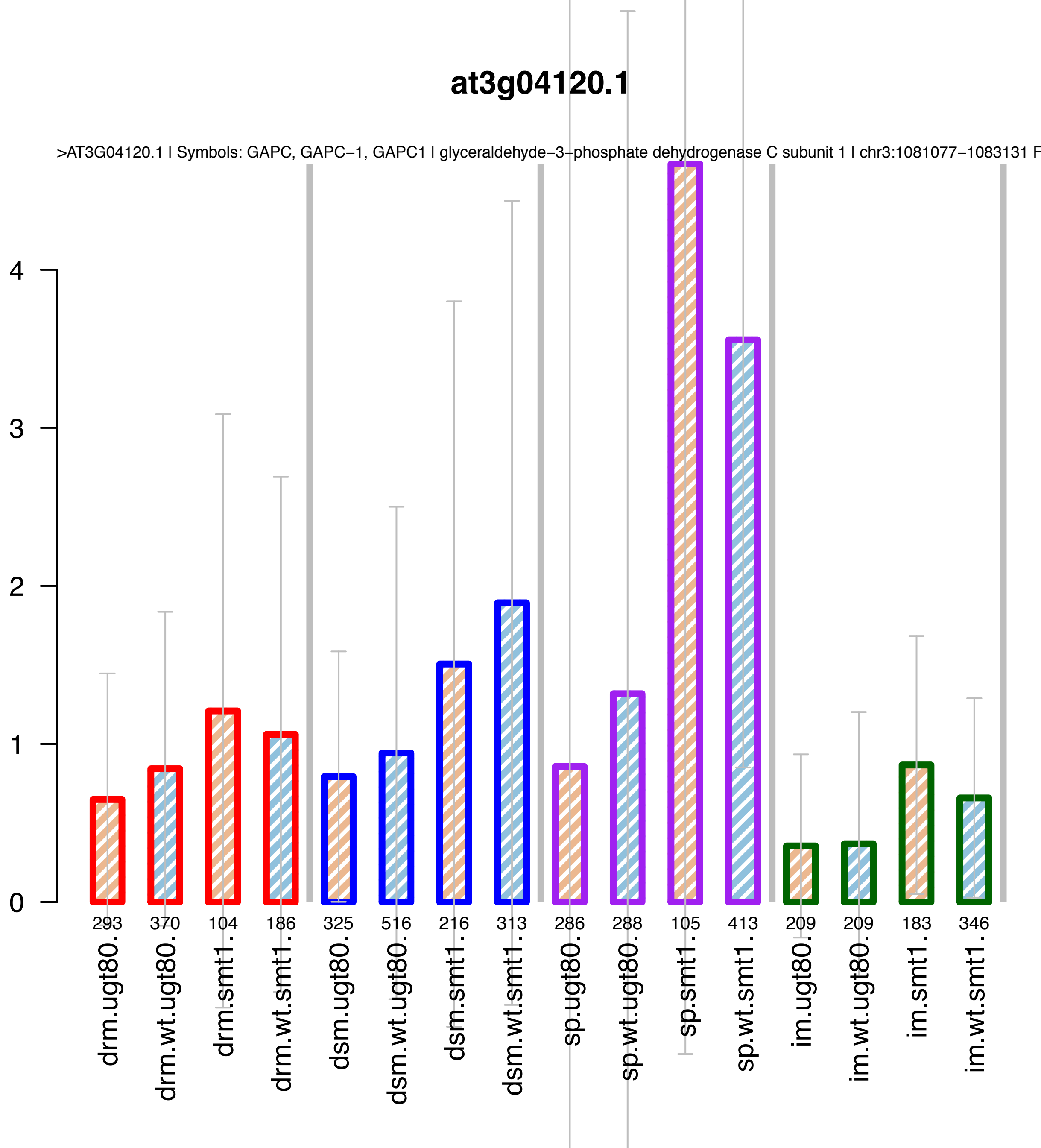

at3g04120.1
>AT3G04120.1 | Symbols: GAPC, GAPC−1, GAPC1 | glyceraldehyde−3−phosphate dehydrogenase C subunit 1 | chr3:1081077−1083131 F
0
1
2
3
4
293
370
104
186
325
516
216
313
286
288
105
413
209
209
183
346
drm.ugt80..
drm.wt.ugt80..
drm.smt1..
drm.wt.smt1..
dsm.ugt80..
dsm.wt.ugt80..
dsm.smt1..
dsm.wt.smt1..
sp.ugt80..
sp.wt.ugt80..
sp.smt1..
sp.wt.smt1..
im.ugt80..
im.wt.ugt80..
im.smt1..
im.wt.smt1..

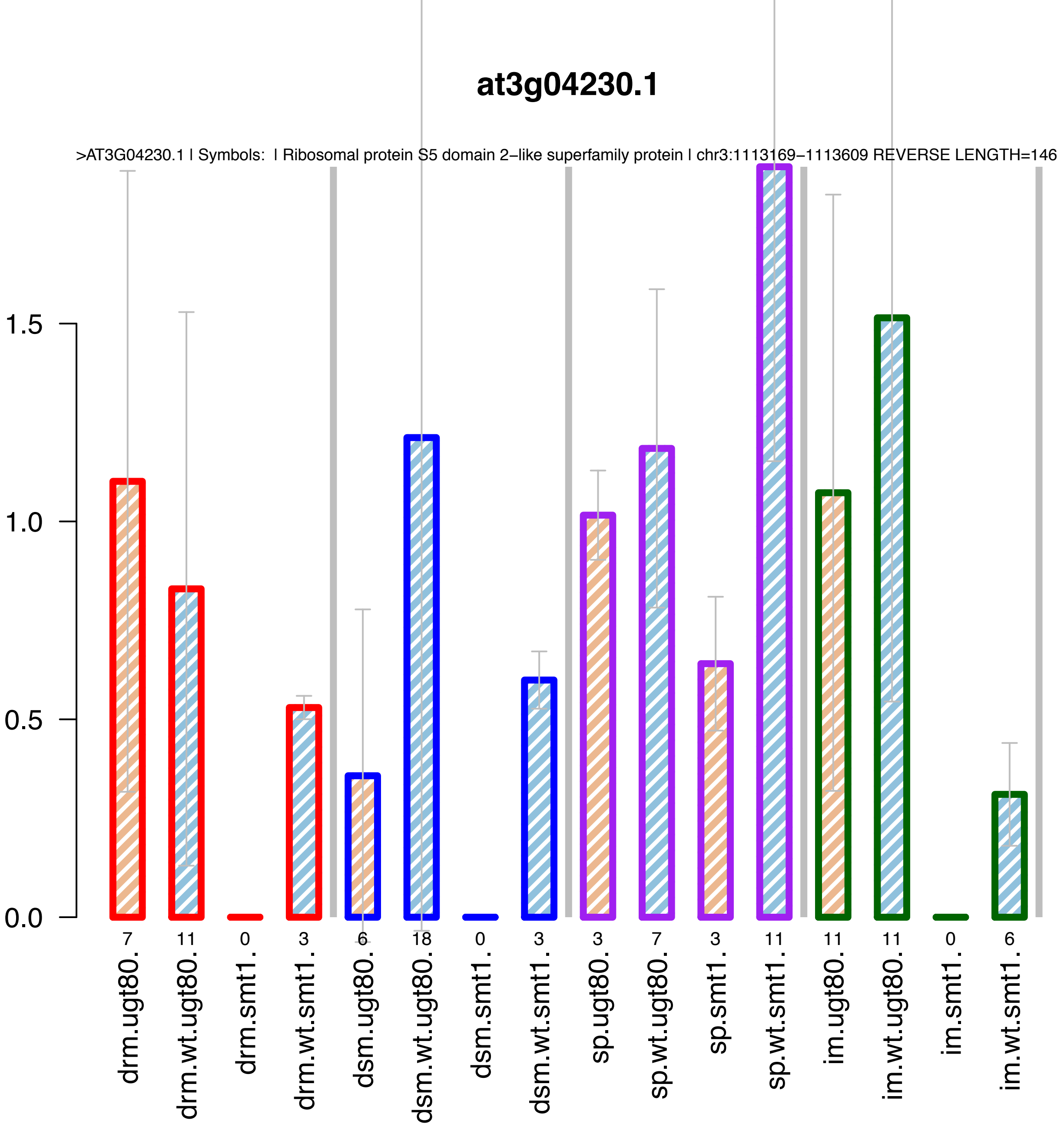
at3g04230.1
>AT3G04230.1 | Symbols: | Ribosomal protein S5 domain 2-like superfamily protein | chr3:1113169-1113609 REVERSE LENGTH=146
0.0
0.5
1.0
1.5
7
11
0
3
6
18
0
3
3
7
3
11
11
11
0
6
drm.ugt80.
drm.wt.ugt80.
drm.smt1.
drm.wt.smt1.
dsm.ugt80.
dsm.wt.ugt80.
dsm.smt1.
dsm.wt.smt1.
sp.ugt80.
sp.wt.ugt80.
sp.smt1.
sp.wt.smt1.
im.ugt80.
im.wt.ugt80.
im.smt1.
im.wt.smt1.

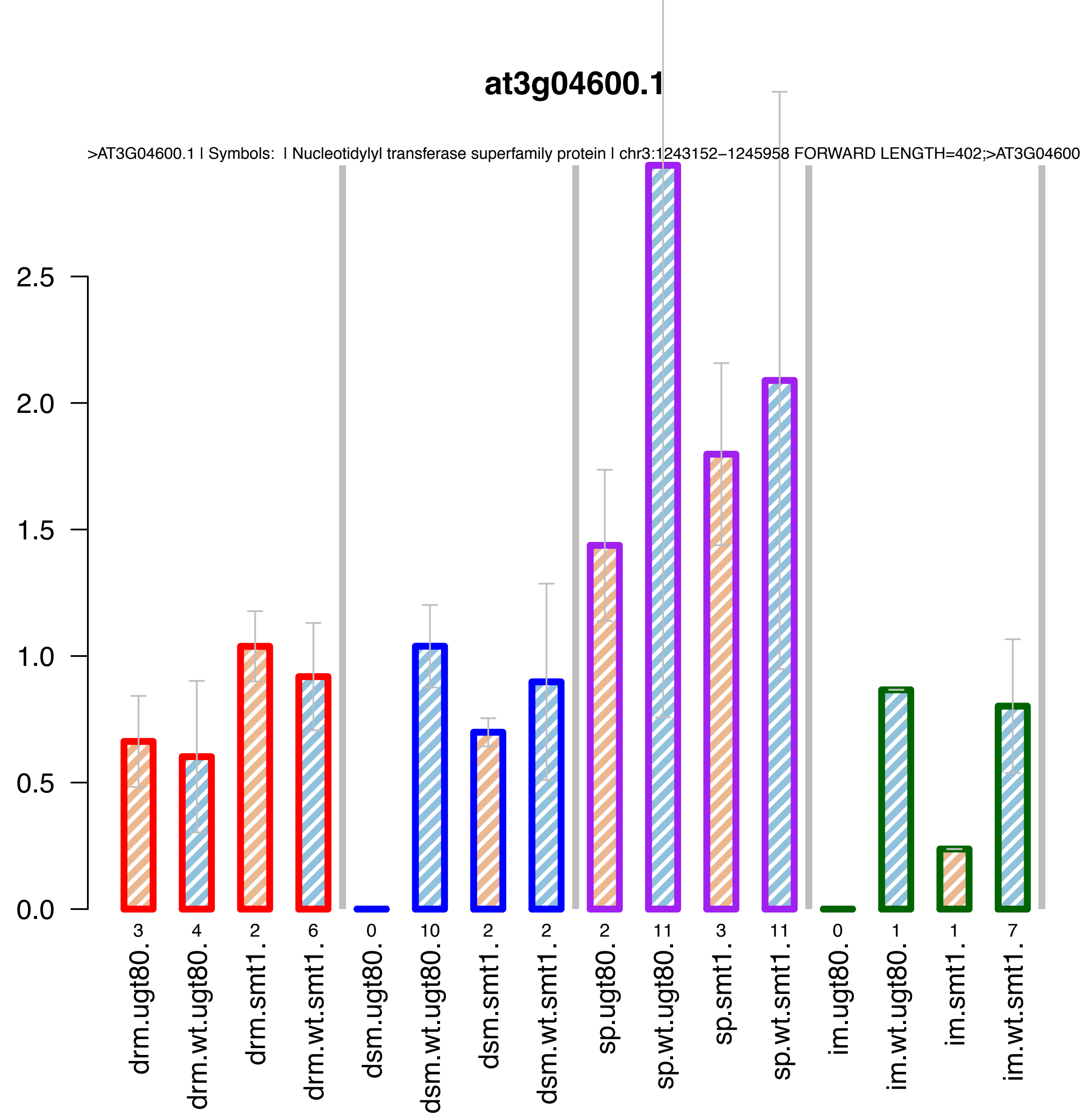

at3g04600.1
>AT3G04600.1 | Symbols: | Nucleotidylyl transferase superfamily protein | chr3:1243152-1245958 FORWARD LENGTH=402;>AT3G04600
2.5
2.0
1.5
1.0
0.5
0.0
3
4
2
6
0
10
2
2
2
11
3
11
0
1
1
7
drm.ugt80.
drm.wt.ugt80.
drm.smt1.
drm.wt.smt1.
dsm.ugt80.
dsm.wt.ugt80.
dsm.smt1.
dsm.wt.smt1.
sp.ugt80.
sp.wt.ugt80.
sp.smt1.
sp.wt.smt1.
im.ugt80.
im.wt.ugt80.
im.smt1.
im.wt.smt1.

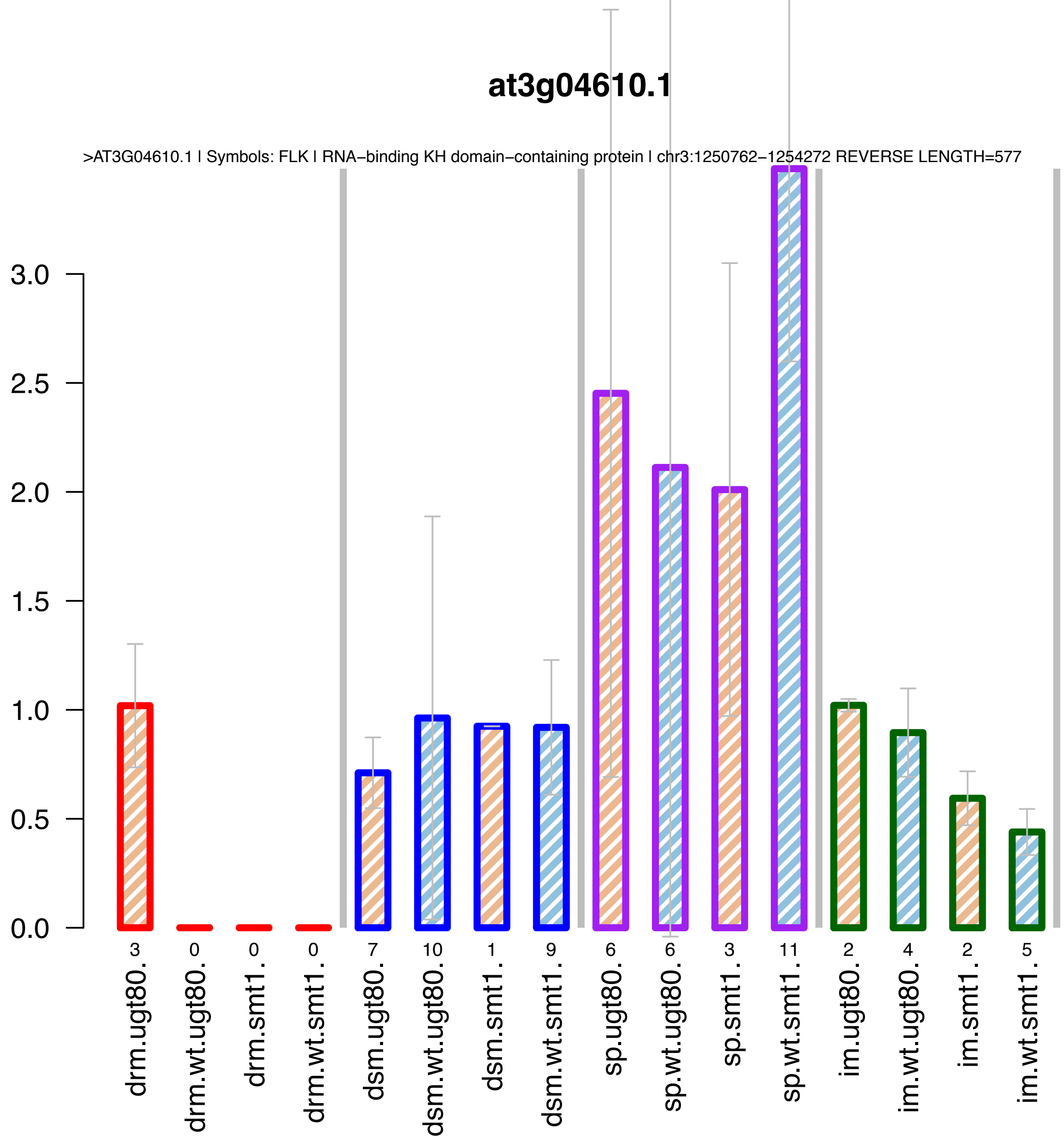
at3g04610.1
>AT3G04610.1 | Symbols: FLK | RNA−binding KH domain−containing protein | chr3:1250762−1254272 REVERSE LENGTH=577
0.0
0.5
1.0
1.5
2.0
2.5
3.0
3
0
0
0
7
10
1
9
6
6
3
11
2
4
2
5
drm.ugt80.
drm.wt.ugt80.
drm.smt1.
drm.wt.smt1.
dsm.ugt80.
dsm.wt.ugt80.
dsm.smt1.
dsm.wt.smt1.
sp.ugt80.
sp.wt.ugt80.
sp.smt1.
sp.wt.smt1.
im.ugt80.
im.wt.ugt80.
im.smt1.
im.wt.smt1.

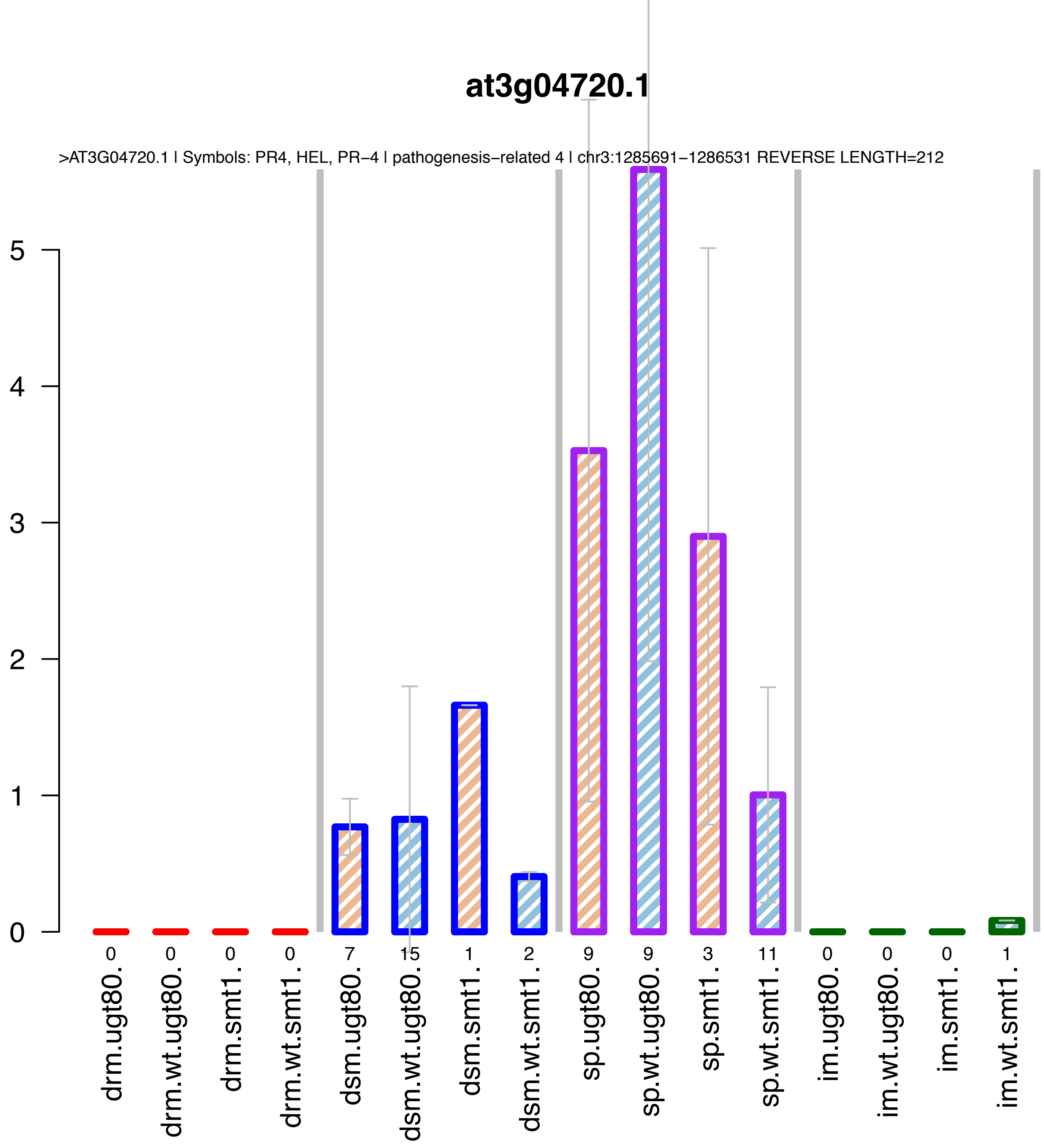

at3g04720.1
>AT3G04720.1 | Symbols: PR4, HEL, PR−4 | pathogenesis−related 4 | chr3:1285691−1286531 REVERSE LENGTH=212
0
1
2
3
4
5
0
0
0
0
7
15
1
2
9
9
3
11
0
0
0
1
drm.ugt80.
drm.wt.ugt80.
drm.smt1.
drm.wt.smt1.
dsm.ugt80.
dsm.wt.ugt80.
dsm.smt1.
dsm.wt.smt1.
sp.ugt80.
sp.wt.ugt80.
sp.smt1.
sp.wt.smt1.
im.ugt80.
im.wt.ugt80.
im.smt1.
im.wt.smt1.

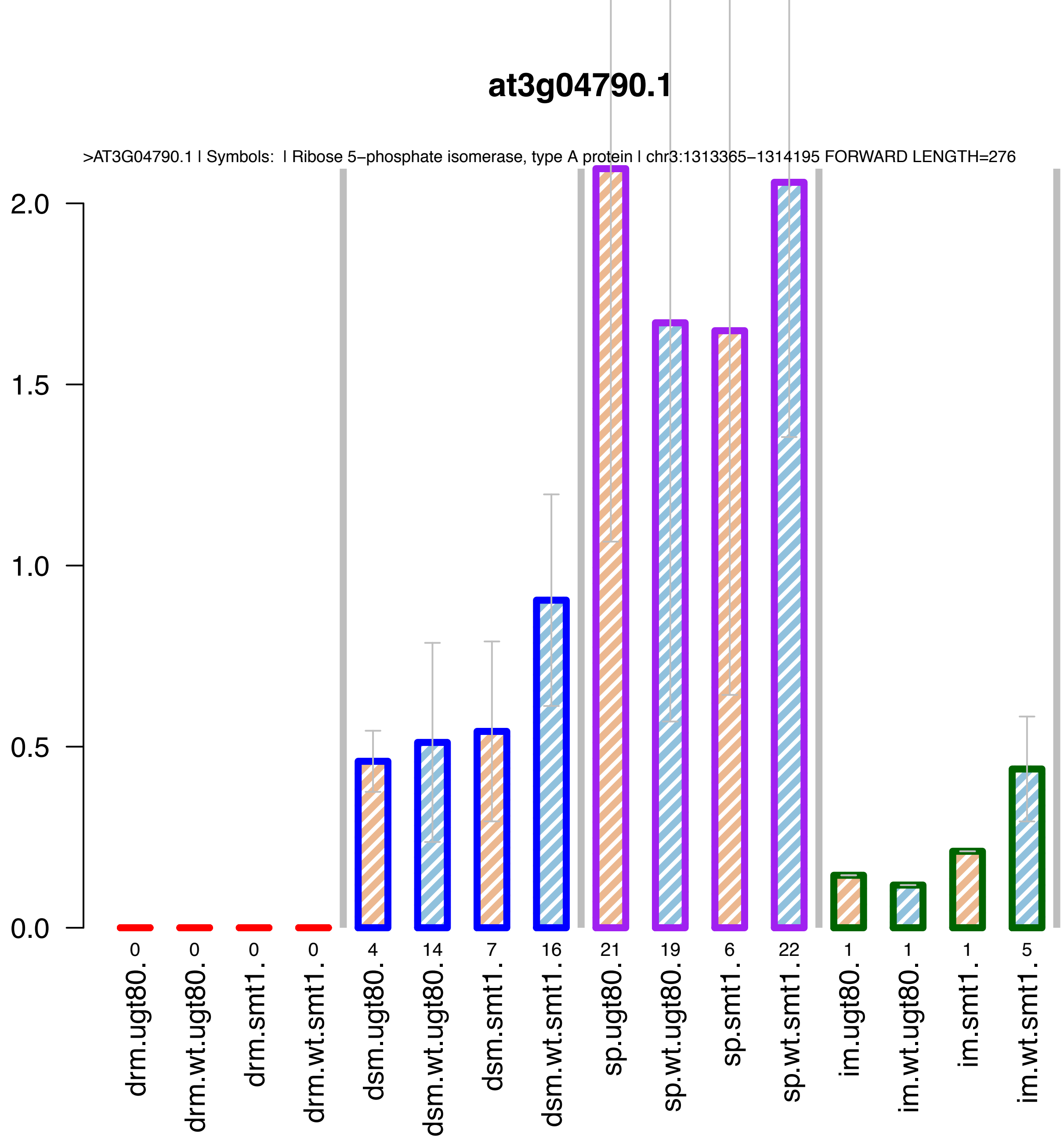

at3g04790.1
>AT3G04790.1 | Symbols: | Ribose 5−phosphate isomerase, type A protein | chr3:1313365−1314195 FORWARD LENGTH=276
2.0
1.5
1.0
0.5
0.0
0 0 0 0 4 14 7 16 21 19 6 22 1 1 1 5
drm.ugt80.
drm.wt.ugt80.
drm.smt1.
drm.wt.smt1.
dsm.ugt80.
dsm.wt.ugt80.
dsm.smt1.
dsm.wt.smt1.
sp.ugt80.
sp.wt.ugt80.
sp.smt1.
sp.wt.smt1.
im.ugt80.
im.wt.ugt80.
im.smt1.
im.wt.smt1.

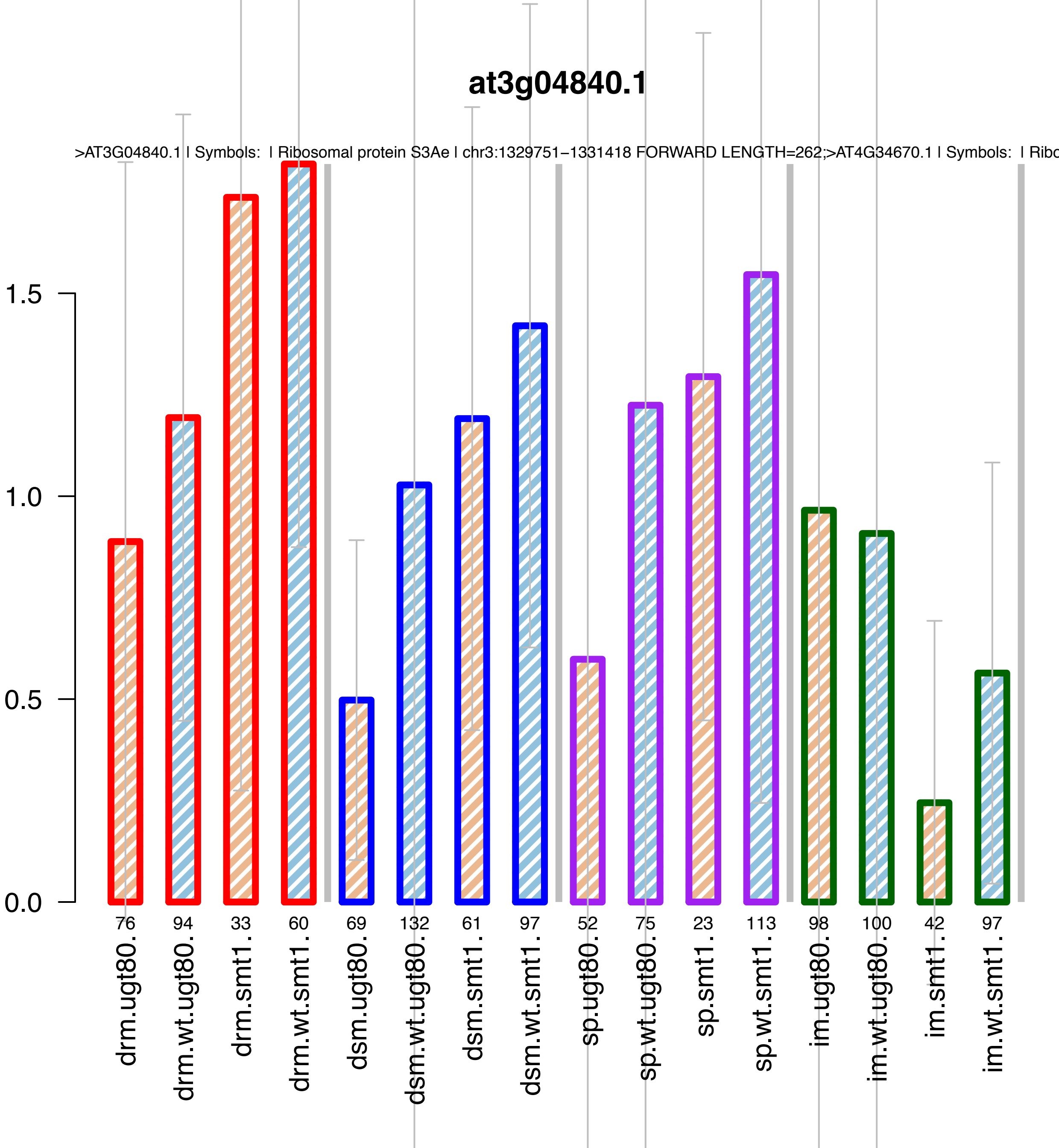

at3g04840.1
>AT3G04840.1 | Symbols: | Ribosomal protein S3Ae | chr3:1329751-1331418 FORWARD LENGTH=262;>AT4G34670.1 | Symbols: | Ribo
0.0
0.5
1.0
1.5
76
94
33
60
69
132
61
97
52
75
23
113
98
100
42
97
drm.ugt80..
drm.wt.ugt80..
drm.smt1..
drm.wt.smt1..
dsm.ugt80..
dsm.wt.ugt80..
dsm.smt1..
dsm.wt.smt1..
sp.ugt80..
sp.wt.ugt80..
sp.smt1..
sp.wt.smt1..
im.ugt80..
im.wt.ugt80..
im.smt1..
im.wt.smt1..

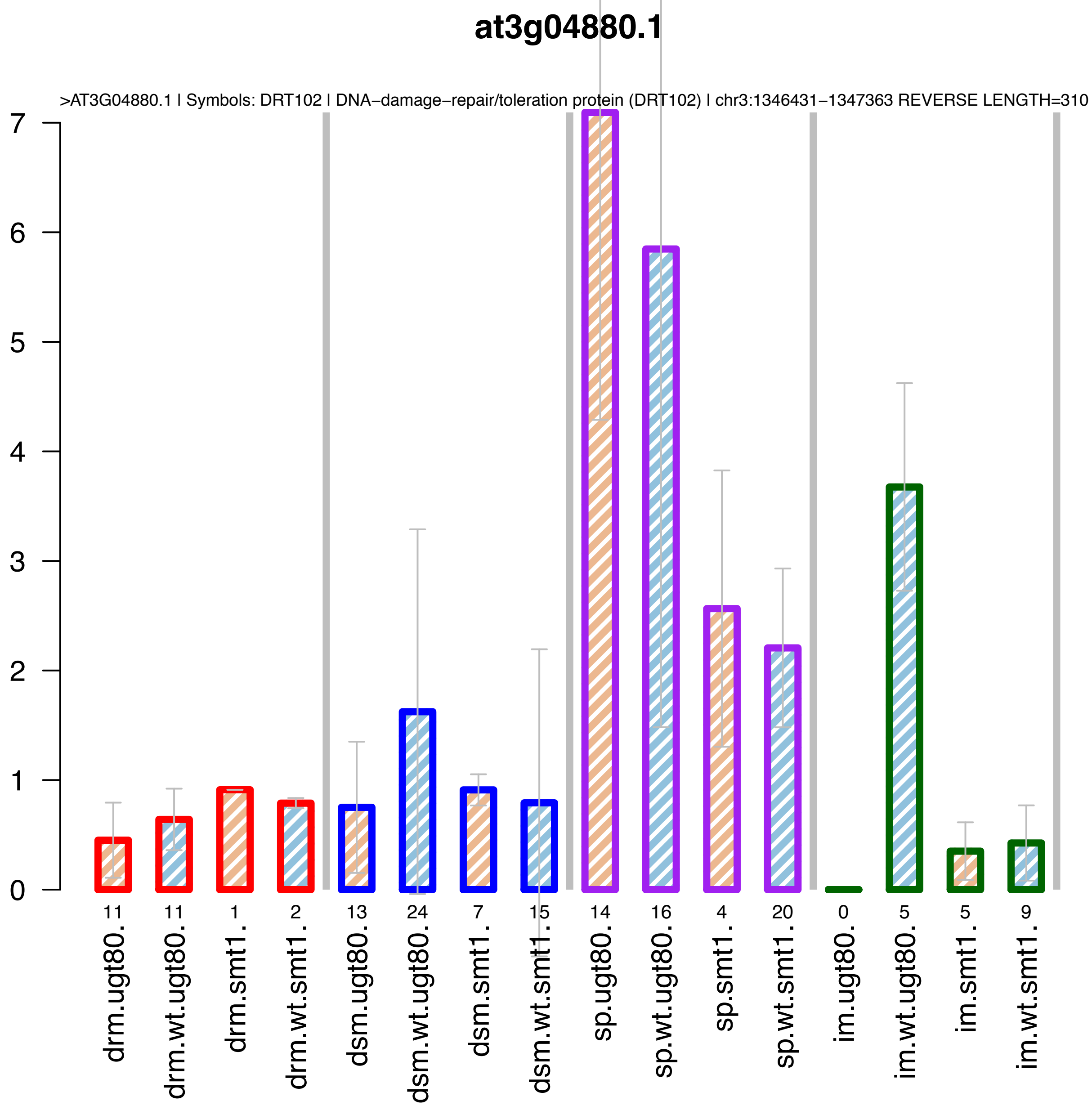

at3g04880.1
>AT3G04880.1 | Symbols: DRT102 | DNA−damage−repair/toleration protein (DRT102) | chr3:1346431−1347363 REVERSE LENGTH=310
0
1
2
3
4
5
6
7
11
11
1
2
13
24
7
15
14
16
4
20
0
5
5
9
drm.ugt80.
drm.wt.ugt80.
drm.smt1.
drm.wt.smt1.
dsm.ugt80.
dsm.wt.ugt80.
dsm.smt1.
dsm.wt.smt1.
sp.ugt80.
sp.wt.ugt80.
sp.smt1.
sp.wt.smt1.
im.ugt80.
im.wt.ugt80.
im.smt1.
im.wt.smt1.

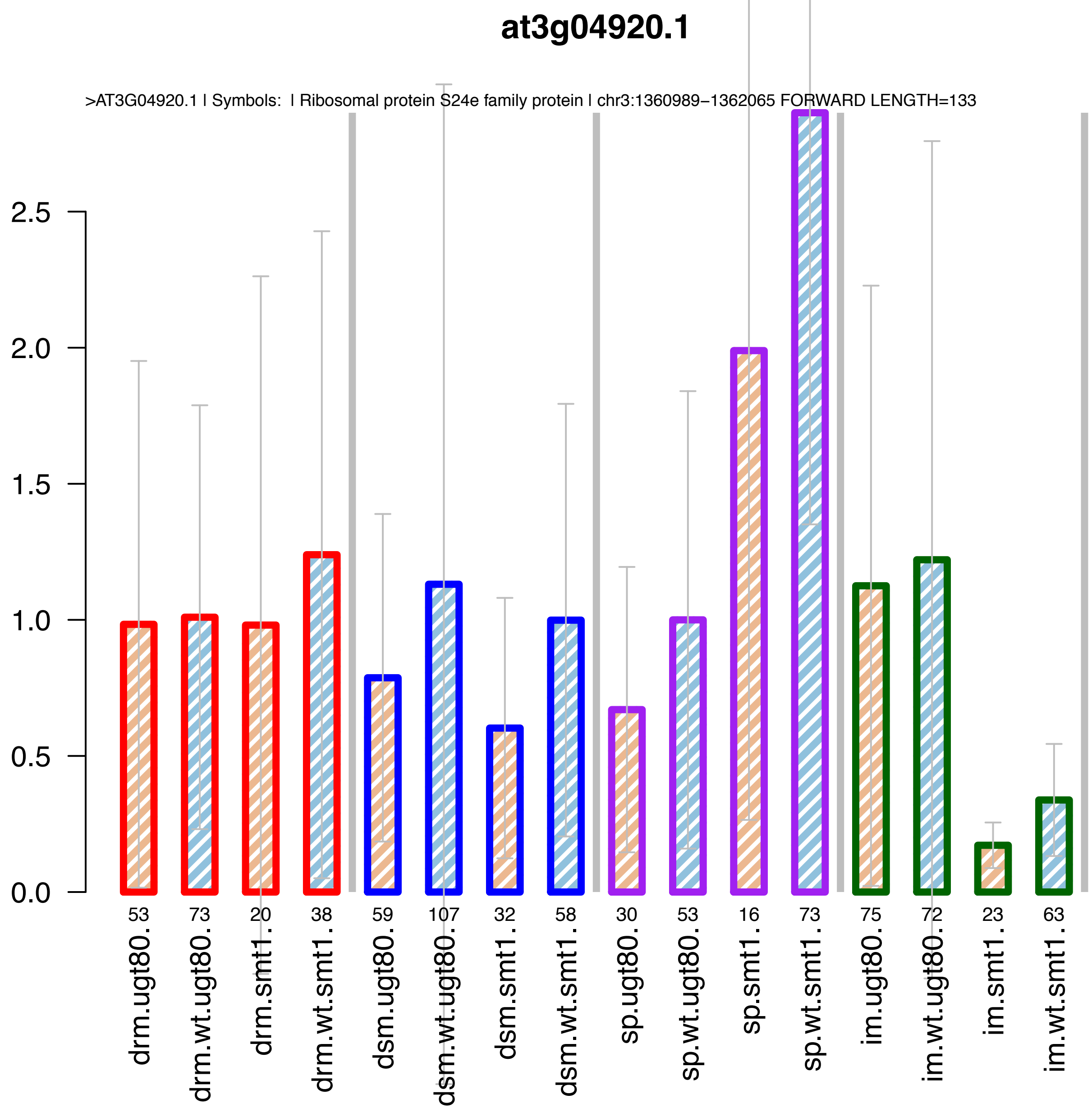

at3g04920.1
>AT3G04920.1 | Symbols: | Ribosomal protein S24e family protein | chr3:1360989-1362065 FORWARD LENGTH=133
0.0
0.5
1.0
1.5
2.0
2.5
53
73
20
38
59
107
32
58
30
53
16
73
75
72
23
63
drm.ugt80..
drm.wt.ugt80..
drm.smt1..
drm.wt.smt1..
dsm.ugt80..
dsm.wt.ugt80..
dsm.smt1..
dsm.wt.smt1..
sp.ugt80..
sp.wt.ugt80..
sp.smt1..
sp.wt.smt1..
im.ugt80..
im.wt.ugt80..
im.smt1..
im.wt.smt1..

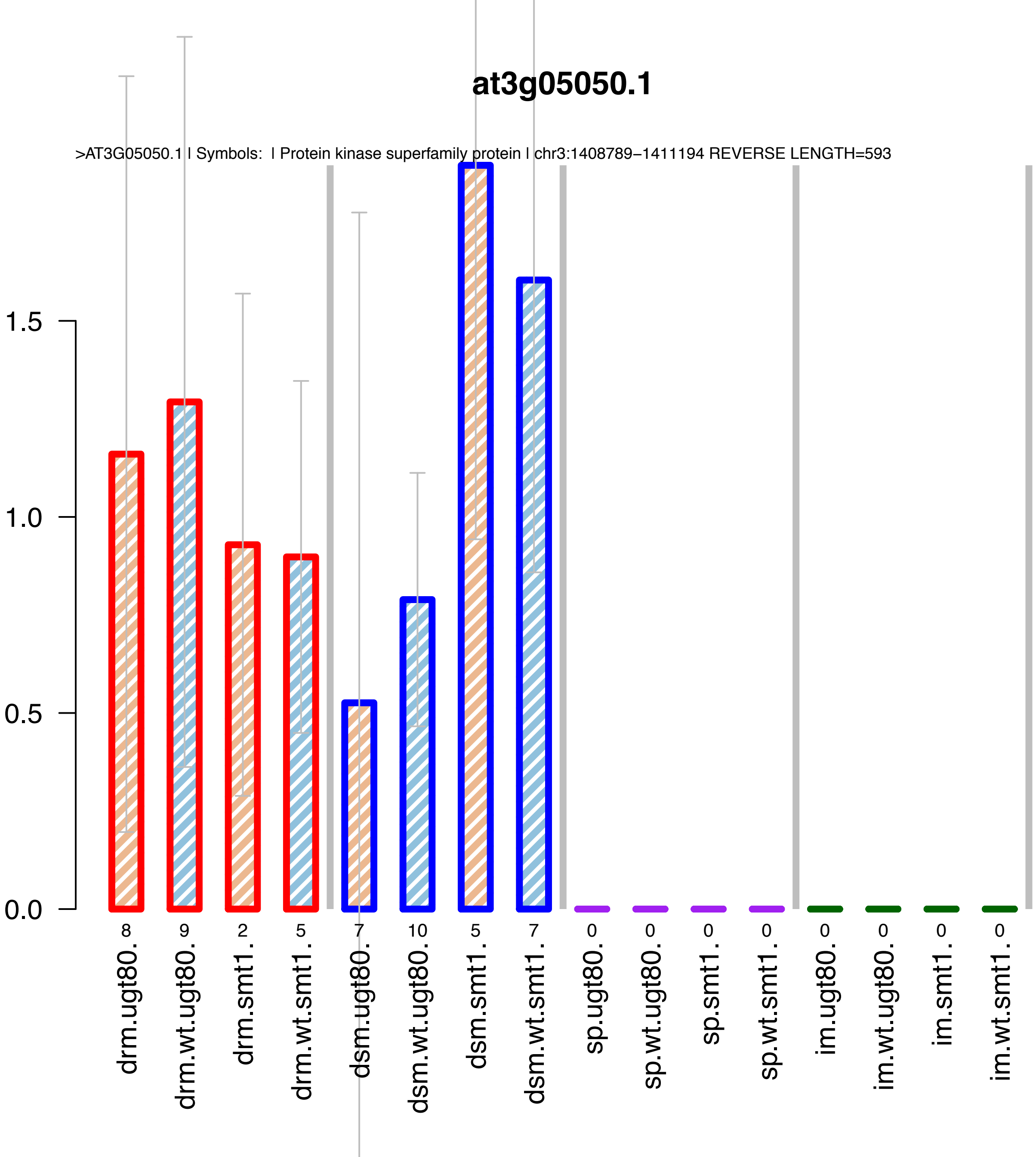
at3g05050.1
>AT3G05050.1 | Symbols: | Protein kinase superfamily protein | chr3:1408789-1411194 REVERSE LENGTH=593
1.5
1.0
0.5
0.0
8
9
2
5
7
10
5
7
0
0
0
0
0
0
0
0
drm.ugt80.
drm.wt.ugt80.
drm.smt1.
drm.wt.smt1.
dsm.ugt80.
dsm.wt.ugt80.
dsm.smt1.
dsm.wt.smt1.
sp.ugt80.
sp.wt.ugt80.
sp.smt1.
sp.wt.smt1.
im.ugt80.
im.wt.ugt80.
im.smt1.
im.wt.smt1.

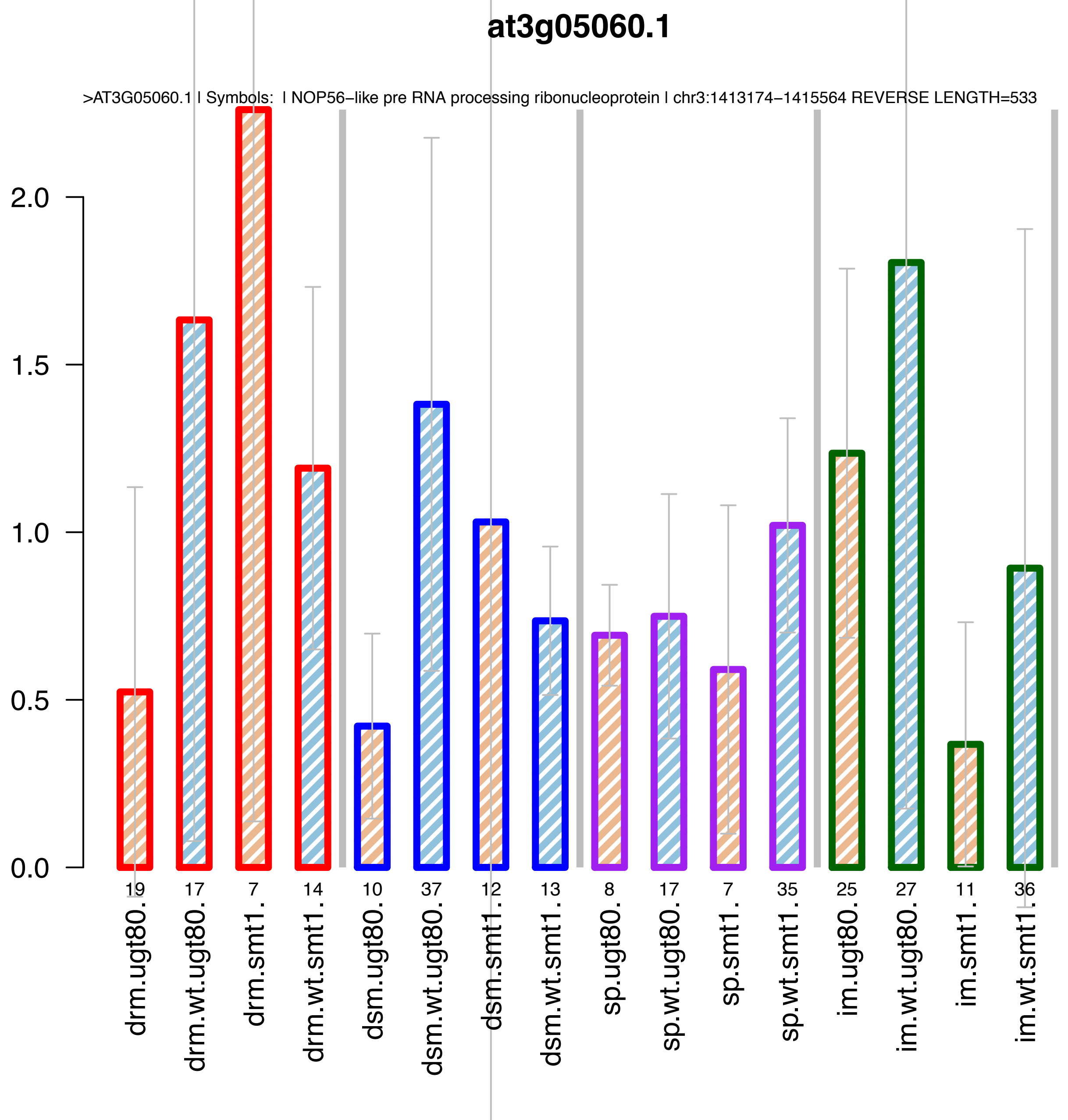

at3g05060.1
>AT3G05060.1 | Symbols: | NOP56-like pre RNA processing ribonucleoprotein | chr3:1413174-1415564 REVERSE LENGTH=533
0.0
0.5
1.0
1.5
2.0
19
17
7
14
10
37
12
13
8
17
7
35
25
27
11
36
drm.ugt80..
drm.wt.ugt80..
drm.smt1..
drm.wt.smt1..
dsm.ugt80..
dsm.wt.ugt80..
dsm.smt1..
dsm.wt.smt1..
sp.ugt80..
sp.wt.ugt80..
sp.smt1..
sp.wt.smt1..
im.ugt80..
im.wt.ugt80..
im.smt1..
im.wt.smt1..

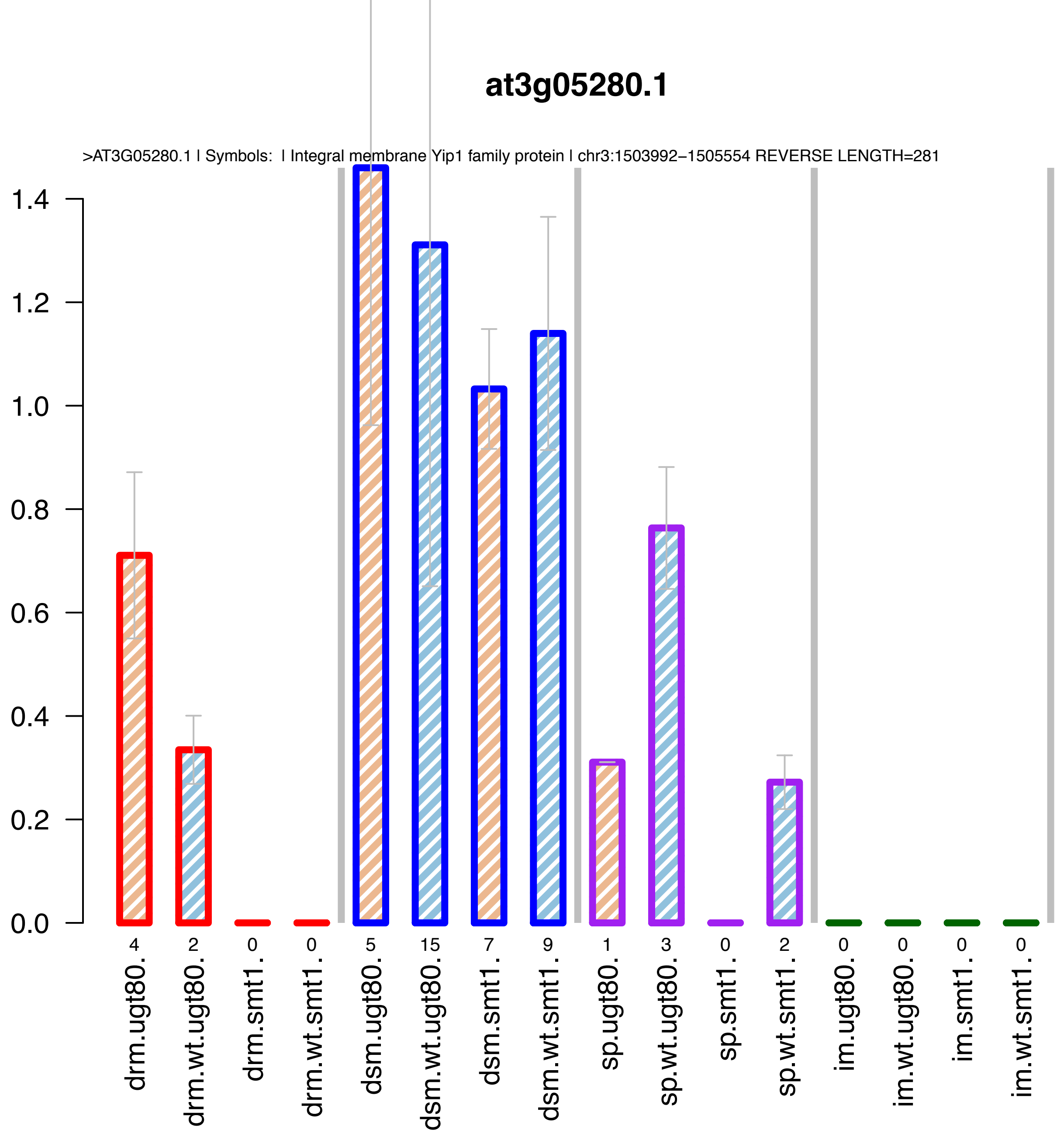

at3g05280.1
>AT3G05280.1 | Symbols: | Integral membrane Yip1 family protein | chr3:1503992-1505554 REVERSE LENGTH=281
1.4
1.2
1.0
0.8
0.6
0.4
0.2
0.0
4
2
0
0
5
15
7
9
1
3
0
2
0
0
0
0
drm.ugt80.
drm.wt.ugt80.
drm.smt1.
drm.wt.smt1.
dsm.ugt80.
dsm.wt.ugt80.
dsm.smt1.
dsm.wt.smt1.
sp.ugt80.
sp.wt.ugt80.
sp.smt1.
sp.wt.smt1.
im.ugt80.
im.wt.ugt80.
im.smt1.
im.wt.smt1.

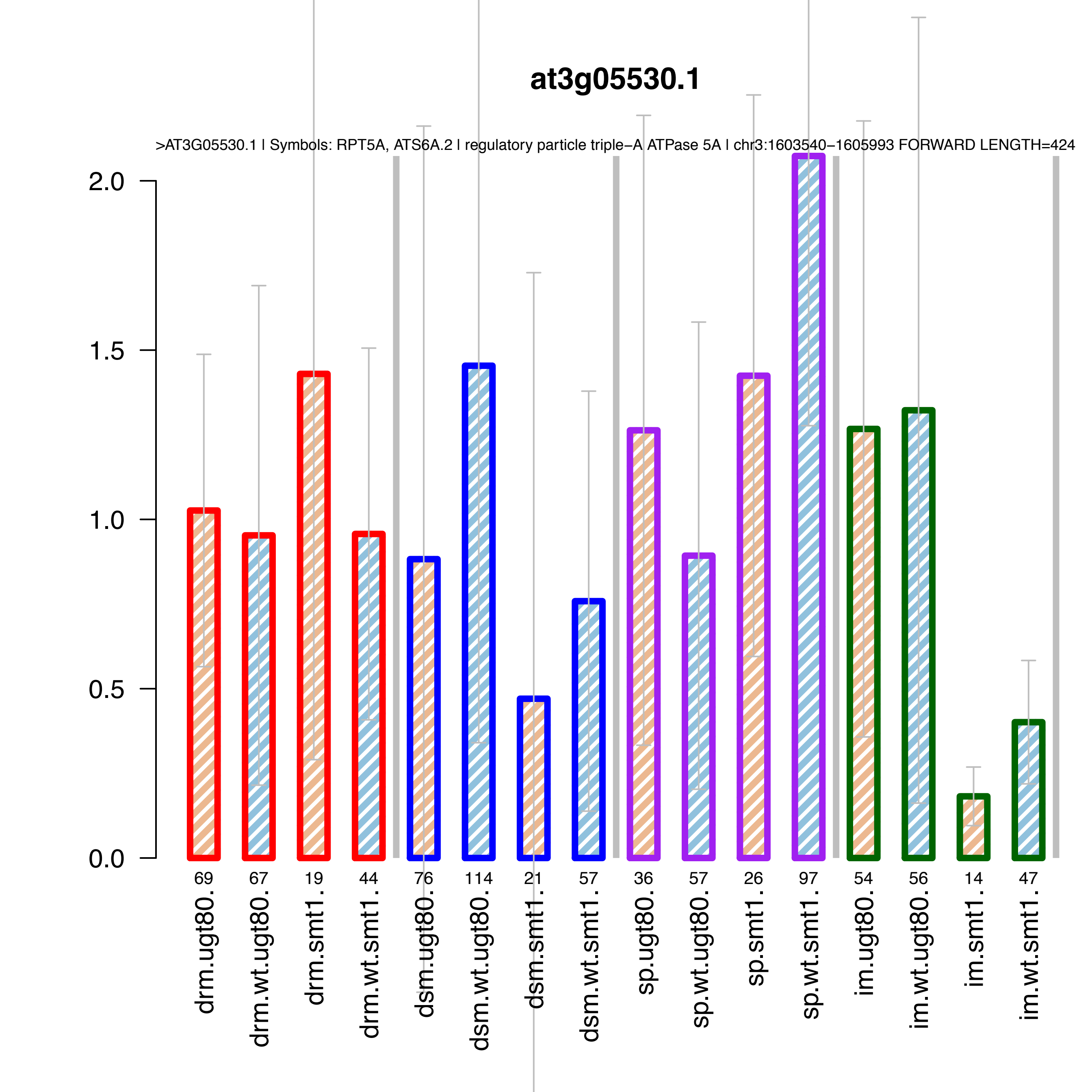

at3g05530.1
>AT3G05530.1 | Symbols: RPT5A, ATS6A.2 | regulatory particle triple−A ATPase 5A | chr3:1603540−1605993 FORWARD LENGTH=424
2.0
1.5
1.0
0.5
0.0
69
67
19
44
76
114
21
57
36
57
26
97
54
56
14
47
drm.ugt80..
drm.wt.ugt80..
drm.smt1..
drm.wt.smt1..
dsm.ugt80..
dsm.wt.ugt80..
dsm.smt1..
dsm.wt.smt1..
sp.ugt80..
sp.wt.ugt80..
sp.smt1..
sp.wt.smt1..
im.ugt80..
im.wt.ugt80..
im.smt1..
im.wt.smt1..

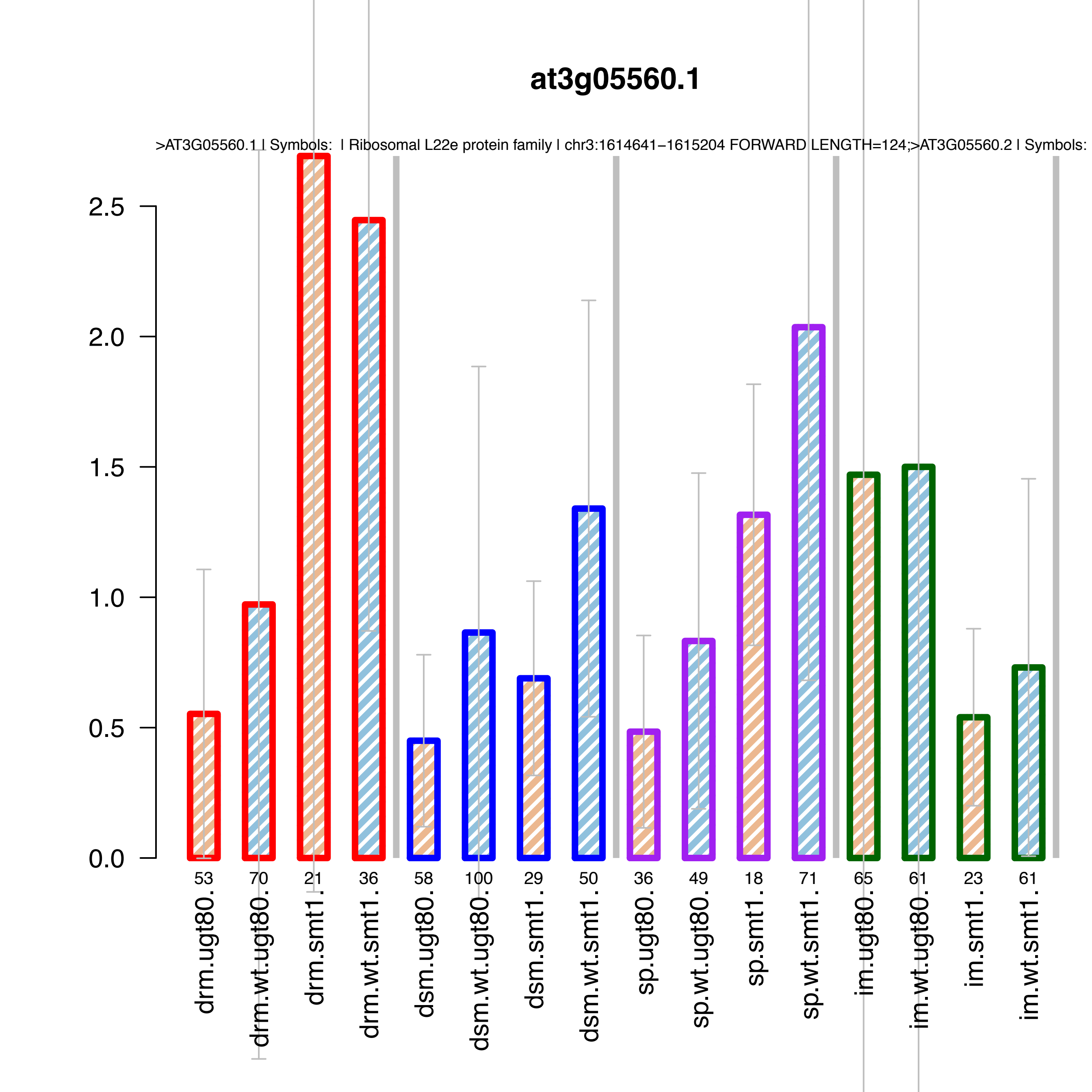
at3g05560.1
>AT3G05560.1 | Symbols: | Ribosomal L22e protein family | chr3:1614641-1615204 FORWARD LENGTH=124;>AT3G05560.2 | Symbols:
0.0
0.5
1.0
1.5
2.0
2.5
53
70
21
36
58
100
29
50
36
49
18
71
65
61
23
61
drm.ugt80..
drm.wt.ugt80..
drm.smt1..
drm.wt.smt1..
dsm.ugt80..
dsm.wt.ugt80..
dsm.smt1..
dsm.wt.smt1..
sp.ugt80..
sp.wt.ugt80..
sp.smt1..
sp.wt.smt1..
im.ugt80..
im.wt.ugt80..
im.smt1..
im.wt.smt1..

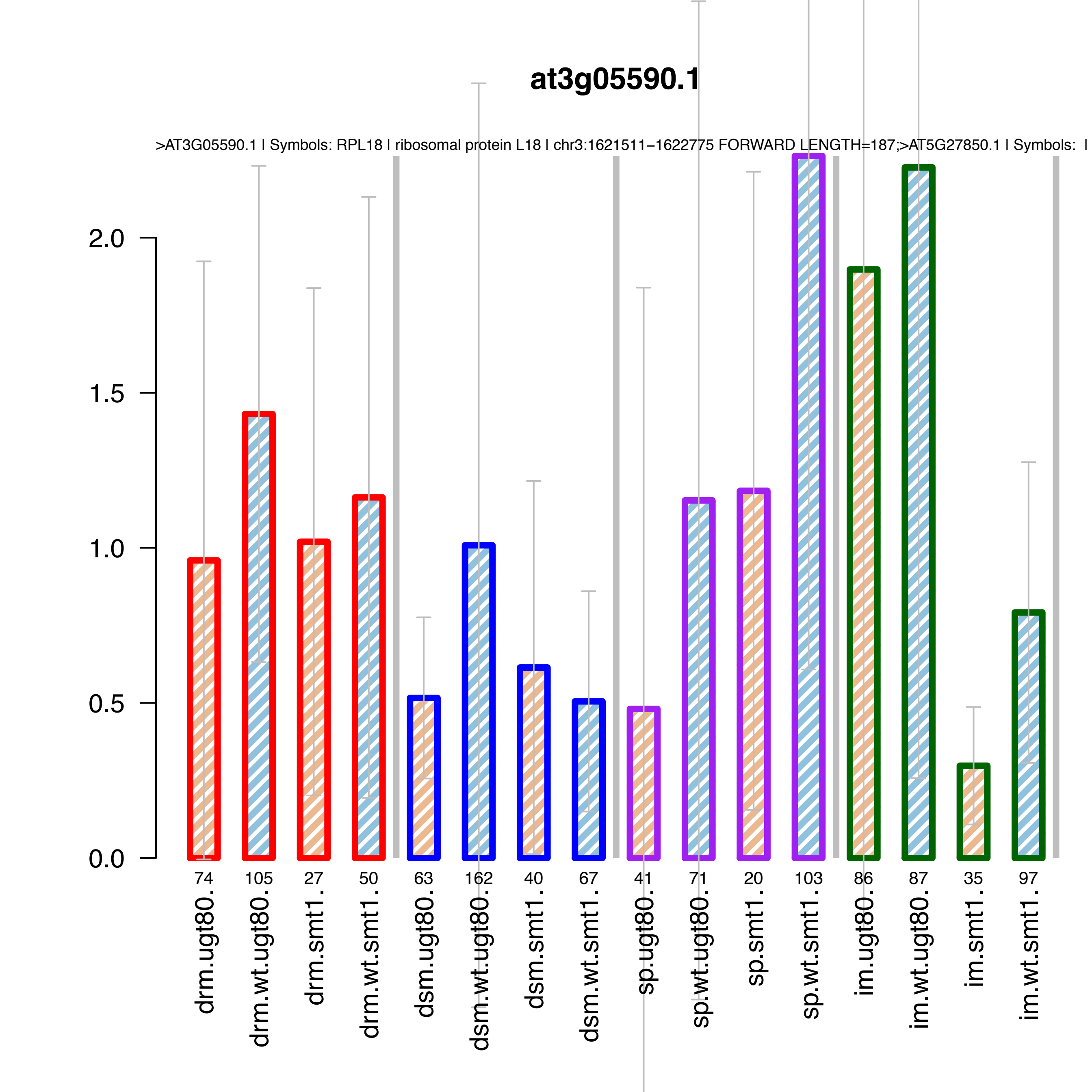

at3g05590.1
>AT3G05590.1 | Symbols: RPL18 | ribosomal protein L18 | chr3:1621511−1622775 FORWARD LENGTH=187;>AT5G27850.1 | Symbols: |
2.0
1.5
1.0
0.5
0.0
74
105
27
50
63
162
40
67
41
71
20
103
86
87
35
97
drm.ugt80..
drm.wt.ugt80..
drm.smt1..
drm.wt.smt1..
dsm.ugt80..
dsm.wt.ugt80..
dsm.smt1..
dsm.wt.smt1..
sp.ugt80..
sp.wt.ugt80..
sp.smt1..
sp.wt.smt1..
im.ugt80..
im.wt.ugt80..
im.smt1..
im.wt.smt1..

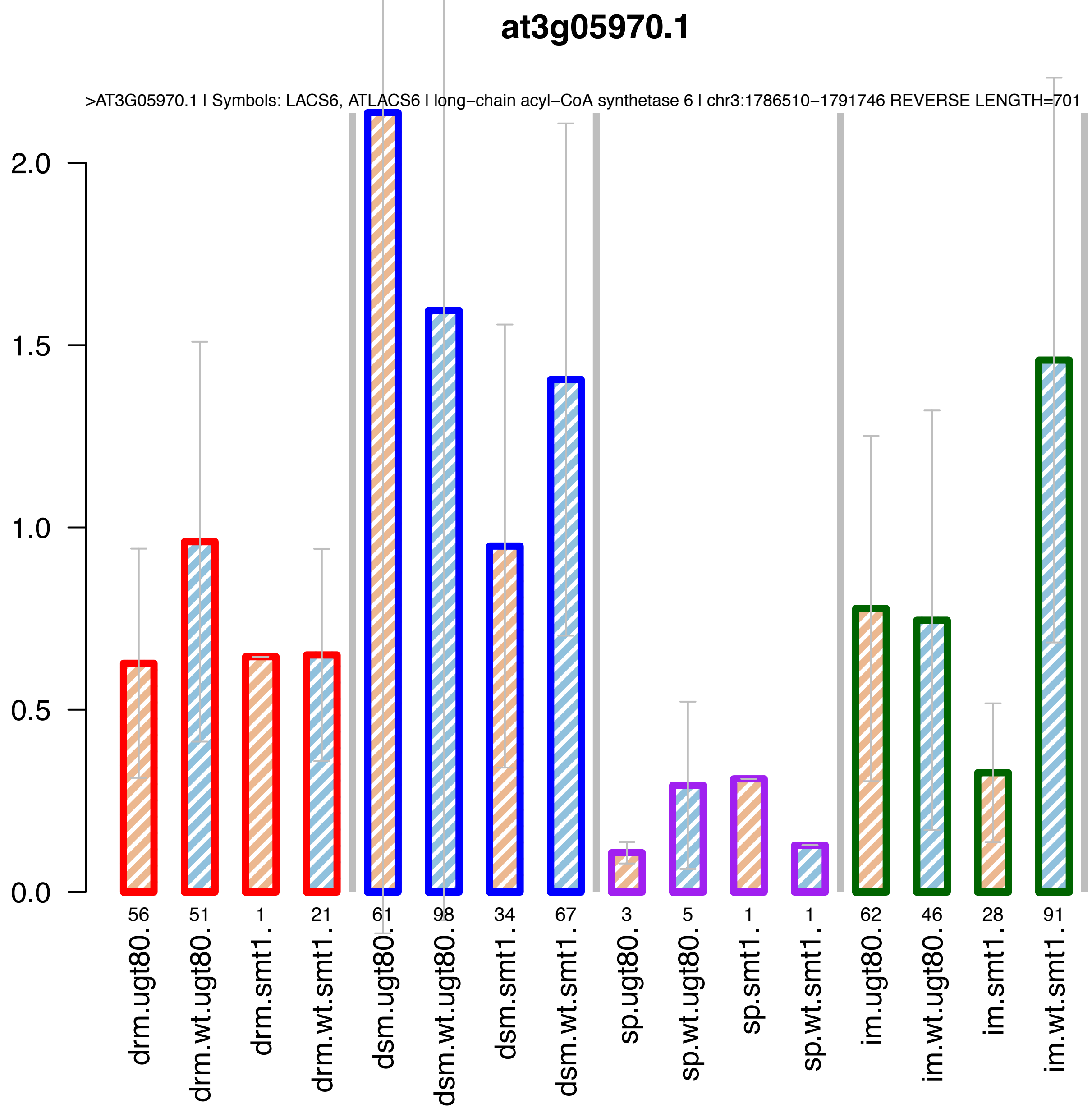

at3g05970.1
>AT3G05970.1 | Symbols: LACS6, ATLACS6 | long−chain acyl−CoA synthetase 6 | chr3:1786510−1791746 REVERSE LENGTH=701
2.0
1.5
1.0
0.5
0.0
56
51
1
21
61
98
34
67
3
5
1
1
62
46
28
91
drm.ugt80..
drm.wt.ugt80..
drm.smt1..
drm.wt.smt1..
dsm.ugt80..
dsm.wt.ugt80..
dsm.smt1..
dsm.wt.smt1..
sp.ugt80..
sp.wt.ugt80..
sp.smt1..
sp.wt.smt1..
im.ugt80..
im.wt.ugt80..
im.smt1..
im.wt.smt1..

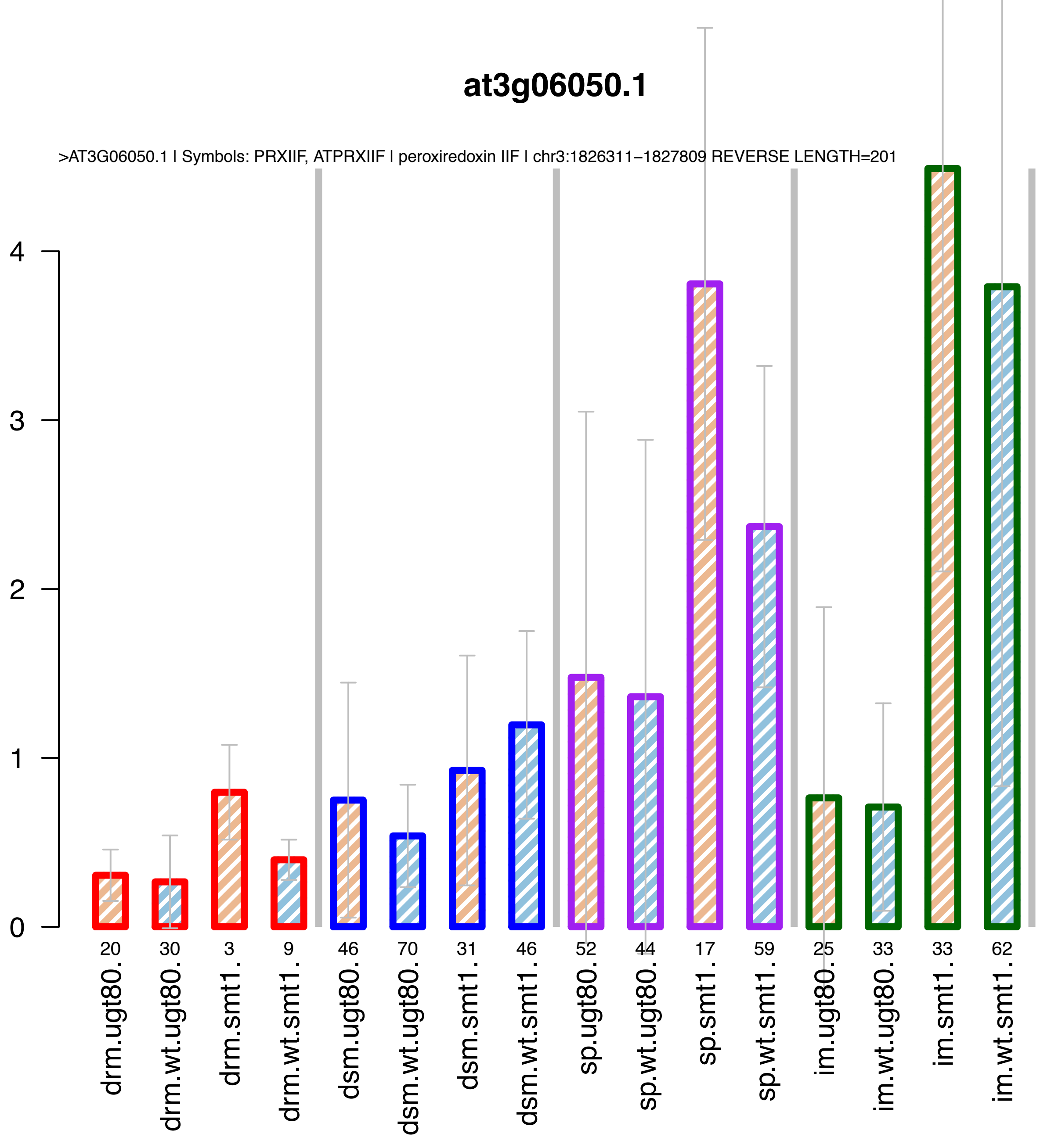
at3g06050.1
>AT3G06050.1 | Symbols: PRXIIF, ATPRXIIF | peroxiredoxin IIF | chr3:1826311−1827809 REVERSE LENGTH=201
0
1
2
3
4
20
30
3
9
46
70
31
46
52
44
17
59
25
33
33
62
drm.ugt80..
drm.wt.ugt80..
drm.smt1..
drm.wt.smt1..
dsm.ugt80..
dsm.wt.ugt80..
dsm.smt1..
dsm.wt.smt1..
sp.ugt80..
sp.wt.ugt80..
sp.smt1..
sp.wt.smt1..
im.ugt80..
im.wt.ugt80..
im.smt1..
im.wt.smt1..

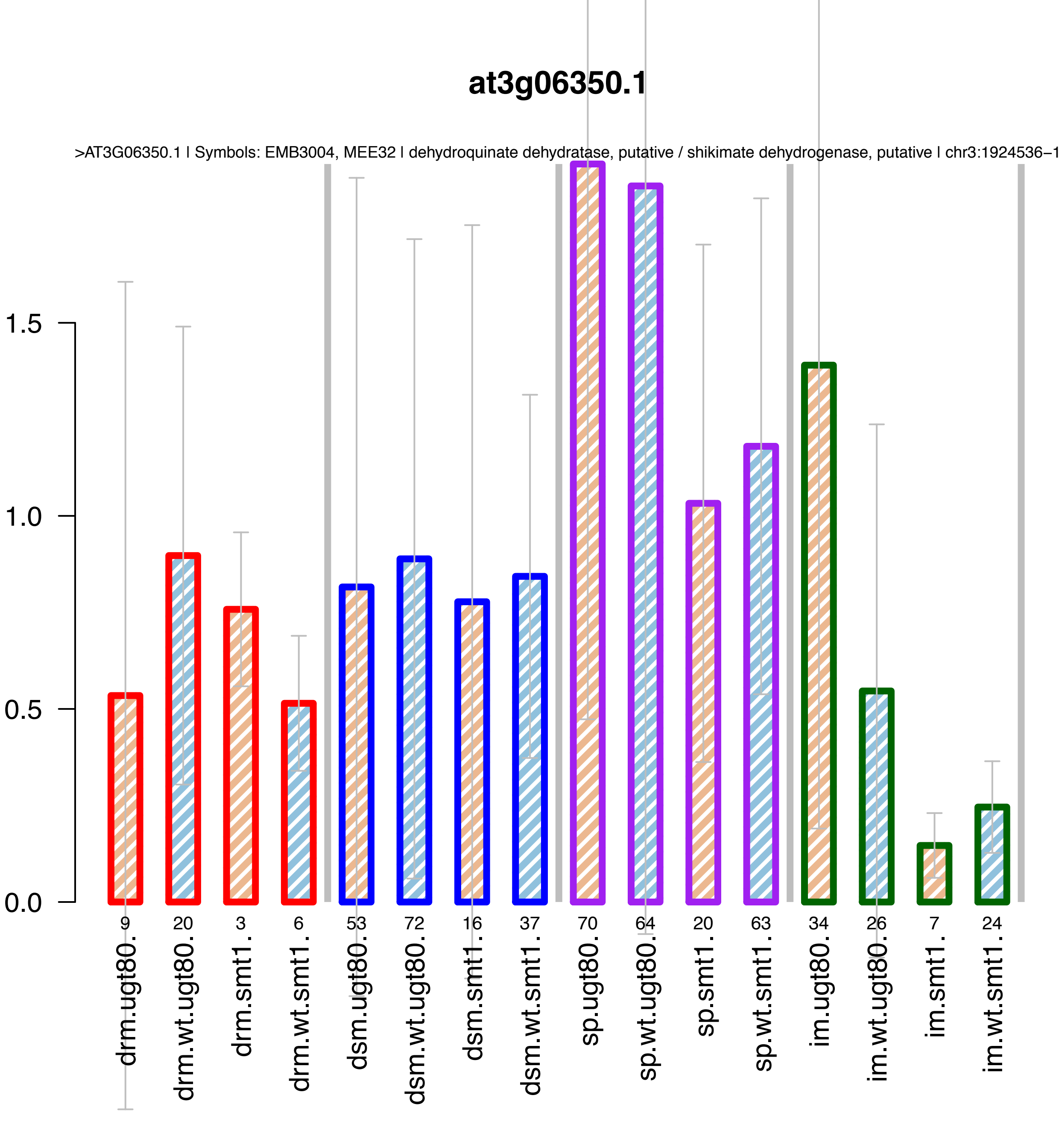

at3g06350.1
>AT3G06350.1 | Symbols: EMB3004, MEE32 | dehydroquinate dehydratase, putative / shikimate dehydrogenase, putative | chr3:1924536–1
1.5
1.0
0.5
0.0
9
20
3
6
53
72
16
37
70
64
20
63
34
26
7
24
drm.ugt80.
drm.wt.ugt80.
drm.smt1.
drm.wt.smt1.
dsm.ugt80.
dsm.wt.ugt80.
dsm.smt1.
dsm.wt.smt1.
sp.ugt80.
sp.wt.ugt80.
sp.smt1.
sp.wt.smt1.
im.ugt80.
im.wt.ugt80.
im.smt1.
im.wt.smt1.

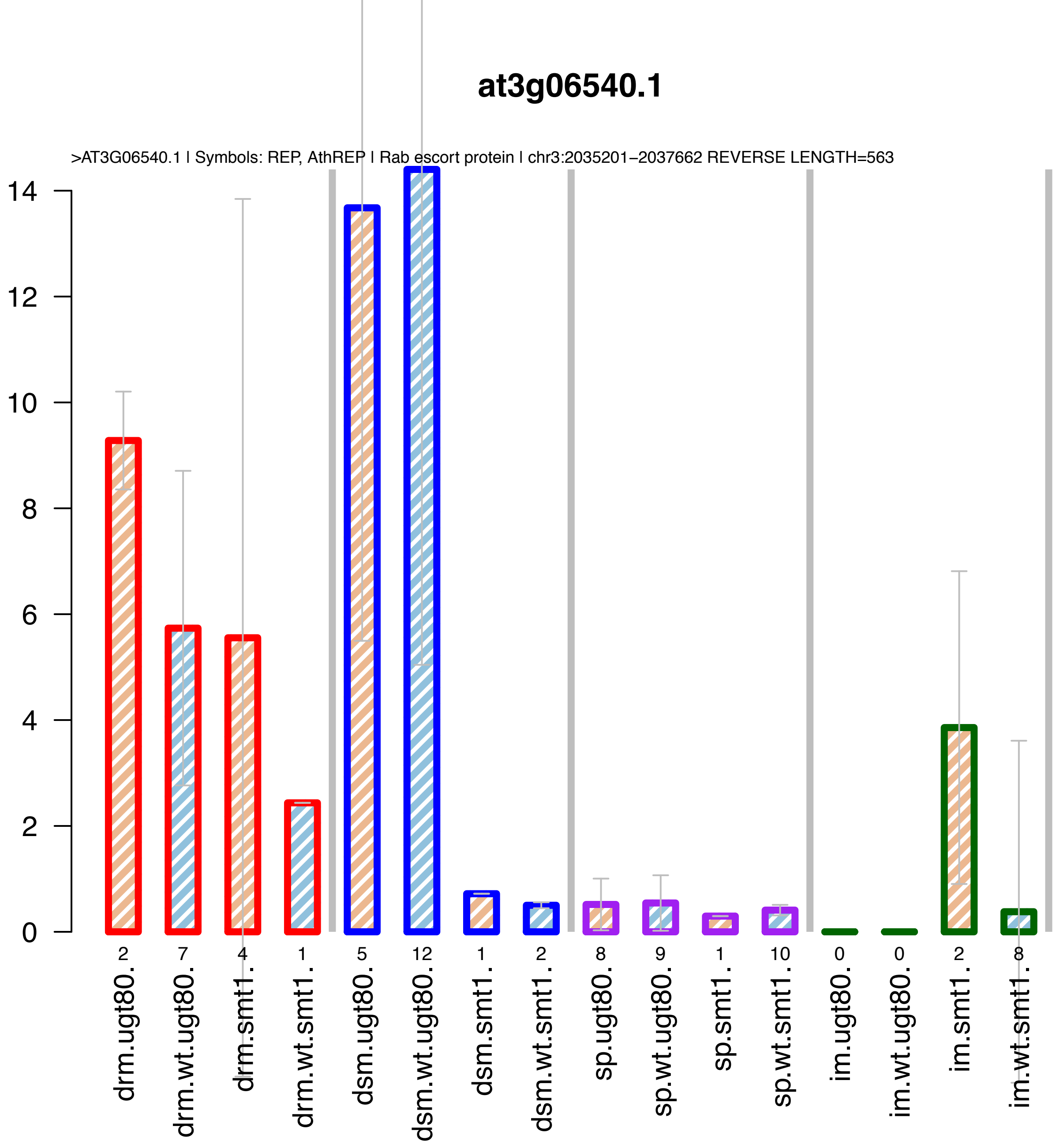

at3g06540.1
>AT3G06540.1 | Symbols: REP, AthREP | Rab escort protein | chr3:2035201–2037662 REVERSE LENGTH=563
0
2
4
6
8
10
12
14
2
7
4
1
5
12
1
2
8
9
1
10
0
0
2
8
drm.ugt80..
drm.wt.ugt80..
drm.smt1..
drm.wt.smt1..
dsm.ugt80..
dsm.wt.ugt80..
dsm.smt1..
dsm.wt.smt1..
sp.ugt80..
sp.wt.ugt80..
sp.smt1..
sp.wt.smt1..
im.ugt80..
im.wt.ugt80..
im.smt1..
im.wt.smt1..

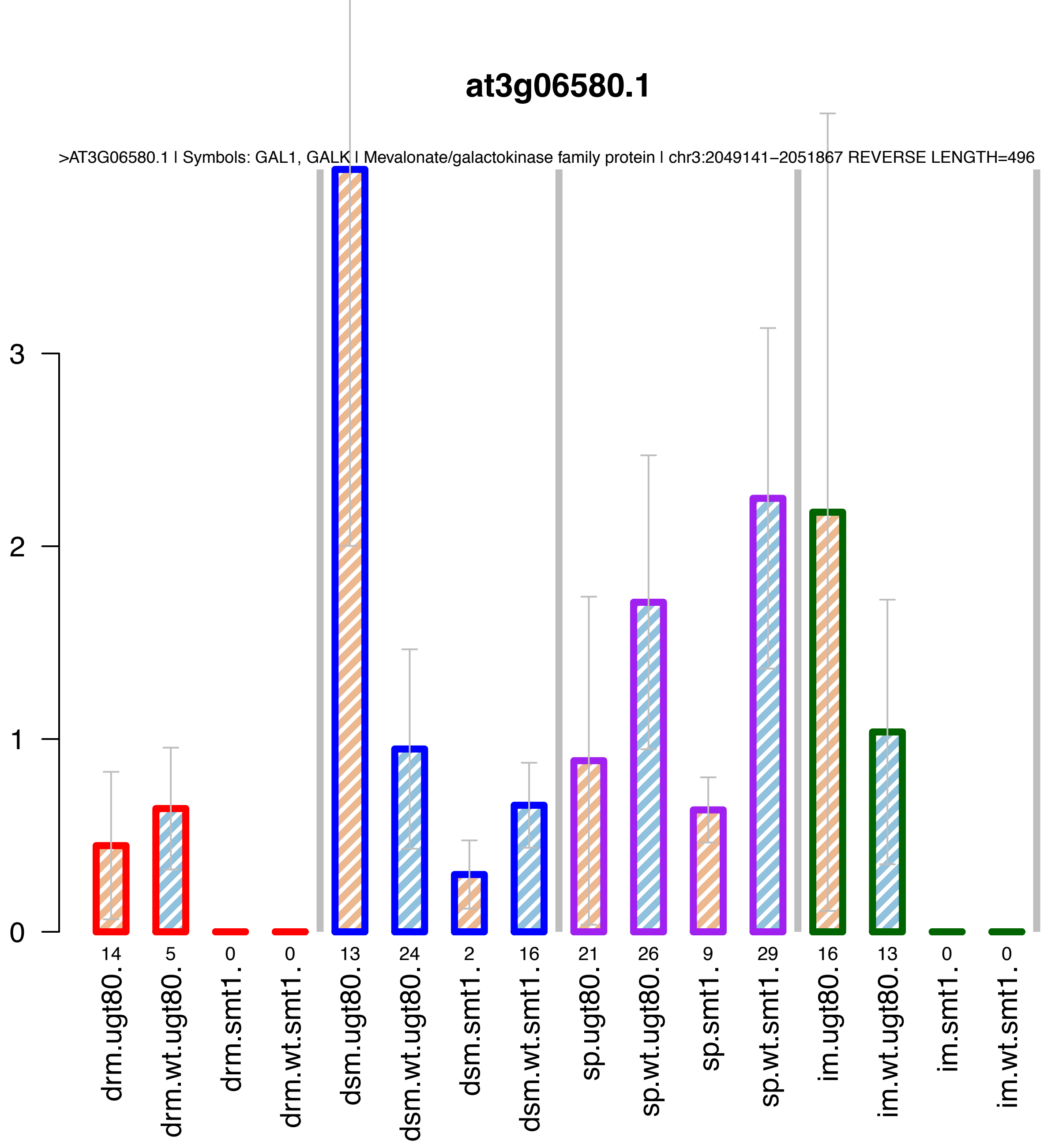
at3g06580.1
>AT3G06580.1 | Symbols: GAL1, GALK | Mevalonate/galactokinase family protein | chr3:2049141-2051867 REVERSE LENGTH=496
0
1
2
3
14
5
0
0
13
24
2
16
21
26
9
29
16
13
0
0
drm.ugt80.
drm.wt.ugt80.
drm.smt1.
drm.wt.smt1.
dsm.ugt80.
dsm.wt.ugt80.
dsm.smt1.
dsm.wt.smt1.
sp.ugt80.
sp.wt.ugt80.
sp.smt1.
sp.wt.smt1.
im.ugt80.
im.wt.ugt80.
im.smt1.
im.wt.smt1.

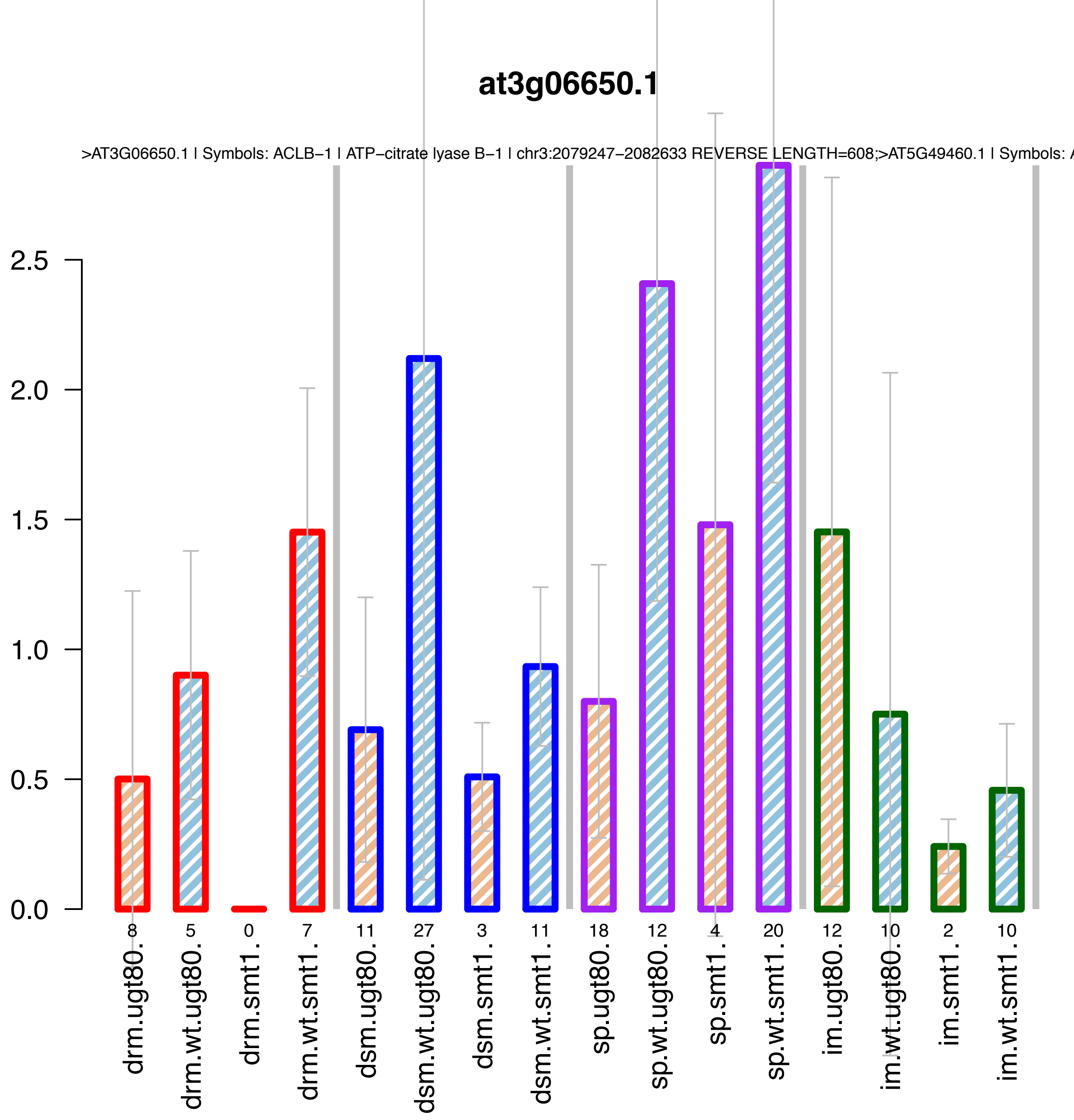

at3g06650.1
>AT3G06650.1 | Symbols: ACLB-1 | ATP-citrate lyase B-1 | chr3:2079247-2082633 REVERSE LENGTH=608;>AT5G49460.1 | Symbols: A
0.0
0.5
1.0
1.5
2.0
2.5
8
5
0
7
11
27
3
11
18
12
4
20
12
10
2
10
drm.ugt80..
drm.wt.ugt80..
drm.smt1..
drm.wt.smt1..
dsm.ugt80..
dsm.wt.ugt80..
dsm.smt1..
dsm.wt.smt1..
sp.ugt80..
sp.wt.ugt80..
sp.smt1..
sp.wt.smt1..
im.ugt80..
im.wt.ugt80..
im.smt1..
im.wt.smt1..

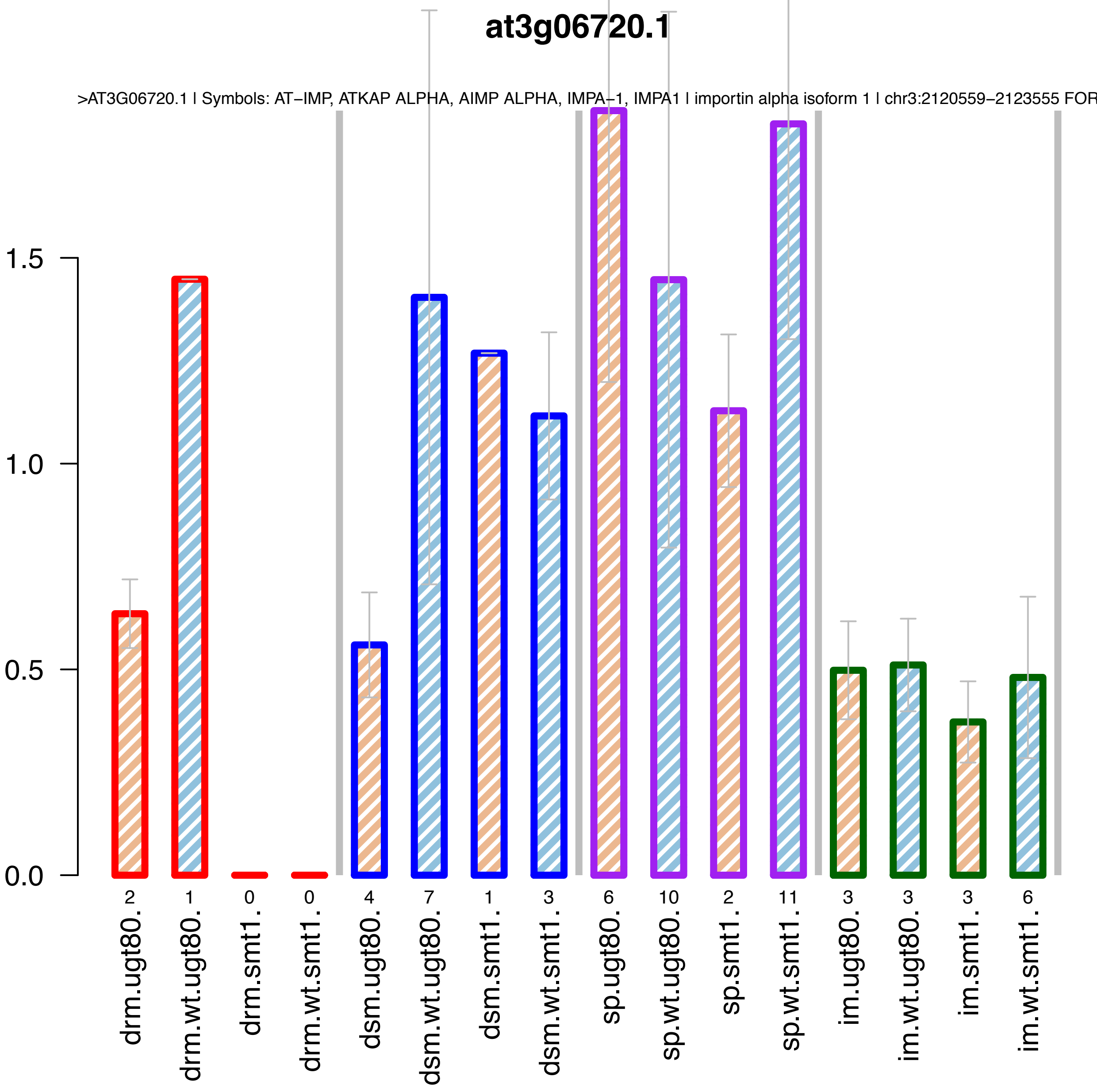

at3g06720.1
>AT3G06720.1 | Symbols: AT−IMP, ATKAP ALPHA, AIMP ALPHA, IMPA−1, IMPA1 | importin alpha isoform 1 | chr3:2120559−2123555 FOR
1.5
1.0
0.5
0.0
2
1
0
0
4
7
1
3
6
10
2
11
3
3
3
6
drm.ugt80.
drm.wt.ugt80.
drm.smt1.
drm.wt.smt1.
dsm.ugt80.
dsm.wt.ugt80.
dsm.smt1.
dsm.wt.smt1.
sp.ugt80.
sp.wt.ugt80.
sp.smt1.
sp.wt.smt1.
im.ugt80.
im.wt.ugt80.
im.smt1.
im.wt.smt1.

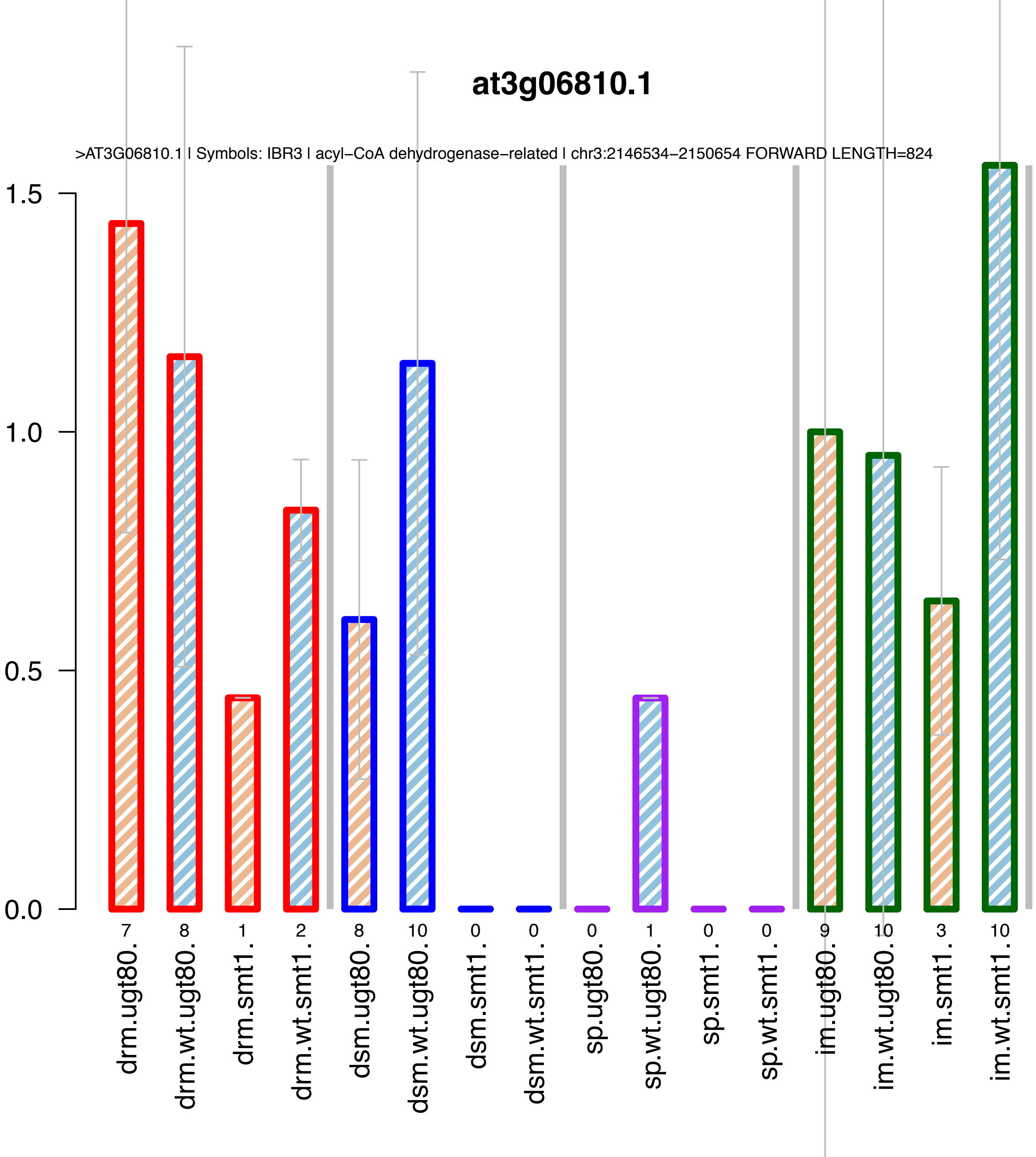
at3g06810.1
>AT3G06810.1 | Symbols: IBR3 | acyl−CoA dehydrogenase−related | chr3:2146534−2150654 FORWARD LENGTH=824
1.5
1.0
0.5
0.0
7
8
1
2
8
10
0
0
0
1
0
0
9
10
3
10
drm.ugt80..
drm.wt.ugt80..
drm.smt1..
drm.wt.smt1..
dsm.ugt80..
dsm.wt.ugt80..
dsm.smt1..
dsm.wt.smt1..
sp.ugt80..
sp.wt.ugt80..
sp.smt1..
sp.wt.smt1..
im.ugt80..
im.wt.ugt80..
im.smt1..
im.wt.smt1..

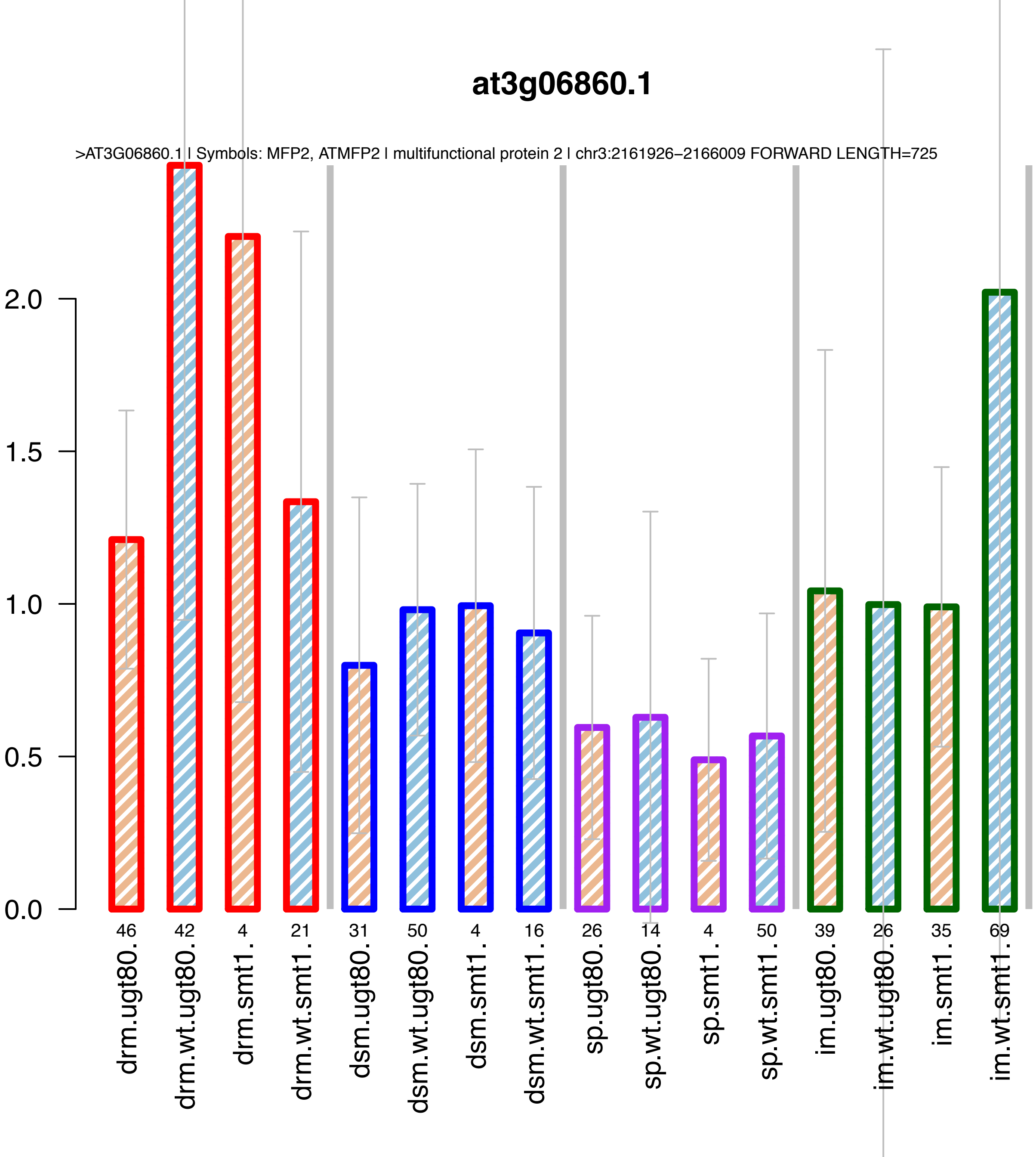

at3g06860.1
>AT3G06860.1 | Symbols: MFP2, ATMFP2 | multifunctional protein 2 | chr3:2161926-2166009 FORWARD LENGTH=725
0.0
0.5
1.0
1.5
2.0
46
42
4
21
31
50
4
16
26
14
4
50
39
26
35
69
drm.ugt80..
drm.wt.ugt80..
drm.smt1..
drm.wt.smt1..
dsm.ugt80..
dsm.wt.ugt80..
dsm.smt1..
dsm.wt.smt1..
sp.ugt80..
sp.wt.ugt80..
sp.smt1..
sp.wt.smt1..
im.ugt80..
im.wt.ugt80..
im.smt1..
im.wt.smt1..

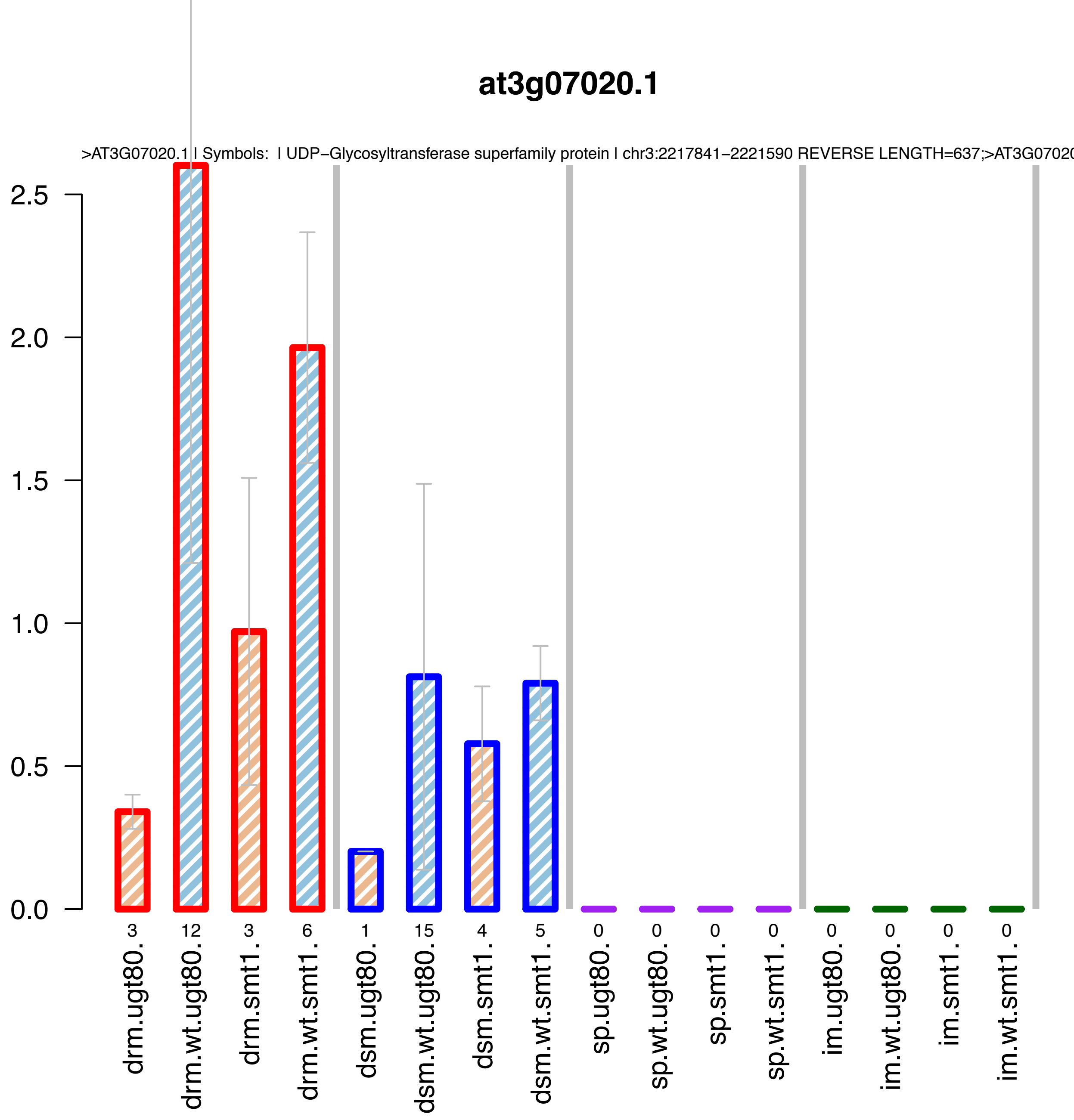

at3g07020.1
>AT3G07020.1 | Symbols: | UDP−Glycosyltransferase superfamily protein | chr3:2217841−2221590 REVERSE LENGTH=637;>AT3G0702
2.5
2.0
1.5
1.0
0.5
0.0
3
12
3
6
1
15
4
5
0
0
0
0
0
0
0
0
drm.ugt80.
drm.wt.ugt80.
drm.smt1.
drm.wt.smt1.
dsm.ugt80.
dsm.wt.ugt80.
dsm.smt1.
dsm.wt.smt1.
sp.ugt80.
sp.wt.ugt80.
sp.smt1.
sp.wt.smt1.
im.ugt80.
im.wt.ugt80.
im.smt1.
im.wt.smt1.

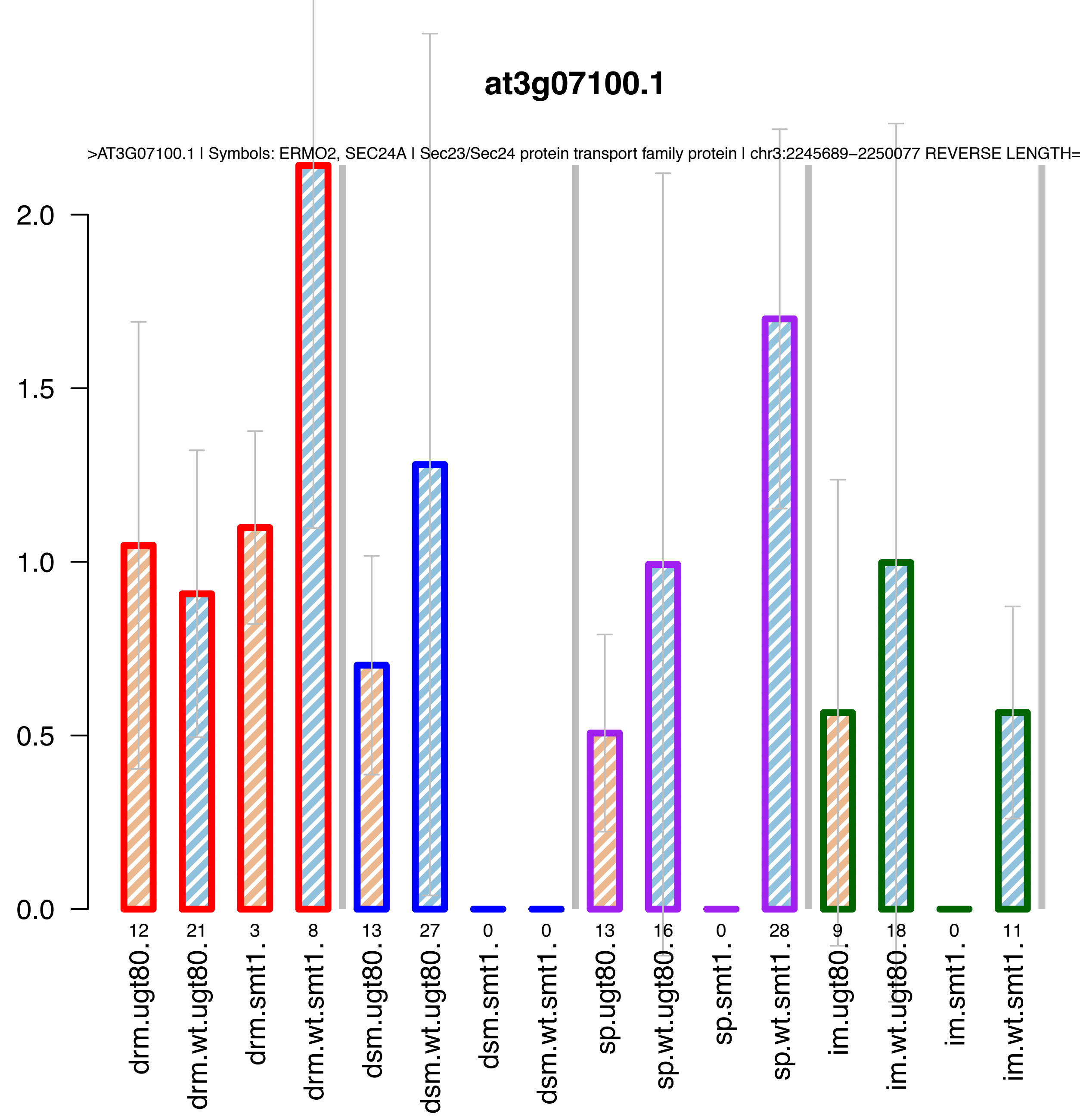

at3g07100.1
>AT3G07100.1 | Symbols: ERMO2, SEC24A | Sec23/Sec24 protein transport family protein | chr3:2245689-2250077 REVERSE LENGTH=
2.0
1.5
1.0
0.5
0.0
12
21
3
8
13
27
0
0
13
16
0
28
9
18
0
11
drm.ugt80.
drm.wt.ugt80.
drm.smt1.
drm.wt.smt1.
dsm.ugt80.
dsm.wt.ugt80.
dsm.smt1.
dsm.wt.smt1.
sp.ugt80.
sp.wt.ugt80.
sp.smt1.
sp.wt.smt1.
im.ugt80.
im.wt.ugt80.
im.smt1.
im.wt.smt1.

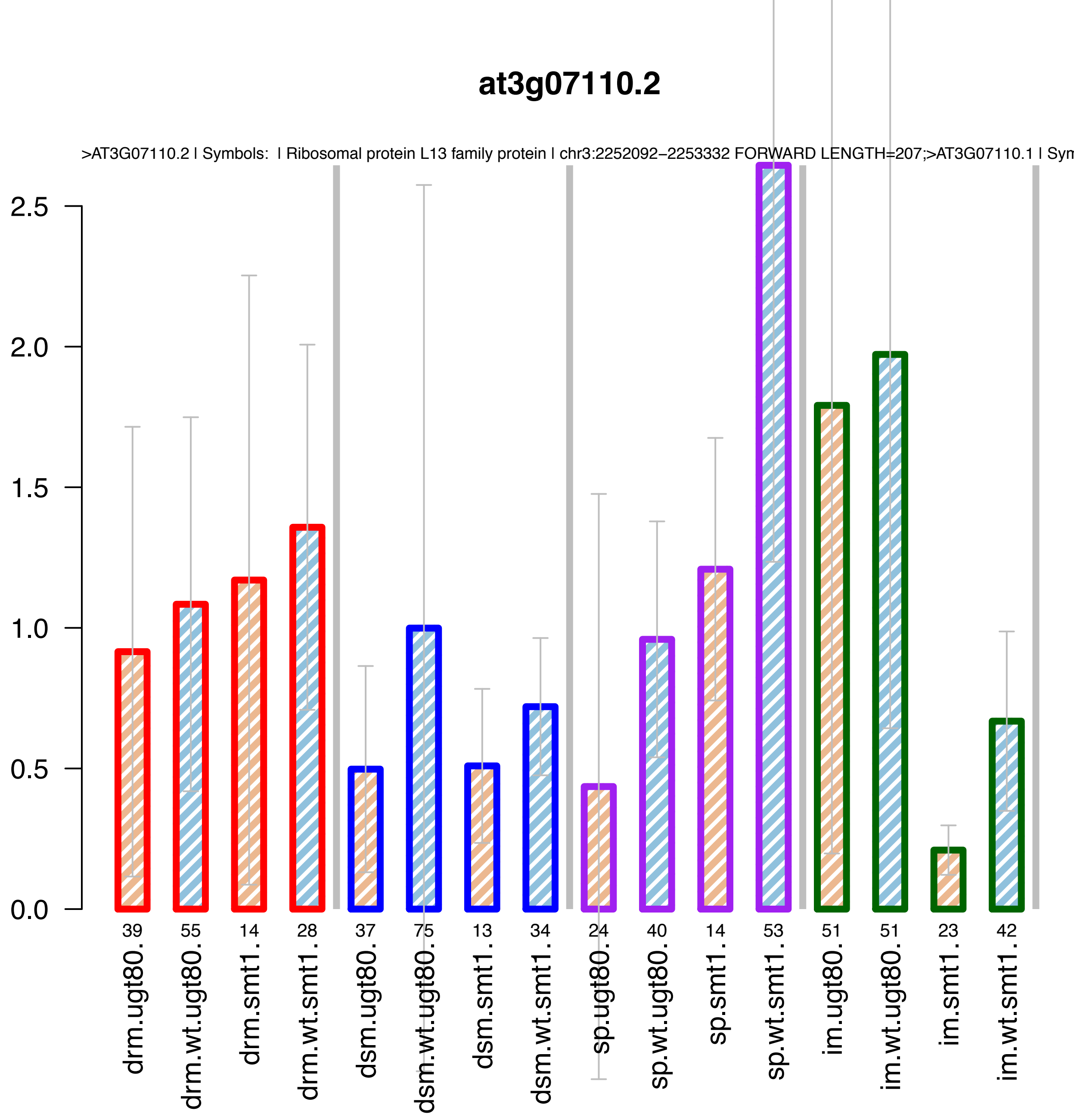
at3g07110.2
>AT3G07110.2 | Symbols: | Ribosomal protein L13 family protein | chr3:2252092−2253332 FORWARD LENGTH=207;>AT3G07110.1 | Sym
0.0
0.5
1.0
1.5
2.0
2.5
39
55
14
28
37
75
13
34
24
40
14
53
51
51
23
42
drm.ugt80..
drm.wt.ugt80..
drm.smt1..
drm.wt.smt1..
dsm.ugt80..
dsm.wt.ugt80..
dsm.smt1..
dsm.wt.smt1..
sp.ugt80..
sp.wt.ugt80..
sp.smt1..
sp.wt.smt1..
im.ugt80..
im.wt.ugt80..
im.smt1..
im.wt.smt1..

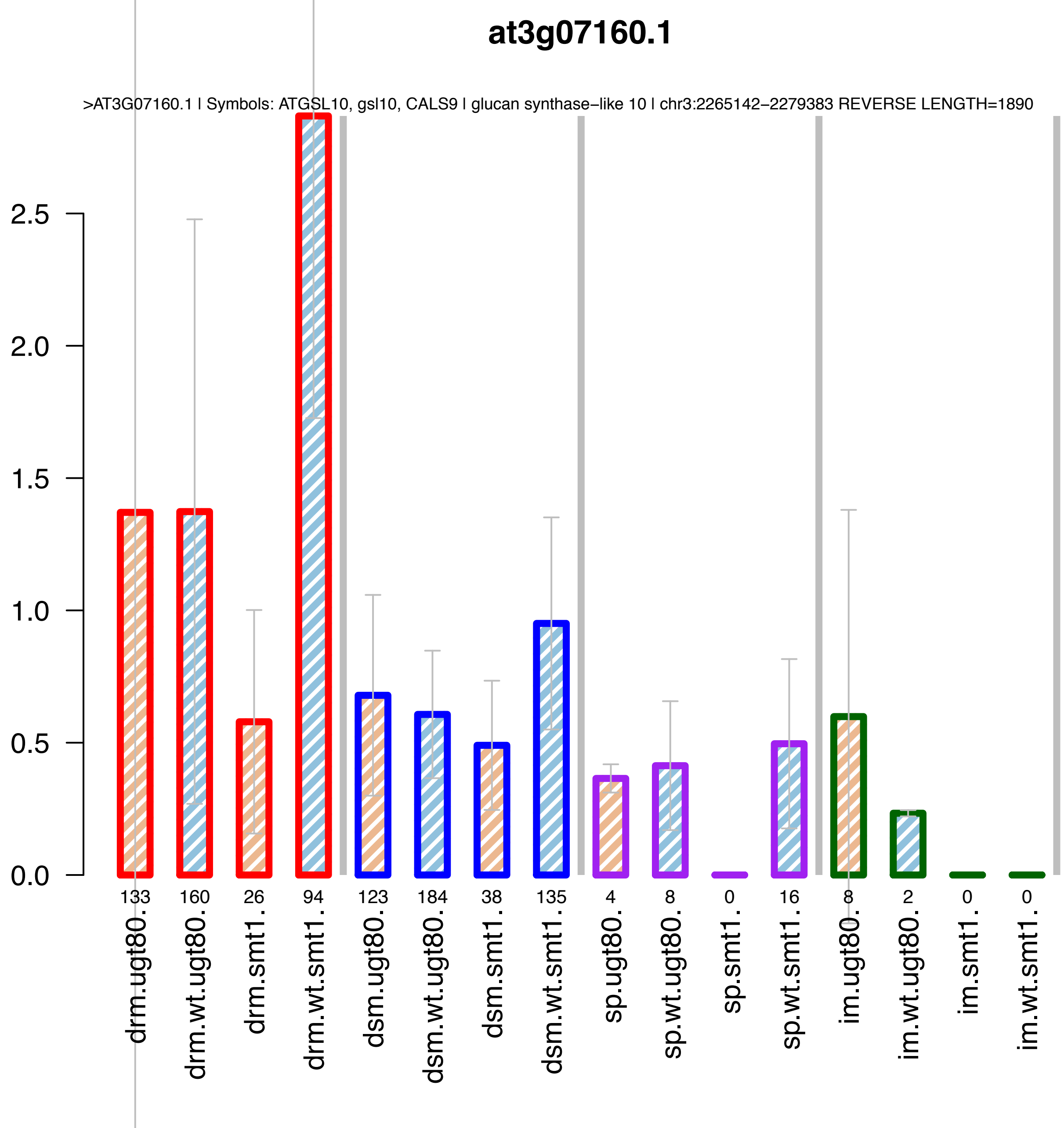

at3g07160.1
>AT3G07160.1 | Symbols: ATGSL10, gsl10, CALS9 | glucan synthase−like 10 | chr3:2265142−2279383 REVERSE LENGTH=1890
0.0
0.5
1.0
1.5
2.0
2.5
133
160
26
94
123
184
38
135
4
8
0
16
8
2
0
0
drm.ugt80..
drm.wt.ugt80..
drm.smt1..
drm.wt.smt1..
dsm.ugt80..
dsm.wt.ugt80..
dsm.smt1..
dsm.wt.smt1..
sp.ugt80..
sp.wt.ugt80..
sp.smt1..
sp.wt.smt1..
im.ugt80..
im.wt.ugt80..
im.smt1..
im.wt.smt1..

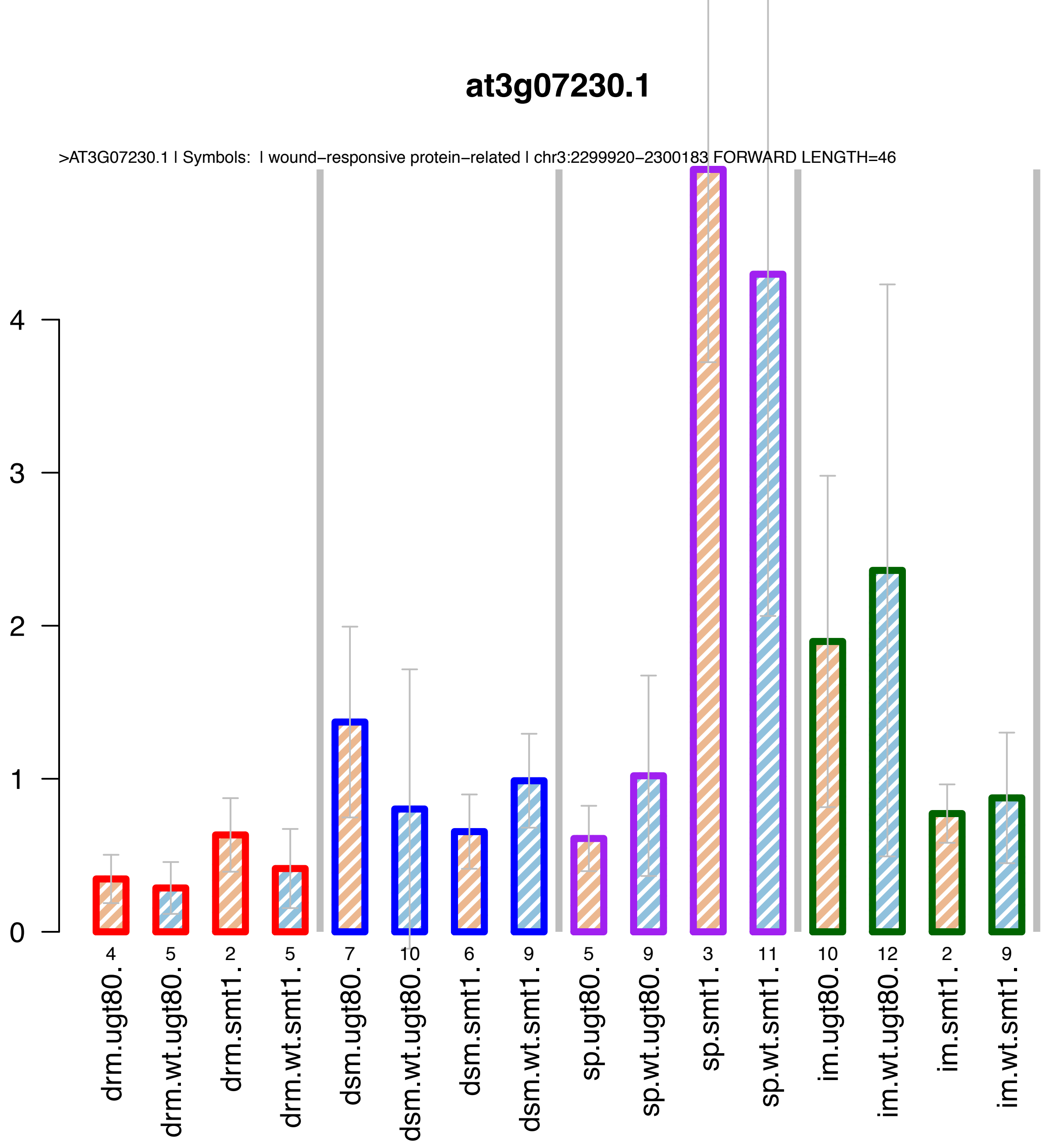
at3g07230.1
>AT3G07230.1 | Symbols: | wound−responsive protein−related | chr3:2299920−2300183 FORWARD LENGTH=46
0
1
2
3
4
4
5
2
5
7
10
6
9
5
9
3
11
10
12
2
9
drm.ugt80.
drm.wt.ugt80.
drm.smt1.
drm.wt.smt1.
dsm.ugt80.
dsm.wt.ugt80.
dsm.smt1.
dsm.wt.smt1.
sp.ugt80.
sp.wt.ugt80.
sp.smt1.
sp.wt.smt1.
im.ugt80.
im.wt.ugt80.
im.smt1.
im.wt.smt1.

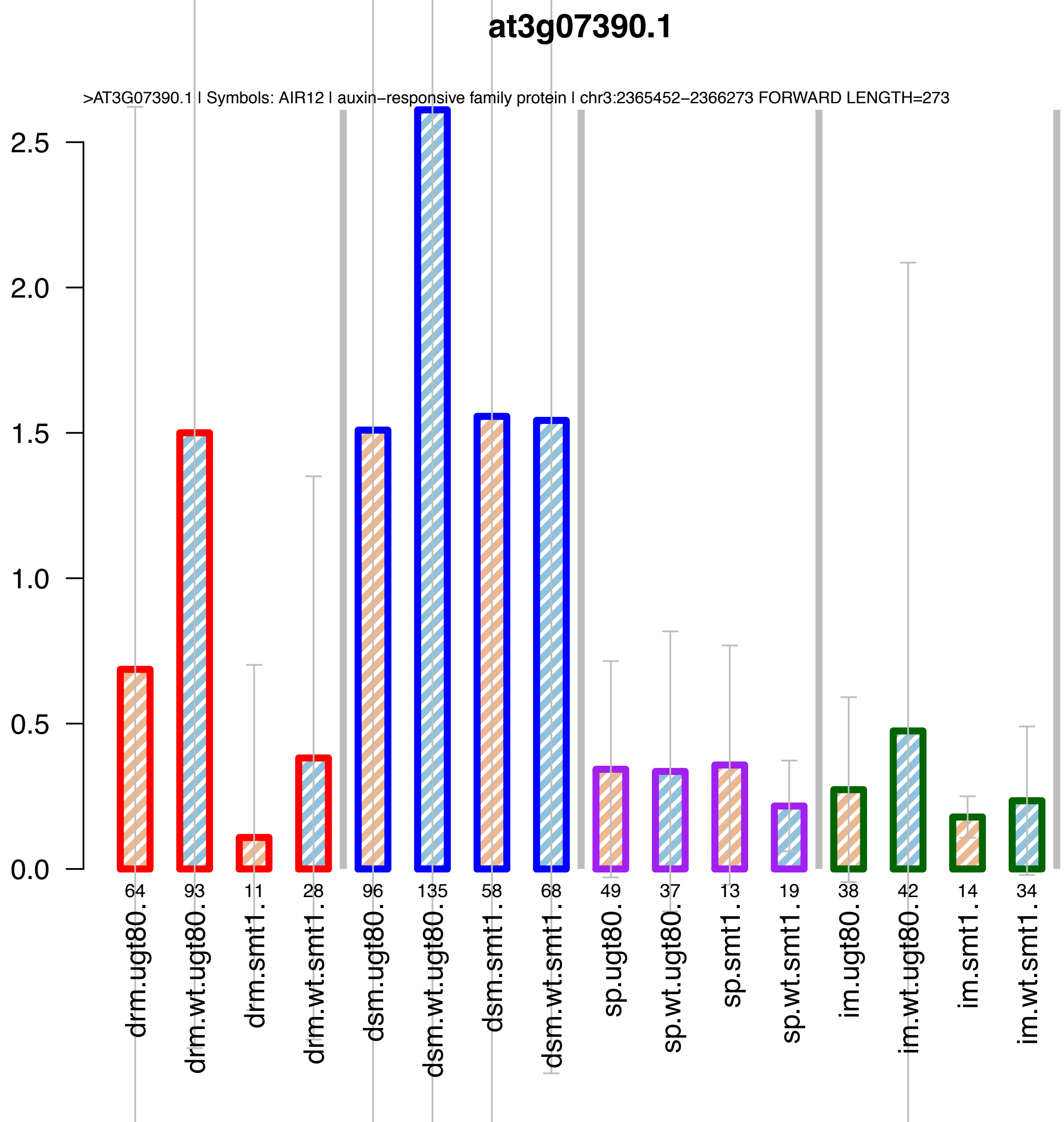

at3g07390.1
>AT3G07390.1 | Symbols: AIR12 | auxin−responsive family protein | chr3:2365452−2366273 FORWARD LENGTH=273
0.0
0.5
1.0
1.5
2.0
2.5
64
93
11
28
96
135
58
68
49
37
13
19
38
42
14
34
drm.ugt80..
drm.wt.ugt80..
drm.smt1..
drm.wt.smt1..
dsm.ugt80..
dsm.wt.ugt80..
dsm.smt1..
dsm.wt.smt1..
sp.ugt80..
sp.wt.ugt80..
sp.smt1..
sp.wt.smt1..
im.ugt80..
im.wt.ugt80..
im.smt1..
im.wt.smt1..

# at3g07480.1

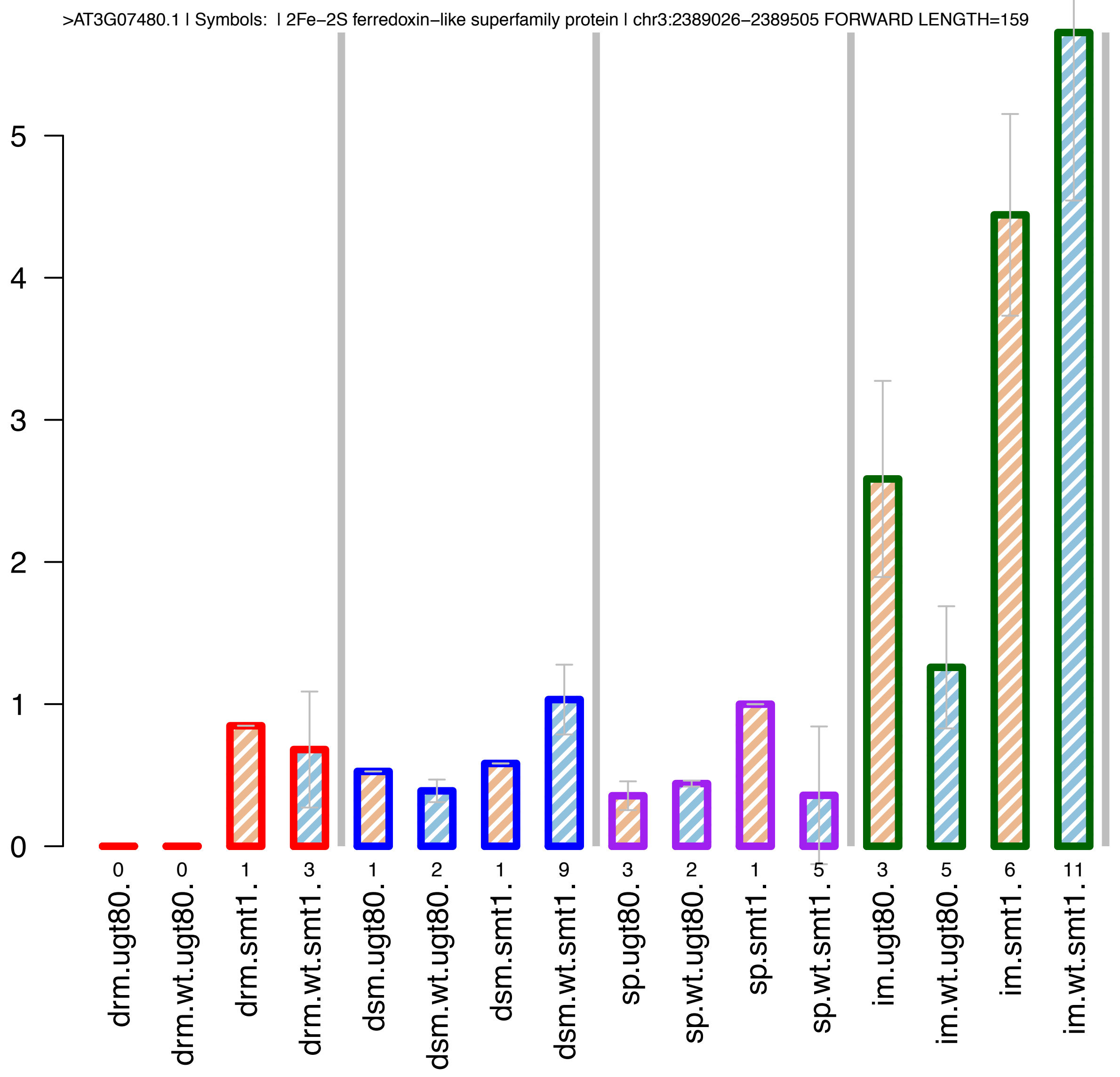

>AT3G07480.1 | Symbols: | 2Fe−2S ferredoxin−like superfamily protein | chr3:2389026−2389505 FORWARD LENGTH=159
0
1
2
3
4
5
0
0
1
3
1
2
1
9
3
2
1
5
3
5
6
11
drm.ugt80.
drm.wt.ugt80.
drm.smt1.
drm.wt.smt1.
dsm.ugt80.
dsm.wt.ugt80.
dsm.smt1.
dsm.wt.smt1.
sp.ugt80.
sp.wt.ugt80.
sp.smt1.
sp.wt.smt1.
im.ugt80.
im.wt.ugt80.
im.smt1.
im.wt.smt1.

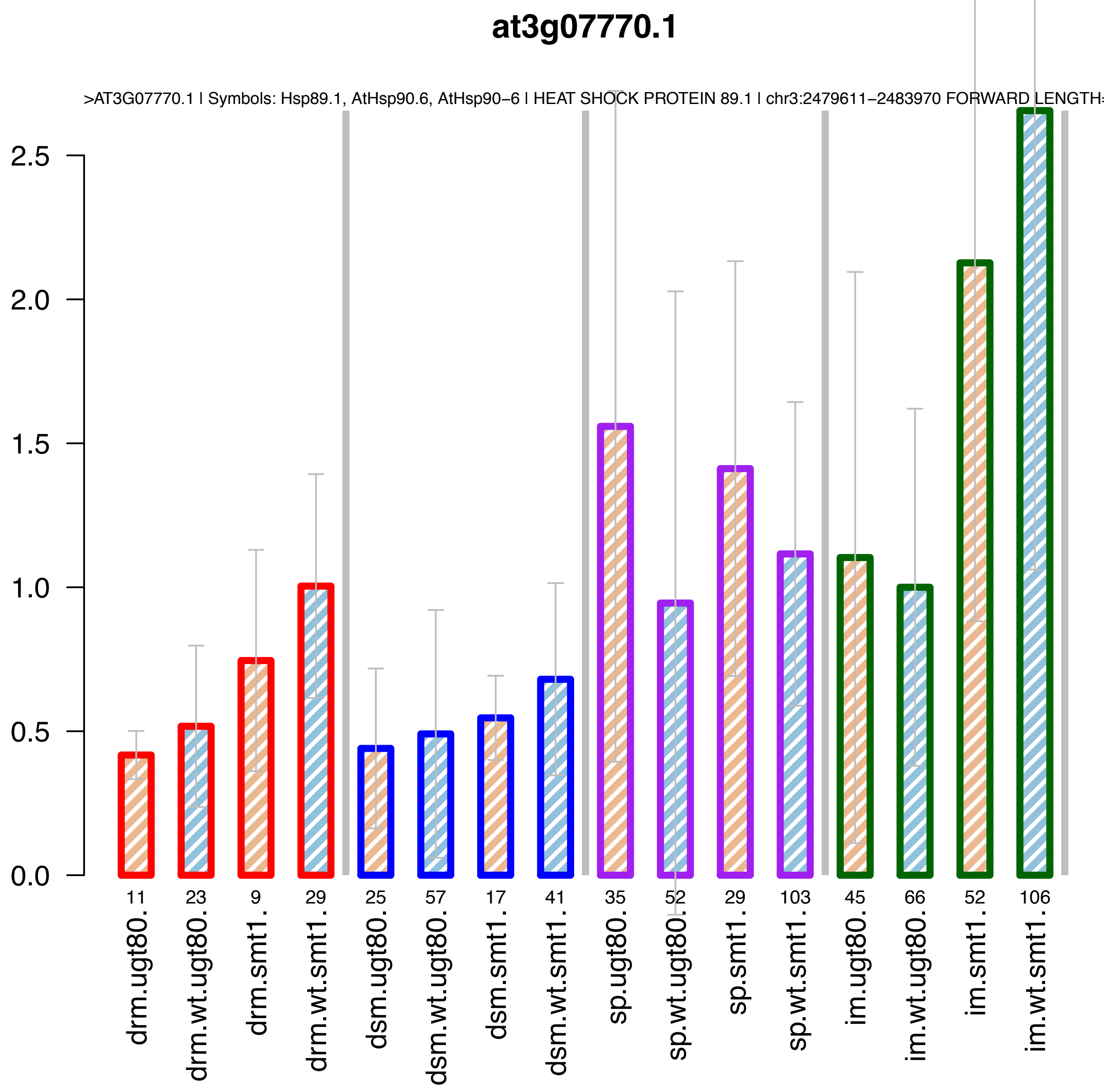

at3g07770.1
>AT3G07770.1 | Symbols: Hsp89.1, AtHsp90.6, AtHsp90-6 | HEAT SHOCK PROTEIN 89.1 | chr3:2479611-2483970 FORWARD LENGTH=
0.0
0.5
1.0
1.5
2.0
2.5
11
23
9
29
25
57
17
41
35
52
29
103
45
66
52
106
drm.ugt80.
drm.wt.ugt80.
drm.smt1.
drm.wt.smt1.
dsm.ugt80.
dsm.wt.ugt80.
dsm.smt1.
dsm.wt.smt1.
sp.ugt80.
sp.wt.ugt80.
sp.smt1.
sp.wt.smt1.
im.ugt80.
im.wt.ugt80.
im.smt1.
im.wt.smt1.

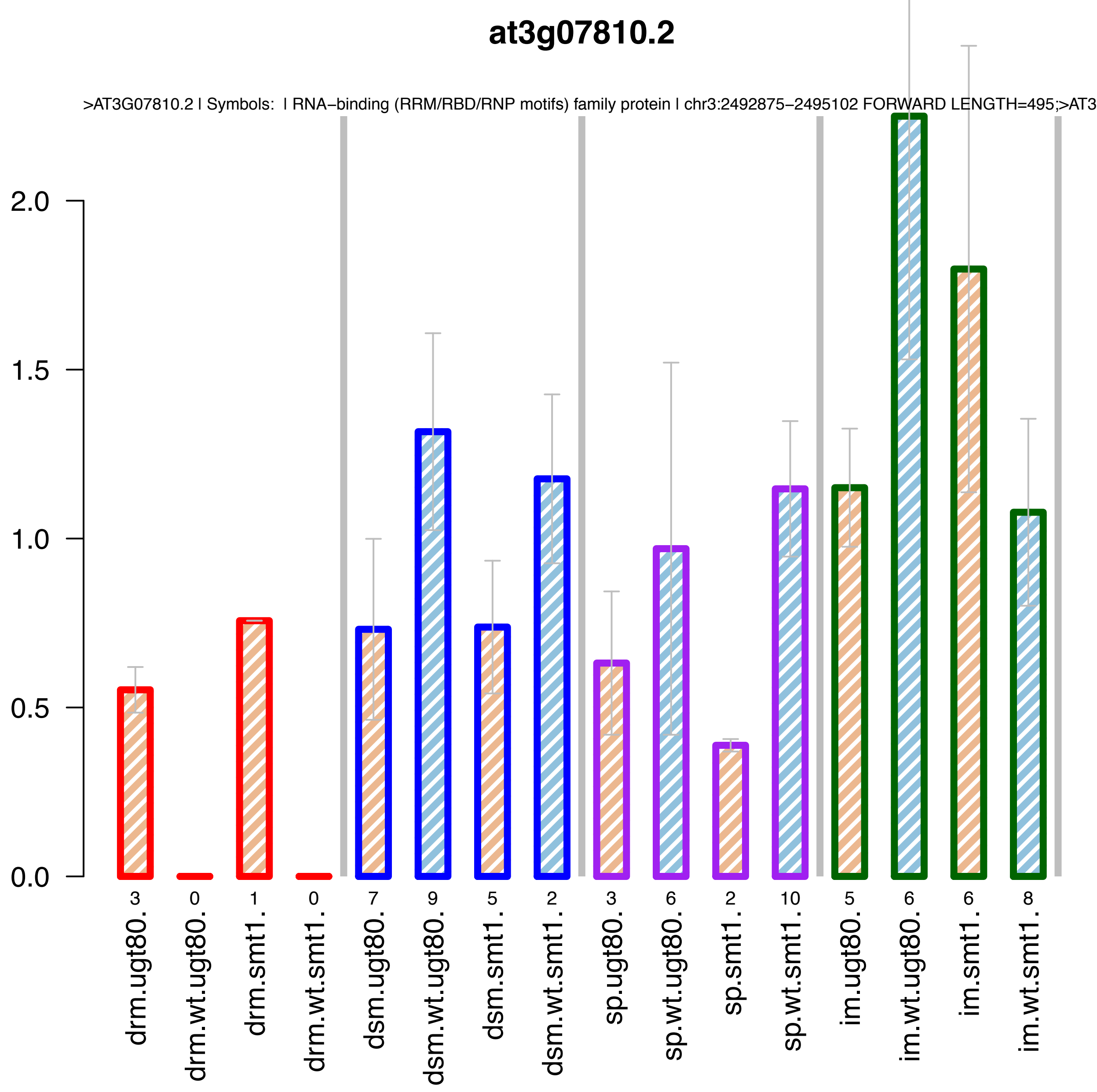

at3g07810.2
>AT3G07810.2 | Symbols: | RNA−binding (RRM/RBD/RNP motifs) family protein | chr3:2492875−2495102 FORWARD LENGTH=495;>AT3
2.0
1.5
1.0
0.5
0.0
3
0
1
0
7
9
5
2
3
6
2
10
5
6
6
8
drm.ugt80.
drm.wt.ugt80.
drm.smt1.
drm.wt.smt1.
dsm.ugt80.
dsm.wt.ugt80.
dsm.smt1.
dsm.wt.smt1.
sp.ugt80.
sp.wt.ugt80.
sp.smt1.
sp.wt.smt1.
im.ugt80.
im.wt.ugt80.
im.smt1.
im.wt.smt1.

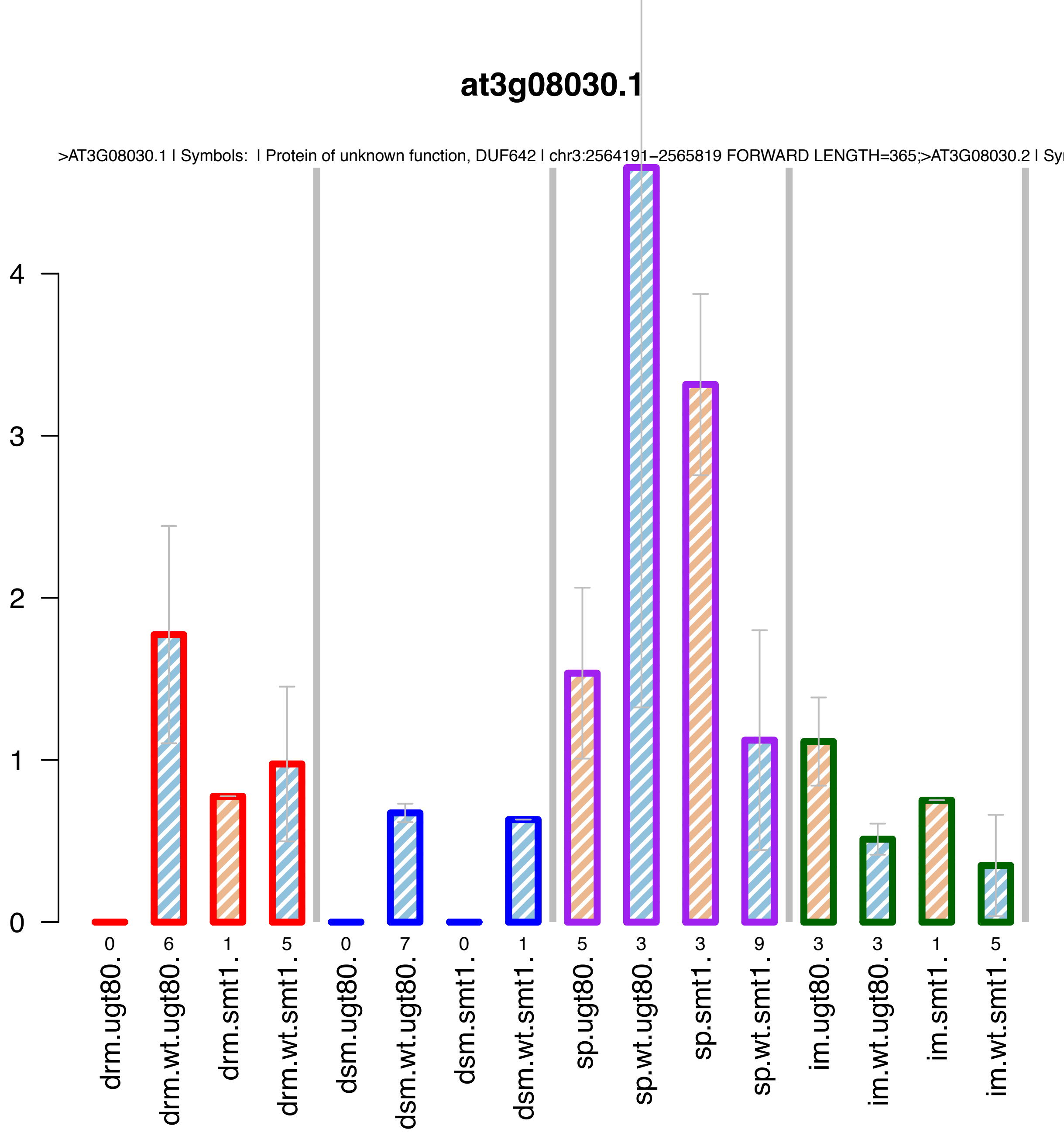

at3g08030.1
>AT3G08030.1 | Symbols: | Protein of unknown function, DUF642 | chr3:2564191–2565819 FORWARD LENGTH=365;>AT3G08030.2 | Sy
0
1
2
3
4
0 6 1 5 0 7 0 1 5 3 3 9 3 3 1 5
drm.ugt80.
drm.wt.ugt80.
drm.smt1.
drm.wt.smt1.
dsm.ugt80.
dsm.wt.ugt80.
dsm.smt1.
dsm.wt.smt1.
sp.ugt80.
sp.wt.ugt80.
sp.smt1.
sp.wt.smt1.
im.ugt80.
im.wt.ugt80.
im.smt1.
im.wt.smt1.

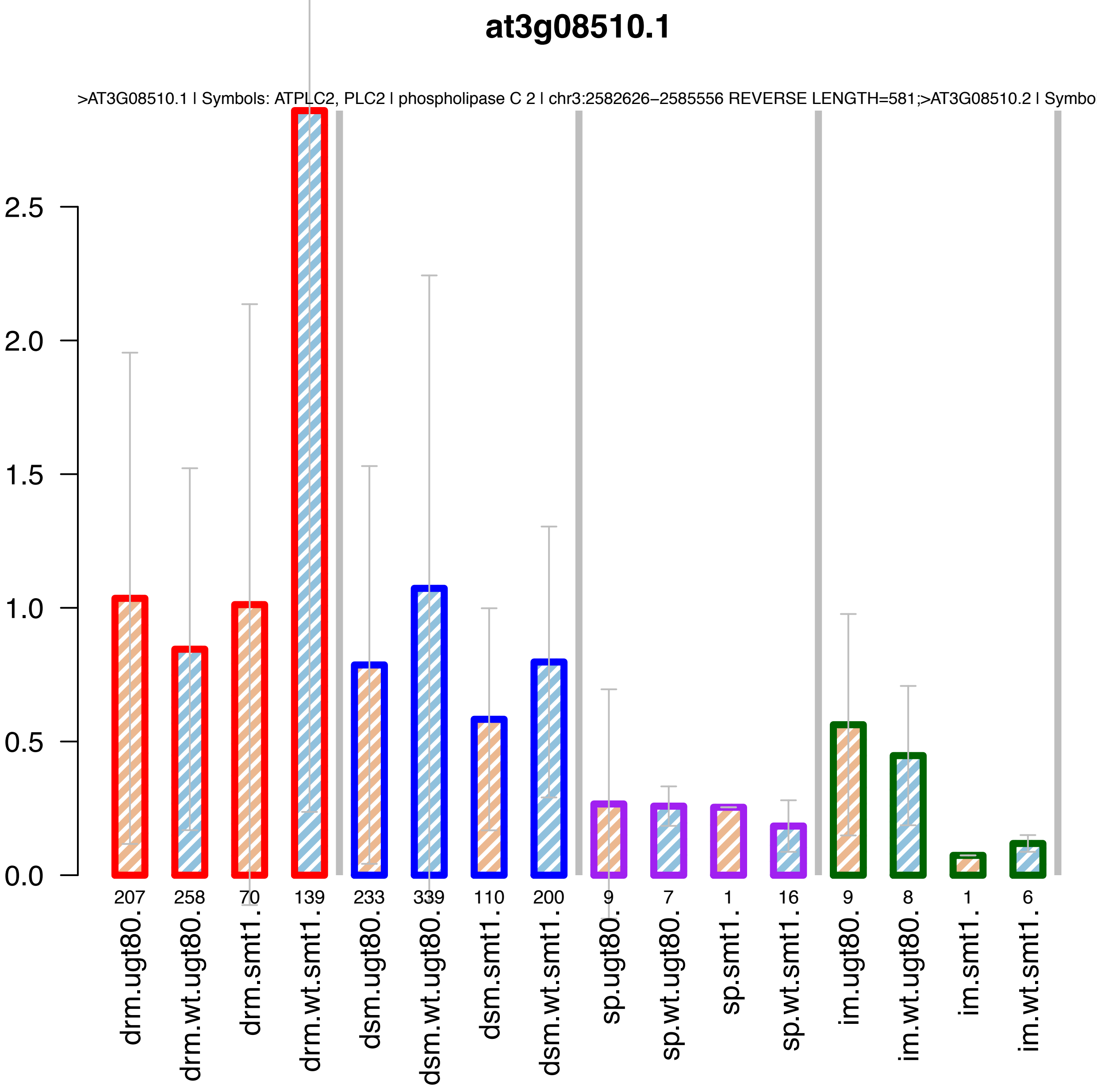
at3g08510.1
>AT3G08510.1 | Symbols: ATPLC2, PLC2 | phospholipase C 2 | chr3:2582626−2585556 REVERSE LENGTH=581;>AT3G08510.2 | Symbo
0.0
0.5
1.0
1.5
2.0
2.5
207
258
70
139
233
339
110
200
9
7
1
16
9
8
1
6
drm.ugt80.
drm.wt.ugt80.
drm.smt1.
drm.wt.smt1.
dsm.ugt80.
dsm.wt.ugt80.
dsm.smt1.
dsm.wt.smt1.
sp.ugt80.
sp.wt.ugt80.
sp.smt1.
sp.wt.smt1.
im.ugt80.
im.wt.ugt80.
im.smt1.
im.wt.smt1.

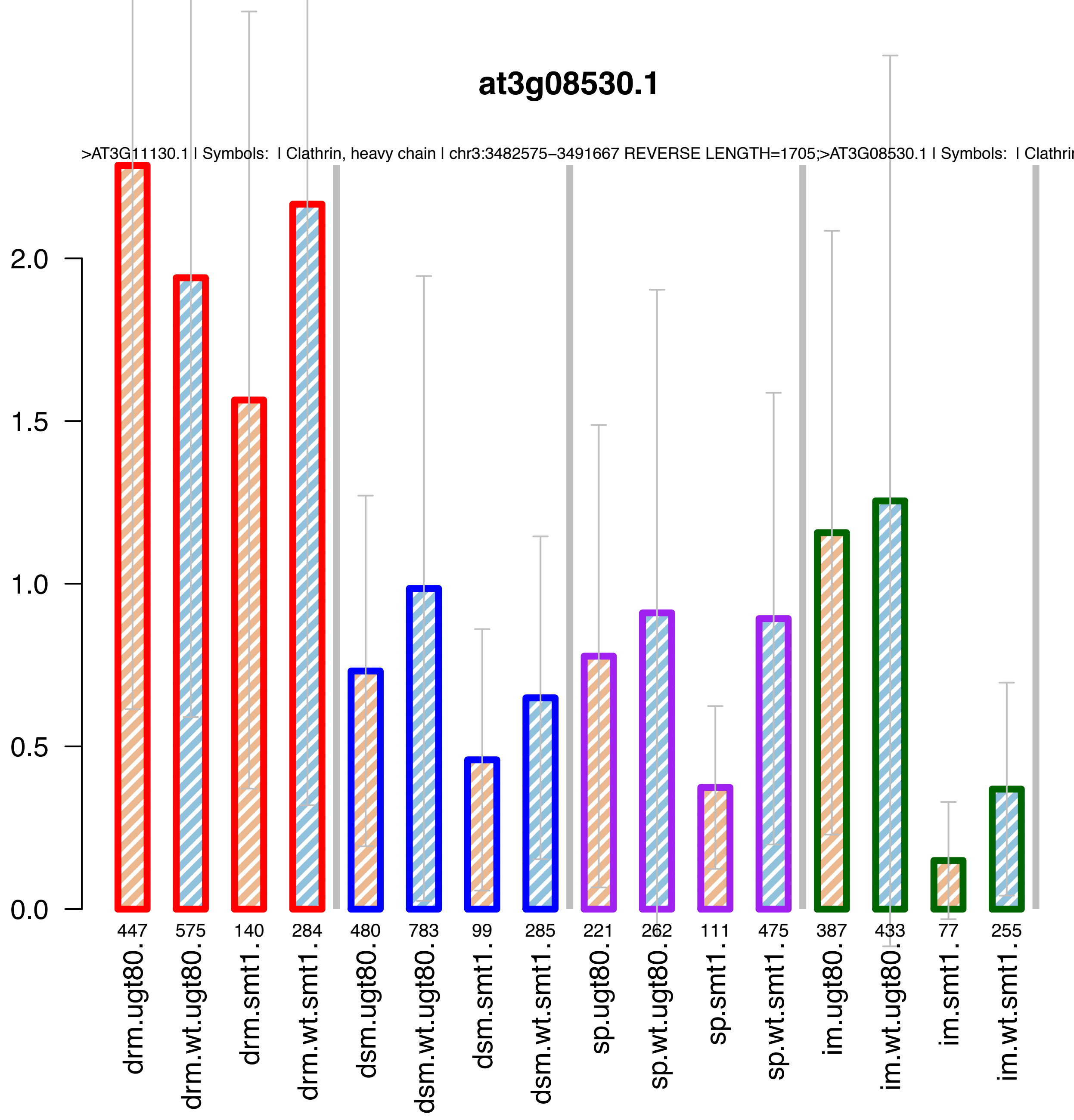
at3g08530.1
>AT3G11130.1 | Symbols: | Clathrin, heavy chain | chr3:3482575–3491667 REVERSE LENGTH=1705;>AT3G08530.1 | Symbols: | Clathri
2.0
1.5
1.0
0.5
0.0
447
575
140
284
480
783
99
285
221
262
111
475
387
433
77
255
drm.ugt80..
drm.wt.ugt80..
drm.smt1..
drm.wt.smt1..
dsm.ugt80..
dsm.wt.ugt80..
dsm.smt1..
dsm.wt.smt1..
sp.ugt80..
sp.wt.ugt80..
sp.smt1..
sp.wt.smt1..
im.ugt80..
im.wt.ugt80..
im.smt1..
im.wt.smt1..

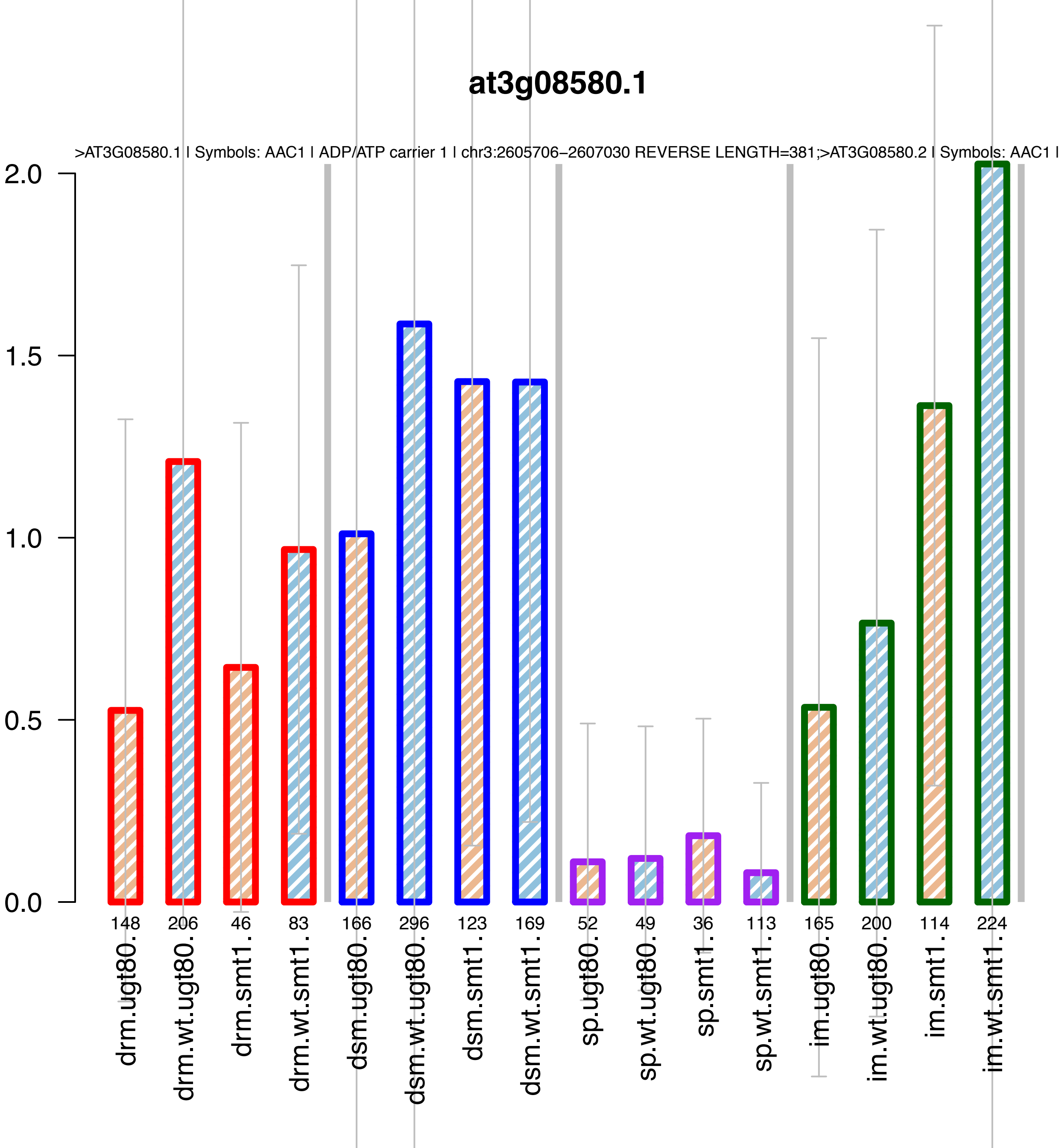

at3g08580.1
>AT3G08580.1 | Symbols: AAC1 | ADP/ATP carrier 1 | chr3:2605706-2607030 REVERSE LENGTH=381;>AT3G08580.2 | Symbols: AAC1 |
2.0
1.5
1.0
0.5
0.0
148
206
46
83
166
296
123
169
52
49
36
113
165
200
114
224
drm.ugt80..
drm.wt.ugt80..
drm.smt1..
drm.wt.smt1..
dsm.ugt80..
dsm.wt.ugt80..
dsm.smt1..
dsm.wt.smt1..
sp.ugt80..
sp.wt.ugt80..
sp.smt1..
sp.wt.smt1..
im.ugt80..
im.wt.ugt80..
im.smt1..
im.wt.smt1..

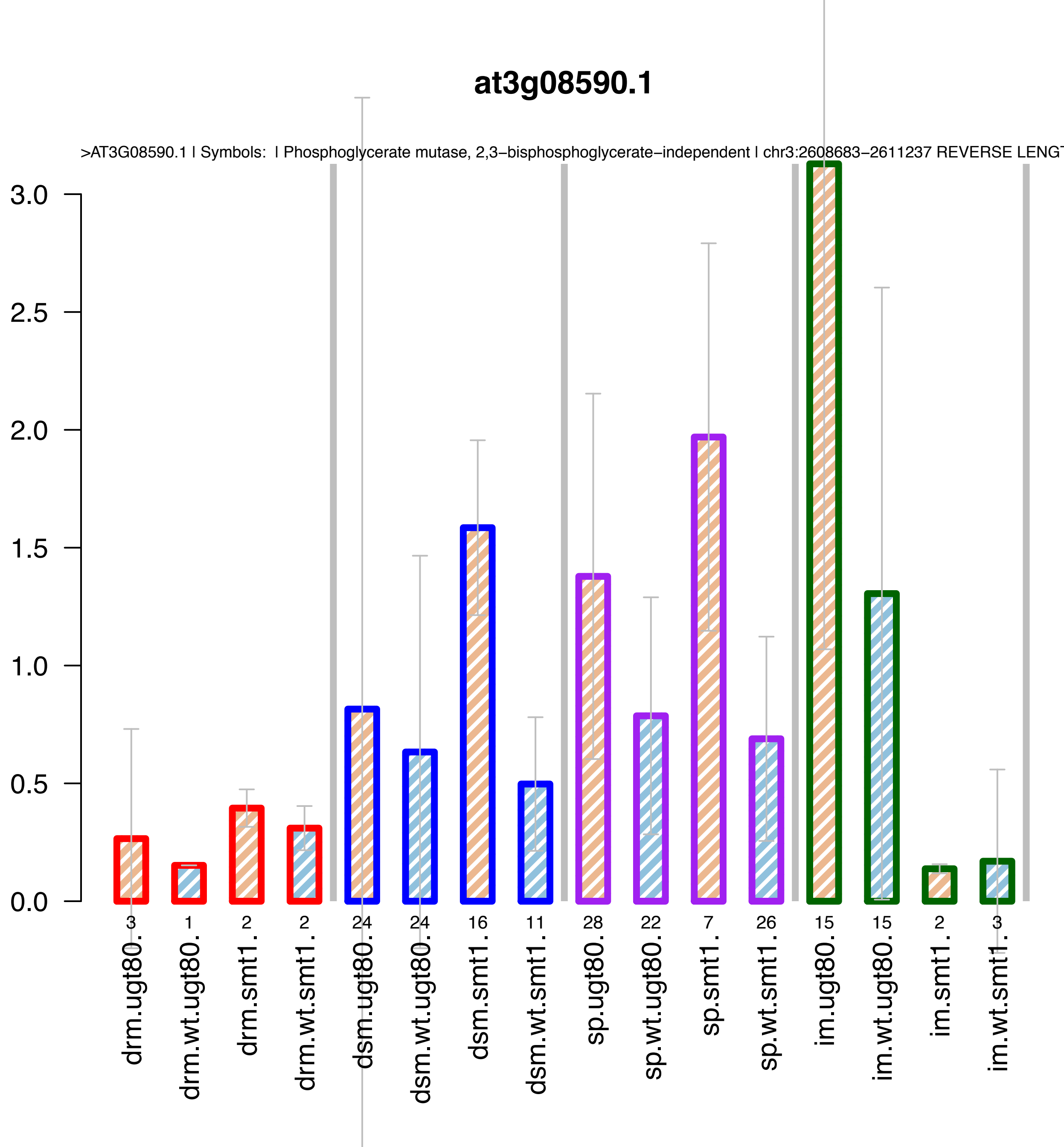
at3g08590.1
>AT3G08590.1 | Symbols: | Phosphoglycerate mutase, 2,3-bisphosphoglycerate-independent | chr3:2608683-2611237 REVERSE LENGT
3.0
2.5
2.0
1.5
1.0
0.5
0.0
3
1
2
2
24
24
16
11
28
22
7
26
15
15
2
3
drm.ugt80..
drm.wt.ugt80..
drm.smt1..
drm.wt.smt1..
dsm.ugt80..
dsm.wt.ugt80..
dsm.smt1..
dsm.wt.smt1..
sp.ugt80..
sp.wt.ugt80..
sp.smt1..
sp.wt.smt1..
im.ugt80..
im.wt.ugt80..
im.smt1..
im.wt.smt1..

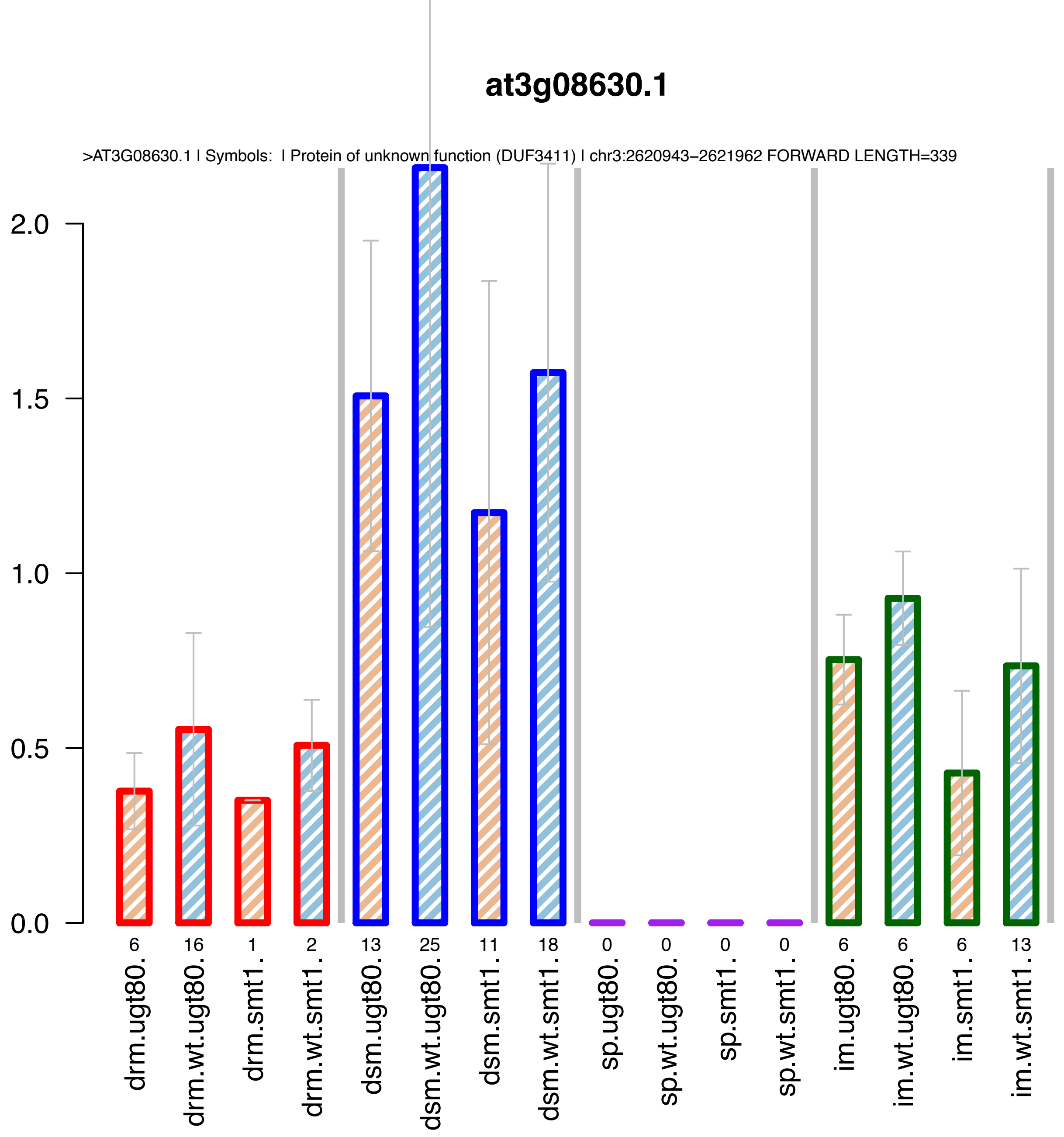

at3g08630.1
>AT3G08630.1 | Symbols: | Protein of unknown function (DUF3411) | chr3:2620943-2621962 FORWARD LENGTH=339
2.0
1.5
1.0
0.5
0.0
6
16
1
2
13
25
11
18
0
0
0
0
6
6
6
13
drm.ugt80.
drm.wt.ugt80.
drm.smt1.
drm.wt.smt1.
dsm.ugt80.
dsm.wt.ugt80.
dsm.smt1.
dsm.wt.smt1.
sp.ugt80.
sp.wt.ugt80.
sp.smt1.
sp.wt.smt1.
im.ugt80.
im.wt.ugt80.
im.smt1.
im.wt.smt1.

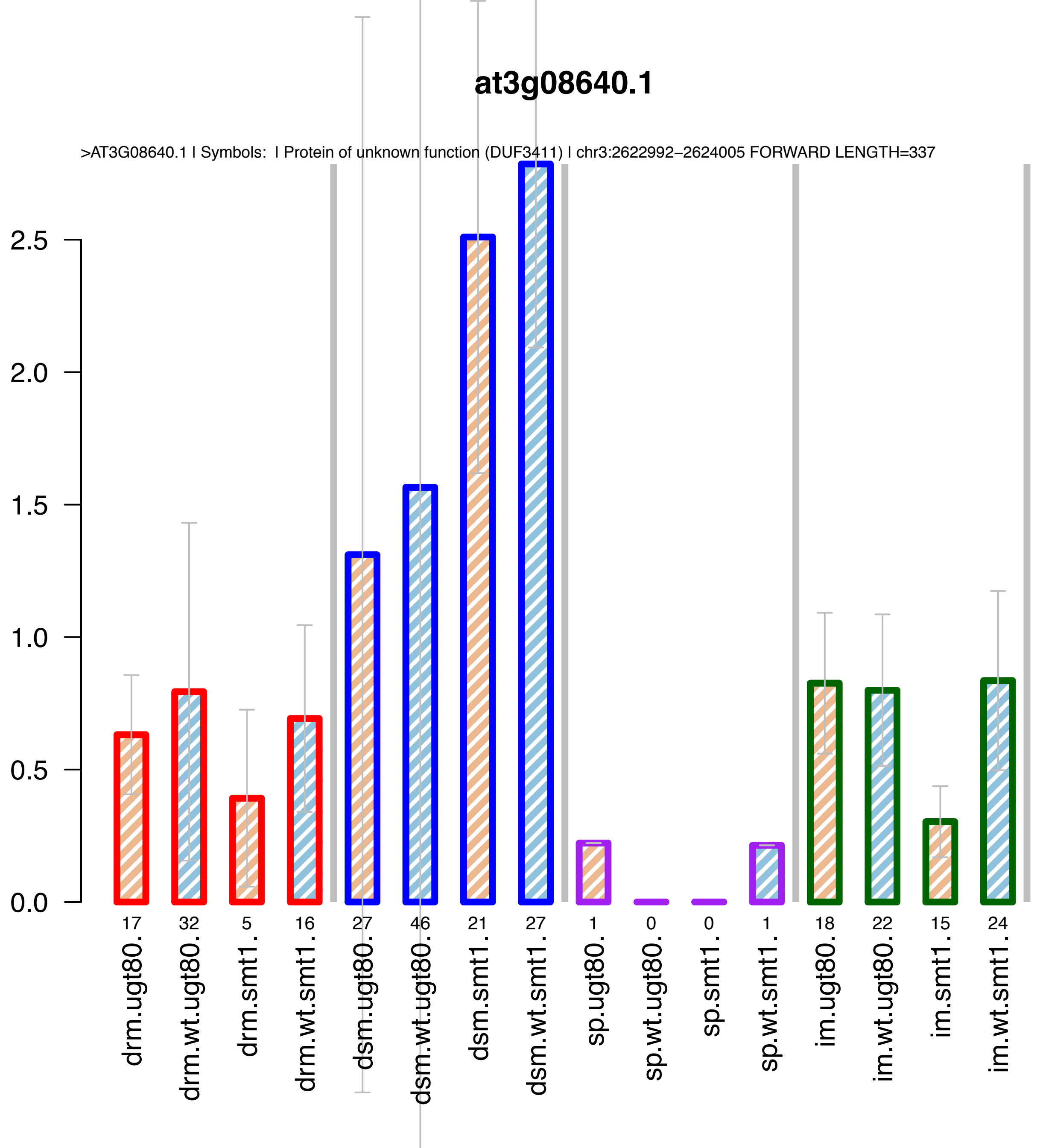

at3g08640.1
>AT3G08640.1 | Symbols: | Protein of unknown function (DUF3411) | chr3:2622992-2624005 FORWARD LENGTH=337
0.0
0.5
1.0
1.5
2.0
2.5
17
32
5
16
27
46
21
27
1
0
0
1
18
22
15
24
drm.ugt80.
drm.wt.ugt80.
drm.smt1.
drm.wt.smt1.
dsm.ugt80.
dsm.wt.ugt80.
dsm.smt1.
dsm.wt.smt1.
sp.ugt80.
sp.wt.ugt80.
sp.smt1.
sp.wt.smt1.
im.ugt80.
im.wt.ugt80.
im.smt1.
im.wt.smt1.

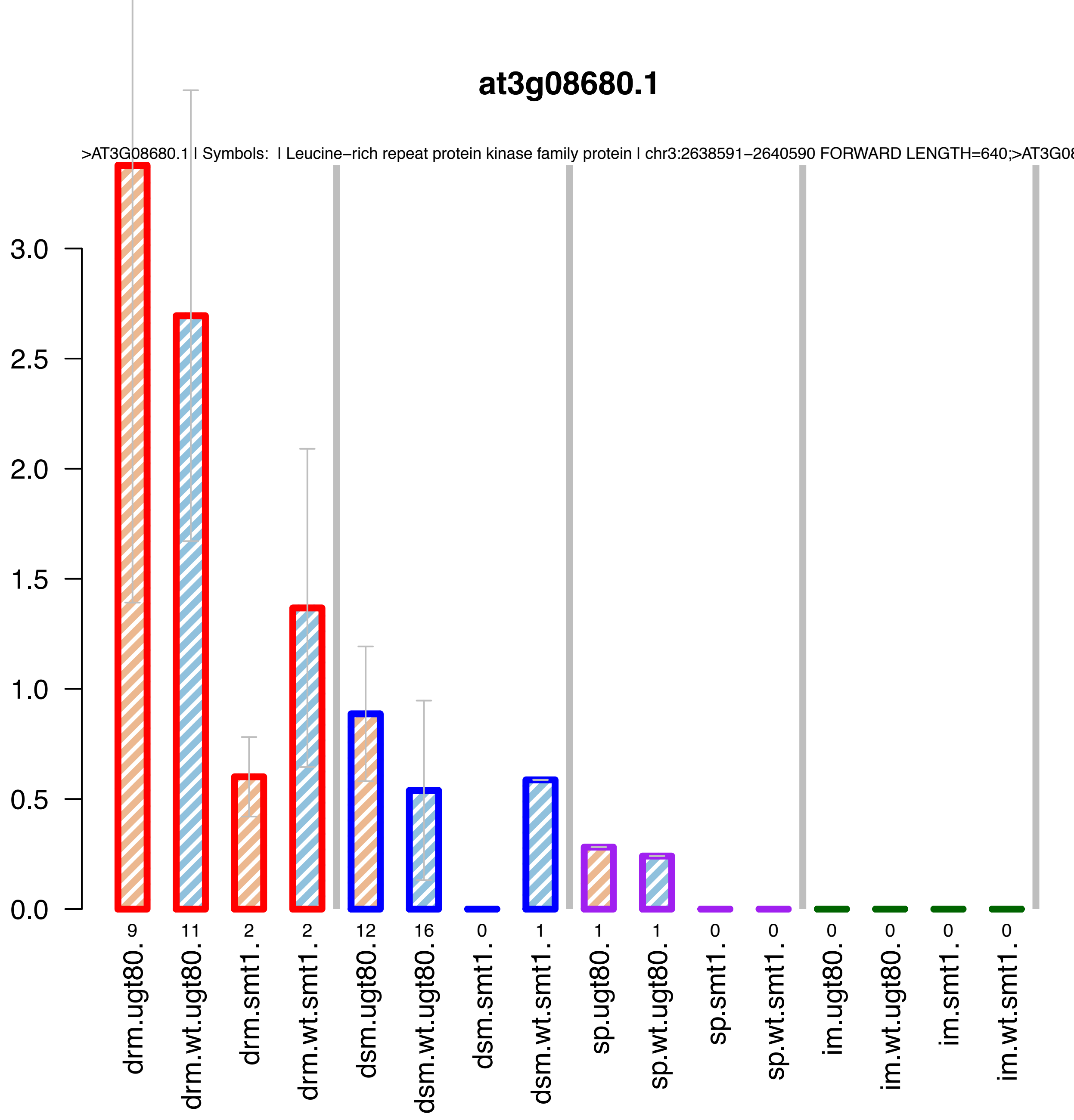

at3g08680.1
>AT3G08680.1 | Symbols: | Leucine−rich repeat protein kinase family protein | chr3:2638591−2640590 FORWARD LENGTH=640;>AT3G08
3.0
2.5
2.0
1.5
1.0
0.5
0.0
9
11
2
2
12
16
0
1
1
1
0
0
0
0
0
0
drm.ugt80.
drm.wt.ugt80.
drm.smt1.
drm.wt.smt1.
dsm.ugt80.
dsm.wt.ugt80.
dsm.smt1.
dsm.wt.smt1.
sp.ugt80.
sp.wt.ugt80.
sp.smt1.
sp.wt.smt1.
im.ugt80.
im.wt.ugt80.
im.smt1.
im.wt.smt1.

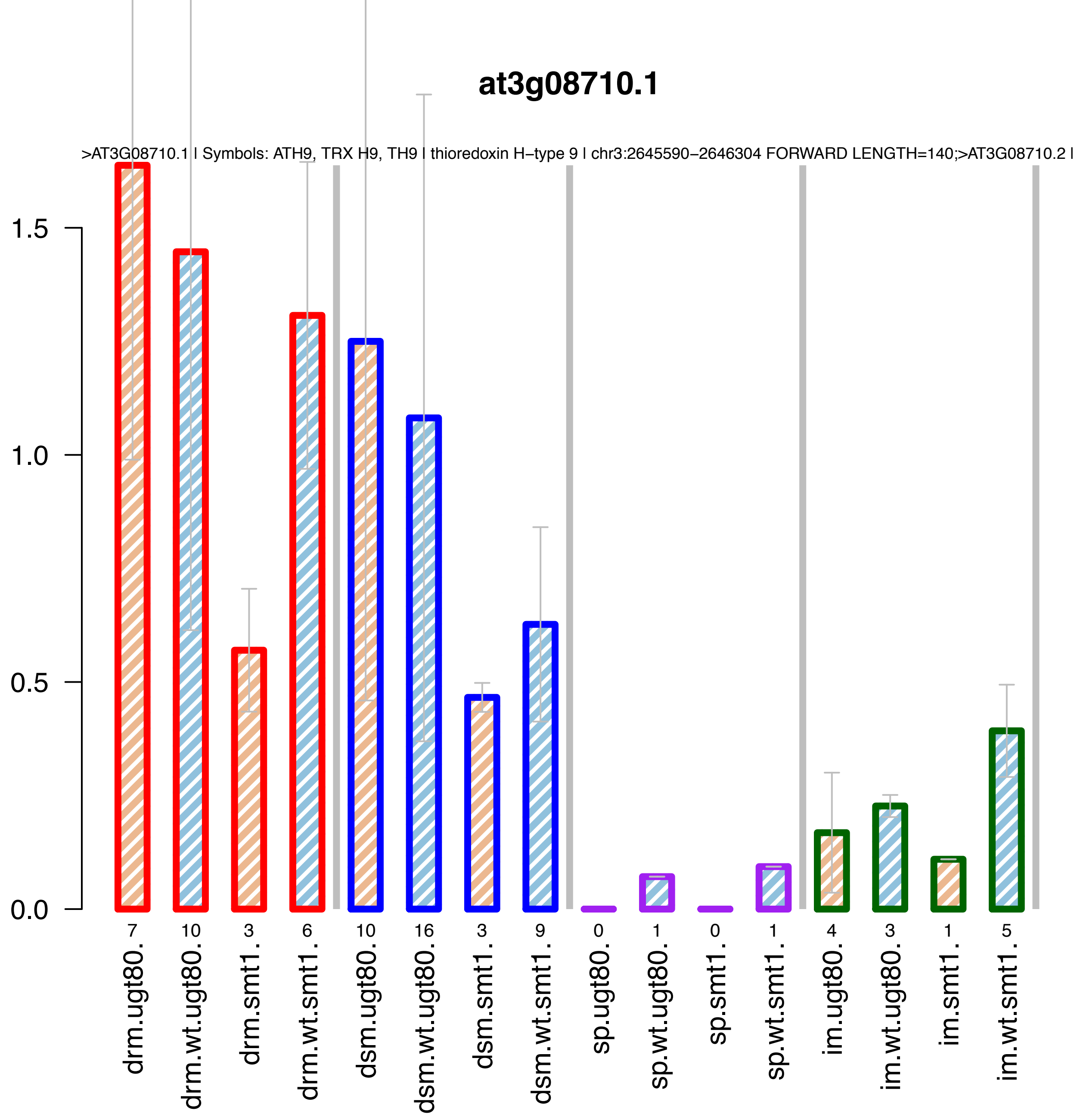

at3g08710.1
>AT3G08710.1 | Symbols: ATH9, TRX H9, TH9 | thioredoxin H-type 9 | chr3:2645590-2646304 FORWARD LENGTH=140;>AT3G08710.2 |
1.5
1.0
0.5
0.0
7
10
3
6
10
16
3
9
0
1
0
1
4
3
1
5
drm.ugt80.
drm.wt.ugt80.
drm.smt1.
drm.wt.smt1.
dsm.ugt80.
dsm.wt.ugt80.
dsm.smt1.
dsm.wt.smt1.
sp.ugt80.
sp.wt.ugt80.
sp.smt1.
sp.wt.smt1.
im.ugt80.
im.wt.ugt80.
im.smt1.
im.wt.smt1.

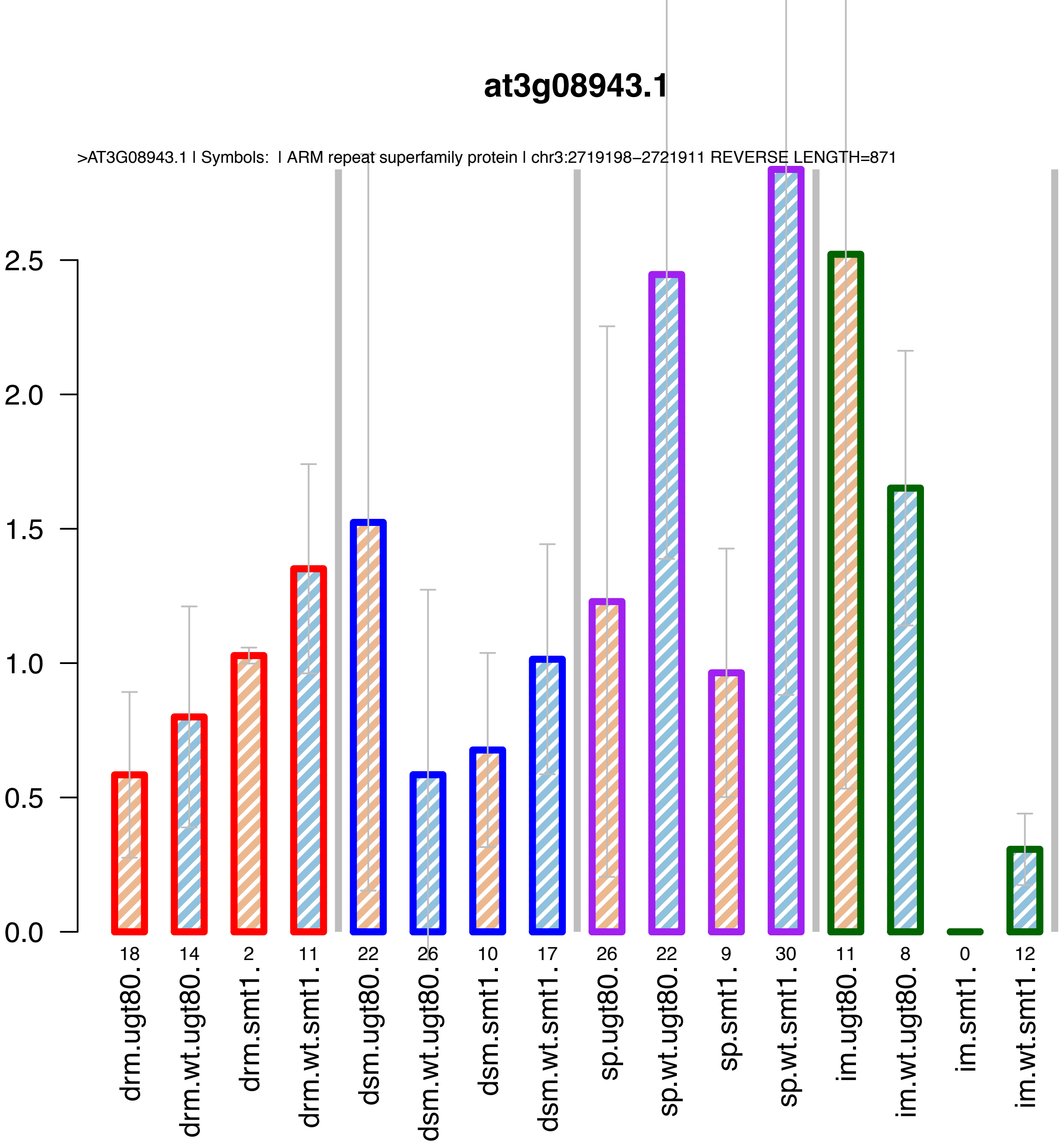
at3g08943.1
>AT3G08943.1 | Symbols: | ARM repeat superfamily protein | chr3:2719198−2721911 REVERSE LENGTH=871
0.0
0.5
1.0
1.5
2.0
2.5
18
14
2
11
22
26
10
17
26
22
9
30
11
8
0
12
drm.ugt80..
drm.wt.ugt80..
drm.smt1..
drm.wt.smt1..
dsm.ugt80..
dsm.wt.ugt80..
dsm.smt1..
dsm.wt.smt1..
sp.ugt80..
sp.wt.ugt80..
sp.smt1..
sp.wt.smt1..
im.ugt80..
im.wt.ugt80..
im.smt1..
im.wt.smt1..

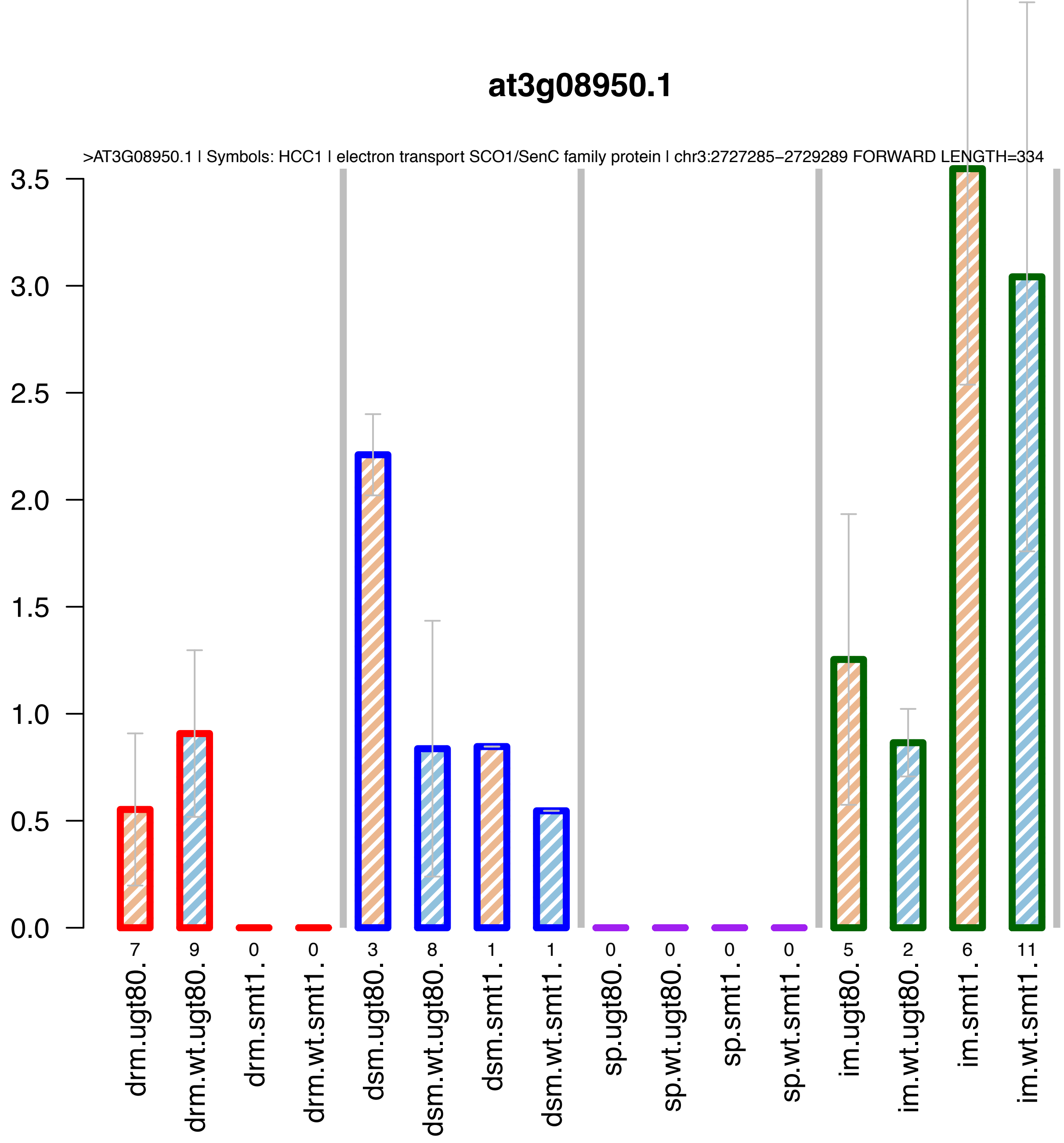

at3g08950.1
>AT3G08950.1 | Symbols: HCC1 | electron transport SCO1/SenC family protein | chr3:2727285-2729289 FORWARD LENGTH=334
3.5
3.0
2.5
2.0
1.5
1.0
0.5
0.0
7
9
0
0
3
8
1
1
0
0
0
0
5
2
6
11
drm.ugt80.
drm.wt.ugt80.
drm.smt1.
drm.wt.smt1.
dsm.ugt80.
dsm.wt.ugt80.
dsm.smt1.
dsm.wt.smt1.
sp.ugt80.
sp.wt.ugt80.
sp.smt1.
sp.wt.smt1.
im.ugt80.
im.wt.ugt80.
im.smt1.
im.wt.smt1.

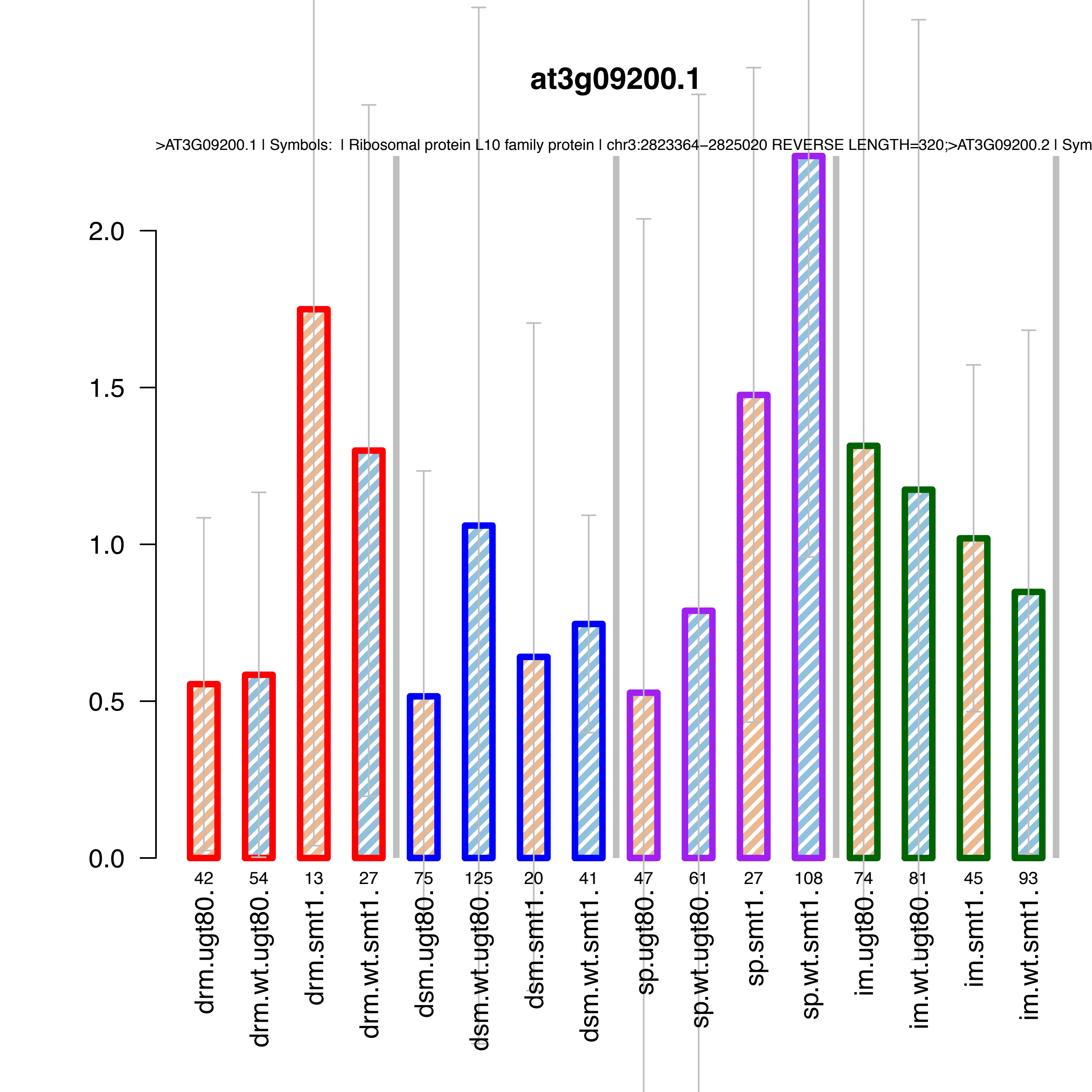

at3g09200.1
>AT3G09200.1 | Symbols: | Ribosomal protein L10 family protein | chr3:2823364–2825020 REVERSE LENGTH=320;>AT3G09200.2 | Sym
2.0
1.5
1.0
0.5
0.0
42
54
13
27
75
125
20
41
47
61
27
108
74
81
45
93
drm.ugt80..
drm.wt.ugt80..
drm.smt1..
drm.wt.smt1..
dsm.ugt80..
dsm.wt.ugt80..
dsm.smt1..
dsm.wt.smt1..
sp.ugt80..
sp.wt.ugt80..
sp.smt1..
sp.wt.smt1..
im.ugt80..
im.wt.ugt80..
im.smt1..
im.wt.smt1..

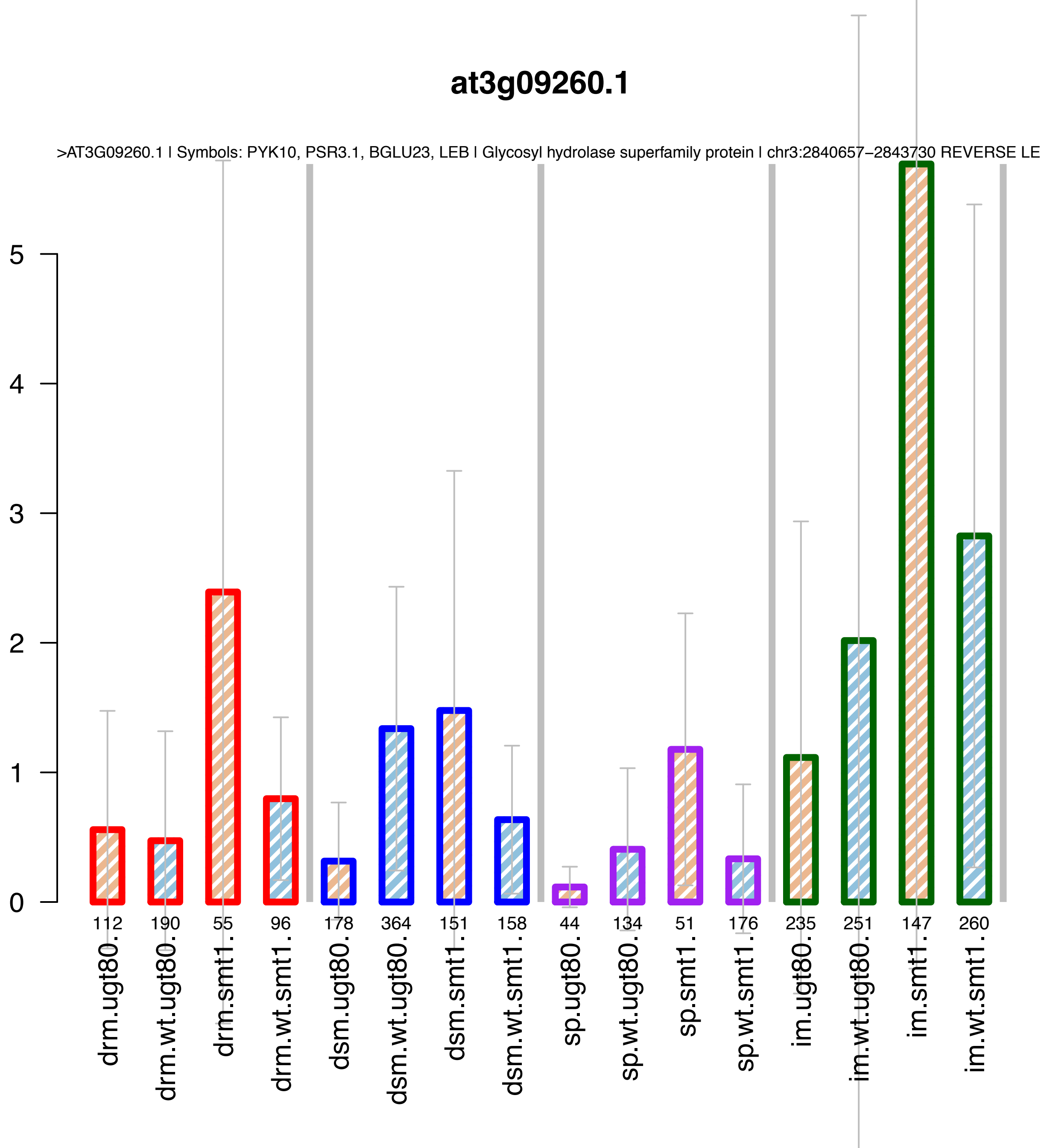
at3g09260.1
>AT3G09260.1 | Symbols: PYK10, PSR3.1, BGLU23, LEB | Glycosyl hydrolase superfamily protein | chr3:2840657−2843730 REVERSE LE
0
1
2
3
4
5
112
190
55
96
178
364
151
158
44
134
51
176
235
251
147
260
drm.ugt80..
drm.wt.ugt80..
drm.smt1..
drm.wt.smt1..
dsm.ugt80..
dsm.wt.ugt80..
dsm.smt1..
dsm.wt.smt1..
sp.ugt80..
sp.wt.ugt80..
sp.smt1..
sp.wt.smt1..
im.ugt80..
im.wt.ugt80..
im.smt1..
im.wt.smt1..

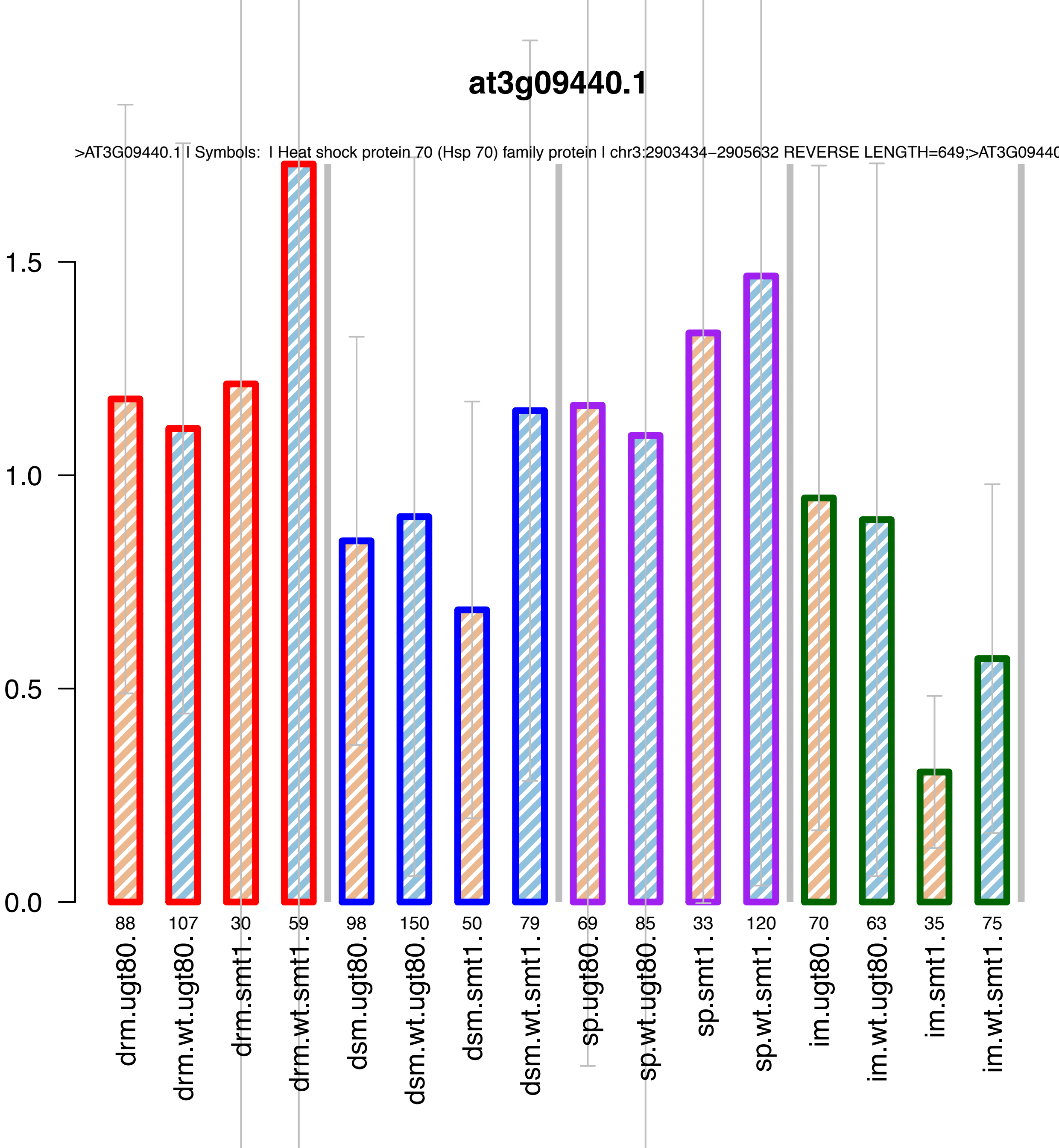
at3g09440.1
>AT3G09440.1 | Symbols: | Heat shock protein 70 (Hsp 70) family protein | chr3:2903434-2905632 REVERSE LENGTH=649;>AT3G09440
1.5
1.0
0.5
0.0
88
107
30
59
98
150
50
79
69
85
33
120
70
63
35
75
drm.ugt80..
drm.wt.ugt80..
drm.smt1..
drm.wt.smt1..
dsm.ugt80..
dsm.wt.ugt80..
dsm.smt1..
dsm.wt.smt1..
sp.ugt80..
sp.wt.ugt80..
sp.smt1..
sp.wt.smt1..
im.ugt80..
im.wt.ugt80..
im.smt1..
im.wt.smt1..

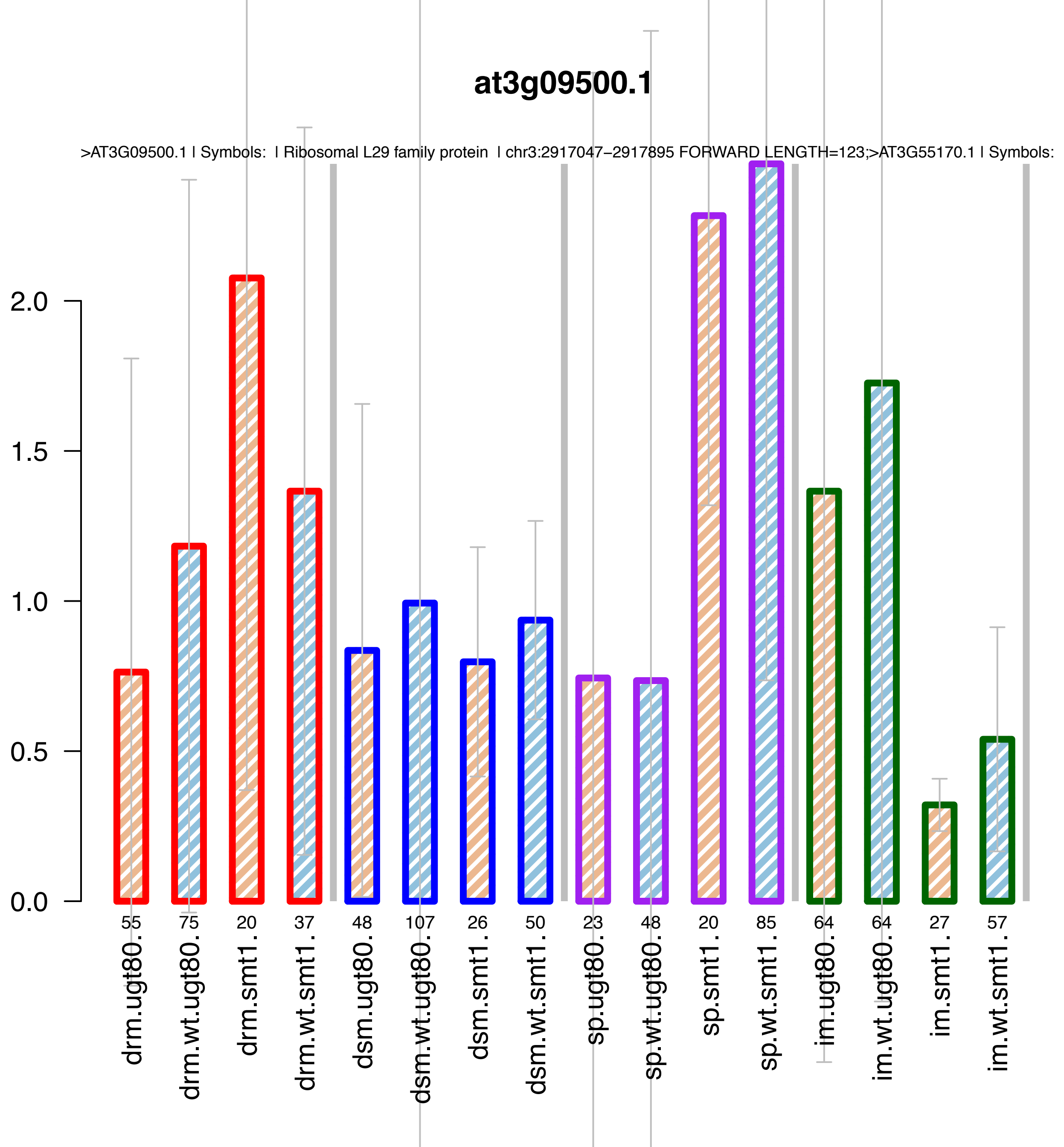

at3g09500.1
>AT3G09500.1 | Symbols: | Ribosomal L29 family protein | chr3:2917047-2917895 FORWARD LENGTH=123;>AT3G55170.1 | Symbols:
0.0
0.5
1.0
1.5
2.0
55
75
20
37
48
107
26
50
23
48
20
85
64
64
27
57
drm.ugt80..
drm.wt.ugt80..
drm.smt1..
drm.wt.smt1..
dsm.ugt80..
dsm.wt.ugt80..
dsm.smt1..
dsm.wt.smt1..
sp.ugt80..
sp.wt.ugt80..
sp.smt1..
sp.wt.smt1..
im.ugt80..
im.wt.ugt80..
im.smt1..
im.wt.smt1..

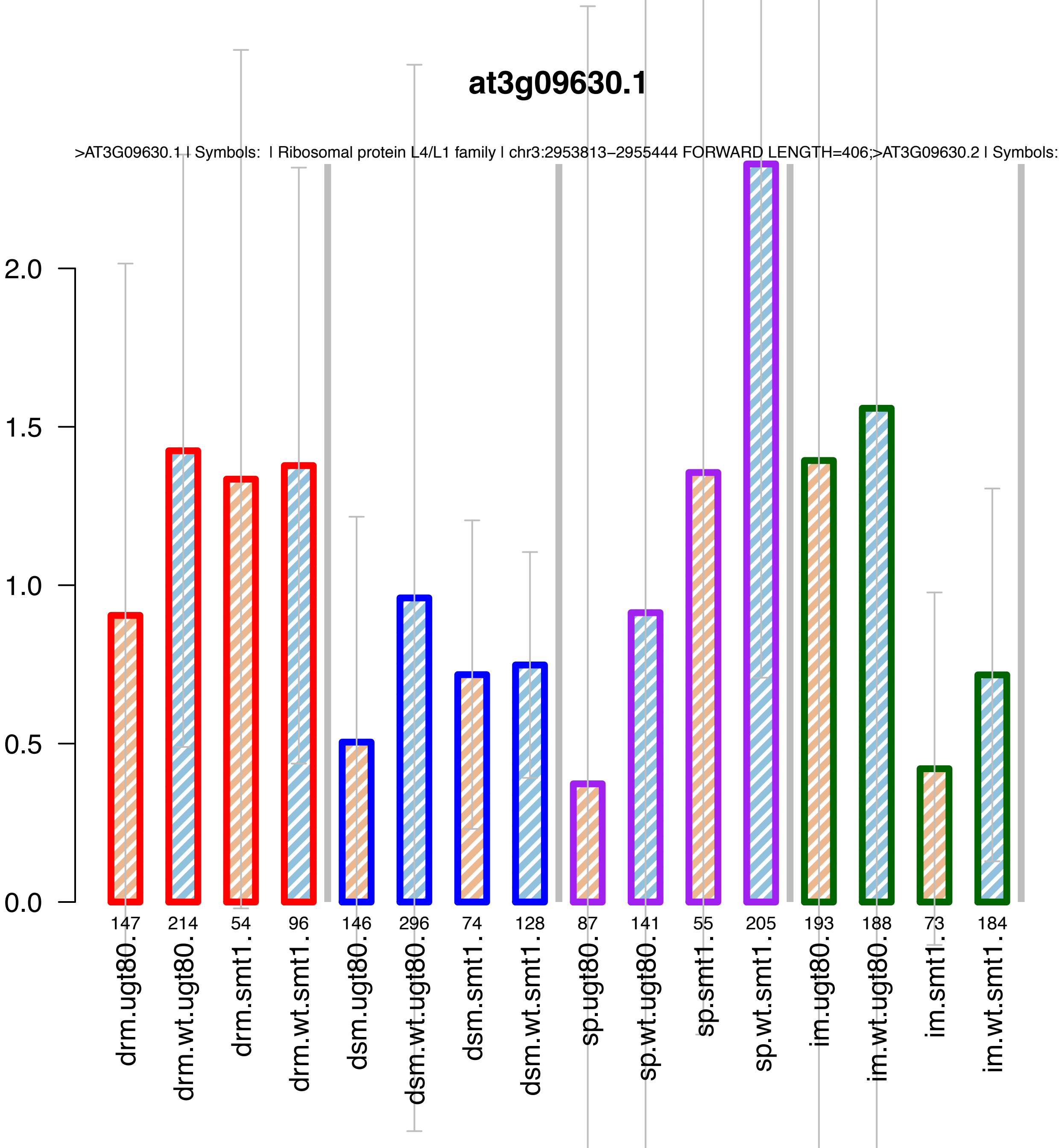
at3g09630.1
>AT3G09630.1 | Symbols: | Ribosomal protein L4/L1 family | chr3:2953813-2955444 FORWARD LENGTH=406;>AT3G09630.2 | Symbols:
0.0
0.5
1.0
1.5
2.0
147
214
54
96
146
296
74
128
87
141
55
205
193
188
73
184
drm.ugt80..
drm.wt.ugt80..
drm.smt1..
drm.wt.smt1..
dsm.ugt80..
dsm.wt.ugt80..
dsm.smt1..
dsm.wt.smt1..
sp.ugt80..
sp.wt.ugt80..
sp.smt1..
sp.wt.smt1..
im.ugt80..
im.wt.ugt80..
im.smt1..
im.wt.smt1..

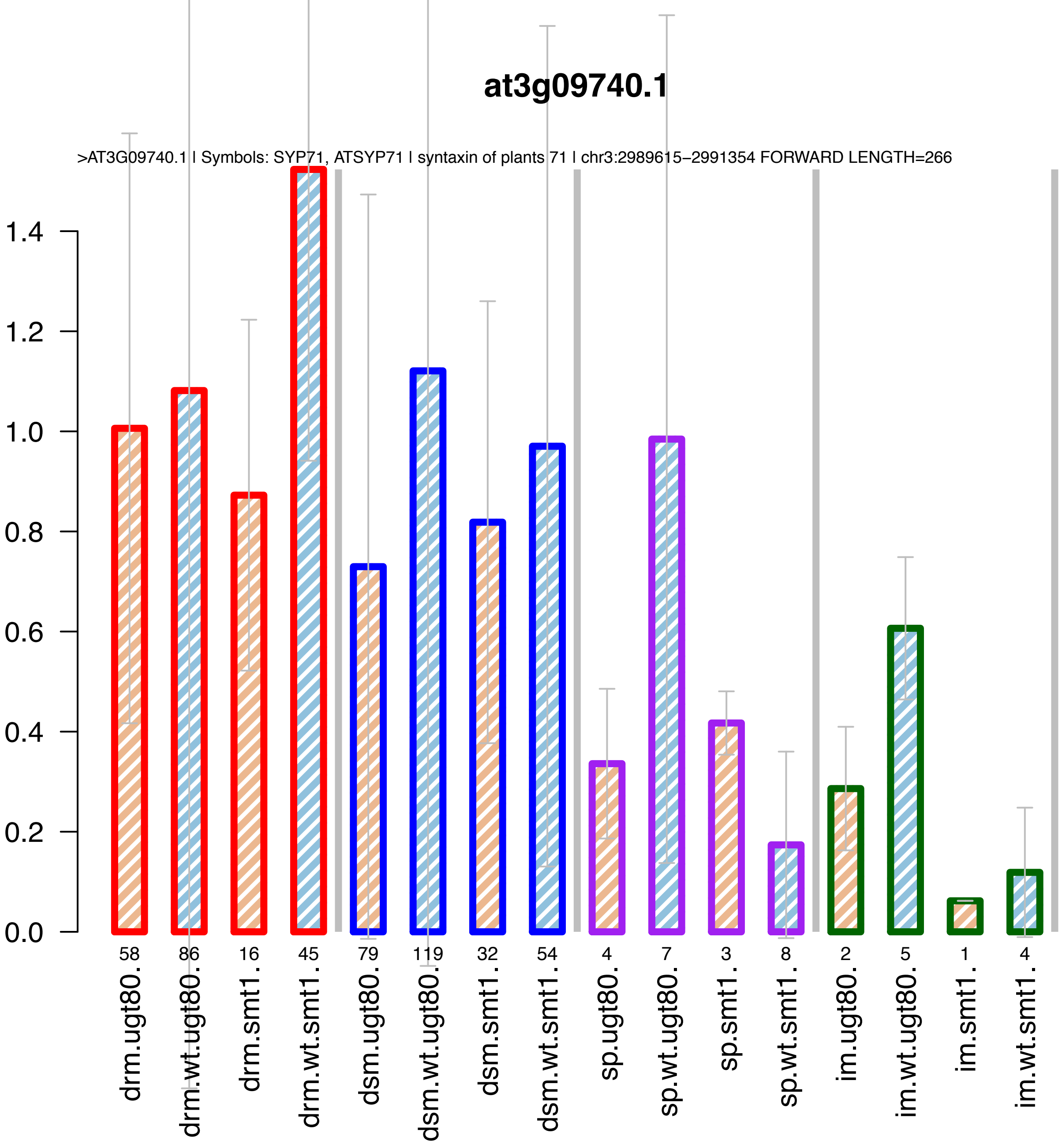
at3g09740.1
>AT3G09740.1 | Symbols: SYP71, ATSYP71 | syntaxin of plants 71 | chr3:2989615-2991354 FORWARD LENGTH=266
0.0
0.2
0.4
0.6
0.8
1.0
1.2
1.4
58
86
16
45
79
119
32
54
4
7
3
8
2
5
1
4
drm.ugt80..
drm.wt.ugt80..
drm.smt1..
drm.wt.smt1..
dsm.ugt80..
dsm.wt.ugt80..
dsm.smt1..
dsm.wt.smt1..
sp.ugt80..
sp.wt.ugt80..
sp.smt1..
sp.wt.smt1..
im.ugt80..
im.wt.ugt80..
im.smt1..
im.wt.smt1..

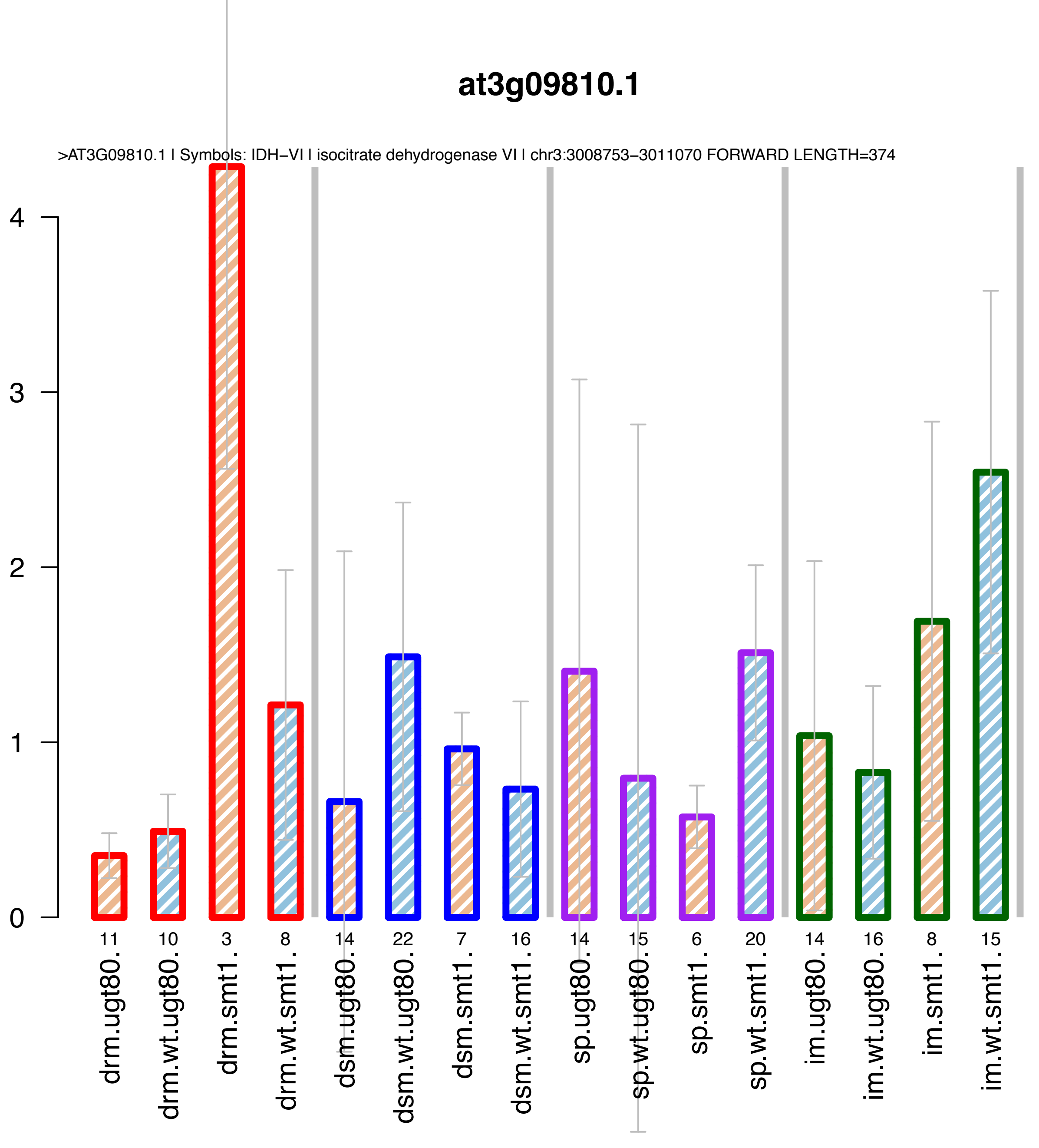

at3g09810.1
>AT3G09810.1 | Symbols: IDH-VI | isocitrate dehydrogenase VI | chr3:3008753-3011070 FORWARD LENGTH=374
0
1
2
3
4
11
10
3
8
14
22
7
16
14
15
6
20
14
16
8
15
drm.ugt80.
drm.wt.ugt80.
drm.smt1.
drm.wt.smt1.
dsm.ugt80.
dsm.wt.ugt80.
dsm.smt1.
dsm.wt.smt1.
sp.ugt80.
sp.wt.ugt80.
sp.smt1.
sp.wt.smt1.
im.ugt80.
im.wt.ugt80.
im.smt1.
im.wt.smt1.

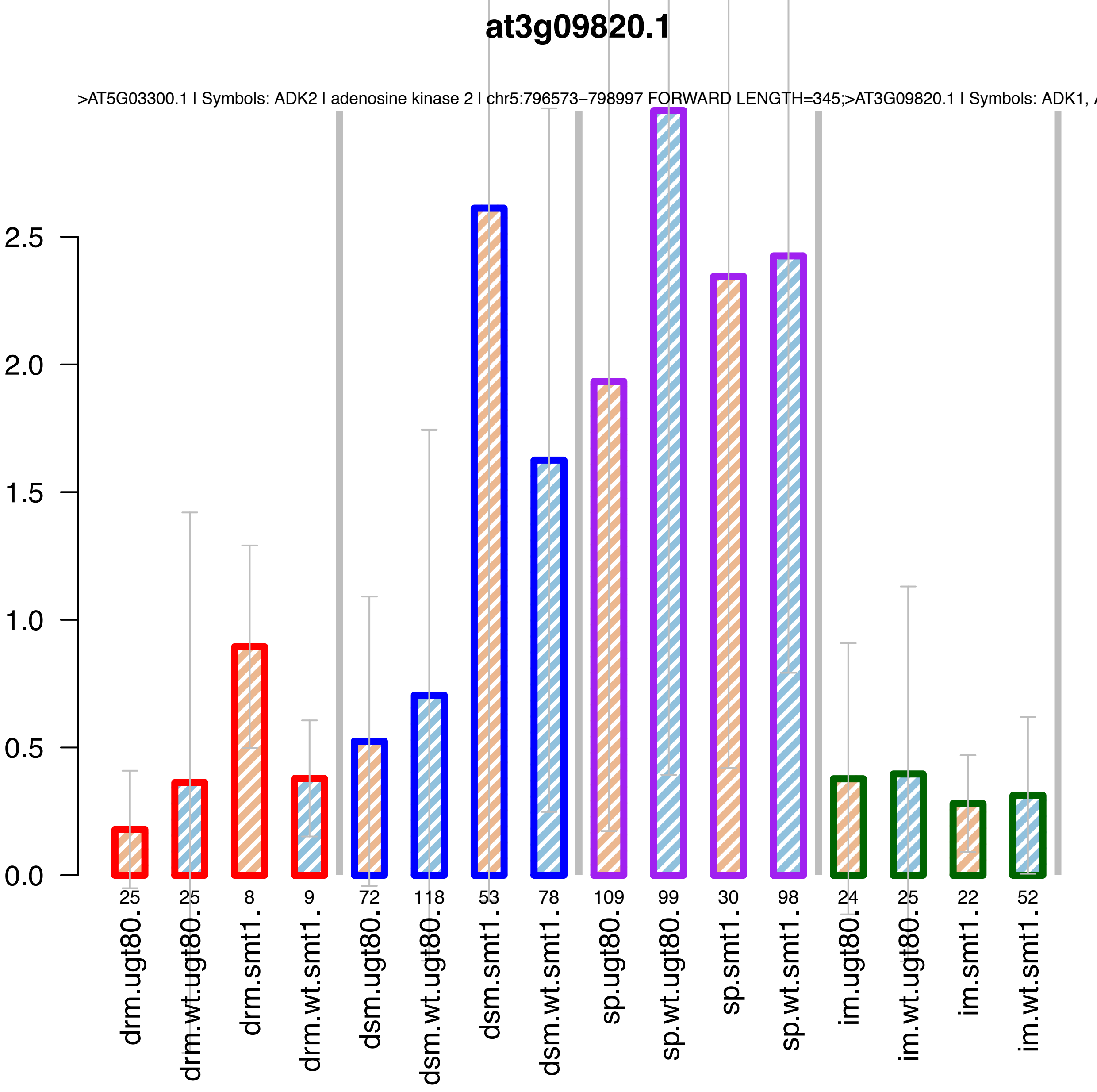

at3g09820.1
>AT5G03300.1 | Symbols: ADK2 | adenosine kinase 2 | chr5:796573−798997 FORWARD LENGTH=345;>AT3G09820.1 | Symbols: ADK1, A
0.0
0.5
1.0
1.5
2.0
2.5
25
25
8
9
72
118
53
78
109
99
30
98
24
25
22
52
drm.ugt80..
drm.wt.ugt80..
drm.smt1..
drm.wt.smt1..
dsm.ugt80..
dsm.wt.ugt80..
dsm.smt1..
dsm.wt.smt1..
sp.ugt80..
sp.wt.ugt80..
sp.smt1..
sp.wt.smt1..
im.ugt80..
im.wt.ugt80..
im.smt1..
im.wt.smt1..

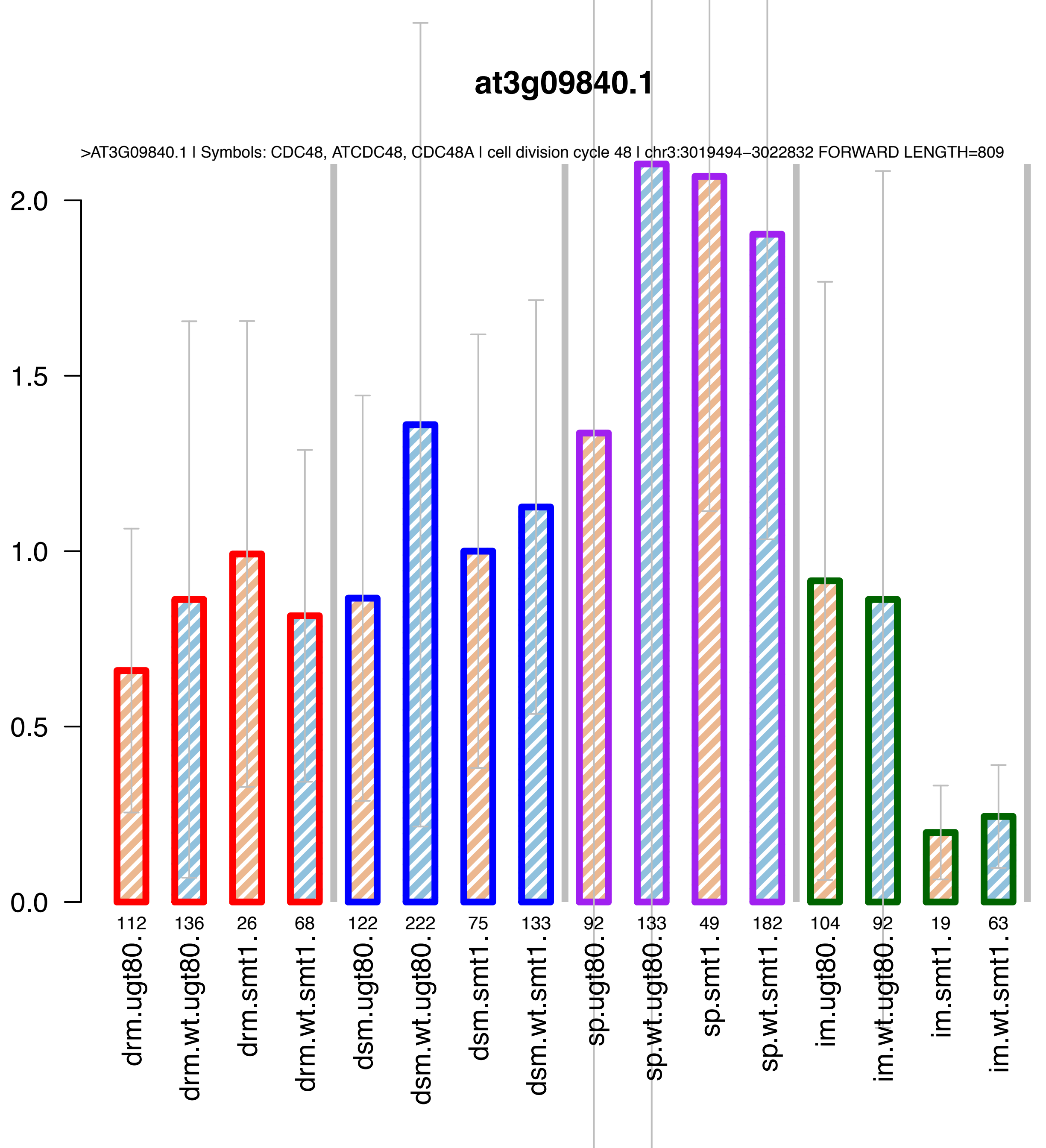

at3g09840.1
>AT3G09840.1 | Symbols: CDC48, ATCDC48, CDC48A | cell division cycle 48 | chr3:3019494–3022832 FORWARD LENGTH=809
2.0
1.5
1.0
0.5
0.0
112
136
26
68
122
222
75
133
92
133
49
182
104
92
19
63
drm.ugt80..
drm.wt.ugt80..
drm.smt1..
drm.wt.smt1..
dsm.ugt80..
dsm.wt.ugt80..
dsm.smt1..
dsm.wt.smt1..
sp.ugt80..
sp.wt.ugt80..
sp.smt1..
sp.wt.smt1..
im.ugt80..
im.wt.ugt80..
im.smt1..
im.wt.smt1..

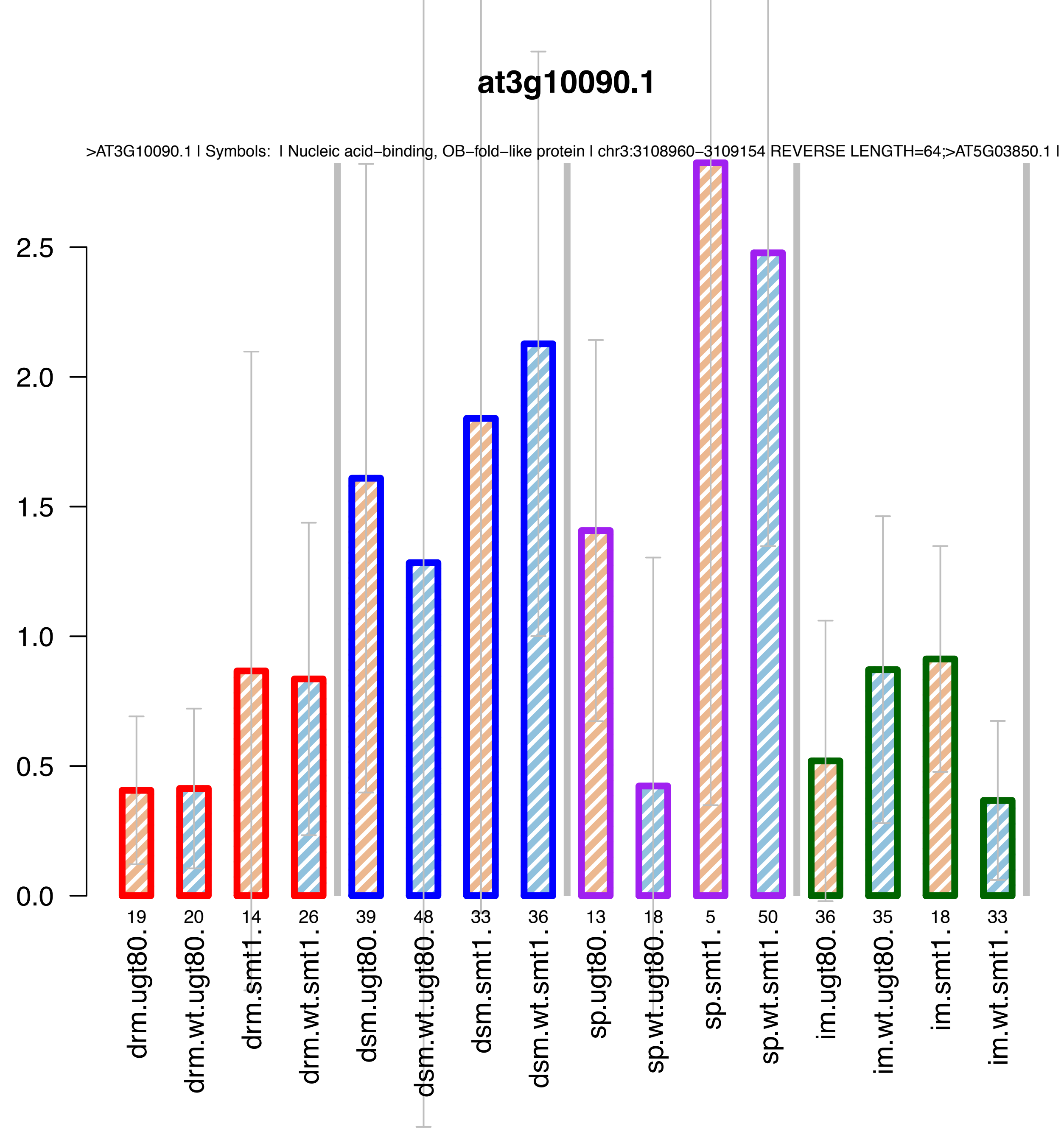
at3g10090.1
>AT3G10090.1 | Symbols: | Nucleic acid–binding, OB–fold–like protein | chr3:3108960–3109154 REVERSE LENGTH=64;>AT5G03850.1 |
0.0
0.5
1.0
1.5
2.0
2.5
19
20
14
26
39
48
33
36
13
18
5
50
36
35
18
33
drm.ugt80..
drm.wt.ugt80..
drm.smt1..
drm.wt.smt1..
dsm.ugt80..
dsm.wt.ugt80..
dsm.smt1..
dsm.wt.smt1..
sp.ugt80..
sp.wt.ugt80..
sp.smt1..
sp.wt.smt1..
im.ugt80..
im.wt.ugt80..
im.smt1..
im.wt.smt1..

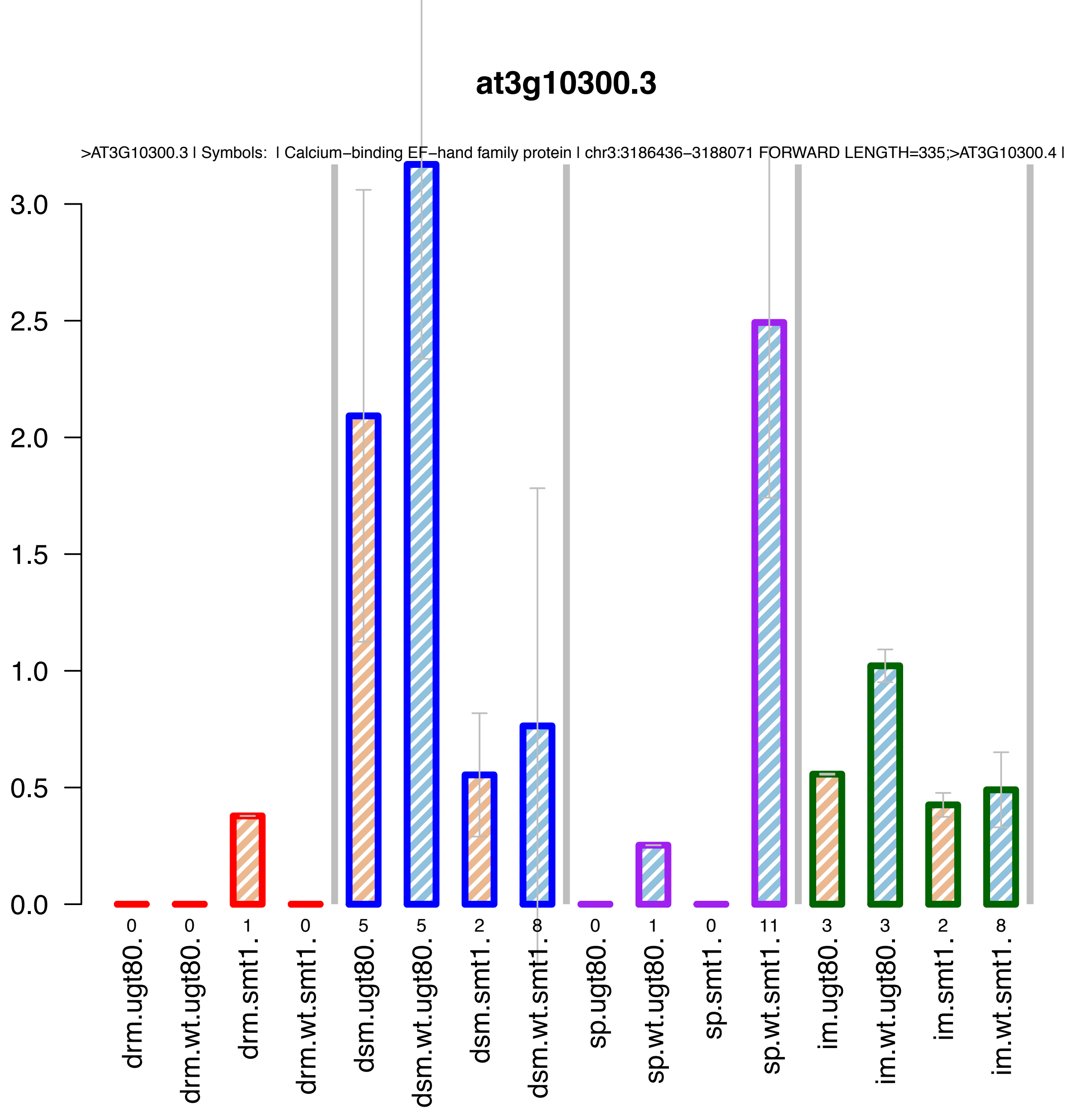

at3g10300.3
>AT3G10300.3 | Symbols: | Calcium−binding EF−hand family protein | chr3:3186436−3188071 FORWARD LENGTH=335;>AT3G10300.4 |
3.0
2.5
2.0
1.5
1.0
0.5
0.0
0
0
1
0
5
5
2
8
0
1
0
11
3
3
2
8
drm.ugt80.
drm.wt.ugt80.
drm.smt1.
drm.wt.smt1.
dsm.ugt80.
dsm.wt.ugt80.
dsm.smt1.
dsm.wt.smt1.
sp.ugt80.
sp.wt.ugt80.
sp.smt1.
sp.wt.smt1.
im.ugt80.
im.wt.ugt80.
im.smt1.
im.wt.smt1.

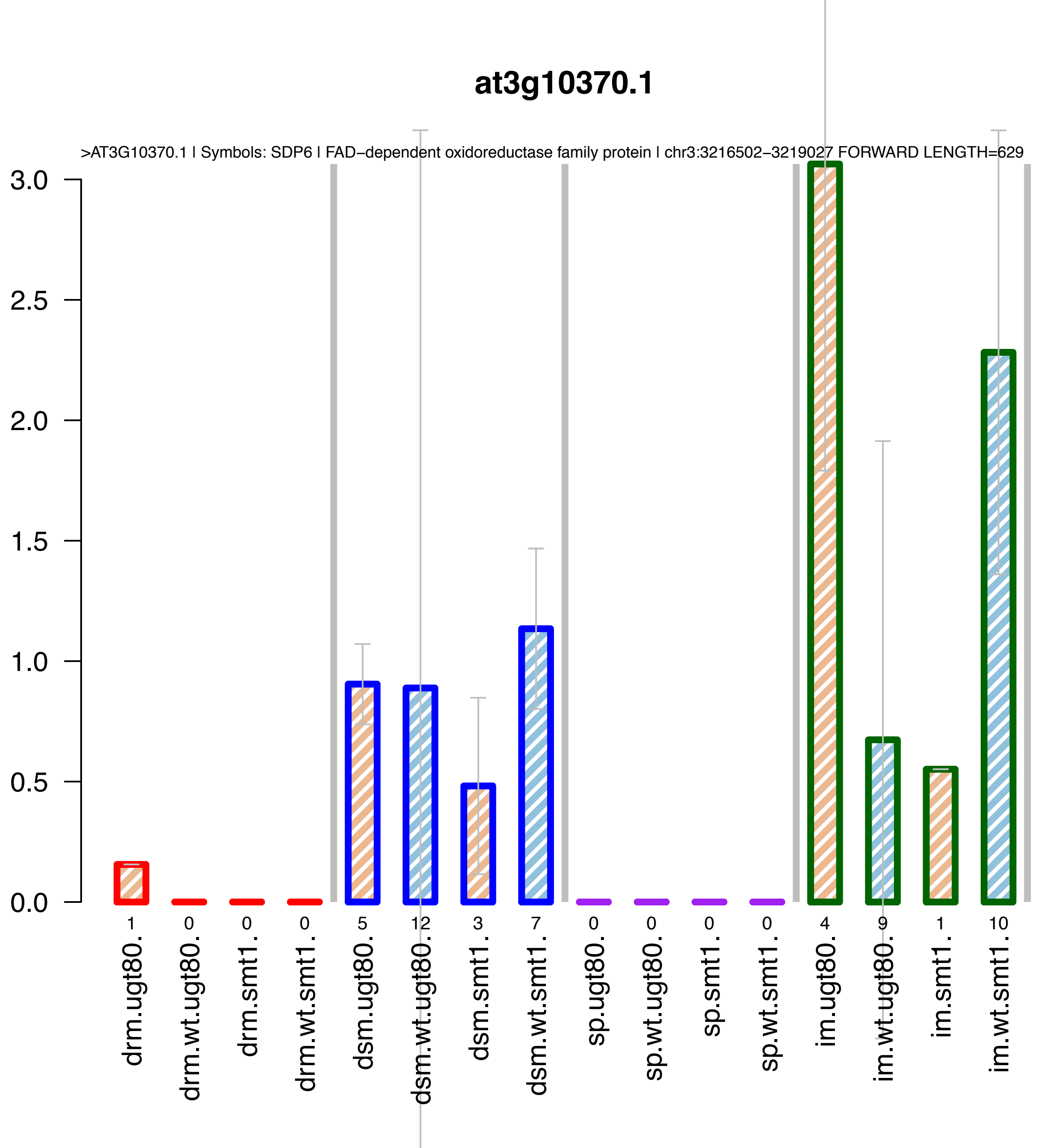

at3g10370.1
>AT3G10370.1 | Symbols: SDP6 | FAD−dependent oxidoreductase family protein | chr3:3216502−3219027 FORWARD LENGTH=629
0.0
0.5
1.0
1.5
2.0
2.5
3.0
1 0 0 0 5 12 3 7 0 0 0 0 4 9 1 10
drm.ugt80..
drm.wt.ugt80..
drm.smt1..
drm.wt.smt1..
dsm.ugt80..
dsm.wt.ugt80..
dsm.smt1..
dsm.wt.smt1..
sp.ugt80..
sp.wt.ugt80..
sp.smt1..
sp.wt.smt1..
im.ugt80..
im.wt.ugt80..
im.smt1..
im.wt.smt1..

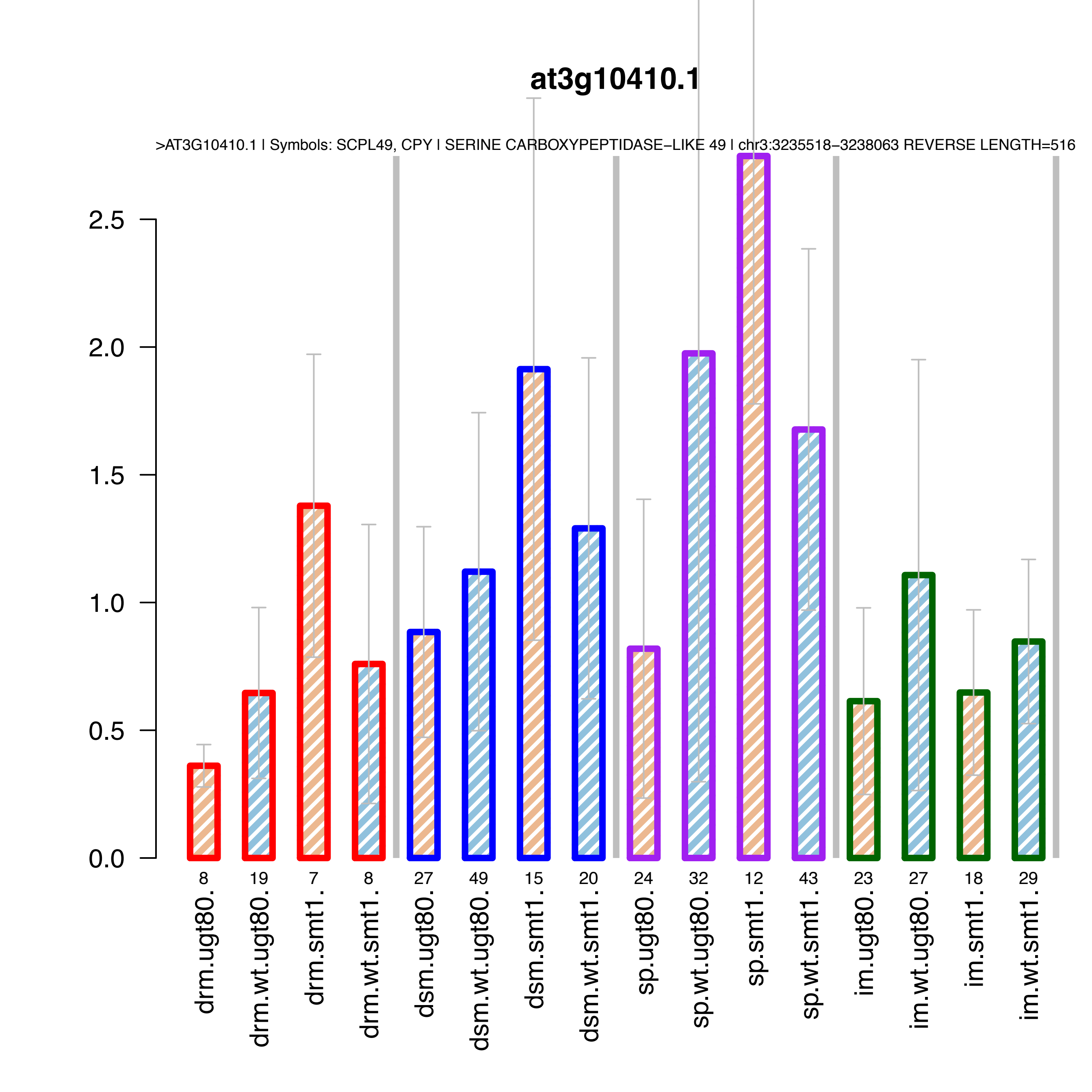

at3g10410.1
>AT3G10410.1 | Symbols: SCPL49, CPY | SERINE CARBOXYPEPTIDASE-LIKE 49 | chr3:3235518-3238063 REVERSE LENGTH=516
0.0
0.5
1.0
1.5
2.0
2.5
8
19
7
8
27
49
15
20
24
32
12
43
23
27
18
29
drm.ugt80..
drm.wt.ugt80..
drm.smt1..
drm.wt.smt1..
dsm.ugt80..
dsm.wt.ugt80..
dsm.smt1..
dsm.wt.smt1..
sp.ugt80..
sp.wt.ugt80..
sp.smt1..
sp.wt.smt1..
im.ugt80..
im.wt.ugt80..
im.smt1..
im.wt.smt1..

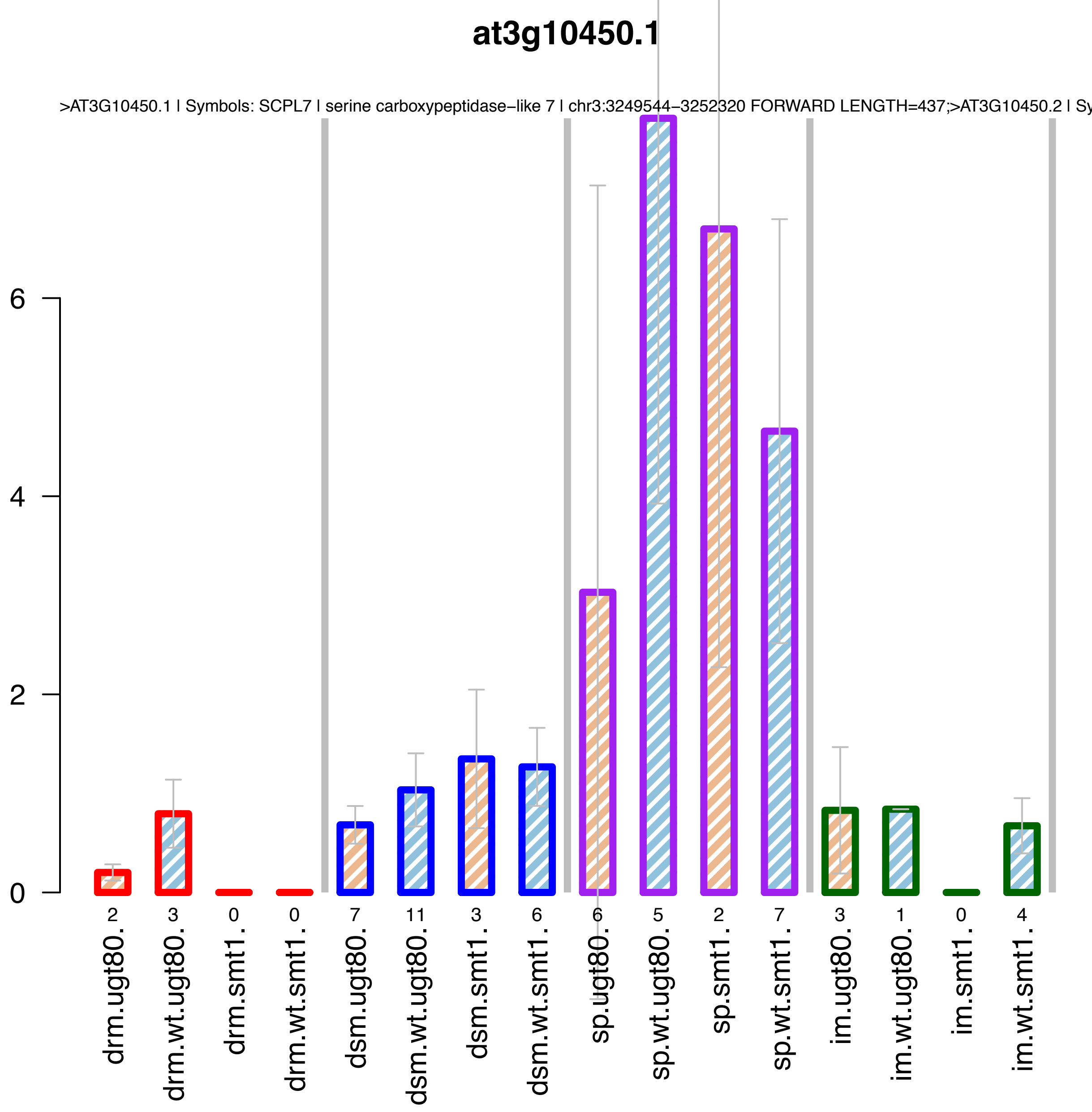

at3g10450.1
>AT3G10450.1 | Symbols: SCPL7 | serine carboxypeptidase–like 7 | chr3:3249544–3252320 FORWARD LENGTH=437;>AT3G10450.2 | Sy
0
2
4
6
2
3
0
0
7
11
3
6
6
5
2
7
3
1
0
4
drm.ugt80.
drm.wt.ugt80.
drm.smt1.
drm.wt.smt1.
dsm.ugt80.
dsm.wt.ugt80.
dsm.smt1.
dsm.wt.smt1.
sp.ugt80.
sp.wt.ugt80.
sp.smt1.
sp.wt.smt1.
im.ugt80.
im.wt.ugt80.
im.smt1.
im.wt.smt1.

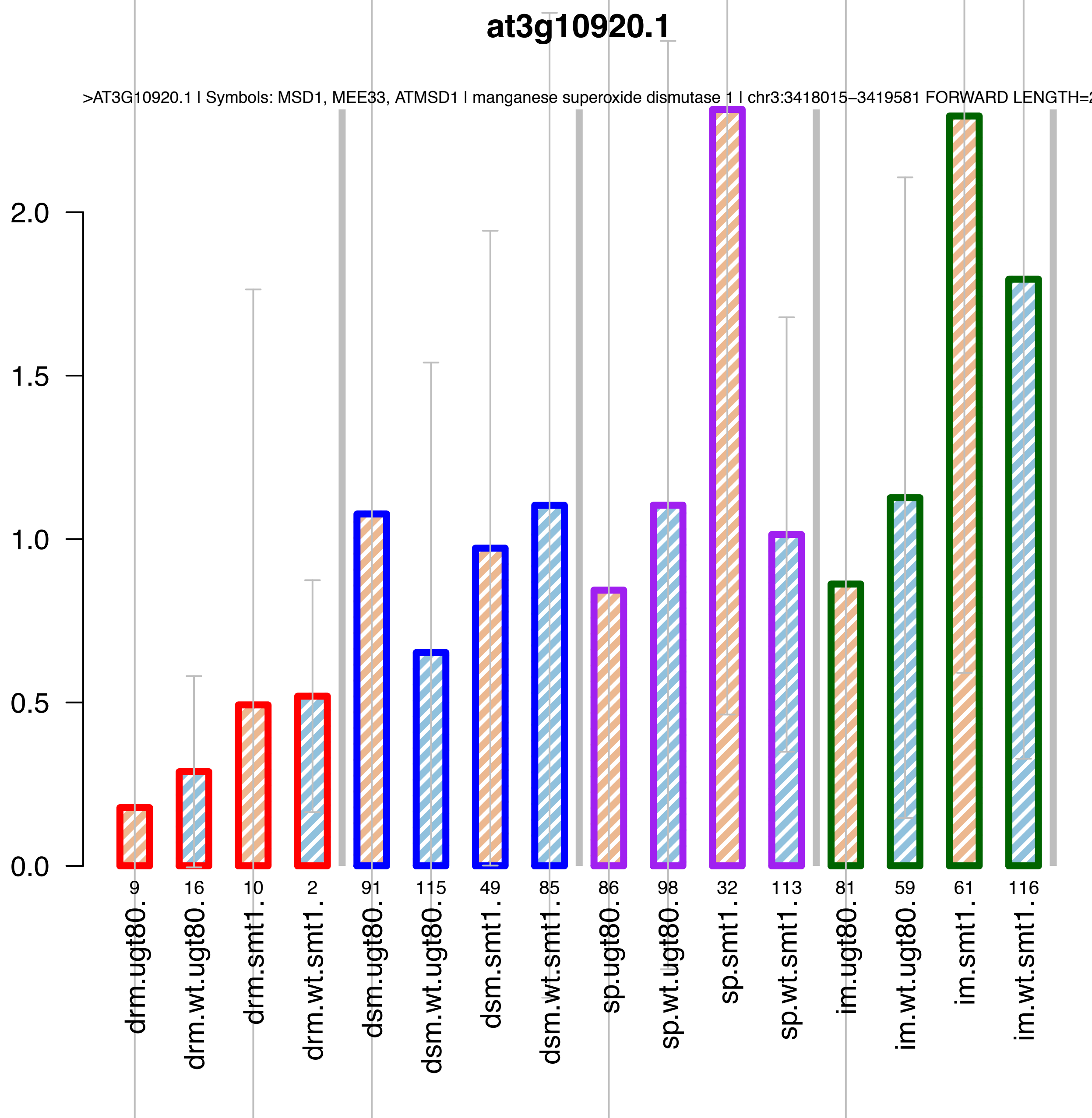

at3g10920.1
>AT3G10920.1 | Symbols: MSD1, MEE33, ATMSD1 | manganese superoxide dismutase 1 | chr3:3418015-3419581 FORWARD LENGTH=
2.0
1.5
1.0
0.5
0.0
9
16
10
2
91
115
49
85
86
98
32
113
81
59
61
116
drm.ugt80..
drm.wt.ugt80..
drm.smt1..
drm.wt.smt1..
dsm.ugt80..
dsm.wt.ugt80..
dsm.smt1..
dsm.wt.smt1..
sp.ugt80..
sp.wt.ugt80..
sp.smt1..
sp.wt.smt1..
im.ugt80..
im.wt.ugt80..
im.smt1..
im.wt.smt1..

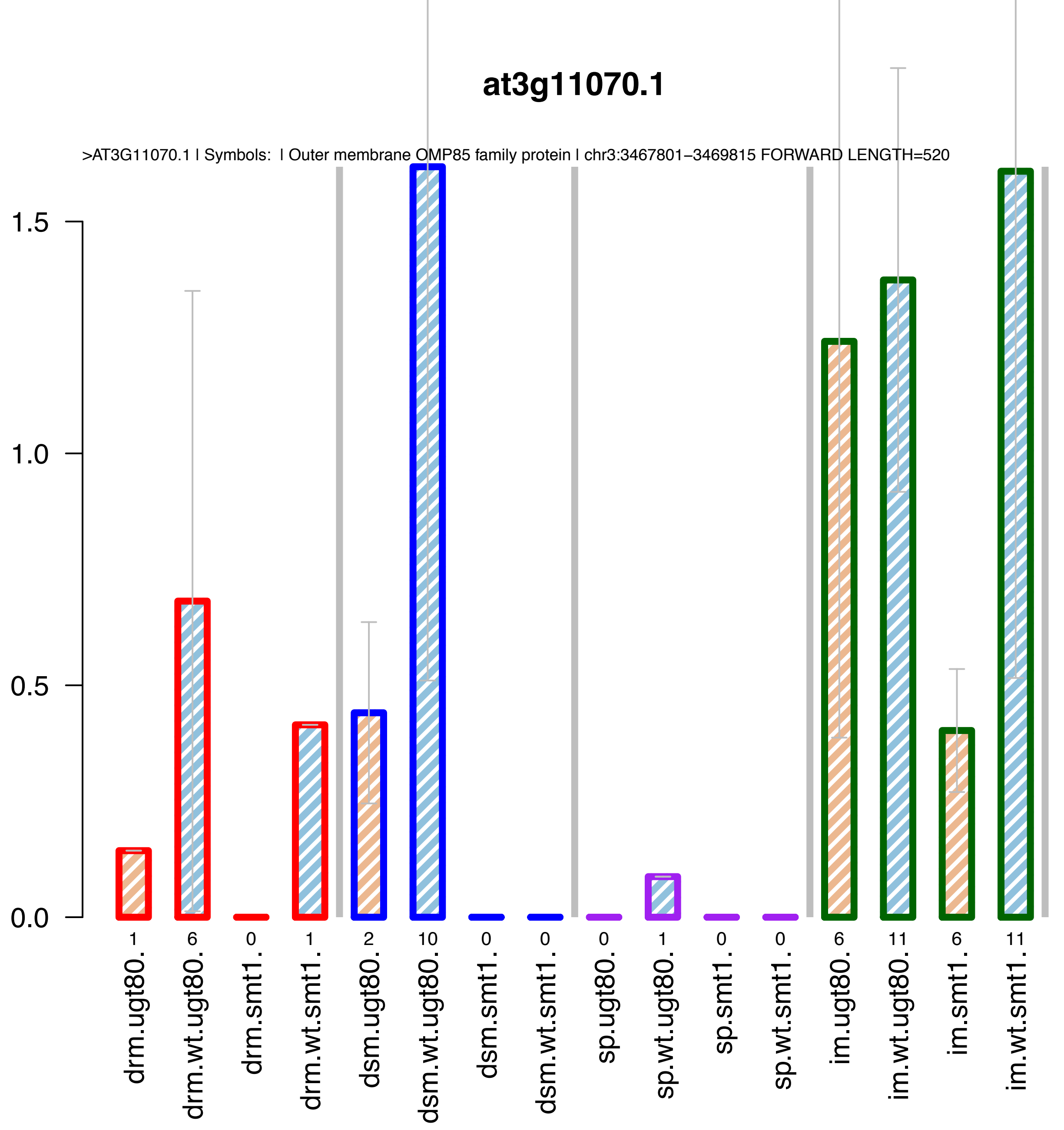
at3g11070.1
>AT3G11070.1 | Symbols: | Outer membrane OMP85 family protein | chr3:3467801-3469815 FORWARD LENGTH=520
1.5
1.0
0.5
0.0
1
6
0
1
2
10
0
0
0
1
0
0
6
11
6
11
drm.ugt80..
drm.wt.ugt80..
drm.smt1..
drm.wt.smt1..
dsm.ugt80..
dsm.wt.ugt80..
dsm.smt1..
dsm.wt.smt1..
sp.ugt80..
sp.wt.ugt80..
sp.smt1..
sp.wt.smt1..
im.ugt80..
im.wt.ugt80..
im.smt1..
im.wt.smt1..

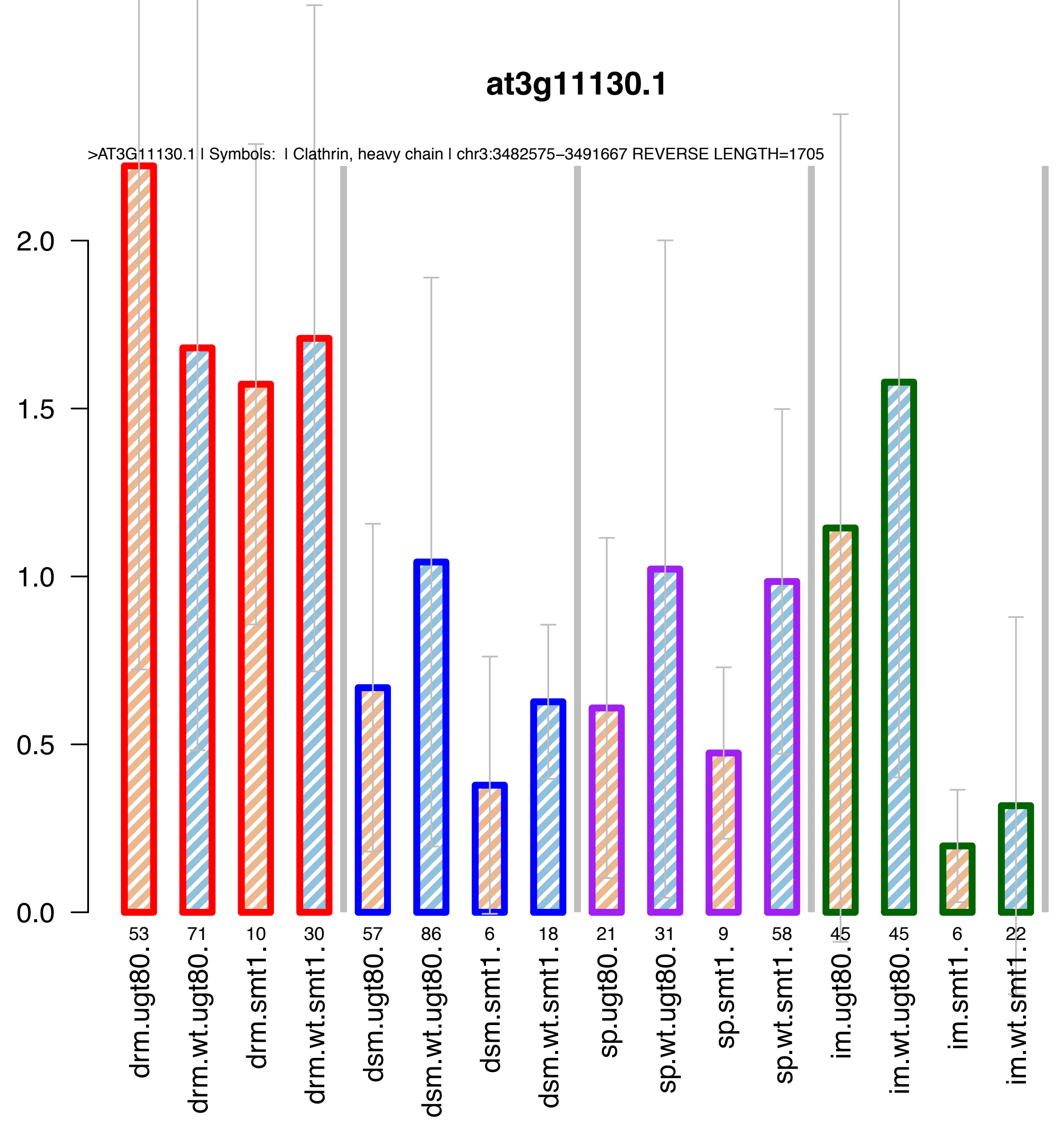
at3g11130.1
>AT3G11130.1 | Symbols: | Clathrin, heavy chain | chr3:3482575-3491667 REVERSE LENGTH=1705
2.0
1.5
1.0
0.5
0.0
53
71
10
30
57
86
6
18
21
31
9
58
45
45
6
22
drm.ugt80..
drm.wt.ugt80..
drm.smt1..
drm.wt.smt1..
dsm.ugt80..
dsm.wt.ugt80..
dsm.smt1..
dsm.wt.smt1..
sp.ugt80..
sp.wt.ugt80..
sp.smt1..
sp.wt.smt1..
im.ugt80..
im.wt.ugt80..
im.smt1..
im.wt.smt1..

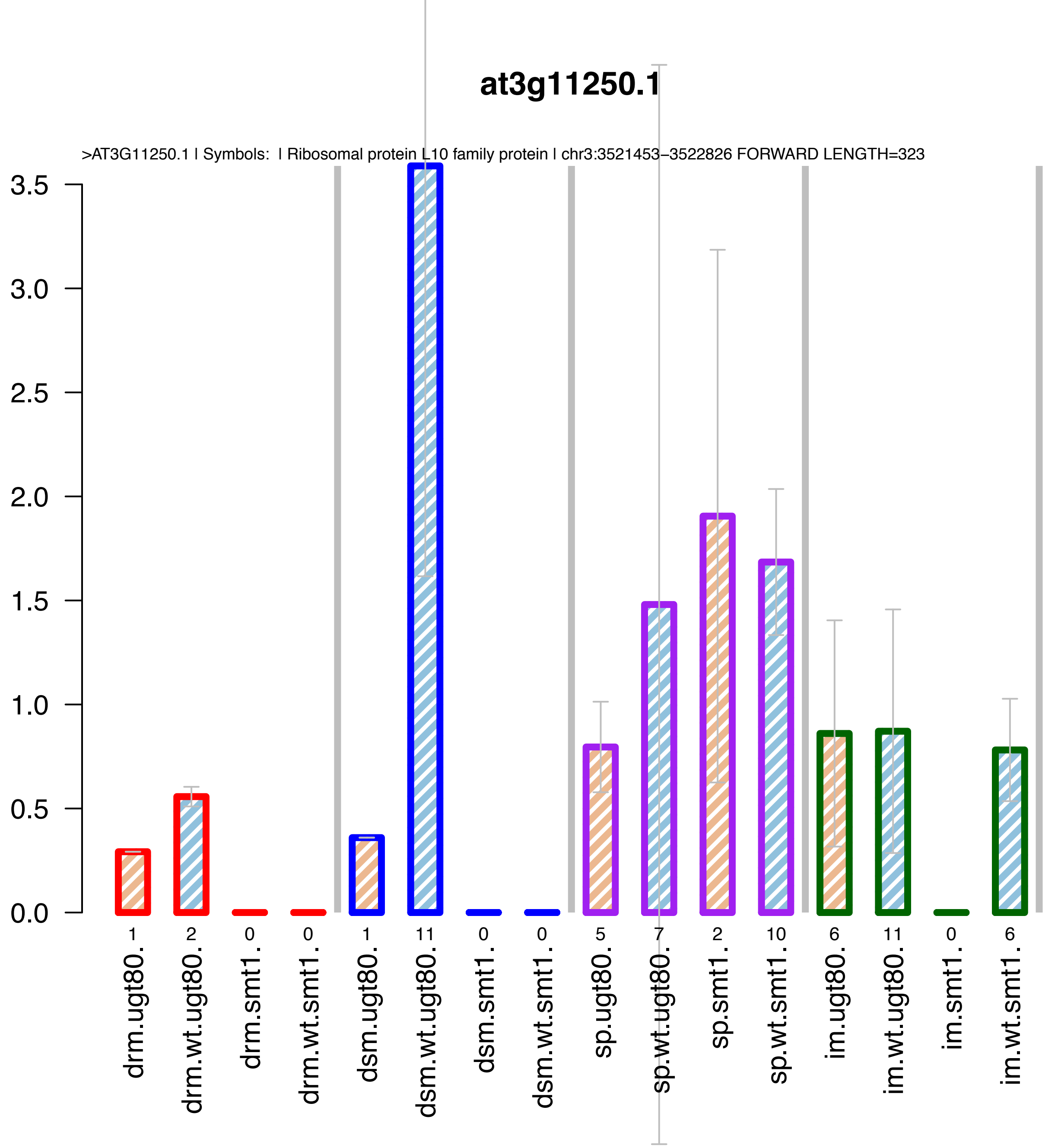

at3g11250.1
>AT3G11250.1 | Symbols: | Ribosomal protein L10 family protein | chr3:3521453–3522826 FORWARD LENGTH=323
3.5
3.0
2.5
2.0
1.5
1.0
0.5
0.0
1
2
0
0
1
11
0
0
5
7
2
10
6
11
0
6
drm.ugt80.
drm.wt.ugt80.
drm.smt1.
drm.wt.smt1.
dsm.ugt80.
dsm.wt.ugt80.
dsm.smt1.
dsm.wt.smt1.
sp.ugt80.
sp.wt.ugt80.
sp.smt1.
sp.wt.smt1.
im.ugt80.
im.wt.ugt80.
im.smt1.
im.wt.smt1.

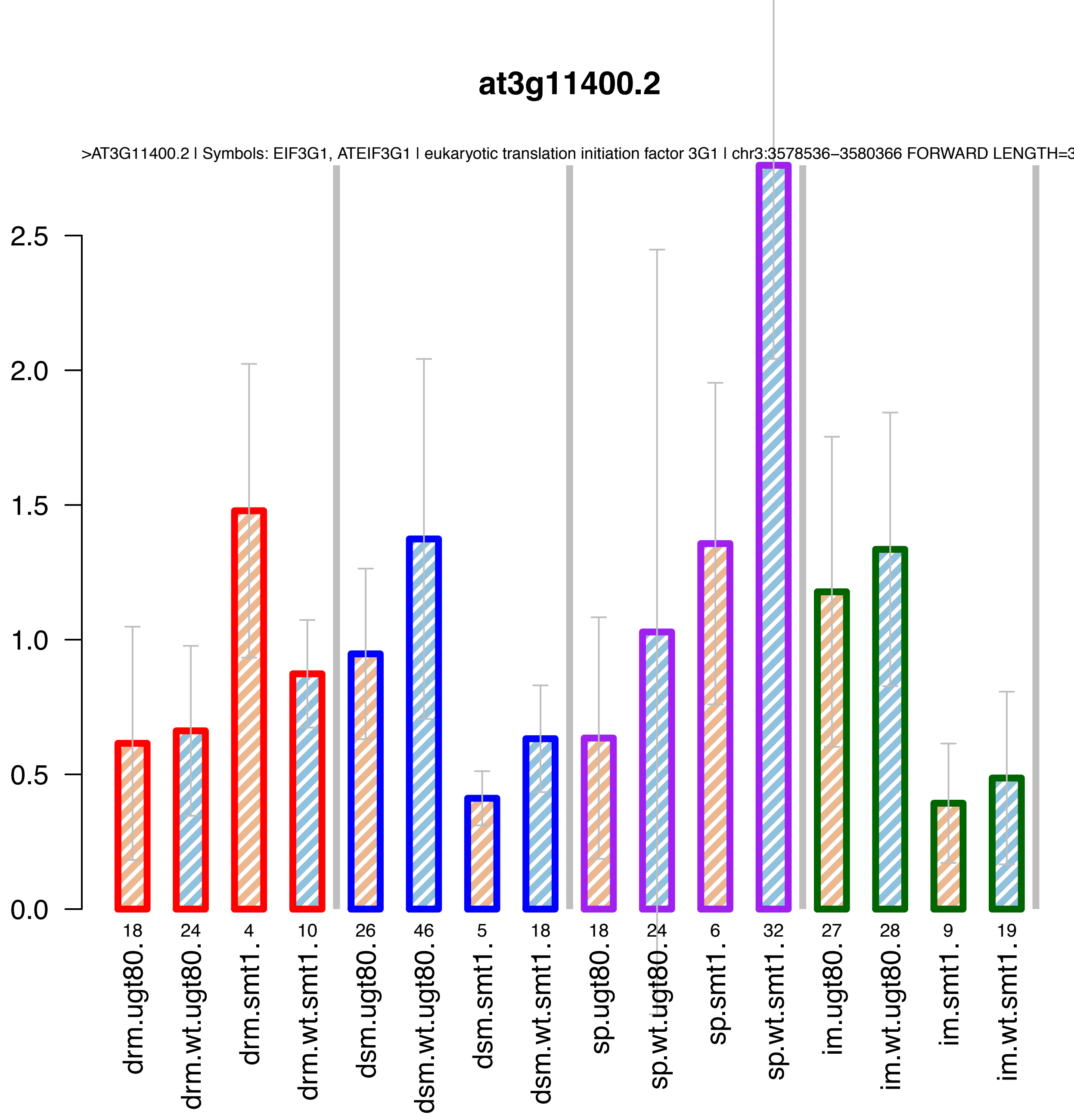

at3g11400.2
>AT3G11400.2 | Symbols: EIF3G1, ATEIF3G1 | eukaryotic translation initiation factor 3G1 | chr3:3578536-3580366 FORWARD LENGTH=3
0.0
0.5
1.0
1.5
2.0
2.5
18
24
4
10
26
46
5
18
18
24
6
32
27
28
9
19
drm.ugt80..
drm.wt.ugt80..
drm.smt1..
drm.wt.smt1..
dsm.ugt80..
dsm.wt.ugt80..
dsm.smt1..
dsm.wt.smt1..
sp.ugt80..
sp.wt.ugt80..
sp.smt1..
sp.wt.smt1..
im.ugt80..
im.wt.ugt80..
im.smt1..
im.wt.smt1..

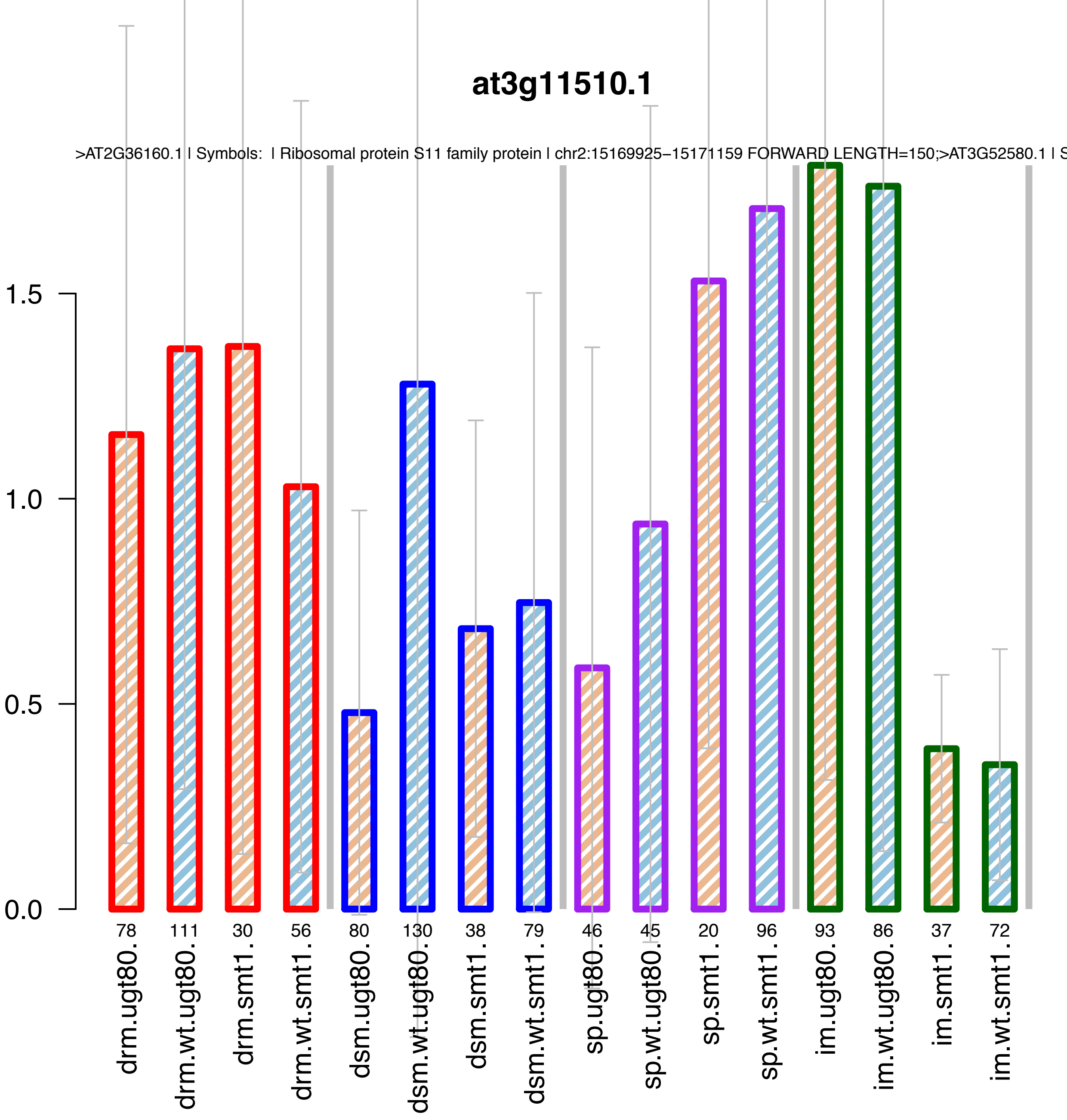

at3g11510.1
>AT2G36160.1 | Symbols: | Ribosomal protein S11 family protein | chr2:15169925-15171159 FORWARD LENGTH=150;>AT3G52580.1 | S
0.0
0.5
1.0
1.5
78
111
30
56
80
130
38
79
46
45
20
96
93
86
37
72
drm.ugt80..
drm.wt.ugt80..
drm.smt1..
drm.wt.smt1..
dsm.ugt80..
dsm.wt.ugt80..
dsm.smt1..
dsm.wt.smt1..
sp.ugt80..
sp.wt.ugt80..
sp.smt1..
sp.wt.smt1..
im.ugt80..
im.wt.ugt80..
im.smt1..
im.wt.smt1..

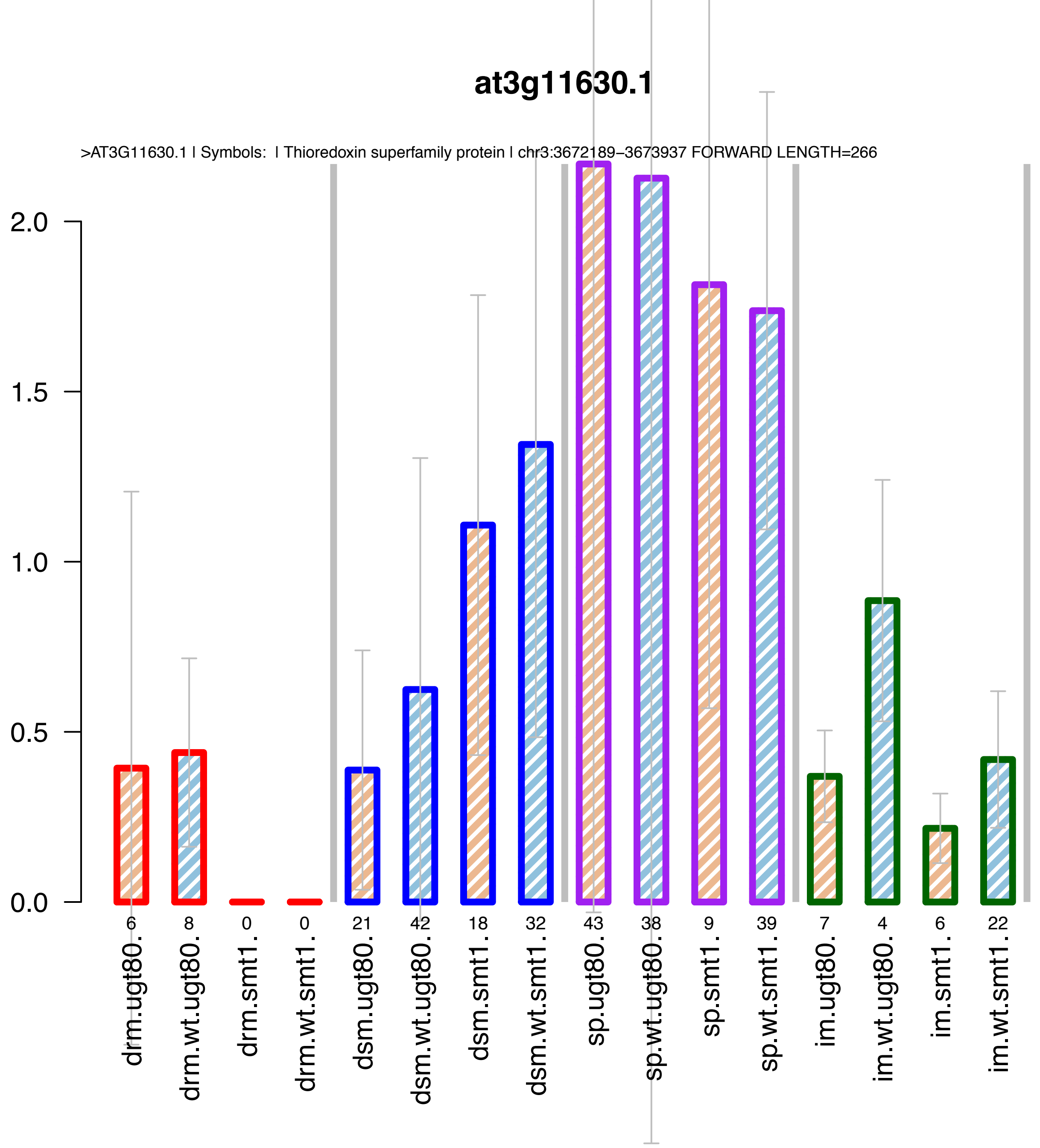

at3g11630.1
>AT3G11630.1 | Symbols: | Thioredoxin superfamily protein | chr3:3672189-3673937 FORWARD LENGTH=266
2.0
1.5
1.0
0.5
0.0
6
8
0
0
21
42
18
32
43
38
9
39
7
4
6
22
drm.ugt80..
drm.wt.ugt80..
drm.smt1..
drm.wt.smt1..
dsm.ugt80..
dsm.wt.ugt80..
dsm.smt1..
dsm.wt.smt1..
sp.ugt80..
sp.wt.ugt80..
sp.smt1..
sp.wt.smt1..
im.ugt80..
im.wt.ugt80..
im.smt1..
im.wt.smt1..

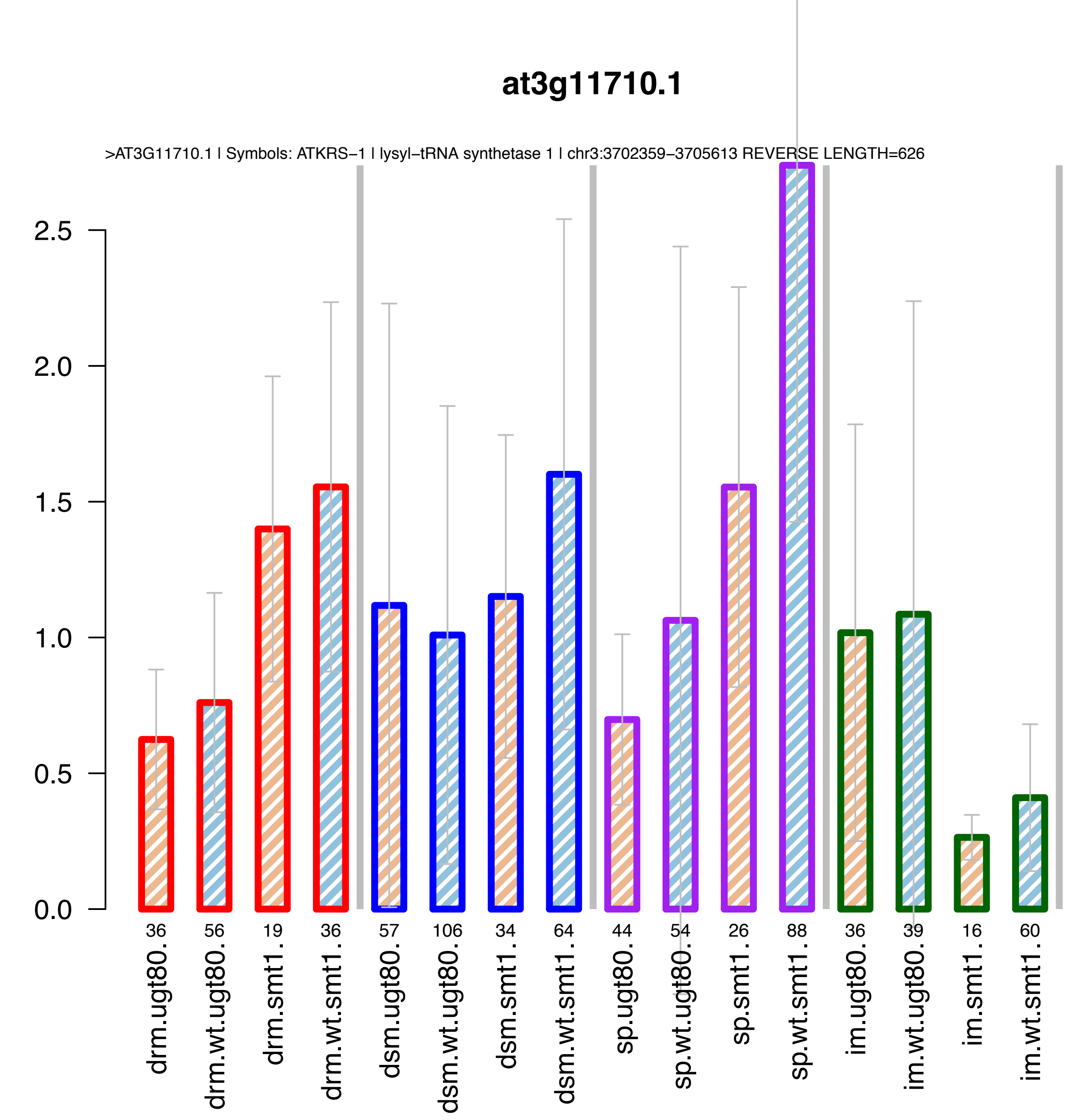
at3g11710.1
>AT3G11710.1 | Symbols: ATKRS−1 | lysyl−tRNA synthetase 1 | chr3:3702359−3705613 REVERSE LENGTH=626
0.0
0.5
1.0
1.5
2.0
2.5
36
56
19
36
57
106
34
64
44
54
26
88
36
39
16
60
drm.ugt80..
drm.wt.ugt80..
drm.smt1..
drm.wt.smt1..
dsm.ugt80..
dsm.wt.ugt80..
dsm.smt1..
dsm.wt.smt1..
sp.ugt80..
sp.wt.ugt80..
sp.smt1..
sp.wt.smt1..
im.ugt80..
im.wt.ugt80..
im.smt1..
im.wt.smt1..

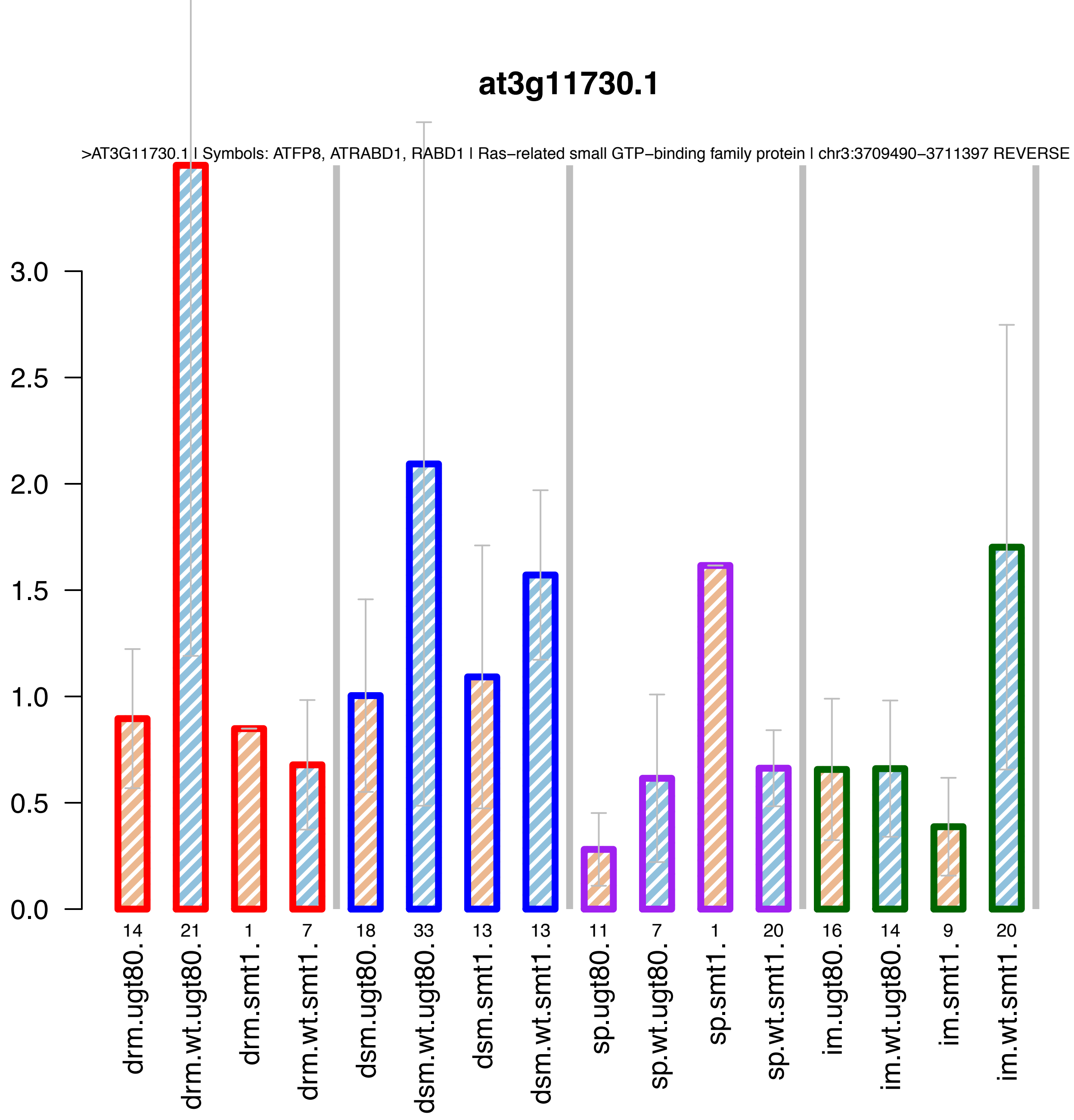
at3g11730.1
>AT3G11730.1 | Symbols: ATFP8, ATRABD1, RABD1 | Ras−related small GTP−binding family protein | chr3:3709490−3711397 REVERSE
0.0
0.5
1.0
1.5
2.0
2.5
3.0
14
21
1
7
18
33
13
13
11
7
1
20
16
14
9
20
drm.ugt80.
drm.wt.ugt80.
drm.smt1.
drm.wt.smt1.
dsm.ugt80.
dsm.wt.ugt80.
dsm.smt1.
dsm.wt.smt1.
sp.ugt80.
sp.wt.ugt80.
sp.smt1.
sp.wt.smt1.
im.ugt80.
im.wt.ugt80.
im.smt1.
im.wt.smt1.

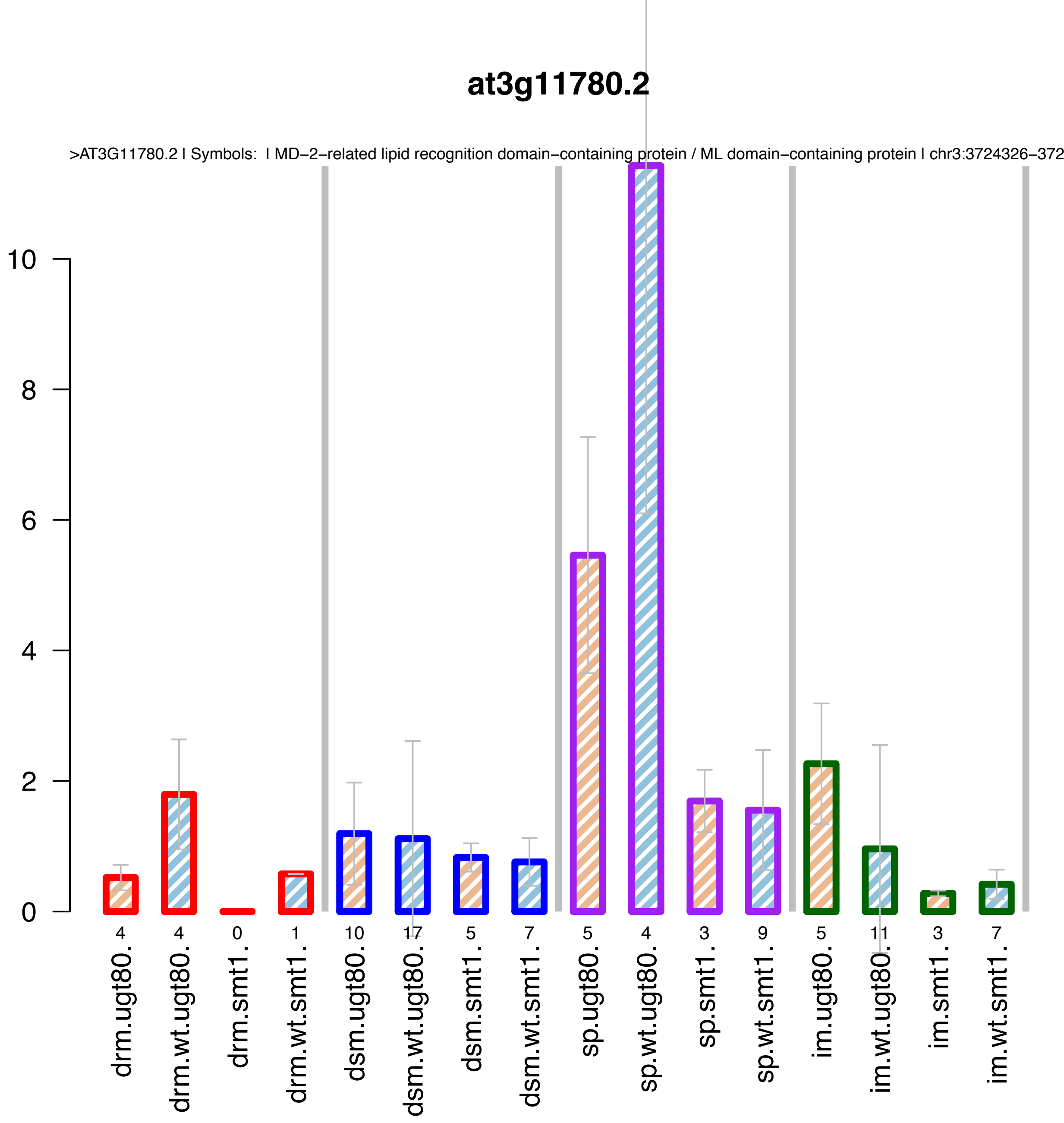
at3g11780.2
>AT3G11780.2 | Symbols: | MD−2−related lipid recognition domain−containing protein / ML domain−containing protein | chr3:3724326−372
0
2
4
6
8
10
4
4
0
1
10
17
5
7
5
4
3
9
5
11
3
7
drm.ugt80.
drm.wt.ugt80.
drm.smt1.
drm.wt.smt1.
dsm.ugt80.
dsm.wt.ugt80.
dsm.smt1.
dsm.wt.smt1.
sp.ugt80.
sp.wt.ugt80.
sp.smt1.
sp.wt.smt1.
im.ugt80.
im.wt.ugt80.
im.smt1.
im.wt.smt1.

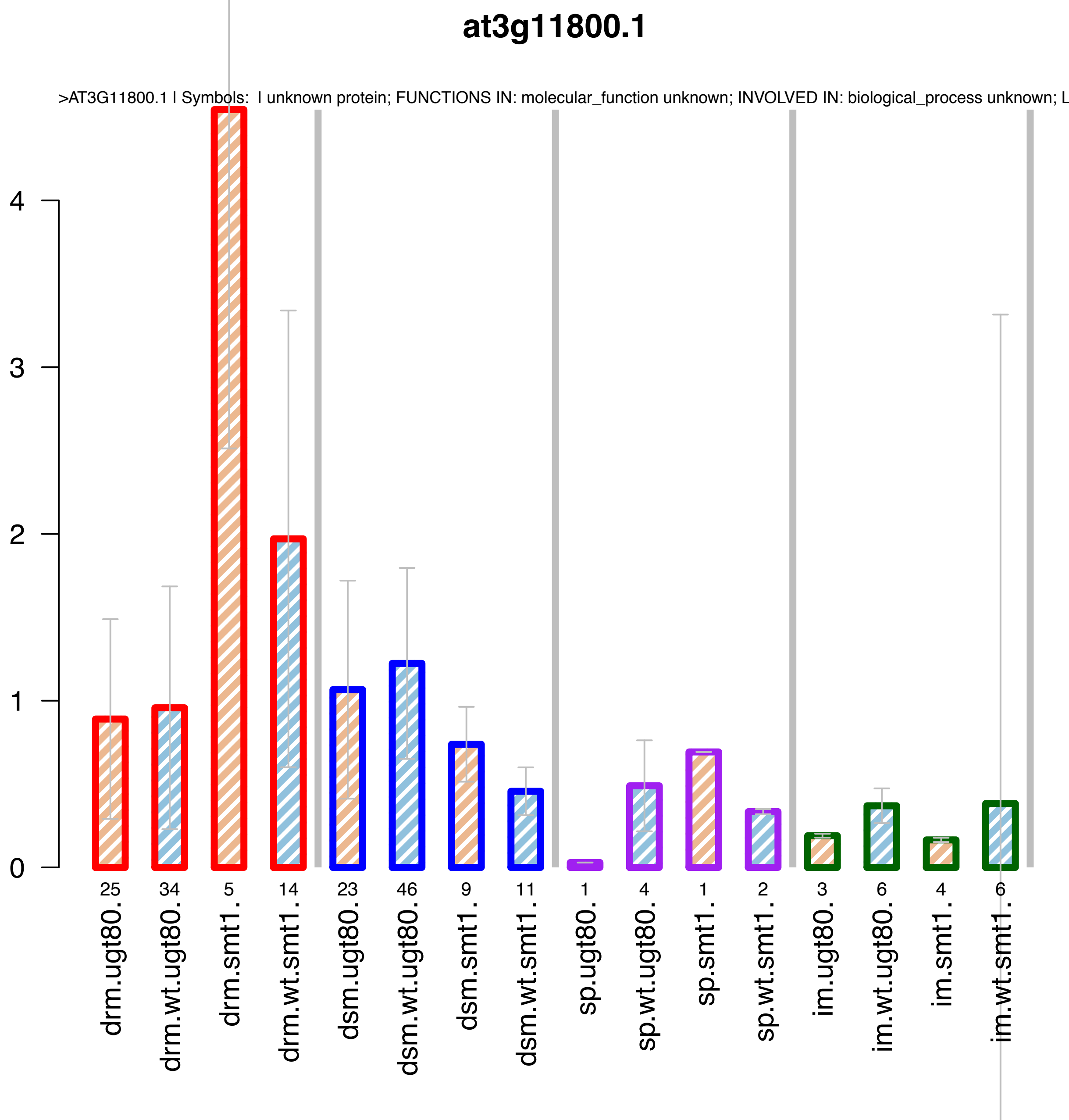

at3g11800.1
>AT3G11800.1 | Symbols: | unknown protein; FUNCTIONS IN: molecular_function unknown; INVOLVED IN: biological_process unknown; L
0
1
2
3
4
25
34
5
14
23
46
9
11
1
4
1
2
3
6
4
6
drm.ugt80.
drm.wt.ugt80.
drm.smt1.
drm.wt.smt1.
dsm.ugt80.
dsm.wt.ugt80.
dsm.smt1.
dsm.wt.smt1.
sp.ugt80.
sp.wt.ugt80.
sp.smt1.
sp.wt.smt1.
im.ugt80.
im.wt.ugt80.
im.smt1.
im.wt.smt1.

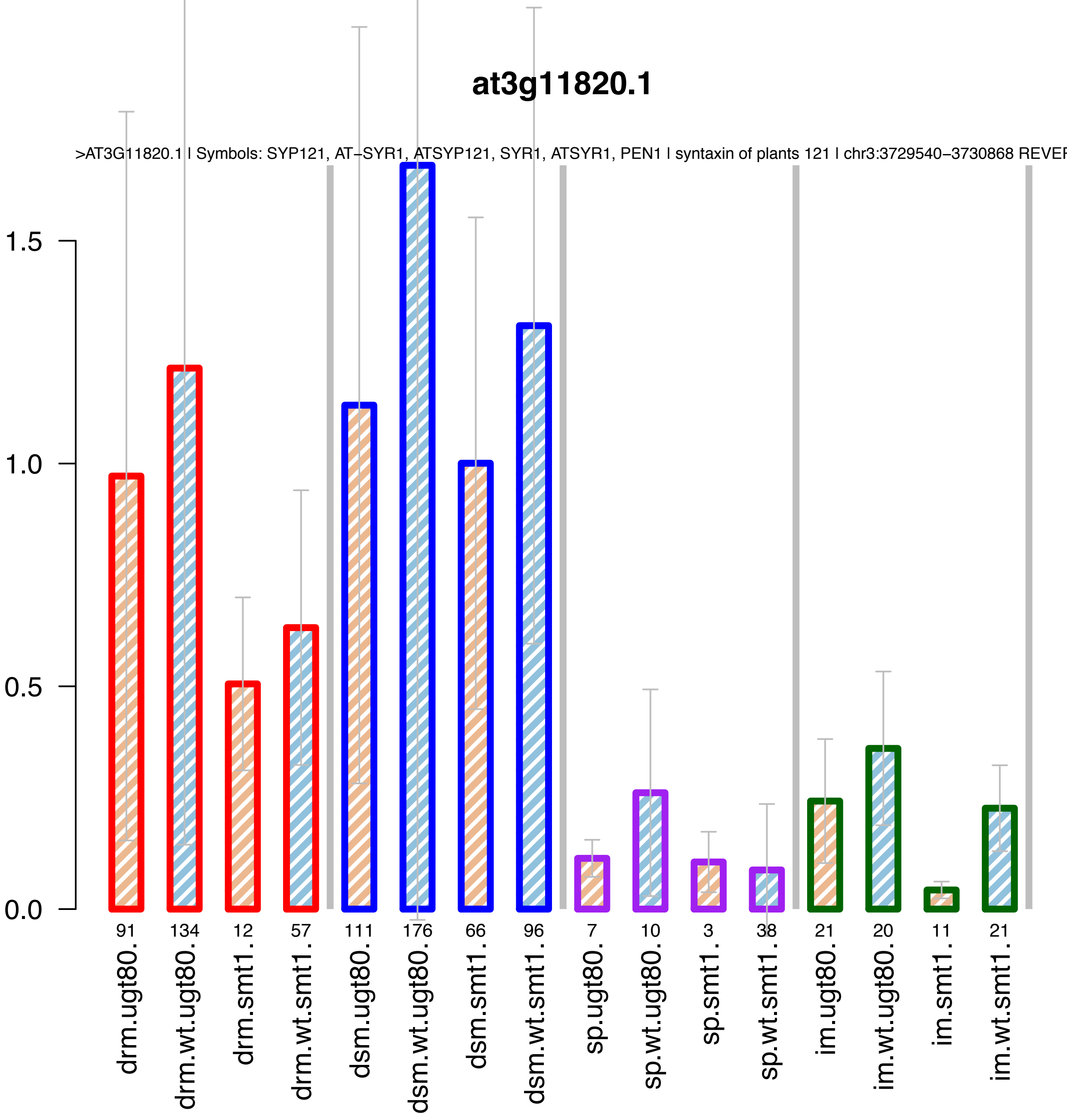

at3g11820.1
>AT3G11820.1 | Symbols: SYP121, AT−SYR1, ATSYP121, SYR1, ATSYR1, PEN1 | syntaxin of plants 121 | chr3:3729540−3730868 REVER
0.0
0.5
1.0
1.5
91
134
12
57
111
176
66
96
7
10
3
38
21
20
11
21
drm.ugt80.
drm.wt.ugt80.
drm.smt1.
drm.wt.smt1.
dsm.ugt80.
dsm.wt.ugt80.
dsm.smt1.
dsm.wt.smt1.
sp.ugt80.
sp.wt.ugt80.
sp.smt1.
sp.wt.smt1.
im.ugt80.
im.wt.ugt80.
im.smt1.
im.wt.smt1.

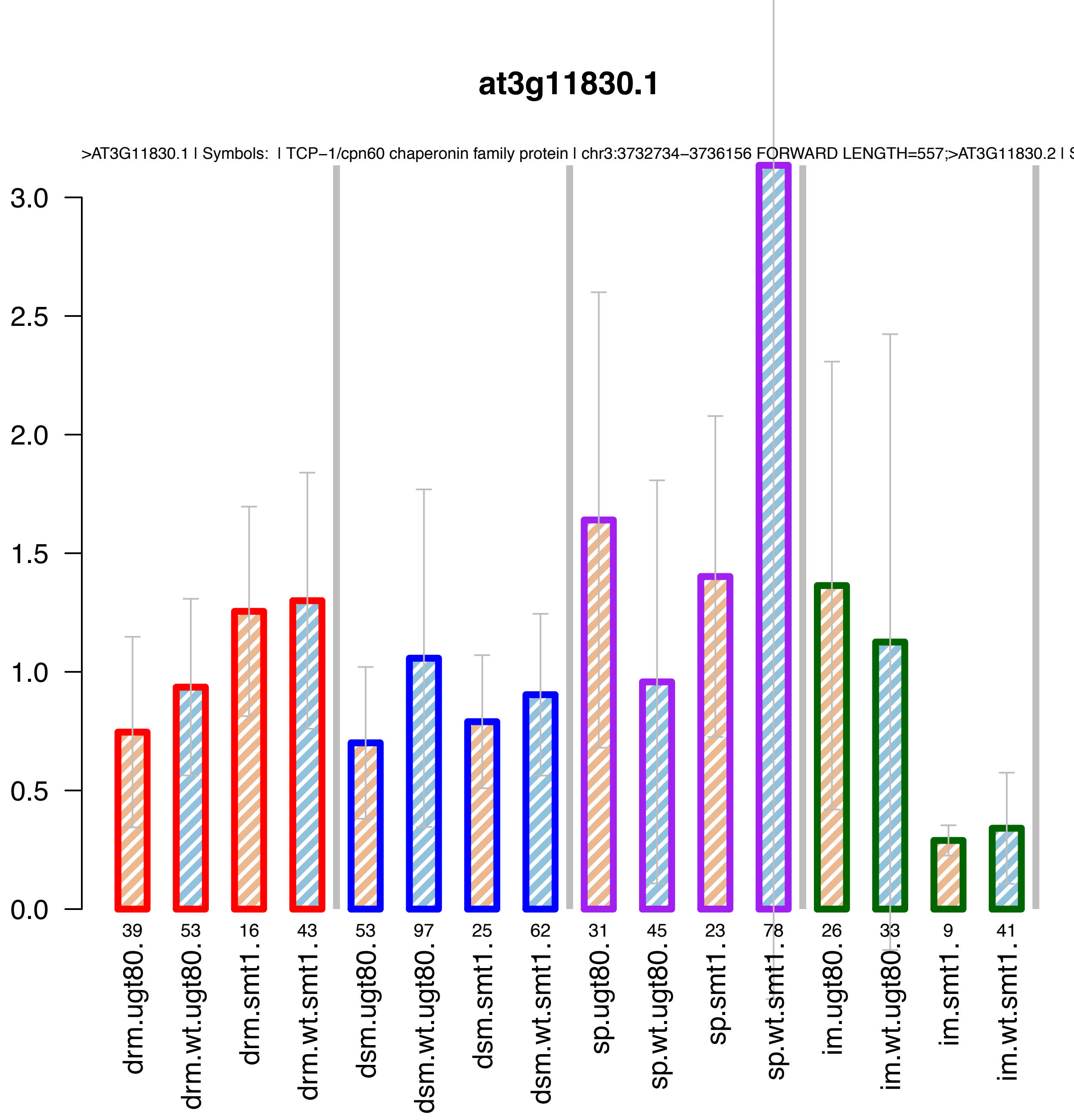

at3g11830.1
>AT3G11830.1 | Symbols: | TCP-1/cpn60 chaperonin family protein | chr3:3732734-3736156 FORWARD LENGTH=557;>AT3G11830.2 | S
3.0
2.5
2.0
1.5
1.0
0.5
0.0
39
53
16
43
53
97
25
62
31
45
23
78
26
33
9
41
drm.ugt80.
drm.wt.ugt80.
drm.smt1.
drm.wt.smt1.
dsm.ugt80.
dsm.wt.ugt80.
dsm.smt1.
dsm.wt.smt1.
sp.ugt80.
sp.wt.ugt80.
sp.smt1.
sp.wt.smt1.
im.ugt80.
im.wt.ugt80.
im.smt1.
im.wt.smt1.

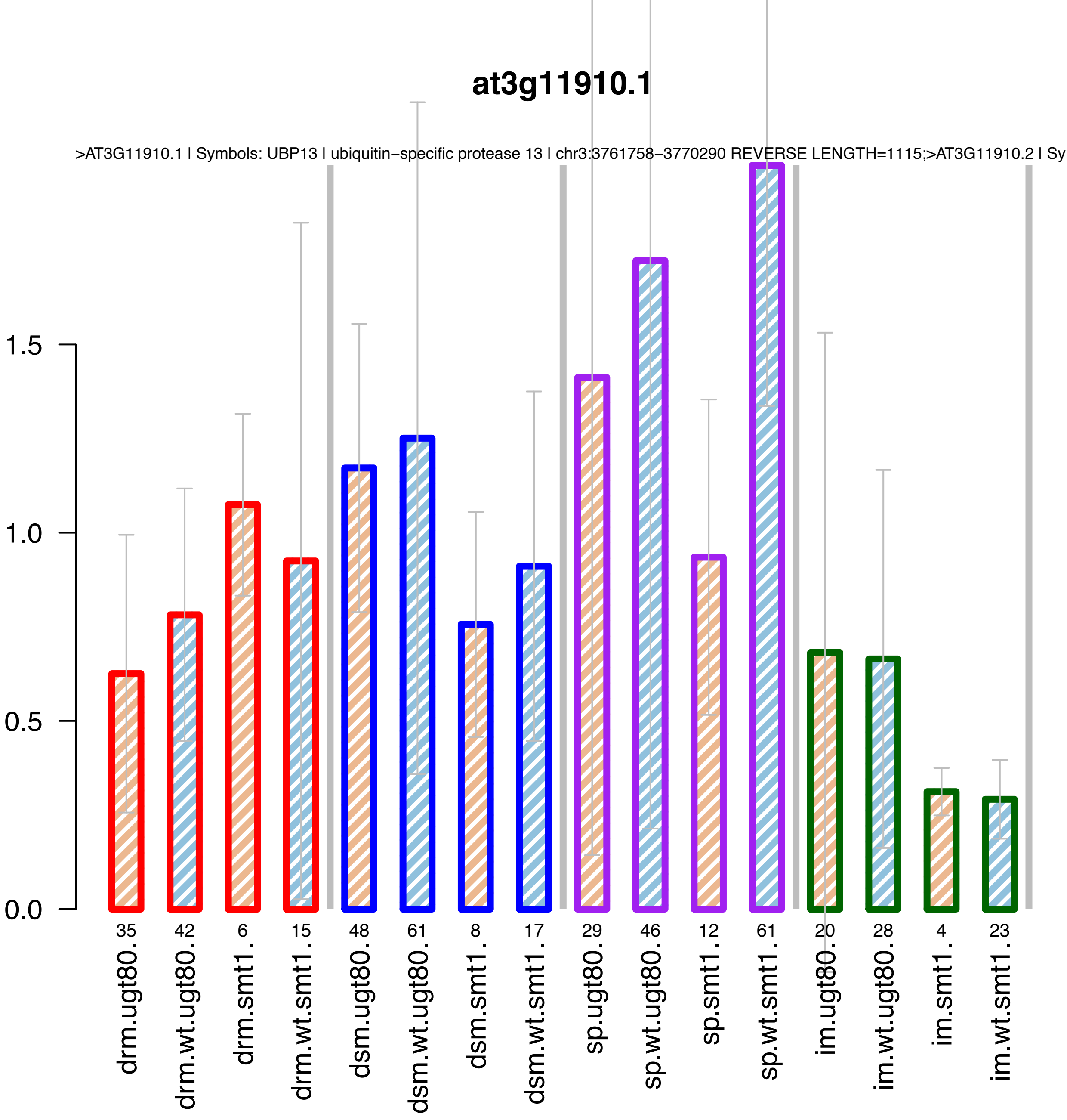
at3g11910.1
>AT3G11910.1 | Symbols: UBP13 | ubiquitin−specific protease 13 | chr3:3761758−3770290 REVERSE LENGTH=1115;>AT3G11910.2 | Sy
0.0
0.5
1.0
1.5
35
42
6
15
48
61
8
17
29
46
12
61
20
28
4
23
drm.ugt80.
drm.wt.ugt80.
drm.smt1.
drm.wt.smt1.
dsm.ugt80.
dsm.wt.ugt80.
dsm.smt1.
dsm.wt.smt1.
sp.ugt80.
sp.wt.ugt80.
sp.smt1.
sp.wt.smt1.
im.ugt80.
im.wt.ugt80.
im.smt1.
im.wt.smt1.

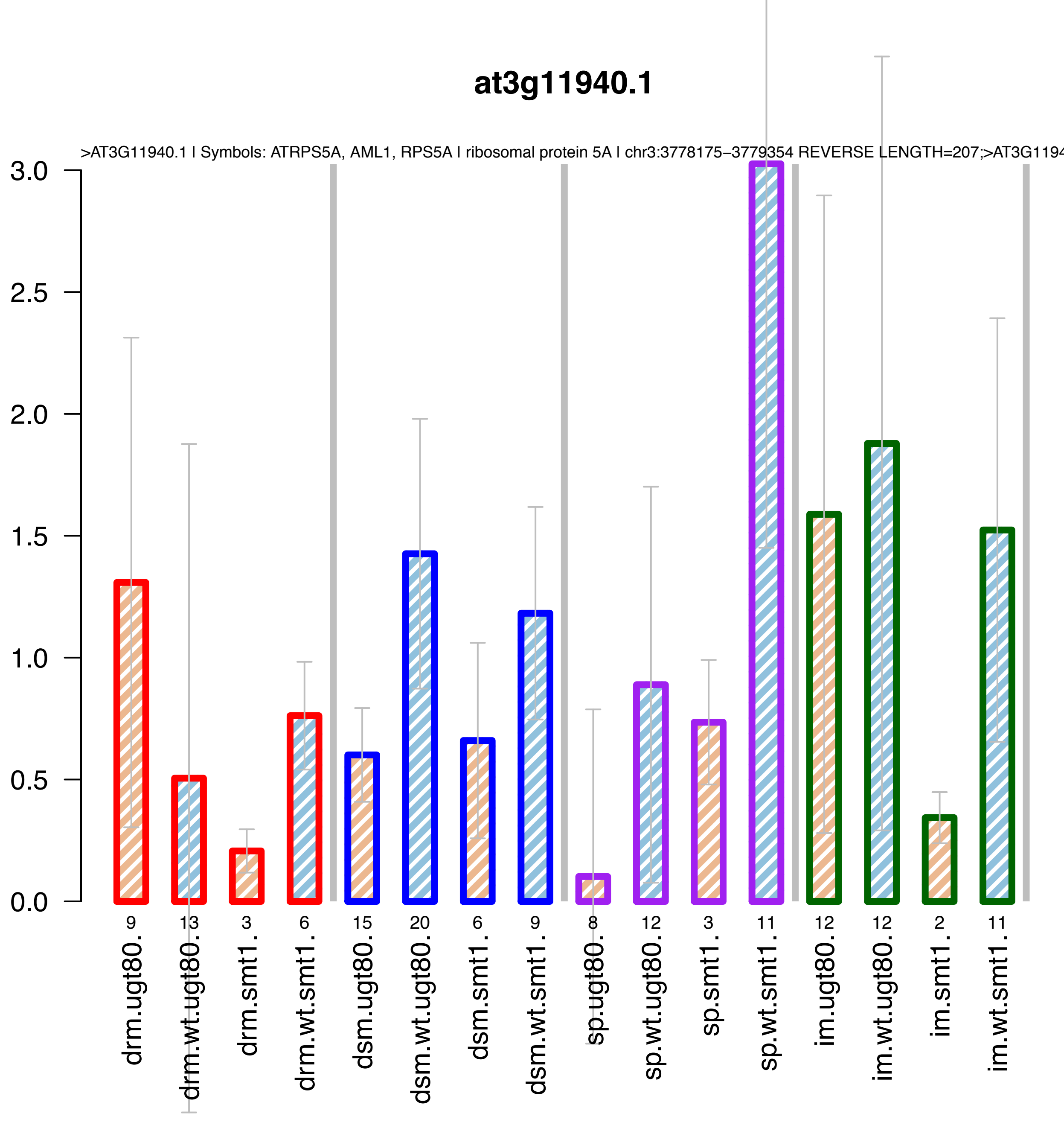

at3g11940.1
>AT3G11940.1 | Symbols: ATRPS5A, AML1, RPS5A | ribosomal protein 5A | chr3:3778175−3779354 REVERSE LENGTH=207;>AT3G1194
3.0
2.5
2.0
1.5
1.0
0.5
0.0
9
13
3
6
15
20
6
9
8
12
3
11
12
12
2
11
drm.ugt80.
drm.wt.ugt80.
drm.smt1.
drm.wt.smt1.
dsm.ugt80.
dsm.wt.ugt80.
dsm.smt1.
dsm.wt.smt1.
sp.ugt80.
sp.wt.ugt80.
sp.smt1.
sp.wt.smt1.
im.ugt80.
im.wt.ugt80.
im.smt1.
im.wt.smt1.

# at3g12050.1

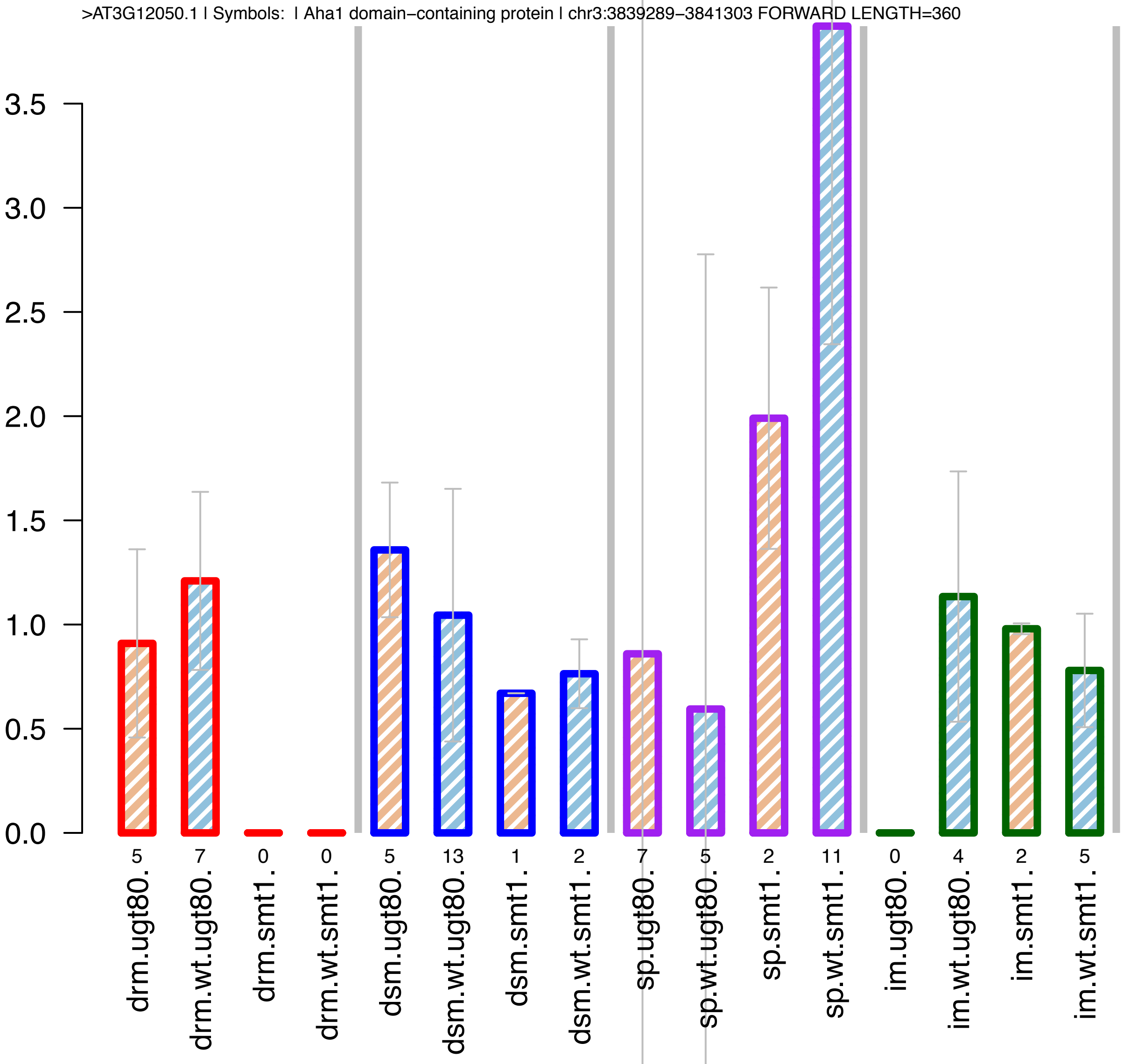

>AT3G12050.1 | Symbols: | Aha1 domain−containing protein | chr3:3839289−3841303 FORWARD LENGTH=360
3.5
3.0
2.5
2.0
1.5
1.0
0.5
0.0
5
7
0
0
5
13
1
2
7
5
2
11
0
4
2
5
drm.ugt80.
drm.wt.ugt80.
drm.smt1.
drm.wt.smt1.
dsm.ugt80.
dsm.wt.ugt80.
dsm.smt1.
dsm.wt.smt1.
sp.ugt80.
sp.wt.ugt80.
sp.smt1.
sp.wt.smt1.
im.ugt80.
im.wt.ugt80.
im.smt1.
im.wt.smt1.

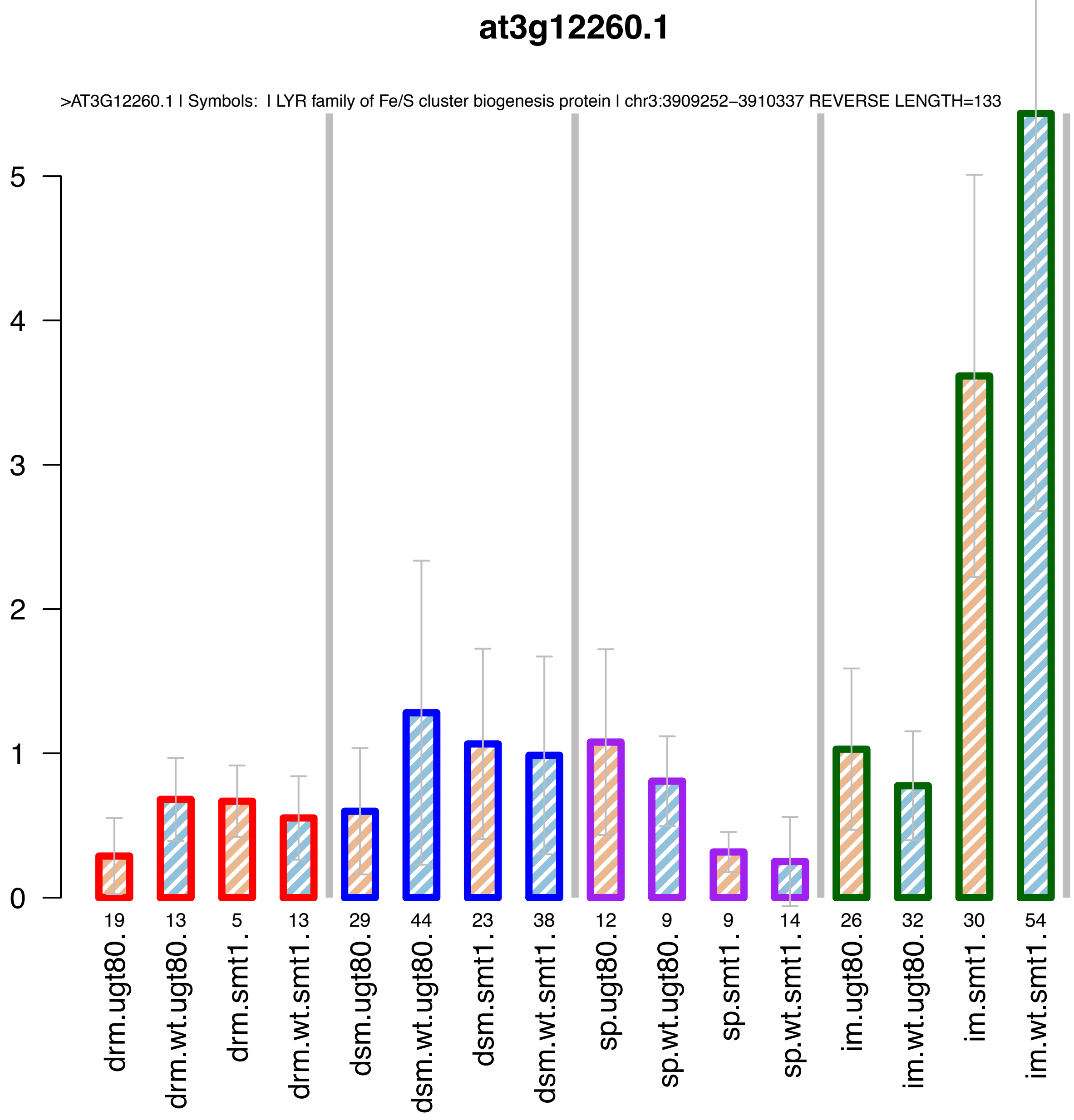

at3g12260.1
>AT3G12260.1 | Symbols: | LYR family of Fe/S cluster biogenesis protein | chr3:3909252–3910337 REVERSE LENGTH=133
0
1
2
3
4
5
19
13
5
13
29
44
23
38
12
9
9
14
26
32
30
54
drm.ugt80.
drm.wt.ugt80.
drm.smt1.
drm.wt.smt1.
dsm.ugt80.
dsm.wt.ugt80.
dsm.smt1.
dsm.wt.smt1.
sp.ugt80.
sp.wt.ugt80.
sp.smt1.
sp.wt.smt1.
im.ugt80.
im.wt.ugt80.
im.smt1.
im.wt.smt1.

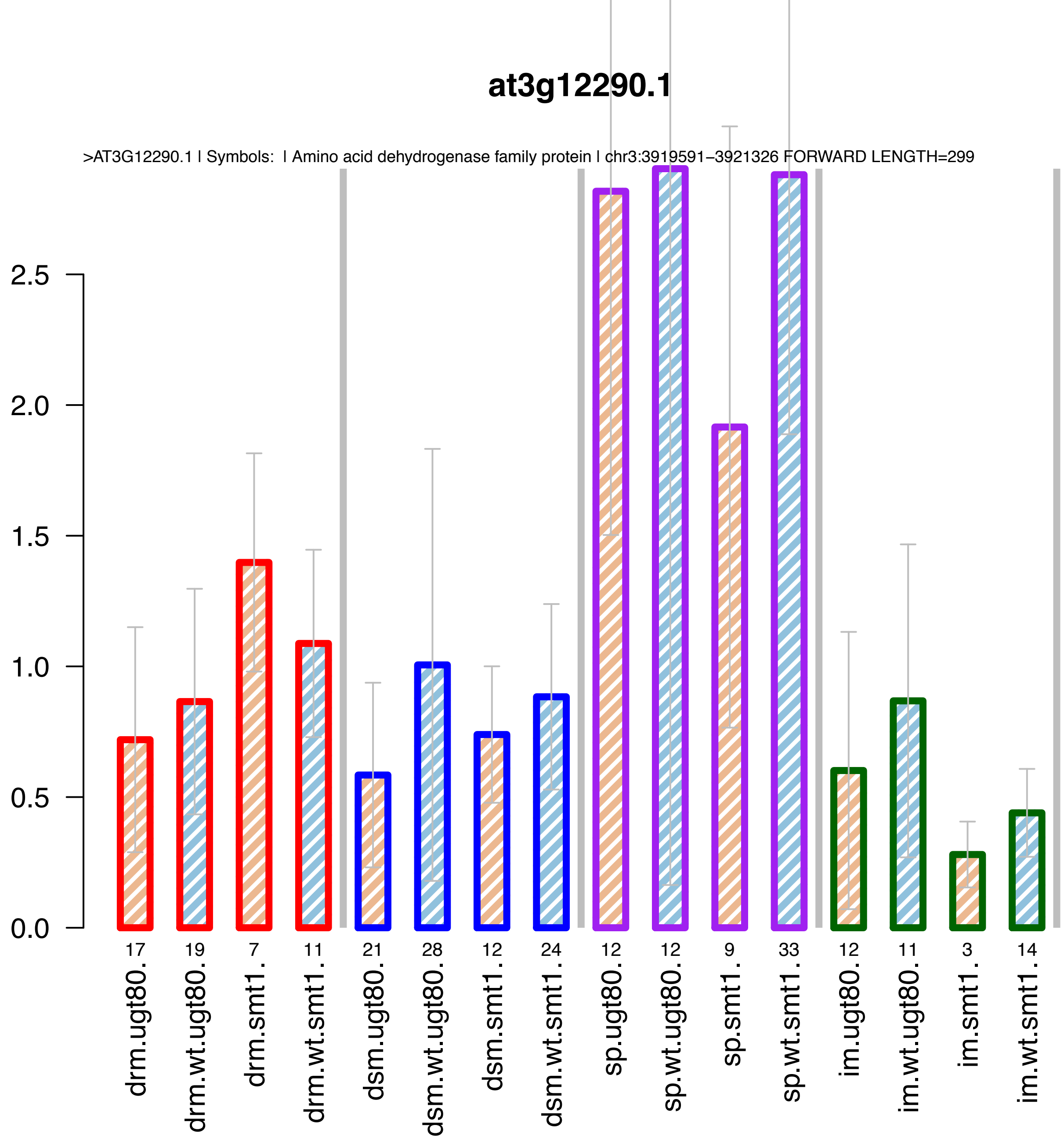
at3g12290.1
>AT3G12290.1 | Symbols: | Amino acid dehydrogenase family protein | chr3:3919591–3921326 FORWARD LENGTH=299
0.0
0.5
1.0
1.5
2.0
2.5
17
19
7
11
21
28
12
24
12
12
9
33
12
11
3
14
drm.ugt80..
drm.wt.ugt80..
drm.smt1..
drm.wt.smt1..
dsm.ugt80..
dsm.wt.ugt80..
dsm.smt1..
dsm.wt.smt1..
sp.ugt80..
sp.wt.ugt80..
sp.smt1..
sp.wt.smt1..
im.ugt80..
im.wt.ugt80..
im.smt1..
im.wt.smt1..

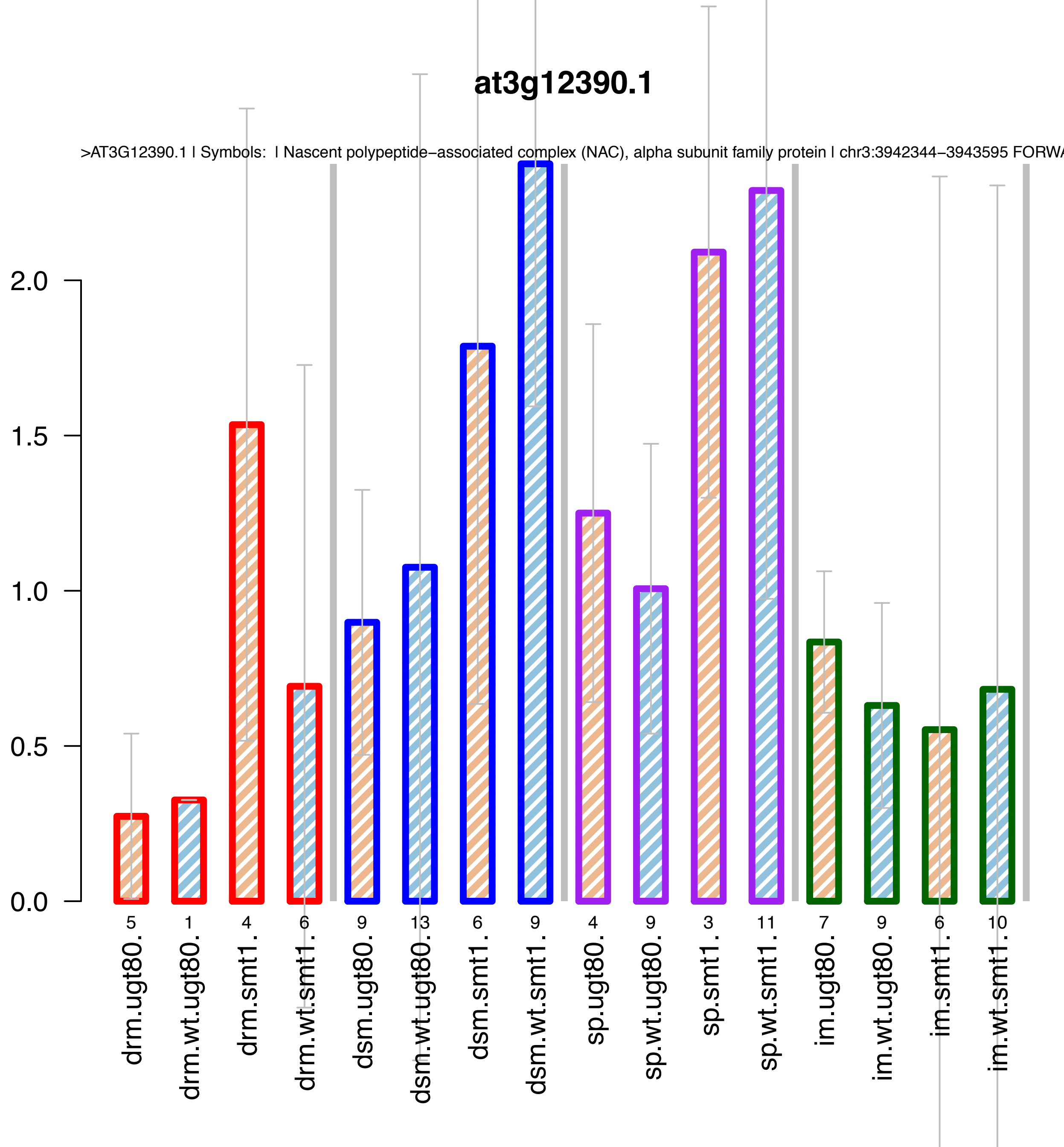

at3g12390.1
>AT3G12390.1 | Symbols: | Nascent polypeptide−associated complex (NAC), alpha subunit family protein | chr3:3942344−3943595 FORWA
0.0
0.5
1.0
1.5
2.0
5
1
4
6
9
13
6
9
4
9
3
11
7
9
6
10
drm.ugt80..
drm.wt.ugt80..
drm.smt1..
drm.wt.smt1..
dsm.ugt80..
dsm.wt.ugt80..
dsm.smt1..
dsm.wt.smt1..
sp.ugt80..
sp.wt.ugt80..
sp.smt1..
sp.wt.smt1..
im.ugt80..
im.wt.ugt80..
im.smt1..
im.wt.smt1..

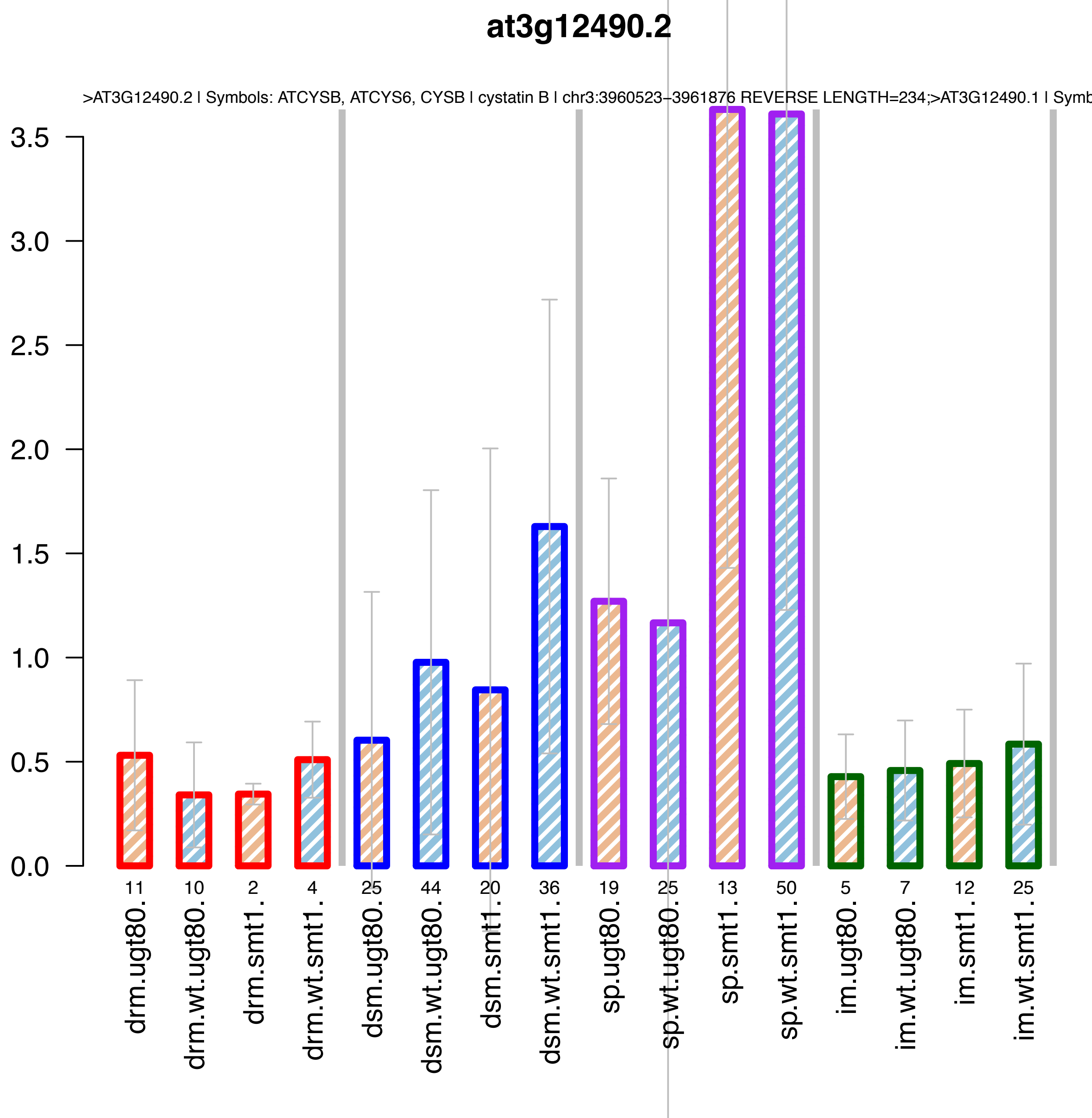

at3g12490.2
>AT3G12490.2 | Symbols: ATCYSB, ATCYS6, CYSB | cystatin B | chr3:3960523−3961876 REVERSE LENGTH=234;>AT3G12490.1 | Symb
3.5
3.0
2.5
2.0
1.5
1.0
0.5
0.0
11
10
2
4
25
44
20
36
19
25
13
50
5
7
12
25
drm.ugt80.
drm.wt.ugt80.
drm.smt1.
drm.wt.smt1.
dsm.ugt80.
dsm.wt.ugt80.
dsm.smt1.
dsm.wt.smt1.
sp.ugt80.
sp.wt.ugt80.
sp.smt1.
sp.wt.smt1.
im.ugt80.
im.wt.ugt80.
im.smt1.
im.wt.smt1.

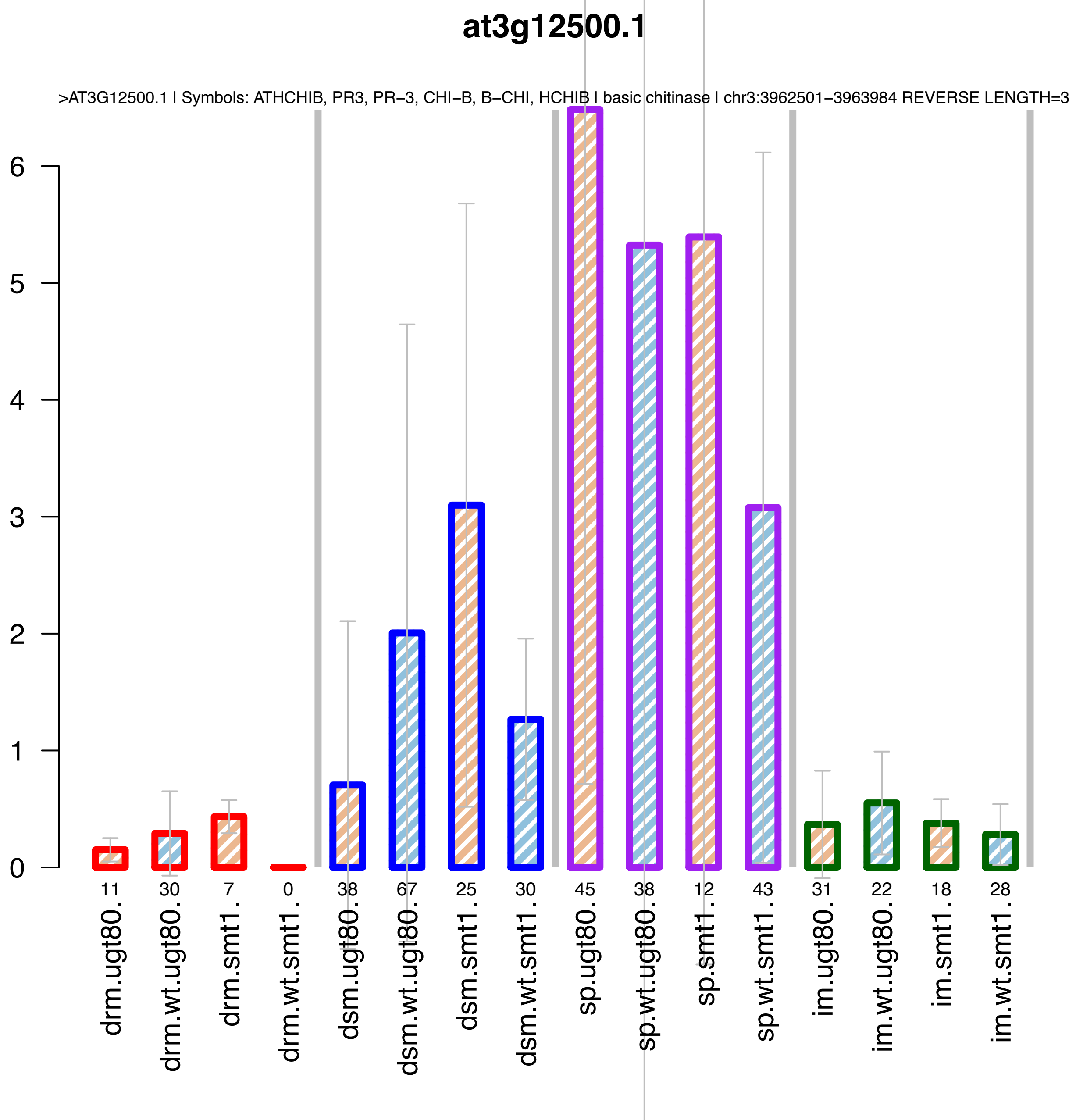

at3g12500.1
>AT3G12500.1 | Symbols: ATHCHIB, PR3, PR−3, CHI−B, B−CHI, HCHIB | basic chitinase | chr3:3962501−3963984 REVERSE LENGTH=3
0
1
2
3
4
5
6
11
30
7
0
38
67
25
30
45
38
12
43
31
22
18
28
drm.ugt80.
drm.wt.ugt80.
drm.smt1.
drm.wt.smt1.
dsm.ugt80.
dsm.wt.ugt80.
dsm.smt1.
dsm.wt.smt1.
sp.ugt80.
sp.wt.ugt80.
sp.smt1.
sp.wt.smt1.
im.ugt80.
im.wt.ugt80.
im.smt1.
im.wt.smt1.

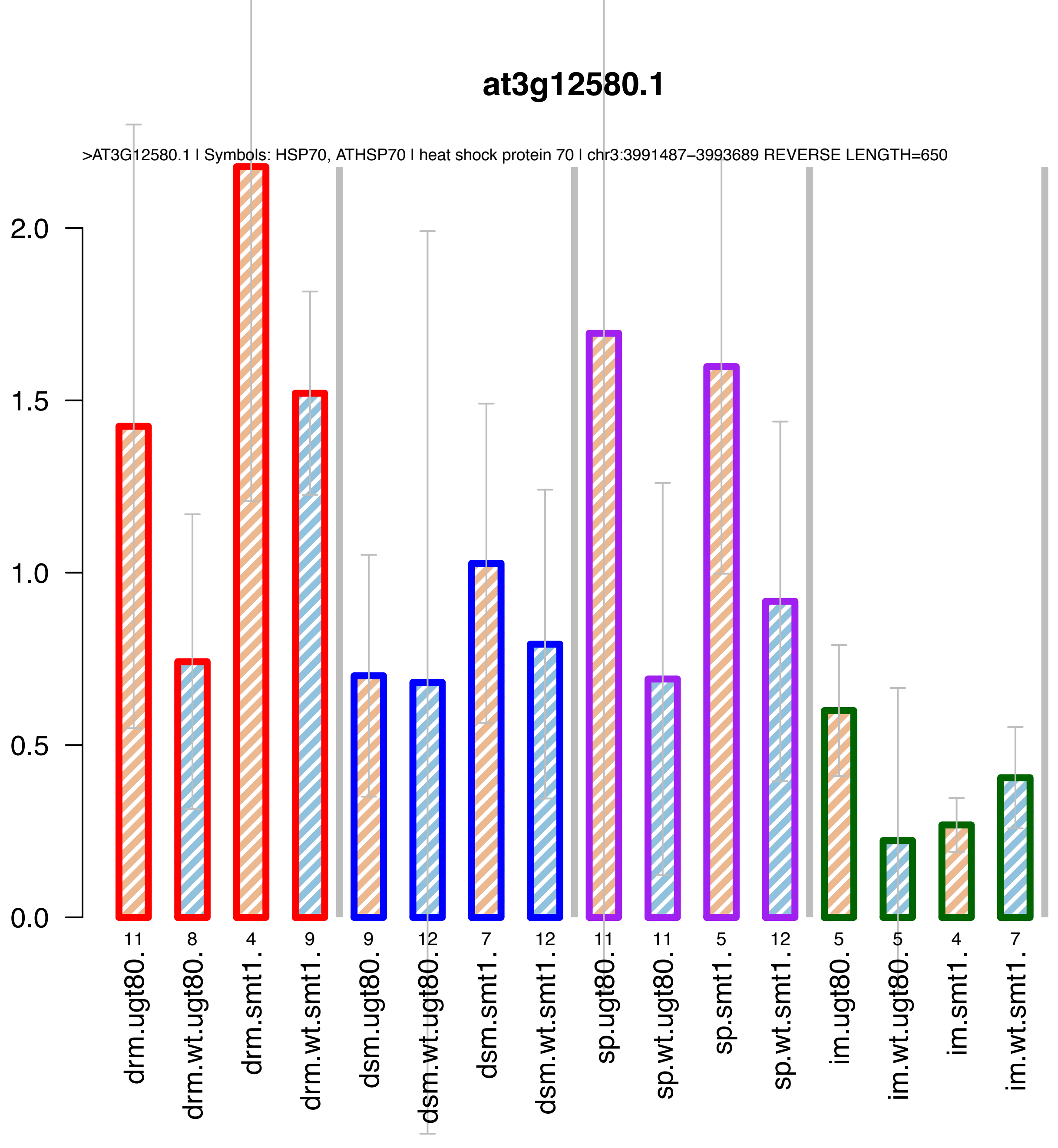

at3g12580.1
>AT3G12580.1 | Symbols: HSP70, ATHSP70 | heat shock protein 70 | chr3:3991487–3993689 REVERSE LENGTH=650
2.0
1.5
1.0
0.5
0.0
11
8
4
9
9
12
7
12
11
11
5
12
5
5
4
7
drm.ugt80.
drm.wt.ugt80.
drm.smt1.
drm.wt.smt1.
dsm.ugt80.
dsm.wt.ugt80.
dsm.smt1.
dsm.wt.smt1.
sp.ugt80.
sp.wt.ugt80.
sp.smt1.
sp.wt.smt1.
im.ugt80.
im.wt.ugt80.
im.smt1.
im.wt.smt1.

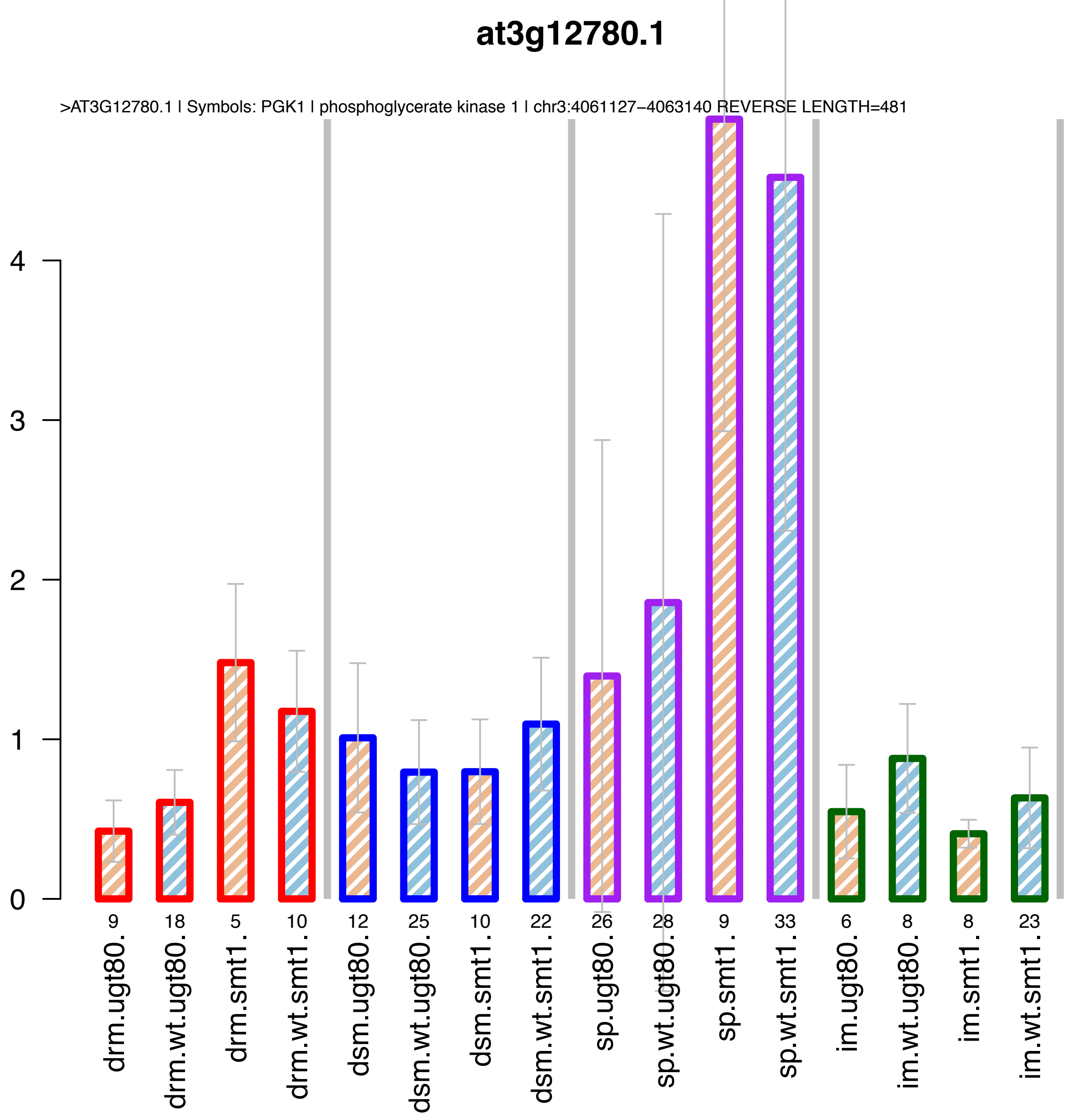

at3g12780.1
>AT3G12780.1 | Symbols: PGK1 | phosphoglycerate kinase 1 | chr3:4061127−4063140 REVERSE LENGTH=481
0
1
2
3
4
9
18
5
10
12
25
10
22
26
28
9
33
6
8
8
23
drm.ugt80.
drm.wt.ugt80.
drm.smt1.
drm.wt.smt1.
dsm.ugt80.
dsm.wt.ugt80.
dsm.smt1.
dsm.wt.smt1.
sp.ugt80.
sp.wt.ugt80.
sp.smt1.
sp.wt.smt1.
im.ugt80.
im.wt.ugt80.
im.smt1.
im.wt.smt1.

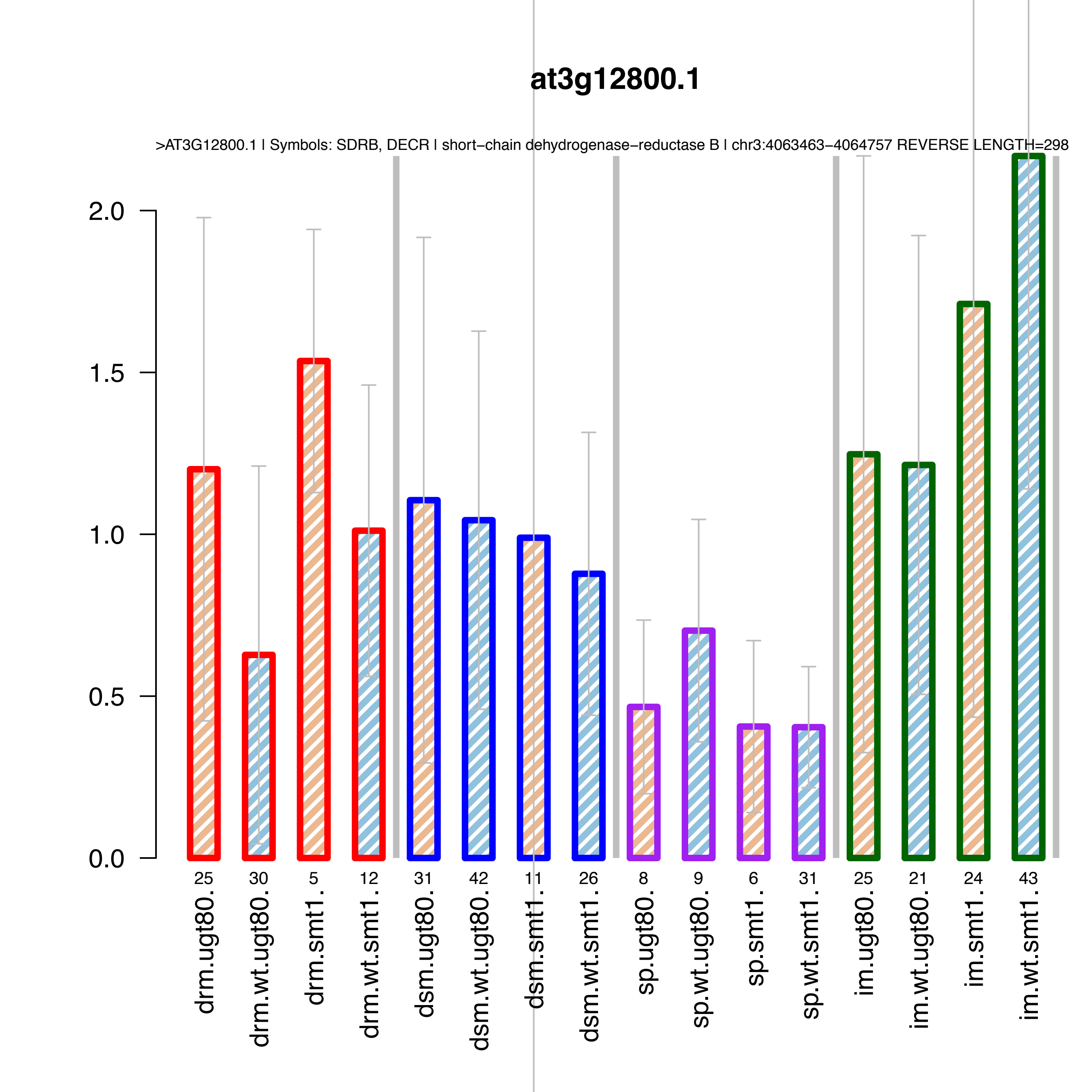

at3g12800.1
>AT3G12800.1 | Symbols: SDRB, DECR | short−chain dehydrogenase−reductase B | chr3:4063463−4064757 REVERSE LENGTH=298
2.0
1.5
1.0
0.5
0.0
25
30
5
12
31
42
11
26
8
9
6
31
25
21
24
43
drm.ugt80..
drm.wt.ugt80..
drm.smt1..
drm.wt.smt1..
dsm.ugt80..
dsm.wt.ugt80..
dsm.smt1..
dsm.wt.smt1..
sp.ugt80..
sp.wt.ugt80..
sp.smt1..
sp.wt.smt1..
im.ugt80..
im.wt.ugt80..
im.smt1..
im.wt.smt1..

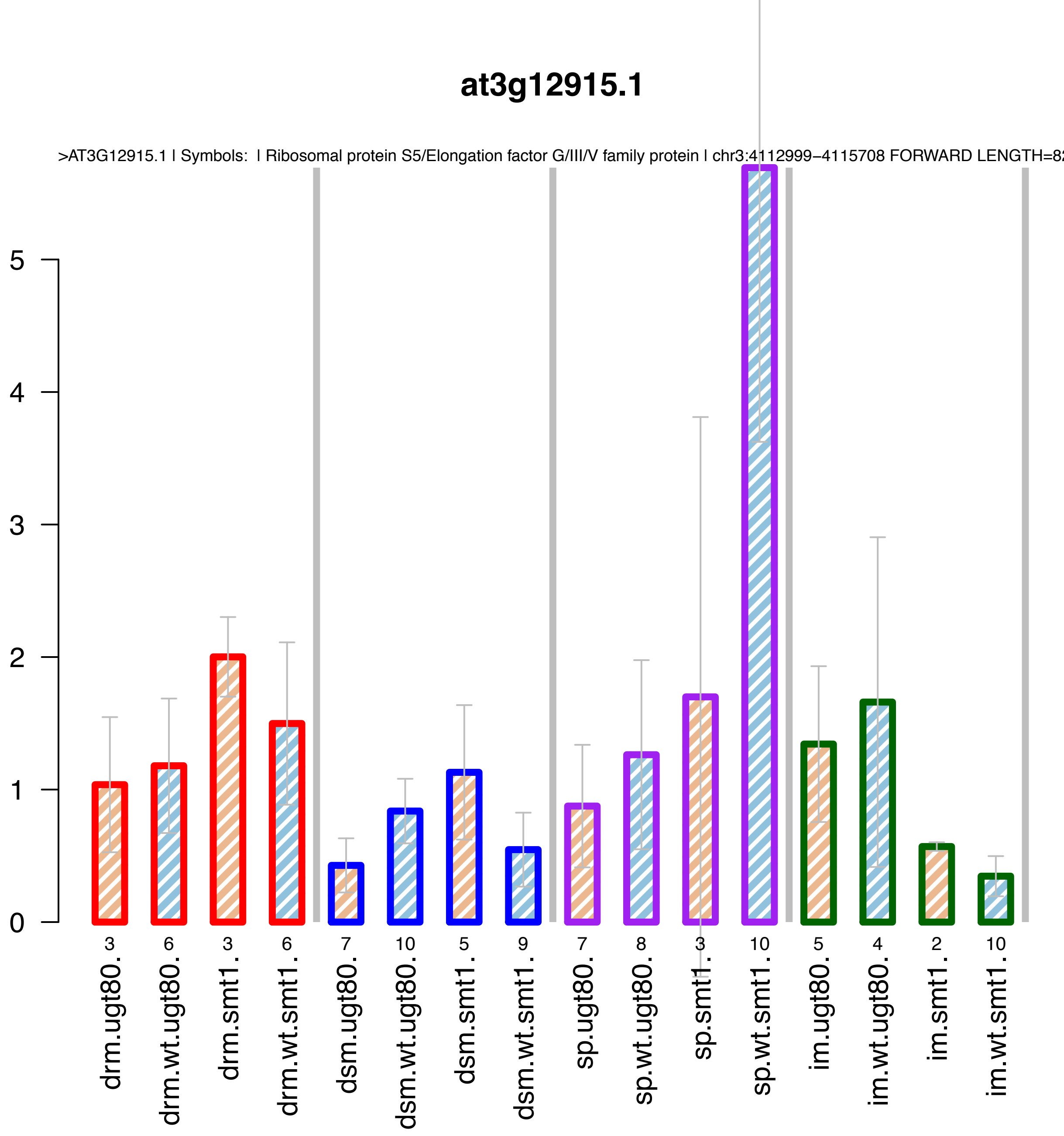

at3g12915.1
>AT3G12915.1 | Symbols: | Ribosomal protein S5/Elongation factor G/III/V family protein | chr3:4112999-4115708 FORWARD LENGTH=8
0
1
2
3
4
5
3
6
3
6
7
10
5
9
7
8
3
10
5
4
2
10
drm.ugt80.
drm.wt.ugt80.
drm.smt1.
drm.wt.smt1.
dsm.ugt80.
dsm.wt.ugt80.
dsm.smt1.
dsm.wt.smt1.
sp.ugt80.
sp.wt.ugt80.
sp.smt1.
sp.wt.smt1.
im.ugt80.
im.wt.ugt80.
im.smt1.
im.wt.smt1.

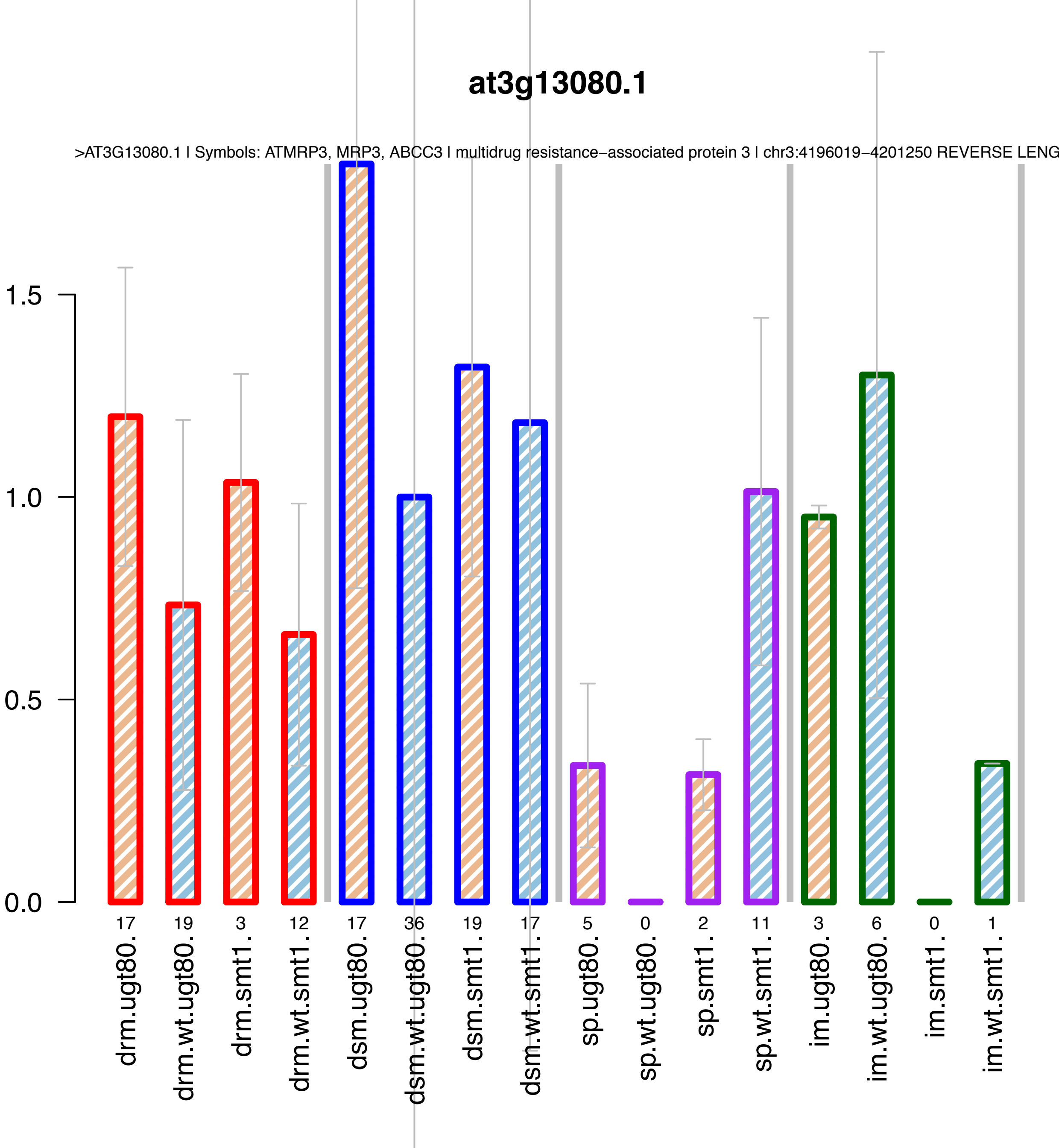

at3g13080.1
>AT3G13080.1 | Symbols: ATMRP3, MRP3, ABCC3 | multidrug resistance-associated protein 3 | chr3:4196019-4201250 REVERSE LENG
0.0
0.5
1.0
1.5
17
19
3
12
17
36
19
17
5
0
2
11
3
6
0
1
drm.ugt80.
drm.wt.ugt80.
drm.smt1.
drm.wt.smt1.
dsm.ugt80.
dsm.wt.ugt80.
dsm.smt1.
dsm.wt.smt1.
sp.ugt80.
sp.wt.ugt80.
sp.smt1.
sp.wt.smt1.
im.ugt80.
im.wt.ugt80.
im.smt1.
im.wt.smt1.

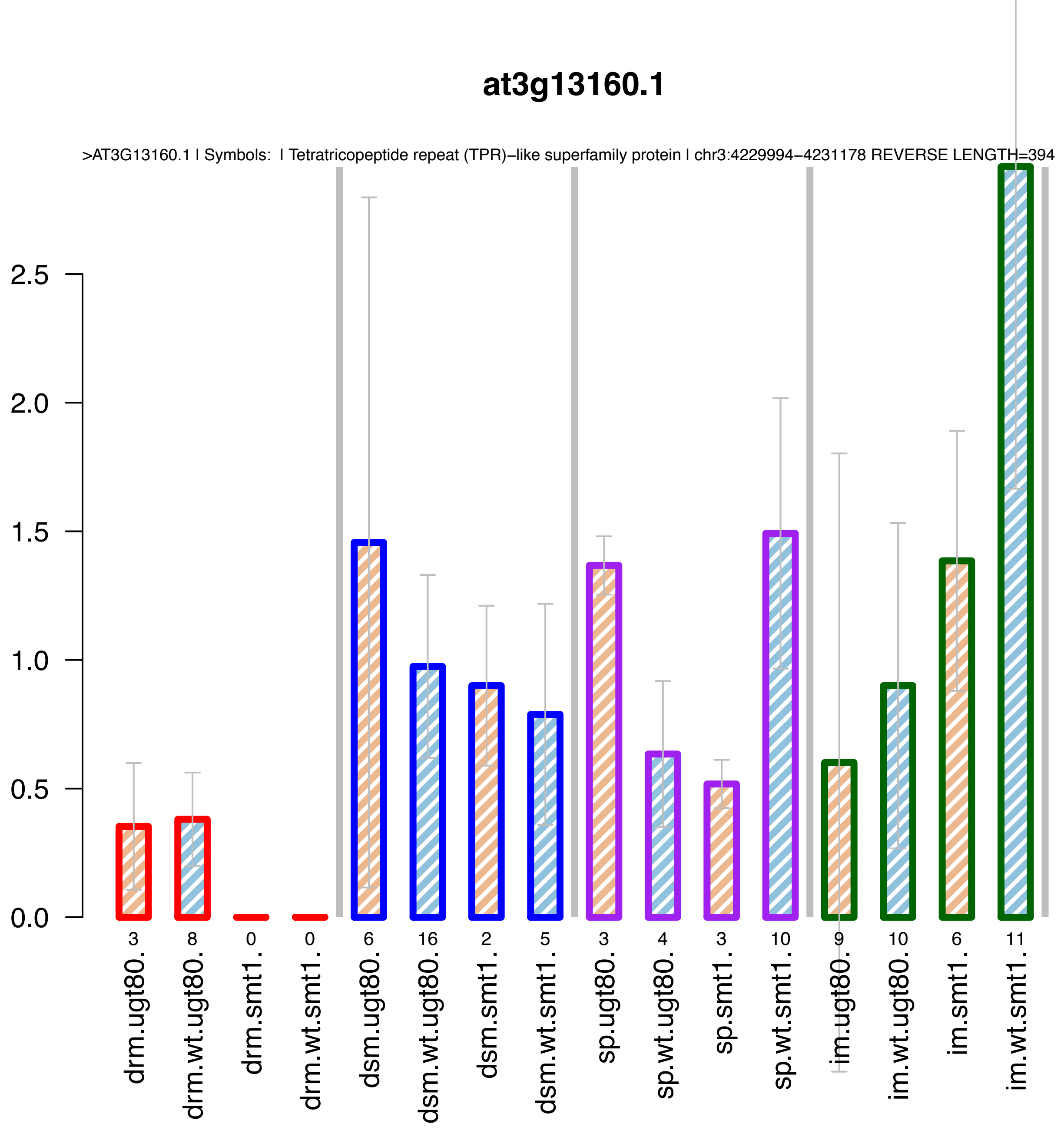

at3g13160.1
>AT3G13160.1 | Symbols: | Tetratricopeptide repeat (TPR)−like superfamily protein | chr3:4229994−4231178 REVERSE LENGTH=394
0.0
0.5
1.0
1.5
2.0
2.5
3
8
0
0
6
16
2
5
3
4
3
10
9
10
6
11
drm.ugt80.
drm.wt.ugt80.
drm.smt1.
drm.wt.smt1.
dsm.ugt80.
dsm.wt.ugt80.
dsm.smt1.
dsm.wt.smt1.
sp.ugt80.
sp.wt.ugt80.
sp.smt1.
sp.wt.smt1.
im.ugt80.
im.wt.ugt80.
im.smt1.
im.wt.smt1.

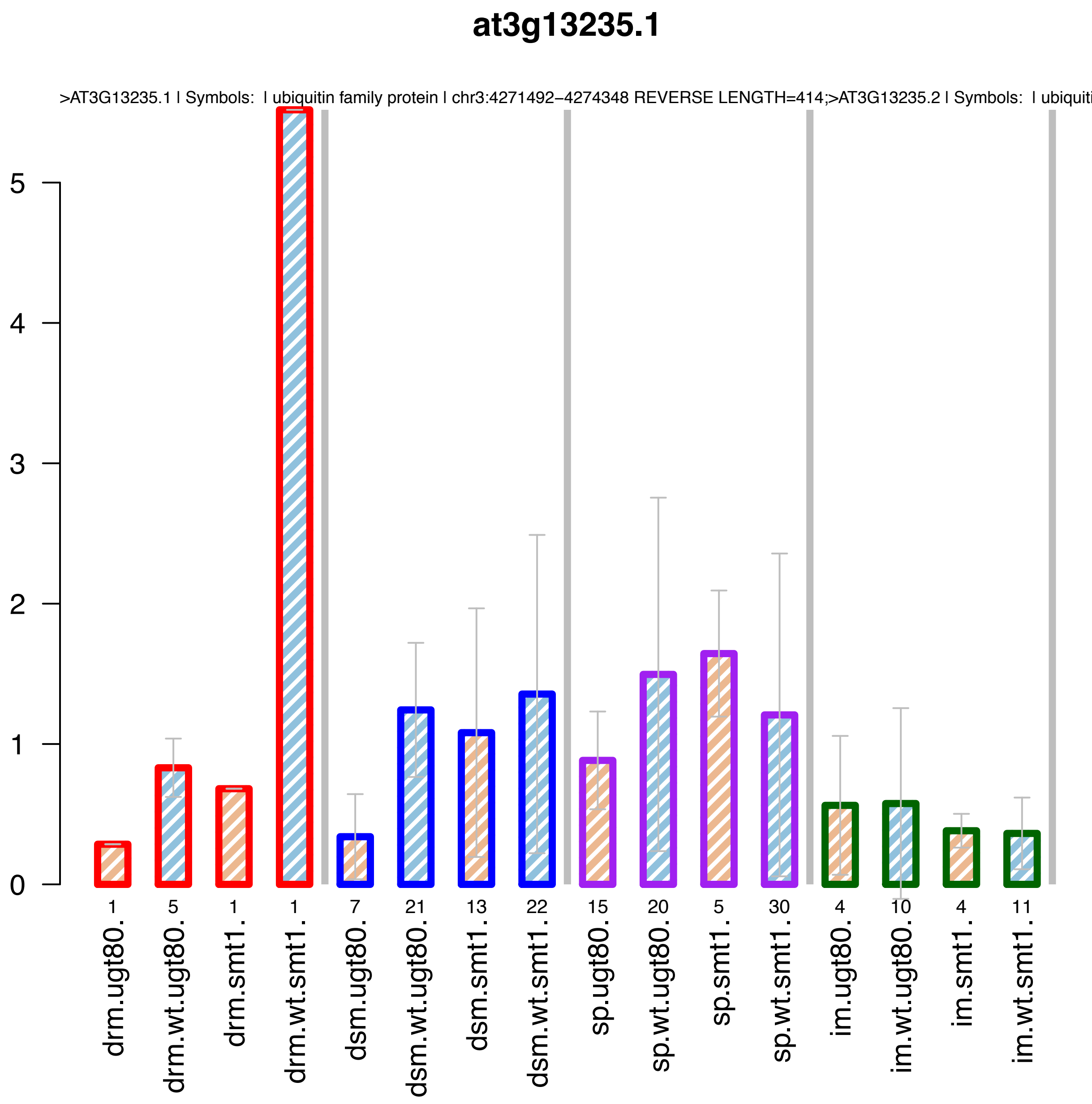

at3g13235.1
>AT3G13235.1 | Symbols: | ubiquitin family protein | chr3:4271492−4274348 REVERSE LENGTH=414;>AT3G13235.2 | Symbols: | ubiquiti
0
1
2
3
4
5
1
5
1
1
7
21
13
22
15
20
5
30
4
10
4
11
drm.ugt80.
drm.wt.ugt80.
drm.smt1.
drm.wt.smt1.
dsm.ugt80.
dsm.wt.ugt80.
dsm.smt1.
dsm.wt.smt1.
sp.ugt80.
sp.wt.ugt80.
sp.smt1.
sp.wt.smt1.
im.ugt80.
im.wt.ugt80.
im.smt1.
im.wt.smt1.

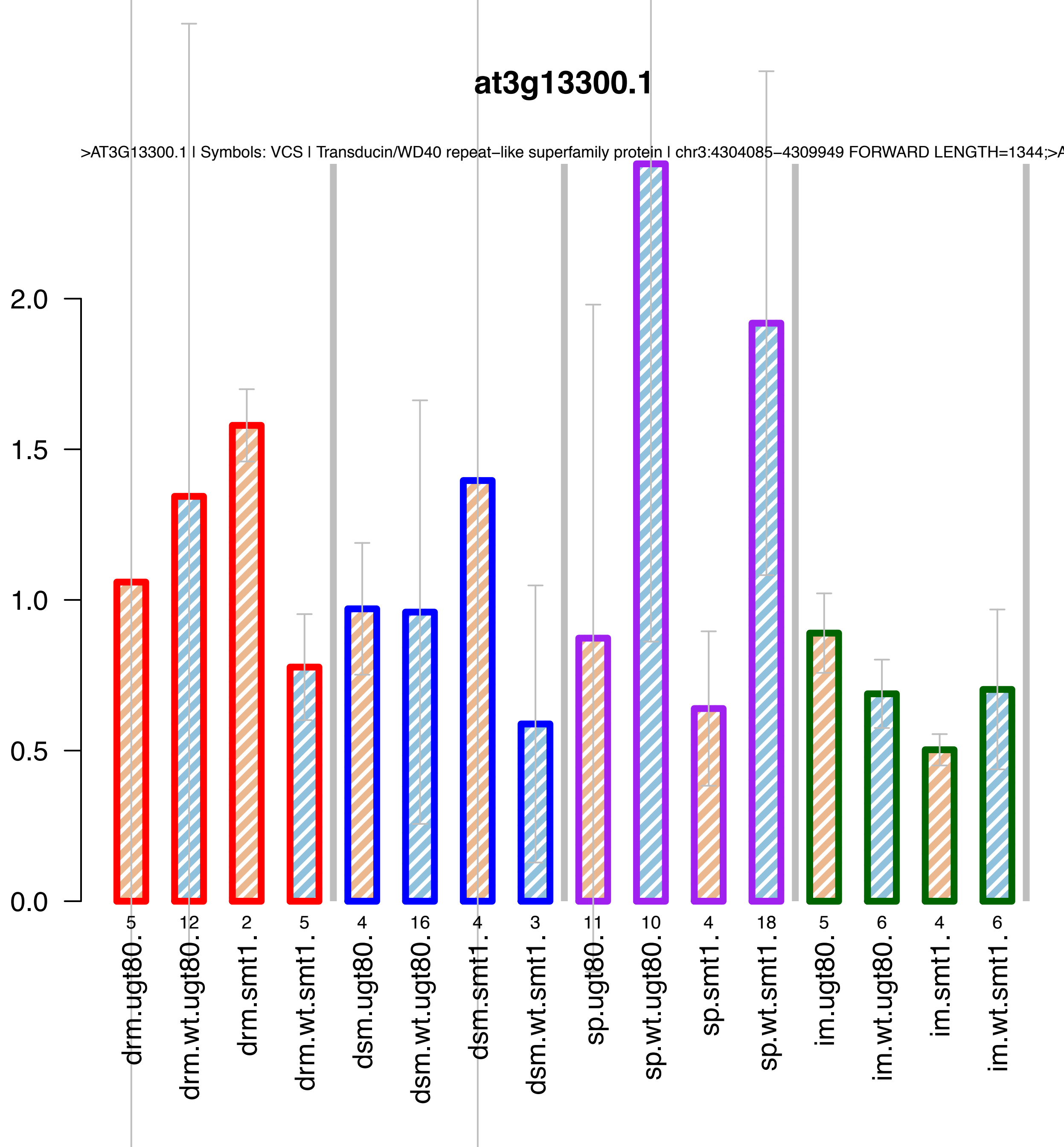

at3g13300.1
>AT3G13300.1 | Symbols: VCS | Transducin/WD40 repeat−like superfamily protein | chr3:4304085−4309949 FORWARD LENGTH=1344;>A
2.0
1.5
1.0
0.5
0.0
5
12
2
5
4
16
4
3
11
10
4
18
5
6
4
6
drm.ugt80..
drm.wt.ugt80..
drm.smt1..
drm.wt.smt1..
dsm.ugt80..
dsm.wt.ugt80..
dsm.smt1..
dsm.wt.smt1..
sp.ugt80..
sp.wt.ugt80..
sp.smt1..
sp.wt.smt1..
im.ugt80..
im.wt.ugt80..
im.smt1..
im.wt.smt1..

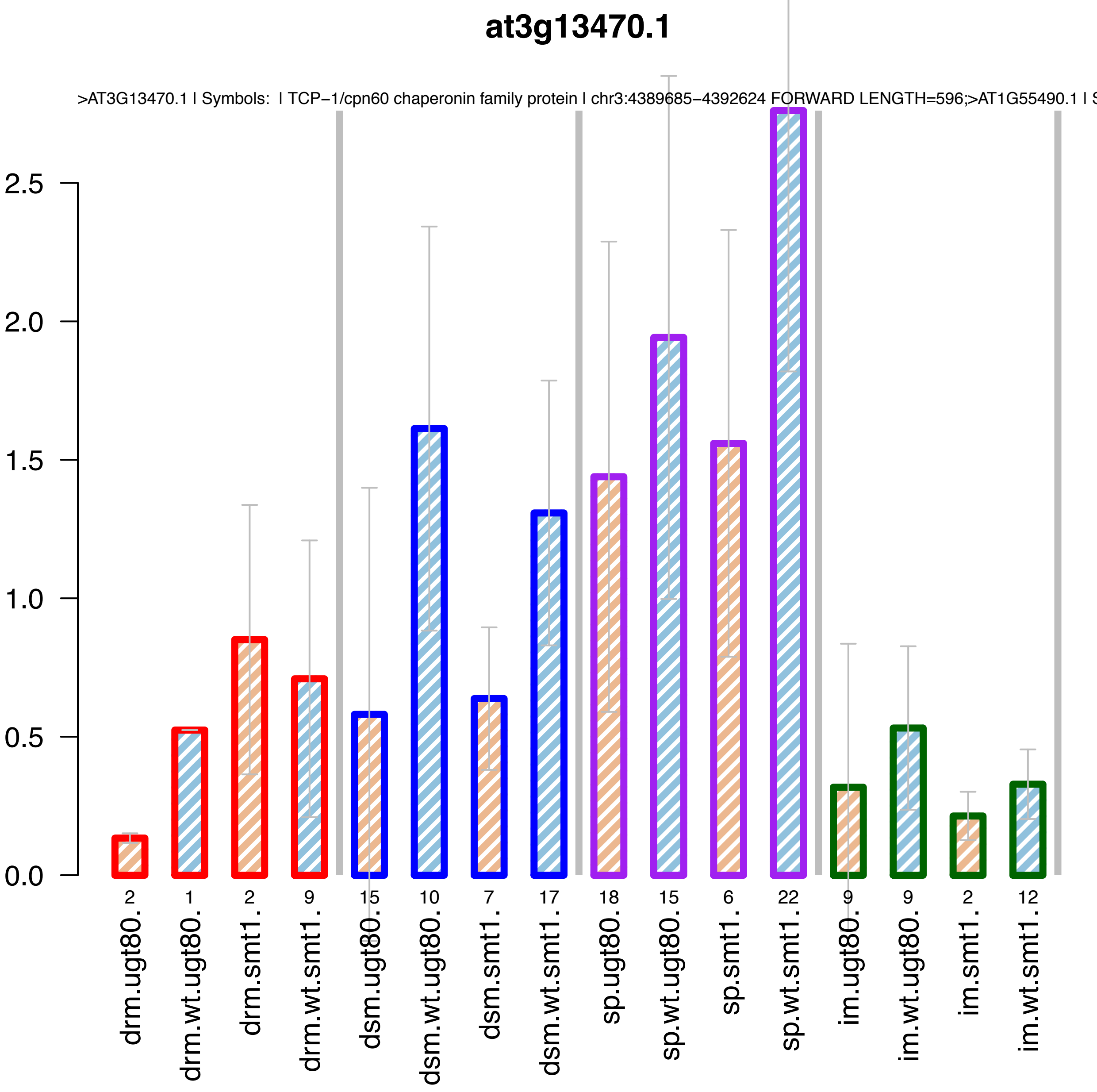

at3g13470.1
>AT3G13470.1 | Symbols: | TCP−1/cpn60 chaperonin family protein | chr3:4389685−4392624 FORWARD LENGTH=596;>AT1G55490.1 |
0.0
0.5
1.0
1.5
2.0
2.5
2
1
2
9
15
10
7
17
18
15
6
22
9
9
2
12
drm.ugt80..
drm.wt.ugt80..
drm.smt1..
drm.wt.smt1..
dsm.ugt80..
dsm.wt.ugt80..
dsm.smt1..
dsm.wt.smt1..
sp.ugt80..
sp.wt.ugt80..
sp.smt1..
sp.wt.smt1..
im.ugt80..
im.wt.ugt80..
im.smt1..
im.wt.smt1..

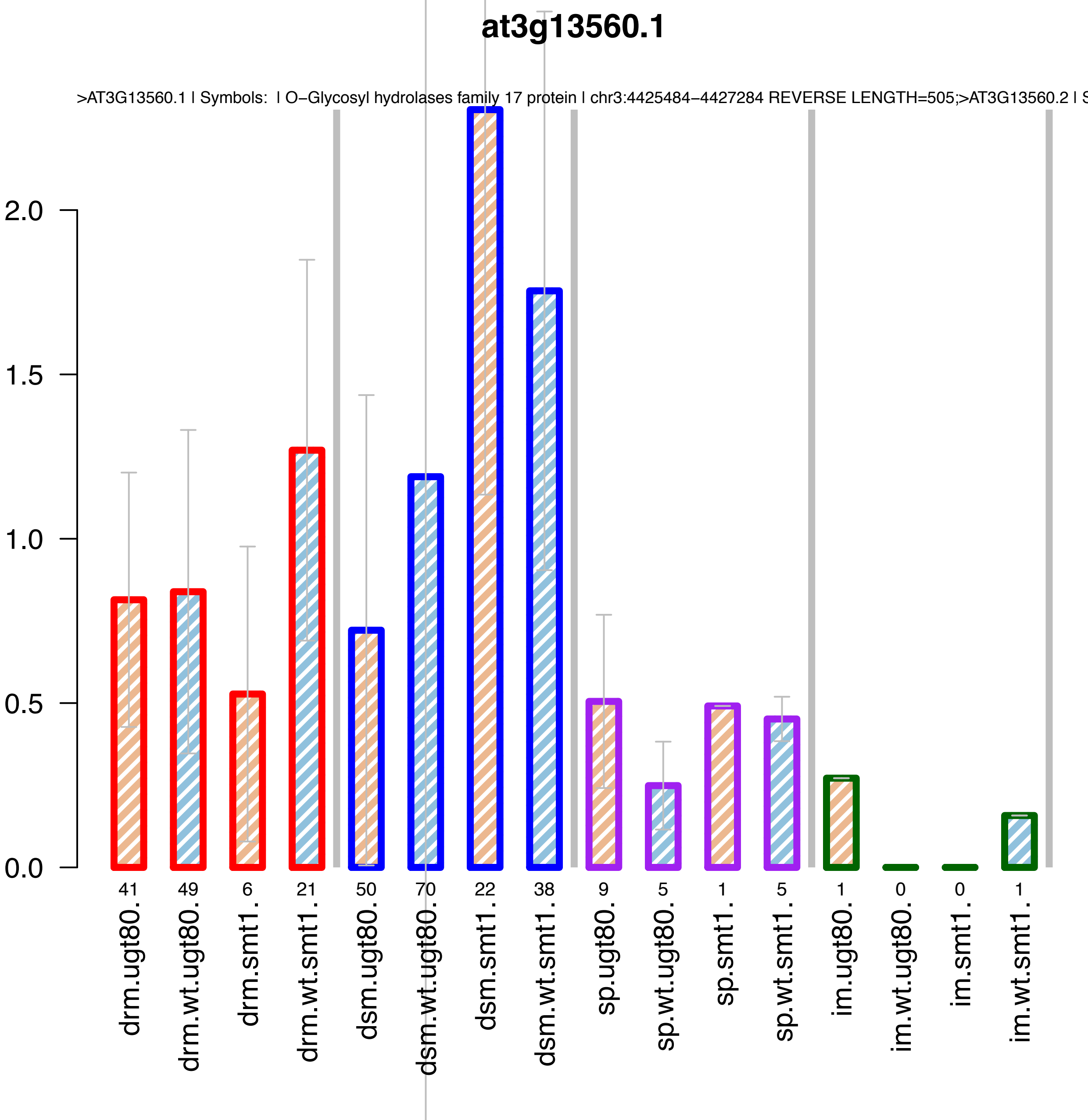

at3g13560.1
>AT3G13560.1 | Symbols: | O−Glycosyl hydrolases family 17 protein | chr3:4425484−4427284 REVERSE LENGTH=505;>AT3G13560.2 | S
2.0
1.5
1.0
0.5
0.0
41
49
6
21
50
70
22
38
9
5
1
5
1
0
0
1
drm.ugt80..
drm.wt.ugt80..
drm.smt1..
drm.wt.smt1..
dsm.ugt80..
dsm.wt.ugt80..
dsm.smt1..
dsm.wt.smt1..
sp.ugt80..
sp.wt.ugt80..
sp.smt1..
sp.wt.smt1..
im.ugt80..
im.wt.ugt80..
im.smt1..
im.wt.smt1..

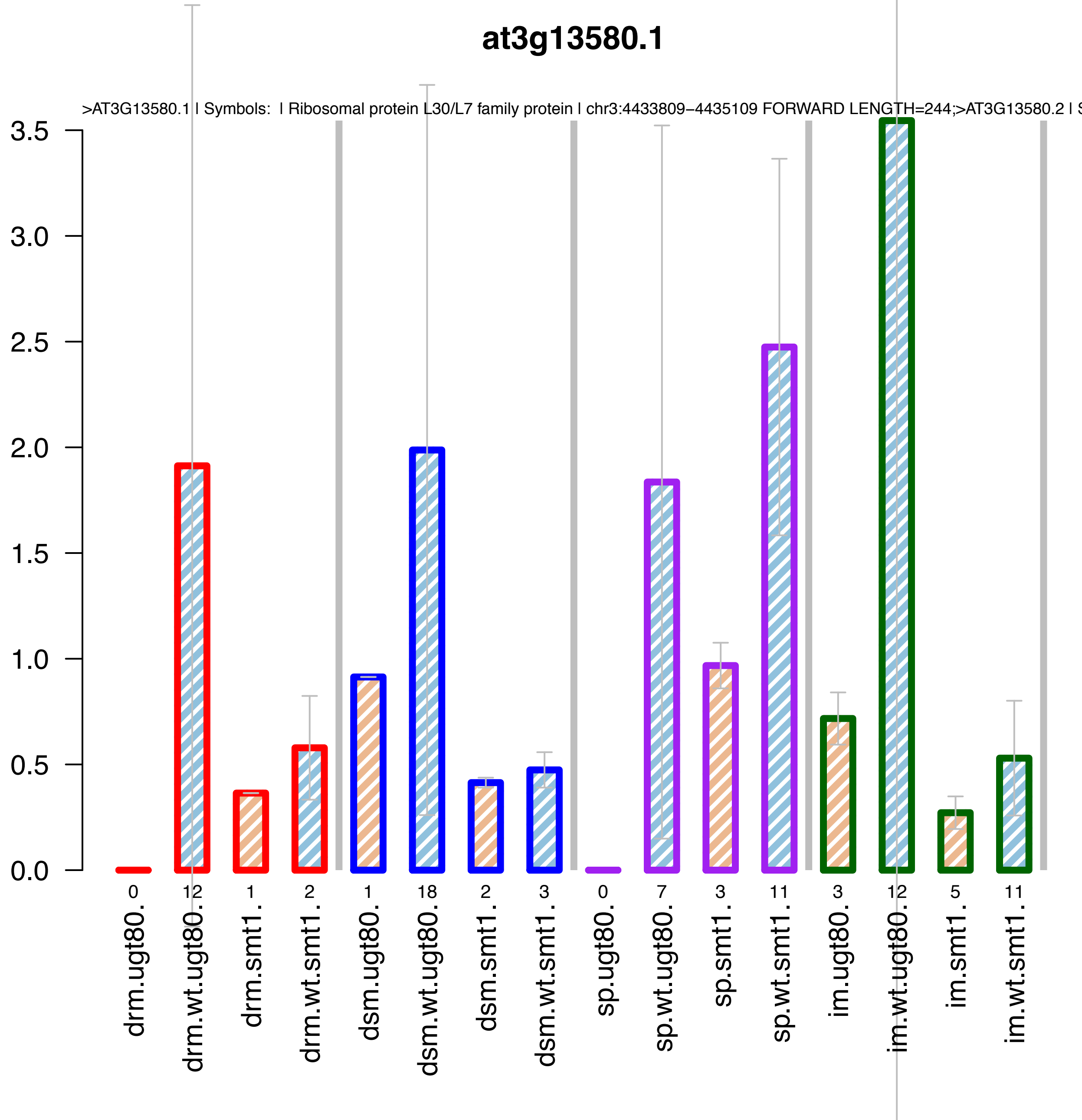

at3g13580.1
>AT3G13580.1 | Symbols: | Ribosomal protein L30/L7 family protein | chr3:4433809-4435109 FORWARD LENGTH=244;>AT3G13580.2 | S
3.5
3.0
2.5
2.0
1.5
1.0
0.5
0.0
0
12
1
2
1
18
2
3
0
7
3
11
3
12
5
11
drm.ugt80.
drm.wt.ugt80.
drm.smt1.
drm.wt.smt1.
dsm.ugt80.
dsm.wt.ugt80.
dsm.smt1.
dsm.wt.smt1.
sp.ugt80.
sp.wt.ugt80.
sp.smt1.
sp.wt.smt1.
im.ugt80.
im.wt.ugt80.
im.smt1.
im.wt.smt1.

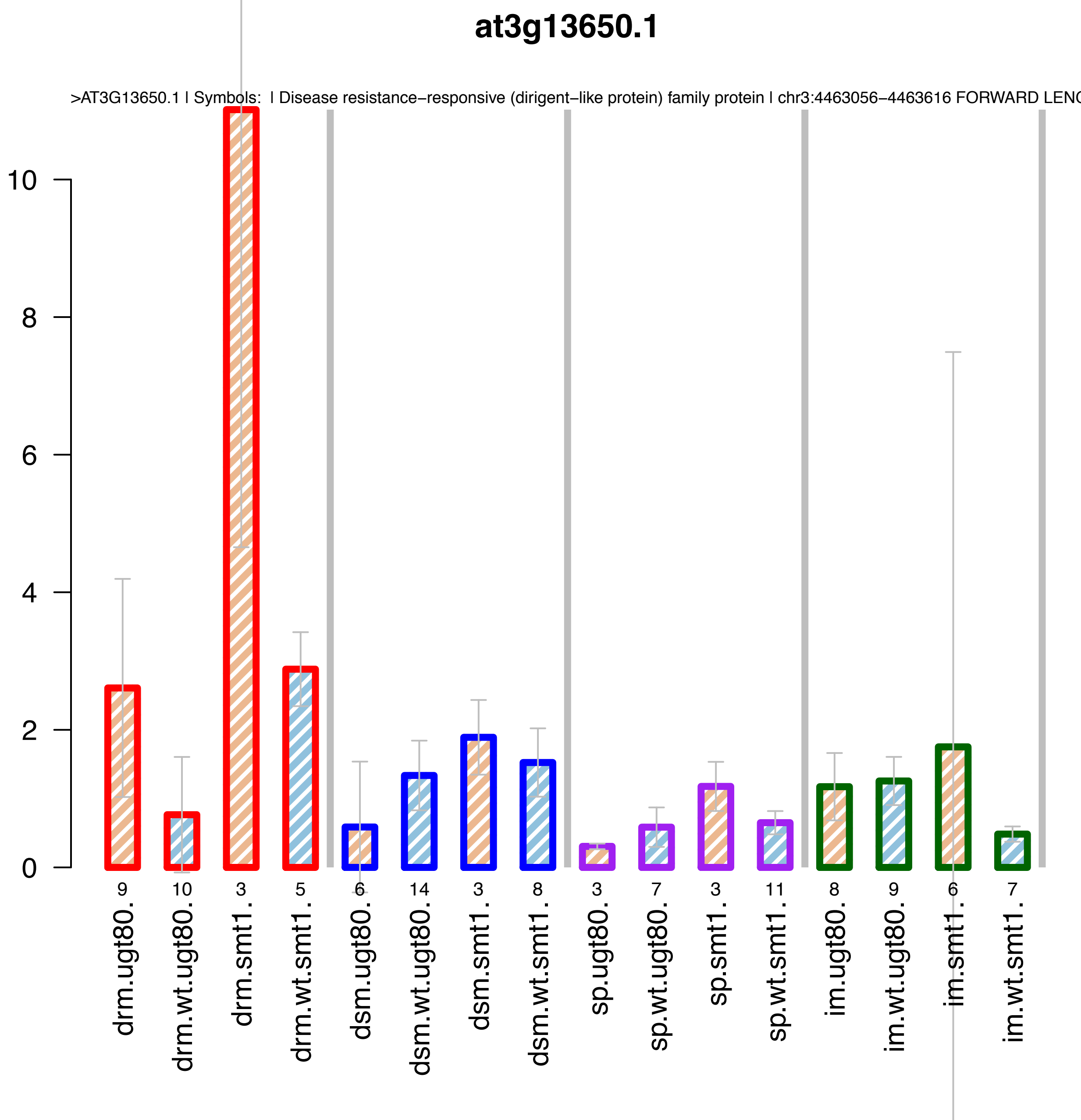

at3g13650.1
>AT3G13650.1 | Symbols: | Disease resistance−responsive (dirigent−like protein) family protein | chr3:4463056−4463616 FORWARD LENG
10
8
6
4
2
0
9
10
3
5
6
14
3
8
3
7
3
11
8
9
6
7
drm.ugt80.
drm.wt.ugt80.
drm.smt1.
drm.wt.smt1.
dsm.ugt80.
dsm.wt.ugt80.
dsm.smt1.
dsm.wt.smt1.
sp.ugt80.
sp.wt.ugt80.
sp.smt1.
sp.wt.smt1.
im.ugt80.
im.wt.ugt80.
im.smt1.
im.wt.smt1.

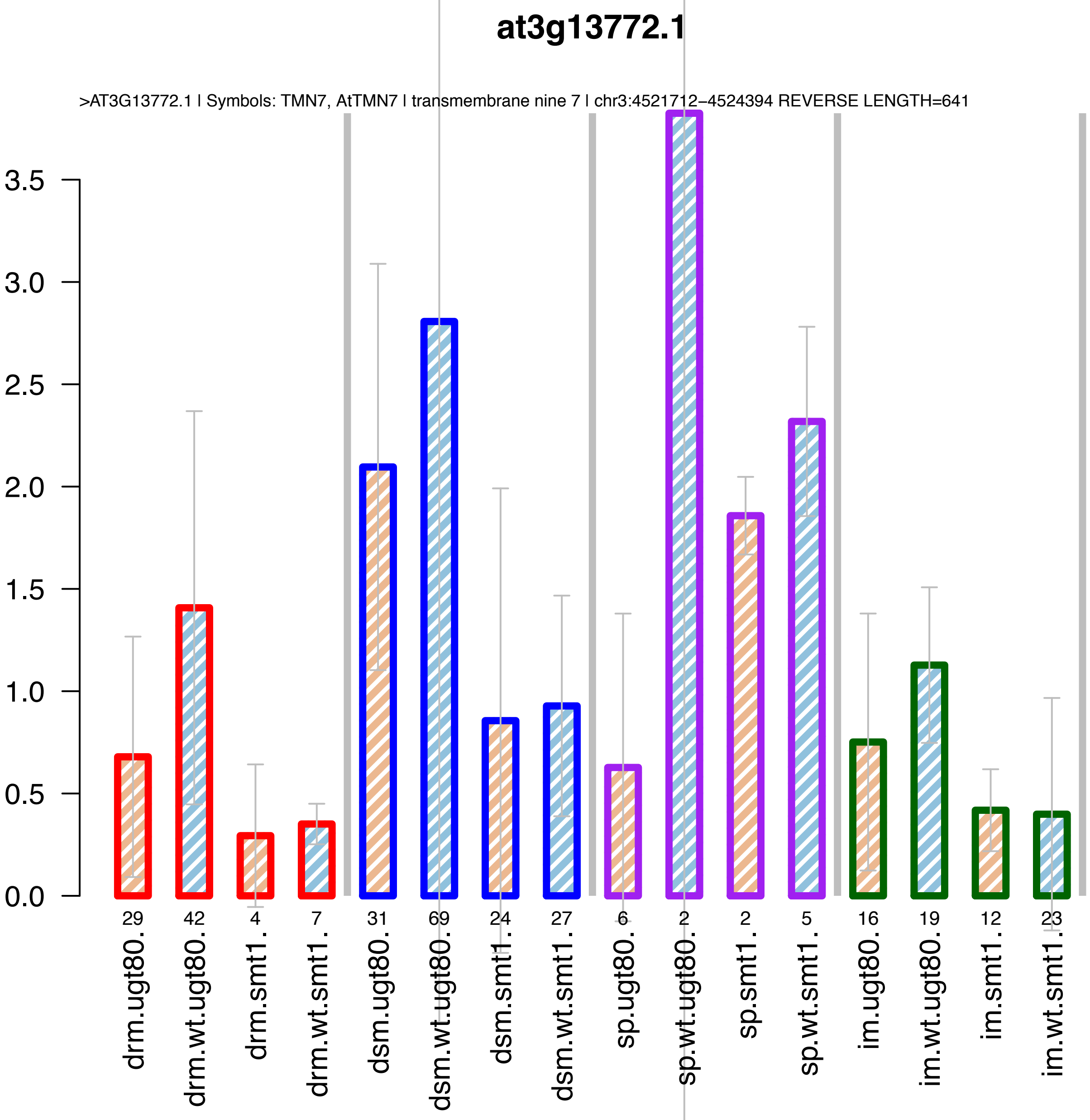

at3g13772.1
>AT3G13772.1 | Symbols: TMN7, AtTMN7 | transmembrane nine 7 | chr3:4521712-4524394 REVERSE LENGTH=641
0.0
0.5
1.0
1.5
2.0
2.5
3.0
3.5
29
42
4
7
31
69
24
27
6
2
2
5
16
19
12
23
drm.ugt80.
drm.wt.ugt80.
drm.smt1.
drm.wt.smt1.
dsm.ugt80.
dsm.wt.ugt80.
dsm.smt1.
dsm.wt.smt1.
sp.ugt80.
sp.wt.ugt80.
sp.smt1.
sp.wt.smt1.
im.ugt80.
im.wt.ugt80.
im.smt1.
im.wt.smt1.

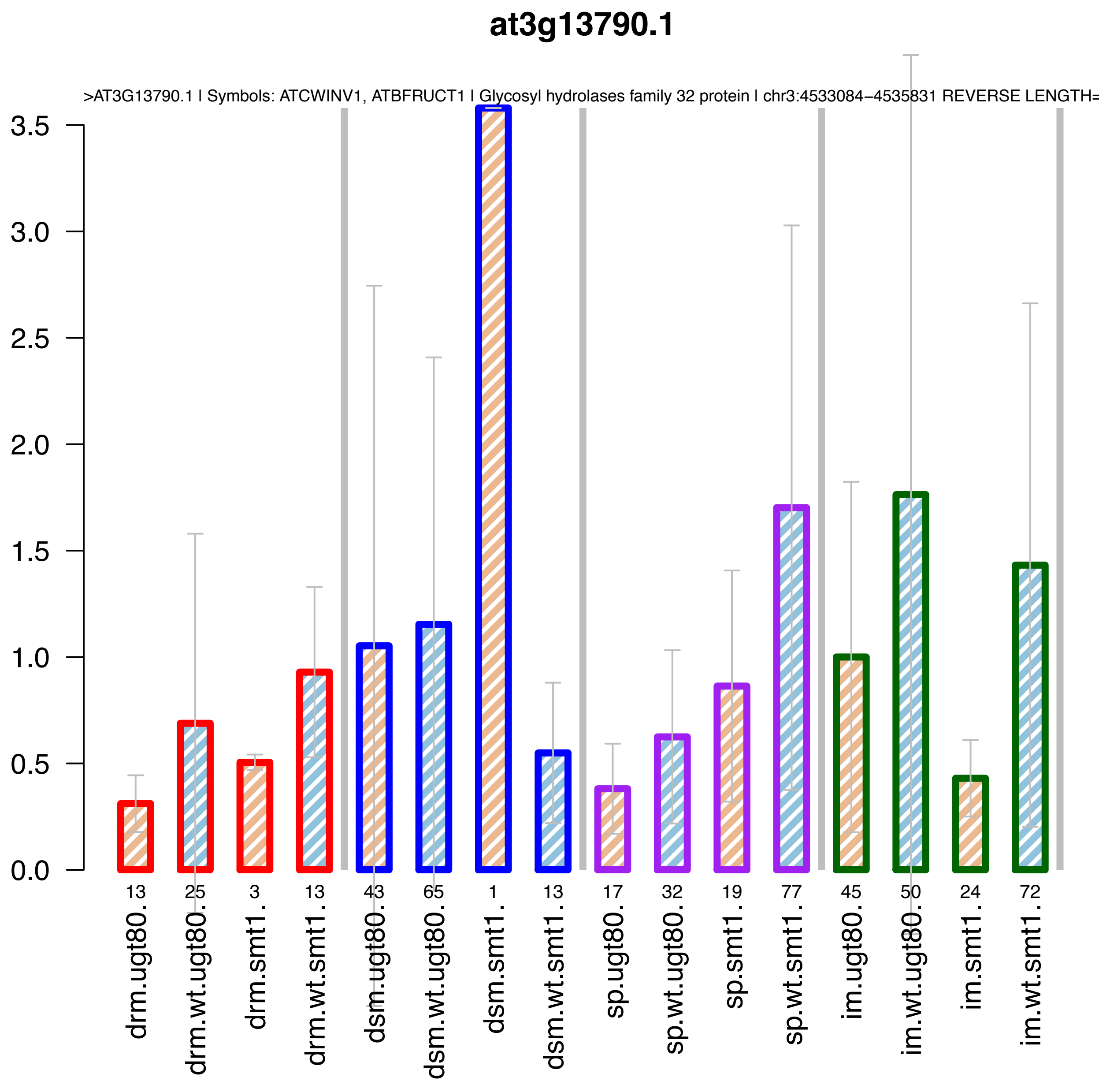
at3g13790.1
>AT3G13790.1 | Symbols: ATCWINV1, ATBFRUCT1 | Glycosyl hydrolases family 32 protein | chr3:4533084-4535831 REVERSE LENGTH=
3.5
3.0
2.5
2.0
1.5
1.0
0.5
0.0
13
25
3
13
43
65
1
13
17
32
19
77
45
50
24
72
drm.ugt80.
drm.wt.ugt80.
drm.smt1.
drm.wt.smt1.
dsm.ugt80.
dsm.wt.ugt80.
dsm.smt1.
dsm.wt.smt1.
sp.ugt80.
sp.wt.ugt80.
sp.smt1.
sp.wt.smt1.
im.ugt80.
im.wt.ugt80.
im.smt1.
im.wt.smt1.

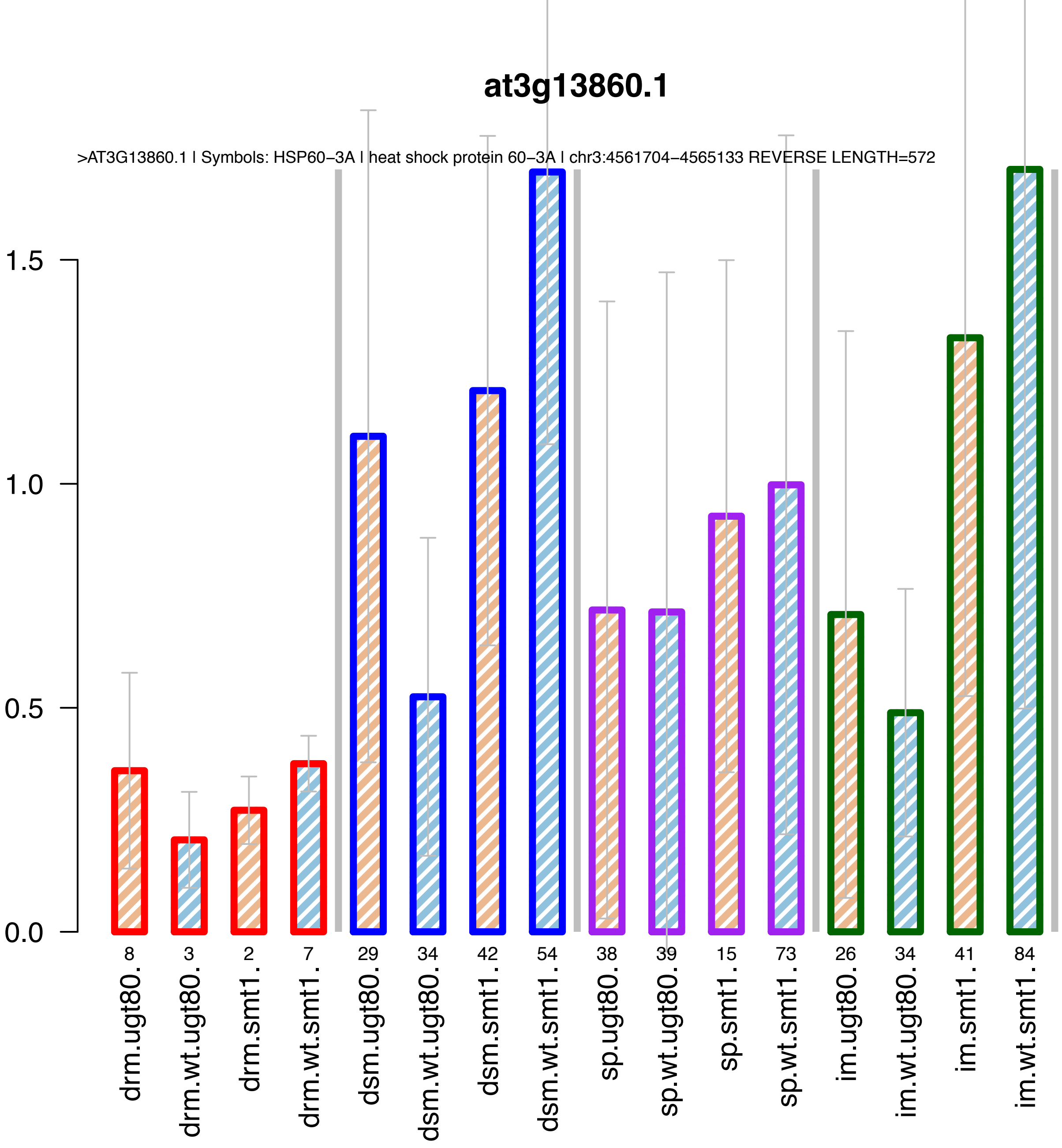

at3g13860.1
>AT3G13860.1 | Symbols: HSP60−3A | heat shock protein 60−3A | chr3:4561704−4565133 REVERSE LENGTH=572
0.0
0.5
1.0
1.5
8
3
2
7
29
34
42
54
38
39
15
73
26
34
41
84
drm.ugt80.
drm.wt.ugt80.
drm.smt1.
drm.wt.smt1.
dsm.ugt80.
dsm.wt.ugt80.
dsm.smt1.
dsm.wt.smt1.
sp.ugt80.
sp.wt.ugt80.
sp.smt1.
sp.wt.smt1.
im.ugt80.
im.wt.ugt80.
im.smt1.
im.wt.smt1.

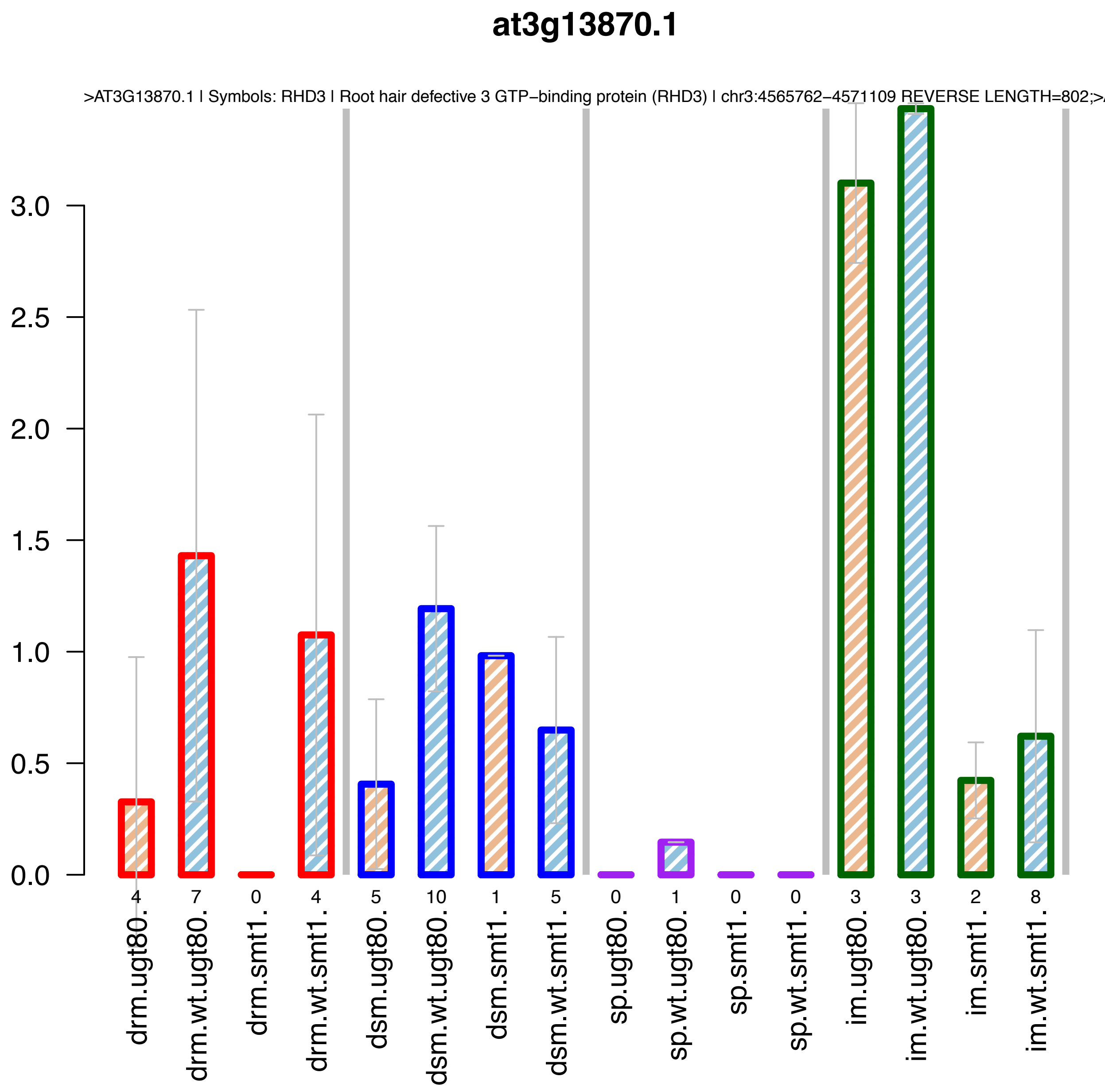
at3g13870.1
>AT3G13870.1 | Symbols: RHD3 | Root hair defective 3 GTP−binding protein (RHD3) | chr3:4565762−4571109 REVERSE LENGTH=802;>
3.0
2.5
2.0
1.5
1.0
0.5
0.0
4
7
0
4
5
10
1
5
0
1
0
0
3
3
2
8
drm.ugt80.
drm.wt.ugt80.
drm.smt1.
drm.wt.smt1.
dsm.ugt80.
dsm.wt.ugt80.
dsm.smt1.
dsm.wt.smt1.
sp.ugt80.
sp.wt.ugt80.
sp.smt1.
sp.wt.smt1.
im.ugt80.
im.wt.ugt80.
im.smt1.
im.wt.smt1.

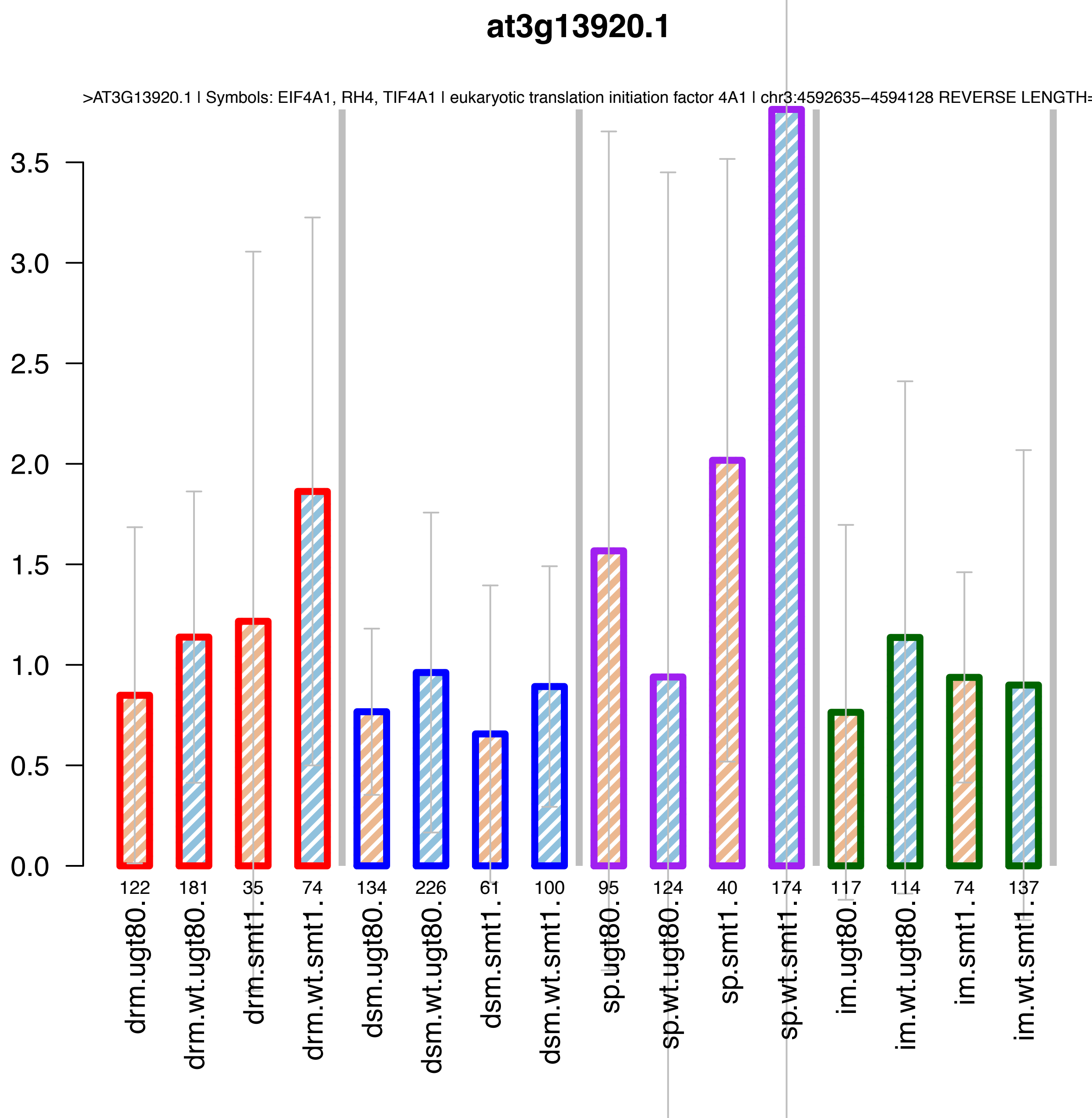

at3g13920.1
>AT3G13920.1 | Symbols: EIF4A1, RH4, TIF4A1 | eukaryotic translation initiation factor 4A1 | chr3:4592635-4594128 REVERSE LENGTH=
0.0
0.5
1.0
1.5
2.0
2.5
3.0
3.5
122
181
35
74
134
226
61
100
95
124
40
174
117
114
74
137
drm.ugt80..
drm.wt.ugt80..
drm.smt1..
drm.wt.smt1..
dsm.ugt80..
dsm.wt.ugt80..
dsm.smt1..
dsm.wt.smt1..
sp.ugt80..
sp.wt.ugt80..
sp.smt1..
sp.wt.smt1..
im.ugt80..
im.wt.ugt80..
im.smt1..
im.wt.smt1..

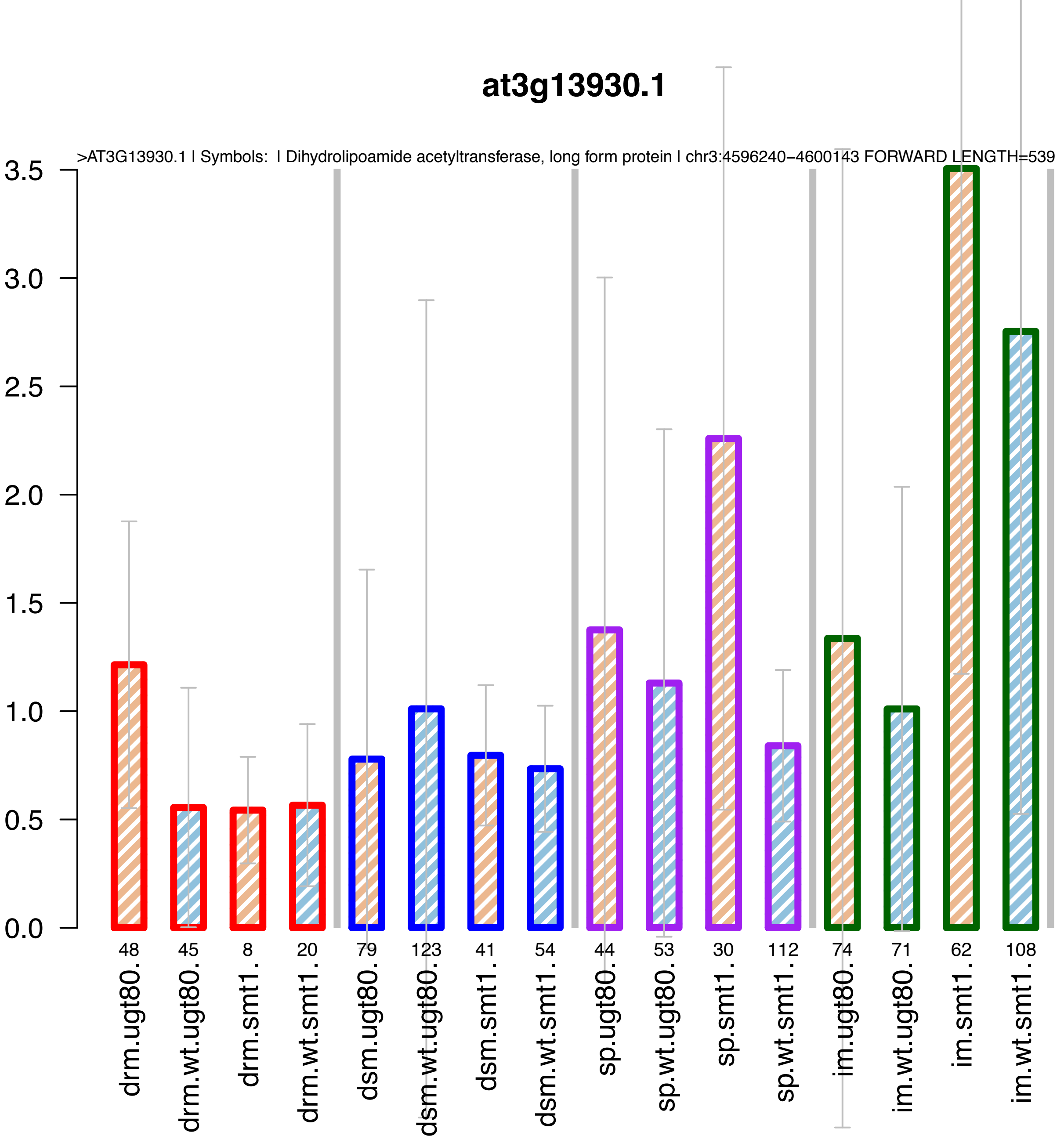

at3g13930.1
>AT3G13930.1 | Symbols: | Dihydrolipoamide acetyltransferase, long form protein | chr3:4596240-4600143 FORWARD LENGTH=539
0.0
0.5
1.0
1.5
2.0
2.5
3.0
3.5
48
45
8
20
79
123
41
54
44
53
30
112
74
71
62
108
drm.ugt80..
drm.wt.ugt80..
drm.smt1..
drm.wt.smt1..
dsm.ugt80..
dsm.wt.ugt80..
dsm.smt1..
dsm.wt.smt1..
sp.ugt80..
sp.wt.ugt80..
sp.smt1..
sp.wt.smt1..
im.ugt80..
im.wt.ugt80..
im.smt1..
im.wt.smt1..

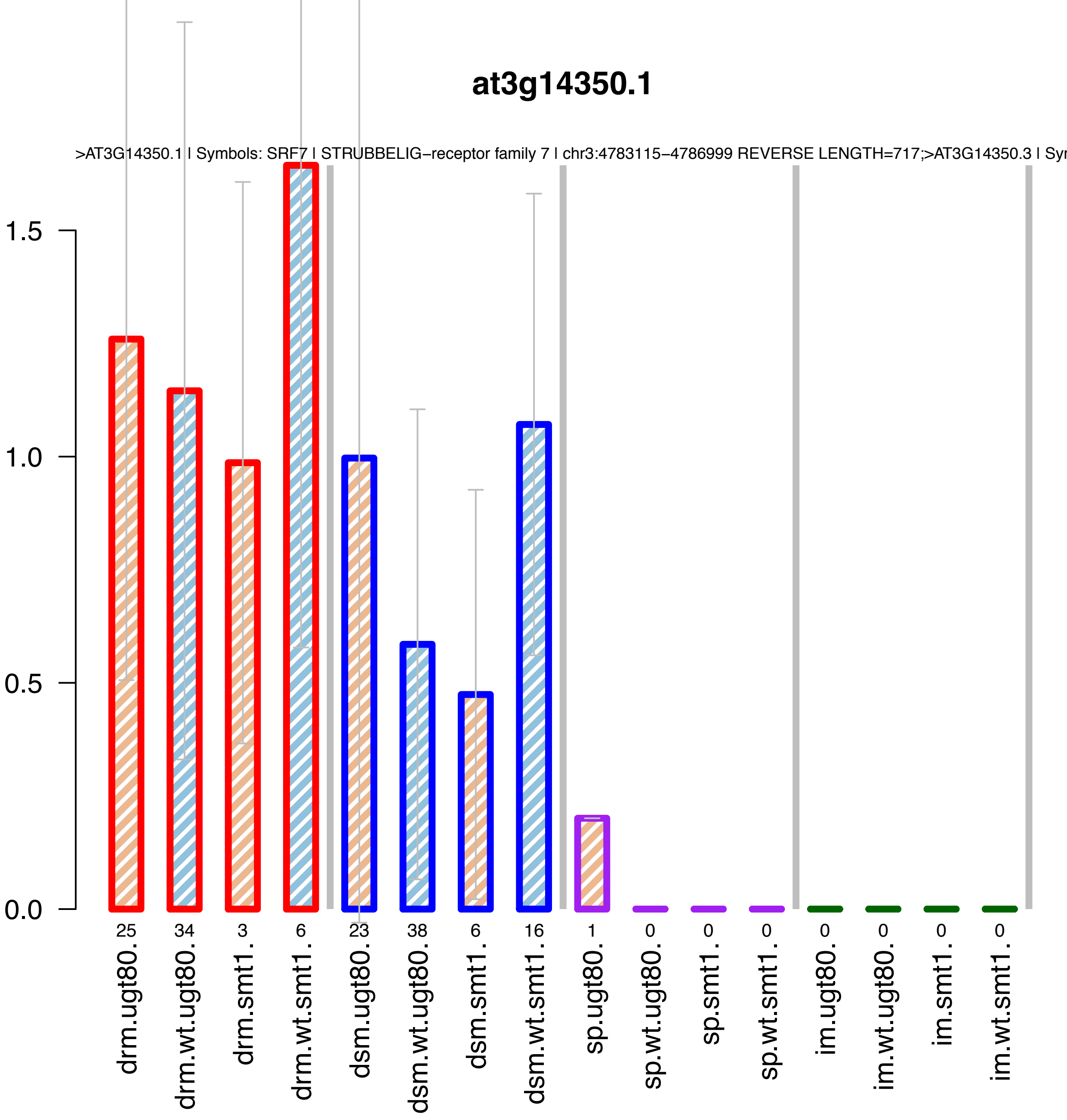

at3g14350.1
>AT3G14350.1 | Symbols: SRF7 | STRUBBELIG-receptor family 7 | chr3:4783115-4786999 REVERSE LENGTH=717;>AT3G14350.3 | Syr
1.5
1.0
0.5
0.0
25
34
3
6
23
38
6
16
1
0
0
0
0
0
0
0
drm.ugt80.
drm.wt.ugt80.
drm.smt1.
drm.wt.smt1.
dsm.ugt80.
dsm.wt.ugt80.
dsm.smt1.
dsm.wt.smt1.
sp.ugt80.
sp.wt.ugt80.
sp.smt1.
sp.wt.smt1.
im.ugt80.
im.wt.ugt80.
im.smt1.
im.wt.smt1.

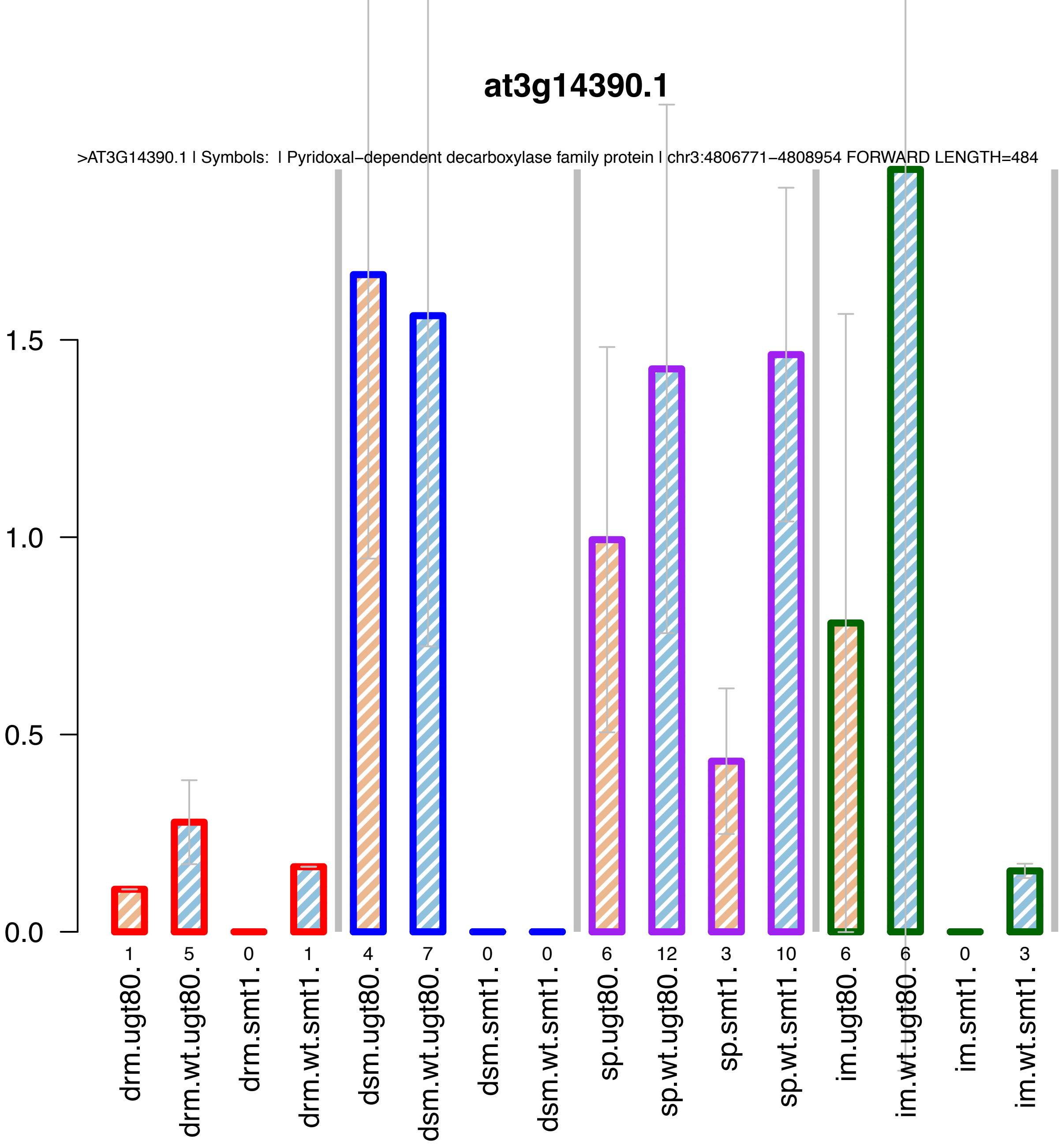

at3g14390.1
>AT3G14390.1 | Symbols: | Pyridoxal−dependent decarboxylase family protein | chr3:4806771−4808954 FORWARD LENGTH=484
0.0
0.5
1.0
1.5
1
5
0
1
4
7
0
0
6
12
3
10
6
6
0
3
drm.ugt80..
drm.wt.ugt80..
drm.smt1..
drm.wt.smt1..
dsm.ugt80..
dsm.wt.ugt80..
dsm.smt1..
dsm.wt.smt1..
sp.ugt80..
sp.wt.ugt80..
sp.smt1..
sp.wt.smt1..
im.ugt80..
im.wt.ugt80..
im.smt1..
im.wt.smt1..

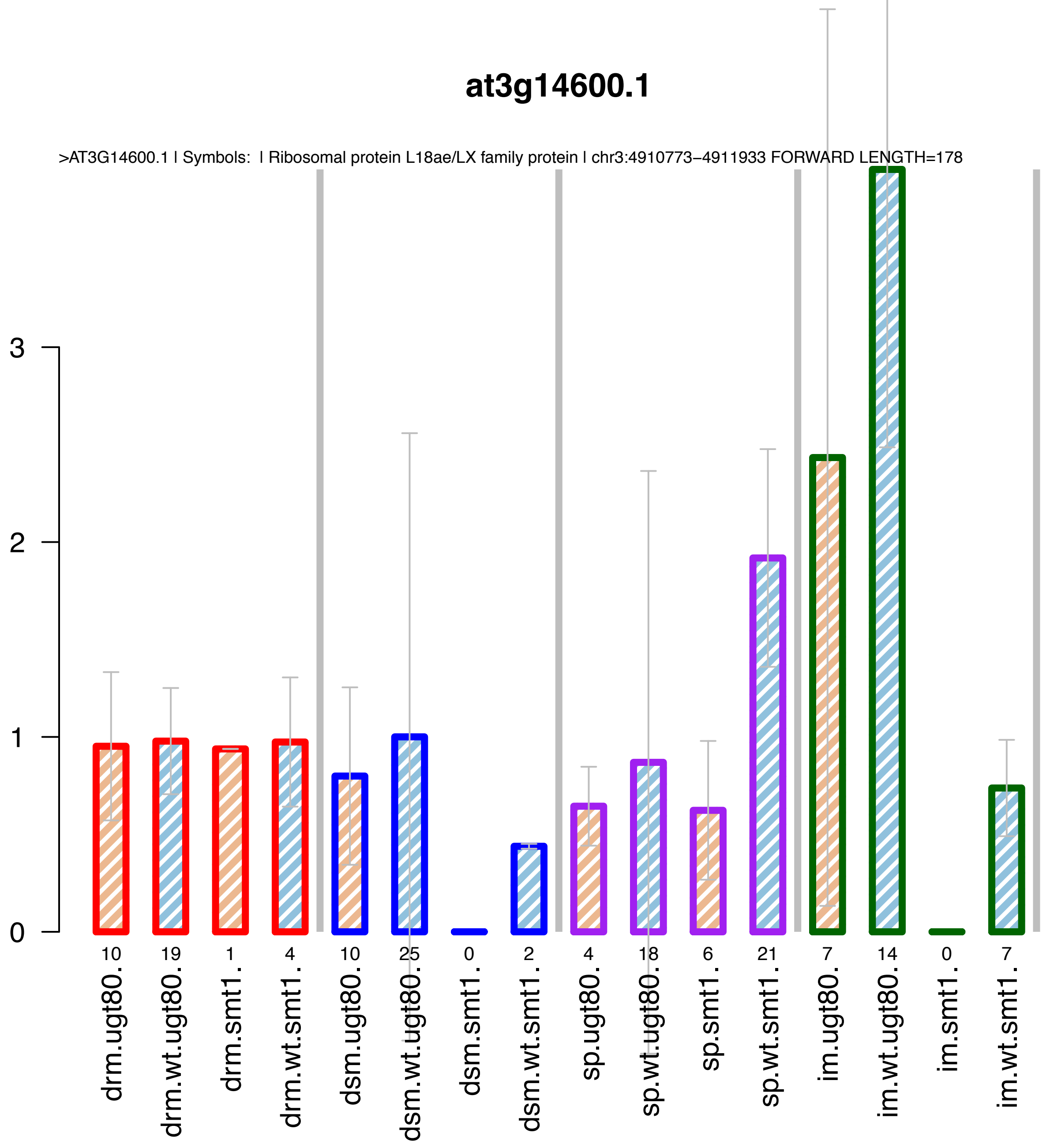

at3g14600.1
>AT3G14600.1 | Symbols: | Ribosomal protein L18ae/LX family protein | chr3:4910773-4911933 FORWARD LENGTH=178
0
1
2
3
10
19
1
4
10
25
0
2
4
18
6
21
7
14
0
7
drm.ugt80.
drm.wt.ugt80.
drm.smt1.
drm.wt.smt1.
dsm.ugt80.
dsm.wt.ugt80.
dsm.smt1.
dsm.wt.smt1.
sp.ugt80.
sp.wt.ugt80.
sp.smt1.
sp.wt.smt1.
im.ugt80.
im.wt.ugt80.
im.smt1.
im.wt.smt1.

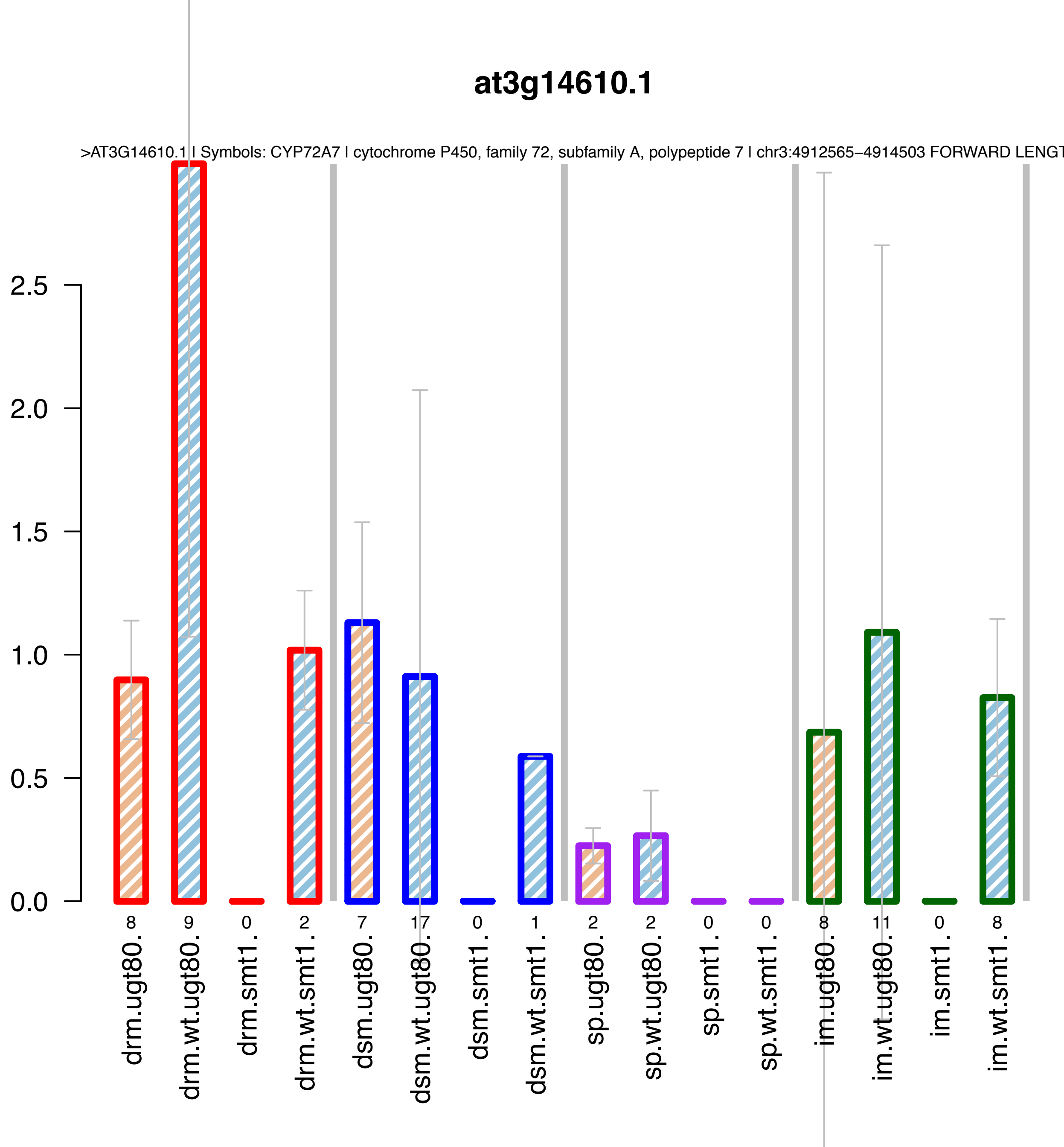
at3g14610.1
>AT3G14610.1 | Symbols: CYP72A7 | cytochrome P450, family 72, subfamily A, polypeptide 7 | chr3:4912565-4914503 FORWARD LENGT
0.0
0.5
1.0
1.5
2.0
2.5
8
9
0
2
7
17
0
1
2
2
0
0
8
11
0
8
drm.ugt80.
drm.wt.ugt80.
drm.smt1.
drm.wt.smt1.
dsm.ugt80.
dsm.wt.ugt80.
dsm.smt1.
dsm.wt.smt1.
sp.ugt80.
sp.wt.ugt80.
sp.smt1.
sp.wt.smt1.
im.ugt80.
im.wt.ugt80.
im.smt1.
im.wt.smt1.

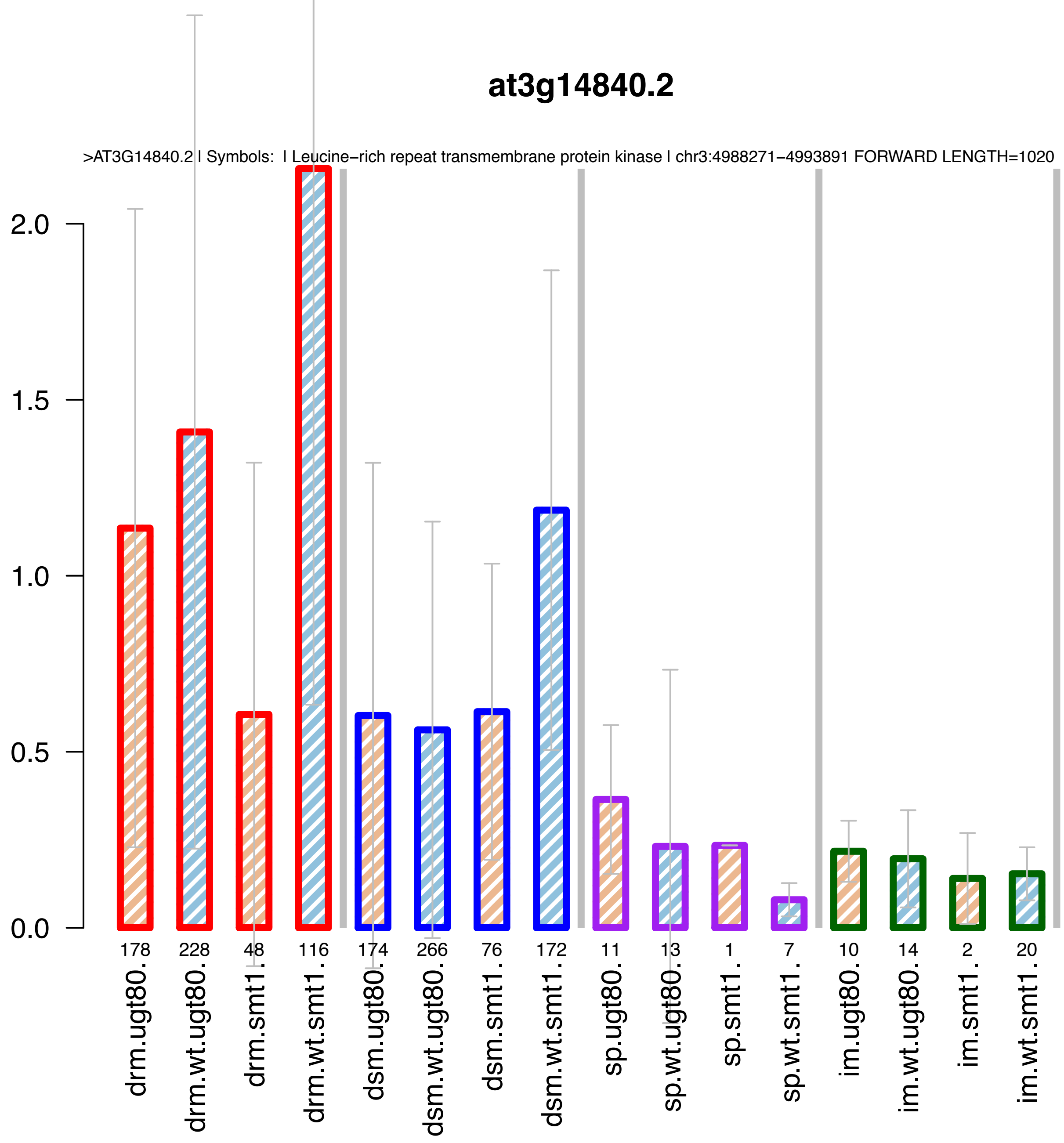
at3g14840.2
>AT3G14840.2 | Symbols: | Leucine−rich repeat transmembrane protein kinase | chr3:4988271−4993891 FORWARD LENGTH=1020
2.0
1.5
1.0
0.5
0.0
178
228
48
116
174
266
76
172
11
13
1
7
10
14
2
20
drm.ugt80..
drm.wt.ugt80..
drm.smt1..
drm.wt.smt1..
dsm.ugt80..
dsm.wt.ugt80..
dsm.smt1..
dsm.wt.smt1..
sp.ugt80..
sp.wt.ugt80..
sp.smt1..
sp.wt.smt1..
im.ugt80..
im.wt.ugt80..
im.smt1..
im.wt.smt1..

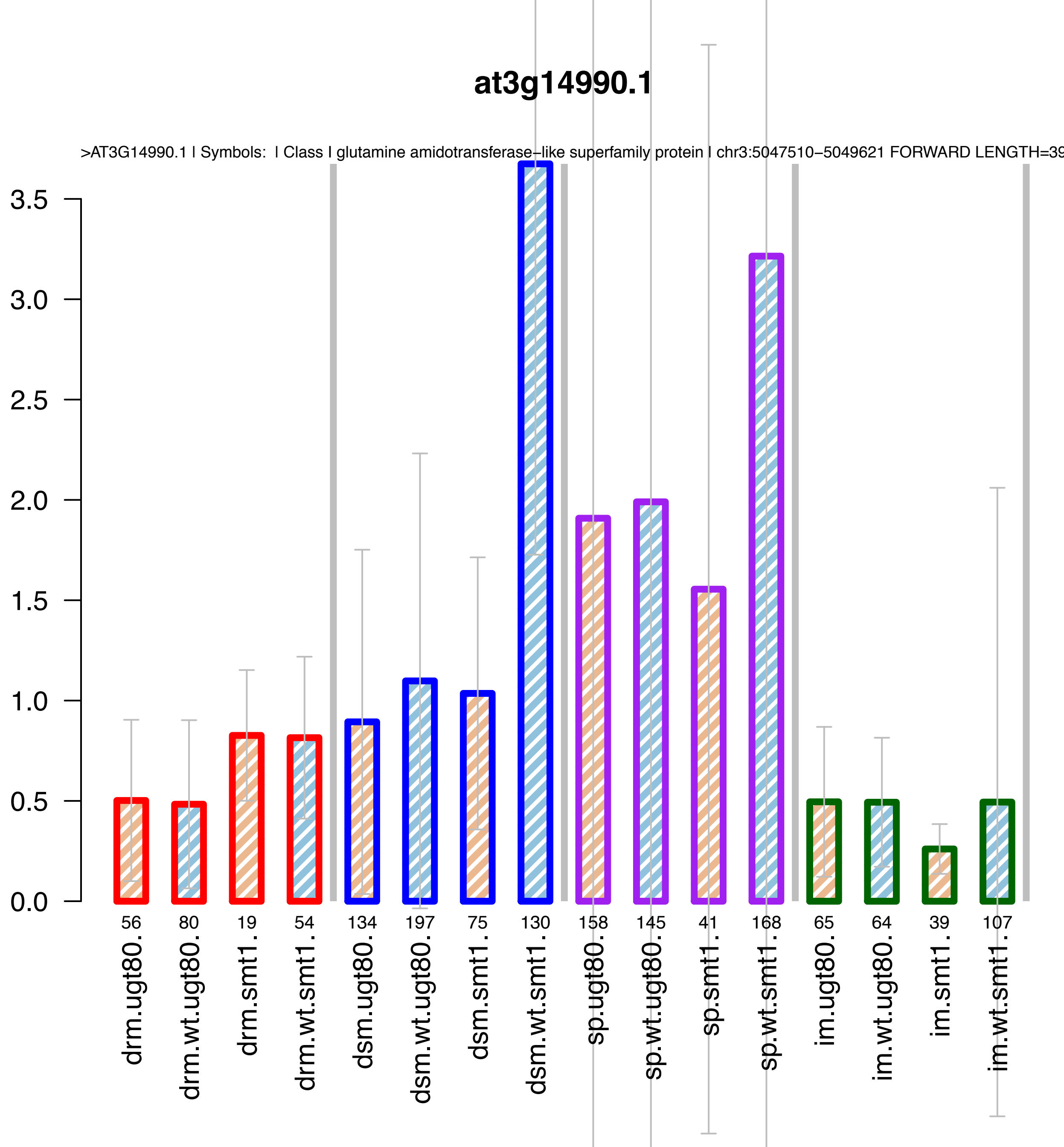
at3g14990.1
>AT3G14990.1 | Symbols: | Class I glutamine amidotransferase−like superfamily protein | chr3:5047510−5049621 FORWARD LENGTH=39
3.5
3.0
2.5
2.0
1.5
1.0
0.5
0.0
56
80
19
54
134
197
75
130
158
145
41
168
65
64
39
107
drm.ugt80..
drm.wt.ugt80..
drm.smt1..
drm.wt.smt1..
dsm.ugt80..
dsm.wt.ugt80..
dsm.smt1..
dsm.wt.smt1..
sp.ugt80..
sp.wt.ugt80..
sp.smt1..
sp.wt.smt1..
im.ugt80..
im.wt.ugt80..
im.smt1..
im.wt.smt1..

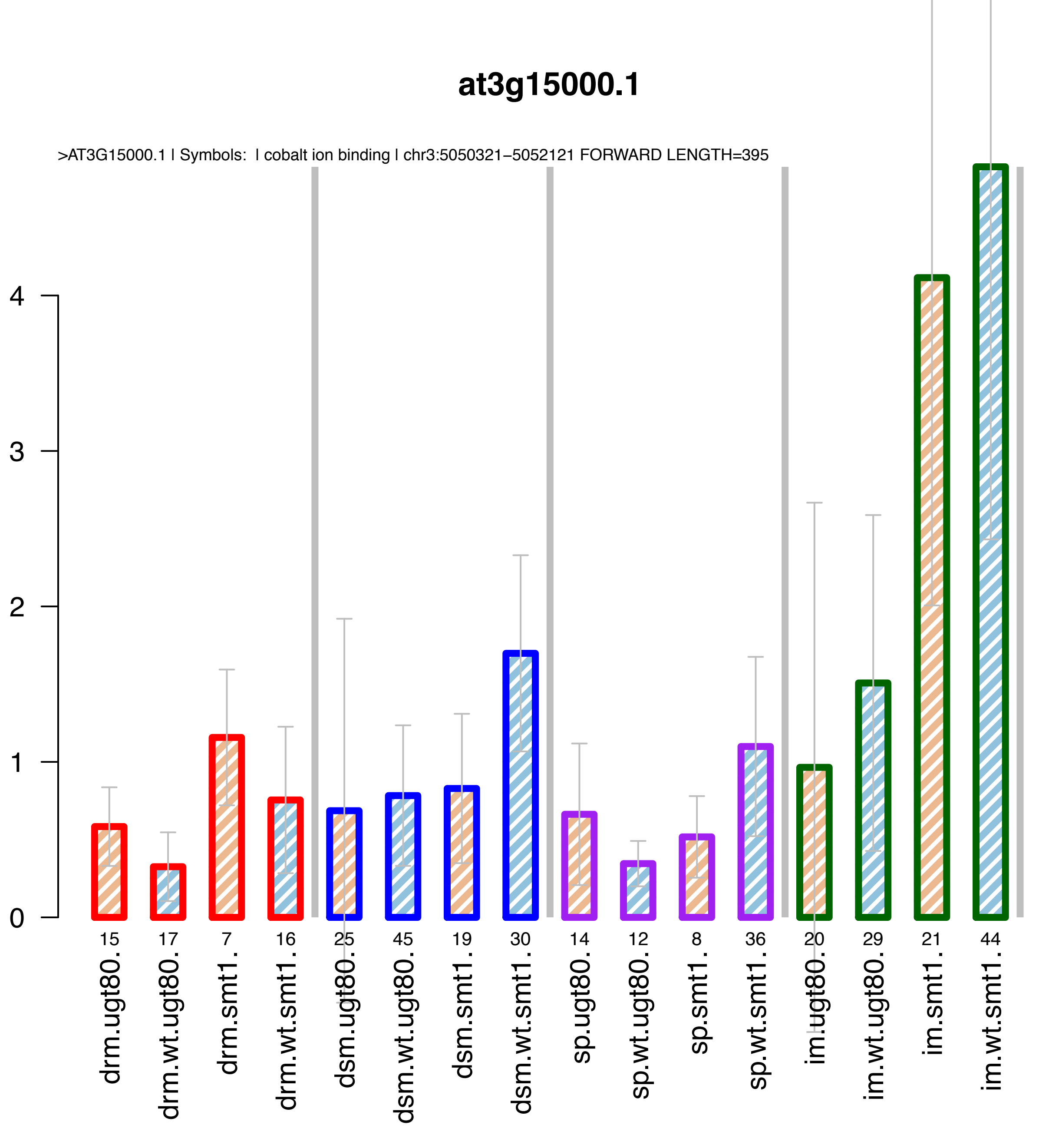

at3g15000.1
>AT3G15000.1 | Symbols: | cobalt ion binding | chr3:5050321-5052121 FORWARD LENGTH=395
0
1
2
3
4
15
17
7
16
25
45
19
30
14
12
8
36
20
29
21
44
drm.ugt80.
drm.wt.ugt80.
drm.smt1.
drm.wt.smt1.
dsm.ugt80.
dsm.wt.ugt80.
dsm.smt1.
dsm.wt.smt1.
sp.ugt80.
sp.wt.ugt80.
sp.smt1.
sp.wt.smt1.
im.ugt80.
im.wt.ugt80.
im.smt1.
im.wt.smt1.

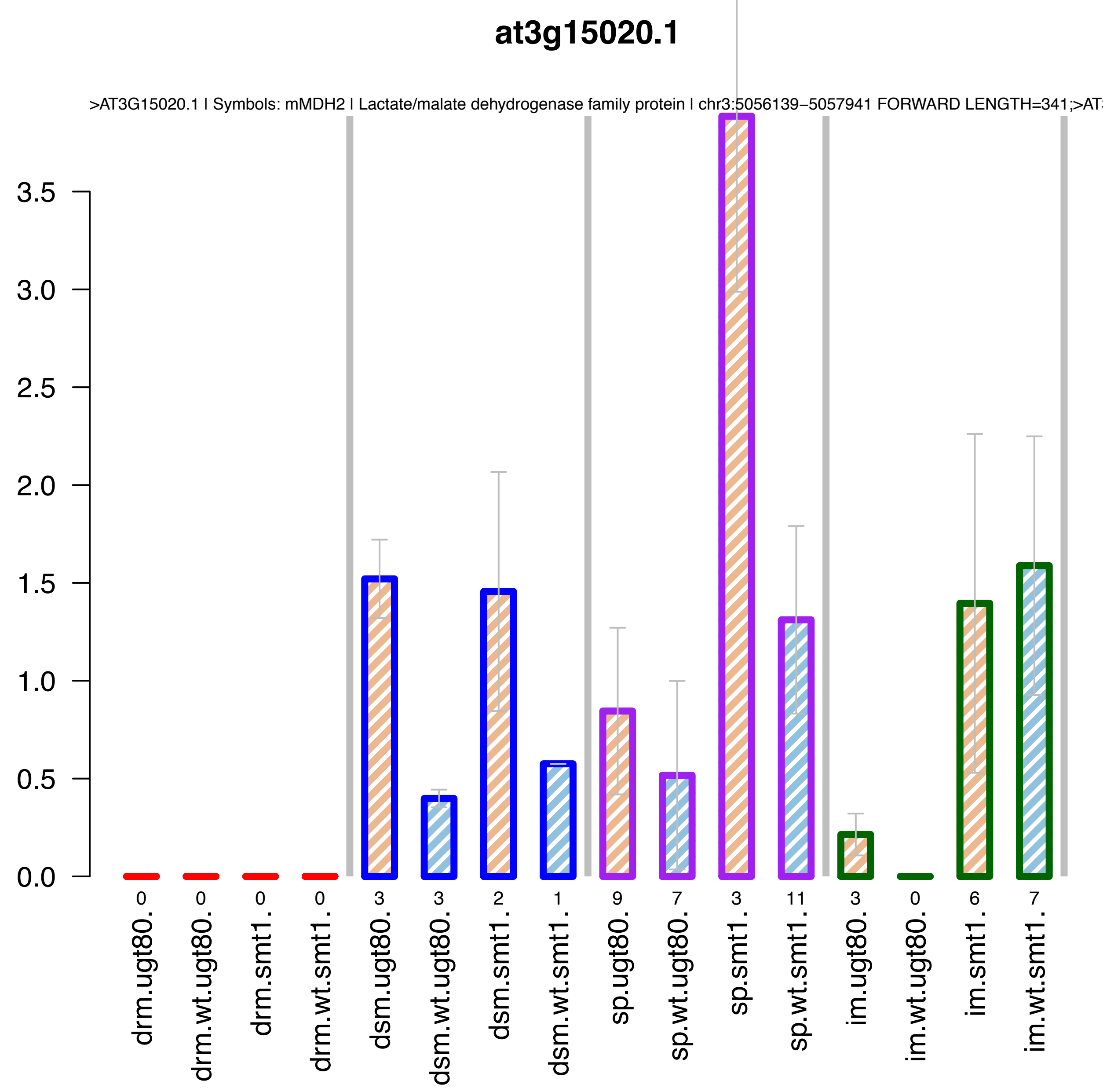
at3g15020.1
>AT3G15020.1 | Symbols: mMDH2 | Lactate/malate dehydrogenase family protein | chr3:5056139−5057941 FORWARD LENGTH=341;>AT
0.0
0.5
1.0
1.5
2.0
2.5
3.0
3.5
0
0
0
0
3
3
2
1
9
7
3
11
3
0
6
7
drm.ugt80.
drm.wt.ugt80.
drm.smt1.
drm.wt.smt1.
dsm.ugt80.
dsm.wt.ugt80.
dsm.smt1.
dsm.wt.smt1.
sp.ugt80.
sp.wt.ugt80.
sp.smt1.
sp.wt.smt1.
im.ugt80.
im.wt.ugt80.
im.smt1.
im.wt.smt1.

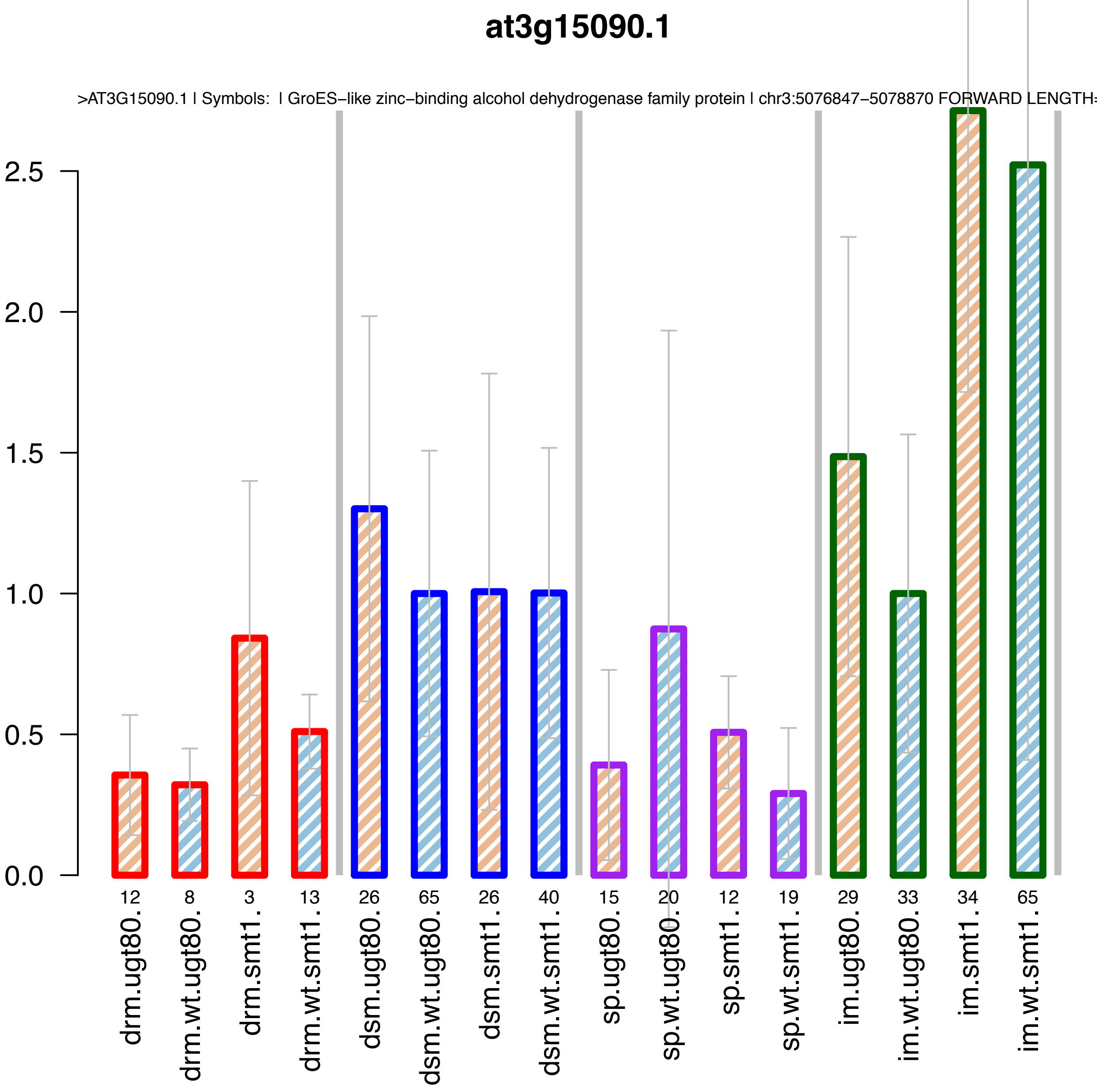

at3g15090.1
>AT3G15090.1 | Symbols: | GroES−like zinc−binding alcohol dehydrogenase family protein | chr3:5076847−5078870 FORWARD LENGTH=
0.0
0.5
1.0
1.5
2.0
2.5
12
8
3
13
26
65
26
40
15
20
12
19
29
33
34
65
drm.ugt80..
drm.wt.ugt80..
drm.smt1..
drm.wt.smt1..
dsm.ugt80..
dsm.wt.ugt80..
dsm.smt1..
dsm.wt.smt1..
sp.ugt80..
sp.wt.ugt80..
sp.smt1..
sp.wt.smt1..
im.ugt80..
im.wt.ugt80..
im.smt1..
im.wt.smt1..

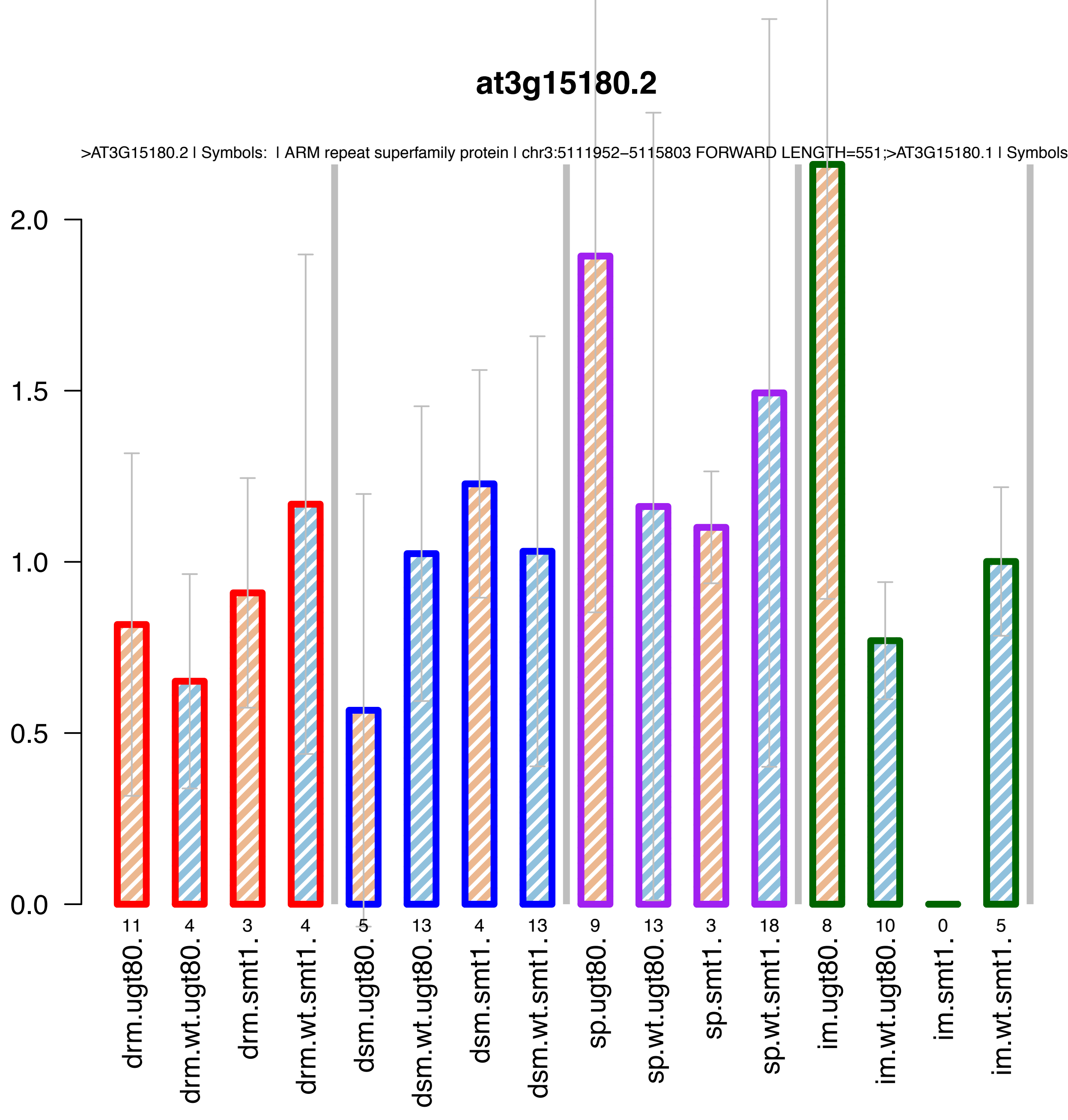

at3g15180.2
>AT3G15180.2 | Symbols: | ARM repeat superfamily protein | chr3:5111952–5115803 FORWARD LENGTH=551;>AT3G15180.1 | Symbols
2.0
1.5
1.0
0.5
0.0
11
4
3
4
5
13
4
13
9
13
3
18
8
10
0
5
drm.ugt80..
drm.wt.ugt80..
drm.smt1..
drm.wt.smt1..
dsm.ugt80..
dsm.wt.ugt80..
dsm.smt1..
dsm.wt.smt1..
sp.ugt80..
sp.wt.ugt80..
sp.smt1..
sp.wt.smt1..
im.ugt80..
im.wt.ugt80..
im.smt1..
im.wt.smt1..

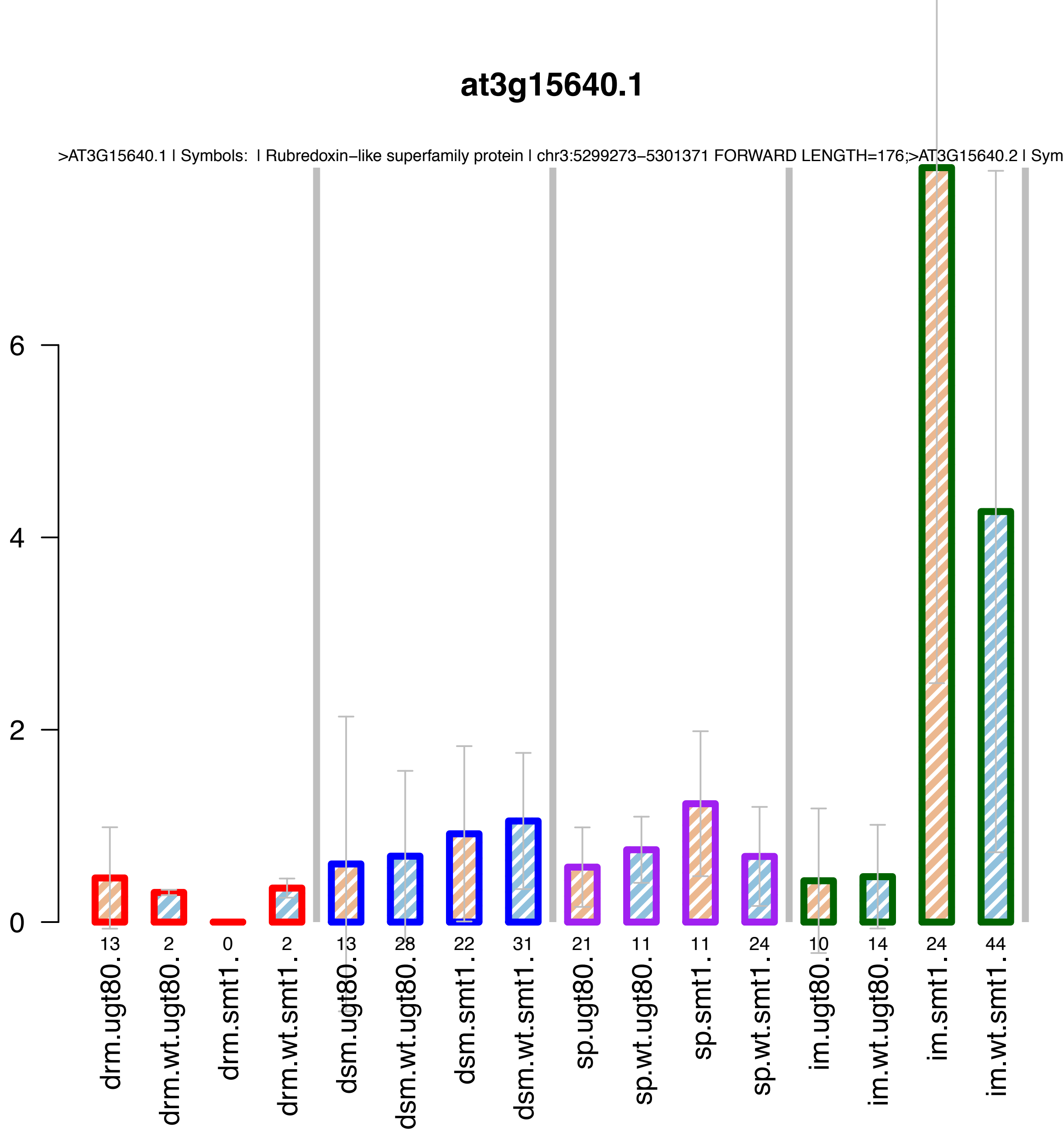

at3g15640.1
>AT3G15640.1 | Symbols: | Rubredoxin−like superfamily protein | chr3:5299273−5301371 FORWARD LENGTH=176;>AT3G15640.2 | Sym
0
2
4
6
13
2
0
2
13
28
22
31
21
11
11
24
10
14
24
44
drm.ugt80..
drm.wt.ugt80..
drm.smt1..
drm.wt.smt1..
dsm.ugt80..
dsm.wt.ugt80..
dsm.smt1..
dsm.wt.smt1..
sp.ugt80..
sp.wt.ugt80..
sp.smt1..
sp.wt.smt1..
im.ugt80..
im.wt.ugt80..
im.smt1..
im.wt.smt1..

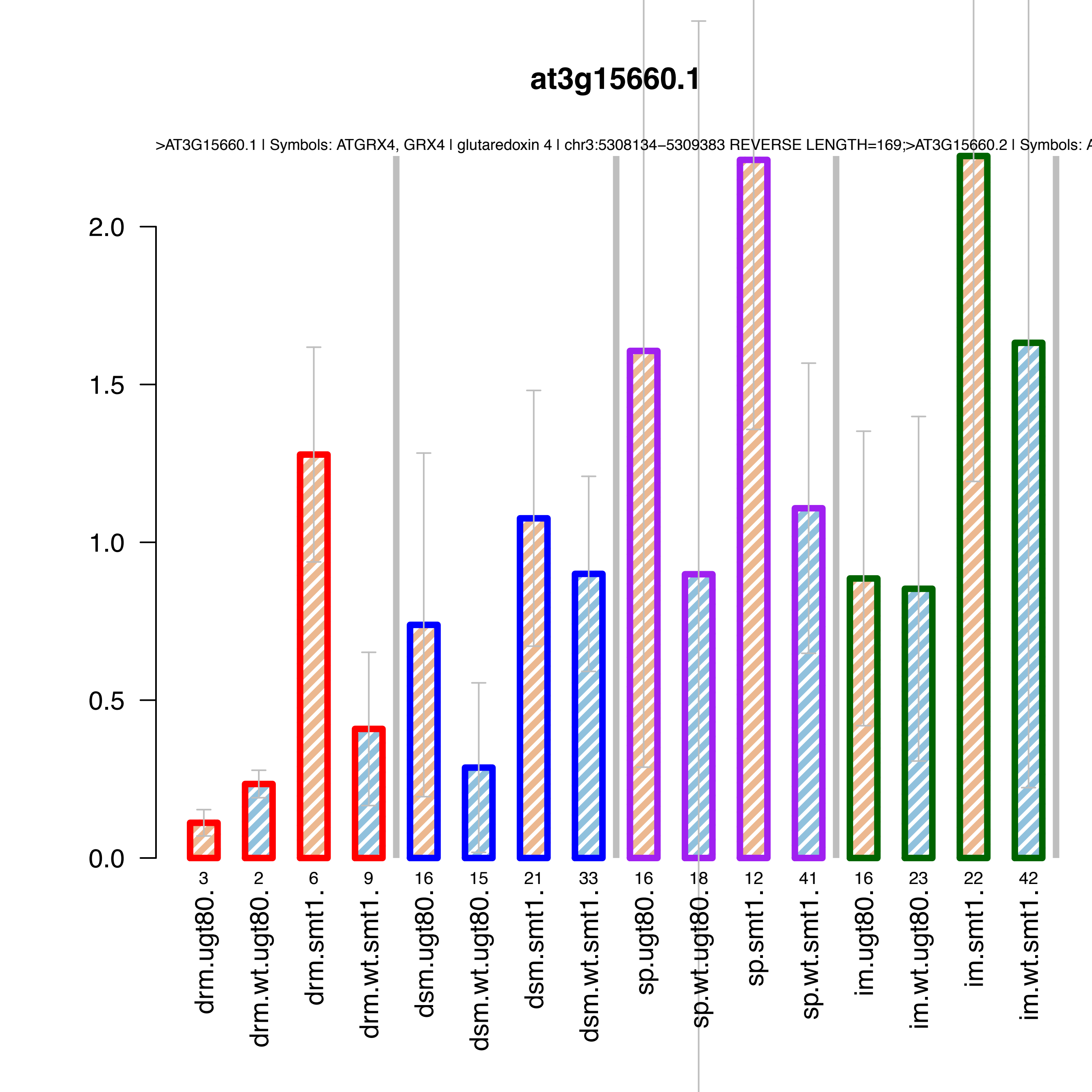

at3g15660.1
>AT3G15660.1 | Symbols: ATGRX4, GRX4 | glutaredoxin 4 | chr3:5308134–5309383 REVERSE LENGTH=169;>AT3G15660.2 | Symbols: A
2.0
1.5
1.0
0.5
0.0
3
2
6
9
16
15
21
33
16
18
12
41
16
23
22
42
drm.ugt80..
drm.wt.ugt80..
drm.smt1..
drm.wt.smt1..
dsm.ugt80..
dsm.wt.ugt80..
dsm.smt1..
dsm.wt.smt1..
sp.ugt80..
sp.wt.ugt80..
sp.smt1..
sp.wt.smt1..
im.ugt80..
im.wt.ugt80..
im.smt1..
im.wt.smt1..

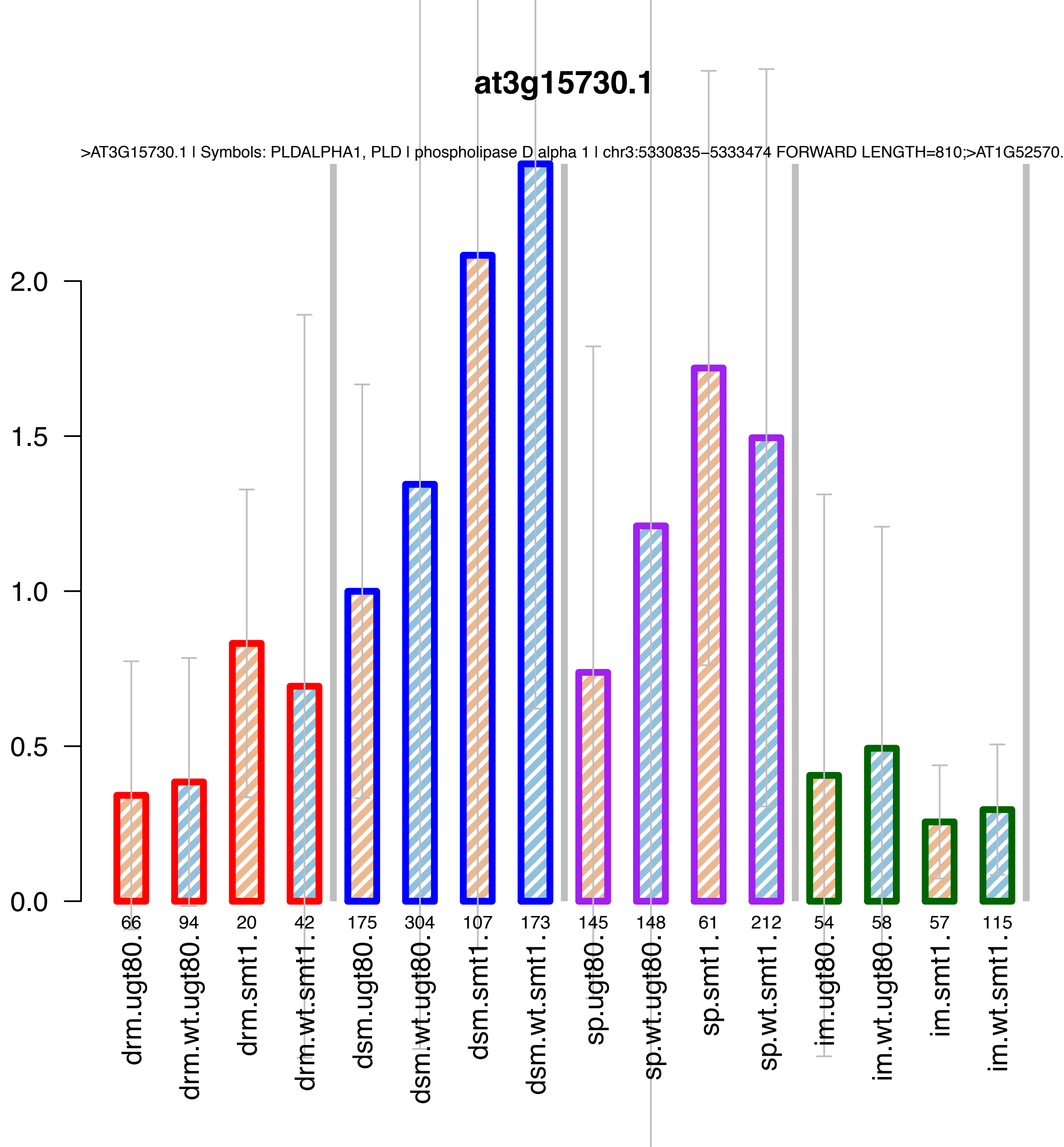

at3g15730.1
>AT3G15730.1 | Symbols: PLDALPHA1, PLD | phospholipase D alpha 1 | chr3:5330835-5333474 FORWARD LENGTH=810;>AT1G52570.
0.0
0.5
1.0
1.5
2.0
66
94
20
42
175
304
107
173
145
148
61
212
54
58
57
115
drm.ugt80..
drm.wt.ugt80..
drm.smt1..
drm.wt.smt1..
dsm.ugt80..
dsm.wt.ugt80..
dsm.smt1..
dsm.wt.smt1..
sp.ugt80..
sp.wt.ugt80..
sp.smt1..
sp.wt.smt1..
im.ugt80..
im.wt.ugt80..
im.smt1..
im.wt.smt1..

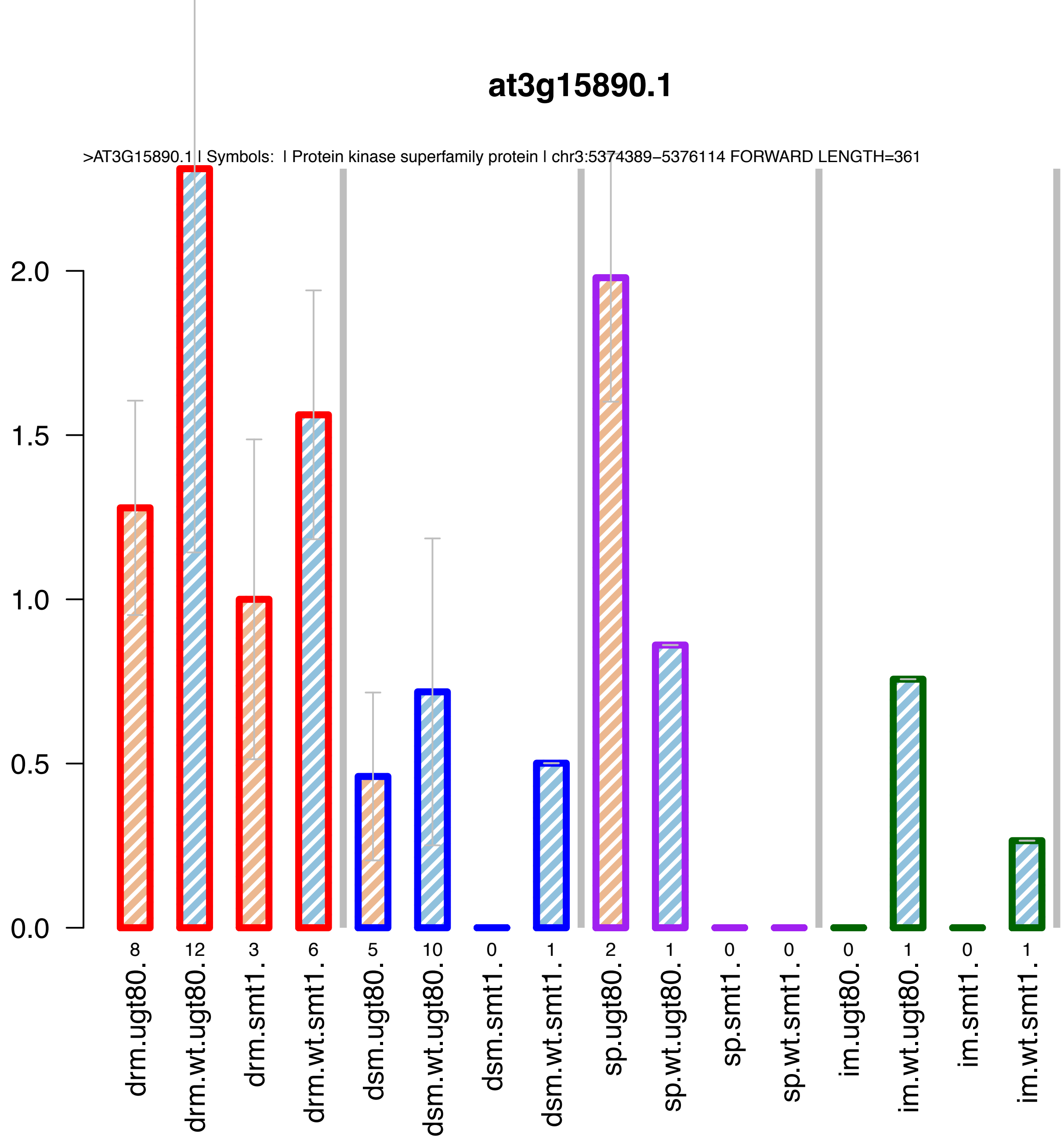

at3g15890.1
>AT3G15890.1 | Symbols: | Protein kinase superfamily protein | chr3:5374389-5376114 FORWARD LENGTH=361
0.0
0.5
1.0
1.5
2.0
8
12
3
6
5
10
0
1
2
1
0
0
0
1
0
1
drm.ugt80.
drm.wt.ugt80.
drm.smt1.
drm.wt.smt1.
dsm.ugt80.
dsm.wt.ugt80.
dsm.smt1.
dsm.wt.smt1.
sp.ugt80.
sp.wt.ugt80.
sp.smt1.
sp.wt.smt1.
im.ugt80.
im.wt.ugt80.
im.smt1.
im.wt.smt1.

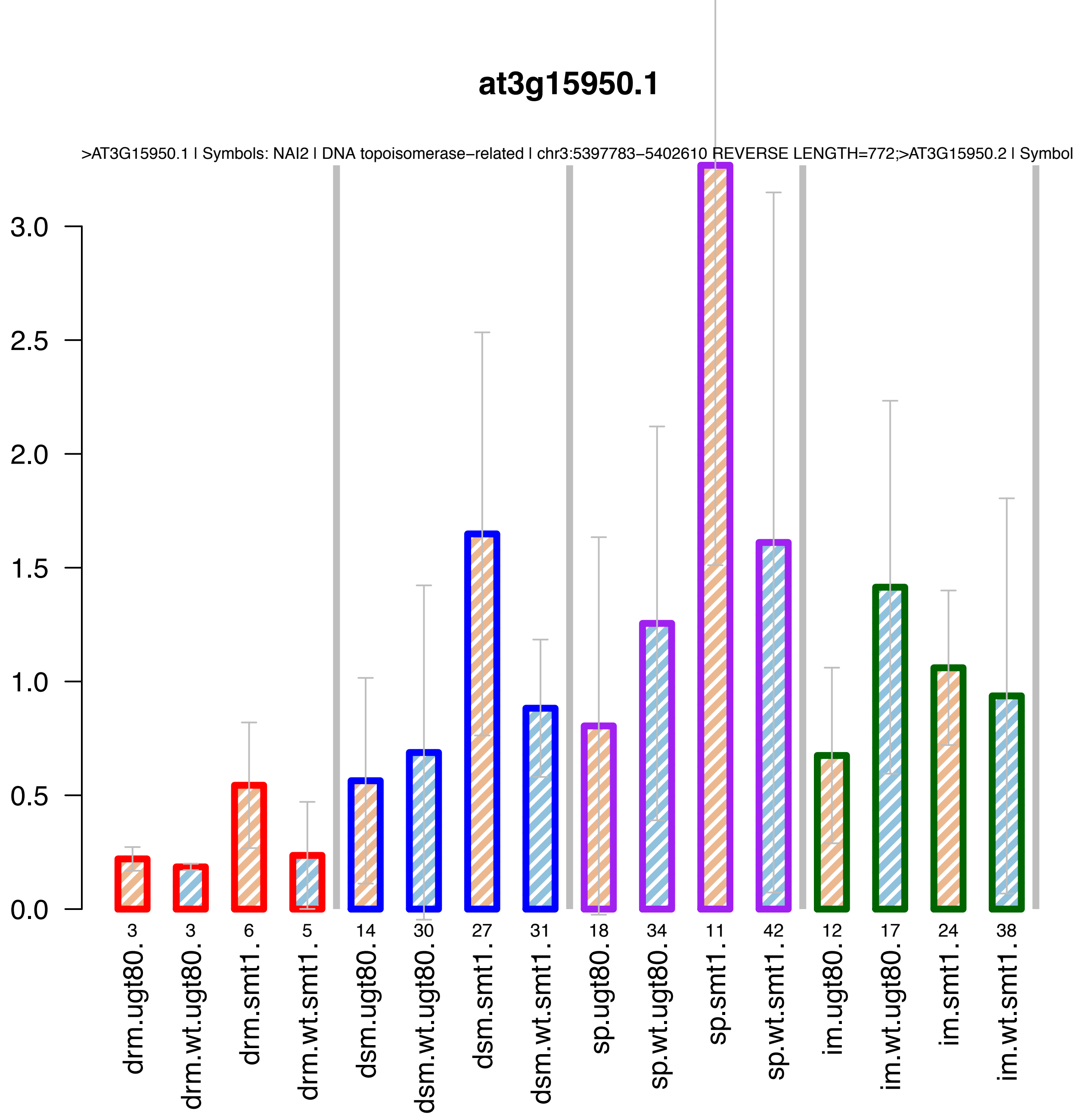
at3g15950.1
>AT3G15950.1 | Symbols: NAI2 | DNA topoisomerase−related | chr3:5397783−5402610 REVERSE LENGTH=772;>AT3G15950.2 | Symbol
3.0
2.5
2.0
1.5
1.0
0.5
0.0
3
3
6
5
14
30
27
31
18
34
11
42
12
17
24
38
drm.ugt80.
drm.wt.ugt80.
drm.smt1.
drm.wt.smt1.
dsm.ugt80.
dsm.wt.ugt80.
dsm.smt1.
dsm.wt.smt1.
sp.ugt80.
sp.wt.ugt80.
sp.smt1.
sp.wt.smt1.
im.ugt80.
im.wt.ugt80.
im.smt1.
im.wt.smt1.

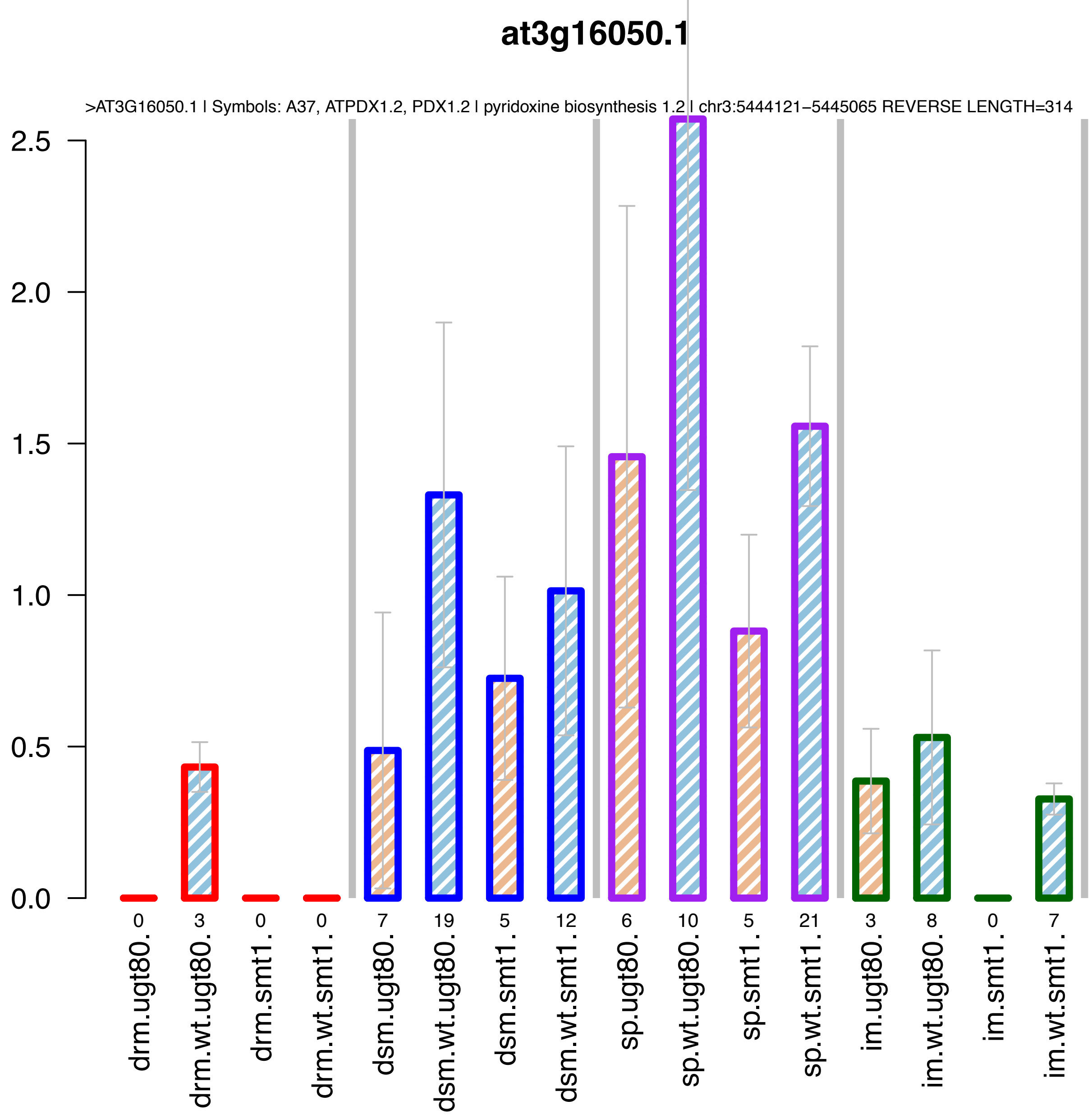

at3g16050.1
>AT3G16050.1 | Symbols: A37, ATPDX1.2, PDX1.2 | pyridoxine biosynthesis 1.2 | chr3:5444121–5445065 REVERSE LENGTH=314
0.0
0.5
1.0
1.5
2.0
2.5
0 3 0 0 7 19 5 12 6 10 5 21 3 8 0 7
drm.ugt80.
drm.wt.ugt80.
drm.smt1.
drm.wt.smt1.
dsm.ugt80.
dsm.wt.ugt80.
dsm.smt1.
dsm.wt.smt1.
sp.ugt80.
sp.wt.ugt80.
sp.smt1.
sp.wt.smt1.
im.ugt80.
im.wt.ugt80.
im.smt1.
im.wt.smt1.

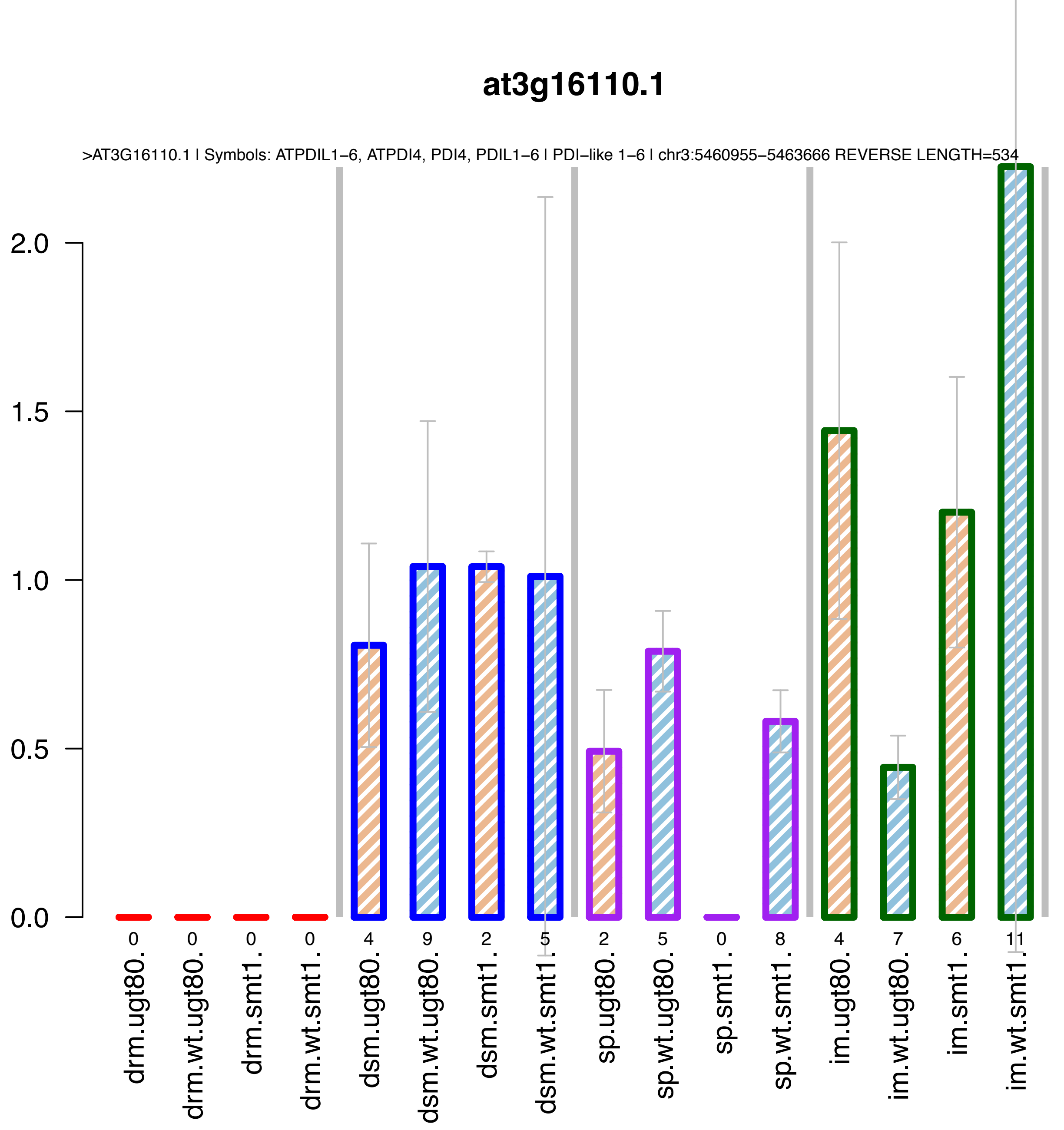

at3g16110.1
>AT3G16110.1 | Symbols: ATPDIL1−6, ATPDI4, PDI4, PDIL1−6 | PDI−like 1−6 | chr3:5460955−5463666 REVERSE LENGTH=534
0.0
0.5
1.0
1.5
2.0
0 0 0 0 4 9 2 5 2 5 0 8 4 7 6 11
drm.ugt80.
drm.wt.ugt80.
drm.smt1.
drm.wt.smt1.
dsm.ugt80.
dsm.wt.ugt80.
dsm.smt1.
dsm.wt.smt1.
sp.ugt80.
sp.wt.ugt80.
sp.smt1.
sp.wt.smt1.
im.ugt80.
im.wt.ugt80.
im.smt1.
im.wt.smt1.

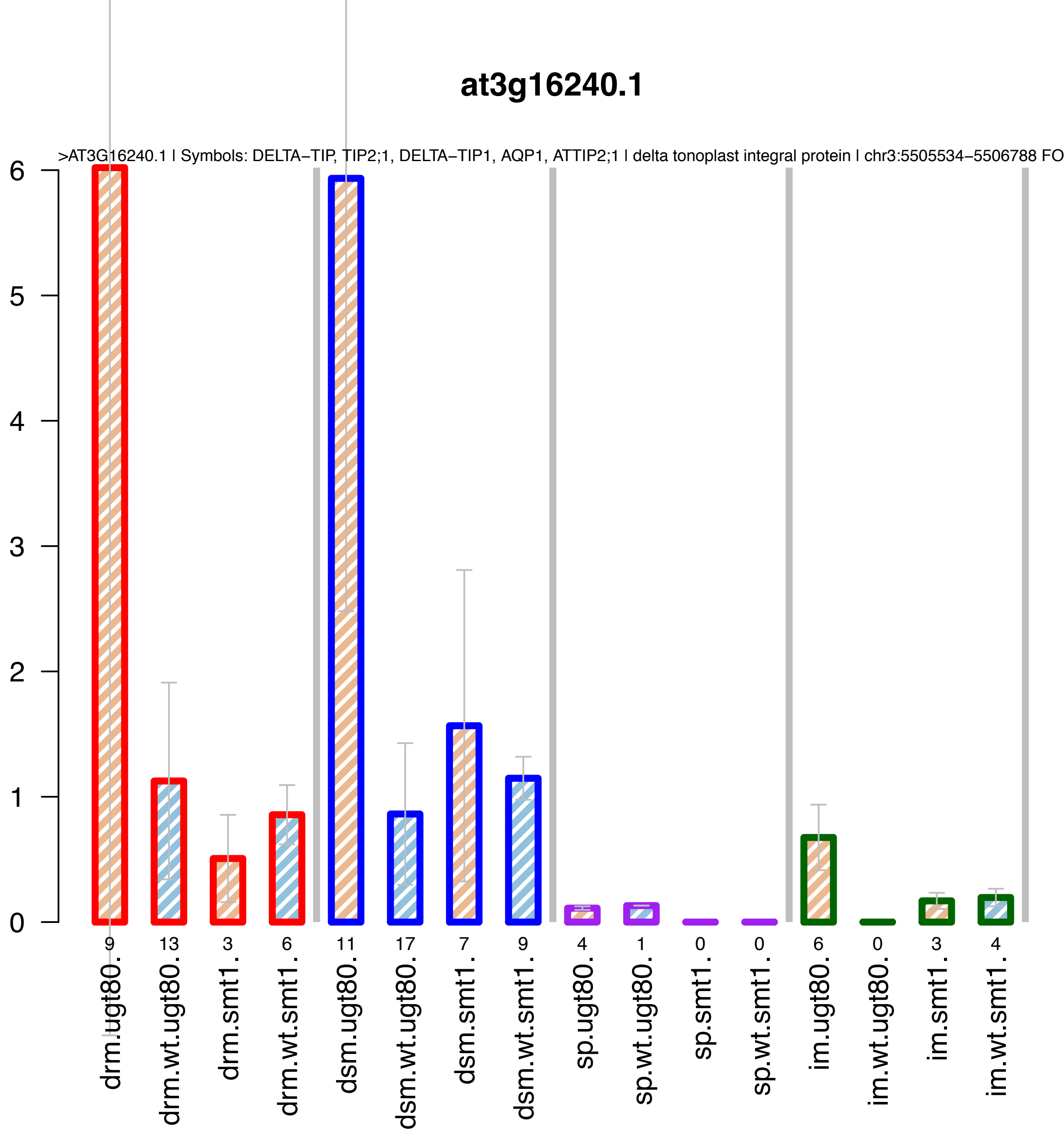

at3g16240.1
>AT3G16240.1 | Symbols: DELTA−TIP, TIP2;1, DELTA−TIP1, AQP1, ATTIP2;1 | delta tonoplast integral protein | chr3:5505534−5506788 FO
0
1
2
3
4
5
6
9
13
3
6
11
17
7
9
4
1
0
0
6
0
3
4
drm.ugt80.
drm.wt.ugt80.
drm.smt1.
drm.wt.smt1.
dsm.ugt80.
dsm.wt.ugt80.
dsm.smt1.
dsm.wt.smt1.
sp.ugt80.
sp.wt.ugt80.
sp.smt1.
sp.wt.smt1.
im.ugt80.
im.wt.ugt80.
im.smt1.
im.wt.smt1.

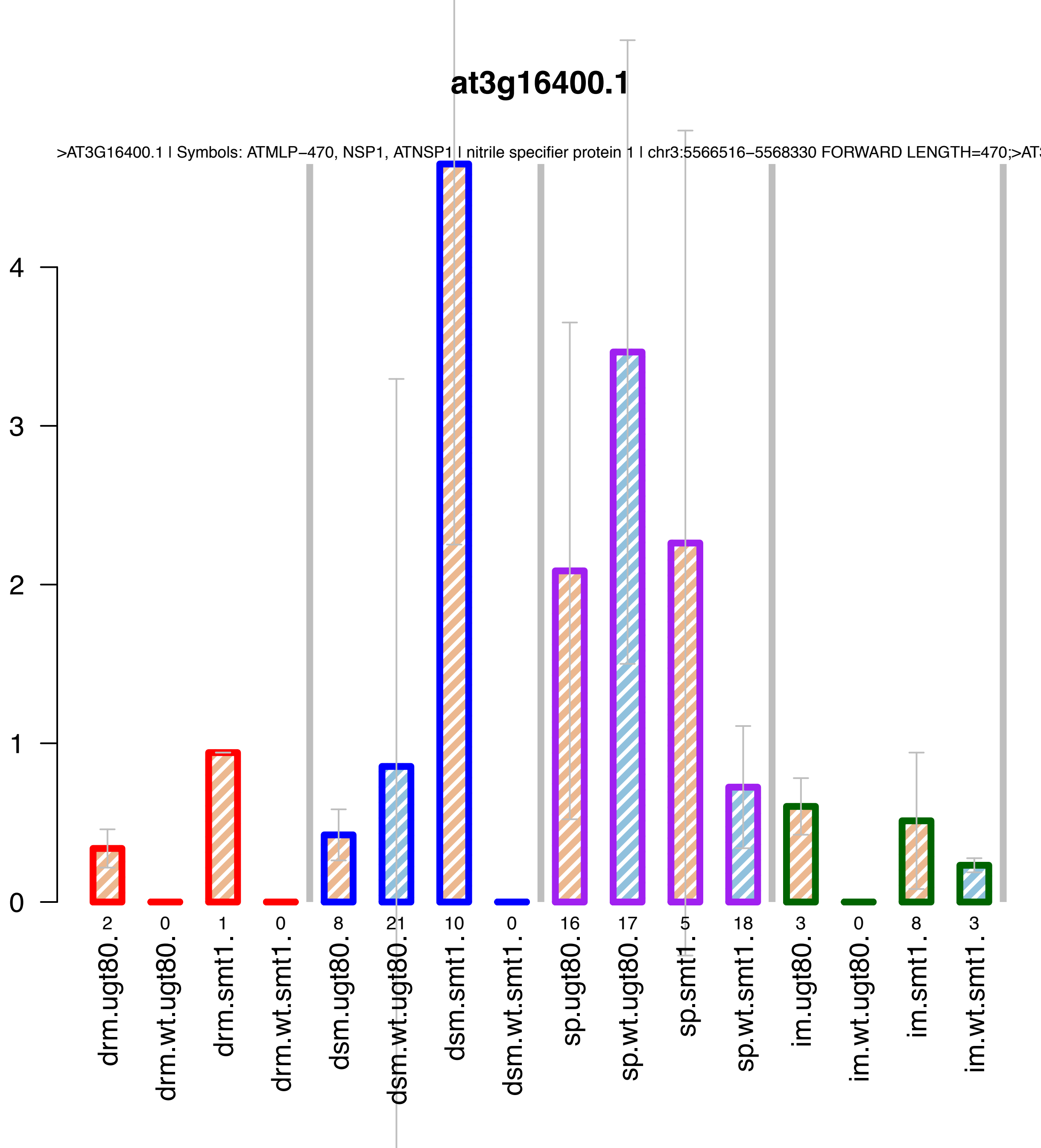

at3g16400.1
>AT3G16400.1 | Symbols: ATMLP−470, NSP1, ATNSP1 | nitrile specifier protein 1 | chr3:5566516−5568330 FORWARD LENGTH=470;>AT
0
1
2
3
4
2
0
1
0
8
21
10
0
16
17
5
18
3
0
8
3
drm.ugt80..
drm.wt.ugt80..
drm.smt1..
drm.wt.smt1..
dsm.ugt80..
dsm.wt.ugt80..
dsm.smt1..
dsm.wt.smt1..
sp.ugt80..
sp.wt.ugt80..
sp.smt1..
sp.wt.smt1..
im.ugt80..
im.wt.ugt80..
im.smt1..
im.wt.smt1..

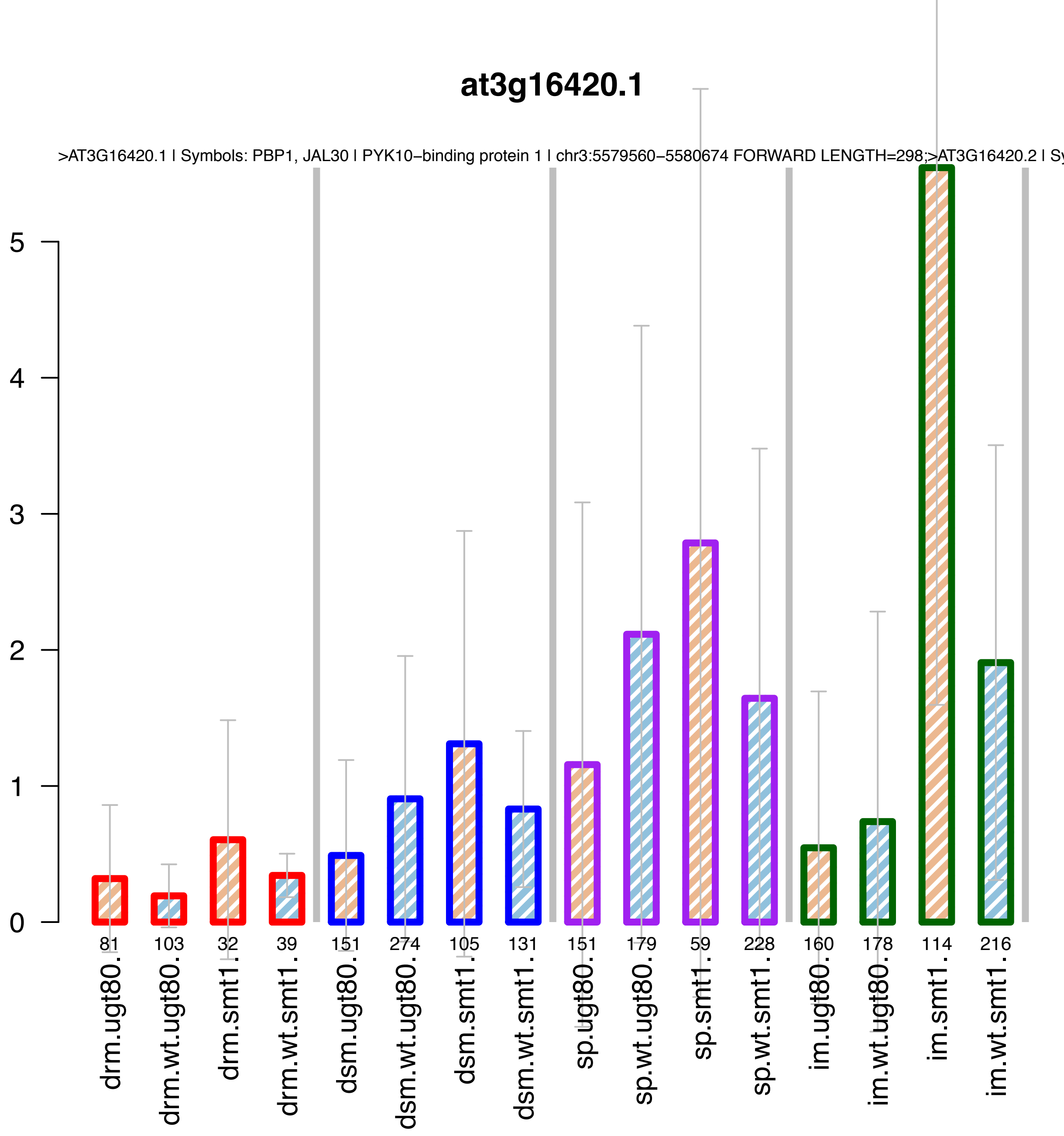

at3g16420.1
>AT3G16420.1 | Symbols: PBP1, JAL30 | PYK10−binding protein 1 | chr3:5579560−5580674 FORWARD LENGTH=298;>AT3G16420.2 | Sy
0
1
2
3
4
5
81
103
32
39
151
274
105
131
151
179
59
228
160
178
114
216
drm.ugt80..
drm.wt.ugt80..
drm.smt1..
drm.wt.smt1..
dsm.ugt80..
dsm.wt.ugt80..
dsm.smt1..
dsm.wt.smt1..
sp.ugt80..
sp.wt.ugt80..
sp.smt1..
sp.wt.smt1..
im.ugt80..
im.wt.ugt80..
im.smt1..
im.wt.smt1..

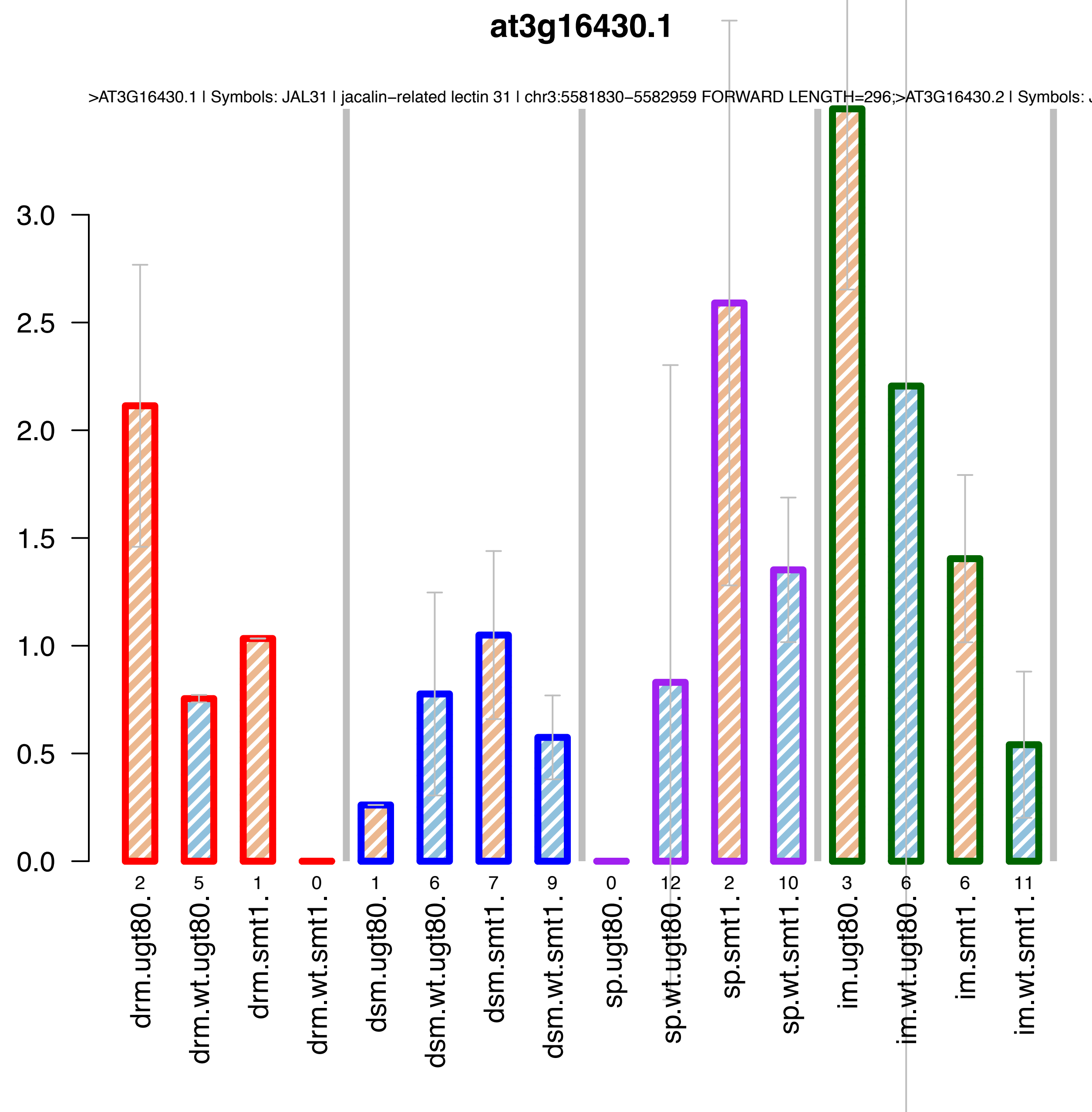

at3g16430.1
>AT3G16430.1 | Symbols: JAL31 | jacalin−related lectin 31 | chr3:5581830−5582959 FORWARD LENGTH=296;>AT3G16430.2 | Symbols: J
0.0
0.5
1.0
1.5
2.0
2.5
3.0
2 5 1 0 1 6 7 9 0 12 2 10 3 6 6 11
drm.ugt80..
drm.wt.ugt80..
drm.smt1..
drm.wt.smt1..
dsm.ugt80..
dsm.wt.ugt80..
dsm.smt1..
dsm.wt.smt1..
sp.ugt80..
sp.wt.ugt80..
sp.smt1..
sp.wt.smt1..
im.ugt80..
im.wt.ugt80..
im.smt1..
im.wt.smt1..

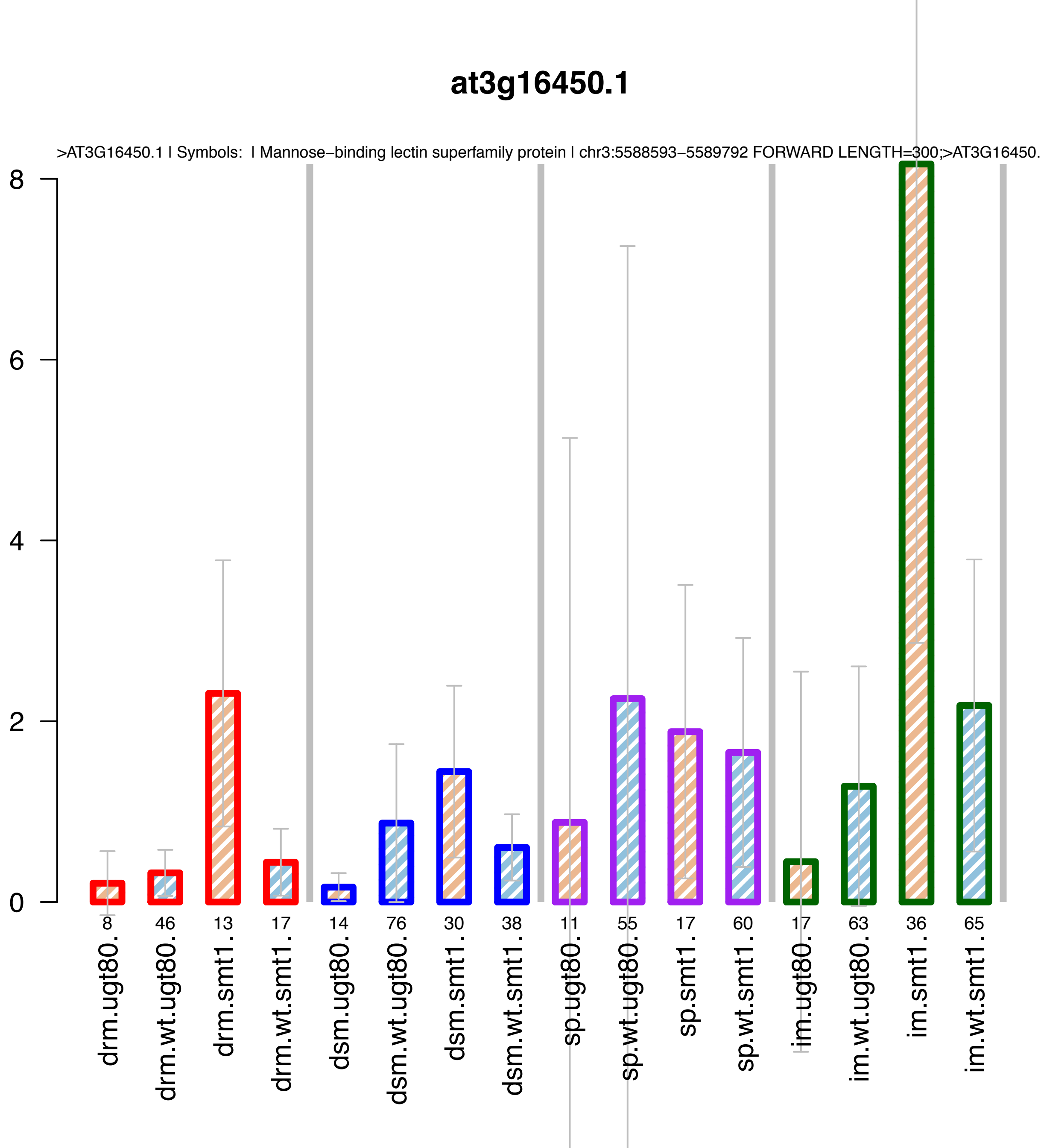

at3g16450.1
>AT3G16450.1 | Symbols: | Mannose−binding lectin superfamily protein | chr3:5588593−5589792 FORWARD LENGTH=300;>AT3G16450.
0
2
4
6
8
8
46
13
17
14
76
30
38
11
55
17
60
17
63
36
65
drm.ugt80.
drm.wt.ugt80.
drm.smt1.
drm.wt.smt1.
dsm.ugt80.
dsm.wt.ugt80.
dsm.smt1.
dsm.wt.smt1.
sp.ugt80.
sp.wt.ugt80.
sp.smt1.
sp.wt.smt1.
im.ugt80.
im.wt.ugt80.
im.smt1.
im.wt.smt1.

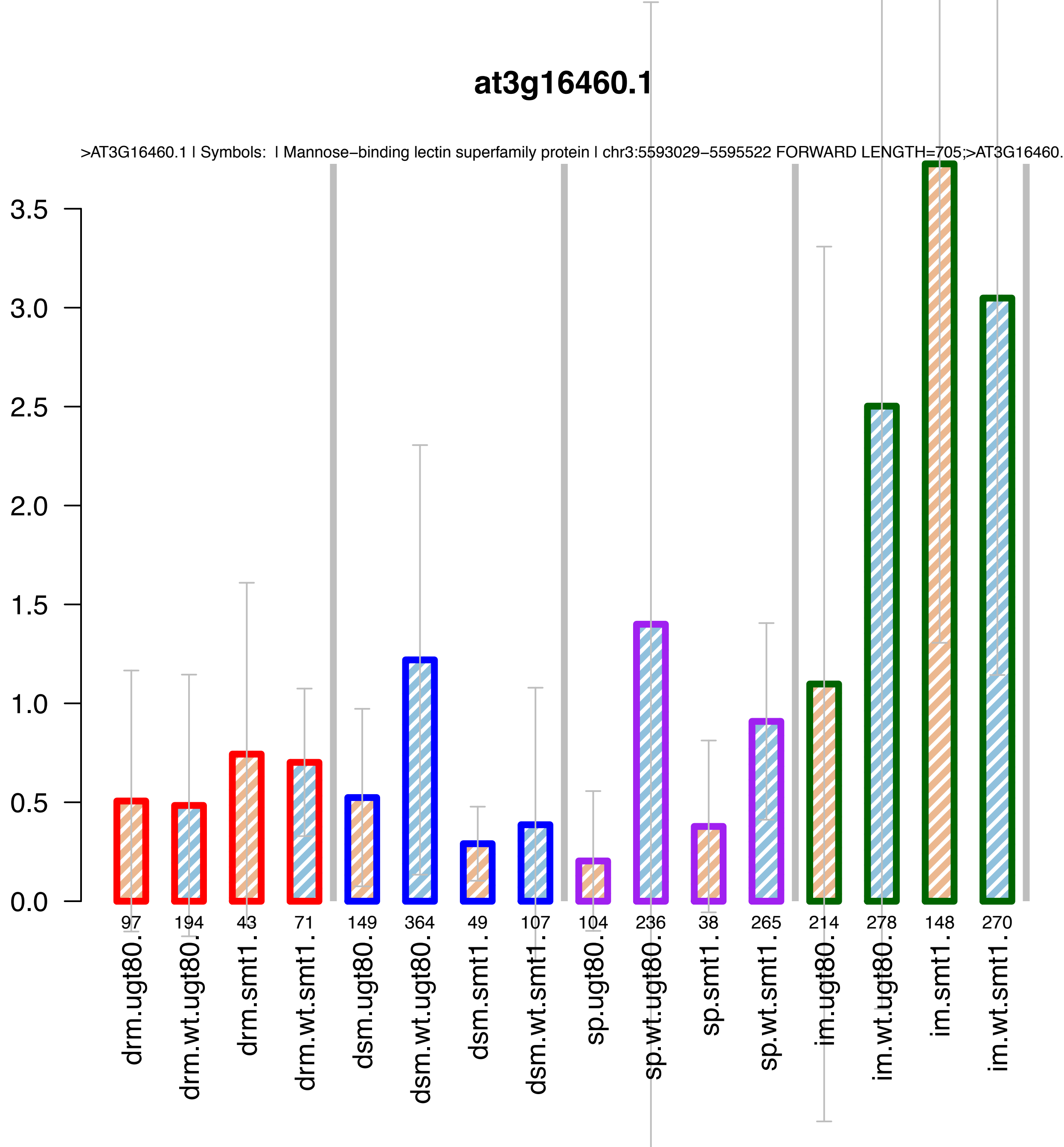
at3g16460.1
>AT3G16460.1 | Symbols: | Mannose−binding lectin superfamily protein | chr3:5593029−5595522 FORWARD LENGTH=705;>AT3G16460.
0.0
0.5
1.0
1.5
2.0
2.5
3.0
3.5
97
194
43
71
149
364
49
107
104
236
38
265
214
278
148
270
drm.ugt80.
drm.wt.ugt80.
drm.smt1.
drm.wt.smt1.
dsm.ugt80.
dsm.wt.ugt80.
dsm.smt1.
dsm.wt.smt1.
sp.ugt80.
sp.wt.ugt80.
sp.smt1.
sp.wt.smt1.
im.ugt80.
im.wt.ugt80.
im.smt1.
im.wt.smt1.

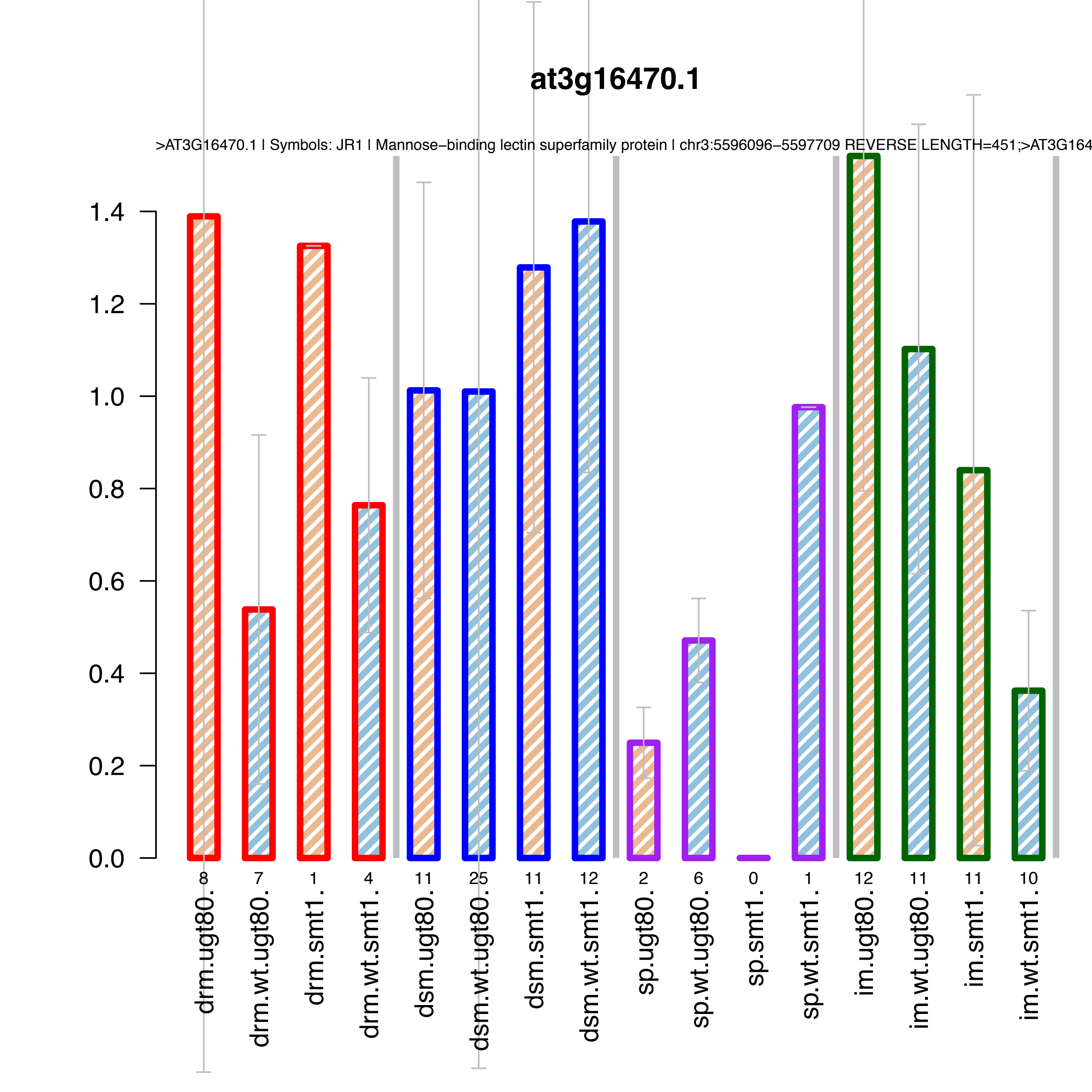

at3g16470.1
>AT3G16470.1 | Symbols: JR1 | Mannose−binding lectin superfamily protein | chr3:5596096−5597709 REVERSE LENGTH=451;>AT3G164
1.4
1.2
1.0
0.8
0.6
0.4
0.2
0.0
8
7
1
4
11
25
11
12
2
6
0
1
12
11
11
10
drm.ugt80.
drm.wt.ugt80.
drm.smt1.
drm.wt.smt1.
dsm.ugt80.
dsm.wt.ugt80.
dsm.smt1.
dsm.wt.smt1.
sp.ugt80.
sp.wt.ugt80.
sp.smt1.
sp.wt.smt1.
im.ugt80.
im.wt.ugt80.
im.smt1.
im.wt.smt1.

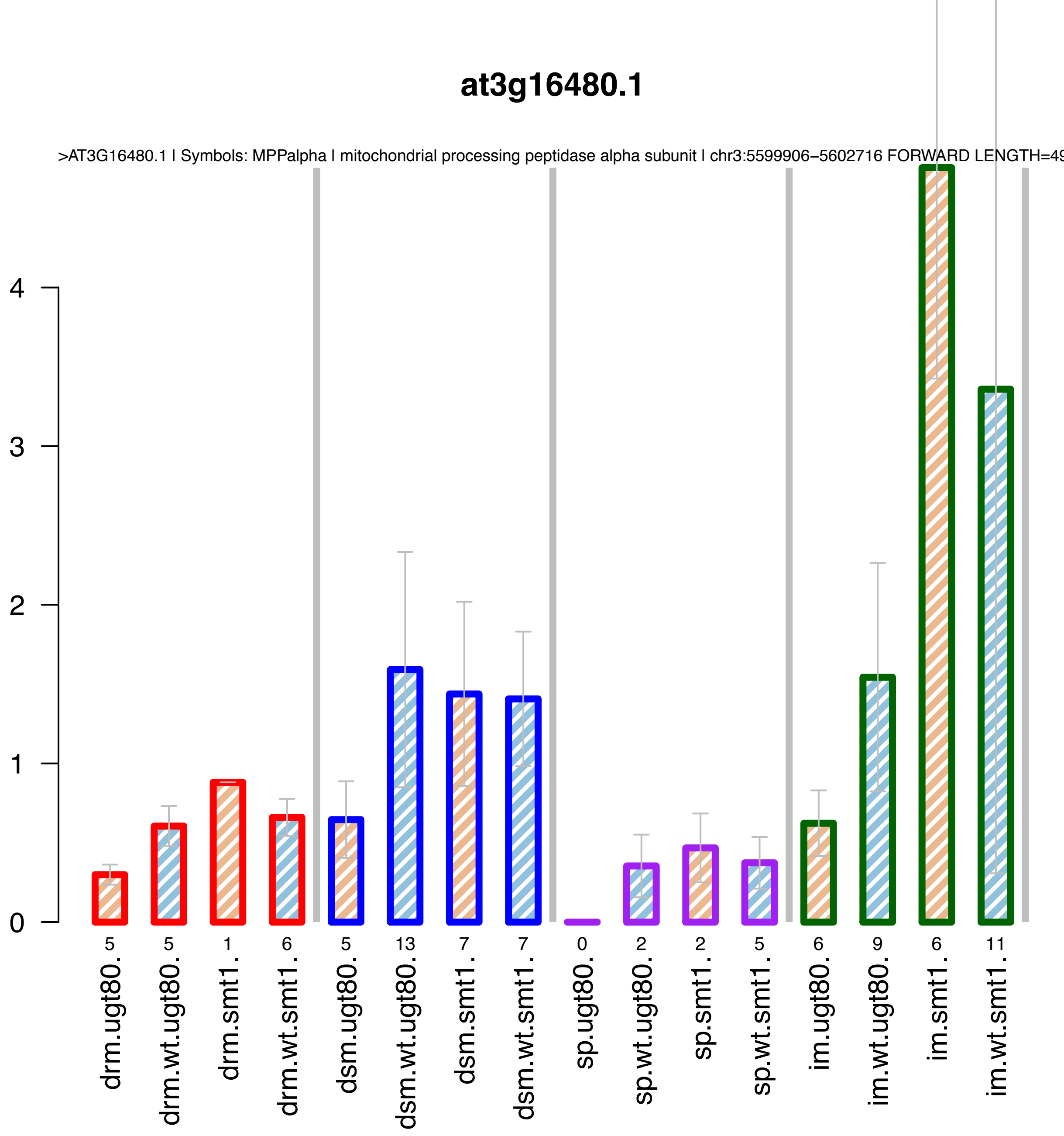
at3g16480.1
>AT3G16480.1 | Symbols: MPPalpha | mitochondrial processing peptidase alpha subunit | chr3:5599906-5602716 FORWARD LENGTH=49
0
1
2
3
4
5 5 1 6 5 13 7 7 0 2 2 5 6 9 6 11
drm.ugt80.
drm.wt.ugt80.
drm.smt1.
drm.wt.smt1.
dsm.ugt80.
dsm.wt.ugt80.
dsm.smt1.
dsm.wt.smt1.
sp.ugt80.
sp.wt.ugt80.
sp.smt1.
sp.wt.smt1.
im.ugt80.
im.wt.ugt80.
im.smt1.
im.wt.smt1.

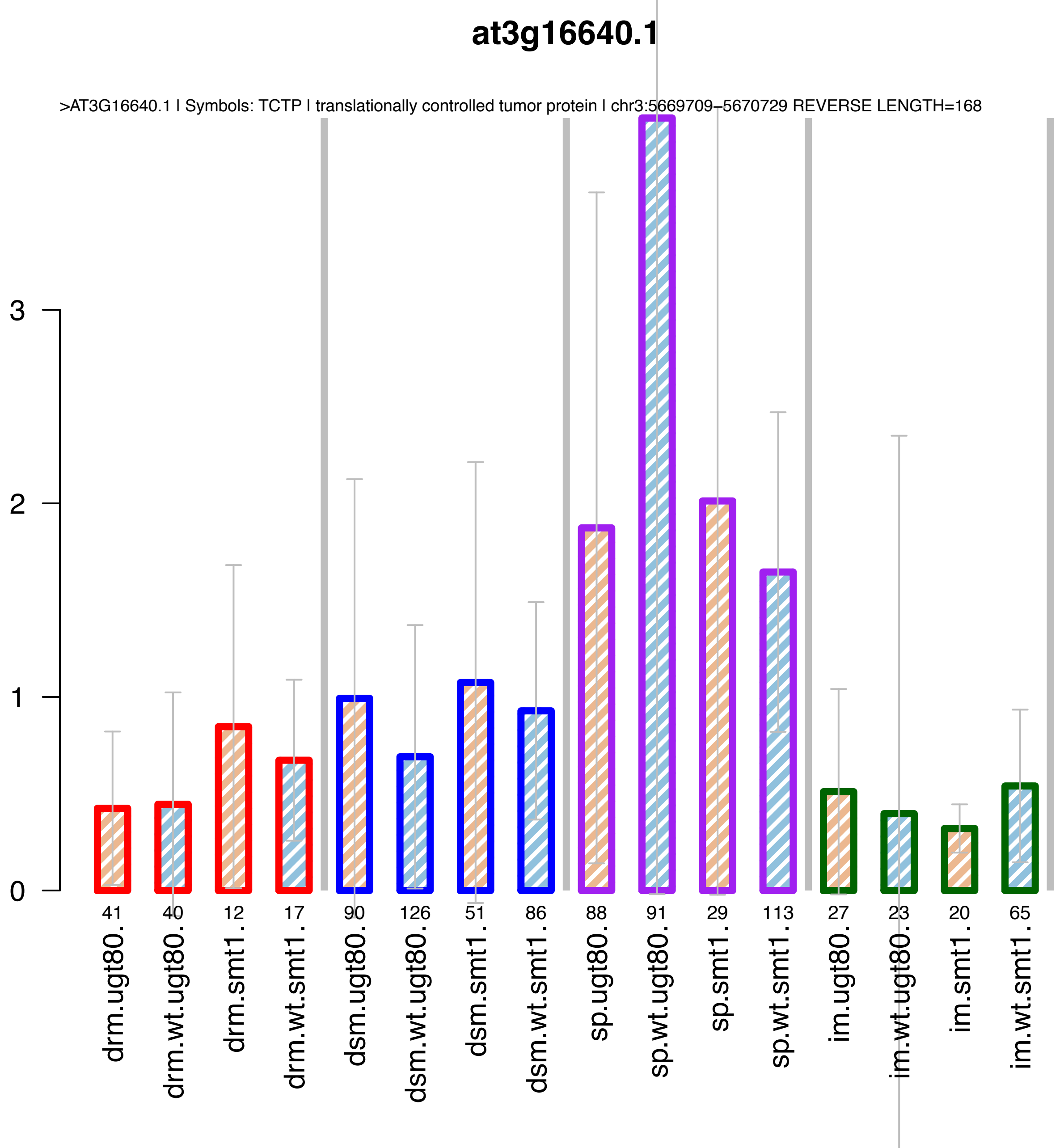

at3g16640.1
>AT3G16640.1 | Symbols: TCTP | translationally controlled tumor protein | chr3:5669709–5670729 REVERSE LENGTH=168
0
1
2
3
41
40
12
17
90
126
51
86
88
91
29
113
27
23
20
65
drm.ugt80..
drm.wt.ugt80..
drm.smt1..
drm.wt.smt1..
dsm.ugt80..
dsm.wt.ugt80..
dsm.smt1..
dsm.wt.smt1..
sp.ugt80..
sp.wt.ugt80..
sp.smt1..
sp.wt.smt1..
im.ugt80..
im.wt.ugt80..
im.smt1..
im.wt.smt1..

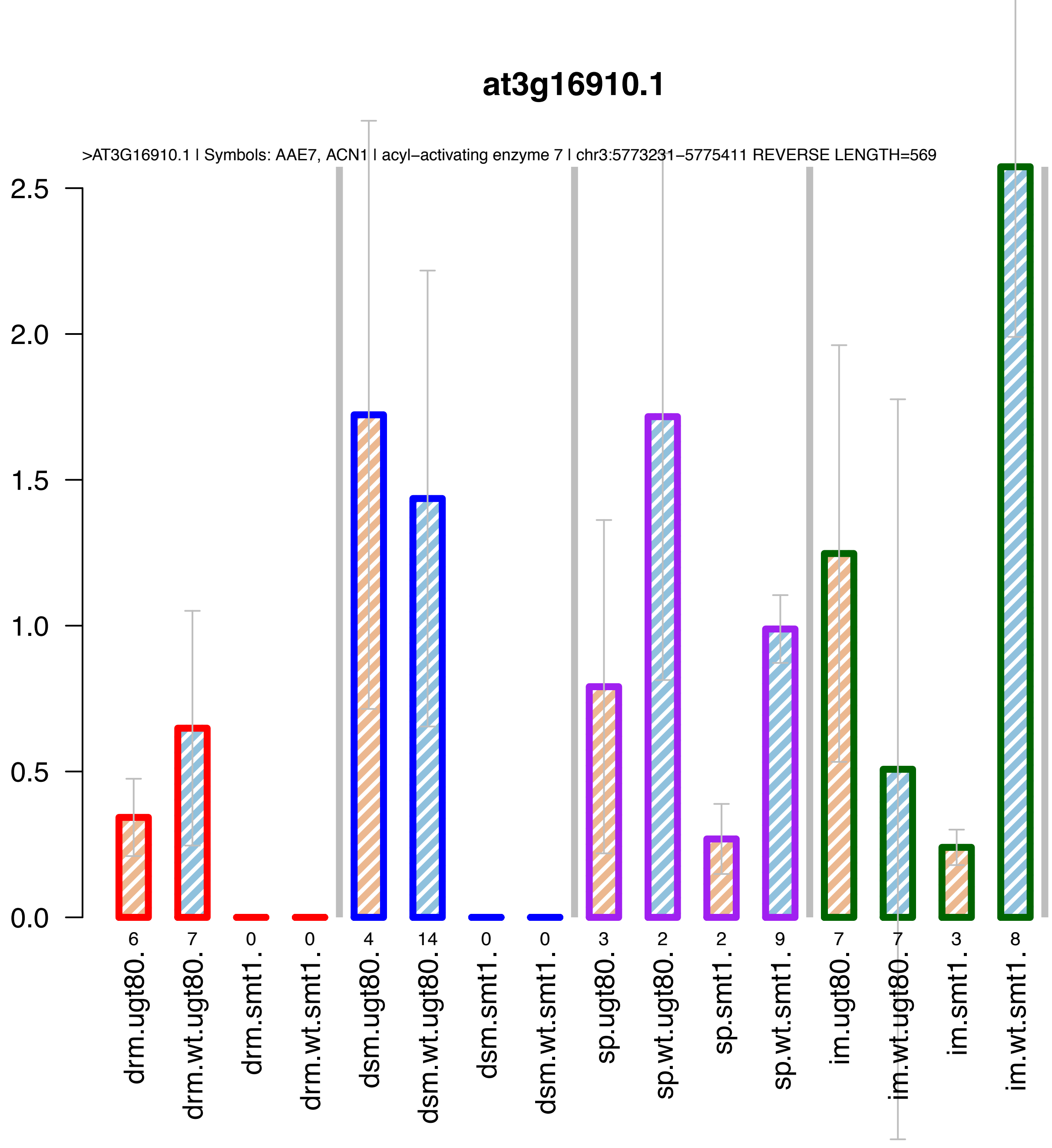

at3g16910.1
>AT3G16910.1 | Symbols: AAE7, ACN1 | acyl−activating enzyme 7 | chr3:5773231−5775411 REVERSE LENGTH=569
0.0
0.5
1.0
1.5
2.0
2.5
6
7
0
0
4
14
0
0
3
2
2
9
7
7
3
8
drm.ugt80.
drm.wt.ugt80.
drm.smt1.
drm.wt.smt1.
dsm.ugt80.
dsm.wt.ugt80.
dsm.smt1.
dsm.wt.smt1.
sp.ugt80.
sp.wt.ugt80.
sp.smt1.
sp.wt.smt1.
im.ugt80.
im.wt.ugt80.
im.smt1.
im.wt.smt1.

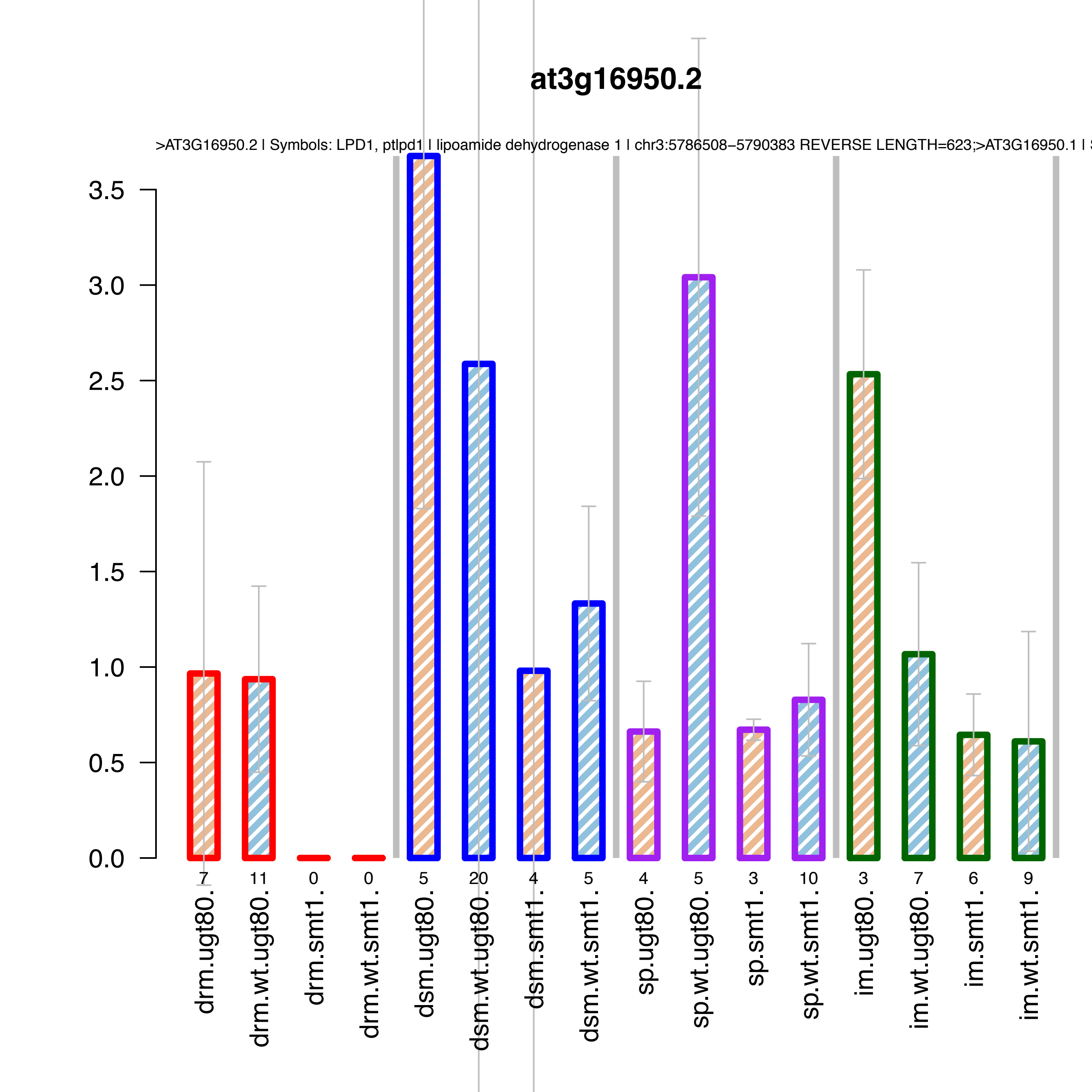
at3g16950.2
>AT3G16950.2 | Symbols: LPD1, ptlpd1 | lipoamide dehydrogenase 1 | chr3:5786508-5790383 REVERSE LENGTH=623;>AT3G16950.1 |
3.5
3.0
2.5
2.0
1.5
1.0
0.5
0.0
7
11
0
0
5
20
4
5
4
5
3
10
3
7
6
9
drm.ugt80.
drm.wt.ugt80.
drm.smt1.
drm.wt.smt1.
dsm.ugt80.
dsm.wt.ugt80.
dsm.smt1.
dsm.wt.smt1.
sp.ugt80.
sp.wt.ugt80.
sp.smt1.
sp.wt.smt1.
im.ugt80.
im.wt.ugt80.
im.smt1.
im.wt.smt1.

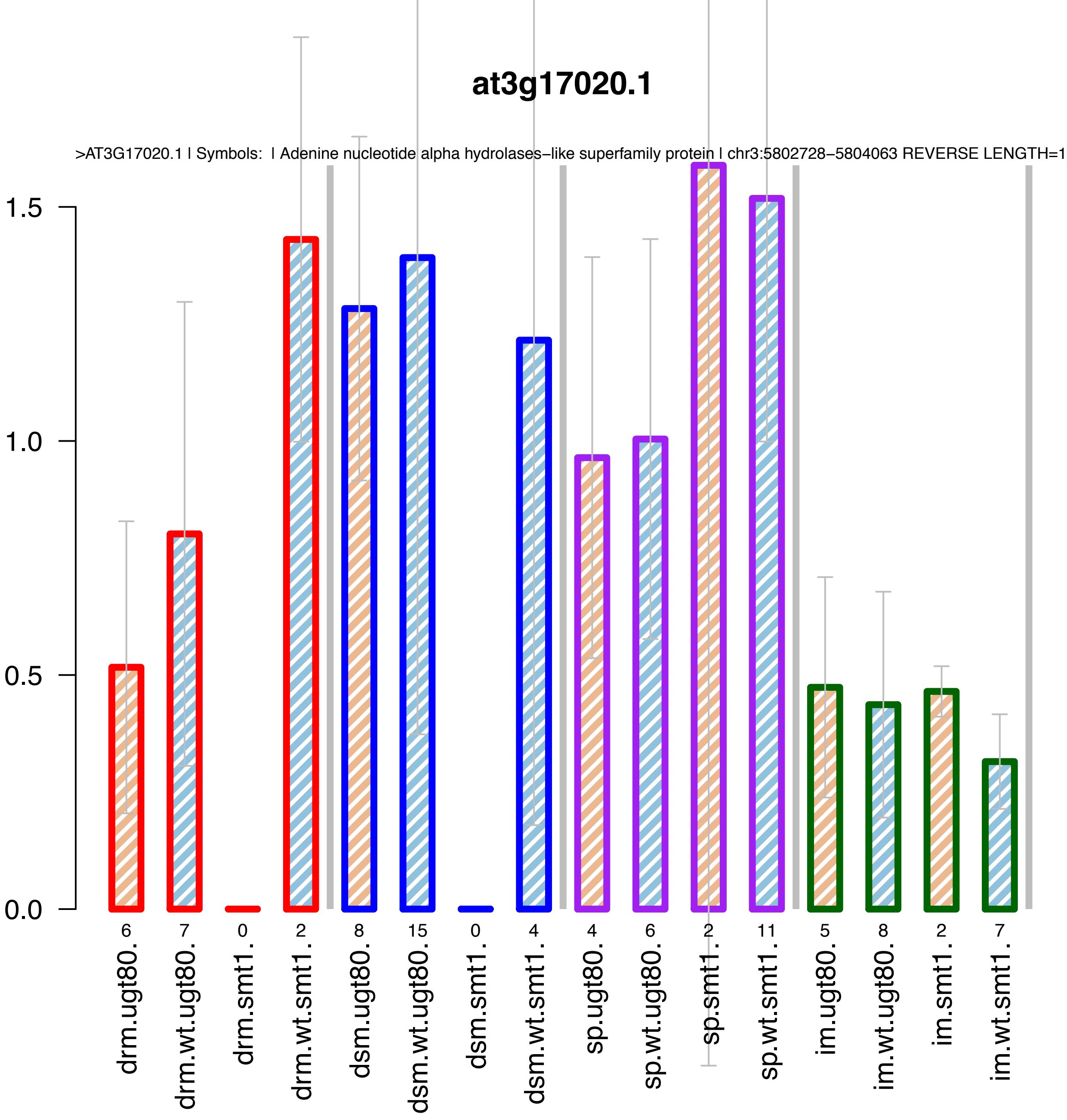
at3g17020.1
>AT3G17020.1 | Symbols: | Adenine nucleotide alpha hydrolases−like superfamily protein | chr3:5802728−5804063 REVERSE LENGTH=1
0.0
0.5
1.0
1.5
6
7
0
2
8
15
0
4
4
6
2
11
5
8
2
7
drm.ugt80..
drm.wt.ugt80..
drm.smt1..
drm.wt.smt1..
dsm.ugt80..
dsm.wt.ugt80..
dsm.smt1..
dsm.wt.smt1..
sp.ugt80..
sp.wt.ugt80..
sp.smt1..
sp.wt.smt1..
im.ugt80..
im.wt.ugt80..
im.smt1..
im.wt.smt1..

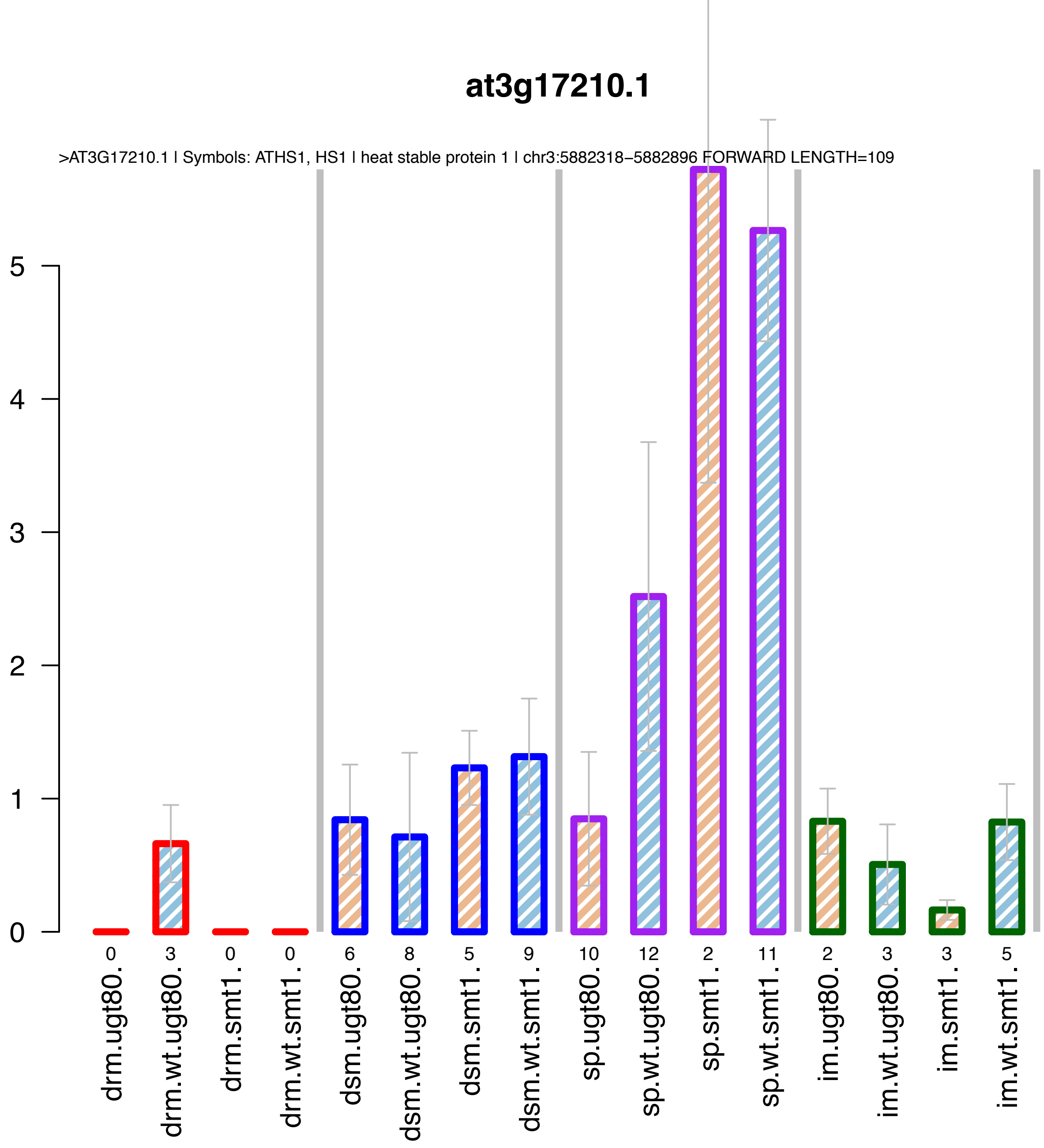

at3g17210.1
>AT3G17210.1 | Symbols: ATHS1, HS1 | heat stable protein 1 | chr3:5882318−5882896 FORWARD LENGTH=109
0
1
2
3
4
5
0
3
0
0
6
8
5
9
10
12
2
11
2
3
3
5
drm.ugt80.
drm.wt.ugt80.
drm.smt1.
drm.wt.smt1.
dsm.ugt80.
dsm.wt.ugt80.
dsm.smt1.
dsm.wt.smt1.
sp.ugt80.
sp.wt.ugt80.
sp.smt1.
sp.wt.smt1.
im.ugt80.
im.wt.ugt80.
im.smt1.
im.wt.smt1.

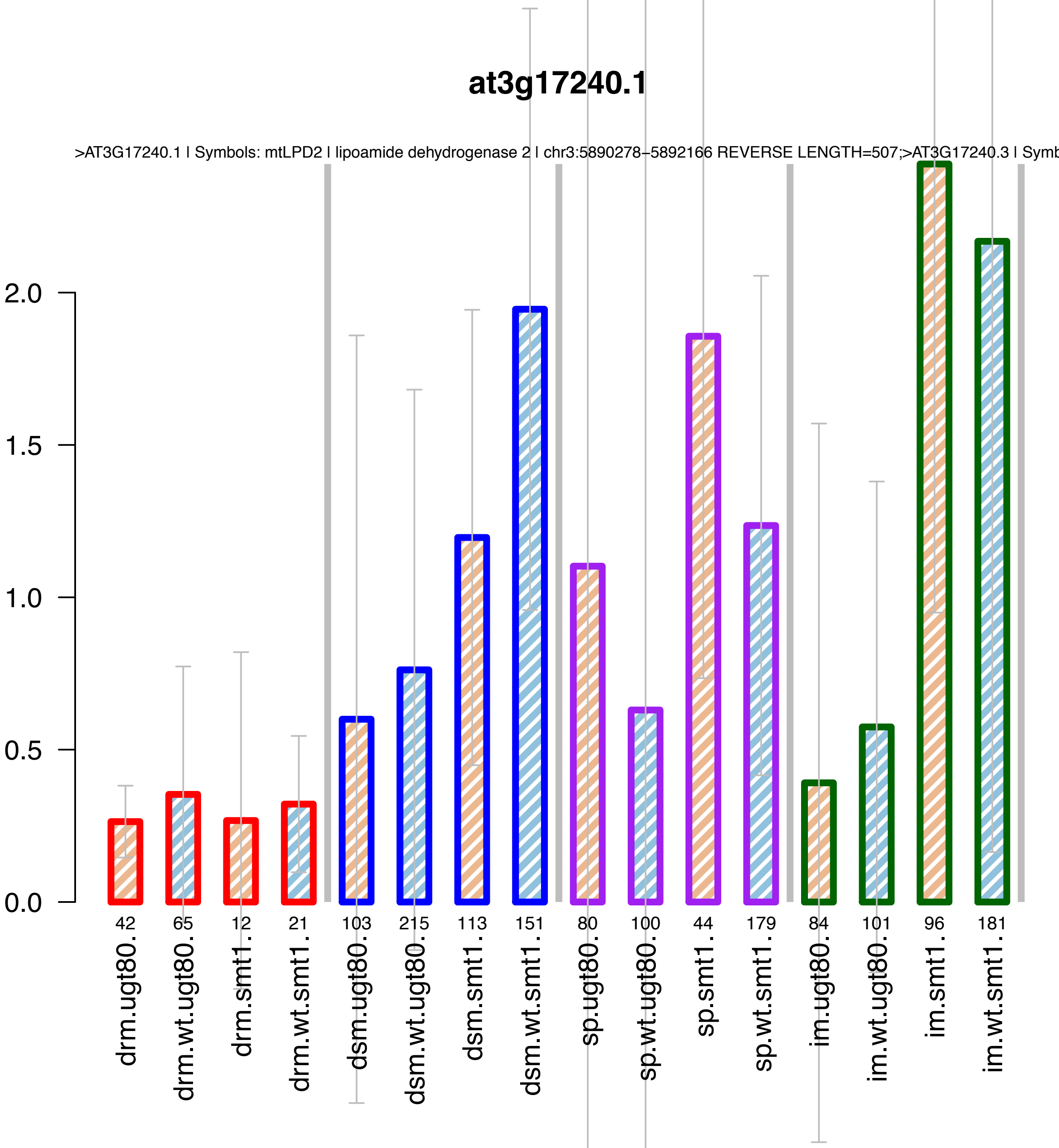
at3g17240.1
>AT3G17240.1 | Symbols: mtLPD2 | lipoamide dehydrogenase 2 | chr3:5890278−5892166 REVERSE LENGTH=507;>AT3G17240.3 | Symb
0.0
0.5
1.0
1.5
2.0
42
65
12
21
103
215
113
151
80
100
44
179
84
101
96
181
drm.ugt80..
drm.wt.ugt80..
drm.smt1..
drm.wt.smt1..
dsm.ugt80..
dsm.wt.ugt80..
dsm.smt1..
dsm.wt.smt1..
sp.ugt80..
sp.wt.ugt80..
sp.smt1..
sp.wt.smt1..
im.ugt80..
im.wt.ugt80..
im.smt1..
im.wt.smt1..

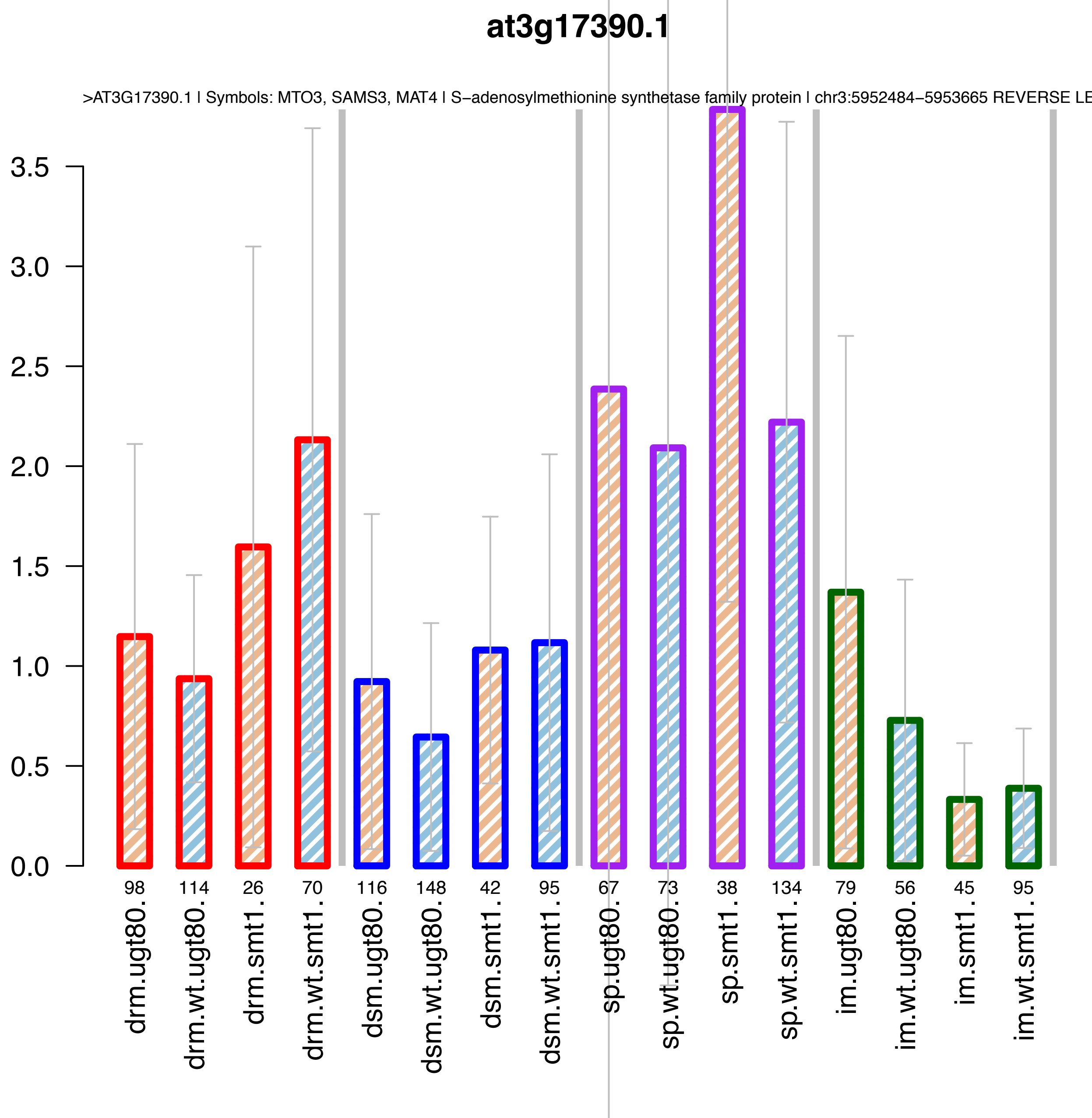

at3g17390.1
>AT3G17390.1 | Symbols: MTO3, SAMS3, MAT4 | S−adenosylmethionine synthetase family protein | chr3:5952484−5953665 REVERSE LE
3.5
3.0
2.5
2.0
1.5
1.0
0.5
0.0
98
114
26
70
116
148
42
95
67
73
38
134
79
56
45
95
drm.ugt80.
drm.wt.ugt80.
drm.smt1.
drm.wt.smt1.
dsm.ugt80.
dsm.wt.ugt80.
dsm.smt1.
dsm.wt.smt1.
sp.ugt80.
sp.wt.ugt80.
sp.smt1.
sp.wt.smt1.
im.ugt80.
im.wt.ugt80.
im.smt1.
im.wt.smt1.

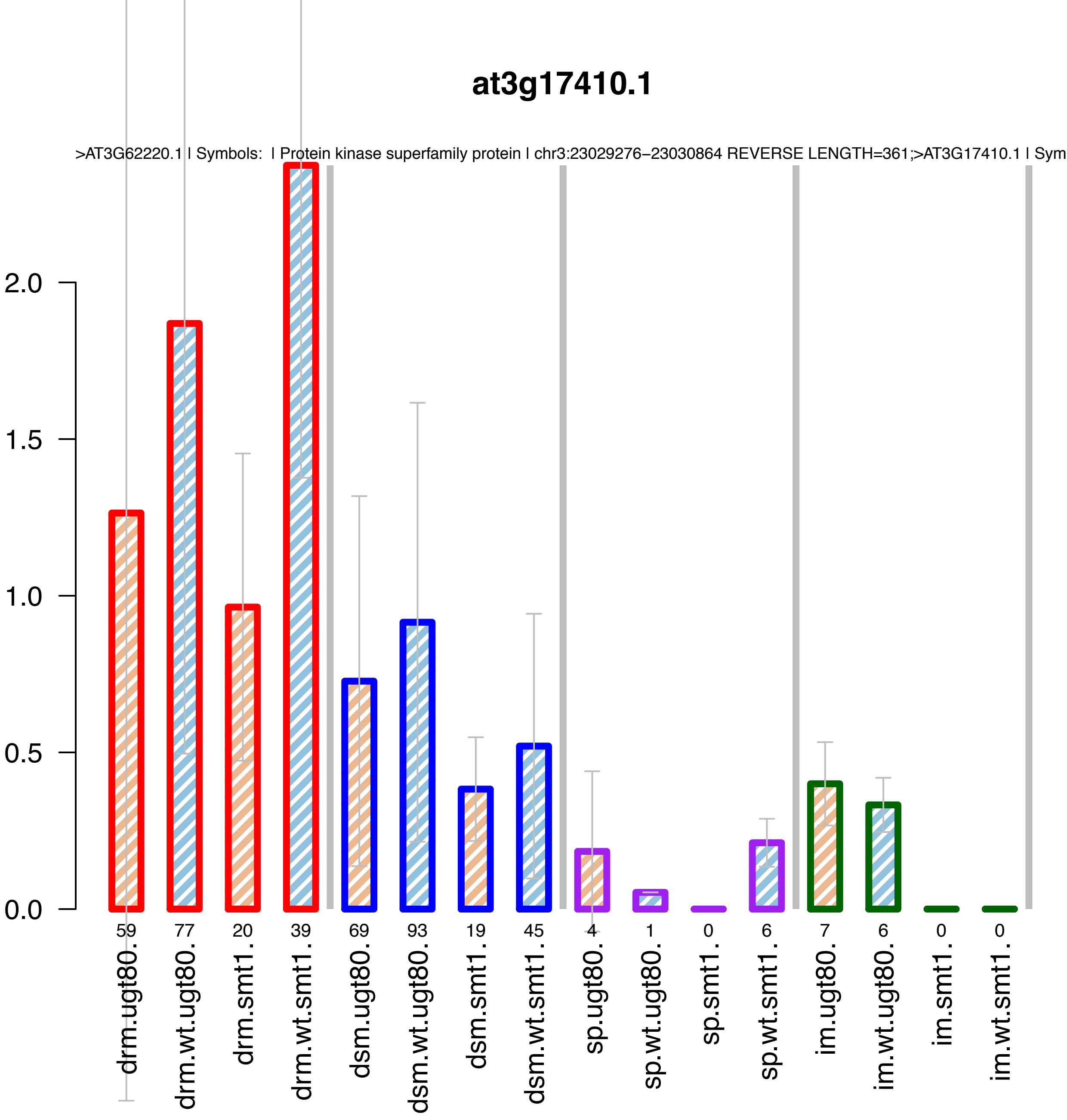

at3g17410.1
>AT3G62220.1 | Symbols: | Protein kinase superfamily protein | chr3:23029276-23030864 REVERSE LENGTH=361;>AT3G17410.1 | Sym
0.0
0.5
1.0
1.5
2.0
59
77
20
39
69
93
19
45
4
1
0
6
7
6
0
0
drm.ugt80.
drm.wt.ugt80.
drm.smt1.
drm.wt.smt1.
dsm.ugt80.
dsm.wt.ugt80.
dsm.smt1.
dsm.wt.smt1.
sp.ugt80.
sp.wt.ugt80.
sp.smt1.
sp.wt.smt1.
im.ugt80.
im.wt.ugt80.
im.smt1.
im.wt.smt1.

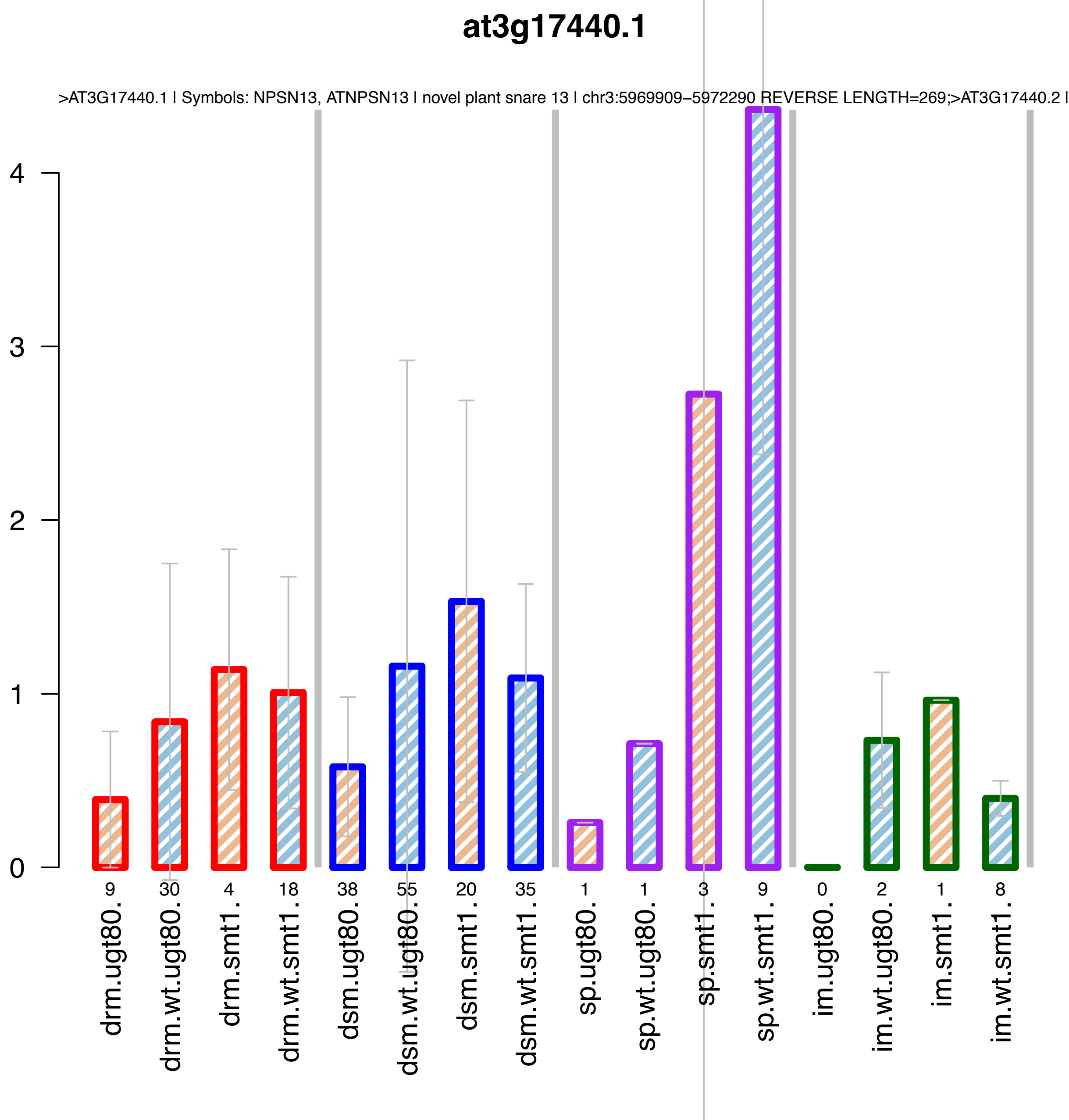

at3g17440.1
>AT3G17440.1 | Symbols: NPSN13, ATNPSN13 | novel plant snare 13 | chr3:5969909-5972290 REVERSE LENGTH=269;>AT3G17440.2 |
0
1
2
3
4
9
30
4
18
38
55
20
35
1
1
3
9
0
2
1
8
drm.ugt80.
drm.wt.ugt80.
drm.smt1.
drm.wt.smt1.
dsm.ugt80.
dsm.wt.ugt80.
dsm.smt1.
dsm.wt.smt1.
sp.ugt80.
sp.wt.ugt80.
sp.smt1.
sp.wt.smt1.
im.ugt80.
im.wt.ugt80.
im.smt1.
im.wt.smt1.

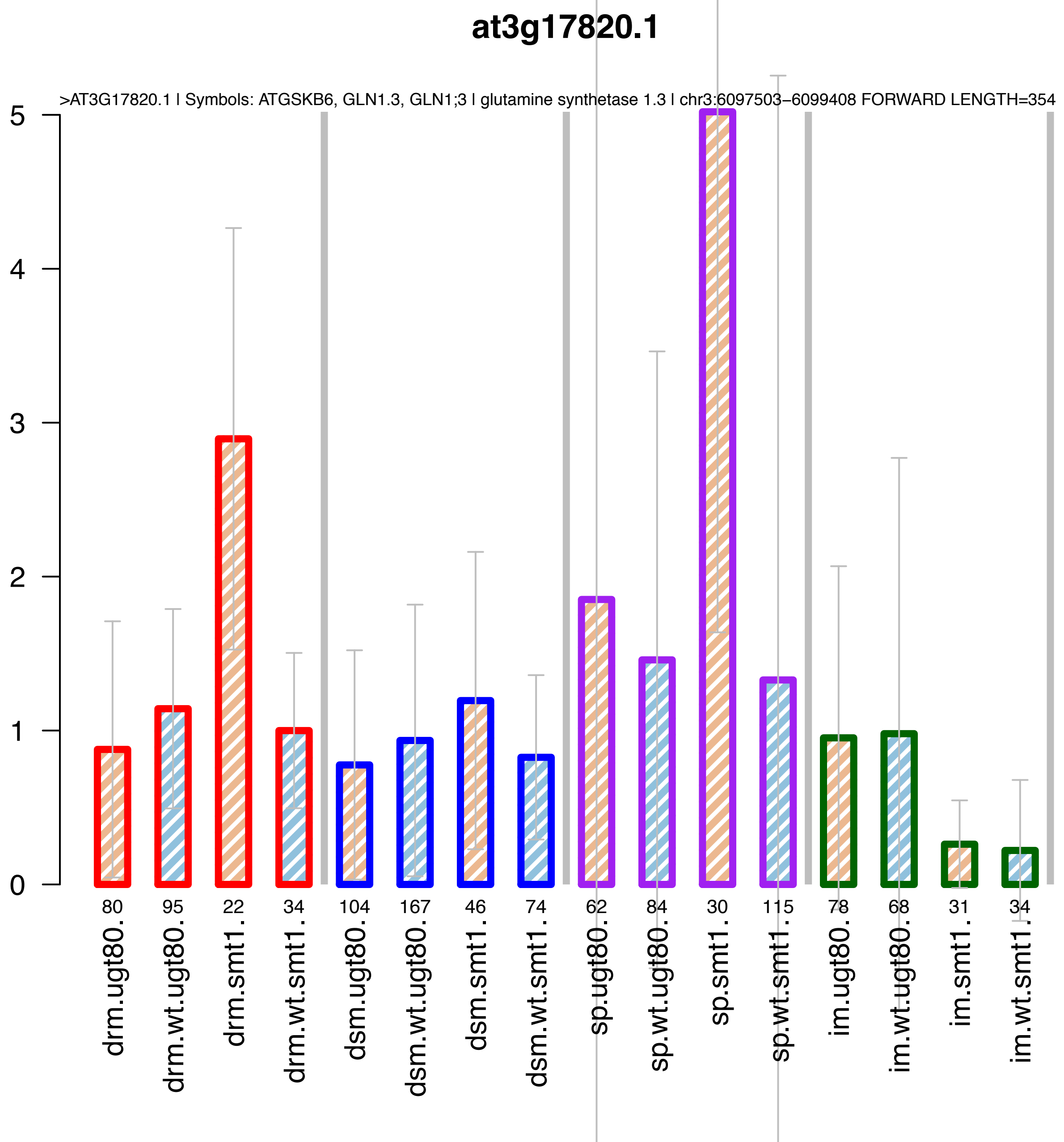

at3g17820.1
>AT3G17820.1 | Symbols: ATGSKB6, GLN1.3, GLN1;3 | glutamine synthetase 1.3 | chr3:6097503-6099408 FORWARD LENGTH=354
0
1
2
3
4
5
80
95
22
34
104
167
46
74
62
84
30
115
78
68
31
34
drm.ugt80.
drm.wt.ugt80.
drm.smt1.
drm.wt.smt1.
dsm.ugt80.
dsm.wt.ugt80.
dsm.smt1.
dsm.wt.smt1.
sp.ugt80.
sp.wt.ugt80.
sp.smt1.
sp.wt.smt1.
im.ugt80.
im.wt.ugt80.
im.smt1.
im.wt.smt1.

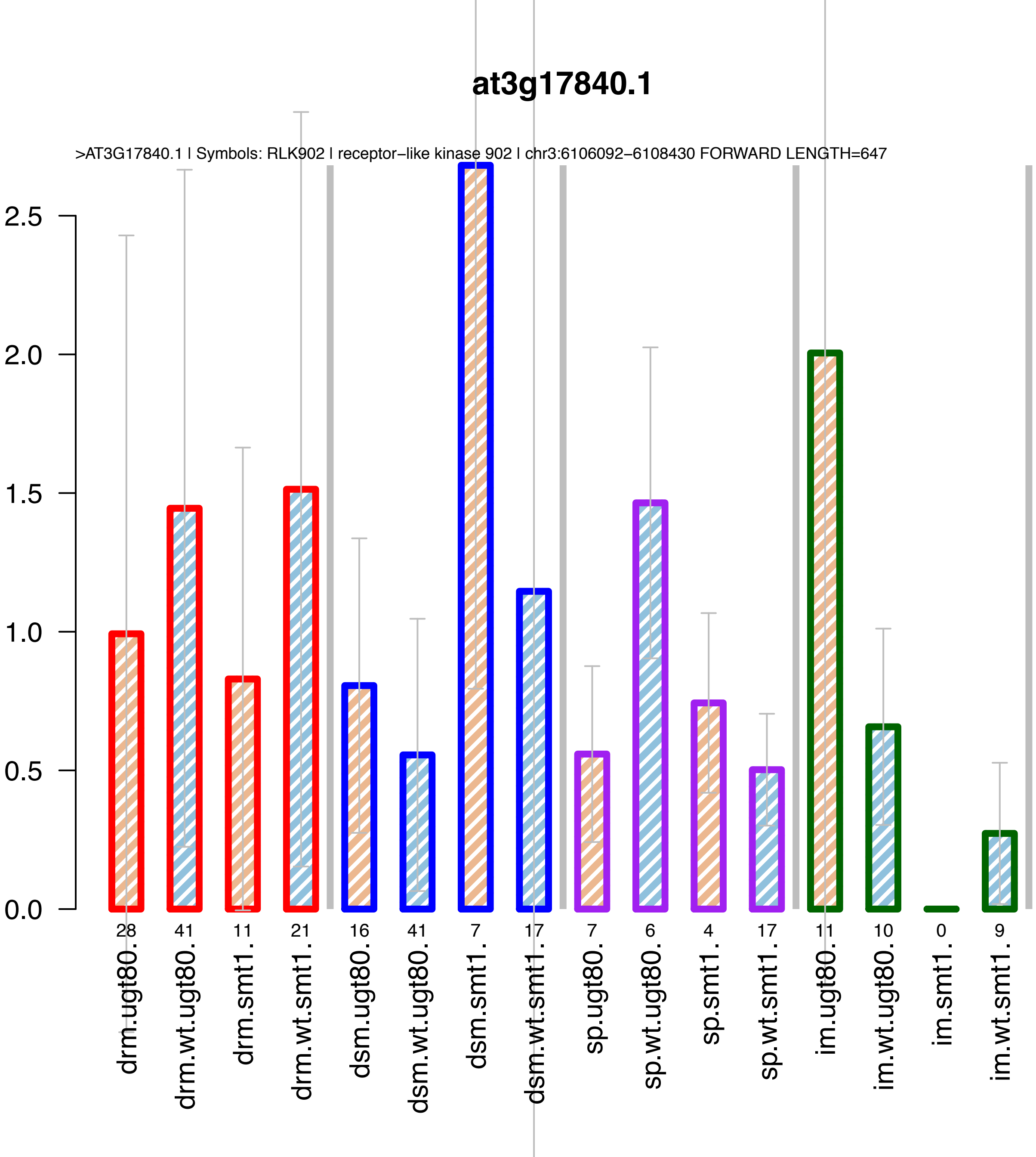

at3g17840.1
>AT3G17840.1 | Symbols: RLK902 | receptor−like kinase 902 | chr3:6106092−6108430 FORWARD LENGTH=647
2.5
2.0
1.5
1.0
0.5
0.0
28
41
11
21
16
41
7
17
7
6
4
17
11
10
0
9
drm.ugt80.
drm.wt.ugt80.
drm.smt1.
drm.wt.smt1.
dsm.ugt80.
dsm.wt.ugt80.
dsm.smt1.
dsm.wt.smt1.
sp.ugt80.
sp.wt.ugt80.
sp.smt1.
sp.wt.smt1.
im.ugt80.
im.wt.ugt80.
im.smt1.
im.wt.smt1.

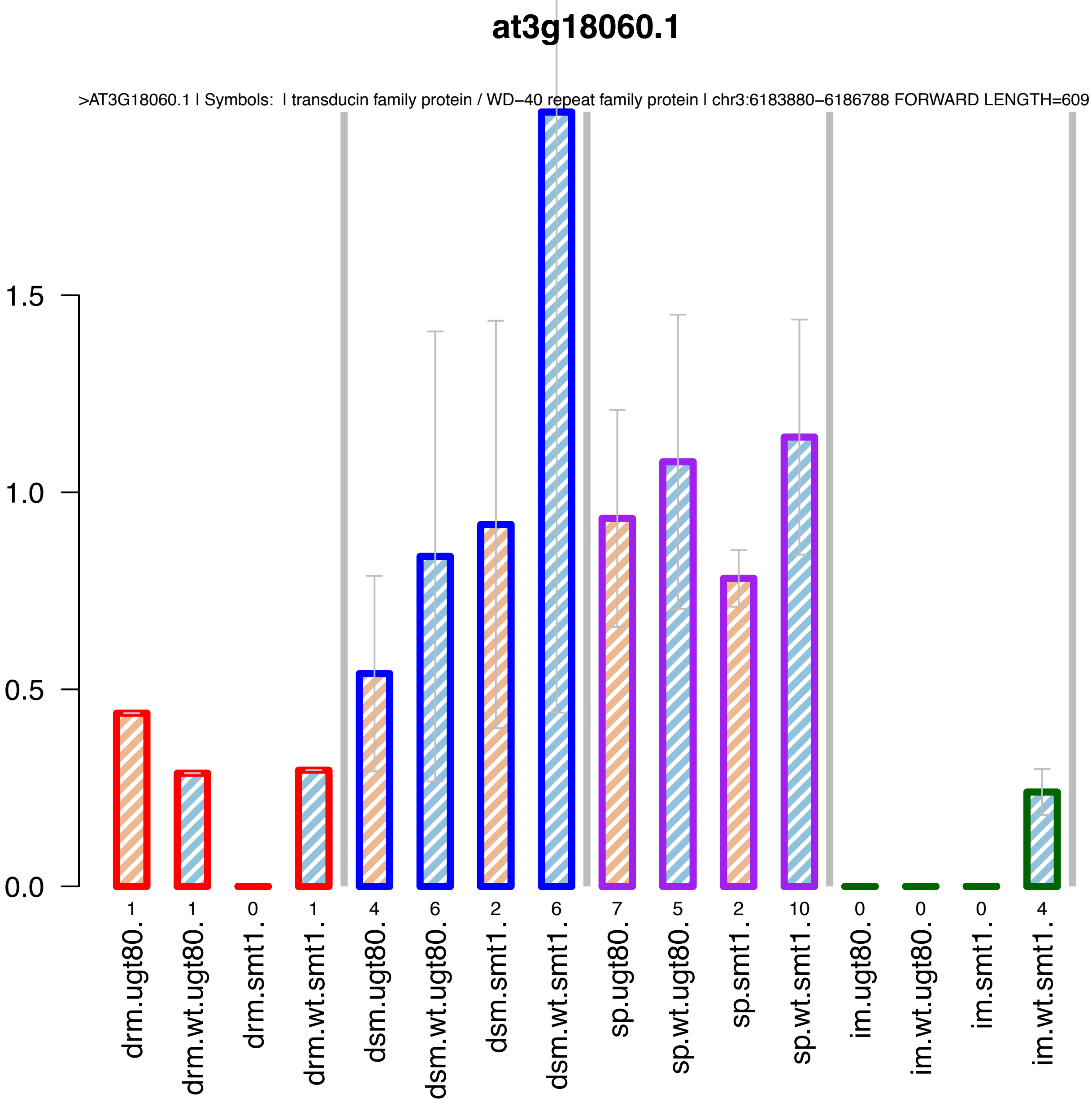
at3g18060.1
>AT3G18060.1 | Symbols: | transducin family protein / WD-40 repeat family protein | chr3:6183880-6186788 FORWARD LENGTH=609
0.0
0.5
1.0
1.5
1
1
0
1
4
6
2
6
7
5
2
10
0
0
0
4
drm.ugt80.
drm.wt.ugt80.
drm.smt1.
drm.wt.smt1.
dsm.ugt80.
dsm.wt.ugt80.
dsm.smt1.
dsm.wt.smt1.
sp.ugt80.
sp.wt.ugt80.
sp.smt1.
sp.wt.smt1.
im.ugt80.
im.wt.ugt80.
im.smt1.
im.wt.smt1.

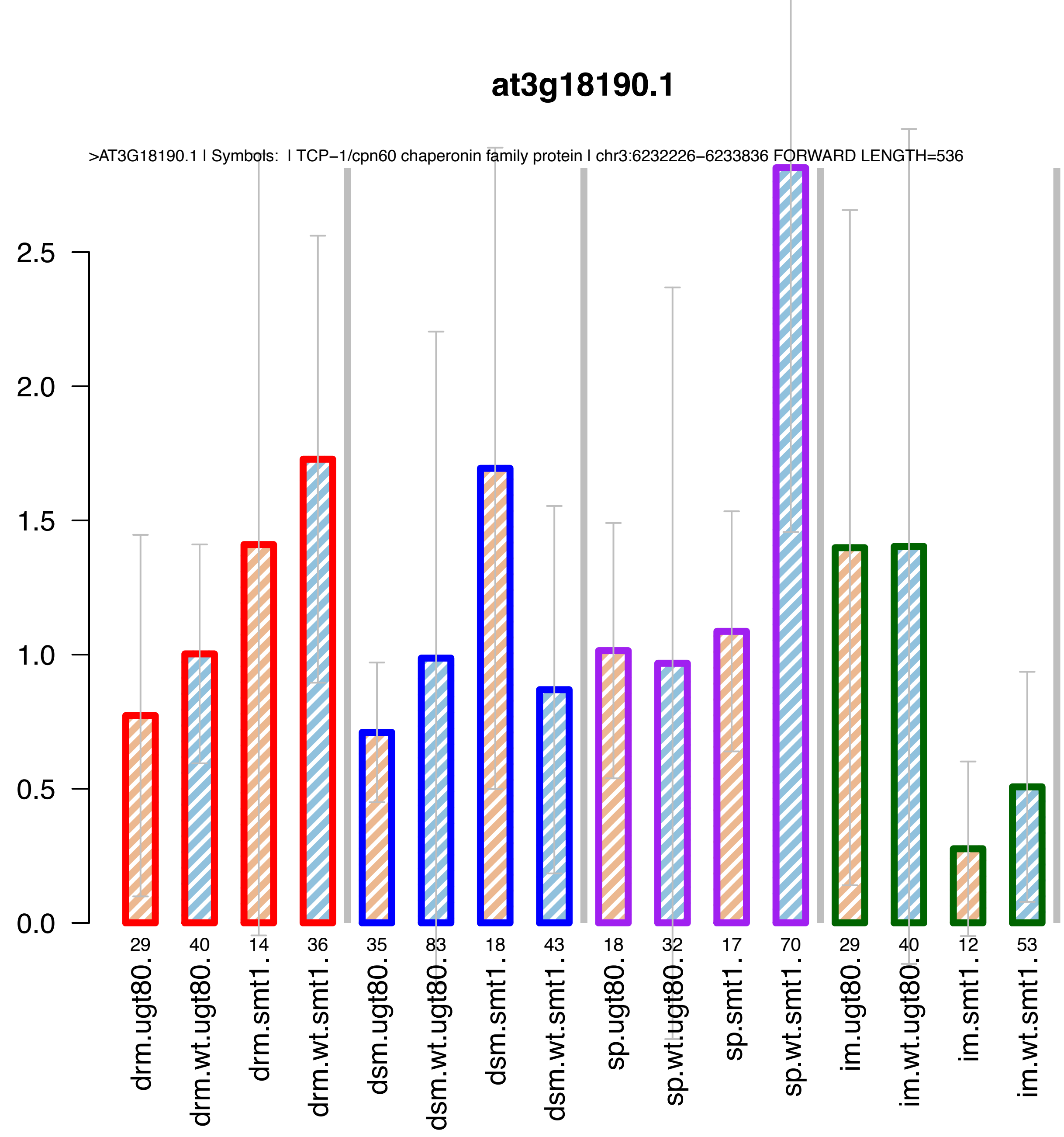

at3g18190.1
>AT3G18190.1 | Symbols: | TCP−1/cpn60 chaperonin family protein | chr3:6232226−6233836 FORWARD LENGTH=536
0.0
0.5
1.0
1.5
2.0
2.5
29
40
14
36
35
83
18
43
18
32
17
70
29
40
12
53
drm.ugt80..
drm.wt.ugt80..
drm.smt1..
drm.wt.smt1..
dsm.ugt80..
dsm.wt.ugt80..
dsm.smt1..
dsm.wt.smt1..
sp.ugt80..
sp.wt.ugt80..
sp.smt1..
sp.wt.smt1..
im.ugt80..
im.wt.ugt80..
im.smt1..
im.wt.smt1..

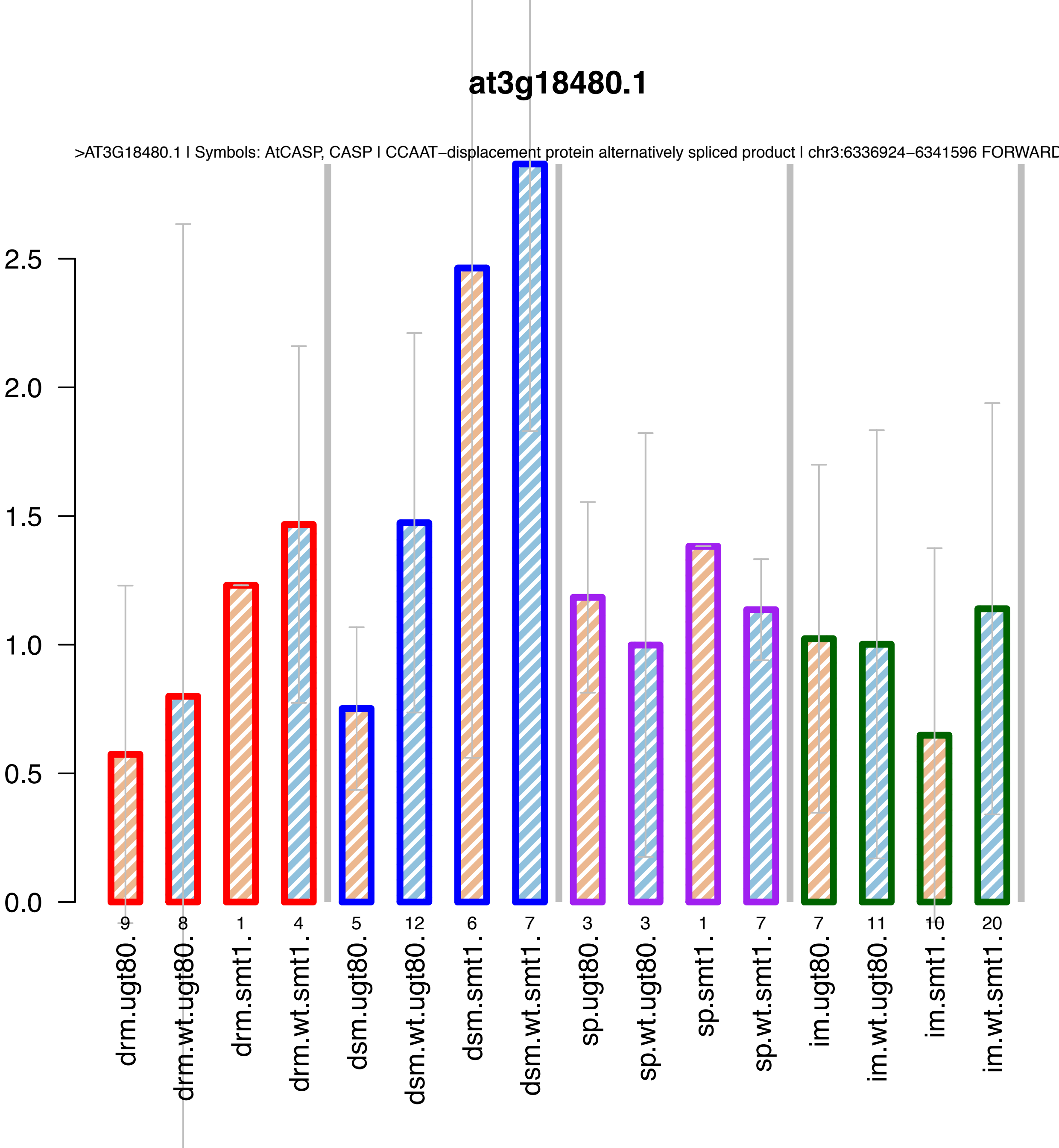

at3g18480.1
>AT3G18480.1 | Symbols: AtCASP, CASP | CCAAT−displacement protein alternatively spliced product | chr3:6336924−6341596 FORWARD
0.0
0.5
1.0
1.5
2.0
2.5
9
8
1
4
5
12
6
7
3
3
1
7
7
11
10
20
drm.ugt80..
drm.wt.ugt80..
drm.smt1..
drm.wt.smt1..
dsm.ugt80..
dsm.wt.ugt80..
dsm.smt1..
dsm.wt.smt1..
sp.ugt80..
sp.wt.ugt80..
sp.smt1..
sp.wt.smt1..
im.ugt80..
im.wt.ugt80..
im.smt1..
im.wt.smt1..

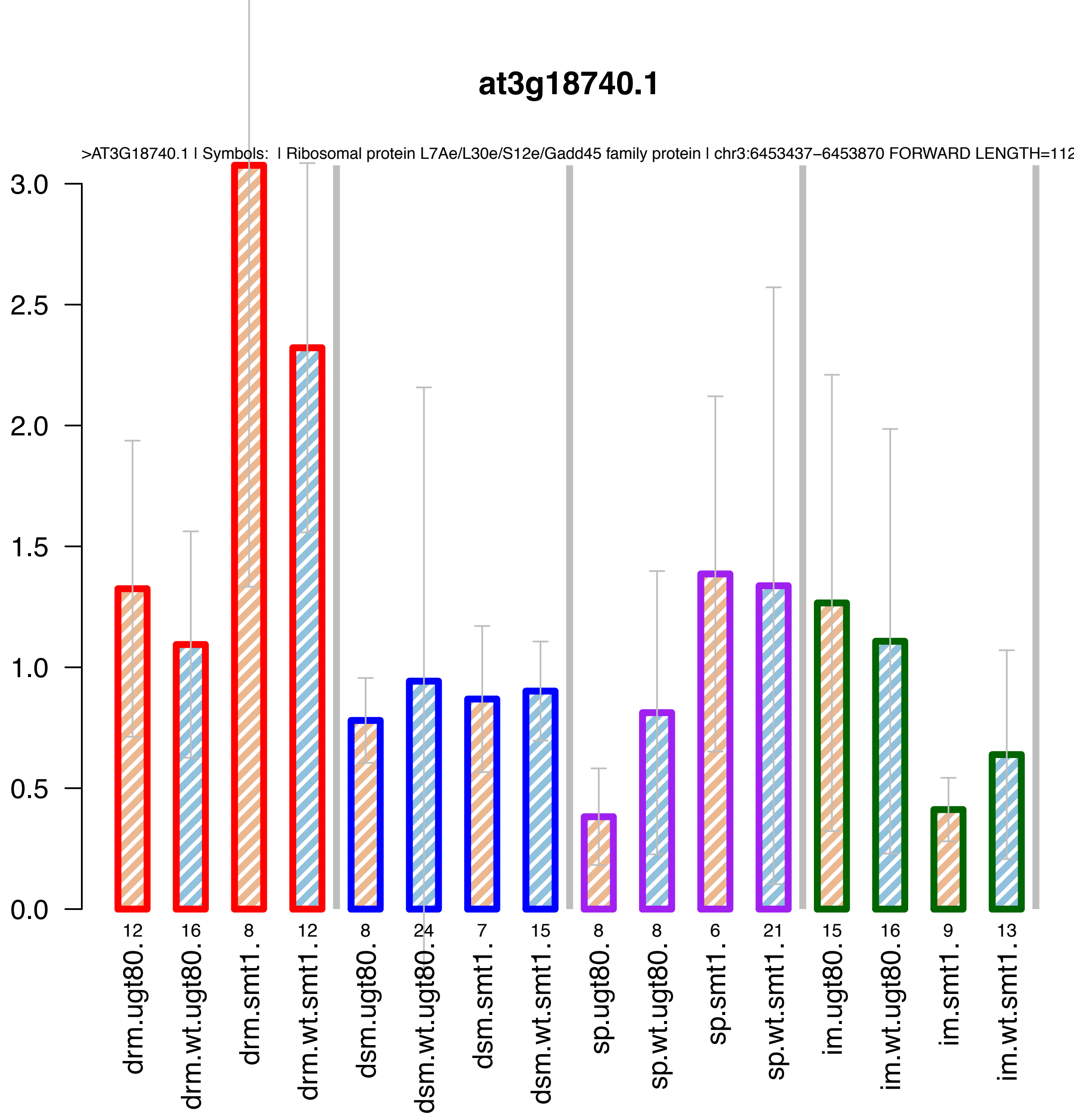
at3g18740.1
>AT3G18740.1 | Symbols: | Ribosomal protein L7Ae/L30e/S12e/Gadd45 family protein | chr3:6453437-6453870 FORWARD LENGTH=112
3.0
2.5
2.0
1.5
1.0
0.5
0.0
12
16
8
12
8
24
7
15
8
8
6
21
15
16
9
13
drm.ugt80..
drm.wt.ugt80..
drm.smt1..
drm.wt.smt1..
dsm.ugt80..
dsm.wt.ugt80..
dsm.smt1..
dsm.wt.smt1..
sp.ugt80..
sp.wt.ugt80..
sp.smt1..
sp.wt.smt1..
im.ugt80..
im.wt.ugt80..
im.smt1..
im.wt.smt1..

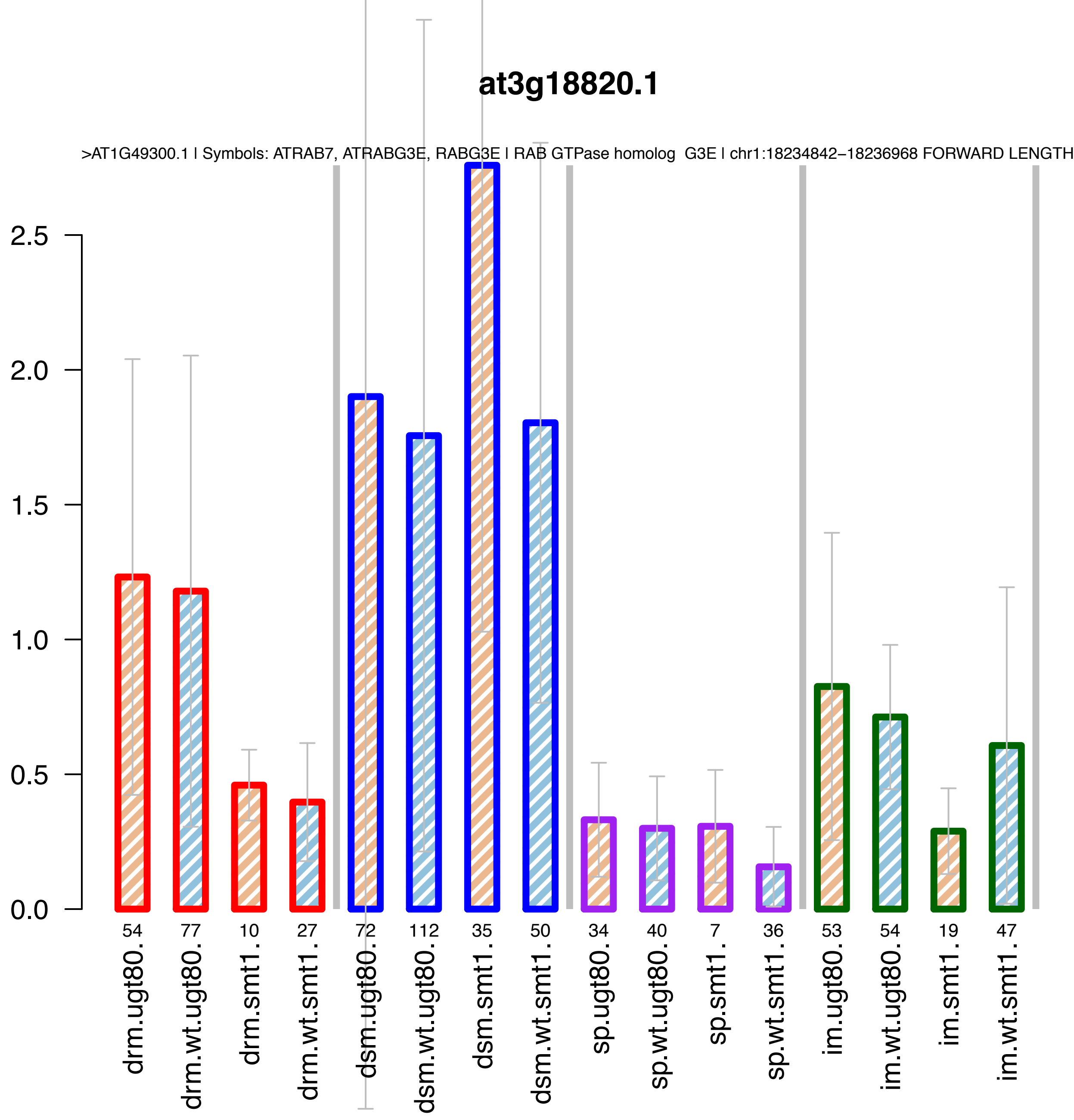

at3g18820.1
>AT1G49300.1 | Symbols: ATRAB7, ATRABG3E, RABG3E | RAB GTPase homolog G3E | chr1:18234842-18236968 FORWARD LENGTH
0.0
0.5
1.0
1.5
2.0
2.5
54
77
10
27
72
112
35
50
34
40
7
36
53
54
19
47
drm.ugt80.
drm.wt.ugt80.
drm.smt1.
drm.wt.smt1.
dsm.ugt80.
dsm.wt.ugt80.
dsm.smt1.
dsm.wt.smt1.
sp.ugt80.
sp.wt.ugt80.
sp.smt1.
sp.wt.smt1.
im.ugt80.
im.wt.ugt80.
im.smt1.
im.wt.smt1.

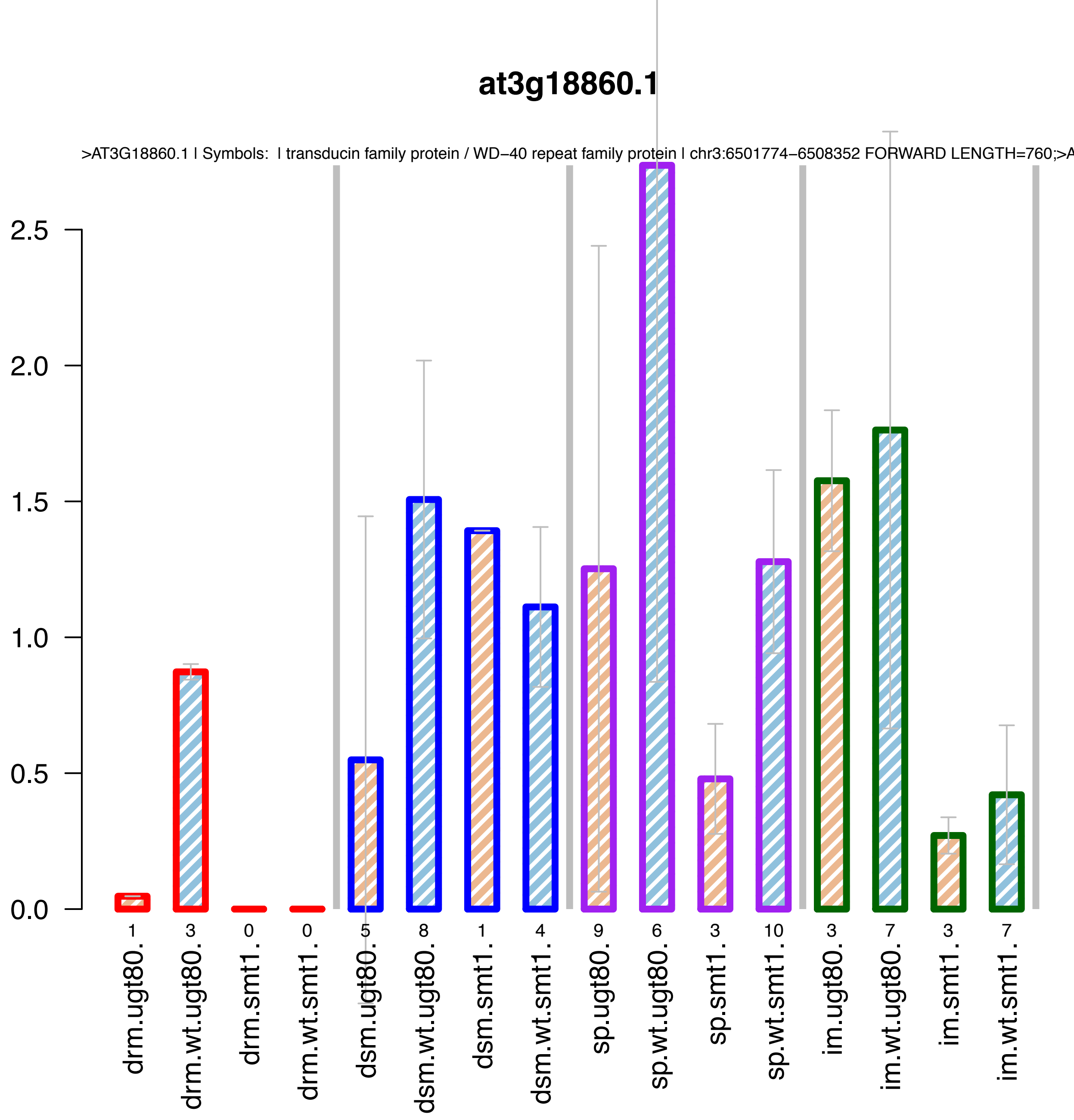

at3g18860.1
>AT3G18860.1 | Symbols: | transducin family protein / WD−40 repeat family protein | chr3:6501774−6508352 FORWARD LENGTH=760;>A
0.0
0.5
1.0
1.5
2.0
2.5
1
3
0
0
5
8
1
4
9
6
3
10
3
7
3
7
drm.ugt80.
drm.wt.ugt80.
drm.smt1.
drm.wt.smt1.
dsm.ugt80.
dsm.wt.ugt80.
dsm.smt1.
dsm.wt.smt1.
sp.ugt80.
sp.wt.ugt80.
sp.smt1.
sp.wt.smt1.
im.ugt80.
im.wt.ugt80.
im.smt1.
im.wt.smt1.

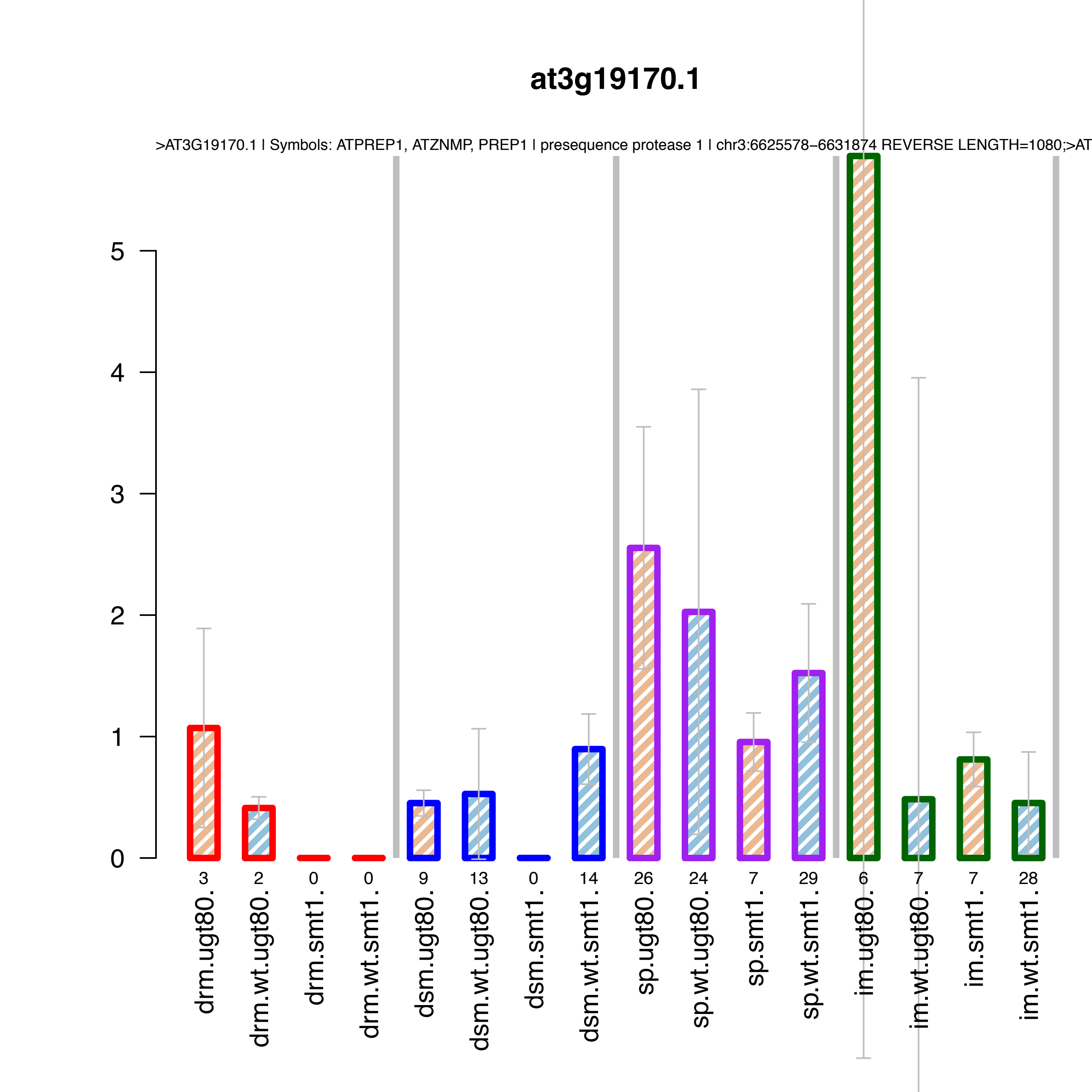
at3g19170.1
>AT3G19170.1 | Symbols: ATPREP1, ATZNMP, PREP1 | presequence protease 1 | chr3:6625578-6631874 REVERSE LENGTH=1080;>AT
0
1
2
3
4
5
3
2
0
0
9
13
0
14
26
24
7
29
6
7
7
28
drm.ugt80.
drm.wt.ugt80.
drm.smt1.
drm.wt.smt1.
dsm.ugt80.
dsm.wt.ugt80.
dsm.smt1.
dsm.wt.smt1.
sp.ugt80.
sp.wt.ugt80.
sp.smt1.
sp.wt.smt1.
im.ugt80.
im.wt.ugt80.
im.smt1.
im.wt.smt1.

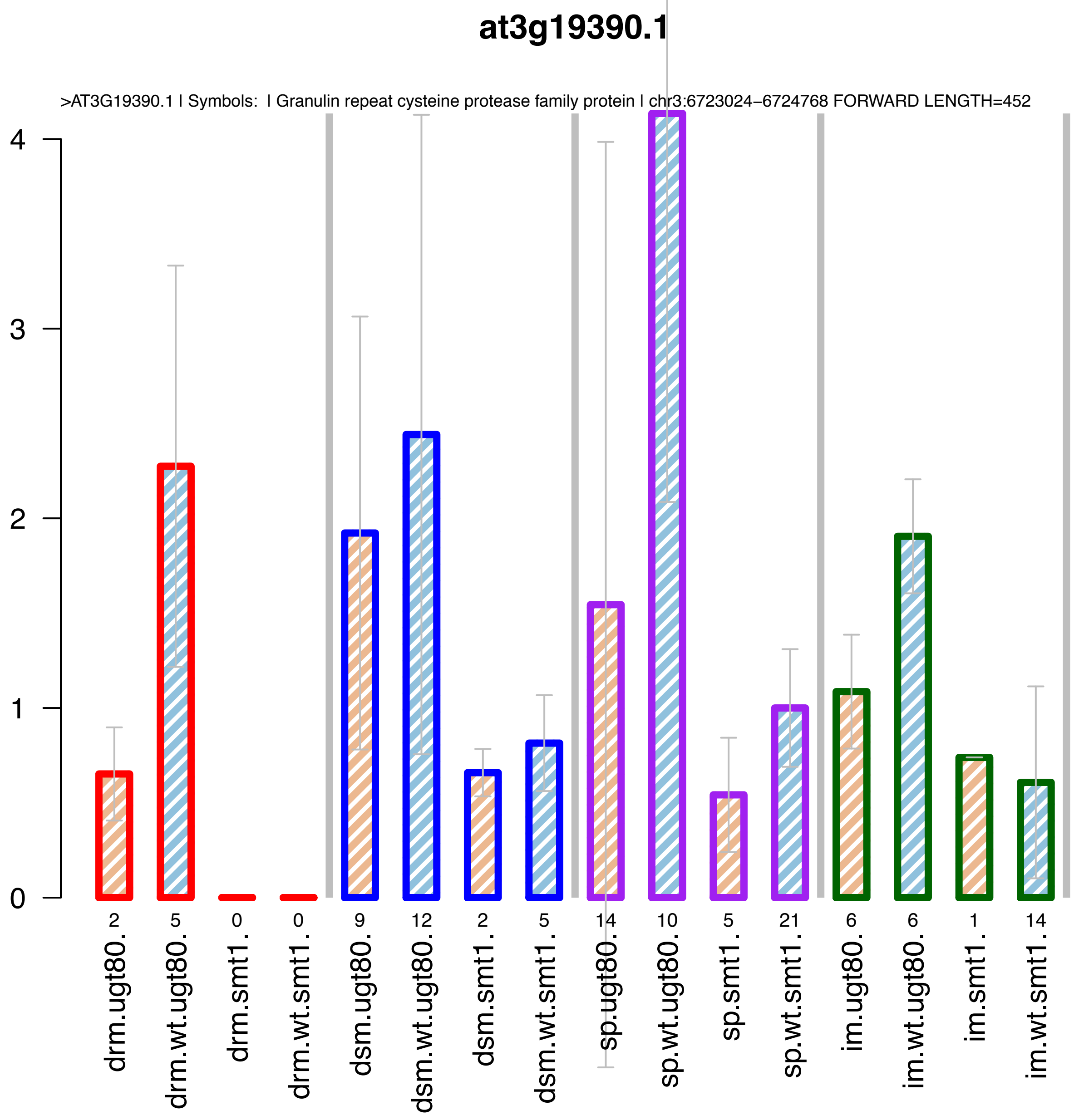

at3g19390.1
>AT3G19390.1 | Symbols: | Granulin repeat cysteine protease family protein | chr3:6723024-6724768 FORWARD LENGTH=452
0
1
2
3
4
2
5
0
0
9
12
2
5
14
10
5
21
6
6
1
14
drm.ugt80..
drm.wt.ugt80..
drm.smt1..
drm.wt.smt1..
dsm.ugt80..
dsm.wt.ugt80..
dsm.smt1..
dsm.wt.smt1..
sp.ugt80..
sp.wt.ugt80..
sp.smt1..
sp.wt.smt1..
im.ugt80..
im.wt.ugt80..
im.smt1..
im.wt.smt1..

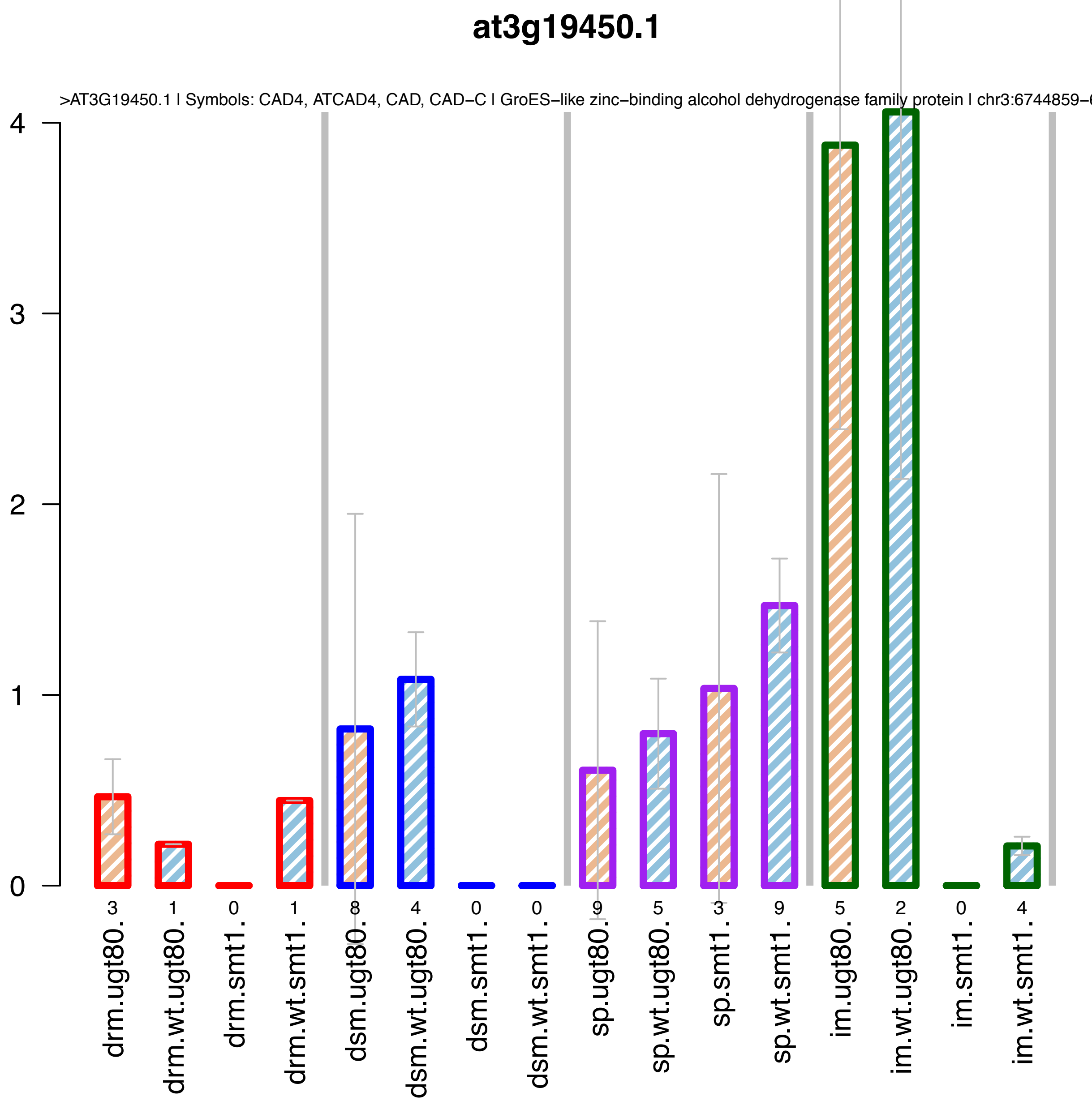

at3g19450.1
>AT3G19450.1 | Symbols: CAD4, ATCAD4, CAD, CAD−C | GroES−like zinc−binding alcohol dehydrogenase family protein | chr3:6744859−6
4
3
2
1
0
3
1
0
1
8
4
0
0
9
5
3
9
5
2
0
4
drm.ugt80.
drm.wt.ugt80.
drm.smt1.
drm.wt.smt1.
dsm.ugt80.
dsm.wt.ugt80.
dsm.smt1.
dsm.wt.smt1.
sp.ugt80.
sp.wt.ugt80.
sp.smt1.
sp.wt.smt1.
im.ugt80.
im.wt.ugt80.
im.smt1.
im.wt.smt1.

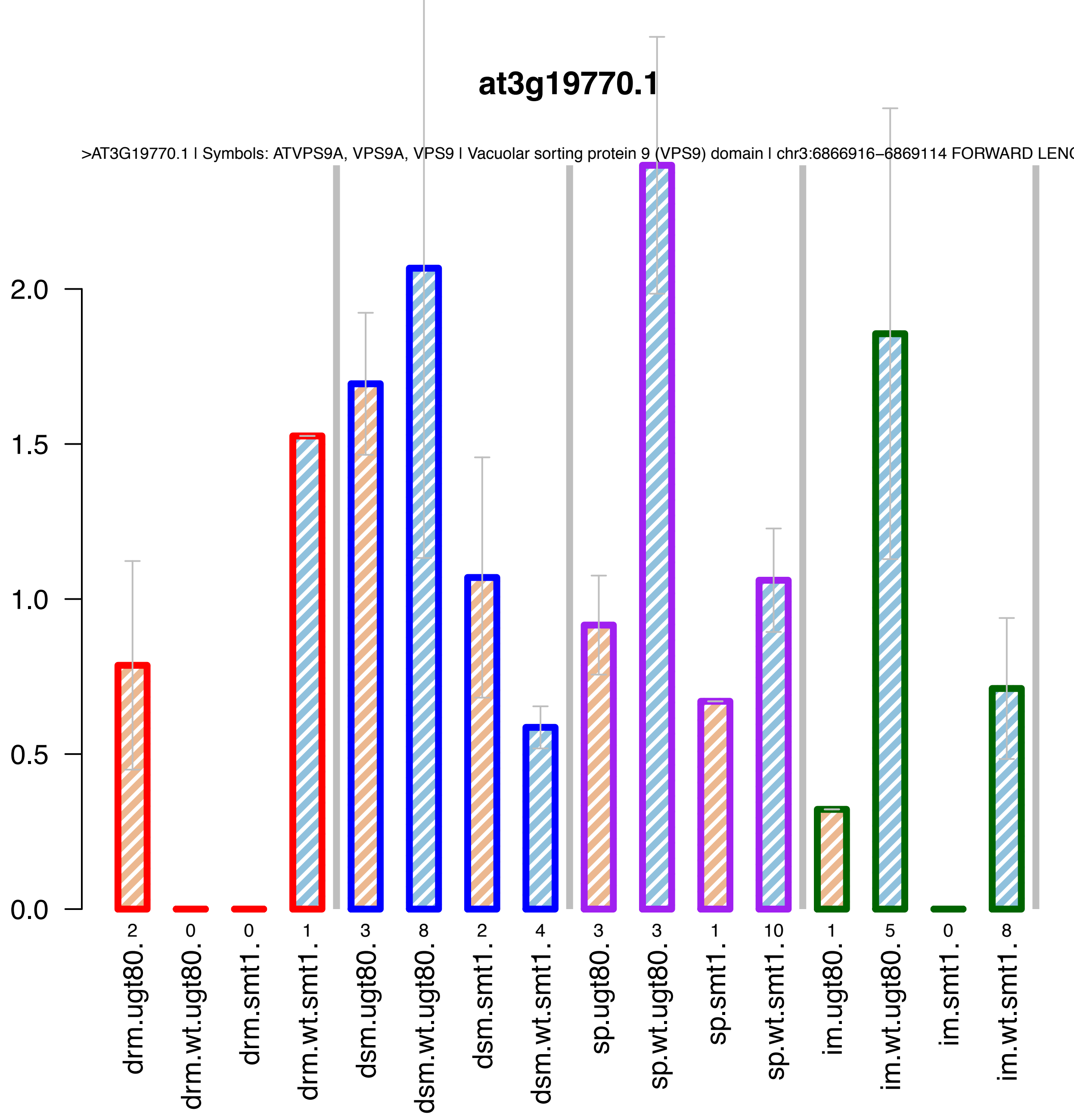

at3g19770.1
>AT3G19770.1 | Symbols: ATVPS9A, VPS9A, VPS9 | Vacuolar sorting protein 9 (VPS9) domain | chr3:6866916-6869114 FORWARD LENG
2.0
1.5
1.0
0.5
0.0
2
0
0
1
3
8
2
4
3
3
1
10
1
5
0
8
drm.ugt80.
drm.wt.ugt80.
drm.smt1.
drm.wt.smt1.
dsm.ugt80.
dsm.wt.ugt80.
dsm.smt1.
dsm.wt.smt1.
sp.ugt80.
sp.wt.ugt80.
sp.smt1.
sp.wt.smt1.
im.ugt80.
im.wt.ugt80.
im.smt1.
im.wt.smt1.

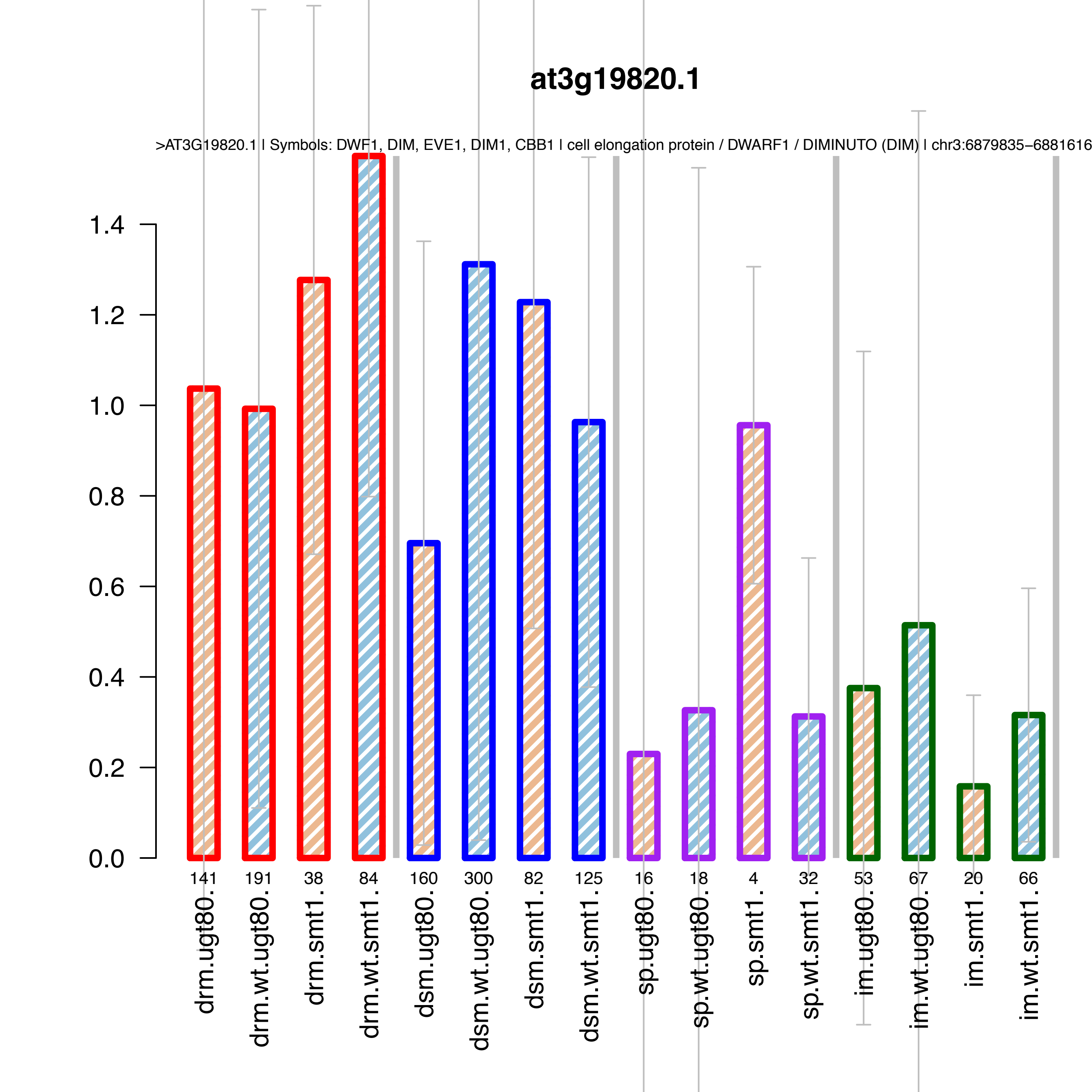
at3g19820.1
>AT3G19820.1 | Symbols: DWF1, DIM, EVE1, DIM1, CBB1 | cell elongation protein / DWARF1 / DIMINUTO (DIM) | chr3:6879835-6881616
0.0
0.2
0.4
0.6
0.8
1.0
1.2
1.4
141
191
38
84
160
300
82
125
16
18
4
32
53
67
20
66
drm.ugt80..
drm.wt.ugt80..
drm.smt1..
drm.wt.smt1..
dsm.ugt80..
dsm.wt.ugt80..
dsm.smt1..
dsm.wt.smt1..
sp.ugt80..
sp.wt.ugt80..
sp.smt1..
sp.wt.smt1..
im.ugt80..
im.wt.ugt80..
im.smt1..
im.wt.smt1..

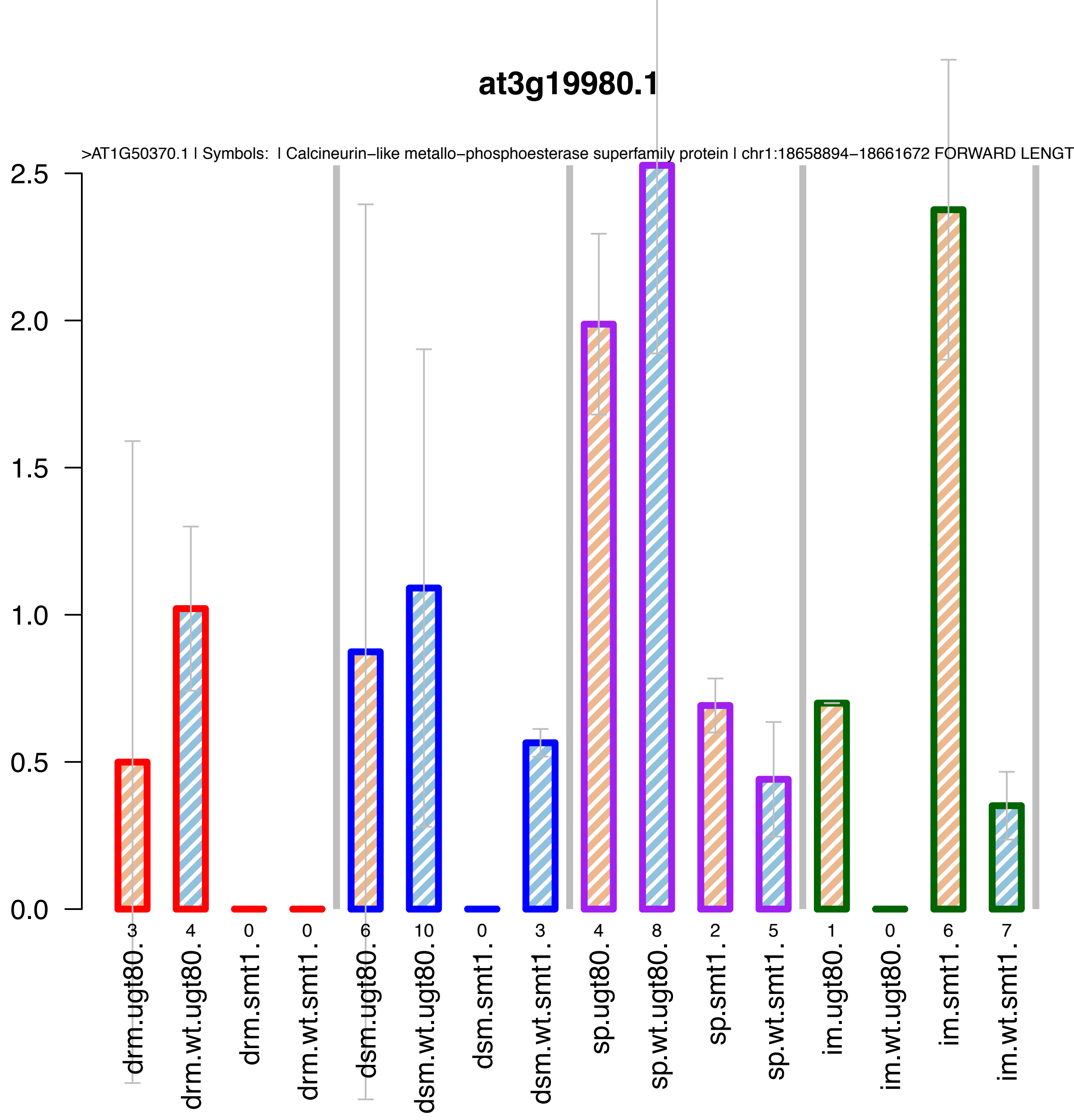

at3g19980.1
>AT1G50370.1 | Symbols: | Calcineurin–like metallo–phosphoesterase superfamily protein | chr1:18658894–18661672 FORWARD LENGT
2.5
2.0
1.5
1.0
0.5
0.0
3
4
0
0
6
10
0
3
4
8
2
5
1
0
6
7
drm.ugt80..
drm.wt.ugt80..
drm.smt1..
drm.wt.smt1..
dsm.ugt80..
dsm.wt.ugt80..
dsm.smt1..
dsm.wt.smt1..
sp.ugt80..
sp.wt.ugt80..
sp.smt1..
sp.wt.smt1..
im.ugt80..
im.wt.ugt80..
im.smt1..
im.wt.smt1..

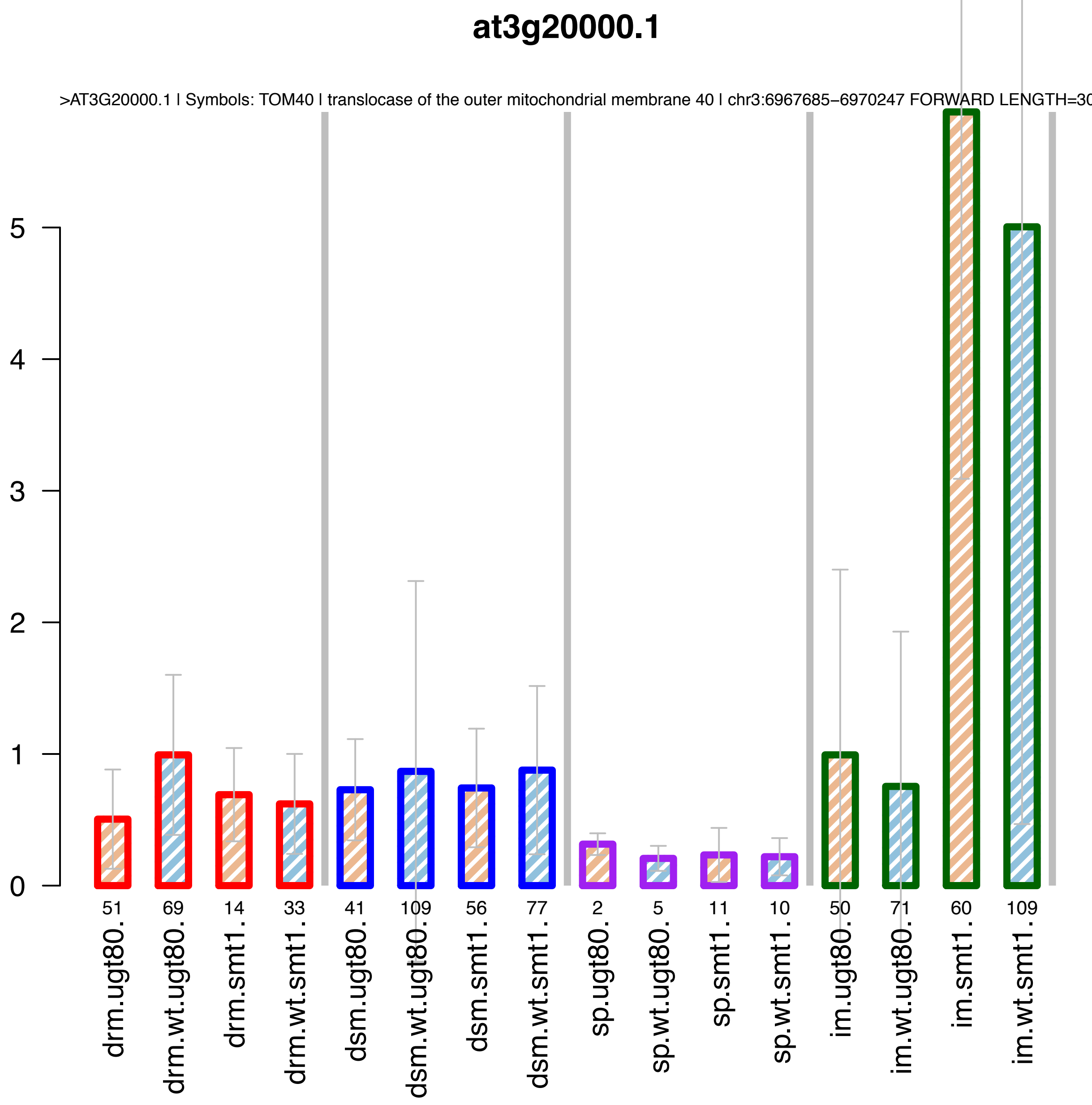

at3g20000.1
>AT3G20000.1 | Symbols: TOM40 | translocase of the outer mitochondrial membrane 40 | chr3:6967685–6970247 FORWARD LENGTH=30
0
1
2
3
4
5
51
69
14
33
41
109
56
77
2
5
11
10
50
71
60
109
drm.ugt80..
drm.wt.ugt80..
drm.smt1..
drm.wt.smt1..
dsm.ugt80..
dsm.wt.ugt80..
dsm.smt1..
dsm.wt.smt1..
sp.ugt80..
sp.wt.ugt80..
sp.smt1..
sp.wt.smt1..
im.ugt80..
im.wt.ugt80..
im.smt1..
im.wt.smt1..

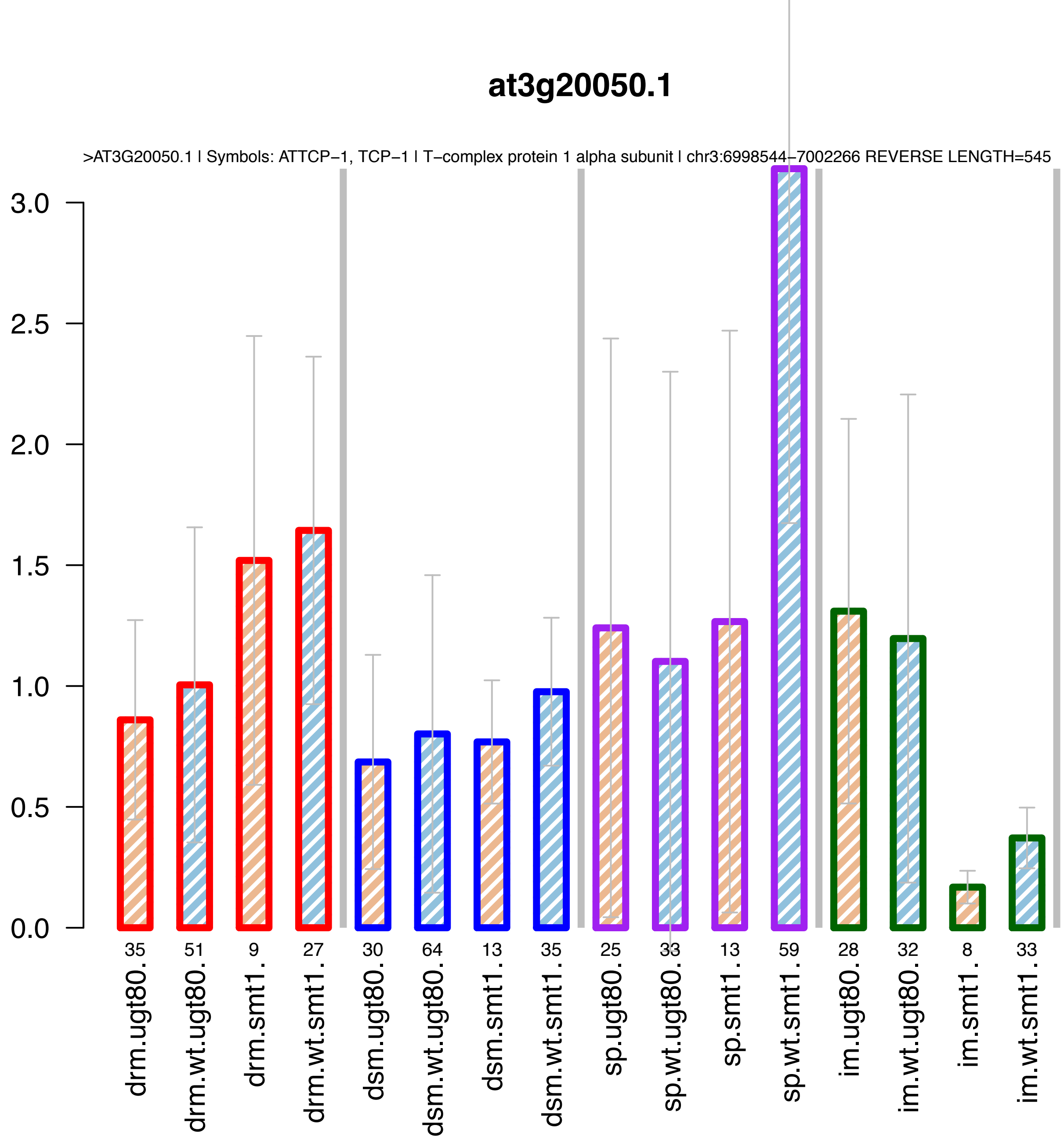
at3g20050.1
>AT3G20050.1 | Symbols: ATTCP-1, TCP-1 | T-complex protein 1 alpha subunit | chr3:6998544-7002266 REVERSE LENGTH=545
3.0
2.5
2.0
1.5
1.0
0.5
0.0
35
51
9
27
30
64
13
35
25
33
13
59
28
32
8
33
drm.ugt80.
drm.wt.ugt80.
drm.smt1.
drm.wt.smt1.
dsm.ugt80.
dsm.wt.ugt80.
dsm.smt1.
dsm.wt.smt1.
sp.ugt80.
sp.wt.ugt80.
sp.smt1.
sp.wt.smt1.
im.ugt80.
im.wt.ugt80.
im.smt1.
im.wt.smt1.

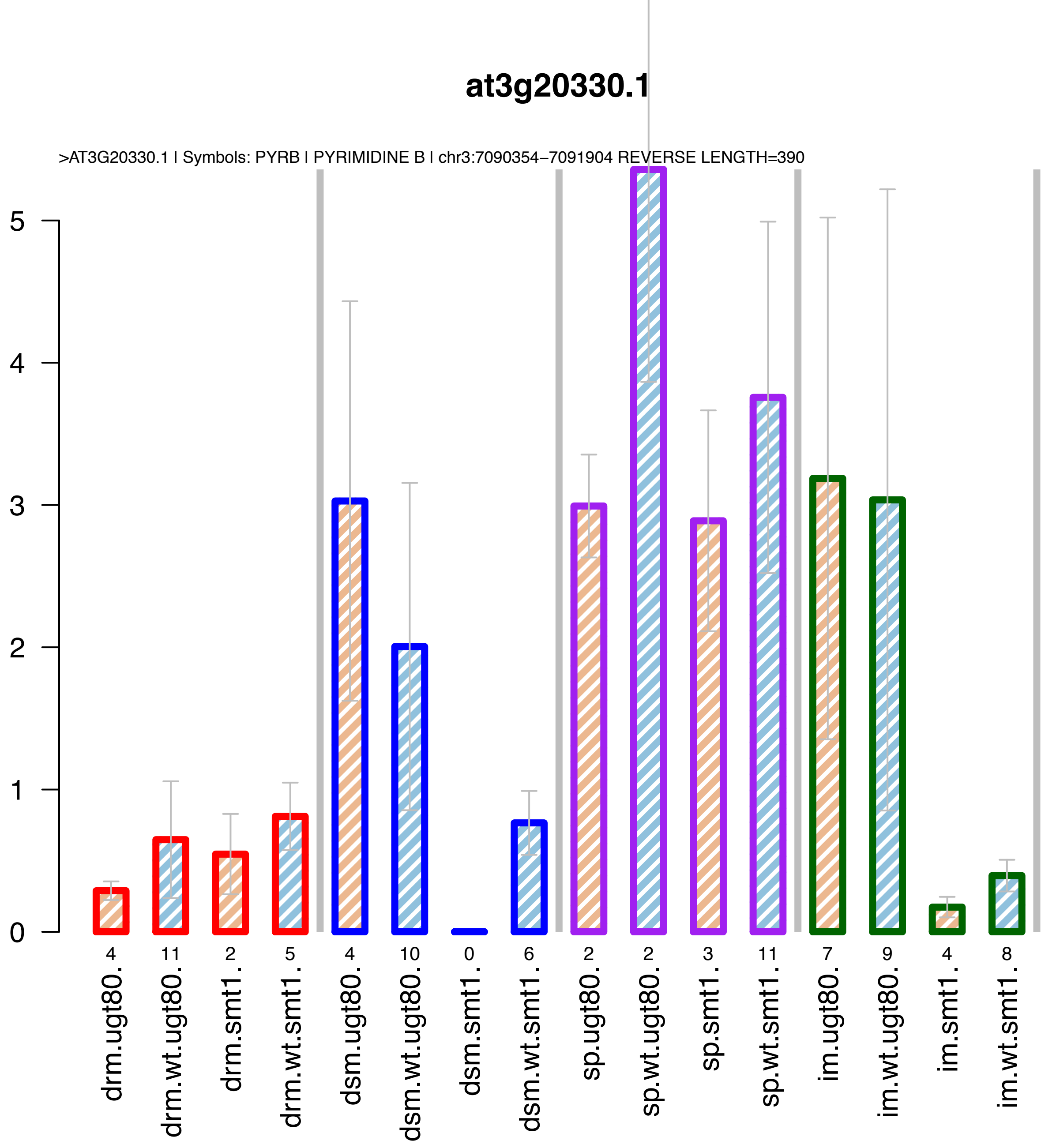
at3g20330.1
>AT3G20330.1 | Symbols: PYRB | PYRIMIDINE B | chr3:7090354-7091904 REVERSE LENGTH=390
0
1
2
3
4
5
4
11
2
5
4
10
0
6
2
2
3
11
7
9
4
8
drm.ugt80.
drm.wt.ugt80.
drm.smt1.
drm.wt.smt1.
dsm.ugt80.
dsm.wt.ugt80.
dsm.smt1.
dsm.wt.smt1.
sp.ugt80.
sp.wt.ugt80.
sp.smt1.
sp.wt.smt1.
im.ugt80.
im.wt.ugt80.
im.smt1.
im.wt.smt1.

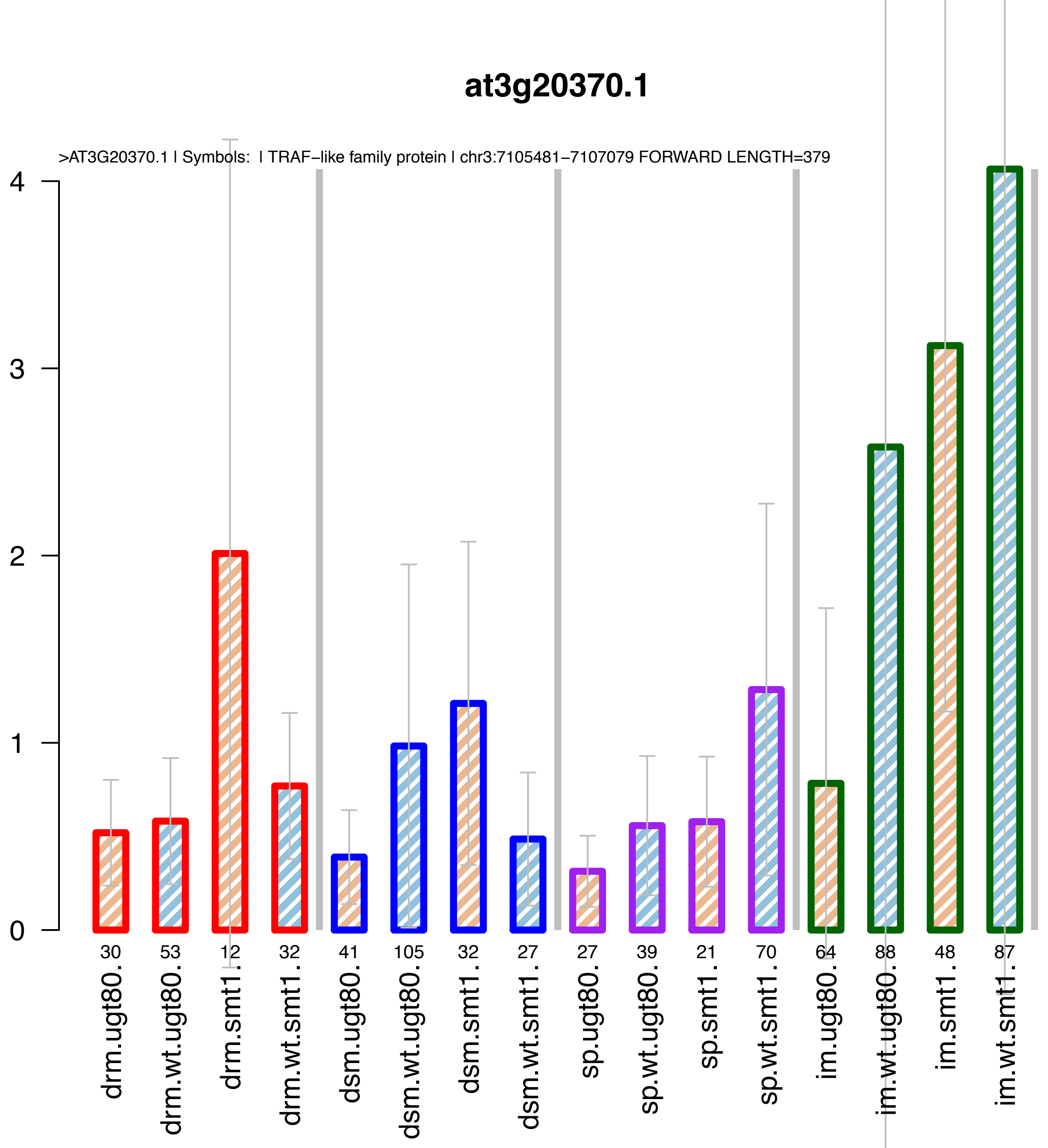

at3g20370.1
>AT3G20370.1 | Symbols: | TRAF−like family protein | chr3:7105481−7107079 FORWARD LENGTH=379
4
3
2
1
0
30
53
12
32
41
105
32
27
27
39
21
70
64
88
48
87
drm.ugt80..
drm.wt.ugt80..
drm.smt1..
drm.wt.smt1..
dsm.ugt80..
dsm.wt.ugt80..
dsm.smt1..
dsm.wt.smt1..
sp.ugt80..
sp.wt.ugt80..
sp.smt1..
sp.wt.smt1..
im.ugt80..
im.wt.ugt80..
im.smt1..
im.wt.smt1..

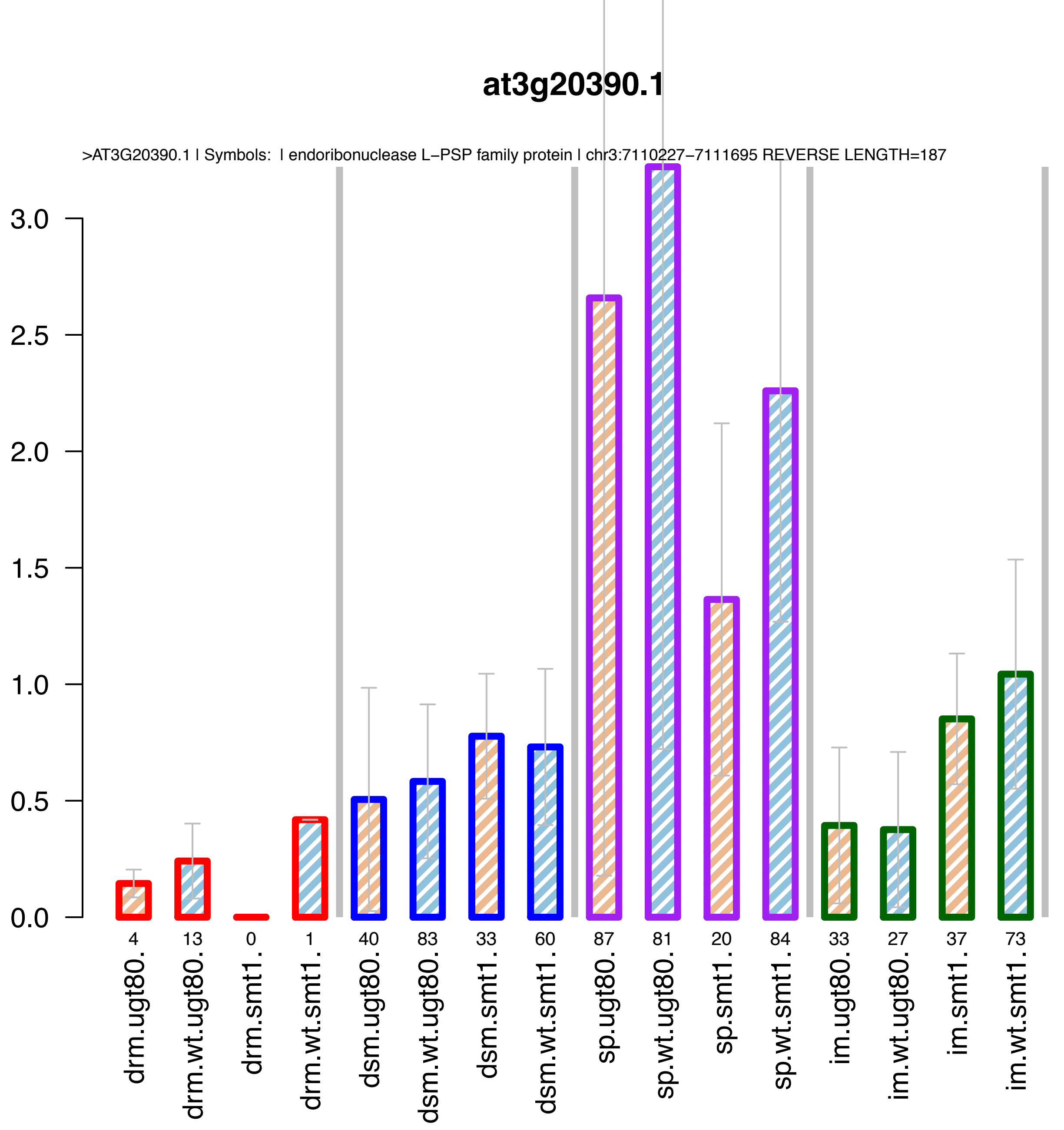

at3g20390.1
>AT3G20390.1 | Symbols: | endoribonuclease L−PSP family protein | chr3:7110227−7111695 REVERSE LENGTH=187
3.0
2.5
2.0
1.5
1.0
0.5
0.0
4
13
0
1
40
83
33
60
87
81
20
84
33
27
37
73
drm.ugt80.
drm.wt.ugt80.
drm.smt1.
drm.wt.smt1.
dsm.ugt80.
dsm.wt.ugt80.
dsm.smt1.
dsm.wt.smt1.
sp.ugt80.
sp.wt.ugt80.
sp.smt1.
sp.wt.smt1.
im.ugt80.
im.wt.ugt80.
im.smt1.
im.wt.smt1.

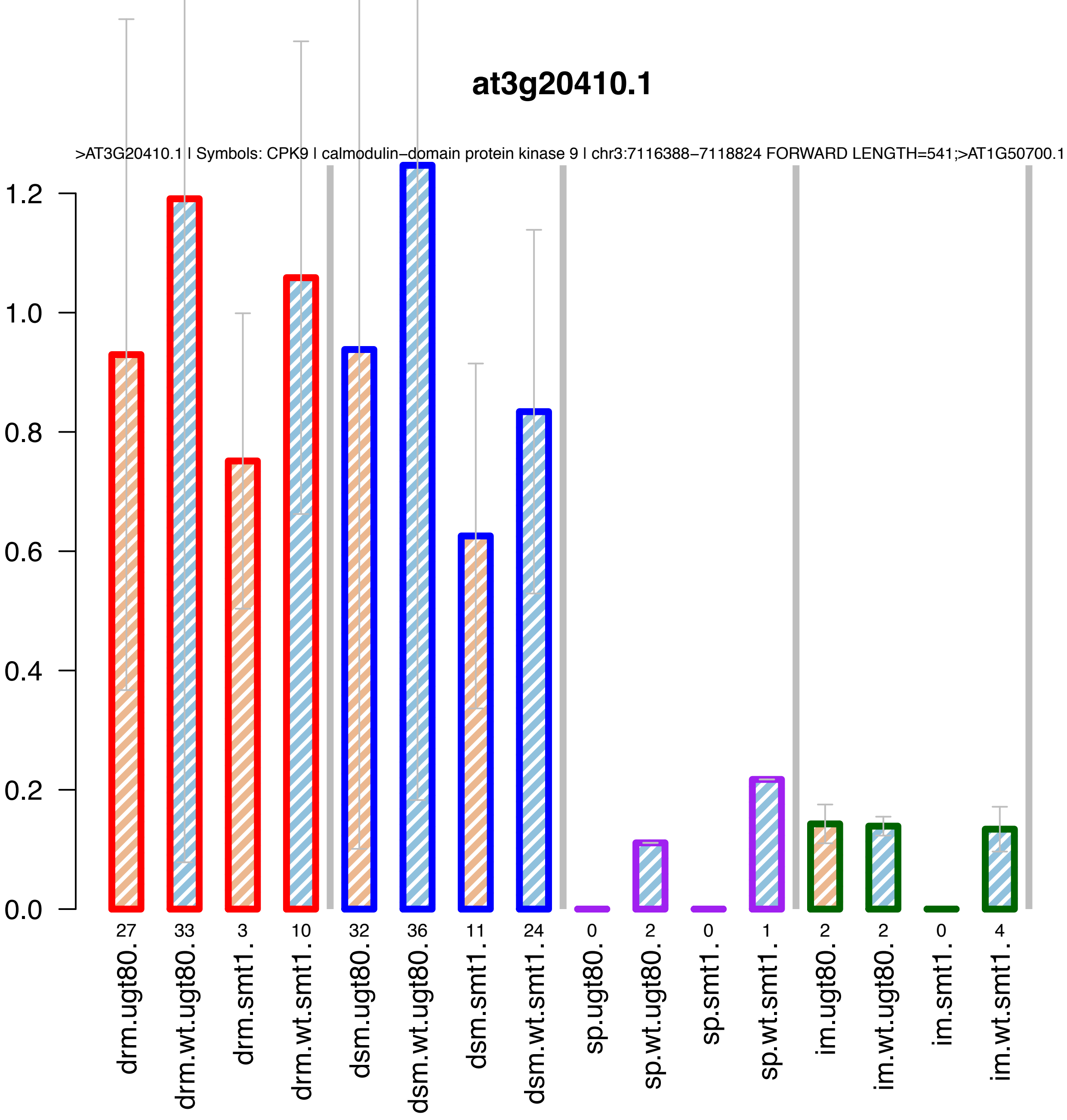

at3g20410.1
>AT3G20410.1 | Symbols: CPK9 | calmodulin−domain protein kinase 9 | chr3:7116388−7118824 FORWARD LENGTH=541;>AT1G50700.1
1.2
1.0
0.8
0.6
0.4
0.2
0.0
27
33
3
10
32
36
11
24
0
2
0
1
2
2
0
4
drm.ugt80..
drm.wt.ugt80..
drm.smt1..
drm.wt.smt1..
dsm.ugt80..
dsm.wt.ugt80..
dsm.smt1..
dsm.wt.smt1..
sp.ugt80..
sp.wt.ugt80..
sp.smt1..
sp.wt.smt1..
im.ugt80..
im.wt.ugt80..
im.smt1..
im.wt.smt1..

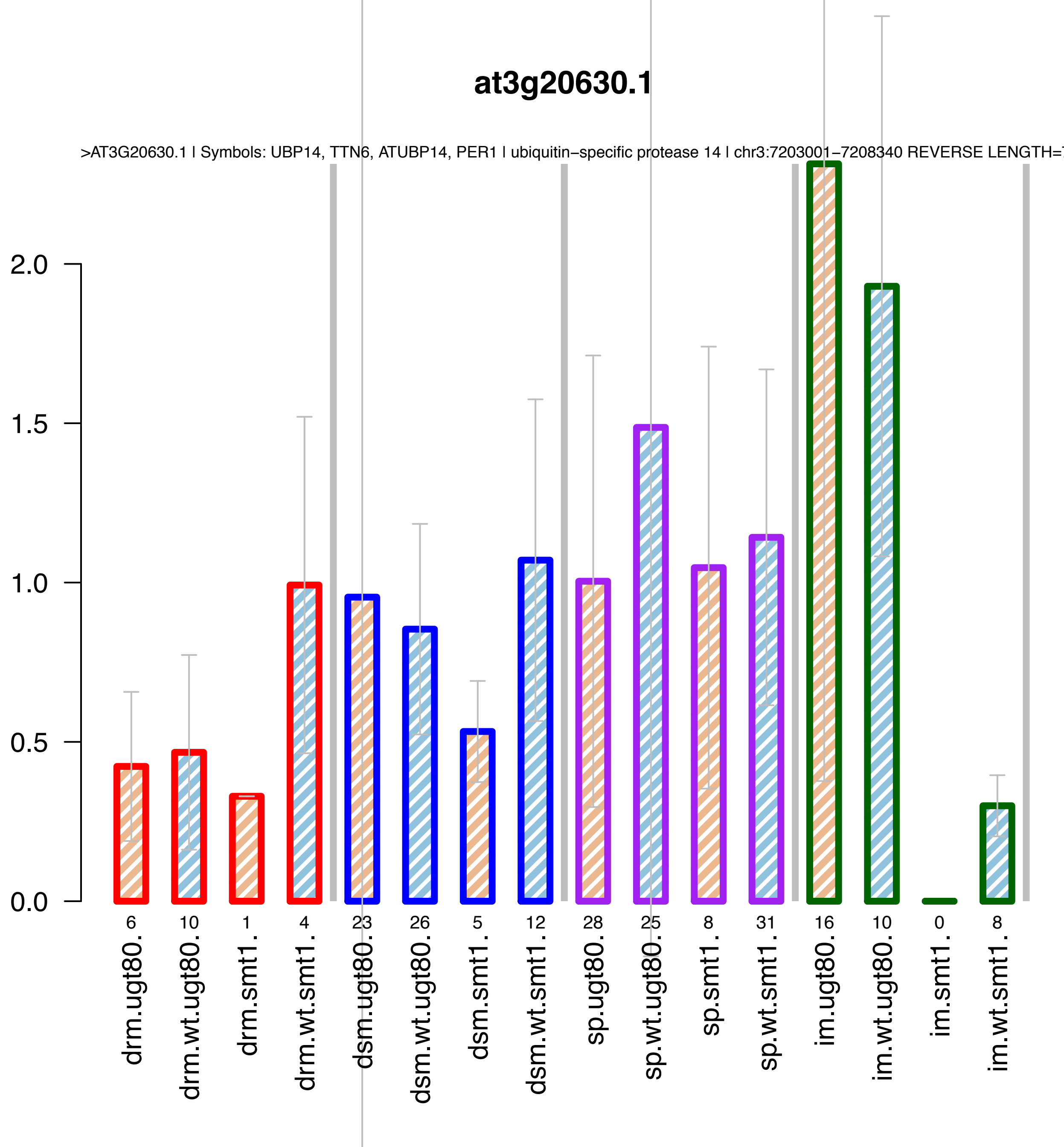

at3g20630.1
>AT3G20630.1 | Symbols: UBP14, TTN6, ATUBP14, PER1 | ubiquitin−specific protease 14 | chr3:7203001−7208340 REVERSE LENGTH=
0.0
0.5
1.0
1.5
2.0
6
10
1
4
23
26
5
12
28
25
8
31
16
10
0
8
drm.ugt80.
drm.wt.ugt80.
drm.smt1.
drm.wt.smt1.
dsm.ugt80.
dsm.wt.ugt80.
dsm.smt1.
dsm.wt.smt1.
sp.ugt80.
sp.wt.ugt80.
sp.smt1.
sp.wt.smt1.
im.ugt80.
im.wt.ugt80.
im.smt1.
im.wt.smt1.

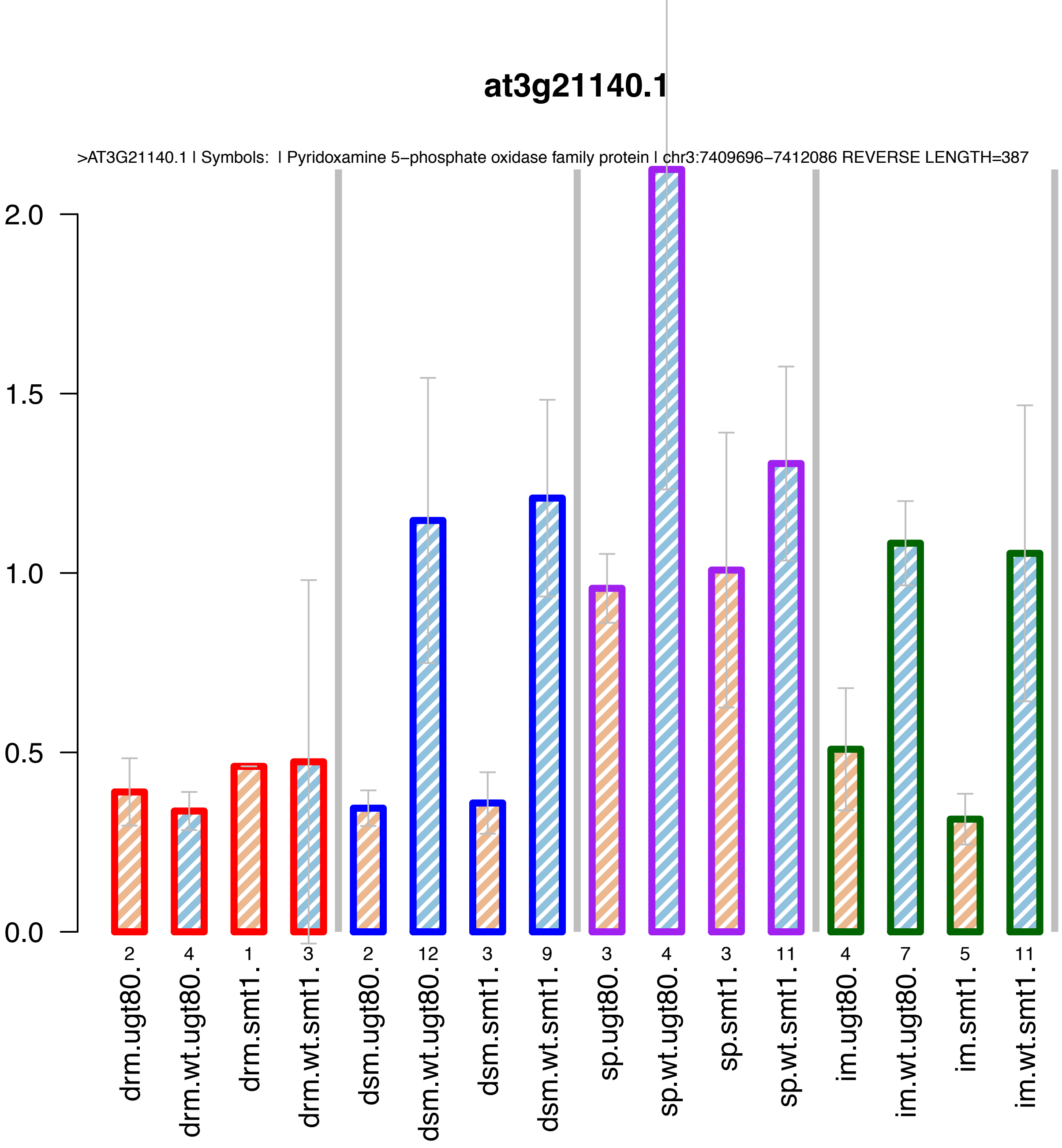

at3g21140.1
>AT3G21140.1 | Symbols: | Pyridoxamine 5−phosphate oxidase family protein | chr3:7409696−7412086 REVERSE LENGTH=387
2.0
1.5
1.0
0.5
0.0
2
4
1
3
2
12
3
9
3
4
3
11
4
7
5
11
drm.ugt80..
drm.wt.ugt80..
drm.smt1..
drm.wt.smt1..
dsm.ugt80..
dsm.wt.ugt80..
dsm.smt1..
dsm.wt.smt1..
sp.ugt80..
sp.wt.ugt80..
sp.smt1..
sp.wt.smt1..
im.ugt80..
im.wt.ugt80..
im.smt1..
im.wt.smt1..

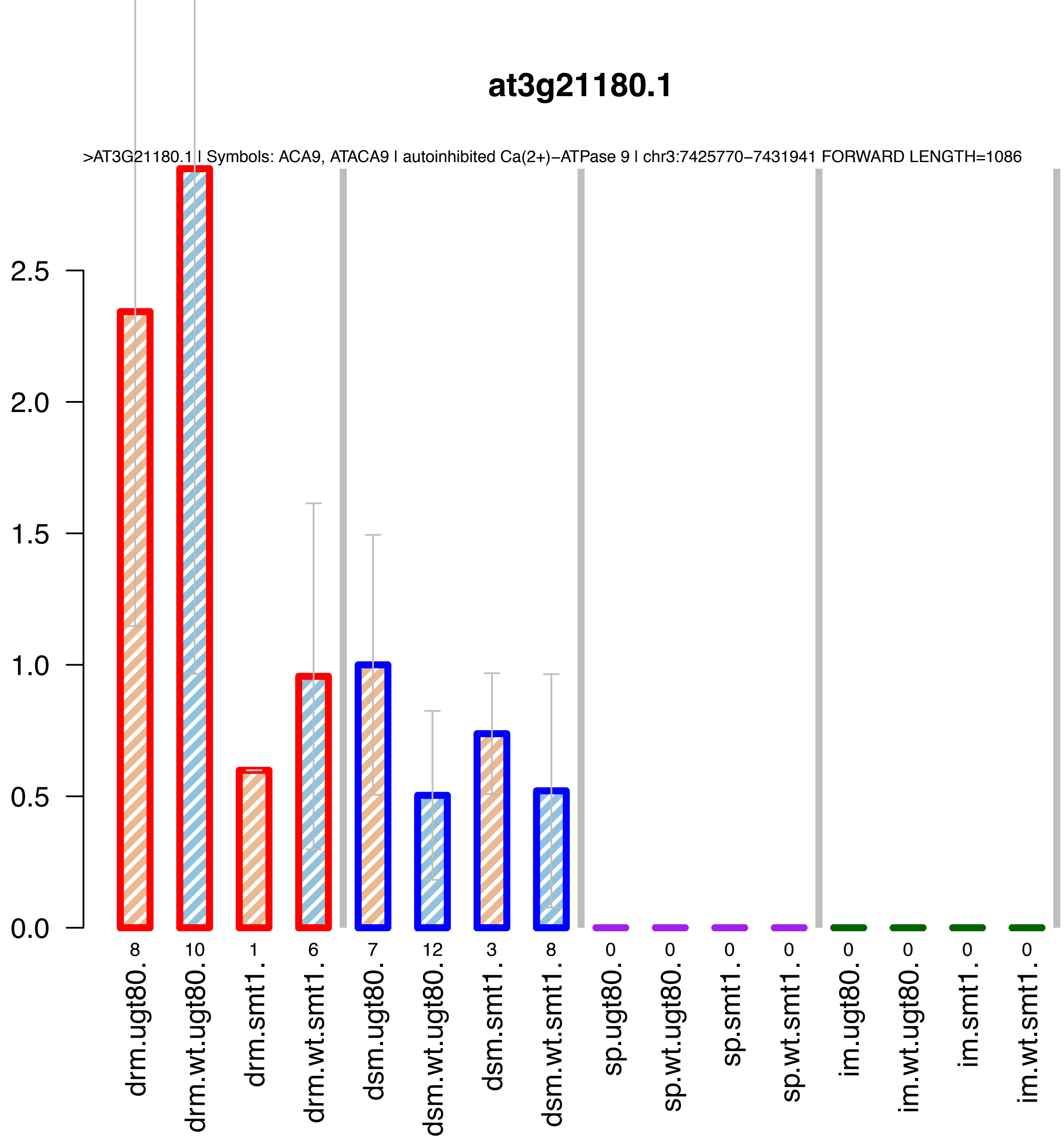

at3g21180.1
>AT3G21180.1 | Symbols: ACA9, ATACA9 | autoinhibited Ca(2+)−ATPase 9 | chr3:7425770−7431941 FORWARD LENGTH=1086
0.0
0.5
1.0
1.5
2.0
2.5
8
10
1
6
7
12
3
8
0
0
0
0
0
0
0
0
drm.ugt80.
drm.wt.ugt80.
drm.smt1.
drm.wt.smt1.
dsm.ugt80.
dsm.wt.ugt80.
dsm.smt1.
dsm.wt.smt1.
sp.ugt80.
sp.wt.ugt80.
sp.smt1.
sp.wt.smt1.
im.ugt80.
im.wt.ugt80.
im.smt1.
im.wt.smt1.

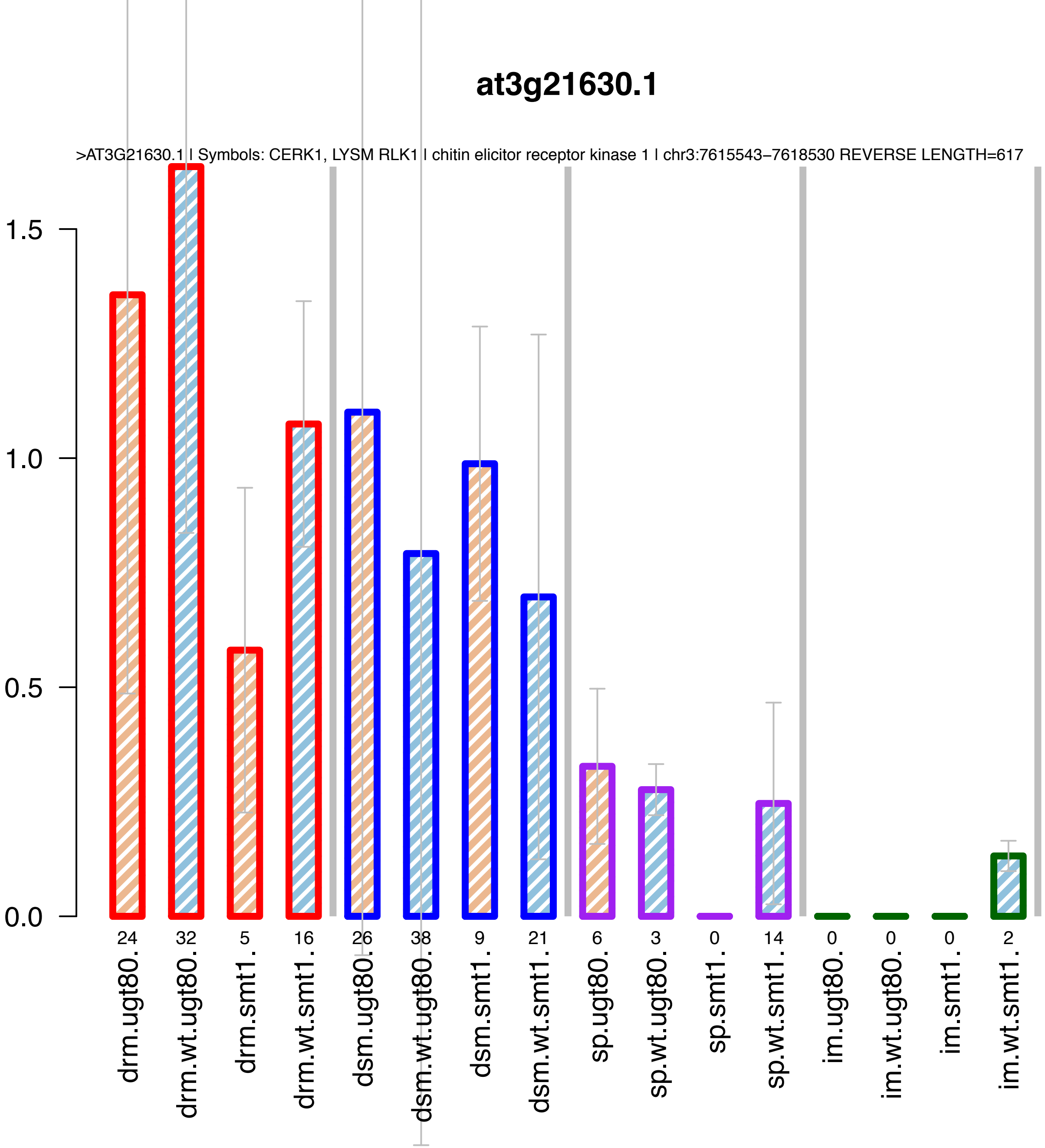
at3g21630.1
>AT3G21630.1 | Symbols: CERK1, LYSM RLK1 | chitin elicitor receptor kinase 1 | chr3:7615543–7618530 REVERSE LENGTH=617
1.5
1.0
0.5
0.0
24
32
5
16
26
38
9
21
6
3
0
14
0
0
0
2
drm.ugt80.
drm.wt.ugt80.
drm.smt1.
drm.wt.smt1.
dsm.ugt80.
dsm.wt.ugt80.
dsm.smt1.
dsm.wt.smt1.
sp.ugt80.
sp.wt.ugt80.
sp.smt1.
sp.wt.smt1.
im.ugt80.
im.wt.ugt80.
im.smt1.
im.wt.smt1.

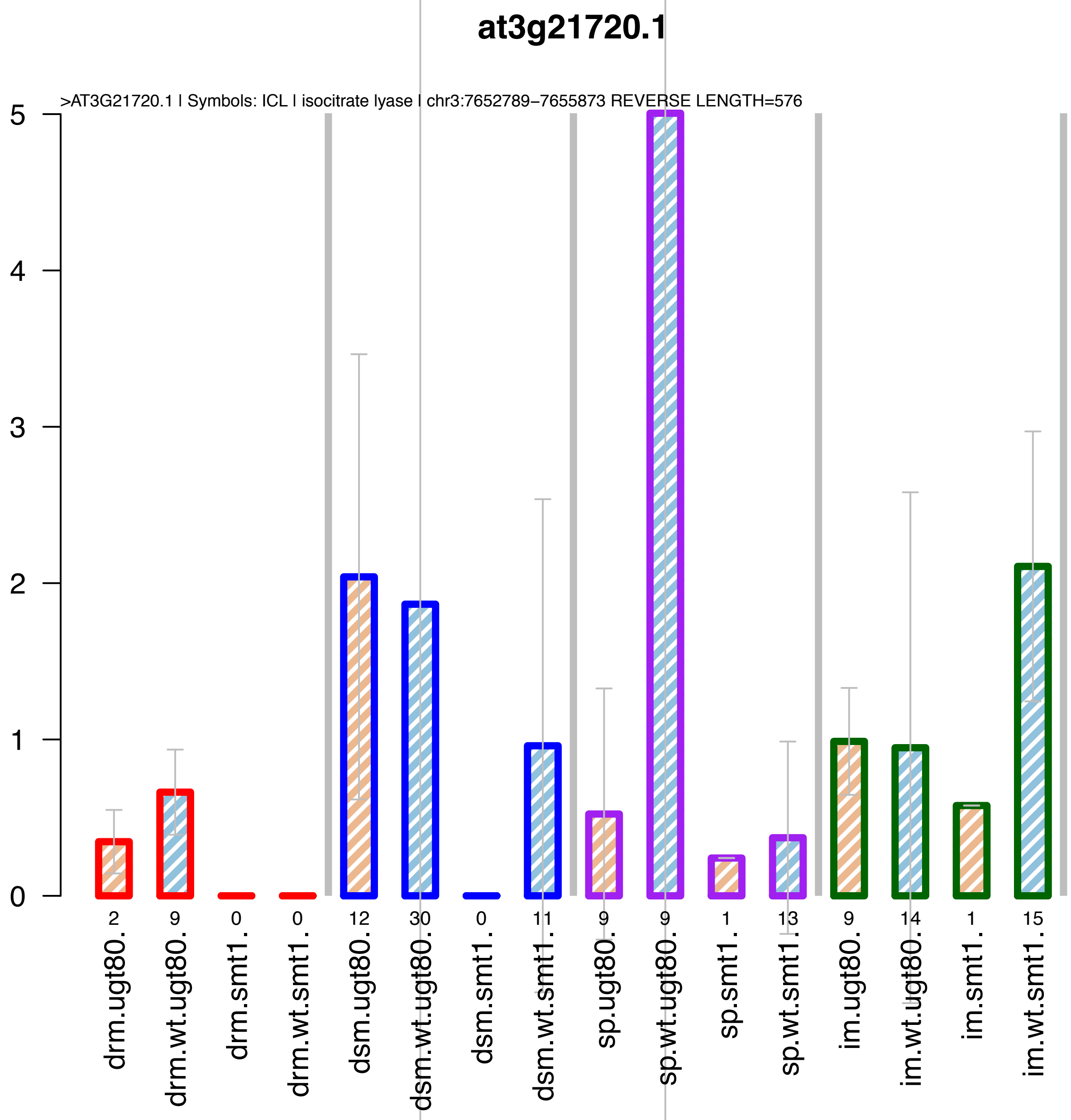

at3g21720.1
>AT3G21720.1 | Symbols: ICL | isocitrate lyase | chr3:7652789-7655873 REVERSE LENGTH=576
0
1
2
3
4
5
2
9
0
0
12
30
0
11
9
9
1
13
9
14
1
15
drm.ugt80..
drm.wt.ugt80..
drm.smt1..
drm.wt.smt1..
dsm.ugt80..
dsm.wt.ugt80..
dsm.smt1..
dsm.wt.smt1..
sp.ugt80..
sp.wt.ugt80..
sp.smt1..
sp.wt.smt1..
im.ugt80..
im.wt.ugt80..
im.smt1..
im.wt.smt1..

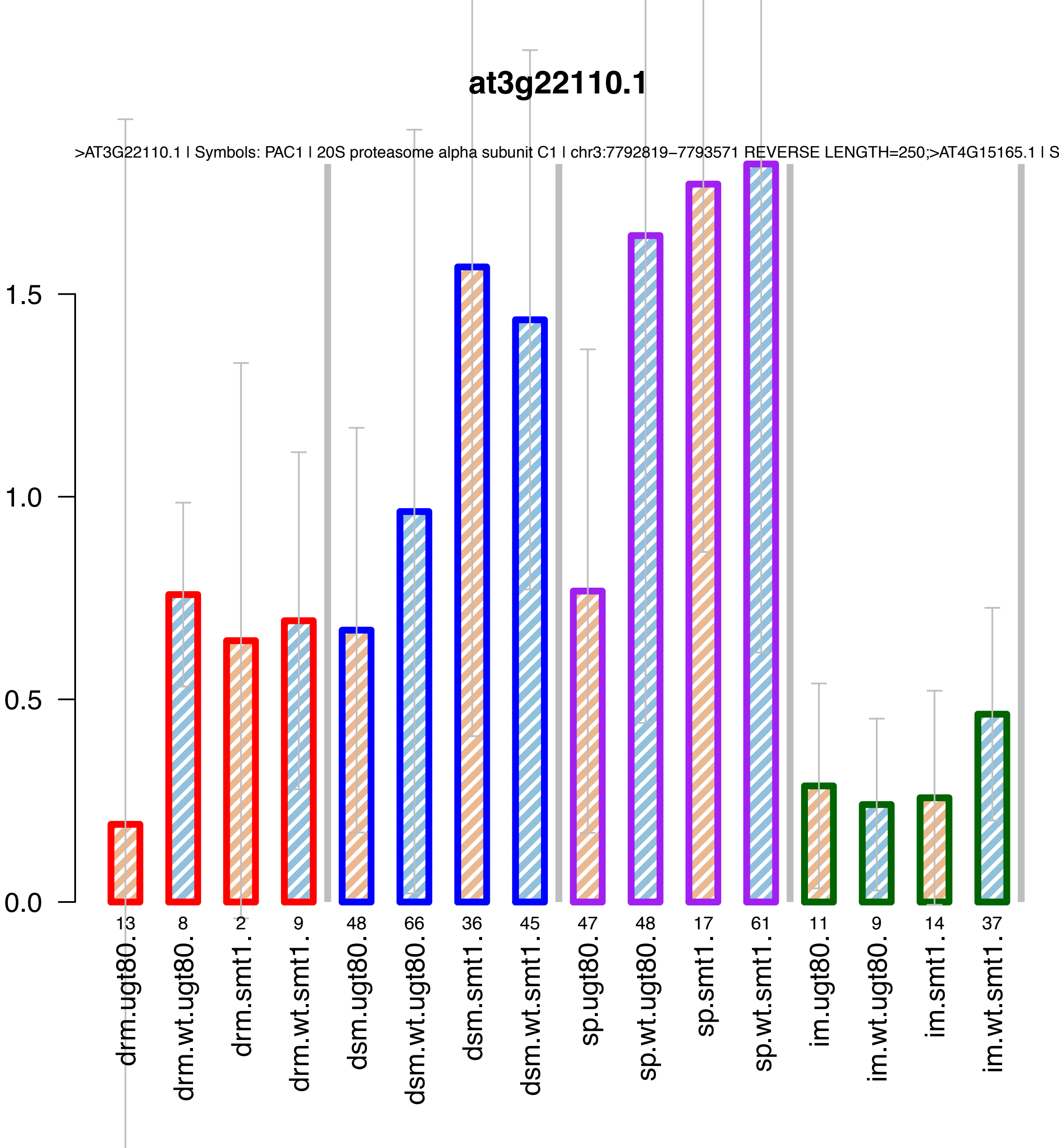

at3g22110.1
>AT3G22110.1 | Symbols: PAC1 | 20S proteasome alpha subunit C1 | chr3:7792819−7793571 REVERSE LENGTH=250;>AT4G15165.1 | S
1.5
1.0
0.5
0.0
13
8
2
9
48
66
36
45
47
48
17
61
11
9
14
37
drm.ugt80..
drm.wt.ugt80..
drm.smt1..
drm.wt.smt1..
dsm.ugt80..
dsm.wt.ugt80..
dsm.smt1..
dsm.wt.smt1..
sp.ugt80..
sp.wt.ugt80..
sp.smt1..
sp.wt.smt1..
im.ugt80..
im.wt.ugt80..
im.smt1..
im.wt.smt1..

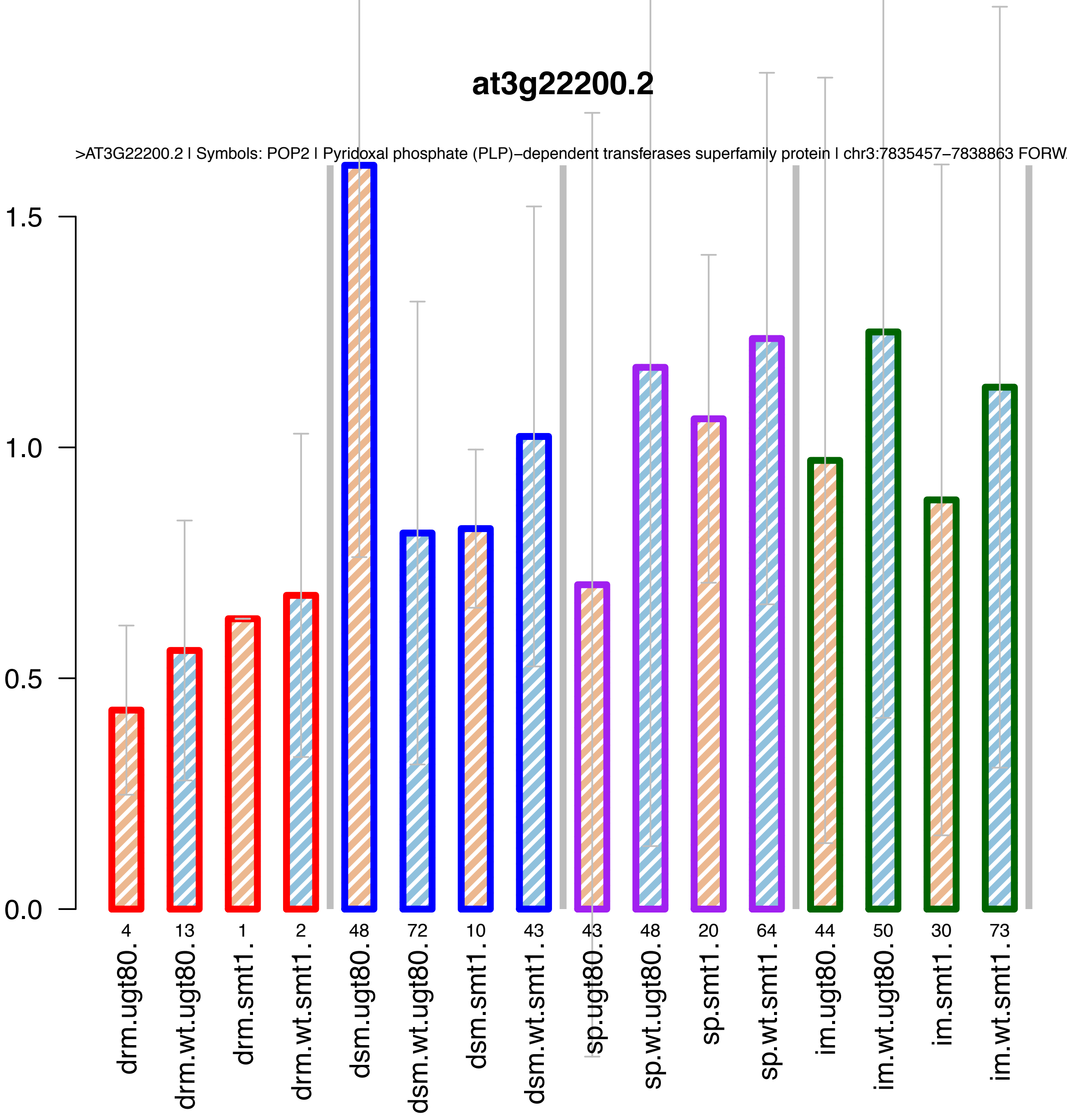

at3g22200.2
>AT3G22200.2 | Symbols: POP2 | Pyridoxal phosphate (PLP)−dependent transferases superfamily protein | chr3:7835457−7838863 FORW
1.5
1.0
0.5
0.0
4
13
1
2
48
72
10
43
43
48
20
64
44
50
30
73
drm.ugt80..
drm.wt.ugt80..
drm.smt1..
drm.wt.smt1..
dsm.ugt80..
dsm.wt.ugt80..
dsm.smt1..
dsm.wt.smt1..
sp.ugt80..
sp.wt.ugt80..
sp.smt1..
sp.wt.smt1..
im.ugt80..
im.wt.ugt80..
im.smt1..
im.wt.smt1..

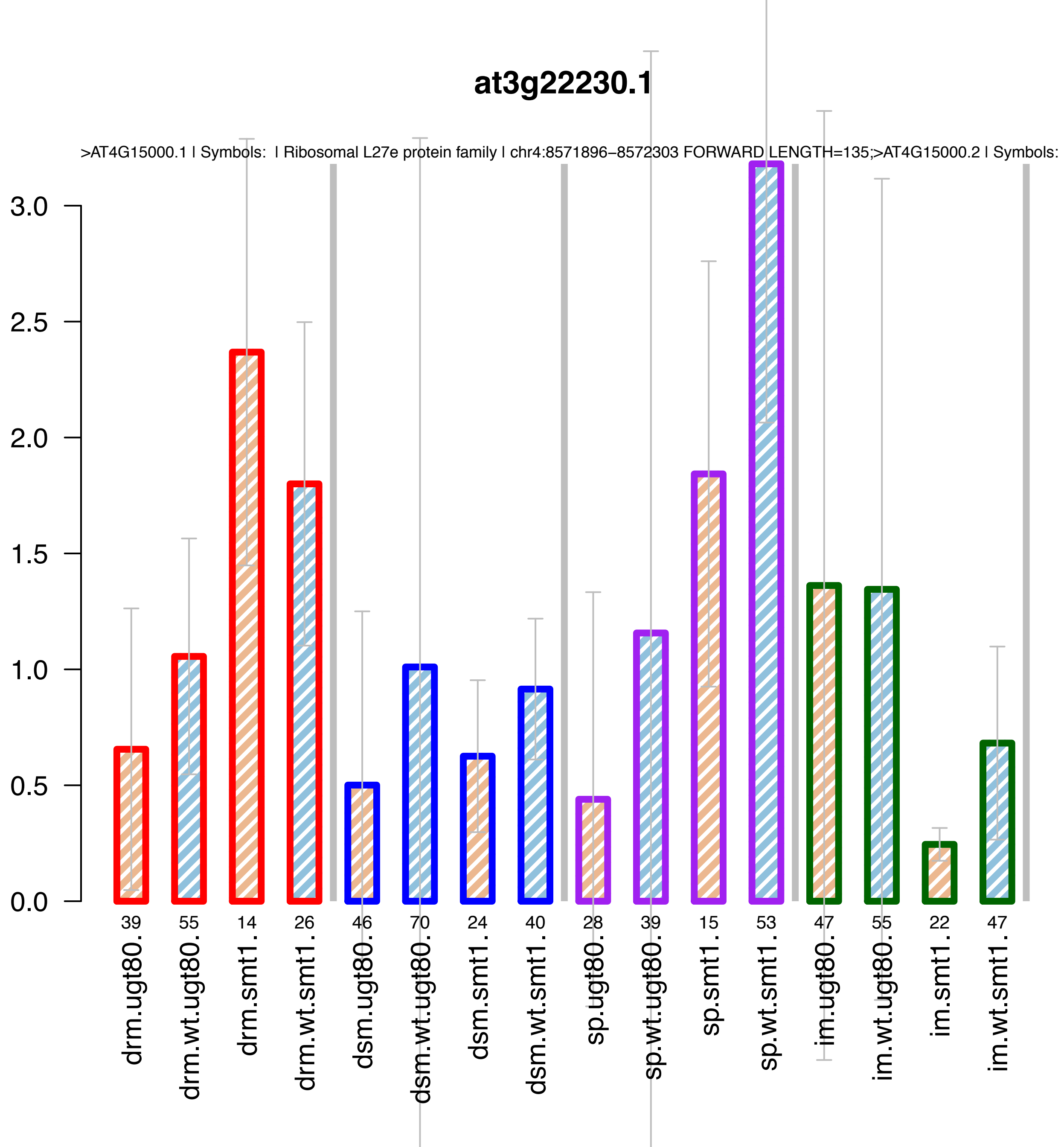

at3g22230.1
>AT4G15000.1 | Symbols: | Ribosomal L27e protein family | chr4:8571896-8572303 FORWARD LENGTH=135;>AT4G15000.2 | Symbols:
0.0
0.5
1.0
1.5
2.0
2.5
3.0
39
55
14
26
46
70
24
40
28
39
15
53
47
55
22
47
drm.ugt80.
drm.wt.ugt80.
drm.smt1.
drm.wt.smt1.
dsm.ugt80.
dsm.wt.ugt80.
dsm.smt1.
dsm.wt.smt1.
sp.ugt80.
sp.wt.ugt80.
sp.smt1.
sp.wt.smt1.
im.ugt80.
im.wt.ugt80.
im.smt1.
im.wt.smt1.

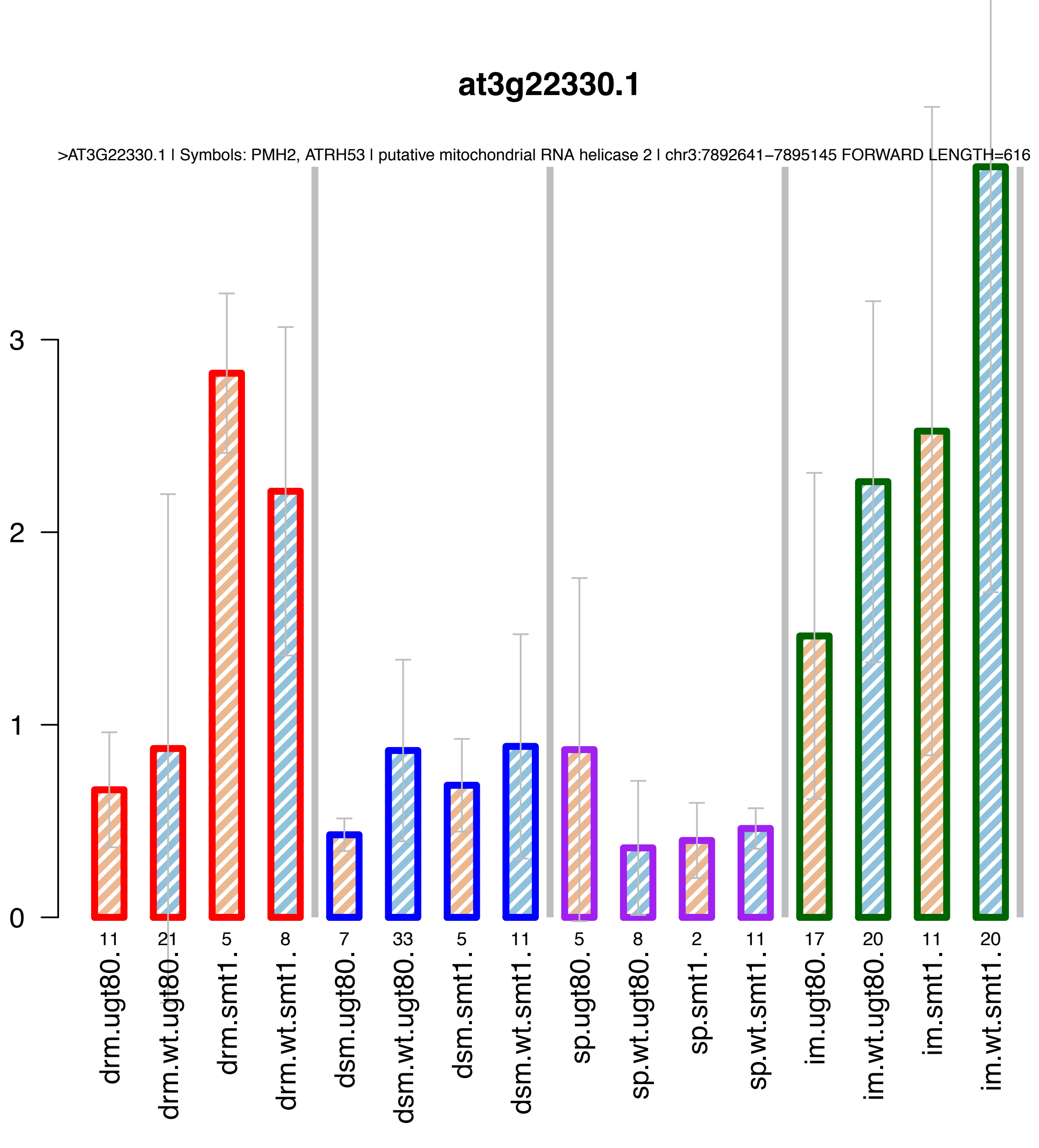

at3g22330.1
>AT3G22330.1 | Symbols: PMH2, ATRH53 | putative mitochondrial RNA helicase 2 | chr3:7892641−7895145 FORWARD LENGTH=616
0
1
2
3
11
21
5
8
7
33
5
11
5
8
2
11
17
20
11
20
drm.ugt80.
drm.wt.ugt80.
drm.smt1.
drm.wt.smt1.
dsm.ugt80.
dsm.wt.ugt80.
dsm.smt1.
dsm.wt.smt1.
sp.ugt80.
sp.wt.ugt80.
sp.smt1.
sp.wt.smt1.
im.ugt80.
im.wt.ugt80.
im.smt1.
im.wt.smt1.

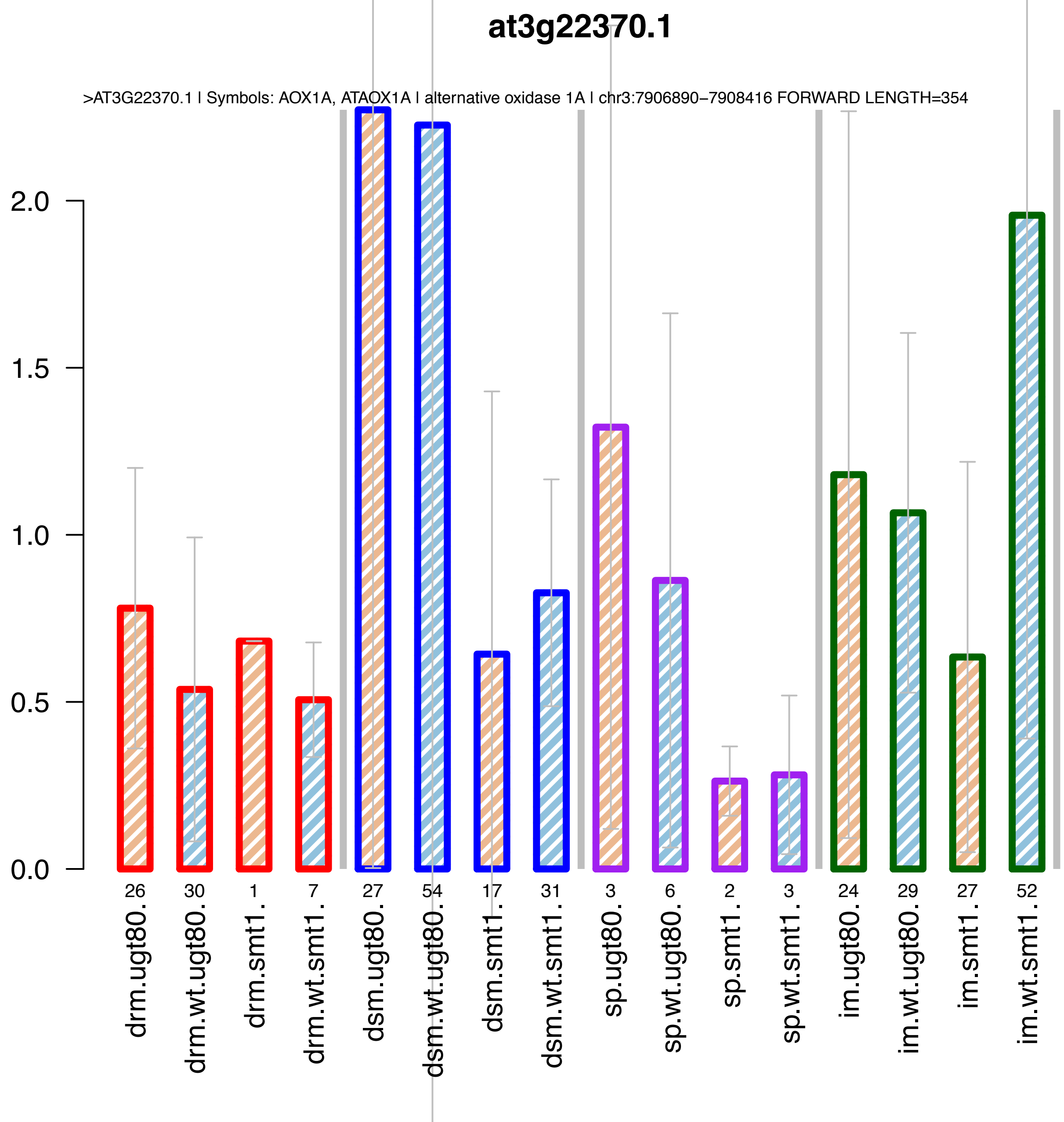
at3g22370.1
>AT3G22370.1 | Symbols: AOX1A, ATAOX1A | alternative oxidase 1A | chr3:7906890-7908416 FORWARD LENGTH=354
0.0
0.5
1.0
1.5
2.0
26
30
1
7
27
54
17
31
3
6
2
3
24
29
27
52
drm.ugt80.
drm.wt.ugt80.
drm.smt1.
drm.wt.smt1.
dsm.ugt80.
dsm.wt.ugt80.
dsm.smt1.
dsm.wt.smt1.
sp.ugt80.
sp.wt.ugt80.
sp.smt1.
sp.wt.smt1.
im.ugt80.
im.wt.ugt80.
im.smt1.
im.wt.smt1.

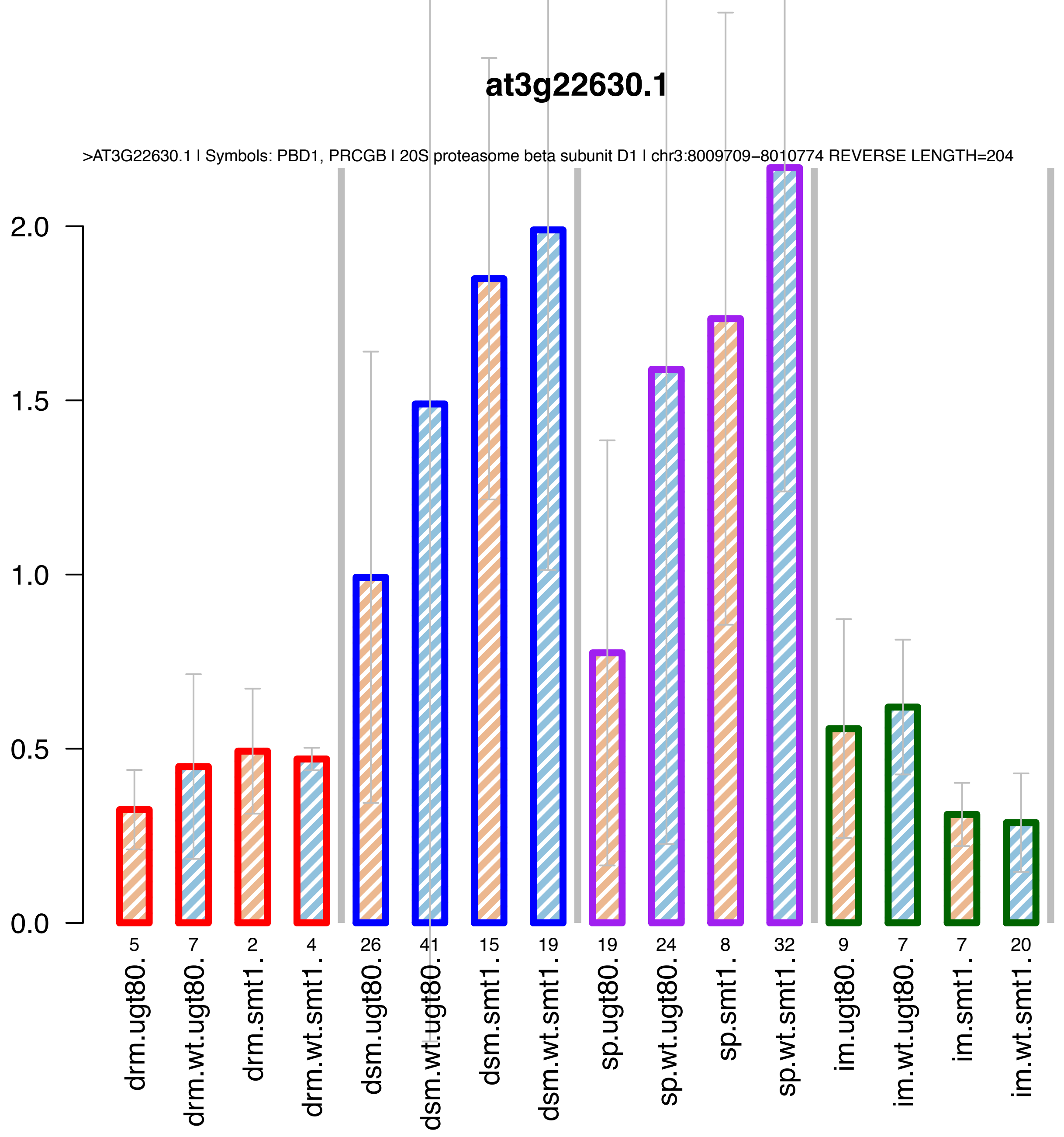

at3g22630.1
>AT3G22630.1 | Symbols: PBD1, PRCGB | 20S proteasome beta subunit D1 | chr3:8009709-8010774 REVERSE LENGTH=204
2.0
1.5
1.0
0.5
0.0
5
7
2
4
26
41
15
19
19
24
8
32
9
7
7
20
drm.ugt80.
drm.wt.ugt80.
drm.smt1.
drm.wt.smt1.
dsm.ugt80.
dsm.wt.ugt80.
dsm.smt1.
dsm.wt.smt1.
sp.ugt80.
sp.wt.ugt80.
sp.smt1.
sp.wt.smt1.
im.ugt80.
im.wt.ugt80.
im.smt1.
im.wt.smt1.

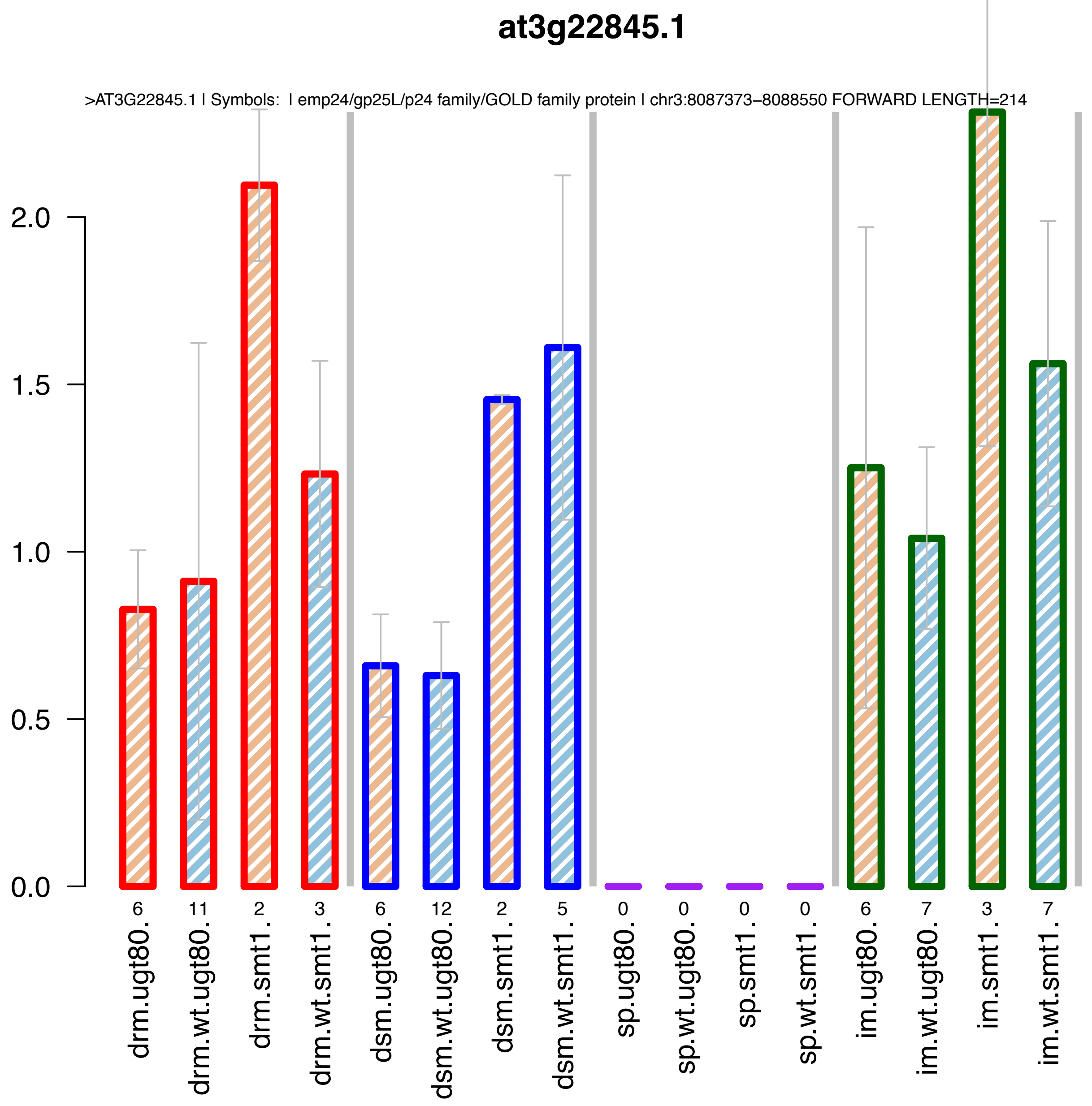

at3g22845.1
>AT3G22845.1 | Symbols: | emp24/gp25L/p24 family/GOLD family protein | chr3:8087373-8088550 FORWARD LENGTH=214
2.0
1.5
1.0
0.5
0.0
6
11
2
3
6
12
2
5
0
0
0
0
6
7
3
7
drm.ugt80.
drm.wt.ugt80.
drm.smt1.
drm.wt.smt1.
dsm.ugt80.
dsm.wt.ugt80.
dsm.smt1.
dsm.wt.smt1.
sp.ugt80.
sp.wt.ugt80.
sp.smt1.
sp.wt.smt1.
im.ugt80.
im.wt.ugt80.
im.smt1.
im.wt.smt1.

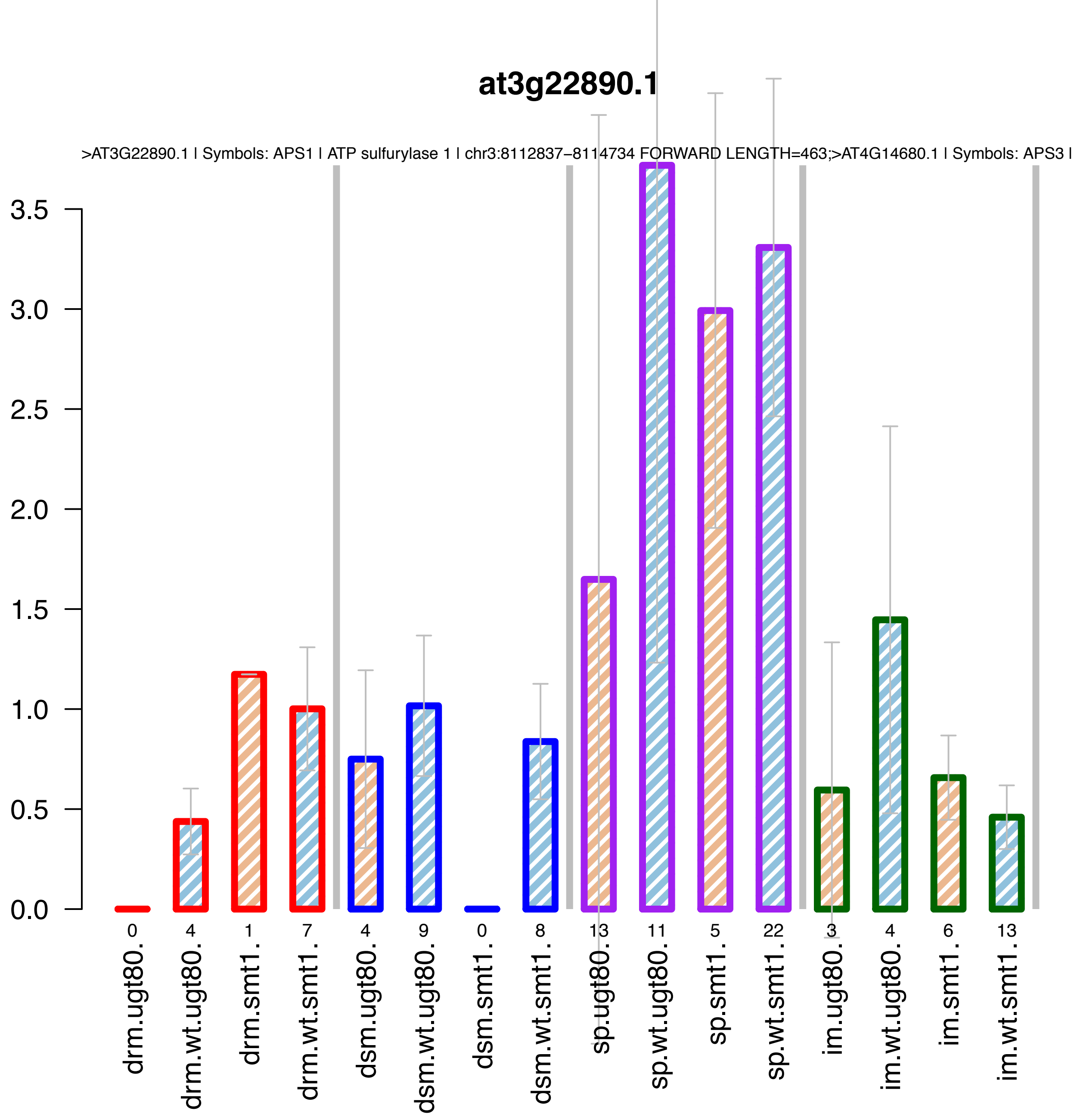

at3g22890.1
>AT3G22890.1 | Symbols: APS1 | ATP sulfurylase 1 | chr3:8112837−8114734 FORWARD LENGTH=463;>AT4G14680.1 | Symbols: APS3 |
3.5
3.0
2.5
2.0
1.5
1.0
0.5
0.0
0
4
1
7
4
9
0
8
13
11
5
22
3
4
6
13
drm.ugt80.
drm.wt.ugt80.
drm.smt1.
drm.wt.smt1.
dsm.ugt80.
dsm.wt.ugt80.
dsm.smt1.
dsm.wt.smt1.
sp.ugt80.
sp.wt.ugt80.
sp.smt1.
sp.wt.smt1.
im.ugt80.
im.wt.ugt80.
im.smt1.
im.wt.smt1.

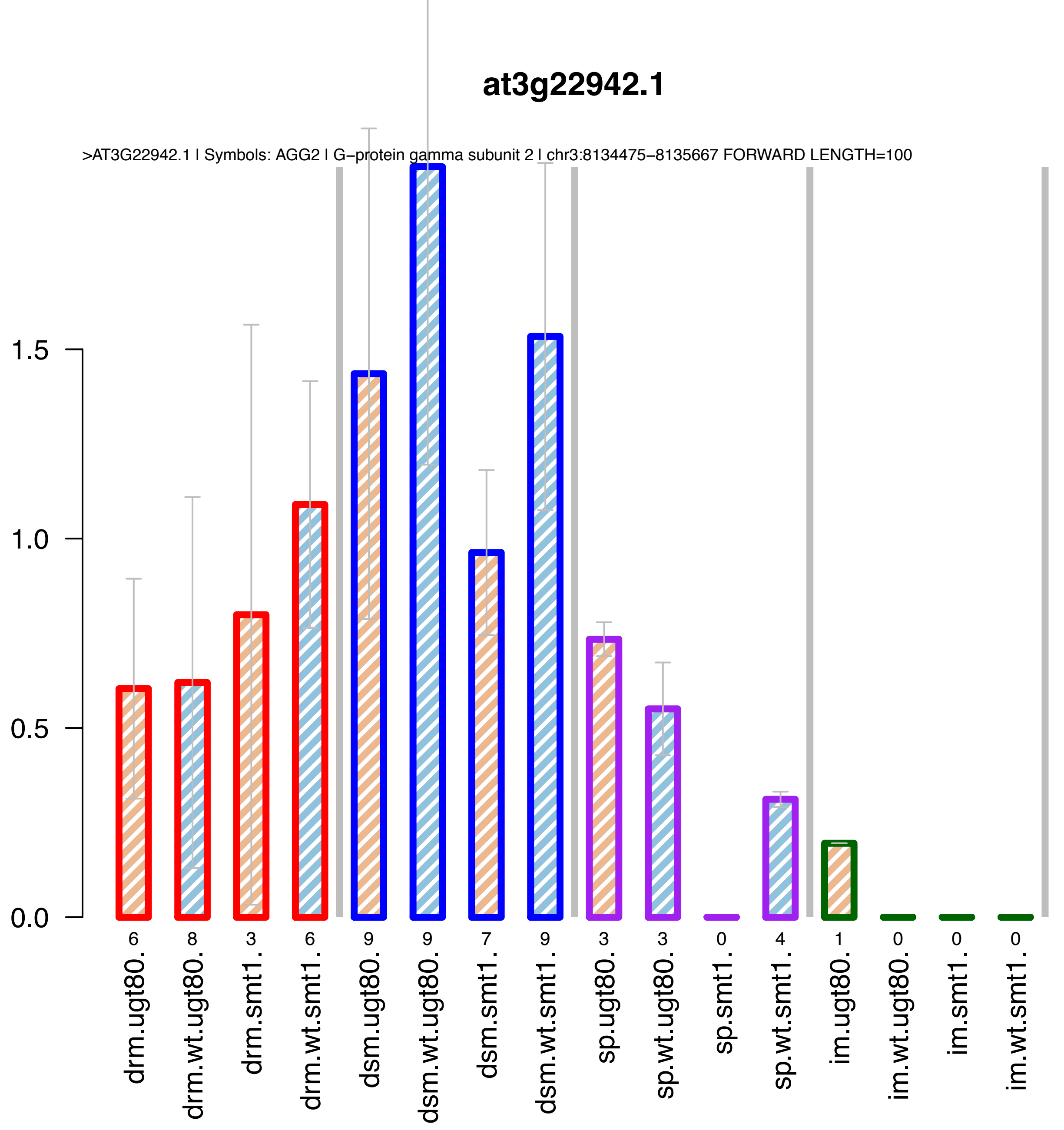
at3g22942.1
>AT3G22942.1 | Symbols: AGG2 | G−protein gamma subunit 2 | chr3:8134475−8135667 FORWARD LENGTH=100
0.0
0.5
1.0
1.5
6
8
3
6
9
9
7
9
3
3
0
4
1
0
0
0
drm.ugt80.
drm.wt.ugt80.
drm.smt1.
drm.wt.smt1.
dsm.ugt80.
dsm.wt.ugt80.
dsm.smt1.
dsm.wt.smt1.
sp.ugt80.
sp.wt.ugt80.
sp.smt1.
sp.wt.smt1.
im.ugt80.
im.wt.ugt80.
im.smt1.
im.wt.smt1.

# at3g22960.1

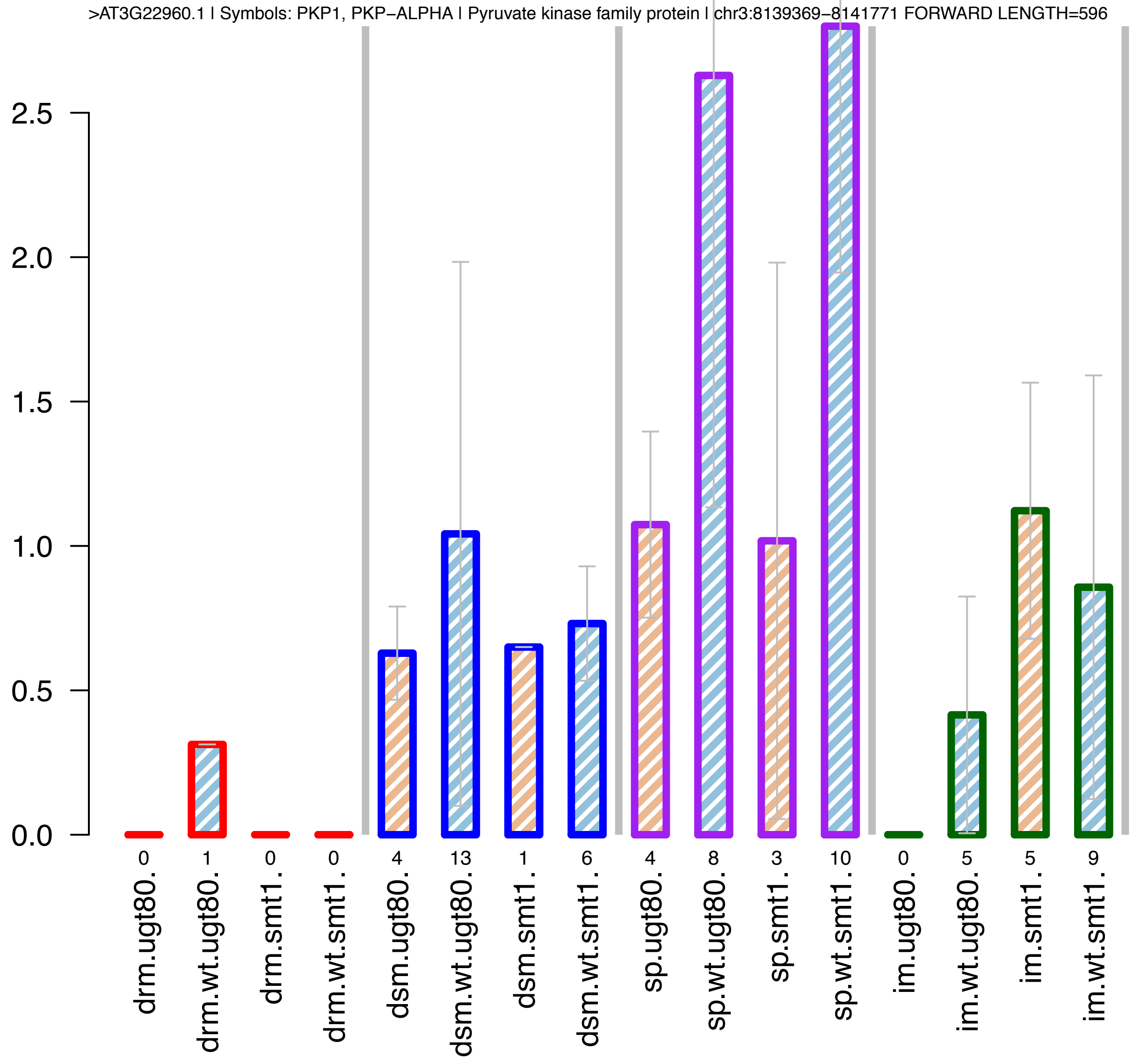

>AT3G22960.1 | Symbols: PKP1, PKP-ALPHA | Pyruvate kinase family protein | chr3:8139369-8141771 FORWARD LENGTH=596
0.0
0.5
1.0
1.5
2.0
2.5
0
1
0
0
4
13
1
6
4
8
3
10
0
5
5
9
drm.ugt80.
drm.wt.ugt80.
drm.smt1.
drm.wt.smt1.
dsm.ugt80.
dsm.wt.ugt80.
dsm.smt1.
dsm.wt.smt1.
sp.ugt80.
sp.wt.ugt80.
sp.smt1.
sp.wt.smt1.
im.ugt80.
im.wt.ugt80.
im.smt1.
im.wt.smt1.

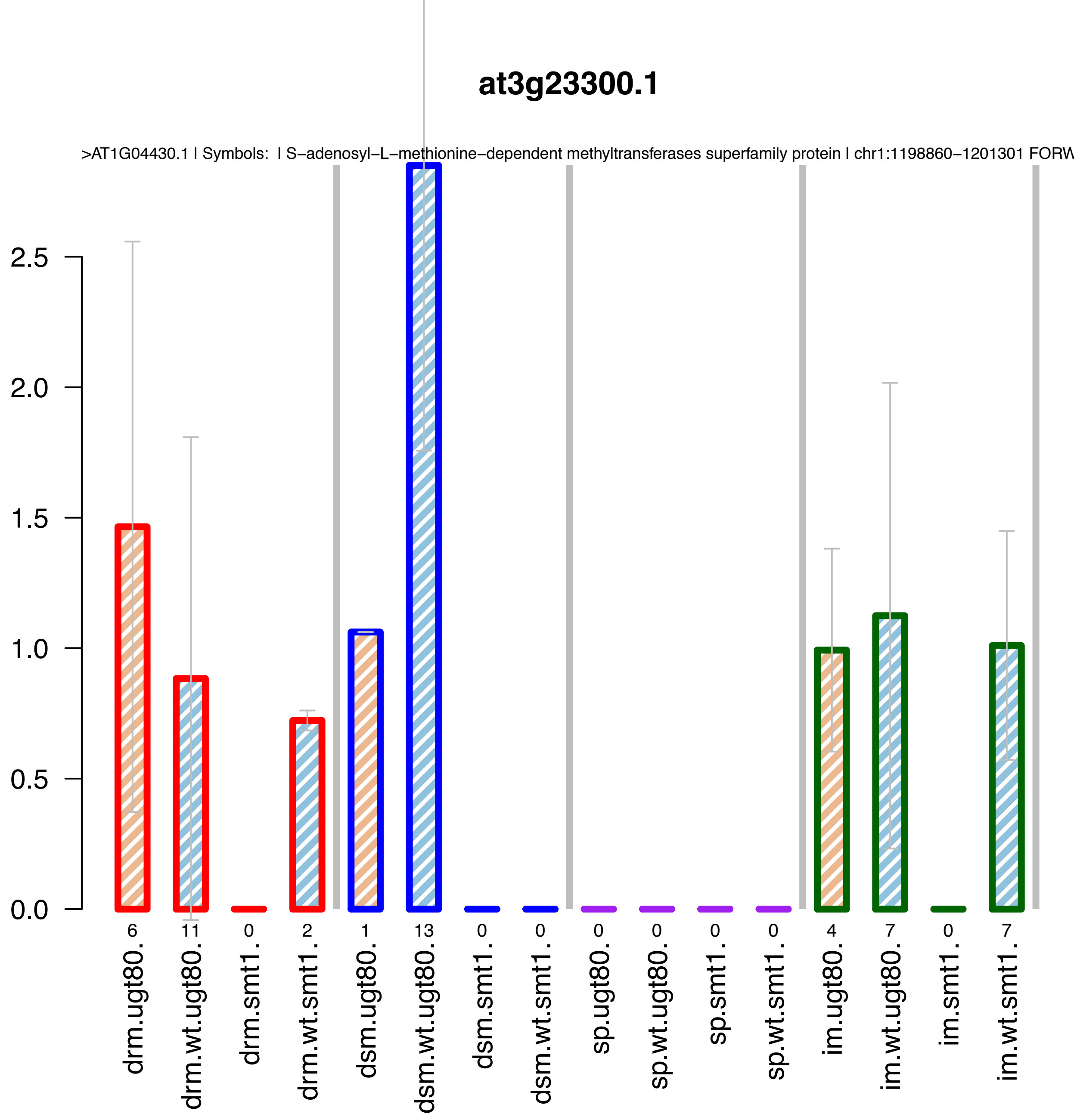

at3g23300.1
>AT1G04430.1 | Symbols: | S–adenosyl–L–methionine–dependent methyltransferases superfamily protein | chr1:1198860–1201301 FORW
0.0
0.5
1.0
1.5
2.0
2.5
6
11
0
2
1
13
0
0
0
0
0
0
4
7
0
7
drm.ugt80.
drm.wt.ugt80.
drm.smt1.
drm.wt.smt1.
dsm.ugt80.
dsm.wt.ugt80.
dsm.smt1.
dsm.wt.smt1.
sp.ugt80.
sp.wt.ugt80.
sp.smt1.
sp.wt.smt1.
im.ugt80.
im.wt.ugt80.
im.smt1.
im.wt.smt1.

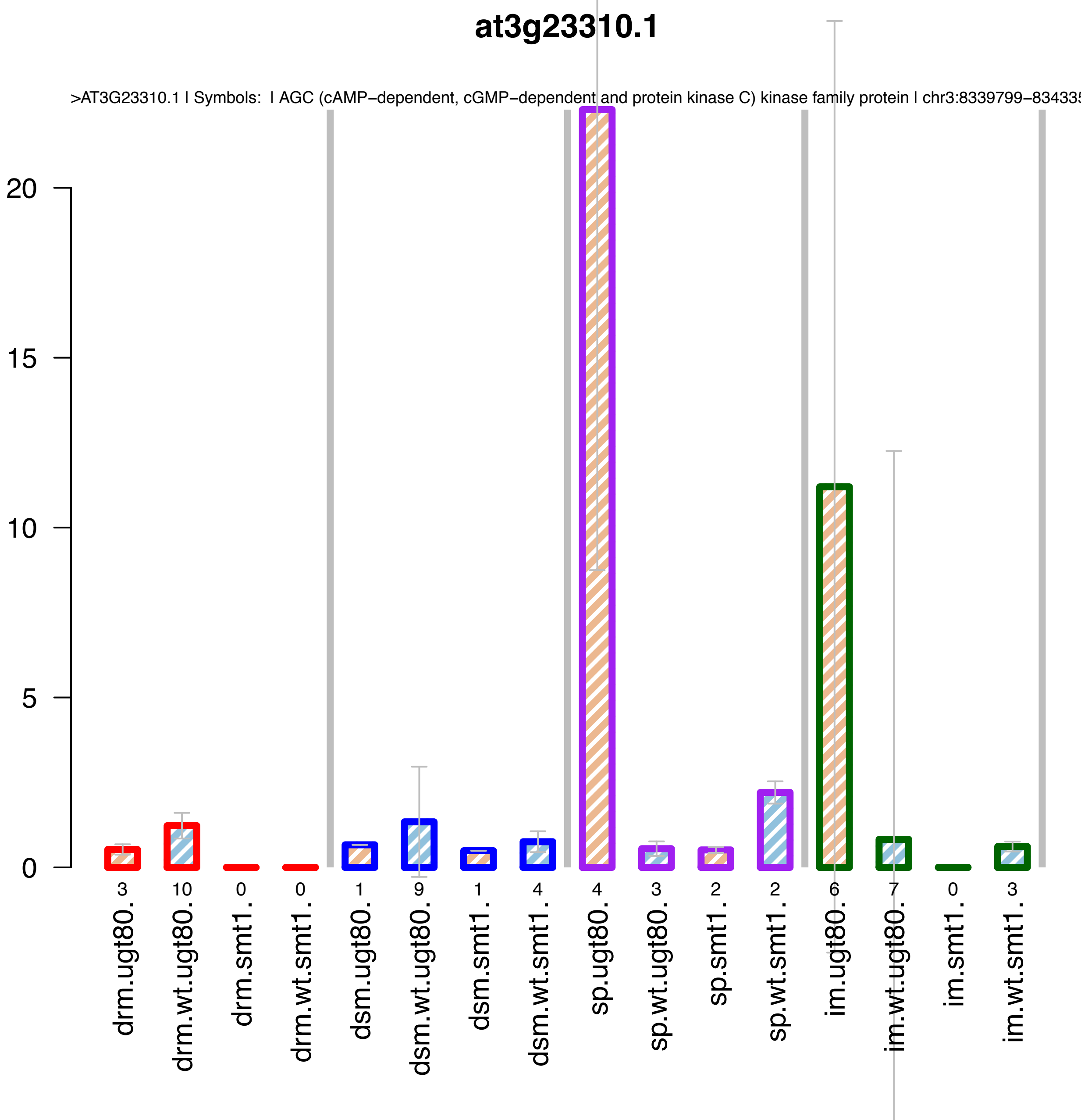

at3g23310.1
>AT3G23310.1 | Symbols: | AGC (cAMP−dependent, cGMP−dependent and protein kinase C) kinase family protein | chr3:8339799−834335
20
15
10
5
0
3
10
0
0
1
9
1
4
4
3
2
2
6
7
0
3
drm.ugt80.
drm.wt.ugt80.
drm.smt1.
drm.wt.smt1.
dsm.ugt80.
dsm.wt.ugt80.
dsm.smt1.
dsm.wt.smt1.
sp.ugt80.
sp.wt.ugt80.
sp.smt1.
sp.wt.smt1.
im.ugt80.
im.wt.ugt80.
im.smt1.
im.wt.smt1.

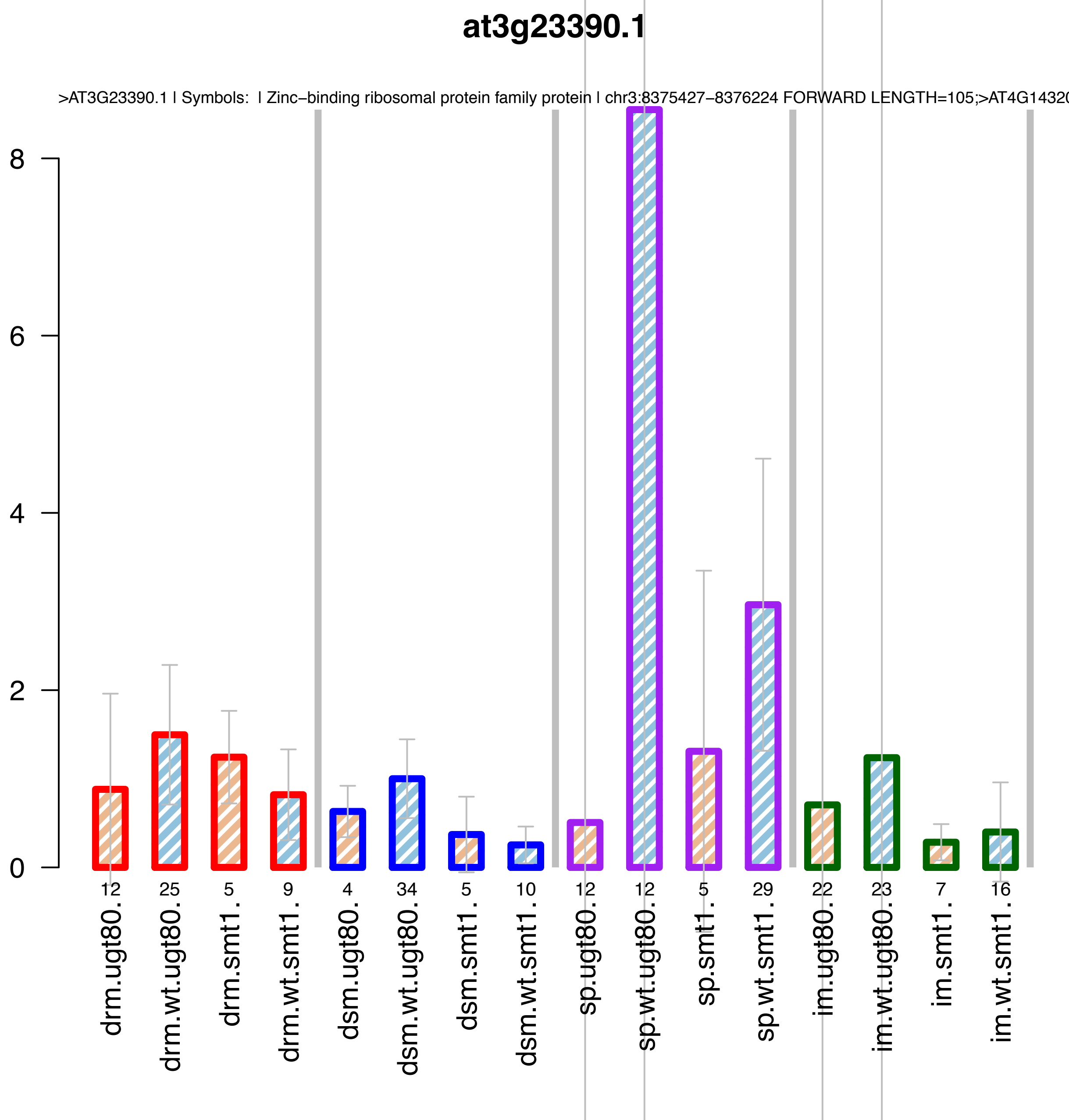

at3g23390.1
>AT3G23390.1 | Symbols: | Zinc−binding ribosomal protein family protein | chr3:8375427−8376224 FORWARD LENGTH=105;>AT4G1432
0
2
4
6
8
12
25
5
9
4
34
5
10
12
12
5
29
22
23
7
16
drm.ugt80..
drm.wt.ugt80..
drm.smt1..
drm.wt.smt1..
dsm.ugt80..
dsm.wt.ugt80..
dsm.smt1..
dsm.wt.smt1..
sp.ugt80..
sp.wt.ugt80..
sp.smt1..
sp.wt.smt1..
im.ugt80..
im.wt.ugt80..
im.smt1..
im.wt.smt1..

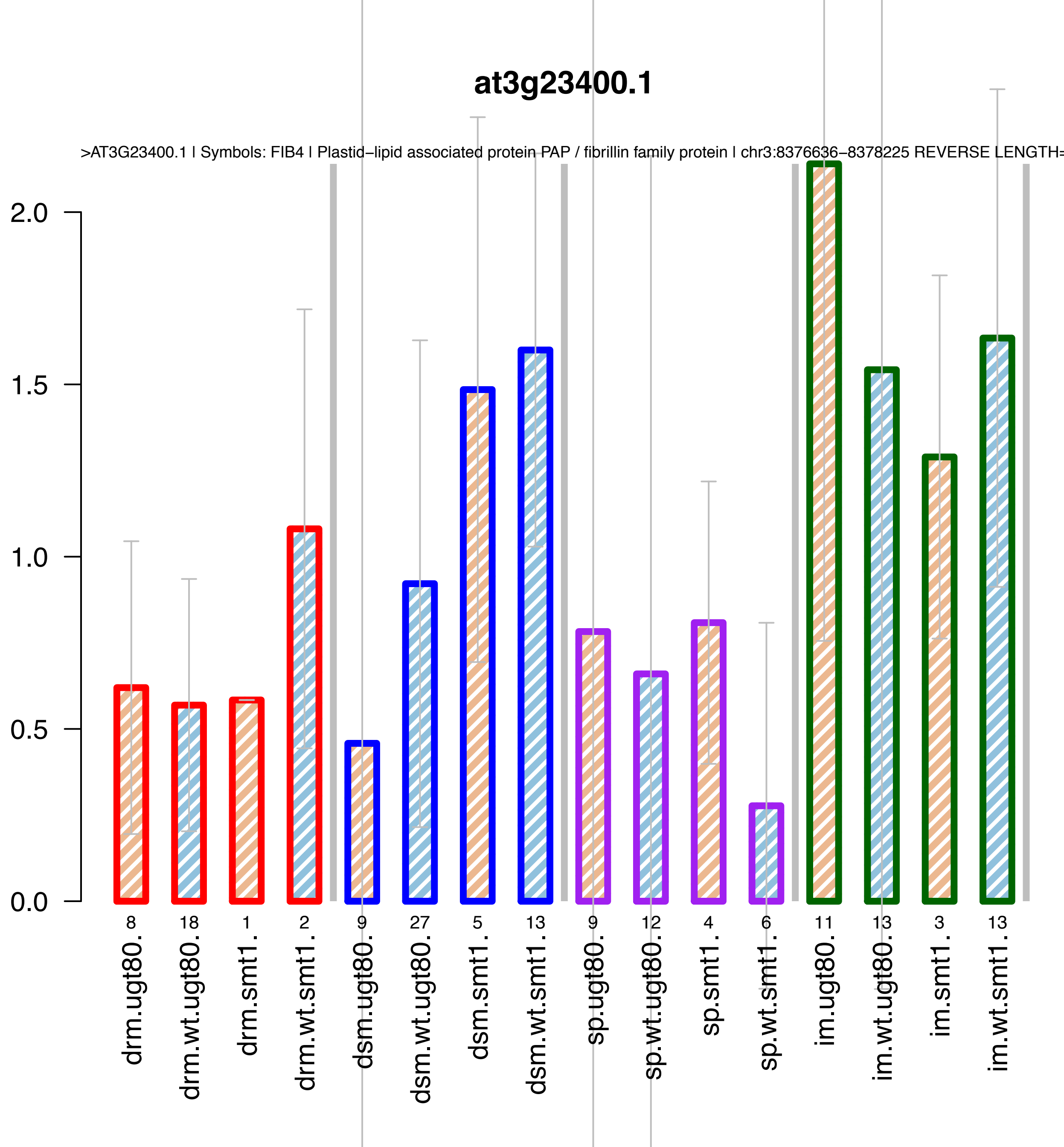
at3g23400.1
>AT3G23400.1 | Symbols: FIB4 | Plastid−lipid associated protein PAP / fibrillin family protein | chr3:8376636−8378225 REVERSE LENGTH=
2.0
1.5
1.0
0.5
0.0
8
18
1
2
9
27
5
13
9
12
4
6
11
13
3
13
drm.ugt80.
drm.wt.ugt80.
drm.smt1.
drm.wt.smt1.
dsm.ugt80.
dsm.wt.ugt80.
dsm.smt1.
dsm.wt.smt1.
sp.ugt80.
sp.wt.ugt80.
sp.smt1.
sp.wt.smt1.
im.ugt80.
im.wt.ugt80.
im.smt1.
im.wt.smt1.

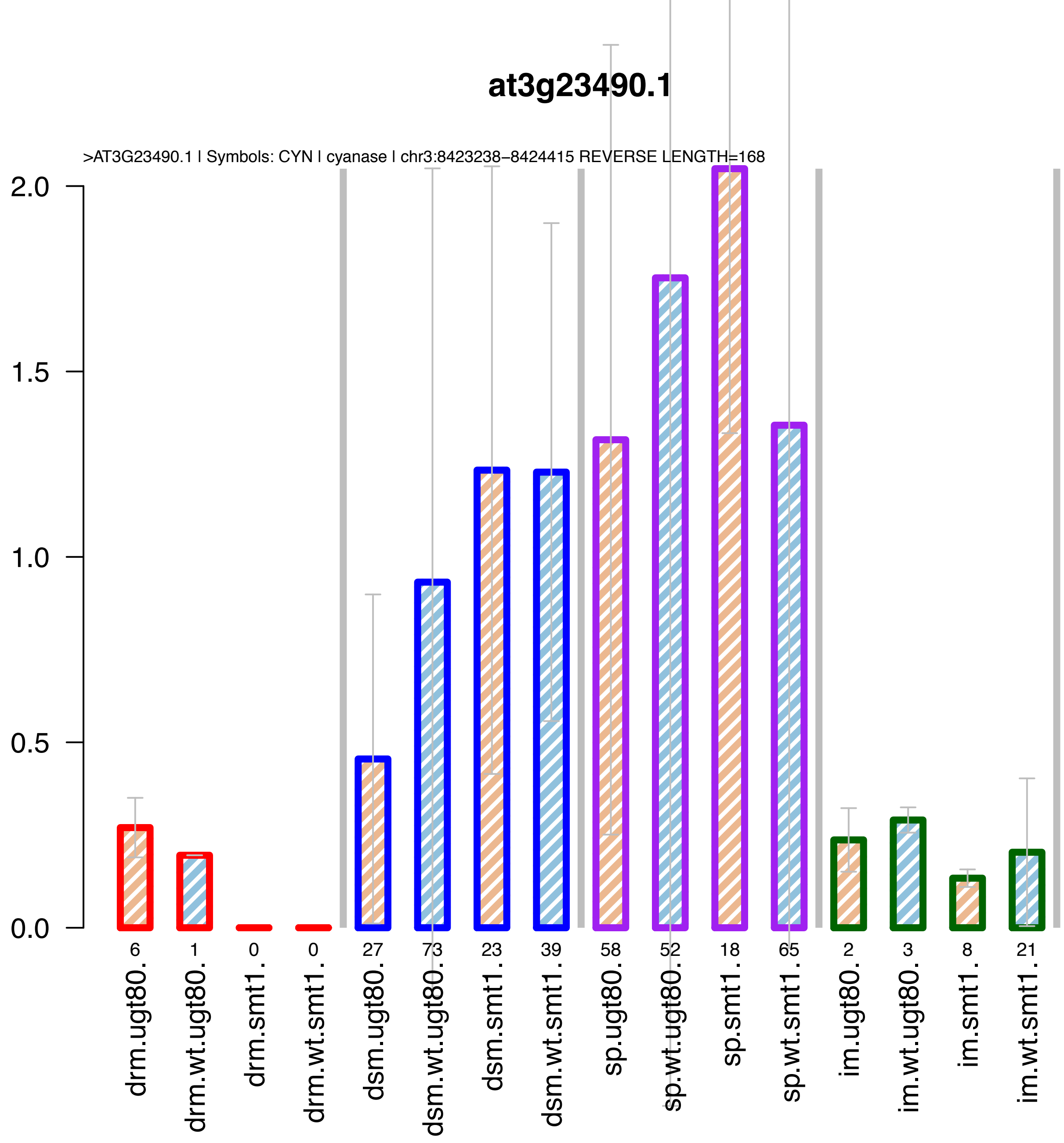

at3g23490.1
>AT3G23490.1 | Symbols: CYN | cyanase | chr3:8423238-8424415 REVERSE LENGTH=168
2.0
1.5
1.0
0.5
0.0
6
1
0
0
27
73
23
39
58
52
18
65
2
3
8
21
drm.ugt80..
drm.wt.ugt80..
drm.smt1..
drm.wt.smt1..
dsm.ugt80..
dsm.wt.ugt80..
dsm.smt1..
dsm.wt.smt1..
sp.ugt80..
sp.wt.ugt80..
sp.smt1..
sp.wt.smt1..
im.ugt80..
im.wt.ugt80..
im.smt1..
im.wt.smt1..

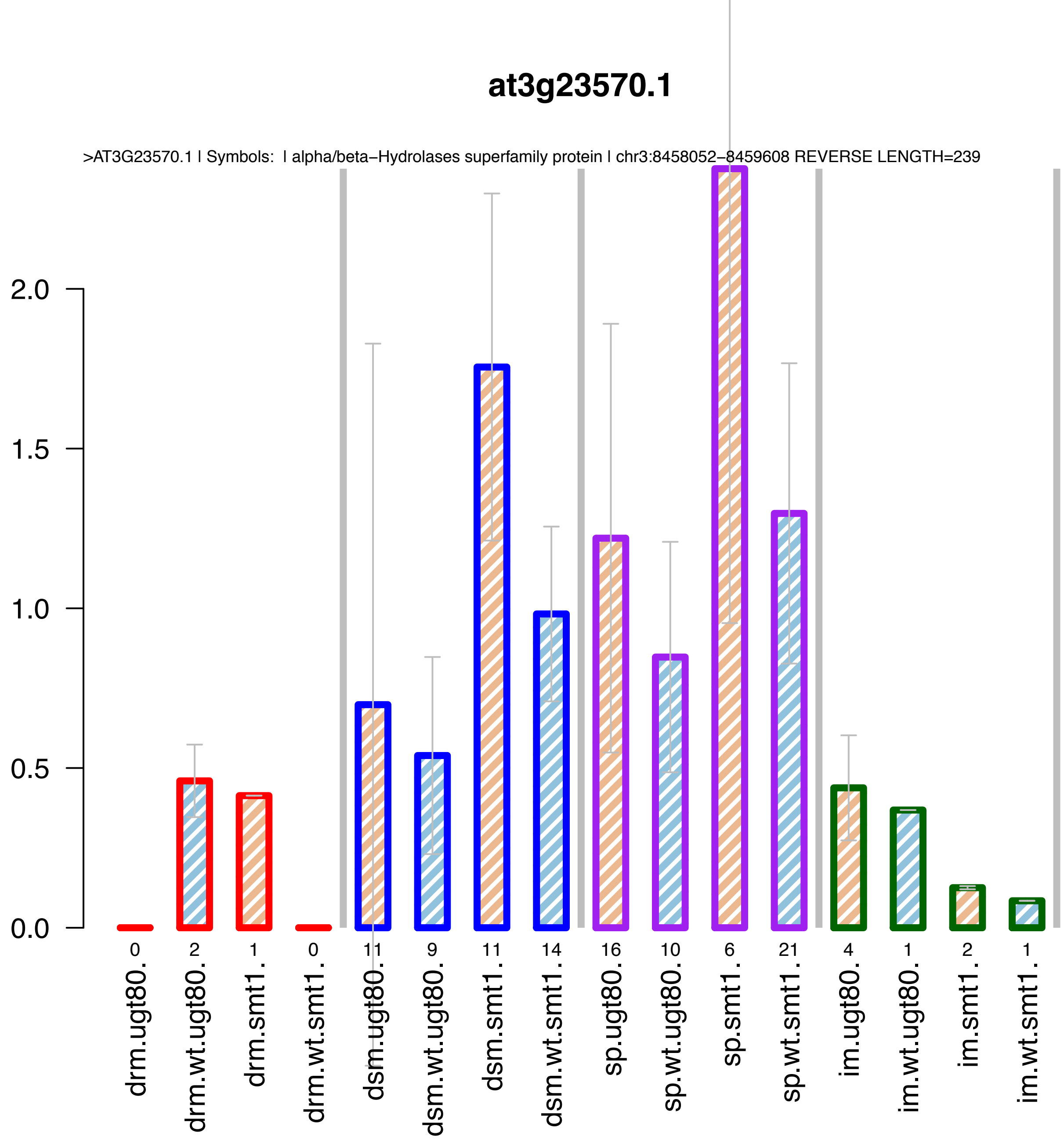

at3g23570.1
>AT3G23570.1 | Symbols: | alpha/beta−Hydrolases superfamily protein | chr3:8458052−8459608 REVERSE LENGTH=239
0.0
0.5
1.0
1.5
2.0
0
2
1
0
11
9
11
14
16
10
6
21
4
1
2
1
drm.ugt80..
drm.wt.ugt80..
drm.smt1..
drm.wt.smt1..
dsm.ugt80..
dsm.wt.ugt80..
dsm.smt1..
dsm.wt.smt1..
sp.ugt80..
sp.wt.ugt80..
sp.smt1..
sp.wt.smt1..
im.ugt80..
im.wt.ugt80..
im.smt1..
im.wt.smt1..

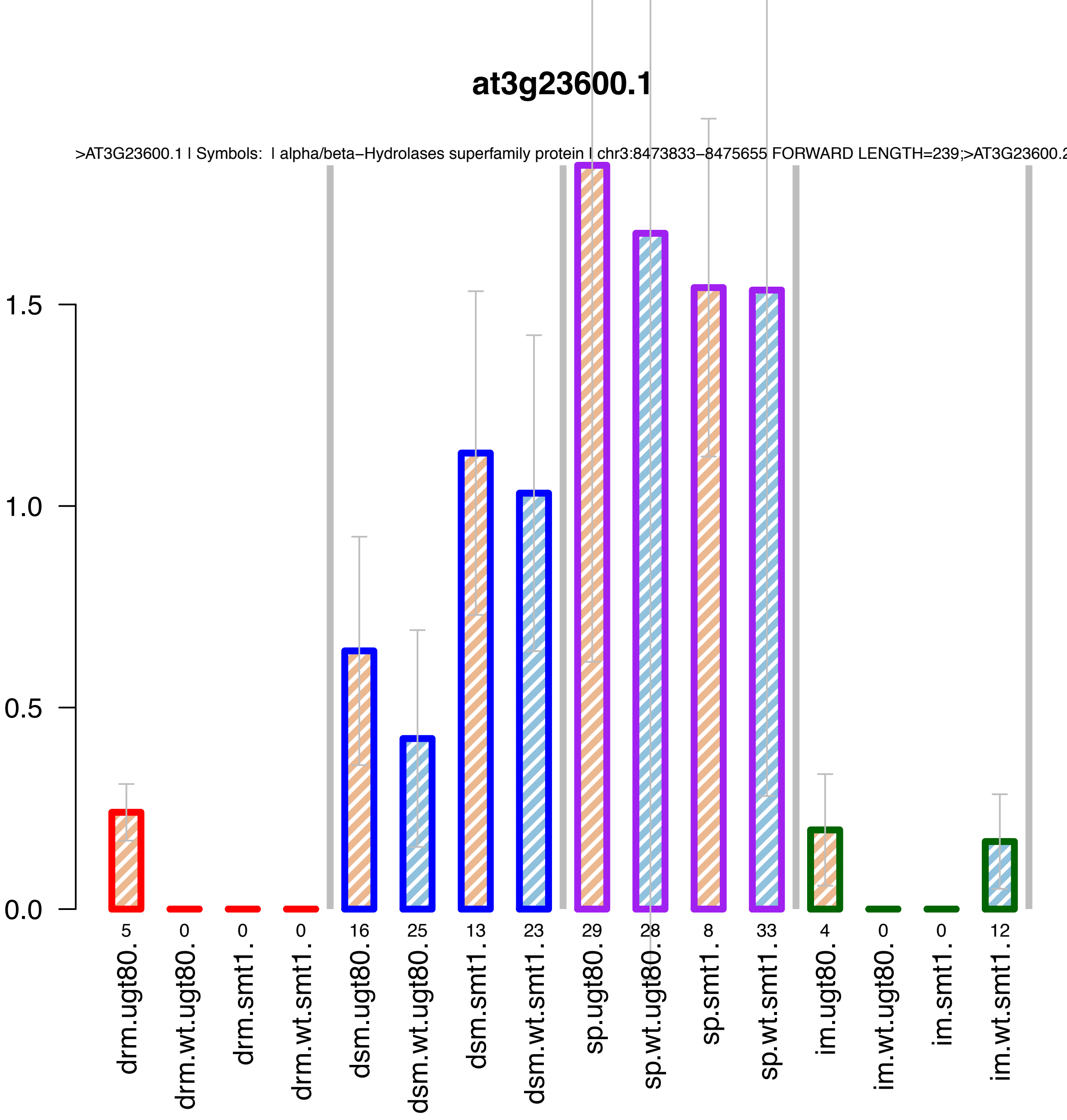

at3g23600.1
>AT3G23600.1 | Symbols: | alpha/beta−Hydrolases superfamily protein | chr3:8473833−8475655 FORWARD LENGTH=239;>AT3G23600.2
0.0
0.5
1.0
1.5
5
0
0
0
16
25
13
23
29
28
8
33
4
0
0
12
drm.ugt80.
drm.wt.ugt80.
drm.smt1.
drm.wt.smt1.
dsm.ugt80.
dsm.wt.ugt80.
dsm.smt1.
dsm.wt.smt1.
sp.ugt80.
sp.wt.ugt80.
sp.smt1.
sp.wt.smt1.
im.ugt80.
im.wt.ugt80.
im.smt1.
im.wt.smt1.

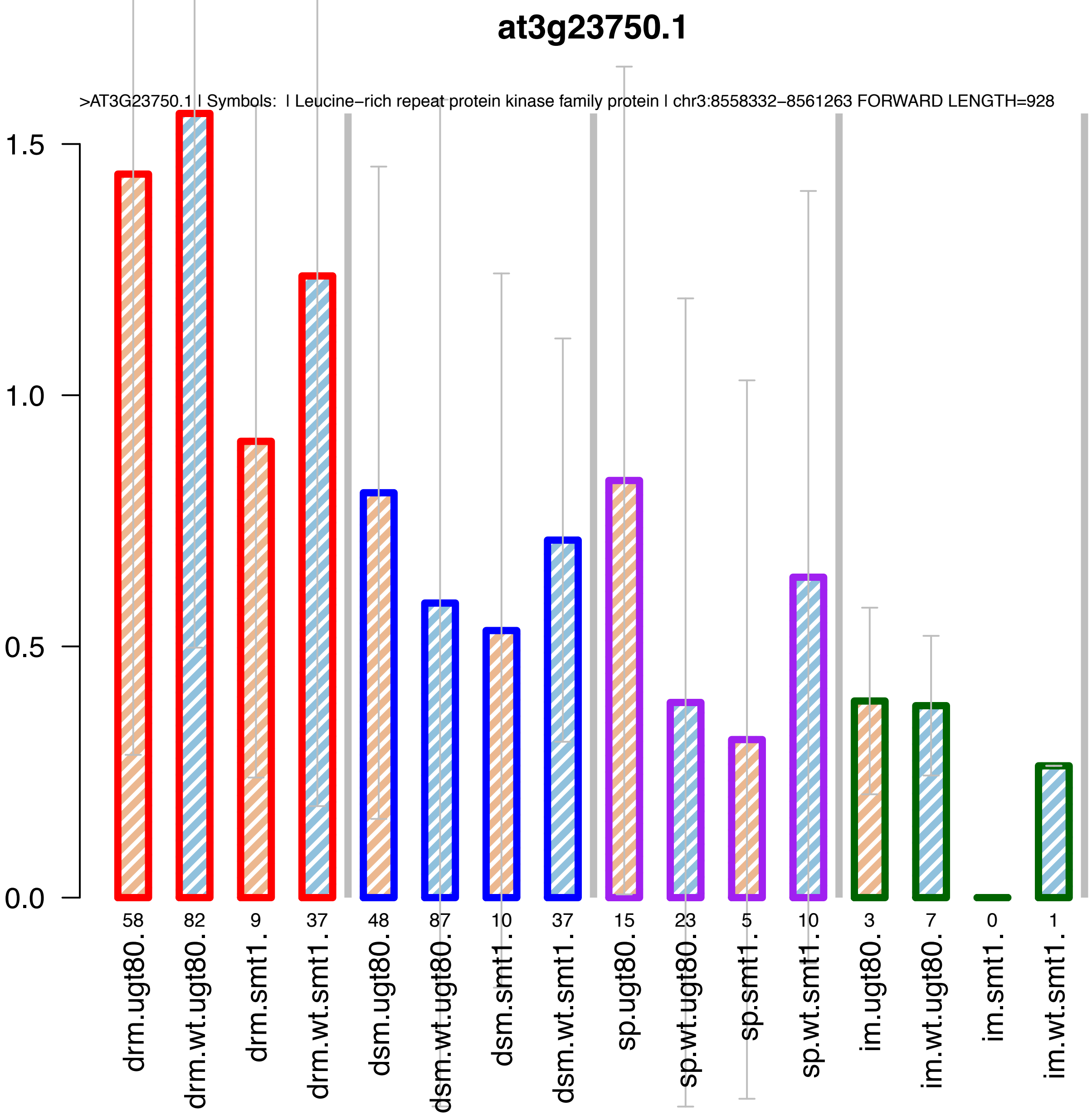

at3g23750.1
>AT3G23750.1 | Symbols: | Leucine−rich repeat protein kinase family protein | chr3:8558332−8561263 FORWARD LENGTH=928
0.0
0.5
1.0
1.5
58
82
9
37
48
87
10
37
15
23
5
10
3
7
0
1
drm.ugt80..
drm.wt.ugt80..
drm.smt1..
drm.wt.smt1..
dsm.ugt80..
dsm.wt.ugt80..
dsm.smt1..
dsm.wt.smt1..
sp.ugt80..
sp.wt.ugt80..
sp.smt1..
sp.wt.smt1..
im.ugt80..
im.wt.ugt80..
im.smt1..
im.wt.smt1..

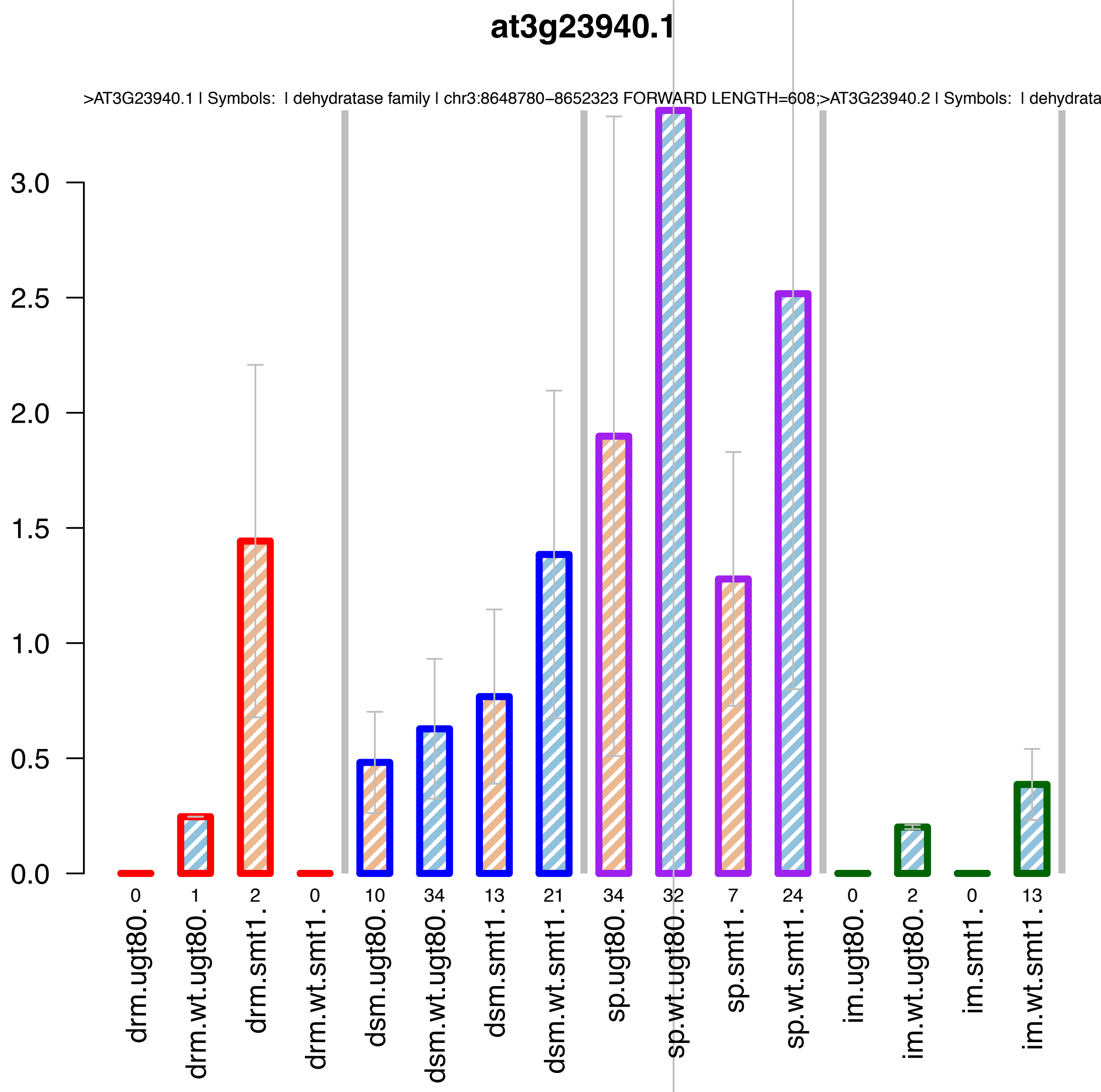
at3g23940.1
>AT3G23940.1 | Symbols: | dehydratase family | chr3:8648780−8652323 FORWARD LENGTH=608;>AT3G23940.2 | Symbols: | dehydrata
3.0
2.5
2.0
1.5
1.0
0.5
0.0
0
1
2
0
10
34
13
21
34
32
7
24
0
2
0
13
drm.ugt80.
drm.wt.ugt80.
drm.smt1.
drm.wt.smt1.
dsm.ugt80.
dsm.wt.ugt80.
dsm.smt1.
dsm.wt.smt1.
sp.ugt80.
sp.wt.ugt80.
sp.smt1.
sp.wt.smt1.
im.ugt80.
im.wt.ugt80.
im.smt1.
im.wt.smt1.

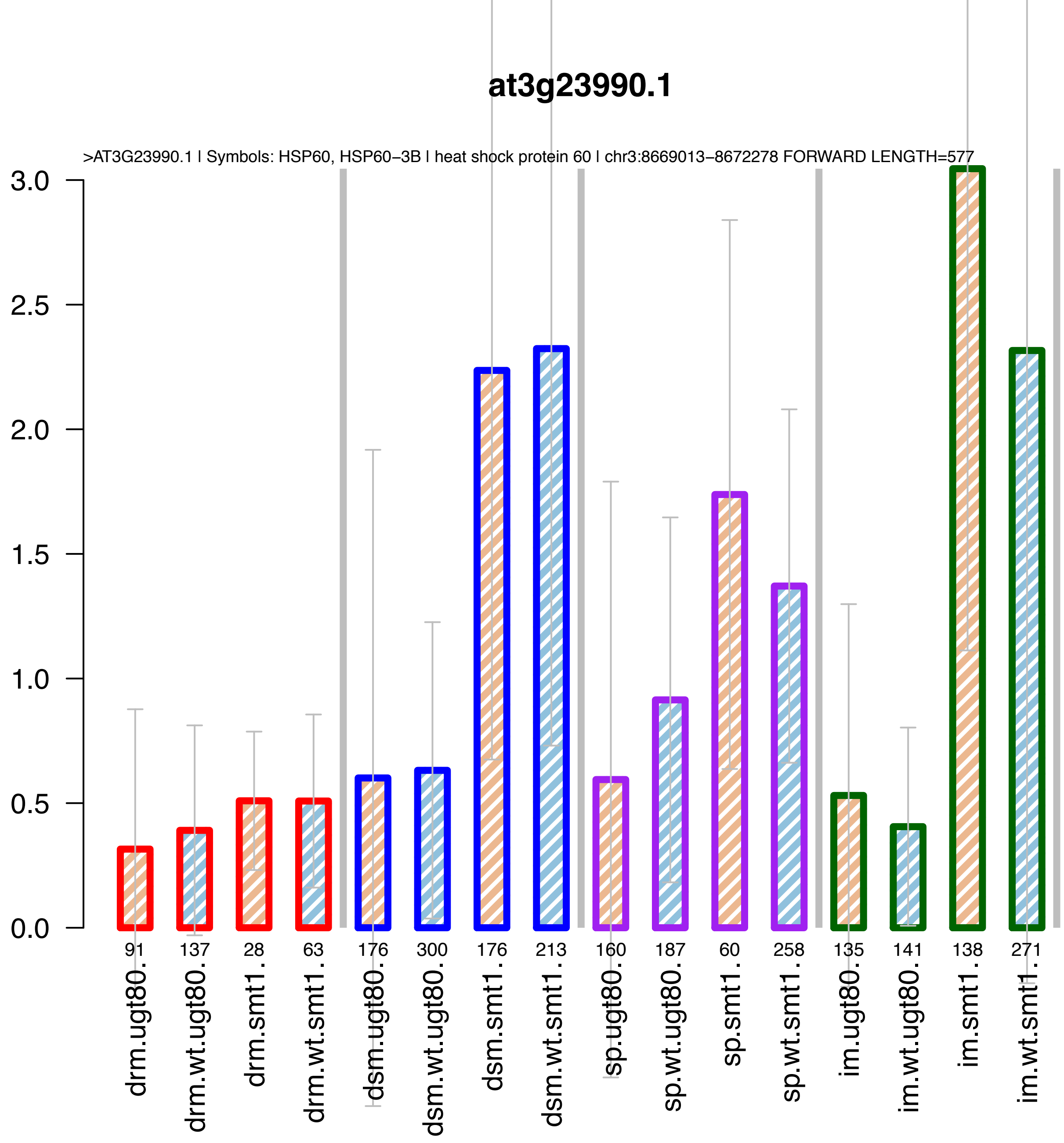
at3g23990.1
>AT3G23990.1 | Symbols: HSP60, HSP60-3B | heat shock protein 60 | chr3:8669013-8672278 FORWARD LENGTH=577
3.0
2.5
2.0
1.5
1.0
0.5
0.0
91
137
28
63
176
300
176
213
160
187
60
258
135
141
138
271
drm.ugt80..
drm.wt.ugt80..
drm.smt1..
drm.wt.smt1..
dsm.ugt80..
dsm.wt.ugt80..
dsm.smt1..
dsm.wt.smt1..
sp.ugt80..
sp.wt.ugt80..
sp.smt1..
sp.wt.smt1..
im.ugt80..
im.wt.ugt80..
im.smt1..
im.wt.smt1..

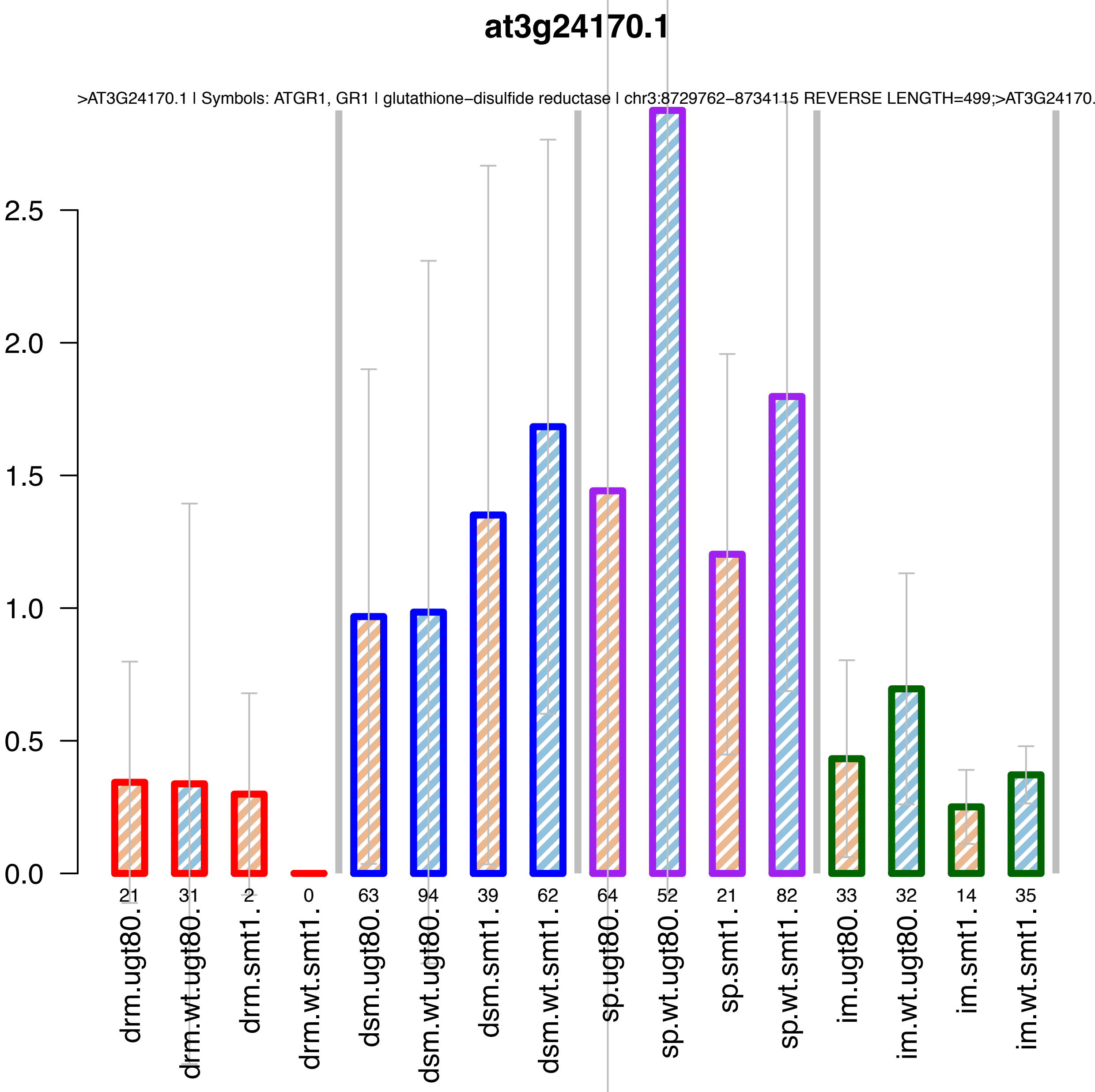
at3g24170.1
>AT3G24170.1 | Symbols: ATGR1, GR1 | glutathione-disulfide reductase | chr3:8729762-8734115 REVERSE LENGTH=499;>AT3G24170.
0.0
0.5
1.0
1.5
2.0
2.5
21
31
2
0
63
94
39
62
64
52
21
82
33
32
14
35
drm.ugt80.
drm.wt.ugt80.
drm.smt1.
drm.wt.smt1.
dsm.ugt80.
dsm.wt.ugt80.
dsm.smt1.
dsm.wt.smt1.
sp.ugt80.
sp.wt.ugt80.
sp.smt1.
sp.wt.smt1.
im.ugt80.
im.wt.ugt80.
im.smt1.
im.wt.smt1.

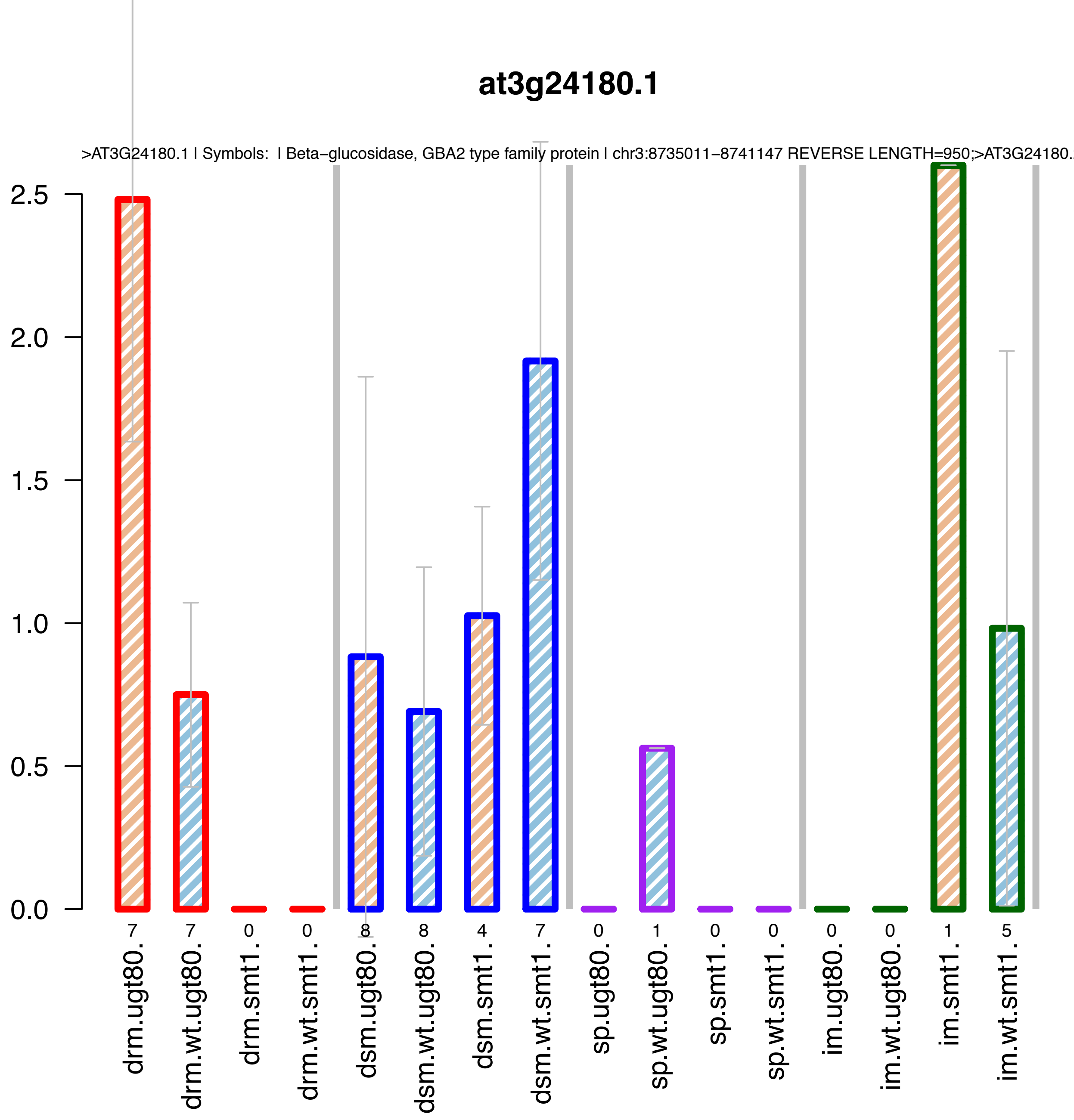

at3g24180.1
>AT3G24180.1 | Symbols: | Beta−glucosidase, GBA2 type family protein | chr3:8735011−8741147 REVERSE LENGTH=950;>AT3G24180.
2.5
2.0
1.5
1.0
0.5
0.0
7
7
0
0
8
8
4
7
0
1
0
0
0
0
1
5
drm.ugt80.
drm.wt.ugt80.
drm.smt1.
drm.wt.smt1.
dsm.ugt80.
dsm.wt.ugt80.
dsm.smt1.
dsm.wt.smt1.
sp.ugt80.
sp.wt.ugt80.
sp.smt1.
sp.wt.smt1.
im.ugt80.
im.wt.ugt80.
im.smt1.
im.wt.smt1.

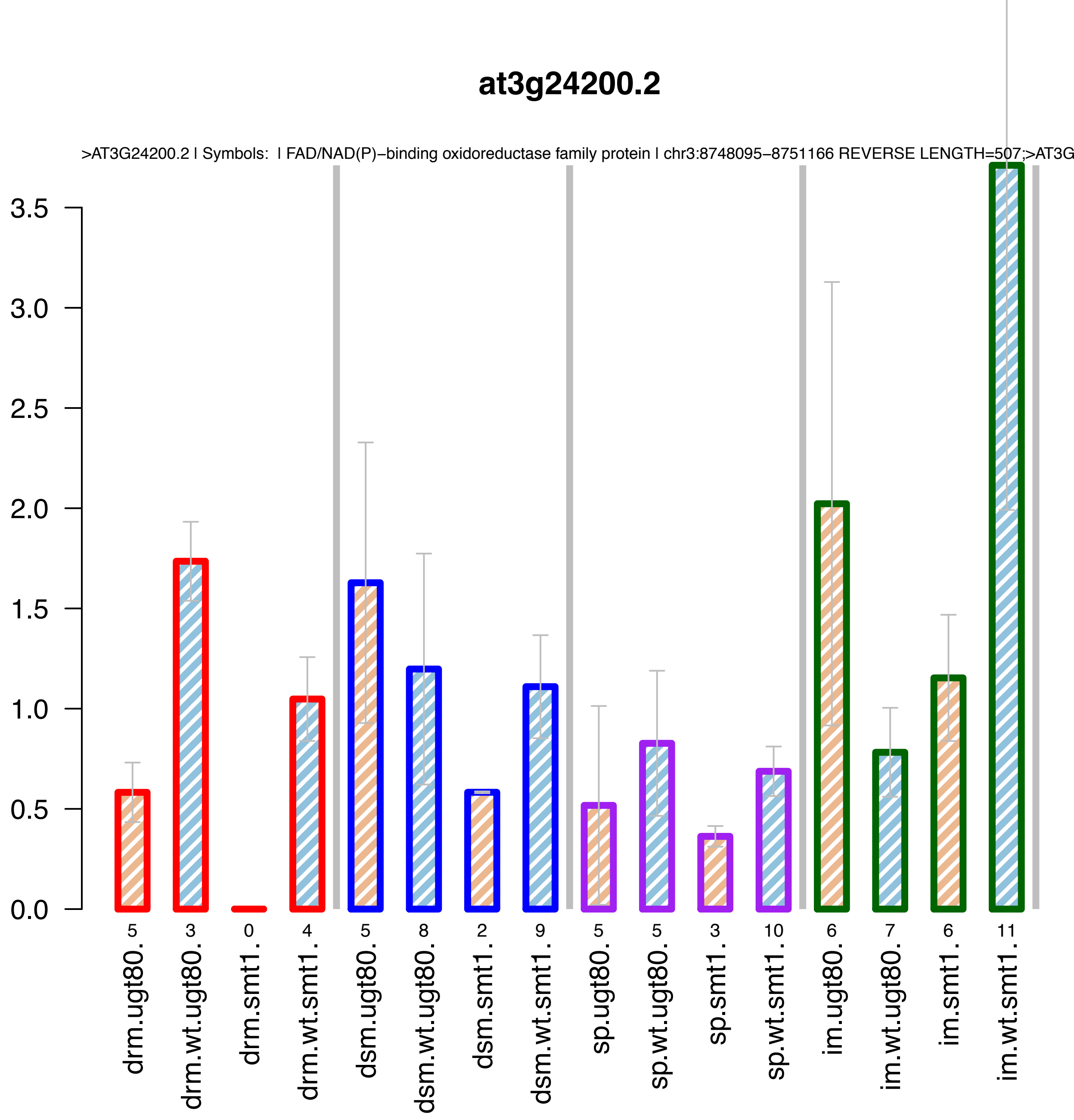

at3g24200.2
>AT3G24200.2 | Symbols: | FAD/NAD(P)−binding oxidoreductase family protein | chr3:8748095−8751166 REVERSE LENGTH=507;>AT3G
0.0
0.5
1.0
1.5
2.0
2.5
3.0
3.5
5
3
0
4
5
8
2
9
5
5
3
10
6
7
6
11
drm.ugt80.
drm.wt.ugt80.
drm.smt1.
drm.wt.smt1.
dsm.ugt80.
dsm.wt.ugt80.
dsm.smt1.
dsm.wt.smt1.
sp.ugt80.
sp.wt.ugt80.
sp.smt1.
sp.wt.smt1.
im.ugt80.
im.wt.ugt80.
im.smt1.
im.wt.smt1.

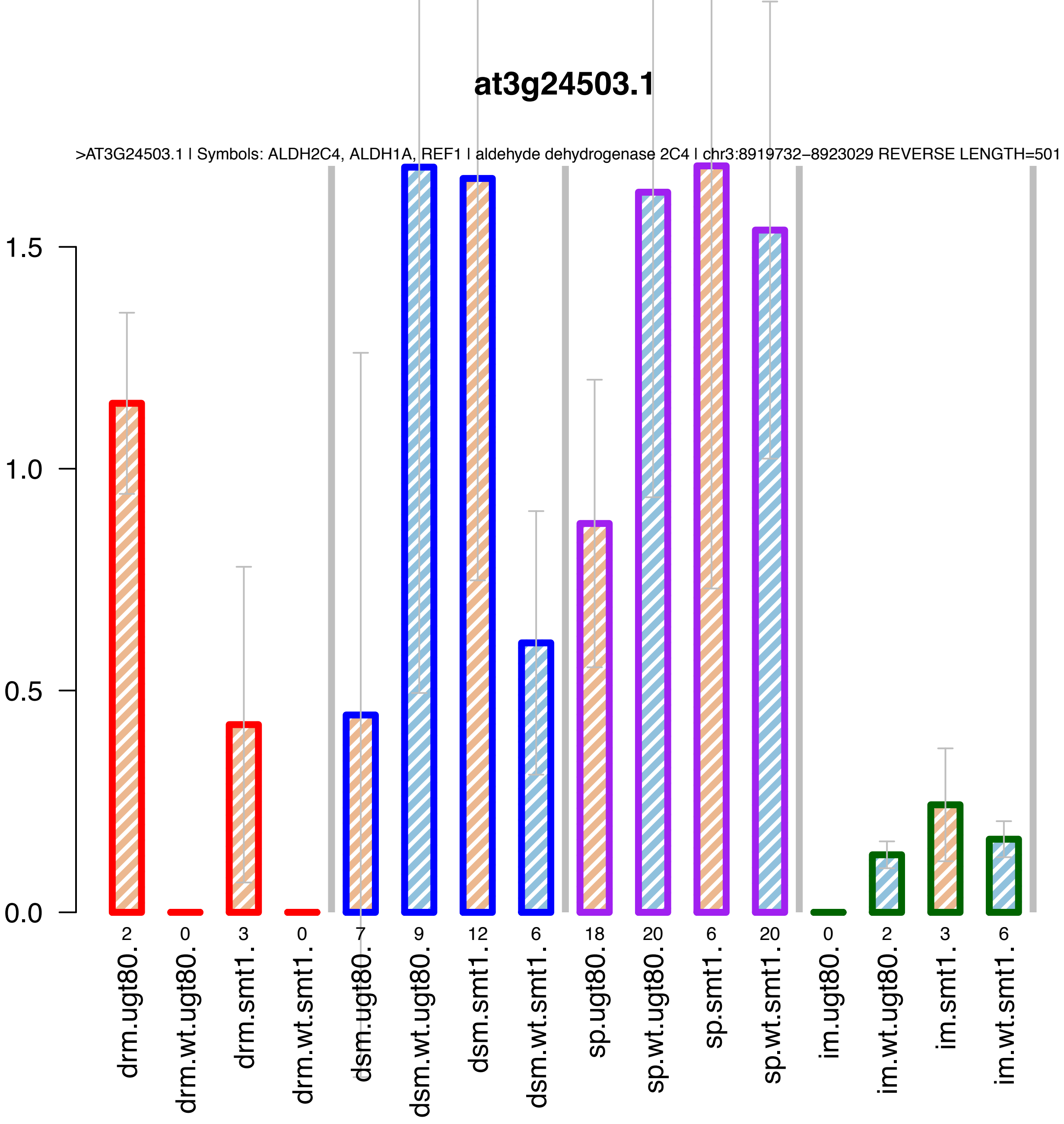

at3g24503.1
>AT3G24503.1 | Symbols: ALDH2C4, ALDH1A, REF1 | aldehyde dehydrogenase 2C4 | chr3:8919732–8923029 REVERSE LENGTH=501
0.0
0.5
1.0
1.5
2
0
3
0
7
9
12
6
18
20
6
20
0
2
3
6
drm.ugt80..
drm.wt.ugt80..
drm.smt1..
drm.wt.smt1..
dsm.ugt80..
dsm.wt.ugt80..
dsm.smt1..
dsm.wt.smt1..
sp.ugt80..
sp.wt.ugt80..
sp.smt1..
sp.wt.smt1..
im.ugt80..
im.wt.ugt80..
im.smt1..
im.wt.smt1..

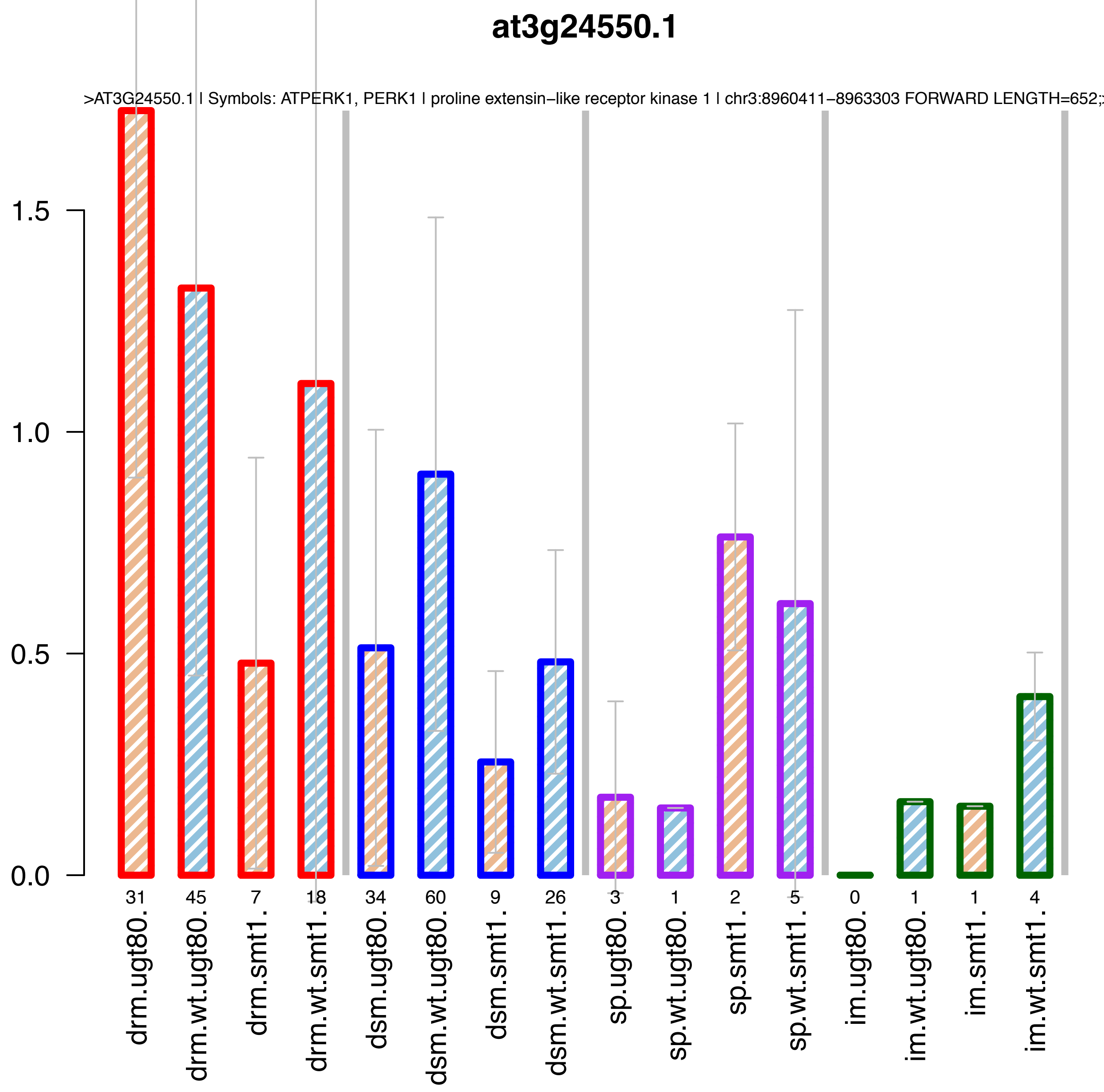

at3g24550.1
>AT3G24550.1 | Symbols: ATPERK1, PERK1 | proline extensin−like receptor kinase 1 | chr3:8960411−8963303 FORWARD LENGTH=652;
0.0
0.5
1.0
1.5
31
45
7
18
34
60
9
26
3
1
2
5
0
1
1
4
drm.ugt80.
drm.wt.ugt80.
drm.smt1.
drm.wt.smt1.
dsm.ugt80.
dsm.wt.ugt80.
dsm.smt1.
dsm.wt.smt1.
sp.ugt80.
sp.wt.ugt80.
sp.smt1.
sp.wt.smt1.
im.ugt80.
im.wt.ugt80.
im.smt1.
im.wt.smt1.

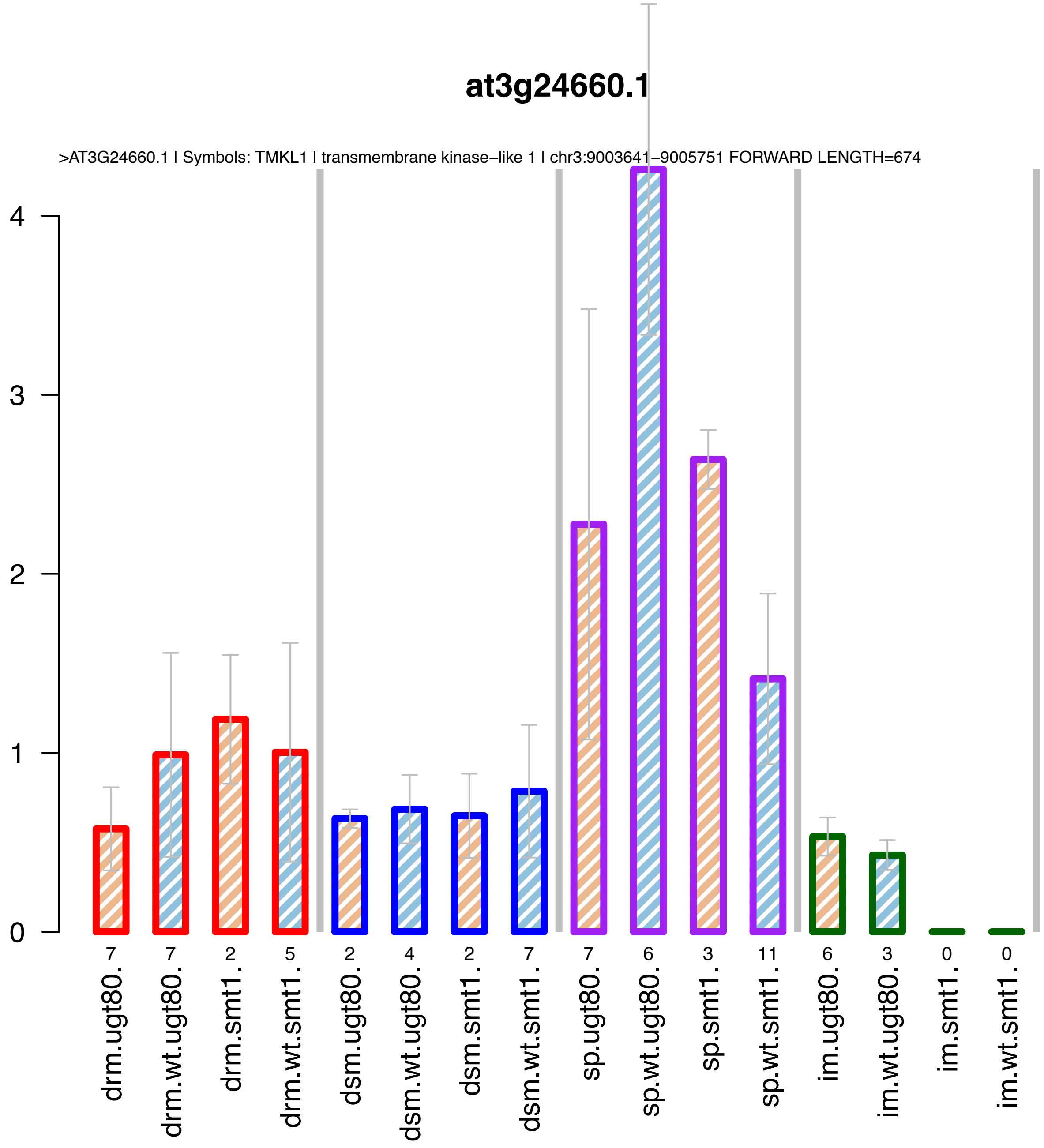
at3g24660.1
>AT3G24660.1 | Symbols: TMKL1 | transmembrane kinase-like 1 | chr3:9003641-9005751 FORWARD LENGTH=674
0
1
2
3
4
7
7
2
5
2
4
2
7
7
6
3
11
6
3
0
0
drm.ugt80.
drm.wt.ugt80.
drm.smt1.
drm.wt.smt1.
dsm.ugt80.
dsm.wt.ugt80.
dsm.smt1.
dsm.wt.smt1.
sp.ugt80.
sp.wt.ugt80.
sp.smt1.
sp.wt.smt1.
im.ugt80.
im.wt.ugt80.
im.smt1.
im.wt.smt1.

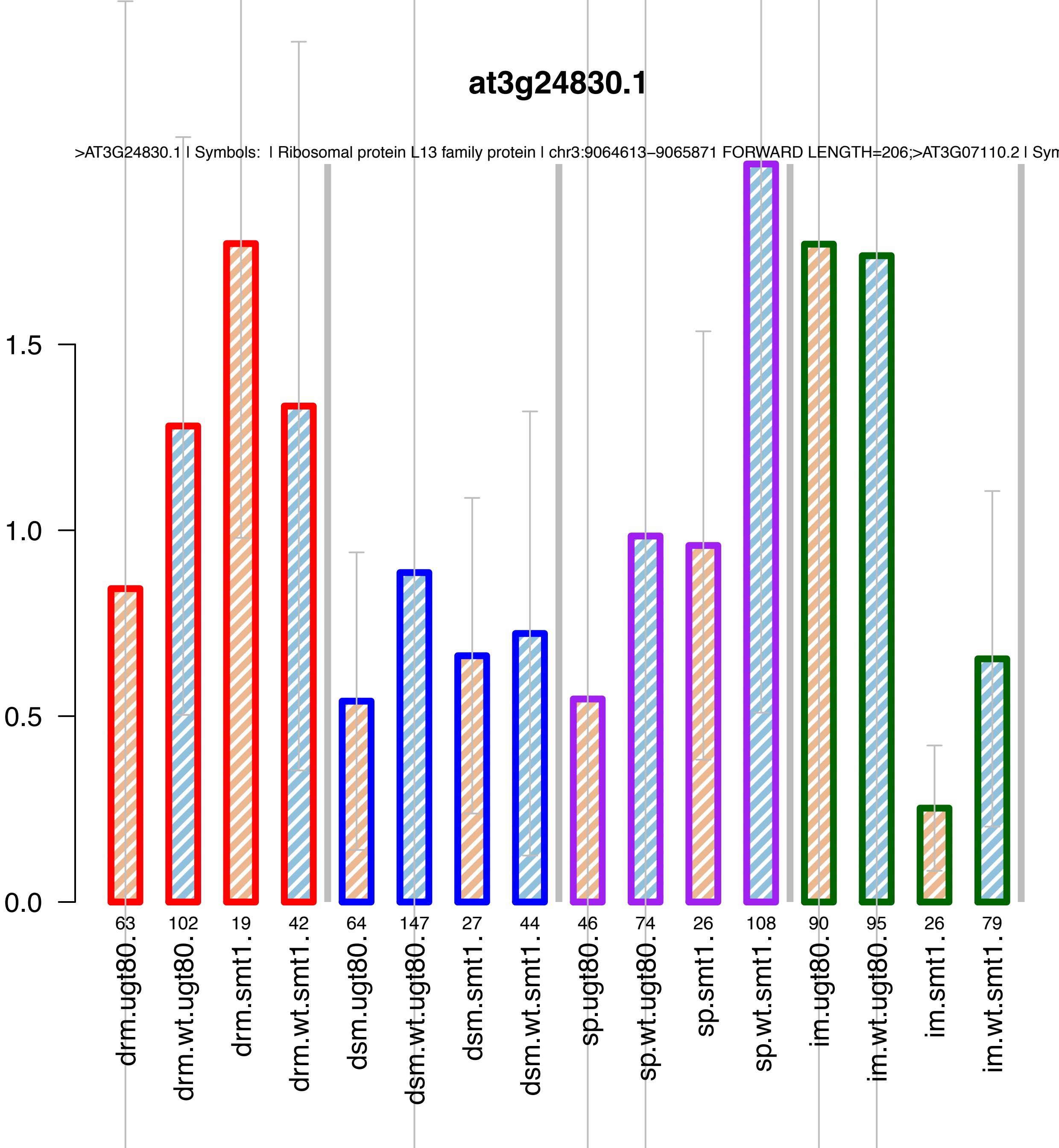

at3g24830.1
>AT3G24830.1 | Symbols: | Ribosomal protein L13 family protein | chr3:9064613-9065871 FORWARD LENGTH=206;>AT3G07110.2 | Sym
0.0
0.5
1.0
1.5
63
102
19
42
64
147
27
44
46
74
26
108
90
95
26
79
drm.ugt80..
drm.wt.ugt80..
drm.smt1..
drm.wt.smt1..
dsm.ugt80..
dsm.wt.ugt80..
dsm.smt1..
dsm.wt.smt1..
sp.ugt80..
sp.wt.ugt80..
sp.smt1..
sp.wt.smt1..
im.ugt80..
im.wt.ugt80..
im.smt1..
im.wt.smt1..

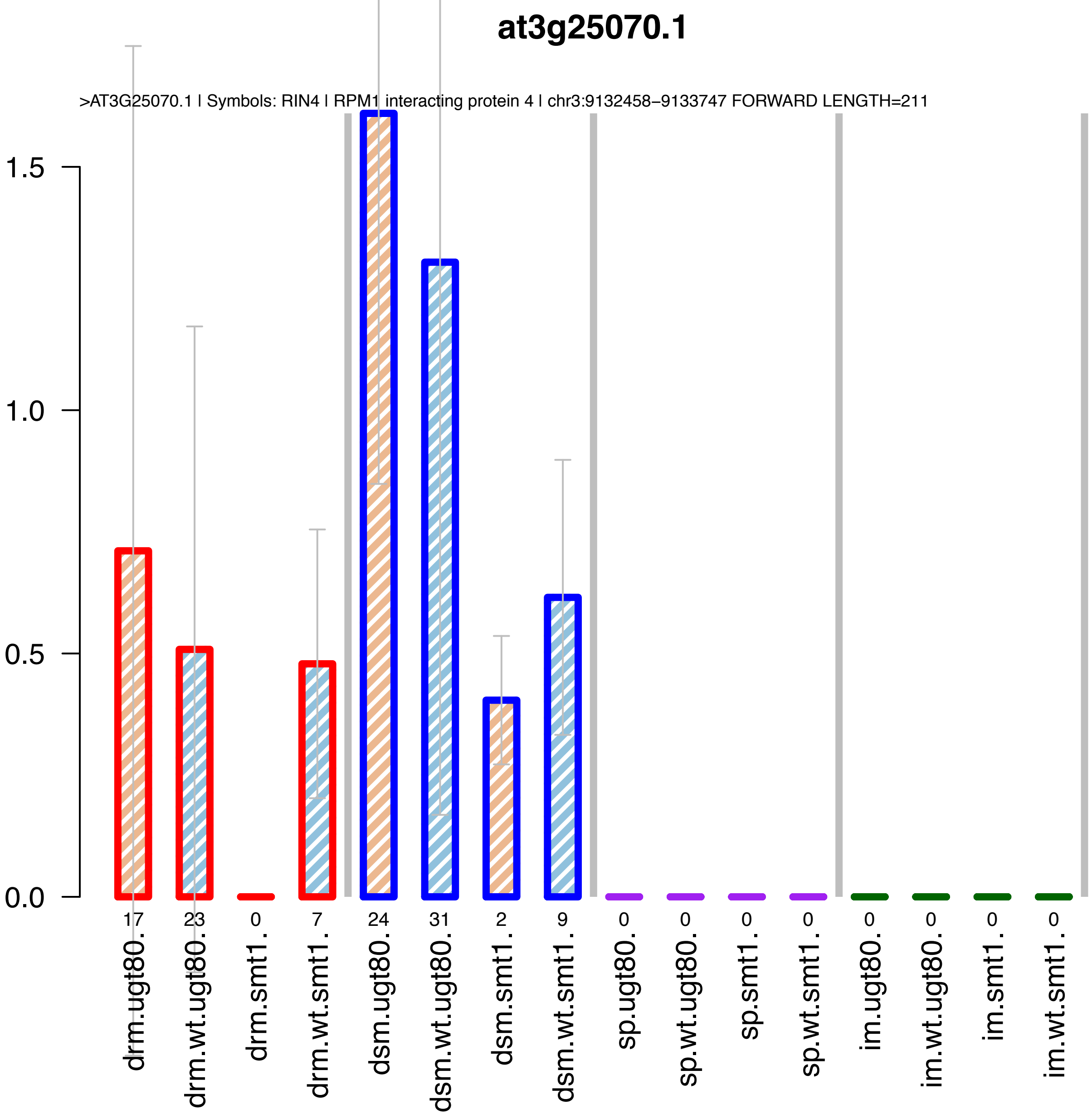
at3g25070.1
>AT3G25070.1 | Symbols: RIN4 | RPM1 interacting protein 4 | chr3:9132458-9133747 FORWARD LENGTH=211
1.5
1.0
0.5
0.0
17
23
0
7
24
31
2
9
0
0
0
0
0
0
0
0
drm.ugt80.
drm.wt.ugt80.
drm.smt1.
drm.wt.smt1.
dsm.ugt80.
dsm.wt.ugt80.
dsm.smt1.
dsm.wt.smt1.
sp.ugt80.
sp.wt.ugt80.
sp.smt1.
sp.wt.smt1.
im.ugt80.
im.wt.ugt80.
im.smt1.
im.wt.smt1.

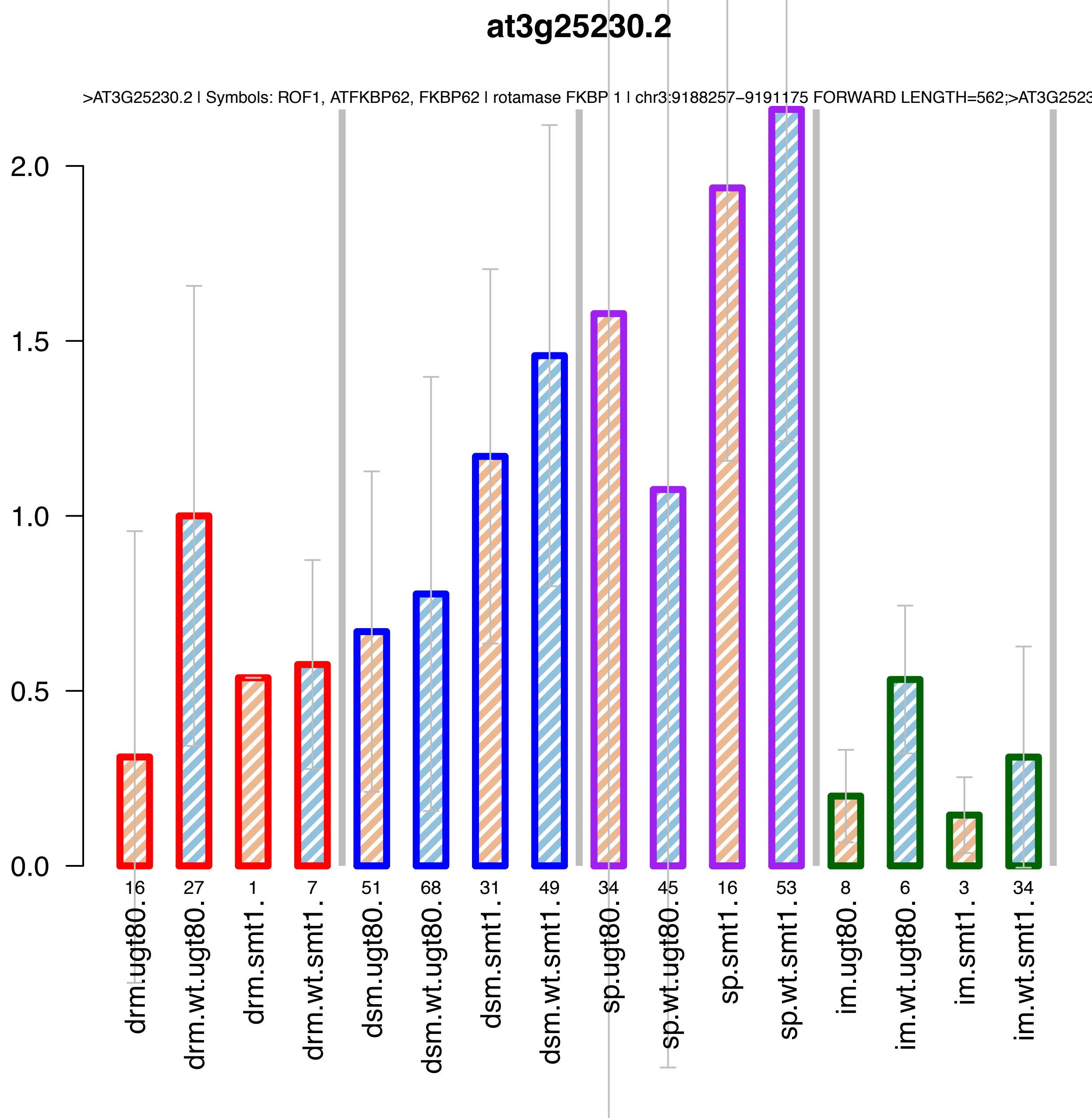

at3g25230.2
>AT3G25230.2 | Symbols: ROF1, ATFKBP62, FKBP62 | rotamase FKBP 1 | chr3:9188257−9191175 FORWARD LENGTH=562;>AT3G2523
2.0
1.5
1.0
0.5
0.0
16
27
1
7
51
68
31
49
34
45
16
53
8
6
3
34
drm.ugt80.
drm.wt.ugt80.
drm.smt1.
drm.wt.smt1.
dsm.ugt80.
dsm.wt.ugt80.
dsm.smt1.
dsm.wt.smt1.
sp.ugt80.
sp.wt.ugt80.
sp.smt1.
sp.wt.smt1.
im.ugt80.
im.wt.ugt80.
im.smt1.
im.wt.smt1.

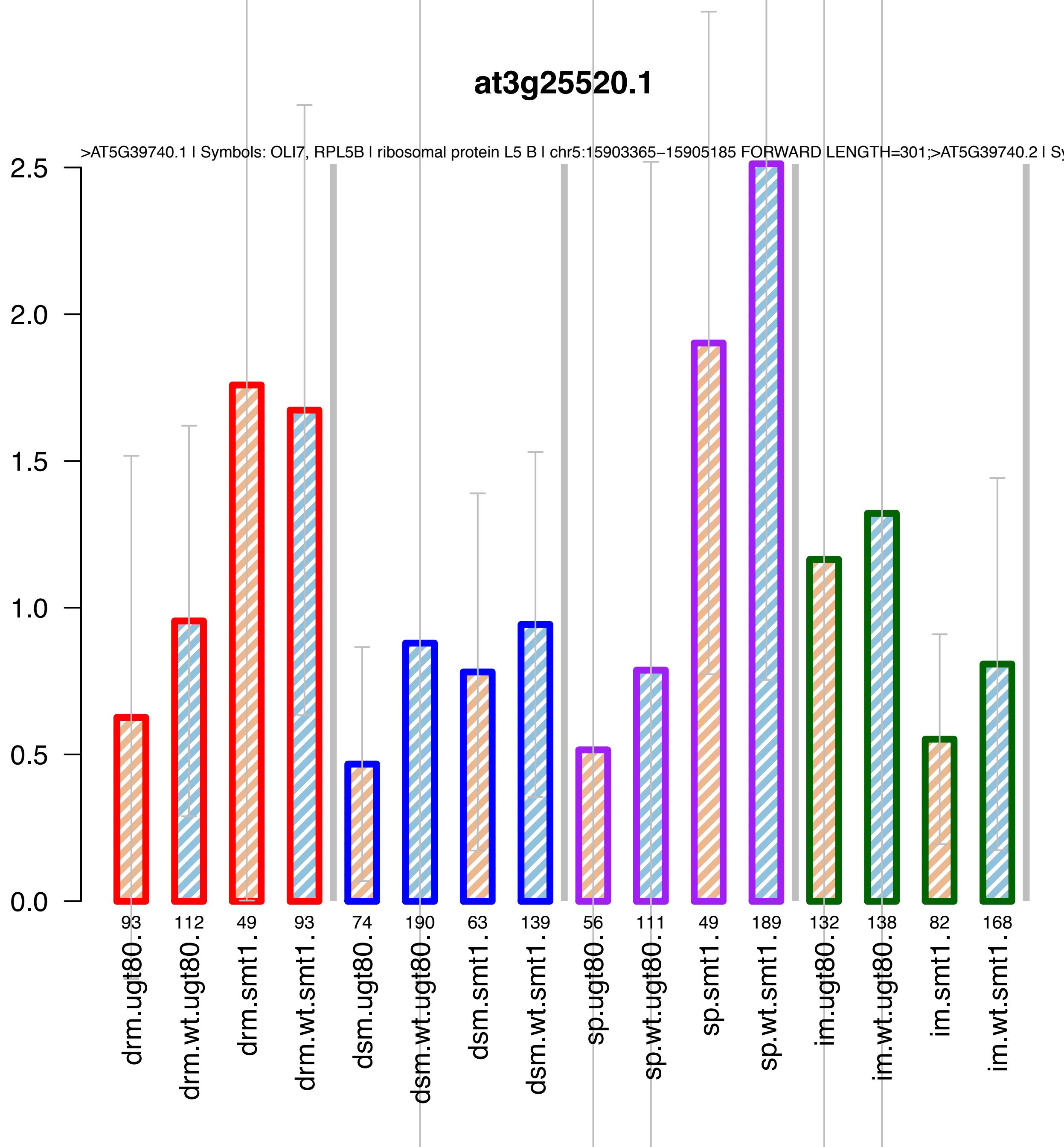
at3g25520.1
>AT5G39740.1 | Symbols: OLI7, RPL5B | ribosomal protein L5 B | chr5:15903365–15905185 FORWARD LENGTH=301;>AT5G39740.2 | Sy
2.5
2.0
1.5
1.0
0.5
0.0
93
112
49
93
74
190
63
139
56
111
49
189
132
138
82
168
drm.ugt80..
drm.wt.ugt80..
drm.smt1..
drm.wt.smt1..
dsm.ugt80..
dsm.wt.ugt80..
dsm.smt1..
dsm.wt.smt1..
sp.ugt80..
sp.wt.ugt80..
sp.smt1..
sp.wt.smt1..
im.ugt80..
im.wt.ugt80..
im.smt1..
im.wt.smt1..

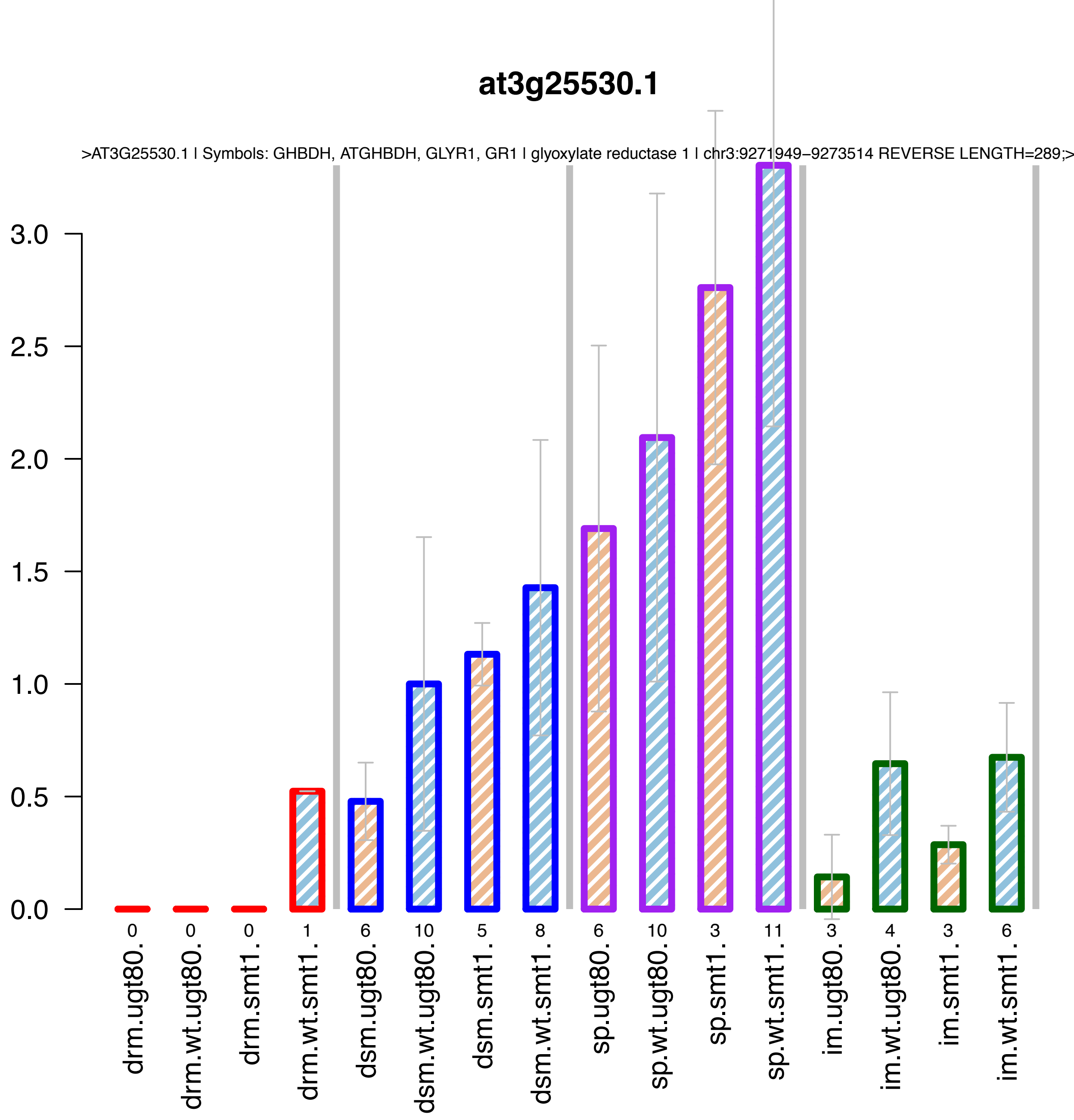

at3g25530.1
>AT3G25530.1 | Symbols: GHBDH, ATGHBDH, GLYR1, GR1 | glyoxylate reductase 1 | chr3:9271949−9273514 REVERSE LENGTH=289;>
3.0
2.5
2.0
1.5
1.0
0.5
0.0
0 0 0 1 6 10 5 8 6 10 3 11 3 4 3 6
drm.ugt80.
drm.wt.ugt80.
drm.smt1.
drm.wt.smt1.
dsm.ugt80.
dsm.wt.ugt80.
dsm.smt1.
dsm.wt.smt1.
sp.ugt80.
sp.wt.ugt80.
sp.smt1.
sp.wt.smt1.
im.ugt80.
im.wt.ugt80.
im.smt1.
im.wt.smt1.

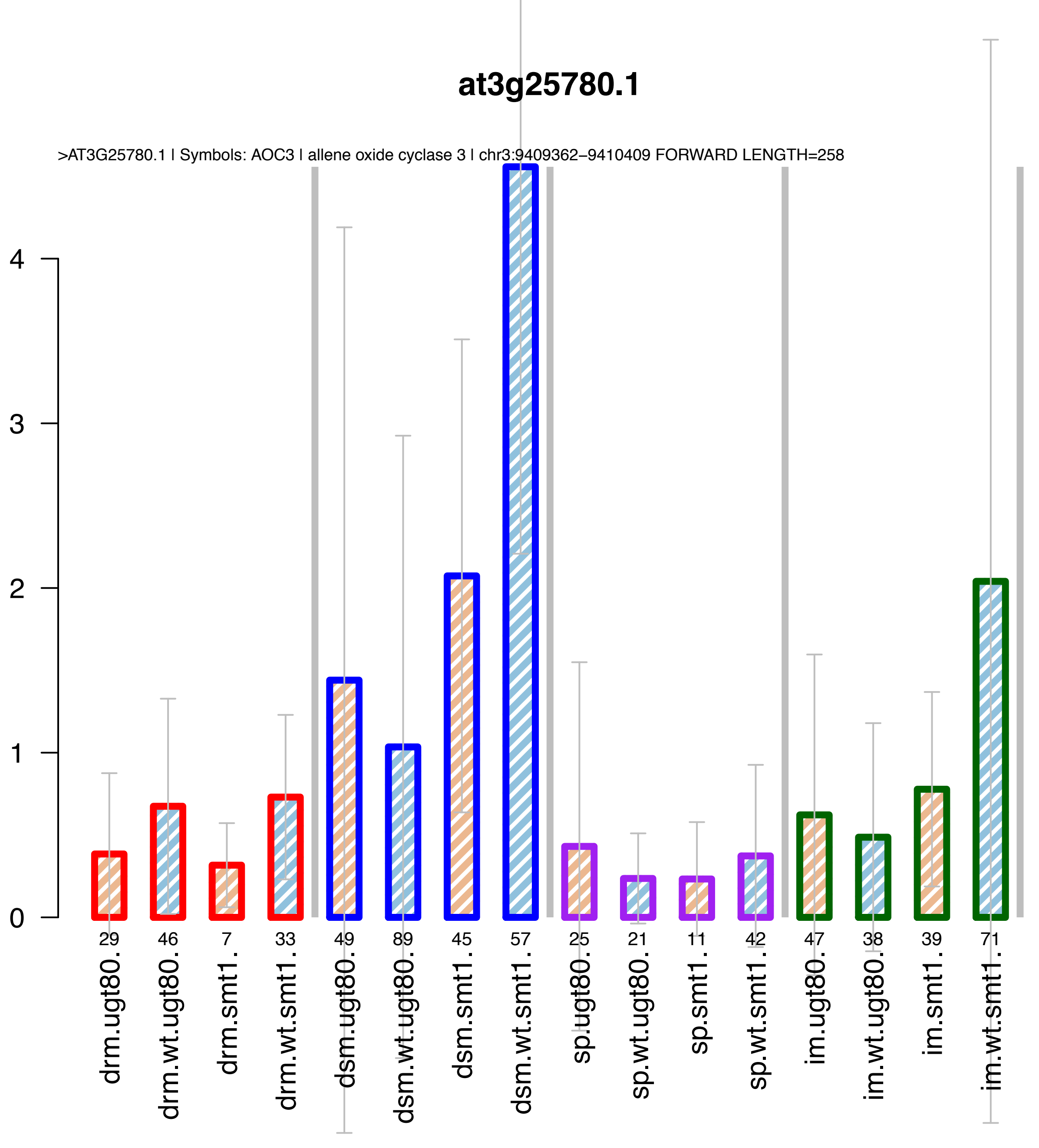

at3g25780.1
>AT3G25780.1 | Symbols: AOC3 | allene oxide cyclase 3 | chr3:9409362-9410409 FORWARD LENGTH=258
0
1
2
3
4
29
46
7
33
49
89
45
57
25
21
11
42
47
38
39
71
drm.ugt80.
drm.wt.ugt80.
drm.smt1.
drm.wt.smt1.
dsm.ugt80.
dsm.wt.ugt80.
dsm.smt1.
dsm.wt.smt1.
sp.ugt80.
sp.wt.ugt80.
sp.smt1.
sp.wt.smt1.
im.ugt80.
im.wt.ugt80.
im.smt1.
im.wt.smt1.

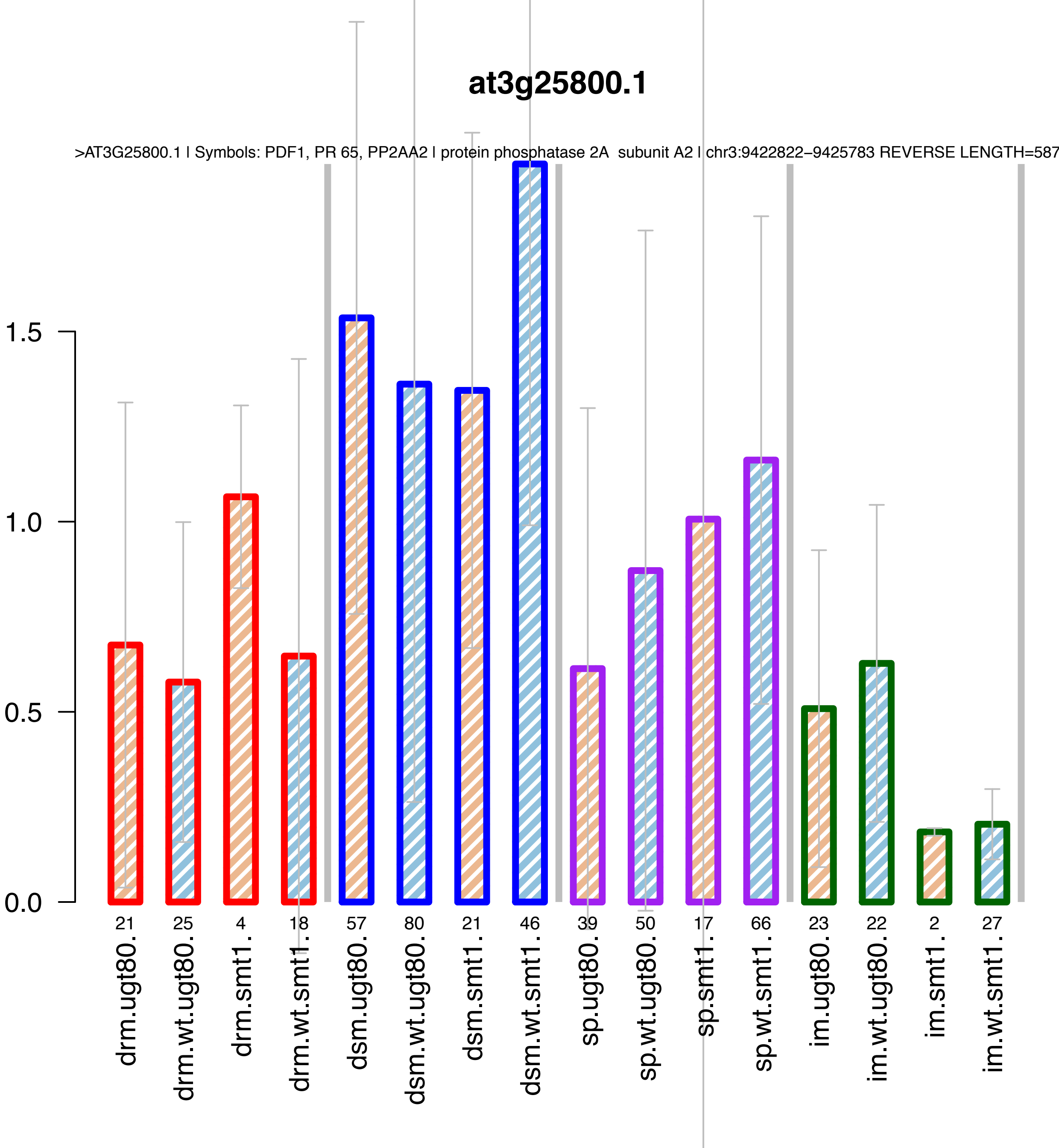

at3g25800.1
>AT3G25800.1 | Symbols: PDF1, PR 65, PP2AA2 | protein phosphatase 2A subunit A2 | chr3:9422822-9425783 REVERSE LENGTH=587
0.0
0.5
1.0
1.5
21
25
4
18
57
80
21
46
39
50
17
66
23
22
2
27
drm.ugt80..
drm.wt.ugt80..
drm.smt1..
drm.wt.smt1..
dsm.ugt80..
dsm.wt.ugt80..
dsm.smt1..
dsm.wt.smt1..
sp.ugt80..
sp.wt.ugt80..
sp.smt1..
sp.wt.smt1..
im.ugt80..
im.wt.ugt80..
im.smt1..
im.wt.smt1..

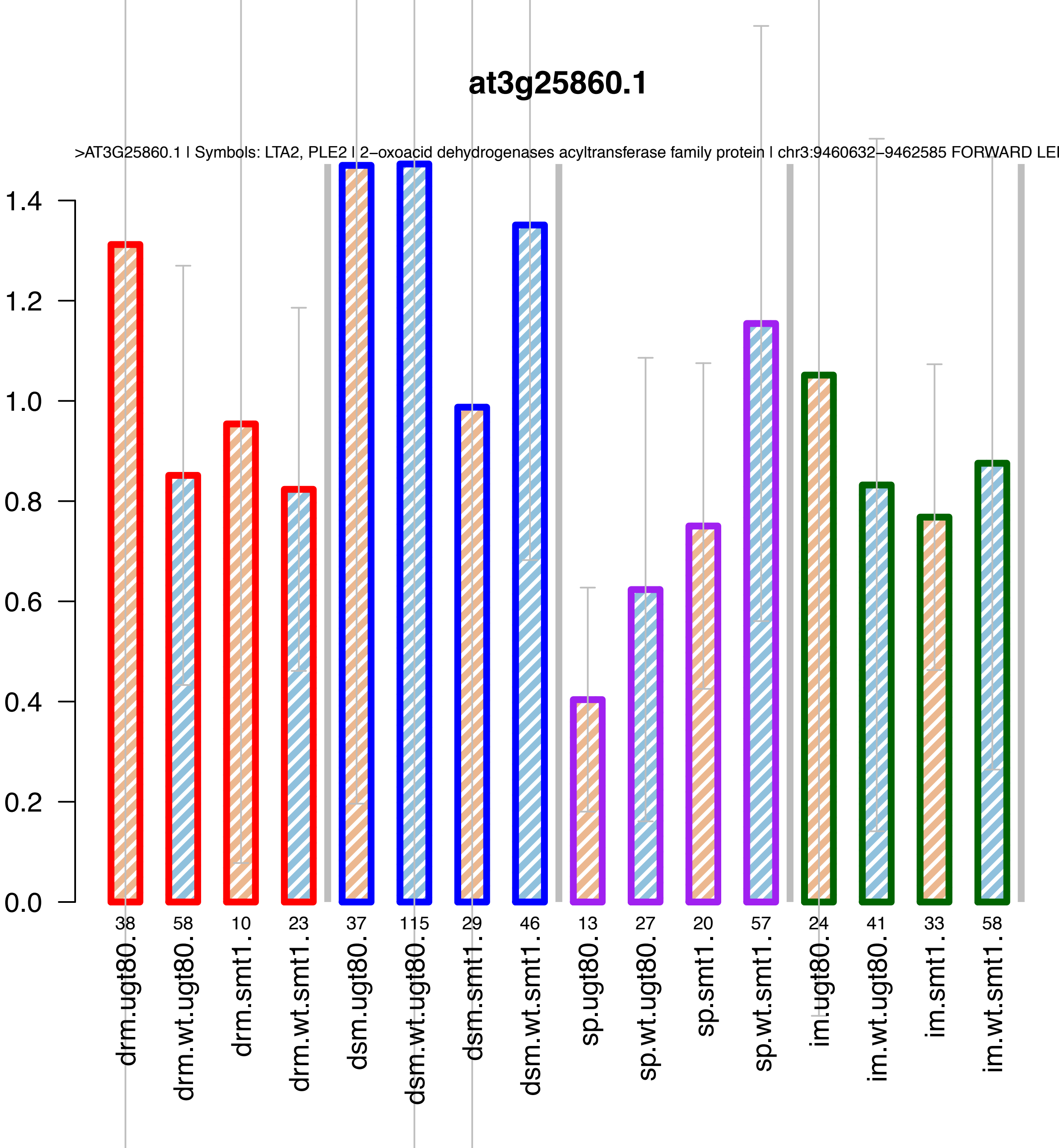

at3g25860.1
>AT3G25860.1 | Symbols: LTA2, PLE2 | 2−oxoacid dehydrogenases acyltransferase family protein | chr3:9460632−9462585 FORWARD LE
1.4
1.2
1.0
0.8
0.6
0.4
0.2
0.0
38
58
10
23
37
115
29
46
13
27
20
57
24
41
33
58
drm.ugt80..
drm.wt.ugt80..
drm.smt1..
drm.wt.smt1..
dsm.ugt80..
dsm.wt.ugt80..
dsm.smt1..
dsm.wt.smt1..
sp.ugt80..
sp.wt.ugt80..
sp.smt1..
sp.wt.smt1..
im.ugt80..
im.wt.ugt80..
im.smt1..
im.wt.smt1..

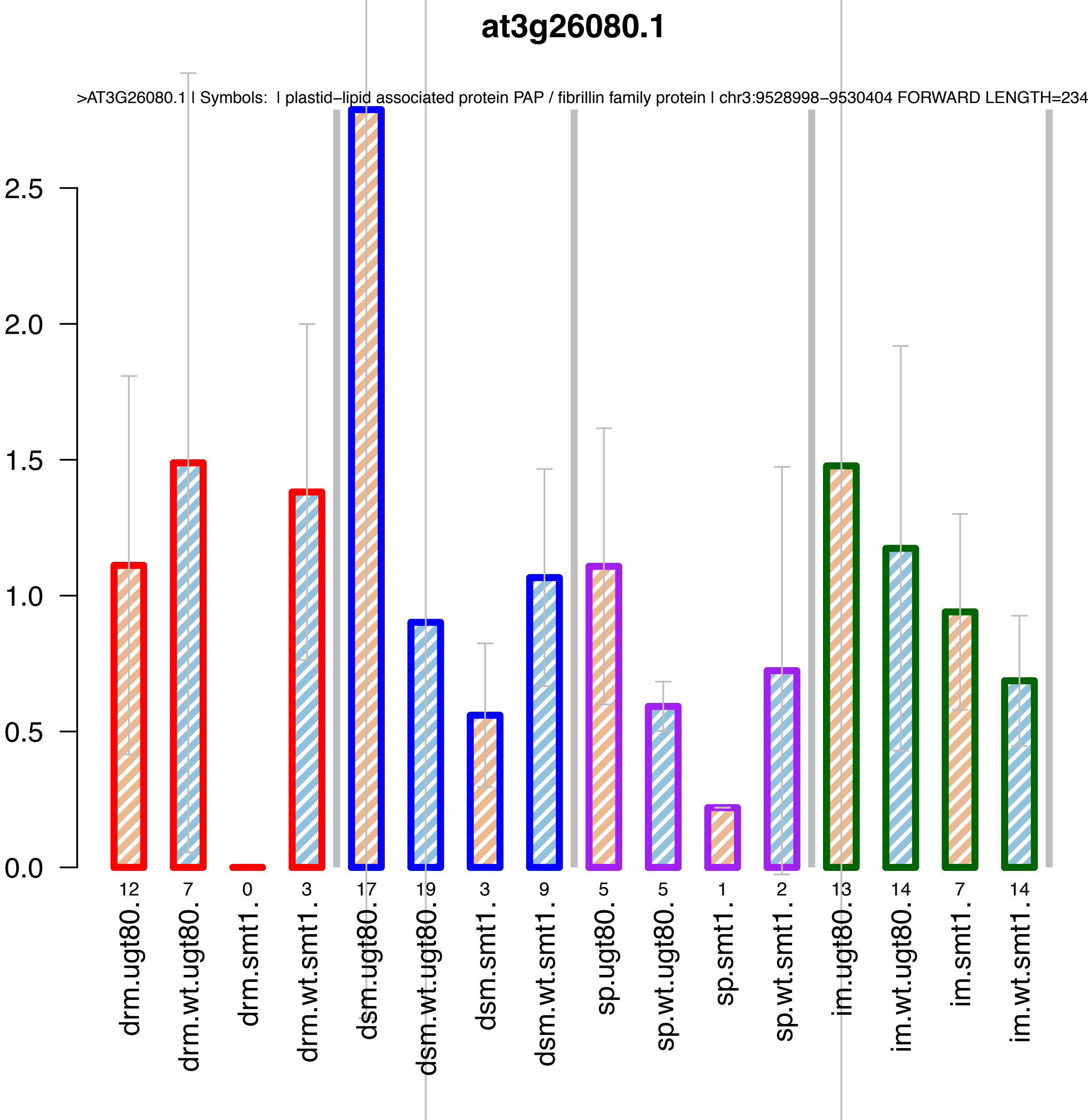

at3g26080.1
>AT3G26080.1 | Symbols: | plastid–lipid associated protein PAP / fibrillin family protein | chr3:9528998–9530404 FORWARD LENGTH=234
0.0
0.5
1.0
1.5
2.0
2.5
12
7
0
3
17
19
3
9
5
5
1
2
13
14
7
14
drm.ugt80..
drm.wt.ugt80..
drm.smt1..
drm.wt.smt1..
dsm.ugt80..
dsm.wt.ugt80..
dsm.smt1..
dsm.wt.smt1..
sp.ugt80..
sp.wt.ugt80..
sp.smt1..
sp.wt.smt1..
im.ugt80..
im.wt.ugt80..
im.smt1..
im.wt.smt1..

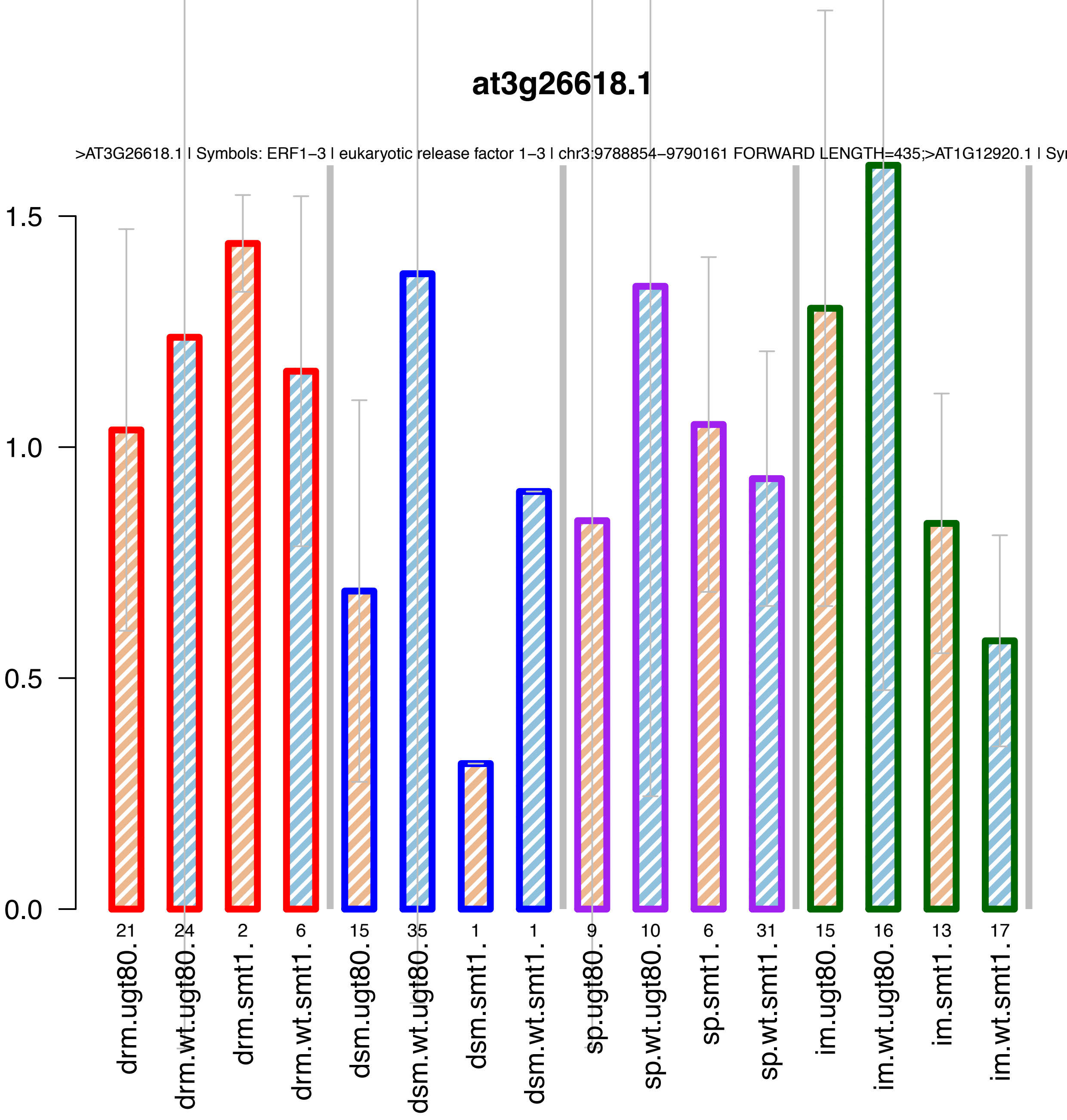
at3g26618.1
>AT3G26618.1 | Symbols: ERF1−3 | eukaryotic release factor 1−3 | chr3:9788854−9790161 FORWARD LENGTH=435;>AT1G12920.1 | Sy
0.0
0.5
1.0
1.5
21
24
2
6
15
35
1
1
9
10
6
31
15
16
13
17
drm.ugt80..
drm.wt.ugt80..
drm.smt1..
drm.wt.smt1..
dsm.ugt80..
dsm.wt.ugt80..
dsm.smt1..
dsm.wt.smt1..
sp.ugt80..
sp.wt.ugt80..
sp.smt1..
sp.wt.smt1..
im.ugt80..
im.wt.ugt80..
im.smt1..
im.wt.smt1..

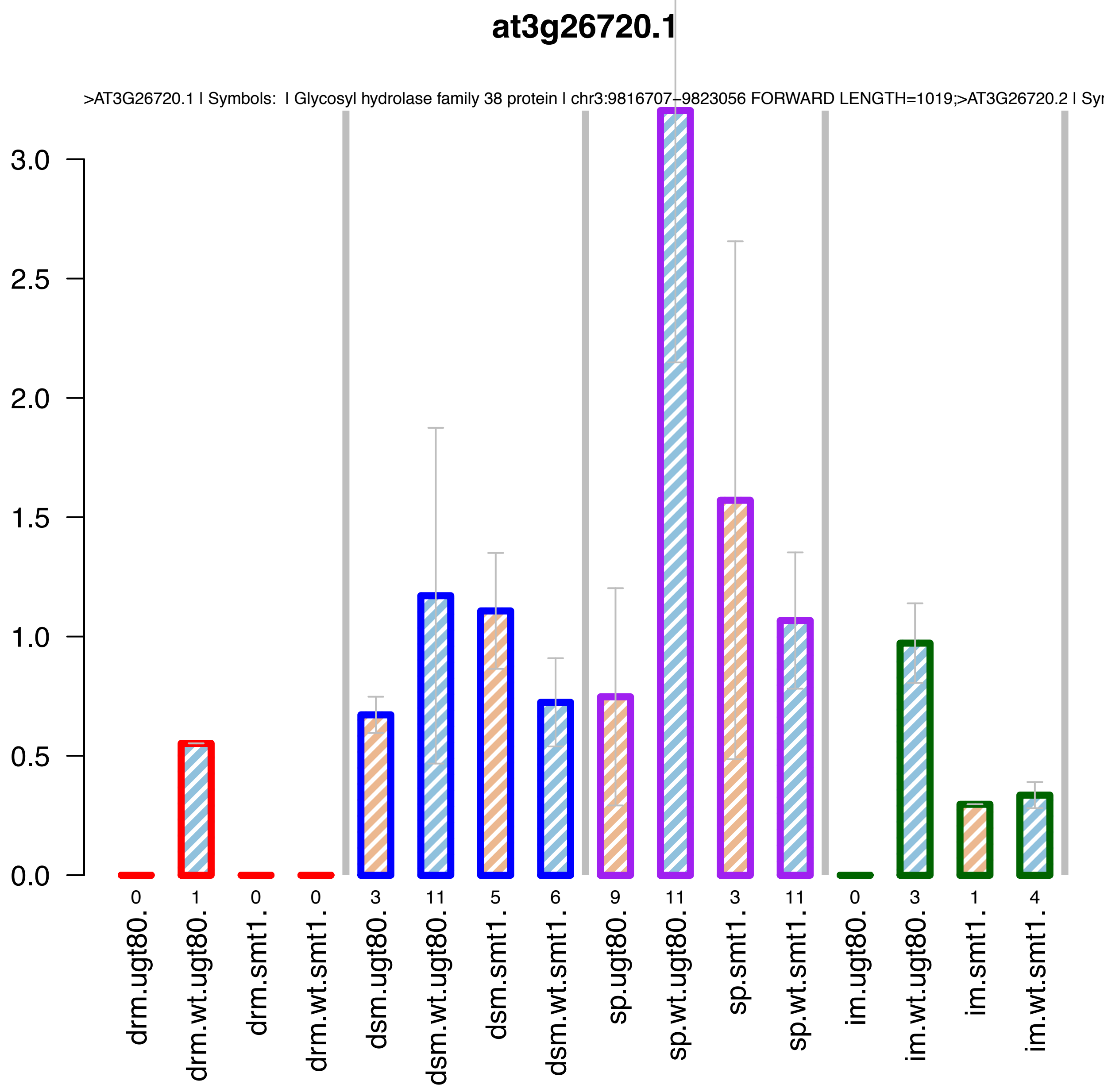

at3g26720.1
>AT3G26720.1 | Symbols: | Glycosyl hydrolase family 38 protein | chr3:9816707-9823056 FORWARD LENGTH=1019;>AT3G26720.2 | Sy
3.0
2.5
2.0
1.5
1.0
0.5
0.0
0
1
0
0
3
11
5
6
9
11
3
11
0
3
1
4
drm.ugt80.
drm.wt.ugt80.
drm.smt1.
drm.wt.smt1.
dsm.ugt80.
dsm.wt.ugt80.
dsm.smt1.
dsm.wt.smt1.
sp.ugt80.
sp.wt.ugt80.
sp.smt1.
sp.wt.smt1.
im.ugt80.
im.wt.ugt80.
im.smt1.
im.wt.smt1.

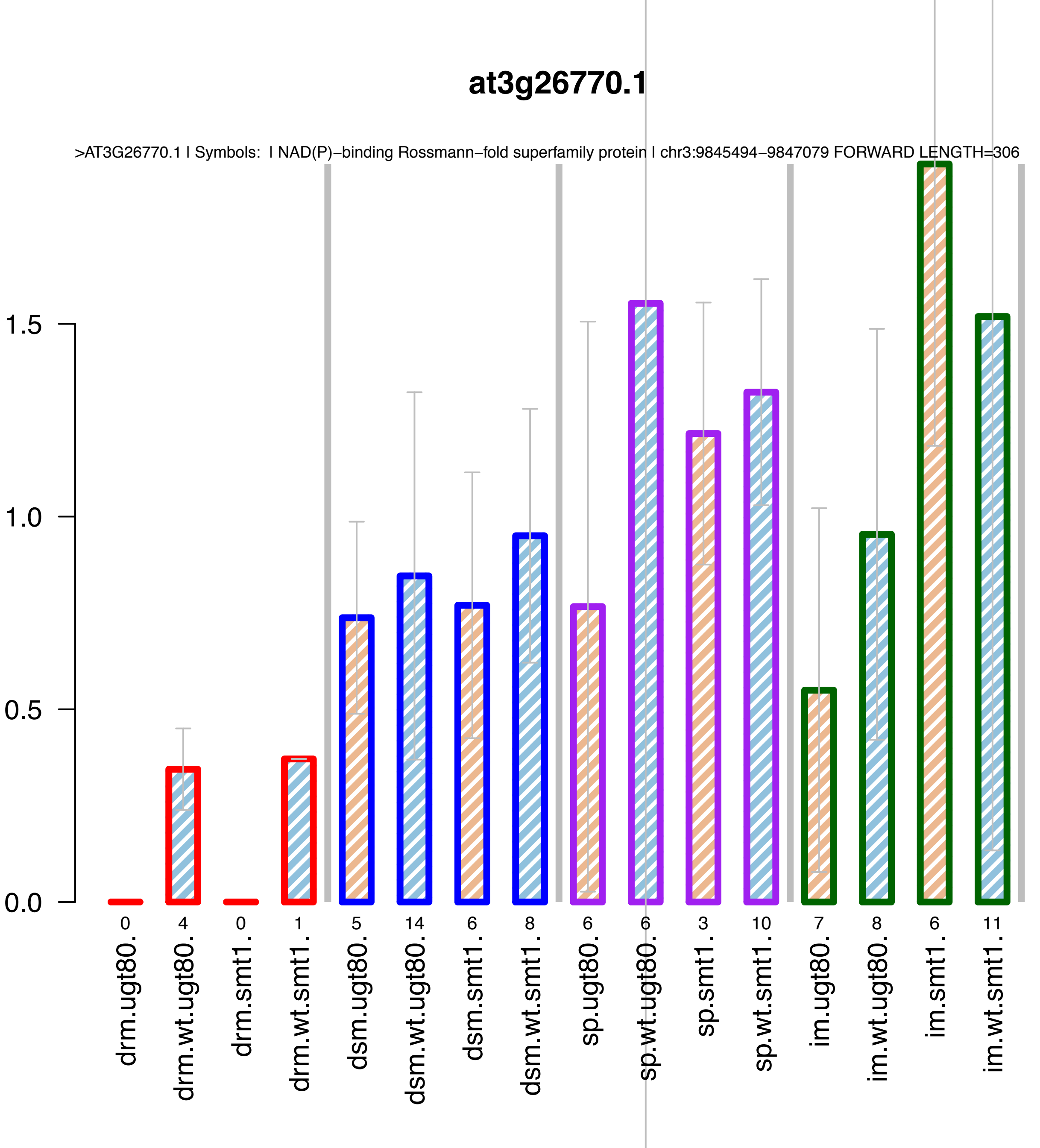

at3g26770.1
>AT3G26770.1 | Symbols: | NAD(P)−binding Rossmann−fold superfamily protein | chr3:9845494−9847079 FORWARD LENGTH=306
0.0
0.5
1.0
1.5
0
4
0
1
5
14
6
8
6
6
3
10
7
8
6
11
drm.ugt80.
drm.wt.ugt80.
drm.smt1.
drm.wt.smt1.
dsm.ugt80.
dsm.wt.ugt80.
dsm.smt1.
dsm.wt.smt1.
sp.ugt80.
sp.wt.ugt80.
sp.smt1.
sp.wt.smt1.
im.ugt80.
im.wt.ugt80.
im.smt1.
im.wt.smt1.

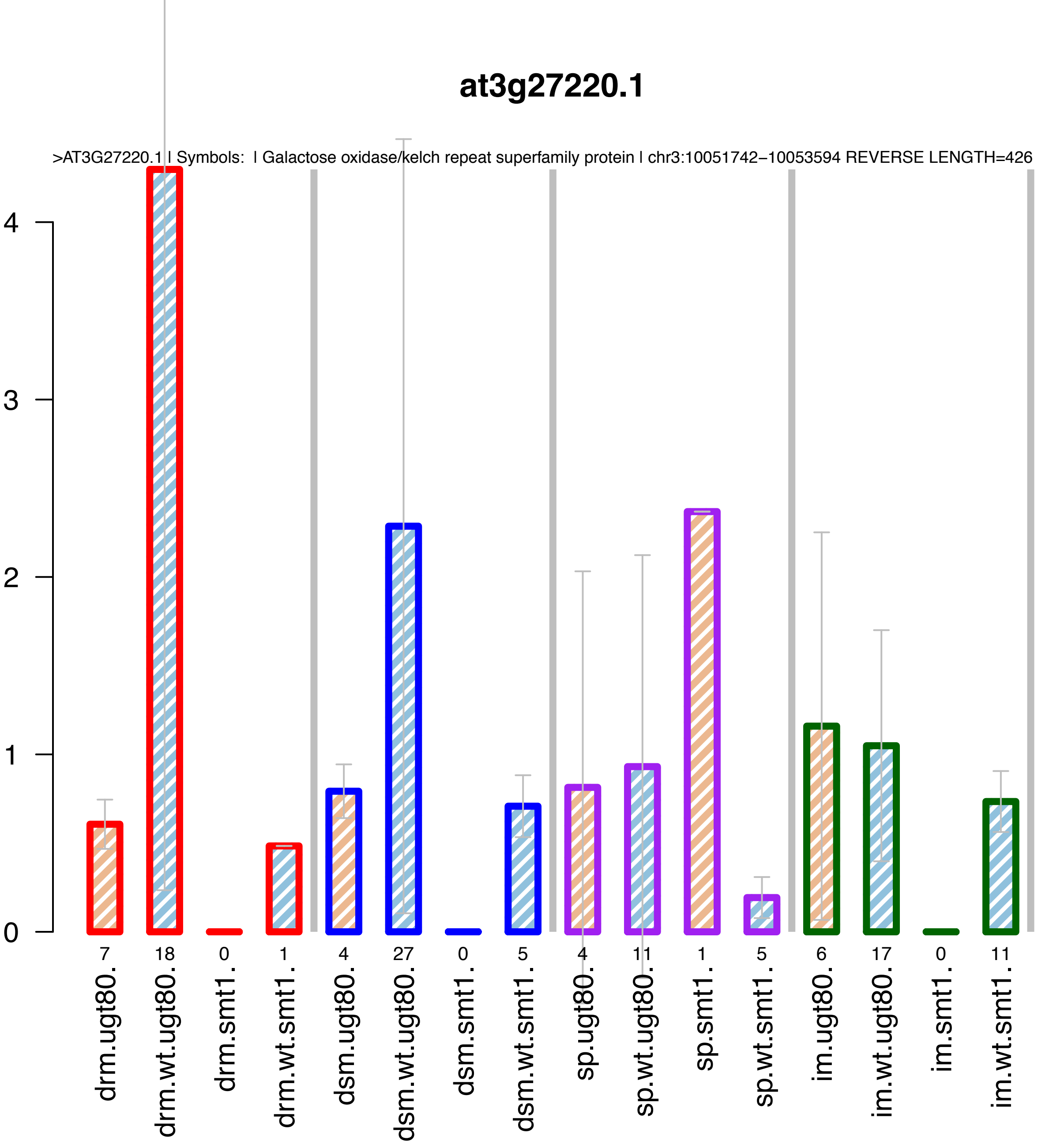

at3g27220.1
>AT3G27220.1 | Symbols: | Galactose oxidase/kelch repeat superfamily protein | chr3:10051742-10053594 REVERSE LENGTH=426
0
1
2
3
4
7
18
0
1
4
27
0
5
4
11
1
5
6
17
0
11
drm.ugt80.
drm.wt.ugt80.
drm.smt1.
drm.wt.smt1.
dsm.ugt80.
dsm.wt.ugt80.
dsm.smt1.
dsm.wt.smt1.
sp.ugt80.
sp.wt.ugt80.
sp.smt1.
sp.wt.smt1.
im.ugt80.
im.wt.ugt80.
im.smt1.
im.wt.smt1.

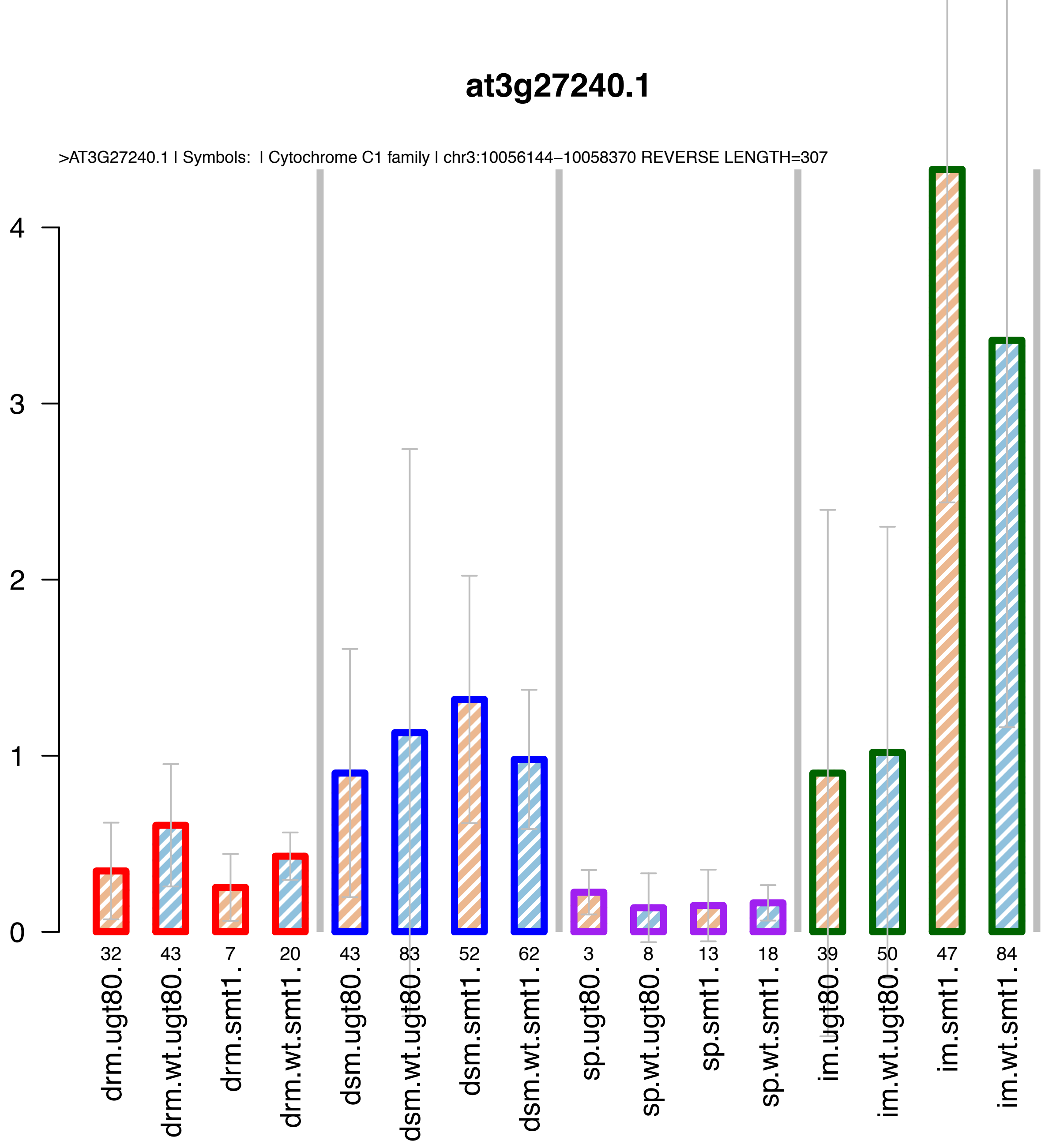
at3g27240.1
>AT3G27240.1 | Symbols: | Cytochrome C1 family | chr3:10056144-10058370 REVERSE LENGTH=307
0
1
2
3
4
32
43
7
20
43
83
52
62
3
8
13
18
39
50
47
84
drm.ugt80..
drm.wt.ugt80..
drm.smt1..
drm.wt.smt1..
dsm.ugt80..
dsm.wt.ugt80..
dsm.smt1..
dsm.wt.smt1..
sp.ugt80..
sp.wt.ugt80..
sp.smt1..
sp.wt.smt1..
im.ugt80..
im.wt.ugt80..
im.smt1..
im.wt.smt1..

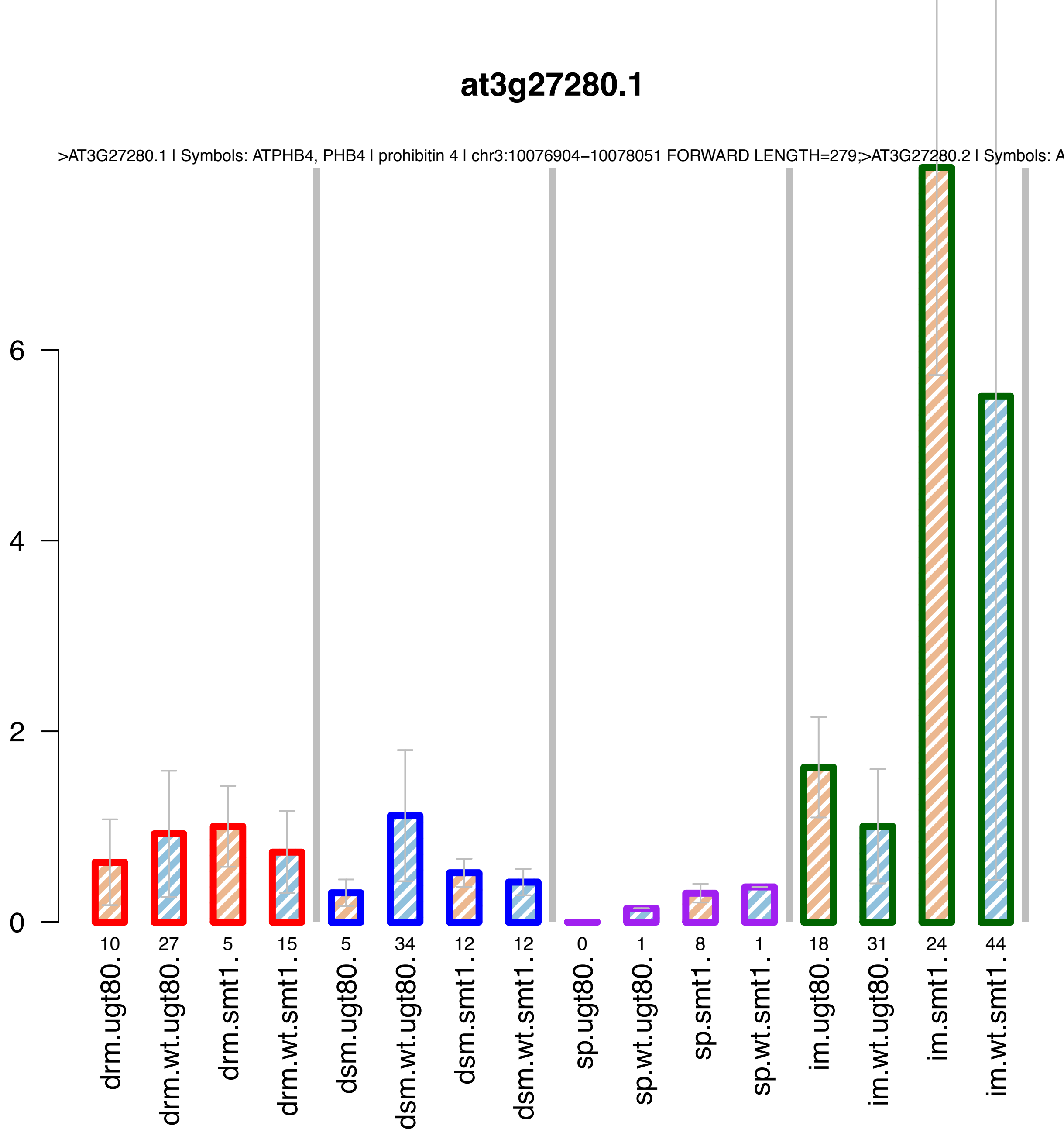

at3g27280.1
>AT3G27280.1 | Symbols: ATPHB4, PHB4 | prohibitin 4 | chr3:10076904–10078051 FORWARD LENGTH=279;>AT3G27280.2 | Symbols: A
0
2
4
6
10
27
5
15
5
34
12
12
0
1
8
1
18
31
24
44
drm.ugt80..
drm.wt.ugt80..
drm.smt1..
drm.wt.smt1..
dsm.ugt80..
dsm.wt.ugt80..
dsm.smt1..
dsm.wt.smt1..
sp.ugt80..
sp.wt.ugt80..
sp.smt1..
sp.wt.smt1..
im.ugt80..
im.wt.ugt80..
im.smt1..
im.wt.smt1..

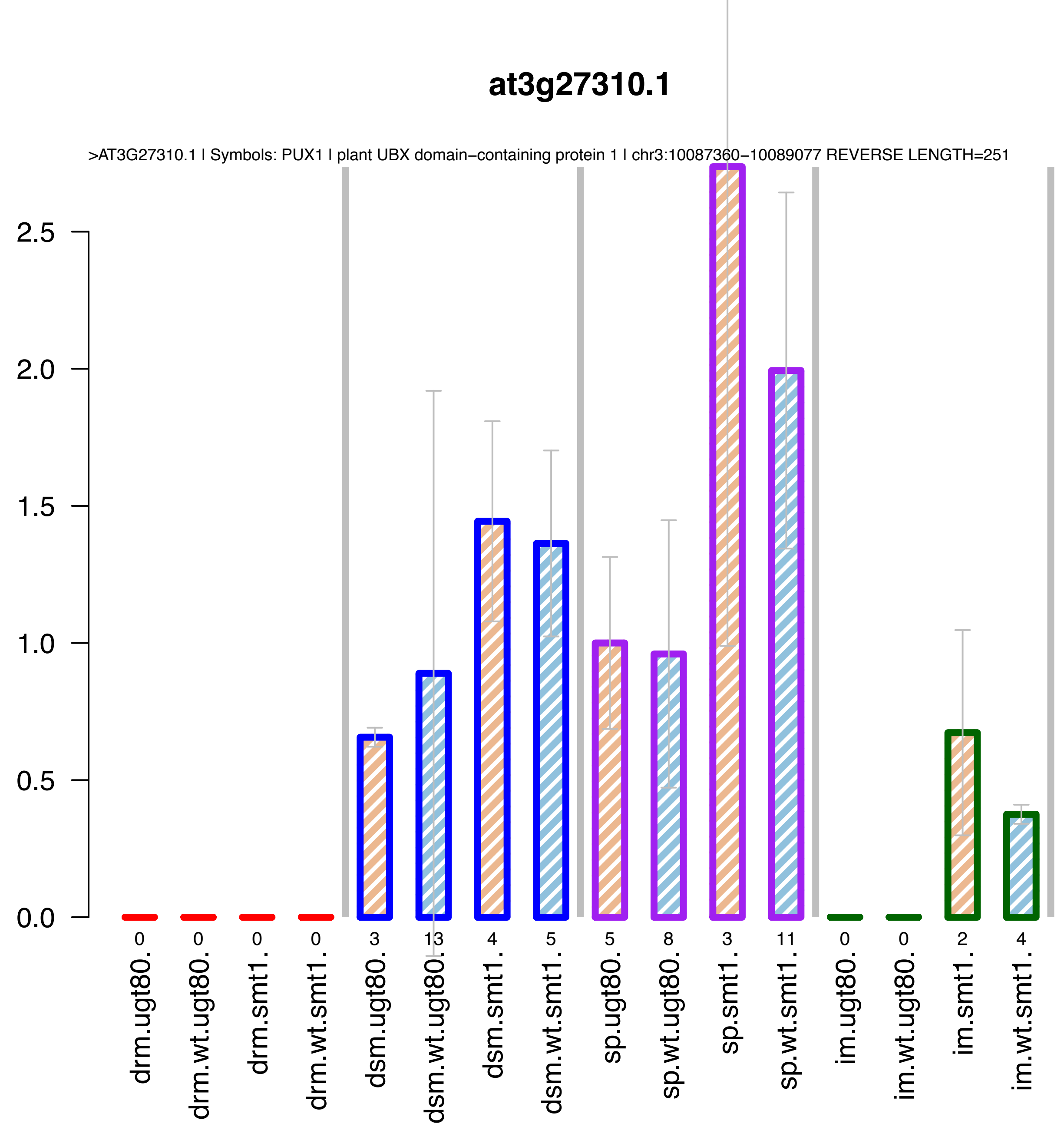
at3g27310.1
>AT3G27310.1 | Symbols: PUX1 | plant UBX domain−containing protein 1 | chr3:10087360−10089077 REVERSE LENGTH=251
0.0
0.5
1.0
1.5
2.0
2.5
0
0
0
0
3
13
4
5
5
8
3
11
0
0
2
4
drm.ugt80.
drm.wt.ugt80.
drm.smt1.
drm.wt.smt1.
dsm.ugt80.
dsm.wt.ugt80.
dsm.smt1.
dsm.wt.smt1.
sp.ugt80.
sp.wt.ugt80.
sp.smt1.
sp.wt.smt1.
im.ugt80.
im.wt.ugt80.
im.smt1.
im.wt.smt1.

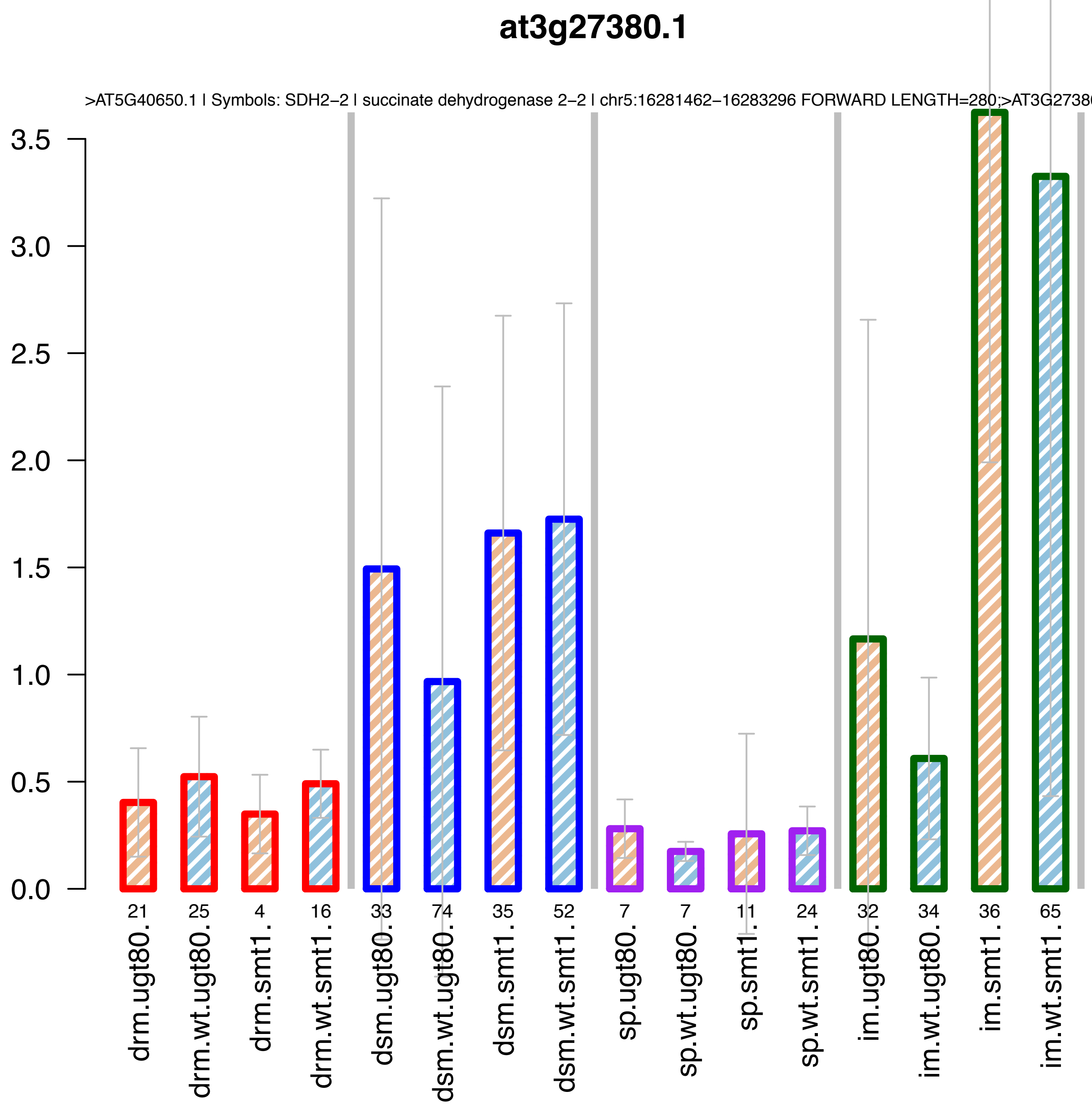

at3g27380.1
>AT5G40650.1 | Symbols: SDH2−2 | succinate dehydrogenase 2−2 | chr5:16281462−16283296 FORWARD LENGTH=280;>AT3G27380.1 |
3.5
3.0
2.5
2.0
1.5
1.0
0.5
0.0
21
25
4
16
33
74
35
52
7
7
11
24
32
34
36
65
drm.ugt80..
drm.wt.ugt80..
drm.smt1..
drm.wt.smt1..
dsm.ugt80..
dsm.wt.ugt80..
dsm.smt1..
dsm.wt.smt1..
sp.ugt80..
sp.wt.ugt80..
sp.smt1..
sp.wt.smt1..
im.ugt80..
im.wt.ugt80..
im.smt1..
im.wt.smt1..

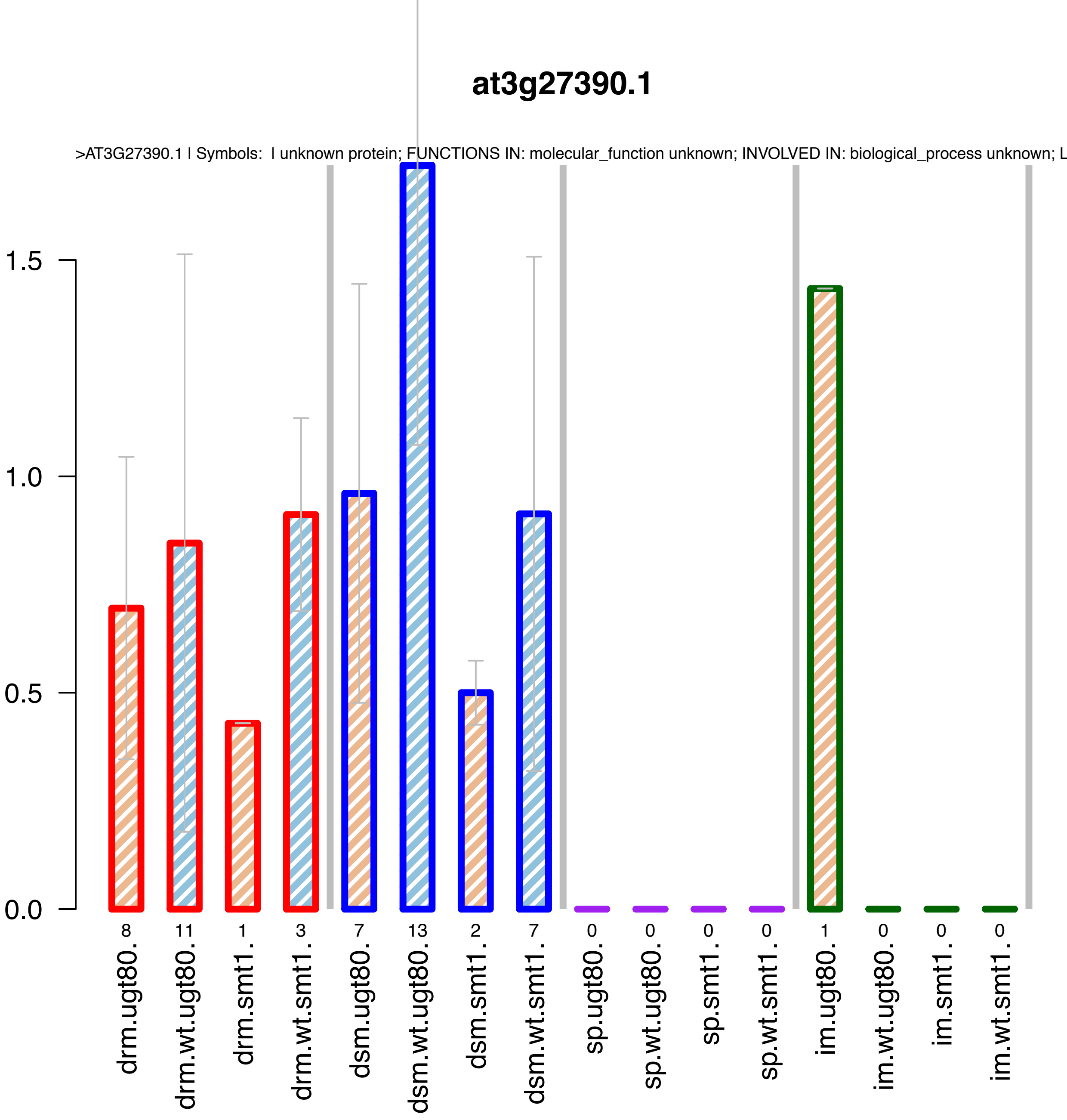

at3g27390.1
>AT3G27390.1 | Symbols: | unknown protein; FUNCTIONS IN: molecular_function unknown; INVOLVED IN: biological_process unknown; L
1.5
1.0
0.5
0.0
8
11
1
3
7
13
2
7
0
0
0
0
1
0
0
0
drm.ugt80.
drm.wt.ugt80.
drm.smt1.
drm.wt.smt1.
dsm.ugt80.
dsm.wt.ugt80.
dsm.smt1.
dsm.wt.smt1.
sp.ugt80.
sp.wt.ugt80.
sp.smt1.
sp.wt.smt1.
im.ugt80.
im.wt.ugt80.
im.smt1.
im.wt.smt1.

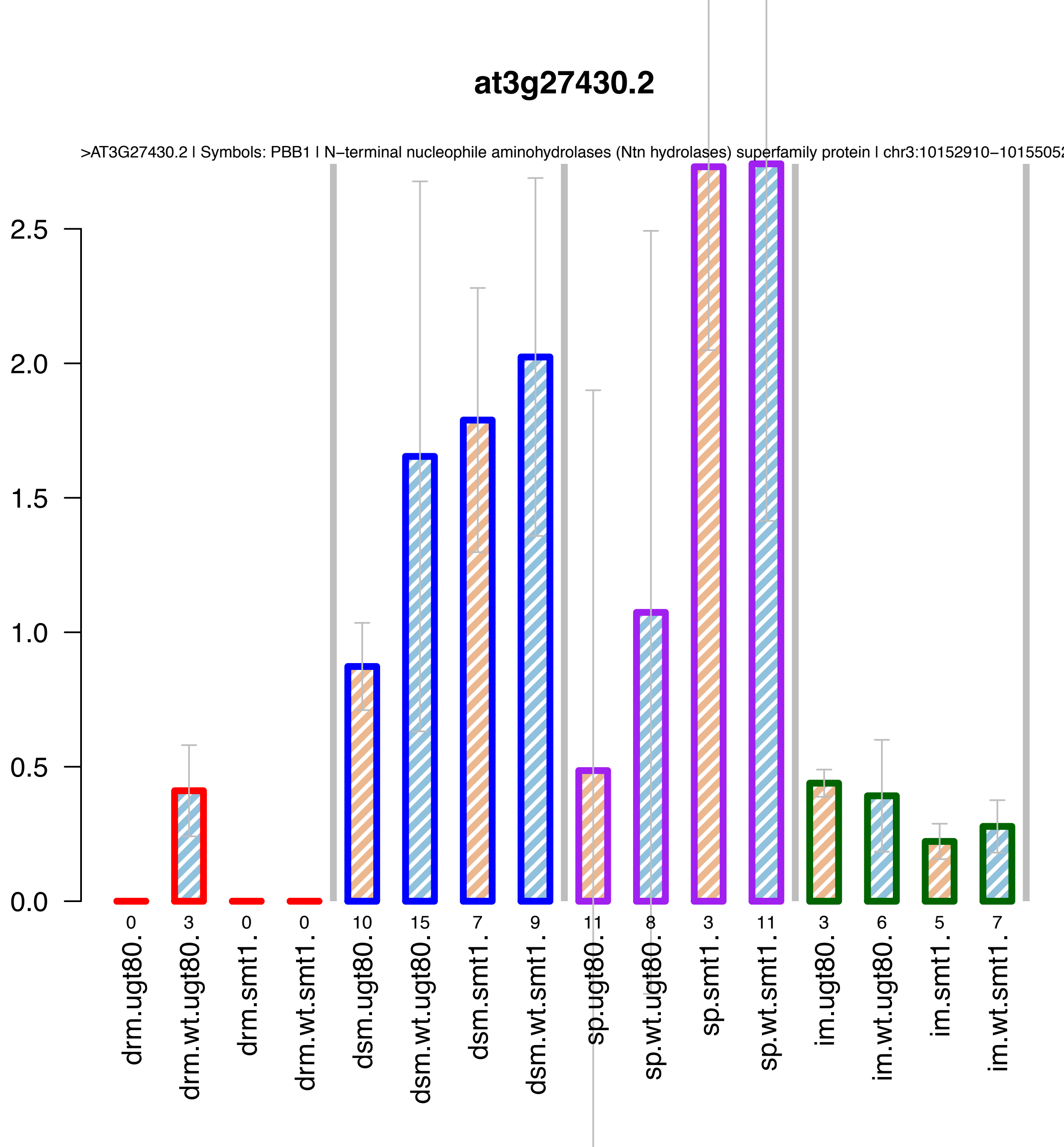

at3g27430.2
>AT3G27430.2 | Symbols: PBB1 | N–terminal nucleophile aminohydrolases (Ntn hydrolases) superfamily protein | chr3:10152910–1015505
0.0
0.5
1.0
1.5
2.0
2.5
0 3 0 0 10 15 7 9 11 8 3 11 3 6 5 7
drm.ugt80.
drm.wt.ugt80.
drm.smt1.
drm.wt.smt1.
dsm.ugt80.
dsm.wt.ugt80.
dsm.smt1.
dsm.wt.smt1.
sp.ugt80.
sp.wt.ugt80.
sp.smt1.
sp.wt.smt1.
im.ugt80.
im.wt.ugt80.
im.smt1.
im.wt.smt1.

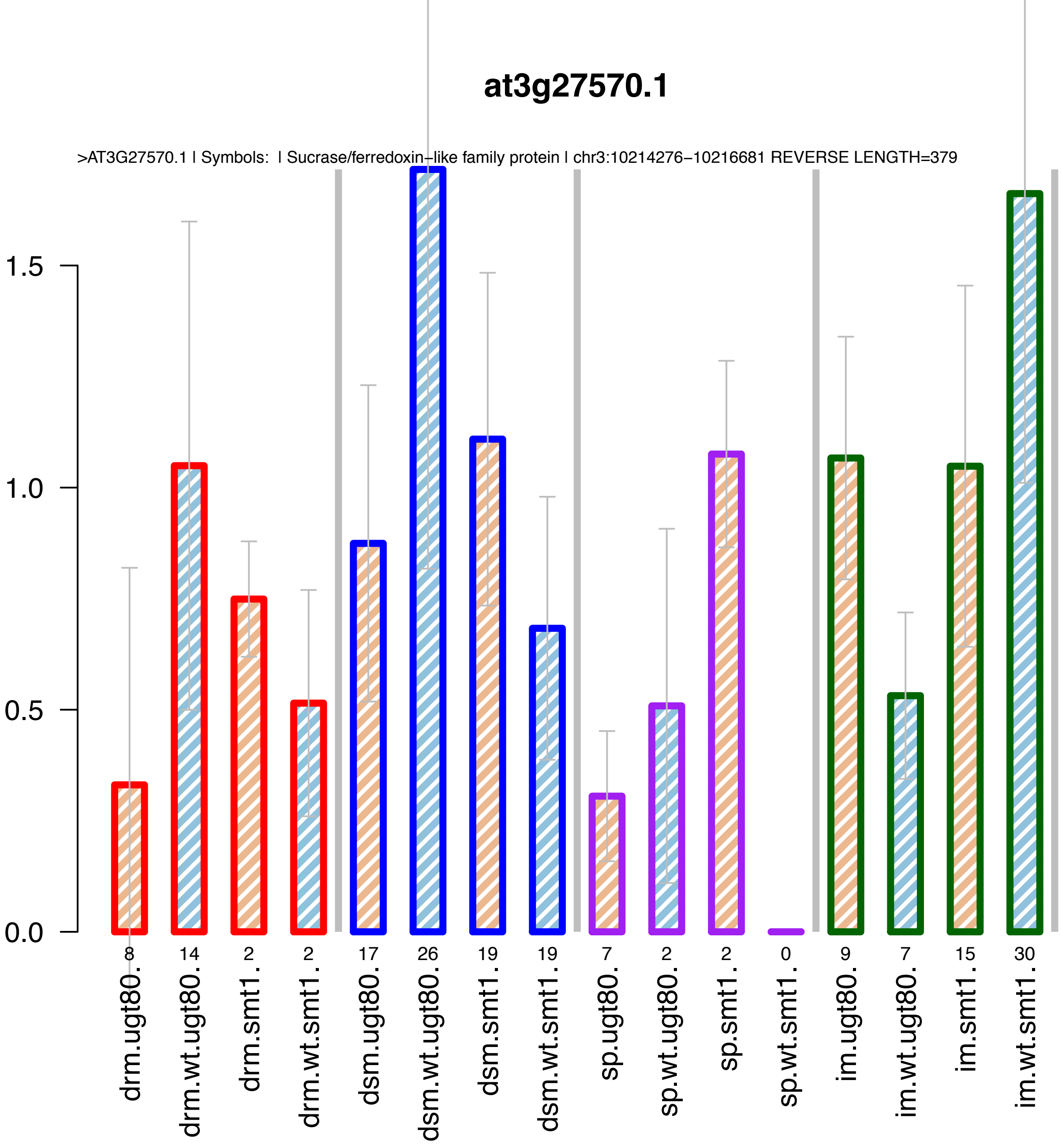

at3g27570.1
>AT3G27570.1 | Symbols: | Sucrase/ferredoxin−like family protein | chr3:10214276−10216681 REVERSE LENGTH=379
1.5
1.0
0.5
0.0
8
14
2
2
17
26
19
19
7
2
2
0
9
7
15
30
drm.ugt80..
drm.wt.ugt80..
drm.smt1..
drm.wt.smt1..
dsm.ugt80..
dsm.wt.ugt80..
dsm.smt1..
dsm.wt.smt1..
sp.ugt80..
sp.wt.ugt80..
sp.smt1..
sp.wt.smt1..
im.ugt80..
im.wt.ugt80..
im.smt1..
im.wt.smt1..

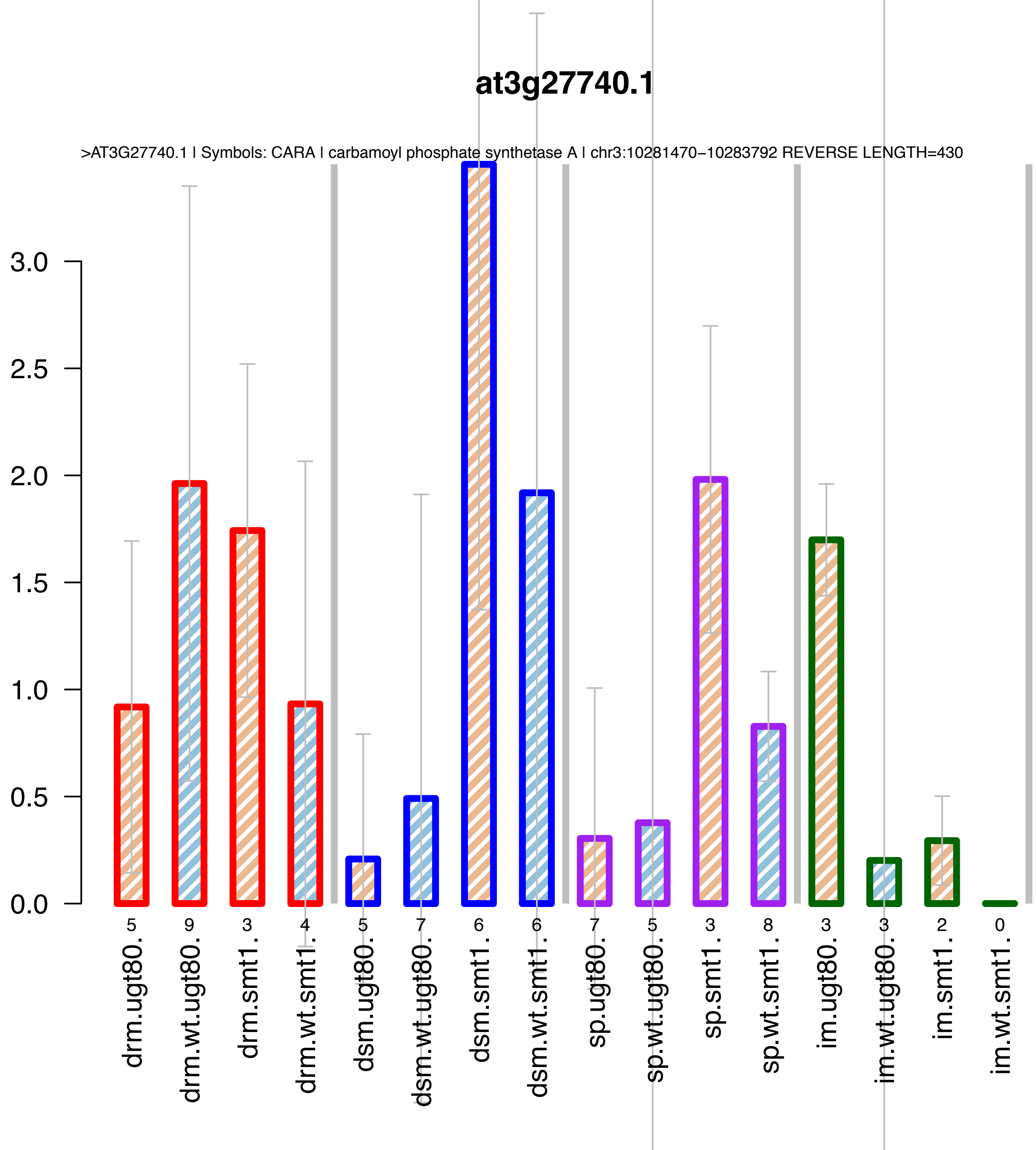

at3g27740.1
>AT3G27740.1 | Symbols: CARA | carbamoyl phosphate synthetase A | chr3:10281470-10283792 REVERSE LENGTH=430
0.0
0.5
1.0
1.5
2.0
2.5
3.0
5
9
3
4
5
7
6
6
7
5
3
8
3
3
2
0
drm.ugt80.
drm.wt.ugt80.
drm.smt1.
drm.wt.smt1.
dsm.ugt80.
dsm.wt.ugt80.
dsm.smt1.
dsm.wt.smt1.
sp.ugt80.
sp.wt.ugt80.
sp.smt1.
sp.wt.smt1.
im.ugt80.
im.wt.ugt80.
im.smt1.
im.wt.smt1.

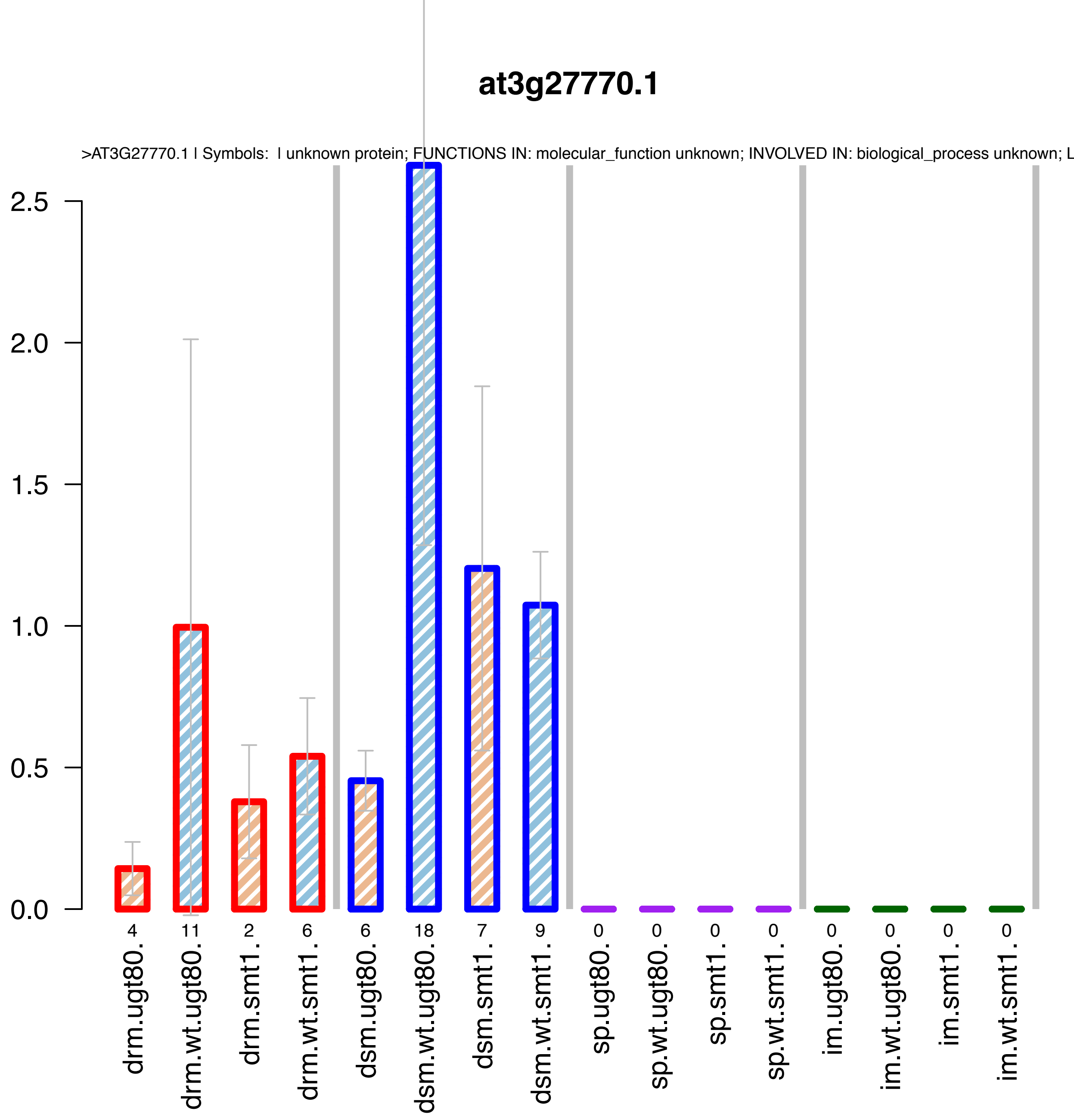

at3g27770.1
>AT3G27770.1 | Symbols: | unknown protein; FUNCTIONS IN: molecular_function unknown; INVOLVED IN: biological_process unknown; L
0.0
0.5
1.0
1.5
2.0
2.5
4
11
2
6
6
18
7
9
0
0
0
0
0
0
0
0
drm.ugt80.
drm.wt.ugt80.
drm.smt1.
drm.wt.smt1.
dsm.ugt80.
dsm.wt.ugt80.
dsm.smt1.
dsm.wt.smt1.
sp.ugt80.
sp.wt.ugt80.
sp.smt1.
sp.wt.smt1.
im.ugt80.
im.wt.ugt80.
im.smt1.
im.wt.smt1.

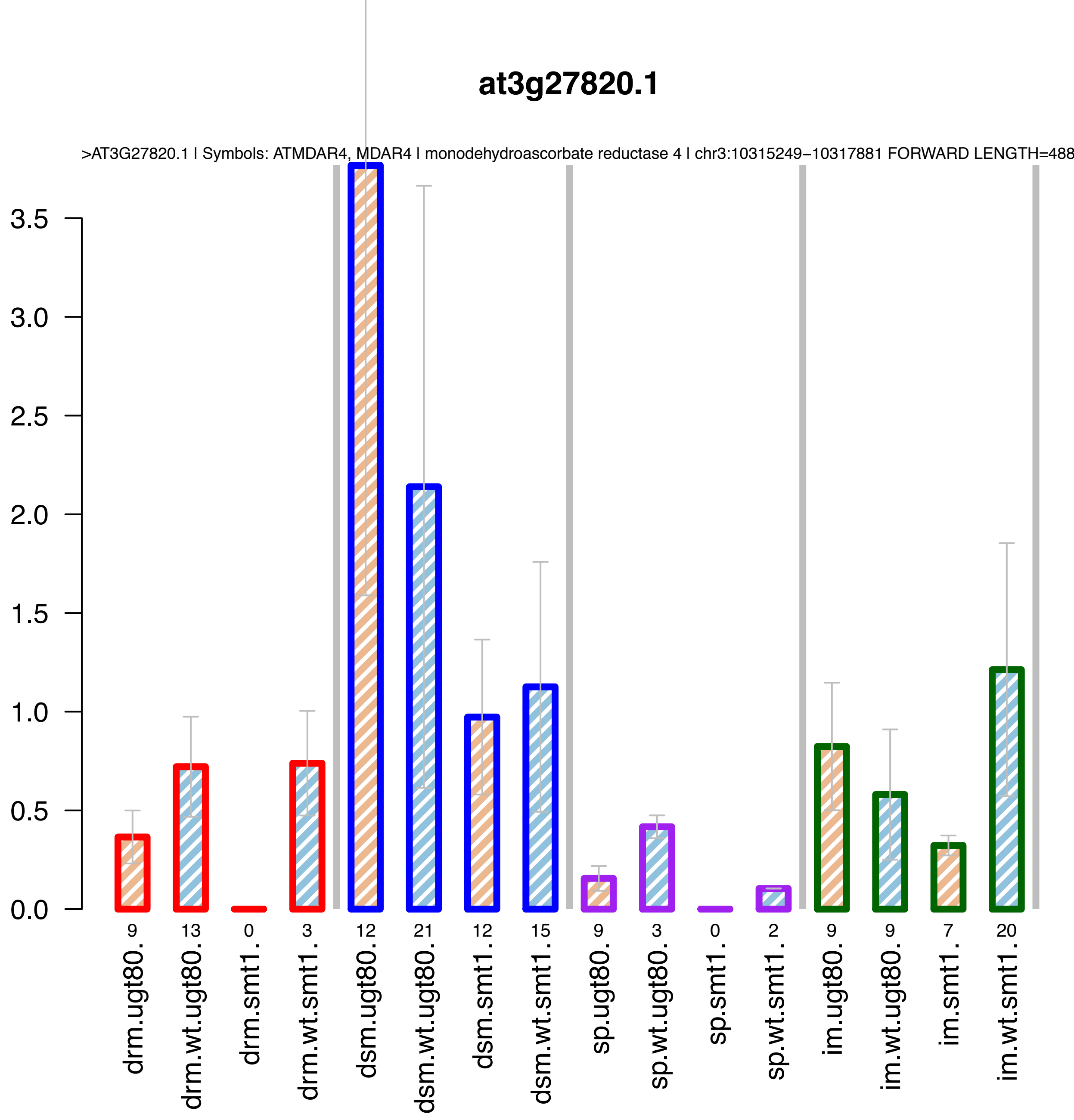
at3g27820.1
>AT3G27820.1 | Symbols: ATMDAR4, MDAR4 | monodehydroascorbate reductase 4 | chr3:10315249-10317881 FORWARD LENGTH=488
3.5
3.0
2.5
2.0
1.5
1.0
0.5
0.0
9
13
0
3
12
21
12
15
9
3
0
2
9
9
7
20
drm.ugt80.
drm.wt.ugt80.
drm.smt1.
drm.wt.smt1.
dsm.ugt80.
dsm.wt.ugt80.
dsm.smt1.
dsm.wt.smt1.
sp.ugt80.
sp.wt.ugt80.
sp.smt1.
sp.wt.smt1.
im.ugt80.
im.wt.ugt80.
im.smt1.
im.wt.smt1.

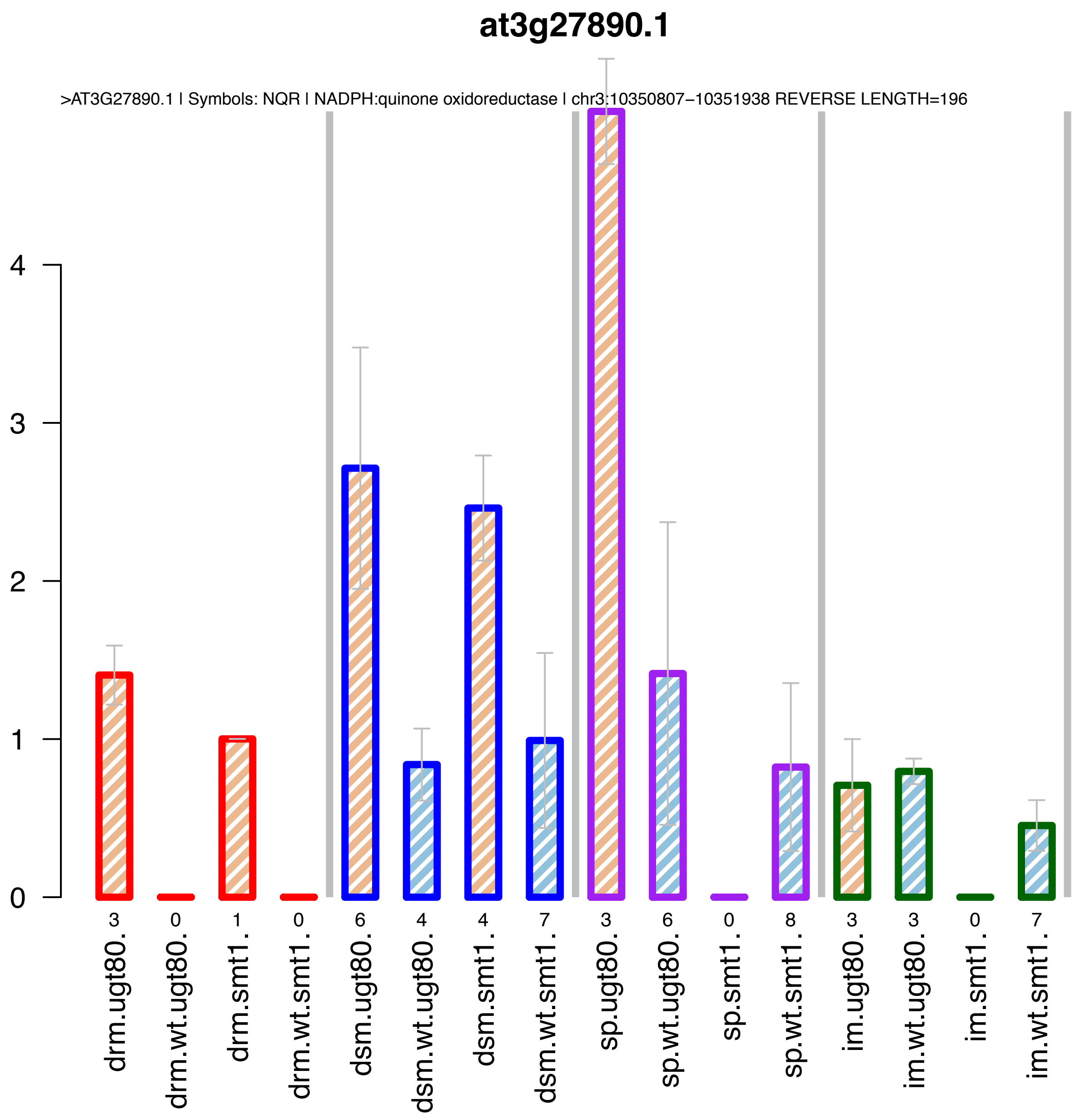

at3g27890.1
>AT3G27890.1 | Symbols: NQR | NADPH:quinone oxidoreductase | chr3:10350807−10351938 REVERSE LENGTH=196
0
1
2
3
4
3
0
1
0
6
4
4
7
3
6
0
8
3
3
0
7
drm.ugt80.
drm.wt.ugt80.
drm.smt1.
drm.wt.smt1.
dsm.ugt80.
dsm.wt.ugt80.
dsm.smt1.
dsm.wt.smt1.
sp.ugt80.
sp.wt.ugt80.
sp.smt1.
sp.wt.smt1.
im.ugt80.
im.wt.ugt80.
im.smt1.
im.wt.smt1.

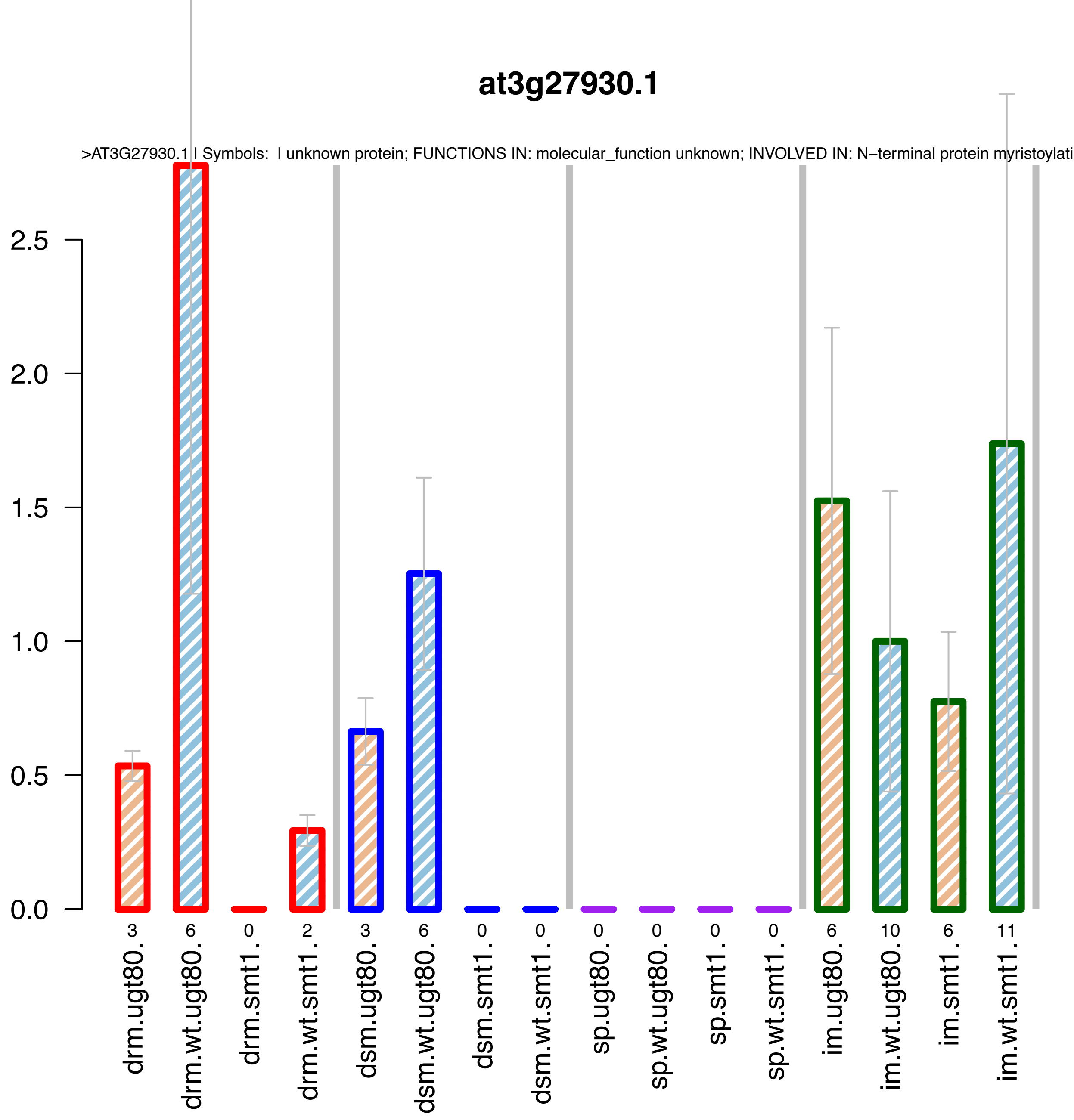
at3g27930.1
>AT3G27930.1 | Symbols: | unknown protein; FUNCTIONS IN: molecular_function unknown; INVOLVED IN: N–terminal protein myristoylati
0.0
0.5
1.0
1.5
2.0
2.5
3
6
0
2
3
6
0
0
0
0
0
0
6
10
6
11
drm.ugt80.
drm.wt.ugt80.
drm.smt1.
drm.wt.smt1.
dsm.ugt80.
dsm.wt.ugt80.
dsm.smt1.
dsm.wt.smt1.
sp.ugt80.
sp.wt.ugt80.
sp.smt1.
sp.wt.smt1.
im.ugt80.
im.wt.ugt80.
im.smt1.
im.wt.smt1.

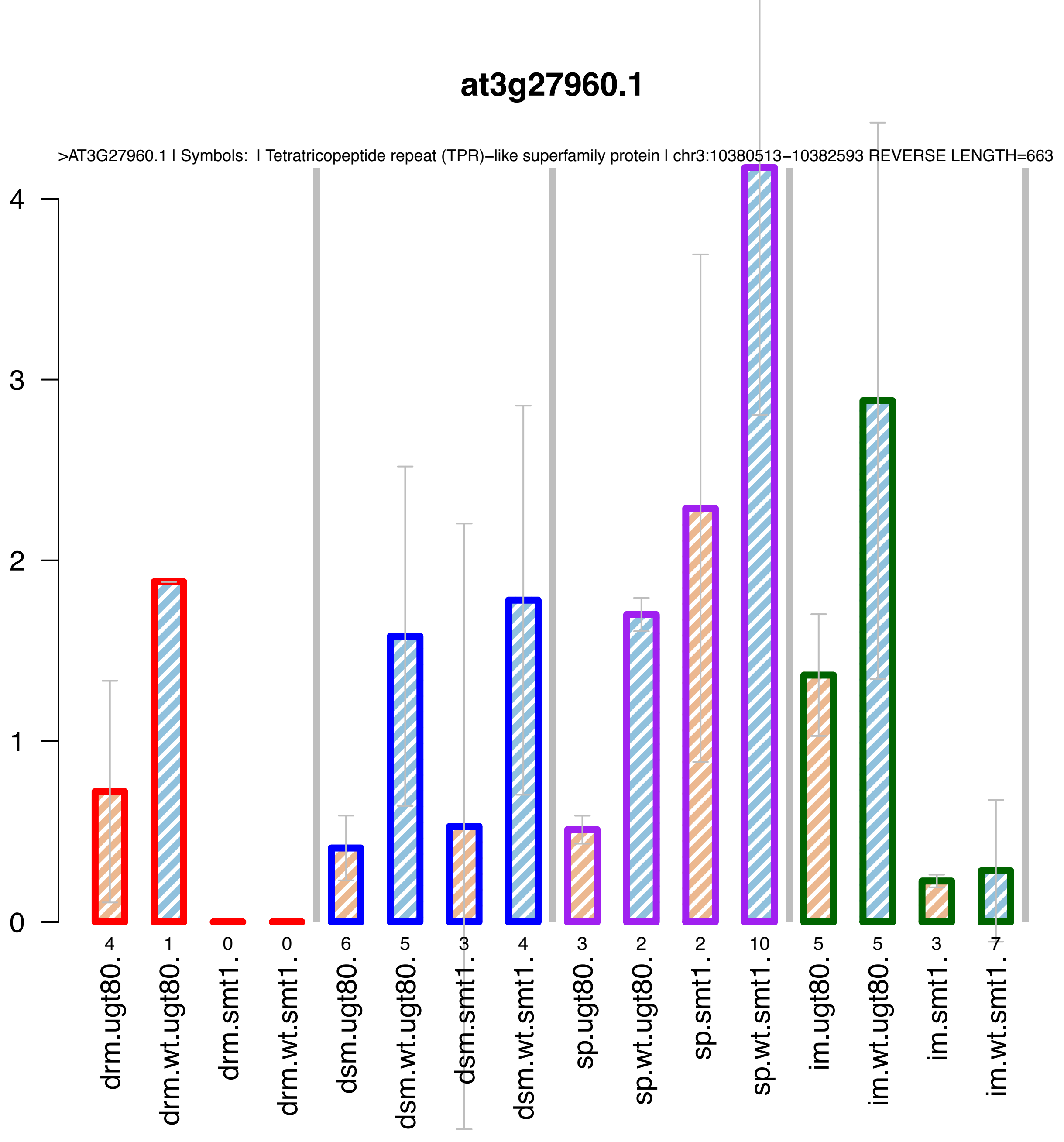

at3g27960.1
>AT3G27960.1 | Symbols: | Tetratricopeptide repeat (TPR)−like superfamily protein | chr3:10380513−10382593 REVERSE LENGTH=663
0
1
2
3
4
4
1
0
0
6
5
3
4
3
2
2
10
5
5
3
7
drm.ugt80..
drm.wt.ugt80..
drm.smt1..
drm.wt.smt1..
dsm.ugt80..
dsm.wt.ugt80..
dsm.smt1..
dsm.wt.smt1..
sp.ugt80..
sp.wt.ugt80..
sp.smt1..
sp.wt.smt1..
im.ugt80..
im.wt.ugt80..
im.smt1..
im.wt.smt1..

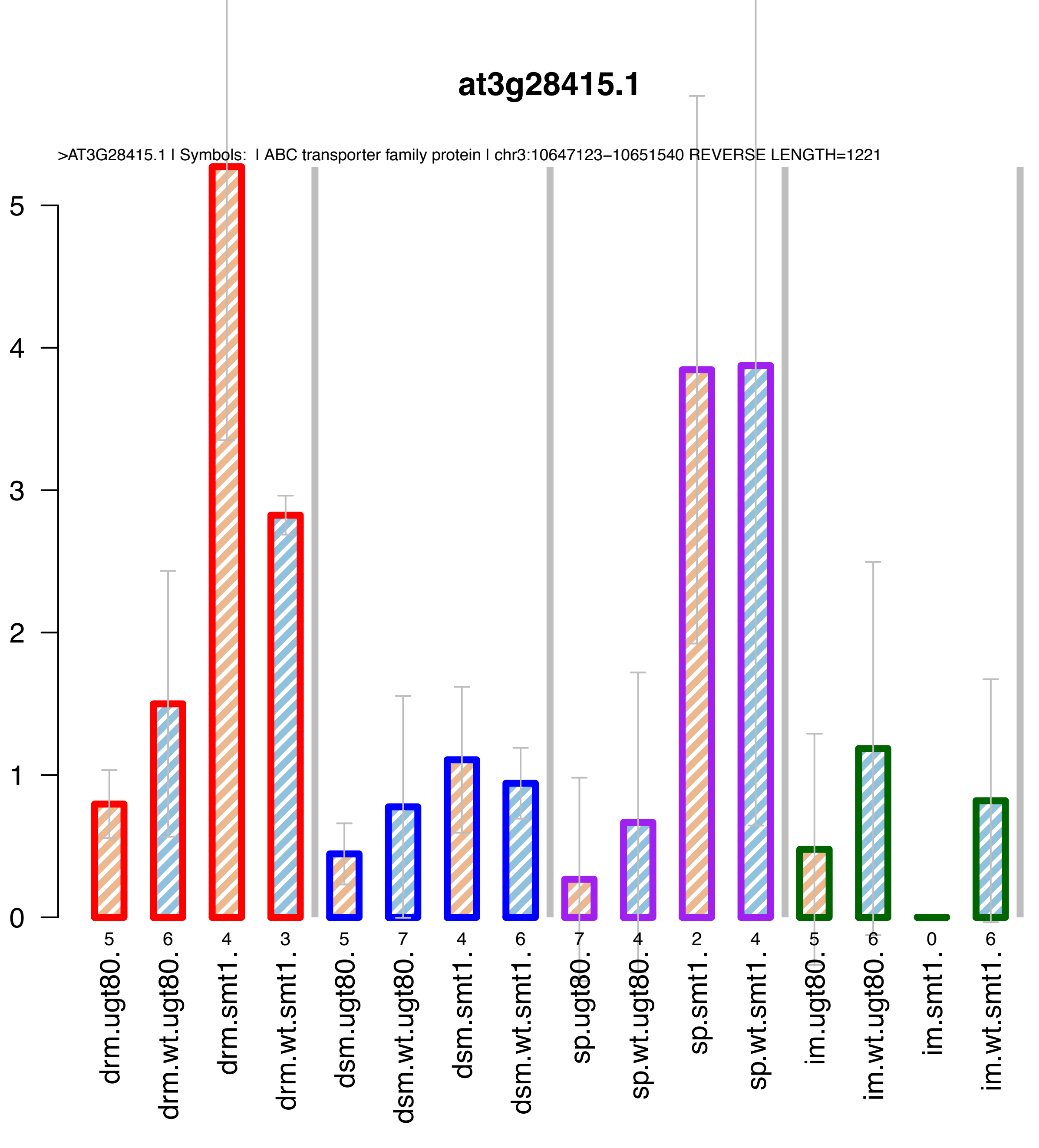

at3g28415.1
>AT3G28415.1 | Symbols: | ABC transporter family protein | chr3:10647123-10651540 REVERSE LENGTH=1221
0
1
2
3
4
5
5
6
4
3
5
7
4
6
7
4
2
4
5
6
0
6
drm.ugt80.
drm.wt.ugt80.
drm.smt1.
drm.wt.smt1.
dsm.ugt80.
dsm.wt.ugt80.
dsm.smt1.
dsm.wt.smt1.
sp.ugt80.
sp.wt.ugt80.
sp.smt1.
sp.wt.smt1.
im.ugt80.
im.wt.ugt80.
im.smt1.
im.wt.smt1.

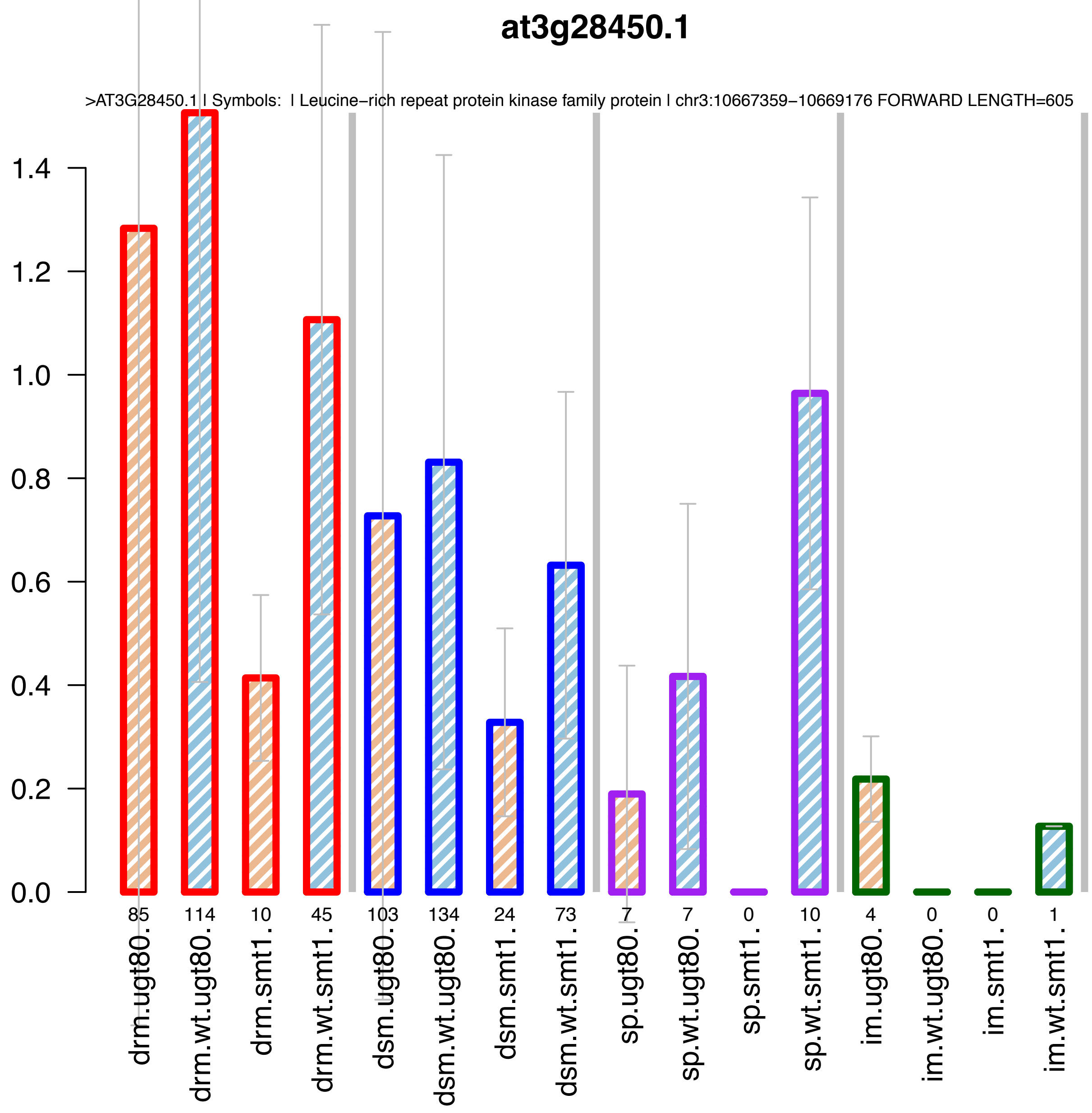

at3g28450.1
>AT3G28450.1 | Symbols: | Leucine−rich repeat protein kinase family protein | chr3:10667359−10669176 FORWARD LENGTH=605
1.4
1.2
1.0
0.8
0.6
0.4
0.2
0.0
85
114
10
45
103
134
24
73
7
7
0
10
4
0
0
1
drm.ugt80..
drm.wt.ugt80..
drm.smt1..
drm.wt.smt1..
dsm.ugt80..
dsm.wt.ugt80..
dsm.smt1..
dsm.wt.smt1..
sp.ugt80..
sp.wt.ugt80..
sp.smt1..
sp.wt.smt1..
im.ugt80..
im.wt.ugt80..
im.smt1..
im.wt.smt1..

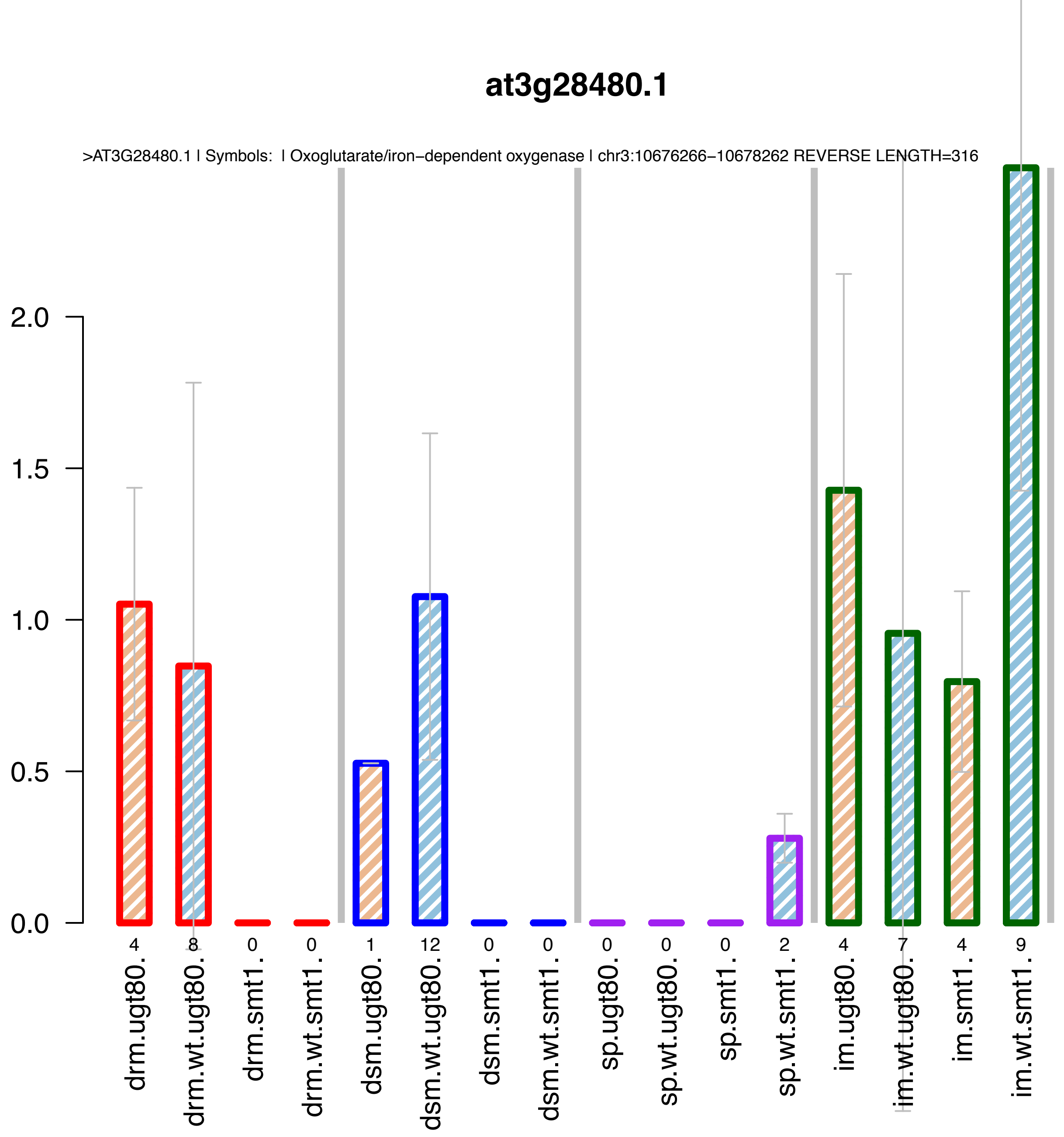

at3g28480.1
>AT3G28480.1 | Symbols: | Oxoglutarate/iron−dependent oxygenase | chr3:10676266−10678262 REVERSE LENGTH=316
0.0
0.5
1.0
1.5
2.0
4
8
0
0
1
12
0
0
0
0
0
2
4
7
4
9
drm.ugt80.
drm.wt.ugt80.
drm.smt1.
drm.wt.smt1.
dsm.ugt80.
dsm.wt.ugt80.
dsm.smt1.
dsm.wt.smt1.
sp.ugt80.
sp.wt.ugt80.
sp.smt1.
sp.wt.smt1.
im.ugt80.
im.wt.ugt80.
im.smt1.
im.wt.smt1.

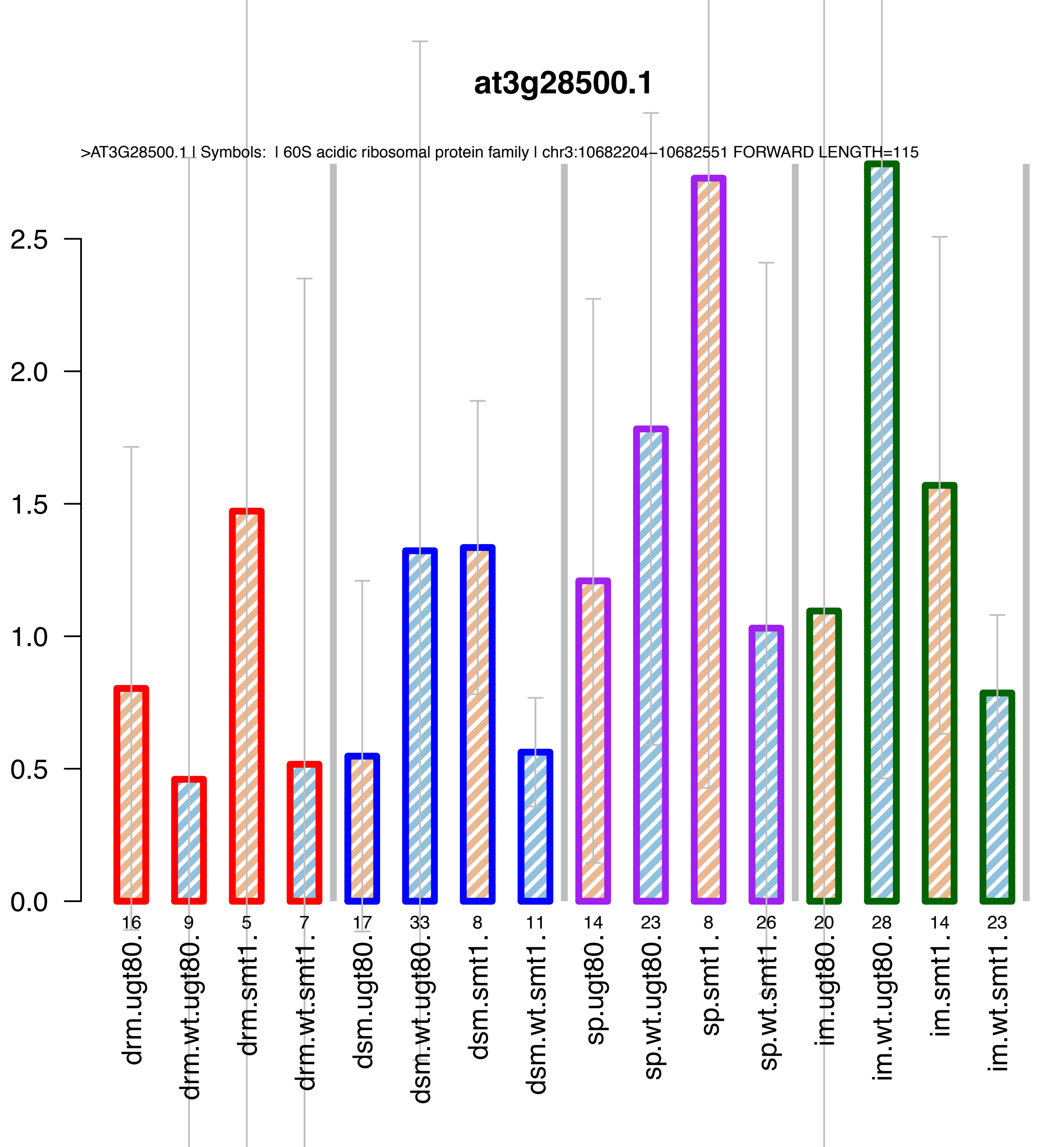
at3g28500.1
>AT3G28500.1 | Symbols: | 60S acidic ribosomal protein family | chr3:10682204-10682551 FORWARD LENGTH=115
0.0
0.5
1.0
1.5
2.0
2.5
16
9
5
7
17
33
8
11
14
23
8
26
20
28
14
23
drm.ugt80.
drm.wt.ugt80.
drm.smt1.
drm.wt.smt1.
dsm.ugt80.
dsm.wt.ugt80.
dsm.smt1.
dsm.wt.smt1.
sp.ugt80.
sp.wt.ugt80.
sp.smt1.
sp.wt.smt1.
im.ugt80.
im.wt.ugt80.
im.smt1.
im.wt.smt1.

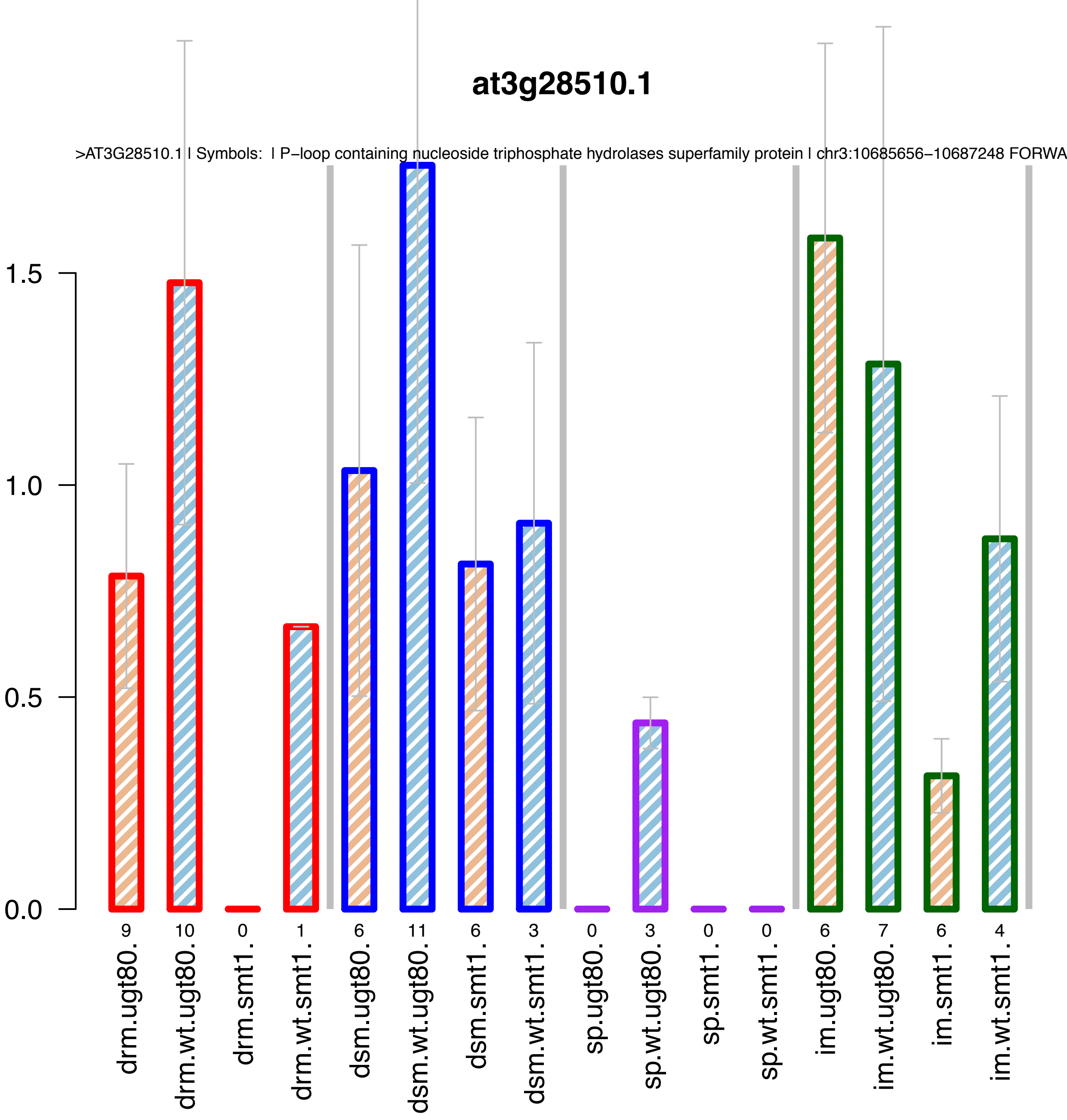
at3g28510.1
>AT3G28510.1 | Symbols: | P–loop containing nucleoside triphosphate hydrolases superfamily protein | chr3:10685656–10687248 FORWA
1.5
1.0
0.5
0.0
9
10
0
1
6
11
6
3
0
3
0
0
6
7
6
4
drm.ugt80.
drm.wt.ugt80.
drm.smt1.
drm.wt.smt1.
dsm.ugt80.
dsm.wt.ugt80.
dsm.smt1.
dsm.wt.smt1.
sp.ugt80.
sp.wt.ugt80.
sp.smt1.
sp.wt.smt1.
im.ugt80.
im.wt.ugt80.
im.smt1.
im.wt.smt1.

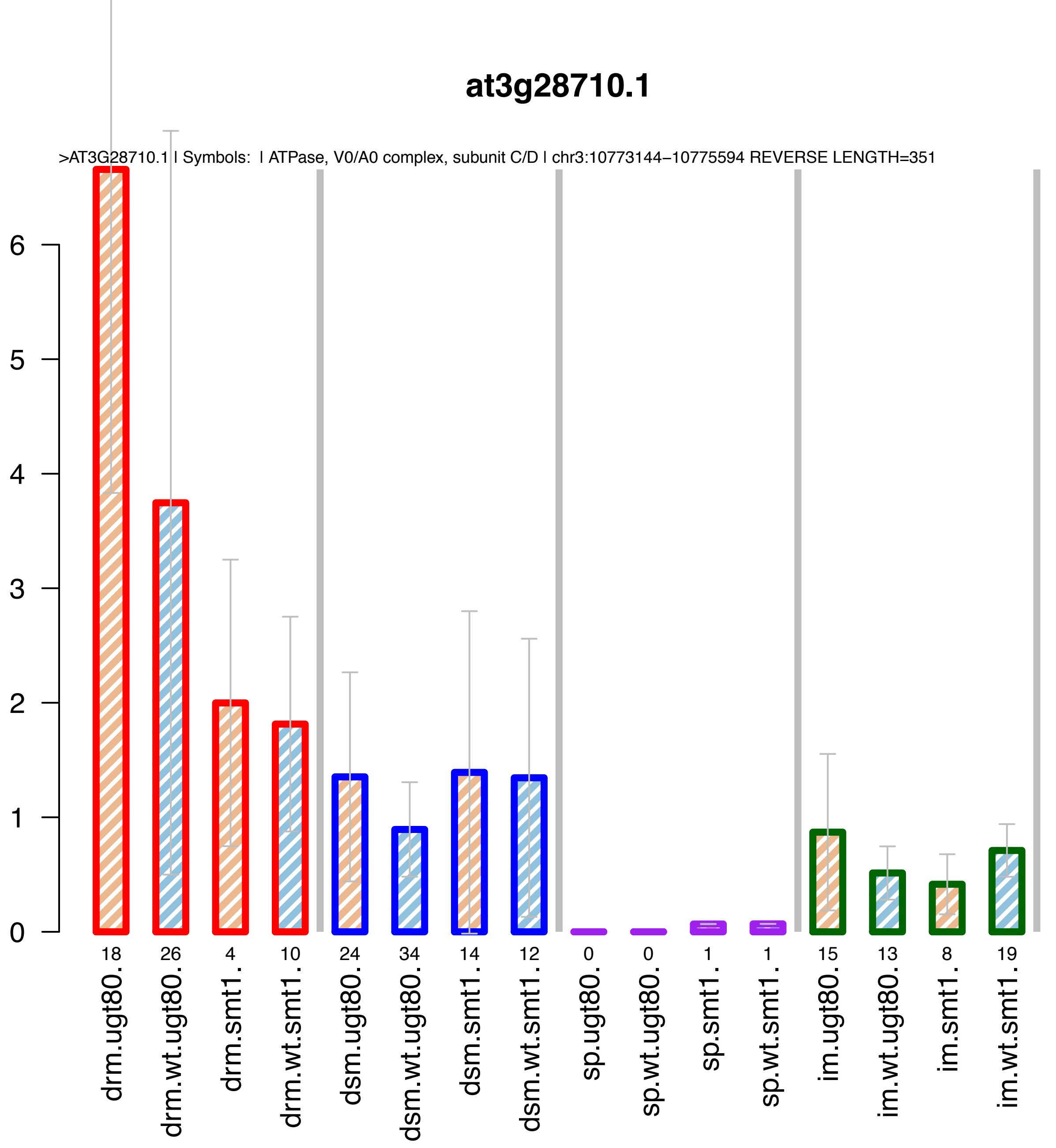
at3g28710.1
>AT3G28710.1 | Symbols: | ATPase, V0/A0 complex, subunit C/D | chr3:10773144-10775594 REVERSE LENGTH=351
0
1
2
3
4
5
6
18
26
4
10
24
34
14
12
0
0
1
1
15
13
8
19
drm.ugt80.
drm.wt.ugt80.
drm.smt1.
drm.wt.smt1.
dsm.ugt80.
dsm.wt.ugt80.
dsm.smt1.
dsm.wt.smt1.
sp.ugt80.
sp.wt.ugt80.
sp.smt1.
sp.wt.smt1.
im.ugt80.
im.wt.ugt80.
im.smt1.
im.wt.smt1.

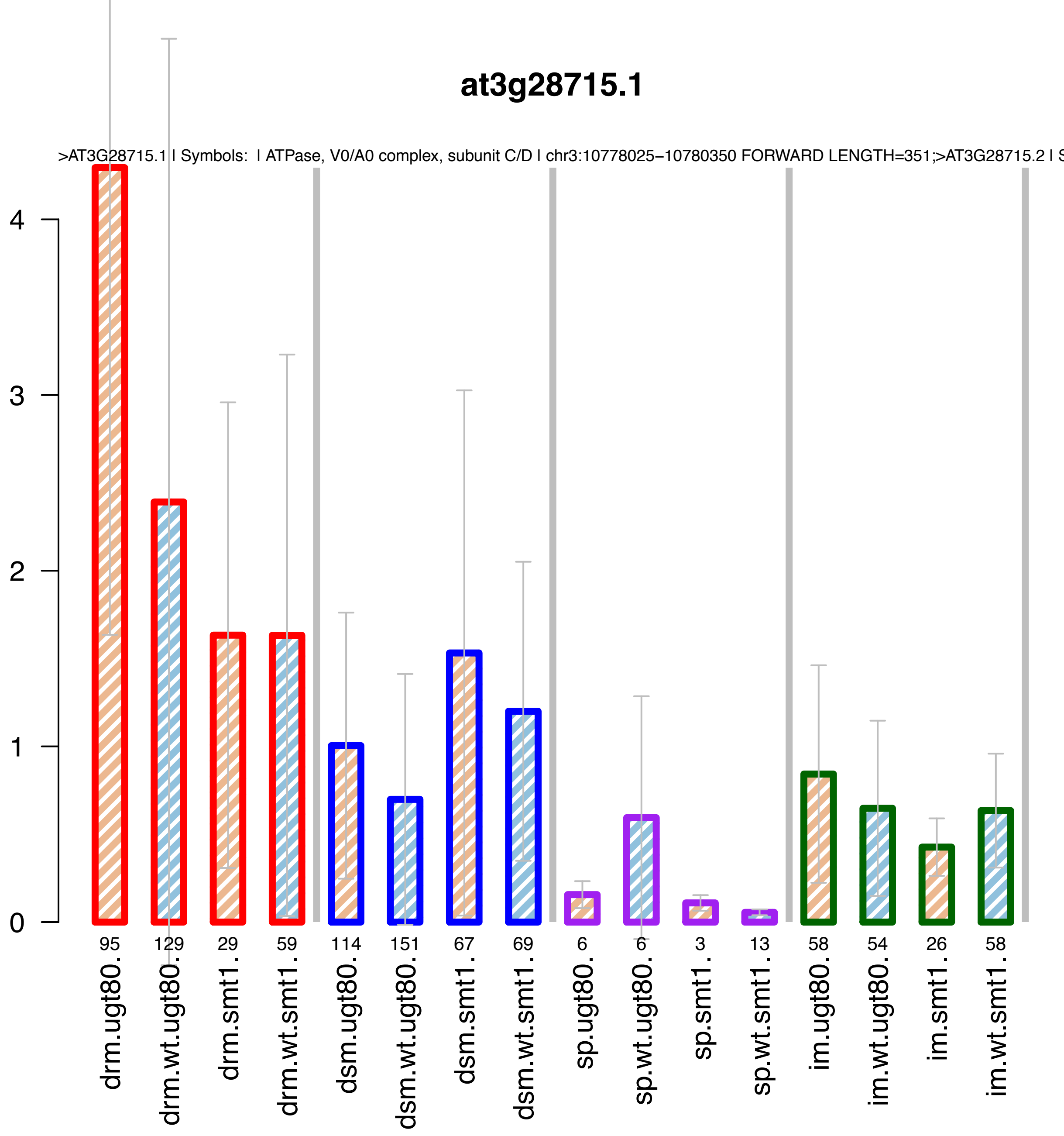

at3g28715.1
>AT3G28715.1 | Symbols: | ATPase, V0/A0 complex, subunit C/D | chr3:10778025-10780350 FORWARD LENGTH=351;>AT3G28715.2 | S
0
1
2
3
4
95
129
29
59
114
151
67
69
6
6
3
13
58
54
26
58
drm.ugt80.
drm.wt.ugt80.
drm.smt1.
drm.wt.smt1.
dsm.ugt80.
dsm.wt.ugt80.
dsm.smt1.
dsm.wt.smt1.
sp.ugt80.
sp.wt.ugt80.
sp.smt1.
sp.wt.smt1.
im.ugt80.
im.wt.ugt80.
im.smt1.
im.wt.smt1.

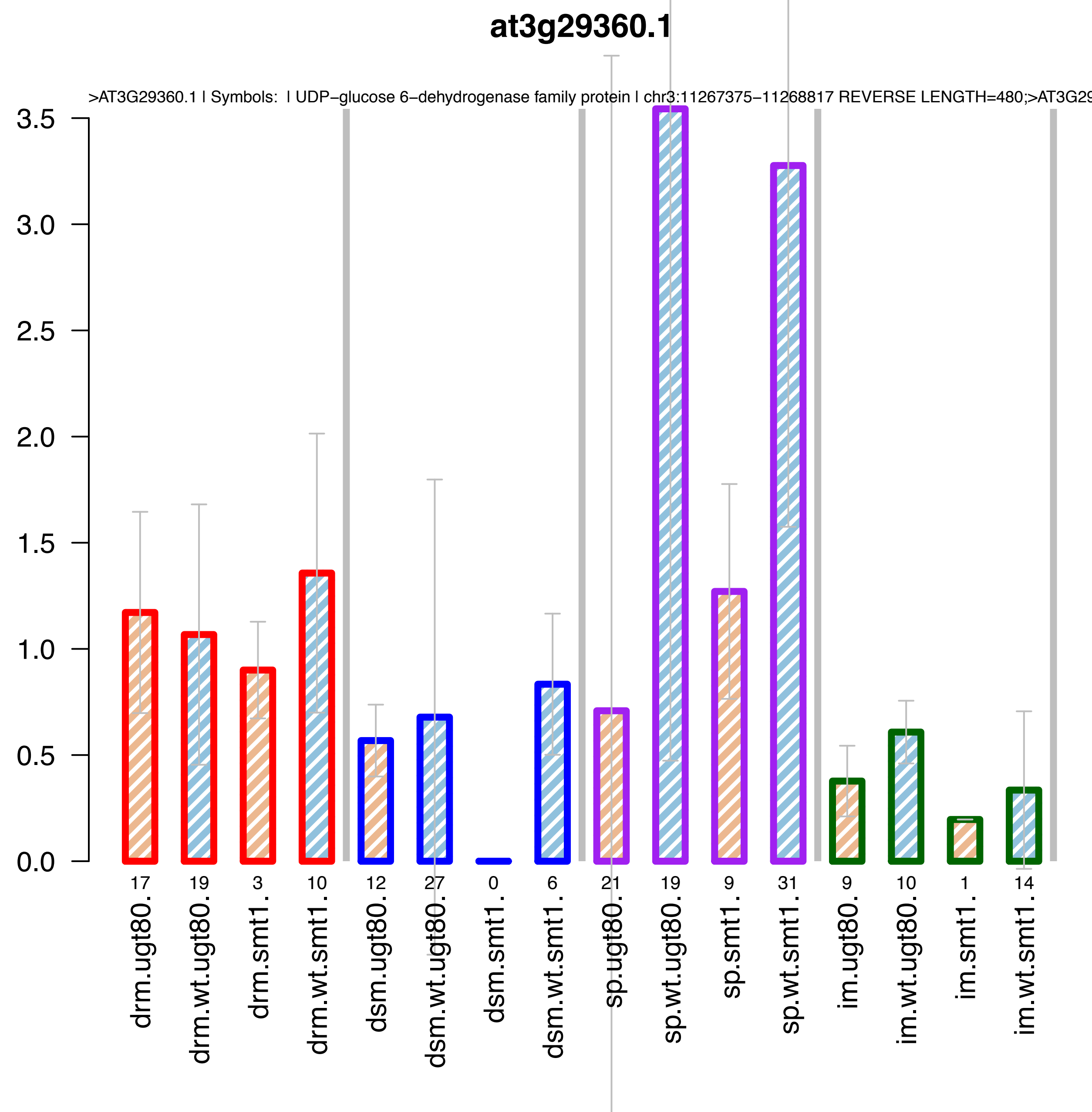

at3g29360.1
>AT3G29360.1 | Symbols: | UDP−glucose 6−dehydrogenase family protein | chr3:11267375−11268817 REVERSE LENGTH=480;>AT3G29
3.5
3.0
2.5
2.0
1.5
1.0
0.5
0.0
17
19
3
10
12
27
0
6
21
19
9
31
9
10
1
14
drm.ugt80..
drm.wt.ugt80..
drm.smt1..
drm.wt.smt1..
dsm.ugt80..
dsm.wt.ugt80..
dsm.smt1..
dsm.wt.smt1..
sp.ugt80..
sp.wt.ugt80..
sp.smt1..
sp.wt.smt1..
im.ugt80..
im.wt.ugt80..
im.smt1..
im.wt.smt1..

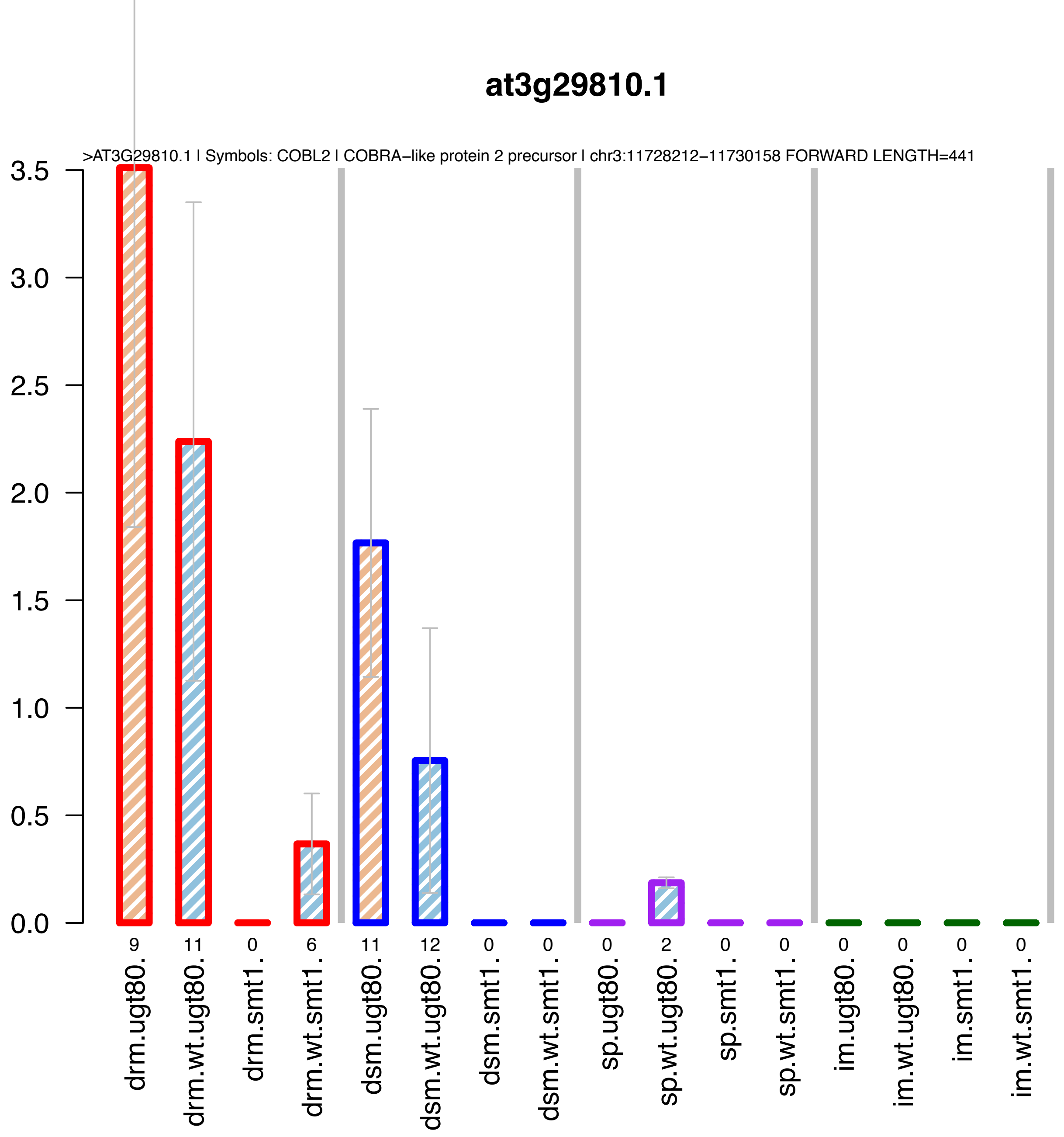

at3g29810.1
>AT3G29810.1 | Symbols: COBL2 | COBRA−like protein 2 precursor | chr3:11728212−11730158 FORWARD LENGTH=441
3.5
3.0
2.5
2.0
1.5
1.0
0.5
0.0
9
11
0
6
11
12
0
0
0
2
0
0
0
0
0
0
drm.ugt80.
drm.wt.ugt80.
drm.smt1.
drm.wt.smt1.
dsm.ugt80.
dsm.wt.ugt80.
dsm.smt1.
dsm.wt.smt1.
sp.ugt80.
sp.wt.ugt80.
sp.smt1.
sp.wt.smt1.
im.ugt80.
im.wt.ugt80.
im.smt1.
im.wt.smt1.

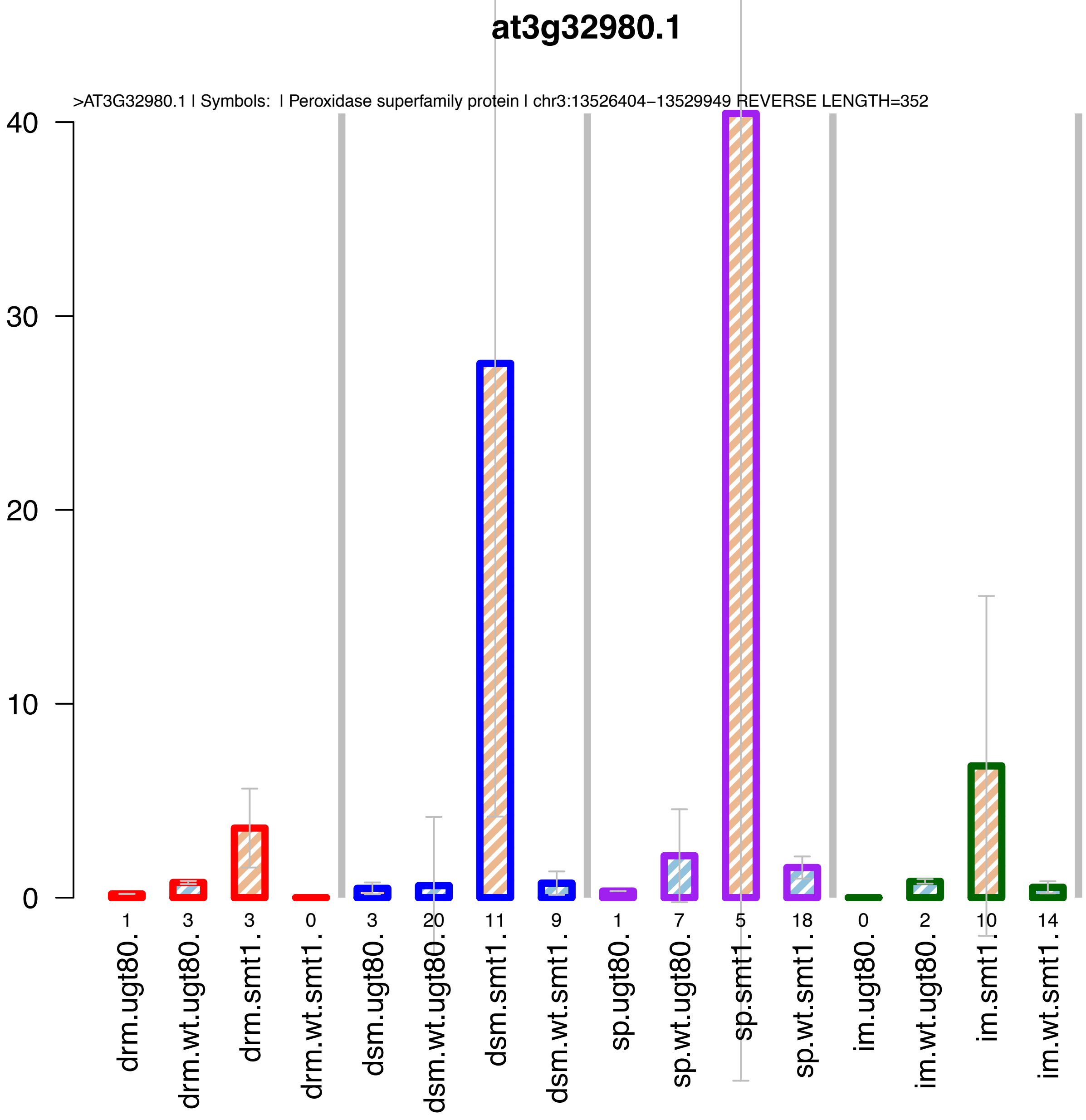

at3g32980.1
>AT3G32980.1 | Symbols: | Peroxidase superfamily protein | chr3:13526404−13529949 REVERSE LENGTH=352
40
30
20
10
0
1
3
3
0
3
20
11
9
1
7
5
18
0
2
10
14
drm.ugt80.
drm.wt.ugt80.
drm.smt1.
drm.wt.smt1.
dsm.ugt80.
dsm.wt.ugt80.
dsm.smt1.
dsm.wt.smt1.
sp.ugt80.
sp.wt.ugt80.
sp.smt1.
sp.wt.smt1.
im.ugt80.
im.wt.ugt80.
im.smt1.
im.wt.smt1.

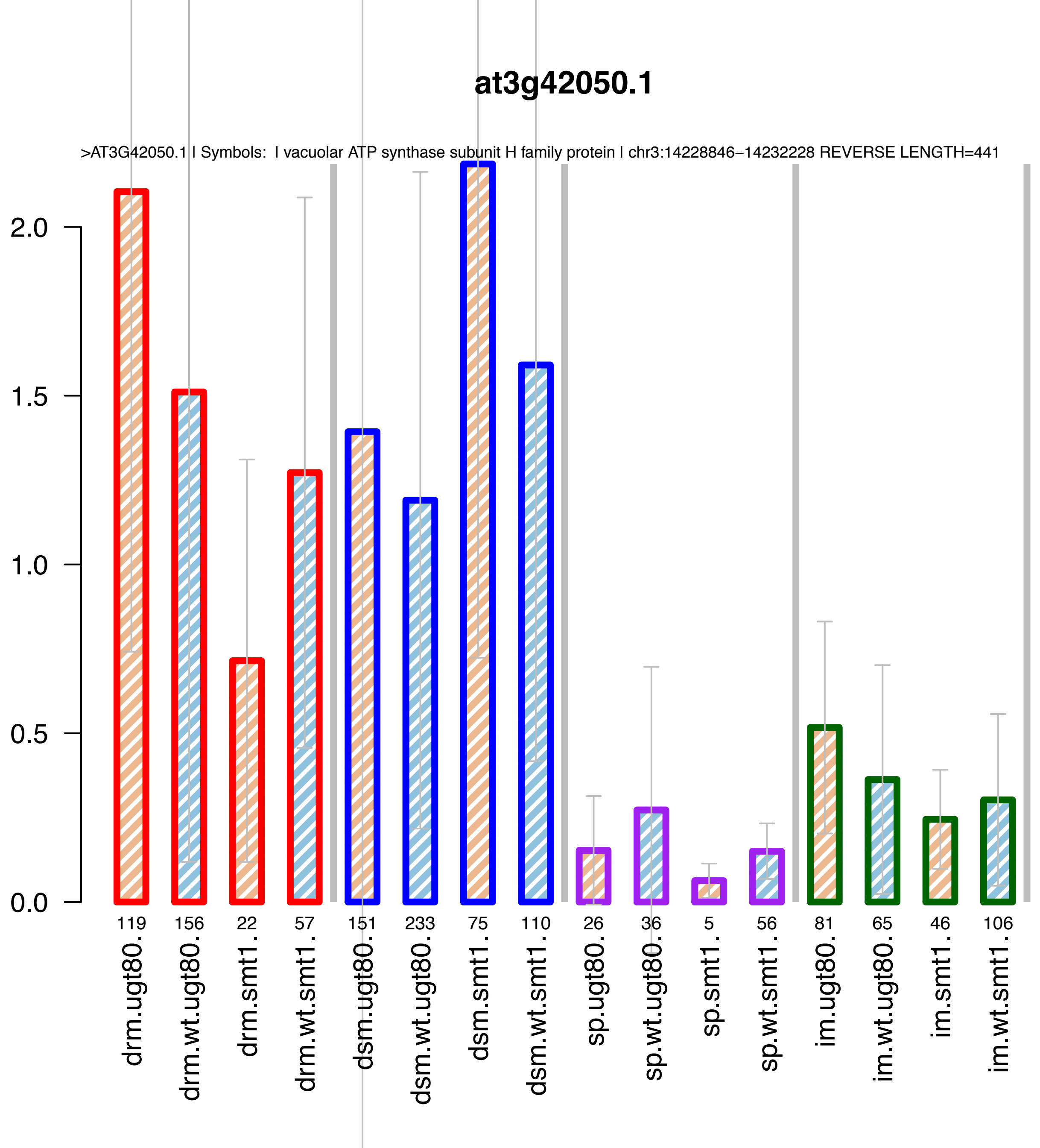

at3g42050.1
>AT3G42050.1 | Symbols: | vacuolar ATP synthase subunit H family protein | chr3:14228846-14232228 REVERSE LENGTH=441
0.0
0.5
1.0
1.5
2.0
119
156
22
57
151
233
75
110
26
36
5
56
81
65
46
106
drm.ugt80..
drm.wt.ugt80..
drm.smt1..
drm.wt.smt1..
dsm.ugt80..
dsm.wt.ugt80..
dsm.smt1..
dsm.wt.smt1..
sp.ugt80..
sp.wt.ugt80..
sp.smt1..
sp.wt.smt1..
im.ugt80..
im.wt.ugt80..
im.smt1..
im.wt.smt1..

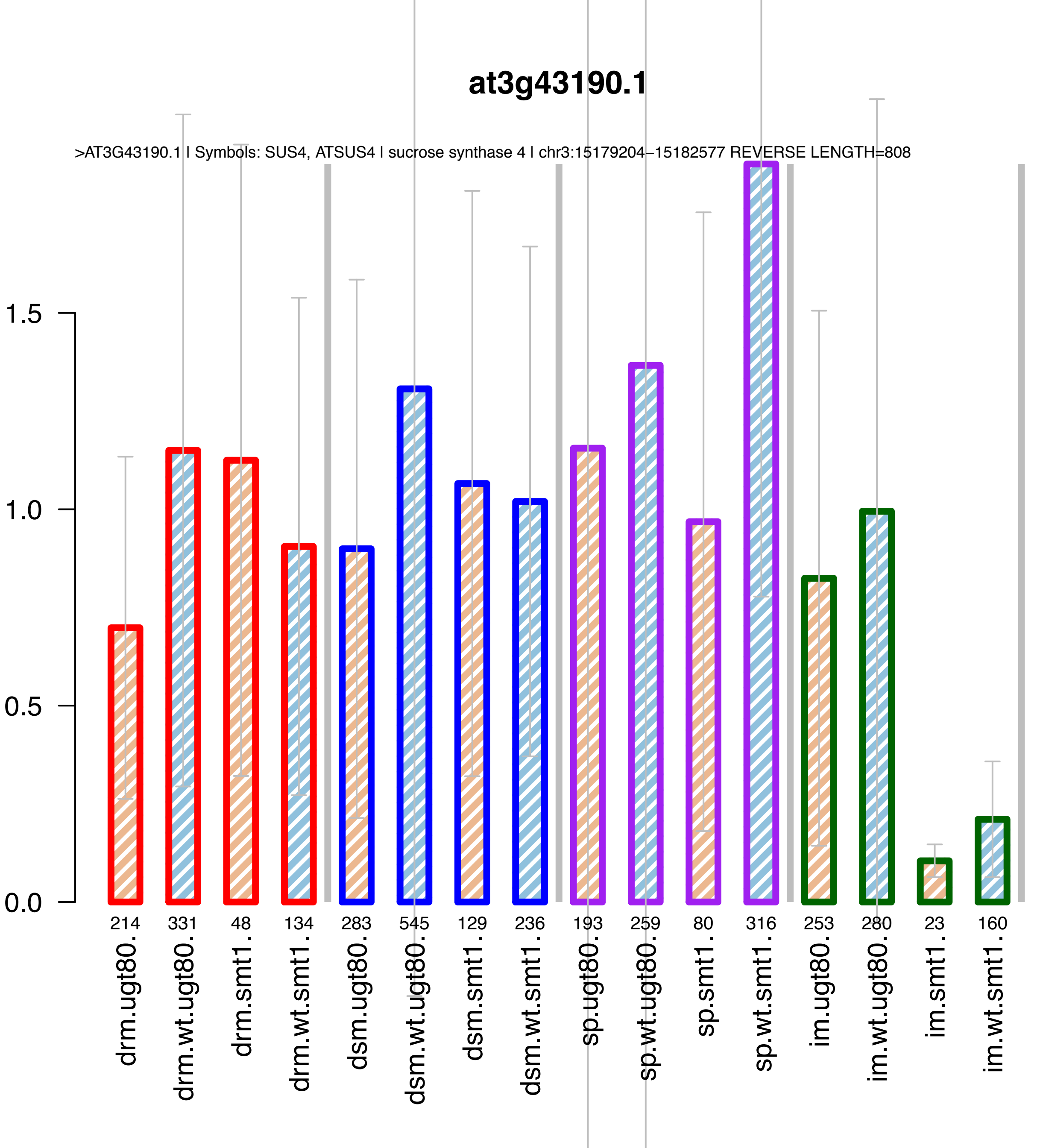

at3g43190.1
>AT3G43190.1 | Symbols: SUS4, ATSUS4 | sucrose synthase 4 | chr3:15179204–15182577 REVERSE LENGTH=808
1.5
1.0
0.5
0.0
214
331
48
134
283
545
129
236
193
259
80
316
253
280
23
160
drm.ugt80..
drm.wt.ugt80..
drm.smt1..
drm.wt.smt1..
dsm.ugt80..
dsm.wt.ugt80..
dsm.smt1..
dsm.wt.smt1..
sp.ugt80..
sp.wt.ugt80..
sp.smt1..
sp.wt.smt1..
im.ugt80..
im.wt.ugt80..
im.smt1..
im.wt.smt1..

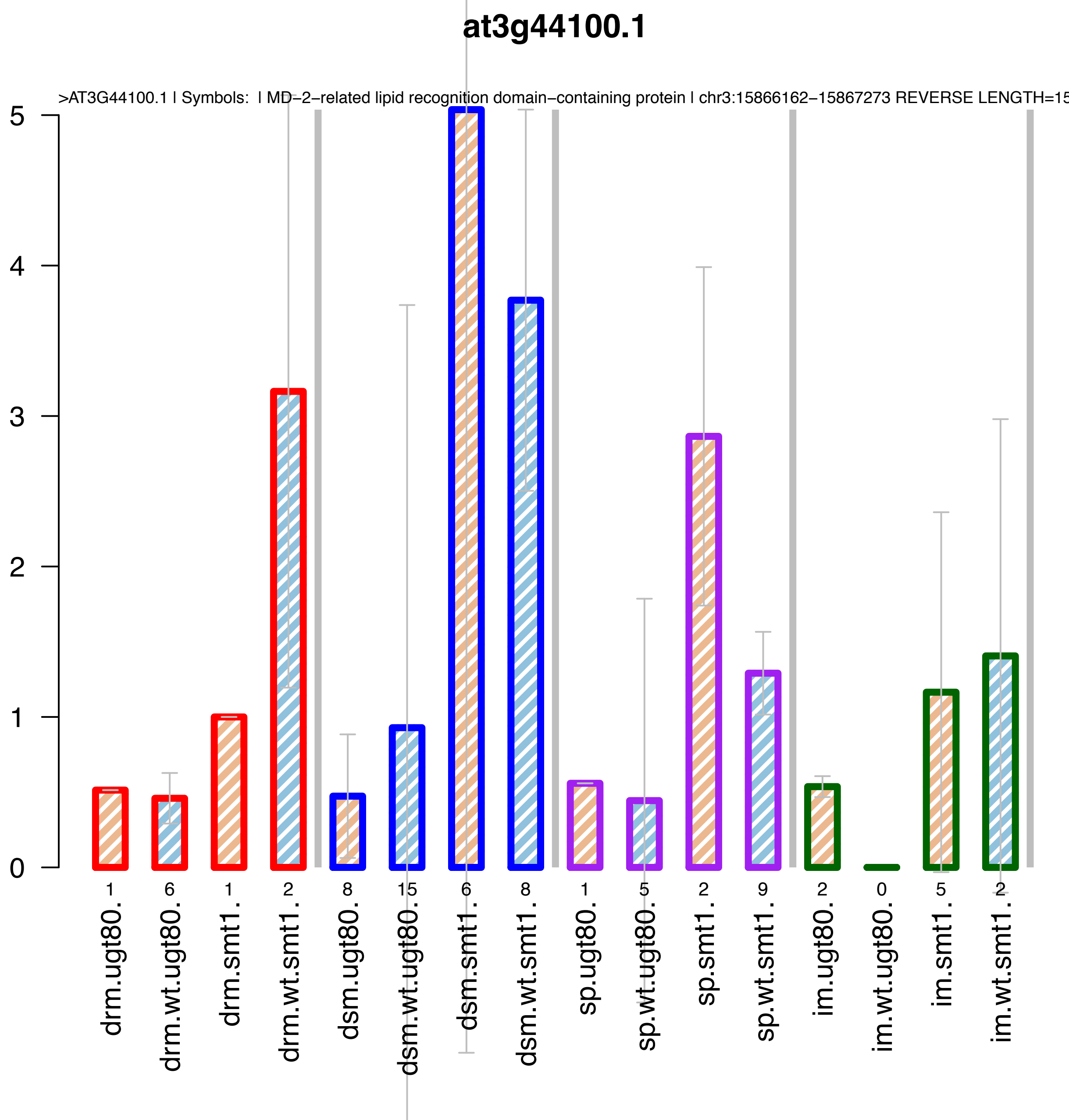

at3g44100.1
>AT3G44100.1 | Symbols: | MD−2−related lipid recognition domain−containing protein | chr3:15866162−15867273 REVERSE LENGTH=15
0
1
2
3
4
5
1
6
1
2
8
15
6
8
1
5
2
9
2
0
5
2
drm.ugt80..
drm.wt.ugt80..
drm.smt1..
drm.wt.smt1..
dsm.ugt80..
dsm.wt.ugt80..
dsm.smt1..
dsm.wt.smt1..
sp.ugt80..
sp.wt.ugt80..
sp.smt1..
sp.wt.smt1..
im.ugt80..
im.wt.ugt80..
im.smt1..
im.wt.smt1..

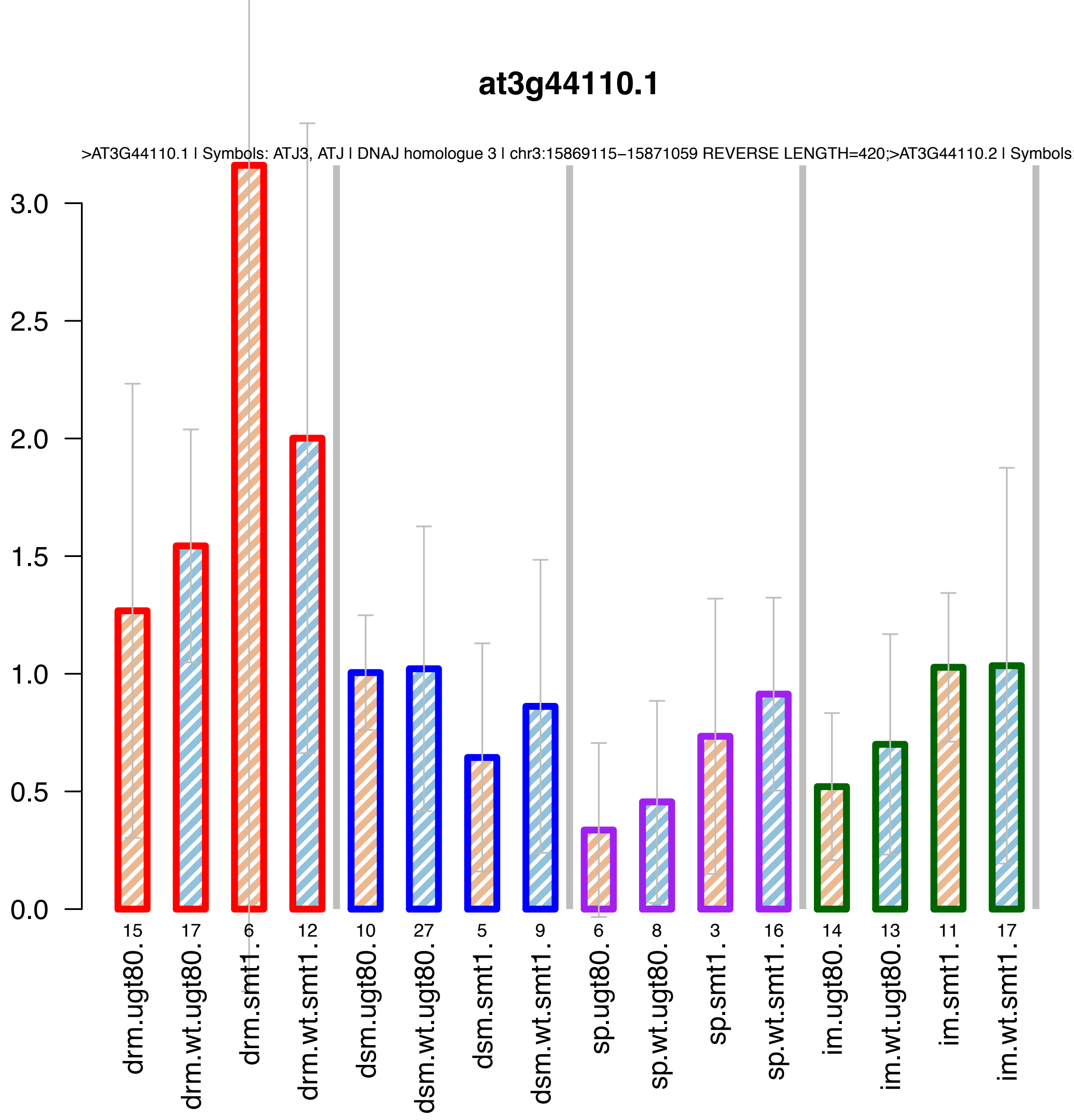

at3g44110.1
>AT3G44110.1 | Symbols: ATJ3, ATJ | DNAJ homologue 3 | chr3:15869115-15871059 REVERSE LENGTH=420;>AT3G44110.2 | Symbols
0.0
0.5
1.0
1.5
2.0
2.5
3.0
15
17
6
12
10
27
5
9
6
8
3
16
14
13
11
17
drm.ugt80.
drm.wt.ugt80.
drm.smt1.
drm.wt.smt1.
dsm.ugt80.
dsm.wt.ugt80.
dsm.smt1.
dsm.wt.smt1.
sp.ugt80.
sp.wt.ugt80.
sp.smt1.
sp.wt.smt1.
im.ugt80.
im.wt.ugt80.
im.smt1.
im.wt.smt1.

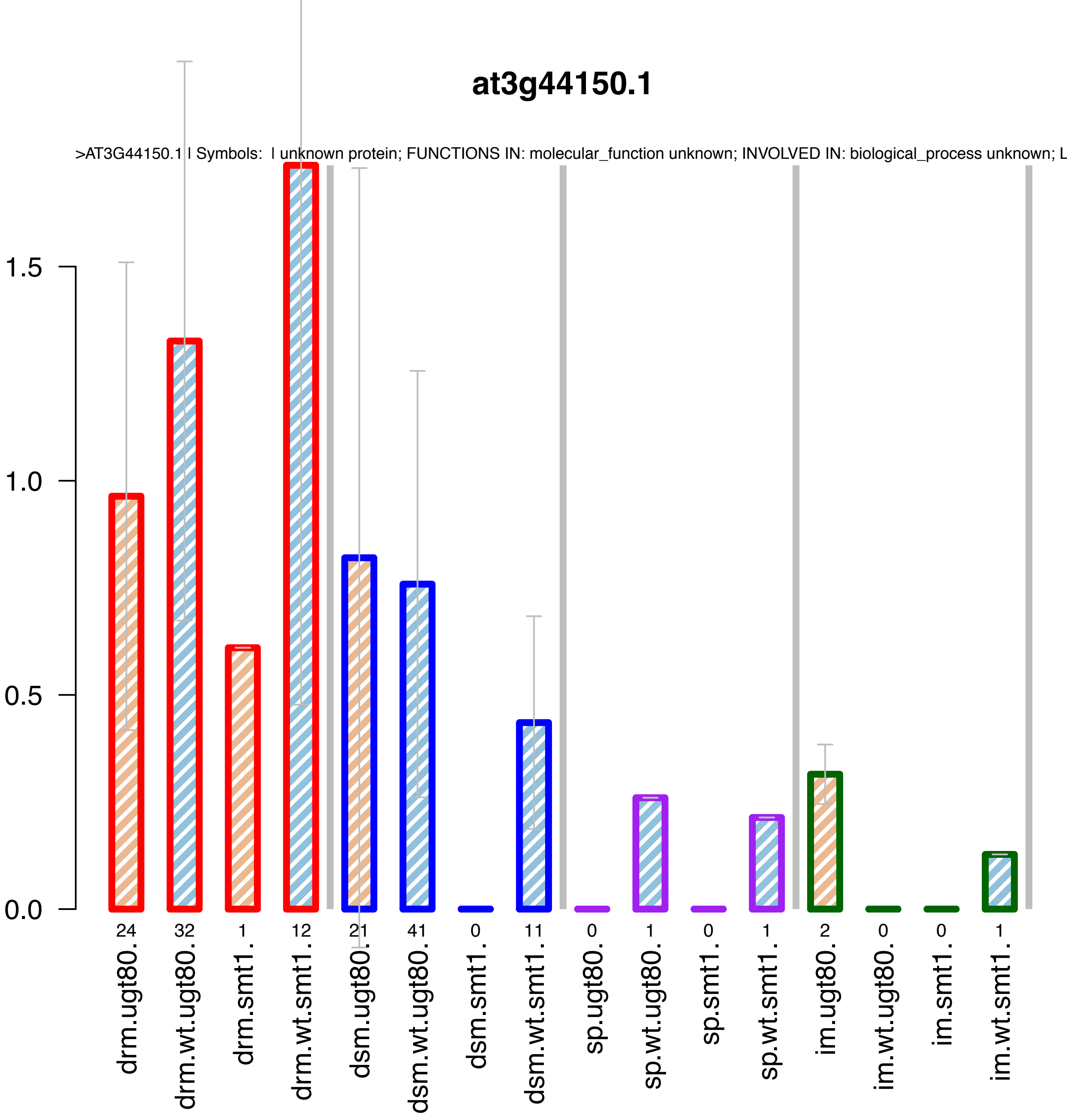

at3g44150.1
>AT3G44150.1 | Symbols: | unknown protein; FUNCTIONS IN: molecular_function unknown; INVOLVED IN: biological_process unknown; L
1.5
1.0
0.5
0.0
24
32
1
12
21
41
0
11
0
1
0
1
2
0
0
1
drm.ugt80.
drm.wt.ugt80.
drm.smt1.
drm.wt.smt1.
dsm.ugt80.
dsm.wt.ugt80.
dsm.smt1.
dsm.wt.smt1.
sp.ugt80.
sp.wt.ugt80.
sp.smt1.
sp.wt.smt1.
im.ugt80.
im.wt.ugt80.
im.smt1.
im.wt.smt1.

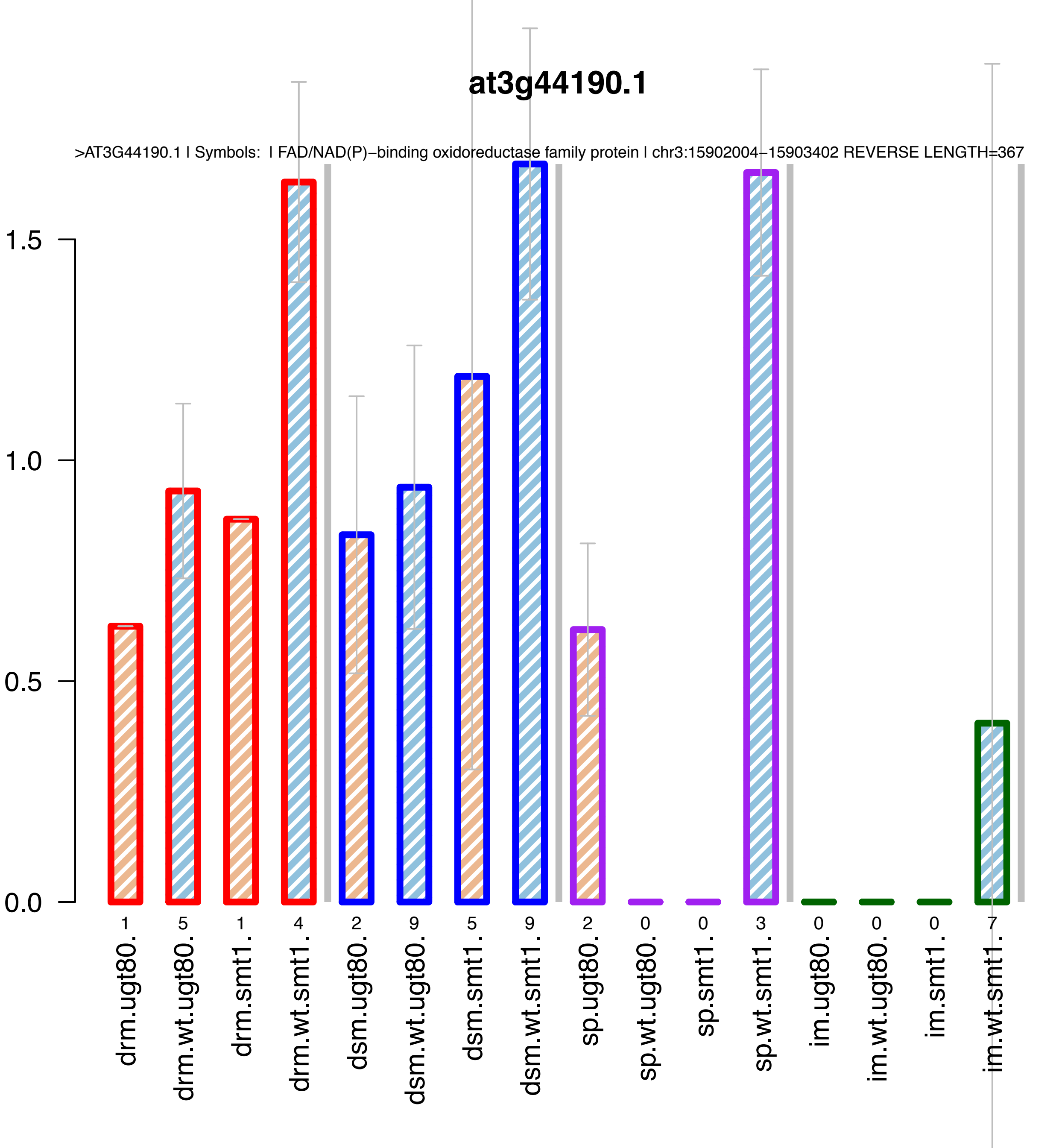

at3g44190.1
>AT3G44190.1 | Symbols: | FAD/NAD(P)−binding oxidoreductase family protein | chr3:15902004−15903402 REVERSE LENGTH=367
1.5
1.0
0.5
0.0
1
5
1
4
2
9
5
9
2
0
0
3
0
0
0
7
drm.ugt80..
drm.wt.ugt80..
drm.smt1..
drm.wt.smt1..
dsm.ugt80..
dsm.wt.ugt80..
dsm.smt1..
dsm.wt.smt1..
sp.ugt80..
sp.wt.ugt80..
sp.smt1..
sp.wt.smt1..
im.ugt80..
im.wt.ugt80..
im.smt1..
im.wt.smt1..

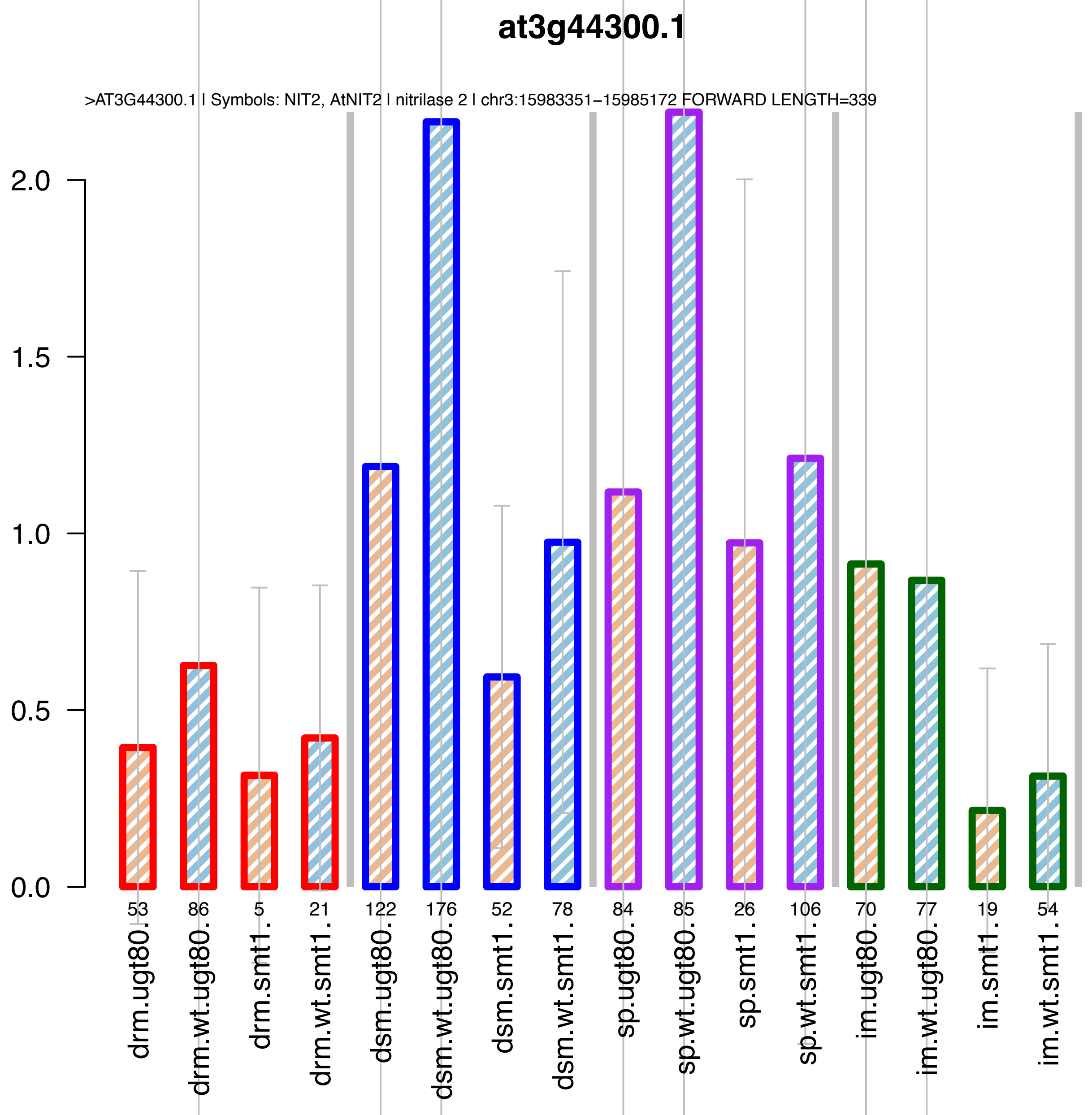
at3g44300.1
>AT3G44300.1 | Symbols: NIT2, AtNIT2 | nitrilase 2 | chr3:15983351-15985172 FORWARD LENGTH=339
2.0
1.5
1.0
0.5
0.0
53
86
5
21
122
176
52
78
84
85
26
106
70
77
19
54
drm.ugt80.
drm.wt.ugt80.
drm.smt1.
drm.wt.smt1.
dsm.ugt80.
dsm.wt.ugt80.
dsm.smt1.
dsm.wt.smt1.
sp.ugt80.
sp.wt.ugt80.
sp.smt1.
sp.wt.smt1.
im.ugt80.
im.wt.ugt80.
im.smt1.
im.wt.smt1.

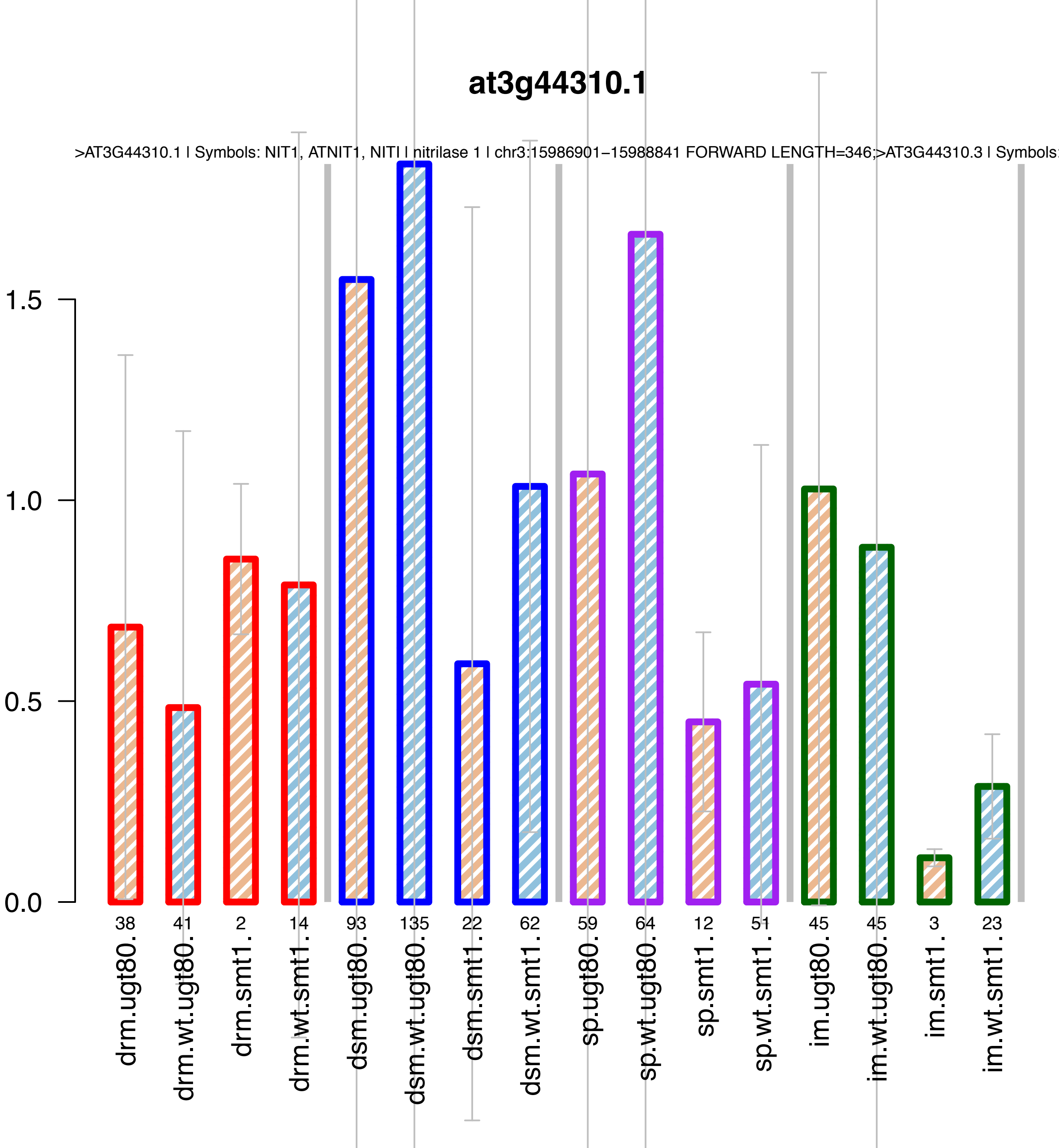

at3g44310.1
>AT3G44310.1 | Symbols: NIT1, ATNIT1, NITI | nitrilase 1 | chr3:15986901-15988841 FORWARD LENGTH=346;>AT3G44310.3 | Symbols:
0.0
0.5
1.0
1.5
38
41
2
14
93
135
22
62
59
64
12
51
45
45
3
23
drm.ugt80..
drm.wt.ugt80..
drm.smt1..
drm.wt.smt1..
dsm.ugt80..
dsm.wt.ugt80..
dsm.smt1..
dsm.wt.smt1..
sp.ugt80..
sp.wt.ugt80..
sp.smt1..
sp.wt.smt1..
im.ugt80..
im.wt.ugt80..
im.smt1..
im.wt.smt1..

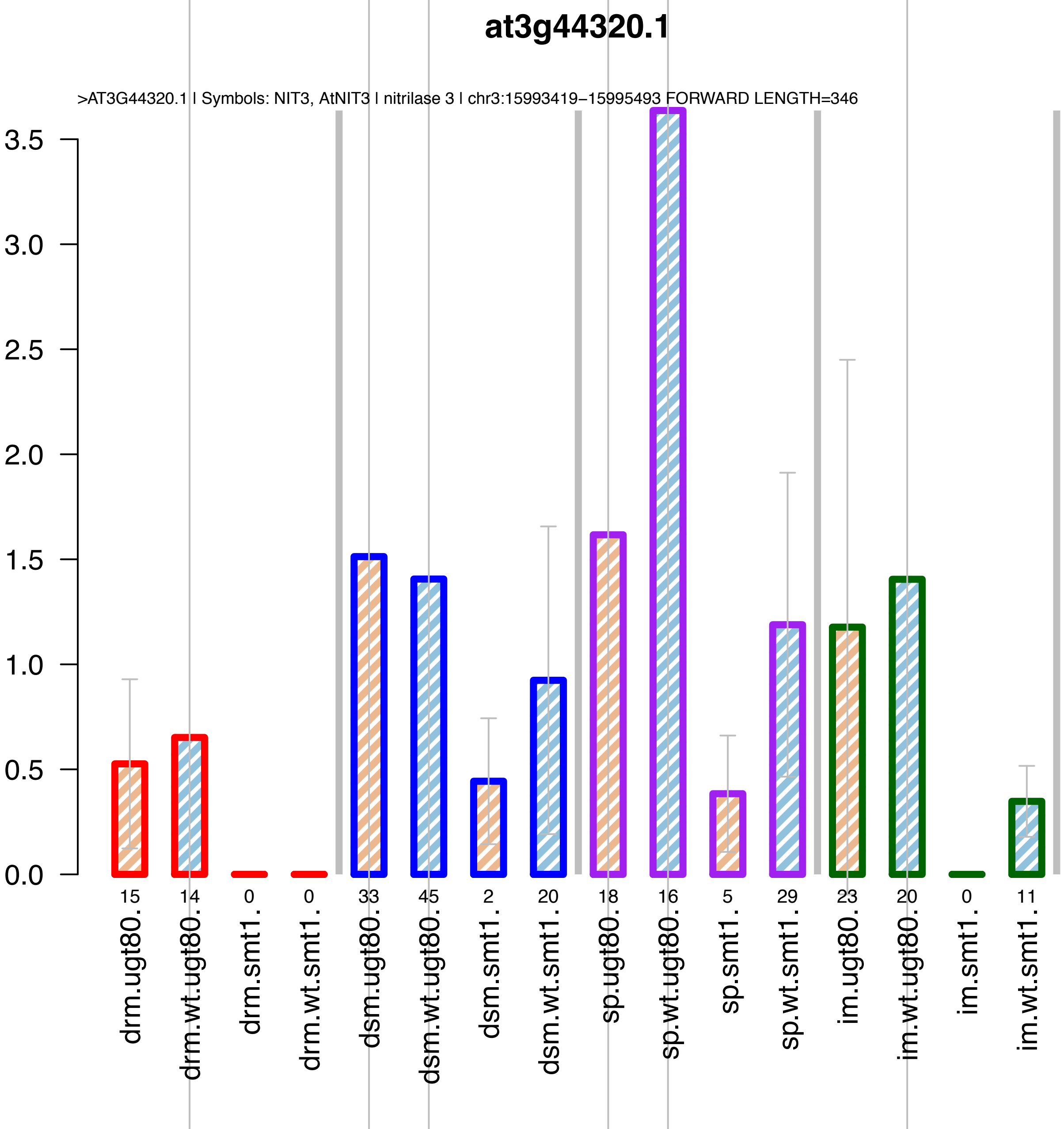

at3g44320.1
>AT3G44320.1 | Symbols: NIT3, AtNIT3 | nitrilase 3 | chr3:15993419-15995493 FORWARD LENGTH=346
3.5
3.0
2.5
2.0
1.5
1.0
0.5
0.0
15
14
0
0
33
45
2
20
18
16
5
29
23
20
0
11
drm.ugt80.
drm.wt.ugt80.
drm.smt1.
drm.wt.smt1.
dsm.ugt80.
dsm.wt.ugt80.
dsm.smt1.
dsm.wt.smt1.
sp.ugt80.
sp.wt.ugt80.
sp.smt1.
sp.wt.smt1.
im.ugt80.
im.wt.ugt80.
im.smt1.
im.wt.smt1.

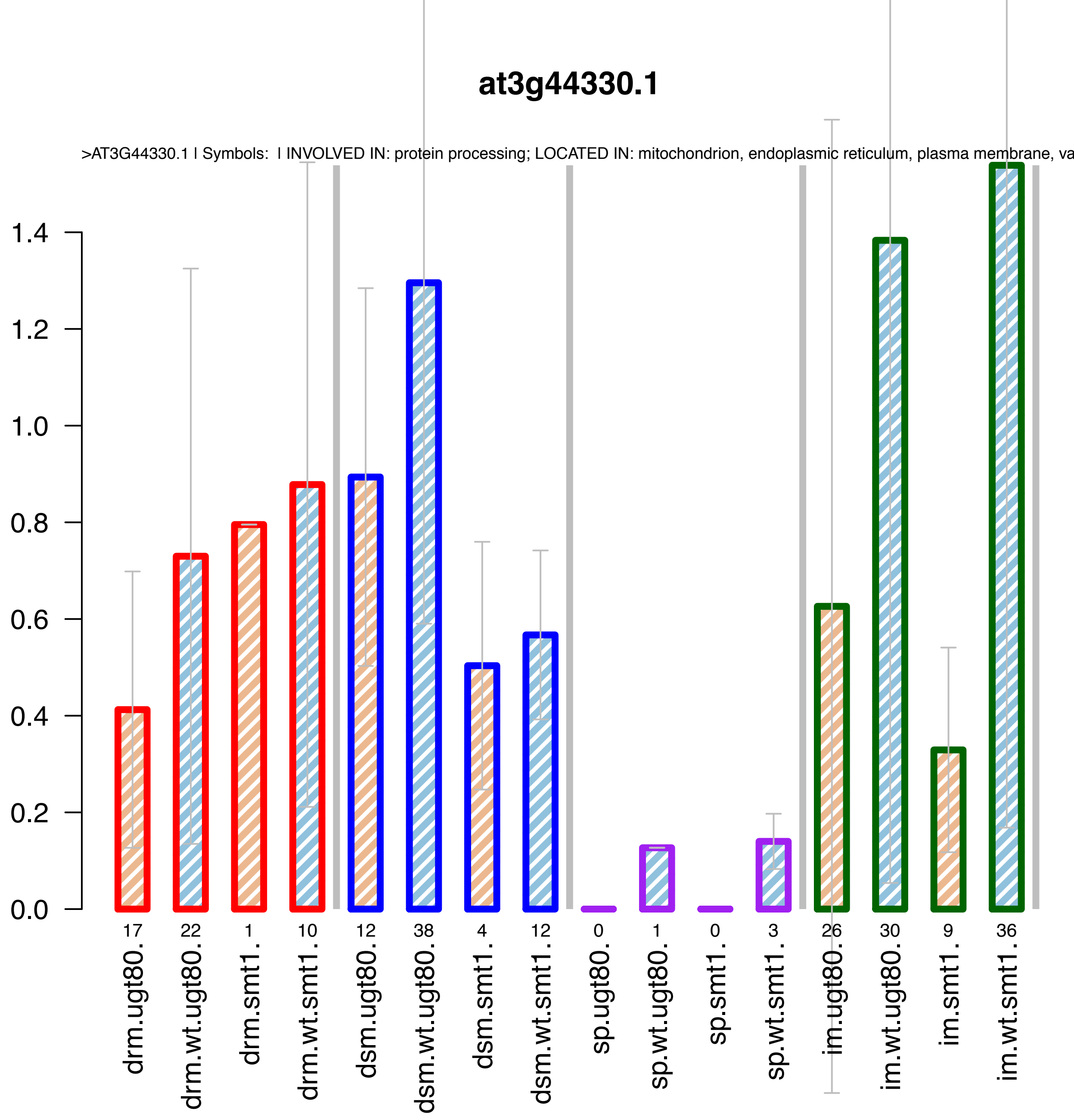

at3g44330.1
>AT3G44330.1 | Symbols: | INVOLVED IN: protein processing; LOCATED IN: mitochondrion, endoplasmic reticulum, plasma membrane, va
1.4
1.2
1.0
0.8
0.6
0.4
0.2
0.0
17
22
1
10
12
38
4
12
0
1
0
3
26
30
9
36
drm.ugt80..
drm.wt.ugt80..
drm.smt1..
drm.wt.smt1..
dsm.ugt80..
dsm.wt.ugt80..
dsm.smt1..
dsm.wt.smt1..
sp.ugt80..
sp.wt.ugt80..
sp.smt1..
sp.wt.smt1..
im.ugt80..
im.wt.ugt80..
im.smt1..
im.wt.smt1..

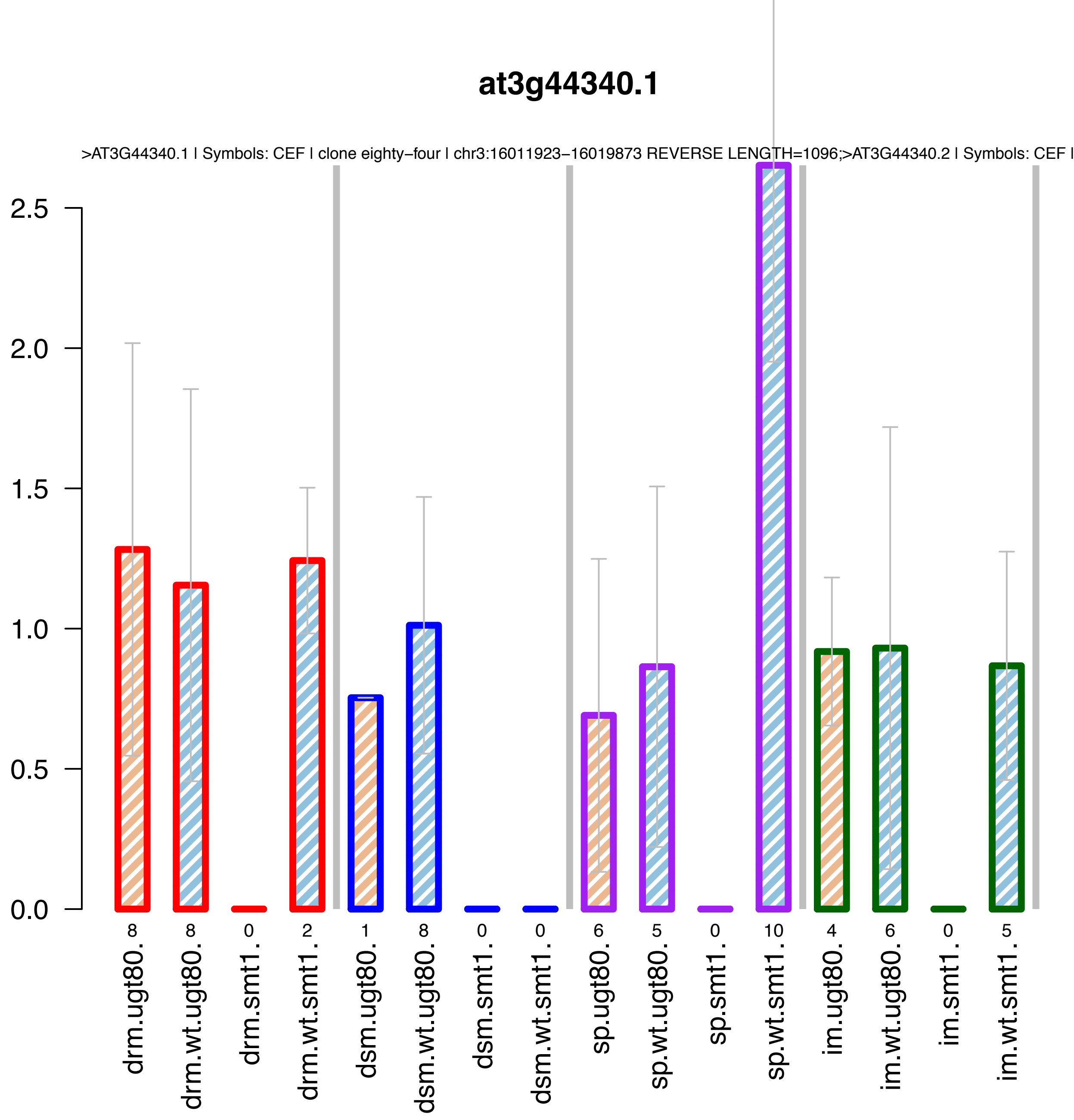
at3g44340.1
>AT3G44340.1 | Symbols: CEF | clone eighty-four | chr3:16011923-16019873 REVERSE LENGTH=1096;>AT3G44340.2 | Symbols: CEF |
0.0
0.5
1.0
1.5
2.0
2.5
8
8
0
2
1
8
0
0
6
5
0
10
4
6
0
5
drm.ugt80.
drm.wt.ugt80.
drm.smt1.
drm.wt.smt1.
dsm.ugt80.
dsm.wt.ugt80.
dsm.smt1.
dsm.wt.smt1.
sp.ugt80.
sp.wt.ugt80.
sp.smt1.
sp.wt.smt1.
im.ugt80.
im.wt.ugt80.
im.smt1.
im.wt.smt1.

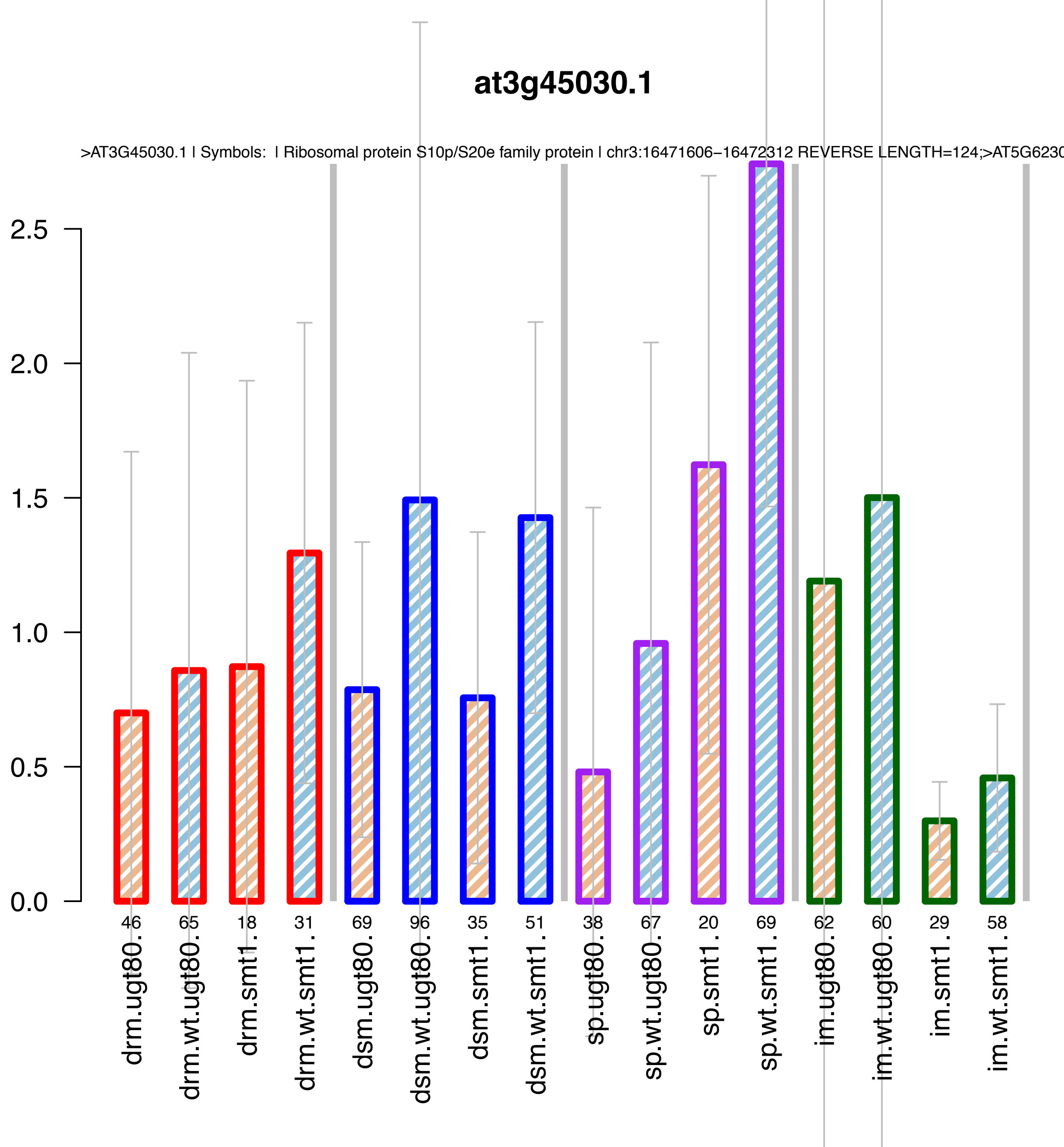

at3g45030.1
>AT3G45030.1 | Symbols: | Ribosomal protein S10p/S20e family protein | chr3:16471606-16472312 REVERSE LENGTH=124;>AT5G6230
2.5
2.0
1.5
1.0
0.5
0.0
46
65
18
31
69
96
35
51
38
67
20
69
62
60
29
58
drm.ugt80..
drm.wt.ugt80..
drm.smt1..
drm.wt.smt1..
dsm.ugt80..
dsm.wt.ugt80..
dsm.smt1..
dsm.wt.smt1..
sp.ugt80..
sp.wt.ugt80..
sp.smt1..
sp.wt.smt1..
im.ugt80..
im.wt.ugt80..
im.smt1..
im.wt.smt1..

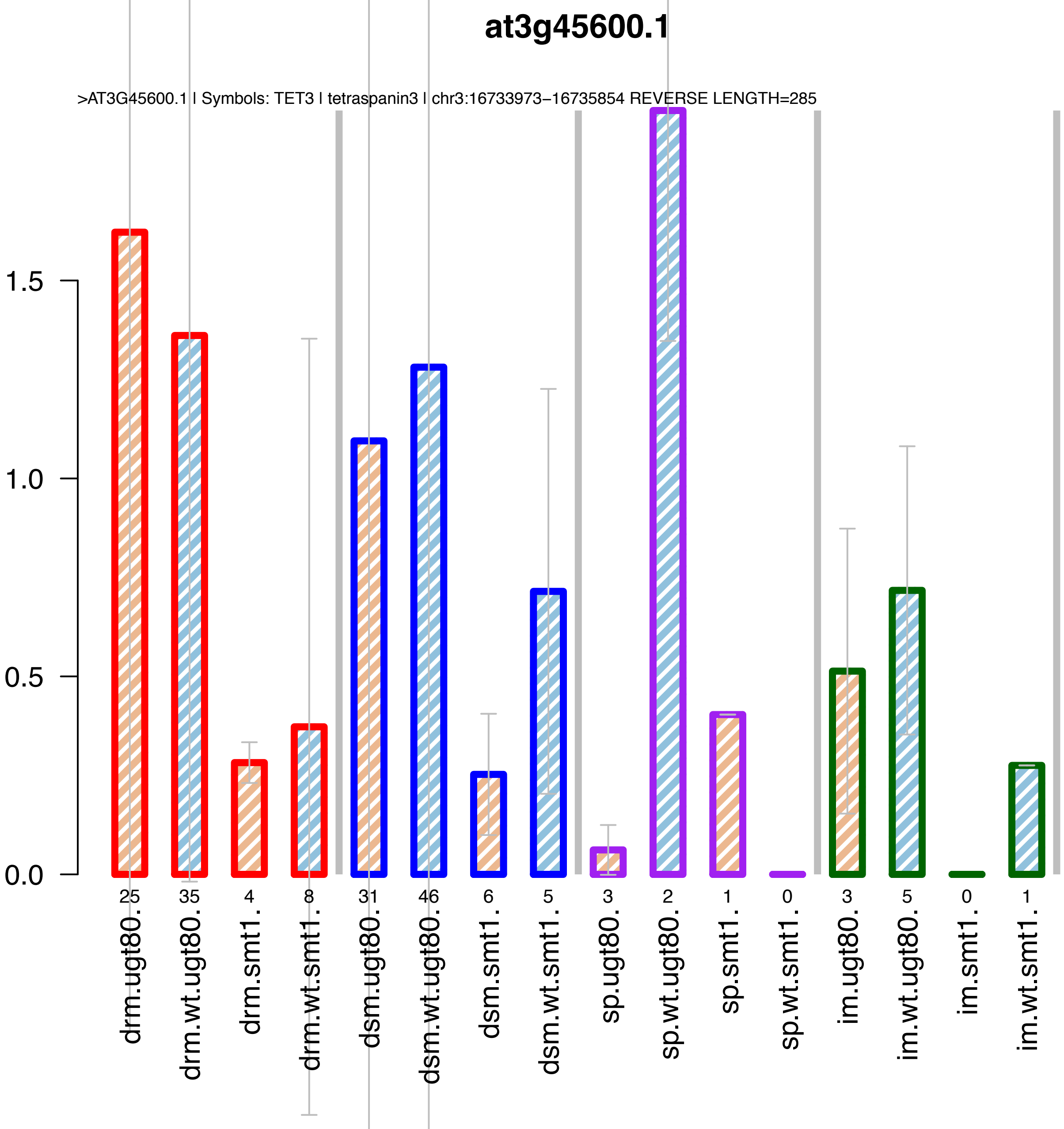

at3g45600.1
>AT3G45600.1 | Symbols: TET3 | tetraspanin3 | chr3:16733973-16735854 REVERSE LENGTH=285
0.0
0.5
1.0
1.5
25
35
4
8
31
46
6
5
3
2
1
0
3
5
0
1
drm.ugt80..
drm.wt.ugt80..
drm.smt1..
drm.wt.smt1..
dsm.ugt80..
dsm.wt.ugt80..
dsm.smt1..
dsm.wt.smt1..
sp.ugt80..
sp.wt.ugt80..
sp.smt1..
sp.wt.smt1..
im.ugt80..
im.wt.ugt80..
im.smt1..
im.wt.smt1..

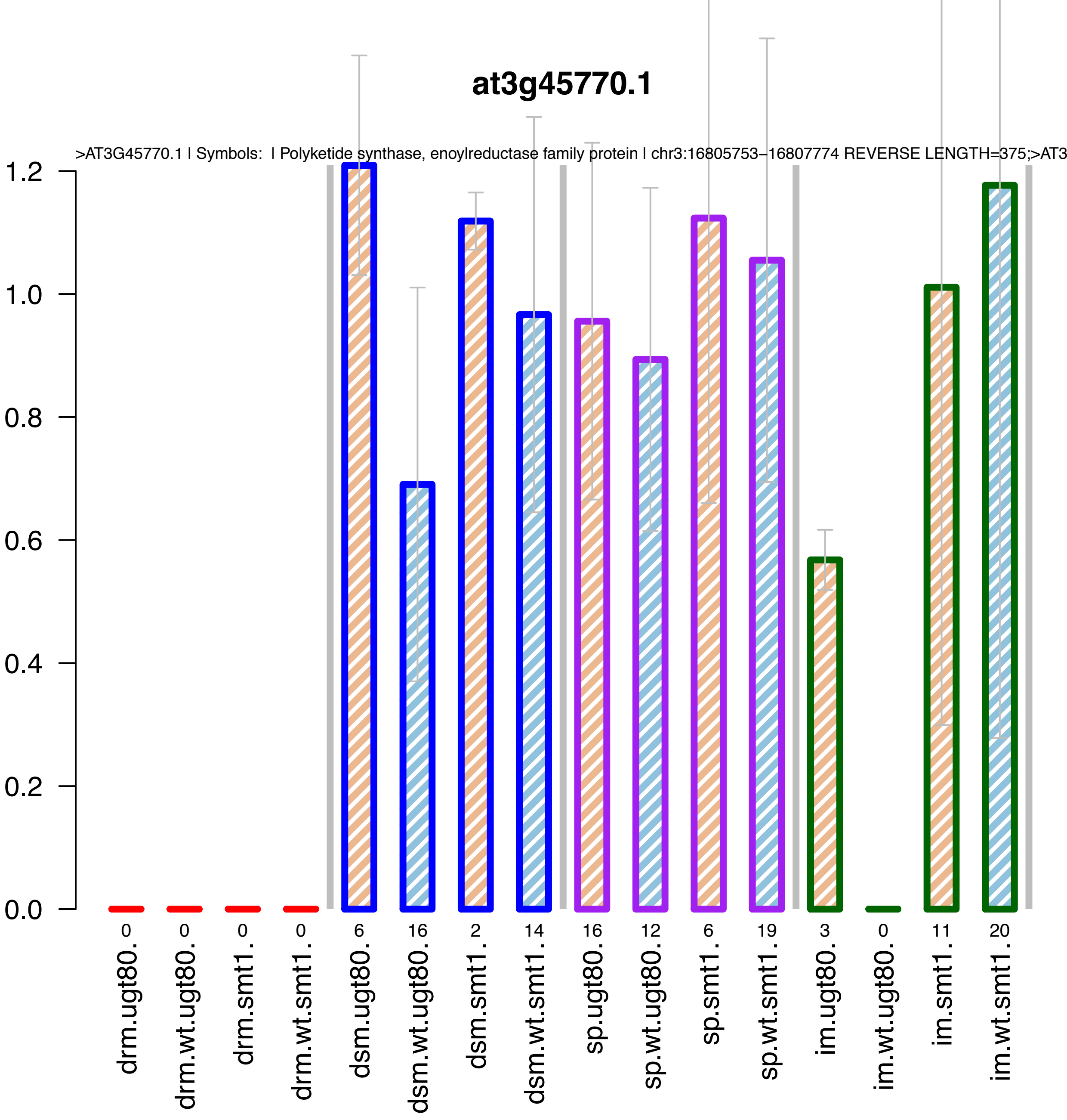

at3g45770.1
>AT3G45770.1 | Symbols: | Polyketide synthase, enoylreductase family protein | chr3:16805753–16807774 REVERSE LENGTH=375;>AT3
1.2
1.0
0.8
0.6
0.4
0.2
0.0
0
0
0
0
6
16
2
14
16
12
6
19
3
0
11
20
drm.ugt80.
drm.wt.ugt80.
drm.smt1.
drm.wt.smt1.
dsm.ugt80.
dsm.wt.ugt80.
dsm.smt1.
dsm.wt.smt1.
sp.ugt80.
sp.wt.ugt80.
sp.smt1.
sp.wt.smt1.
im.ugt80.
im.wt.ugt80.
im.smt1.
im.wt.smt1.

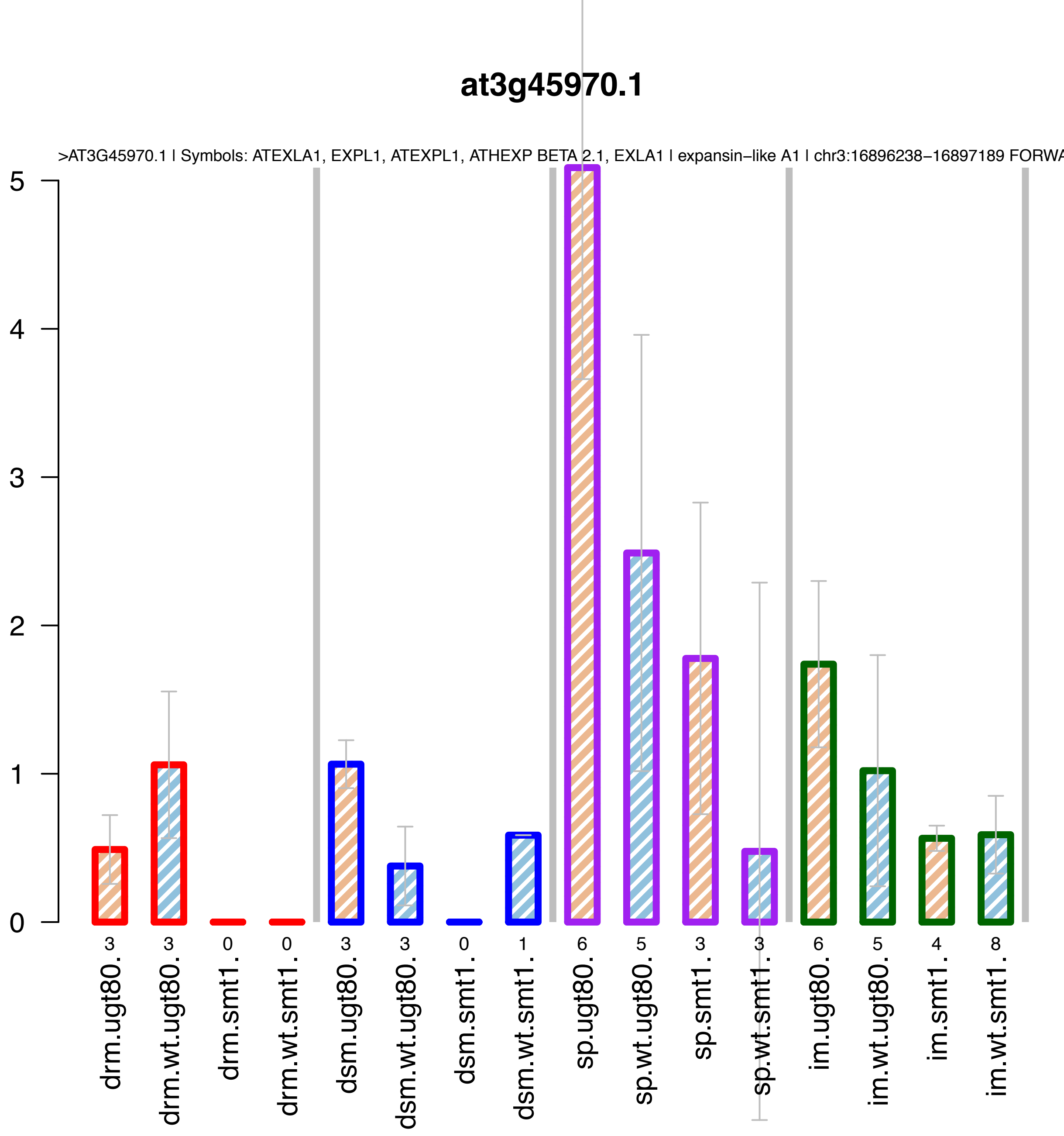
at3g45970.1
>AT3G45970.1 | Symbols: ATEXLA1, EXPL1, ATEXPL1, ATHEXP BETA 2.1, EXLA1 | expansin-like A1 | chr3:16896238-16897189 FORWA
0
1
2
3
4
5
3
3
0
0
3
3
0
1
6
5
3
3
6
5
4
8
drm.ugt80.
drm.wt.ugt80.
drm.smt1.
drm.wt.smt1.
dsm.ugt80.
dsm.wt.ugt80.
dsm.smt1.
dsm.wt.smt1.
sp.ugt80.
sp.wt.ugt80.
sp.smt1.
sp.wt.smt1.
im.ugt80.
im.wt.ugt80.
im.smt1.
im.wt.smt1.

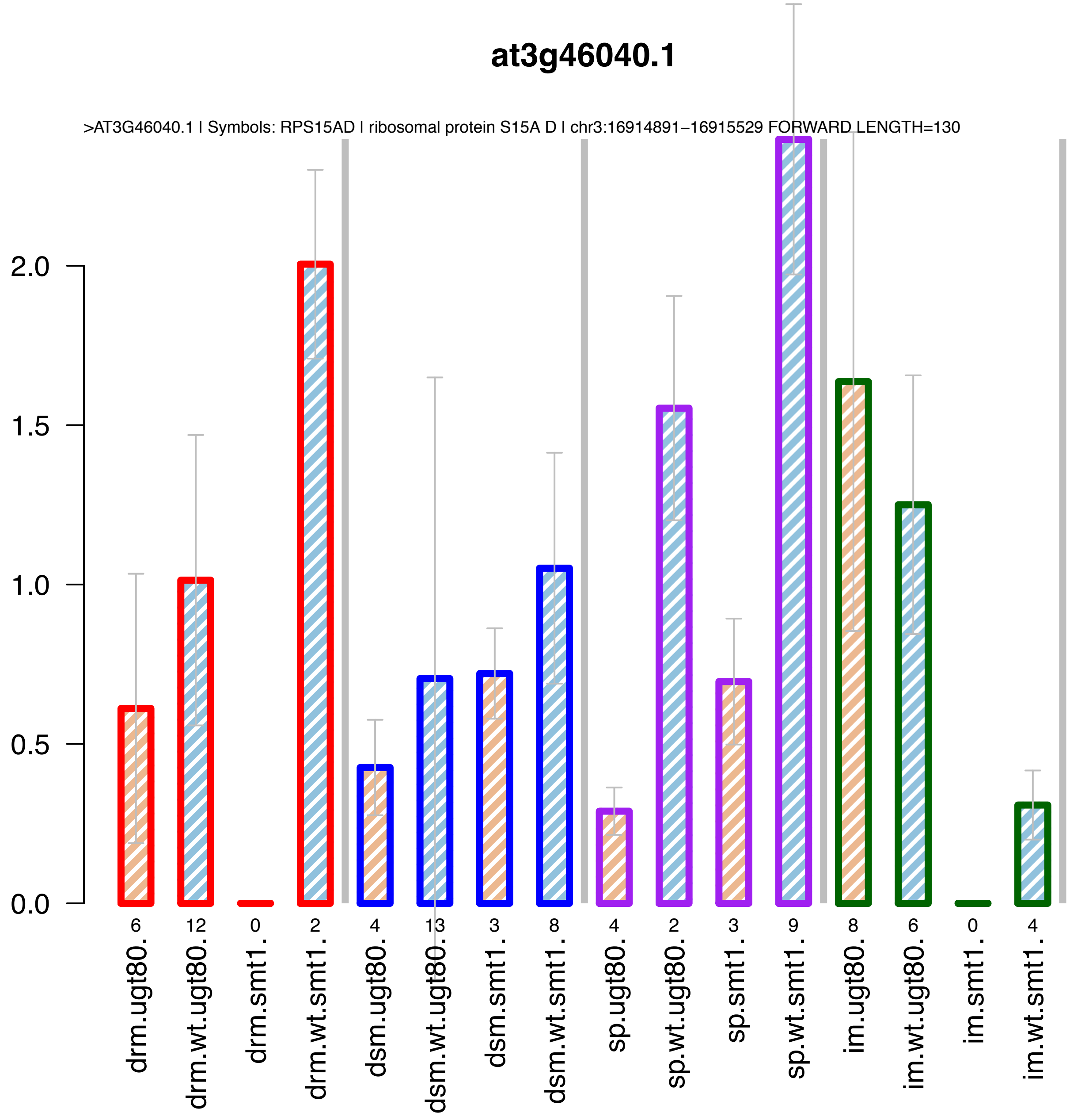
at3g46040.1
>AT3G46040.1 | Symbols: RPS15AD | ribosomal protein S15A D | chr3:16914891−16915529 FORWARD LENGTH=130
2.0
1.5
1.0
0.5
0.0
6
12
0
2
4
13
3
8
4
2
3
9
8
6
0
4
drm.ugt80.
drm.wt.ugt80.
drm.smt1.
drm.wt.smt1.
dsm.ugt80.
dsm.wt.ugt80.
dsm.smt1.
dsm.wt.smt1.
sp.ugt80.
sp.wt.ugt80.
sp.smt1.
sp.wt.smt1.
im.ugt80.
im.wt.ugt80.
im.smt1.
im.wt.smt1.

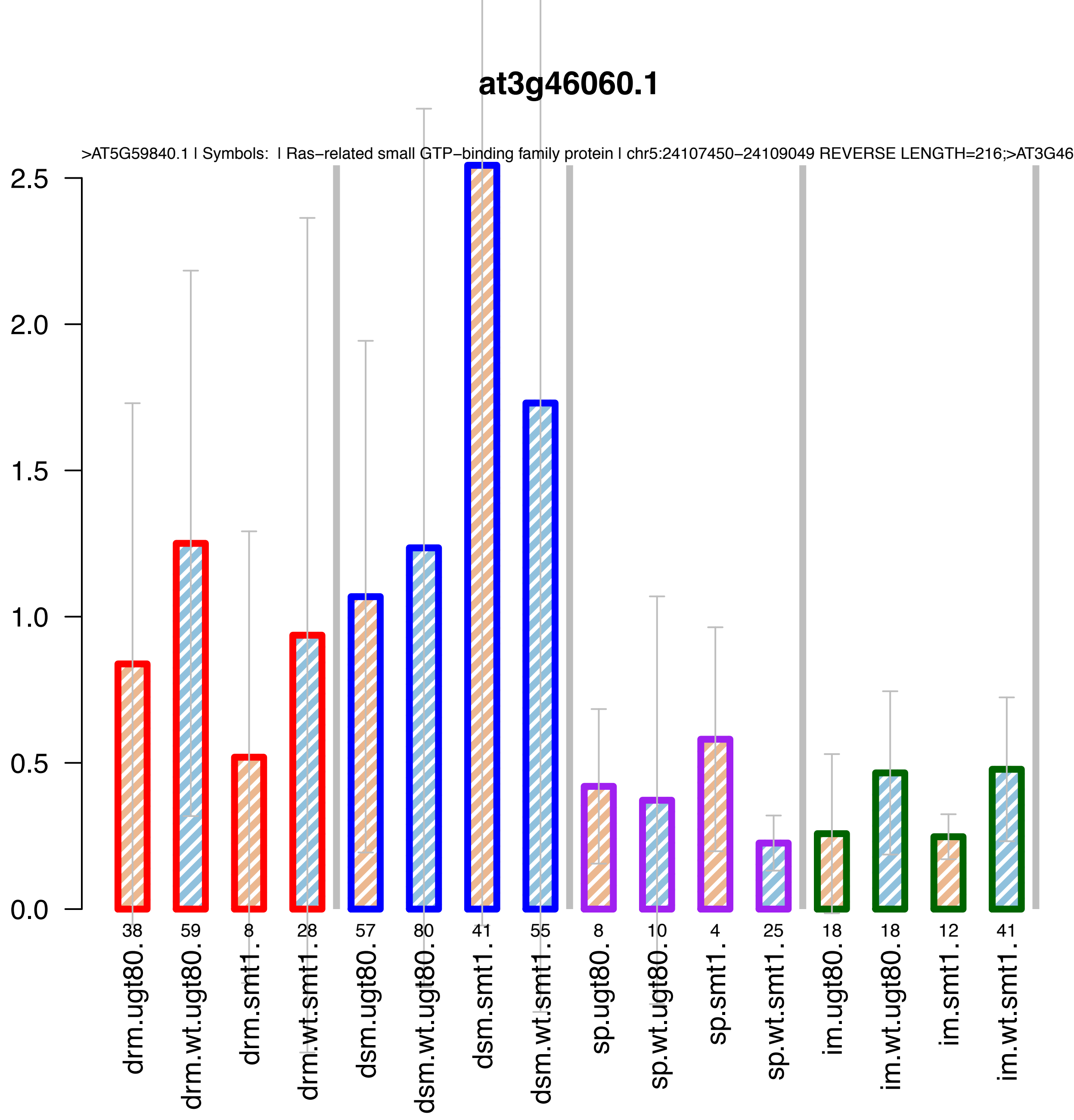

at3g46060.1
>AT5G59840.1 | Symbols: | Ras−related small GTP−binding family protein | chr5:24107450−24109049 REVERSE LENGTH=216;>AT3G46
2.5
2.0
1.5
1.0
0.5
0.0
38
59
8
28
57
80
41
55
8
10
4
25
18
18
12
41
drm.ugt80.
drm.wt.ugt80.
drm.smt1.
drm.wt.smt1.
dsm.ugt80.
dsm.wt.ugt80.
dsm.smt1.
dsm.wt.smt1.
sp.ugt80.
sp.wt.ugt80.
sp.smt1.
sp.wt.smt1.
im.ugt80.
im.wt.ugt80.
im.smt1.
im.wt.smt1.

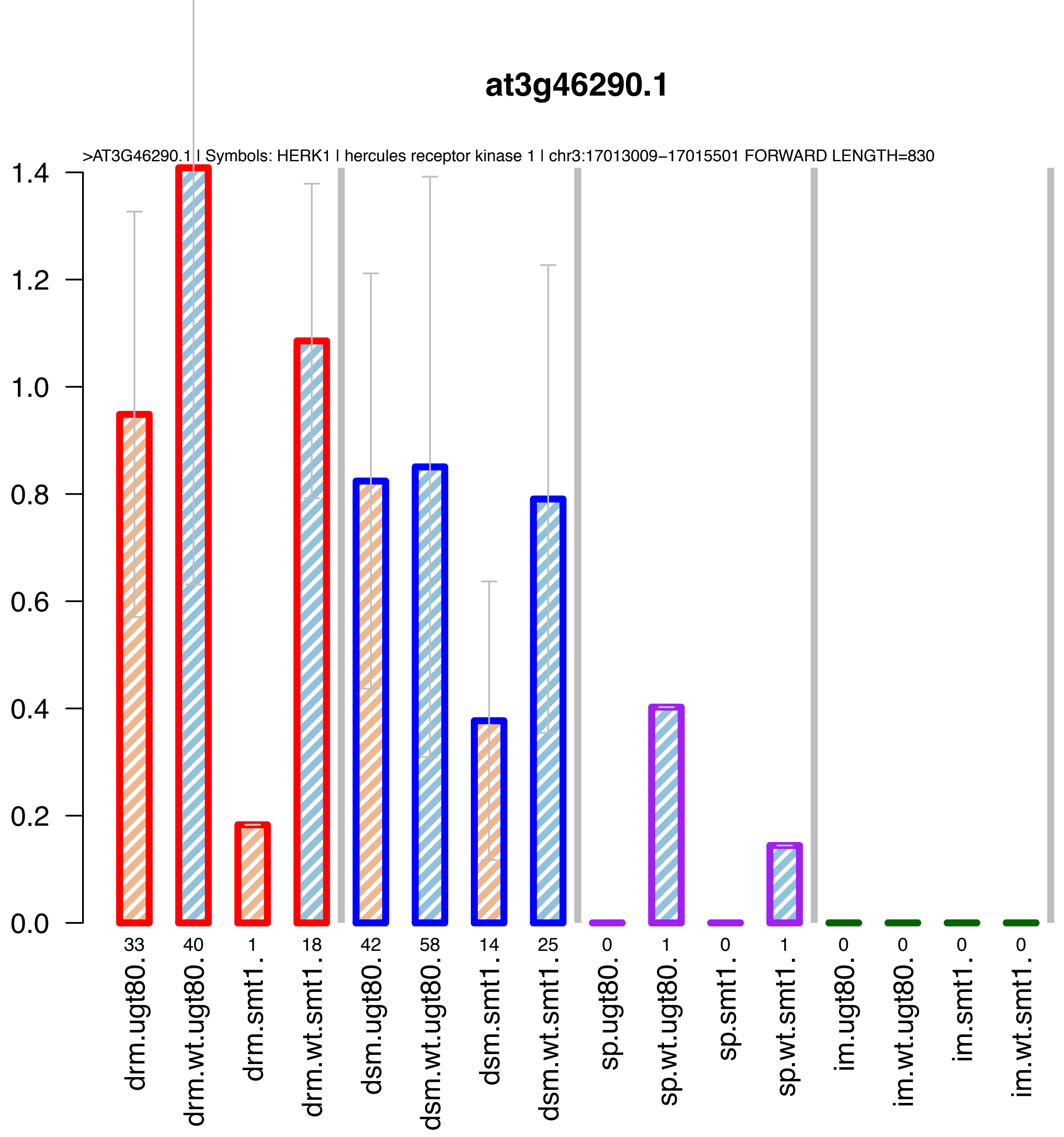
at3g46290.1
>AT3G46290.1 | Symbols: HERK1 | hercules receptor kinase 1 | chr3:17013009-17015501 FORWARD LENGTH=830
1.4
1.2
1.0
0.8
0.6
0.4
0.2
0.0
33
40
1
18
42
58
14
25
0
1
0
1
0
0
0
0
drm.ugt80.
drm.wt.ugt80.
drm.smt1.
drm.wt.smt1.
dsm.ugt80.
dsm.wt.ugt80.
dsm.smt1.
dsm.wt.smt1.
sp.ugt80.
sp.wt.ugt80.
sp.smt1.
sp.wt.smt1.
im.ugt80.
im.wt.ugt80.
im.smt1.
im.wt.smt1.

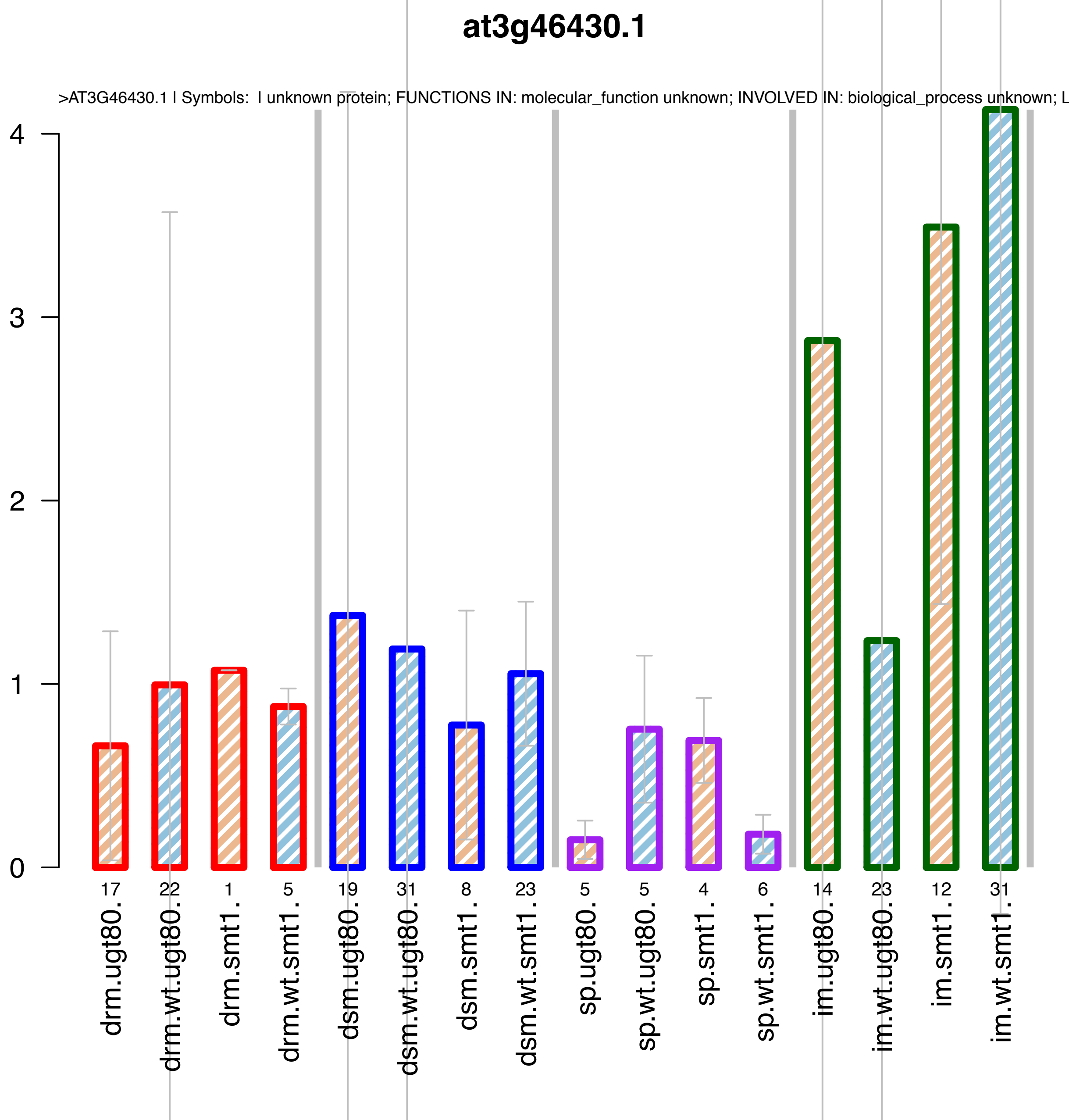
at3g46430.1
>AT3G46430.1 | Symbols: | unknown protein; FUNCTIONS IN: molecular_function unknown; INVOLVED IN: biological_process unknown; L
0
1
2
3
4
17
22
1
5
19
31
8
23
5
5
4
6
14
23
12
31
drm.ugt80.
drm.wt.ugt80.
drm.smt1.
drm.wt.smt1.
dsm.ugt80.
dsm.wt.ugt80.
dsm.smt1.
dsm.wt.smt1.
sp.ugt80.
sp.wt.ugt80.
sp.smt1.
sp.wt.smt1.
im.ugt80.
im.wt.ugt80.
im.smt1.
im.wt.smt1.

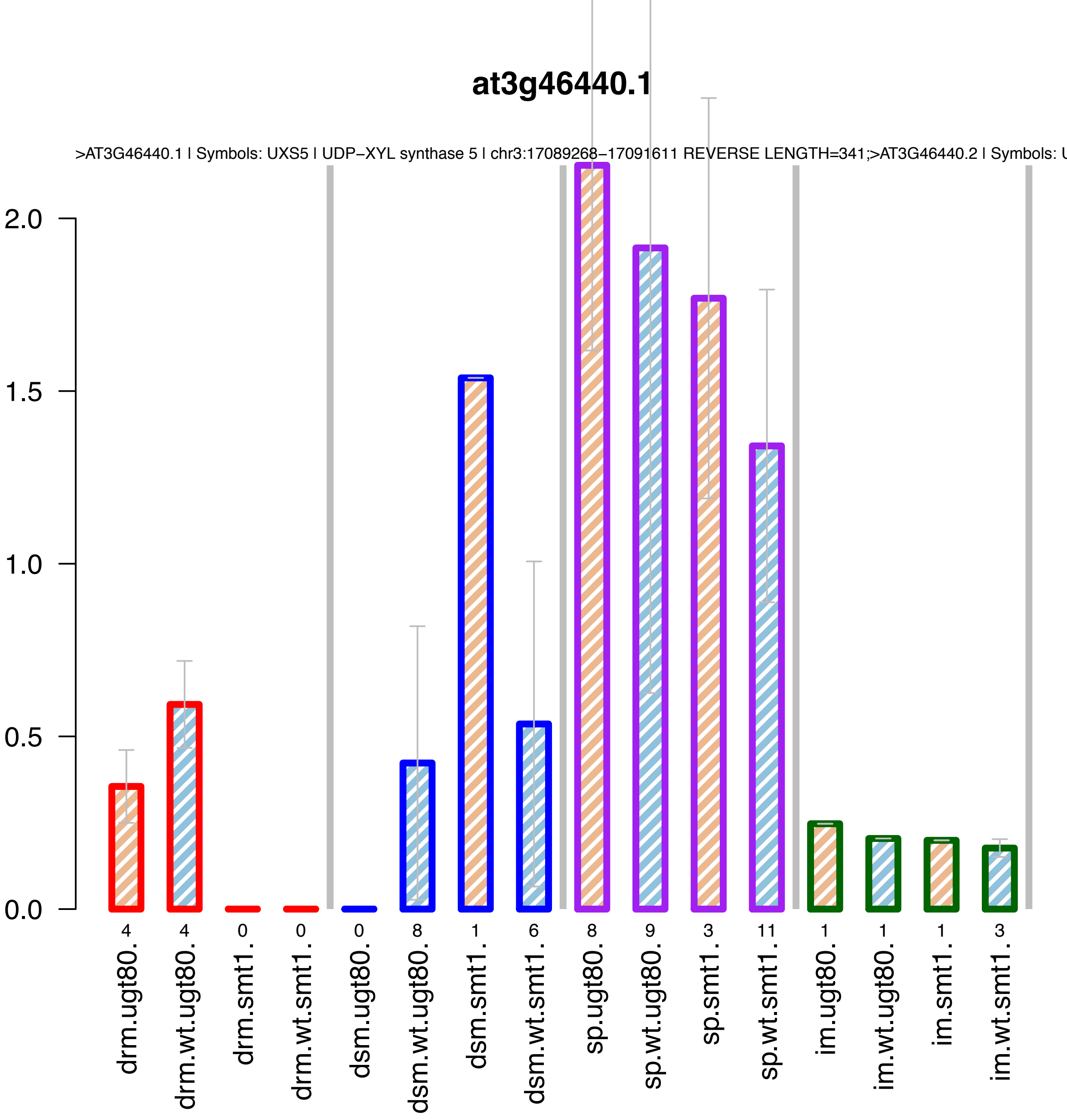
at3g46440.1
>AT3G46440.1 | Symbols: UXS5 | UDP−XYL synthase 5 | chr3:17089268−17091611 REVERSE LENGTH=341;>AT3G46440.2 | Symbols: U
2.0
1.5
1.0
0.5
0.0
4
4
0
0
0
8
1
6
8
9
3
11
1
1
1
3
drm.ugt80.
drm.wt.ugt80.
drm.smt1.
drm.wt.smt1.
dsm.ugt80.
dsm.wt.ugt80.
dsm.smt1.
dsm.wt.smt1.
sp.ugt80.
sp.wt.ugt80.
sp.smt1.
sp.wt.smt1.
im.ugt80.
im.wt.ugt80.
im.smt1.
im.wt.smt1.

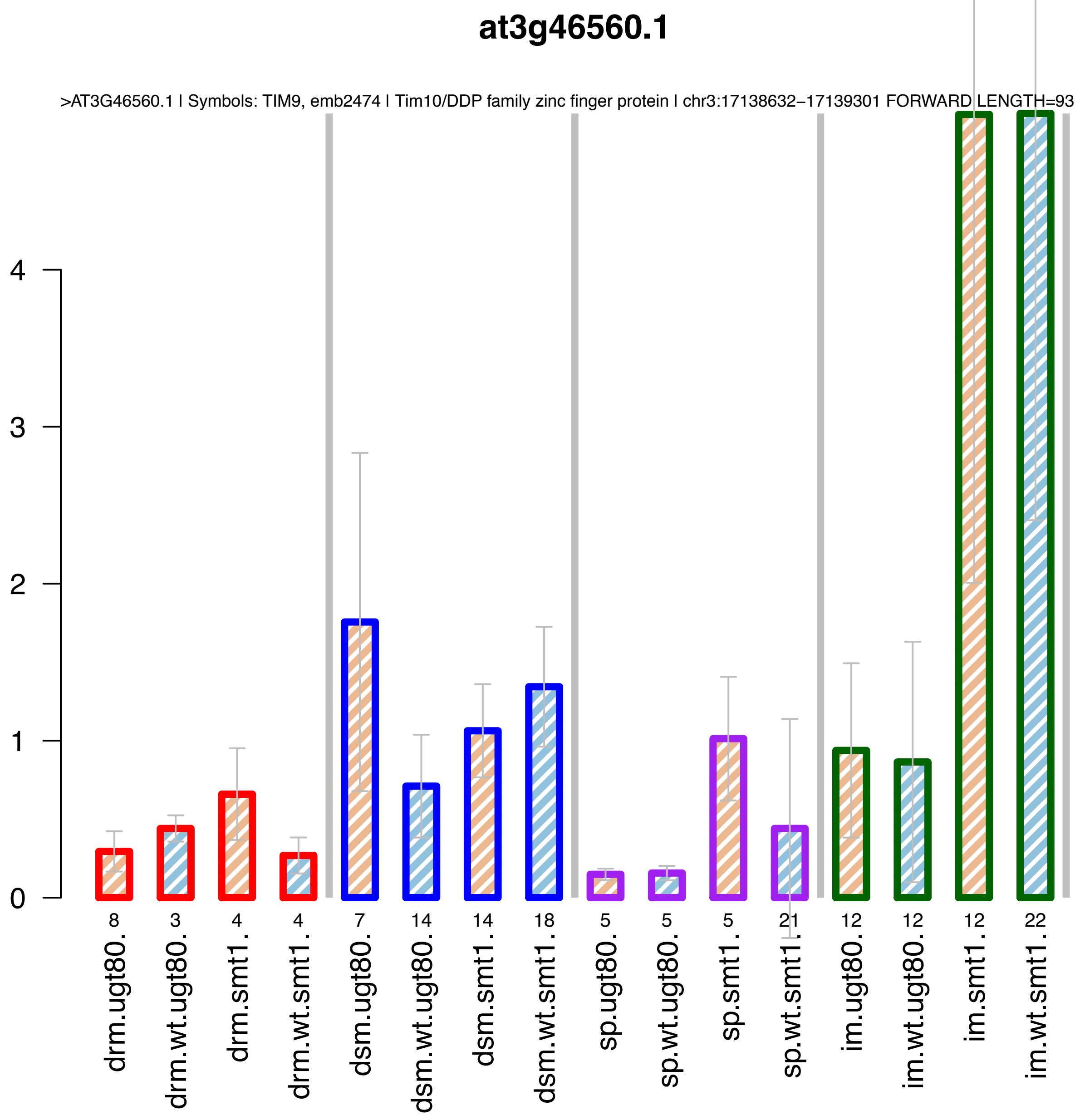

at3g46560.1
>AT3G46560.1 | Symbols: TIM9, emb2474 | Tim10/DDP family zinc finger protein | chr3:17138632−17139301 FORWARD LENGTH=93
0
1
2
3
4
8
3
4
4
7
14
14
18
5
5
5
21
12
12
12
22
drm.ugt80..
drm.wt.ugt80..
drm.smt1..
drm.wt.smt1..
dsm.ugt80..
dsm.wt.ugt80..
dsm.smt1..
dsm.wt.smt1..
sp.ugt80..
sp.wt.ugt80..
sp.smt1..
sp.wt.smt1..
im.ugt80..
im.wt.ugt80..
im.smt1..
im.wt.smt1..

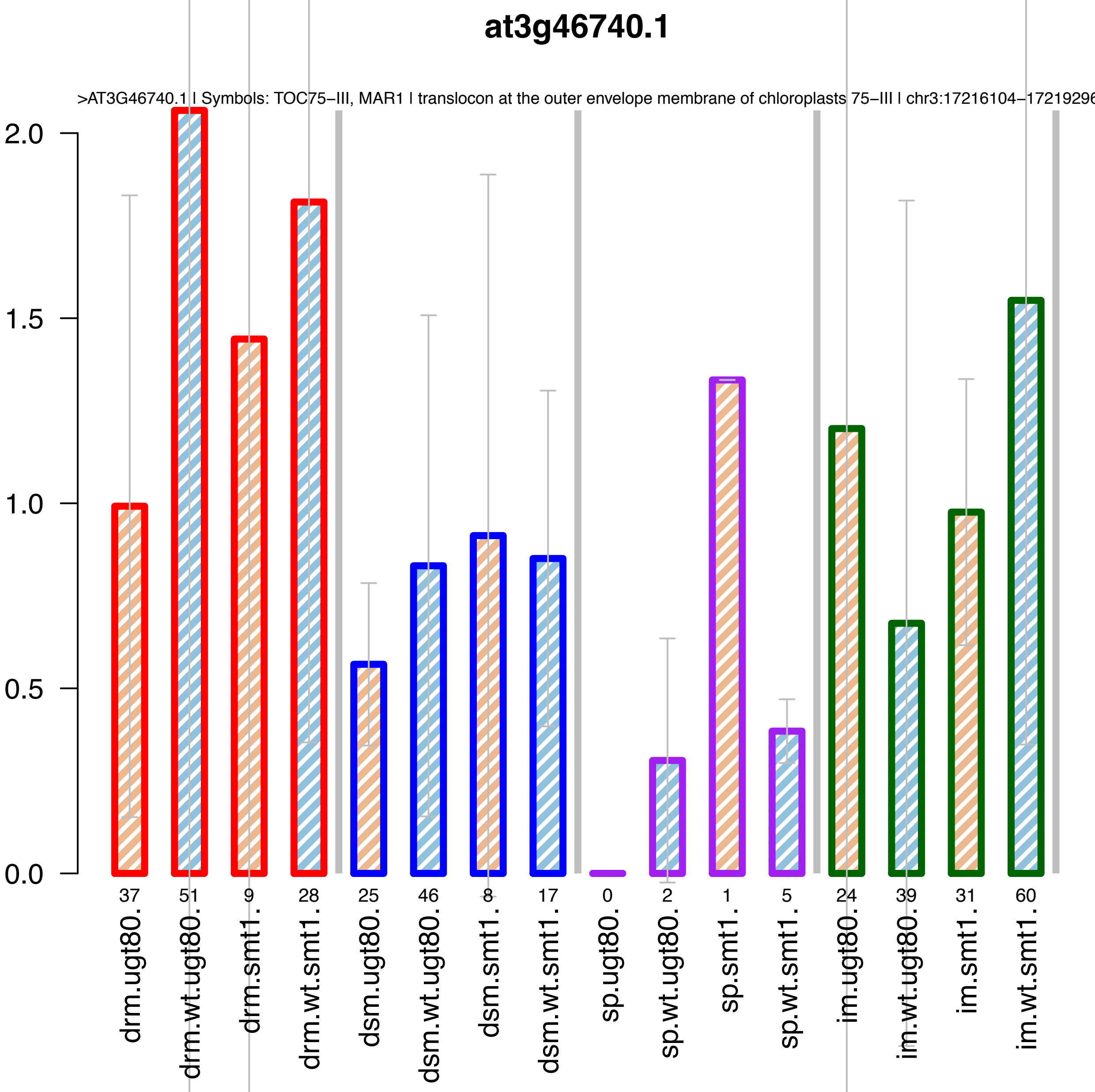

at3g46740.1
>AT3G46740.1 | Symbols: TOC75−III, MAR1 | translocon at the outer envelope membrane of chloroplasts 75−III | chr3:17216104−17219296
2.0
1.5
1.0
0.5
0.0
37
51
9
28
25
46
8
17
0
2
1
5
24
39
31
60
drm.ugt80.
drm.wt.ugt80.
drm.smt1.
drm.wt.smt1.
dsm.ugt80.
dsm.wt.ugt80.
dsm.smt1.
dsm.wt.smt1.
sp.ugt80.
sp.wt.ugt80.
sp.smt1.
sp.wt.smt1.
im.ugt80.
im.wt.ugt80.
im.smt1.
im.wt.smt1.

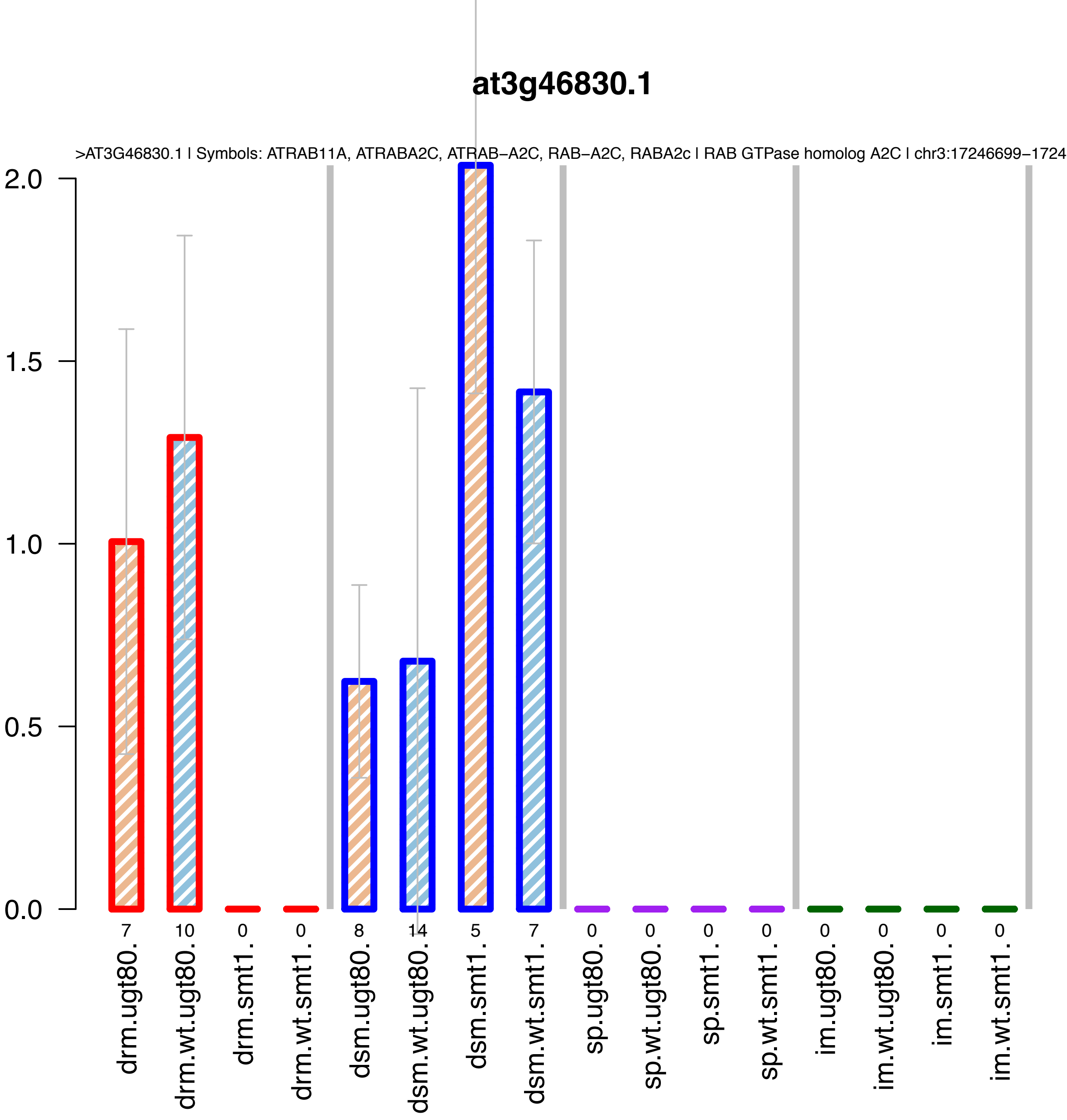

at3g46830.1
>AT3G46830.1 | Symbols: ATRAB11A, ATRABA2C, ATRAB-A2C, RAB-A2C, RABA2c | RAB GTPase homolog A2C | chr3:17246699-1724
2.0
1.5
1.0
0.5
0.0
7
10
0
0
8
14
5
7
0
0
0
0
0
0
0
0
drm.ugt80.
drm.wt.ugt80.
drm.smt1.
drm.wt.smt1.
dsm.ugt80.
dsm.wt.ugt80.
dsm.smt1.
dsm.wt.smt1.
sp.ugt80.
sp.wt.ugt80.
sp.smt1.
sp.wt.smt1.
im.ugt80.
im.wt.ugt80.
im.smt1.
im.wt.smt1.

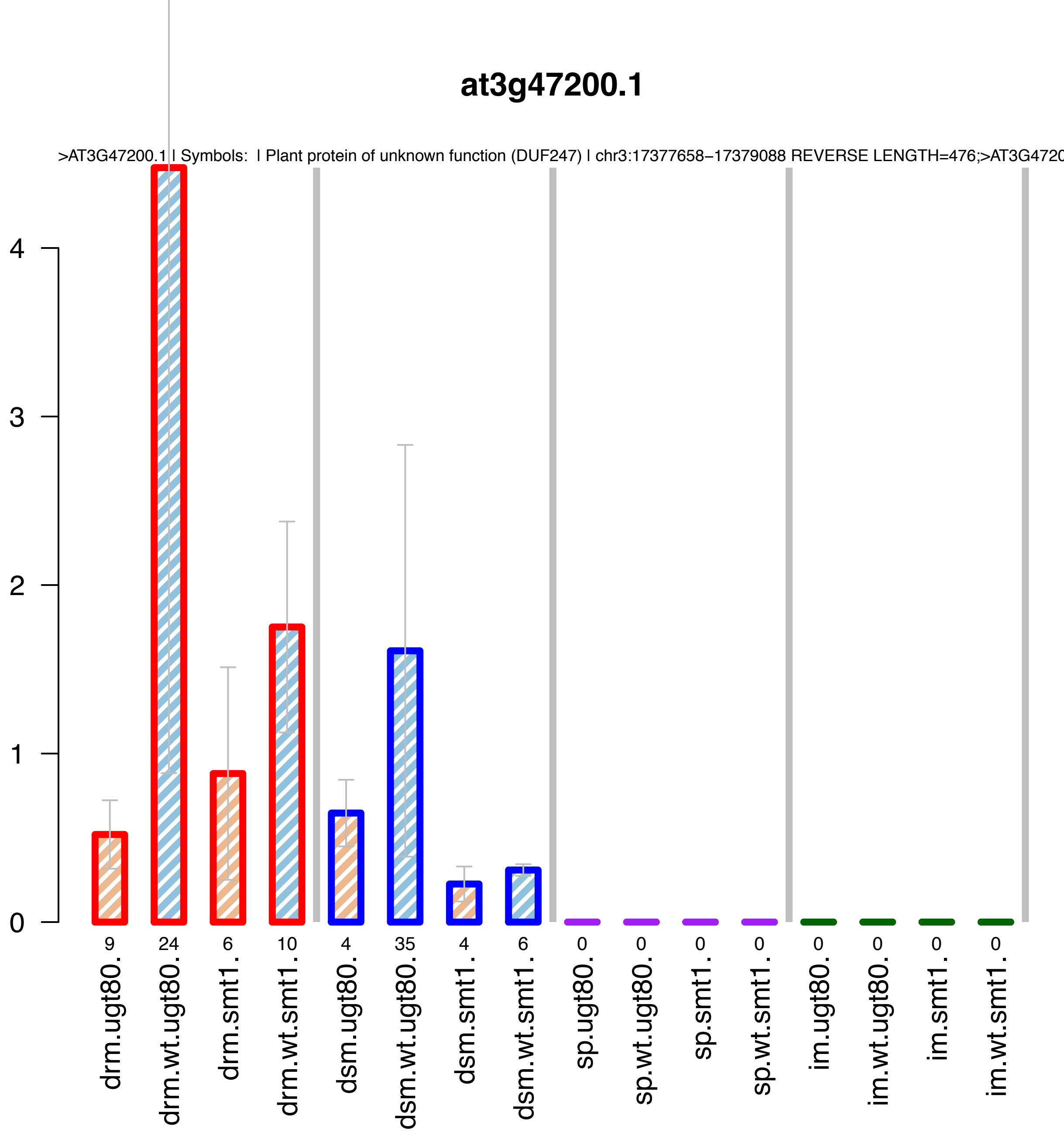

at3g47200.1
>AT3G47200.1 | Symbols: | Plant protein of unknown function (DUF247) | chr3:17377658-17379088 REVERSE LENGTH=476;>AT3G4720
0
1
2
3
4
9
24
6
10
4
35
4
6
0
0
0
0
0
0
0
0
drm.ugt80.
drm.wt.ugt80.
drm.smt1.
drm.wt.smt1.
dsm.ugt80.
dsm.wt.ugt80.
dsm.smt1.
dsm.wt.smt1.
sp.ugt80.
sp.wt.ugt80.
sp.smt1.
sp.wt.smt1.
im.ugt80.
im.wt.ugt80.
im.smt1.
im.wt.smt1.

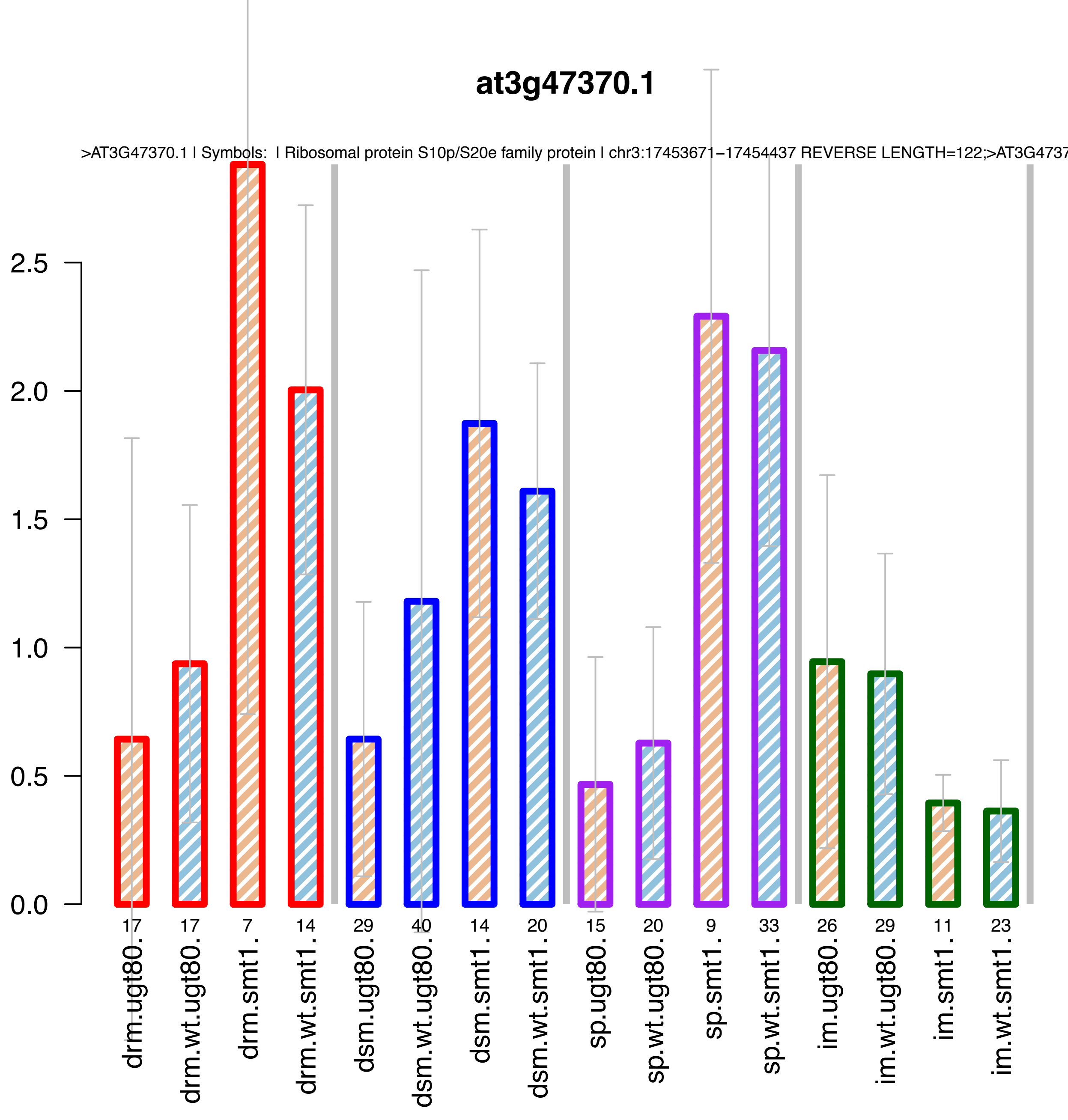

at3g47370.1
>AT3G47370.1 | Symbols: | Ribosomal protein S10p/S20e family protein | chr3:17453671-17454437 REVERSE LENGTH=122;>AT3G4737
2.5
2.0
1.5
1.0
0.5
0.0
17
17
7
14
29
40
14
20
15
20
9
33
26
29
11
23
drm.ugt80.
drm.wt.ugt80.
drm.smt1.
drm.wt.smt1.
dsm.ugt80.
dsm.wt.ugt80.
dsm.smt1.
dsm.wt.smt1.
sp.ugt80.
sp.wt.ugt80.
sp.smt1.
sp.wt.smt1.
im.ugt80.
im.wt.ugt80.
im.smt1.
im.wt.smt1.

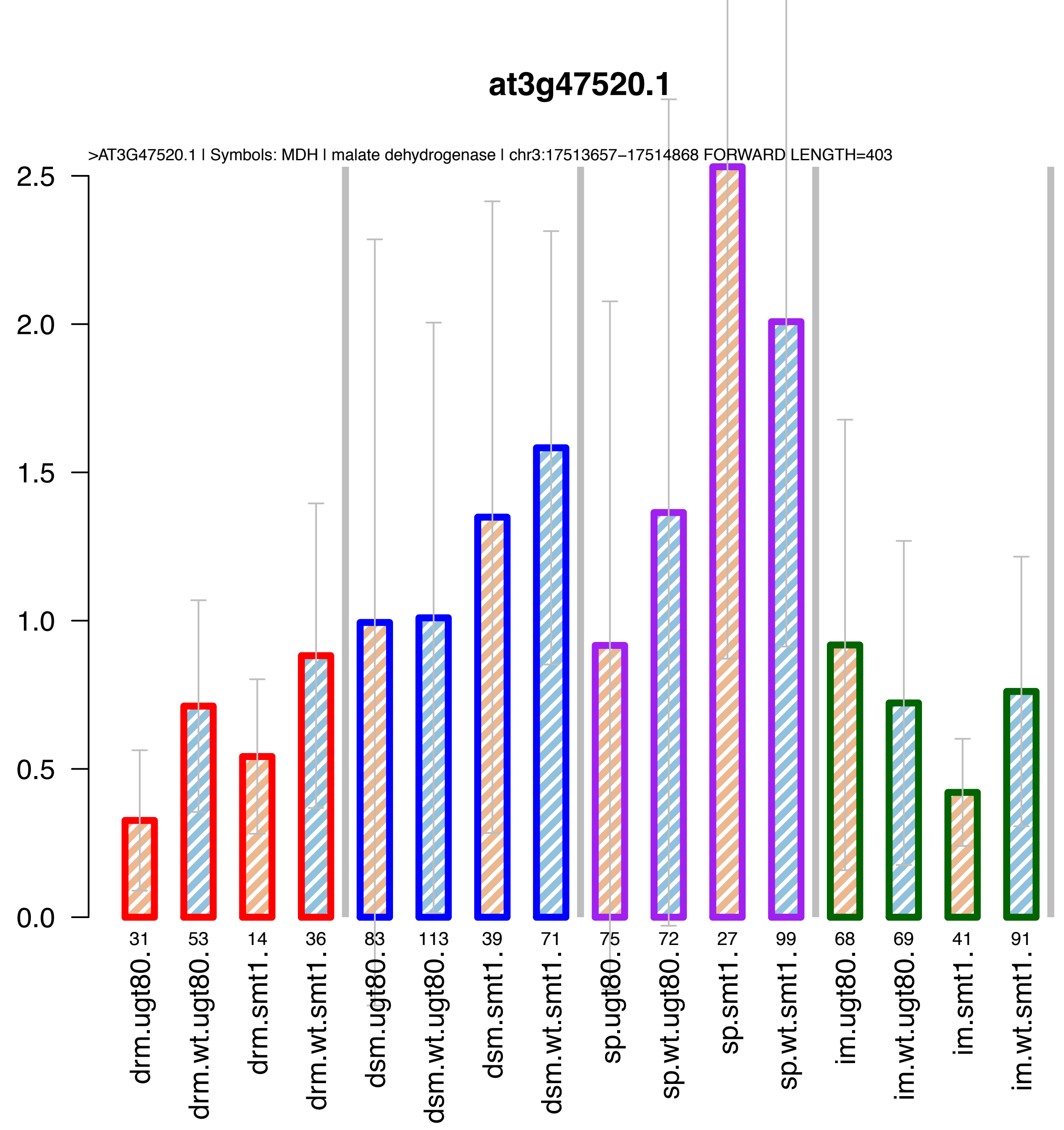

at3g47520.1
>AT3G47520.1 | Symbols: MDH | malate dehydrogenase | chr3:17513657−17514868 FORWARD LENGTH=403
2.5
2.0
1.5
1.0
0.5
0.0
31
53
14
36
83
113
39
71
75
72
27
99
68
69
41
91
drm.ugt80..
drm.wt.ugt80..
drm.smt1..
drm.wt.smt1..
dsm.ugt80..
dsm.wt.ugt80..
dsm.smt1..
dsm.wt.smt1..
sp.ugt80..
sp.wt.ugt80..
sp.smt1..
sp.wt.smt1..
im.ugt80..
im.wt.ugt80..
im.smt1..
im.wt.smt1..

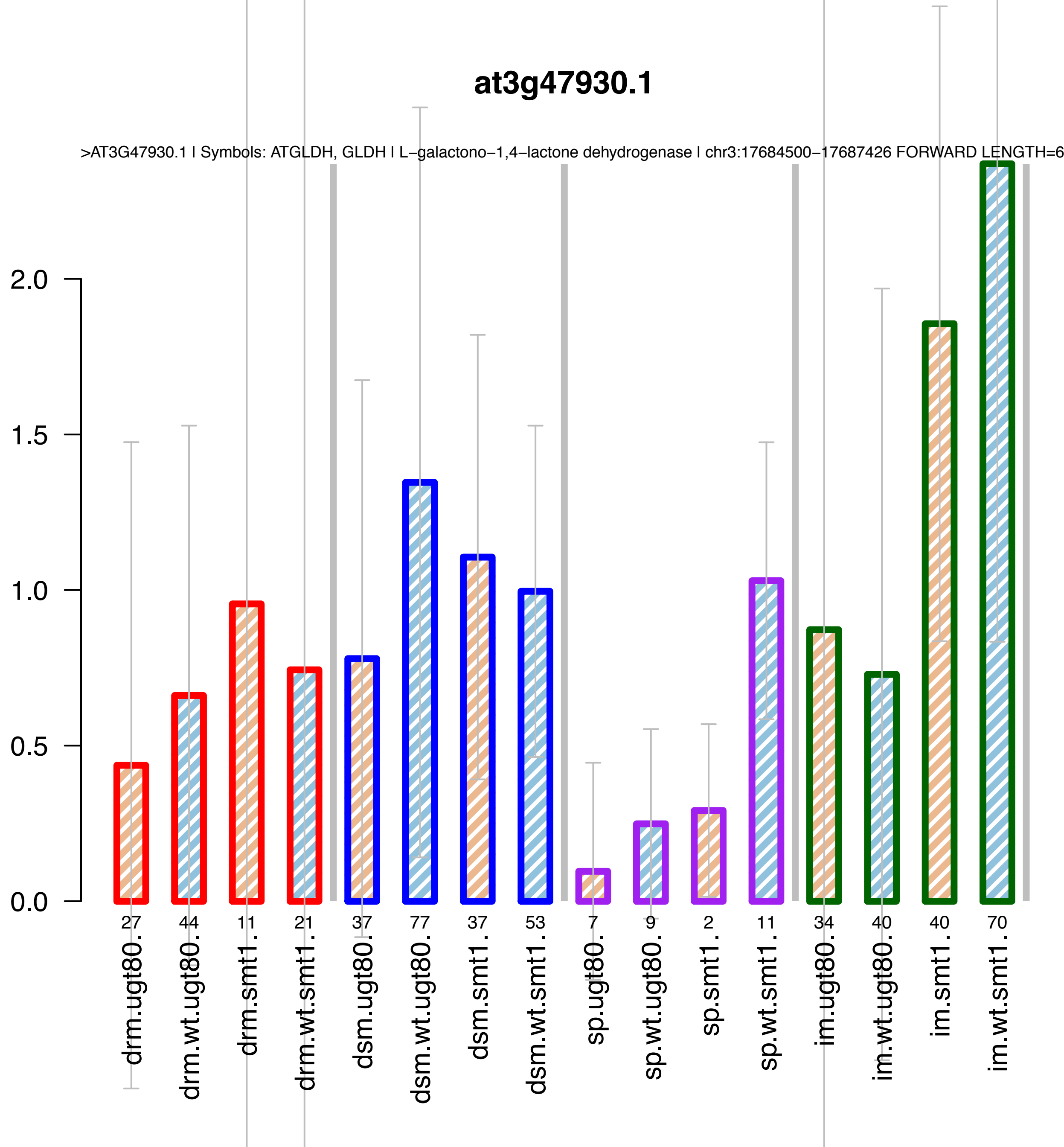
at3g47930.1
>AT3G47930.1 | Symbols: ATGLDH, GLDH | L−galactono−1,4−lactone dehydrogenase | chr3:17684500−17687426 FORWARD LENGTH=6
2.0
1.5
1.0
0.5
0.0
27
44
11
21
37
77
37
53
7
9
2
11
34
40
40
70
drm.ugt80.
drm.wt.ugt80.
drm.smt1.
drm.wt.smt1.
dsm.ugt80.
dsm.wt.ugt80.
dsm.smt1.
dsm.wt.smt1.
sp.ugt80.
sp.wt.ugt80.
sp.smt1.
sp.wt.smt1.
im.ugt80.
im.wt.ugt80.
im.smt1.
im.wt.smt1.

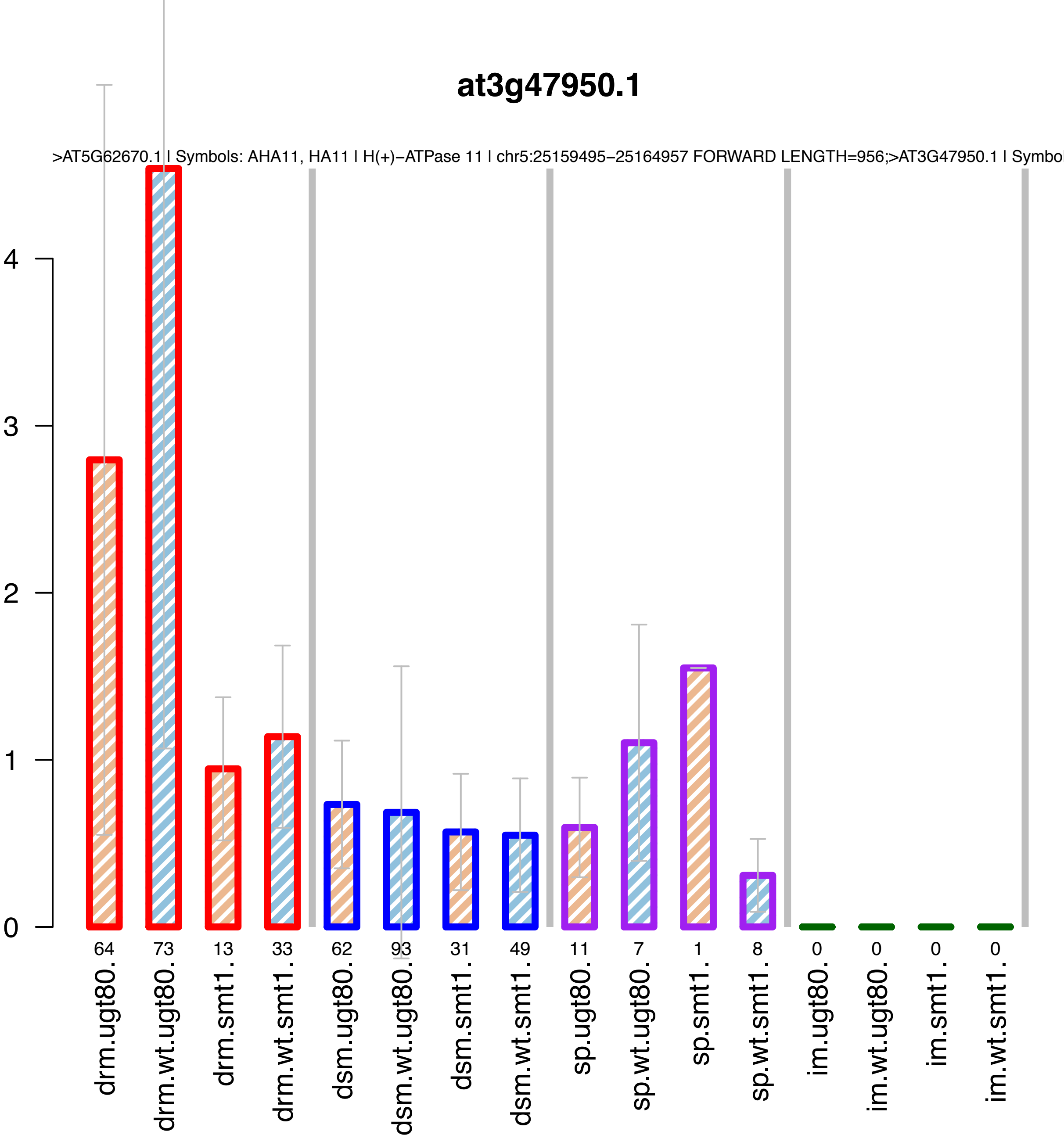

at3g47950.1
>AT5G62670.1 | Symbols: AHA11, HA11 | H(+)−ATPase 11 | chr5:25159495−25164957 FORWARD LENGTH=956;>AT3G47950.1 | Symbo
0
1
2
3
4
64
73
13
33
62
93
31
49
11
7
1
8
0
0
0
0
drm.ugt80.
drm.wt.ugt80.
drm.smt1.
drm.wt.smt1.
dsm.ugt80.
dsm.wt.ugt80.
dsm.smt1.
dsm.wt.smt1.
sp.ugt80.
sp.wt.ugt80.
sp.smt1.
sp.wt.smt1.
im.ugt80.
im.wt.ugt80.
im.smt1.
im.wt.smt1.

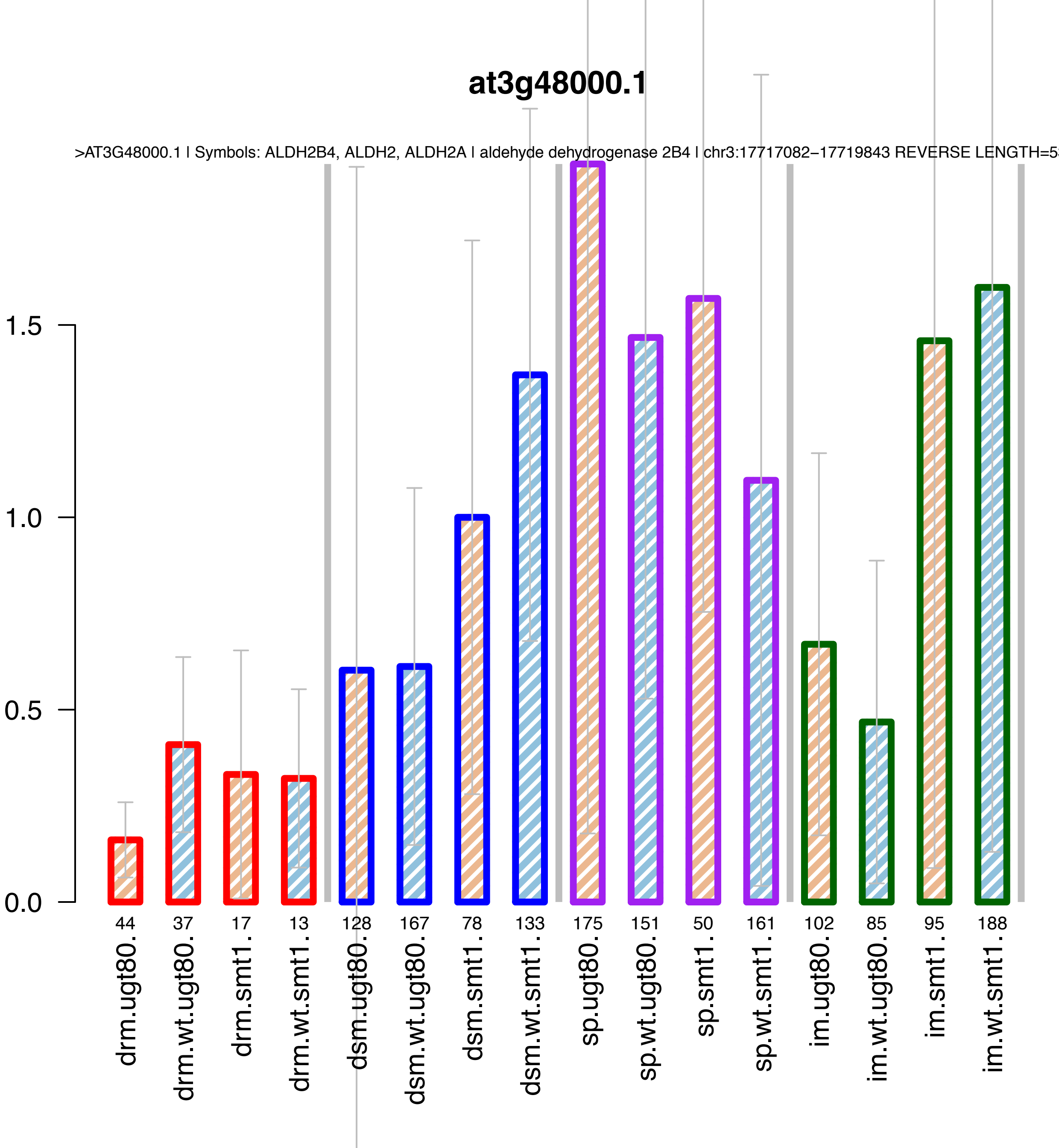
at3g48000.1
>AT3G48000.1 | Symbols: ALDH2B4, ALDH2, ALDH2A | aldehyde dehydrogenase 2B4 | chr3:17717082-17719843 REVERSE LENGTH=5
0.0
0.5
1.0
1.5
44
37
17
13
128
167
78
133
175
151
50
161
102
85
95
188
drm.ugt80..
drm.wt.ugt80..
drm.smt1..
drm.wt.smt1..
dsm.ugt80..
dsm.wt.ugt80..
dsm.smt1..
dsm.wt.smt1..
sp.ugt80..
sp.wt.ugt80..
sp.smt1..
sp.wt.smt1..
im.ugt80..
im.wt.ugt80..
im.smt1..
im.wt.smt1..

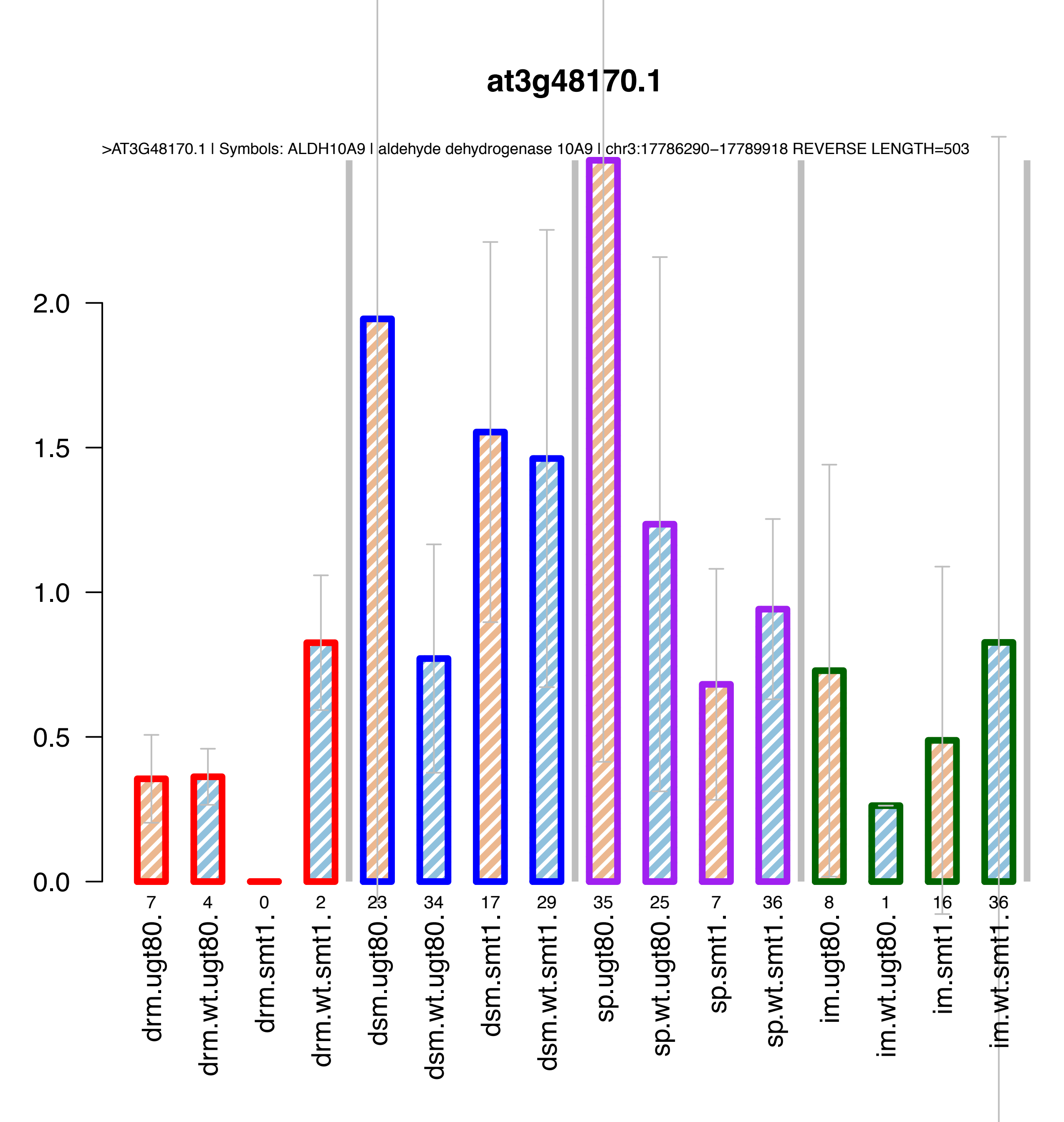

at3g48170.1
>AT3G48170.1 | Symbols: ALDH10A9 | aldehyde dehydrogenase 10A9 | chr3:17786290-17789918 REVERSE LENGTH=503
0.0
0.5
1.0
1.5
2.0
7
4
0
2
23
34
17
29
35
25
7
36
8
1
16
36
drm.ugt80.
drm.wt.ugt80.
drm.smt1.
drm.wt.smt1.
dsm.ugt80.
dsm.wt.ugt80.
dsm.smt1.
dsm.wt.smt1.
sp.ugt80.
sp.wt.ugt80.
sp.smt1.
sp.wt.smt1.
im.ugt80.
im.wt.ugt80.
im.smt1.
im.wt.smt1.

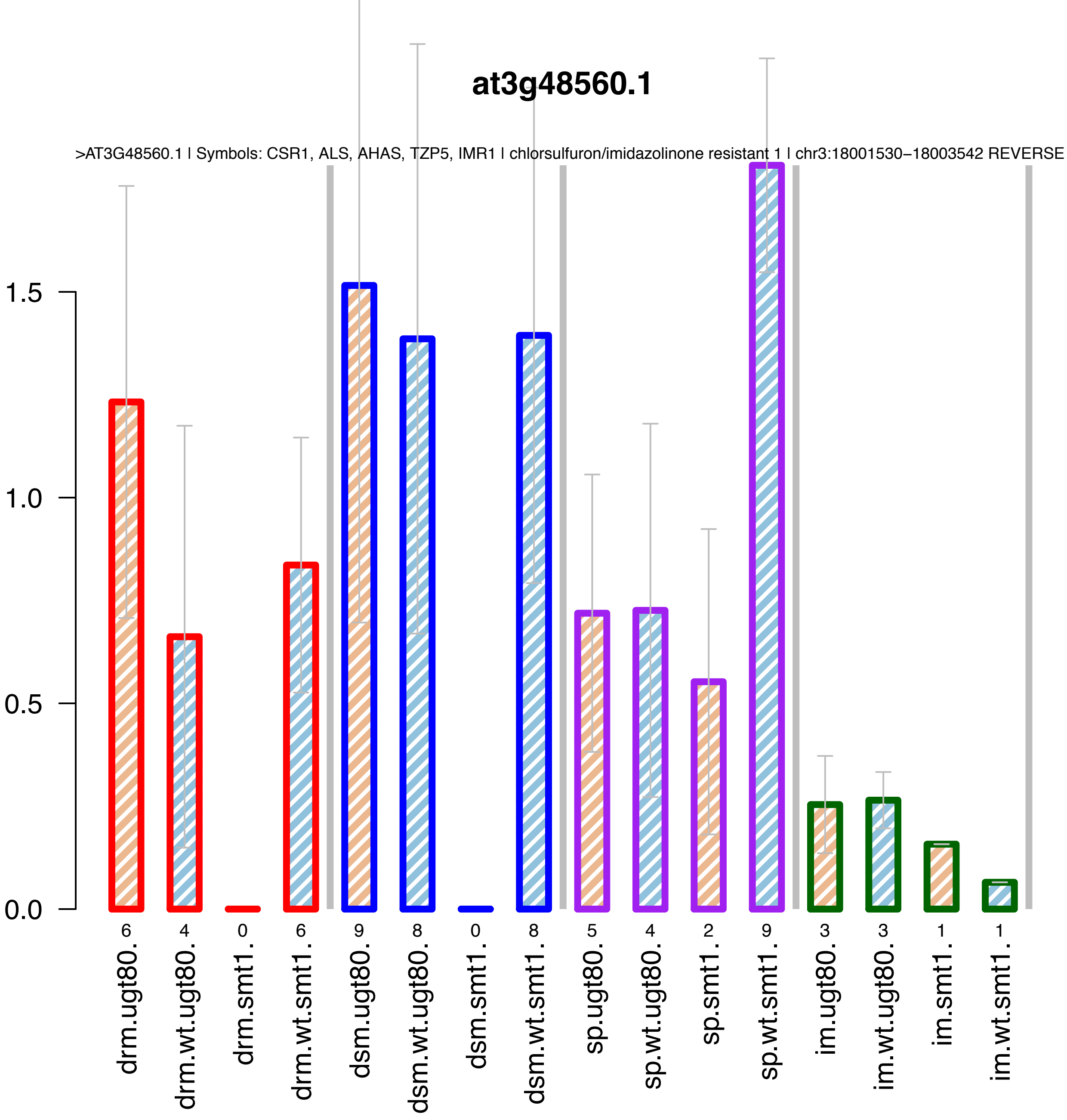

at3g48560.1
>AT3G48560.1 | Symbols: CSR1, ALS, AHAS, TZP5, IMR1 | chlorsulfuron/imidazolinone resistant 1 | chr3:18001530−18003542 REVERSE
0.0
0.5
1.0
1.5
6
4
0
6
9
8
0
8
5
4
2
9
3
3
1
1
drm.ugt80.
drm.wt.ugt80.
drm.smt1.
drm.wt.smt1.
dsm.ugt80.
dsm.wt.ugt80.
dsm.smt1.
dsm.wt.smt1.
sp.ugt80.
sp.wt.ugt80.
sp.smt1.
sp.wt.smt1.
im.ugt80.
im.wt.ugt80.
im.smt1.
im.wt.smt1.

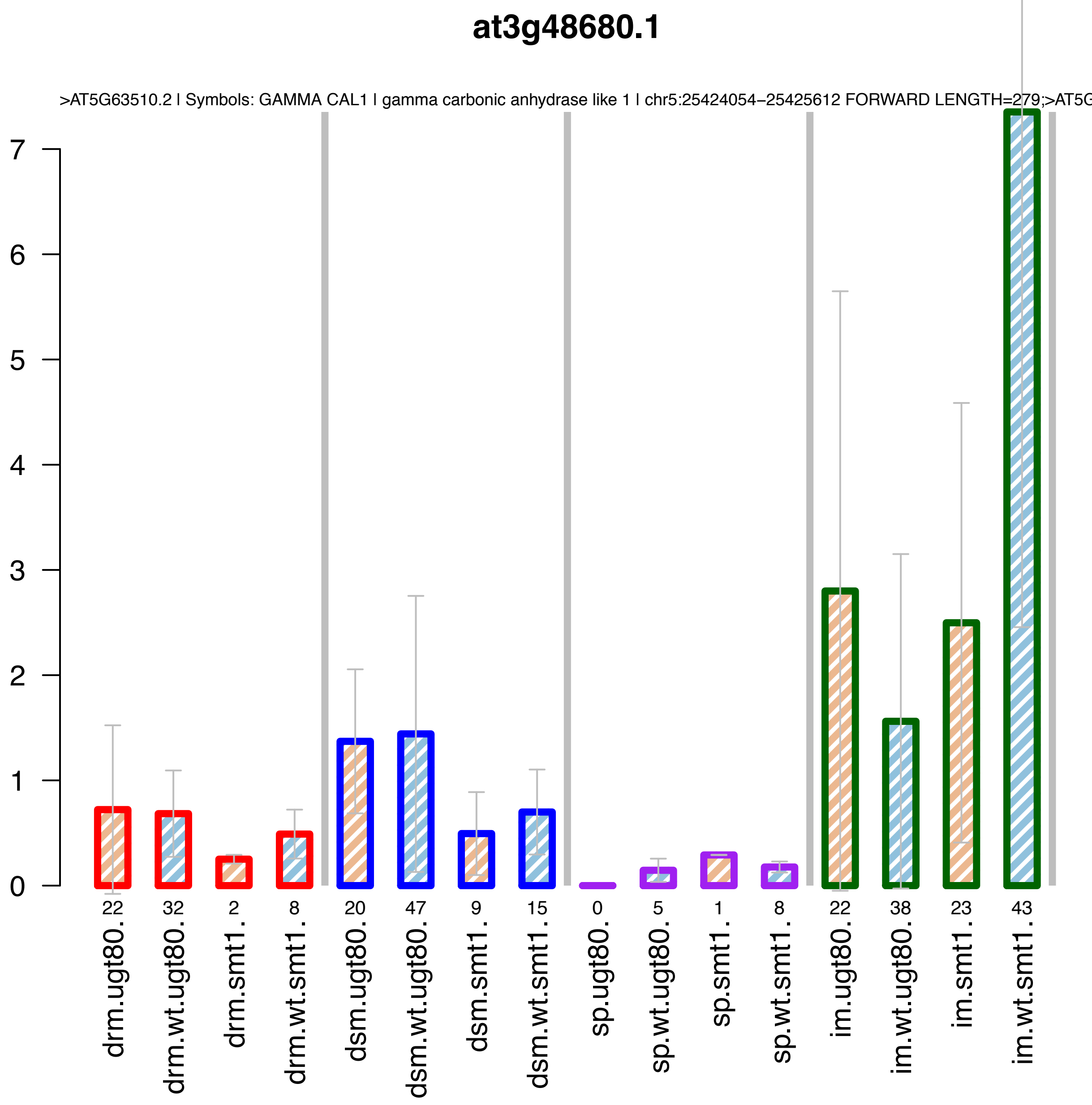

at3g48680.1
>AT5G63510.2 | Symbols: GAMMA CAL1 | gamma carbonic anhydrase like 1 | chr5:25424054-25425612 FORWARD LENGTH=279;>AT5G
0
1
2
3
4
5
6
7
22
32
2
8
20
47
9
15
0
5
1
8
22
38
23
43
drm.ugt80..
drm.wt.ugt80..
drm.smt1..
drm.wt.smt1..
dsm.ugt80..
dsm.wt.ugt80..
dsm.smt1..
dsm.wt.smt1..
sp.ugt80..
sp.wt.ugt80..
sp.smt1..
sp.wt.smt1..
im.ugt80..
im.wt.ugt80..
im.smt1..
im.wt.smt1..

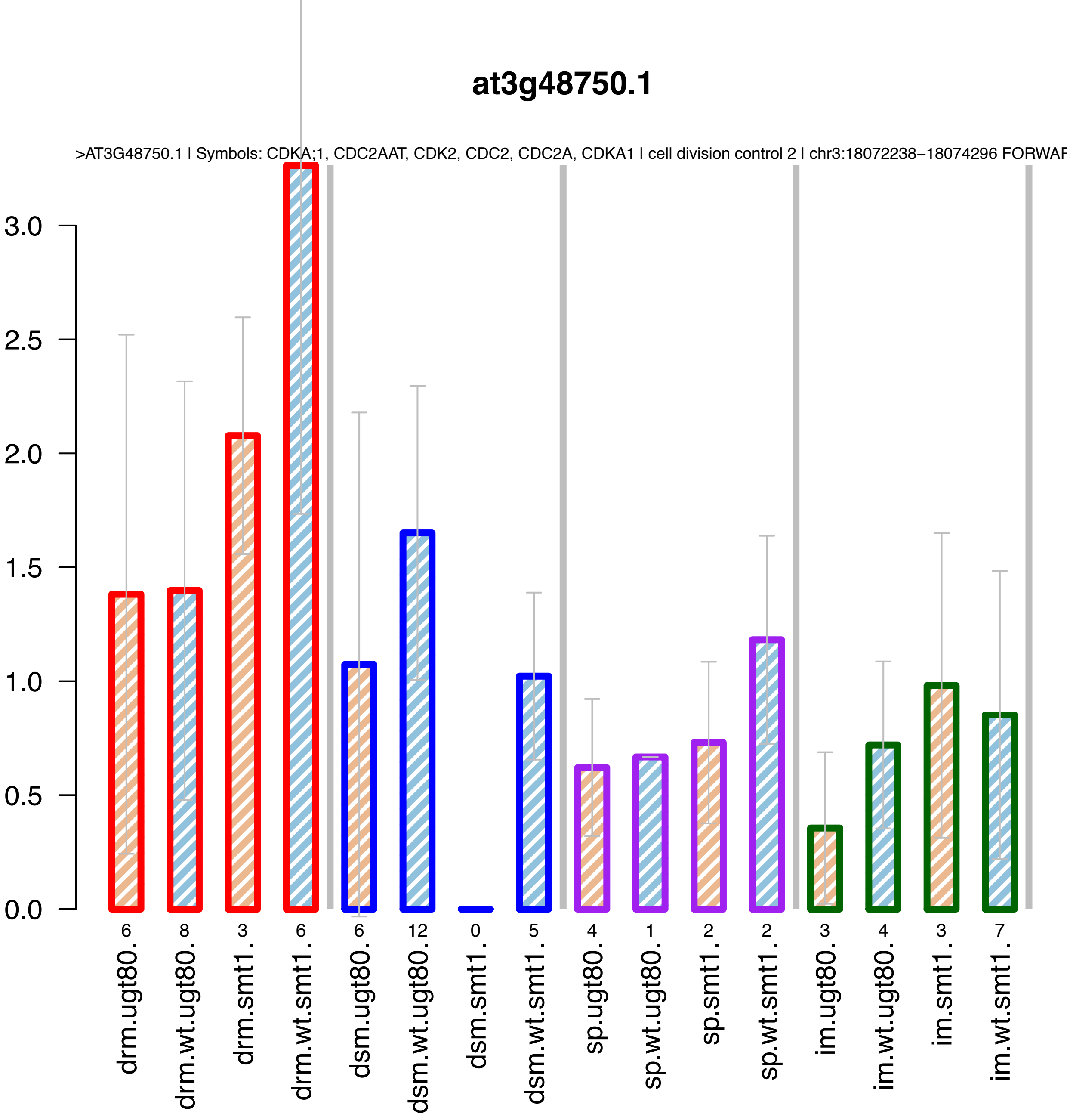

at3g48750.1
>AT3G48750.1 | Symbols: CDKA;1, CDC2AAT, CDK2, CDC2, CDC2A, CDKA1 | cell division control 2 | chr3:18072238-18074296 FORWAR
0.0
0.5
1.0
1.5
2.0
2.5
3.0
6
8
3
6
6
12
0
5
4
1
2
2
3
4
3
7
drm.ugt80.
drm.wt.ugt80.
drm.smt1.
drm.wt.smt1.
dsm.ugt80.
dsm.wt.ugt80.
dsm.smt1.
dsm.wt.smt1.
sp.ugt80.
sp.wt.ugt80.
sp.smt1.
sp.wt.smt1.
im.ugt80.
im.wt.ugt80.
im.smt1.
im.wt.smt1.

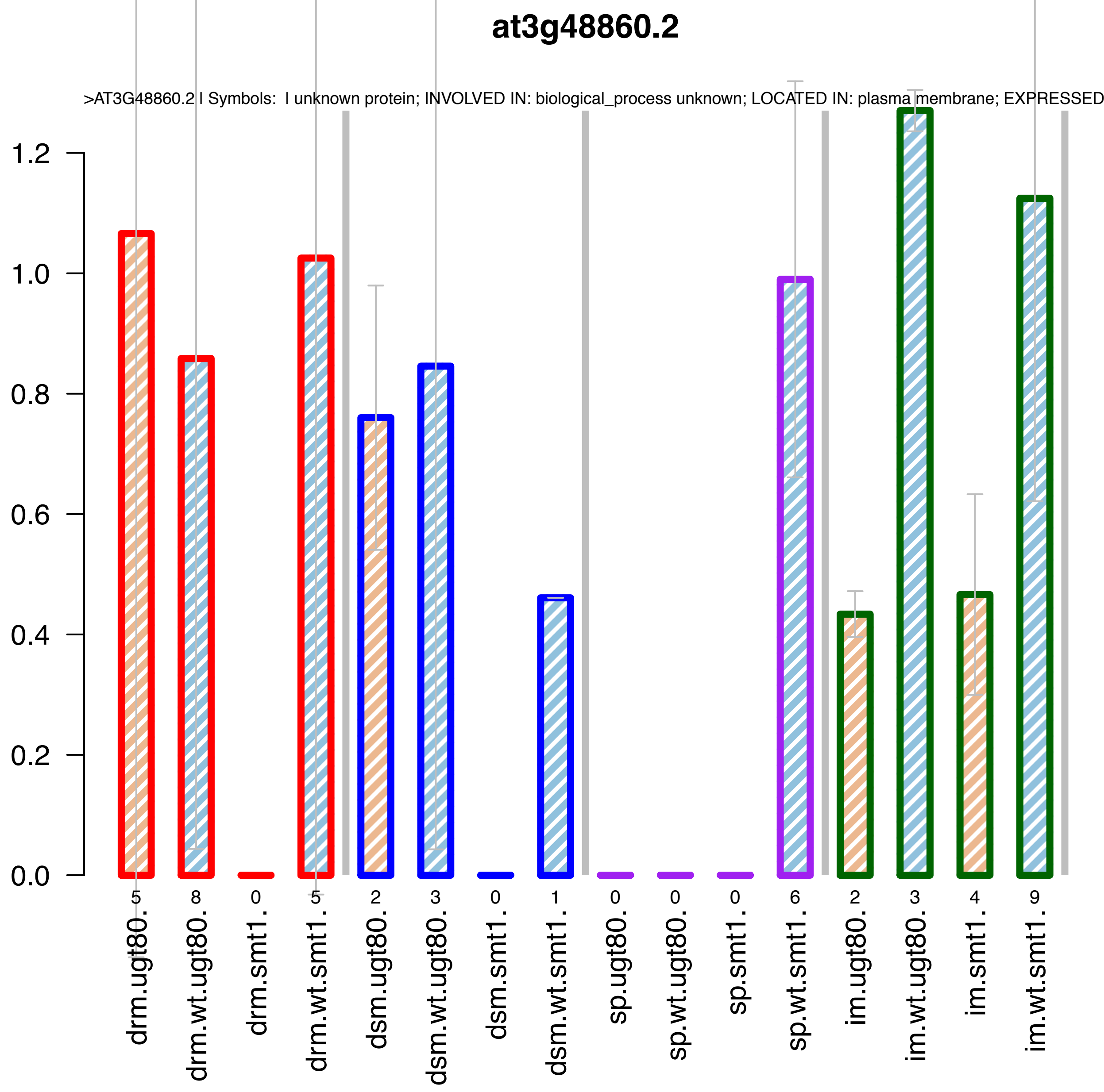

at3g48860.2
>AT3G48860.2 | Symbols: | unknown protein; INVOLVED IN: biological_process unknown; LOCATED IN: plasma membrane; EXPRESSED
1.2
1.0
0.8
0.6
0.4
0.2
0.0
5
8
0
5
2
3
0
1
0
0
0
6
2
3
4
9
drm.ugt80..
drm.wt.ugt80..
drm.smt1..
drm.wt.smt1..
dsm.ugt80..
dsm.wt.ugt80..
dsm.smt1..
dsm.wt.smt1..
sp.ugt80..
sp.wt.ugt80..
sp.smt1..
sp.wt.smt1..
im.ugt80..
im.wt.ugt80..
im.smt1..
im.wt.smt1..

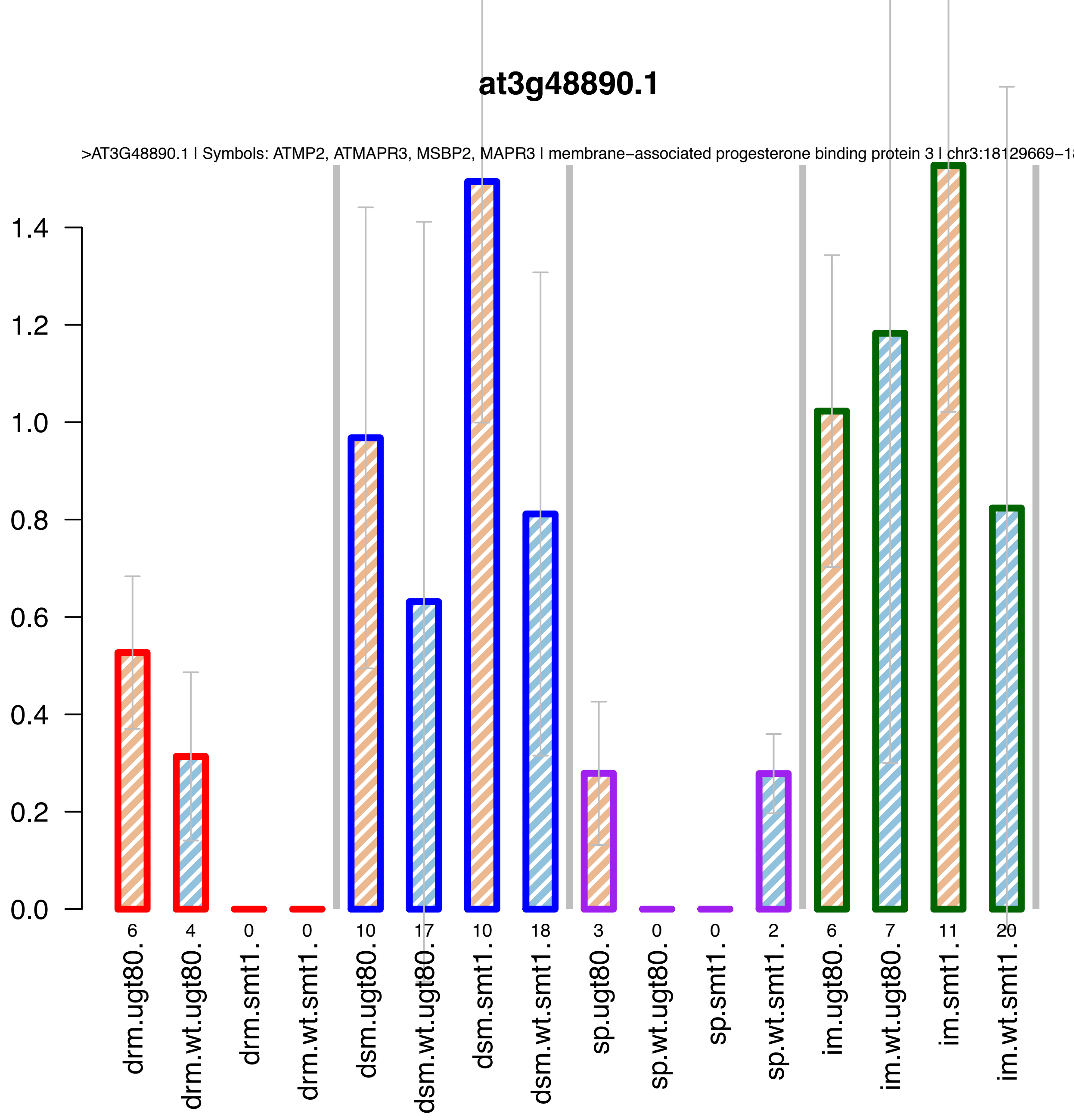

at3g48890.1
>AT3G48890.1 | Symbols: ATMP2, ATMAPR3, MSBP2, MAPR3 | membrane−associated progesterone binding protein 3 | chr3:18129669−1
0.0
0.2
0.4
0.6
0.8
1.0
1.2
1.4
6
4
0
0
10
17
10
18
3
0
0
2
6
7
11
20
drm.ugt80.
drm.wt.ugt80.
drm.smt1.
drm.wt.smt1.
dsm.ugt80.
dsm.wt.ugt80.
dsm.smt1.
dsm.wt.smt1.
sp.ugt80.
sp.wt.ugt80.
sp.smt1.
sp.wt.smt1.
im.ugt80.
im.wt.ugt80.
im.smt1.
im.wt.smt1.

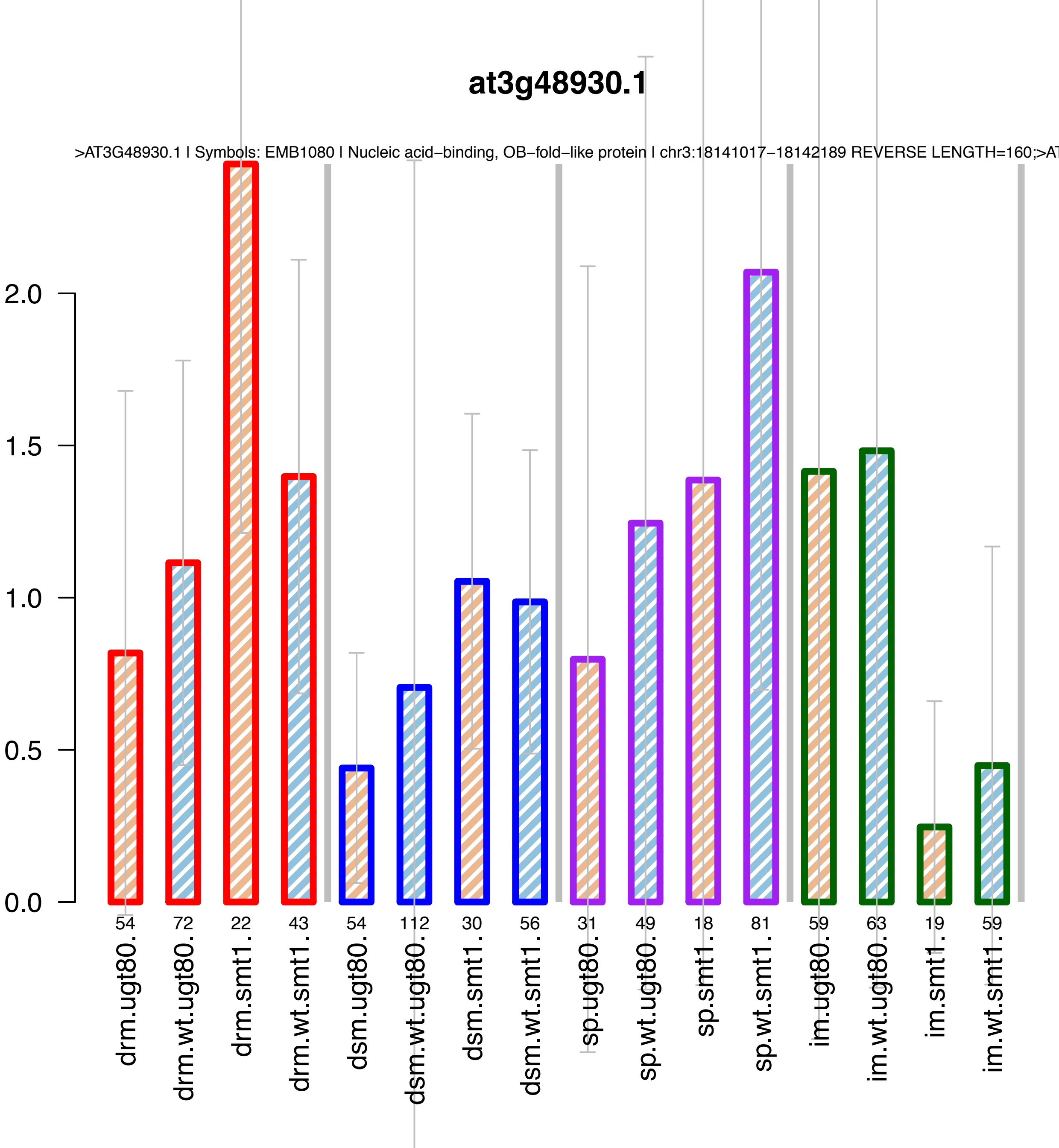

at3g48930.1
>AT3G48930.1 | Symbols: EMB1080 | Nucleic acid-binding, OB-fold-like protein | chr3:18141017-18142189 REVERSE LENGTH=160;>AT
2.0
1.5
1.0
0.5
0.0
54
72
22
43
54
112
30
56
31
49
18
81
59
63
19
59
drm.ugt80..
drm.wt.ugt80..
drm.smt1..
drm.wt.smt1..
dsm.ugt80..
dsm.wt.ugt80..
dsm.smt1..
dsm.wt.smt1..
sp.ugt80..
sp.wt.ugt80..
sp.smt1..
sp.wt.smt1..
im.ugt80..
im.wt.ugt80..
im.smt1..
im.wt.smt1..

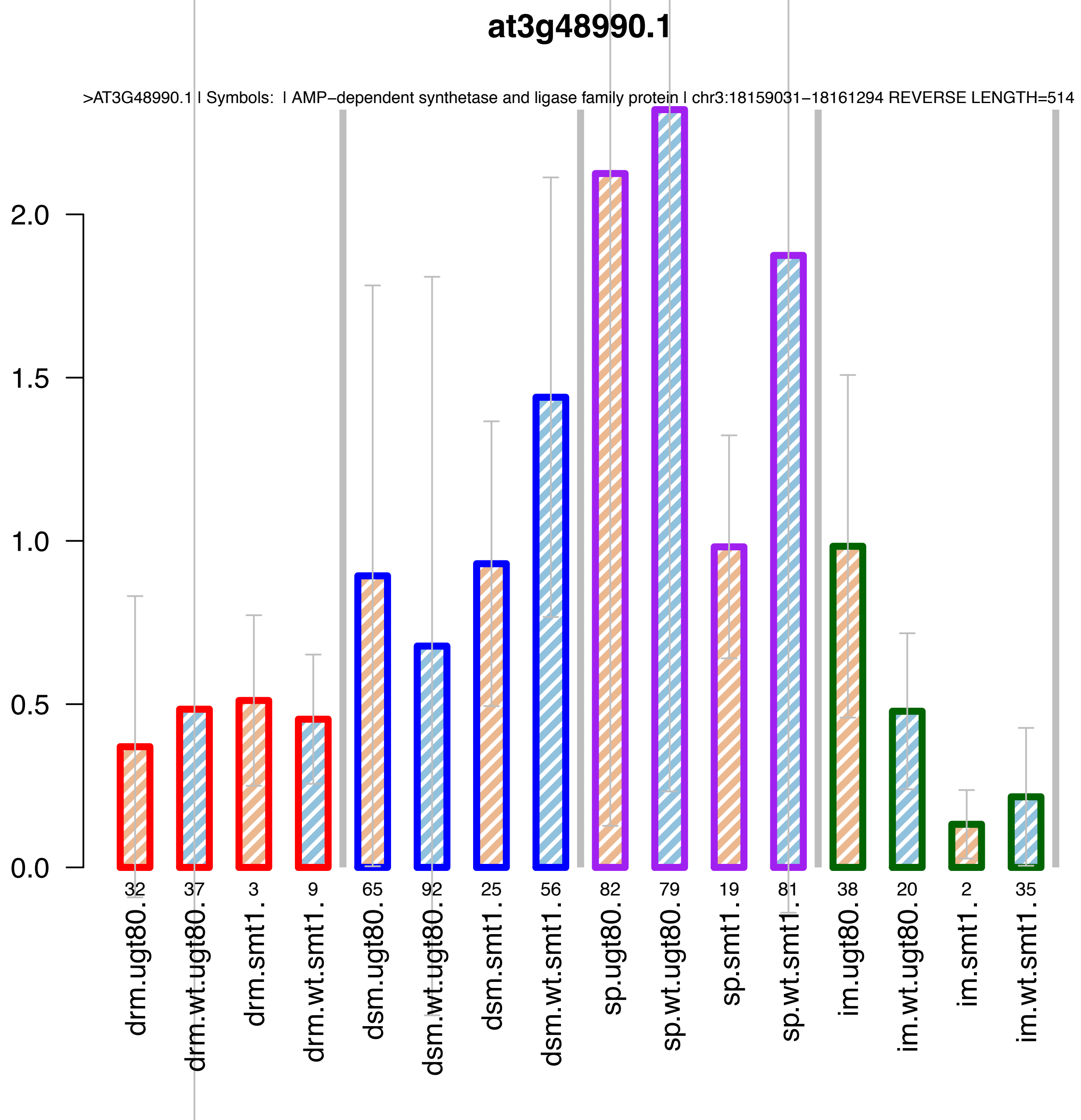

at3g48990.1
>AT3G48990.1 | Symbols: | AMP−dependent synthetase and ligase family protein | chr3:18159031−18161294 REVERSE LENGTH=514
2.0
1.5
1.0
0.5
0.0
32
37
3
9
65
92
25
56
82
79
19
81
38
20
2
35
drm.ugt80..
drm.wt.ugt80..
drm.smt1..
drm.wt.smt1..
dsm.ugt80..
dsm.wt.ugt80..
dsm.smt1..
dsm.wt.smt1..
sp.ugt80..
sp.wt.ugt80..
sp.smt1..
sp.wt.smt1..
im.ugt80..
im.wt.ugt80..
im.smt1..
im.wt.smt1..

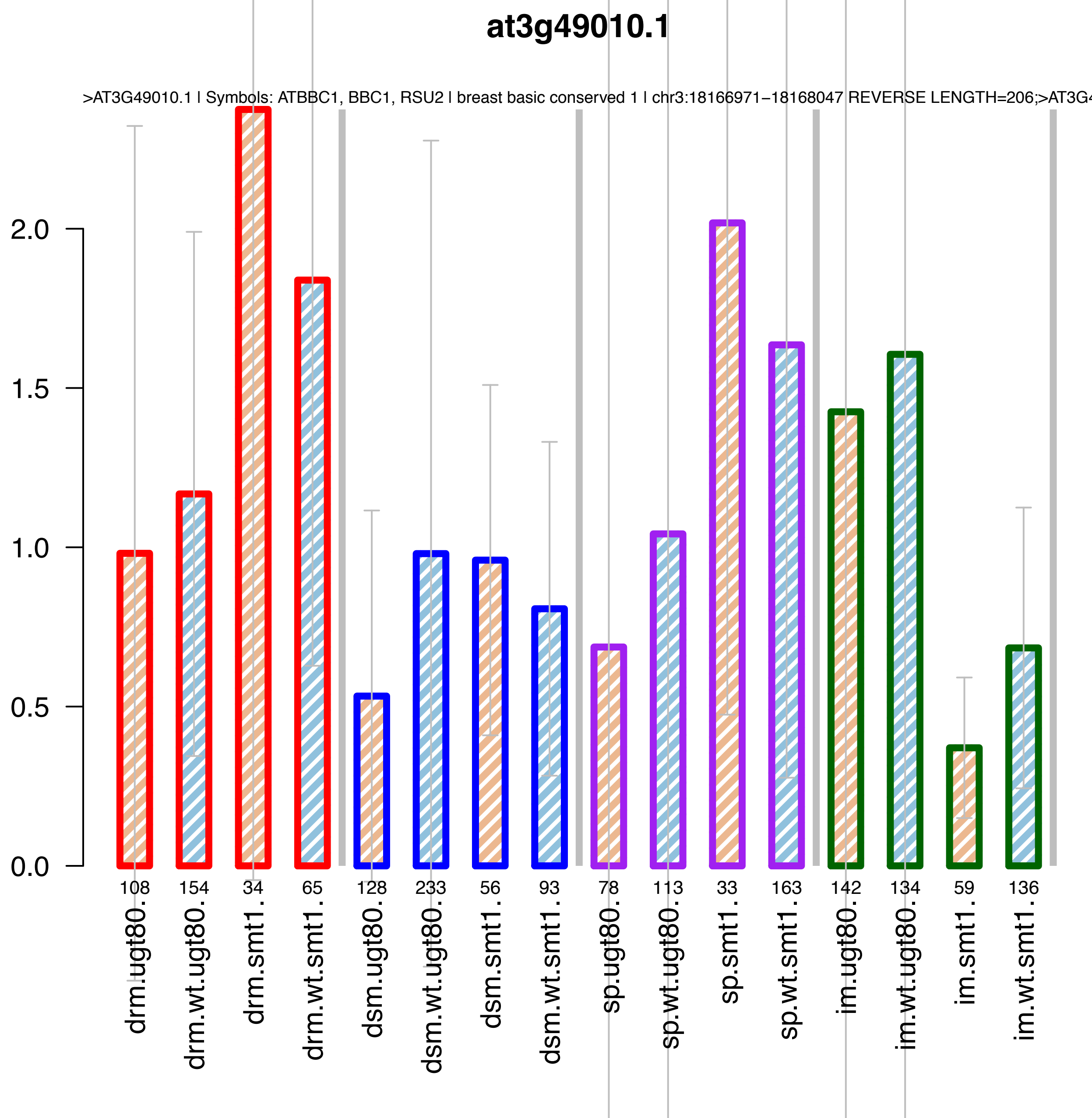
at3g49010.1
>AT3G49010.1 | Symbols: ATBBC1, BBC1, RSU2 | breast basic conserved 1 | chr3:18166971–18168047 REVERSE LENGTH=206;>AT3G4
2.0
1.5
1.0
0.5
0.0
108
154
34
65
128
233
56
93
78
113
33
163
142
134
59
136
drm.ugt80..
drm.wt.ugt80..
drm.smt1..
drm.wt.smt1..
dsm.ugt80..
dsm.wt.ugt80..
dsm.smt1..
dsm.wt.smt1..
sp.ugt80..
sp.wt.ugt80..
sp.smt1..
sp.wt.smt1..
im.ugt80..
im.wt.ugt80..
im.smt1..
im.wt.smt1..

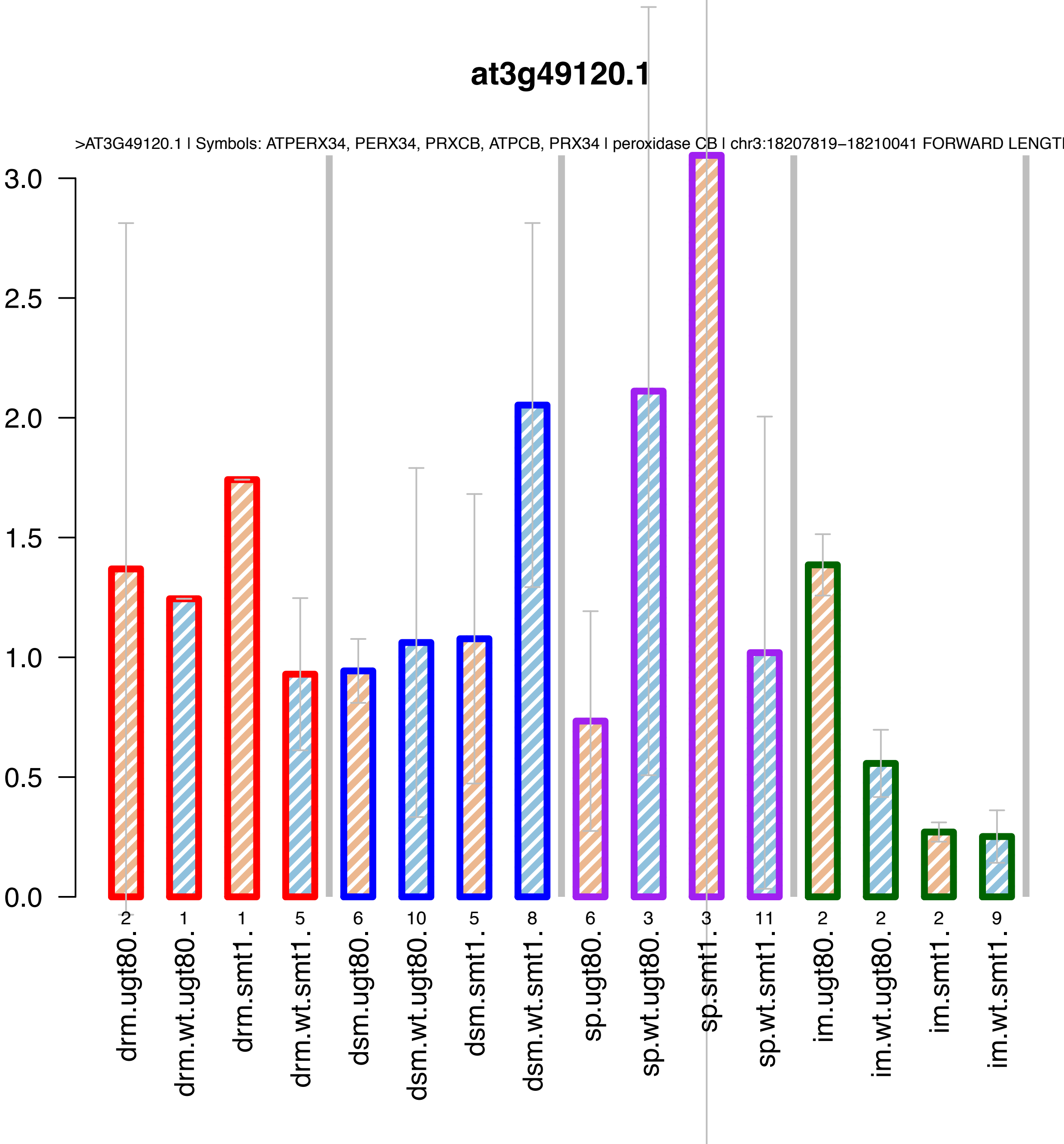

at3g49120.1
>AT3G49120.1 | Symbols: ATPERX34, PERX34, PRXCB, ATPCB, PRX34 | peroxidase CB | chr3:18207819-18210041 FORWARD LENGT
3.0
2.5
2.0
1.5
1.0
0.5
0.0
2
1
1
5
6
10
5
8
6
3
3
11
2
2
2
9
drm.ugt80.
drm.wt.ugt80.
drm.smt1.
drm.wt.smt1.
dsm.ugt80.
dsm.wt.ugt80.
dsm.smt1.
dsm.wt.smt1.
sp.ugt80.
sp.wt.ugt80.
sp.smt1.
sp.wt.smt1.
im.ugt80.
im.wt.ugt80.
im.smt1.
im.wt.smt1.

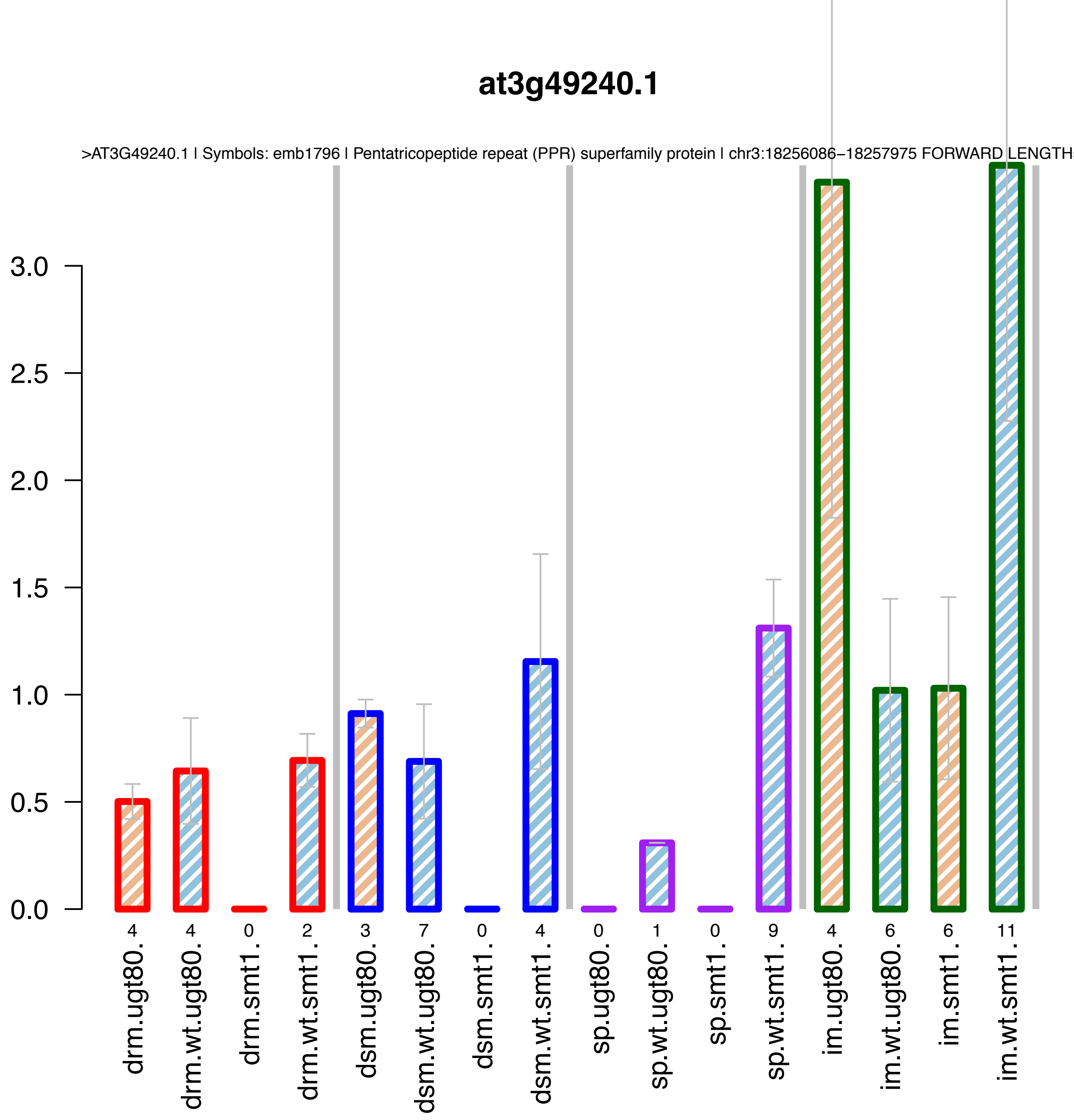

at3g49240.1
>AT3G49240.1 | Symbols: emb1796 | Pentatricopeptide repeat (PPR) superfamily protein | chr3:18256086-18257975 FORWARD LENGTH
0.0
0.5
1.0
1.5
2.0
2.5
3.0
4 4 0 2 3 7 0 4 0 1 0 9 4 6 6 11
drm.ugt80.
drm.wt.ugt80.
drm.smt1.
drm.wt.smt1.
dsm.ugt80.
dsm.wt.ugt80.
dsm.smt1.
dsm.wt.smt1.
sp.ugt80.
sp.wt.ugt80.
sp.smt1.
sp.wt.smt1.
im.ugt80.
im.wt.ugt80.
im.smt1.
im.wt.smt1.

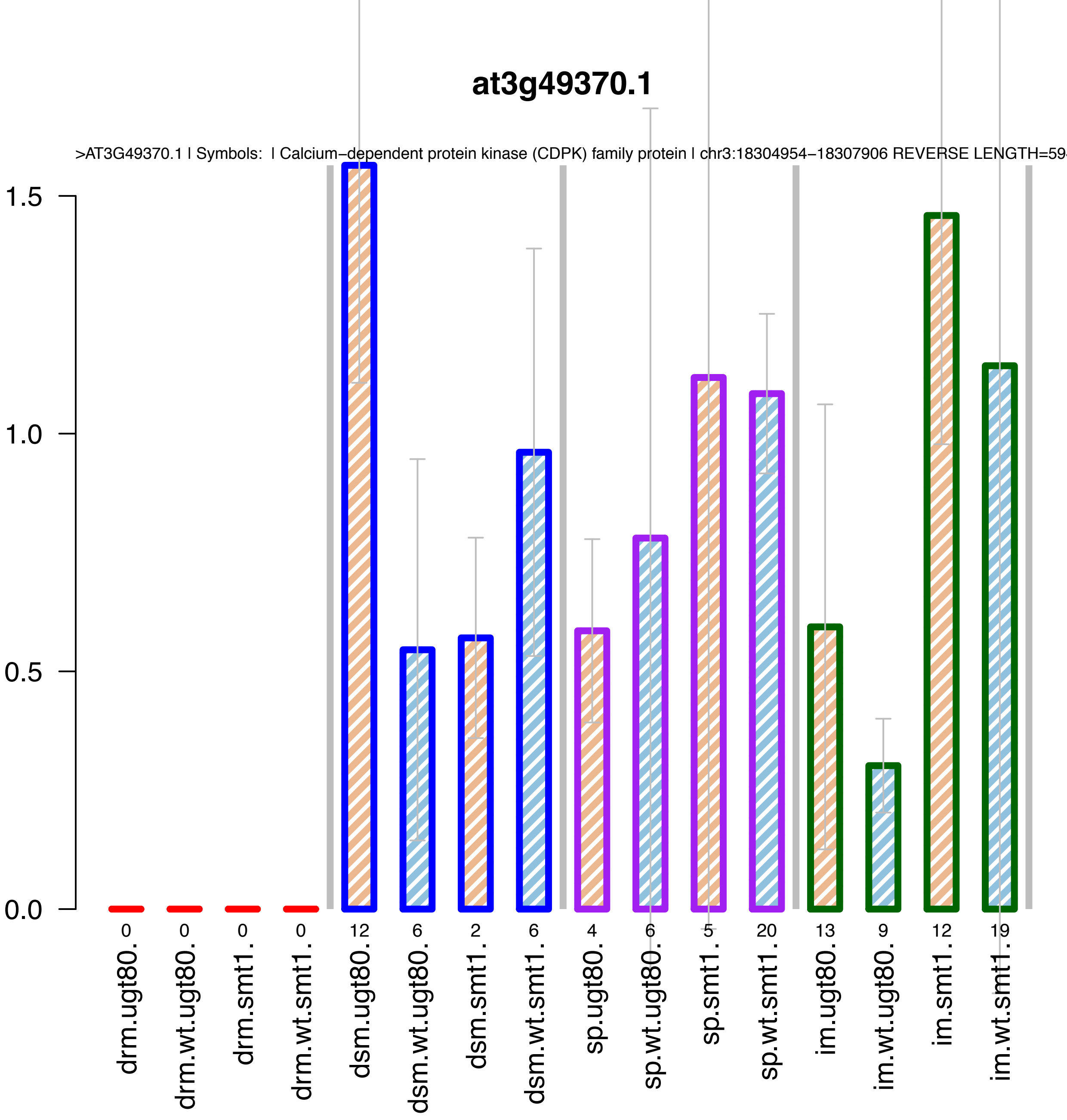
at3g49370.1
>AT3G49370.1 | Symbols: | Calcium−dependent protein kinase (CDPK) family protein | chr3:18304954−18307906 REVERSE LENGTH=59
1.5
1.0
0.5
0.0
0
0
0
0
12
6
2
6
4
6
5
20
13
9
12
19
drm.ugt80.
drm.wt.ugt80.
drm.smt1.
drm.wt.smt1.
dsm.ugt80.
dsm.wt.ugt80.
dsm.smt1.
dsm.wt.smt1.
sp.ugt80.
sp.wt.ugt80.
sp.smt1.
sp.wt.smt1.
im.ugt80.
im.wt.ugt80.
im.smt1.
im.wt.smt1.

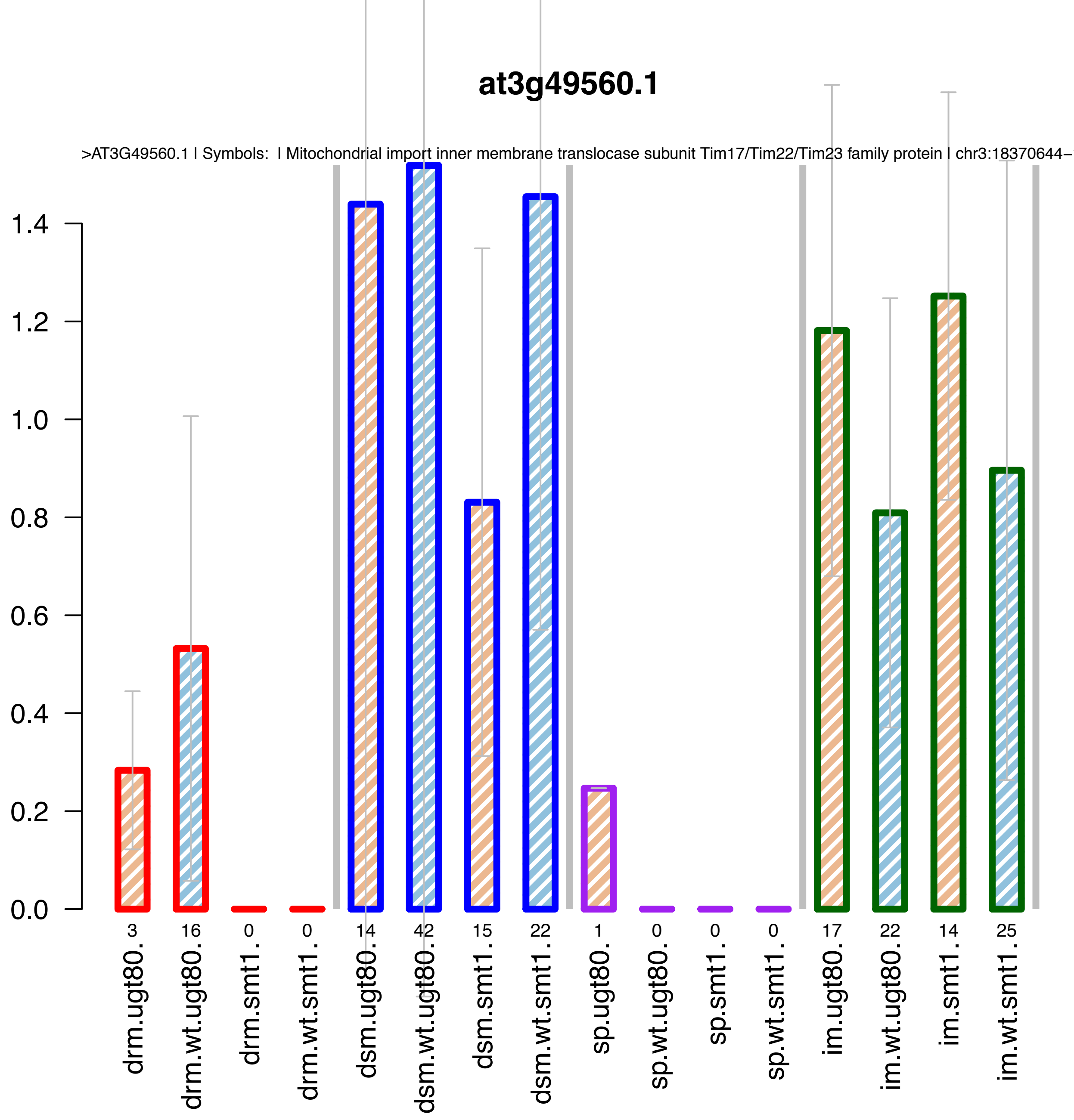
at3g49560.1
>AT3G49560.1 | Symbols: | Mitochondrial import inner membrane translocase subunit Tim17/Tim22/Tim23 family protein | chr3:18370644–
0.0
0.2
0.4
0.6
0.8
1.0
1.2
1.4
3
16
0
0
14
42
15
22
1
0
0
0
17
22
14
25
drm.ugt80..
drm.wt.ugt80..
drm.smt1..
drm.wt.smt1..
dsm.ugt80..
dsm.wt.ugt80..
dsm.smt1..
dsm.wt.smt1..
sp.ugt80..
sp.wt.ugt80..
sp.smt1..
sp.wt.smt1..
im.ugt80..
im.wt.ugt80..
im.smt1..
im.wt.smt1..

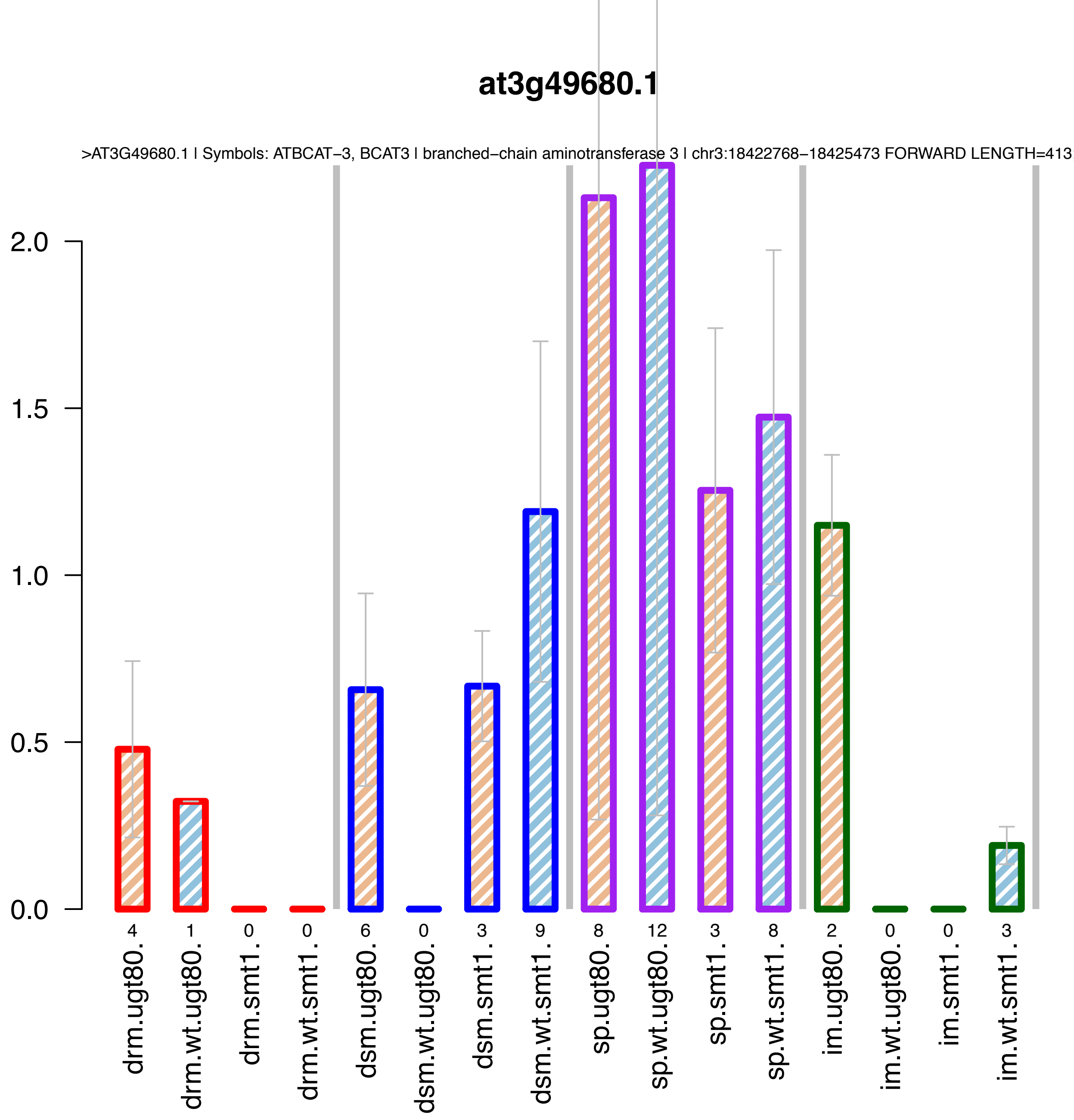

at3g49680.1
>AT3G49680.1 | Symbols: ATBCAT-3, BCAT3 | branched-chain aminotransferase 3 | chr3:18422768-18425473 FORWARD LENGTH=413
2.0
1.5
1.0
0.5
0.0
4
1
0
0
6
0
3
9
8
12
3
8
2
0
0
3
drm.ugt80.
drm.wt.ugt80.
drm.smt1.
drm.wt.smt1.
dsm.ugt80.
dsm.wt.ugt80.
dsm.smt1.
dsm.wt.smt1.
sp.ugt80.
sp.wt.ugt80.
sp.smt1.
sp.wt.smt1.
im.ugt80.
im.wt.ugt80.
im.smt1.
im.wt.smt1.

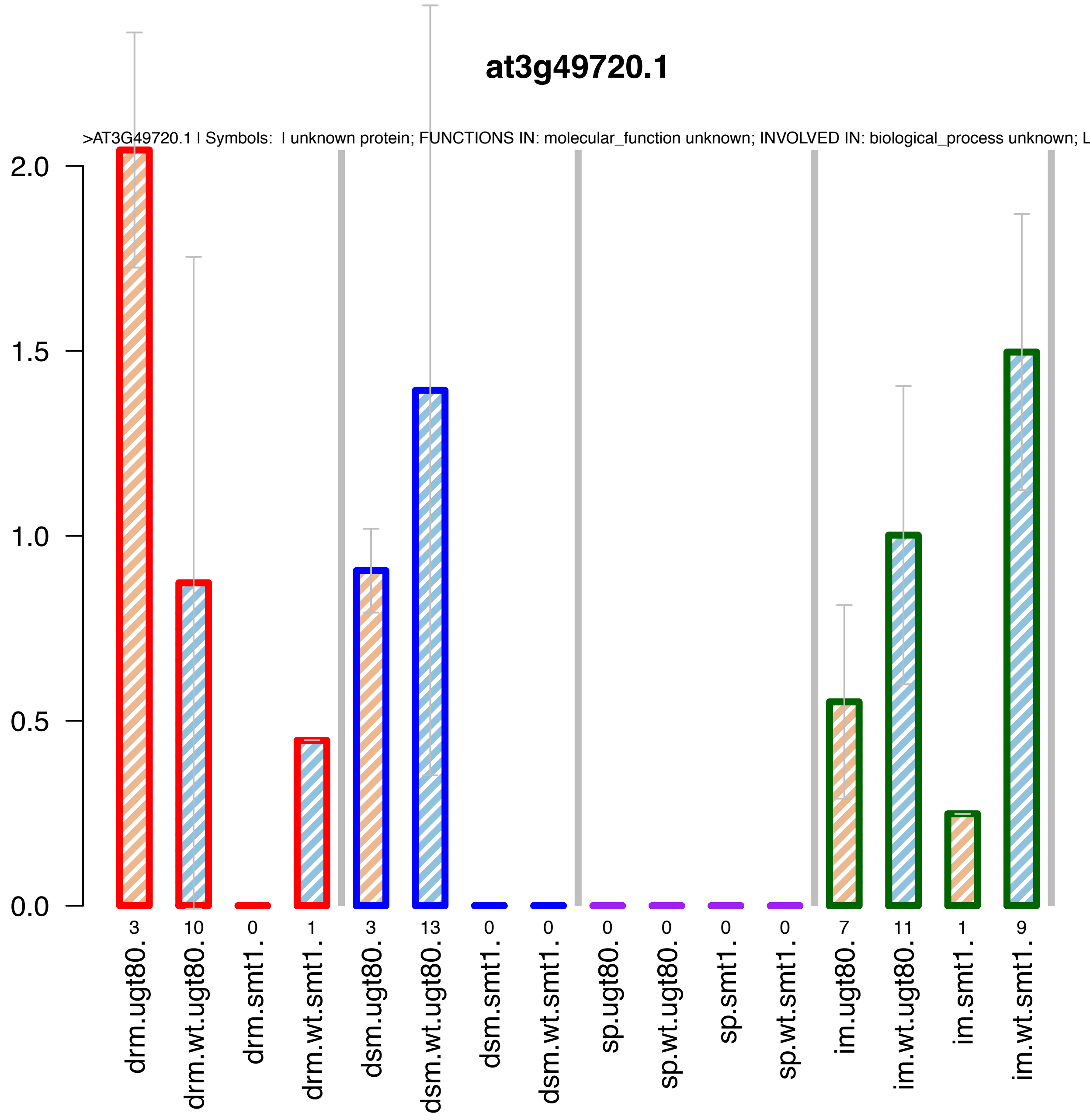

at3g49720.1
>AT3G49720.1 | Symbols: | unknown protein; FUNCTIONS IN: molecular_function unknown; INVOLVED IN: biological_process unknown; L
2.0
1.5
1.0
0.5
0.0
3
10
0
1
3
13
0
0
0
0
0
0
7
11
1
9
drm.ugt80.
drm.wt.ugt80.
drm.smt1.
drm.wt.smt1.
dsm.ugt80.
dsm.wt.ugt80.
dsm.smt1.
dsm.wt.smt1.
sp.ugt80.
sp.wt.ugt80.
sp.smt1.
sp.wt.smt1.
im.ugt80.
im.wt.ugt80.
im.smt1.
im.wt.smt1.

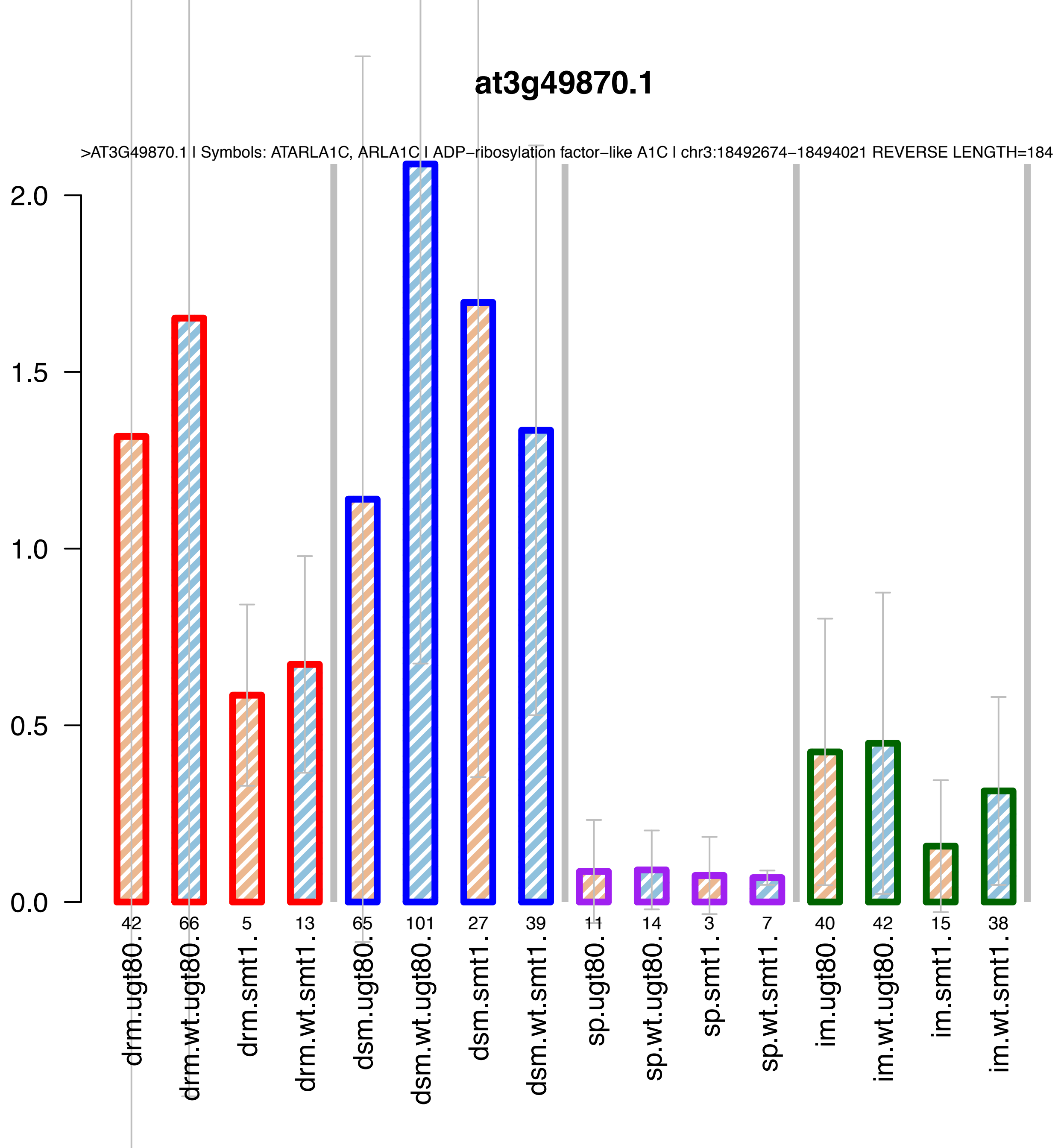
at3g49870.1
>AT3G49870.1 | Symbols: ATARLA1C, ARLA1C | ADP−ribosylation factor−like A1C | chr3:18492674−18494021 REVERSE LENGTH=184
2.0
1.5
1.0
0.5
0.0
42
66
5
13
65
101
27
39
11
14
3
7
40
42
15
38
drm.ugt80..
drm.wt.ugt80..
drm.smt1..
drm.wt.smt1..
dsm.ugt80..
dsm.wt.ugt80..
dsm.smt1..
dsm.wt.smt1..
sp.ugt80..
sp.wt.ugt80..
sp.smt1..
sp.wt.smt1..
im.ugt80..
im.wt.ugt80..
im.smt1..
im.wt.smt1..

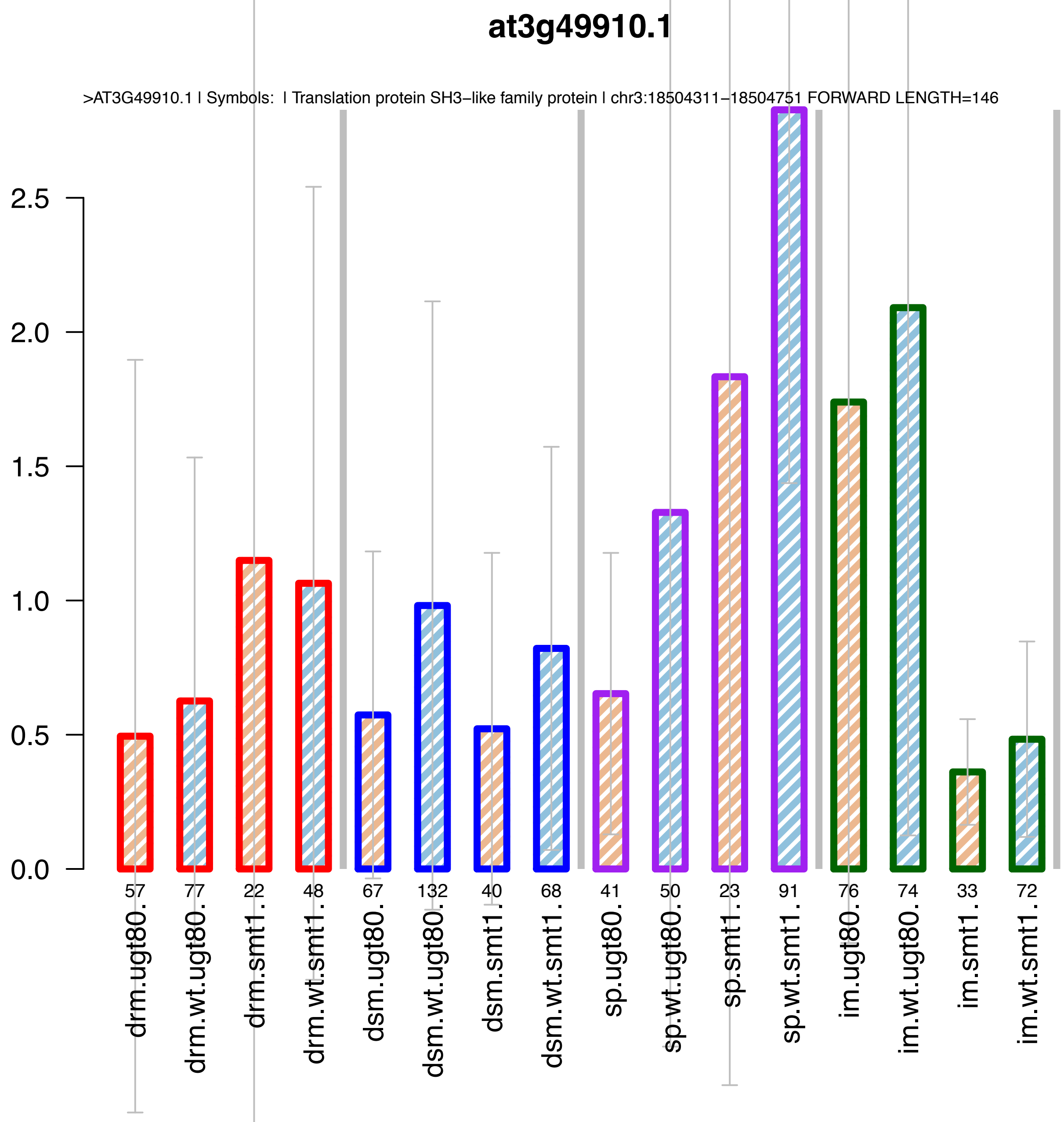

at3g49910.1
>AT3G49910.1 | Symbols: | Translation protein SH3−like family protein | chr3:18504311−18504751 FORWARD LENGTH=146
0.0
0.5
1.0
1.5
2.0
2.5
57
77
22
48
67
132
40
68
41
50
23
91
76
74
33
72
drm.ugt80..
drm.wt.ugt80..
drm.smt1..
drm.wt.smt1..
dsm.ugt80..
dsm.wt.ugt80..
dsm.smt1..
dsm.wt.smt1..
sp.ugt80..
sp.wt.ugt80..
sp.smt1..
sp.wt.smt1..
im.ugt80..
im.wt.ugt80..
im.smt1..
im.wt.smt1..

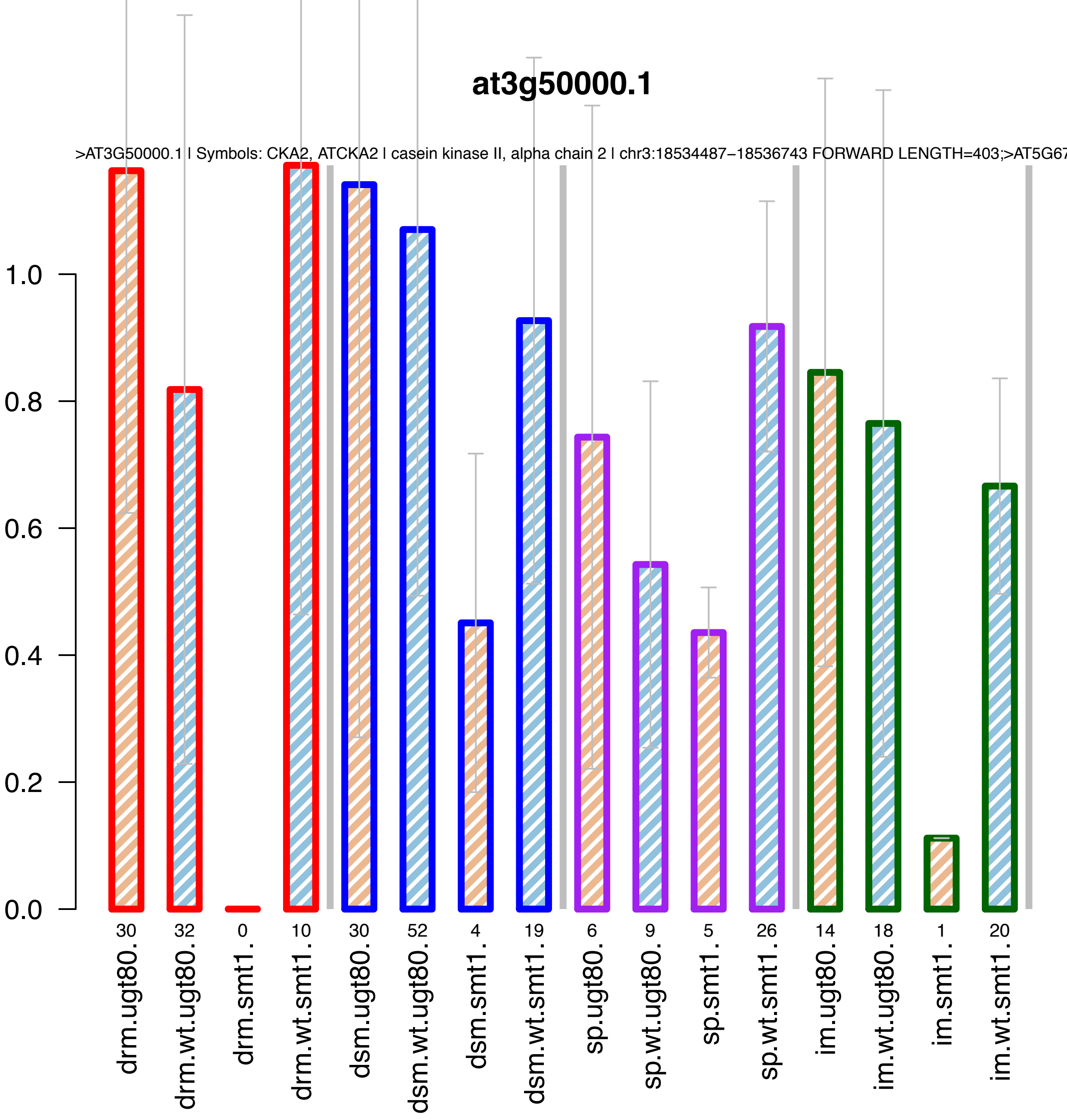

at3g50000.1
>AT3G50000.1 | Symbols: CKA2, ATCKA2 | casein kinase II, alpha chain 2 | chr3:18534487−18536743 FORWARD LENGTH=403;>AT5G67
1.0
0.8
0.6
0.4
0.2
0.0
30
32
0
10
30
52
4
19
6
9
5
26
14
18
1
20
drm.ugt80..
drm.wt.ugt80..
drm.smt1..
drm.wt.smt1..
dsm.ugt80..
dsm.wt.ugt80..
dsm.smt1..
dsm.wt.smt1..
sp.ugt80..
sp.wt.ugt80..
sp.smt1..
sp.wt.smt1..
im.ugt80..
im.wt.ugt80..
im.smt1..
im.wt.smt1..

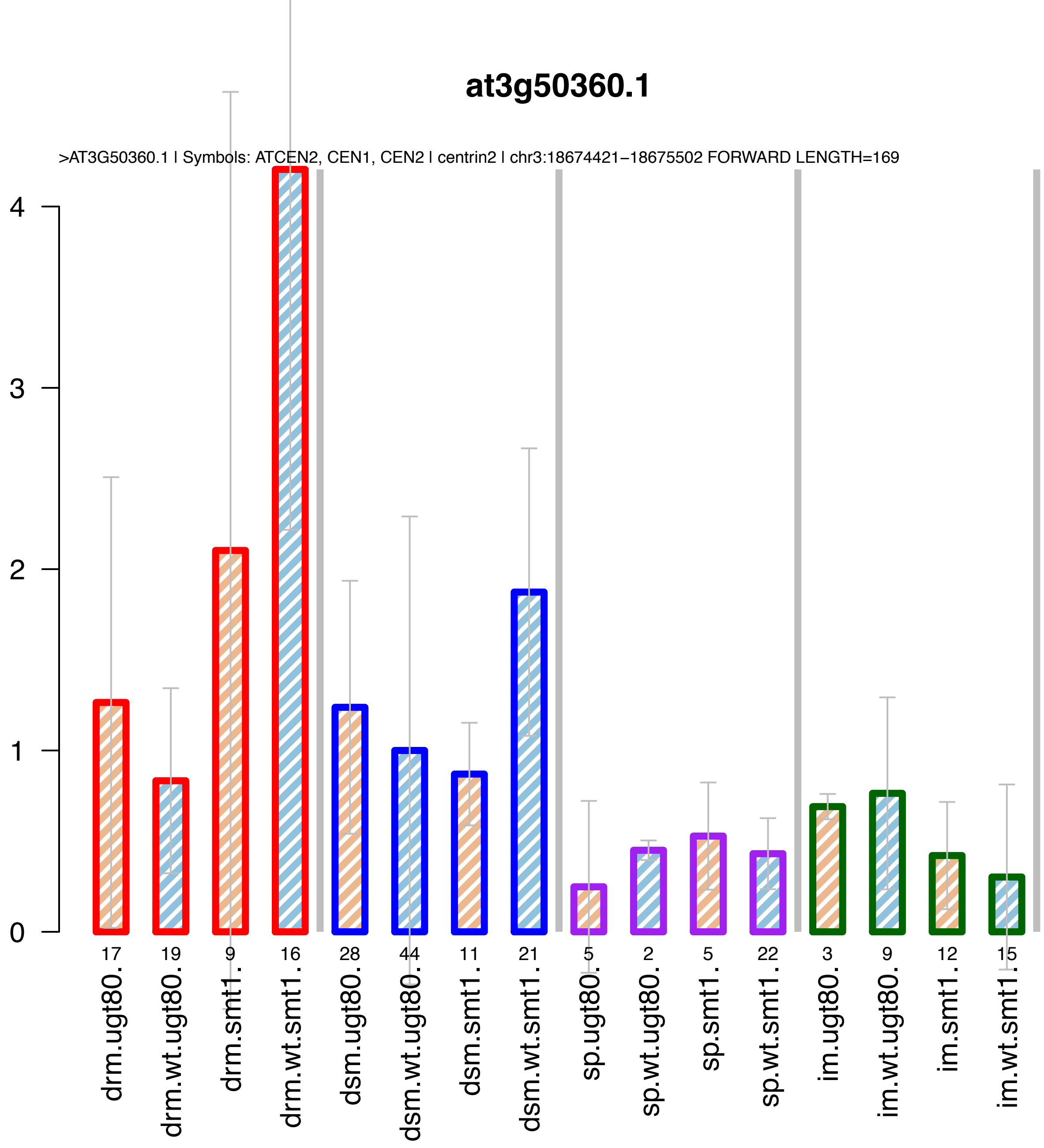

at3g50360.1
>AT3G50360.1 | Symbols: ATCEN2, CEN1, CEN2 | centrin2 | chr3:18674421–18675502 FORWARD LENGTH=169
0
1
2
3
4
17
19
9
16
28
44
11
21
5
2
5
22
3
9
12
15
drm.ugt80.
drm.wt.ugt80.
drm.smt1.
drm.wt.smt1.
dsm.ugt80.
dsm.wt.ugt80.
dsm.smt1.
dsm.wt.smt1.
sp.ugt80.
sp.wt.ugt80.
sp.smt1.
sp.wt.smt1.
im.ugt80.
im.wt.ugt80.
im.smt1.
im.wt.smt1.

# at3g50930.1

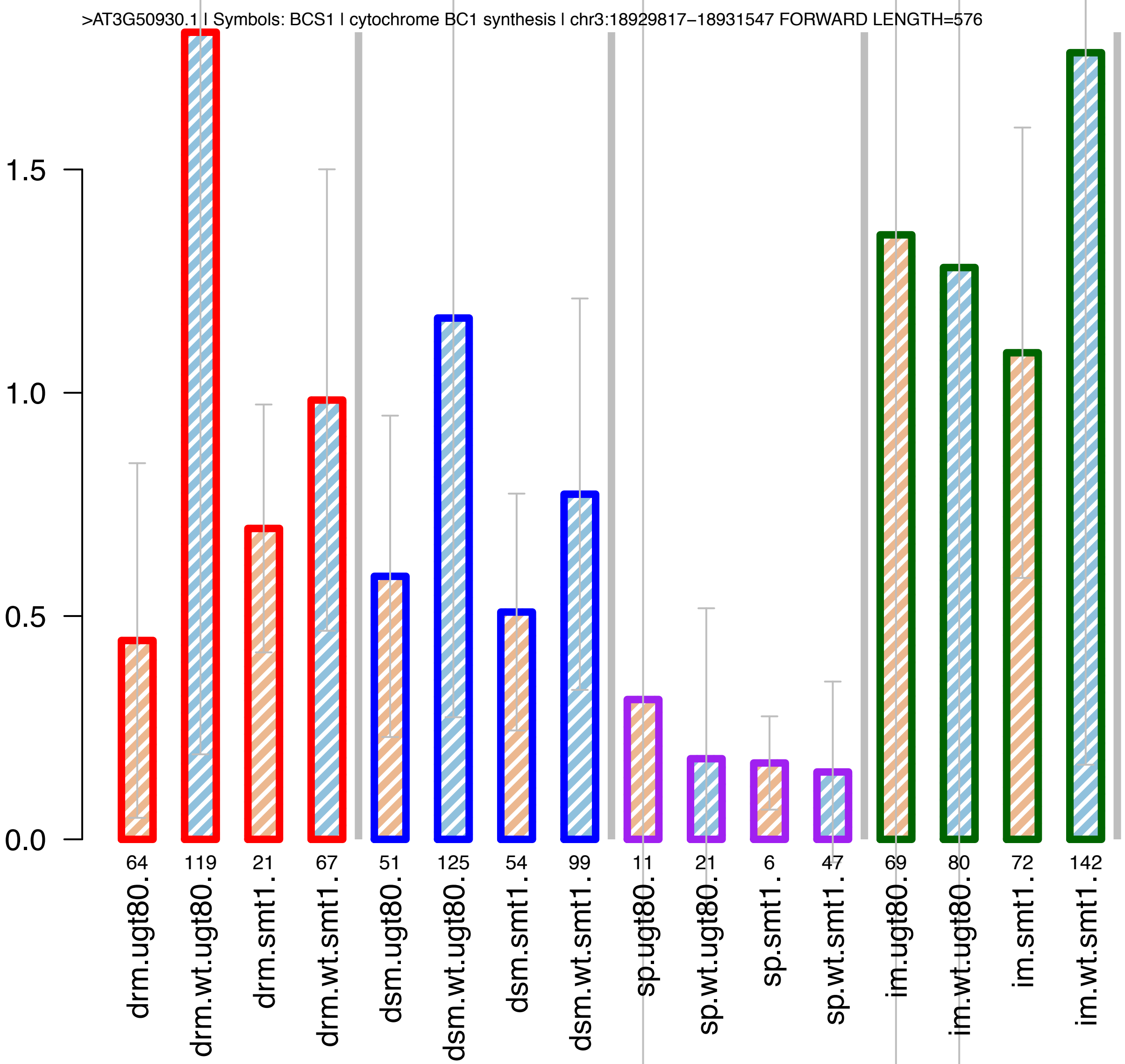

>AT3G50930.1 | Symbols: BCS1 | cytochrome BC1 synthesis | chr3:18929817−18931547 FORWARD LENGTH=576
1.5
1.0
0.5
0.0
64
119
21
67
51
125
54
99
11
21
6
47
69
80
72
142
drm.ugt80..
drm.wt.ugt80..
drm.smt1..
drm.wt.smt1..
dsm.ugt80..
dsm.wt.ugt80..
dsm.smt1..
dsm.wt.smt1..
sp.ugt80..
sp.wt.ugt80..
sp.smt1..
sp.wt.smt1..
im.ugt80..
im.wt.ugt80..
im.smt1..
im.wt.smt1..

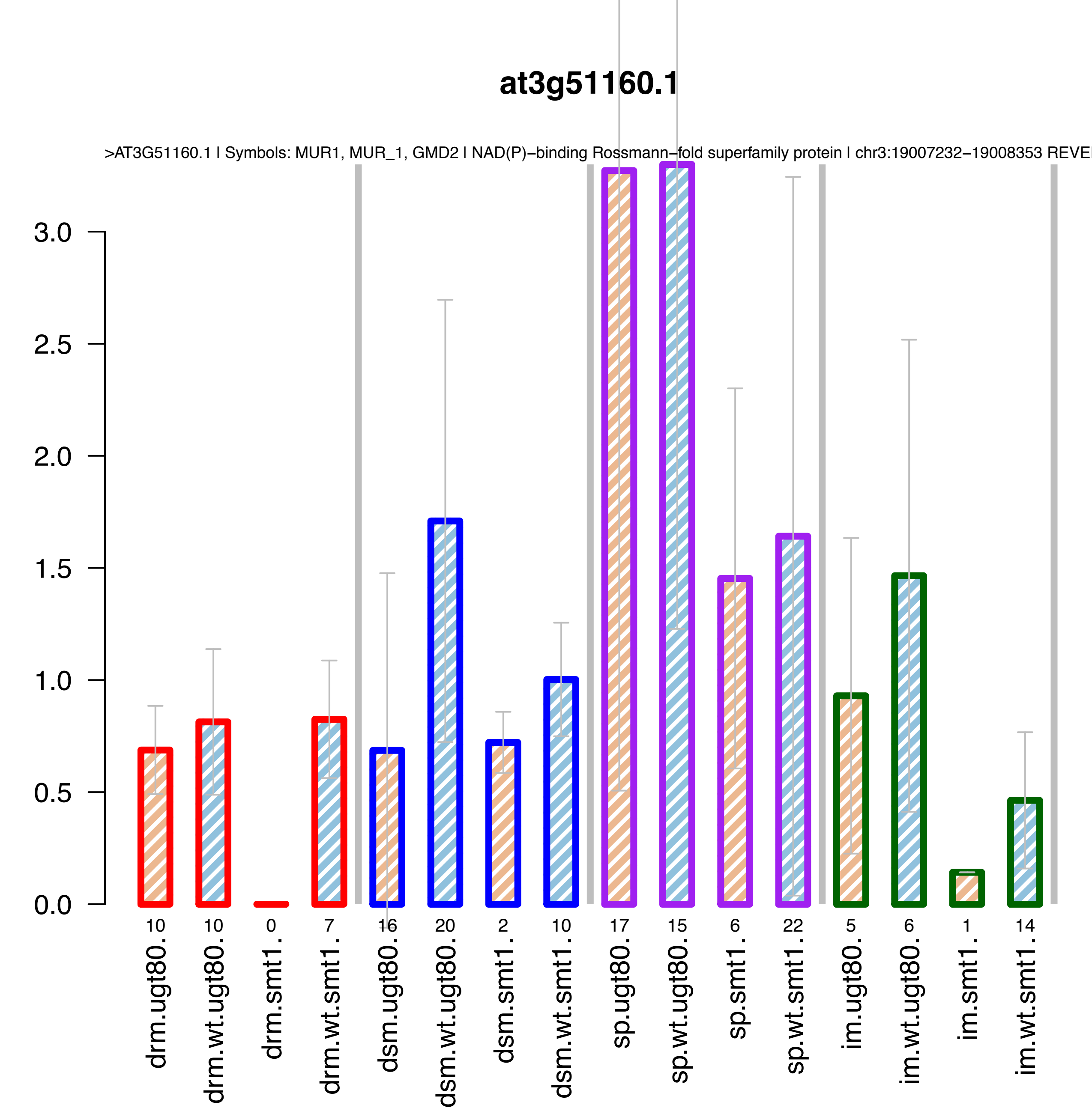

at3g51160.1
>AT3G51160.1 | Symbols: MUR1, MUR_1, GMD2 | NAD(P)−binding Rossmann−fold superfamily protein | chr3:19007232−19008353 REVE
3.0
2.5
2.0
1.5
1.0
0.5
0.0
10
10
0
7
16
20
2
10
17
15
6
22
5
6
1
14
drm.ugt80.
drm.wt.ugt80.
drm.smt1.
drm.wt.smt1.
dsm.ugt80.
dsm.wt.ugt80.
dsm.smt1.
dsm.wt.smt1.
sp.ugt80.
sp.wt.ugt80.
sp.smt1.
sp.wt.smt1.
im.ugt80.
im.wt.ugt80.
im.smt1.
im.wt.smt1.

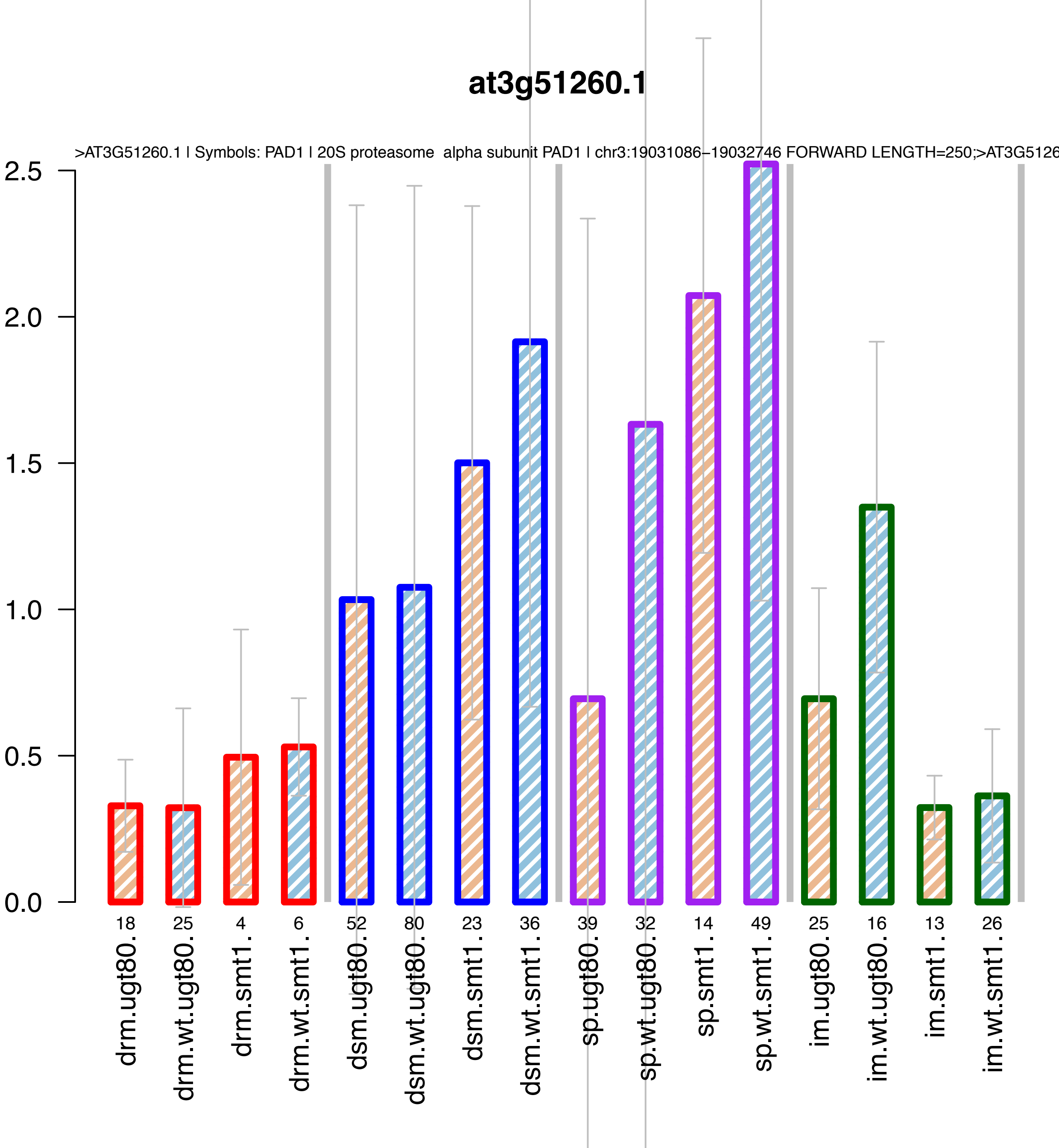

at3g51260.1
>AT3G51260.1 | Symbols: PAD1 | 20S proteasome alpha subunit PAD1 | chr3:19031086-19032746 FORWARD LENGTH=250;>AT3G5126
2.5
2.0
1.5
1.0
0.5
0.0
18
25
4
6
52
80
23
36
39
32
14
49
25
16
13
26
drm.ugt80..
drm.wt.ugt80..
drm.smt1..
drm.wt.smt1..
dsm.ugt80..
dsm.wt.ugt80..
dsm.smt1..
dsm.wt.smt1..
sp.ugt80..
sp.wt.ugt80..
sp.smt1..
sp.wt.smt1..
im.ugt80..
im.wt.ugt80..
im.smt1..
im.wt.smt1..

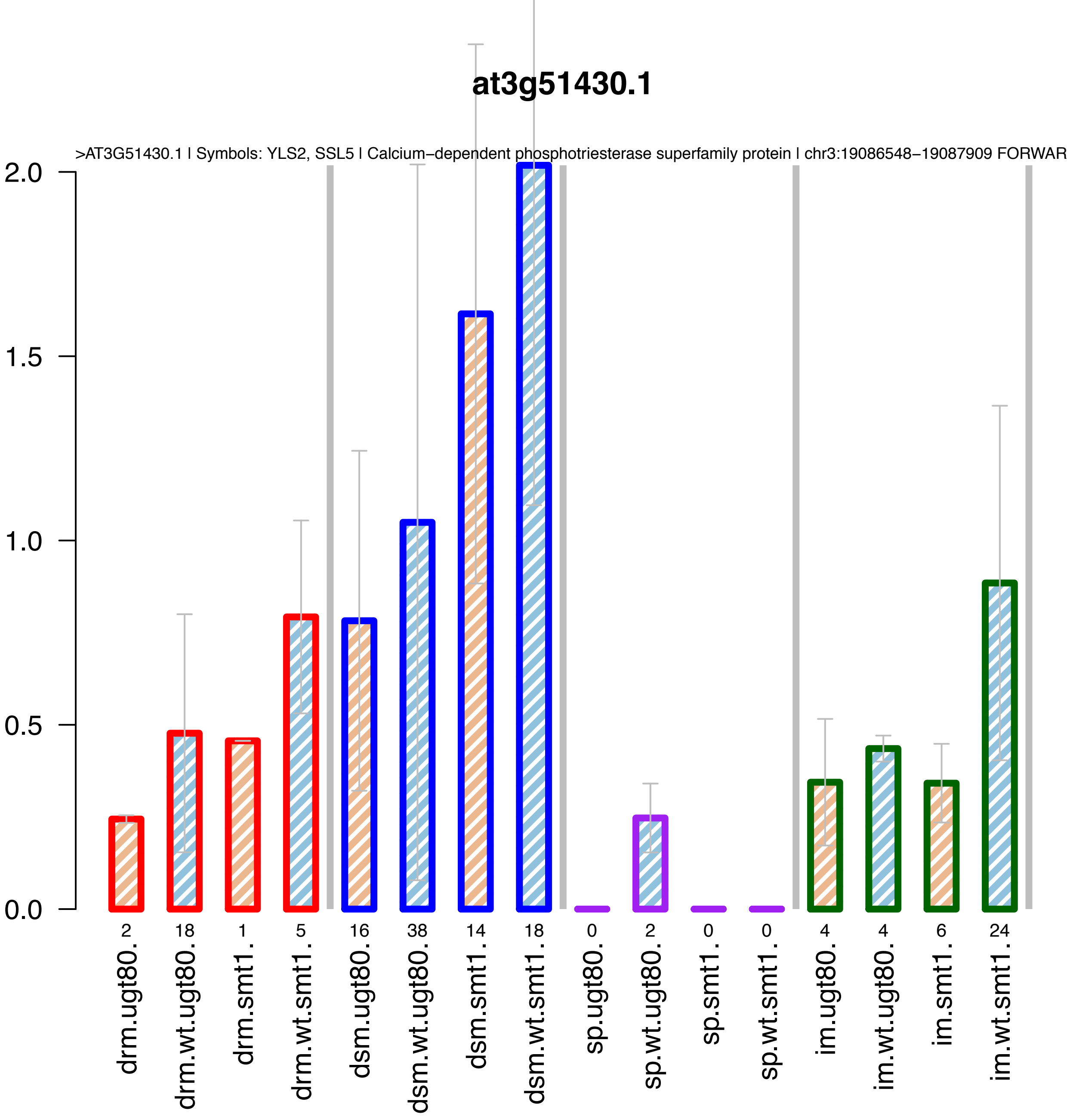

at3g51430.1
>AT3G51430.1 | Symbols: YLS2, SSL5 | Calcium−dependent phosphotriesterase superfamily protein | chr3:19086548−19087909 FORWAR
2.0
1.5
1.0
0.5
0.0
2
18
1
5
16
38
14
18
0
2
0
0
4
4
6
24
drm.ugt80..
drm.wt.ugt80..
drm.smt1..
drm.wt.smt1..
dsm.ugt80..
dsm.wt.ugt80..
dsm.smt1..
dsm.wt.smt1..
sp.ugt80..
sp.wt.ugt80..
sp.smt1..
sp.wt.smt1..
im.ugt80..
im.wt.ugt80..
im.smt1..
im.wt.smt1..

# at3g51550.1

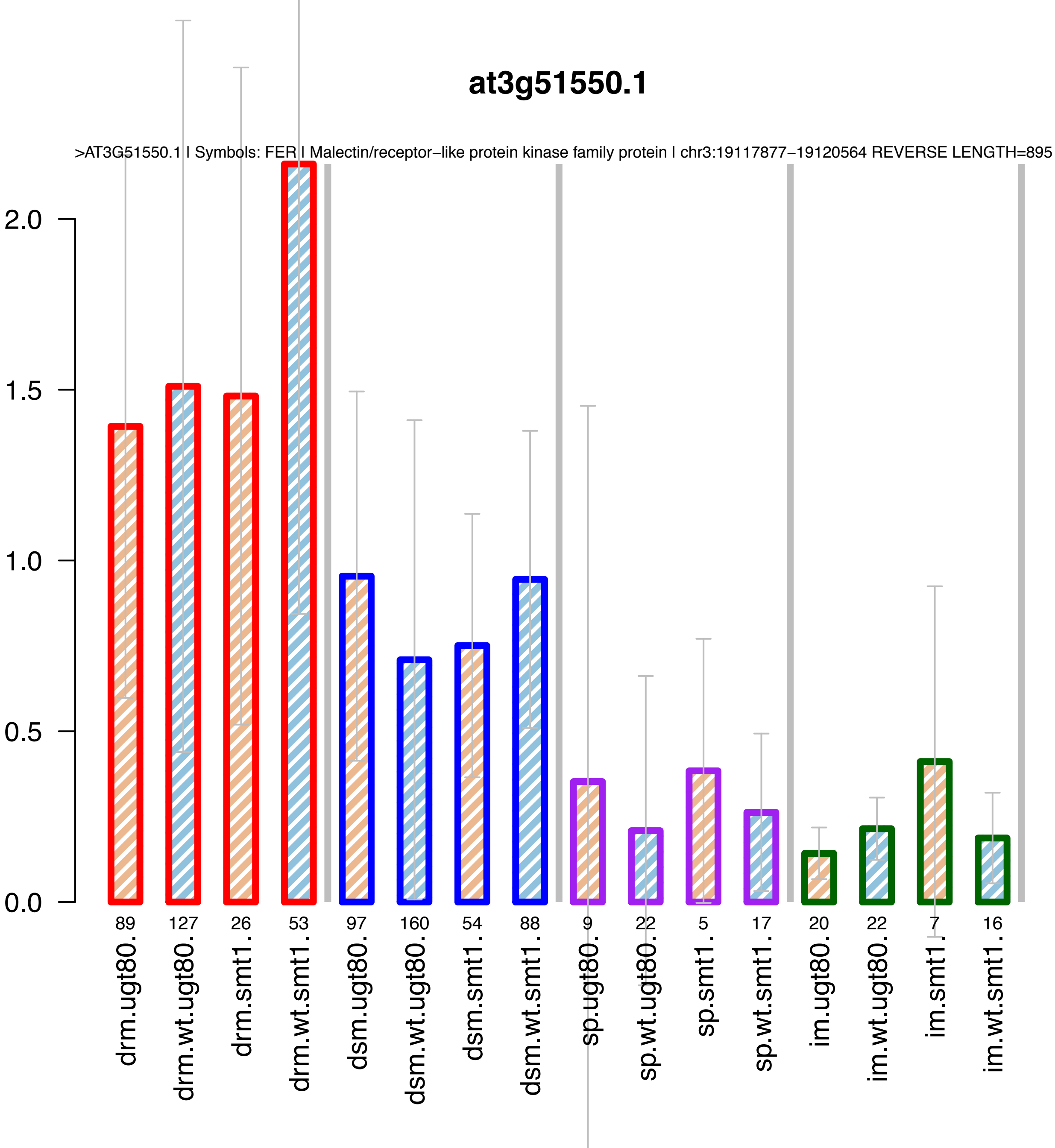

>AT3G51550.1 | Symbols: FER | Malectin/receptor-like protein kinase family protein | chr3:19117877-19120564 REVERSE LENGTH=895
2.0
1.5
1.0
0.5
0.0
89
127
26
53
97
160
54
88
9
22
5
17
20
22
7
16
drm.ugt80.
drm.wt.ugt80.
drm.smt1.
drm.wt.smt1.
dsm.ugt80.
dsm.wt.ugt80.
dsm.smt1.
dsm.wt.smt1.
sp.ugt80.
sp.wt.ugt80.
sp.smt1.
sp.wt.smt1.
im.ugt80.
im.wt.ugt80.
im.smt1.
im.wt.smt1.

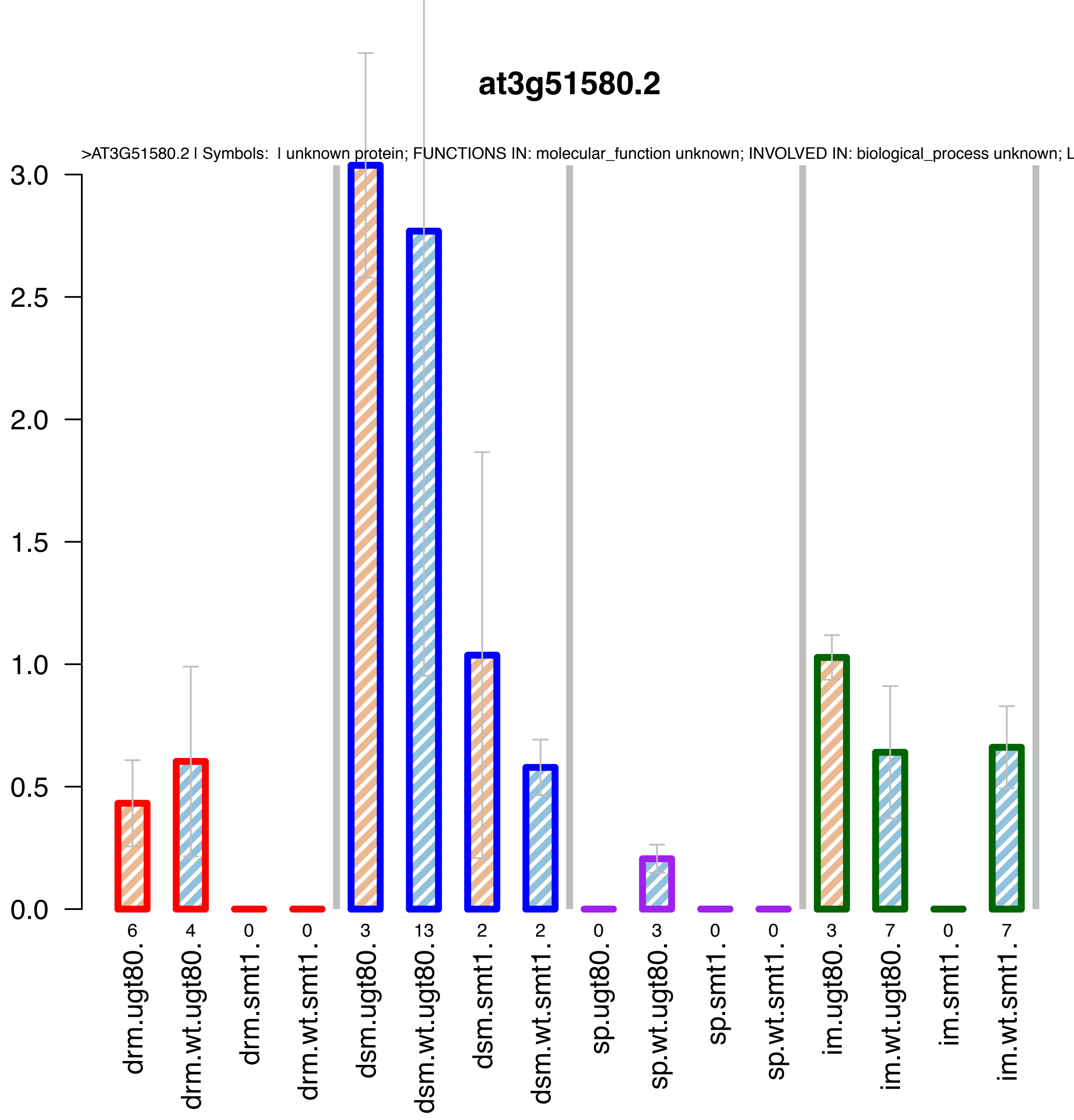

at3g51580.2
>AT3G51580.2 | Symbols: | unknown protein; FUNCTIONS IN: molecular_function unknown; INVOLVED IN: biological_process unknown; L
3.0
2.5
2.0
1.5
1.0
0.5
0.0
6
4
0
0
3
13
2
2
0
3
0
0
3
7
0
7
drm.ugt80.
drm.wt.ugt80.
drm.smt1.
drm.wt.smt1.
dsm.ugt80.
dsm.wt.ugt80.
dsm.smt1.
dsm.wt.smt1.
sp.ugt80.
sp.wt.ugt80.
sp.smt1.
sp.wt.smt1.
im.ugt80.
im.wt.ugt80.
im.smt1.
im.wt.smt1.

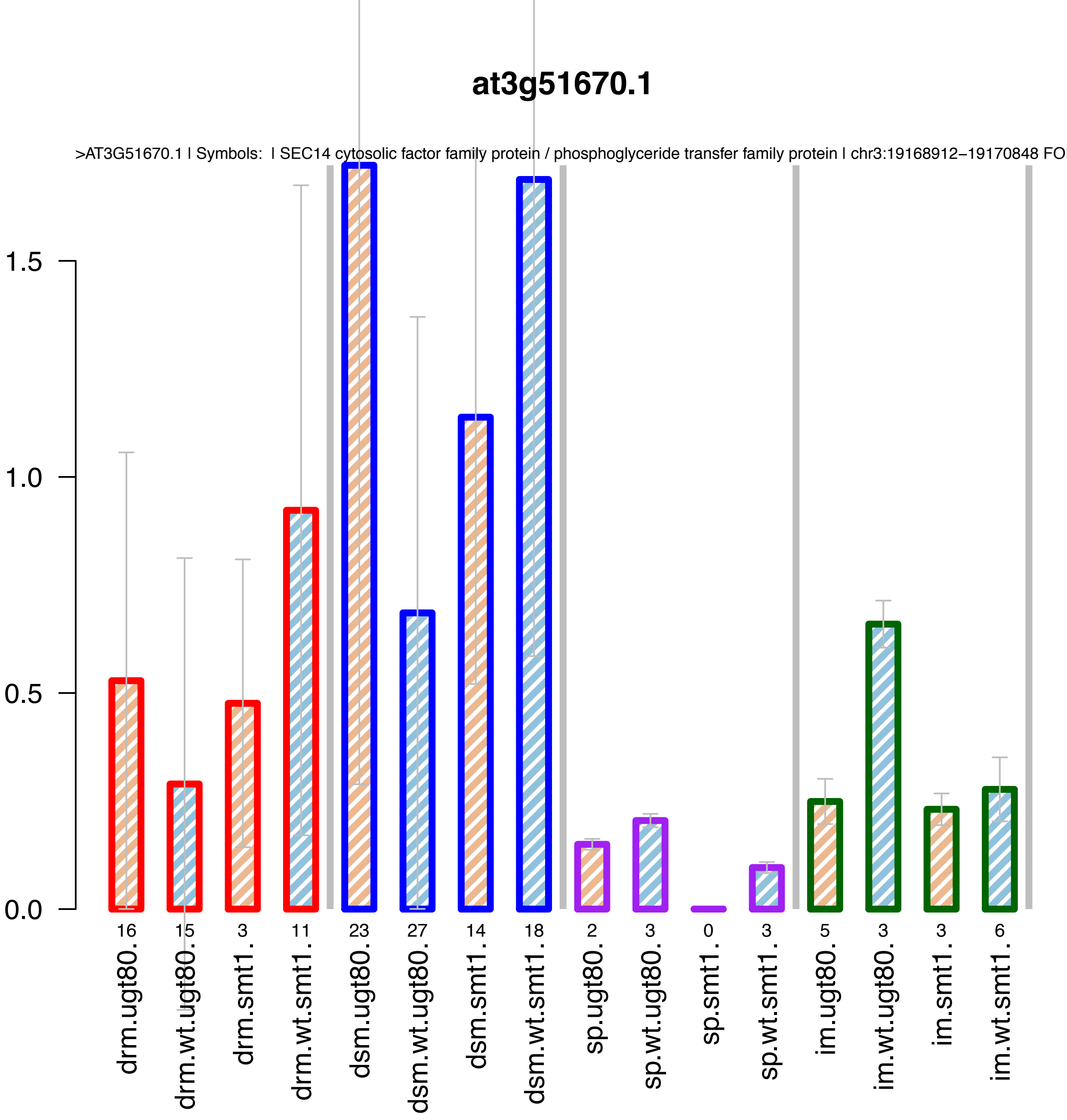
at3g51670.1
>AT3G51670.1 | Symbols: | SEC14 cytosolic factor family protein / phosphoglyceride transfer family protein | chr3:19168912-19170848 FO
1.5
1.0
0.5
0.0
16
15
3
11
23
27
14
18
2
3
0
3
5
3
3
6
drm.ugt80..
drm.wt.ugt80..
drm.smt1..
drm.wt.smt1..
dsm.ugt80..
dsm.wt.ugt80..
dsm.smt1..
dsm.wt.smt1..
sp.ugt80..
sp.wt.ugt80..
sp.smt1..
sp.wt.smt1..
im.ugt80..
im.wt.ugt80..
im.smt1..
im.wt.smt1..

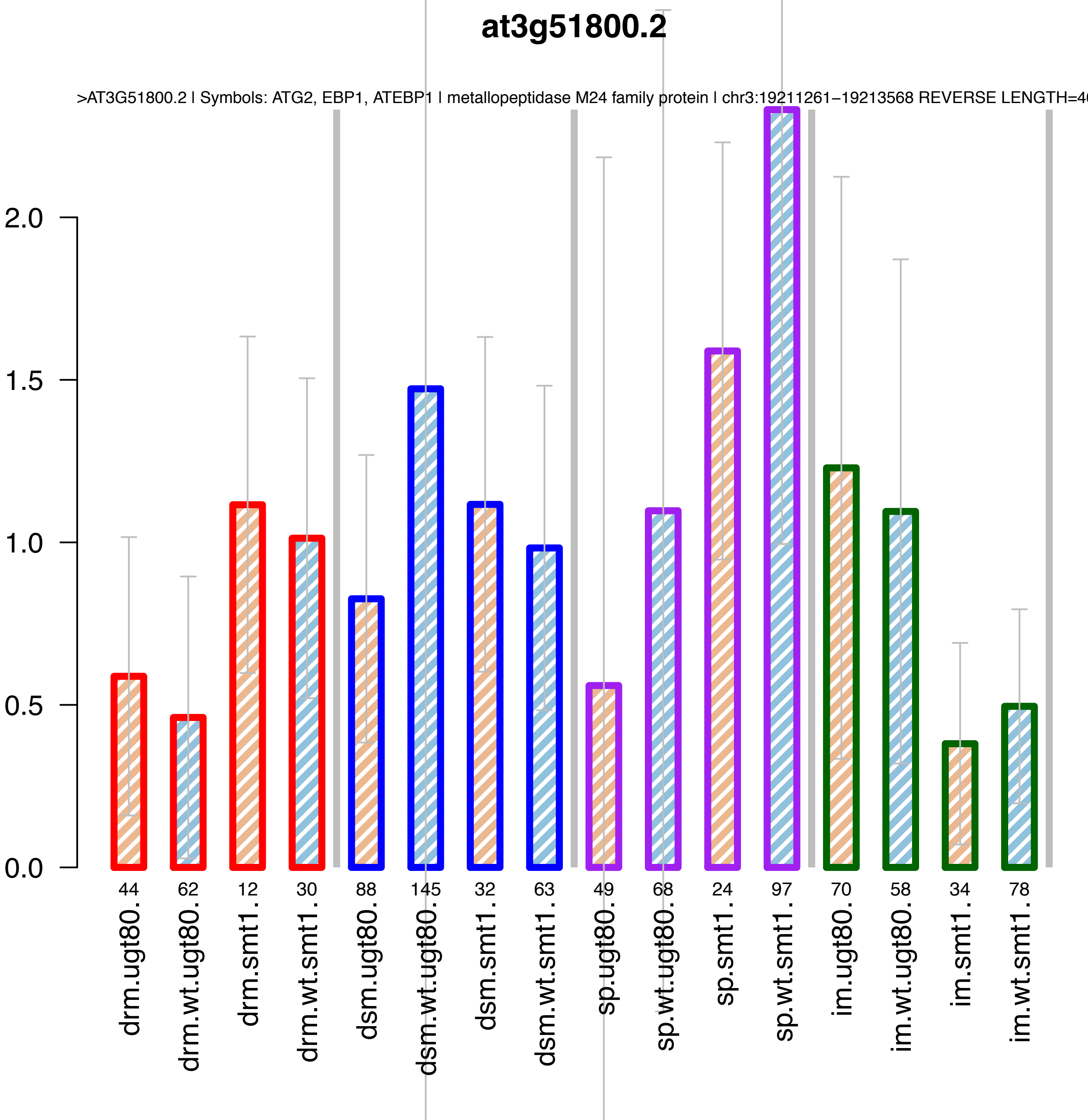

at3g51800.2
>AT3G51800.2 | Symbols: ATG2, EBP1, ATEBP1 | metallopeptidase M24 family protein | chr3:19211261-19213568 REVERSE LENGTH=4
0.0
0.5
1.0
1.5
2.0
44
62
12
30
88
145
32
63
49
68
24
97
70
58
34
78
drm.ugt80..
drm.wt.ugt80..
drm.smt1..
drm.wt.smt1..
dsm.ugt80..
dsm.wt.ugt80..
dsm.smt1..
dsm.wt.smt1..
sp.ugt80..
sp.wt.ugt80..
sp.smt1..
sp.wt.smt1..
im.ugt80..
im.wt.ugt80..
im.smt1..
im.wt.smt1..

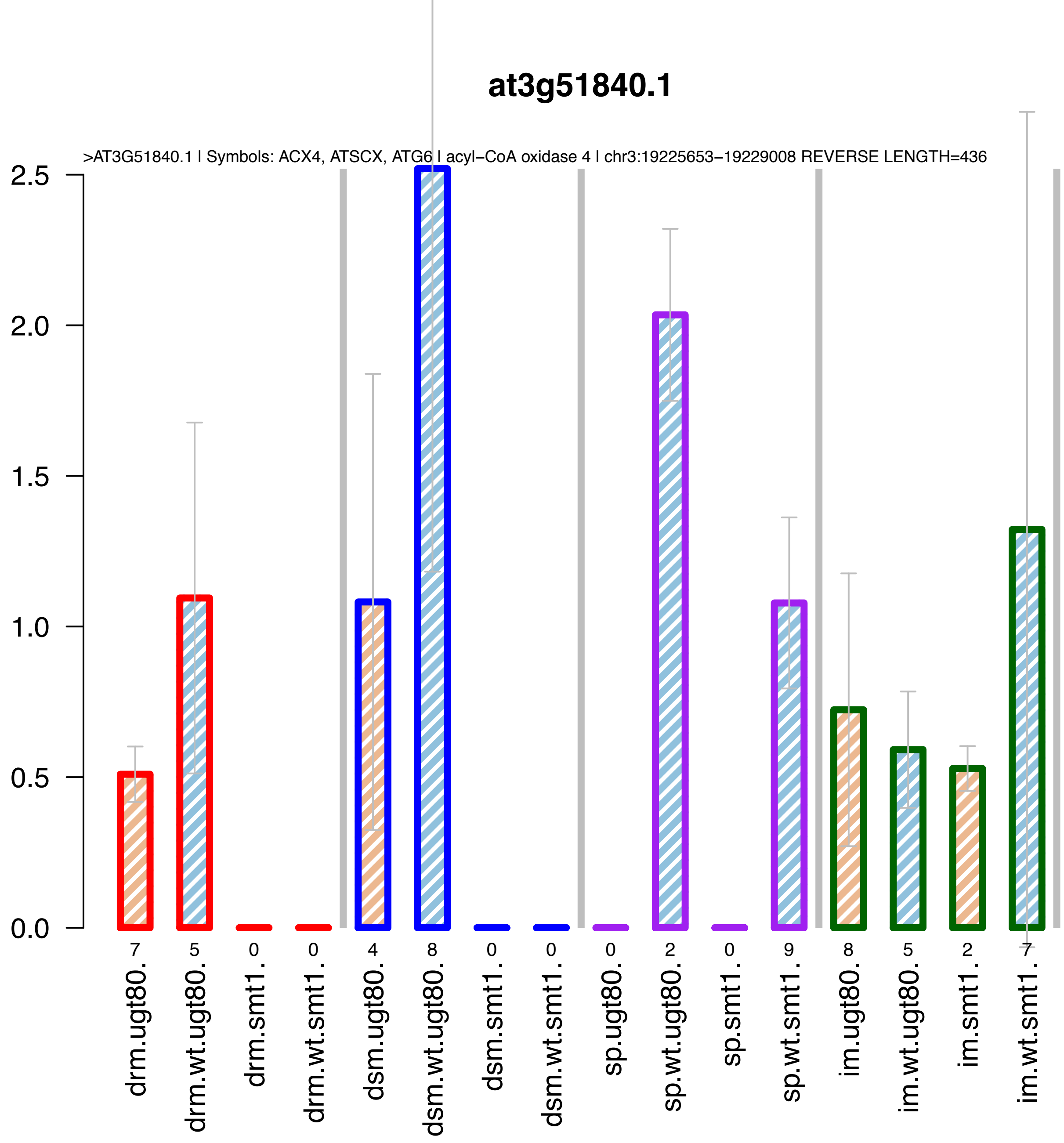

at3g51840.1
>AT3G51840.1 | Symbols: ACX4, ATSCX, ATG6 | acyl−CoA oxidase 4 | chr3:19225653−19229008 REVERSE LENGTH=436
2.5
2.0
1.5
1.0
0.5
0.0
7
5
0
0
4
8
0
0
0
2
0
9
8
5
2
7
drm.ugt80.
drm.wt.ugt80.
drm.smt1.
drm.wt.smt1.
dsm.ugt80.
dsm.wt.ugt80.
dsm.smt1.
dsm.wt.smt1.
sp.ugt80.
sp.wt.ugt80.
sp.smt1.
sp.wt.smt1.
im.ugt80.
im.wt.ugt80.
im.smt1.
im.wt.smt1.

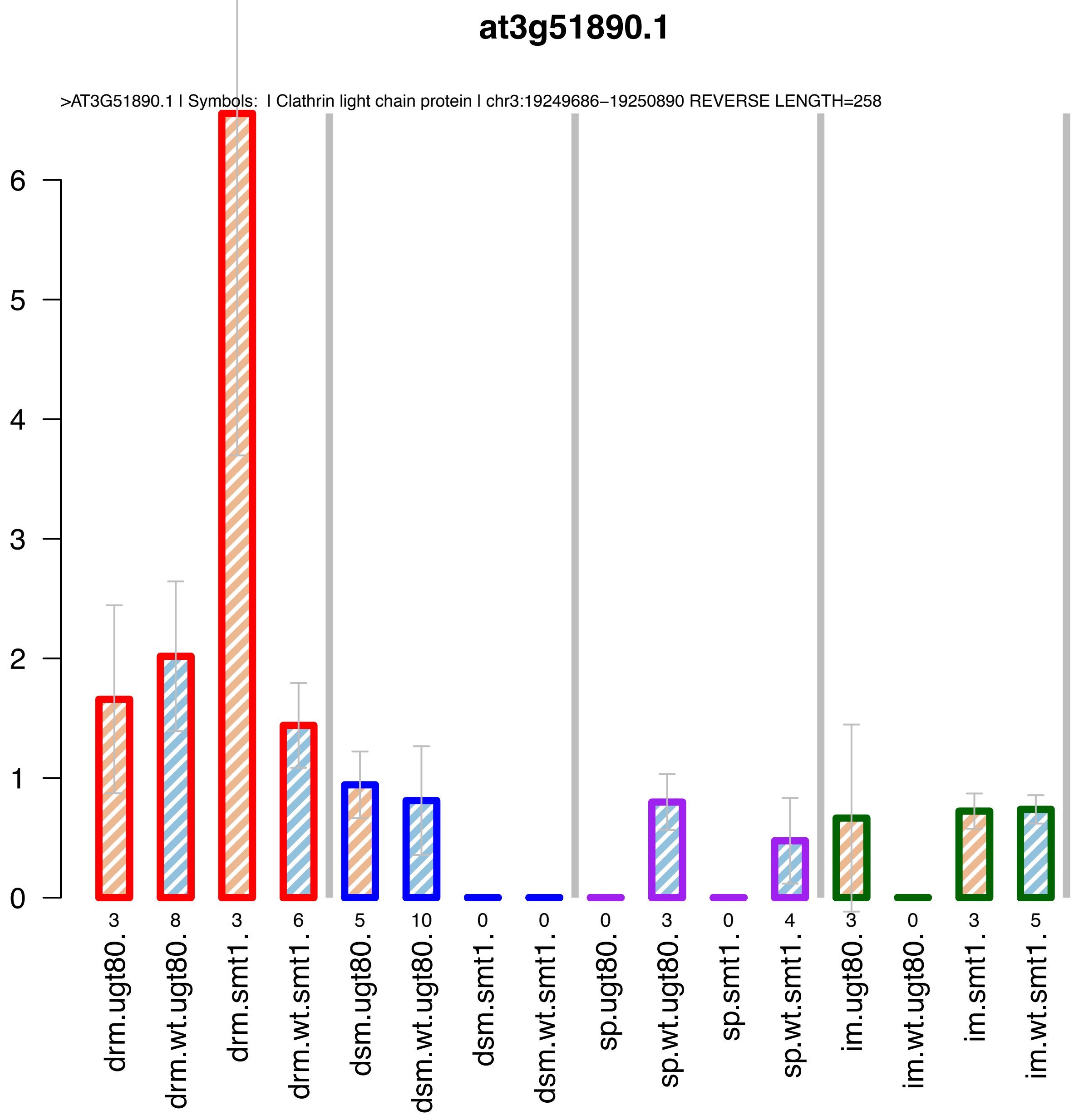
at3g51890.1
>AT3G51890.1 | Symbols: | Clathrin light chain protein | chr3:19249686-19250890 REVERSE LENGTH=258
0
1
2
3
4
5
6
3
8
3
6
5
10
0
0
0
3
0
4
3
0
3
5
drm.ugt80..
drm.wt.ugt80..
drm.smt1..
drm.wt.smt1..
dsm.ugt80..
dsm.wt.ugt80..
dsm.smt1..
dsm.wt.smt1..
sp.ugt80..
sp.wt.ugt80..
sp.smt1..
sp.wt.smt1..
im.ugt80..
im.wt.ugt80..
im.smt1..
im.wt.smt1..

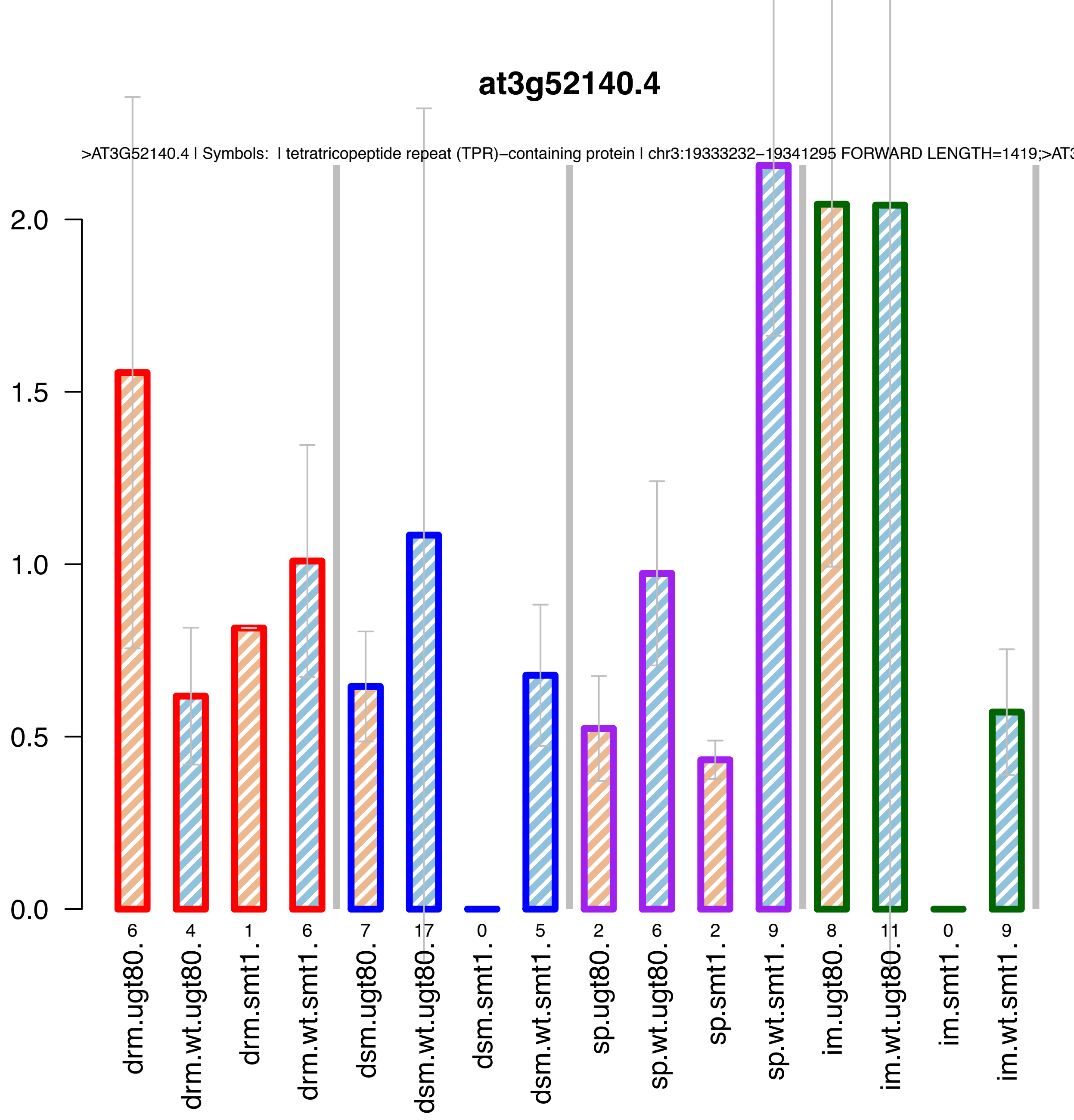

at3g52140.4
>AT3G52140.4 | Symbols: | tetratricopeptide repeat (TPR)−containing protein | chr3:19333232−19341295 FORWARD LENGTH=1419;>AT3
0.0
0.5
1.0
1.5
2.0
6
4
1
6
7
17
0
5
2
6
2
9
8
11
0
9
drm.ugt80.
drm.wt.ugt80.
drm.smt1.
drm.wt.smt1.
dsm.ugt80.
dsm.wt.ugt80.
dsm.smt1.
dsm.wt.smt1.
sp.ugt80.
sp.wt.ugt80.
sp.smt1.
sp.wt.smt1.
im.ugt80.
im.wt.ugt80.
im.smt1.
im.wt.smt1.

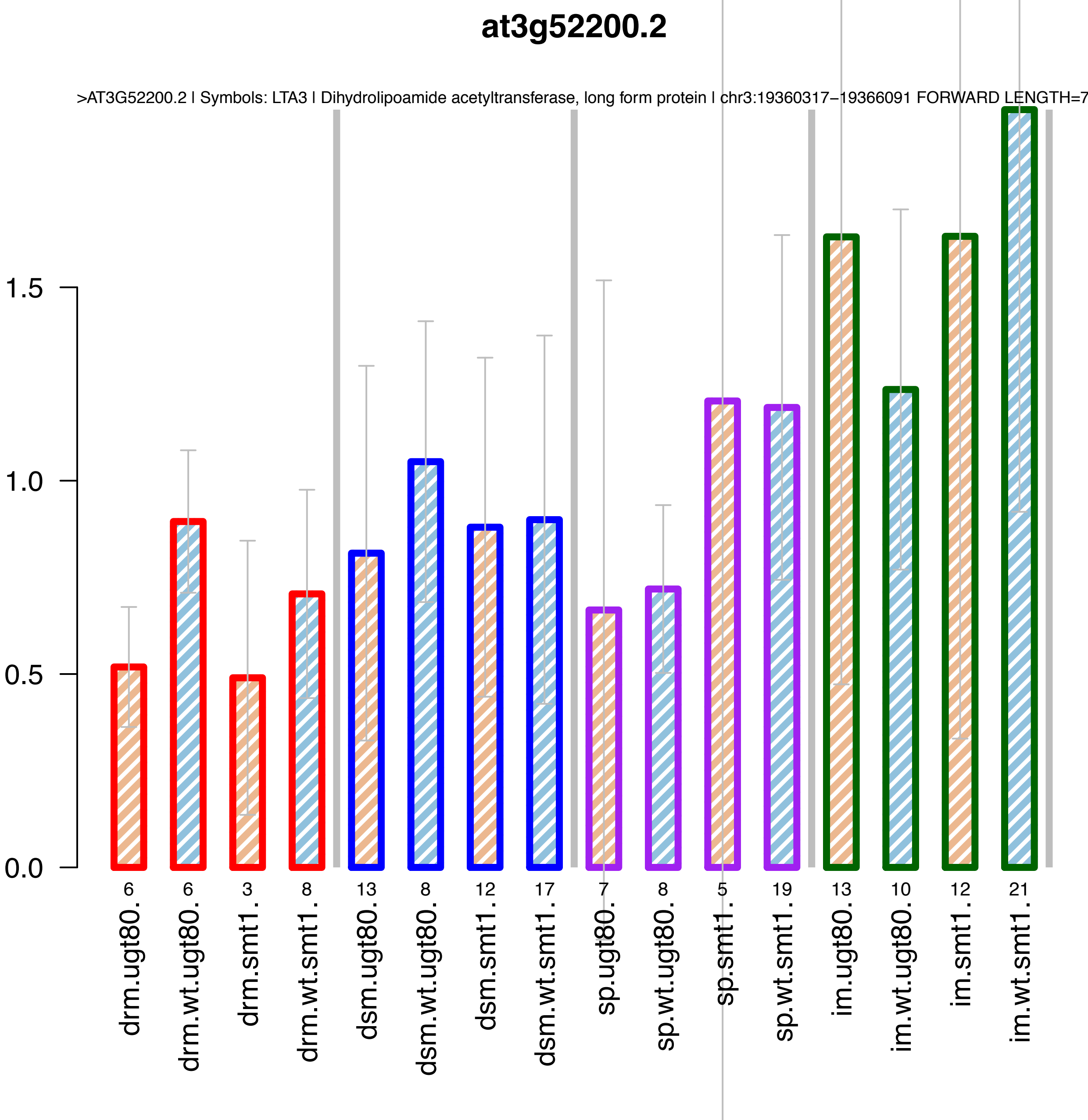

at3g52200.2
>AT3G52200.2 | Symbols: LTA3 | Dihydrolipoamide acetyltransferase, long form protein | chr3:19360317-19366091 FORWARD LENGTH=7
0.0
0.5
1.0
1.5
6
6
3
8
13
8
12
17
7
8
5
19
13
10
12
21
drm.ugt80..
drm.wt.ugt80..
drm.smt1..
drm.wt.smt1..
dsm.ugt80..
dsm.wt.ugt80..
dsm.smt1..
dsm.wt.smt1..
sp.ugt80..
sp.wt.ugt80..
sp.smt1..
sp.wt.smt1..
im.ugt80..
im.wt.ugt80..
im.smt1..
im.wt.smt1..

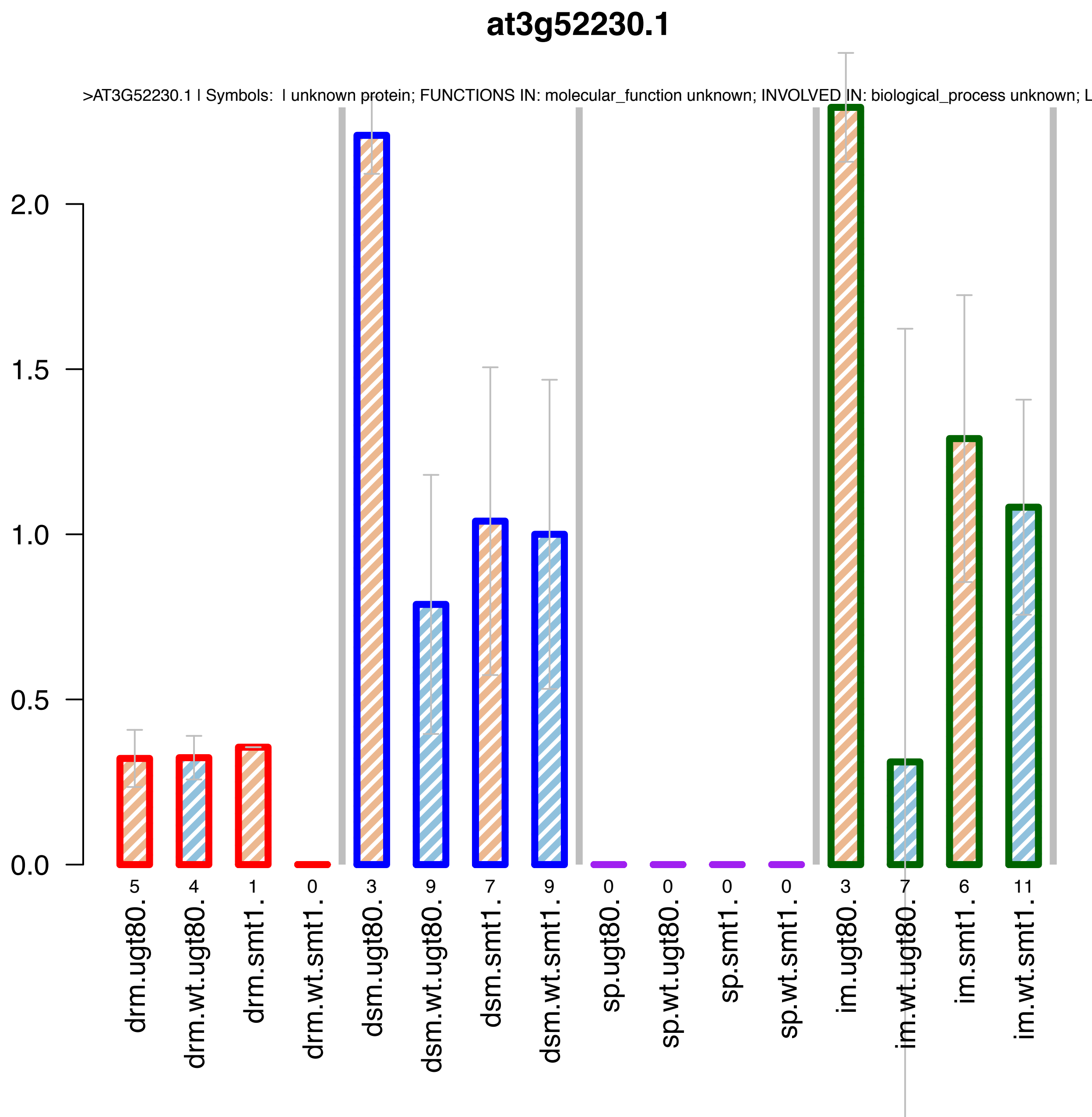

at3g52230.1
>AT3G52230.1 | Symbols: | unknown protein; FUNCTIONS IN: molecular_function unknown; INVOLVED IN: biological_process unknown; L
2.0
1.5
1.0
0.5
0.0
5
4
1
0
3
9
7
9
0
0
0
0
3
7
6
11
drm.ugt80.
drm.wt.ugt80.
drm.smt1.
drm.wt.smt1.
dsm.ugt80.
dsm.wt.ugt80.
dsm.smt1.
dsm.wt.smt1.
sp.ugt80.
sp.wt.ugt80.
sp.smt1.
sp.wt.smt1.
im.ugt80.
im.wt.ugt80.
im.smt1.
im.wt.smt1.

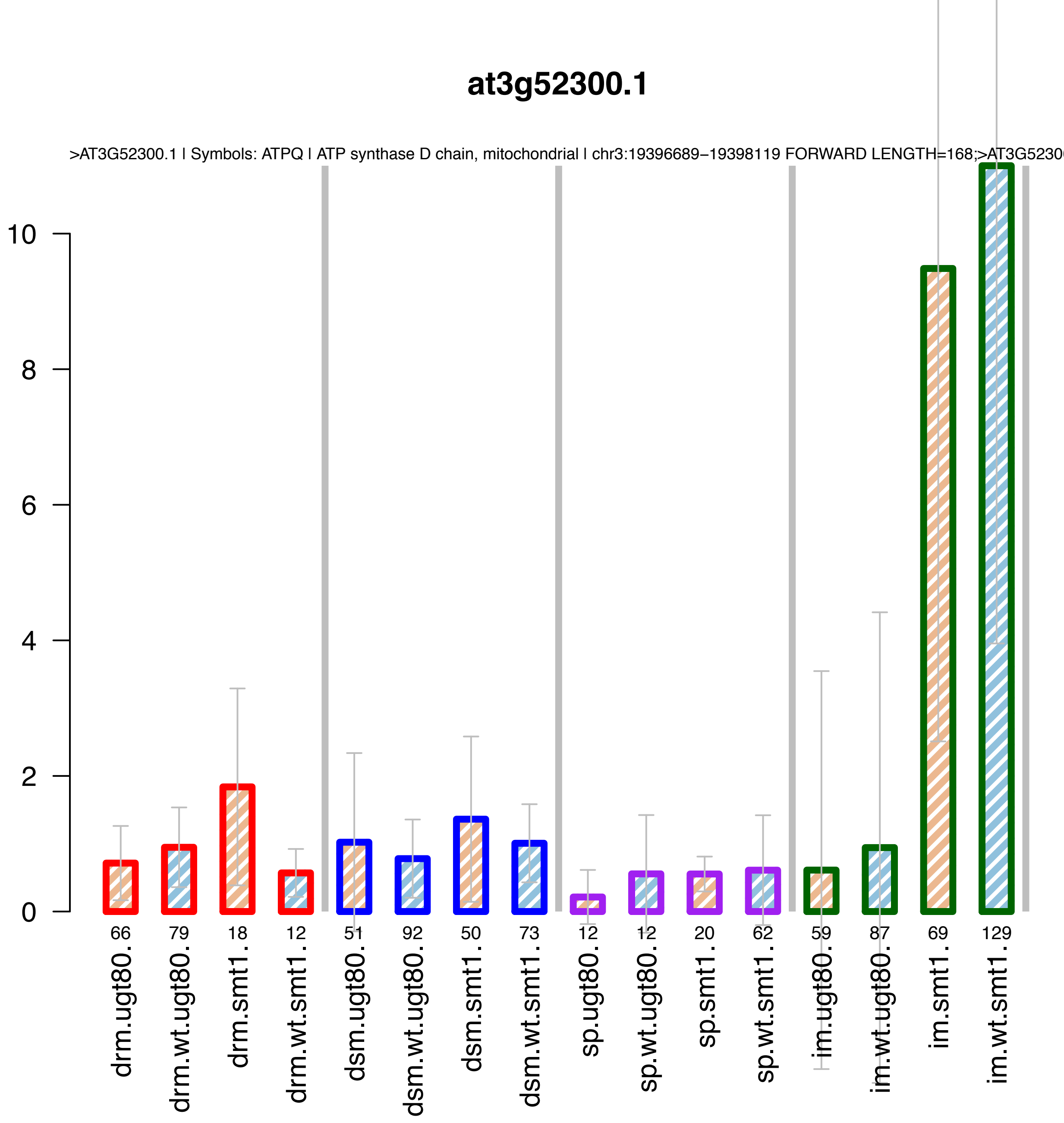

at3g52300.1
>AT3G52300.1 | Symbols: ATPQ | ATP synthase D chain, mitochondrial | chr3:19396689-19398119 FORWARD LENGTH=168;>AT3G5230
0
2
4
6
8
10
66
79
18
12
51
92
50
73
12
12
20
62
59
87
69
129
drm.ugt80.
drm.wt.ugt80.
drm.smt1.
drm.wt.smt1.
dsm.ugt80.
dsm.wt.ugt80.
dsm.smt1.
dsm.wt.smt1.
sp.ugt80.
sp.wt.ugt80.
sp.smt1.
sp.wt.smt1.
im.ugt80.
im.wt.ugt80.
im.smt1.
im.wt.smt1.

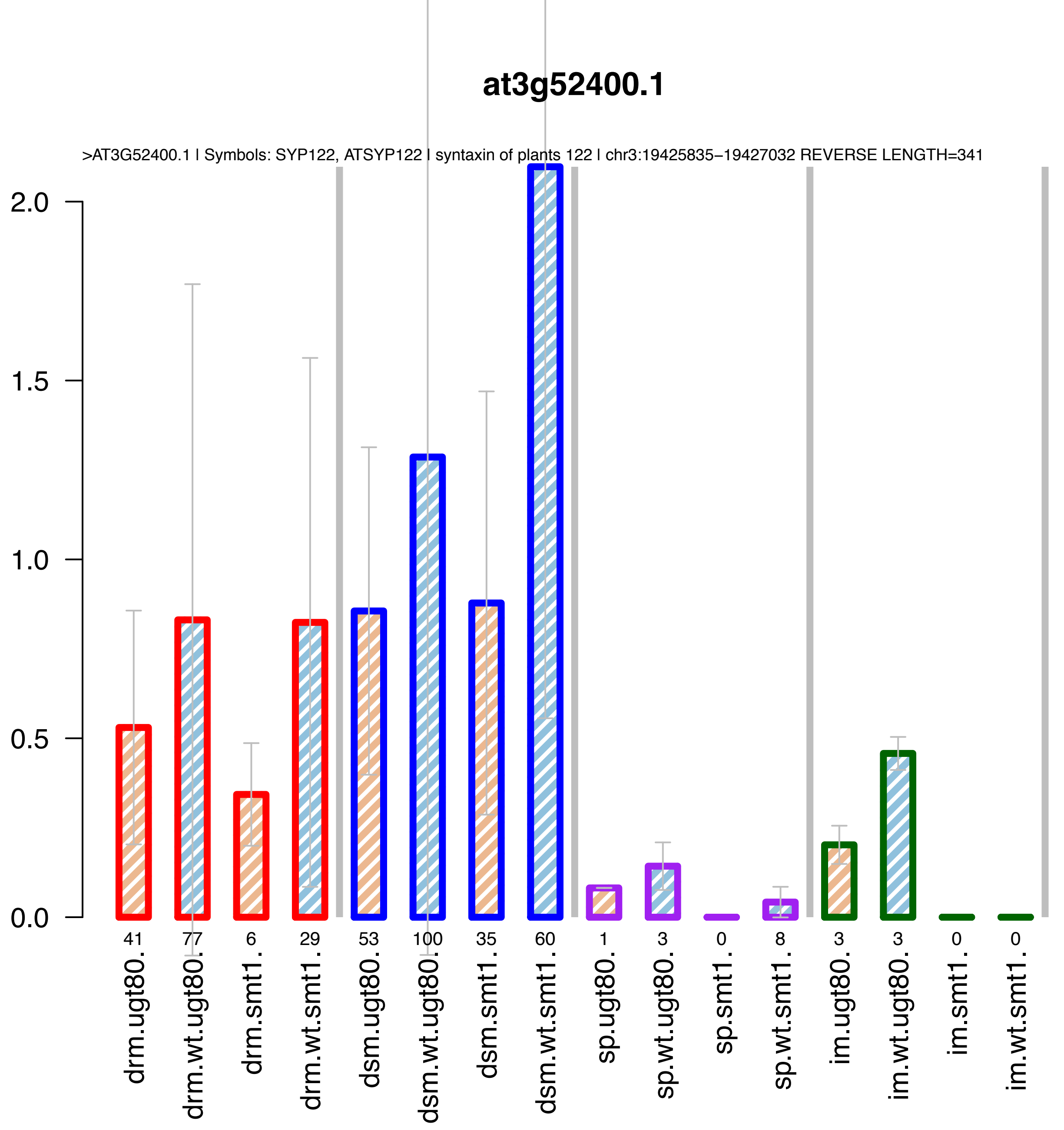

at3g52400.1
>AT3G52400.1 | Symbols: SYP122, ATSYP122 | syntaxin of plants 122 | chr3:19425835-19427032 REVERSE LENGTH=341
2.0
1.5
1.0
0.5
0.0
41
77
6
29
53
100
35
60
1
3
0
8
3
3
0
0
drm.ugt80.
drm.wt.ugt80.
drm.smt1.
drm.wt.smt1.
dsm.ugt80.
dsm.wt.ugt80.
dsm.smt1.
dsm.wt.smt1.
sp.ugt80.
sp.wt.ugt80.
sp.smt1.
sp.wt.smt1.
im.ugt80.
im.wt.ugt80.
im.smt1.
im.wt.smt1.

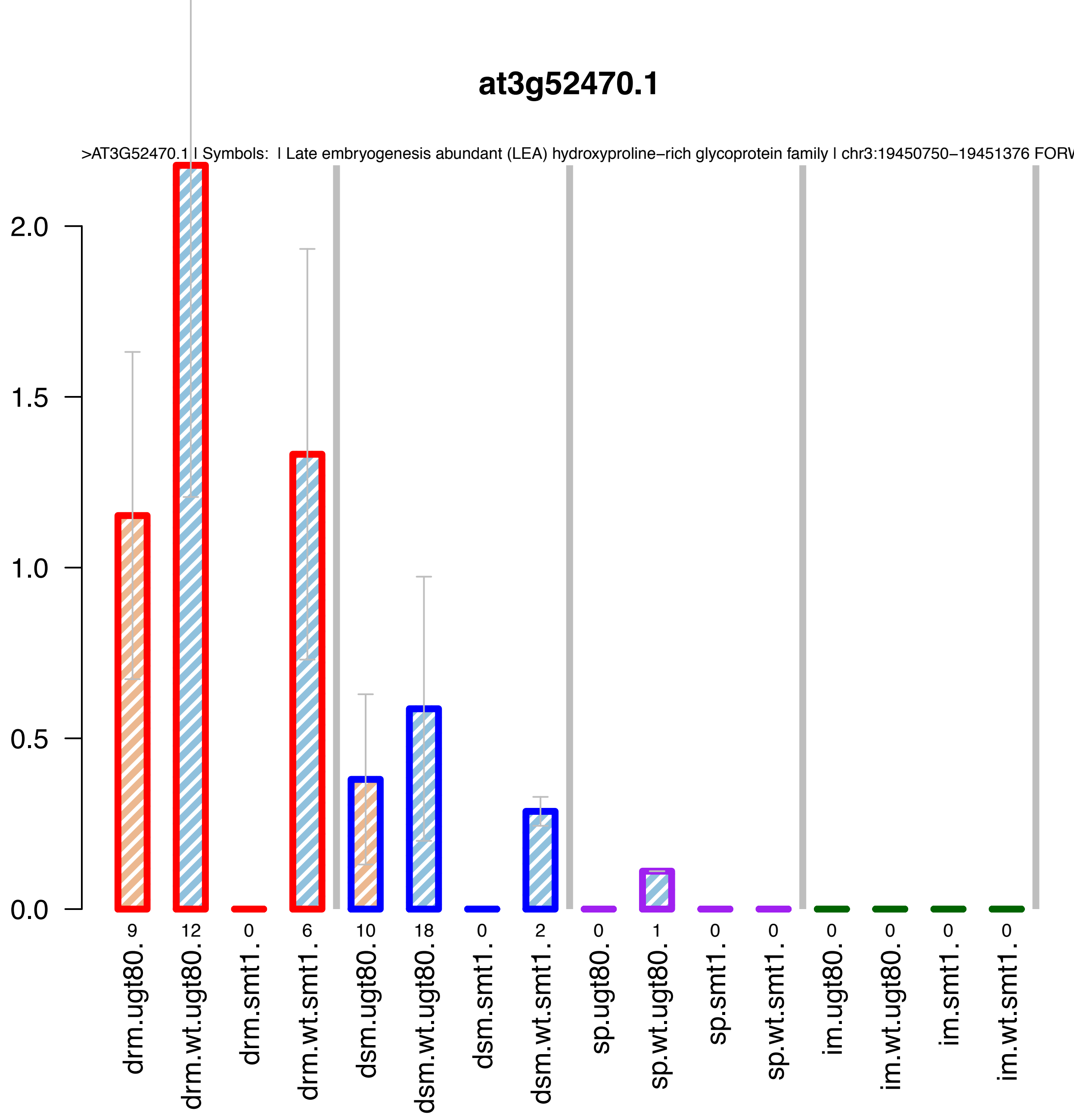

at3g52470.1
>AT3G52470.1 | Symbols: | Late embryogenesis abundant (LEA) hydroxyproline−rich glycoprotein family | chr3:19450750−19451376 FORW
0.0
0.5
1.0
1.5
2.0
9
12
0
6
10
18
0
2
0
1
0
0
0
0
0
0
drm.ugt80.
drm.wt.ugt80.
drm.smt1.
drm.wt.smt1.
dsm.ugt80.
dsm.wt.ugt80.
dsm.smt1.
dsm.wt.smt1.
sp.ugt80.
sp.wt.ugt80.
sp.smt1.
sp.wt.smt1.
im.ugt80.
im.wt.ugt80.
im.smt1.
im.wt.smt1.

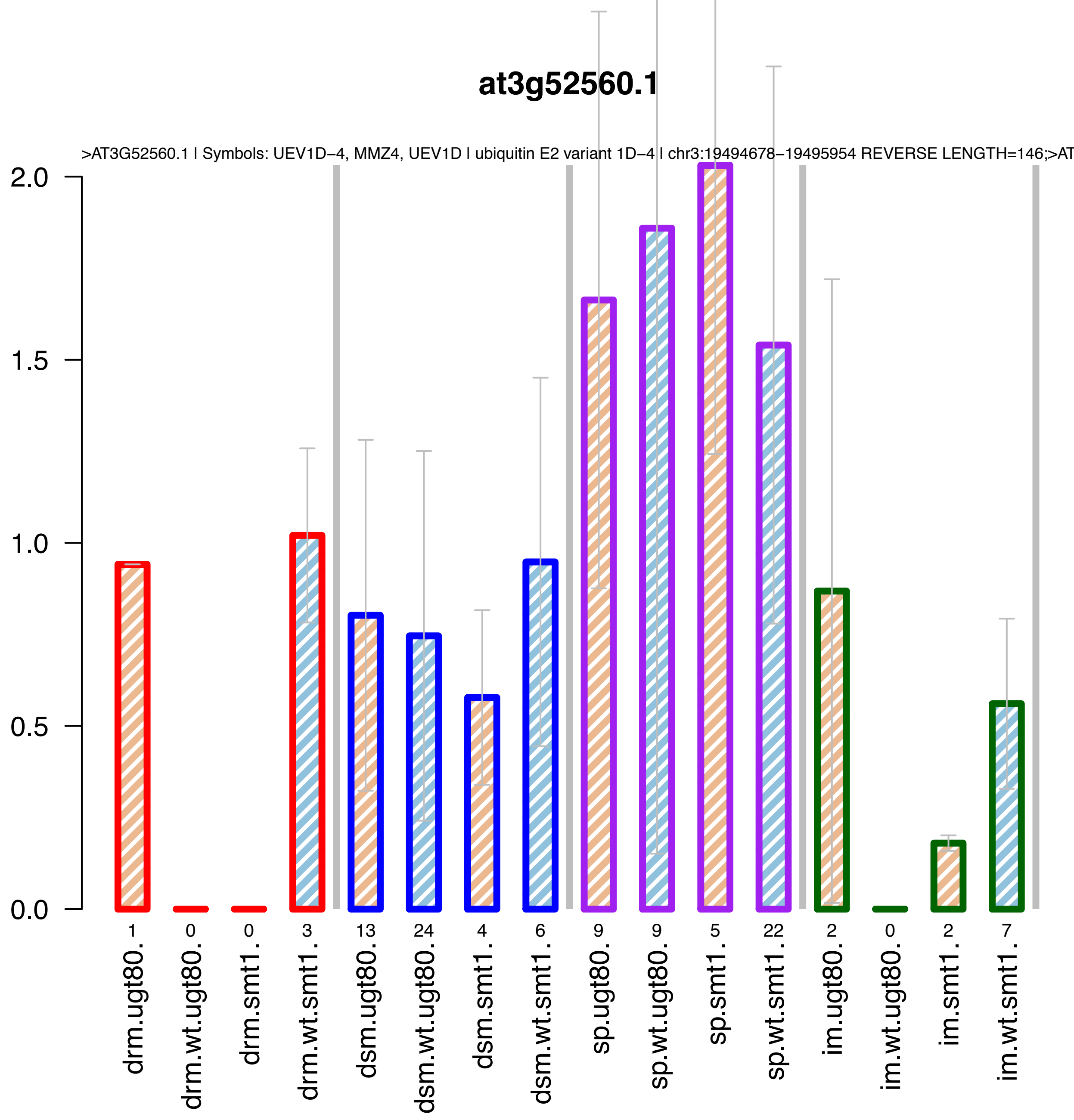
at3g52560.1
>AT3G52560.1 | Symbols: UEV1D−4, MMZ4, UEV1D | ubiquitin E2 variant 1D−4 | chr3:19494678−19495954 REVERSE LENGTH=146;>AT
2.0
1.5
1.0
0.5
0.0
1 0 0 3 13 24 4 6 9 9 5 22 2 0 2 7
drm.ugt80..
drm.wt.ugt80..
drm.smt1..
drm.wt.smt1..
dsm.ugt80..
dsm.wt.ugt80..
dsm.smt1..
dsm.wt.smt1..
sp.ugt80..
sp.wt.ugt80..
sp.smt1..
sp.wt.smt1..
im.ugt80..
im.wt.ugt80..
im.smt1..
im.wt.smt1..

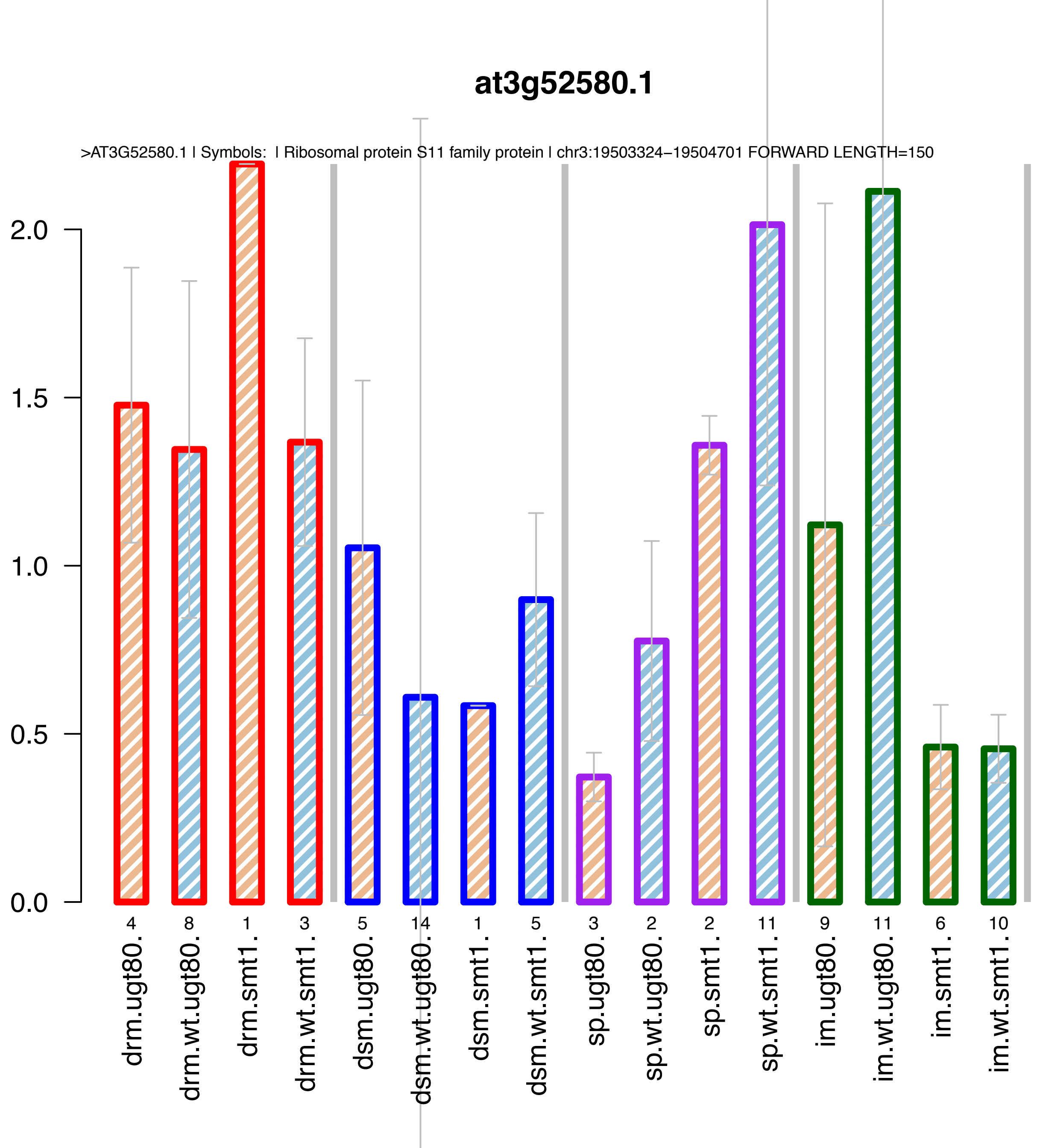

at3g52580.1
>AT3G52580.1 | Symbols: | Ribosomal protein S11 family protein | chr3:19503324-19504701 FORWARD LENGTH=150
0.0
0.5
1.0
1.5
2.0
4
8
1
3
5
14
1
5
3
2
2
11
9
11
6
10
drm.ugt80..
drm.wt.ugt80..
drm.smt1..
drm.wt.smt1..
dsm.ugt80..
dsm.wt.ugt80..
dsm.smt1..
dsm.wt.smt1..
sp.ugt80..
sp.wt.ugt80..
sp.smt1..
sp.wt.smt1..
im.ugt80..
im.wt.ugt80..
im.smt1..
im.wt.smt1..

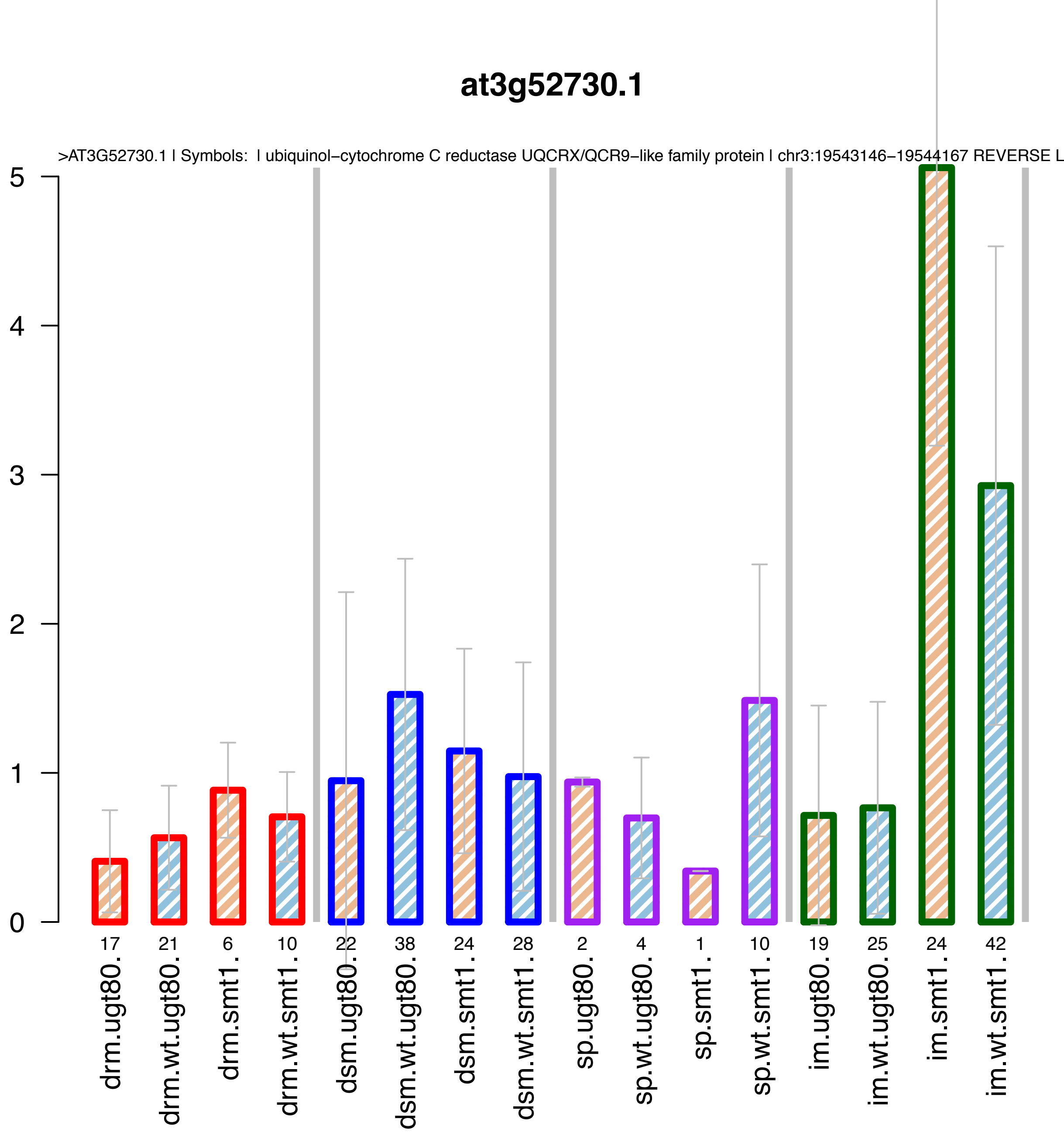

at3g52730.1
>AT3G52730.1 | Symbols: | ubiquinol−cytochrome C reductase UQCRX/QCR9−like family protein | chr3:19543146−19544167 REVERSE L
0
1
2
3
4
5
17
21
6
10
22
38
24
28
2
4
1
10
19
25
24
42
drm.ugt80..
drm.wt.ugt80..
drm.smt1..
drm.wt.smt1..
dsm.ugt80..
dsm.wt.ugt80..
dsm.smt1..
dsm.wt.smt1..
sp.ugt80..
sp.wt.ugt80..
sp.smt1..
sp.wt.smt1..
im.ugt80..
im.wt.ugt80..
im.smt1..
im.wt.smt1..

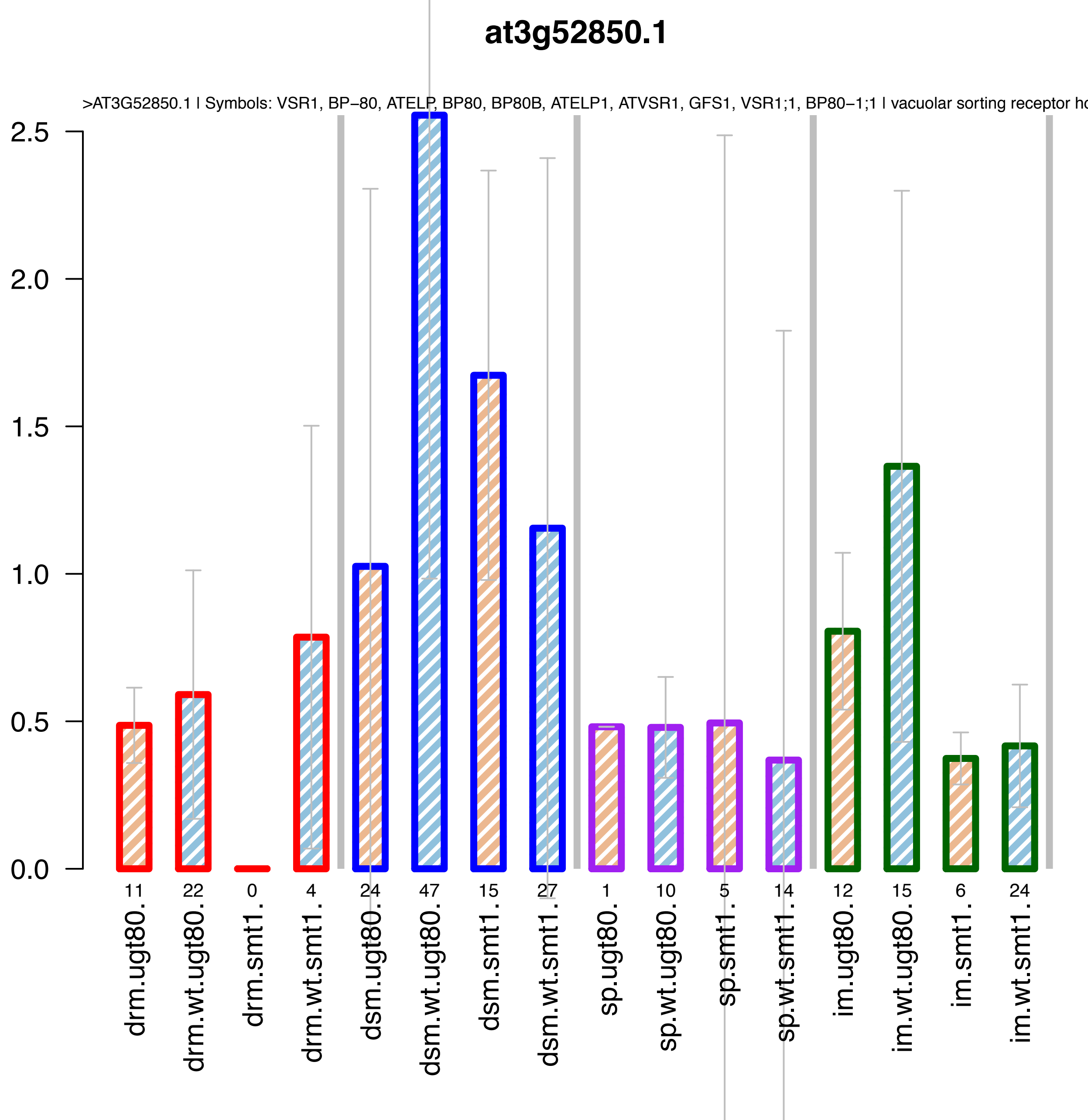

at3g52850.1
>AT3G52850.1 | Symbols: VSR1, BP−80, ATELP, BP80, BP80B, ATELP1, ATVSR1, GFS1, VSR1;1, BP80−1;1 | vacuolar sorting receptor ho
0.0
0.5
1.0
1.5
2.0
2.5
11
22
0
4
24
47
15
27
1
10
5
14
12
15
6
24
drm.ugt80.
drm.wt.ugt80.
drm.smt1.
drm.wt.smt1.
dsm.ugt80.
dsm.wt.ugt80.
dsm.smt1.
dsm.wt.smt1.
sp.ugt80.
sp.wt.ugt80.
sp.smt1.
sp.wt.smt1.
im.ugt80.
im.wt.ugt80.
im.smt1.
im.wt.smt1.

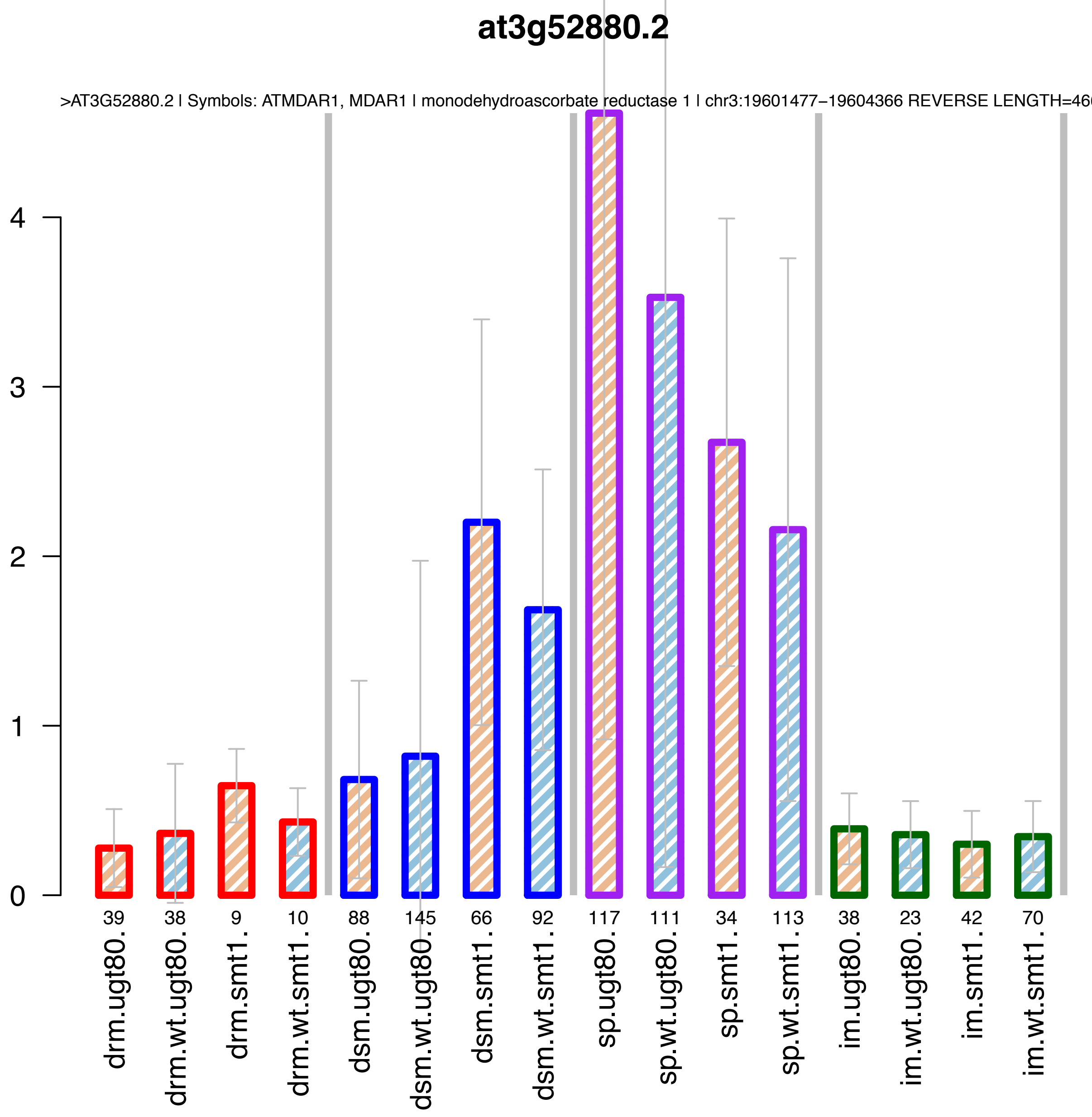

at3g52880.2
>AT3G52880.2 | Symbols: ATMDAR1, MDAR1 | monodehydroascorbate reductase 1 | chr3:19601477−19604366 REVERSE LENGTH=466;
0
1
2
3
4
39
38
9
10
88
145
66
92
117
111
34
113
38
23
42
70
drm.ugt80.
drm.wt.ugt80.
drm.smt1.
drm.wt.smt1.
dsm.ugt80.
dsm.wt.ugt80.
dsm.smt1.
dsm.wt.smt1.
sp.ugt80.
sp.wt.ugt80.
sp.smt1.
sp.wt.smt1.
im.ugt80.
im.wt.ugt80.
im.smt1.
im.wt.smt1.

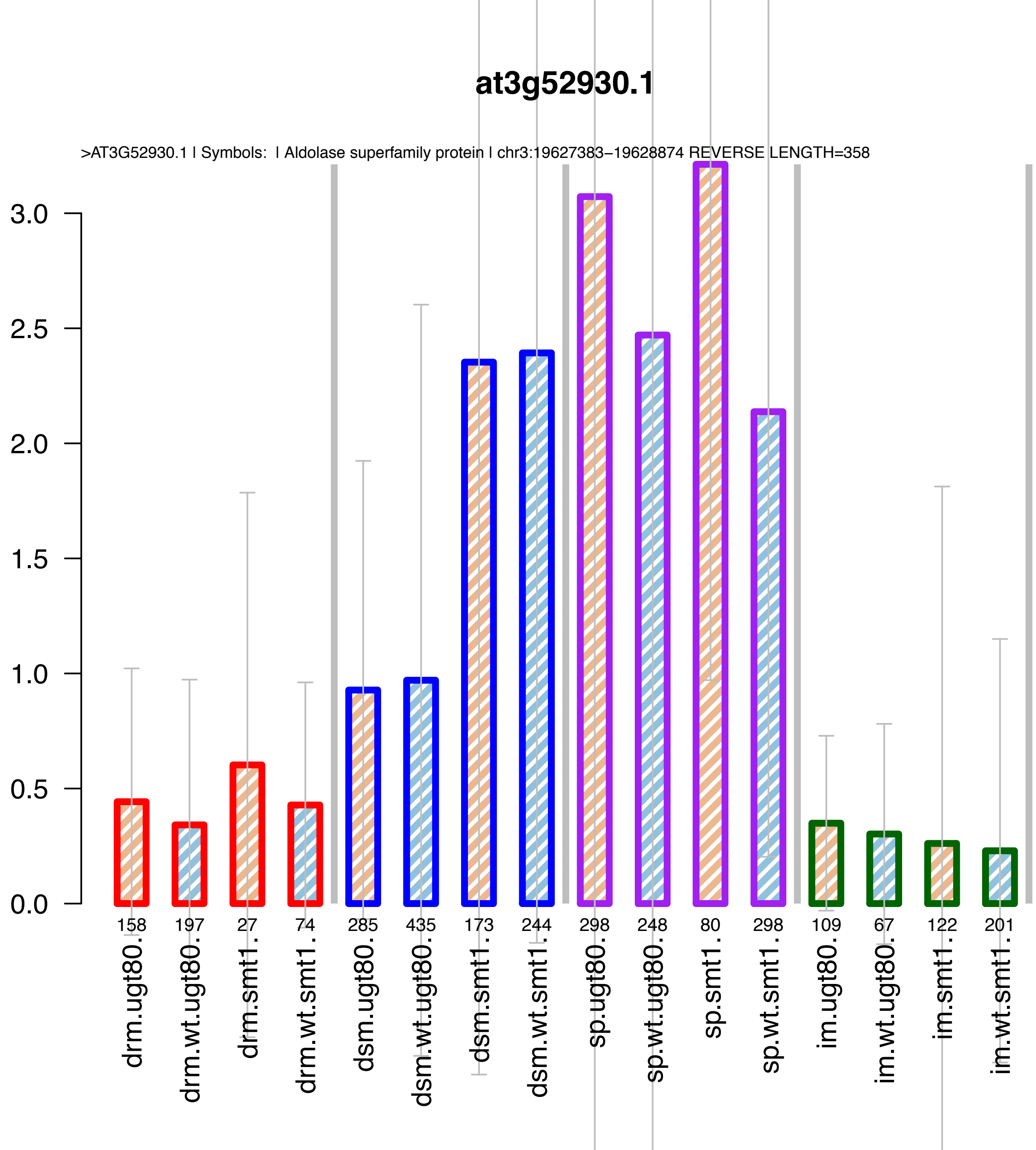

at3g52930.1
>AT3G52930.1 | Symbols: | Aldolase superfamily protein | chr3:19627383−19628874 REVERSE LENGTH=358
3.0
2.5
2.0
1.5
1.0
0.5
0.0
158
197
27
74
285
435
173
244
298
248
80
298
109
67
122
201
drm.ugt80.
drm.wt.ugt80.
drm.smt1.
drm.wt.smt1.
dsm.ugt80.
dsm.wt.ugt80.
dsm.smt1.
dsm.wt.smt1.
sp.ugt80.
sp.wt.ugt80.
sp.smt1.
sp.wt.smt1.
im.ugt80.
im.wt.ugt80.
im.smt1.
im.wt.smt1.

# at3g52960.1

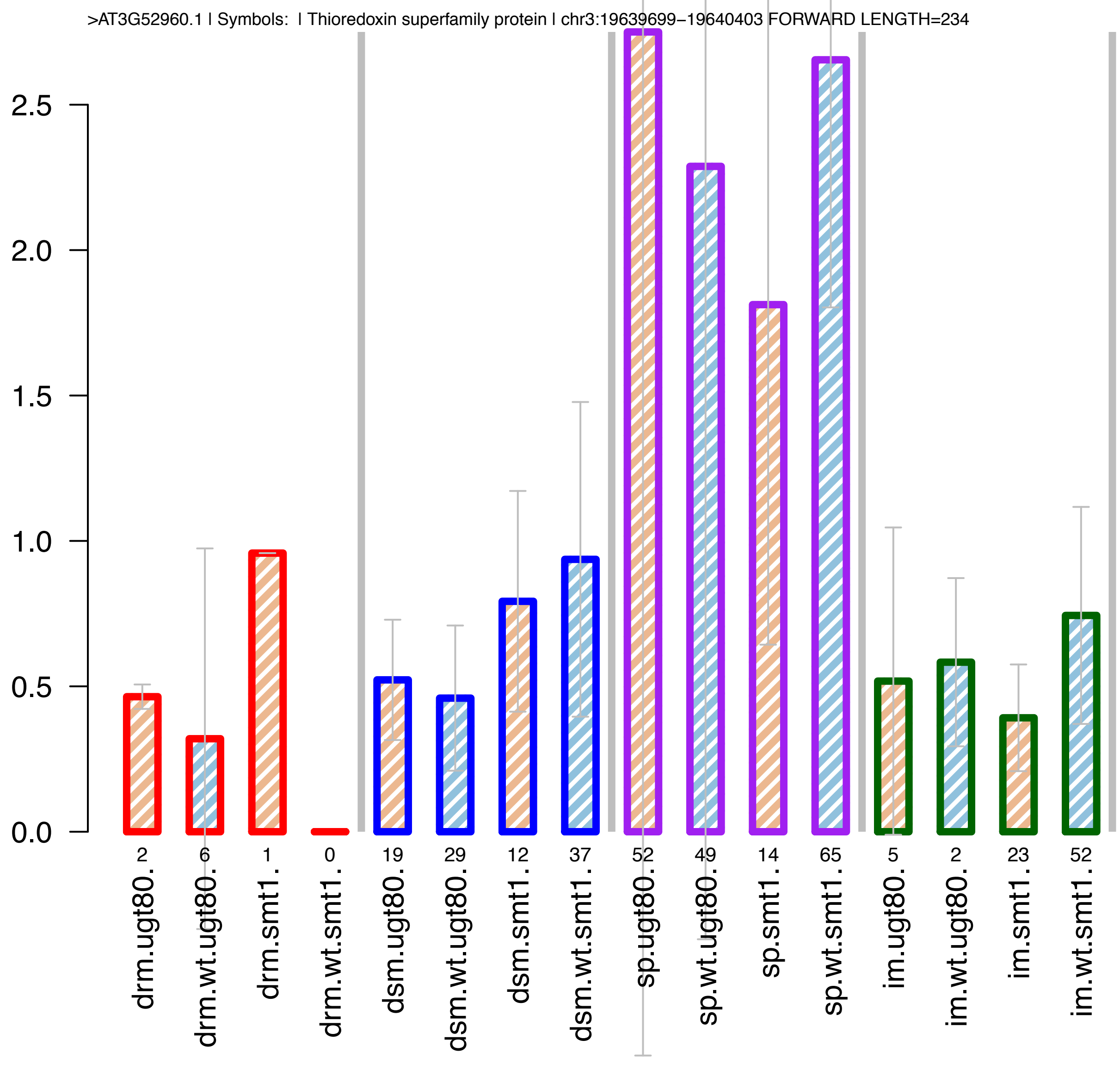

>AT3G52960.1 | Symbols: | Thioredoxin superfamily protein | chr3:19639699-19640403 FORWARD LENGTH=234
0.0
0.5
1.0
1.5
2.0
2.5
2
6
1
0
19
29
12
37
52
49
14
65
5
2
23
52
drm.ugt80..
drm.wt.ugt80..
drm.smt1..
drm.wt.smt1..
dsm.ugt80..
dsm.wt.ugt80..
dsm.smt1..
dsm.wt.smt1..
sp.ugt80..
sp.wt.ugt80..
sp.smt1..
sp.wt.smt1..
im.ugt80..
im.wt.ugt80..
im.smt1..
im.wt.smt1..

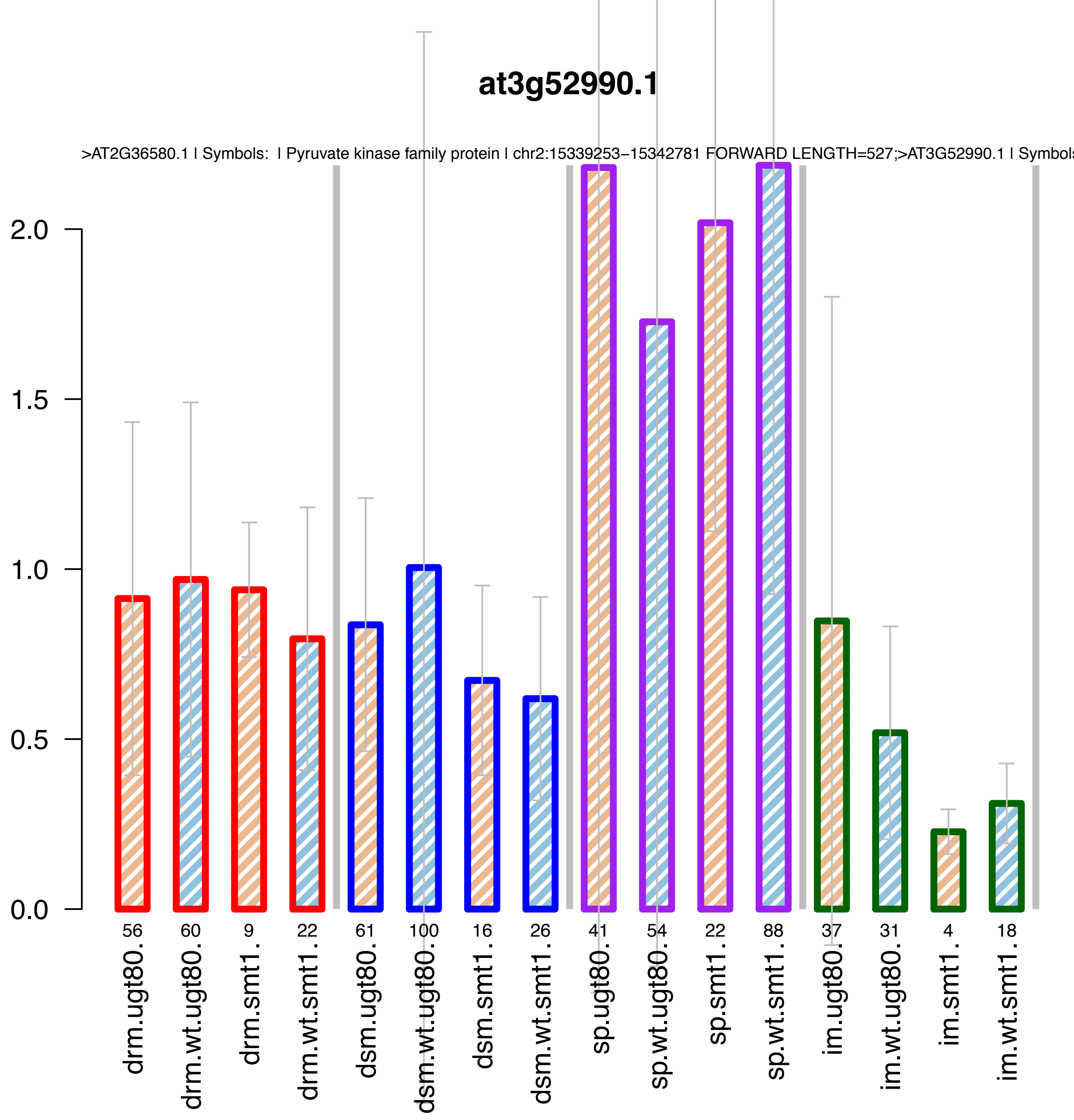

at3g52990.1
>AT2G36580.1 | Symbols: | Pyruvate kinase family protein | chr2:15339253-15342781 FORWARD LENGTH=527;>AT3G52990.1 | Symbols
2.0
1.5
1.0
0.5
0.0
56
60
9
22
61
100
16
26
41
54
22
88
37
31
4
18
drm.ugt80.
drm.wt.ugt80.
drm.smt1.
drm.wt.smt1.
dsm.ugt80.
dsm.wt.ugt80.
dsm.smt1.
dsm.wt.smt1.
sp.ugt80.
sp.wt.ugt80.
sp.smt1.
sp.wt.smt1.
im.ugt80.
im.wt.ugt80.
im.smt1.
im.wt.smt1.

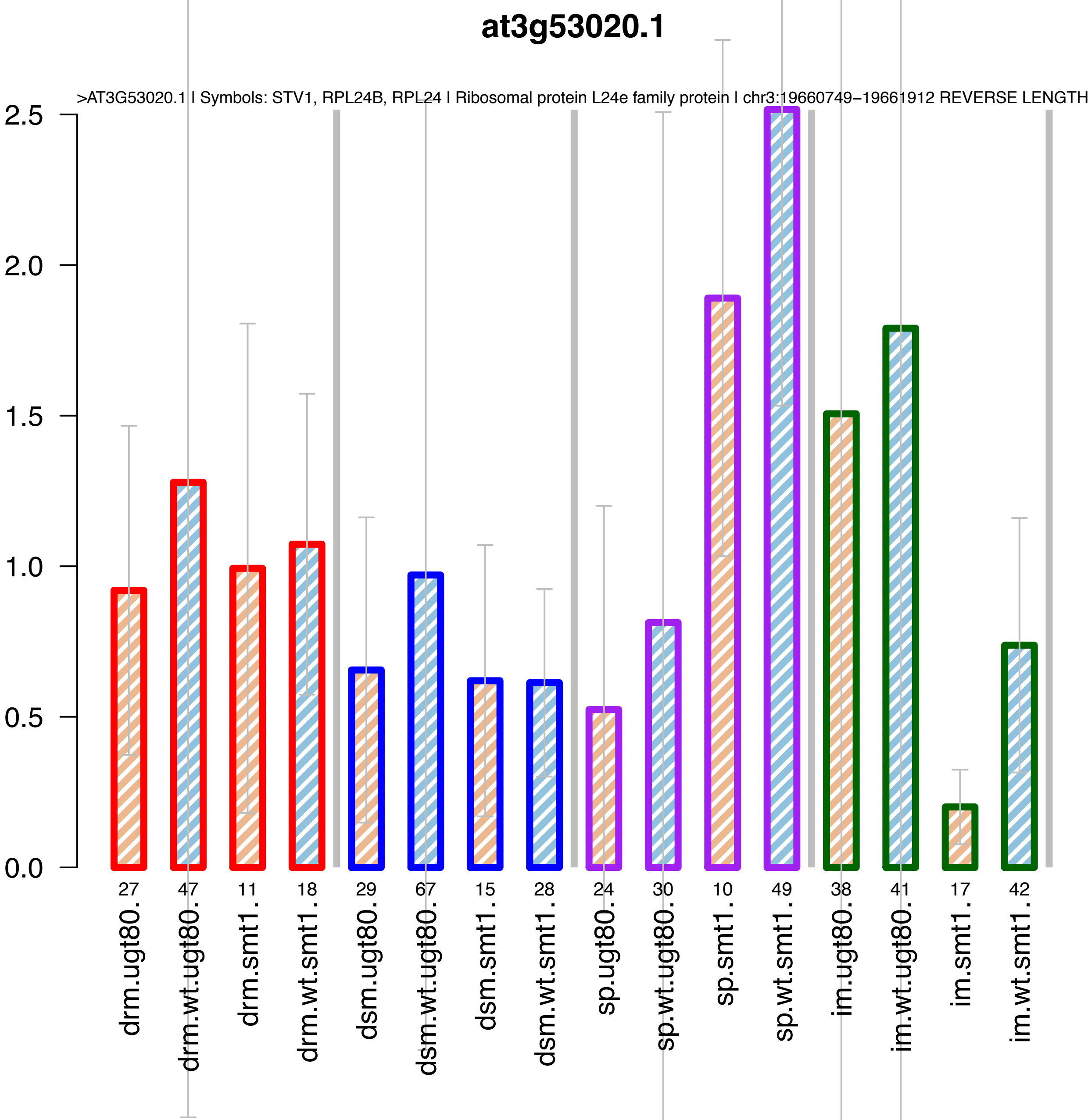

at3g53020.1
>AT3G53020.1 | Symbols: STV1, RPL24B, RPL24 | Ribosomal protein L24e family protein | chr3:19660749-19661912 REVERSE LENGTH
2.5
2.0
1.5
1.0
0.5
0.0
27
47
11
18
29
67
15
28
24
30
10
49
38
41
17
42
drm.ugt80..
drm.wt.ugt80..
drm.smt1..
drm.wt.smt1..
dsm.ugt80..
dsm.wt.ugt80..
dsm.smt1..
dsm.wt.smt1..
sp.ugt80..
sp.wt.ugt80..
sp.smt1..
sp.wt.smt1..
im.ugt80..
im.wt.ugt80..
im.smt1..
im.wt.smt1..

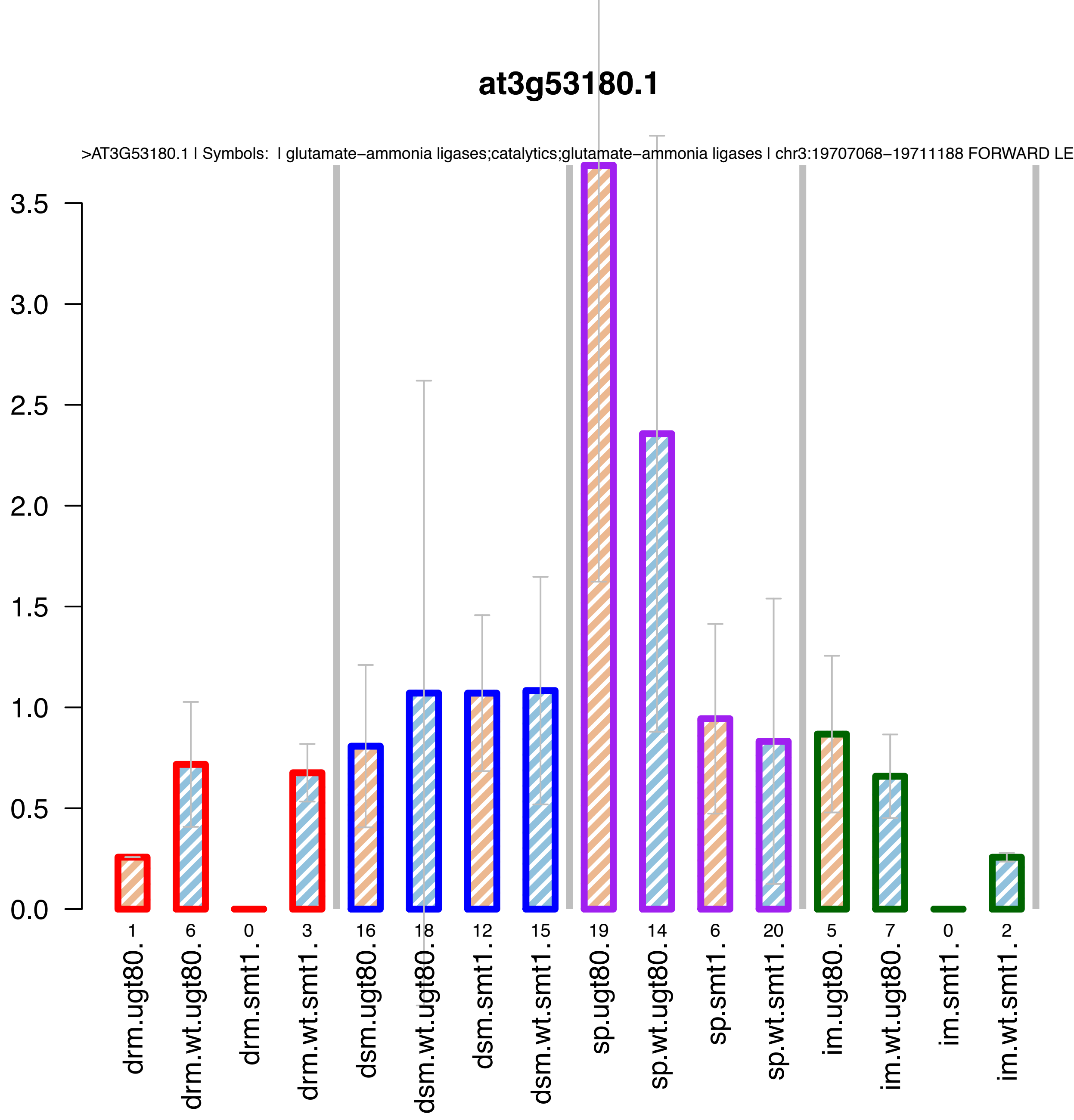

at3g53180.1
>AT3G53180.1 | Symbols: | glutamate–ammonia ligases;catalytics;glutamate–ammonia ligases | chr3:19707068–19711188 FORWARD LE
3.5
3.0
2.5
2.0
1.5
1.0
0.5
0.0
1
6
0
3
16
18
12
15
19
14
6
20
5
7
0
2
drm.ugt80.
drm.wt.ugt80.
drm.smt1.
drm.wt.smt1.
dsm.ugt80.
dsm.wt.ugt80.
dsm.smt1.
dsm.wt.smt1.
sp.ugt80.
sp.wt.ugt80.
sp.smt1.
sp.wt.smt1.
im.ugt80.
im.wt.ugt80.
im.smt1.
im.wt.smt1.

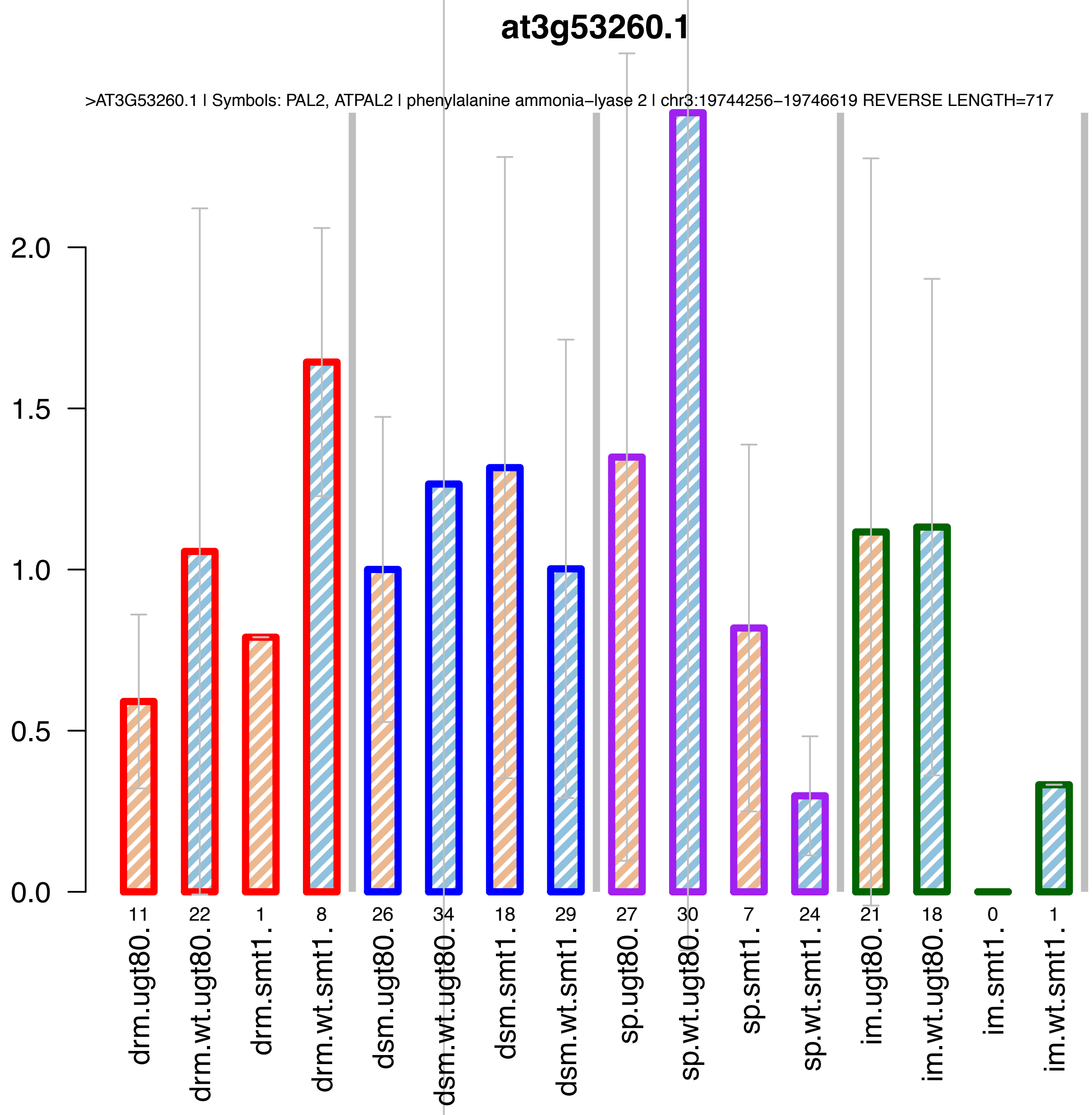

at3g53260.1
>AT3G53260.1 | Symbols: PAL2, ATPAL2 | phenylalanine ammonia−lyase 2 | chr3:19744256−19746619 REVERSE LENGTH=717
0.0
0.5
1.0
1.5
2.0
11
22
1
8
26
34
18
29
27
30
7
24
21
18
0
1
drm.ugt80..
drm.wt.ugt80..
drm.smt1..
drm.wt.smt1..
dsm.ugt80..
dsm.wt.ugt80..
dsm.smt1..
dsm.wt.smt1..
sp.ugt80..
sp.wt.ugt80..
sp.smt1..
sp.wt.smt1..
im.ugt80..
im.wt.ugt80..
im.smt1..
im.wt.smt1..

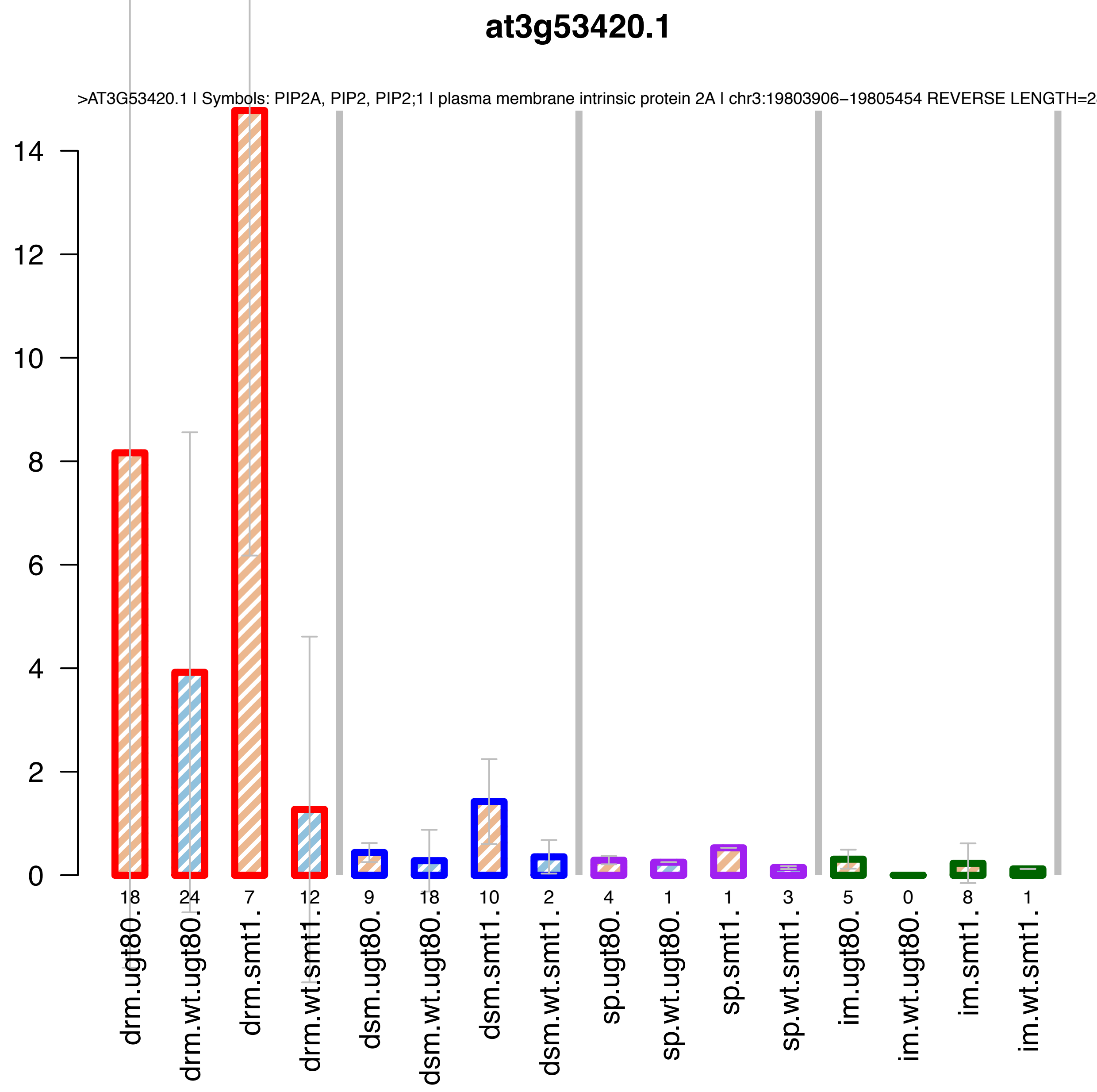

at3g53420.1
>AT3G53420.1 | Symbols: PIP2A, PIP2, PIP2;1 | plasma membrane intrinsic protein 2A | chr3:19803906-19805454 REVERSE LENGTH=2
0
2
4
6
8
10
12
14
18
24
7
12
9
18
10
2
4
1
1
3
5
0
8
1
drm.ugt80.
drm.wt.ugt80.
drm.smt1.
drm.wt.smt1.
dsm.ugt80.
dsm.wt.ugt80.
dsm.smt1.
dsm.wt.smt1.
sp.ugt80.
sp.wt.ugt80.
sp.smt1.
sp.wt.smt1.
im.ugt80.
im.wt.ugt80.
im.smt1.
im.wt.smt1.

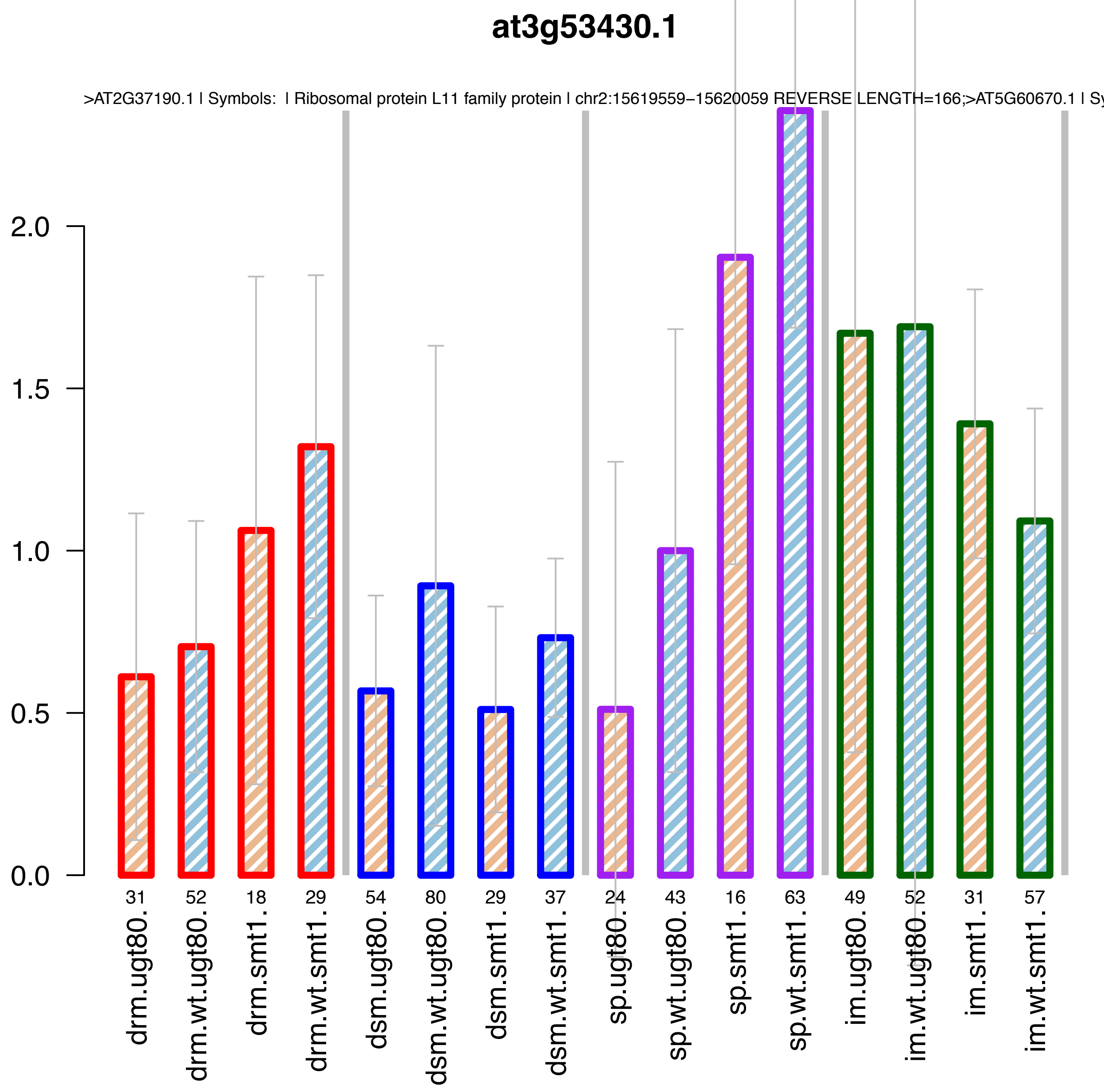

at3g53430.1
>AT2G37190.1 | Symbols: | Ribosomal protein L11 family protein | chr2:15619559-15620059 REVERSE LENGTH=166;>AT5G60670.1 | Sy
0.0
0.5
1.0
1.5
2.0
31
52
18
29
54
80
29
37
24
43
16
63
49
52
31
57
drm.ugt80..
drm.wt.ugt80..
drm.smt1..
drm.wt.smt1..
dsm.ugt80..
dsm.wt.ugt80..
dsm.smt1..
dsm.wt.smt1..
sp.ugt80..
sp.wt.ugt80..
sp.smt1..
sp.wt.smt1..
im.ugt80..
im.wt.ugt80..
im.smt1..
im.wt.smt1..

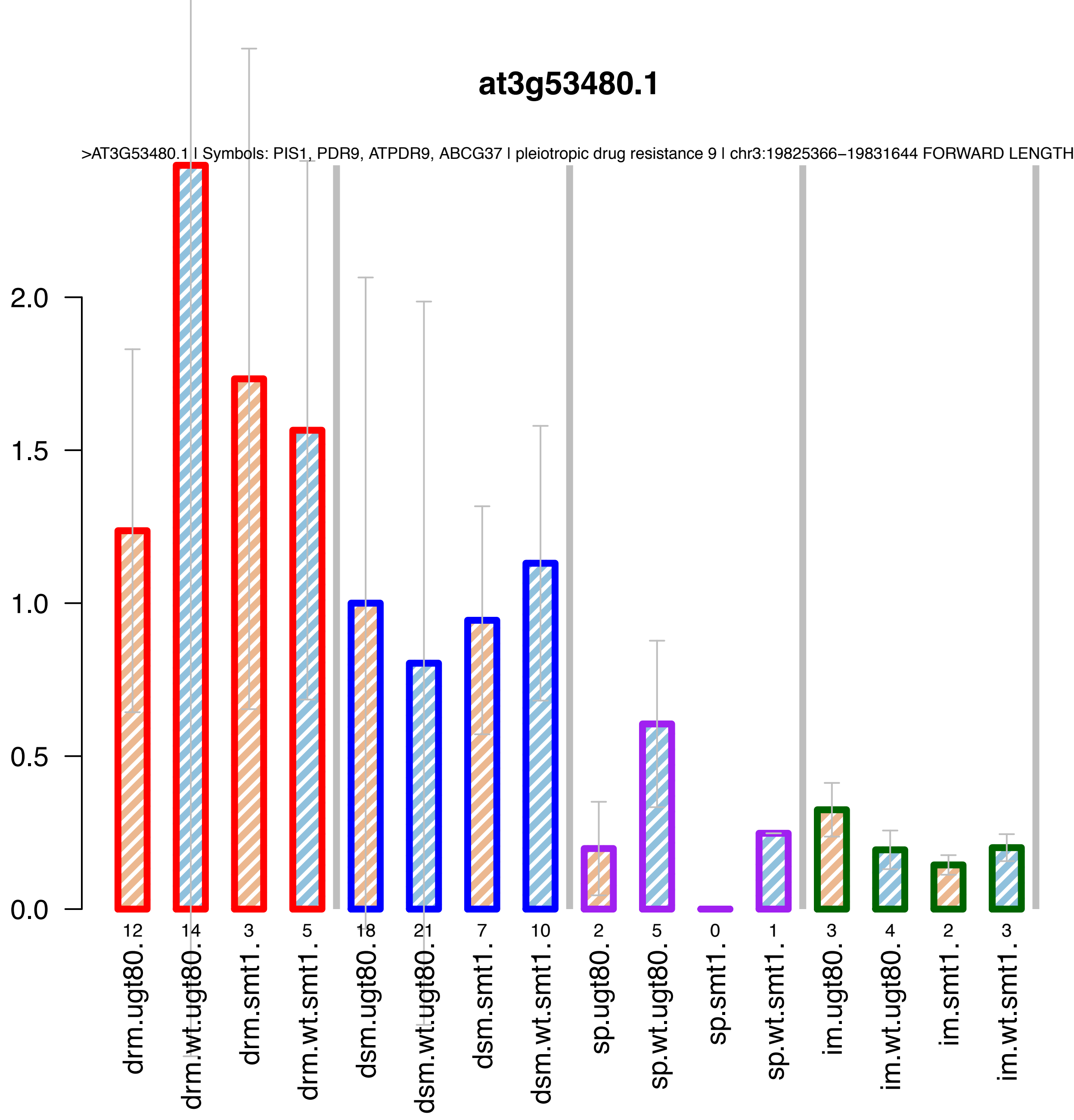

at3g53480.1
>AT3G53480.1 | Symbols: PIS1, PDR9, ATPDR9, ABCG37 | pleiotropic drug resistance 9 | chr3:19825366-19831644 FORWARD LENGTH
0.0
0.5
1.0
1.5
2.0
12
14
3
5
18
21
7
10
2
5
0
1
3
4
2
3
drm.ugt80..
drm.wt.ugt80..
drm.smt1..
drm.wt.smt1..
dsm.ugt80..
dsm.wt.ugt80..
dsm.smt1..
dsm.wt.smt1..
sp.ugt80..
sp.wt.ugt80..
sp.smt1..
sp.wt.smt1..
im.ugt80..
im.wt.ugt80..
im.smt1..
im.wt.smt1..

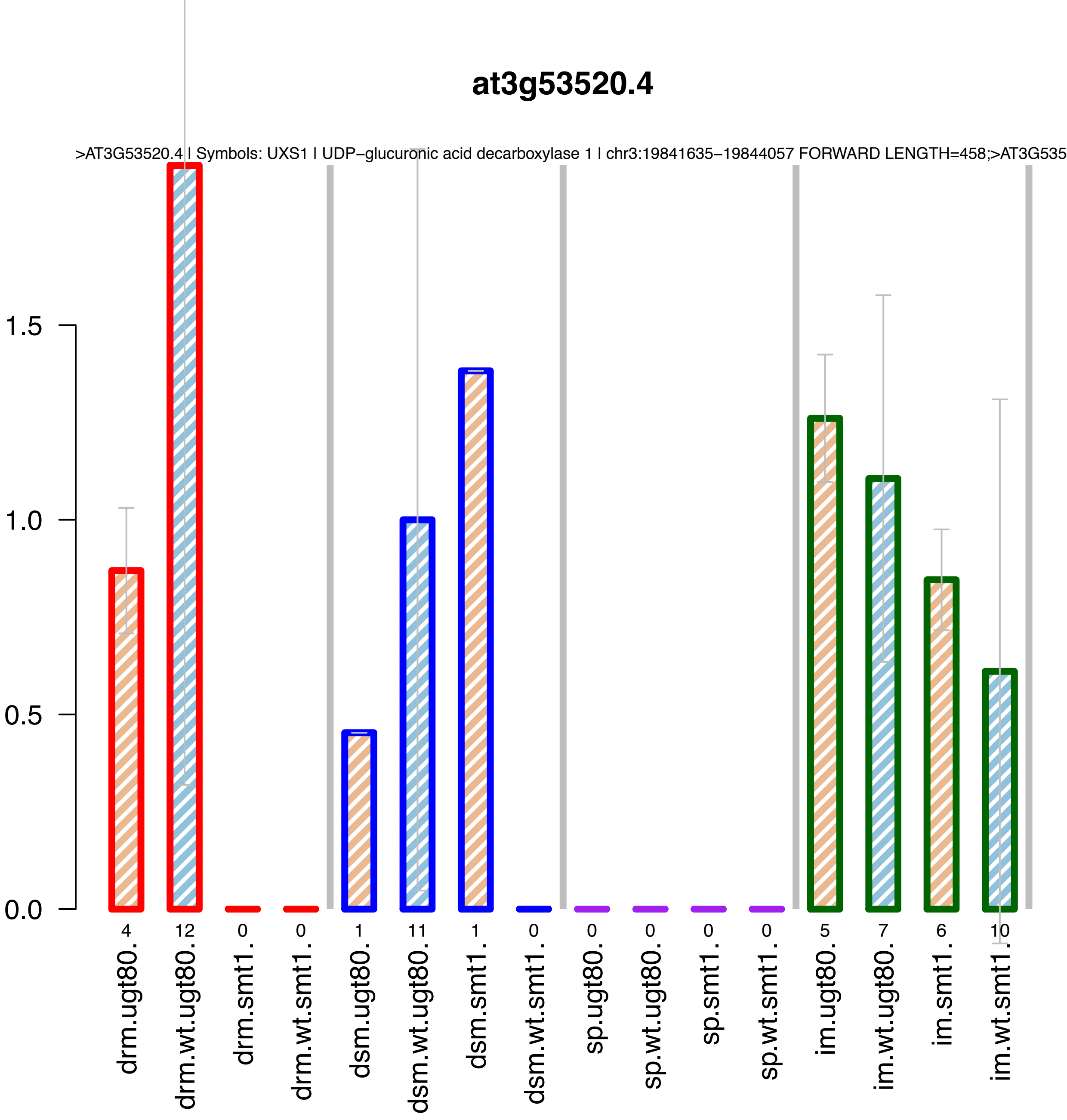

at3g53520.4
>AT3G53520.4 | Symbols: UXS1 | UDP−glucuronic acid decarboxylase 1 | chr3:19841635−19844057 FORWARD LENGTH=458;>AT3G535
0.0
0.5
1.0
1.5
4
12
0
0
1
11
1
0
0
0
0
0
5
7
6
10
drm.ugt80.
drm.wt.ugt80.
drm.smt1.
drm.wt.smt1.
dsm.ugt80.
dsm.wt.ugt80.
dsm.smt1.
dsm.wt.smt1.
sp.ugt80.
sp.wt.ugt80.
sp.smt1.
sp.wt.smt1.
im.ugt80.
im.wt.ugt80.
im.smt1.
im.wt.smt1.

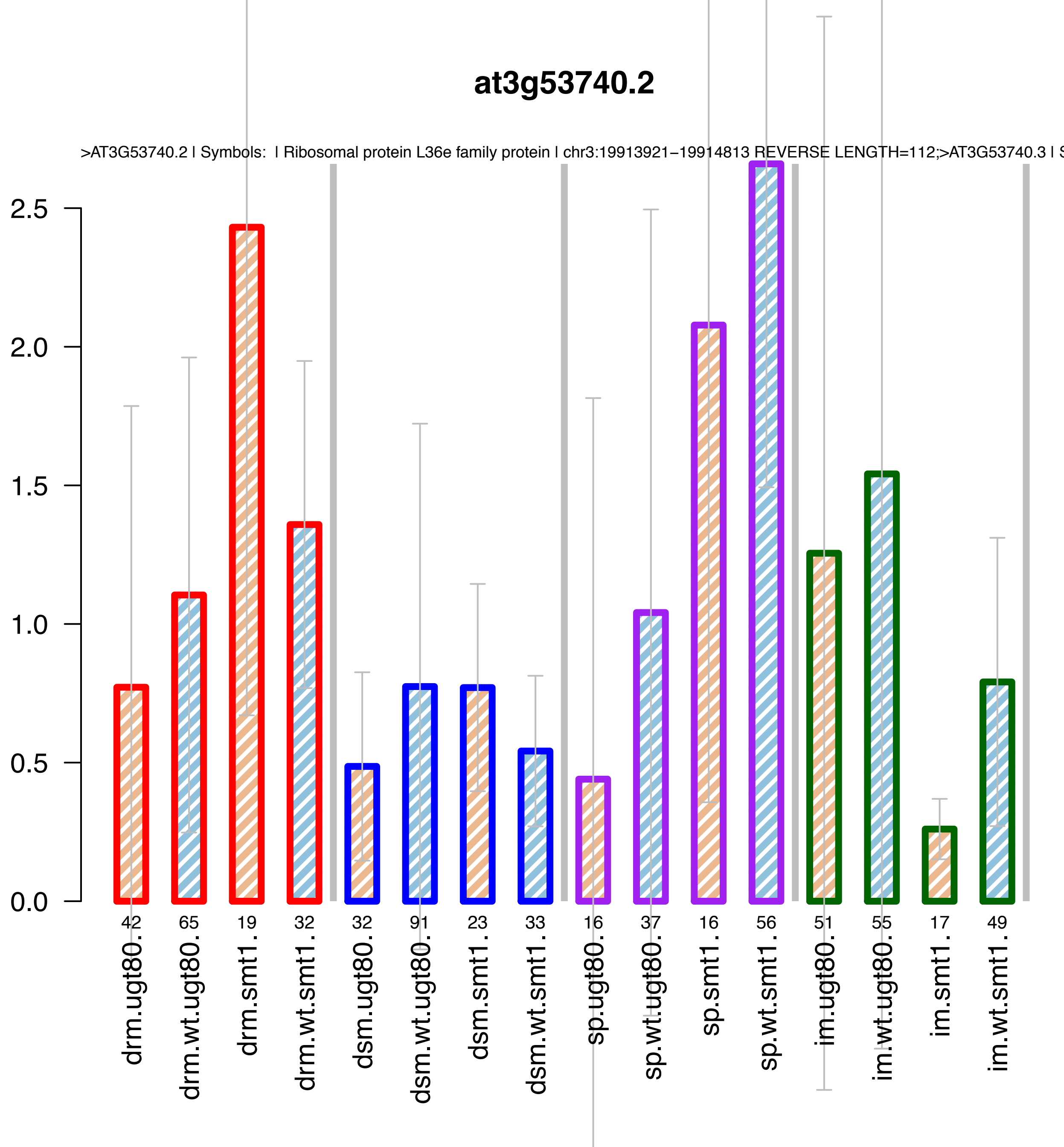

at3g53740.2
>AT3G53740.2 | Symbols: | Ribosomal protein L36e family protein | chr3:19913921−19914813 REVERSE LENGTH=112;>AT3G53740.3 | S
0.0
0.5
1.0
1.5
2.0
2.5
42
65
19
32
32
91
23
33
16
37
16
56
51
55
17
49
drm.ugt80..
drm.wt.ugt80..
drm.smt1..
drm.wt.smt1..
dsm.ugt80..
dsm.wt.ugt80..
dsm.smt1..
dsm.wt.smt1..
sp.ugt80..
sp.wt.ugt80..
sp.smt1..
sp.wt.smt1..
im.ugt80..
im.wt.ugt80..
im.smt1..
im.wt.smt1..

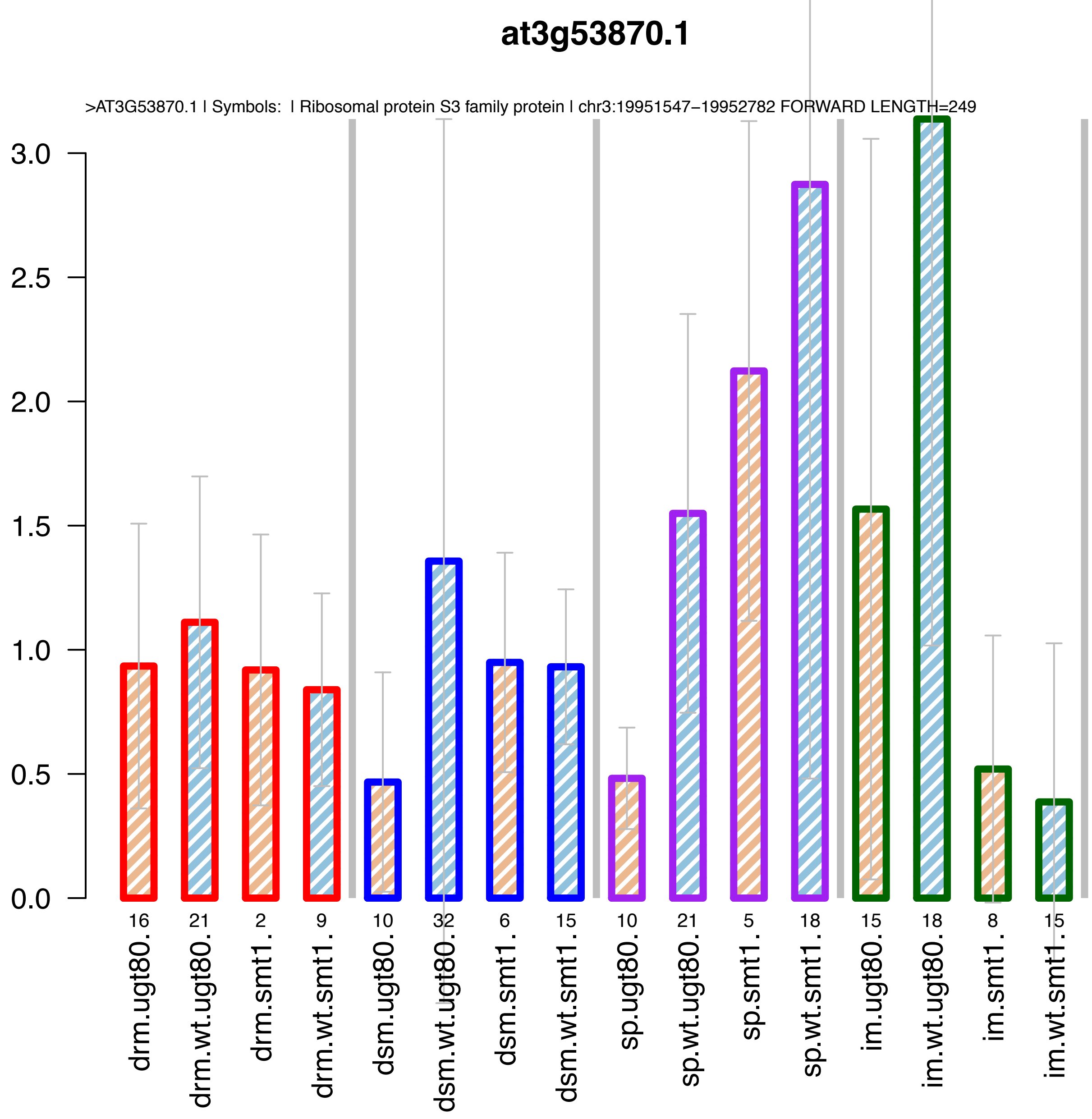
at3g53870.1
>AT3G53870.1 | Symbols: | Ribosomal protein S3 family protein | chr3:19951547-19952782 FORWARD LENGTH=249
0.0
0.5
1.0
1.5
2.0
2.5
3.0
16
21
2
9
10
32
6
15
10
21
5
18
15
18
8
15
drm.ugt80..
drm.wt.ugt80..
drm.smt1..
drm.wt.smt1..
dsm.ugt80..
dsm.wt.ugt80..
dsm.smt1..
dsm.wt.smt1..
sp.ugt80..
sp.wt.ugt80..
sp.smt1..
sp.wt.smt1..
im.ugt80..
im.wt.ugt80..
im.smt1..
im.wt.smt1..

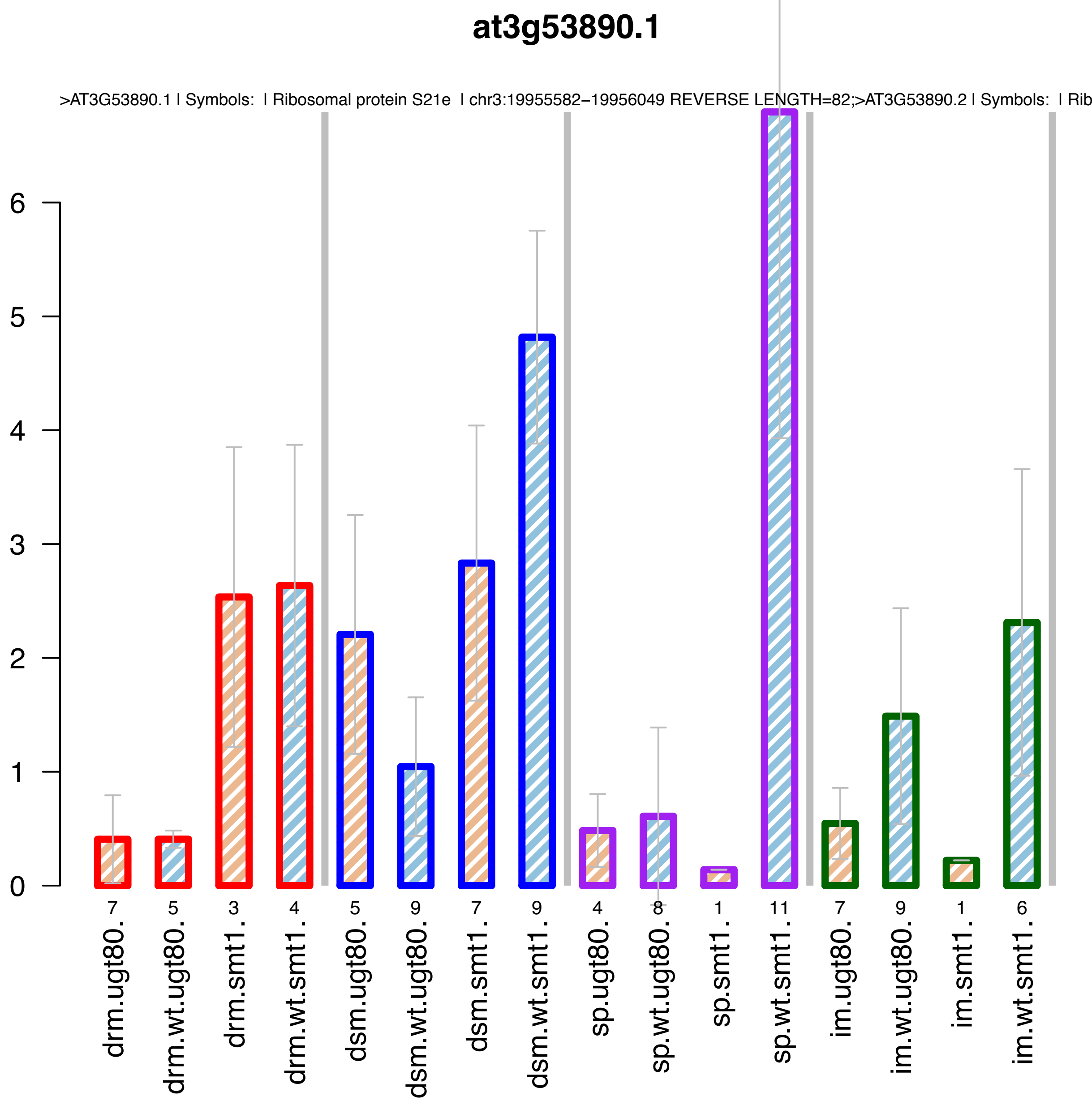

at3g53890.1
>AT3G53890.1 | Symbols: | Ribosomal protein S21e | chr3:19955582–19956049 REVERSE LENGTH=82;>AT3G53890.2 | Symbols: | Rib
0
1
2
3
4
5
6
7
5
3
4
5
9
7
9
4
8
1
11
7
9
1
6
drm.ugt80.
drm.wt.ugt80.
drm.smt1.
drm.wt.smt1.
dsm.ugt80.
dsm.wt.ugt80.
dsm.smt1.
dsm.wt.smt1.
sp.ugt80.
sp.wt.ugt80.
sp.smt1.
sp.wt.smt1.
im.ugt80.
im.wt.ugt80.
im.smt1.
im.wt.smt1.

# at3g53970.1

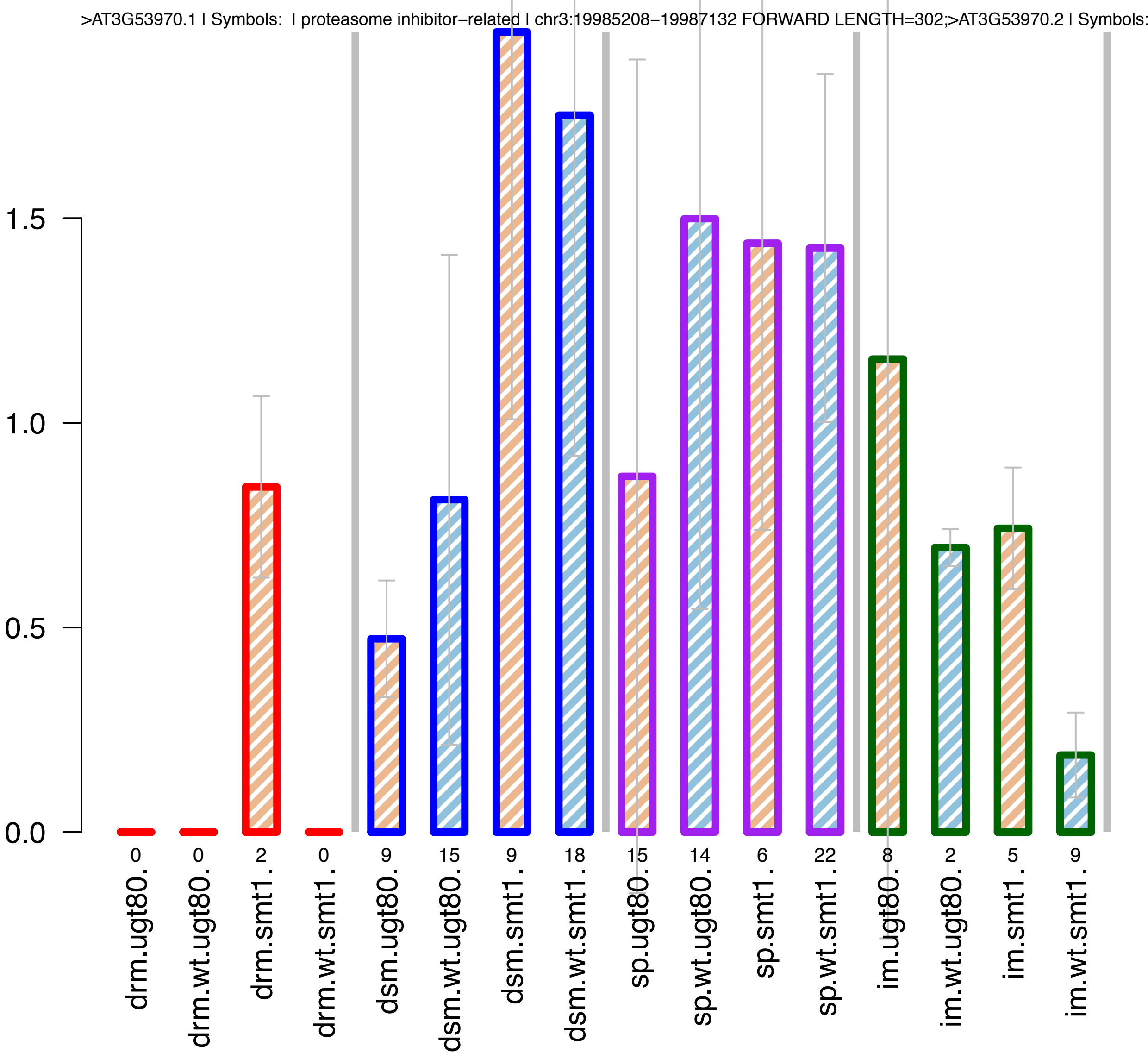

>AT3G53970.1 | Symbols: | proteasome inhibitor−related | chr3:19985208−19987132 FORWARD LENGTH=302;>AT3G53970.2 | Symbols:
1.5
1.0
0.5
0.0
0
0
2
0
9
15
9
18
15
14
6
22
8
2
5
9
drm.ugt80.
drm.wt.ugt80.
drm.smt1.
drm.wt.smt1.
dsm.ugt80.
dsm.wt.ugt80.
dsm.smt1.
dsm.wt.smt1.
sp.ugt80.
sp.wt.ugt80.
sp.smt1.
sp.wt.smt1.
im.ugt80.
im.wt.ugt80.
im.smt1.
im.wt.smt1.

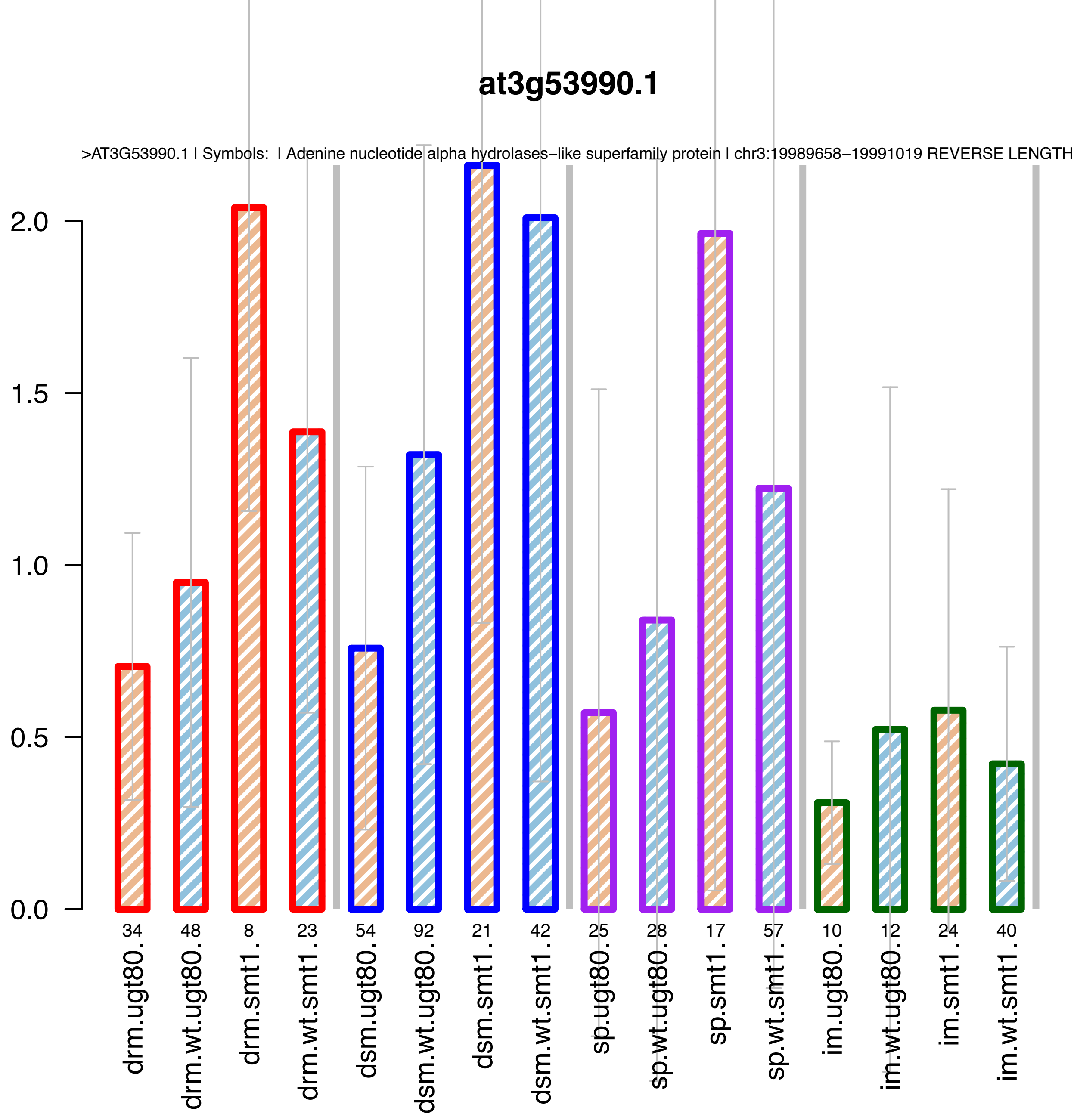

at3g53990.1
>AT3G53990.1 | Symbols: | Adenine nucleotide alpha hydrolases−like superfamily protein | chr3:19989658−19991019 REVERSE LENGTH
2.0
1.5
1.0
0.5
0.0
34
48
8
23
54
92
21
42
25
28
17
57
10
12
24
40
drm.ugt80..
drm.wt.ugt80..
drm.smt1..
drm.wt.smt1..
dsm.ugt80..
dsm.wt.ugt80..
dsm.smt1..
dsm.wt.smt1..
sp.ugt80..
sp.wt.ugt80..
sp.smt1..
sp.wt.smt1..
im.ugt80..
im.wt.ugt80..
im.smt1..
im.wt.smt1..

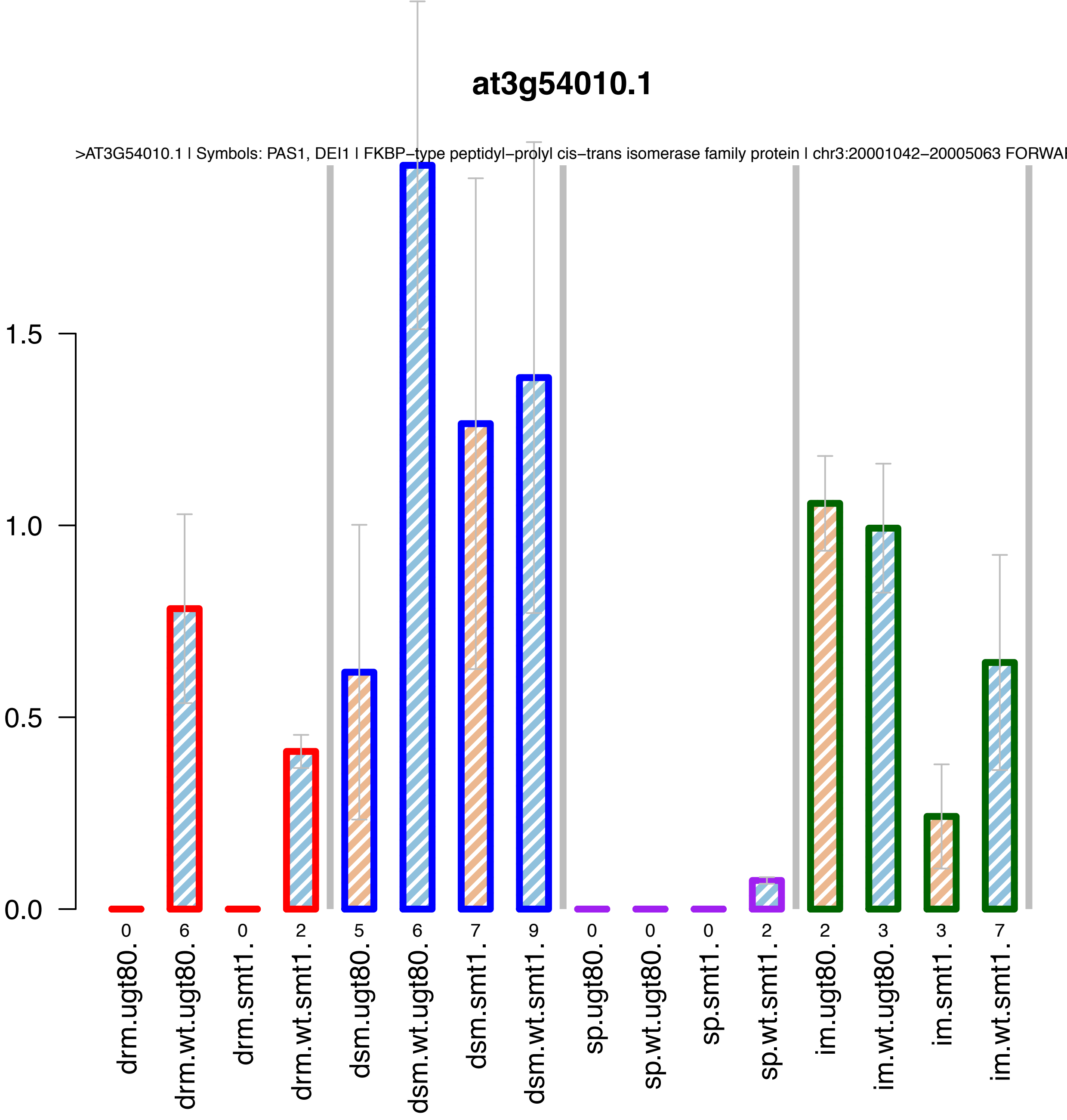

at3g54010.1
>AT3G54010.1 | Symbols: PAS1, DEI1 | FKBP–type peptidyl–prolyl cis–trans isomerase family protein | chr3:20001042–20005063 FORWA
0.0
0.5
1.0
1.5
0
6
0
2
5
6
7
9
0
0
0
2
2
3
3
7
drm.ugt80.
drm.wt.ugt80.
drm.smt1.
drm.wt.smt1.
dsm.ugt80.
dsm.wt.ugt80.
dsm.smt1.
dsm.wt.smt1.
sp.ugt80.
sp.wt.ugt80.
sp.smt1.
sp.wt.smt1.
im.ugt80.
im.wt.ugt80.
im.smt1.
im.wt.smt1.

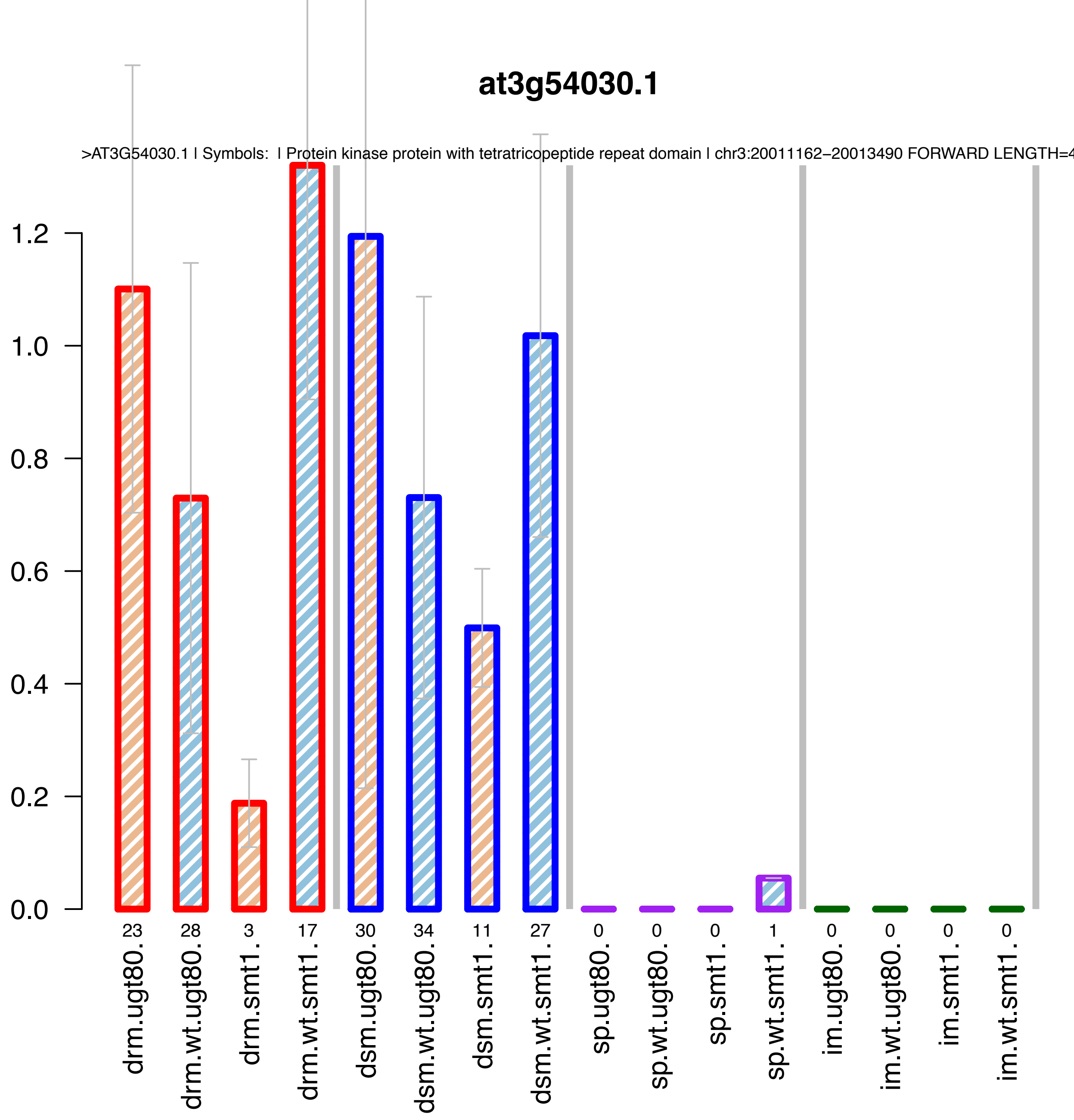

at3g54030.1
>AT3G54030.1 | Symbols: | Protein kinase protein with tetratricopeptide repeat domain | chr3:20011162-20013490 FORWARD LENGTH=4
1.2
1.0
0.8
0.6
0.4
0.2
0.0
23
28
3
17
30
34
11
27
0
0
0
1
0
0
0
0
drm.ugt80.
drm.wt.ugt80.
drm.smt1.
drm.wt.smt1.
dsm.ugt80.
dsm.wt.ugt80.
dsm.smt1.
dsm.wt.smt1.
sp.ugt80.
sp.wt.ugt80.
sp.smt1.
sp.wt.smt1.
im.ugt80.
im.wt.ugt80.
im.smt1.
im.wt.smt1.

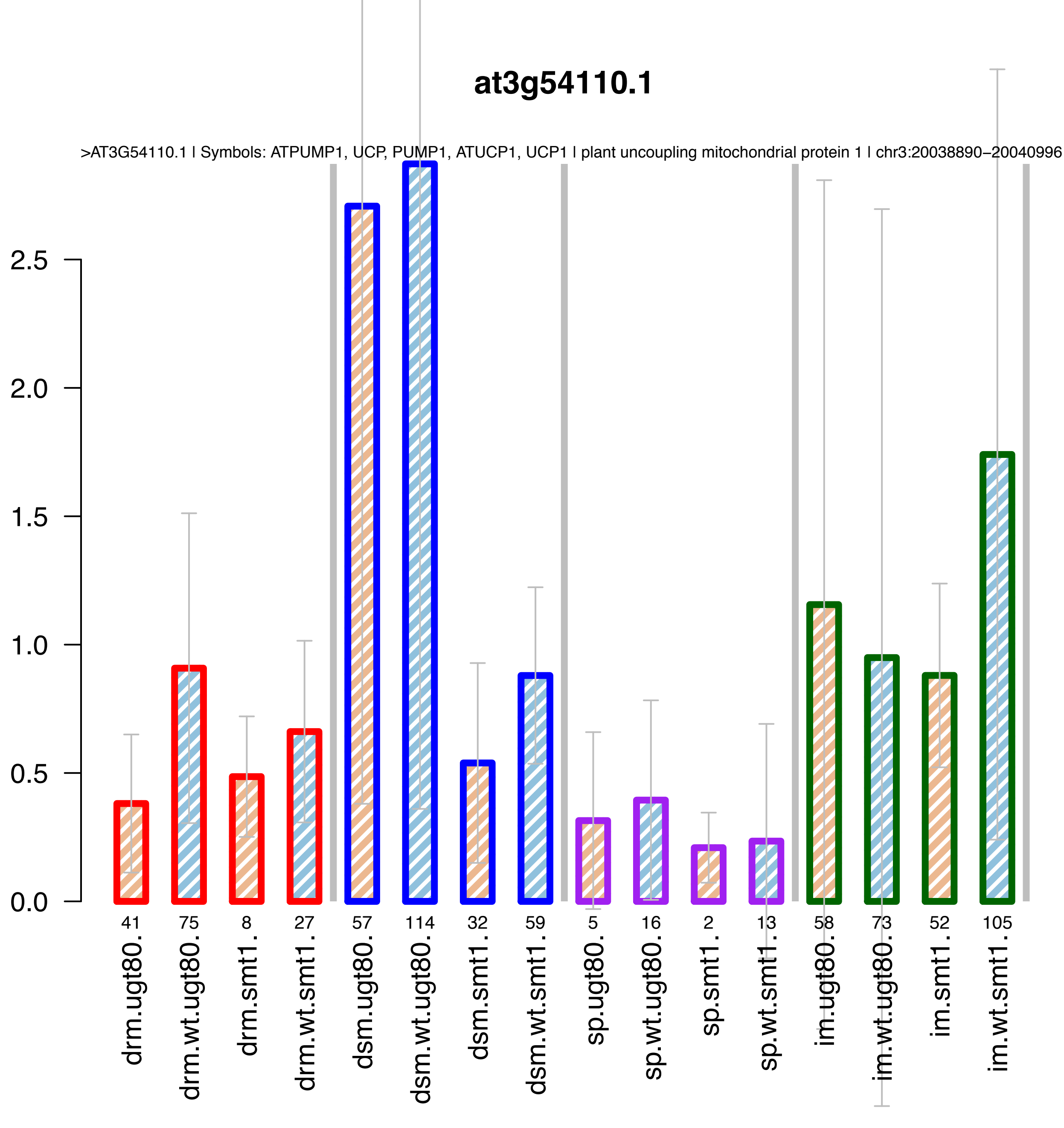

at3g54110.1
>AT3G54110.1 | Symbols: ATPUMP1, UCP, PUMP1, ATUCP1, UCP1 | plant uncoupling mitochondrial protein 1 | chr3:20038890-20040996
0.0
0.5
1.0
1.5
2.0
2.5
41
75
8
27
57
114
32
59
5
16
2
13
58
73
52
105
drm.ugt80..
drm.wt.ugt80..
drm.smt1..
drm.wt.smt1..
dsm.ugt80..
dsm.wt.ugt80..
dsm.smt1..
dsm.wt.smt1..
sp.ugt80..
sp.wt.ugt80..
sp.smt1..
sp.wt.smt1..
im.ugt80..
im.wt.ugt80..
im.smt1..
im.wt.smt1..

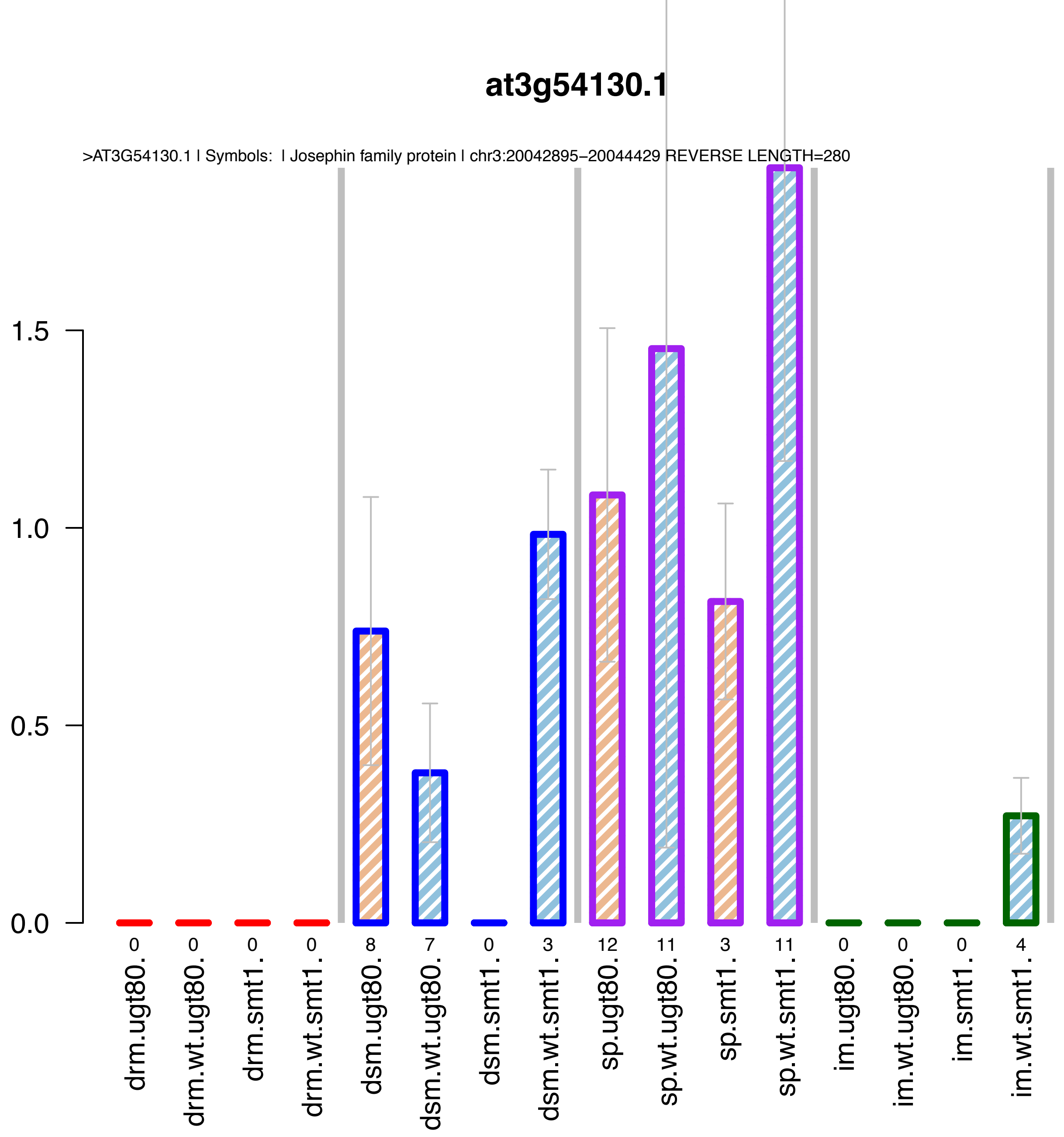

at3g54130.1
>AT3G54130.1 | Symbols: | Josephin family protein | chr3:20042895-20044429 REVERSE LENGTH=280
1.5
1.0
0.5
0.0
0
0
0
0
8
7
0
3
12
11
3
11
0
0
0
4
drm.ugt80.
drm.wt.ugt80.
drm.smt1.
drm.wt.smt1.
dsm.ugt80.
dsm.wt.ugt80.
dsm.smt1.
dsm.wt.smt1.
sp.ugt80.
sp.wt.ugt80.
sp.smt1.
sp.wt.smt1.
im.ugt80.
im.wt.ugt80.
im.smt1.
im.wt.smt1.

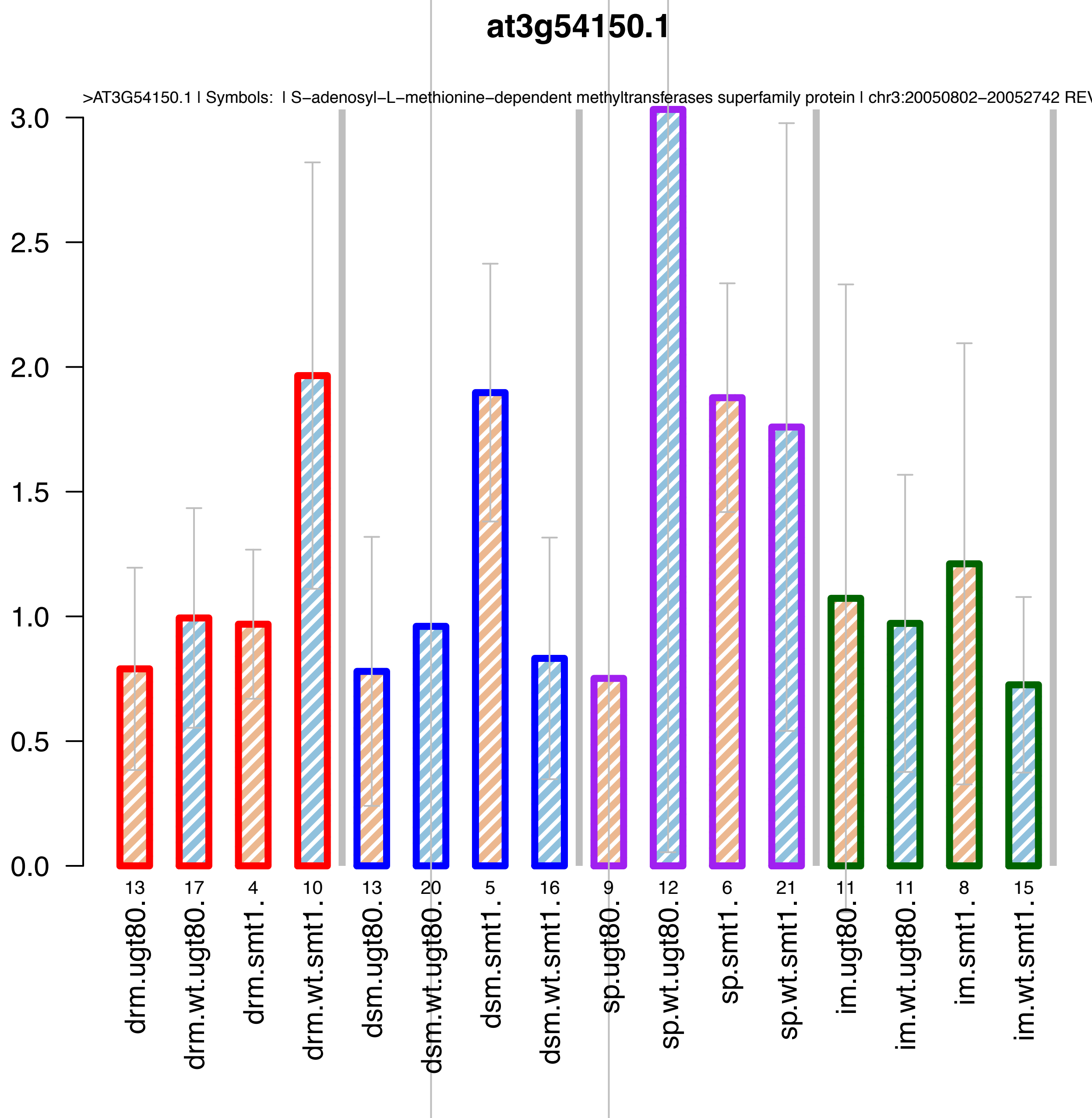

at3g54150.1
>AT3G54150.1 | Symbols: | S–adenosyl–L–methionine–dependent methyltransferases superfamily protein | chr3:20050802–20052742 REV
3.0
2.5
2.0
1.5
1.0
0.5
0.0
13
17
4
10
13
20
5
16
9
12
6
21
11
11
8
15
drm.ugt80.
drm.wt.ugt80.
drm.smt1.
drm.wt.smt1.
dsm.ugt80.
dsm.wt.ugt80.
dsm.smt1.
dsm.wt.smt1.
sp.ugt80.
sp.wt.ugt80.
sp.smt1.
sp.wt.smt1.
im.ugt80.
im.wt.ugt80.
im.smt1.
im.wt.smt1.

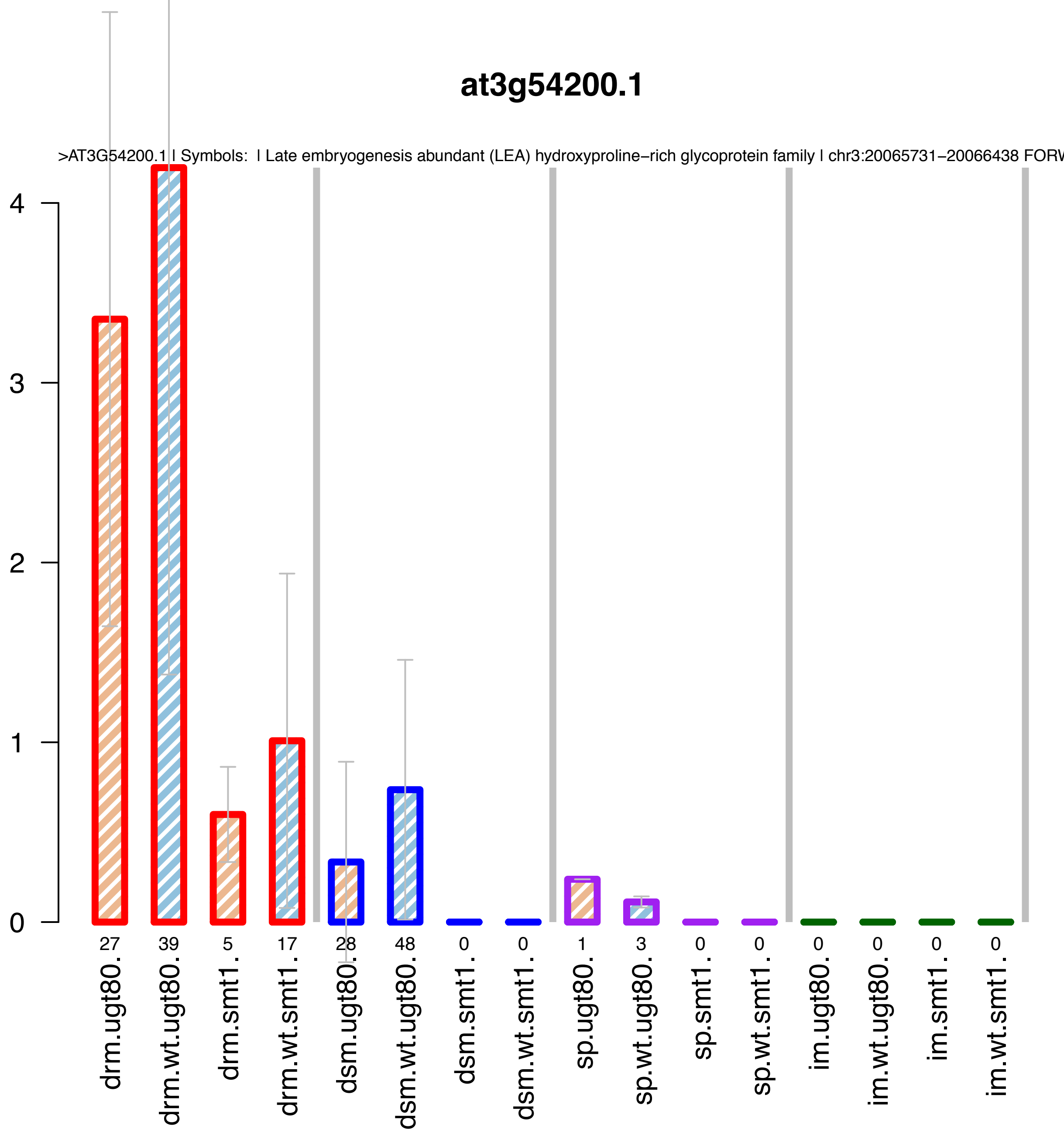
at3g54200.1
>AT3G54200.1 | Symbols: | Late embryogenesis abundant (LEA) hydroxyproline−rich glycoprotein family | chr3:20065731−20066438 FORW
0
1
2
3
4
27
39
5
17
28
48
0
0
1
3
0
0
0
0
0
0
drm.ugt80.
drm.wt.ugt80.
drm.smt1.
drm.wt.smt1.
dsm.ugt80.
dsm.wt.ugt80.
dsm.smt1.
dsm.wt.smt1.
sp.ugt80.
sp.wt.ugt80.
sp.smt1.
sp.wt.smt1.
im.ugt80.
im.wt.ugt80.
im.smt1.
im.wt.smt1.

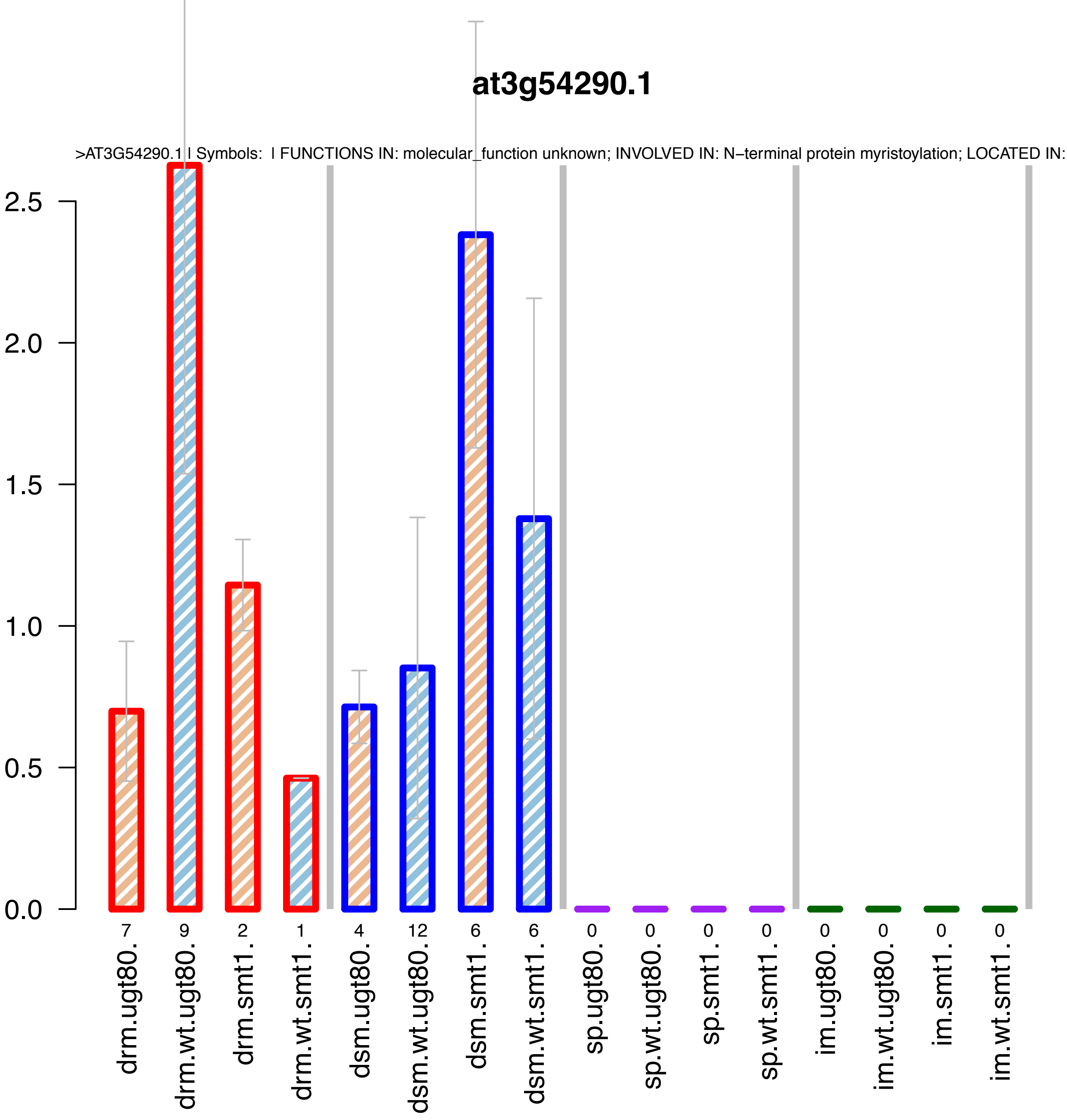
at3g54290.1
>AT3G54290.1 | Symbols: | FUNCTIONS IN: molecular_function unknown; INVOLVED IN: N–terminal protein myristoylation; LOCATED IN:
0.0
0.5
1.0
1.5
2.0
2.5
7 9 2 1 4 12 6 6 0 0 0 0 0 0 0 0
drm.ugt80.
drm.wt.ugt80.
drm.smt1.
drm.wt.smt1.
dsm.ugt80.
dsm.wt.ugt80.
dsm.smt1.
dsm.wt.smt1.
sp.ugt80.
sp.wt.ugt80.
sp.smt1.
sp.wt.smt1.
im.ugt80.
im.wt.ugt80.
im.smt1.
im.wt.smt1.

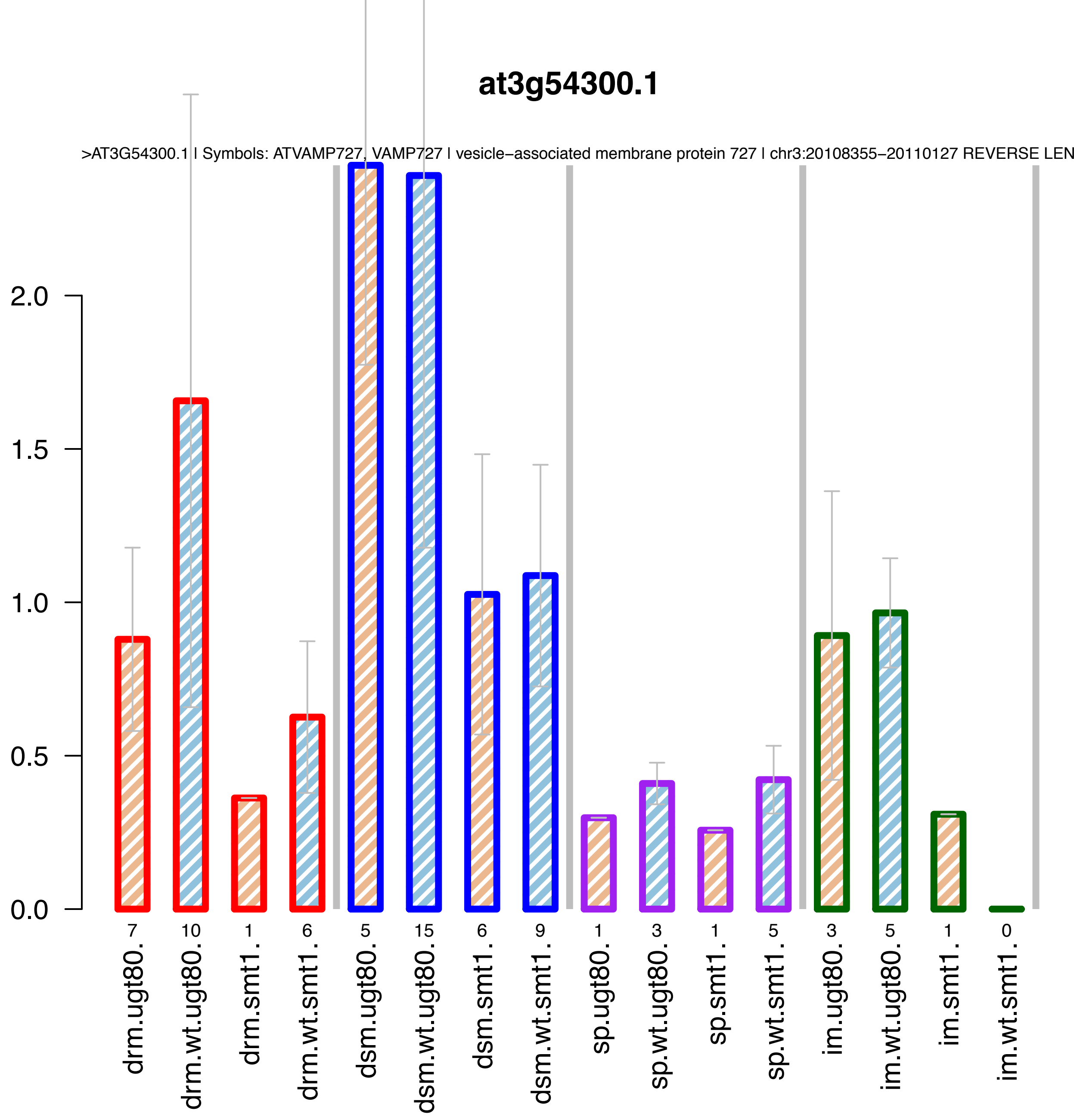

at3g54300.1
>AT3G54300.1 | Symbols: ATVAMP727, VAMP727 | vesicle-associated membrane protein 727 | chr3:20108355-20110127 REVERSE LEN
0.0
0.5
1.0
1.5
2.0
7
10
1
6
5
15
6
9
1
3
1
5
3
5
1
0
drm.ugt80.
drm.wt.ugt80.
drm.smt1.
drm.wt.smt1.
dsm.ugt80.
dsm.wt.ugt80.
dsm.smt1.
dsm.wt.smt1.
sp.ugt80.
sp.wt.ugt80.
sp.smt1.
sp.wt.smt1.
im.ugt80.
im.wt.ugt80.
im.smt1.
im.wt.smt1.

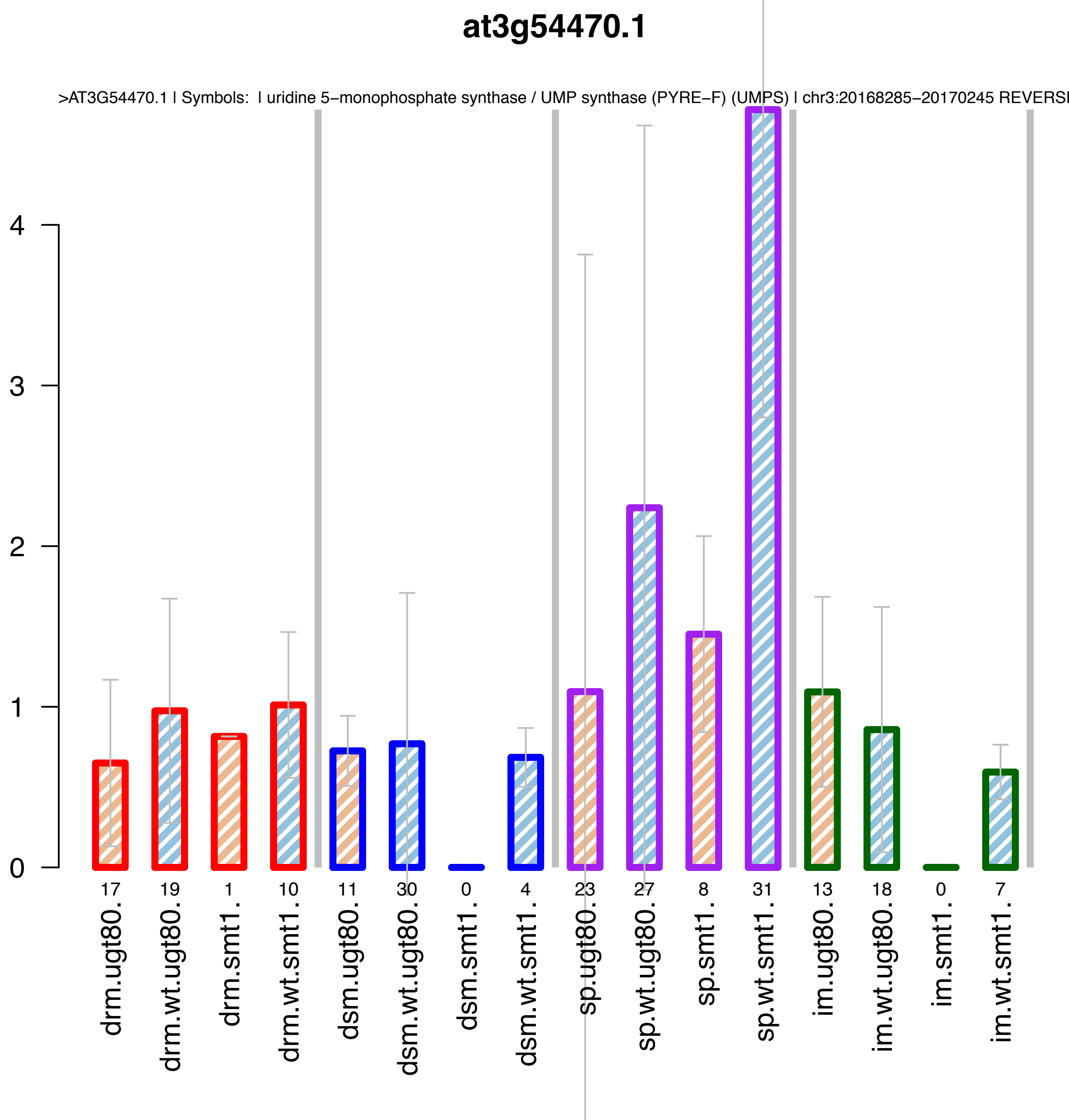

at3g54470.1
>AT3G54470.1 | Symbols: | uridine 5−monophosphate synthase / UMP synthase (PYRE−F) (UMPS) | chr3:20168285−20170245 REVERSE
0
1
2
3
4
17
19
1
10
11
30
0
4
23
27
8
31
13
18
0
7
drm.ugt80.
drm.wt.ugt80.
drm.smt1.
drm.wt.smt1.
dsm.ugt80.
dsm.wt.ugt80.
dsm.smt1.
dsm.wt.smt1.
sp.ugt80.
sp.wt.ugt80.
sp.smt1.
sp.wt.smt1.
im.ugt80.
im.wt.ugt80.
im.smt1.
im.wt.smt1.

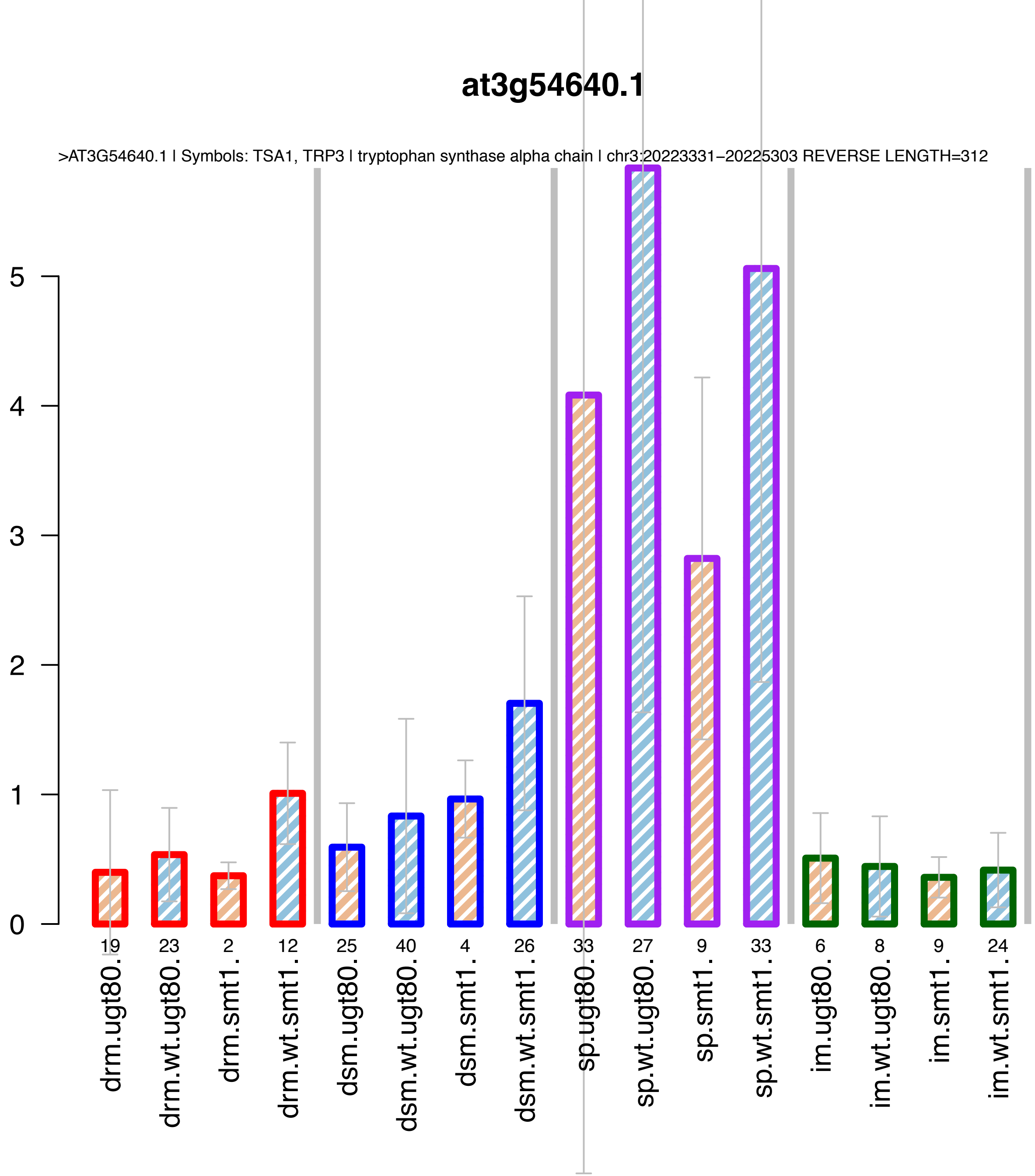

at3g54640.1
>AT3G54640.1 | Symbols: TSA1, TRP3 | tryptophan synthase alpha chain | chr3:20223331−20225303 REVERSE LENGTH=312
0
1
2
3
4
5
19
23
2
12
25
40
4
26
33
27
9
33
6
8
9
24
drm.ugt80..
drm.wt.ugt80..
drm.smt1..
drm.wt.smt1..
dsm.ugt80..
dsm.wt.ugt80..
dsm.smt1..
dsm.wt.smt1..
sp.ugt80..
sp.wt.ugt80..
sp.smt1..
sp.wt.smt1..
im.ugt80..
im.wt.ugt80..
im.smt1..
im.wt.smt1..

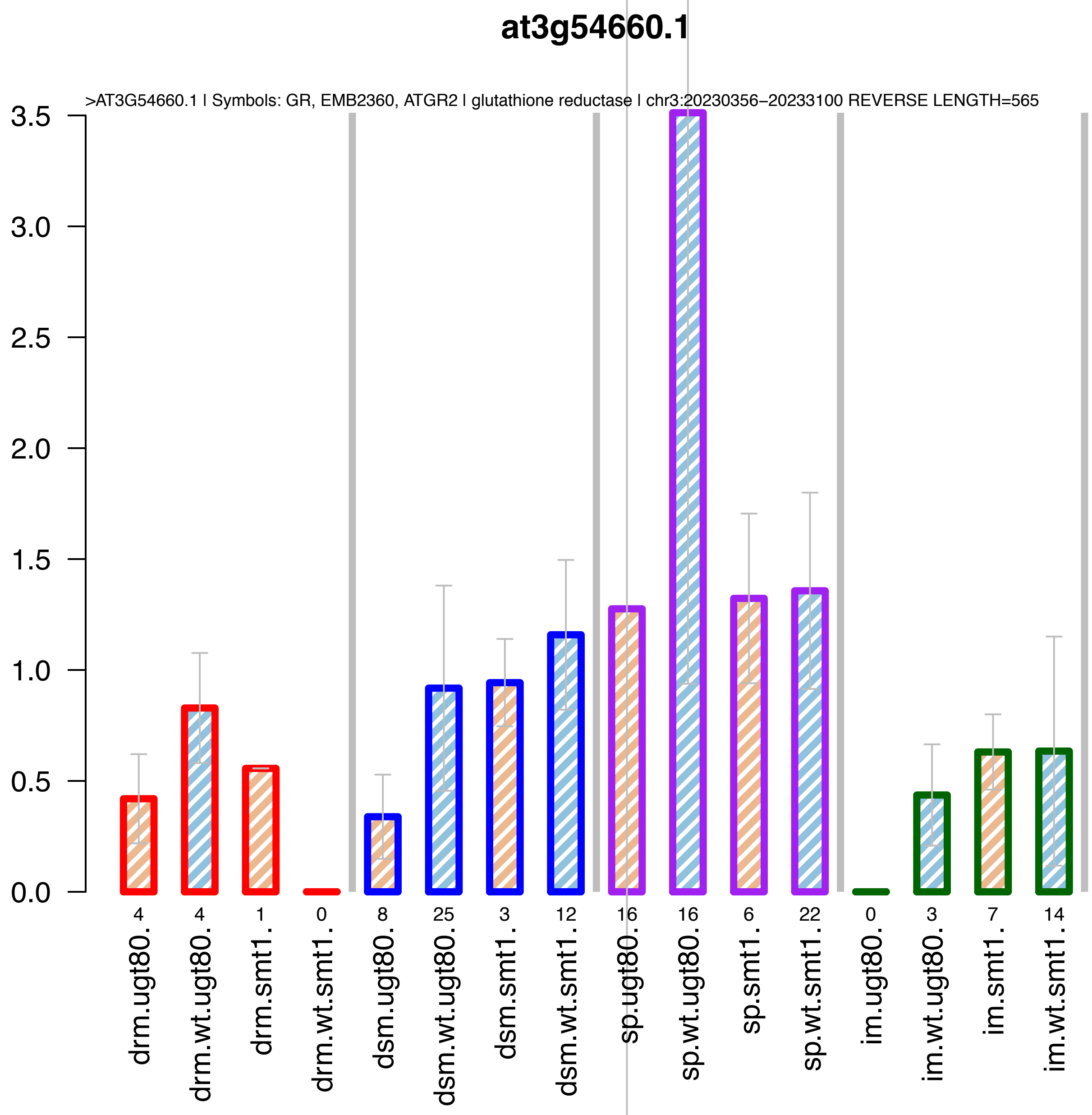

at3g54660.1
>AT3G54660.1 | Symbols: GR, EMB2360, ATGR2 | glutathione reductase | chr3:20230356-20233100 REVERSE LENGTH=565
3.5
3.0
2.5
2.0
1.5
1.0
0.5
0.0
4
4
1
0
8
25
3
12
16
16
6
22
0
3
7
14
drm.ugt80..
drm.wt.ugt80..
drm.smt1..
drm.wt.smt1..
dsm.ugt80..
dsm.wt.ugt80..
dsm.smt1..
dsm.wt.smt1..
sp.ugt80..
sp.wt.ugt80..
sp.smt1..
sp.wt.smt1..
im.ugt80..
im.wt.ugt80..
im.smt1..
im.wt.smt1..

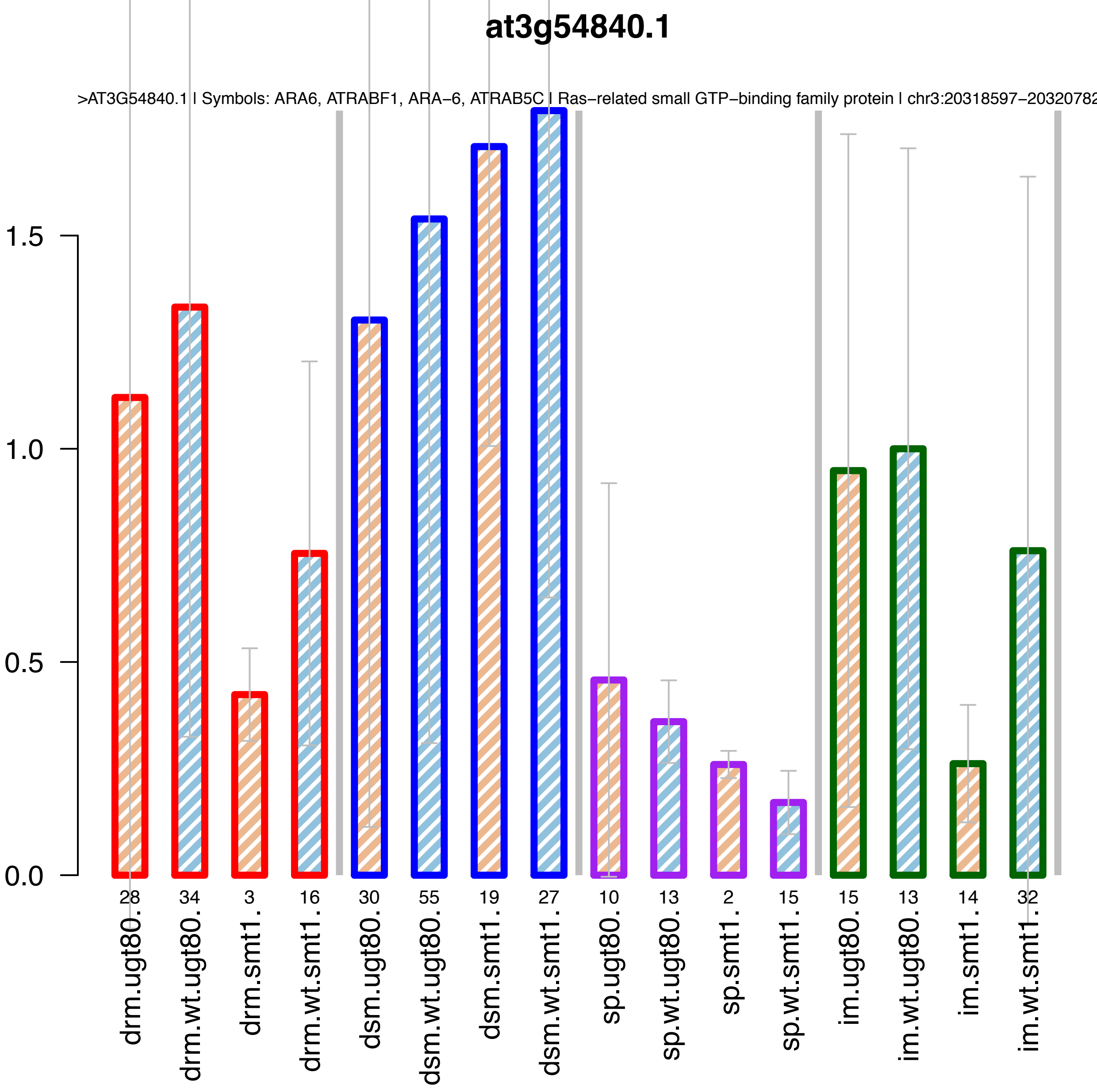

at3g54840.1
>AT3G54840.1 | Symbols: ARA6, ATRABF1, ARA−6, ATRAB5C | Ras−related small GTP−binding family protein | chr3:20318597−20320782
1.5
1.0
0.5
0.0
28
34
3
16
30
55
19
27
10
13
2
15
15
13
14
32
drm.ugt80..
drm.wt.ugt80..
drm.smt1..
drm.wt.smt1..
dsm.ugt80..
dsm.wt.ugt80..
dsm.smt1..
dsm.wt.smt1..
sp.ugt80..
sp.wt.ugt80..
sp.smt1..
sp.wt.smt1..
im.ugt80..
im.wt.ugt80..
im.smt1..
im.wt.smt1..

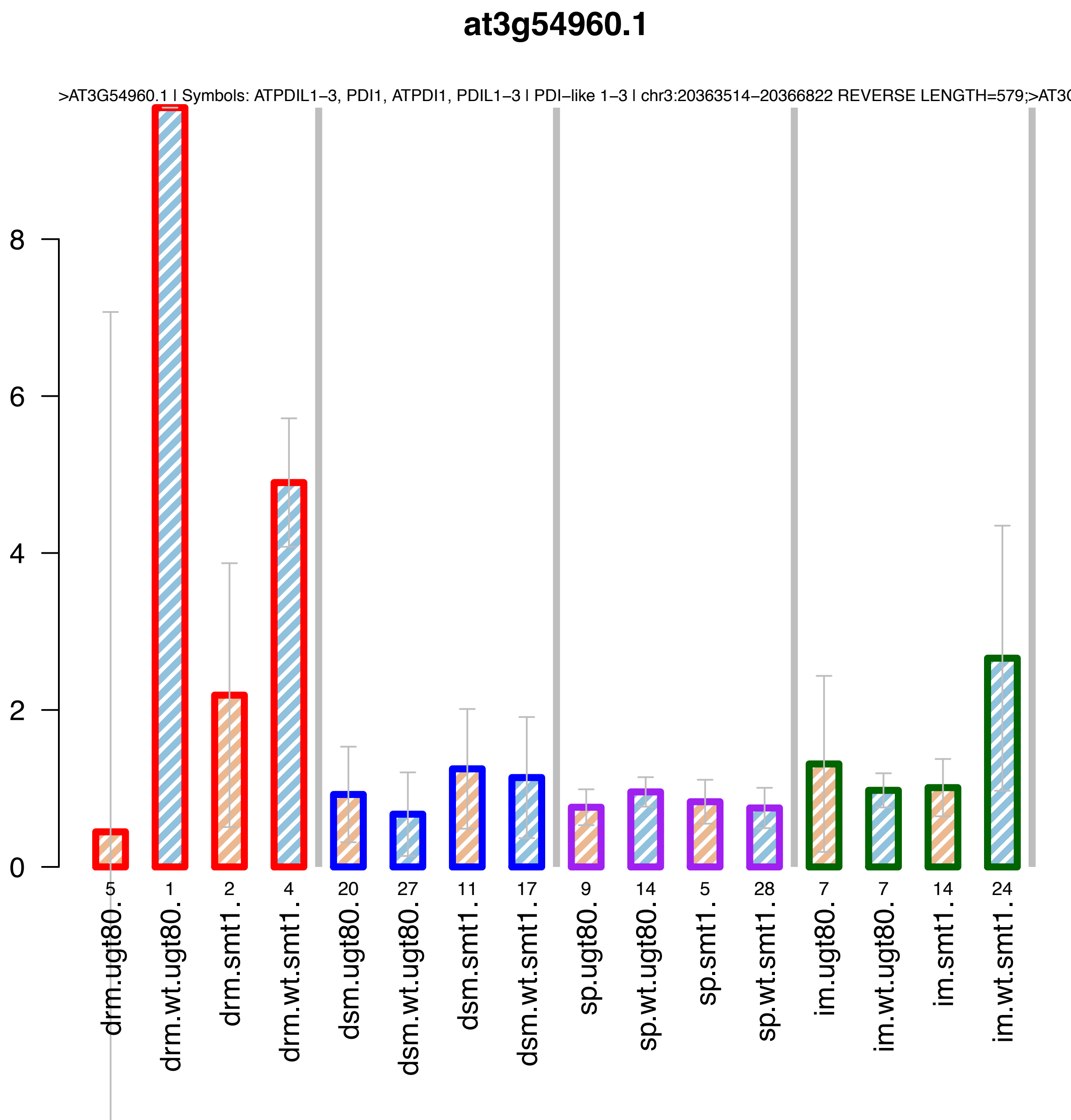

at3g54960.1
>AT3G54960.1 | Symbols: ATPDIL1−3, PDI1, ATPDI1, PDIL1−3 | PDI−like 1−3 | chr3:20363514−20366822 REVERSE LENGTH=579;>AT3
0
2
4
6
8
5
1
2
4
20
27
11
17
9
14
5
28
7
7
14
24
drm.ugt80.
drm.wt.ugt80.
drm.smt1.
drm.wt.smt1.
dsm.ugt80.
dsm.wt.ugt80.
dsm.smt1.
dsm.wt.smt1.
sp.ugt80.
sp.wt.ugt80.
sp.smt1.
sp.wt.smt1.
im.ugt80.
im.wt.ugt80.
im.smt1.
im.wt.smt1.

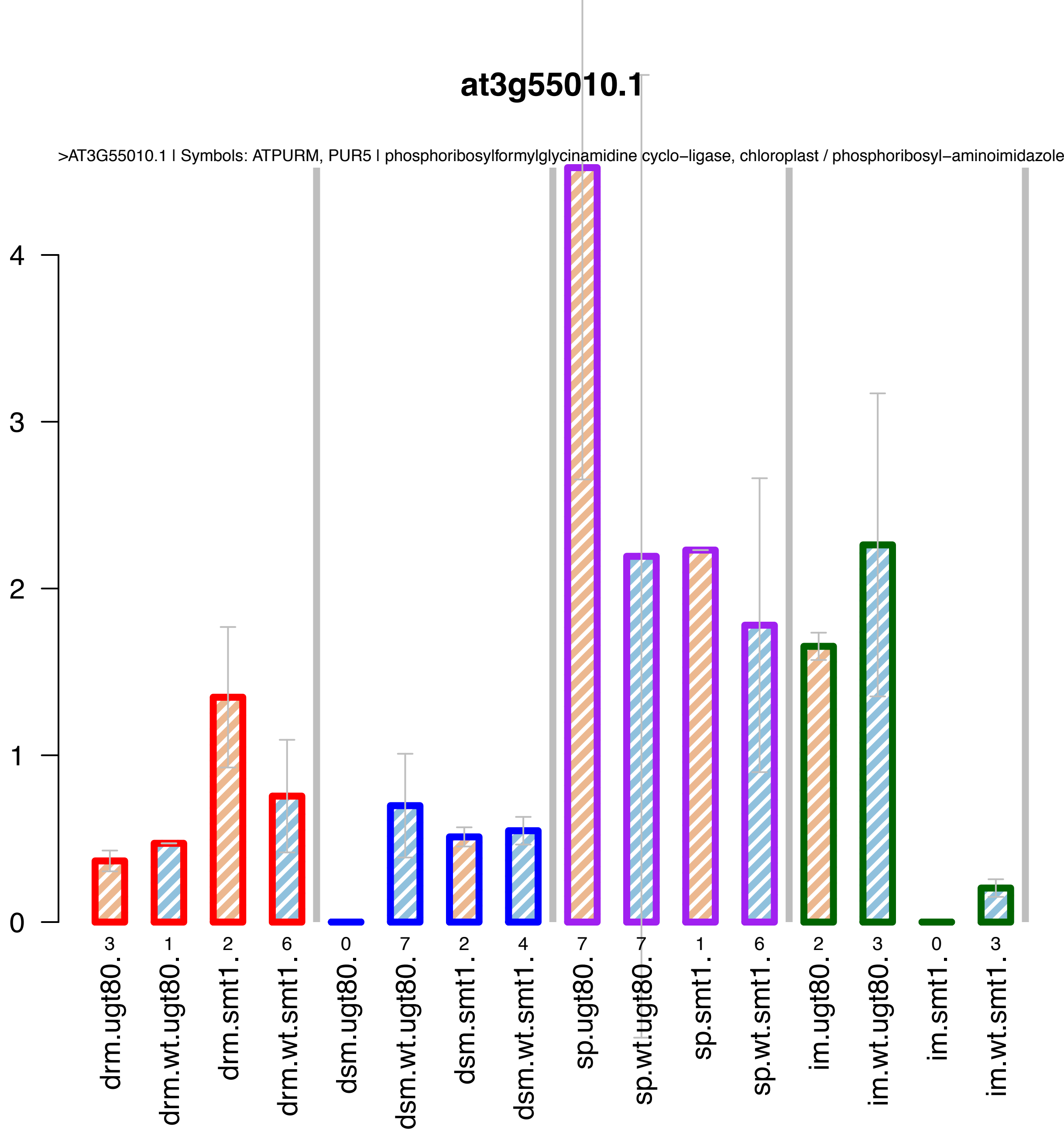

at3g55010.1
>AT3G55010.1 | Symbols: ATPURM, PUR5 | phosphoribosylformylglycinamidine cyclo−ligase, chloroplast / phosphoribosyl−aminoimidazole
0
1
2
3
4
3
1
2
6
0
7
2
4
7
7
1
6
2
3
0
3
drm.ugt80..
drm.wt.ugt80..
drm.smt1..
drm.wt.smt1..
dsm.ugt80..
dsm.wt.ugt80..
dsm.smt1..
dsm.wt.smt1..
sp.ugt80..
sp.wt.ugt80..
sp.smt1..
sp.wt.smt1..
im.ugt80..
im.wt.ugt80..
im.smt1..
im.wt.smt1..

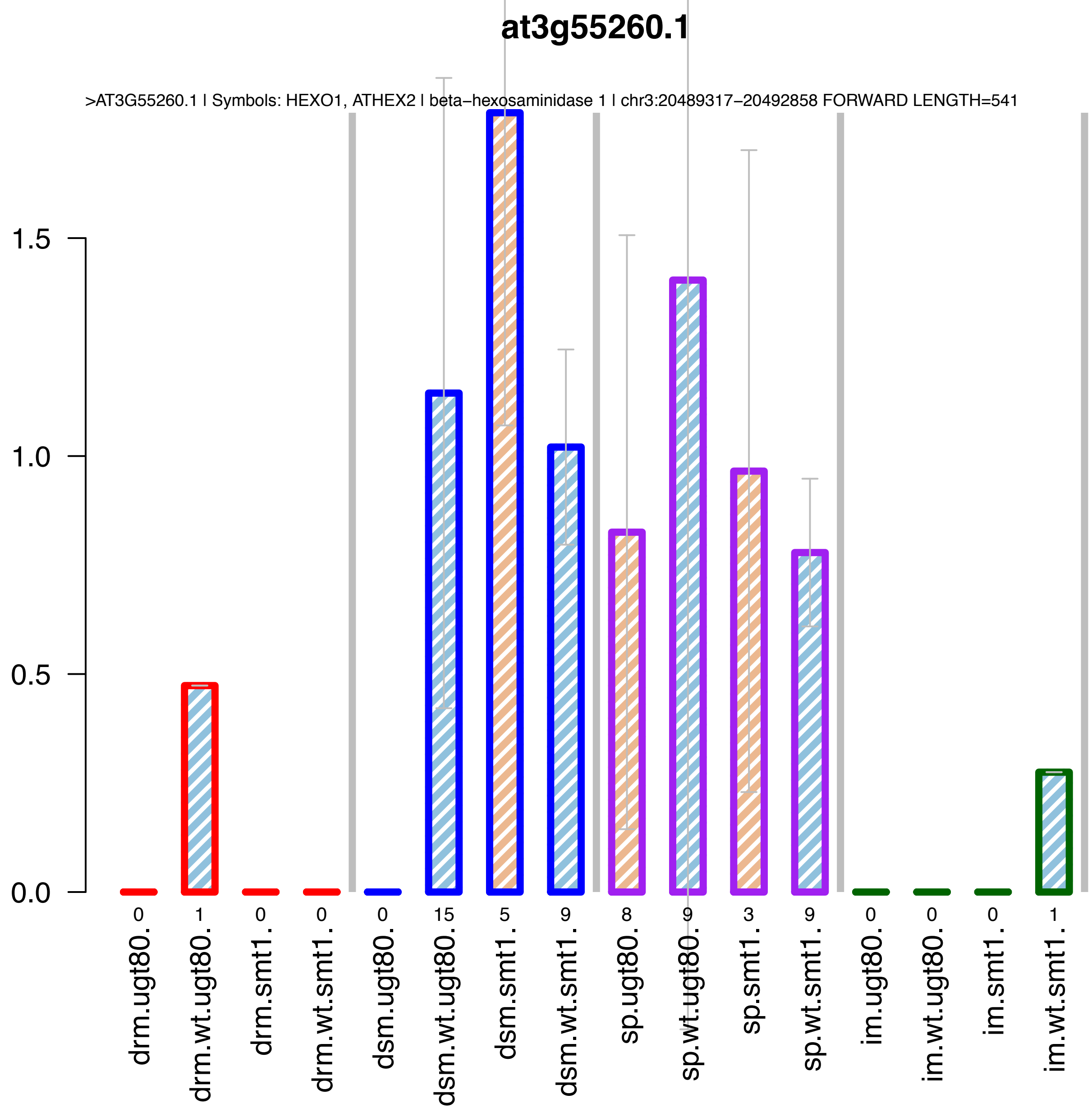

at3g55260.1
>AT3G55260.1 | Symbols: HEXO1, ATHEX2 | beta−hexosaminidase 1 | chr3:20489317−20492858 FORWARD LENGTH=541
0.0
0.5
1.0
1.5
0
1
0
0
0
15
5
9
8
9
3
9
0
0
0
1
drm.ugt80.
drm.wt.ugt80.
drm.smt1.
drm.wt.smt1.
dsm.ugt80.
dsm.wt.ugt80.
dsm.smt1.
dsm.wt.smt1.
sp.ugt80.
sp.wt.ugt80.
sp.smt1.
sp.wt.smt1.
im.ugt80.
im.wt.ugt80.
im.smt1.
im.wt.smt1.

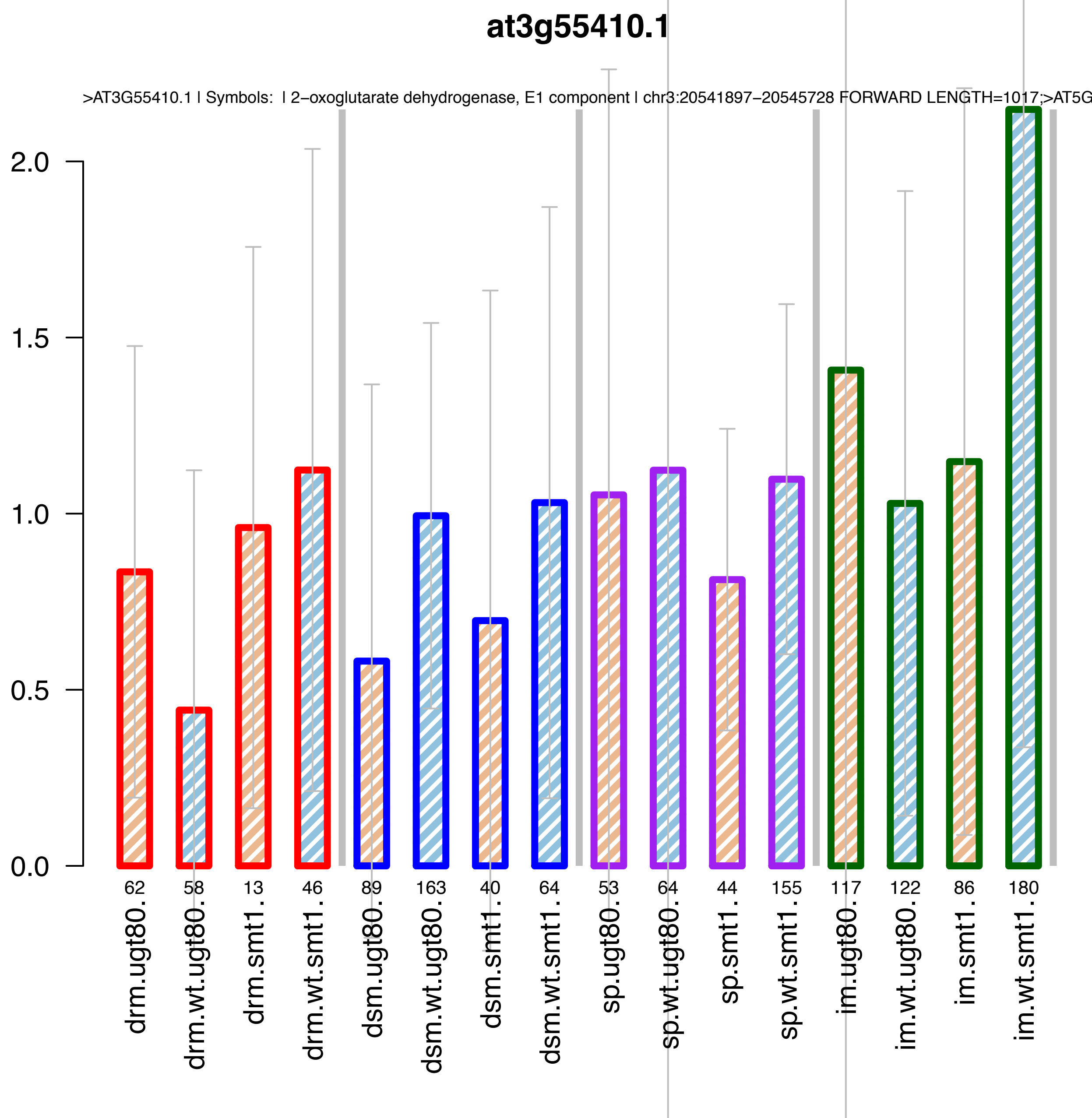

at3g55410.1
>AT3G55410.1 | Symbols: | 2−oxoglutarate dehydrogenase, E1 component | chr3:20541897−20545728 FORWARD LENGTH=1017;>AT5G
0.0
0.5
1.0
1.5
2.0
62
58
13
46
89
163
40
64
53
64
44
155
117
122
86
180
drm.ugt80..
drm.wt.ugt80..
drm.smt1..
drm.wt.smt1..
dsm.ugt80..
dsm.wt.ugt80..
dsm.smt1..
dsm.wt.smt1..
sp.ugt80..
sp.wt.ugt80..
sp.smt1..
sp.wt.smt1..
im.ugt80..
im.wt.ugt80..
im.smt1..
im.wt.smt1..

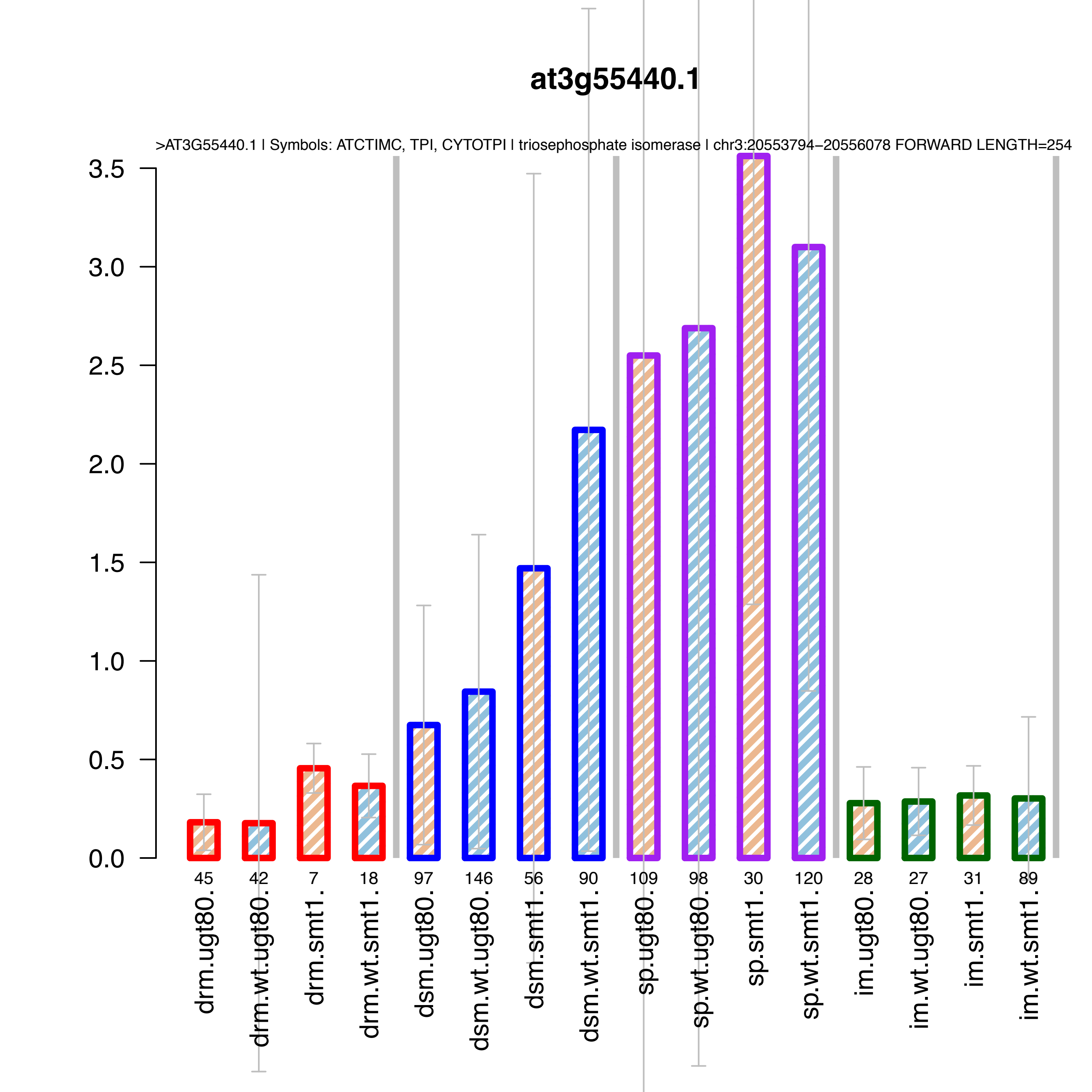

at3g55440.1
>AT3G55440.1 | Symbols: ATCTIMC, TPI, CYTOTPI | triosephosphate isomerase | chr3:20553794−20556078 FORWARD LENGTH=254
3.5
3.0
2.5
2.0
1.5
1.0
0.5
0.0
45
42
7
18
97
146
56
90
109
98
30
120
28
27
31
89
drm.ugt80.
drm.wt.ugt80.
drm.smt1.
drm.wt.smt1.
dsm.ugt80.
dsm.wt.ugt80.
dsm.smt1.
dsm.wt.smt1.
sp.ugt80.
sp.wt.ugt80.
sp.smt1.
sp.wt.smt1.
im.ugt80.
im.wt.ugt80.
im.smt1.
im.wt.smt1.

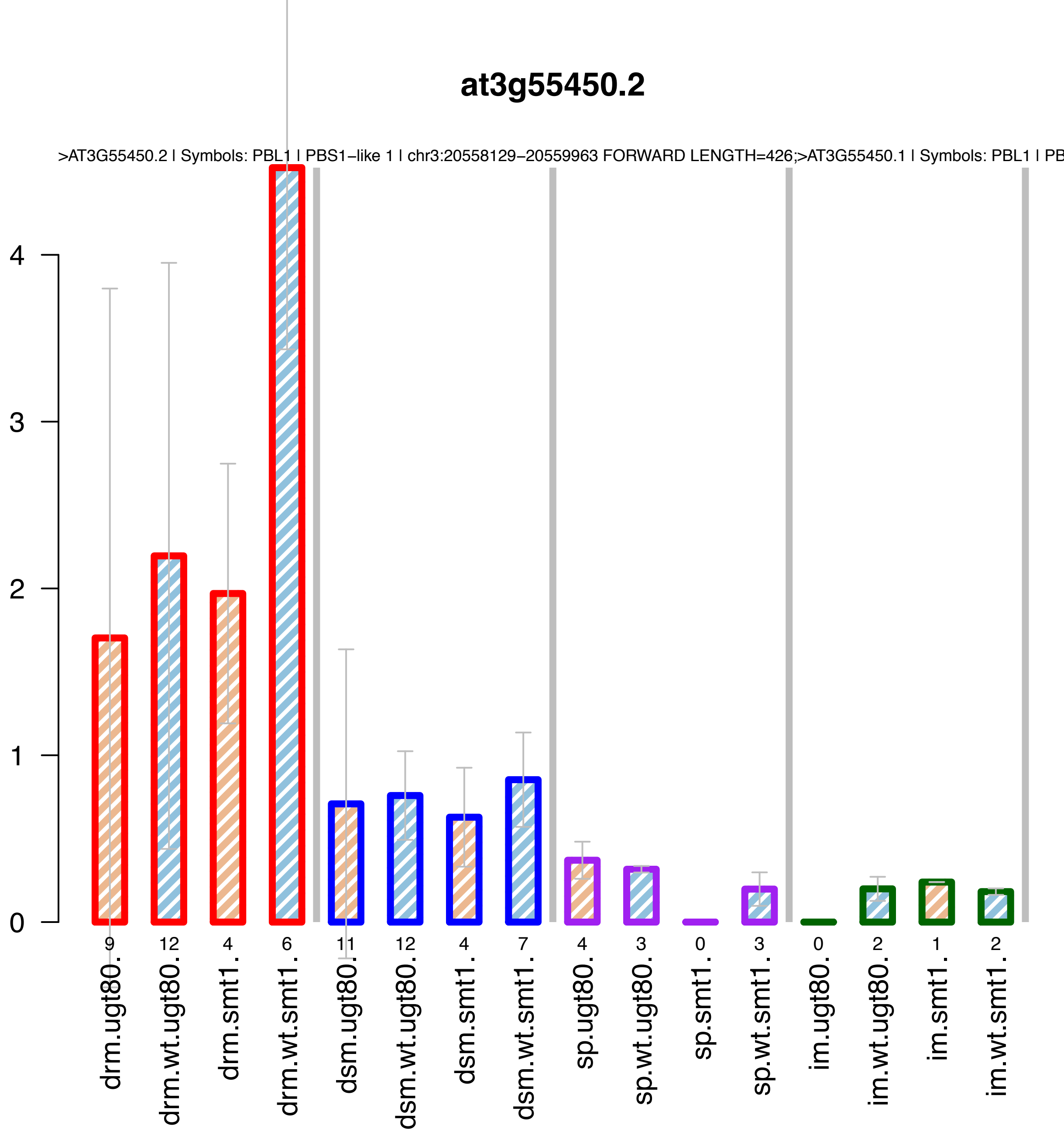

at3g55450.2
>AT3G55450.2 | Symbols: PBL1 | PBS1−like 1 | chr3:20558129−20559963 FORWARD LENGTH=426;>AT3G55450.1 | Symbols: PBL1 | PB
0
1
2
3
4
9
12
4
6
11
12
4
7
4
3
0
3
0
2
1
2
drm.ugt80..
drm.wt.ugt80..
drm.smt1..
drm.wt.smt1..
dsm.ugt80..
dsm.wt.ugt80..
dsm.smt1..
dsm.wt.smt1..
sp.ugt80..
sp.wt.ugt80..
sp.smt1..
sp.wt.smt1..
im.ugt80..
im.wt.ugt80..
im.smt1..
im.wt.smt1..

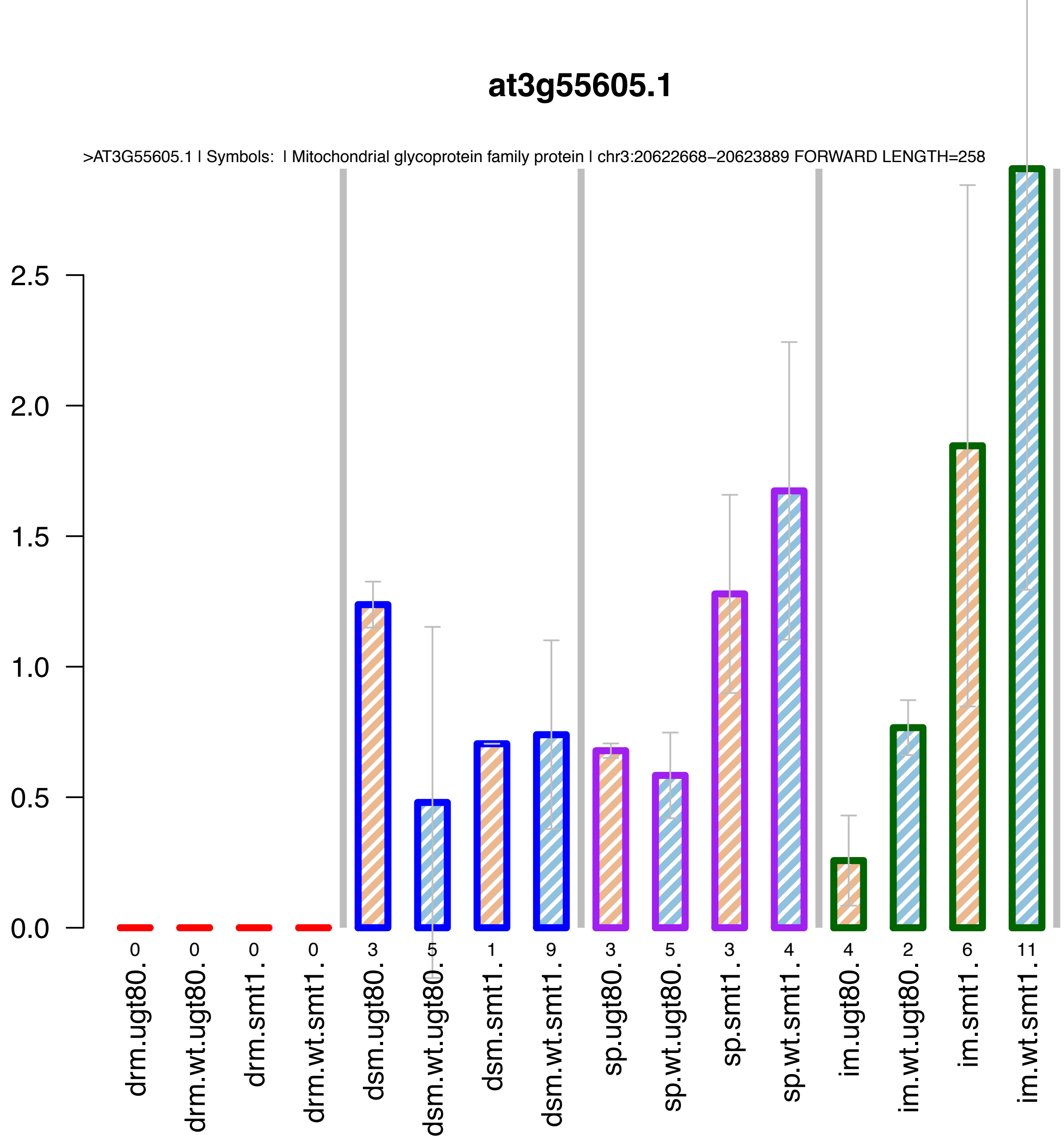

at3g55605.1
>AT3G55605.1 | Symbols: | Mitochondrial glycoprotein family protein | chr3:20622668-20623889 FORWARD LENGTH=258
0.0
0.5
1.0
1.5
2.0
2.5
0
0
0
0
3
5
1
9
3
5
3
4
4
2
6
11
drm.ugt80.
drm.wt.ugt80.
drm.smt1.
drm.wt.smt1.
dsm.ugt80.
dsm.wt.ugt80.
dsm.smt1.
dsm.wt.smt1.
sp.ugt80.
sp.wt.ugt80.
sp.smt1.
sp.wt.smt1.
im.ugt80.
im.wt.ugt80.
im.smt1.
im.wt.smt1.

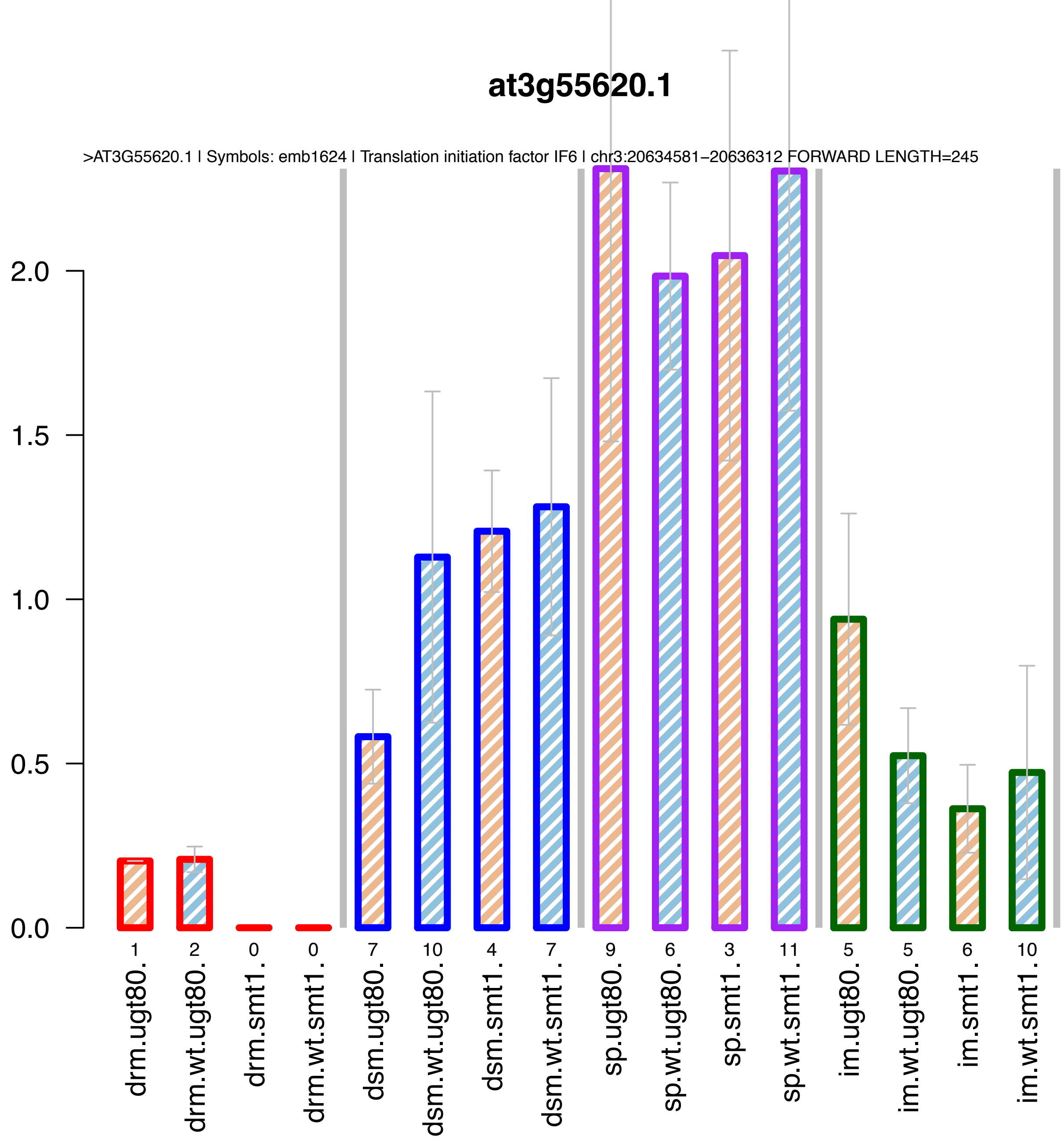

at3g55620.1
>AT3G55620.1 | Symbols: emb1624 | Translation initiation factor IF6 | chr3:20634581−20636312 FORWARD LENGTH=245
0.0
0.5
1.0
1.5
2.0
1
2
0
0
7
10
4
7
9
6
3
11
5
5
6
10
drm.ugt80.
drm.wt.ugt80.
drm.smt1.
drm.wt.smt1.
dsm.ugt80.
dsm.wt.ugt80.
dsm.smt1.
dsm.wt.smt1.
sp.ugt80.
sp.wt.ugt80.
sp.smt1.
sp.wt.smt1.
im.ugt80.
im.wt.ugt80.
im.smt1.
im.wt.smt1.

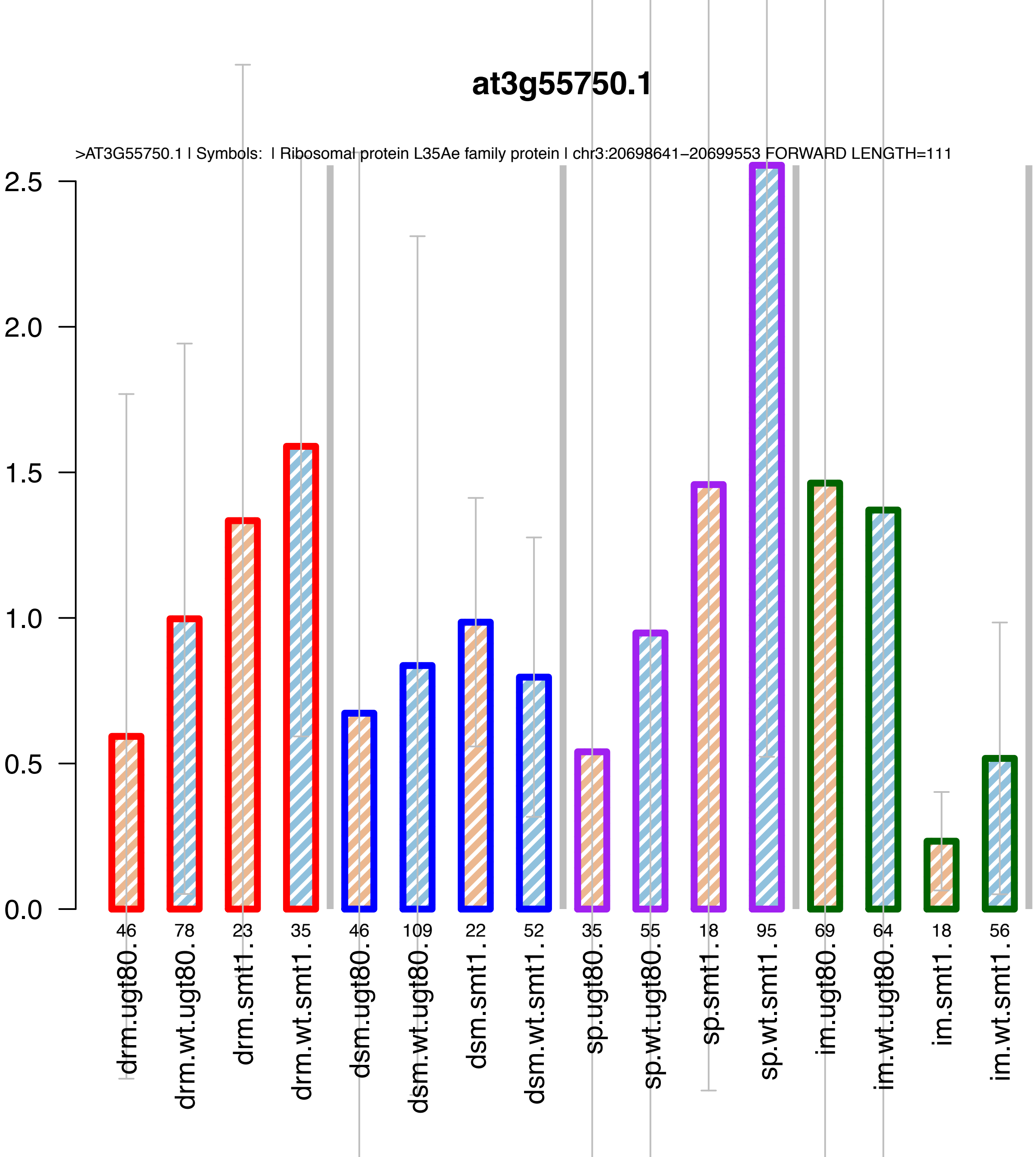

at3g55750.1
>AT3G55750.1 | Symbols: | Ribosomal protein L35Ae family protein | chr3:20698641-20699553 FORWARD LENGTH=111
2.5
2.0
1.5
1.0
0.5
0.0
46
78
23
35
46
109
22
52
35
55
18
95
69
64
18
56
drm.ugt80..
drm.wt.ugt80..
drm.smt1..
drm.wt.smt1..
dsm.ugt80..
dsm.wt.ugt80..
dsm.smt1..
dsm.wt.smt1..
sp.ugt80..
sp.wt.ugt80..
sp.smt1..
sp.wt.smt1..
im.ugt80..
im.wt.ugt80..
im.smt1..
im.wt.smt1..

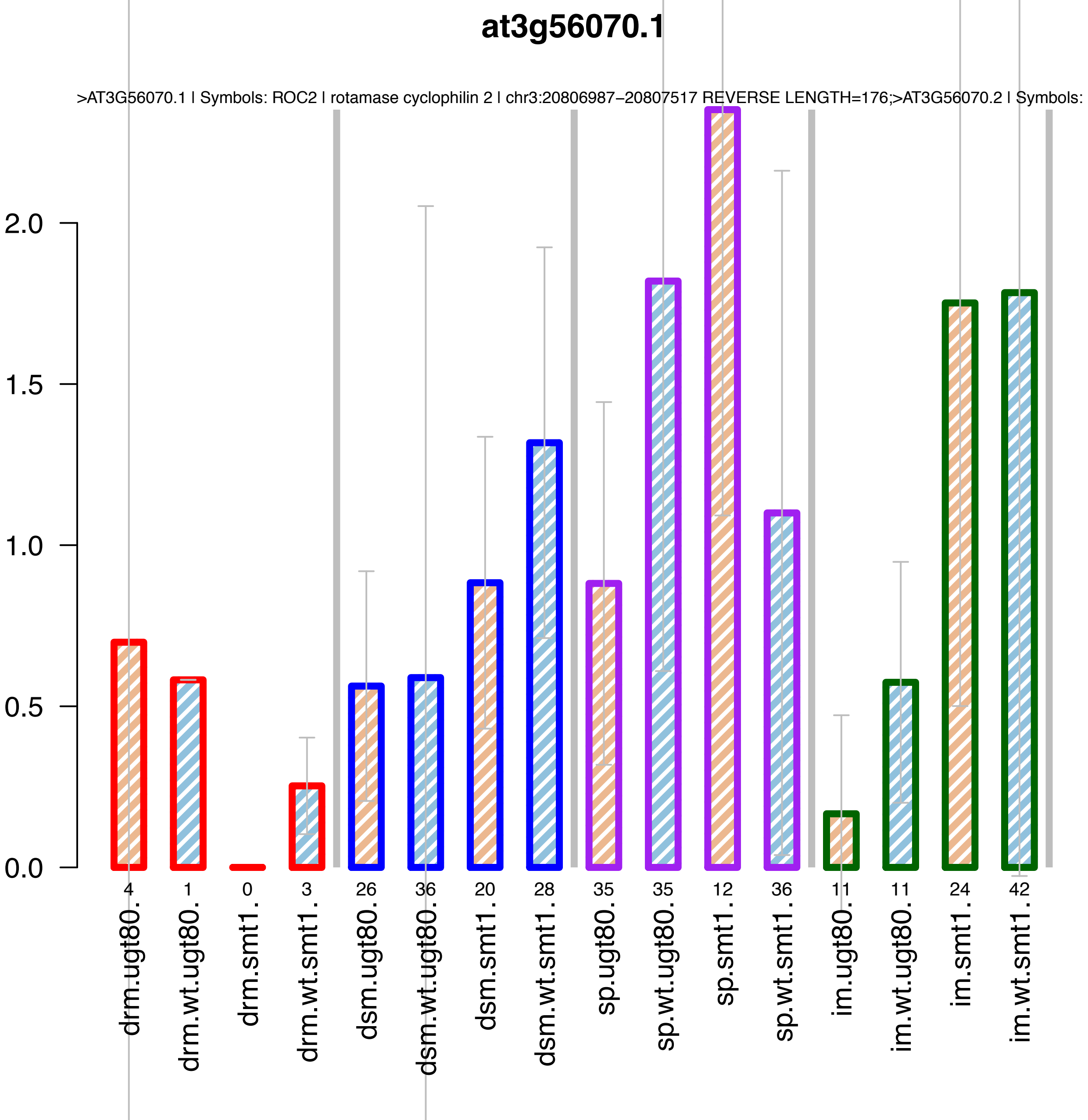
at3g56070.1
>AT3G56070.1 | Symbols: ROC2 | rotamase cyclophilin 2 | chr3:20806987-20807517 REVERSE LENGTH=176;>AT3G56070.2 | Symbols:
0.0
0.5
1.0
1.5
2.0
4
1
0
3
26
36
20
28
35
35
12
36
11
11
24
42
drm.ugt80..
drm.wt.ugt80..
drm.smt1..
drm.wt.smt1..
dsm.ugt80..
dsm.wt.ugt80..
dsm.smt1..
dsm.wt.smt1..
sp.ugt80..
sp.wt.ugt80..
sp.smt1..
sp.wt.smt1..
im.ugt80..
im.wt.ugt80..
im.smt1..
im.wt.smt1..

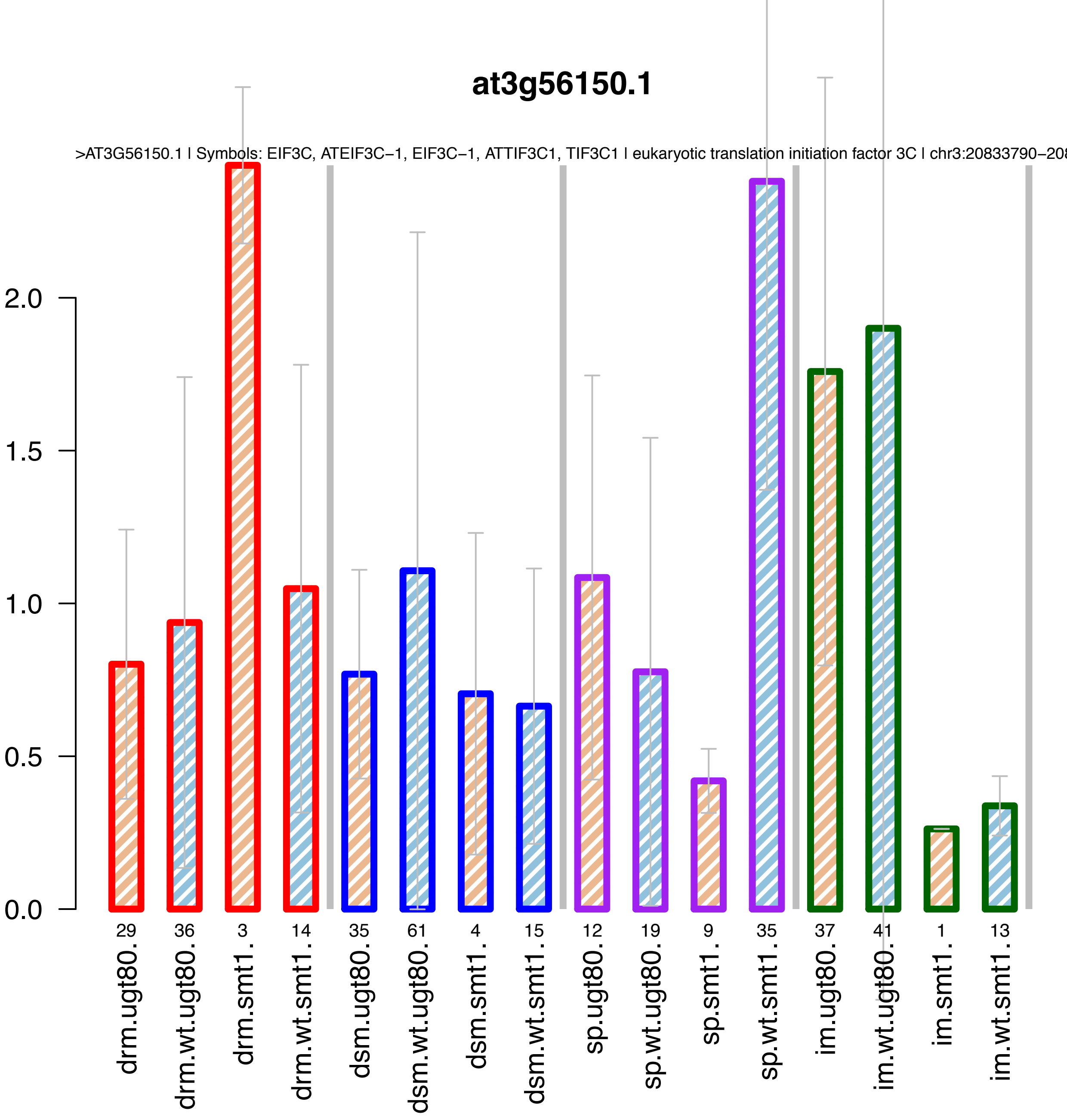

at3g56150.1
>AT3G56150.1 | Symbols: EIF3C, ATEIF3C−1, EIF3C−1, ATTIF3C1, TIF3C1 | eukaryotic translation initiation factor 3C | chr3:20833790−208
2.0
1.5
1.0
0.5
0.0
29
36
3
14
35
61
4
15
12
19
9
35
37
41
1
13
drm.ugt80..
drm.wt.ugt80..
drm.smt1..
drm.wt.smt1..
dsm.ugt80..
dsm.wt.ugt80..
dsm.smt1..
dsm.wt.smt1..
sp.ugt80..
sp.wt.ugt80..
sp.smt1..
sp.wt.smt1..
im.ugt80..
im.wt.ugt80..
im.smt1..
im.wt.smt1..

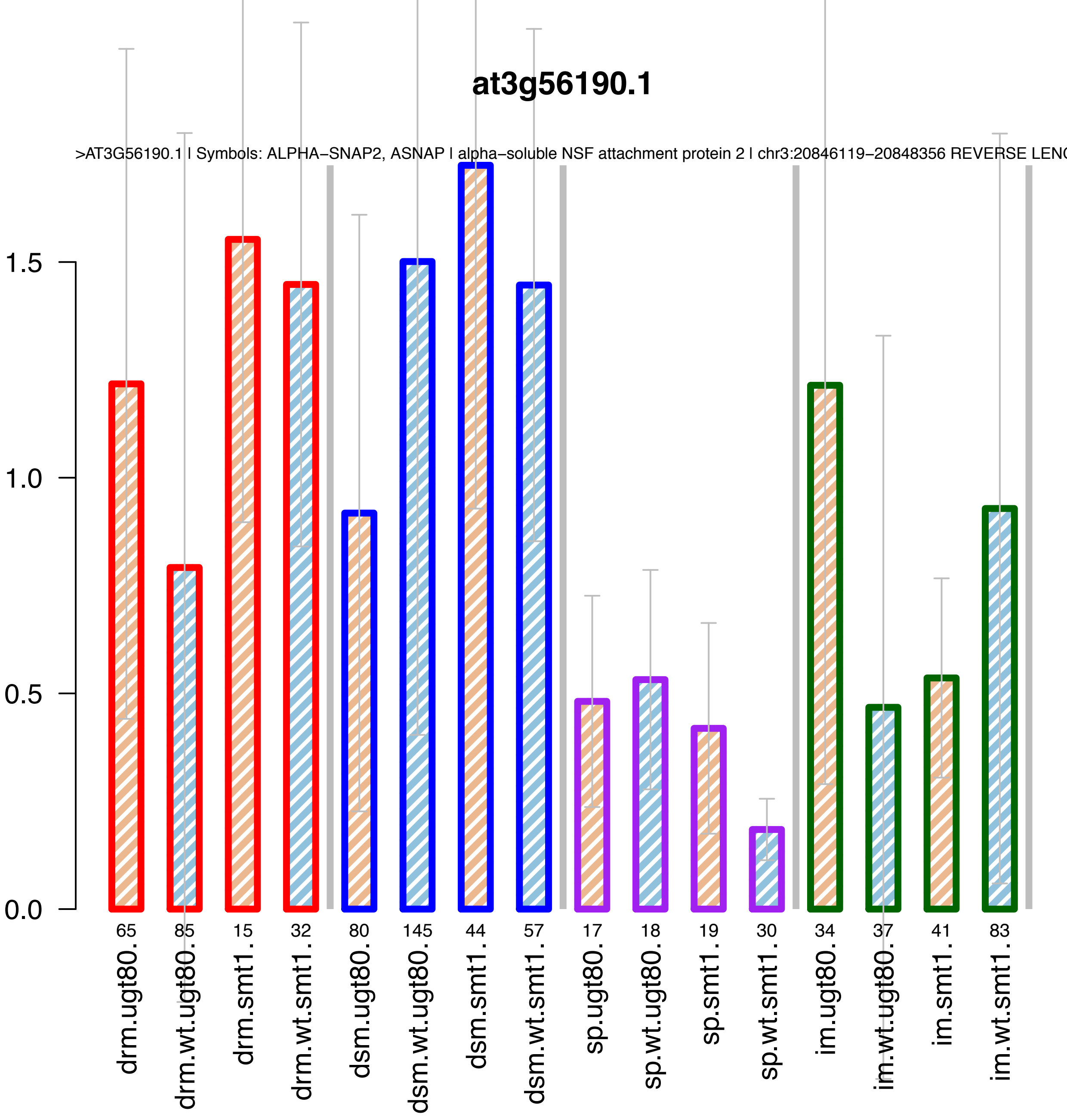

at3g56190.1
>AT3G56190.1 | Symbols: ALPHA−SNAP2, ASNAP | alpha−soluble NSF attachment protein 2 | chr3:20846119−20848356 REVERSE LENG
0.0
0.5
1.0
1.5
65
85
15
32
80
145
44
57
17
18
19
30
34
37
41
83
drm.ugt80.
drm.wt.ugt80.
drm.smt1.
drm.wt.smt1.
dsm.ugt80.
dsm.wt.ugt80.
dsm.smt1.
dsm.wt.smt1.
sp.ugt80.
sp.wt.ugt80.
sp.smt1.
sp.wt.smt1.
im.ugt80.
im.wt.ugt80.
im.smt1.
im.wt.smt1.

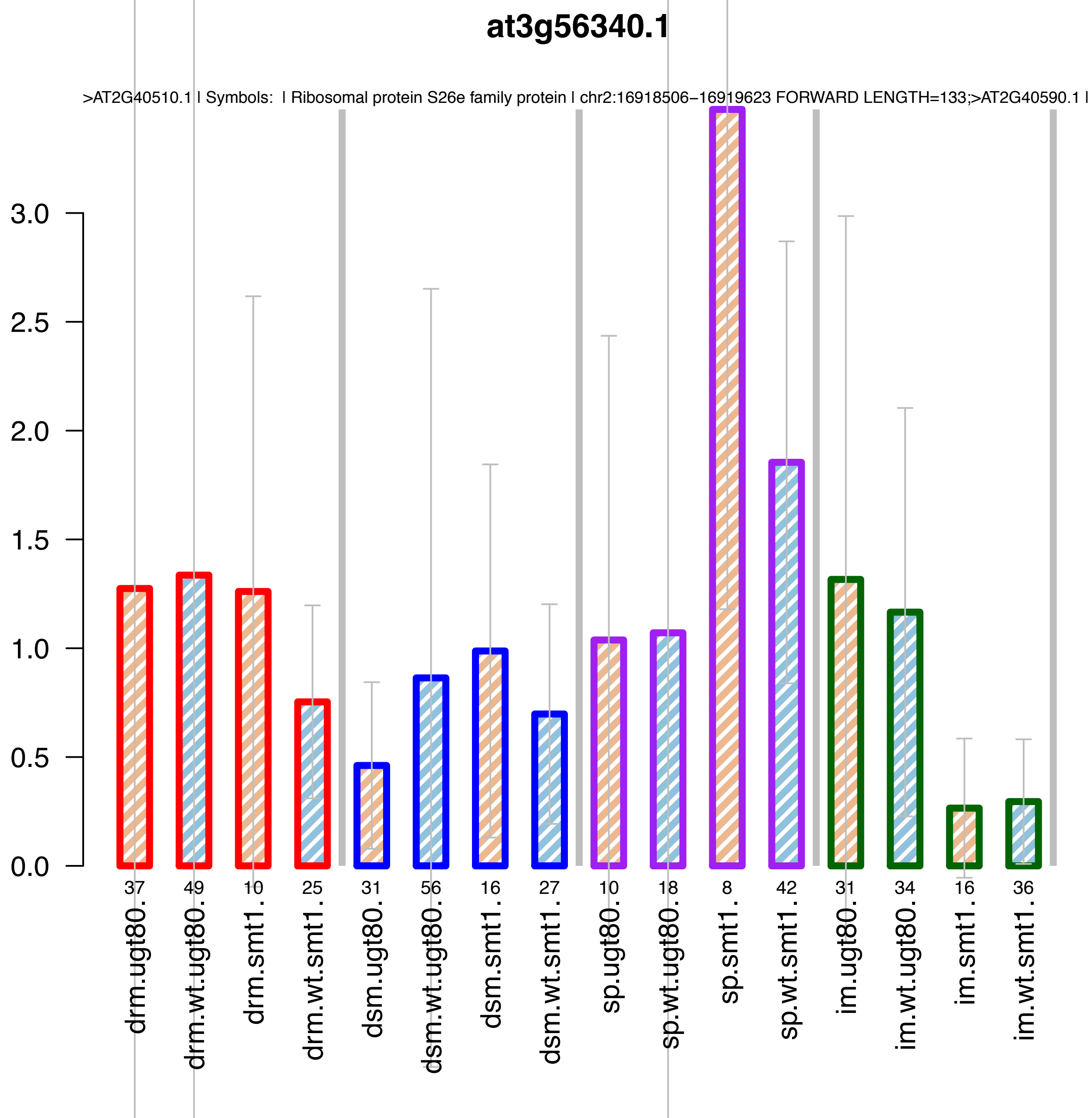
at3g56340.1
>AT2G40510.1 | Symbols: | Ribosomal protein S26e family protein | chr2:16918506-16919623 FORWARD LENGTH=133;>AT2G40590.1 |
0.0
0.5
1.0
1.5
2.0
2.5
3.0
37
49
10
25
31
56
16
27
10
18
8
42
31
34
16
36
drm.ugt80.
drm.wt.ugt80.
drm.smt1.
drm.wt.smt1.
dsm.ugt80.
dsm.wt.ugt80.
dsm.smt1.
dsm.wt.smt1.
sp.ugt80.
sp.wt.ugt80.
sp.smt1.
sp.wt.smt1.
im.ugt80.
im.wt.ugt80.
im.smt1.
im.wt.smt1.

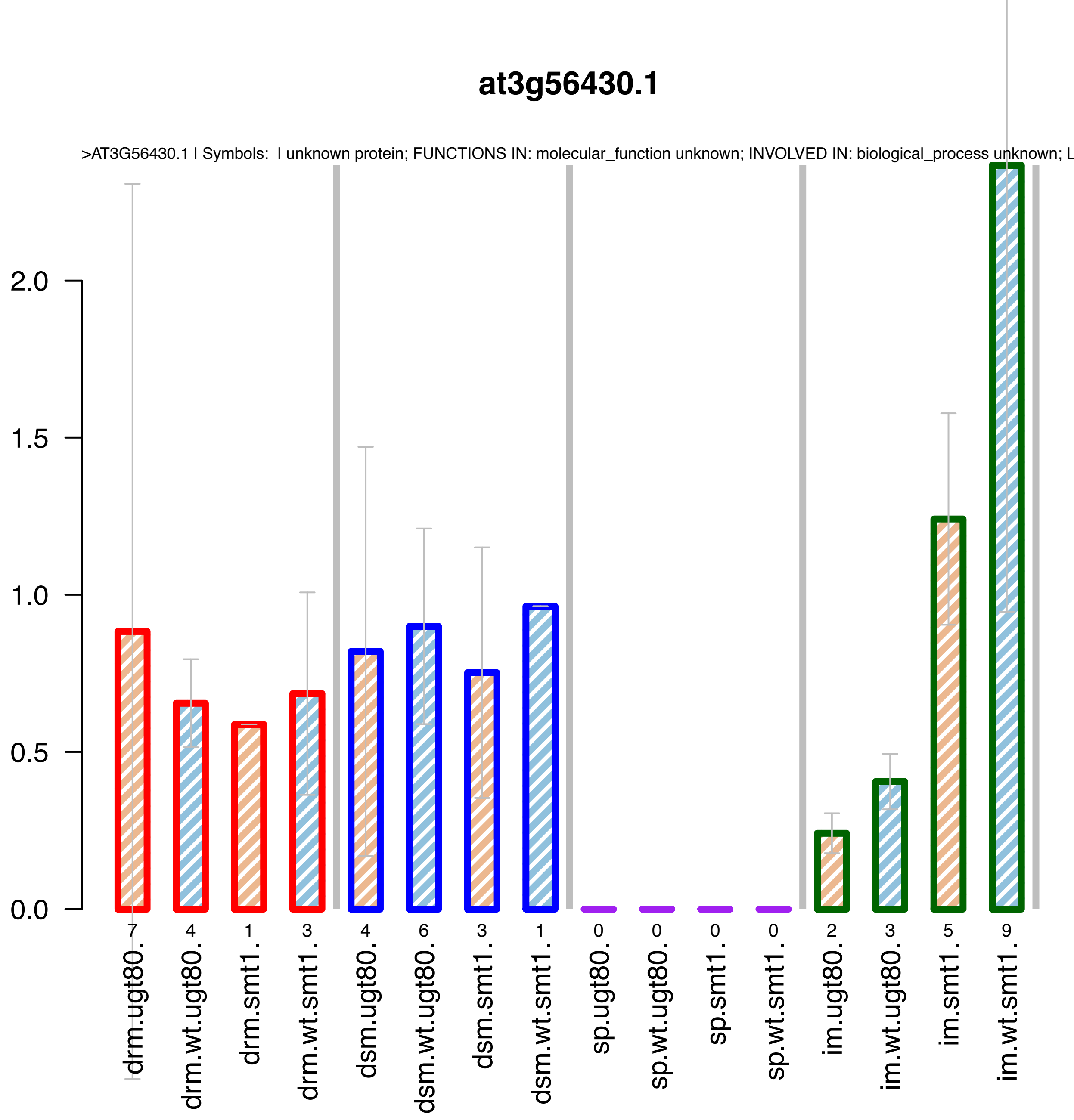
at3g56430.1
>AT3G56430.1 | Symbols: | unknown protein; FUNCTIONS IN: molecular_function unknown; INVOLVED IN: biological_process unknown; L
0.0
0.5
1.0
1.5
2.0
7
4
1
3
4
6
3
1
0
0
0
0
2
3
5
9
drm.ugt80.
drm.wt.ugt80.
drm.smt1.
drm.wt.smt1.
dsm.ugt80.
dsm.wt.ugt80.
dsm.smt1.
dsm.wt.smt1.
sp.ugt80.
sp.wt.ugt80.
sp.smt1.
sp.wt.smt1.
im.ugt80.
im.wt.ugt80.
im.smt1.
im.wt.smt1.

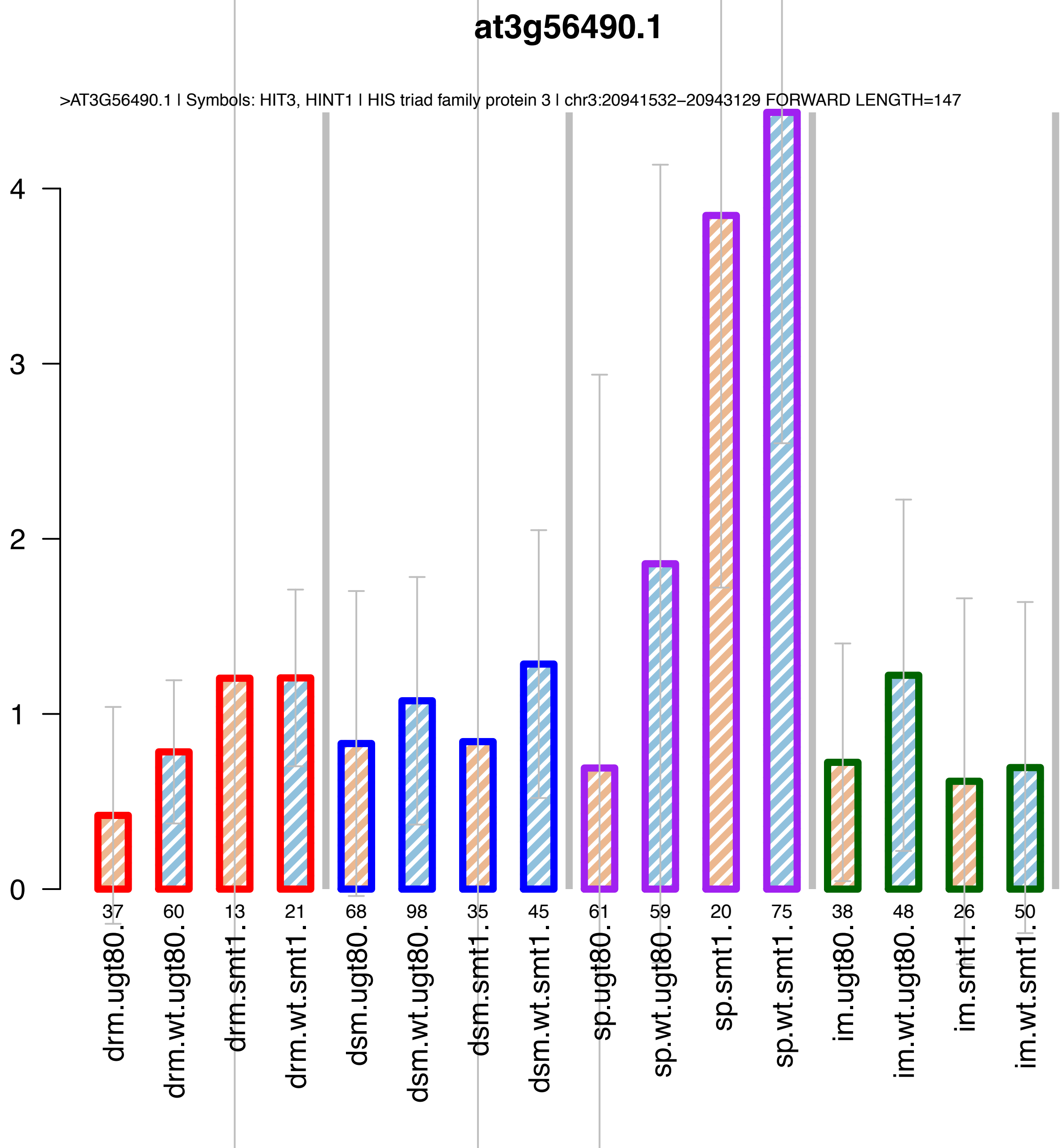

at3g56490.1
>AT3G56490.1 | Symbols: HIT3, HINT1 | HIS triad family protein 3 | chr3:20941532-20943129 FORWARD LENGTH=147
0
1
2
3
4
37
60
13
21
68
98
35
45
61
59
20
75
38
48
26
50
drm.ugt80..
drm.wt.ugt80..
drm.smt1..
drm.wt.smt1..
dsm.ugt80..
dsm.wt.ugt80..
dsm.smt1..
dsm.wt.smt1..
sp.ugt80..
sp.wt.ugt80..
sp.smt1..
sp.wt.smt1..
im.ugt80..
im.wt.ugt80..
im.smt1..
im.wt.smt1..

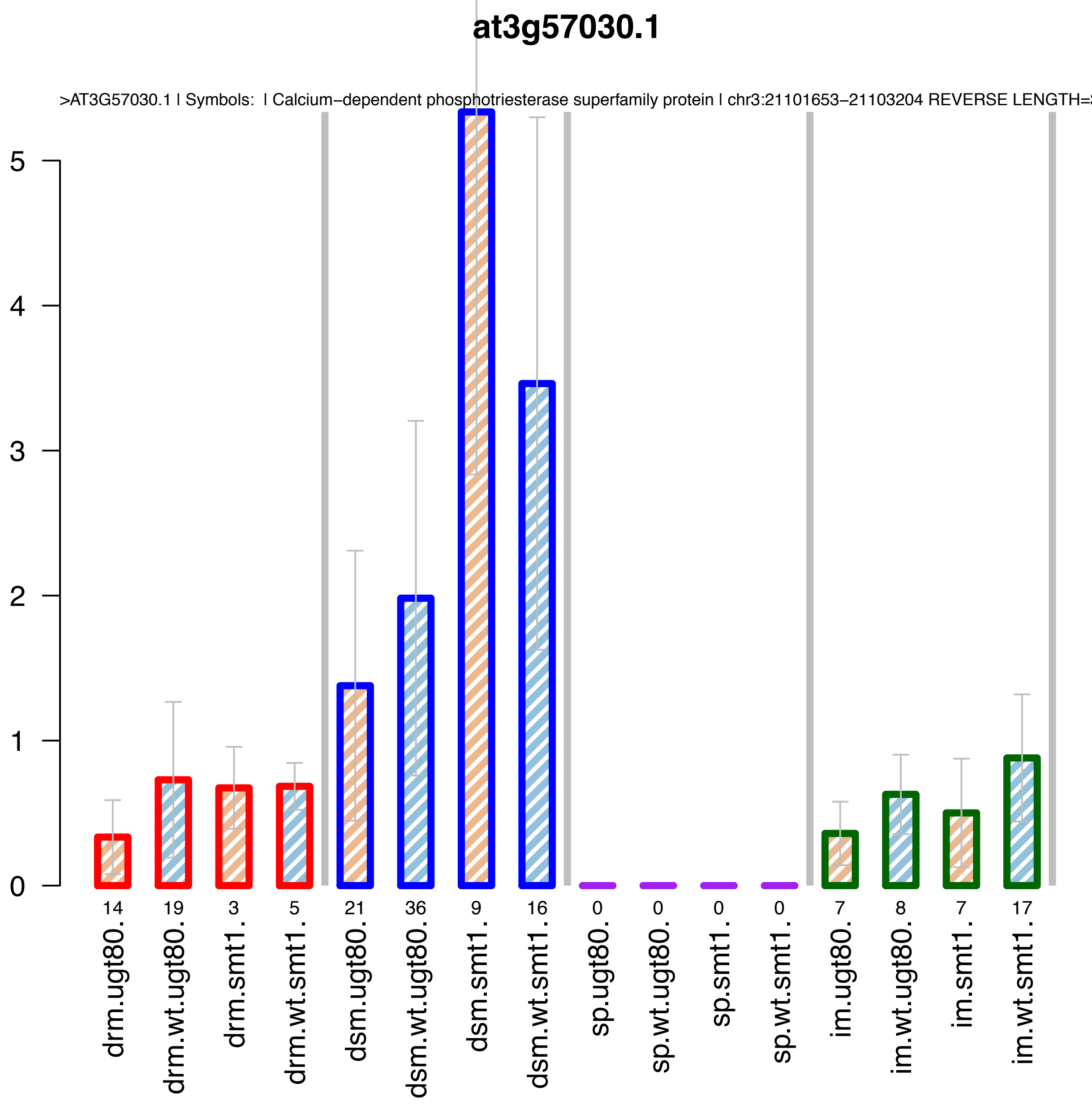

at3g57030.1
>AT3G57030.1 | Symbols: | Calcium−dependent phosphotriesterase superfamily protein | chr3:21101653−21103204 REVERSE LENGTH=
0
1
2
3
4
5
14
19
3
5
21
36
9
16
0
0
0
0
7
8
7
17
drm.ugt80.
drm.wt.ugt80.
drm.smt1.
drm.wt.smt1.
dsm.ugt80.
dsm.wt.ugt80.
dsm.smt1.
dsm.wt.smt1.
sp.ugt80.
sp.wt.ugt80.
sp.smt1.
sp.wt.smt1.
im.ugt80.
im.wt.ugt80.
im.smt1.
im.wt.smt1.

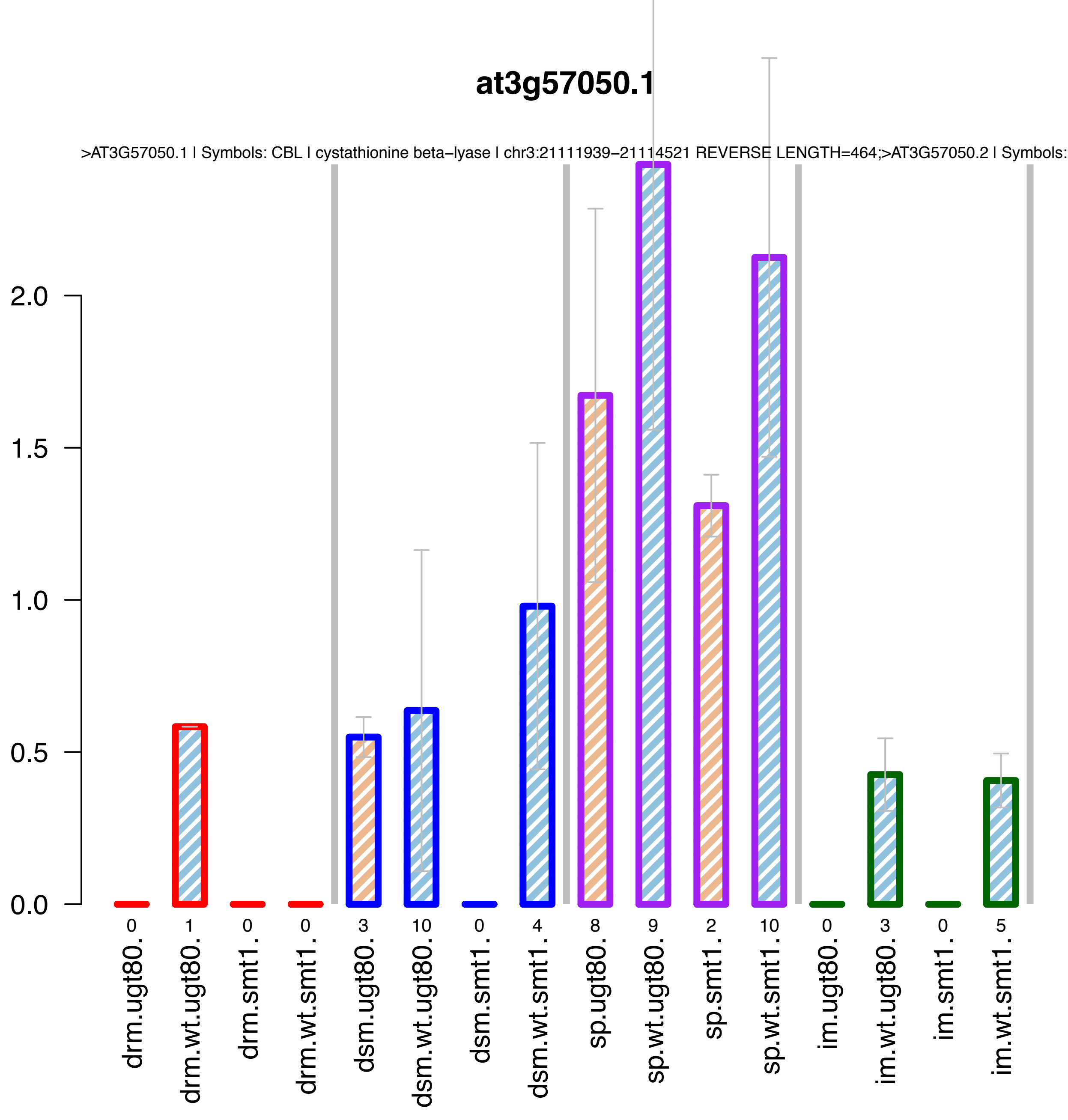

at3g57050.1
>AT3G57050.1 | Symbols: CBL | cystathionine beta−lyase | chr3:21111939−21114521 REVERSE LENGTH=464;>AT3G57050.2 | Symbols:
2.0
1.5
1.0
0.5
0.0
0
1
0
0
3
10
0
4
8
9
2
10
0
3
0
5
drm.ugt80.
drm.wt.ugt80.
drm.smt1.
drm.wt.smt1.
dsm.ugt80.
dsm.wt.ugt80.
dsm.smt1.
dsm.wt.smt1.
sp.ugt80.
sp.wt.ugt80.
sp.smt1.
sp.wt.smt1.
im.ugt80.
im.wt.ugt80.
im.smt1.
im.wt.smt1.

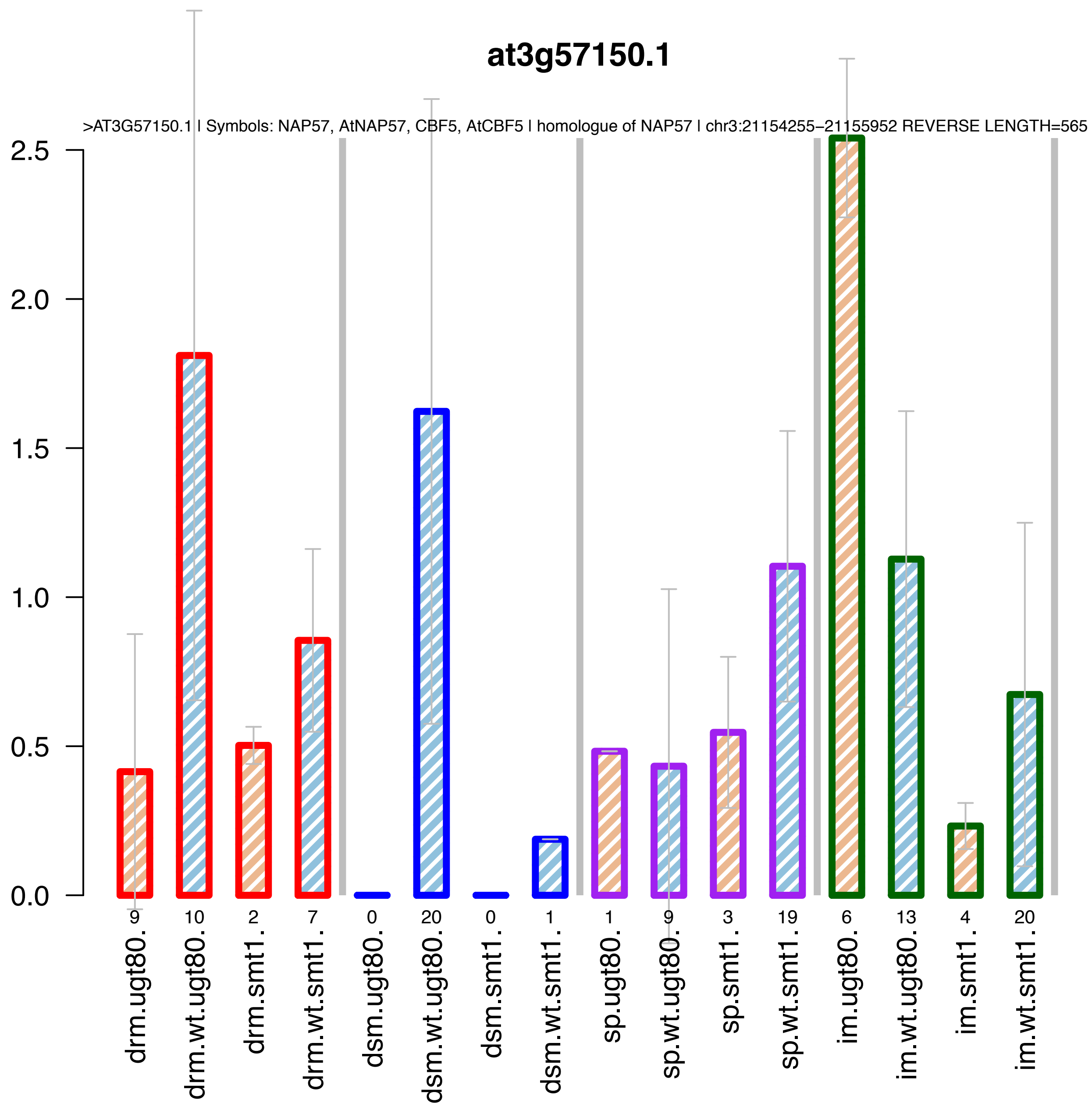

at3g57150.1
>AT3G57150.1 | Symbols: NAP57, AtNAP57, CBF5, AtCBF5 | homologue of NAP57 | chr3:21154255-21155952 REVERSE LENGTH=565
2.5
2.0
1.5
1.0
0.5
0.0
9
10
2
7
0
20
0
1
1
9
3
19
6
13
4
20
drm.ugt80.
drm.wt.ugt80.
drm.smt1.
drm.wt.smt1.
dsm.ugt80.
dsm.wt.ugt80.
dsm.smt1.
dsm.wt.smt1.
sp.ugt80.
sp.wt.ugt80.
sp.smt1.
sp.wt.smt1.
im.ugt80.
im.wt.ugt80.
im.smt1.
im.wt.smt1.

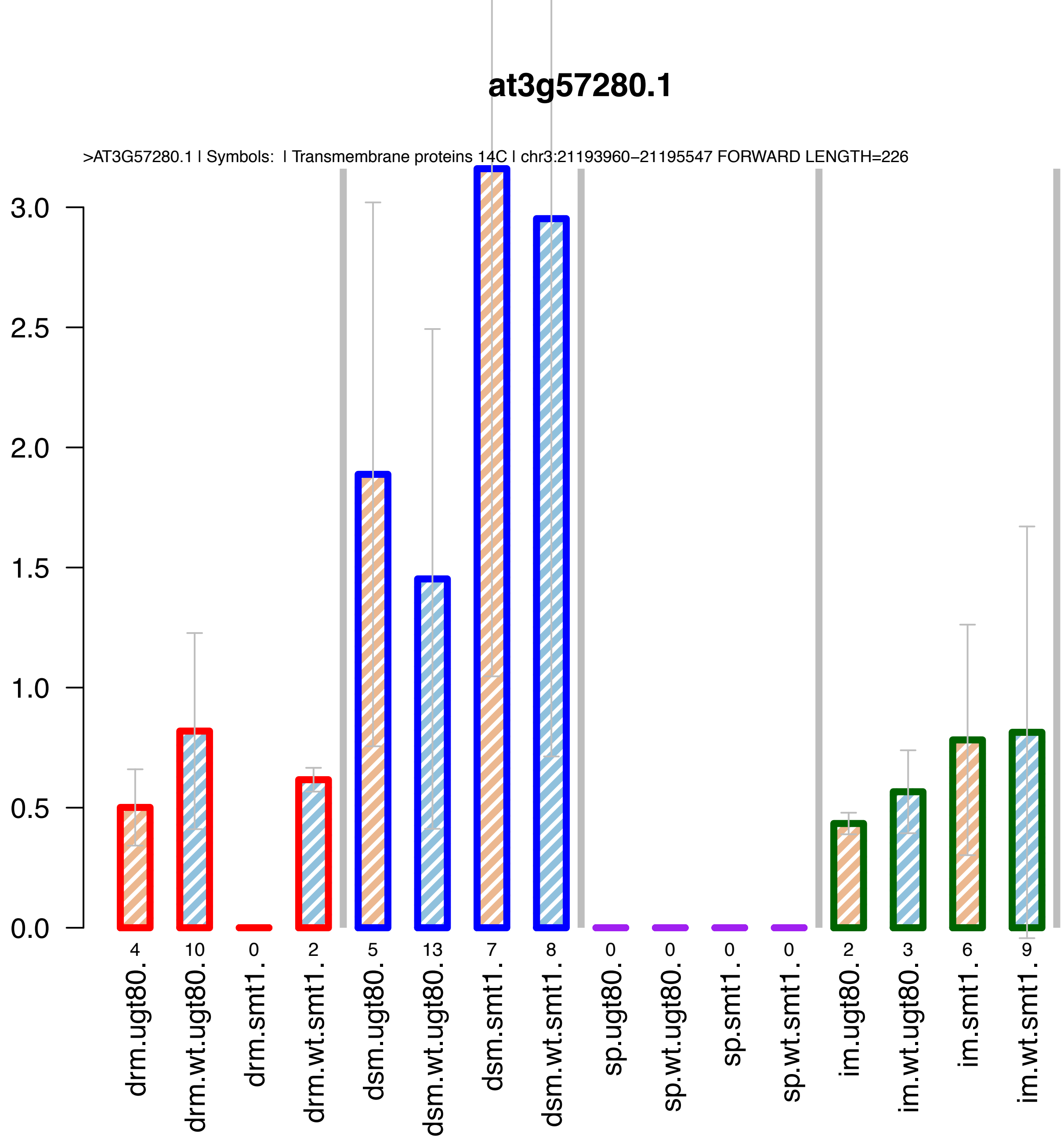

at3g57280.1
>AT3G57280.1 | Symbols: | Transmembrane proteins 14C | chr3:21193960−21195547 FORWARD LENGTH=226
3.0
2.5
2.0
1.5
1.0
0.5
0.0
4
10
0
2
5
13
7
8
0
0
0
0
2
3
6
9
drm.ugt80.
drm.wt.ugt80.
drm.smt1.
drm.wt.smt1.
dsm.ugt80.
dsm.wt.ugt80.
dsm.smt1.
dsm.wt.smt1.
sp.ugt80.
sp.wt.ugt80.
sp.smt1.
sp.wt.smt1.
im.ugt80.
im.wt.ugt80.
im.smt1.
im.wt.smt1.

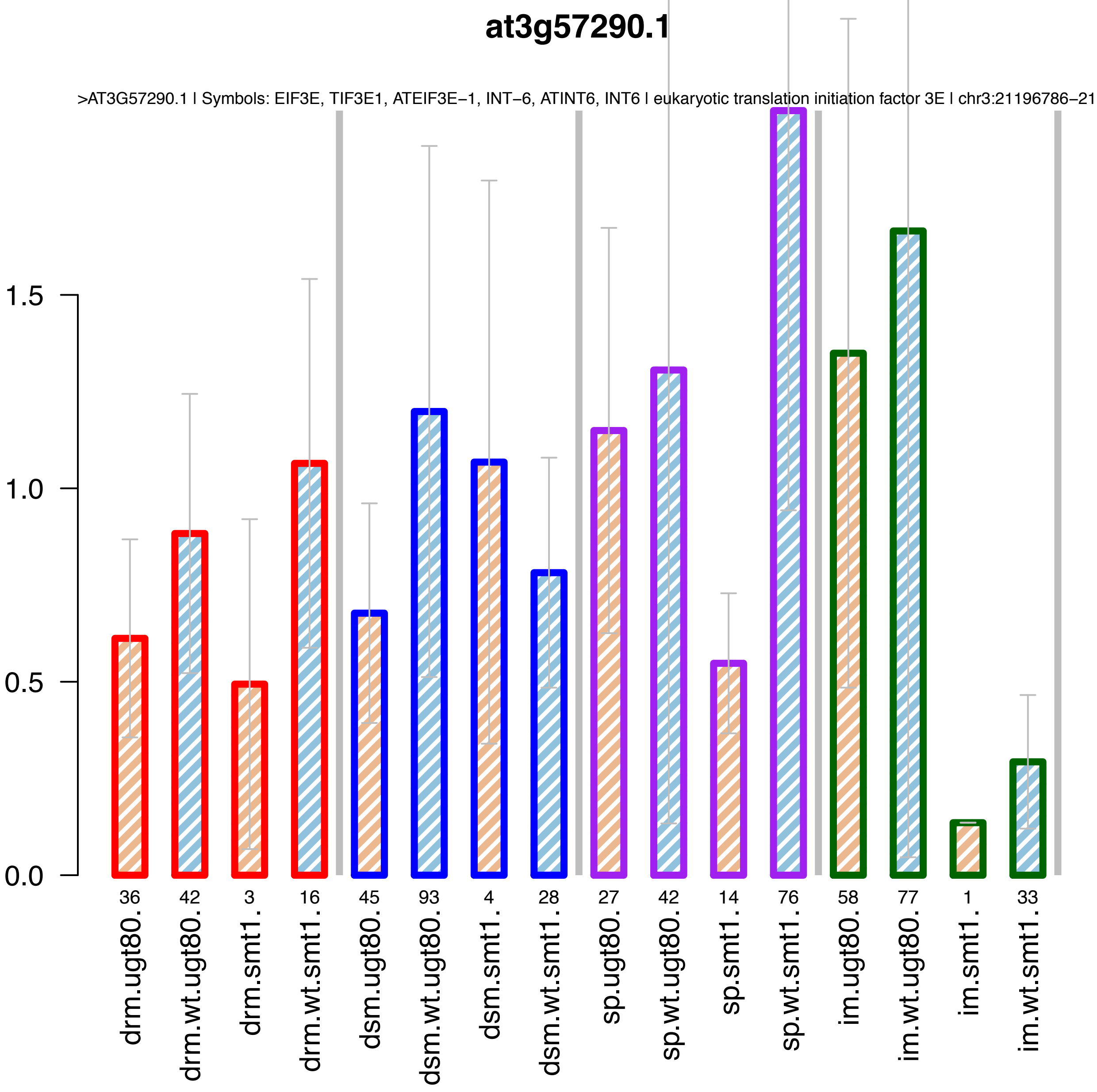
at3g57290.1
>AT3G57290.1 | Symbols: EIF3E, TIF3E1, ATEIF3E−1, INT−6, ATINT6, INT6 | eukaryotic translation initiation factor 3E | chr3:21196786−21
0.0
0.5
1.0
1.5
36
42
3
16
45
93
4
28
27
42
14
76
58
77
1
33
drm.ugt80..
drm.wt.ugt80..
drm.smt1..
drm.wt.smt1..
dsm.ugt80..
dsm.wt.ugt80..
dsm.smt1..
dsm.wt.smt1..
sp.ugt80..
sp.wt.ugt80..
sp.smt1..
sp.wt.smt1..
im.ugt80..
im.wt.ugt80..
im.smt1..
im.wt.smt1..

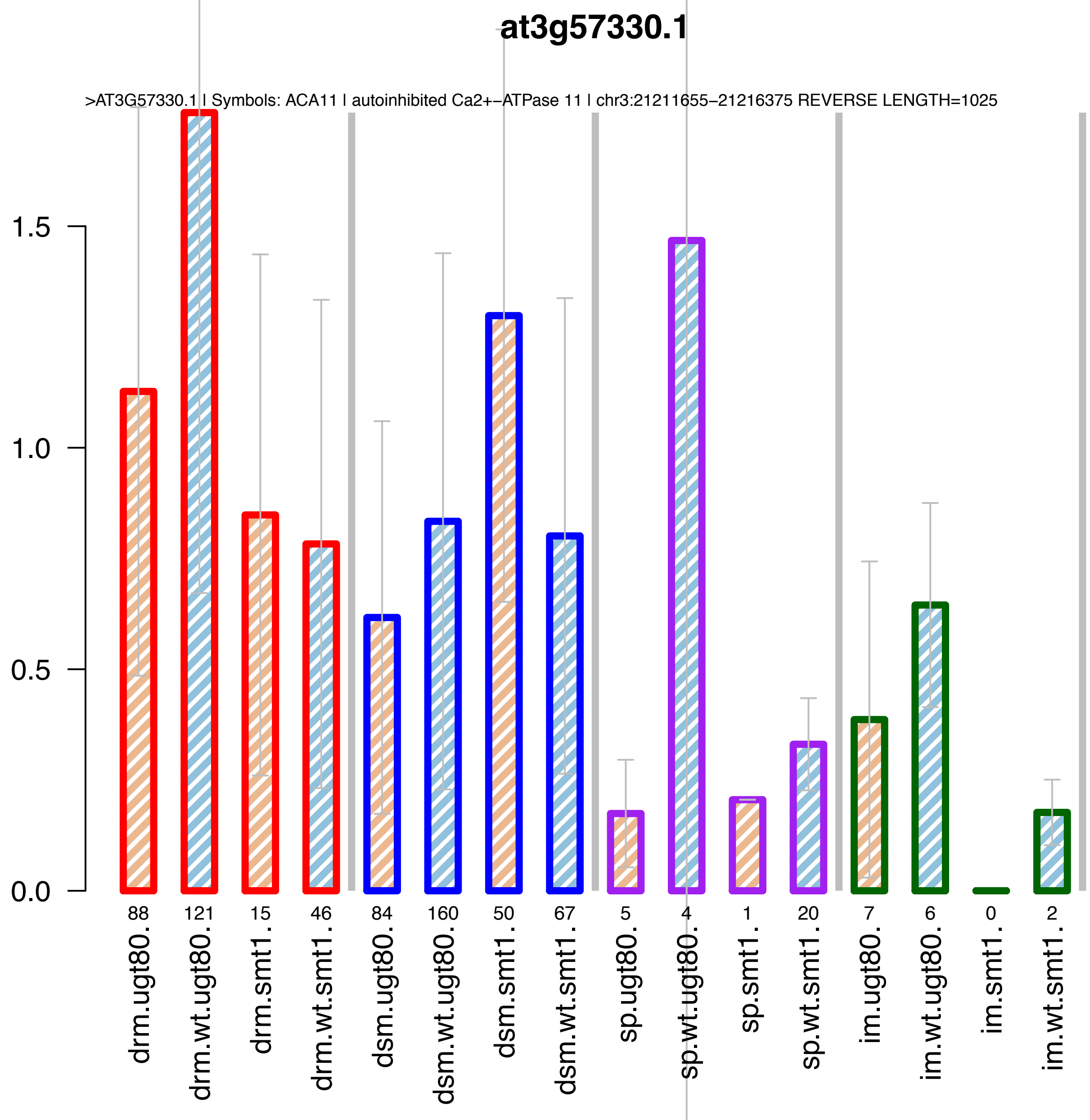

at3g57330.1
>AT3G57330.1 | Symbols: ACA11 | autoinhibited Ca2+-ATPase 11 | chr3:21211655-21216375 REVERSE LENGTH=1025
1.5
1.0
0.5
0.0
88
121
15
46
84
160
50
67
5
4
1
20
7
6
0
2
drm.ugt80.
drm.wt.ugt80.
drm.smt1.
drm.wt.smt1.
dsm.ugt80.
dsm.wt.ugt80.
dsm.smt1.
dsm.wt.smt1.
sp.ugt80.
sp.wt.ugt80.
sp.smt1.
sp.wt.smt1.
im.ugt80.
im.wt.ugt80.
im.smt1.
im.wt.smt1.

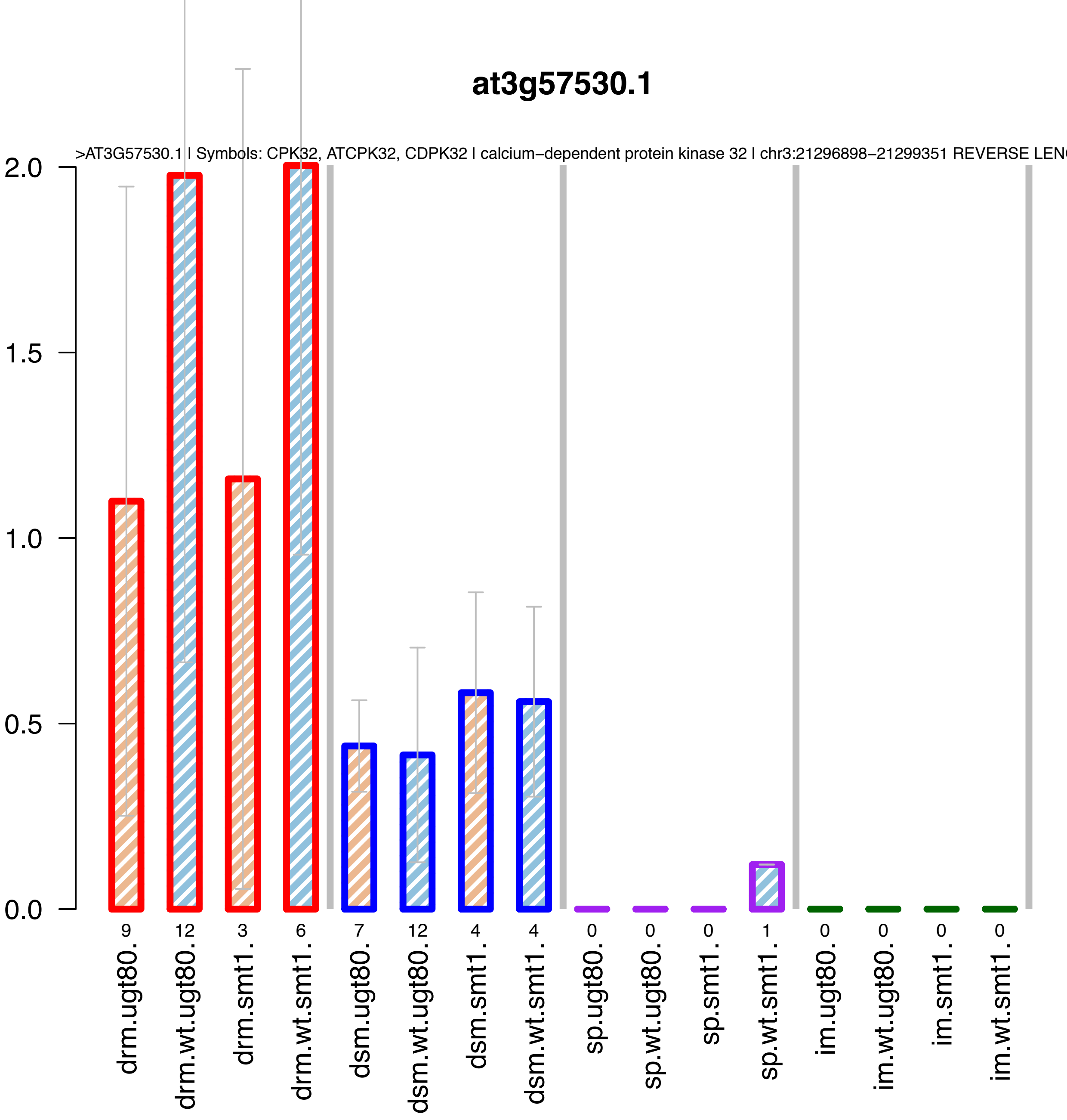
at3g57530.1
>AT3G57530.1 | Symbols: CPK32, ATCPK32, CDPK32 | calcium−dependent protein kinase 32 | chr3:21296898−21299351 REVERSE LEN
2.0
1.5
1.0
0.5
0.0
9
12
3
6
7
12
4
4
0
0
0
1
0
0
0
0
drm.ugt80.
drm.wt.ugt80.
drm.smt1.
drm.wt.smt1.
dsm.ugt80.
dsm.wt.ugt80.
dsm.smt1.
dsm.wt.smt1.
sp.ugt80.
sp.wt.ugt80.
sp.smt1.
sp.wt.smt1.
im.ugt80.
im.wt.ugt80.
im.smt1.
im.wt.smt1.

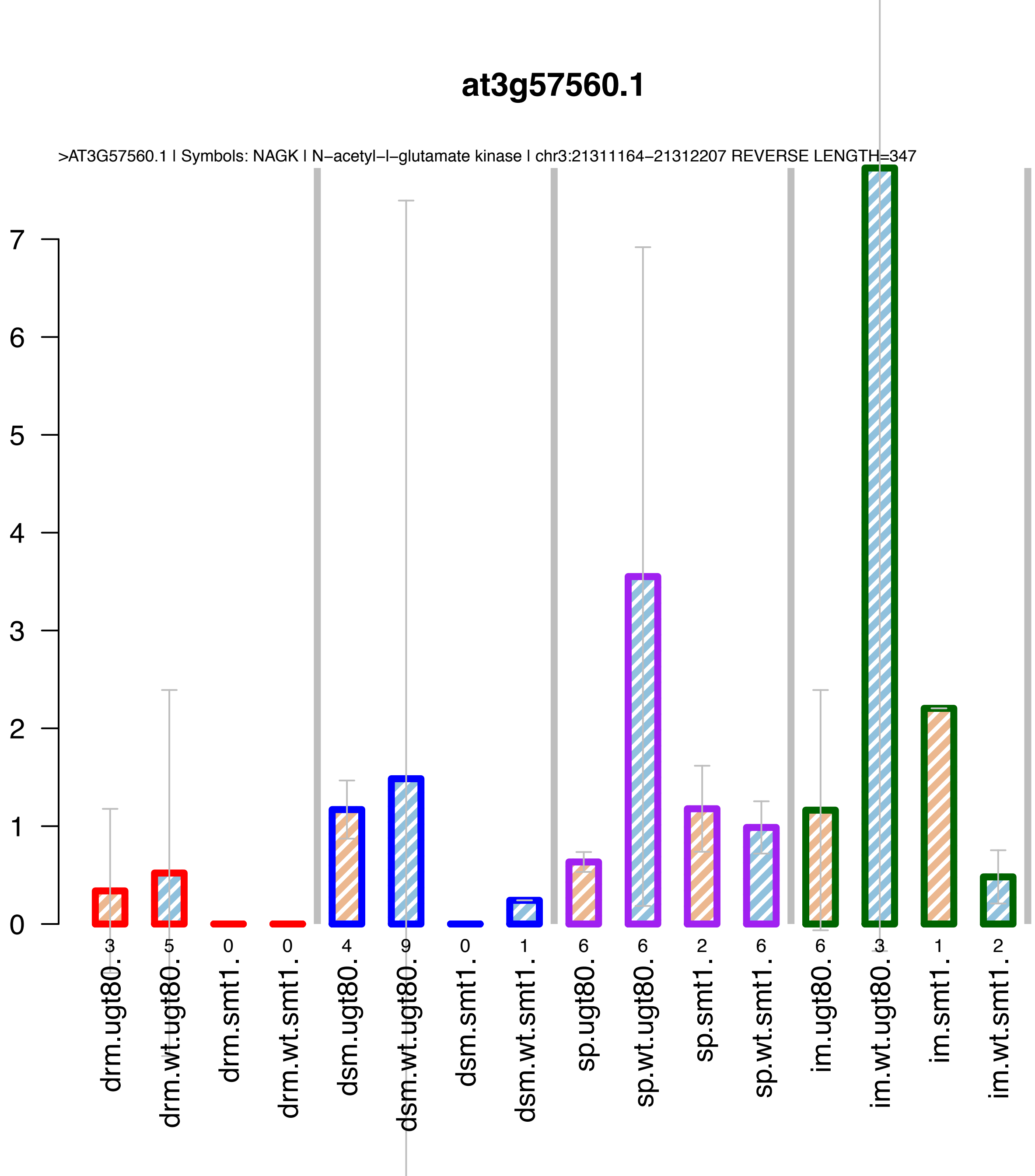

at3g57560.1
>AT3G57560.1 | Symbols: NAGK | N−acetyl−l−glutamate kinase | chr3:21311164−21312207 REVERSE LENGTH=347
0
1
2
3
4
5
6
7
3
5
0
0
4
9
0
1
6
6
2
6
6
3
1
2
drm.ugt80.
drm.wt.ugt80.
drm.smt1.
drm.wt.smt1.
dsm.ugt80.
dsm.wt.ugt80.
dsm.smt1.
dsm.wt.smt1.
sp.ugt80.
sp.wt.ugt80.
sp.smt1.
sp.wt.smt1.
im.ugt80.
im.wt.ugt80.
im.smt1.
im.wt.smt1.

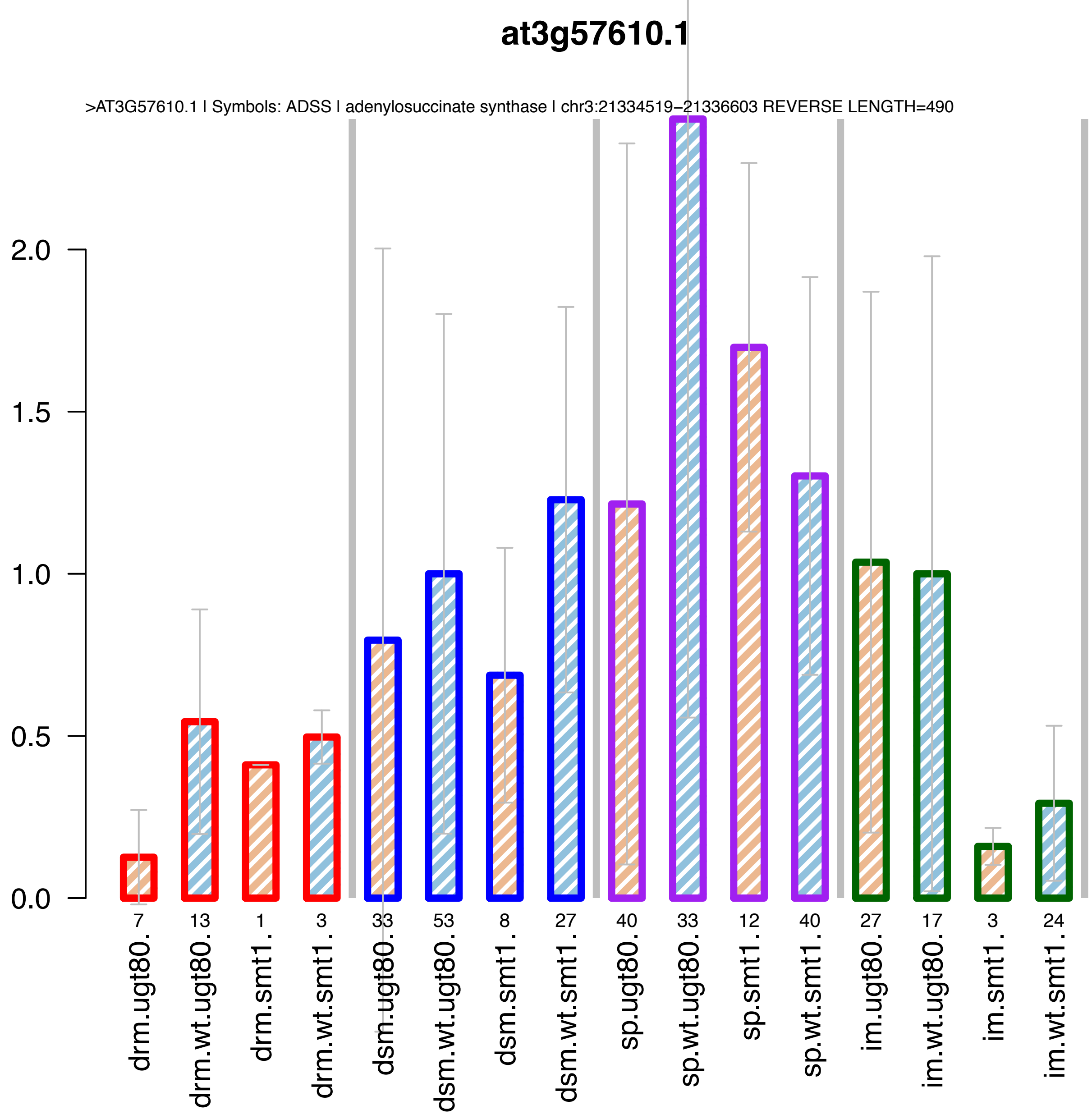
at3g57610.1
>AT3G57610.1 | Symbols: ADSS | adenylosuccinate synthase | chr3:21334519–21336603 REVERSE LENGTH=490
2.0
1.5
1.0
0.5
0.0
7
13
1
3
33
53
8
27
40
33
12
40
27
17
3
24
drm.ugt80.
drm.wt.ugt80.
drm.smt1.
drm.wt.smt1.
dsm.ugt80.
dsm.wt.ugt80.
dsm.smt1.
dsm.wt.smt1.
sp.ugt80.
sp.wt.ugt80.
sp.smt1.
sp.wt.smt1.
im.ugt80.
im.wt.ugt80.
im.smt1.
im.wt.smt1.

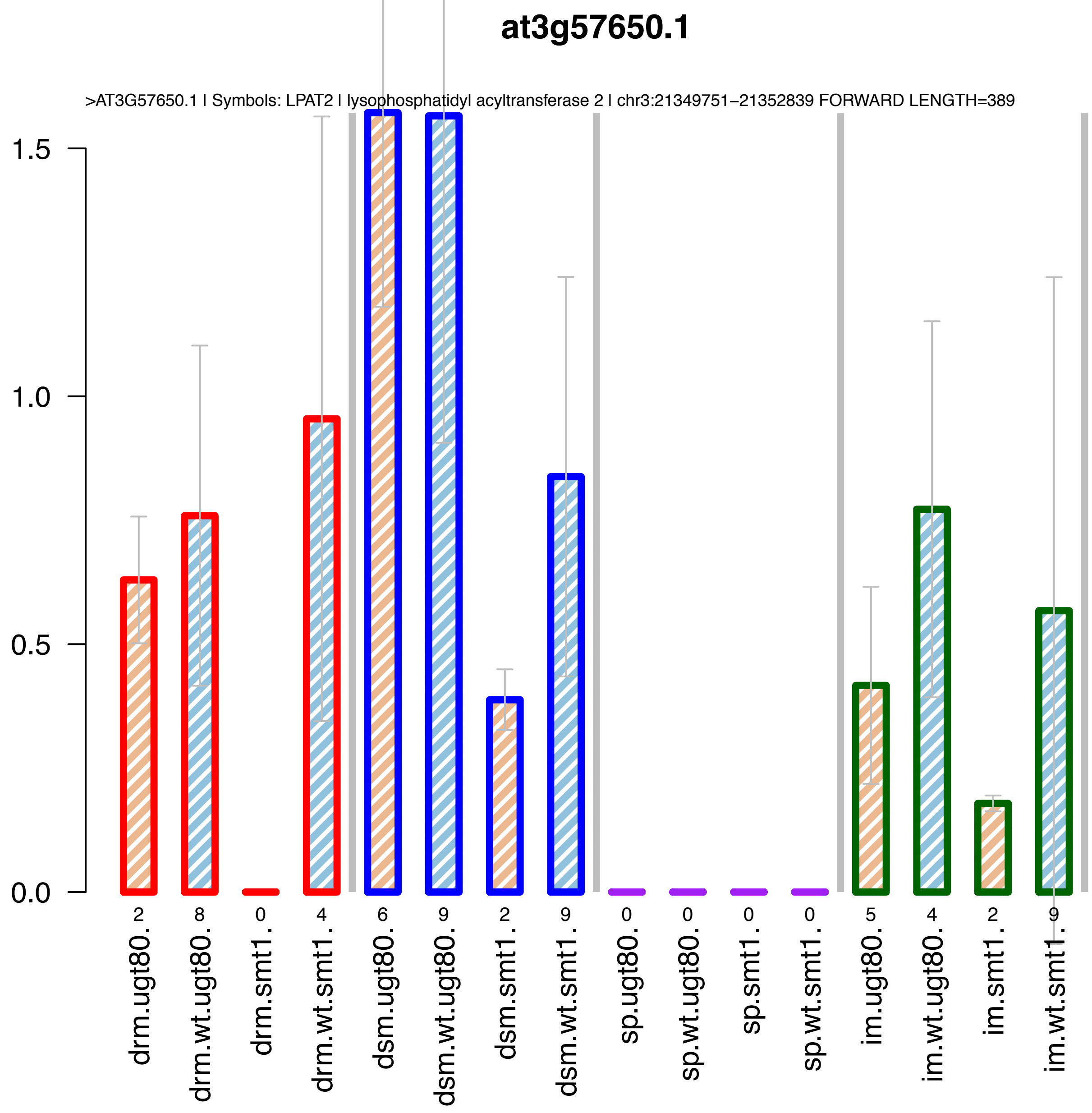
at3g57650.1
>AT3G57650.1 | Symbols: LPAT2 | lysophosphatidyl acyltransferase 2 | chr3:21349751–21352839 FORWARD LENGTH=389
1.5
1.0
0.5
0.0
2
8
0
4
6
9
2
9
0
0
0
0
5
4
2
9
drm.ugt80..
drm.wt.ugt80..
drm.smt1..
drm.wt.smt1..
dsm.ugt80..
dsm.wt.ugt80..
dsm.smt1..
dsm.wt.smt1..
sp.ugt80..
sp.wt.ugt80..
sp.smt1..
sp.wt.smt1..
im.ugt80..
im.wt.ugt80..
im.smt1..
im.wt.smt1..

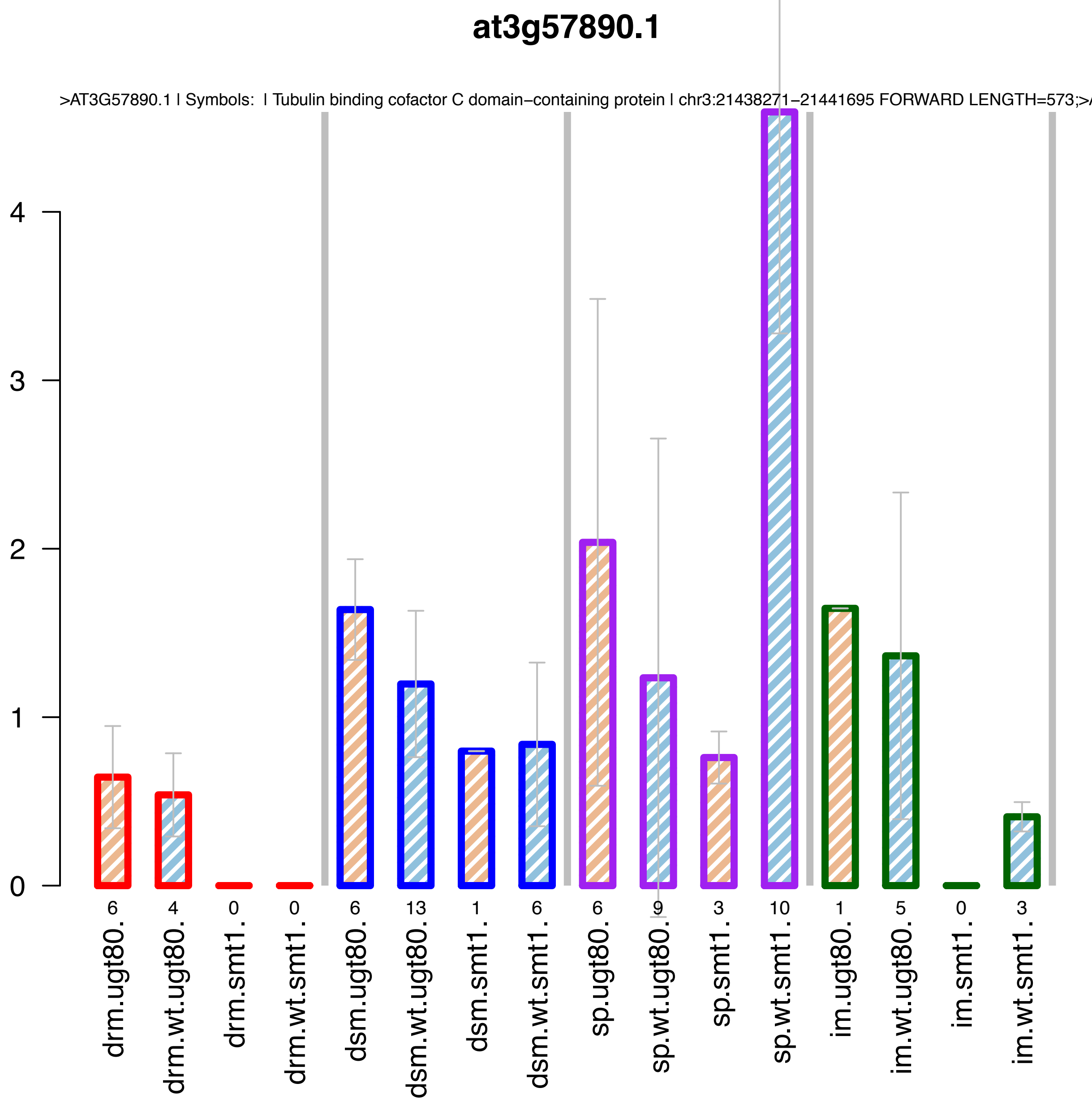
at3g57890.1
>AT3G57890.1 | Symbols: | Tubulin binding cofactor C domain−containing protein | chr3:21438271−21441695 FORWARD LENGTH=573;>
0
1
2
3
4
6
4
0
0
6
13
1
6
6
9
3
10
1
5
0
3
drm.ugt80.
drm.wt.ugt80.
drm.smt1.
drm.wt.smt1.
dsm.ugt80.
dsm.wt.ugt80.
dsm.smt1.
dsm.wt.smt1.
sp.ugt80.
sp.wt.ugt80.
sp.smt1.
sp.wt.smt1.
im.ugt80.
im.wt.ugt80.
im.smt1.
im.wt.smt1.

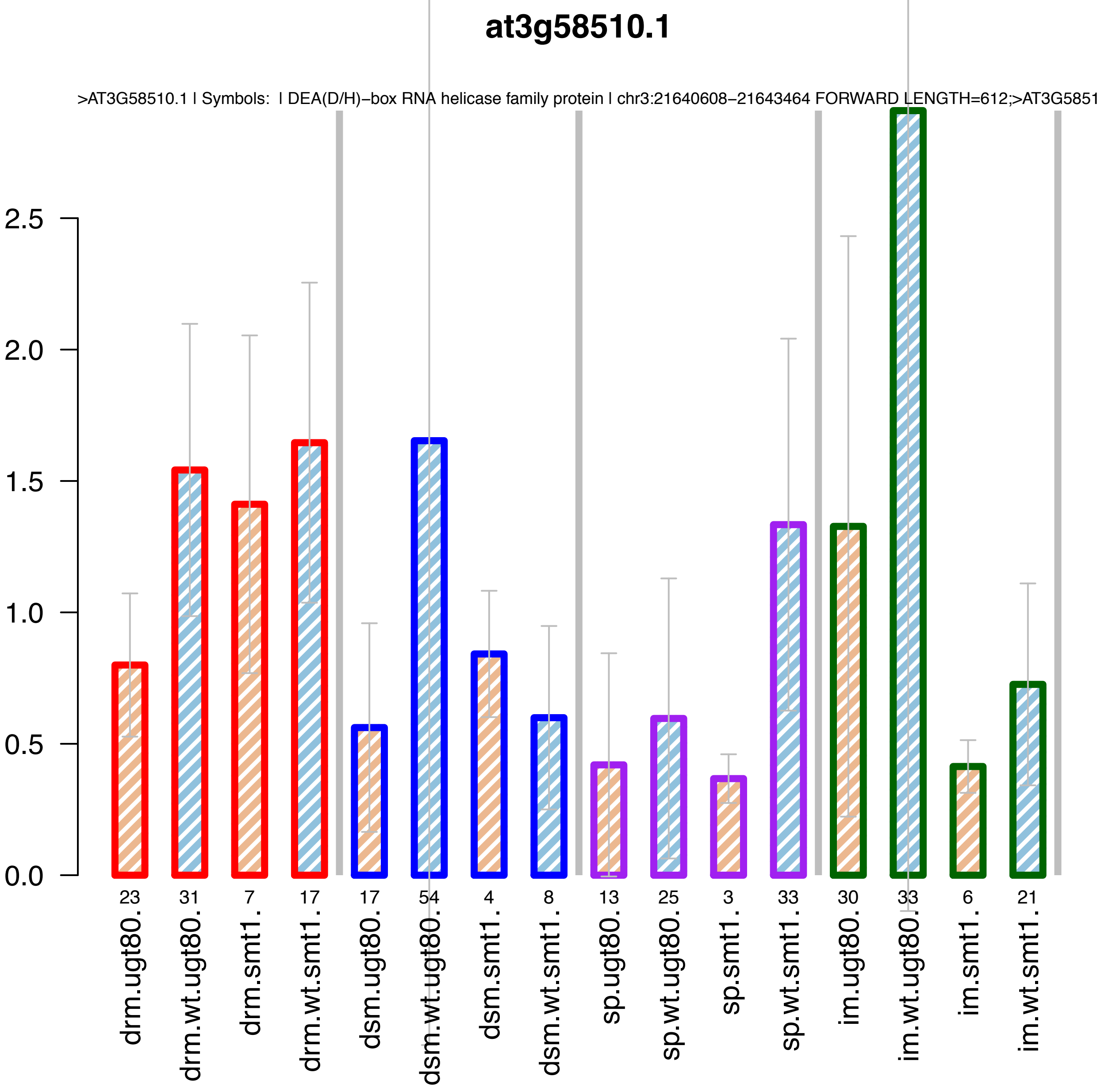

at3g58510.1
>AT3G58510.1 | Symbols: | DEA(D/H)-box RNA helicase family protein | chr3:21640608-21643464 FORWARD LENGTH=612;>AT3G5851
0.0
0.5
1.0
1.5
2.0
2.5
23
31
7
17
17
54
4
8
13
25
3
33
30
33
6
21
drm.ugt80..
drm.wt.ugt80..
drm.smt1..
drm.wt.smt1..
dsm.ugt80..
dsm.wt.ugt80..
dsm.smt1..
dsm.wt.smt1..
sp.ugt80..
sp.wt.ugt80..
sp.smt1..
sp.wt.smt1..
im.ugt80..
im.wt.ugt80..
im.smt1..
im.wt.smt1..

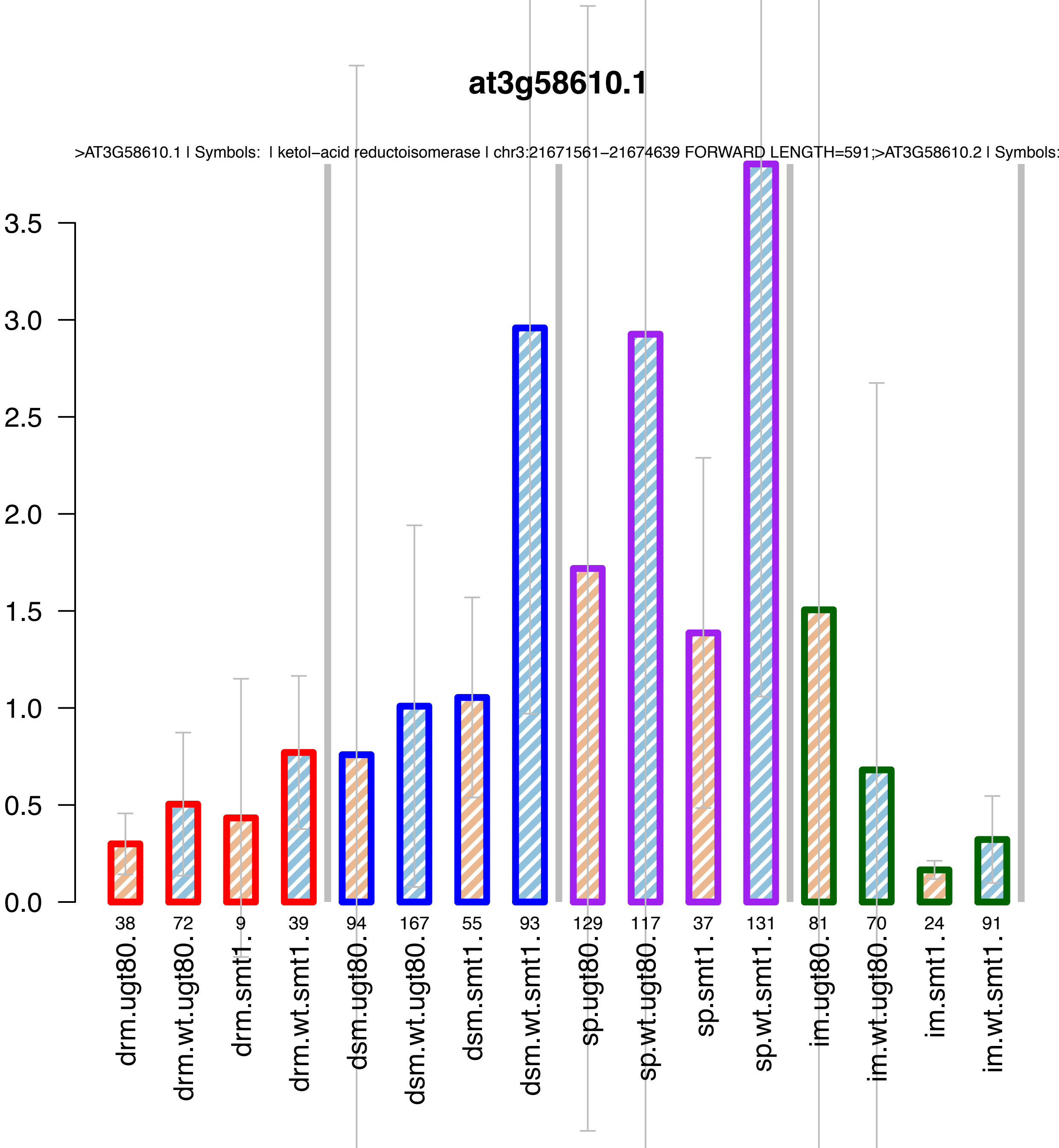
at3g58610.1
>AT3G58610.1 | Symbols: | ketol−acid reductoisomerase | chr3:21671561−21674639 FORWARD LENGTH=591;>AT3G58610.2 | Symbols:
3.5
3.0
2.5
2.0
1.5
1.0
0.5
0.0
38
72
9
39
94
167
55
93
129
117
37
131
81
70
24
91
drm.ugt80.
drm.wt.ugt80.
drm.smt1.
drm.wt.smt1.
dsm.ugt80.
dsm.wt.ugt80.
dsm.smt1.
dsm.wt.smt1.
sp.ugt80.
sp.wt.ugt80.
sp.smt1.
sp.wt.smt1.
im.ugt80.
im.wt.ugt80.
im.smt1.
im.wt.smt1.

# at3g58700.1

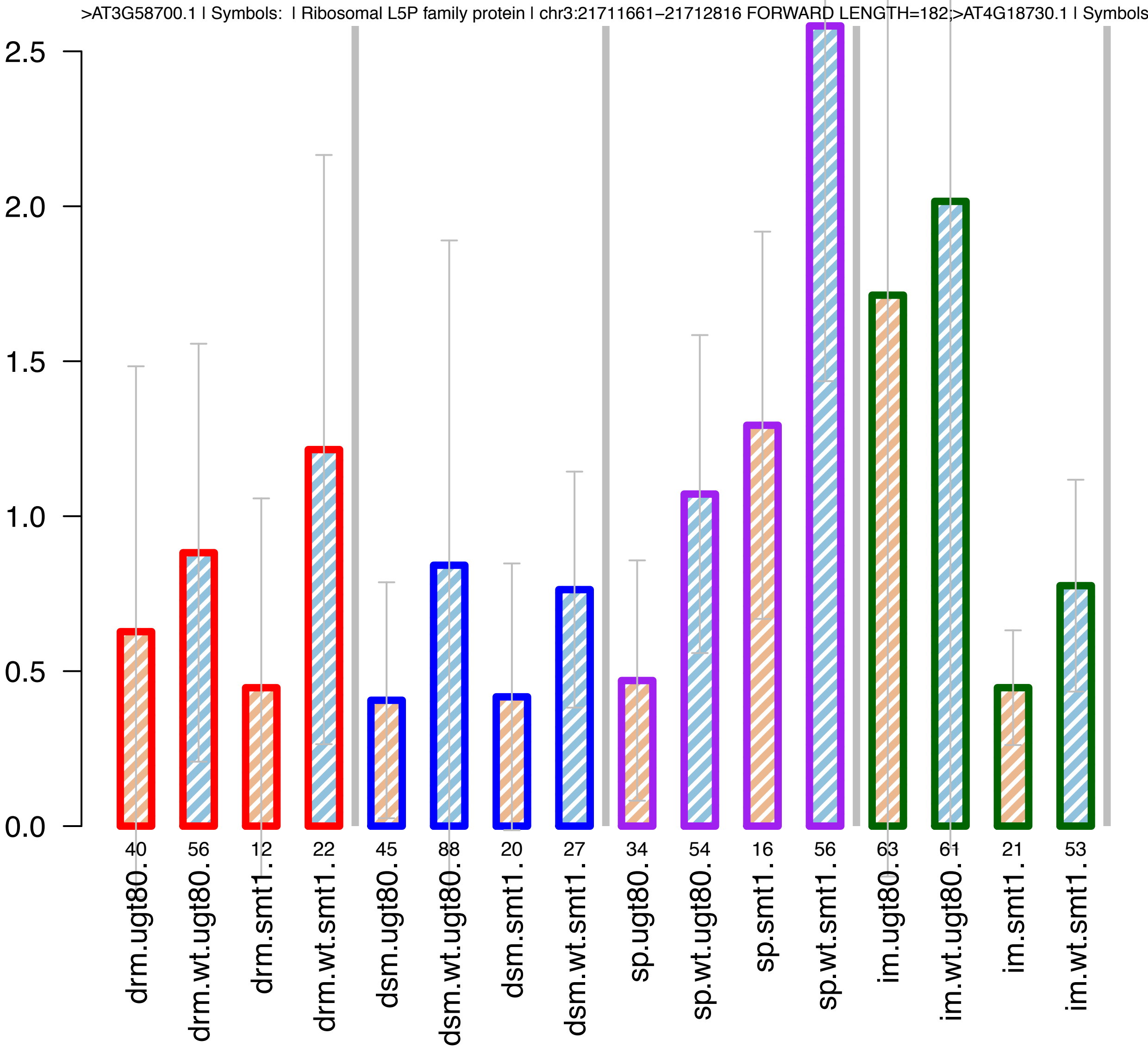
>AT3G58700.1 | Symbols: | Ribosomal L5P family protein | chr3:21711661–21712816 FORWARD LENGTH=182;>AT4G18730.1 | Symbols
2.5
2.0
1.5
1.0
0.5
0.0
40
56
12
22
45
88
20
27
34
54
16
56
63
61
21
53
drm.ugt80..
drm.wt.ugt80..
drm.smt1..
drm.wt.smt1..
dsm.ugt80..
dsm.wt.ugt80..
dsm.smt1..
dsm.wt.smt1..
sp.ugt80..
sp.wt.ugt80..
sp.smt1..
sp.wt.smt1..
im.ugt80..
im.wt.ugt80..
im.smt1..
im.wt.smt1..

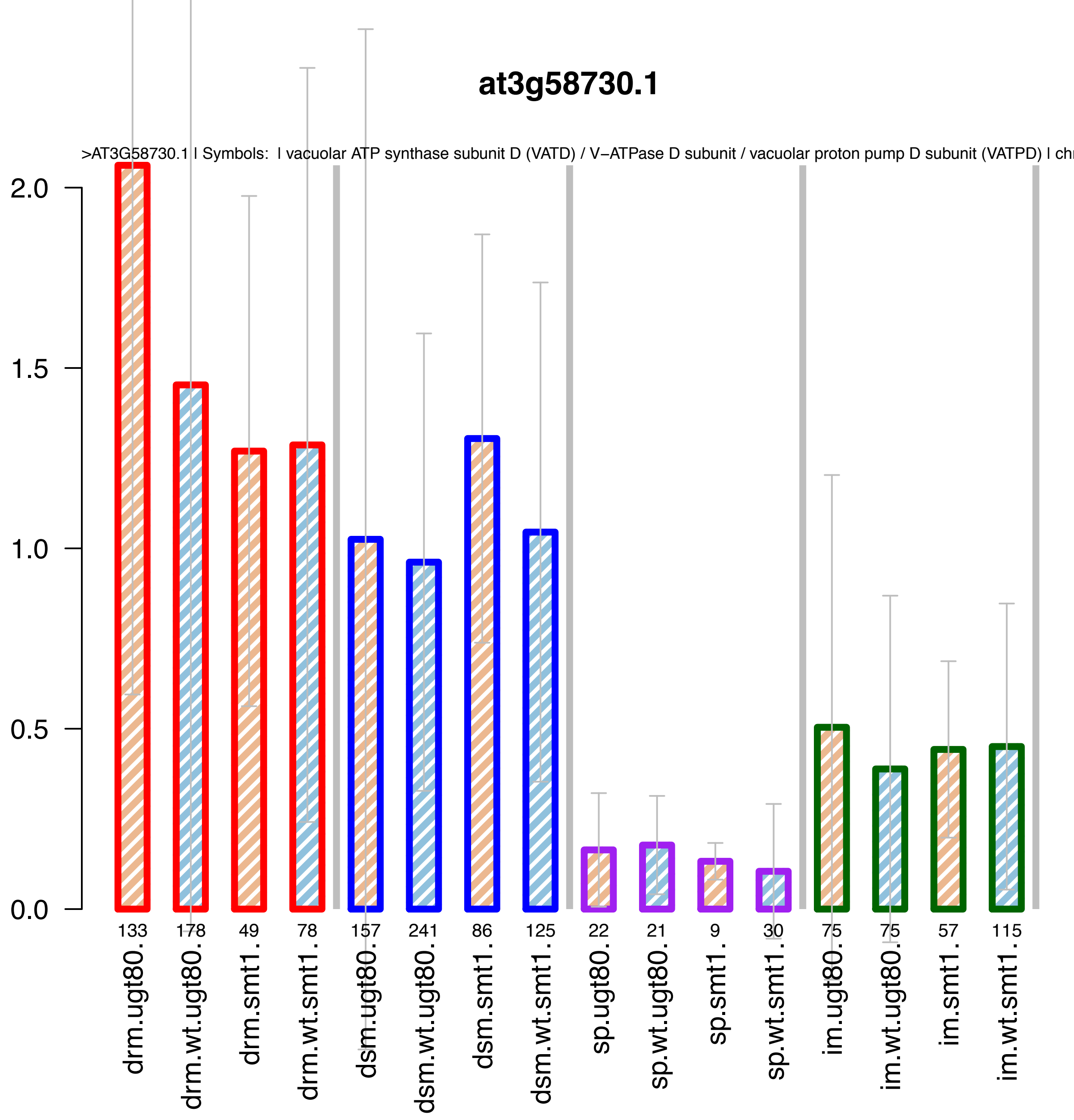

at3g58730.1
>AT3G58730.1 | Symbols: | vacuolar ATP synthase subunit D (VATD) / V−ATPase D subunit / vacuolar proton pump D subunit (VATPD) | ch
2.0
1.5
1.0
0.5
0.0
133
178
49
78
157
241
86
125
22
21
9
30
75
75
57
115
drm.ugt80..
drm.wt.ugt80..
drm.smt1..
drm.wt.smt1..
dsm.ugt80..
dsm.wt.ugt80..
dsm.smt1..
dsm.wt.smt1..
sp.ugt80..
sp.wt.ugt80..
sp.smt1..
sp.wt.smt1..
im.ugt80..
im.wt.ugt80..
im.smt1..
im.wt.smt1..

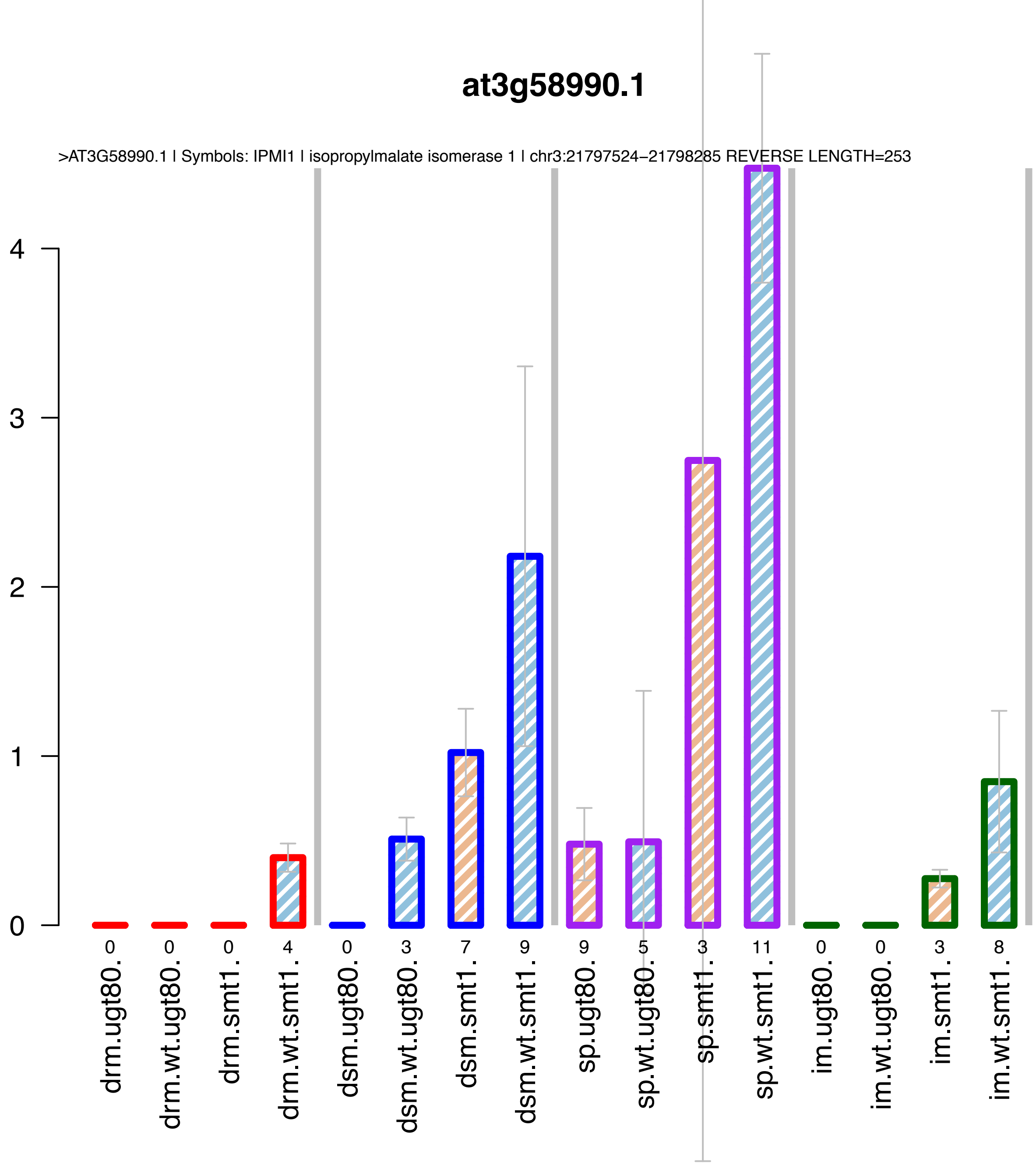

at3g58990.1
>AT3G58990.1 | Symbols: IPMI1 | isopropylmalate isomerase 1 | chr3:21797524−21798285 REVERSE LENGTH=253
0
1
2
3
4
0 0 0 4 0 3 7 9 9 5 3 11 0 0 3 8
drm.ugt80.
drm.wt.ugt80.
drm.smt1.
drm.wt.smt1.
dsm.ugt80.
dsm.wt.ugt80.
dsm.smt1.
dsm.wt.smt1.
sp.ugt80.
sp.wt.ugt80.
sp.smt1.
sp.wt.smt1.
im.ugt80.
im.wt.ugt80.
im.smt1.
im.wt.smt1.

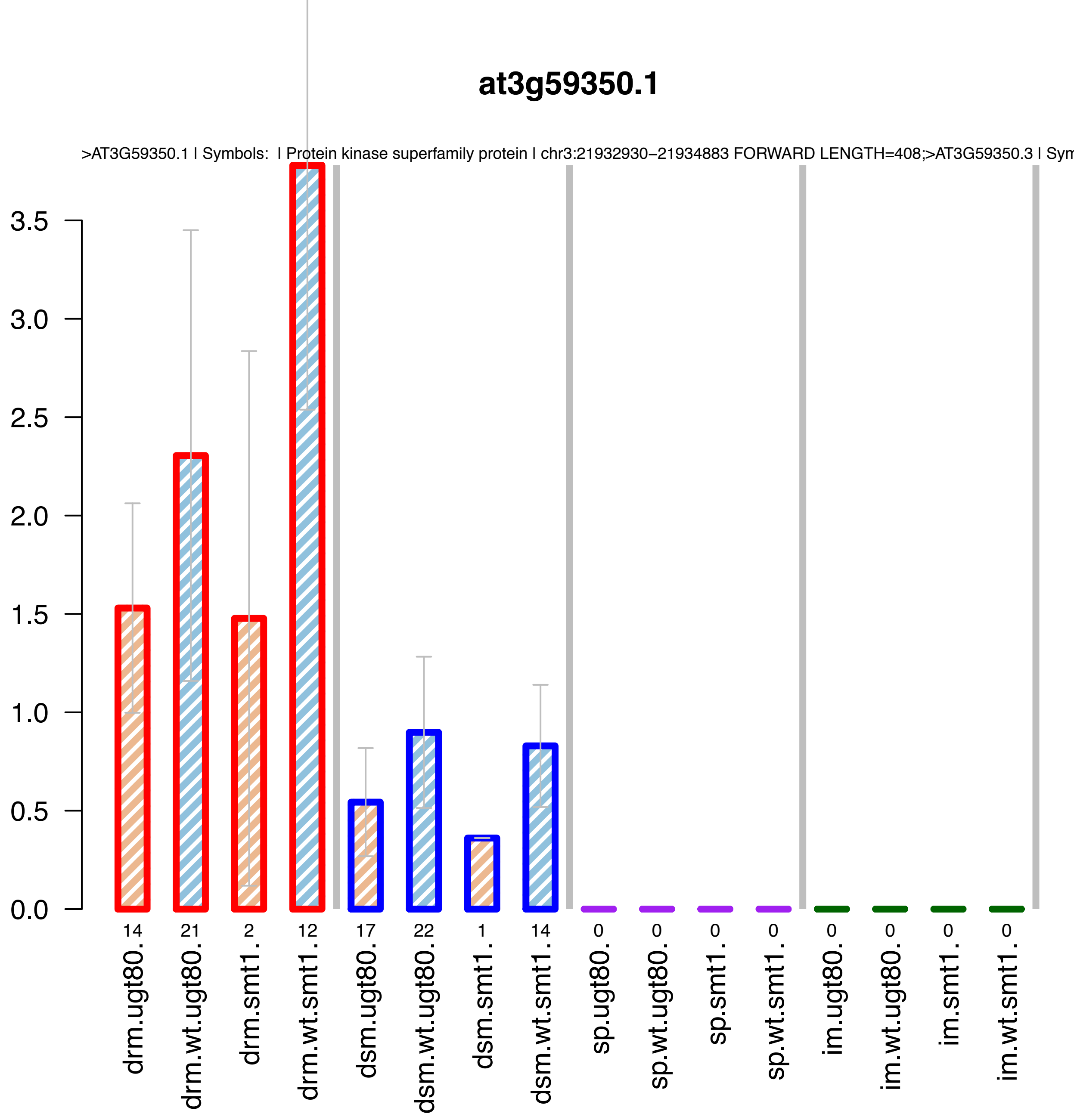
at3g59350.1
>AT3G59350.1 | Symbols: | Protein kinase superfamily protein | chr3:21932930-21934883 FORWARD LENGTH=408;>AT3G59350.3 | Sym
3.5
3.0
2.5
2.0
1.5
1.0
0.5
0.0
14
21
2
12
17
22
1
14
0
0
0
0
0
0
0
0
drm.ugt80..
drm.wt.ugt80..
drm.smt1..
drm.wt.smt1..
dsm.ugt80..
dsm.wt.ugt80..
dsm.smt1..
dsm.wt.smt1..
sp.ugt80..
sp.wt.ugt80..
sp.smt1..
sp.wt.smt1..
im.ugt80..
im.wt.ugt80..
im.smt1..
im.wt.smt1..

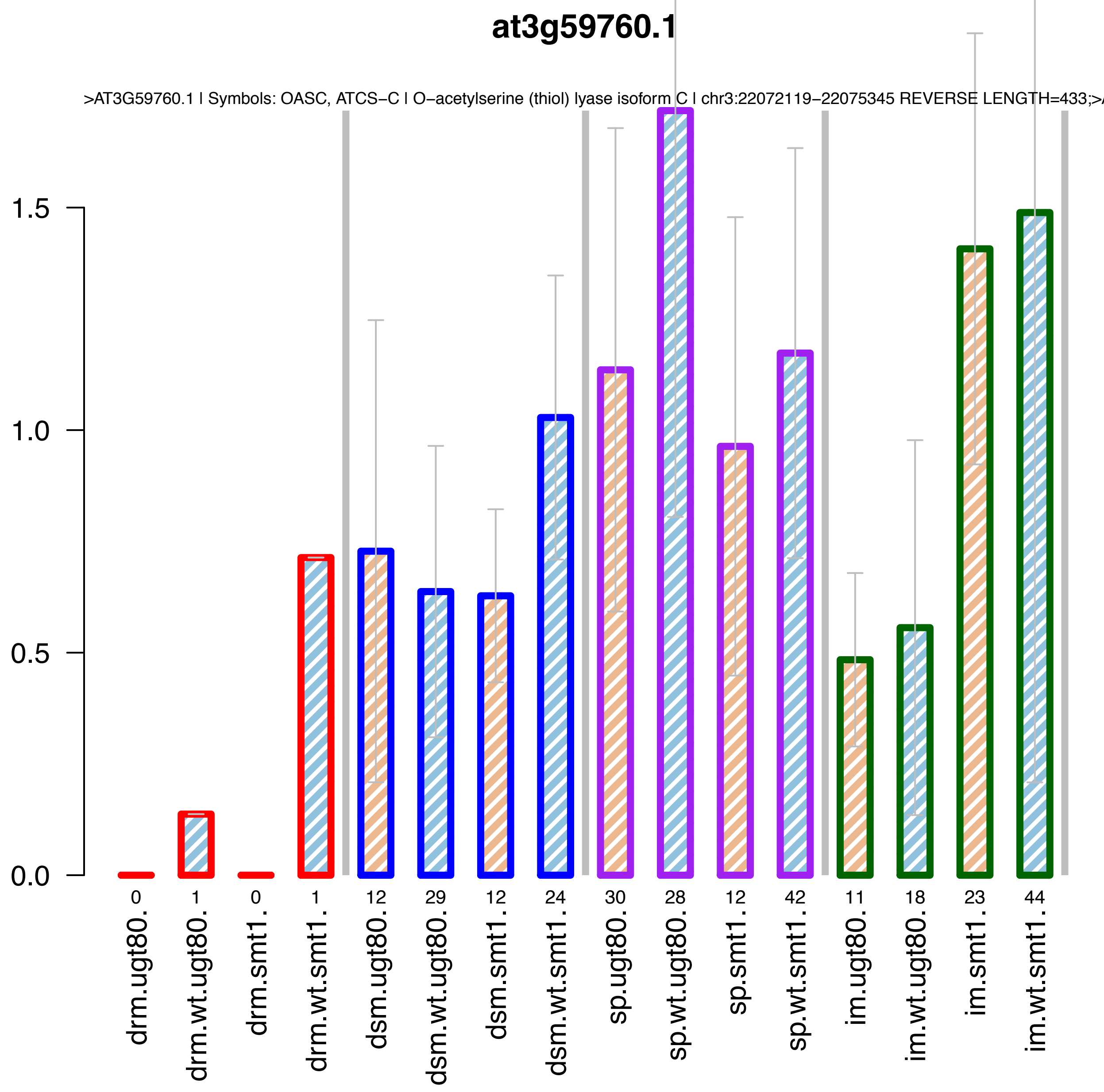

at3g59760.1
>AT3G59760.1 | Symbols: OASC, ATCS−C | O−acetylserine (thiol) lyase isoform C | chr3:22072119−22075345 REVERSE LENGTH=433;>
1.5
1.0
0.5
0.0
0
1
0
1
12
29
12
24
30
28
12
42
11
18
23
44
drm.ugt80.
drm.wt.ugt80.
drm.smt1.
drm.wt.smt1.
dsm.ugt80.
dsm.wt.ugt80.
dsm.smt1.
dsm.wt.smt1.
sp.ugt80.
sp.wt.ugt80.
sp.smt1.
sp.wt.smt1.
im.ugt80.
im.wt.ugt80.
im.smt1.
im.wt.smt1.

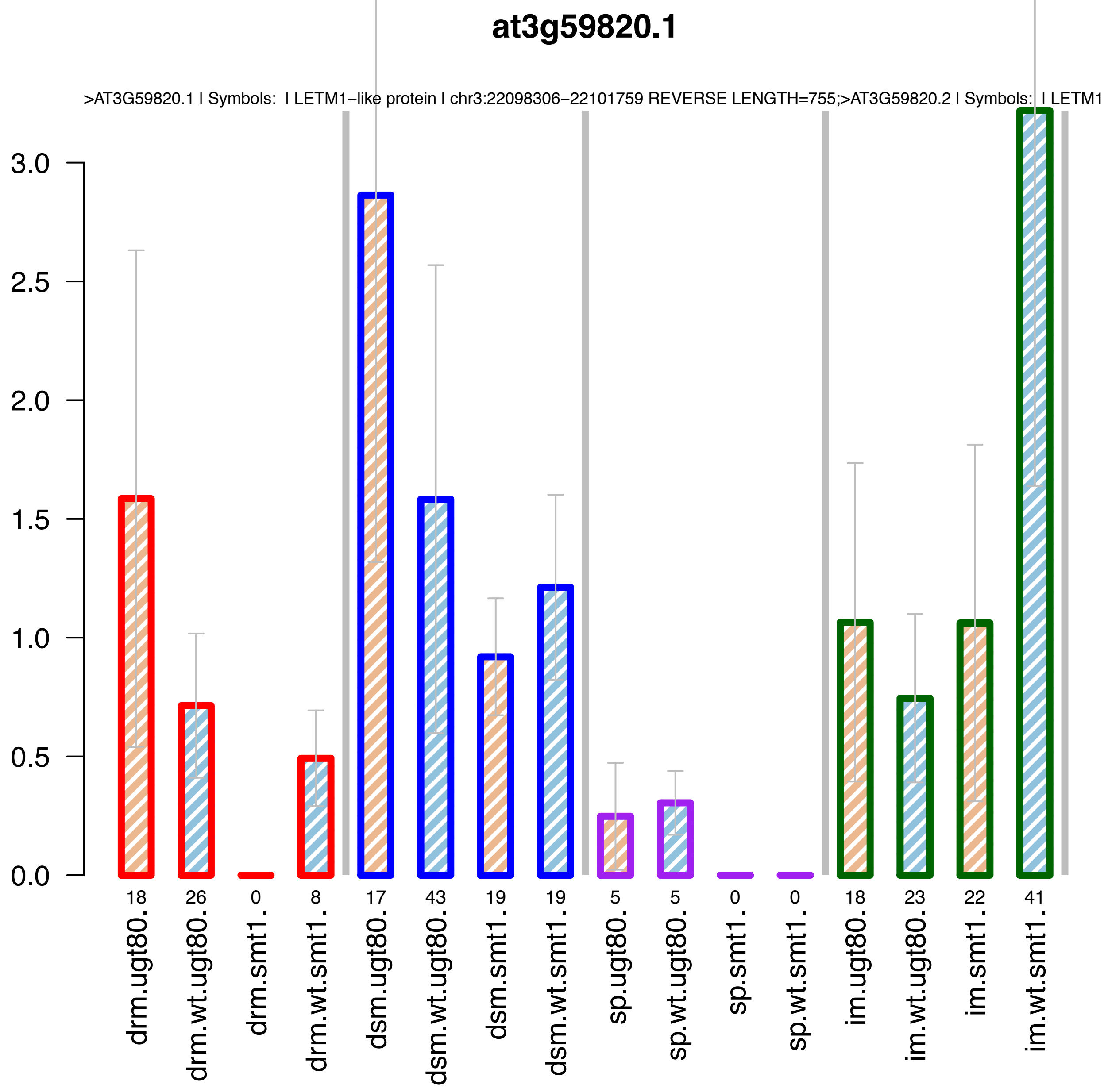

at3g59820.1
>AT3G59820.1 | Symbols: | LETM1−like protein | chr3:22098306−22101759 REVERSE LENGTH=755;>AT3G59820.2 | Symbols: | LETM1
3.0
2.5
2.0
1.5
1.0
0.5
0.0
18
26
0
8
17
43
19
19
5
5
0
0
18
23
22
41
drm.ugt80..
drm.wt.ugt80..
drm.smt1..
drm.wt.smt1..
dsm.ugt80..
dsm.wt.ugt80..
dsm.smt1..
dsm.wt.smt1..
sp.ugt80..
sp.wt.ugt80..
sp.smt1..
sp.wt.smt1..
im.ugt80..
im.wt.ugt80..
im.smt1..
im.wt.smt1..

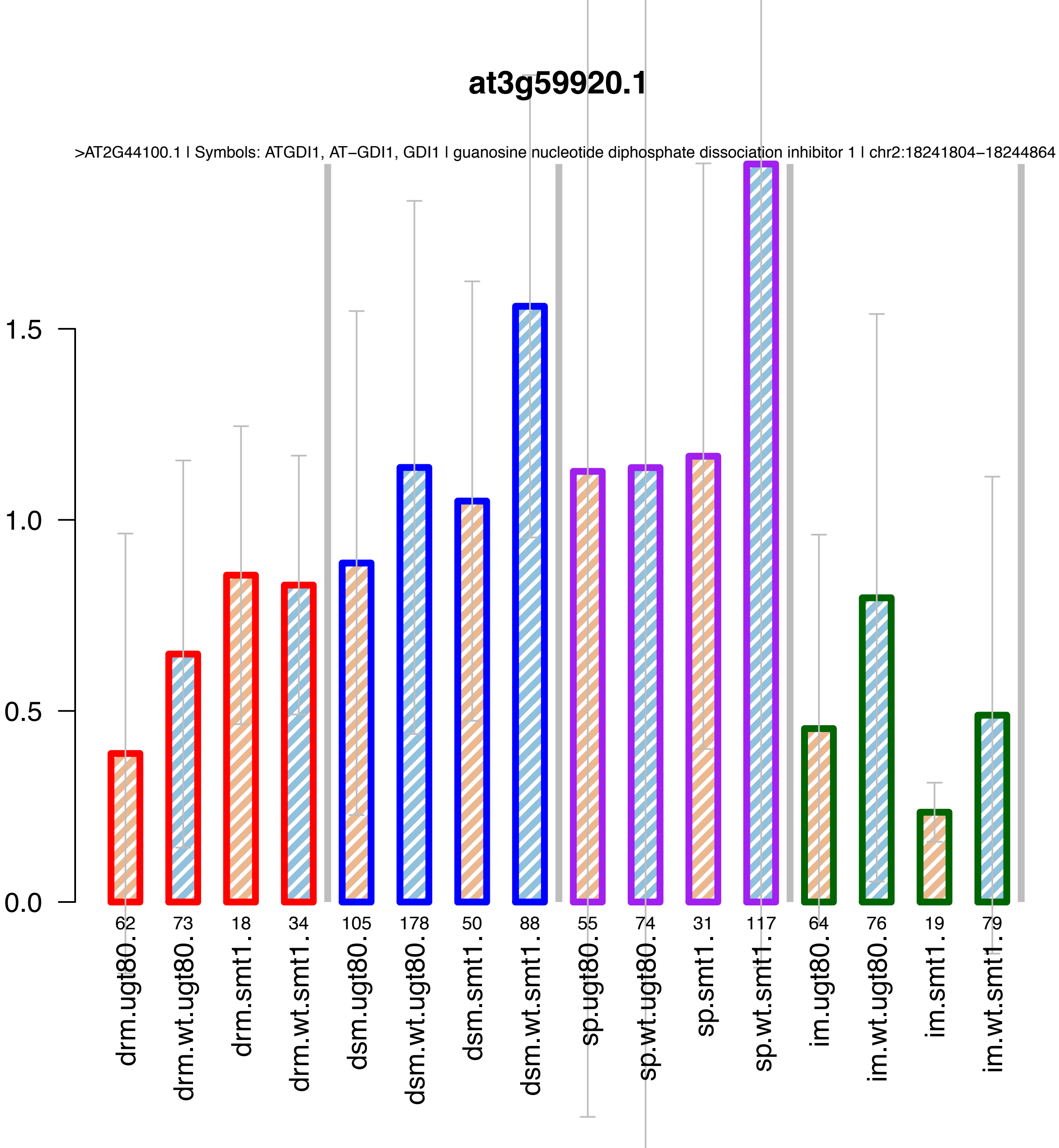

at3g59920.1
>AT2G44100.1 | Symbols: ATGDI1, AT−GDI1, GDI1 | guanosine nucleotide diphosphate dissociation inhibitor 1 | chr2:18241804−18244864
0.0
0.5
1.0
1.5
62
73
18
34
105
178
50
88
55
74
31
117
64
76
19
79
drm.ugt80..
drm.wt.ugt80..
drm.smt1..
drm.wt.smt1..
dsm.ugt80..
dsm.wt.ugt80..
dsm.smt1..
dsm.wt.smt1..
sp.ugt80..
sp.wt.ugt80..
sp.smt1..
sp.wt.smt1..
im.ugt80..
im.wt.ugt80..
im.smt1..
im.wt.smt1..

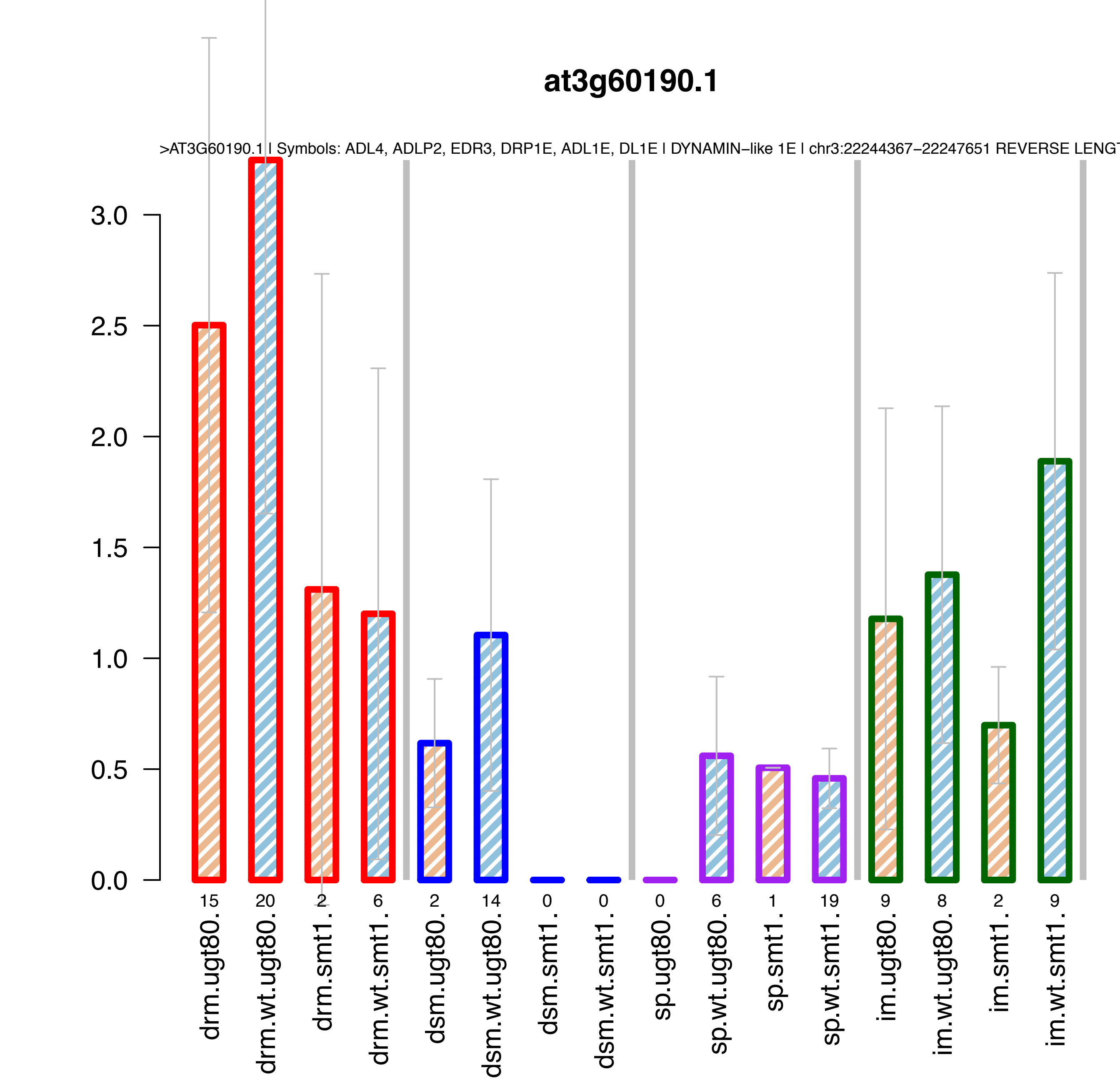

at3g60190.1
>AT3G60190.1 | Symbols: ADL4, ADLP2, EDR3, DRP1E, ADL1E, DL1E | DYNAMIN−like 1E | chr3:22244367−22247651 REVERSE LENG
0.0
0.5
1.0
1.5
2.0
2.5
3.0
15
20
2
6
2
14
0
0
0
6
1
19
9
8
2
9
drm.ugt80..
drm.wt.ugt80..
drm.smt1..
drm.wt.smt1..
dsm.ugt80..
dsm.wt.ugt80..
dsm.smt1..
dsm.wt.smt1..
sp.ugt80..
sp.wt.ugt80..
sp.smt1..
sp.wt.smt1..
im.ugt80..
im.wt.ugt80..
im.smt1..
im.wt.smt1..

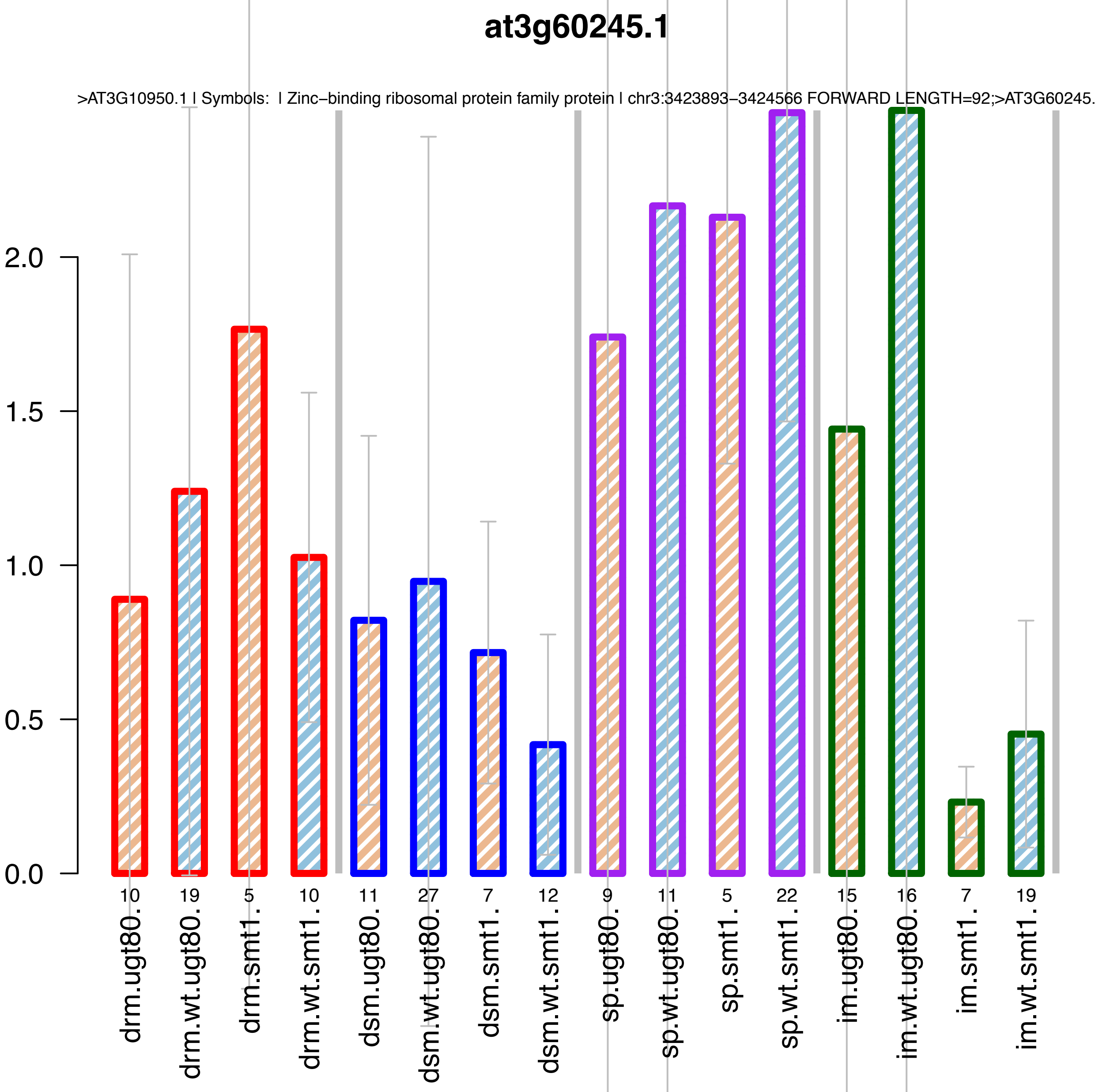
at3g60245.1
>AT3G10950.1 | Symbols: | Zinc–binding ribosomal protein family protein | chr3:3423893–3424566 FORWARD LENGTH=92;>AT3G60245.
0.0
0.5
1.0
1.5
2.0
10
19
5
10
11
27
7
12
9
11
5
22
15
16
7
19
drm.ugt80..
drm.wt.ugt80..
drm.smt1..
drm.wt.smt1..
dsm.ugt80..
dsm.wt.ugt80..
dsm.smt1..
dsm.wt.smt1..
sp.ugt80..
sp.wt.ugt80..
sp.smt1..
sp.wt.smt1..
im.ugt80..
im.wt.ugt80..
im.smt1..
im.wt.smt1..

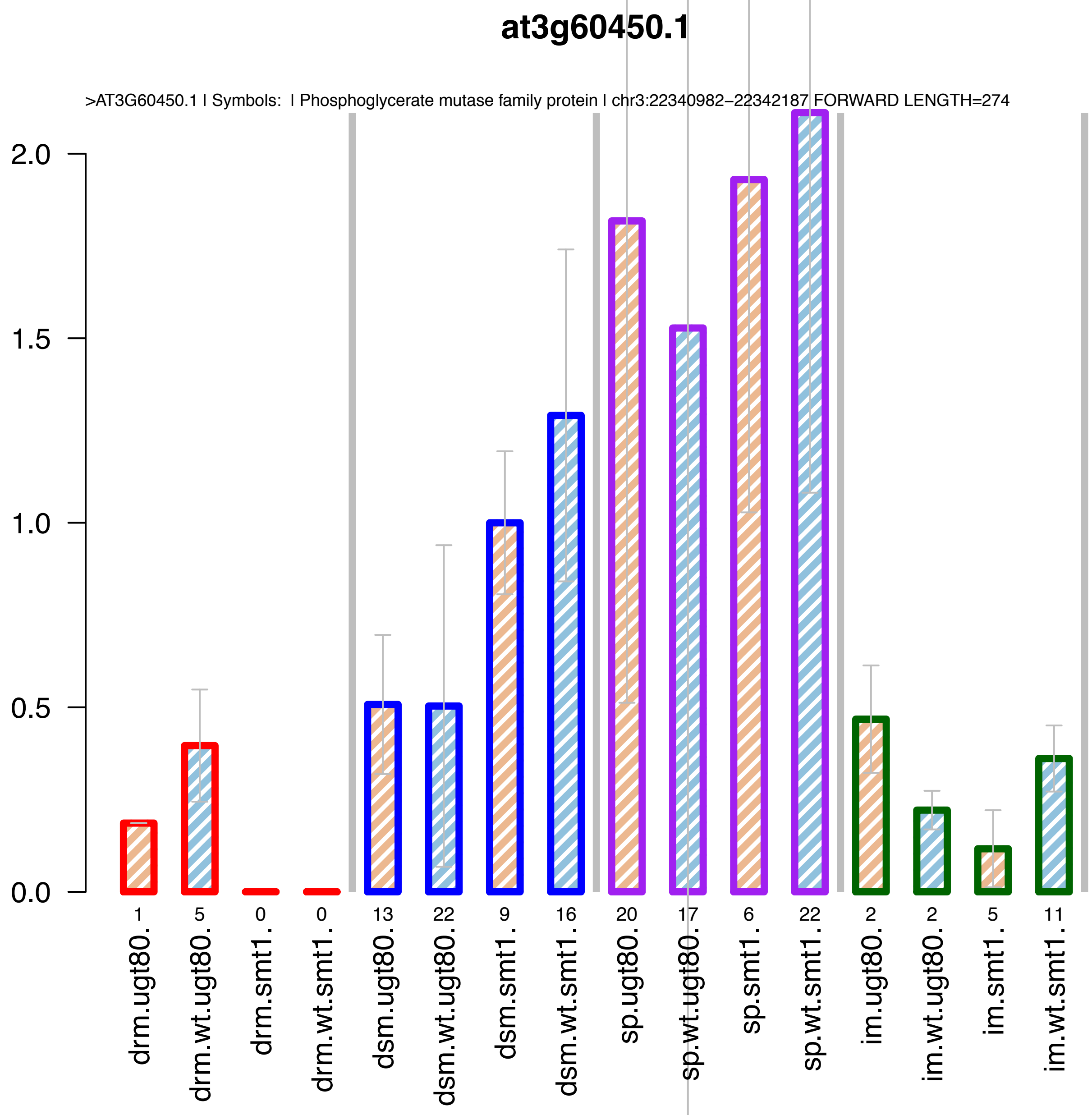

at3g60450.1
>AT3G60450.1 | Symbols: | Phosphoglycerate mutase family protein | chr3:22340982−22342187 FORWARD LENGTH=274
2.0
1.5
1.0
0.5
0.0
1
5
0
0
13
22
9
16
20
17
6
22
2
2
5
11
drm.ugt80.
drm.wt.ugt80.
drm.smt1.
drm.wt.smt1.
dsm.ugt80.
dsm.wt.ugt80.
dsm.smt1.
dsm.wt.smt1.
sp.ugt80.
sp.wt.ugt80.
sp.smt1.
sp.wt.smt1.
im.ugt80.
im.wt.ugt80.
im.smt1.
im.wt.smt1.

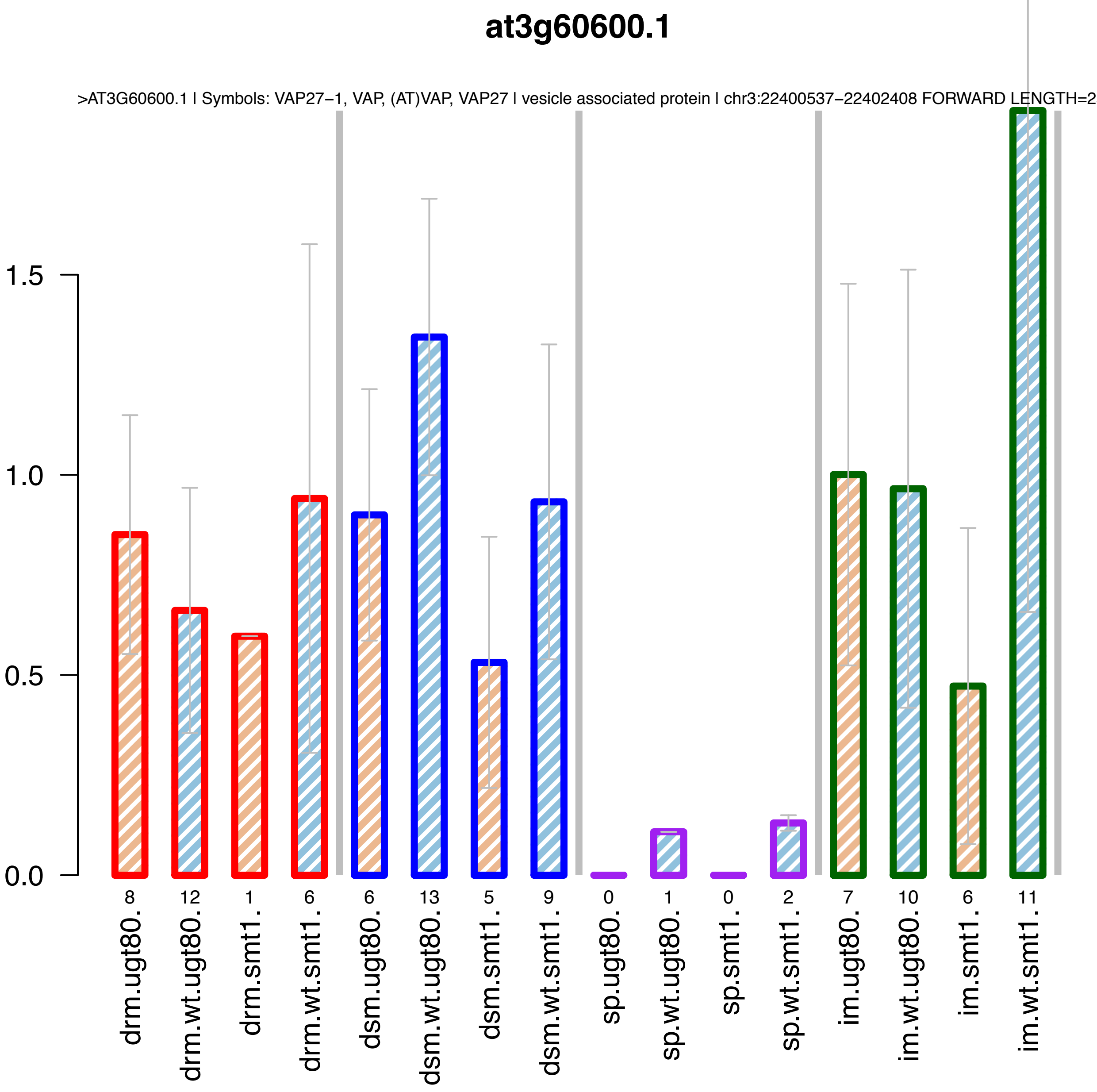

at3g60600.1
>AT3G60600.1 | Symbols: VAP27−1, VAP, (AT)VAP, VAP27 | vesicle associated protein | chr3:22400537−22402408 FORWARD LENGTH=2
1.5
1.0
0.5
0.0
8
12
1
6
6
13
5
9
0
1
0
2
7
10
6
11
drm.ugt80.
drm.wt.ugt80.
drm.smt1.
drm.wt.smt1.
dsm.ugt80.
dsm.wt.ugt80.
dsm.smt1.
dsm.wt.smt1.
sp.ugt80.
sp.wt.ugt80.
sp.smt1.
sp.wt.smt1.
im.ugt80.
im.wt.ugt80.
im.smt1.
im.wt.smt1.

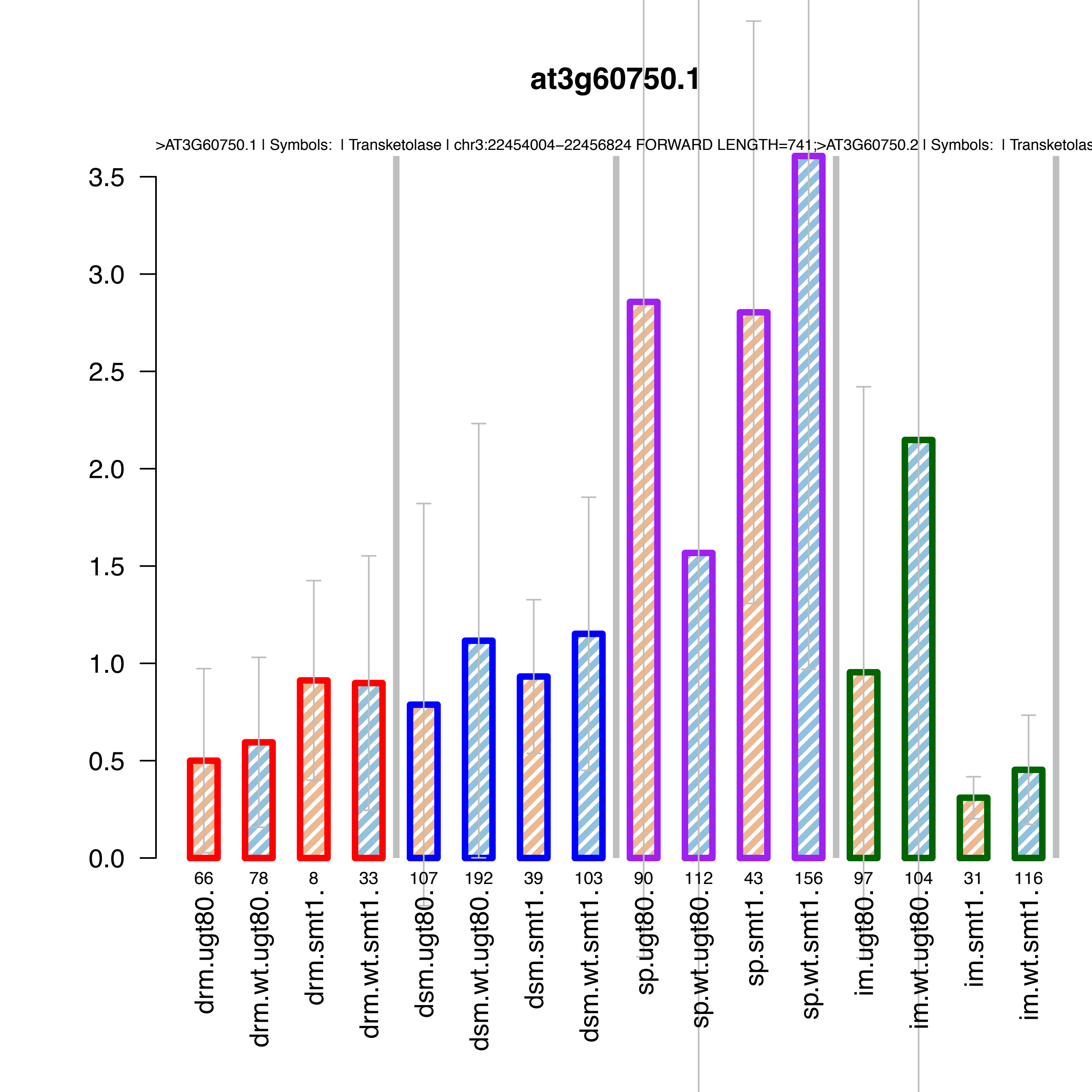

at3g60750.1
>AT3G60750.1 | Symbols: | Transketolase | chr3:22454004-22456824 FORWARD LENGTH=741;>AT3G60750.2 | Symbols: | Transketolas
3.5
3.0
2.5
2.0
1.5
1.0
0.5
0.0
66
78
8
33
107
192
39
103
90
112
43
156
97
104
31
116
drm.ugt80.
drm.wt.ugt80.
drm.smt1.
drm.wt.smt1.
dsm.ugt80.
dsm.wt.ugt80.
dsm.smt1.
dsm.wt.smt1.
sp.ugt80.
sp.wt.ugt80.
sp.smt1.
sp.wt.smt1.
im.ugt80.
im.wt.ugt80.
im.smt1.
im.wt.smt1.

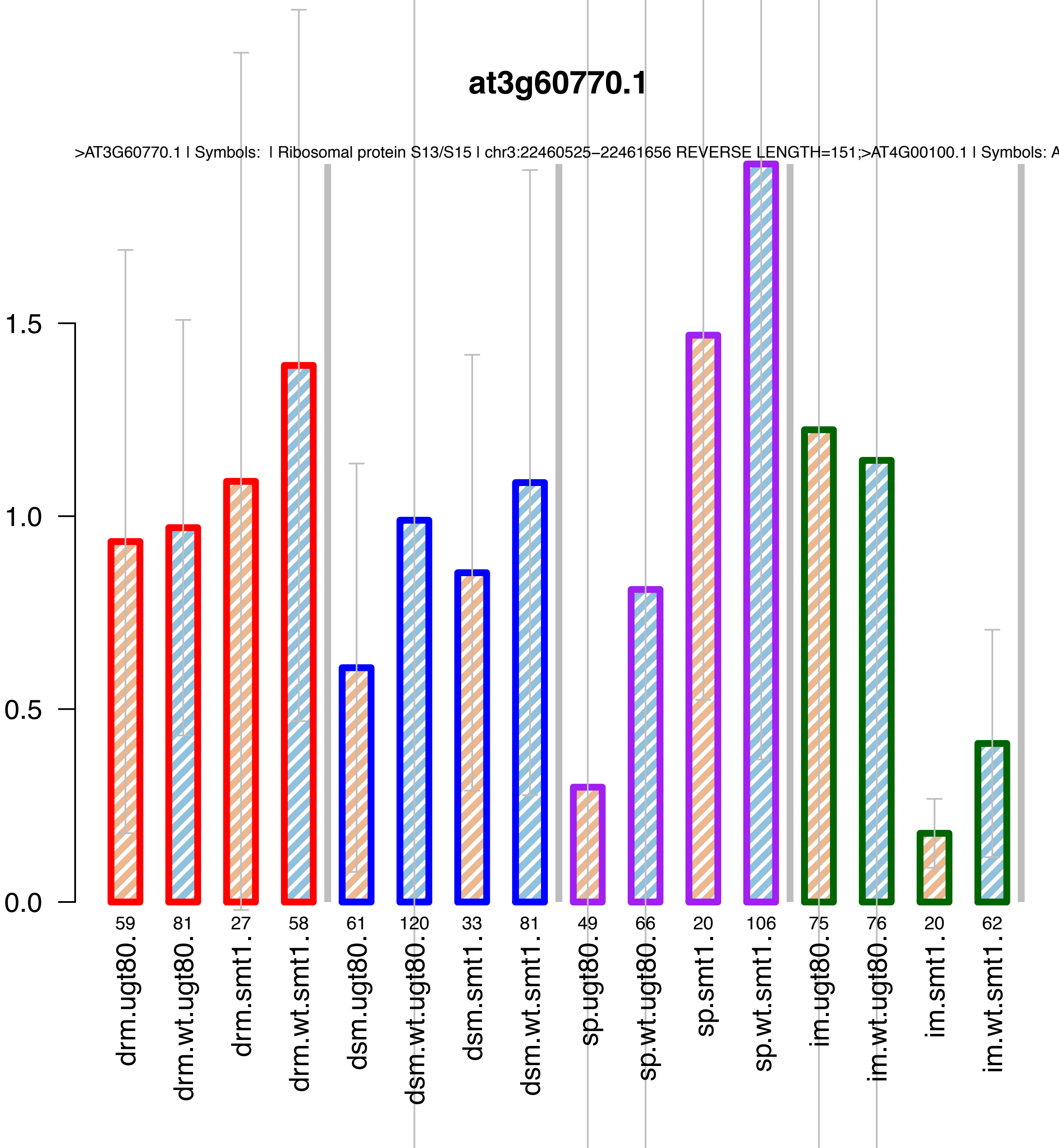

at3g60770.1
>AT3G60770.1 | Symbols: | Ribosomal protein S13/S15 | chr3:22460525-22461656 REVERSE LENGTH=151;>AT4G00100.1 | Symbols: A
1.5
1.0
0.5
0.0
59
81
27
58
61
120
33
81
49
66
20
106
75
76
20
62
drm.ugt80.
drm.wt.ugt80.
drm.smt1.
drm.wt.smt1.
dsm.ugt80.
dsm.wt.ugt80.
dsm.smt1.
dsm.wt.smt1.
sp.ugt80.
sp.wt.ugt80.
sp.smt1.
sp.wt.smt1.
im.ugt80.
im.wt.ugt80.
im.smt1.
im.wt.smt1.

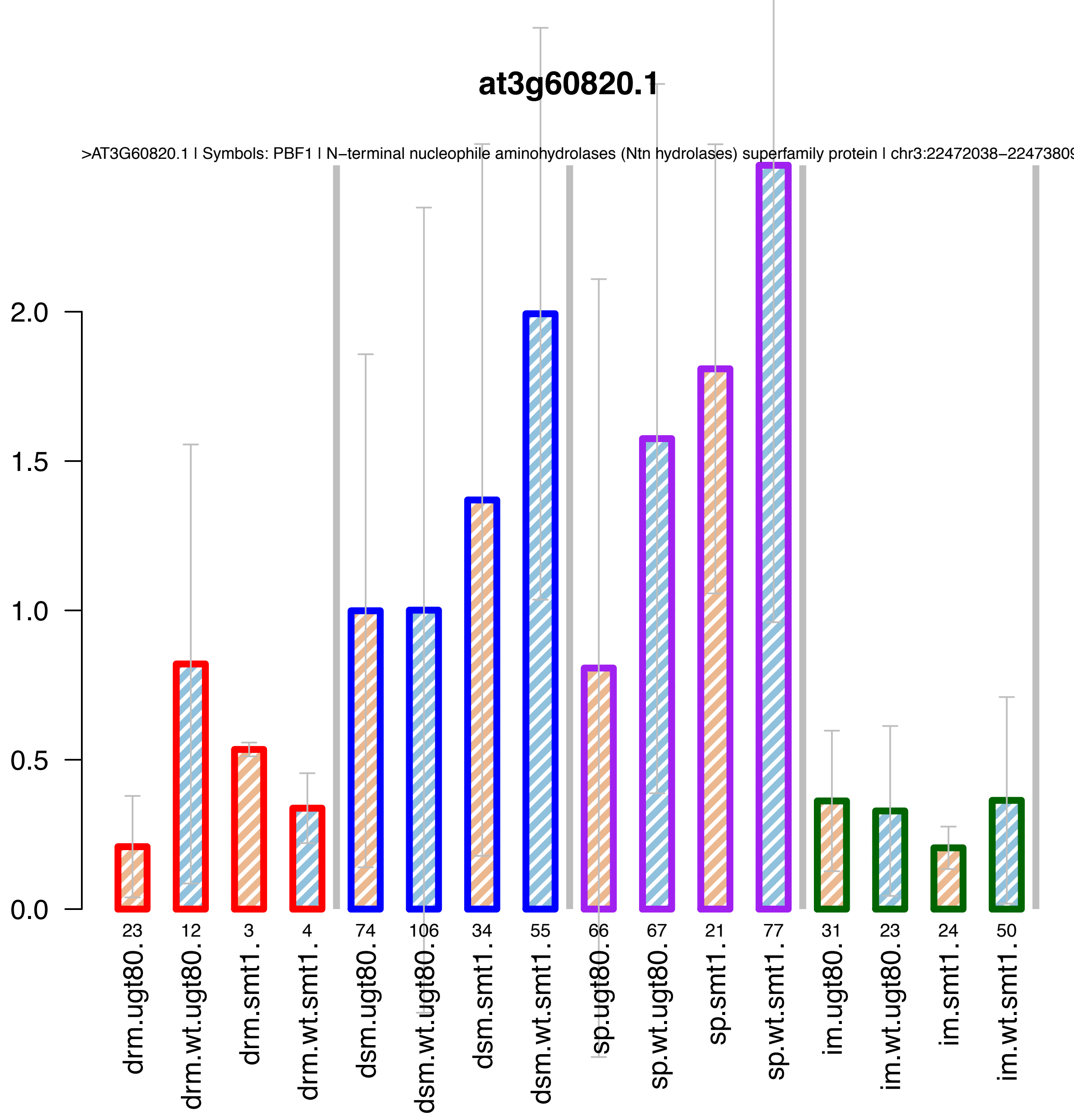

at3g60820.1
>AT3G60820.1 | Symbols: PBF1 | N−terminal nucleophile aminohydrolases (Ntn hydrolases) superfamily protein | chr3:22472038−22473809
0.0
0.5
1.0
1.5
2.0
23
12
3
4
74
106
34
55
66
67
21
77
31
23
24
50
drm.ugt80..
drm.wt.ugt80..
drm.smt1..
drm.wt.smt1..
dsm.ugt80..
dsm.wt.ugt80..
dsm.smt1..
dsm.wt.smt1..
sp.ugt80..
sp.wt.ugt80..
sp.smt1..
sp.wt.smt1..
im.ugt80..
im.wt.ugt80..
im.smt1..
im.wt.smt1..

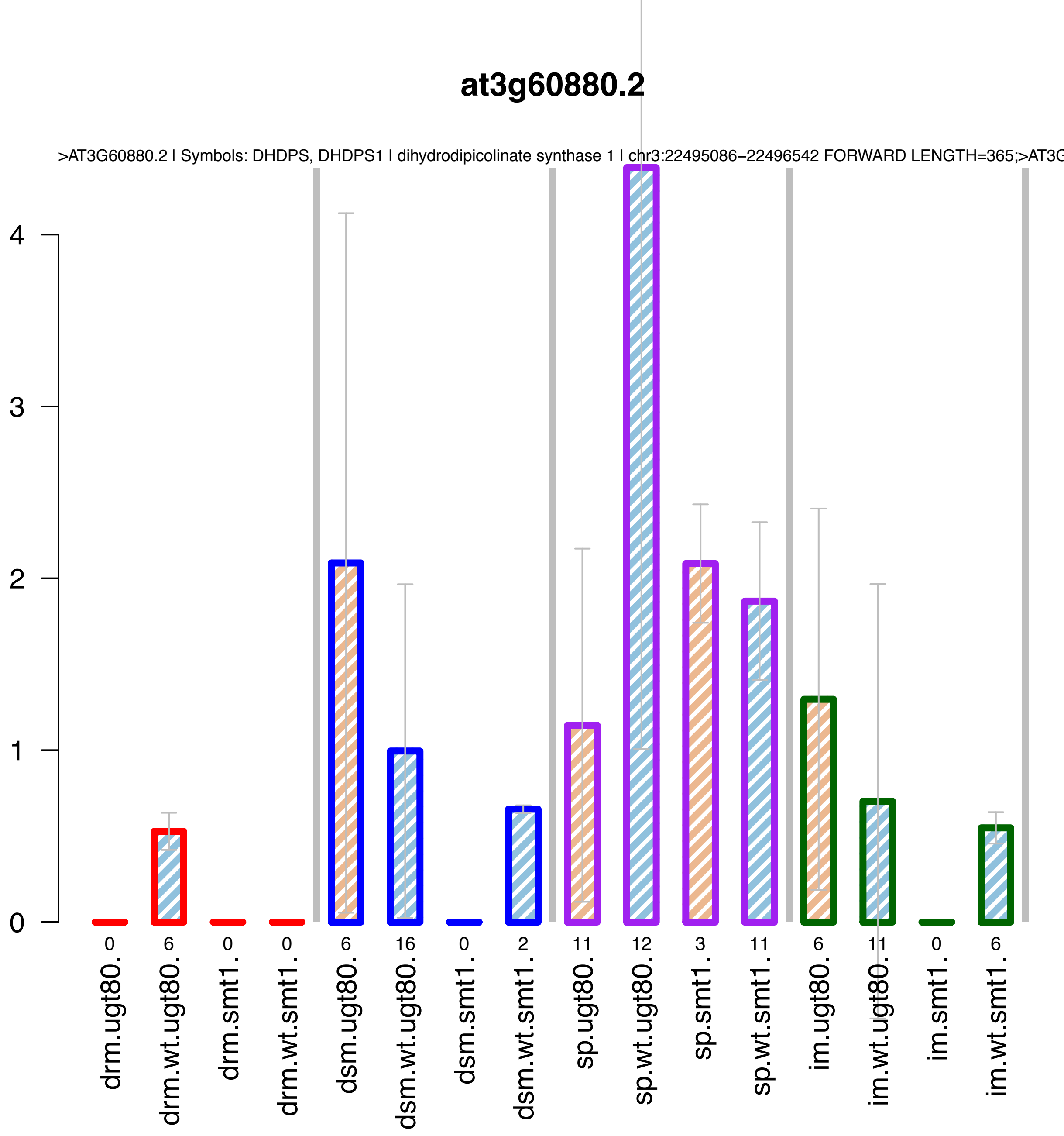

at3g60880.2
>AT3G60880.2 | Symbols: DHDPS, DHDPS1 | dihydrodipicolinate synthase 1 | chr3:22495086–22496542 FORWARD LENGTH=365;>AT3G
0
1
2
3
4
0
6
0
0
6
16
0
2
11
12
3
11
6
11
0
6
drm.ugt80..
drm.wt.ugt80..
drm.smt1..
drm.wt.smt1..
dsm.ugt80..
dsm.wt.ugt80..
dsm.smt1..
dsm.wt.smt1..
sp.ugt80..
sp.wt.ugt80..
sp.smt1..
sp.wt.smt1..
im.ugt80..
im.wt.ugt80..
im.smt1..
im.wt.smt1..

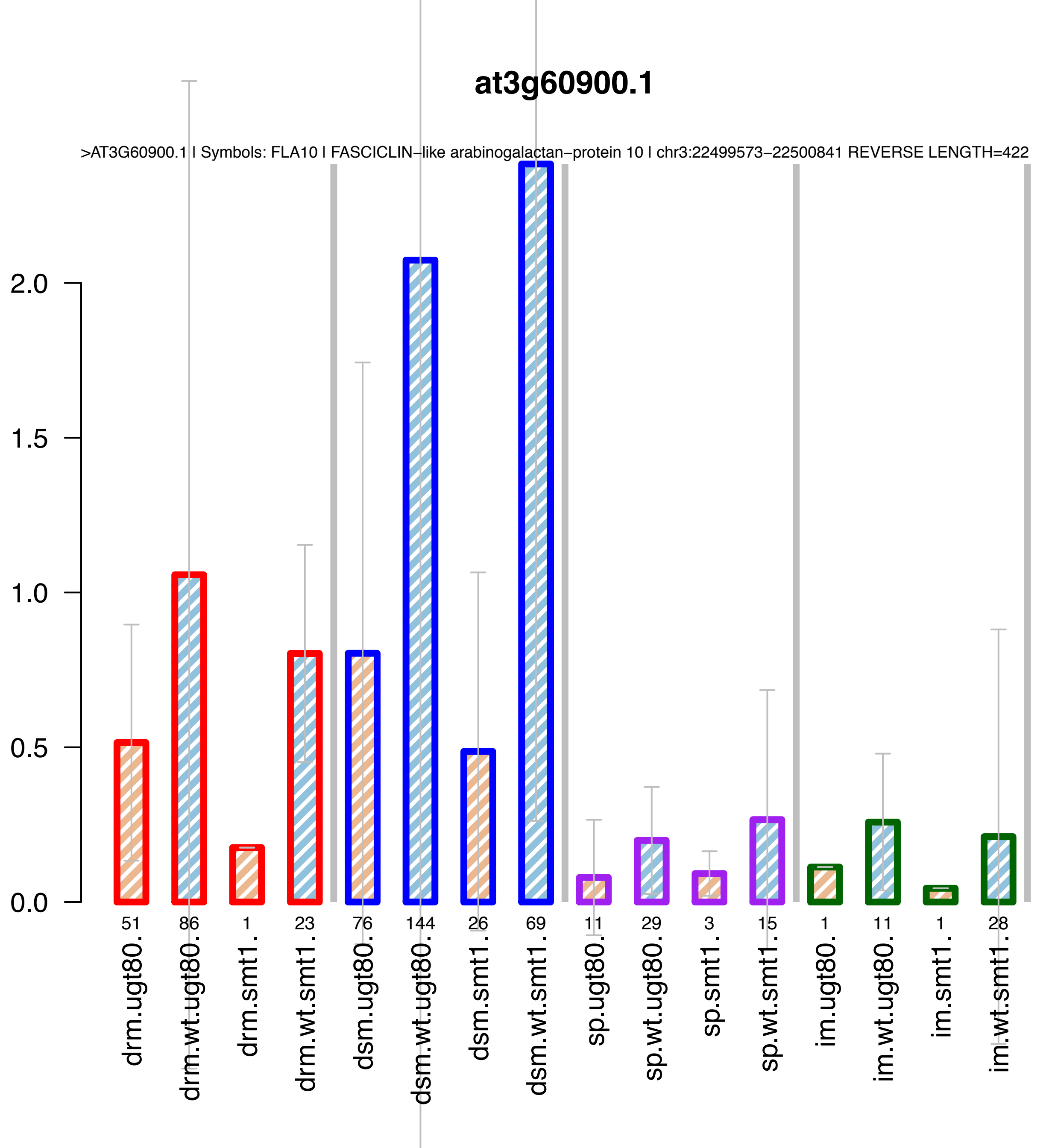

at3g60900.1
>AT3G60900.1 | Symbols: FLA10 | FASCICLIN−like arabinogalactan−protein 10 | chr3:22499573−22500841 REVERSE LENGTH=422
2.0
1.5
1.0
0.5
0.0
51
86
1
23
76
144
26
69
11
29
3
15
1
11
1
28
drm.ugt80..
drm.wt.ugt80..
drm.smt1..
drm.wt.smt1..
dsm.ugt80..
dsm.wt.ugt80..
dsm.smt1..
dsm.wt.smt1..
sp.ugt80..
sp.wt.ugt80..
sp.smt1..
sp.wt.smt1..
im.ugt80..
im.wt.ugt80..
im.smt1..
im.wt.smt1..

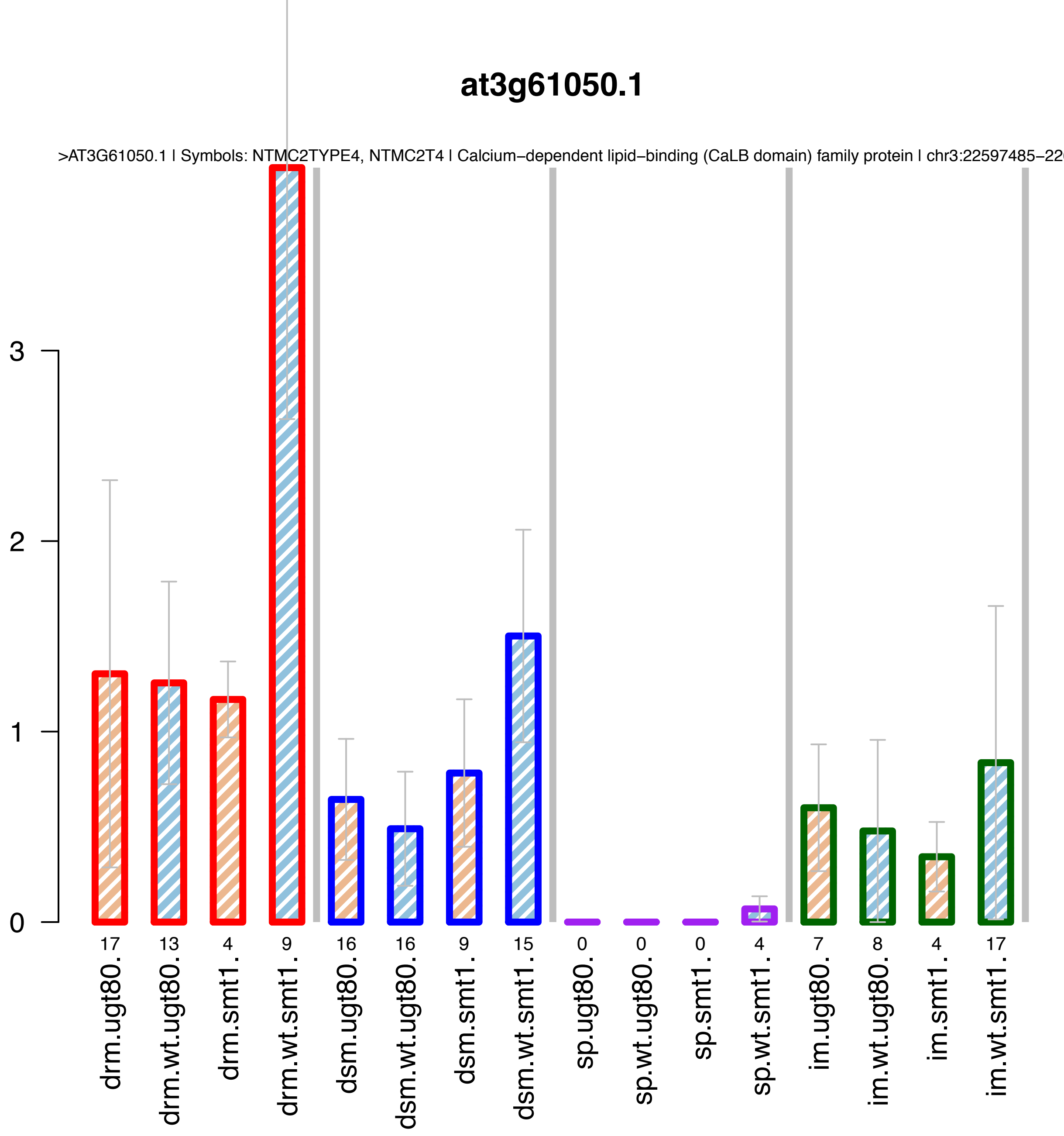

at3g61050.1
>AT3G61050.1 | Symbols: NTMC2TYPE4, NTMC2T4 | Calcium−dependent lipid−binding (CaLB domain) family protein | chr3:22597485−22
3
2
1
0
17
13
4
9
16
16
9
15
0
0
0
4
7
8
4
17
drm.ugt80.
drm.wt.ugt80.
drm.smt1.
drm.wt.smt1.
dsm.ugt80.
dsm.wt.ugt80.
dsm.smt1.
dsm.wt.smt1.
sp.ugt80.
sp.wt.ugt80.
sp.smt1.
sp.wt.smt1.
im.ugt80.
im.wt.ugt80.
im.smt1.
im.wt.smt1.

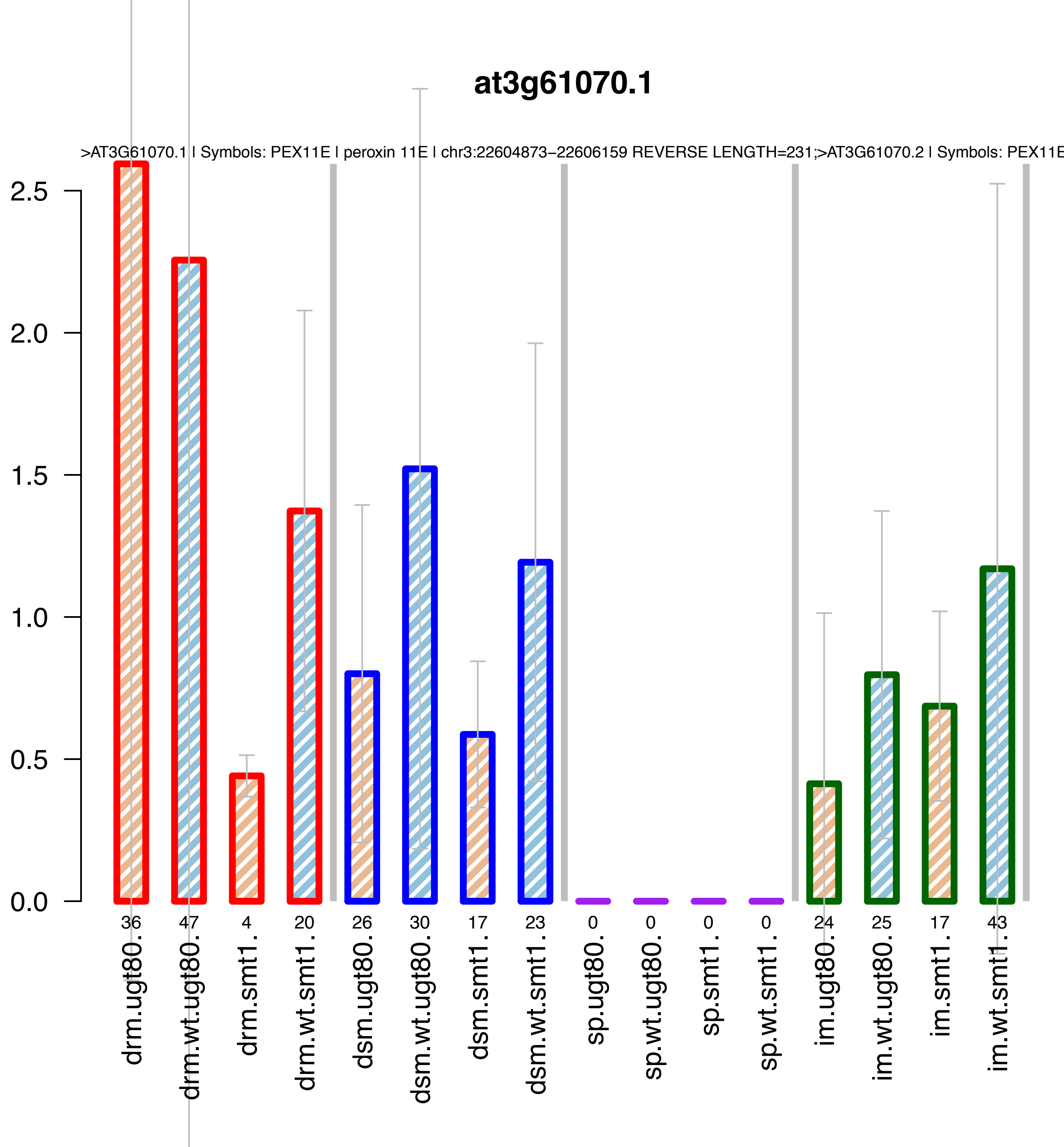

at3g61070.1
>AT3G61070.1 | Symbols: PEX11E | peroxin 11E | chr3:22604873−22606159 REVERSE LENGTH=231;>AT3G61070.2 | Symbols: PEX11E
0.0
0.5
1.0
1.5
2.0
2.5
36
47
4
20
26
30
17
23
0
0
0
0
24
25
17
43
drm.ugt80.
drm.wt.ugt80.
drm.smt1.
drm.wt.smt1.
dsm.ugt80.
dsm.wt.ugt80.
dsm.smt1.
dsm.wt.smt1.
sp.ugt80.
sp.wt.ugt80.
sp.smt1.
sp.wt.smt1.
im.ugt80.
im.wt.ugt80.
im.smt1.
im.wt.smt1.

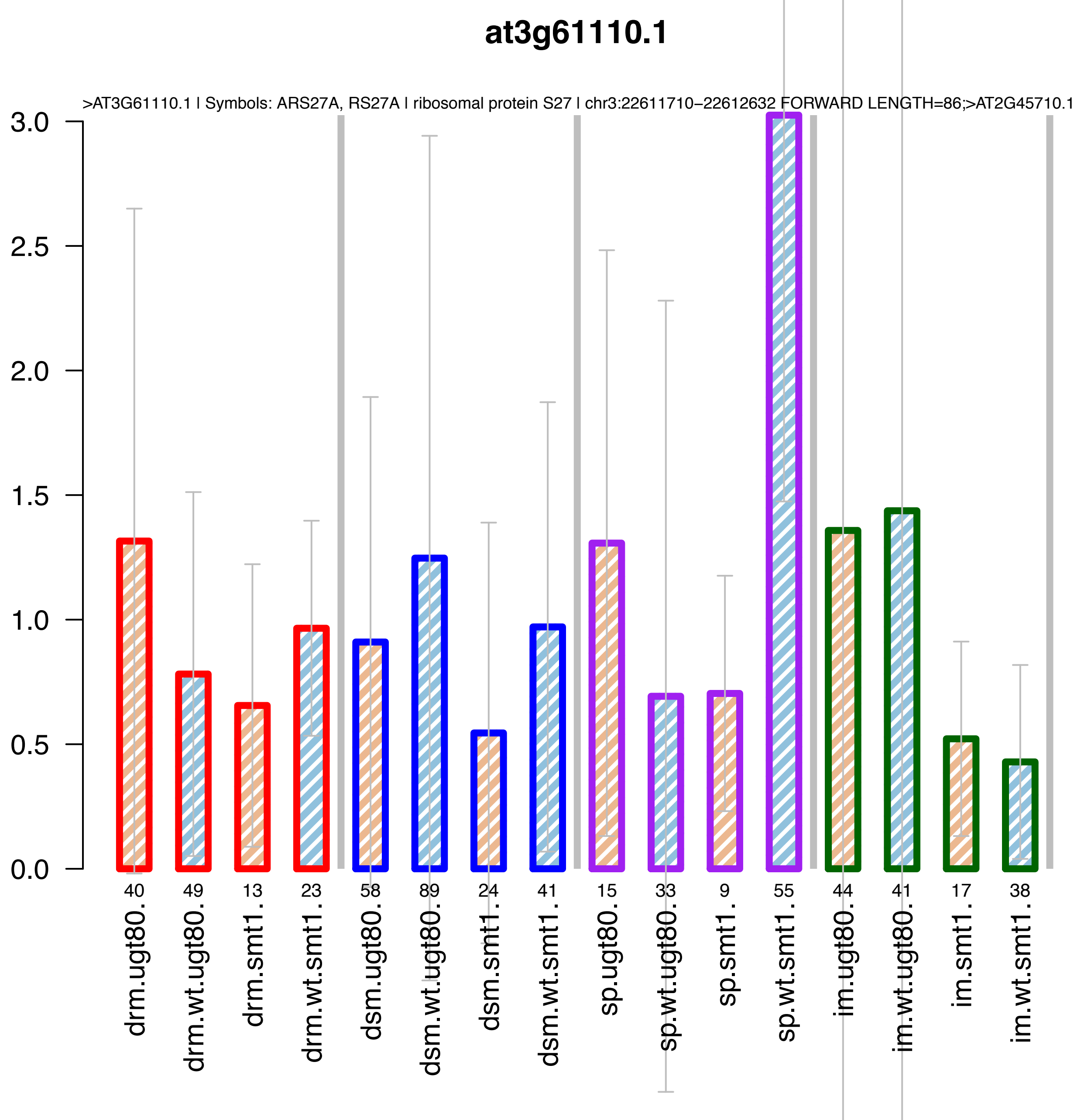

at3g61110.1
>AT3G61110.1 | Symbols: ARS27A, RS27A | ribosomal protein S27 | chr3:22611710-22612632 FORWARD LENGTH=86;>AT2G45710.1 |
3.0
2.5
2.0
1.5
1.0
0.5
0.0
40
49
13
23
58
89
24
41
15
33
9
55
44
41
17
38
drm.ugt80..
drm.wt.ugt80..
drm.smt1..
drm.wt.smt1..
dsm.ugt80..
dsm.wt.ugt80..
dsm.smt1..
dsm.wt.smt1..
sp.ugt80..
sp.wt.ugt80..
sp.smt1..
sp.wt.smt1..
im.ugt80..
im.wt.ugt80..
im.smt1..
im.wt.smt1..

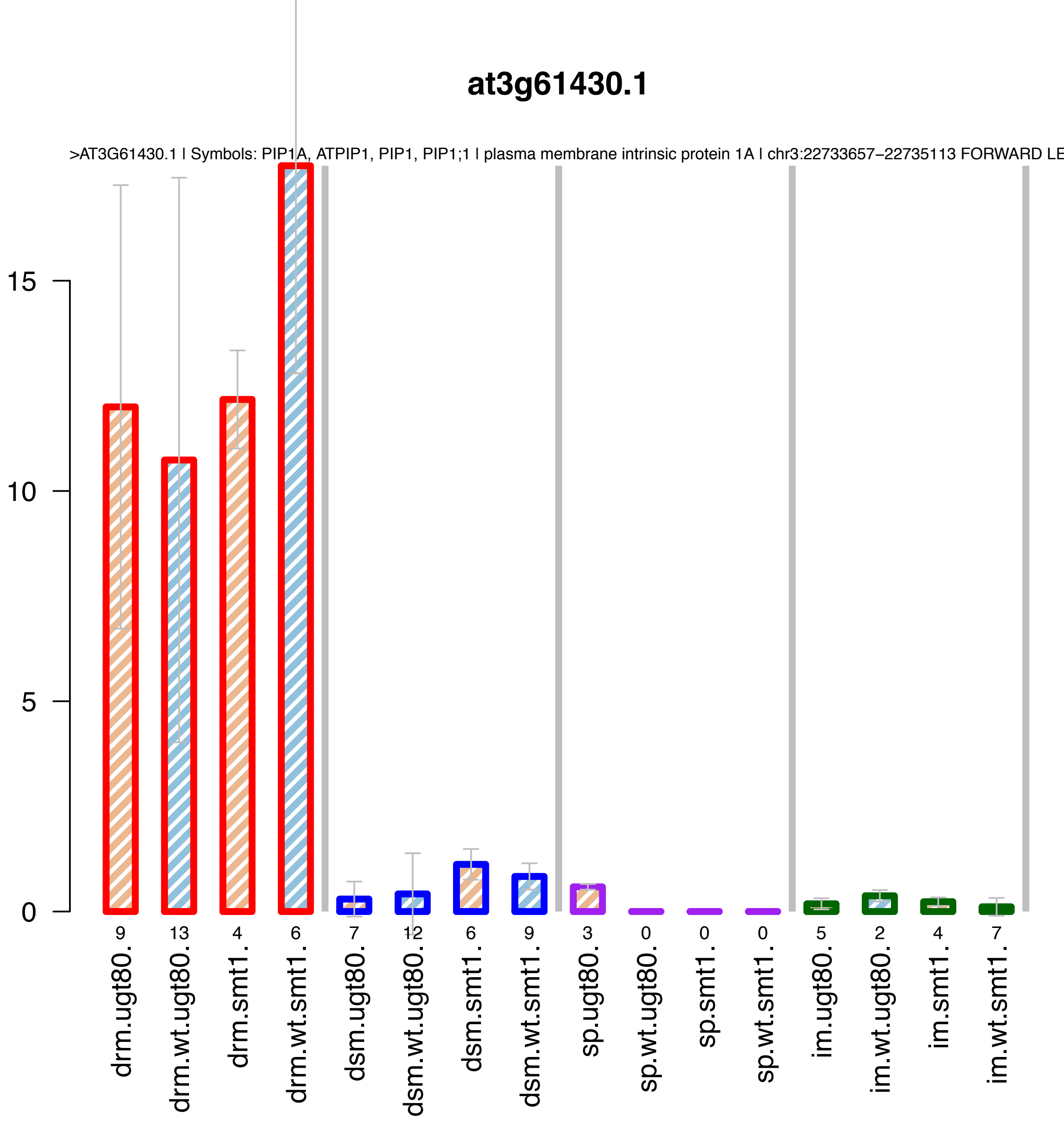

at3g61430.1
>AT3G61430.1 | Symbols: PIP1A, ATPIP1, PIP1, PIP1;1 | plasma membrane intrinsic protein 1A | chr3:22733657-22735113 FORWARD LE
15
10
5
0
9
13
4
6
7
12
6
9
3
0
0
0
5
2
4
7
drm.ugt80.
drm.wt.ugt80.
drm.smt1.
drm.wt.smt1.
dsm.ugt80.
dsm.wt.ugt80.
dsm.smt1.
dsm.wt.smt1.
sp.ugt80.
sp.wt.ugt80.
sp.smt1.
sp.wt.smt1.
im.ugt80.
im.wt.ugt80.
im.smt1.
im.wt.smt1.

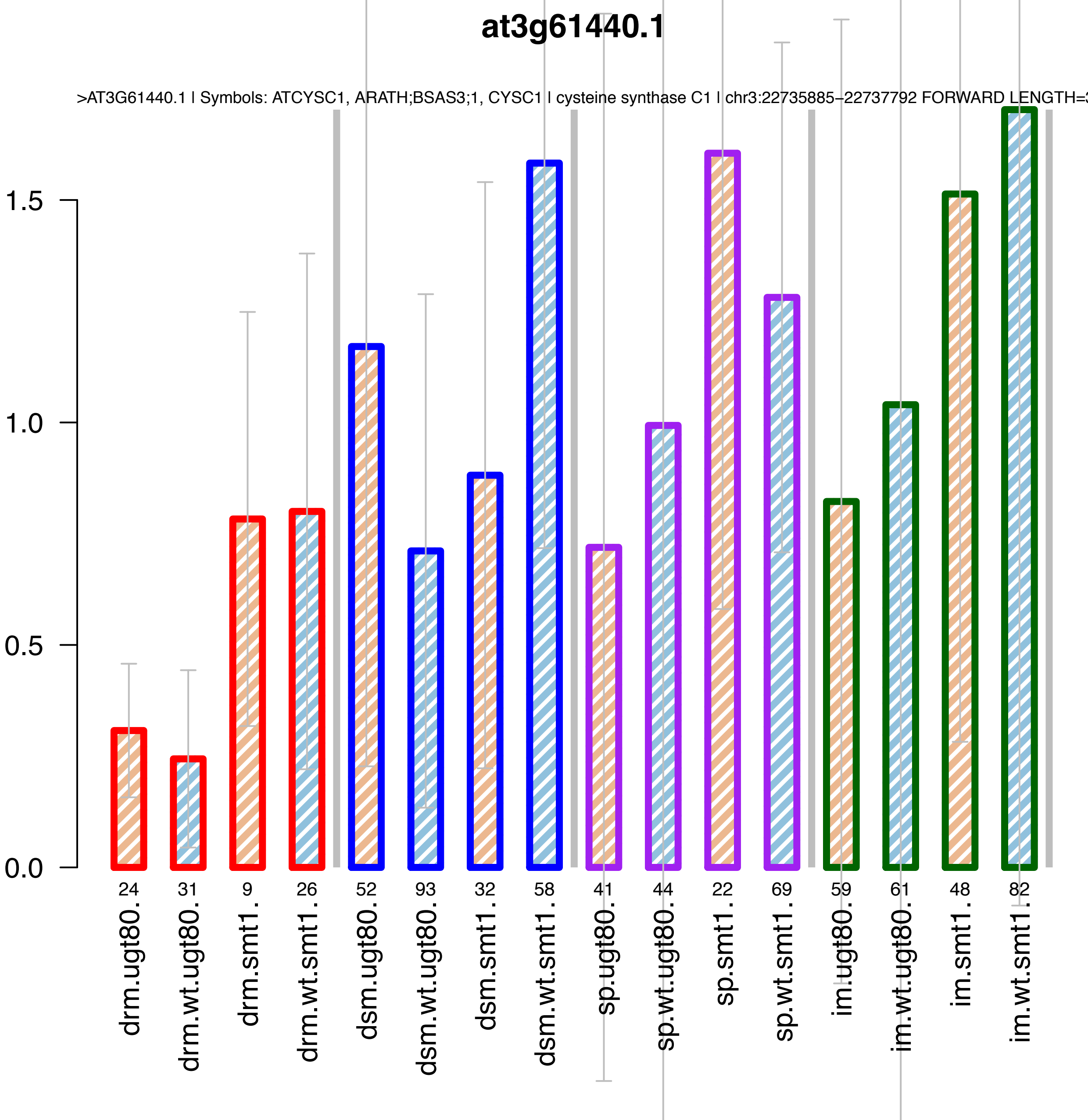

at3g61440.1
>AT3G61440.1 | Symbols: ATCYSC1, ARATH;BSAS3;1, CYSC1 | cysteine synthase C1 | chr3:22735885-22737792 FORWARD LENGTH=3
1.5
1.0
0.5
0.0
24
31
9
26
52
93
32
58
41
44
22
69
59
61
48
82
drm.ugt80.
drm.wt.ugt80.
drm.smt1.
drm.wt.smt1.
dsm.ugt80.
dsm.wt.ugt80.
dsm.smt1.
dsm.wt.smt1.
sp.ugt80.
sp.wt.ugt80.
sp.smt1.
sp.wt.smt1.
im.ugt80.
im.wt.ugt80.
im.smt1.
im.wt.smt1.

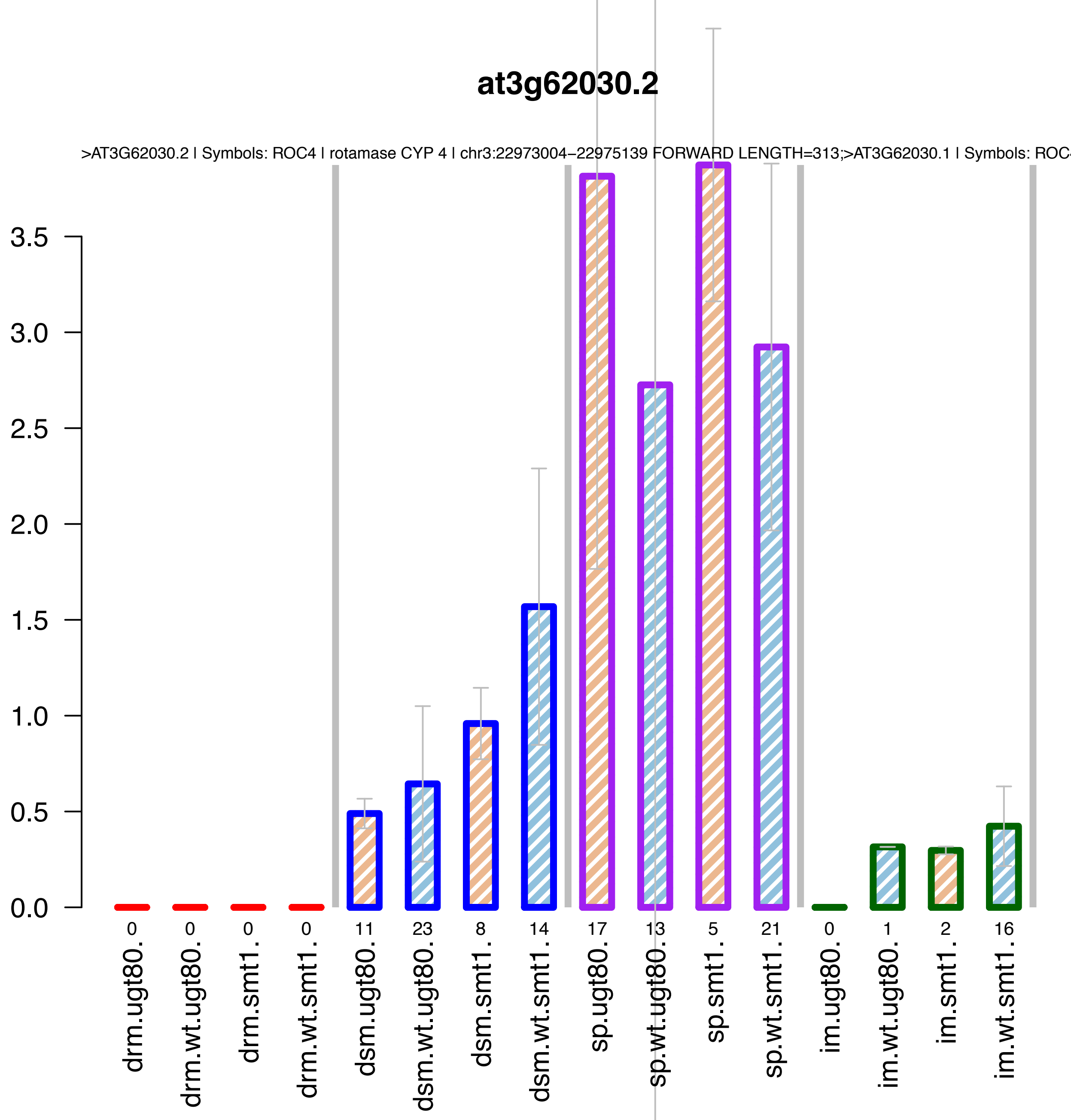

at3g62030.2
>AT3G62030.2 | Symbols: ROC4 | rotamase CYP 4 | chr3:22973004−22975139 FORWARD LENGTH=313;>AT3G62030.1 | Symbols: ROC
3.5
3.0
2.5
2.0
1.5
1.0
0.5
0.0
0 0 0 0 11 23 8 14 17 13 5 21 0 1 2 16
drm.ugt80.
drm.wt.ugt80.
drm.smt1.
drm.wt.smt1.
dsm.ugt80.
dsm.wt.ugt80.
dsm.smt1.
dsm.wt.smt1.
sp.ugt80.
sp.wt.ugt80.
sp.smt1.
sp.wt.smt1.
im.ugt80.
im.wt.ugt80.
im.smt1.
im.wt.smt1.

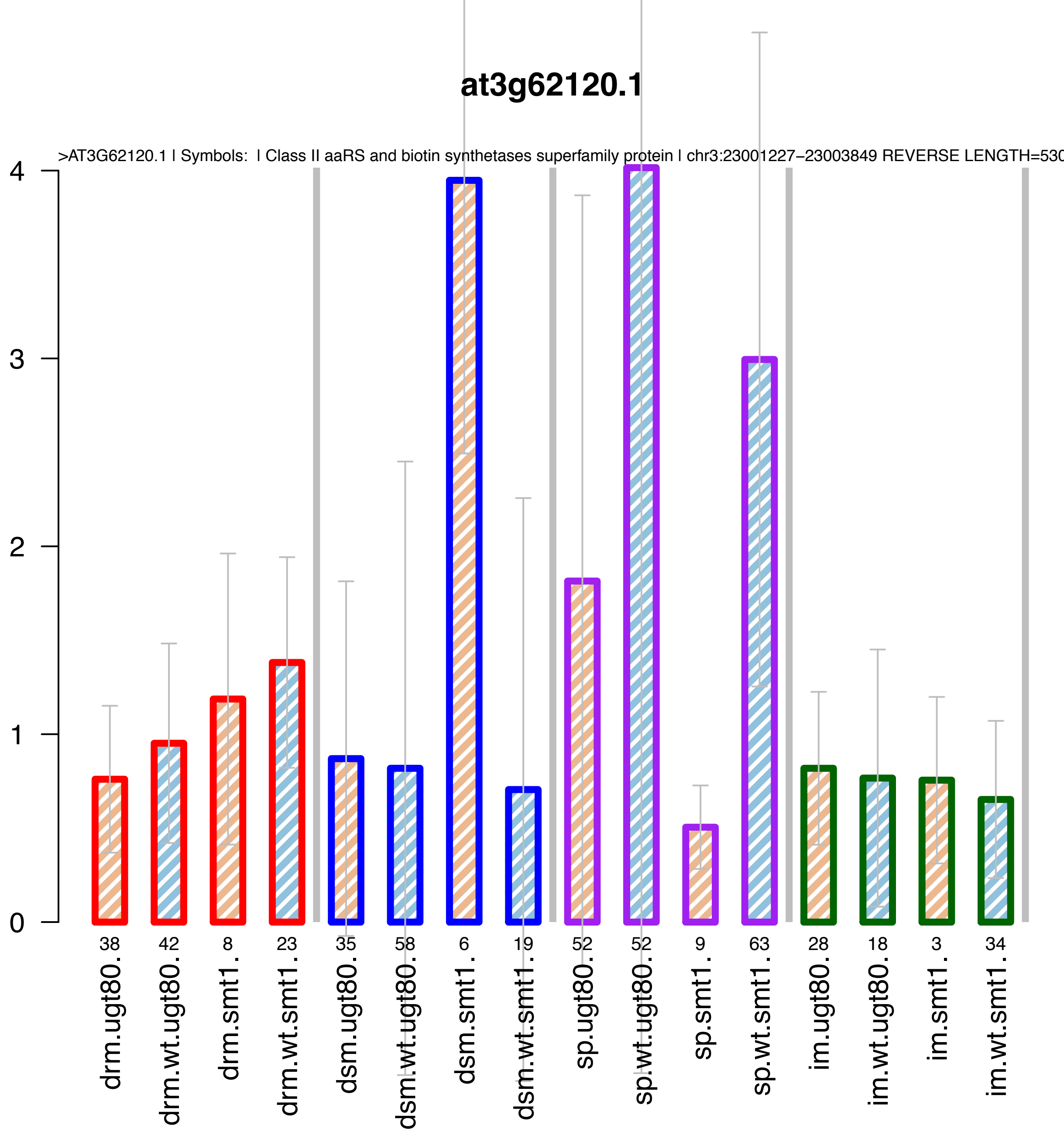

at3g62120.1
>AT3G62120.1 | Symbols: | Class II aaRS and biotin synthetases superfamily protein | chr3:23001227–23003849 REVERSE LENGTH=530
0
1
2
3
4
38
42
8
23
35
58
6
19
52
52
9
63
28
18
3
34
drm.ugt80..
drm.wt.ugt80..
drm.smt1..
drm.wt.smt1..
dsm.ugt80..
dsm.wt.ugt80..
dsm.smt1..
dsm.wt.smt1..
sp.ugt80..
sp.wt.ugt80..
sp.smt1..
sp.wt.smt1..
im.ugt80..
im.wt.ugt80..
im.smt1..
im.wt.smt1..

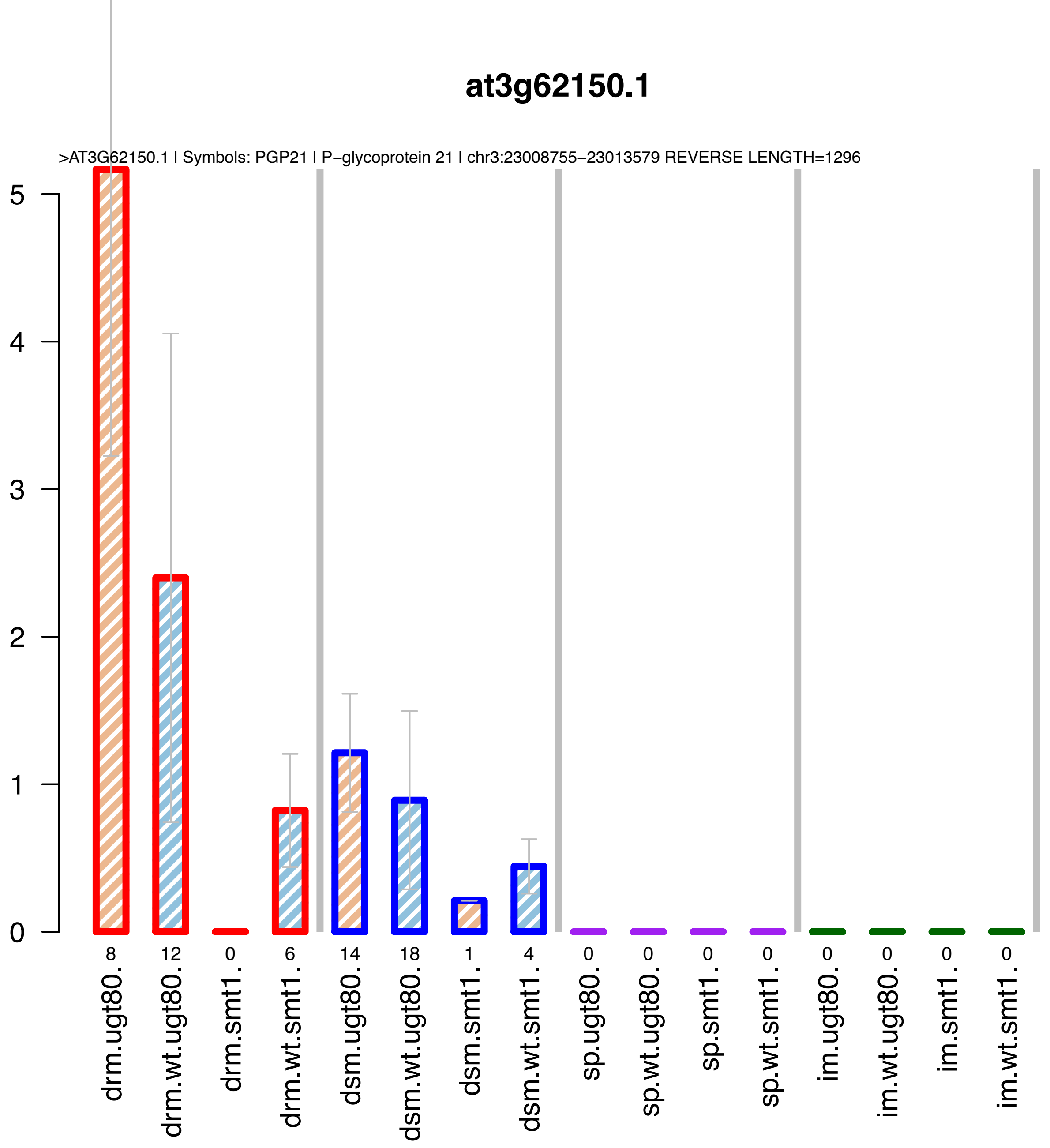

at3g62150.1
>AT3G62150.1 | Symbols: PGP21 | P−glycoprotein 21 | chr3:23008755−23013579 REVERSE LENGTH=1296
0
1
2
3
4
5
8
12
0
6
14
18
1
4
0
0
0
0
0
0
0
0
drm.ugt80.
drm.wt.ugt80.
drm.smt1.
drm.wt.smt1.
dsm.ugt80.
dsm.wt.ugt80.
dsm.smt1.
dsm.wt.smt1.
sp.ugt80.
sp.wt.ugt80.
sp.smt1.
sp.wt.smt1.
im.ugt80.
im.wt.ugt80.
im.smt1.
im.wt.smt1.

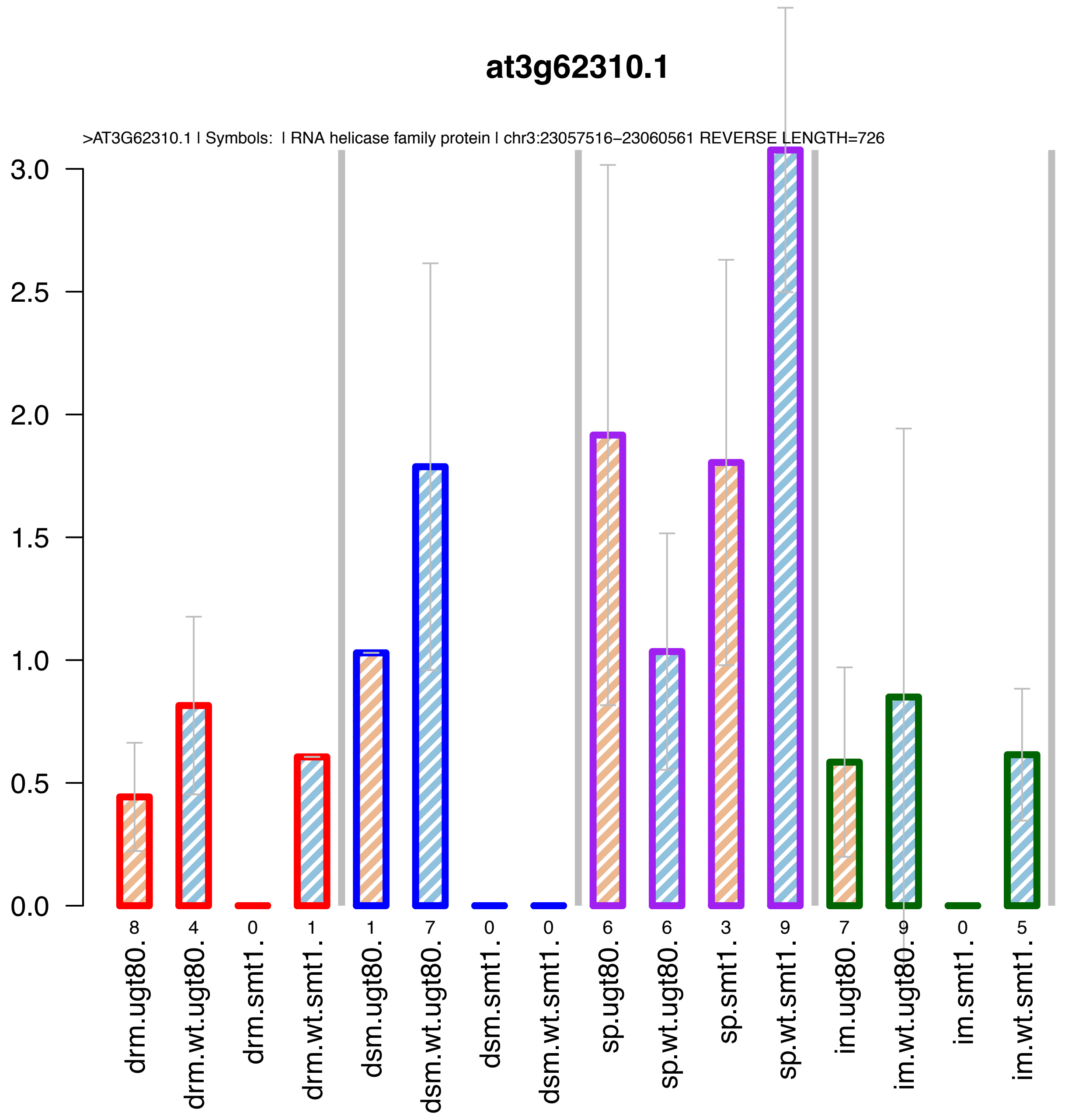

at3g62310.1
>AT3G62310.1 | Symbols: | RNA helicase family protein | chr3:23057516-23060561 REVERSE LENGTH=726
3.0
2.5
2.0
1.5
1.0
0.5
0.0
8
4
0
1
1
7
0
0
6
6
3
9
7
9
0
5
drm.ugt80.
drm.wt.ugt80.
drm.smt1.
drm.wt.smt1.
dsm.ugt80.
dsm.wt.ugt80.
dsm.smt1.
dsm.wt.smt1.
sp.ugt80.
sp.wt.ugt80.
sp.smt1.
sp.wt.smt1.
im.ugt80.
im.wt.ugt80.
im.smt1.
im.wt.smt1.

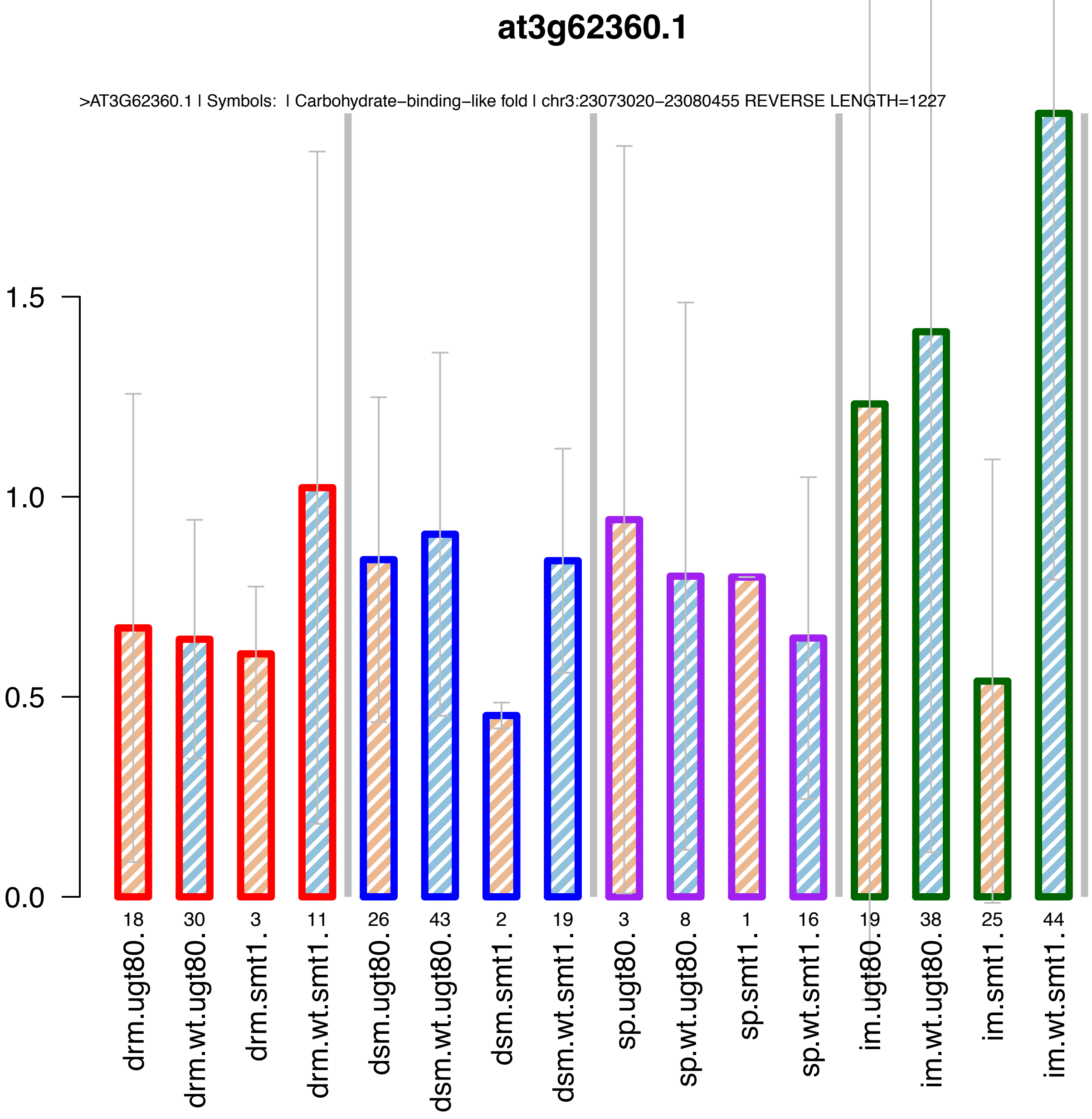

at3g62360.1
>AT3G62360.1 | Symbols: | Carbohydrate−binding−like fold | chr3:23073020−23080455 REVERSE LENGTH=1227
0.0
0.5
1.0
1.5
18
30
3
11
26
43
2
19
3
8
1
16
19
38
25
44
drm.ugt80..
drm.wt.ugt80..
drm.smt1..
drm.wt.smt1..
dsm.ugt80..
dsm.wt.ugt80..
dsm.smt1..
dsm.wt.smt1..
sp.ugt80..
sp.wt.ugt80..
sp.smt1..
sp.wt.smt1..
im.ugt80..
im.wt.ugt80..
im.smt1..
im.wt.smt1..

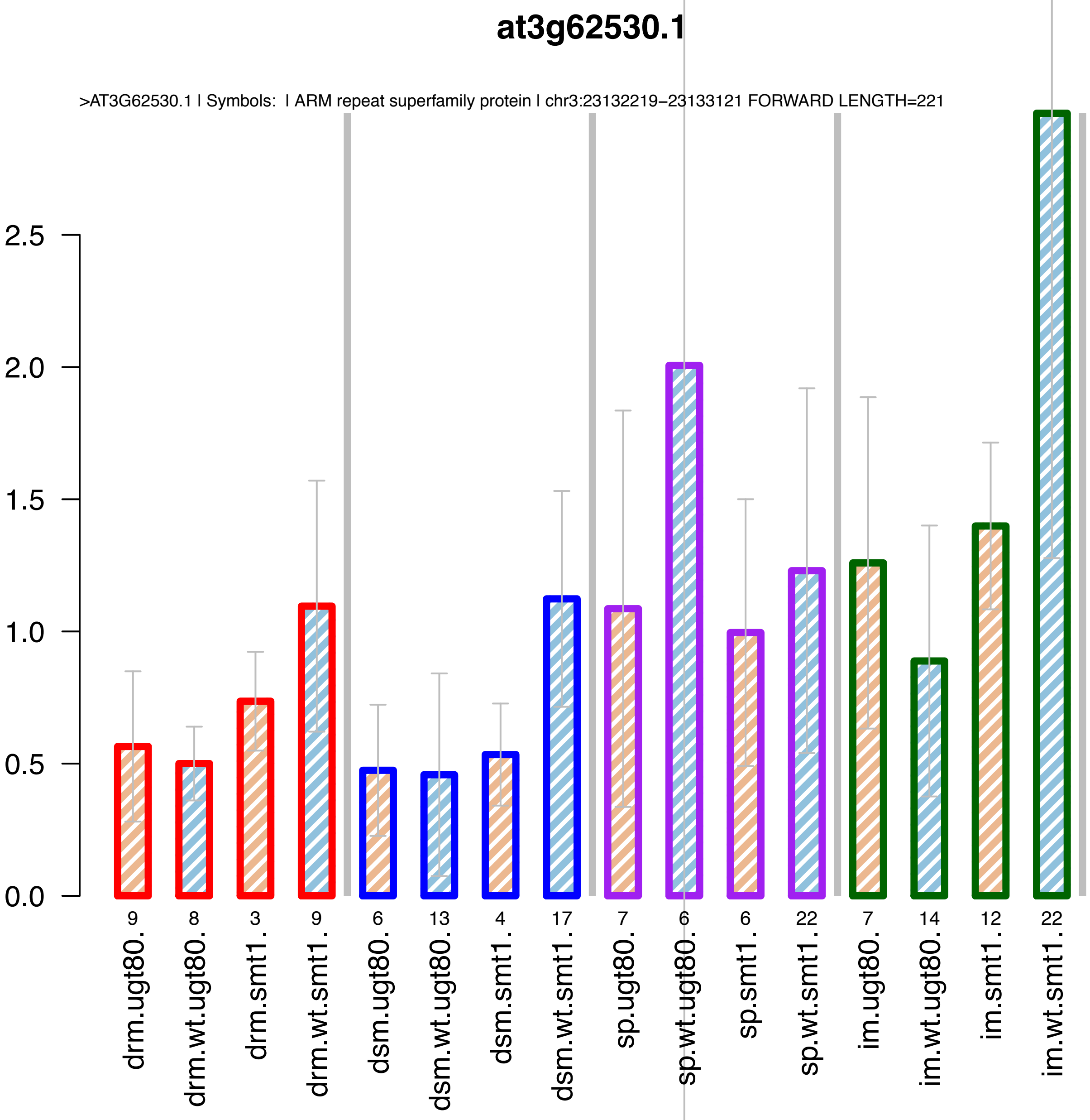

at3g62530.1
>AT3G62530.1 | Symbols: | ARM repeat superfamily protein | chr3:23132219−23133121 FORWARD LENGTH=221
2.5
2.0
1.5
1.0
0.5
0.0
9
8
3
9
6
13
4
17
7
6
6
22
7
14
12
22
drm.ugt80.
drm.wt.ugt80.
drm.smt1.
drm.wt.smt1.
dsm.ugt80.
dsm.wt.ugt80.
dsm.smt1.
dsm.wt.smt1.
sp.ugt80.
sp.wt.ugt80.
sp.smt1.
sp.wt.smt1.
im.ugt80.
im.wt.ugt80.
im.smt1.
im.wt.smt1.

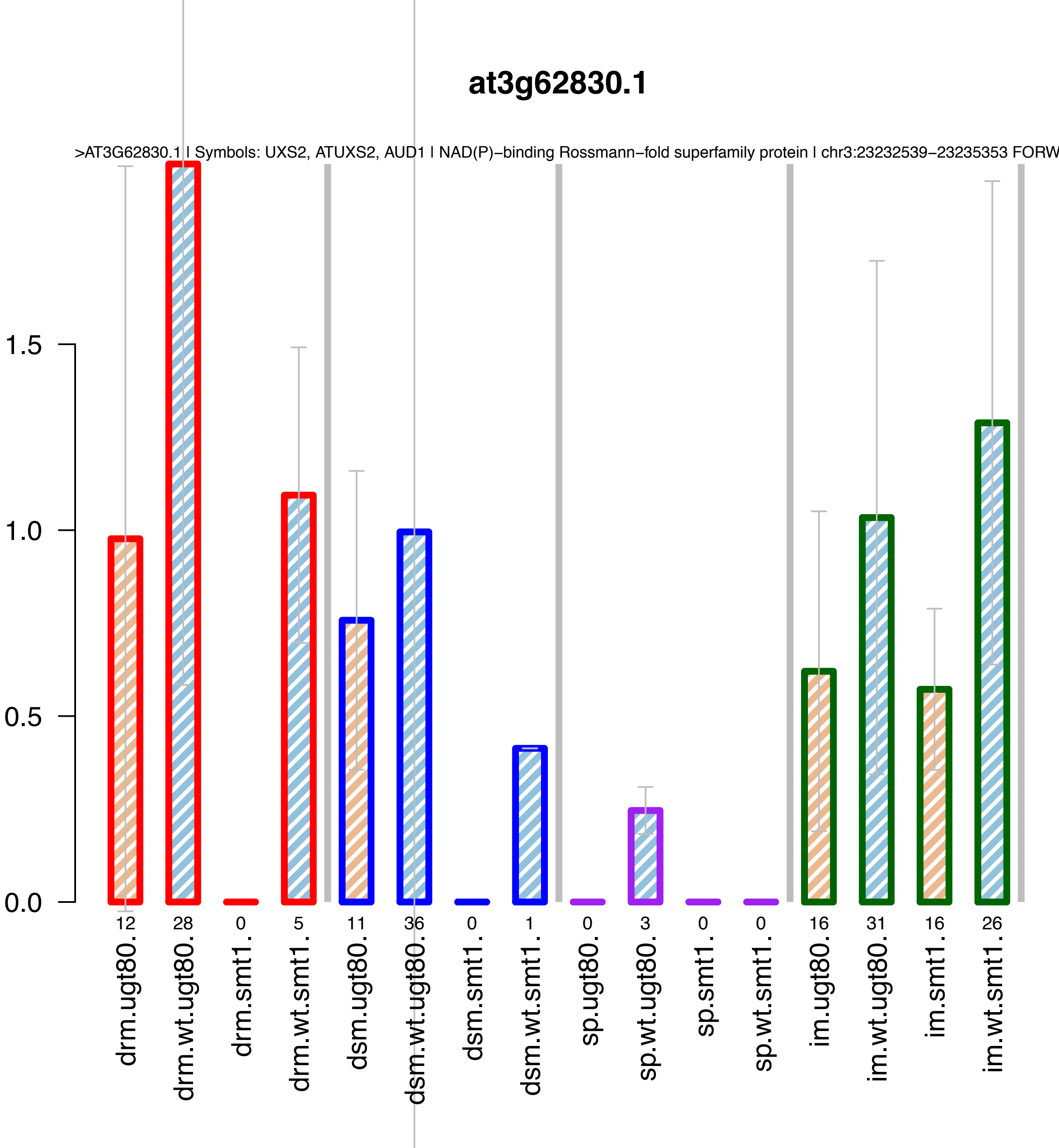

at3g62830.1
>AT3G62830.1 | Symbols: UXS2, ATUXS2, AUD1 | NAD(P)−binding Rossmann−fold superfamily protein | chr3:23232539−23235353 FORW
1.5
1.0
0.5
0.0
12
28
0
5
11
36
0
1
0
3
0
0
16
31
16
26
drm.ugt80.
drm.wt.ugt80.
drm.smt1.
drm.wt.smt1.
dsm.ugt80.
dsm.wt.ugt80.
dsm.smt1.
dsm.wt.smt1.
sp.ugt80.
sp.wt.ugt80.
sp.smt1.
sp.wt.smt1.
im.ugt80.
im.wt.ugt80.
im.smt1.
im.wt.smt1.

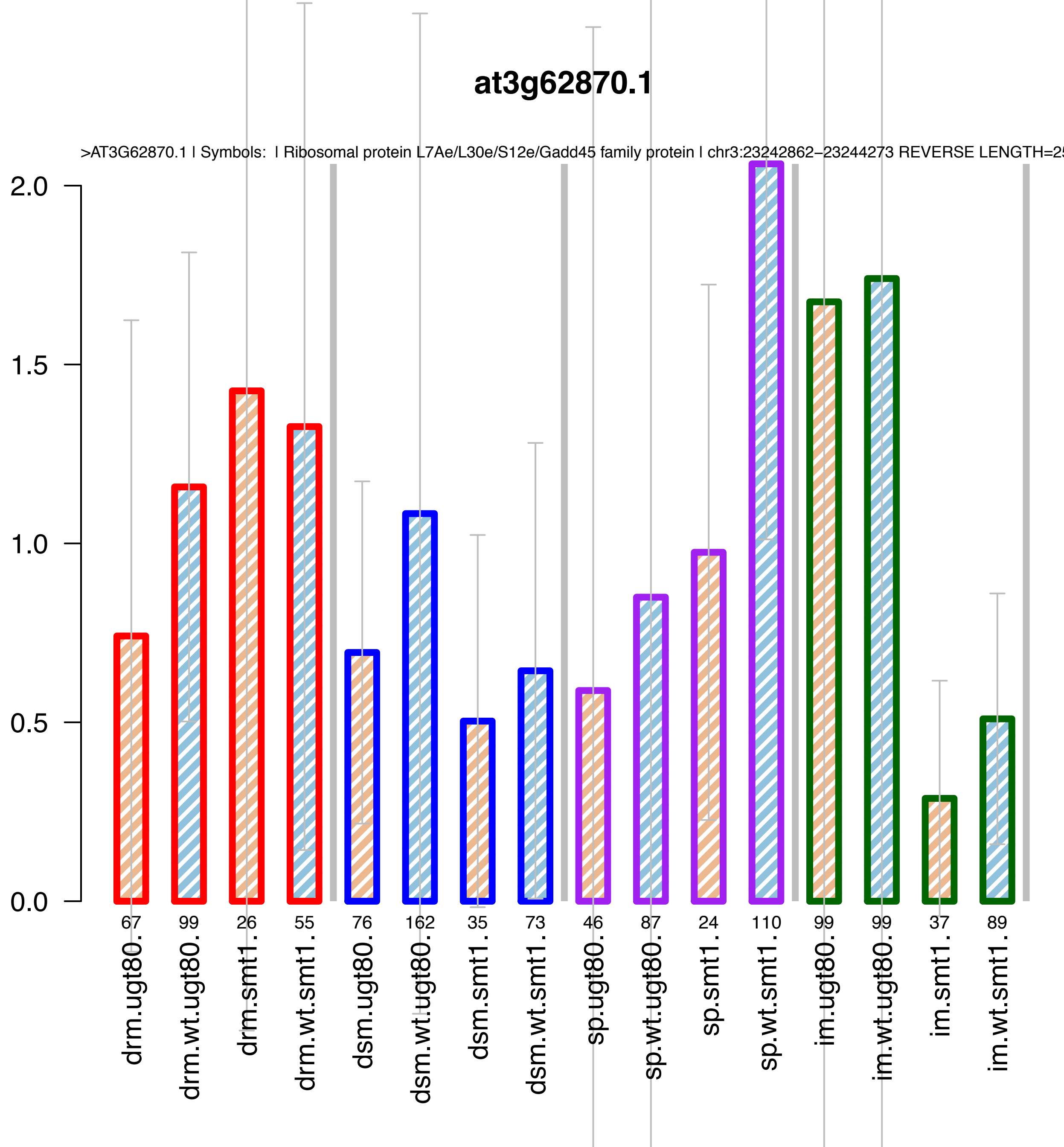

at3g62870.1
>AT3G62870.1 | Symbols: | Ribosomal protein L7Ae/L30e/S12e/Gadd45 family protein | chr3:23242862−23244273 REVERSE LENGTH=2
2.0
1.5
1.0
0.5
0.0
67
99
26
55
76
162
35
73
46
87
24
110
99
99
37
89
drm.ugt80..
drm.wt.ugt80..
drm.smt1..
drm.wt.smt1..
dsm.ugt80..
dsm.wt.ugt80..
dsm.smt1..
dsm.wt.smt1..
sp.ugt80..
sp.wt.ugt80..
sp.smt1..
sp.wt.smt1..
im.ugt80..
im.wt.ugt80..
im.smt1..
im.wt.smt1..

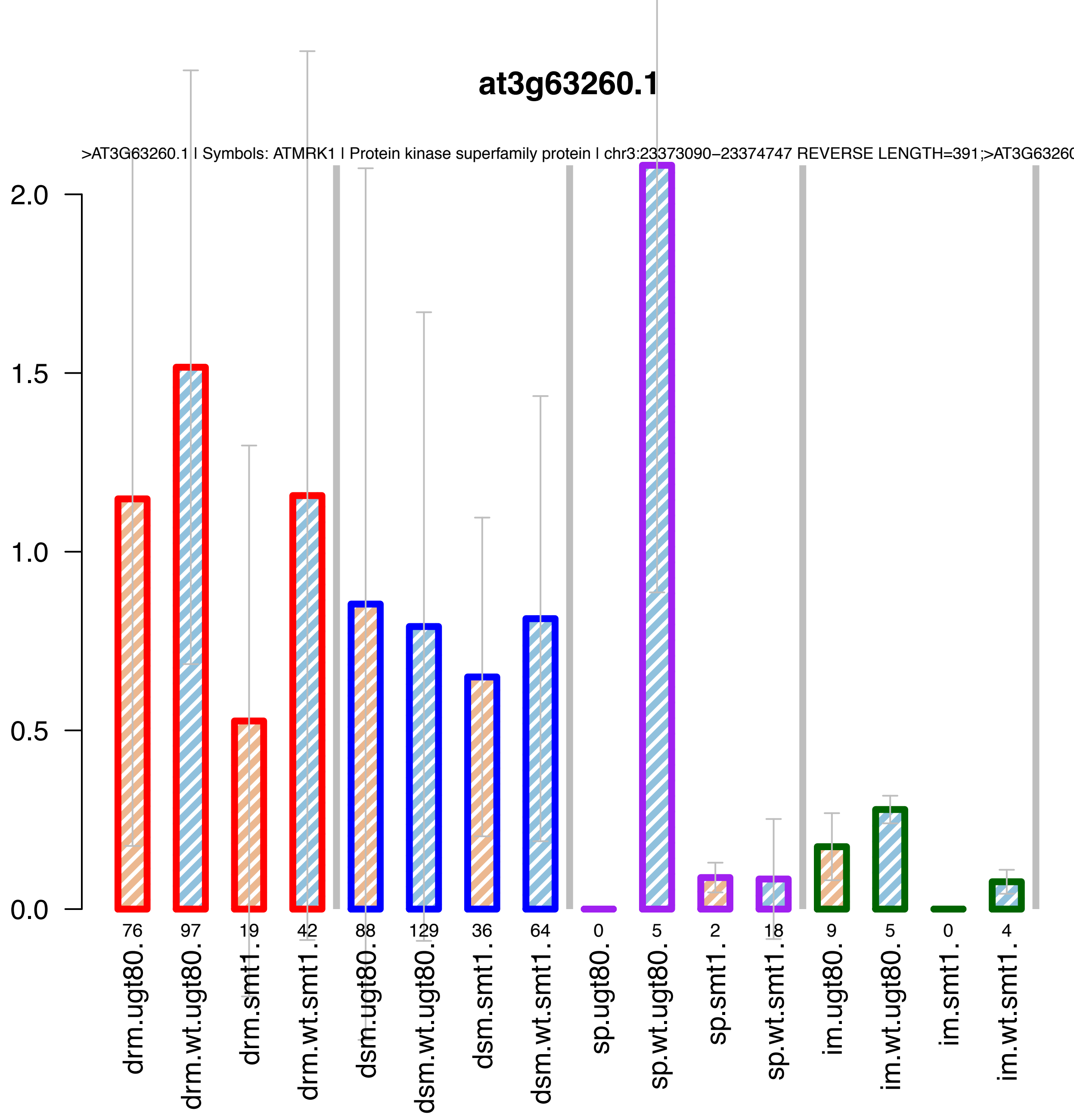

at3g63260.1
>AT3G63260.1 | Symbols: ATMRK1 | Protein kinase superfamily protein | chr3:23373090-23374747 REVERSE LENGTH=391;>AT3G63260
2.0
1.5
1.0
0.5
0.0
76
97
19
42
88
129
36
64
0
5
2
18
9
5
0
4
drm.ugt80.
drm.wt.ugt80.
drm.smt1.
drm.wt.smt1.
dsm.ugt80.
dsm.wt.ugt80.
dsm.smt1.
dsm.wt.smt1.
sp.ugt80.
sp.wt.ugt80.
sp.smt1.
sp.wt.smt1.
im.ugt80.
im.wt.ugt80.
im.smt1.
im.wt.smt1.

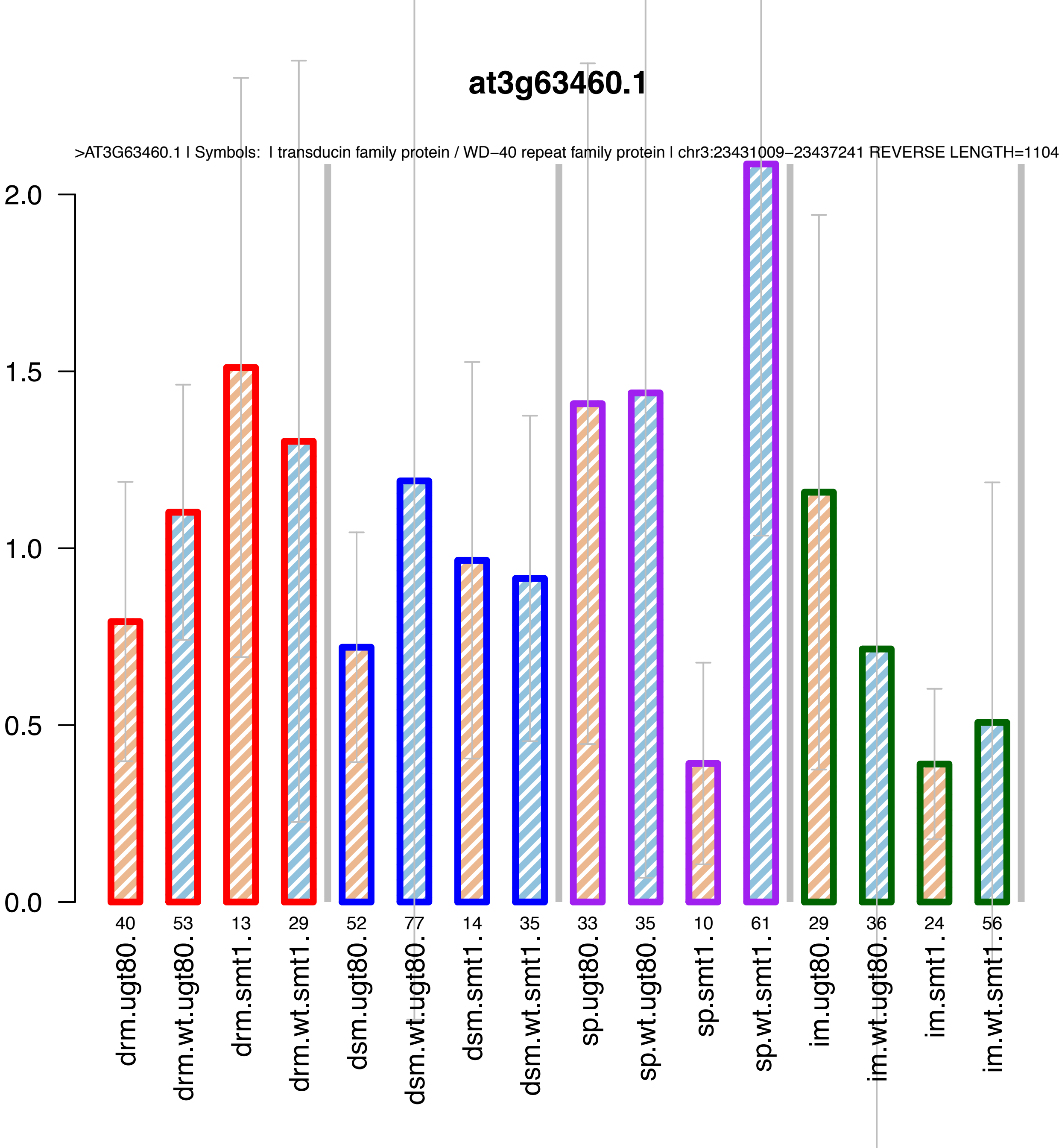
at3g63460.1
>AT3G63460.1 | Symbols: | transducin family protein / WD−40 repeat family protein | chr3:23431009−23437241 REVERSE LENGTH=1104
2.0
1.5
1.0
0.5
0.0
40
53
13
29
52
77
14
35
33
35
10
61
29
36
24
56
drm.ugt80..
drm.wt.ugt80..
drm.smt1..
drm.wt.smt1..
dsm.ugt80..
dsm.wt.ugt80..
dsm.smt1..
dsm.wt.smt1..
sp.ugt80..
sp.wt.ugt80..
sp.smt1..
sp.wt.smt1..
im.ugt80..
im.wt.ugt80..
im.smt1..
im.wt.smt1..

# at3g63520.1

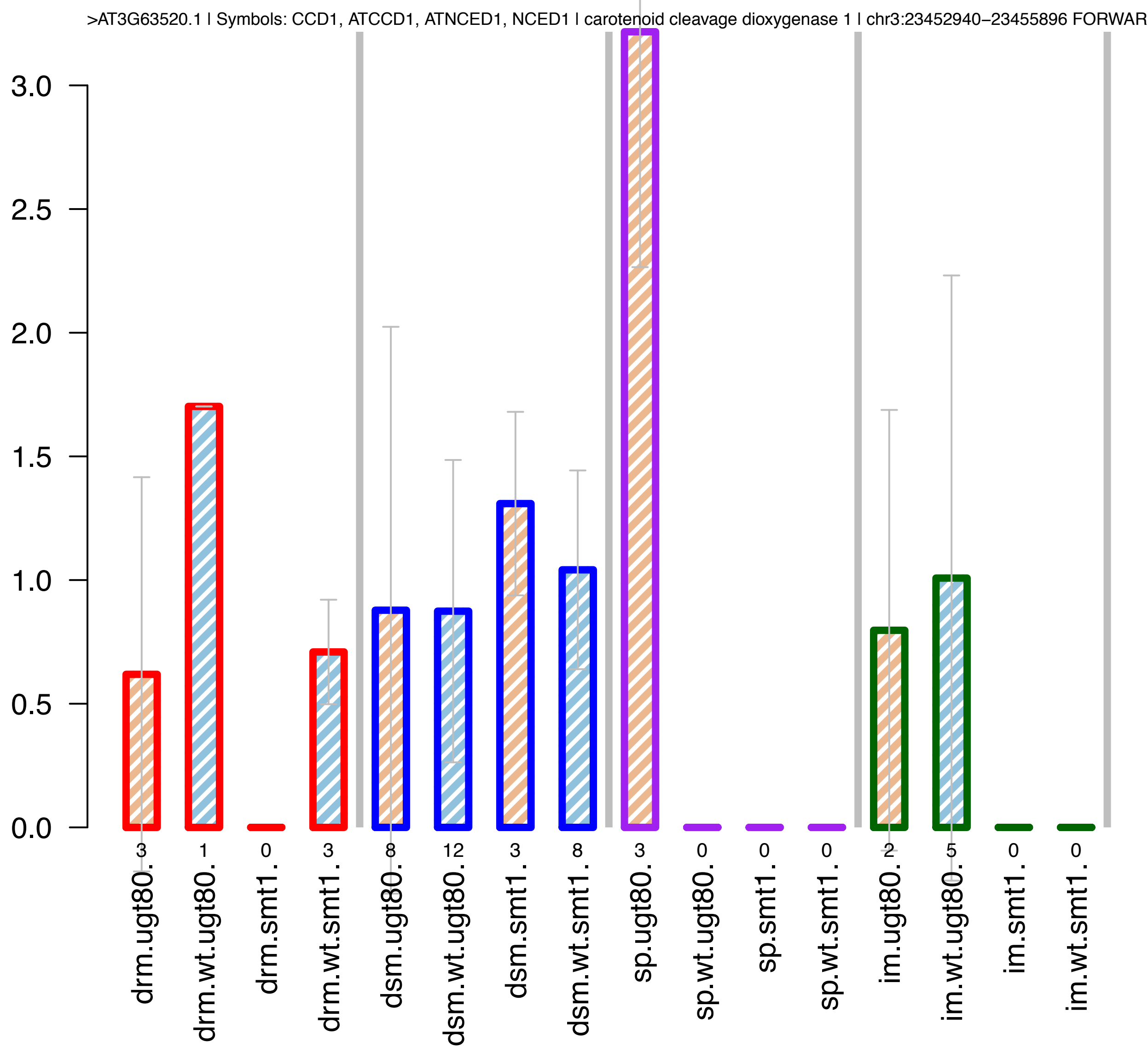

>AT3G63520.1 | Symbols: CCD1, ATCCD1, ATNCED1, NCED1 | carotenoid cleavage dioxygenase 1 | chr3:23452940–23455896 FORWAR
3.0
2.5
2.0
1.5
1.0
0.5
0.0
3
1
0
3
8
12
3
8
3
0
0
0
2
5
0
0
drm.ugt80.
drm.wt.ugt80.
drm.smt1.
drm.wt.smt1.
dsm.ugt80.
dsm.wt.ugt80.
dsm.smt1.
dsm.wt.smt1.
sp.ugt80.
sp.wt.ugt80.
sp.smt1.
sp.wt.smt1.
im.ugt80.
im.wt.ugt80.
im.smt1.
im.wt.smt1.

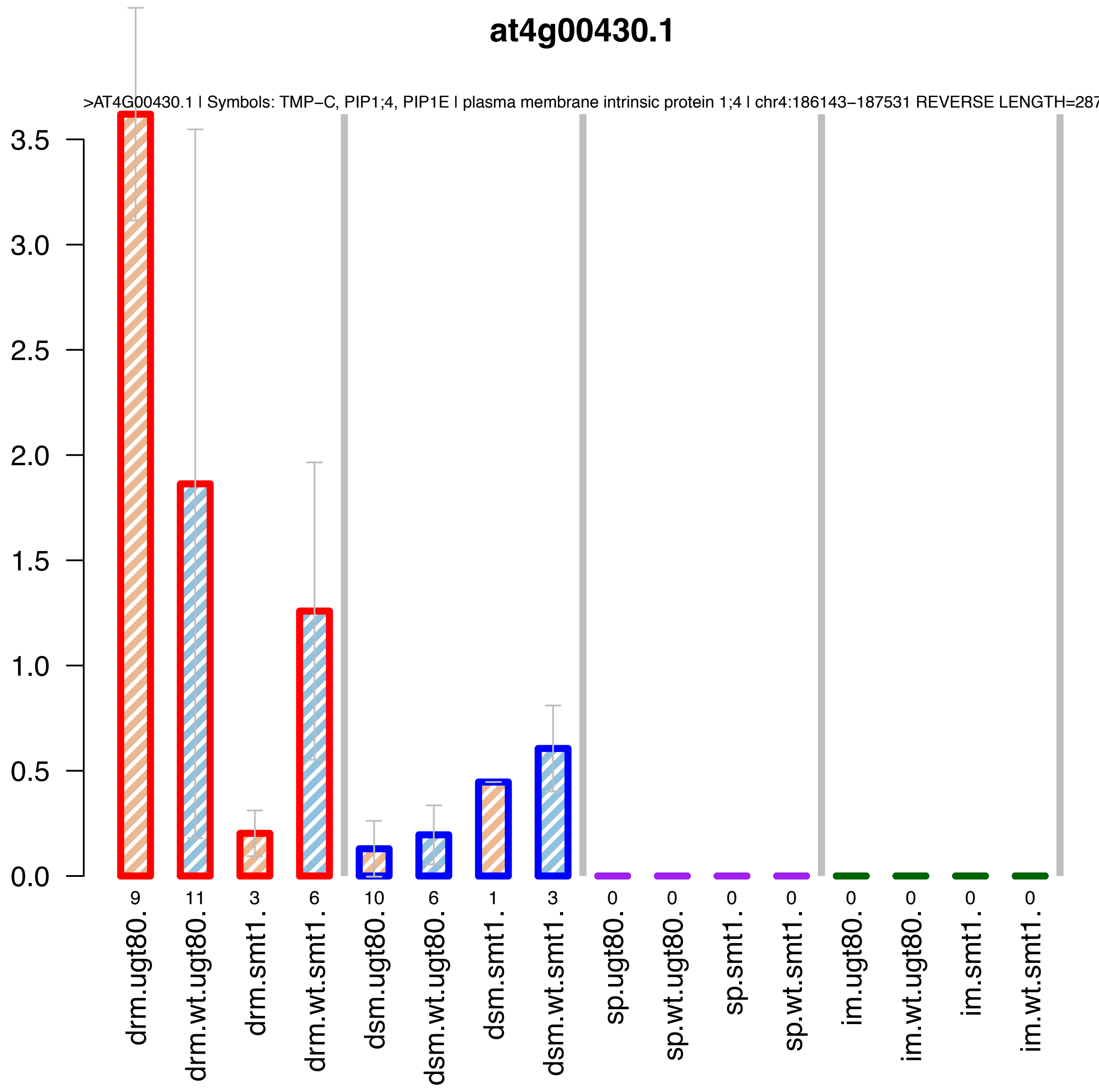

at4g00430.1
>AT4G00430.1 | Symbols: TMP−C, PIP1;4, PIP1E | plasma membrane intrinsic protein 1;4 | chr4:186143−187531 REVERSE LENGTH=287
0.0
0.5
1.0
1.5
2.0
2.5
3.0
3.5
9
11
3
6
10
6
1
3
0
0
0
0
0
0
0
0
drm.ugt80.
drm.wt.ugt80.
drm.smt1.
drm.wt.smt1.
dsm.ugt80.
dsm.wt.ugt80.
dsm.smt1.
dsm.wt.smt1.
sp.ugt80.
sp.wt.ugt80.
sp.smt1.
sp.wt.smt1.
im.ugt80.
im.wt.ugt80.
im.smt1.
im.wt.smt1.

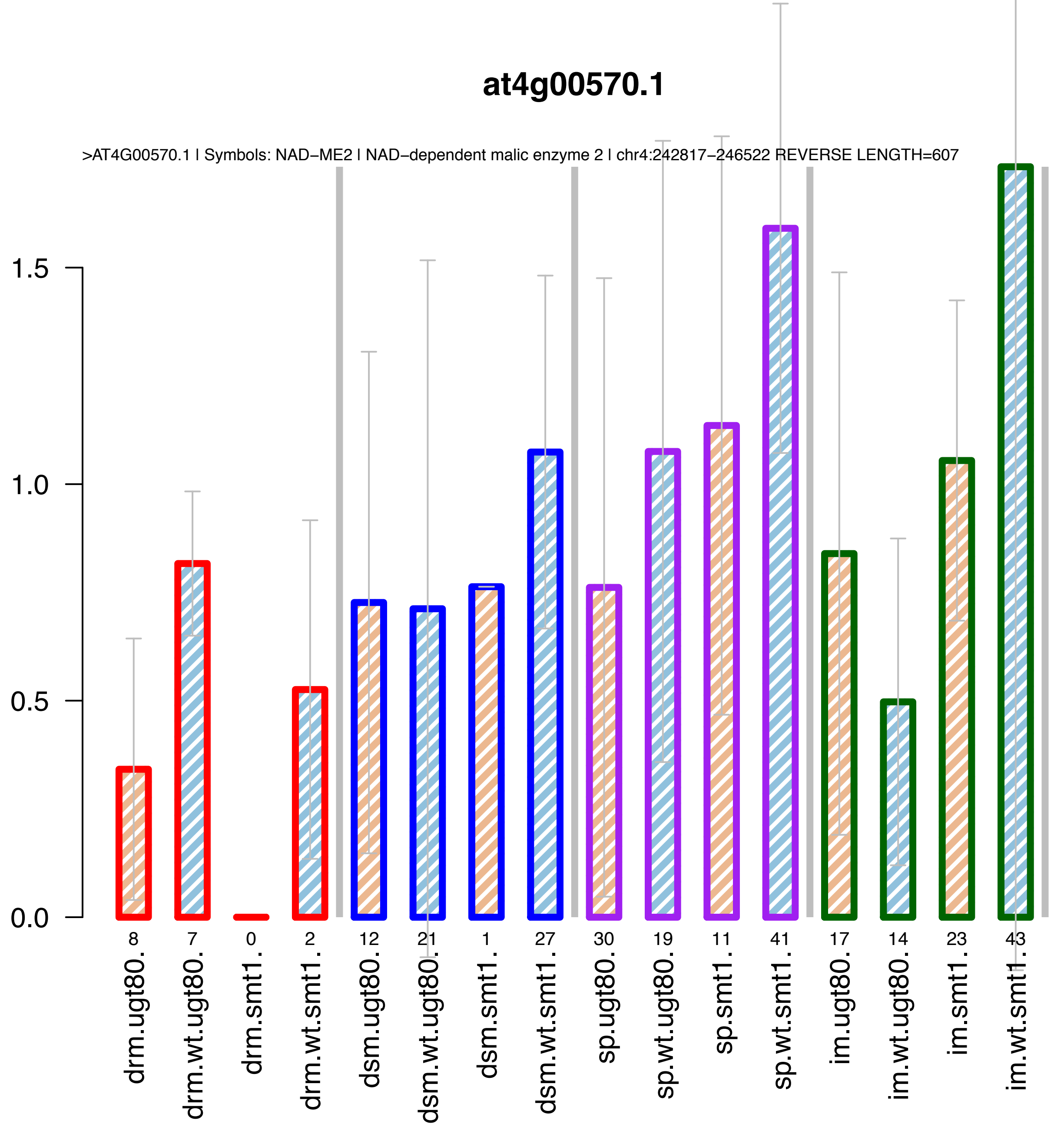

at4g00570.1
>AT4G00570.1 | Symbols: NAD−ME2 | NAD−dependent malic enzyme 2 | chr4:242817−246522 REVERSE LENGTH=607
1.5
1.0
0.5
0.0
8
7
0
2
12
21
1
27
30
19
11
41
17
14
23
43
drm.ugt80..
drm.wt.ugt80..
drm.smt1..
drm.wt.smt1..
dsm.ugt80..
dsm.wt.ugt80..
dsm.smt1..
dsm.wt.smt1..
sp.ugt80..
sp.wt.ugt80..
sp.smt1..
sp.wt.smt1..
im.ugt80..
im.wt.ugt80..
im.smt1..
im.wt.smt1..

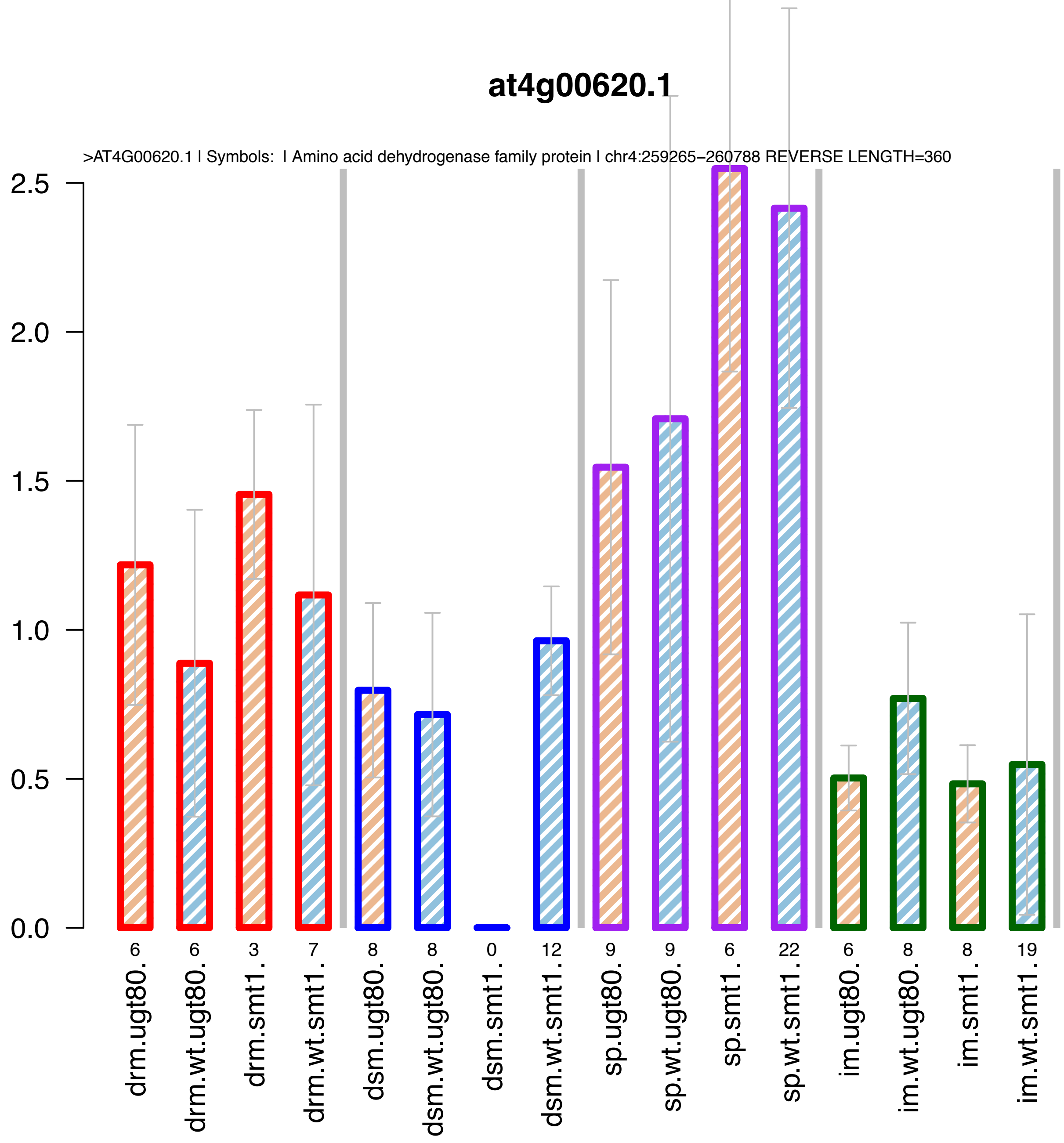
at4g00620.1
>AT4G00620.1 | Symbols: | Amino acid dehydrogenase family protein | chr4:259265-260788 REVERSE LENGTH=360
0.0
0.5
1.0
1.5
2.0
2.5
6
6
3
7
8
8
0
12
9
9
6
22
6
8
8
19
drm.ugt80..
drm.wt.ugt80..
drm.smt1..
drm.wt.smt1..
dsm.ugt80..
dsm.wt.ugt80..
dsm.smt1..
dsm.wt.smt1..
sp.ugt80..
sp.wt.ugt80..
sp.smt1..
sp.wt.smt1..
im.ugt80..
im.wt.ugt80..
im.smt1..
im.wt.smt1..

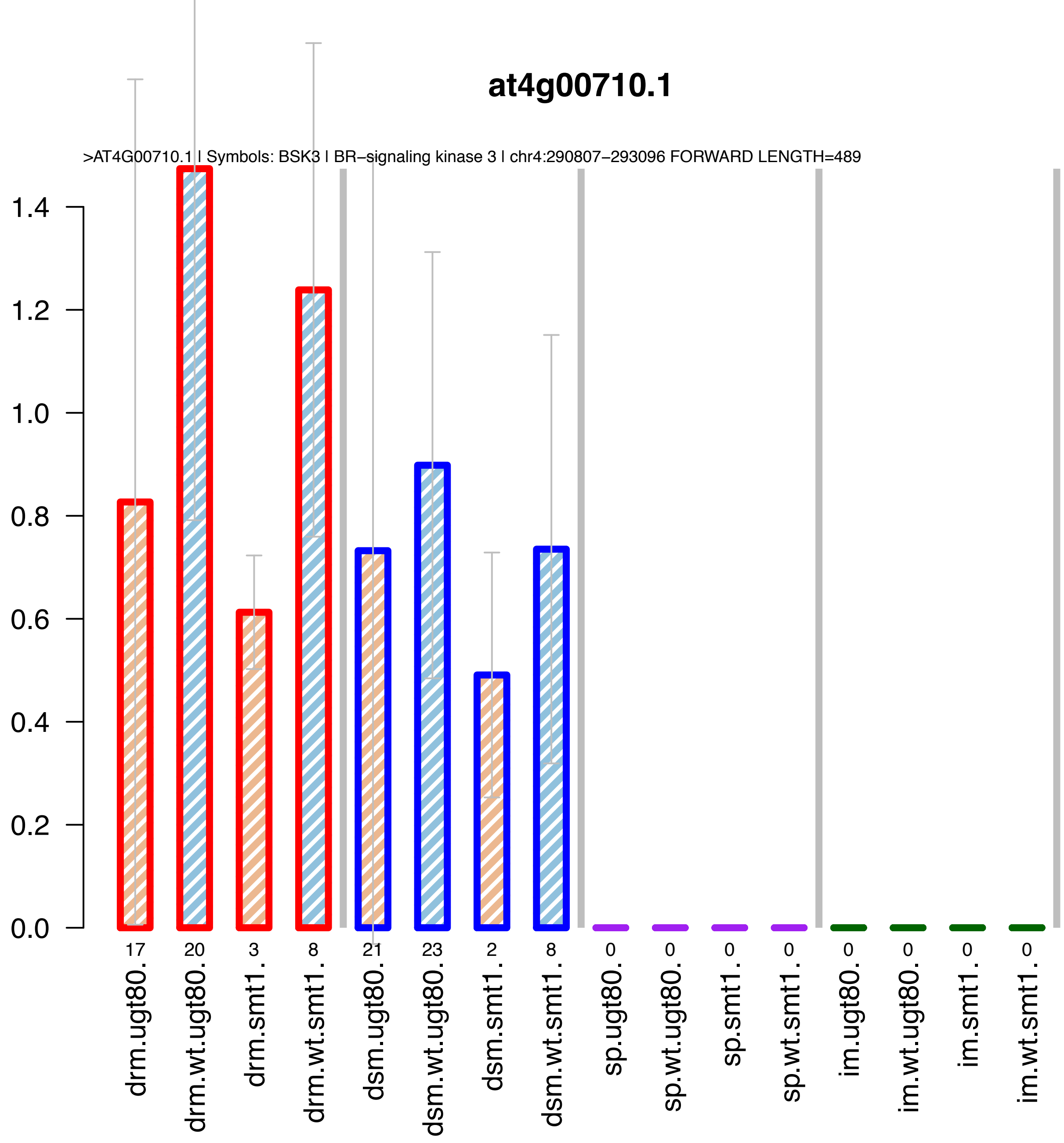

at4g00710.1
>AT4G00710.1 | Symbols: BSK3 | BR−signaling kinase 3 | chr4:290807−293096 FORWARD LENGTH=489
1.4
1.2
1.0
0.8
0.6
0.4
0.2
0.0
17
20
3
8
21
23
2
8
0
0
0
0
0
0
0
0
drm.ugt80.
drm.wt.ugt80.
drm.smt1.
drm.wt.smt1.
dsm.ugt80.
dsm.wt.ugt80.
dsm.smt1.
dsm.wt.smt1.
sp.ugt80.
sp.wt.ugt80.
sp.smt1.
sp.wt.smt1.
im.ugt80.
im.wt.ugt80.
im.smt1.
im.wt.smt1.

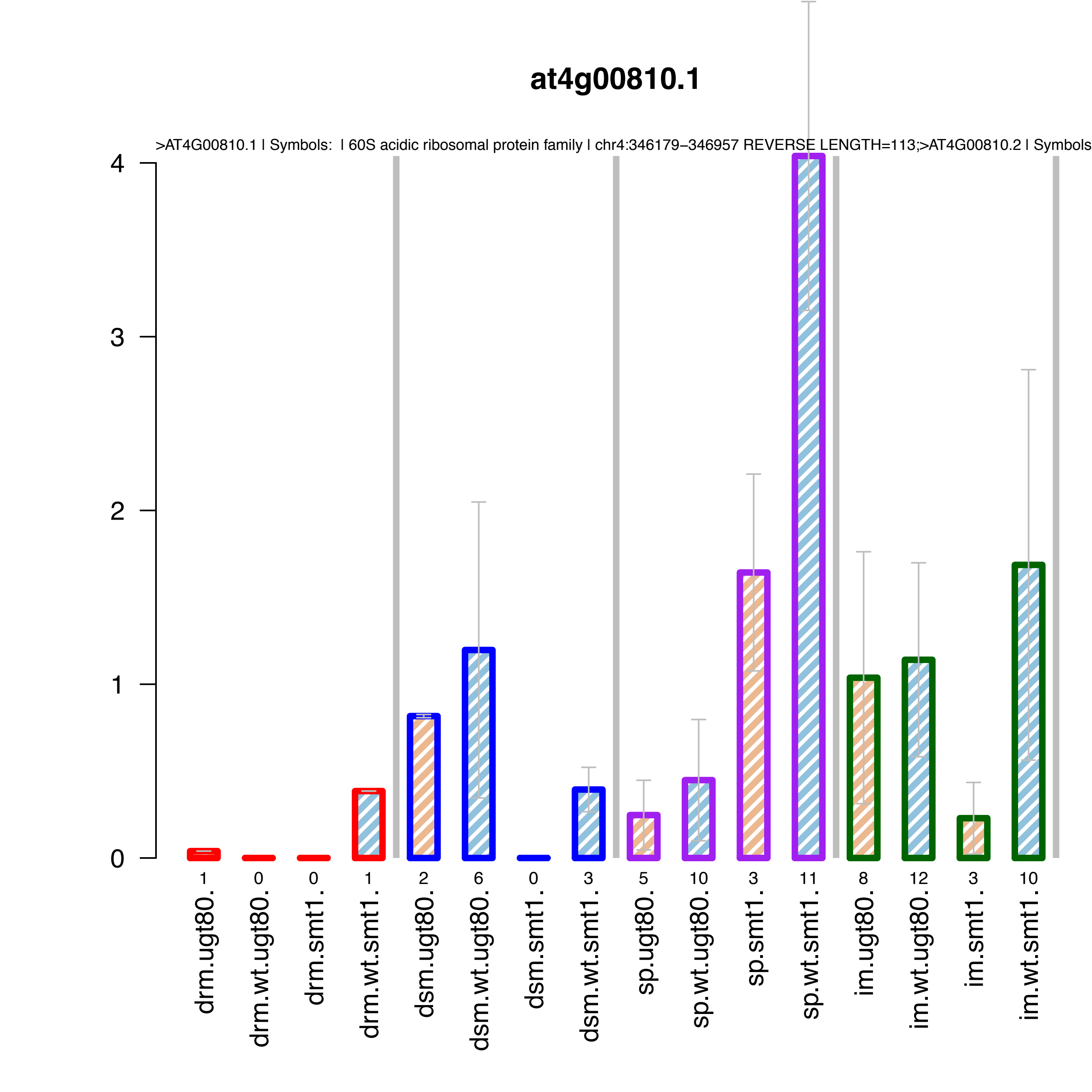

at4g00810.1
>AT4G00810.1 | Symbols: | 60S acidic ribosomal protein family | chr4:346179-346957 REVERSE LENGTH=113;>AT4G00810.2 | Symbols
4
3
2
1
0
1
0
0
1
2
6
0
3
5
10
3
11
8
12
3
10
drm.ugt80.
drm.wt.ugt80.
drm.smt1.
drm.wt.smt1.
dsm.ugt80.
dsm.wt.ugt80.
dsm.smt1.
dsm.wt.smt1.
sp.ugt80.
sp.wt.ugt80.
sp.smt1.
sp.wt.smt1.
im.ugt80.
im.wt.ugt80.
im.smt1.
im.wt.smt1.

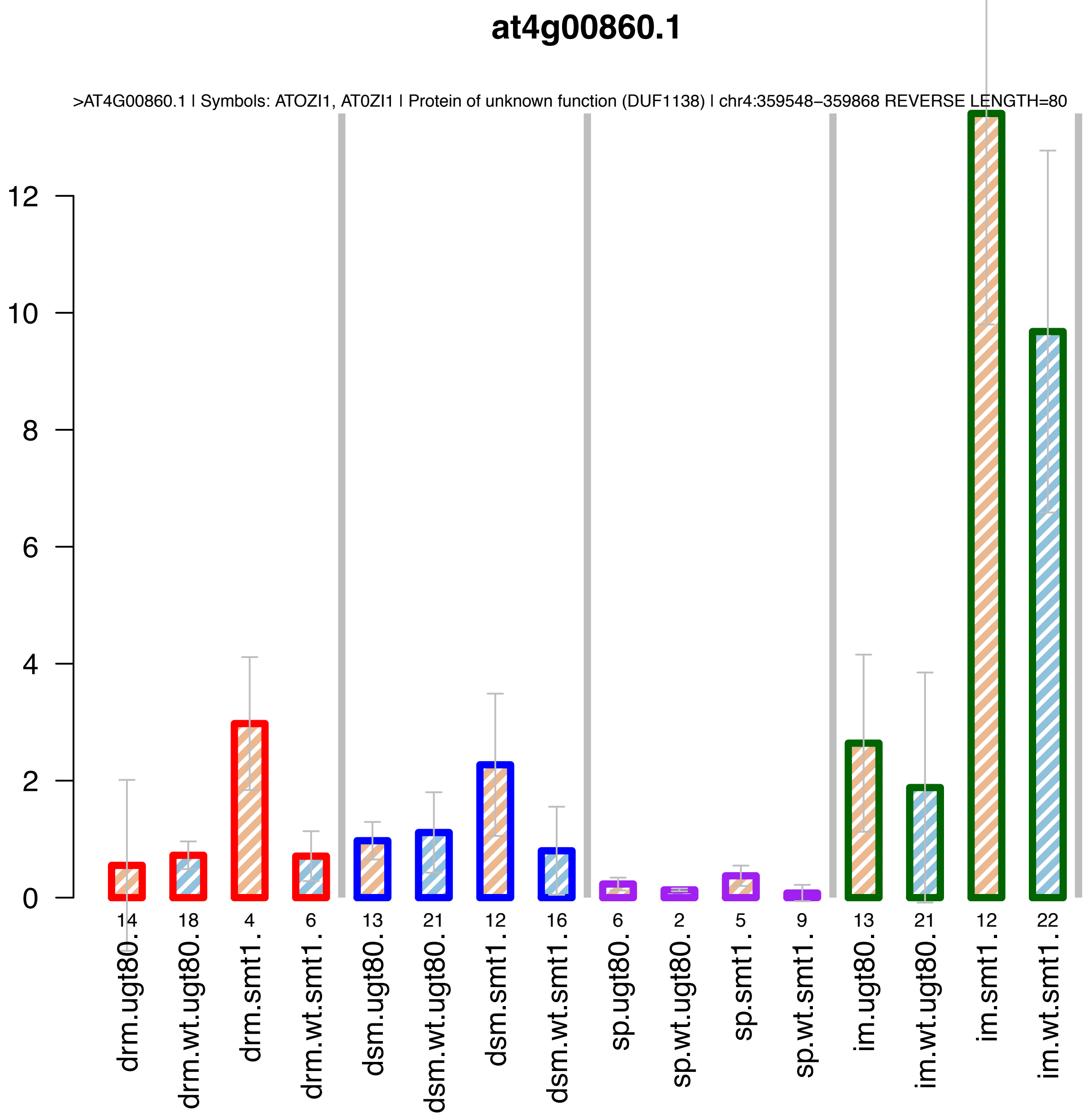

at4g00860.1
>AT4G00860.1 | Symbols: ATOZI1, AT0ZI1 | Protein of unknown function (DUF1138) | chr4:359548–359868 REVERSE LENGTH=80
0
2
4
6
8
10
12
14
18
4
6
13
21
12
16
6
2
5
9
13
21
12
22
drm.ugt80..
drm.wt.ugt80..
drm.smt1..
drm.wt.smt1..
dsm.ugt80..
dsm.wt.ugt80..
dsm.smt1..
dsm.wt.smt1..
sp.ugt80..
sp.wt.ugt80..
sp.smt1..
sp.wt.smt1..
im.ugt80..
im.wt.ugt80..
im.smt1..
im.wt.smt1..

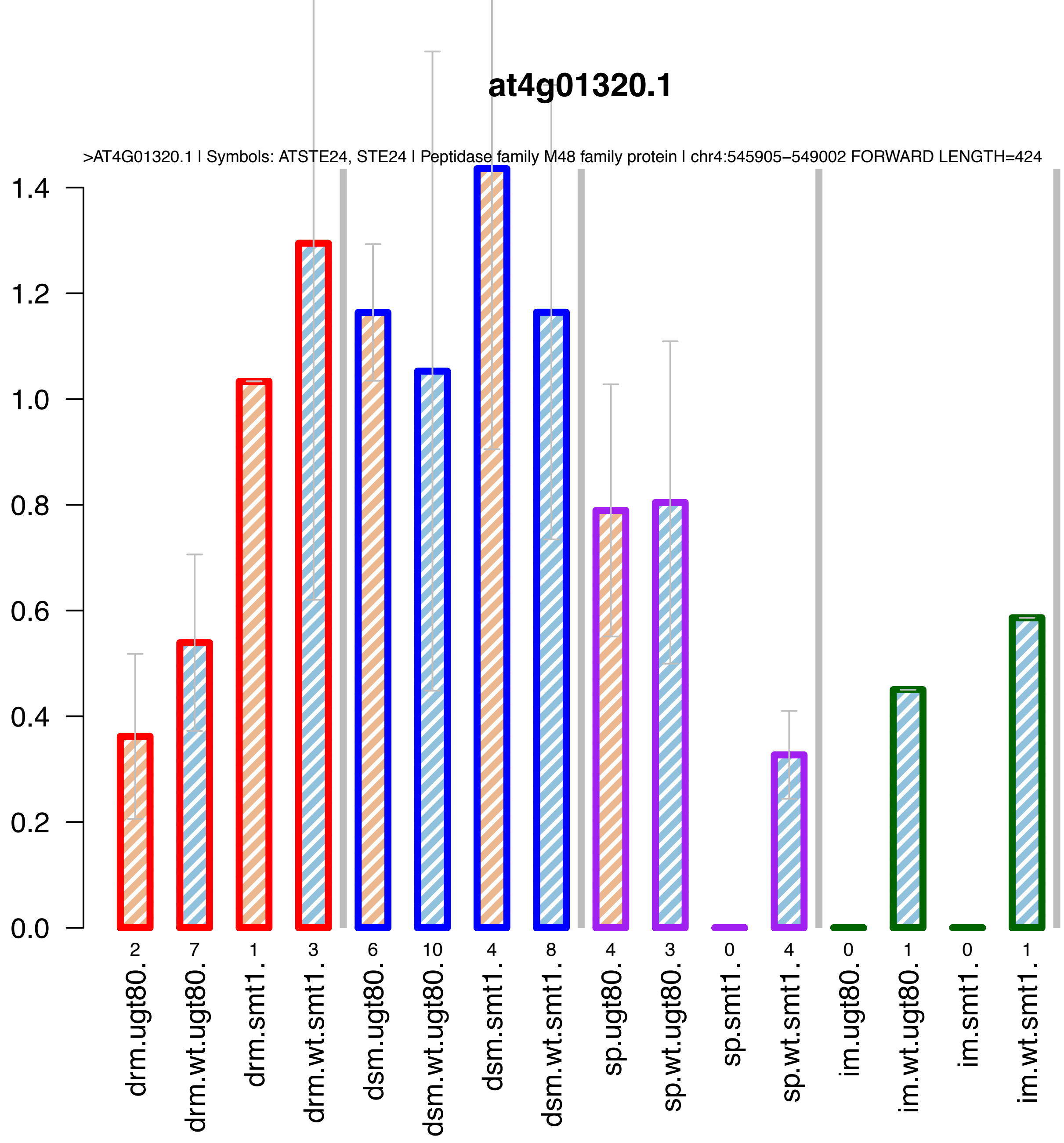

at4g01320.1
>AT4G01320.1 | Symbols: ATSTE24, STE24 | Peptidase family M48 family protein | chr4:545905-549002 FORWARD LENGTH=424
0.0
0.2
0.4
0.6
0.8
1.0
1.2
1.4
2 7 1 3 6 10 4 8 4 3 0 4 0 1 0 1
drm.ugt80.
drm.wt.ugt80.
drm.smt1.
drm.wt.smt1.
dsm.ugt80.
dsm.wt.ugt80.
dsm.smt1.
dsm.wt.smt1.
sp.ugt80.
sp.wt.ugt80.
sp.smt1.
sp.wt.smt1.
im.ugt80.
im.wt.ugt80.
im.smt1.
im.wt.smt1.

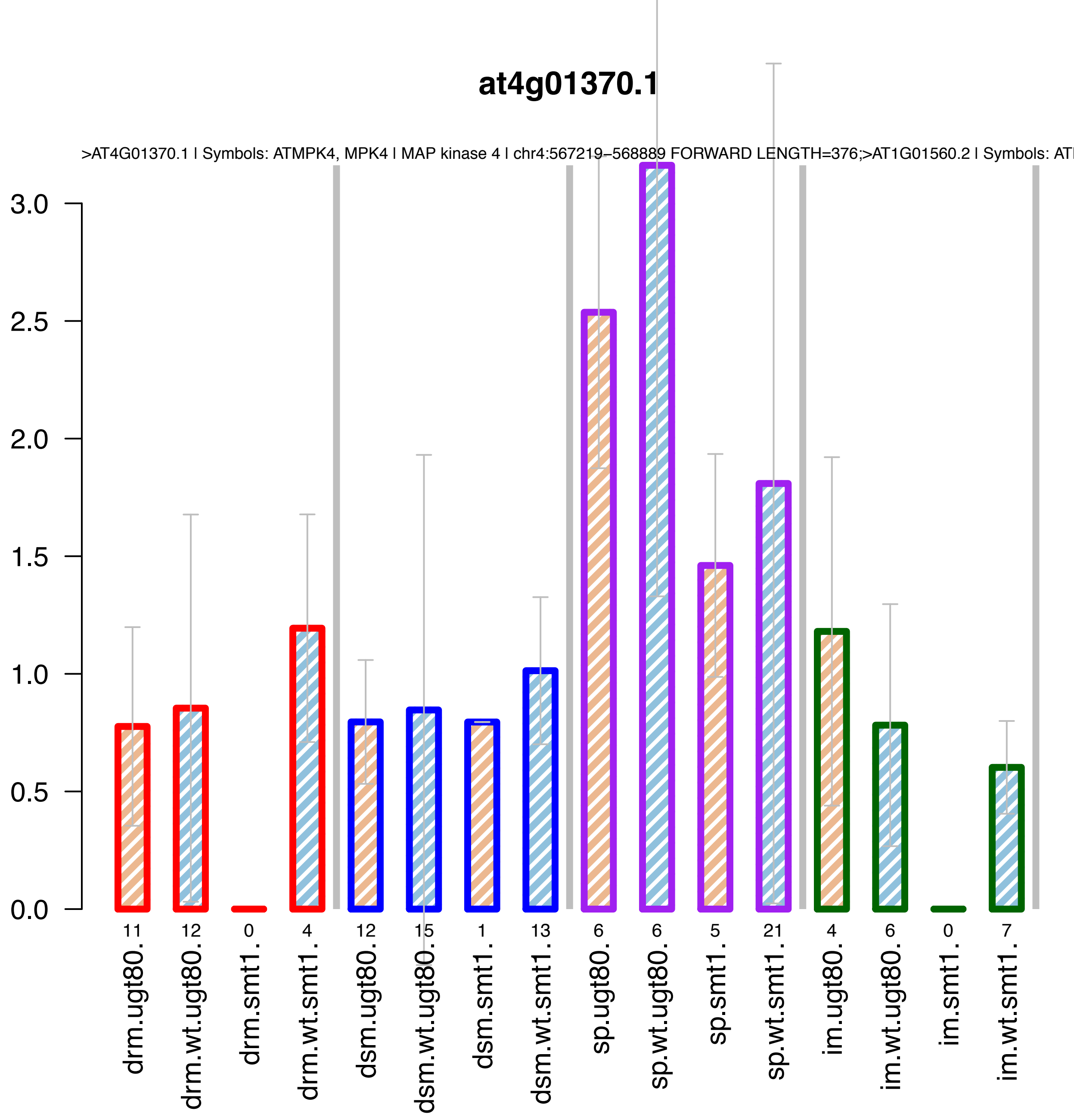

at4g01370.1
>AT4G01370.1 | Symbols: ATMPK4, MPK4 | MAP kinase 4 | chr4:567219-568889 FORWARD LENGTH=376;>AT1G01560.2 | Symbols: AT
3.0
2.5
2.0
1.5
1.0
0.5
0.0
11
12
0
4
12
15
1
13
6
6
5
21
4
6
0
7
drm.ugt80..
drm.wt.ugt80..
drm.smt1..
drm.wt.smt1..
dsm.ugt80..
dsm.wt.ugt80..
dsm.smt1..
dsm.wt.smt1..
sp.ugt80..
sp.wt.ugt80..
sp.smt1..
sp.wt.smt1..
im.ugt80..
im.wt.ugt80..
im.smt1..
im.wt.smt1..

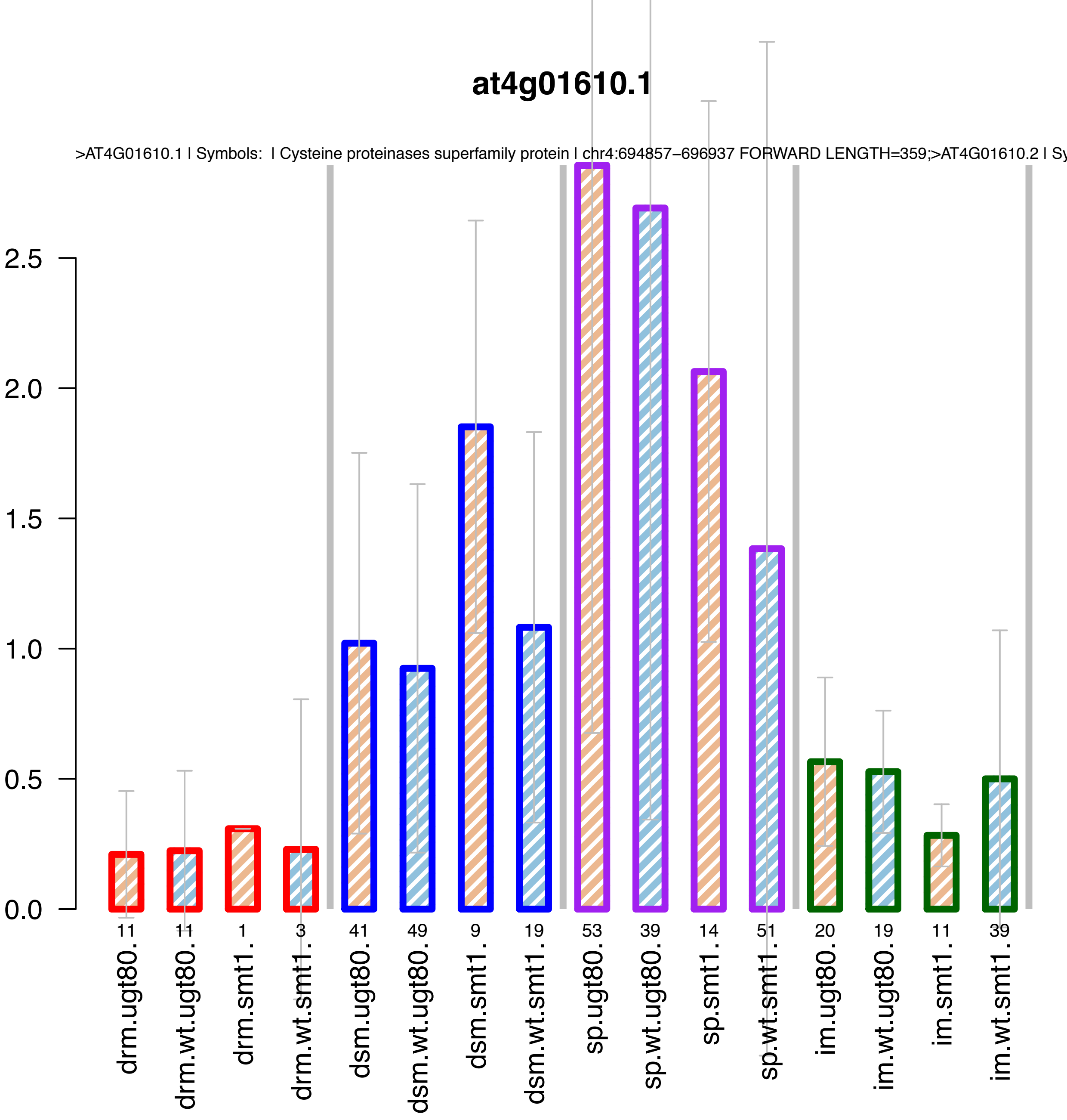

at4g01610.1
>AT4G01610.1 | Symbols: | Cysteine proteinases superfamily protein | chr4:694857−696937 FORWARD LENGTH=359;>AT4G01610.2 | Sy
0.0
0.5
1.0
1.5
2.0
2.5
11
11
1
3
41
49
9
19
53
39
14
51
20
19
11
39
drm.ugt80..
drm.wt.ugt80..
drm.smt1..
drm.wt.smt1..
dsm.ugt80..
dsm.wt.ugt80..
dsm.smt1..
dsm.wt.smt1..
sp.ugt80..
sp.wt.ugt80..
sp.smt1..
sp.wt.smt1..
im.ugt80..
im.wt.ugt80..
im.smt1..
im.wt.smt1..

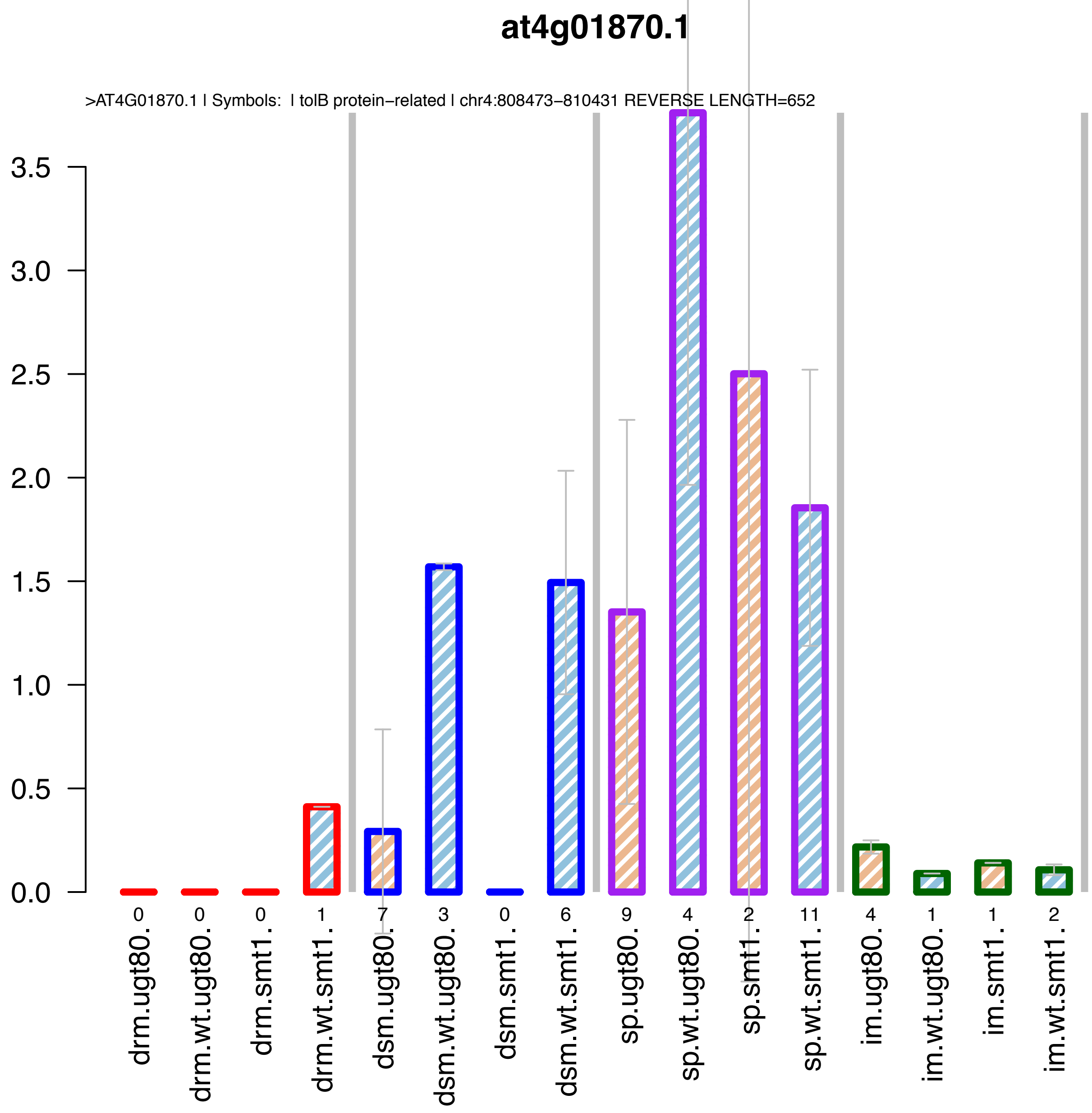
at4g01870.1
>AT4G01870.1 | Symbols: | tolB protein−related | chr4:808473−810431 REVERSE LENGTH=652
0.0
0.5
1.0
1.5
2.0
2.5
3.0
3.5
0 0 0 1 7 3 0 6 9 4 2 11 4 1 1 2
drm.ugt80.
drm.wt.ugt80.
drm.smt1.
drm.wt.smt1.
dsm.ugt80.
dsm.wt.ugt80.
dsm.smt1.
dsm.wt.smt1.
sp.ugt80.
sp.wt.ugt80.
sp.smt1.
sp.wt.smt1.
im.ugt80.
im.wt.ugt80.
im.smt1.
im.wt.smt1.

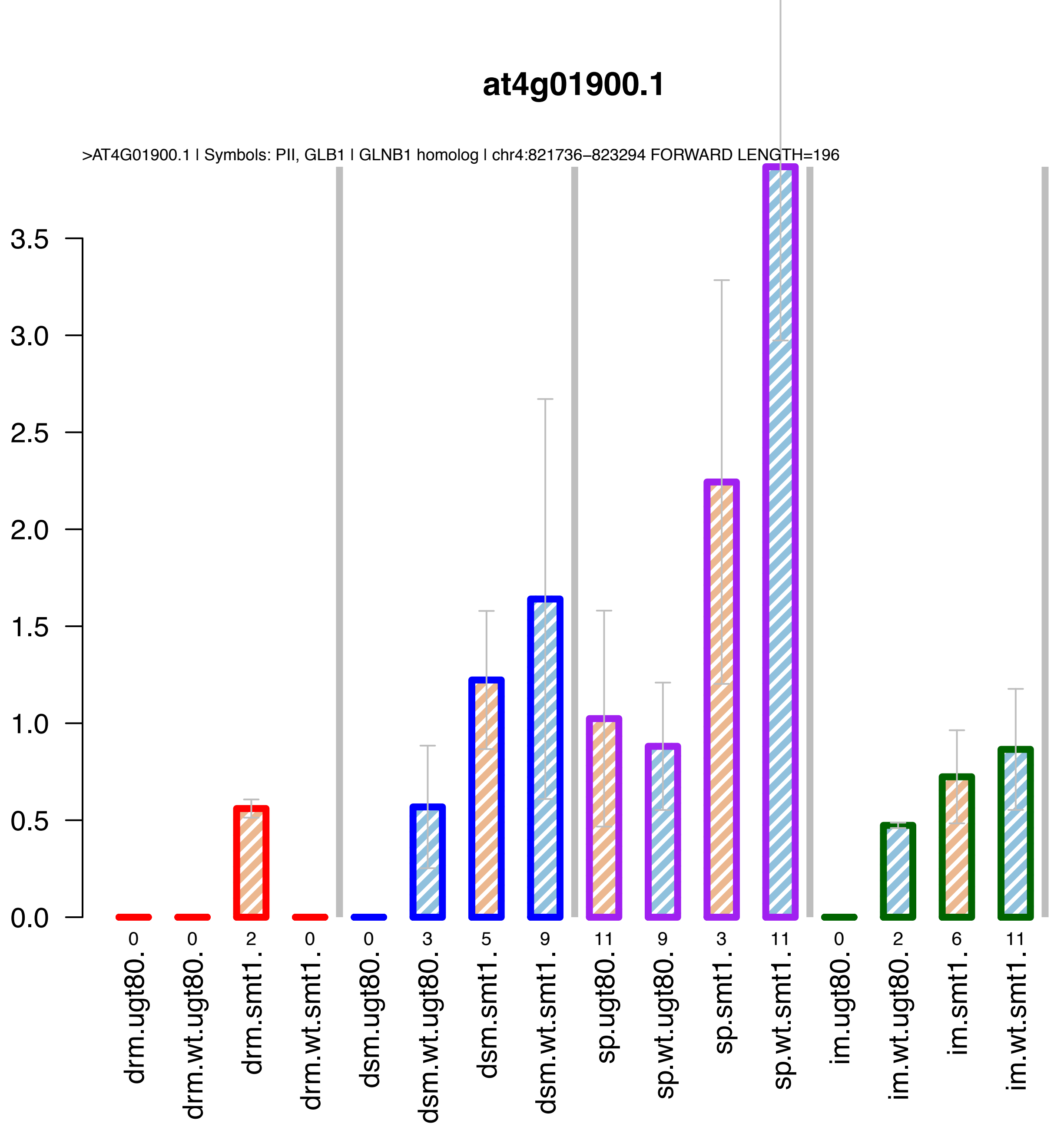

at4g01900.1
>AT4G01900.1 | Symbols: PII, GLB1 | GLNB1 homolog | chr4:821736–823294 FORWARD LENGTH=196
3.5
3.0
2.5
2.0
1.5
1.0
0.5
0.0
0
0
2
0
0
3
5
9
11
9
3
11
0
2
6
11
drm.ugt80.
drm.wt.ugt80.
drm.smt1.
drm.wt.smt1.
dsm.ugt80.
dsm.wt.ugt80.
dsm.smt1.
dsm.wt.smt1.
sp.ugt80.
sp.wt.ugt80.
sp.smt1.
sp.wt.smt1.
im.ugt80.
im.wt.ugt80.
im.smt1.
im.wt.smt1.

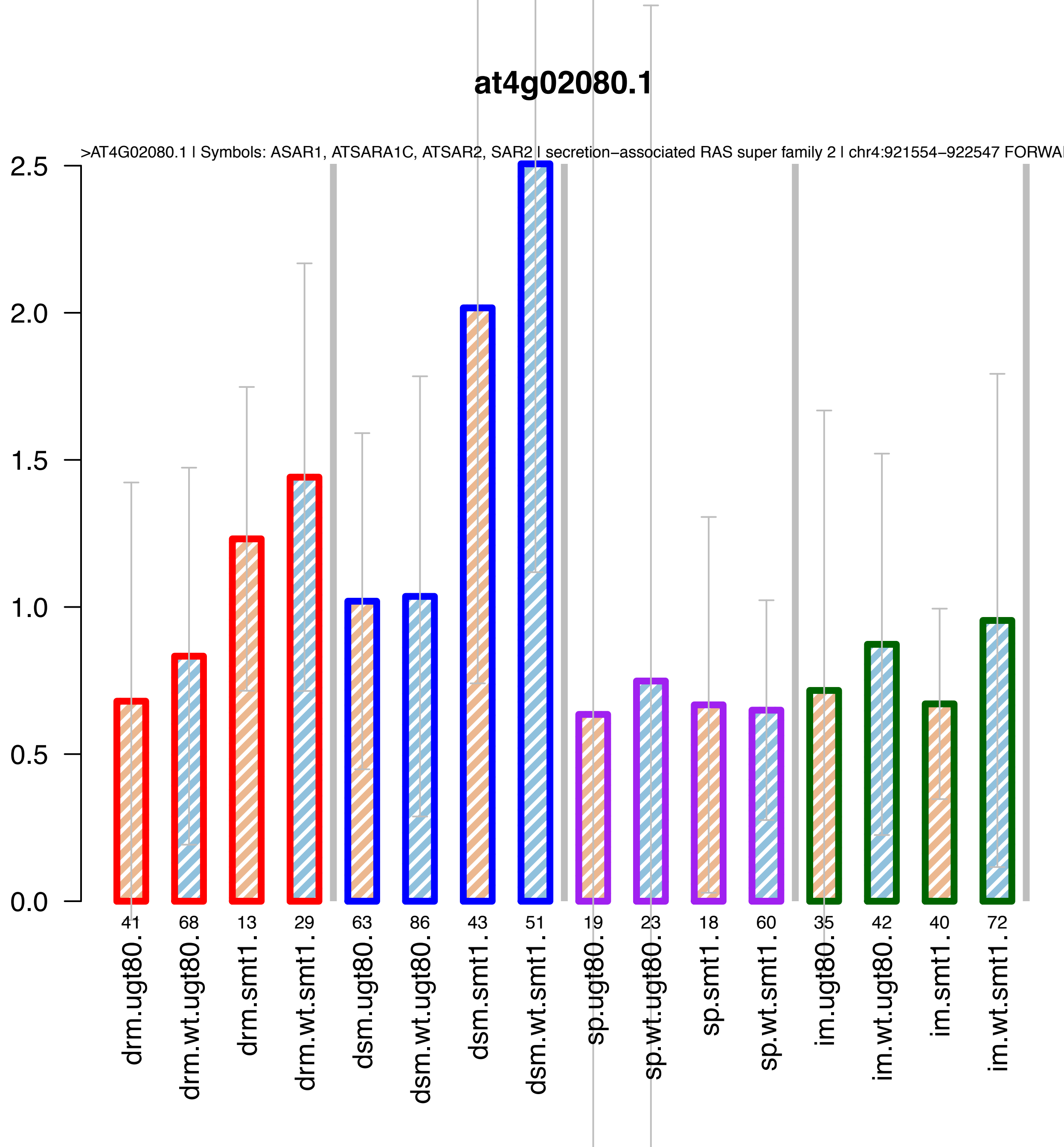

at4g02080.1
>AT4G02080.1 | Symbols: ASAR1, ATSARA1C, ATSAR2, SAR2 | secretion-associated RAS super family 2 | chr4:921554-922547 FORWA
0.0
0.5
1.0
1.5
2.0
2.5
41
68
13
29
63
86
43
51
19
23
18
60
35
42
40
72
drm.ugt80..
drm.wt.ugt80..
drm.smt1..
drm.wt.smt1..
dsm.ugt80..
dsm.wt.ugt80..
dsm.smt1..
dsm.wt.smt1..
sp.ugt80..
sp.wt.ugt80..
sp.smt1..
sp.wt.smt1..
im.ugt80..
im.wt.ugt80..
im.smt1..
im.wt.smt1..

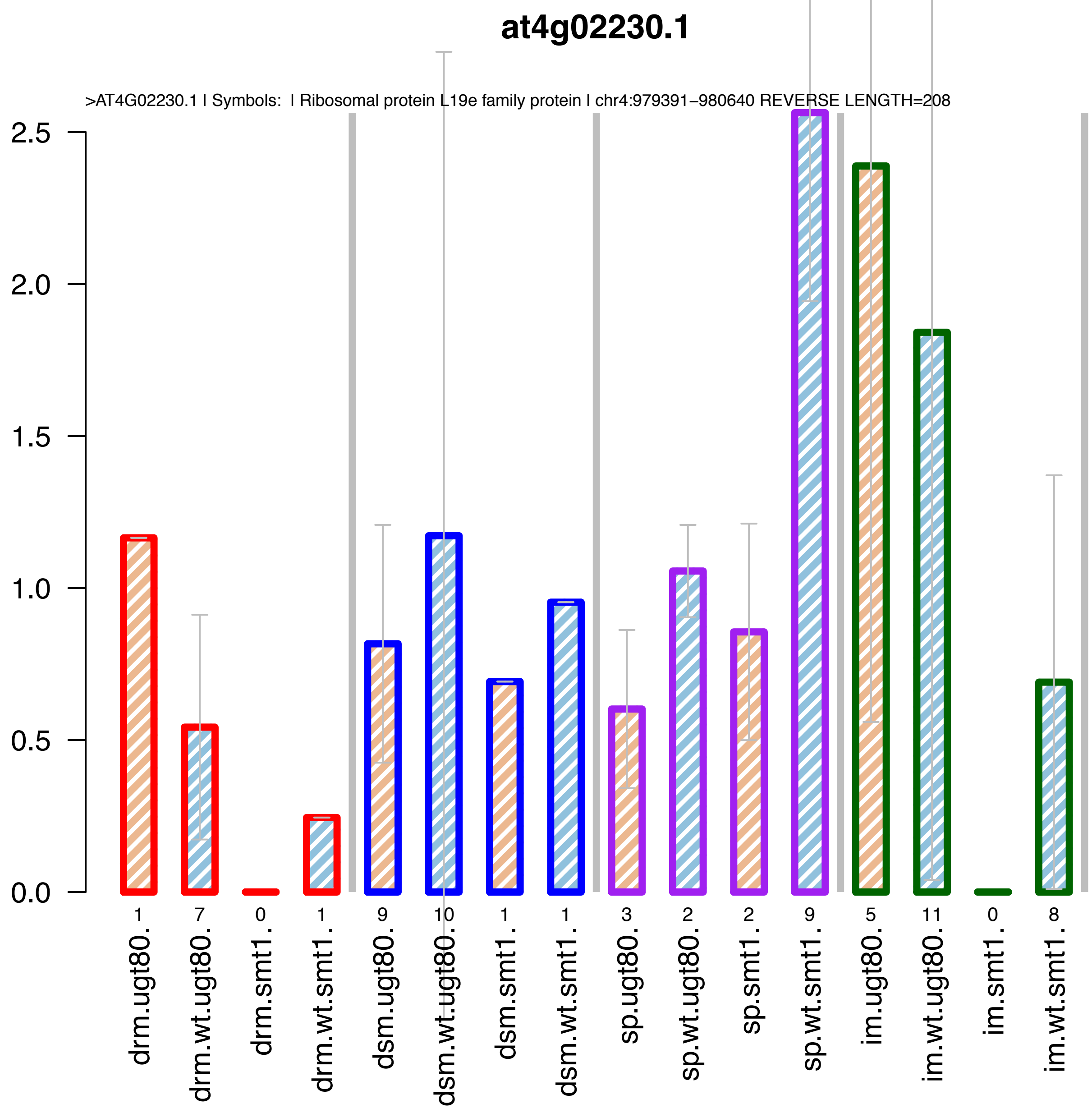

at4g02230.1
>AT4G02230.1 | Symbols: | Ribosomal protein L19e family protein | chr4:979391−980640 REVERSE LENGTH=208
2.5
2.0
1.5
1.0
0.5
0.0
1
7
0
1
9
10
1
1
3
2
2
9
5
11
0
8
drm.ugt80.
drm.wt.ugt80.
drm.smt1.
drm.wt.smt1.
dsm.ugt80.
dsm.wt.ugt80.
dsm.smt1.
dsm.wt.smt1.
sp.ugt80.
sp.wt.ugt80.
sp.smt1.
sp.wt.smt1.
im.ugt80.
im.wt.ugt80.
im.smt1.
im.wt.smt1.

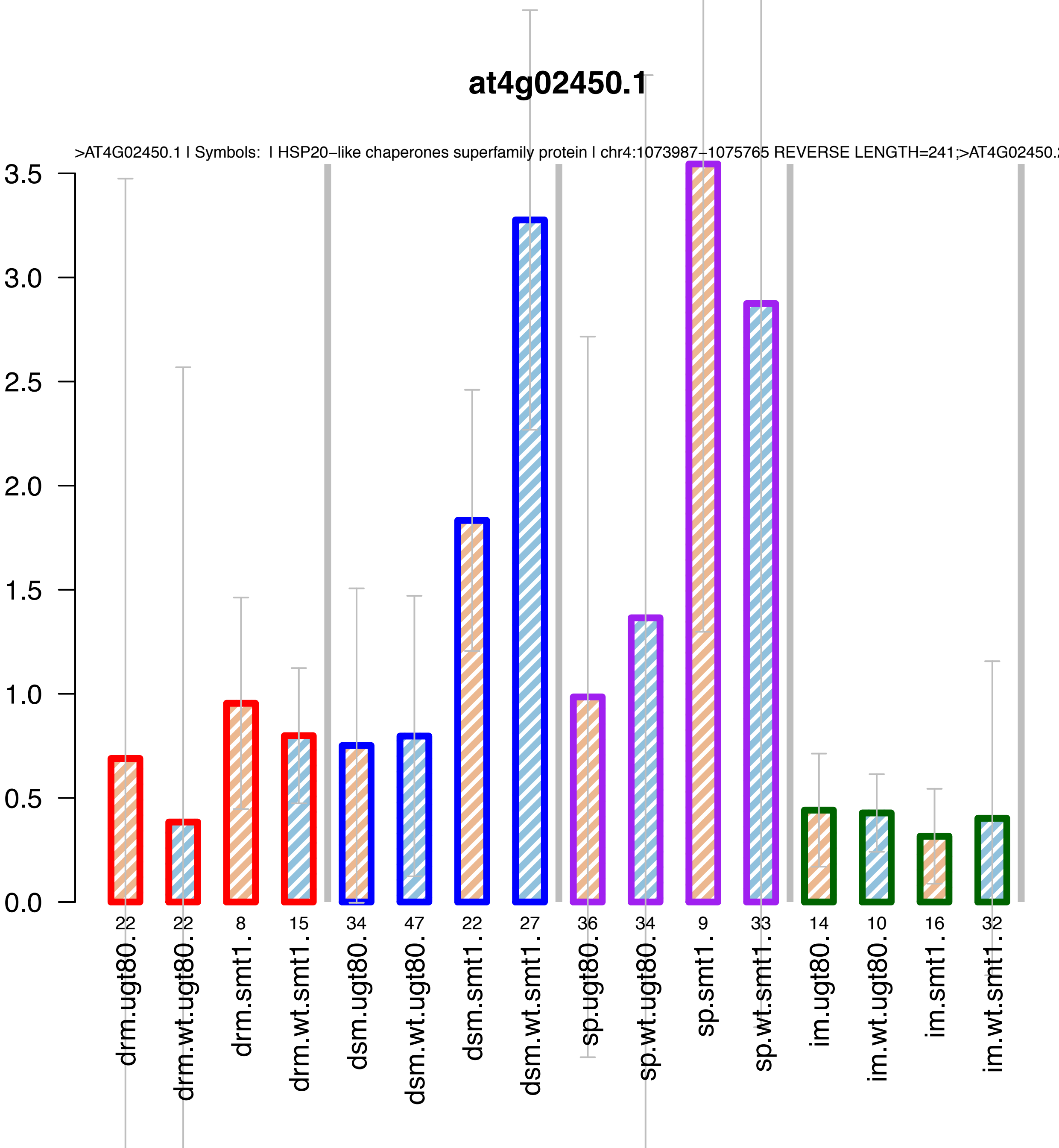

at4g02450.1
>AT4G02450.1 | Symbols: | HSP20–like chaperones superfamily protein | chr4:1073987–1075765 REVERSE LENGTH=241;>AT4G02450.2
3.5
3.0
2.5
2.0
1.5
1.0
0.5
0.0
22
22
8
15
34
47
22
27
36
34
9
33
14
10
16
32
drm.ugt80..
drm.wt.ugt80..
drm.smt1..
drm.wt.smt1..
dsm.ugt80..
dsm.wt.ugt80..
dsm.smt1..
dsm.wt.smt1..
sp.ugt80..
sp.wt.ugt80..
sp.smt1..
sp.wt.smt1..
im.ugt80..
im.wt.ugt80..
im.smt1..
im.wt.smt1..

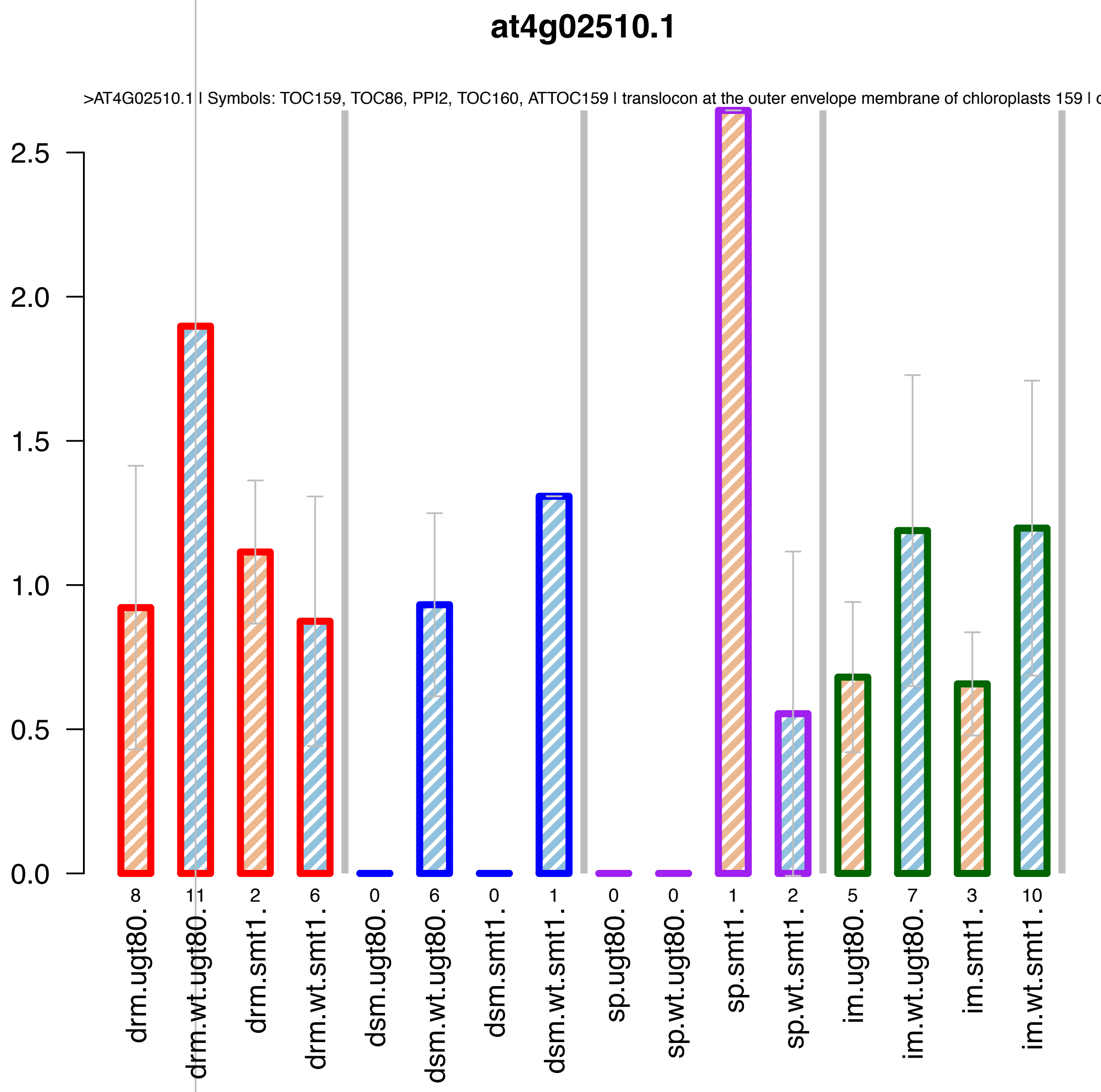

at4g02510.1
>AT4G02510.1 | Symbols: TOC159, TOC86, PPI2, TOC160, ATTOC159 | translocon at the outer envelope membrane of chloroplasts 159 | c
2.5
2.0
1.5
1.0
0.5
0.0
8
11
2
6
0
6
0
1
0
0
1
2
5
7
3
10
drm.ugt80..
drm.wt.ugt80..
drm.smt1..
drm.wt.smt1..
dsm.ugt80..
dsm.wt.ugt80..
dsm.smt1..
dsm.wt.smt1..
sp.ugt80..
sp.wt.ugt80..
sp.smt1..
sp.wt.smt1..
im.ugt80..
im.wt.ugt80..
im.smt1..
im.wt.smt1..

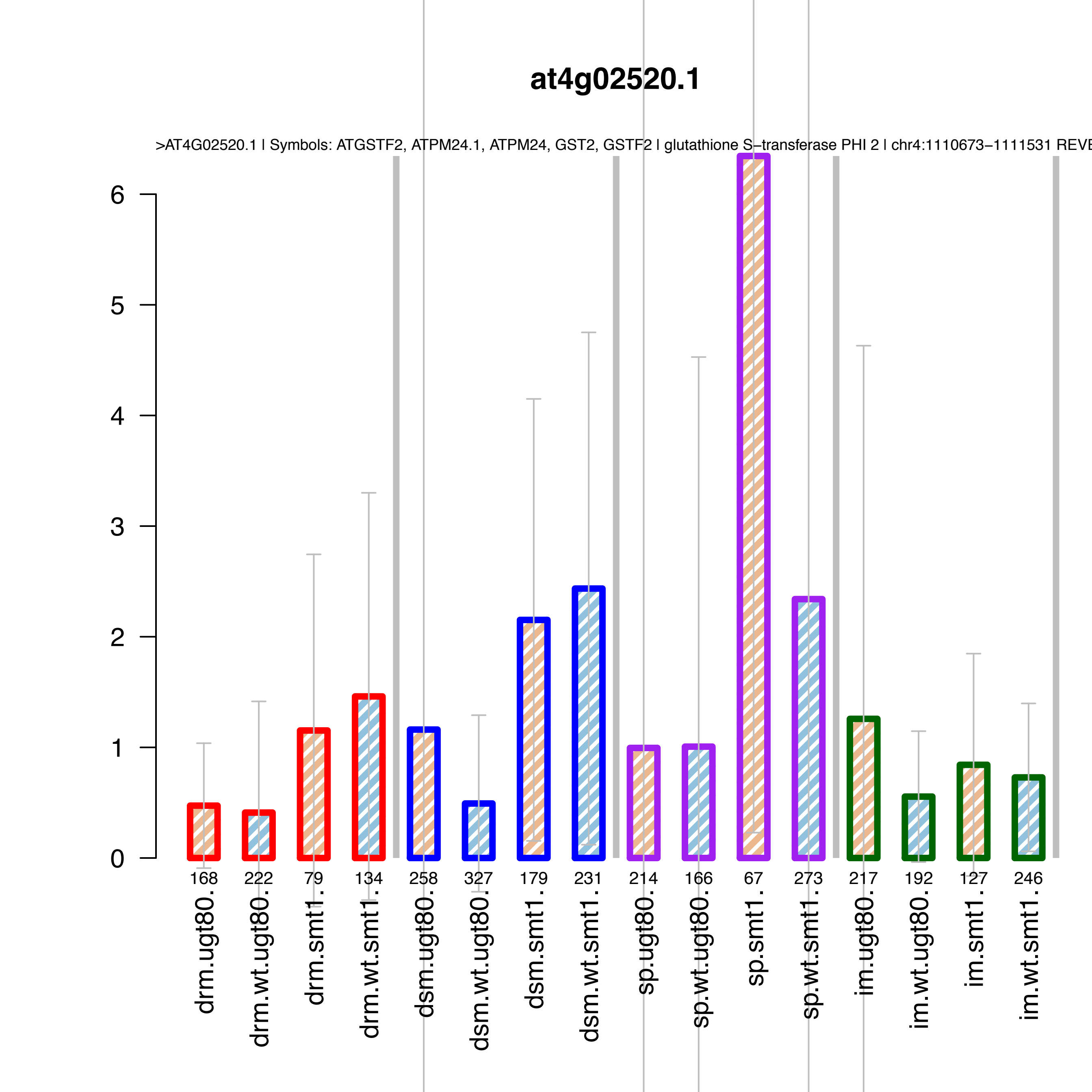

at4g02520.1
>AT4G02520.1 | Symbols: ATGSTF2, ATPM24.1, ATPM24, GST2, GSTF2 | glutathione S−transferase PHI 2 | chr4:1110673−1111531 REVE
0
1
2
3
4
5
6
168
222
79
134
258
327
179
231
214
166
67
273
217
192
127
246
drm.ugt80..
drm.wt.ugt80..
drm.smt1..
drm.wt.smt1..
dsm.ugt80..
dsm.wt.ugt80..
dsm.smt1..
dsm.wt.smt1..
sp.ugt80..
sp.wt.ugt80..
sp.smt1..
sp.wt.smt1..
im.ugt80..
im.wt.ugt80..
im.smt1..
im.wt.smt1..

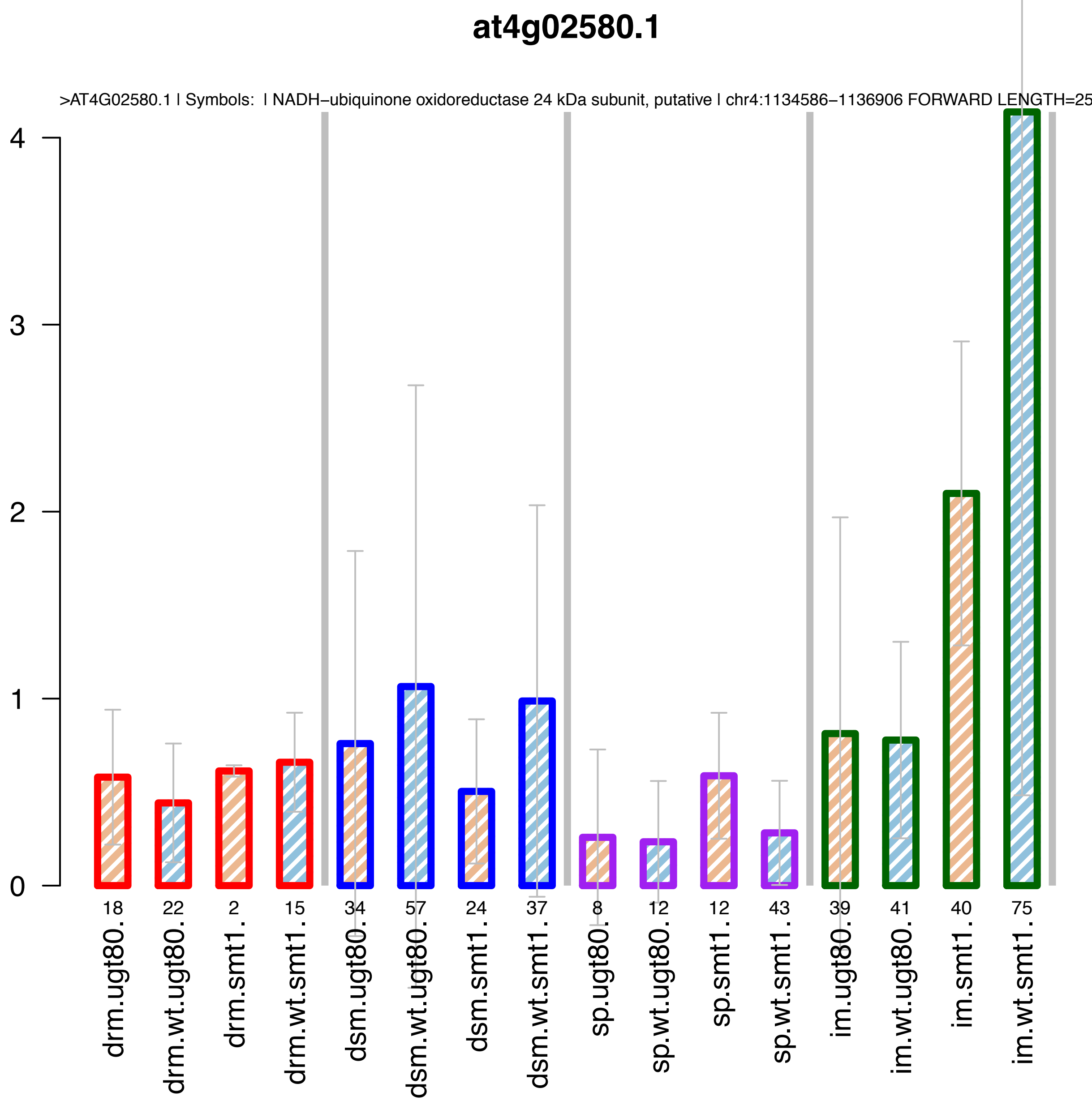

at4g02580.1
>AT4G02580.1 | Symbols: | NADH−ubiquinone oxidoreductase 24 kDa subunit, putative | chr4:1134586−1136906 FORWARD LENGTH=25
0
1
2
3
4
18
22
2
15
34
57
24
37
8
12
12
43
39
41
40
75
drm.ugt80.
drm.wt.ugt80.
drm.smt1.
drm.wt.smt1.
dsm.ugt80.
dsm.wt.ugt80.
dsm.smt1.
dsm.wt.smt1.
sp.ugt80.
sp.wt.ugt80.
sp.smt1.
sp.wt.smt1.
im.ugt80.
im.wt.ugt80.
im.smt1.
im.wt.smt1.

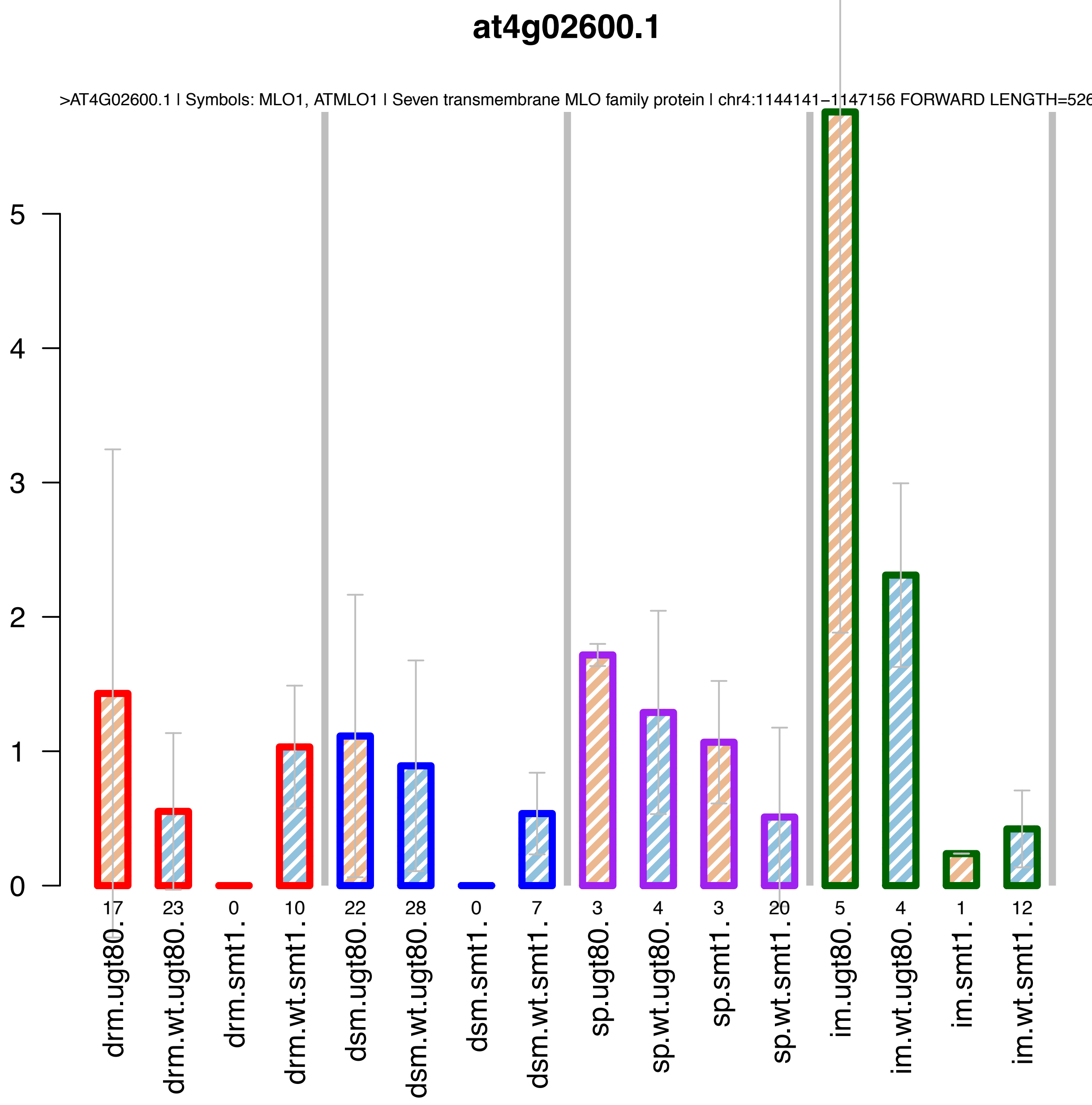

at4g02600.1
>AT4G02600.1 | Symbols: MLO1, ATMLO1 | Seven transmembrane MLO family protein | chr4:1144141-1147156 FORWARD LENGTH=526
0
1
2
3
4
5
17
23
0
10
22
28
0
7
3
4
3
20
5
4
1
12
drm.ugt80..
drm.wt.ugt80..
drm.smt1..
drm.wt.smt1..
dsm.ugt80..
dsm.wt.ugt80..
dsm.smt1..
dsm.wt.smt1..
sp.ugt80..
sp.wt.ugt80..
sp.smt1..
sp.wt.smt1..
im.ugt80..
im.wt.ugt80..
im.smt1..
im.wt.smt1..

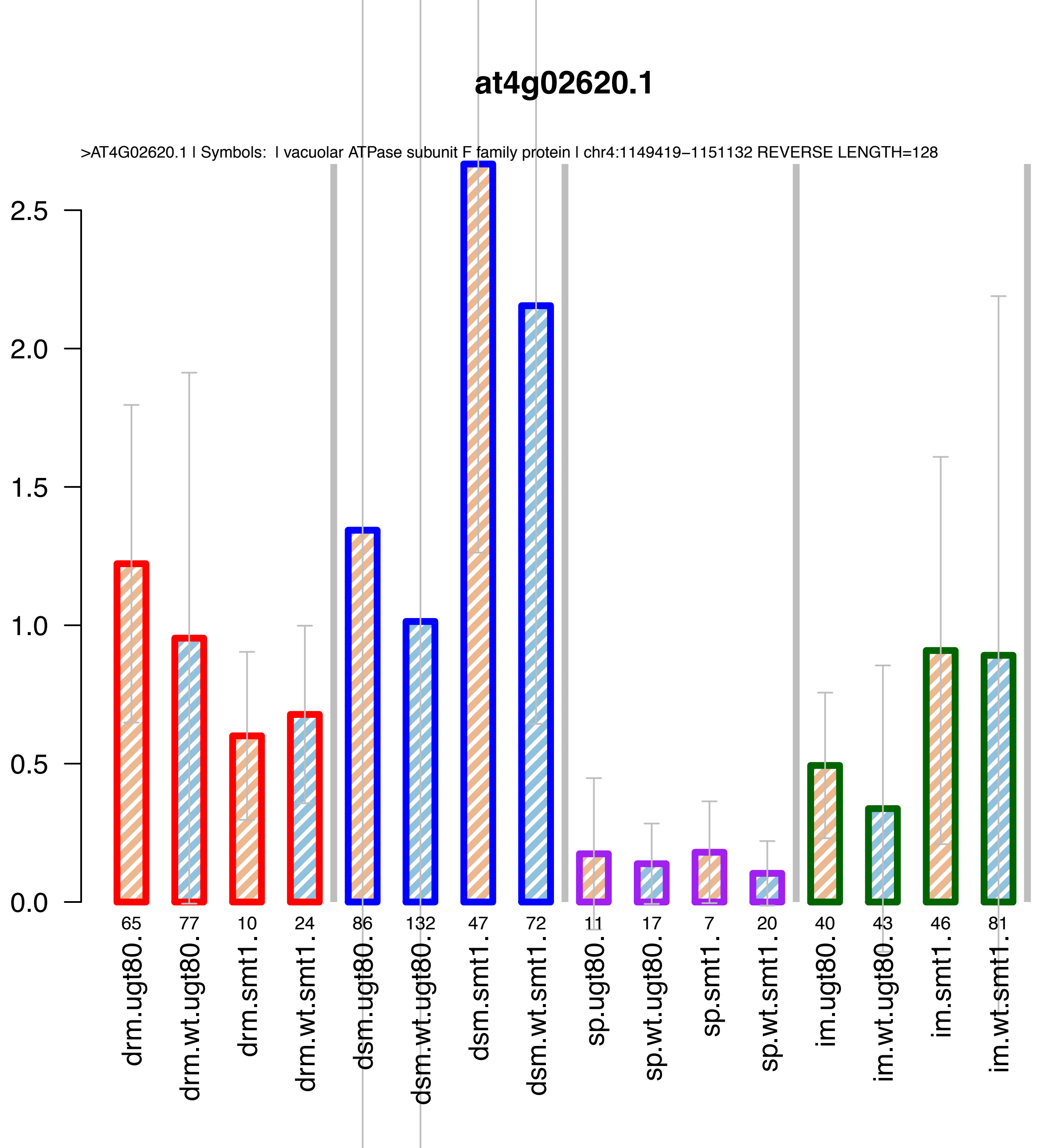
at4g02620.1
>AT4G02620.1 | Symbols: | vacuolar ATPase subunit F family protein | chr4:1149419-1151132 REVERSE LENGTH=128
0.0
0.5
1.0
1.5
2.0
2.5
65
77
10
24
86
132
47
72
11
17
7
20
40
43
46
81
drm.ugt80..
drm.wt.ugt80..
drm.smt1..
drm.wt.smt1..
dsm.ugt80..
dsm.wt.ugt80..
dsm.smt1..
dsm.wt.smt1..
sp.ugt80..
sp.wt.ugt80..
sp.smt1..
sp.wt.smt1..
im.ugt80..
im.wt.ugt80..
im.smt1..
im.wt.smt1..

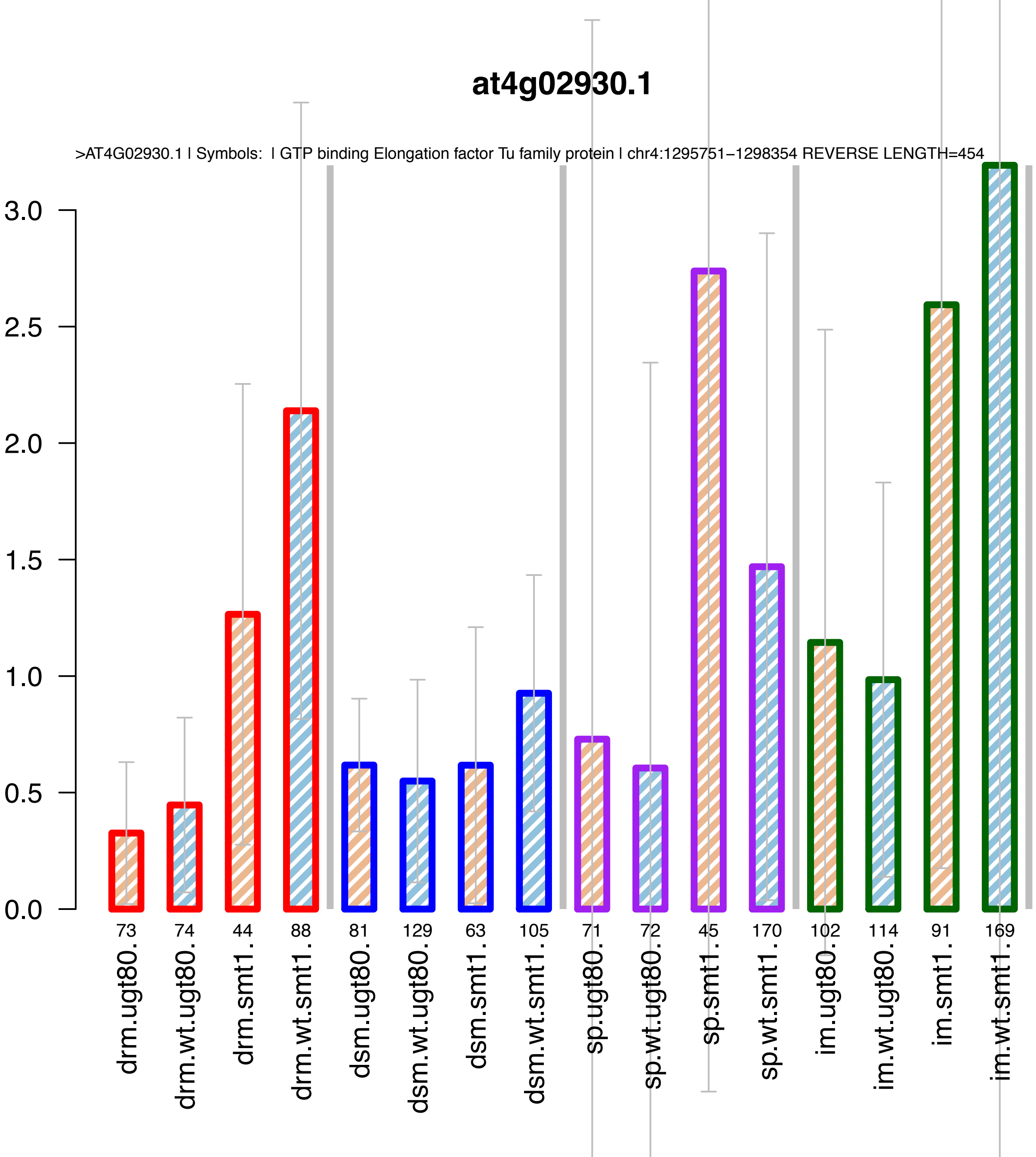

at4g02930.1
>AT4G02930.1 | Symbols: | GTP binding Elongation factor Tu family protein | chr4:1295751−1298354 REVERSE LENGTH=454
3.0
2.5
2.0
1.5
1.0
0.5
0.0
73
74
44
88
81
129
63
105
71
72
45
170
102
114
91
169
drm.ugt80..
drm.wt.ugt80..
drm.smt1..
drm.wt.smt1..
dsm.ugt80..
dsm.wt.ugt80..
dsm.smt1..
dsm.wt.smt1..
sp.ugt80..
sp.wt.ugt80..
sp.smt1..
sp.wt.smt1..
im.ugt80..
im.wt.ugt80..
im.smt1..
im.wt.smt1..

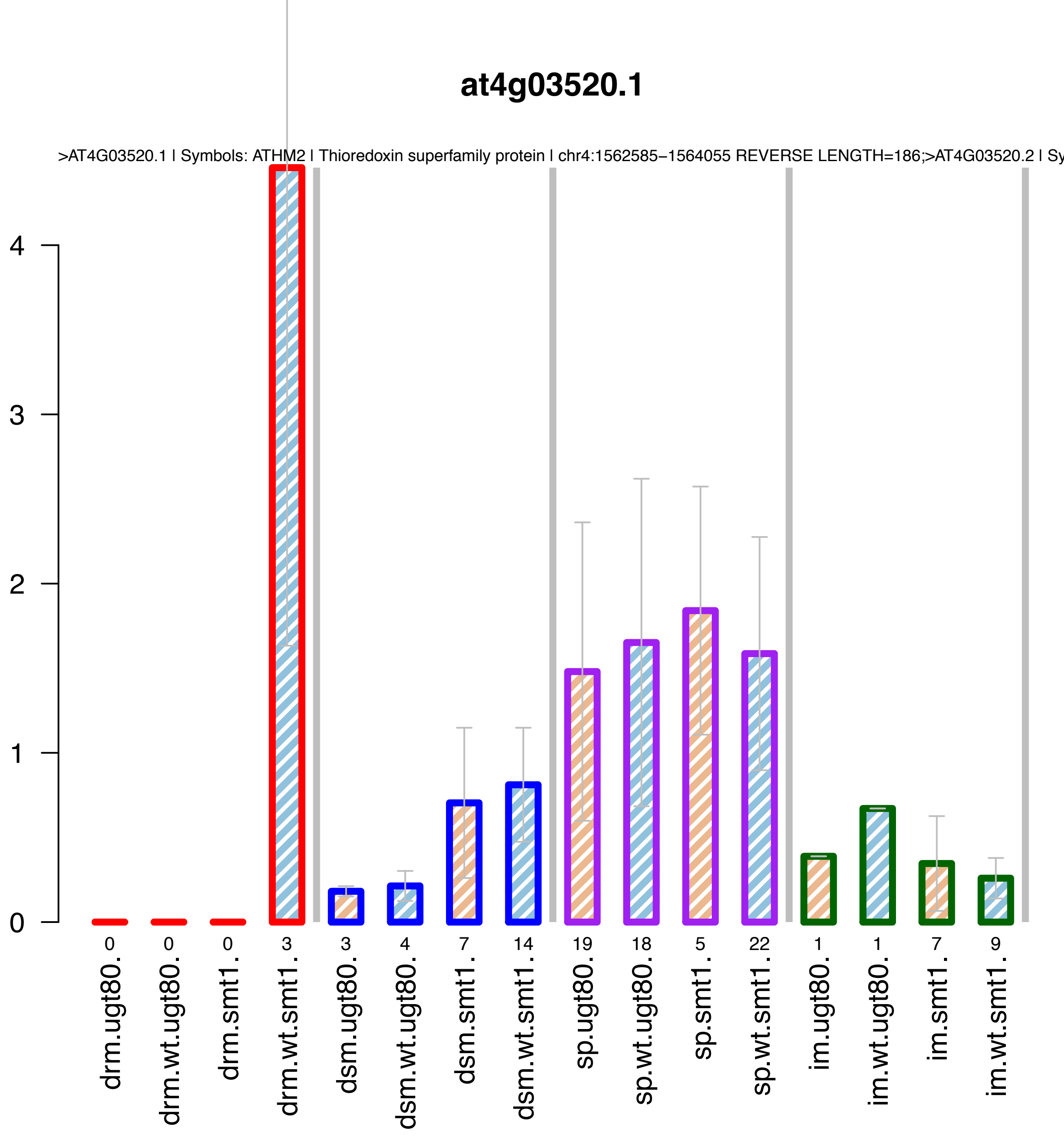

at4g03520.1
>AT4G03520.1 | Symbols: ATHM2 | Thioredoxin superfamily protein | chr4:1562585−1564055 REVERSE LENGTH=186;>AT4G03520.2 | Sy
0
1
2
3
4
0 0 0 3 3 4 7 14 19 18 5 22 1 1 7 9
drm.ugt80.
drm.wt.ugt80.
drm.smt1.
drm.wt.smt1.
dsm.ugt80.
dsm.wt.ugt80.
dsm.smt1.
dsm.wt.smt1.
sp.ugt80.
sp.wt.ugt80.
sp.smt1.
sp.wt.smt1.
im.ugt80.
im.wt.ugt80.
im.smt1.
im.wt.smt1.

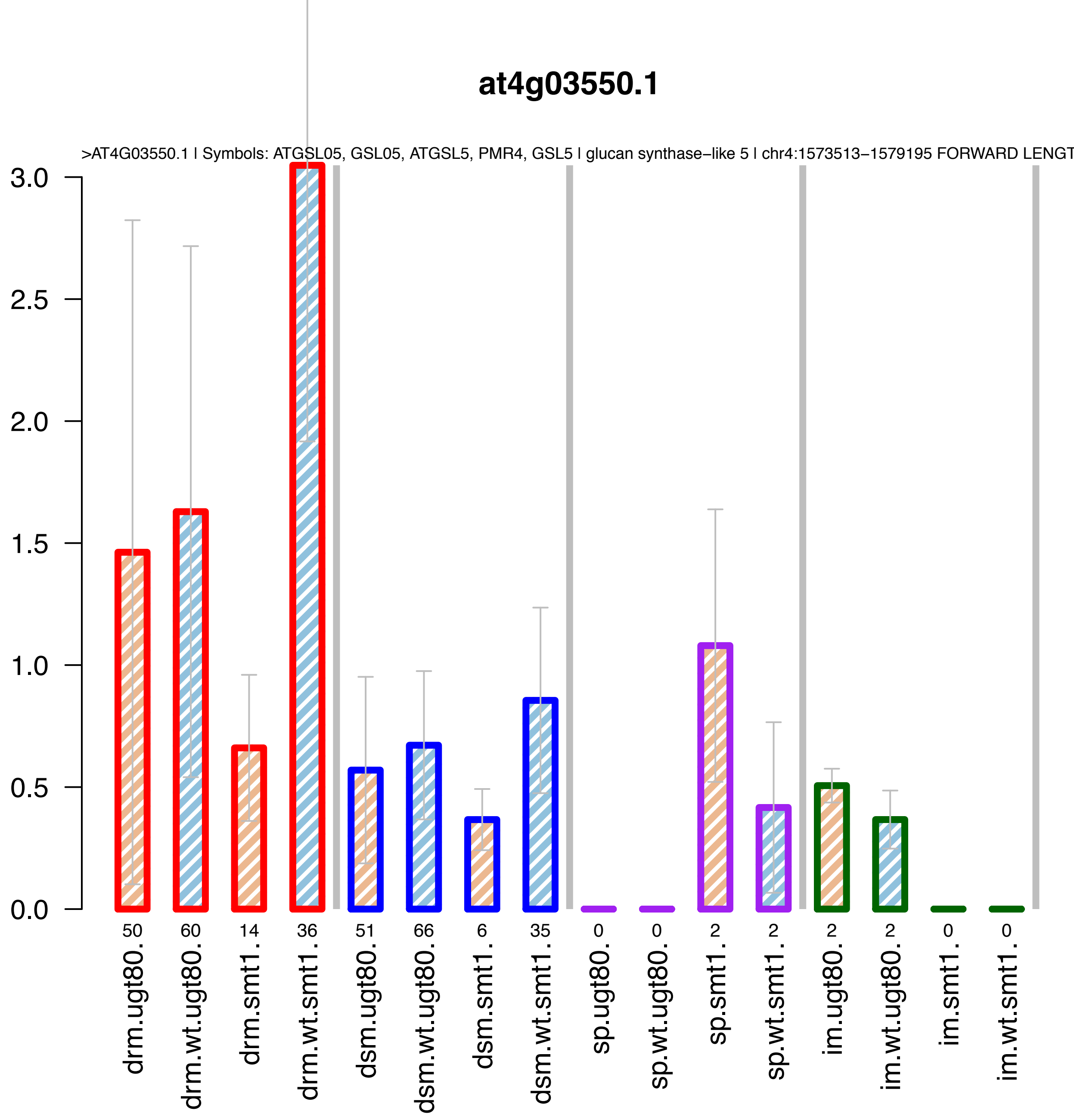

at4g03550.1
>AT4G03550.1 | Symbols: ATGSL05, GSL05, ATGSL5, PMR4, GSL5 | glucan synthase-like 5 | chr4:1573513-1579195 FORWARD LENGT
3.0
2.5
2.0
1.5
1.0
0.5
0.0
50
60
14
36
51
66
6
35
0
0
2
2
2
2
0
0
drm.ugt80.
drm.wt.ugt80.
drm.smt1.
drm.wt.smt1.
dsm.ugt80.
dsm.wt.ugt80.
dsm.smt1.
dsm.wt.smt1.
sp.ugt80.
sp.wt.ugt80.
sp.smt1.
sp.wt.smt1.
im.ugt80.
im.wt.ugt80.
im.smt1.
im.wt.smt1.

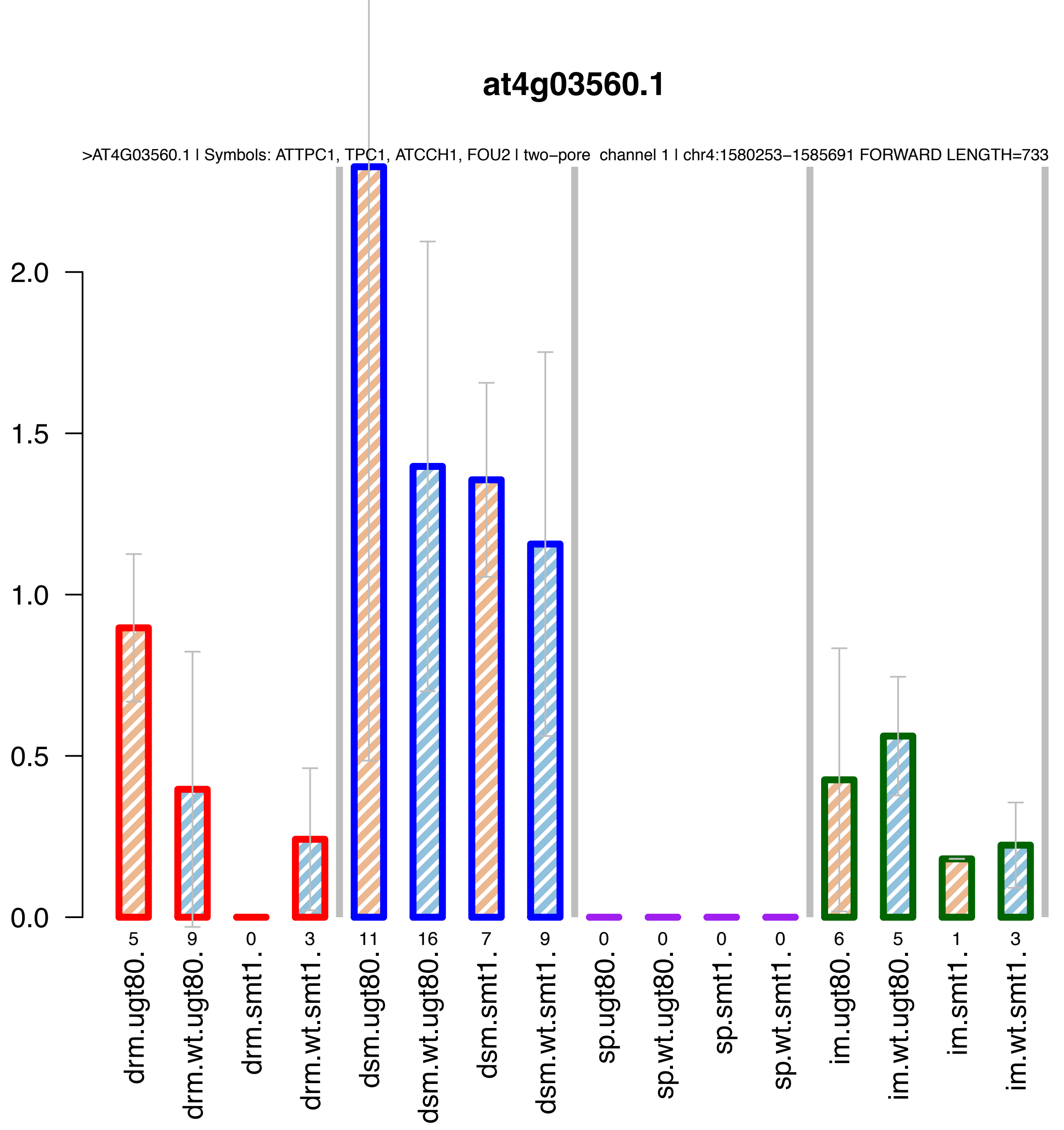
at4g03560.1
>AT4G03560.1 | Symbols: ATTPC1, TPC1, ATCCH1, FOU2 | two-pore channel 1 | chr4:1580253-1585691 FORWARD LENGTH=733
2.0
1.5
1.0
0.5
0.0
5
9
0
3
11
16
7
9
0
0
0
0
6
5
1
3
drm.ugt80.
drm.wt.ugt80.
drm.smt1.
drm.wt.smt1.
dsm.ugt80.
dsm.wt.ugt80.
dsm.smt1.
dsm.wt.smt1.
sp.ugt80.
sp.wt.ugt80.
sp.smt1.
sp.wt.smt1.
im.ugt80.
im.wt.ugt80.
im.smt1.
im.wt.smt1.

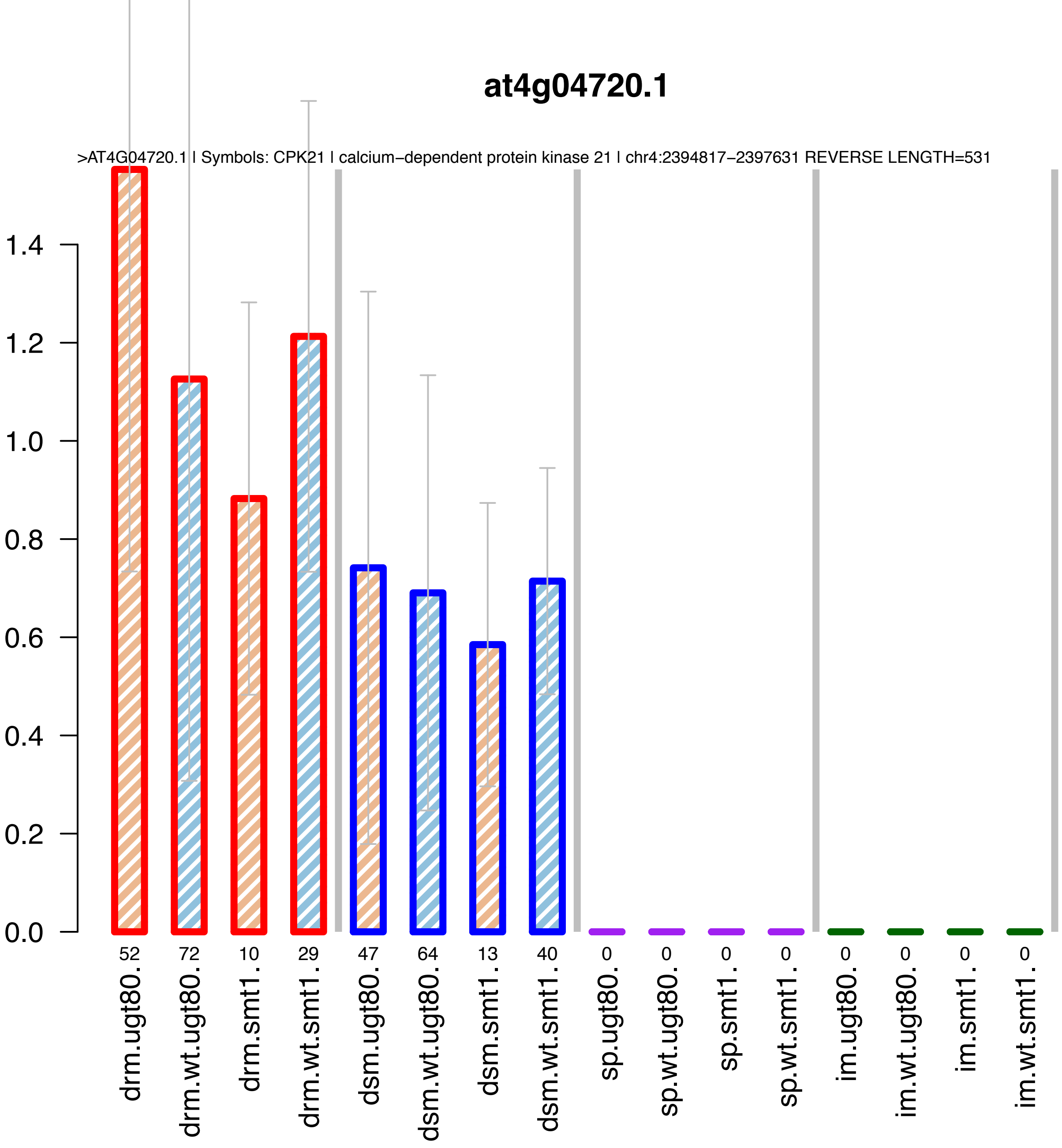

at4g04720.1
>AT4G04720.1 | Symbols: CPK21 | calcium−dependent protein kinase 21 | chr4:2394817−2397631 REVERSE LENGTH=531
0.0
0.2
0.4
0.6
0.8
1.0
1.2
1.4
52
72
10
29
47
64
13
40
0
0
0
0
0
0
0
0
drm.ugt80.
drm.wt.ugt80.
drm.smt1.
drm.wt.smt1.
dsm.ugt80.
dsm.wt.ugt80.
dsm.smt1.
dsm.wt.smt1.
sp.ugt80.
sp.wt.ugt80.
sp.smt1.
sp.wt.smt1.
im.ugt80.
im.wt.ugt80.
im.smt1.
im.wt.smt1.

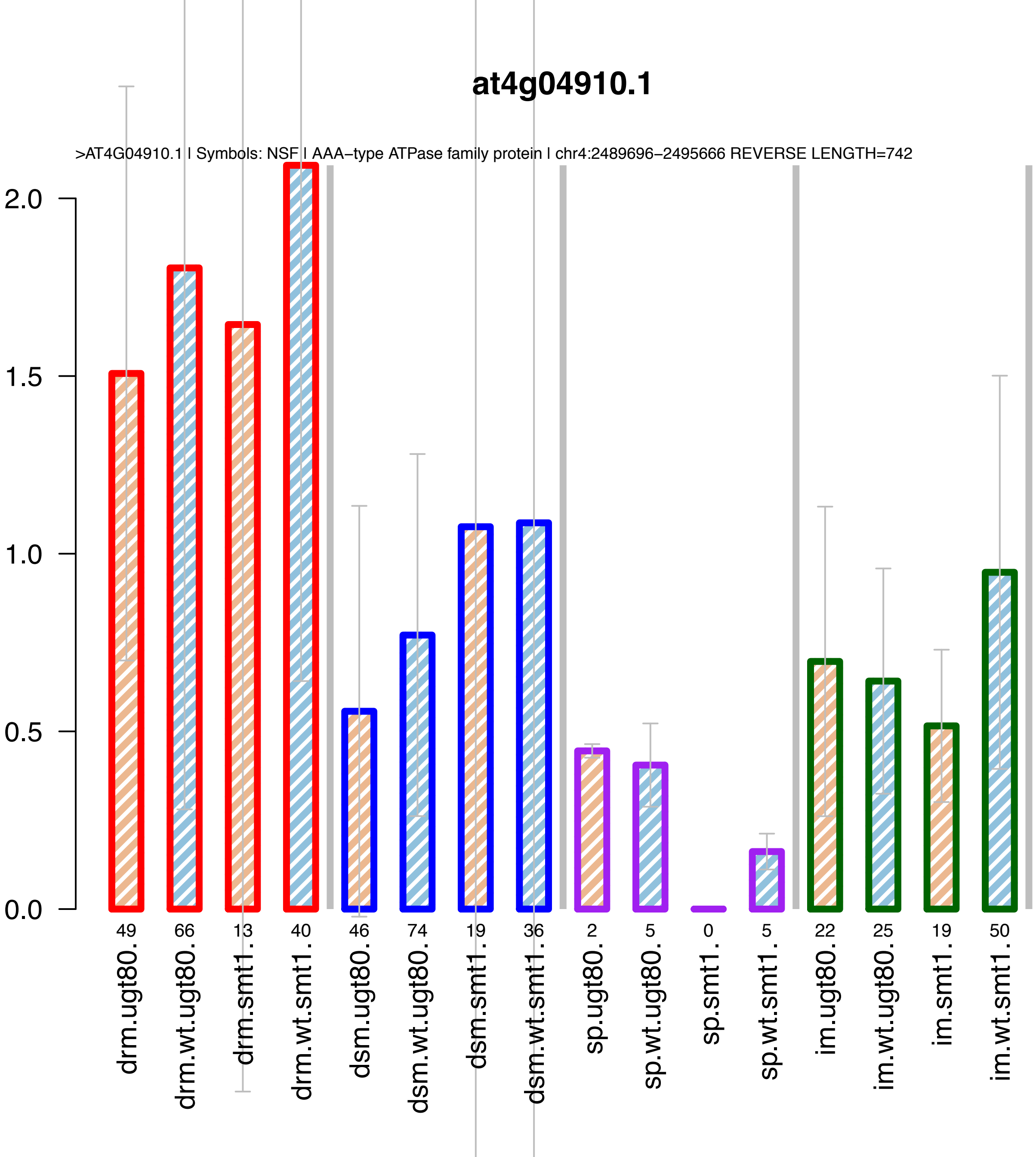

at4g04910.1
>AT4G04910.1 | Symbols: NSF | AAA−type ATPase family protein | chr4:2489696−2495666 REVERSE LENGTH=742
2.0
1.5
1.0
0.5
0.0
49
66
13
40
46
74
19
36
2
5
0
5
22
25
19
50
drm.ugt80.
drm.wt.ugt80.
drm.smt1.
drm.wt.smt1.
dsm.ugt80.
dsm.wt.ugt80.
dsm.smt1.
dsm.wt.smt1.
sp.ugt80.
sp.wt.ugt80.
sp.smt1.
sp.wt.smt1.
im.ugt80.
im.wt.ugt80.
im.smt1.
im.wt.smt1.

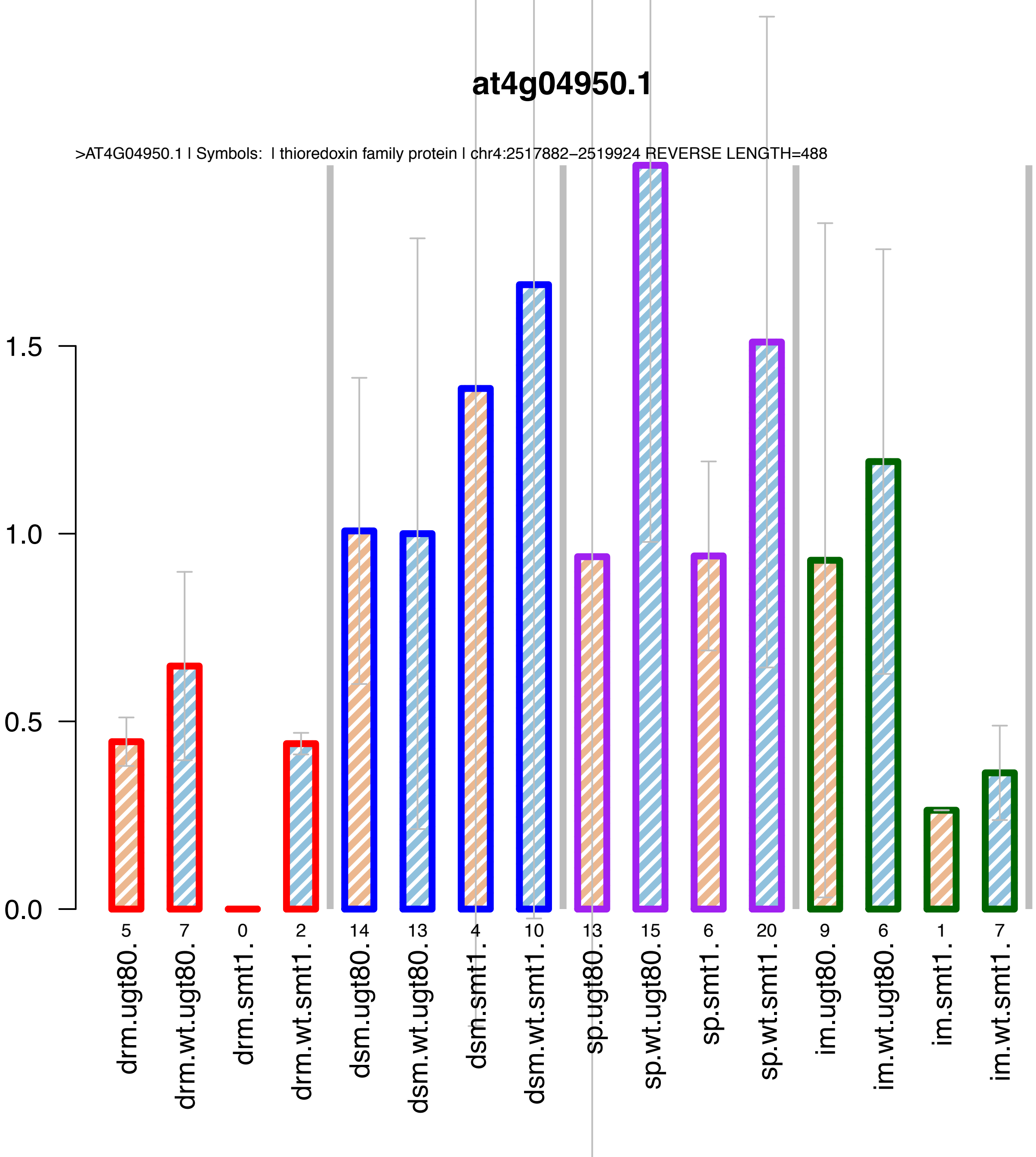

at4g04950.1
>AT4G04950.1 | Symbols: | thioredoxin family protein | chr4:2517882−2519924 REVERSE LENGTH=488
0.0
0.5
1.0
1.5
5
7
0
2
14
13
4
10
13
15
6
20
9
6
1
7
drm.ugt80..
drm.wt.ugt80..
drm.smt1..
drm.wt.smt1..
dsm.ugt80..
dsm.wt.ugt80..
dsm.smt1..
dsm.wt.smt1..
sp.ugt80..
sp.wt.ugt80..
sp.smt1..
sp.wt.smt1..
im.ugt80..
im.wt.ugt80..
im.smt1..
im.wt.smt1..

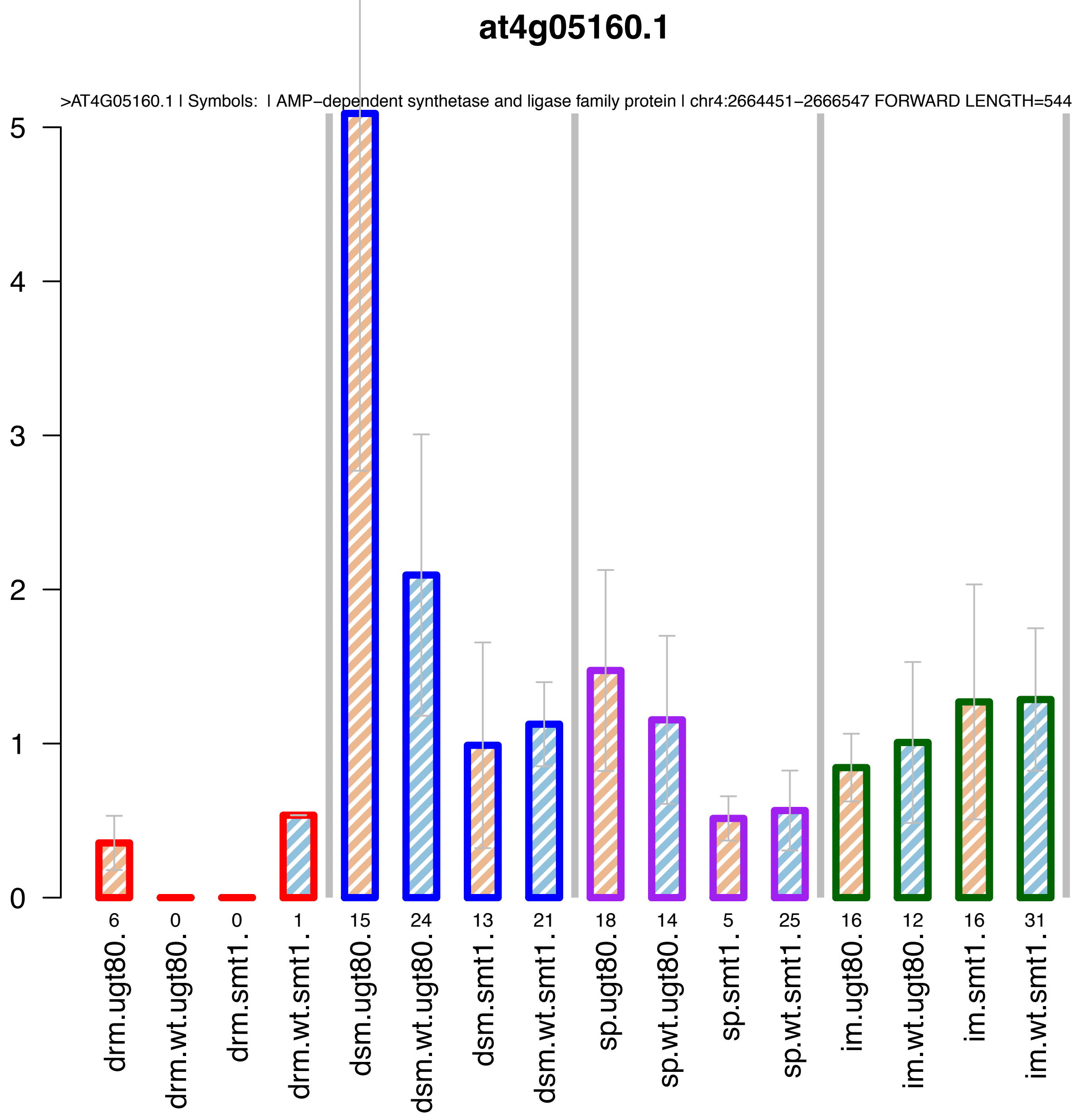

at4g05160.1
>AT4G05160.1 | Symbols: | AMP−dependent synthetase and ligase family protein | chr4:2664451−2666547 FORWARD LENGTH=544
0
1
2
3
4
5
6
0
0
1
15
24
13
21
18
14
5
25
16
12
16
31
drm.ugt80..
drm.wt.ugt80..
drm.smt1..
drm.wt.smt1..
dsm.ugt80..
dsm.wt.ugt80..
dsm.smt1..
dsm.wt.smt1..
sp.ugt80..
sp.wt.ugt80..
sp.smt1..
sp.wt.smt1..
im.ugt80..
im.wt.ugt80..
im.smt1..
im.wt.smt1..

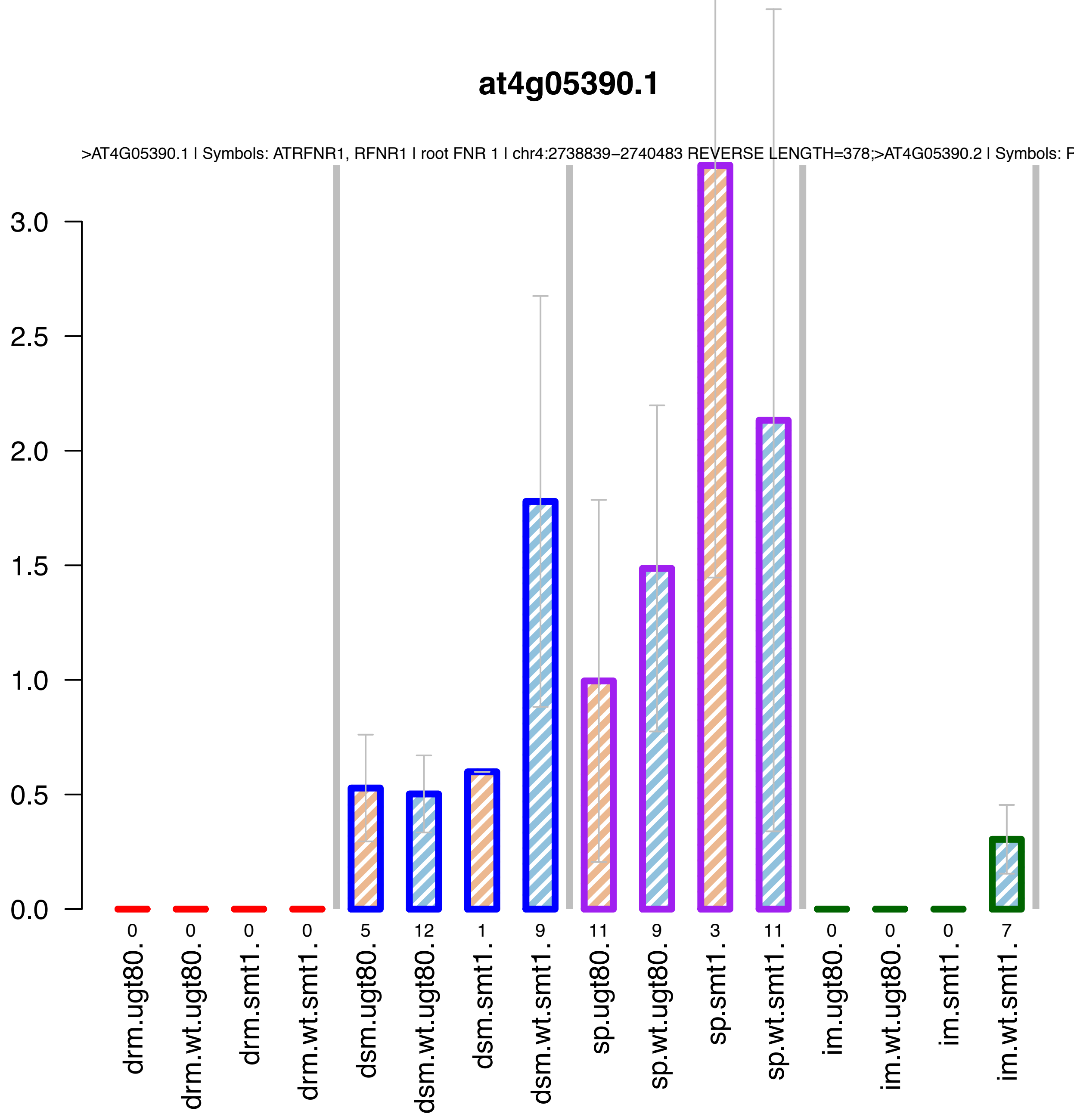

at4g05390.1
>AT4G05390.1 | Symbols: ATRFNR1, RFNR1 | root FNR 1 | chr4:2738839−2740483 REVERSE LENGTH=378;>AT4G05390.2 | Symbols: F
3.0
2.5
2.0
1.5
1.0
0.5
0.0
0
0
0
0
5
12
1
9
11
9
3
11
0
0
0
7
drm.ugt80.
drm.wt.ugt80.
drm.smt1.
drm.wt.smt1.
dsm.ugt80.
dsm.wt.ugt80.
dsm.smt1.
dsm.wt.smt1.
sp.ugt80.
sp.wt.ugt80.
sp.smt1.
sp.wt.smt1.
im.ugt80.
im.wt.ugt80.
im.smt1.
im.wt.smt1.

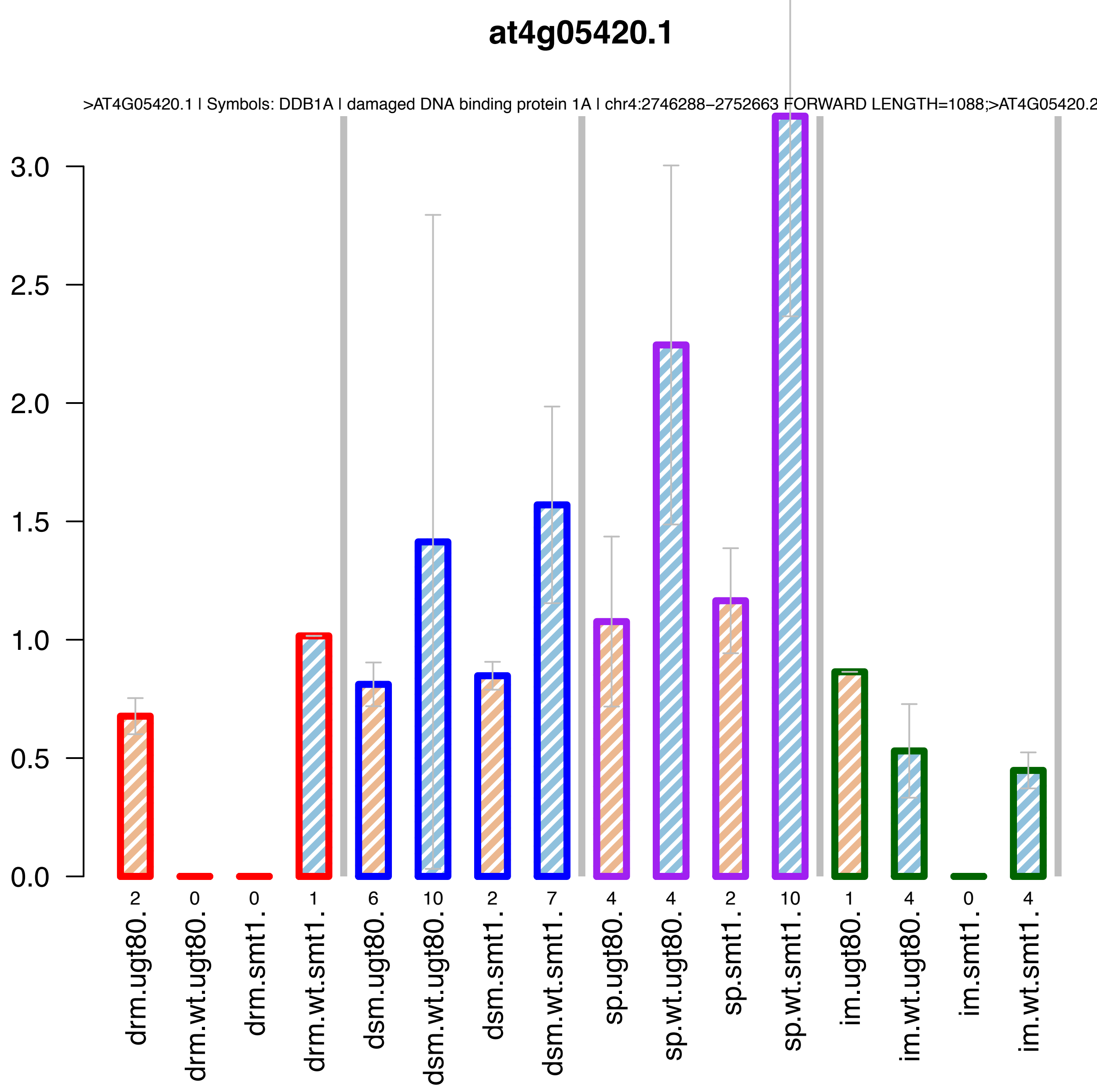
at4g05420.1
>AT4G05420.1 | Symbols: DDB1A | damaged DNA binding protein 1A | chr4:2746288-2752663 FORWARD LENGTH=1088;>AT4G05420.2
3.0
2.5
2.0
1.5
1.0
0.5
0.0
2
0
0
1
6
10
2
7
4
4
2
10
1
4
0
4
drm.ugt80.
drm.wt.ugt80.
drm.smt1.
drm.wt.smt1.
dsm.ugt80.
dsm.wt.ugt80.
dsm.smt1.
dsm.wt.smt1.
sp.ugt80.
sp.wt.ugt80.
sp.smt1.
sp.wt.smt1.
im.ugt80.
im.wt.ugt80.
im.smt1.
im.wt.smt1.

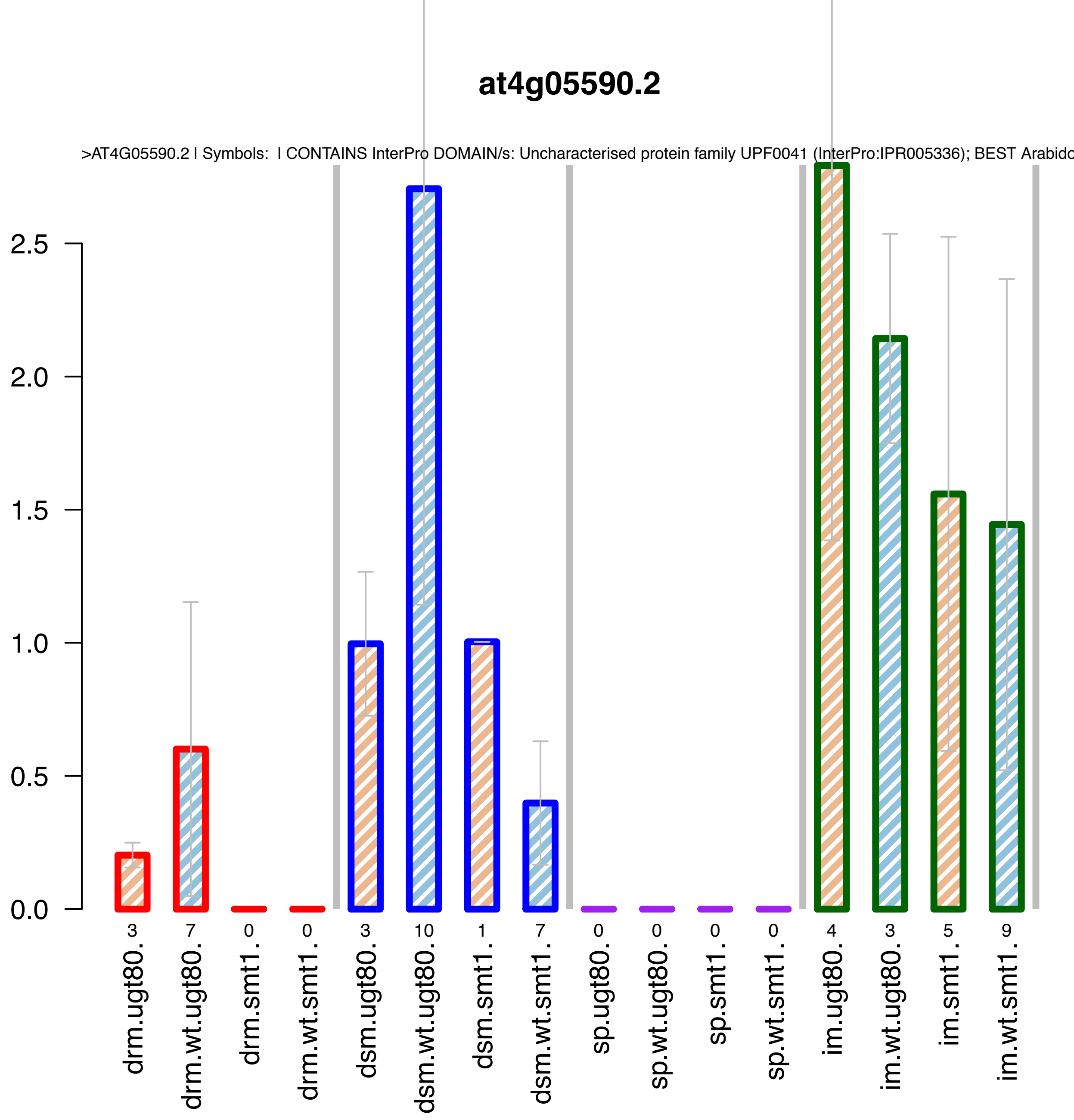

at4g05590.2
>AT4G05590.2 | Symbols: | CONTAINS InterPro DOMAIN/s: Uncharacterised protein family UPF0041 (InterPro:IPR005336); BEST Arabido
0.0
0.5
1.0
1.5
2.0
2.5
3
7
0
0
3
10
1
7
0
0
0
0
4
3
5
9
drm.ugt80.
drm.wt.ugt80.
drm.smt1.
drm.wt.smt1.
dsm.ugt80.
dsm.wt.ugt80.
dsm.smt1.
dsm.wt.smt1.
sp.ugt80.
sp.wt.ugt80.
sp.smt1.
sp.wt.smt1.
im.ugt80.
im.wt.ugt80.
im.smt1.
im.wt.smt1.

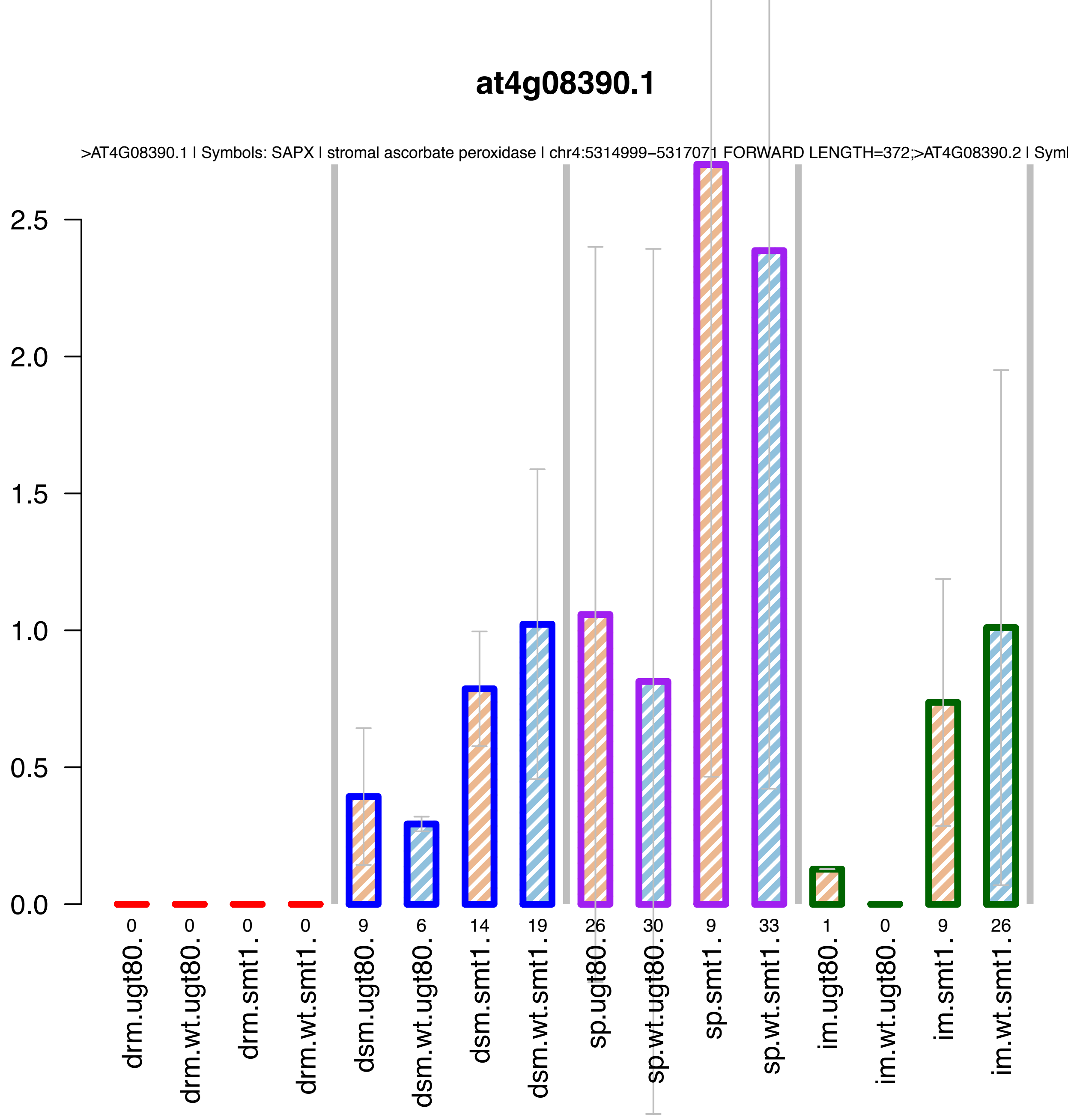

at4g08390.1
>AT4G08390.1 | Symbols: SAPX | stromal ascorbate peroxidase | chr4:5314999-5317071 FORWARD LENGTH=372;>AT4G08390.2 | Syml
2.5
2.0
1.5
1.0
0.5
0.0
0
0
0
0
9
6
14
19
26
30
9
33
1
0
9
26
drm.ugt80.
drm.wt.ugt80.
drm.smt1.
drm.wt.smt1.
dsm.ugt80.
dsm.wt.ugt80.
dsm.smt1.
dsm.wt.smt1.
sp.ugt80.
sp.wt.ugt80.
sp.smt1.
sp.wt.smt1.
im.ugt80.
im.wt.ugt80.
im.smt1.
im.wt.smt1.

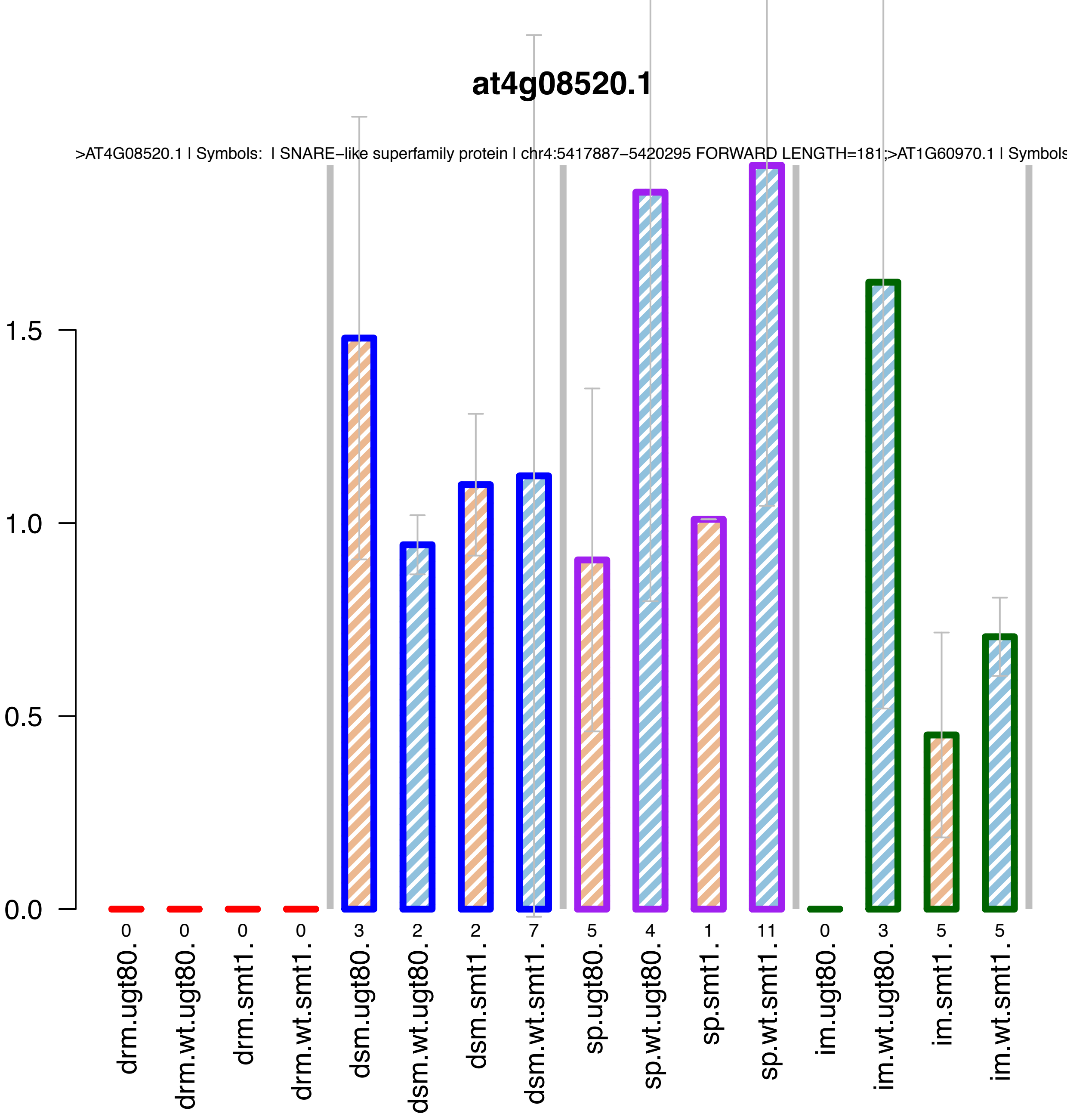
at4g08520.1
>AT4G08520.1 | Symbols: | SNARE−like superfamily protein | chr4:5417887−5420295 FORWARD LENGTH=181;>AT1G60970.1 | Symbols
1.5
1.0
0.5
0.0
0
0
0
0
3
2
2
7
5
4
1
11
0
3
5
5
drm.ugt80.
drm.wt.ugt80.
drm.smt1.
drm.wt.smt1.
dsm.ugt80.
dsm.wt.ugt80.
dsm.smt1.
dsm.wt.smt1.
sp.ugt80.
sp.wt.ugt80.
sp.smt1.
sp.wt.smt1.
im.ugt80.
im.wt.ugt80.
im.smt1.
im.wt.smt1.

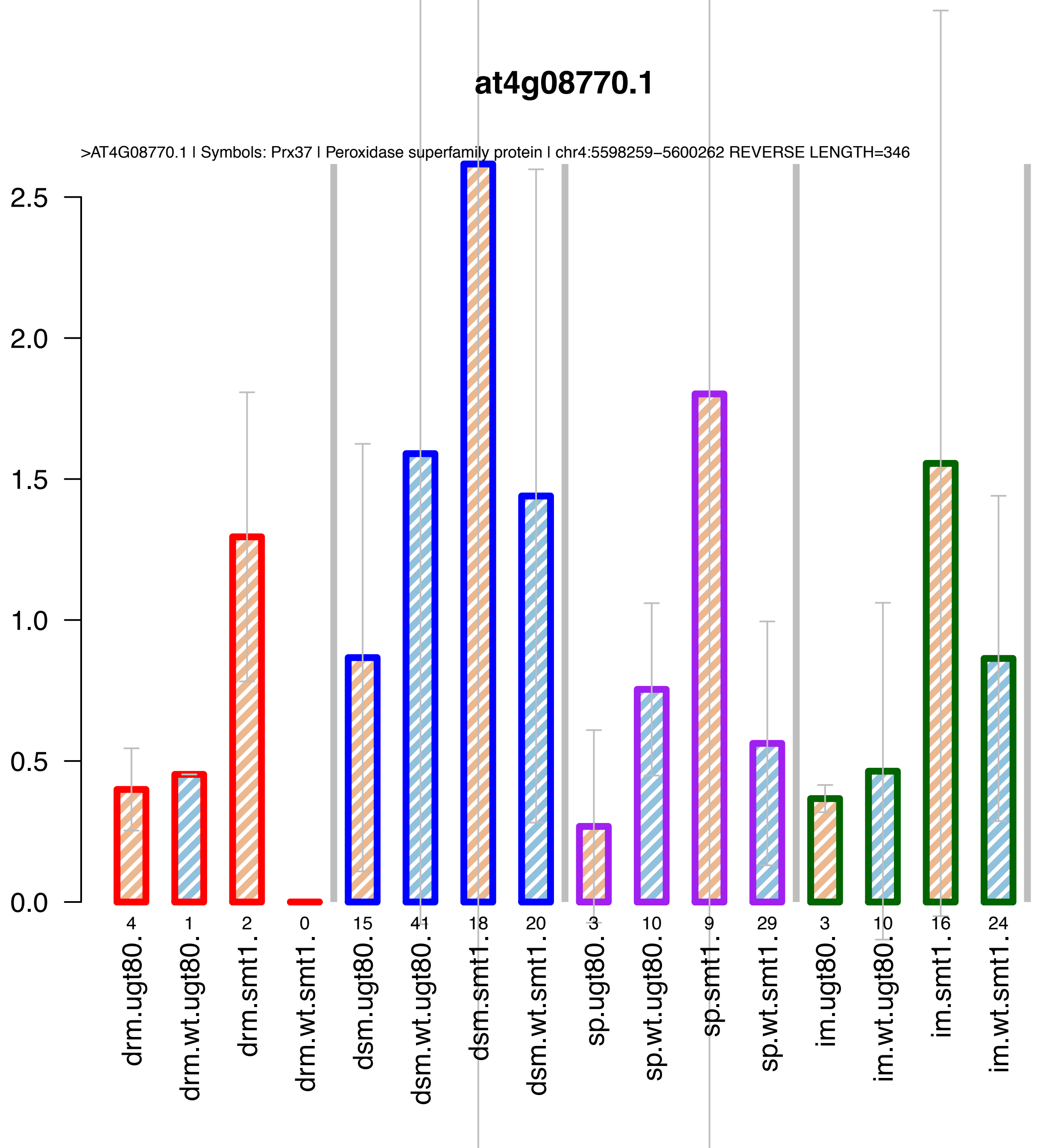

at4g08770.1
>AT4G08770.1 | Symbols: Prx37 | Peroxidase superfamily protein | chr4:5598259−5600262 REVERSE LENGTH=346
0.0
0.5
1.0
1.5
2.0
2.5
4
1
2
0
15
41
18
20
3
10
9
29
3
10
16
24
drm.ugt80.
drm.wt.ugt80.
drm.smt1.
drm.wt.smt1.
dsm.ugt80.
dsm.wt.ugt80.
dsm.smt1.
dsm.wt.smt1.
sp.ugt80.
sp.wt.ugt80.
sp.smt1.
sp.wt.smt1.
im.ugt80.
im.wt.ugt80.
im.smt1.
im.wt.smt1.

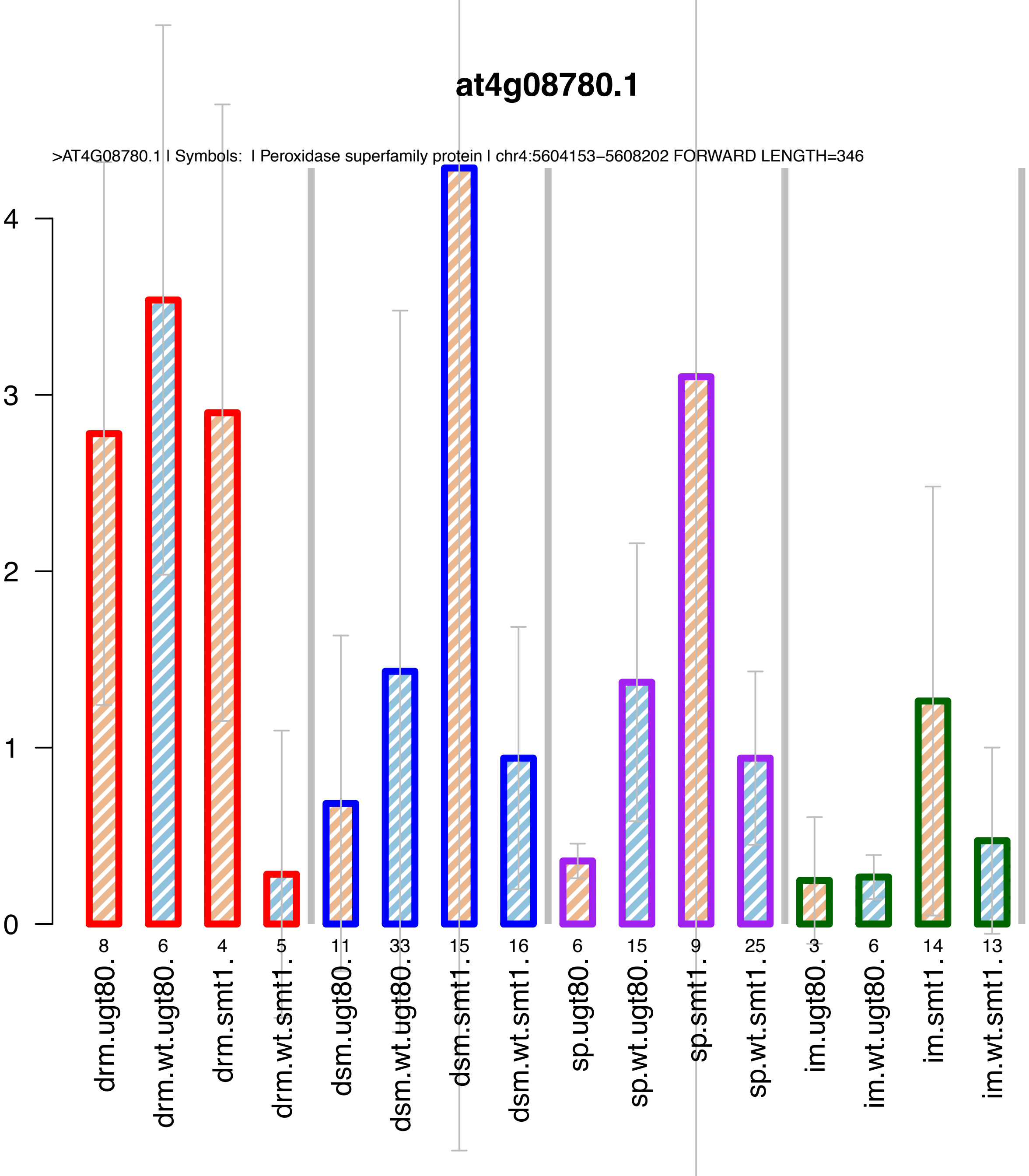

at4g08780.1
>AT4G08780.1 | Symbols: | Peroxidase superfamily protein | chr4:5604153-5608202 FORWARD LENGTH=346
0
1
2
3
4
8
6
4
5
11
33
15
16
6
15
9
25
3
6
14
13
drm.ugt80..
drm.wt.ugt80..
drm.smt1..
drm.wt.smt1..
dsm.ugt80..
dsm.wt.ugt80..
dsm.smt1..
dsm.wt.smt1..
sp.ugt80..
sp.wt.ugt80..
sp.smt1..
sp.wt.smt1..
im.ugt80..
im.wt.ugt80..
im.smt1..
im.wt.smt1..

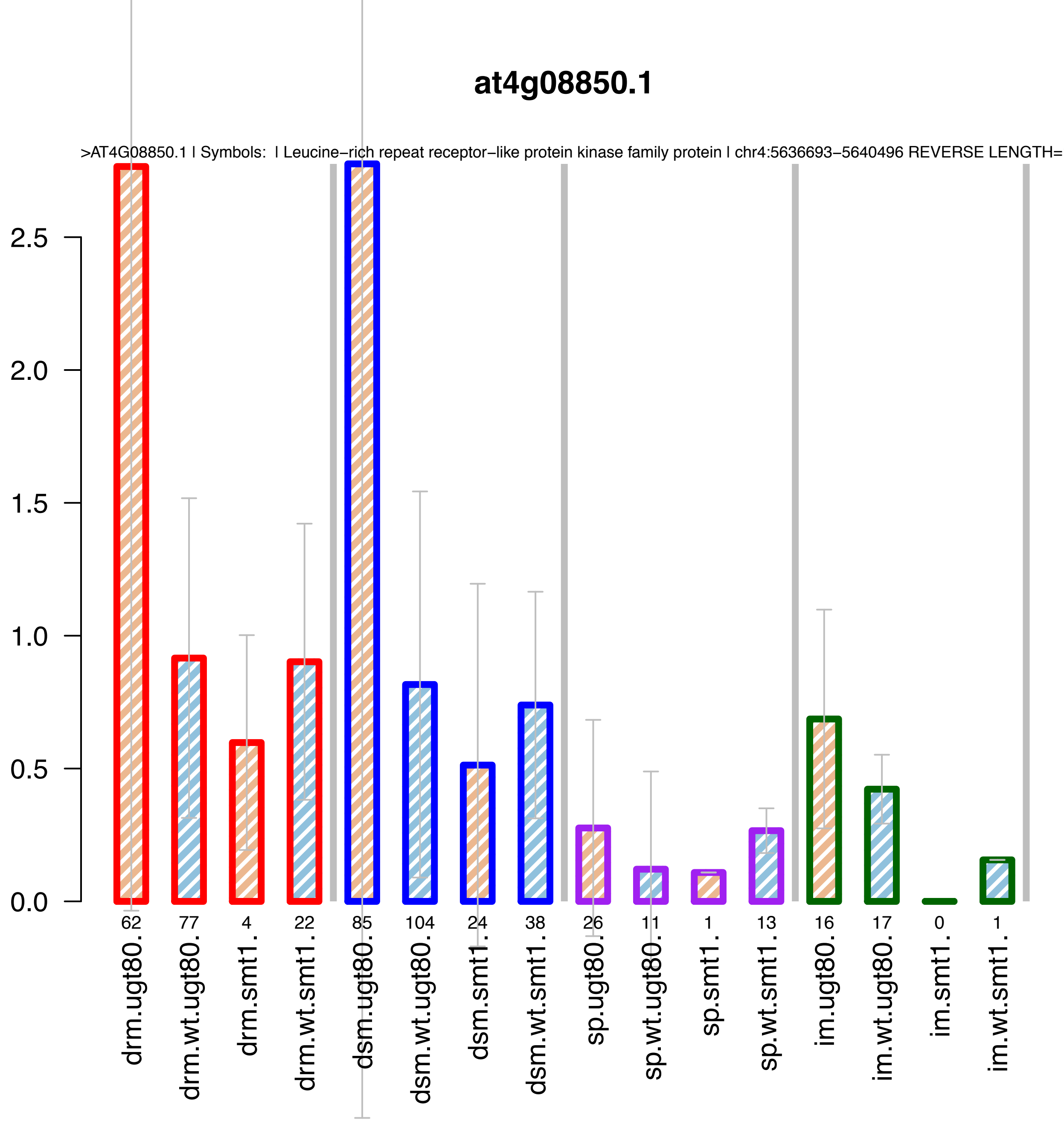

at4g08850.1
>AT4G08850.1 | Symbols: | Leucine−rich repeat receptor−like protein kinase family protein | chr4:5636693−5640496 REVERSE LENGTH=
0.0
0.5
1.0
1.5
2.0
2.5
62
77
4
22
85
104
24
38
26
11
1
13
16
17
0
1
drm.ugt80..
drm.wt.ugt80..
drm.smt1..
drm.wt.smt1..
dsm.ugt80..
dsm.wt.ugt80..
dsm.smt1..
dsm.wt.smt1..
sp.ugt80..
sp.wt.ugt80..
sp.smt1..
sp.wt.smt1..
im.ugt80..
im.wt.ugt80..
im.smt1..
im.wt.smt1..

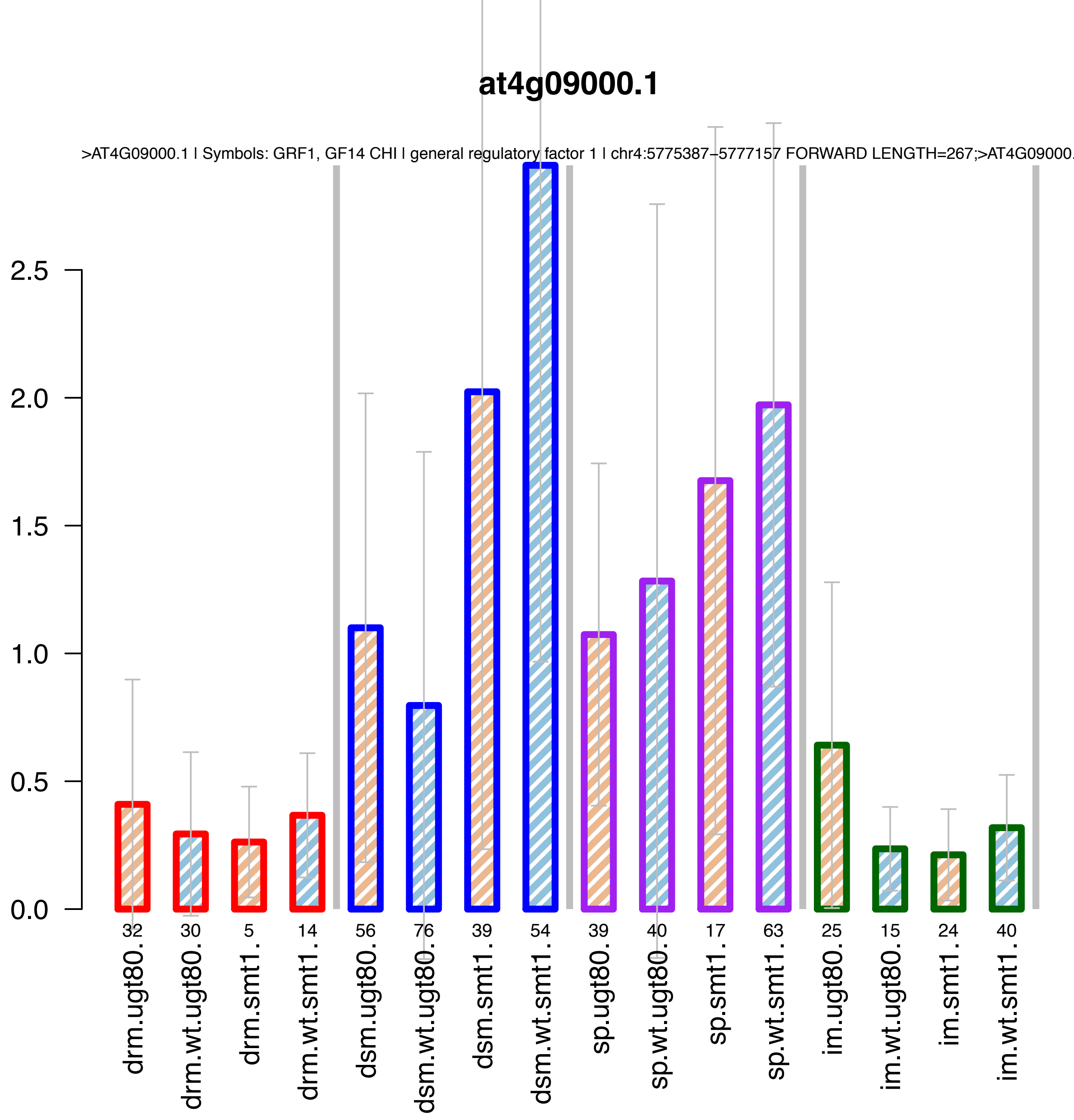

at4g09000.1
>AT4G09000.1 | Symbols: GRF1, GF14 CHI | general regulatory factor 1 | chr4:5775387−5777157 FORWARD LENGTH=267;>AT4G09000.
0.0
0.5
1.0
1.5
2.0
2.5
32
30
5
14
56
76
39
54
39
40
17
63
25
15
24
40
drm.ugt80..
drm.wt.ugt80..
drm.smt1..
drm.wt.smt1..
dsm.ugt80..
dsm.wt.ugt80..
dsm.smt1..
dsm.wt.smt1..
sp.ugt80..
sp.wt.ugt80..
sp.smt1..
sp.wt.smt1..
im.ugt80..
im.wt.ugt80..
im.smt1..
im.wt.smt1..

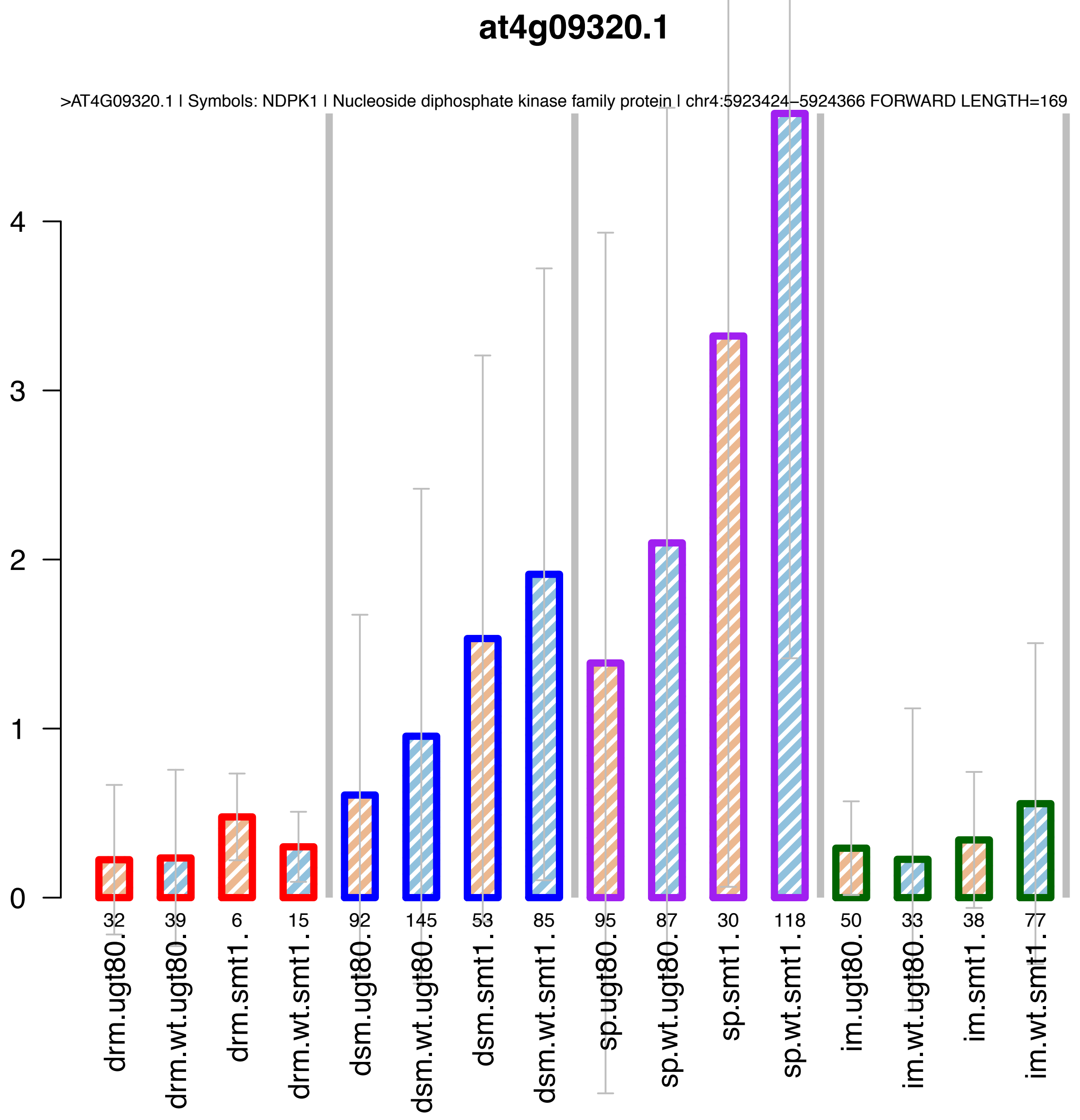

at4g09320.1
>AT4G09320.1 | Symbols: NDPK1 | Nucleoside diphosphate kinase family protein | chr4:5923424–5924366 FORWARD LENGTH=169
0
1
2
3
4
32
39
6
15
92
145
53
85
95
87
30
118
50
33
38
77
drm.ugt80.
drm.wt.ugt80.
drm.smt1.
drm.wt.smt1.
dsm.ugt80.
dsm.wt.ugt80.
dsm.smt1.
dsm.wt.smt1.
sp.ugt80.
sp.wt.ugt80.
sp.smt1.
sp.wt.smt1.
im.ugt80.
im.wt.ugt80.
im.smt1.
im.wt.smt1.

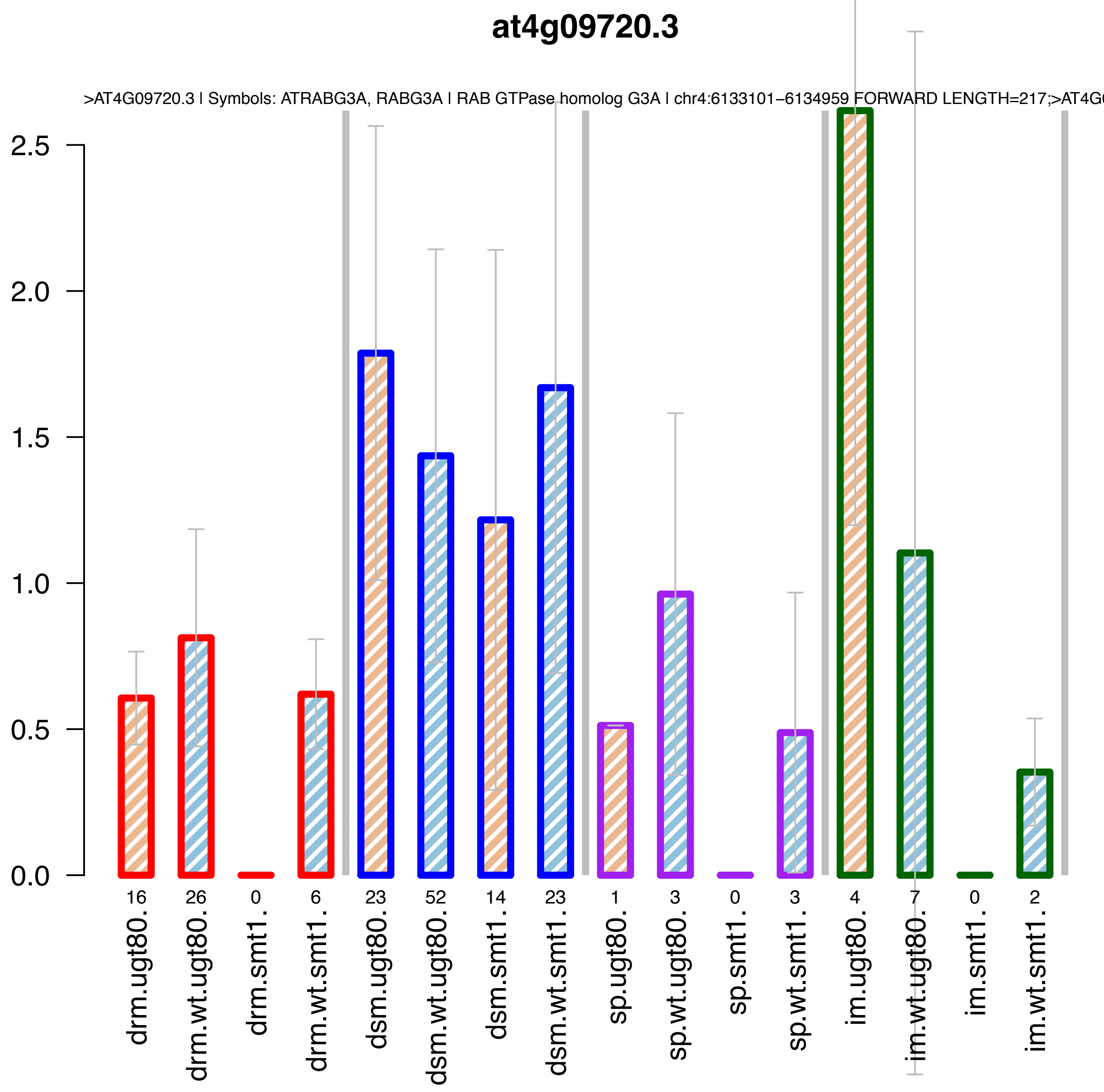

at4g09720.3
>AT4G09720.3 | Symbols: ATRABG3A, RABG3A | RAB GTPase homolog G3A | chr4:6133101-6134959 FORWARD LENGTH=217;>AT4G
2.5
2.0
1.5
1.0
0.5
0.0
16
26
0
6
23
52
14
23
1
3
0
3
4
7
0
2
drm.ugt80..
drm.wt.ugt80..
drm.smt1..
drm.wt.smt1..
dsm.ugt80..
dsm.wt.ugt80..
dsm.smt1..
dsm.wt.smt1..
sp.ugt80..
sp.wt.ugt80..
sp.smt1..
sp.wt.smt1..
im.ugt80..
im.wt.ugt80..
im.smt1..
im.wt.smt1..

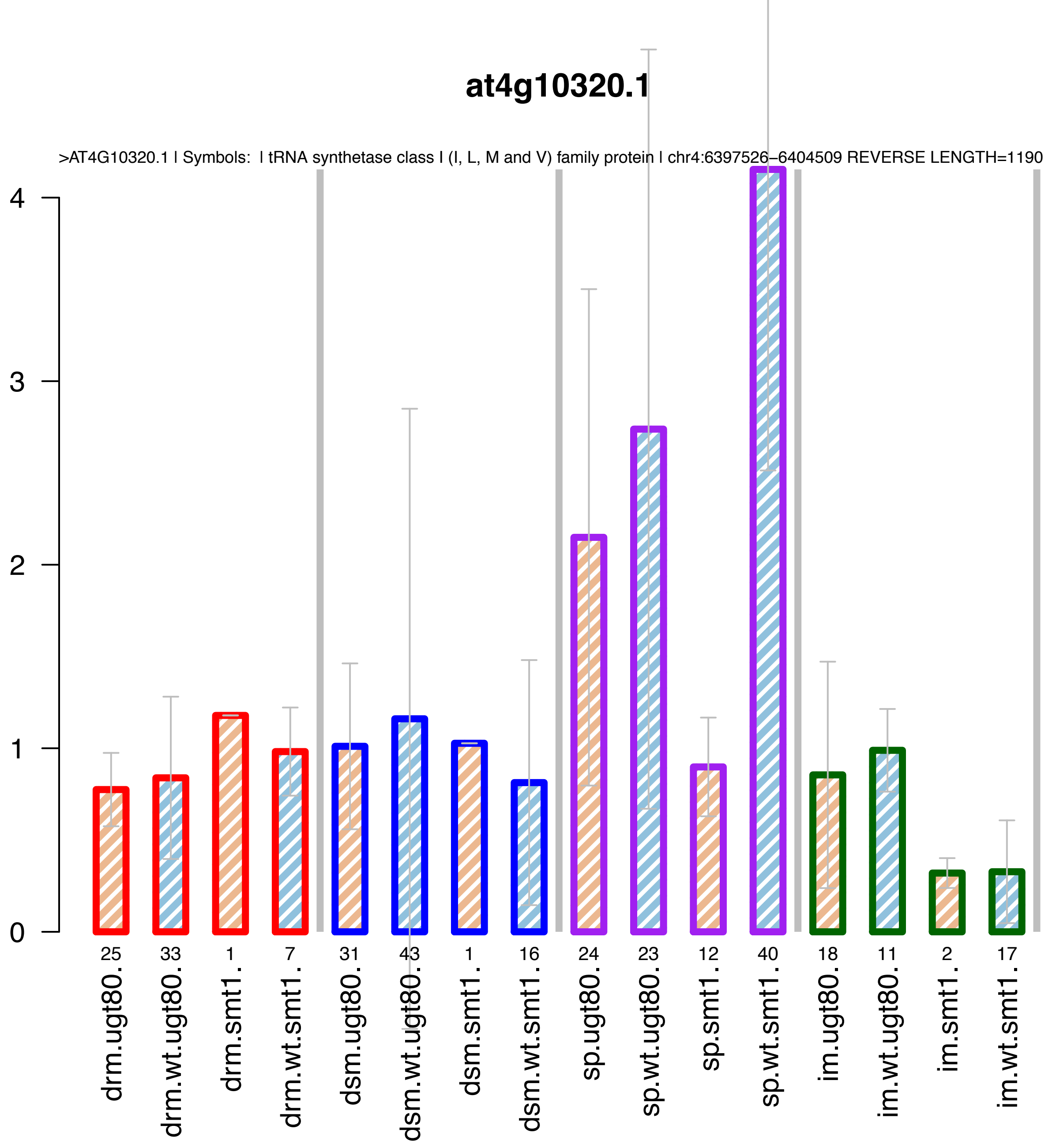

at4g10320.1
>AT4G10320.1 | Symbols: | tRNA synthetase class I (I, L, M and V) family protein | chr4:6397526-6404509 REVERSE LENGTH=1190
0
1
2
3
4
25
33
1
7
31
43
1
16
24
23
12
40
18
11
2
17
drm.ugt80..
drm.wt.ugt80..
drm.smt1..
drm.wt.smt1..
dsm.ugt80..
dsm.wt.ugt80..
dsm.smt1..
dsm.wt.smt1..
sp.ugt80..
sp.wt.ugt80..
sp.smt1..
sp.wt.smt1..
im.ugt80..
im.wt.ugt80..
im.smt1..
im.wt.smt1..

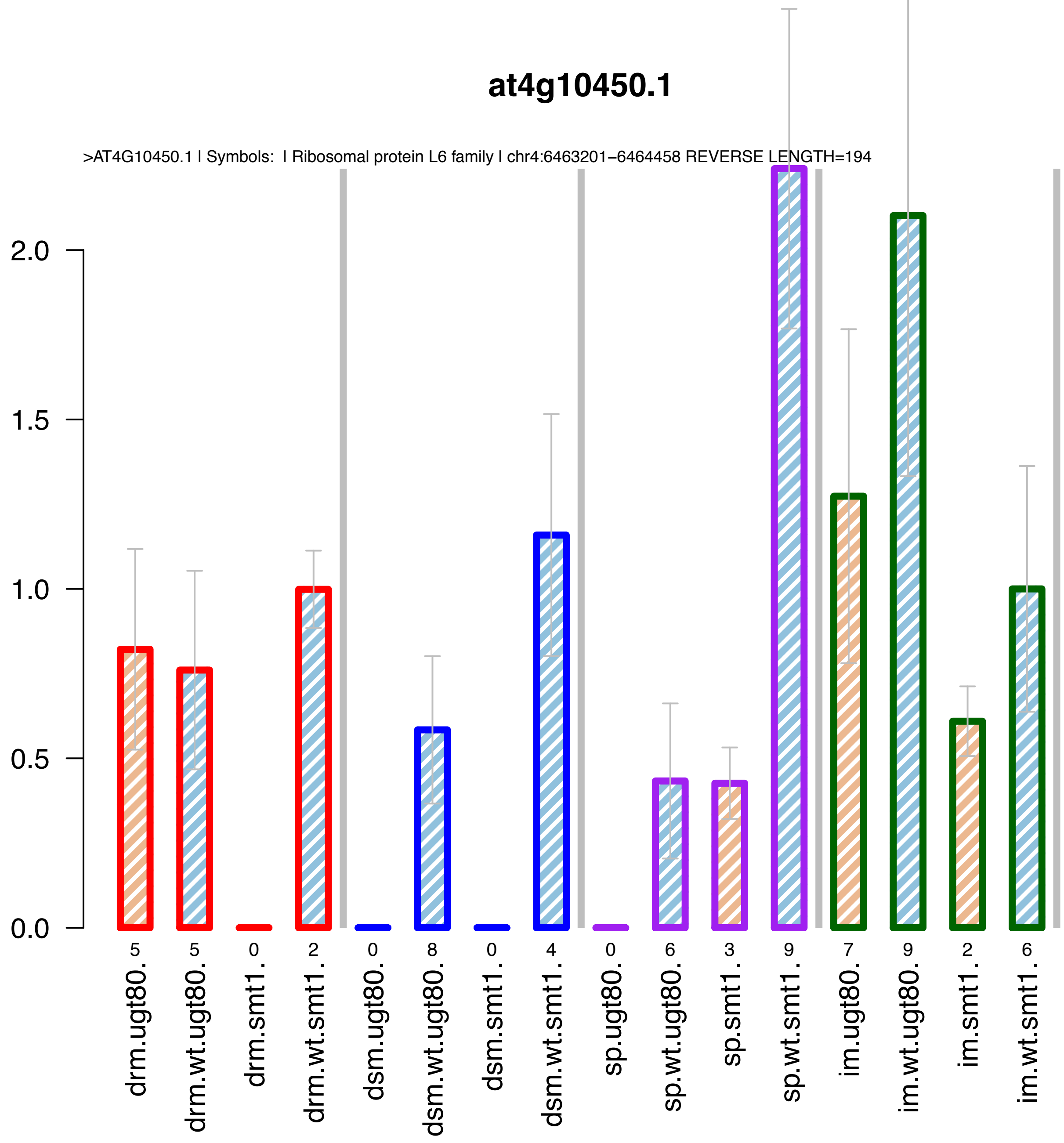
at4g10450.1
>AT4G10450.1 | Symbols: | Ribosomal protein L6 family | chr4:6463201−6464458 REVERSE LENGTH=194
0.0
0.5
1.0
1.5
2.0
5
5
0
2
0
8
0
4
0
6
3
9
7
9
2
6
drm.ugt80.
drm.wt.ugt80.
drm.smt1.
drm.wt.smt1.
dsm.ugt80.
dsm.wt.ugt80.
dsm.smt1.
dsm.wt.smt1.
sp.ugt80.
sp.wt.ugt80.
sp.smt1.
sp.wt.smt1.
im.ugt80.
im.wt.ugt80.
im.smt1.
im.wt.smt1.

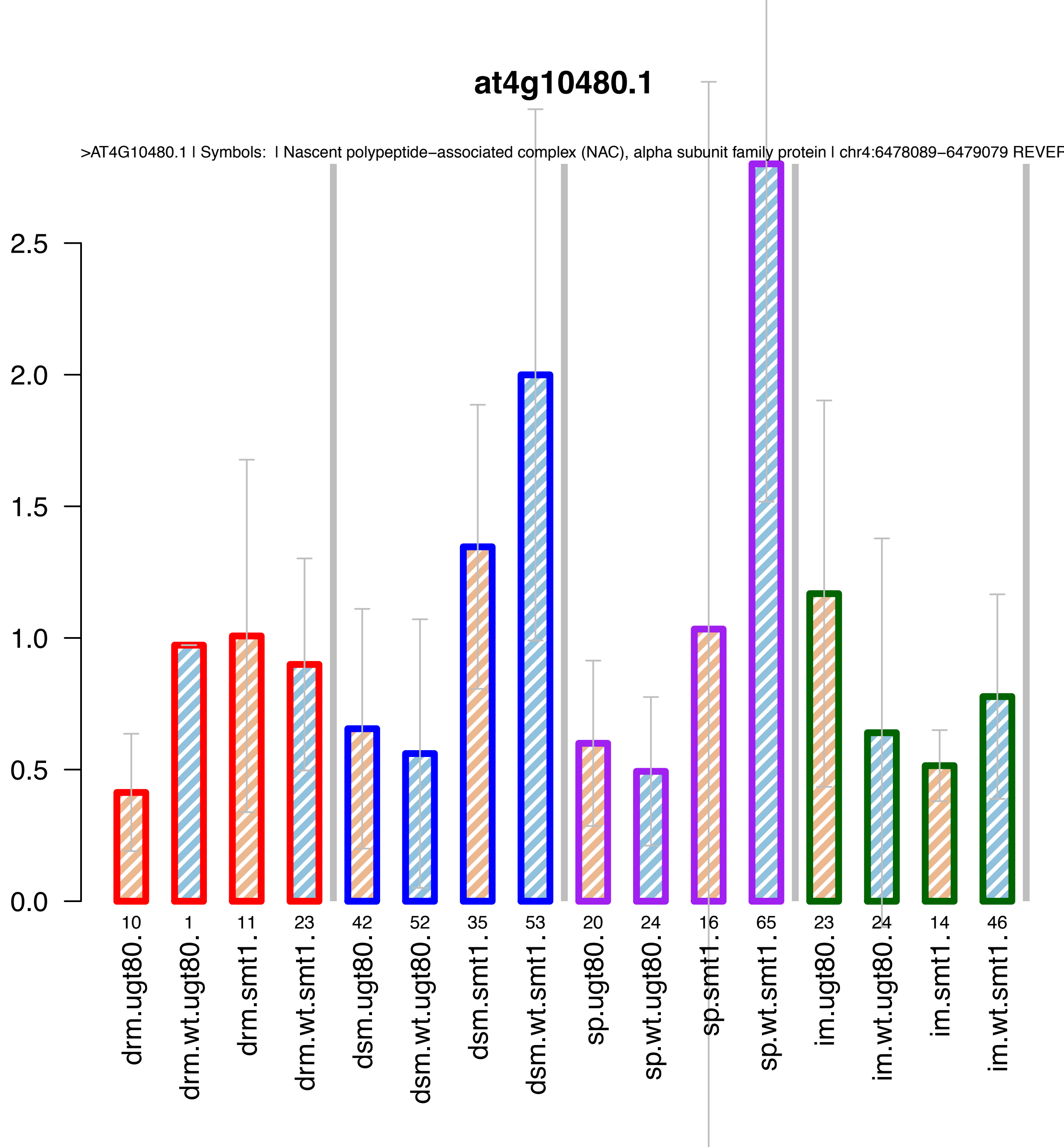
at4g10480.1
>AT4G10480.1 | Symbols: | Nascent polypeptide−associated complex (NAC), alpha subunit family protein | chr4:6478089−6479079 REVEF
2.5
2.0
1.5
1.0
0.5
0.0
10
1
11
23
42
52
35
53
20
24
16
65
23
24
14
46
drm.ugt80..
drm.wt.ugt80..
drm.smt1..
drm.wt.smt1..
dsm.ugt80..
dsm.wt.ugt80..
dsm.smt1..
dsm.wt.smt1..
sp.ugt80..
sp.wt.ugt80..
sp.smt1..
sp.wt.smt1..
im.ugt80..
im.wt.ugt80..
im.smt1..
im.wt.smt1..

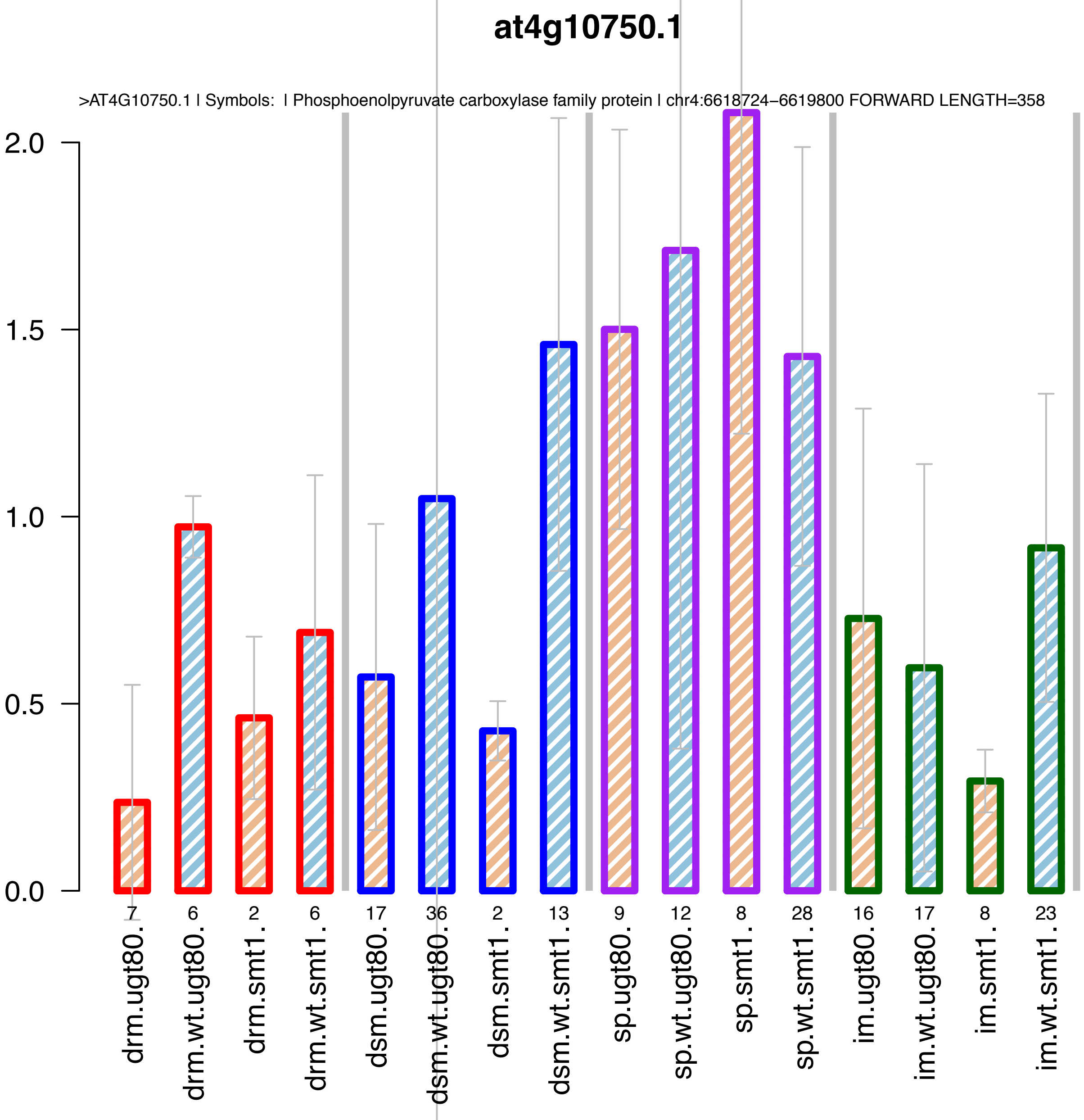

at4g10750.1
>AT4G10750.1 | Symbols: | Phosphoenolpyruvate carboxylase family protein | chr4:6618724-6619800 FORWARD LENGTH=358
2.0
1.5
1.0
0.5
0.0
7
6
2
6
17
36
2
13
9
12
8
28
16
17
8
23
drm.ugt80..
drm.wt.ugt80..
drm.smt1..
drm.wt.smt1..
dsm.ugt80..
dsm.wt.ugt80..
dsm.smt1..
dsm.wt.smt1..
sp.ugt80..
sp.wt.ugt80..
sp.smt1..
sp.wt.smt1..
im.ugt80..
im.wt.ugt80..
im.smt1..
im.wt.smt1..

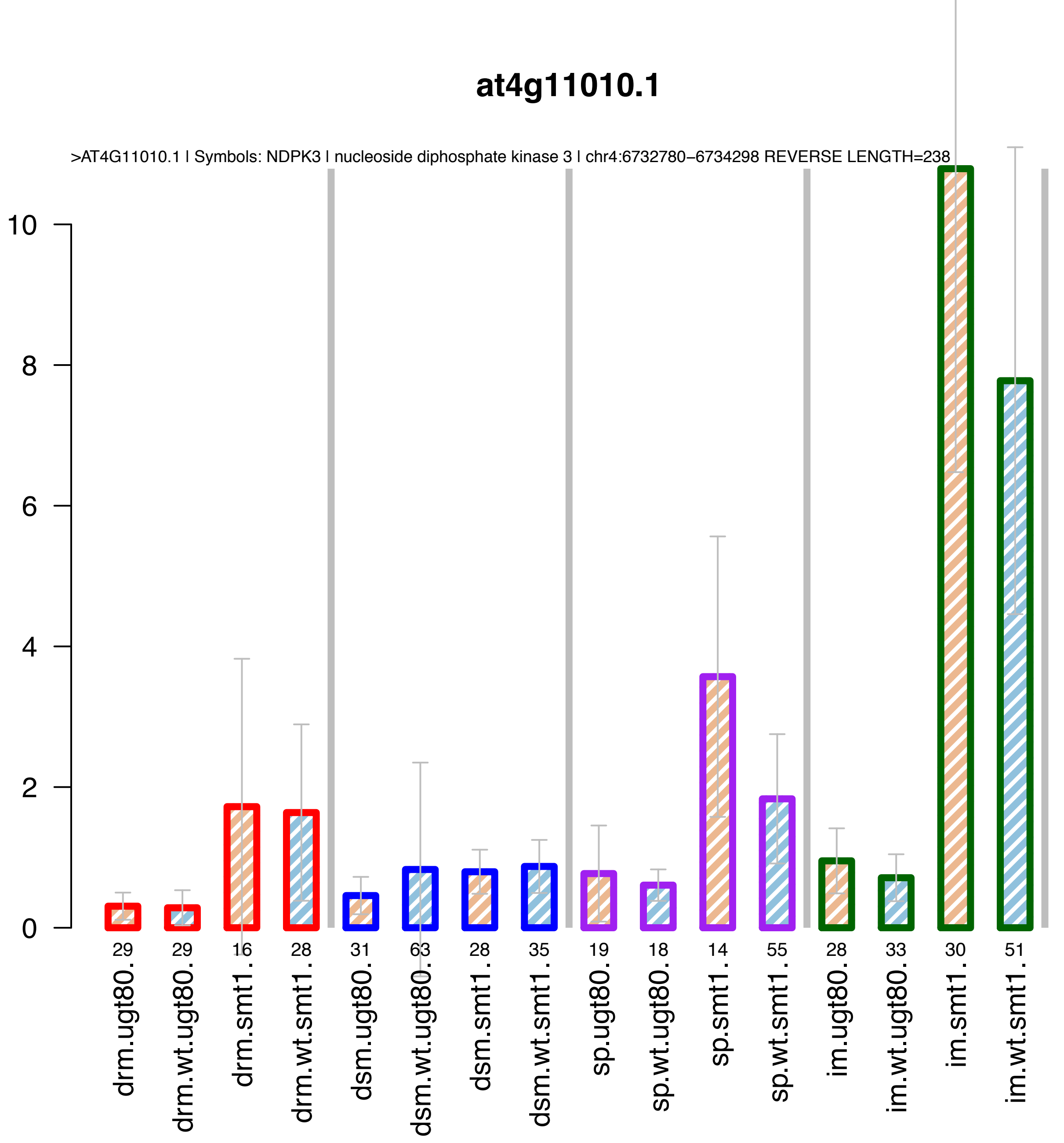

at4g11010.1
>AT4G11010.1 | Symbols: NDPK3 | nucleoside diphosphate kinase 3 | chr4:6732780−6734298 REVERSE LENGTH=238
0
2
4
6
8
10
29
29
16
28
31
63
28
35
19
18
14
55
28
33
30
51
drm.ugt80..
drm.wt.ugt80..
drm.smt1..
drm.wt.smt1..
dsm.ugt80..
dsm.wt.ugt80..
dsm.smt1..
dsm.wt.smt1..
sp.ugt80..
sp.wt.ugt80..
sp.smt1..
sp.wt.smt1..
im.ugt80..
im.wt.ugt80..
im.smt1..
im.wt.smt1..

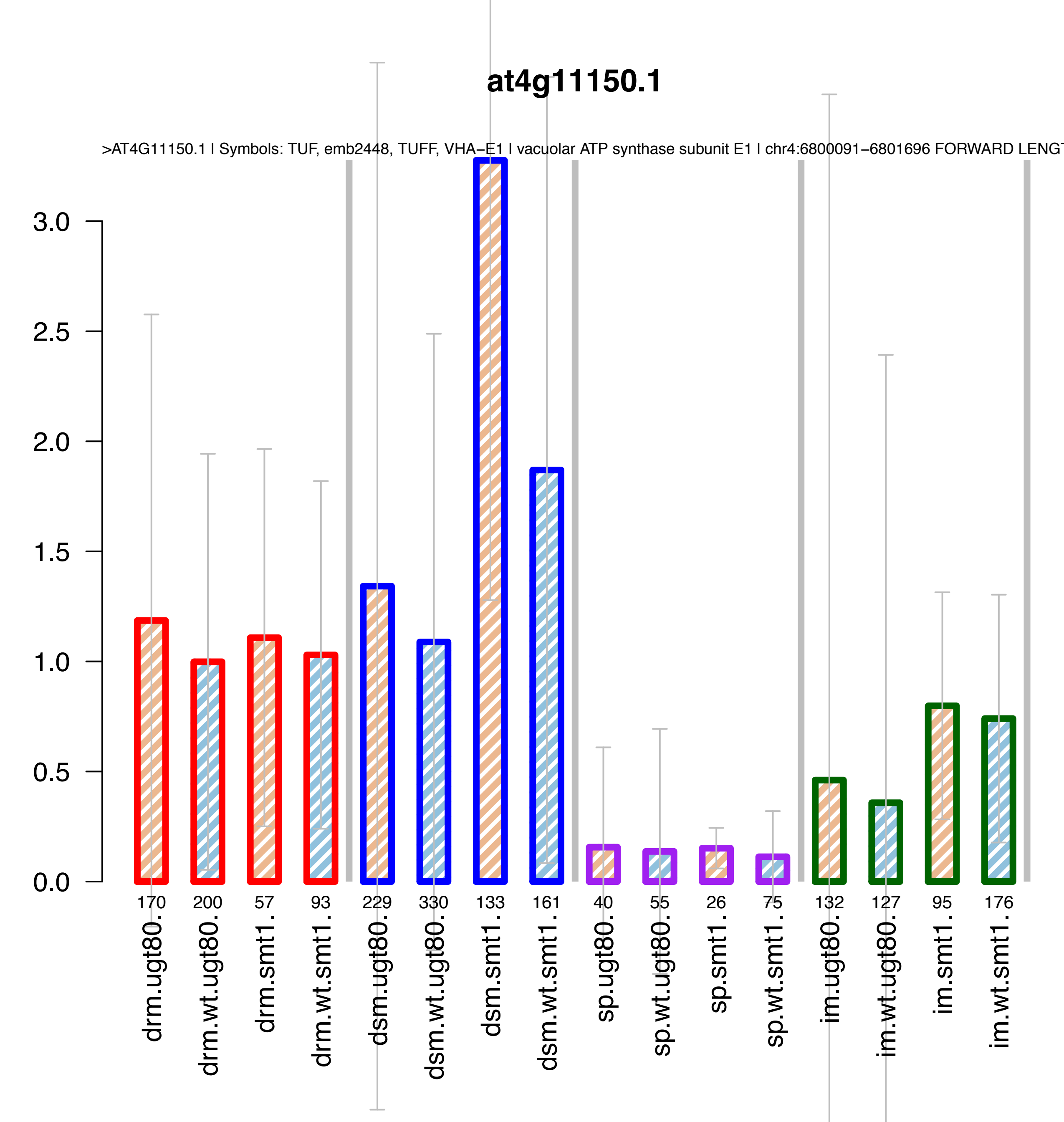

at4g11150.1
>AT4G11150.1 | Symbols: TUF, emb2448, TUFF, VHA−E1 | vacuolar ATP synthase subunit E1 | chr4:6800091−6801696 FORWARD LENG
3.0
2.5
2.0
1.5
1.0
0.5
0.0
170
200
57
93
229
330
133
161
40
55
26
75
132
127
95
176
drm.ugt80..
drm.wt.ugt80..
drm.smt1..
drm.wt.smt1..
dsm.ugt80..
dsm.wt.ugt80..
dsm.smt1..
dsm.wt.smt1..
sp.ugt80..
sp.wt.ugt80..
sp.smt1..
sp.wt.smt1..
im.ugt80..
im.wt.ugt80..
im.smt1..
im.wt.smt1..

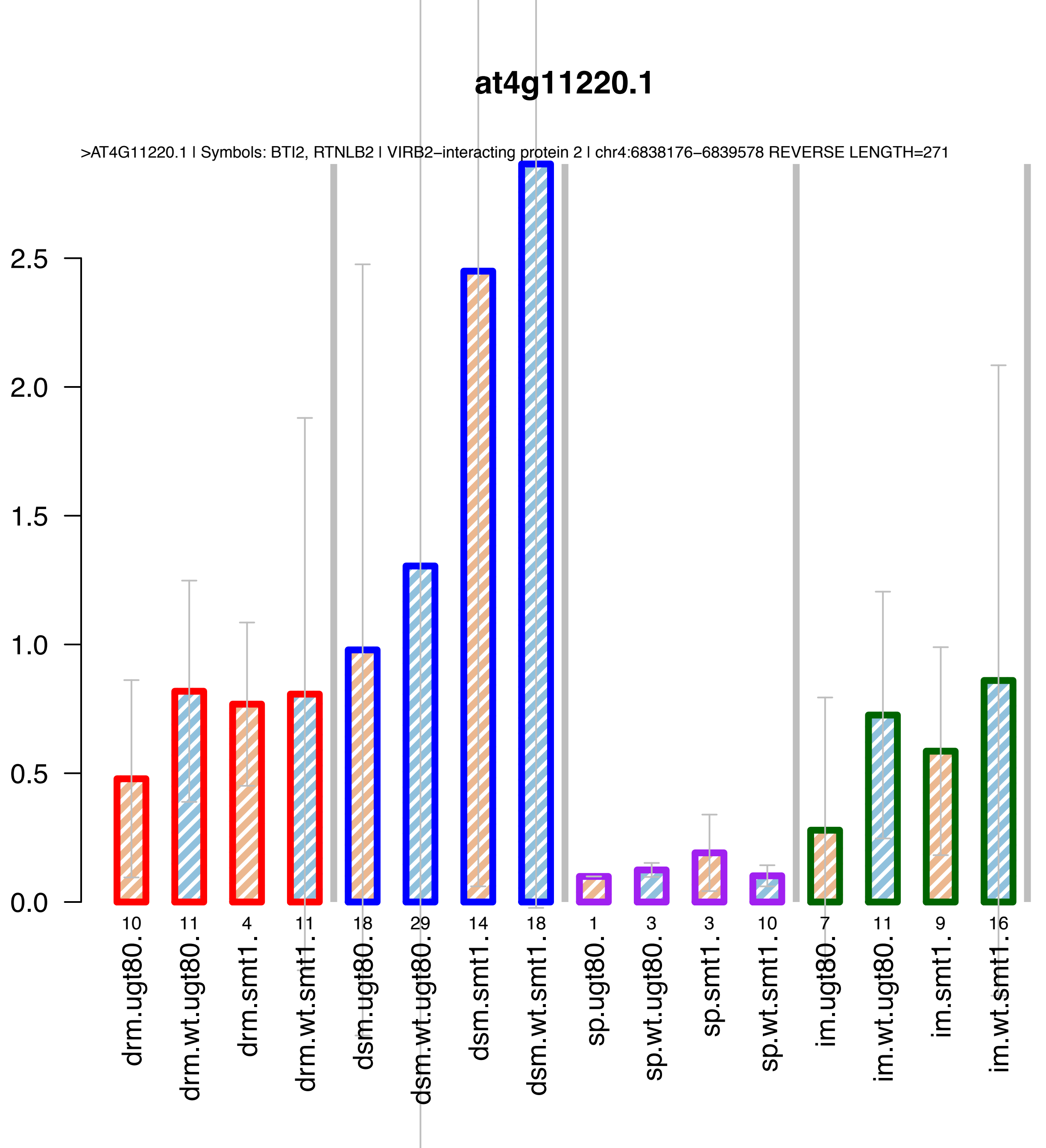

at4g11220.1
>AT4G11220.1 | Symbols: BTI2, RTNLB2 | VIRB2−interacting protein 2 | chr4:6838176−6839578 REVERSE LENGTH=271
0.0
0.5
1.0
1.5
2.0
2.5
10
11
4
11
18
29
14
18
1
3
3
10
7
11
9
16
drm.ugt80..
drm.wt.ugt80..
drm.smt1..
drm.wt.smt1..
dsm.ugt80..
dsm.wt.ugt80..
dsm.smt1..
dsm.wt.smt1..
sp.ugt80..
sp.wt.ugt80..
sp.smt1..
sp.wt.smt1..
im.ugt80..
im.wt.ugt80..
im.smt1..
im.wt.smt1..

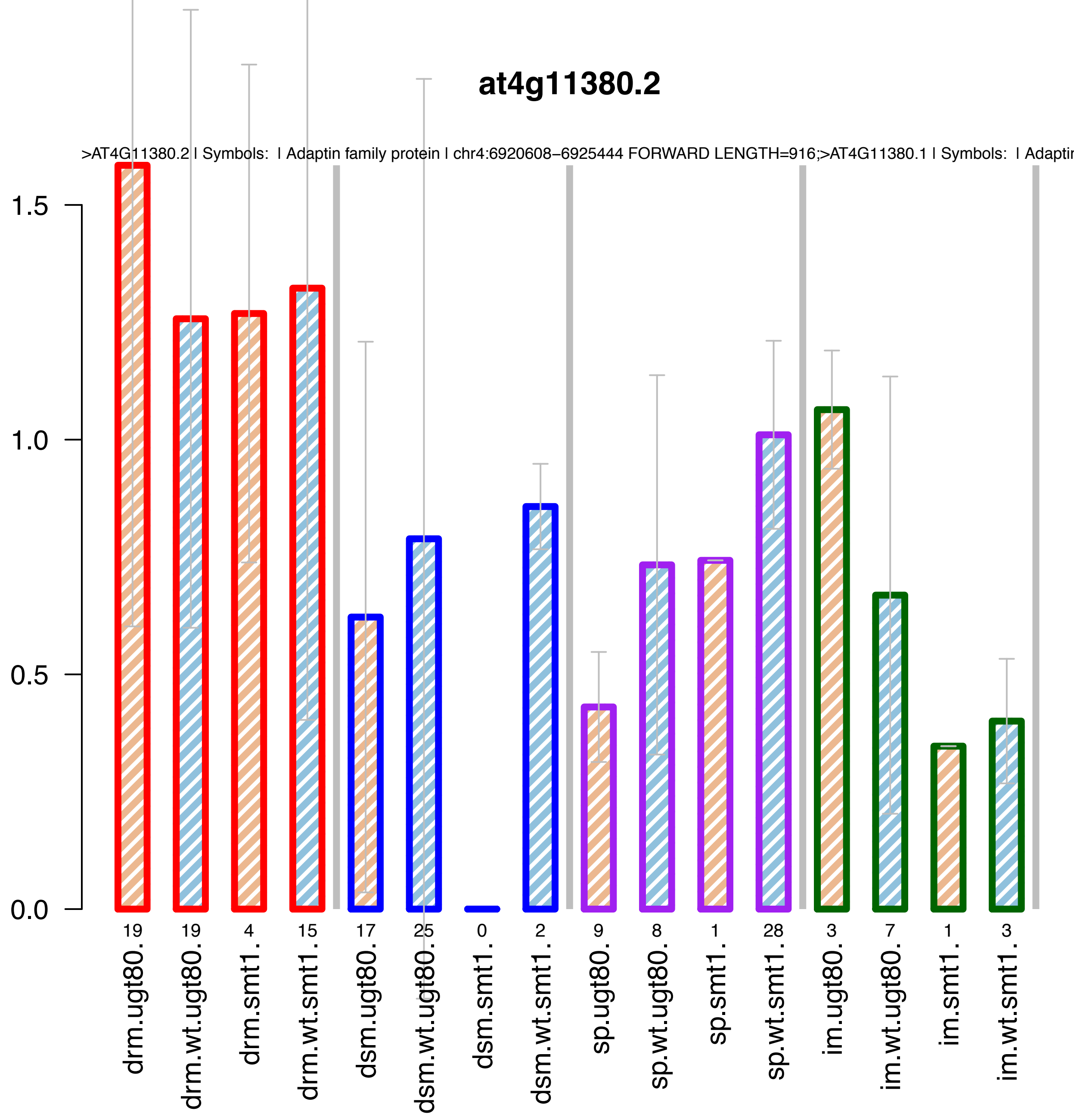

at4g11380.2
>AT4G11380.2 | Symbols: | Adaptin family protein | chr4:6920608-6925444 FORWARD LENGTH=916;>AT4G11380.1 | Symbols: | Adaptin
1.5
1.0
0.5
0.0
19
19
4
15
17
25
0
2
9
8
1
28
3
7
1
3
drm.ugt80.
drm.wt.ugt80.
drm.smt1.
drm.wt.smt1.
dsm.ugt80.
dsm.wt.ugt80.
dsm.smt1.
dsm.wt.smt1.
sp.ugt80.
sp.wt.ugt80.
sp.smt1.
sp.wt.smt1.
im.ugt80.
im.wt.ugt80.
im.smt1.
im.wt.smt1.

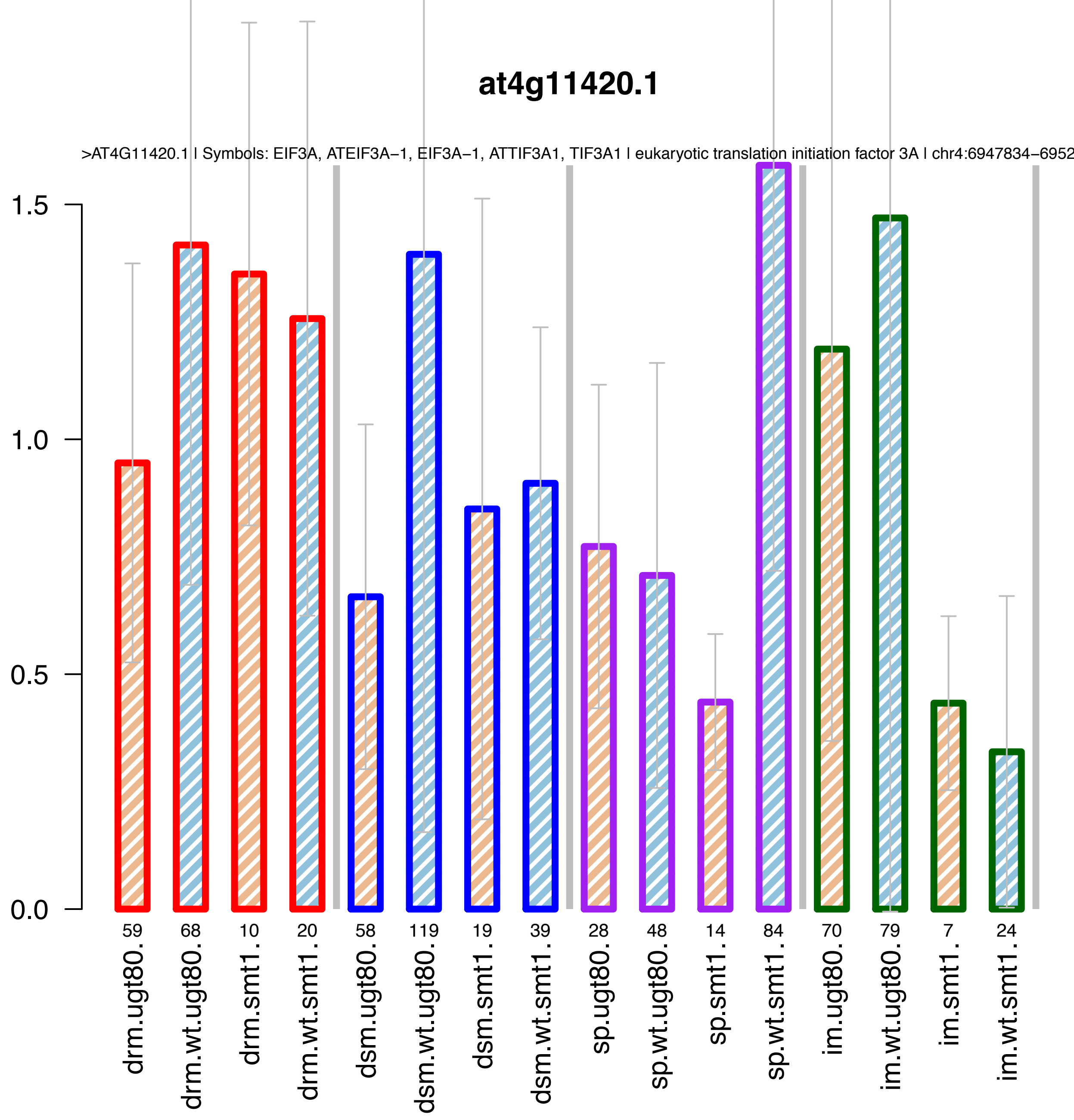

at4g11420.1
>AT4G11420.1 | Symbols: EIF3A, ATEIF3A−1, EIF3A−1, ATTIF3A1, TIF3A1 | eukaryotic translation initiation factor 3A | chr4:6947834−6952
1.5
1.0
0.5
0.0
59
68
10
20
58
119
19
39
28
48
14
84
70
79
7
24
drm.ugt80.
drm.wt.ugt80.
drm.smt1.
drm.wt.smt1.
dsm.ugt80.
dsm.wt.ugt80.
dsm.smt1.
dsm.wt.smt1.
sp.ugt80.
sp.wt.ugt80.
sp.smt1.
sp.wt.smt1.
im.ugt80.
im.wt.ugt80.
im.smt1.
im.wt.smt1.

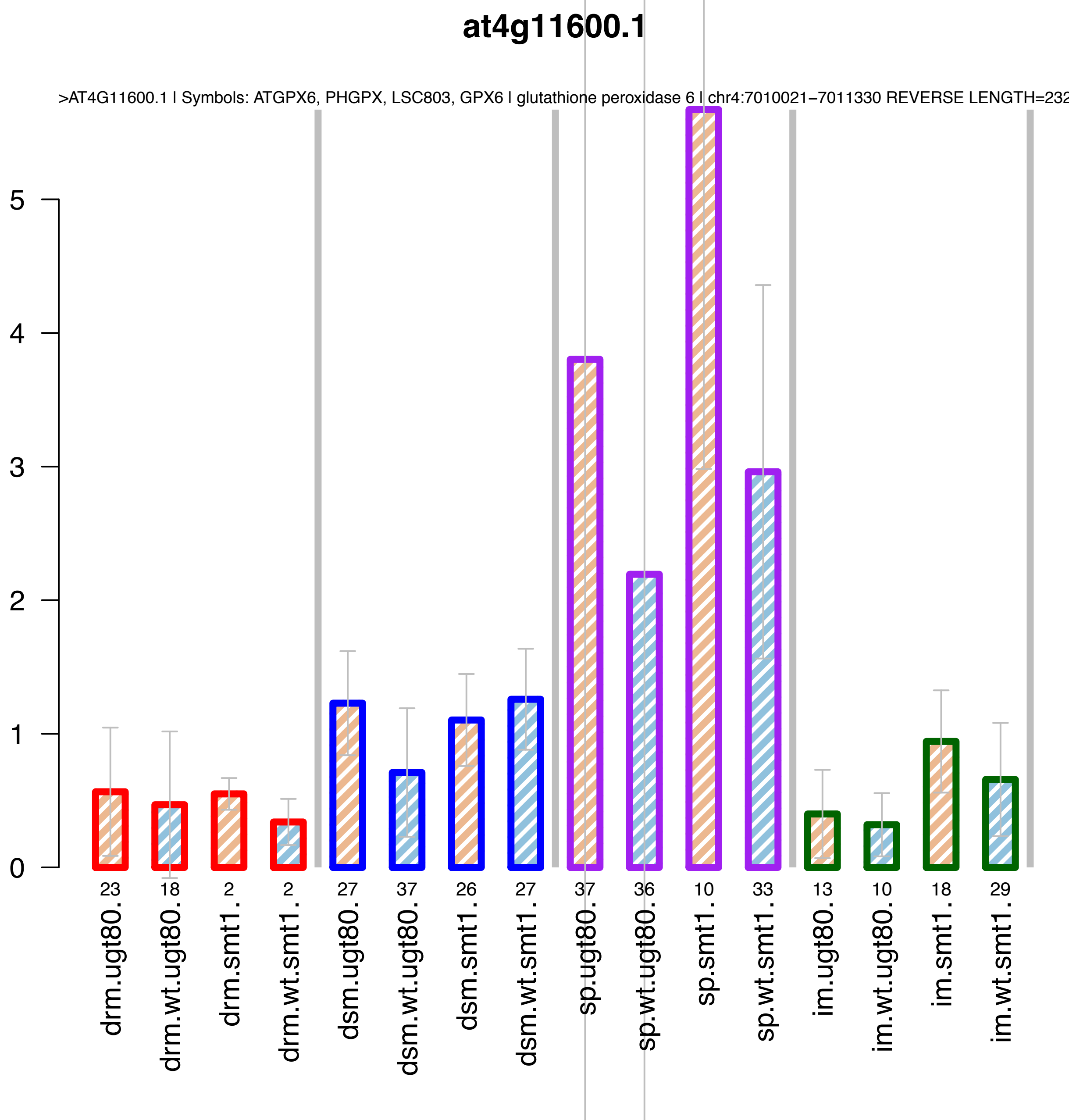

at4g11600.1
>AT4G11600.1 | Symbols: ATGPX6, PHGPX, LSC803, GPX6 | glutathione peroxidase 6 | chr4:7010021−7011330 REVERSE LENGTH=232
0
1
2
3
4
5
23
18
2
2
27
37
26
27
37
36
10
33
13
10
18
29
drm.ugt80.
drm.wt.ugt80.
drm.smt1.
drm.wt.smt1.
dsm.ugt80.
dsm.wt.ugt80.
dsm.smt1.
dsm.wt.smt1.
sp.ugt80.
sp.wt.ugt80.
sp.smt1.
sp.wt.smt1.
im.ugt80.
im.wt.ugt80.
im.smt1.
im.wt.smt1.

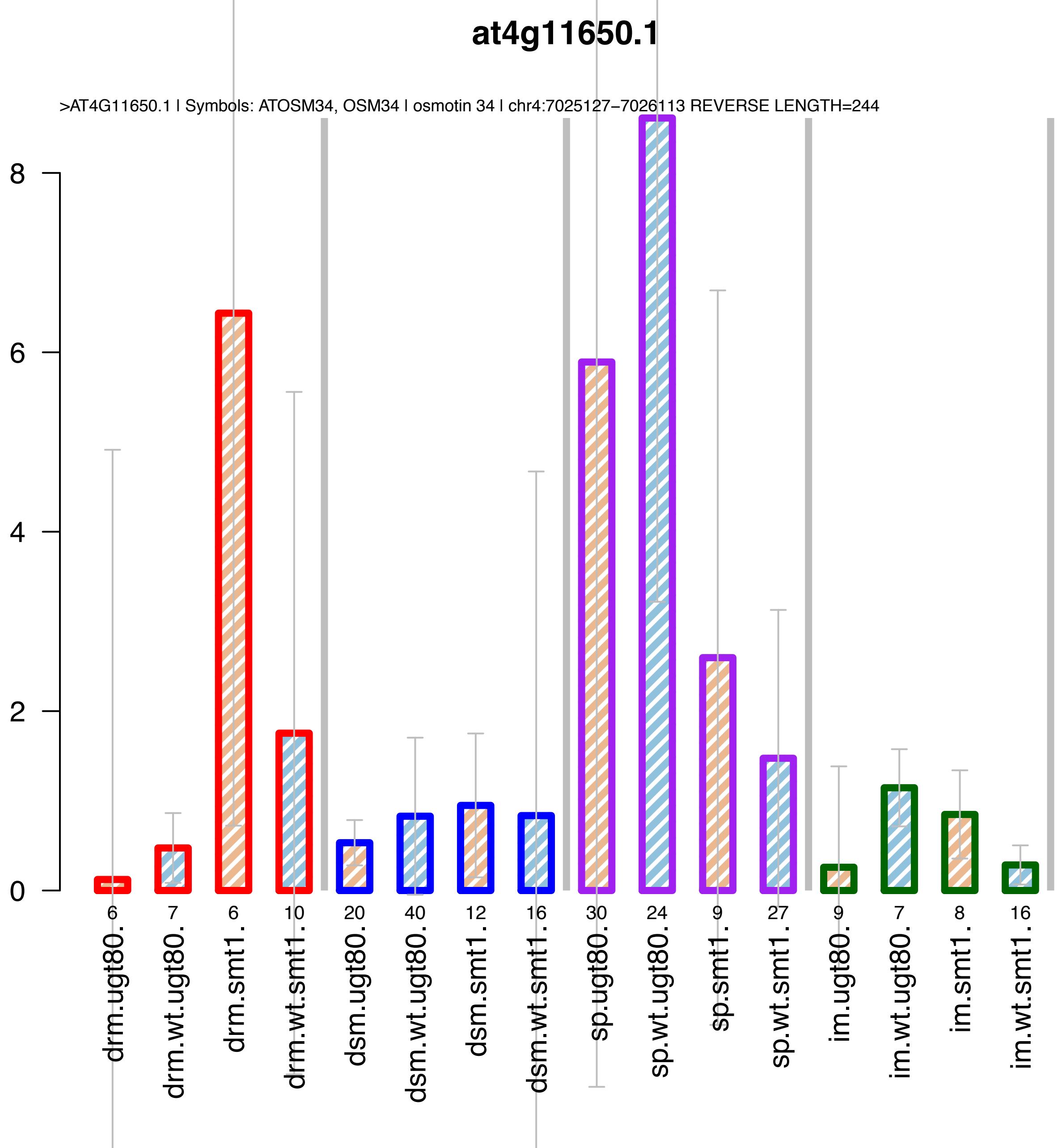
at4g11650.1
>AT4G11650.1 | Symbols: ATOSM34, OSM34 | osmotin 34 | chr4:7025127-7026113 REVERSE LENGTH=244
0
2
4
6
8
6
7
6
10
20
40
12
16
30
24
9
27
9
7
8
16
drm.ugt80.
drm.wt.ugt80.
drm.smt1.
drm.wt.smt1.
dsm.ugt80.
dsm.wt.ugt80.
dsm.smt1.
dsm.wt.smt1.
sp.ugt80.
sp.wt.ugt80.
sp.smt1.
sp.wt.smt1.
im.ugt80.
im.wt.ugt80.
im.smt1.
im.wt.smt1.

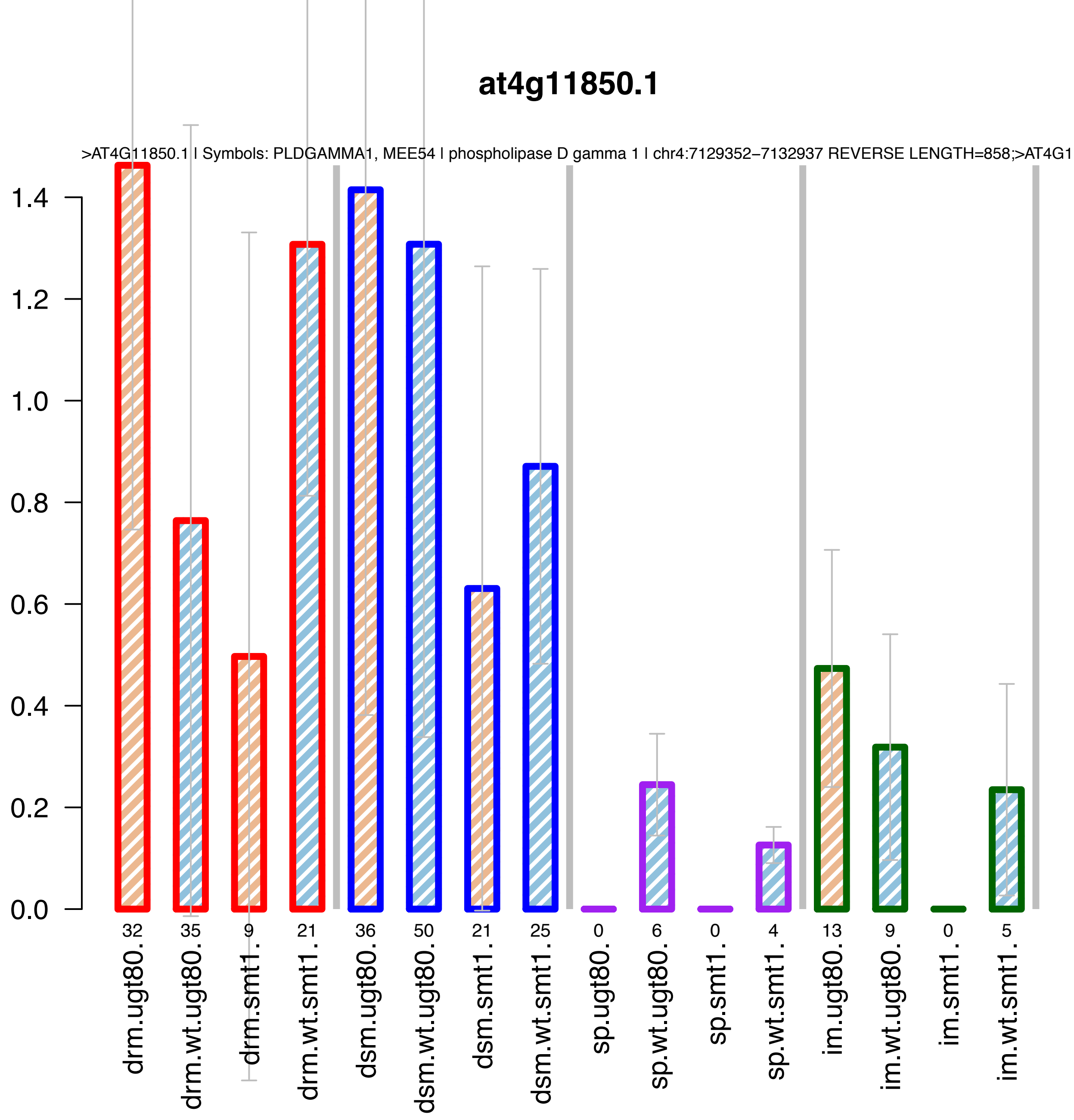

at4g11850.1
>AT4G11850.1 | Symbols: PLDGAMMA1, MEE54 | phospholipase D gamma 1 | chr4:7129352-7132937 REVERSE LENGTH=858;>AT4G1
1.4
1.2
1.0
0.8
0.6
0.4
0.2
0.0
32
35
9
21
36
50
21
25
0
6
0
4
13
9
0
5
drm.ugt80.
drm.wt.ugt80.
drm.smt1.
drm.wt.smt1.
dsm.ugt80.
dsm.wt.ugt80.
dsm.smt1.
dsm.wt.smt1.
sp.ugt80.
sp.wt.ugt80.
sp.smt1.
sp.wt.smt1.
im.ugt80.
im.wt.ugt80.
im.smt1.
im.wt.smt1.

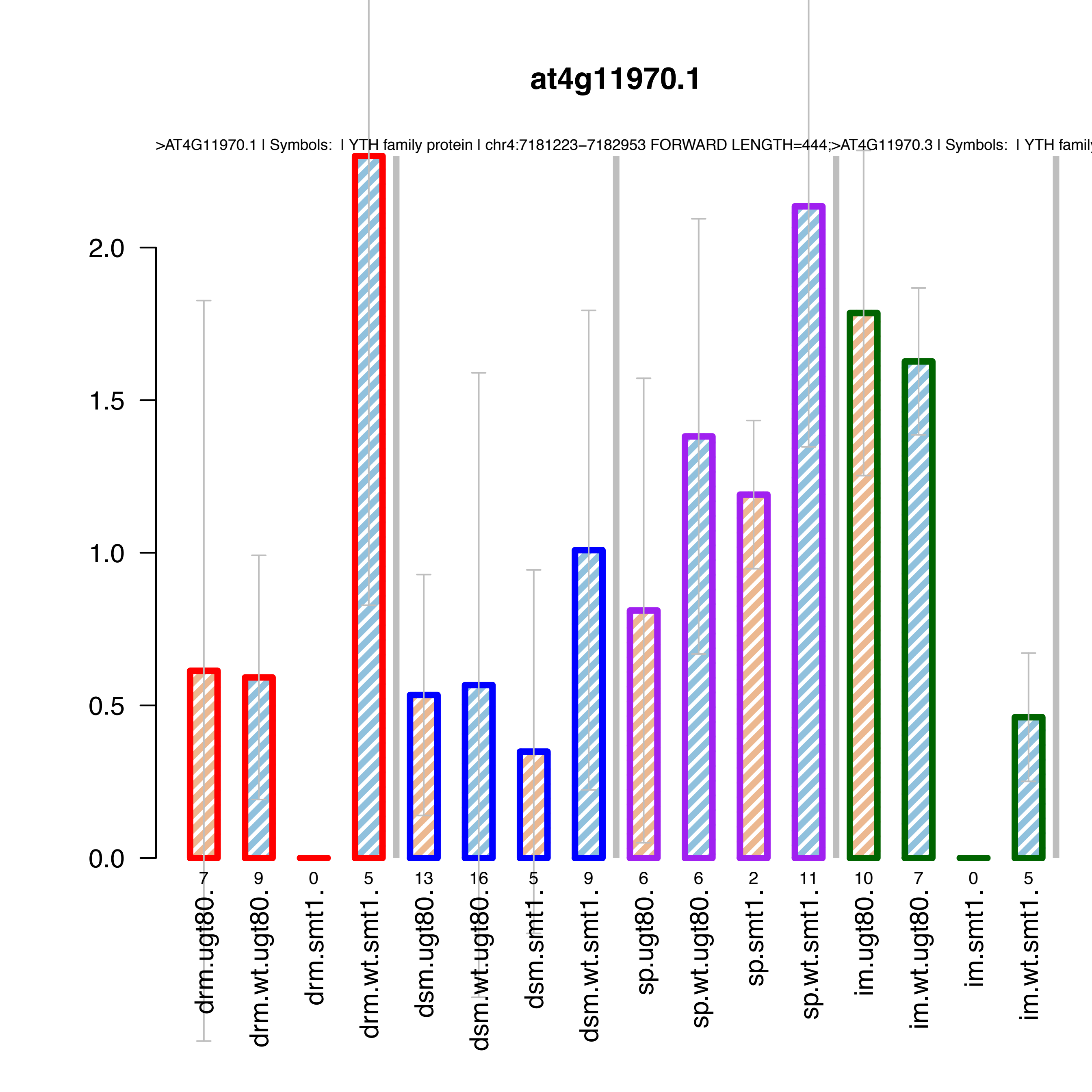

at4g11970.1
>AT4G11970.1 | Symbols: | YTH family protein | chr4:7181223-7182953 FORWARD LENGTH=444;>AT4G11970.3 | Symbols: | YTH family
2.0
1.5
1.0
0.5
0.0
7
9
0
5
13
16
5
9
6
6
2
11
10
7
0
5
drm.ugt80.
drm.wt.ugt80.
drm.smt1.
drm.wt.smt1.
dsm.ugt80.
dsm.wt.ugt80.
dsm.smt1.
dsm.wt.smt1.
sp.ugt80.
sp.wt.ugt80.
sp.smt1.
sp.wt.smt1.
im.ugt80.
im.wt.ugt80.
im.smt1.
im.wt.smt1.

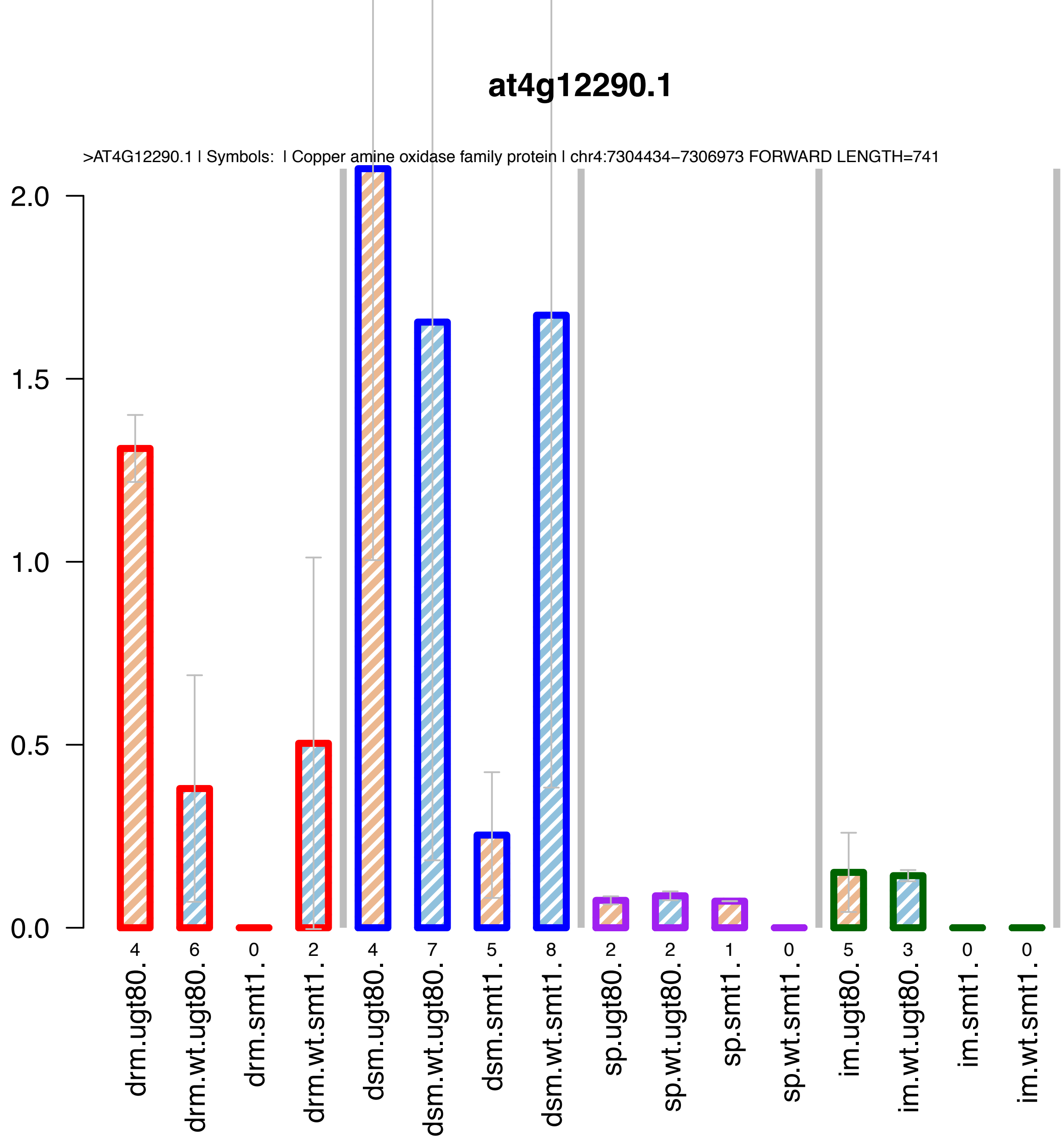

at4g12290.1
>AT4G12290.1 | Symbols: | Copper amine oxidase family protein | chr4:7304434-7306973 FORWARD LENGTH=741
2.0
1.5
1.0
0.5
0.0
4
6
0
2
4
7
5
8
2
2
1
0
5
3
0
0
drm.ugt80..
drm.wt.ugt80..
drm.smt1..
drm.wt.smt1..
dsm.ugt80..
dsm.wt.ugt80..
dsm.smt1..
dsm.wt.smt1..
sp.ugt80..
sp.wt.ugt80..
sp.smt1..
sp.wt.smt1..
im.ugt80..
im.wt.ugt80..
im.smt1..
im.wt.smt1..

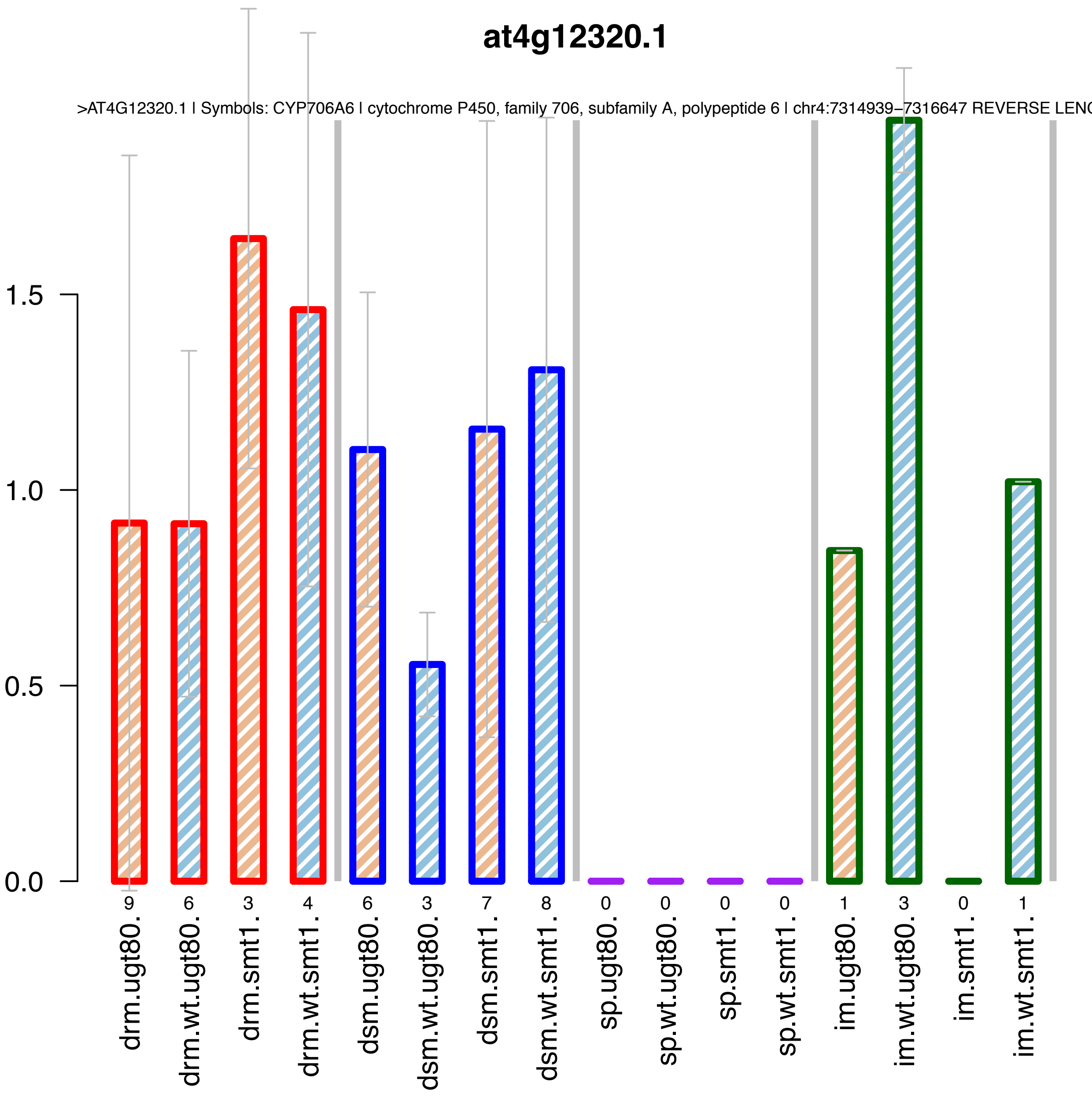

at4g12320.1
>AT4G12320.1 | Symbols: CYP706A6 | cytochrome P450, family 706, subfamily A, polypeptide 6 | chr4:7314939-7316647 REVERSE LENG
0.0
0.5
1.0
1.5
9
6
3
4
6
3
7
8
0
0
0
0
1
3
0
1
drm.ugt80.
drm.wt.ugt80.
drm.smt1.
drm.wt.smt1.
dsm.ugt80.
dsm.wt.ugt80.
dsm.smt1.
dsm.wt.smt1.
sp.ugt80.
sp.wt.ugt80.
sp.smt1.
sp.wt.smt1.
im.ugt80.
im.wt.ugt80.
im.smt1.
im.wt.smt1.

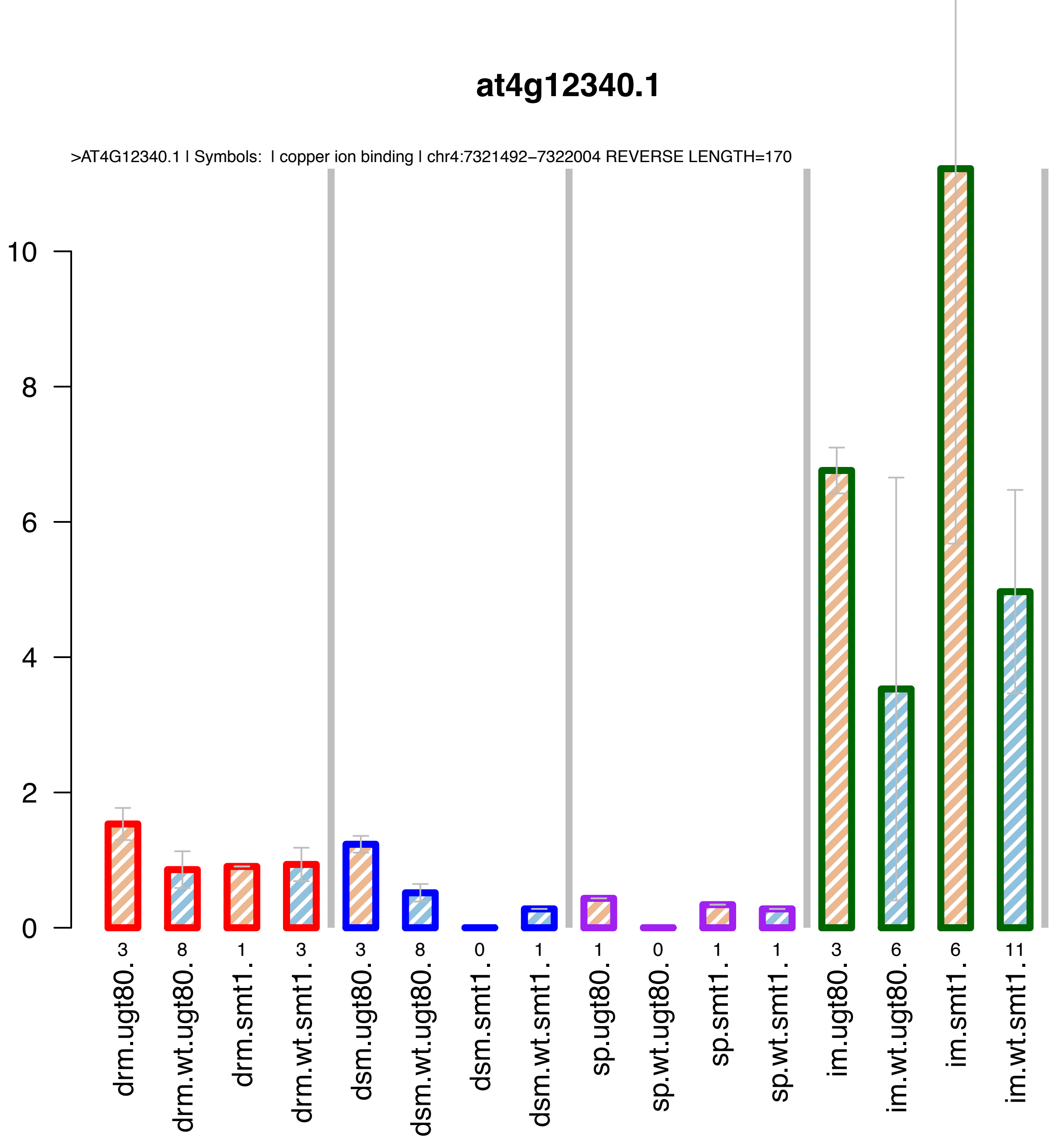

at4g12340.1
>AT4G12340.1 | Symbols: | copper ion binding | chr4:7321492–7322004 REVERSE LENGTH=170
0
2
4
6
8
10
3
8
1
3
3
8
0
1
1
0
1
1
3
6
6
11
drm.ugt80..
drm.wt.ugt80..
drm.smt1..
drm.wt.smt1..
dsm.ugt80..
dsm.wt.ugt80..
dsm.smt1..
dsm.wt.smt1..
sp.ugt80..
sp.wt.ugt80..
sp.smt1..
sp.wt.smt1..
im.ugt80..
im.wt.ugt80..
im.smt1..
im.wt.smt1..

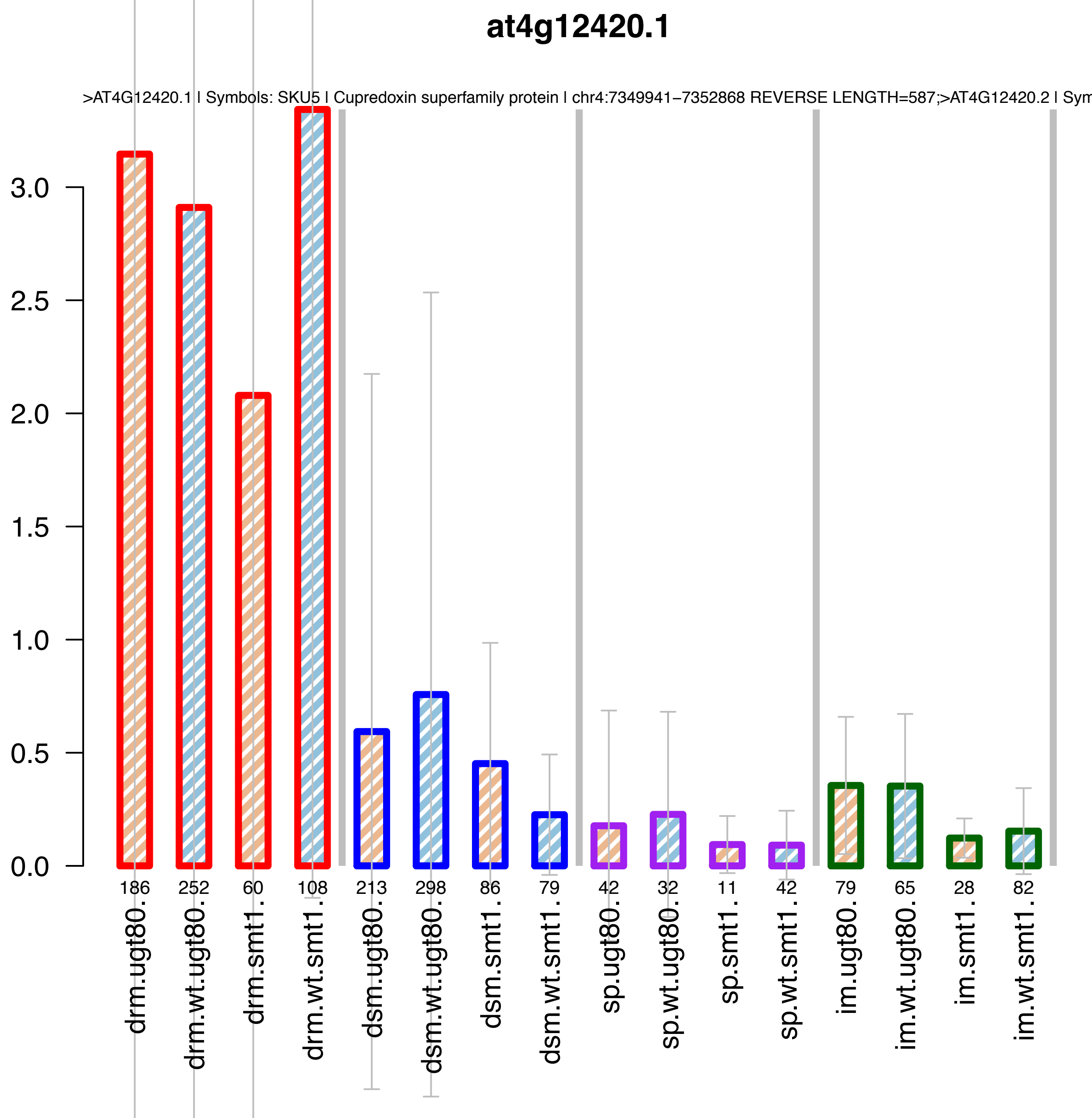

at4g12420.1
>AT4G12420.1 | Symbols: SKU5 | Cupredoxin superfamily protein | chr4:7349941-7352868 REVERSE LENGTH=587;>AT4G12420.2 | Sym
3.0
2.5
2.0
1.5
1.0
0.5
0.0
186
252
60
108
213
298
86
79
42
32
11
42
79
65
28
82
drm.ugt80..
drm.wt.ugt80..
drm.smt1..
drm.wt.smt1..
dsm.ugt80..
dsm.wt.ugt80..
dsm.smt1..
dsm.wt.smt1..
sp.ugt80..
sp.wt.ugt80..
sp.smt1..
sp.wt.smt1..
im.ugt80..
im.wt.ugt80..
im.smt1..
im.wt.smt1..

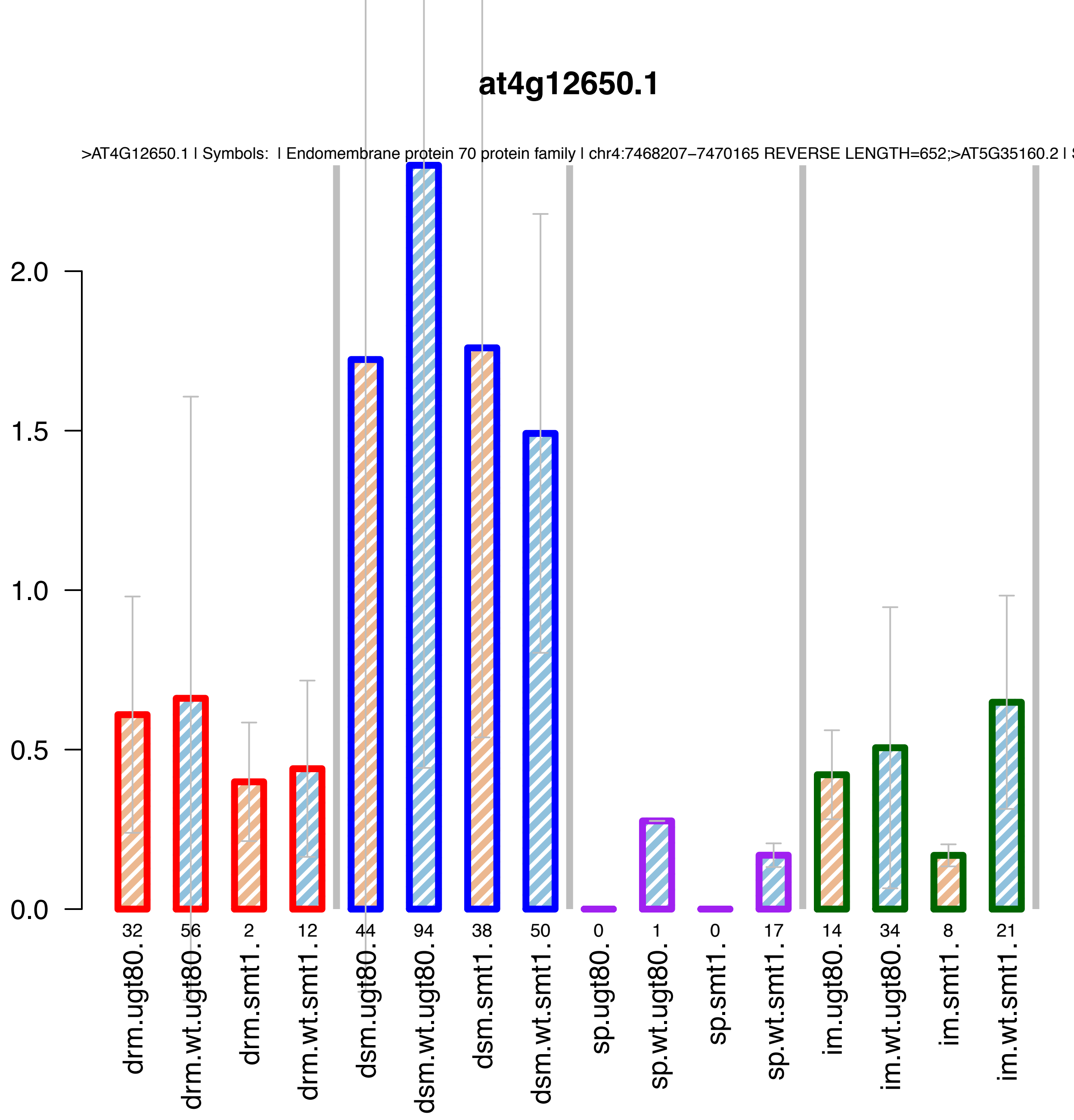

at4g12650.1
>AT4G12650.1 | Symbols: | Endomembrane protein 70 protein family | chr4:7468207-7470165 REVERSE LENGTH=652;>AT5G35160.2 |
2.0
1.5
1.0
0.5
0.0
32
56
2
12
44
94
38
50
0
1
0
17
14
34
8
21
drm.ugt80..
drm.wt.ugt80..
drm.smt1..
drm.wt.smt1..
dsm.ugt80..
dsm.wt.ugt80..
dsm.smt1..
dsm.wt.smt1..
sp.ugt80..
sp.wt.ugt80..
sp.smt1..
sp.wt.smt1..
im.ugt80..
im.wt.ugt80..
im.smt1..
im.wt.smt1..

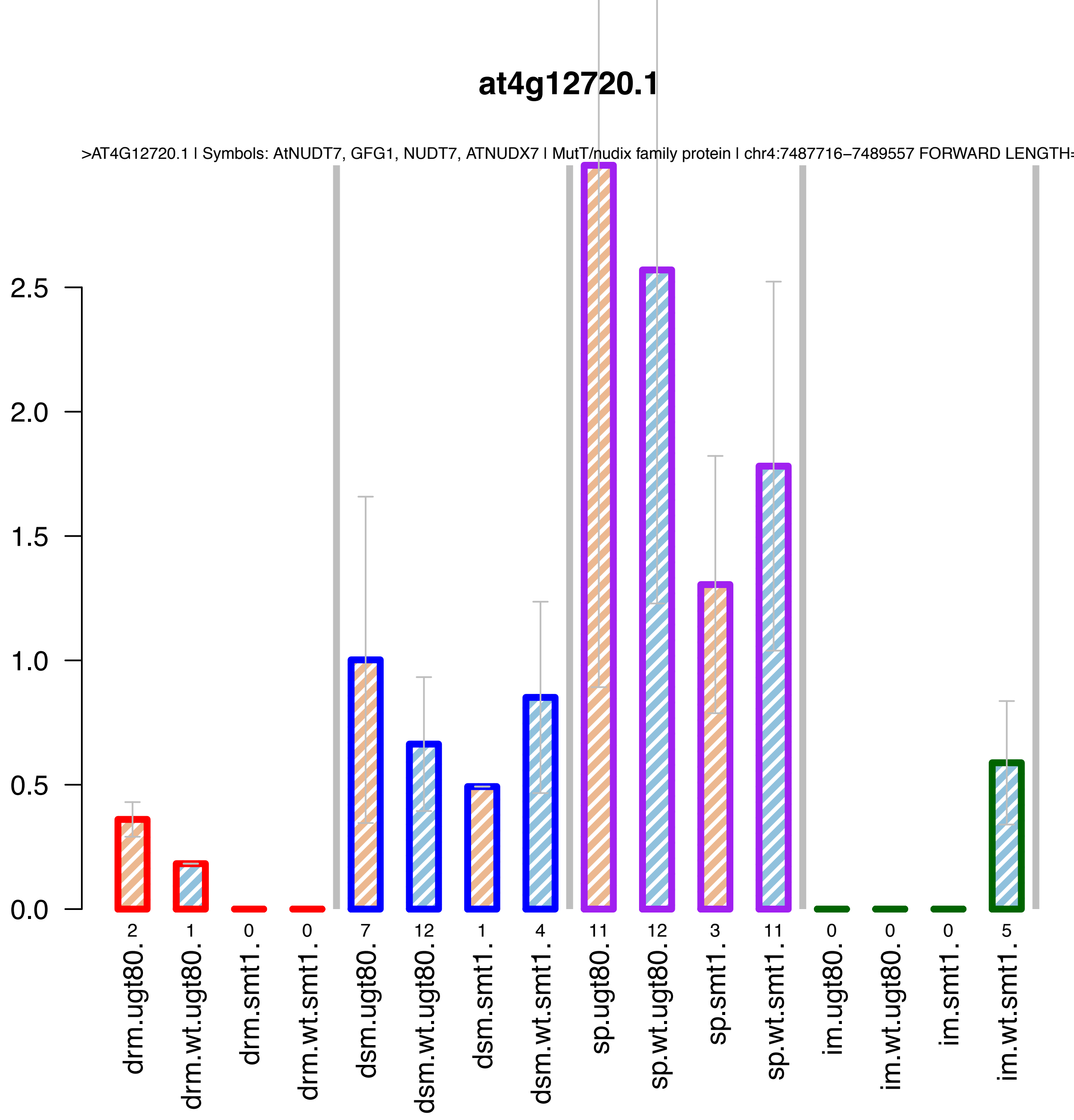

at4g12720.1
>AT4G12720.1 | Symbols: AtNUDT7, GFG1, NUDT7, ATNUDX7 | MutT/nudix family protein | chr4:7487716-7489557 FORWARD LENGTH=
0.0
0.5
1.0
1.5
2.0
2.5
2
1
0
0
7
12
1
4
11
12
3
11
0
0
0
5
drm.ugt80.
drm.wt.ugt80.
drm.smt1.
drm.wt.smt1.
dsm.ugt80.
dsm.wt.ugt80.
dsm.smt1.
dsm.wt.smt1.
sp.ugt80.
sp.wt.ugt80.
sp.smt1.
sp.wt.smt1.
im.ugt80.
im.wt.ugt80.
im.smt1.
im.wt.smt1.

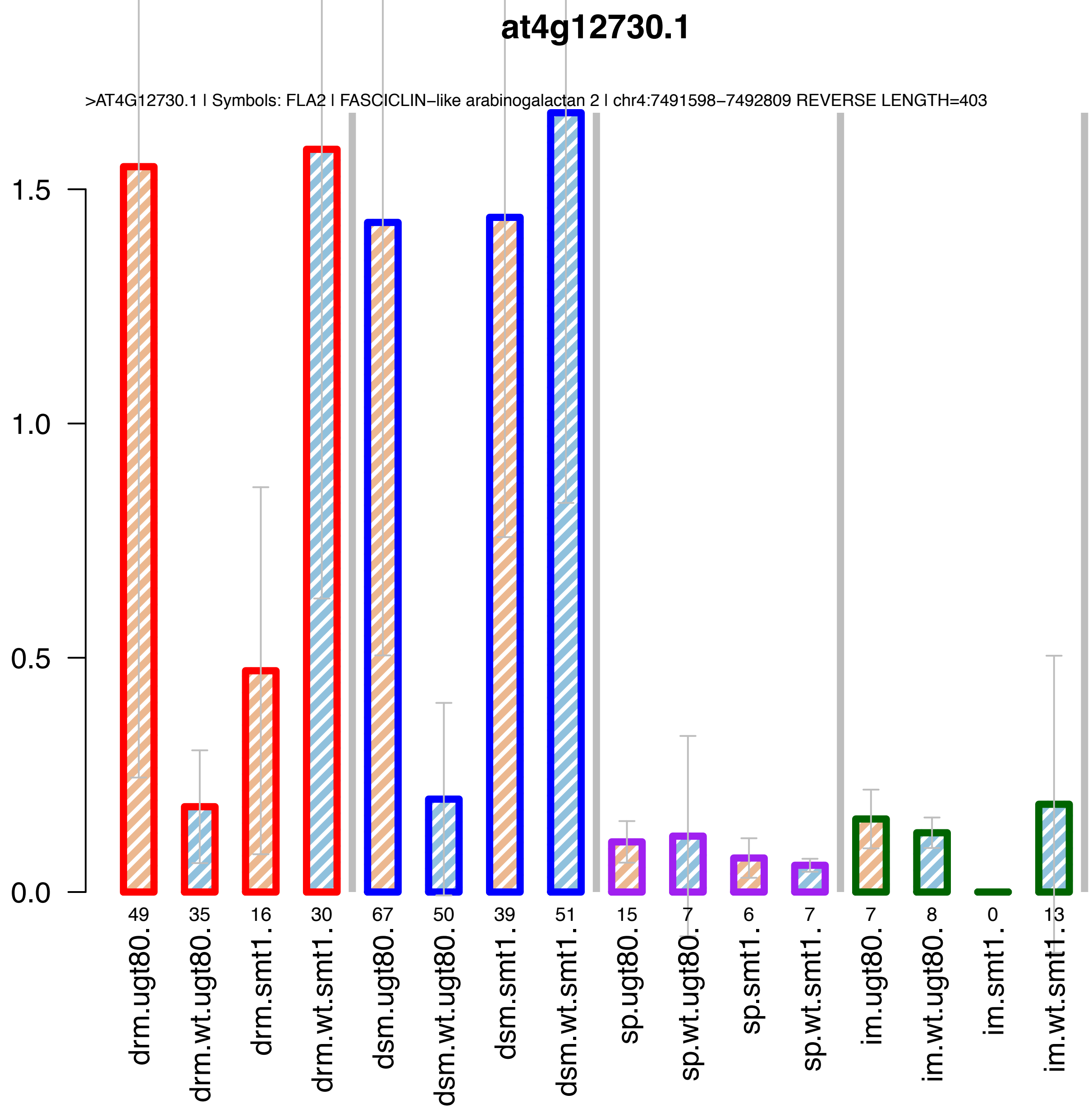
at4g12730.1
>AT4G12730.1 | Symbols: FLA2 | FASCICLIN−like arabinogalactan 2 | chr4:7491598−7492809 REVERSE LENGTH=403
1.5
1.0
0.5
0.0
49
35
16
30
67
50
39
51
15
7
6
7
7
8
0
13
drm.ugt80.
drm.wt.ugt80.
drm.smt1.
drm.wt.smt1.
dsm.ugt80.
dsm.wt.ugt80.
dsm.smt1.
dsm.wt.smt1.
sp.ugt80.
sp.wt.ugt80.
sp.smt1.
sp.wt.smt1.
im.ugt80.
im.wt.ugt80.
im.smt1.
im.wt.smt1.

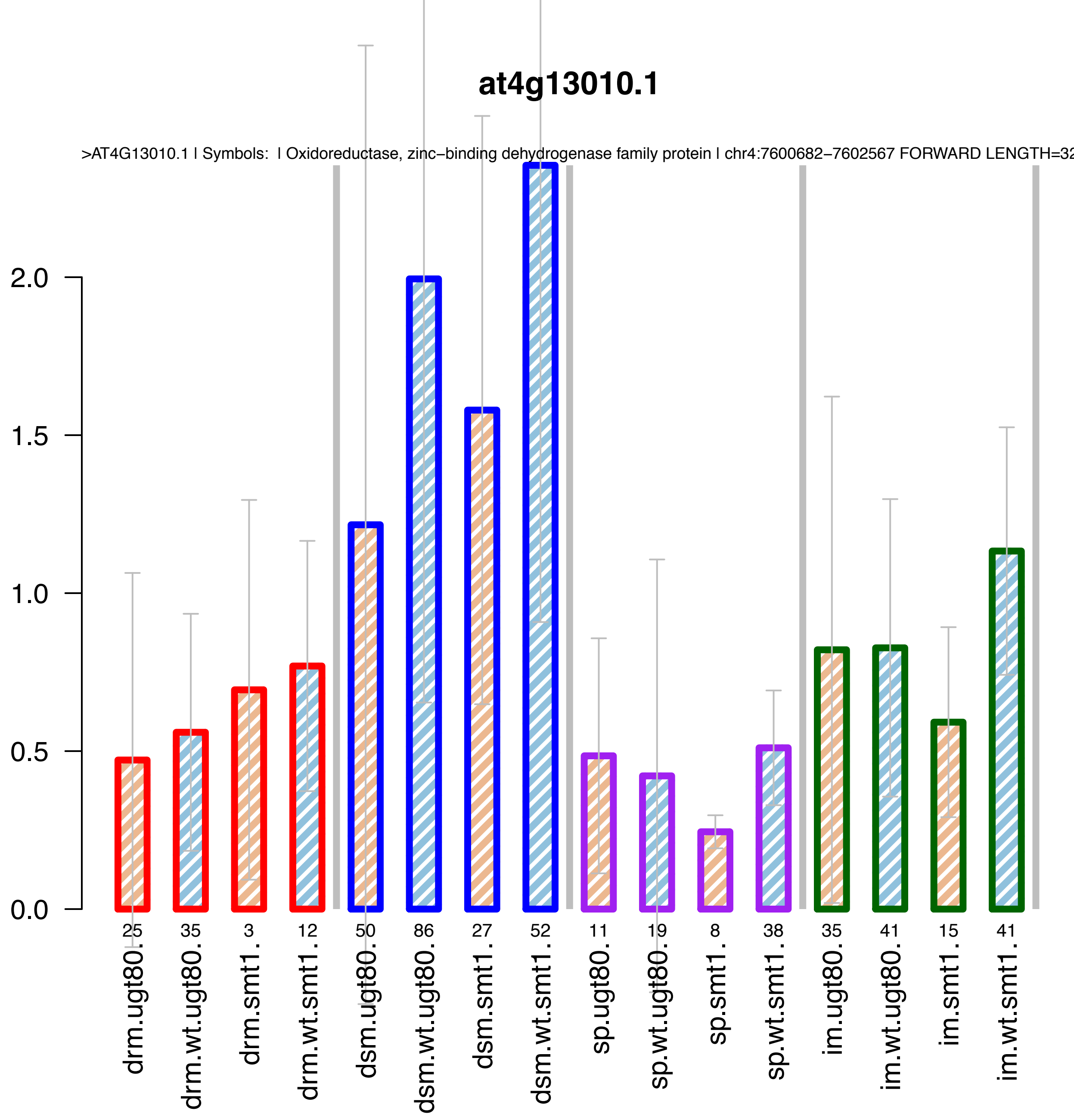

at4g13010.1
>AT4G13010.1 | Symbols: | Oxidoreductase, zinc−binding dehydrogenase family protein | chr4:7600682−7602567 FORWARD LENGTH=32
2.0
1.5
1.0
0.5
0.0
25
35
3
12
50
86
27
52
11
19
8
38
35
41
15
41
drm.ugt80.
drm.wt.ugt80.
drm.smt1.
drm.wt.smt1.
dsm.ugt80.
dsm.wt.ugt80.
dsm.smt1.
dsm.wt.smt1.
sp.ugt80.
sp.wt.ugt80.
sp.smt1.
sp.wt.smt1.
im.ugt80.
im.wt.ugt80.
im.smt1.
im.wt.smt1.

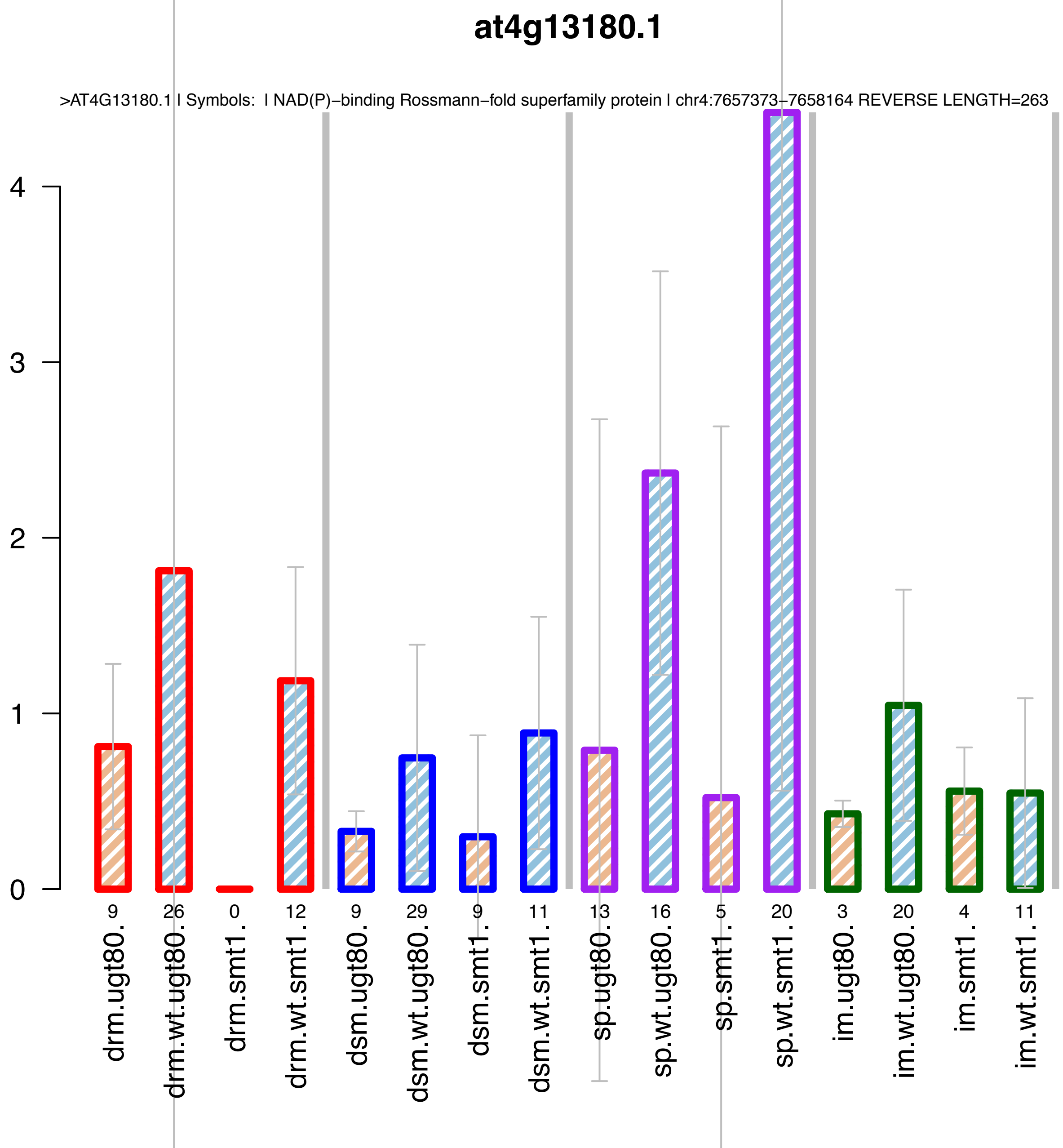

at4g13180.1
>AT4G13180.1 | Symbols: | NAD(P)–binding Rossmann–fold superfamily protein | chr4:7657373–7658164 REVERSE LENGTH=263
0
1
2
3
4
9
26
0
12
9
29
9
11
13
16
5
20
3
20
4
11
drm.ugt80.
drm.wt.ugt80.
drm.smt1.
drm.wt.smt1.
dsm.ugt80.
dsm.wt.ugt80.
dsm.smt1.
dsm.wt.smt1.
sp.ugt80.
sp.wt.ugt80.
sp.smt1.
sp.wt.smt1.
im.ugt80.
im.wt.ugt80.
im.smt1.
im.wt.smt1.

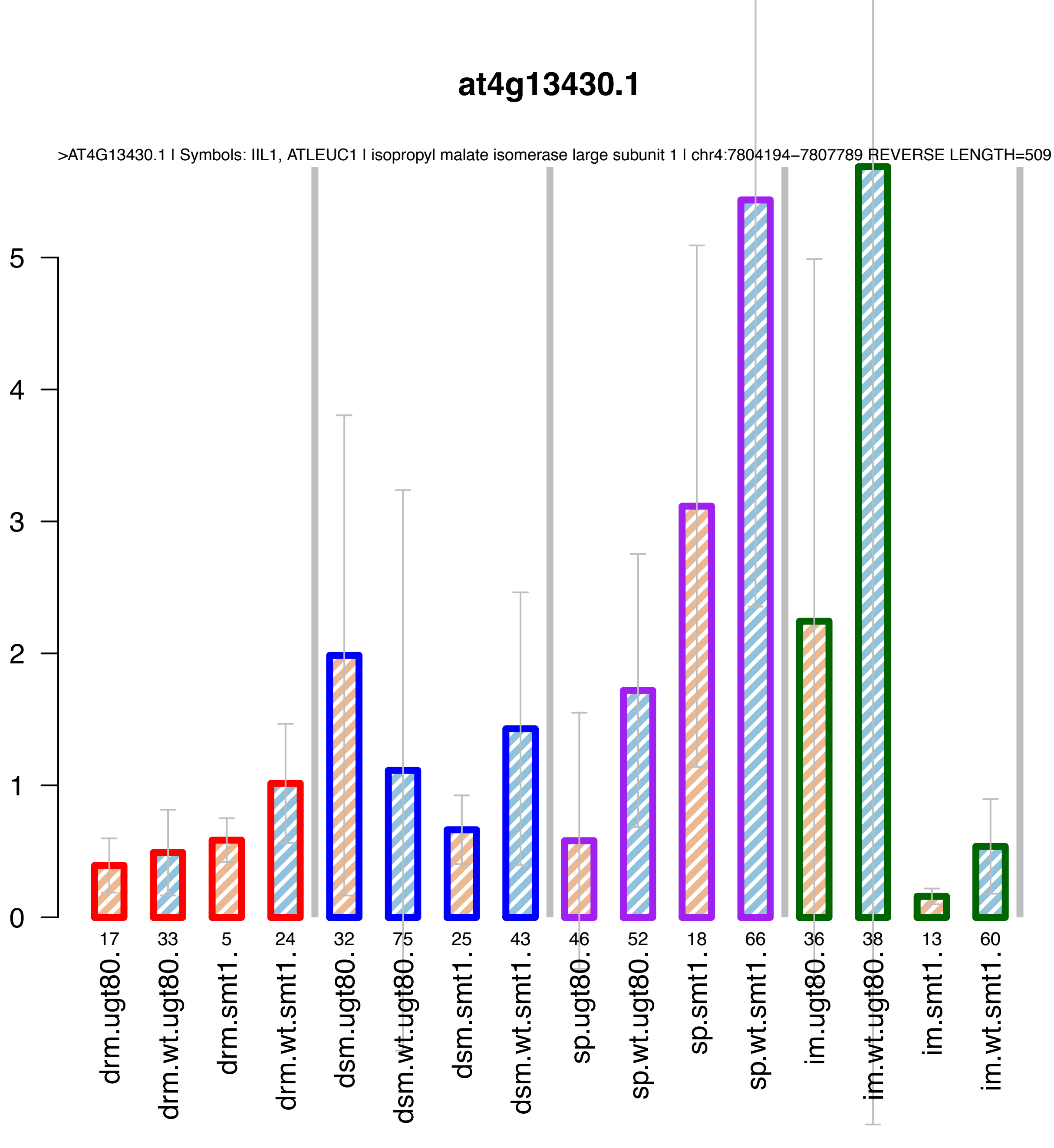

at4g13430.1
>AT4G13430.1 | Symbols: IIL1, ATLEUC1 | isopropyl malate isomerase large subunit 1 | chr4:7804194−7807789 REVERSE LENGTH=509
0
1
2
3
4
5
17
33
5
24
32
75
25
43
46
52
18
66
36
38
13
60
drm.ugt80.
drm.wt.ugt80.
drm.smt1.
drm.wt.smt1.
dsm.ugt80.
dsm.wt.ugt80.
dsm.smt1.
dsm.wt.smt1.
sp.ugt80.
sp.wt.ugt80.
sp.smt1.
sp.wt.smt1.
im.ugt80.
im.wt.ugt80.
im.smt1.
im.wt.smt1.

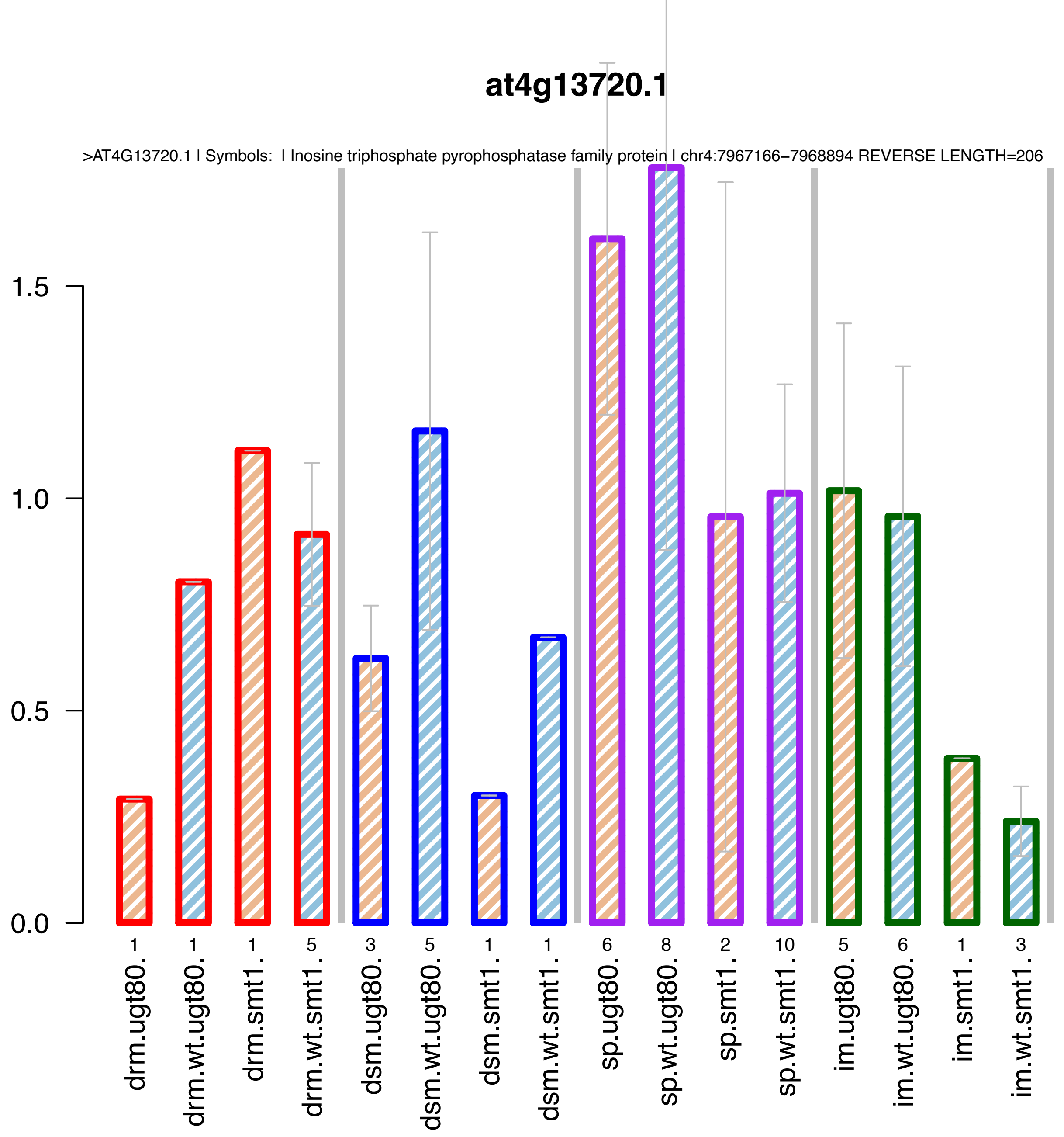

at4g13720.1
>AT4G13720.1 | Symbols: | Inosine triphosphate pyrophosphatase family protein | chr4:7967166-7968894 REVERSE LENGTH=206
1.5
1.0
0.5
0.0
1
1
1
5
3
5
1
1
6
8
2
10
5
6
1
3
drm.ugt80..
drm.wt.ugt80..
drm.smt1..
drm.wt.smt1..
dsm.ugt80..
dsm.wt.ugt80..
dsm.smt1..
dsm.wt.smt1..
sp.ugt80..
sp.wt.ugt80..
sp.smt1..
sp.wt.smt1..
im.ugt80..
im.wt.ugt80..
im.smt1..
im.wt.smt1..

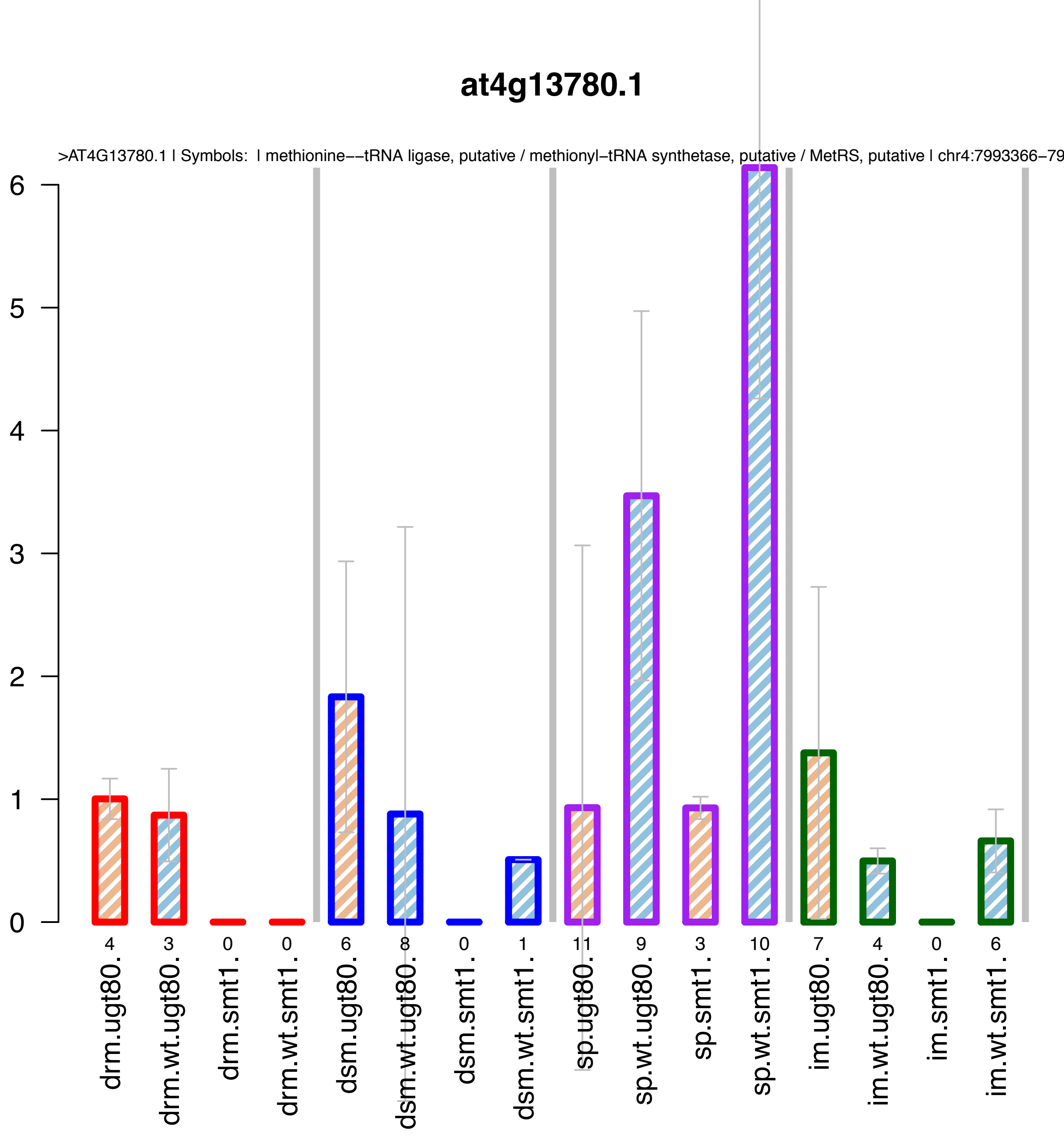

at4g13780.1
>AT4G13780.1 | Symbols: | methionine--tRNA ligase, putative / methionyl-tRNA synthetase, putative / MetRS, putative | chr4:7993366-79
0
1
2
3
4
5
6
4
3
0
0
6
8
0
1
11
9
3
10
7
4
0
6
drm.ugt80.
drm.wt.ugt80.
drm.smt1.
drm.wt.smt1.
dsm.ugt80.
dsm.wt.ugt80.
dsm.smt1.
dsm.wt.smt1.
sp.ugt80.
sp.wt.ugt80.
sp.smt1.
sp.wt.smt1.
im.ugt80.
im.wt.ugt80.
im.smt1.
im.wt.smt1.

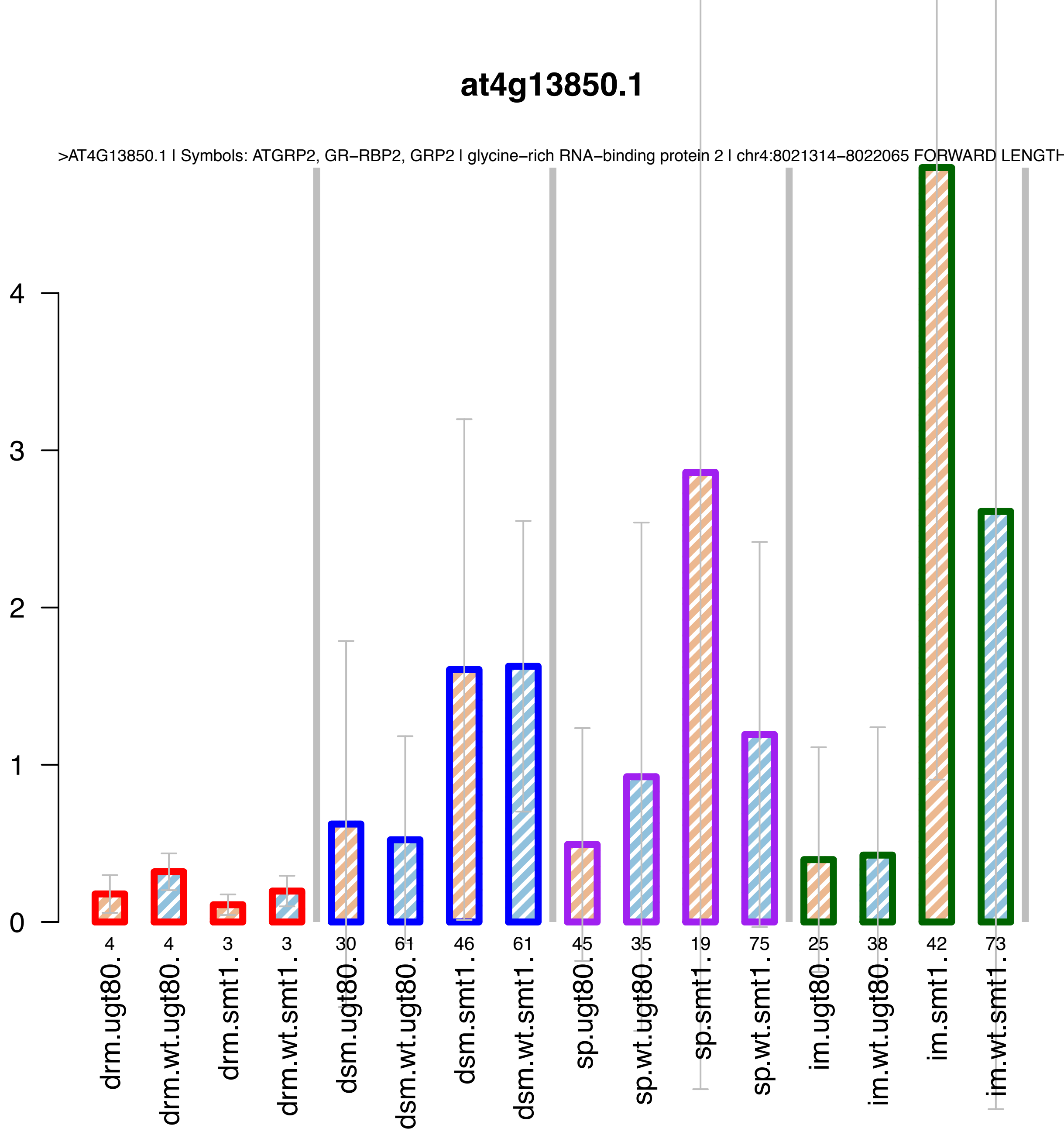

at4g13850.1
>AT4G13850.1 | Symbols: ATGRP2, GR−RBP2, GRP2 | glycine−rich RNA−binding protein 2 | chr4:8021314−8022065 FORWARD LENGTH
0
1
2
3
4
4
4
3
3
30
61
46
61
45
35
19
75
25
38
42
73
drm.ugt80..
drm.wt.ugt80..
drm.smt1..
drm.wt.smt1..
dsm.ugt80..
dsm.wt.ugt80..
dsm.smt1..
dsm.wt.smt1..
sp.ugt80..
sp.wt.ugt80..
sp.smt1..
sp.wt.smt1..
im.ugt80..
im.wt.ugt80..
im.smt1..
im.wt.smt1..

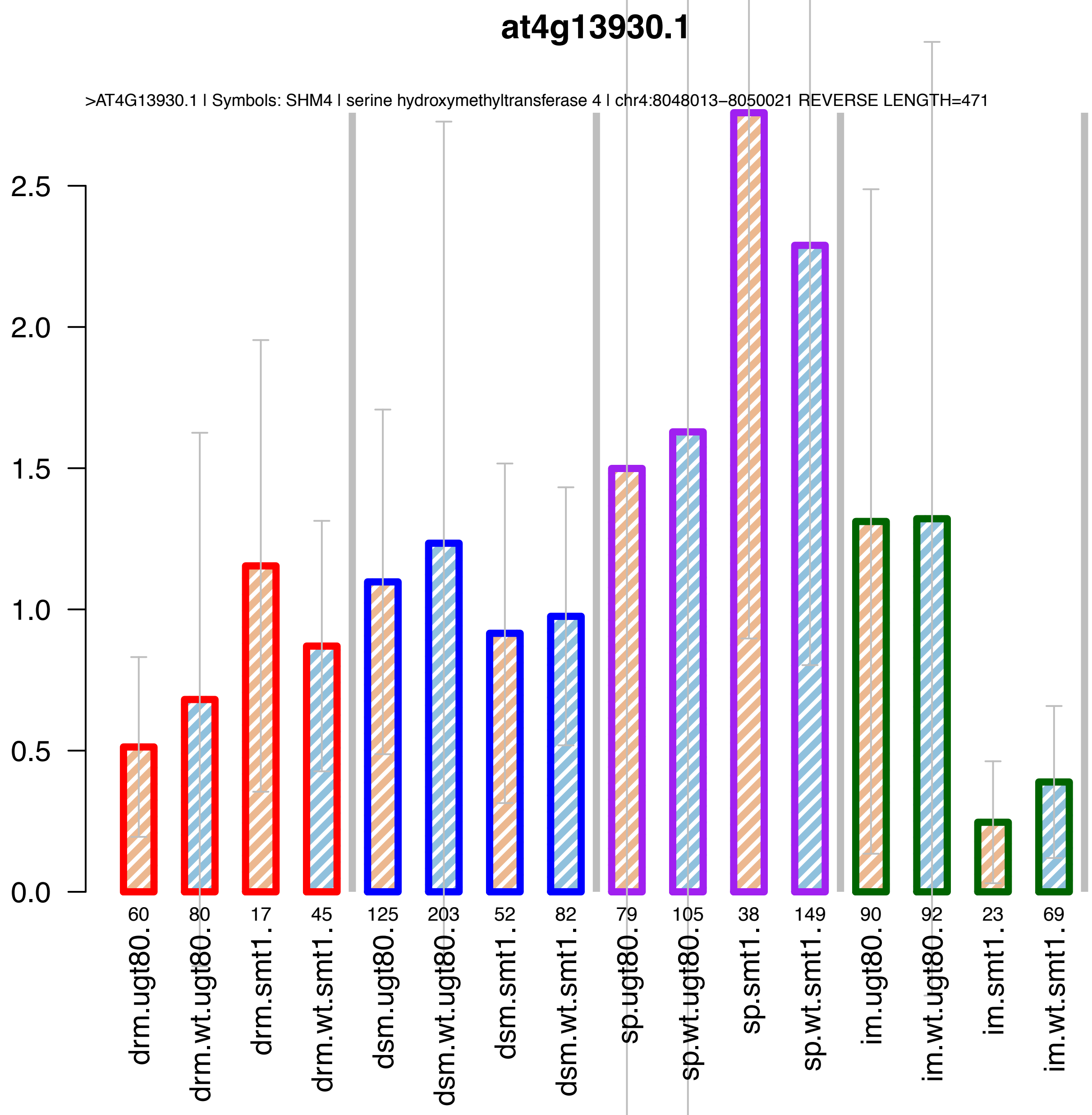

at4g13930.1
>AT4G13930.1 | Symbols: SHM4 | serine hydroxymethyltransferase 4 | chr4:8048013-8050021 REVERSE LENGTH=471
0.0
0.5
1.0
1.5
2.0
2.5
60
80
17
45
125
203
52
82
79
105
38
149
90
92
23
69
drm.ugt80..
drm.wt.ugt80..
drm.smt1..
drm.wt.smt1..
dsm.ugt80..
dsm.wt.ugt80..
dsm.smt1..
dsm.wt.smt1..
sp.ugt80..
sp.wt.ugt80..
sp.smt1..
sp.wt.smt1..
im.ugt80..
im.wt.ugt80..
im.smt1..
im.wt.smt1..

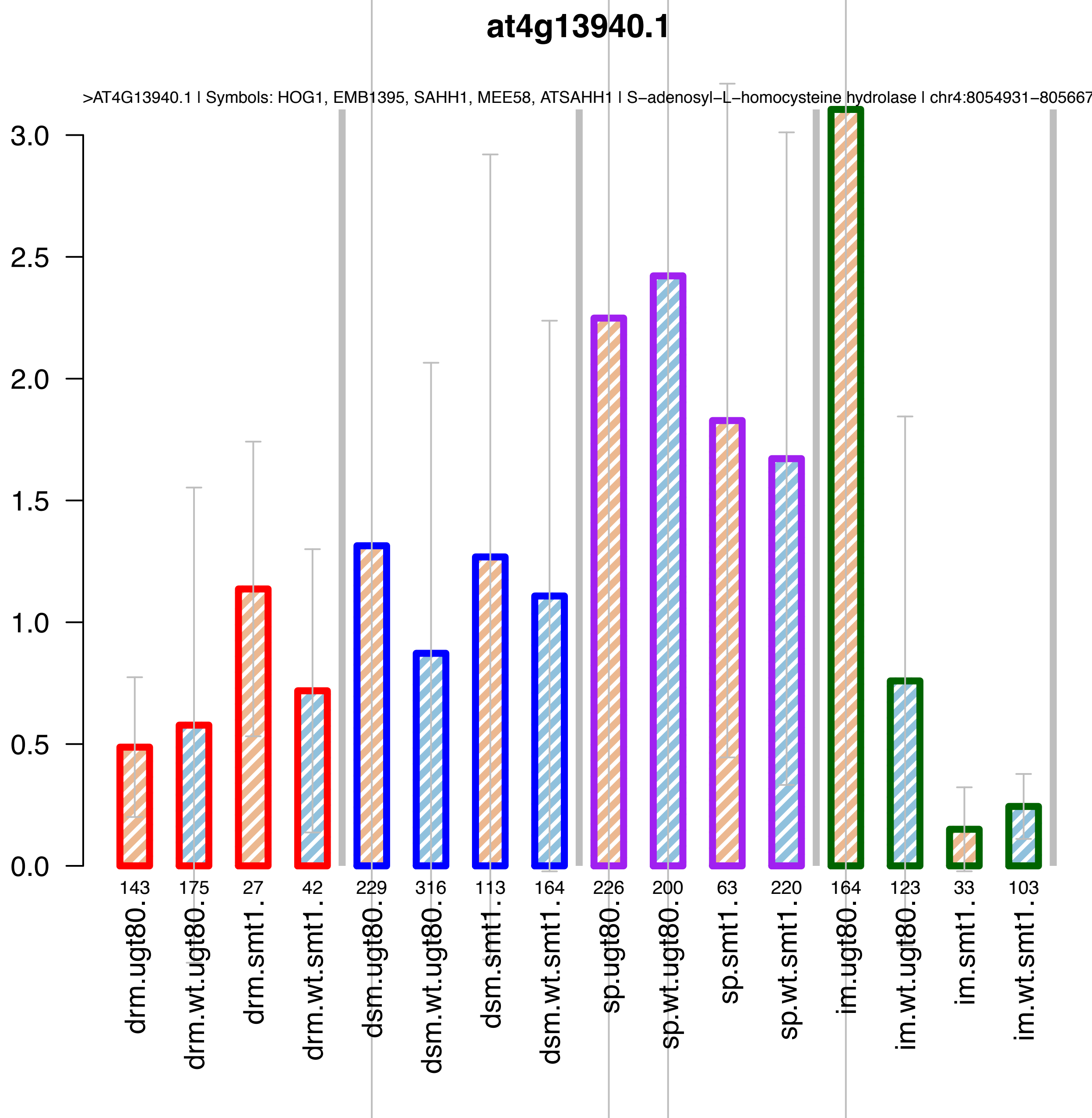

at4g13940.1
>AT4G13940.1 | Symbols: HOG1, EMB1395, SAHH1, MEE58, ATSAHH1 | S-adenosyl-L-homocysteine hydrolase | chr4:8054931-805667
3.0
2.5
2.0
1.5
1.0
0.5
0.0
143
175
27
42
229
316
113
164
226
200
63
220
164
123
33
103
drm.ugt80..
drm.wt.ugt80..
drm.smt1..
drm.wt.smt1..
dsm.ugt80..
dsm.wt.ugt80..
dsm.smt1..
dsm.wt.smt1..
sp.ugt80..
sp.wt.ugt80..
sp.smt1..
sp.wt.smt1..
im.ugt80..
im.wt.ugt80..
im.smt1..
im.wt.smt1..

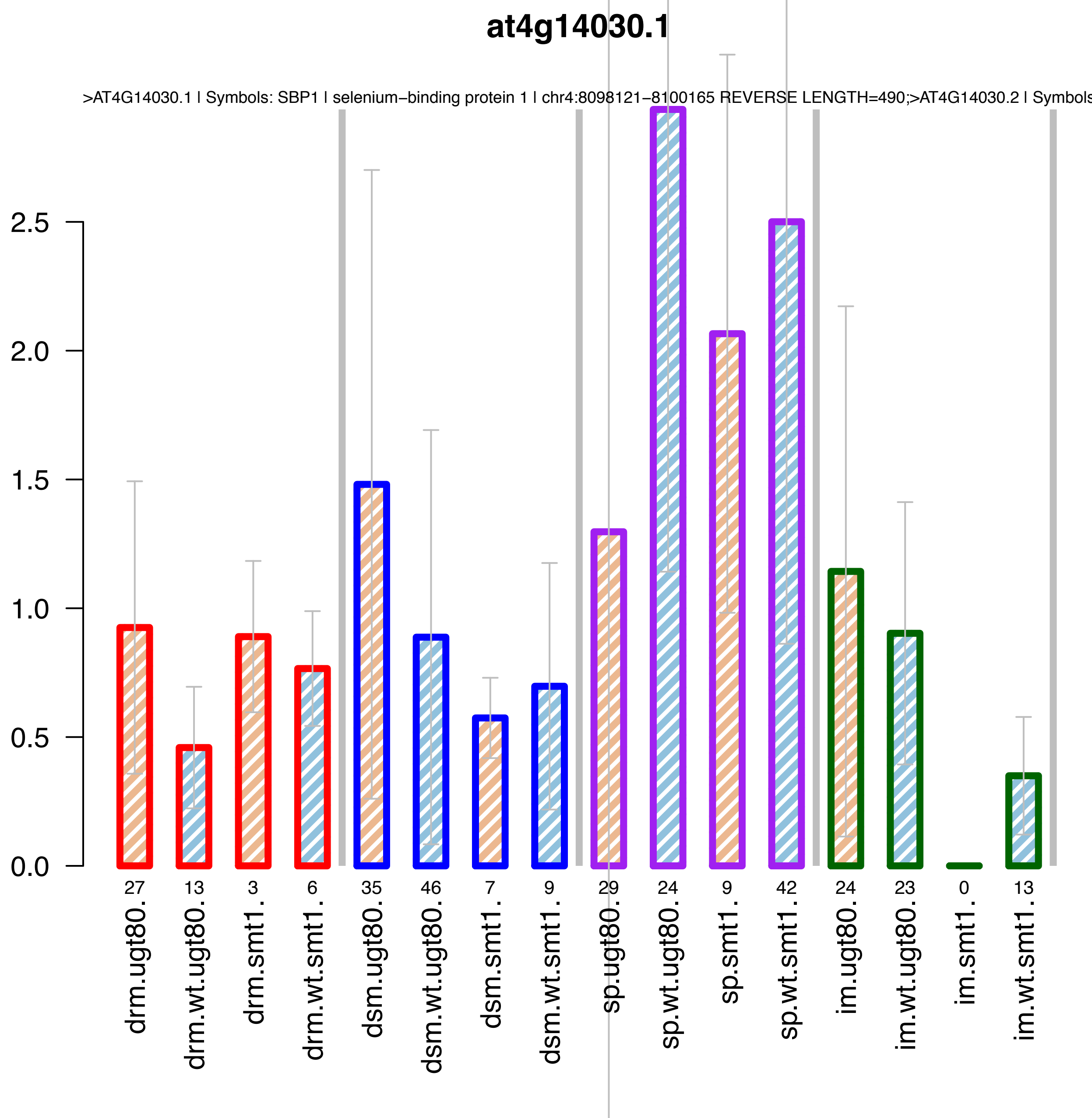

at4g14030.1
>AT4G14030.1 | Symbols: SBP1 | selenium−binding protein 1 | chr4:8098121−8100165 REVERSE LENGTH=490;>AT4G14030.2 | Symbols
2.5
2.0
1.5
1.0
0.5
0.0
27
13
3
6
35
46
7
9
29
24
9
42
24
23
0
13
drm.ugt80.
drm.wt.ugt80.
drm.smt1.
drm.wt.smt1.
dsm.ugt80.
dsm.wt.ugt80.
dsm.smt1.
dsm.wt.smt1.
sp.ugt80.
sp.wt.ugt80.
sp.smt1.
sp.wt.smt1.
im.ugt80.
im.wt.ugt80.
im.smt1.
im.wt.smt1.

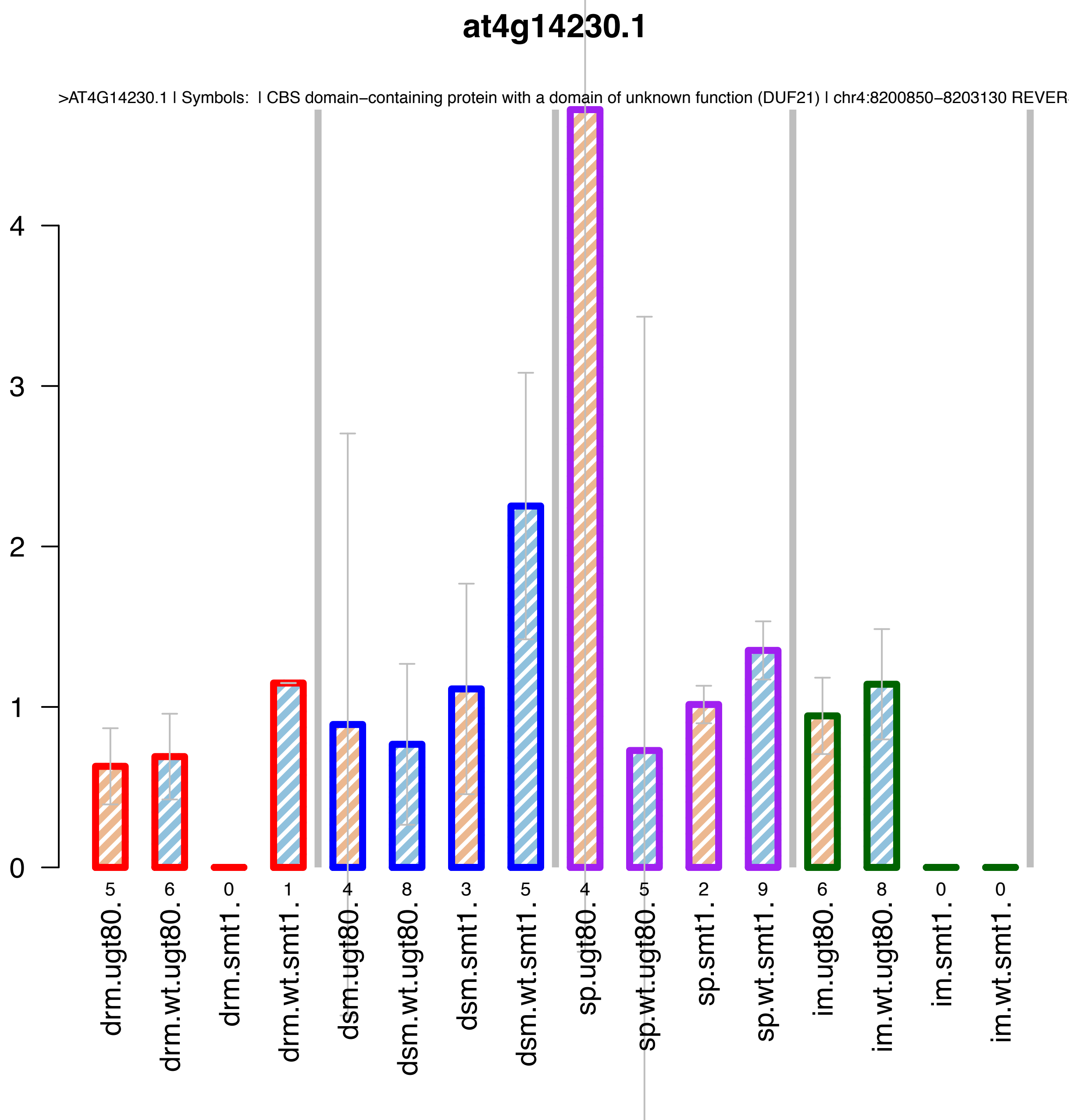

at4g14230.1
>AT4G14230.1 | Symbols: | CBS domain−containing protein with a domain of unknown function (DUF21) | chr4:8200850−8203130 REVER
0
1
2
3
4
5
6
0
1
4
8
3
5
4
5
2
9
6
8
0
0
drm.ugt80.
drm.wt.ugt80.
drm.smt1.
drm.wt.smt1.
dsm.ugt80.
dsm.wt.ugt80.
dsm.smt1.
dsm.wt.smt1.
sp.ugt80.
sp.wt.ugt80.
sp.smt1.
sp.wt.smt1.
im.ugt80.
im.wt.ugt80.
im.smt1.
im.wt.smt1.

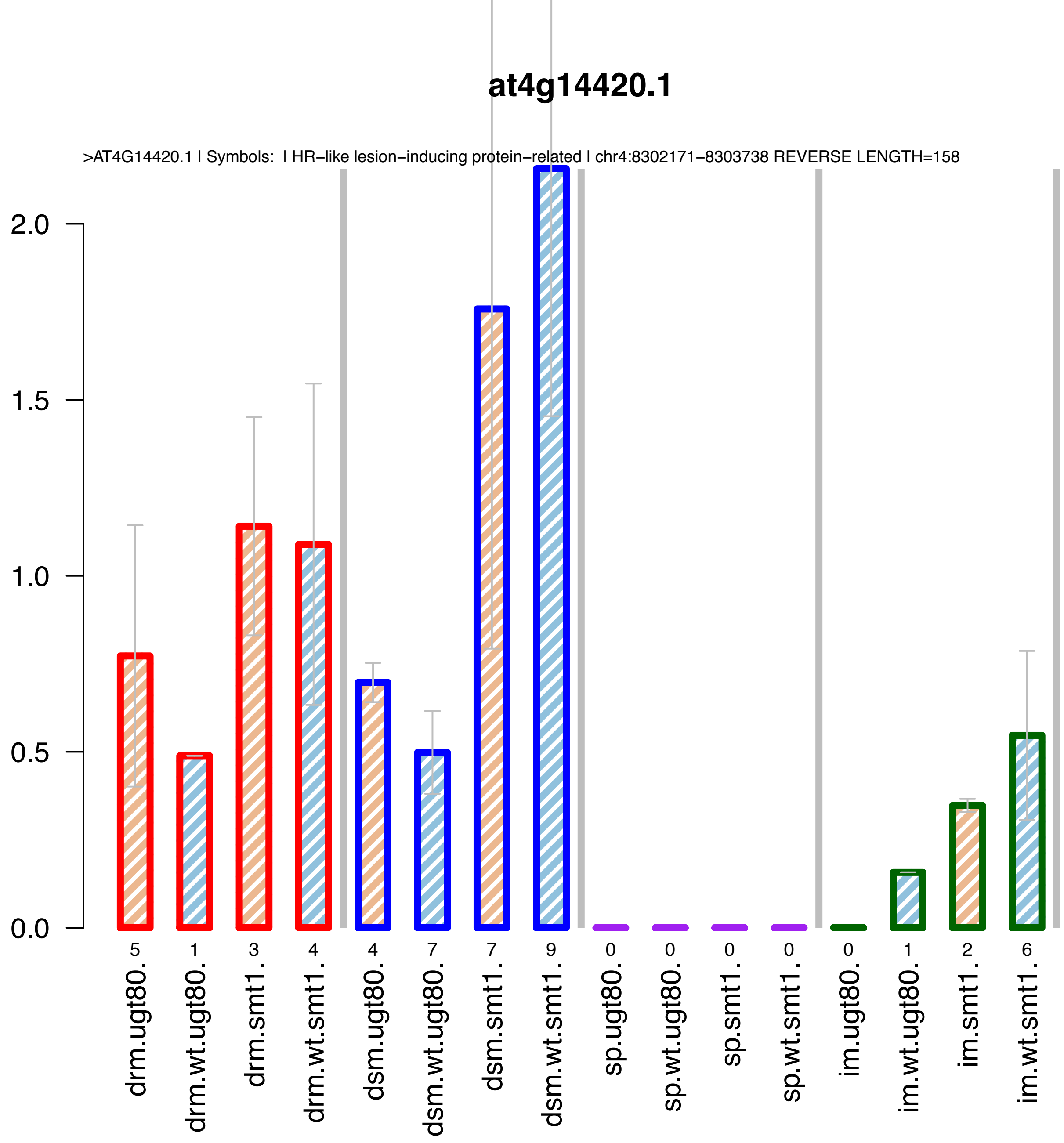
at4g14420.1
>AT4G14420.1 | Symbols: | HR–like lesion–inducing protein–related | chr4:8302171–8303738 REVERSE LENGTH=158
0.0
0.5
1.0
1.5
2.0
5
1
3
4
4
7
7
9
0
0
0
0
0
1
2
6
drm.ugt80.
drm.wt.ugt80.
drm.smt1.
drm.wt.smt1.
dsm.ugt80.
dsm.wt.ugt80.
dsm.smt1.
dsm.wt.smt1.
sp.ugt80.
sp.wt.ugt80.
sp.smt1.
sp.wt.smt1.
im.ugt80.
im.wt.ugt80.
im.smt1.
im.wt.smt1.

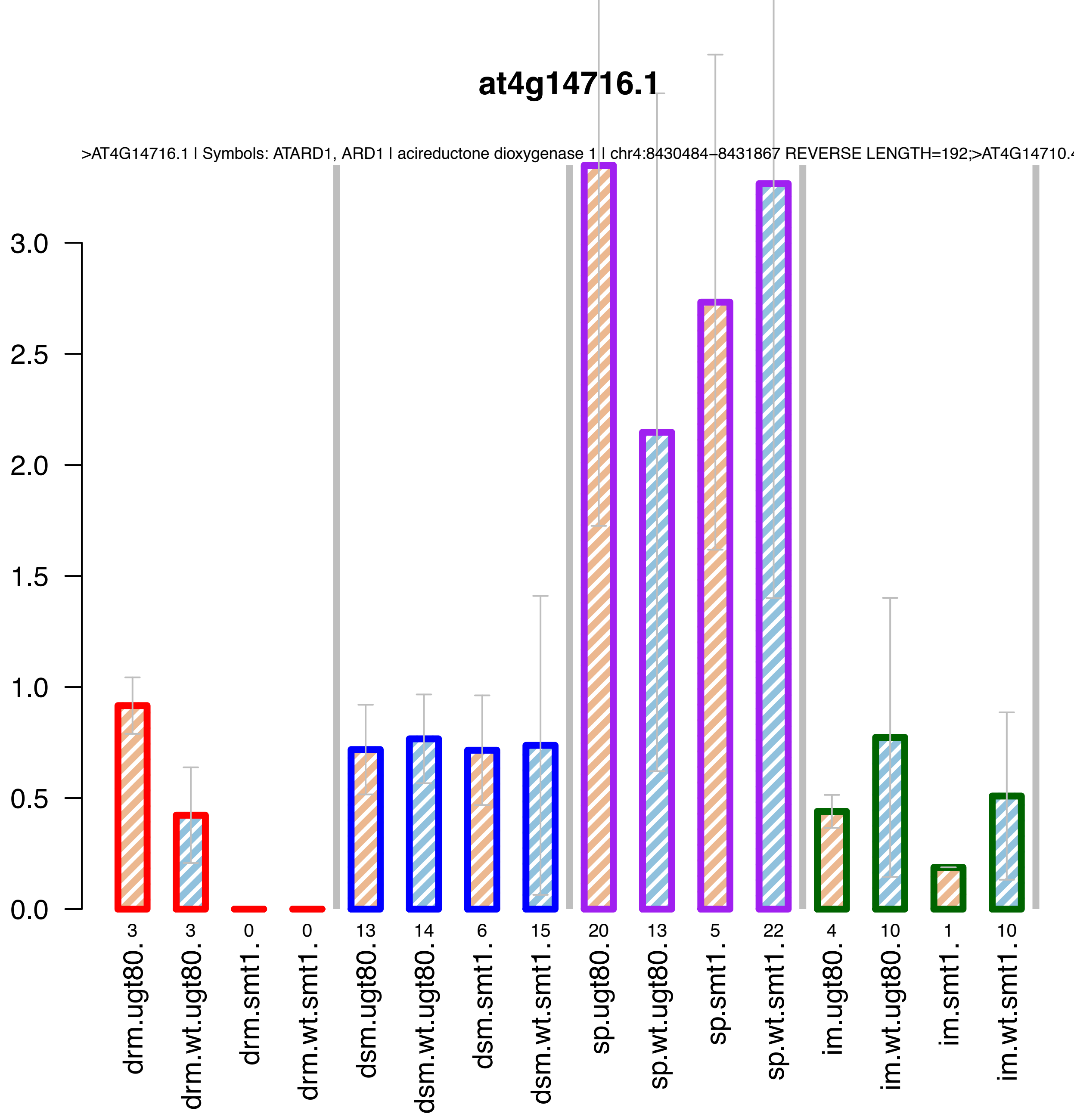

at4g14716.1
>AT4G14716.1 | Symbols: ATARD1, ARD1 | acireductone dioxygenase 1 | chr4:8430484-8431867 REVERSE LENGTH=192;>AT4G14710.4
3.0
2.5
2.0
1.5
1.0
0.5
0.0
3
3
0
0
13
14
6
15
20
13
5
22
4
10
1
10
drm.ugt80.
drm.wt.ugt80.
drm.smt1.
drm.wt.smt1.
dsm.ugt80.
dsm.wt.ugt80.
dsm.smt1.
dsm.wt.smt1.
sp.ugt80.
sp.wt.ugt80.
sp.smt1.
sp.wt.smt1.
im.ugt80.
im.wt.ugt80.
im.smt1.
im.wt.smt1.

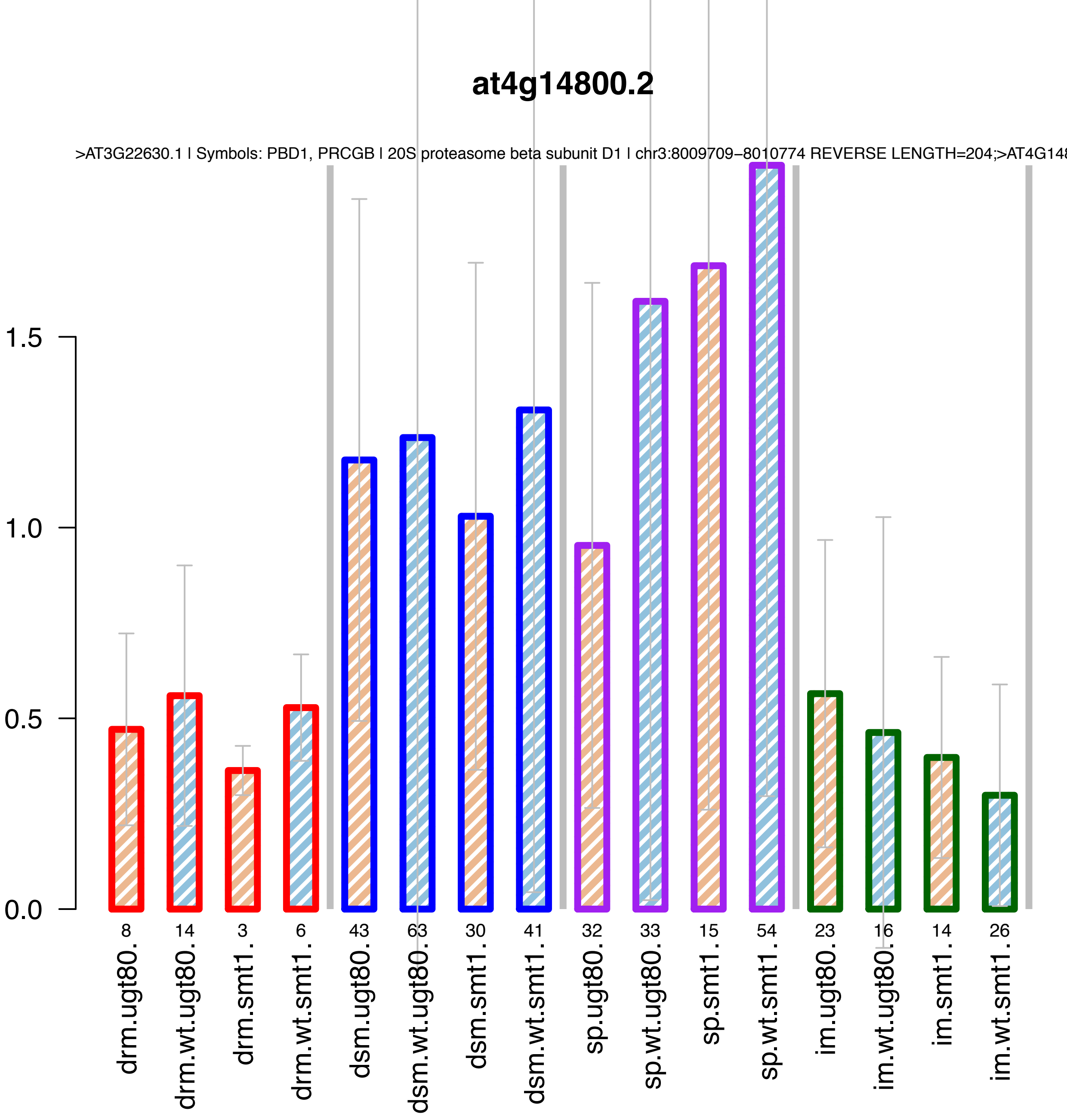

at4g14800.2
>AT3G22630.1 | Symbols: PBD1, PRCGB | 20S proteasome beta subunit D1 | chr3:8009709−8010774 REVERSE LENGTH=204;>AT4G148
0.0
0.5
1.0
1.5
8
14
3
6
43
63
30
41
32
33
15
54
23
16
14
26
drm.ugt80.
drm.wt.ugt80.
drm.smt1.
drm.wt.smt1.
dsm.ugt80.
dsm.wt.ugt80.
dsm.smt1.
dsm.wt.smt1.
sp.ugt80.
sp.wt.ugt80.
sp.smt1.
sp.wt.smt1.
im.ugt80.
im.wt.ugt80.
im.smt1.
im.wt.smt1.

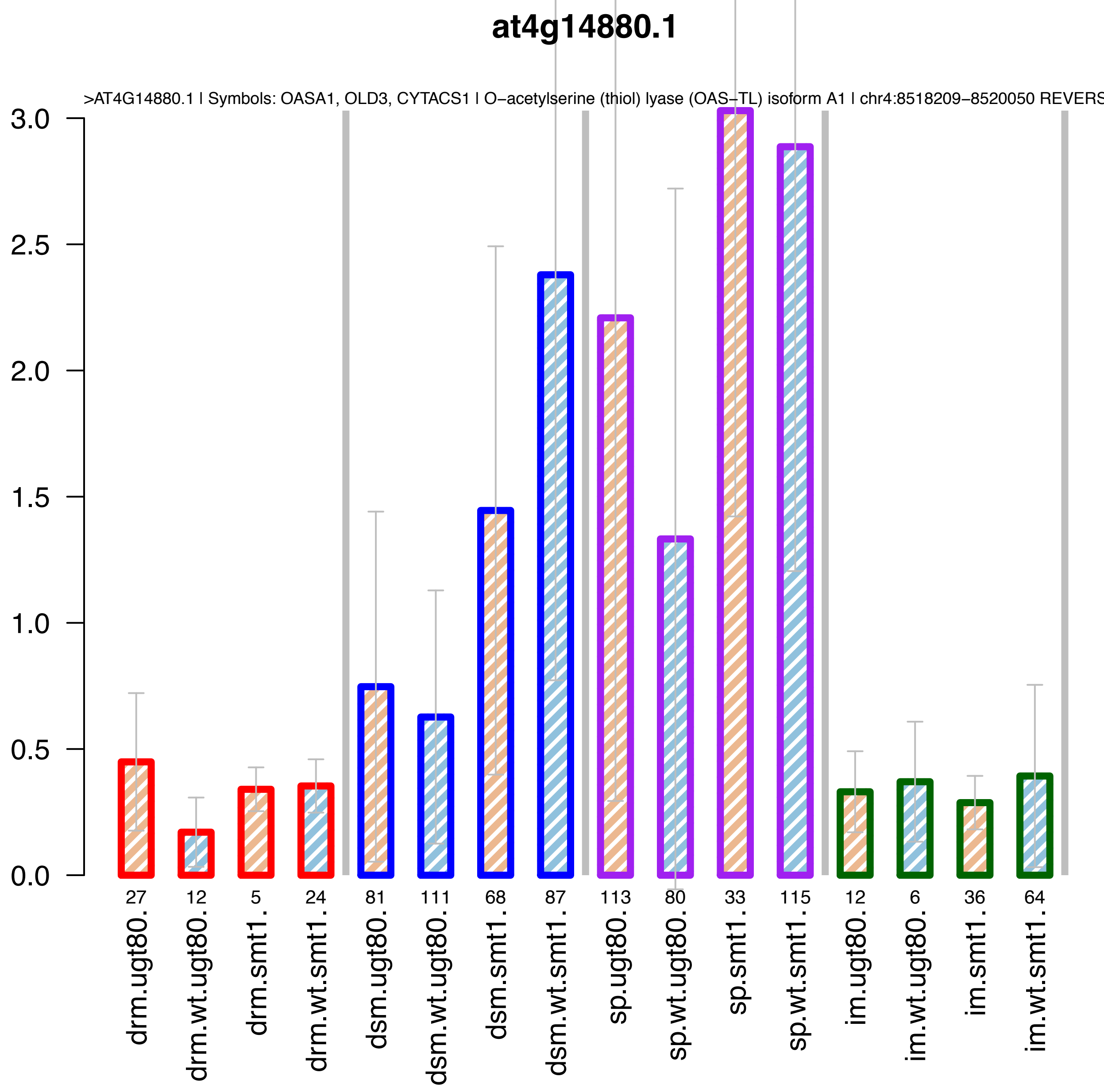
at4g14880.1
>AT4G14880.1 | Symbols: OASA1, OLD3, CYTACS1 | O−acetylserine (thiol) lyase (OAS−TL) isoform A1 | chr4:8518209−8520050 REVERS
3.0
2.5
2.0
1.5
1.0
0.5
0.0
27
12
5
24
81
111
68
87
113
80
33
115
12
6
36
64
drm.ugt80..
drm.wt.ugt80..
drm.smt1..
drm.wt.smt1..
dsm.ugt80..
dsm.wt.ugt80..
dsm.smt1..
dsm.wt.smt1..
sp.ugt80..
sp.wt.ugt80..
sp.smt1..
sp.wt.smt1..
im.ugt80..
im.wt.ugt80..
im.smt1..
im.wt.smt1..

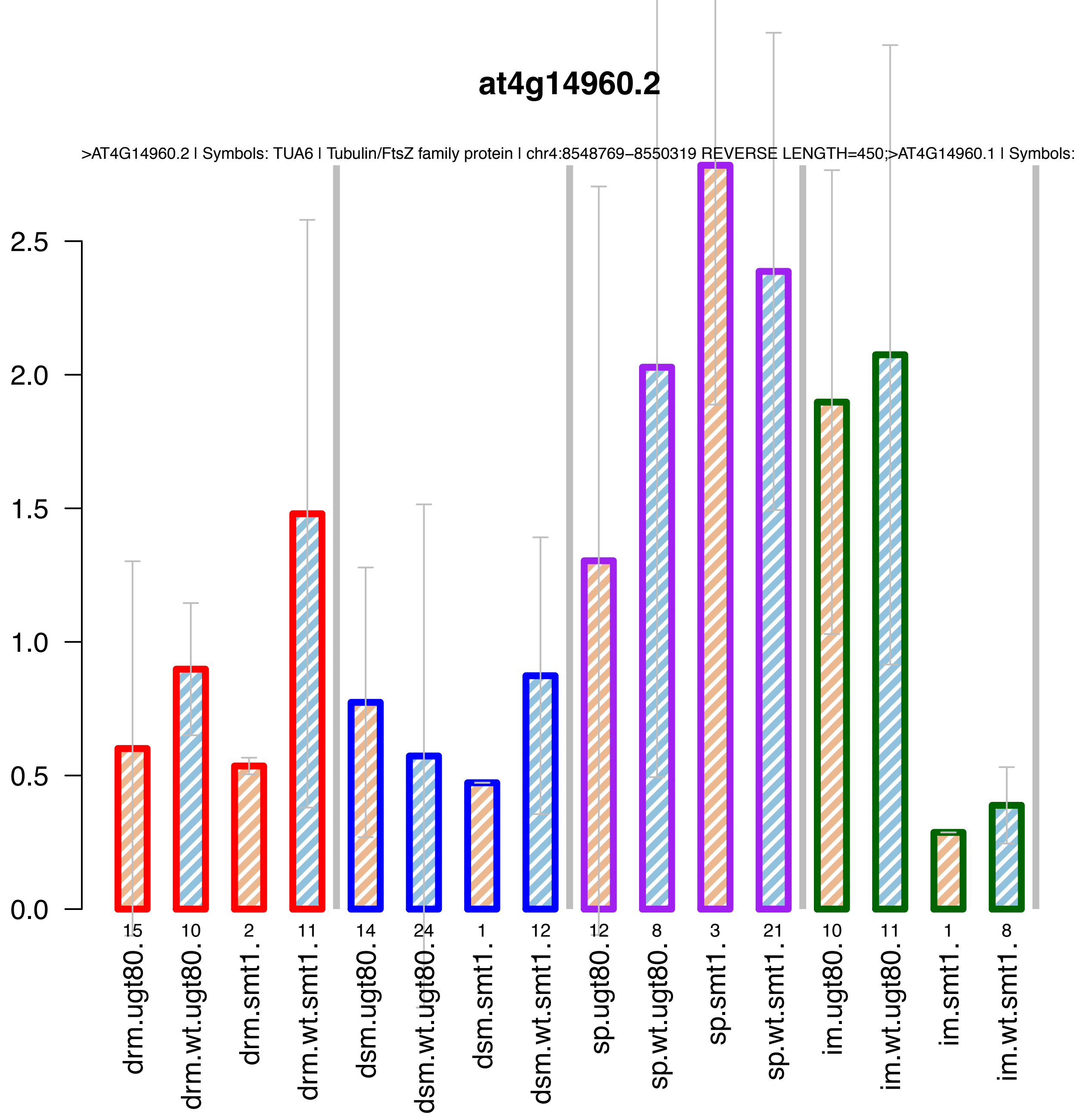

at4g14960.2
>AT4G14960.2 | Symbols: TUA6 | Tubulin/FtsZ family protein | chr4:8548769-8550319 REVERSE LENGTH=450;>AT4G14960.1 | Symbols:
2.5
2.0
1.5
1.0
0.5
0.0
15
10
2
11
14
24
1
12
12
8
3
21
10
11
1
8
drm.ugt80..
drm.wt.ugt80..
drm.smt1..
drm.wt.smt1..
dsm.ugt80..
dsm.wt.ugt80..
dsm.smt1..
dsm.wt.smt1..
sp.ugt80..
sp.wt.ugt80..
sp.smt1..
sp.wt.smt1..
im.ugt80..
im.wt.ugt80..
im.smt1..
im.wt.smt1..

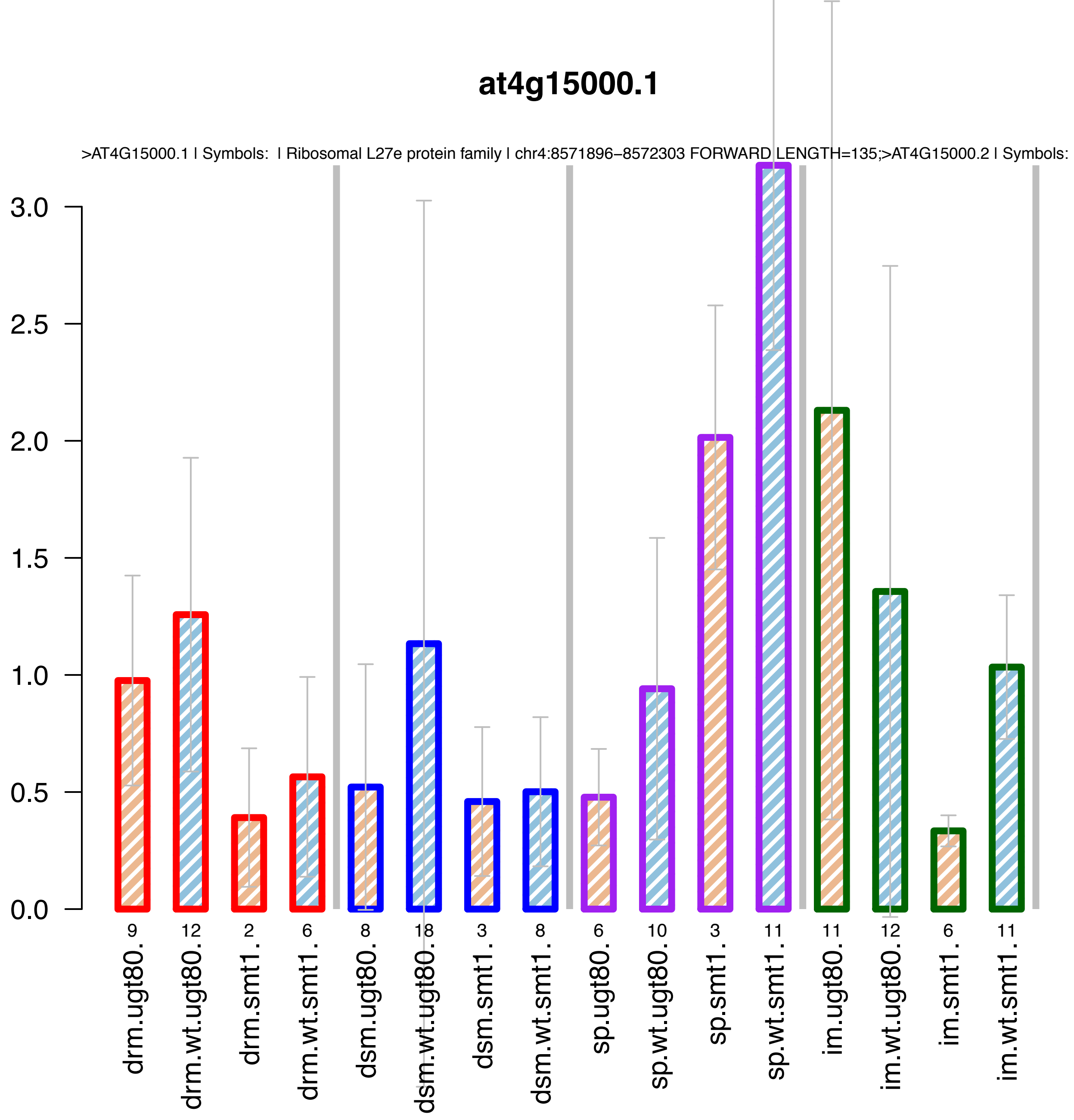

at4g15000.1
>AT4G15000.1 | Symbols: | Ribosomal L27e protein family | chr4:8571896-8572303 FORWARD LENGTH=135;>AT4G15000.2 | Symbols:
0.0
0.5
1.0
1.5
2.0
2.5
3.0
9
12
2
6
8
18
3
8
6
10
3
11
11
12
6
11
drm.ugt80..
drm.wt.ugt80..
drm.smt1..
drm.wt.smt1..
dsm.ugt80..
dsm.wt.ugt80..
dsm.smt1..
dsm.wt.smt1..
sp.ugt80..
sp.wt.ugt80..
sp.smt1..
sp.wt.smt1..
im.ugt80..
im.wt.ugt80..
im.smt1..
im.wt.smt1..

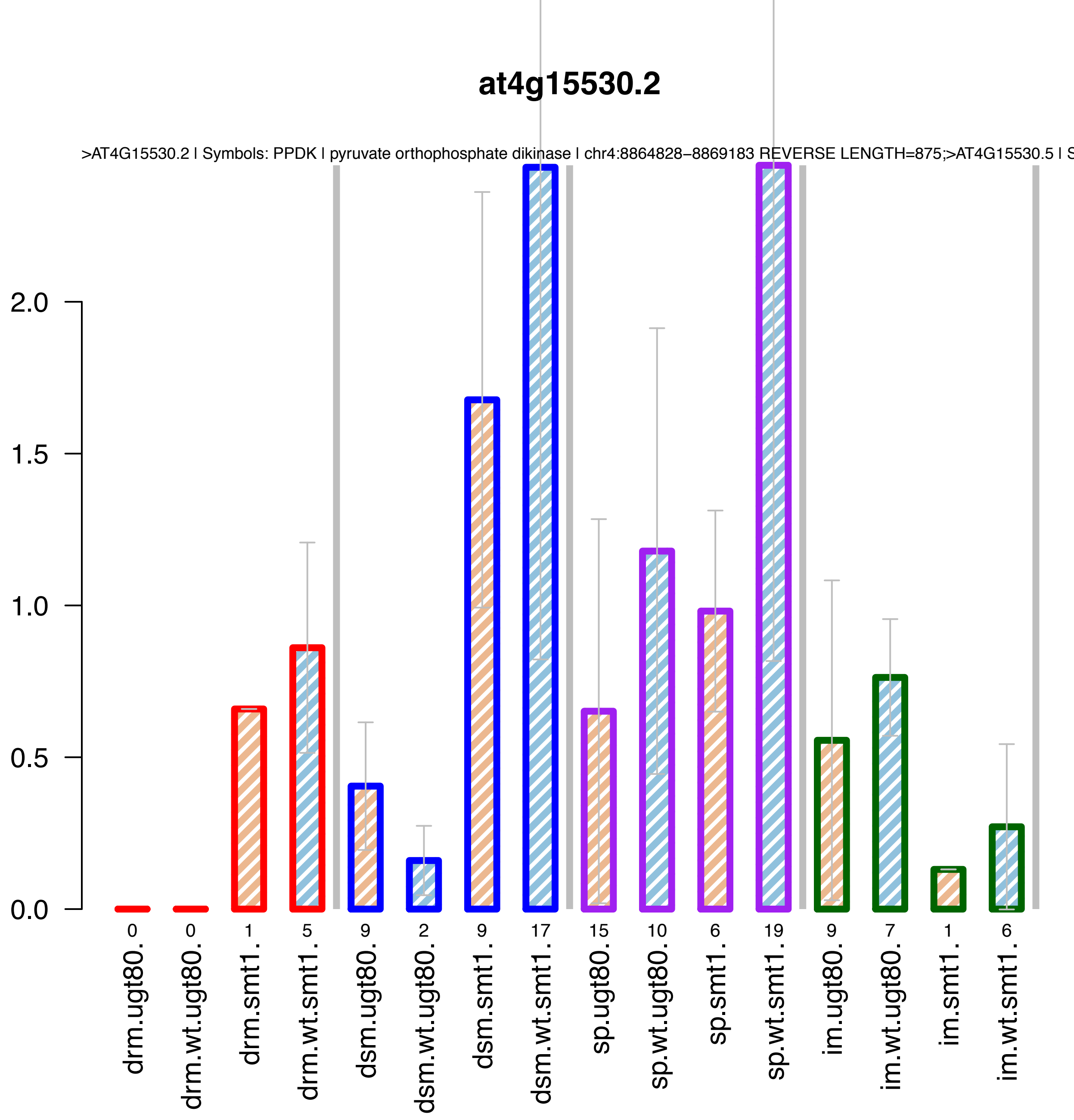

at4g15530.2
>AT4G15530.2 | Symbols: PPDK | pyruvate orthophosphate dikinase | chr4:8864828–8869183 REVERSE LENGTH=875;>AT4G15530.5 | S
0.0
0.5
1.0
1.5
2.0
0
0
1
5
9
2
9
17
15
10
6
19
9
7
1
6
drm.ugt80.
drm.wt.ugt80.
drm.smt1.
drm.wt.smt1.
dsm.ugt80.
dsm.wt.ugt80.
dsm.smt1.
dsm.wt.smt1.
sp.ugt80.
sp.wt.ugt80.
sp.smt1.
sp.wt.smt1.
im.ugt80.
im.wt.ugt80.
im.smt1.
im.wt.smt1.

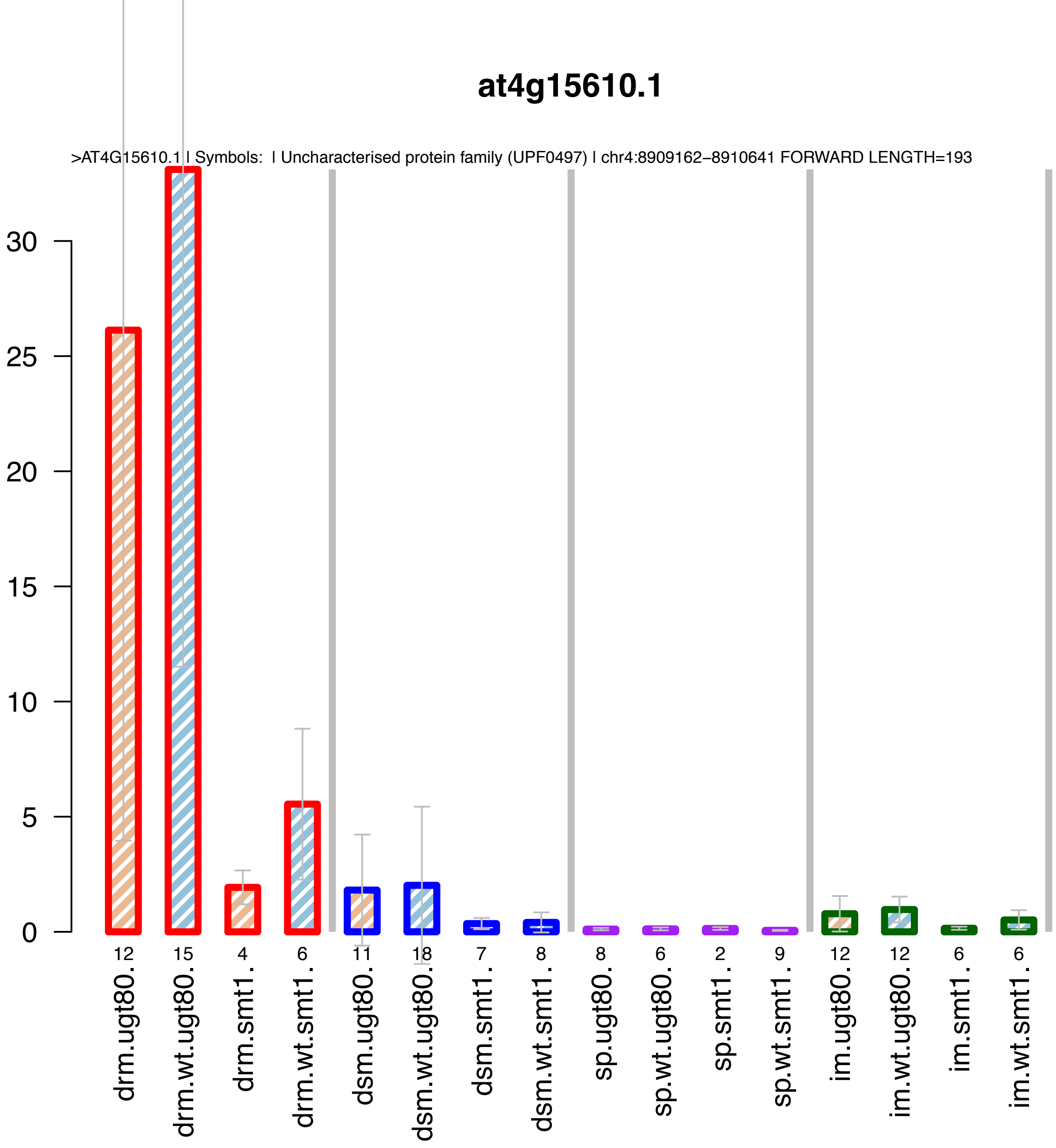

at4g15610.1
>AT4G15610.1 | Symbols: | Uncharacterised protein family (UPF0497) | chr4:8909162-8910641 FORWARD LENGTH=193
0
5
10
15
20
25
30
12
15
4
6
11
18
7
8
8
6
2
9
12
12
6
6
drm.ugt80..
drm.wt.ugt80..
drm.smt1..
drm.wt.smt1..
dsm.ugt80..
dsm.wt.ugt80..
dsm.smt1..
dsm.wt.smt1..
sp.ugt80..
sp.wt.ugt80..
sp.smt1..
sp.wt.smt1..
im.ugt80..
im.wt.ugt80..
im.smt1..
im.wt.smt1..

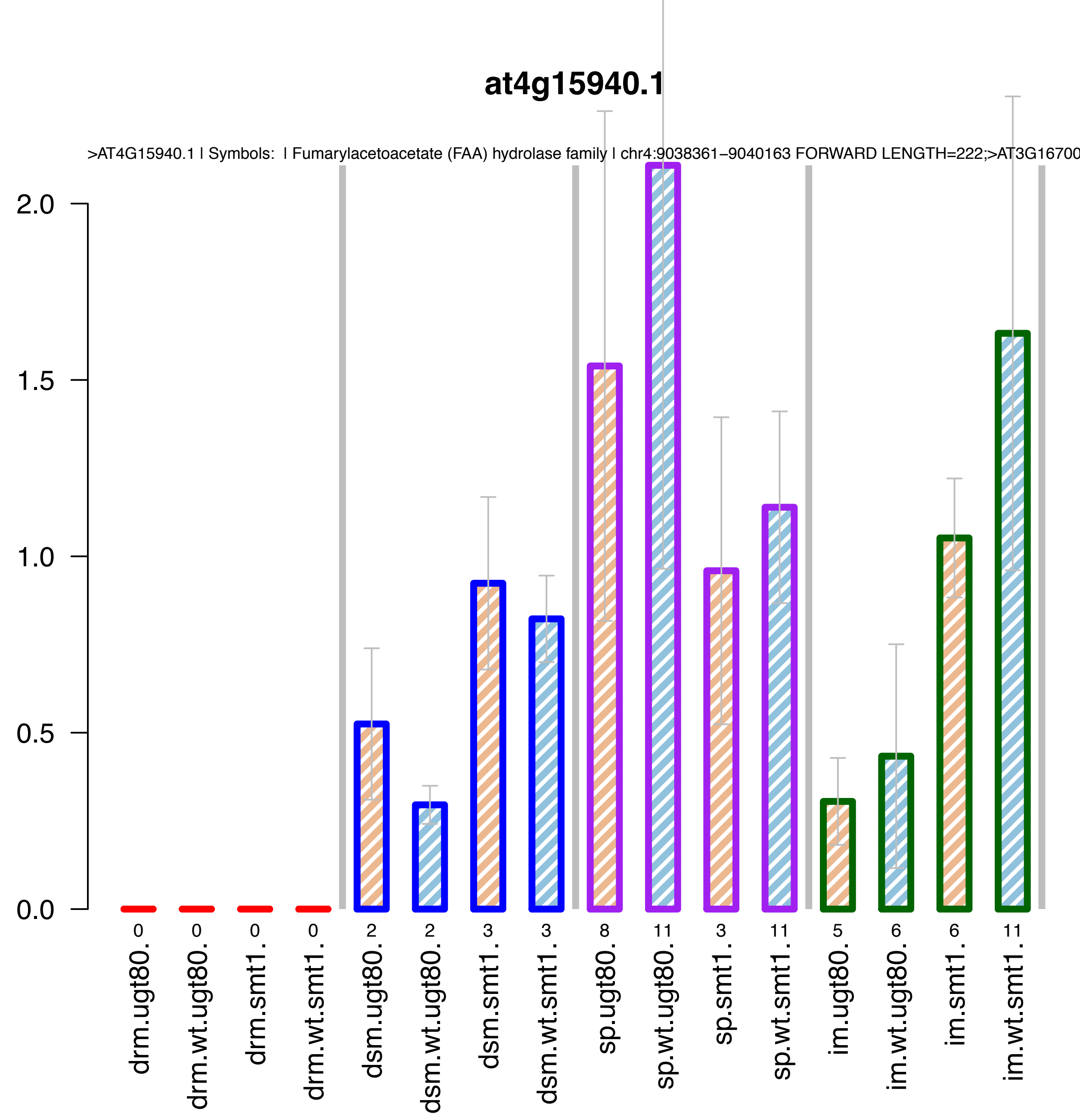

at4g15940.1
>AT4G15940.1 | Symbols: | Fumarylacetoacetate (FAA) hydrolase family | chr4:9038361-9040163 FORWARD LENGTH=222;>AT3G16700
2.0
1.5
1.0
0.5
0.0
0 0 0 0 2 2 3 3 8 11 3 11 5 6 6 11
drm.ugt80.
drm.wt.ugt80.
drm.smt1.
drm.wt.smt1.
dsm.ugt80.
dsm.wt.ugt80.
dsm.smt1.
dsm.wt.smt1.
sp.ugt80.
sp.wt.ugt80.
sp.smt1.
sp.wt.smt1.
im.ugt80.
im.wt.ugt80.
im.smt1.
im.wt.smt1.

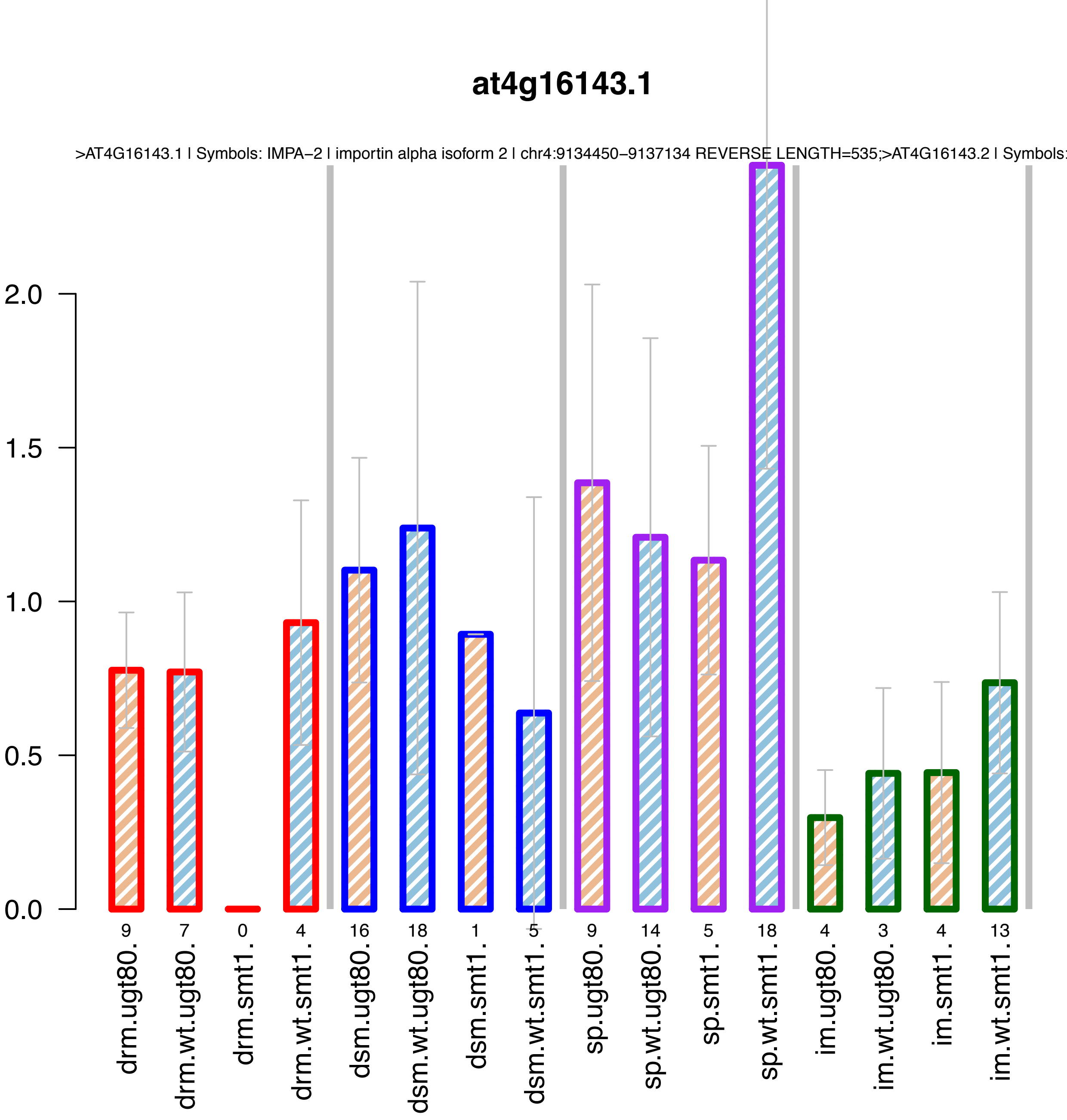

at4g16143.1
>AT4G16143.1 | Symbols: IMPA−2 | importin alpha isoform 2 | chr4:9134450−9137134 REVERSE LENGTH=535;>AT4G16143.2 | Symbols:
2.0
1.5
1.0
0.5
0.0
9
7
0
4
16
18
1
5
9
14
5
18
4
3
4
13
drm.ugt80.
drm.wt.ugt80.
drm.smt1.
drm.wt.smt1.
dsm.ugt80.
dsm.wt.ugt80.
dsm.smt1.
dsm.wt.smt1.
sp.ugt80.
sp.wt.ugt80.
sp.smt1.
sp.wt.smt1.
im.ugt80.
im.wt.ugt80.
im.smt1.
im.wt.smt1.

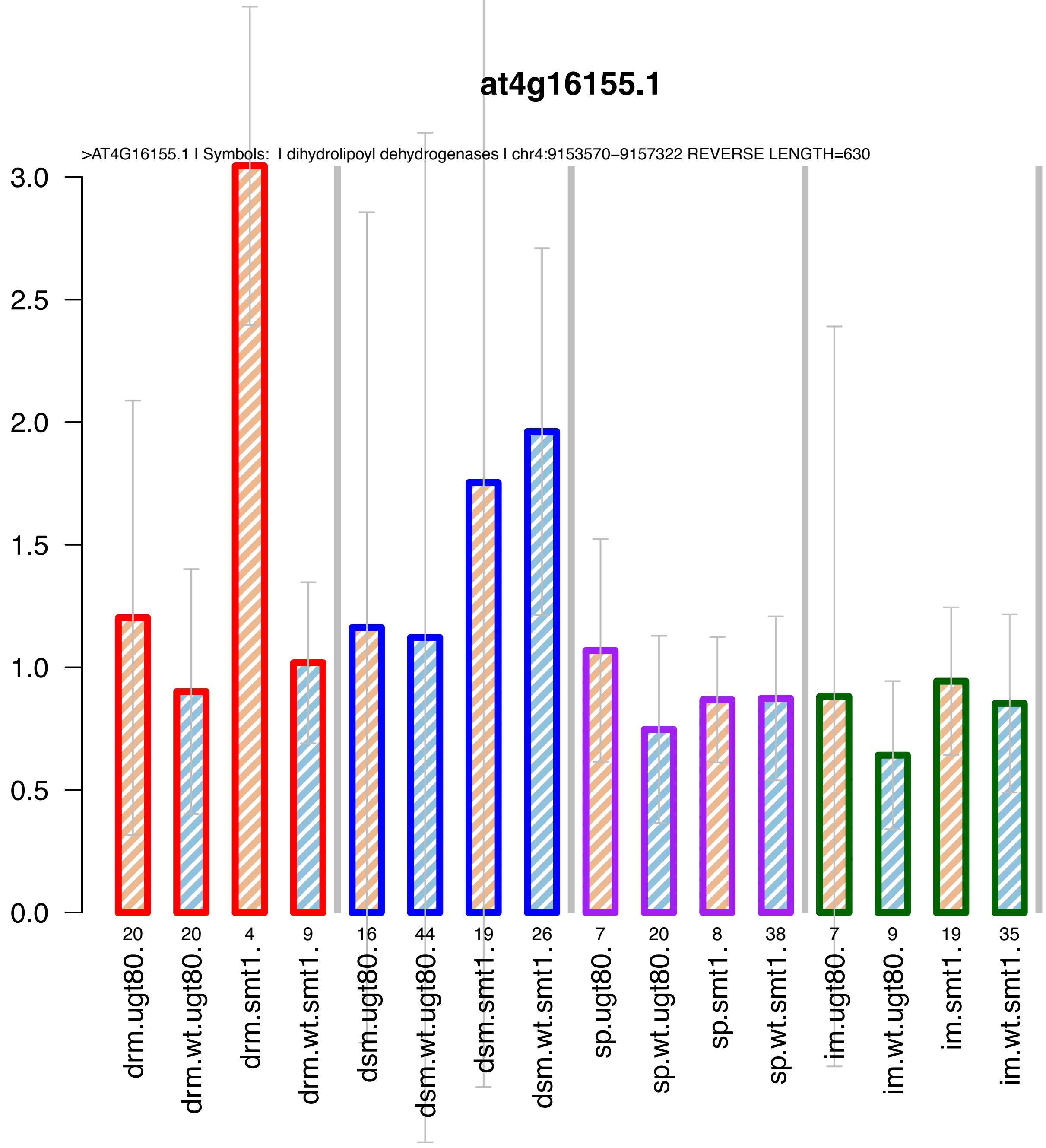

at4g16155.1
>AT4G16155.1 | Symbols: | dihydrolipoyl dehydrogenases | chr4:9153570-9157322 REVERSE LENGTH=630
3.0
2.5
2.0
1.5
1.0
0.5
0.0
20
20
4
9
16
44
19
26
7
20
8
38
7
9
19
35
drm.ugt80..
drm.wt.ugt80..
drm.smt1..
drm.wt.smt1..
dsm.ugt80..
dsm.wt.ugt80..
dsm.smt1..
dsm.wt.smt1..
sp.ugt80..
sp.wt.ugt80..
sp.smt1..
sp.wt.smt1..
im.ugt80..
im.wt.ugt80..
im.smt1..
im.wt.smt1..

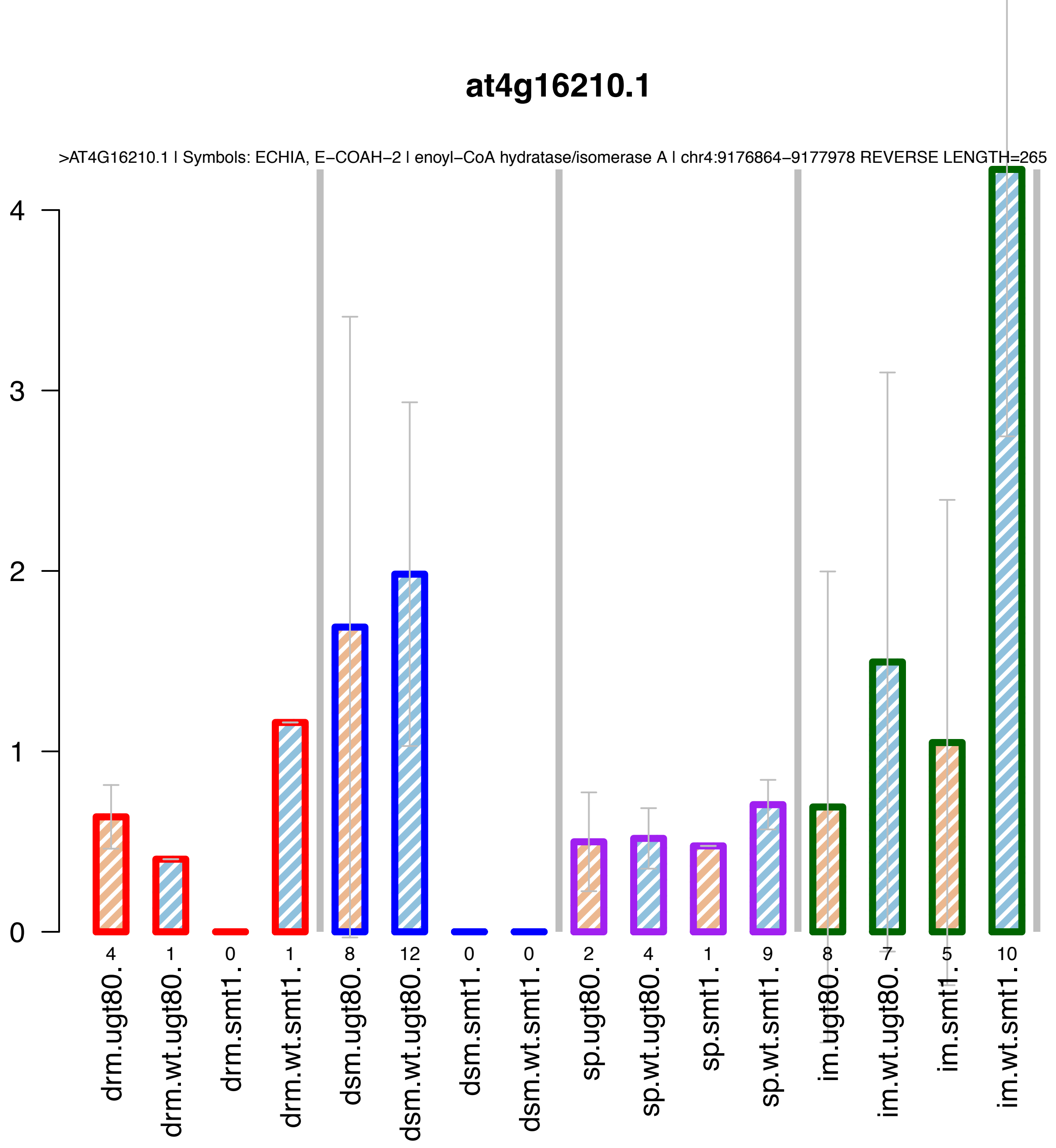

at4g16210.1
>AT4G16210.1 | Symbols: ECHIA, E−COAH−2 | enoyl−CoA hydratase/isomerase A | chr4:9176864-9177978 REVERSE LENGTH=265
0
1
2
3
4
4
1
0
1
8
12
0
0
2
4
1
9
8
7
5
10
drm.ugt80.
drm.wt.ugt80.
drm.smt1.
drm.wt.smt1.
dsm.ugt80.
dsm.wt.ugt80.
dsm.smt1.
dsm.wt.smt1.
sp.ugt80.
sp.wt.ugt80.
sp.smt1.
sp.wt.smt1.
im.ugt80.
im.wt.ugt80.
im.smt1.
im.wt.smt1.

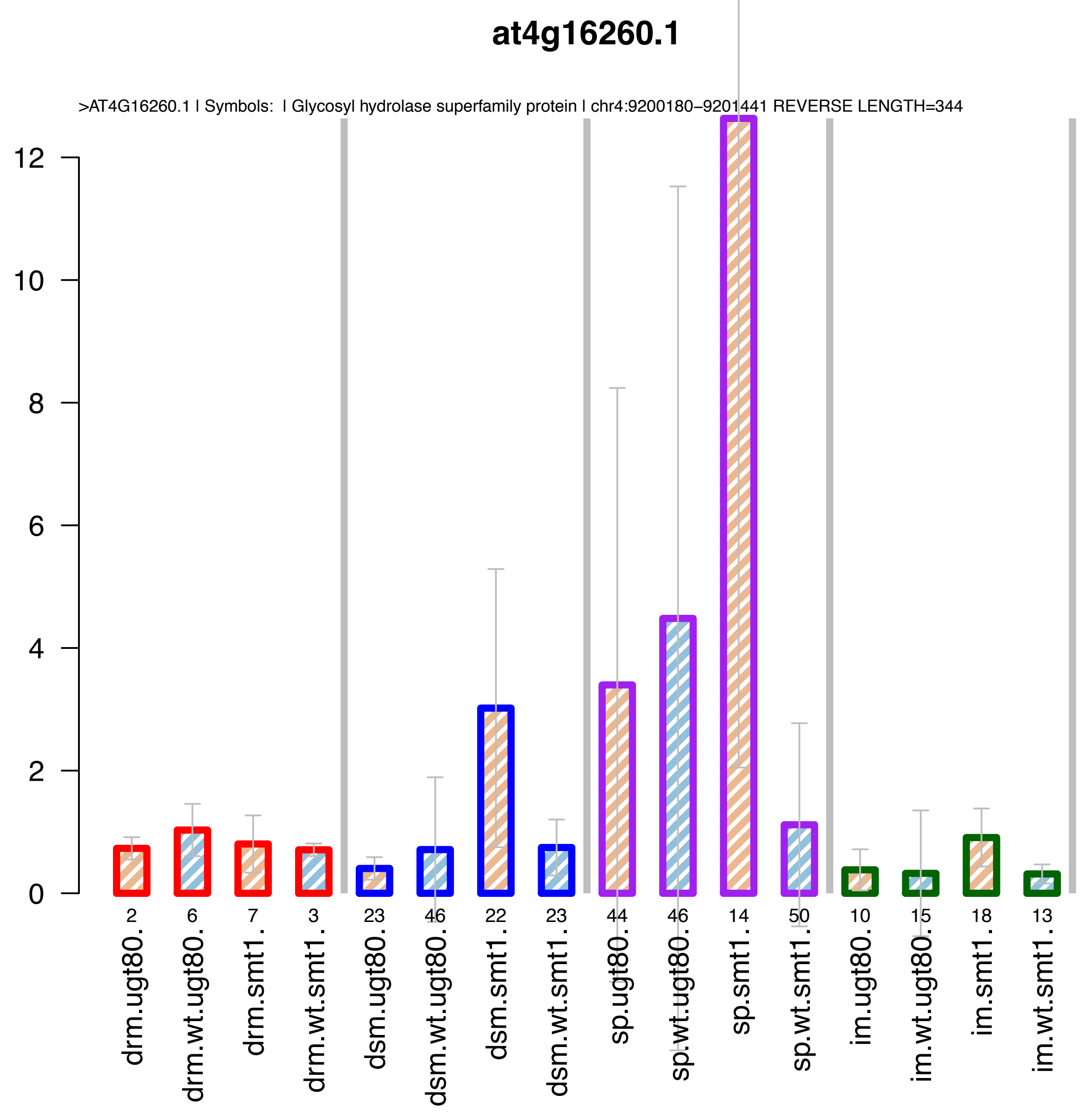

at4g16260.1
>AT4G16260.1 | Symbols: | Glycosyl hydrolase superfamily protein | chr4:9200180-9201441 REVERSE LENGTH=344
0
2
4
6
8
10
12
2
6
7
3
23
46
22
23
44
46
14
50
10
15
18
13
drm.ugt80..
drm.wt.ugt80..
drm.smt1..
drm.wt.smt1..
dsm.ugt80..
dsm.wt.ugt80..
dsm.smt1..
dsm.wt.smt1..
sp.ugt80..
sp.wt.ugt80..
sp.smt1..
sp.wt.smt1..
im.ugt80..
im.wt.ugt80..
im.smt1..
im.wt.smt1..

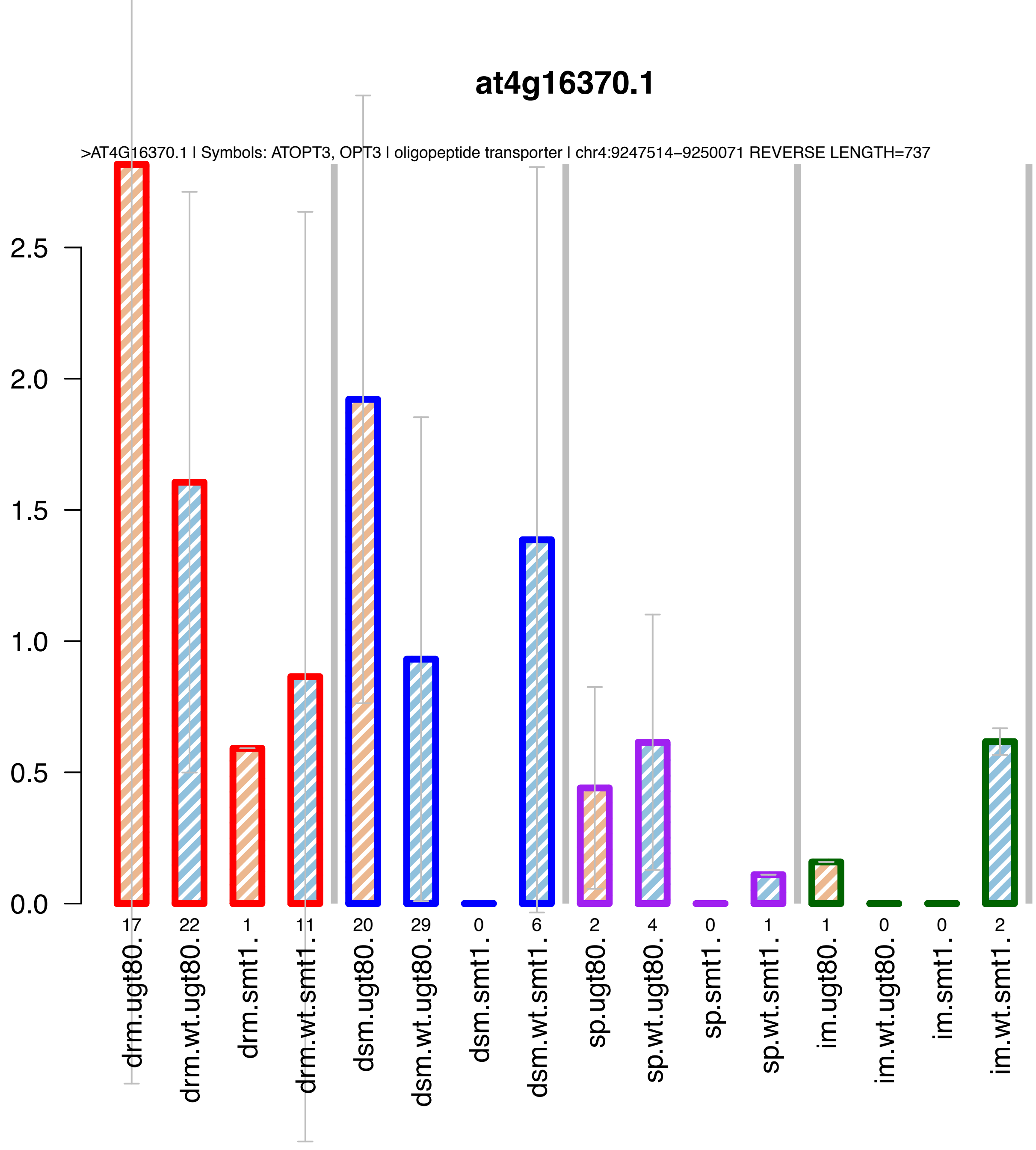

at4g16370.1
>AT4G16370.1 | Symbols: ATOPT3, OPT3 | oligopeptide transporter | chr4:9247514-9250071 REVERSE LENGTH=737
0.0
0.5
1.0
1.5
2.0
2.5
17
22
1
11
20
29
0
6
2
4
0
1
1
0
0
2
drm.ugt80..
drm.wt.ugt80..
drm.smt1..
drm.wt.smt1..
dsm.ugt80..
dsm.wt.ugt80..
dsm.smt1..
dsm.wt.smt1..
sp.ugt80..
sp.wt.ugt80..
sp.smt1..
sp.wt.smt1..
im.ugt80..
im.wt.ugt80..
im.smt1..
im.wt.smt1..

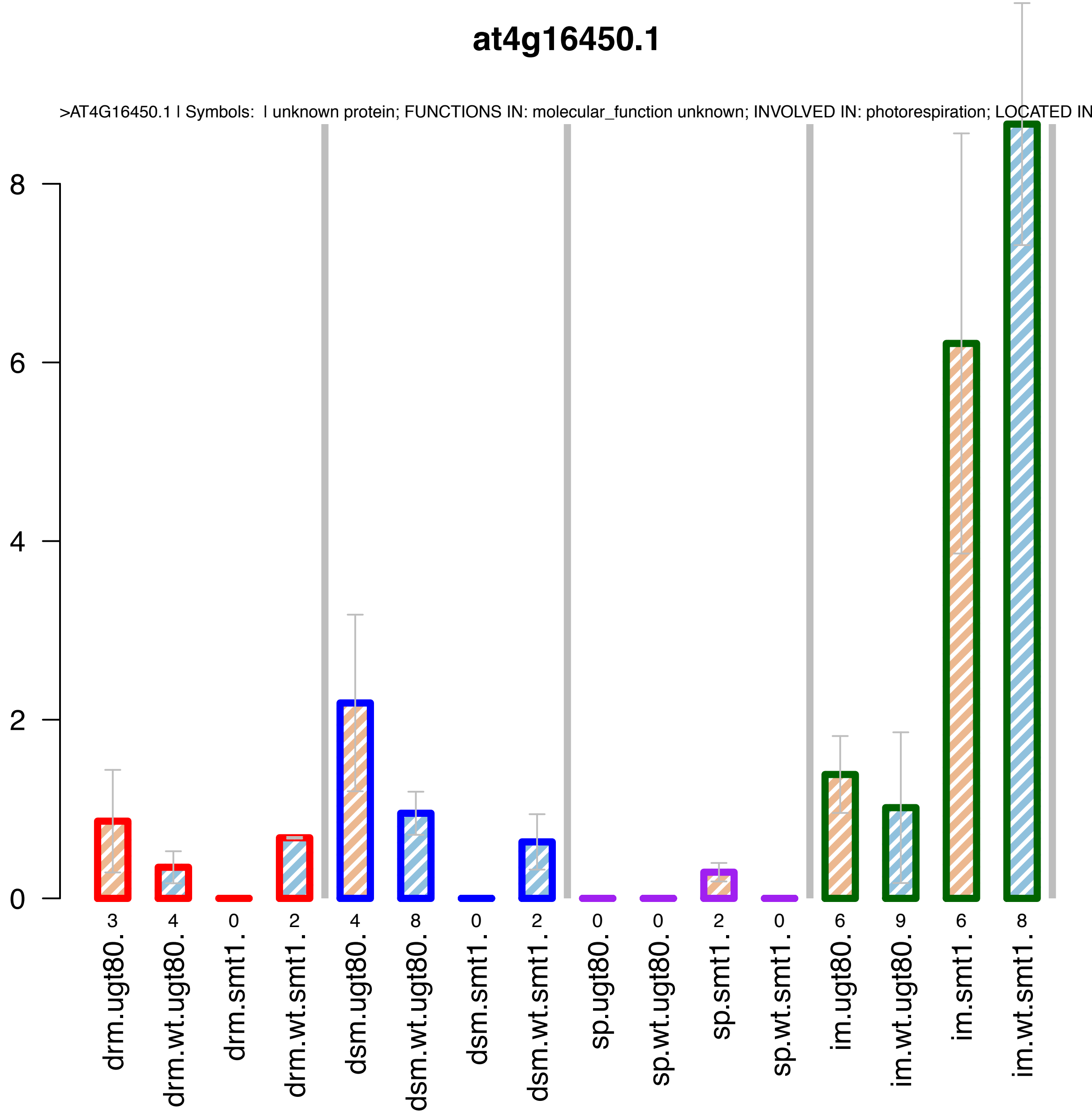

at4g16450.1
>AT4G16450.1 | Symbols: | unknown protein; FUNCTIONS IN: molecular_function unknown; INVOLVED IN: photorespiration; LOCATED IN
0
2
4
6
8
3
4
0
2
4
8
0
2
0
0
2
0
6
9
6
8
drm.ugt80.
drm.wt.ugt80.
drm.smt1.
drm.wt.smt1.
dsm.ugt80.
dsm.wt.ugt80.
dsm.smt1.
dsm.wt.smt1.
sp.ugt80.
sp.wt.ugt80.
sp.smt1.
sp.wt.smt1.
im.ugt80.
im.wt.ugt80.
im.smt1.
im.wt.smt1.

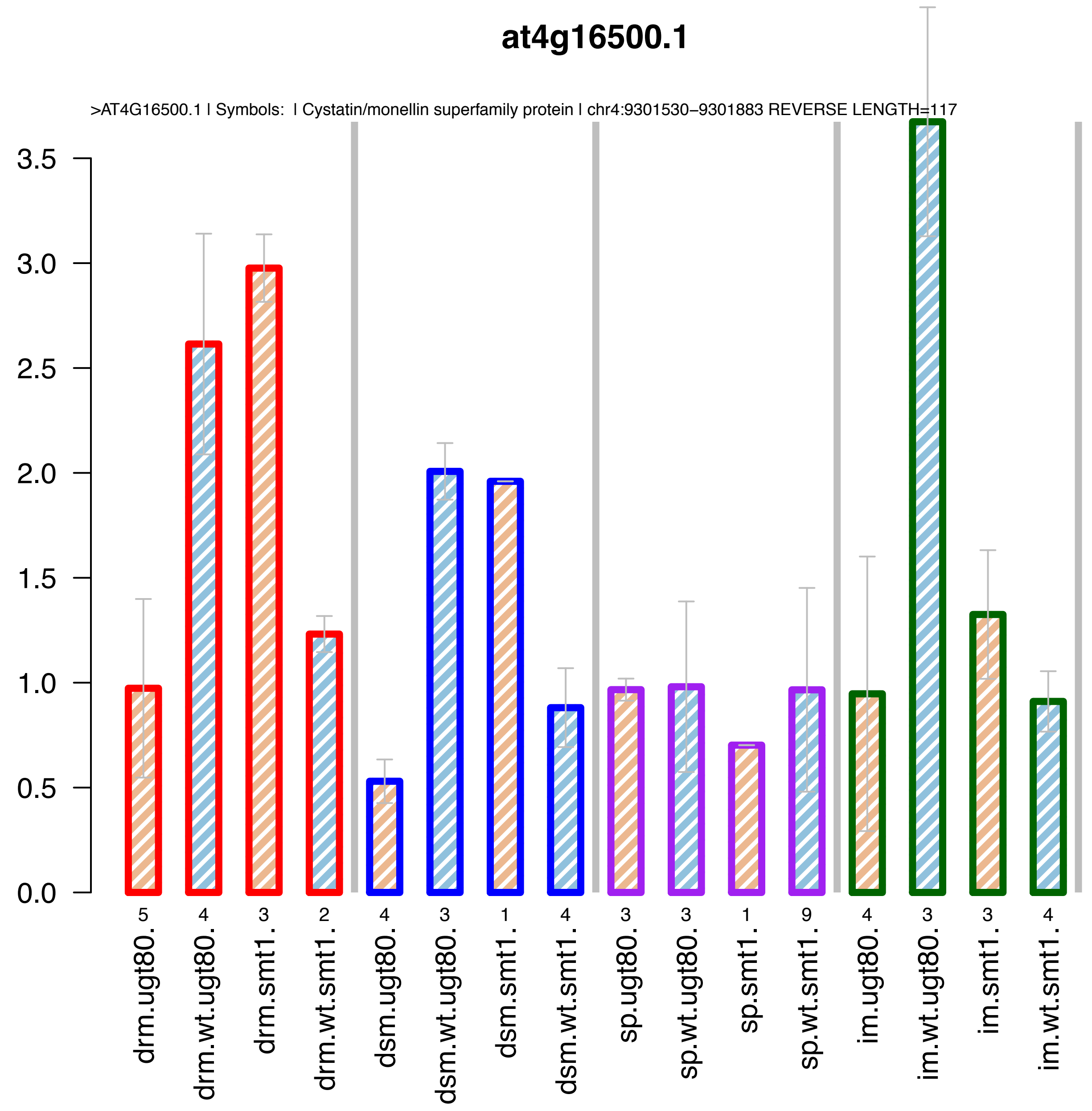

at4g16500.1
>AT4G16500.1 | Symbols: | Cystatin/monellin superfamily protein | chr4:9301530-9301883 REVERSE LENGTH=117
0.0
0.5
1.0
1.5
2.0
2.5
3.0
3.5
5
4
3
2
4
3
1
4
3
3
1
9
4
3
3
4
drm.ugt80..
drm.wt.ugt80..
drm.smt1..
drm.wt.smt1..
dsm.ugt80..
dsm.wt.ugt80..
dsm.smt1..
dsm.wt.smt1..
sp.ugt80..
sp.wt.ugt80..
sp.smt1..
sp.wt.smt1..
im.ugt80..
im.wt.ugt80..
im.smt1..
im.wt.smt1..

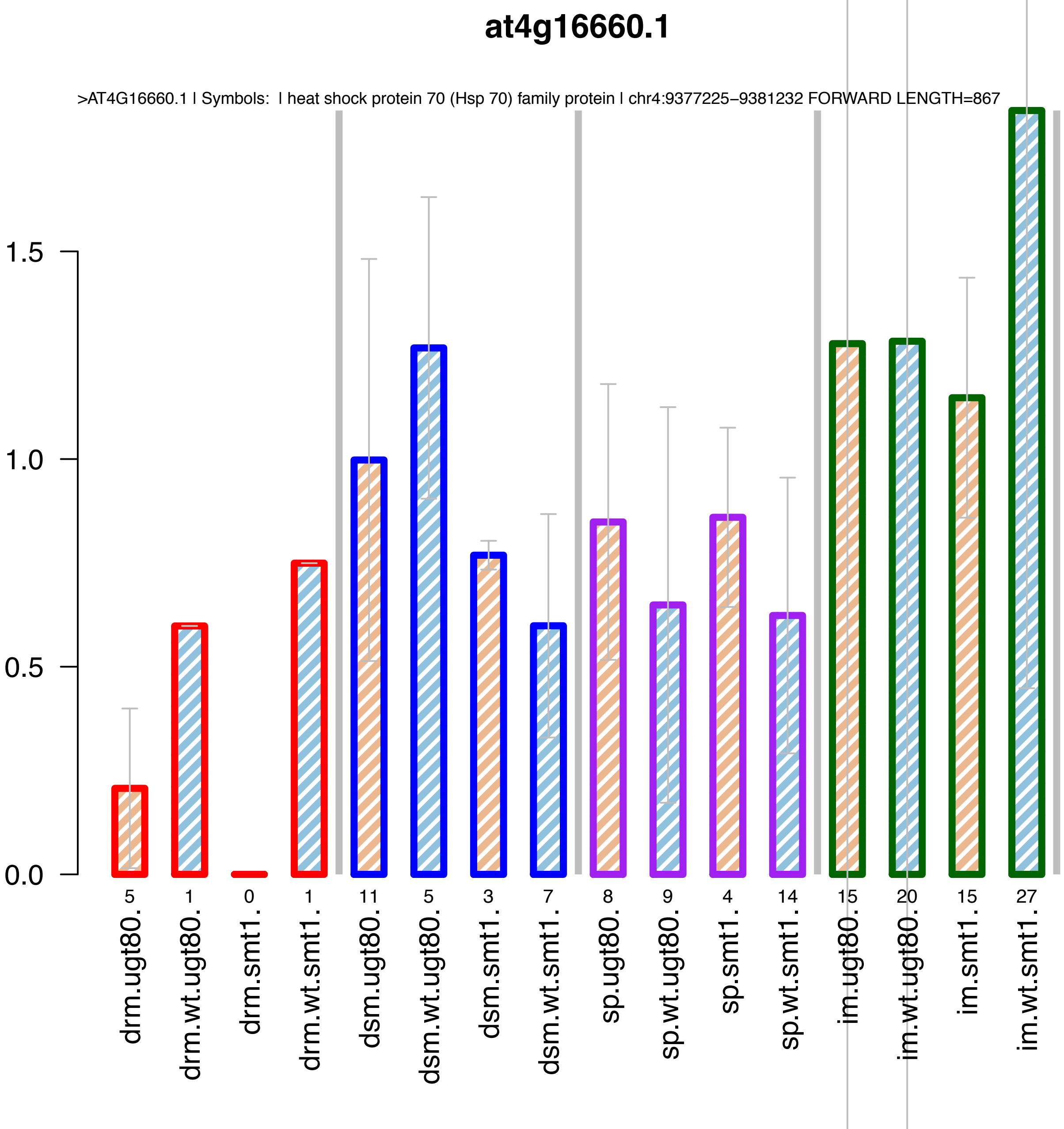

at4g16660.1
>AT4G16660.1 | Symbols: | heat shock protein 70 (Hsp 70) family protein | chr4:9377225-9381232 FORWARD LENGTH=867
1.5
1.0
0.5
0.0
5
1
0
1
11
5
3
7
8
9
4
14
15
20
15
27
drm.ugt80.
drm.wt.ugt80.
drm.smt1.
drm.wt.smt1.
dsm.ugt80.
dsm.wt.ugt80.
dsm.smt1.
dsm.wt.smt1.
sp.ugt80.
sp.wt.ugt80.
sp.smt1.
sp.wt.smt1.
im.ugt80.
im.wt.ugt80.
im.smt1.
im.wt.smt1.

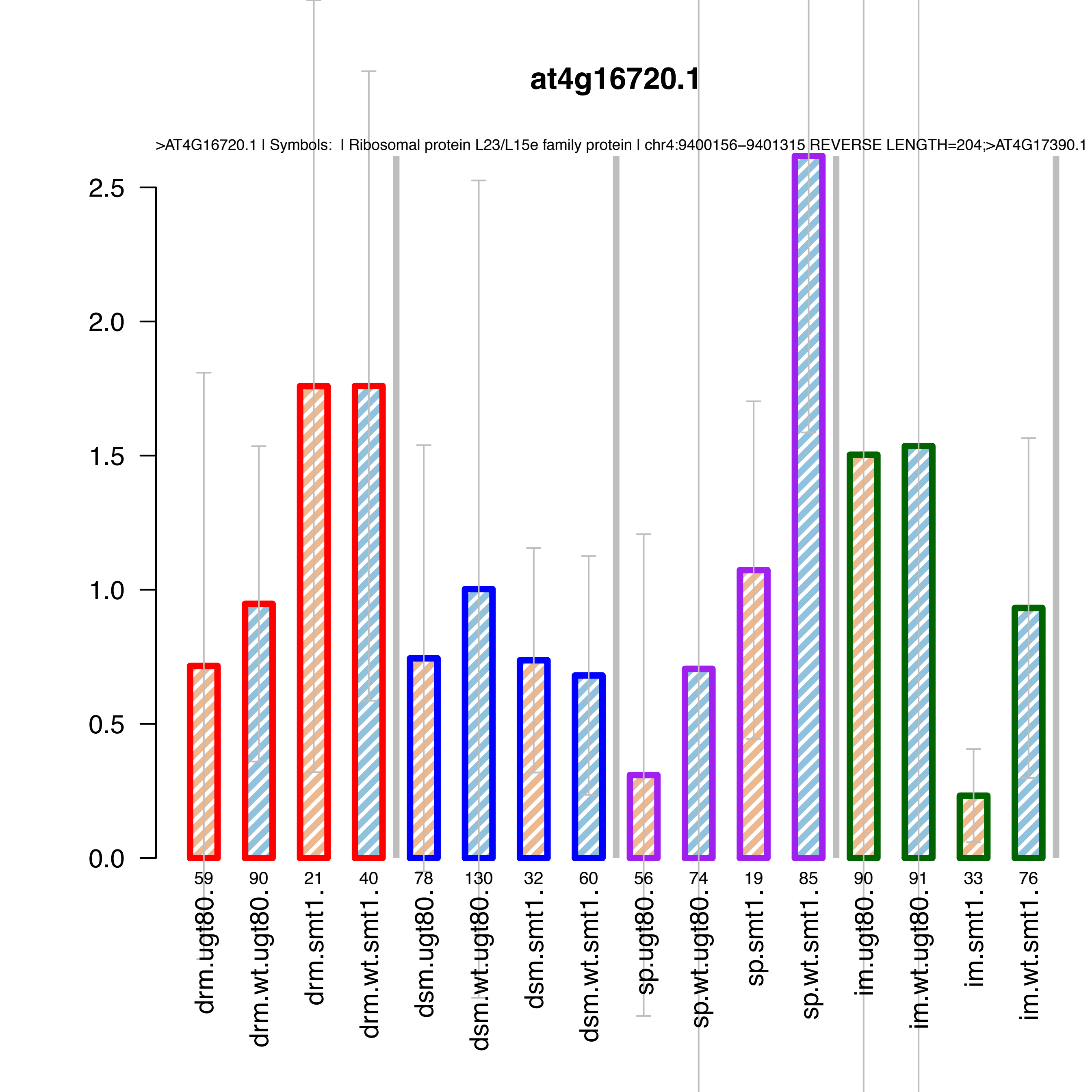

at4g16720.1
>AT4G16720.1 | Symbols: | Ribosomal protein L23/L15e family protein | chr4:9400156-9401315 REVERSE LENGTH=204;>AT4G17390.1
2.5
2.0
1.5
1.0
0.5
0.0
59
90
21
40
78
130
32
60
56
74
19
85
90
91
33
76
drm.ugt80..
drm.wt.ugt80..
drm.smt1..
drm.wt.smt1..
dsm.ugt80..
dsm.wt.ugt80..
dsm.smt1..
dsm.wt.smt1..
sp.ugt80..
sp.wt.ugt80..
sp.smt1..
sp.wt.smt1..
im.ugt80..
im.wt.ugt80..
im.smt1..
im.wt.smt1..

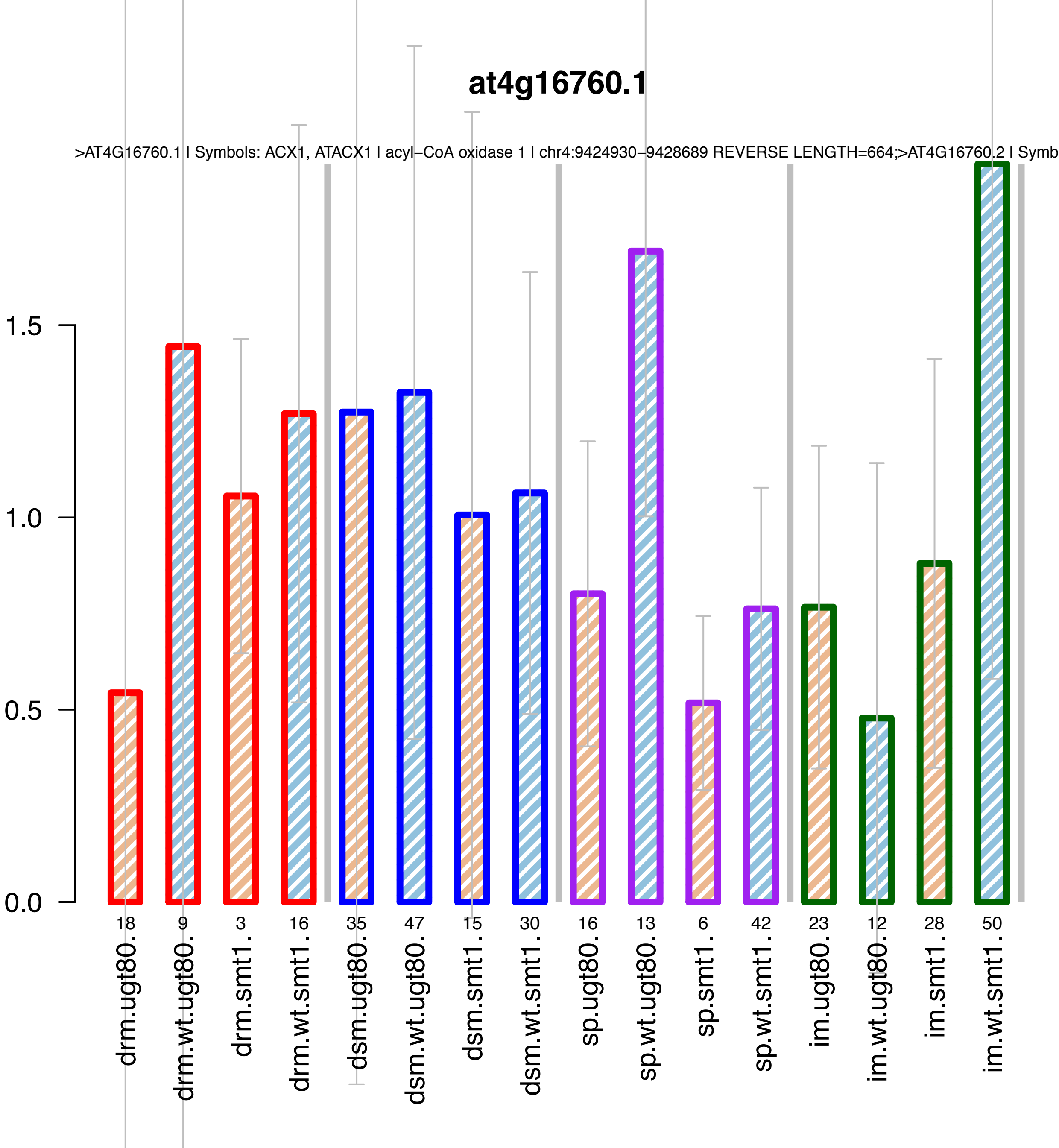

at4g16760.1
>AT4G16760.1 | Symbols: ACX1, ATACX1 | acyl−CoA oxidase 1 | chr4:9424930−9428689 REVERSE LENGTH=664;>AT4G16760.2 | Symb
1.5
1.0
0.5
0.0
18
9
3
16
35
47
15
30
16
13
6
42
23
12
28
50
drm.ugt80..
drm.wt.ugt80..
drm.smt1..
drm.wt.smt1..
dsm.ugt80..
dsm.wt.ugt80..
dsm.smt1..
dsm.wt.smt1..
sp.ugt80..
sp.wt.ugt80..
sp.smt1..
sp.wt.smt1..
im.ugt80..
im.wt.ugt80..
im.smt1..
im.wt.smt1..

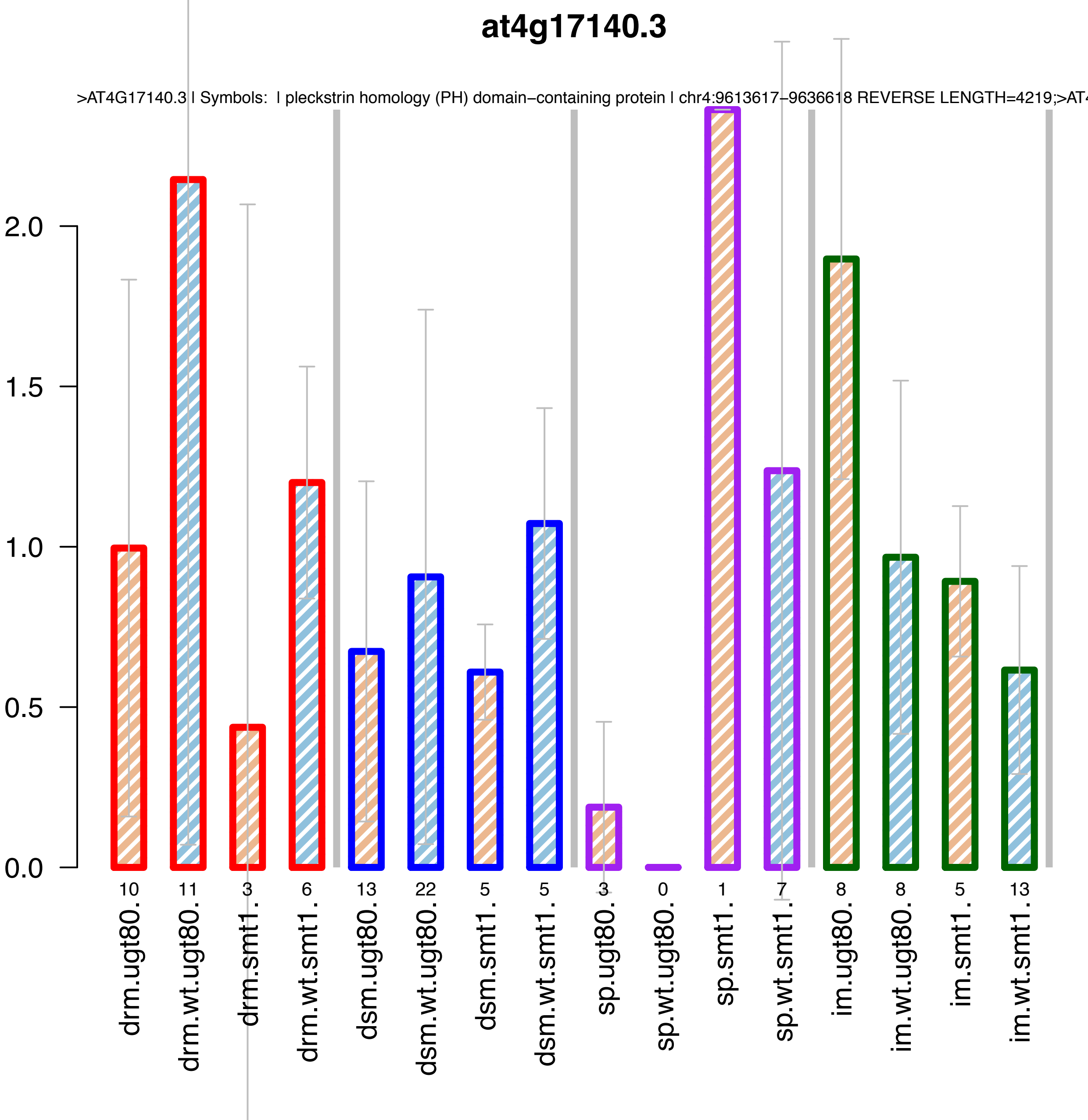

at4g17140.3
>AT4G17140.3 | Symbols: | pleckstrin homology (PH) domain−containing protein | chr4:9613617−9636618 REVERSE LENGTH=4219;>AT
2.0
1.5
1.0
0.5
0.0
10
11
3
6
13
22
5
5
3
0
1
7
8
8
5
13
drm.ugt80..
drm.wt.ugt80..
drm.smt1..
drm.wt.smt1..
dsm.ugt80..
dsm.wt.ugt80..
dsm.smt1..
dsm.wt.smt1..
sp.ugt80..
sp.wt.ugt80..
sp.smt1..
sp.wt.smt1..
im.ugt80..
im.wt.ugt80..
im.smt1..
im.wt.smt1..

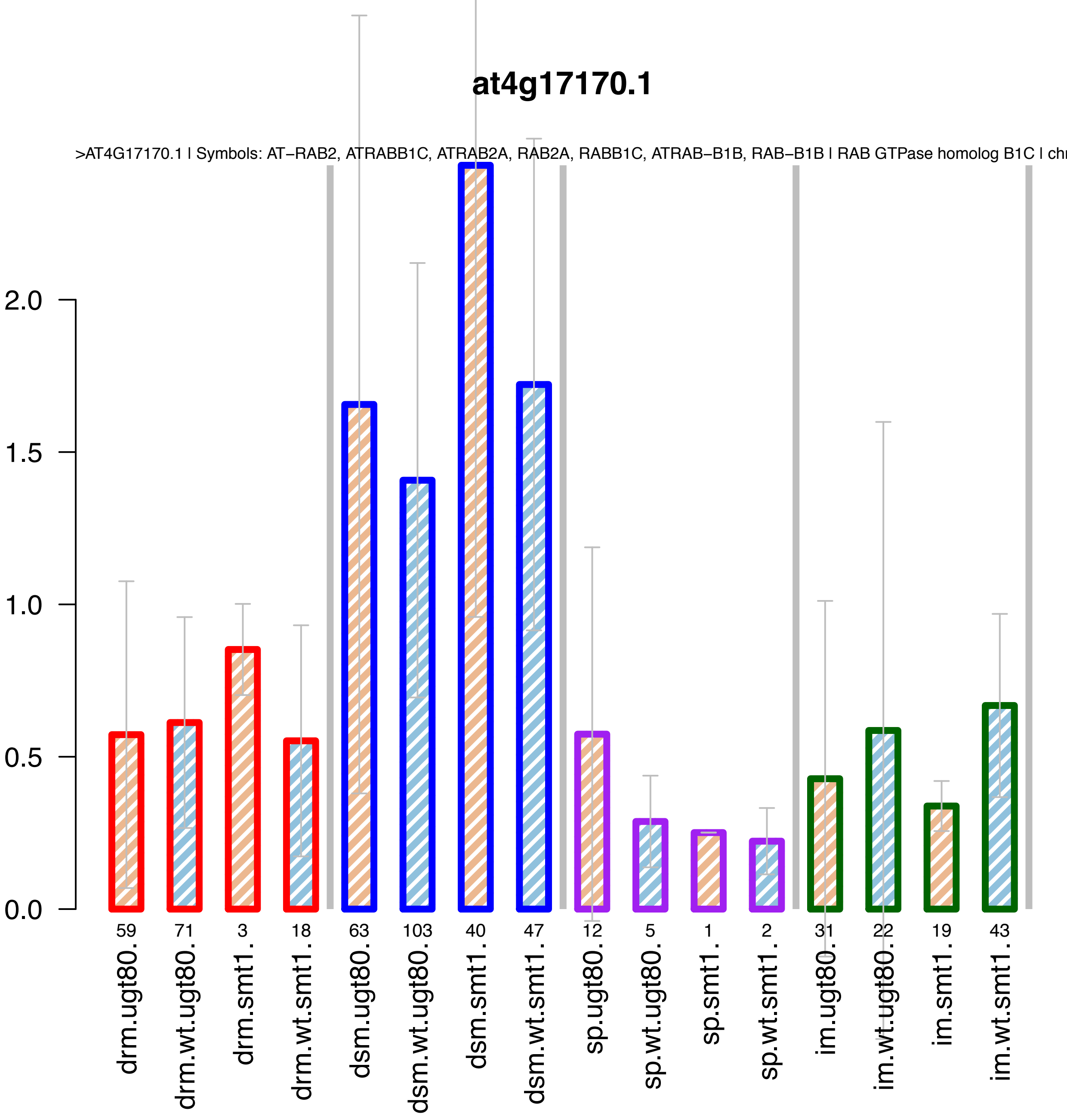

at4g17170.1
>AT4G17170.1 | Symbols: AT−RAB2, ATRABB1C, ATRAB2A, RAB2A, RABB1C, ATRAB−B1B, RAB−B1B | RAB GTPase homolog B1C | chr
2.0
1.5
1.0
0.5
0.0
59
71
3
18
63
103
40
47
12
5
1
2
31
22
19
43
drm.ugt80.
drm.wt.ugt80.
drm.smt1.
drm.wt.smt1.
dsm.ugt80.
dsm.wt.ugt80.
dsm.smt1.
dsm.wt.smt1.
sp.ugt80.
sp.wt.ugt80.
sp.smt1.
sp.wt.smt1.
im.ugt80.
im.wt.ugt80.
im.smt1.
im.wt.smt1.

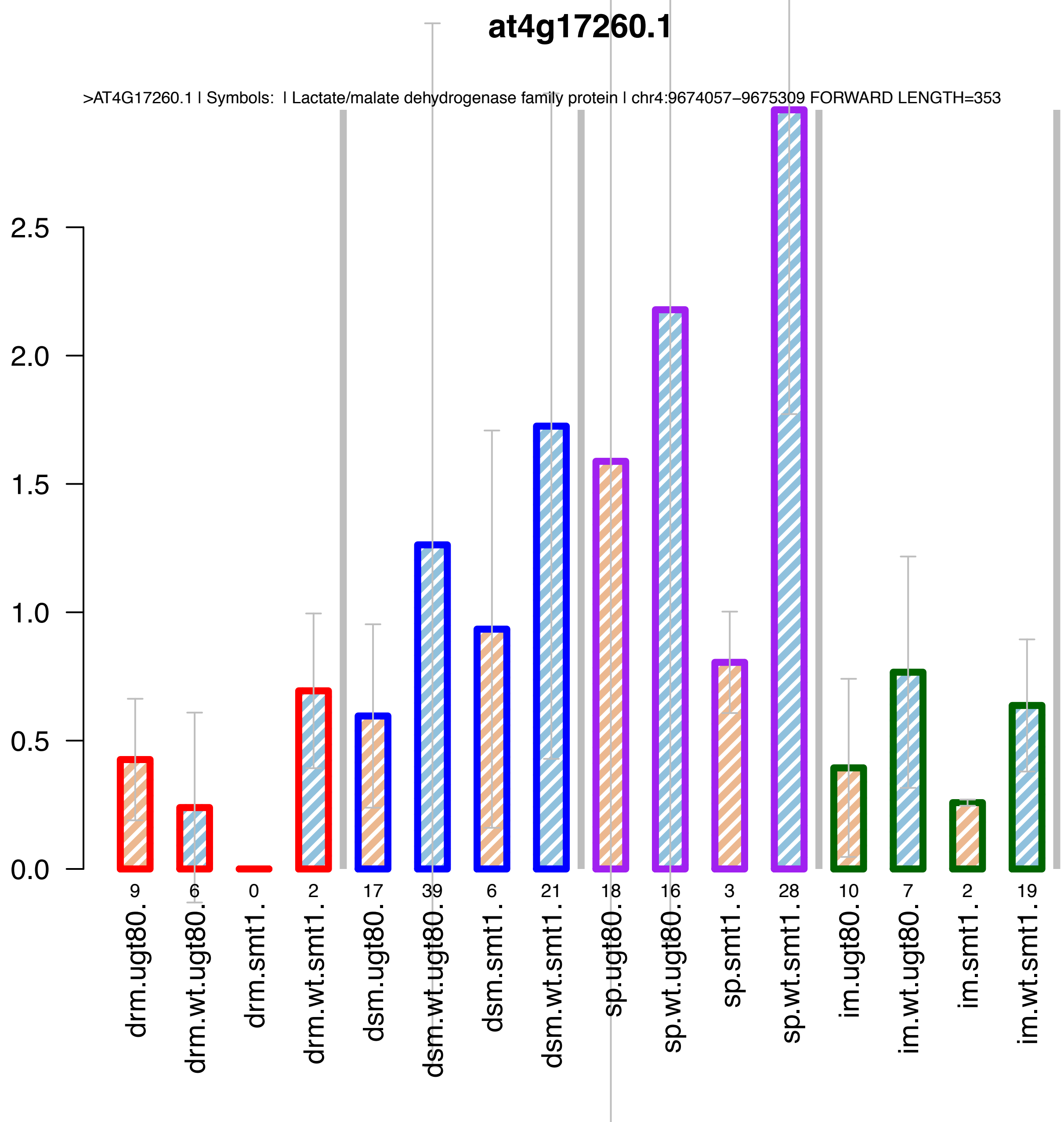

at4g17260.1
>AT4G17260.1 | Symbols: | Lactate/malate dehydrogenase family protein | chr4:9674057-9675309 FORWARD LENGTH=353
0.0
0.5
1.0
1.5
2.0
2.5
9
6
0
2
17
39
6
21
18
16
3
28
10
7
2
19
drm.ugt80.
drm.wt.ugt80.
drm.smt1.
drm.wt.smt1.
dsm.ugt80.
dsm.wt.ugt80.
dsm.smt1.
dsm.wt.smt1.
sp.ugt80.
sp.wt.ugt80.
sp.smt1.
sp.wt.smt1.
im.ugt80.
im.wt.ugt80.
im.smt1.
im.wt.smt1.

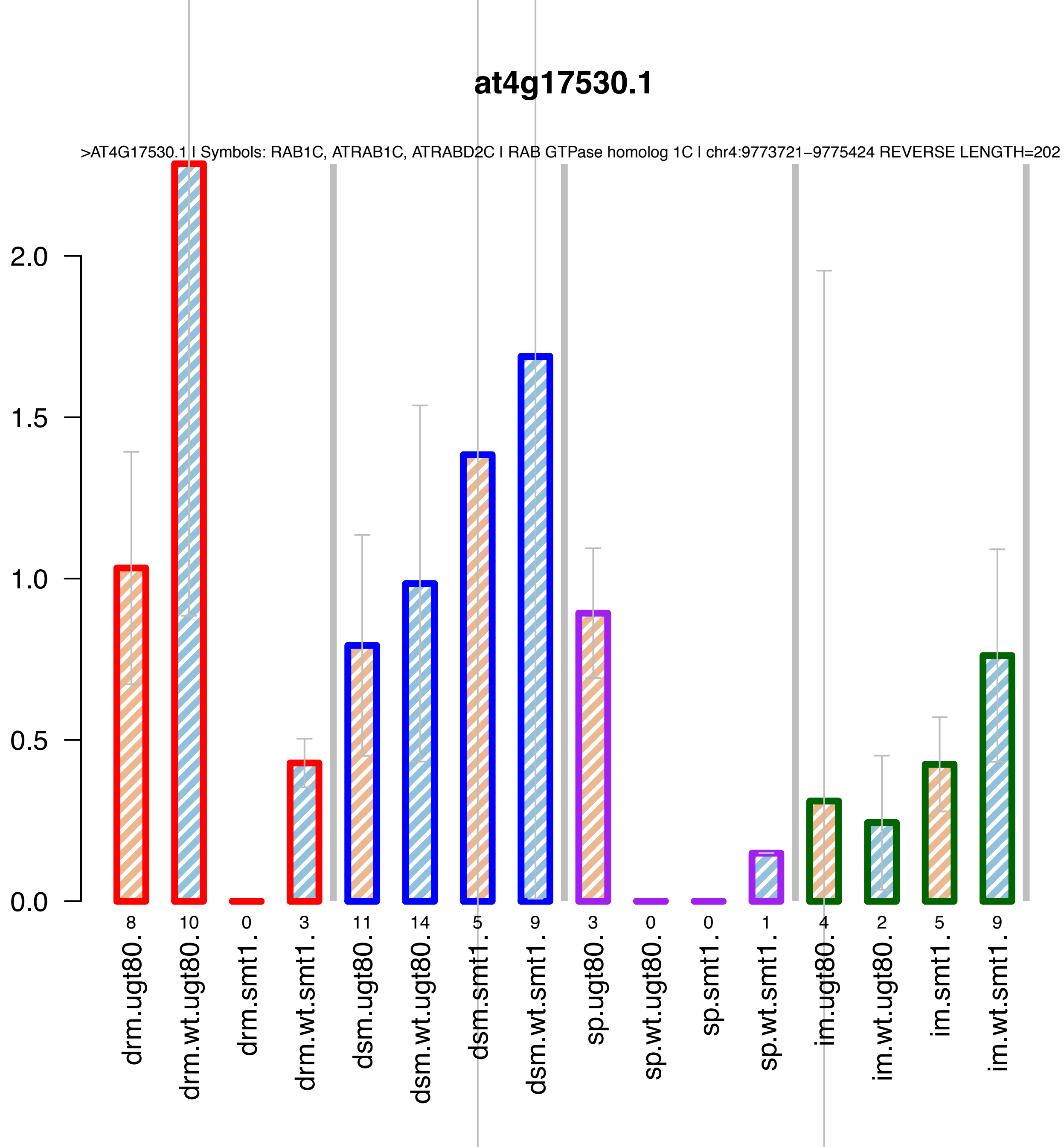

at4g17530.1
>AT4G17530.1 | Symbols: RAB1C, ATRAB1C, ATRABD2C | RAB GTPase homolog 1C | chr4:9773721−9775424 REVERSE LENGTH=202
0.0
0.5
1.0
1.5
2.0
8
10
0
3
11
14
5
9
3
0
0
1
4
2
5
9
drm.ugt80.
drm.wt.ugt80.
drm.smt1.
drm.wt.smt1.
dsm.ugt80.
dsm.wt.ugt80.
dsm.smt1.
dsm.wt.smt1.
sp.ugt80.
sp.wt.ugt80.
sp.smt1.
sp.wt.smt1.
im.ugt80.
im.wt.ugt80.
im.smt1.
im.wt.smt1.

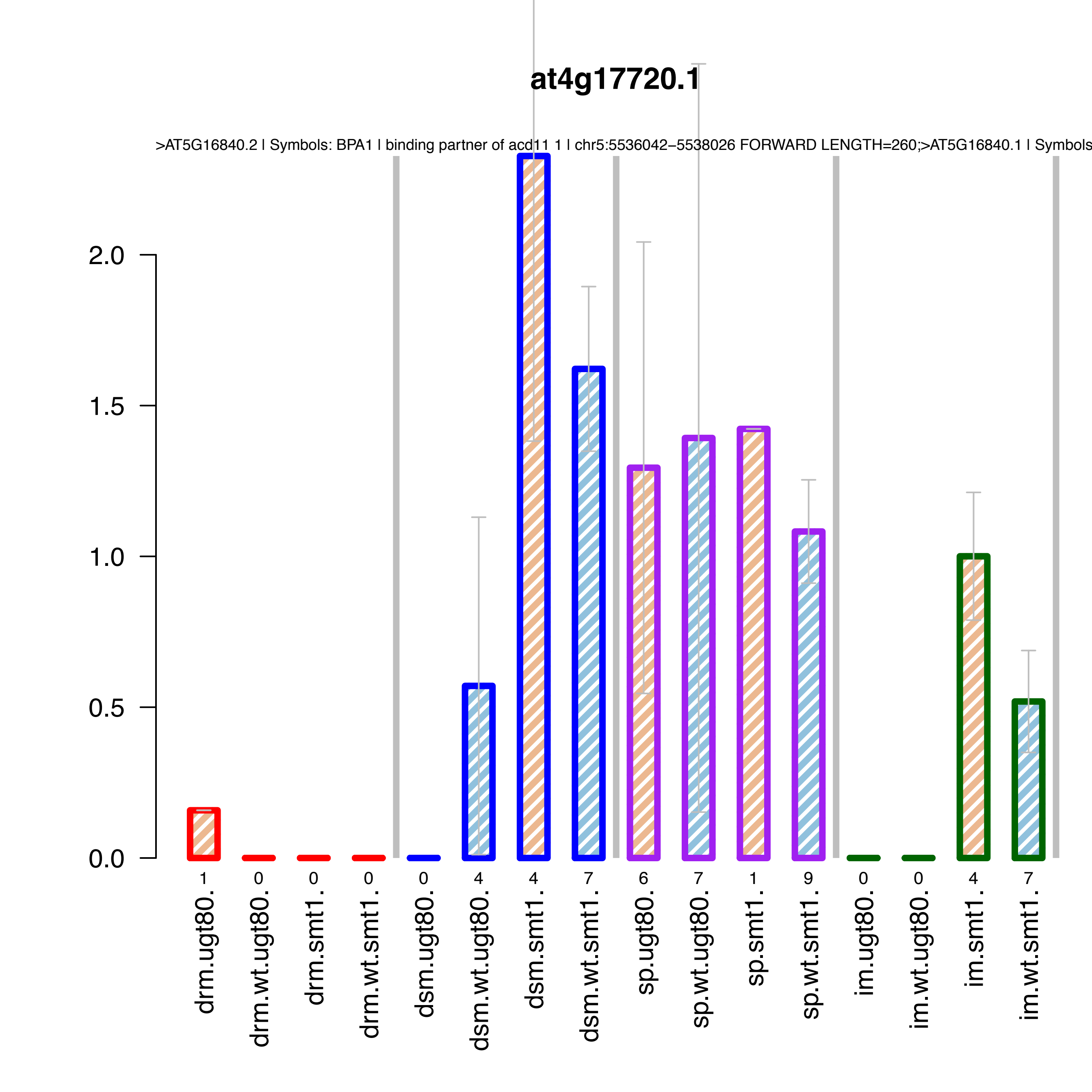

at4g17720.1
>AT5G16840.2 | Symbols: BPA1 | binding partner of acd11 1 | chr5:5536042-5538026 FORWARD LENGTH=260;>AT5G16840.1 | Symbols
2.0
1.5
1.0
0.5
0.0
1
0
0
0
0
4
4
7
6
7
1
9
0
0
4
7
drm.ugt80.
drm.wt.ugt80.
drm.smt1.
drm.wt.smt1.
dsm.ugt80.
dsm.wt.ugt80.
dsm.smt1.
dsm.wt.smt1.
sp.ugt80.
sp.wt.ugt80.
sp.smt1.
sp.wt.smt1.
im.ugt80.
im.wt.ugt80.
im.smt1.
im.wt.smt1.

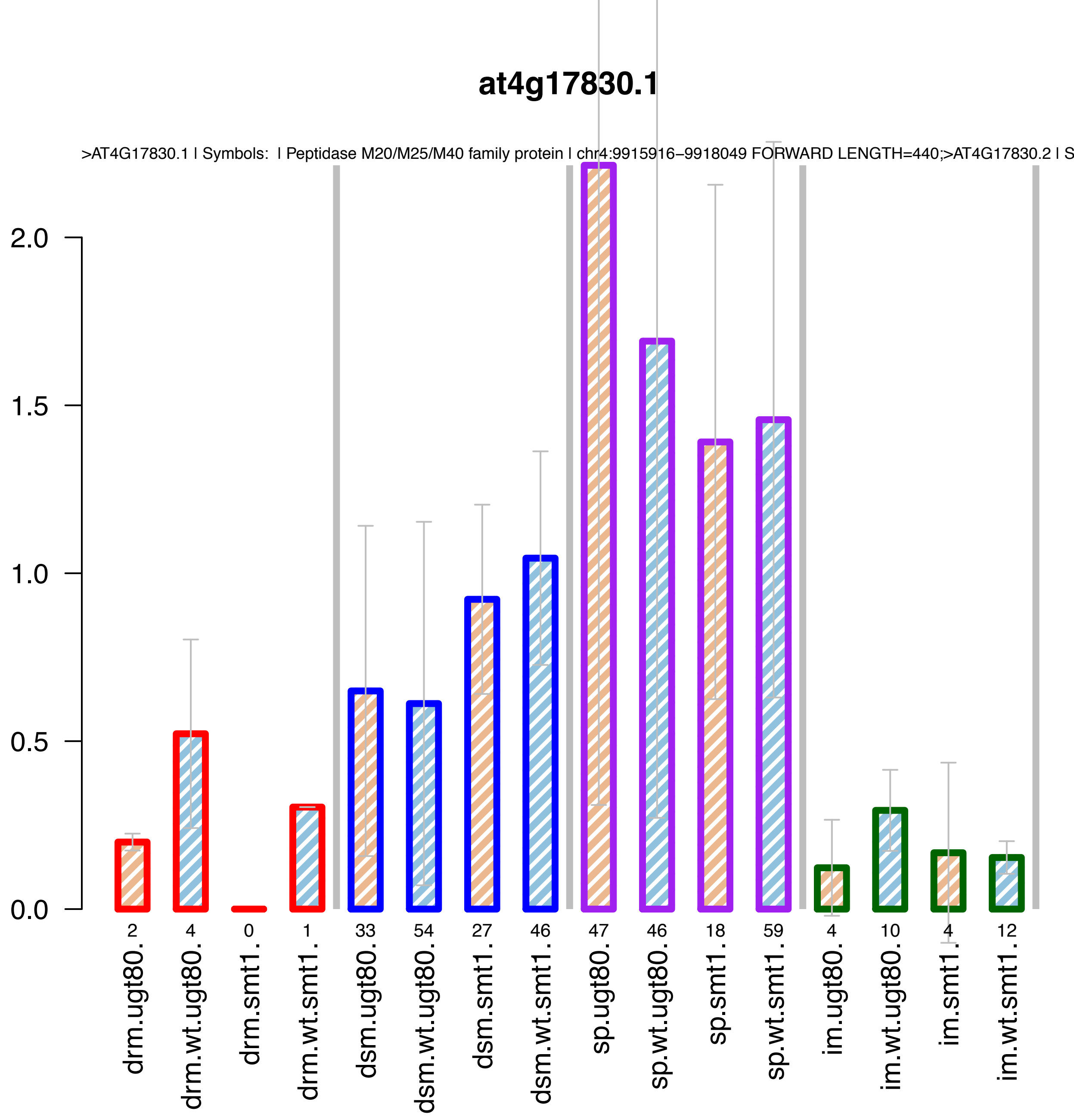

at4g17830.1
>AT4G17830.1 | Symbols: | Peptidase M20/M25/M40 family protein | chr4:9915916-9918049 FORWARD LENGTH=440;>AT4G17830.2 | S
2.0
1.5
1.0
0.5
0.0
2
4
0
1
33
54
27
46
47
46
18
59
4
10
4
12
drm.ugt80.
drm.wt.ugt80.
drm.smt1.
drm.wt.smt1.
dsm.ugt80.
dsm.wt.ugt80.
dsm.smt1.
dsm.wt.smt1.
sp.ugt80.
sp.wt.ugt80.
sp.smt1.
sp.wt.smt1.
im.ugt80.
im.wt.ugt80.
im.smt1.
im.wt.smt1.

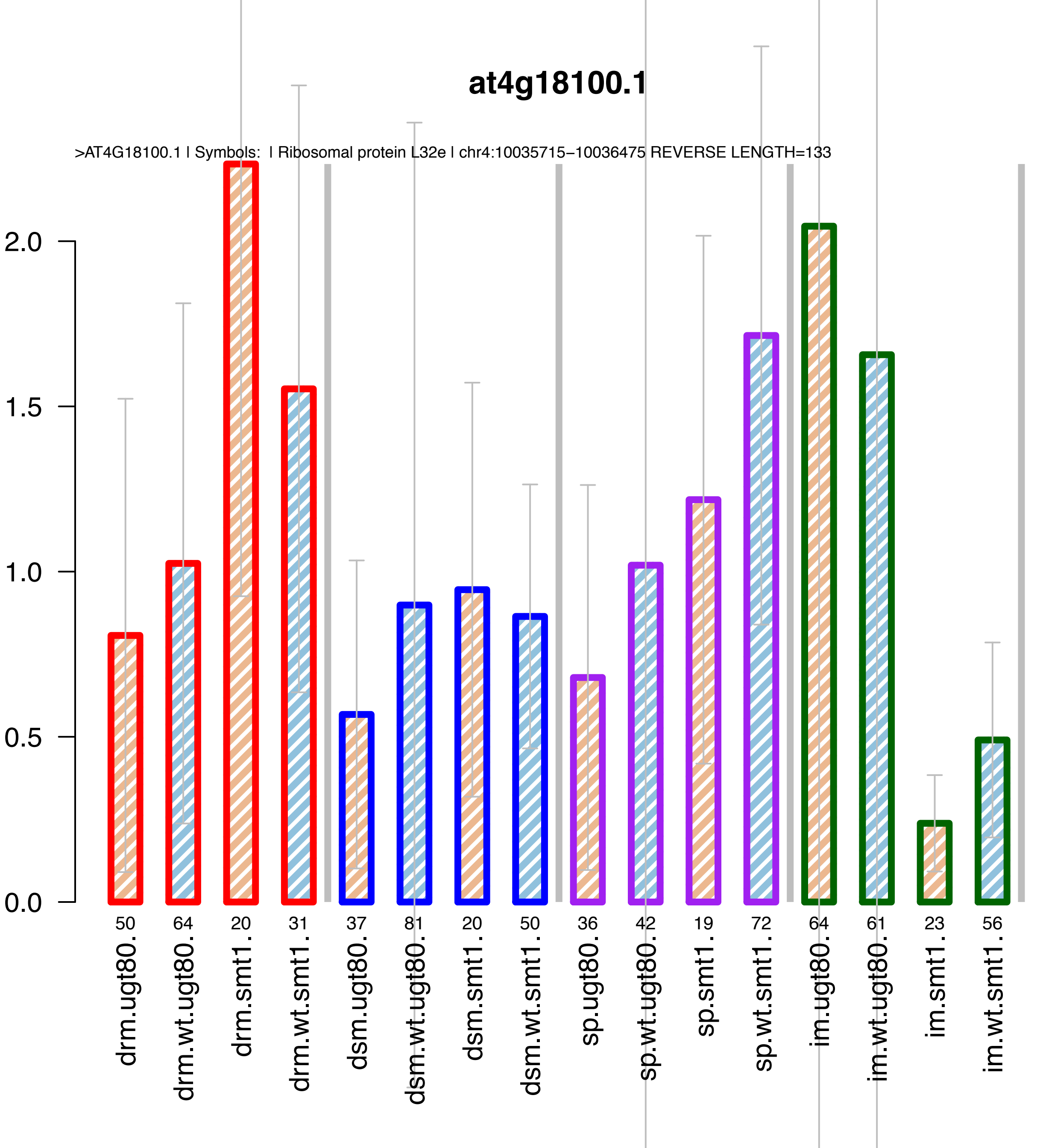

at4g18100.1
>AT4G18100.1 | Symbols: | Ribosomal protein L32e | chr4:10035715-10036475 REVERSE LENGTH=133
2.0
1.5
1.0
0.5
0.0
50
64
20
31
37
81
20
50
36
42
19
72
64
61
23
56
drm.ugt80..
drm.wt.ugt80..
drm.smt1..
drm.wt.smt1..
dsm.ugt80..
dsm.wt.ugt80..
dsm.smt1..
dsm.wt.smt1..
sp.ugt80..
sp.wt.ugt80..
sp.smt1..
sp.wt.smt1..
im.ugt80..
im.wt.ugt80..
im.smt1..
im.wt.smt1..

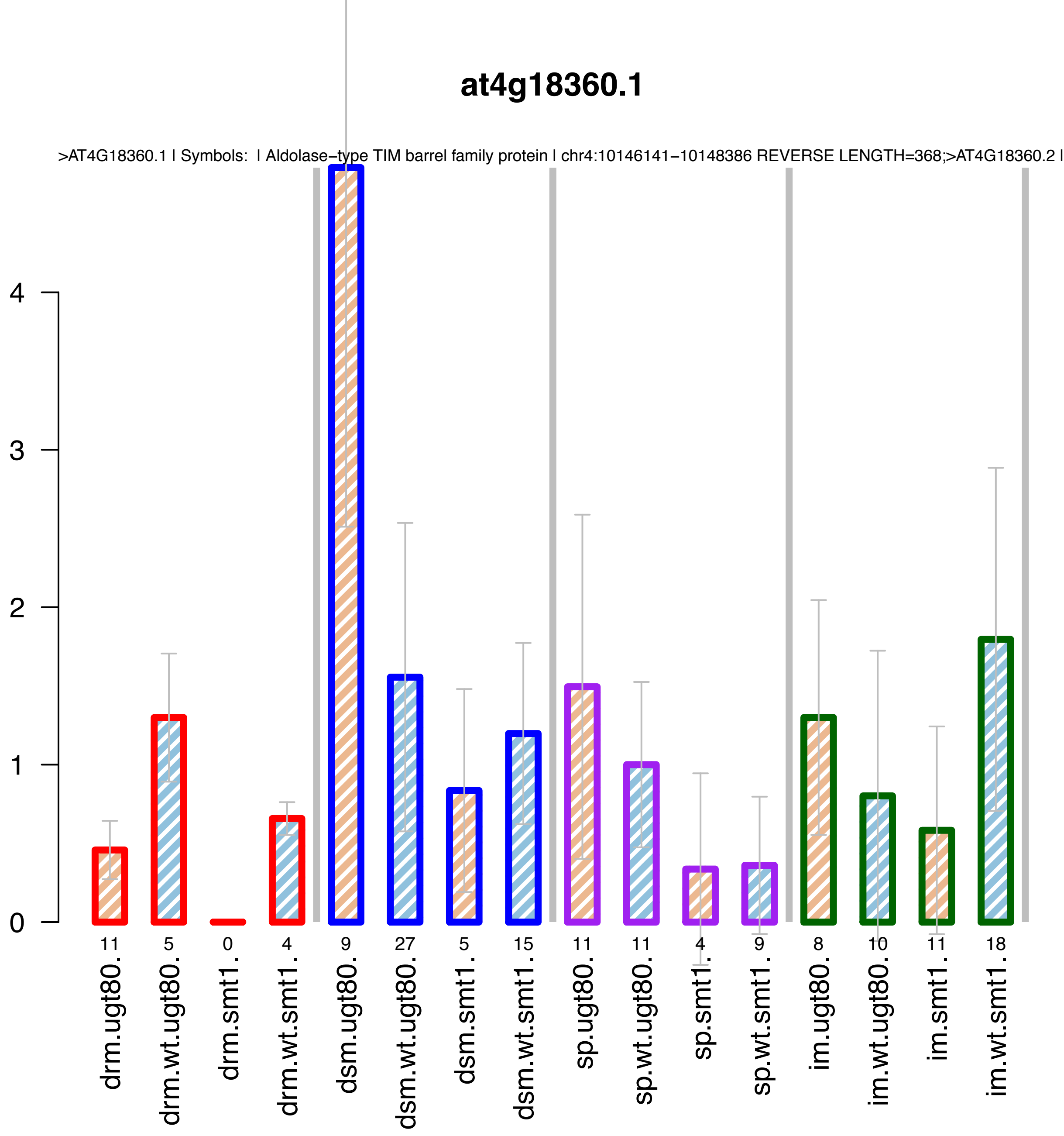
at4g18360.1
>AT4G18360.1 | Symbols: | Aldolase–type TIM barrel family protein | chr4:10146141–10148386 REVERSE LENGTH=368;>AT4G18360.2 |
0
1
2
3
4
11
5
0
4
9
27
5
15
11
11
4
9
8
10
11
18
drm.ugt80.
drm.wt.ugt80.
drm.smt1.
drm.wt.smt1.
dsm.ugt80.
dsm.wt.ugt80.
dsm.smt1.
dsm.wt.smt1.
sp.ugt80.
sp.wt.ugt80.
sp.smt1.
sp.wt.smt1.
im.ugt80.
im.wt.ugt80.
im.smt1.
im.wt.smt1.

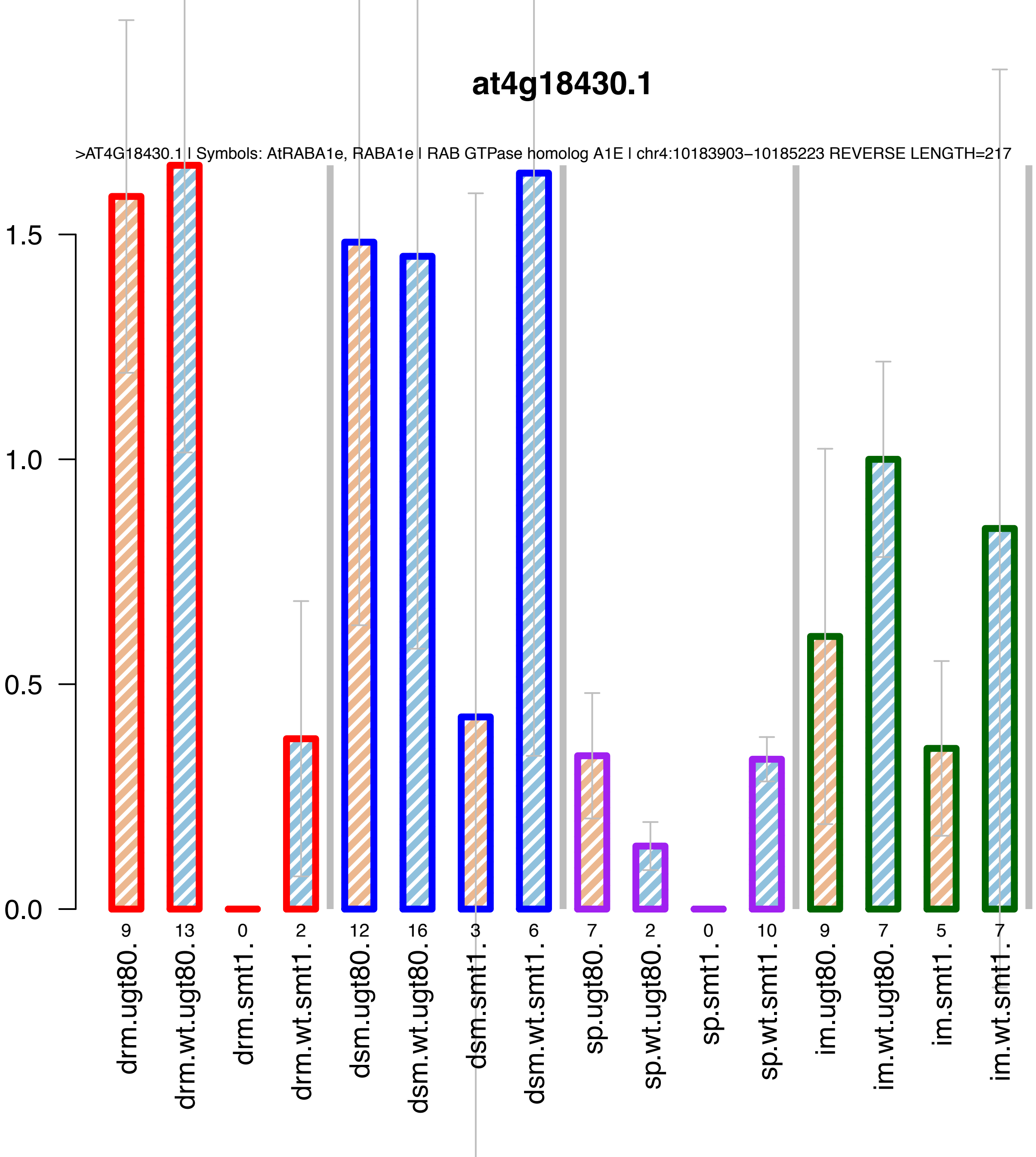
at4g18430.1
>AT4G18430.1 | Symbols: AtRABA1e, RABA1e | RAB GTPase homolog A1E | chr4:10183903-10185223 REVERSE LENGTH=217
1.5
1.0
0.5
0.0
9
13
0
2
12
16
3
6
7
2
0
10
9
7
5
7
drm.ugt80..
drm.wt.ugt80..
drm.smt1..
drm.wt.smt1..
dsm.ugt80..
dsm.wt.ugt80..
dsm.smt1..
dsm.wt.smt1..
sp.ugt80..
sp.wt.ugt80..
sp.smt1..
sp.wt.smt1..
im.ugt80..
im.wt.ugt80..
im.smt1..
im.wt.smt1..

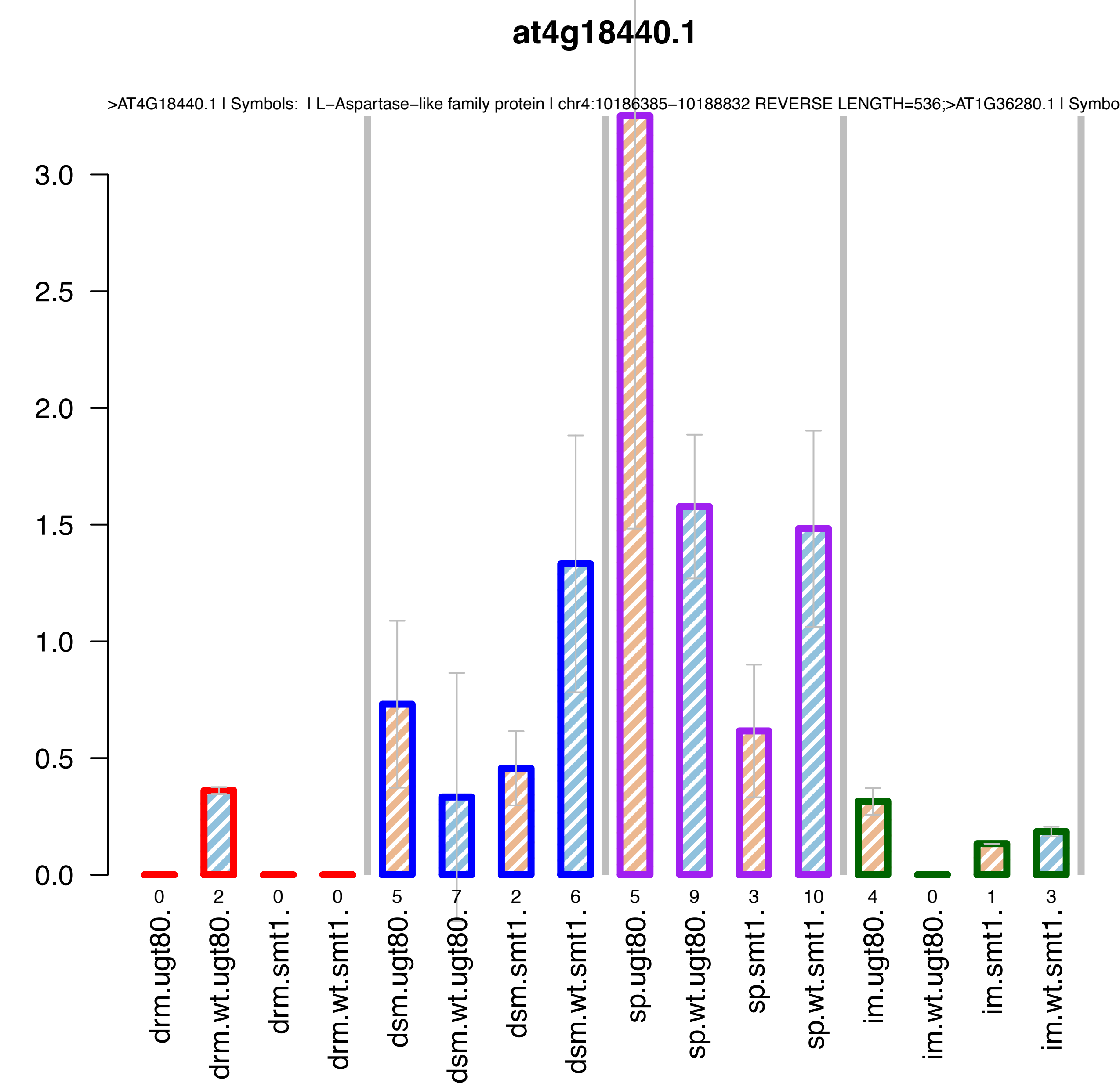

at4g18440.1
>AT4G18440.1 | Symbols: | L−Aspartase−like family protein | chr4:10186385−10188832 REVERSE LENGTH=536;>AT1G36280.1 | Symbo
3.0
2.5
2.0
1.5
1.0
0.5
0.0
0
2
0
0
5
7
2
6
5
9
3
10
4
0
1
3
drm.ugt80.
drm.wt.ugt80.
drm.smt1.
drm.wt.smt1.
dsm.ugt80.
dsm.wt.ugt80.
dsm.smt1.
dsm.wt.smt1.
sp.ugt80.
sp.wt.ugt80.
sp.smt1.
sp.wt.smt1.
im.ugt80.
im.wt.ugt80.
im.smt1.
im.wt.smt1.

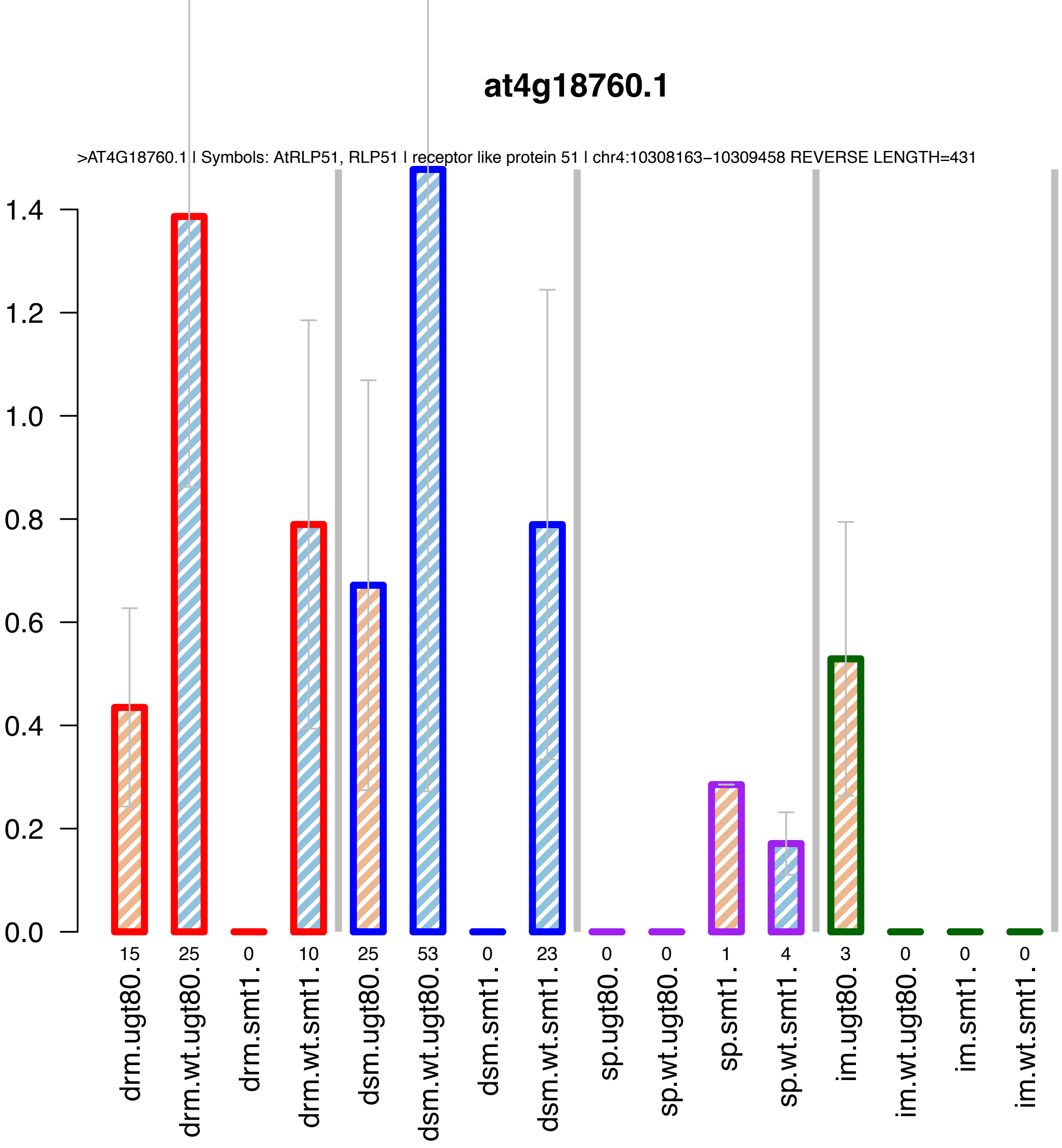
at4g18760.1
>AT4G18760.1 | Symbols: AtRLP51, RLP51 | receptor like protein 51 | chr4:10308163−10309458 REVERSE LENGTH=431
1.4
1.2
1.0
0.8
0.6
0.4
0.2
0.0
15
25
0
10
25
53
0
23
0
0
1
4
3
0
0
0
drm.ugt80.
drm.wt.ugt80.
drm.smt1.
drm.wt.smt1.
dsm.ugt80.
dsm.wt.ugt80.
dsm.smt1.
dsm.wt.smt1.
sp.ugt80.
sp.wt.ugt80.
sp.smt1.
sp.wt.smt1.
im.ugt80.
im.wt.ugt80.
im.smt1.
im.wt.smt1.

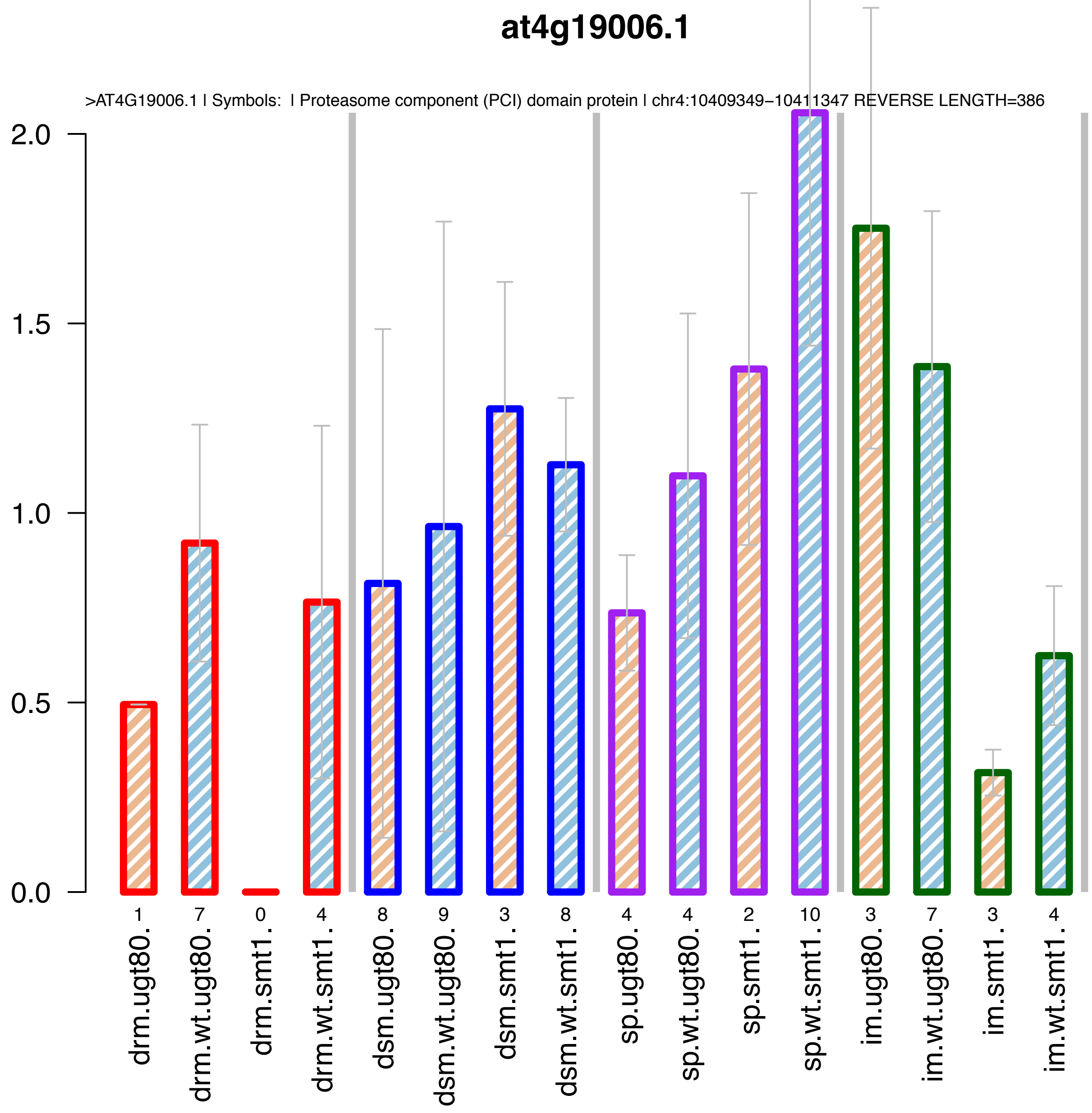

at4g19006.1
>AT4G19006.1 | Symbols: | Proteasome component (PCI) domain protein | chr4:10409349-10411347 REVERSE LENGTH=386
2.0
1.5
1.0
0.5
0.0
1
7
0
4
8
9
3
8
4
4
2
10
3
7
3
4
drm.ugt80.
drm.wt.ugt80.
drm.smt1.
drm.wt.smt1.
dsm.ugt80.
dsm.wt.ugt80.
dsm.smt1.
dsm.wt.smt1.
sp.ugt80.
sp.wt.ugt80.
sp.smt1.
sp.wt.smt1.
im.ugt80.
im.wt.ugt80.
im.smt1.
im.wt.smt1.

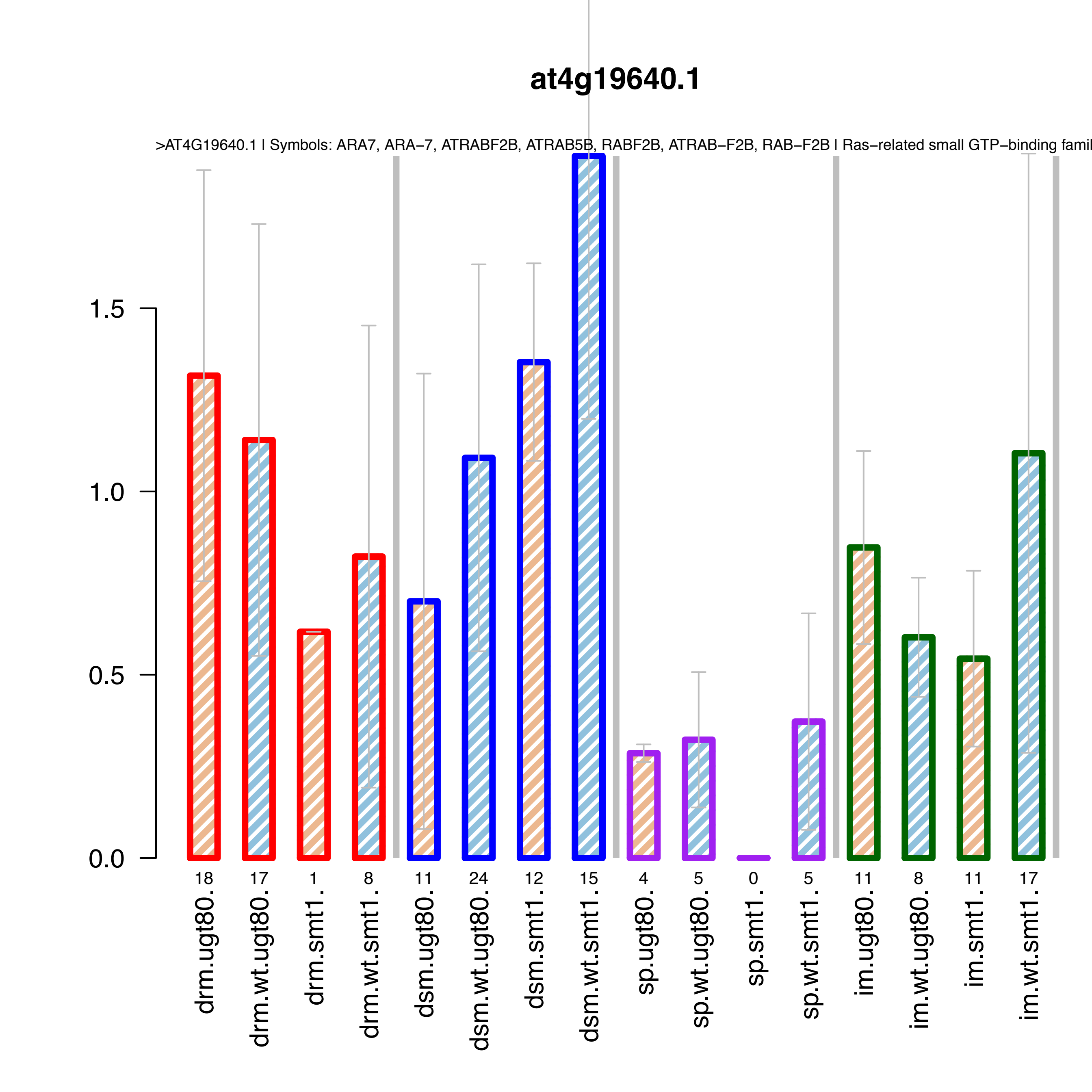

at4g19640.1
>AT4G19640.1 | Symbols: ARA7, ARA−7, ATRABF2B, ATRAB5B, RABF2B, ATRAB−F2B, RAB−F2B | Ras−related small GTP−binding famil
1.5
1.0
0.5
0.0
18
17
1
8
11
24
12
15
4
5
0
5
11
8
11
17
drm.ugt80.
drm.wt.ugt80.
drm.smt1.
drm.wt.smt1.
dsm.ugt80.
dsm.wt.ugt80.
dsm.smt1.
dsm.wt.smt1.
sp.ugt80.
sp.wt.ugt80.
sp.smt1.
sp.wt.smt1.
im.ugt80.
im.wt.ugt80.
im.smt1.
im.wt.smt1.

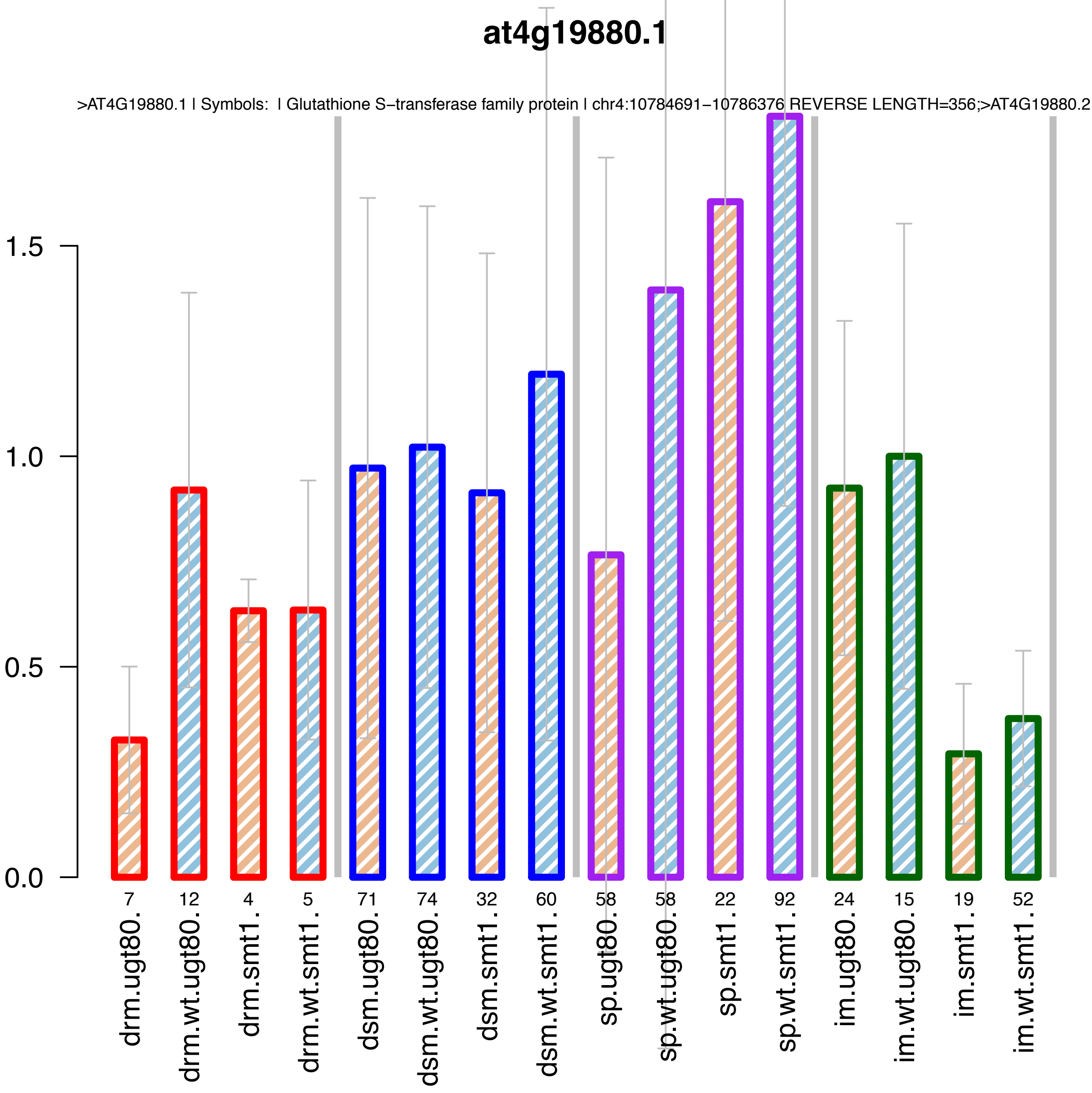

at4g19880.1
>AT4G19880.1 | Symbols: | Glutathione S−transferase family protein | chr4:10784691−10786376 REVERSE LENGTH=356;>AT4G19880.2
0.0
0.5
1.0
1.5
7
12
4
5
71
74
32
60
58
58
22
92
24
15
19
52
drm.ugt80..
drm.wt.ugt80..
drm.smt1..
drm.wt.smt1..
dsm.ugt80..
dsm.wt.ugt80..
dsm.smt1..
dsm.wt.smt1..
sp.ugt80..
sp.wt.ugt80..
sp.smt1..
sp.wt.smt1..
im.ugt80..
im.wt.ugt80..
im.smt1..
im.wt.smt1..

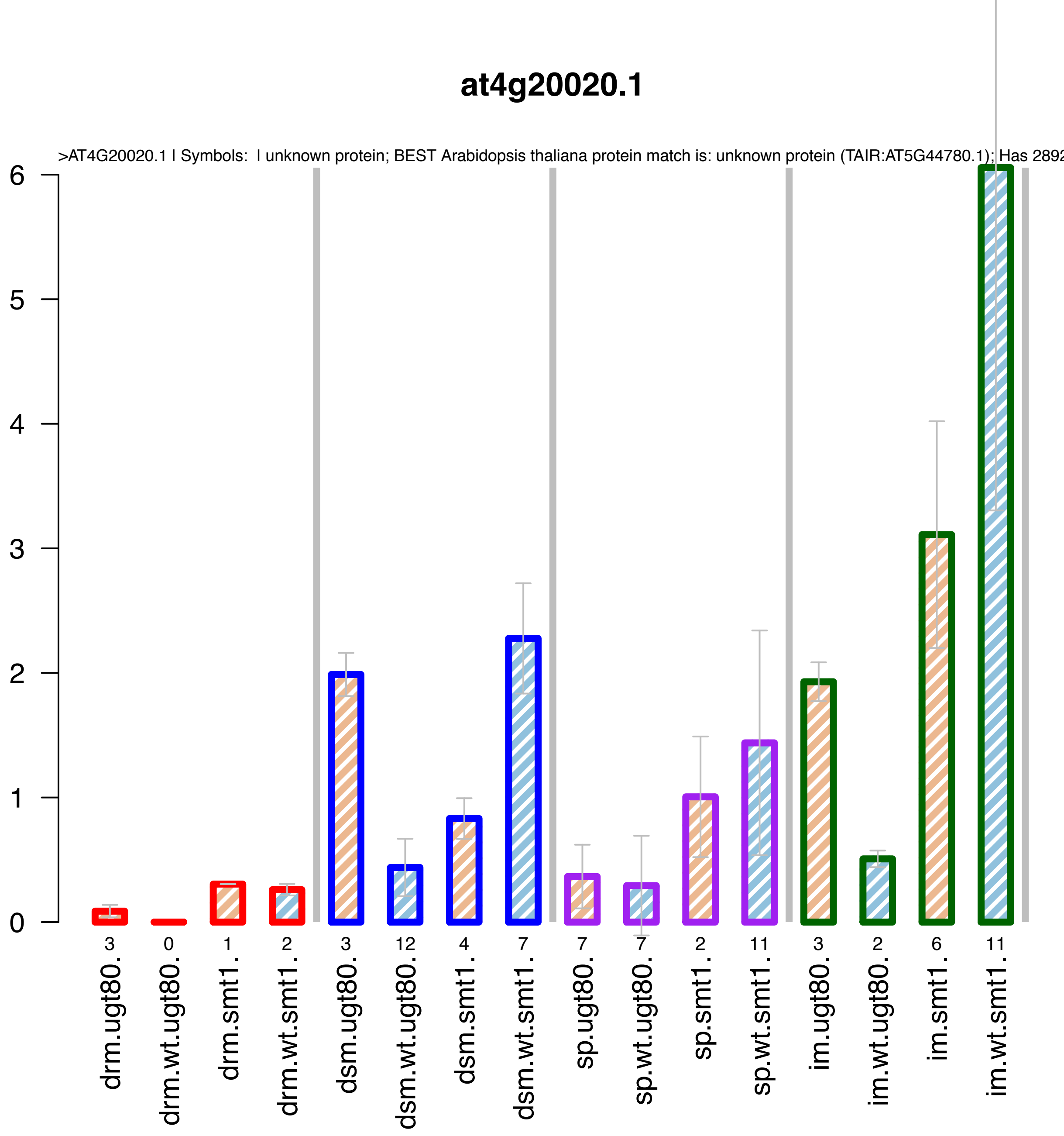

at4g20020.1
>AT4G20020.1 | Symbols: | unknown protein; BEST Arabidopsis thaliana protein match is: unknown protein (TAIR:AT5G44780.1); Has 2892
0
1
2
3
4
5
6
3
0
1
2
3
12
4
7
7
7
2
11
3
2
6
11
drm.ugt80.
drm.wt.ugt80.
drm.smt1.
drm.wt.smt1.
dsm.ugt80.
dsm.wt.ugt80.
dsm.smt1.
dsm.wt.smt1.
sp.ugt80.
sp.wt.ugt80.
sp.smt1.
sp.wt.smt1.
im.ugt80.
im.wt.ugt80.
im.smt1.
im.wt.smt1.

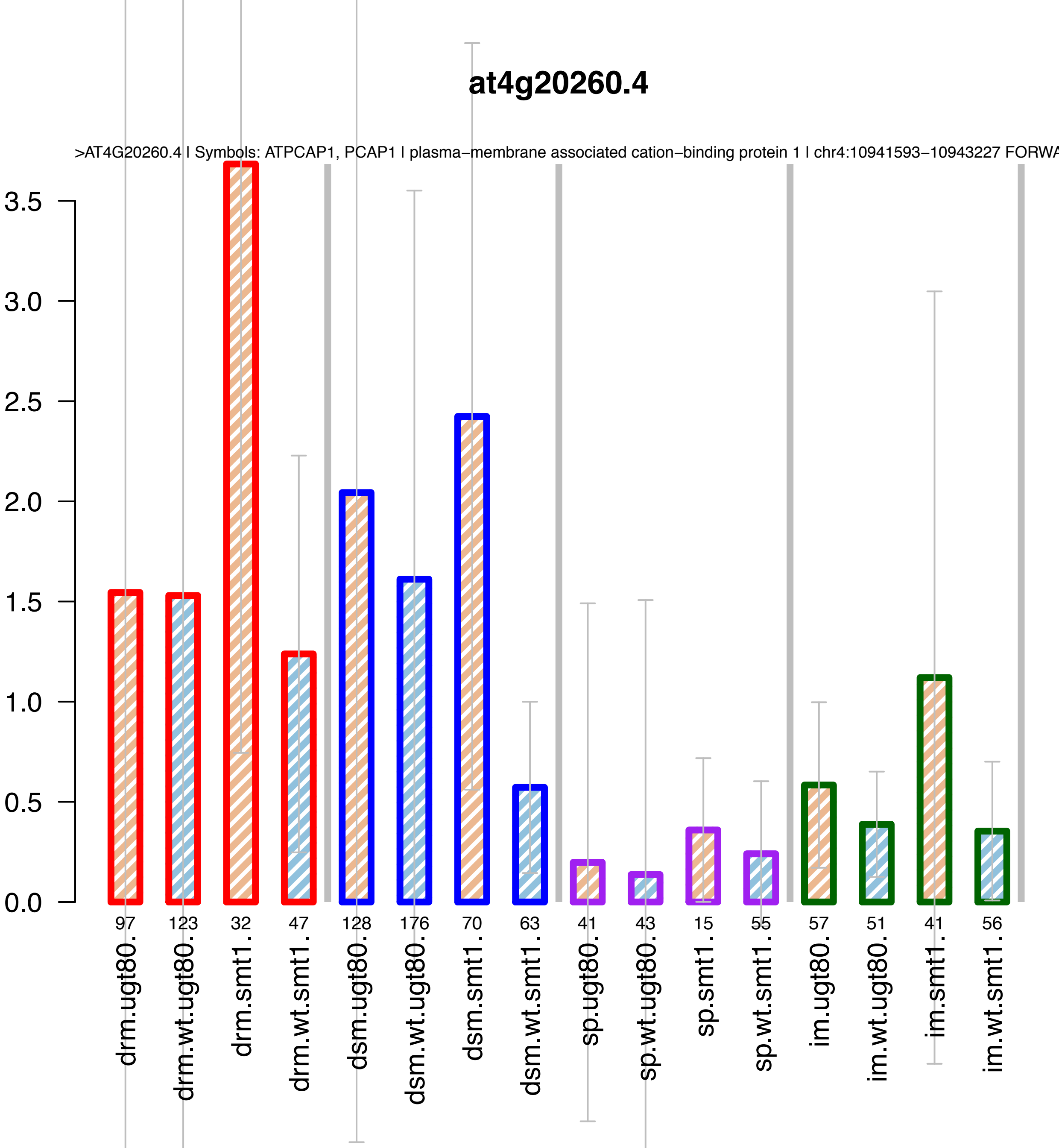

at4g20260.4
>AT4G20260.4 | Symbols: ATPCAP1, PCAP1 | plasma−membrane associated cation−binding protein 1 | chr4:10941593−10943227 FORWA
3.5
3.0
2.5
2.0
1.5
1.0
0.5
0.0
97
123
32
47
128
176
70
63
41
43
15
55
57
51
41
56
drm.ugt80..
drm.wt.ugt80..
drm.smt1..
drm.wt.smt1..
dsm.ugt80..
dsm.wt.ugt80..
dsm.smt1..
dsm.wt.smt1..
sp.ugt80..
sp.wt.ugt80..
sp.smt1..
sp.wt.smt1..
im.ugt80..
im.wt.ugt80..
im.smt1..
im.wt.smt1..

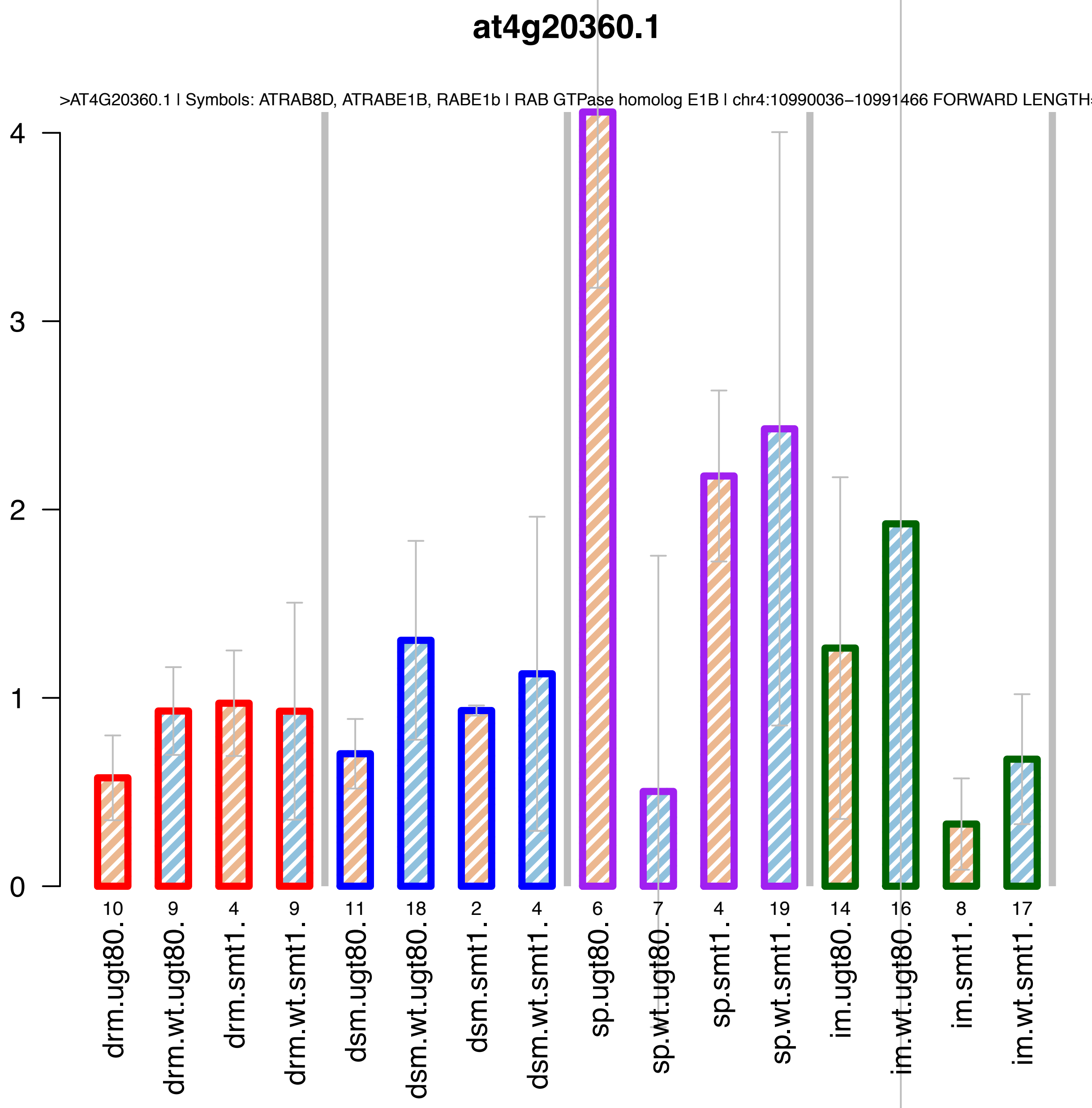

at4g20360.1
>AT4G20360.1 | Symbols: ATRAB8D, ATRABE1B, RABE1b | RAB GTPase homolog E1B | chr4:10990036-10991466 FORWARD LENGTH=
4
3
2
1
0
10
9
4
9
11
18
2
4
6
7
4
19
14
16
8
17
drm.ugt80..
drm.wt.ugt80..
drm.smt1..
drm.wt.smt1..
dsm.ugt80..
dsm.wt.ugt80..
dsm.smt1..
dsm.wt.smt1..
sp.ugt80..
sp.wt.ugt80..
sp.smt1..
sp.wt.smt1..
im.ugt80..
im.wt.ugt80..
im.smt1..
im.wt.smt1..

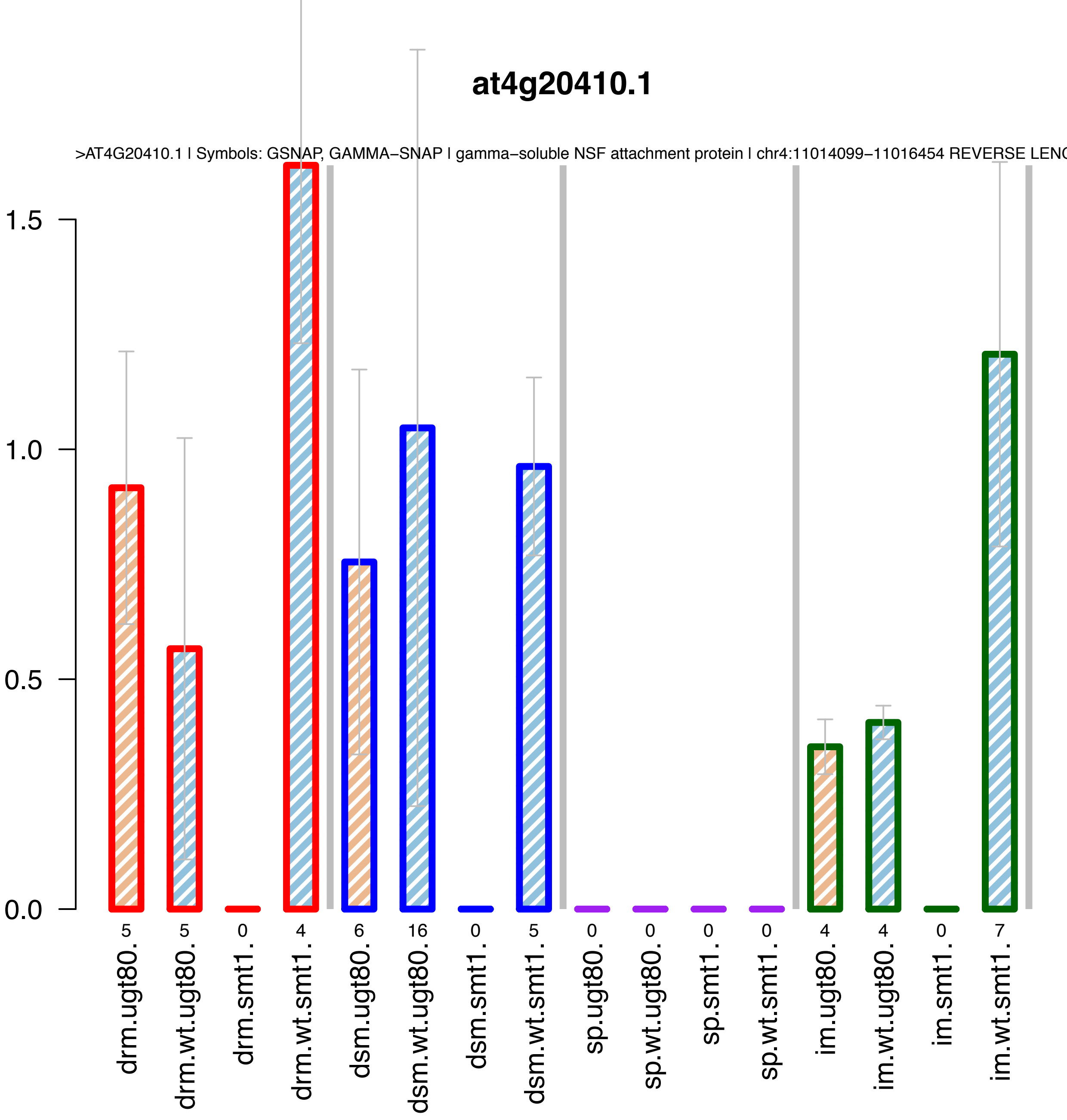

at4g20410.1
>AT4G20410.1 | Symbols: GSNAP, GAMMA-SNAP | gamma-soluble NSF attachment protein | chr4:11014099-11016454 REVERSE LENG
1.5
1.0
0.5
0.0
5
5
0
4
6
16
0
5
0
0
0
0
4
4
0
7
drm.ugt80.
drm.wt.ugt80.
drm.smt1.
drm.wt.smt1.
dsm.ugt80.
dsm.wt.ugt80.
dsm.smt1.
dsm.wt.smt1.
sp.ugt80.
sp.wt.ugt80.
sp.smt1.
sp.wt.smt1.
im.ugt80.
im.wt.ugt80.
im.smt1.
im.wt.smt1.

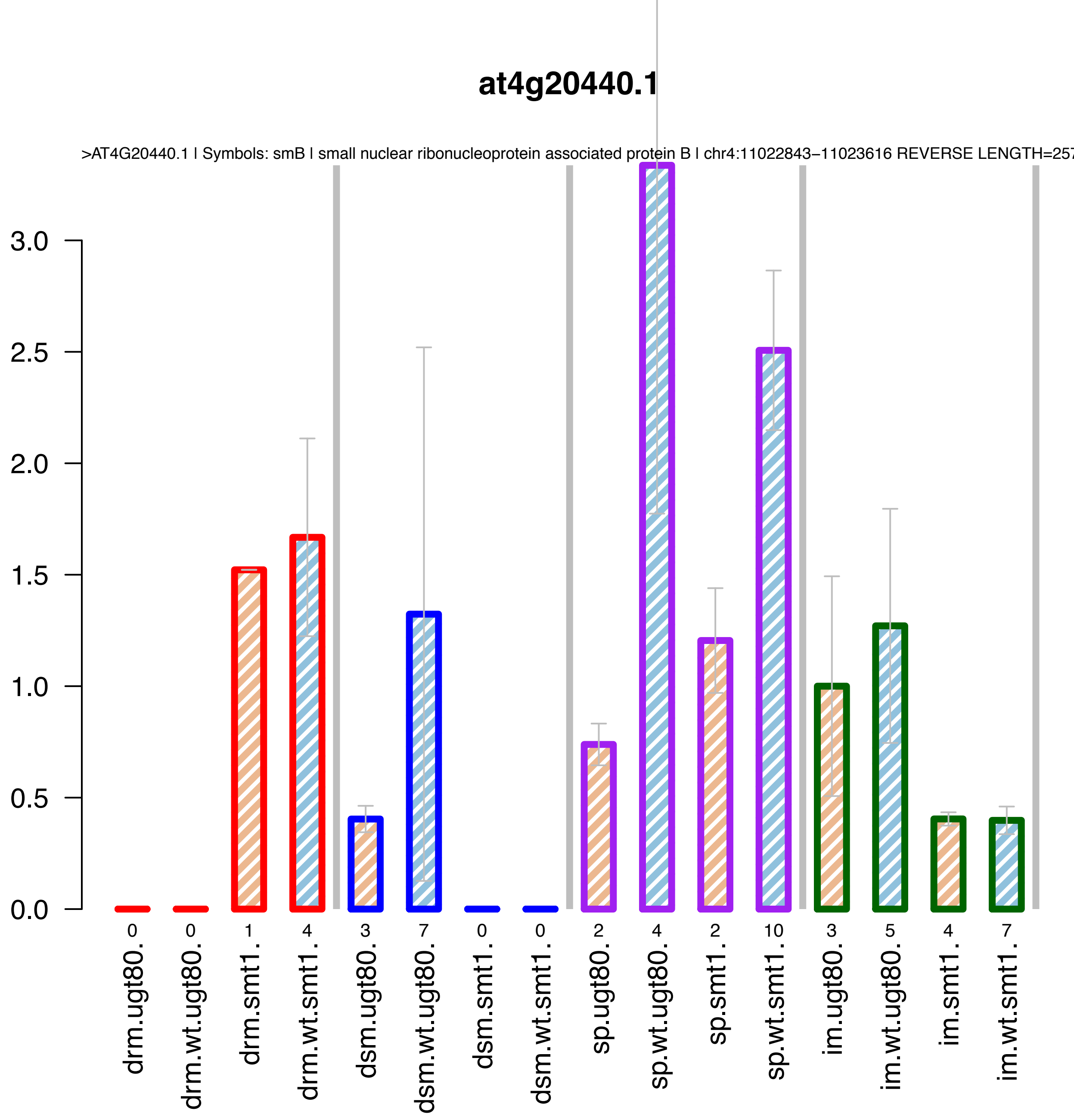

at4g20440.1
>AT4G20440.1 | Symbols: smB | small nuclear ribonucleoprotein associated protein B | chr4:11022843-11023616 REVERSE LENGTH=257
3.0
2.5
2.0
1.5
1.0
0.5
0.0
0
0
1
4
3
7
0
0
2
4
2
10
3
5
4
7
drm.ugt80.
drm.wt.ugt80.
drm.smt1.
drm.wt.smt1.
dsm.ugt80.
dsm.wt.ugt80.
dsm.smt1.
dsm.wt.smt1.
sp.ugt80.
sp.wt.ugt80.
sp.smt1.
sp.wt.smt1.
im.ugt80.
im.wt.ugt80.
im.smt1.
im.wt.smt1.

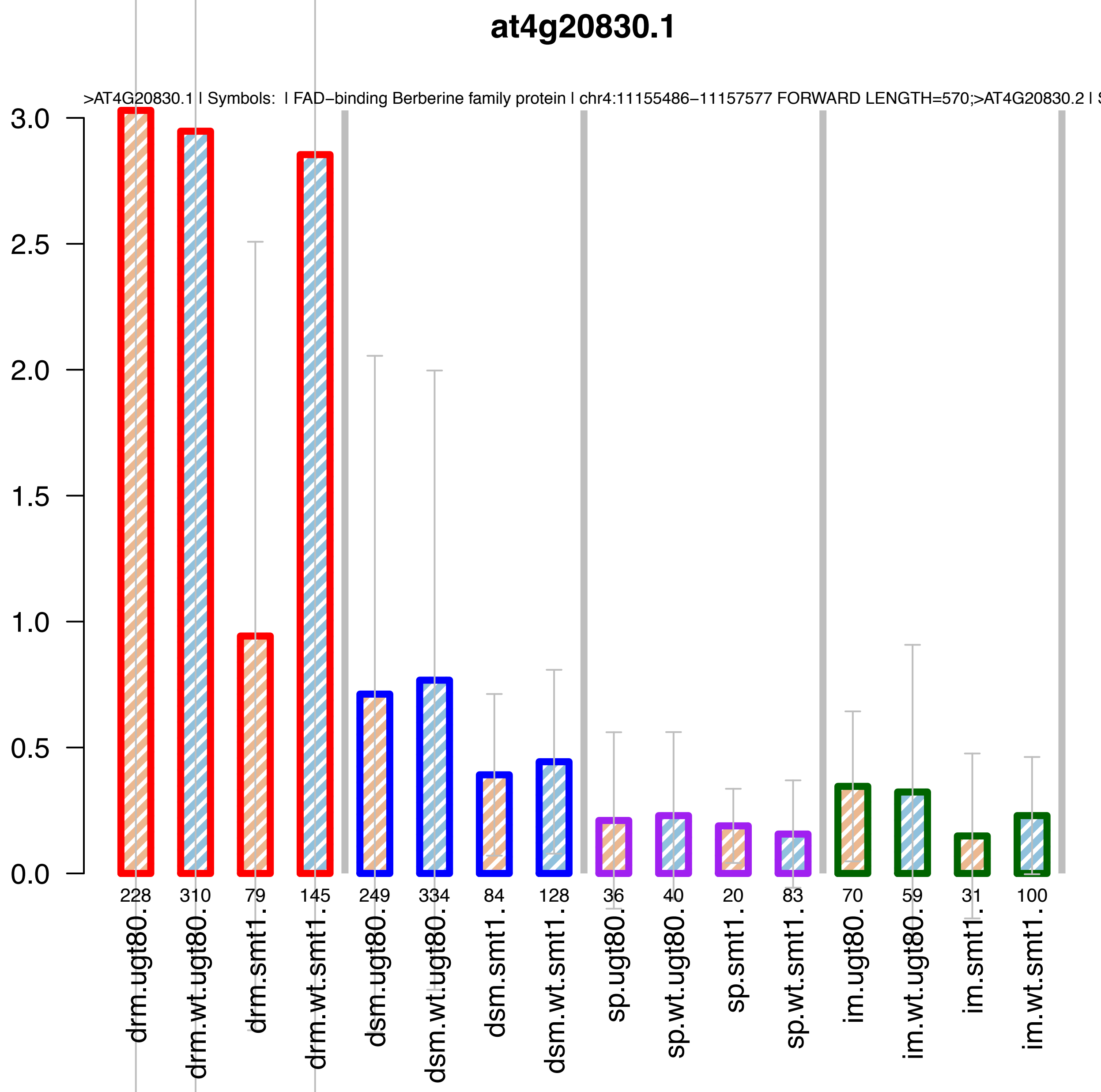

at4g20830.1
>AT4G20830.1 | Symbols: | FAD−binding Berberine family protein | chr4:11155486−11157577 FORWARD LENGTH=570;>AT4G20830.2 |
3.0
2.5
2.0
1.5
1.0
0.5
0.0
228
310
79
145
249
334
84
128
36
40
20
83
70
59
31
100
drm.ugt80.
drm.wt.ugt80.
drm.smt1.
drm.wt.smt1.
dsm.ugt80.
dsm.wt.ugt80.
dsm.smt1.
dsm.wt.smt1.
sp.ugt80.
sp.wt.ugt80.
sp.smt1.
sp.wt.smt1.
im.ugt80.
im.wt.ugt80.
im.smt1.
im.wt.smt1.

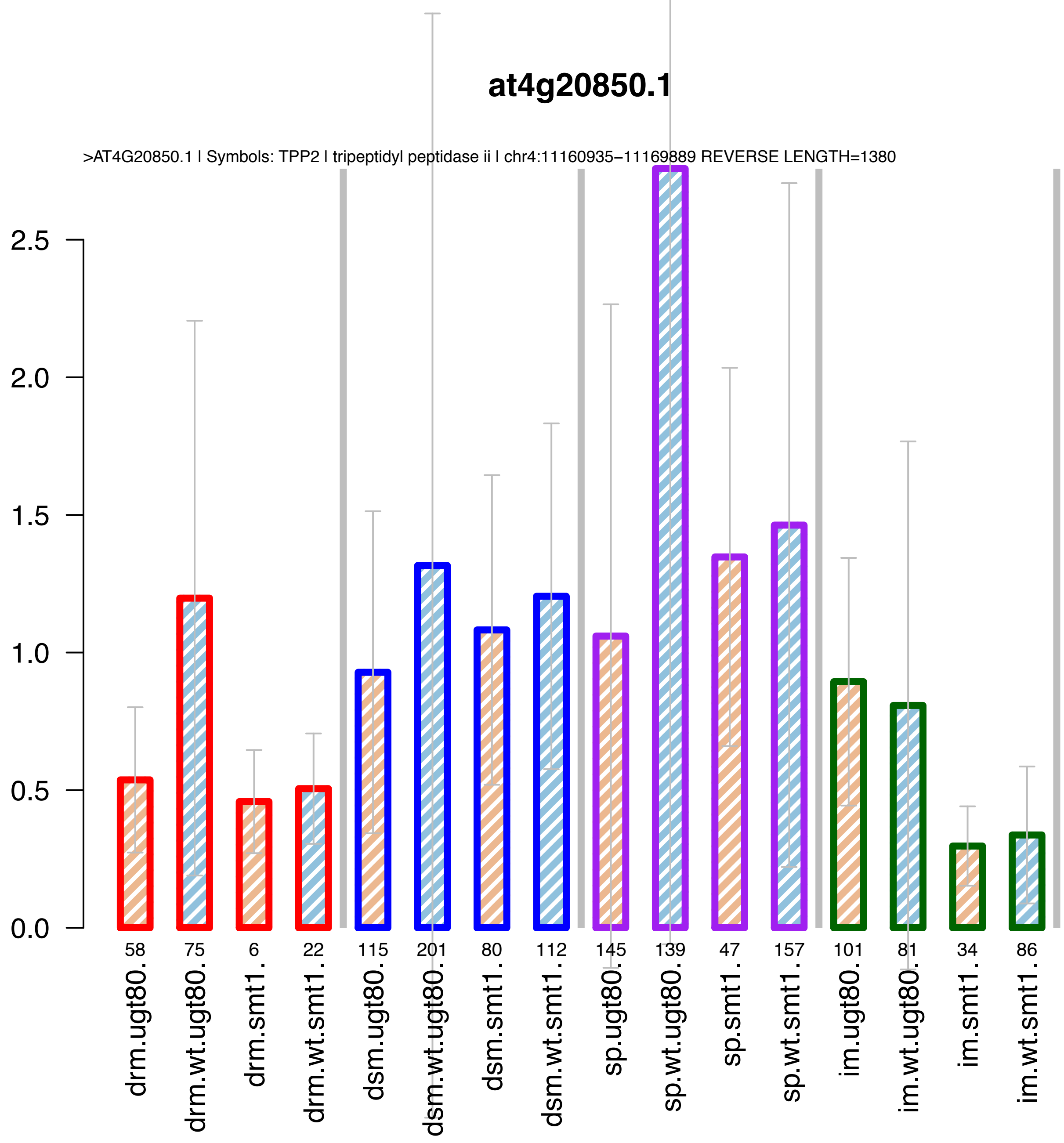

at4g20850.1
>AT4G20850.1 | Symbols: TPP2 | tripeptidyl peptidase ii | chr4:11160935-11169889 REVERSE LENGTH=1380
0.0
0.5
1.0
1.5
2.0
2.5
58
75
6
22
115
201
80
112
145
139
47
157
101
81
34
86
drm.ugt80..
drm.wt.ugt80..
drm.smt1..
drm.wt.smt1..
dsm.ugt80..
dsm.wt.ugt80..
dsm.smt1..
dsm.wt.smt1..
sp.ugt80..
sp.wt.ugt80..
sp.smt1..
sp.wt.smt1..
im.ugt80..
im.wt.ugt80..
im.smt1..
im.wt.smt1..

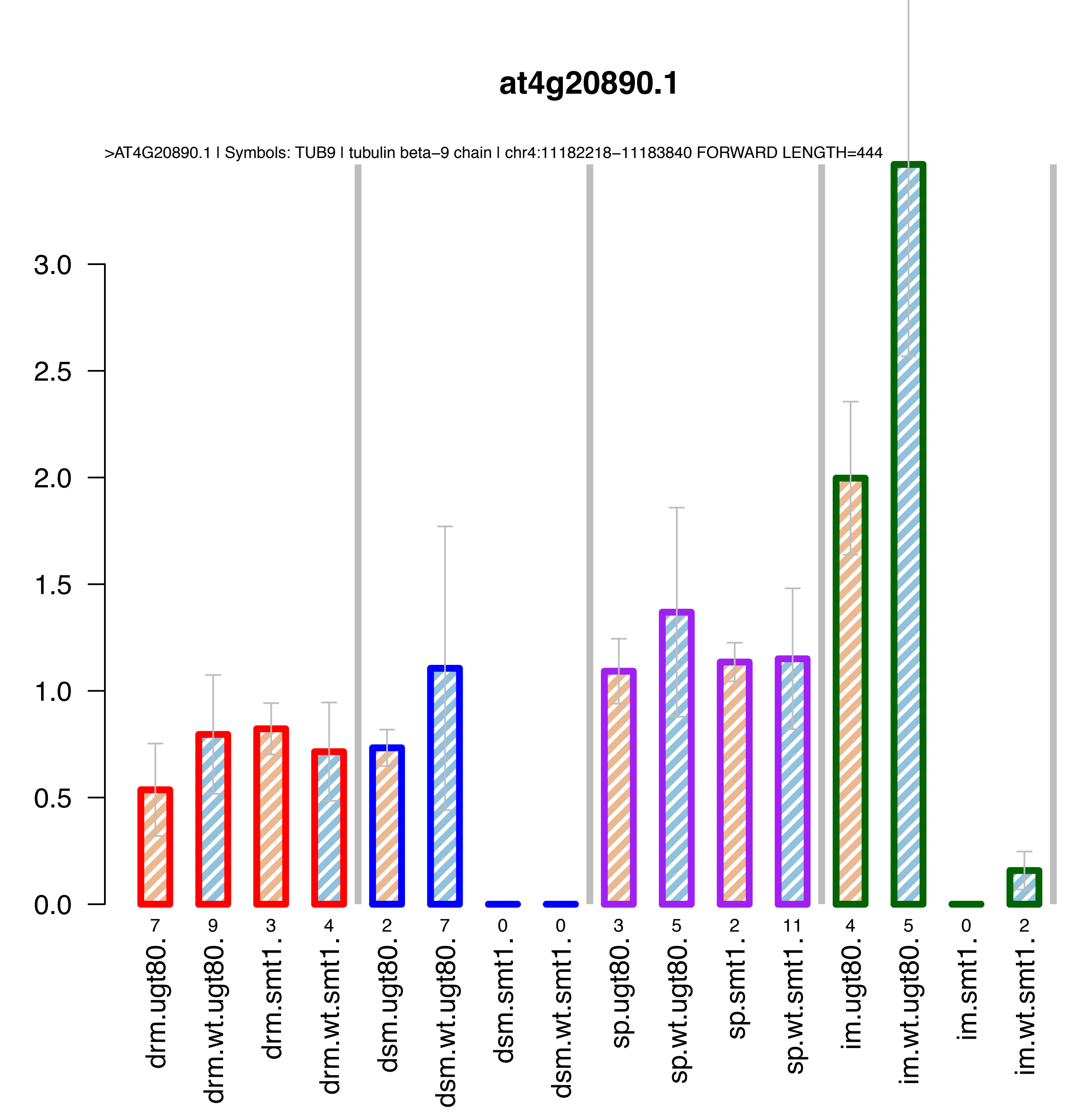
at4g20890.1
>AT4G20890.1 | Symbols: TUB9 | tubulin beta−9 chain | chr4:11182218−11183840 FORWARD LENGTH=444
3.0
2.5
2.0
1.5
1.0
0.5
0.0
7
9
3
4
2
7
0
0
3
5
2
11
4
5
0
2
drm.ugt80.
drm.wt.ugt80.
drm.smt1.
drm.wt.smt1.
dsm.ugt80.
dsm.wt.ugt80.
dsm.smt1.
dsm.wt.smt1.
sp.ugt80.
sp.wt.ugt80.
sp.smt1.
sp.wt.smt1.
im.ugt80.
im.wt.ugt80.
im.smt1.
im.wt.smt1.

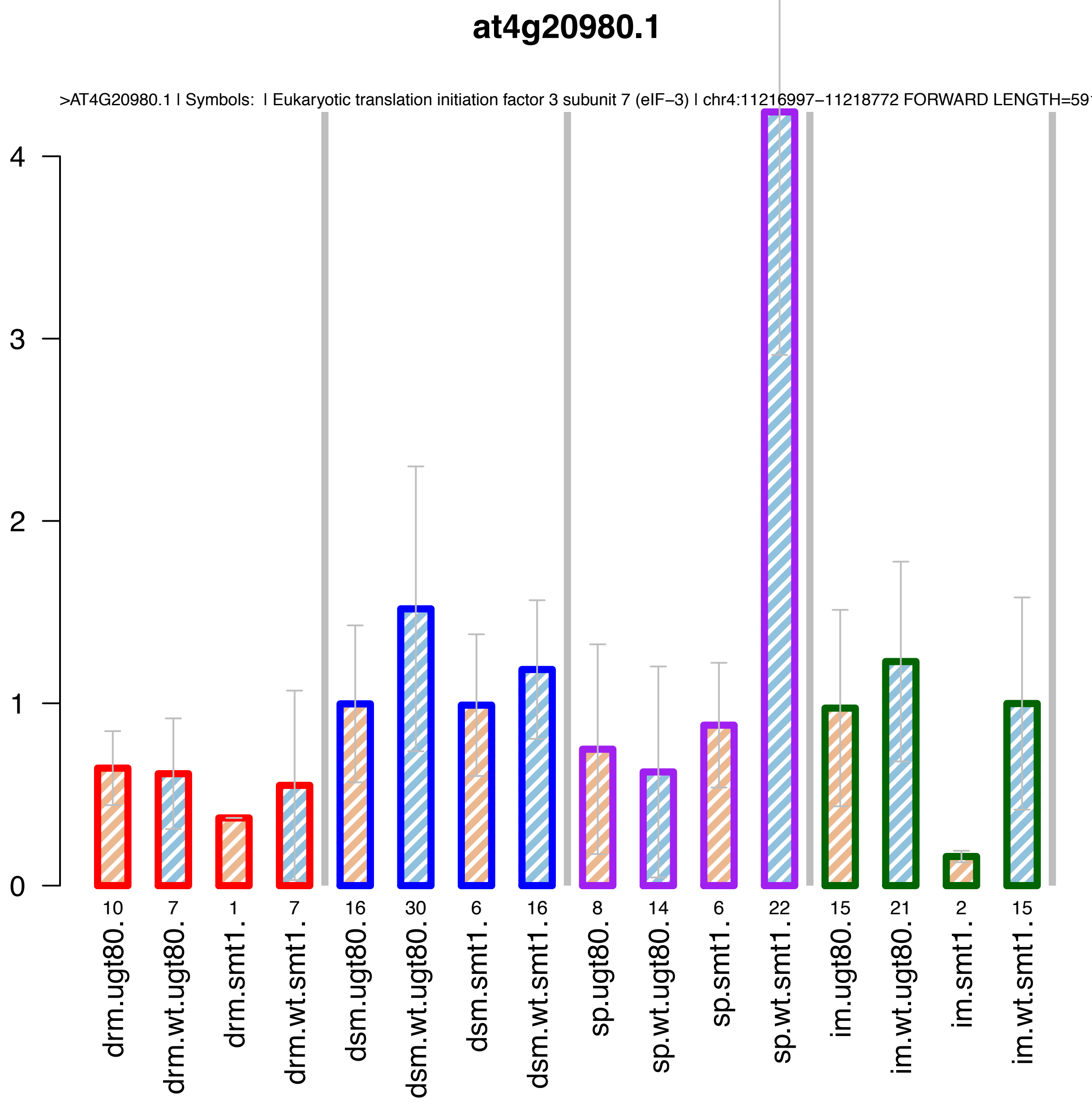

at4g20980.1
>AT4G20980.1 | Symbols: | Eukaryotic translation initiation factor 3 subunit 7 (eIF−3) | chr4:11216997−11218772 FORWARD LENGTH=591
0
1
2
3
4
10
7
1
7
16
30
6
16
8
14
6
22
15
21
2
15
drm.ugt80.
drm.wt.ugt80.
drm.smt1.
drm.wt.smt1.
dsm.ugt80.
dsm.wt.ugt80.
dsm.smt1.
dsm.wt.smt1.
sp.ugt80.
sp.wt.ugt80.
sp.smt1.
sp.wt.smt1.
im.ugt80.
im.wt.ugt80.
im.smt1.
im.wt.smt1.

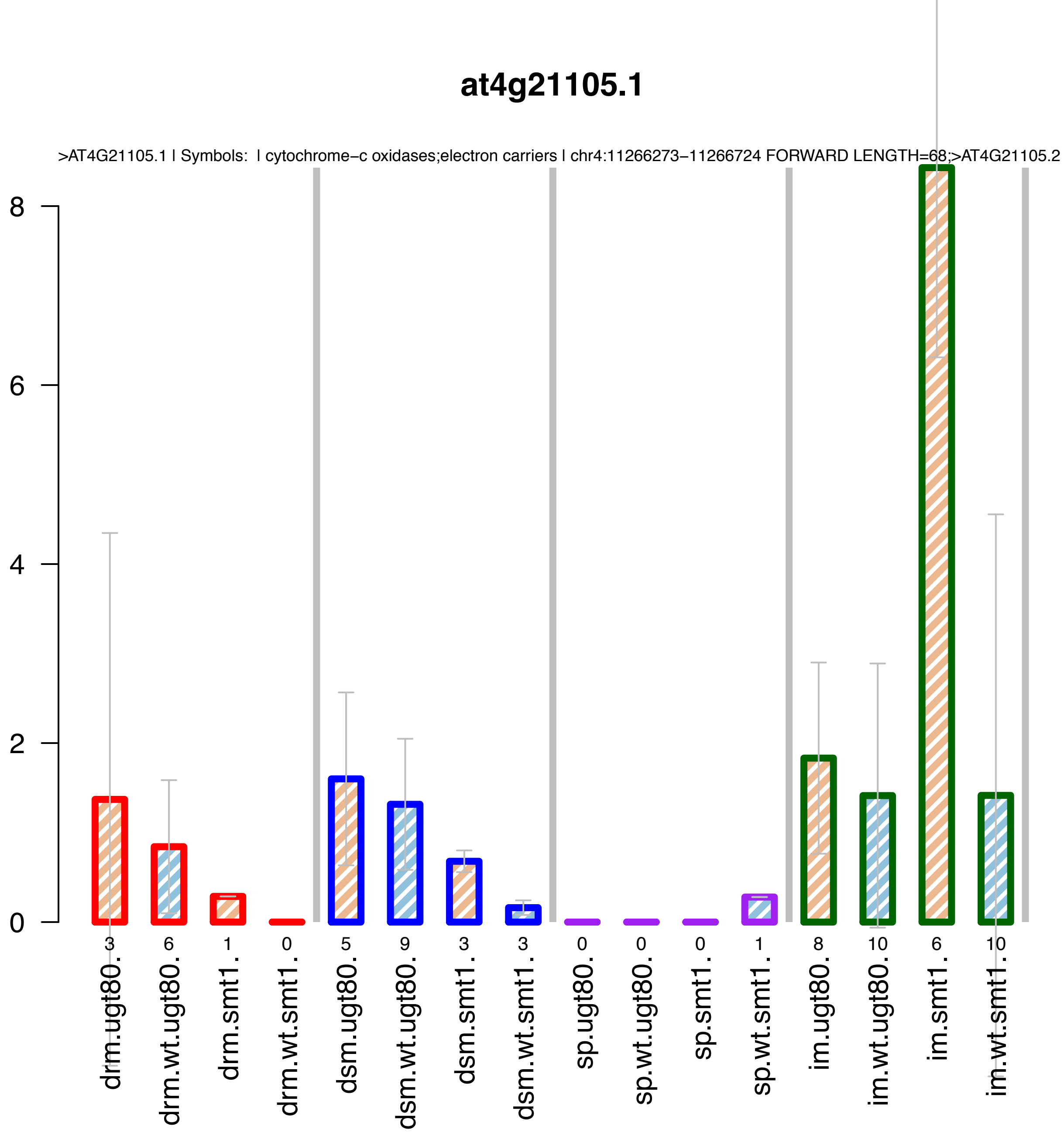

at4g21105.1
>AT4G21105.1 | Symbols: | cytochrome-c oxidases;electron carriers | chr4:11266273-11266724 FORWARD LENGTH=68;>AT4G21105.2
8
6
4
2
0
3
6
1
0
5
9
3
3
0
0
0
1
8
10
6
10
drm.ugt80.
drm.wt.ugt80.
drm.smt1.
drm.wt.smt1.
dsm.ugt80.
dsm.wt.ugt80.
dsm.smt1.
dsm.wt.smt1.
sp.ugt80.
sp.wt.ugt80.
sp.smt1.
sp.wt.smt1.
im.ugt80.
im.wt.ugt80.
im.smt1.
im.wt.smt1.

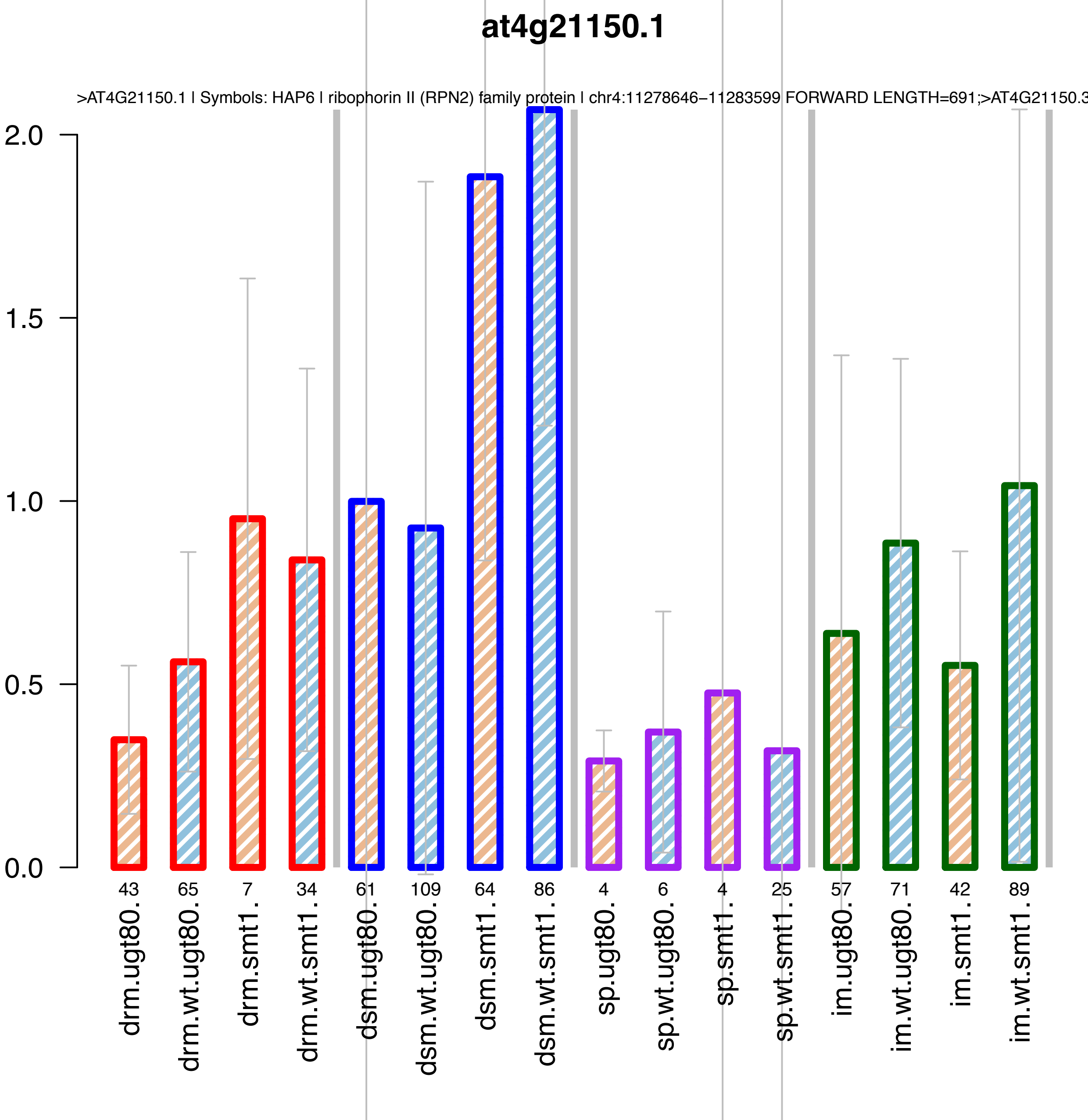

at4g21150.1
>AT4G21150.1 | Symbols: HAP6 | ribophorin II (RPN2) family protein | chr4:11278646-11283599 FORWARD LENGTH=691;>AT4G21150.3
2.0
1.5
1.0
0.5
0.0
43
65
7
34
61
109
64
86
4
6
4
25
57
71
42
89
drm.ugt80.
drm.wt.ugt80.
drm.smt1.
drm.wt.smt1.
dsm.ugt80.
dsm.wt.ugt80.
dsm.smt1.
dsm.wt.smt1.
sp.ugt80.
sp.wt.ugt80.
sp.smt1.
sp.wt.smt1.
im.ugt80.
im.wt.ugt80.
im.smt1.
im.wt.smt1.

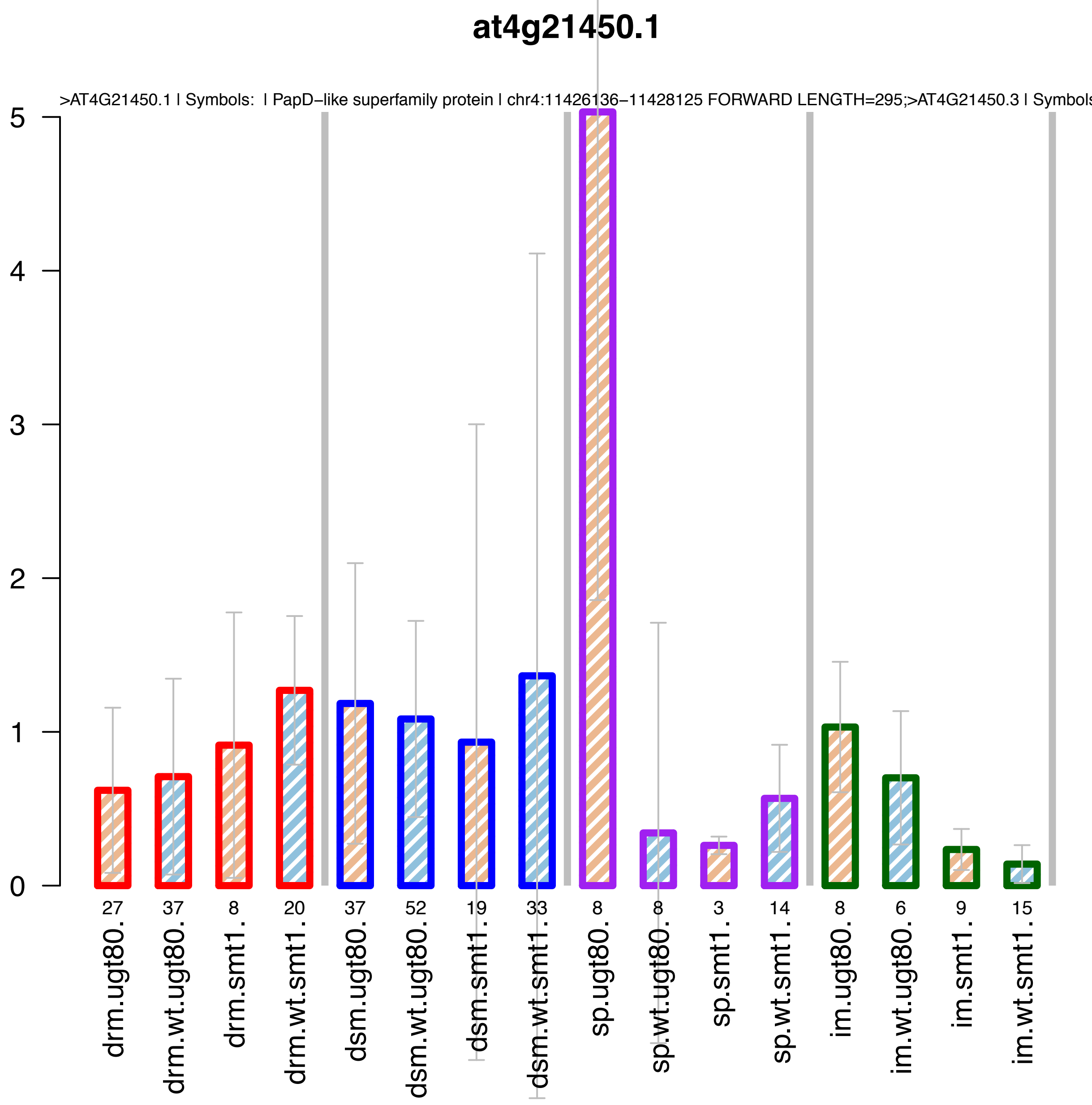

at4g21450.1
>AT4G21450.1 | Symbols: | PapD−like superfamily protein | chr4:11426136−11428125 FORWARD LENGTH=295;>AT4G21450.3 | Symbols
0
1
2
3
4
5
27
37
8
20
37
52
19
33
8
8
3
14
8
6
9
15
drm.ugt80.
drm.wt.ugt80.
drm.smt1.
drm.wt.smt1.
dsm.ugt80.
dsm.wt.ugt80.
dsm.smt1.
dsm.wt.smt1.
sp.ugt80.
sp.wt.ugt80.
sp.smt1.
sp.wt.smt1.
im.ugt80.
im.wt.ugt80.
im.smt1.
im.wt.smt1.

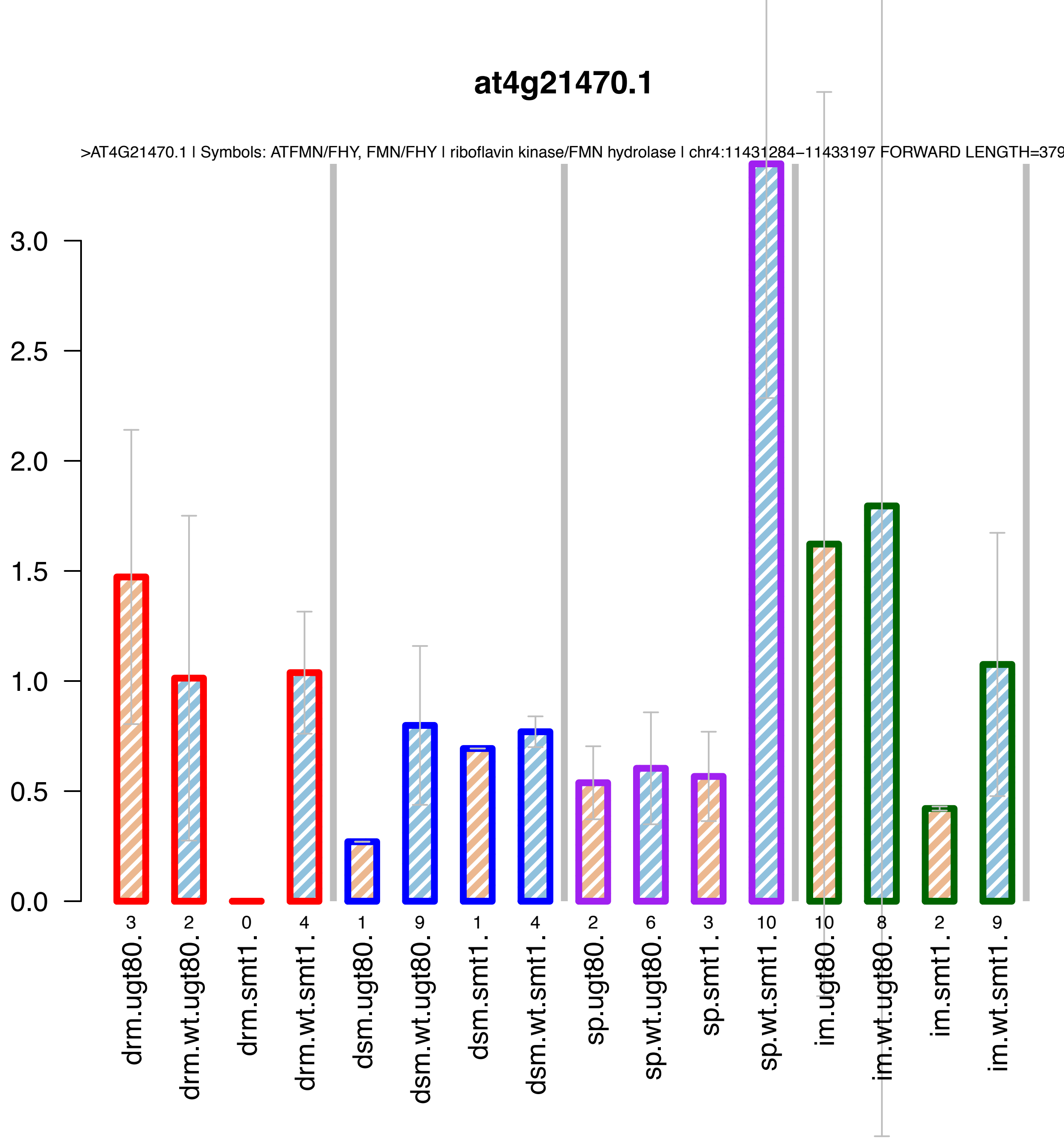

at4g21470.1
>AT4G21470.1 | Symbols: ATFMN/FHY, FMN/FHY | riboflavin kinase/FMN hydrolase | chr4:11431284-11433197 FORWARD LENGTH=379
3.0
2.5
2.0
1.5
1.0
0.5
0.0
3
2
0
4
1
9
1
4
2
6
3
10
10
8
2
9
drm.ugt80.
drm.wt.ugt80.
drm.smt1.
drm.wt.smt1.
dsm.ugt80.
dsm.wt.ugt80.
dsm.smt1.
dsm.wt.smt1.
sp.ugt80.
sp.wt.ugt80.
sp.smt1.
sp.wt.smt1.
im.ugt80.
im.wt.ugt80.
im.smt1.
im.wt.smt1.

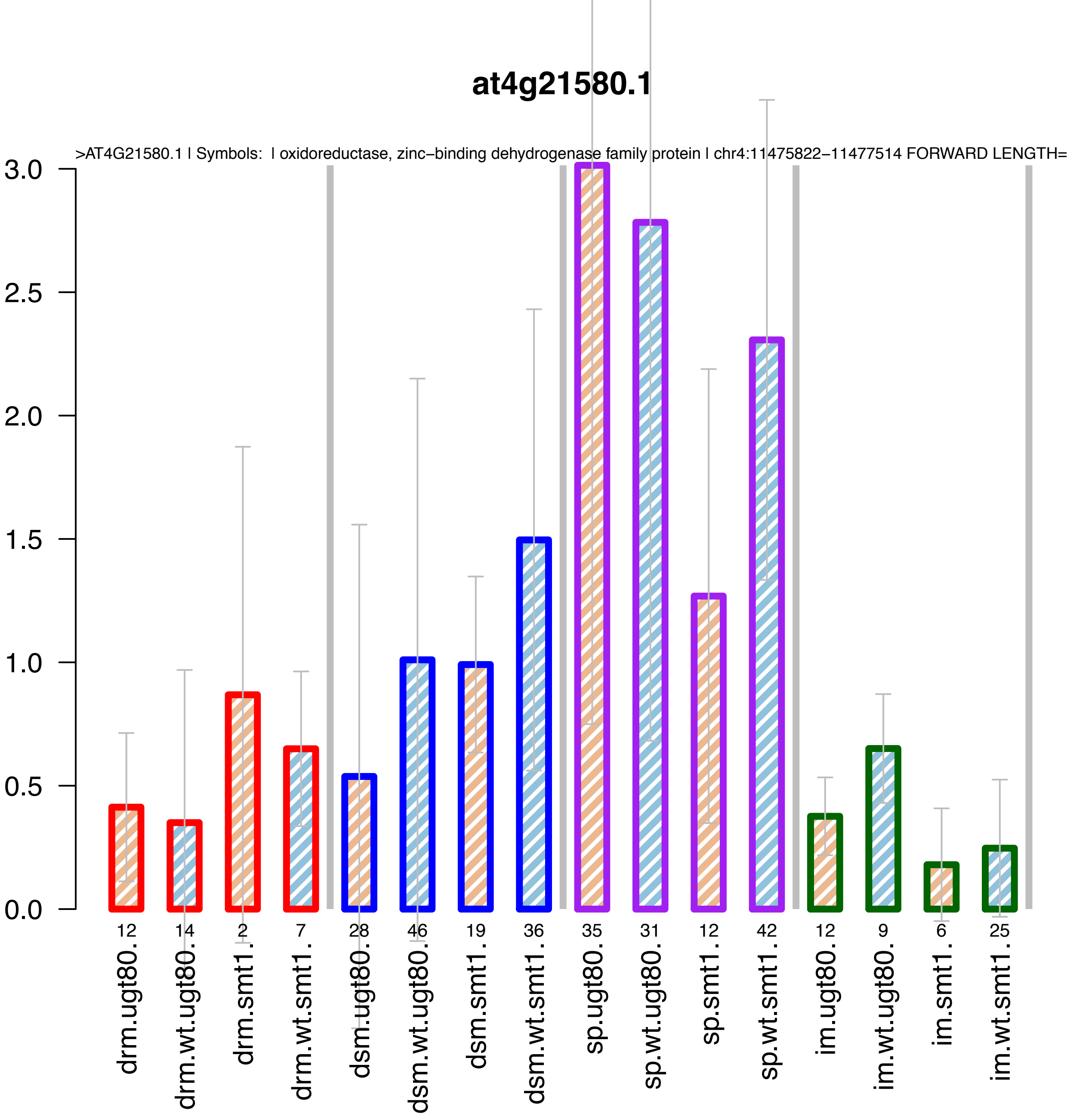

at4g21580.1
>AT4G21580.1 | Symbols: | oxidoreductase, zinc−binding dehydrogenase family protein | chr4:11475822−11477514 FORWARD LENGTH=
3.0
2.5
2.0
1.5
1.0
0.5
0.0
12
14
2
7
28
46
19
36
35
31
12
42
12
9
6
25
drm.ugt80..
drm.wt.ugt80..
drm.smt1..
drm.wt.smt1..
dsm.ugt80..
dsm.wt.ugt80..
dsm.smt1..
dsm.wt.smt1..
sp.ugt80..
sp.wt.ugt80..
sp.smt1..
sp.wt.smt1..
im.ugt80..
im.wt.ugt80..
im.smt1..
im.wt.smt1..

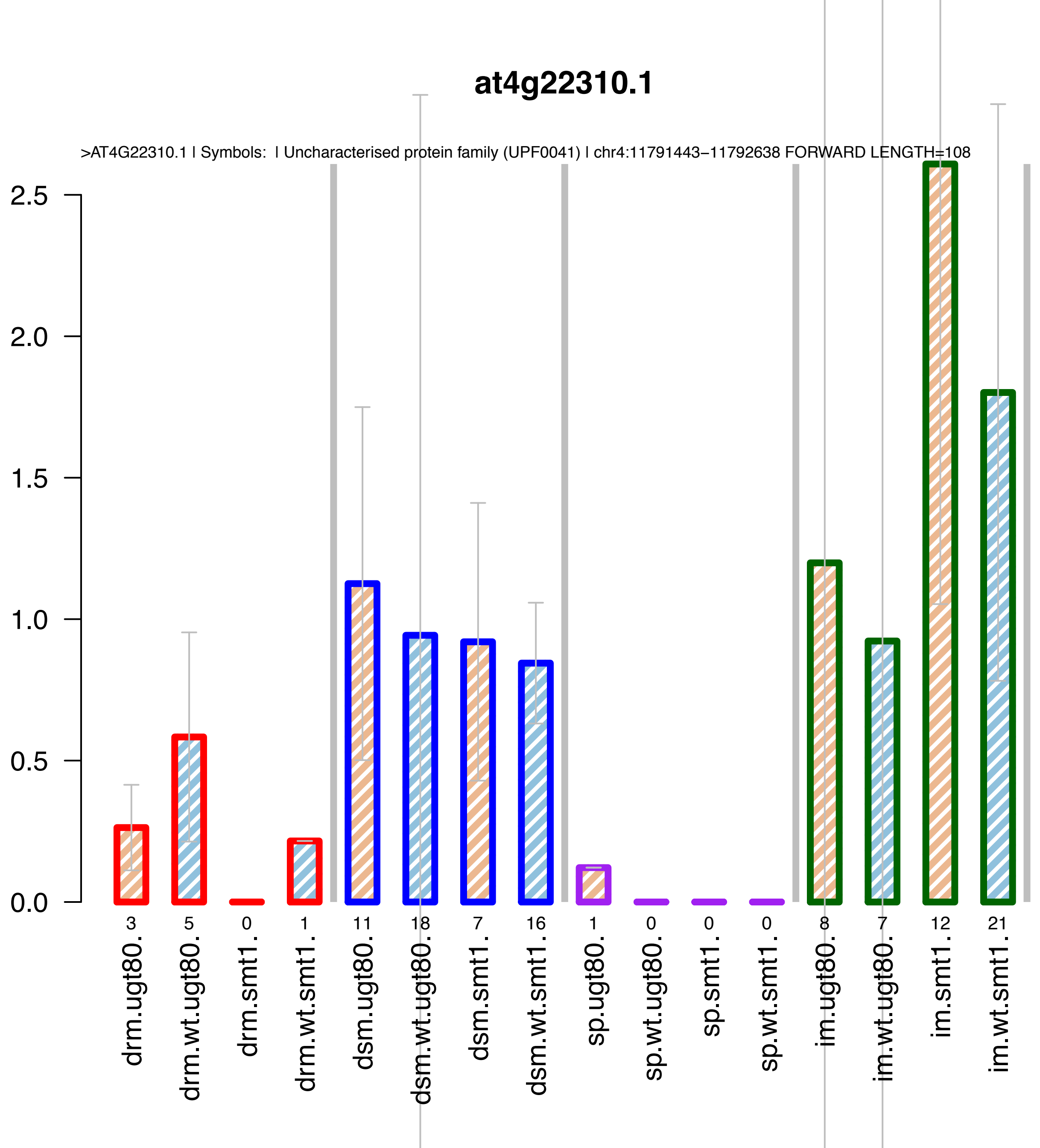

at4g22310.1
>AT4G22310.1 | Symbols: | Uncharacterised protein family (UPF0041) | chr4:11791443-11792638 FORWARD LENGTH=108
2.5
2.0
1.5
1.0
0.5
0.0
3
5
0
1
11
18
7
16
1
0
0
0
8
7
12
21
drm.ugt80.
drm.wt.ugt80.
drm.smt1.
drm.wt.smt1.
dsm.ugt80.
dsm.wt.ugt80.
dsm.smt1.
dsm.wt.smt1.
sp.ugt80.
sp.wt.ugt80.
sp.smt1.
sp.wt.smt1.
im.ugt80.
im.wt.ugt80.
im.smt1.
im.wt.smt1.

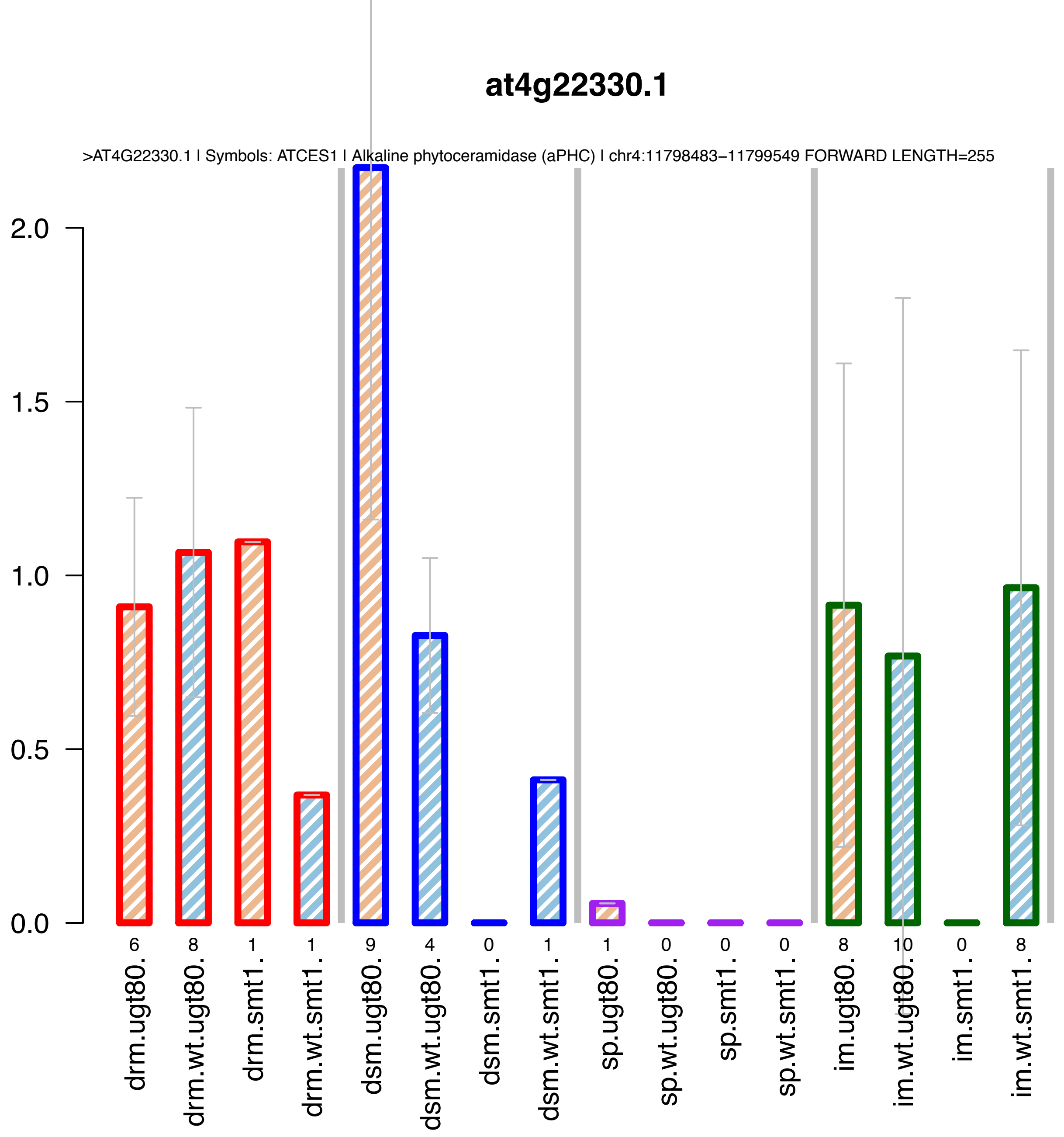

at4g22330.1
>AT4G22330.1 | Symbols: ATCES1 | Alkaline phytoceramidase (aPHC) | chr4:11798483-11799549 FORWARD LENGTH=255
2.0
1.5
1.0
0.5
0.0
6
8
1
1
9
4
0
1
1
0
0
0
8
10
0
8
drm.ugt80.
drm.wt.ugt80.
drm.smt1.
drm.wt.smt1.
dsm.ugt80.
dsm.wt.ugt80.
dsm.smt1.
dsm.wt.smt1.
sp.ugt80.
sp.wt.ugt80.
sp.smt1.
sp.wt.smt1.
im.ugt80.
im.wt.ugt80.
im.smt1.
im.wt.smt1.

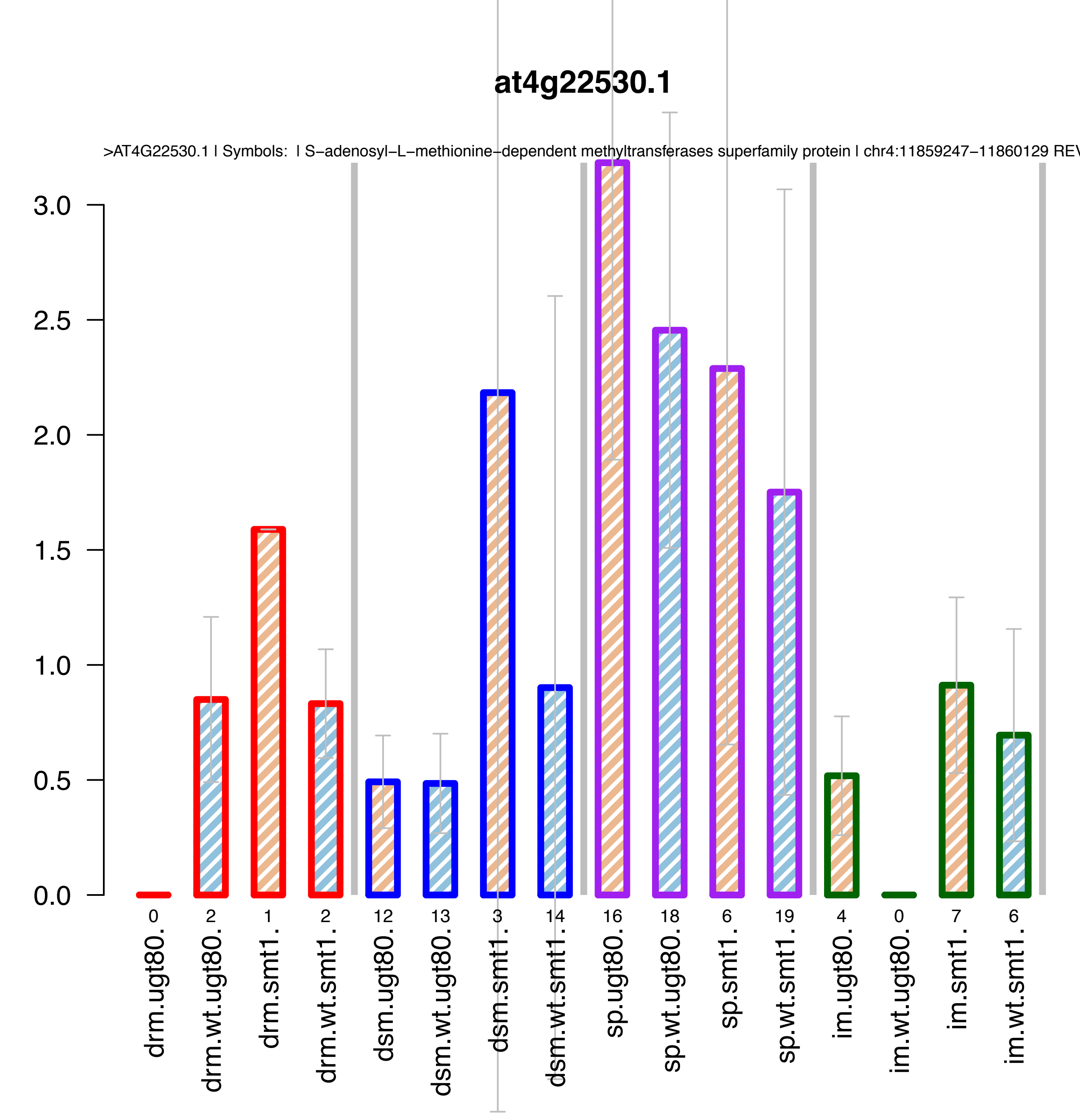

at4g22530.1
>AT4G22530.1 | Symbols: | S–adenosyl–L–methionine–dependent methyltransferases superfamily protein | chr4:11859247–11860129 REV
3.0
2.5
2.0
1.5
1.0
0.5
0.0
0
2
1
2
12
13
3
14
16
18
6
19
4
0
7
6
drm.ugt80.
drm.wt.ugt80.
drm.smt1.
drm.wt.smt1.
dsm.ugt80.
dsm.wt.ugt80.
dsm.smt1.
dsm.wt.smt1.
sp.ugt80.
sp.wt.ugt80.
sp.smt1.
sp.wt.smt1.
im.ugt80.
im.wt.ugt80.
im.smt1.
im.wt.smt1.

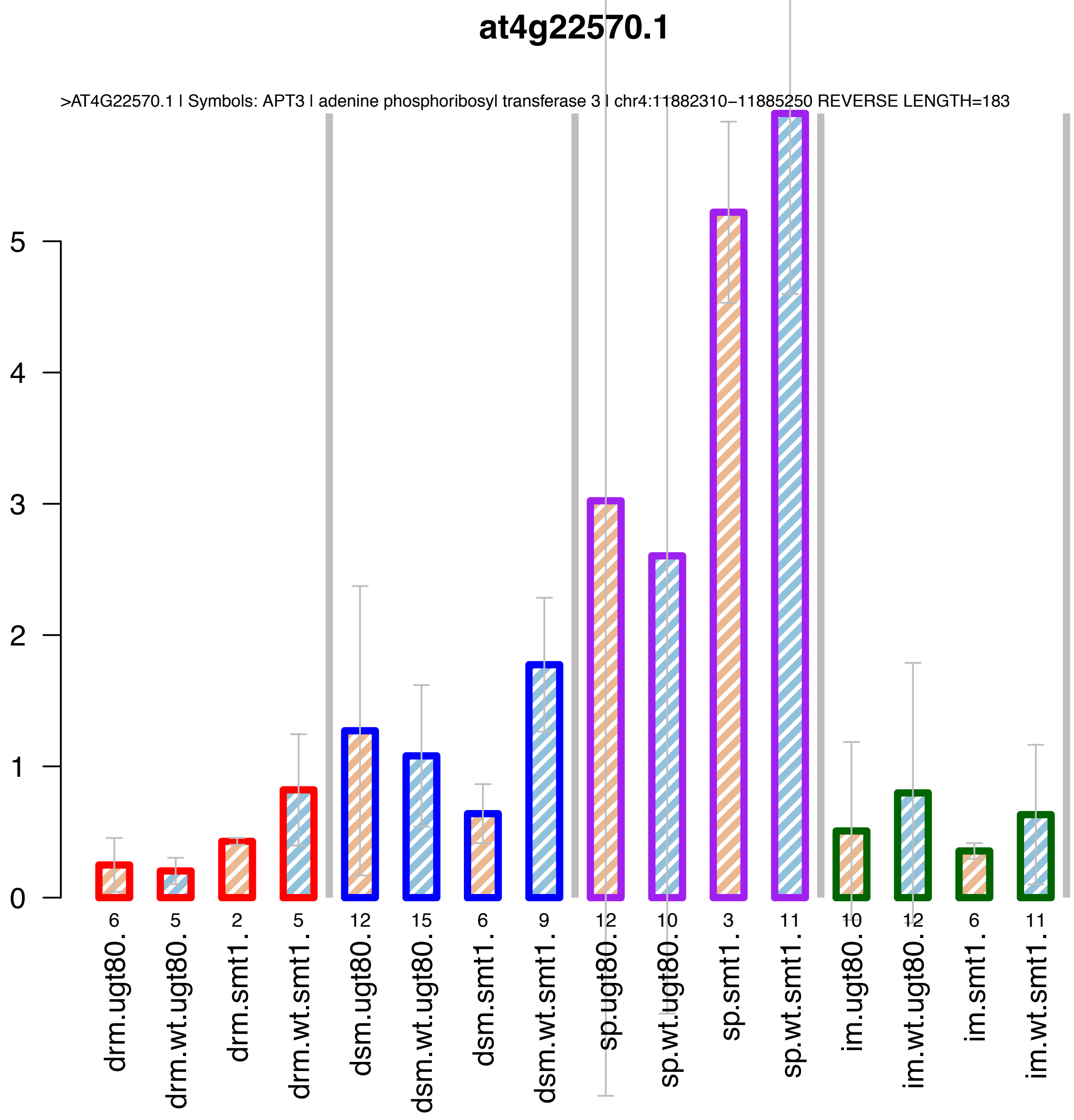
at4g22570.1
>AT4G22570.1 | Symbols: APT3 | adenine phosphoribosyl transferase 3 | chr4:11882310−11885250 REVERSE LENGTH=183
0
1
2
3
4
5
6
5
2
5
12
15
6
9
12
10
3
11
10
12
6
11
drm.ugt80.
drm.wt.ugt80.
drm.smt1.
drm.wt.smt1.
dsm.ugt80.
dsm.wt.ugt80.
dsm.smt1.
dsm.wt.smt1.
sp.ugt80.
sp.wt.ugt80.
sp.smt1.
sp.wt.smt1.
im.ugt80.
im.wt.ugt80.
im.smt1.
im.wt.smt1.

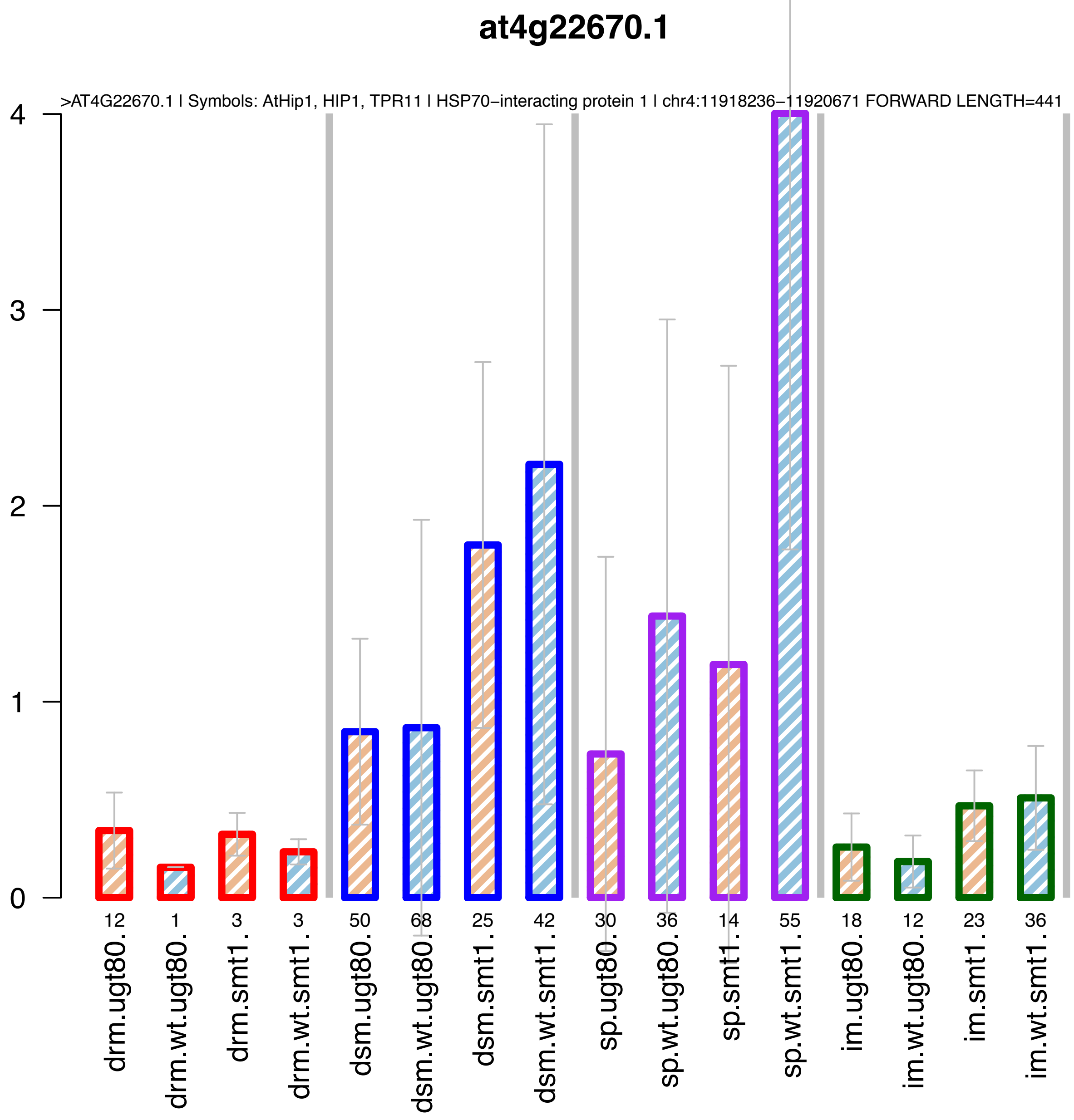

at4g22670.1
>AT4G22670.1 | Symbols: AtHip1, HIP1, TPR11 | HSP70−interacting protein 1 | chr4:11918236−11920671 FORWARD LENGTH=441
4
3
2
1
0
12
1
3
3
50
68
25
42
30
36
14
55
18
12
23
36
drm.ugt80.
drm.wt.ugt80.
drm.smt1.
drm.wt.smt1.
dsm.ugt80.
dsm.wt.ugt80.
dsm.smt1.
dsm.wt.smt1.
sp.ugt80.
sp.wt.ugt80.
sp.smt1.
sp.wt.smt1.
im.ugt80.
im.wt.ugt80.
im.smt1.
im.wt.smt1.

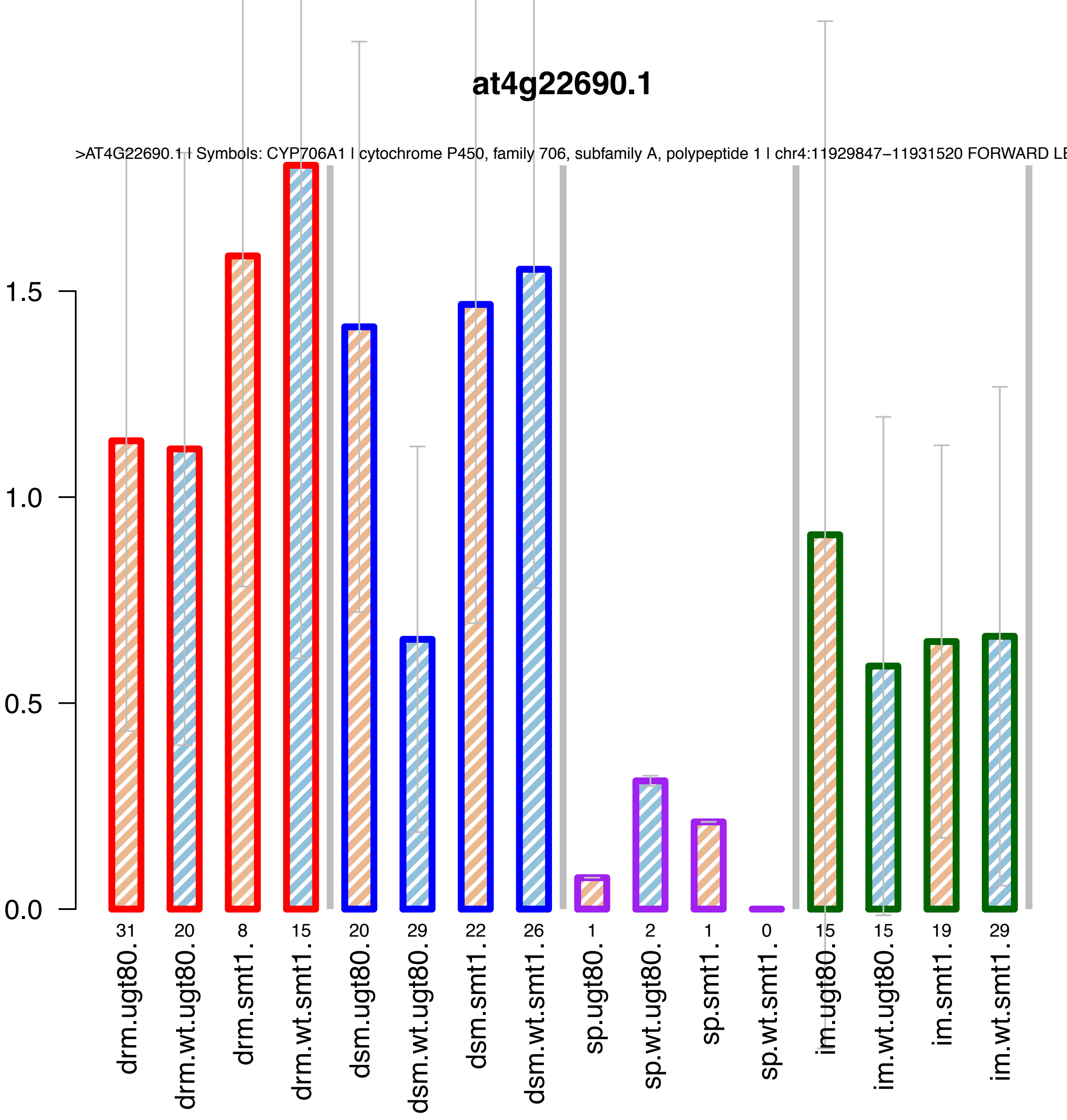

at4g22690.1
>AT4G22690.1 | Symbols: CYP706A1 | cytochrome P450, family 706, subfamily A, polypeptide 1 | chr4:11929847-11931520 FORWARD LE
1.5
1.0
0.5
0.0
31
20
8
15
20
29
22
26
1
2
1
0
15
15
19
29
drm.ugt80.
drm.wt.ugt80.
drm.smt1.
drm.wt.smt1.
dsm.ugt80.
dsm.wt.ugt80.
dsm.smt1.
dsm.wt.smt1.
sp.ugt80.
sp.wt.ugt80.
sp.smt1.
sp.wt.smt1.
im.ugt80.
im.wt.ugt80.
im.smt1.
im.wt.smt1.

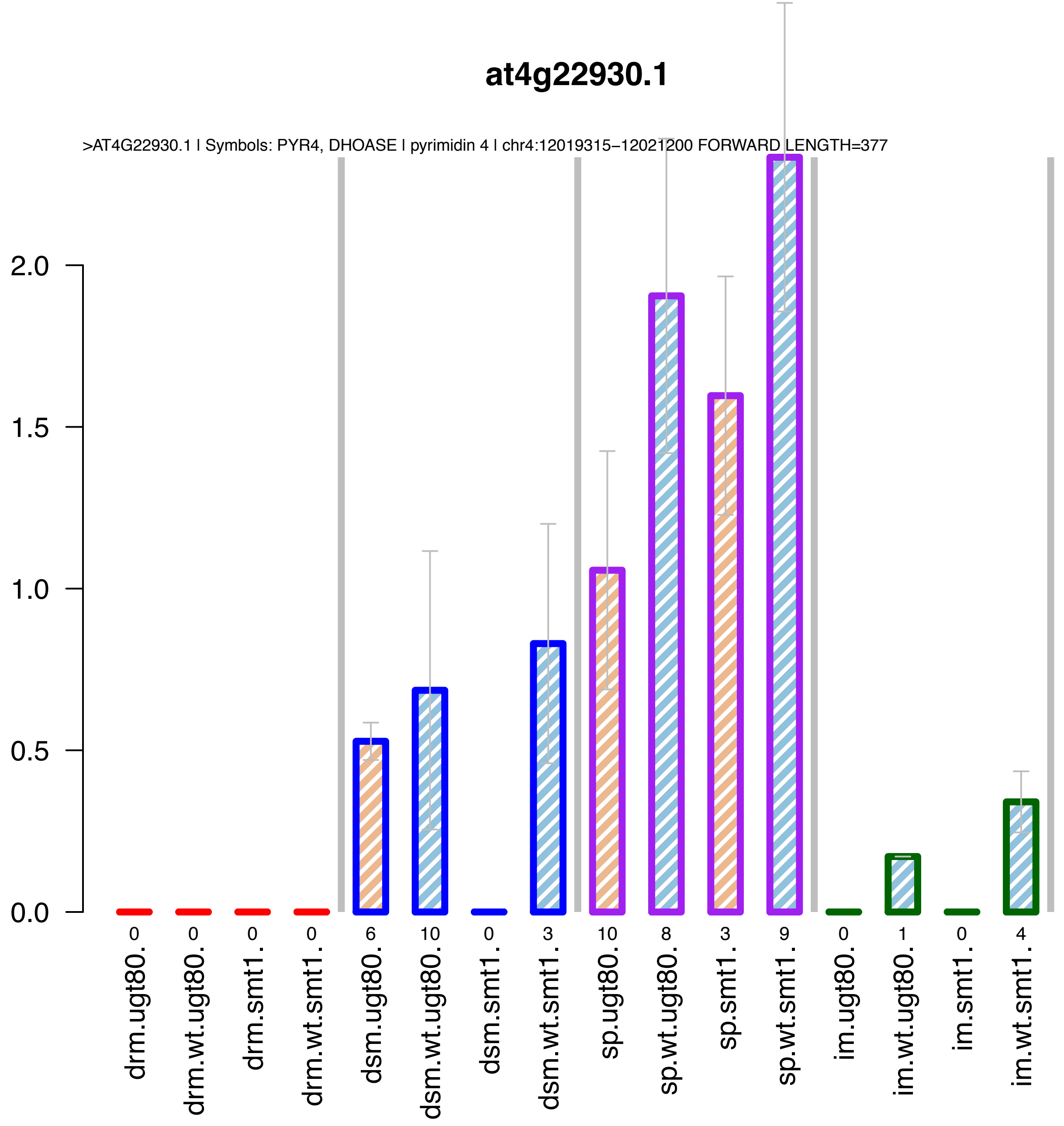

at4g22930.1
>AT4G22930.1 | Symbols: PYR4, DHOASE | pyrimidin 4 | chr4:12019315-12021200 FORWARD LENGTH=377
2.0
1.5
1.0
0.5
0.0
0
0
0
0
6
10
0
3
10
8
3
9
0
1
0
4
drm.ugt80.
drm.wt.ugt80.
drm.smt1.
drm.wt.smt1.
dsm.ugt80.
dsm.wt.ugt80.
dsm.smt1.
dsm.wt.smt1.
sp.ugt80.
sp.wt.ugt80.
sp.smt1.
sp.wt.smt1.
im.ugt80.
im.wt.ugt80.
im.smt1.
im.wt.smt1.

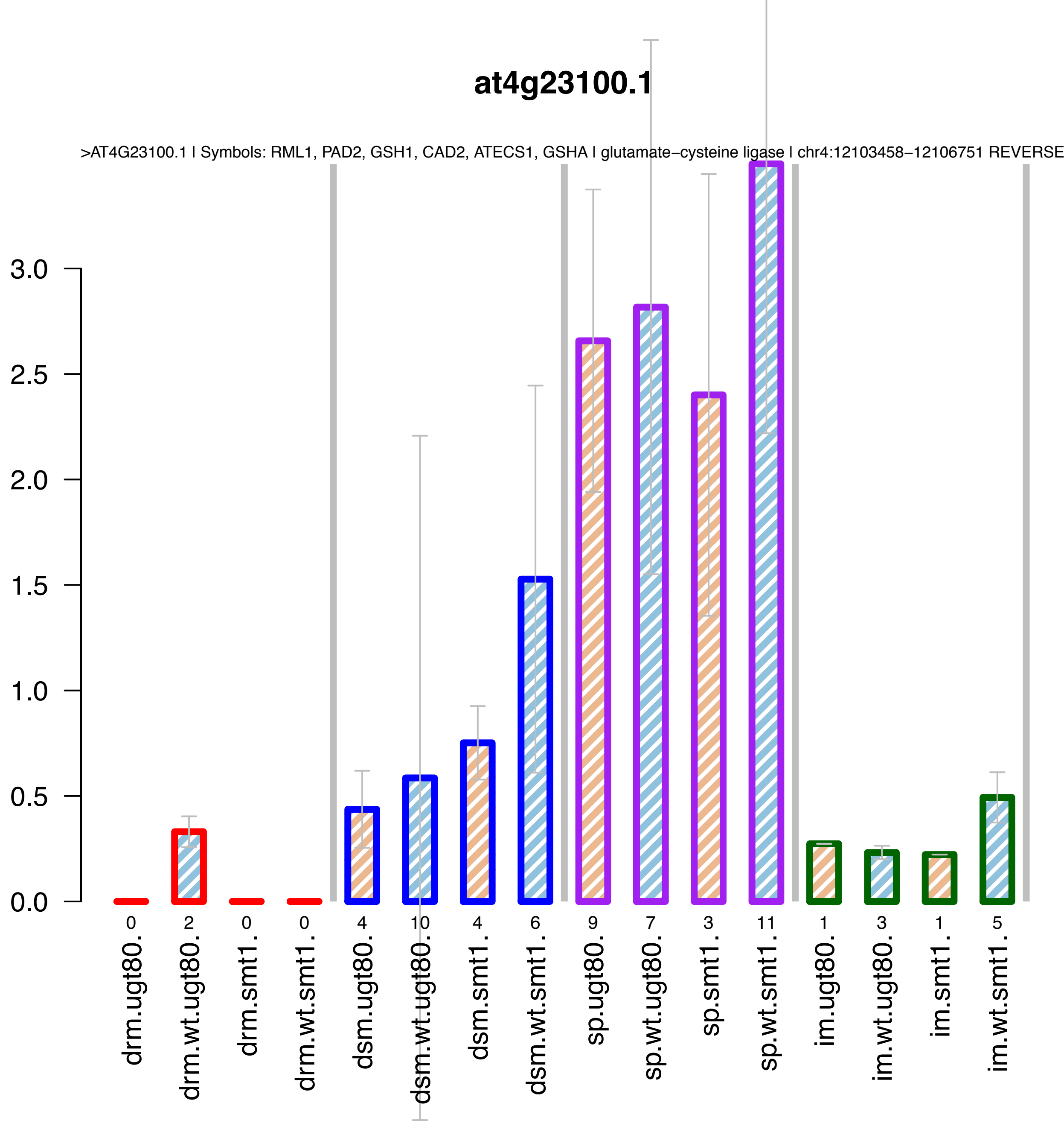

at4g23100.1
>AT4G23100.1 | Symbols: RML1, PAD2, GSH1, CAD2, ATECS1, GSHA | glutamate−cysteine ligase | chr4:12103458−12106751 REVERSE
3.0
2.5
2.0
1.5
1.0
0.5
0.0
0
2
0
0
4
10
4
6
9
7
3
11
1
3
1
5
drm.ugt80.
drm.wt.ugt80.
drm.smt1.
drm.wt.smt1.
dsm.ugt80.
dsm.wt.ugt80.
dsm.smt1.
dsm.wt.smt1.
sp.ugt80.
sp.wt.ugt80.
sp.smt1.
sp.wt.smt1.
im.ugt80.
im.wt.ugt80.
im.smt1.
im.wt.smt1.

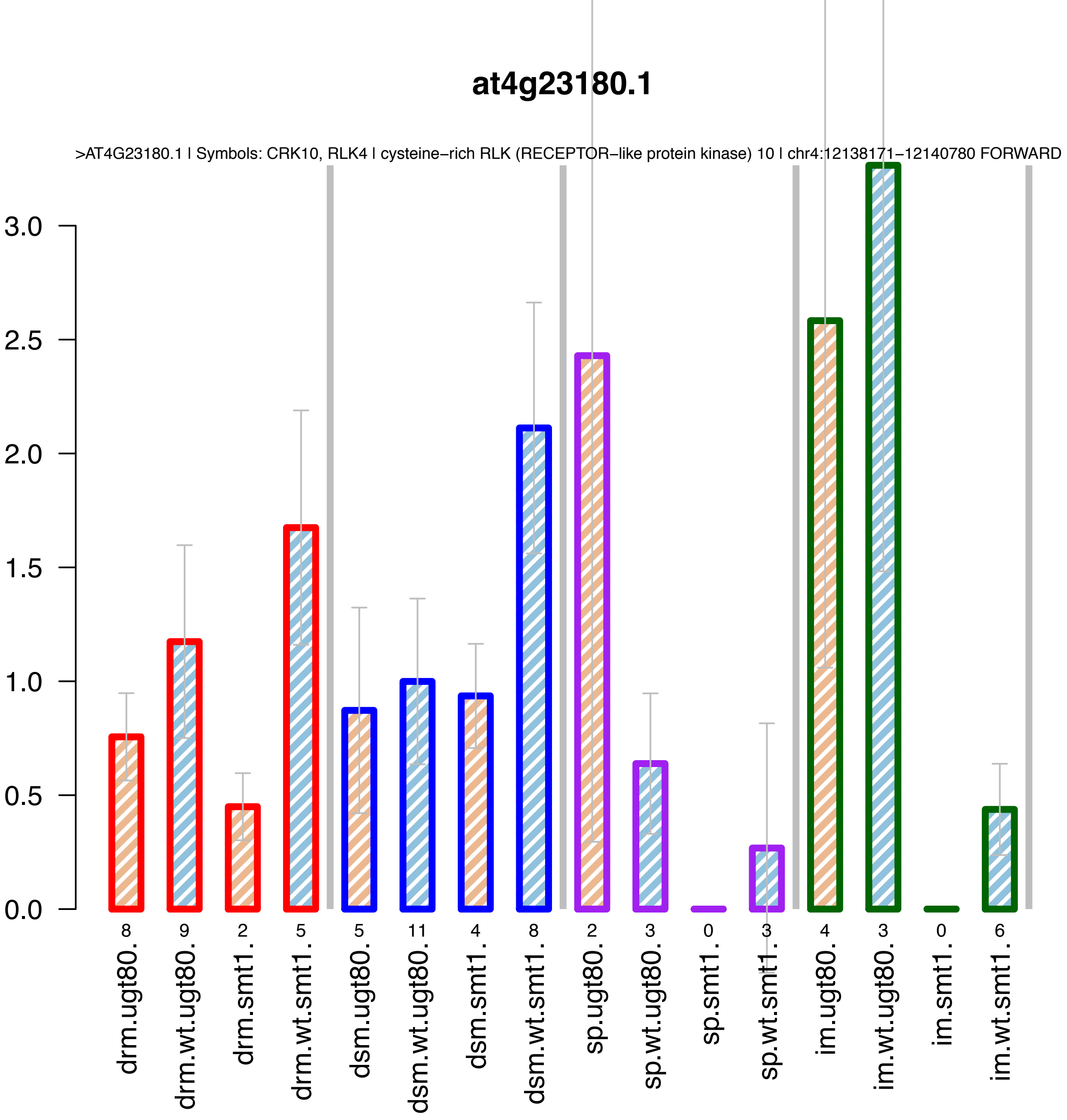

at4g23180.1
>AT4G23180.1 | Symbols: CRK10, RLK4 | cysteine−rich RLK (RECEPTOR−like protein kinase) 10 | chr4:12138171−12140780 FORWARD
3.0
2.5
2.0
1.5
1.0
0.5
0.0
8
9
2
5
5
11
4
8
2
3
0
3
4
3
0
6
drm.ugt80..
drm.wt.ugt80..
drm.smt1..
drm.wt.smt1..
dsm.ugt80..
dsm.wt.ugt80..
dsm.smt1..
dsm.wt.smt1..
sp.ugt80..
sp.wt.ugt80..
sp.smt1..
sp.wt.smt1..
im.ugt80..
im.wt.ugt80..
im.smt1..
im.wt.smt1..

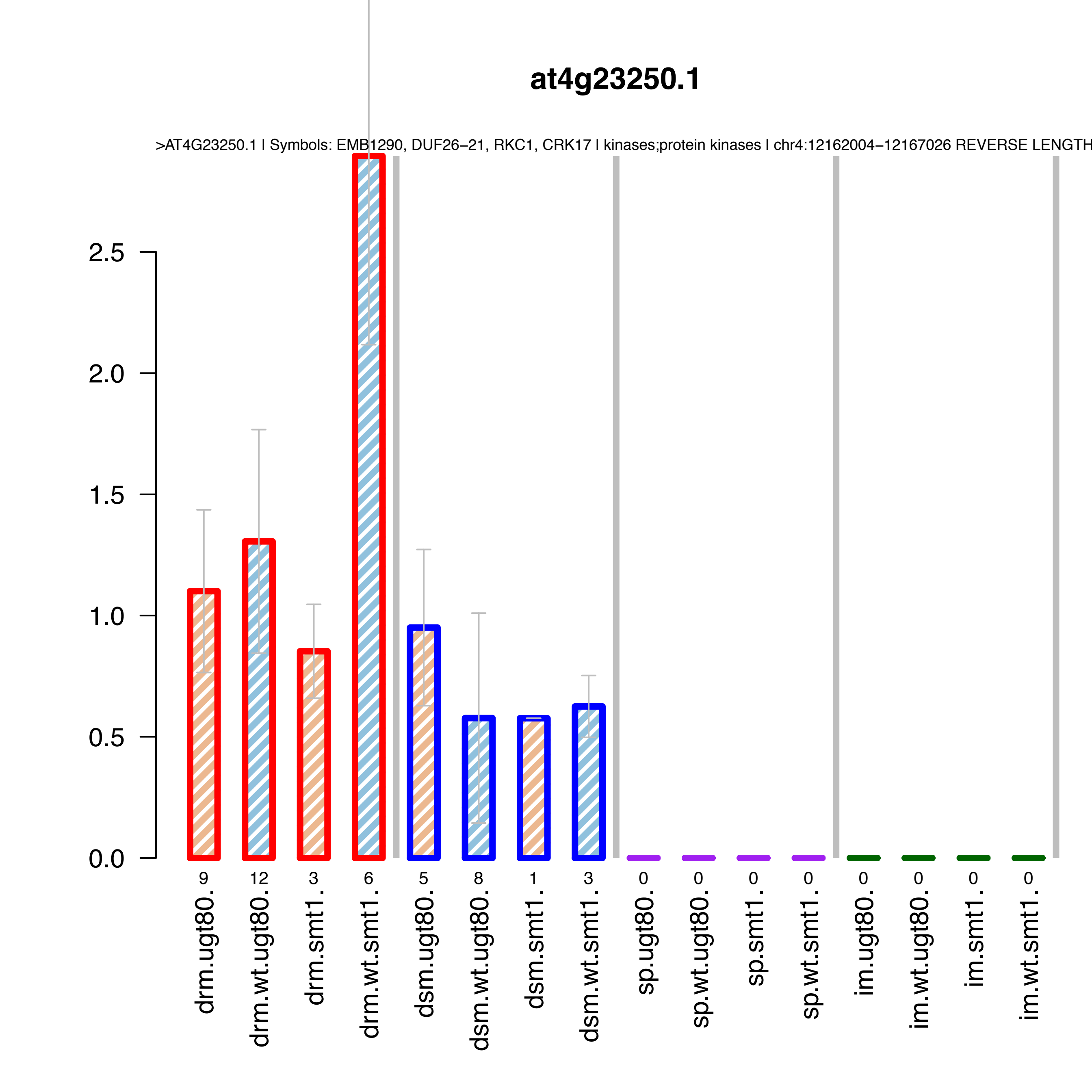

at4g23250.1
>AT4G23250.1 | Symbols: EMB1290, DUF26-21, RKC1, CRK17 | kinases;protein kinases | chr4:12162004-12167026 REVERSE LENGTH
0.0
0.5
1.0
1.5
2.0
2.5
9
12
3
6
5
8
1
3
0
0
0
0
0
0
0
0
drm.ugt80.
drm.wt.ugt80.
drm.smt1.
drm.wt.smt1.
dsm.ugt80.
dsm.wt.ugt80.
dsm.smt1.
dsm.wt.smt1.
sp.ugt80.
sp.wt.ugt80.
sp.smt1.
sp.wt.smt1.
im.ugt80.
im.wt.ugt80.
im.smt1.
im.wt.smt1.

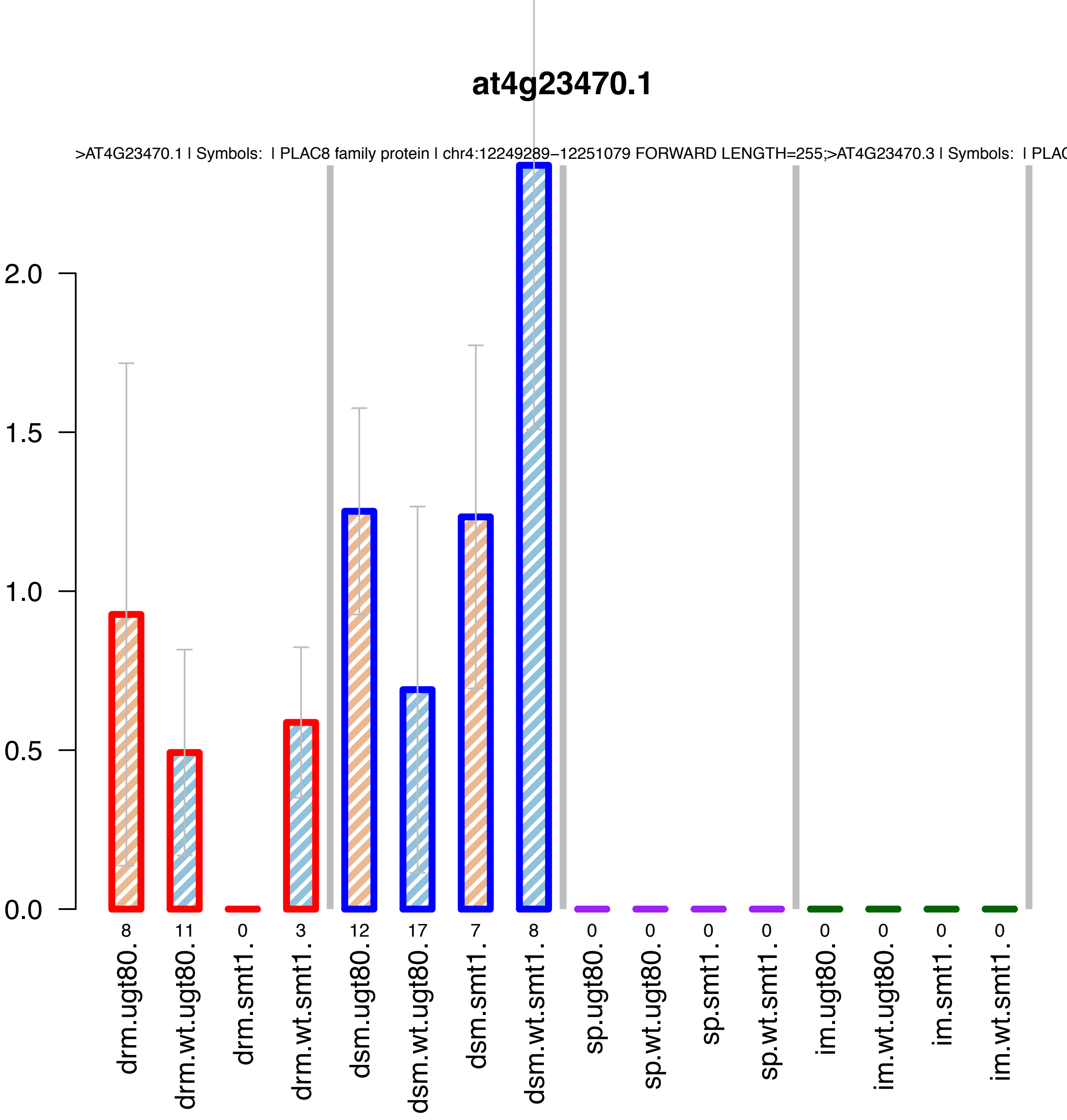

at4g23470.1
>AT4G23470.1 | Symbols: | PLAC8 family protein | chr4:12249289−12251079 FORWARD LENGTH=255;>AT4G23470.3 | Symbols: | PLAC
2.0
1.5
1.0
0.5
0.0
8
11
0
3
12
17
7
8
0
0
0
0
0
0
0
0
drm.ugt80.
drm.wt.ugt80.
drm.smt1.
drm.wt.smt1.
dsm.ugt80.
dsm.wt.ugt80.
dsm.smt1.
dsm.wt.smt1.
sp.ugt80.
sp.wt.ugt80.
sp.smt1.
sp.wt.smt1.
im.ugt80.
im.wt.ugt80.
im.smt1.
im.wt.smt1.

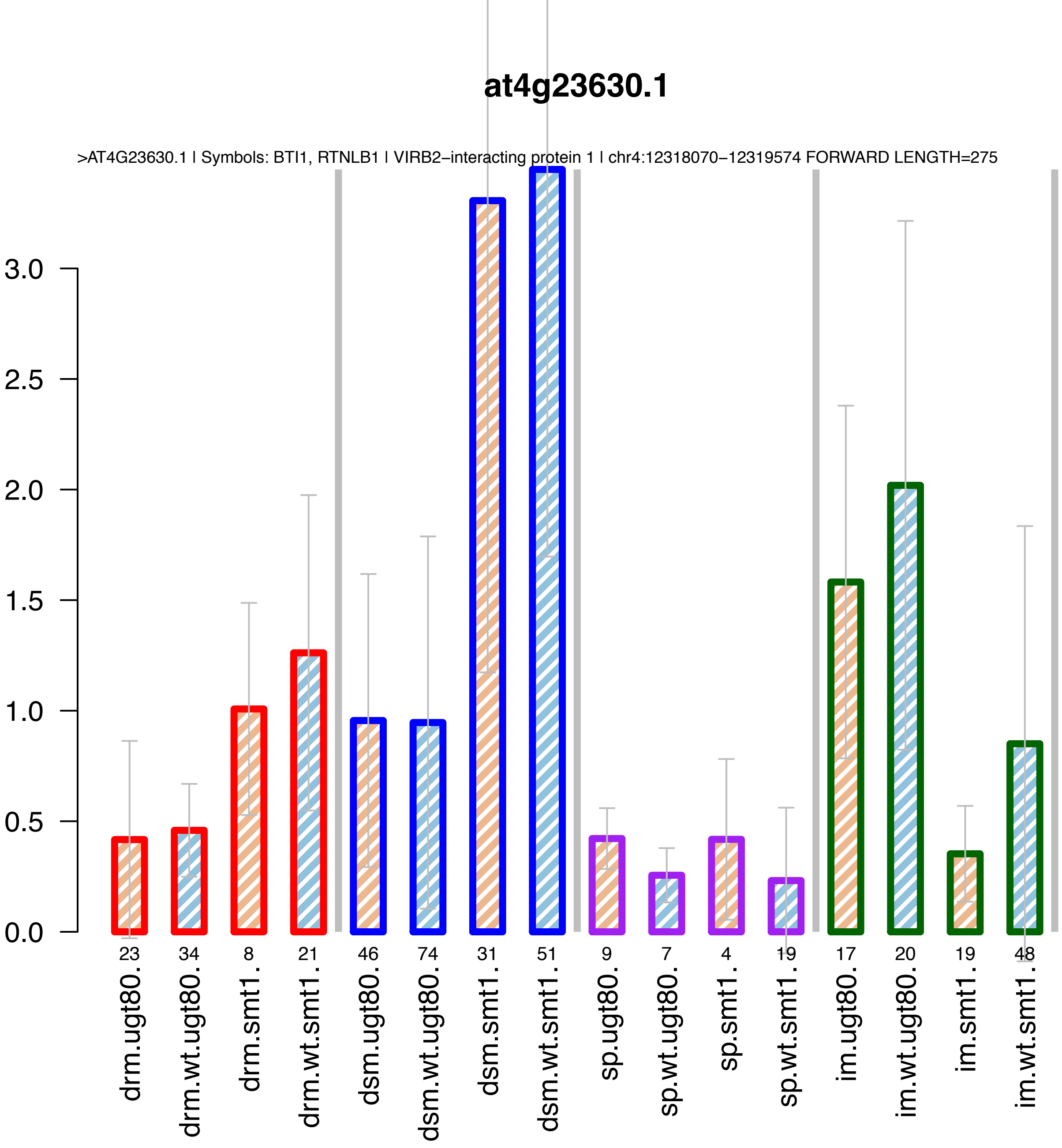

at4g23630.1
>AT4G23630.1 | Symbols: BTI1, RTNLB1 | VIRB2−interacting protein 1 | chr4:12318070−12319574 FORWARD LENGTH=275
0.0
0.5
1.0
1.5
2.0
2.5
3.0
23
34
8
21
46
74
31
51
9
7
4
19
17
20
19
48
drm.ugt80..
drm.wt.ugt80..
drm.smt1..
drm.wt.smt1..
dsm.ugt80..
dsm.wt.ugt80..
dsm.smt1..
dsm.wt.smt1..
sp.ugt80..
sp.wt.ugt80..
sp.smt1..
sp.wt.smt1..
im.ugt80..
im.wt.ugt80..
im.smt1..
im.wt.smt1..

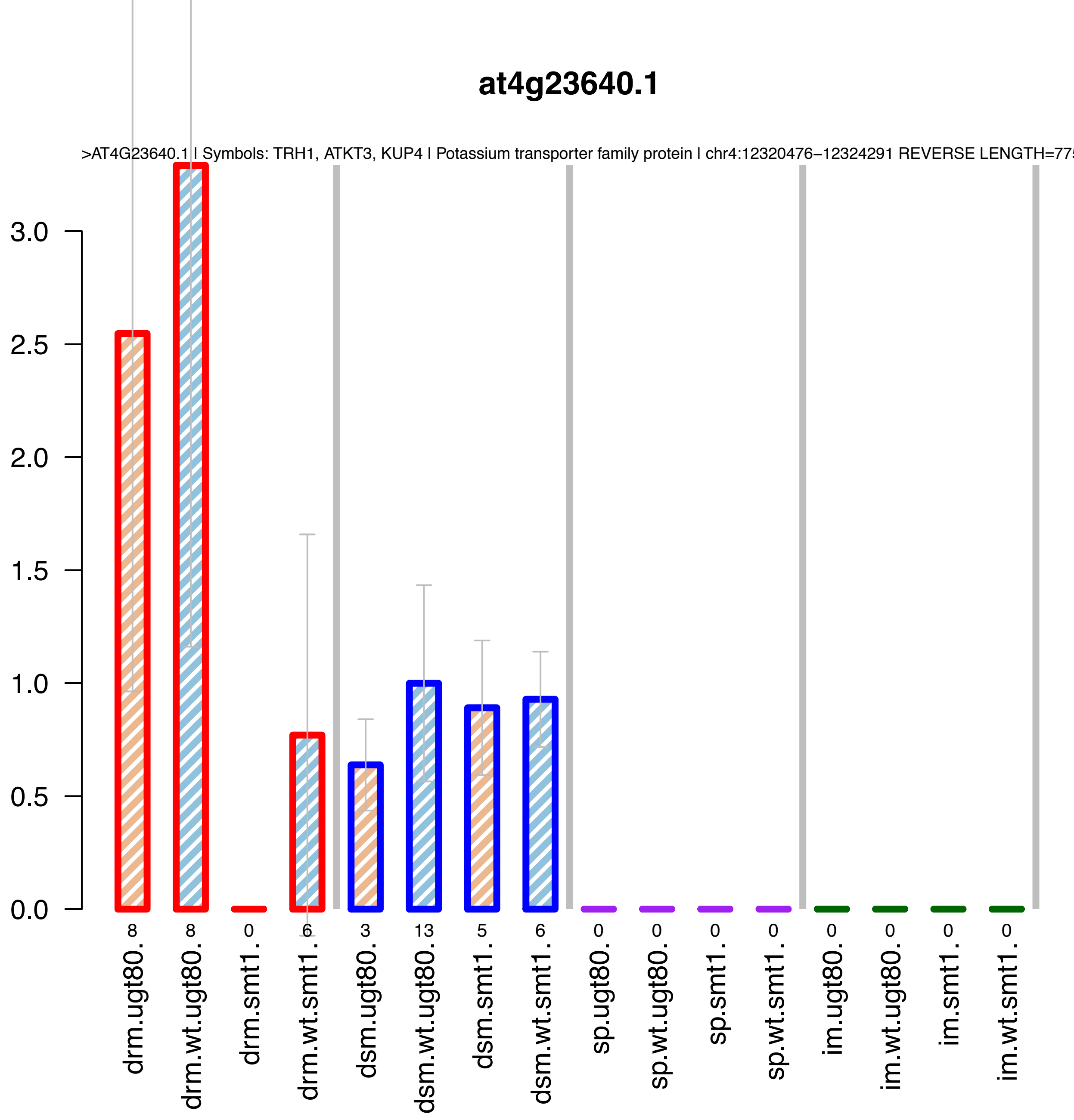

at4g23640.1
>AT4G23640.1 | Symbols: TRH1, ATKT3, KUP4 | Potassium transporter family protein | chr4:12320476–12324291 REVERSE LENGTH=775
3.0
2.5
2.0
1.5
1.0
0.5
0.0
8
8
0
6
3
13
5
6
0
0
0
0
0
0
0
0
drm.ugt80.
drm.wt.ugt80.
drm.smt1.
drm.wt.smt1.
dsm.ugt80.
dsm.wt.ugt80.
dsm.smt1.
dsm.wt.smt1.
sp.ugt80.
sp.wt.ugt80.
sp.smt1.
sp.wt.smt1.
im.ugt80.
im.wt.ugt80.
im.smt1.
im.wt.smt1.

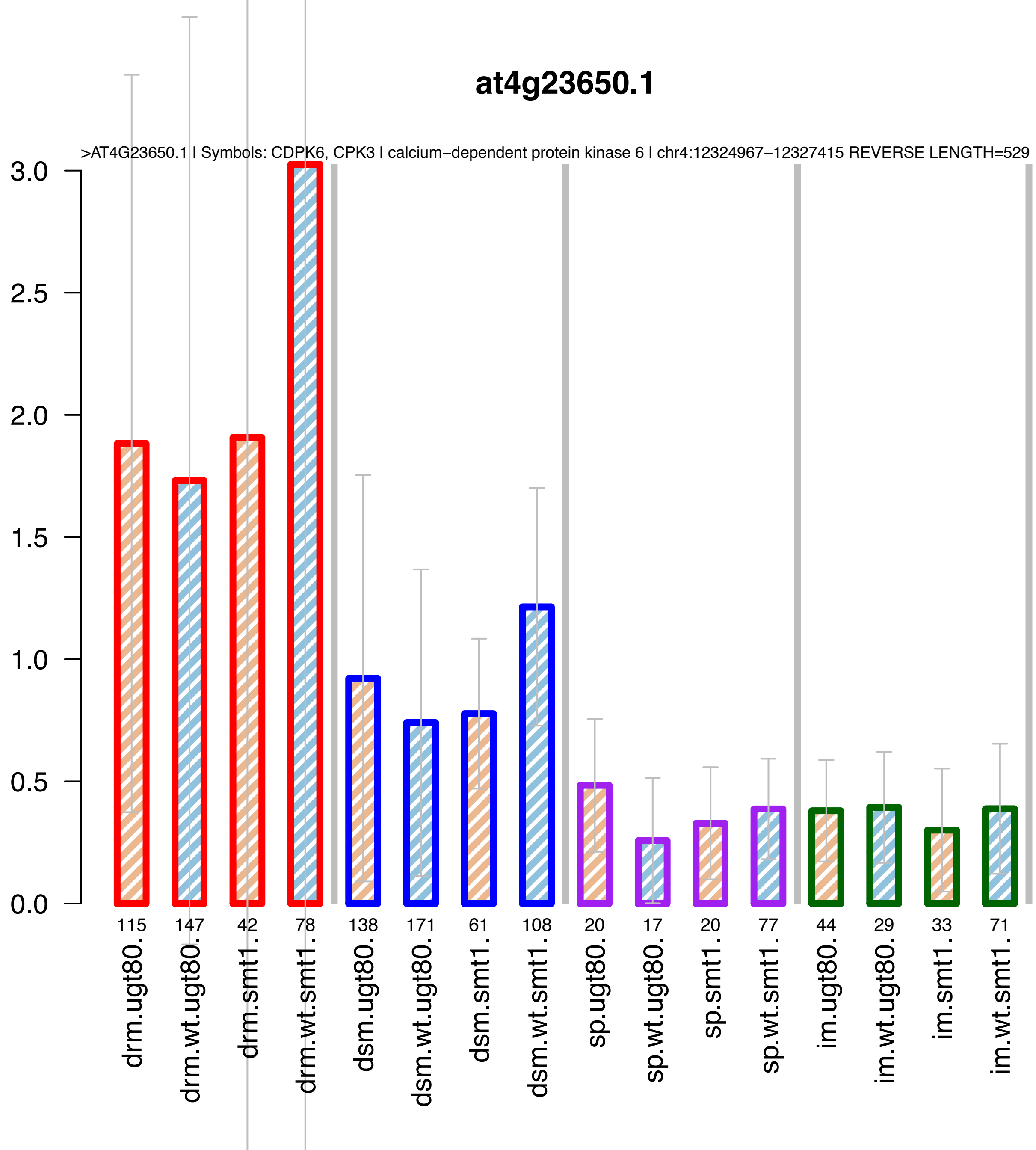

at4g23650.1
>AT4G23650.1 | Symbols: CDPK6, CPK3 | calcium-dependent protein kinase 6 | chr4:12324967-12327415 REVERSE LENGTH=529
3.0
2.5
2.0
1.5
1.0
0.5
0.0
115
147
42
78
138
171
61
108
20
17
20
77
44
29
33
71
drm.ugt80..
drm.wt.ugt80..
drm.smt1..
drm.wt.smt1..
dsm.ugt80..
dsm.wt.ugt80..
dsm.smt1..
dsm.wt.smt1..
sp.ugt80..
sp.wt.ugt80..
sp.smt1..
sp.wt.smt1..
im.ugt80..
im.wt.ugt80..
im.smt1..
im.wt.smt1..

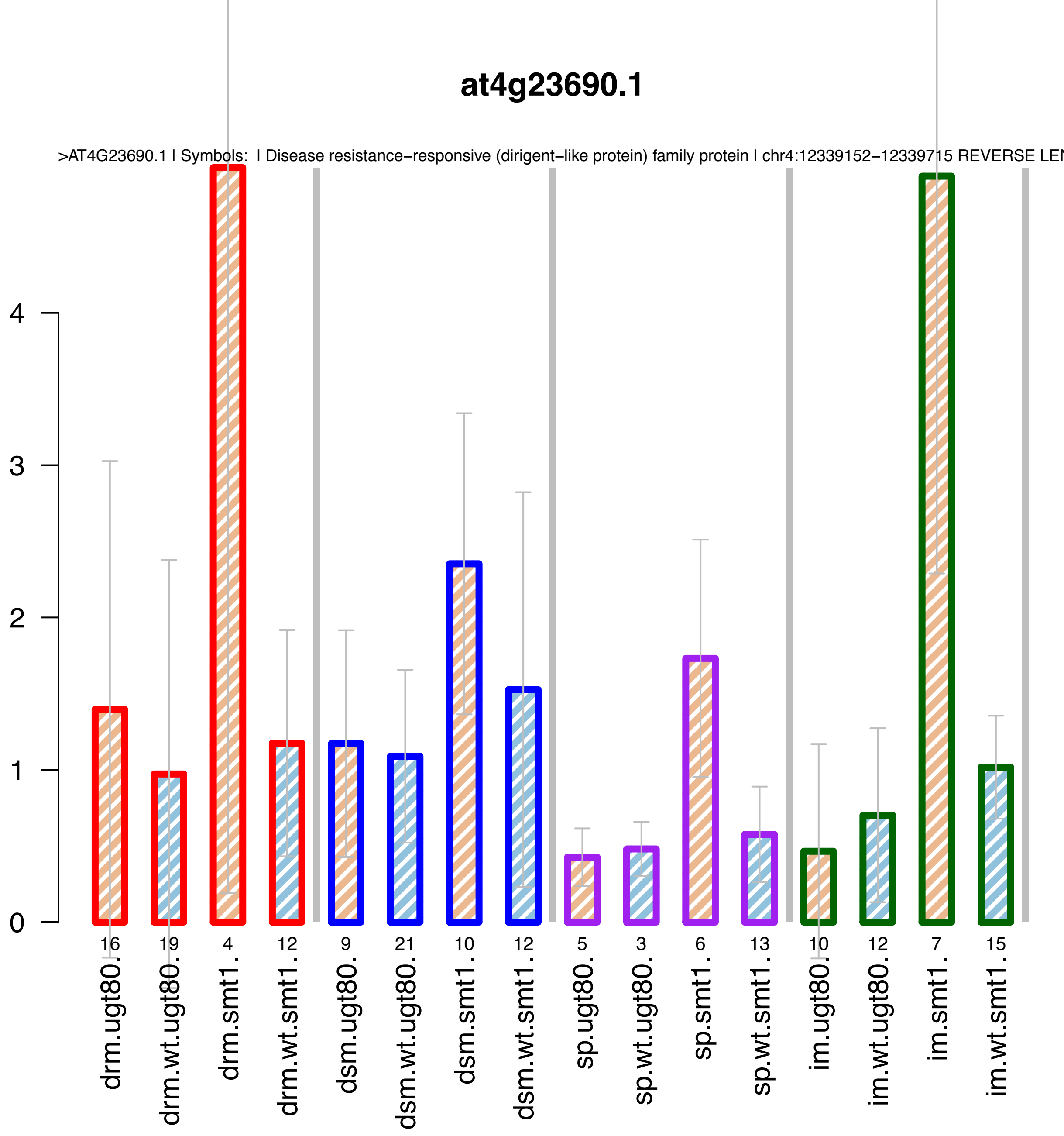

at4g23690.1
>AT4G23690.1 | Symbols: | Disease resistance−responsive (dirigent−like protein) family protein | chr4:12339152−12339715 REVERSE LEN
0
1
2
3
4
16
19
4
12
9
21
10
12
5
3
6
13
10
12
7
15
drm.ugt80.
drm.wt.ugt80.
drm.smt1.
drm.wt.smt1.
dsm.ugt80.
dsm.wt.ugt80.
dsm.smt1.
dsm.wt.smt1.
sp.ugt80.
sp.wt.ugt80.
sp.smt1.
sp.wt.smt1.
im.ugt80.
im.wt.ugt80.
im.smt1.
im.wt.smt1.

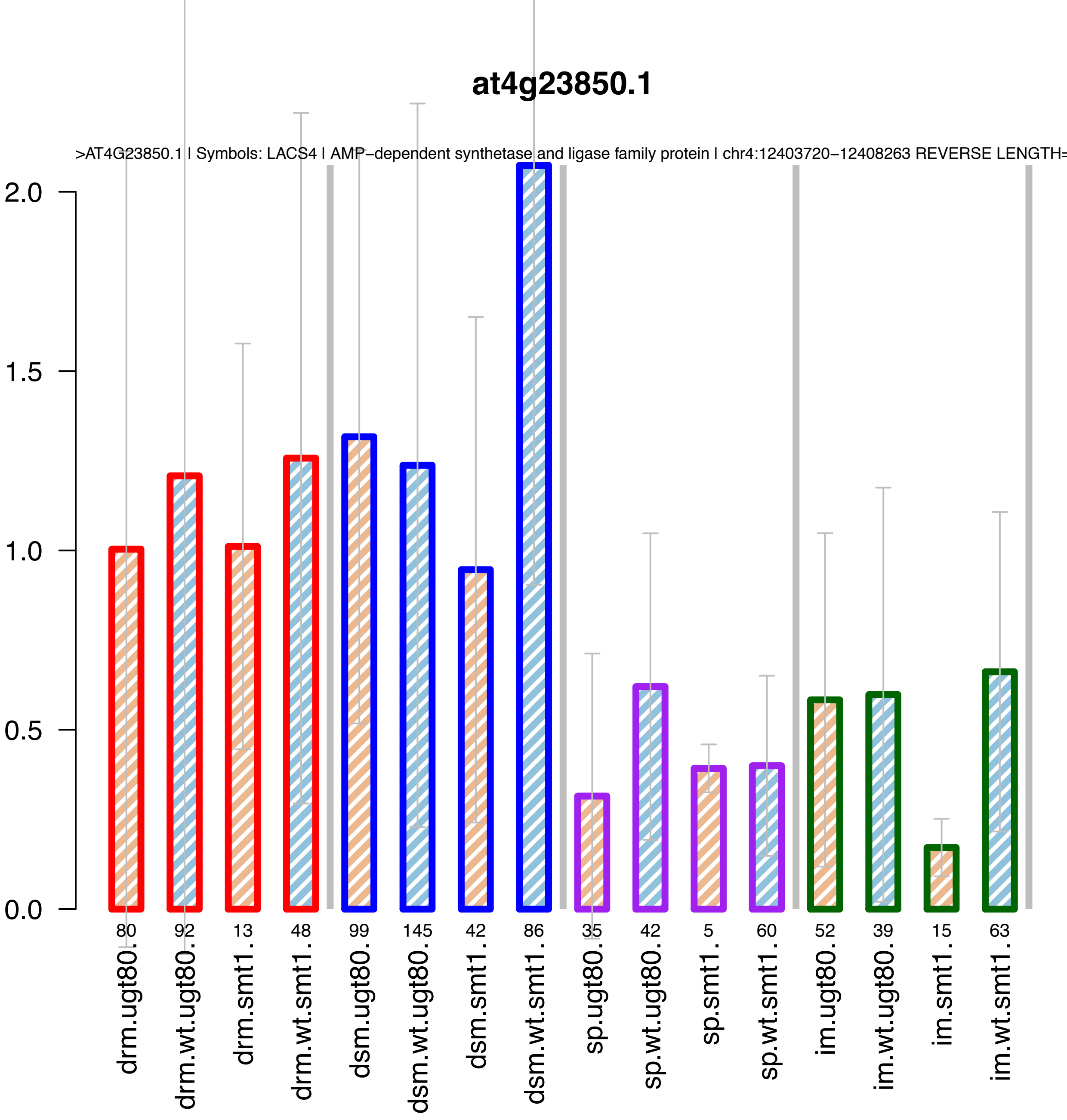
at4g23850.1
>AT4G23850.1 | Symbols: LACS4 | AMP−dependent synthetase and ligase family protein | chr4:12403720−12408263 REVERSE LENGTH=
2.0
1.5
1.0
0.5
0.0
80
92
13
48
99
145
42
86
35
42
5
60
52
39
15
63
drm.ugt80.
drm.wt.ugt80.
drm.smt1.
drm.wt.smt1.
dsm.ugt80.
dsm.wt.ugt80.
dsm.smt1.
dsm.wt.smt1.
sp.ugt80.
sp.wt.ugt80.
sp.smt1.
sp.wt.smt1.
im.ugt80.
im.wt.ugt80.
im.smt1.
im.wt.smt1.

# at4g23885.1

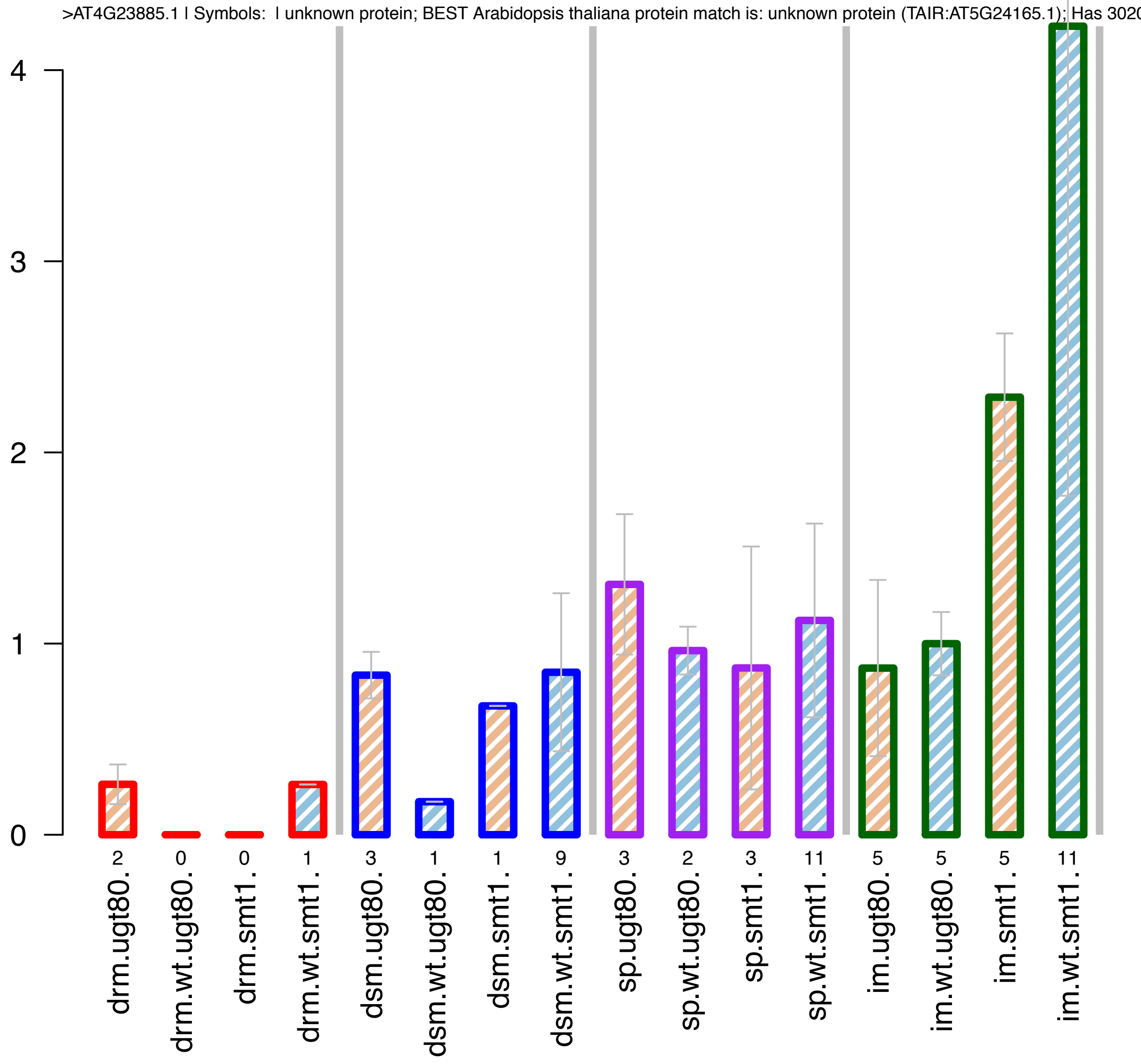

>AT4G23885.1 | Symbols: | unknown protein; BEST Arabidopsis thaliana protein match is: unknown protein (TAIR:AT5G24165.1); Has 3020
4
3
2
1
0
2
0
0
1
3
1
1
9
3
2
3
11
5
5
5
11
drm.ugt80.
drm.wt.ugt80.
drm.smt1.
drm.wt.smt1.
dsm.ugt80.
dsm.wt.ugt80.
dsm.smt1.
dsm.wt.smt1.
sp.ugt80.
sp.wt.ugt80.
sp.smt1.
sp.wt.smt1.
im.ugt80.
im.wt.ugt80.
im.smt1.
im.wt.smt1.

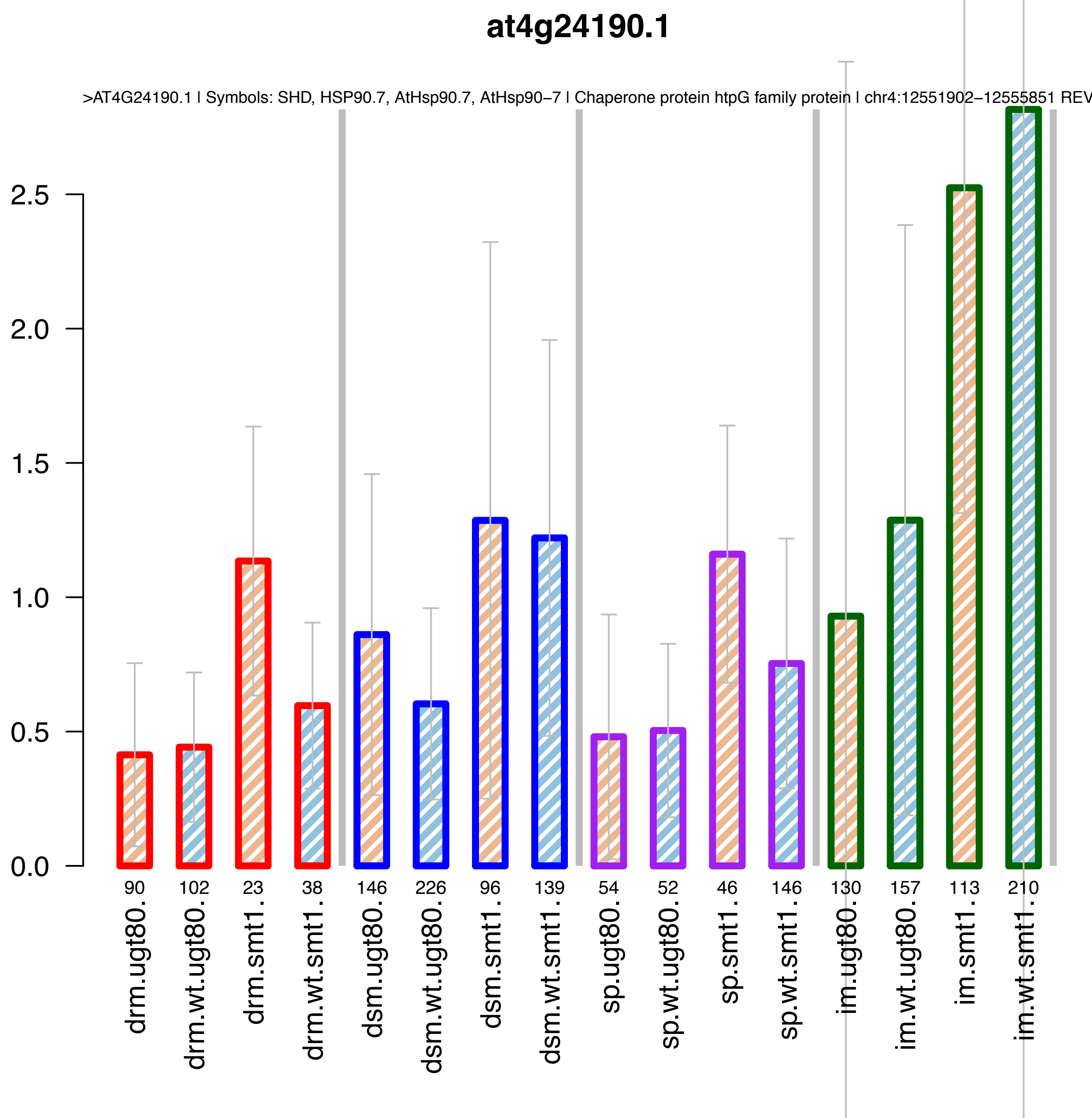

at4g24190.1
>AT4G24190.1 | Symbols: SHD, HSP90.7, AtHsp90.7, AtHsp90−7 | Chaperone protein htpG family protein | chr4:12551902−12555851 REV
0.0
0.5
1.0
1.5
2.0
2.5
90
102
23
38
146
226
96
139
54
52
46
146
130
157
113
210
drm.ugt80..
drm.wt.ugt80..
drm.smt1..
drm.wt.smt1..
dsm.ugt80..
dsm.wt.ugt80..
dsm.smt1..
dsm.wt.smt1..
sp.ugt80..
sp.wt.ugt80..
sp.smt1..
sp.wt.smt1..
im.ugt80..
im.wt.ugt80..
im.smt1..
im.wt.smt1..

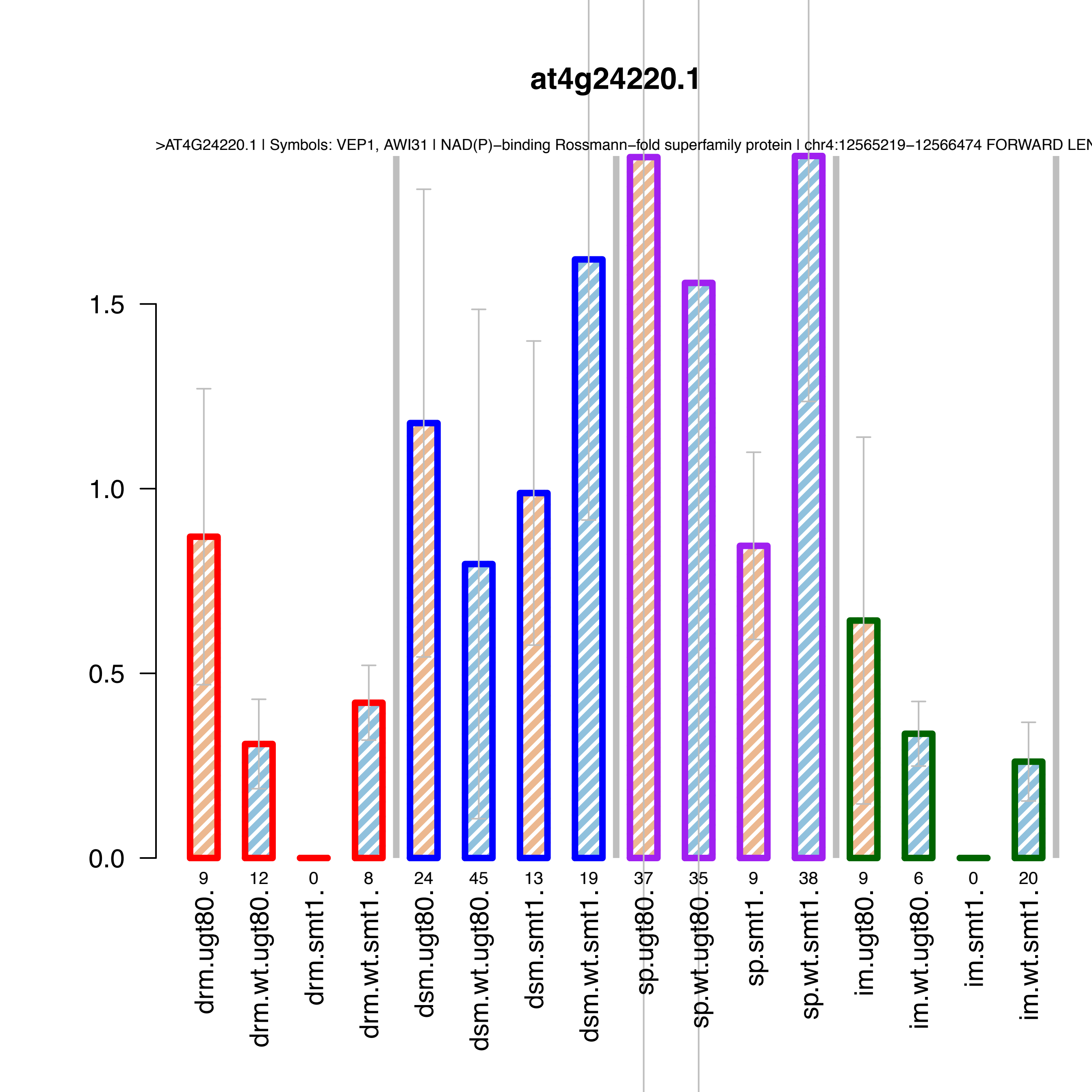

at4g24220.1
>AT4G24220.1 | Symbols: VEP1, AWI31 | NAD(P)−binding Rossmann−fold superfamily protein | chr4:12565219−12566474 FORWARD LEN
1.5
1.0
0.5
0.0
9
12
0
8
24
45
13
19
37
35
9
38
9
6
0
20
drm.ugt80.
drm.wt.ugt80.
drm.smt1.
drm.wt.smt1.
dsm.ugt80.
dsm.wt.ugt80.
dsm.smt1.
dsm.wt.smt1.
sp.ugt80.
sp.wt.ugt80.
sp.smt1.
sp.wt.smt1.
im.ugt80.
im.wt.ugt80.
im.smt1.
im.wt.smt1.

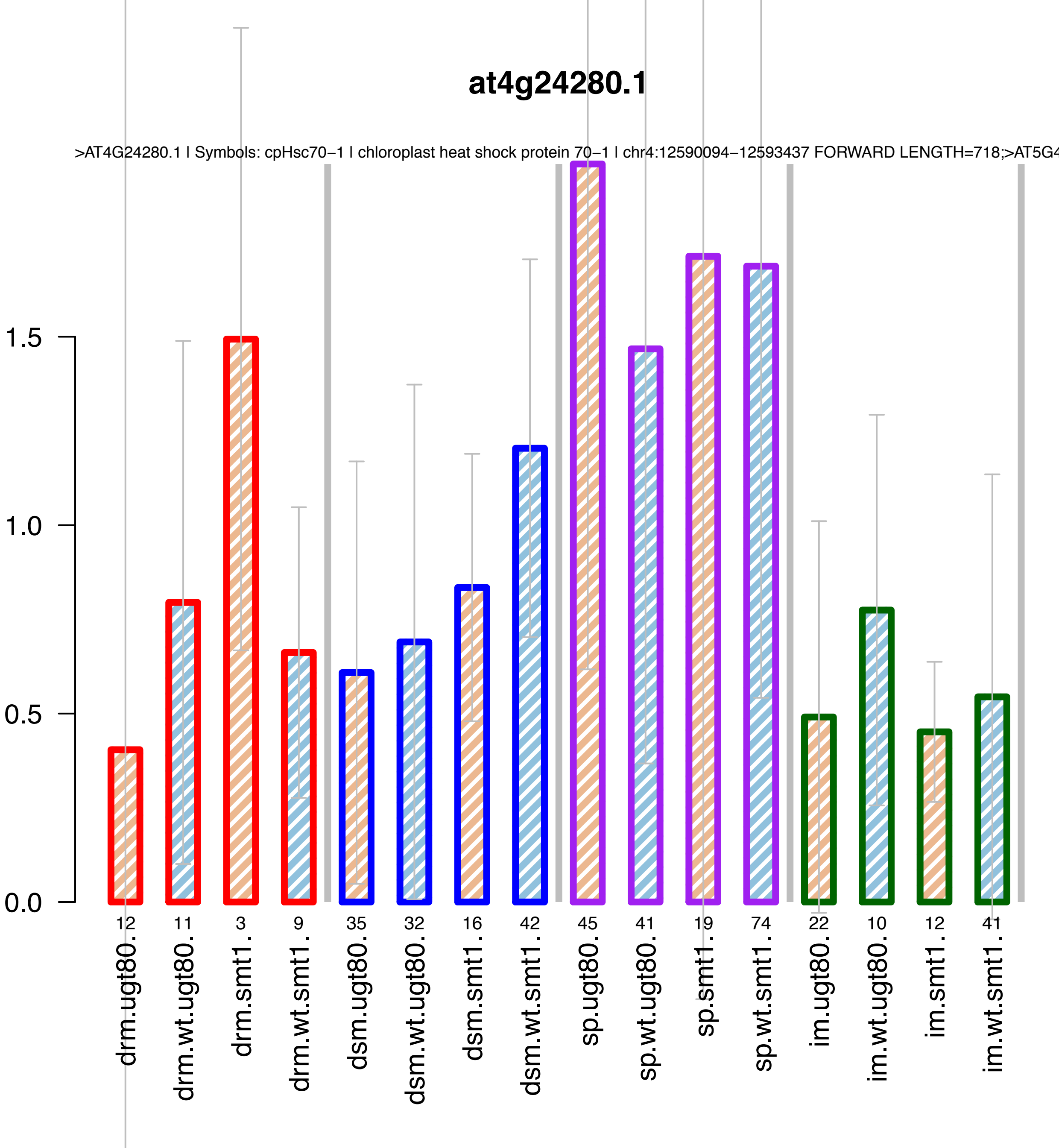

at4g24280.1
>AT4G24280.1 | Symbols: cpHsc70−1 | chloroplast heat shock protein 70−1 | chr4:12590094−12593437 FORWARD LENGTH=718;>AT5G4
0.0
0.5
1.0
1.5
12
11
3
9
35
32
16
42
45
41
19
74
22
10
12
41
drm.ugt80..
drm.wt.ugt80..
drm.smt1..
drm.wt.smt1..
dsm.ugt80..
dsm.wt.ugt80..
dsm.smt1..
dsm.wt.smt1..
sp.ugt80..
sp.wt.ugt80..
sp.smt1..
sp.wt.smt1..
im.ugt80..
im.wt.ugt80..
im.smt1..
im.wt.smt1..

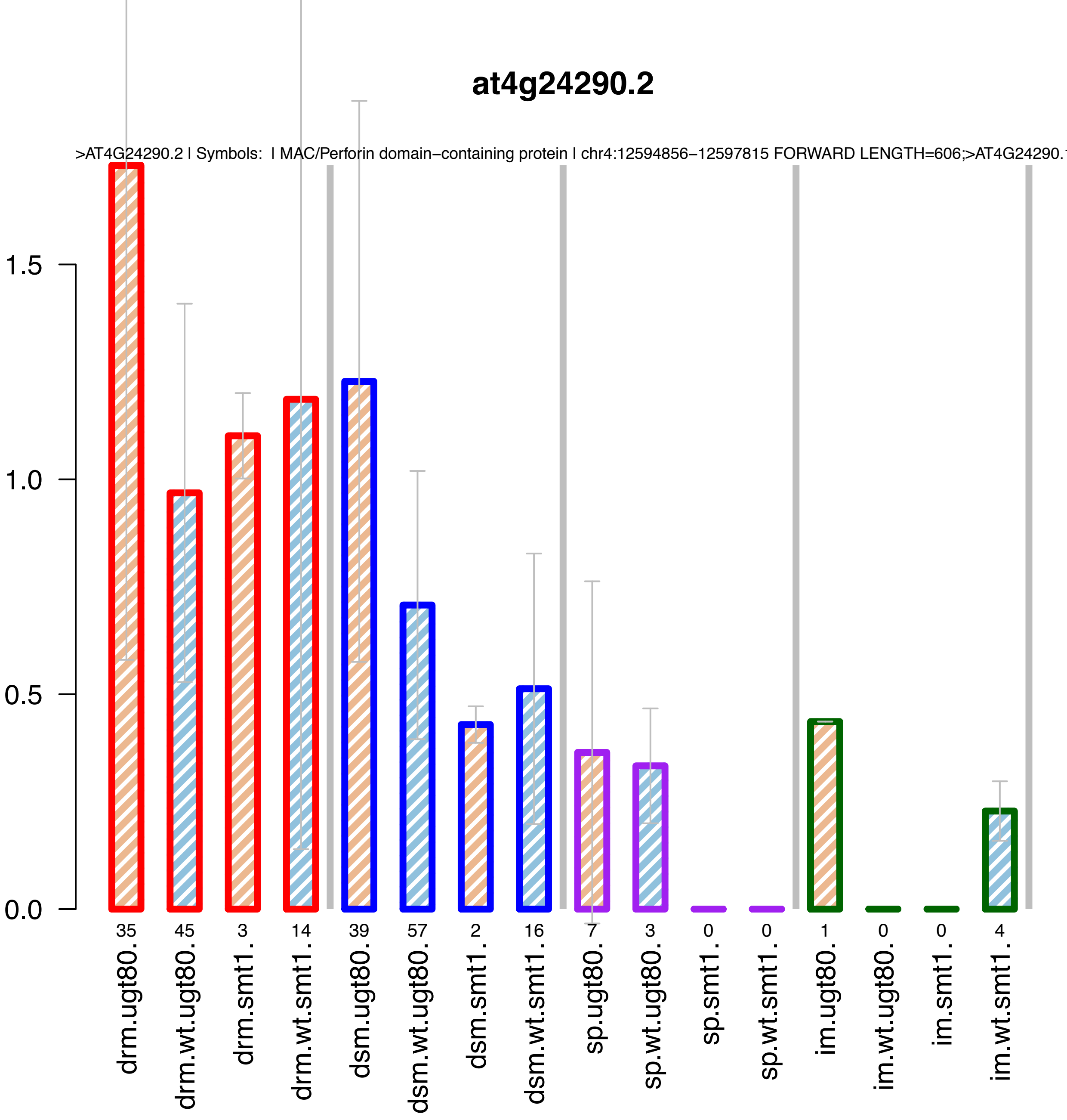

at4g24290.2
>AT4G24290.2 | Symbols: | MAC/Perforin domain−containing protein | chr4:12594856−12597815 FORWARD LENGTH=606;>AT4G24290.
1.5
1.0
0.5
0.0
35
45
3
14
39
57
2
16
7
3
0
0
1
0
0
4
drm.ugt80..
drm.wt.ugt80..
drm.smt1..
drm.wt.smt1..
dsm.ugt80..
dsm.wt.ugt80..
dsm.smt1..
dsm.wt.smt1..
sp.ugt80..
sp.wt.ugt80..
sp.smt1..
sp.wt.smt1..
im.ugt80..
im.wt.ugt80..
im.smt1..
im.wt.smt1..

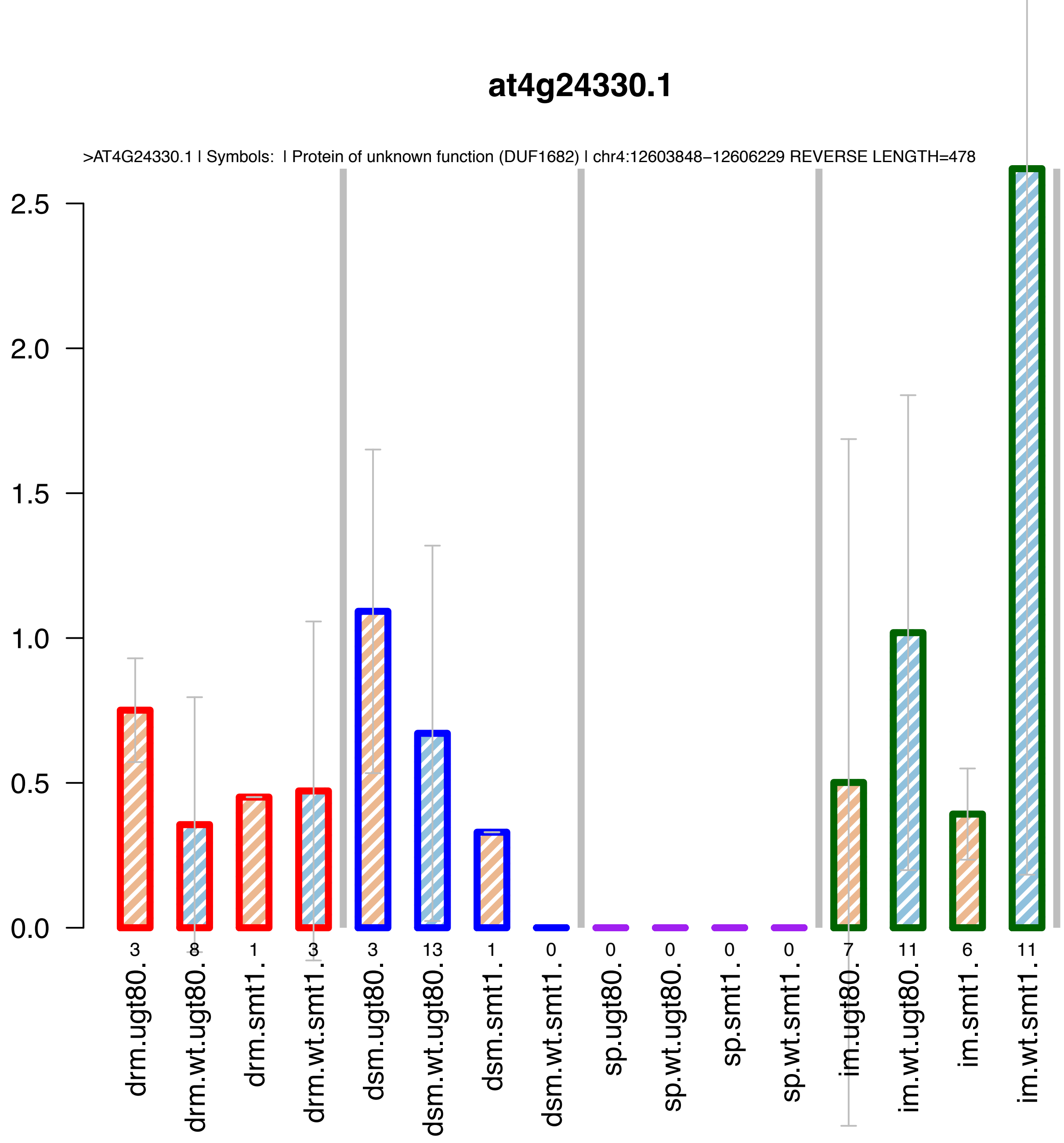
at4g24330.1
>AT4G24330.1 | Symbols: | Protein of unknown function (DUF1682) | chr4:12603848-12606229 REVERSE LENGTH=478
0.0
0.5
1.0
1.5
2.0
2.5
3
8
1
3
3
13
1
0
0
0
0
0
7
11
6
11
drm.ugt80.
drm.wt.ugt80.
drm.smt1.
drm.wt.smt1.
dsm.ugt80.
dsm.wt.ugt80.
dsm.smt1.
dsm.wt.smt1.
sp.ugt80.
sp.wt.ugt80.
sp.smt1.
sp.wt.smt1.
im.ugt80.
im.wt.ugt80.
im.smt1.
im.wt.smt1.

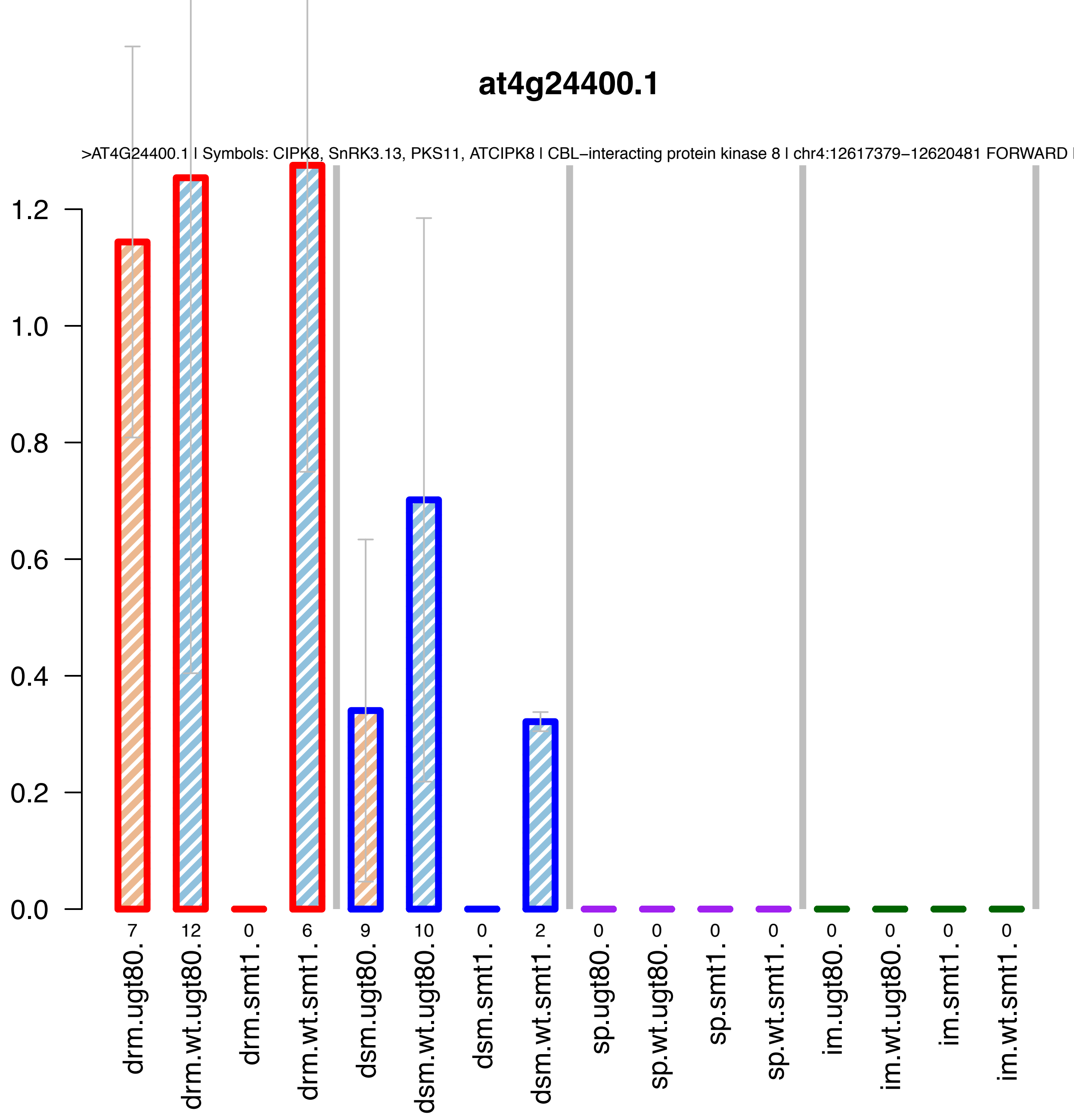
at4g24400.1
>AT4G24400.1 | Symbols: CIPK8, SnRK3.13, PKS11, ATCIPK8 | CBL−interacting protein kinase 8 | chr4:12617379−12620481 FORWARD
0.0
0.2
0.4
0.6
0.8
1.0
1.2
7
12
0
6
9
10
0
2
0
0
0
0
0
0
0
0
drm.ugt80.
drm.wt.ugt80.
drm.smt1.
drm.wt.smt1.
dsm.ugt80.
dsm.wt.ugt80.
dsm.smt1.
dsm.wt.smt1.
sp.ugt80.
sp.wt.ugt80.
sp.smt1.
sp.wt.smt1.
im.ugt80.
im.wt.ugt80.
im.smt1.
im.wt.smt1.

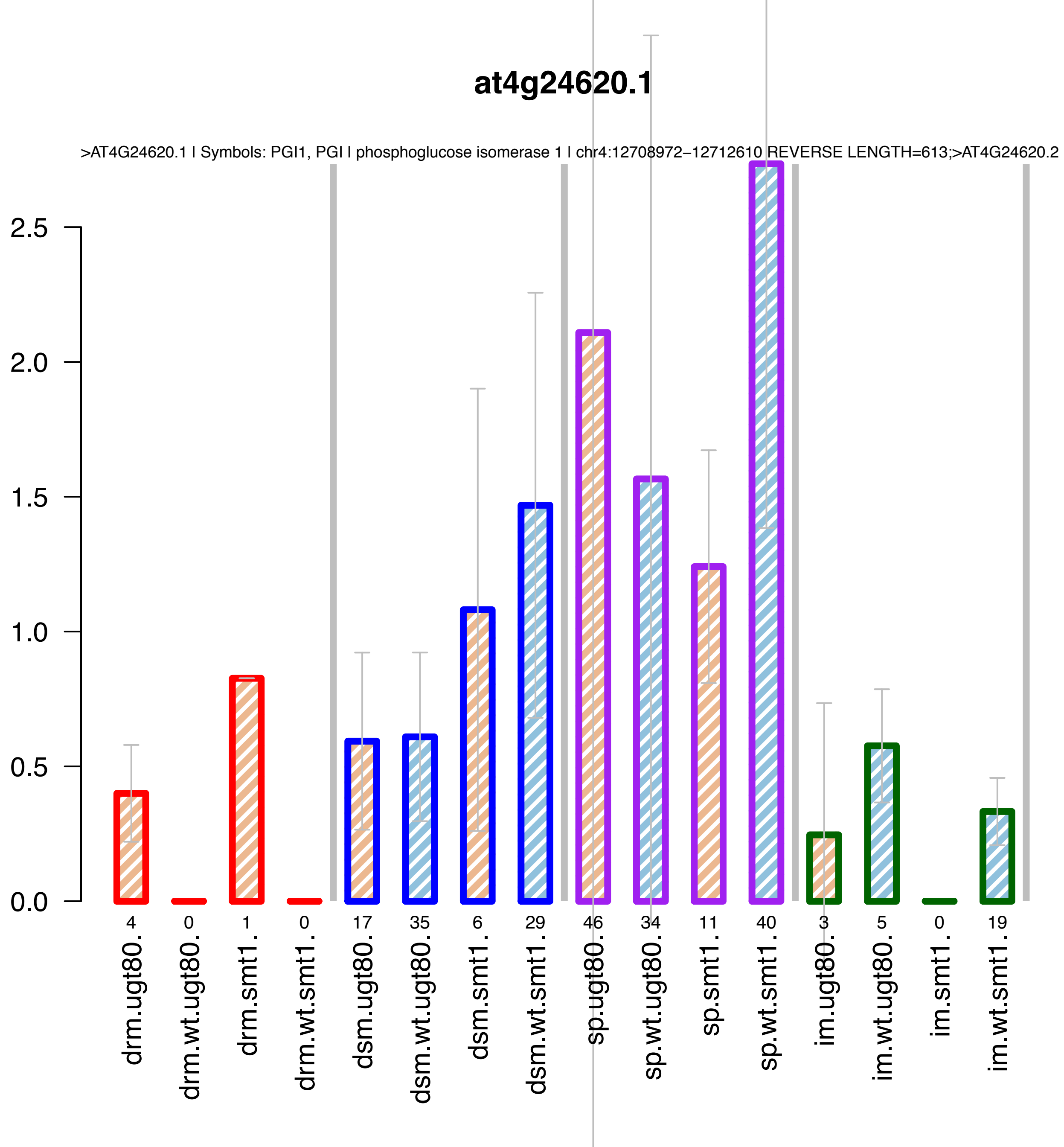

at4g24620.1
>AT4G24620.1 | Symbols: PGI1, PGI | phosphoglucose isomerase 1 | chr4:12708972−12712610 REVERSE LENGTH=613;>AT4G24620.2
0.0
0.5
1.0
1.5
2.0
2.5
4
0
1
0
17
35
6
29
46
34
11
40
3
5
0
19
drm.ugt80..
drm.wt.ugt80..
drm.smt1..
drm.wt.smt1..
dsm.ugt80..
dsm.wt.ugt80..
dsm.smt1..
dsm.wt.smt1..
sp.ugt80..
sp.wt.ugt80..
sp.smt1..
sp.wt.smt1..
im.ugt80..
im.wt.ugt80..
im.smt1..
im.wt.smt1..

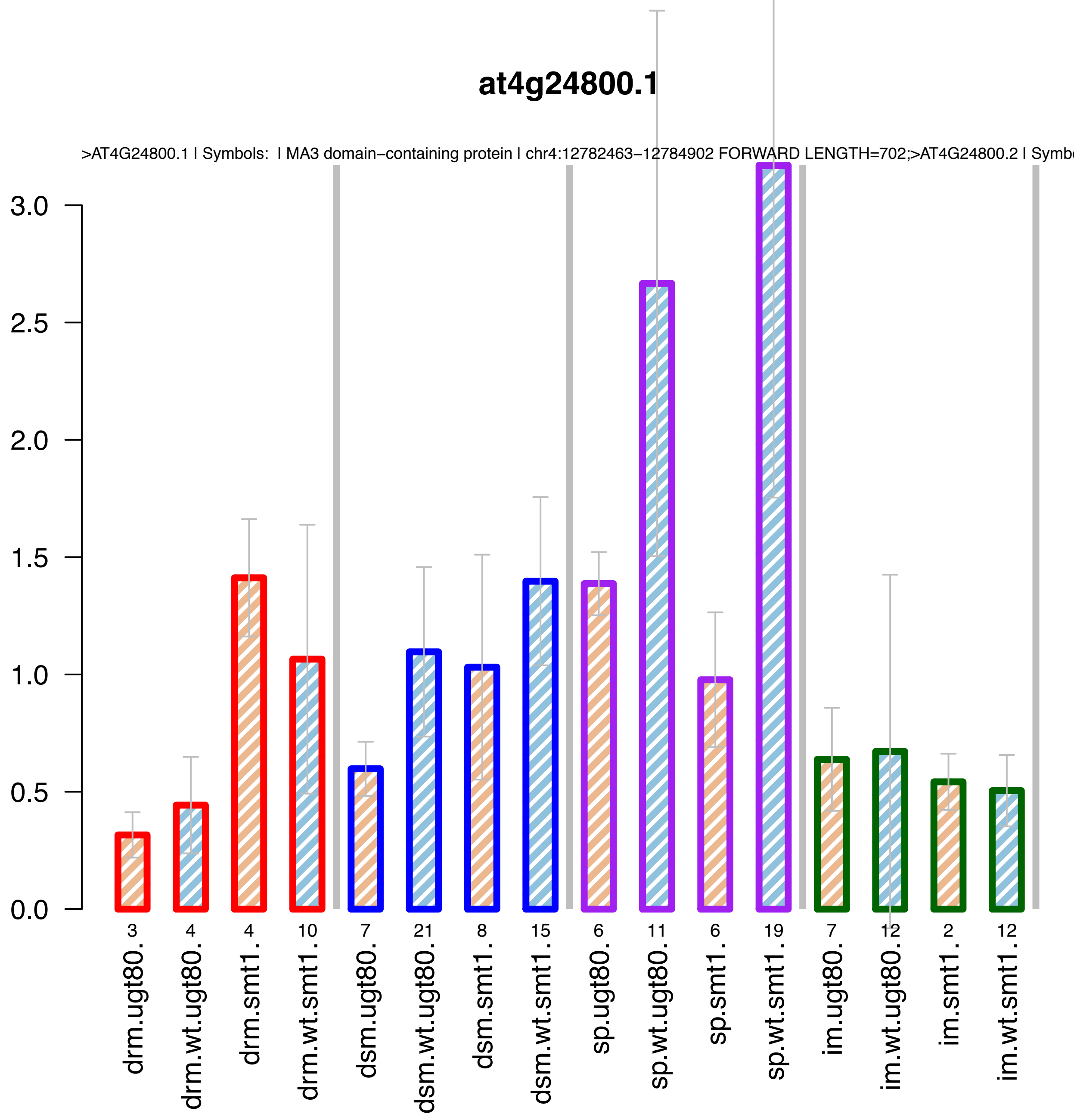

at4g24800.1
>AT4G24800.1 | Symbols: | MA3 domain−containing protein | chr4:12782463−12784902 FORWARD LENGTH=702;>AT4G24800.2 | Symb
3.0
2.5
2.0
1.5
1.0
0.5
0.0
3
4
4
10
7
21
8
15
6
11
6
19
7
12
2
12
drm.ugt80.
drm.wt.ugt80.
drm.smt1.
drm.wt.smt1.
dsm.ugt80.
dsm.wt.ugt80.
dsm.smt1.
dsm.wt.smt1.
sp.ugt80.
sp.wt.ugt80.
sp.smt1.
sp.wt.smt1.
im.ugt80.
im.wt.ugt80.
im.smt1.
im.wt.smt1.

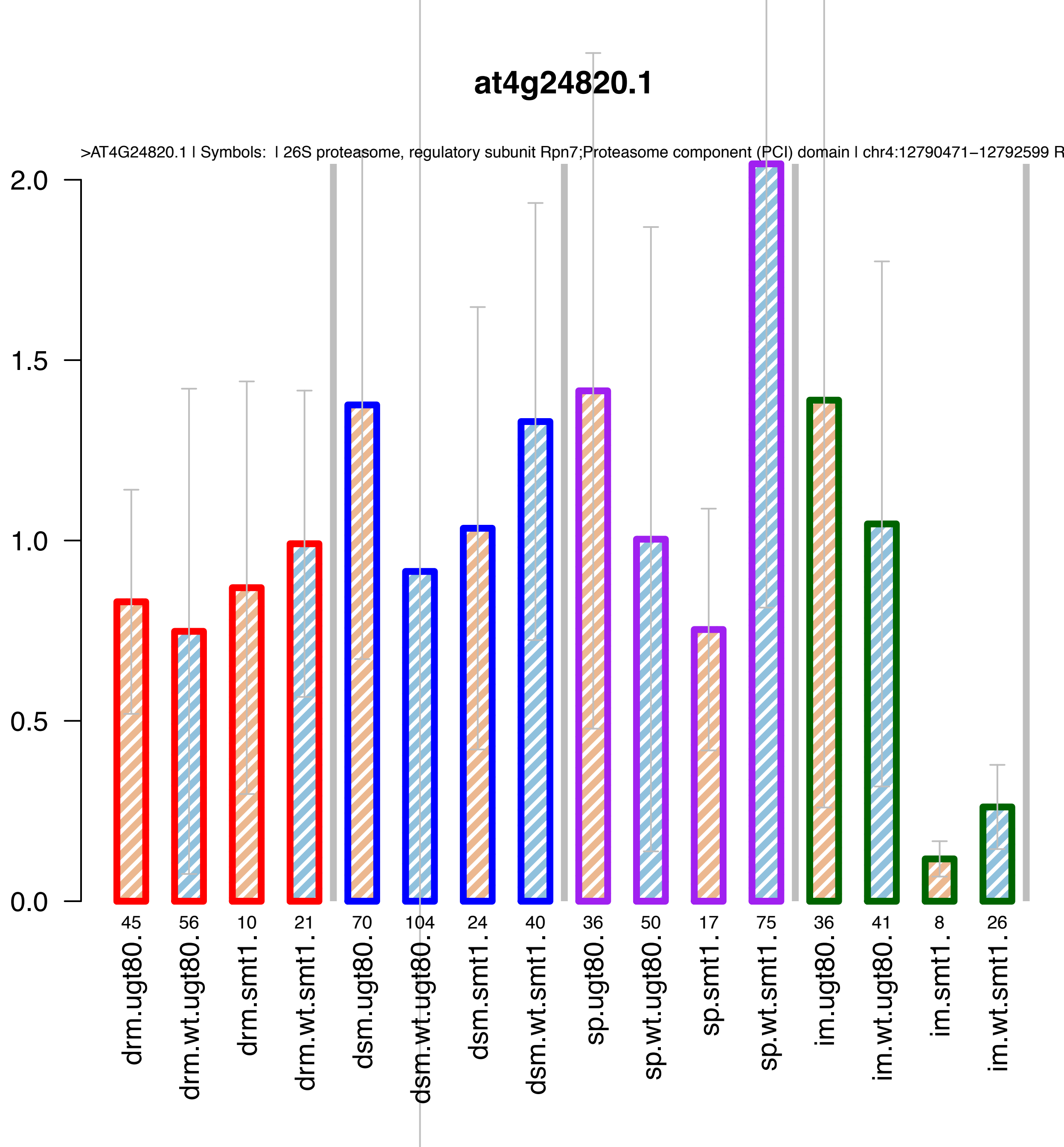

at4g24820.1
>AT4G24820.1 | Symbols: | 26S proteasome, regulatory subunit Rpn7;Proteasome component (PCI) domain | chr4:12790471–12792599 R
2.0
1.5
1.0
0.5
0.0
45
56
10
21
70
104
24
40
36
50
17
75
36
41
8
26
drm.ugt80..
drm.wt.ugt80..
drm.smt1..
drm.wt.smt1..
dsm.ugt80..
dsm.wt.ugt80..
dsm.smt1..
dsm.wt.smt1..
sp.ugt80..
sp.wt.ugt80..
sp.smt1..
sp.wt.smt1..
im.ugt80..
im.wt.ugt80..
im.smt1..
im.wt.smt1..

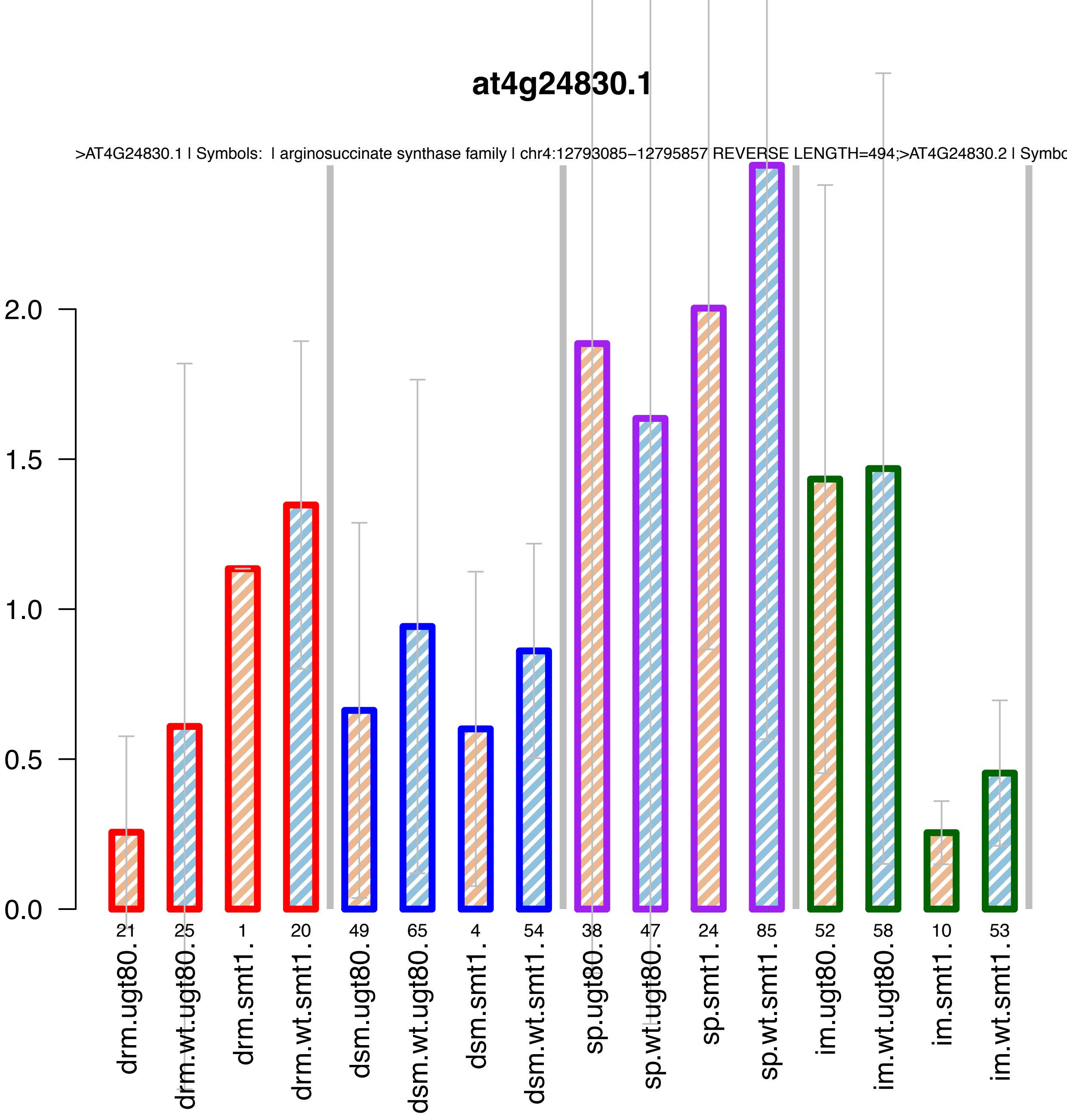

at4g24830.1
>AT4G24830.1 | Symbols: | arginosuccinate synthase family | chr4:12793085−12795857 REVERSE LENGTH=494;>AT4G24830.2 | Symbo
2.0
1.5
1.0
0.5
0.0
21
25
1
20
49
65
4
54
38
47
24
85
52
58
10
53
drm.ugt80..
drm.wt.ugt80..
drm.smt1..
drm.wt.smt1..
dsm.ugt80..
dsm.wt.ugt80..
dsm.smt1..
dsm.wt.smt1..
sp.ugt80..
sp.wt.ugt80..
sp.smt1..
sp.wt.smt1..
im.ugt80..
im.wt.ugt80..
im.smt1..
im.wt.smt1..

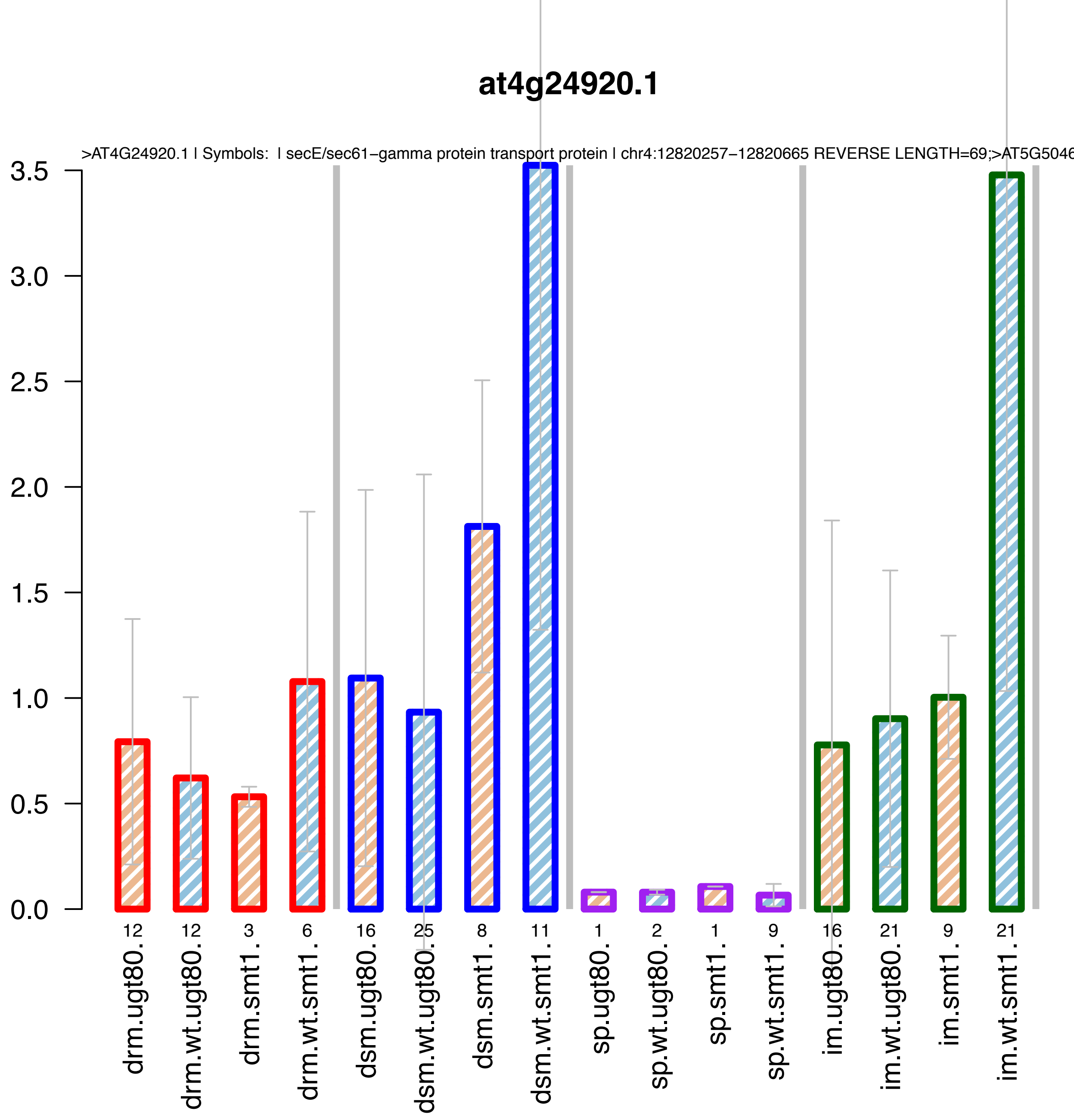
at4g24920.1
>AT4G24920.1 | Symbols: | secE/sec61−gamma protein transport protein | chr4:12820257−12820665 REVERSE LENGTH=69;>AT5G5046
3.5
3.0
2.5
2.0
1.5
1.0
0.5
0.0
12
12
3
6
16
25
8
11
1
2
1
9
16
21
9
21
drm.ugt80..
drm.wt.ugt80..
drm.smt1..
drm.wt.smt1..
dsm.ugt80..
dsm.wt.ugt80..
dsm.smt1..
dsm.wt.smt1..
sp.ugt80..
sp.wt.ugt80..
sp.smt1..
sp.wt.smt1..
im.ugt80..
im.wt.ugt80..
im.smt1..
im.wt.smt1..

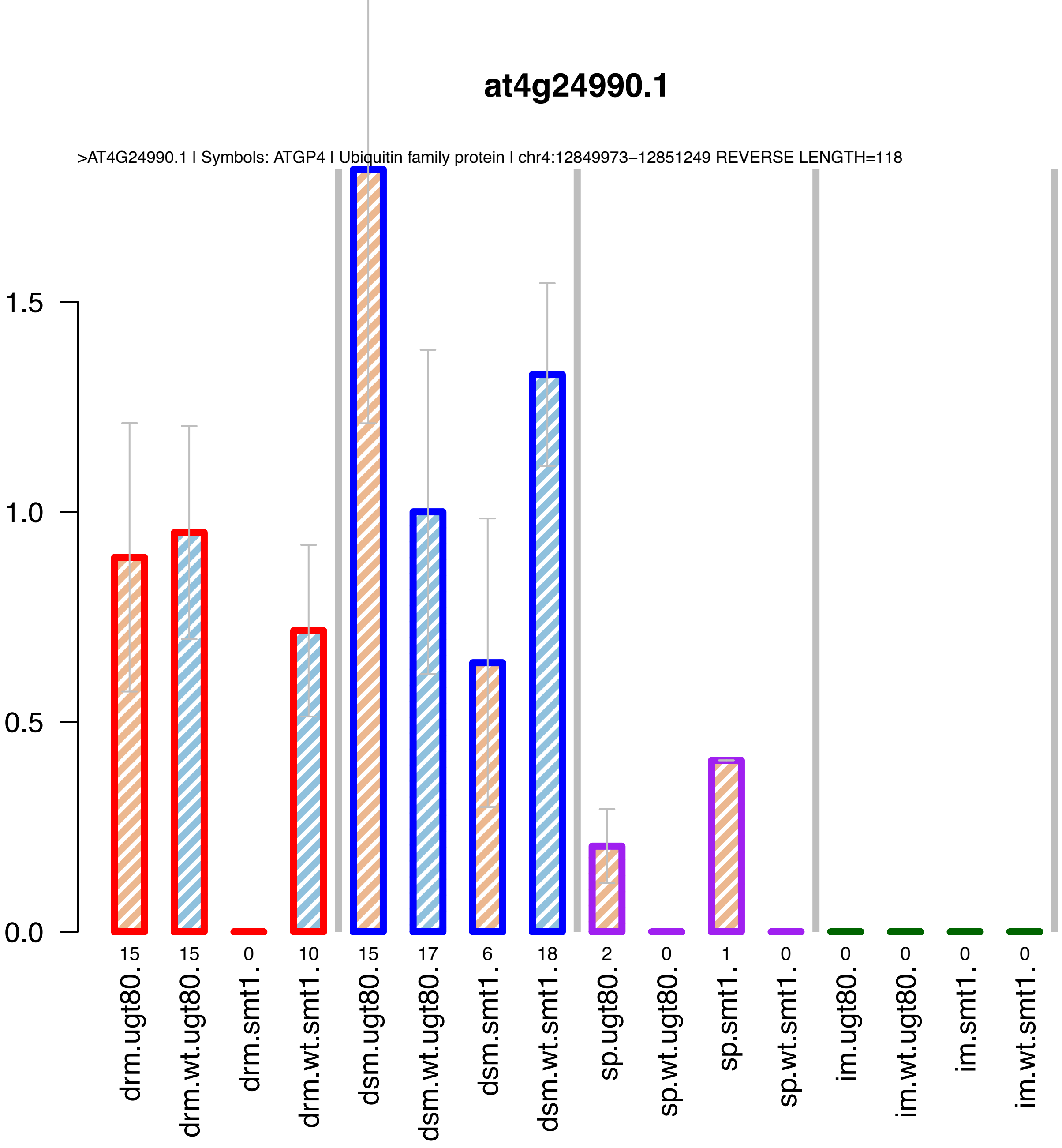

at4g24990.1
>AT4G24990.1 | Symbols: ATGP4 | Ubiquitin family protein | chr4:12849973-12851249 REVERSE LENGTH=118
1.5
1.0
0.5
0.0
15
15
0
10
15
17
6
18
2
0
1
0
0
0
0
0
drm.ugt80.
drm.wt.ugt80.
drm.smt1.
drm.wt.smt1.
dsm.ugt80.
dsm.wt.ugt80.
dsm.smt1.
dsm.wt.smt1.
sp.ugt80.
sp.wt.ugt80.
sp.smt1.
sp.wt.smt1.
im.ugt80.
im.wt.ugt80.
im.smt1.
im.wt.smt1.

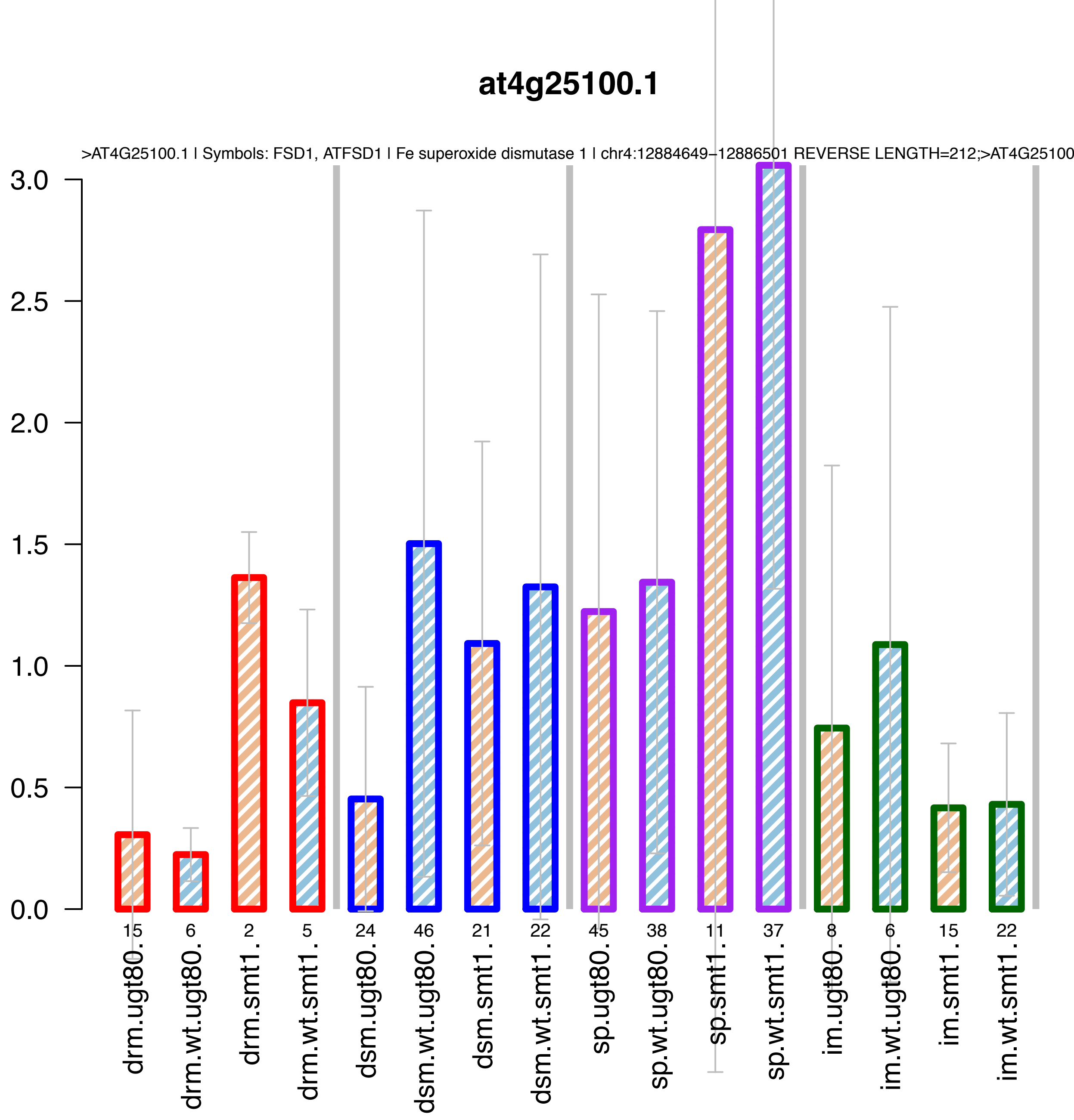

at4g25100.1
>AT4G25100.1 | Symbols: FSD1, ATFSD1 | Fe superoxide dismutase 1 | chr4:12884649–12886501 REVERSE LENGTH=212;>AT4G25100
3.0
2.5
2.0
1.5
1.0
0.5
0.0
15
6
2
5
24
46
21
22
45
38
11
37
8
6
15
22
drm.ugt80..
drm.wt.ugt80..
drm.smt1..
drm.wt.smt1..
dsm.ugt80..
dsm.wt.ugt80..
dsm.smt1..
dsm.wt.smt1..
sp.ugt80..
sp.wt.ugt80..
sp.smt1..
sp.wt.smt1..
im.ugt80..
im.wt.ugt80..
im.smt1..
im.wt.smt1..

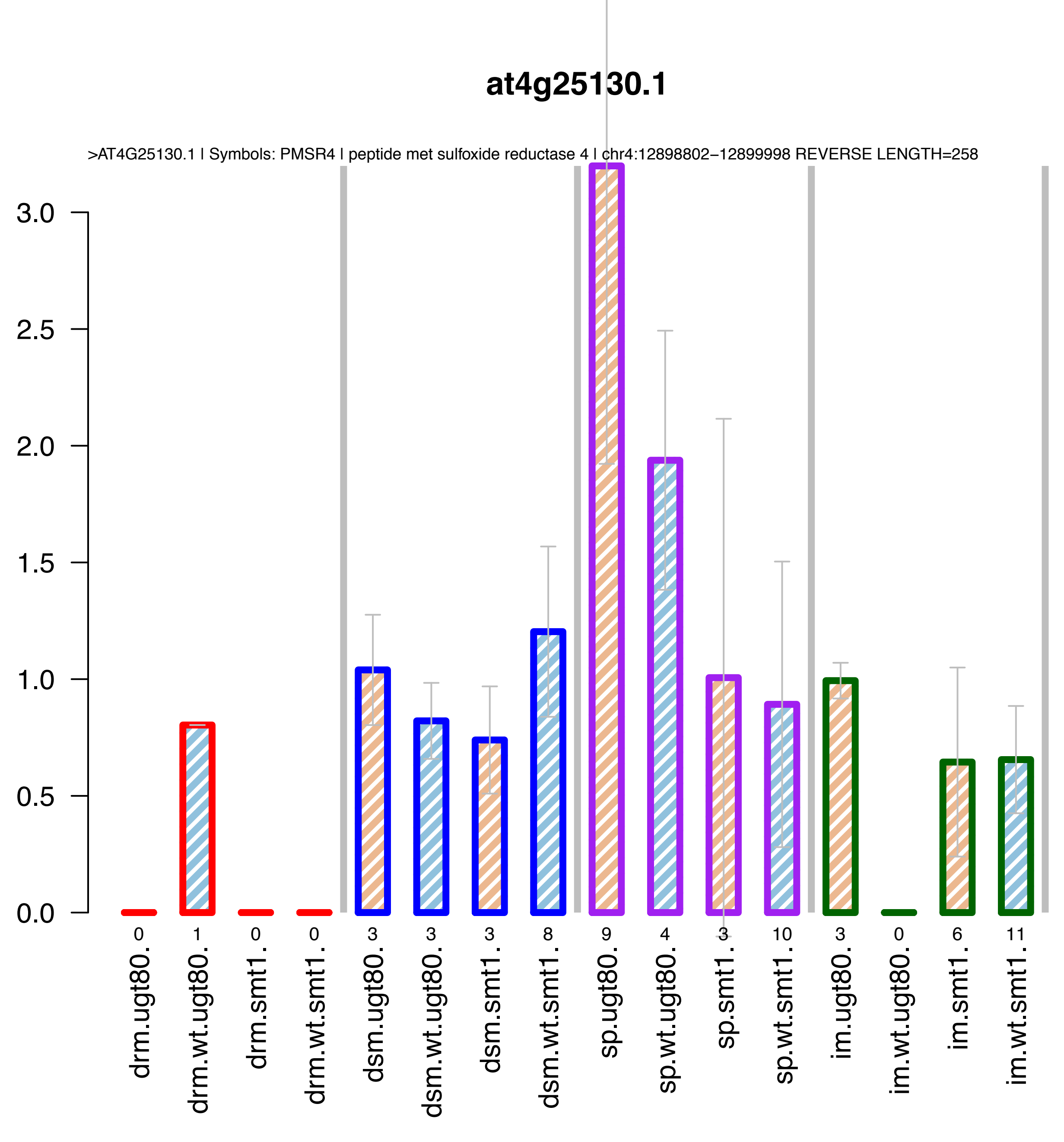

at4g25130.1
>AT4G25130.1 | Symbols: PMSR4 | peptide met sulfoxide reductase 4 | chr4:12898802-12899998 REVERSE LENGTH=258
0.0
0.5
1.0
1.5
2.0
2.5
3.0
0 1 0 0 3 3 3 8 9 4 3 10 3 0 6 11
drm.ugt80.
drm.wt.ugt80.
drm.smt1.
drm.wt.smt1.
dsm.ugt80.
dsm.wt.ugt80.
dsm.smt1.
dsm.wt.smt1.
sp.ugt80.
sp.wt.ugt80.
sp.smt1.
sp.wt.smt1.
im.ugt80.
im.wt.ugt80.
im.smt1.
im.wt.smt1.

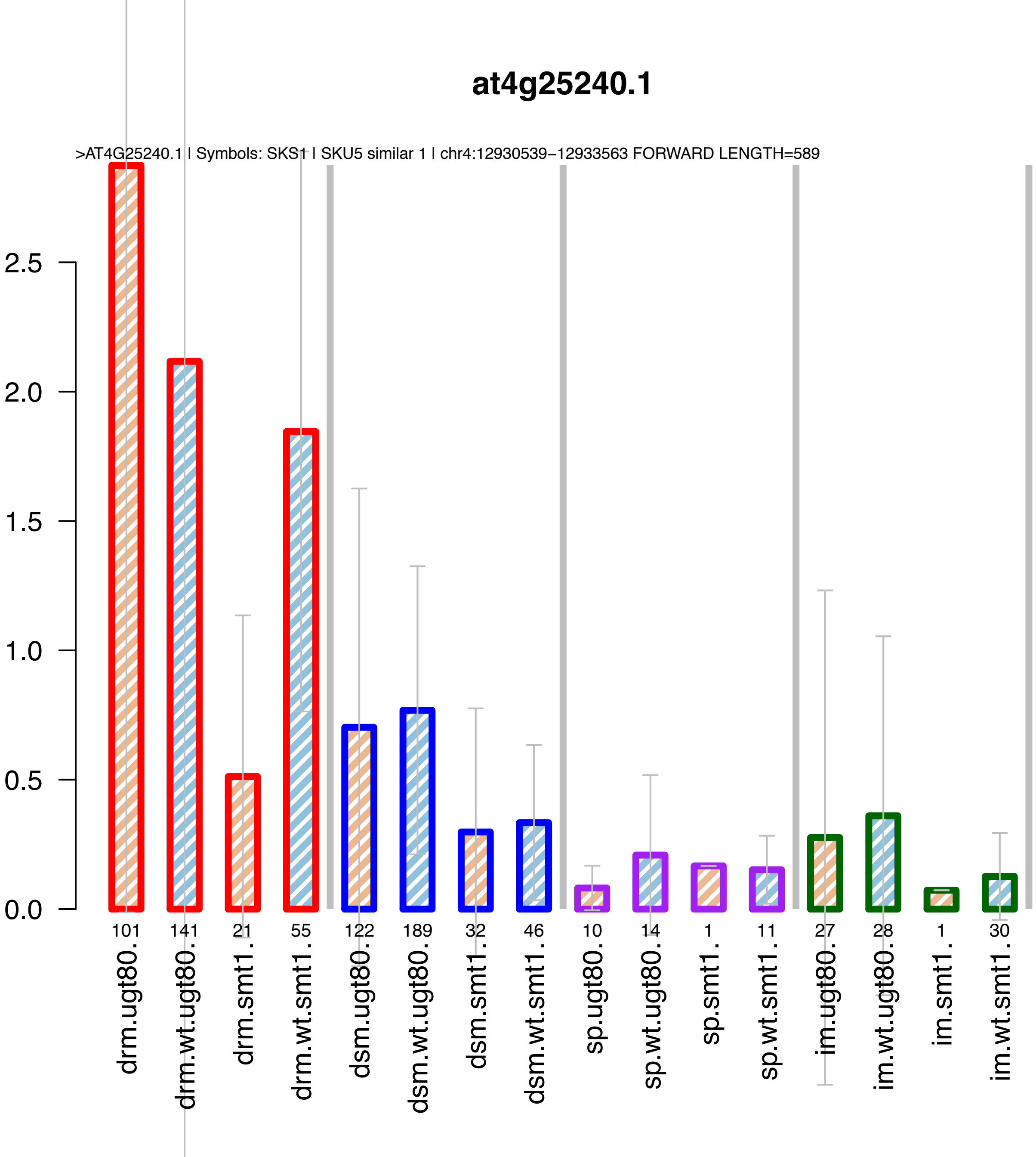

at4g25240.1
>AT4G25240.1 | Symbols: SKS1 | SKU5 similar 1 | chr4:12930539-12933563 FORWARD LENGTH=589
0.0
0.5
1.0
1.5
2.0
2.5
101
141
21
55
122
189
32
46
10
14
1
11
27
28
1
30
drm.ugt80..
drm.wt.ugt80..
drm.smt1..
drm.wt.smt1..
dsm.ugt80..
dsm.wt.ugt80..
dsm.smt1..
dsm.wt.smt1..
sp.ugt80..
sp.wt.ugt80..
sp.smt1..
sp.wt.smt1..
im.ugt80..
im.wt.ugt80..
im.smt1..
im.wt.smt1..

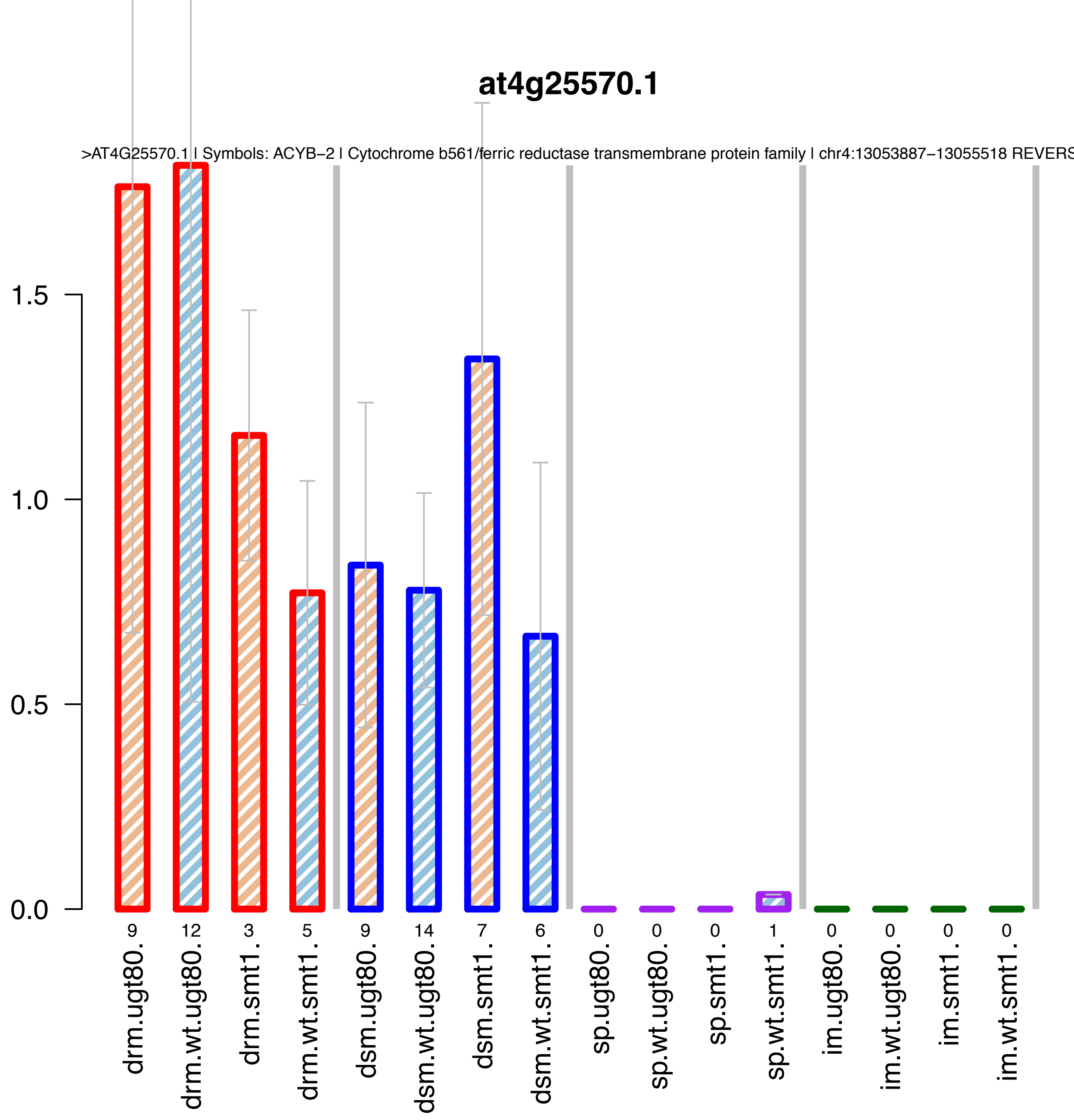

at4g25570.1
>AT4G25570.1 | Symbols: ACYB-2 | Cytochrome b561/ferric reductase transmembrane protein family | chr4:13053887-13055518 REVERS
1.5
1.0
0.5
0.0
9
12
3
5
9
14
7
6
0
0
0
1
0
0
0
0
drm.ugt80.
drm.wt.ugt80.
drm.smt1.
drm.wt.smt1.
dsm.ugt80.
dsm.wt.ugt80.
dsm.smt1.
dsm.wt.smt1.
sp.ugt80.
sp.wt.ugt80.
sp.smt1.
sp.wt.smt1.
im.ugt80.
im.wt.ugt80.
im.smt1.
im.wt.smt1.

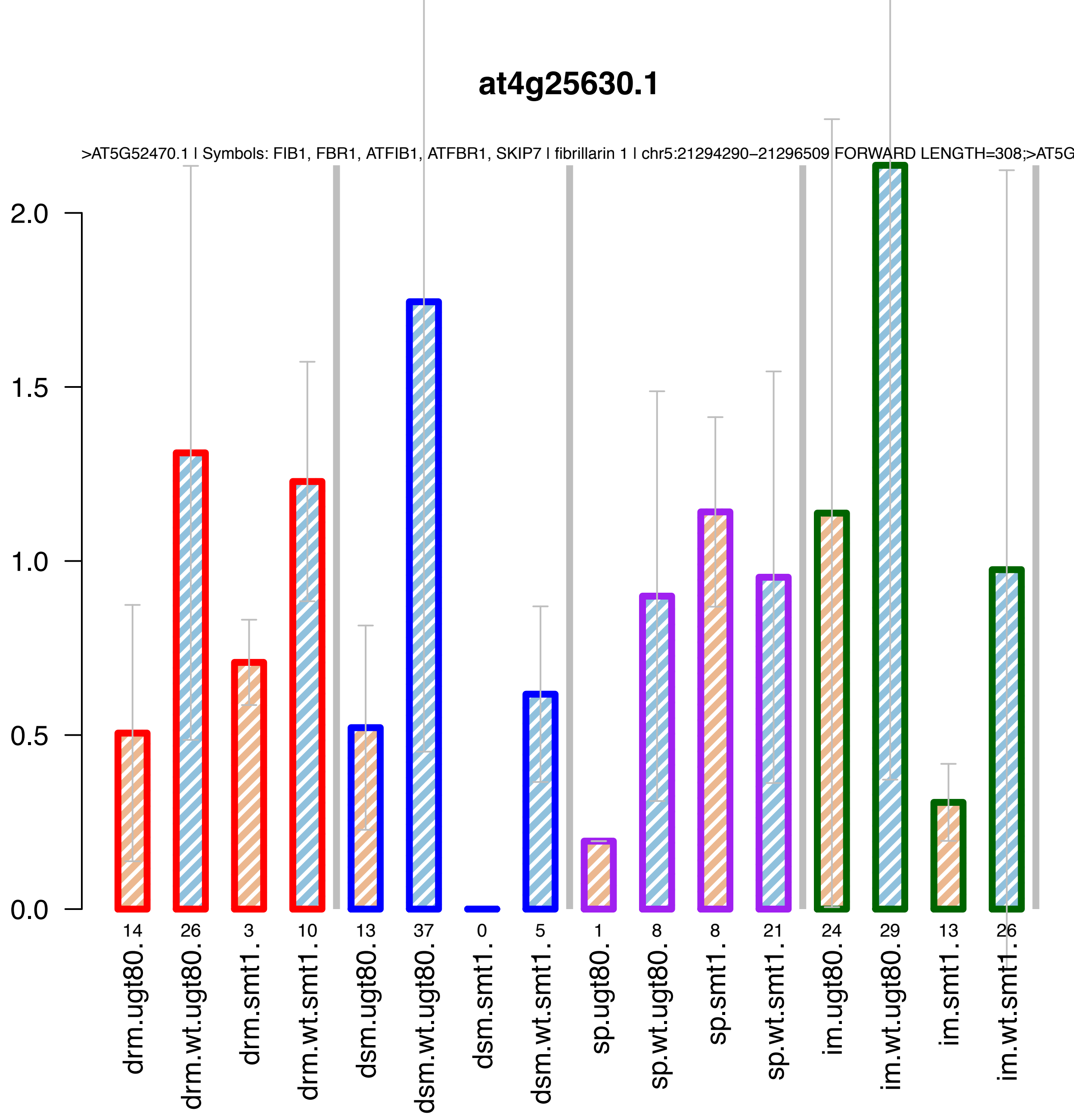

at4g25630.1
>AT5G52470.1 | Symbols: FIB1, FBR1, ATFIB1, ATFBR1, SKIP7 | fibrillarin 1 | chr5:21294290-21296509 FORWARD LENGTH=308;>AT5G
2.0
1.5
1.0
0.5
0.0
14
26
3
10
13
37
0
5
1
8
8
21
24
29
13
26
drm.ugt80..
drm.wt.ugt80..
drm.smt1..
drm.wt.smt1..
dsm.ugt80..
dsm.wt.ugt80..
dsm.smt1..
dsm.wt.smt1..
sp.ugt80..
sp.wt.ugt80..
sp.smt1..
sp.wt.smt1..
im.ugt80..
im.wt.ugt80..
im.smt1..
im.wt.smt1..

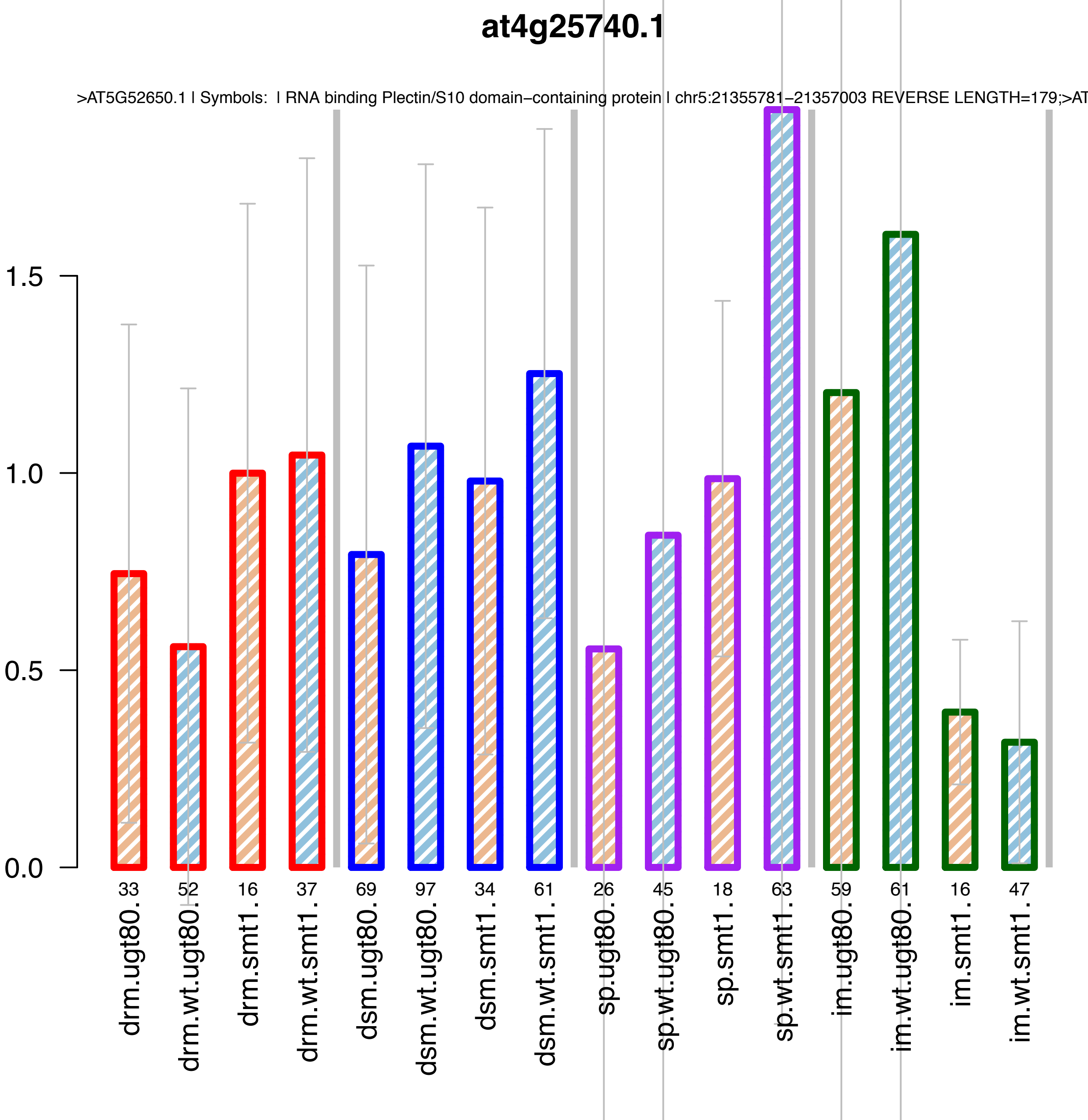

at4g25740.1
>AT5G52650.1 | Symbols: | RNA binding Plectin/S10 domain−containing protein | chr5:21355781−21357003 REVERSE LENGTH=179;>AT
0.0
0.5
1.0
1.5
33
52
16
37
69
97
34
61
26
45
18
63
59
61
16
47
drm.ugt80..
drm.wt.ugt80..
drm.smt1..
drm.wt.smt1..
dsm.ugt80..
dsm.wt.ugt80..
dsm.smt1..
dsm.wt.smt1..
sp.ugt80..
sp.wt.ugt80..
sp.smt1..
sp.wt.smt1..
im.ugt80..
im.wt.ugt80..
im.smt1..
im.wt.smt1..

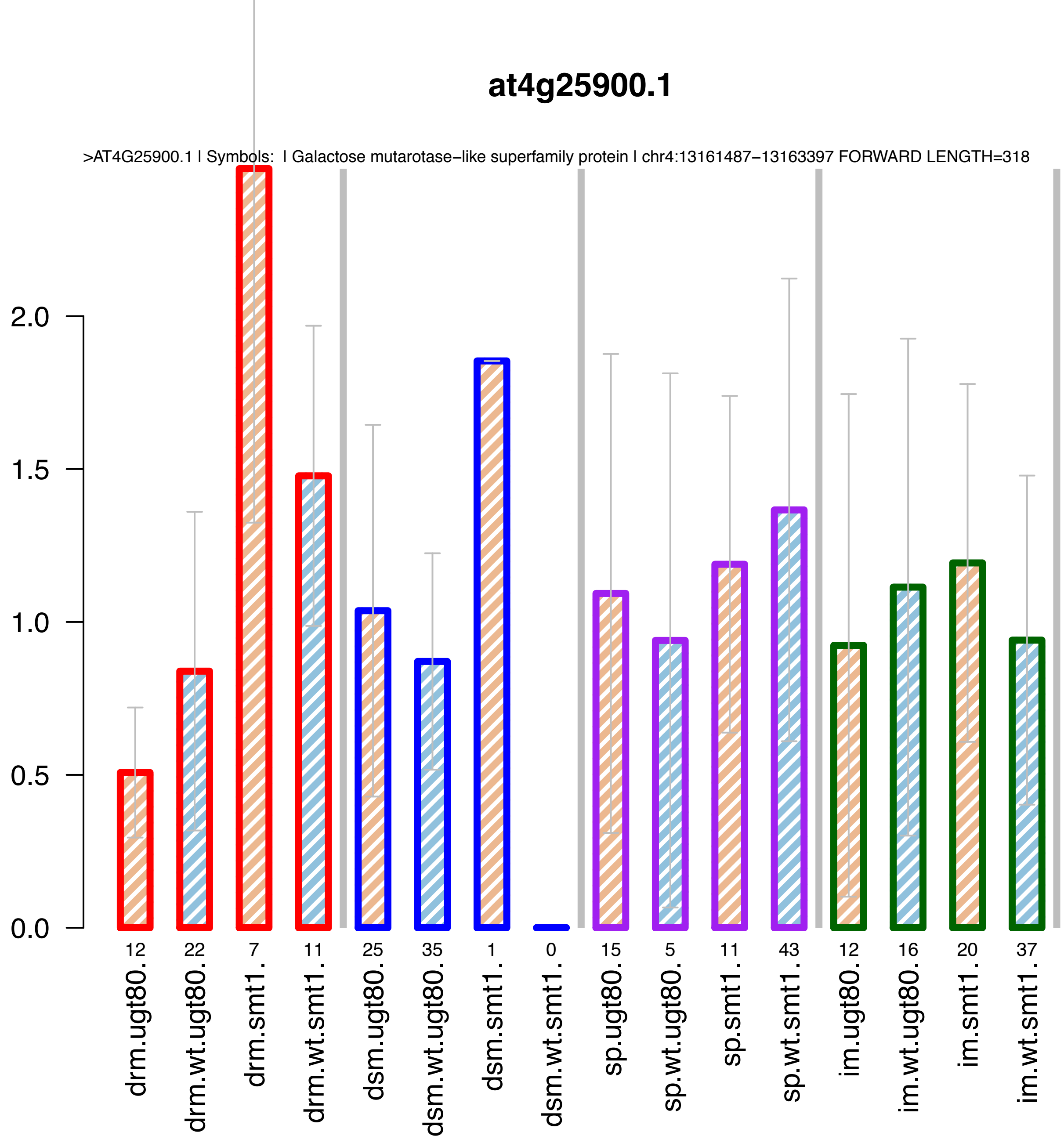

at4g25900.1
>AT4G25900.1 | Symbols: | Galactose mutarotase-like superfamily protein | chr4:13161487-13163397 FORWARD LENGTH=318
0.0
0.5
1.0
1.5
2.0
12
22
7
11
25
35
1
0
15
5
11
43
12
16
20
37
drm.ugt80.
drm.wt.ugt80.
drm.smt1.
drm.wt.smt1.
dsm.ugt80.
dsm.wt.ugt80.
dsm.smt1.
dsm.wt.smt1.
sp.ugt80.
sp.wt.ugt80.
sp.smt1.
sp.wt.smt1.
im.ugt80.
im.wt.ugt80.
im.smt1.
im.wt.smt1.

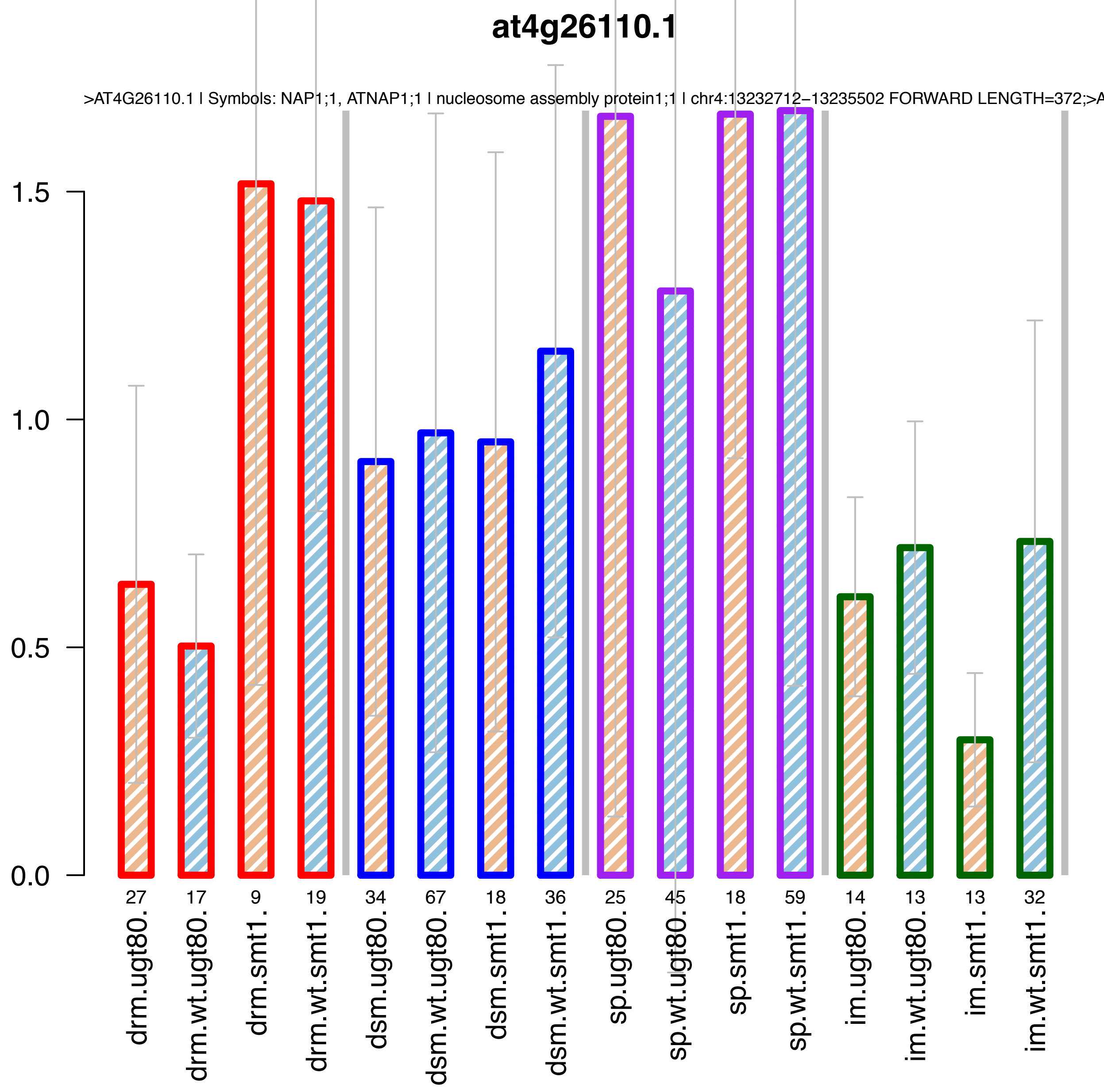

at4g26110.1
>AT4G26110.1 | Symbols: NAP1;1, ATNAP1;1 | nucleosome assembly protein1;1 | chr4:13232712–13235502 FORWARD LENGTH=372;>A
0.0
0.5
1.0
1.5
27
17
9
19
34
67
18
36
25
45
18
59
14
13
13
32
drm.ugt80..
drm.wt.ugt80..
drm.smt1..
drm.wt.smt1..
dsm.ugt80..
dsm.wt.ugt80..
dsm.smt1..
dsm.wt.smt1..
sp.ugt80..
sp.wt.ugt80..
sp.smt1..
sp.wt.smt1..
im.ugt80..
im.wt.ugt80..
im.smt1..
im.wt.smt1..

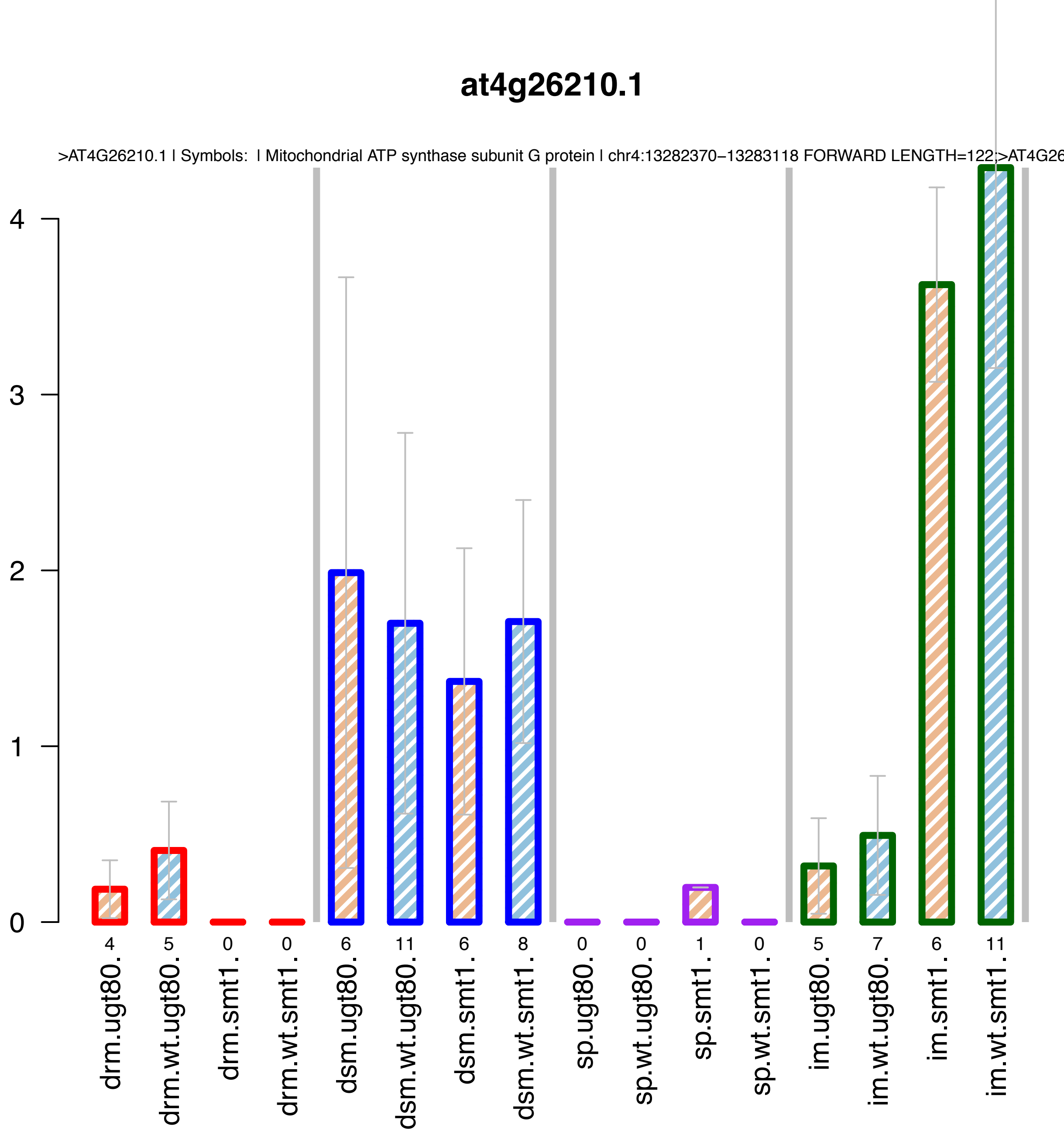
at4g26210.1
>AT4G26210.1 | Symbols: | Mitochondrial ATP synthase subunit G protein | chr4:13282370−13283118 FORWARD LENGTH=122;>AT4G26
4
3
2
1
0
4
5
0
0
6
11
6
8
0
0
1
0
5
7
6
11
drm.ugt80.
drm.wt.ugt80.
drm.smt1.
drm.wt.smt1.
dsm.ugt80.
dsm.wt.ugt80.
dsm.smt1.
dsm.wt.smt1.
sp.ugt80.
sp.wt.ugt80.
sp.smt1.
sp.wt.smt1.
im.ugt80.
im.wt.ugt80.
im.smt1.
im.wt.smt1.

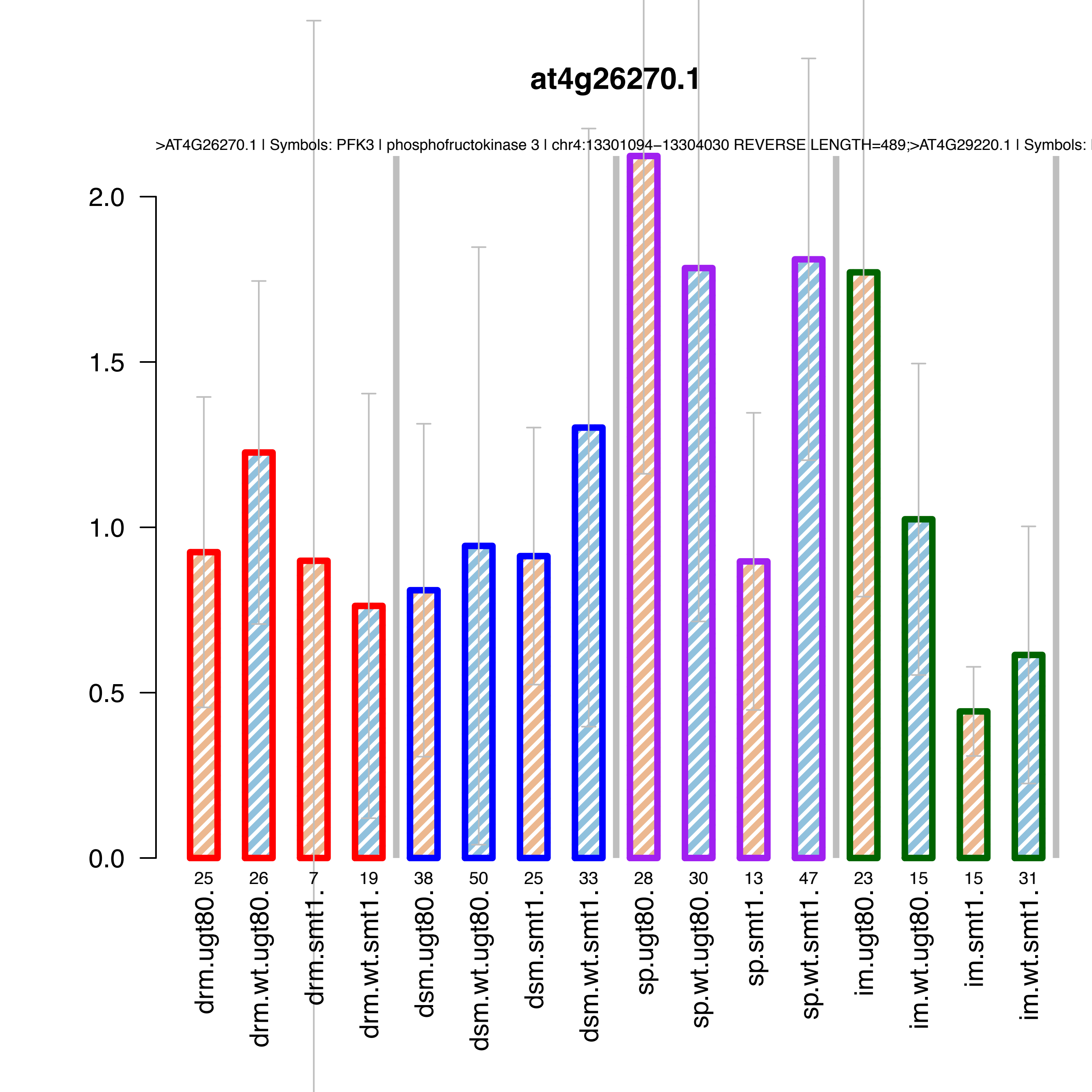

at4g26270.1
>AT4G26270.1 | Symbols: PFK3 | phosphofructokinase 3 | chr4:13301094–13304030 REVERSE LENGTH=489;>AT4G29220.1 | Symbols:
2.0
1.5
1.0
0.5
0.0
25
26
7
19
38
50
25
33
28
30
13
47
23
15
15
31
drm.ugt80..
drm.wt.ugt80..
drm.smt1..
drm.wt.smt1..
dsm.ugt80..
dsm.wt.ugt80..
dsm.smt1..
dsm.wt.smt1..
sp.ugt80..
sp.wt.ugt80..
sp.smt1..
sp.wt.smt1..
im.ugt80..
im.wt.ugt80..
im.smt1..
im.wt.smt1..

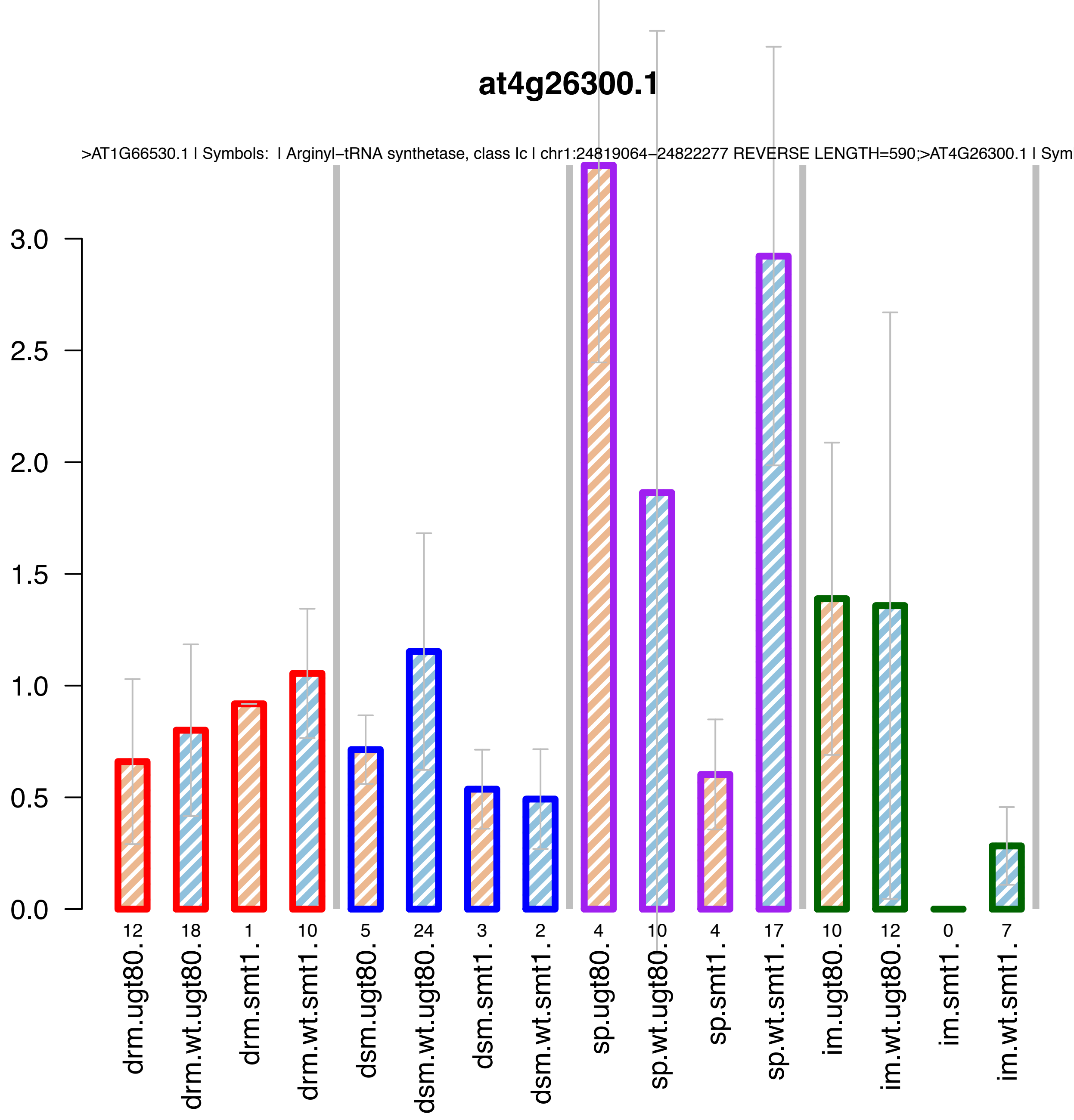

at4g26300.1
>AT1G66530.1 | Symbols: | Arginyl–tRNA synthetase, class Ic | chr1:24819064–24822277 REVERSE LENGTH=590;>AT4G26300.1 | Sym
0.0
0.5
1.0
1.5
2.0
2.5
3.0
12
18
1
10
5
24
3
2
4
10
4
17
10
12
0
7
drm.ugt80..
drm.wt.ugt80..
drm.smt1..
drm.wt.smt1..
dsm.ugt80..
dsm.wt.ugt80..
dsm.smt1..
dsm.wt.smt1..
sp.ugt80..
sp.wt.ugt80..
sp.smt1..
sp.wt.smt1..
im.ugt80..
im.wt.ugt80..
im.smt1..
im.wt.smt1..

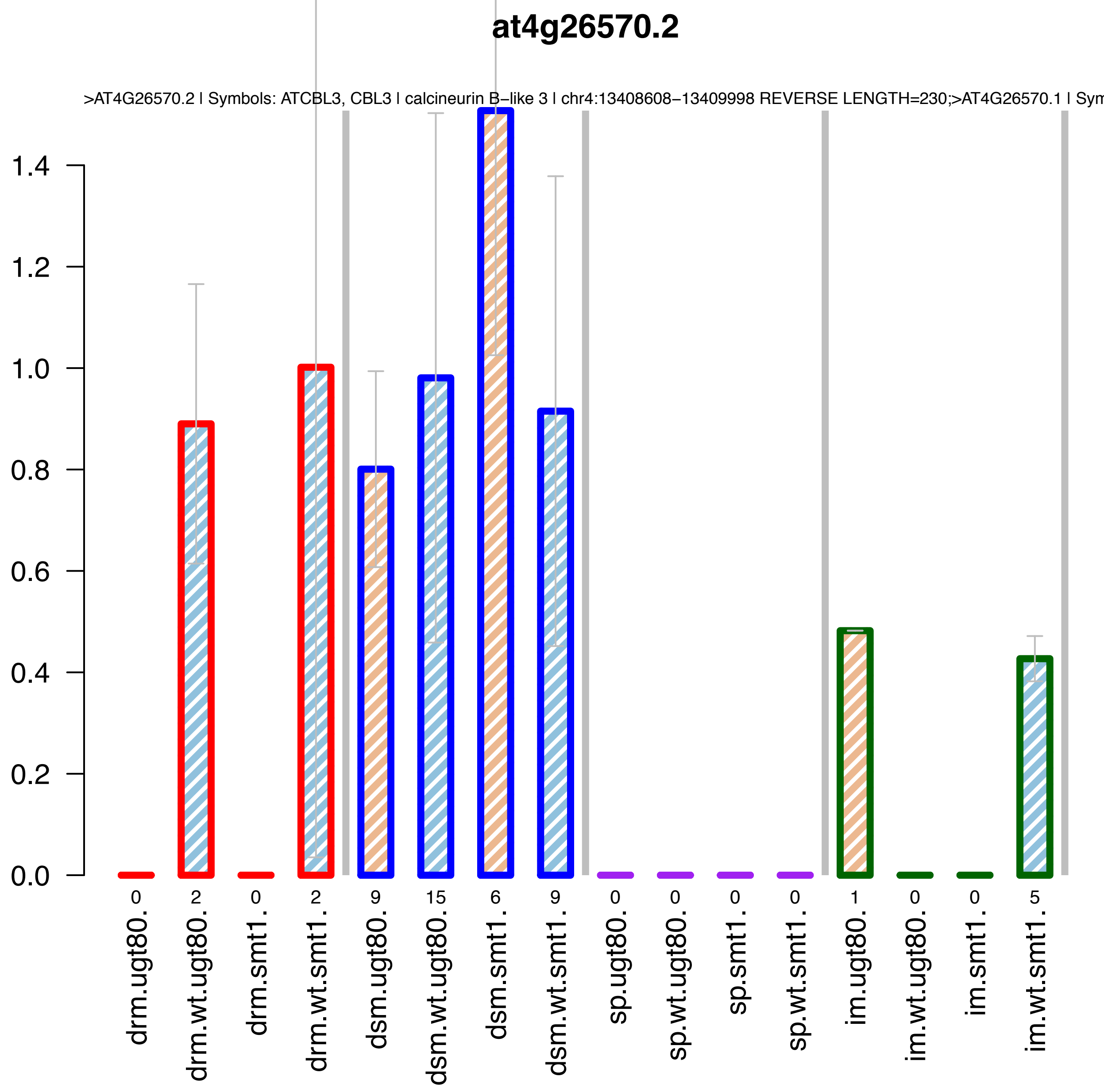

at4g26570.2
>AT4G26570.2 | Symbols: ATCBL3, CBL3 | calcineurin B−like 3 | chr4:13408608−13409998 REVERSE LENGTH=230;>AT4G26570.1 | Sym
1.4
1.2
1.0
0.8
0.6
0.4
0.2
0.0
0
2
0
2
9
15
6
9
0
0
0
0
1
0
0
5
drm.ugt80.
drm.wt.ugt80.
drm.smt1.
drm.wt.smt1.
dsm.ugt80.
dsm.wt.ugt80.
dsm.smt1.
dsm.wt.smt1.
sp.ugt80.
sp.wt.ugt80.
sp.smt1.
sp.wt.smt1.
im.ugt80.
im.wt.ugt80.
im.smt1.
im.wt.smt1.

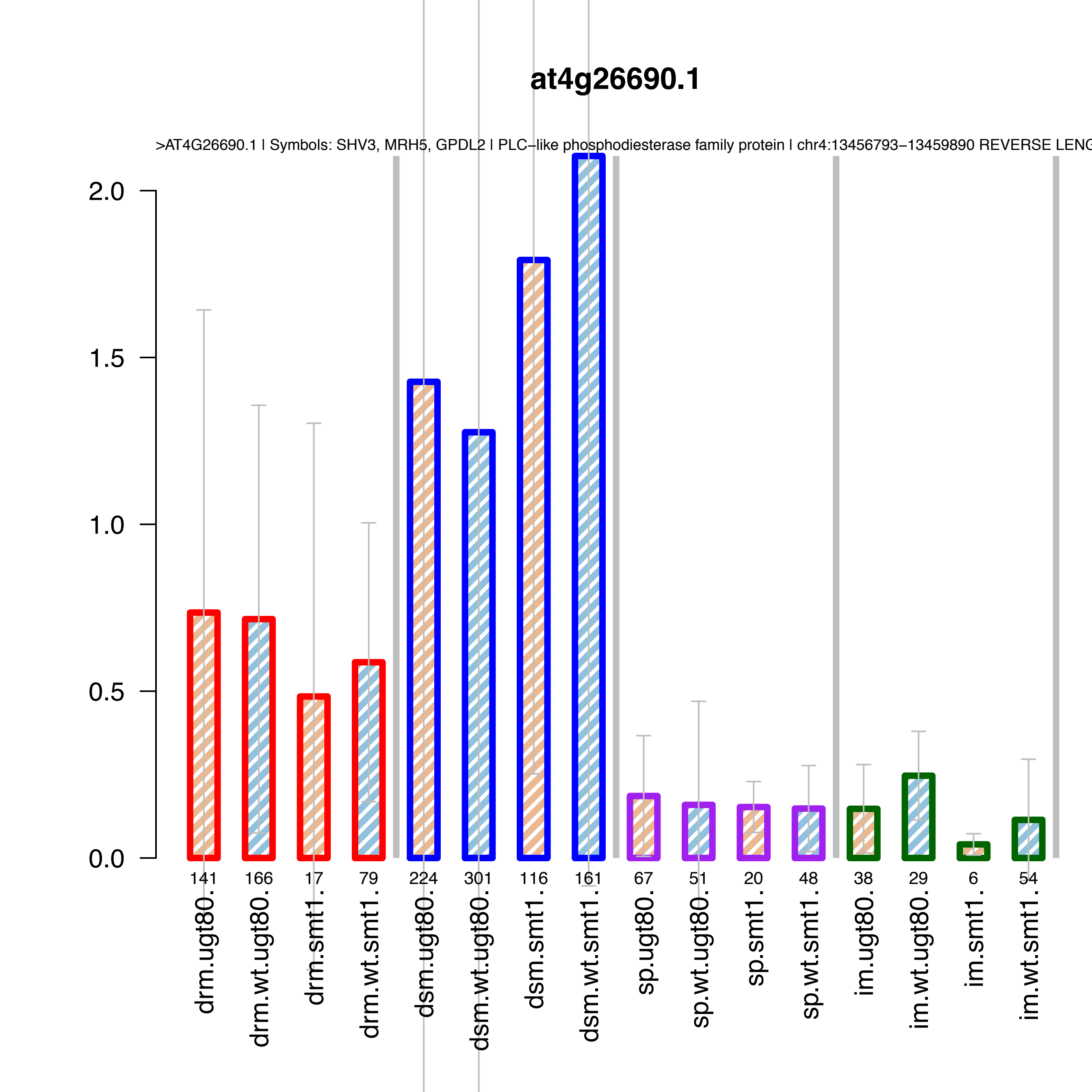
at4g26690.1
>AT4G26690.1 | Symbols: SHV3, MRH5, GPDL2 | PLC−like phosphodiesterase family protein | chr4:13456793−13459890 REVERSE LENG
2.0
1.5
1.0
0.5
0.0
141
166
17
79
224
301
116
161
67
51
20
48
38
29
6
54
drm.ugt80.
drm.wt.ugt80.
drm.smt1.
drm.wt.smt1.
dsm.ugt80.
dsm.wt.ugt80.
dsm.smt1.
dsm.wt.smt1.
sp.ugt80.
sp.wt.ugt80.
sp.smt1.
sp.wt.smt1.
im.ugt80.
im.wt.ugt80.
im.smt1.
im.wt.smt1.

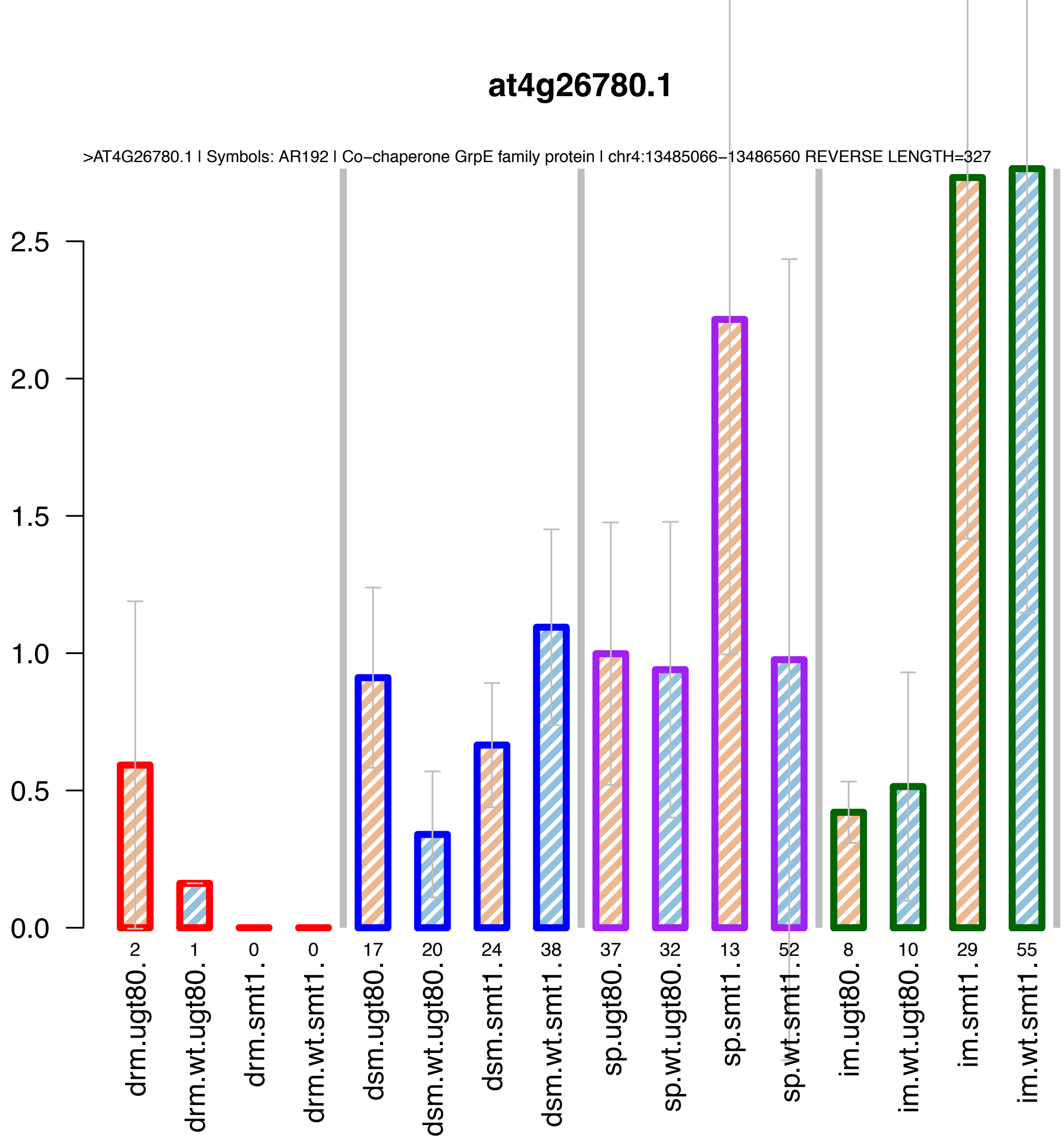

at4g26780.1
>AT4G26780.1 | Symbols: AR192 | Co−chaperone GrpE family protein | chr4:13485066−13486560 REVERSE LENGTH=327
0.0
0.5
1.0
1.5
2.0
2.5
2
1
0
0
17
20
24
38
37
32
13
52
8
10
29
55
drm.ugt80.
drm.wt.ugt80.
drm.smt1.
drm.wt.smt1.
dsm.ugt80.
dsm.wt.ugt80.
dsm.smt1.
dsm.wt.smt1.
sp.ugt80.
sp.wt.ugt80.
sp.smt1.
sp.wt.smt1.
im.ugt80.
im.wt.ugt80.
im.smt1.
im.wt.smt1.

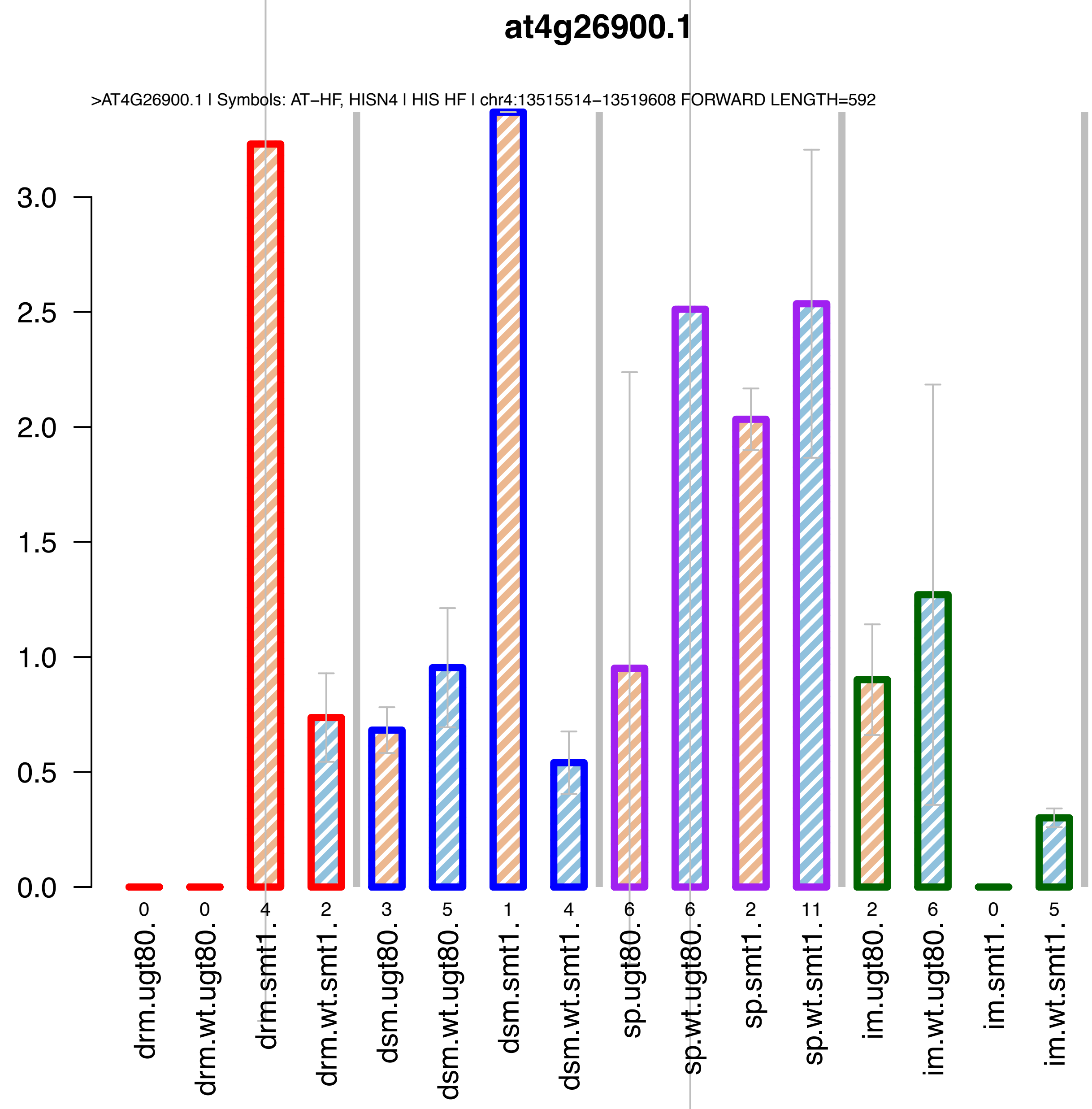

at4g26900.1
>AT4G26900.1 | Symbols: AT−HF, HISN4 | HIS HF | chr4:13515514−13519608 FORWARD LENGTH=592
3.0
2.5
2.0
1.5
1.0
0.5
0.0
0
0
4
2
3
5
1
4
6
6
2
11
2
6
0
5
drm.ugt80.
drm.wt.ugt80.
drm.smt1.
drm.wt.smt1.
dsm.ugt80.
dsm.wt.ugt80.
dsm.smt1.
dsm.wt.smt1.
sp.ugt80.
sp.wt.ugt80.
sp.smt1.
sp.wt.smt1.
im.ugt80.
im.wt.ugt80.
im.smt1.
im.wt.smt1.

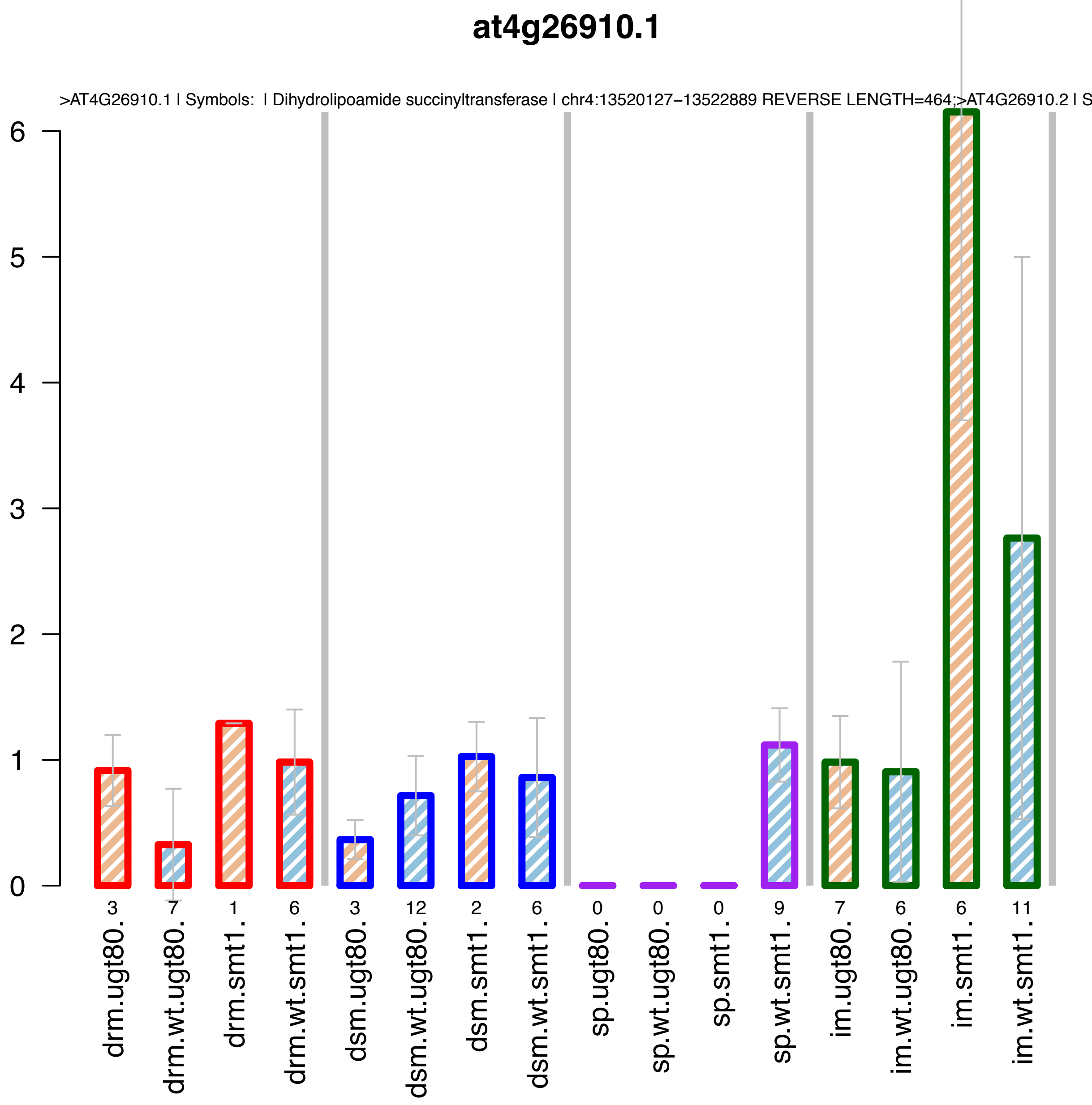

at4g26910.1
>AT4G26910.1 | Symbols: | Dihydrolipoamide succinyltransferase | chr4:13520127-13522889 REVERSE LENGTH=464;>AT4G26910.2 | S
0
1
2
3
4
5
6
3
7
1
6
3
12
2
6
0
0
0
9
7
6
6
11
drm.ugt80.
drm.wt.ugt80.
drm.smt1.
drm.wt.smt1.
dsm.ugt80.
dsm.wt.ugt80.
dsm.smt1.
dsm.wt.smt1.
sp.ugt80.
sp.wt.ugt80.
sp.smt1.
sp.wt.smt1.
im.ugt80.
im.wt.ugt80.
im.smt1.
im.wt.smt1.

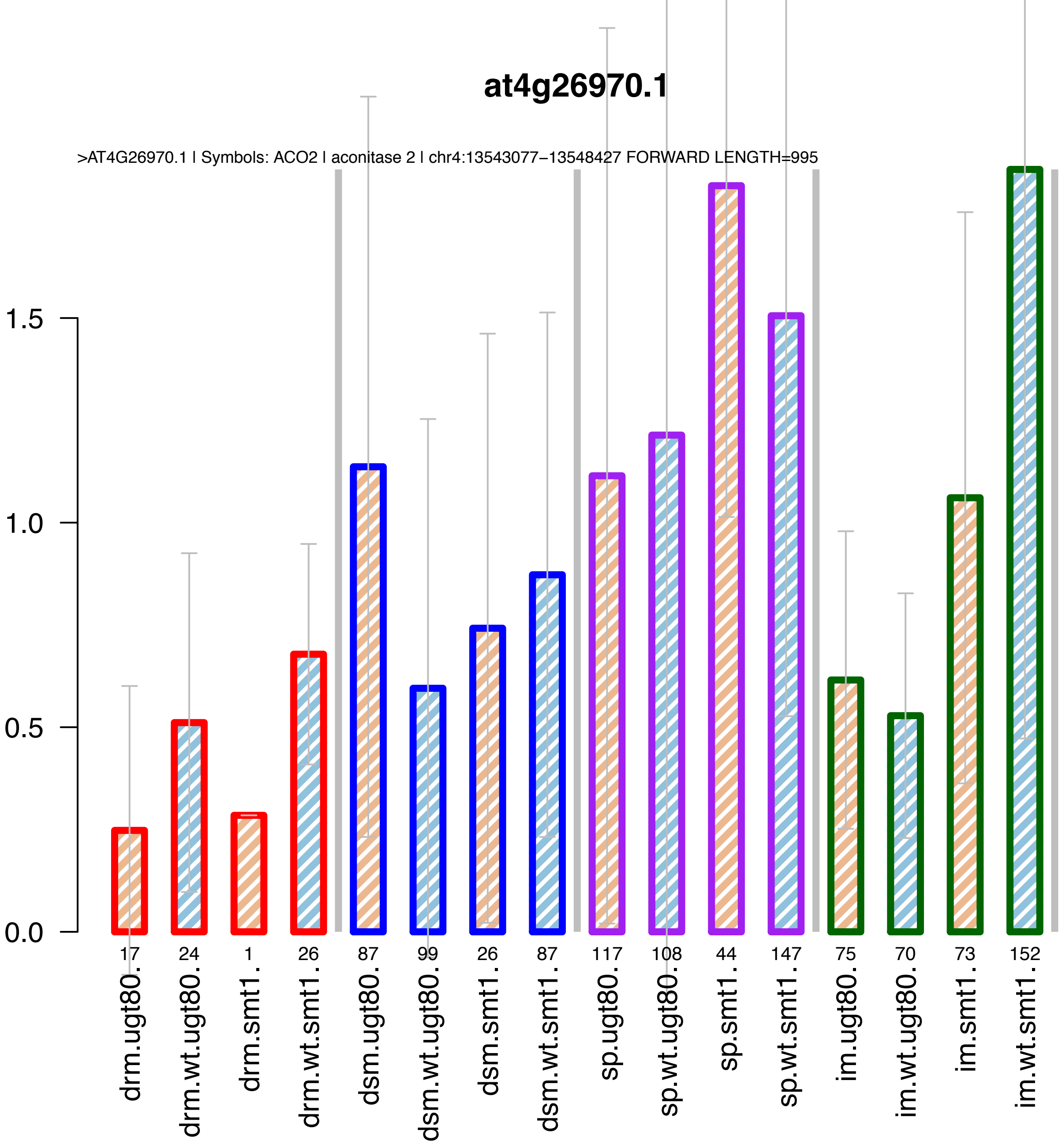

at4g26970.1
>AT4G26970.1 | Symbols: ACO2 | aconitase 2 | chr4:13543077–13548427 FORWARD LENGTH=995
0.0
0.5
1.0
1.5
17
24
1
26
87
99
26
87
117
108
44
147
75
70
73
152
drm.ugt80..
drm.wt.ugt80..
drm.smt1..
drm.wt.smt1..
dsm.ugt80..
dsm.wt.ugt80..
dsm.smt1..
dsm.wt.smt1..
sp.ugt80..
sp.wt.ugt80..
sp.smt1..
sp.wt.smt1..
im.ugt80..
im.wt.ugt80..
im.smt1..
im.wt.smt1..

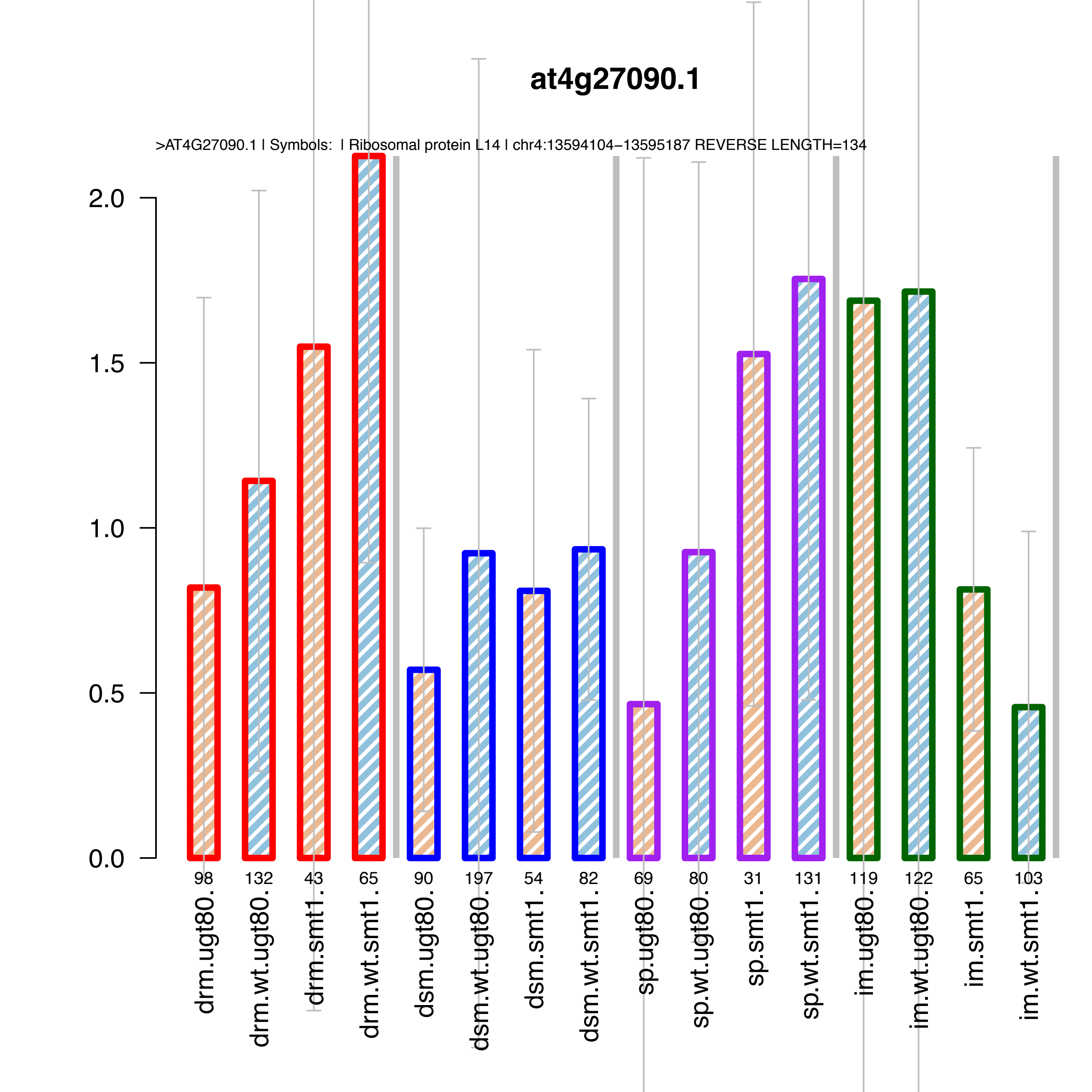

at4g27090.1
>AT4G27090.1 | Symbols: | Ribosomal protein L14 | chr4:13594104-13595187 REVERSE LENGTH=134
2.0
1.5
1.0
0.5
0.0
98
132
43
65
90
197
54
82
69
80
31
131
119
122
65
103
drm.ugt80..
drm.wt.ugt80..
drm.smt1..
drm.wt.smt1..
dsm.ugt80..
dsm.wt.ugt80..
dsm.smt1..
dsm.wt.smt1..
sp.ugt80..
sp.wt.ugt80..
sp.smt1..
sp.wt.smt1..
im.ugt80..
im.wt.ugt80..
im.smt1..
im.wt.smt1..

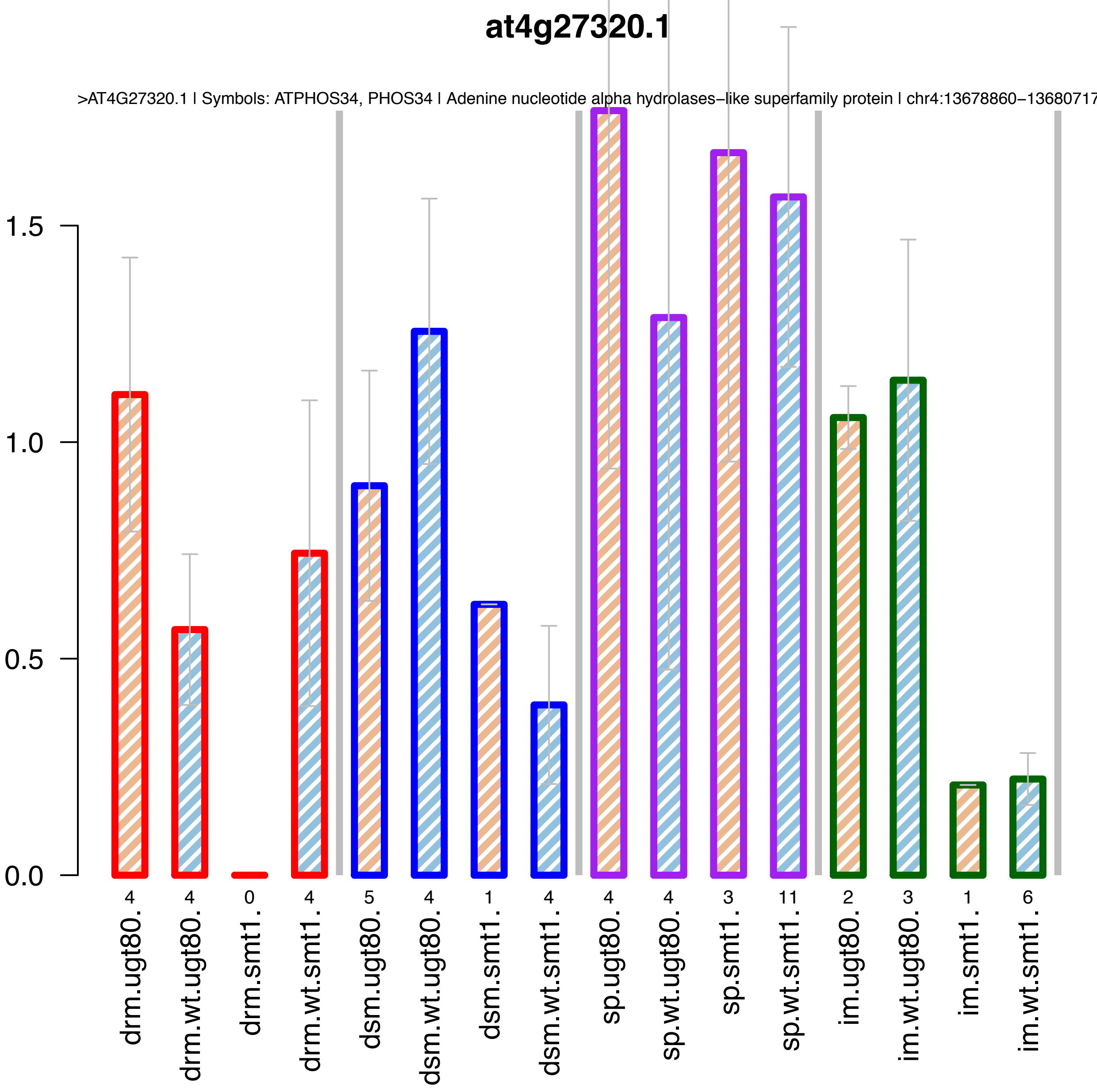
at4g27320.1
>AT4G27320.1 | Symbols: ATPHOS34, PHOS34 | Adenine nucleotide alpha hydrolases−like superfamily protein | chr4:13678860−13680717
0.0
0.5
1.0
1.5
4
4
0
4
5
4
1
4
4
4
3
11
2
3
1
6
drm.ugt80.
drm.wt.ugt80.
drm.smt1.
drm.wt.smt1.
dsm.ugt80.
dsm.wt.ugt80.
dsm.smt1.
dsm.wt.smt1.
sp.ugt80.
sp.wt.ugt80.
sp.smt1.
sp.wt.smt1.
im.ugt80.
im.wt.ugt80.
im.smt1.
im.wt.smt1.

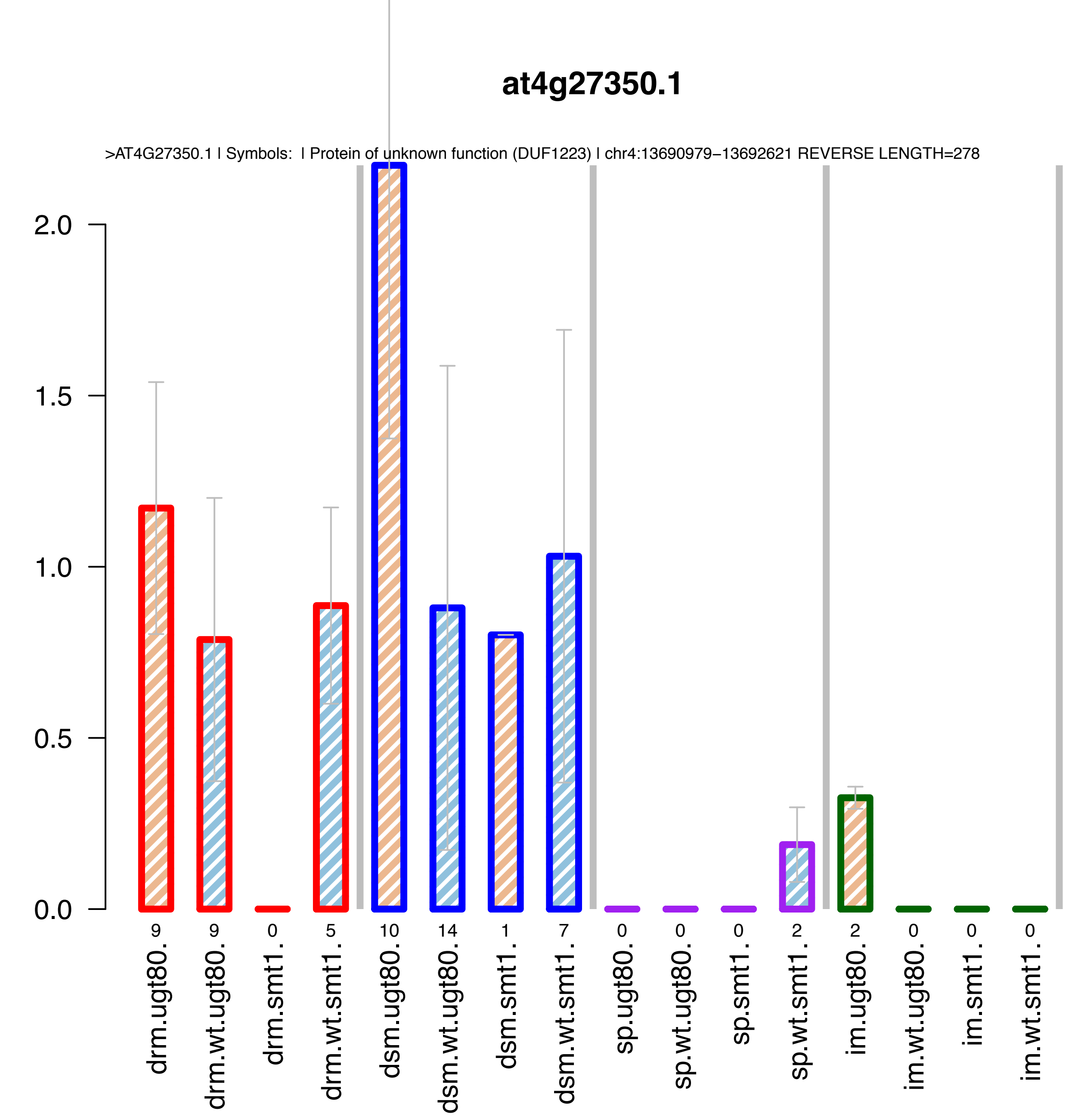

at4g27350.1
>AT4G27350.1 | Symbols: | Protein of unknown function (DUF1223) | chr4:13690979-13692621 REVERSE LENGTH=278
2.0
1.5
1.0
0.5
0.0
9
9
0
5
10
14
1
7
0
0
0
2
2
0
0
0
drm.ugt80.
drm.wt.ugt80.
drm.smt1.
drm.wt.smt1.
dsm.ugt80.
dsm.wt.ugt80.
dsm.smt1.
dsm.wt.smt1.
sp.ugt80.
sp.wt.ugt80.
sp.smt1.
sp.wt.smt1.
im.ugt80.
im.wt.ugt80.
im.smt1.
im.wt.smt1.

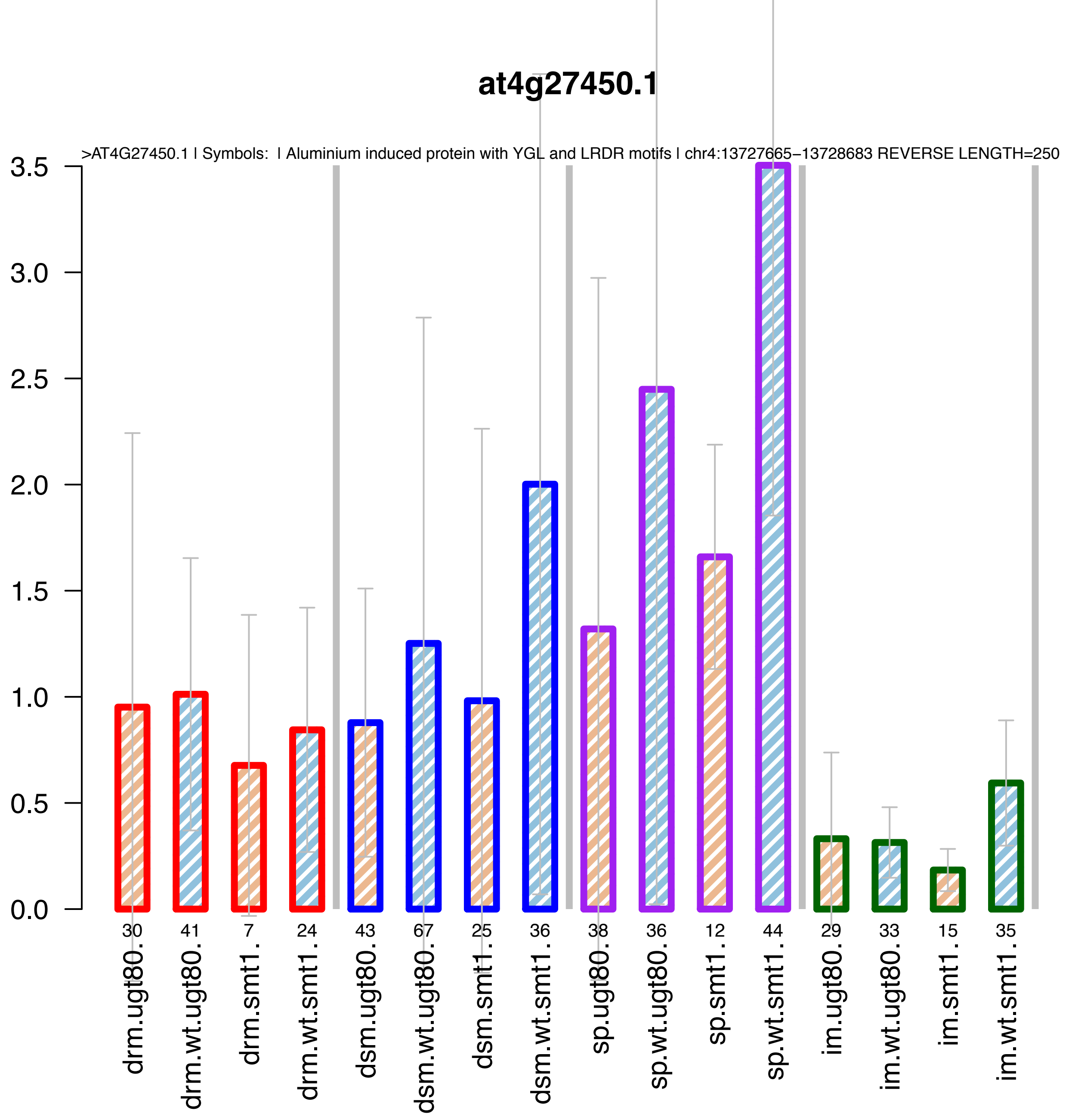

at4g27450.1
>AT4G27450.1 | Symbols: | Aluminium induced protein with YGL and LRDR motifs | chr4:13727665–13728683 REVERSE LENGTH=250
0.0
0.5
1.0
1.5
2.0
2.5
3.0
3.5
30
41
7
24
43
67
25
36
38
36
12
44
29
33
15
35
drm.ugt80..
drm.wt.ugt80..
drm.smt1..
drm.wt.smt1..
dsm.ugt80..
dsm.wt.ugt80..
dsm.smt1..
dsm.wt.smt1..
sp.ugt80..
sp.wt.ugt80..
sp.smt1..
sp.wt.smt1..
im.ugt80..
im.wt.ugt80..
im.smt1..
im.wt.smt1..

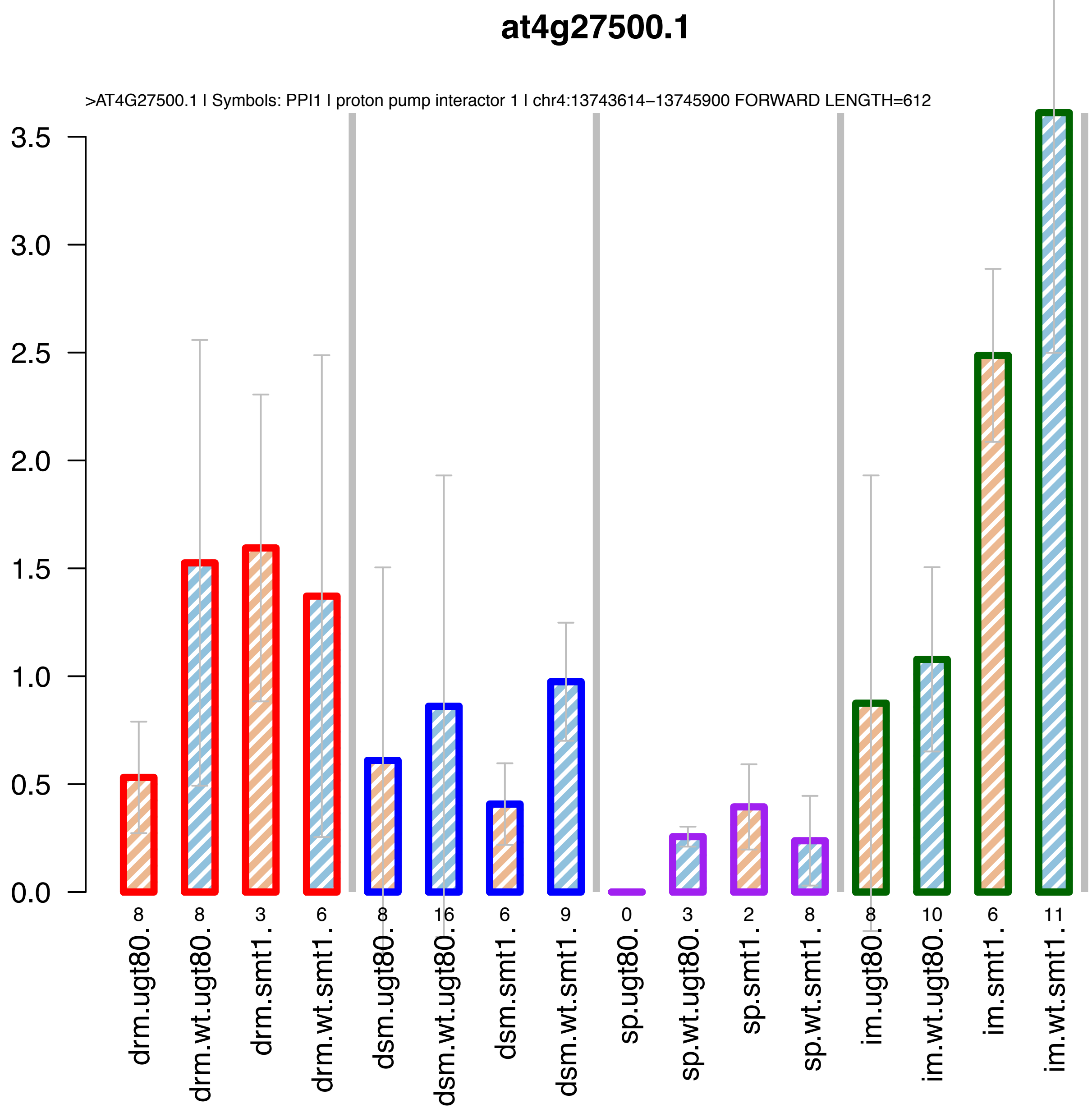

at4g27500.1
>AT4G27500.1 | Symbols: PPI1 | proton pump interactor 1 | chr4:13743614–13745900 FORWARD LENGTH=612
3.5
3.0
2.5
2.0
1.5
1.0
0.5
0.0
8
8
3
6
8
16
6
9
0
3
2
8
8
10
6
11
drm.ugt80.
drm.wt.ugt80.
drm.smt1.
drm.wt.smt1.
dsm.ugt80.
dsm.wt.ugt80.
dsm.smt1.
dsm.wt.smt1.
sp.ugt80.
sp.wt.ugt80.
sp.smt1.
sp.wt.smt1.
im.ugt80.
im.wt.ugt80.
im.smt1.
im.wt.smt1.

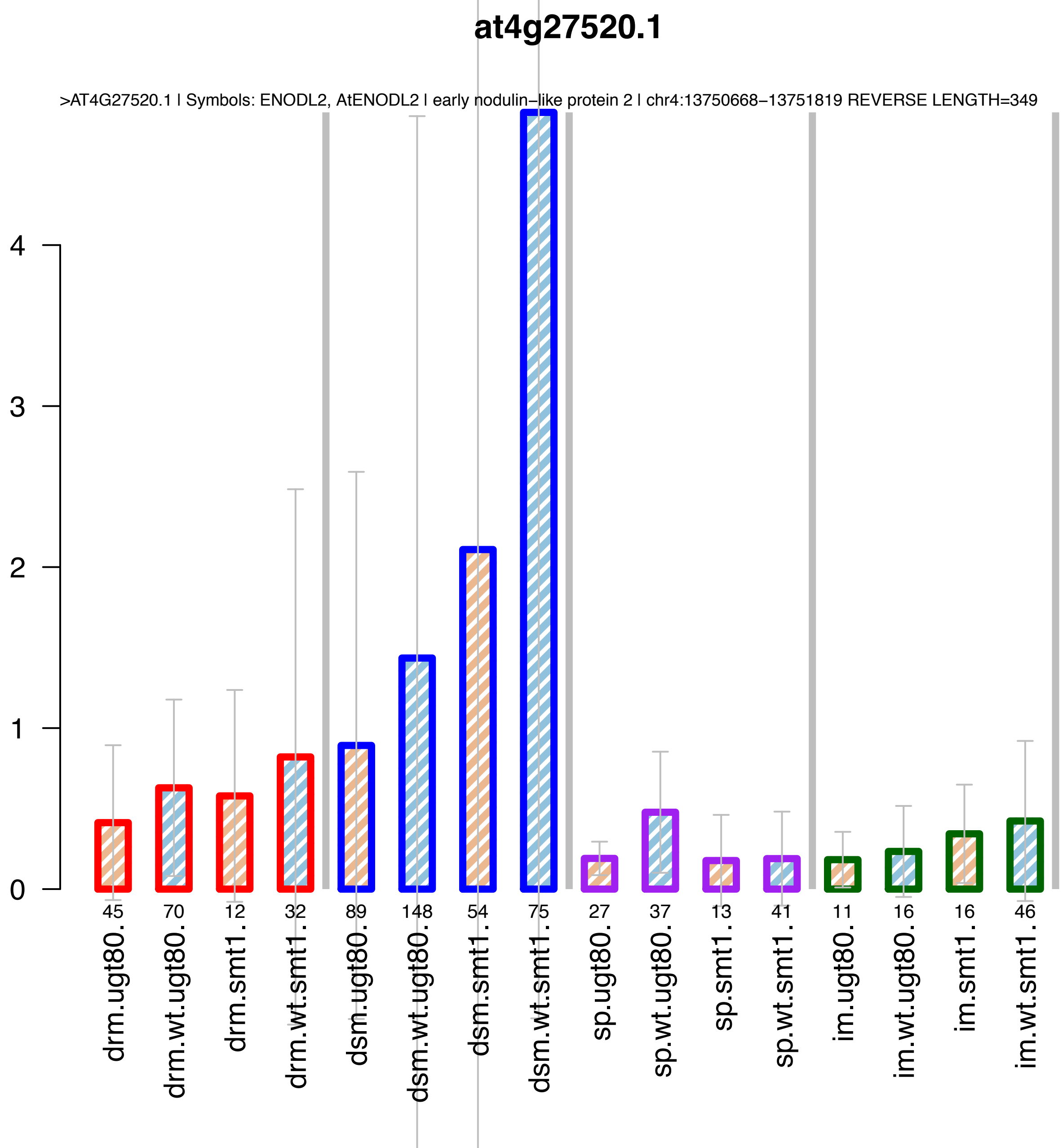

at4g27520.1
>AT4G27520.1 | Symbols: ENODL2, AtENODL2 | early nodulin−like protein 2 | chr4:13750668−13751819 REVERSE LENGTH=349
0
1
2
3
4
45
70
12
32
89
148
54
75
27
37
13
41
11
16
16
46
drm.ugt80..
drm.wt.ugt80..
drm.smt1..
drm.wt.smt1..
dsm.ugt80..
dsm.wt.ugt80..
dsm.smt1..
dsm.wt.smt1..
sp.ugt80..
sp.wt.ugt80..
sp.smt1..
sp.wt.smt1..
im.ugt80..
im.wt.ugt80..
im.smt1..
im.wt.smt1..

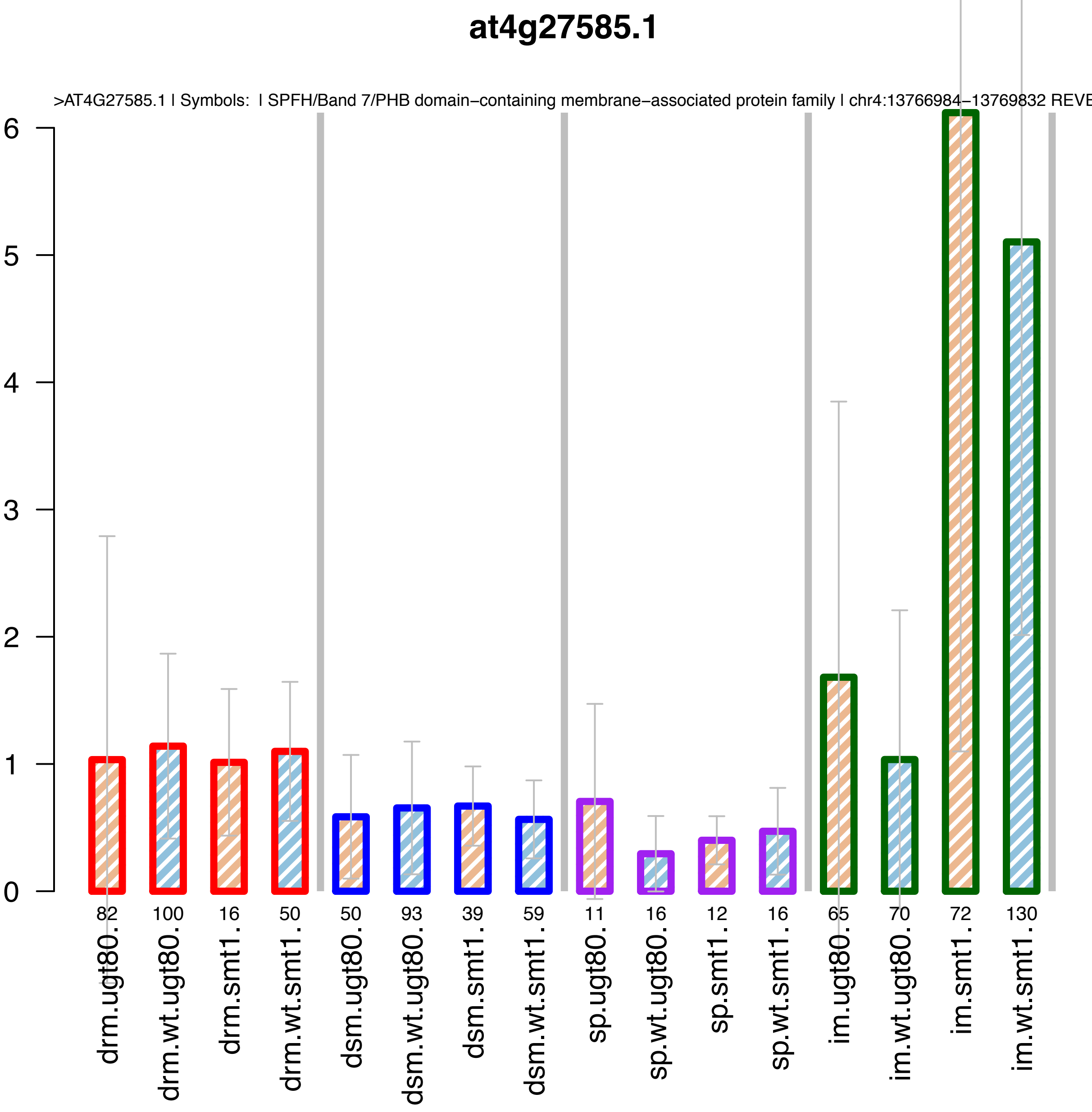

at4g27585.1
>AT4G27585.1 | Symbols: | SPFH/Band 7/PHB domain−containing membrane−associated protein family | chr4:13766984−13769832 REVE
0
1
2
3
4
5
6
82
100
16
50
50
93
39
59
11
16
12
16
65
70
72
130
drm.ugt80.
drm.wt.ugt80.
drm.smt1.
drm.wt.smt1.
dsm.ugt80.
dsm.wt.ugt80.
dsm.smt1.
dsm.wt.smt1.
sp.ugt80.
sp.wt.ugt80.
sp.smt1.
sp.wt.smt1.
im.ugt80.
im.wt.ugt80.
im.smt1.
im.wt.smt1.

# at4g27690.1

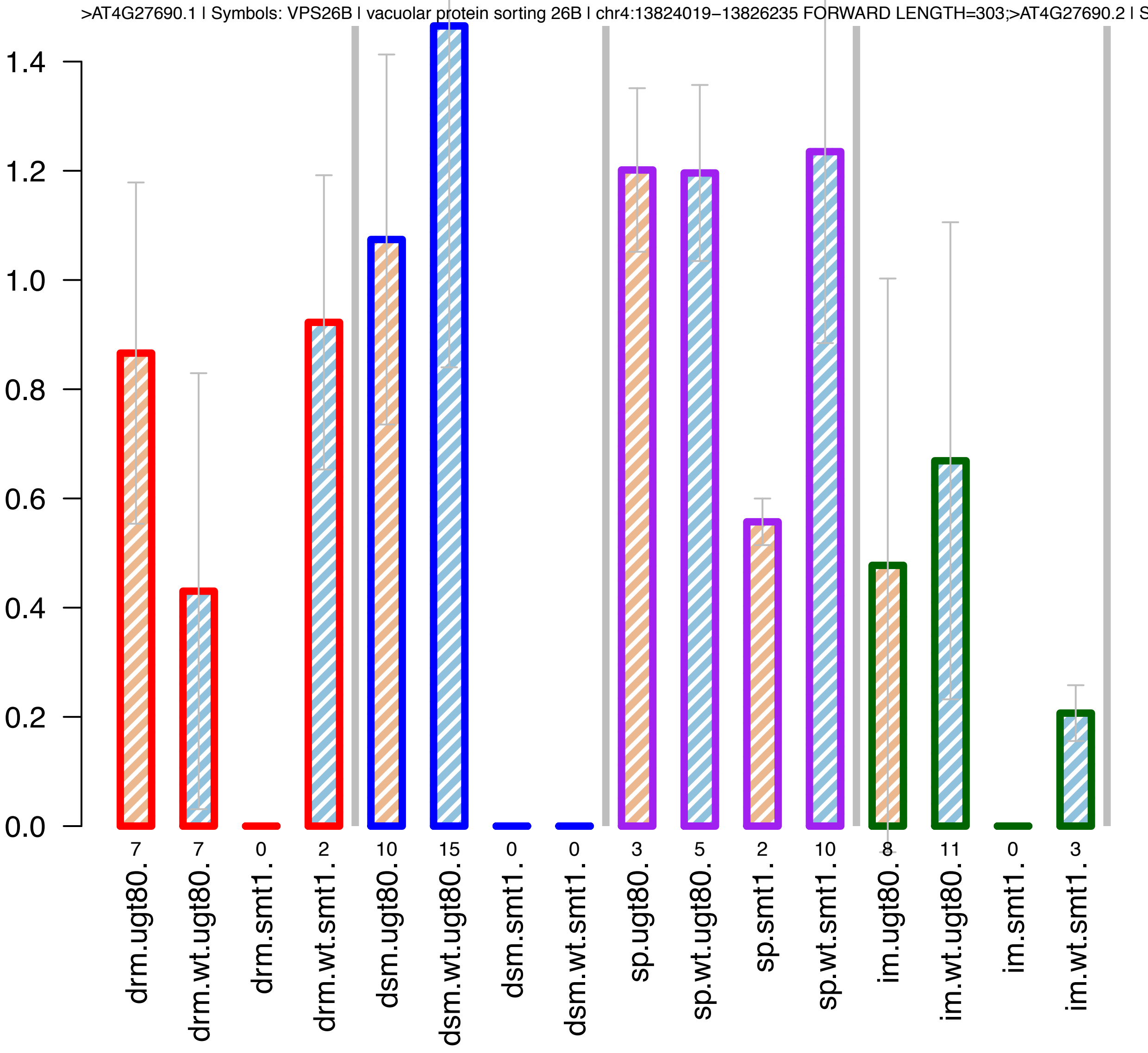
>AT4G27690.1 | Symbols: VPS26B | vacuolar protein sorting 26B | chr4:13824019-13826235 FORWARD LENGTH=303;>AT4G27690.2 | S
1.4
1.2
1.0
0.8
0.6
0.4
0.2
0.0
7
7
0
2
10
15
0
0
3
5
2
10
8
11
0
3
drm.ugt80.
drm.wt.ugt80.
drm.smt1.
drm.wt.smt1.
dsm.ugt80.
dsm.wt.ugt80.
dsm.smt1.
dsm.wt.smt1.
sp.ugt80.
sp.wt.ugt80.
sp.smt1.
sp.wt.smt1.
im.ugt80.
im.wt.ugt80.
im.smt1.
im.wt.smt1.

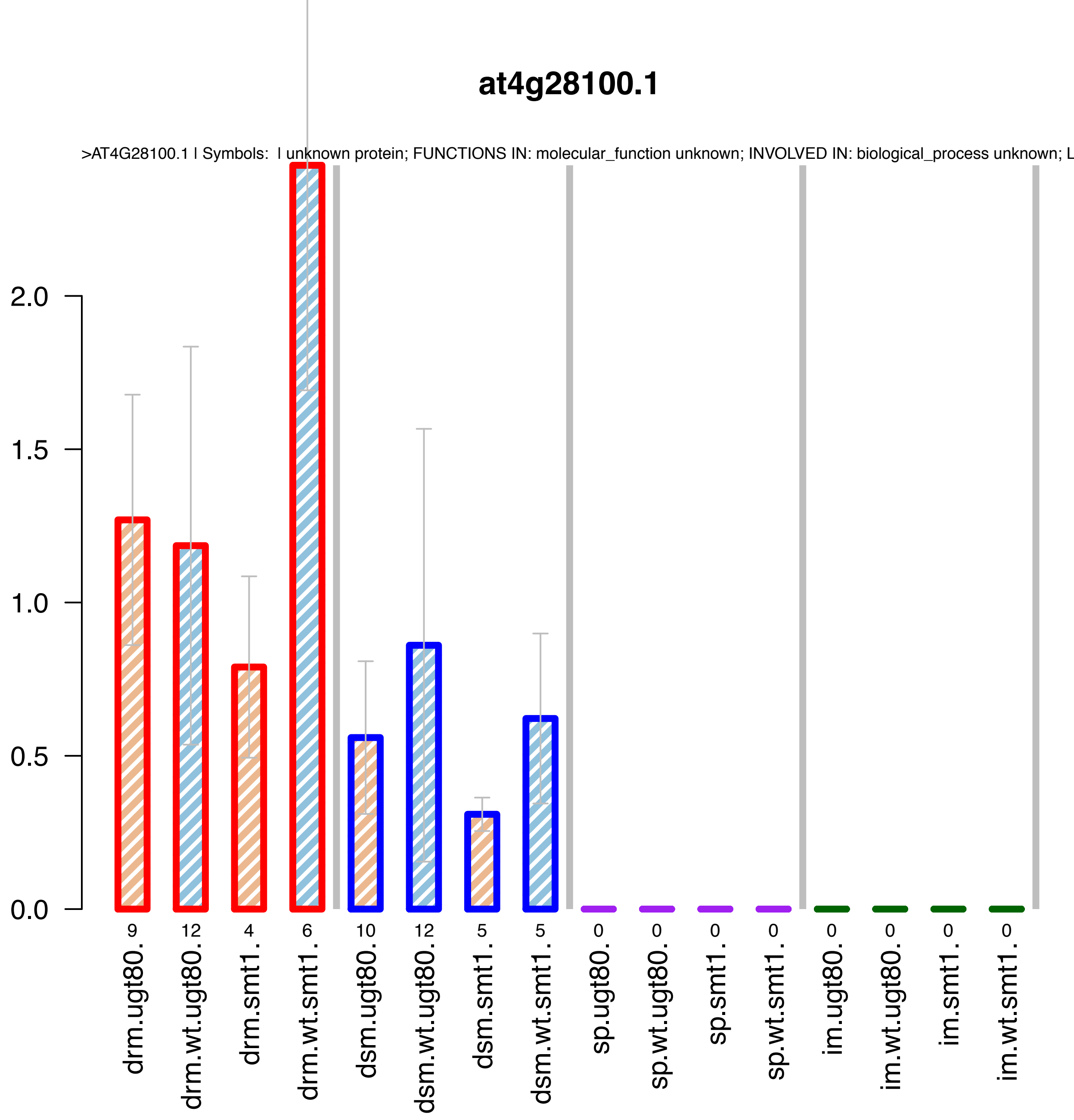
at4g28100.1
>AT4G28100.1 | Symbols: | unknown protein; FUNCTIONS IN: molecular_function unknown; INVOLVED IN: biological_process unknown; L
0.0
0.5
1.0
1.5
2.0
9
12
4
6
10
12
5
5
0
0
0
0
0
0
0
0
drm.ugt80.
drm.wt.ugt80.
drm.smt1.
drm.wt.smt1.
dsm.ugt80.
dsm.wt.ugt80.
dsm.smt1.
dsm.wt.smt1.
sp.ugt80.
sp.wt.ugt80.
sp.smt1.
sp.wt.smt1.
im.ugt80.
im.wt.ugt80.
im.smt1.
im.wt.smt1.

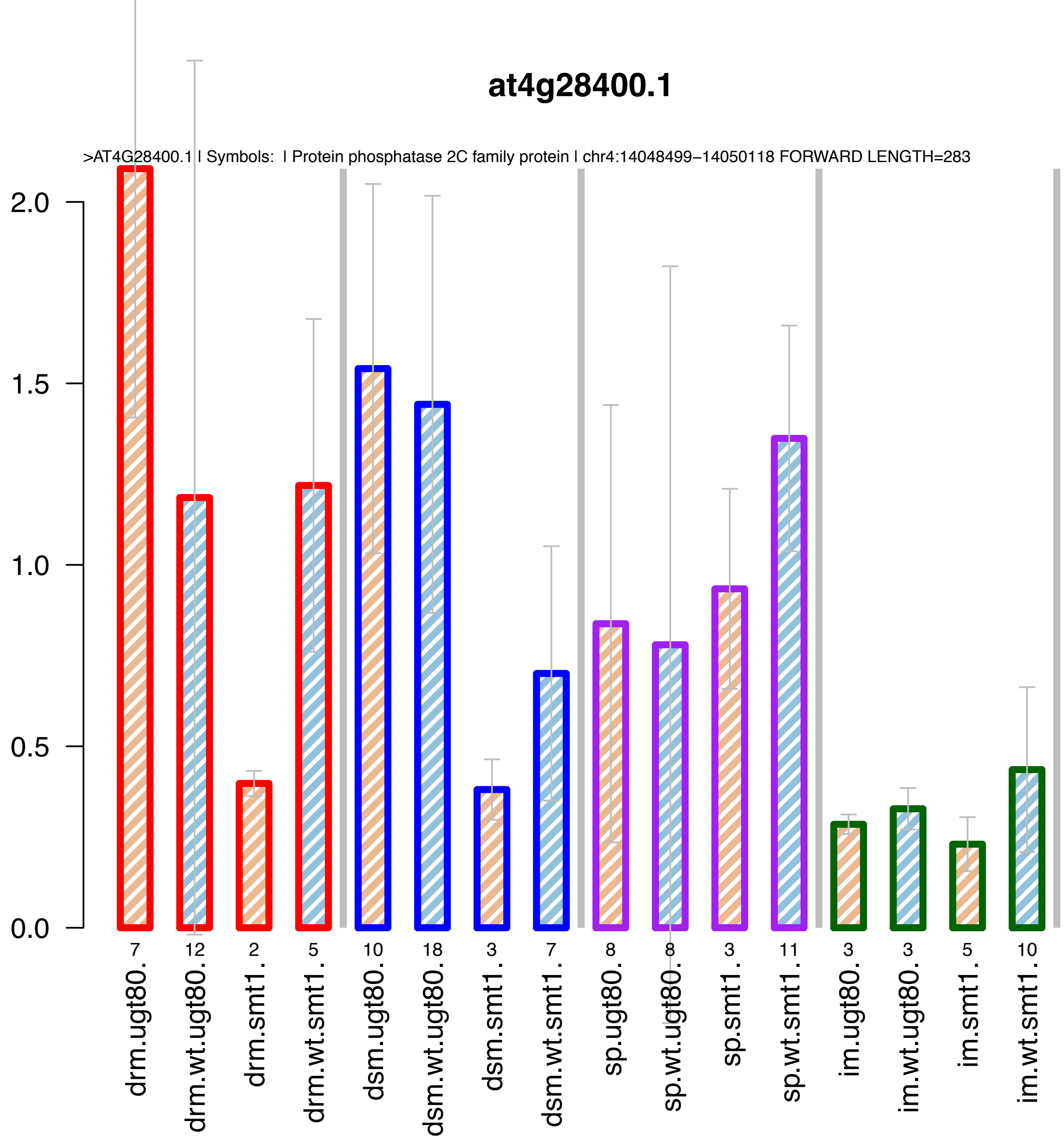
at4g28400.1
>AT4G28400.1 | Symbols: | Protein phosphatase 2C family protein | chr4:14048499-14050118 FORWARD LENGTH=283
2.0
1.5
1.0
0.5
0.0
7
12
2
5
10
18
3
7
8
8
3
11
3
3
5
10
drm.ugt80..
drm.wt.ugt80..
drm.smt1..
drm.wt.smt1..
dsm.ugt80..
dsm.wt.ugt80..
dsm.smt1..
dsm.wt.smt1..
sp.ugt80..
sp.wt.ugt80..
sp.smt1..
sp.wt.smt1..
im.ugt80..
im.wt.ugt80..
im.smt1..
im.wt.smt1..

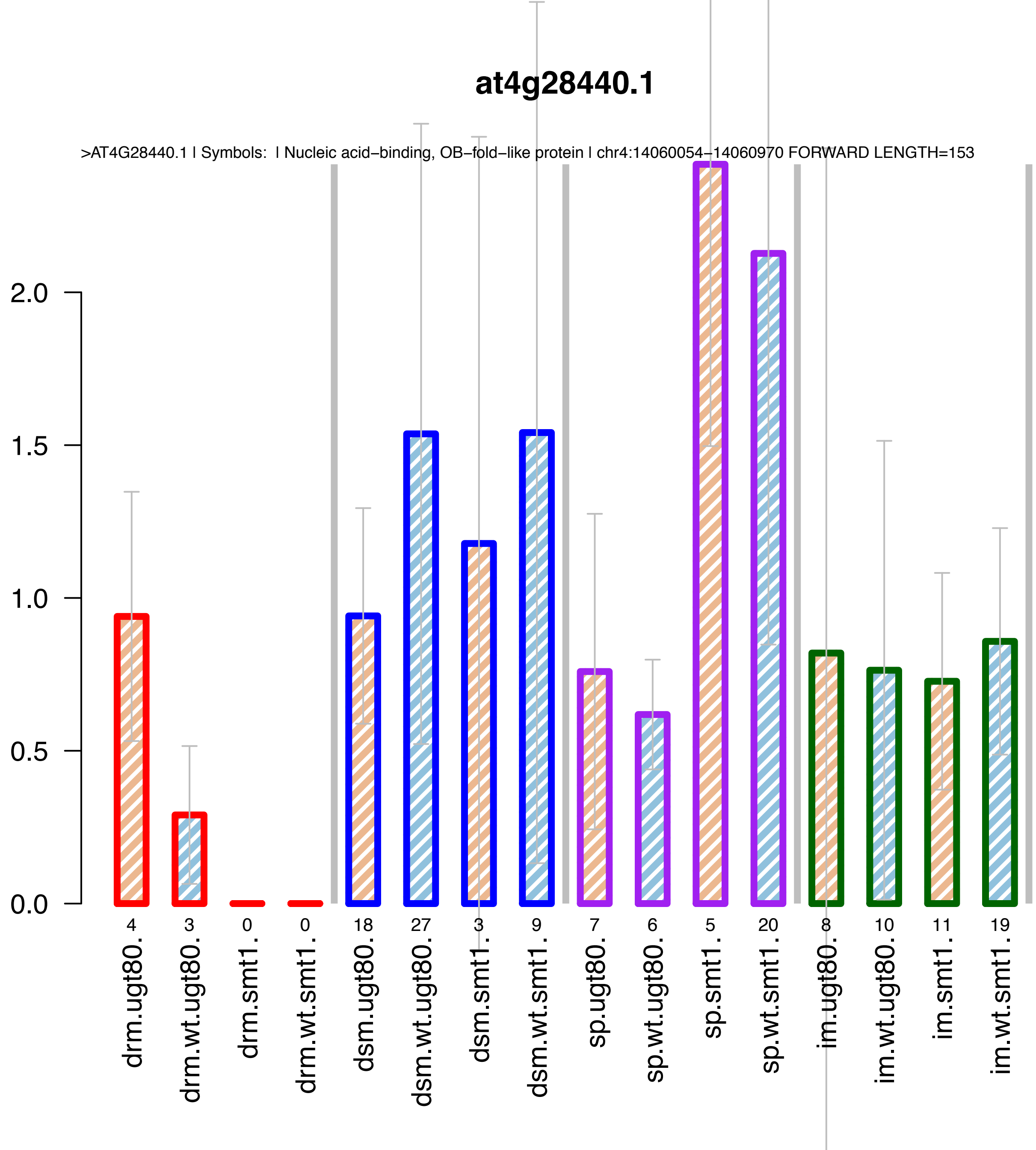

at4g28440.1
>AT4G28440.1 | Symbols: | Nucleic acid–binding, OB–fold–like protein | chr4:14060054–14060970 FORWARD LENGTH=153
2.0
1.5
1.0
0.5
0.0
4
3
0
0
18
27
3
9
7
6
5
20
8
10
11
19
drm.ugt80.
drm.wt.ugt80.
drm.smt1.
drm.wt.smt1.
dsm.ugt80.
dsm.wt.ugt80.
dsm.smt1.
dsm.wt.smt1.
sp.ugt80.
sp.wt.ugt80.
sp.smt1.
sp.wt.smt1.
im.ugt80.
im.wt.ugt80.
im.smt1.
im.wt.smt1.

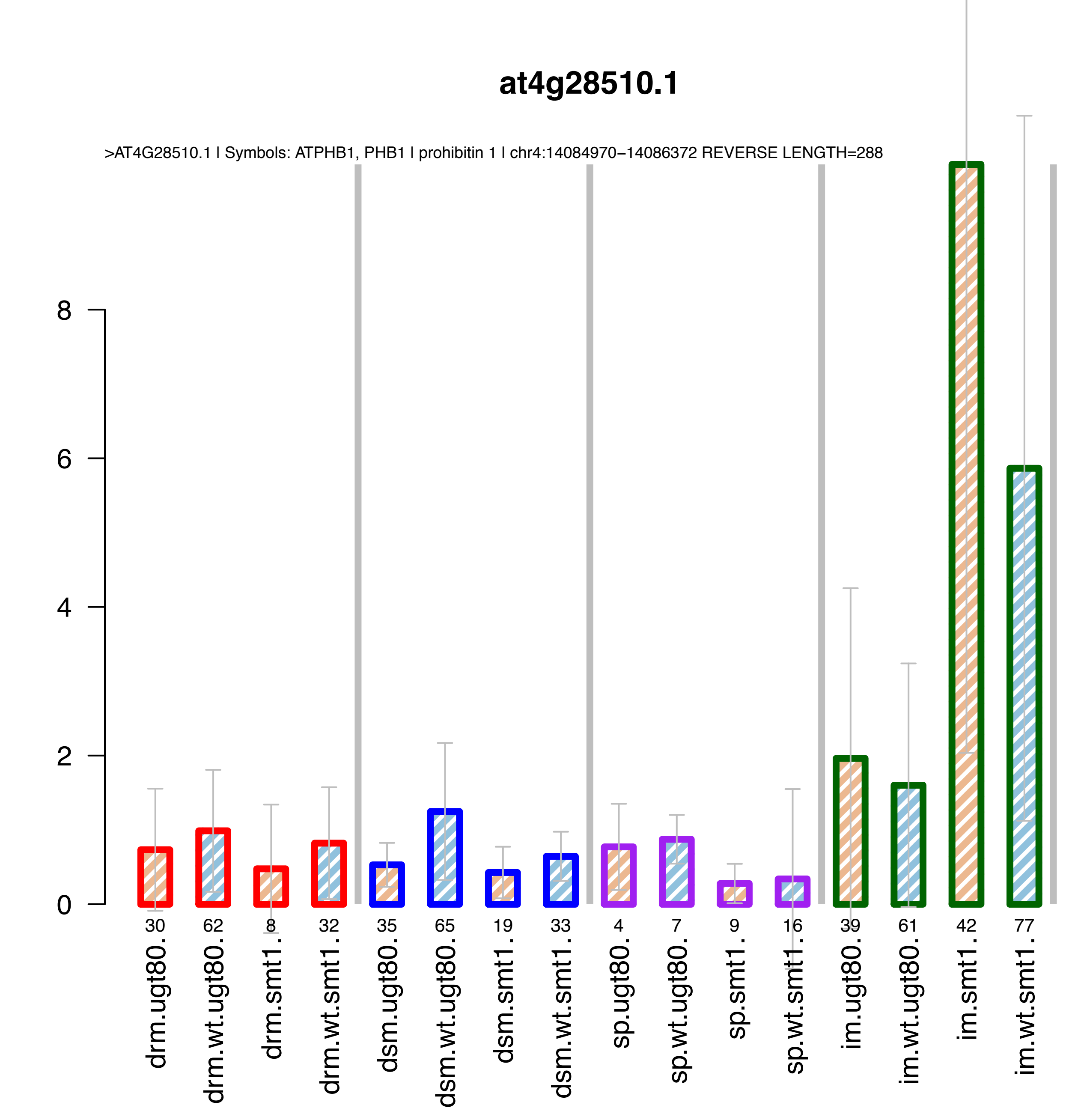

at4g28510.1
>AT4G28510.1 | Symbols: ATPHB1, PHB1 | prohibitin 1 | chr4:14084970–14086372 REVERSE LENGTH=288
0
2
4
6
8
30
62
8
32
35
65
19
33
4
7
9
16
39
61
42
77
drm.ugt80.
drm.wt.ugt80.
drm.smt1.
drm.wt.smt1.
dsm.ugt80.
dsm.wt.ugt80.
dsm.smt1.
dsm.wt.smt1.
sp.ugt80.
sp.wt.ugt80.
sp.smt1.
sp.wt.smt1.
im.ugt80.
im.wt.ugt80.
im.smt1.
im.wt.smt1.

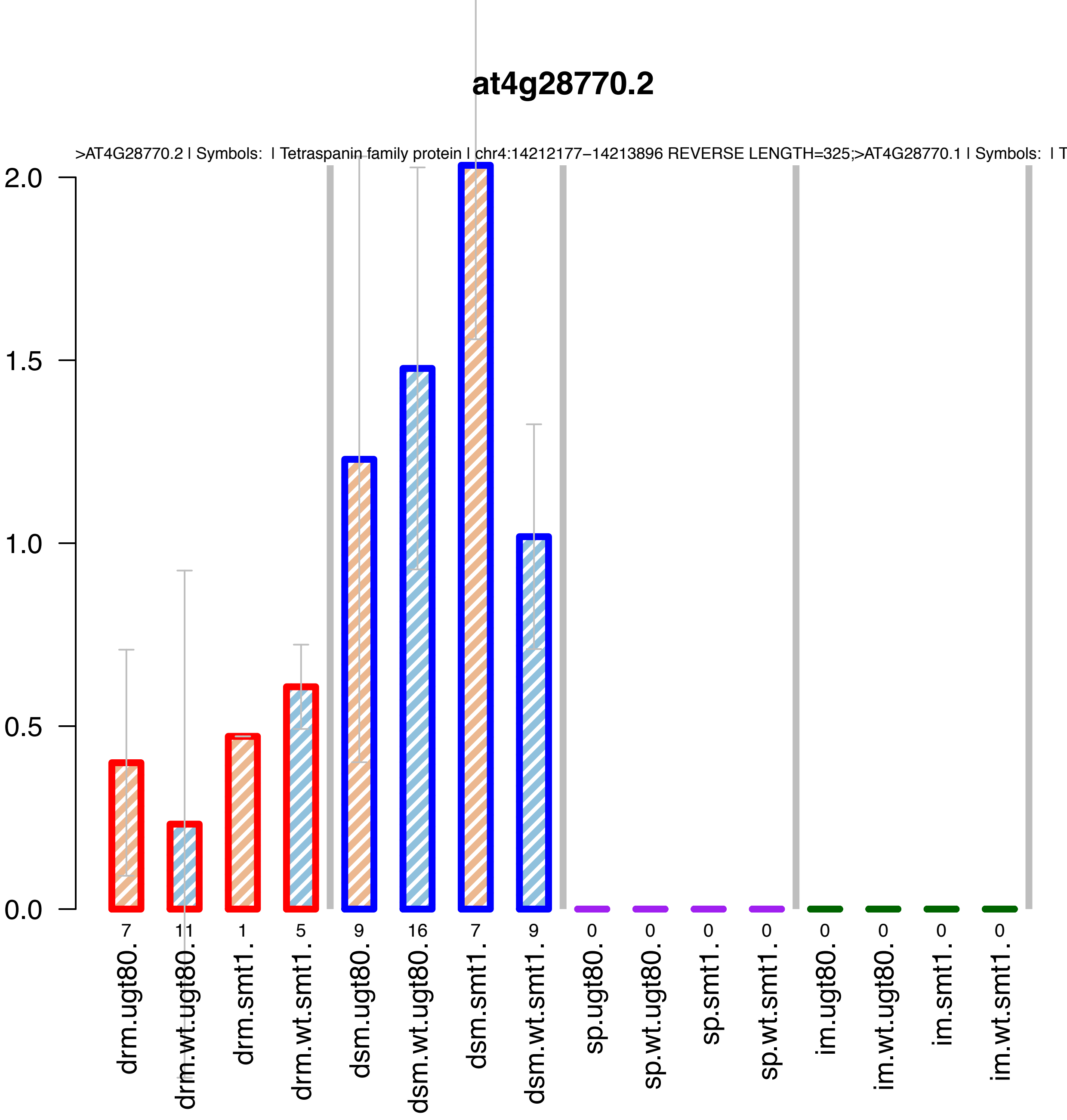

at4g28770.2
>AT4G28770.2 | Symbols: | Tetraspanin family protein | chr4:14212177-14213896 REVERSE LENGTH=325;>AT4G28770.1 | Symbols: | T
2.0
1.5
1.0
0.5
0.0
7
11
1
5
9
16
7
9
0
0
0
0
0
0
0
0
drm.ugt80.
drm.wt.ugt80.
drm.smt1.
drm.wt.smt1.
dsm.ugt80.
dsm.wt.ugt80.
dsm.smt1.
dsm.wt.smt1.
sp.ugt80.
sp.wt.ugt80.
sp.smt1.
sp.wt.smt1.
im.ugt80.
im.wt.ugt80.
im.smt1.
im.wt.smt1.

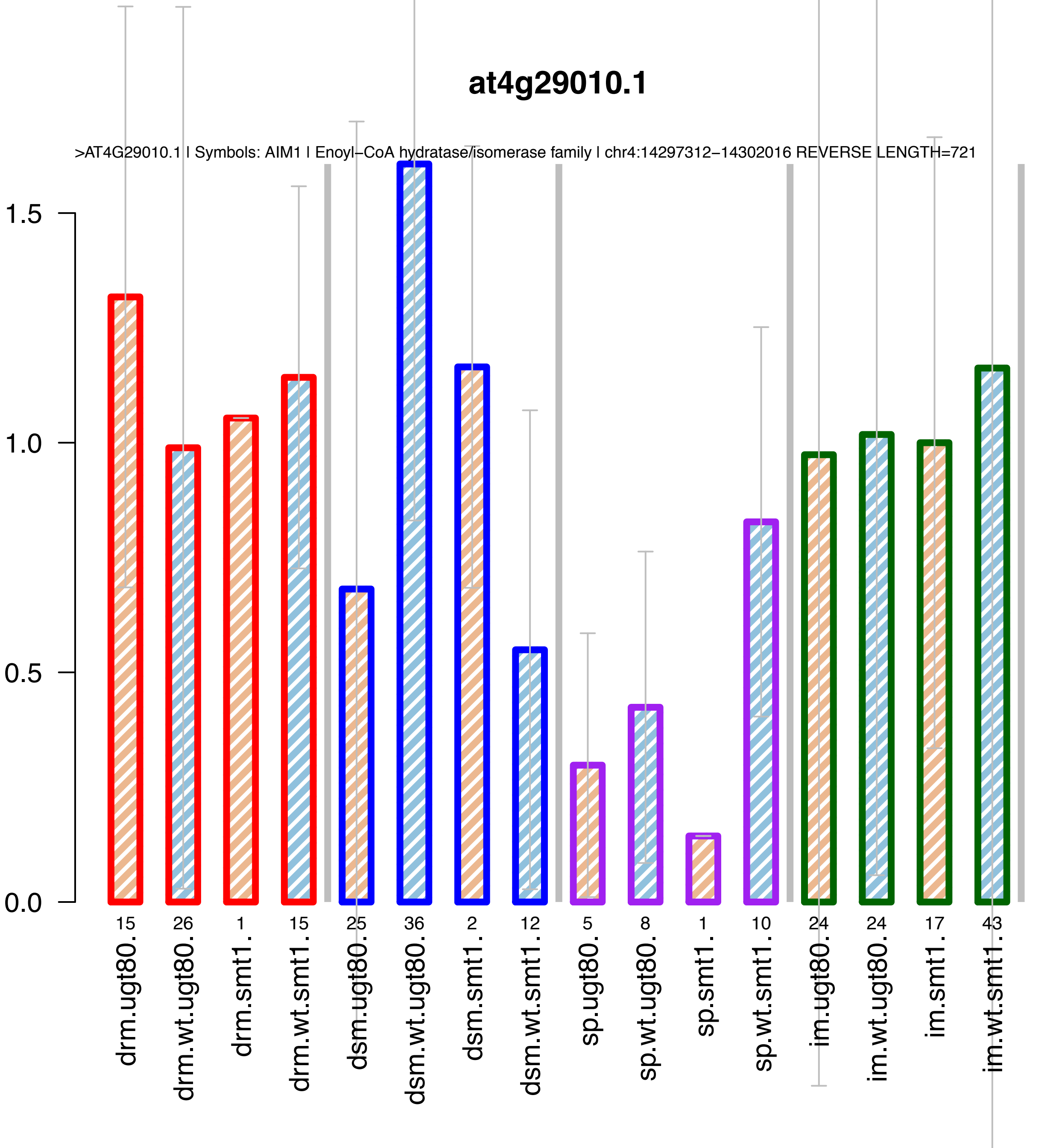

at4g29010.1
>AT4G29010.1 | Symbols: AIM1 | Enoyl−CoA hydratase/isomerase family | chr4:14297312−14302016 REVERSE LENGTH=721
1.5
1.0
0.5
0.0
15
26
1
15
25
36
2
12
5
8
1
10
24
24
17
43
drm.ugt80..
drm.wt.ugt80..
drm.smt1..
drm.wt.smt1..
dsm.ugt80..
dsm.wt.ugt80..
dsm.smt1..
dsm.wt.smt1..
sp.ugt80..
sp.wt.ugt80..
sp.smt1..
sp.wt.smt1..
im.ugt80..
im.wt.ugt80..
im.smt1..
im.wt.smt1..

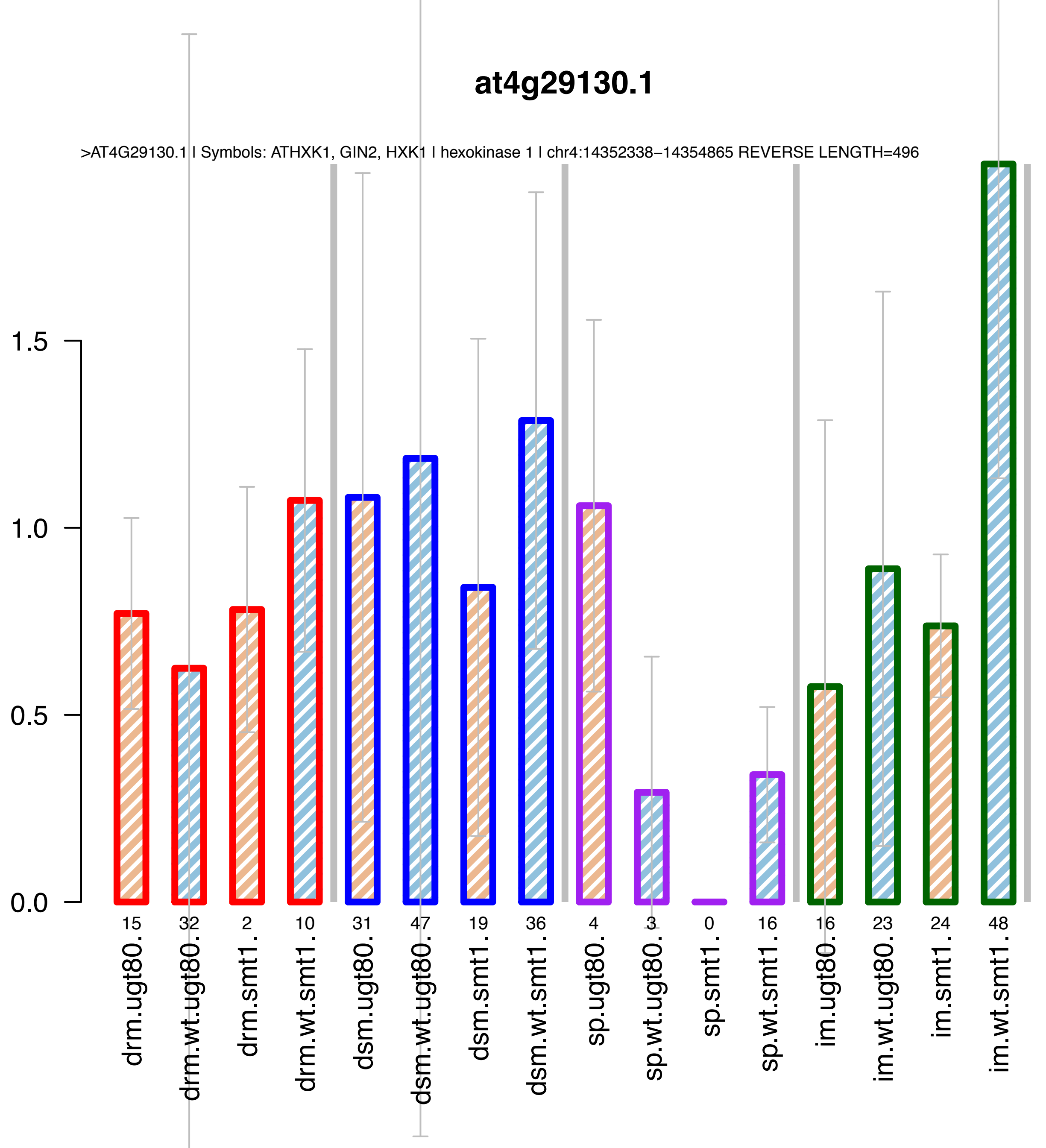

at4g29130.1
>AT4G29130.1 | Symbols: ATHXK1, GIN2, HXK1 | hexokinase 1 | chr4:14352338−14354865 REVERSE LENGTH=496
1.5
1.0
0.5
0.0
15
32
2
10
31
47
19
36
4
3
0
16
16
23
24
48
drm.ugt80..
drm.wt.ugt80..
drm.smt1..
drm.wt.smt1..
dsm.ugt80..
dsm.wt.ugt80..
dsm.smt1..
dsm.wt.smt1..
sp.ugt80..
sp.wt.ugt80..
sp.smt1..
sp.wt.smt1..
im.ugt80..
im.wt.ugt80..
im.smt1..
im.wt.smt1..

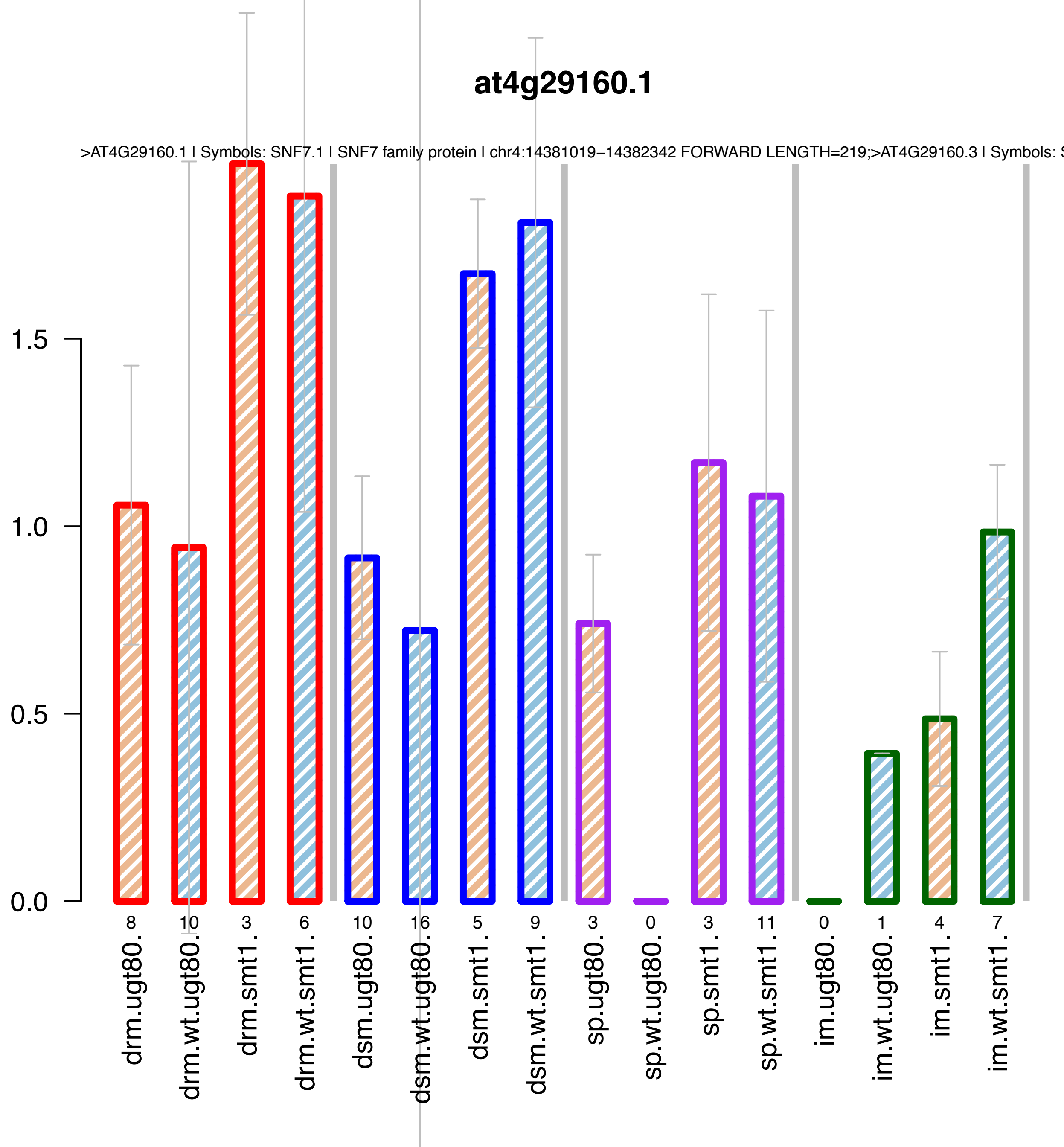

at4g29160.1
>AT4G29160.1 | Symbols: SNF7.1 | SNF7 family protein | chr4:14381019-14382342 FORWARD LENGTH=219;>AT4G29160.3 | Symbols: S
0.0
0.5
1.0
1.5
8
10
3
6
10
16
5
9
3
0
3
11
0
1
4
7
drm.ugt80..
drm.wt.ugt80..
drm.smt1..
drm.wt.smt1..
dsm.ugt80..
dsm.wt.ugt80..
dsm.smt1..
dsm.wt.smt1..
sp.ugt80..
sp.wt.ugt80..
sp.smt1..
sp.wt.smt1..
im.ugt80..
im.wt.ugt80..
im.smt1..
im.wt.smt1..

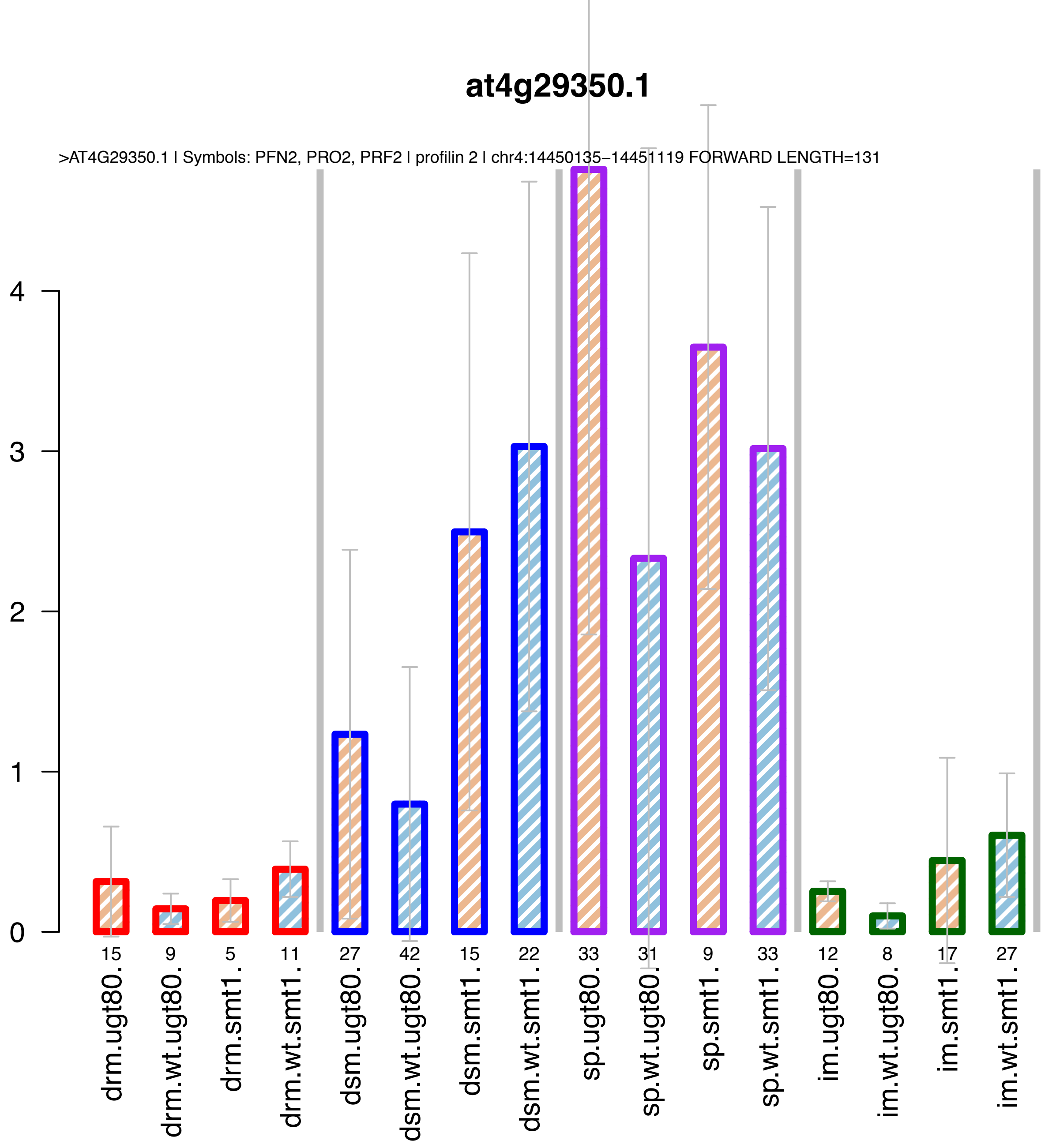

at4g29350.1
>AT4G29350.1 | Symbols: PFN2, PRO2, PRF2 | profilin 2 | chr4:14450135–14451119 FORWARD LENGTH=131
0
1
2
3
4
15
9
5
11
27
42
15
22
33
31
9
33
12
8
17
27
drm.ugt80..
drm.wt.ugt80..
drm.smt1..
drm.wt.smt1..
dsm.ugt80..
dsm.wt.ugt80..
dsm.smt1..
dsm.wt.smt1..
sp.ugt80..
sp.wt.ugt80..
sp.smt1..
sp.wt.smt1..
im.ugt80..
im.wt.ugt80..
im.smt1..
im.wt.smt1..

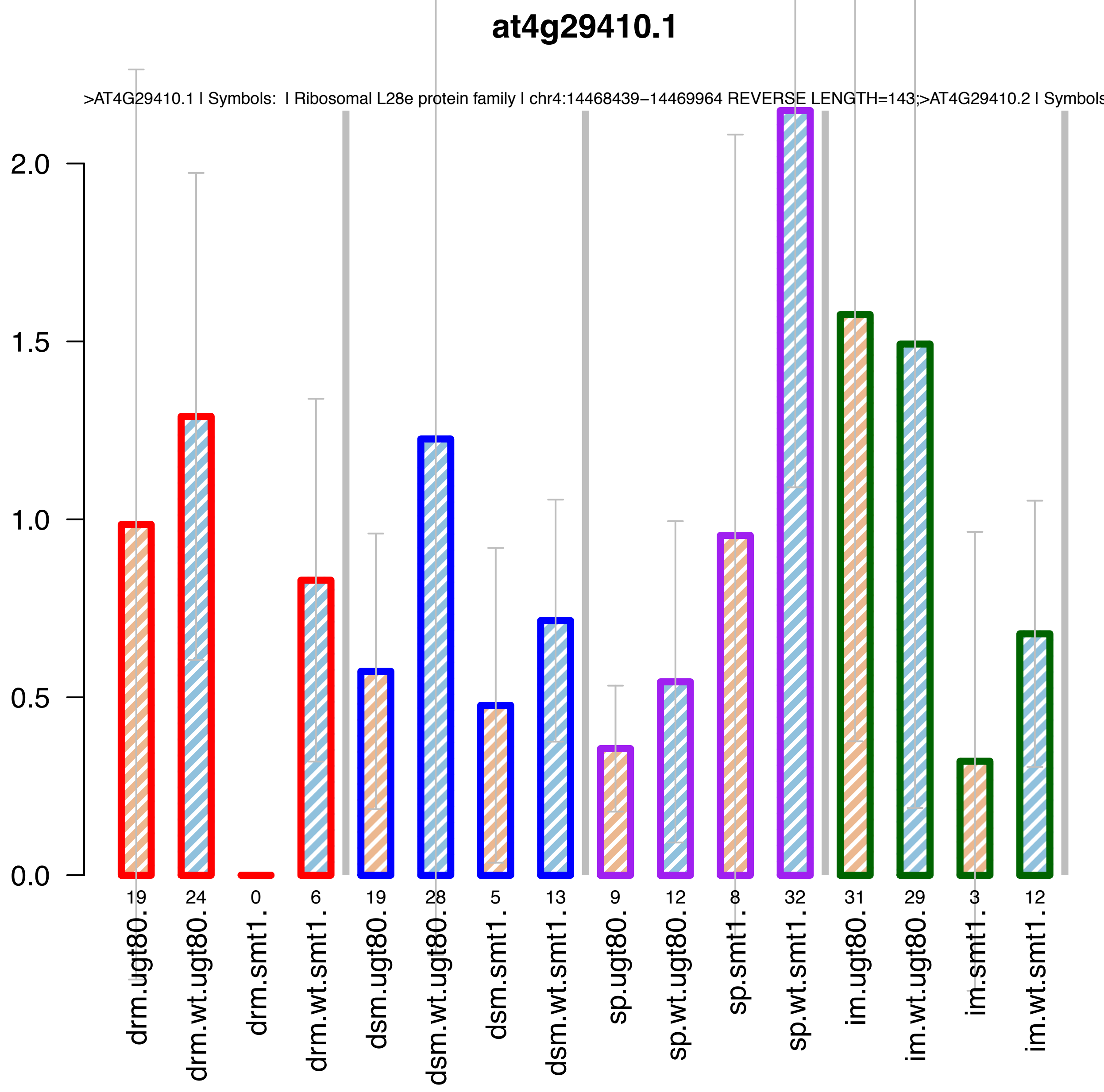

at4g29410.1
>AT4G29410.1 | Symbols: | Ribosomal L28e protein family | chr4:14468439-14469964 REVERSE LENGTH=143;>AT4G29410.2 | Symbols
2.0
1.5
1.0
0.5
0.0
19
24
0
6
19
28
5
13
9
12
8
32
31
29
3
12
drm.ugt80..
drm.wt.ugt80..
drm.smt1..
drm.wt.smt1..
dsm.ugt80..
dsm.wt.ugt80..
dsm.smt1..
dsm.wt.smt1..
sp.ugt80..
sp.wt.ugt80..
sp.smt1..
sp.wt.smt1..
im.ugt80..
im.wt.ugt80..
im.smt1..
im.wt.smt1..

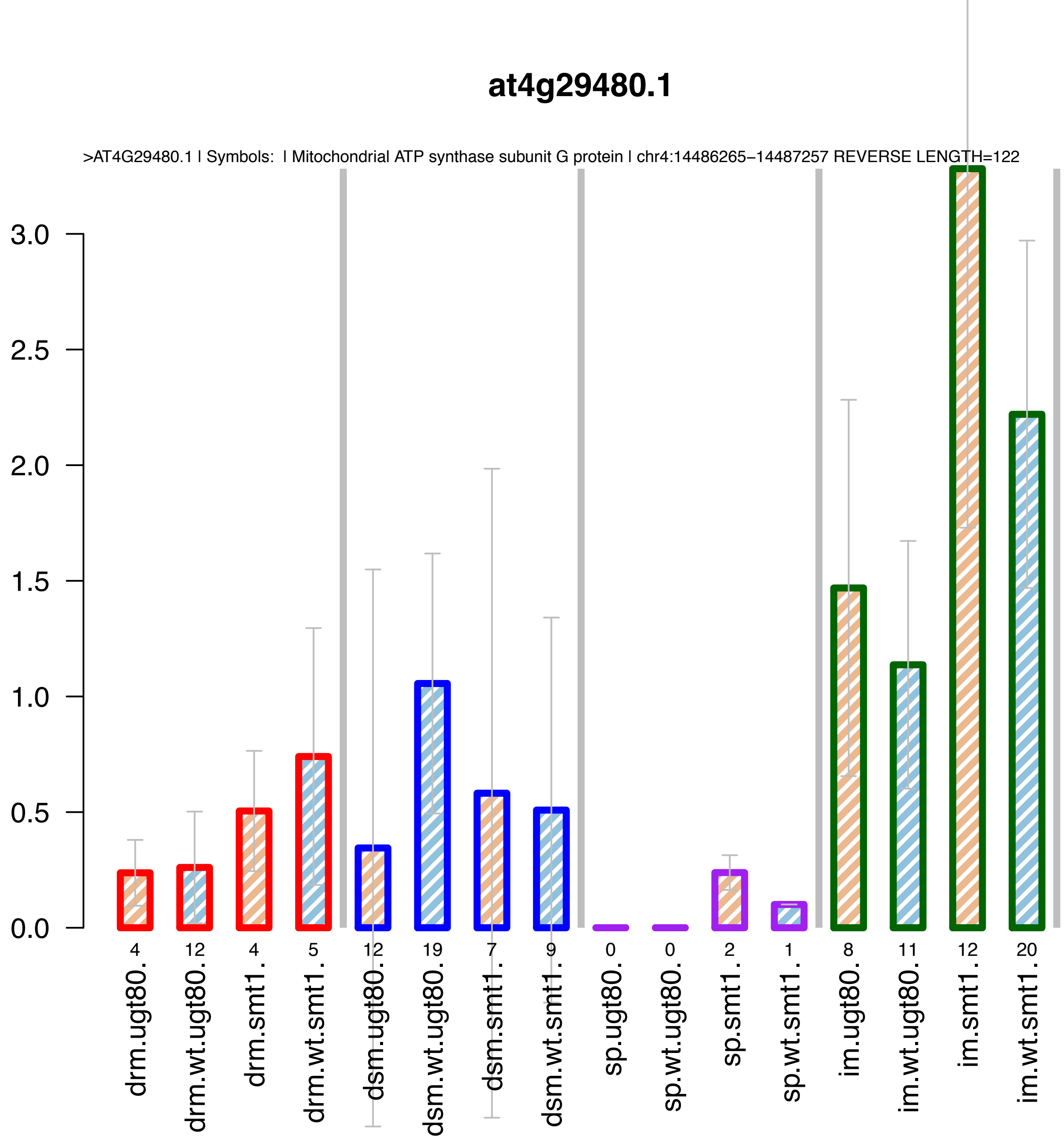

at4g29480.1
>AT4G29480.1 | Symbols: | Mitochondrial ATP synthase subunit G protein | chr4:14486265−14487257 REVERSE LENGTH=122
0.0
0.5
1.0
1.5
2.0
2.5
3.0
4
12
4
5
12
19
7
9
0
0
2
1
8
11
12
20
drm.ugt80.
drm.wt.ugt80.
drm.smt1.
drm.wt.smt1.
dsm.ugt80.
dsm.wt.ugt80.
dsm.smt1.
dsm.wt.smt1.
sp.ugt80.
sp.wt.ugt80.
sp.smt1.
sp.wt.smt1.
im.ugt80.
im.wt.ugt80.
im.smt1.
im.wt.smt1.

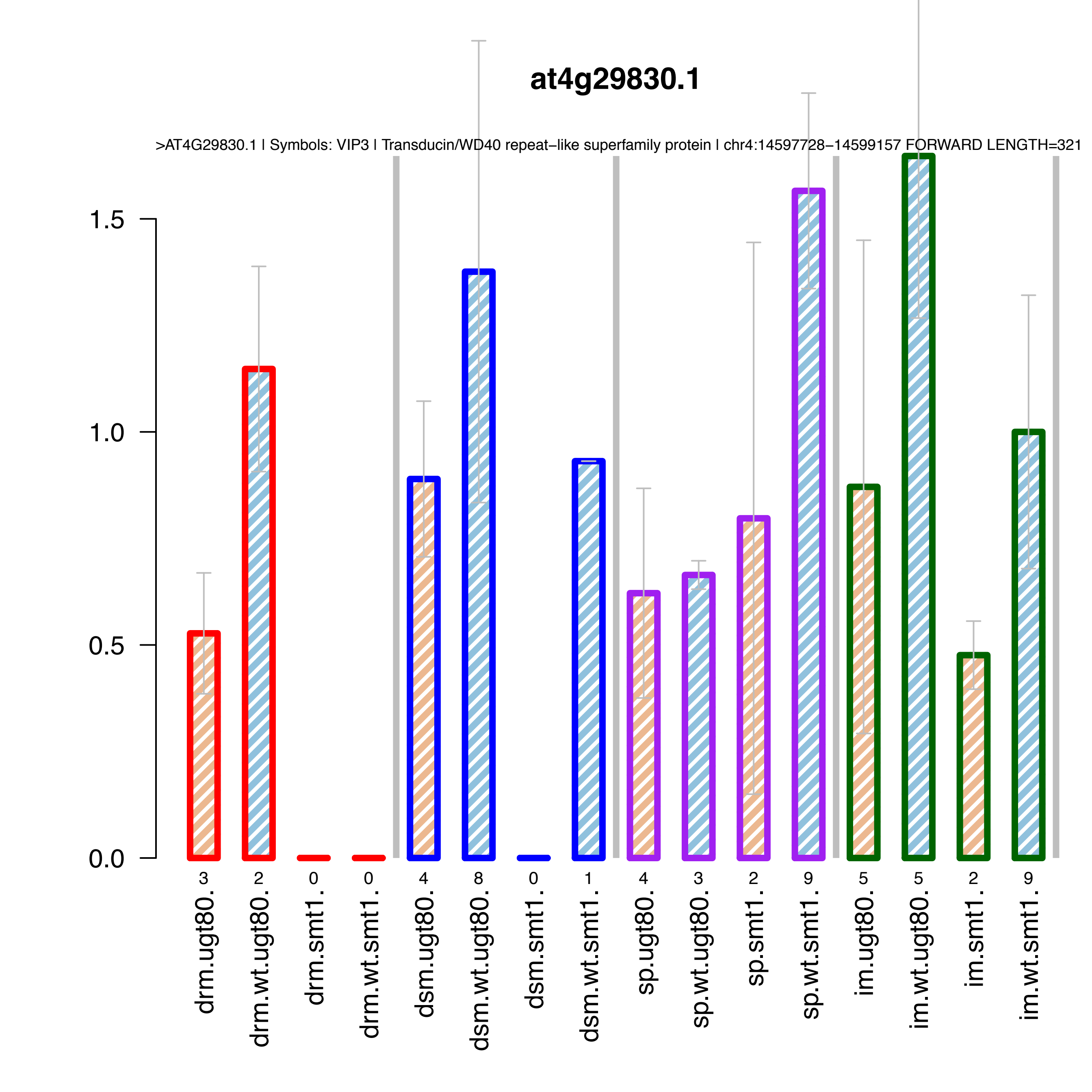

at4g29830.1
>AT4G29830.1 | Symbols: VIP3 | Transducin/WD40 repeat−like superfamily protein | chr4:14597728−14599157 FORWARD LENGTH=321
1.5
1.0
0.5
0.0
3
2
0
0
4
8
0
1
4
3
2
9
5
5
2
9
drm.ugt80.
drm.wt.ugt80.
drm.smt1.
drm.wt.smt1.
dsm.ugt80.
dsm.wt.ugt80.
dsm.smt1.
dsm.wt.smt1.
sp.ugt80.
sp.wt.ugt80.
sp.smt1.
sp.wt.smt1.
im.ugt80.
im.wt.ugt80.
im.smt1.
im.wt.smt1.

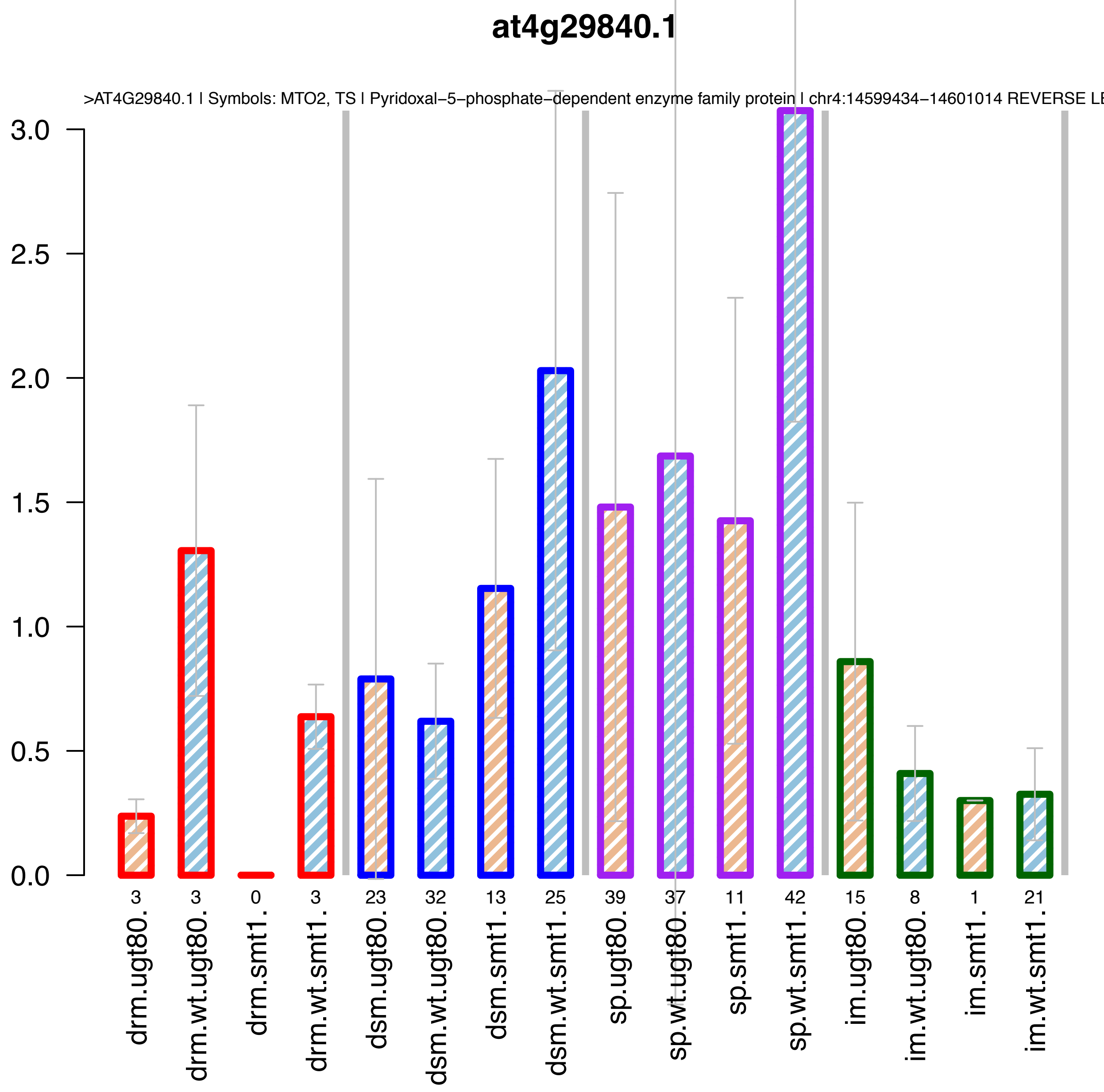

at4g29840.1
>AT4G29840.1 | Symbols: MTO2, TS | Pyridoxal−5−phosphate−dependent enzyme family protein | chr4:14599434−14601014 REVERSE LE
3.0
2.5
2.0
1.5
1.0
0.5
0.0
3
3
0
3
23
32
13
25
39
37
11
42
15
8
1
21
drm.ugt80..
drm.wt.ugt80..
drm.smt1..
drm.wt.smt1..
dsm.ugt80..
dsm.wt.ugt80..
dsm.smt1..
dsm.wt.smt1..
sp.ugt80..
sp.wt.ugt80..
sp.smt1..
sp.wt.smt1..
im.ugt80..
im.wt.ugt80..
im.smt1..
im.wt.smt1..

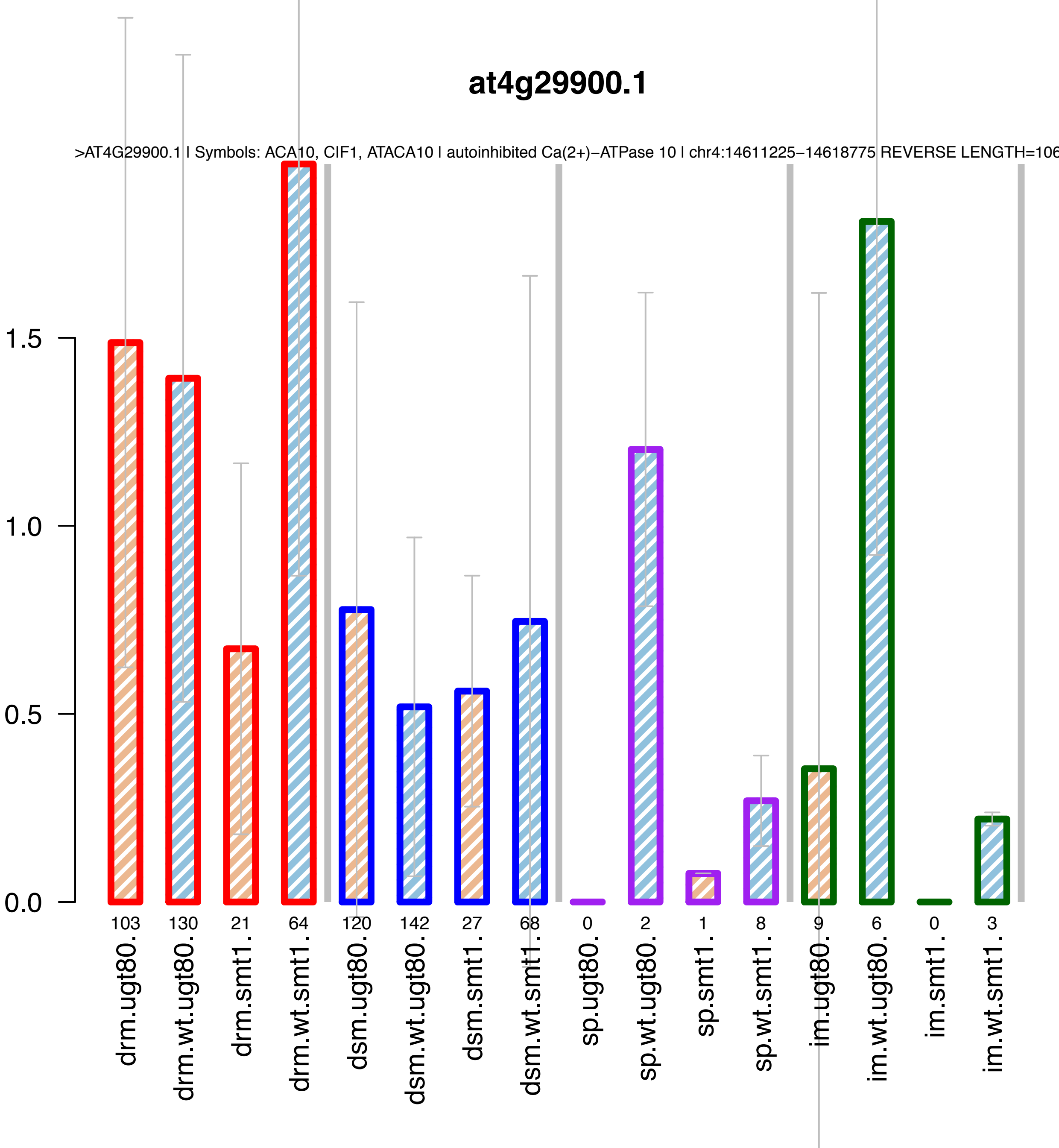

at4g29900.1
>AT4G29900.1 | Symbols: ACA10, CIF1, ATACA10 | autoinhibited Ca(2+)-ATPase 10 | chr4:14611225-14618775 REVERSE LENGTH=106
0.0
0.5
1.0
1.5
103
130
21
64
120
142
27
68
0
2
1
8
9
6
0
3
drm.ugt80..
drm.wt.ugt80..
drm.smt1..
drm.wt.smt1..
dsm.ugt80..
dsm.wt.ugt80..
dsm.smt1..
dsm.wt.smt1..
sp.ugt80..
sp.wt.ugt80..
sp.smt1..
sp.wt.smt1..
im.ugt80..
im.wt.ugt80..
im.smt1..
im.wt.smt1..

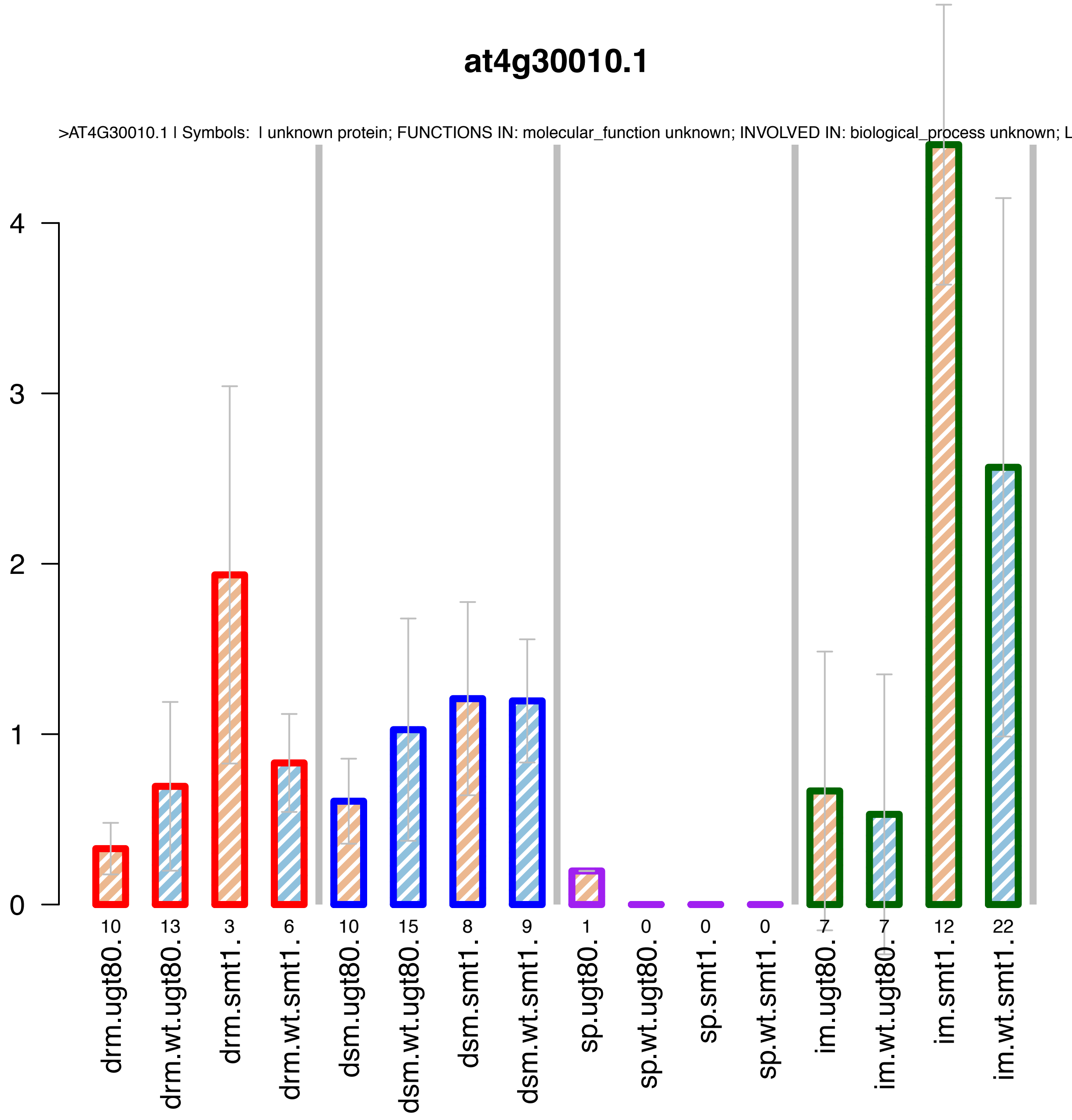

at4g30010.1
>AT4G30010.1 | Symbols: | unknown protein; FUNCTIONS IN: molecular_function unknown; INVOLVED IN: biological_process unknown; L
0
1
2
3
4
10
13
3
6
10
15
8
9
1
0
0
0
7
7
12
22
drm.ugt80.
drm.wt.ugt80.
drm.smt1.
drm.wt.smt1.
dsm.ugt80.
dsm.wt.ugt80.
dsm.smt1.
dsm.wt.smt1.
sp.ugt80.
sp.wt.ugt80.
sp.smt1.
sp.wt.smt1.
im.ugt80.
im.wt.ugt80.
im.smt1.
im.wt.smt1.

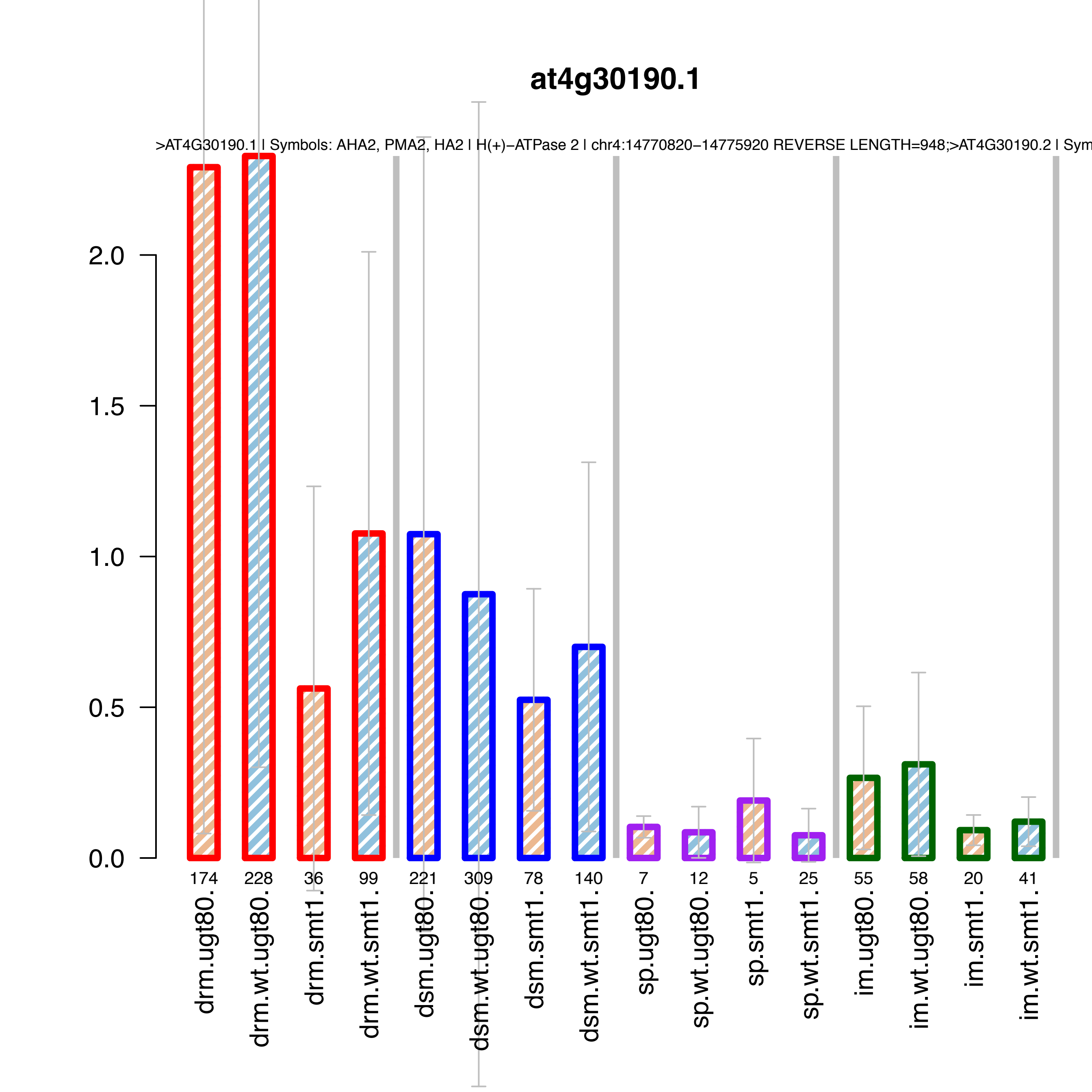

at4g30190.1
>AT4G30190.1 | Symbols: AHA2, PMA2, HA2 | H(+)-ATPase 2 | chr4:14770820-14775920 REVERSE LENGTH=948;>AT4G30190.2 | Sym
0.0
0.5
1.0
1.5
2.0
174
228
36
99
221
309
78
140
7
12
5
25
55
58
20
41
drm.ugt80..
drm.wt.ugt80..
drm.smt1..
drm.wt.smt1..
dsm.ugt80..
dsm.wt.ugt80..
dsm.smt1..
dsm.wt.smt1..
sp.ugt80..
sp.wt.ugt80..
sp.smt1..
sp.wt.smt1..
im.ugt80..
im.wt.ugt80..
im.smt1..
im.wt.smt1..

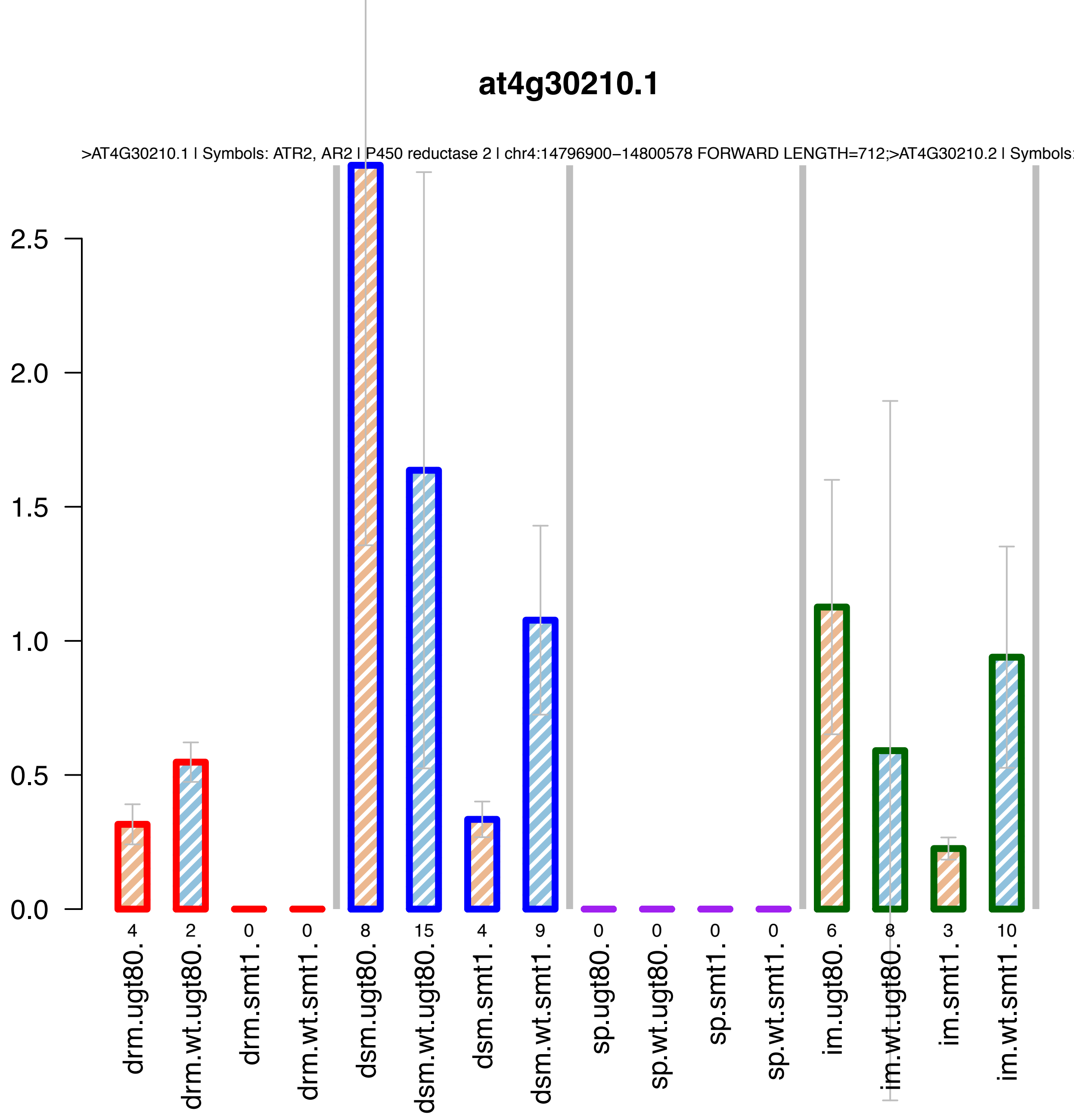

at4g30210.1
>AT4G30210.1 | Symbols: ATR2, AR2 | P450 reductase 2 | chr4:14796900-14800578 FORWARD LENGTH=712;>AT4G30210.2 | Symbols:
2.5
2.0
1.5
1.0
0.5
0.0
4
2
0
0
8
15
4
9
0
0
0
0
6
8
3
10
drm.ugt80.
drm.wt.ugt80.
drm.smt1.
drm.wt.smt1.
dsm.ugt80.
dsm.wt.ugt80.
dsm.smt1.
dsm.wt.smt1.
sp.ugt80.
sp.wt.ugt80.
sp.smt1.
sp.wt.smt1.
im.ugt80.
im.wt.ugt80.
im.smt1.
im.wt.smt1.

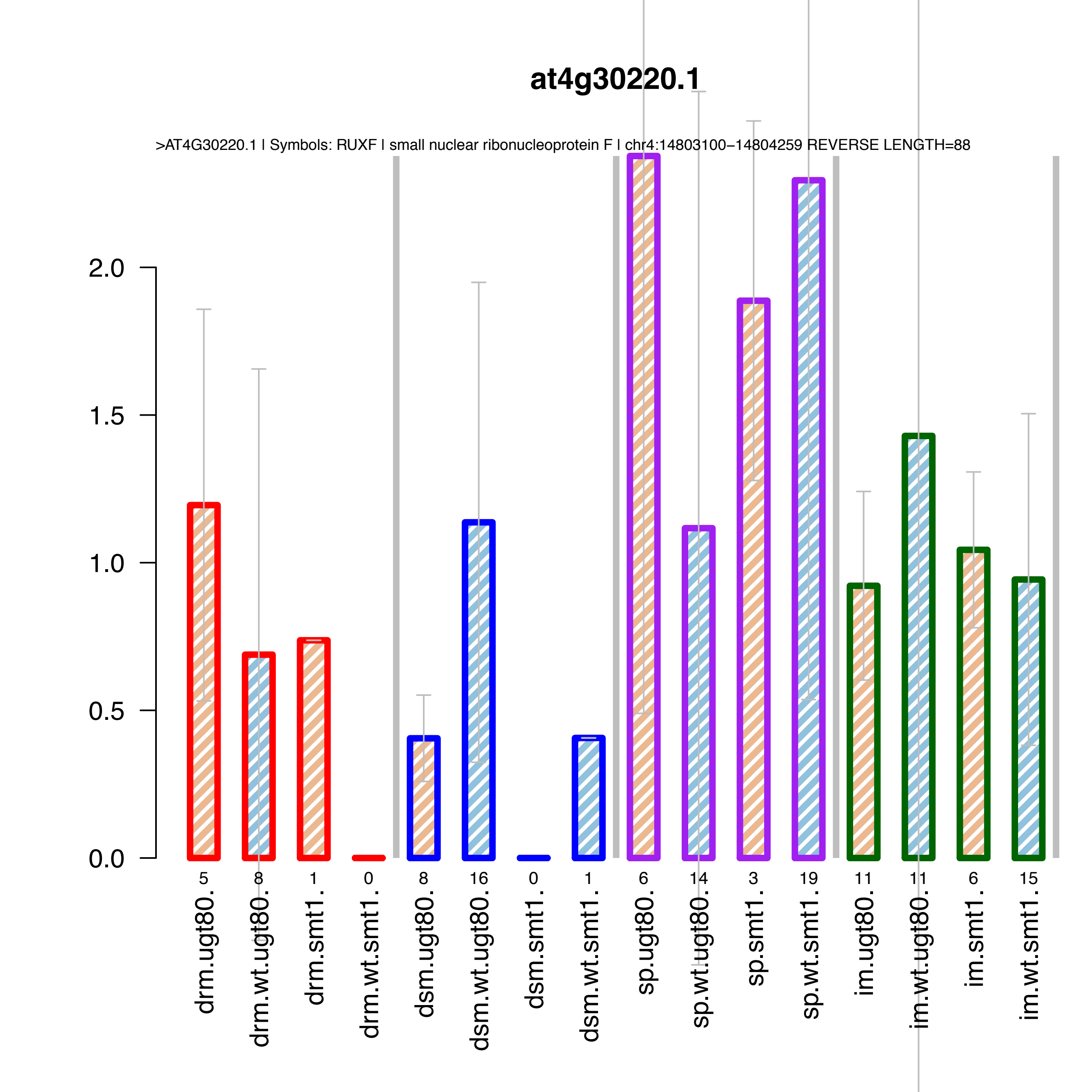
at4g30220.1
>AT4G30220.1 | Symbols: RUXF | small nuclear ribonucleoprotein F | chr4:14803100-14804259 REVERSE LENGTH=88
2.0
1.5
1.0
0.5
0.0
5
8
1
0
8
16
0
1
6
14
3
19
11
11
6
15
drm.ugt80..
drm.wt.ugt80..
drm.smt1..
drm.wt.smt1..
dsm.ugt80..
dsm.wt.ugt80..
dsm.smt1..
dsm.wt.smt1..
sp.ugt80..
sp.wt.ugt80..
sp.smt1..
sp.wt.smt1..
im.ugt80..
im.wt.ugt80..
im.smt1..
im.wt.smt1..

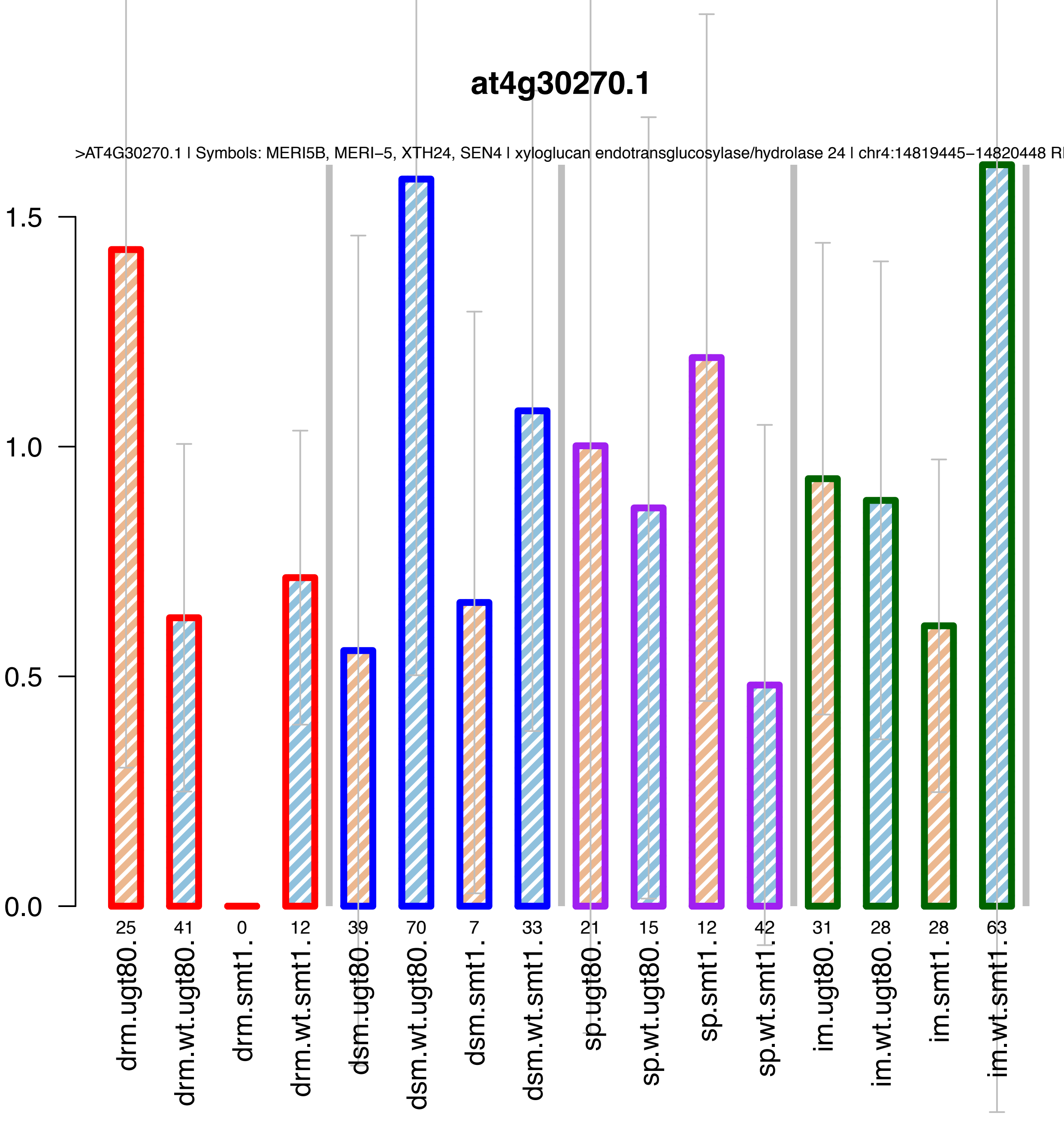

at4g30270.1
>AT4G30270.1 | Symbols: MERI5B, MERI-5, XTH24, SEN4 | xyloglucan endotransglucosylase/hydrolase 24 | chr4:14819445-14820448 R
1.5
1.0
0.5
0.0
25
41
0
12
39
70
7
33
21
15
12
42
31
28
28
63
drm.ugt80..
drm.wt.ugt80..
drm.smt1..
drm.wt.smt1..
dsm.ugt80..
dsm.wt.ugt80..
dsm.smt1..
dsm.wt.smt1..
sp.ugt80..
sp.wt.ugt80..
sp.smt1..
sp.wt.smt1..
im.ugt80..
im.wt.ugt80..
im.smt1..
im.wt.smt1..

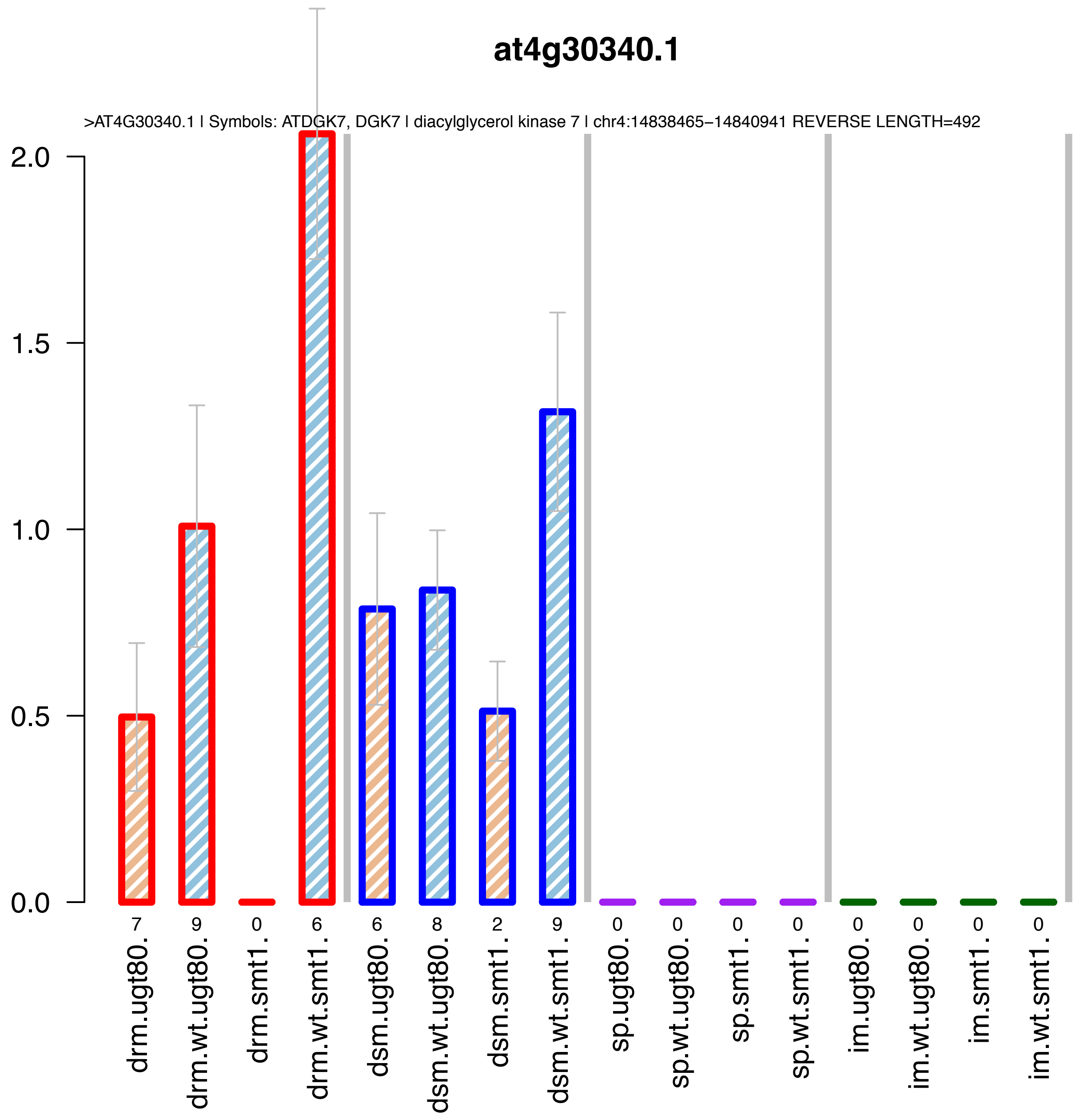

at4g30340.1
>AT4G30340.1 | Symbols: ATDGK7, DGK7 | diacylglycerol kinase 7 | chr4:14838465–14840941 REVERSE LENGTH=492
2.0
1.5
1.0
0.5
0.0
7
9
0
6
6
8
2
9
0
0
0
0
0
0
0
0
drm.ugt80.
drm.wt.ugt80.
drm.smt1.
drm.wt.smt1.
dsm.ugt80.
dsm.wt.ugt80.
dsm.smt1.
dsm.wt.smt1.
sp.ugt80.
sp.wt.ugt80.
sp.smt1.
sp.wt.smt1.
im.ugt80.
im.wt.ugt80.
im.smt1.
im.wt.smt1.

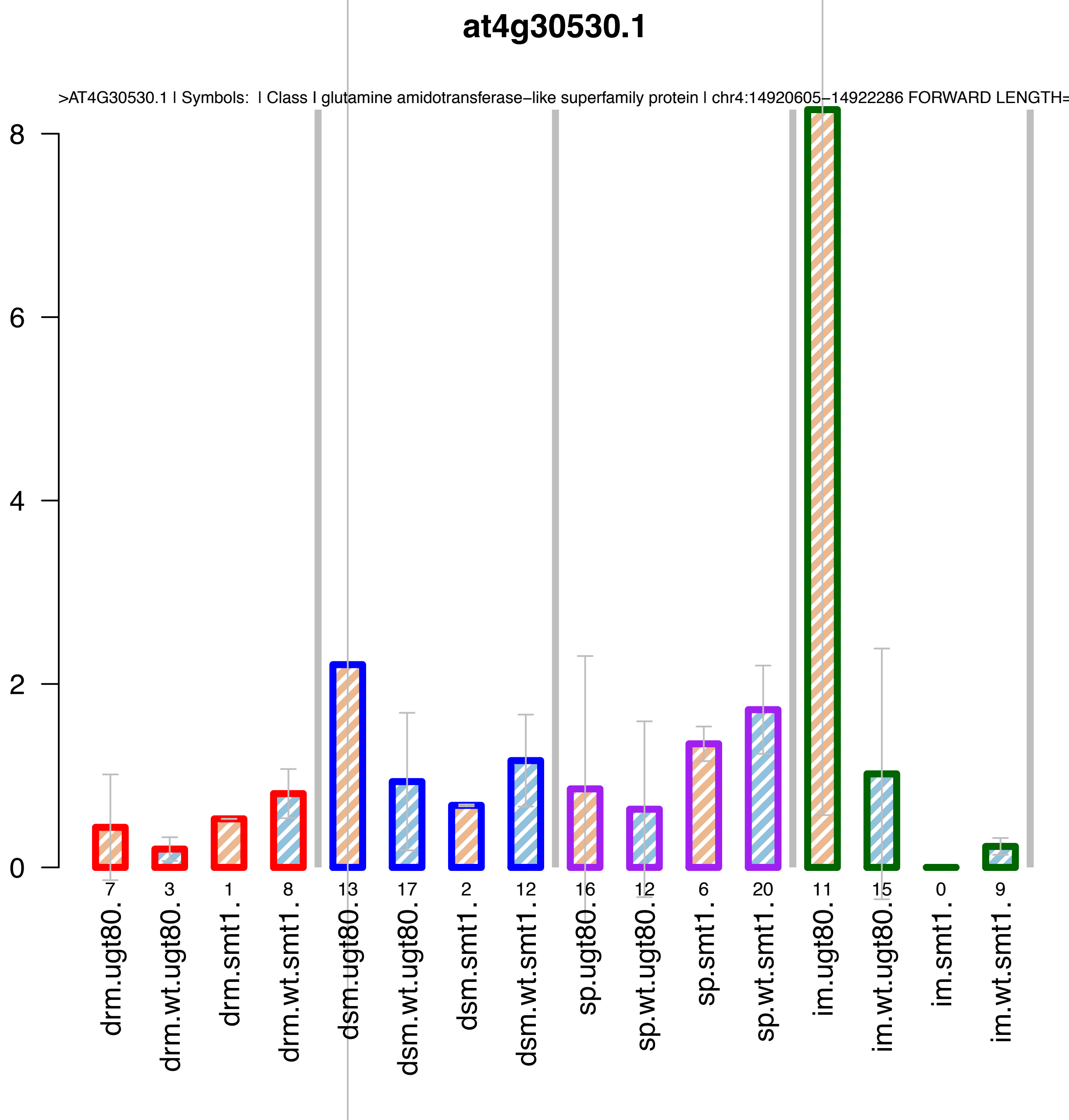

at4g30530.1
>AT4G30530.1 | Symbols: | Class | glutamine amidotransferase−like superfamily protein | chr4:14920605−14922286 FORWARD LENGTH=
0
2
4
6
8
7
3
1
8
13
17
2
12
16
12
6
20
11
15
0
9
drm.ugt80.
drm.wt.ugt80.
drm.smt1.
drm.wt.smt1.
dsm.ugt80.
dsm.wt.ugt80.
dsm.smt1.
dsm.wt.smt1.
sp.ugt80.
sp.wt.ugt80.
sp.smt1.
sp.wt.smt1.
im.ugt80.
im.wt.ugt80.
im.smt1.
im.wt.smt1.

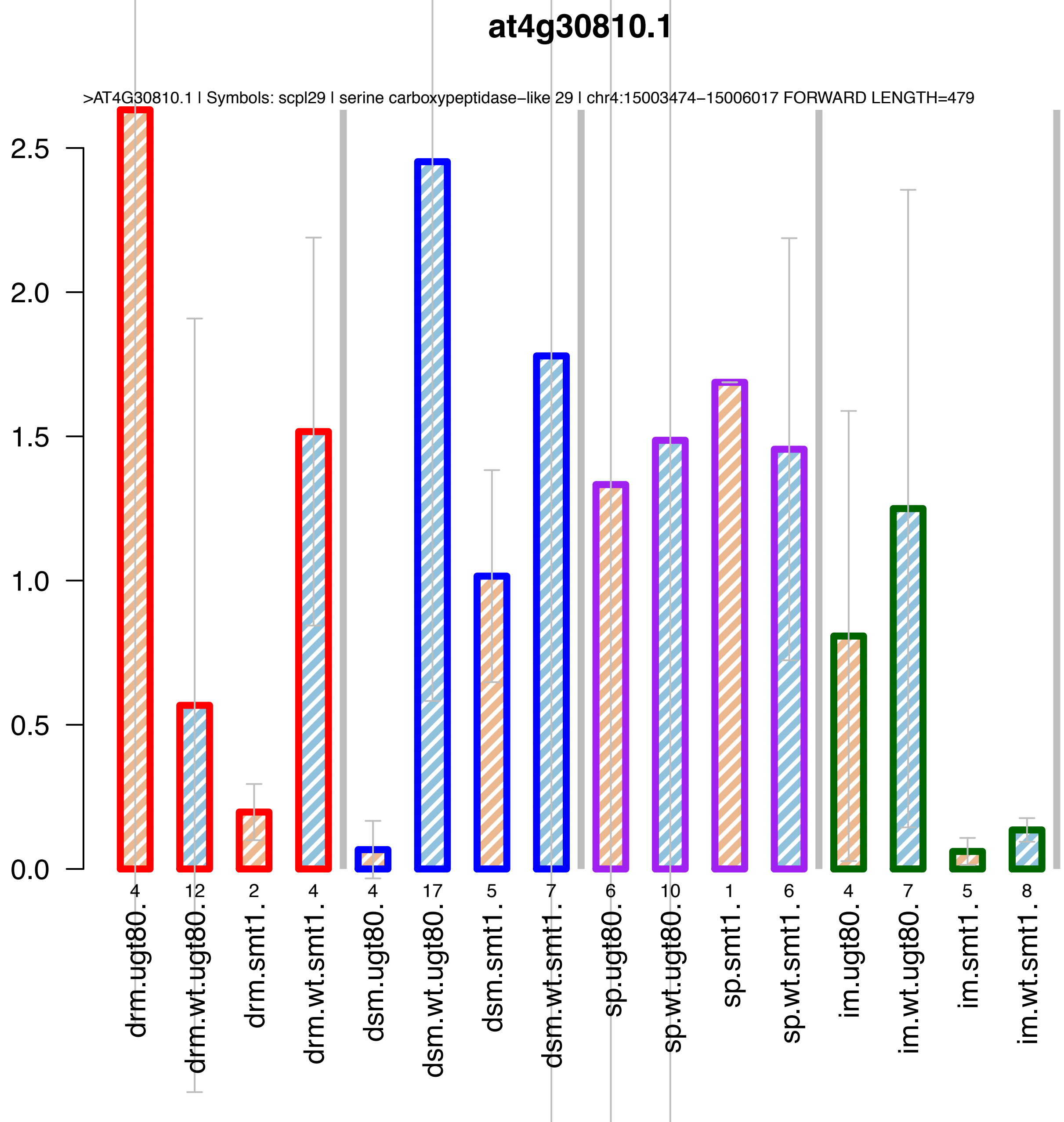

at4g30810.1
>AT4G30810.1 | Symbols: scpl29 | serine carboxypeptidase−like 29 | chr4:15003474−15006017 FORWARD LENGTH=479
2.5
2.0
1.5
1.0
0.5
0.0
4
12
2
4
4
17
5
7
6
10
1
6
4
7
5
8
drm.ugt80..
drm.wt.ugt80..
drm.smt1..
drm.wt.smt1..
dsm.ugt80..
dsm.wt.ugt80..
dsm.smt1..
dsm.wt.smt1..
sp.ugt80..
sp.wt.ugt80..
sp.smt1..
sp.wt.smt1..
im.ugt80..
im.wt.ugt80..
im.smt1..
im.wt.smt1..

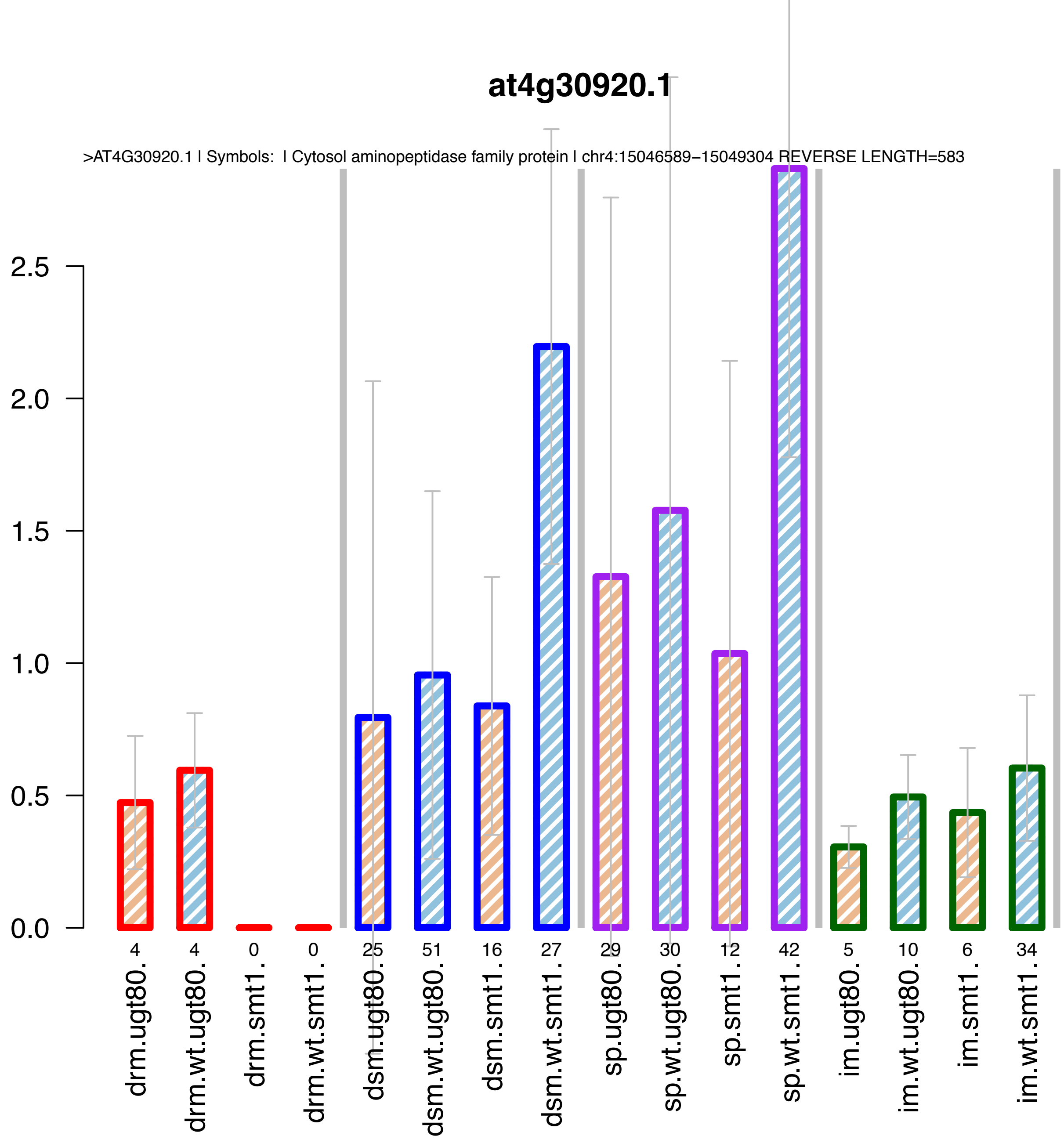

at4g30920.1
>AT4G30920.1 | Symbols: | Cytosol aminopeptidase family protein | chr4:15046589-15049304 REVERSE LENGTH=583
0.0
0.5
1.0
1.5
2.0
2.5
4
4
0
0
25
51
16
27
29
30
12
42
5
10
6
34
drm.ugt80.
drm.wt.ugt80.
drm.smt1.
drm.wt.smt1.
dsm.ugt80.
dsm.wt.ugt80.
dsm.smt1.
dsm.wt.smt1.
sp.ugt80.
sp.wt.ugt80.
sp.smt1.
sp.wt.smt1.
im.ugt80.
im.wt.ugt80.
im.smt1.
im.wt.smt1.

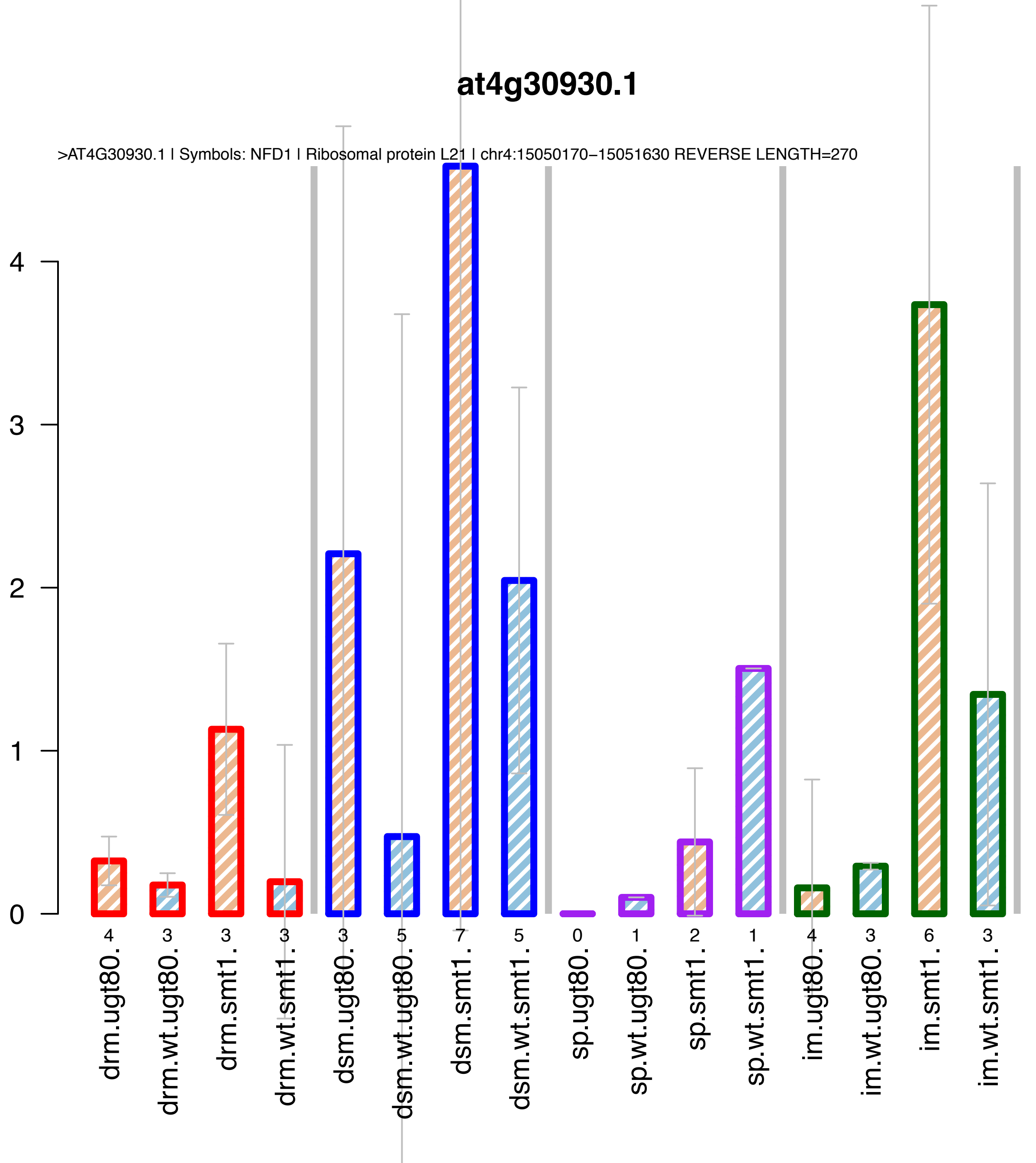
at4g30930.1
>AT4G30930.1 | Symbols: NFD1 | Ribosomal protein L21 | chr4:15050170-15051630 REVERSE LENGTH=270
0
1
2
3
4
4
3
3
3
3
5
7
5
0
1
2
1
4
3
6
3
drm.ugt80..
drm.wt.ugt80..
drm.smt1..
drm.wt.smt1..
dsm.ugt80..
dsm.wt.ugt80..
dsm.smt1..
dsm.wt.smt1..
sp.ugt80..
sp.wt.ugt80..
sp.smt1..
sp.wt.smt1..
im.ugt80..
im.wt.ugt80..
im.smt1..
im.wt.smt1..

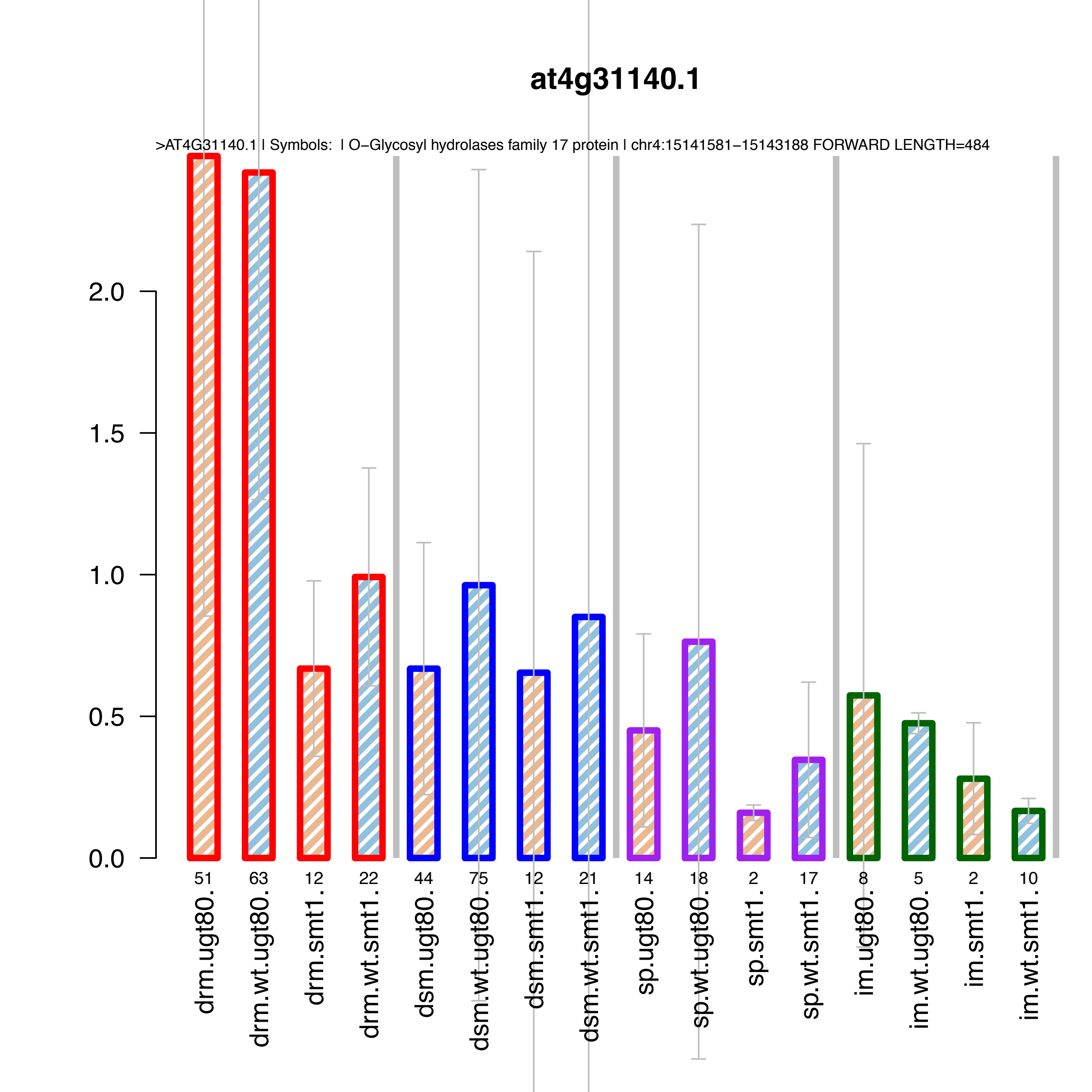

at4g31140.1
>AT4G31140.1 | Symbols: | O−Glycosyl hydrolases family 17 protein | chr4:15141581−15143188 FORWARD LENGTH=484
0.0
0.5
1.0
1.5
2.0
51
63
12
22
44
75
12
21
14
18
2
17
8
5
2
10
drm.ugt80..
drm.wt.ugt80..
drm.smt1..
drm.wt.smt1..
dsm.ugt80..
dsm.wt.ugt80..
dsm.smt1..
dsm.wt.smt1..
sp.ugt80..
sp.wt.ugt80..
sp.smt1..
sp.wt.smt1..
im.ugt80..
im.wt.ugt80..
im.smt1..
im.wt.smt1..

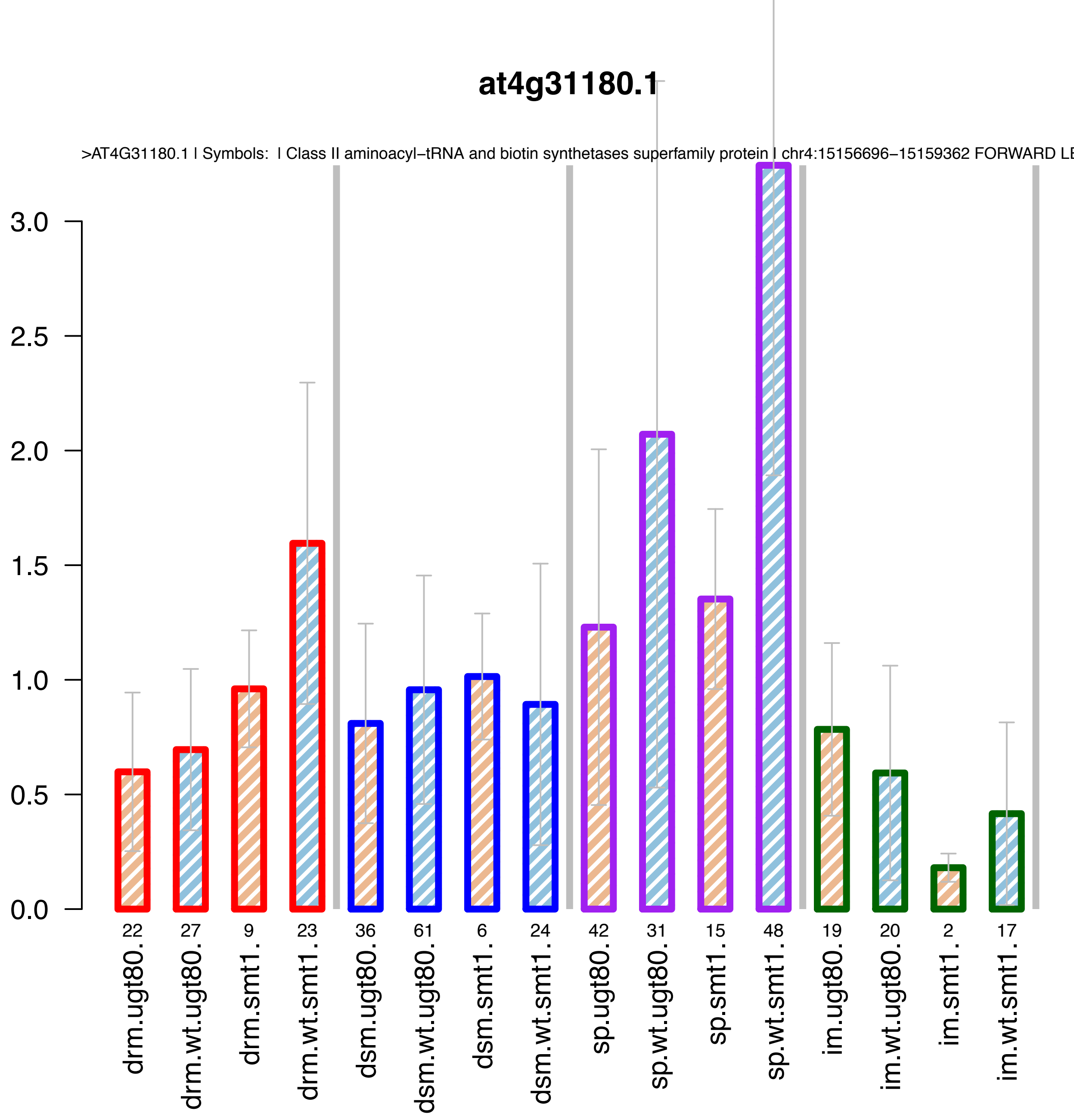

at4g31180.1
>AT4G31180.1 | Symbols: | Class II aminoacyl-tRNA and biotin synthetases superfamily protein | chr4:15156696-15159362 FORWARD LE
3.0
2.5
2.0
1.5
1.0
0.5
0.0
22
27
9
23
36
61
6
24
42
31
15
48
19
20
2
17
drm.ugt80..
drm.wt.ugt80..
drm.smt1..
drm.wt.smt1..
dsm.ugt80..
dsm.wt.ugt80..
dsm.smt1..
dsm.wt.smt1..
sp.ugt80..
sp.wt.ugt80..
sp.smt1..
sp.wt.smt1..
im.ugt80..
im.wt.ugt80..
im.smt1..
im.wt.smt1..

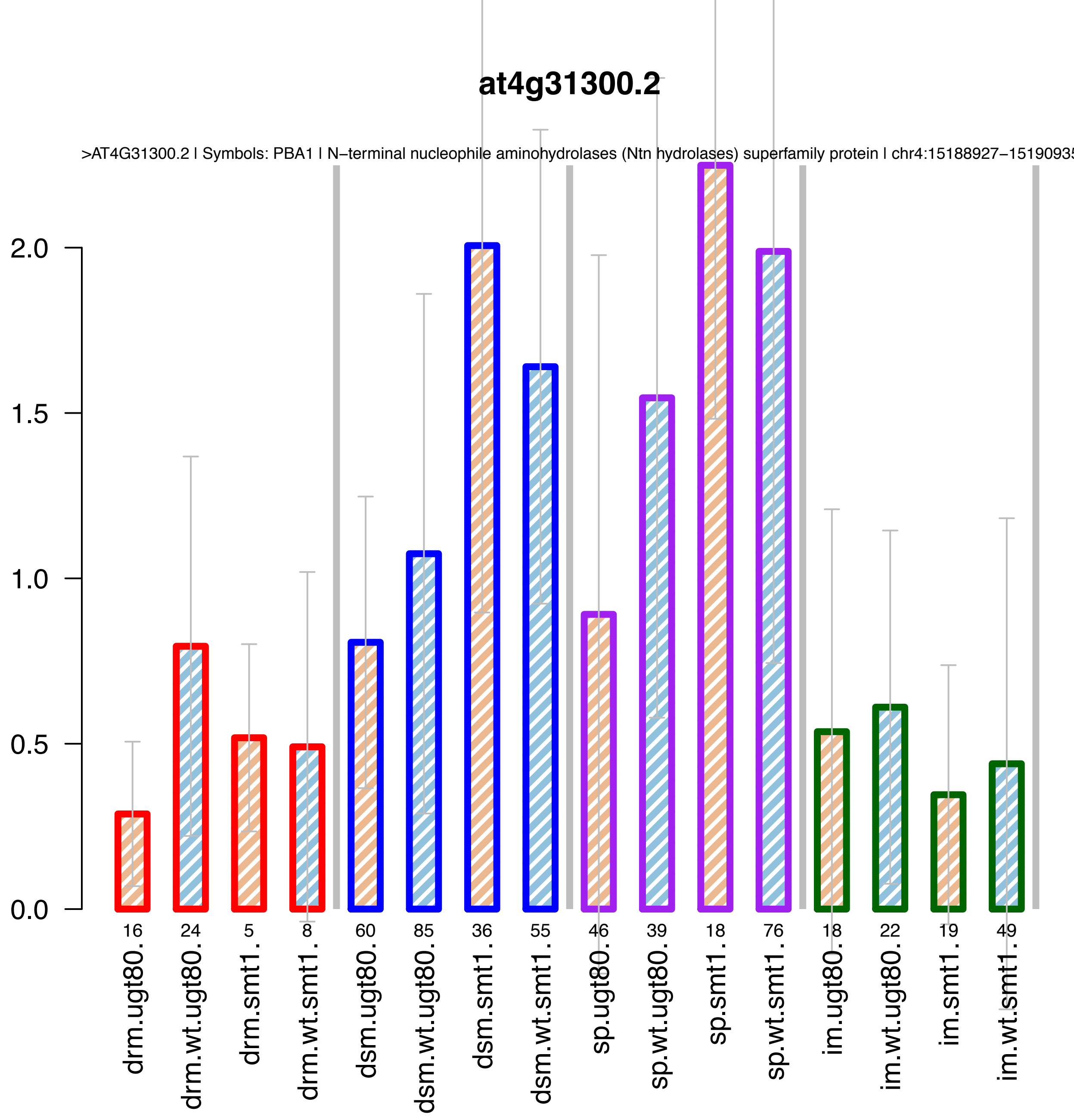
at4g31300.2
>AT4G31300.2 | Symbols: PBA1 | N−terminal nucleophile aminohydrolases (Ntn hydrolases) superfamily protein | chr4:15188927−1519093
2.0
1.5
1.0
0.5
0.0
16
24
5
8
60
85
36
55
46
39
18
76
18
22
19
49
drm.ugt80..
drm.wt.ugt80..
drm.smt1..
drm.wt.smt1..
dsm.ugt80..
dsm.wt.ugt80..
dsm.smt1..
dsm.wt.smt1..
sp.ugt80..
sp.wt.ugt80..
sp.smt1..
sp.wt.smt1..
im.ugt80..
im.wt.ugt80..
im.smt1..
im.wt.smt1..

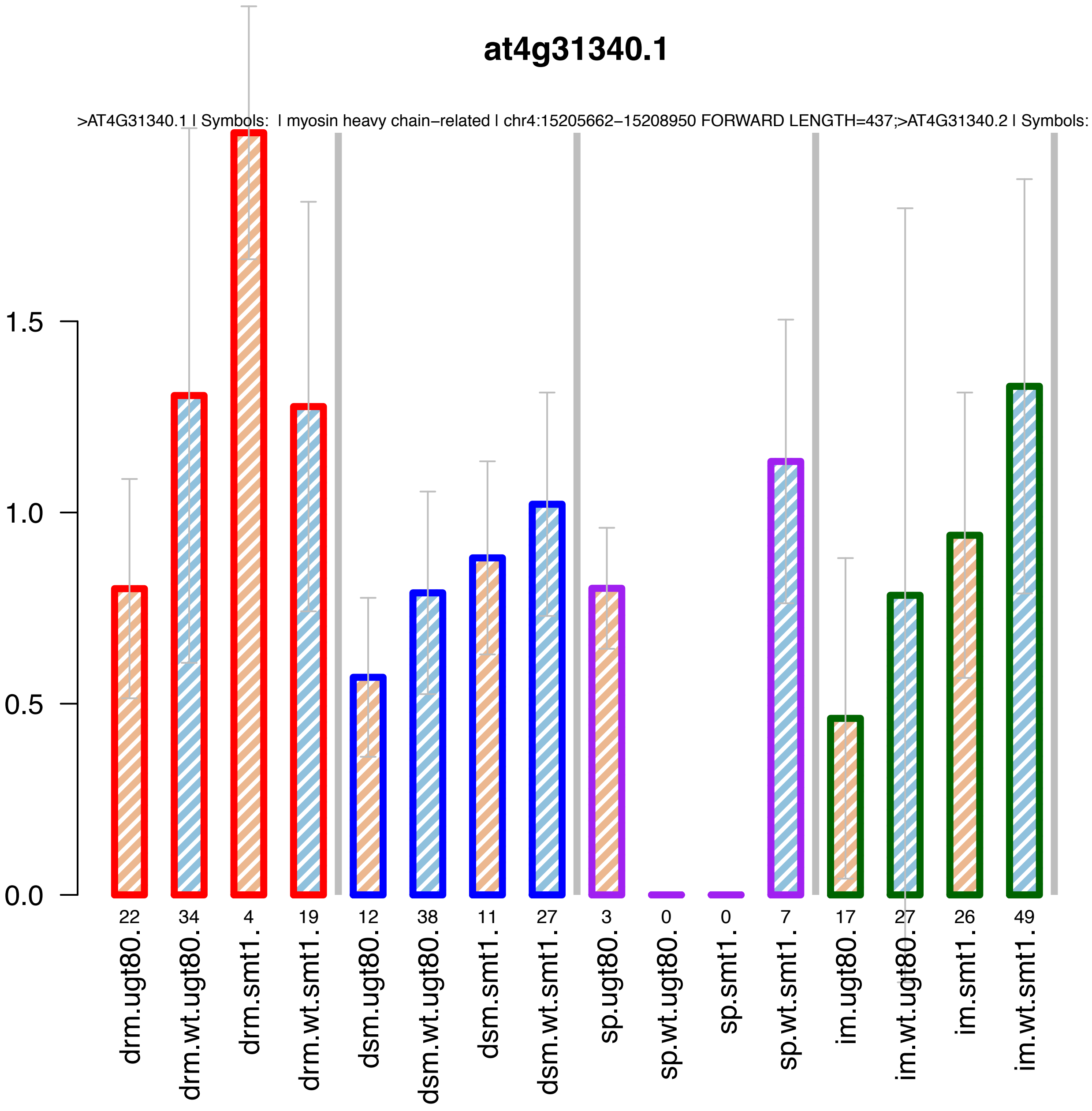

at4g31340.1
>AT4G31340.1 | Symbols: | myosin heavy chain−related | chr4:15205662−15208950 FORWARD LENGTH=437;>AT4G31340.2 | Symbols:
0.0
0.5
1.0
1.5
22
34
4
19
12
38
11
27
3
0
0
7
17
27
26
49
drm.ugt80..
drm.wt.ugt80..
drm.smt1..
drm.wt.smt1..
dsm.ugt80..
dsm.wt.ugt80..
dsm.smt1..
dsm.wt.smt1..
sp.ugt80..
sp.wt.ugt80..
sp.smt1..
sp.wt.smt1..
im.ugt80..
im.wt.ugt80..
im.smt1..
im.wt.smt1..

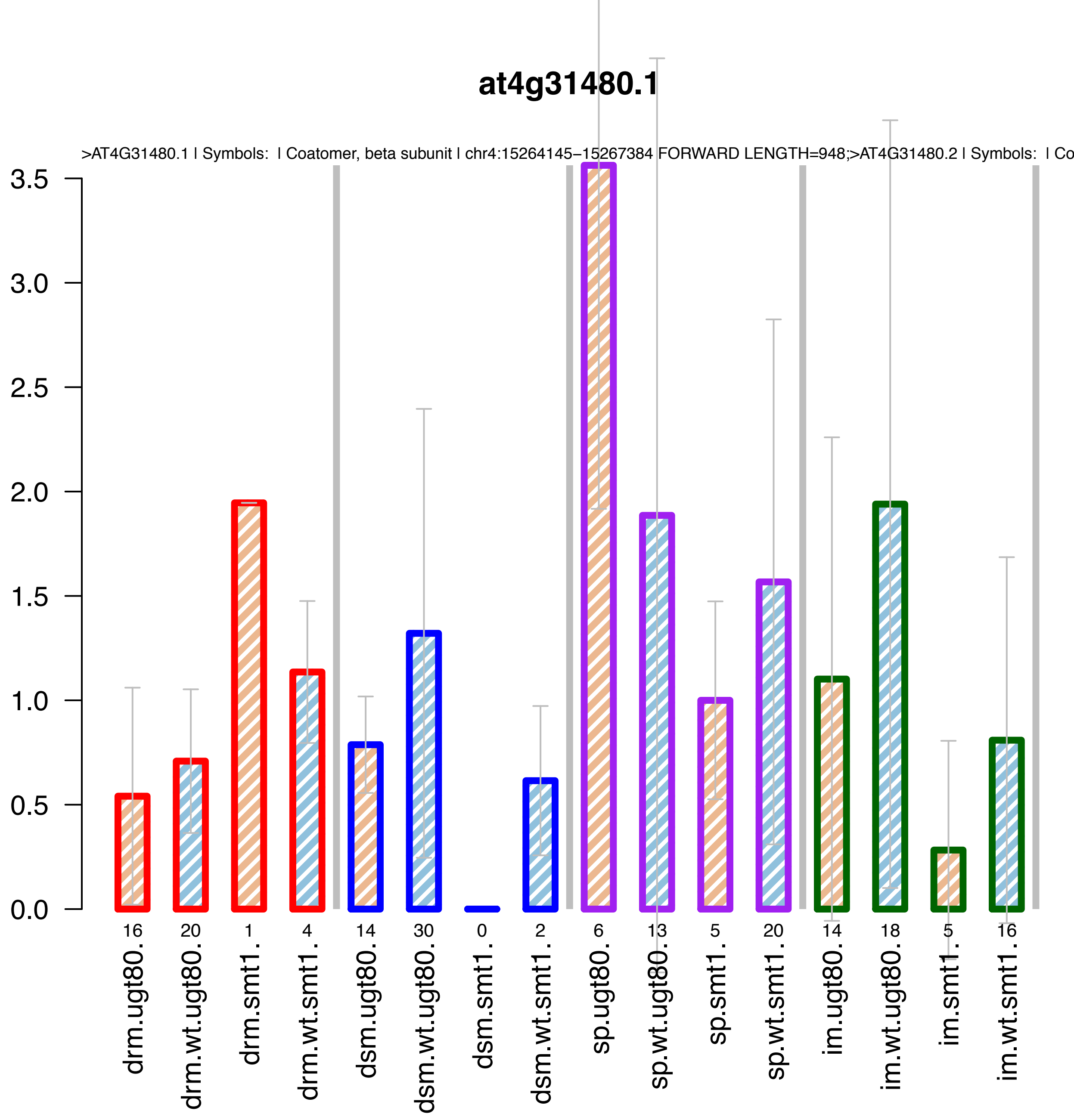

at4g31480.1
>AT4G31480.1 | Symbols: | Coatomer, beta subunit | chr4:15264145-15267384 FORWARD LENGTH=948;>AT4G31480.2 | Symbols: | Co
3.5
3.0
2.5
2.0
1.5
1.0
0.5
0.0
16
20
1
4
14
30
0
2
6
13
5
20
14
18
5
16
drm.ugt80.
drm.wt.ugt80.
drm.smt1.
drm.wt.smt1.
dsm.ugt80.
dsm.wt.ugt80.
dsm.smt1.
dsm.wt.smt1.
sp.ugt80.
sp.wt.ugt80.
sp.smt1.
sp.wt.smt1.
im.ugt80.
im.wt.ugt80.
im.smt1.
im.wt.smt1.

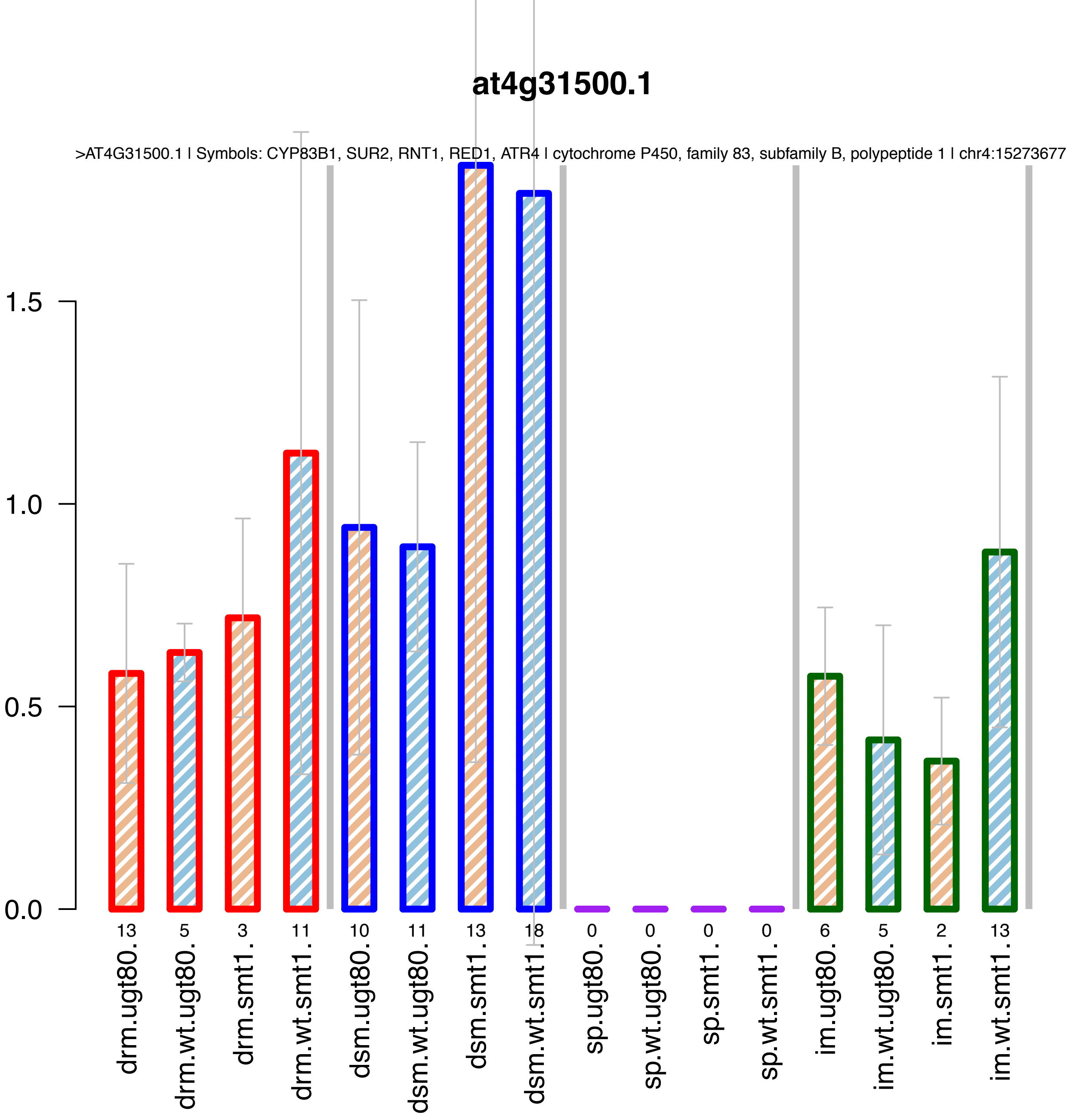

at4g31500.1
>AT4G31500.1 | Symbols: CYP83B1, SUR2, RNT1, RED1, ATR4 | cytochrome P450, family 83, subfamily B, polypeptide 1 | chr4:15273677
1.5
1.0
0.5
0.0
13
5
3
11
10
11
13
18
0
0
0
0
6
5
2
13
drm.ugt80.
drm.wt.ugt80.
drm.smt1.
drm.wt.smt1.
dsm.ugt80.
dsm.wt.ugt80.
dsm.smt1.
dsm.wt.smt1.
sp.ugt80.
sp.wt.ugt80.
sp.smt1.
sp.wt.smt1.
im.ugt80.
im.wt.ugt80.
im.smt1.
im.wt.smt1.

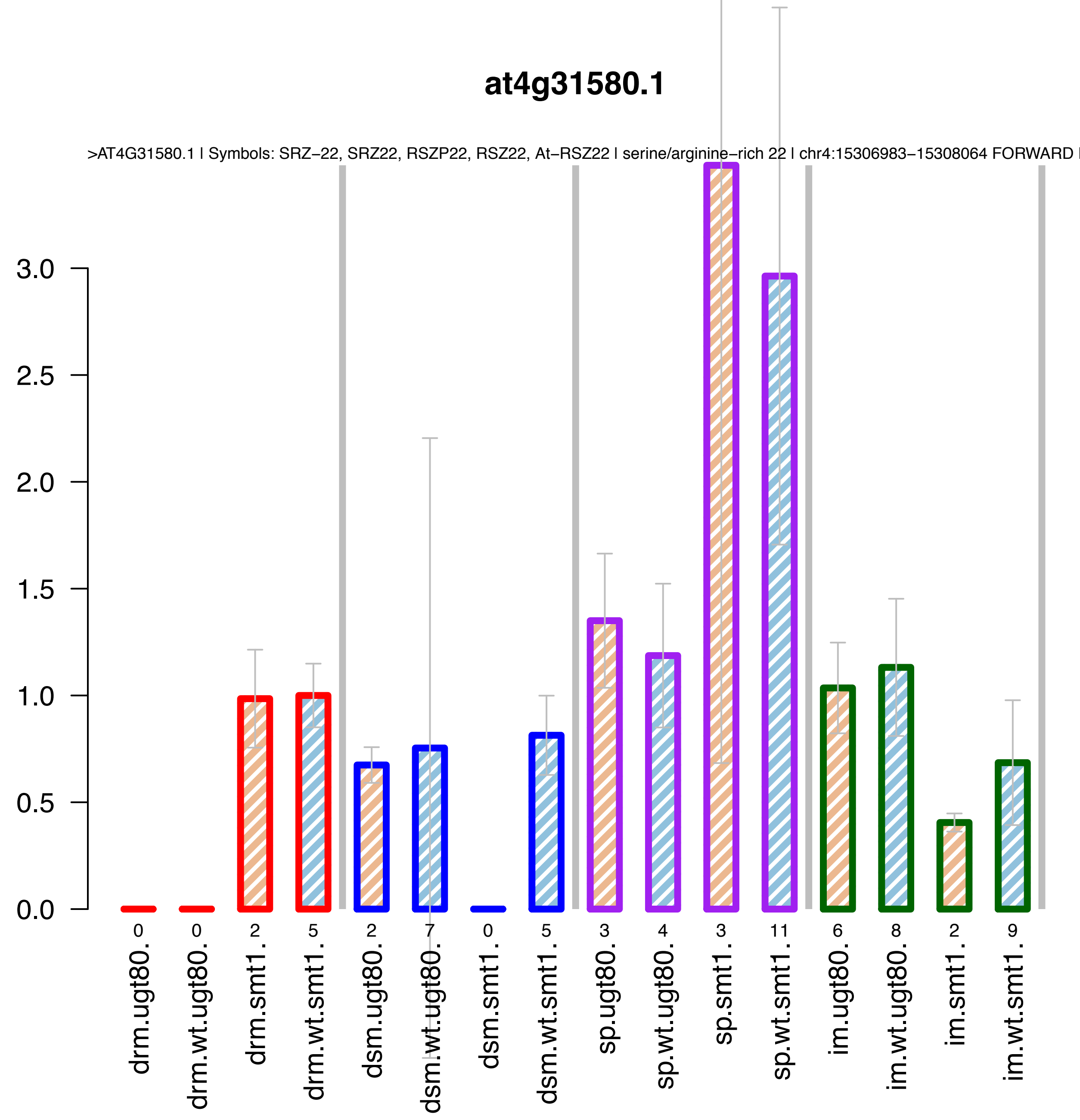

at4g31580.1
>AT4G31580.1 | Symbols: SRZ−22, SRZ22, RSZP22, RSZ22, At−RSZ22 | serine/arginine−rich 22 | chr4:15306983−15308064 FORWARD
0.0
0.5
1.0
1.5
2.0
2.5
3.0
0 0 2 5 2 7 0 5 3 4 3 11 6 8 2 9
drm.ugt80.
drm.wt.ugt80.
drm.smt1.
drm.wt.smt1.
dsm.ugt80.
dsm.wt.ugt80.
dsm.smt1.
dsm.wt.smt1.
sp.ugt80.
sp.wt.ugt80.
sp.smt1.
sp.wt.smt1.
im.ugt80.
im.wt.ugt80.
im.smt1.
im.wt.smt1.

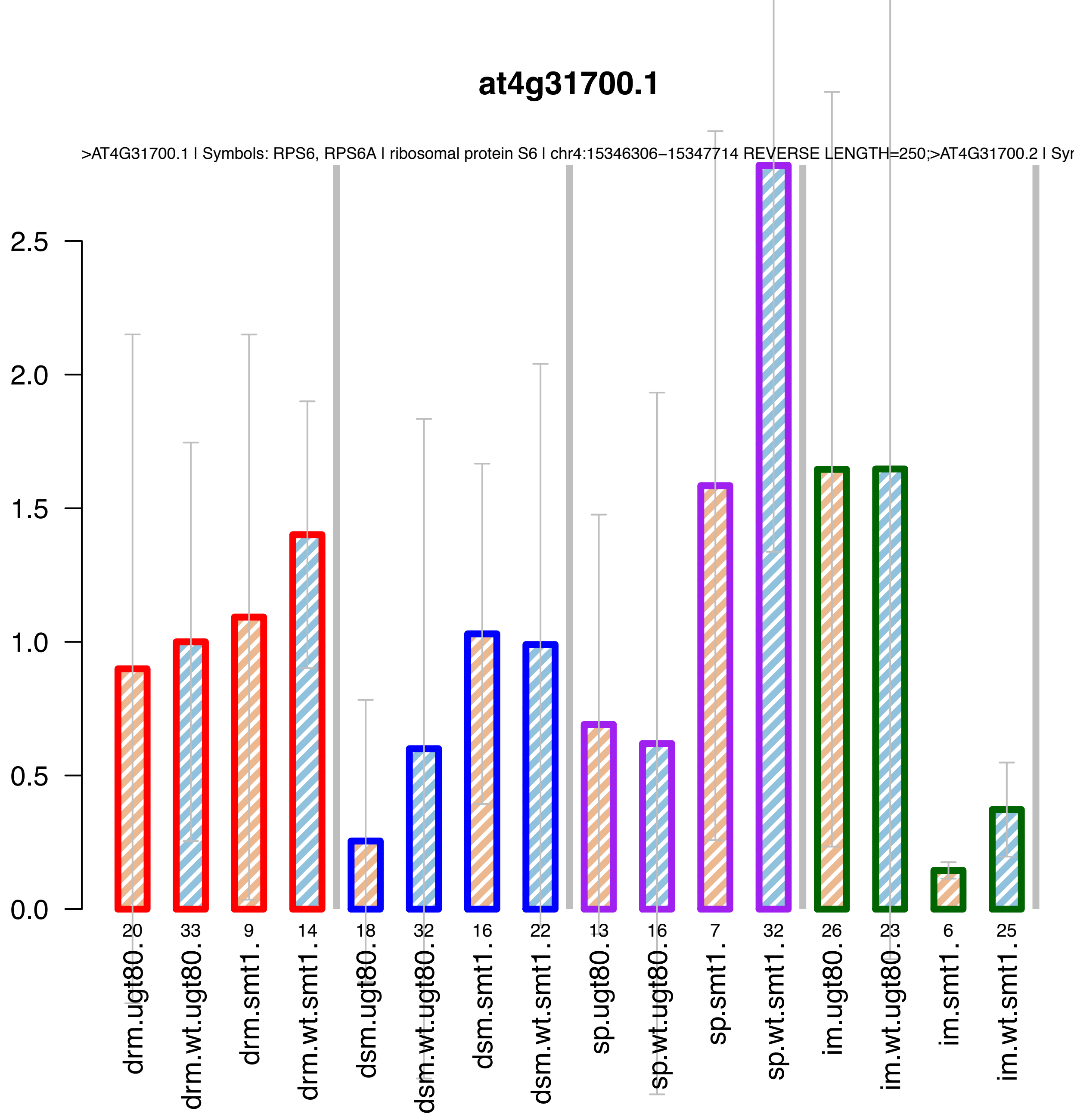

at4g31700.1
>AT4G31700.1 | Symbols: RPS6, RPS6A | ribosomal protein S6 | chr4:15346306−15347714 REVERSE LENGTH=250;>AT4G31700.2 | Sym
0.0
0.5
1.0
1.5
2.0
2.5
20
33
9
14
18
32
16
22
13
16
7
32
26
23
6
25
drm.ugt80..
drm.wt.ugt80..
drm.smt1..
drm.wt.smt1..
dsm.ugt80..
dsm.wt.ugt80..
dsm.smt1..
dsm.wt.smt1..
sp.ugt80..
sp.wt.ugt80..
sp.smt1..
sp.wt.smt1..
im.ugt80..
im.wt.ugt80..
im.smt1..
im.wt.smt1..

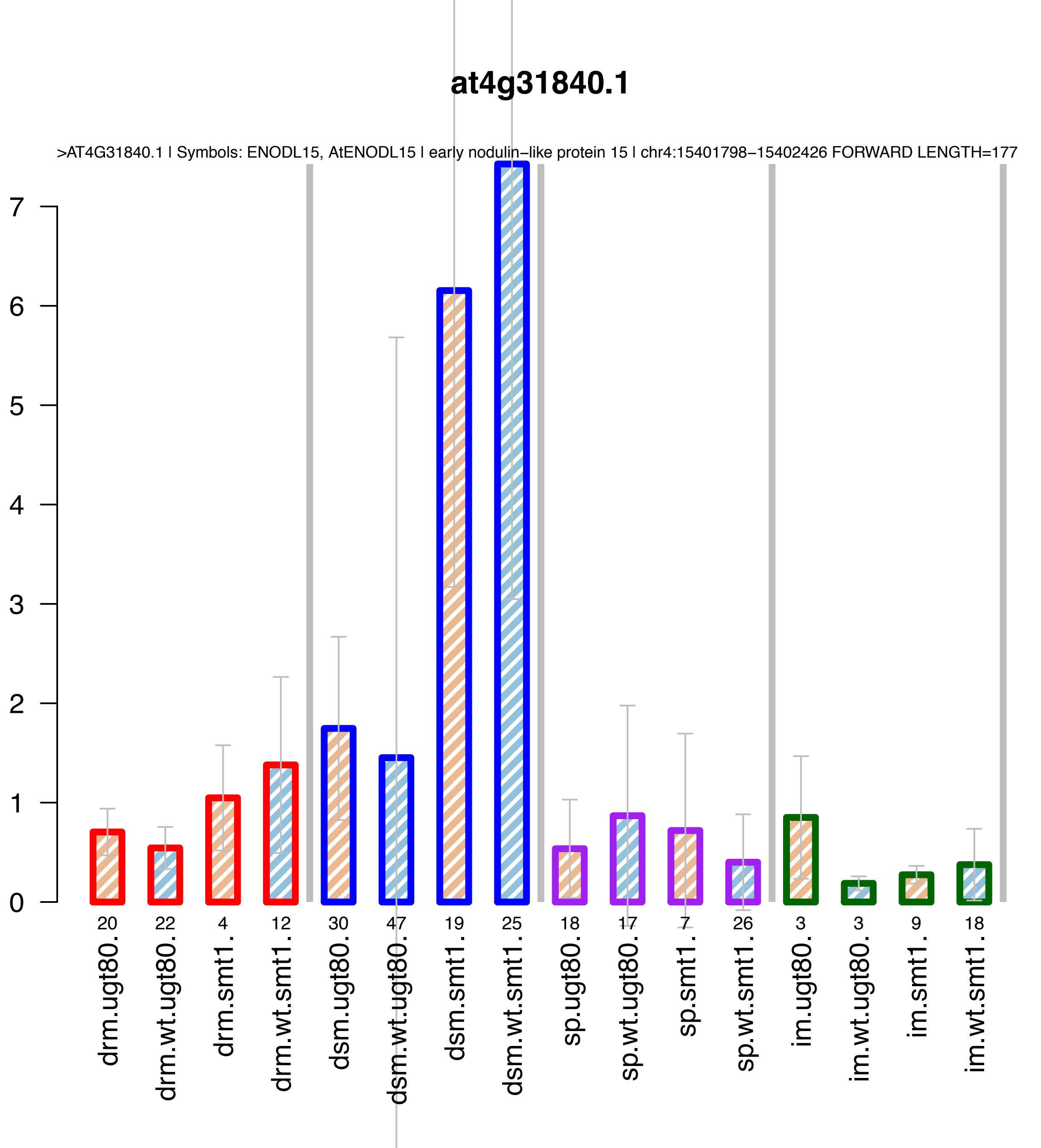

at4g31840.1
>AT4G31840.1 | Symbols: ENODL15, AtENODL15 | early nodulin−like protein 15 | chr4:15401798−15402426 FORWARD LENGTH=177
0
1
2
3
4
5
6
7
20
22
4
12
30
47
19
25
18
17
7
26
3
3
9
18
drm.ugt80.
drm.wt.ugt80.
drm.smt1.
drm.wt.smt1.
dsm.ugt80.
dsm.wt.ugt80.
dsm.smt1.
dsm.wt.smt1.
sp.ugt80.
sp.wt.ugt80.
sp.smt1.
sp.wt.smt1.
im.ugt80.
im.wt.ugt80.
im.smt1.
im.wt.smt1.

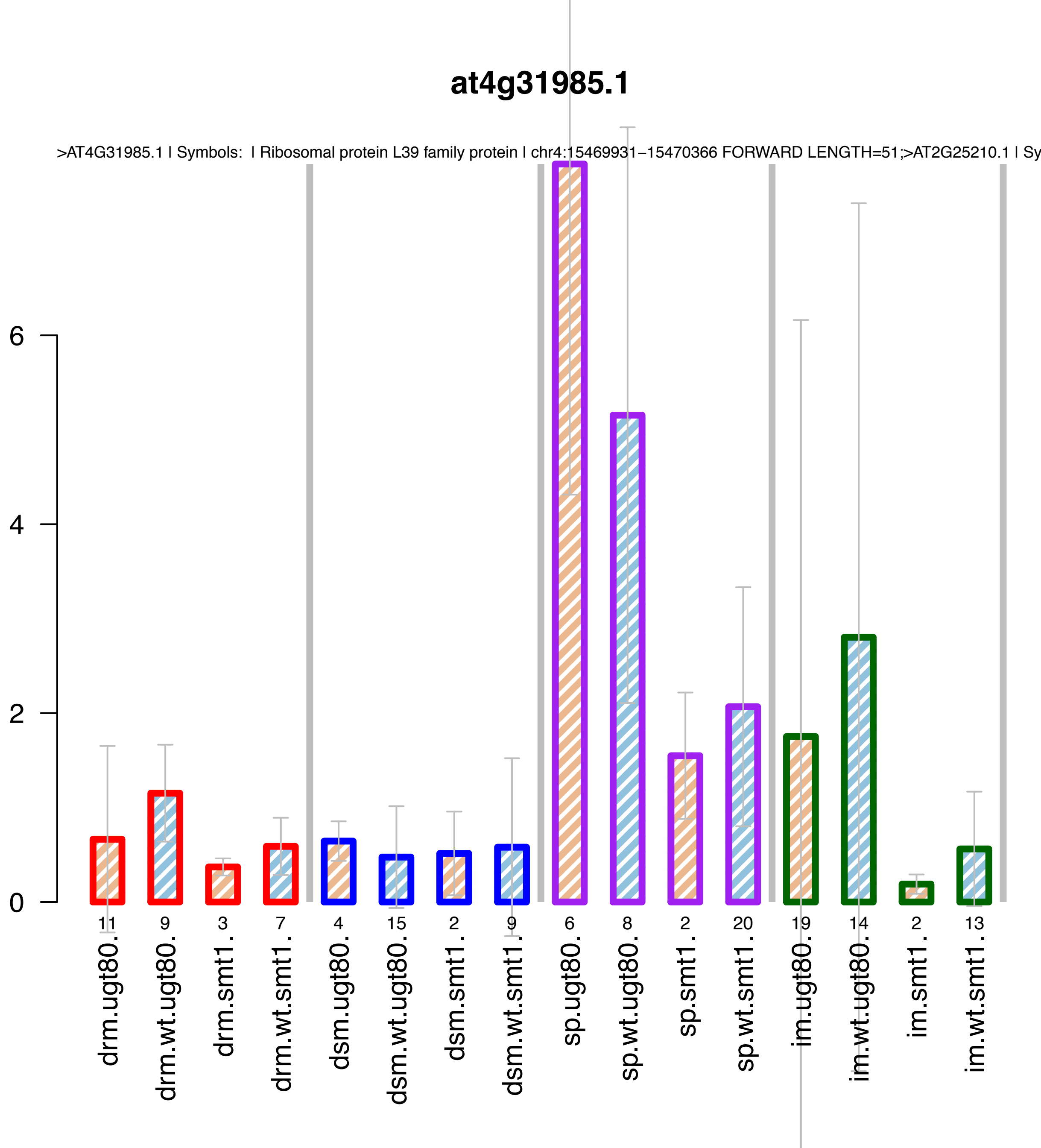

at4g31985.1
>AT4G31985.1 | Symbols: | Ribosomal protein L39 family protein | chr4:15469931−15470366 FORWARD LENGTH=51;>AT2G25210.1 | Sy
0
2
4
6
11
9
3
7
4
15
2
9
6
8
2
20
19
14
2
13
drm.ugt80.
drm.wt.ugt80.
drm.smt1.
drm.wt.smt1.
dsm.ugt80.
dsm.wt.ugt80.
dsm.smt1.
dsm.wt.smt1.
sp.ugt80.
sp.wt.ugt80.
sp.smt1.
sp.wt.smt1.
im.ugt80.
im.wt.ugt80.
im.smt1.
im.wt.smt1.

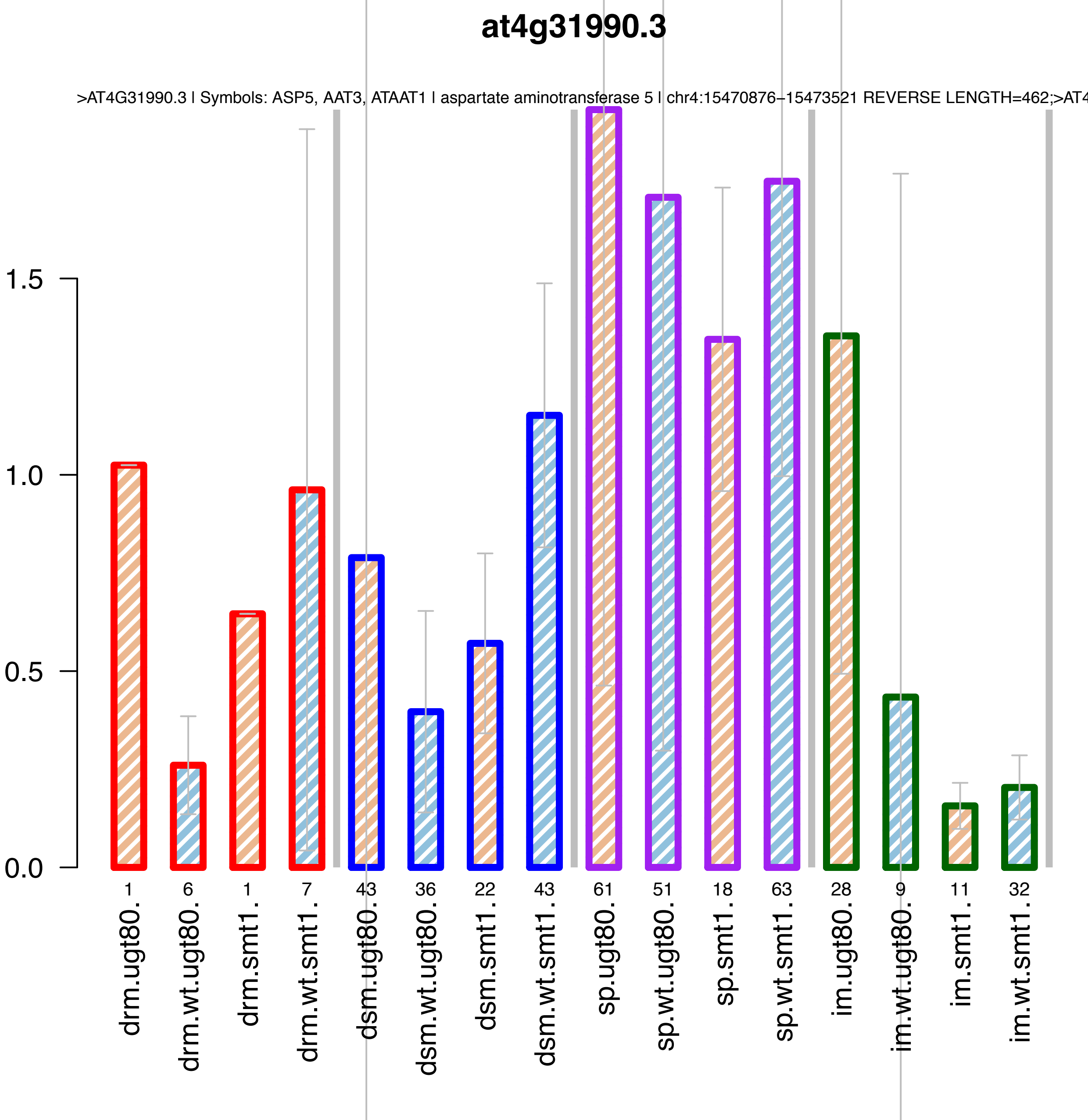

at4g31990.3
>AT4G31990.3 | Symbols: ASP5, AAT3, ATAAT1 | aspartate aminotransferase 5 | chr4:15470876-15473521 REVERSE LENGTH=462;>AT4
0.0
0.5
1.0
1.5
1
6
1
7
43
36
22
43
61
51
18
63
28
9
11
32
drm.ugt80..
drm.wt.ugt80..
drm.smt1..
drm.wt.smt1..
dsm.ugt80..
dsm.wt.ugt80..
dsm.smt1..
dsm.wt.smt1..
sp.ugt80..
sp.wt.ugt80..
sp.smt1..
sp.wt.smt1..
im.ugt80..
im.wt.ugt80..
im.smt1..
im.wt.smt1..

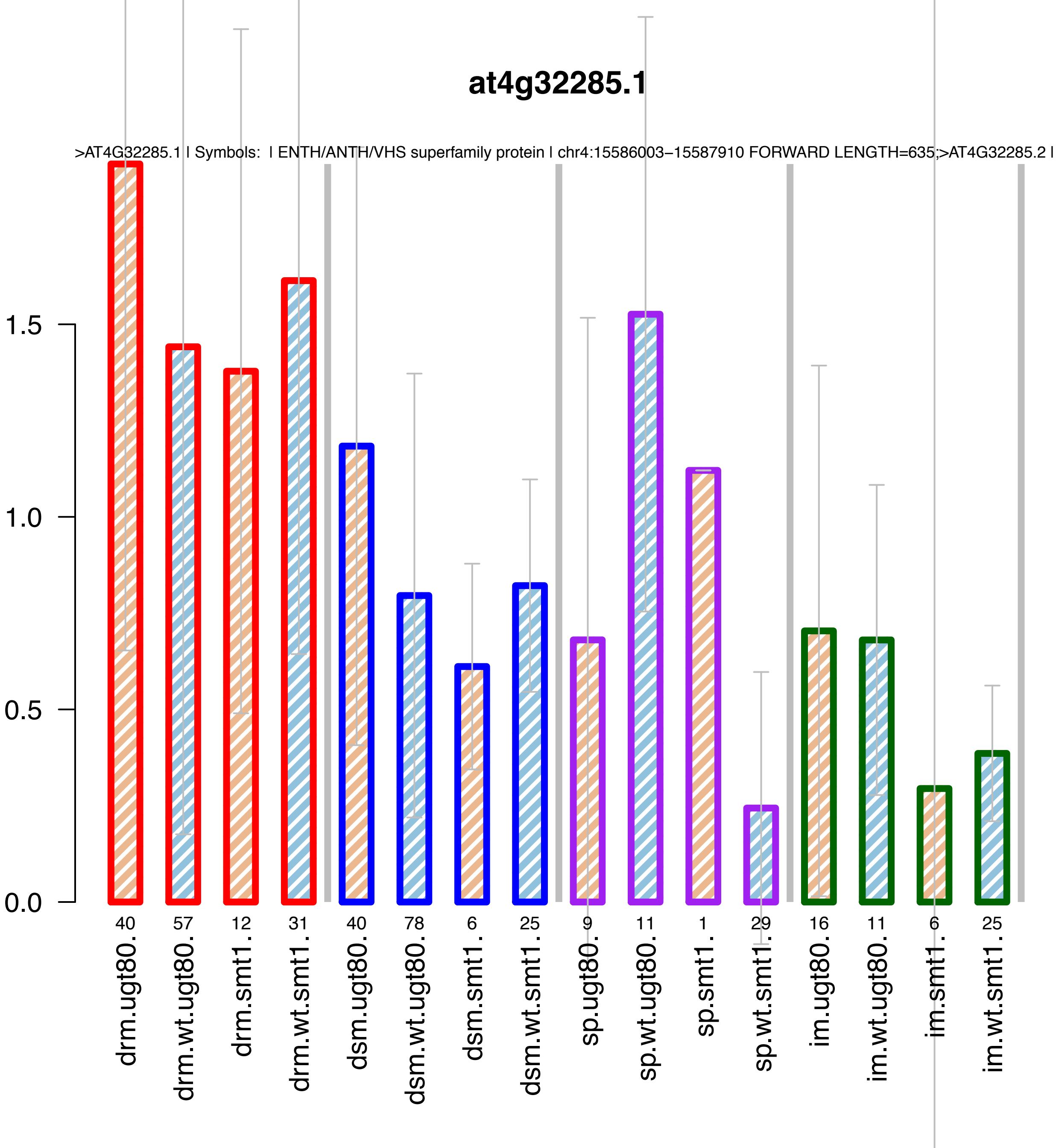

at4g32285.1
>AT4G32285.1 | Symbols: | ENTH/ANTH/VHS superfamily protein | chr4:15586003-15587910 FORWARD LENGTH=635;>AT4G32285.2 |
0.0
0.5
1.0
1.5
40
57
12
31
40
78
6
25
9
11
1
29
16
11
6
25
drm.ugt80..
drm.wt.ugt80..
drm.smt1..
drm.wt.smt1..
dsm.ugt80..
dsm.wt.ugt80..
dsm.smt1..
dsm.wt.smt1..
sp.ugt80..
sp.wt.ugt80..
sp.smt1..
sp.wt.smt1..
im.ugt80..
im.wt.ugt80..
im.smt1..
im.wt.smt1..

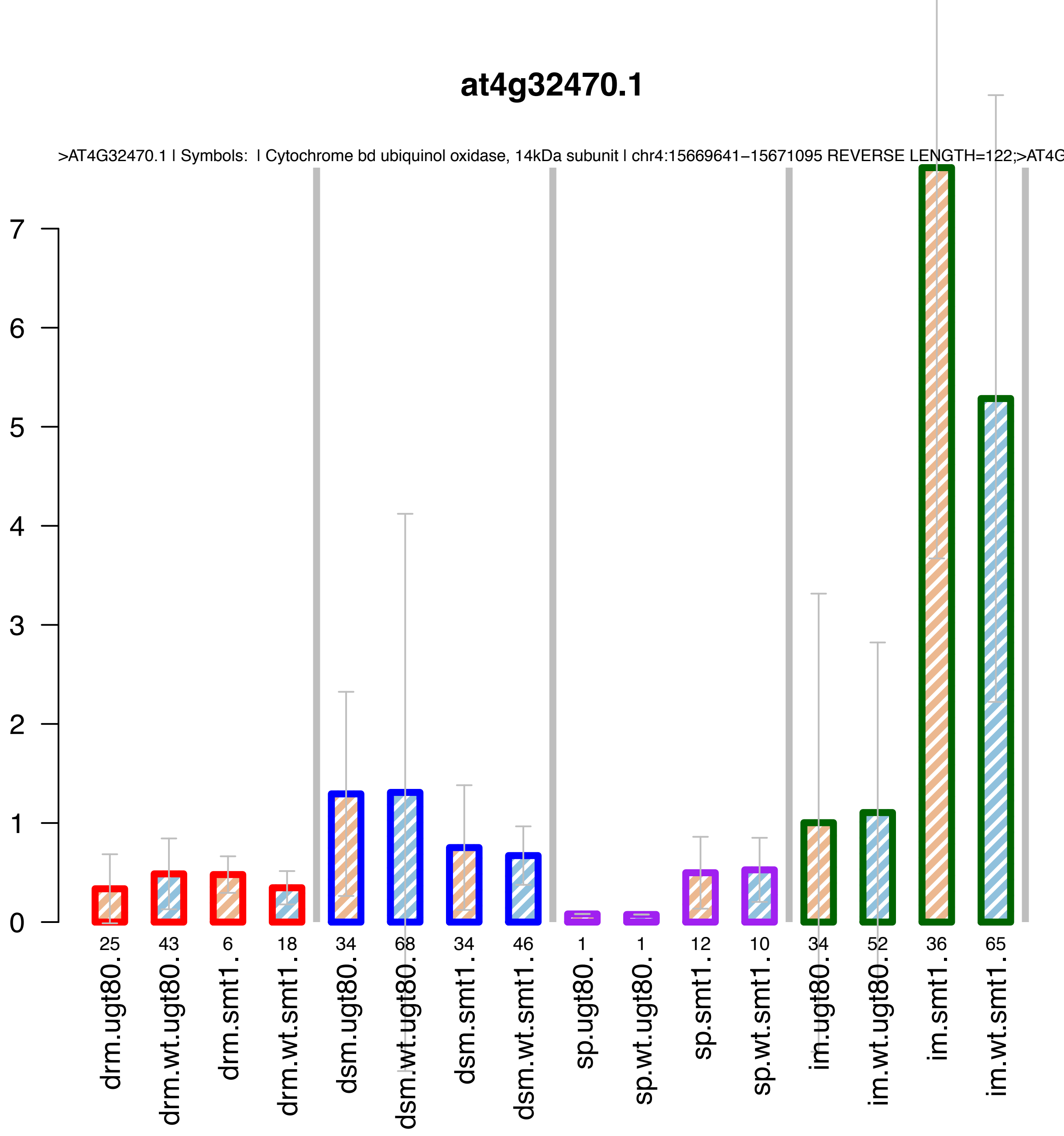

at4g32470.1
>AT4G32470.1 | Symbols: | Cytochrome bd ubiquinol oxidase, 14kDa subunit | chr4:15669641-15671095 REVERSE LENGTH=122;>AT4G
0
1
2
3
4
5
6
7
25
43
6
18
34
68
34
46
1
1
12
10
34
52
36
65
drm.ugt80..
drm.wt.ugt80..
drm.smt1..
drm.wt.smt1..
dsm.ugt80..
dsm.wt.ugt80..
dsm.smt1..
dsm.wt.smt1..
sp.ugt80..
sp.wt.ugt80..
sp.smt1..
sp.wt.smt1..
im.ugt80..
im.wt.ugt80..
im.smt1..
im.wt.smt1..

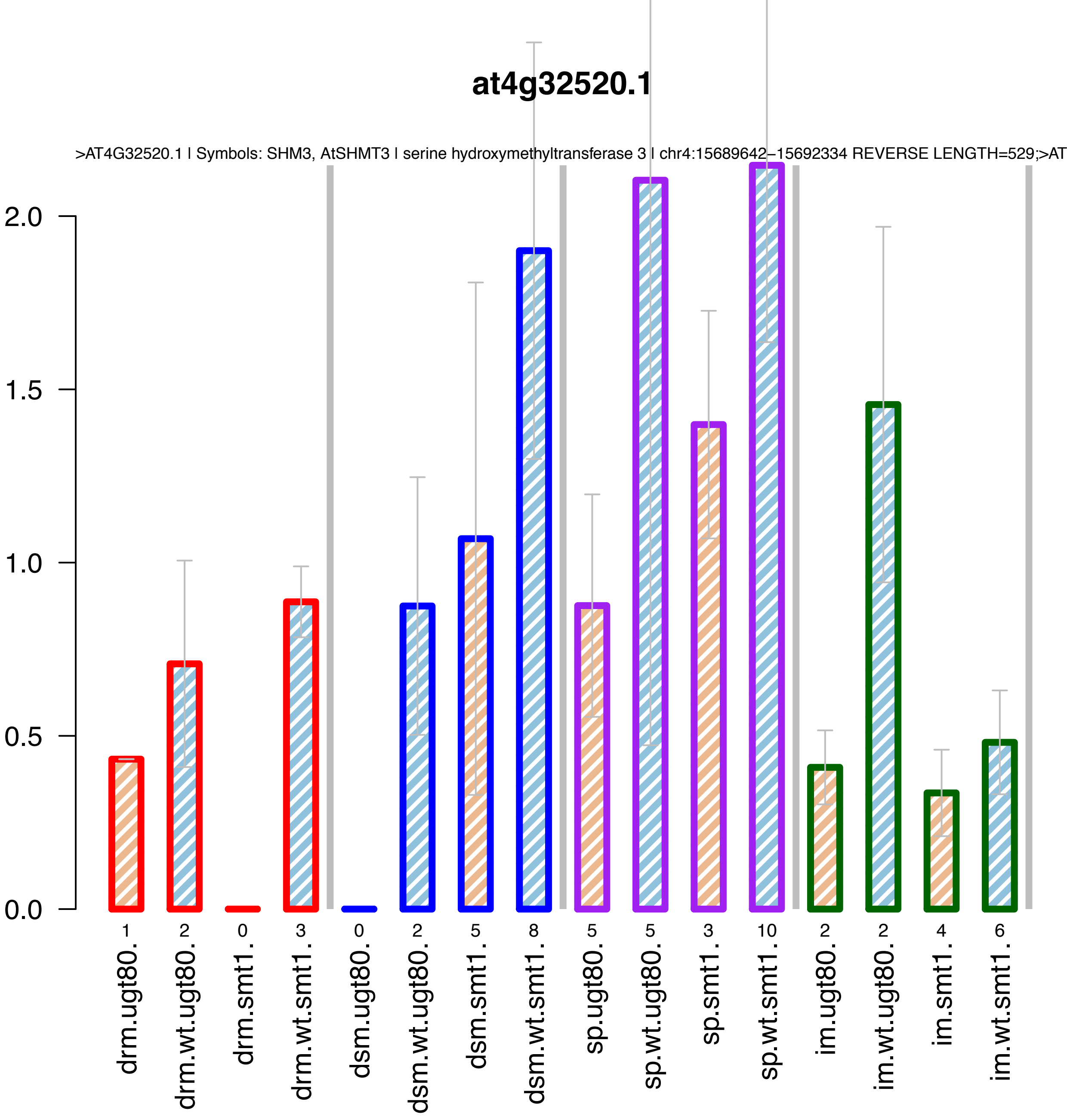

at4g32520.1
>AT4G32520.1 | Symbols: SHM3, AtSHMT3 | serine hydroxymethyltransferase 3 | chr4:15689642–15692334 REVERSE LENGTH=529;>AT
2.0
1.5
1.0
0.5
0.0
1
2
0
3
0
2
5
8
5
5
3
10
2
2
4
6
drm.ugt80..
drm.wt.ugt80..
drm.smt1..
drm.wt.smt1..
dsm.ugt80..
dsm.wt.ugt80..
dsm.smt1..
dsm.wt.smt1..
sp.ugt80..
sp.wt.ugt80..
sp.smt1..
sp.wt.smt1..
im.ugt80..
im.wt.ugt80..
im.smt1..
im.wt.smt1..

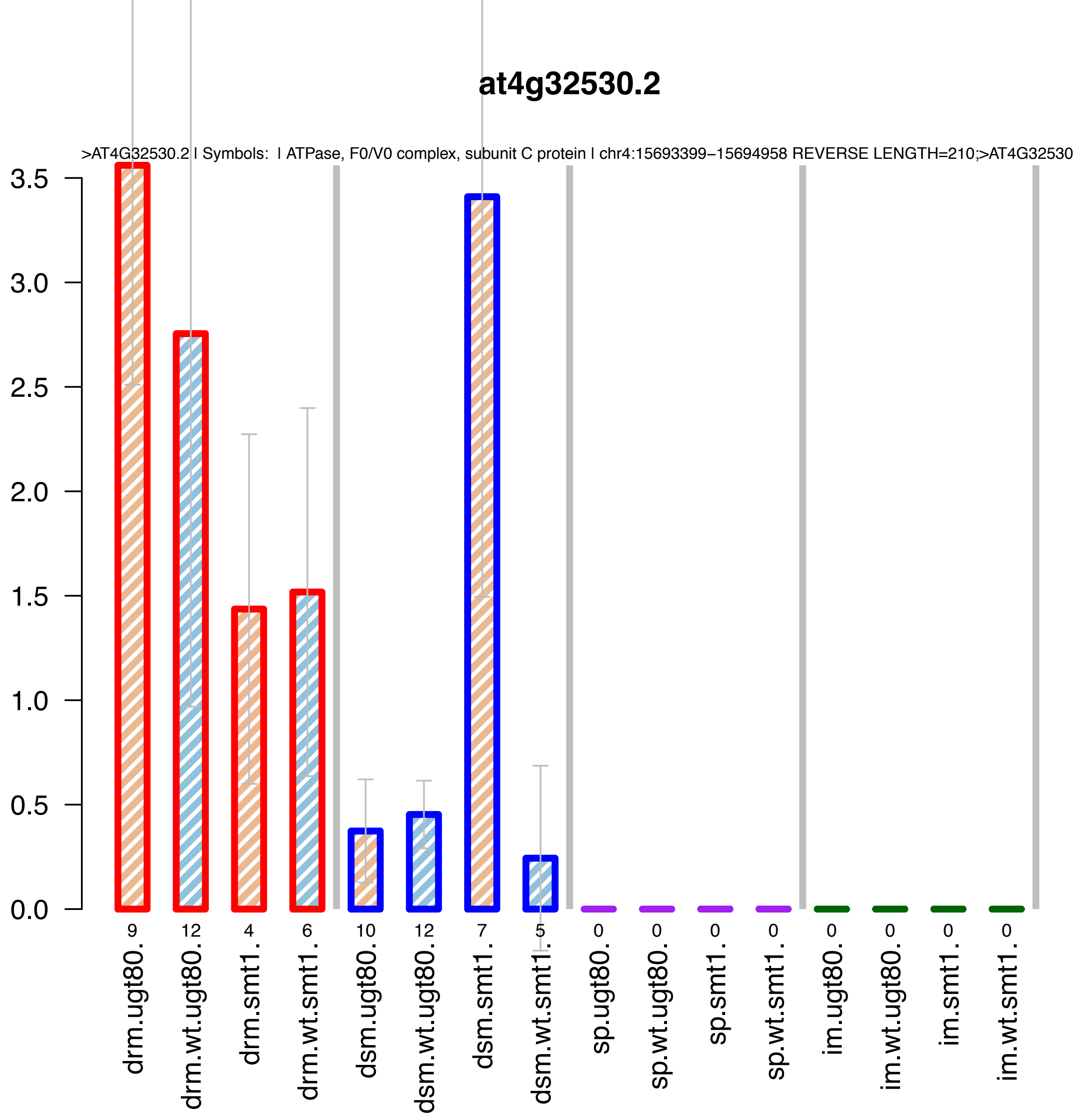

at4g32530.2
>AT4G32530.2 | Symbols: | ATPase, F0/V0 complex, subunit C protein | chr4:15693399-15694958 REVERSE LENGTH=210;>AT4G32530
3.5
3.0
2.5
2.0
1.5
1.0
0.5
0.0
9
12
4
6
10
12
7
5
0
0
0
0
0
0
0
0
drm.ugt80.
drm.wt.ugt80.
drm.smt1.
drm.wt.smt1.
dsm.ugt80.
dsm.wt.ugt80.
dsm.smt1.
dsm.wt.smt1.
sp.ugt80.
sp.wt.ugt80.
sp.smt1.
sp.wt.smt1.
im.ugt80.
im.wt.ugt80.
im.smt1.
im.wt.smt1.

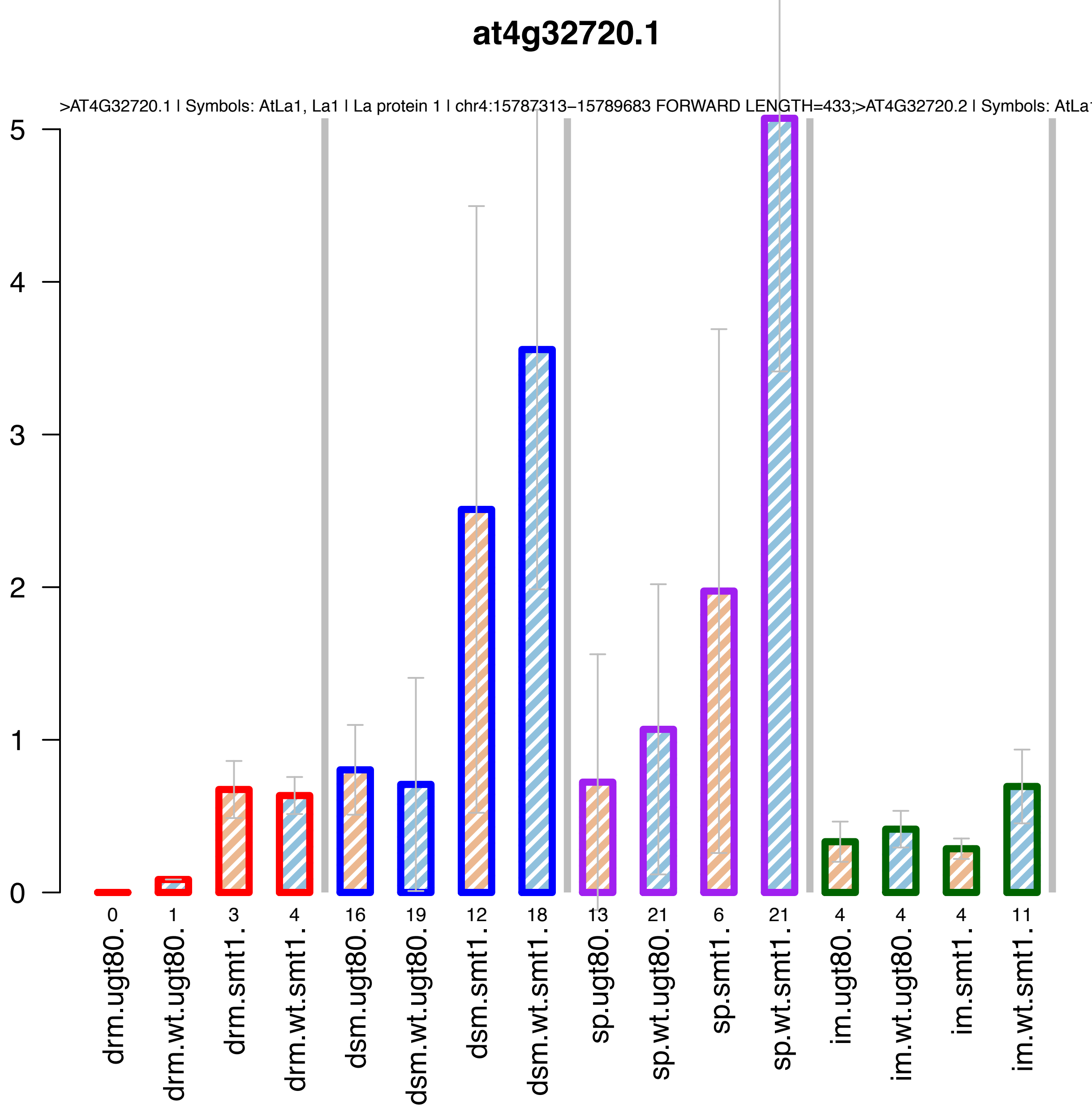

at4g32720.1
>AT4G32720.1 | Symbols: AtLa1, La1 | La protein 1 | chr4:15787313−15789683 FORWARD LENGTH=433;>AT4G32720.2 | Symbols: AtLa1
0
1
2
3
4
5
0
1
3
4
16
19
12
18
13
21
6
21
4
4
4
11
drm.ugt80.
drm.wt.ugt80.
drm.smt1.
drm.wt.smt1.
dsm.ugt80.
dsm.wt.ugt80.
dsm.smt1.
dsm.wt.smt1.
sp.ugt80.
sp.wt.ugt80.
sp.smt1.
sp.wt.smt1.
im.ugt80.
im.wt.ugt80.
im.smt1.
im.wt.smt1.

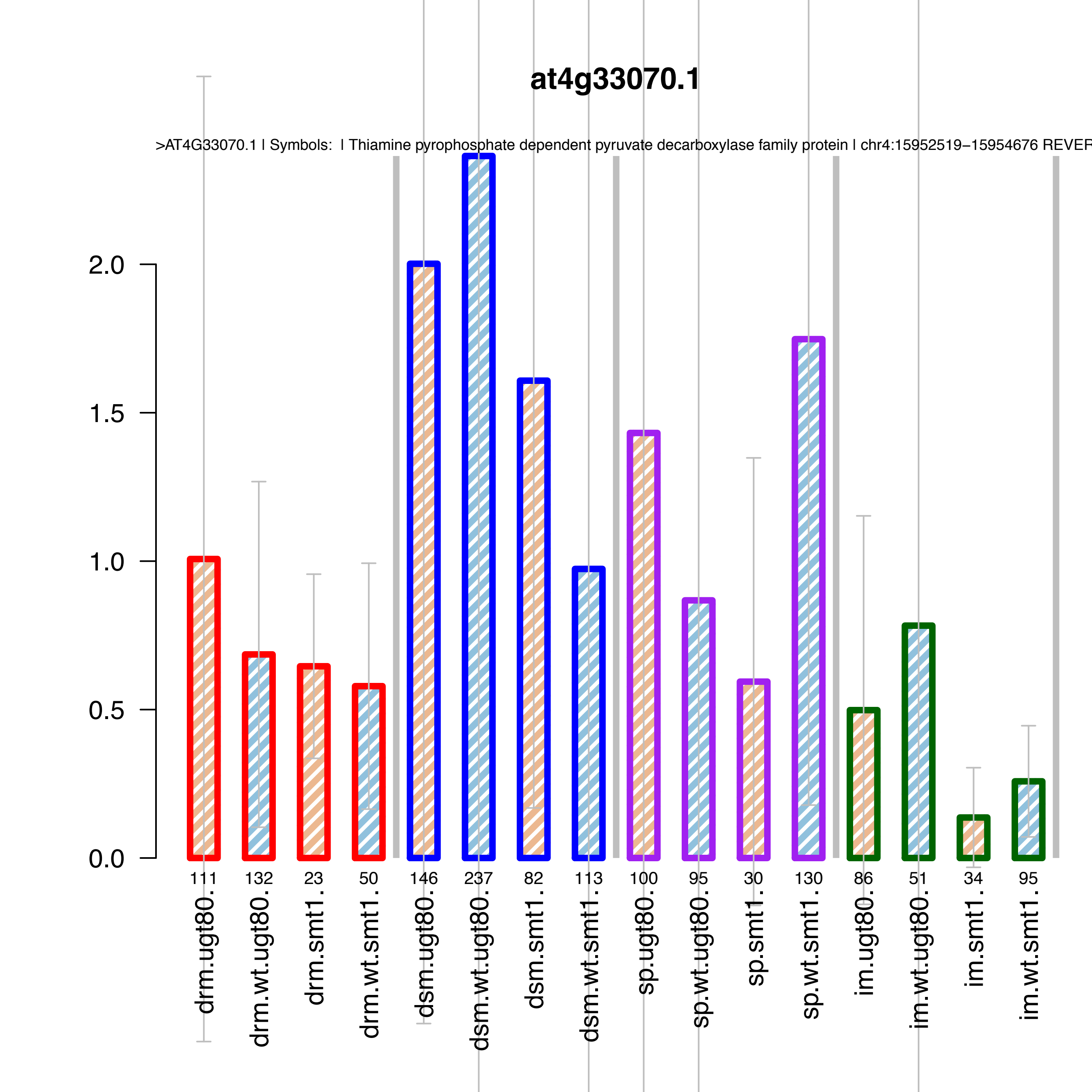

at4g33070.1
>AT4G33070.1 | Symbols: | Thiamine pyrophosphate dependent pyruvate decarboxylase family protein | chr4:15952519-15954676 REVER
2.0
1.5
1.0
0.5
0.0
111
132
23
50
146
237
82
113
100
95
30
130
86
51
34
95
drm.ugt80..
drm.wt.ugt80..
drm.smt1..
drm.wt.smt1..
dsm.ugt80..
dsm.wt.ugt80..
dsm.smt1..
dsm.wt.smt1..
sp.ugt80..
sp.wt.ugt80..
sp.smt1..
sp.wt.smt1..
im.ugt80..
im.wt.ugt80..
im.smt1..
im.wt.smt1..

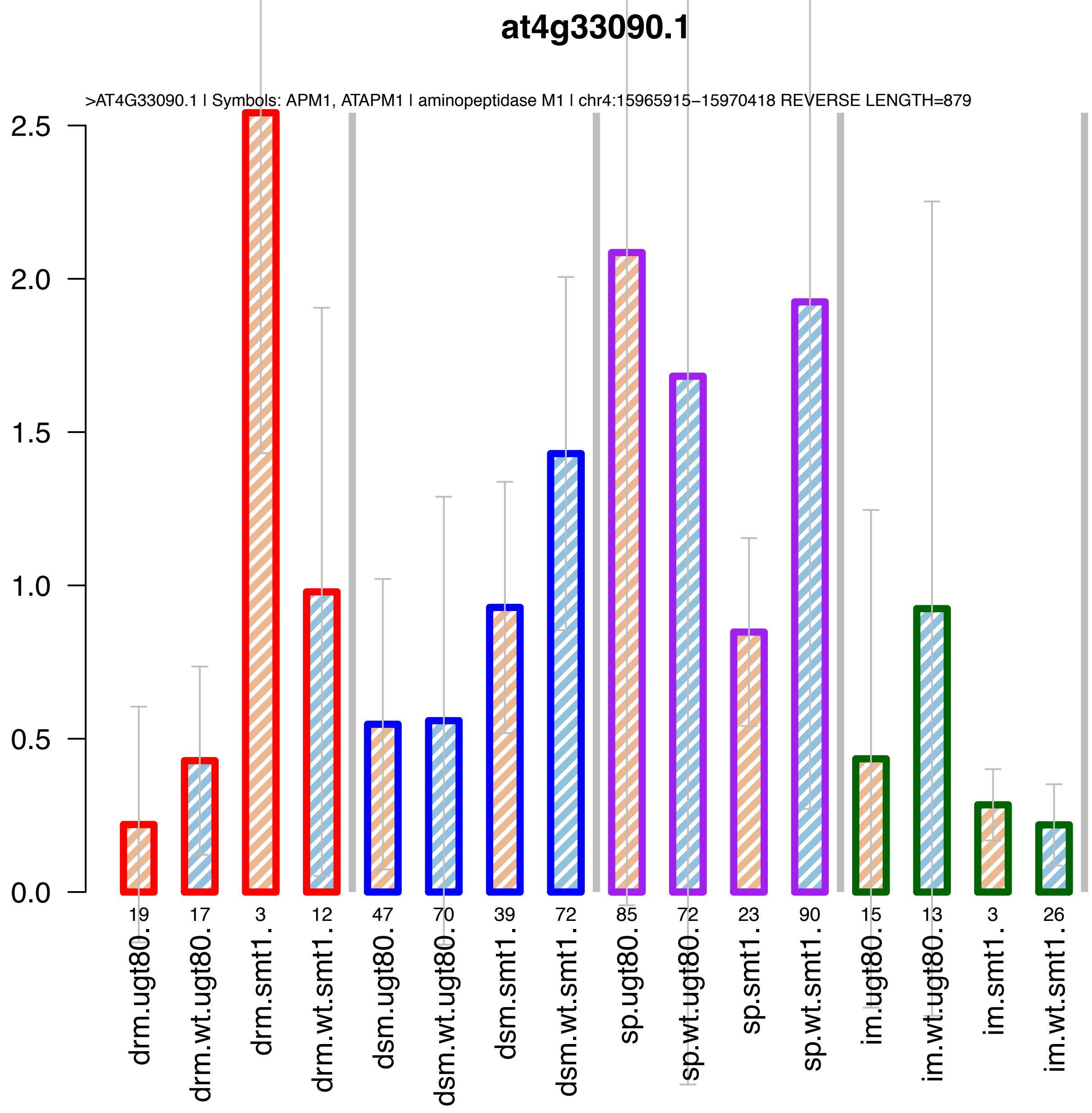

at4g33090.1
>AT4G33090.1 | Symbols: APM1, ATAPM1 | aminopeptidase M1 | chr4:15965915−15970418 REVERSE LENGTH=879
2.5
2.0
1.5
1.0
0.5
0.0
19
17
3
12
47
70
39
72
85
72
23
90
15
13
3
26
drm.ugt80..
drm.wt.ugt80..
drm.smt1..
drm.wt.smt1..
dsm.ugt80..
dsm.wt.ugt80..
dsm.smt1..
dsm.wt.smt1..
sp.ugt80..
sp.wt.ugt80..
sp.smt1..
sp.wt.smt1..
im.ugt80..
im.wt.ugt80..
im.smt1..
im.wt.smt1..

# at4g33350.1

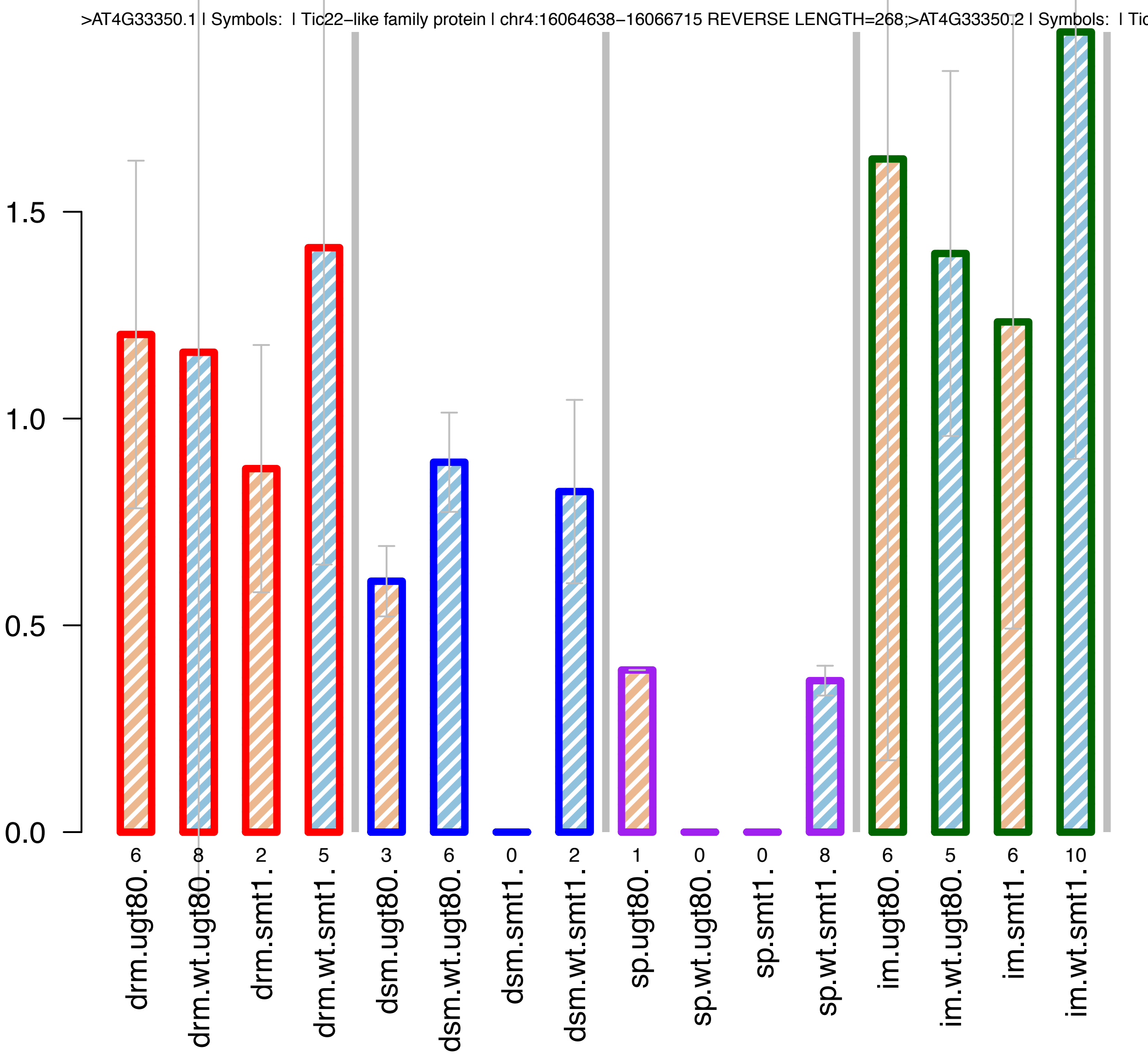

>AT4G33350.1 | Symbols: | Tic22–like family protein | chr4:16064638–16066715 REVERSE LENGTH=268;>AT4G33350.2 | Symbols: | Tic
1.5
1.0
0.5
0.0
6
8
2
5
3
6
0
2
1
0
0
8
6
5
6
10
drm.ugt80.
drm.wt.ugt80.
drm.smt1.
drm.wt.smt1.
dsm.ugt80.
dsm.wt.ugt80.
dsm.smt1.
dsm.wt.smt1.
sp.ugt80.
sp.wt.ugt80.
sp.smt1.
sp.wt.smt1.
im.ugt80.
im.wt.ugt80.
im.smt1.
im.wt.smt1.

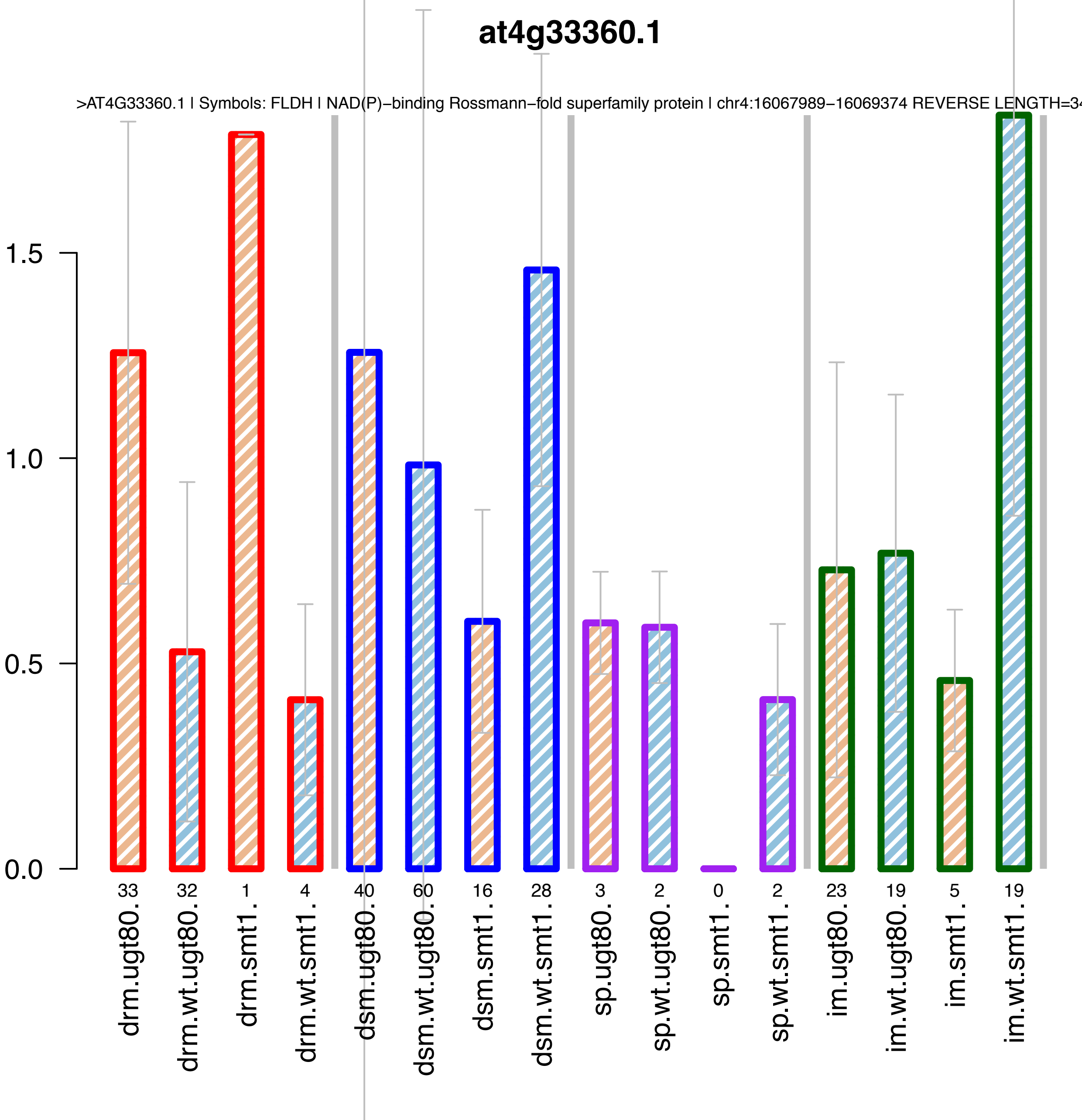

at4g33360.1
>AT4G33360.1 | Symbols: FLDH | NAD(P)-binding Rossmann-fold superfamily protein | chr4:16067989-16069374 REVERSE LENGTH=34
0.0
0.5
1.0
1.5
33
32
1
4
40
60
16
28
3
2
0
2
23
19
5
19
drm.ugt80.
drm.wt.ugt80.
drm.smt1.
drm.wt.smt1.
dsm.ugt80.
dsm.wt.ugt80.
dsm.smt1.
dsm.wt.smt1.
sp.ugt80.
sp.wt.ugt80.
sp.smt1.
sp.wt.smt1.
im.ugt80.
im.wt.ugt80.
im.smt1.
im.wt.smt1.

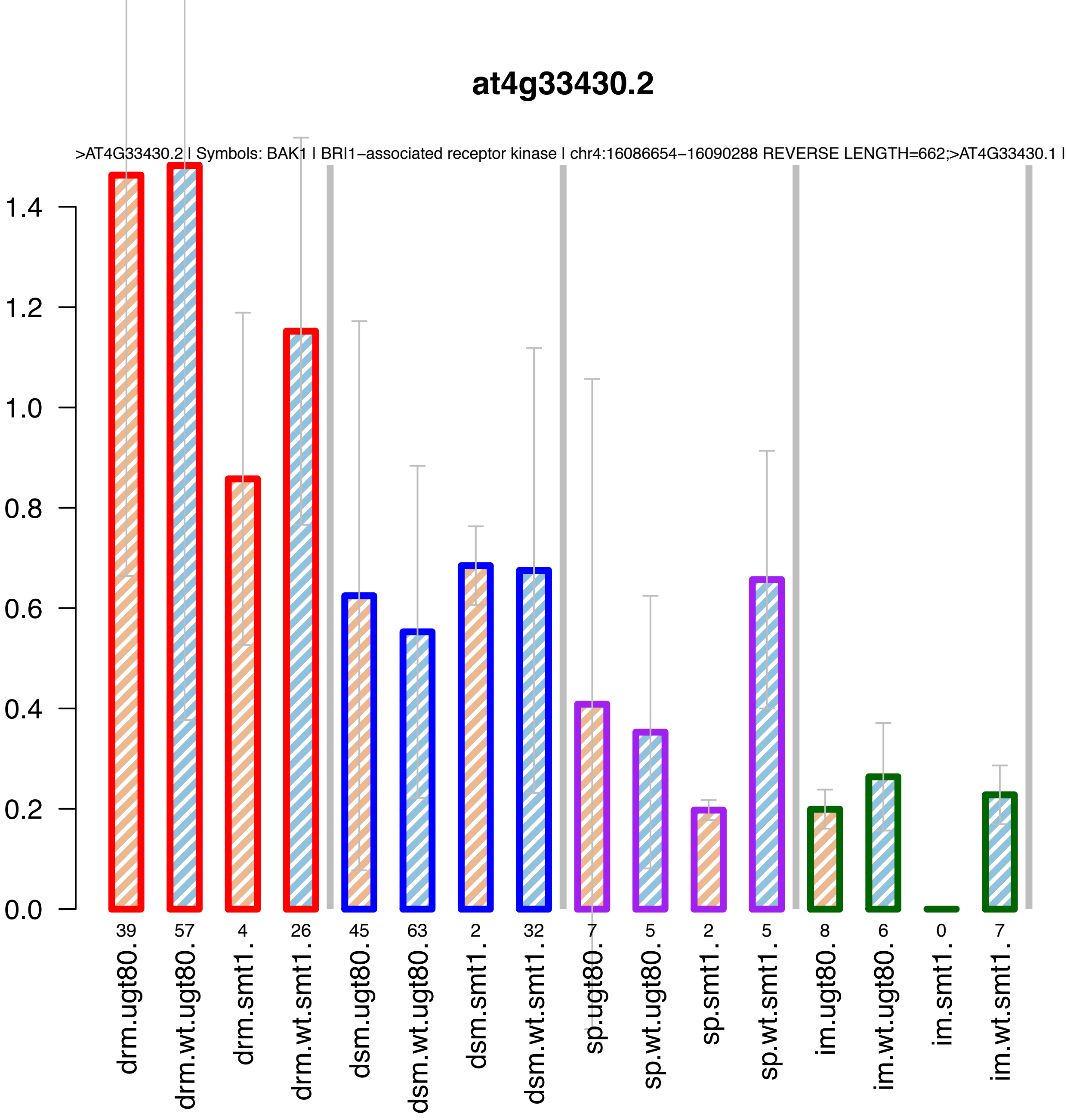

at4g33430.2
>AT4G33430.2 | Symbols: BAK1 | BRI1−associated receptor kinase | chr4:16086654−16090288 REVERSE LENGTH=662;>AT4G33430.1 |
1.4
1.2
1.0
0.8
0.6
0.4
0.2
0.0
39
57
4
26
45
63
2
32
7
5
2
5
8
6
0
7
drm.ugt80.
drm.wt.ugt80.
drm.smt1.
drm.wt.smt1.
dsm.ugt80.
dsm.wt.ugt80.
dsm.smt1.
dsm.wt.smt1.
sp.ugt80.
sp.wt.ugt80.
sp.smt1.
sp.wt.smt1.
im.ugt80.
im.wt.ugt80.
im.smt1.
im.wt.smt1.

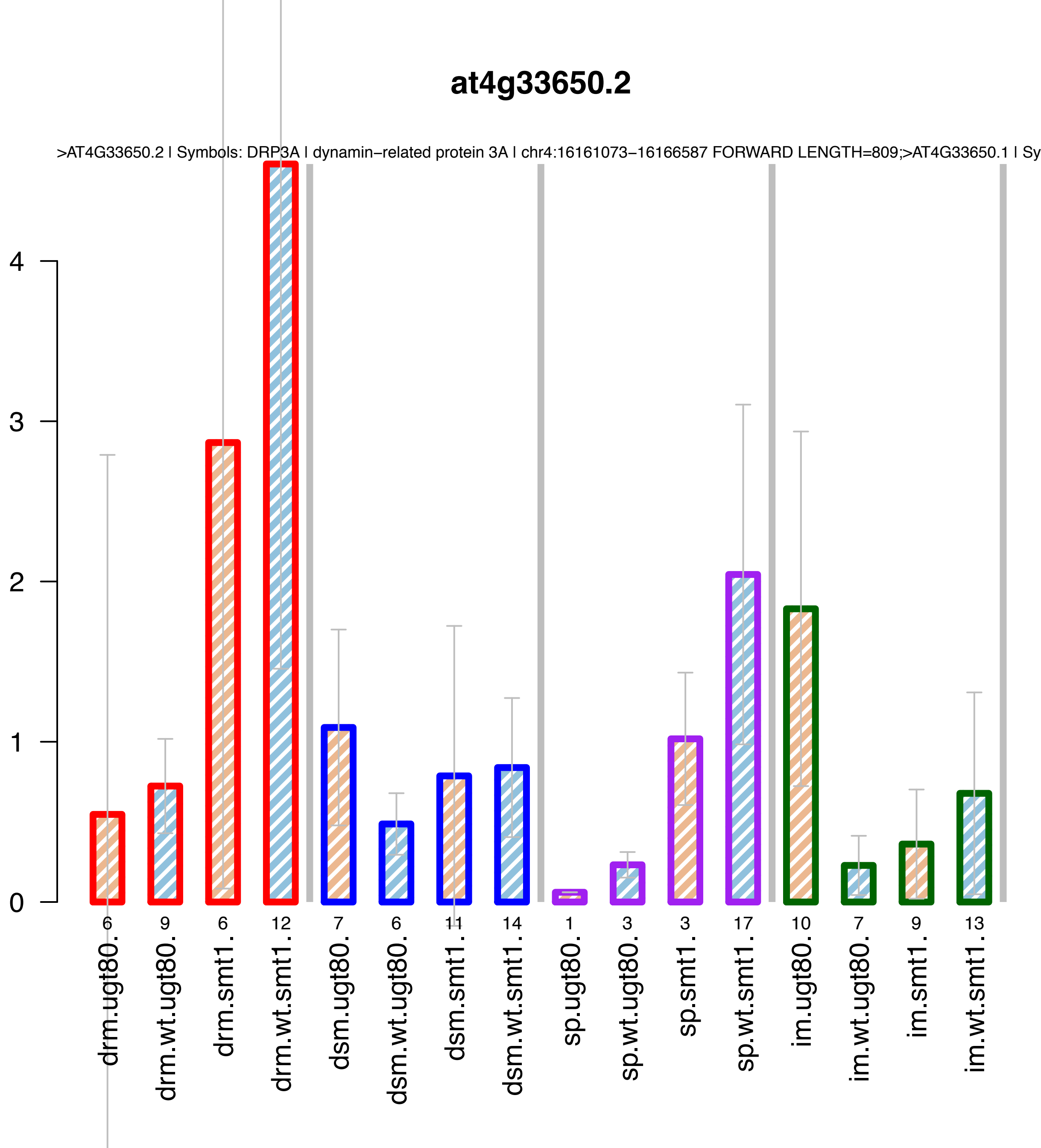

at4g33650.2
>AT4G33650.2 | Symbols: DRP3A | dynamin−related protein 3A | chr4:16161073−16166587 FORWARD LENGTH=809;>AT4G33650.1 | Sy
0
1
2
3
4
6
9
6
12
7
6
11
14
1
3
3
17
10
7
9
13
drm.ugt80.
drm.wt.ugt80.
drm.smt1.
drm.wt.smt1.
dsm.ugt80.
dsm.wt.ugt80.
dsm.smt1.
dsm.wt.smt1.
sp.ugt80.
sp.wt.ugt80.
sp.smt1.
sp.wt.smt1.
im.ugt80.
im.wt.ugt80.
im.smt1.
im.wt.smt1.

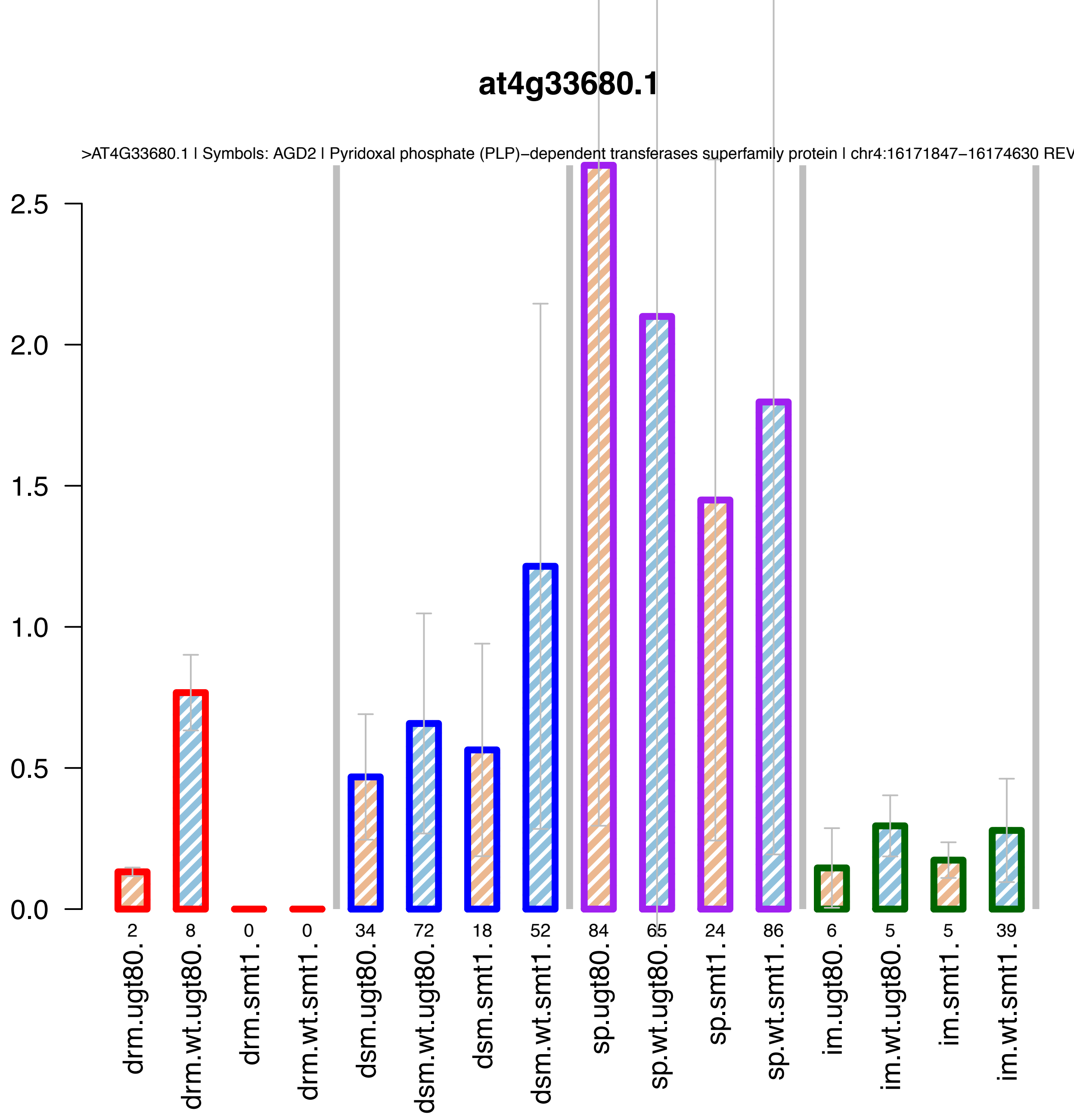

at4g33680.1
>AT4G33680.1 | Symbols: AGD2 | Pyridoxal phosphate (PLP)−dependent transferases superfamily protein | chr4:16171847−16174630 REV
0.0
0.5
1.0
1.5
2.0
2.5
2
8
0
0
34
72
18
52
84
65
24
86
6
5
5
39
drm.ugt80.
drm.wt.ugt80.
drm.smt1.
drm.wt.smt1.
dsm.ugt80.
dsm.wt.ugt80.
dsm.smt1.
dsm.wt.smt1.
sp.ugt80.
sp.wt.ugt80.
sp.smt1.
sp.wt.smt1.
im.ugt80.
im.wt.ugt80.
im.smt1.
im.wt.smt1.

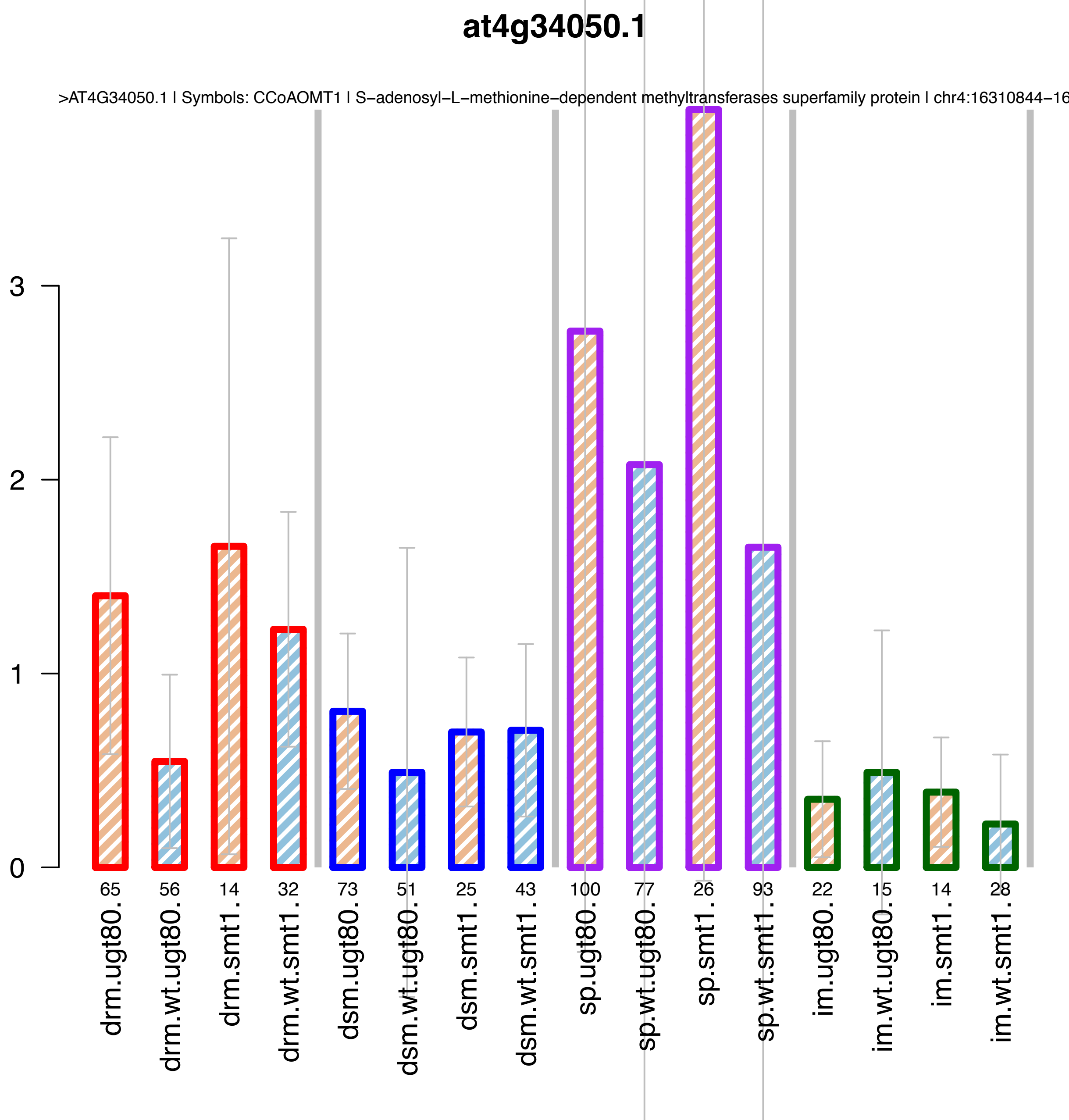

at4g34050.1
>AT4G34050.1 | Symbols: CCoAOMT1 | S−adenosyl−L−methionine−dependent methyltransferases superfamily protein | chr4:16310844−16
0
1
2
3
65
56
14
32
73
51
25
43
100
77
26
93
22
15
14
28
drm.ugt80.
drm.wt.ugt80.
drm.smt1.
drm.wt.smt1.
dsm.ugt80.
dsm.wt.ugt80.
dsm.smt1.
dsm.wt.smt1.
sp.ugt80.
sp.wt.ugt80.
sp.smt1.
sp.wt.smt1.
im.ugt80.
im.wt.ugt80.
im.smt1.
im.wt.smt1.

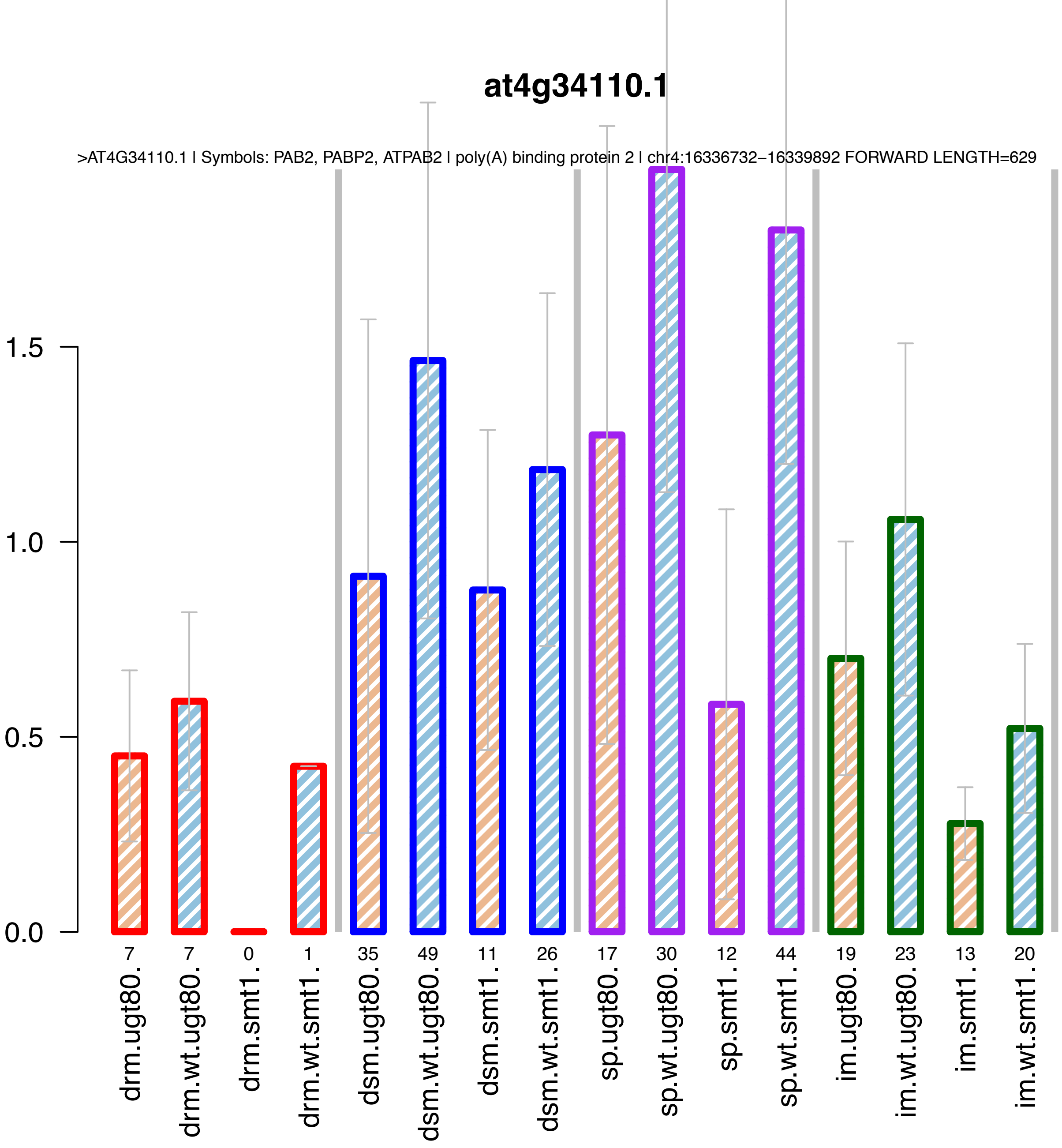

at4g34110.1
>AT4G34110.1 | Symbols: PAB2, PABP2, ATPAB2 | poly(A) binding protein 2 | chr4:16336732–16339892 FORWARD LENGTH=629
1.5
1.0
0.5
0.0
7
7
0
1
35
49
11
26
17
30
12
44
19
23
13
20
drm.ugt80..
drm.wt.ugt80..
drm.smt1..
drm.wt.smt1..
dsm.ugt80..
dsm.wt.ugt80..
dsm.smt1..
dsm.wt.smt1..
sp.ugt80..
sp.wt.ugt80..
sp.smt1..
sp.wt.smt1..
im.ugt80..
im.wt.ugt80..
im.smt1..
im.wt.smt1..

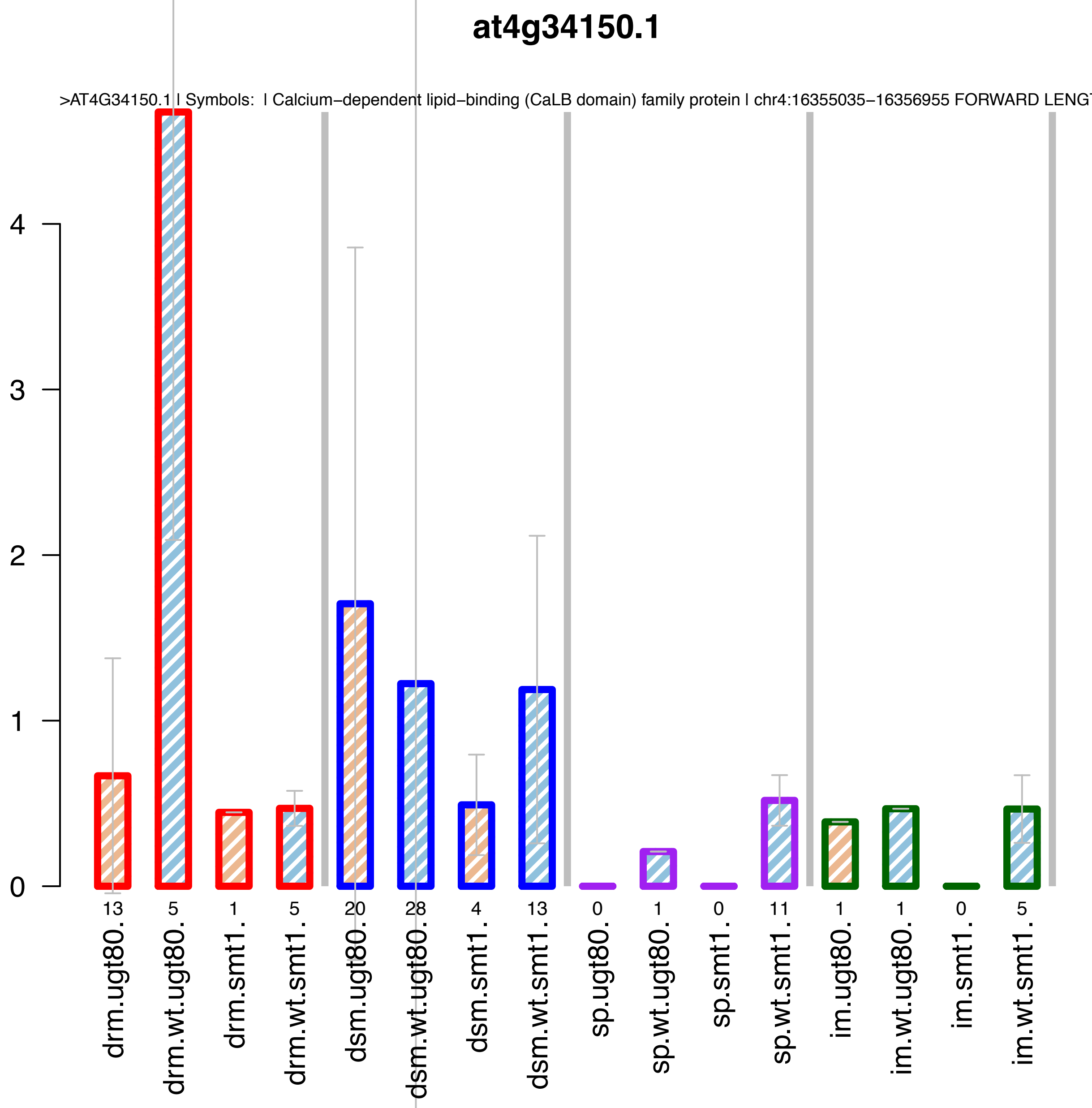

at4g34150.1
>AT4G34150.1 | Symbols: | Calcium−dependent lipid−binding (CaLB domain) family protein | chr4:16355035−16356955 FORWARD LENG
0
1
2
3
4
13
5
1
5
20
28
4
13
0
1
0
11
1
1
0
5
drm.ugt80.
drm.wt.ugt80.
drm.smt1.
drm.wt.smt1.
dsm.ugt80.
dsm.wt.ugt80.
dsm.smt1.
dsm.wt.smt1.
sp.ugt80.
sp.wt.ugt80.
sp.smt1.
sp.wt.smt1.
im.ugt80.
im.wt.ugt80.
im.smt1.
im.wt.smt1.

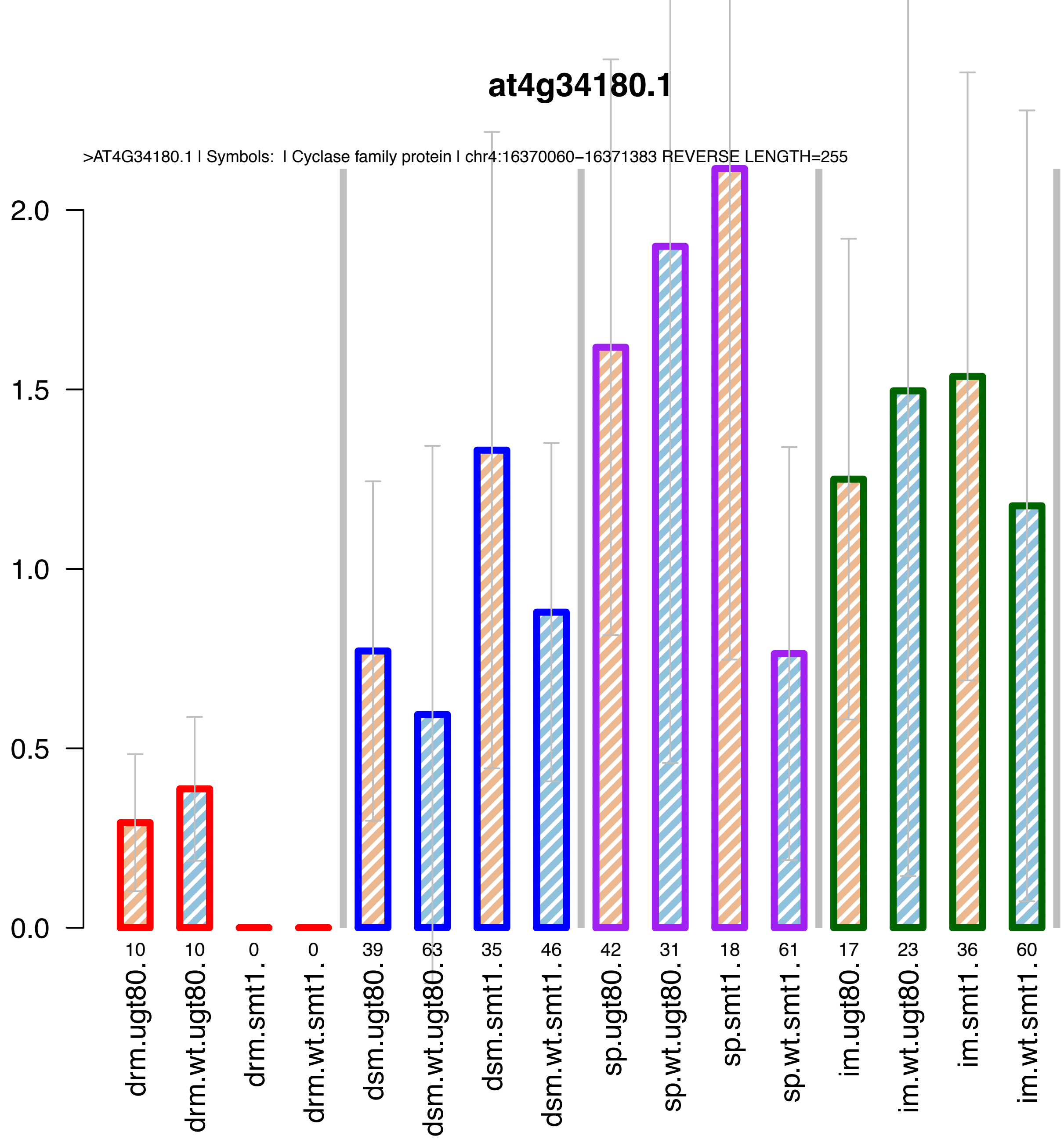

at4g34180.1
>AT4G34180.1 | Symbols: | Cyclase family protein | chr4:16370060-16371383 REVERSE LENGTH=255
2.0
1.5
1.0
0.5
0.0
10
10
0
0
39
63
35
46
42
31
18
61
17
23
36
60
drm.ugt80.
drm.wt.ugt80.
drm.smt1.
drm.wt.smt1.
dsm.ugt80.
dsm.wt.ugt80.
dsm.smt1.
dsm.wt.smt1.
sp.ugt80.
sp.wt.ugt80.
sp.smt1.
sp.wt.smt1.
im.ugt80.
im.wt.ugt80.
im.smt1.
im.wt.smt1.

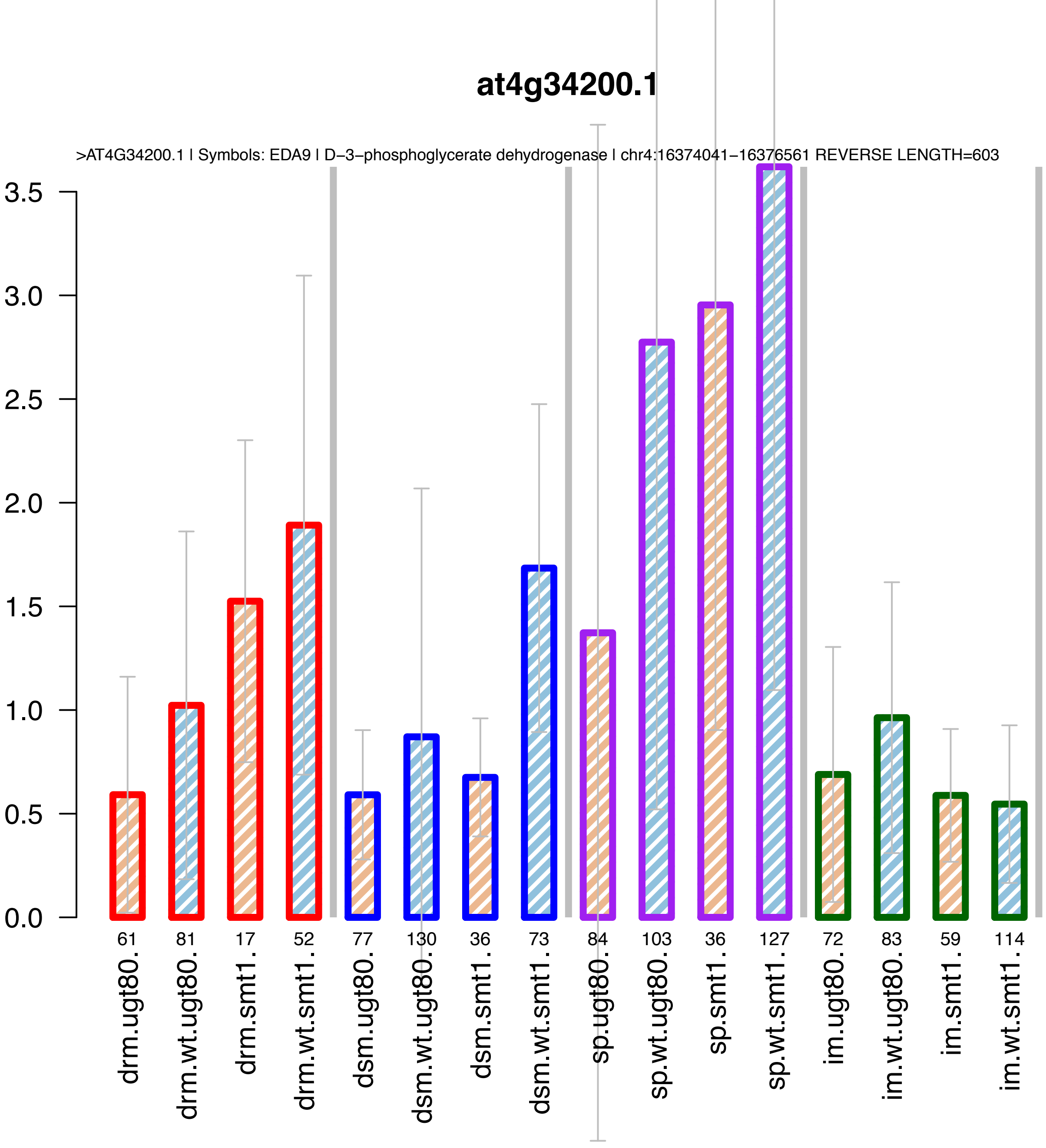

at4g34200.1
>AT4G34200.1 | Symbols: EDA9 | D−3−phosphoglycerate dehydrogenase | chr4:16374041−16376561 REVERSE LENGTH=603
3.5
3.0
2.5
2.0
1.5
1.0
0.5
0.0
61
81
17
52
77
130
36
73
84
103
36
127
72
83
59
114
drm.ugt80.
drm.wt.ugt80.
drm.smt1.
drm.wt.smt1.
dsm.ugt80.
dsm.wt.ugt80.
dsm.smt1.
dsm.wt.smt1.
sp.ugt80.
sp.wt.ugt80.
sp.smt1.
sp.wt.smt1.
im.ugt80.
im.wt.ugt80.
im.smt1.
im.wt.smt1.

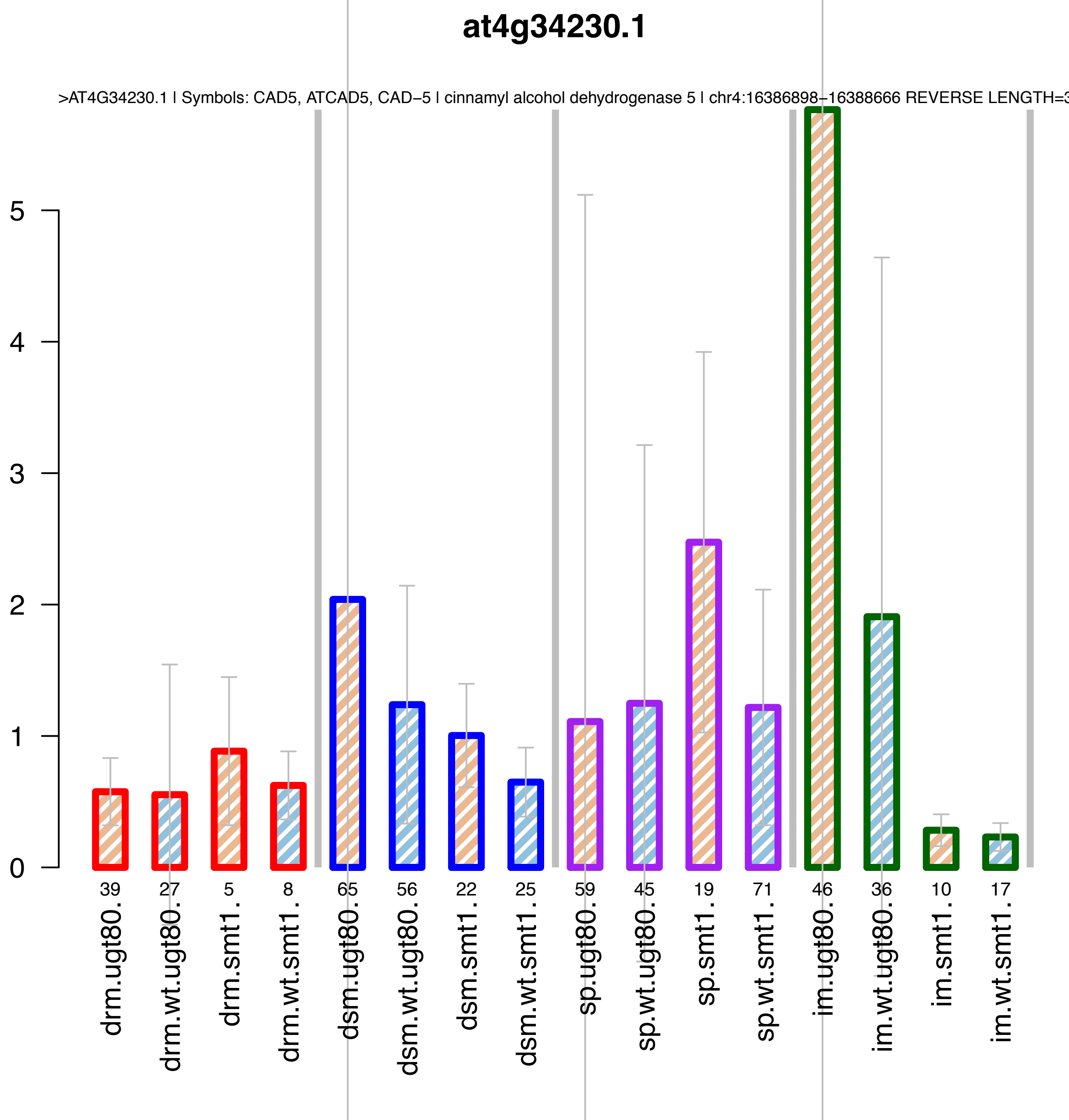

at4g34230.1
>AT4G34230.1 | Symbols: CAD5, ATCAD5, CAD−5 | cinnamyl alcohol dehydrogenase 5 | chr4:16386898−16388666 REVERSE LENGTH=3
0
1
2
3
4
5
39
27
5
8
65
56
22
25
59
45
19
71
46
36
10
17
drm.ugt80.
drm.wt.ugt80.
drm.smt1.
drm.wt.smt1.
dsm.ugt80.
dsm.wt.ugt80.
dsm.smt1.
dsm.wt.smt1.
sp.ugt80.
sp.wt.ugt80.
sp.smt1.
sp.wt.smt1.
im.ugt80.
im.wt.ugt80.
im.smt1.
im.wt.smt1.

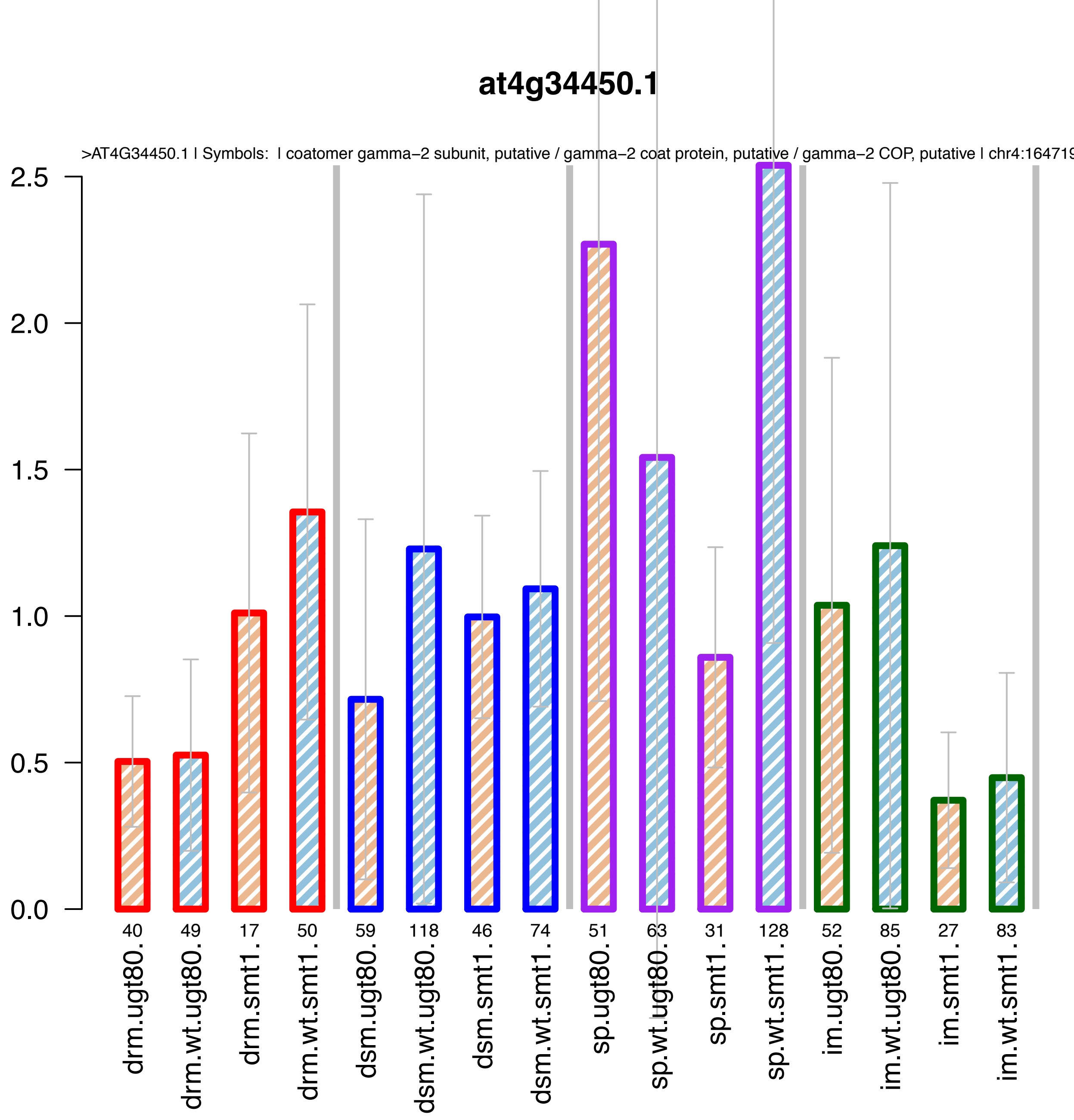

at4g34450.1
>AT4G34450.1 | Symbols: | coatomer gamma−2 subunit, putative / gamma−2 coat protein, putative / gamma−2 COP, putative | chr4:164719
2.5
2.0
1.5
1.0
0.5
0.0
40
49
17
50
59
118
46
74
51
63
31
128
52
85
27
83
drm.ugt80.
drm.wt.ugt80.
drm.smt1.
drm.wt.smt1.
dsm.ugt80.
dsm.wt.ugt80.
dsm.smt1.
dsm.wt.smt1.
sp.ugt80.
sp.wt.ugt80.
sp.smt1.
sp.wt.smt1.
im.ugt80.
im.wt.ugt80.
im.smt1.
im.wt.smt1.

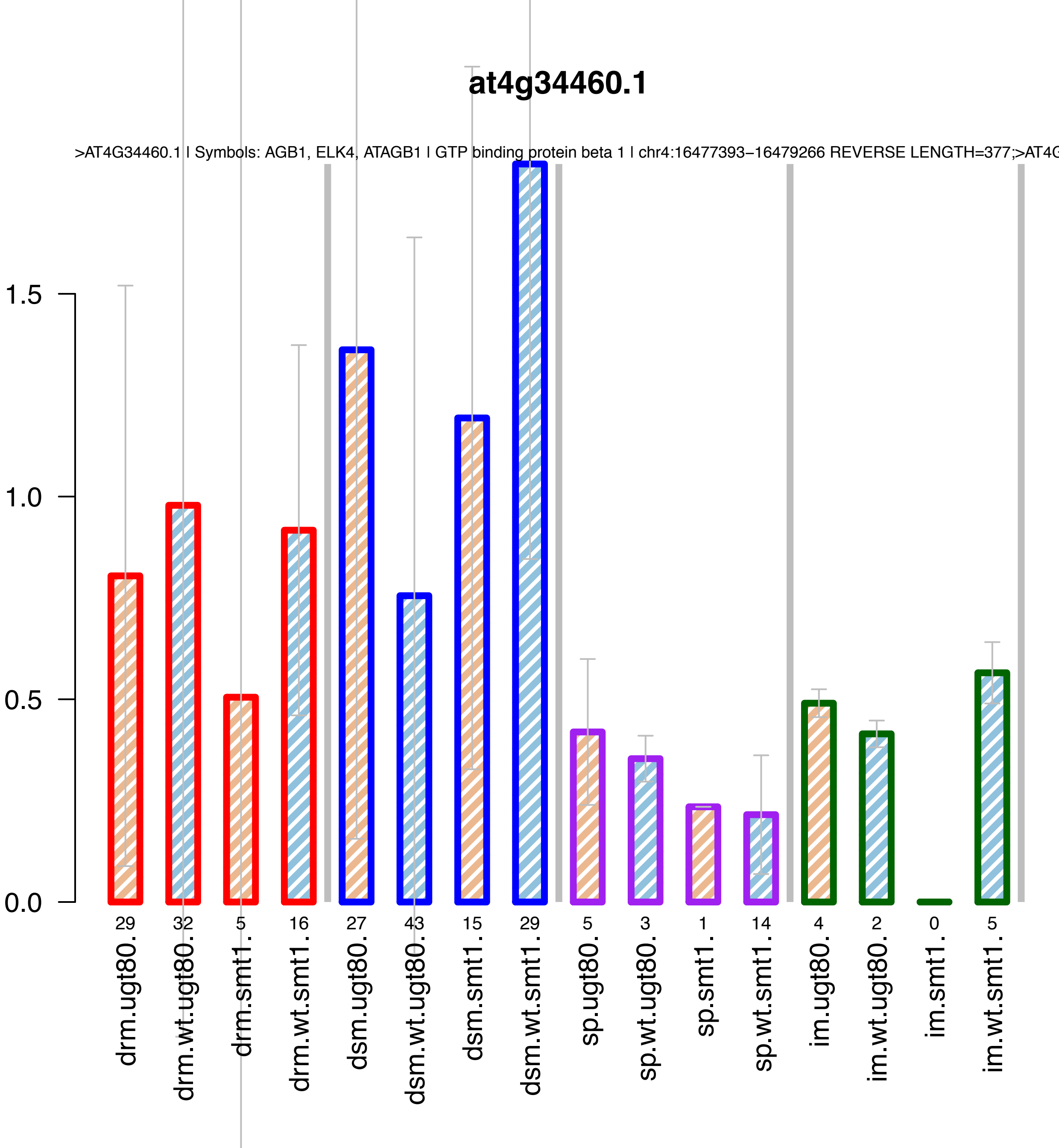

at4g34460.1
>AT4G34460.1 | Symbols: AGB1, ELK4, ATAGB1 | GTP binding protein beta 1 | chr4:16477393-16479266 REVERSE LENGTH=377;>AT4G
1.5
1.0
0.5
0.0
29
32
5
16
27
43
15
29
5
3
1
14
4
2
0
5
drm.ugt80.
drm.wt.ugt80.
drm.smt1.
drm.wt.smt1.
dsm.ugt80.
dsm.wt.ugt80.
dsm.smt1.
dsm.wt.smt1.
sp.ugt80.
sp.wt.ugt80.
sp.smt1.
sp.wt.smt1.
im.ugt80.
im.wt.ugt80.
im.smt1.
im.wt.smt1.

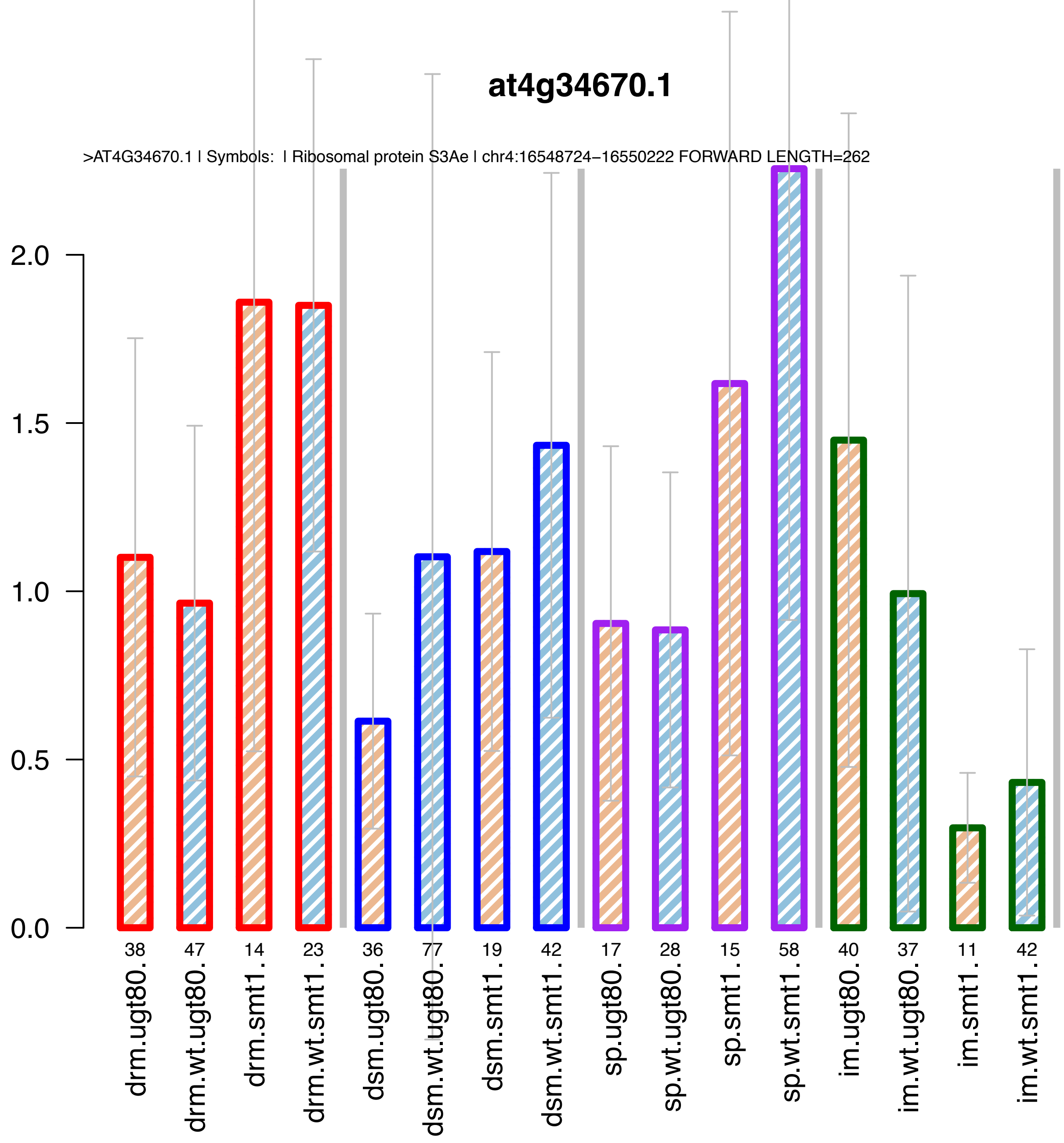
at4g34670.1
>AT4G34670.1 | Symbols: | Ribosomal protein S3Ae | chr4:16548724-16550222 FORWARD LENGTH=262
2.0
1.5
1.0
0.5
0.0
38
47
14
23
36
77
19
42
17
28
15
58
40
37
11
42
drm.ugt80..
drm.wt.ugt80..
drm.smt1..
drm.wt.smt1..
dsm.ugt80..
dsm.wt.ugt80..
dsm.smt1..
dsm.wt.smt1..
sp.ugt80..
sp.wt.ugt80..
sp.smt1..
sp.wt.smt1..
im.ugt80..
im.wt.ugt80..
im.smt1..
im.wt.smt1..

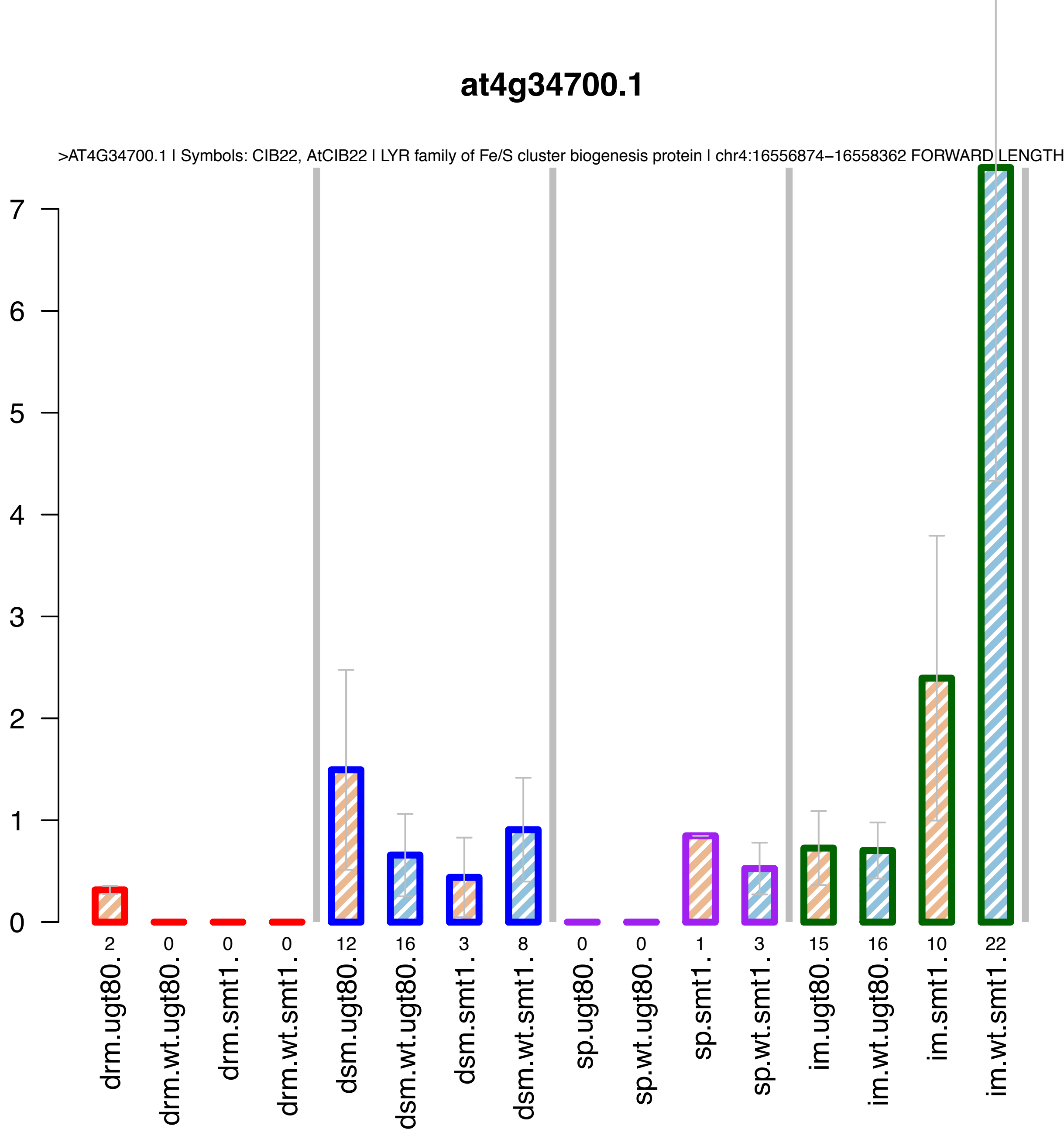

at4g34700.1
>AT4G34700.1 | Symbols: CIB22, AtCIB22 | LYR family of Fe/S cluster biogenesis protein | chr4:16556874-16558362 FORWARD LENGTH
7
6
5
4
3
2
1
0
2
0
0
0
12
16
3
8
0
0
1
3
15
16
10
22
drm.ugt80.
drm.wt.ugt80.
drm.smt1.
drm.wt.smt1.
dsm.ugt80.
dsm.wt.ugt80.
dsm.smt1.
dsm.wt.smt1.
sp.ugt80.
sp.wt.ugt80.
sp.smt1.
sp.wt.smt1.
im.ugt80.
im.wt.ugt80.
im.smt1.
im.wt.smt1.

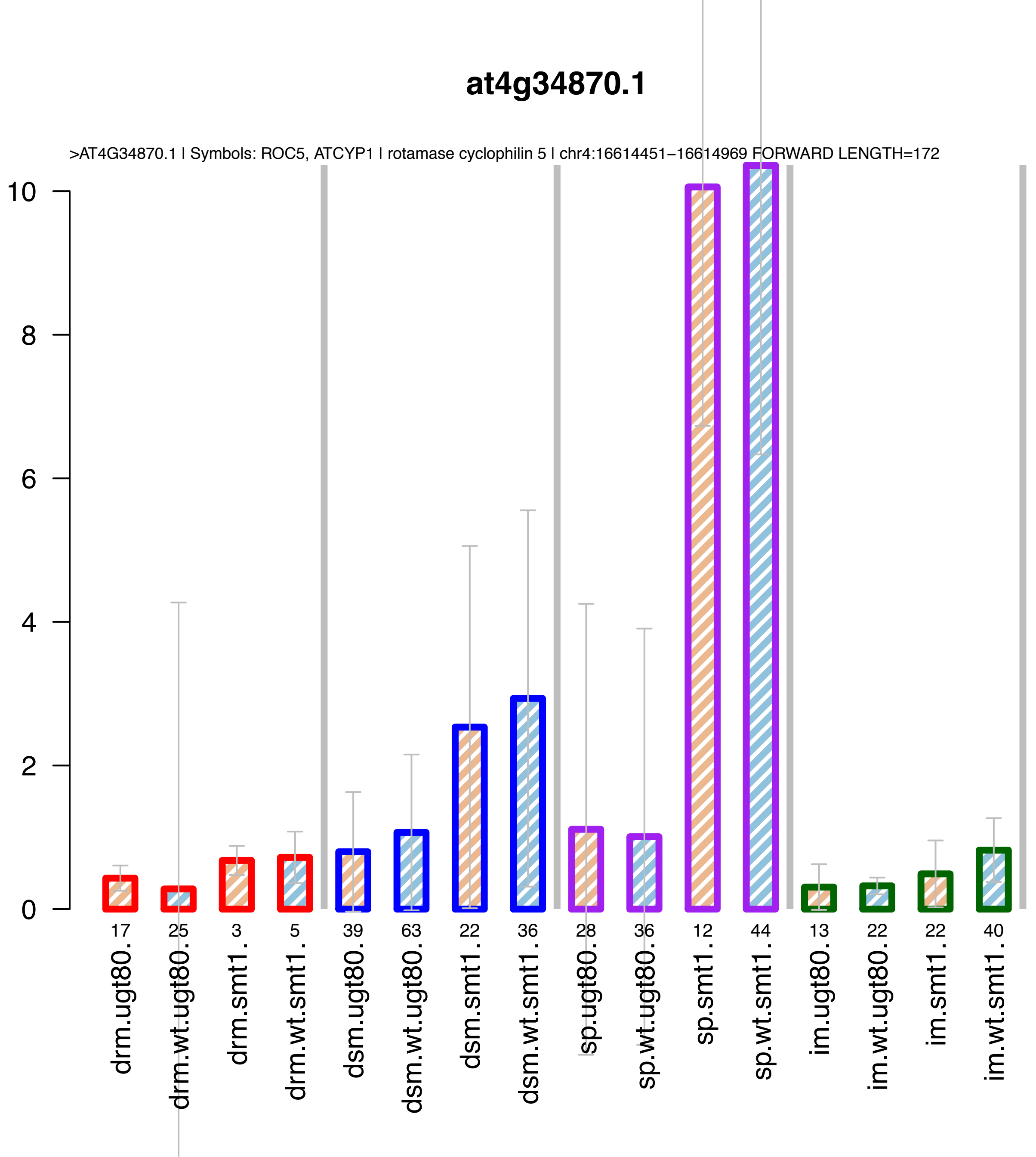

at4g34870.1
>AT4G34870.1 | Symbols: ROC5, ATCYP1 | rotamase cyclophilin 5 | chr4:16614451–16614969 FORWARD LENGTH=172
0
2
4
6
8
10
17
25
3
5
39
63
22
36
28
36
12
44
13
22
22
40
drm.ugt80.
drm.wt.ugt80.
drm.smt1.
drm.wt.smt1.
dsm.ugt80.
dsm.wt.ugt80.
dsm.smt1.
dsm.wt.smt1.
sp.ugt80.
sp.wt.ugt80.
sp.smt1.
sp.wt.smt1.
im.ugt80.
im.wt.ugt80.
im.smt1.
im.wt.smt1.

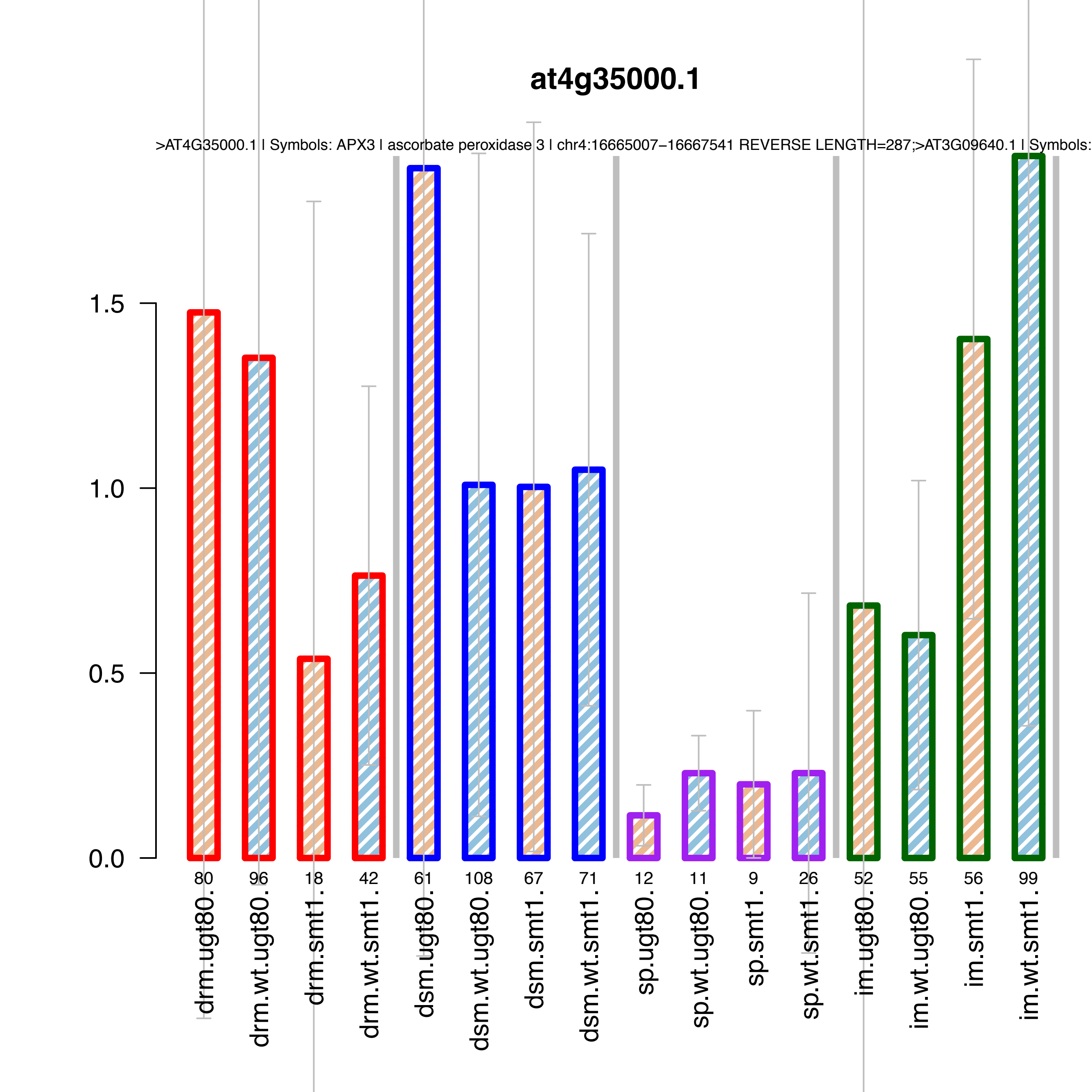

at4g35000.1
>AT4G35000.1 | Symbols: APX3 | ascorbate peroxidase 3 | chr4:16665007−16667541 REVERSE LENGTH=287;>AT3G09640.1 | Symbols:
0.0
0.5
1.0
1.5
80
96
18
42
61
108
67
71
12
11
9
26
52
55
56
99
drm.ugt80..
drm.wt.ugt80..
drm.smt1..
drm.wt.smt1..
dsm.ugt80..
dsm.wt.ugt80..
dsm.smt1..
dsm.wt.smt1..
sp.ugt80..
sp.wt.ugt80..
sp.smt1..
sp.wt.smt1..
im.ugt80..
im.wt.ugt80..
im.smt1..
im.wt.smt1..

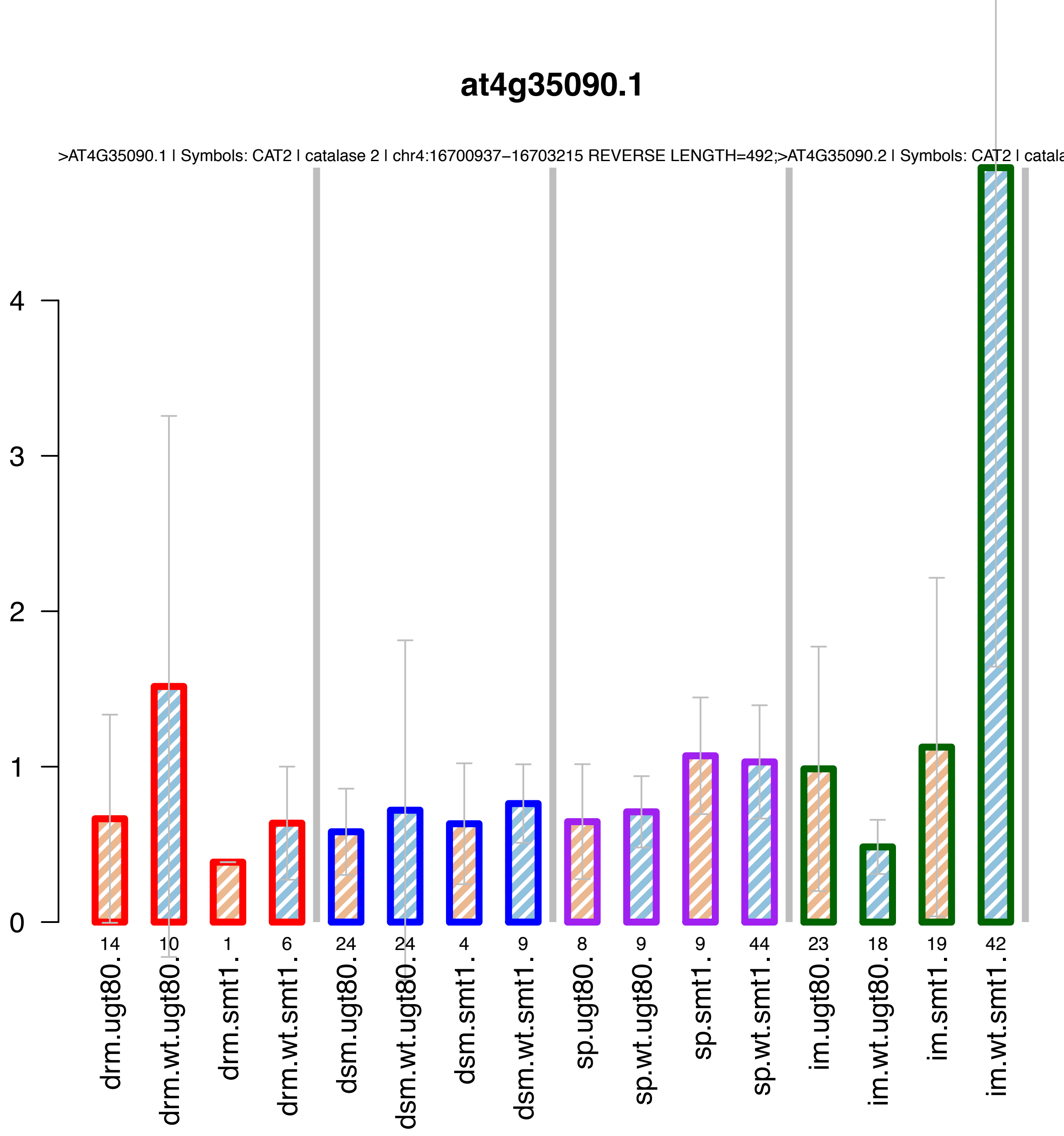

at4g35090.1
>AT4G35090.1 | Symbols: CAT2 | catalase 2 | chr4:16700937-16703215 REVERSE LENGTH=492;>AT4G35090.2 | Symbols: CAT2 | catala
0
1
2
3
4
14
10
1
6
24
24
4
9
8
9
9
44
23
18
19
42
drm.ugt80..
drm.wt.ugt80..
drm.smt1..
drm.wt.smt1..
dsm.ugt80..
dsm.wt.ugt80..
dsm.smt1..
dsm.wt.smt1..
sp.ugt80..
sp.wt.ugt80..
sp.smt1..
sp.wt.smt1..
im.ugt80..
im.wt.ugt80..
im.smt1..
im.wt.smt1..

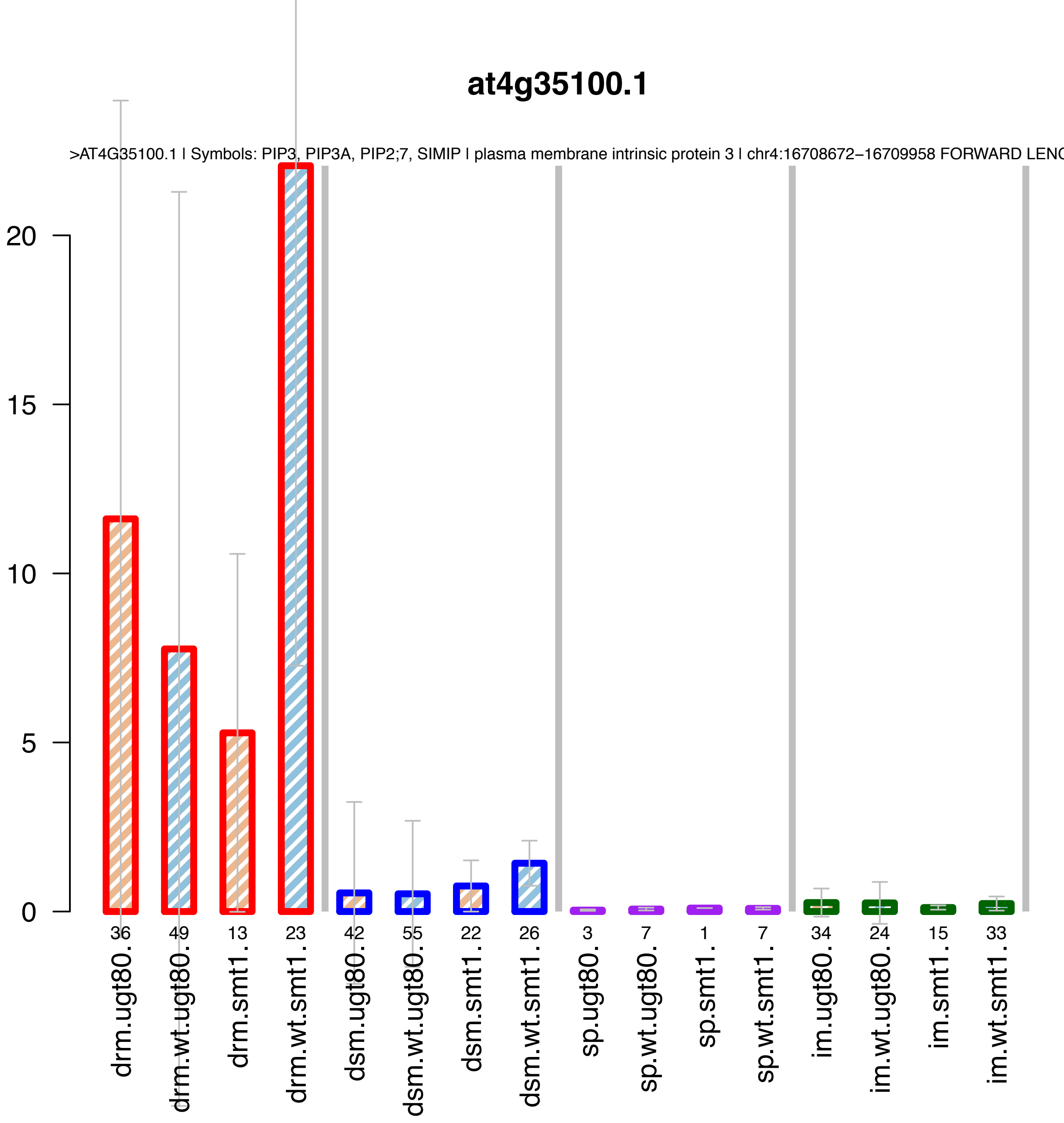

at4g35100.1
>AT4G35100.1 | Symbols: PIP3, PIP3A, PIP2;7, SIMIP | plasma membrane intrinsic protein 3 | chr4:16708672–16709958 FORWARD LENG
0
5
10
15
20
36
49
13
23
42
55
22
26
3
7
1
7
34
24
15
33
drm.ugt80.
drm.wt.ugt80.
drm.smt1.
drm.wt.smt1.
dsm.ugt80.
dsm.wt.ugt80.
dsm.smt1.
dsm.wt.smt1.
sp.ugt80.
sp.wt.ugt80.
sp.smt1.
sp.wt.smt1.
im.ugt80.
im.wt.ugt80.
im.smt1.
im.wt.smt1.

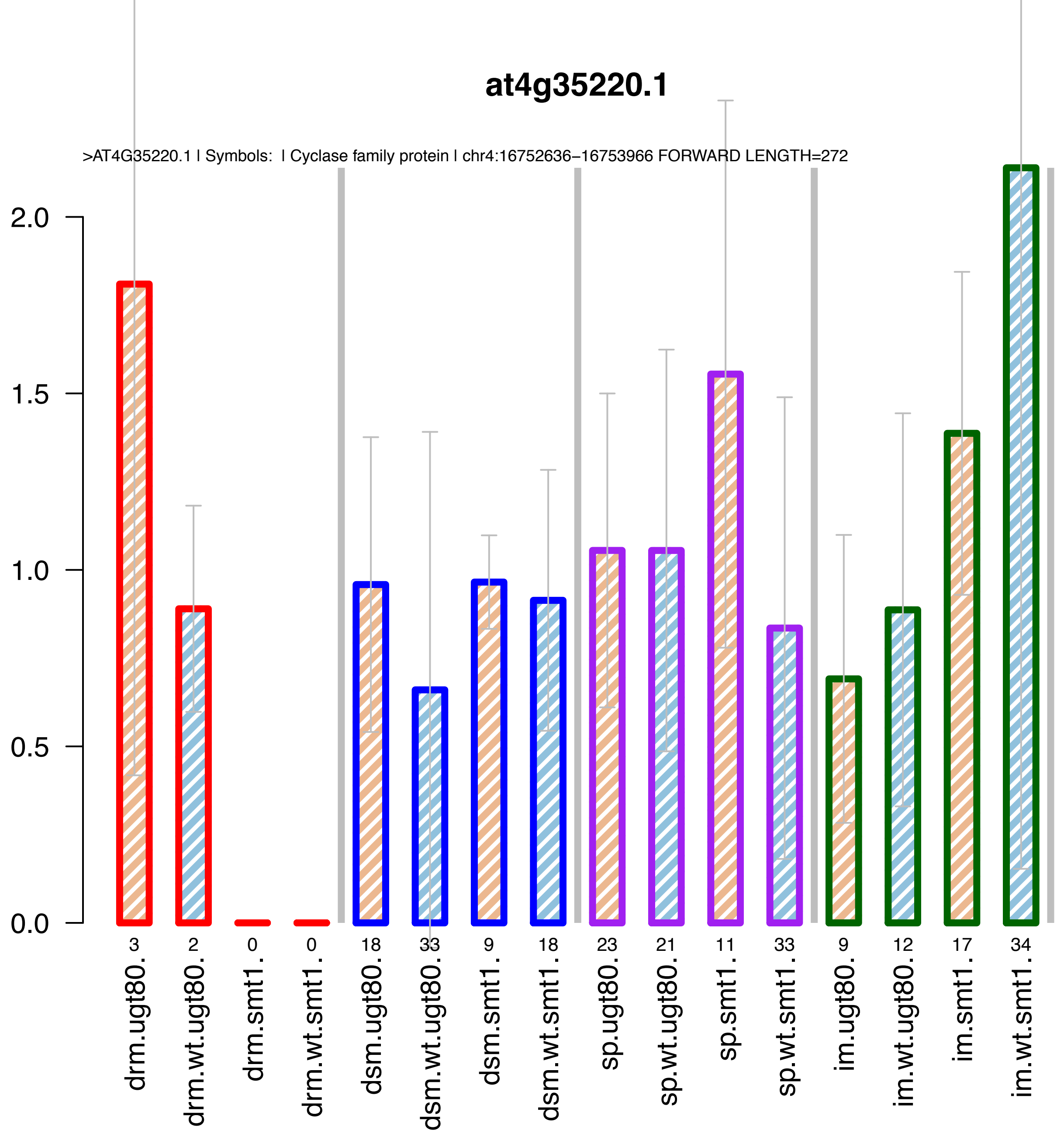

at4g35220.1
>AT4G35220.1 | Symbols: | Cyclase family protein | chr4:16752636-16753966 FORWARD LENGTH=272
2.0
1.5
1.0
0.5
0.0
3
2
0
0
18
33
9
18
23
21
11
33
9
12
17
34
drm.ugt80.
drm.wt.ugt80.
drm.smt1.
drm.wt.smt1.
dsm.ugt80.
dsm.wt.ugt80.
dsm.smt1.
dsm.wt.smt1.
sp.ugt80.
sp.wt.ugt80.
sp.smt1.
sp.wt.smt1.
im.ugt80.
im.wt.ugt80.
im.smt1.
im.wt.smt1.

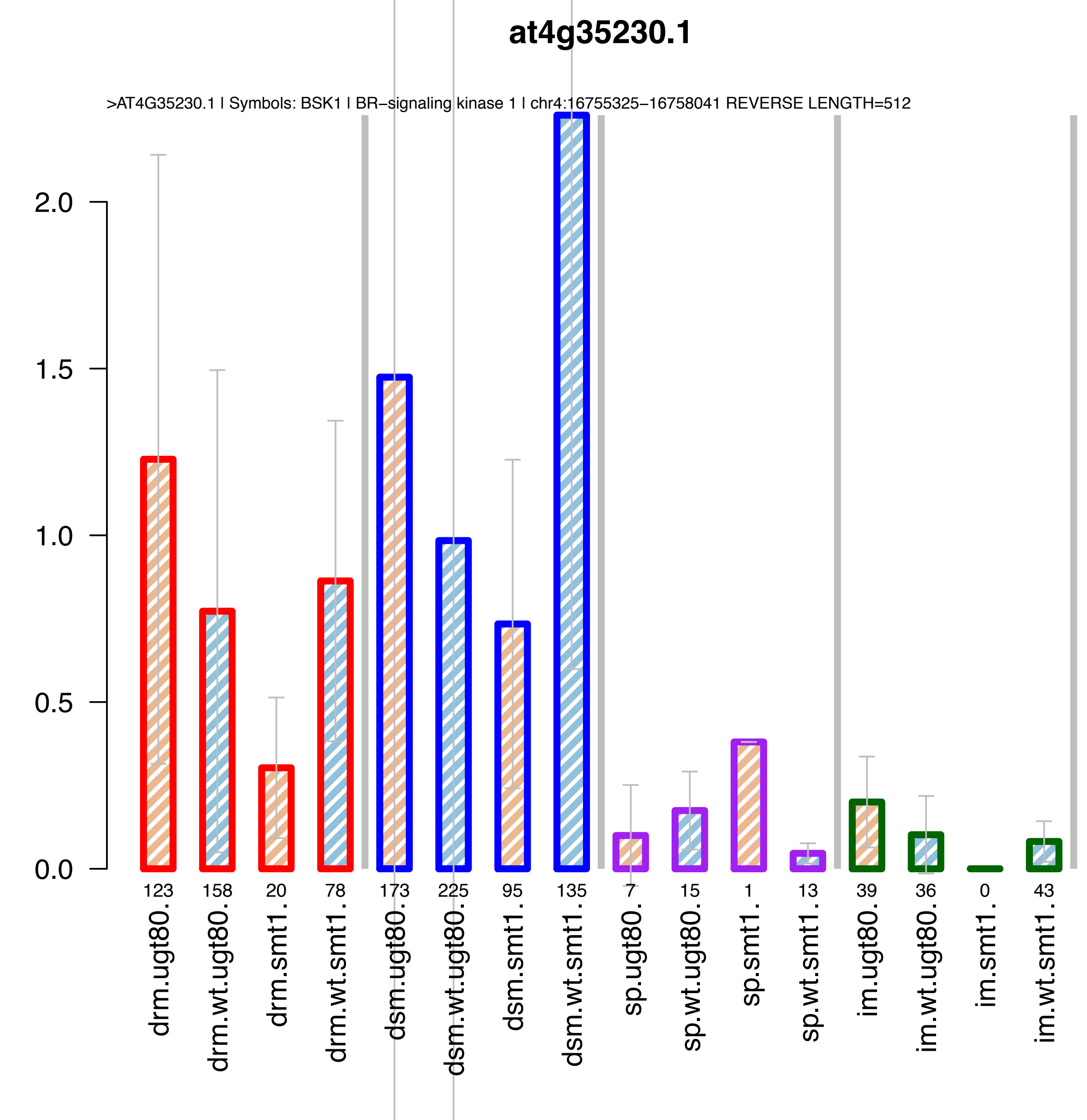

at4g35230.1
>AT4G35230.1 | Symbols: BSK1 | BR−signaling kinase 1 | chr4:16755325−16758041 REVERSE LENGTH=512
2.0
1.5
1.0
0.5
0.0
123
158
20
78
173
225
95
135
7
15
1
13
39
36
0
43
drm.ugt80..
drm.wt.ugt80..
drm.smt1..
drm.wt.smt1..
dsm.ugt80..
dsm.wt.ugt80..
dsm.smt1..
dsm.wt.smt1..
sp.ugt80..
sp.wt.ugt80..
sp.smt1..
sp.wt.smt1..
im.ugt80..
im.wt.ugt80..
im.smt1..
im.wt.smt1..

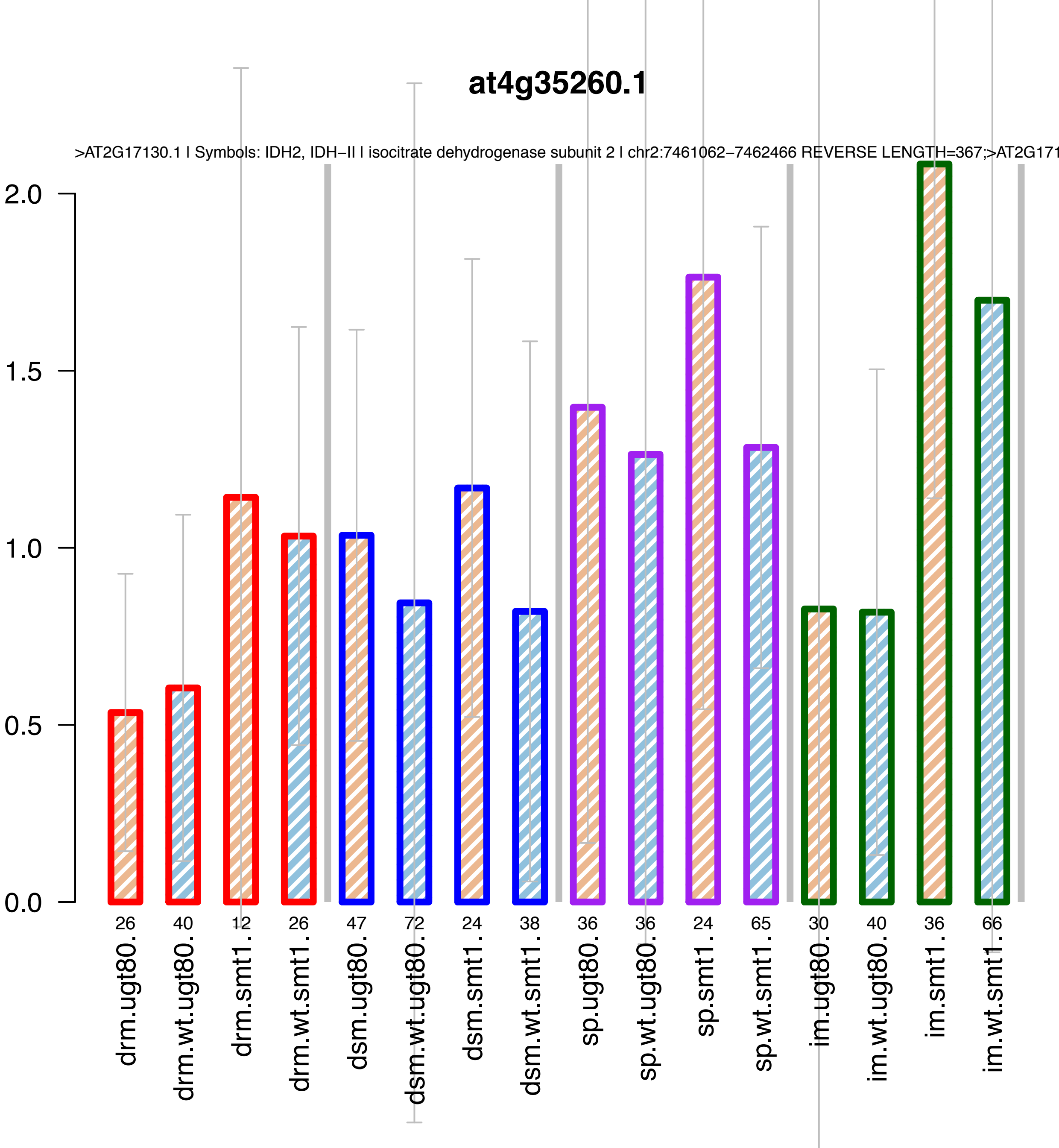
at4g35260.1
>AT2G17130.1 | Symbols: IDH2, IDH−II | isocitrate dehydrogenase subunit 2 | chr2:7461062−7462466 REVERSE LENGTH=367;>AT2G171
2.0
1.5
1.0
0.5
0.0
26
40
12
26
47
72
24
38
36
36
24
65
30
40
36
66
drm.ugt80.
drm.wt.ugt80.
drm.smt1.
drm.wt.smt1.
dsm.ugt80.
dsm.wt.ugt80.
dsm.smt1.
dsm.wt.smt1.
sp.ugt80.
sp.wt.ugt80.
sp.smt1.
sp.wt.smt1.
im.ugt80.
im.wt.ugt80.
im.smt1.
im.wt.smt1.

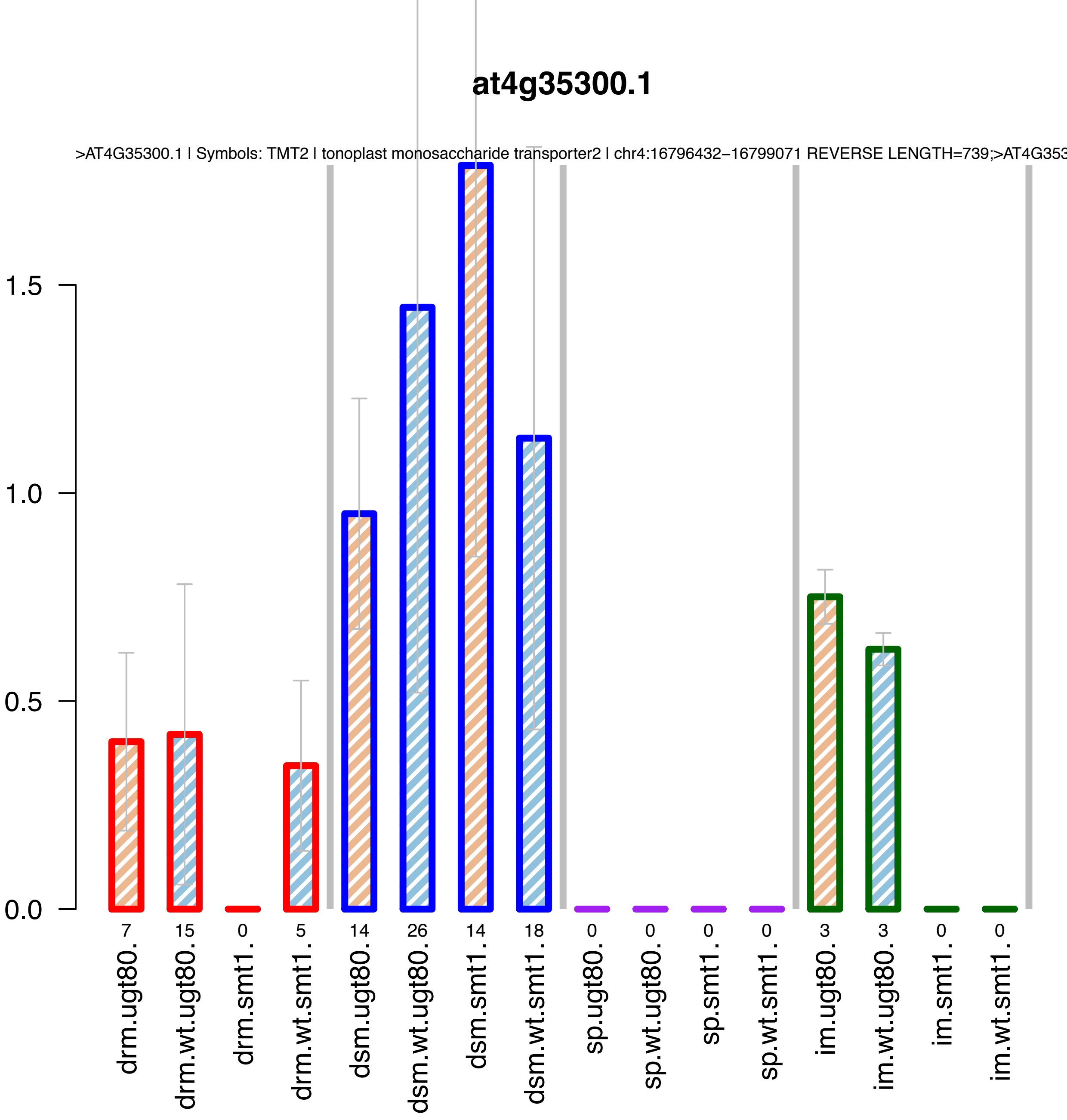

at4g35300.1
>AT4G35300.1 | Symbols: TMT2 | tonoplast monosaccharide transporter2 | chr4:16796432-16799071 REVERSE LENGTH=739;>AT4G353
1.5
1.0
0.5
0.0
7
15
0
5
14
26
14
18
0
0
0
0
3
3
0
0
drm.ugt80.
drm.wt.ugt80.
drm.smt1.
drm.wt.smt1.
dsm.ugt80.
dsm.wt.ugt80.
dsm.smt1.
dsm.wt.smt1.
sp.ugt80.
sp.wt.ugt80.
sp.smt1.
sp.wt.smt1.
im.ugt80.
im.wt.ugt80.
im.smt1.
im.wt.smt1.

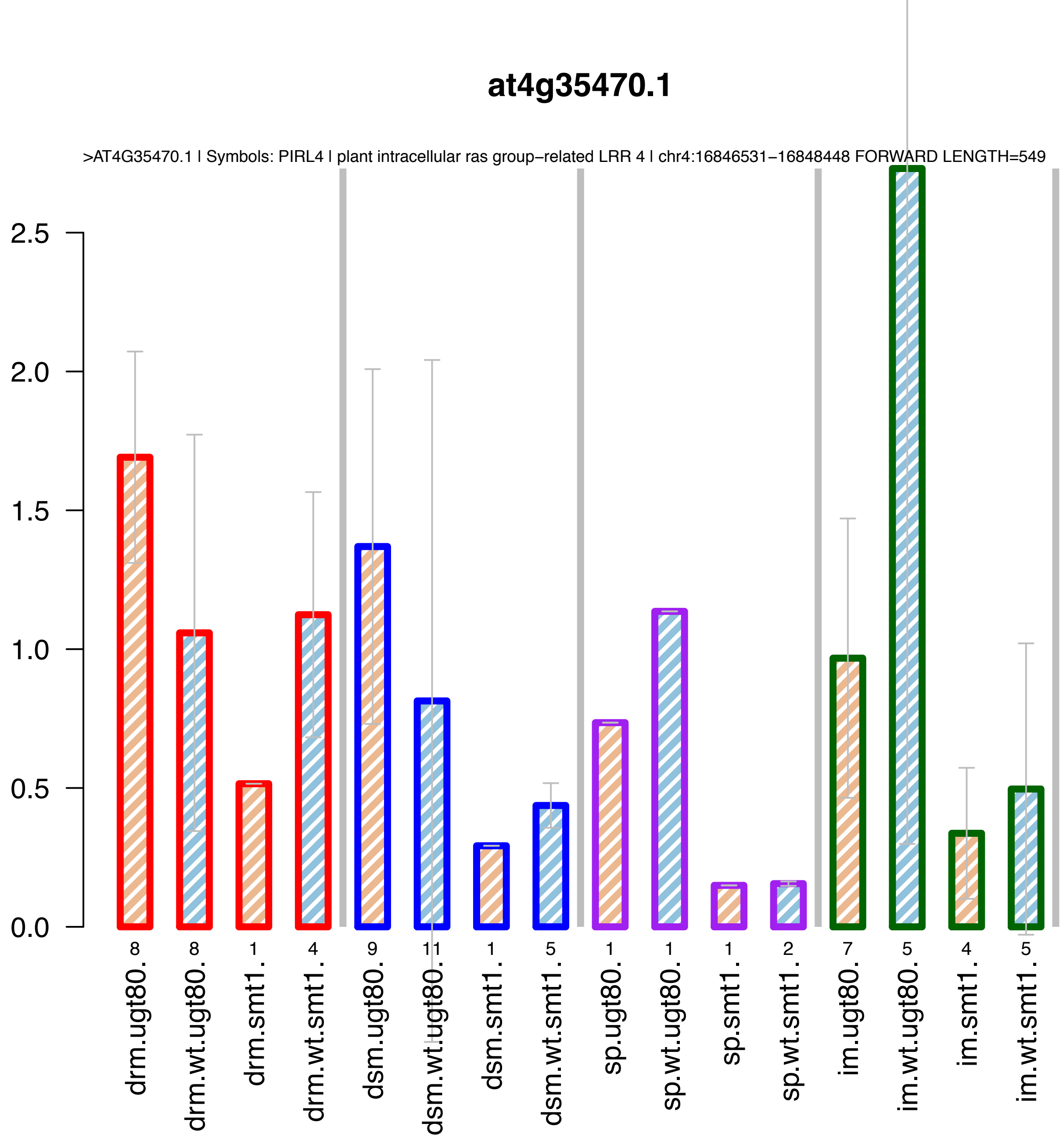

at4g35470.1
>AT4G35470.1 | Symbols: PIRL4 | plant intracellular ras group−related LRR 4 | chr4:16846531−16848448 FORWARD LENGTH=549
0.0
0.5
1.0
1.5
2.0
2.5
8
8
1
4
9
11
1
5
1
1
1
2
7
5
4
5
drm.ugt80..
drm.wt.ugt80..
drm.smt1..
drm.wt.smt1..
dsm.ugt80..
dsm.wt.ugt80..
dsm.smt1..
dsm.wt.smt1..
sp.ugt80..
sp.wt.ugt80..
sp.smt1..
sp.wt.smt1..
im.ugt80..
im.wt.ugt80..
im.smt1..
im.wt.smt1..

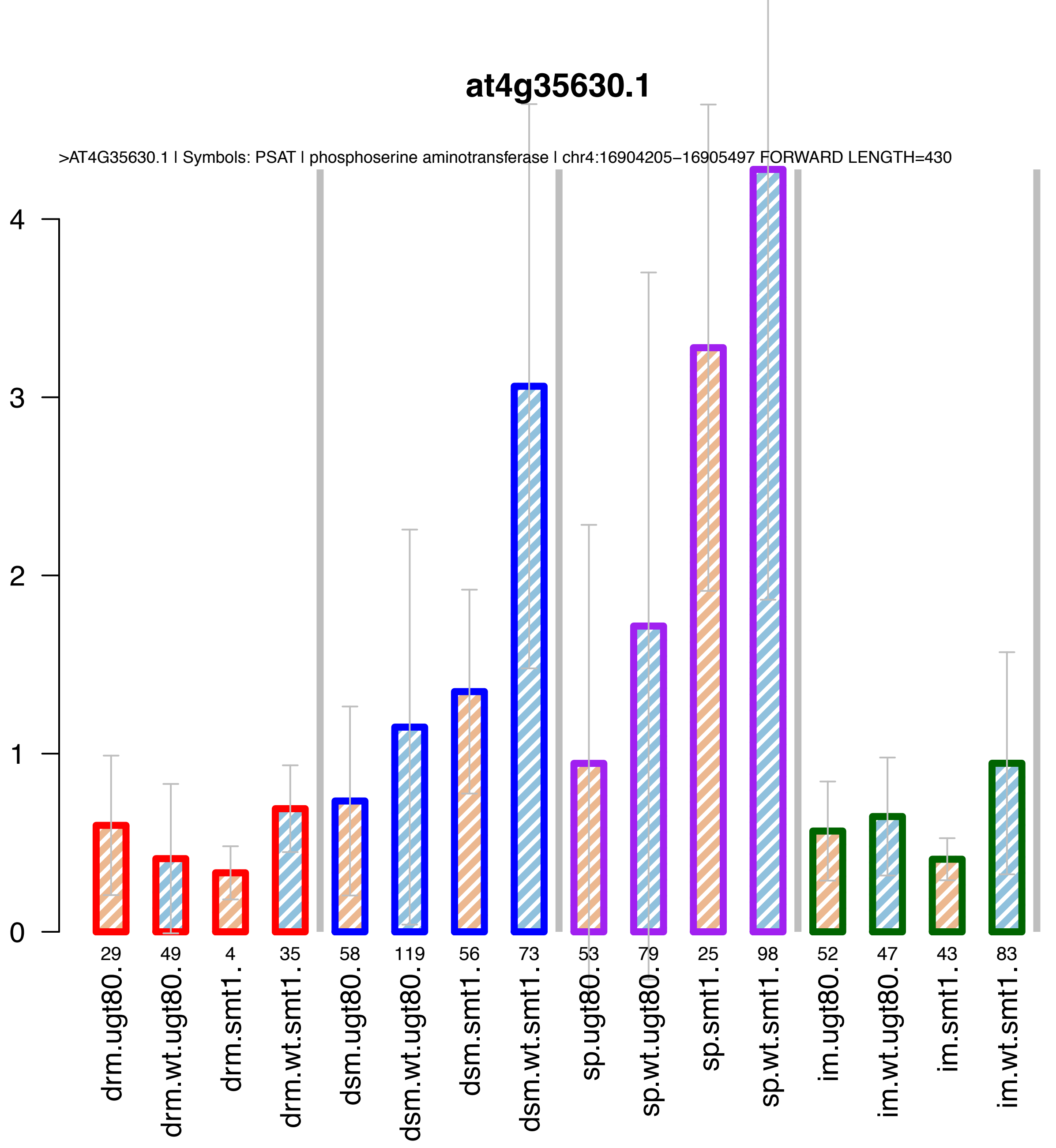

at4g35630.1
>AT4G35630.1 | Symbols: PSAT | phosphoserine aminotransferase | chr4:16904205-16905497 FORWARD LENGTH=430
0
1
2
3
4
29
49
4
35
58
119
56
73
53
79
25
98
52
47
43
83
drm.ugt80.
drm.wt.ugt80.
drm.smt1.
drm.wt.smt1.
dsm.ugt80.
dsm.wt.ugt80.
dsm.smt1.
dsm.wt.smt1.
sp.ugt80.
sp.wt.ugt80.
sp.smt1.
sp.wt.smt1.
im.ugt80.
im.wt.ugt80.
im.smt1.
im.wt.smt1.

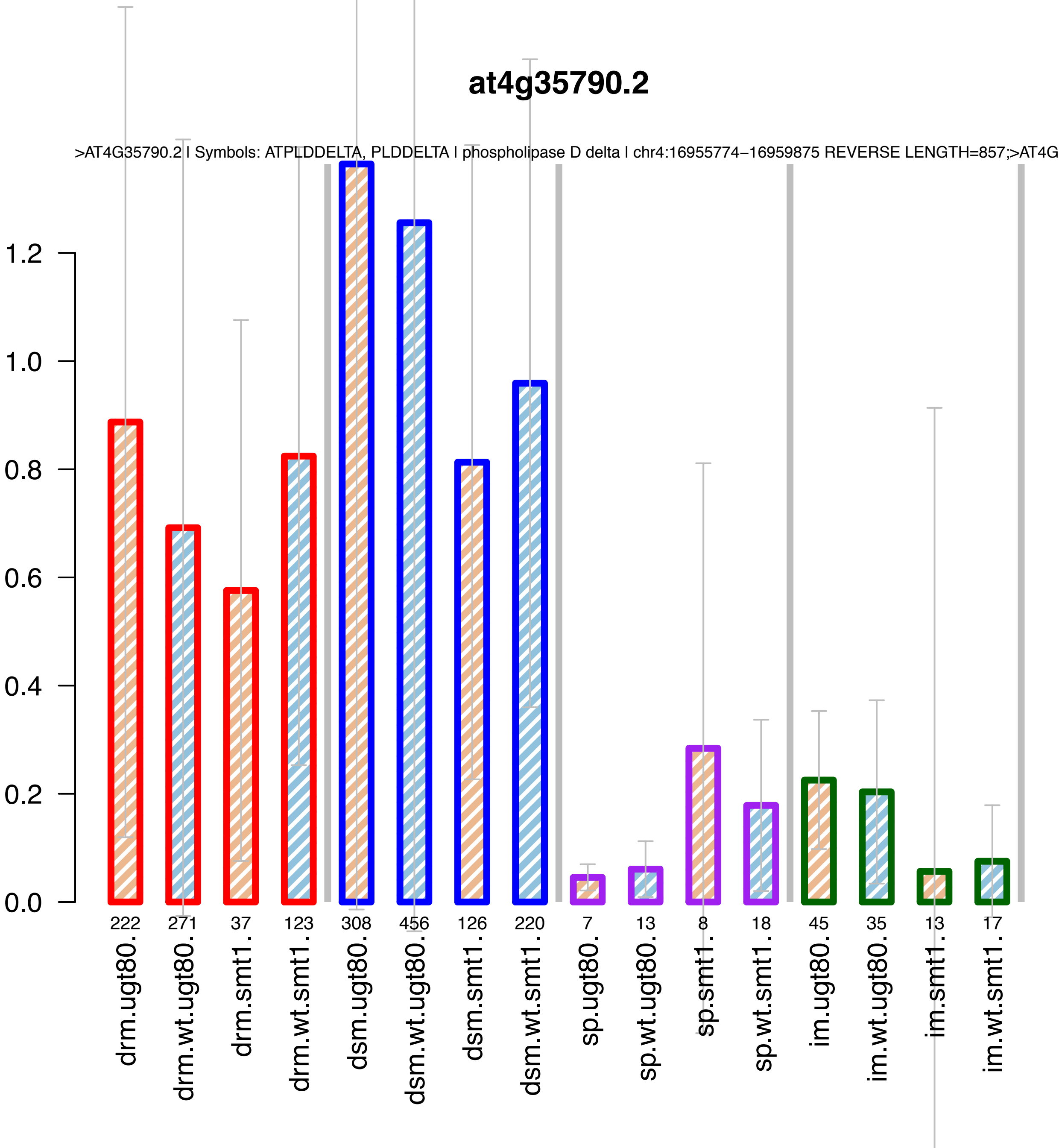

at4g35790.2
>AT4G35790.2 | Symbols: ATPLDDELTA, PLDDELTA | phospholipase D delta | chr4:16955774-16959875 REVERSE LENGTH=857;>AT4G
0.0
0.2
0.4
0.6
0.8
1.0
1.2
222
271
37
123
308
456
126
220
7
13
8
18
45
35
13
17
drm.ugt80..
drm.wt.ugt80..
drm.smt1..
drm.wt.smt1..
dsm.ugt80..
dsm.wt.ugt80..
dsm.smt1..
dsm.wt.smt1..
sp.ugt80..
sp.wt.ugt80..
sp.smt1..
sp.wt.smt1..
im.ugt80..
im.wt.ugt80..
im.smt1..
im.wt.smt1..

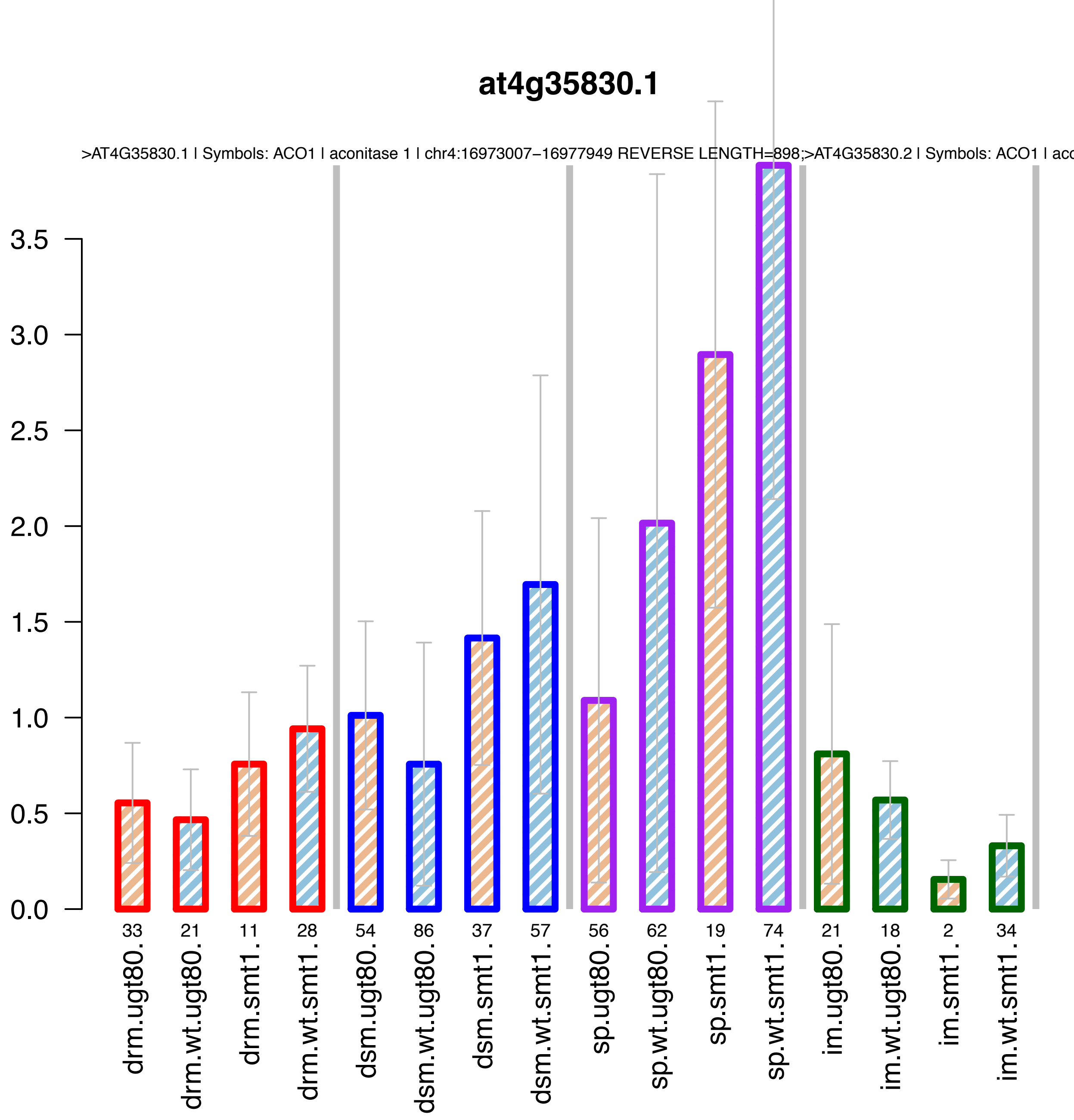

at4g35830.1
>AT4G35830.1 | Symbols: ACO1 | aconitase 1 | chr4:16973007-16977949 REVERSE LENGTH=898;>AT4G35830.2 | Symbols: ACO1 | aco
0.0
0.5
1.0
1.5
2.0
2.5
3.0
3.5
33
21
11
28
54
86
37
57
56
62
19
74
21
18
2
34
drm.ugt80.
drm.wt.ugt80.
drm.smt1.
drm.wt.smt1.
dsm.ugt80.
dsm.wt.ugt80.
dsm.smt1.
dsm.wt.smt1.
sp.ugt80.
sp.wt.ugt80.
sp.smt1.
sp.wt.smt1.
im.ugt80.
im.wt.ugt80.
im.smt1.
im.wt.smt1.

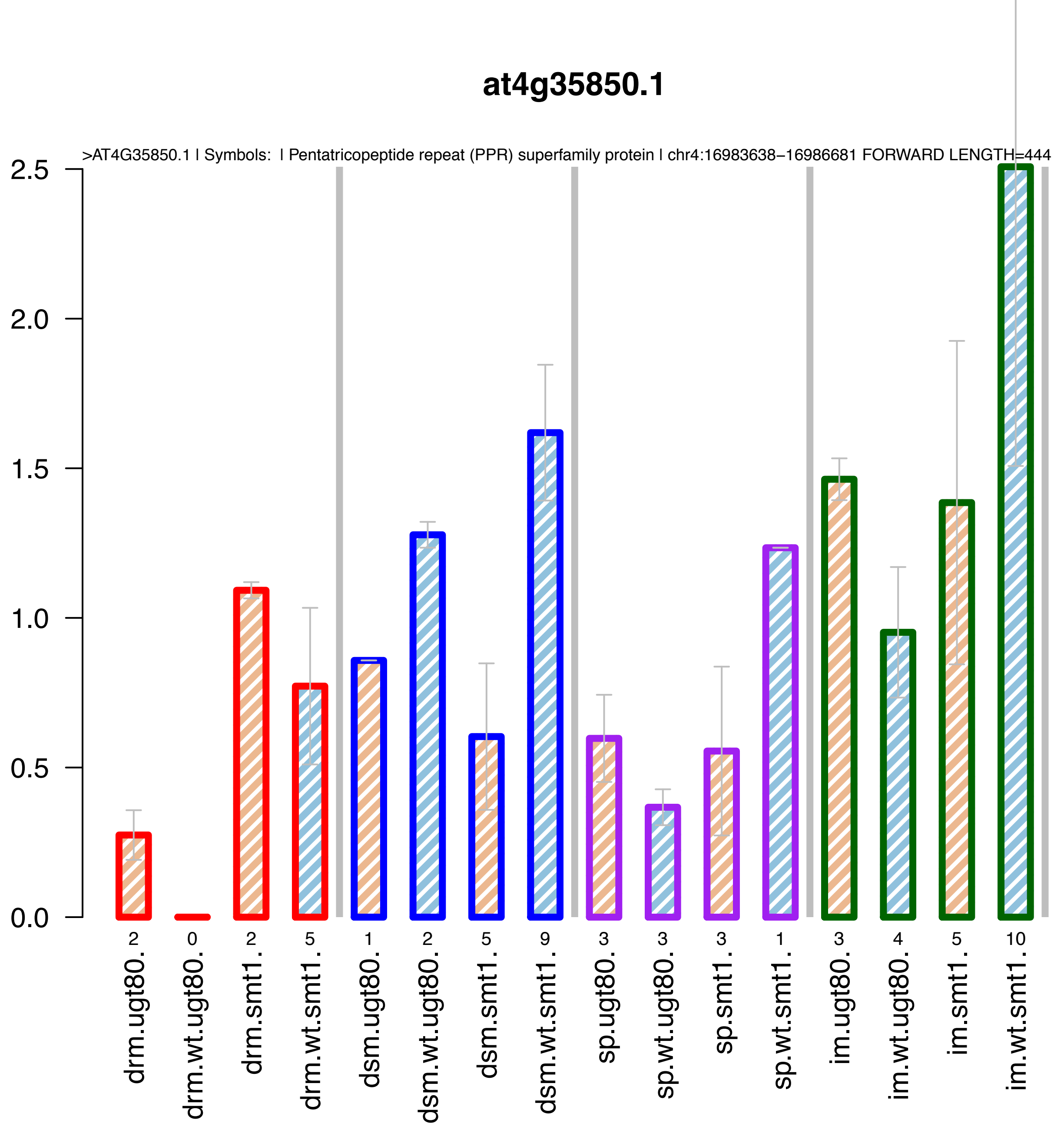

at4g35850.1
>AT4G35850.1 | Symbols: | Pentatricopeptide repeat (PPR) superfamily protein | chr4:16983638-16986681 FORWARD LENGTH=444
2.5
2.0
1.5
1.0
0.5
0.0
2
0
2
5
1
2
5
9
3
3
3
1
3
4
5
10
drm.ugt80..
drm.wt.ugt80..
drm.smt1..
drm.wt.smt1..
dsm.ugt80..
dsm.wt.ugt80..
dsm.smt1..
dsm.wt.smt1..
sp.ugt80..
sp.wt.ugt80..
sp.smt1..
sp.wt.smt1..
im.ugt80..
im.wt.ugt80..
im.smt1..
im.wt.smt1..

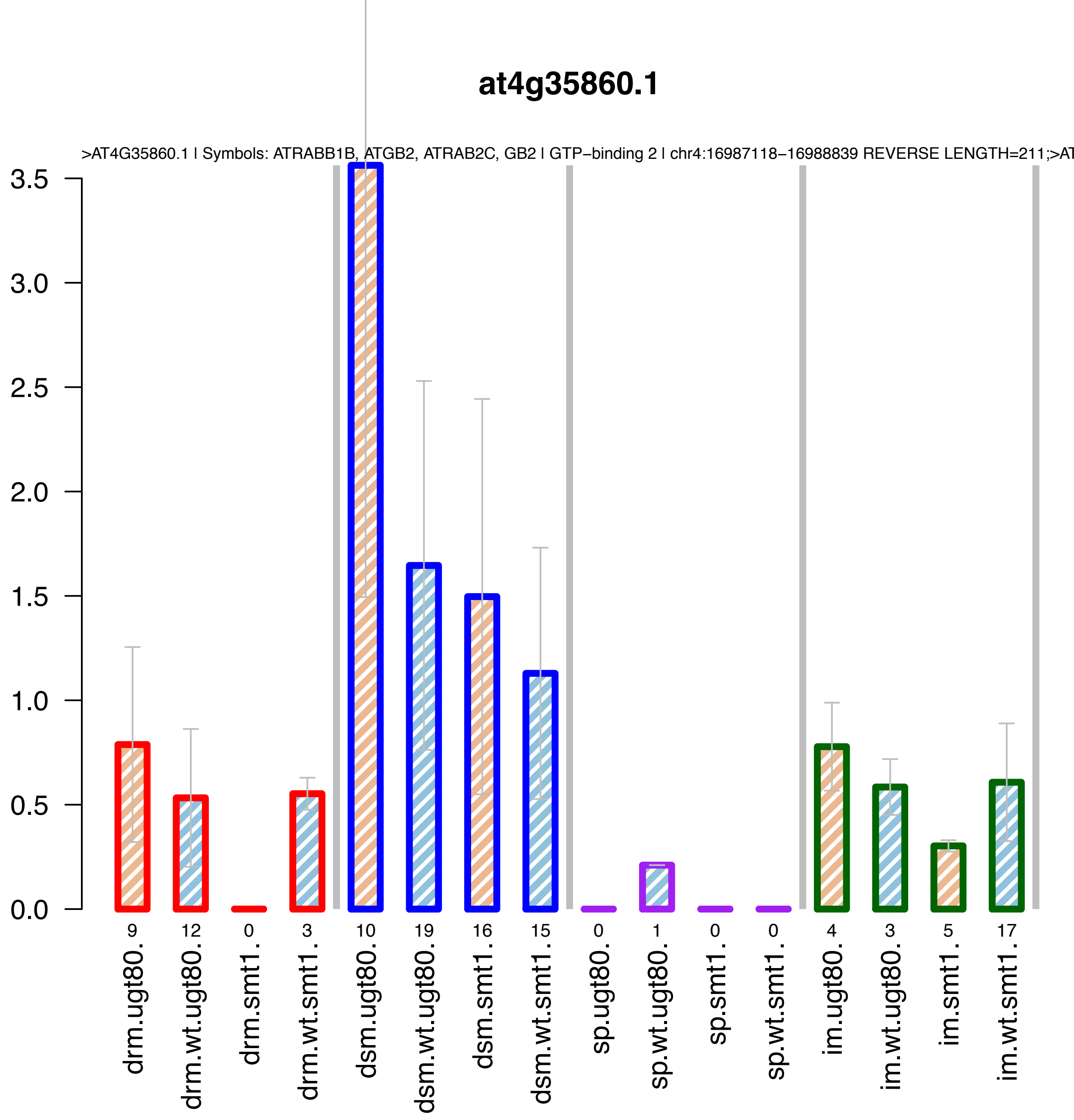

at4g35860.1
>AT4G35860.1 | Symbols: ATRABB1B, ATGB2, ATRAB2C, GB2 | GTP−binding 2 | chr4:16987118−16988839 REVERSE LENGTH=211;>AT
0.0
0.5
1.0
1.5
2.0
2.5
3.0
3.5
9
12
0
3
10
19
16
15
0
1
0
0
4
3
5
17
drm.ugt80.
drm.wt.ugt80.
drm.smt1.
drm.wt.smt1.
dsm.ugt80.
dsm.wt.ugt80.
dsm.smt1.
dsm.wt.smt1.
sp.ugt80.
sp.wt.ugt80.
sp.smt1.
sp.wt.smt1.
im.ugt80.
im.wt.ugt80.
im.smt1.
im.wt.smt1.

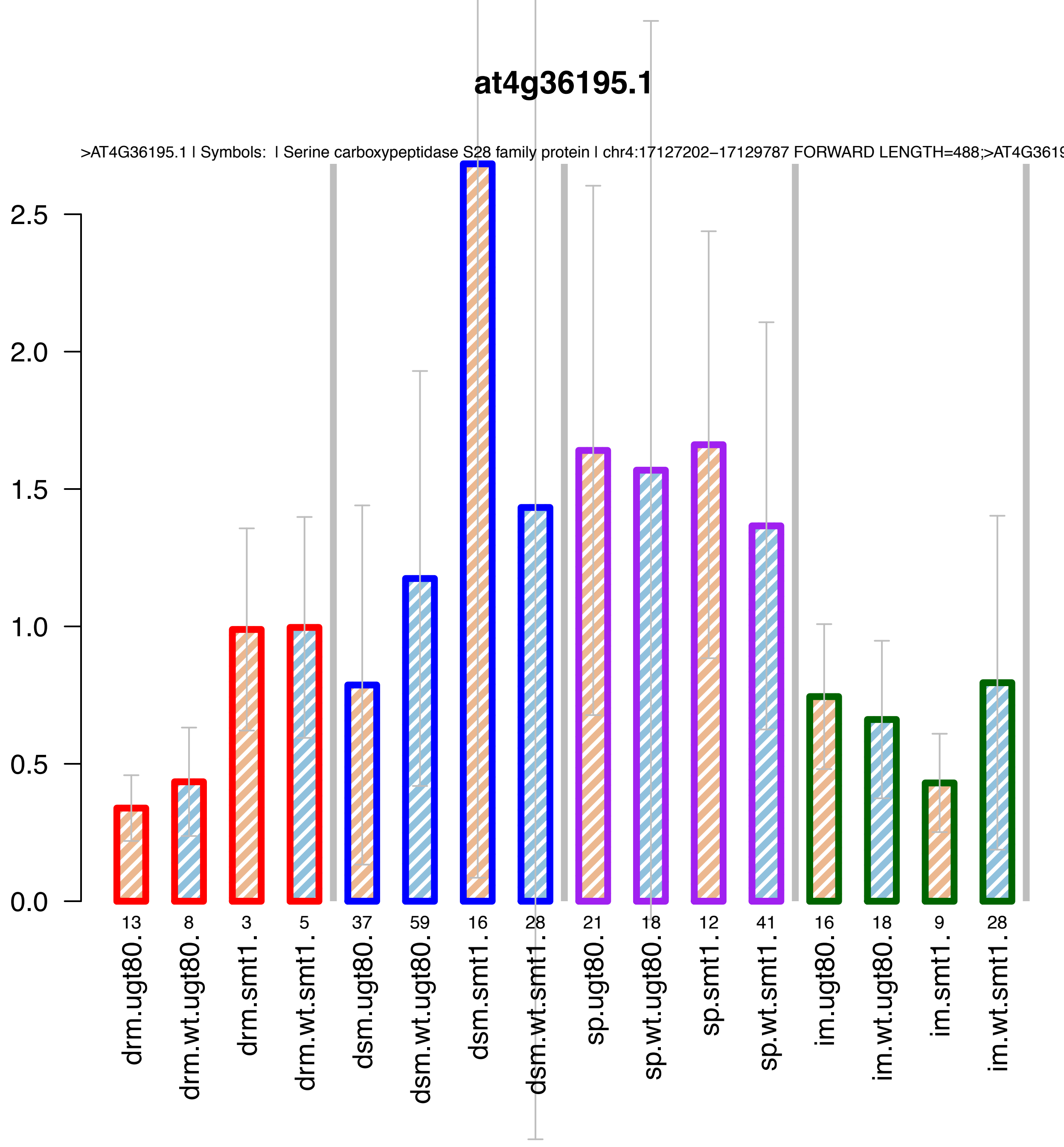

at4g36195.1
>AT4G36195.1 | Symbols: | Serine carboxypeptidase S28 family protein | chr4:17127202-17129787 FORWARD LENGTH=488;>AT4G3619
0.0
0.5
1.0
1.5
2.0
2.5
13
8
3
5
37
59
16
28
21
18
12
41
16
18
9
28
drm.ugt80..
drm.wt.ugt80..
drm.smt1..
drm.wt.smt1..
dsm.ugt80..
dsm.wt.ugt80..
dsm.smt1..
dsm.wt.smt1..
sp.ugt80..
sp.wt.ugt80..
sp.smt1..
sp.wt.smt1..
im.ugt80..
im.wt.ugt80..
im.smt1..
im.wt.smt1..

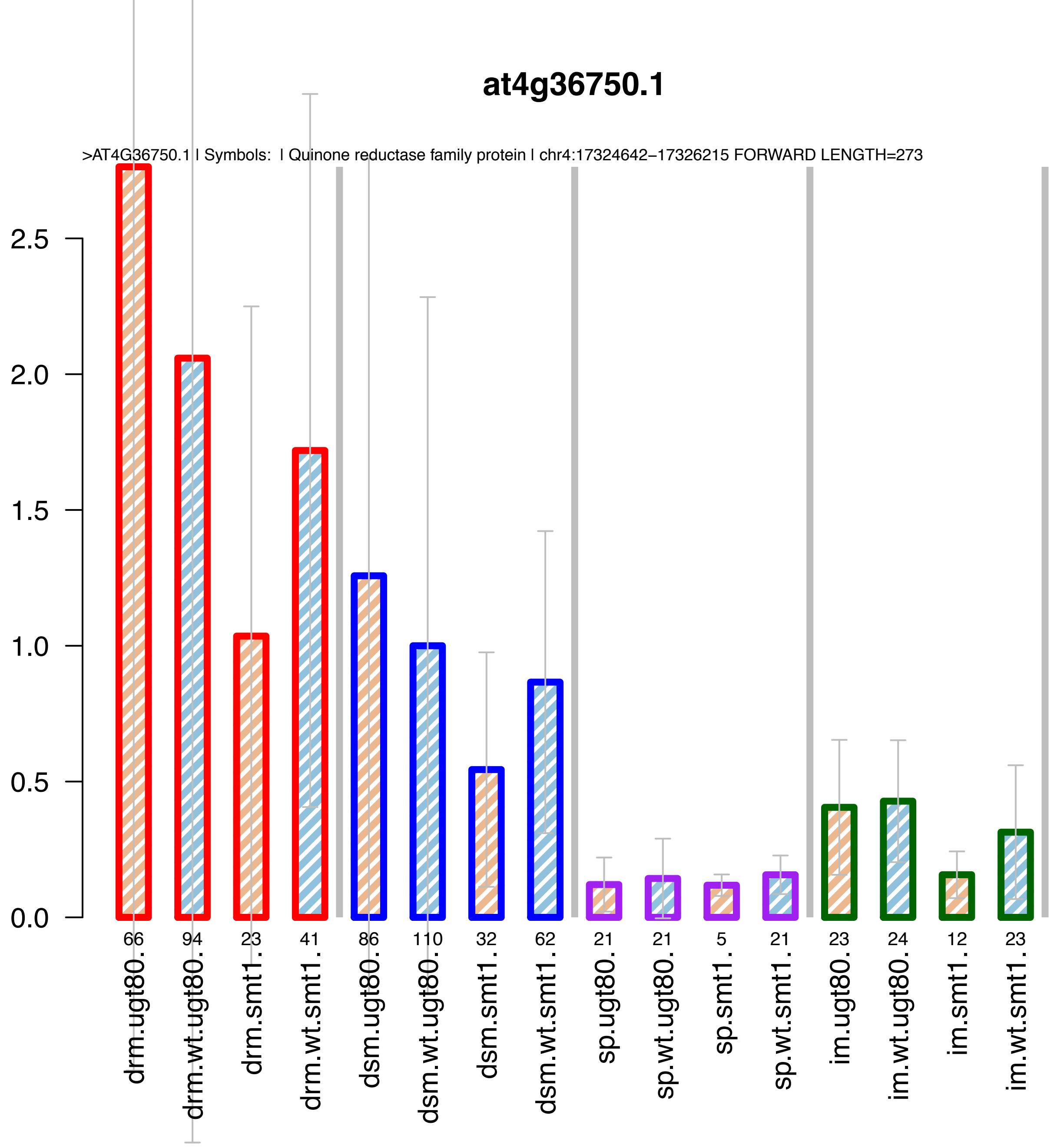
at4g36750.1
>AT4G36750.1 | Symbols: | Quinone reductase family protein | chr4:17324642-17326215 FORWARD LENGTH=273
0.0
0.5
1.0
1.5
2.0
2.5
66
94
23
41
86
110
32
62
21
21
5
21
23
24
12
23
drm.ugt80..
drm.wt.ugt80..
drm.smt1..
drm.wt.smt1..
dsm.ugt80..
dsm.wt.ugt80..
dsm.smt1..
dsm.wt.smt1..
sp.ugt80..
sp.wt.ugt80..
sp.smt1..
sp.wt.smt1..
im.ugt80..
im.wt.ugt80..
im.smt1..
im.wt.smt1..

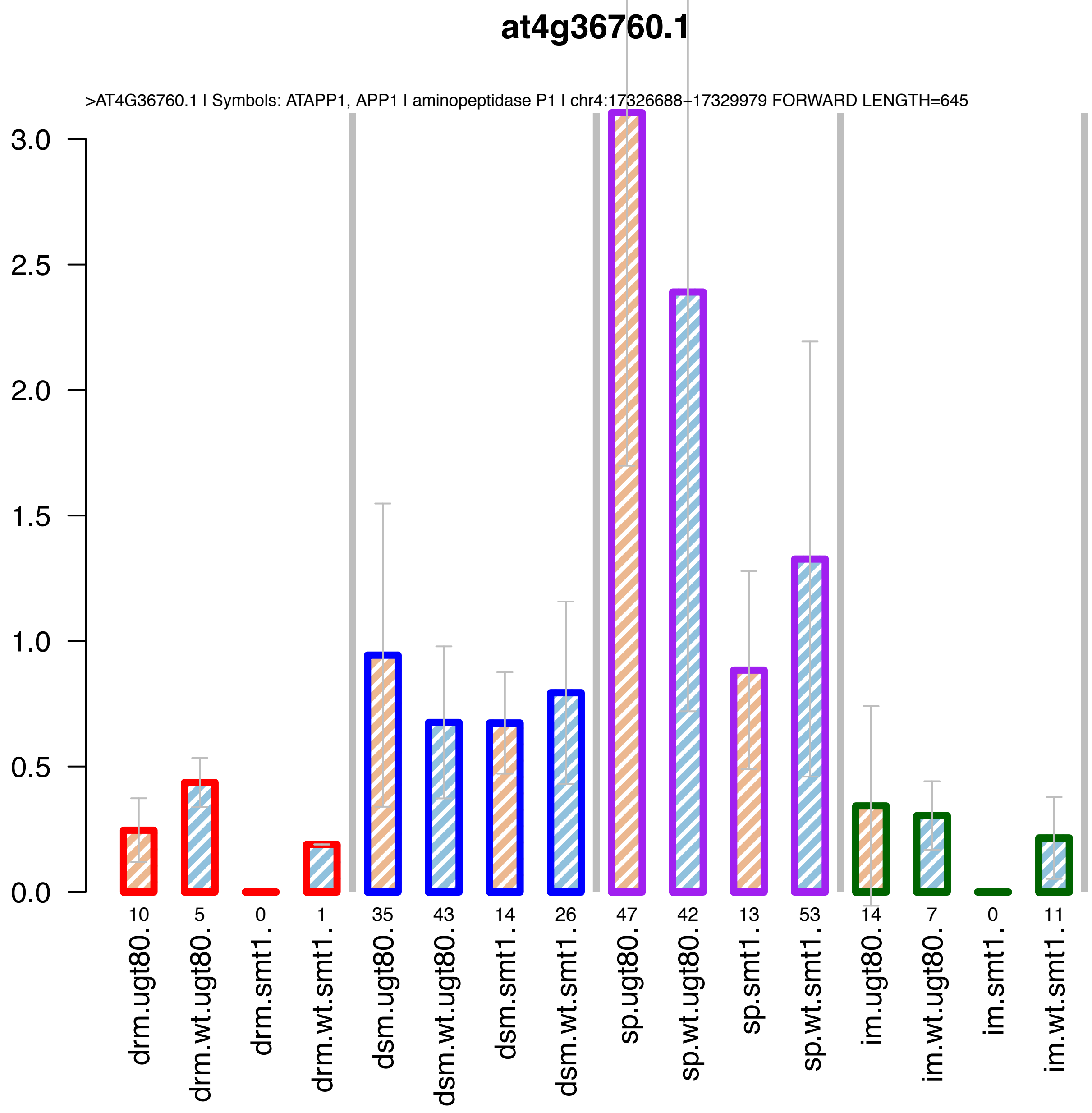

at4g36760.1
>AT4G36760.1 | Symbols: ATAPP1, APP1 | aminopeptidase P1 | chr4:17326688-17329979 FORWARD LENGTH=645
3.0
2.5
2.0
1.5
1.0
0.5
0.0
10
5
0
1
35
43
14
26
47
42
13
53
14
7
0
11
drm.ugt80..
drm.wt.ugt80..
drm.smt1..
drm.wt.smt1..
dsm.ugt80..
dsm.wt.ugt80..
dsm.smt1..
dsm.wt.smt1..
sp.ugt80..
sp.wt.ugt80..
sp.smt1..
sp.wt.smt1..
im.ugt80..
im.wt.ugt80..
im.smt1..
im.wt.smt1..

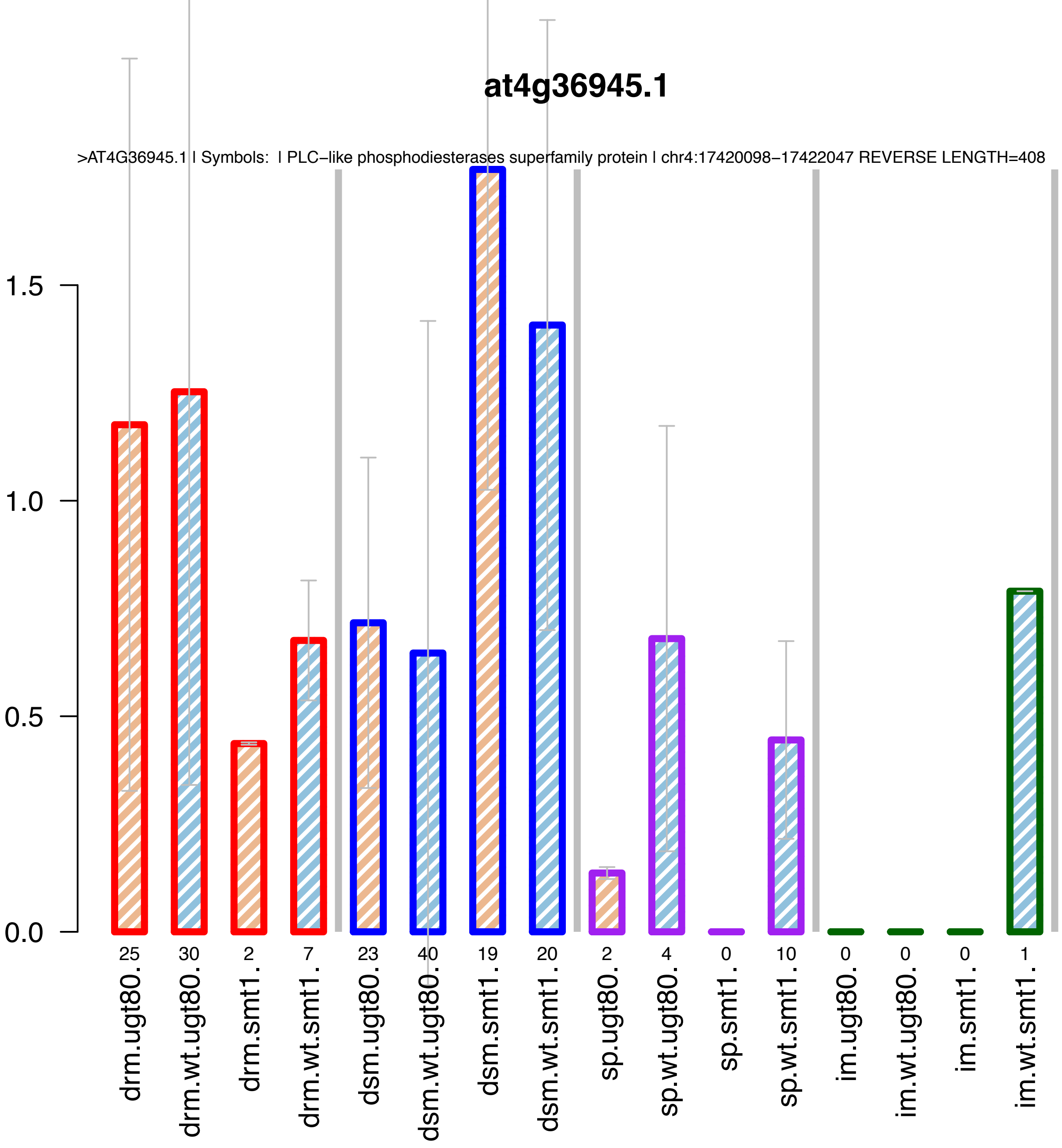

at4g36945.1
>AT4G36945.1 | Symbols: | PLC−like phosphodiesterases superfamily protein | chr4:17420098−17422047 REVERSE LENGTH=408
0.0
0.5
1.0
1.5
25
30
2
7
23
40
19
20
2
4
0
10
0
0
0
1
drm.ugt80..
drm.wt.ugt80..
drm.smt1..
drm.wt.smt1..
dsm.ugt80..
dsm.wt.ugt80..
dsm.smt1..
dsm.wt.smt1..
sp.ugt80..
sp.wt.ugt80..
sp.smt1..
sp.wt.smt1..
im.ugt80..
im.wt.ugt80..
im.smt1..
im.wt.smt1..

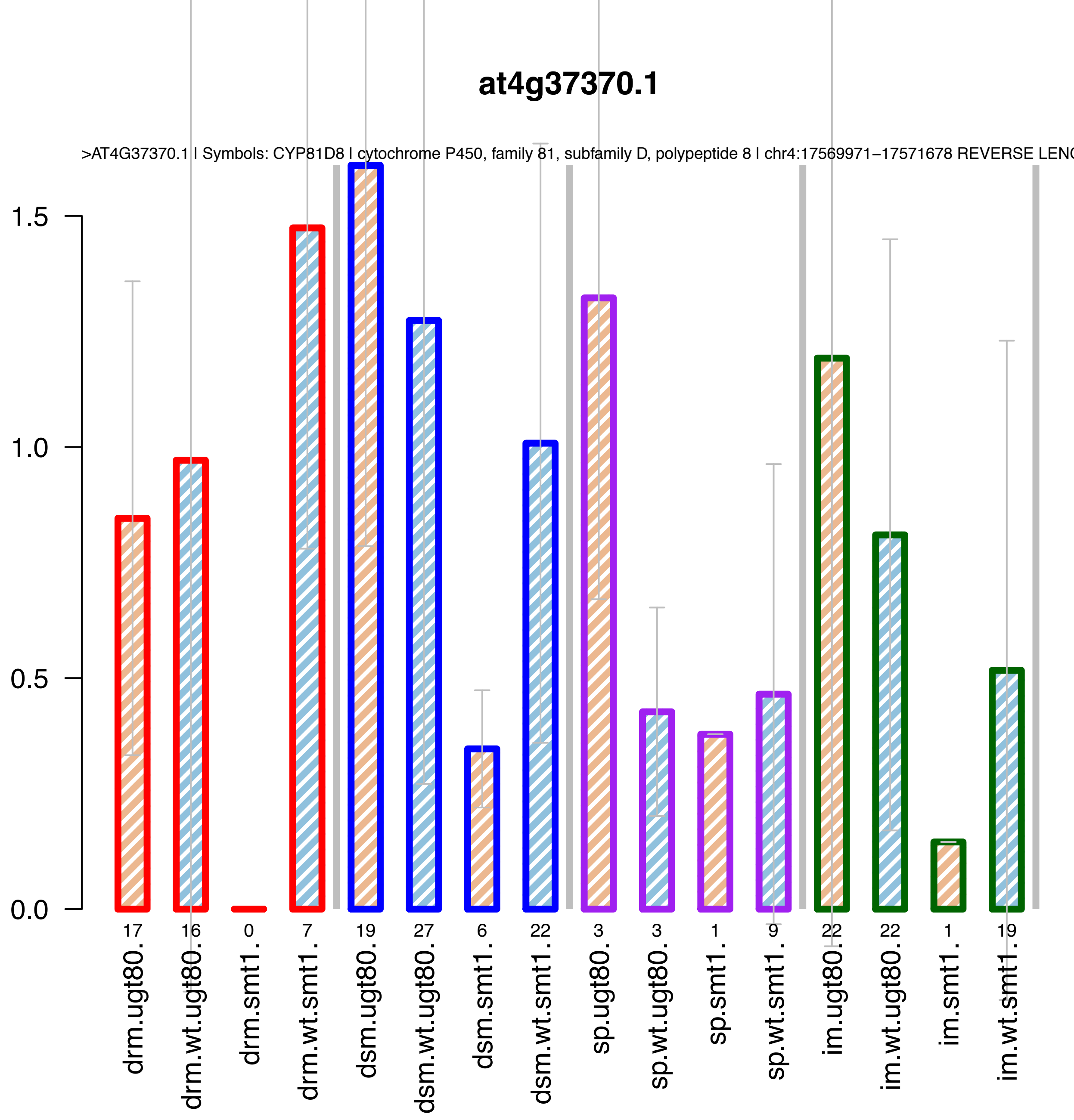

at4g37370.1
>AT4G37370.1 | Symbols: CYP81D8 | cytochrome P450, family 81, subfamily D, polypeptide 8 | chr4:17569971−17571678 REVERSE LENG
1.5
1.0
0.5
0.0
17
16
0
7
19
27
6
22
3
3
1
9
22
22
1
19
drm.ugt80.
drm.wt.ugt80.
drm.smt1.
drm.wt.smt1.
dsm.ugt80.
dsm.wt.ugt80.
dsm.smt1.
dsm.wt.smt1.
sp.ugt80.
sp.wt.ugt80.
sp.smt1.
sp.wt.smt1.
im.ugt80.
im.wt.ugt80.
im.smt1.
im.wt.smt1.

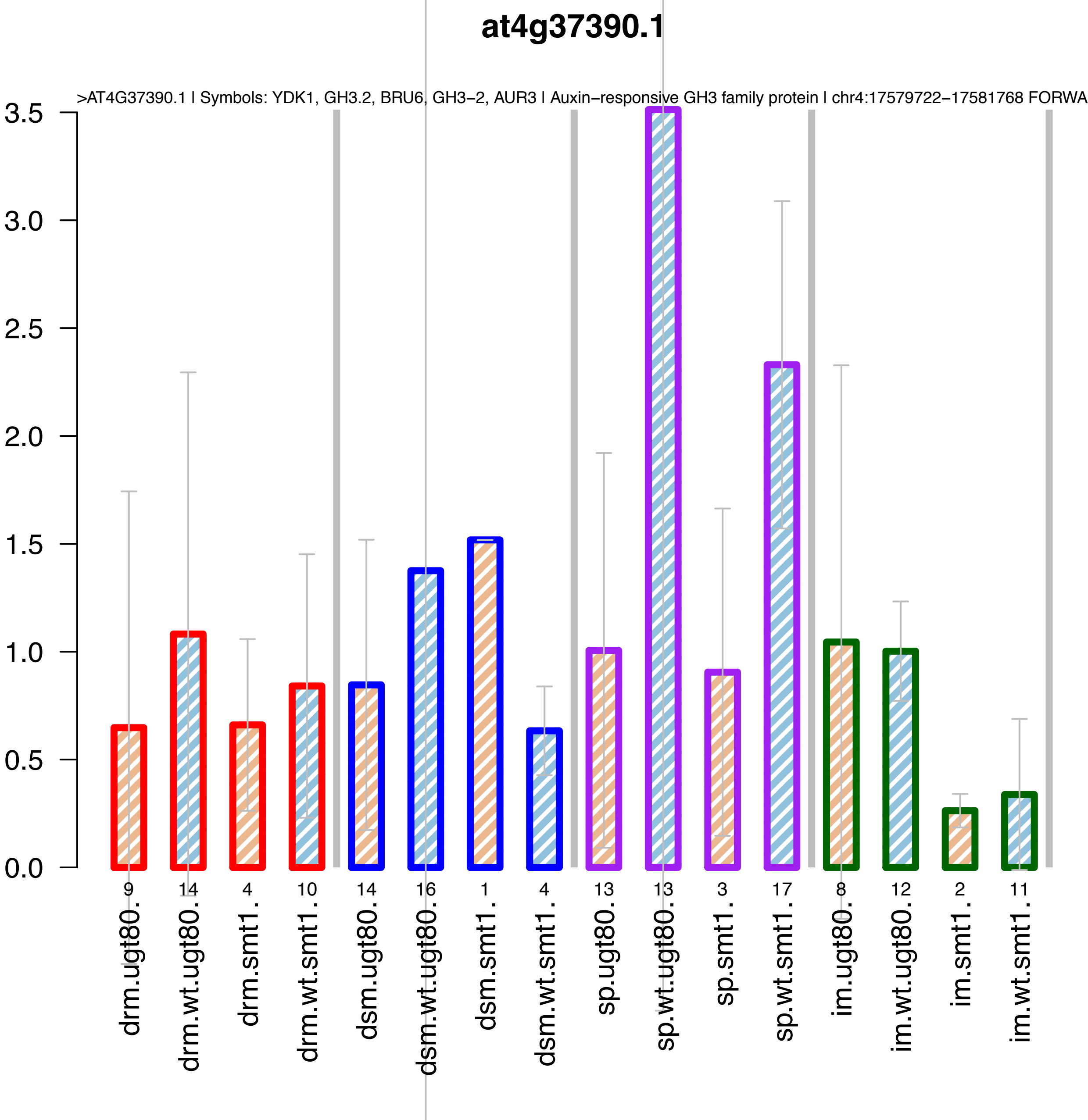
at4g37390.1
>AT4G37390.1 | Symbols: YDK1, GH3.2, BRU6, GH3-2, AUR3 | Auxin-responsive GH3 family protein | chr4:17579722-17581768 FORWA
3.5
3.0
2.5
2.0
1.5
1.0
0.5
0.0
9
14
4
10
14
16
1
4
13
13
3
17
8
12
2
11
drm.ugt80..
drm.wt.ugt80..
drm.smt1..
drm.wt.smt1..
dsm.ugt80..
dsm.wt.ugt80..
dsm.smt1..
dsm.wt.smt1..
sp.ugt80..
sp.wt.ugt80..
sp.smt1..
sp.wt.smt1..
im.ugt80..
im.wt.ugt80..
im.smt1..
im.wt.smt1..

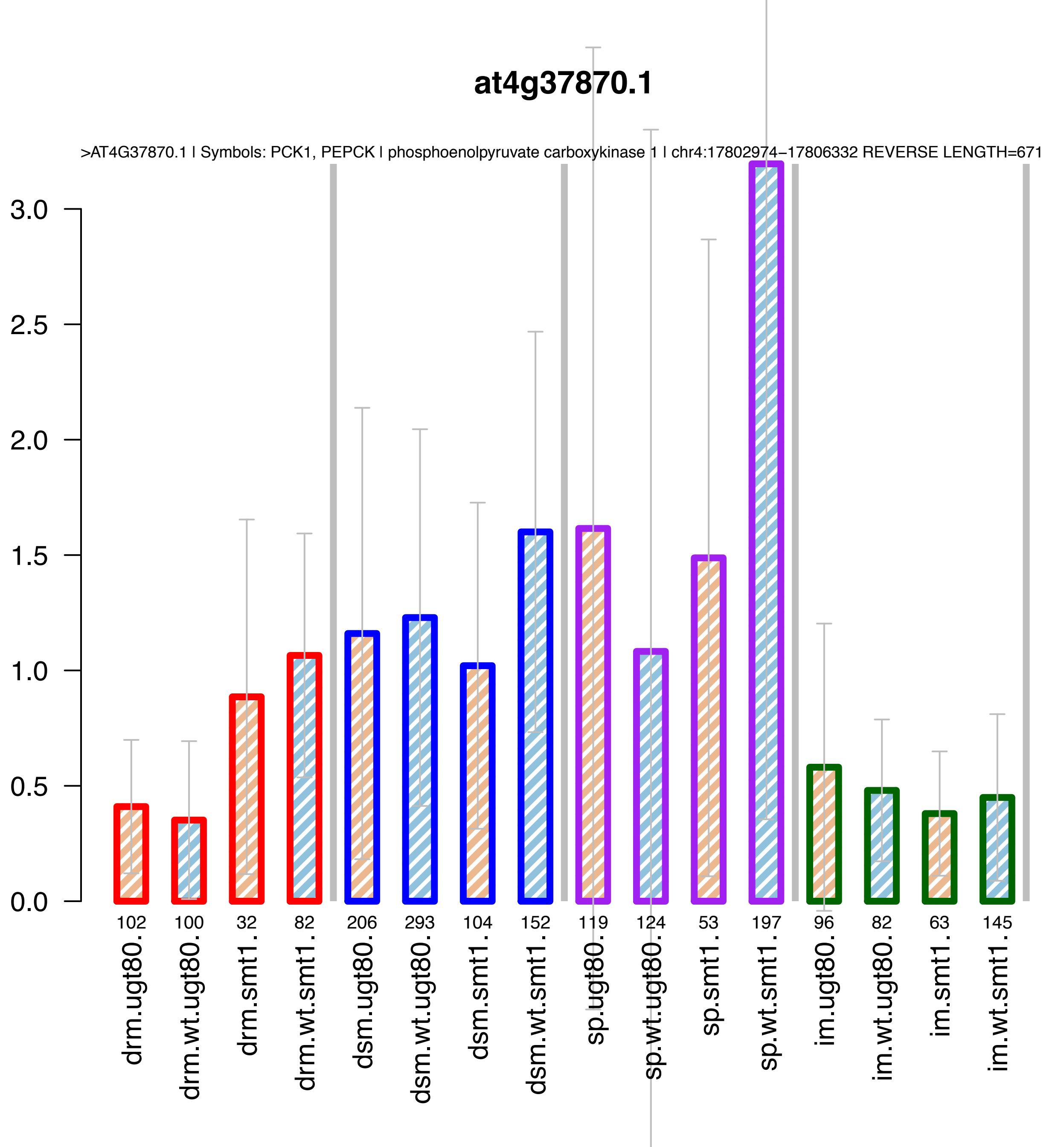

at4g37870.1
>AT4G37870.1 | Symbols: PCK1, PEPCK | phosphoenolpyruvate carboxykinase 1 | chr4:17802974–17806332 REVERSE LENGTH=671
3.0
2.5
2.0
1.5
1.0
0.5
0.0
102
100
32
82
206
293
104
152
119
124
53
197
96
82
63
145
drm.ugt80..
drm.wt.ugt80..
drm.smt1..
drm.wt.smt1..
dsm.ugt80..
dsm.wt.ugt80..
dsm.smt1..
dsm.wt.smt1..
sp.ugt80..
sp.wt.ugt80..
sp.smt1..
sp.wt.smt1..
im.ugt80..
im.wt.ugt80..
im.smt1..
im.wt.smt1..

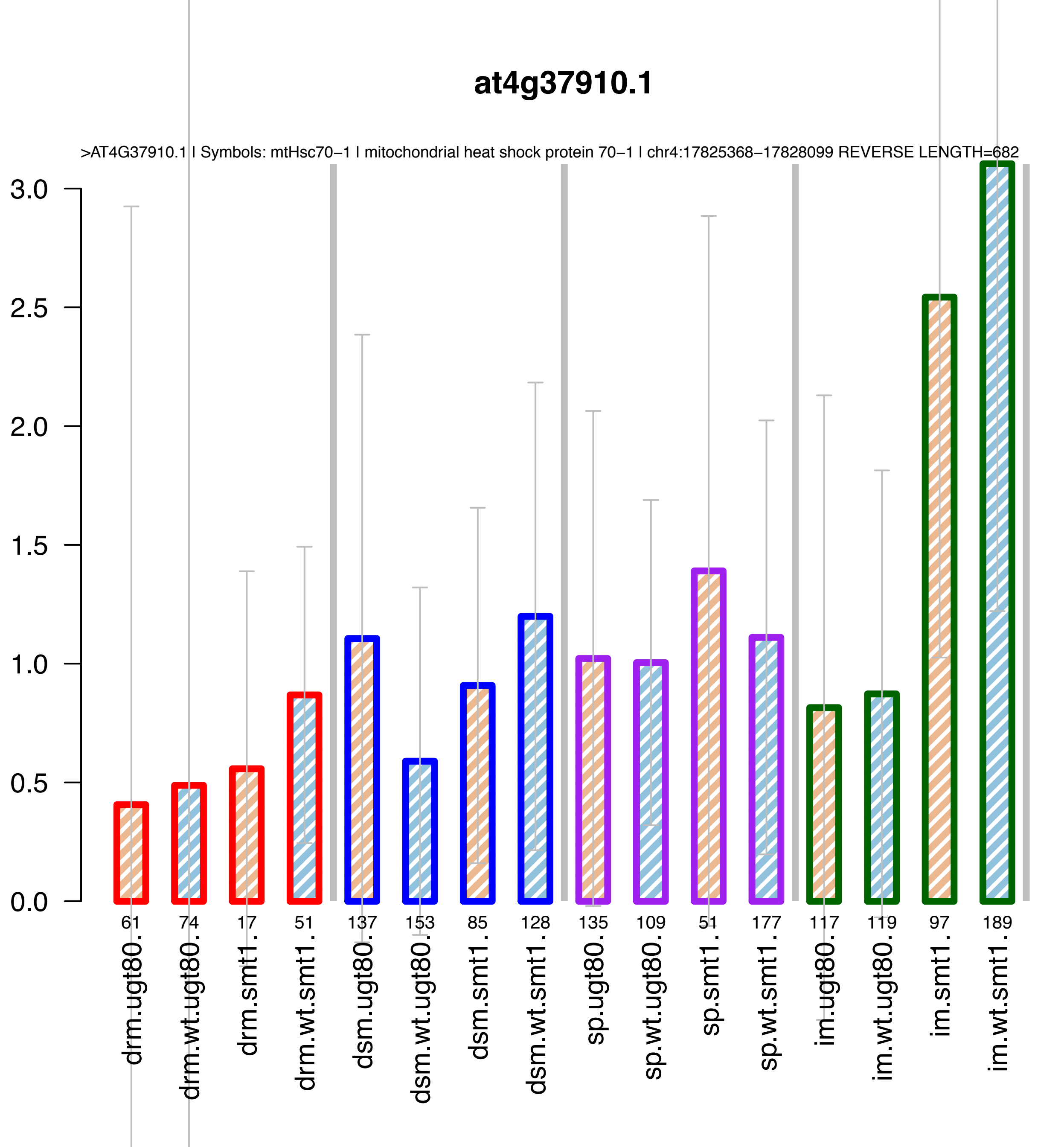

at4g37910.1
>AT4G37910.1 | Symbols: mtHsc70−1 | mitochondrial heat shock protein 70−1 | chr4:17825368−17828099 REVERSE LENGTH=682
3.0
2.5
2.0
1.5
1.0
0.5
0.0
61
74
17
51
137
153
85
128
135
109
51
177
117
119
97
189
drm.ugt80..
drm.wt.ugt80..
drm.smt1..
drm.wt.smt1..
dsm.ugt80..
dsm.wt.ugt80..
dsm.smt1..
dsm.wt.smt1..
sp.ugt80..
sp.wt.ugt80..
sp.smt1..
sp.wt.smt1..
im.ugt80..
im.wt.ugt80..
im.smt1..
im.wt.smt1..

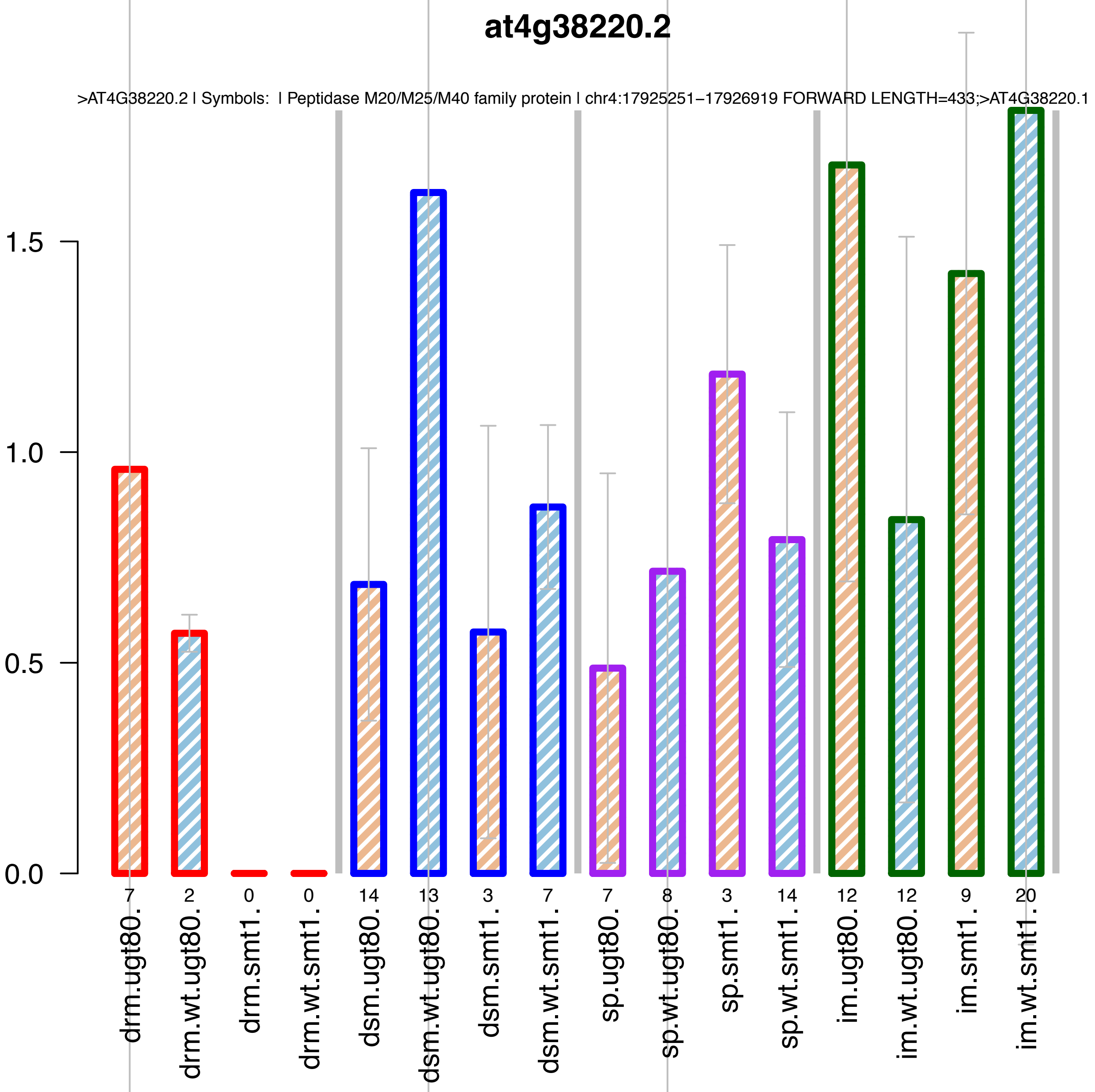
at4g38220.2
>AT4G38220.2 | Symbols: | Peptidase M20/M25/M40 family protein | chr4:17925251-17926919 FORWARD LENGTH=433;>AT4G38220.1
0.0
0.5
1.0
1.5
7
2
0
0
14
13
3
7
7
8
3
14
12
12
9
20
drm.ugt80..
drm.wt.ugt80..
drm.smt1..
drm.wt.smt1..
dsm.ugt80..
dsm.wt.ugt80..
dsm.smt1..
dsm.wt.smt1..
sp.ugt80..
sp.wt.ugt80..
sp.smt1..
sp.wt.smt1..
im.ugt80..
im.wt.ugt80..
im.smt1..
im.wt.smt1..

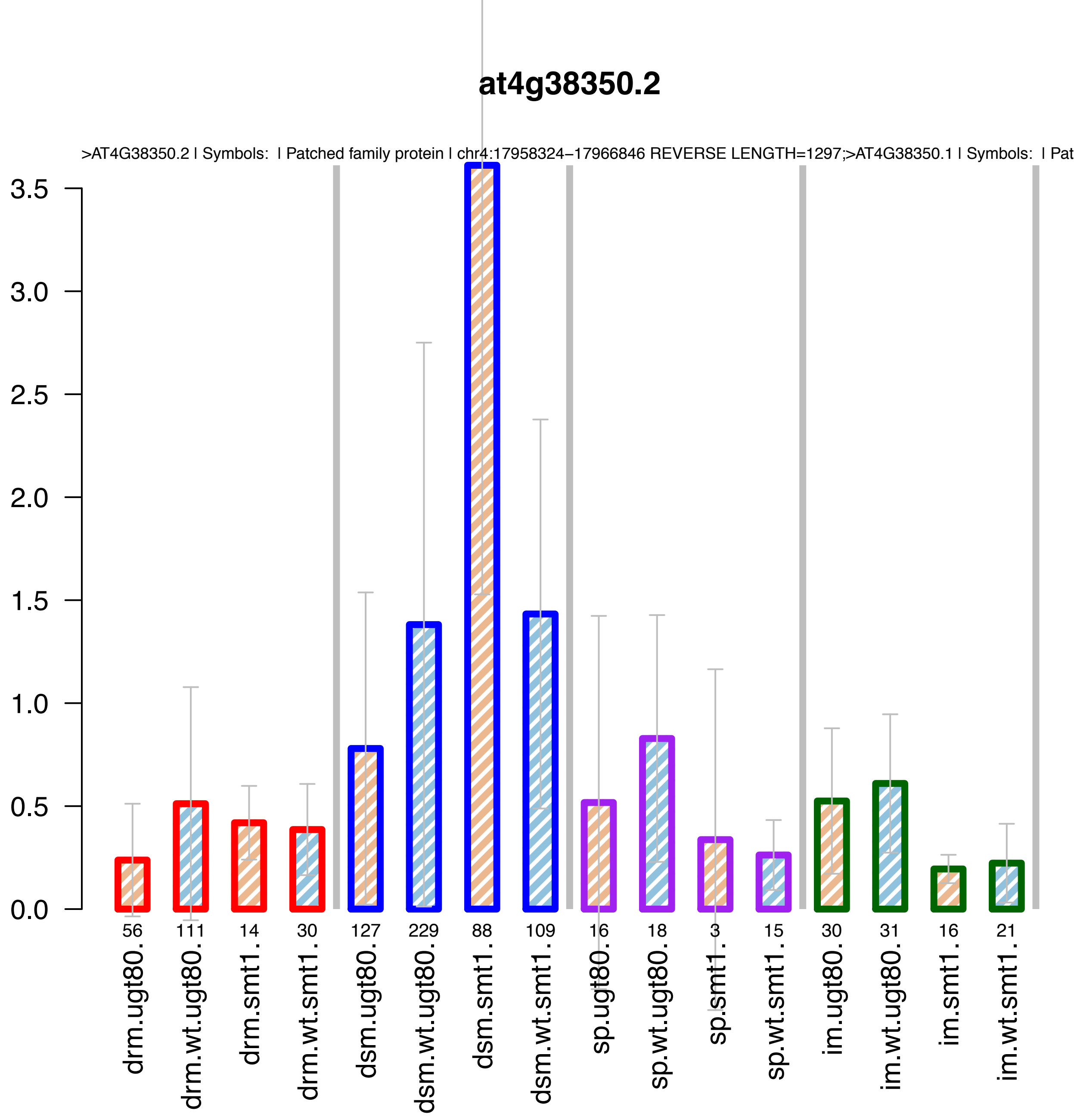

at4g38350.2
>AT4G38350.2 | Symbols: | Patched family protein | chr4:17958324–17966846 REVERSE LENGTH=1297;>AT4G38350.1 | Symbols: | Pat
0.0
0.5
1.0
1.5
2.0
2.5
3.0
3.5
56
111
14
30
127
229
88
109
16
18
3
15
30
31
16
21
drm.ugt80.
drm.wt.ugt80.
drm.smt1.
drm.wt.smt1.
dsm.ugt80.
dsm.wt.ugt80.
dsm.smt1.
dsm.wt.smt1.
sp.ugt80.
sp.wt.ugt80.
sp.smt1.
sp.wt.smt1.
im.ugt80.
im.wt.ugt80.
im.smt1.
im.wt.smt1.

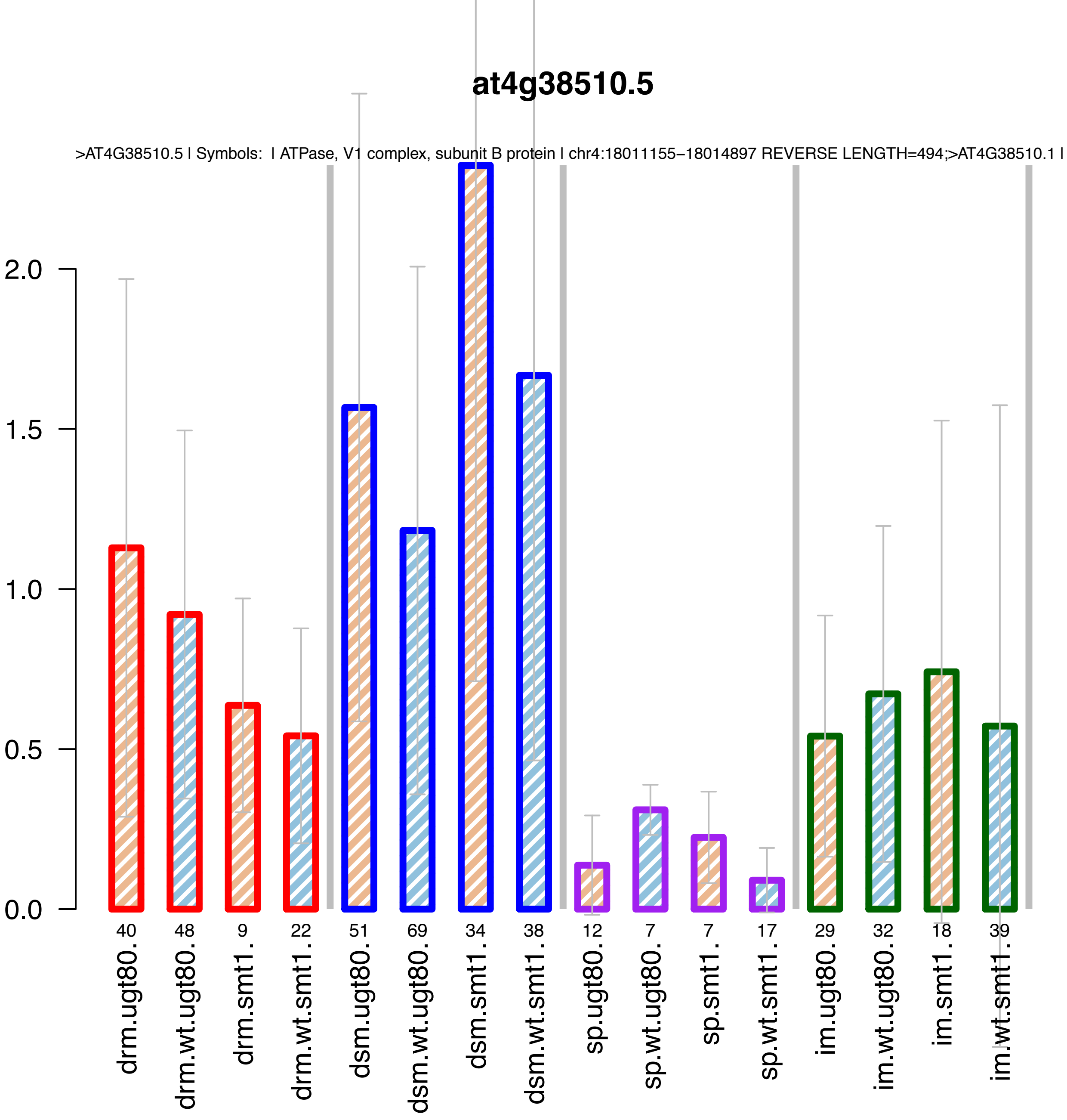

at4g38510.5
>AT4G38510.5 | Symbols: | ATPase, V1 complex, subunit B protein | chr4:18011155-18014897 REVERSE LENGTH=494;>AT4G38510.1 |
2.0
1.5
1.0
0.5
0.0
40
48
9
22
51
69
34
38
12
7
7
17
29
32
18
39
drm.ugt80..
drm.wt.ugt80..
drm.smt1..
drm.wt.smt1..
dsm.ugt80..
dsm.wt.ugt80..
dsm.smt1..
dsm.wt.smt1..
sp.ugt80..
sp.wt.ugt80..
sp.smt1..
sp.wt.smt1..
im.ugt80..
im.wt.ugt80..
im.smt1..
im.wt.smt1..

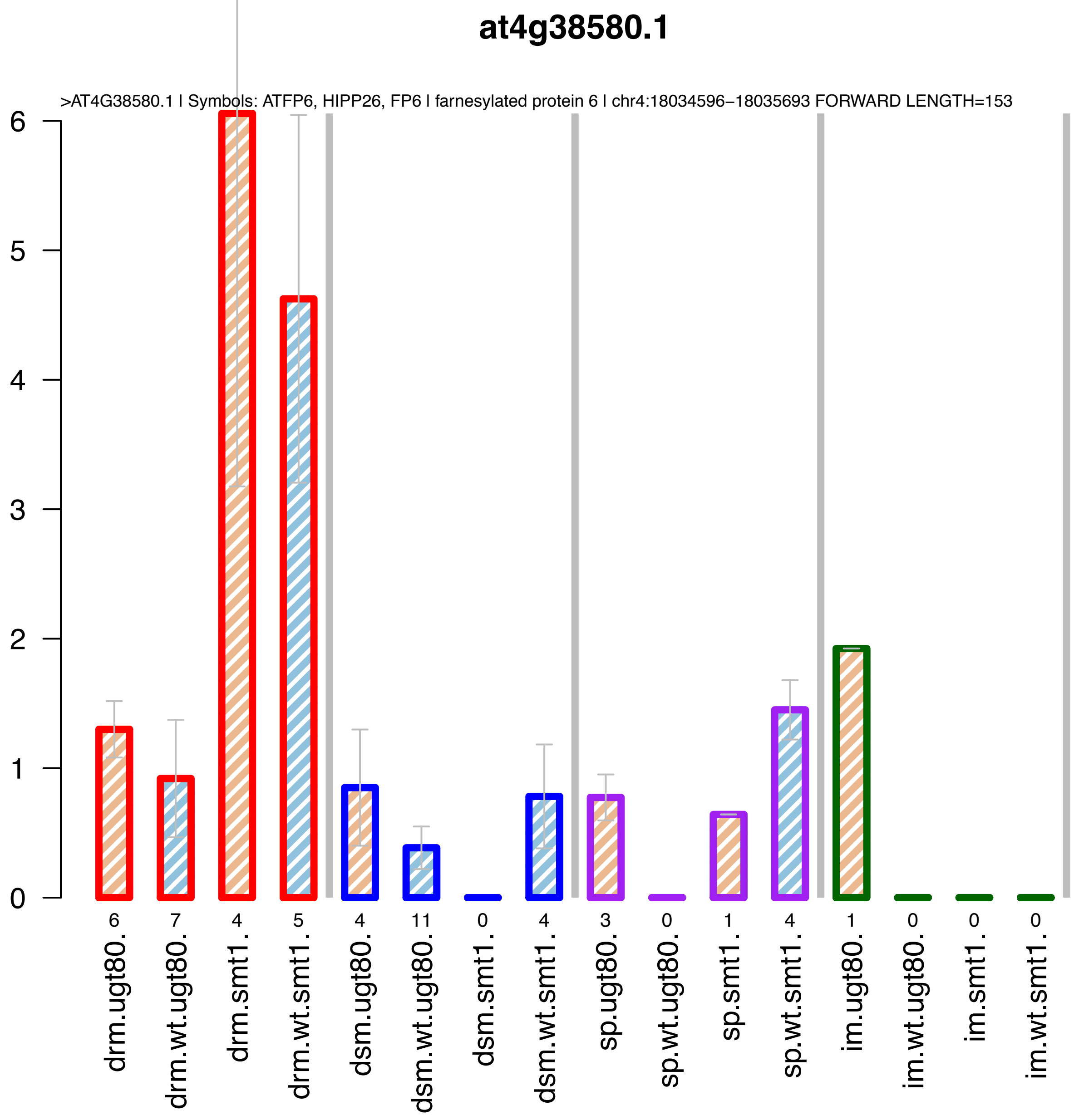

at4g38580.1
>AT4G38580.1 | Symbols: ATFP6, HIPP26, FP6 | farnesylated protein 6 | chr4:18034596−18035693 FORWARD LENGTH=153
0
1
2
3
4
5
6
6
7
4
5
4
11
0
4
3
0
1
4
1
0
0
0
drm.ugt80.
drm.wt.ugt80.
drm.smt1.
drm.wt.smt1.
dsm.ugt80.
dsm.wt.ugt80.
dsm.smt1.
dsm.wt.smt1.
sp.ugt80.
sp.wt.ugt80.
sp.smt1.
sp.wt.smt1.
im.ugt80.
im.wt.ugt80.
im.smt1.
im.wt.smt1.

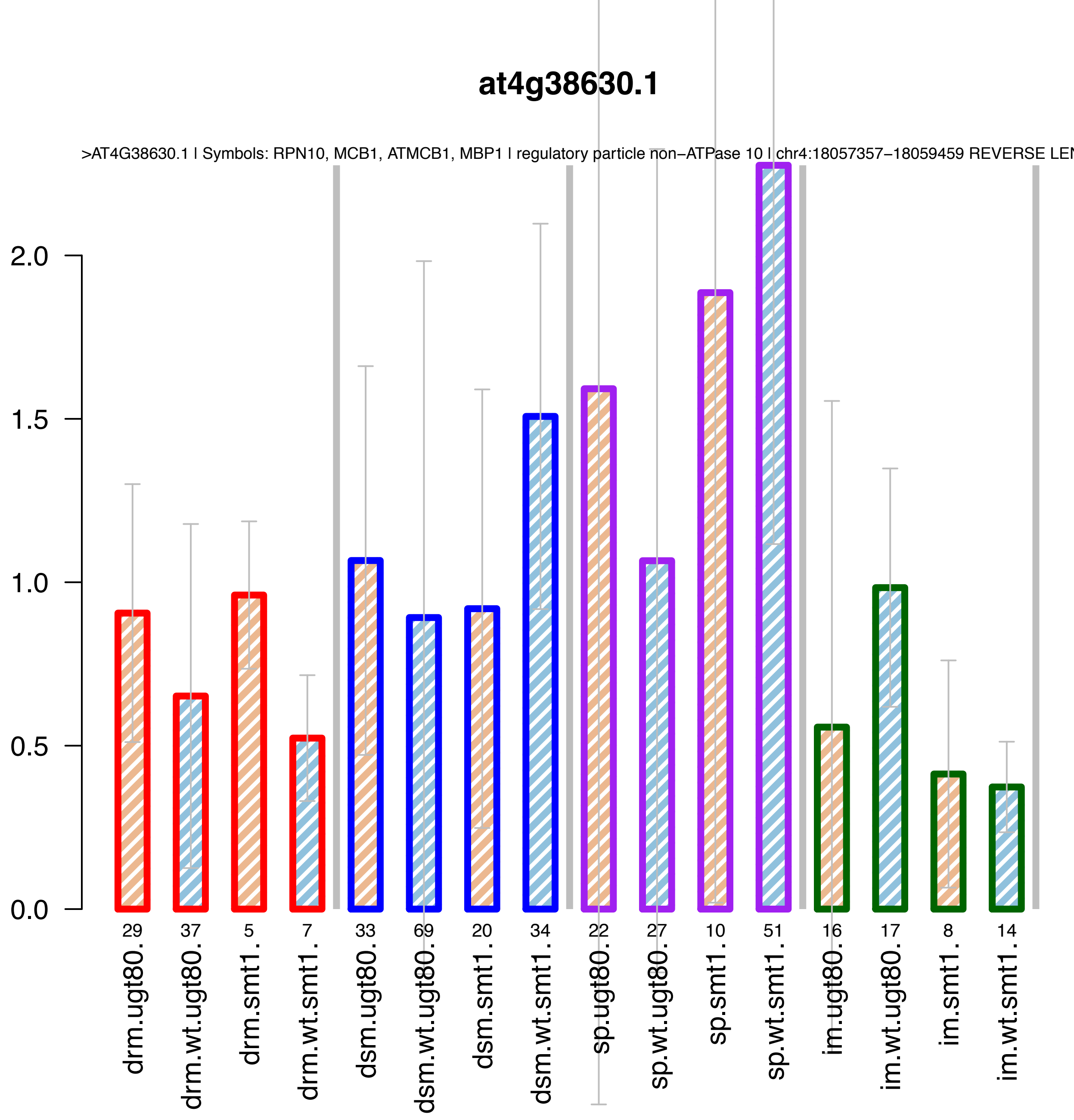

at4g38630.1
>AT4G38630.1 | Symbols: RPN10, MCB1, ATMCB1, MBP1 | regulatory particle non−ATPase 10 | chr4:18057357−18059459 REVERSE LEN
2.0
1.5
1.0
0.5
0.0
29
37
5
7
33
69
20
34
22
27
10
51
16
17
8
14
drm.ugt80.
drm.wt.ugt80.
drm.smt1.
drm.wt.smt1.
dsm.ugt80.
dsm.wt.ugt80.
dsm.smt1.
dsm.wt.smt1.
sp.ugt80.
sp.wt.ugt80.
sp.smt1.
sp.wt.smt1.
im.ugt80.
im.wt.ugt80.
im.smt1.
im.wt.smt1.

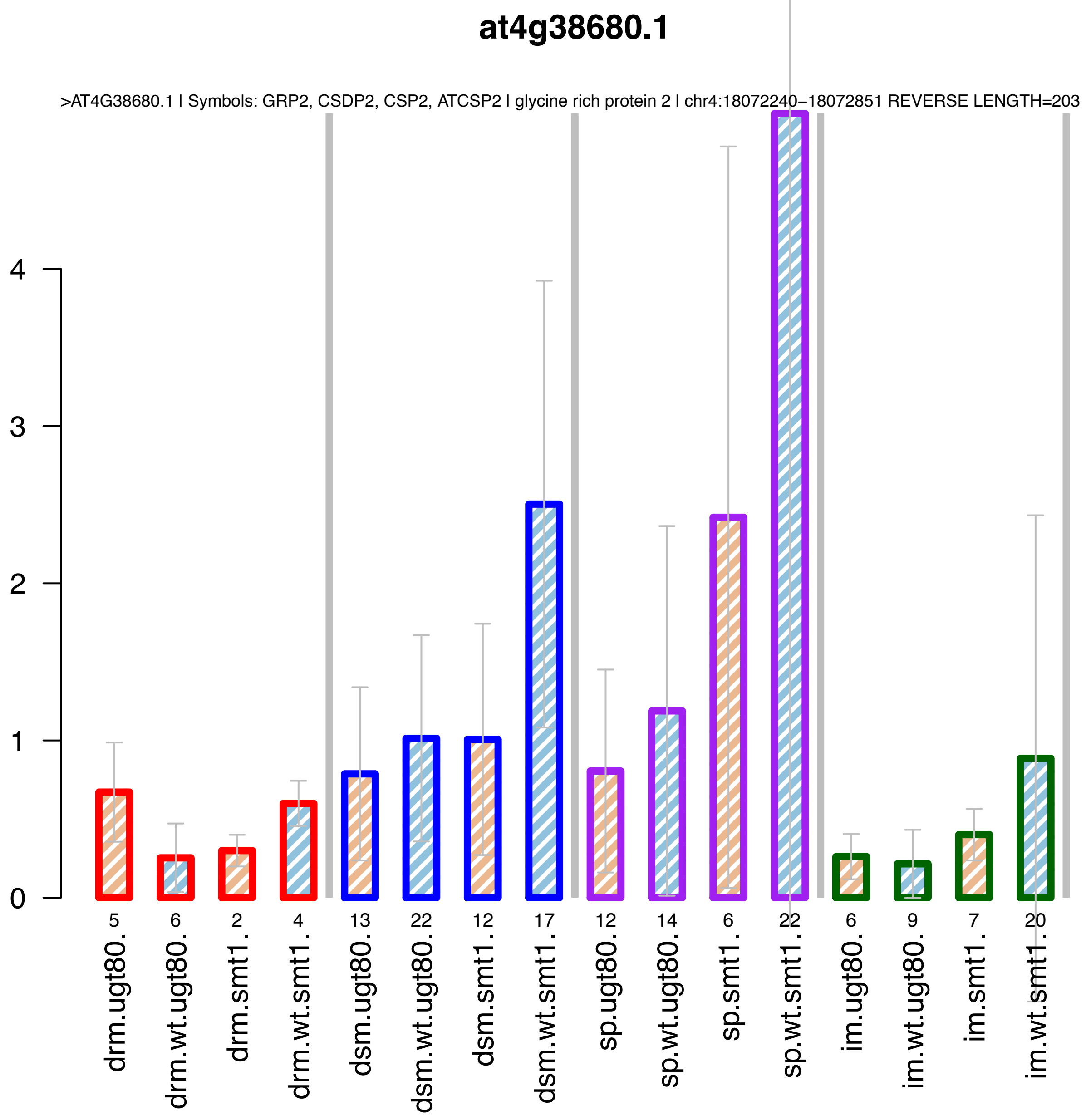

at4g38680.1
>AT4G38680.1 | Symbols: GRP2, CSDP2, CSP2, ATCSP2 | glycine rich protein 2 | chr4:18072240−18072851 REVERSE LENGTH=203
0
1
2
3
4
5
6
2
4
13
22
12
17
12
14
6
22
6
9
7
20
drm.ugt80.
drm.wt.ugt80.
drm.smt1.
drm.wt.smt1.
dsm.ugt80.
dsm.wt.ugt80.
dsm.smt1.
dsm.wt.smt1.
sp.ugt80.
sp.wt.ugt80.
sp.smt1.
sp.wt.smt1.
im.ugt80.
im.wt.ugt80.
im.smt1.
im.wt.smt1.

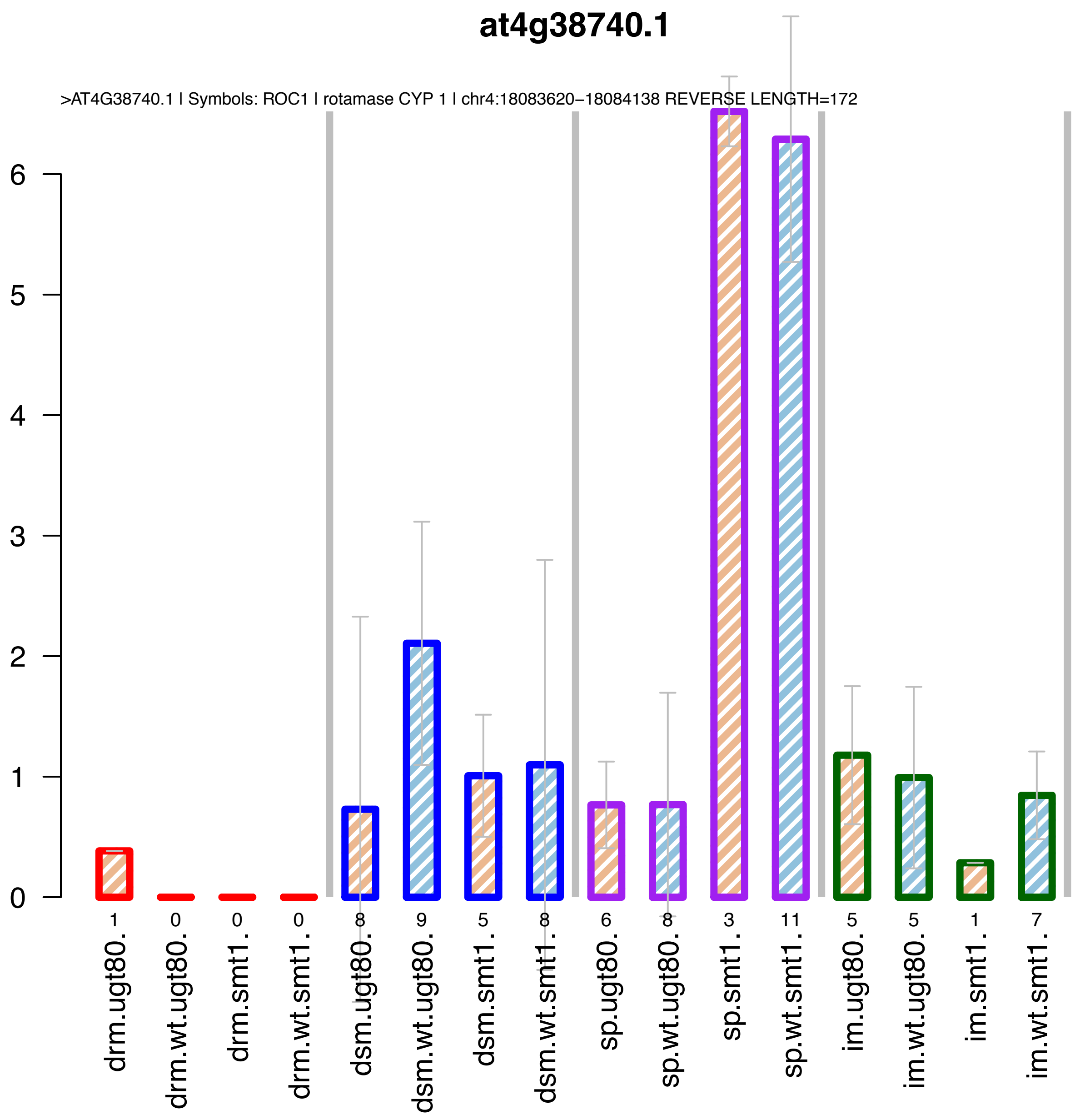
at4g38740.1
>AT4G38740.1 | Symbols: ROC1 | rotamase CYP 1 | chr4:18083620-18084138 REVERSE LENGTH=172
0
1
2
3
4
5
6
1
0
0
0
8
9
5
8
6
8
3
11
5
5
1
7
drm.ugt80.
drm.wt.ugt80.
drm.smt1.
drm.wt.smt1.
dsm.ugt80.
dsm.wt.ugt80.
dsm.smt1.
dsm.wt.smt1.
sp.ugt80.
sp.wt.ugt80.
sp.smt1.
sp.wt.smt1.
im.ugt80.
im.wt.ugt80.
im.smt1.
im.wt.smt1.

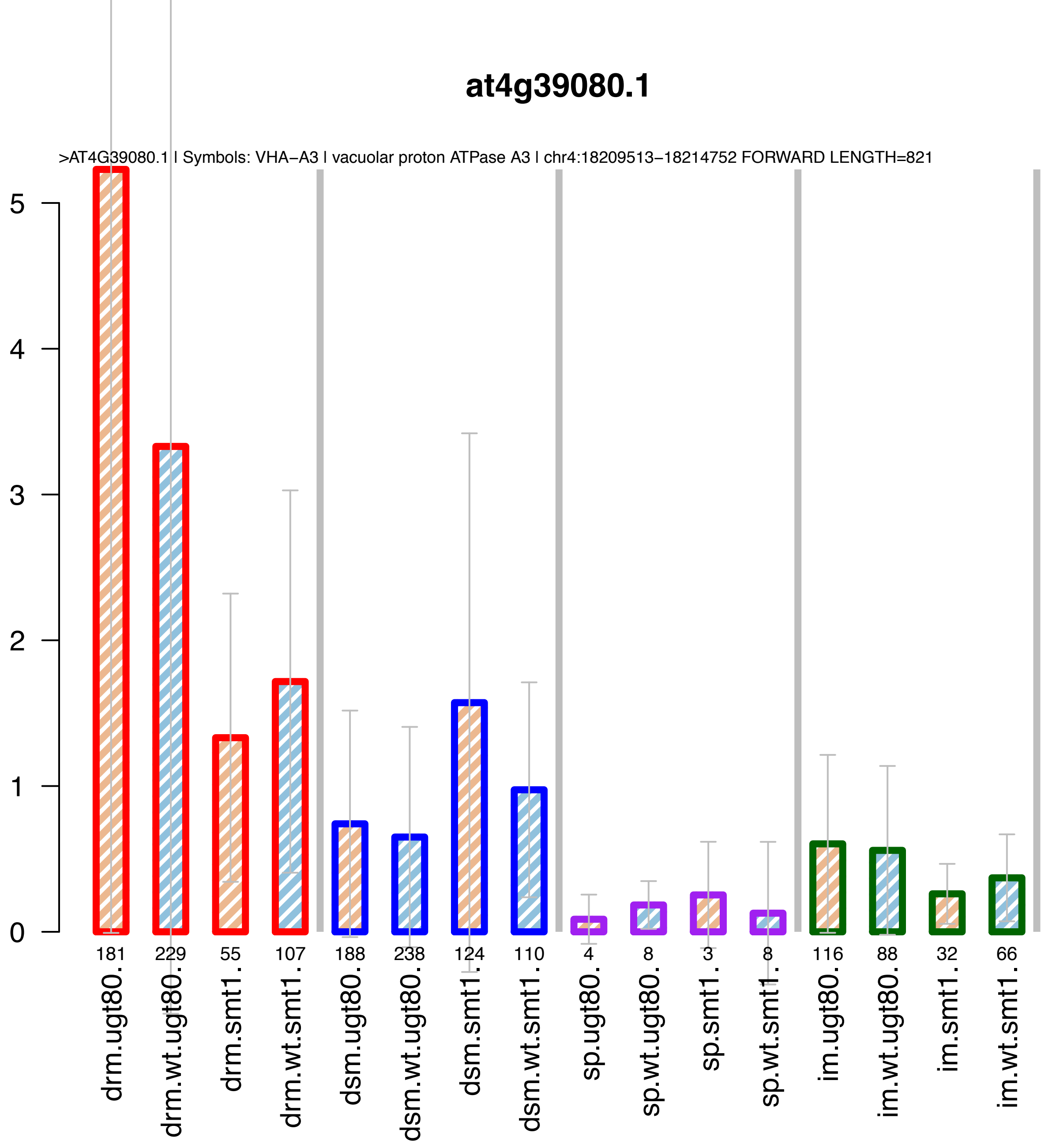

at4g39080.1
>AT4G39080.1 | Symbols: VHA−A3 | vacuolar proton ATPase A3 | chr4:18209513−18214752 FORWARD LENGTH=821
5
4
3
2
1
0
181
229
55
107
188
238
124
110
4
8
3
8
116
88
32
66
drm.ugt80..
drm.wt.ugt80..
drm.smt1..
drm.wt.smt1..
dsm.ugt80..
dsm.wt.ugt80..
dsm.smt1..
dsm.wt.smt1..
sp.ugt80..
sp.wt.ugt80..
sp.smt1..
sp.wt.smt1..
im.ugt80..
im.wt.ugt80..
im.smt1..
im.wt.smt1..

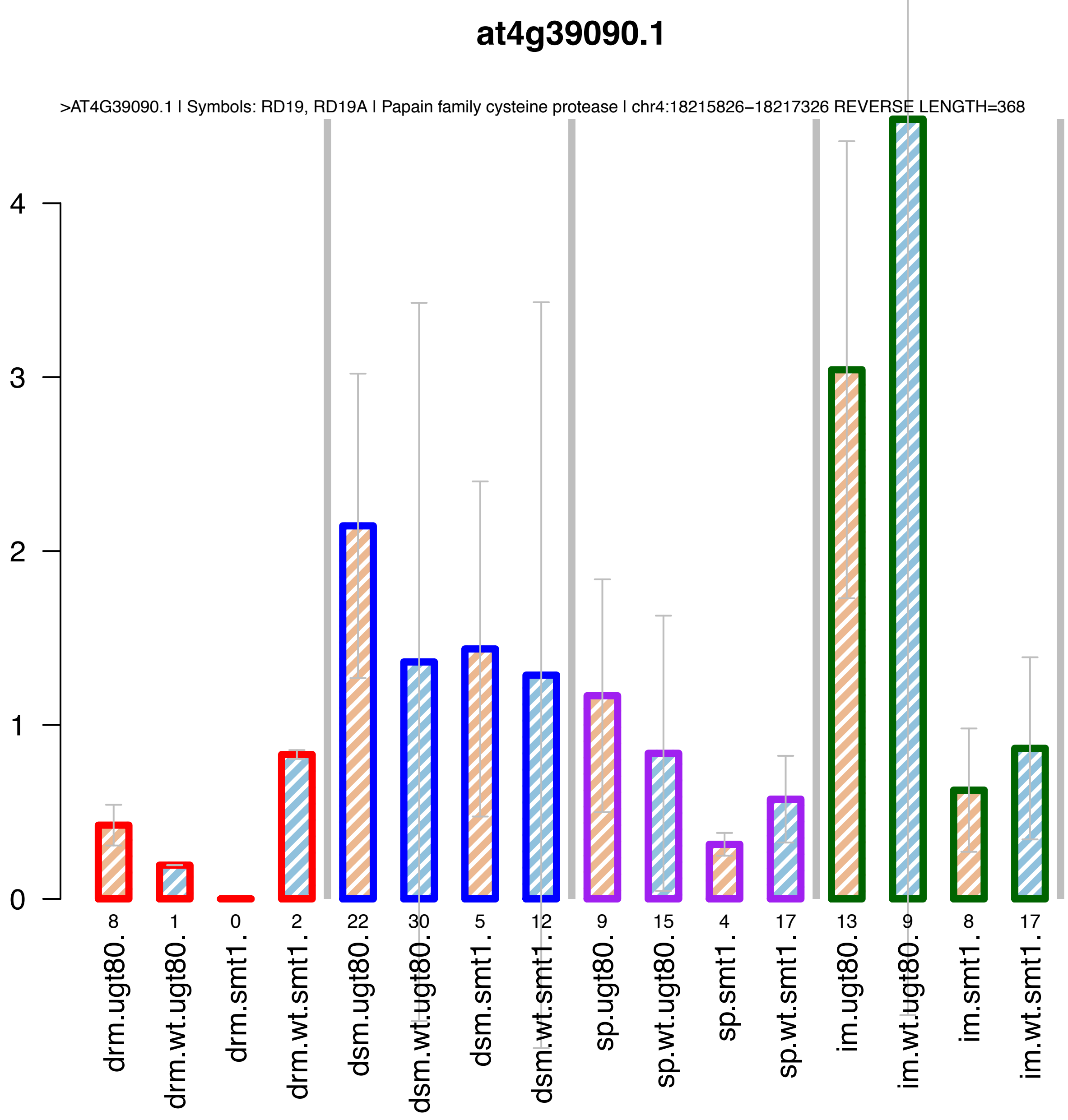

at4g39090.1
>AT4G39090.1 | Symbols: RD19, RD19A | Papain family cysteine protease | chr4:18215826-18217326 REVERSE LENGTH=368
0
1
2
3
4
8
1
0
2
22
30
5
12
9
15
4
17
13
9
8
17
drm.ugt80.
drm.wt.ugt80.
drm.smt1.
drm.wt.smt1.
dsm.ugt80.
dsm.wt.ugt80.
dsm.smt1.
dsm.wt.smt1.
sp.ugt80.
sp.wt.ugt80.
sp.smt1.
sp.wt.smt1.
im.ugt80.
im.wt.ugt80.
im.smt1.
im.wt.smt1.

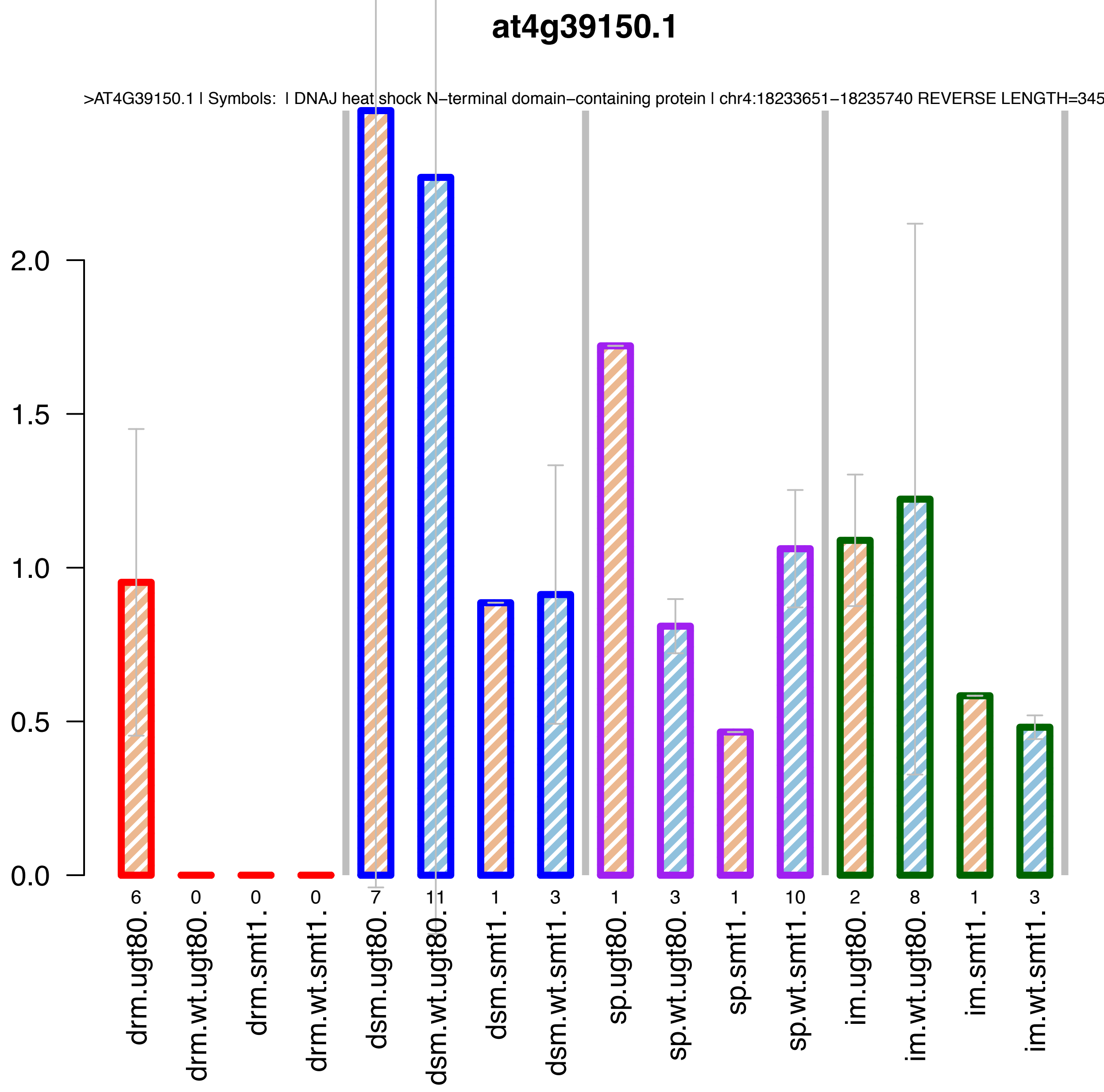
at4g39150.1
>AT4G39150.1 | Symbols: | DNAJ heat shock N−terminal domain−containing protein | chr4:18233651−18235740 REVERSE LENGTH=345
2.0
1.5
1.0
0.5
0.0
6
0
0
0
7
11
1
3
1
3
1
10
2
8
1
3
drm.ugt80.
drm.wt.ugt80.
drm.smt1.
drm.wt.smt1.
dsm.ugt80.
dsm.wt.ugt80.
dsm.smt1.
dsm.wt.smt1.
sp.ugt80.
sp.wt.ugt80.
sp.smt1.
sp.wt.smt1.
im.ugt80.
im.wt.ugt80.
im.smt1.
im.wt.smt1.

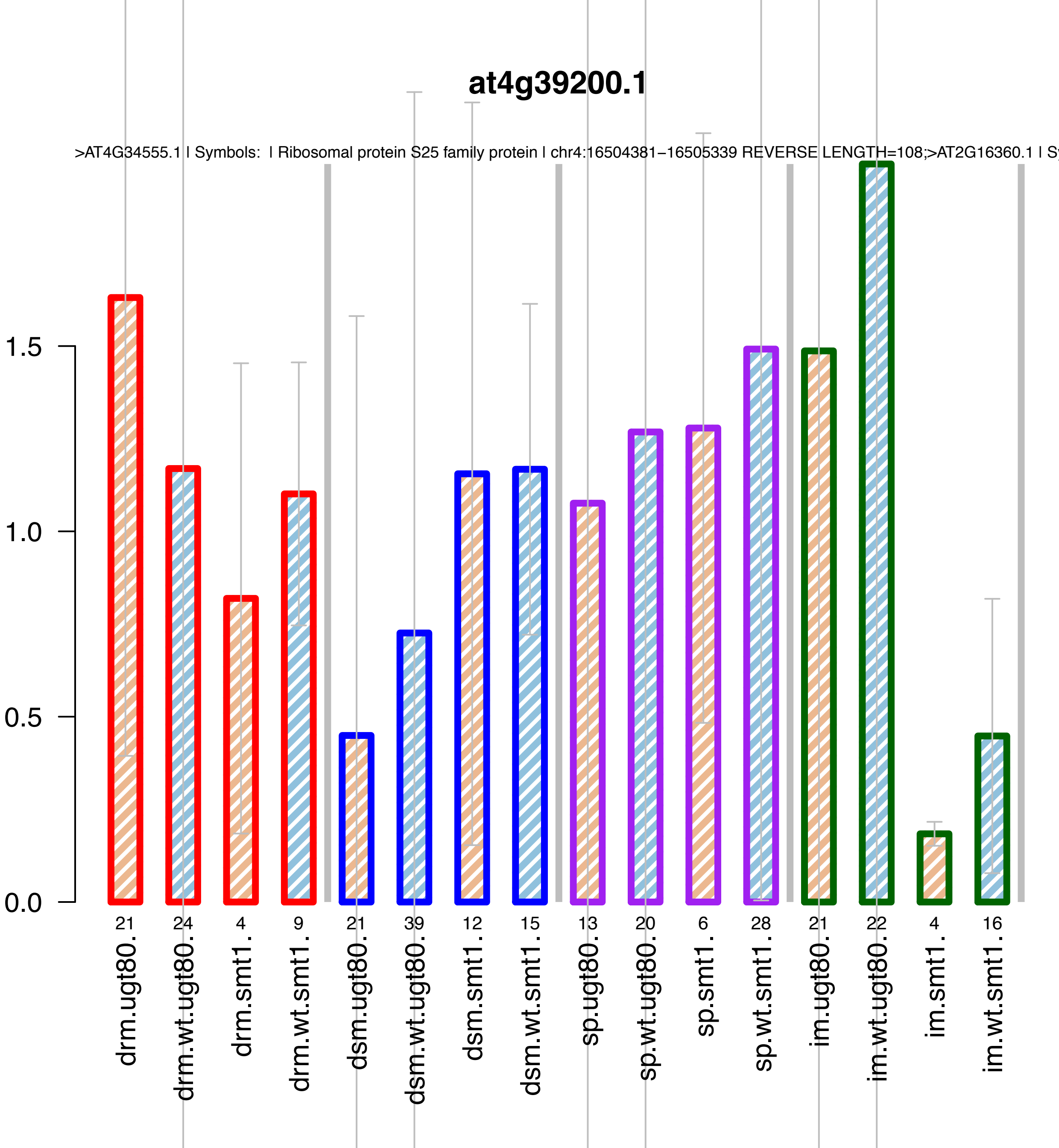

at4g39200.1
>AT4G34555.1 | Symbols: | Ribosomal protein S25 family protein | chr4:16504381-16505339 REVERSE LENGTH=108;>AT2G16360.1 | S
0.0
0.5
1.0
1.5
21
24
4
9
21
39
12
15
13
20
6
28
21
22
4
16
drm.ugt80..
drm.wt.ugt80..
drm.smt1..
drm.wt.smt1..
dsm.ugt80..
dsm.wt.ugt80..
dsm.smt1..
dsm.wt.smt1..
sp.ugt80..
sp.wt.ugt80..
sp.smt1..
sp.wt.smt1..
im.ugt80..
im.wt.ugt80..
im.smt1..
im.wt.smt1..

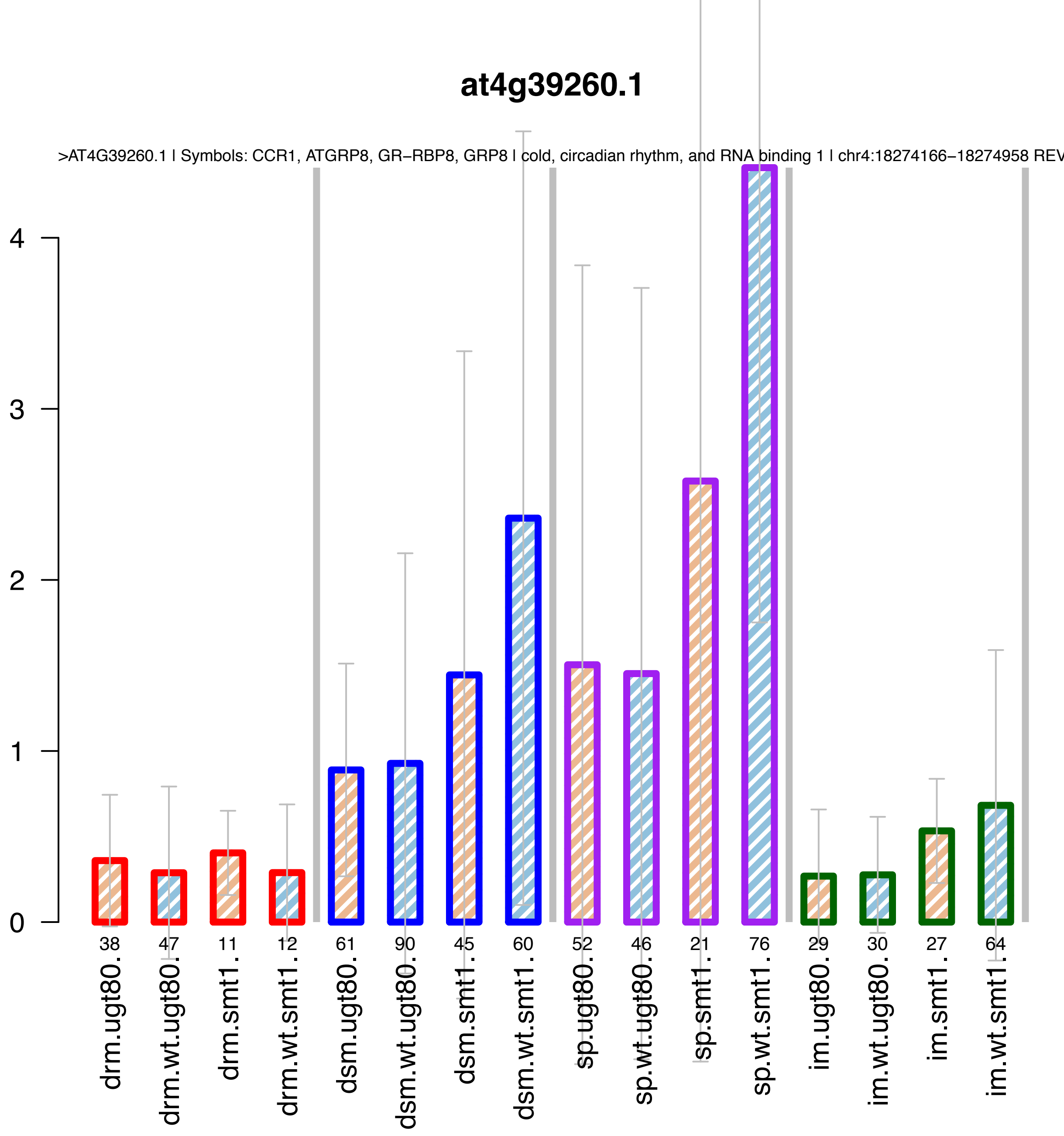

at4g39260.1
>AT4G39260.1 | Symbols: CCR1, ATGRP8, GR−RBP8, GRP8 | cold, circadian rhythm, and RNA binding 1 | chr4:18274166−18274958 REV
0
1
2
3
4
38
47
11
12
61
90
45
60
52
46
21
76
29
30
27
64
drm.ugt80..
drm.wt.ugt80..
drm.smt1..
drm.wt.smt1..
dsm.ugt80..
dsm.wt.ugt80..
dsm.smt1..
dsm.wt.smt1..
sp.ugt80..
sp.wt.ugt80..
sp.smt1..
sp.wt.smt1..
im.ugt80..
im.wt.ugt80..
im.smt1..
im.wt.smt1..

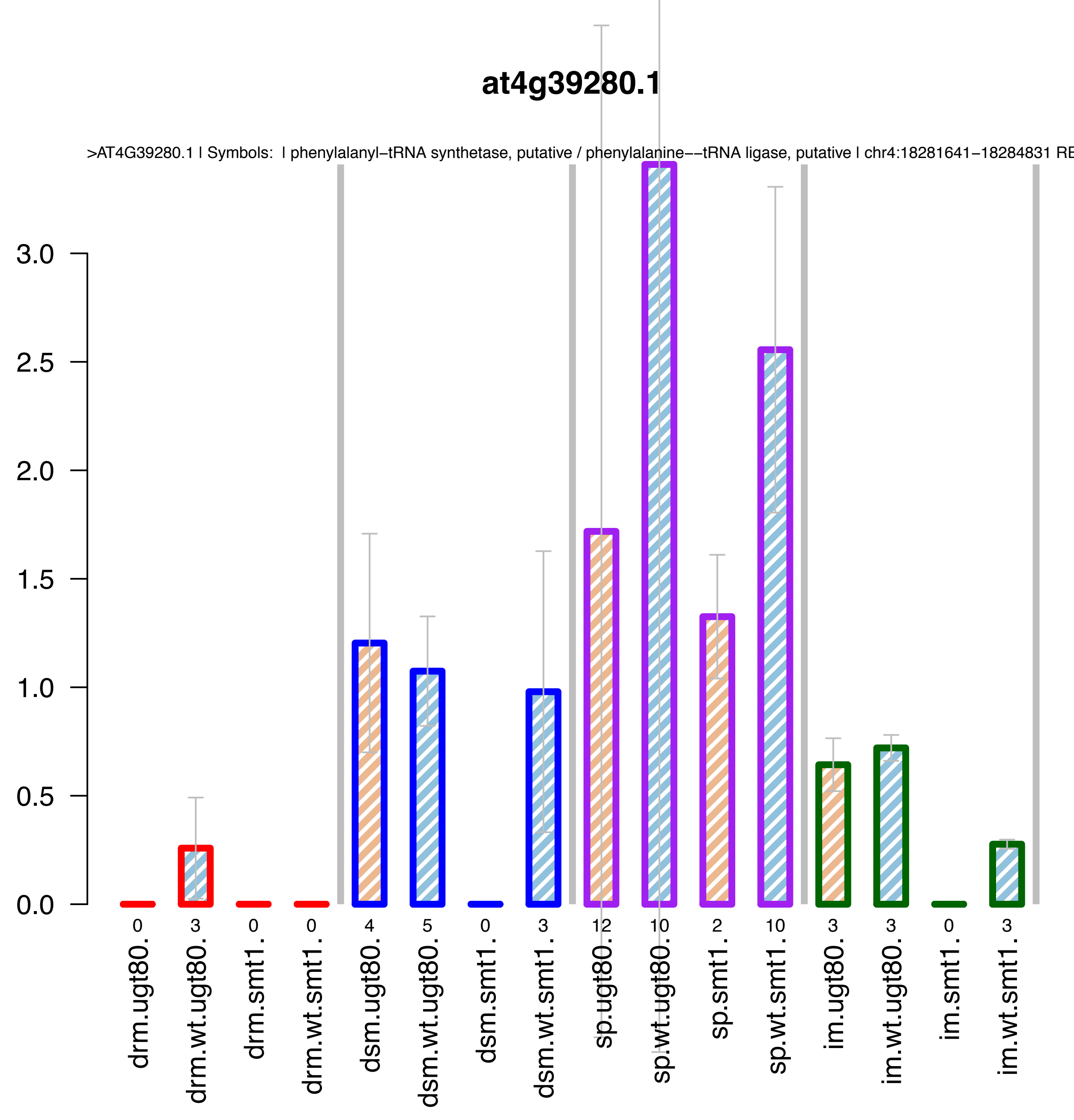

at4g39280.1
>AT4G39280.1 | Symbols: | phenylalanyl–tRNA synthetase, putative / phenylalanine––tRNA ligase, putative | chr4:18281641–18284831 RE
3.0
2.5
2.0
1.5
1.0
0.5
0.0
0
3
0
0
4
5
0
3
12
10
2
10
3
3
0
3
drm.ugt80.
drm.wt.ugt80.
drm.smt1.
drm.wt.smt1.
dsm.ugt80.
dsm.wt.ugt80.
dsm.smt1.
dsm.wt.smt1.
sp.ugt80.
sp.wt.ugt80.
sp.smt1.
sp.wt.smt1.
im.ugt80.
im.wt.ugt80.
im.smt1.
im.wt.smt1.

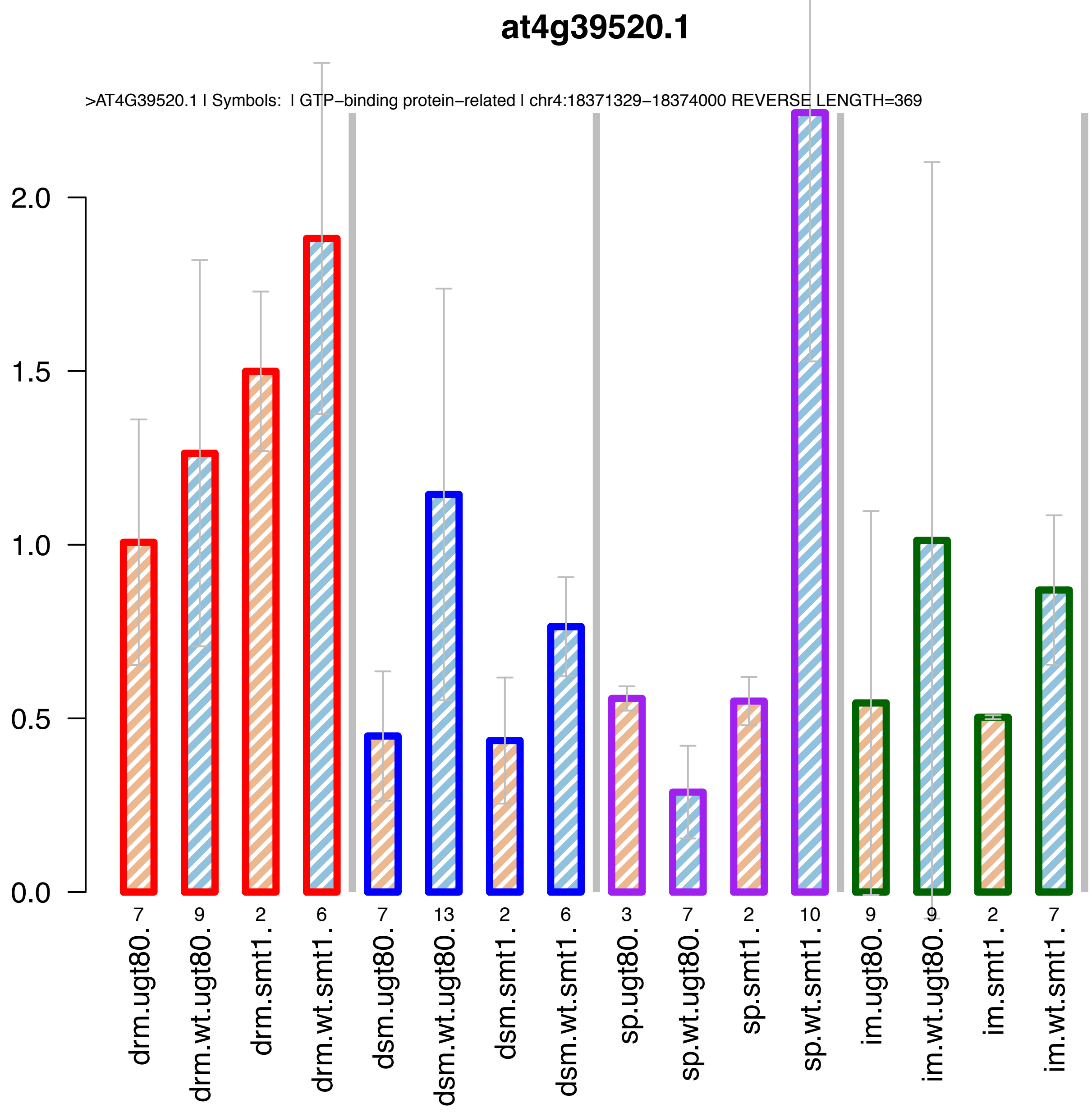

at4g39520.1
>AT4G39520.1 | Symbols: | GTP−binding protein−related | chr4:18371329−18374000 REVERSE LENGTH=369
2.0
1.5
1.0
0.5
0.0
7
9
2
6
7
13
2
6
3
7
2
10
9
9
2
7
drm.ugt80..
drm.wt.ugt80..
drm.smt1..
drm.wt.smt1..
dsm.ugt80..
dsm.wt.ugt80..
dsm.smt1..
dsm.wt.smt1..
sp.ugt80..
sp.wt.ugt80..
sp.smt1..
sp.wt.smt1..
im.ugt80..
im.wt.ugt80..
im.smt1..
im.wt.smt1..

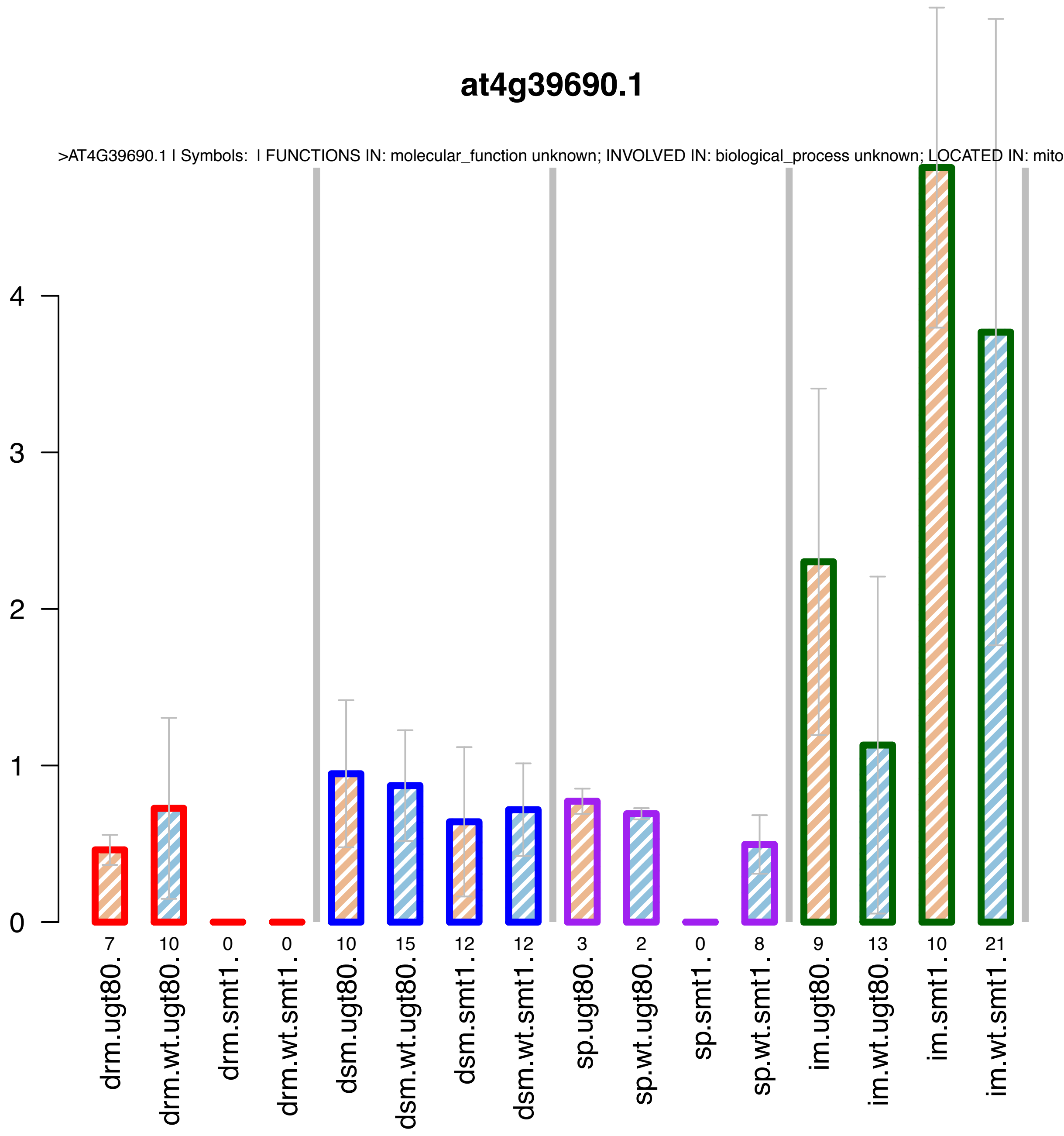

at4g39690.1
>AT4G39690.1 | Symbols: | FUNCTIONS IN: molecular_function unknown; INVOLVED IN: biological_process unknown; LOCATED IN: mito
0
1
2
3
4
7
10
0
0
10
15
12
12
3
2
0
8
9
13
10
21
drm.ugt80.
drm.wt.ugt80.
drm.smt1.
drm.wt.smt1.
dsm.ugt80.
dsm.wt.ugt80.
dsm.smt1.
dsm.wt.smt1.
sp.ugt80.
sp.wt.ugt80.
sp.smt1.
sp.wt.smt1.
im.ugt80.
im.wt.ugt80.
im.smt1.
im.wt.smt1.

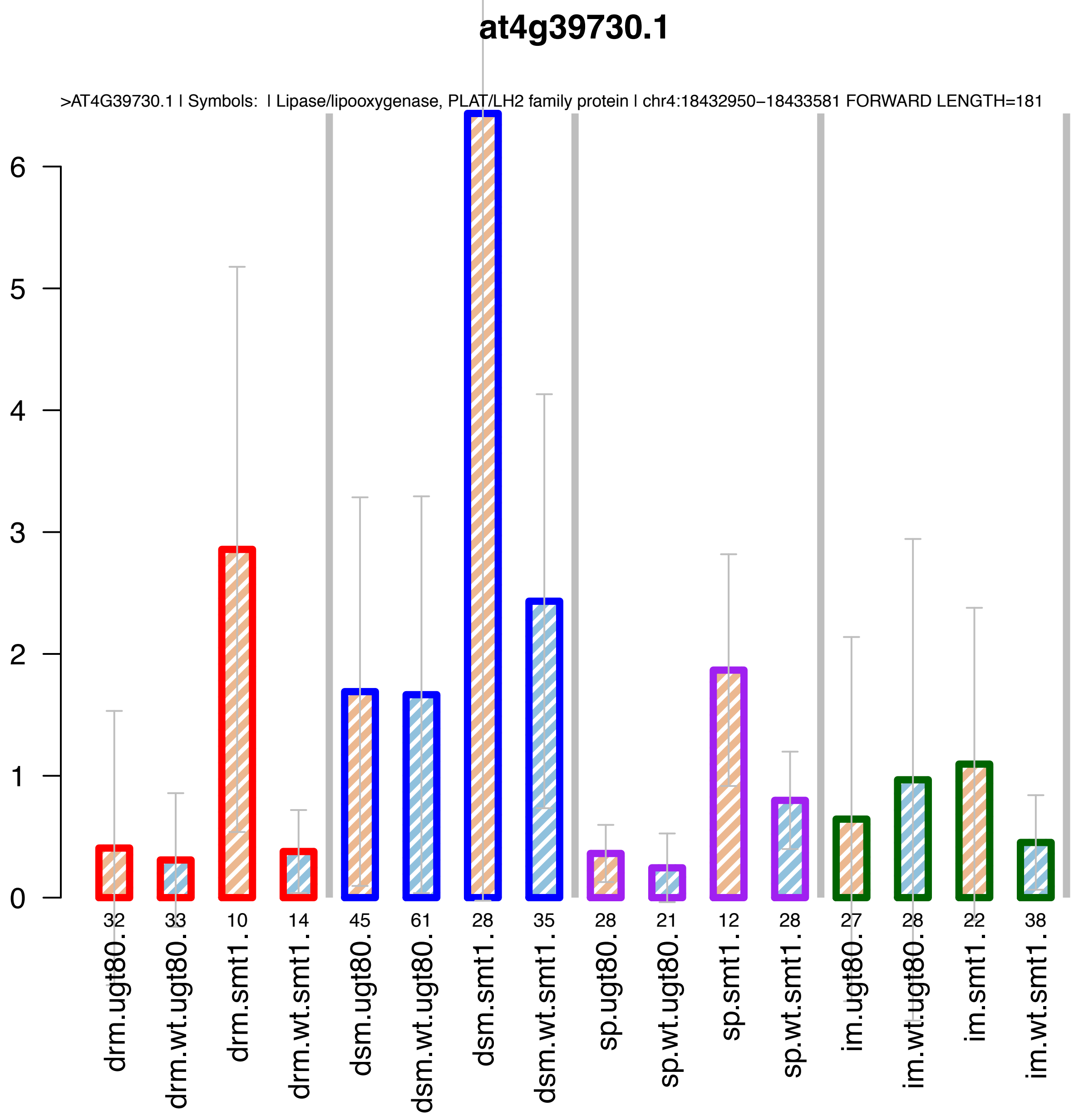

at4g39730.1
>AT4G39730.1 | Symbols: | Lipase/lipooxygenase, PLAT/LH2 family protein | chr4:18432950−18433581 FORWARD LENGTH=181
0
1
2
3
4
5
6
32
33
10
14
45
61
28
35
28
21
12
28
27
28
22
38
drm.ugt80.
drm.wt.ugt80.
drm.smt1.
drm.wt.smt1.
dsm.ugt80.
dsm.wt.ugt80.
dsm.smt1.
dsm.wt.smt1.
sp.ugt80.
sp.wt.ugt80.
sp.smt1.
sp.wt.smt1.
im.ugt80.
im.wt.ugt80.
im.smt1.
im.wt.smt1.

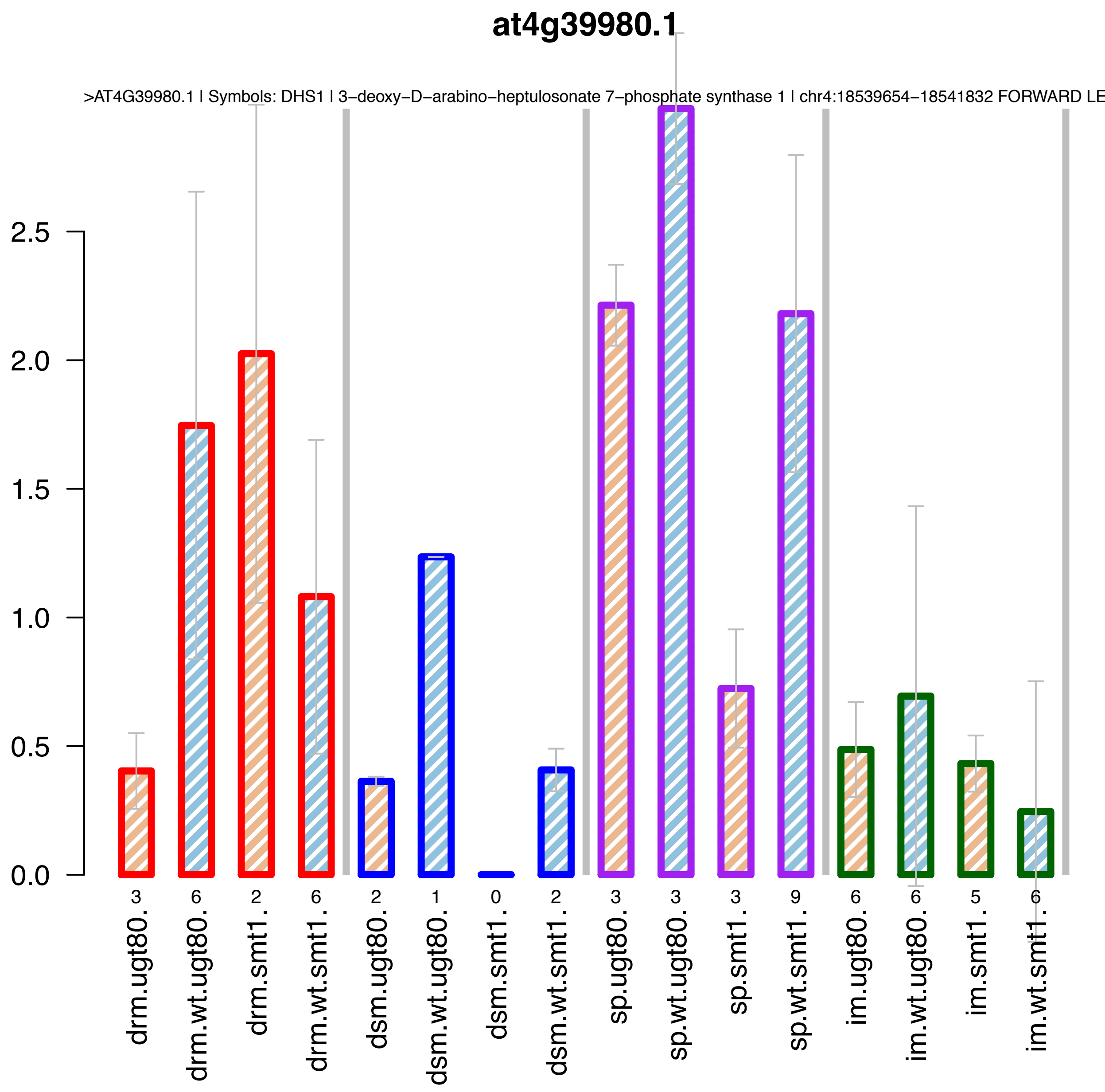
at4g39980.1
>AT4G39980.1 | Symbols: DHS1 | 3−deoxy−D−arabino−heptulosonate 7−phosphate synthase 1 | chr4:18539654−18541832 FORWARD LE
0.0
0.5
1.0
1.5
2.0
2.5
3
6
2
6
2
1
0
2
3
3
3
9
6
6
5
6
drm.ugt80..
drm.wt.ugt80..
drm.smt1..
drm.wt.smt1..
dsm.ugt80..
dsm.wt.ugt80..
dsm.smt1..
dsm.wt.smt1..
sp.ugt80..
sp.wt.ugt80..
sp.smt1..
sp.wt.smt1..
im.ugt80..
im.wt.ugt80..
im.smt1..
im.wt.smt1..

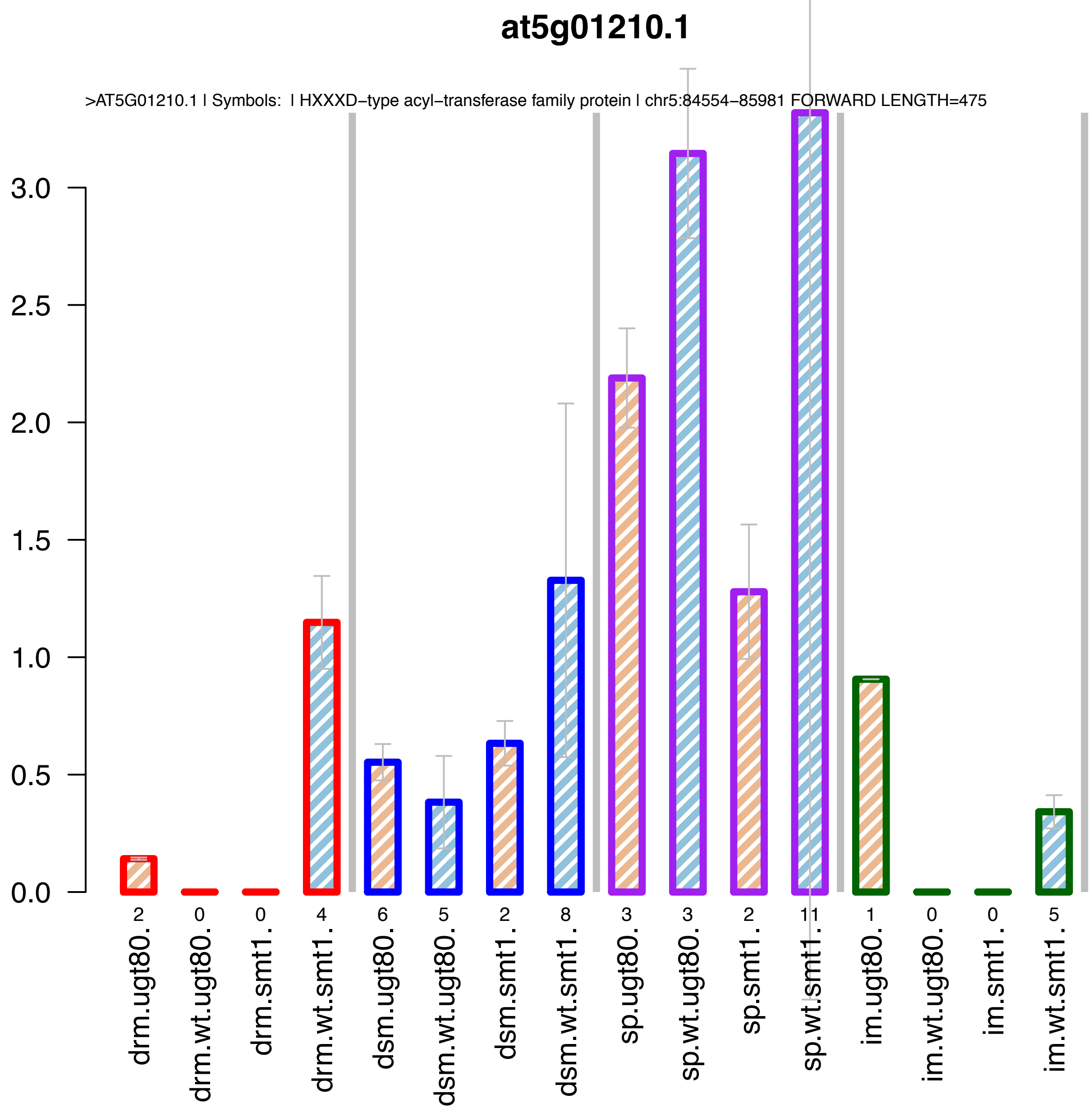
at5g01210.1
>AT5G01210.1 | Symbols: | HXXXD−type acyl−transferase family protein | chr5:84554−85981 FORWARD LENGTH=475
0.0
0.5
1.0
1.5
2.0
2.5
3.0
2
0
0
4
6
5
2
8
3
3
2
11
1
0
0
5
drm.ugt80.
drm.wt.ugt80.
drm.smt1.
drm.wt.smt1.
dsm.ugt80.
dsm.wt.ugt80.
dsm.smt1.
dsm.wt.smt1.
sp.ugt80.
sp.wt.ugt80.
sp.smt1.
sp.wt.smt1.
im.ugt80.
im.wt.ugt80.
im.smt1.
im.wt.smt1.

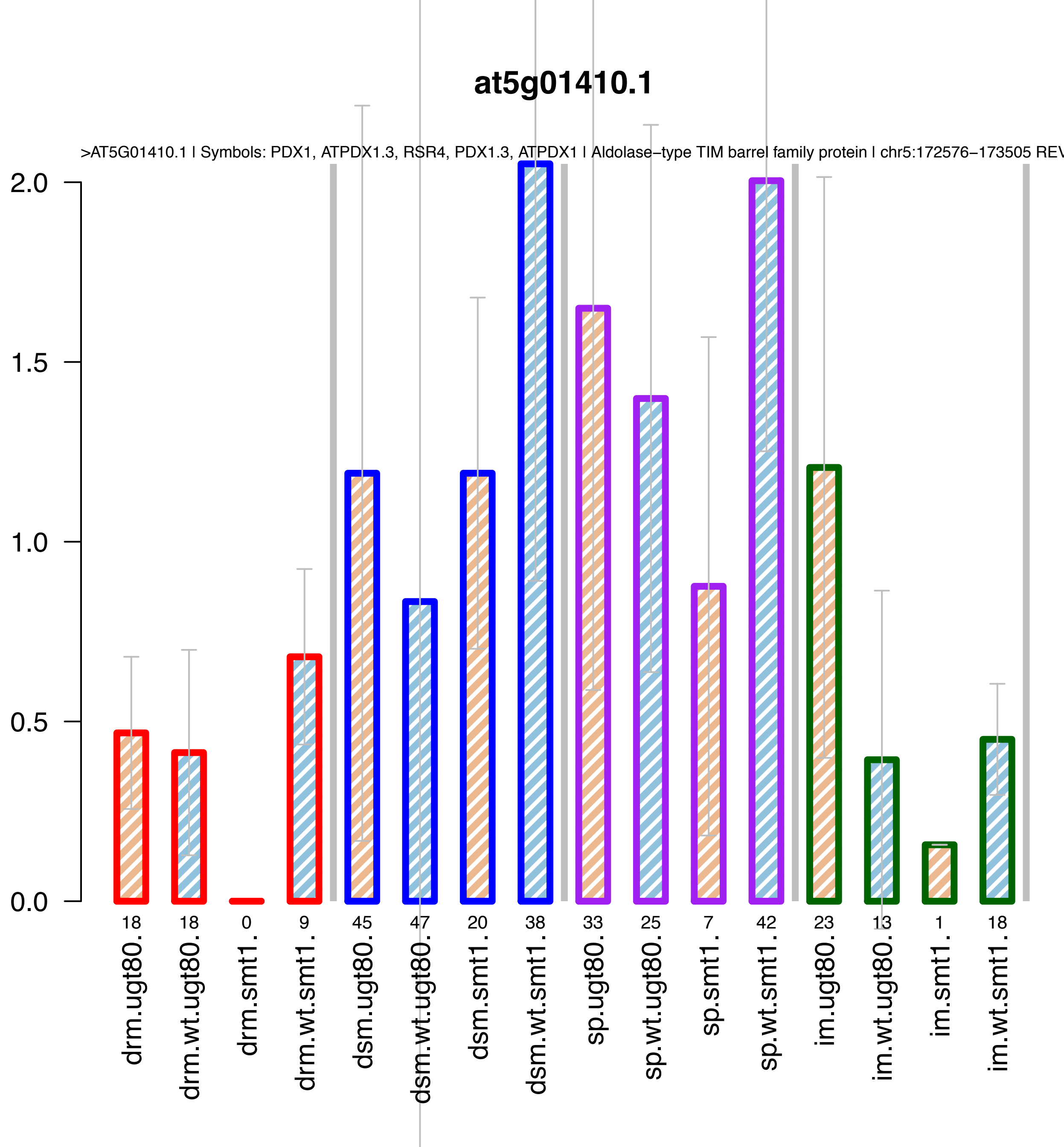

at5g01410.1
>AT5G01410.1 | Symbols: PDX1, ATPDX1.3, RSR4, PDX1.3, ATPDX1 | Aldolase−type TIM barrel family protein | chr5:172576−173505 REV
2.0
1.5
1.0
0.5
0.0
18
18
0
9
45
47
20
38
33
25
7
42
23
13
1
18
drm.ugt80..
drm.wt.ugt80..
drm.smt1..
drm.wt.smt1..
dsm.ugt80..
dsm.wt.ugt80..
dsm.smt1..
dsm.wt.smt1..
sp.ugt80..
sp.wt.ugt80..
sp.smt1..
sp.wt.smt1..
im.ugt80..
im.wt.ugt80..
im.smt1..
im.wt.smt1..

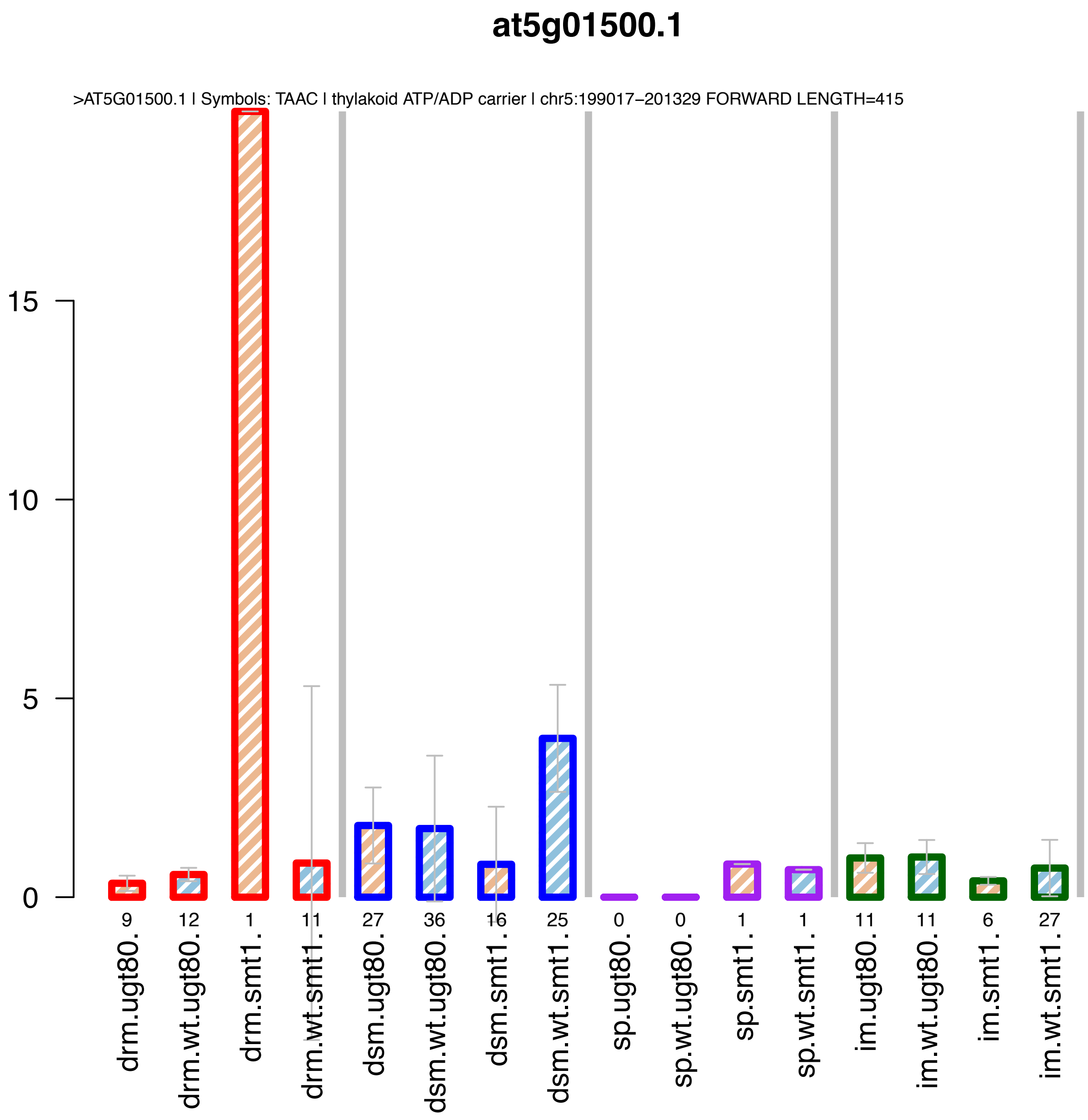

at5g01500.1
>AT5G01500.1 | Symbols: TAAC | thylakoid ATP/ADP carrier | chr5:199017-201329 FORWARD LENGTH=415
0
5
10
15
9
12
1
11
27
36
16
25
0
0
1
1
11
11
6
27
drm.ugt80..
drm.wt.ugt80..
drm.smt1..
drm.wt.smt1..
dsm.ugt80..
dsm.wt.ugt80..
dsm.smt1..
dsm.wt.smt1..
sp.ugt80..
sp.wt.ugt80..
sp.smt1..
sp.wt.smt1..
im.ugt80..
im.wt.ugt80..
im.smt1..
im.wt.smt1..

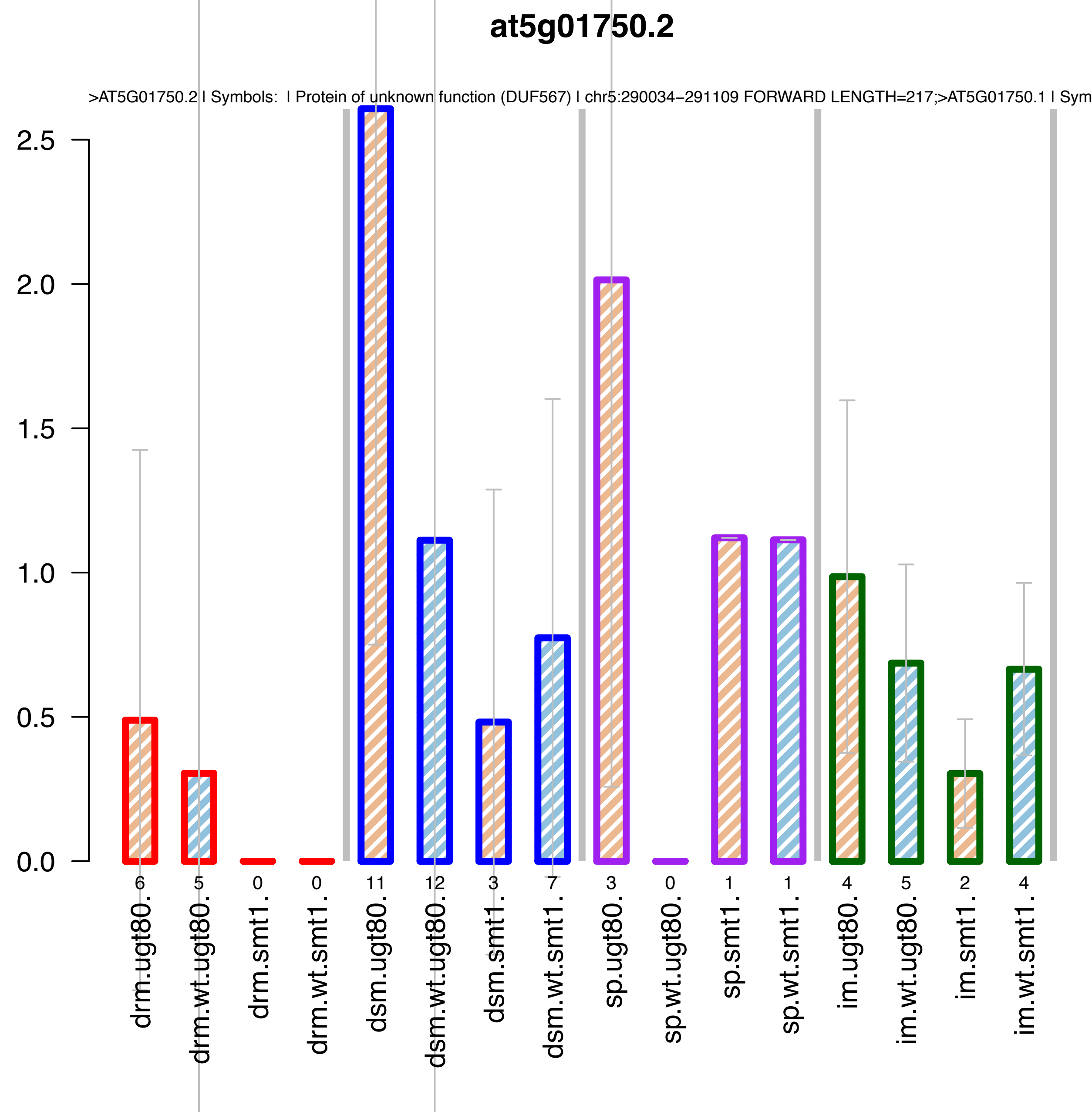

at5g01750.2
>AT5G01750.2 | Symbols: | Protein of unknown function (DUF567) | chr5:290034-291109 FORWARD LENGTH=217;>AT5G01750.1 | Sym
2.5
2.0
1.5
1.0
0.5
0.0
6
5
0
0
11
12
3
7
3
0
1
1
4
5
2
4
drm.ugt80.
drm.wt.ugt80.
drm.smt1.
drm.wt.smt1.
dsm.ugt80.
dsm.wt.ugt80.
dsm.smt1.
dsm.wt.smt1.
sp.ugt80.
sp.wt.ugt80.
sp.smt1.
sp.wt.smt1.
im.ugt80.
im.wt.ugt80.
im.smt1.
im.wt.smt1.

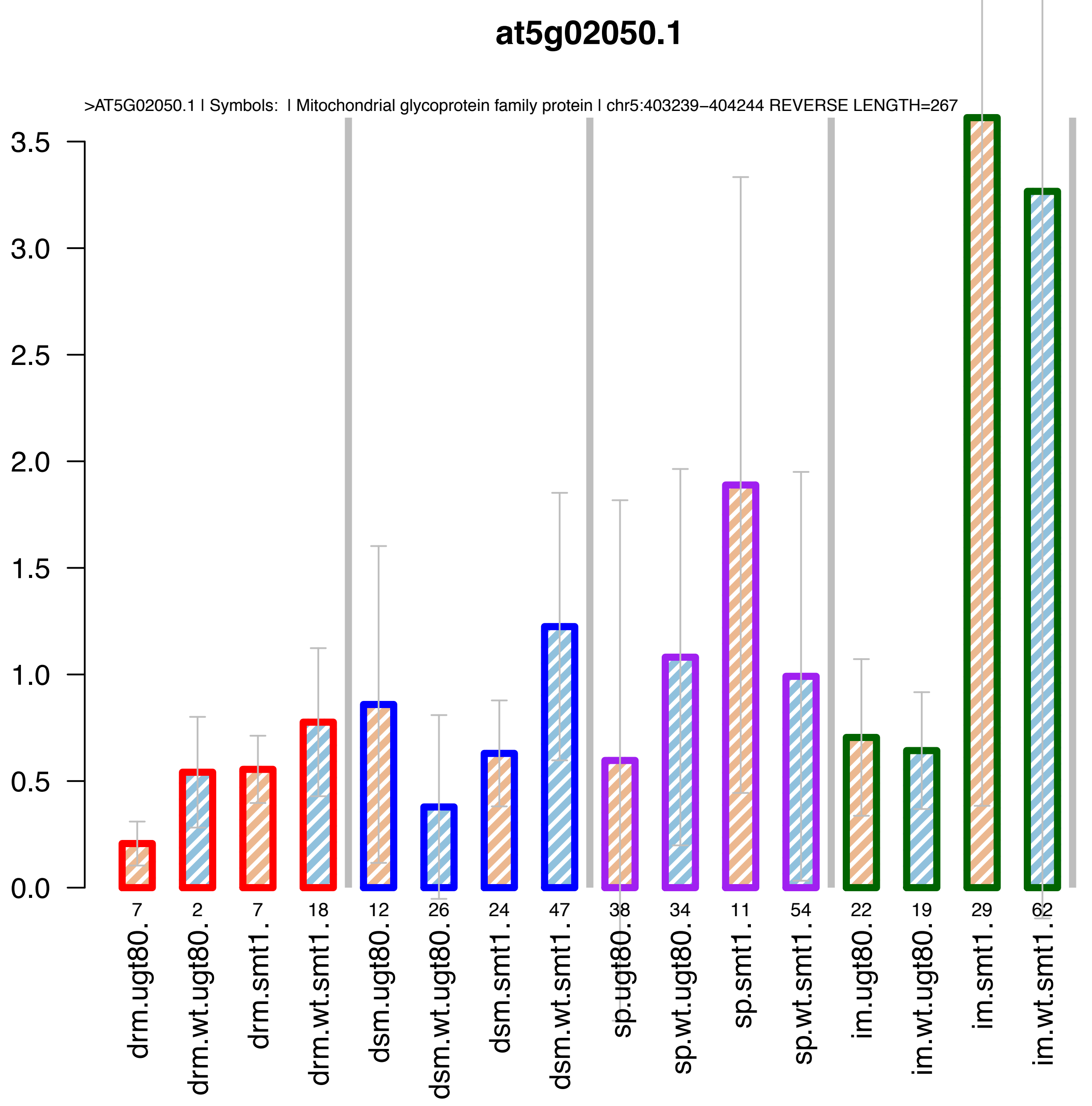

at5g02050.1
>AT5G02050.1 | Symbols: | Mitochondrial glycoprotein family protein | chr5:403239-404244 REVERSE LENGTH=267
0.0
0.5
1.0
1.5
2.0
2.5
3.0
3.5
7
2
7
18
12
26
24
47
38
34
11
54
22
19
29
62
drm.ugt80..
drm.wt.ugt80..
drm.smt1..
drm.wt.smt1..
dsm.ugt80..
dsm.wt.ugt80..
dsm.smt1..
dsm.wt.smt1..
sp.ugt80..
sp.wt.ugt80..
sp.smt1..
sp.wt.smt1..
im.ugt80..
im.wt.ugt80..
im.smt1..
im.wt.smt1..

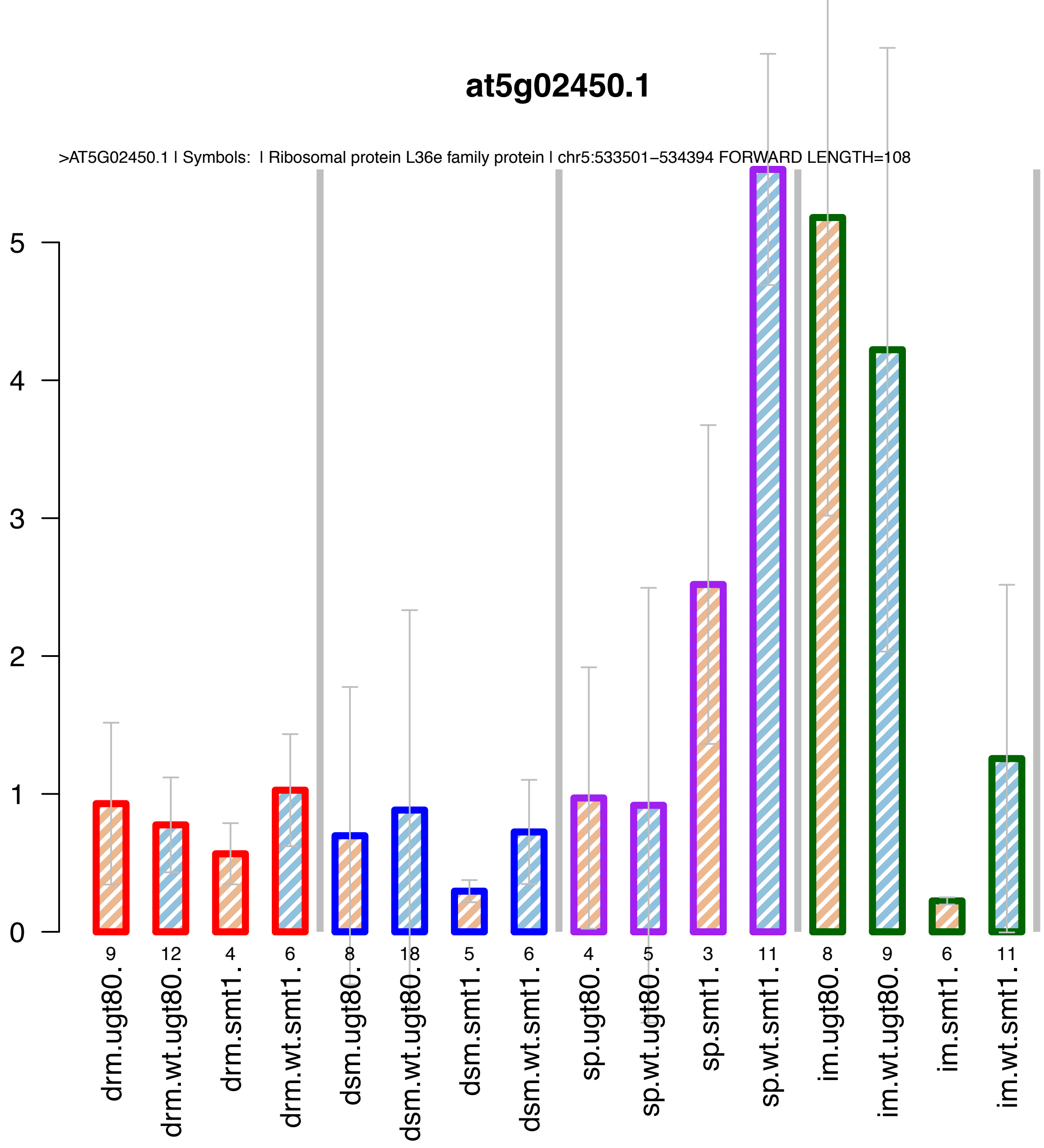

at5g02450.1
>AT5G02450.1 | Symbols: | Ribosomal protein L36e family protein | chr5:533501–534394 FORWARD LENGTH=108
0
1
2
3
4
5
9
12
4
6
8
18
5
6
4
5
3
11
8
9
6
11
drm.ugt80.
drm.wt.ugt80.
drm.smt1.
drm.wt.smt1.
dsm.ugt80.
dsm.wt.ugt80.
dsm.smt1.
dsm.wt.smt1.
sp.ugt80.
sp.wt.ugt80.
sp.smt1.
sp.wt.smt1.
im.ugt80.
im.wt.ugt80.
im.smt1.
im.wt.smt1.

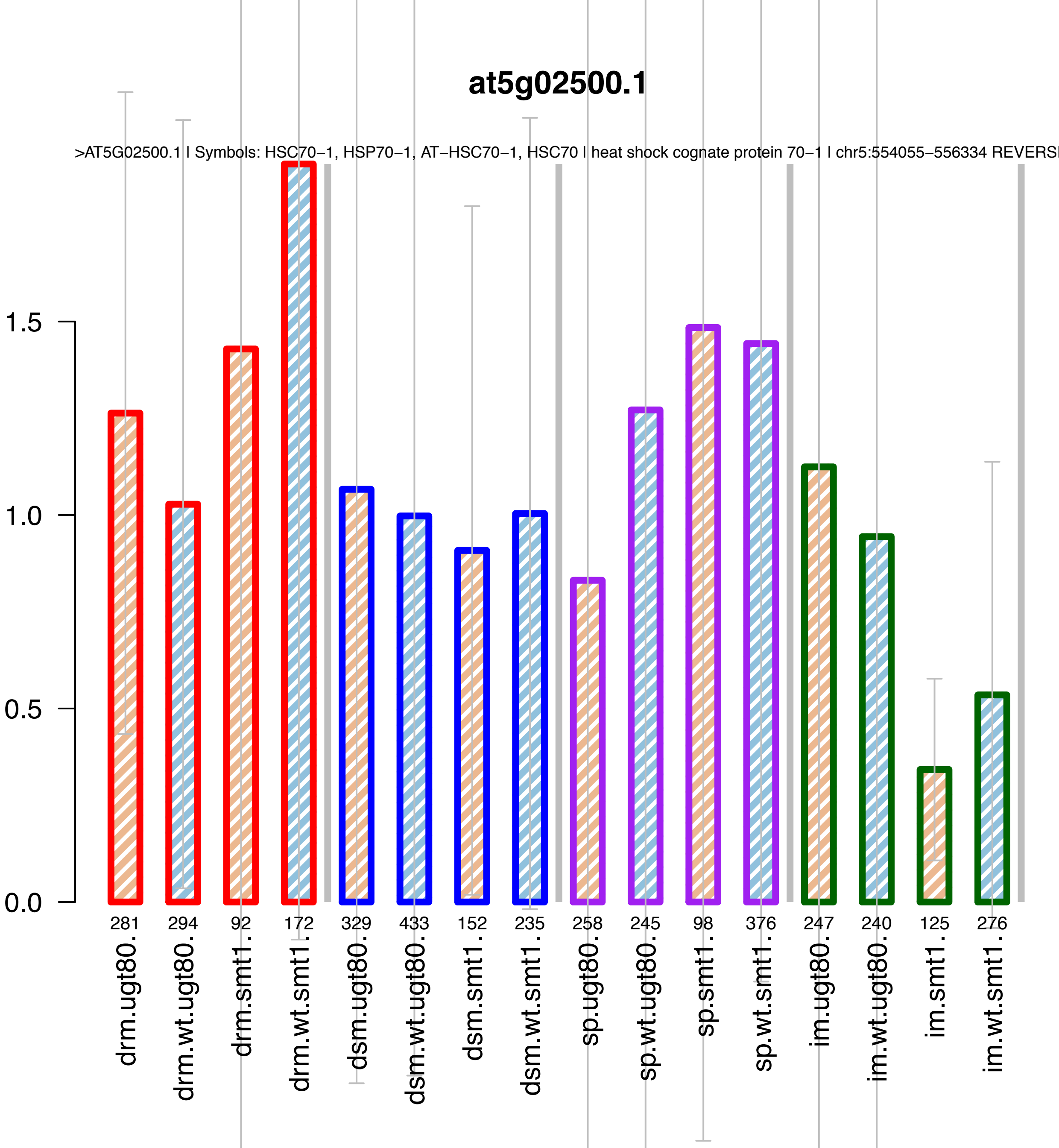
at5g02500.1
>AT5G02500.1 | Symbols: HSC70−1, HSP70−1, AT−HSC70−1, HSC70 | heat shock cognate protein 70−1 | chr5:554055−556334 REVERSE
0.0
0.5
1.0
1.5
281
294
92
172
329
433
152
235
258
245
98
376
247
240
125
276
drm.ugt80..
drm.wt.ugt80..
drm.smt1..
drm.wt.smt1..
dsm.ugt80..
dsm.wt.ugt80..
dsm.smt1..
dsm.wt.smt1..
sp.ugt80..
sp.wt.ugt80..
sp.smt1..
sp.wt.smt1..
im.ugt80..
im.wt.ugt80..
im.smt1..
im.wt.smt1..

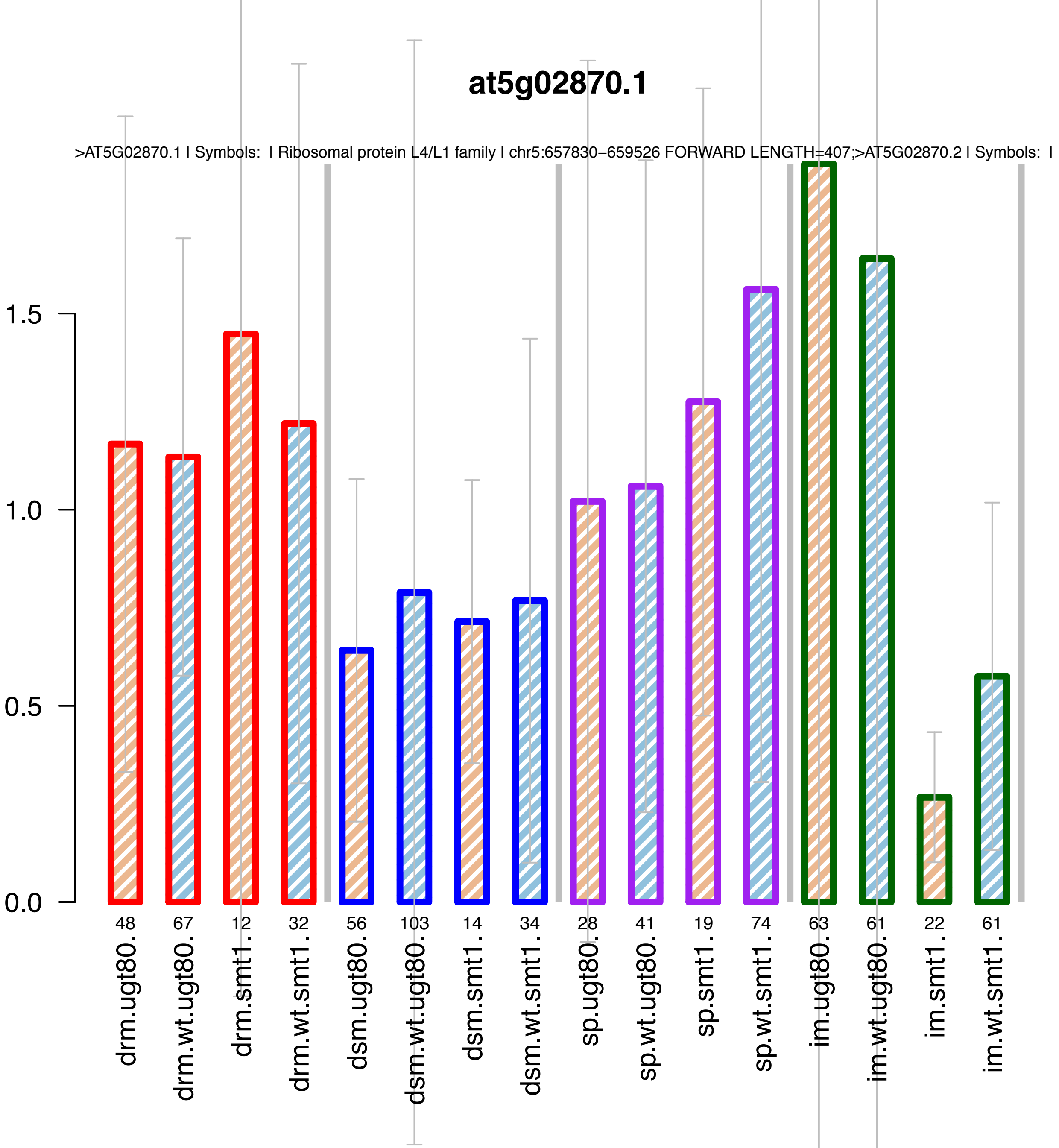

at5g02870.1
>AT5G02870.1 | Symbols: | Ribosomal protein L4/L1 family | chr5:657830-659526 FORWARD LENGTH=407;>AT5G02870.2 | Symbols: |
0.0
0.5
1.0
1.5
48
67
12
32
56
103
14
34
28
41
19
74
63
61
22
61
drm.ugt80..
drm.wt.ugt80..
drm.smt1..
drm.wt.smt1..
dsm.ugt80..
dsm.wt.ugt80..
dsm.smt1..
dsm.wt.smt1..
sp.ugt80..
sp.wt.ugt80..
sp.smt1..
sp.wt.smt1..
im.ugt80..
im.wt.ugt80..
im.smt1..
im.wt.smt1..

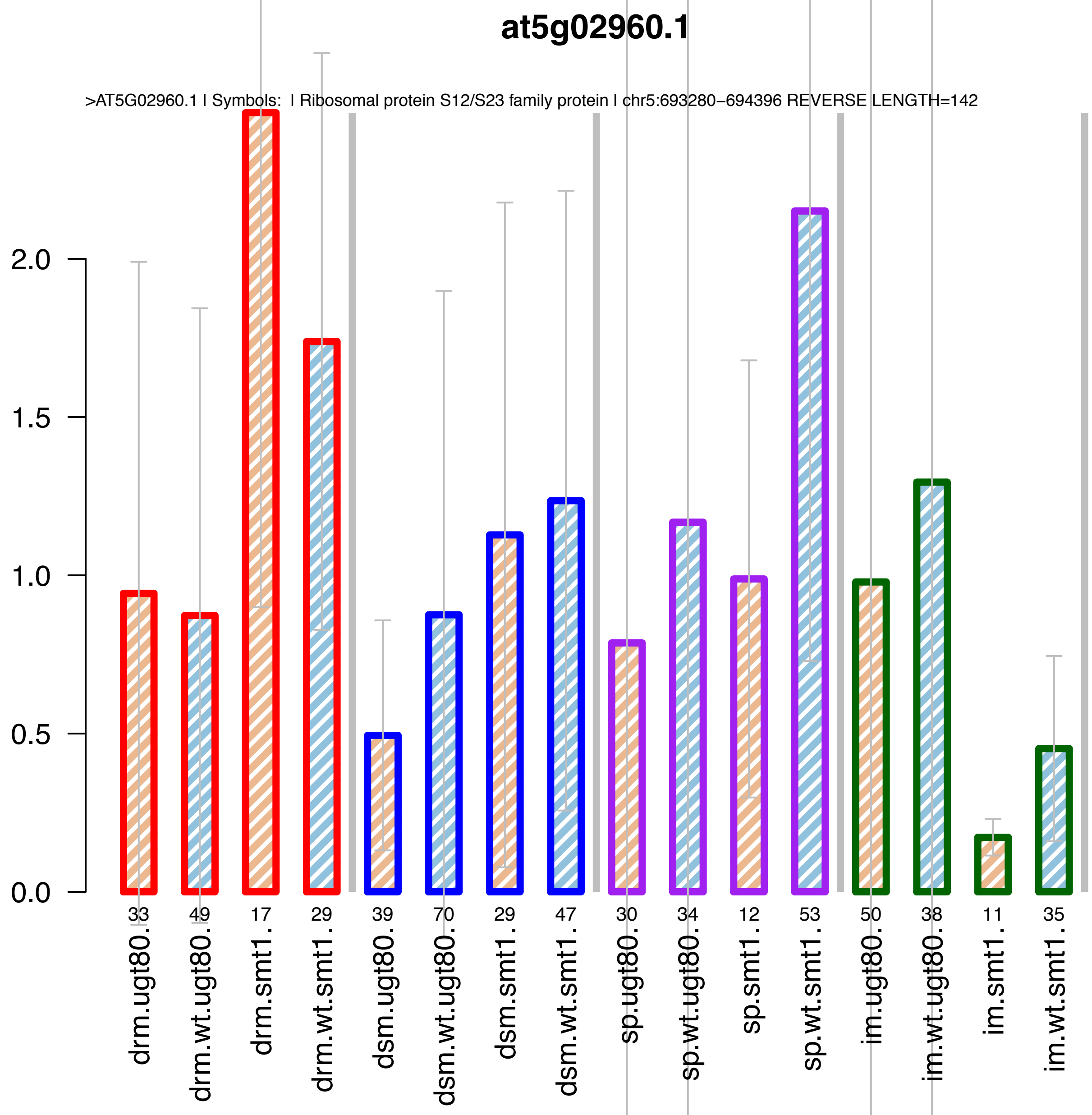

at5g02960.1
>AT5G02960.1 | Symbols: | Ribosomal protein S12/S23 family protein | chr5:693280-694396 REVERSE LENGTH=142
2.0
1.5
1.0
0.5
0.0
33
49
17
29
39
70
29
47
30
34
12
53
50
38
11
35
drm.ugt80..
drm.wt.ugt80..
drm.smt1..
drm.wt.smt1..
dsm.ugt80..
dsm.wt.ugt80..
dsm.smt1..
dsm.wt.smt1..
sp.ugt80..
sp.wt.ugt80..
sp.smt1..
sp.wt.smt1..
im.ugt80..
im.wt.ugt80..
im.smt1..
im.wt.smt1..

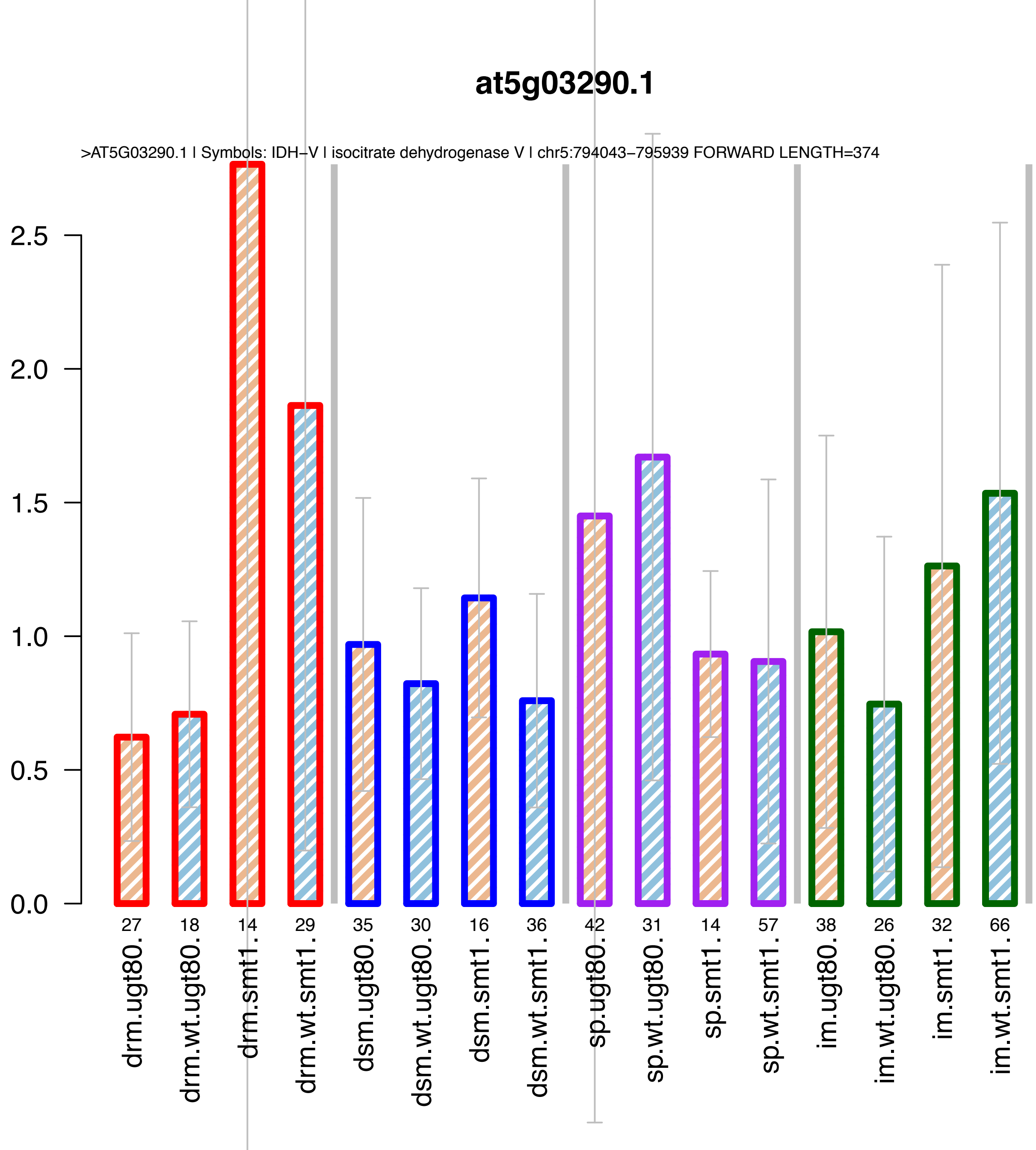

at5g03290.1
>AT5G03290.1 | Symbols: IDH−V | isocitrate dehydrogenase V | chr5:794043−795939 FORWARD LENGTH=374
0.0
0.5
1.0
1.5
2.0
2.5
27 18 14 29 35 30 16 36 42 31 14 57 38 26 32 66
drm.ugt80..
drm.wt.ugt80..
drm.smt1..
drm.wt.smt1..
dsm.ugt80..
dsm.wt.ugt80..
dsm.smt1..
dsm.wt.smt1..
sp.ugt80..
sp.wt.ugt80..
sp.smt1..
sp.wt.smt1..
im.ugt80..
im.wt.ugt80..
im.smt1..
im.wt.smt1..

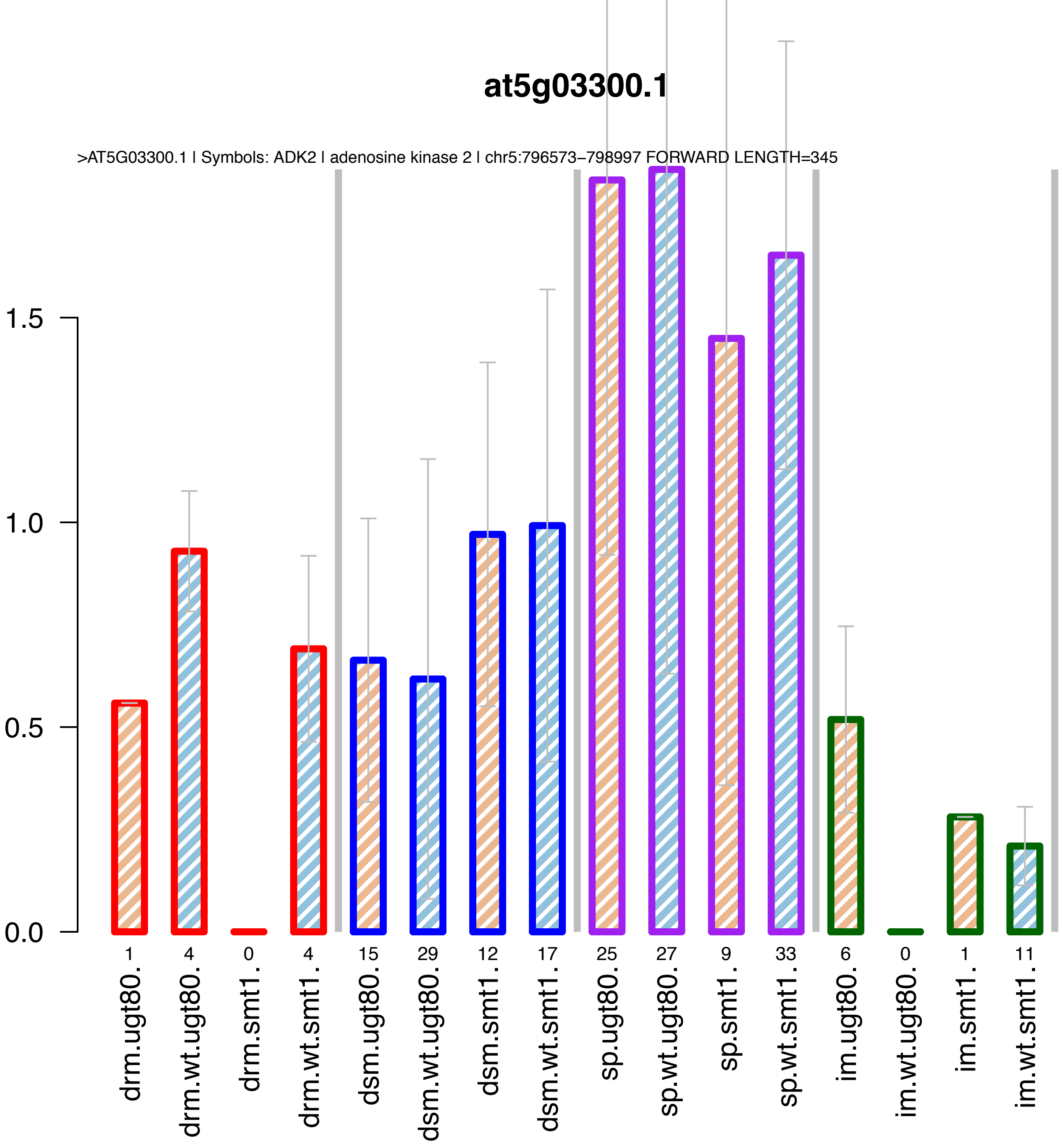

at5g03300.1
>AT5G03300.1 | Symbols: ADK2 | adenosine kinase 2 | chr5:796573−798997 FORWARD LENGTH=345
0.0
0.5
1.0
1.5
1
4
0
4
15
29
12
17
25
27
9
33
6
0
1
11
drm.ugt80.
drm.wt.ugt80.
drm.smt1.
drm.wt.smt1.
dsm.ugt80.
dsm.wt.ugt80.
dsm.smt1.
dsm.wt.smt1.
sp.ugt80.
sp.wt.ugt80.
sp.smt1.
sp.wt.smt1.
im.ugt80.
im.wt.ugt80.
im.smt1.
im.wt.smt1.

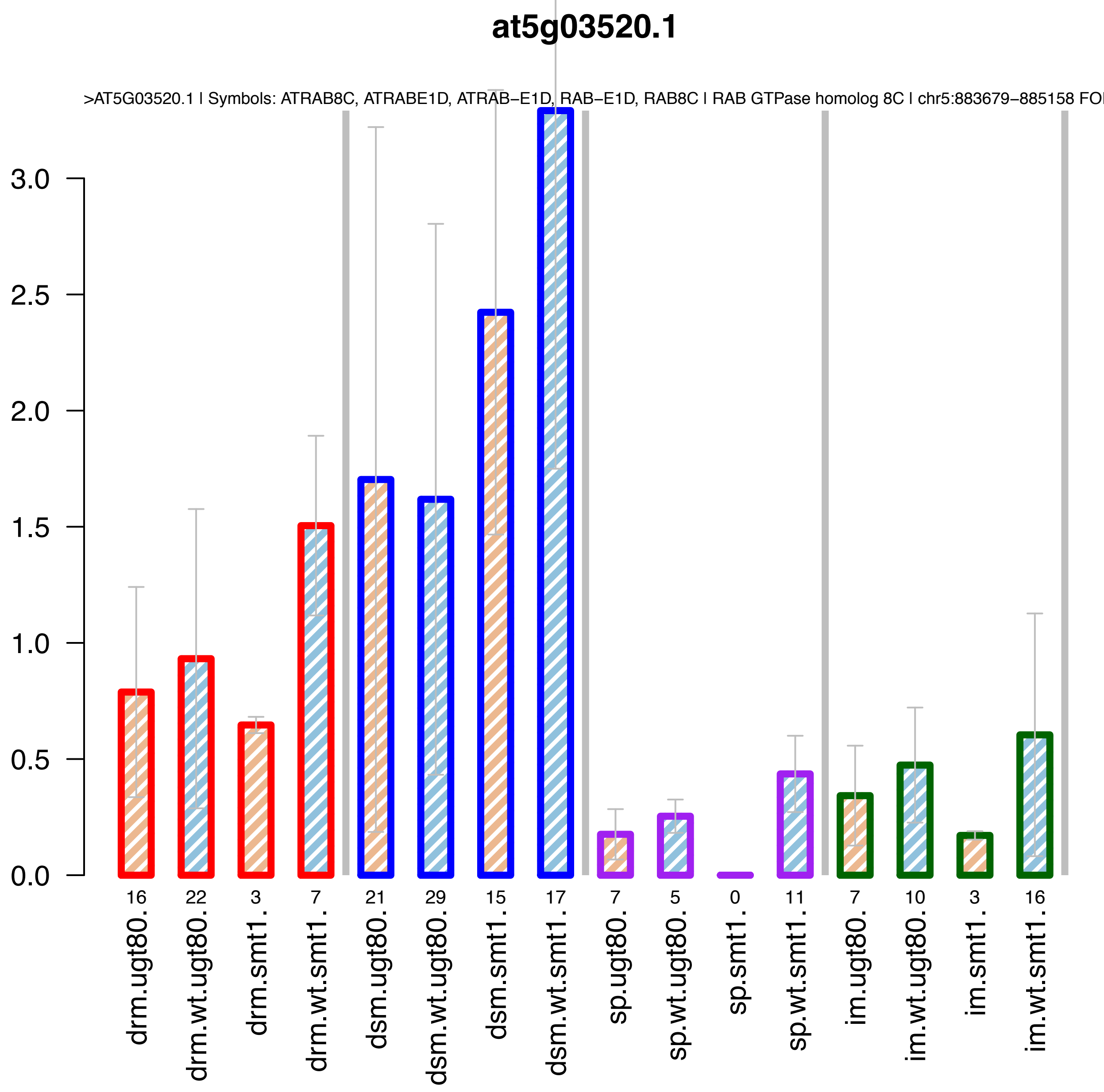

at5g03520.1
>AT5G03520.1 | Symbols: ATRAB8C, ATRABE1D, ATRAB−E1D, RAB−E1D, RAB8C | RAB GTPase homolog 8C | chr5:883679−885158 FO
3.0
2.5
2.0
1.5
1.0
0.5
0.0
16
22
3
7
21
29
15
17
7
5
0
11
7
10
3
16
drm.ugt80.
drm.wt.ugt80.
drm.smt1.
drm.wt.smt1.
dsm.ugt80.
dsm.wt.ugt80.
dsm.smt1.
dsm.wt.smt1.
sp.ugt80.
sp.wt.ugt80.
sp.smt1.
sp.wt.smt1.
im.ugt80.
im.wt.ugt80.
im.smt1.
im.wt.smt1.

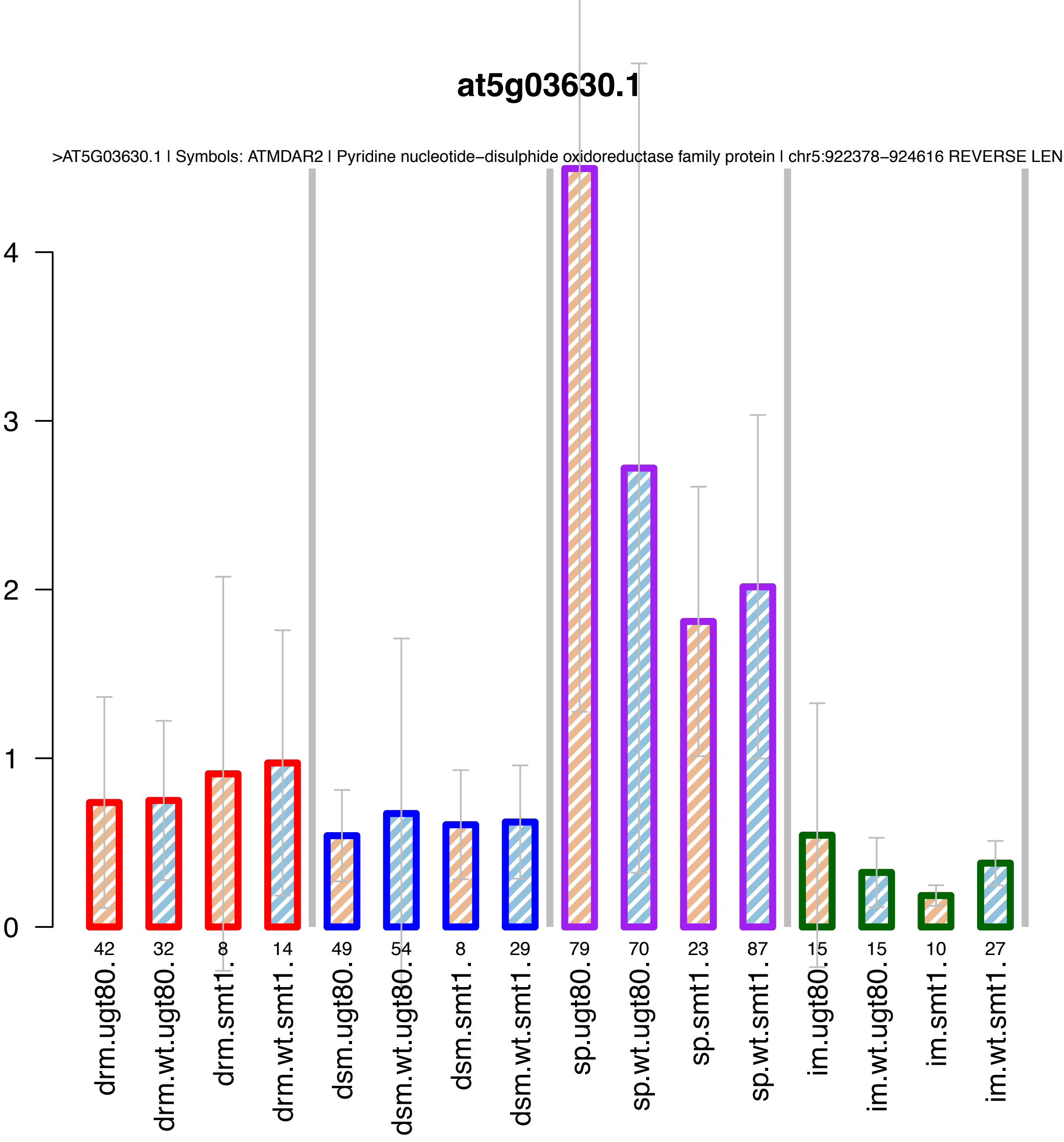

at5g03630.1
>AT5G03630.1 | Symbols: ATMDAR2 | Pyridine nucleotide−disulphide oxidoreductase family protein | chr5:922378−924616 REVERSE LEN
4
3
2
1
0
42
32
8
14
49
54
8
29
79
70
23
87
15
15
10
27
drm.ugt80..
drm.wt.ugt80..
drm.smt1..
drm.wt.smt1..
dsm.ugt80..
dsm.wt.ugt80..
dsm.smt1..
dsm.wt.smt1..
sp.ugt80..
sp.wt.ugt80..
sp.smt1..
sp.wt.smt1..
im.ugt80..
im.wt.ugt80..
im.smt1..
im.wt.smt1..

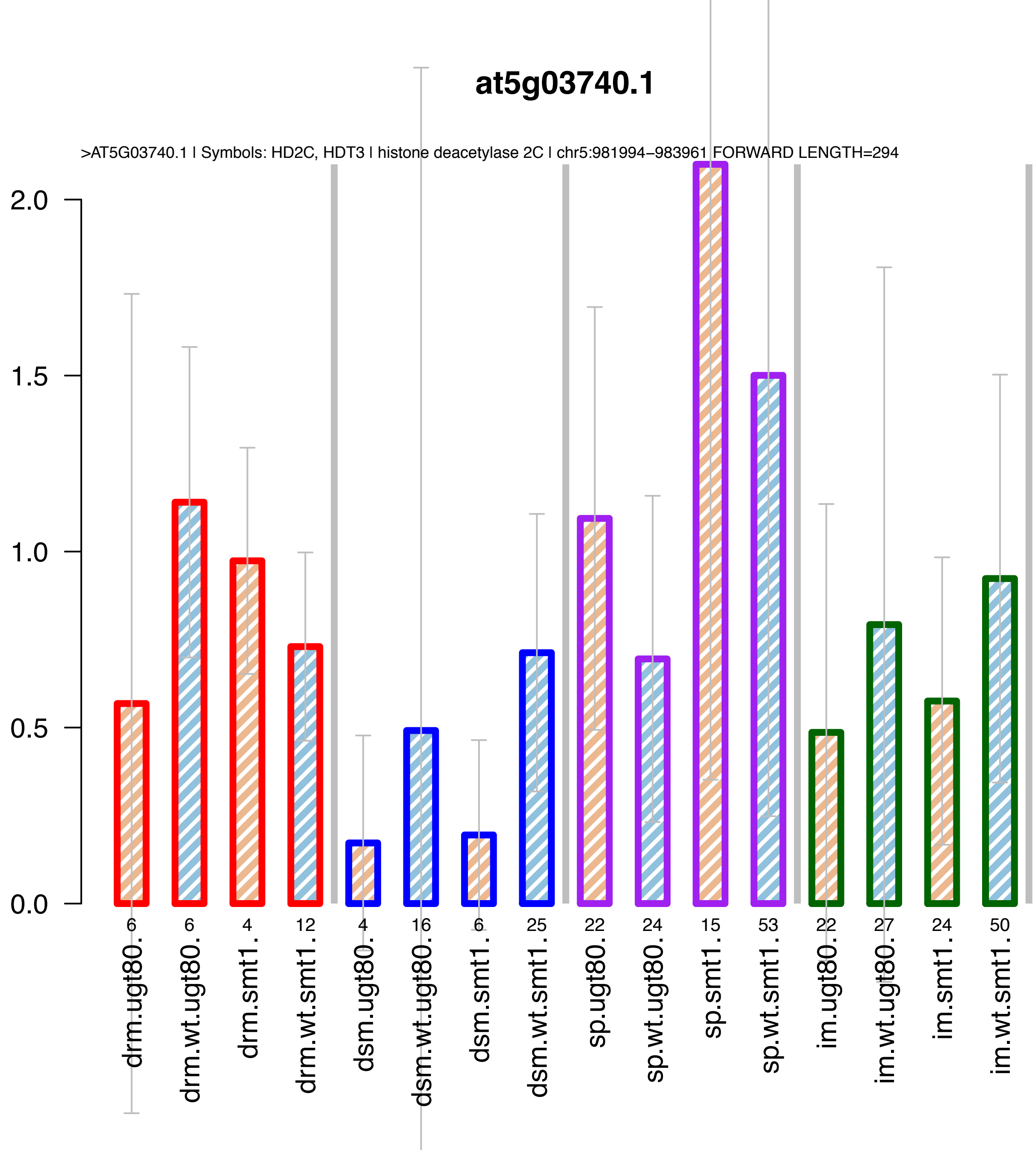

at5g03740.1
>AT5G03740.1 | Symbols: HD2C, HDT3 | histone deacetylase 2C | chr5:981994-983961 FORWARD LENGTH=294
2.0
1.5
1.0
0.5
0.0
6
6
4
12
4
16
6
25
22
24
15
53
22
27
24
50
drm.ugt80..
drm.wt.ugt80..
drm.smt1..
drm.wt.smt1..
dsm.ugt80..
dsm.wt.ugt80..
dsm.smt1..
dsm.wt.smt1..
sp.ugt80..
sp.wt.ugt80..
sp.smt1..
sp.wt.smt1..
im.ugt80..
im.wt.ugt80..
im.smt1..
im.wt.smt1..

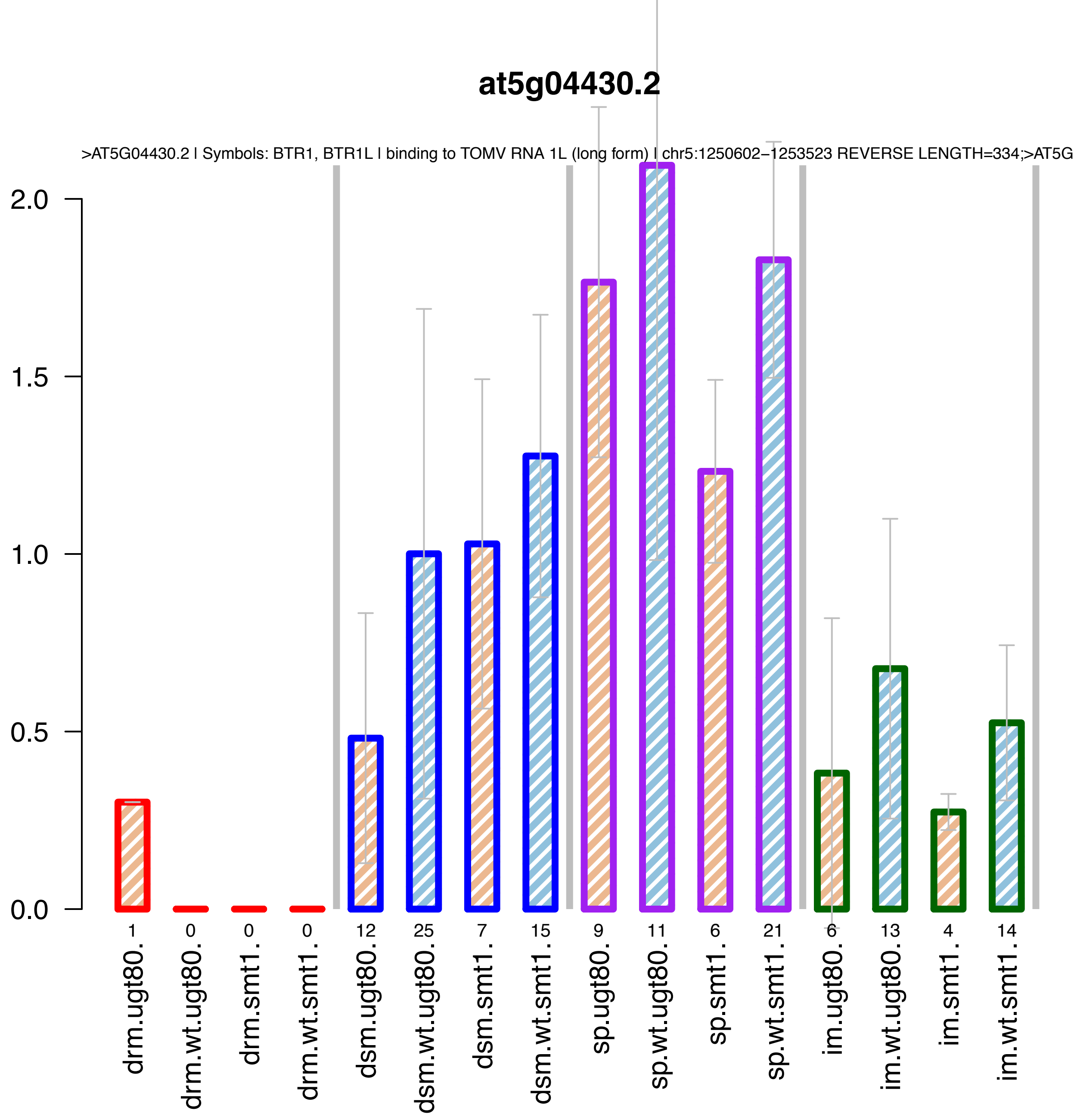

at5g04430.2
>AT5G04430.2 | Symbols: BTR1, BTR1L | binding to TOMV RNA 1L (long form) | chr5:1250602–1253523 REVERSE LENGTH=334;>AT5G
2.0
1.5
1.0
0.5
0.0
1
0
0
0
12
25
7
15
9
11
6
21
6
13
4
14
drm.ugt80.
drm.wt.ugt80.
drm.smt1.
drm.wt.smt1.
dsm.ugt80.
dsm.wt.ugt80.
dsm.smt1.
dsm.wt.smt1.
sp.ugt80.
sp.wt.ugt80.
sp.smt1.
sp.wt.smt1.
im.ugt80.
im.wt.ugt80.
im.smt1.
im.wt.smt1.

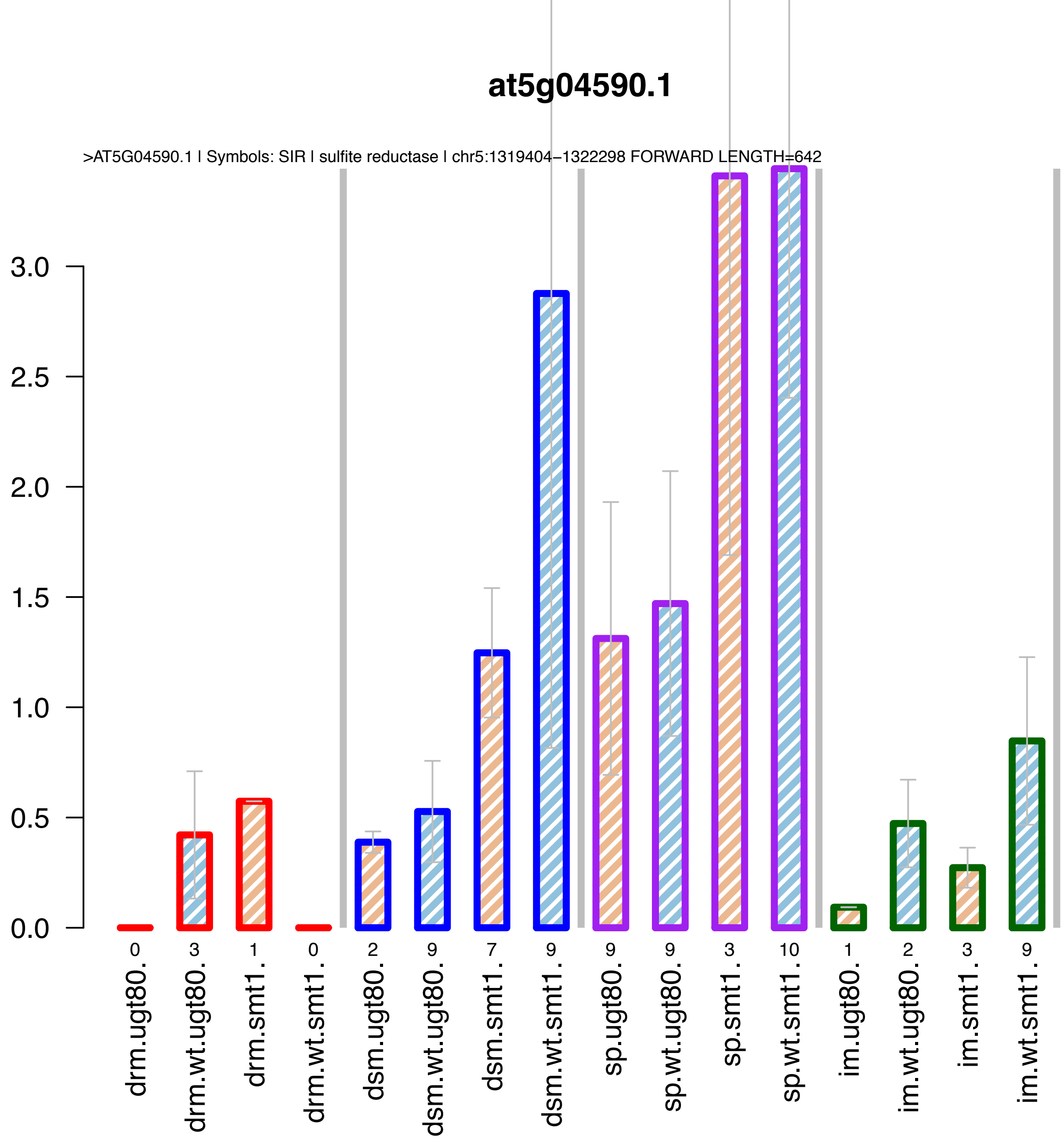

at5g04590.1
>AT5G04590.1 | Symbols: SIR | sulfite reductase | chr5:1319404–1322298 FORWARD LENGTH=642
3.0
2.5
2.0
1.5
1.0
0.5
0.0
0
3
1
0
2
9
7
9
9
9
3
10
1
2
3
9
drm.ugt80.
drm.wt.ugt80.
drm.smt1.
drm.wt.smt1.
dsm.ugt80.
dsm.wt.ugt80.
dsm.smt1.
dsm.wt.smt1.
sp.ugt80.
sp.wt.ugt80.
sp.smt1.
sp.wt.smt1.
im.ugt80.
im.wt.ugt80.
im.smt1.
im.wt.smt1.

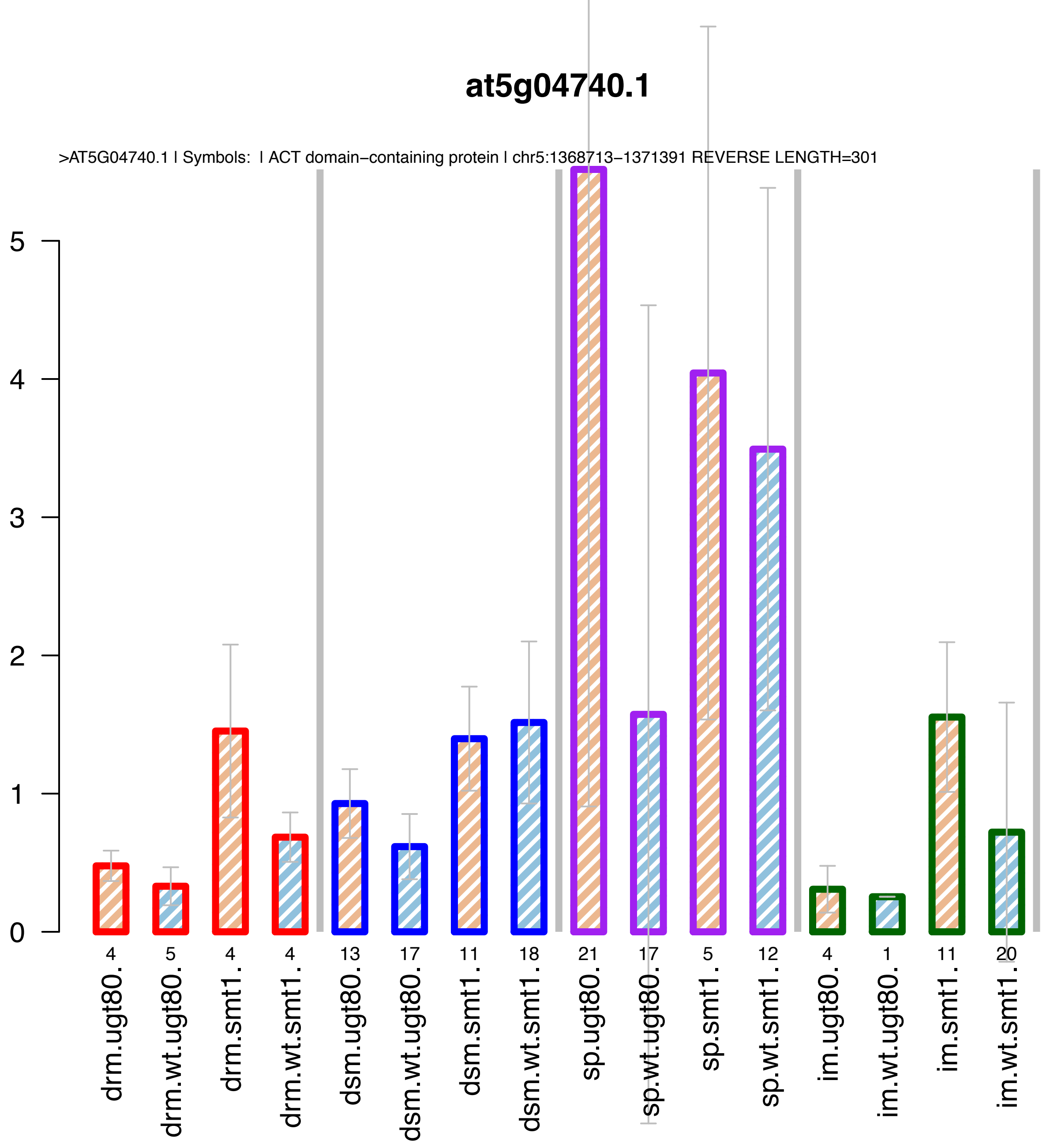

at5g04740.1
>AT5G04740.1 | Symbols: | ACT domain-containing protein | chr5:1368713-1371391 REVERSE LENGTH=301
0
1
2
3
4
5
4
5
4
4
13
17
11
18
21
17
5
12
4
1
11
20
drm.ugt80..
drm.wt.ugt80..
drm.smt1..
drm.wt.smt1..
dsm.ugt80..
dsm.wt.ugt80..
dsm.smt1..
dsm.wt.smt1..
sp.ugt80..
sp.wt.ugt80..
sp.smt1..
sp.wt.smt1..
im.ugt80..
im.wt.ugt80..
im.smt1..
im.wt.smt1..

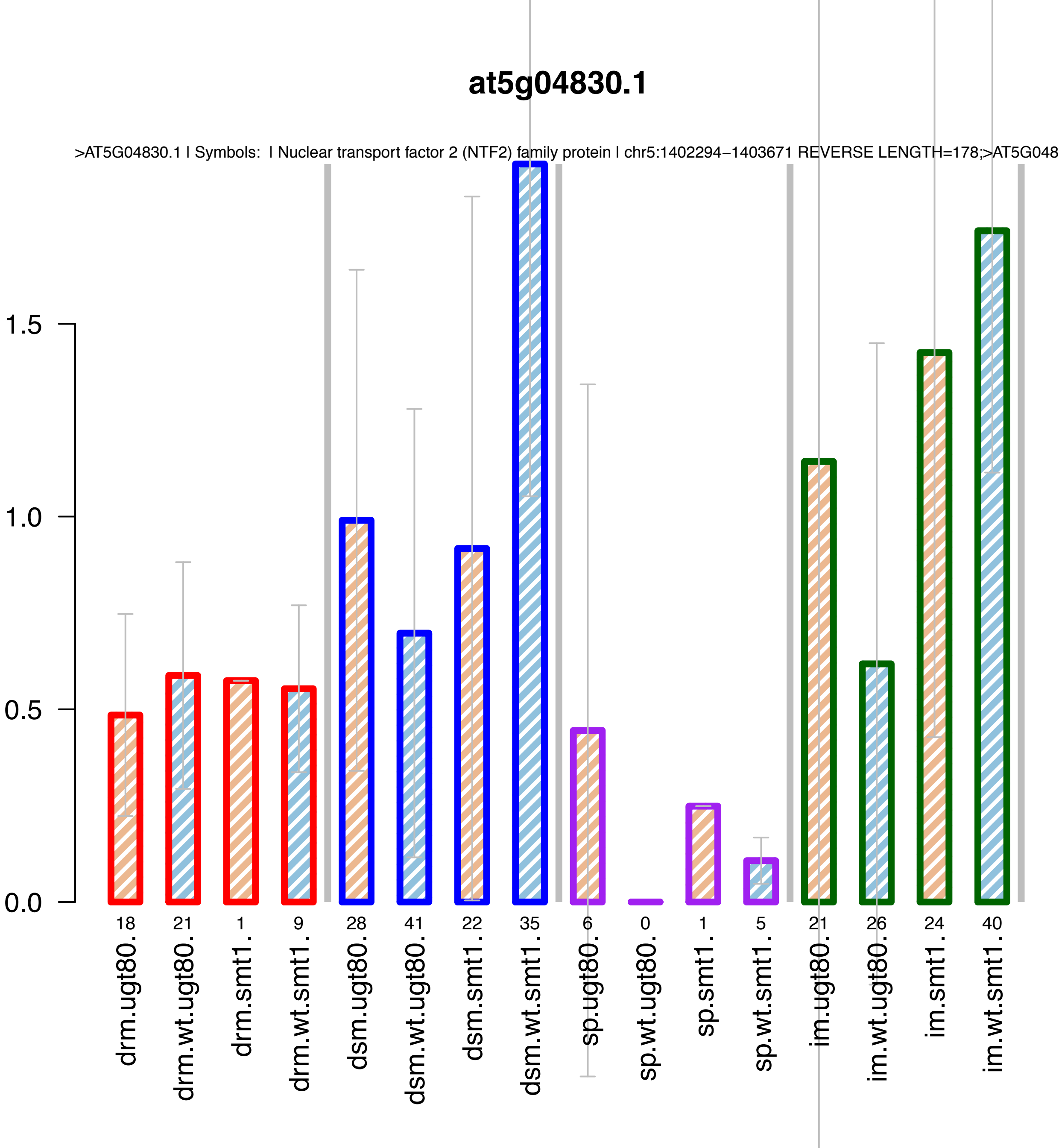

at5g04830.1
>AT5G04830.1 | Symbols: | Nuclear transport factor 2 (NTF2) family protein | chr5:1402294-1403671 REVERSE LENGTH=178;>AT5G048
1.5
1.0
0.5
0.0
18
21
1
9
28
41
22
35
6
0
1
5
21
26
24
40
drm.ugt80..
drm.wt.ugt80..
drm.smt1..
drm.wt.smt1..
dsm.ugt80..
dsm.wt.ugt80..
dsm.smt1..
dsm.wt.smt1..
sp.ugt80..
sp.wt.ugt80..
sp.smt1..
sp.wt.smt1..
im.ugt80..
im.wt.ugt80..
im.smt1..
im.wt.smt1..

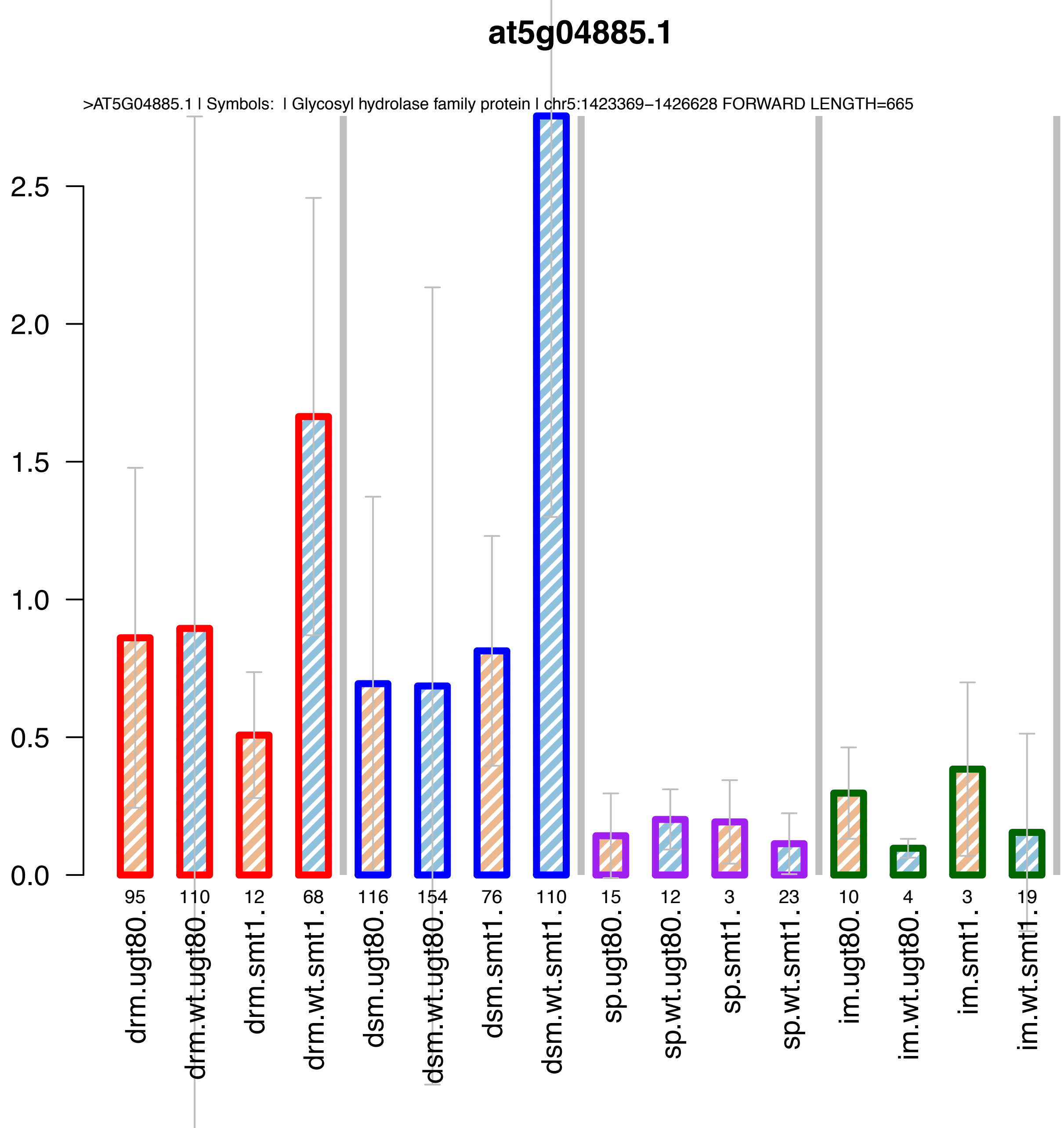
at5g04885.1
>AT5G04885.1 | Symbols: | Glycosyl hydrolase family protein | chr5:1423369-1426628 FORWARD LENGTH=665
0.0
0.5
1.0
1.5
2.0
2.5
95
110
12
68
116
154
76
110
15
12
3
23
10
4
3
19
drm.ugt80..
drm.wt.ugt80..
drm.smt1..
drm.wt.smt1..
dsm.ugt80..
dsm.wt.ugt80..
dsm.smt1..
dsm.wt.smt1..
sp.ugt80..
sp.wt.ugt80..
sp.smt1..
sp.wt.smt1..
im.ugt80..
im.wt.ugt80..
im.smt1..
im.wt.smt1..

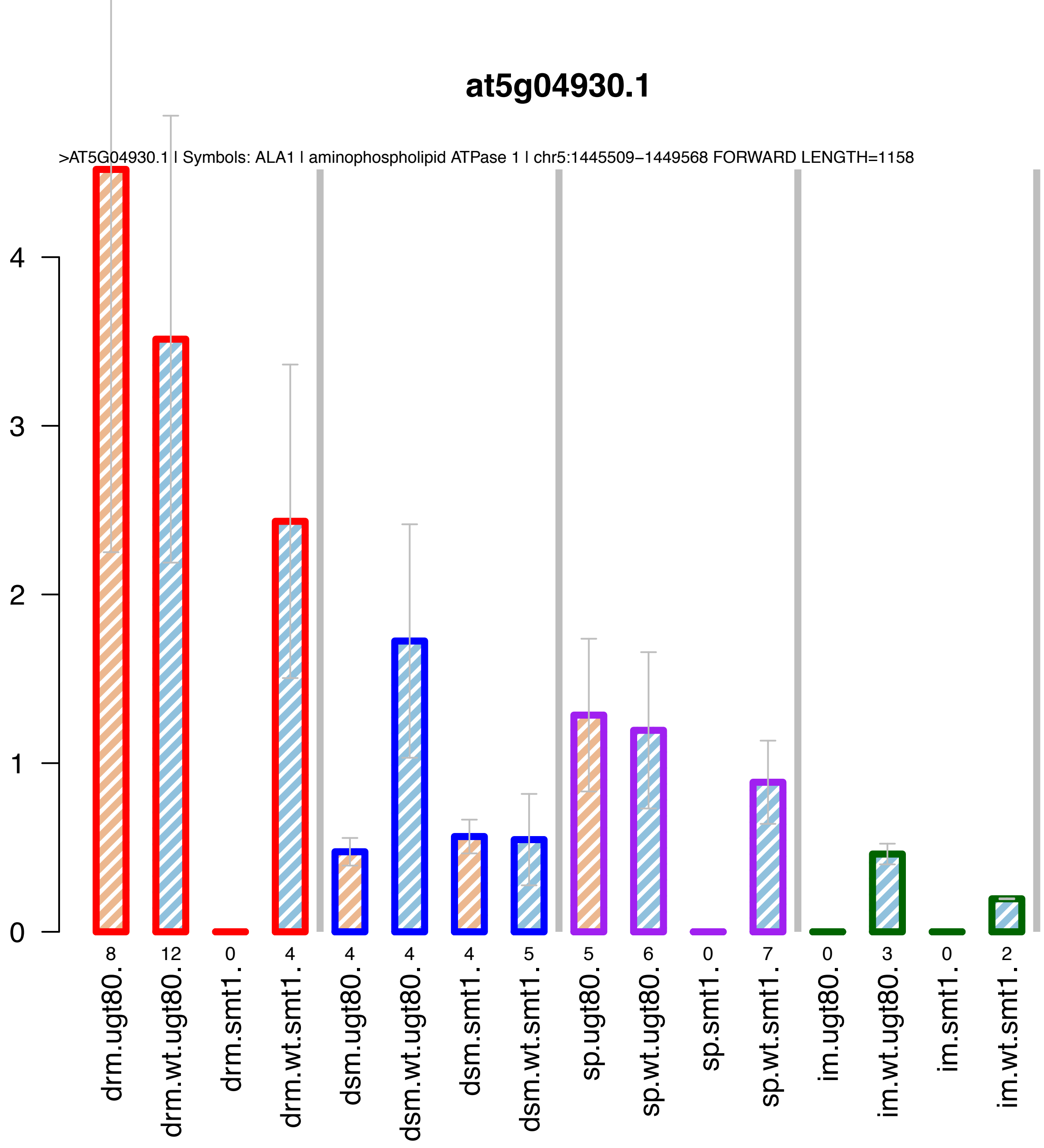

at5g04930.1
>AT5G04930.1 | Symbols: ALA1 | aminophospholipid ATPase 1 | chr5:1445509−1449568 FORWARD LENGTH=1158
0
1
2
3
4
8
12
0
4
4
4
4
5
5
6
0
7
0
3
0
2
drm.ugt80.
drm.wt.ugt80.
drm.smt1.
drm.wt.smt1.
dsm.ugt80.
dsm.wt.ugt80.
dsm.smt1.
dsm.wt.smt1.
sp.ugt80.
sp.wt.ugt80.
sp.smt1.
sp.wt.smt1.
im.ugt80.
im.wt.ugt80.
im.smt1.
im.wt.smt1.

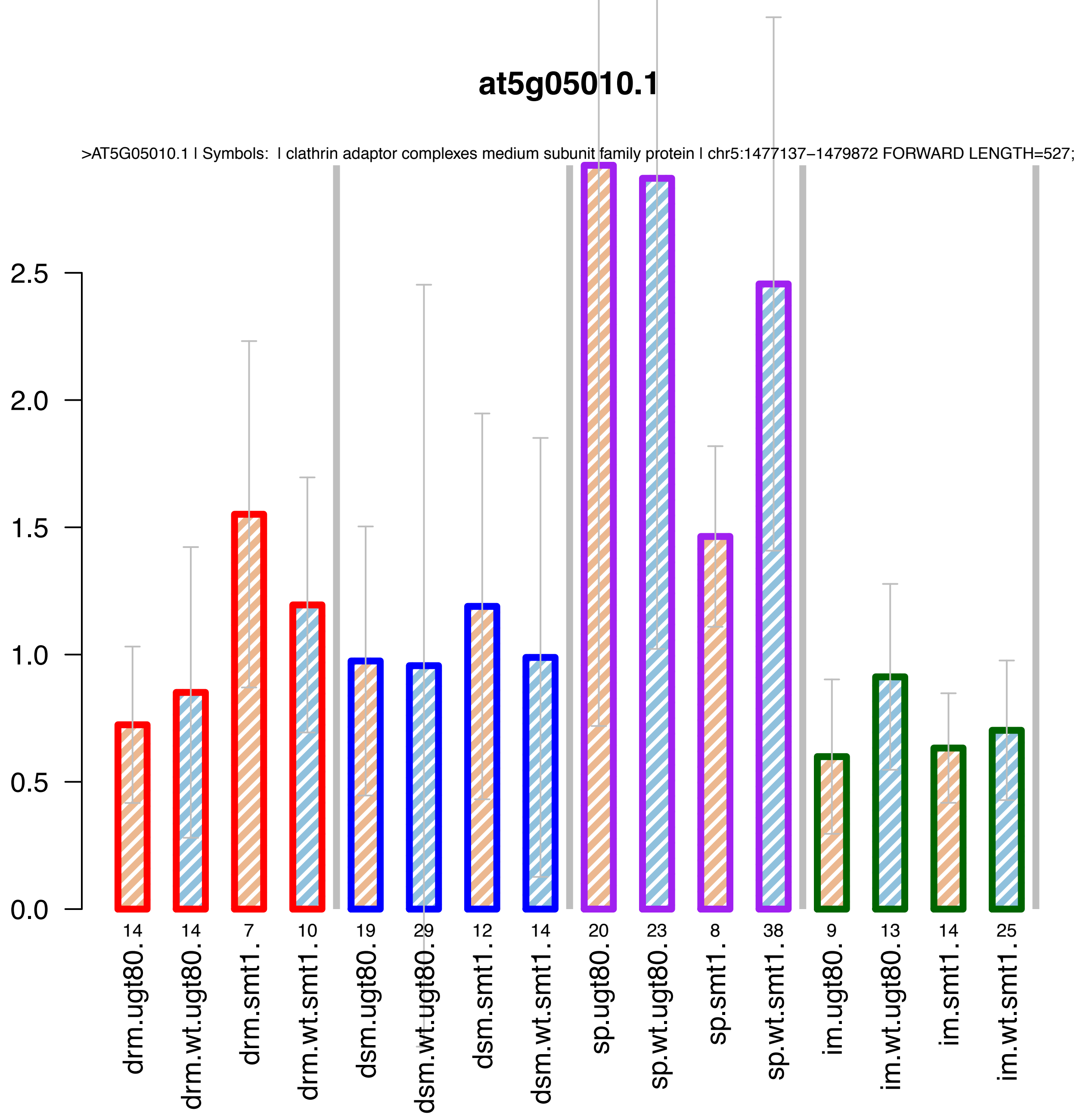

at5g05010.1
>AT5G05010.1 | Symbols: | clathrin adaptor complexes medium subunit family protein | chr5:1477137−1479872 FORWARD LENGTH=527;
0.0
0.5
1.0
1.5
2.0
2.5
14
14
7
10
19
29
12
14
20
23
8
38
9
13
14
25
drm.ugt80..
drm.wt.ugt80..
drm.smt1..
drm.wt.smt1..
dsm.ugt80..
dsm.wt.ugt80..
dsm.smt1..
dsm.wt.smt1..
sp.ugt80..
sp.wt.ugt80..
sp.smt1..
sp.wt.smt1..
im.ugt80..
im.wt.ugt80..
im.smt1..
im.wt.smt1..

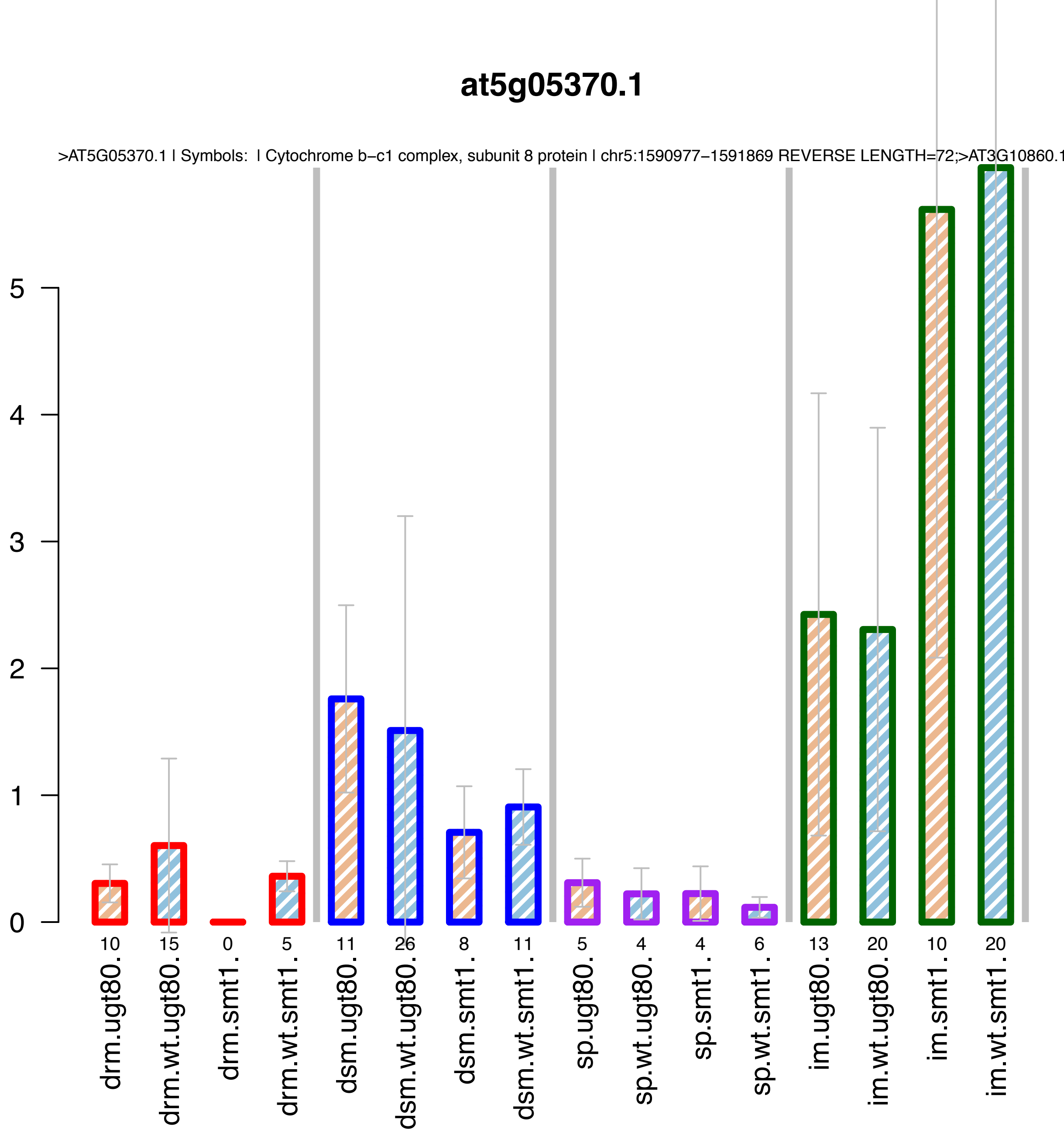

at5g05370.1
>AT5G05370.1 | Symbols: | Cytochrome b−c1 complex, subunit 8 protein | chr5:1590977−1591869 REVERSE LENGTH=72;>AT3G10860.1
0
1
2
3
4
5
10
15
0
5
11
26
8
11
5
4
4
6
13
20
10
20
drm.ugt80.
drm.wt.ugt80.
drm.smt1.
drm.wt.smt1.
dsm.ugt80.
dsm.wt.ugt80.
dsm.smt1.
dsm.wt.smt1.
sp.ugt80.
sp.wt.ugt80.
sp.smt1.
sp.wt.smt1.
im.ugt80.
im.wt.ugt80.
im.smt1.
im.wt.smt1.

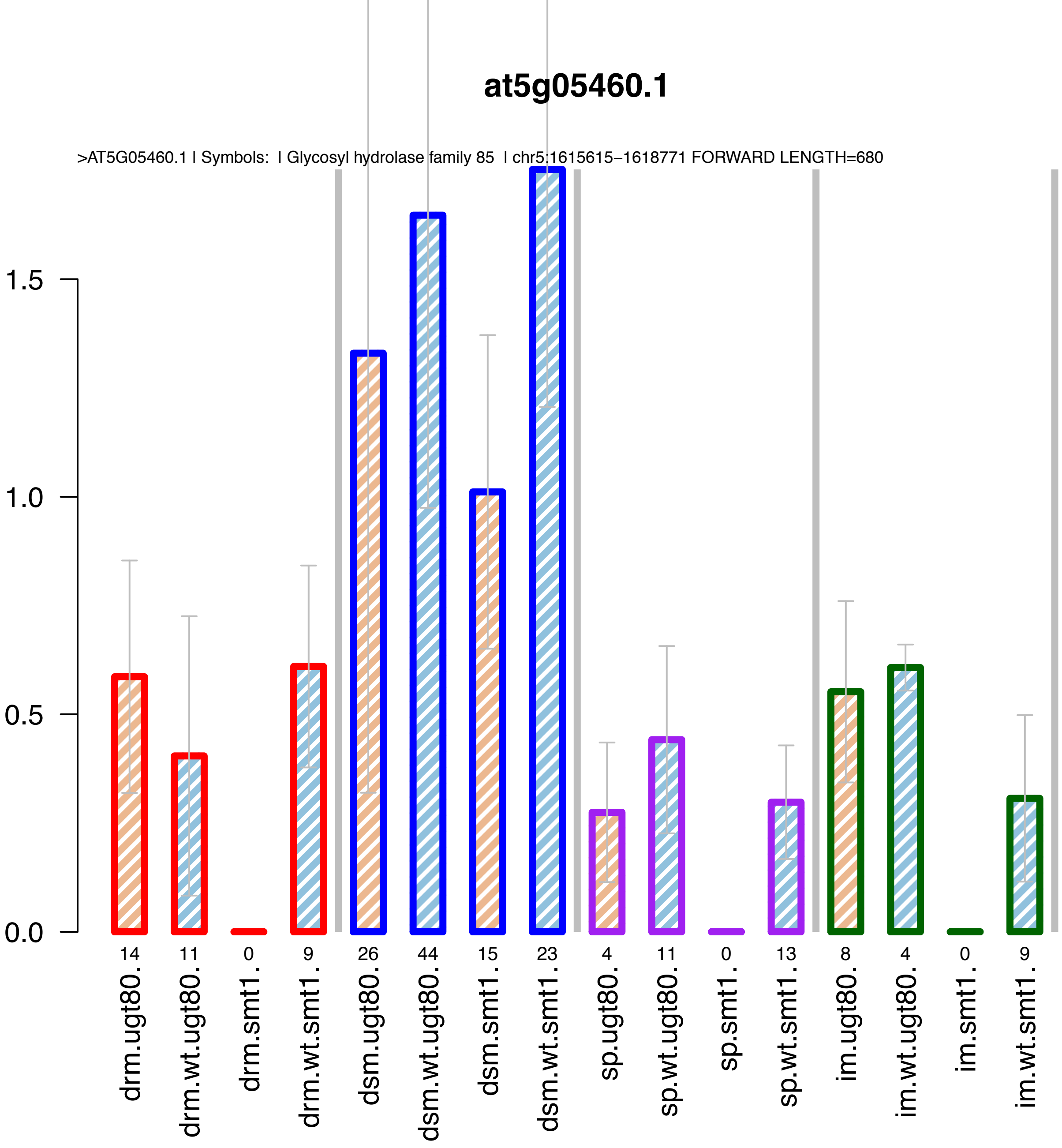
at5g05460.1
>AT5G05460.1 | Symbols: | Glycosyl hydrolase family 85 | chr5:1615615-1618771 FORWARD LENGTH=680
0.0
0.5
1.0
1.5
14
11
0
9
26
44
15
23
4
11
0
13
8
4
0
9
drm.ugt80.
drm.wt.ugt80.
drm.smt1.
drm.wt.smt1.
dsm.ugt80.
dsm.wt.ugt80.
dsm.smt1.
dsm.wt.smt1.
sp.ugt80.
sp.wt.ugt80.
sp.smt1.
sp.wt.smt1.
im.ugt80.
im.wt.ugt80.
im.smt1.
im.wt.smt1.

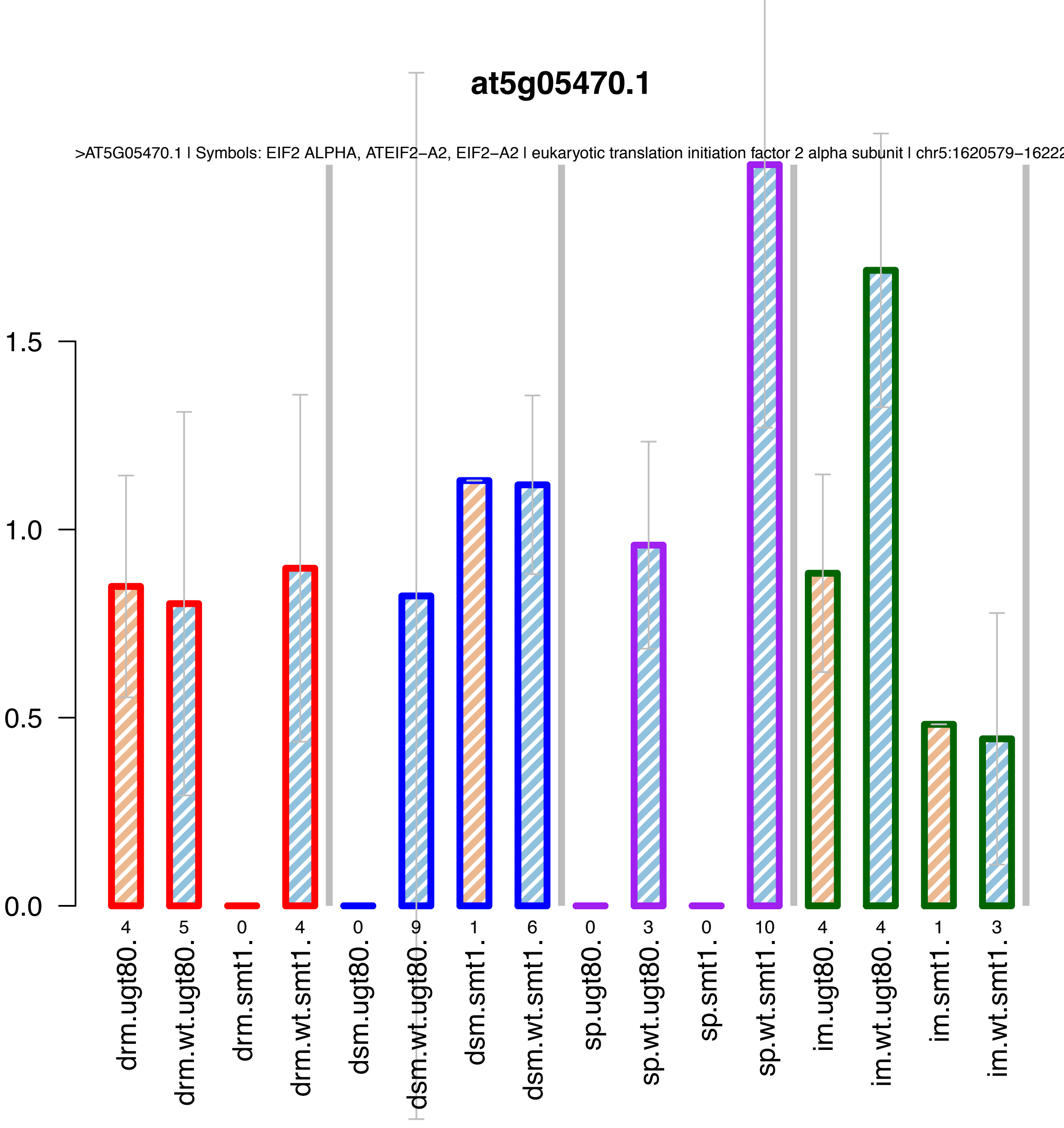

at5g05470.1
>AT5G05470.1 | Symbols: EIF2 ALPHA, ATEIF2−A2, EIF2−A2 | eukaryotic translation initiation factor 2 alpha subunit | chr5:1620579−16222
0.0
0.5
1.0
1.5
4
5
0
4
0
9
1
6
0
3
0
10
4
4
1
3
drm.ugt80..
drm.wt.ugt80..
drm.smt1..
drm.wt.smt1..
dsm.ugt80..
dsm.wt.ugt80..
dsm.smt1..
dsm.wt.smt1..
sp.ugt80..
sp.wt.ugt80..
sp.smt1..
sp.wt.smt1..
im.ugt80..
im.wt.ugt80..
im.smt1..
im.wt.smt1..

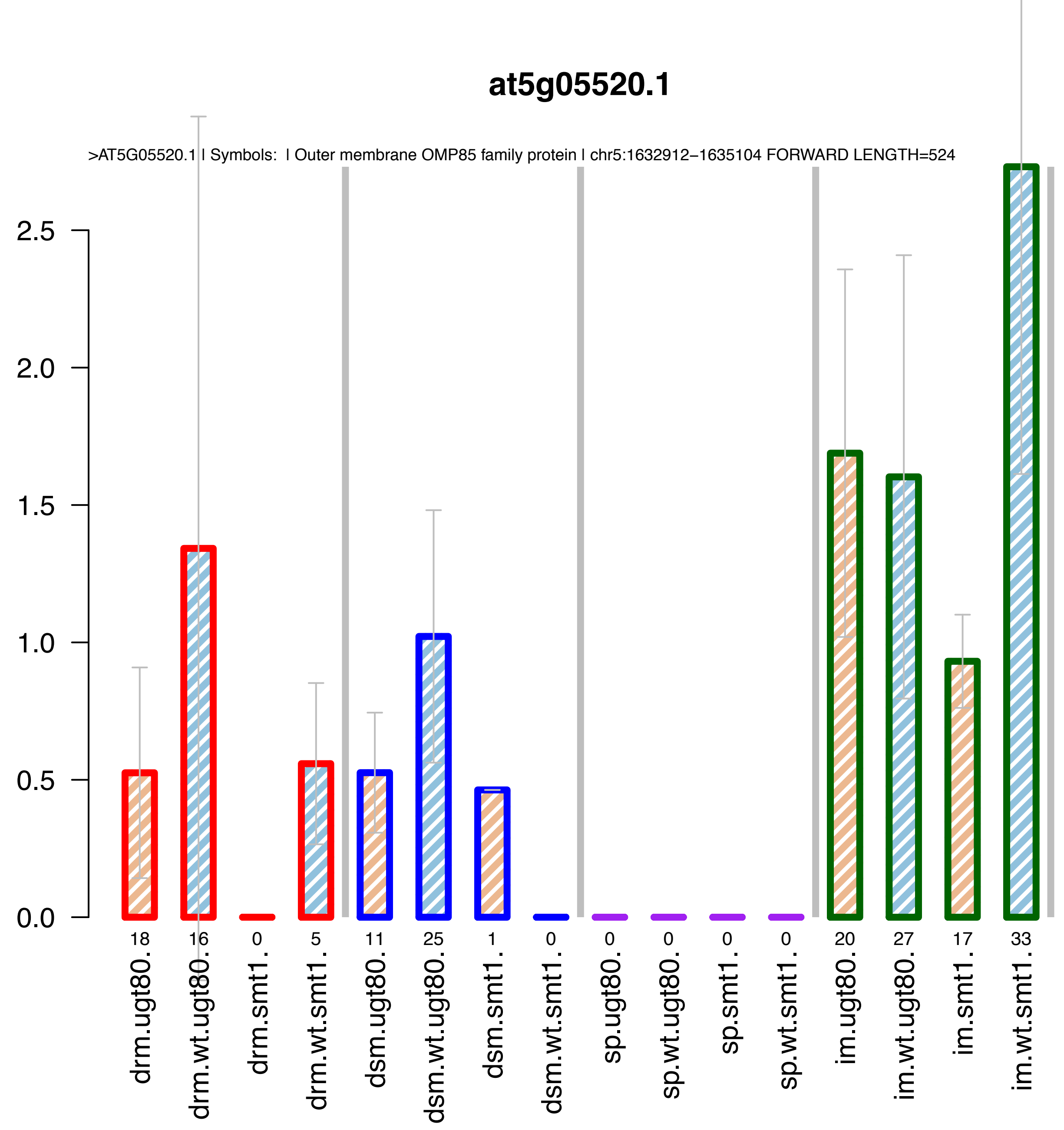
at5g05520.1
>AT5G05520.1 | Symbols: | Outer membrane OMP85 family protein | chr5:1632912-1635104 FORWARD LENGTH=524
0.0
0.5
1.0
1.5
2.0
2.5
18
16
0
5
11
25
1
0
0
0
0
0
20
27
17
33
drm.ugt80.
drm.wt.ugt80.
drm.smt1.
drm.wt.smt1.
dsm.ugt80.
dsm.wt.ugt80.
dsm.smt1.
dsm.wt.smt1.
sp.ugt80.
sp.wt.ugt80.
sp.smt1.
sp.wt.smt1.
im.ugt80.
im.wt.ugt80.
im.smt1.
im.wt.smt1.

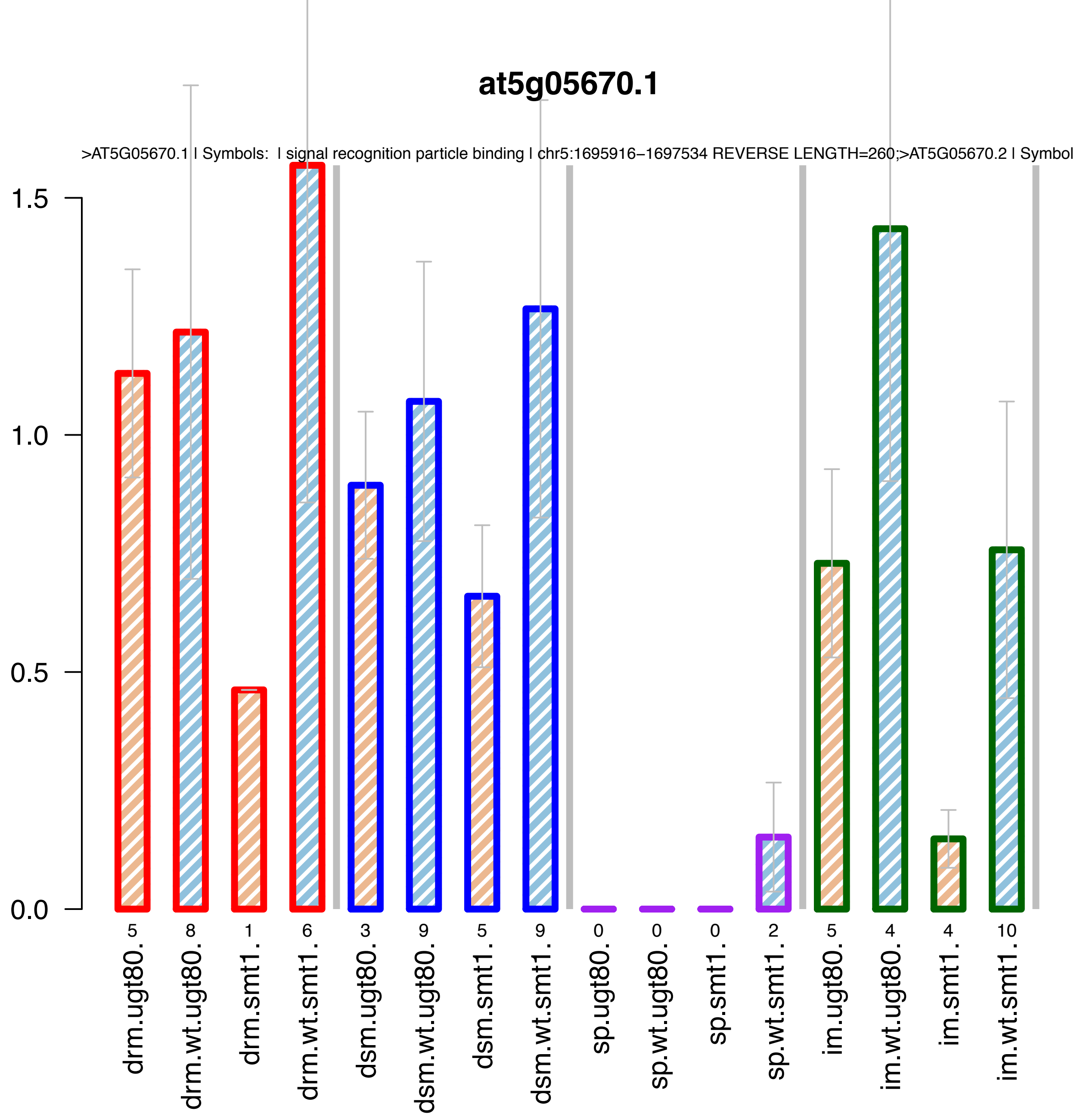
at5g05670.1
>AT5G05670.1 | Symbols: | signal recognition particle binding | chr5:1695916-1697534 REVERSE LENGTH=260;>AT5G05670.2 | Symbol
1.5
1.0
0.5
0.0
5
8
1
6
3
9
5
9
0
0
0
2
5
4
4
10
drm.ugt80.
drm.wt.ugt80.
drm.smt1.
drm.wt.smt1.
dsm.ugt80.
dsm.wt.ugt80.
dsm.smt1.
dsm.wt.smt1.
sp.ugt80.
sp.wt.ugt80.
sp.smt1.
sp.wt.smt1.
im.ugt80.
im.wt.ugt80.
im.smt1.
im.wt.smt1.

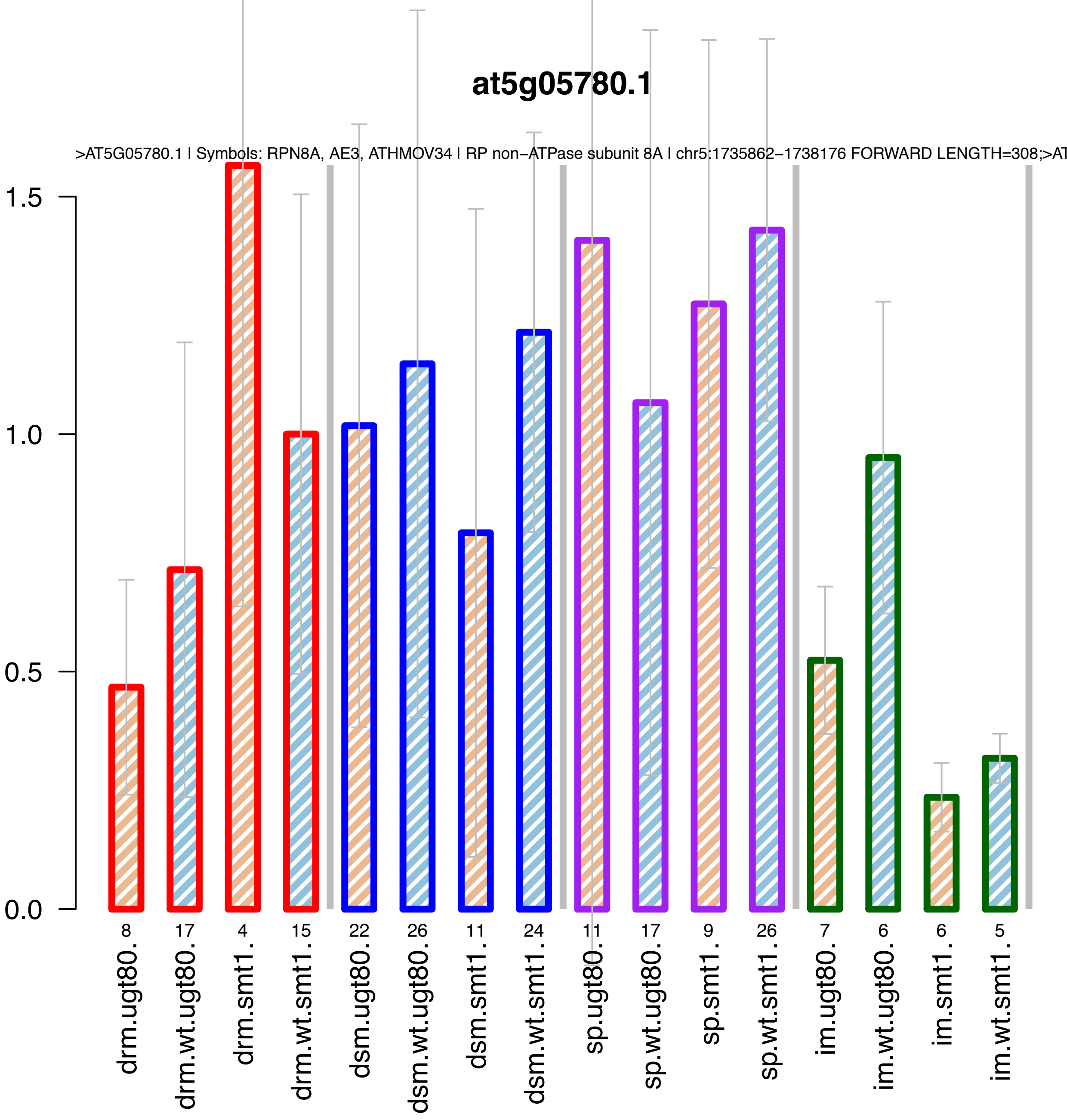

at5g05780.1
>AT5G05780.1 | Symbols: RPN8A, AE3, ATHMOV34 | RP non−ATPase subunit 8A | chr5:1735862−1738176 FORWARD LENGTH=308;>AT
1.5
1.0
0.5
0.0
8
17
4
15
22
26
11
24
11
17
9
26
7
6
6
5
drm.ugt80.
drm.wt.ugt80.
drm.smt1.
drm.wt.smt1.
dsm.ugt80.
dsm.wt.ugt80.
dsm.smt1.
dsm.wt.smt1.
sp.ugt80.
sp.wt.ugt80.
sp.smt1.
sp.wt.smt1.
im.ugt80.
im.wt.ugt80.
im.smt1.
im.wt.smt1.

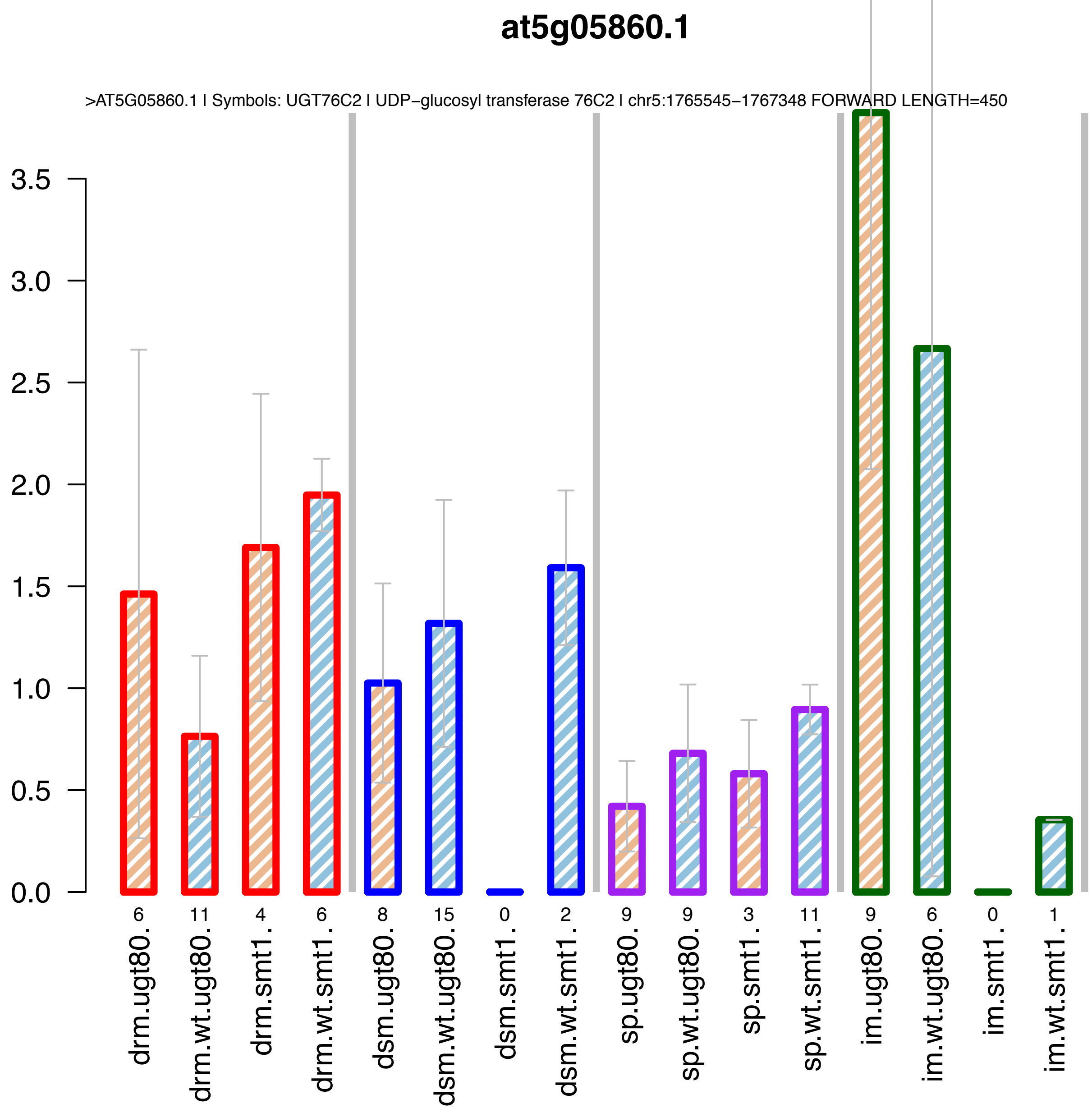

at5g05860.1
>AT5G05860.1 | Symbols: UGT76C2 | UDP−glucosyl transferase 76C2 | chr5:1765545−1767348 FORWARD LENGTH=450
0.0
0.5
1.0
1.5
2.0
2.5
3.0
3.5
6
11
4
6
8
15
0
2
9
9
3
11
9
6
0
1
drm.ugt80.
drm.wt.ugt80.
drm.smt1.
drm.wt.smt1.
dsm.ugt80.
dsm.wt.ugt80.
dsm.smt1.
dsm.wt.smt1.
sp.ugt80.
sp.wt.ugt80.
sp.smt1.
sp.wt.smt1.
im.ugt80.
im.wt.ugt80.
im.smt1.
im.wt.smt1.

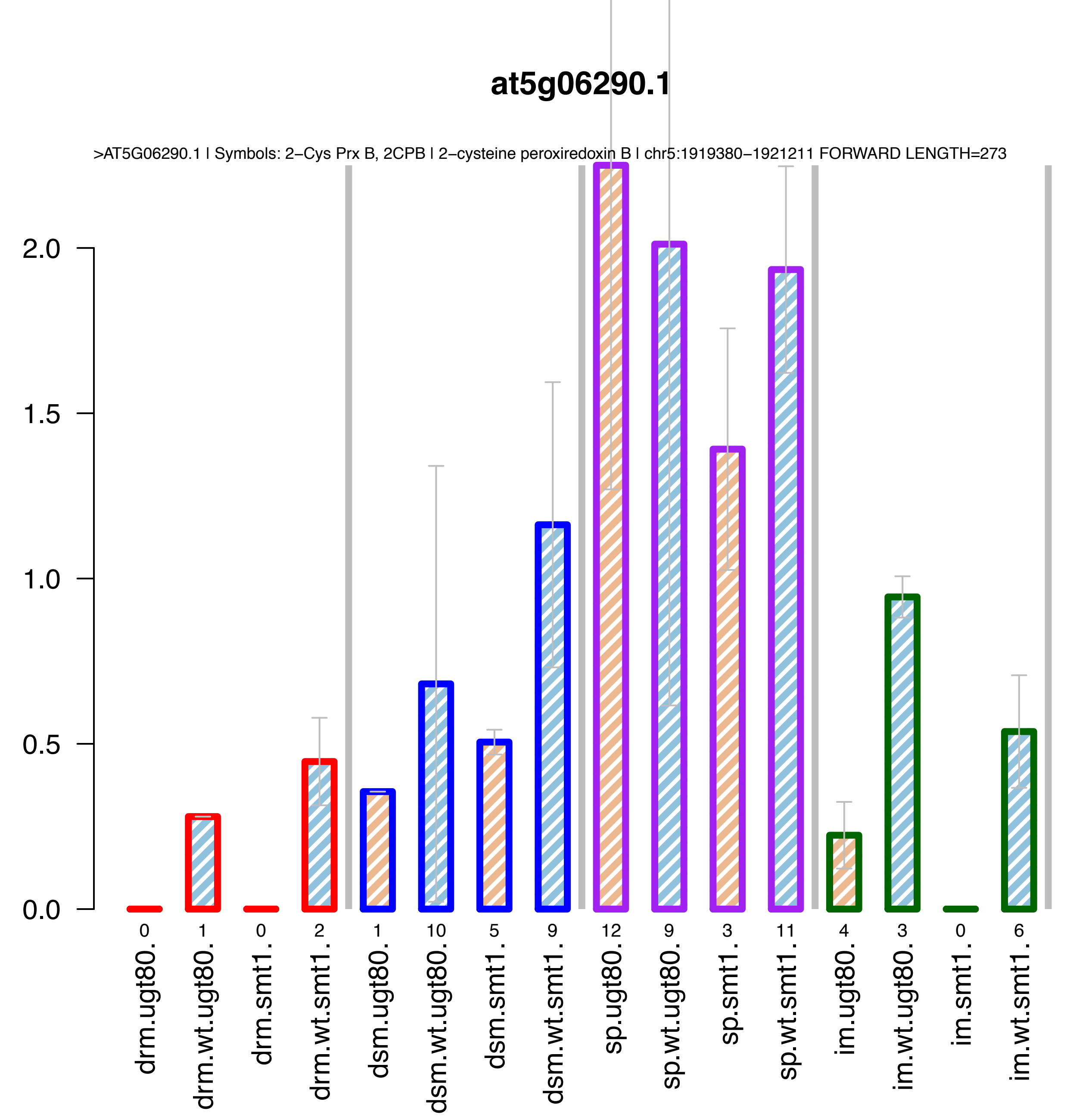

at5g06290.1
>AT5G06290.1 | Symbols: 2−Cys Prx B, 2CPB | 2−cysteine peroxiredoxin B | chr5:1919380−1921211 FORWARD LENGTH=273
2.0
1.5
1.0
0.5
0.0
0
1
0
2
1
10
5
9
12
9
3
11
4
3
0
6
drm.ugt80.
drm.wt.ugt80.
drm.smt1.
drm.wt.smt1.
dsm.ugt80.
dsm.wt.ugt80.
dsm.smt1.
dsm.wt.smt1.
sp.ugt80.
sp.wt.ugt80.
sp.smt1.
sp.wt.smt1.
im.ugt80.
im.wt.ugt80.
im.smt1.
im.wt.smt1.

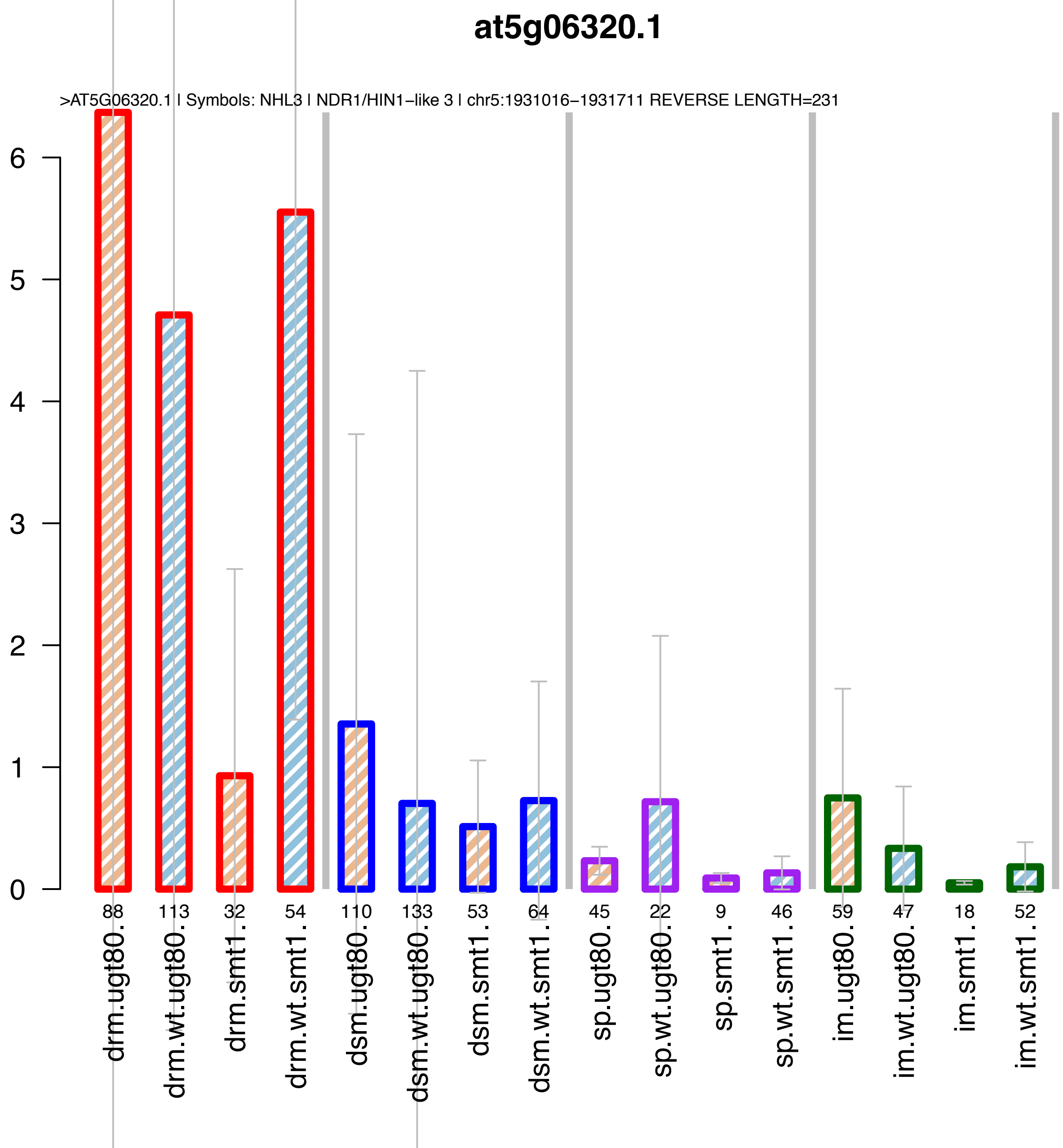

at5g06320.1
>AT5G06320.1 | Symbols: NHL3 | NDR1/HIN1-like 3 | chr5:1931016-1931711 REVERSE LENGTH=231
0
1
2
3
4
5
6
88
113
32
54
110
133
53
64
45
22
9
46
59
47
18
52
drm.ugt80..
drm.wt.ugt80..
drm.smt1..
drm.wt.smt1..
dsm.ugt80..
dsm.wt.ugt80..
dsm.smt1..
dsm.wt.smt1..
sp.ugt80..
sp.wt.ugt80..
sp.smt1..
sp.wt.smt1..
im.ugt80..
im.wt.ugt80..
im.smt1..
im.wt.smt1..

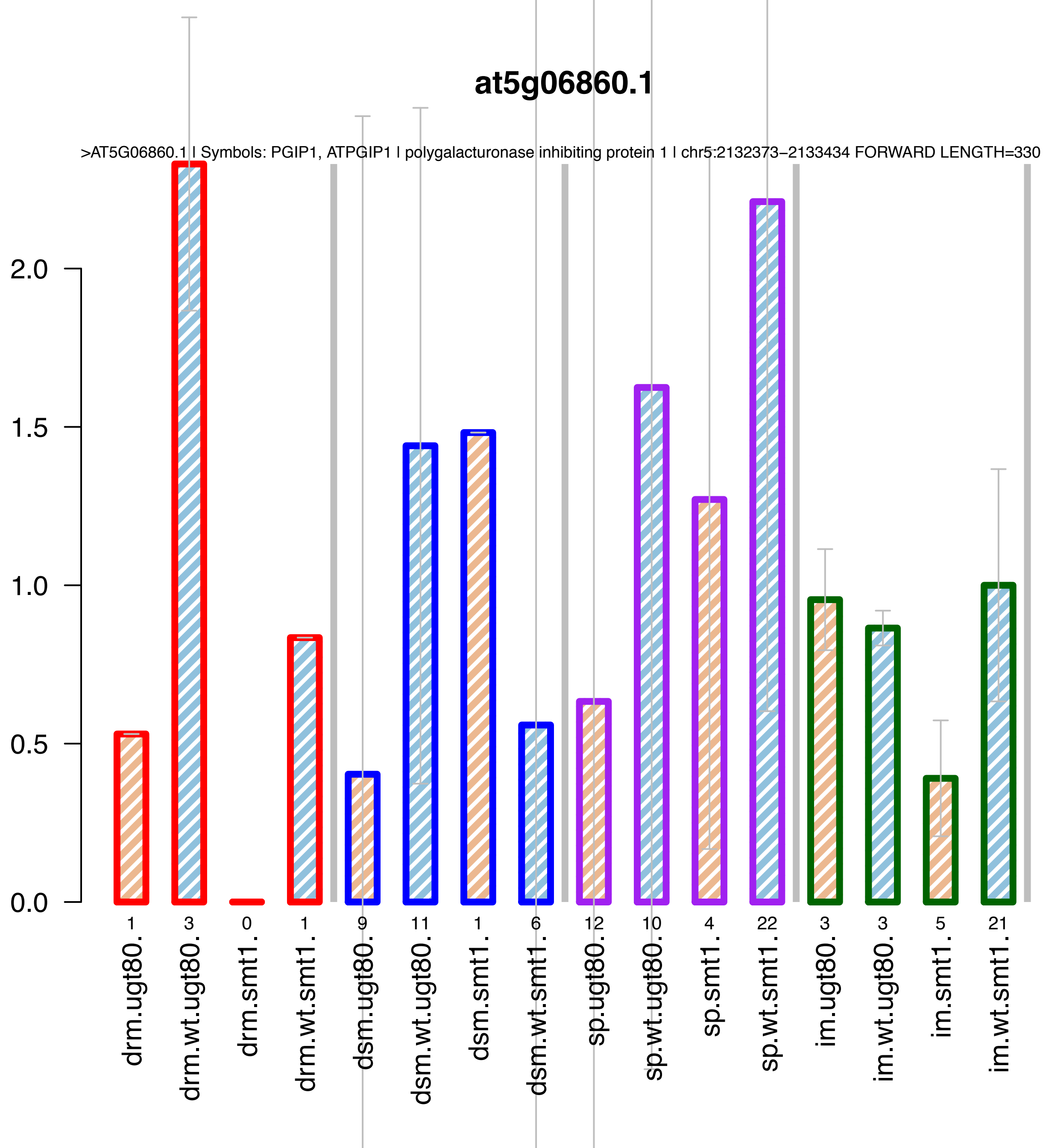

at5g06860.1
>AT5G06860.1 | Symbols: PGIP1, ATPGIP1 | polygalacturonase inhibiting protein 1 | chr5:2132373–2133434 FORWARD LENGTH=330
0.0
0.5
1.0
1.5
2.0
1
3
0
1
9
11
1
6
12
10
4
22
3
3
5
21
drm.ugt80..
drm.wt.ugt80..
drm.smt1..
drm.wt.smt1..
dsm.ugt80..
dsm.wt.ugt80..
dsm.smt1..
dsm.wt.smt1..
sp.ugt80..
sp.wt.ugt80..
sp.smt1..
sp.wt.smt1..
im.ugt80..
im.wt.ugt80..
im.smt1..
im.wt.smt1..

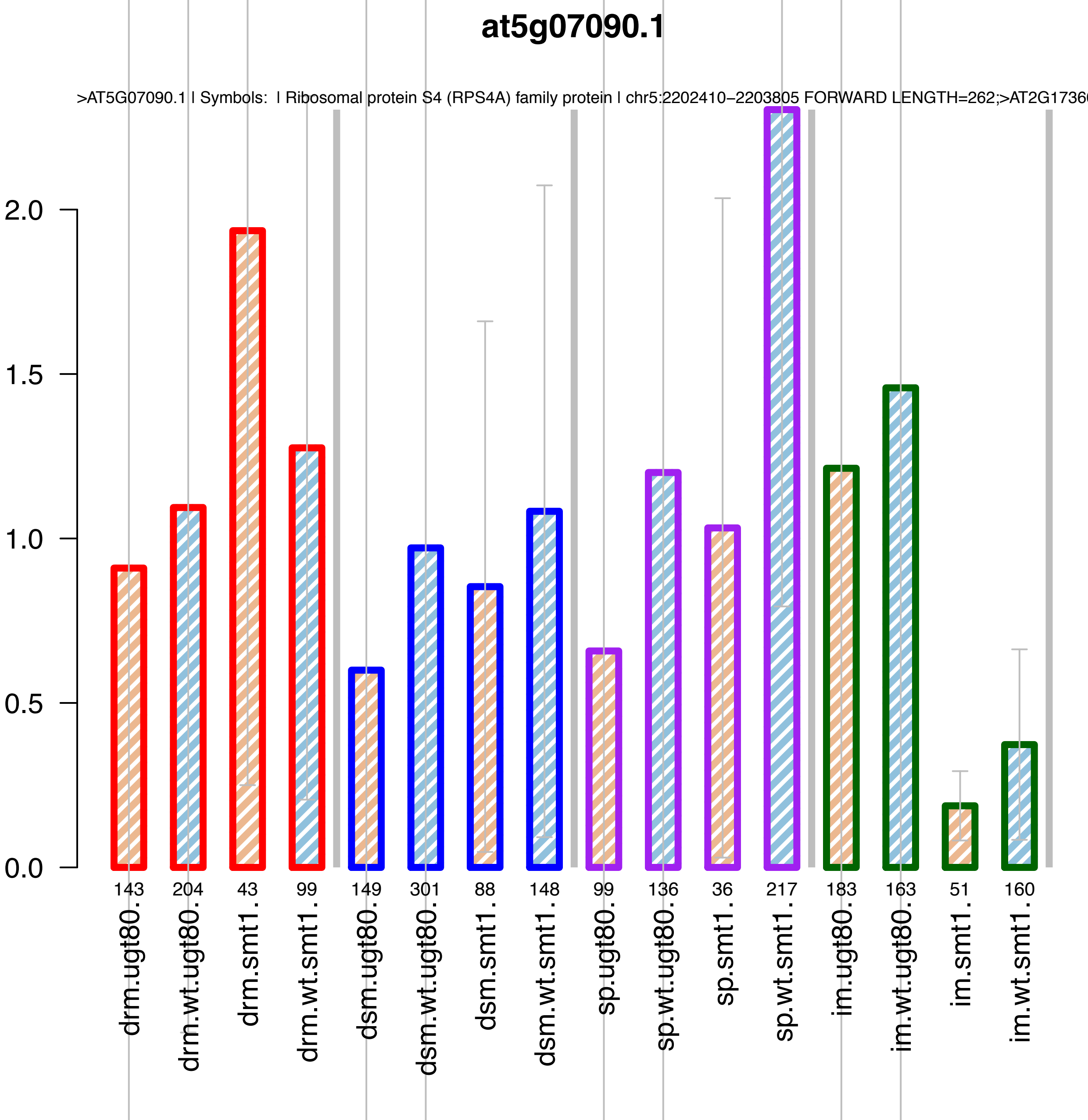

at5g07090.1
>AT5G07090.1 | Symbols: | Ribosomal protein S4 (RPS4A) family protein | chr5:2202410-2203805 FORWARD LENGTH=262;>AT2G1736
2.0
1.5
1.0
0.5
0.0
143
204
43
99
149
301
88
148
99
136
36
217
183
163
51
160
drm.ugt80..
drm.wt.ugt80..
drm.smt1..
drm.wt.smt1..
dsm.ugt80..
dsm.wt.ugt80..
dsm.smt1..
dsm.wt.smt1..
sp.ugt80..
sp.wt.ugt80..
sp.smt1..
sp.wt.smt1..
im.ugt80..
im.wt.ugt80..
im.smt1..
im.wt.smt1..

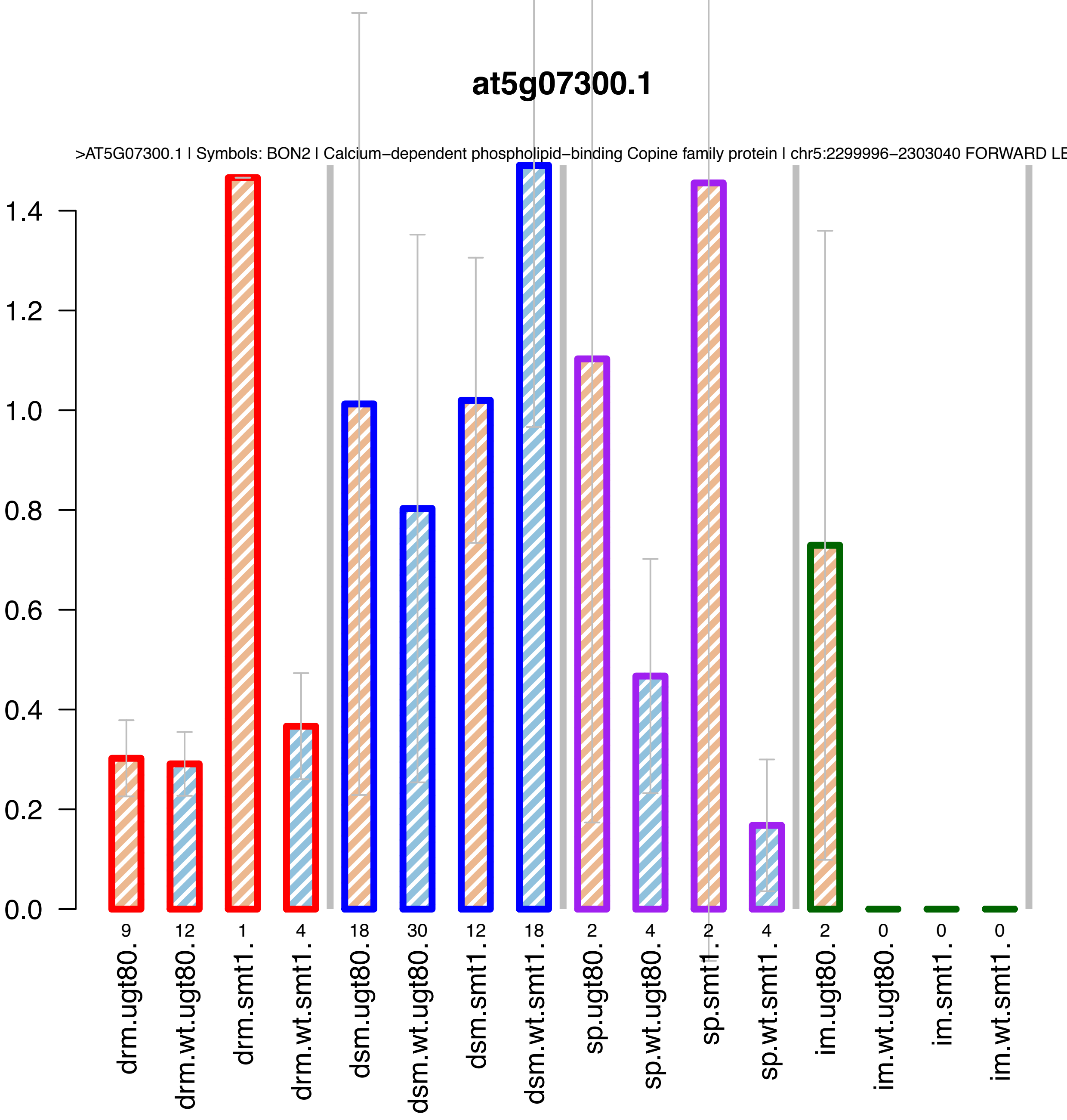

at5g07300.1
>AT5G07300.1 | Symbols: BON2 | Calcium−dependent phospholipid−binding Copine family protein | chr5:2299996−2303040 FORWARD LE
1.4
1.2
1.0
0.8
0.6
0.4
0.2
0.0
9
12
1
4
18
30
12
18
2
4
2
4
2
0
0
0
drm.ugt80..
drm.wt.ugt80..
drm.smt1..
drm.wt.smt1..
dsm.ugt80..
dsm.wt.ugt80..
dsm.smt1..
dsm.wt.smt1..
sp.ugt80..
sp.wt.ugt80..
sp.smt1..
sp.wt.smt1..
im.ugt80..
im.wt.ugt80..
im.smt1..
im.wt.smt1..

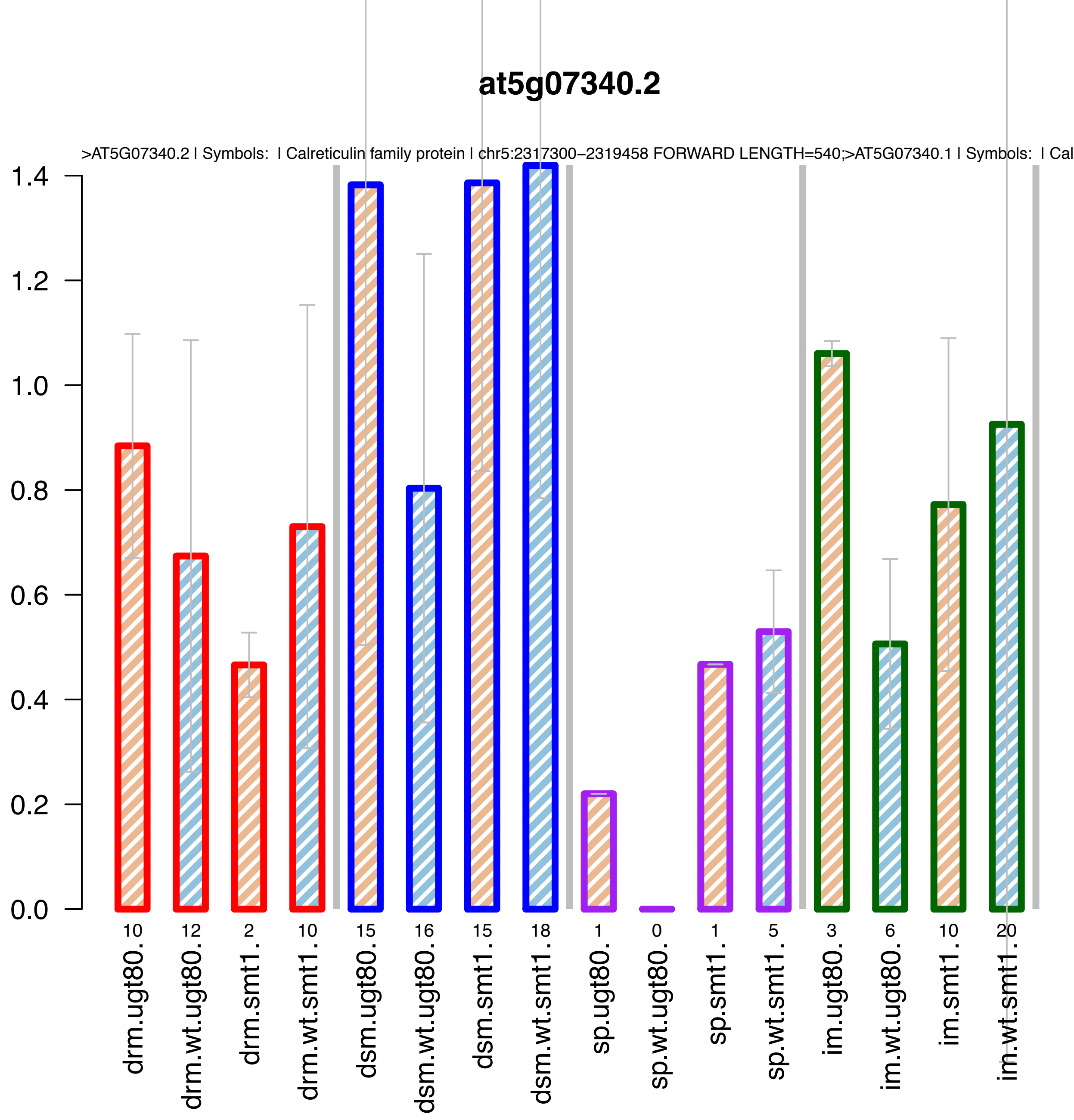

at5g07340.2
>AT5G07340.2 | Symbols: | Calreticulin family protein | chr5:2317300-2319458 FORWARD LENGTH=540;>AT5G07340.1 | Symbols: | Cal
1.4
1.2
1.0
0.8
0.6
0.4
0.2
0.0
10
12
2
10
15
16
15
18
1
0
1
5
3
6
10
20
drm.ugt80..
drm.wt.ugt80..
drm.smt1..
drm.wt.smt1..
dsm.ugt80..
dsm.wt.ugt80..
dsm.smt1..
dsm.wt.smt1..
sp.ugt80..
sp.wt.ugt80..
sp.smt1..
sp.wt.smt1..
im.ugt80..
im.wt.ugt80..
im.smt1..
im.wt.smt1..

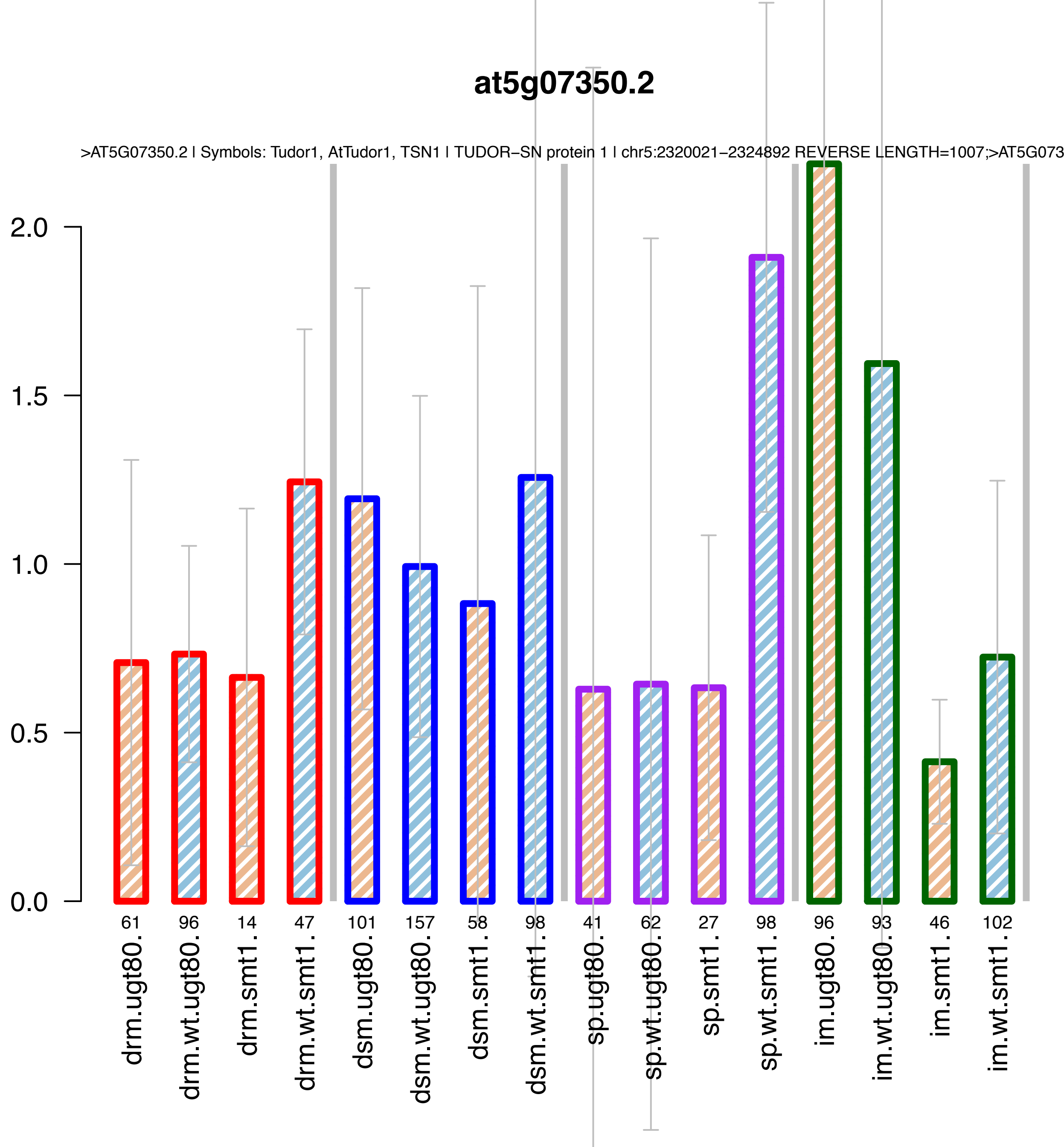
at5g07350.2
>AT5G07350.2 | Symbols: Tudor1, AtTudor1, TSN1 | TUDOR−SN protein 1 | chr5:2320021−2324892 REVERSE LENGTH=1007;>AT5G073
2.0
1.5
1.0
0.5
0.0
61
96
14
47
101
157
58
98
41
62
27
98
96
93
46
102
drm.ugt80..
drm.wt.ugt80..
drm.smt1..
drm.wt.smt1..
dsm.ugt80..
dsm.wt.ugt80..
dsm.smt1..
dsm.wt.smt1..
sp.ugt80..
sp.wt.ugt80..
sp.smt1..
sp.wt.smt1..
im.ugt80..
im.wt.ugt80..
im.smt1..
im.wt.smt1..

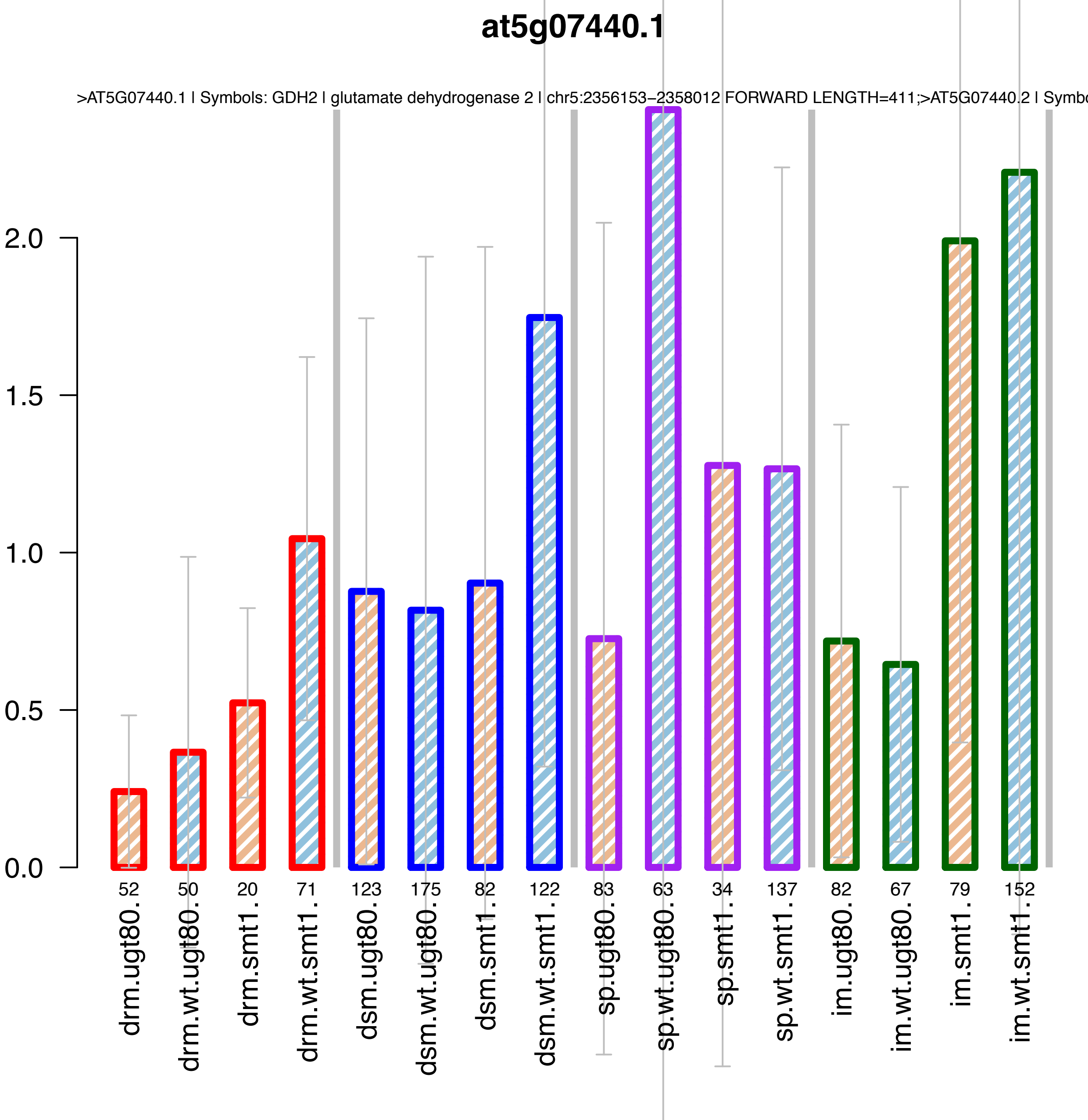

at5g07440.1
>AT5G07440.1 | Symbols: GDH2 | glutamate dehydrogenase 2 | chr5:2356153–2358012 FORWARD LENGTH=411;>AT5G07440.2 | Symbo
2.0
1.5
1.0
0.5
0.0
52
50
20
71
123
175
82
122
83
63
34
137
82
67
79
152
drm.ugt80..
drm.wt.ugt80..
drm.smt1..
drm.wt.smt1..
dsm.ugt80..
dsm.wt.ugt80..
dsm.smt1..
dsm.wt.smt1..
sp.ugt80..
sp.wt.ugt80..
sp.smt1..
sp.wt.smt1..
im.ugt80..
im.wt.ugt80..
im.smt1..
im.wt.smt1..

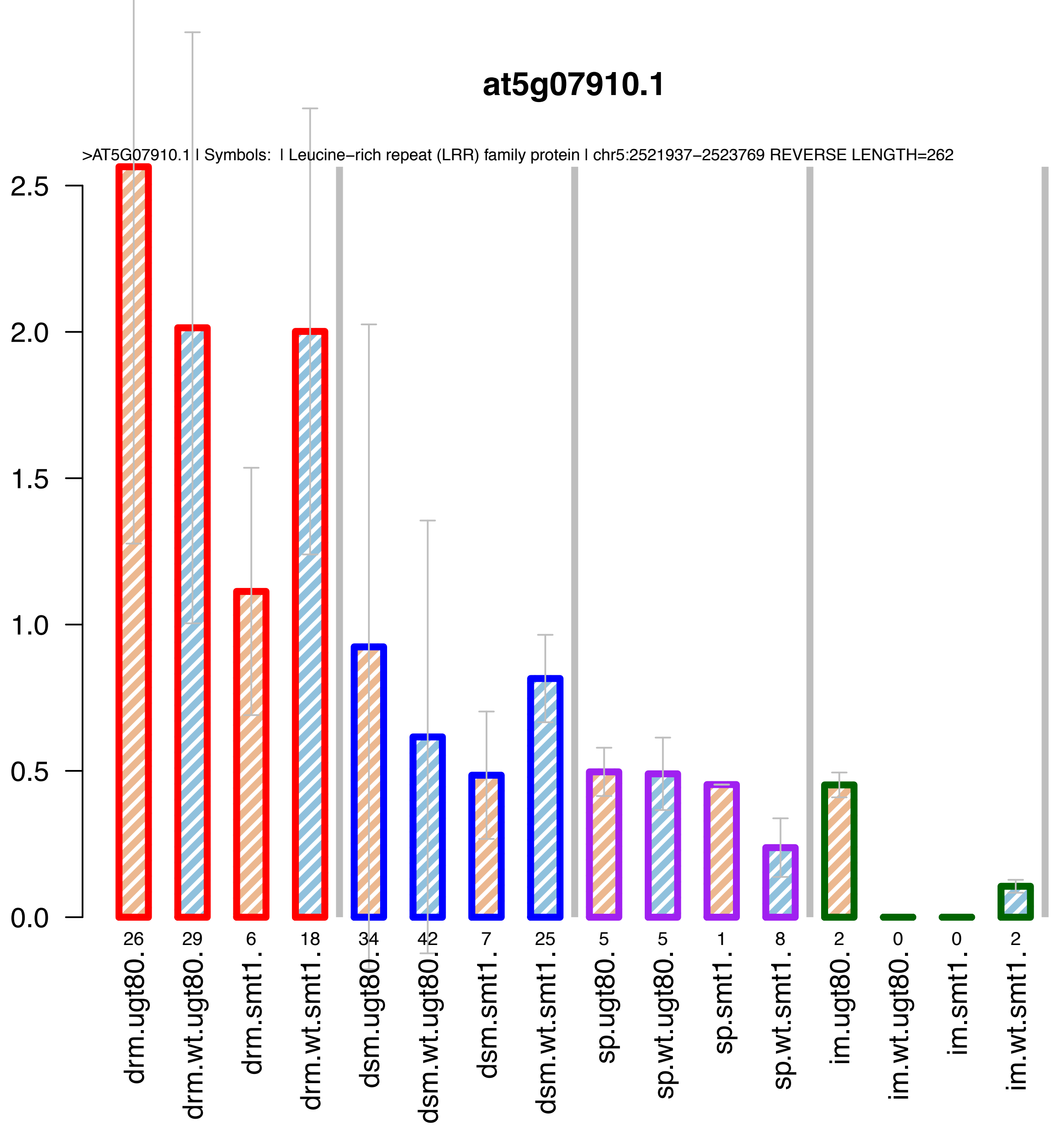
at5g07910.1
>AT5G07910.1 | Symbols: | Leucine−rich repeat (LRR) family protein | chr5:2521937−2523769 REVERSE LENGTH=262
2.5
2.0
1.5
1.0
0.5
0.0
26
29
6
18
34
42
7
25
5
5
1
8
2
0
0
2
drm.ugt80..
drm.wt.ugt80..
drm.smt1..
drm.wt.smt1..
dsm.ugt80..
dsm.wt.ugt80..
dsm.smt1..
dsm.wt.smt1..
sp.ugt80..
sp.wt.ugt80..
sp.smt1..
sp.wt.smt1..
im.ugt80..
im.wt.ugt80..
im.smt1..
im.wt.smt1..

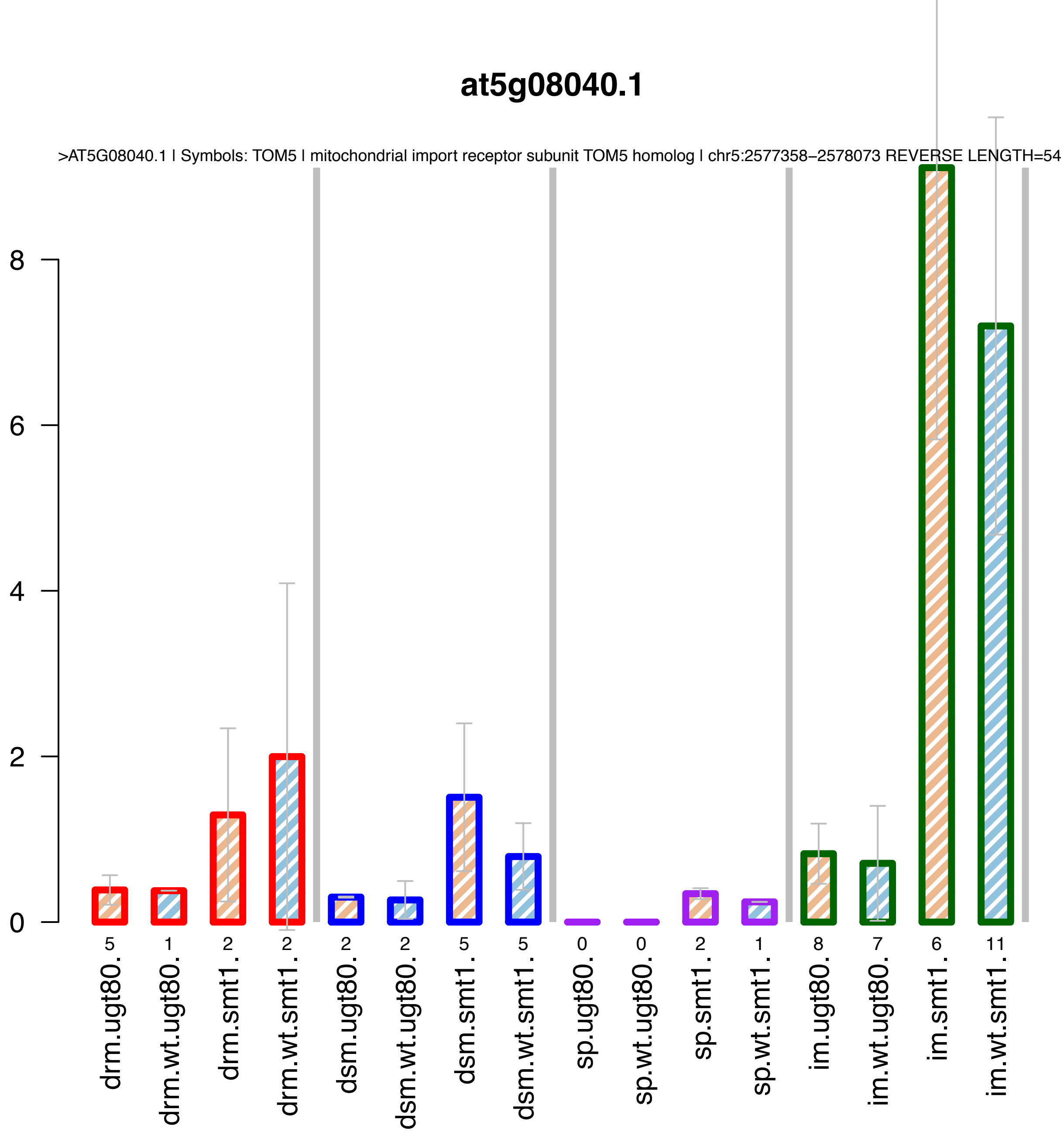

at5g08040.1
>AT5G08040.1 | Symbols: TOM5 | mitochondrial import receptor subunit TOM5 homolog | chr5:2577358−2578073 REVERSE LENGTH=54
0
2
4
6
8
5
1
2
2
2
2
5
5
0
0
2
1
8
7
6
11
drm.ugt80.
drm.wt.ugt80.
drm.smt1.
drm.wt.smt1.
dsm.ugt80.
dsm.wt.ugt80.
dsm.smt1.
dsm.wt.smt1.
sp.ugt80.
sp.wt.ugt80.
sp.smt1.
sp.wt.smt1.
im.ugt80.
im.wt.ugt80.
im.smt1.
im.wt.smt1.

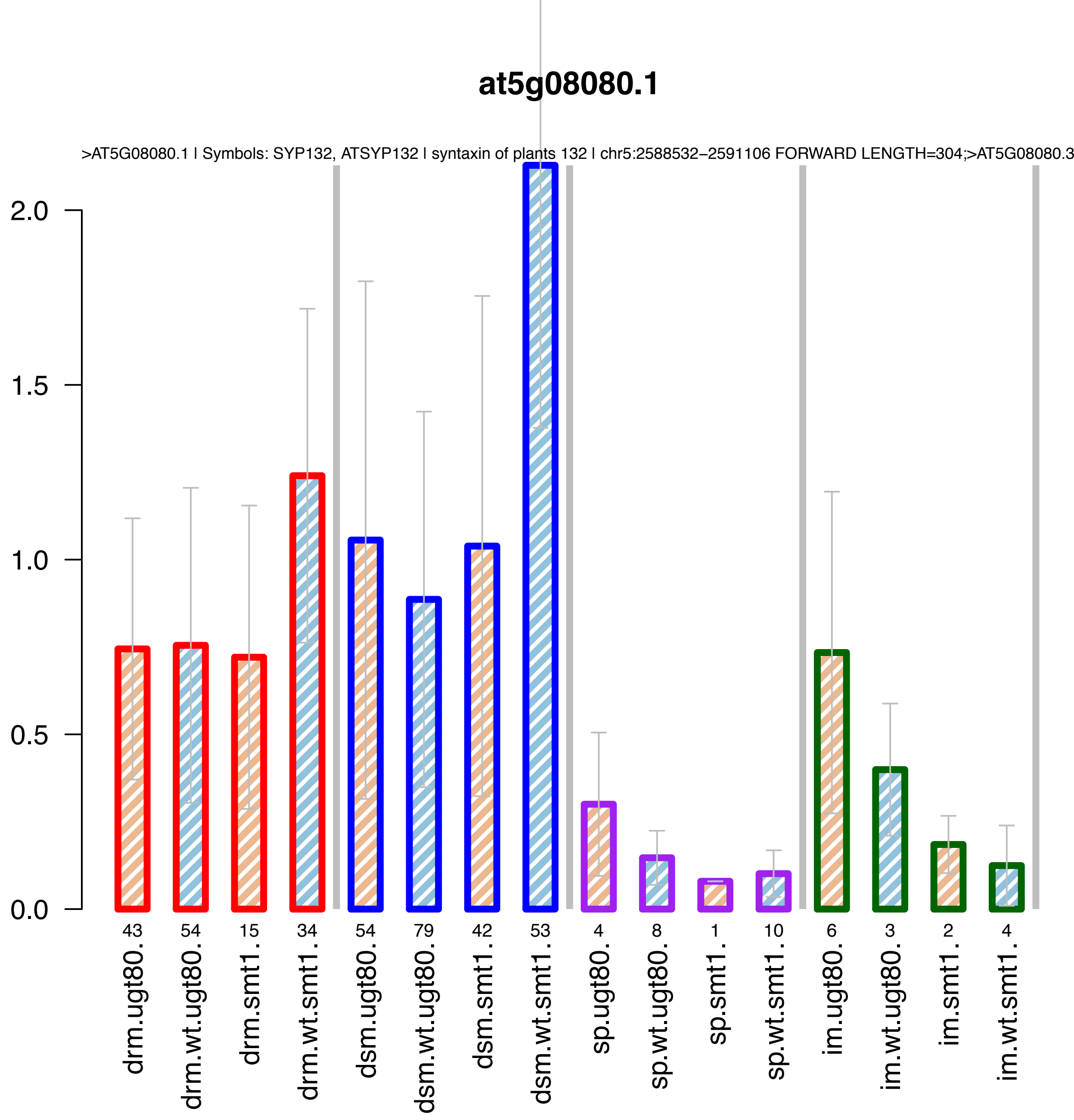

at5g08080.1
>AT5G08080.1 | Symbols: SYP132, ATSYP132 | syntaxin of plants 132 | chr5:2588532–2591106 FORWARD LENGTH=304;>AT5G08080.3
2.0
1.5
1.0
0.5
0.0
43
54
15
34
54
79
42
53
4
8
1
10
6
3
2
4
drm.ugt80..
drm.wt.ugt80..
drm.smt1..
drm.wt.smt1..
dsm.ugt80..
dsm.wt.ugt80..
dsm.smt1..
dsm.wt.smt1..
sp.ugt80..
sp.wt.ugt80..
sp.smt1..
sp.wt.smt1..
im.ugt80..
im.wt.ugt80..
im.smt1..
im.wt.smt1..

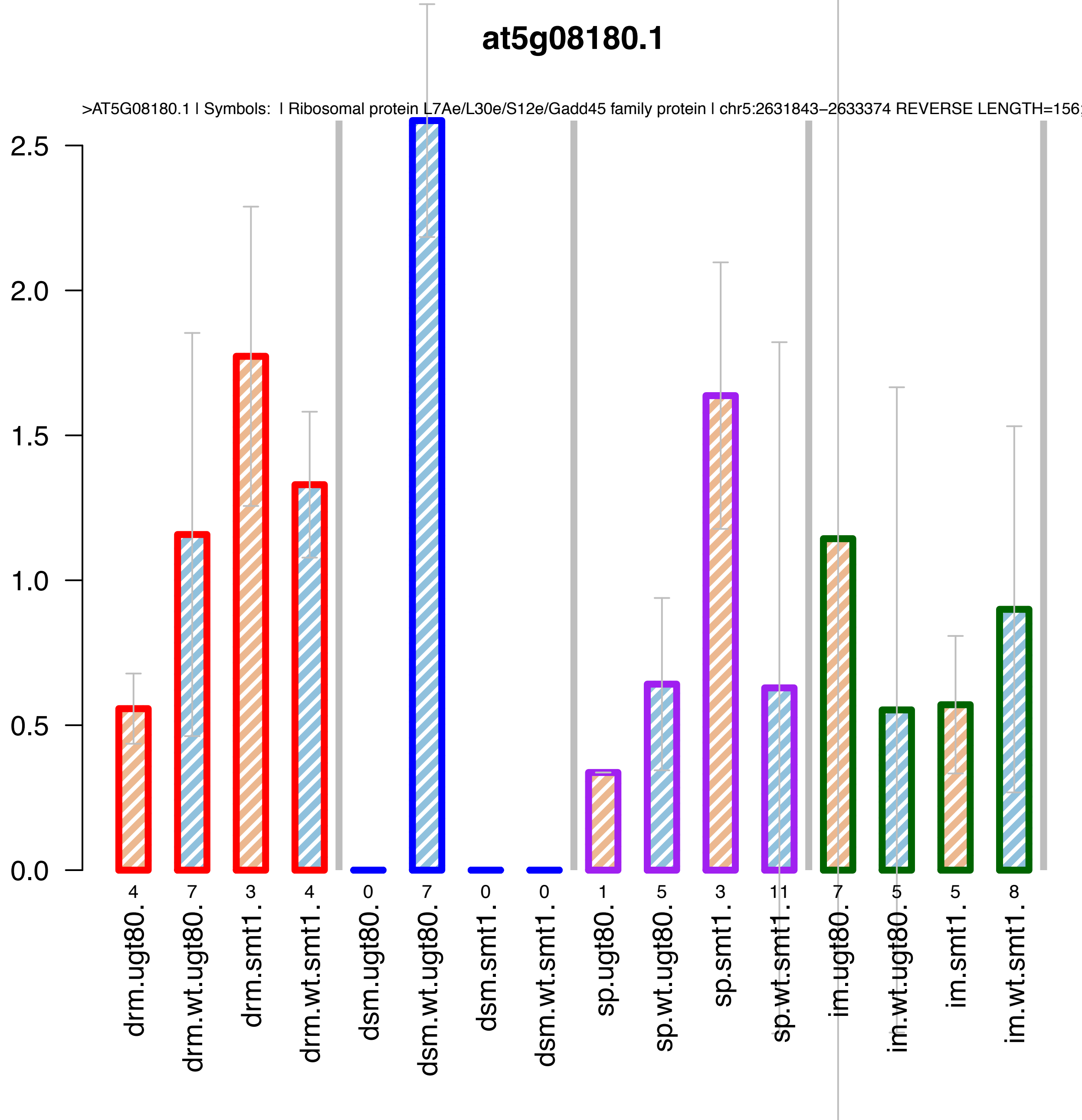

at5g08180.1
>AT5G08180.1 | Symbols: | Ribosomal protein L7Ae/L30e/S12e/Gadd45 family protein | chr5:2631843-2633374 REVERSE LENGTH=156
2.5
2.0
1.5
1.0
0.5
0.0
4
7
3
4
0
7
0
0
1
5
3
11
7
5
5
8
drm.ugt80.
drm.wt.ugt80.
drm.smt1.
drm.wt.smt1.
dsm.ugt80.
dsm.wt.ugt80.
dsm.smt1.
dsm.wt.smt1.
sp.ugt80.
sp.wt.ugt80.
sp.smt1.
sp.wt.smt1.
im.ugt80.
im.wt.ugt80.
im.smt1.
im.wt.smt1.

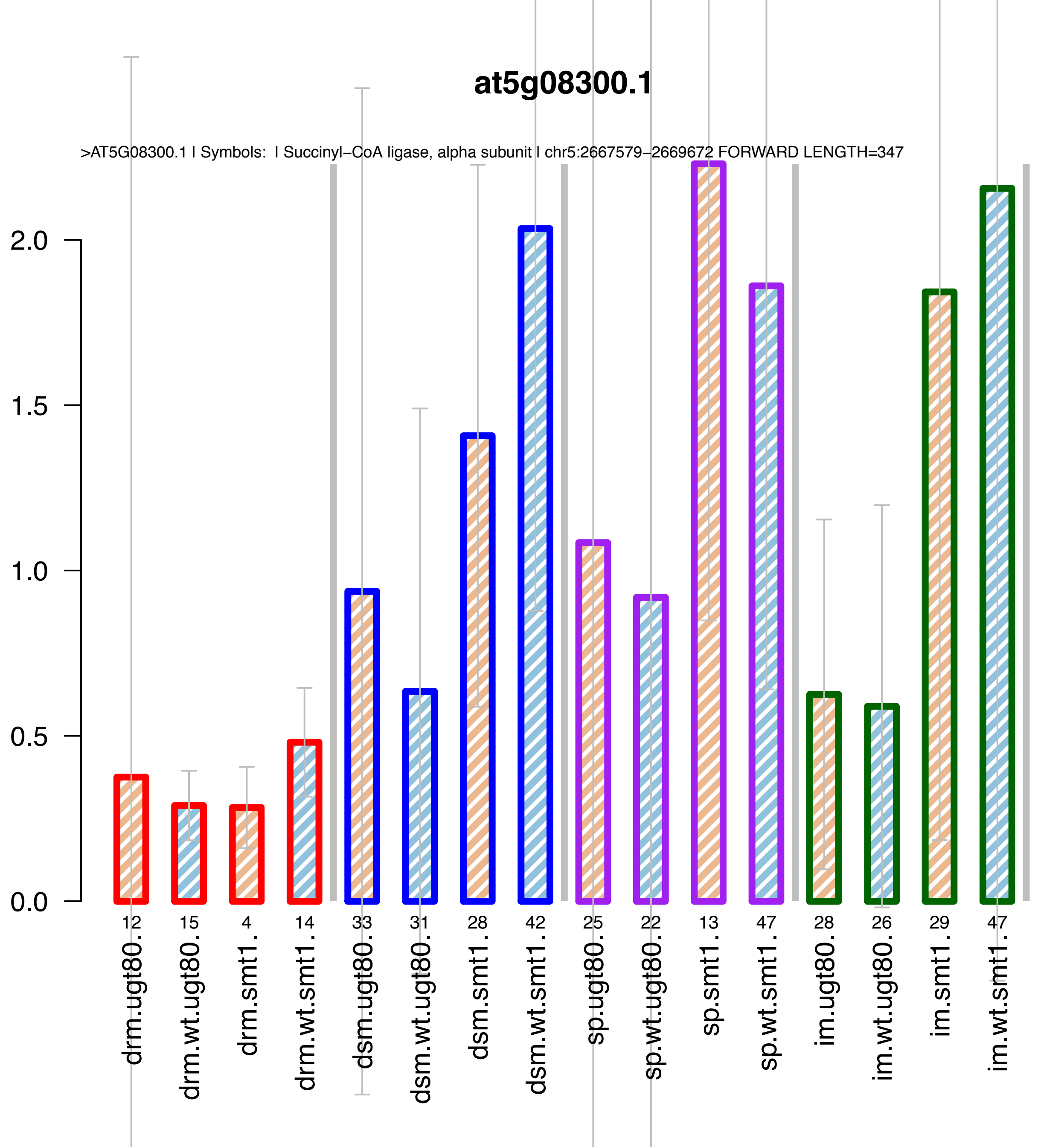

at5g08300.1
>AT5G08300.1 | Symbols: | Succinyl−CoA ligase, alpha subunit | chr5:2667579−2669672 FORWARD LENGTH=347
0.0
0.5
1.0
1.5
2.0
12
15
4
14
33
31
28
42
25
22
13
47
28
26
29
47
drm.ugt80..
drm.wt.ugt80..
drm.smt1..
drm.wt.smt1..
dsm.ugt80..
dsm.wt.ugt80..
dsm.smt1..
dsm.wt.smt1..
sp.ugt80..
sp.wt.ugt80..
sp.smt1..
sp.wt.smt1..
im.ugt80..
im.wt.ugt80..
im.smt1..
im.wt.smt1..

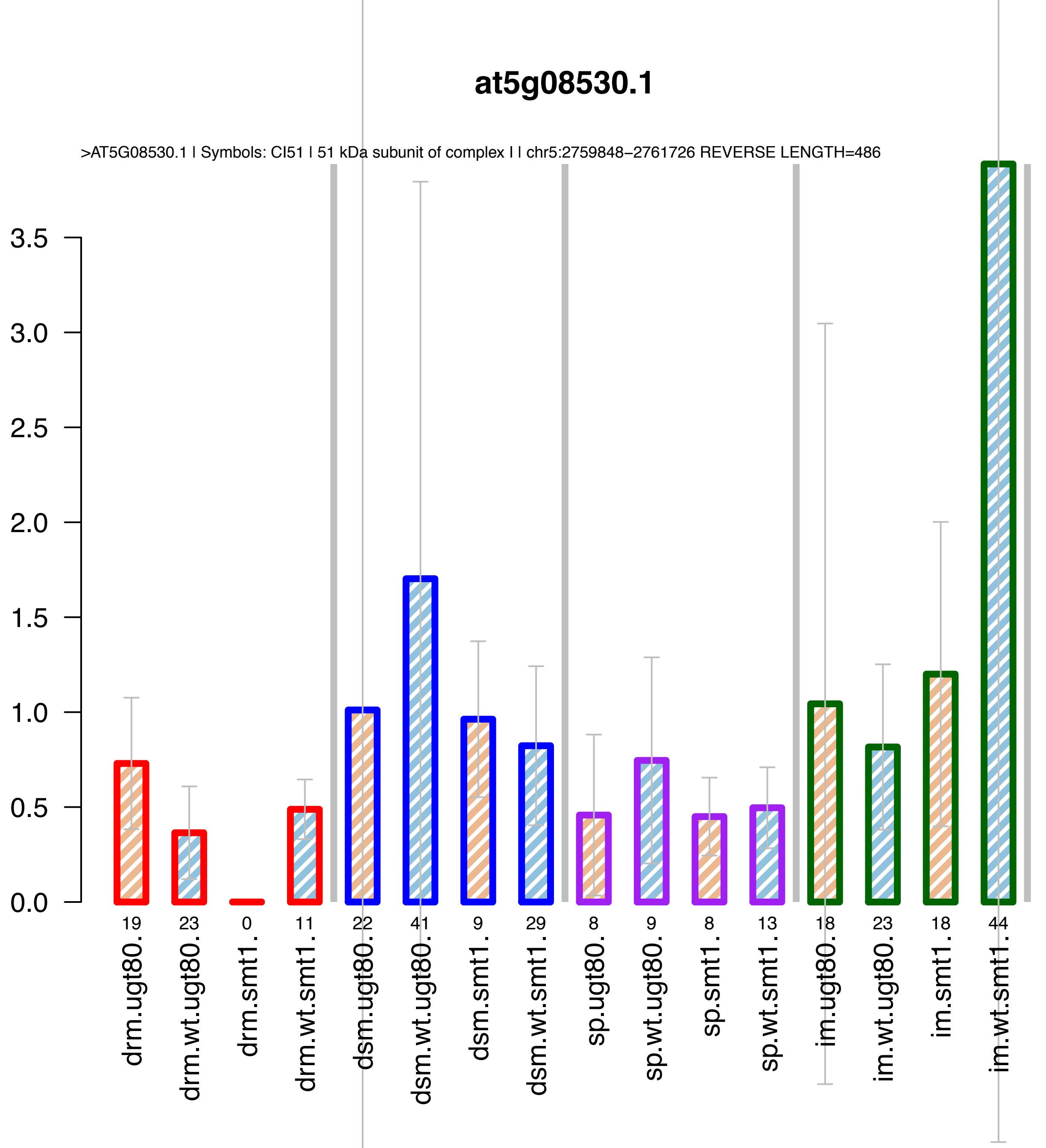

at5g08530.1
>AT5G08530.1 | Symbols: CI51 | 51 kDa subunit of complex I | chr5:2759848–2761726 REVERSE LENGTH=486
0.0
0.5
1.0
1.5
2.0
2.5
3.0
3.5
19
23
0
11
22
41
9
29
8
9
8
13
18
23
18
44
drm.ugt80.
drm.wt.ugt80.
drm.smt1.
drm.wt.smt1.
dsm.ugt80.
dsm.wt.ugt80.
dsm.smt1.
dsm.wt.smt1.
sp.ugt80.
sp.wt.ugt80.
sp.smt1.
sp.wt.smt1.
im.ugt80.
im.wt.ugt80.
im.smt1.
im.wt.smt1.

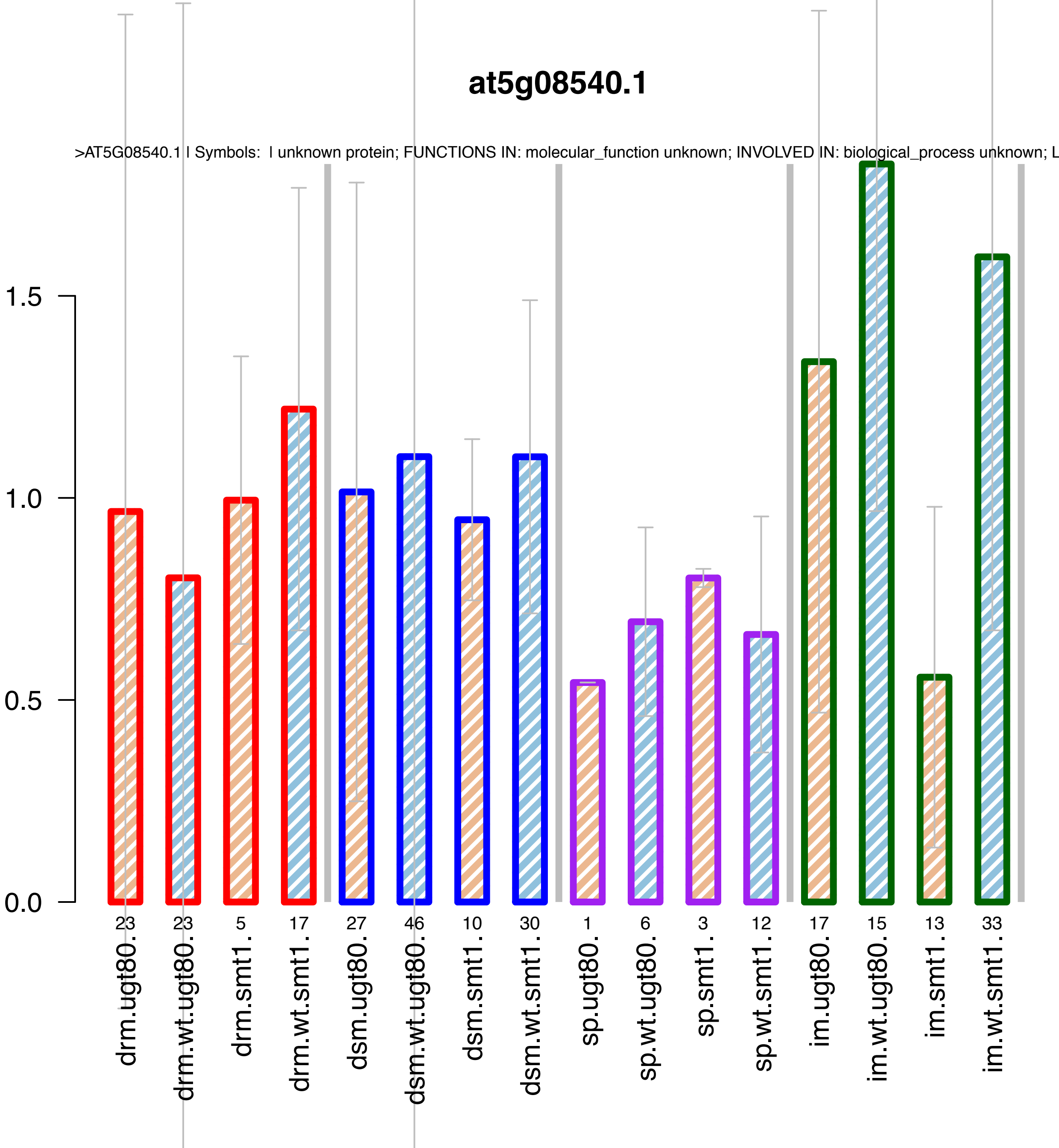

at5g08540.1
>AT5G08540.1 | Symbols: | unknown protein; FUNCTIONS IN: molecular_function unknown; INVOLVED IN: biological_process unknown; L
1.5
1.0
0.5
0.0
23
23
5
17
27
46
10
30
1
6
3
12
17
15
13
33
drm.ugt80..
drm.wt.ugt80..
drm.smt1..
drm.wt.smt1..
dsm.ugt80..
dsm.wt.ugt80..
dsm.smt1..
dsm.wt.smt1..
sp.ugt80..
sp.wt.ugt80..
sp.smt1..
sp.wt.smt1..
im.ugt80..
im.wt.ugt80..
im.smt1..
im.wt.smt1..

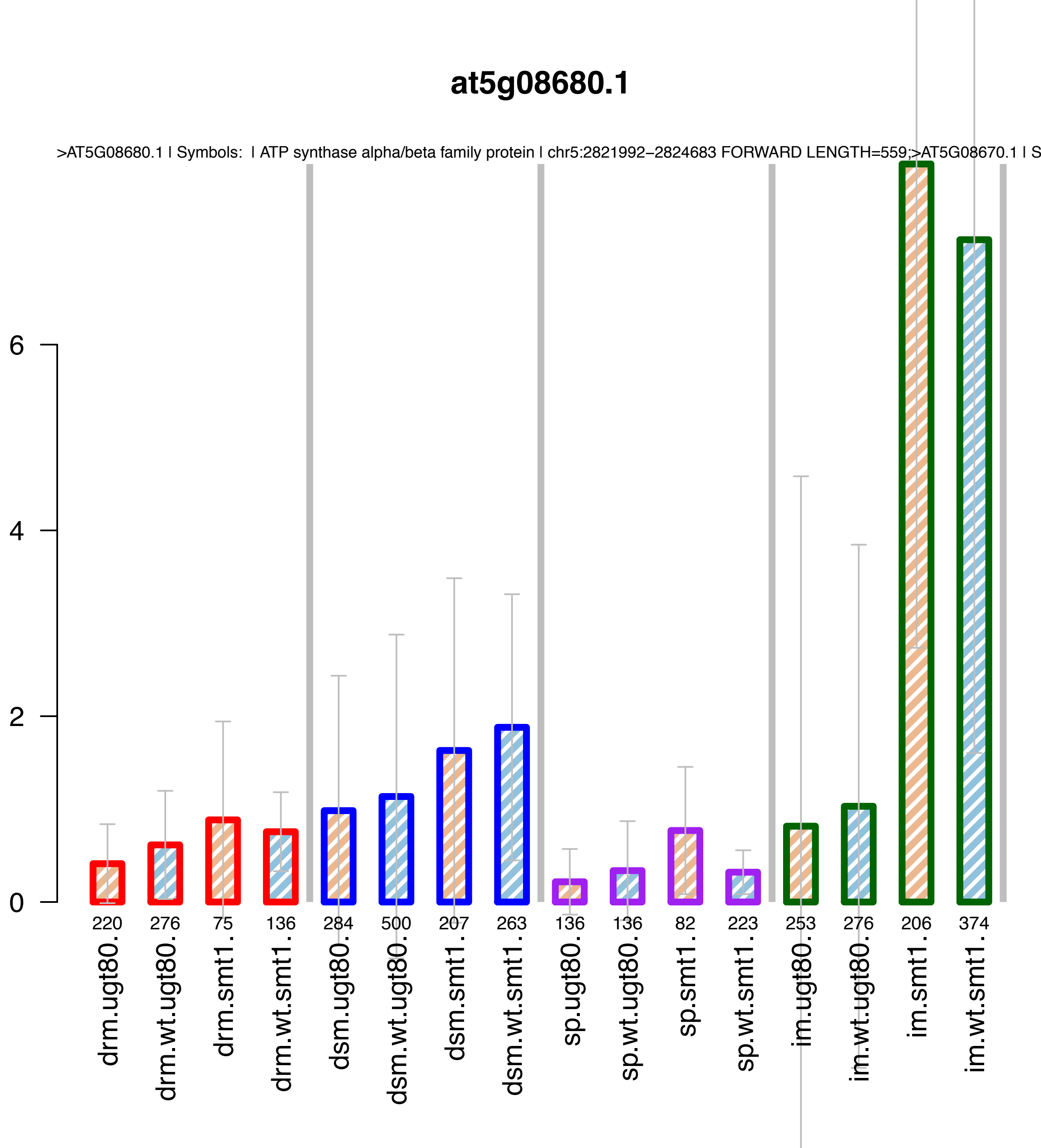

at5g08680.1
>AT5G08680.1 | Symbols: | ATP synthase alpha/beta family protein | chr5:2821992–2824683 FORWARD LENGTH=559;>AT5G08670.1 | S
6
4
2
0
220
276
75
136
284
500
207
263
136
136
82
223
253
276
206
374
drm.ugt80..
drm.wt.ugt80..
drm.smt1..
drm.wt.smt1..
dsm.ugt80..
dsm.wt.ugt80..
dsm.smt1..
dsm.wt.smt1..
sp.ugt80..
sp.wt.ugt80..
sp.smt1..
sp.wt.smt1..
im.ugt80..
im.wt.ugt80..
im.smt1..
im.wt.smt1..

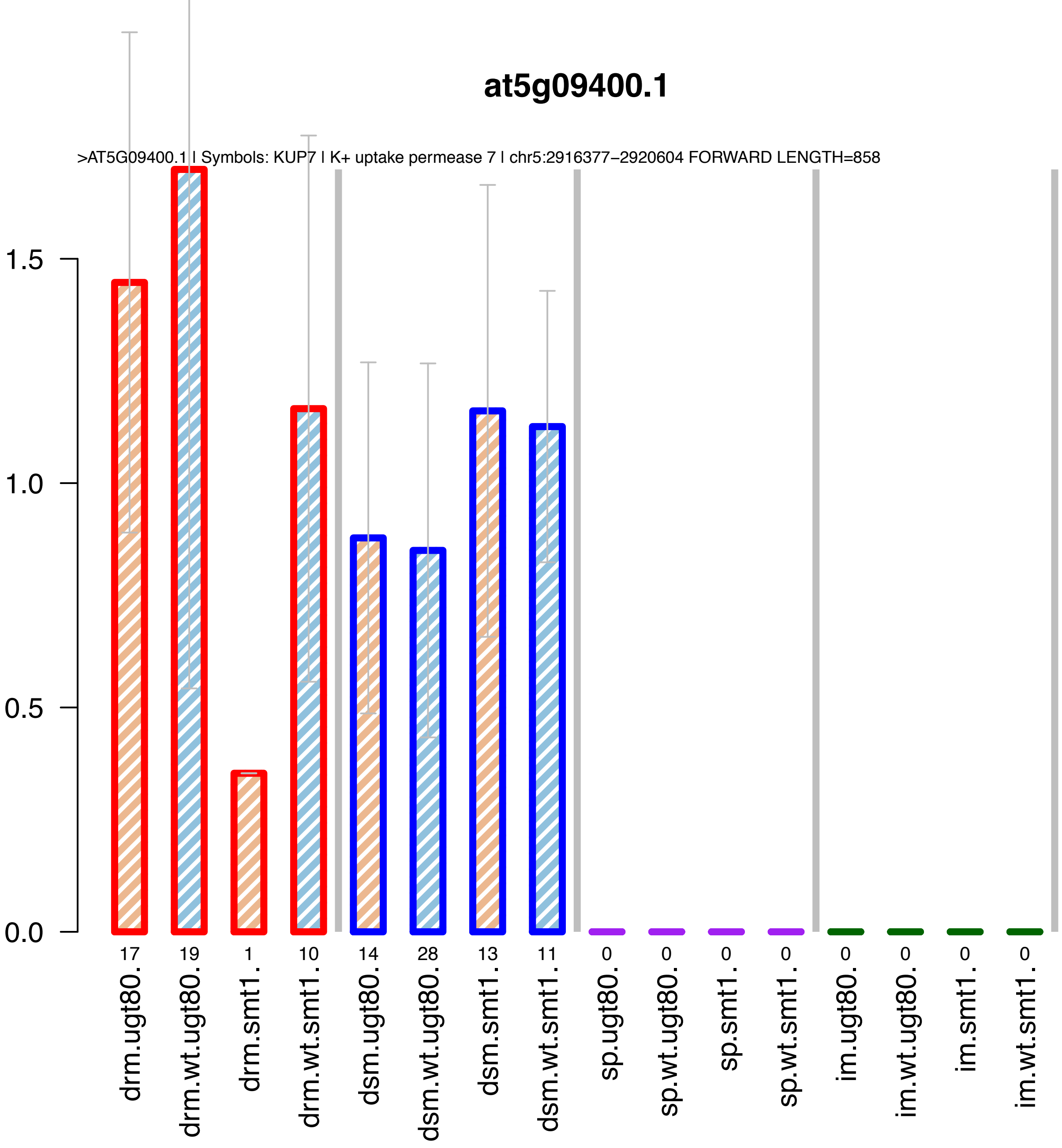

at5g09400.1
>AT5G09400.1 | Symbols: KUP7 | K+ uptake permease 7 | chr5:2916377−2920604 FORWARD LENGTH=858
1.5
1.0
0.5
0.0
17
19
1
10
14
28
13
11
0
0
0
0
0
0
0
0
drm.ugt80.
drm.wt.ugt80.
drm.smt1.
drm.wt.smt1.
dsm.ugt80.
dsm.wt.ugt80.
dsm.smt1.
dsm.wt.smt1.
sp.ugt80.
sp.wt.ugt80.
sp.smt1.
sp.wt.smt1.
im.ugt80.
im.wt.ugt80.
im.smt1.
im.wt.smt1.

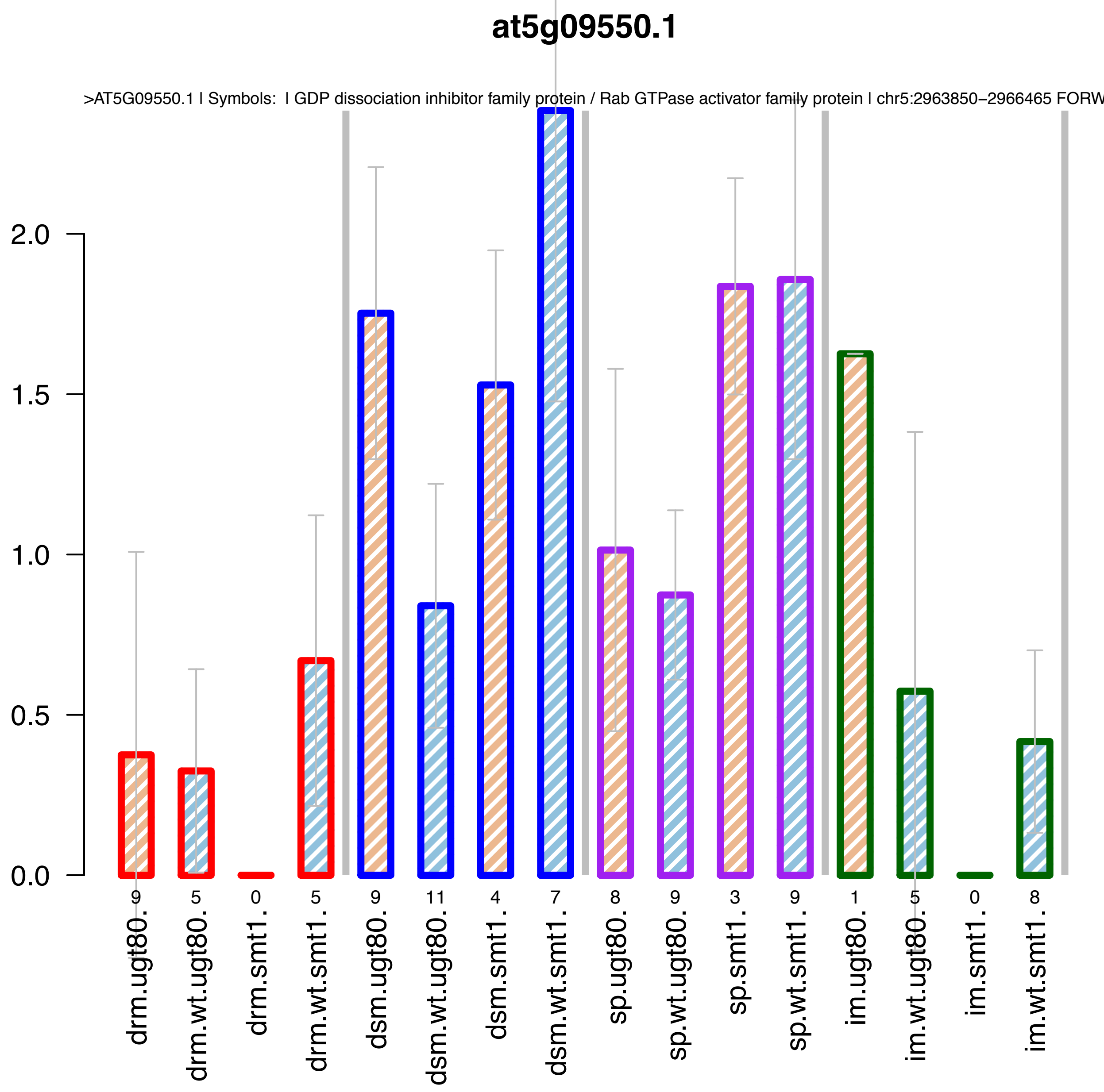

at5g09550.1
>AT5G09550.1 | Symbols: | GDP dissociation inhibitor family protein / Rab GTPase activator family protein | chr5:2963850-2966465 FORW
0.0
0.5
1.0
1.5
2.0
9
5
0
5
9
11
4
7
8
9
3
9
1
5
0
8
drm.ugt80.
drm.wt.ugt80.
drm.smt1.
drm.wt.smt1.
dsm.ugt80.
dsm.wt.ugt80.
dsm.smt1.
dsm.wt.smt1.
sp.ugt80.
sp.wt.ugt80.
sp.smt1.
sp.wt.smt1.
im.ugt80.
im.wt.ugt80.
im.smt1.
im.wt.smt1.

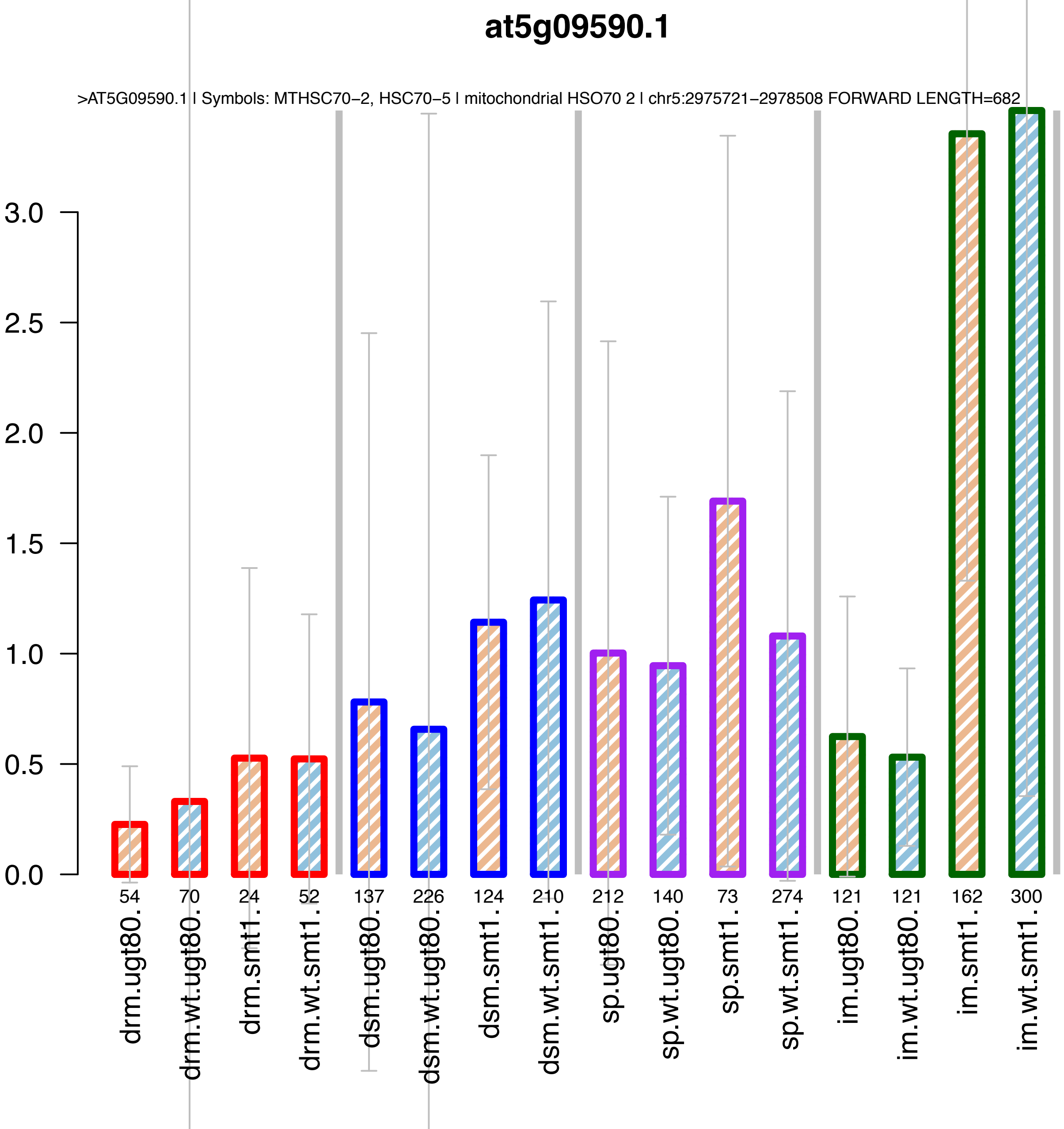

at5g09590.1
>AT5G09590.1 | Symbols: MTHSC70−2, HSC70−5 | mitochondrial HSO70 2 | chr5:2975721−2978508 FORWARD LENGTH=682
3.0
2.5
2.0
1.5
1.0
0.5
0.0
54
70
24
52
137
226
124
210
212
140
73
274
121
121
162
300
drm.ugt80..
drm.wt.ugt80..
drm.smt1..
drm.wt.smt1..
dsm.ugt80..
dsm.wt.ugt80..
dsm.smt1..
dsm.wt.smt1..
sp.ugt80..
sp.wt.ugt80..
sp.smt1..
sp.wt.smt1..
im.ugt80..
im.wt.ugt80..
im.smt1..
im.wt.smt1..

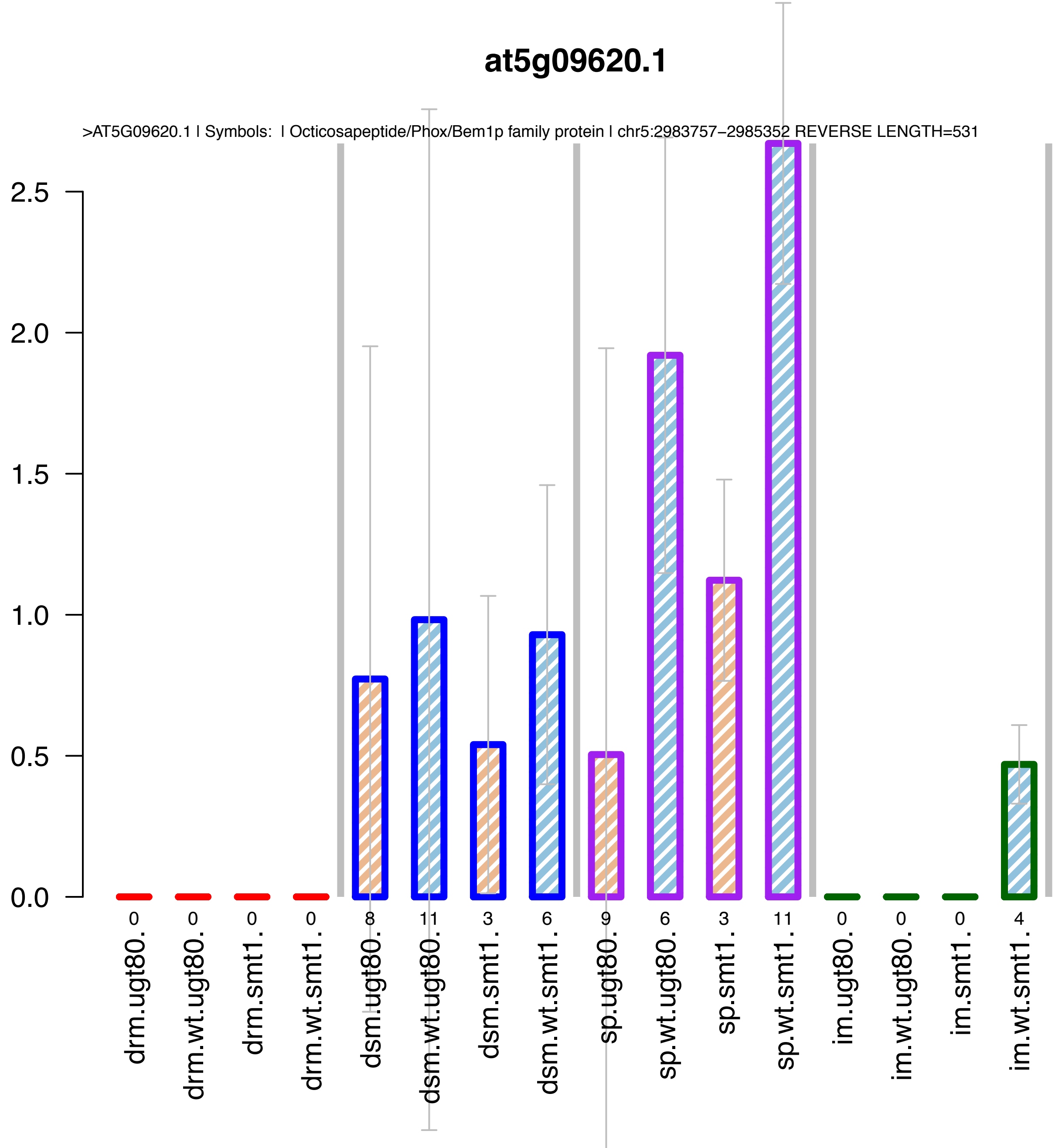

at5g09620.1
>AT5G09620.1 | Symbols: | Octicosapeptide/Phox/Bem1p family protein | chr5:2983757−2985352 REVERSE LENGTH=531
2.5
2.0
1.5
1.0
0.5
0.0
0
0
0
0
8
11
3
6
9
6
3
11
0
0
0
4
drm.ugt80.
drm.wt.ugt80.
drm.smt1.
drm.wt.smt1.
dsm.ugt80.
dsm.wt.ugt80.
dsm.smt1.
dsm.wt.smt1.
sp.ugt80.
sp.wt.ugt80.
sp.smt1.
sp.wt.smt1.
im.ugt80.
im.wt.ugt80.
im.smt1.
im.wt.smt1.

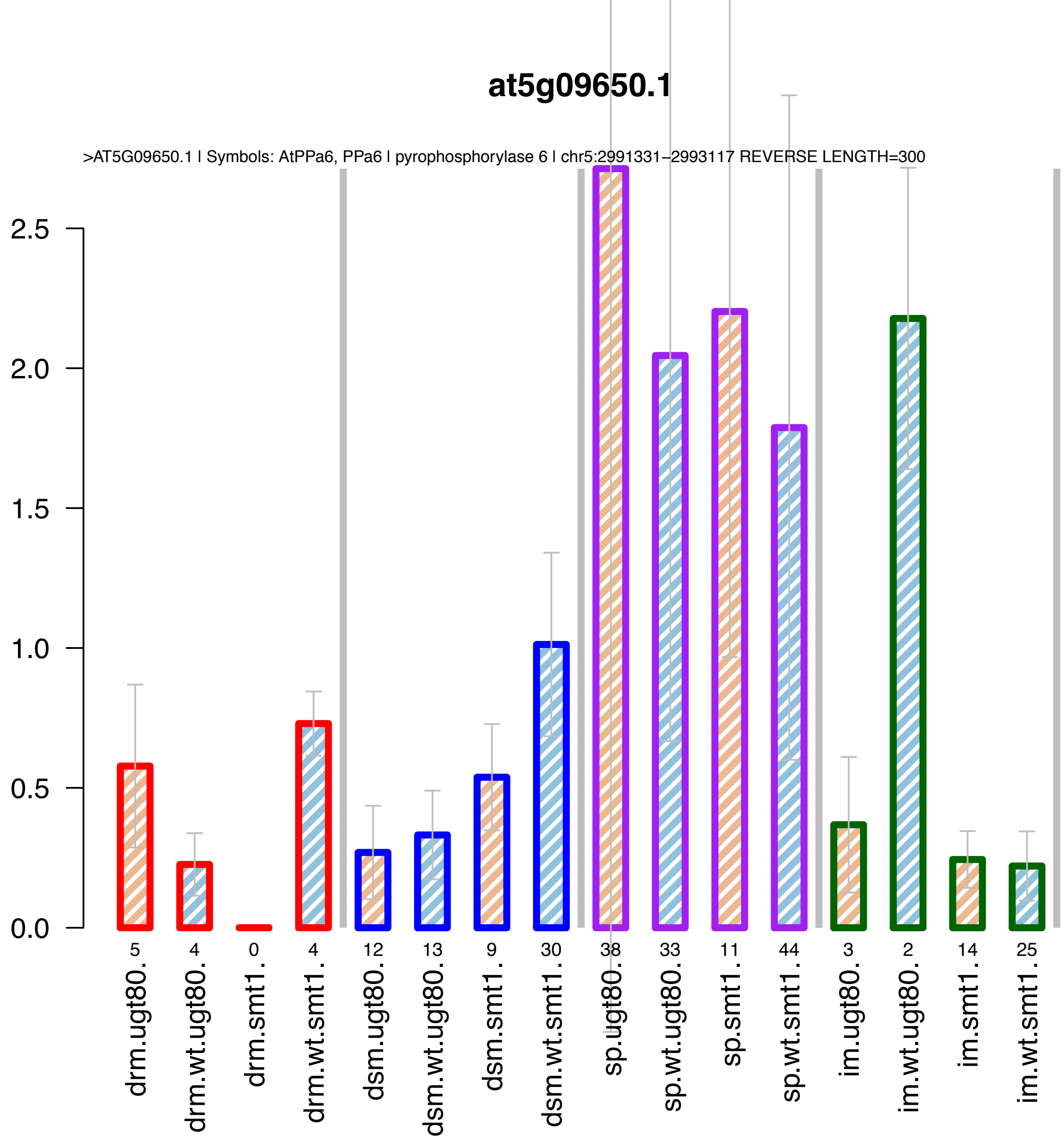

at5g09650.1
>AT5G09650.1 | Symbols: AtPPa6, PPa6 | pyrophosphorylase 6 | chr5:2991331–2993117 REVERSE LENGTH=300
0.0
0.5
1.0
1.5
2.0
2.5
5
4
0
4
12
13
9
30
38
33
11
44
3
2
14
25
drm.ugt80.
drm.wt.ugt80.
drm.smt1.
drm.wt.smt1.
dsm.ugt80.
dsm.wt.ugt80.
dsm.smt1.
dsm.wt.smt1.
sp.ugt80.
sp.wt.ugt80.
sp.smt1.
sp.wt.smt1.
im.ugt80.
im.wt.ugt80.
im.smt1.
im.wt.smt1.

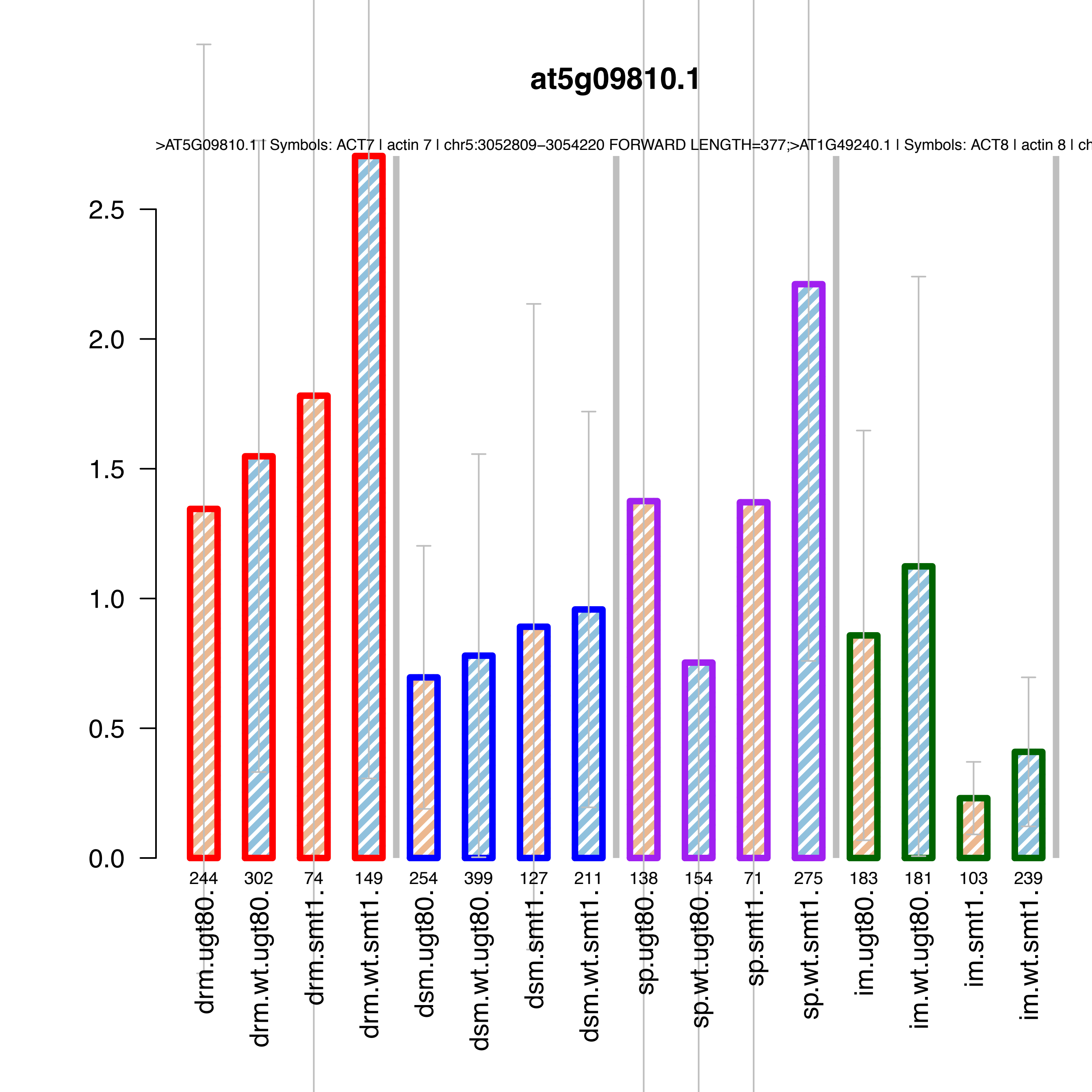

at5g09810.1
>AT5G09810.1 | Symbols: ACT7 | actin 7 | chr5:3052809−3054220 FORWARD LENGTH=377;>AT1G49240.1 | Symbols: ACT8 | actin 8 | ch
2.5
2.0
1.5
1.0
0.5
0.0
244
302
74
149
254
399
127
211
138
154
71
275
183
181
103
239
drm.ugt80..
drm.wt.ugt80..
drm.smt1..
drm.wt.smt1..
dsm.ugt80..
dsm.wt.ugt80..
dsm.smt1..
dsm.wt.smt1..
sp.ugt80..
sp.wt.ugt80..
sp.smt1..
sp.wt.smt1..
im.ugt80..
im.wt.ugt80..
im.smt1..
im.wt.smt1..

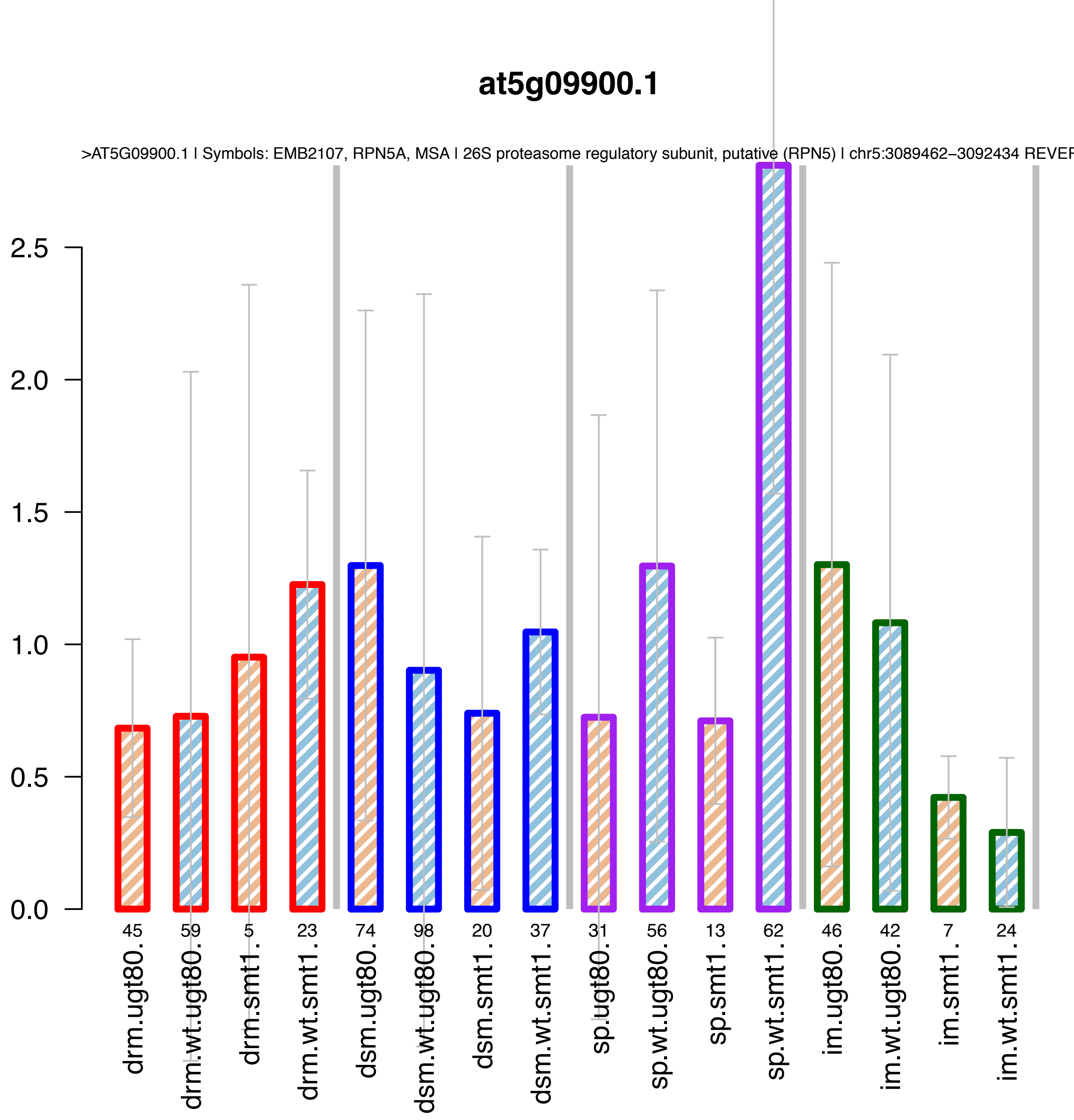

at5g09900.1
>AT5G09900.1 | Symbols: EMB2107, RPN5A, MSA | 26S proteasome regulatory subunit, putative (RPN5) | chr5:3089462–3092434 REVER
0.0
0.5
1.0
1.5
2.0
2.5
45
59
5
23
74
98
20
37
31
56
13
62
46
42
7
24
drm.ugt80.
drm.wt.ugt80.
drm.smt1.
drm.wt.smt1.
dsm.ugt80.
dsm.wt.ugt80.
dsm.smt1.
dsm.wt.smt1.
sp.ugt80.
sp.wt.ugt80.
sp.smt1.
sp.wt.smt1.
im.ugt80.
im.wt.ugt80.
im.smt1.
im.wt.smt1.

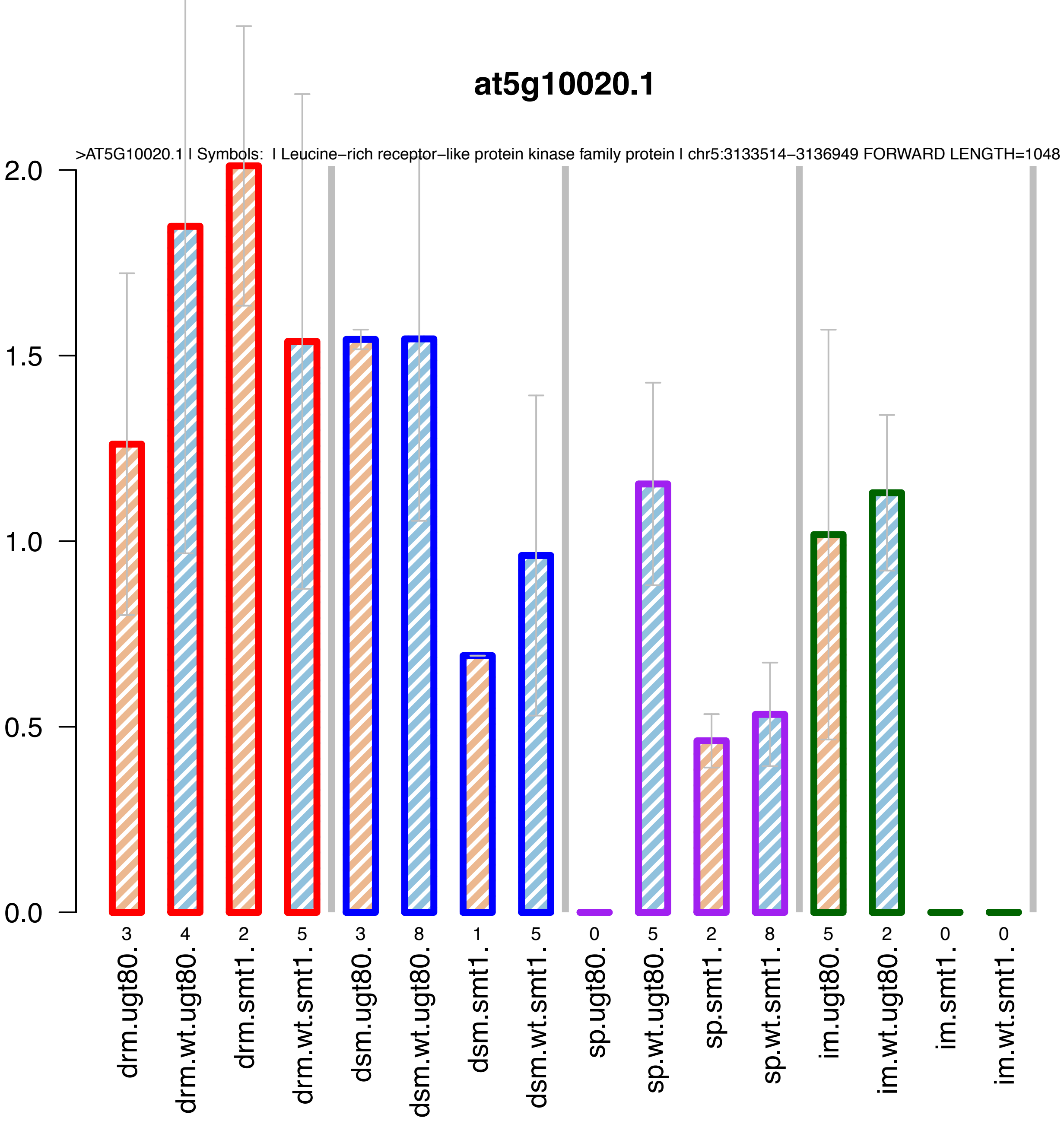

at5g10020.1
>AT5G10020.1 | Symbols: | Leucine−rich receptor−like protein kinase family protein | chr5:3133514−3136949 FORWARD LENGTH=1048
2.0
1.5
1.0
0.5
0.0
3
4
2
5
3
8
1
5
0
5
2
8
5
2
0
0
drm.ugt80.
drm.wt.ugt80.
drm.smt1.
drm.wt.smt1.
dsm.ugt80.
dsm.wt.ugt80.
dsm.smt1.
dsm.wt.smt1.
sp.ugt80.
sp.wt.ugt80.
sp.smt1.
sp.wt.smt1.
im.ugt80.
im.wt.ugt80.
im.smt1.
im.wt.smt1.

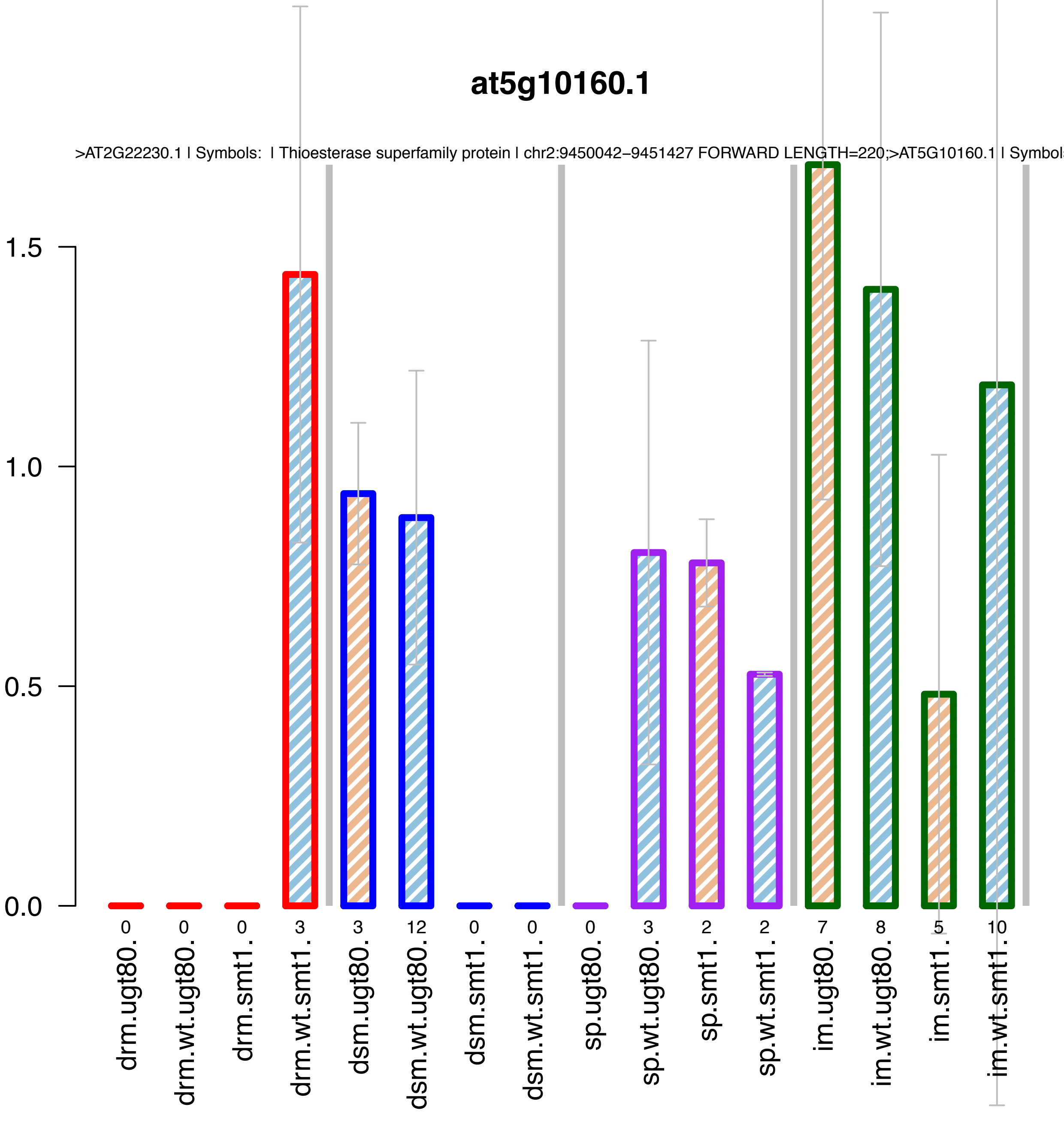

at5g10160.1
>AT2G22230.1 | Symbols: | Thioesterase superfamily protein | chr2:9450042-9451427 FORWARD LENGTH=220;>AT5G10160.1 | Symbol
1.5
1.0
0.5
0.0
0
0
0
3
3
12
0
0
0
3
2
2
7
8
5
10
drm.ugt80.
drm.wt.ugt80.
drm.smt1.
drm.wt.smt1.
dsm.ugt80.
dsm.wt.ugt80.
dsm.smt1.
dsm.wt.smt1.
sp.ugt80.
sp.wt.ugt80.
sp.smt1.
sp.wt.smt1.
im.ugt80.
im.wt.ugt80.
im.smt1.
im.wt.smt1.

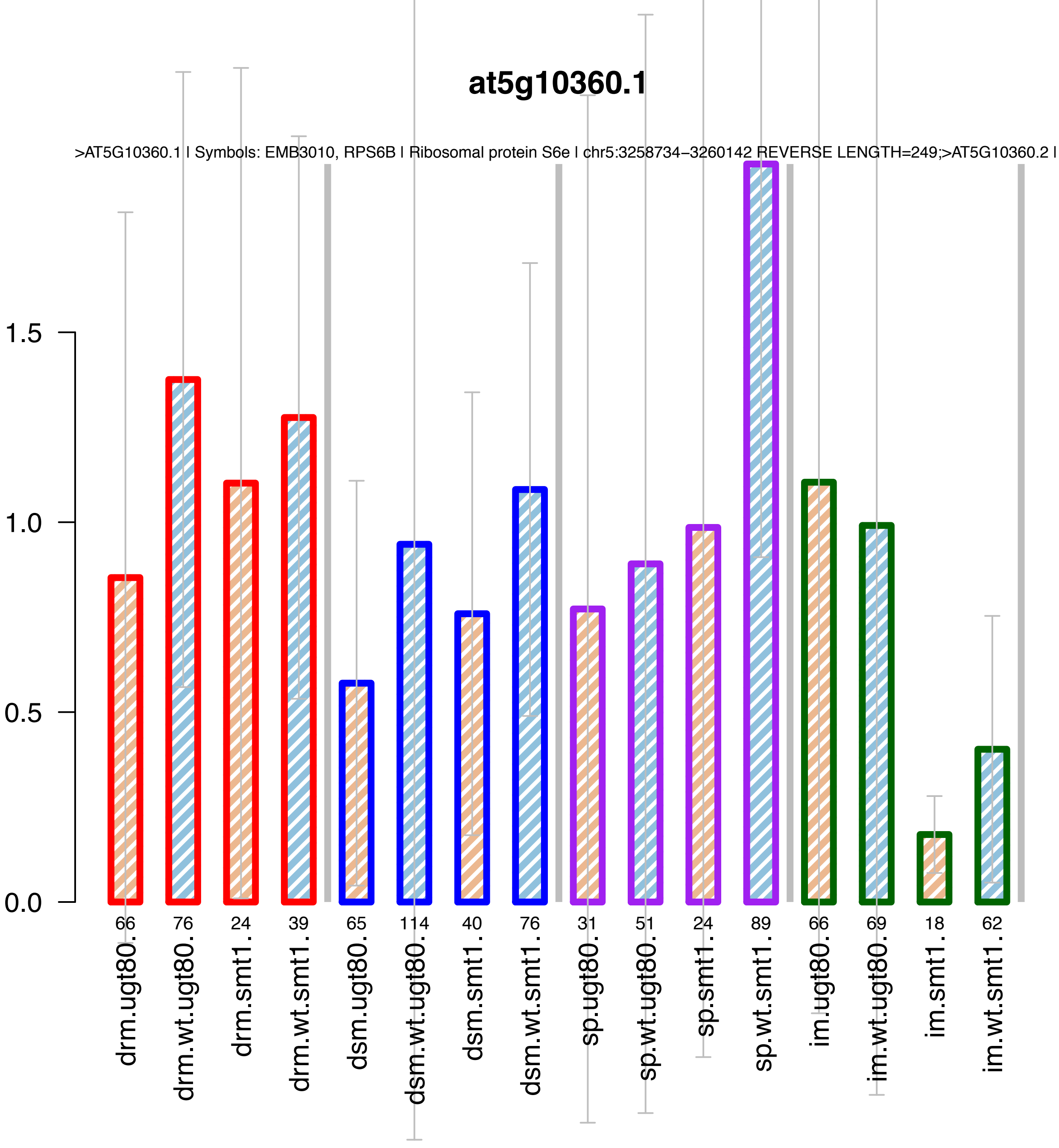

at5g10360.1
>AT5G10360.1 | Symbols: EMB3010, RPS6B | Ribosomal protein S6e | chr5:3258734–3260142 REVERSE LENGTH=249;>AT5G10360.2 |
1.5
1.0
0.5
0.0
66
76
24
39
65
114
40
76
31
51
24
89
66
69
18
62
drm.ugt80.
drm.wt.ugt80.
drm.smt1.
drm.wt.smt1.
dsm.ugt80.
dsm.wt.ugt80.
dsm.smt1.
dsm.wt.smt1.
sp.ugt80.
sp.wt.ugt80.
sp.smt1.
sp.wt.smt1.
im.ugt80.
im.wt.ugt80.
im.smt1.
im.wt.smt1.

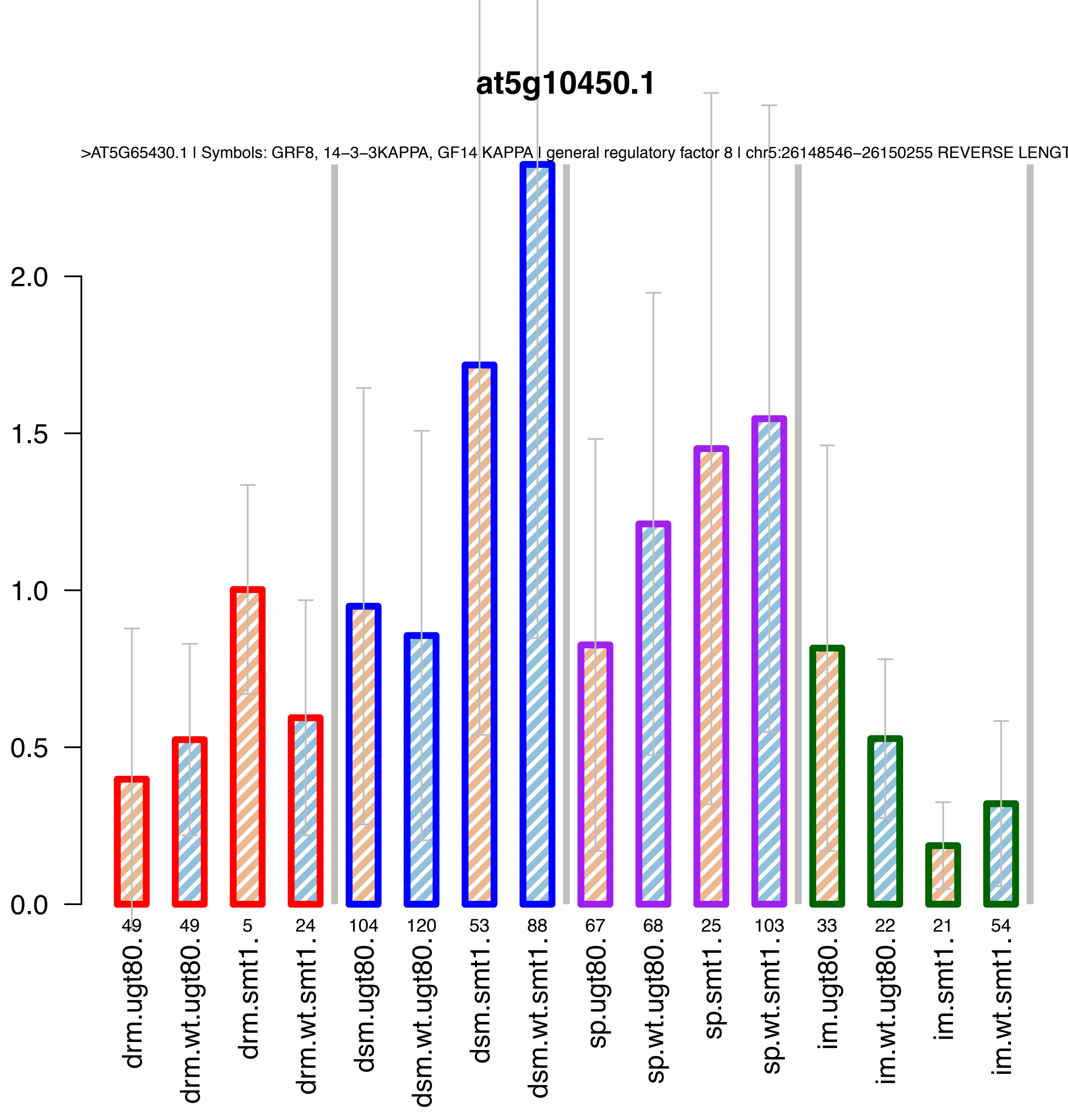

at5g10450.1
>AT5G65430.1 | Symbols: GRF8, 14-3-3KAPPA, GF14 KAPPA | general regulatory factor 8 | chr5:26148546-26150255 REVERSE LENGT
0.0
0.5
1.0
1.5
2.0
49
49
5
24
104
120
53
88
67
68
25
103
33
22
21
54
drm.ugt80.
drm.wt.ugt80.
drm.smt1.
drm.wt.smt1.
dsm.ugt80.
dsm.wt.ugt80.
dsm.smt1.
dsm.wt.smt1.
sp.ugt80.
sp.wt.ugt80.
sp.smt1.
sp.wt.smt1.
im.ugt80.
im.wt.ugt80.
im.smt1.
im.wt.smt1.

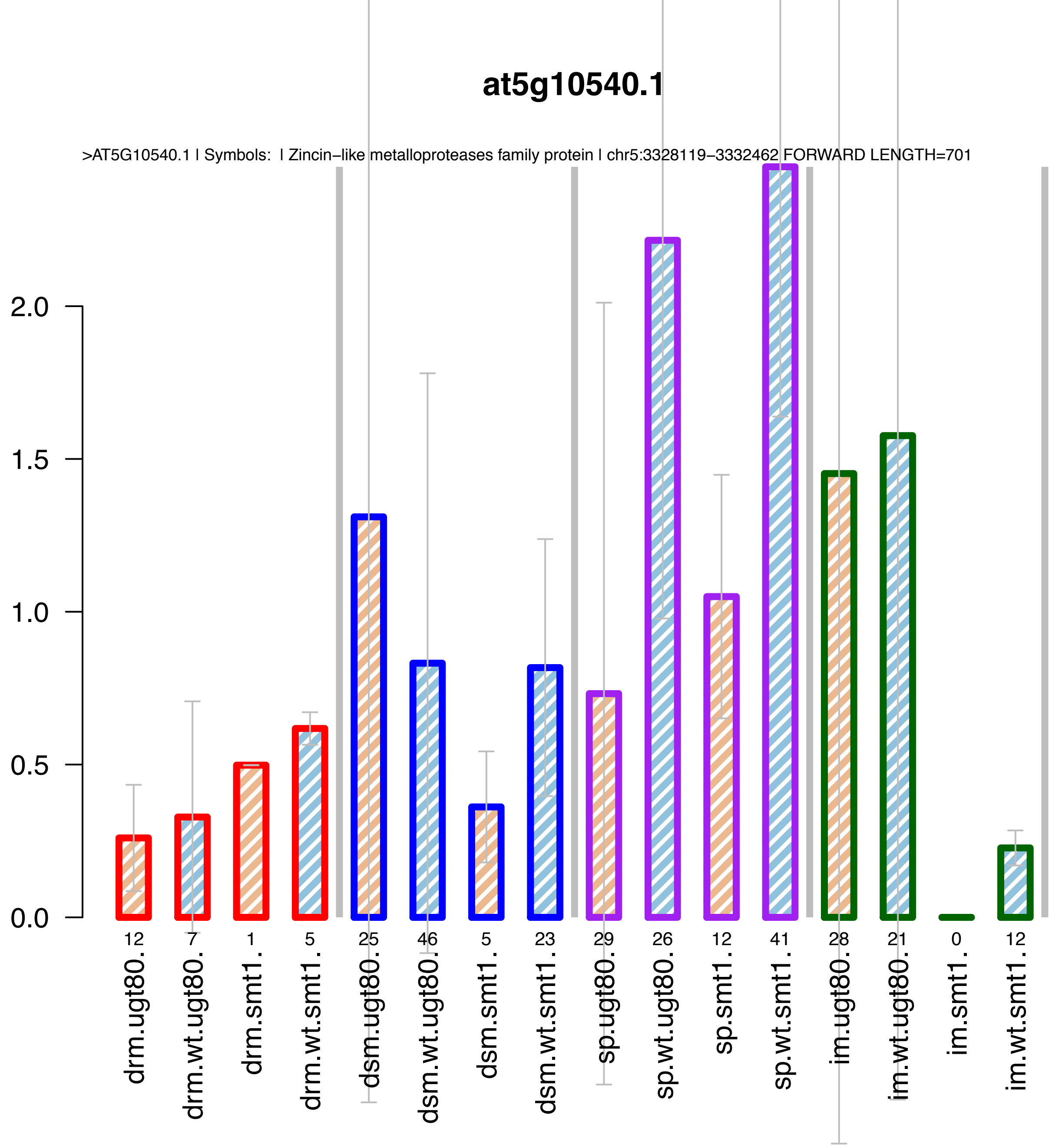

at5g10540.1
>AT5G10540.1 | Symbols: | Zincin–like metalloproteases family protein | chr5:3328119–3332462 FORWARD LENGTH=701
2.0
1.5
1.0
0.5
0.0
12
7
1
5
25
46
5
23
29
26
12
41
28
21
0
12
drm.ugt80..
drm.wt.ugt80..
drm.smt1..
drm.wt.smt1..
dsm.ugt80..
dsm.wt.ugt80..
dsm.smt1..
dsm.wt.smt1..
sp.ugt80..
sp.wt.ugt80..
sp.smt1..
sp.wt.smt1..
im.ugt80..
im.wt.ugt80..
im.smt1..
im.wt.smt1..

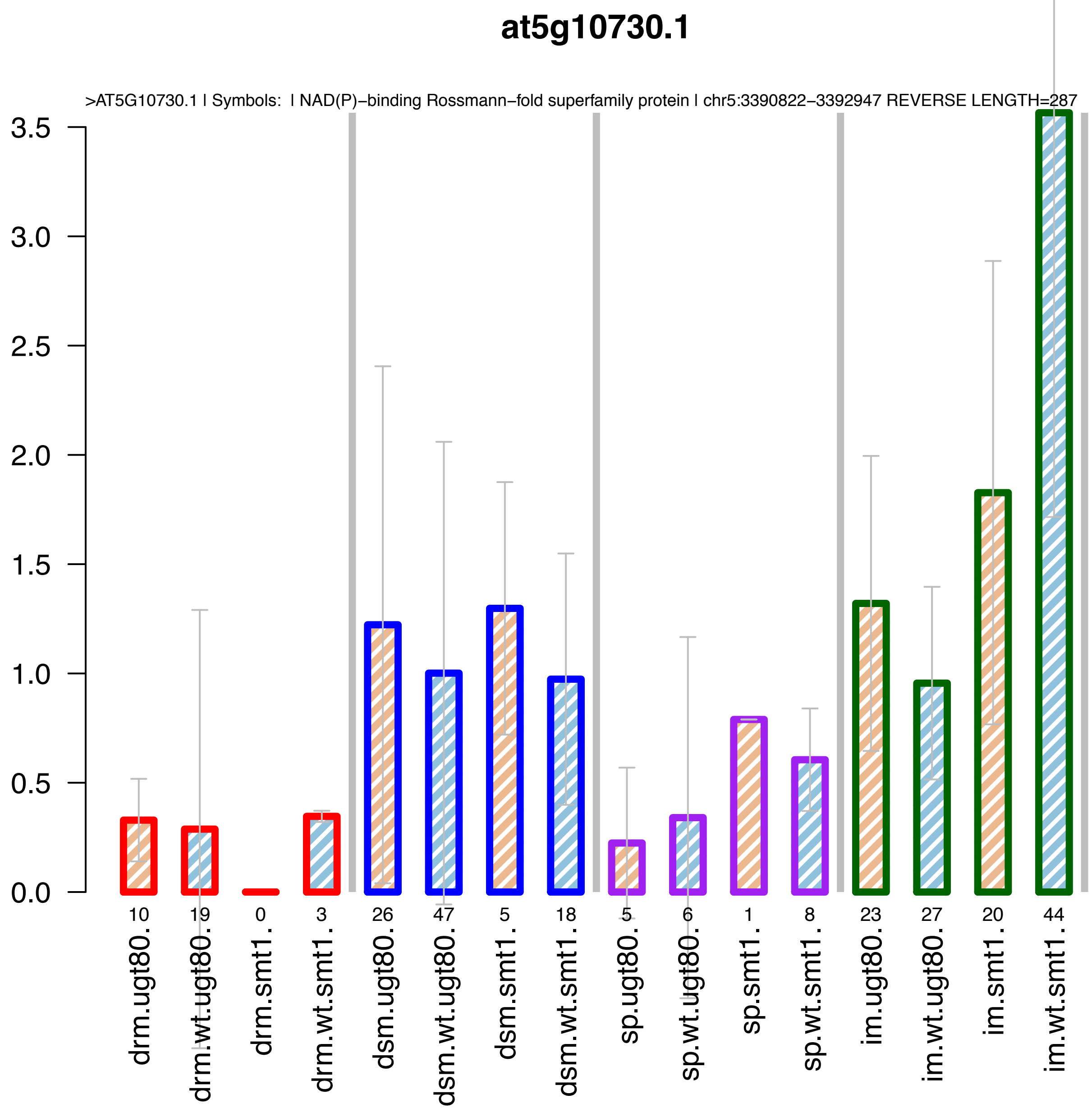

at5g10730.1
>AT5G10730.1 | Symbols: | NAD(P)−binding Rossmann−fold superfamily protein | chr5:3390822−3392947 REVERSE LENGTH=287
0.0
0.5
1.0
1.5
2.0
2.5
3.0
3.5
10
19
0
3
26
47
5
18
5
6
1
8
23
27
20
44
drm.ugt80.
drm.wt.ugt80.
drm.smt1.
drm.wt.smt1.
dsm.ugt80.
dsm.wt.ugt80.
dsm.smt1.
dsm.wt.smt1.
sp.ugt80.
sp.wt.ugt80.
sp.smt1.
sp.wt.smt1.
im.ugt80.
im.wt.ugt80.
im.smt1.
im.wt.smt1.

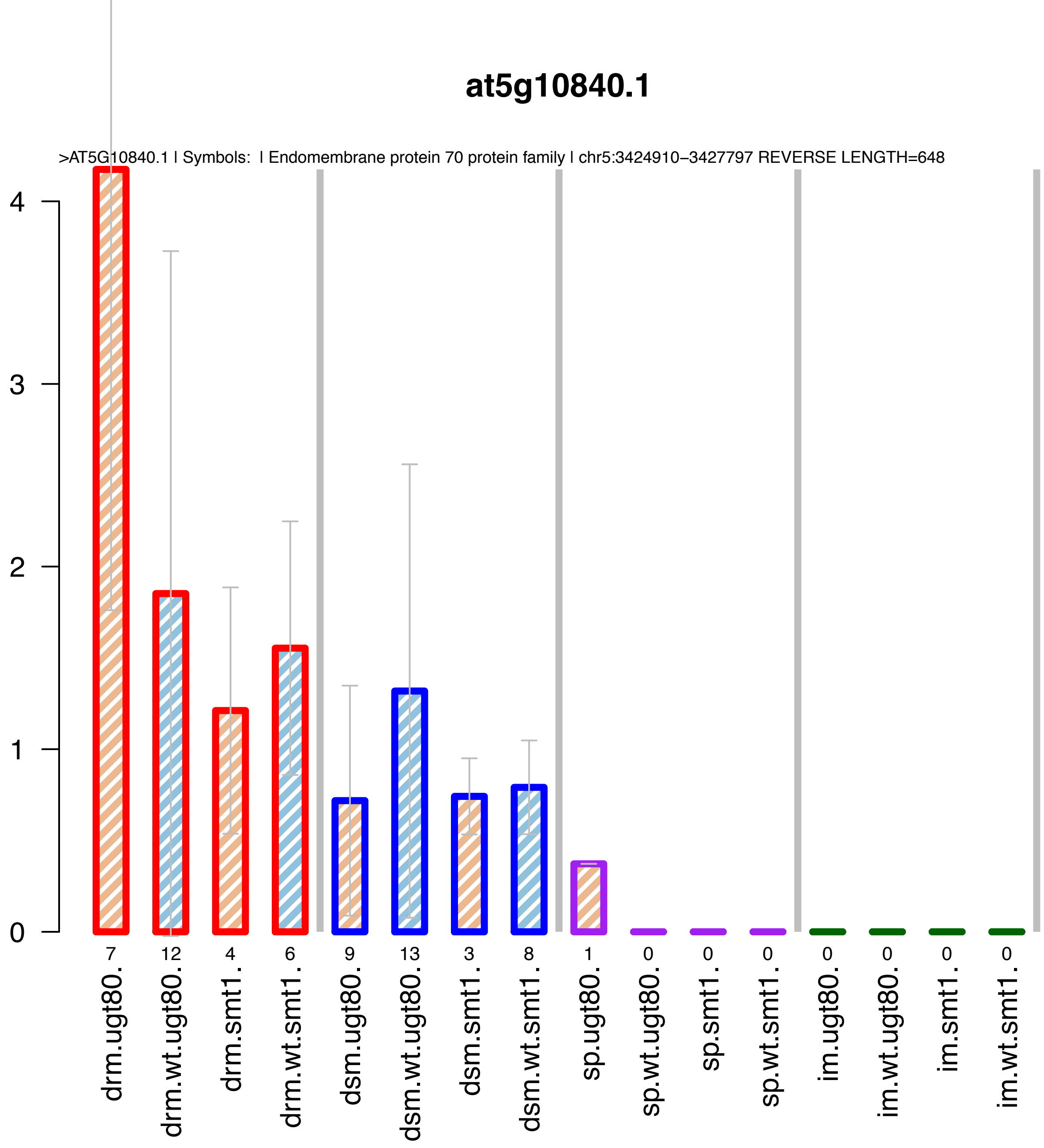

at5g10840.1
>AT5G10840.1 | Symbols: | Endomembrane protein 70 protein family | chr5:3424910-3427797 REVERSE LENGTH=648
0
1
2
3
4
7
12
4
6
9
13
3
8
1
0
0
0
0
0
0
0
drm.ugt80.
drm.wt.ugt80.
drm.smt1.
drm.wt.smt1.
dsm.ugt80.
dsm.wt.ugt80.
dsm.smt1.
dsm.wt.smt1.
sp.ugt80.
sp.wt.ugt80.
sp.smt1.
sp.wt.smt1.
im.ugt80.
im.wt.ugt80.
im.smt1.
im.wt.smt1.

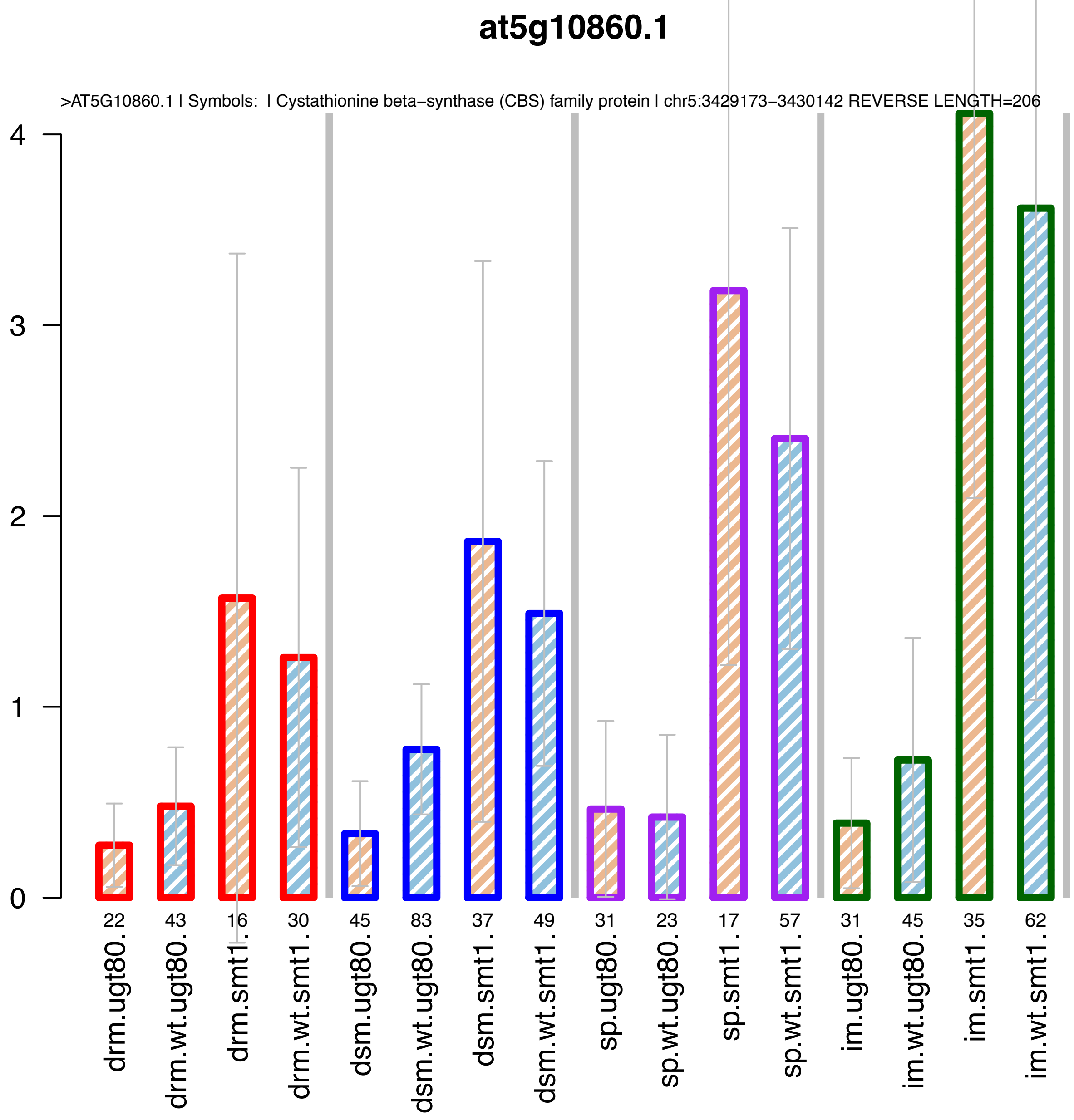

at5g10860.1
>AT5G10860.1 | Symbols: | Cystathionine beta−synthase (CBS) family protein | chr5:3429173−3430142 REVERSE LENGTH=206
0
1
2
3
4
22
43
16
30
45
83
37
49
31
23
17
57
31
45
35
62
drm.ugt80..
drm.wt.ugt80..
drm.smt1..
drm.wt.smt1..
dsm.ugt80..
dsm.wt.ugt80..
dsm.smt1..
dsm.wt.smt1..
sp.ugt80..
sp.wt.ugt80..
sp.smt1..
sp.wt.smt1..
im.ugt80..
im.wt.ugt80..
im.smt1..
im.wt.smt1..

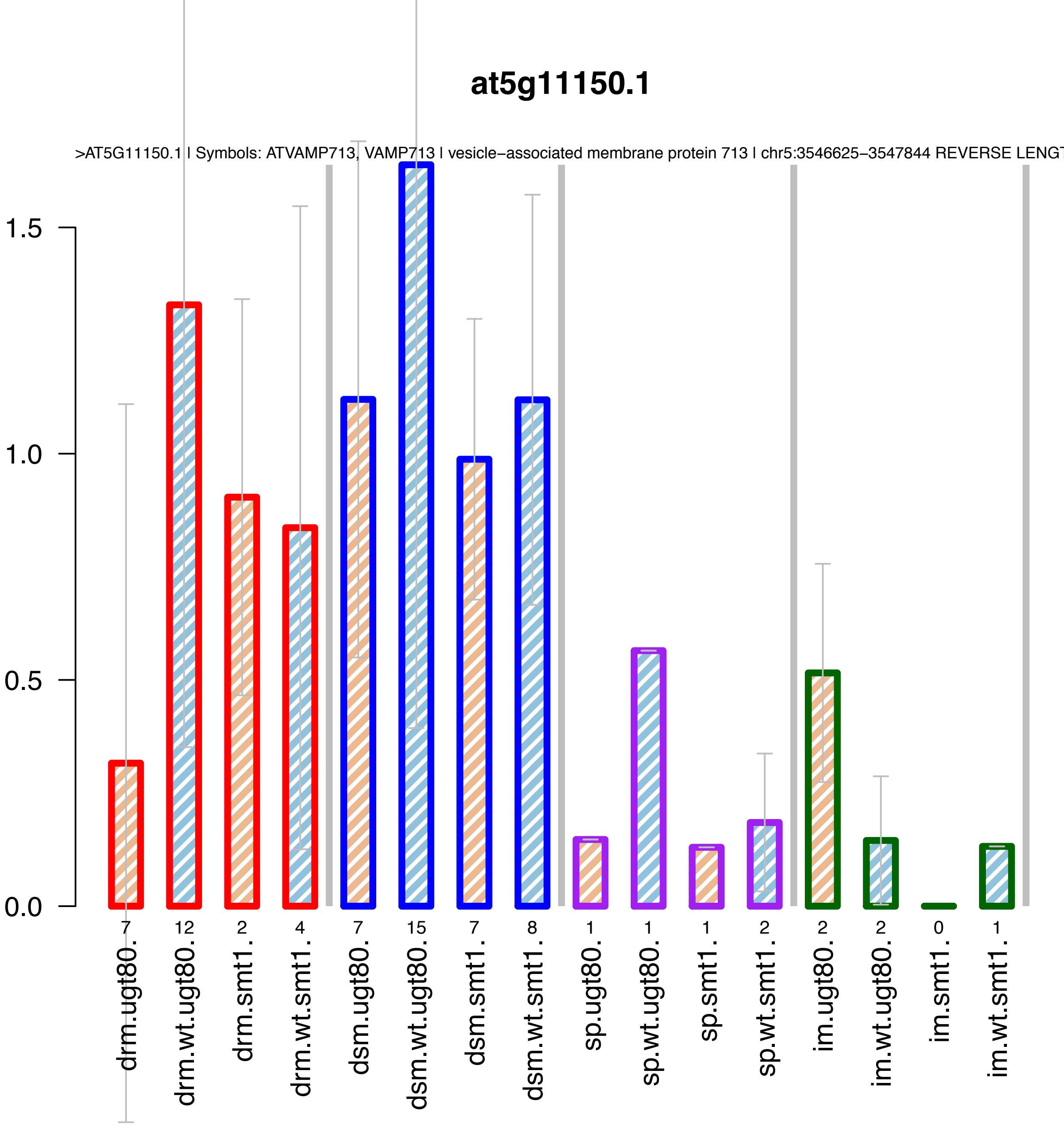

at5g11150.1
>AT5G11150.1 | Symbols: ATVAMP713, VAMP713 | vesicle-associated membrane protein 713 | chr5:3546625-3547844 REVERSE LENGT
1.5
1.0
0.5
0.0
7
12
2
4
7
15
7
8
1
1
1
2
2
2
0
1
drm.ugt80..
drm.wt.ugt80..
drm.smt1..
drm.wt.smt1..
dsm.ugt80..
dsm.wt.ugt80..
dsm.smt1..
dsm.wt.smt1..
sp.ugt80..
sp.wt.ugt80..
sp.smt1..
sp.wt.smt1..
im.ugt80..
im.wt.ugt80..
im.smt1..
im.wt.smt1..

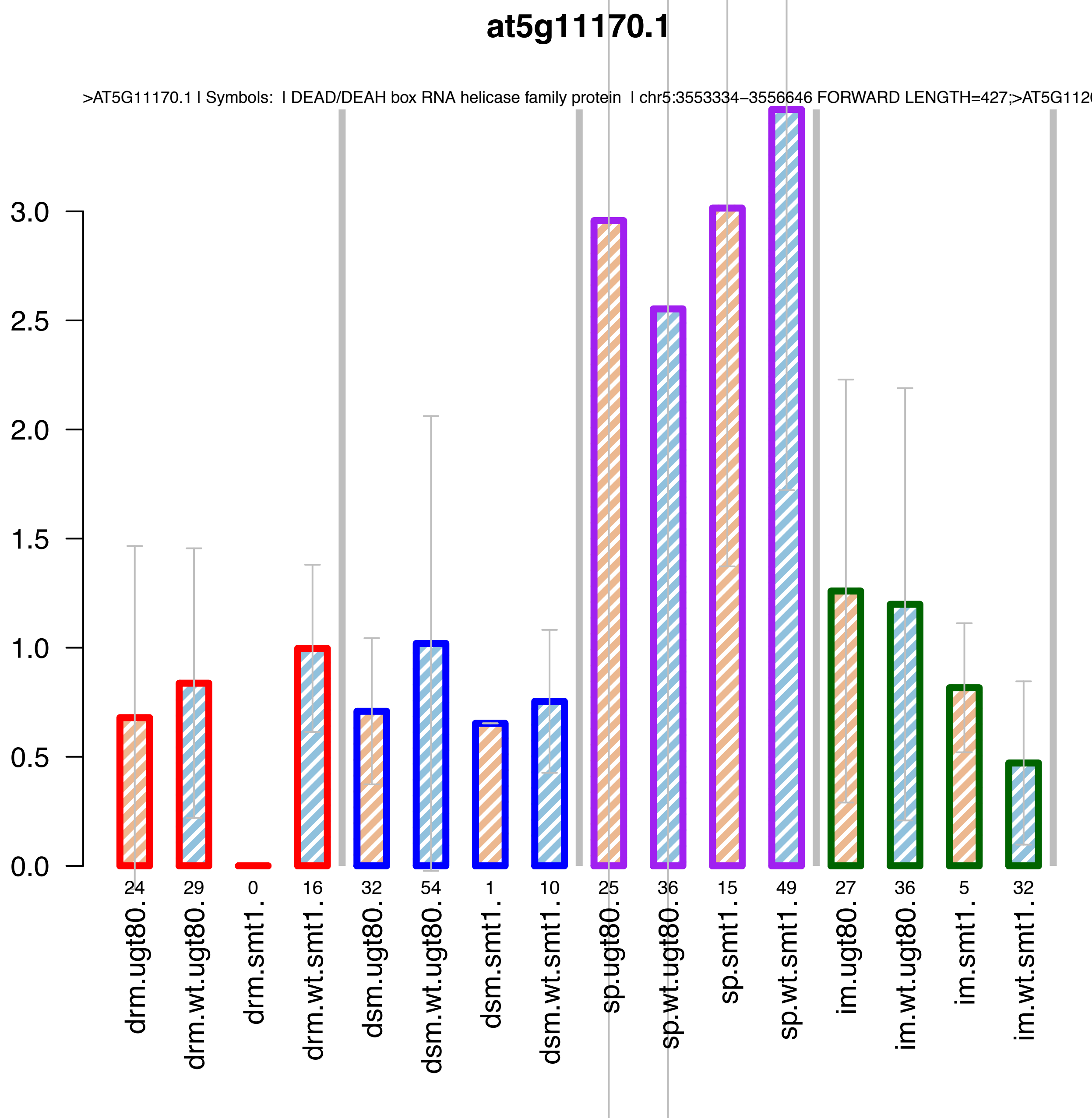

at5g11170.1
>AT5G11170.1 | Symbols: | DEAD/DEAH box RNA helicase family protein | chr5:3553334-3556646 FORWARD LENGTH=427;>AT5G112
0.0
0.5
1.0
1.5
2.0
2.5
3.0
24
29
0
16
32
54
1
10
25
36
15
49
27
36
5
32
drm.ugt80.
drm.wt.ugt80.
drm.smt1.
drm.wt.smt1.
dsm.ugt80.
dsm.wt.ugt80.
dsm.smt1.
dsm.wt.smt1.
sp.ugt80.
sp.wt.ugt80.
sp.smt1.
sp.wt.smt1.
im.ugt80.
im.wt.ugt80.
im.smt1.
im.wt.smt1.

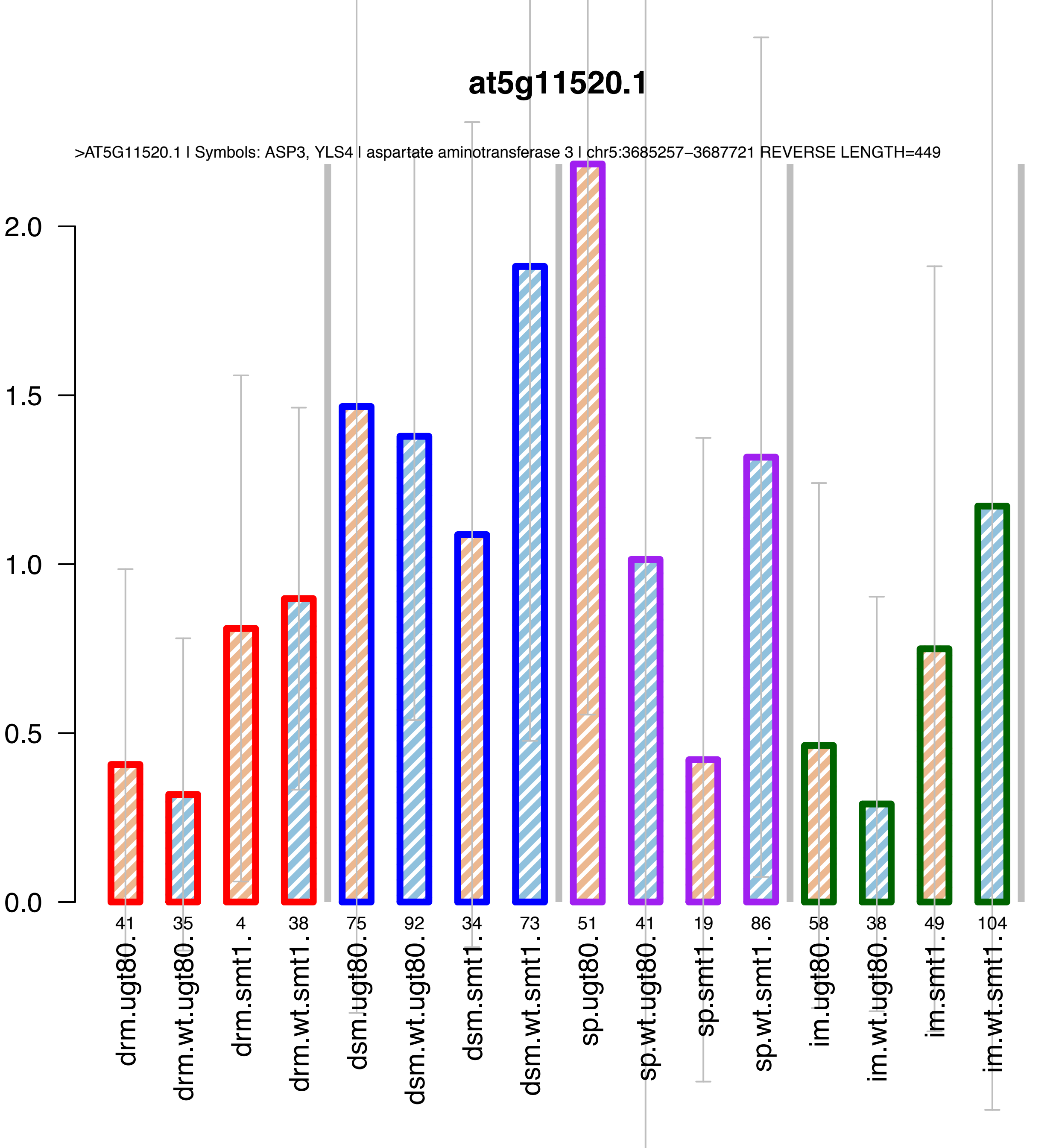

at5g11520.1
>AT5G11520.1 | Symbols: ASP3, YLS4 | aspartate aminotransferase 3 | chr5:3685257−3687721 REVERSE LENGTH=449
2.0
1.5
1.0
0.5
0.0
41
35
4
38
75
92
34
73
51
41
19
86
58
38
49
104
drm.ugt80..
drm.wt.ugt80..
drm.smt1..
drm.wt.smt1..
dsm.ugt80..
dsm.wt.ugt80..
dsm.smt1..
dsm.wt.smt1..
sp.ugt80..
sp.wt.ugt80..
sp.smt1..
sp.wt.smt1..
im.ugt80..
im.wt.ugt80..
im.smt1..
im.wt.smt1..

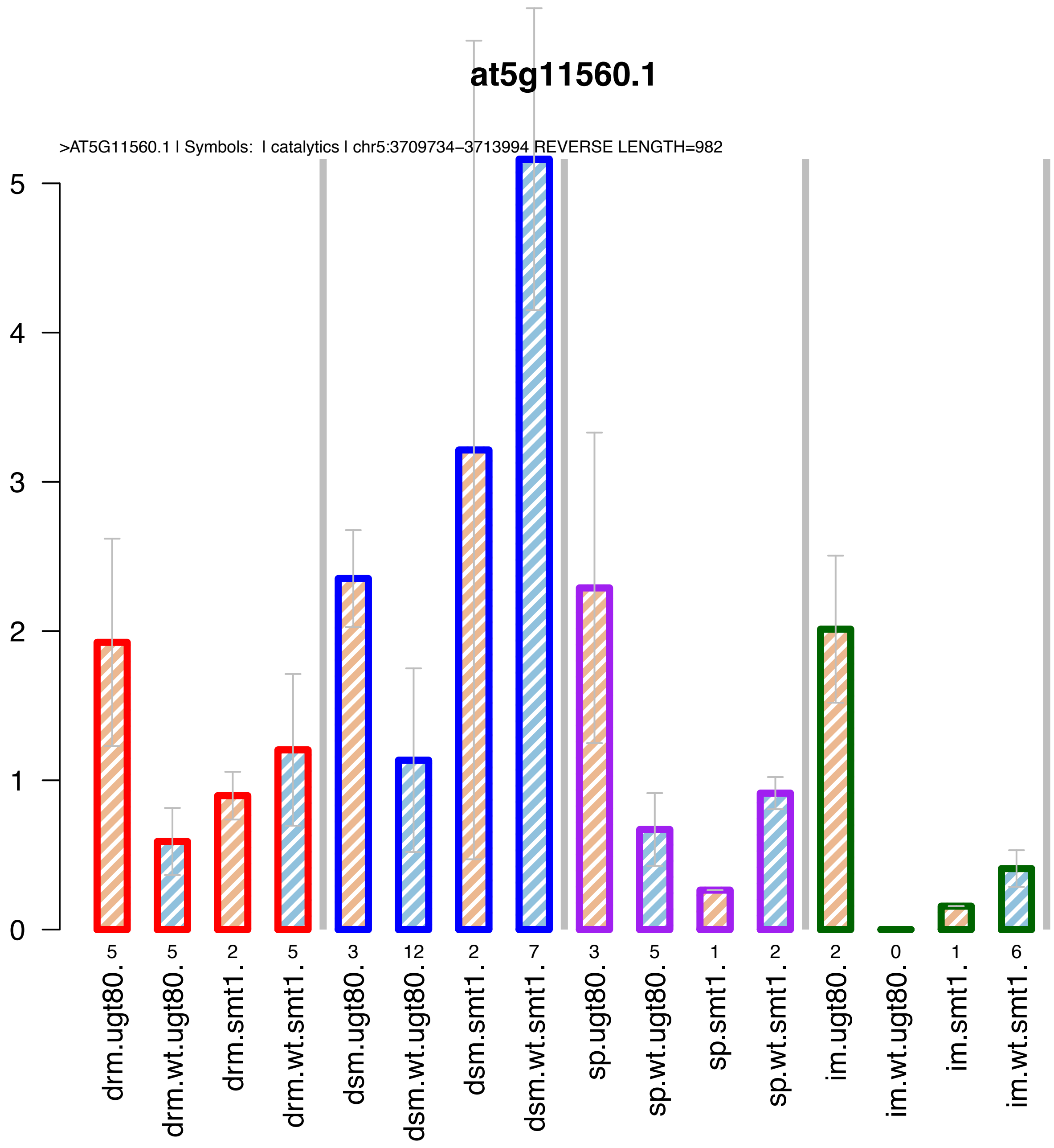

at5g11560.1
>AT5G11560.1 | Symbols: | catalytics | chr5:3709734–3713994 REVERSE LENGTH=982
0
1
2
3
4
5
5
5
2
5
3
12
2
7
3
5
1
2
2
0
1
6
drm.ugt80..
drm.wt.ugt80..
drm.smt1..
drm.wt.smt1..
dsm.ugt80..
dsm.wt.ugt80..
dsm.smt1..
dsm.wt.smt1..
sp.ugt80..
sp.wt.ugt80..
sp.smt1..
sp.wt.smt1..
im.ugt80..
im.wt.ugt80..
im.smt1..
im.wt.smt1..

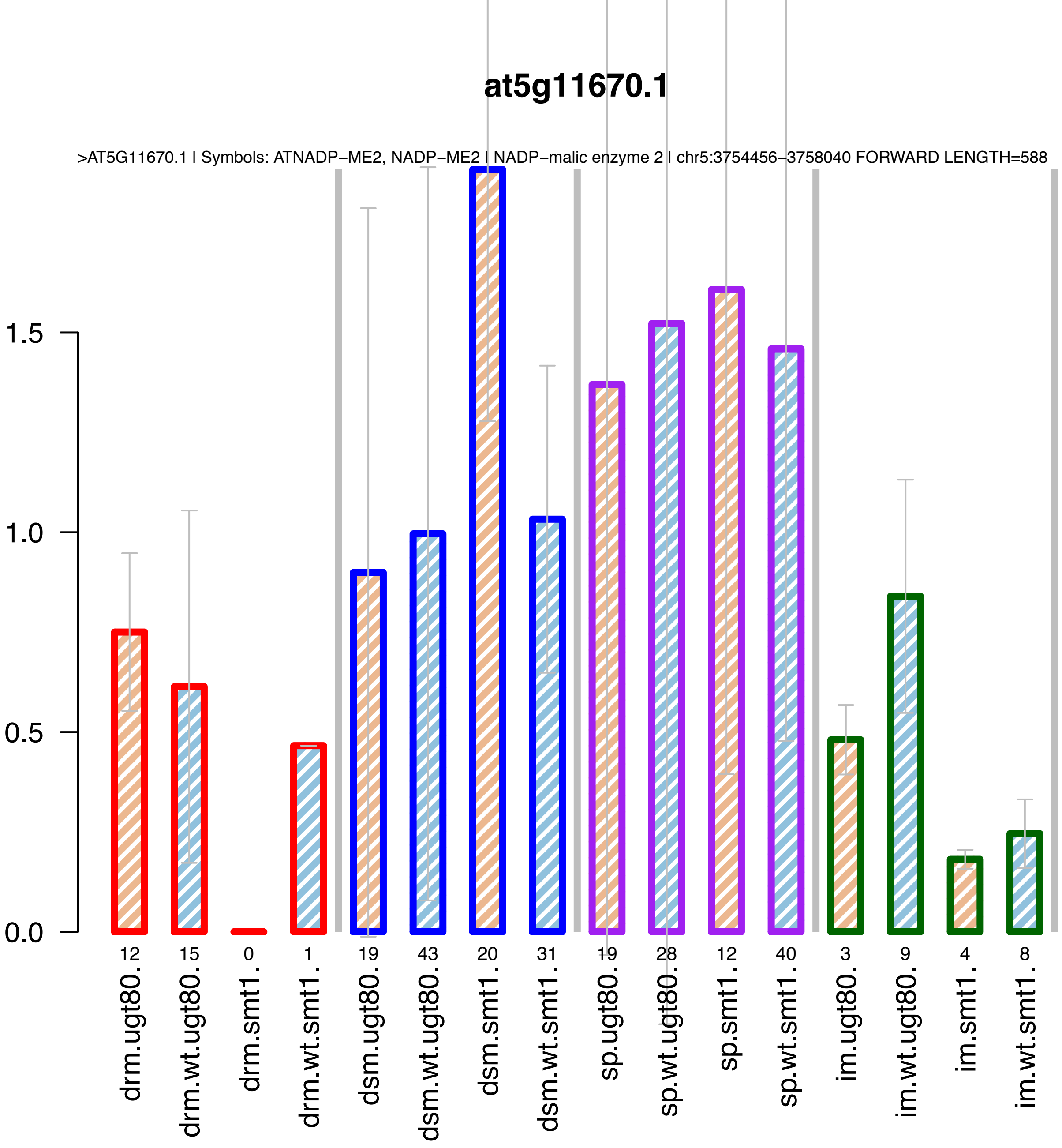
at5g11670.1
>AT5G11670.1 | Symbols: ATNADP−ME2, NADP−ME2 | NADP−malic enzyme 2 | chr5:3754456−3758040 FORWARD LENGTH=588
0.0
0.5
1.0
1.5
12
15
0
1
19
43
20
31
19
28
12
40
3
9
4
8
drm.ugt80..
drm.wt.ugt80..
drm.smt1..
drm.wt.smt1..
dsm.ugt80..
dsm.wt.ugt80..
dsm.smt1..
dsm.wt.smt1..
sp.ugt80..
sp.wt.ugt80..
sp.smt1..
sp.wt.smt1..
im.ugt80..
im.wt.ugt80..
im.smt1..
im.wt.smt1..

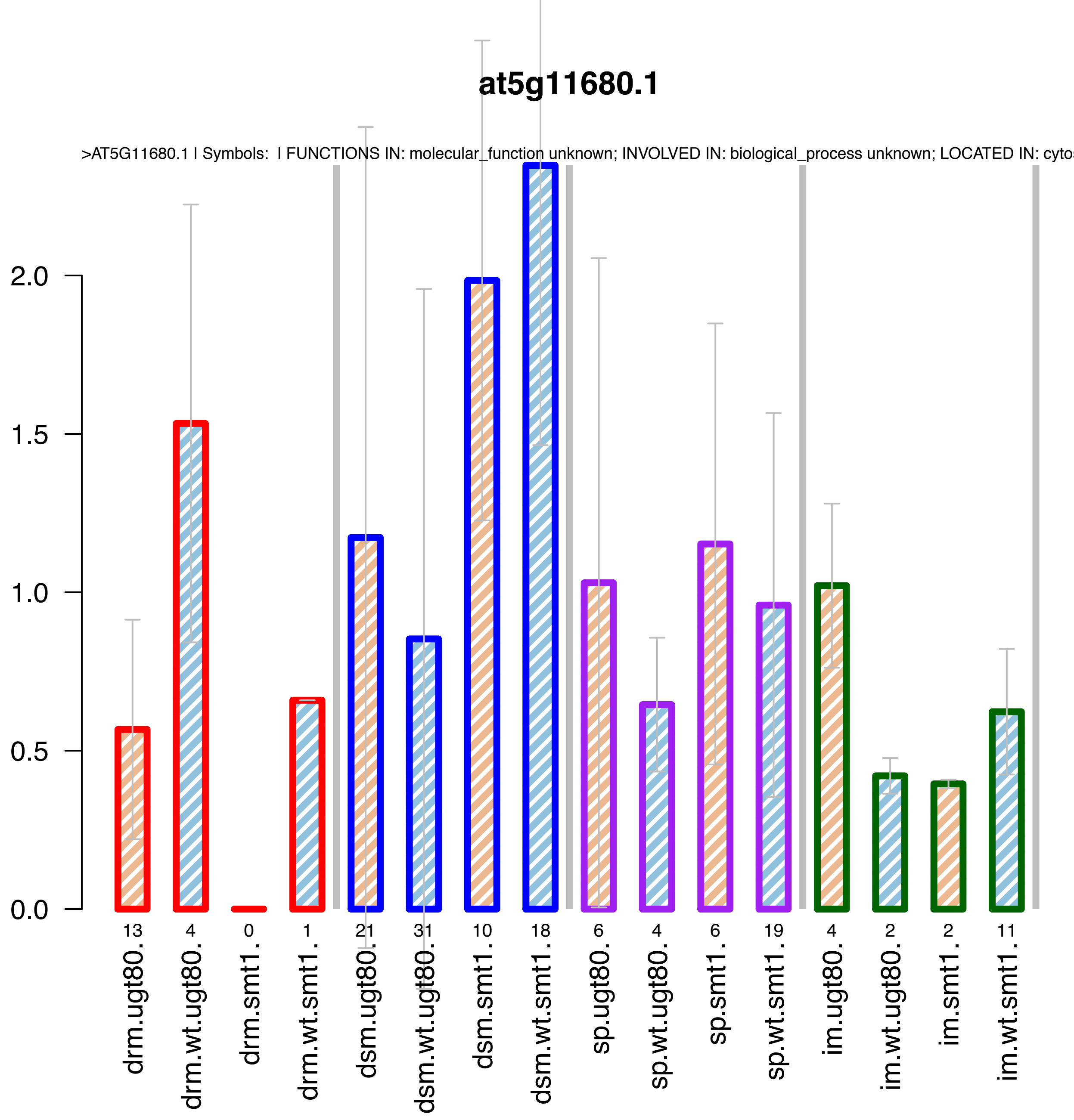

at5g11680.1
>AT5G11680.1 | Symbols: | FUNCTIONS IN: molecular_function unknown; INVOLVED IN: biological_process unknown; LOCATED IN: cyto
2.0
1.5
1.0
0.5
0.0
13
4
0
1
21
31
10
18
6
4
6
19
4
2
2
11
drm.ugt80.
drm.wt.ugt80.
drm.smt1.
drm.wt.smt1.
dsm.ugt80.
dsm.wt.ugt80.
dsm.smt1.
dsm.wt.smt1.
sp.ugt80.
sp.wt.ugt80.
sp.smt1.
sp.wt.smt1.
im.ugt80.
im.wt.ugt80.
im.smt1.
im.wt.smt1.

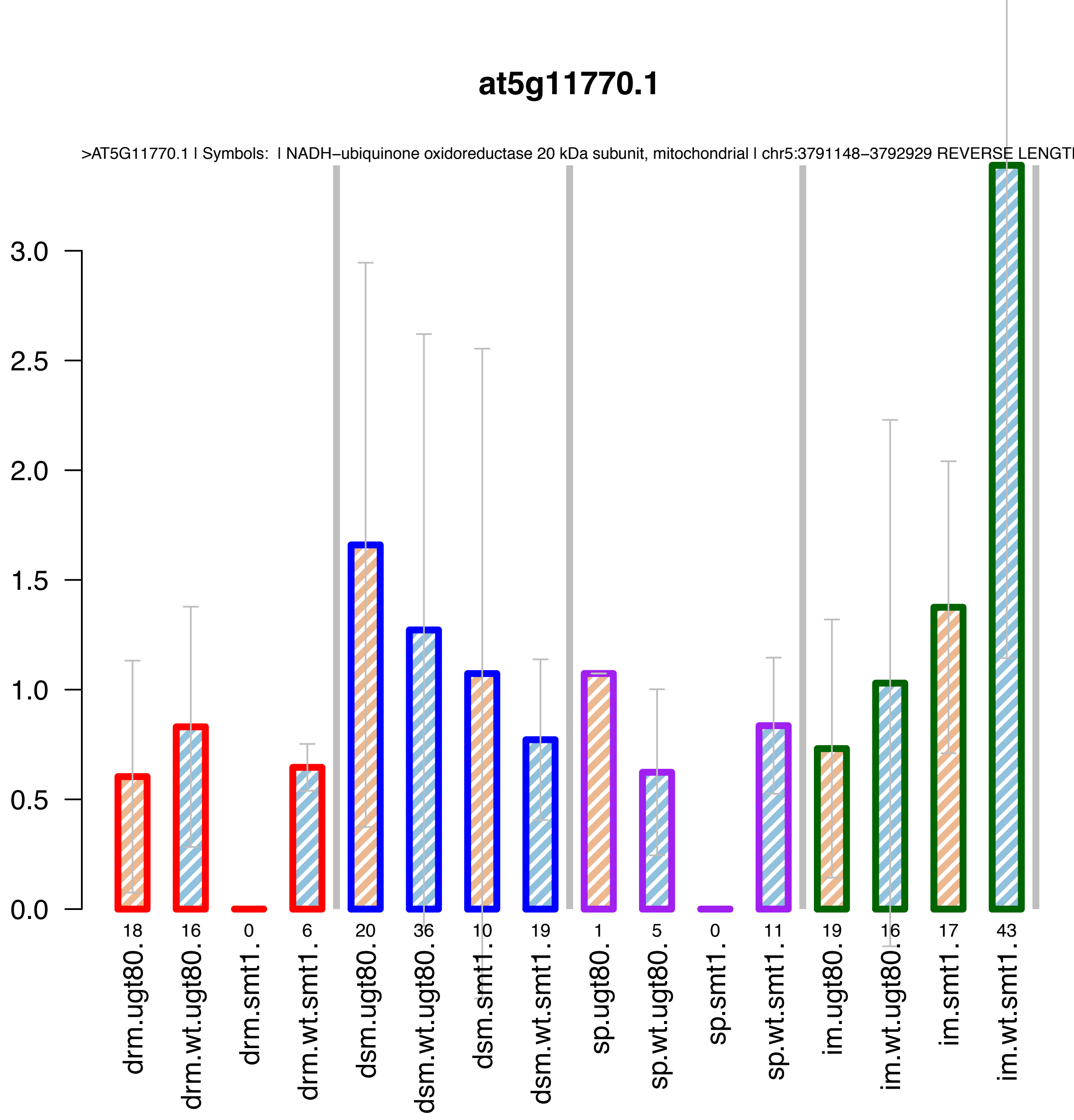

at5g11770.1
>AT5G11770.1 | Symbols: | NADH–ubiquinone oxidoreductase 20 kDa subunit, mitochondrial | chr5:3791148–3792929 REVERSE LENGTH
0.0
0.5
1.0
1.5
2.0
2.5
3.0
18
16
0
6
20
36
10
19
1
5
0
11
19
16
17
43
drm.ugt80.
drm.wt.ugt80.
drm.smt1.
drm.wt.smt1.
dsm.ugt80.
dsm.wt.ugt80.
dsm.smt1.
dsm.wt.smt1.
sp.ugt80.
sp.wt.ugt80.
sp.smt1.
sp.wt.smt1.
im.ugt80.
im.wt.ugt80.
im.smt1.
im.wt.smt1.

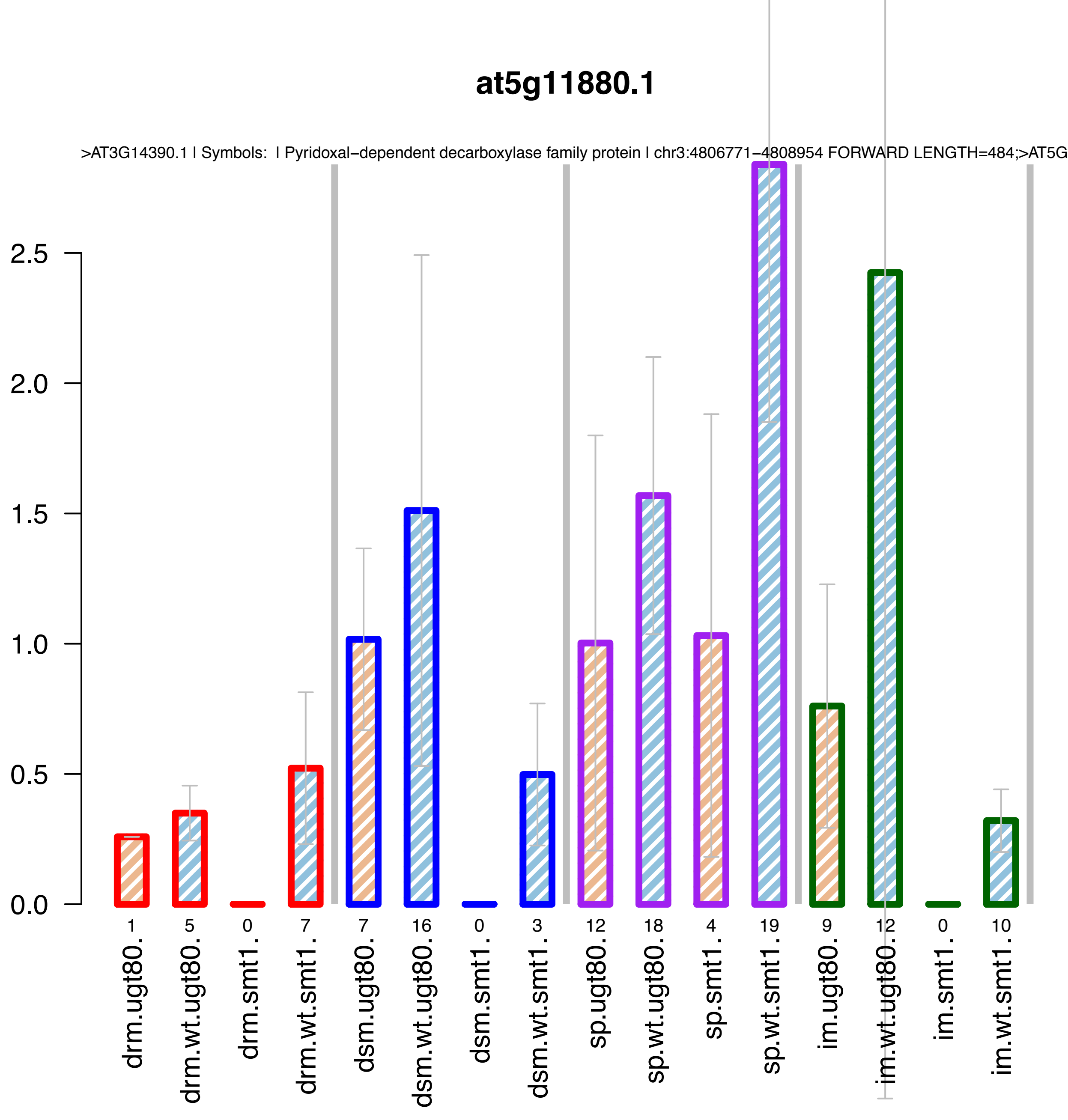
at5g11880.1
>AT3G14390.1 | Symbols: | Pyridoxal−dependent decarboxylase family protein | chr3:4806771−4808954 FORWARD LENGTH=484;>AT5G
0.0
0.5
1.0
1.5
2.0
2.5
1
5
0
7
7
16
0
3
12
18
4
19
9
12
0
10
drm.ugt80.
drm.wt.ugt80.
drm.smt1.
drm.wt.smt1.
dsm.ugt80.
dsm.wt.ugt80.
dsm.smt1.
dsm.wt.smt1.
sp.ugt80.
sp.wt.ugt80.
sp.smt1.
sp.wt.smt1.
im.ugt80.
im.wt.ugt80.
im.smt1.
im.wt.smt1.

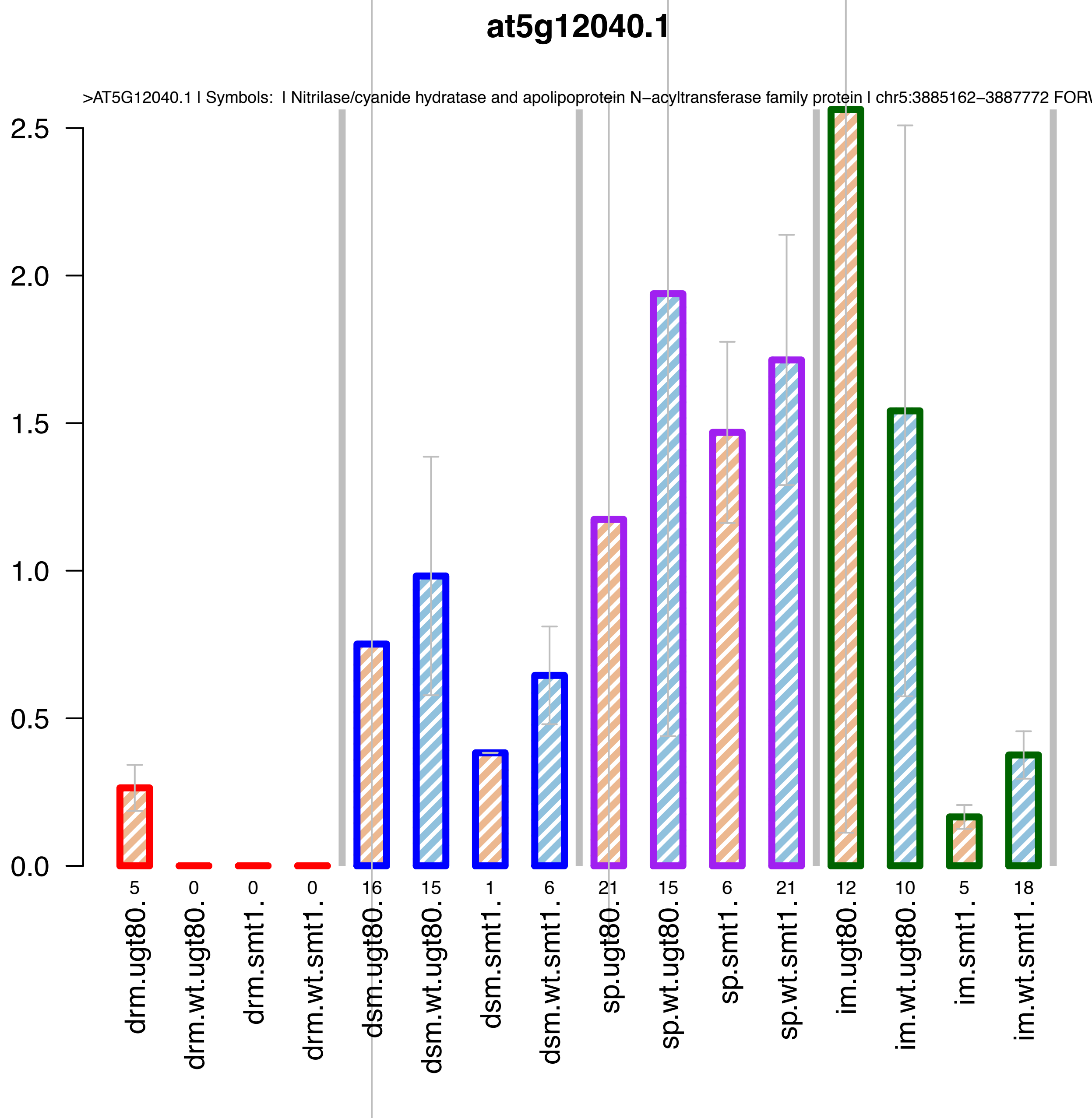

at5g12040.1
>AT5G12040.1 | Symbols: | Nitrilase/cyanide hydratase and apolipoprotein N−acyltransferase family protein | chr5:3885162−3887772 FORW
0.0
0.5
1.0
1.5
2.0
2.5
5
0
0
0
16
15
1
6
21
15
6
21
12
10
5
18
drm.ugt80..
drm.wt.ugt80..
drm.smt1..
drm.wt.smt1..
dsm.ugt80..
dsm.wt.ugt80..
dsm.smt1..
dsm.wt.smt1..
sp.ugt80..
sp.wt.ugt80..
sp.smt1..
sp.wt.smt1..
im.ugt80..
im.wt.ugt80..
im.smt1..
im.wt.smt1..

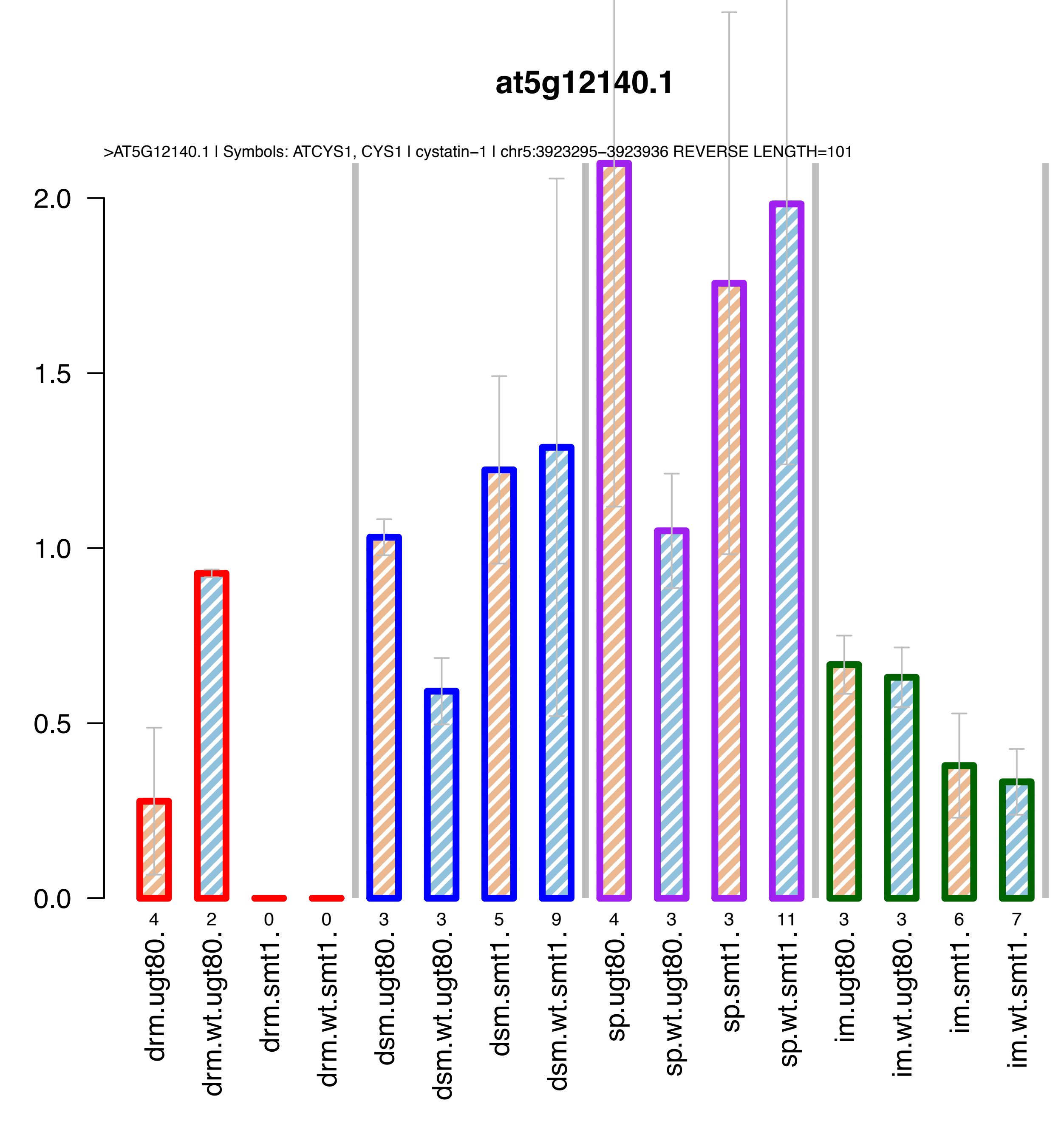

at5g12140.1
>AT5G12140.1 | Symbols: ATCYS1, CYS1 | cystatin−1 | chr5:3923295−3923936 REVERSE LENGTH=101
2.0
1.5
1.0
0.5
0.0
4
2
0
0
3
3
5
9
4
3
3
11
3
3
6
7
drm.ugt80..
drm.wt.ugt80..
drm.smt1..
drm.wt.smt1..
dsm.ugt80..
dsm.wt.ugt80..
dsm.smt1..
dsm.wt.smt1..
sp.ugt80..
sp.wt.ugt80..
sp.smt1..
sp.wt.smt1..
im.ugt80..
im.wt.ugt80..
im.smt1..
im.wt.smt1..

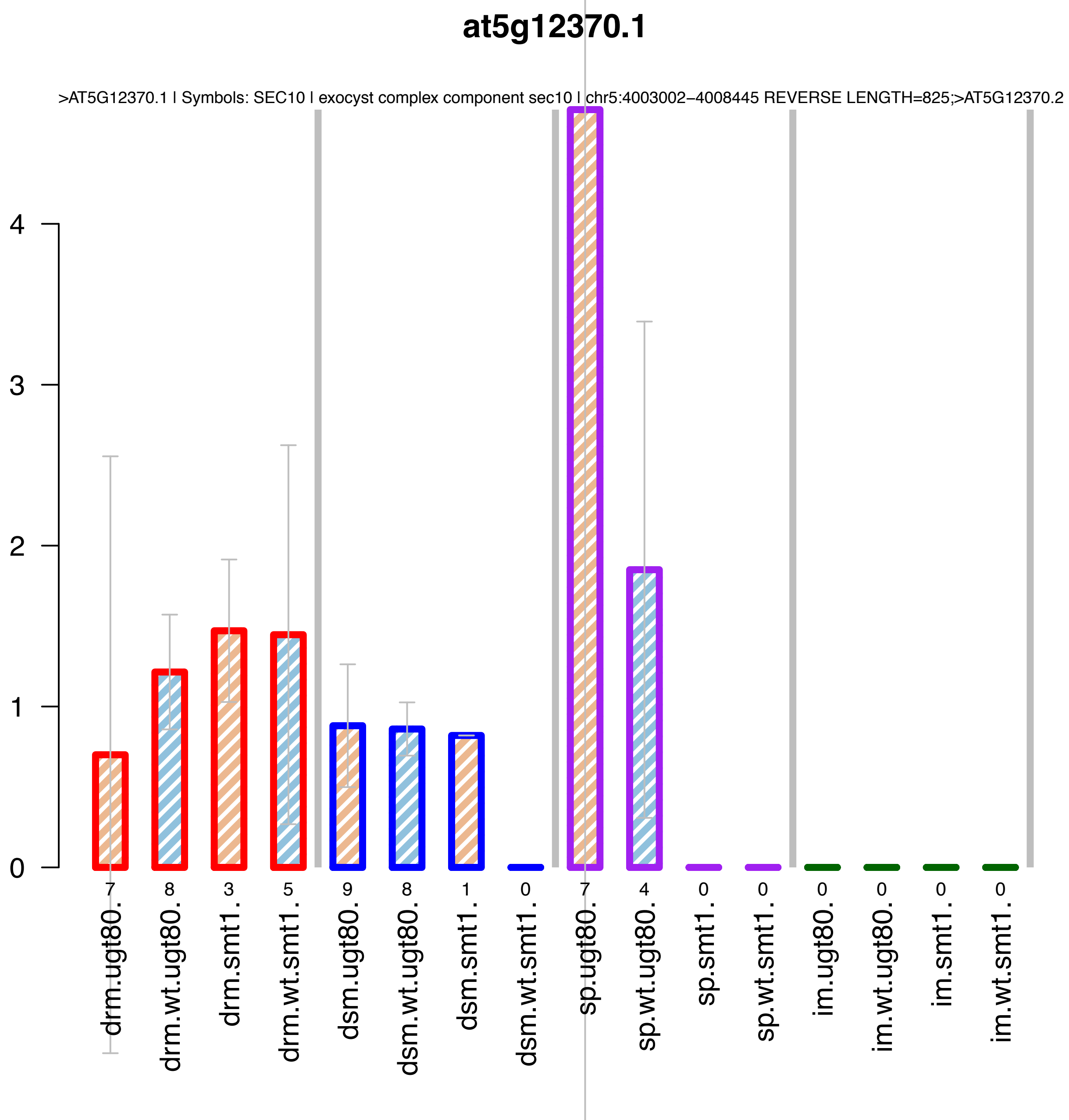

at5g12370.1
>AT5G12370.1 | Symbols: SEC10 | exocyst complex component sec10 | chr5:4003002−4008445 REVERSE LENGTH=825;>AT5G12370.2
0
1
2
3
4
7
8
3
5
9
8
1
0
7
4
0
0
0
0
0
0
drm.ugt80.
drm.wt.ugt80.
drm.smt1.
drm.wt.smt1.
dsm.ugt80.
dsm.wt.ugt80.
dsm.smt1.
dsm.wt.smt1.
sp.ugt80.
sp.wt.ugt80.
sp.smt1.
sp.wt.smt1.
im.ugt80.
im.wt.ugt80.
im.smt1.
im.wt.smt1.

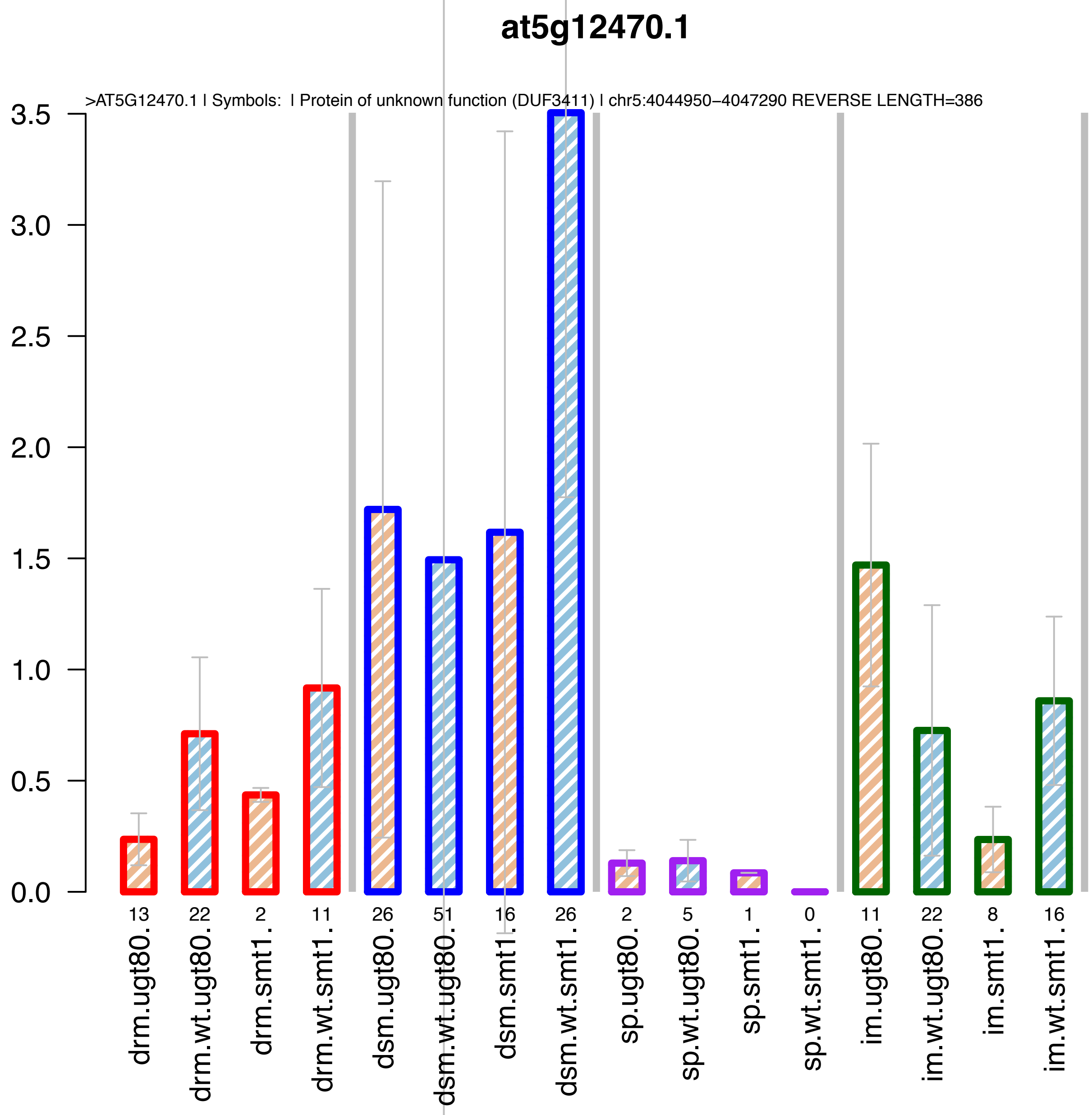

at5g12470.1
>AT5G12470.1 | Symbols: | Protein of unknown function (DUF3411) | chr5:4044950-4047290 REVERSE LENGTH=386
3.5
3.0
2.5
2.0
1.5
1.0
0.5
0.0
13
22
2
11
26
51
16
26
2
5
1
0
11
22
8
16
drm.ugt80..
drm.wt.ugt80..
drm.smt1..
drm.wt.smt1..
dsm.ugt80..
dsm.wt.ugt80..
dsm.smt1..
dsm.wt.smt1..
sp.ugt80..
sp.wt.ugt80..
sp.smt1..
sp.wt.smt1..
im.ugt80..
im.wt.ugt80..
im.smt1..
im.wt.smt1..

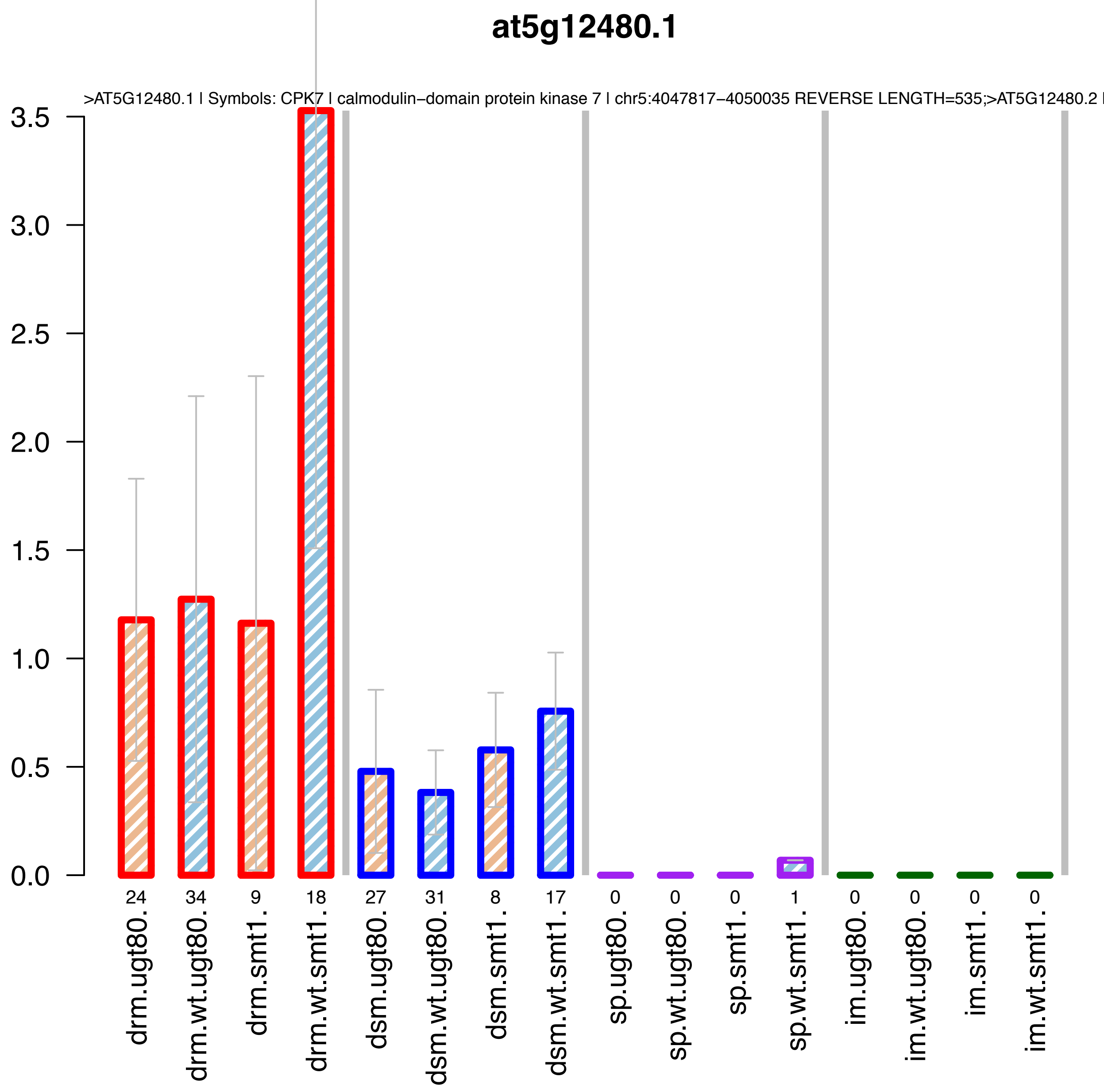

at5g12480.1
>AT5G12480.1 | Symbols: CPK7 | calmodulin–domain protein kinase 7 | chr5:4047817–4050035 REVERSE LENGTH=535;>AT5G12480.2
0.0
0.5
1.0
1.5
2.0
2.5
3.0
3.5
24
34
9
18
27
31
8
17
0
0
0
1
0
0
0
0
drm.ugt80.
drm.wt.ugt80.
drm.smt1.
drm.wt.smt1.
dsm.ugt80.
dsm.wt.ugt80.
dsm.smt1.
dsm.wt.smt1.
sp.ugt80.
sp.wt.ugt80.
sp.smt1.
sp.wt.smt1.
im.ugt80.
im.wt.ugt80.
im.smt1.
im.wt.smt1.

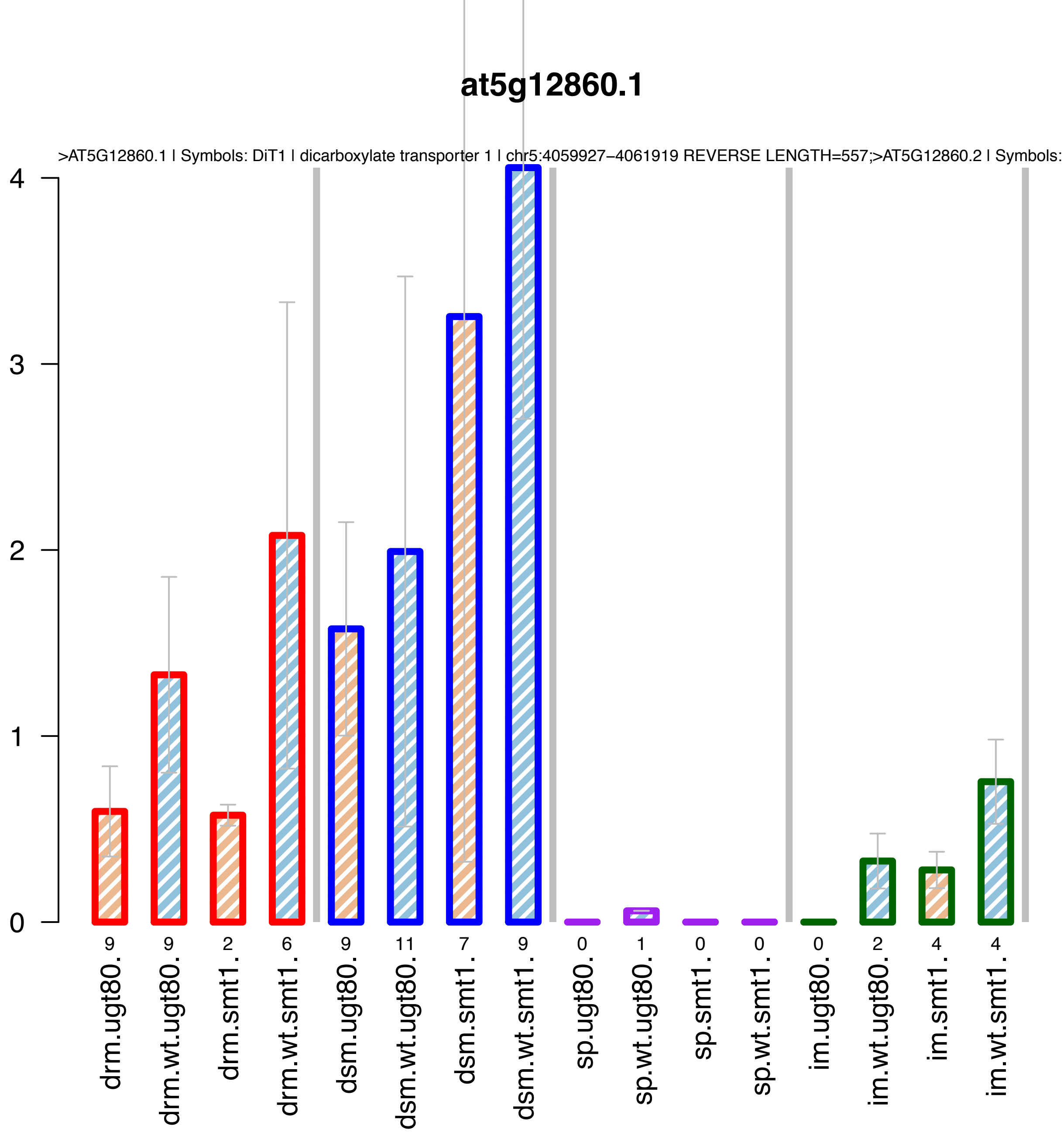

at5g12860.1
>AT5G12860.1 | Symbols: DiT1 | dicarboxylate transporter 1 | chr5:4059927-4061919 REVERSE LENGTH=557;>AT5G12860.2 | Symbols:
0
1
2
3
4
9
9
2
6
9
11
7
9
0
1
0
0
0
2
4
4
drm.ugt80.
drm.wt.ugt80.
drm.smt1.
drm.wt.smt1.
dsm.ugt80.
dsm.wt.ugt80.
dsm.smt1.
dsm.wt.smt1.
sp.ugt80.
sp.wt.ugt80.
sp.smt1.
sp.wt.smt1.
im.ugt80.
im.wt.ugt80.
im.smt1.
im.wt.smt1.

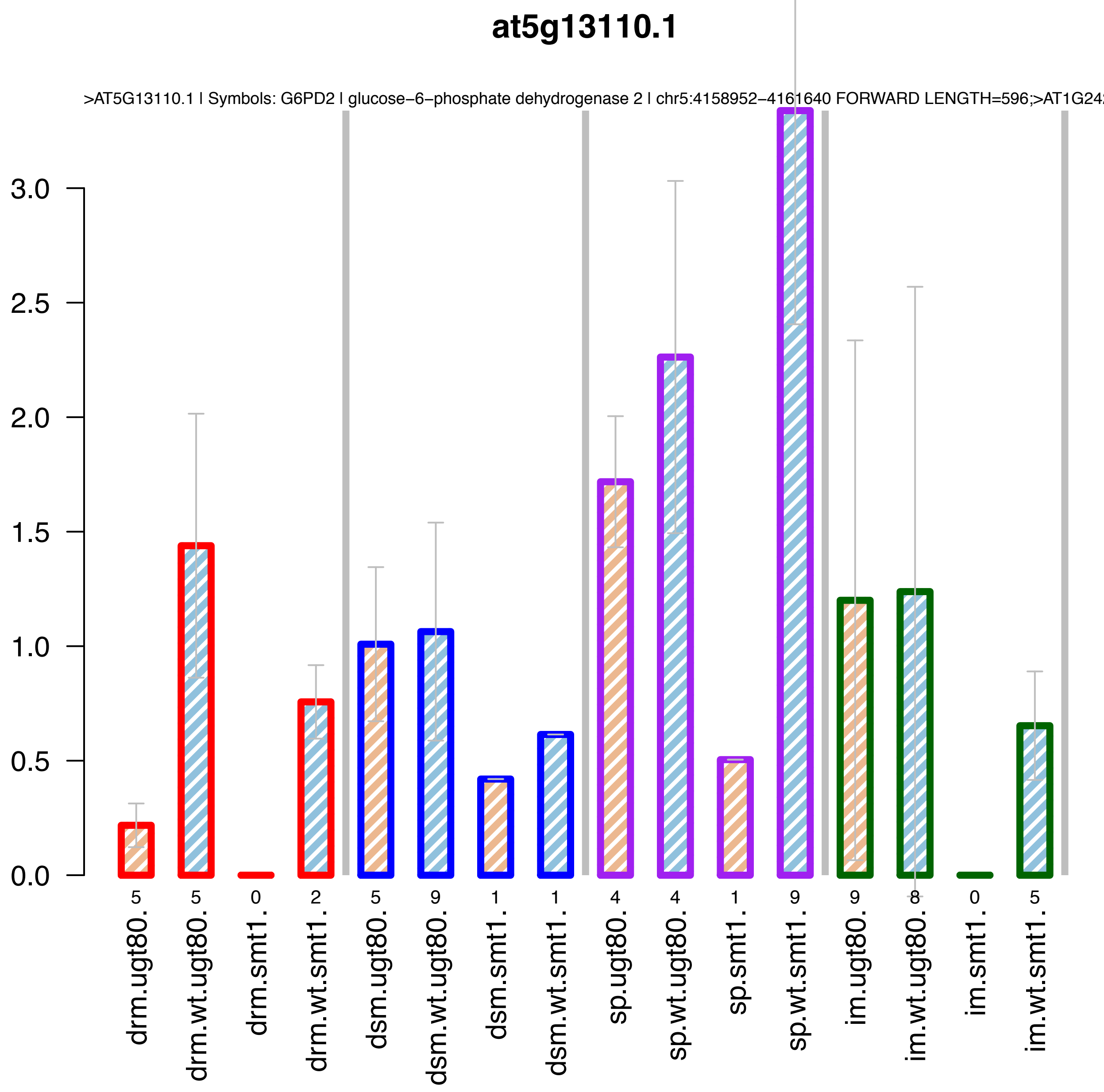

at5g13110.1
>AT5G13110.1 | Symbols: G6PD2 | glucose−6−phosphate dehydrogenase 2 | chr5:4158952−4161640 FORWARD LENGTH=596;>AT1G24
0.0
0.5
1.0
1.5
2.0
2.5
3.0
5
5
0
2
5
9
1
1
4
4
1
9
9
8
0
5
drm.ugt80.
drm.wt.ugt80.
drm.smt1.
drm.wt.smt1.
dsm.ugt80.
dsm.wt.ugt80.
dsm.smt1.
dsm.wt.smt1.
sp.ugt80.
sp.wt.ugt80.
sp.smt1.
sp.wt.smt1.
im.ugt80.
im.wt.ugt80.
im.smt1.
im.wt.smt1.

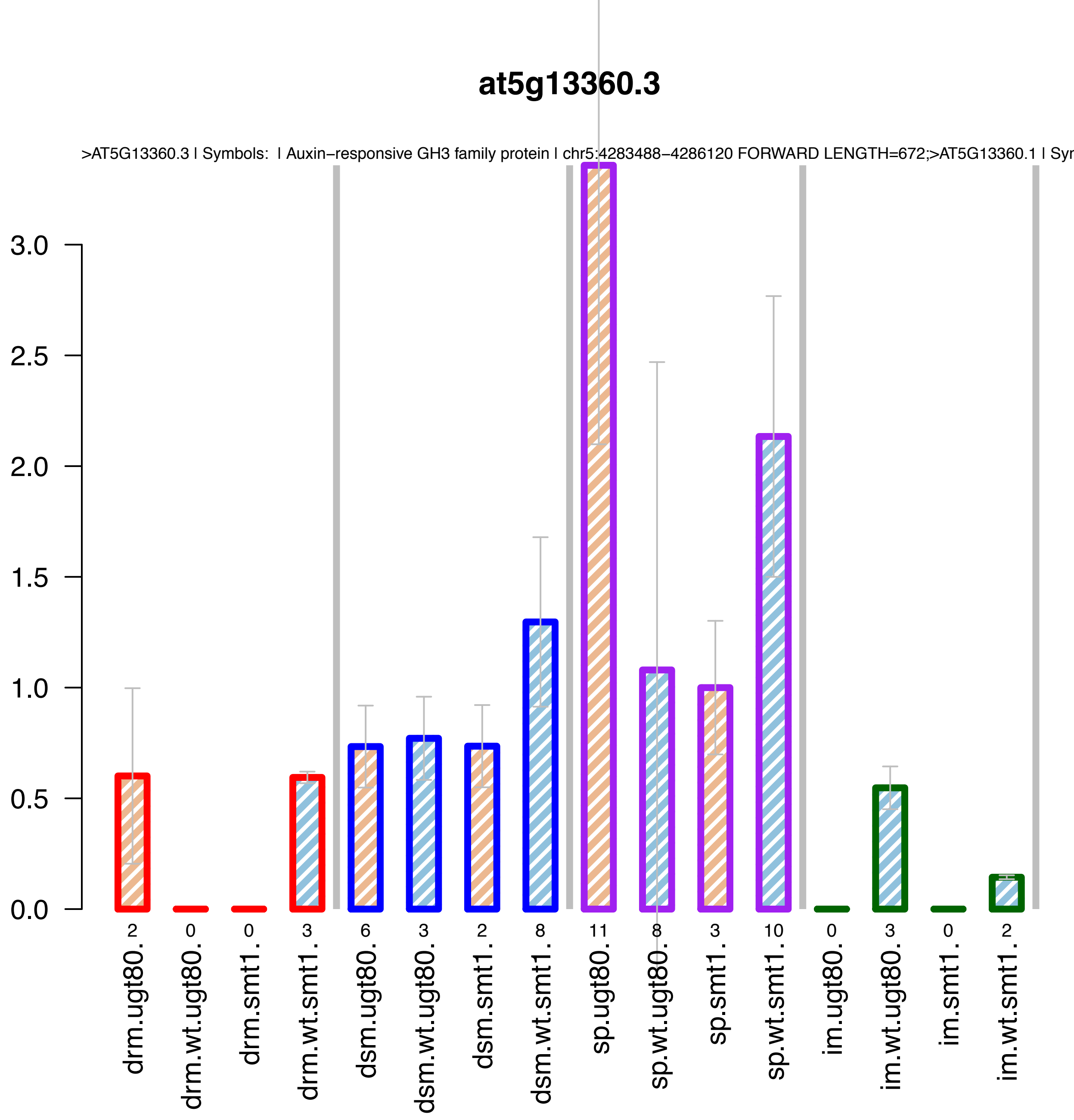

at5g13360.3
>AT5G13360.3 | Symbols: | Auxin−responsive GH3 family protein | chr5:4283488−4286120 FORWARD LENGTH=672;>AT5G13360.1 | Sym
3.0
2.5
2.0
1.5
1.0
0.5
0.0
2
0
0
3
6
3
2
8
11
8
3
10
0
3
0
2
drm.ugt80.
drm.wt.ugt80.
drm.smt1.
drm.wt.smt1.
dsm.ugt80.
dsm.wt.ugt80.
dsm.smt1.
dsm.wt.smt1.
sp.ugt80.
sp.wt.ugt80.
sp.smt1.
sp.wt.smt1.
im.ugt80.
im.wt.ugt80.
im.smt1.
im.wt.smt1.

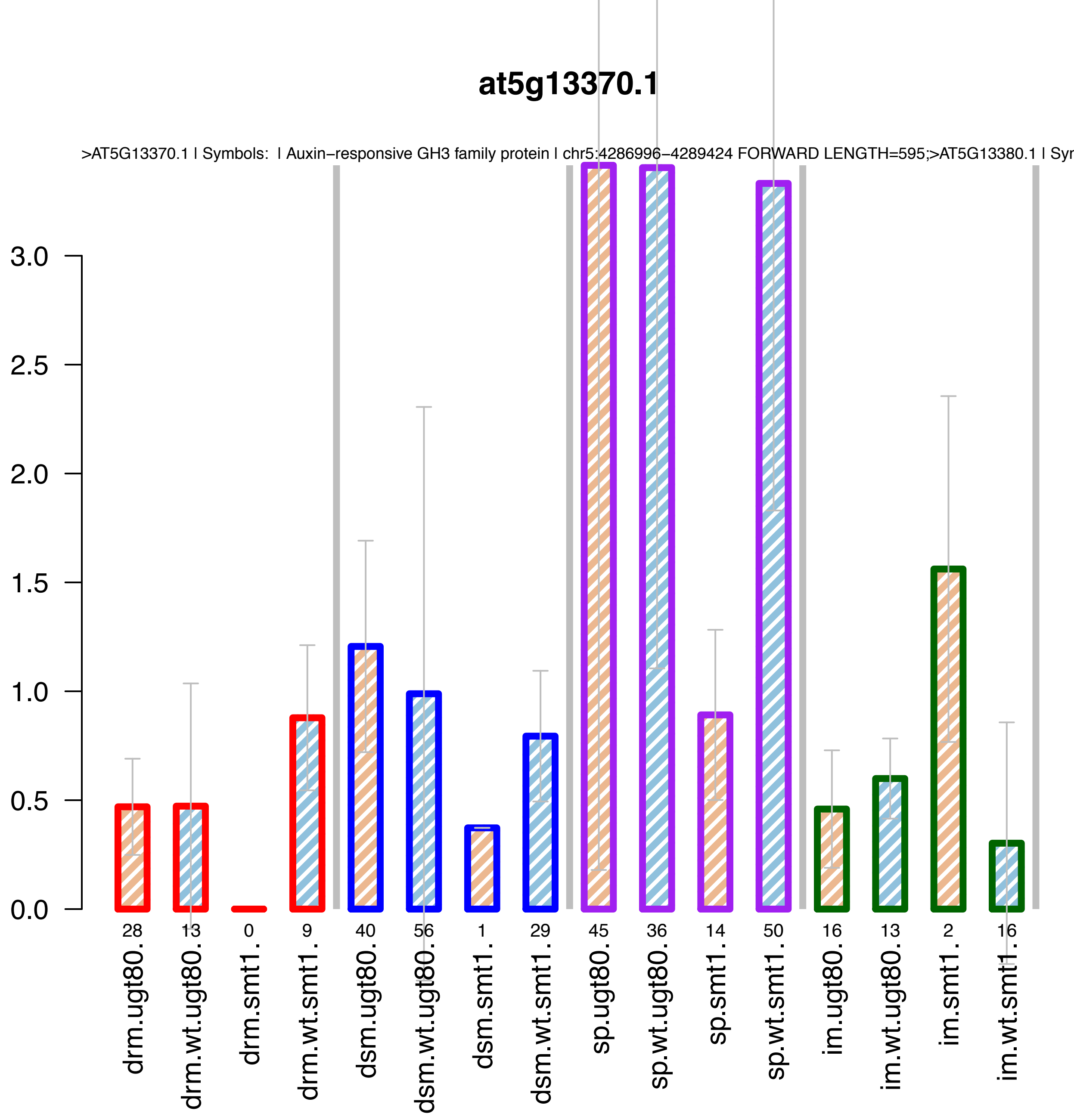

at5g13370.1
>AT5G13370.1 | Symbols: | Auxin−responsive GH3 family protein | chr5:4286996−4289424 FORWARD LENGTH=595;>AT5G13380.1 | Syn
3.0
2.5
2.0
1.5
1.0
0.5
0.0
28
13
0
9
40
56
1
29
45
36
14
50
16
13
2
16
drm.ugt80..
drm.wt.ugt80..
drm.smt1..
drm.wt.smt1..
dsm.ugt80..
dsm.wt.ugt80..
dsm.smt1..
dsm.wt.smt1..
sp.ugt80..
sp.wt.ugt80..
sp.smt1..
sp.wt.smt1..
im.ugt80..
im.wt.ugt80..
im.smt1..
im.wt.smt1..

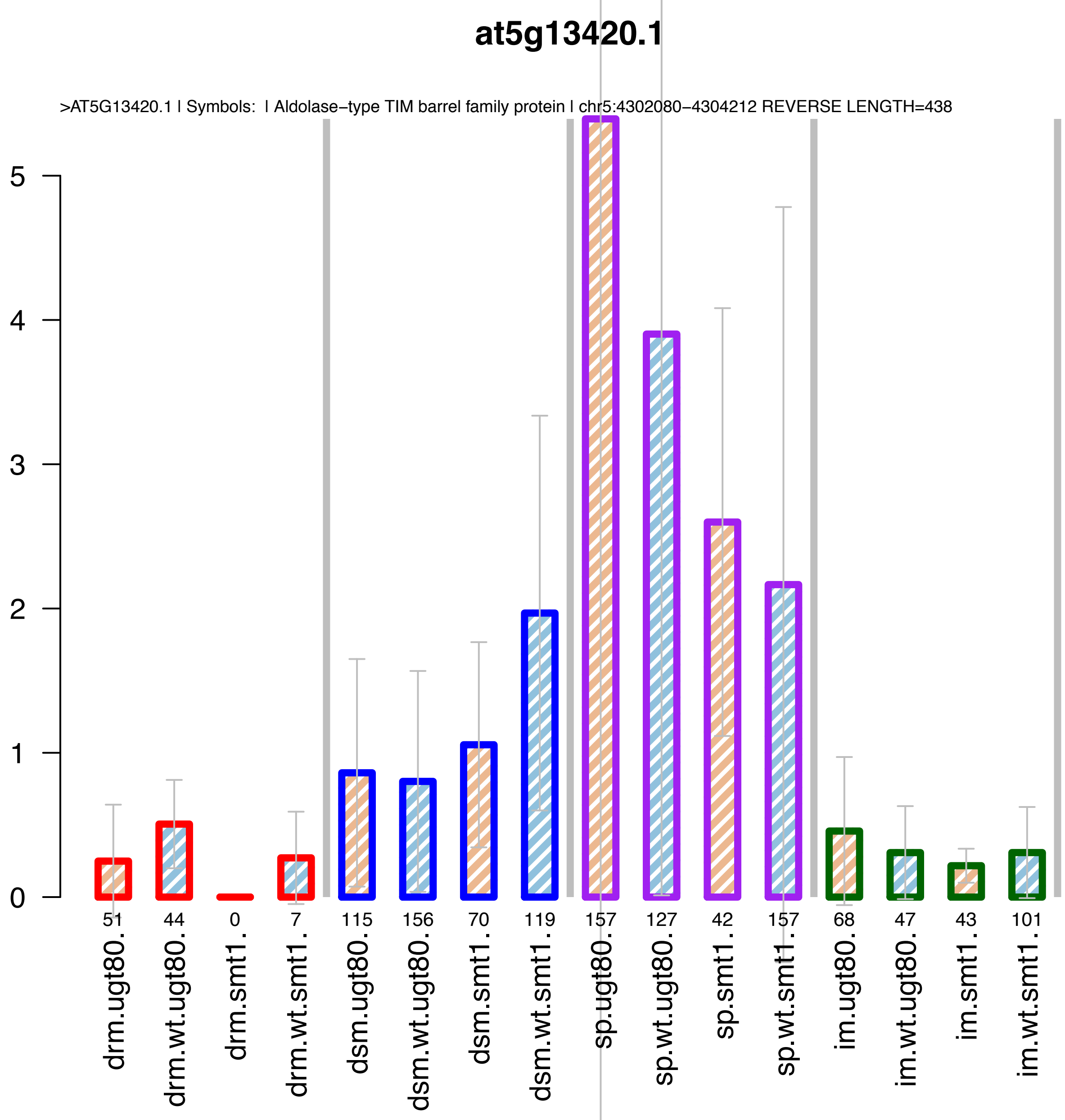

at5g13420.1
>AT5G13420.1 | Symbols: | Aldolase−type TIM barrel family protein | chr5:4302080−4304212 REVERSE LENGTH=438
0
1
2
3
4
5
51
44
0
7
115
156
70
119
157
127
42
157
68
47
43
101
drm.ugt80.
drm.wt.ugt80.
drm.smt1.
drm.wt.smt1.
dsm.ugt80.
dsm.wt.ugt80.
dsm.smt1.
dsm.wt.smt1.
sp.ugt80.
sp.wt.ugt80.
sp.smt1.
sp.wt.smt1.
im.ugt80.
im.wt.ugt80.
im.smt1.
im.wt.smt1.

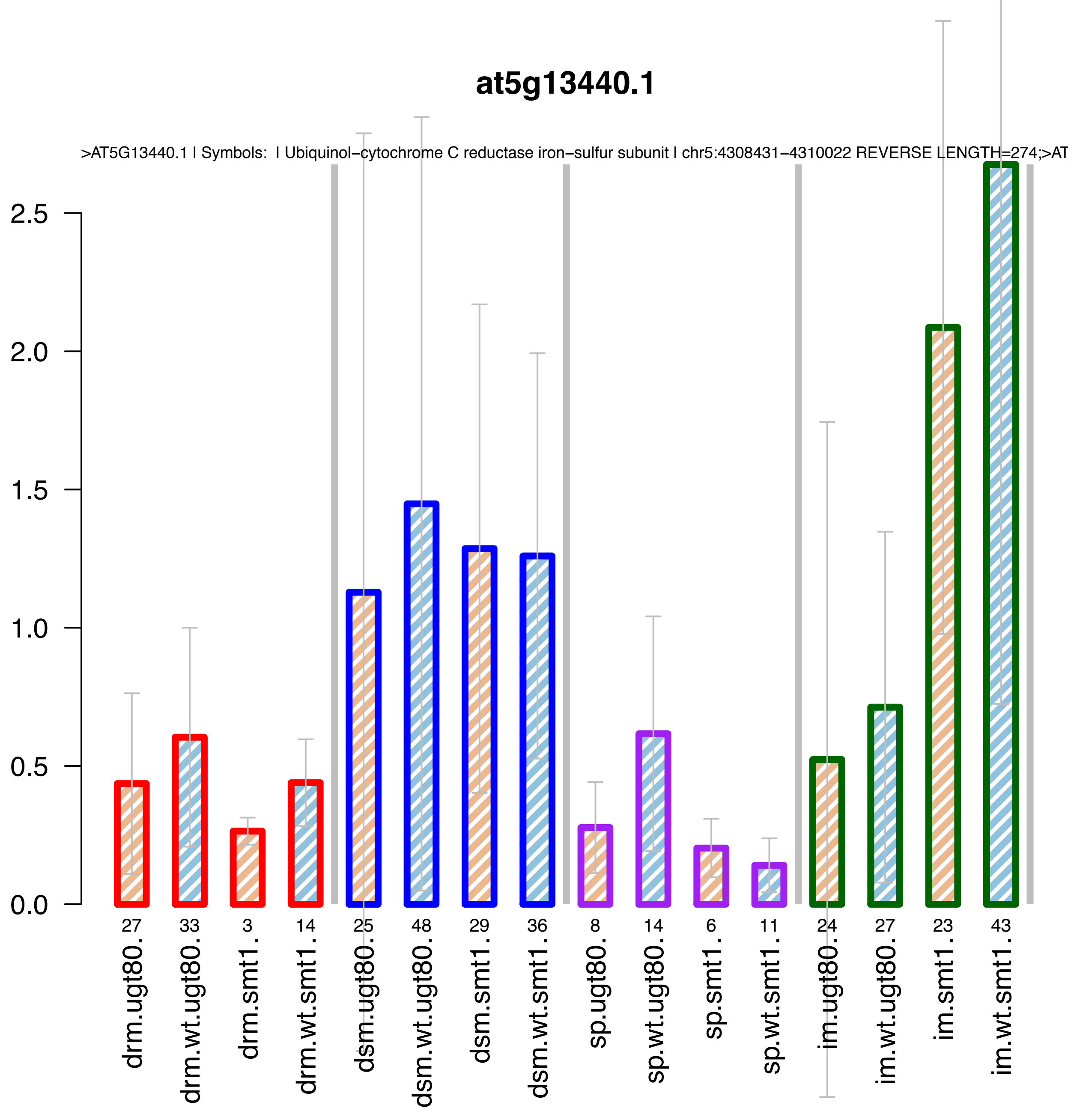

at5g13440.1
>AT5G13440.1 | Symbols: | Ubiquinol−cytochrome C reductase iron−sulfur subunit | chr5:4308431−4310022 REVERSE LENGTH=274;>AT
0.0
0.5
1.0
1.5
2.0
2.5
27
33
3
14
25
48
29
36
8
14
6
11
24
27
23
43
drm.ugt80.
drm.wt.ugt80.
drm.smt1.
drm.wt.smt1.
dsm.ugt80.
dsm.wt.ugt80.
dsm.smt1.
dsm.wt.smt1.
sp.ugt80.
sp.wt.ugt80.
sp.smt1.
sp.wt.smt1.
im.ugt80.
im.wt.ugt80.
im.smt1.
im.wt.smt1.

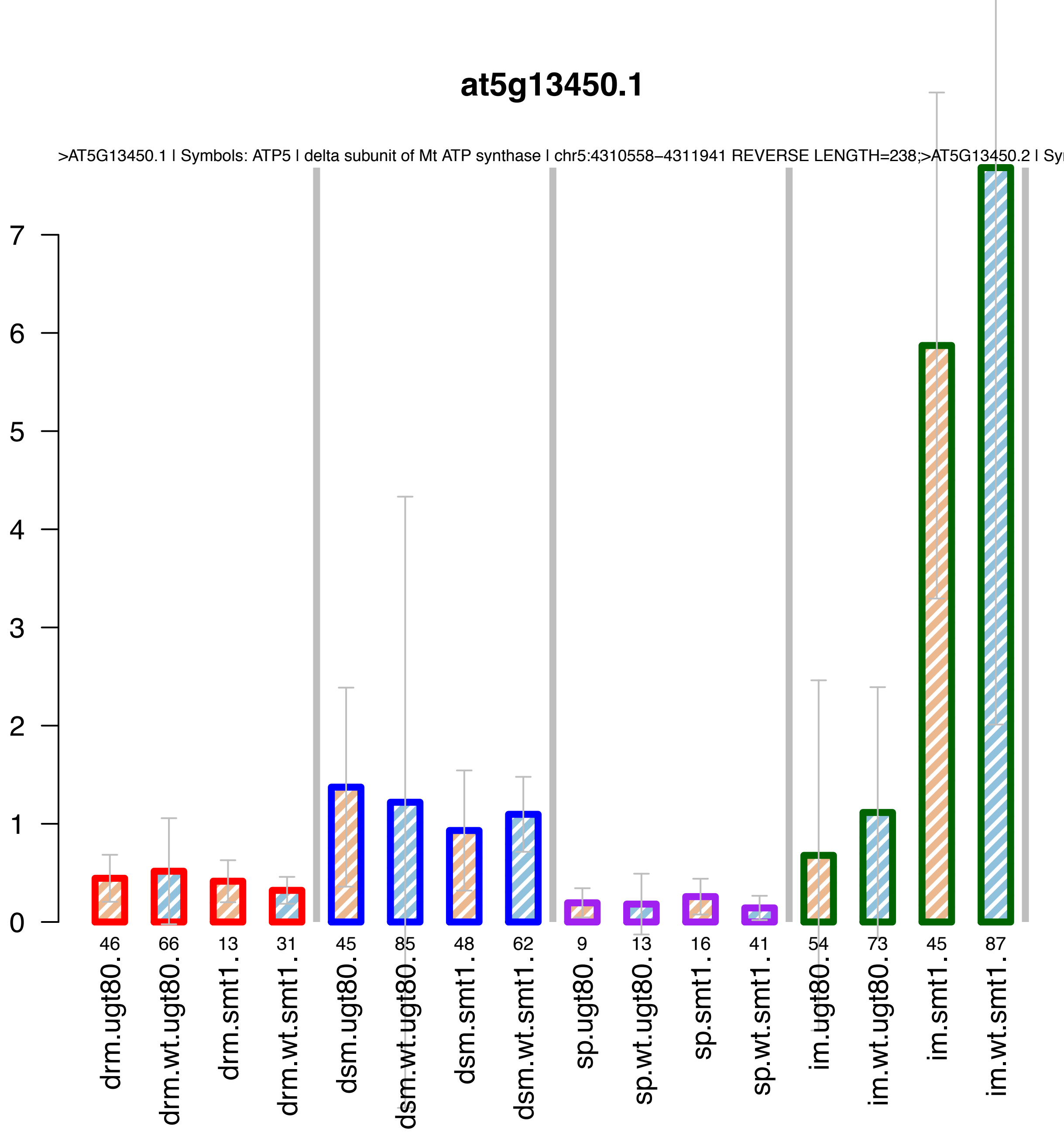

at5g13450.1
>AT5G13450.1 | Symbols: ATP5 | delta subunit of Mt ATP synthase | chr5:4310558-4311941 REVERSE LENGTH=238;>AT5G13450.2 | Sy
0
1
2
3
4
5
6
7
46
66
13
31
45
85
48
62
9
13
16
41
54
73
45
87
drm.ugt80.
drm.wt.ugt80.
drm.smt1.
drm.wt.smt1.
dsm.ugt80.
dsm.wt.ugt80.
dsm.smt1.
dsm.wt.smt1.
sp.ugt80.
sp.wt.ugt80.
sp.smt1.
sp.wt.smt1.
im.ugt80.
im.wt.ugt80.
im.smt1.
im.wt.smt1.

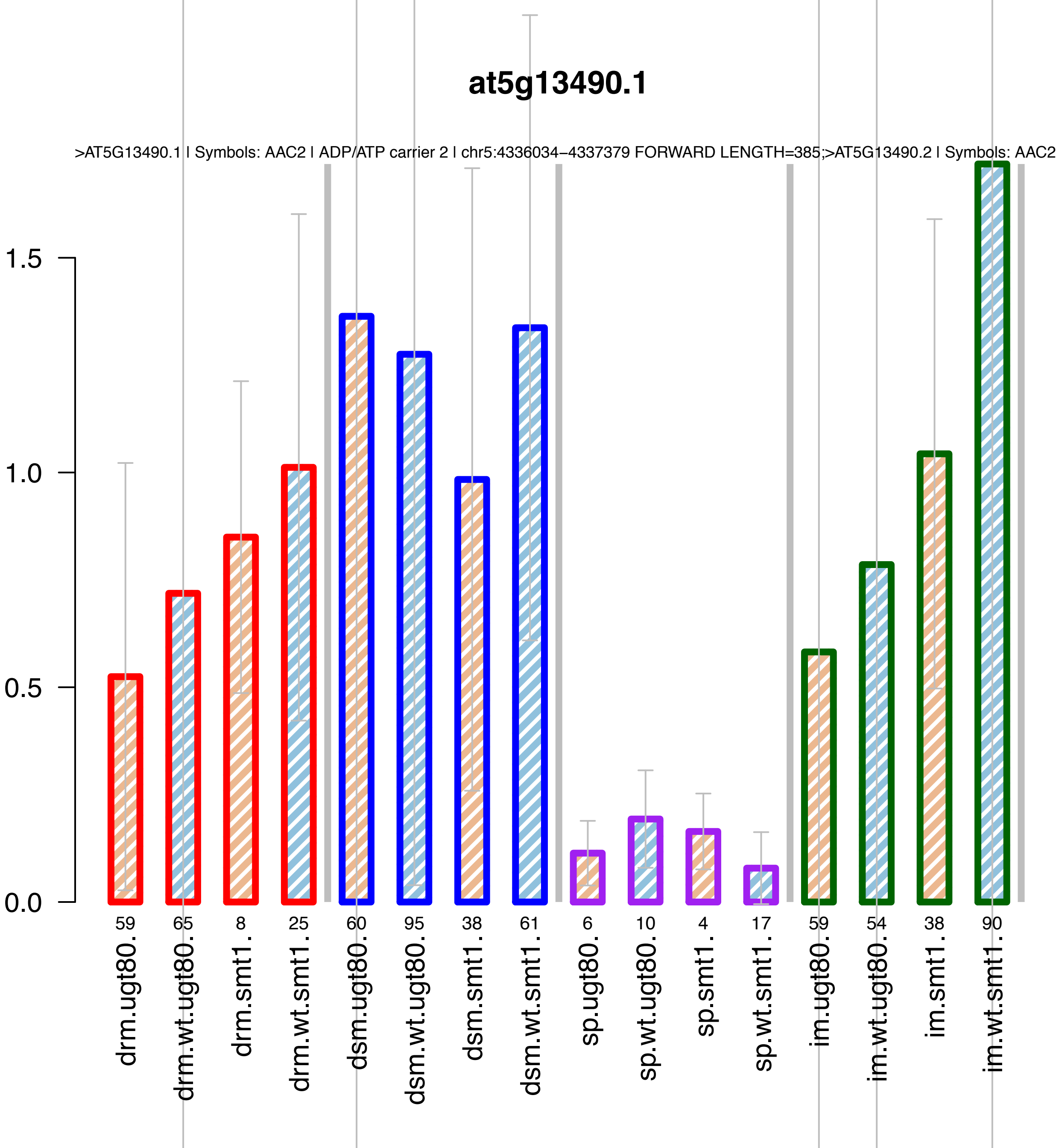

at5g13490.1
>AT5G13490.1 | Symbols: AAC2 | ADP/ATP carrier 2 | chr5:4336034-4337379 FORWARD LENGTH=385;>AT5G13490.2 | Symbols: AAC2
1.5
1.0
0.5
0.0
59
65
8
25
60
95
38
61
6
10
4
17
59
54
38
90
drm.ugt80..
drm.wt.ugt80..
drm.smt1..
drm.wt.smt1..
dsm.ugt80..
dsm.wt.ugt80..
dsm.smt1..
dsm.wt.smt1..
sp.ugt80..
sp.wt.ugt80..
sp.smt1..
sp.wt.smt1..
im.ugt80..
im.wt.ugt80..
im.smt1..
im.wt.smt1..

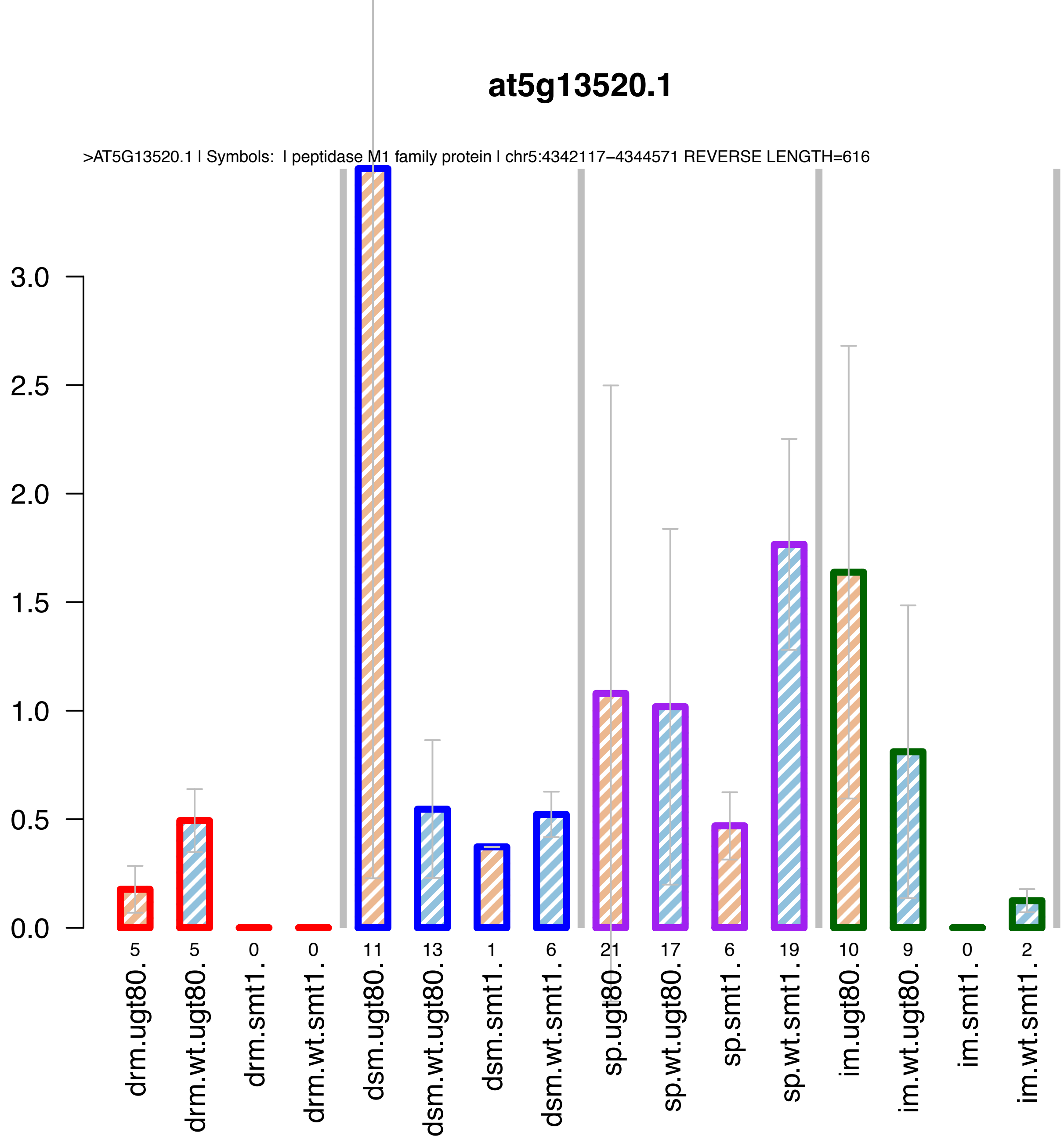

at5g13520.1
>AT5G13520.1 | Symbols: | peptidase M1 family protein | chr5:4342117-4344571 REVERSE LENGTH=616
3.0
2.5
2.0
1.5
1.0
0.5
0.0
5
5
0
0
11
13
1
6
21
17
6
19
10
9
0
2
drm.ugt80.
drm.wt.ugt80.
drm.smt1.
drm.wt.smt1.
dsm.ugt80.
dsm.wt.ugt80.
dsm.smt1.
dsm.wt.smt1.
sp.ugt80.
sp.wt.ugt80.
sp.smt1.
sp.wt.smt1.
im.ugt80.
im.wt.ugt80.
im.smt1.
im.wt.smt1.

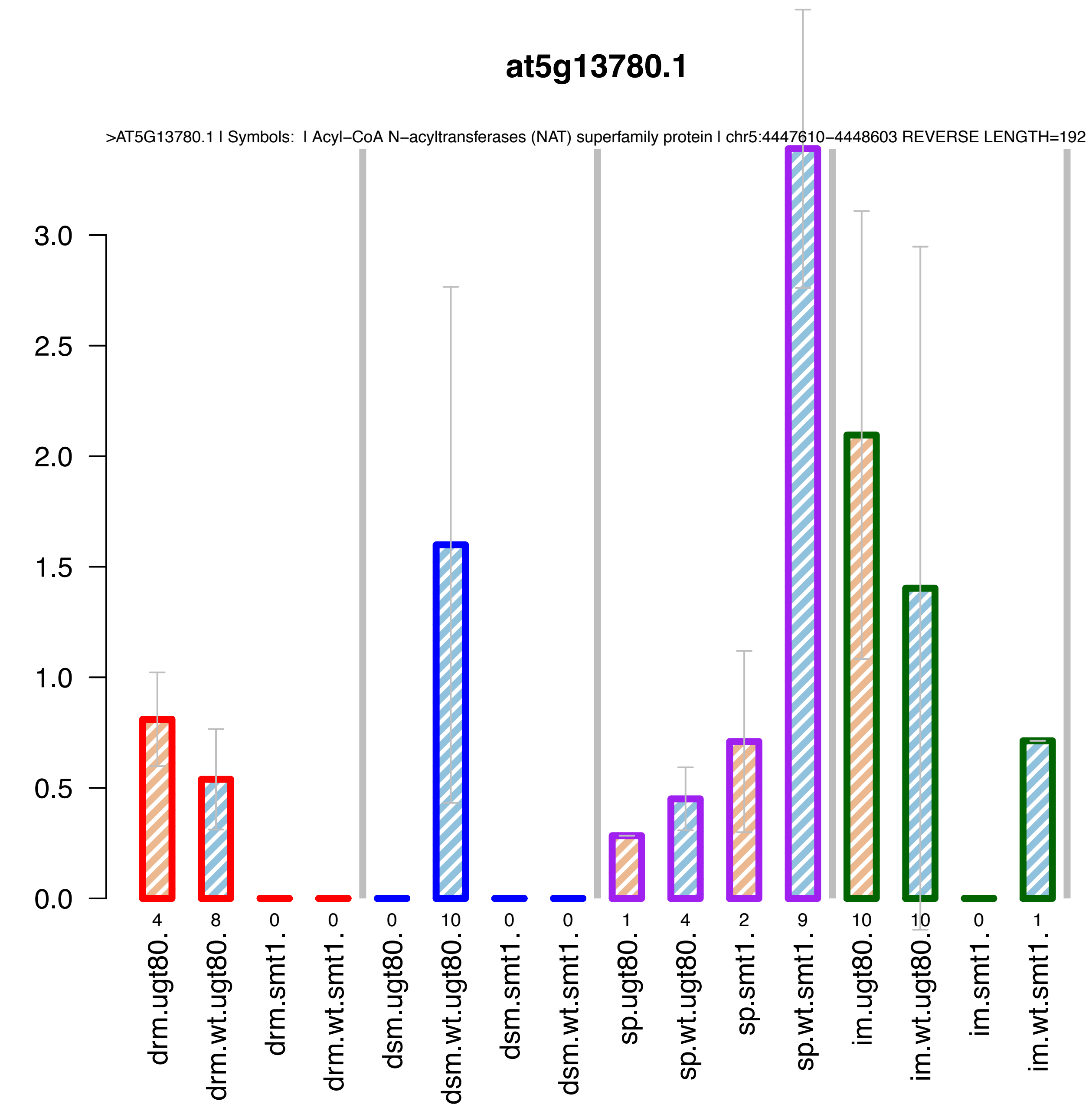

at5g13780.1
>AT5G13780.1 | Symbols: | Acyl−CoA N−acyltransferases (NAT) superfamily protein | chr5:4447610−4448603 REVERSE LENGTH=192
0.0
0.5
1.0
1.5
2.0
2.5
3.0
4
8
0
0
0
10
0
0
1
4
2
9
10
10
0
1
drm.ugt80..
drm.wt.ugt80..
drm.smt1..
drm.wt.smt1..
dsm.ugt80..
dsm.wt.ugt80..
dsm.smt1..
dsm.wt.smt1..
sp.ugt80..
sp.wt.ugt80..
sp.smt1..
sp.wt.smt1..
im.ugt80..
im.wt.ugt80..
im.smt1..
im.wt.smt1..

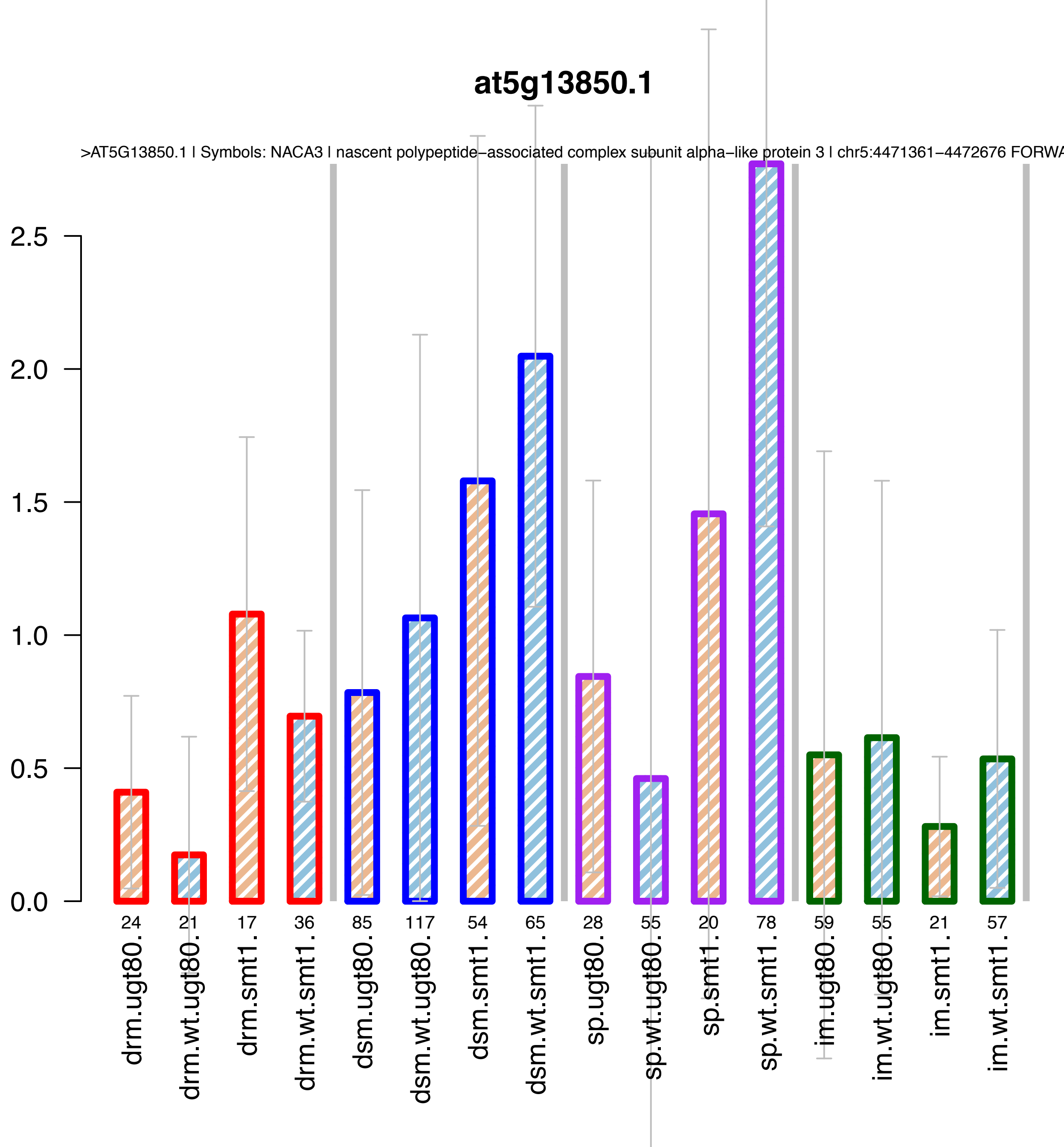

at5g13850.1
>AT5G13850.1 | Symbols: NACA3 | nascent polypeptide−associated complex subunit alpha−like protein 3 | chr5:4471361−4472676 FORWA
0.0
0.5
1.0
1.5
2.0
2.5
24
21
17
36
85
117
54
65
28
55
20
78
59
55
21
57
drm.ugt80..
drm.wt.ugt80..
drm.smt1..
drm.wt.smt1..
dsm.ugt80..
dsm.wt.ugt80..
dsm.smt1..
dsm.wt.smt1..
sp.ugt80..
sp.wt.ugt80..
sp.smt1..
sp.wt.smt1..
im.ugt80..
im.wt.ugt80..
im.smt1..
im.wt.smt1..

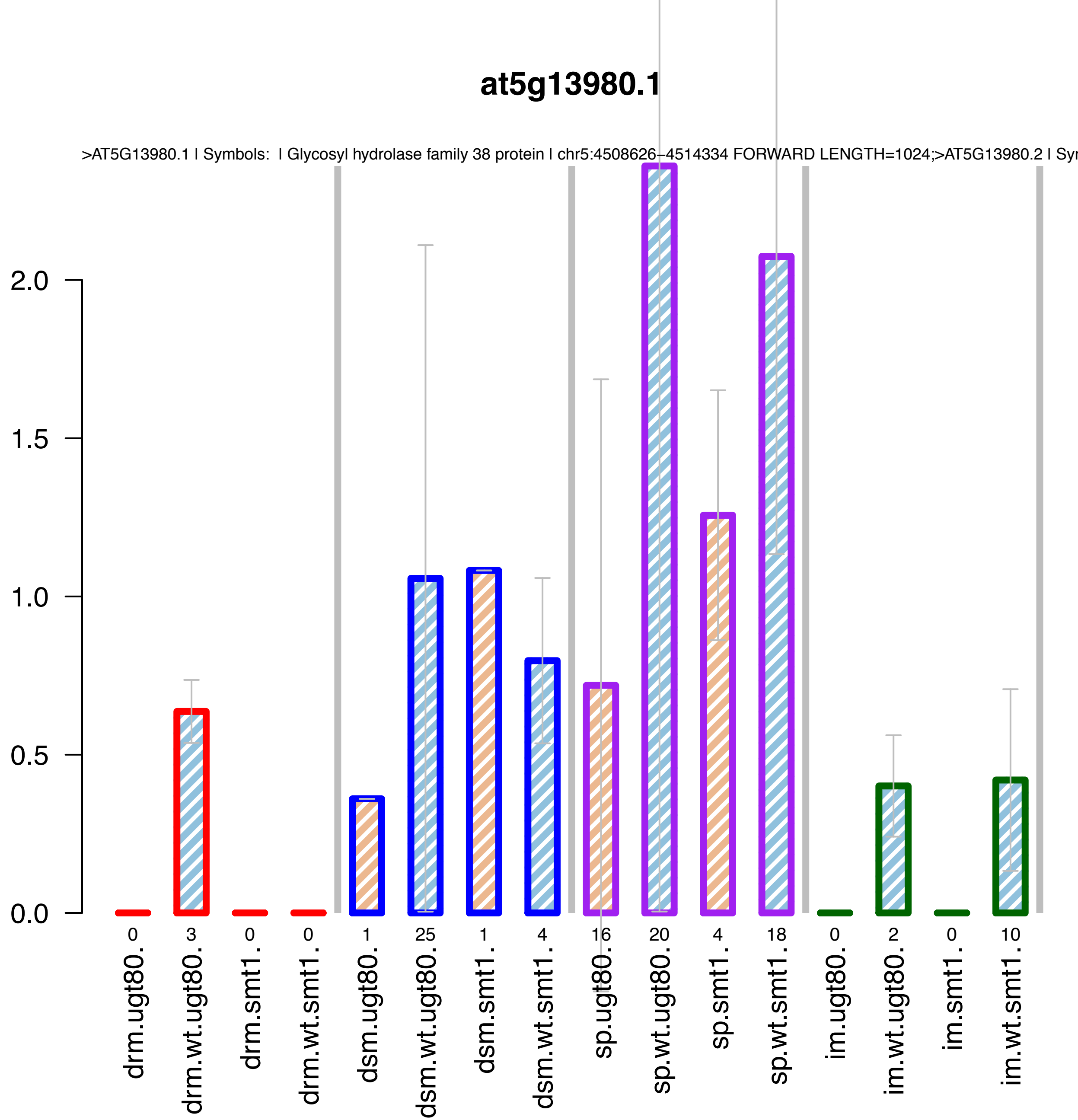
at5g13980.1
>AT5G13980.1 | Symbols: | Glycosyl hydrolase family 38 protein | chr5:4508626-4514334 FORWARD LENGTH=1024;>AT5G13980.2 | Sy
2.0
1.5
1.0
0.5
0.0
0
3
0
0
1
25
1
4
16
20
4
18
0
2
0
10
drm.ugt80.
drm.wt.ugt80.
drm.smt1.
drm.wt.smt1.
dsm.ugt80.
dsm.wt.ugt80.
dsm.smt1.
dsm.wt.smt1.
sp.ugt80.
sp.wt.ugt80.
sp.smt1.
sp.wt.smt1.
im.ugt80.
im.wt.ugt80.
im.smt1.
im.wt.smt1.

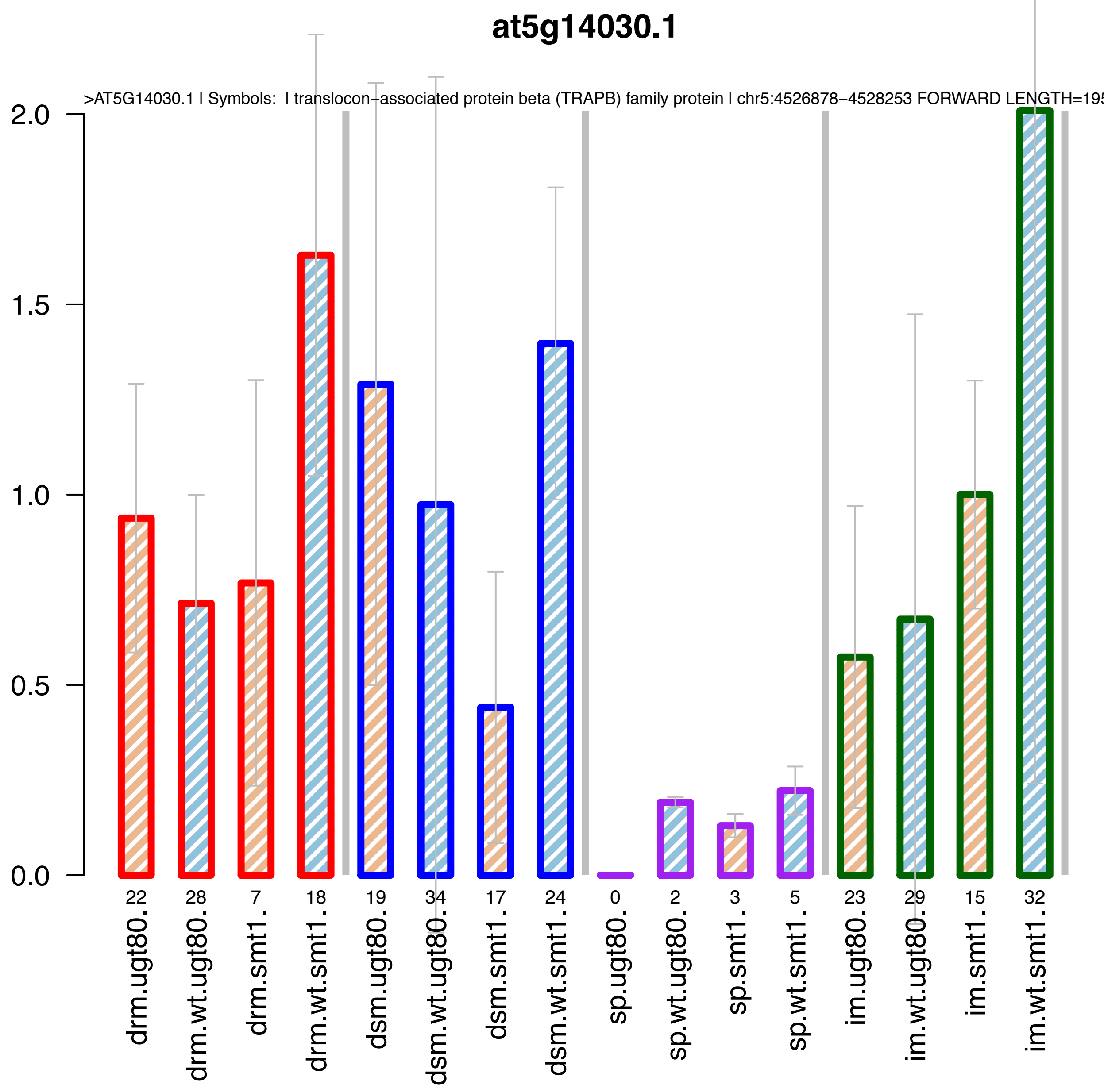

at5g14030.1
>AT5G14030.1 | Symbols: | translocon−associated protein beta (TRAPB) family protein | chr5:4526878−4528253 FORWARD LENGTH=195
2.0
1.5
1.0
0.5
0.0
22
28
7
18
19
34
17
24
0
2
3
5
23
29
15
32
drm.ugt80..
drm.wt.ugt80..
drm.smt1..
drm.wt.smt1..
dsm.ugt80..
dsm.wt.ugt80..
dsm.smt1..
dsm.wt.smt1..
sp.ugt80..
sp.wt.ugt80..
sp.smt1..
sp.wt.smt1..
im.ugt80..
im.wt.ugt80..
im.smt1..
im.wt.smt1..

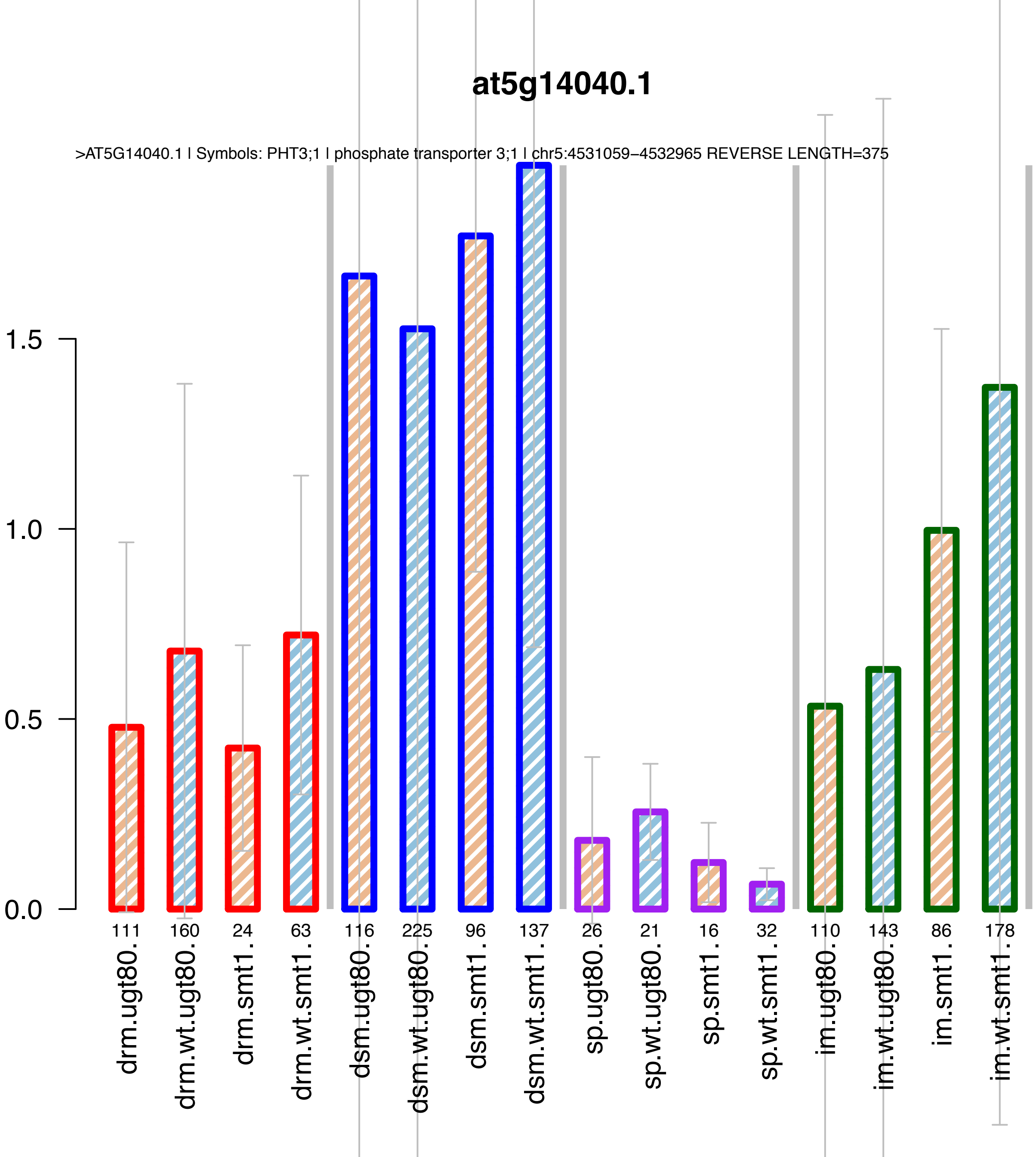
at5g14040.1
>AT5G14040.1 | Symbols: PHT3;1 | phosphate transporter 3;1 | chr5:4531059-4532965 REVERSE LENGTH=375
0.0
0.5
1.0
1.5
111
160
24
63
116
225
96
137
26
21
16
32
110
143
86
178
drm.ugt80..
drm.wt.ugt80..
drm.smt1..
drm.wt.smt1..
dsm.ugt80..
dsm.wt.ugt80..
dsm.smt1..
dsm.wt.smt1..
sp.ugt80..
sp.wt.ugt80..
sp.smt1..
sp.wt.smt1..
im.ugt80..
im.wt.ugt80..
im.smt1..
im.wt.smt1..

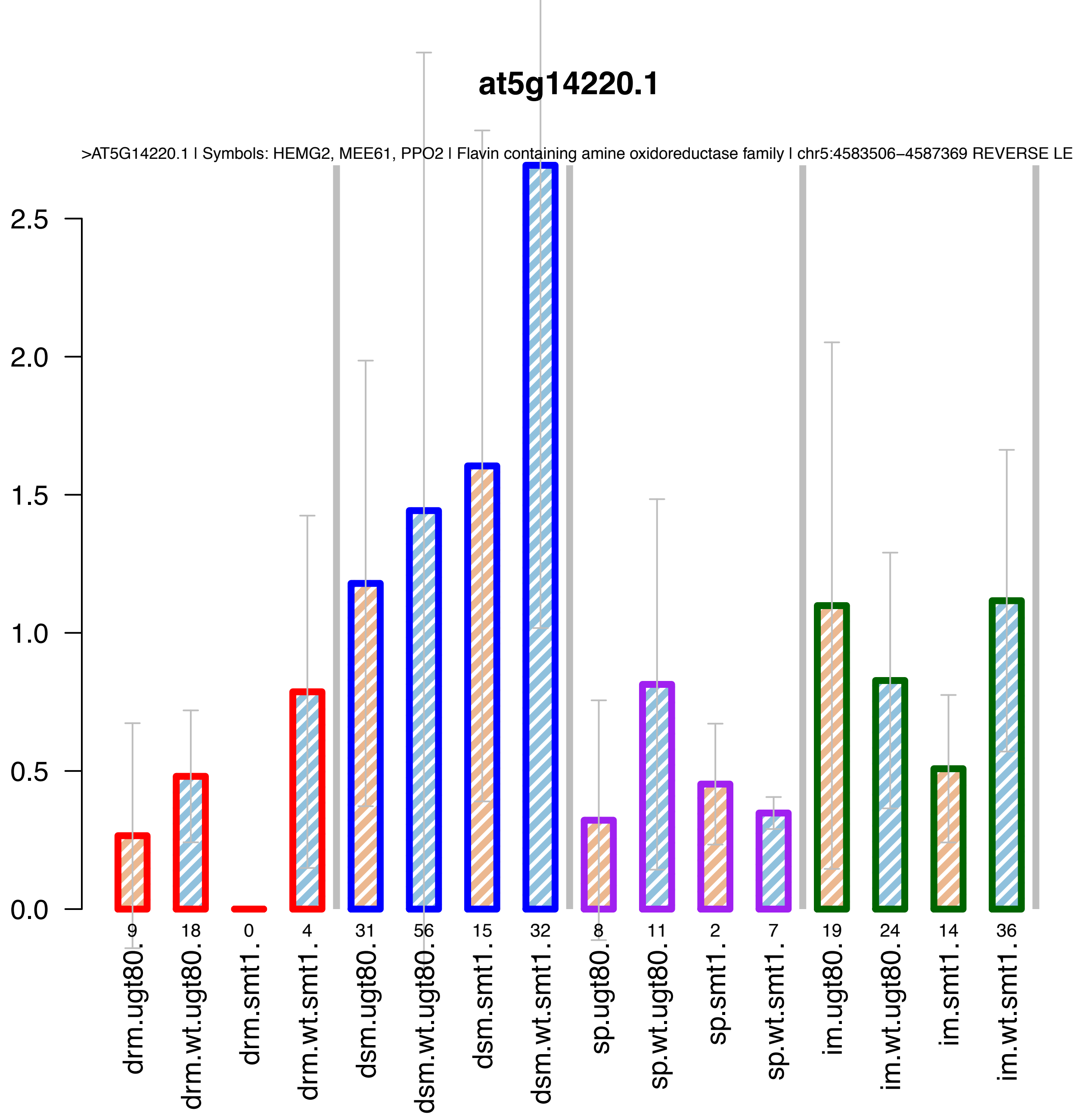

at5g14220.1
>AT5G14220.1 | Symbols: HEMG2, MEE61, PPO2 | Flavin containing amine oxidoreductase family | chr5:4583506-4587369 REVERSE LE
0.0
0.5
1.0
1.5
2.0
2.5
9
18
0
4
31
56
15
32
8
11
2
7
19
24
14
36
drm.ugt80.
drm.wt.ugt80.
drm.smt1.
drm.wt.smt1.
dsm.ugt80.
dsm.wt.ugt80.
dsm.smt1.
dsm.wt.smt1.
sp.ugt80.
sp.wt.ugt80.
sp.smt1.
sp.wt.smt1.
im.ugt80.
im.wt.ugt80.
im.smt1.
im.wt.smt1.

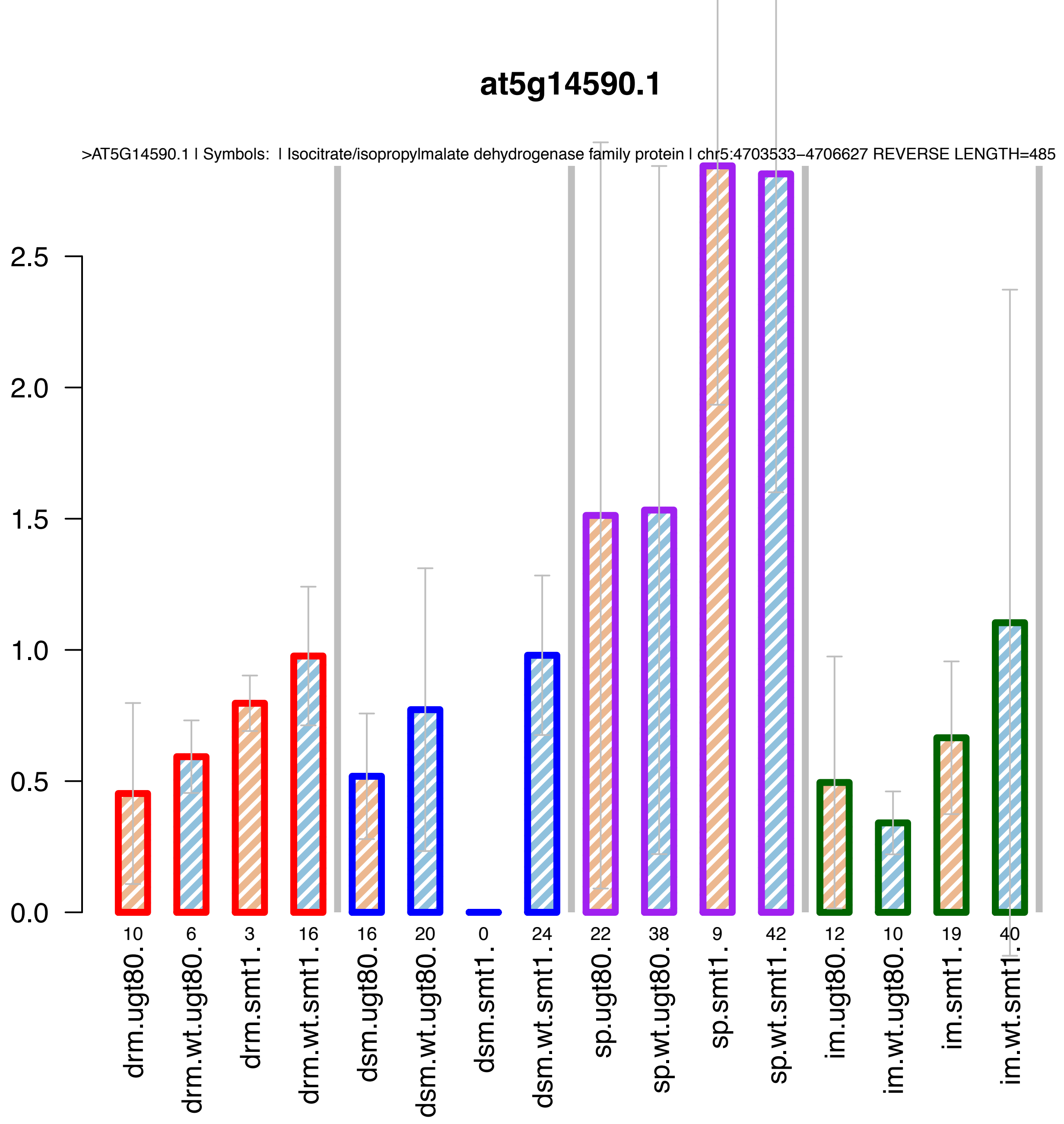

at5g14590.1
>AT5G14590.1 | Symbols: | Isocitrate/isopropylmalate dehydrogenase family protein | chr5:4703533-4706627 REVERSE LENGTH=485
0.0
0.5
1.0
1.5
2.0
2.5
10
6
3
16
16
20
0
24
22
38
9
42
12
10
19
40
drm.ugt80.
drm.wt.ugt80.
drm.smt1.
drm.wt.smt1.
dsm.ugt80.
dsm.wt.ugt80.
dsm.smt1.
dsm.wt.smt1.
sp.ugt80.
sp.wt.ugt80.
sp.smt1.
sp.wt.smt1.
im.ugt80.
im.wt.ugt80.
im.smt1.
im.wt.smt1.

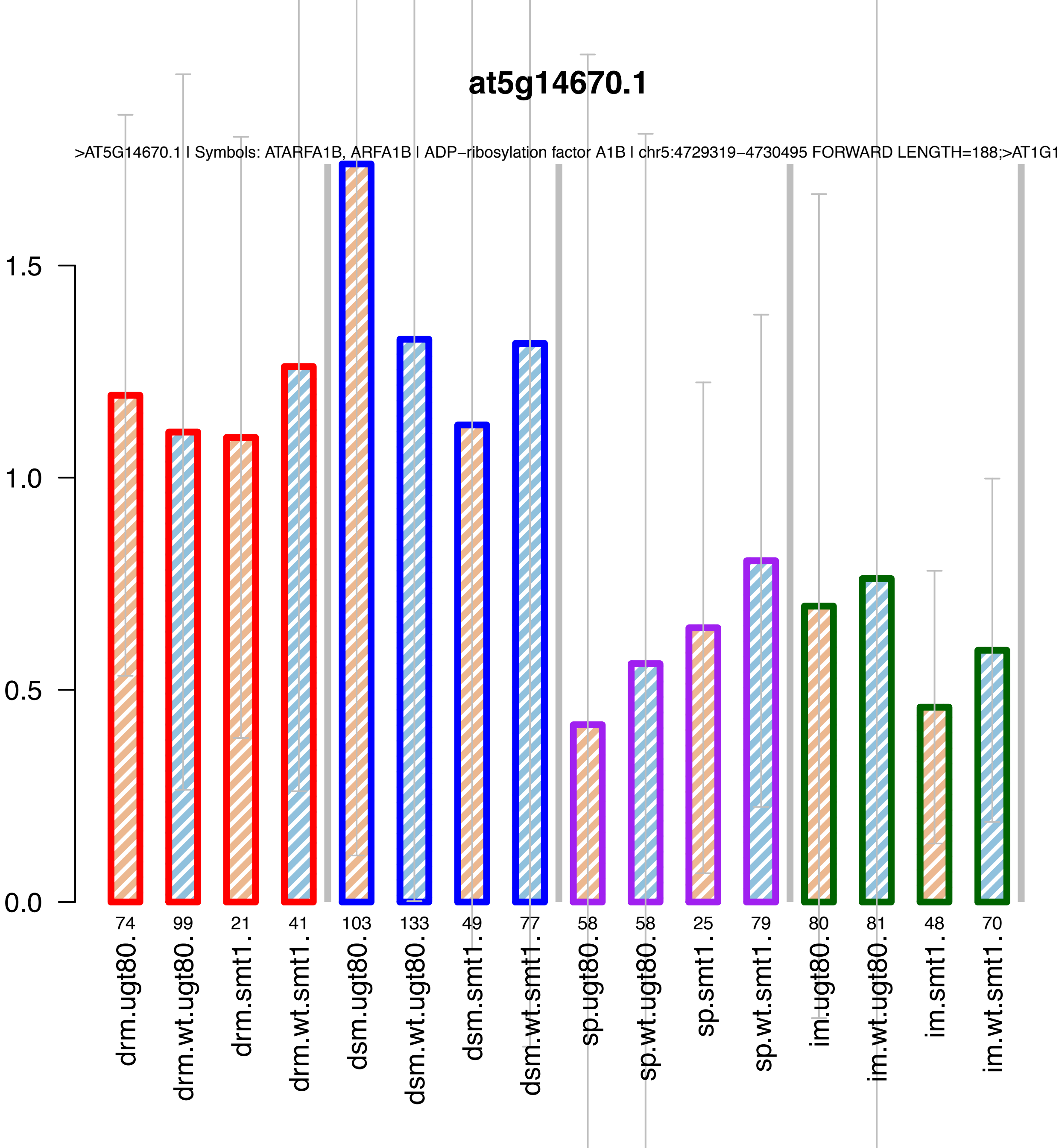
at5g14670.1
>AT5G14670.1 | Symbols: ATARFA1B, ARFA1B | ADP−ribosylation factor A1B | chr5:4729319−4730495 FORWARD LENGTH=188;>AT1G1
1.5
1.0
0.5
0.0
74
99
21
41
103
133
49
77
58
58
25
79
80
81
48
70
drm.ugt80..
drm.wt.ugt80..
drm.smt1..
drm.wt.smt1..
dsm.ugt80..
dsm.wt.ugt80..
dsm.smt1..
dsm.wt.smt1..
sp.ugt80..
sp.wt.ugt80..
sp.smt1..
sp.wt.smt1..
im.ugt80..
im.wt.ugt80..
im.smt1..
im.wt.smt1..

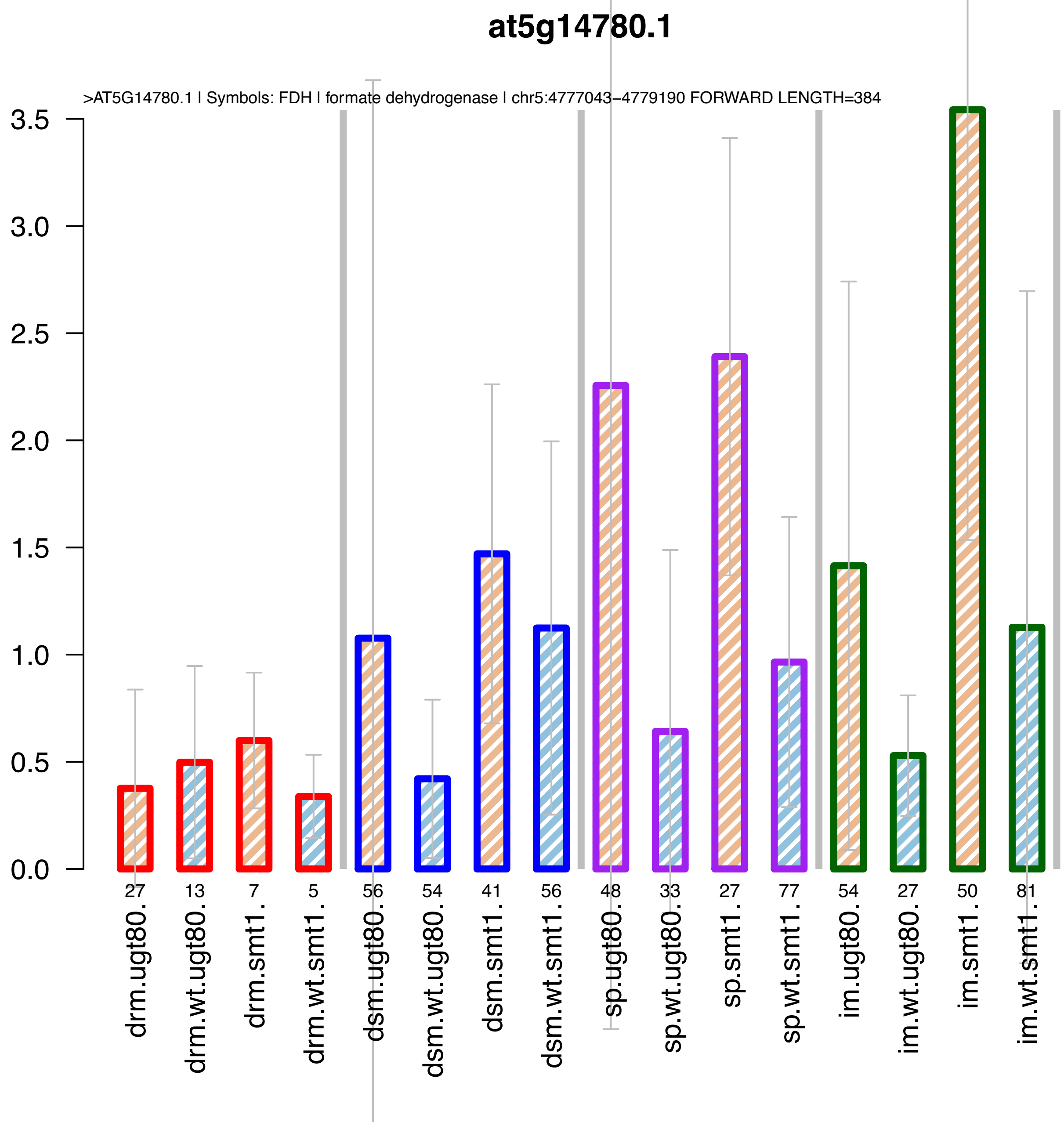

at5g14780.1
>AT5G14780.1 | Symbols: FDH | formate dehydrogenase | chr5:4777043-4779190 FORWARD LENGTH=384
0.0
0.5
1.0
1.5
2.0
2.5
3.0
3.5
27
13
7
5
56
54
41
56
48
33
27
77
54
27
50
81
drm.ugt80..
drm.wt.ugt80..
drm.smt1..
drm.wt.smt1..
dsm.ugt80..
dsm.wt.ugt80..
dsm.smt1..
dsm.wt.smt1..
sp.ugt80..
sp.wt.ugt80..
sp.smt1..
sp.wt.smt1..
im.ugt80..
im.wt.ugt80..
im.smt1..
im.wt.smt1..

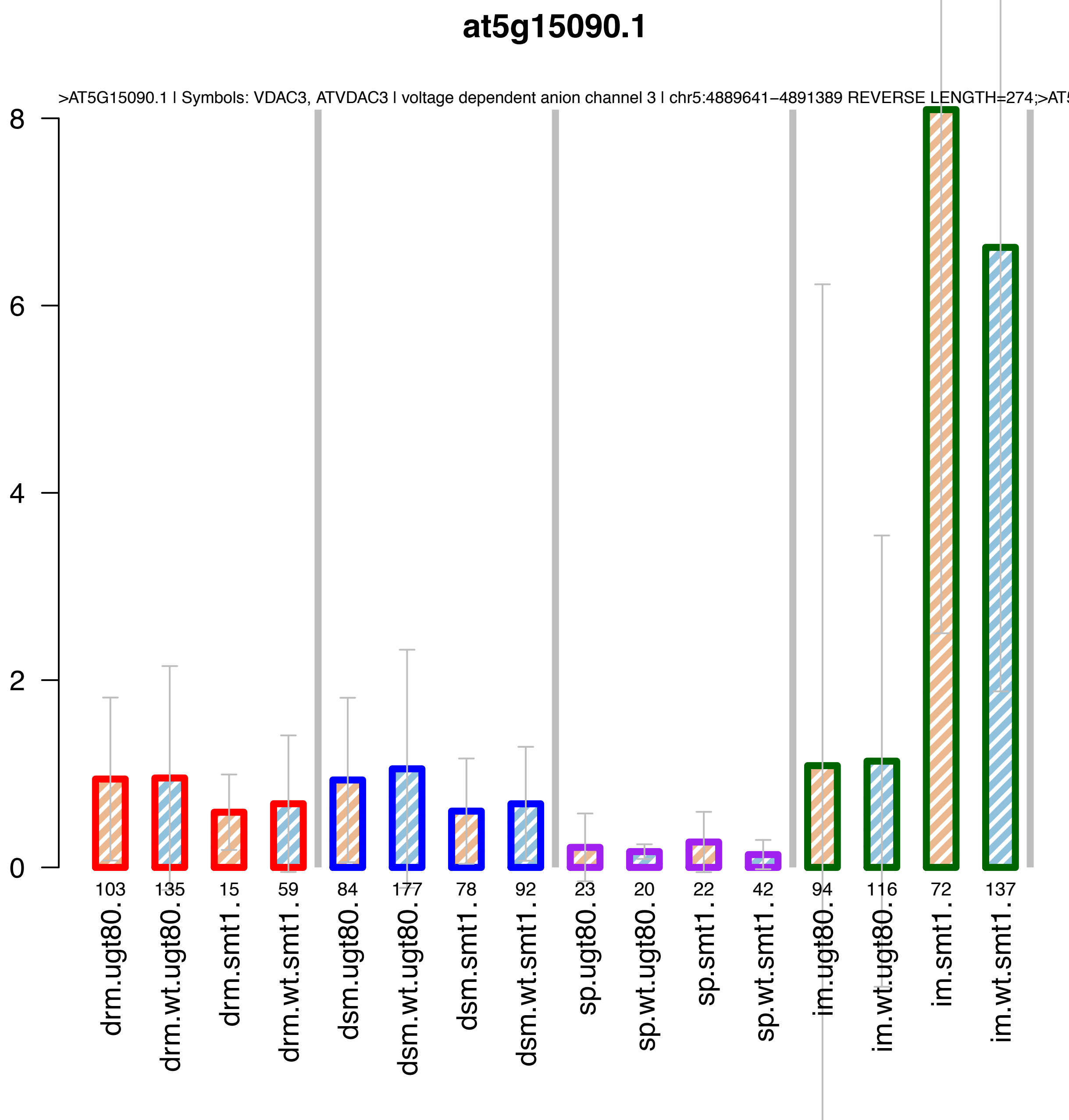
at5g15090.1
>AT5G15090.1 | Symbols: VDAC3, ATVDAC3 | voltage dependent anion channel 3 | chr5:4889641−4891389 REVERSE LENGTH=274;>AT5
0
2
4
6
8
103
135
15
59
84
177
78
92
23
20
22
42
94
116
72
137
drm.ugt80..
drm.wt.ugt80..
drm.smt1..
drm.wt.smt1..
dsm.ugt80..
dsm.wt.ugt80..
dsm.smt1..
dsm.wt.smt1..
sp.ugt80..
sp.wt.ugt80..
sp.smt1..
sp.wt.smt1..
im.ugt80..
im.wt.ugt80..
im.smt1..
im.wt.smt1..

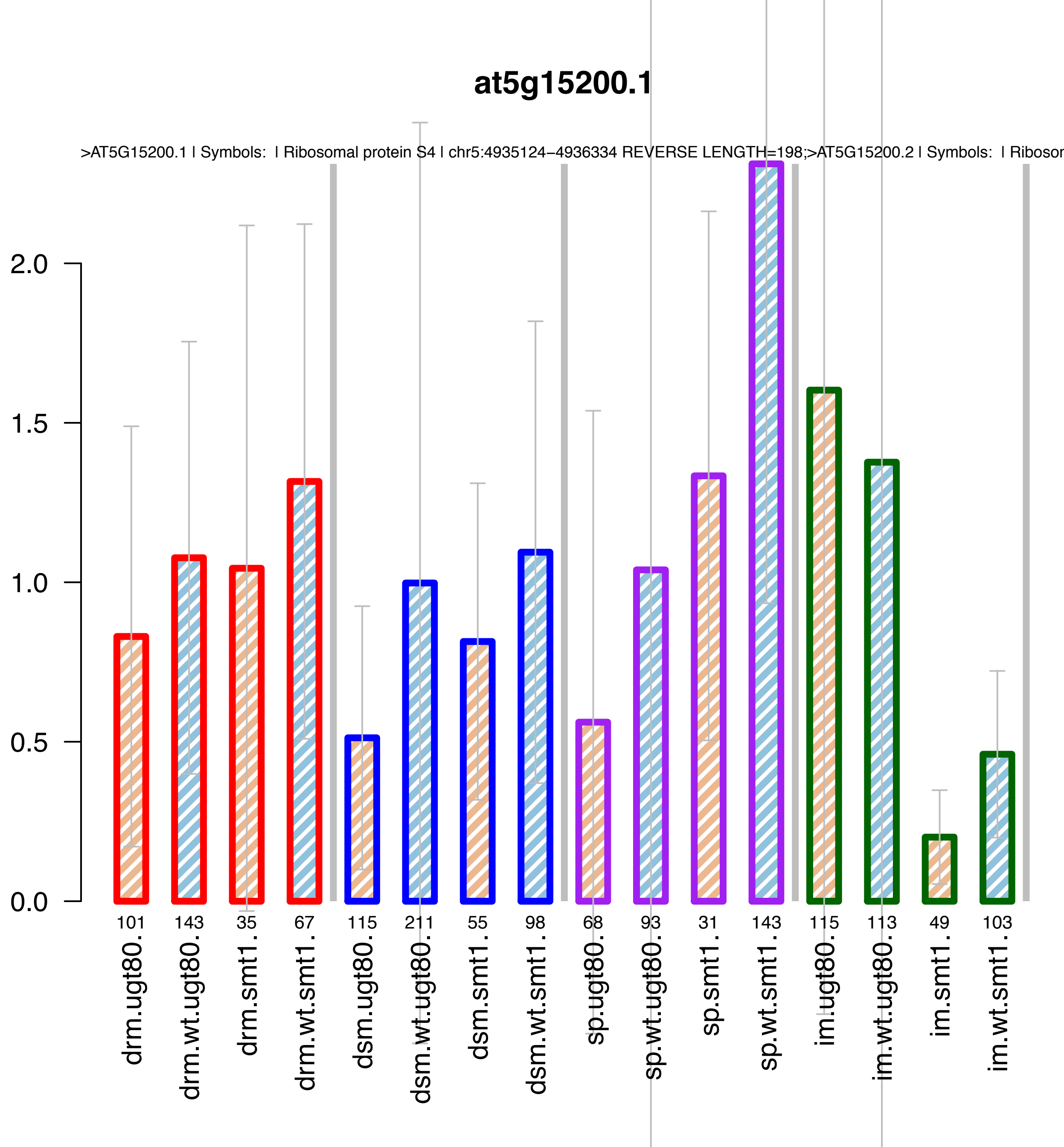

at5g15200.1
>AT5G15200.1 | Symbols: | Ribosomal protein S4 | chr5:4935124−4936334 REVERSE LENGTH=198;>AT5G15200.2 | Symbols: | Riboso
2.0
1.5
1.0
0.5
0.0
101
143
35
67
115
211
55
98
68
93
31
143
115
113
49
103
drm.ugt80..
drm.wt.ugt80..
drm.smt1..
drm.wt.smt1..
dsm.ugt80..
dsm.wt.ugt80..
dsm.smt1..
dsm.wt.smt1..
sp.ugt80..
sp.wt.ugt80..
sp.smt1..
sp.wt.smt1..
im.ugt80..
im.wt.ugt80..
im.smt1..
im.wt.smt1..

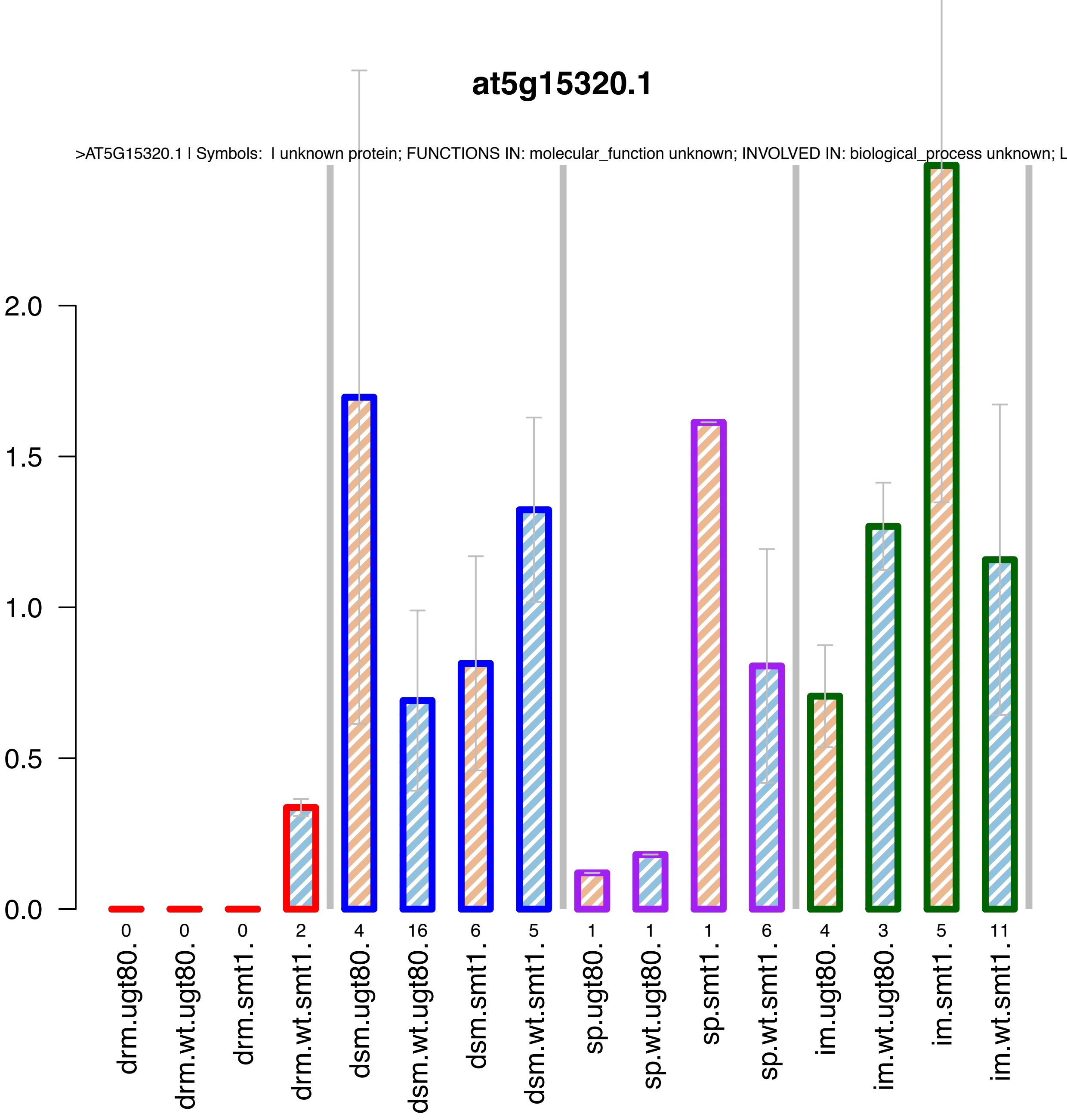

at5g15320.1
>AT5G15320.1 | Symbols: | unknown protein; FUNCTIONS IN: molecular_function unknown; INVOLVED IN: biological_process unknown; L
2.0
1.5
1.0
0.5
0.0
0
0
0
2
4
16
6
5
1
1
1
6
4
3
5
11
drm.ugt80.
drm.wt.ugt80.
drm.smt1.
drm.wt.smt1.
dsm.ugt80.
dsm.wt.ugt80.
dsm.smt1.
dsm.wt.smt1.
sp.ugt80.
sp.wt.ugt80.
sp.smt1.
sp.wt.smt1.
im.ugt80.
im.wt.ugt80.
im.smt1.
im.wt.smt1.

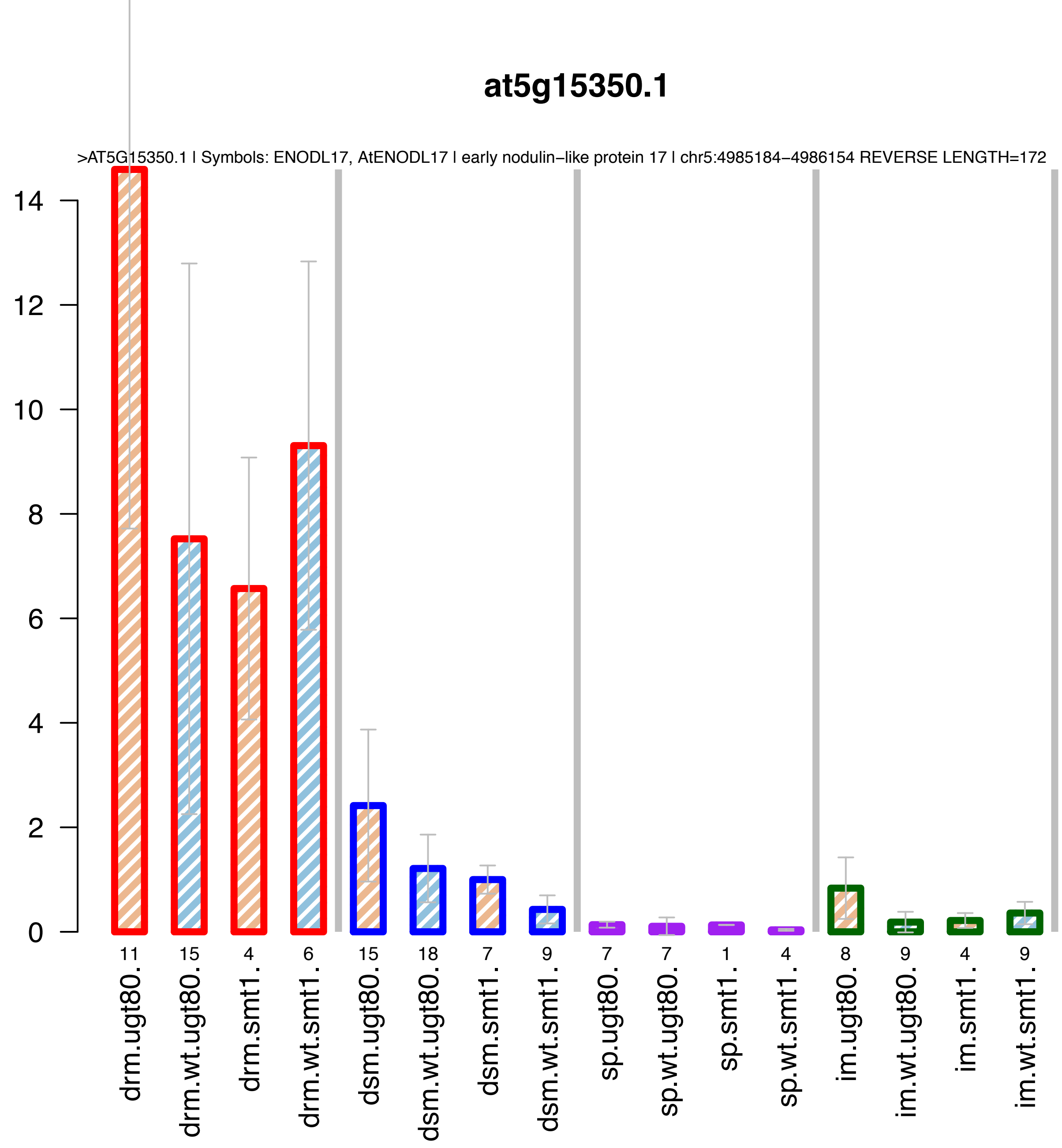

at5g15350.1
>AT5G15350.1 | Symbols: ENODL17, AtENODL17 | early nodulin−like protein 17 | chr5:4985184−4986154 REVERSE LENGTH=172
0
2
4
6
8
10
12
14
11
15
4
6
15
18
7
9
7
7
1
4
8
9
4
9
drm.ugt80.
drm.wt.ugt80.
drm.smt1.
drm.wt.smt1.
dsm.ugt80.
dsm.wt.ugt80.
dsm.smt1.
dsm.wt.smt1.
sp.ugt80.
sp.wt.ugt80.
sp.smt1.
sp.wt.smt1.
im.ugt80.
im.wt.ugt80.
im.smt1.
im.wt.smt1.

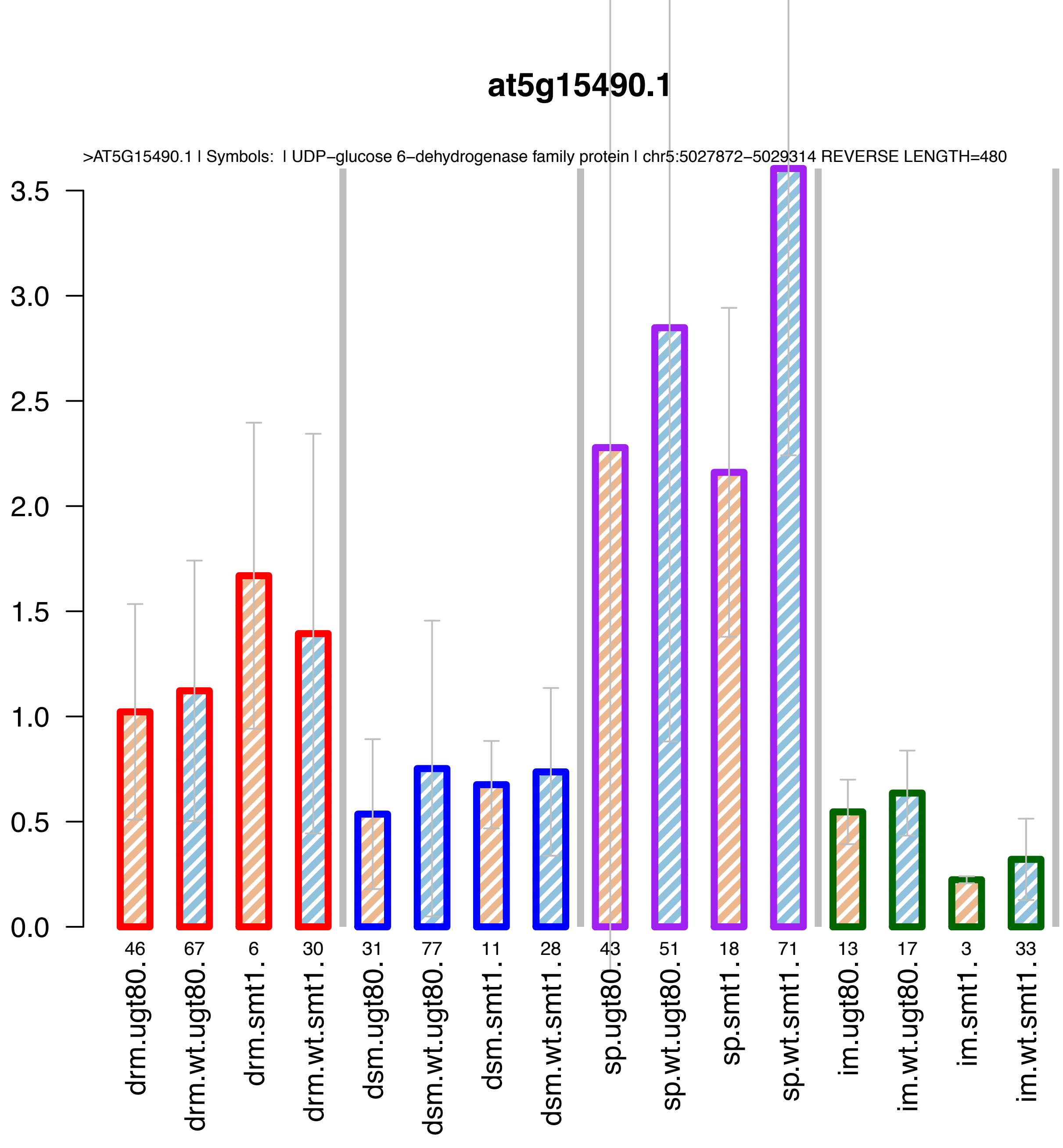

at5g15490.1
>AT5G15490.1 | Symbols: | UDP−glucose 6−dehydrogenase family protein | chr5:5027872−5029314 REVERSE LENGTH=480
3.5
3.0
2.5
2.0
1.5
1.0
0.5
0.0
46
67
6
30
31
77
11
28
43
51
18
71
13
17
3
33
drm.ugt80..
drm.wt.ugt80..
drm.smt1..
drm.wt.smt1..
dsm.ugt80..
dsm.wt.ugt80..
dsm.smt1..
dsm.wt.smt1..
sp.ugt80..
sp.wt.ugt80..
sp.smt1..
sp.wt.smt1..
im.ugt80..
im.wt.ugt80..
im.smt1..
im.wt.smt1..

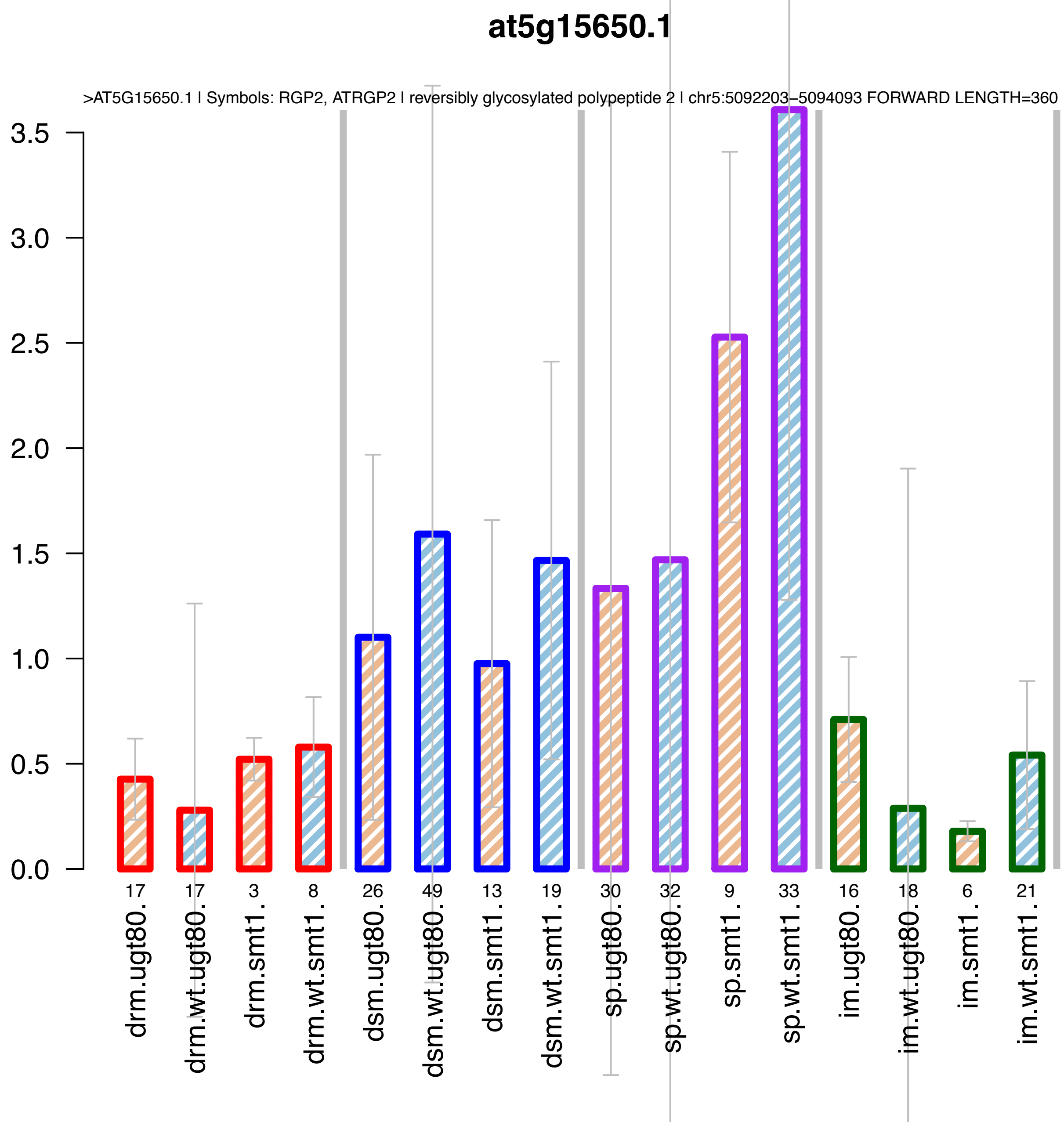
at5g15650.1
>AT5G15650.1 | Symbols: RGP2, ATRGP2 | reversibly glycosylated polypeptide 2 | chr5:5092203–5094093 FORWARD LENGTH=360
3.5
3.0
2.5
2.0
1.5
1.0
0.5
0.0
17
17
3
8
26
49
13
19
30
32
9
33
16
18
6
21
drm.ugt80.
drm.wt.ugt80.
drm.smt1.
drm.wt.smt1.
dsm.ugt80.
dsm.wt.ugt80.
dsm.smt1.
dsm.wt.smt1.
sp.ugt80.
sp.wt.ugt80.
sp.smt1.
sp.wt.smt1.
im.ugt80.
im.wt.ugt80.
im.smt1.
im.wt.smt1.

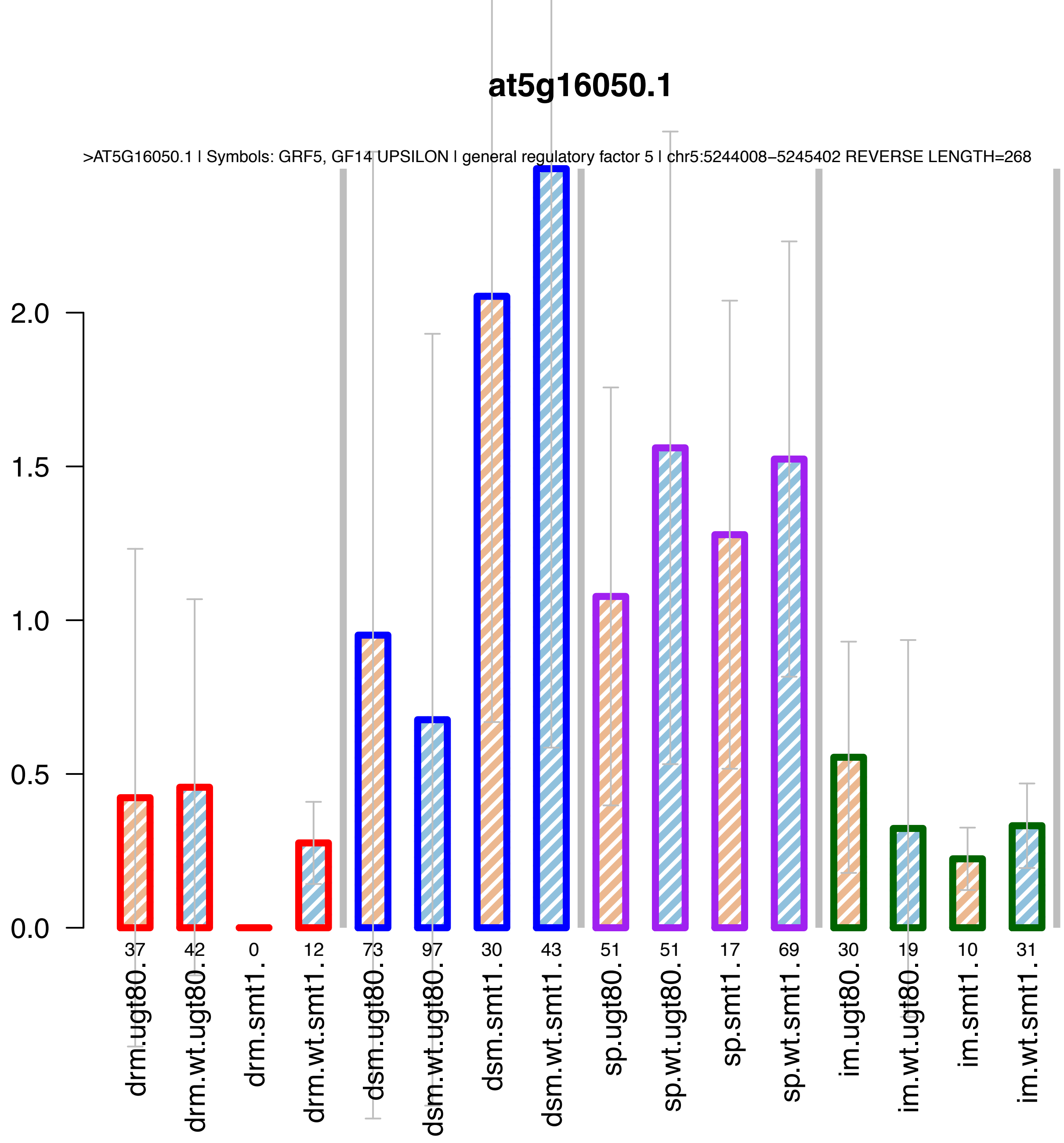
at5g16050.1
>AT5G16050.1 | Symbols: GRF5, GF14 UPSILON | general regulatory factor 5 | chr5:5244008-5245402 REVERSE LENGTH=268
0.0
0.5
1.0
1.5
2.0
37
42
0
12
73
97
30
43
51
51
17
69
30
19
10
31
drm.ugt80.
drm.wt.ugt80.
drm.smt1.
drm.wt.smt1.
dsm.ugt80.
dsm.wt.ugt80.
dsm.smt1.
dsm.wt.smt1.
sp.ugt80.
sp.wt.ugt80.
sp.smt1.
sp.wt.smt1.
im.ugt80.
im.wt.ugt80.
im.smt1.
im.wt.smt1.

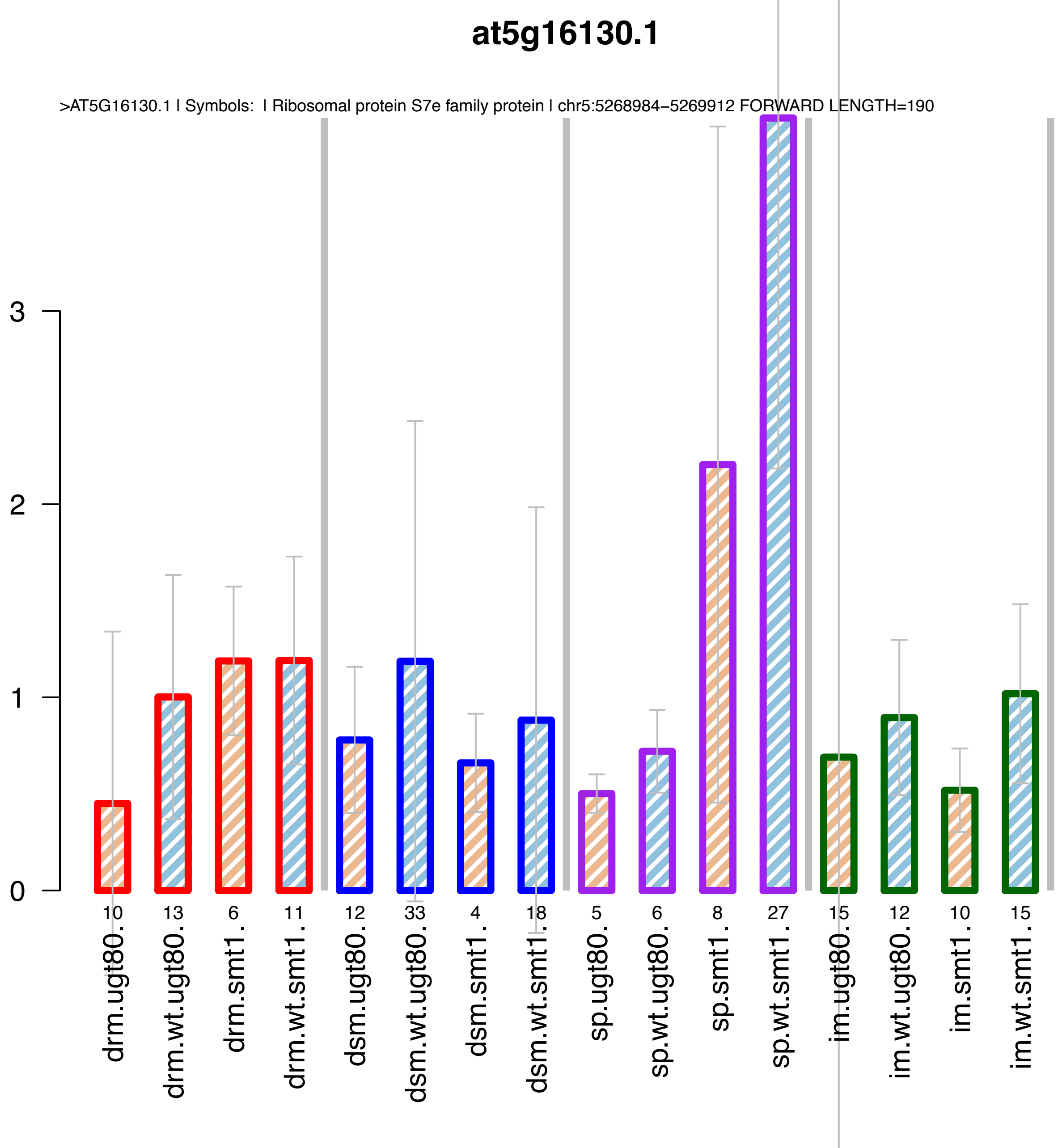

at5g16130.1
>AT5G16130.1 | Symbols: | Ribosomal protein S7e family protein | chr5:5268984-5269912 FORWARD LENGTH=190
0
1
2
3
10
13
6
11
12
33
4
18
5
6
8
27
15
12
10
15
drm.ugt80.
drm.wt.ugt80.
drm.smt1.
drm.wt.smt1.
dsm.ugt80.
dsm.wt.ugt80.
dsm.smt1.
dsm.wt.smt1.
sp.ugt80.
sp.wt.ugt80.
sp.smt1.
sp.wt.smt1.
im.ugt80.
im.wt.ugt80.
im.smt1.
im.wt.smt1.

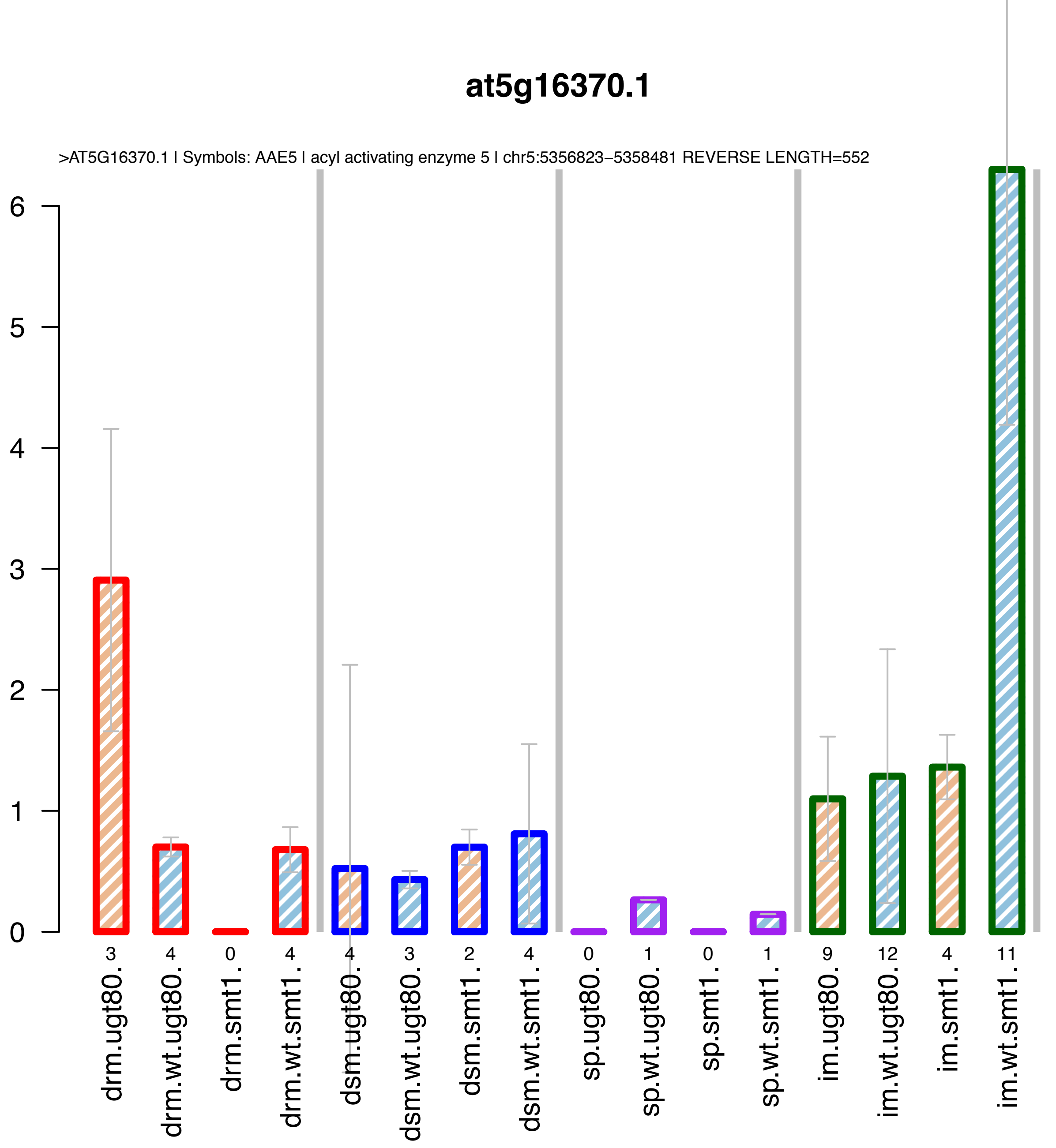
at5g16370.1
>AT5G16370.1 | Symbols: AAE5 | acyl activating enzyme 5 | chr5:5356823–5358481 REVERSE LENGTH=552
0
1
2
3
4
5
6
3
4
0
4
4
3
2
4
0
1
0
1
9
12
4
11
drm.ugt80.
drm.wt.ugt80.
drm.smt1.
drm.wt.smt1.
dsm.ugt80.
dsm.wt.ugt80.
dsm.smt1.
dsm.wt.smt1.
sp.ugt80.
sp.wt.ugt80.
sp.smt1.
sp.wt.smt1.
im.ugt80.
im.wt.ugt80.
im.smt1.
im.wt.smt1.

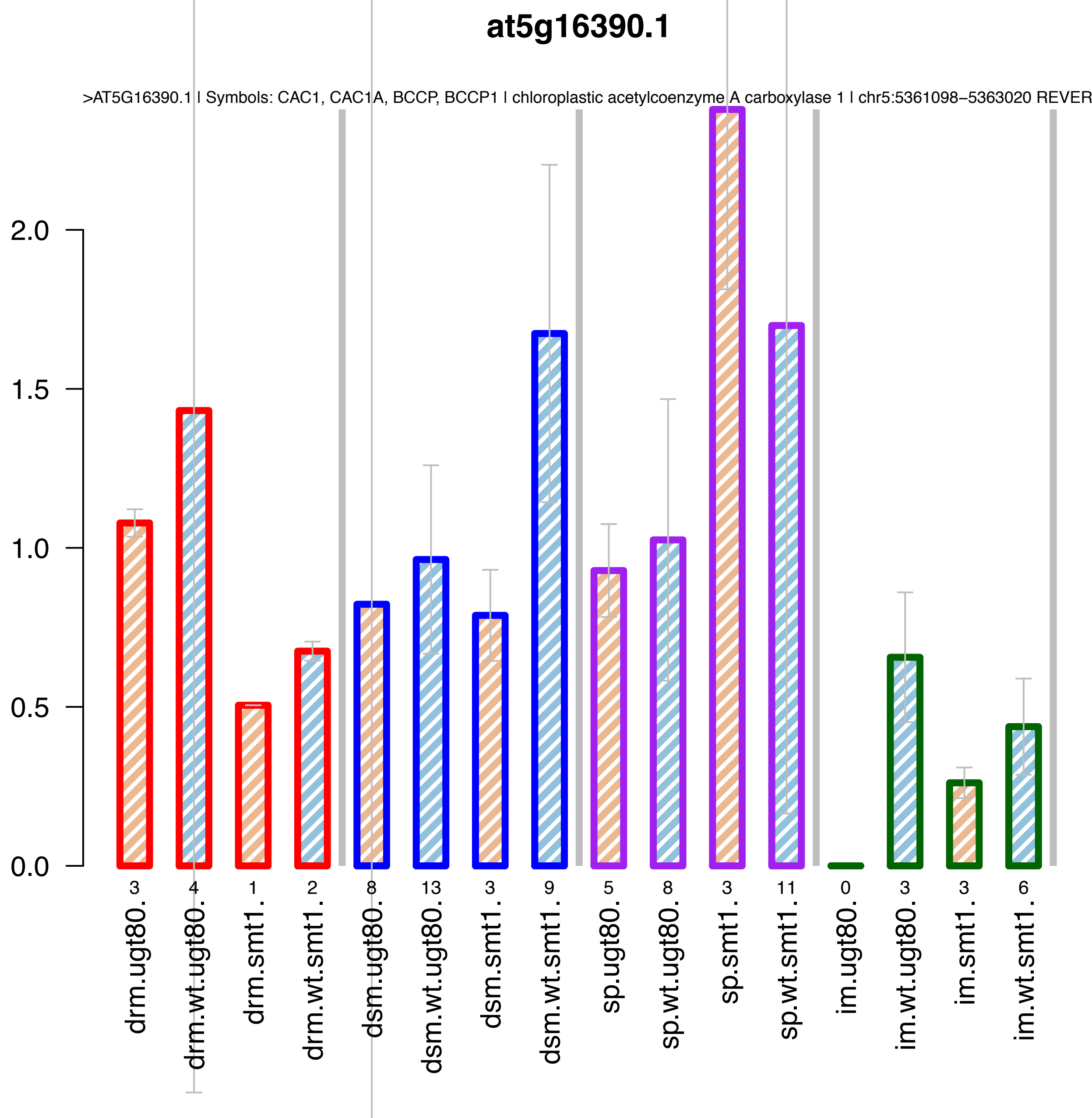

at5g16390.1
>AT5G16390.1 | Symbols: CAC1, CAC1A, BCCP, BCCP1 | chloroplastic acetylcoenzyme A carboxylase 1 | chr5:5361098-5363020 REVER
2.0
1.5
1.0
0.5
0.0
3
4
1
2
8
13
3
9
5
8
3
11
0
3
3
6
drm.ugt80.
drm.wt.ugt80.
drm.smt1.
drm.wt.smt1.
dsm.ugt80.
dsm.wt.ugt80.
dsm.smt1.
dsm.wt.smt1.
sp.ugt80.
sp.wt.ugt80.
sp.smt1.
sp.wt.smt1.
im.ugt80.
im.wt.ugt80.
im.smt1.
im.wt.smt1.

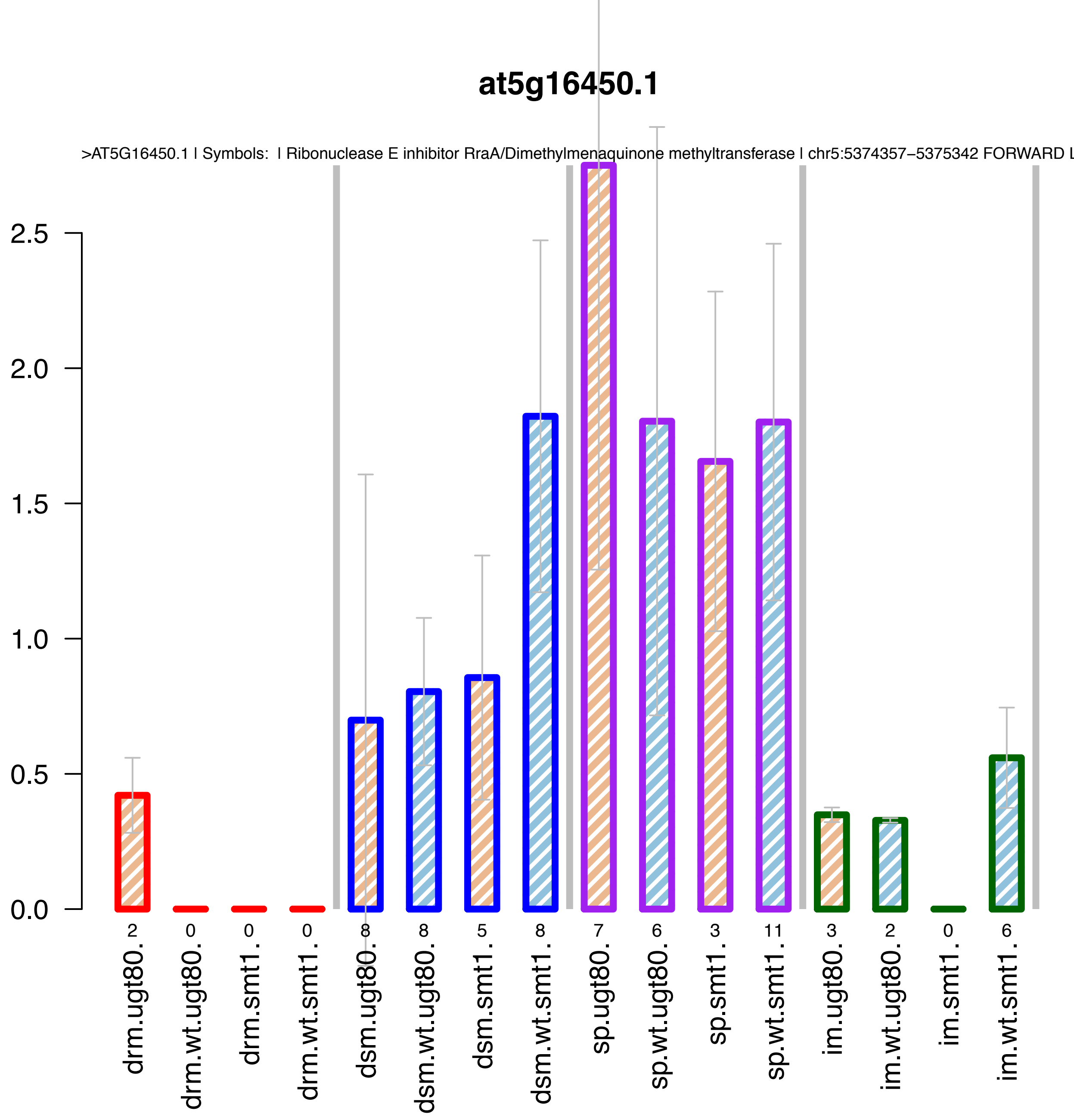

at5g16450.1
>AT5G16450.1 | Symbols: | Ribonuclease E inhibitor RraA/Dimethylmenaquinone methyltransferase | chr5:5374357-5375342 FORWARD L
0.0
0.5
1.0
1.5
2.0
2.5
2
0
0
0
8
8
5
8
7
6
3
11
3
2
0
6
drm.ugt80.
drm.wt.ugt80.
drm.smt1.
drm.wt.smt1.
dsm.ugt80.
dsm.wt.ugt80.
dsm.smt1.
dsm.wt.smt1.
sp.ugt80.
sp.wt.ugt80.
sp.smt1.
sp.wt.smt1.
im.ugt80.
im.wt.ugt80.
im.smt1.
im.wt.smt1.

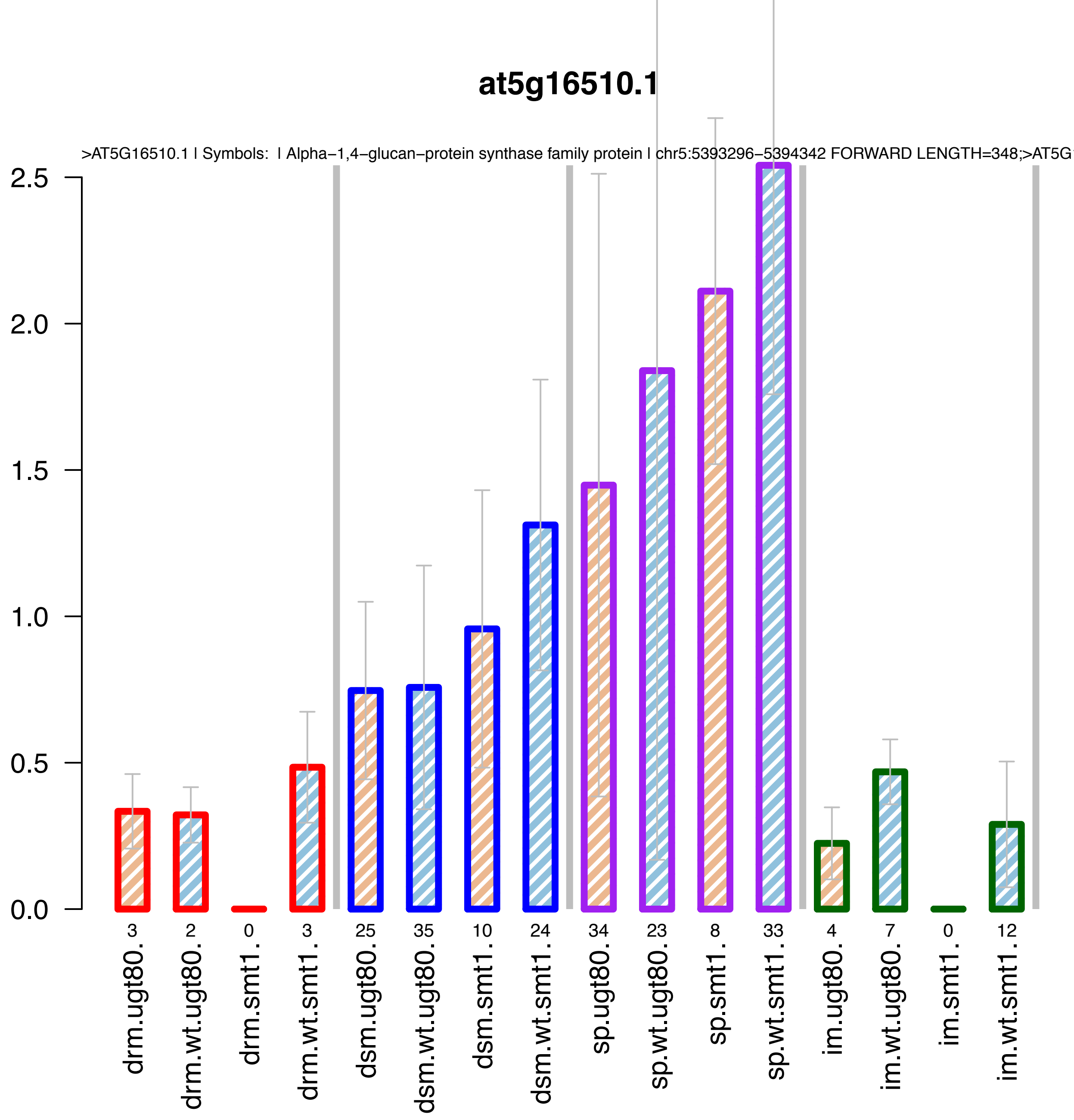

at5g16510.1
>AT5G16510.1 | Symbols: | Alpha−1,4−glucan−protein synthase family protein | chr5:5393296−5394342 FORWARD LENGTH=348;>AT5G
2.5
2.0
1.5
1.0
0.5
0.0
3
2
0
3
25
35
10
24
34
23
8
33
4
7
0
12
drm.ugt80.
drm.wt.ugt80.
drm.smt1.
drm.wt.smt1.
dsm.ugt80.
dsm.wt.ugt80.
dsm.smt1.
dsm.wt.smt1.
sp.ugt80.
sp.wt.ugt80.
sp.smt1.
sp.wt.smt1.
im.ugt80.
im.wt.ugt80.
im.smt1.
im.wt.smt1.

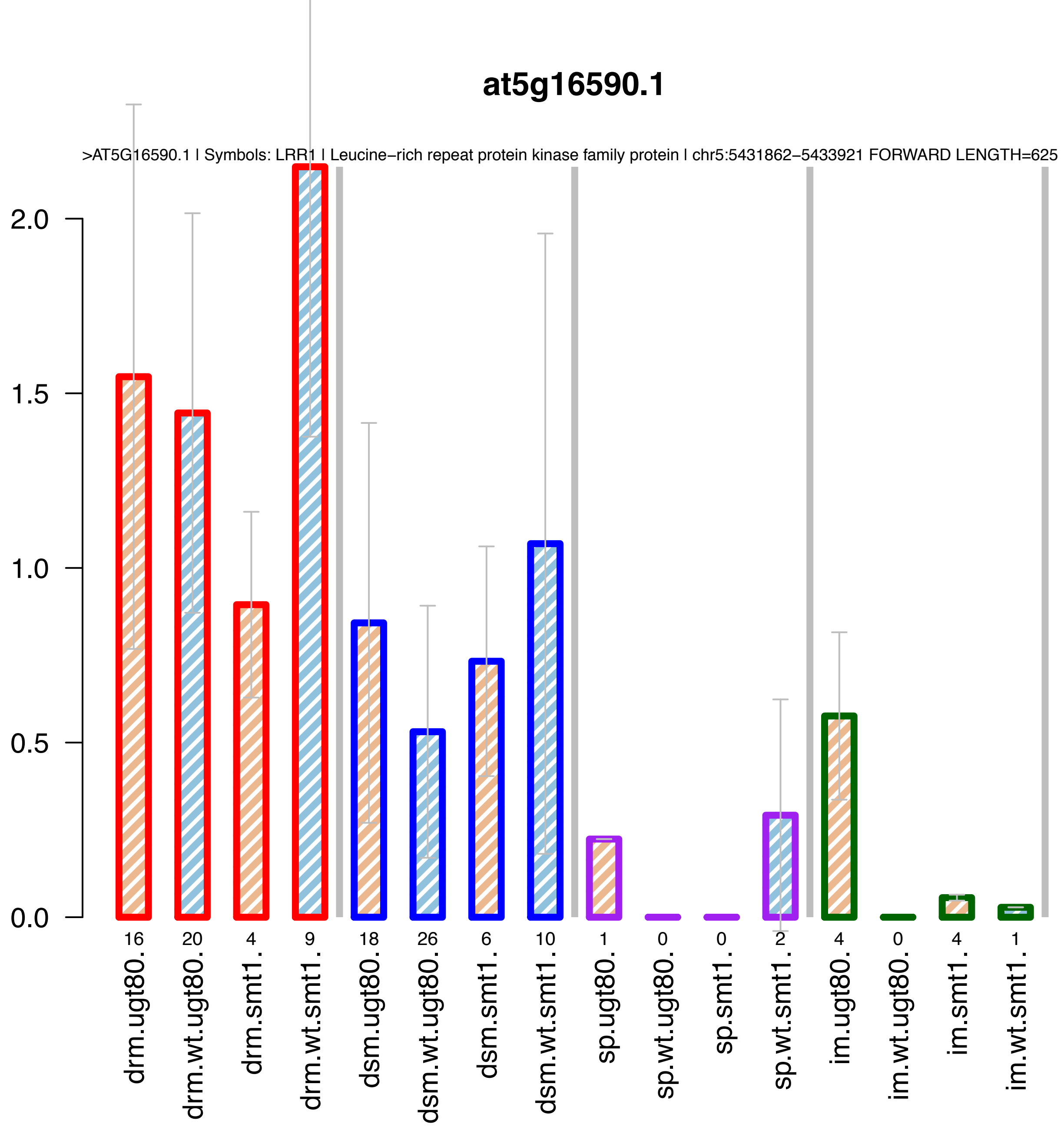

at5g16590.1
>AT5G16590.1 | Symbols: LRR1 | Leucine−rich repeat protein kinase family protein | chr5:5431862−5433921 FORWARD LENGTH=625
2.0
1.5
1.0
0.5
0.0
16
20
4
9
18
26
6
10
1
0
0
2
4
0
4
1
drm.ugt80.
drm.wt.ugt80.
drm.smt1.
drm.wt.smt1.
dsm.ugt80.
dsm.wt.ugt80.
dsm.smt1.
dsm.wt.smt1.
sp.ugt80.
sp.wt.ugt80.
sp.smt1.
sp.wt.smt1.
im.ugt80.
im.wt.ugt80.
im.smt1.
im.wt.smt1.

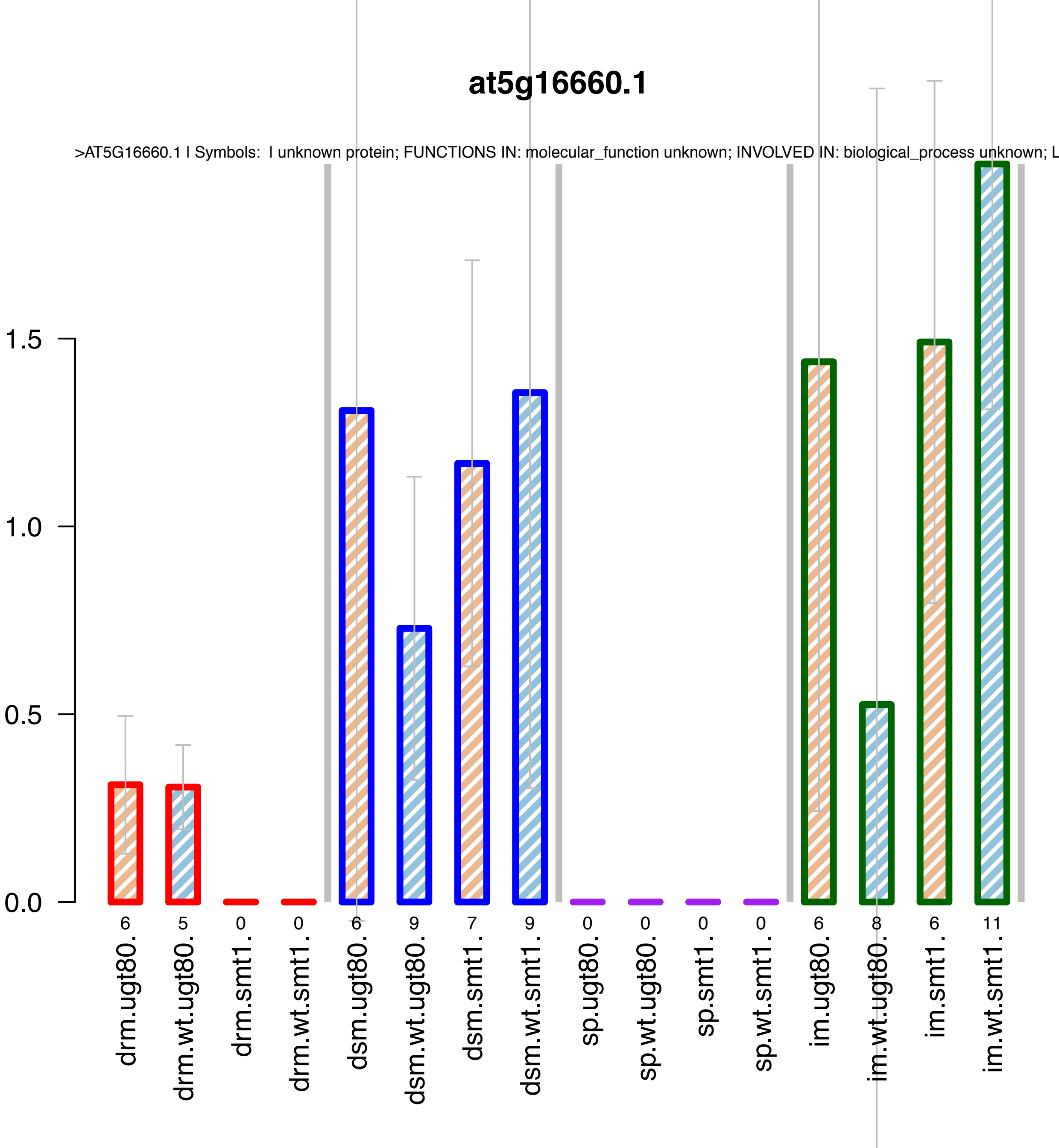

at5g16660.1
>AT5G16660.1 | Symbols: | unknown protein; FUNCTIONS IN: molecular_function unknown; INVOLVED IN: biological_process unknown; L
0.0
0.5
1.0
1.5
6
5
0
0
6
9
7
9
0
0
0
0
6
8
6
11
drm.ugt80.
drm.wt.ugt80.
drm.smt1.
drm.wt.smt1.
dsm.ugt80.
dsm.wt.ugt80.
dsm.smt1.
dsm.wt.smt1.
sp.ugt80.
sp.wt.ugt80.
sp.smt1.
sp.wt.smt1.
im.ugt80.
im.wt.ugt80.
im.smt1.
im.wt.smt1.

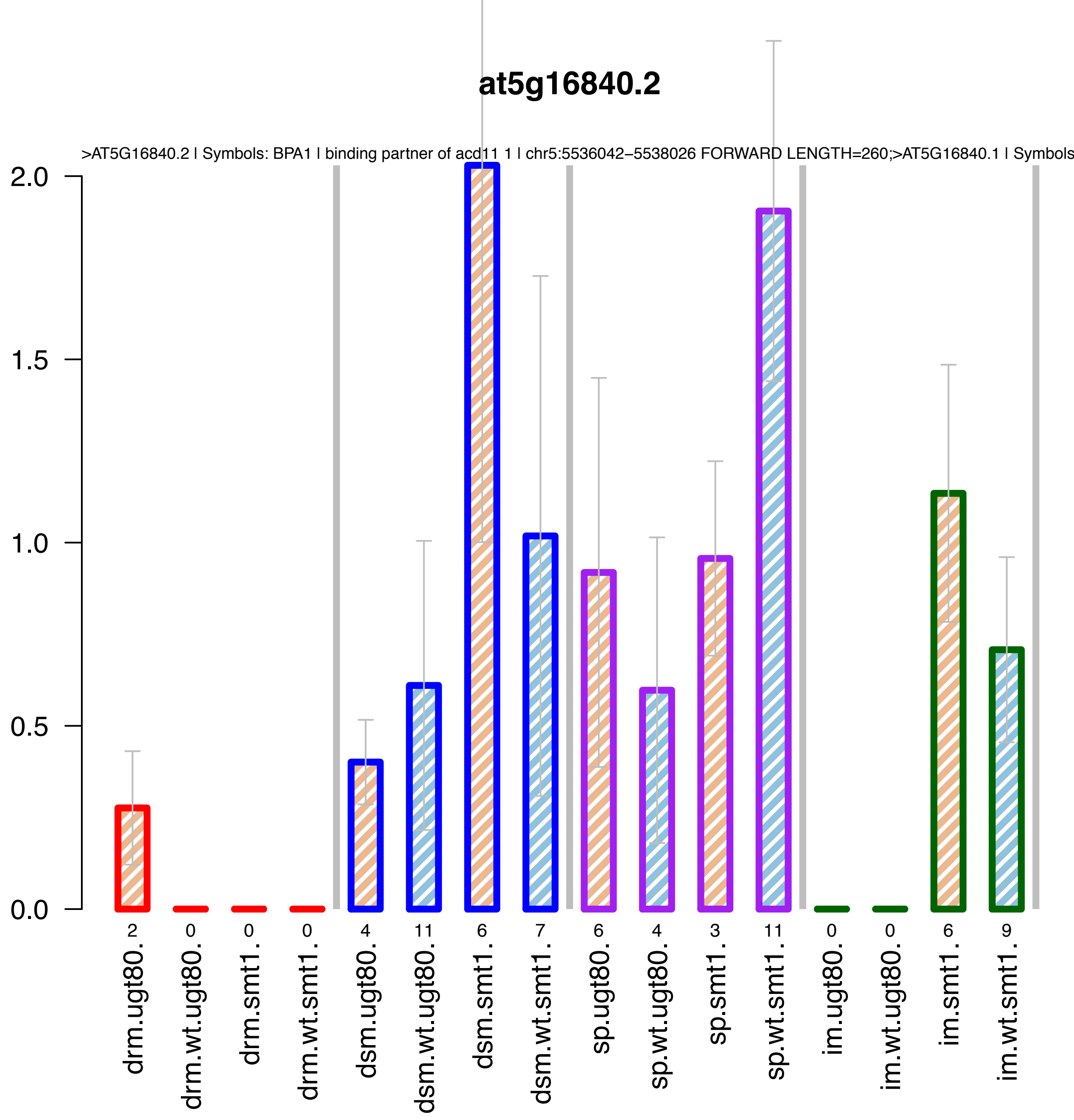

at5g16840.2
>AT5G16840.2 | Symbols: BPA1 | binding partner of acd11 1 | chr5:5536042–5538026 FORWARD LENGTH=260;>AT5G16840.1 | Symbols
2.0
1.5
1.0
0.5
0.0
2
0
0
0
4
11
6
7
6
4
3
11
0
0
6
9
drm.ugt80.
drm.wt.ugt80.
drm.smt1.
drm.wt.smt1.
dsm.ugt80.
dsm.wt.ugt80.
dsm.smt1.
dsm.wt.smt1.
sp.ugt80.
sp.wt.ugt80.
sp.smt1.
sp.wt.smt1.
im.ugt80.
im.wt.ugt80.
im.smt1.
im.wt.smt1.

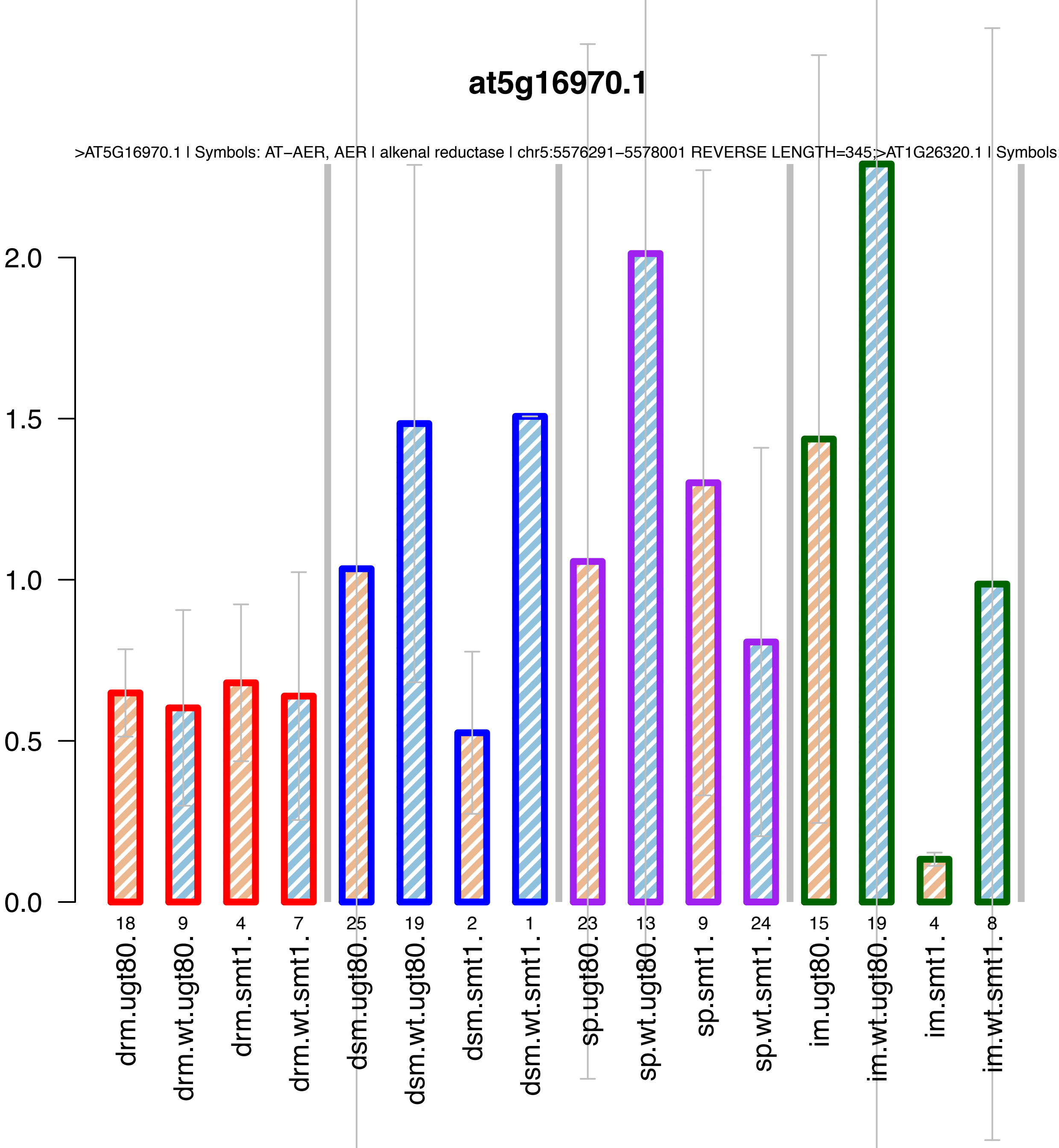

at5g16970.1
>AT5G16970.1 | Symbols: AT−AER, AER | alkenal reductase | chr5:5576291−5578001 REVERSE LENGTH=345;>AT1G26320.1 | Symbols
0.0
0.5
1.0
1.5
2.0
18
9
4
7
25
19
2
1
23
13
9
24
15
19
4
8
drm.ugt80..
drm.wt.ugt80..
drm.smt1..
drm.wt.smt1..
dsm.ugt80..
dsm.wt.ugt80..
dsm.smt1..
dsm.wt.smt1..
sp.ugt80..
sp.wt.ugt80..
sp.smt1..
sp.wt.smt1..
im.ugt80..
im.wt.ugt80..
im.smt1..
im.wt.smt1..

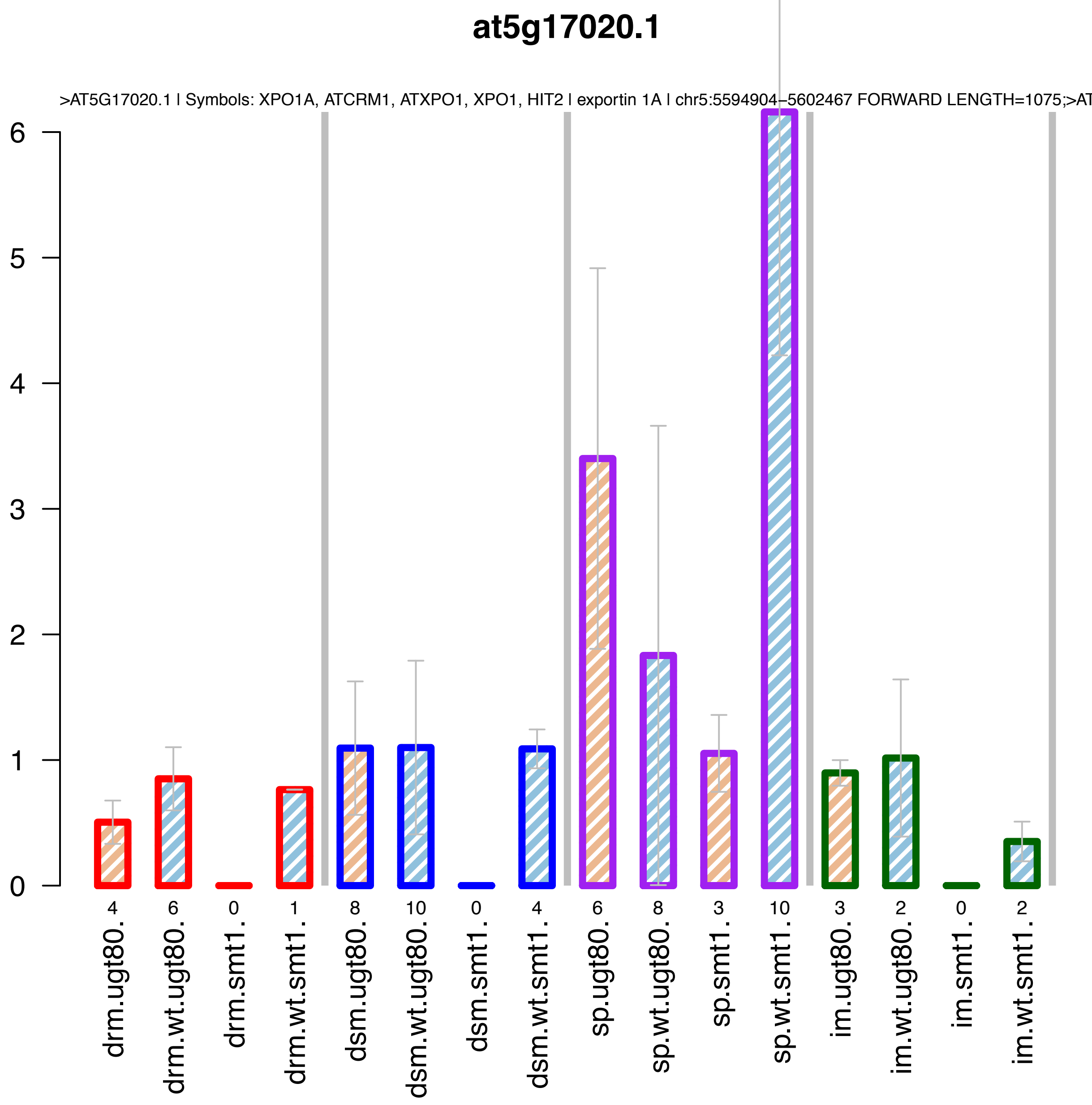

at5g17020.1
>AT5G17020.1 | Symbols: XPO1A, ATCRM1, ATXPO1, XPO1, HIT2 | exportin 1A | chr5:5594904–5602467 FORWARD LENGTH=1075;>AT
6
5
4
3
2
1
0
4
6
0
1
8
10
0
4
6
8
3
10
3
2
0
2
drm.ugt80.
drm.wt.ugt80.
drm.smt1.
drm.wt.smt1.
dsm.ugt80.
dsm.wt.ugt80.
dsm.smt1.
dsm.wt.smt1.
sp.ugt80.
sp.wt.ugt80.
sp.smt1.
sp.wt.smt1.
im.ugt80.
im.wt.ugt80.
im.smt1.
im.wt.smt1.

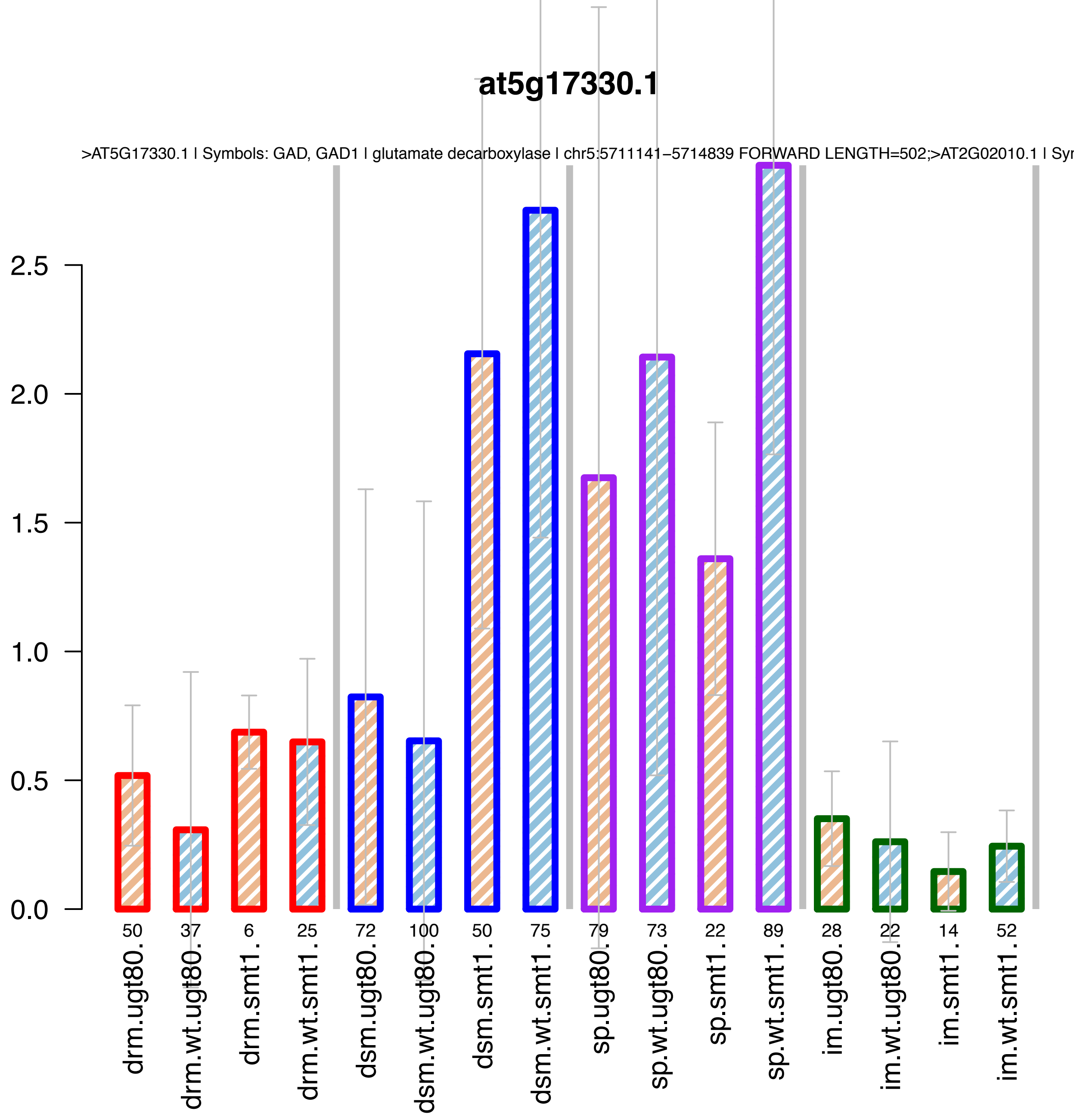
at5g17330.1
>AT5G17330.1 | Symbols: GAD, GAD1 | glutamate decarboxylase | chr5:5711141-5714839 FORWARD LENGTH=502;>AT2G02010.1 | Sy
0.0
0.5
1.0
1.5
2.0
2.5
50
37
6
25
72
100
50
75
79
73
22
89
28
22
14
52
drm.ugt80.
drm.wt.ugt80.
drm.smt1.
drm.wt.smt1.
dsm.ugt80.
dsm.wt.ugt80.
dsm.smt1.
dsm.wt.smt1.
sp.ugt80.
sp.wt.ugt80.
sp.smt1.
sp.wt.smt1.
im.ugt80.
im.wt.ugt80.
im.smt1.
im.wt.smt1.

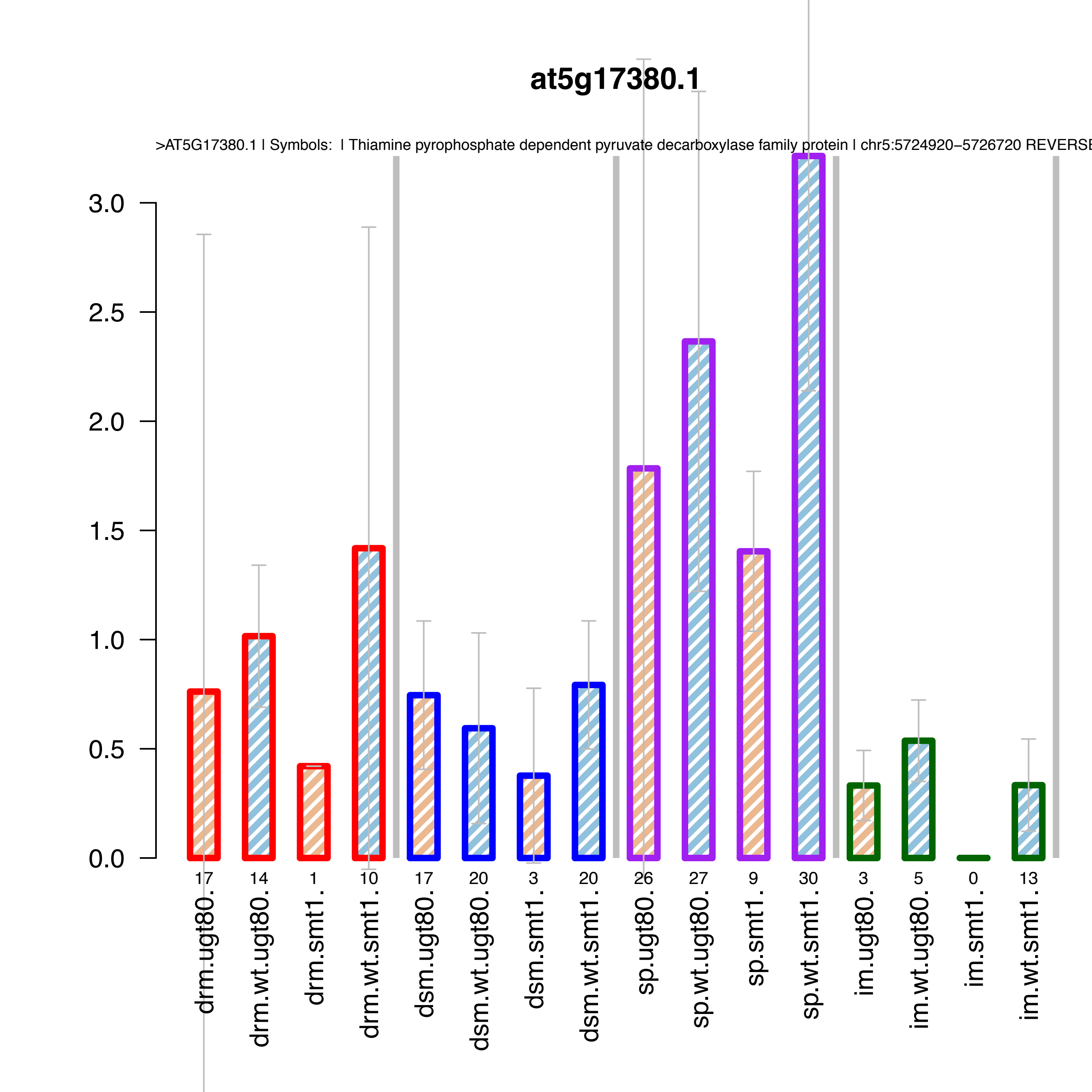
at5g17380.1
>AT5G17380.1 | Symbols: | Thiamine pyrophosphate dependent pyruvate decarboxylase family protein | chr5:5724920−5726720 REVERSE
0.0
0.5
1.0
1.5
2.0
2.5
3.0
17
14
1
10
17
20
3
20
26
27
9
30
3
5
0
13
drm.ugt80.
drm.wt.ugt80.
drm.smt1.
drm.wt.smt1.
dsm.ugt80.
dsm.wt.ugt80.
dsm.smt1.
dsm.wt.smt1.
sp.ugt80.
sp.wt.ugt80.
sp.smt1.
sp.wt.smt1.
im.ugt80.
im.wt.ugt80.
im.smt1.
im.wt.smt1.

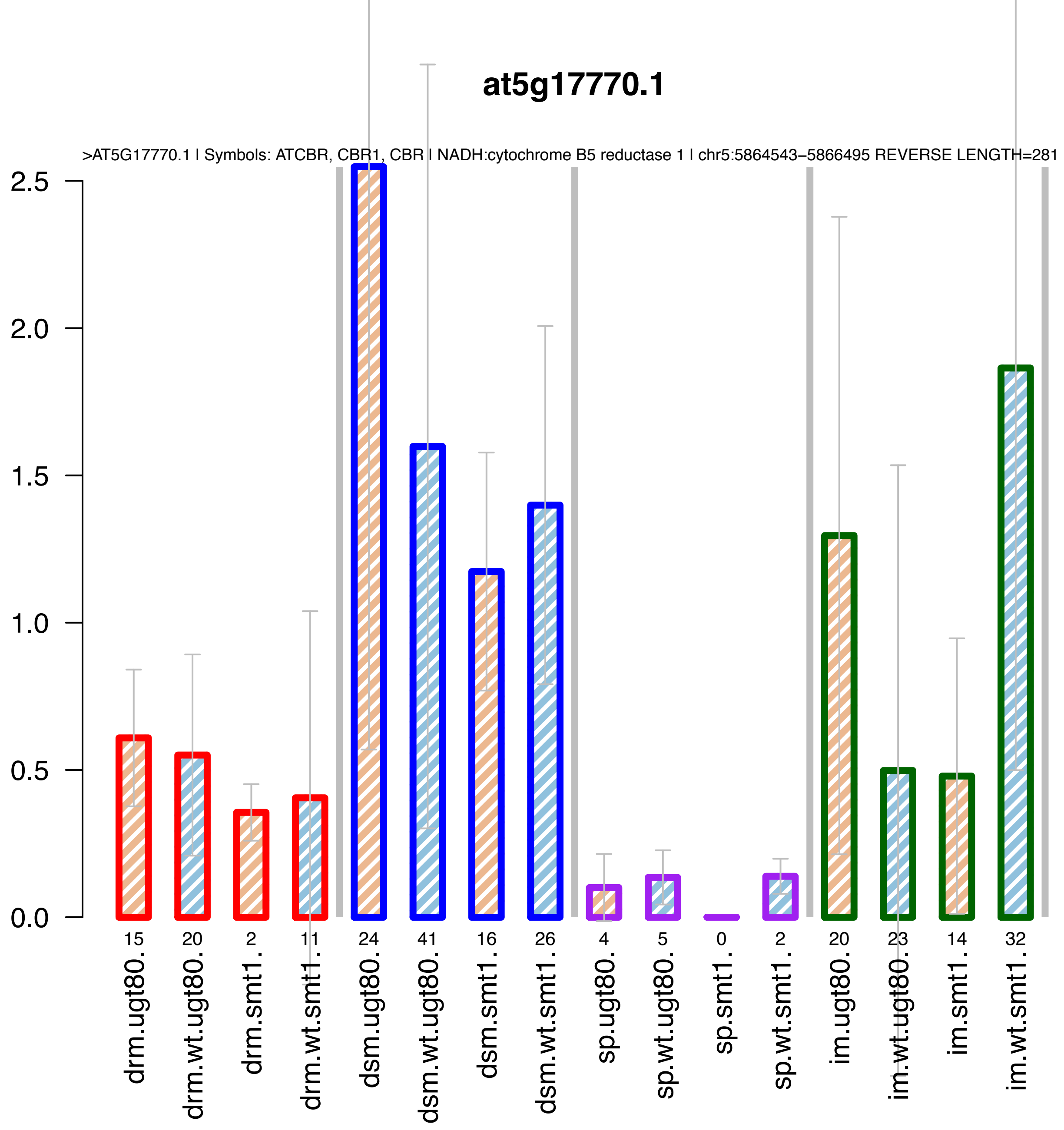

at5g17770.1
>AT5G17770.1 | Symbols: ATCBR, CBR1, CBR | NADH:cytochrome B5 reductase 1 | chr5:5864543-5866495 REVERSE LENGTH=281
0.0
0.5
1.0
1.5
2.0
2.5
15
20
2
11
24
41
16
26
4
5
0
2
20
23
14
32
drm.ugt80..
drm.wt.ugt80..
drm.smt1..
drm.wt.smt1..
dsm.ugt80..
dsm.wt.ugt80..
dsm.smt1..
dsm.wt.smt1..
sp.ugt80..
sp.wt.ugt80..
sp.smt1..
sp.wt.smt1..
im.ugt80..
im.wt.ugt80..
im.smt1..
im.wt.smt1..

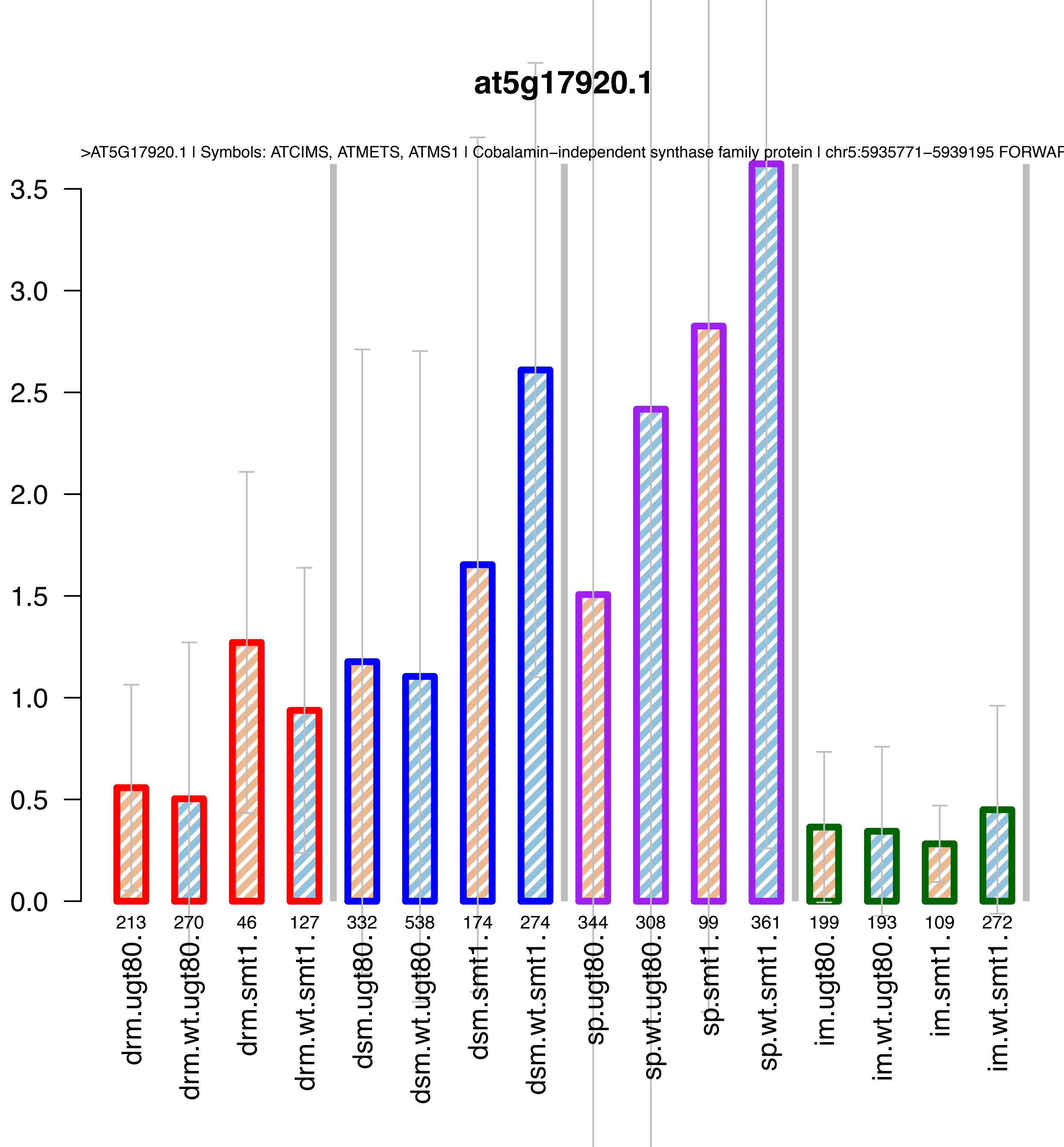

at5g17920.1
>AT5G17920.1 | Symbols: ATCIMS, ATMETS, ATMS1 | Cobalamin−independent synthase family protein | chr5:5935771−5939195 FORWAR
0.0
0.5
1.0
1.5
2.0
2.5
3.0
3.5
213
270
46
127
332
538
174
274
344
308
99
361
199
193
109
272
drm.ugt80..
drm.wt.ugt80..
drm.smt1..
drm.wt.smt1..
dsm.ugt80..
dsm.wt.ugt80..
dsm.smt1..
dsm.wt.smt1..
sp.ugt80..
sp.wt.ugt80..
sp.smt1..
sp.wt.smt1..
im.ugt80..
im.wt.ugt80..
im.smt1..
im.wt.smt1..

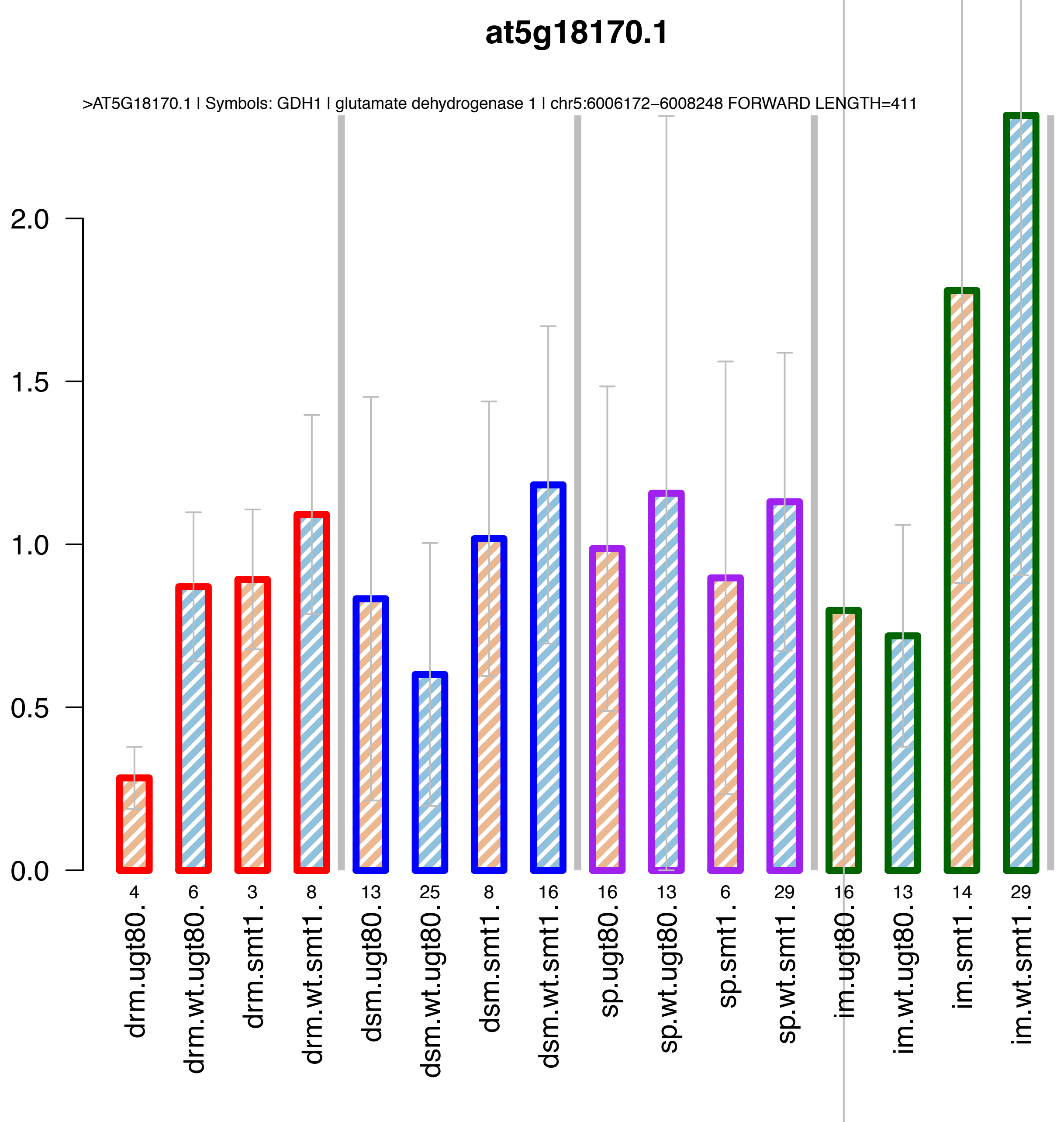

at5g18170.1
>AT5G18170.1 | Symbols: GDH1 | glutamate dehydrogenase 1 | chr5:6006172–6008248 FORWARD LENGTH=411
2.0
1.5
1.0
0.5
0.0
4
6
3
8
13
25
8
16
16
13
6
29
16
13
14
29
drm.ugt80..
drm.wt.ugt80..
drm.smt1..
drm.wt.smt1..
dsm.ugt80..
dsm.wt.ugt80..
dsm.smt1..
dsm.wt.smt1..
sp.ugt80..
sp.wt.ugt80..
sp.smt1..
sp.wt.smt1..
im.ugt80..
im.wt.ugt80..
im.smt1..
im.wt.smt1..

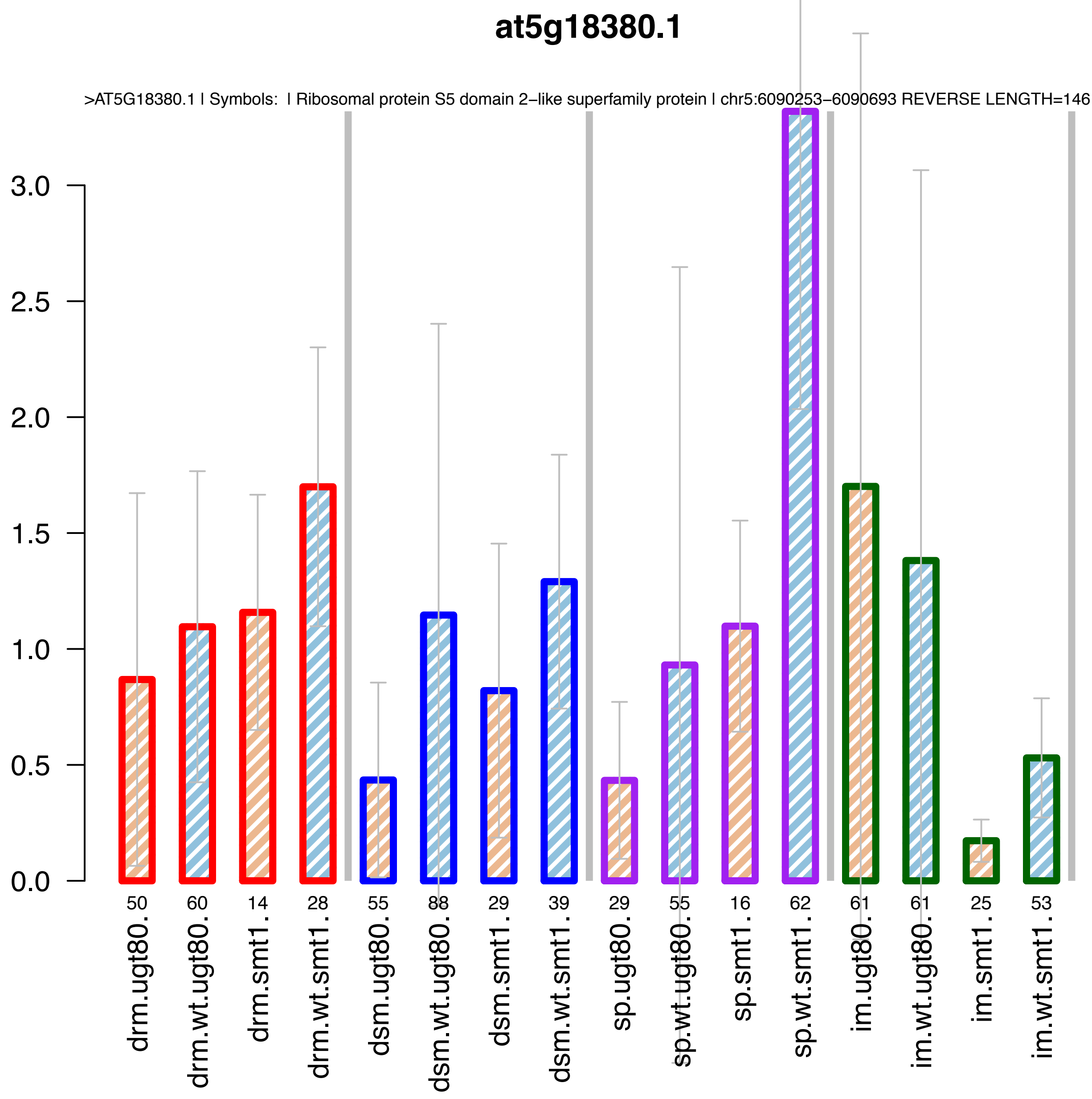

at5g18380.1
>AT5G18380.1 | Symbols: | Ribosomal protein S5 domain 2-like superfamily protein | chr5:6090253-6090693 REVERSE LENGTH=146;>A
3.0
2.5
2.0
1.5
1.0
0.5
0.0
50
60
14
28
55
88
29
39
29
55
16
62
61
61
25
53
drm.ugt80..
drm.wt.ugt80..
drm.smt1..
drm.wt.smt1..
dsm.ugt80..
dsm.wt.ugt80..
dsm.smt1..
dsm.wt.smt1..
sp.ugt80..
sp.wt.ugt80..
sp.smt1..
sp.wt.smt1..
im.ugt80..
im.wt.ugt80..
im.smt1..
im.wt.smt1..

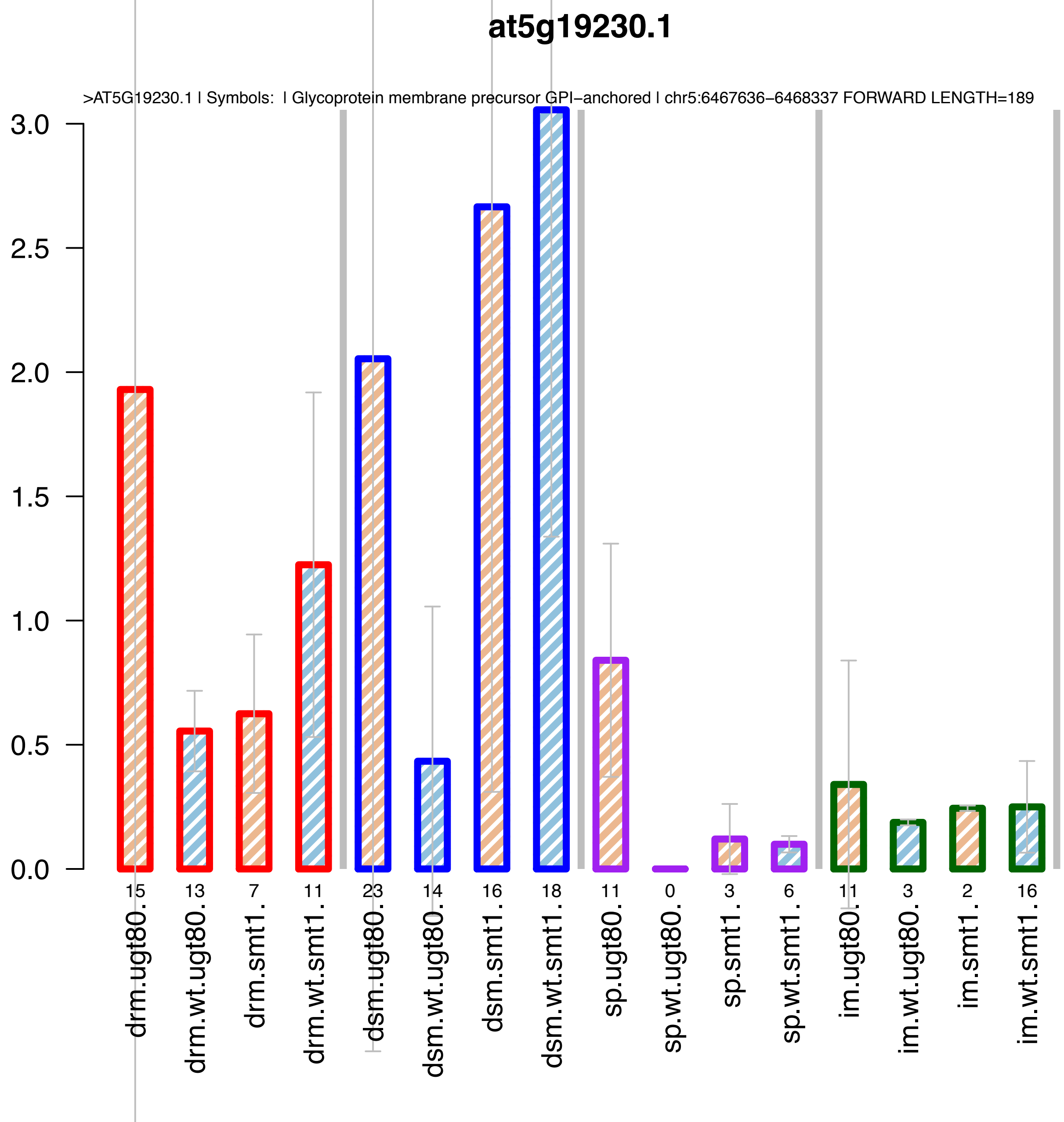

at5g19230.1
>AT5G19230.1 | Symbols: | Glycoprotein membrane precursor GPI−anchored | chr5:6467636−6468337 FORWARD LENGTH=189
3.0
2.5
2.0
1.5
1.0
0.5
0.0
15
13
7
11
23
14
16
18
11
0
3
6
11
3
2
16
drm.ugt80..
drm.wt.ugt80..
drm.smt1..
drm.wt.smt1..
dsm.ugt80..
dsm.wt.ugt80..
dsm.smt1..
dsm.wt.smt1..
sp.ugt80..
sp.wt.ugt80..
sp.smt1..
sp.wt.smt1..
im.ugt80..
im.wt.ugt80..
im.smt1..
im.wt.smt1..

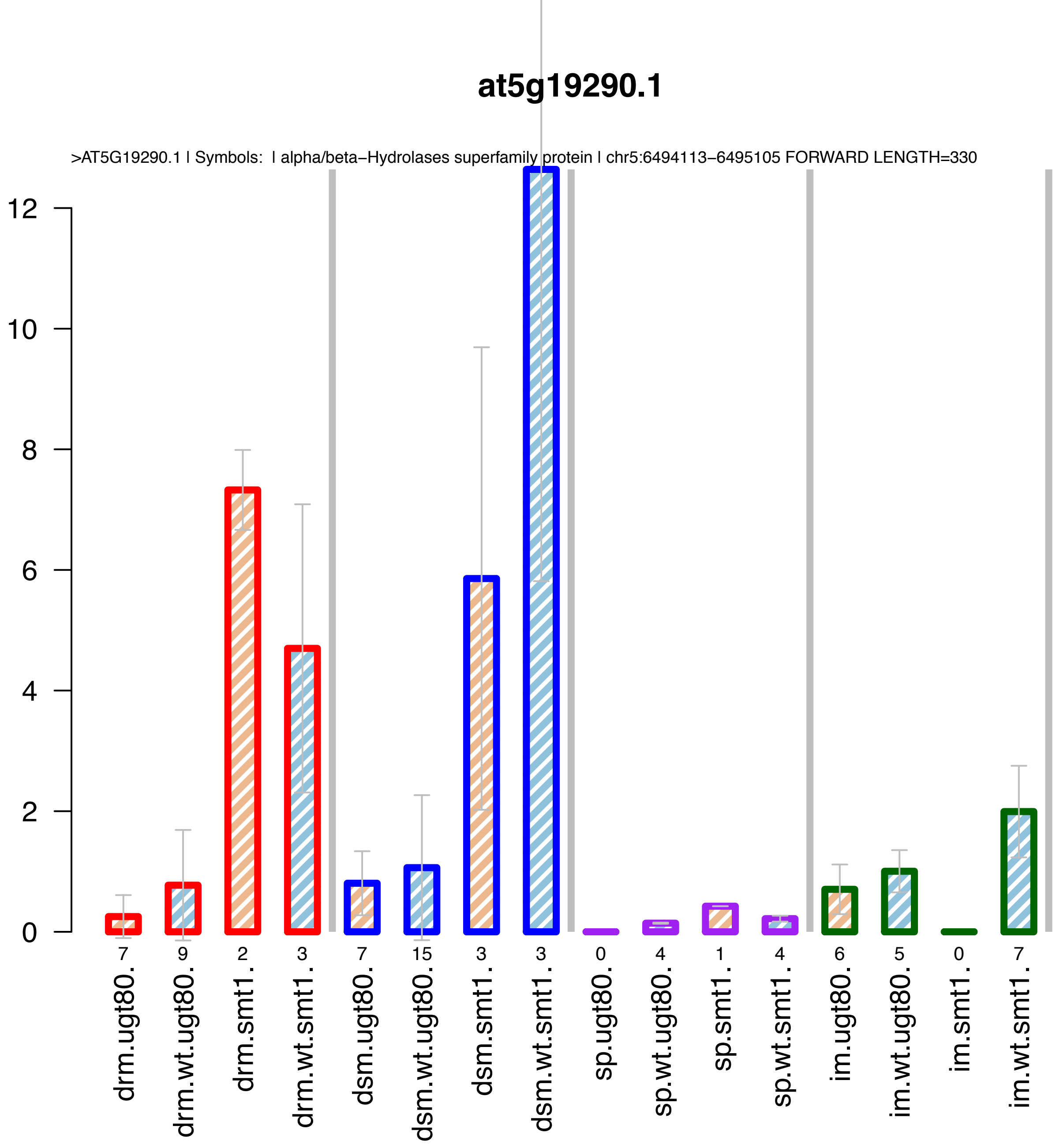

at5g19290.1
>AT5G19290.1 | Symbols: | alpha/beta−Hydrolases superfamily protein | chr5:6494113−6495105 FORWARD LENGTH=330
0
2
4
6
8
10
12
7
9
2
3
7
15
3
3
0
4
1
4
6
5
0
7
drm.ugt80.
drm.wt.ugt80.
drm.smt1.
drm.wt.smt1.
dsm.ugt80.
dsm.wt.ugt80.
dsm.smt1.
dsm.wt.smt1.
sp.ugt80.
sp.wt.ugt80.
sp.smt1.
sp.wt.smt1.
im.ugt80.
im.wt.ugt80.
im.smt1.
im.wt.smt1.

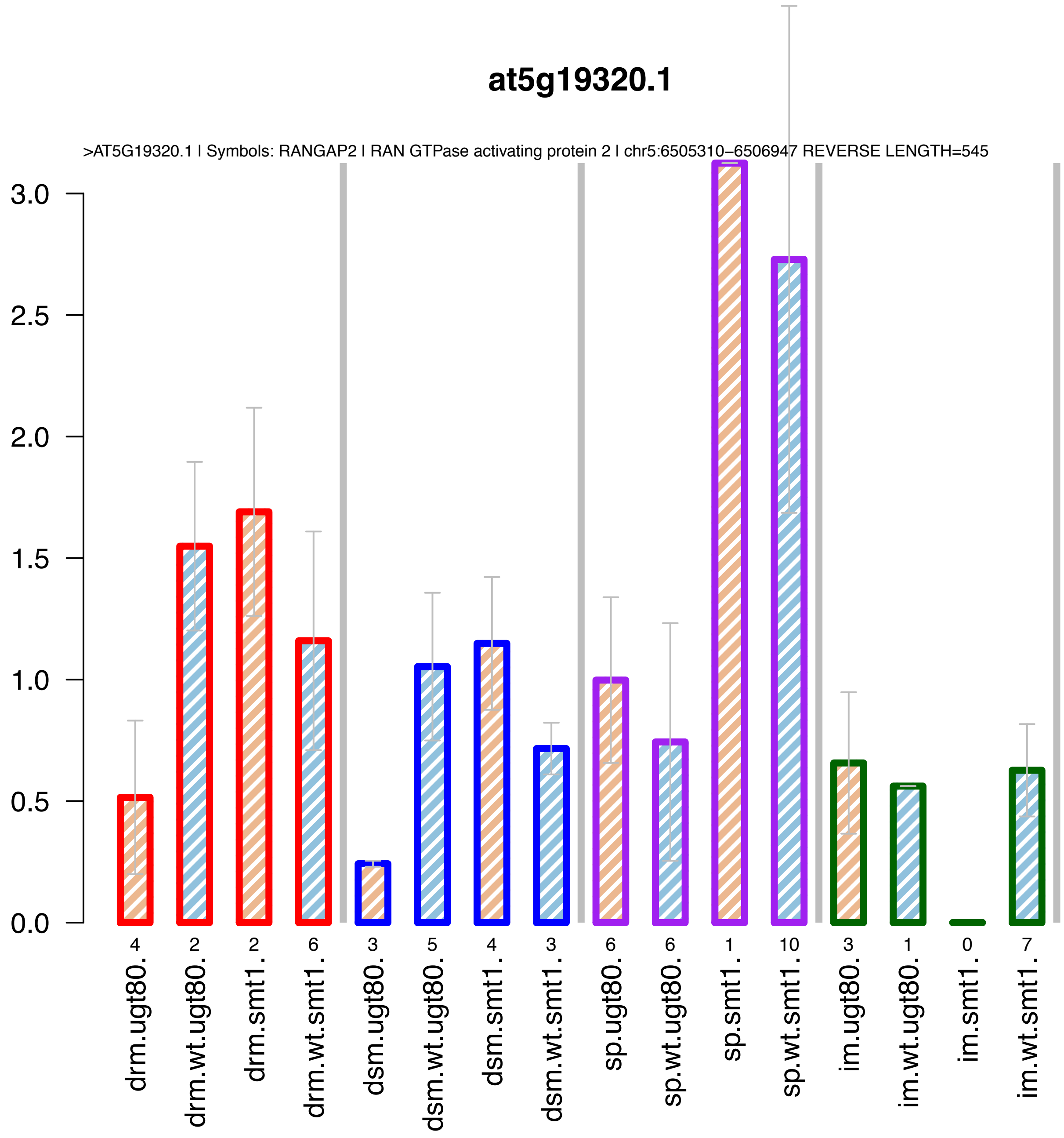
at5g19320.1
>AT5G19320.1 | Symbols: RANGAP2 | RAN GTPase activating protein 2 | chr5:6505310–6506947 REVERSE LENGTH=545
3.0
2.5
2.0
1.5
1.0
0.5
0.0
4
2
2
6
3
5
4
3
6
6
1
10
3
1
0
7
drm.ugt80..
drm.wt.ugt80..
drm.smt1..
drm.wt.smt1..
dsm.ugt80..
dsm.wt.ugt80..
dsm.smt1..
dsm.wt.smt1..
sp.ugt80..
sp.wt.ugt80..
sp.smt1..
sp.wt.smt1..
im.ugt80..
im.wt.ugt80..
im.smt1..
im.wt.smt1..

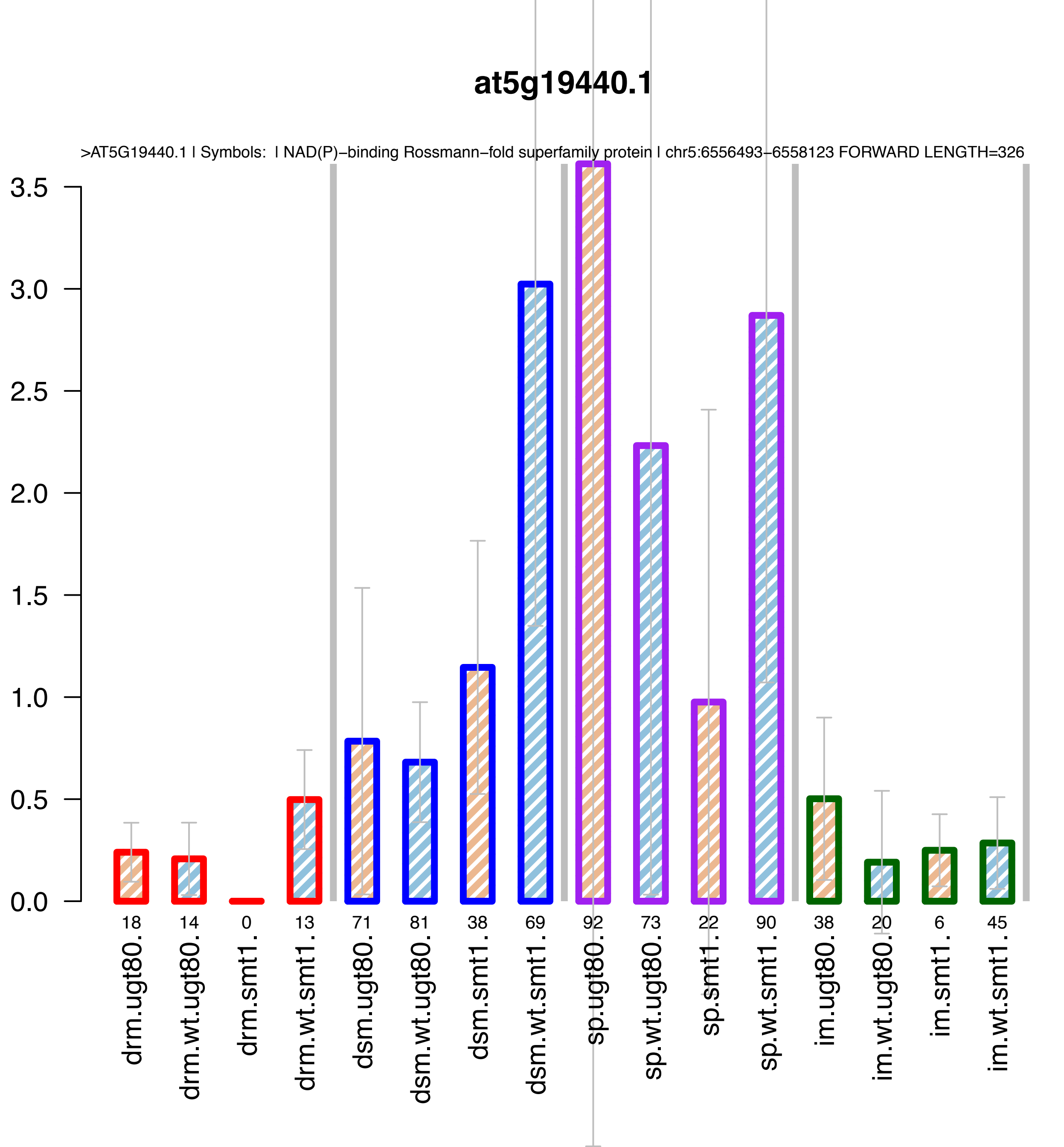

at5g19440.1
>AT5G19440.1 | Symbols: | NAD(P)−binding Rossmann−fold superfamily protein | chr5:6556493−6558123 FORWARD LENGTH=326
3.5
3.0
2.5
2.0
1.5
1.0
0.5
0.0
18
14
0
13
71
81
38
69
92
73
22
90
38
20
6
45
drm.ugt80.
drm.wt.ugt80.
drm.smt1.
drm.wt.smt1.
dsm.ugt80.
dsm.wt.ugt80.
dsm.smt1.
dsm.wt.smt1.
sp.ugt80.
sp.wt.ugt80.
sp.smt1.
sp.wt.smt1.
im.ugt80.
im.wt.ugt80.
im.smt1.
im.wt.smt1.

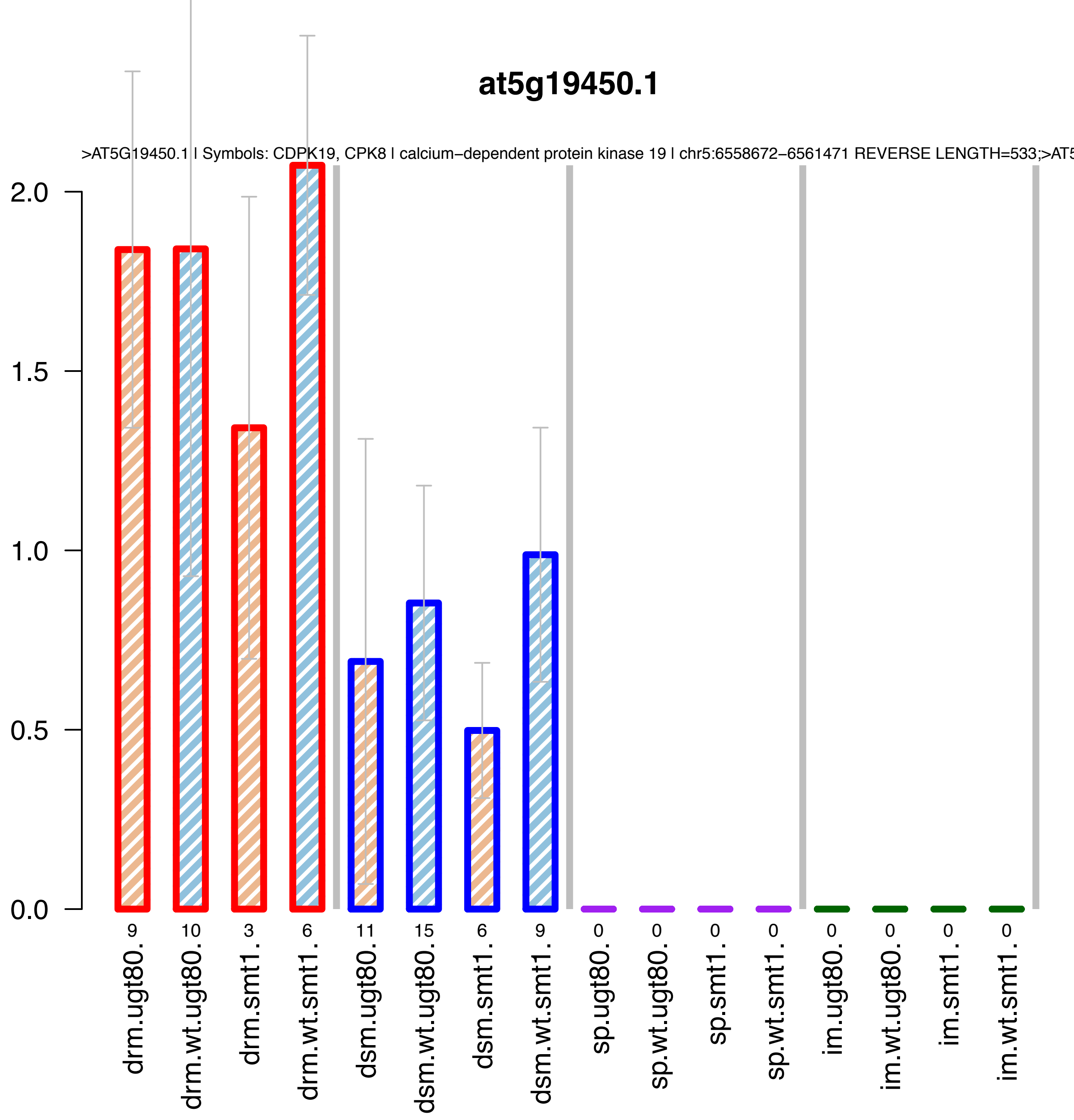

at5g19450.1
>AT5G19450.1 | Symbols: CDPK19, CPK8 | calcium−dependent protein kinase 19 | chr5:6558672−6561471 REVERSE LENGTH=533;>AT5
2.0
1.5
1.0
0.5
0.0
9
10
3
6
11
15
6
9
0
0
0
0
0
0
0
0
drm.ugt80.
drm.wt.ugt80.
drm.smt1.
drm.wt.smt1.
dsm.ugt80.
dsm.wt.ugt80.
dsm.smt1.
dsm.wt.smt1.
sp.ugt80.
sp.wt.ugt80.
sp.smt1.
sp.wt.smt1.
im.ugt80.
im.wt.ugt80.
im.smt1.
im.wt.smt1.

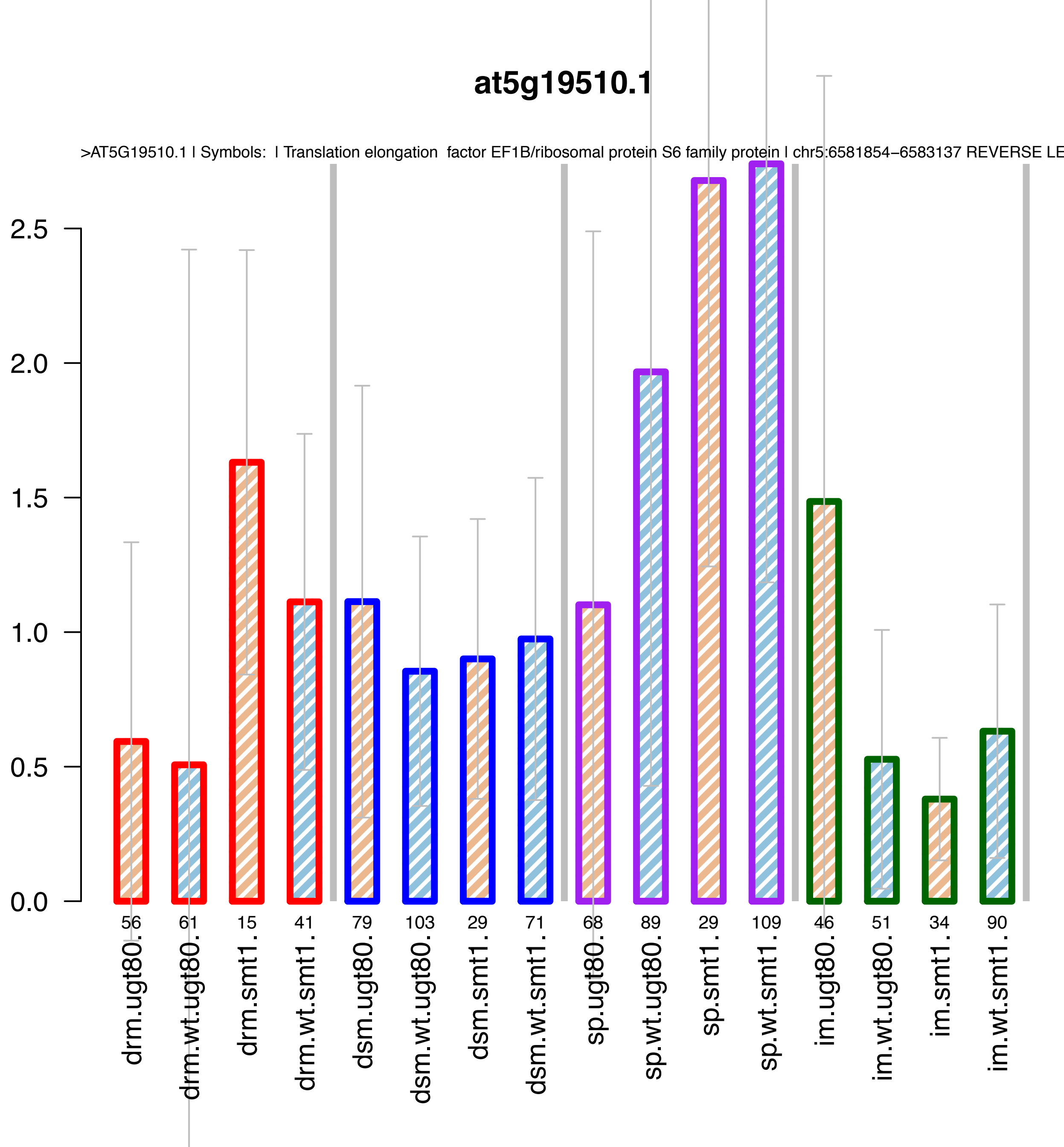

at5g19510.1
>AT5G19510.1 | Symbols: | Translation elongation factor EF1B/ribosomal protein S6 family protein | chr5:6581854-6583137 REVERSE LE
0.0
0.5
1.0
1.5
2.0
2.5
56
61
15
41
79
103
29
71
68
89
29
109
46
51
34
90
drm.ugt80.
drm.wt.ugt80.
drm.smt1.
drm.wt.smt1.
dsm.ugt80.
dsm.wt.ugt80.
dsm.smt1.
dsm.wt.smt1.
sp.ugt80.
sp.wt.ugt80.
sp.smt1.
sp.wt.smt1.
im.ugt80.
im.wt.ugt80.
im.smt1.
im.wt.smt1.

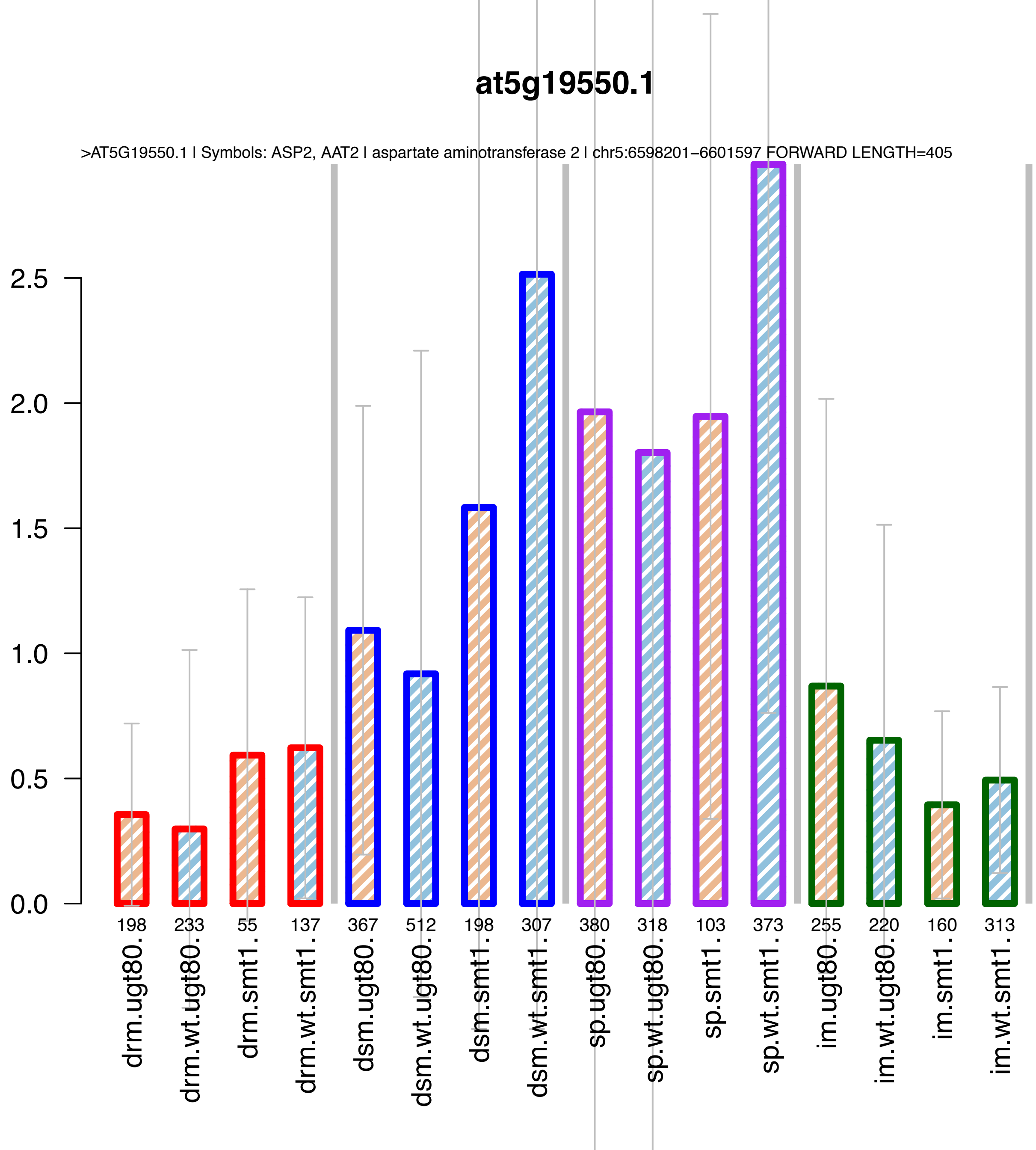

at5g19550.1
>AT5G19550.1 | Symbols: ASP2, AAT2 | aspartate aminotransferase 2 | chr5:6598201−6601597 FORWARD LENGTH=405
0.0
0.5
1.0
1.5
2.0
2.5
198
233
55
137
367
512
198
307
380
318
103
373
255
220
160
313
drm.ugt80..
drm.wt.ugt80..
drm.smt1..
drm.wt.smt1..
dsm.ugt80..
dsm.wt.ugt80..
dsm.smt1..
dsm.wt.smt1..
sp.ugt80..
sp.wt.ugt80..
sp.smt1..
sp.wt.smt1..
im.ugt80..
im.wt.ugt80..
im.smt1..
im.wt.smt1..

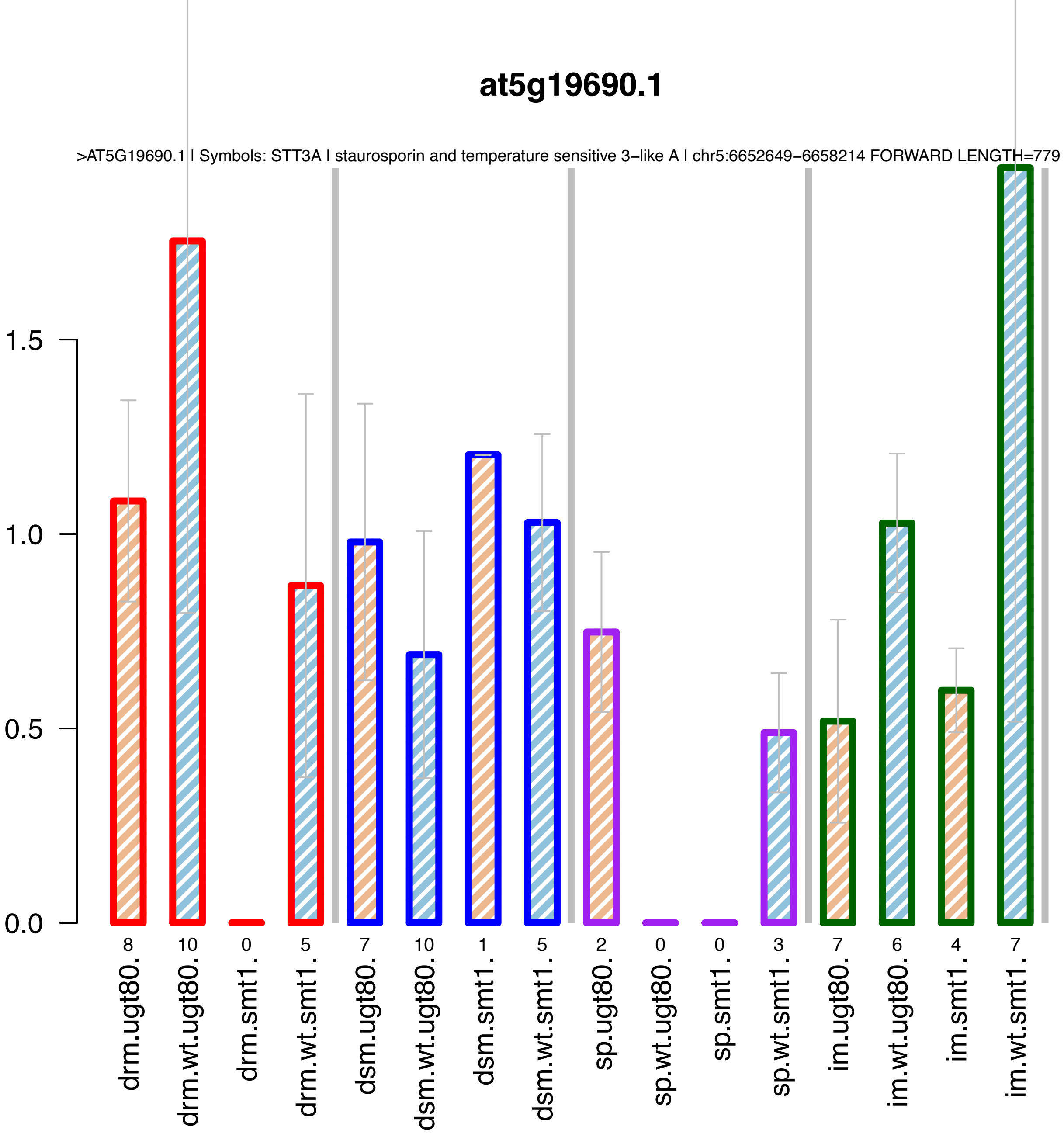

at5g19690.1
>AT5G19690.1 | Symbols: STT3A | staurosporin and temperature sensitive 3−like A | chr5:6652649−6658214 FORWARD LENGTH=779
0.0
0.5
1.0
1.5
8
10
0
5
7
10
1
5
2
0
0
3
7
6
4
7
drm.ugt80.
drm.wt.ugt80.
drm.smt1.
drm.wt.smt1.
dsm.ugt80.
dsm.wt.ugt80.
dsm.smt1.
dsm.wt.smt1.
sp.ugt80.
sp.wt.ugt80.
sp.smt1.
sp.wt.smt1.
im.ugt80.
im.wt.ugt80.
im.smt1.
im.wt.smt1.

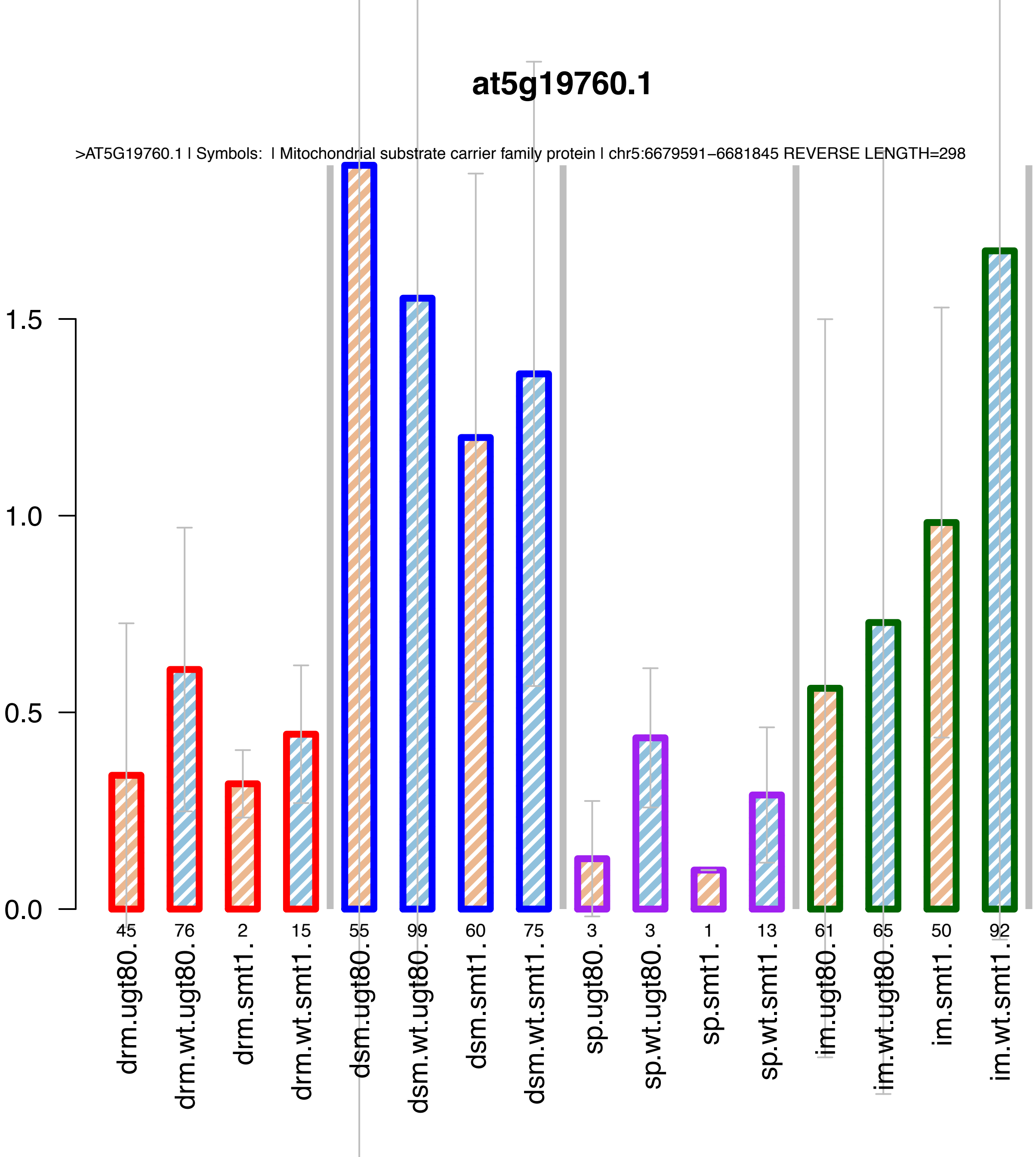
at5g19760.1
>AT5G19760.1 | Symbols: | Mitochondrial substrate carrier family protein | chr5:6679591-6681845 REVERSE LENGTH=298
0.0
0.5
1.0
1.5
45
76
2
15
55
99
60
75
3
3
1
13
61
65
50
92
drm.ugt80..
drm.wt.ugt80..
drm.smt1..
drm.wt.smt1..
dsm.ugt80..
dsm.wt.ugt80..
dsm.smt1..
dsm.wt.smt1..
sp.ugt80..
sp.wt.ugt80..
sp.smt1..
sp.wt.smt1..
im.ugt80..
im.wt.ugt80..
im.smt1..
im.wt.smt1..

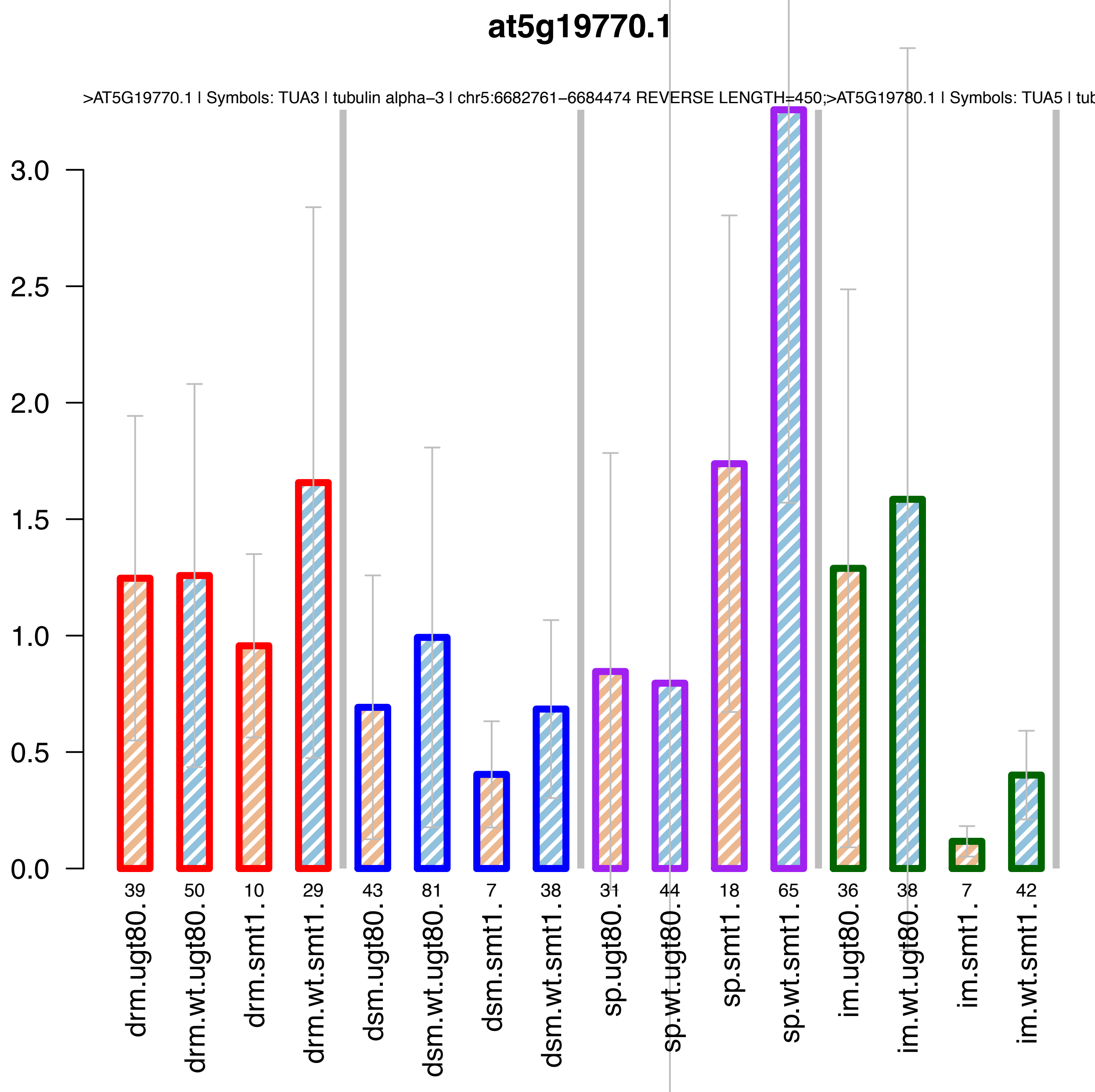

at5g19770.1
>AT5G19770.1 | Symbols: TUA3 | tubulin alpha−3 | chr5:6682761−6684474 REVERSE LENGTH=450;>AT5G19780.1 | Symbols: TUA5 | tub
3.0
2.5
2.0
1.5
1.0
0.5
0.0
39
50
10
29
43
81
7
38
31
44
18
65
36
38
7
42
drm.ugt80.
drm.wt.ugt80.
drm.smt1.
drm.wt.smt1.
dsm.ugt80.
dsm.wt.ugt80.
dsm.smt1.
dsm.wt.smt1.
sp.ugt80.
sp.wt.ugt80.
sp.smt1.
sp.wt.smt1.
im.ugt80.
im.wt.ugt80.
im.smt1.
im.wt.smt1.

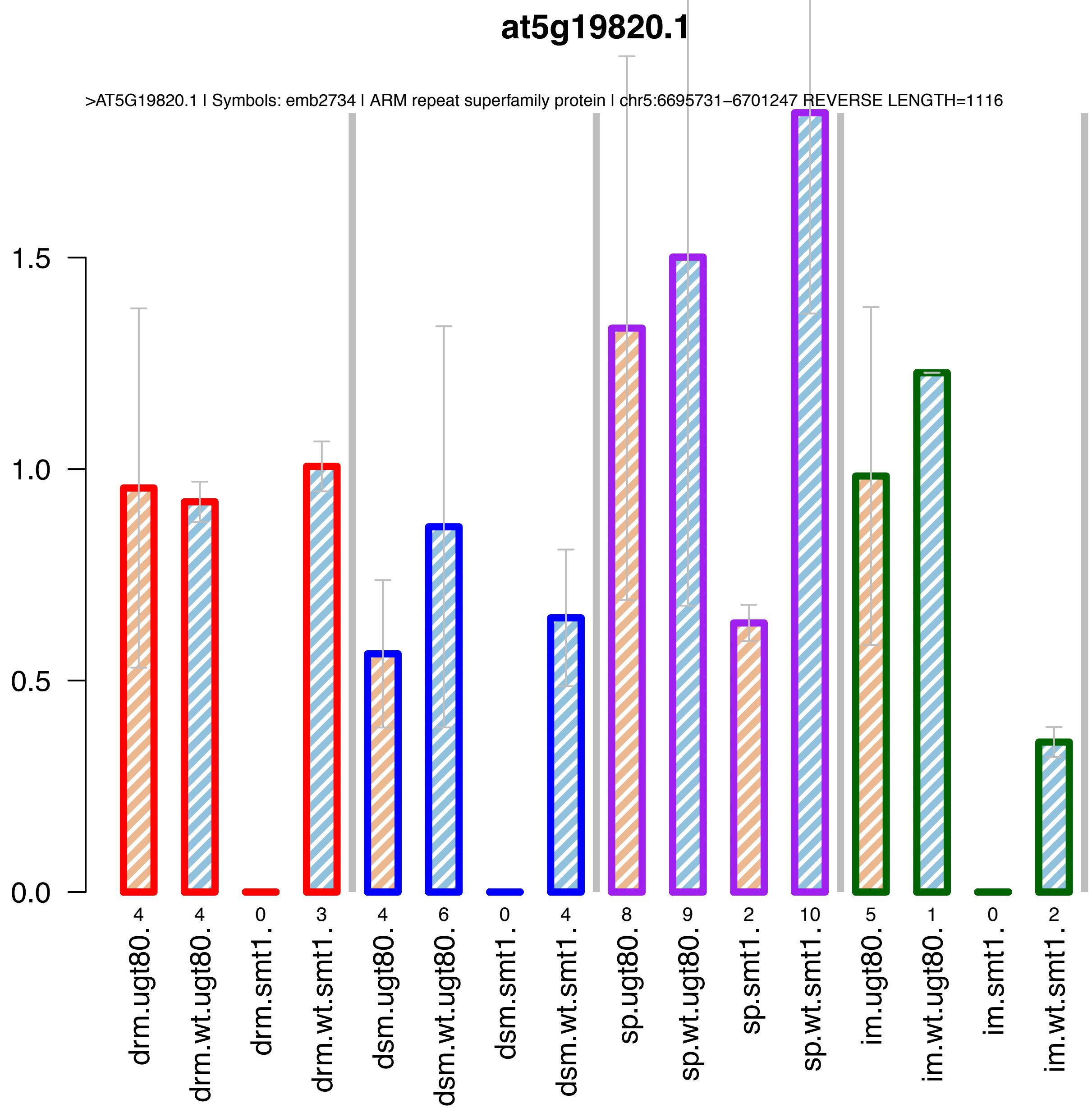
at5g19820.1
>AT5G19820.1 | Symbols: emb2734 | ARM repeat superfamily protein | chr5:6695731-6701247 REVERSE LENGTH=1116
0.0
0.5
1.0
1.5
4
4
0
3
4
6
0
4
8
9
2
10
5
1
0
2
drm.ugt80.
drm.wt.ugt80.
drm.smt1.
drm.wt.smt1.
dsm.ugt80.
dsm.wt.ugt80.
dsm.smt1.
dsm.wt.smt1.
sp.ugt80.
sp.wt.ugt80.
sp.smt1.
sp.wt.smt1.
im.ugt80.
im.wt.ugt80.
im.smt1.
im.wt.smt1.

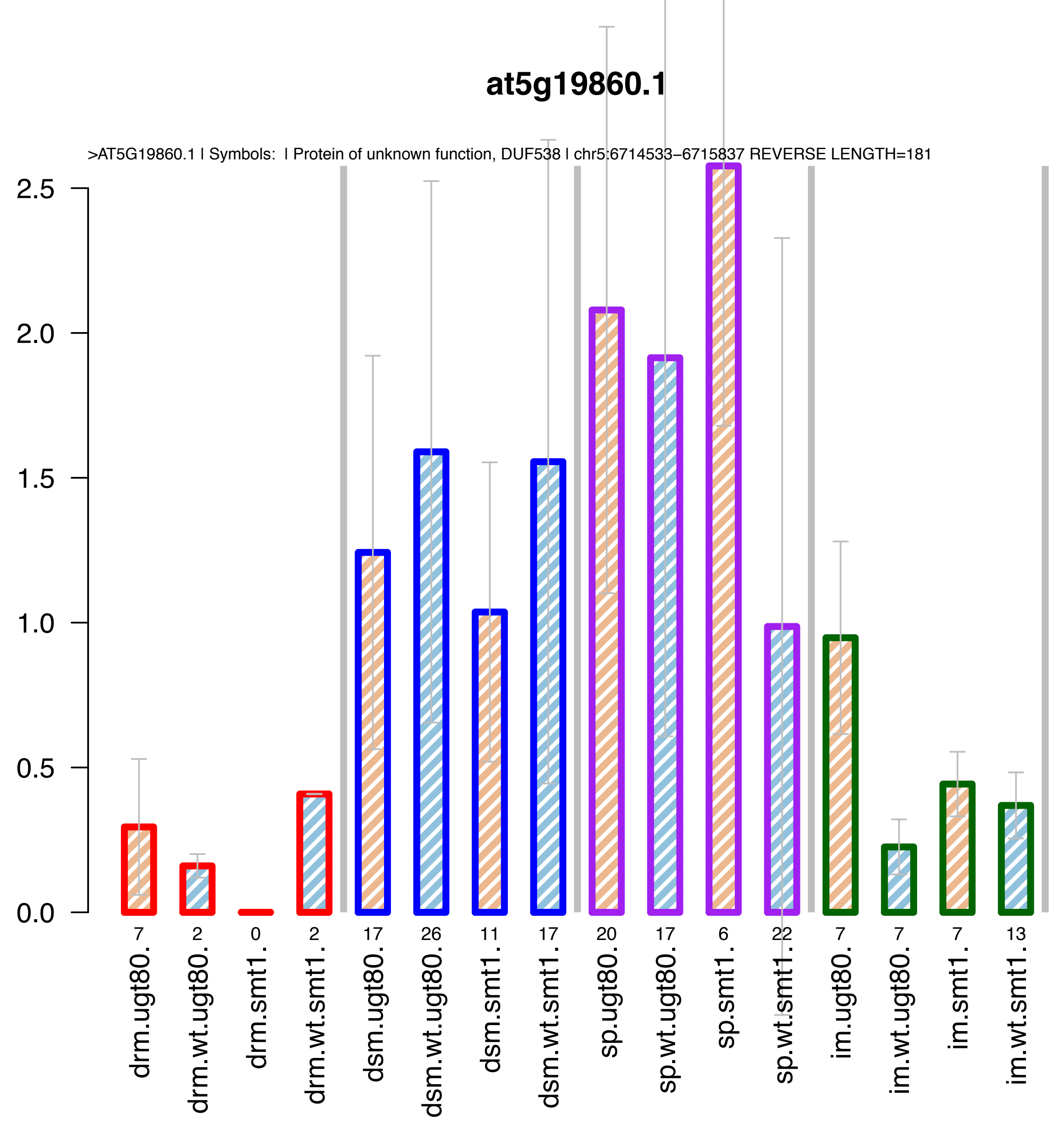

at5g19860.1
>AT5G19860.1 | Symbols: | Protein of unknown function, DUF538 | chr5:6714533-6715837 REVERSE LENGTH=181
0.0
0.5
1.0
1.5
2.0
2.5
7
2
0
2
17
26
11
17
20
17
6
22
7
7
7
13
drm.ugt80.
drm.wt.ugt80.
drm.smt1.
drm.wt.smt1.
dsm.ugt80.
dsm.wt.ugt80.
dsm.smt1.
dsm.wt.smt1.
sp.ugt80.
sp.wt.ugt80.
sp.smt1.
sp.wt.smt1.
im.ugt80.
im.wt.ugt80.
im.smt1.
im.wt.smt1.

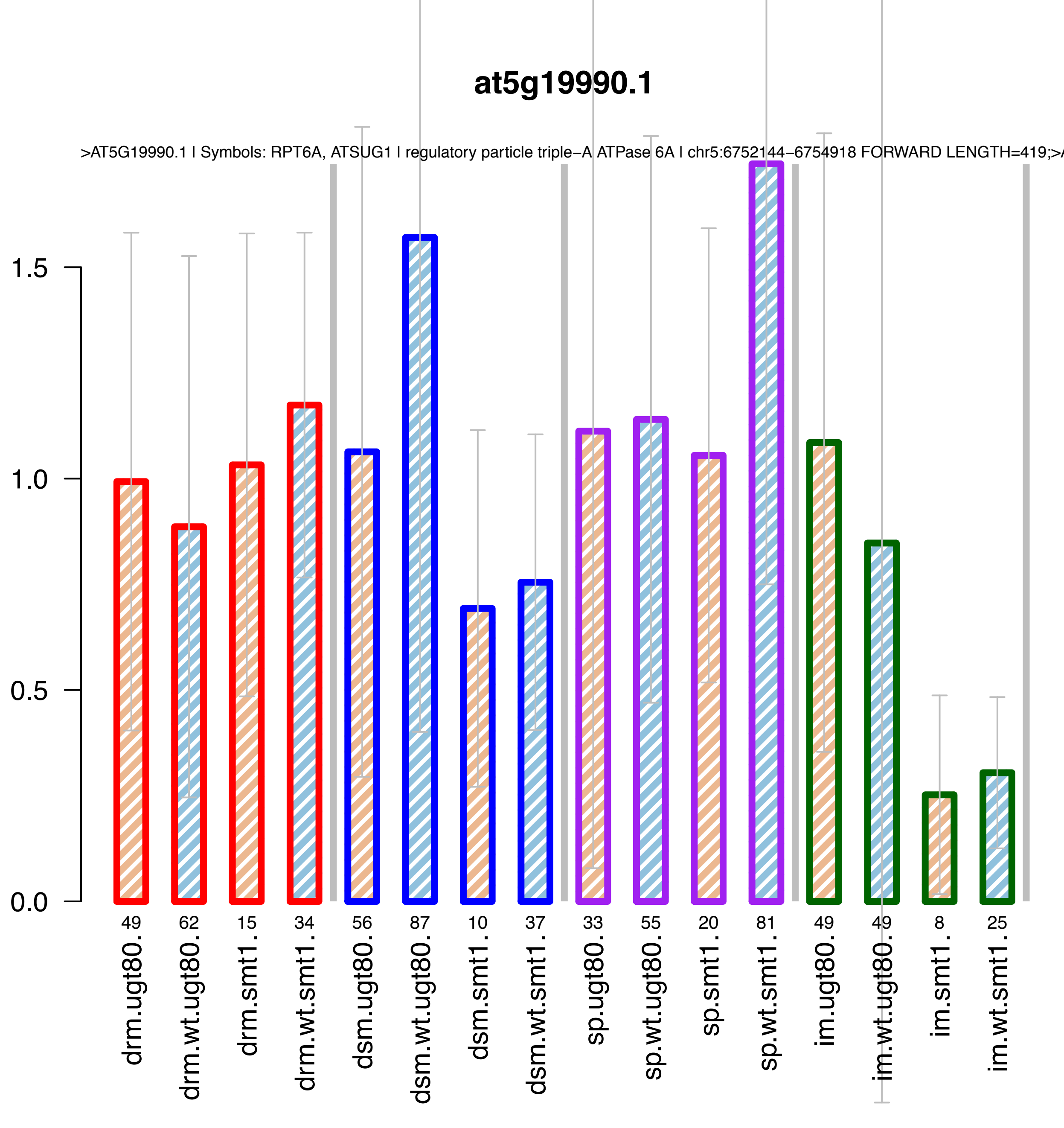

at5g19990.1
>AT5G19990.1 | Symbols: RPT6A, ATSUG1 | regulatory particle triple−A ATPase 6A | chr5:6752144−6754918 FORWARD LENGTH=419;>A
0.0
0.5
1.0
1.5
49
62
15
34
56
87
10
37
33
55
20
81
49
49
8
25
drm.ugt80..
drm.wt.ugt80..
drm.smt1..
drm.wt.smt1..
dsm.ugt80..
dsm.wt.ugt80..
dsm.smt1..
dsm.wt.smt1..
sp.ugt80..
sp.wt.ugt80..
sp.smt1..
sp.wt.smt1..
im.ugt80..
im.wt.ugt80..
im.smt1..
im.wt.smt1..

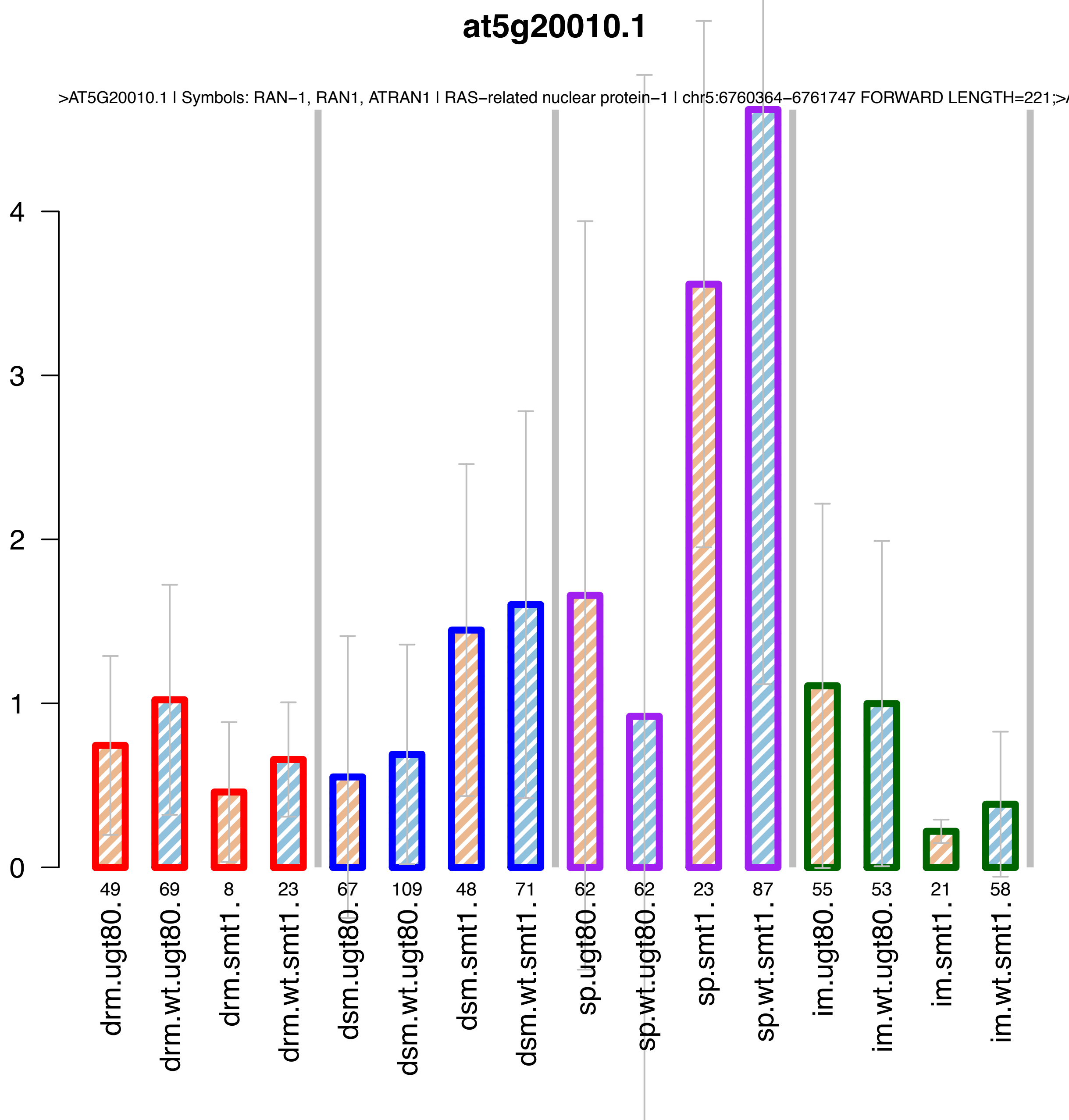

at5g20010.1
>AT5G20010.1 | Symbols: RAN−1, RAN1, ATRAN1 | RAS−related nuclear protein−1 | chr5:6760364−6761747 FORWARD LENGTH=221;>
0
1
2
3
4
49
69
8
23
67
109
48
71
62
62
23
87
55
53
21
58
drm.ugt80.
drm.wt.ugt80.
drm.smt1.
drm.wt.smt1.
dsm.ugt80.
dsm.wt.ugt80.
dsm.smt1.
dsm.wt.smt1.
sp.ugt80.
sp.wt.ugt80.
sp.smt1.
sp.wt.smt1.
im.ugt80.
im.wt.ugt80.
im.smt1.
im.wt.smt1.

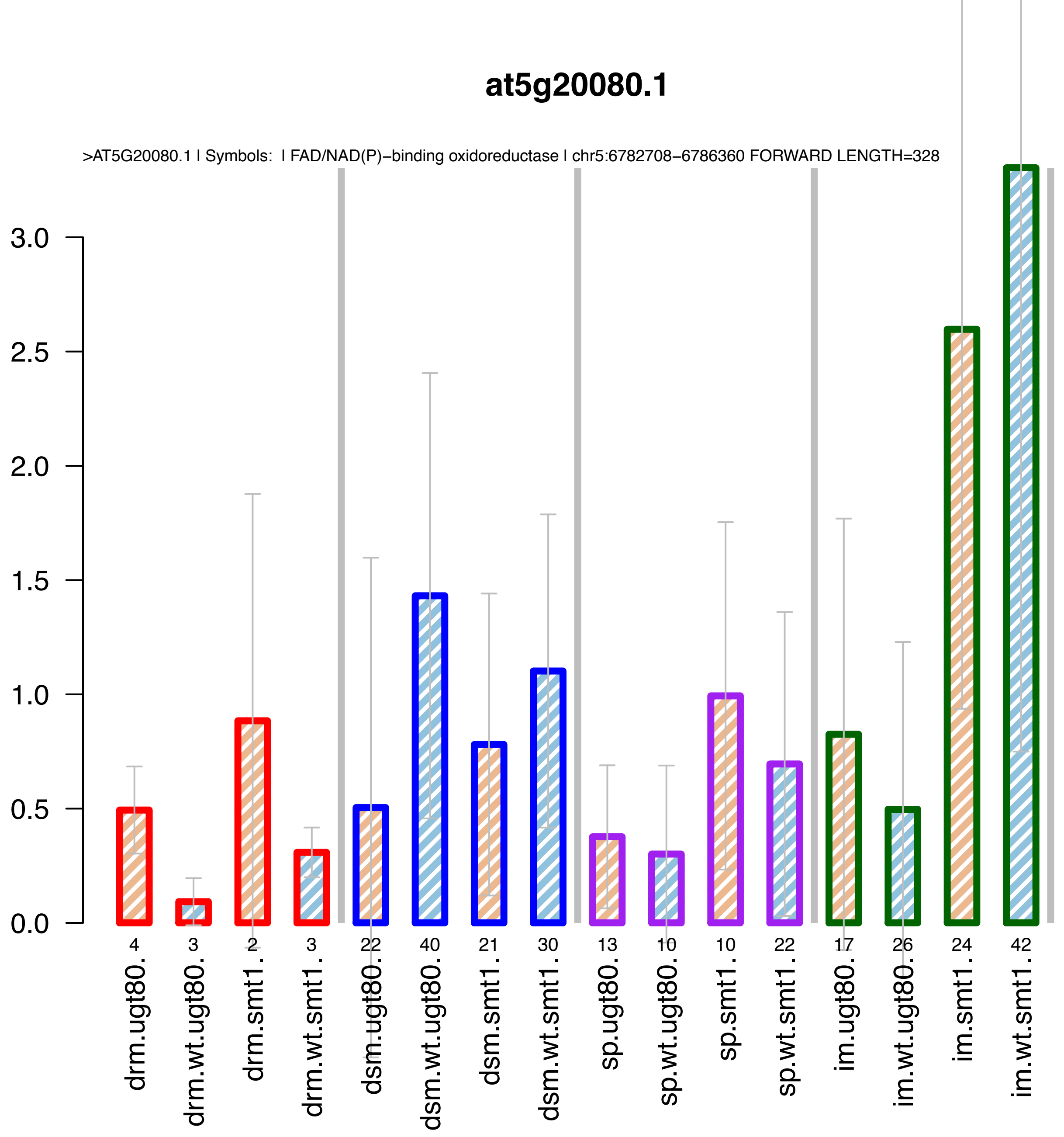

at5g20080.1
>AT5G20080.1 | Symbols: | FAD/NAD(P)−binding oxidoreductase | chr5:6782708−6786360 FORWARD LENGTH=328
3.0
2.5
2.0
1.5
1.0
0.5
0.0
4
3
2
3
22
40
21
30
13
10
10
22
17
26
24
42
drm.ugt80..
drm.wt.ugt80..
drm.smt1..
drm.wt.smt1..
dsm.ugt80..
dsm.wt.ugt80..
dsm.smt1..
dsm.wt.smt1..
sp.ugt80..
sp.wt.ugt80..
sp.smt1..
sp.wt.smt1..
im.ugt80..
im.wt.ugt80..
im.smt1..
im.wt.smt1..

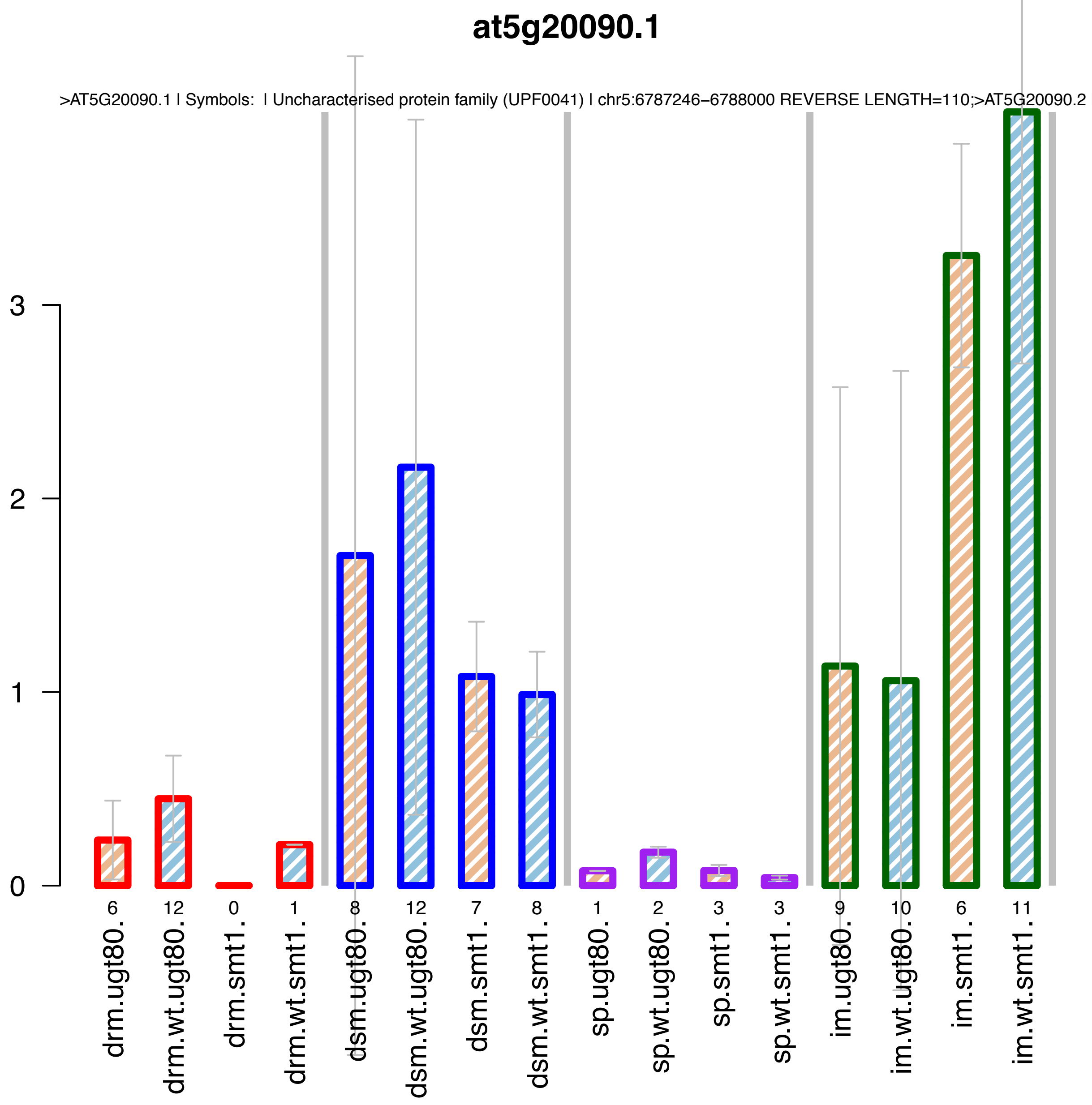

at5g20090.1
>AT5G20090.1 | Symbols: | Uncharacterised protein family (UPF0041) | chr5:6787246-6788000 REVERSE LENGTH=110;>AT5G20090.2
0
1
2
3
6
12
0
1
8
12
7
8
1
2
3
3
9
10
6
11
drm.ugt80..
drm.wt.ugt80..
drm.smt1..
drm.wt.smt1..
dsm.ugt80..
dsm.wt.ugt80..
dsm.smt1..
dsm.wt.smt1..
sp.ugt80..
sp.wt.ugt80..
sp.smt1..
sp.wt.smt1..
im.ugt80..
im.wt.ugt80..
im.smt1..
im.wt.smt1..

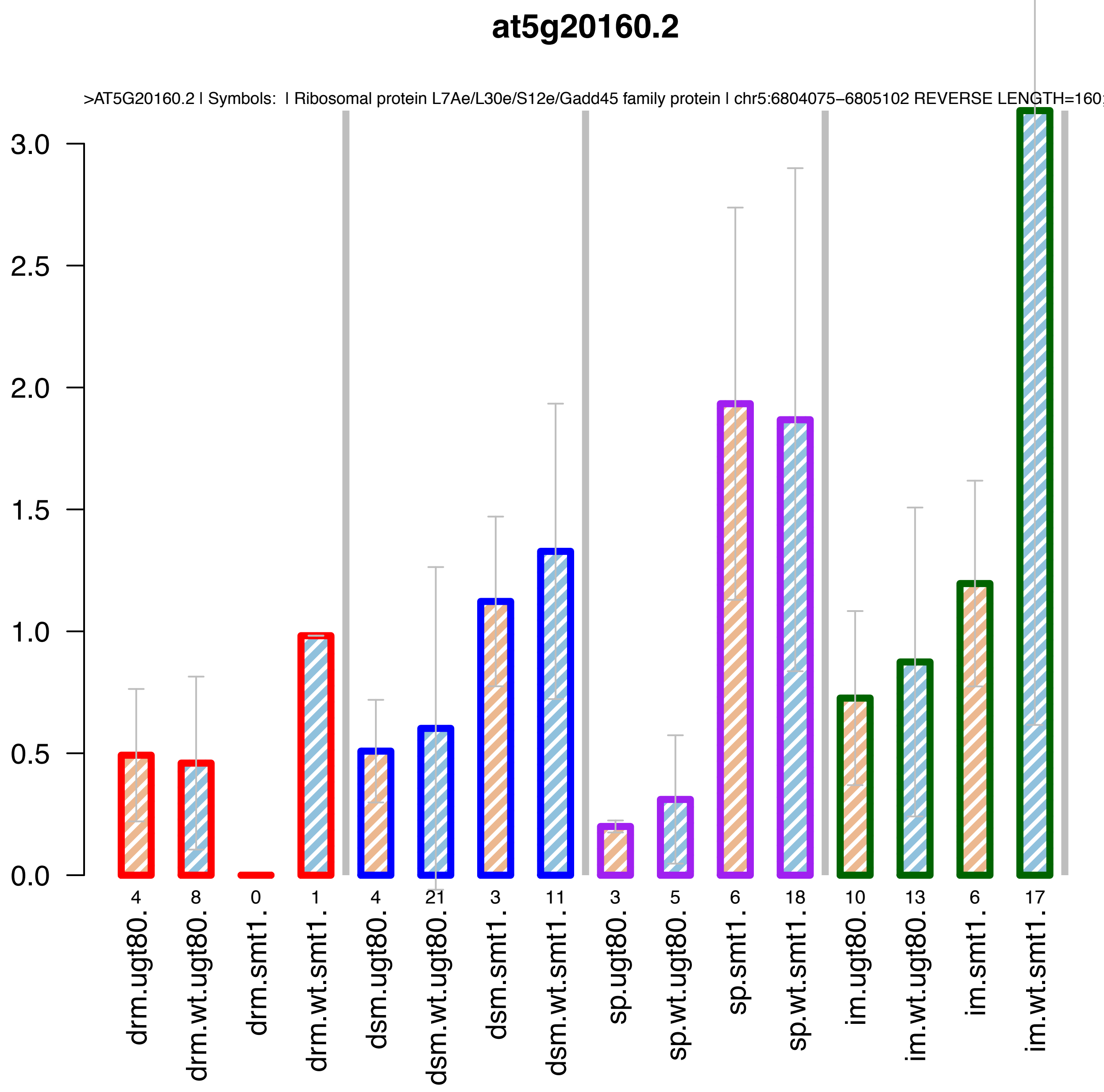

at5g20160.2
>AT5G20160.2 | Symbols: | Ribosomal protein L7Ae/L30e/S12e/Gadd45 family protein | chr5:6804075-6805102 REVERSE LENGTH=160
3.0
2.5
2.0
1.5
1.0
0.5
0.0
4
8
0
1
4
21
3
11
3
5
6
18
10
13
6
17
drm.ugt80.
drm.wt.ugt80.
drm.smt1.
drm.wt.smt1.
dsm.ugt80.
dsm.wt.ugt80.
dsm.smt1.
dsm.wt.smt1.
sp.ugt80.
sp.wt.ugt80.
sp.smt1.
sp.wt.smt1.
im.ugt80.
im.wt.ugt80.
im.smt1.
im.wt.smt1.

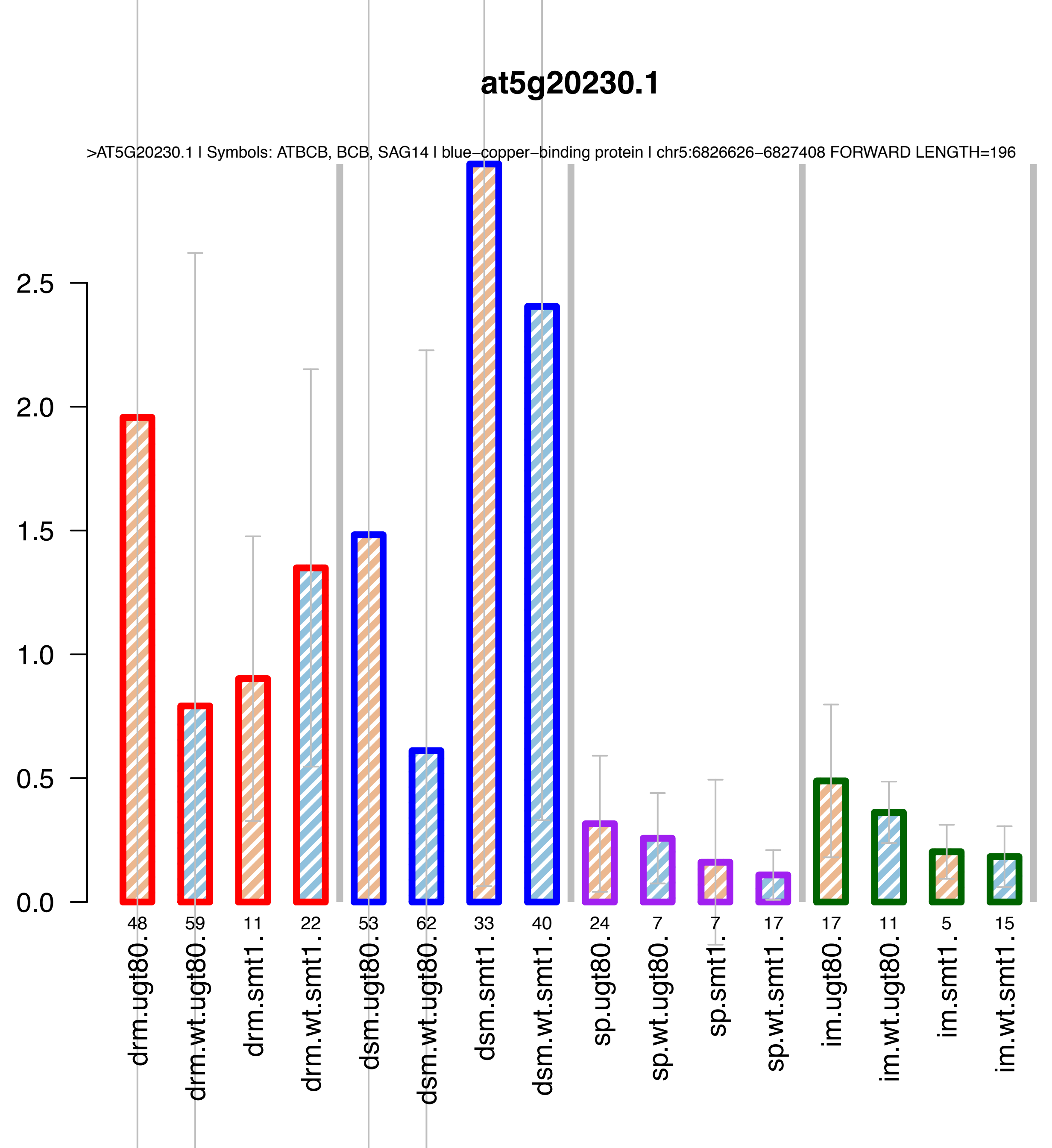
at5g20230.1
>AT5G20230.1 | Symbols: ATBCB, BCB, SAG14 | blue−copper−binding protein | chr5:6826626−6827408 FORWARD LENGTH=196
0.0
0.5
1.0
1.5
2.0
2.5
48
59
11
22
53
62
33
40
24
7
7
17
17
11
5
15
drm.ugt80..
drm.wt.ugt80..
drm.smt1..
drm.wt.smt1..
dsm.ugt80..
dsm.wt.ugt80..
dsm.smt1..
dsm.wt.smt1..
sp.ugt80..
sp.wt.ugt80..
sp.smt1..
sp.wt.smt1..
im.ugt80..
im.wt.ugt80..
im.smt1..
im.wt.smt1..

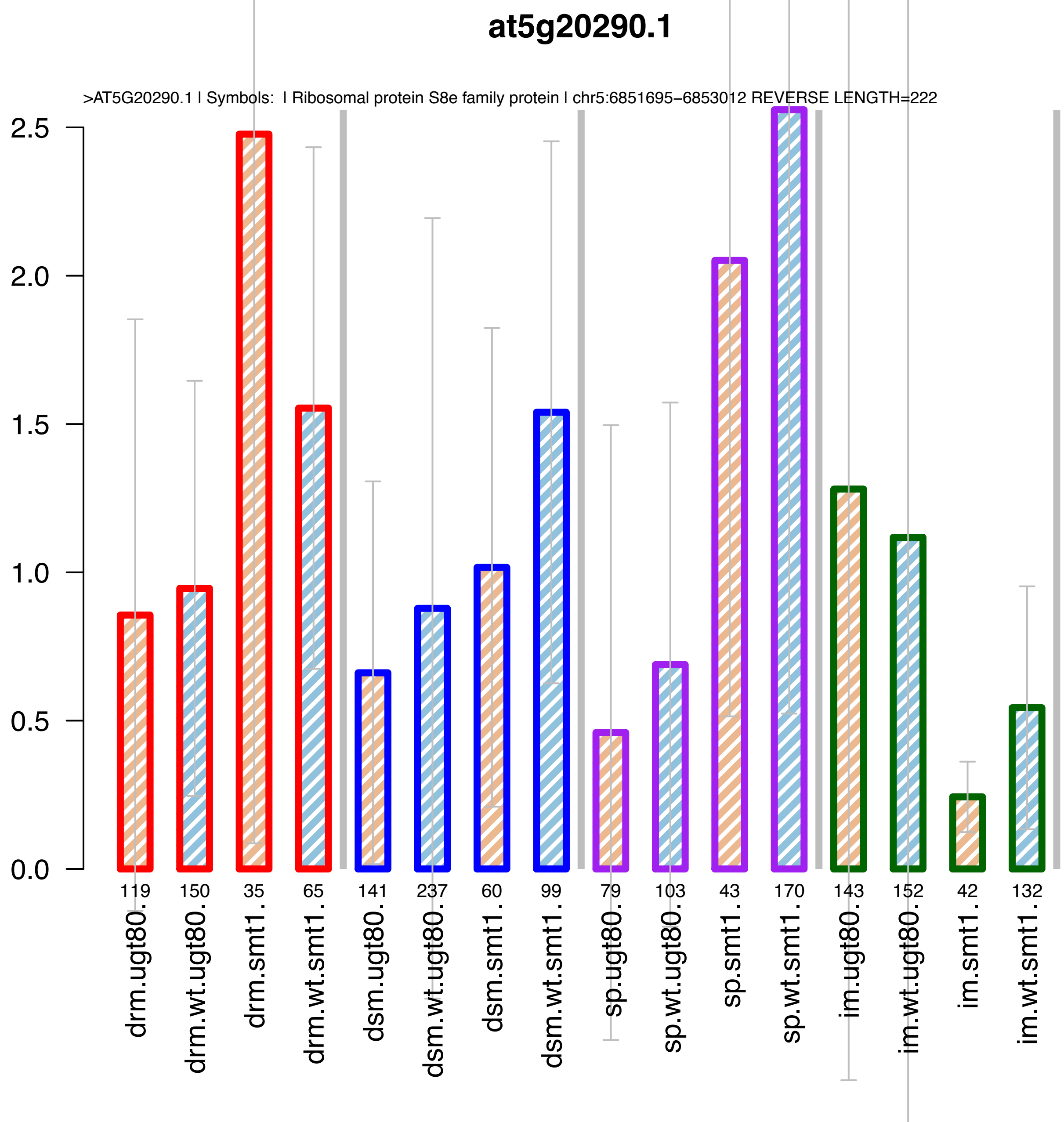

at5g20290.1
>AT5G20290.1 | Symbols: | Ribosomal protein S8e family protein | chr5:6851695-6853012 REVERSE LENGTH=222
2.5
2.0
1.5
1.0
0.5
0.0
119
150
35
65
141
237
60
99
79
103
43
170
143
152
42
132
drm.ugt80..
drm.wt.ugt80..
drm.smt1..
drm.wt.smt1..
dsm.ugt80..
dsm.wt.ugt80..
dsm.smt1..
dsm.wt.smt1..
sp.ugt80..
sp.wt.ugt80..
sp.smt1..
sp.wt.smt1..
im.ugt80..
im.wt.ugt80..
im.smt1..
im.wt.smt1..

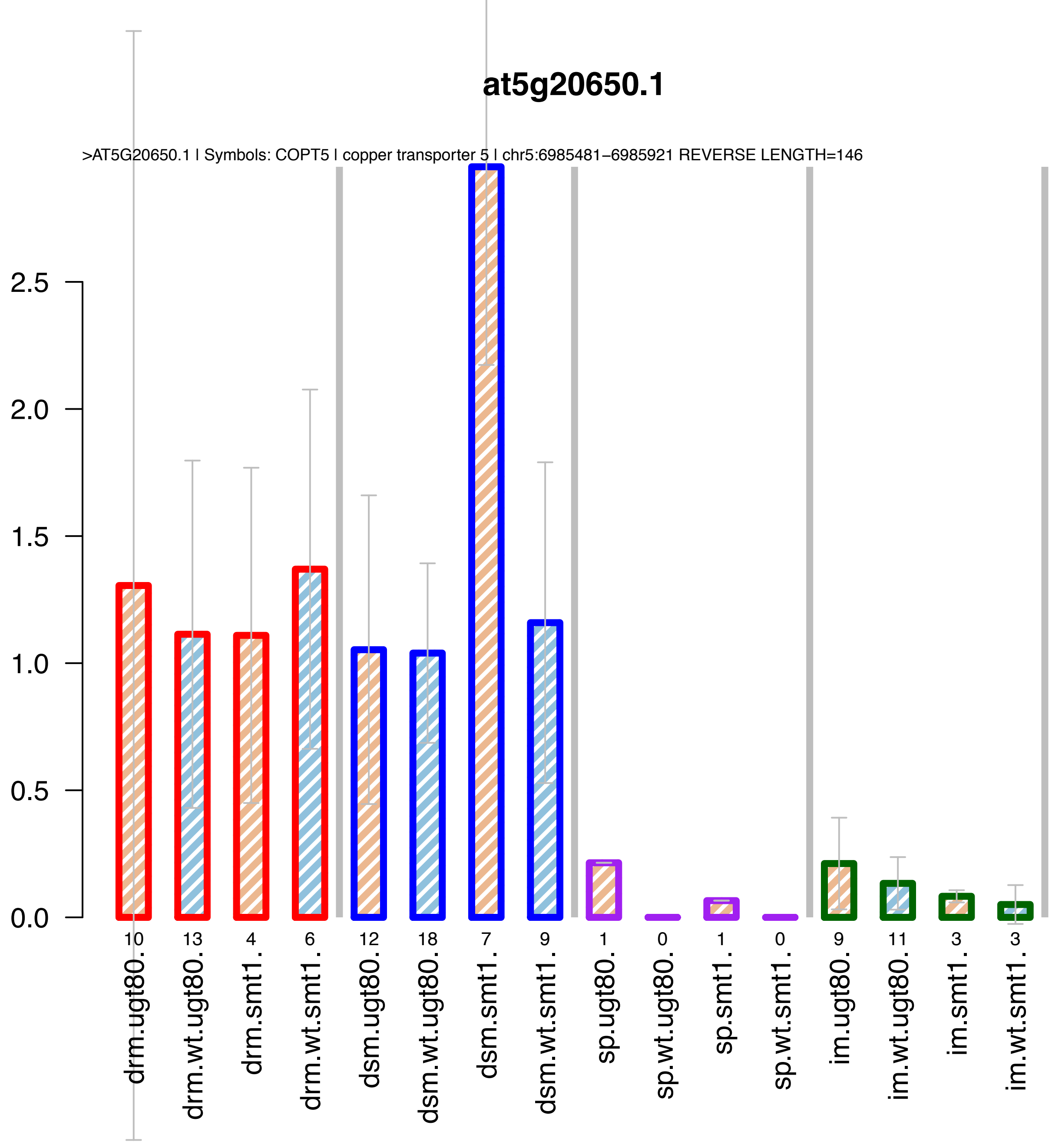
at5g20650.1
>AT5G20650.1 | Symbols: COPT5 | copper transporter 5 | chr5:6985481–6985921 REVERSE LENGTH=146
0.0
0.5
1.0
1.5
2.0
2.5
10
13
4
6
12
18
7
9
1
0
1
0
9
11
3
3
drm.ugt80.
drm.wt.ugt80.
drm.smt1.
drm.wt.smt1.
dsm.ugt80.
dsm.wt.ugt80.
dsm.smt1.
dsm.wt.smt1.
sp.ugt80.
sp.wt.ugt80.
sp.smt1.
sp.wt.smt1.
im.ugt80.
im.wt.ugt80.
im.smt1.
im.wt.smt1.

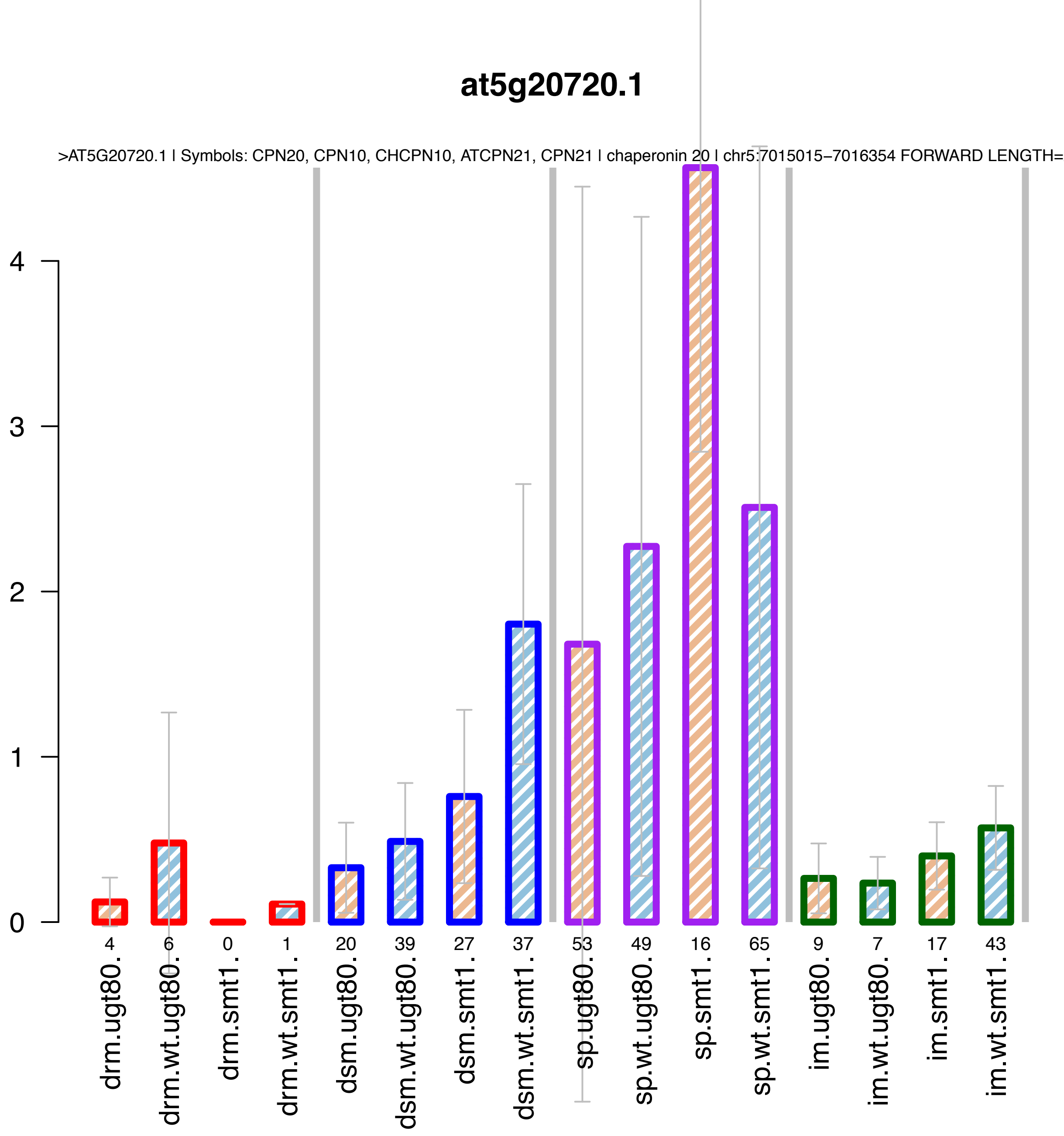
at5g20720.1
>AT5G20720.1 | Symbols: CPN20, CPN10, CHCPN10, ATCPN21, CPN21 | chaperonin 20 | chr5:7015015-7016354 FORWARD LENGTH=
4
3
2
1
0
4
6
0
1
20
39
27
37
53
49
16
65
9
7
17
43
drm.ugt80.
drm.wt.ugt80.
drm.smt1.
drm.wt.smt1.
dsm.ugt80.
dsm.wt.ugt80.
dsm.smt1.
dsm.wt.smt1.
sp.ugt80.
sp.wt.ugt80.
sp.smt1.
sp.wt.smt1.
im.ugt80.
im.wt.ugt80.
im.smt1.
im.wt.smt1.

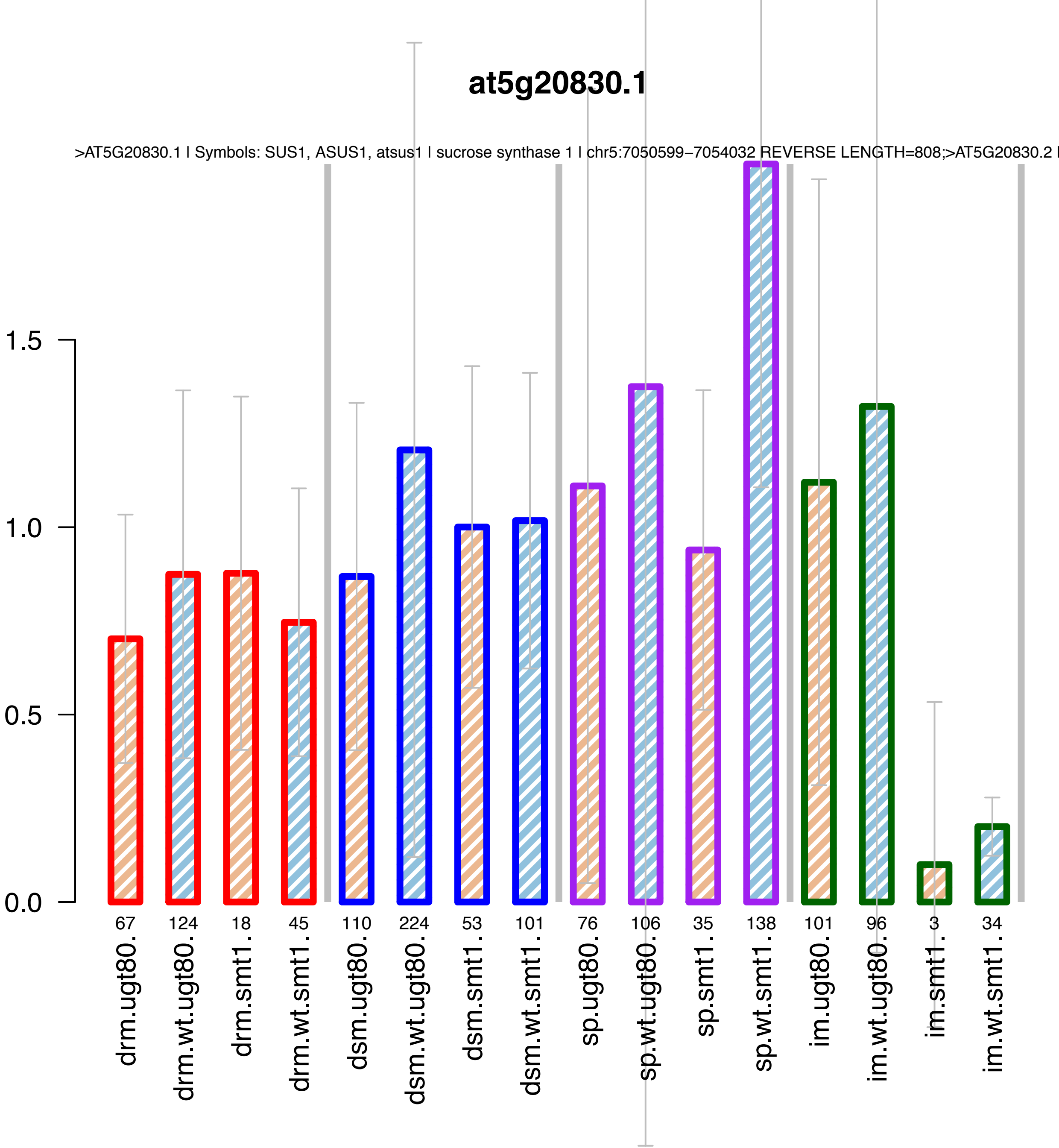

at5g20830.1
>AT5G20830.1 | Symbols: SUS1, ASUS1, atsus1 | sucrose synthase 1 | chr5:7050599-7054032 REVERSE LENGTH=808;>AT5G20830.2 |
0.0
0.5
1.0
1.5
67
124
18
45
110
224
53
101
76
106
35
138
101
96
3
34
drm.ugt80..
drm.wt.ugt80..
drm.smt1..
drm.wt.smt1..
dsm.ugt80..
dsm.wt.ugt80..
dsm.smt1..
dsm.wt.smt1..
sp.ugt80..
sp.wt.ugt80..
sp.smt1..
sp.wt.smt1..
im.ugt80..
im.wt.ugt80..
im.smt1..
im.wt.smt1..

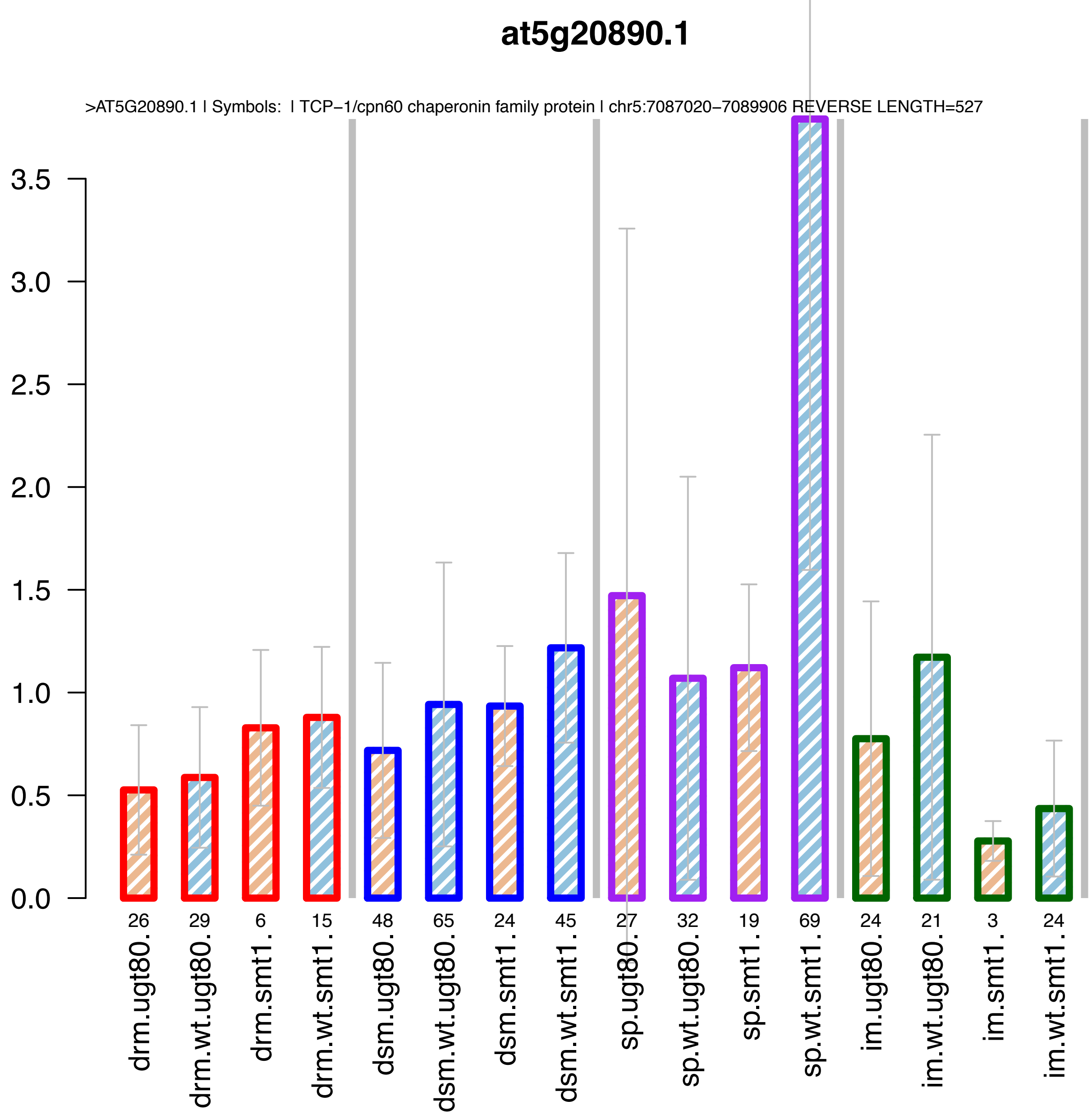

at5g20890.1
>AT5G20890.1 | Symbols: | TCP−1/cpn60 chaperonin family protein | chr5:7087020−7089906 REVERSE LENGTH=527
0.0
0.5
1.0
1.5
2.0
2.5
3.0
3.5
26
29
6
15
48
65
24
45
27
32
19
69
24
21
3
24
drm.ugt80.
drm.wt.ugt80.
drm.smt1.
drm.wt.smt1.
dsm.ugt80.
dsm.wt.ugt80.
dsm.smt1.
dsm.wt.smt1.
sp.ugt80.
sp.wt.ugt80.
sp.smt1.
sp.wt.smt1.
im.ugt80.
im.wt.ugt80.
im.smt1.
im.wt.smt1.

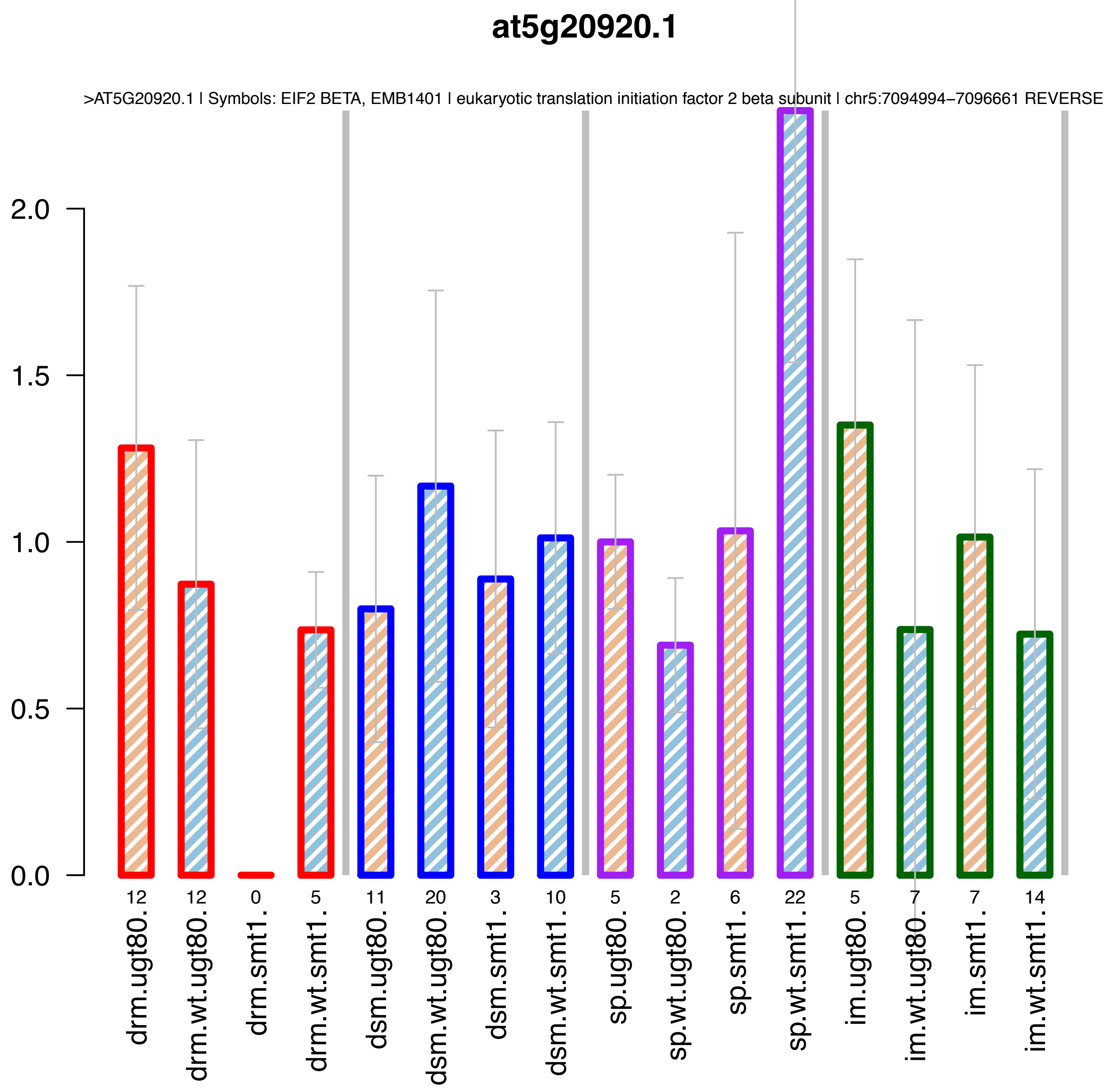

at5g20920.1
>AT5G20920.1 | Symbols: EIF2 BETA, EMB1401 | eukaryotic translation initiation factor 2 beta subunit | chr5:7094994-7096661 REVERSE
2.0
1.5
1.0
0.5
0.0
12
12
0
5
11
20
3
10
5
2
6
22
5
7
7
14
drm.ugt80.
drm.wt.ugt80.
drm.smt1.
drm.wt.smt1.
dsm.ugt80.
dsm.wt.ugt80.
dsm.smt1.
dsm.wt.smt1.
sp.ugt80.
sp.wt.ugt80.
sp.smt1.
sp.wt.smt1.
im.ugt80.
im.wt.ugt80.
im.smt1.
im.wt.smt1.

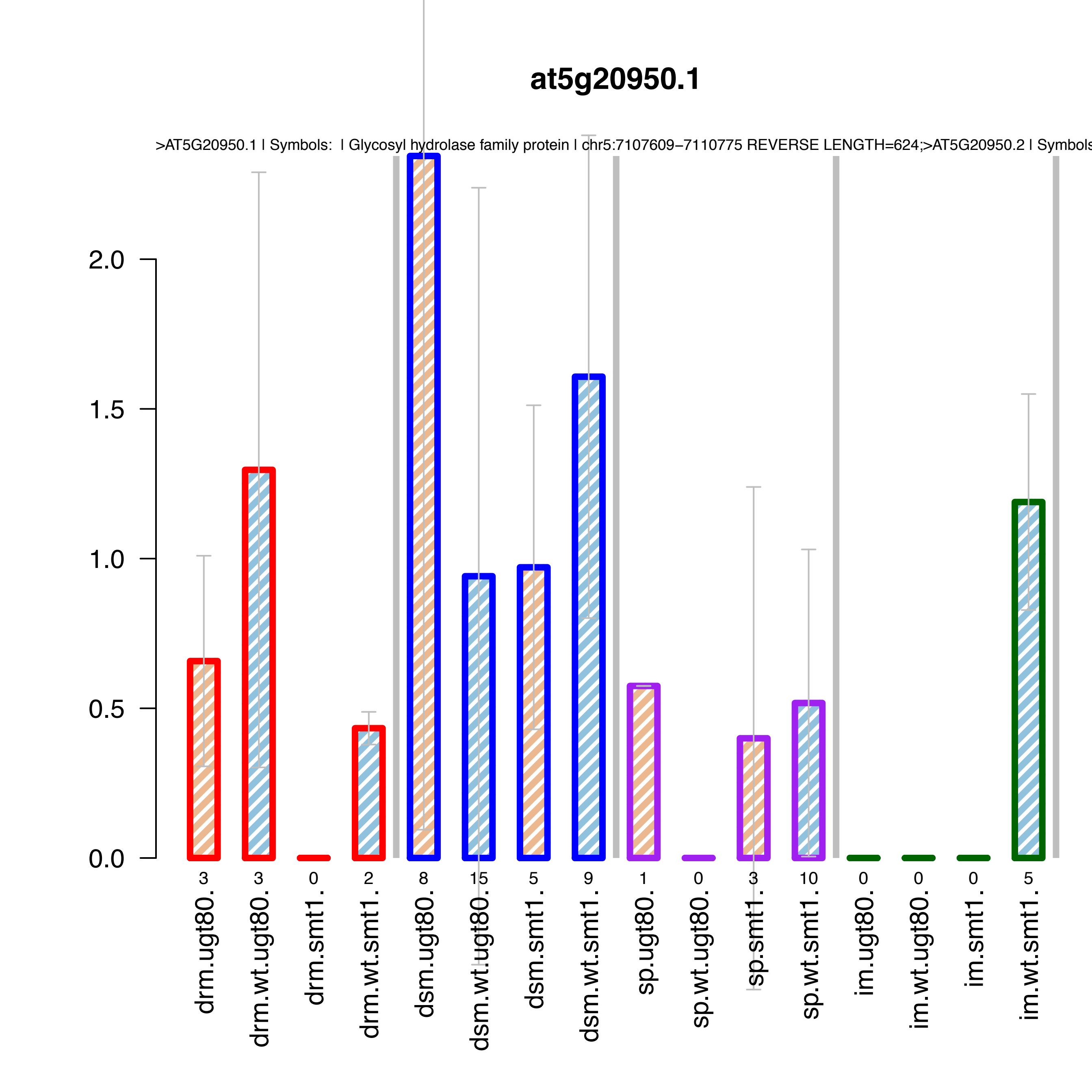

at5g20950.1
>AT5G20950.1 | Symbols: | Glycosyl hydrolase family protein | chr5:7107609−7110775 REVERSE LENGTH=624;>AT5G20950.2 | Symbols
0.0
0.5
1.0
1.5
2.0
3
3
0
2
8
15
5
9
1
0
3
10
0
0
0
5
drm.ugt80.
drm.wt.ugt80.
drm.smt1.
drm.wt.smt1.
dsm.ugt80.
dsm.wt.ugt80.
dsm.smt1.
dsm.wt.smt1.
sp.ugt80.
sp.wt.ugt80.
sp.smt1.
sp.wt.smt1.
im.ugt80.
im.wt.ugt80.
im.smt1.
im.wt.smt1.

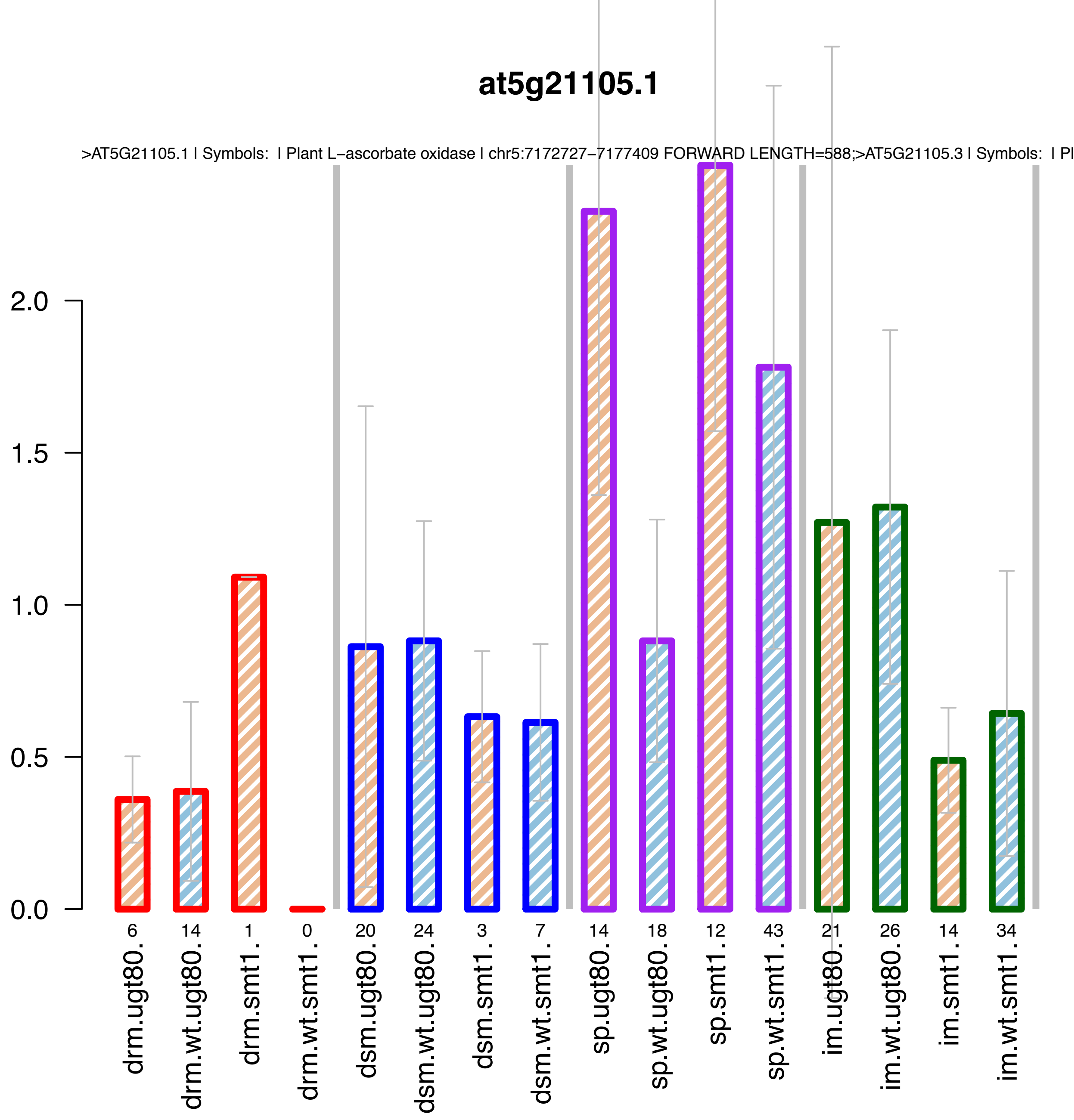

at5g21105.1
>AT5G21105.1 | Symbols: | Plant L-ascorbate oxidase | chr5:7172727-7177409 FORWARD LENGTH=588;>AT5G21105.3 | Symbols: | Pl
2.0
1.5
1.0
0.5
0.0
6
14
1
0
20
24
3
7
14
18
12
43
21
26
14
34
drm.ugt80..
drm.wt.ugt80..
drm.smt1..
drm.wt.smt1..
dsm.ugt80..
dsm.wt.ugt80..
dsm.smt1..
dsm.wt.smt1..
sp.ugt80..
sp.wt.ugt80..
sp.smt1..
sp.wt.smt1..
im.ugt80..
im.wt.ugt80..
im.smt1..
im.wt.smt1..

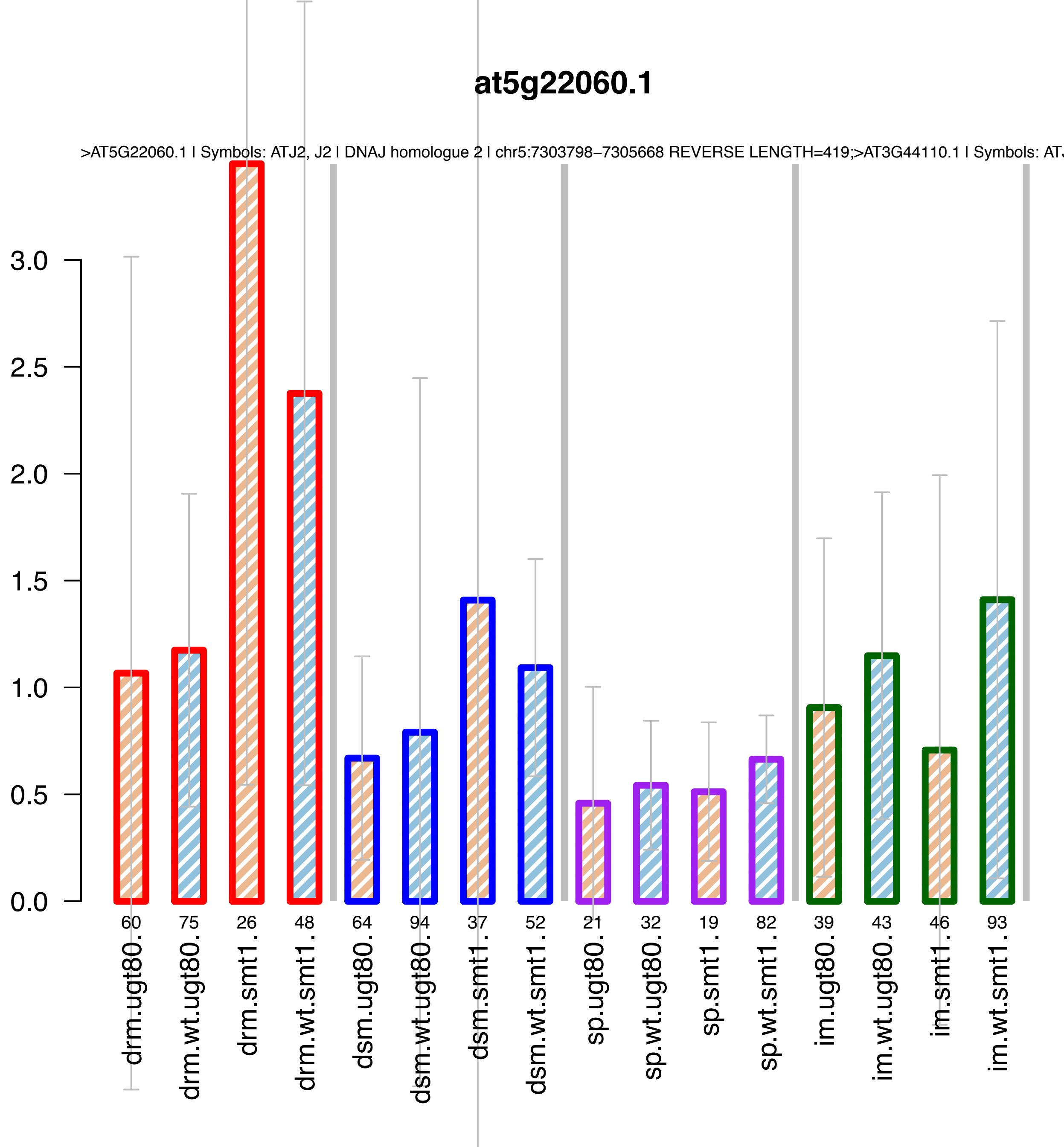

at5g22060.1
>AT5G22060.1 | Symbols: ATJ2, J2 | DNAJ homologue 2 | chr5:7303798−7305668 REVERSE LENGTH=419;>AT3G44110.1 | Symbols: AT
0.0
0.5
1.0
1.5
2.0
2.5
3.0
60
75
26
48
64
94
37
52
21
32
19
82
39
43
46
93
drm.ugt80..
drm.wt.ugt80..
drm.smt1..
drm.wt.smt1..
dsm.ugt80..
dsm.wt.ugt80..
dsm.smt1..
dsm.wt.smt1..
sp.ugt80..
sp.wt.ugt80..
sp.smt1..
sp.wt.smt1..
im.ugt80..
im.wt.ugt80..
im.smt1..
im.wt.smt1..

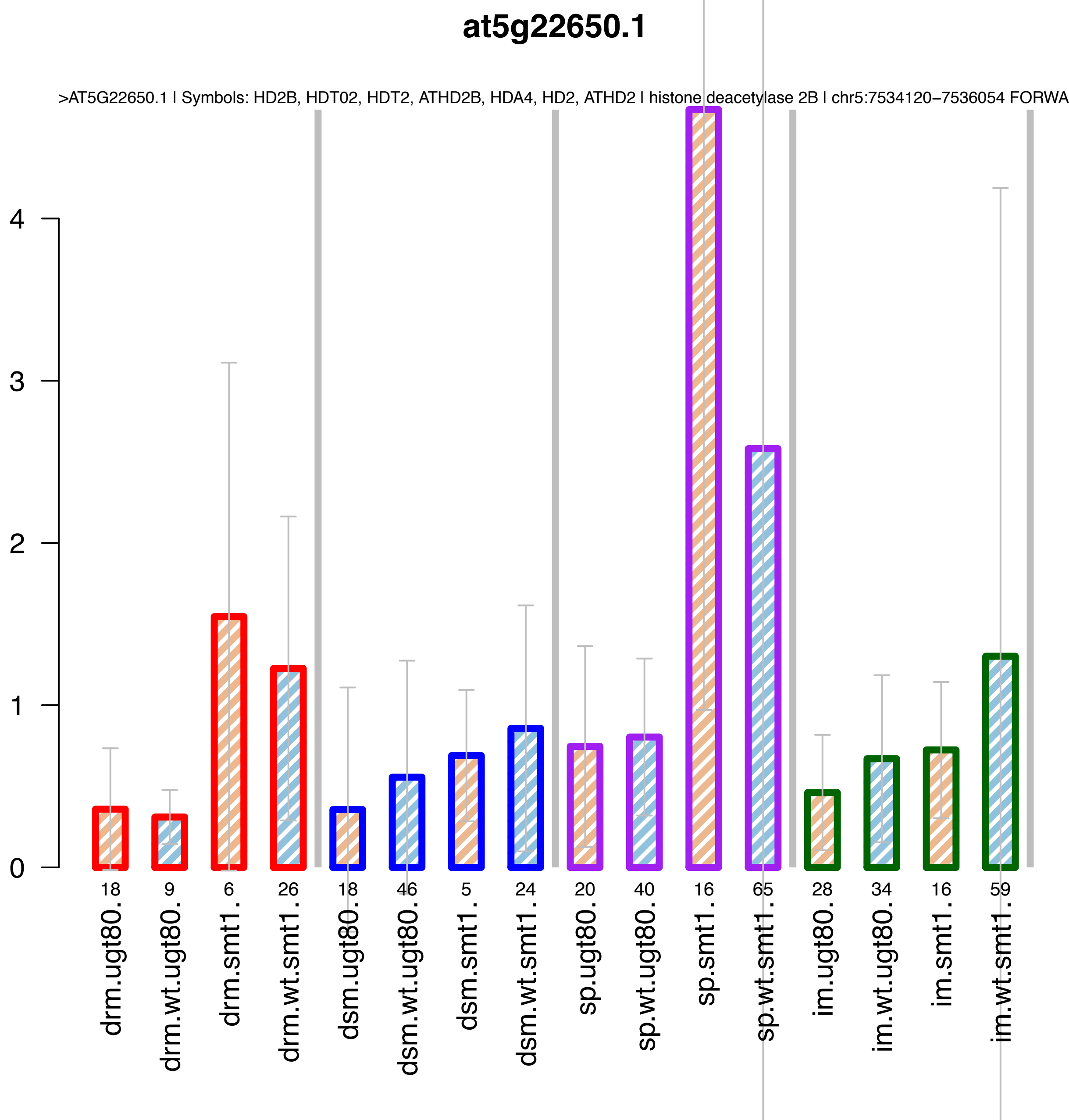

at5g22650.1
>AT5G22650.1 | Symbols: HD2B, HDT02, HDT2, ATHD2B, HDA4, HD2, ATHD2 | histone deacetylase 2B | chr5:7534120-7536054 FORWA
0
1
2
3
4
18
9
6
26
18
46
5
24
20
40
16
65
28
34
16
59
drm.ugt80.
drm.wt.ugt80.
drm.smt1.
drm.wt.smt1.
dsm.ugt80.
dsm.wt.ugt80.
dsm.smt1.
dsm.wt.smt1.
sp.ugt80.
sp.wt.ugt80.
sp.smt1.
sp.wt.smt1.
im.ugt80.
im.wt.ugt80.
im.smt1.
im.wt.smt1.

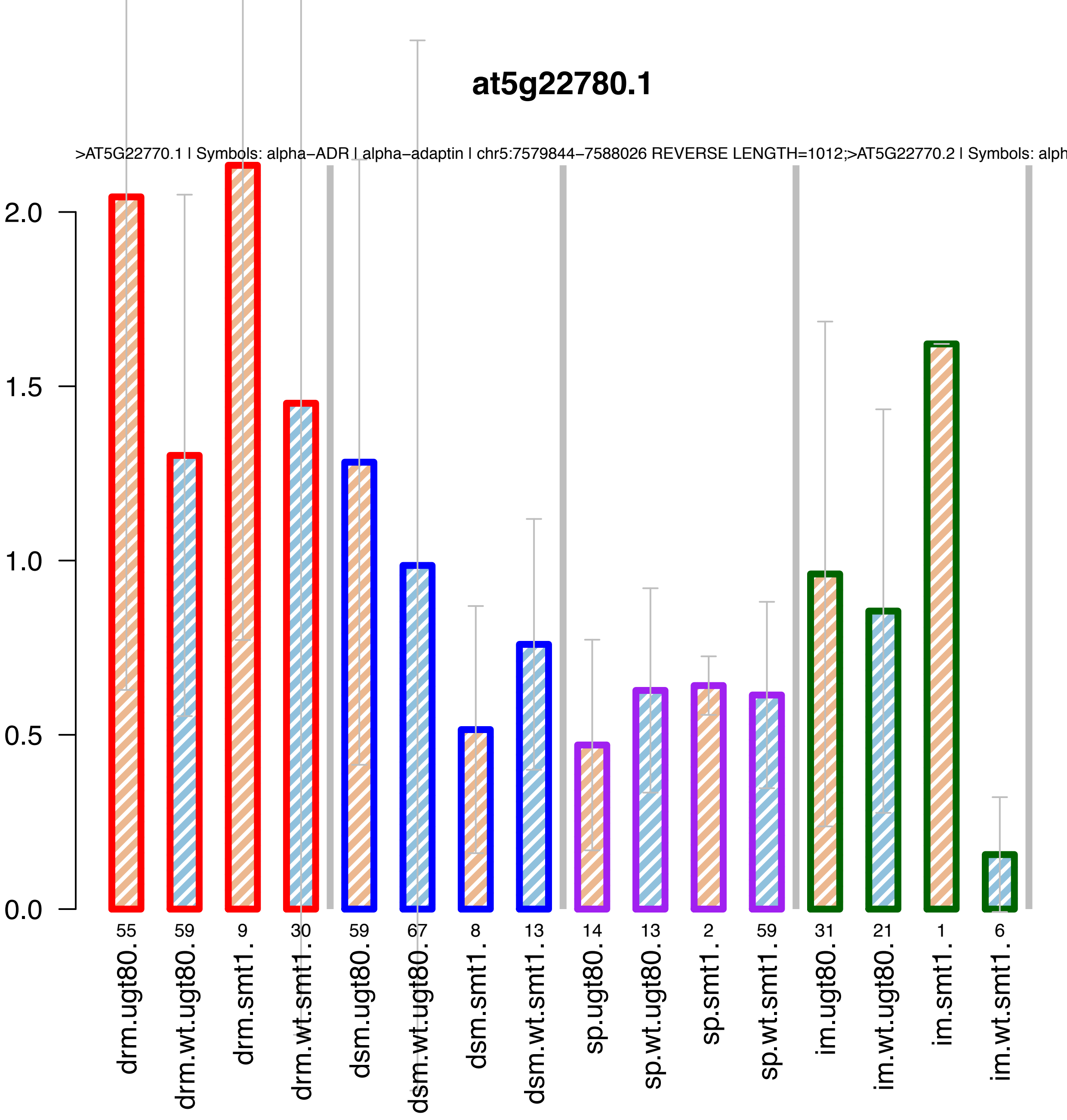

at5g22780.1
>AT5G22770.1 | Symbols: alpha-ADR | alpha-adaptin | chr5:7579844-7588026 REVERSE LENGTH=1012;>AT5G22770.2 | Symbols: alph
2.0
1.5
1.0
0.5
0.0
55
59
9
30
59
67
8
13
14
13
2
59
31
21
1
6
drm.ugt80..
drm.wt.ugt80..
drm.smt1..
drm.wt.smt1..
dsm.ugt80..
dsm.wt.ugt80..
dsm.smt1..
dsm.wt.smt1..
sp.ugt80..
sp.wt.ugt80..
sp.smt1..
sp.wt.smt1..
im.ugt80..
im.wt.ugt80..
im.smt1..
im.wt.smt1..

# at5g22790.1

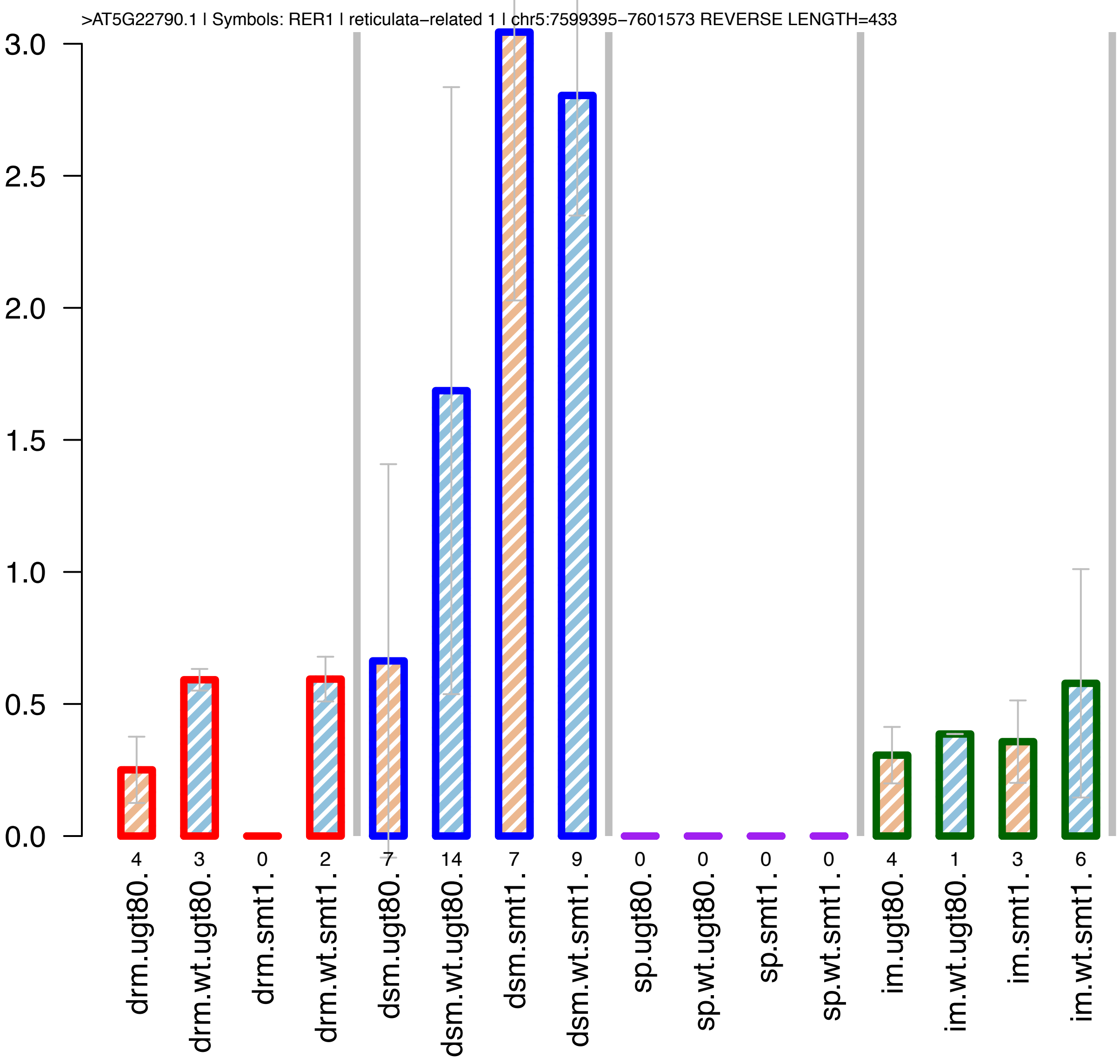

>AT5G22790.1 | Symbols: RER1 | reticulata-related 1 | chr5:7599395-7601573 REVERSE LENGTH=433
3.0
2.5
2.0
1.5
1.0
0.5
0.0
4
3
0
2
7
14
7
9
0
0
0
0
4
1
3
6
drm.ugt80.
drm.wt.ugt80.
drm.smt1.
drm.wt.smt1.
dsm.ugt80.
dsm.wt.ugt80.
dsm.smt1.
dsm.wt.smt1.
sp.ugt80.
sp.wt.ugt80.
sp.smt1.
sp.wt.smt1.
im.ugt80.
im.wt.ugt80.
im.smt1.
im.wt.smt1.

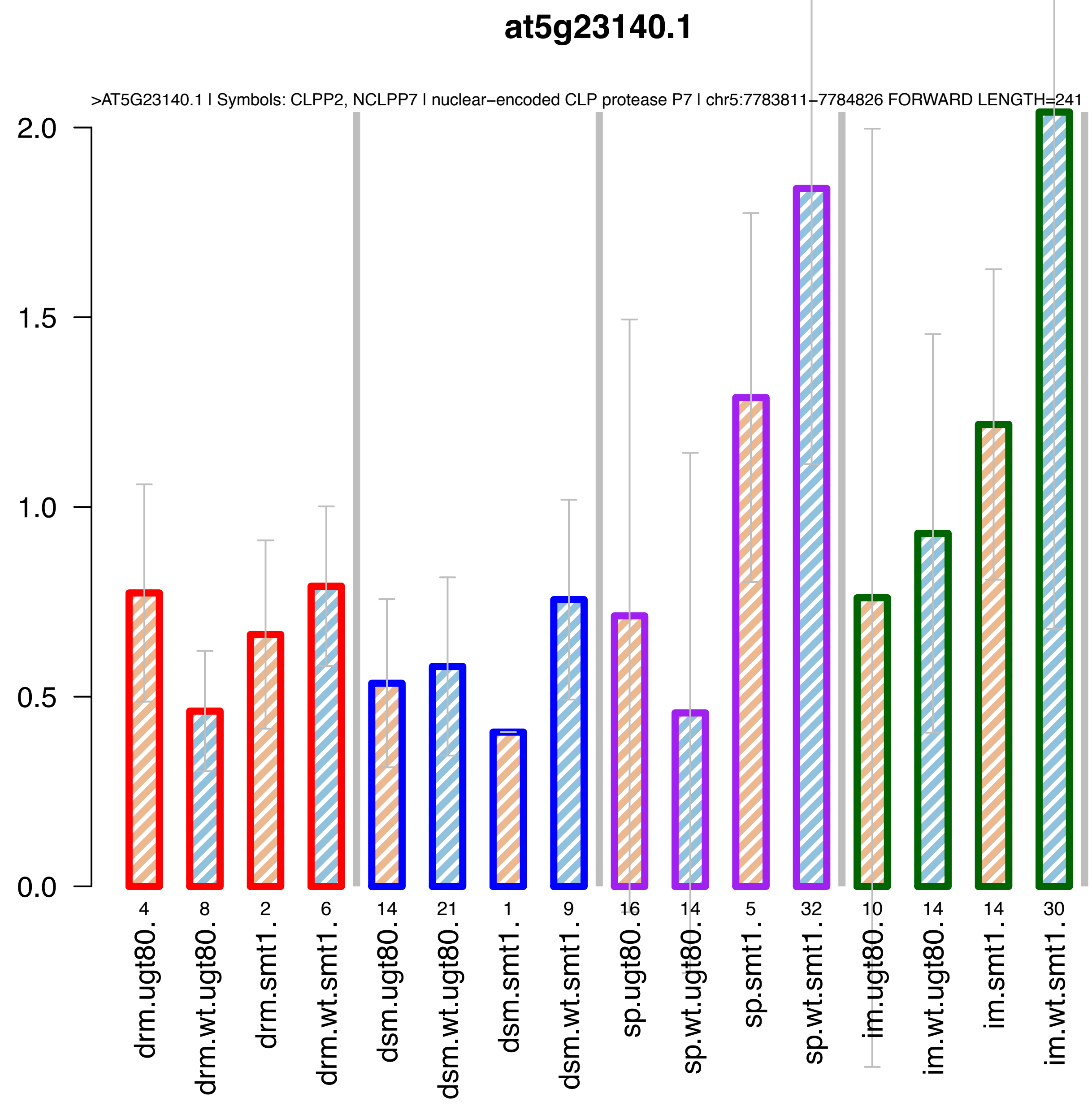

at5g23140.1
>AT5G23140.1 | Symbols: CLPP2, NCLPP7 | nuclear−encoded CLP protease P7 | chr5:7783811−7784826 FORWARD LENGTH=241
2.0
1.5
1.0
0.5
0.0
4
8
2
6
14
21
1
9
16
14
5
32
10
14
14
30
drm.ugt80..
drm.wt.ugt80..
drm.smt1..
drm.wt.smt1..
dsm.ugt80..
dsm.wt.ugt80..
dsm.smt1..
dsm.wt.smt1..
sp.ugt80..
sp.wt.ugt80..
sp.smt1..
sp.wt.smt1..
im.ugt80..
im.wt.ugt80..
im.smt1..
im.wt.smt1..

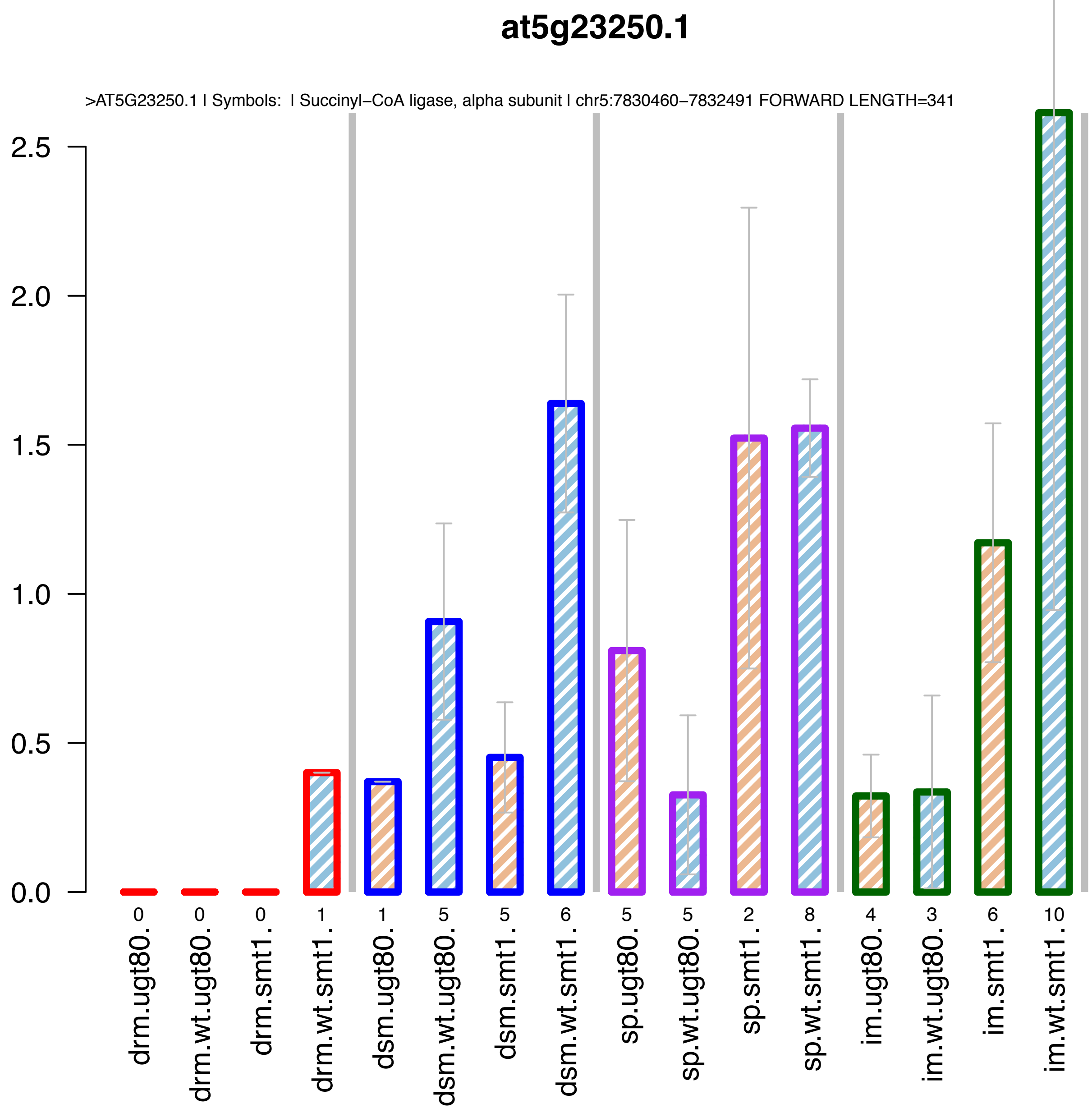
at5g23250.1
>AT5G23250.1 | Symbols: | Succinyl−CoA ligase, alpha subunit | chr5:7830460−7832491 FORWARD LENGTH=341
0.0
0.5
1.0
1.5
2.0
2.5
0
0
0
1
1
5
5
6
5
5
2
8
4
3
6
10
drm.ugt80.
drm.wt.ugt80.
drm.smt1.
drm.wt.smt1.
dsm.ugt80.
dsm.wt.ugt80.
dsm.smt1.
dsm.wt.smt1.
sp.ugt80.
sp.wt.ugt80.
sp.smt1.
sp.wt.smt1.
im.ugt80.
im.wt.ugt80.
im.smt1.
im.wt.smt1.

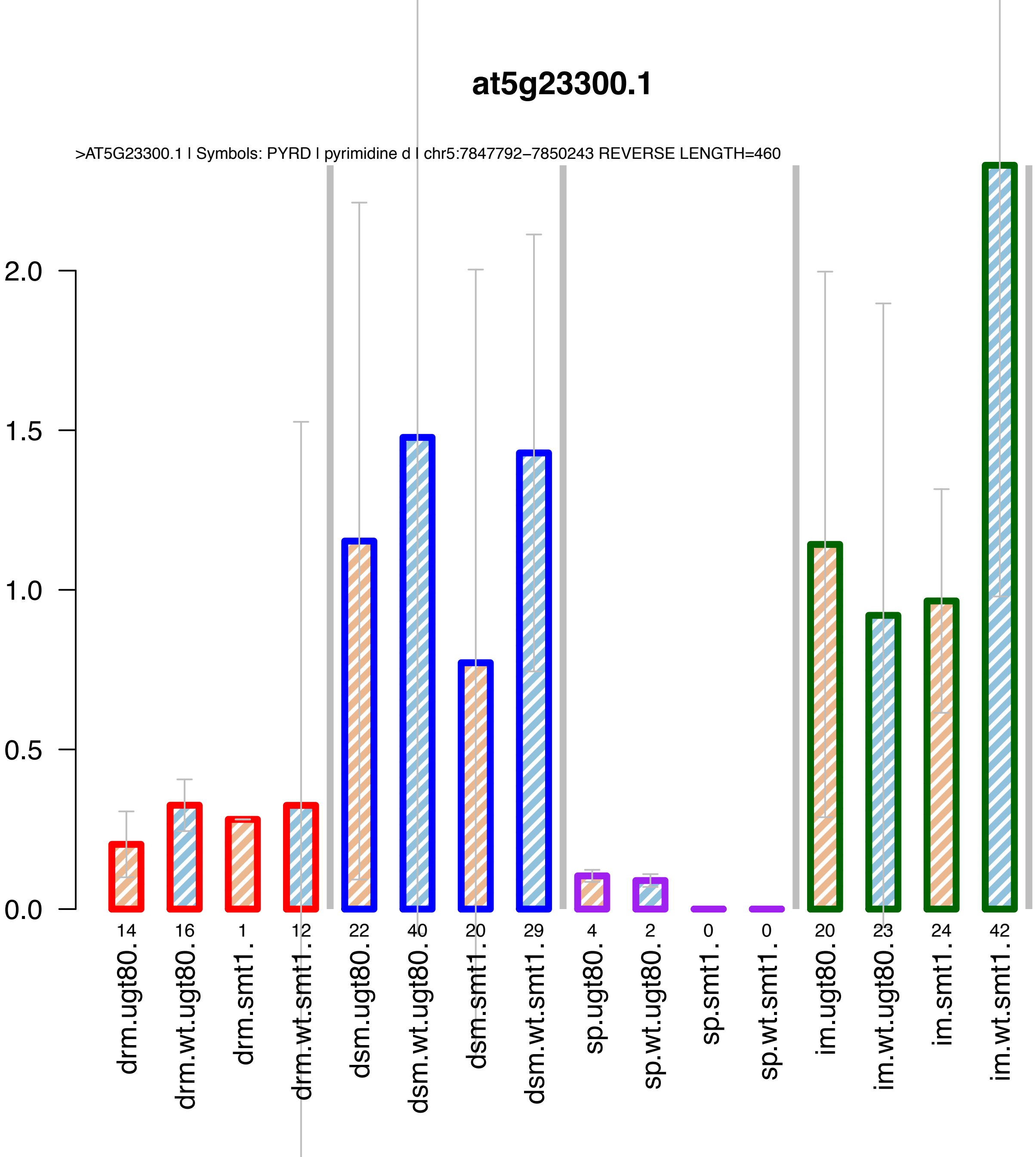
at5g23300.1
>AT5G23300.1 | Symbols: PYRD | pyrimidine d | chr5:7847792−7850243 REVERSE LENGTH=460
2.0
1.5
1.0
0.5
0.0
14
16
1
12
22
40
20
29
4
2
0
0
20
23
24
42
drm.ugt80..
drm.wt.ugt80..
drm.smt1..
drm.wt.smt1..
dsm.ugt80..
dsm.wt.ugt80..
dsm.smt1..
dsm.wt.smt1..
sp.ugt80..
sp.wt.ugt80..
sp.smt1..
sp.wt.smt1..
im.ugt80..
im.wt.ugt80..
im.smt1..
im.wt.smt1..

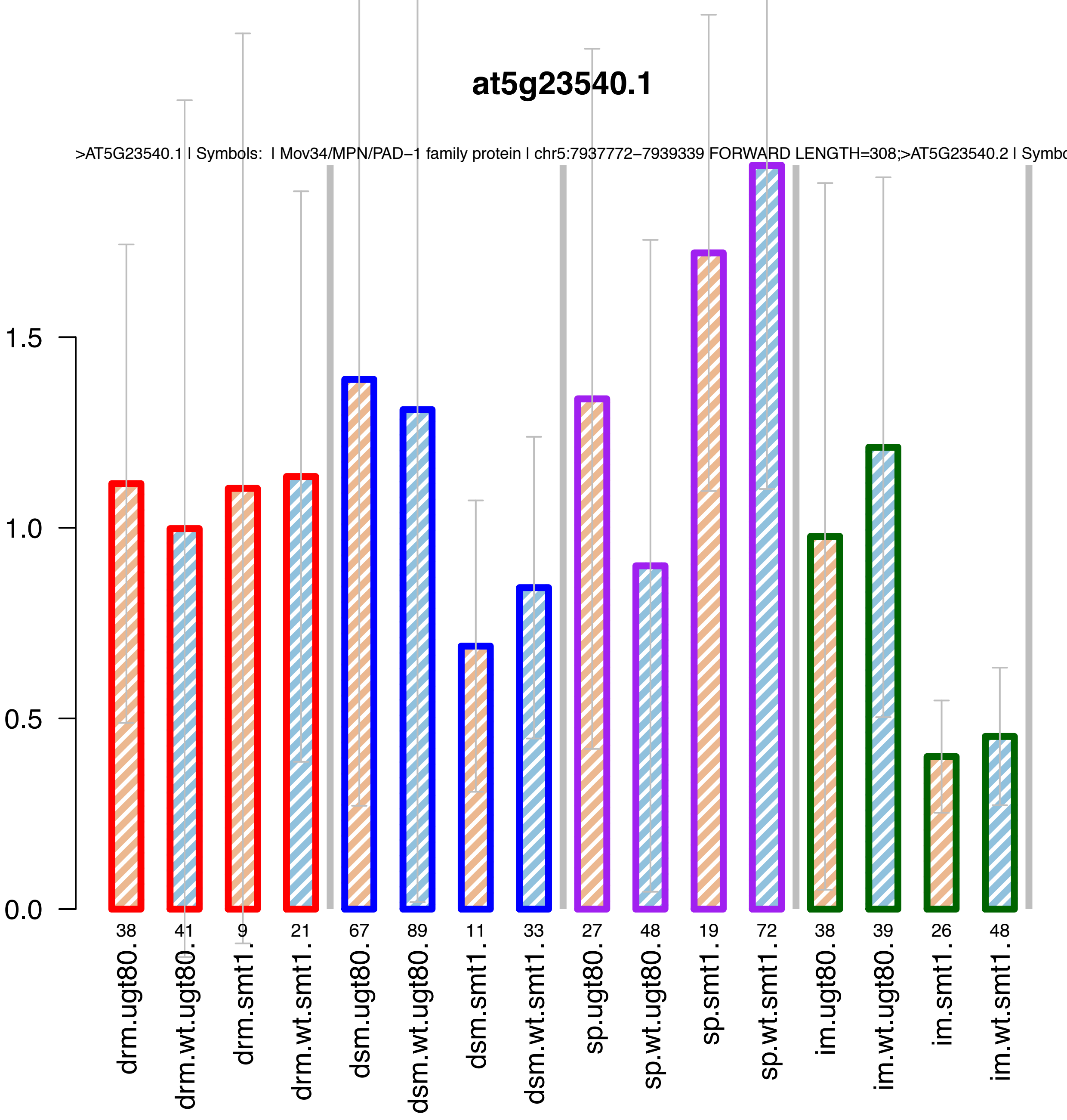

at5g23540.1
>AT5G23540.1 | Symbols: | Mov34/MPN/PAD−1 family protein | chr5:7937772−7939339 FORWARD LENGTH=308;>AT5G23540.2 | Symbo
0.0
0.5
1.0
1.5
38
41
9
21
67
89
11
33
27
48
19
72
38
39
26
48
drm.ugt80.
drm.wt.ugt80.
drm.smt1.
drm.wt.smt1.
dsm.ugt80.
dsm.wt.ugt80.
dsm.smt1.
dsm.wt.smt1.
sp.ugt80.
sp.wt.ugt80.
sp.smt1.
sp.wt.smt1.
im.ugt80.
im.wt.ugt80.
im.smt1.
im.wt.smt1.

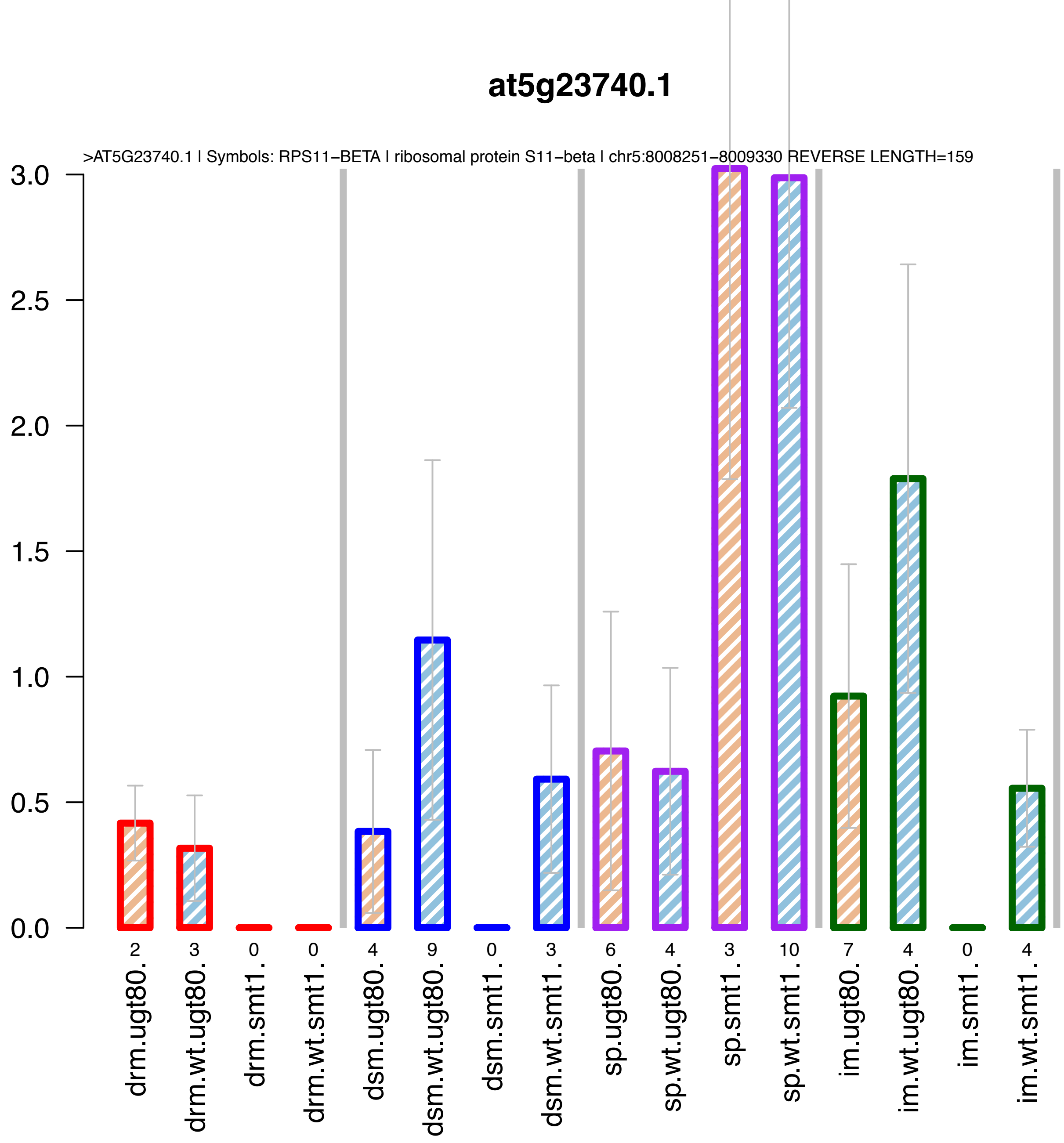
at5g23740.1
>AT5G23740.1 | Symbols: RPS11-BETA | ribosomal protein S11-beta | chr5:8008251-8009330 REVERSE LENGTH=159
0.0
0.5
1.0
1.5
2.0
2.5
3.0
2
3
0
0
4
9
0
3
6
4
3
10
7
4
0
4
drm.ugt80.
drm.wt.ugt80.
drm.smt1.
drm.wt.smt1.
dsm.ugt80.
dsm.wt.ugt80.
dsm.smt1.
dsm.wt.smt1.
sp.ugt80.
sp.wt.ugt80.
sp.smt1.
sp.wt.smt1.
im.ugt80.
im.wt.ugt80.
im.smt1.
im.wt.smt1.

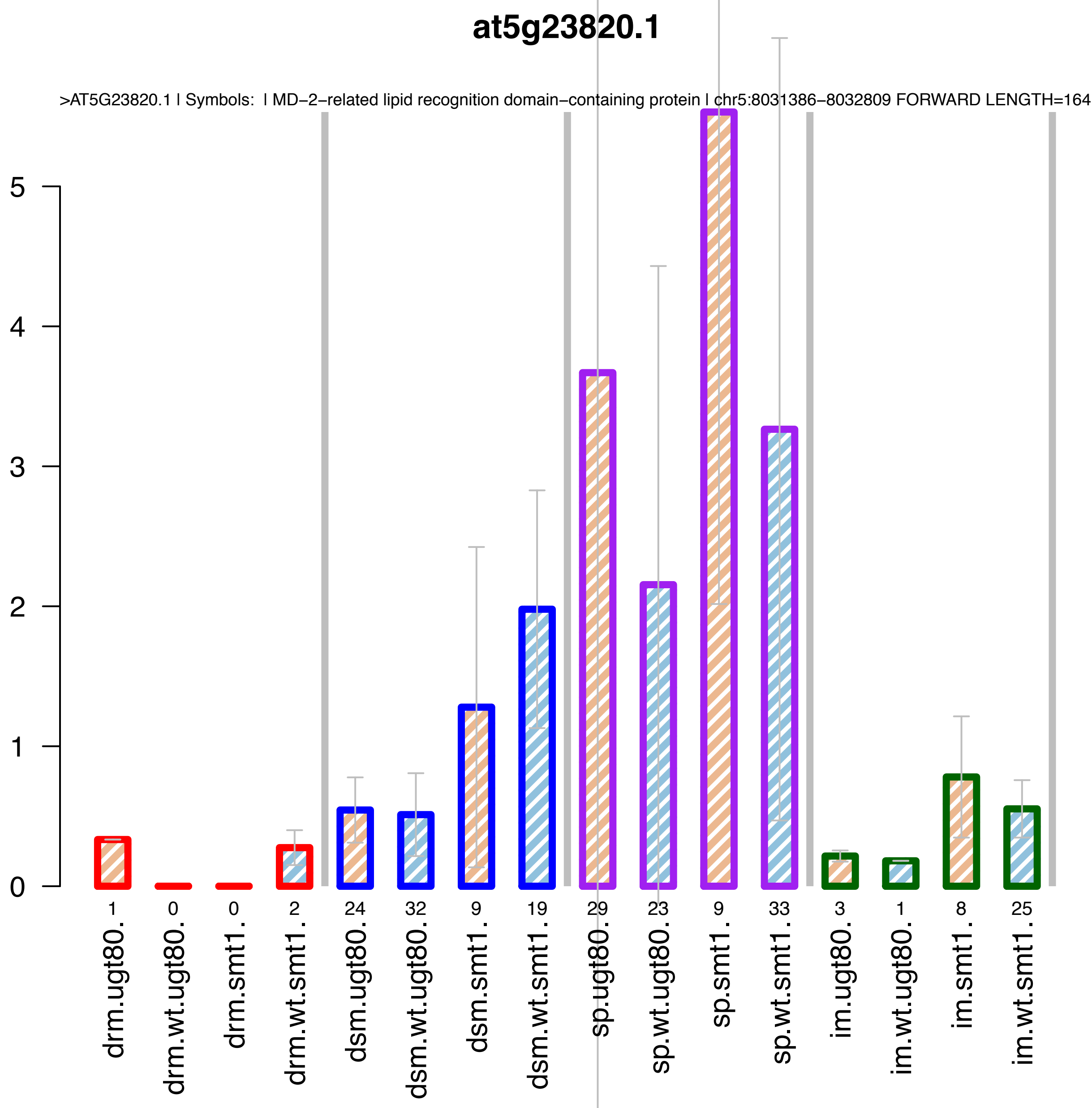

at5g23820.1
>AT5G23820.1 | Symbols: | MD−2−related lipid recognition domain−containing protein | chr5:8031386−8032809 FORWARD LENGTH=164
0
1
2
3
4
5
1
0
0
2
24
32
9
19
29
23
9
33
3
1
8
25
drm.ugt80.
drm.wt.ugt80.
drm.smt1.
drm.wt.smt1.
dsm.ugt80.
dsm.wt.ugt80.
dsm.smt1.
dsm.wt.smt1.
sp.ugt80.
sp.wt.ugt80.
sp.smt1.
sp.wt.smt1.
im.ugt80.
im.wt.ugt80.
im.smt1.
im.wt.smt1.

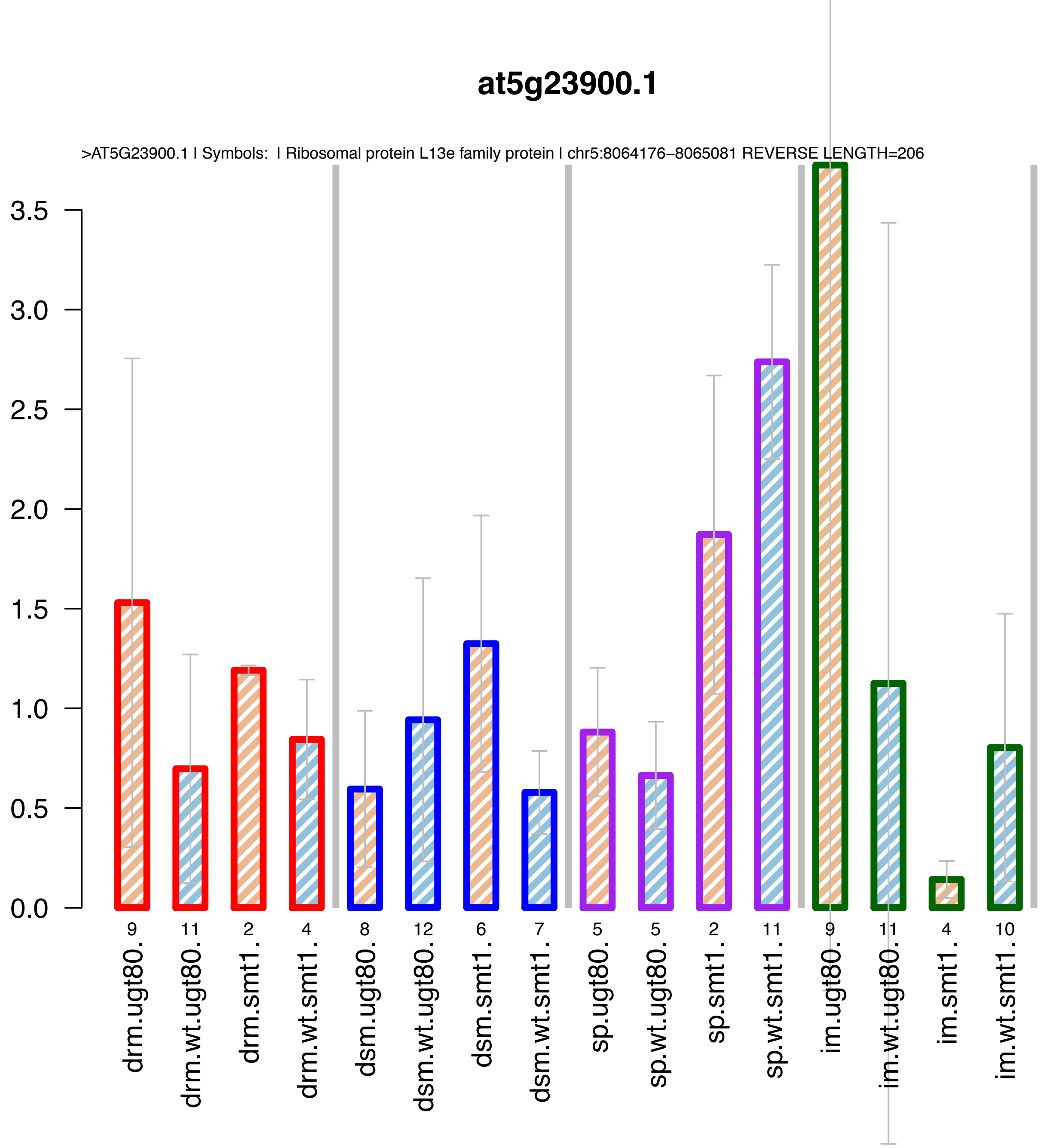

at5g23900.1
>AT5G23900.1 | Symbols: | Ribosomal protein L13e family protein | chr5:8064176-8065081 REVERSE LENGTH=206
3.5
3.0
2.5
2.0
1.5
1.0
0.5
0.0
9
11
2
4
8
12
6
7
5
5
2
11
9
11
4
10
drm.ugt80..
drm.wt.ugt80..
drm.smt1..
drm.wt.smt1..
dsm.ugt80..
dsm.wt.ugt80..
dsm.smt1..
dsm.wt.smt1..
sp.ugt80..
sp.wt.ugt80..
sp.smt1..
sp.wt.smt1..
im.ugt80..
im.wt.ugt80..
im.smt1..
im.wt.smt1..

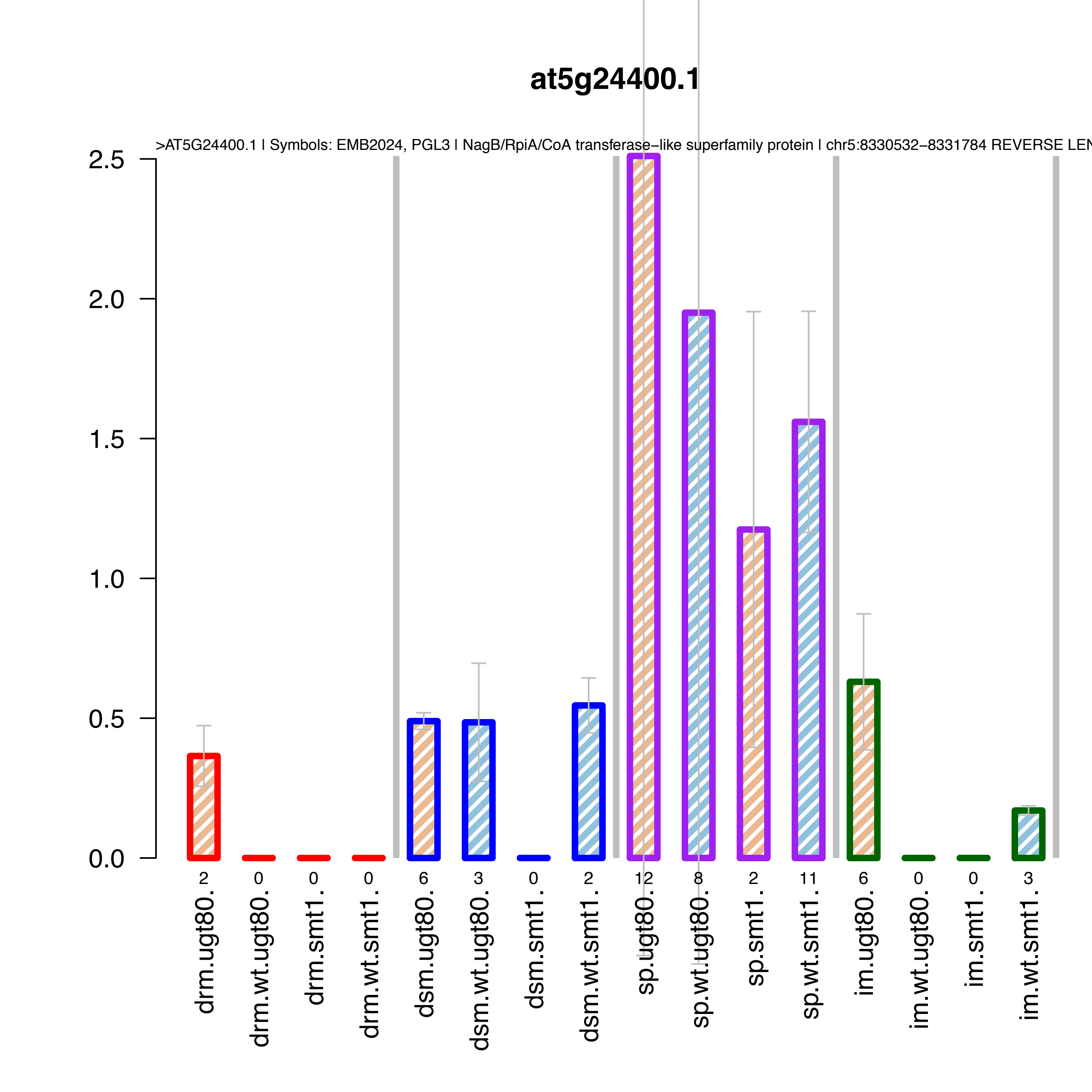

at5g24400.1
>AT5G24400.1 | Symbols: EMB2024, PGL3 | NagB/RpiA/CoA transferase−like superfamily protein | chr5:8330532−8331784 REVERSE LEN
2.5
2.0
1.5
1.0
0.5
0.0
2
0
0
0
6
3
0
2
12
8
2
11
6
0
0
3
drm.ugt80.
drm.wt.ugt80.
drm.smt1.
drm.wt.smt1.
dsm.ugt80.
dsm.wt.ugt80.
dsm.smt1.
dsm.wt.smt1.
sp.ugt80.
sp.wt.ugt80.
sp.smt1.
sp.wt.smt1.
im.ugt80.
im.wt.ugt80.
im.smt1.
im.wt.smt1.

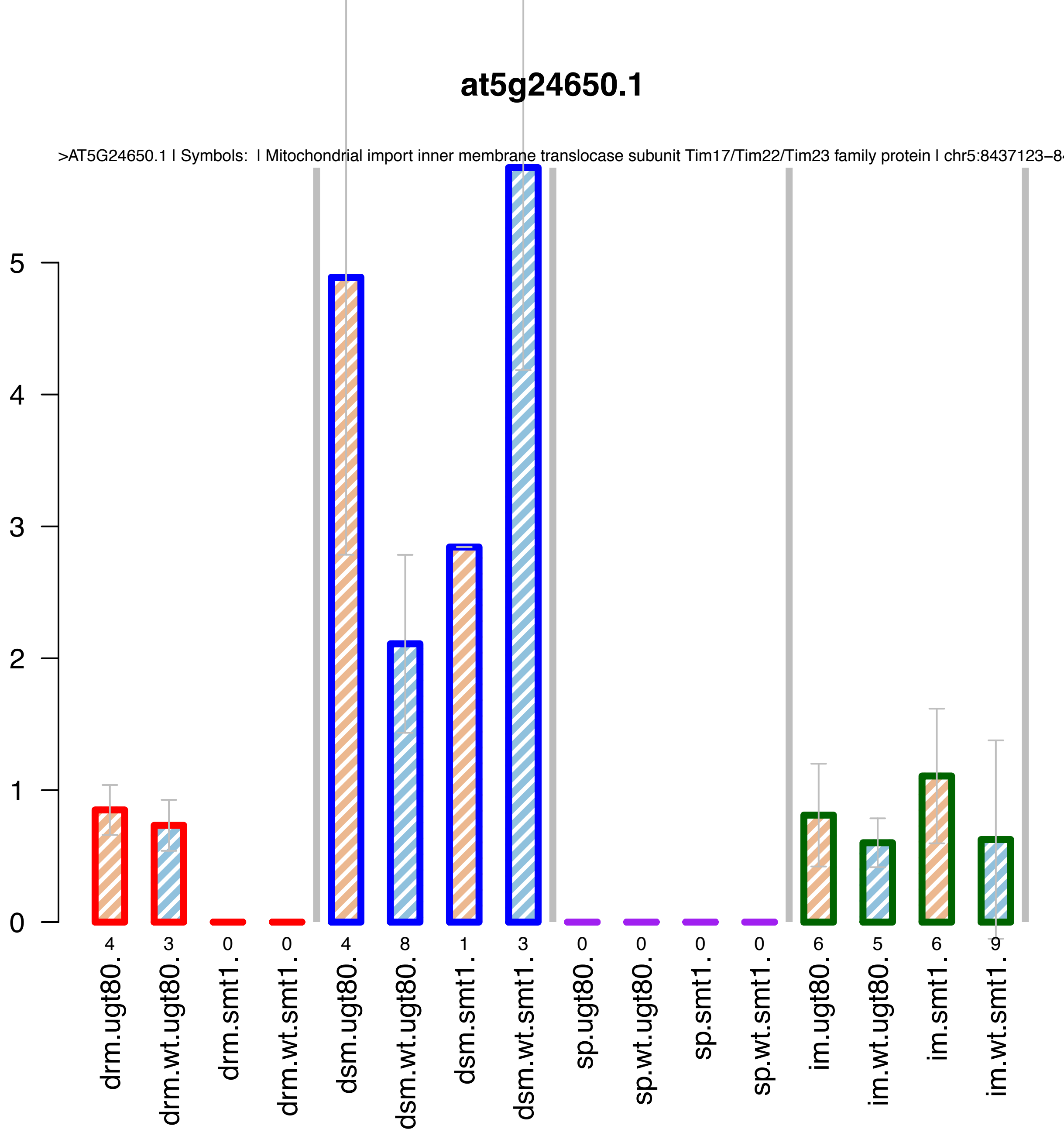

at5g24650.1
>AT5G24650.1 | Symbols: | Mitochondrial import inner membrane translocase subunit Tim17/Tim22/Tim23 family protein | chr5:8437123-8
0
1
2
3
4
5
4
3
0
0
4
8
1
3
0
0
0
0
6
5
6
9
drm.ugt80.
drm.wt.ugt80.
drm.smt1.
drm.wt.smt1.
dsm.ugt80.
dsm.wt.ugt80.
dsm.smt1.
dsm.wt.smt1.
sp.ugt80.
sp.wt.ugt80.
sp.smt1.
sp.wt.smt1.
im.ugt80.
im.wt.ugt80.
im.smt1.
im.wt.smt1.

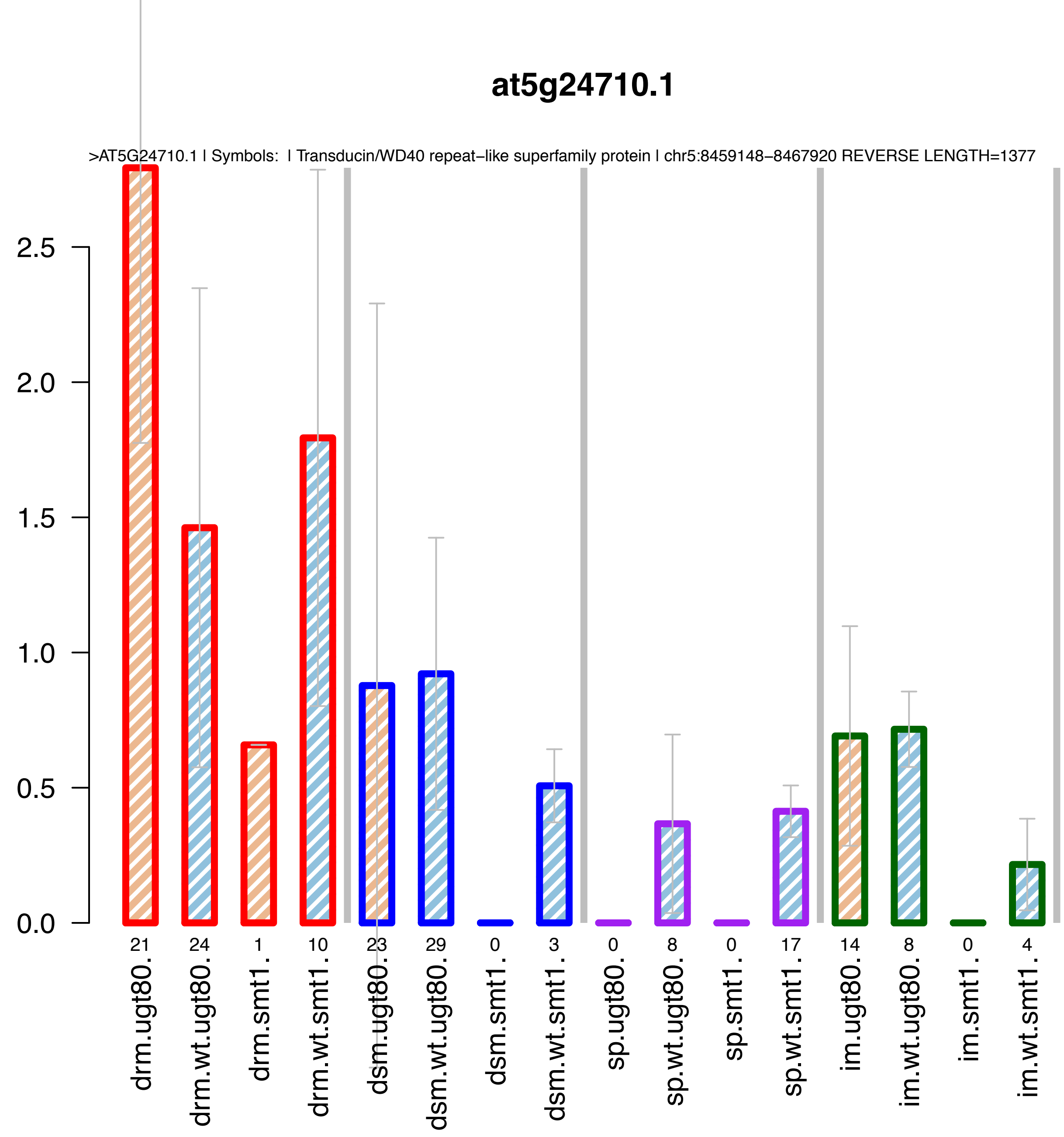

at5g24710.1
>AT5G24710.1 | Symbols: | Transducin/WD40 repeat-like superfamily protein | chr5:8459148-8467920 REVERSE LENGTH=1377
0.0
0.5
1.0
1.5
2.0
2.5
21 24 1 10 23 29 0 3 0 8 0 17 14 8 0 4
drm.ugt80.
drm.wt.ugt80.
drm.smt1.
drm.wt.smt1.
dsm.ugt80.
dsm.wt.ugt80.
dsm.smt1.
dsm.wt.smt1.
sp.ugt80.
sp.wt.ugt80.
sp.smt1.
sp.wt.smt1.
im.ugt80.
im.wt.ugt80.
im.smt1.
im.wt.smt1.

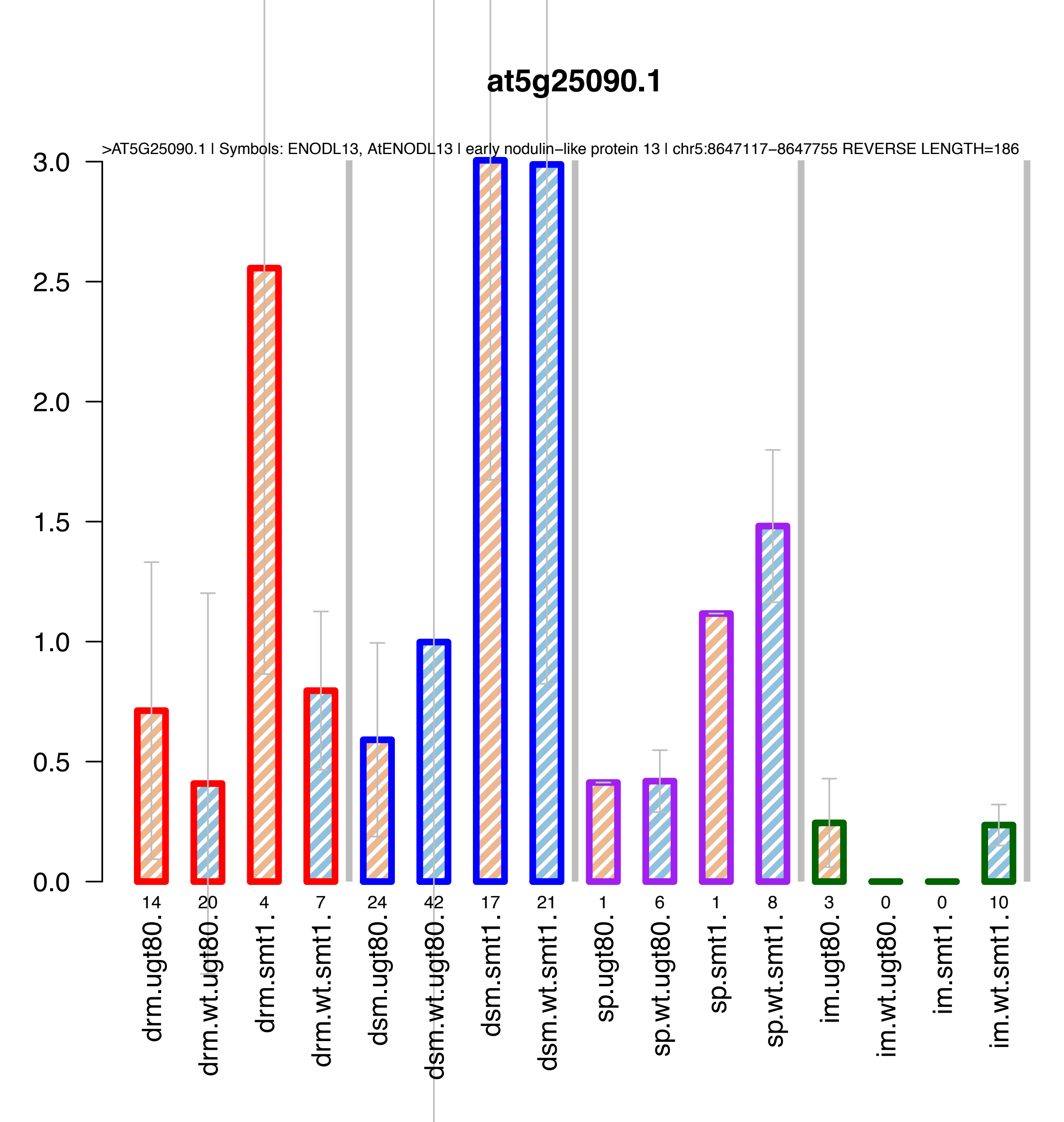

at5g25090.1
>AT5G25090.1 | Symbols: ENODL13, AtENODL13 | early nodulin−like protein 13 | chr5:8647117−8647755 REVERSE LENGTH=186
3.0
2.5
2.0
1.5
1.0
0.5
0.0
14
20
4
7
24
42
17
21
1
6
1
8
3
0
0
10
drm.ugt80.
drm.wt.ugt80.
drm.smt1.
drm.wt.smt1.
dsm.ugt80.
dsm.wt.ugt80.
dsm.smt1.
dsm.wt.smt1.
sp.ugt80.
sp.wt.ugt80.
sp.smt1.
sp.wt.smt1.
im.ugt80.
im.wt.ugt80.
im.smt1.
im.wt.smt1.

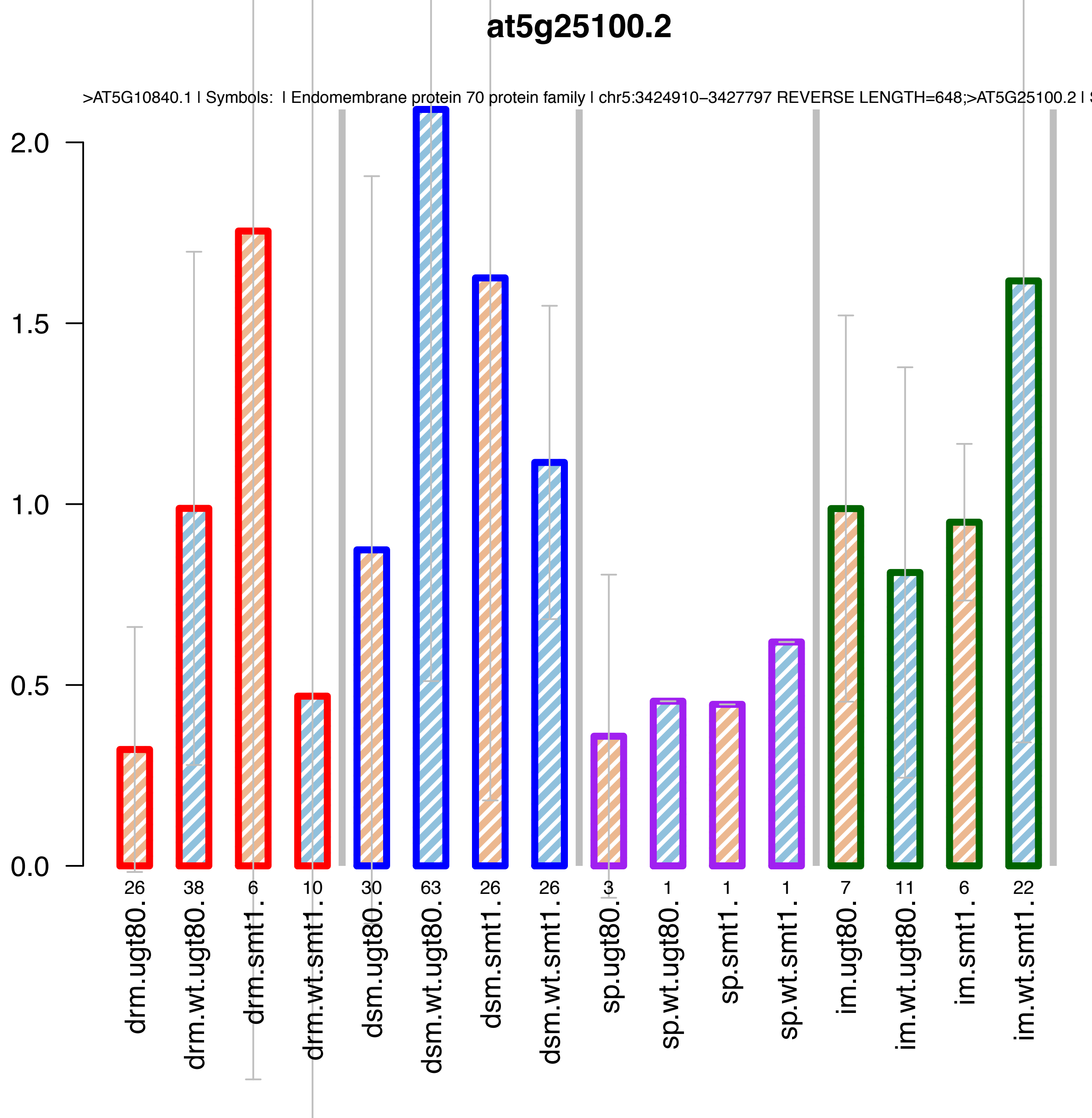

at5g25100.2
>AT5G10840.1 | Symbols: | Endomembrane protein 70 protein family | chr5:3424910-3427797 REVERSE LENGTH=648;>AT5G25100.2 | S
2.0
1.5
1.0
0.5
0.0
26
38
6
10
30
63
26
26
3
1
1
1
7
11
6
22
drm.ugt80.
drm.wt.ugt80.
drm.smt1.
drm.wt.smt1.
dsm.ugt80.
dsm.wt.ugt80.
dsm.smt1.
dsm.wt.smt1.
sp.ugt80.
sp.wt.ugt80.
sp.smt1.
sp.wt.smt1.
im.ugt80.
im.wt.ugt80.
im.smt1.
im.wt.smt1.

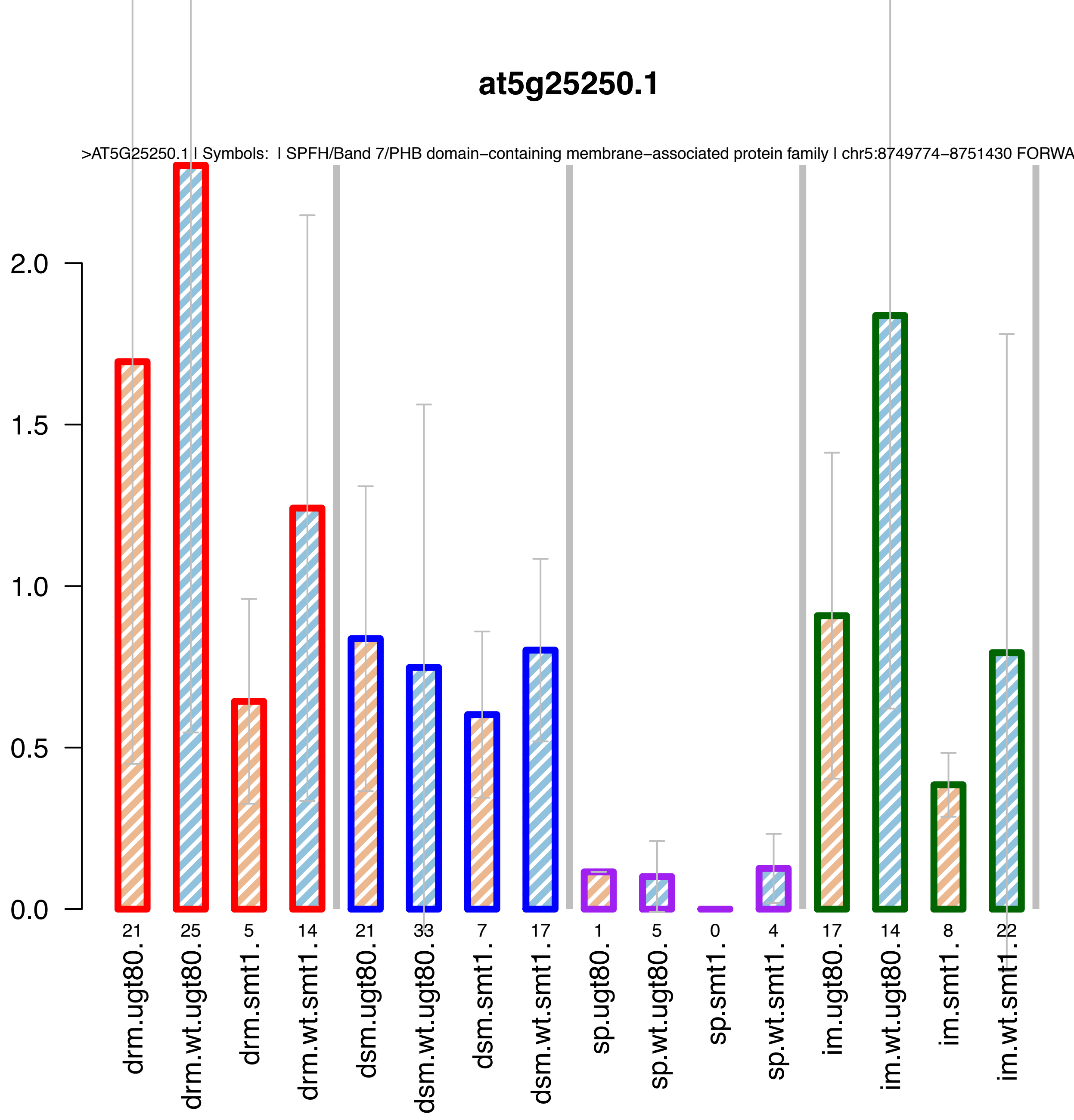

at5g25250.1
>AT5G25250.1 | Symbols: | SPFH/Band 7/PHB domain-containing membrane-associated protein family | chr5:8749774-8751430 FORWA
2.0
1.5
1.0
0.5
0.0
21
25
5
14
21
33
7
17
1
5
0
4
17
14
8
22
drm.ugt80.
drm.wt.ugt80.
drm.smt1.
drm.wt.smt1.
dsm.ugt80.
dsm.wt.ugt80.
dsm.smt1.
dsm.wt.smt1.
sp.ugt80.
sp.wt.ugt80.
sp.smt1.
sp.wt.smt1.
im.ugt80.
im.wt.ugt80.
im.smt1.
im.wt.smt1.

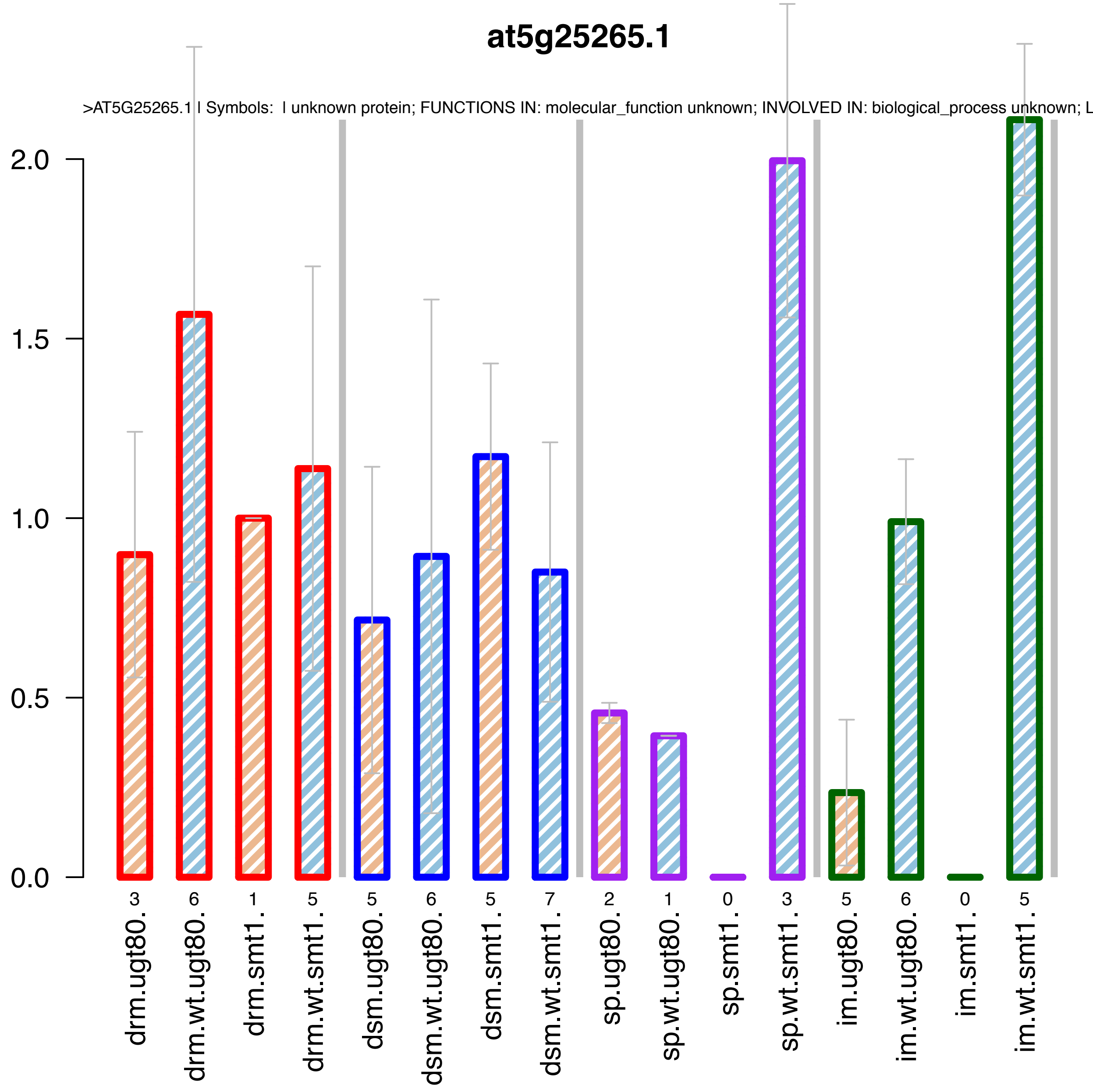

at5g25265.1
>AT5G25265.1 | Symbols: | unknown protein; FUNCTIONS IN: molecular_function unknown; INVOLVED IN: biological_process unknown; L
2.0
1.5
1.0
0.5
0.0
3
6
1
5
5
6
5
7
2
1
0
3
5
6
0
5
drm.ugt80.
drm.wt.ugt80.
drm.smt1.
drm.wt.smt1.
dsm.ugt80.
dsm.wt.ugt80.
dsm.smt1.
dsm.wt.smt1.
sp.ugt80.
sp.wt.ugt80.
sp.smt1.
sp.wt.smt1.
im.ugt80.
im.wt.ugt80.
im.smt1.
im.wt.smt1.

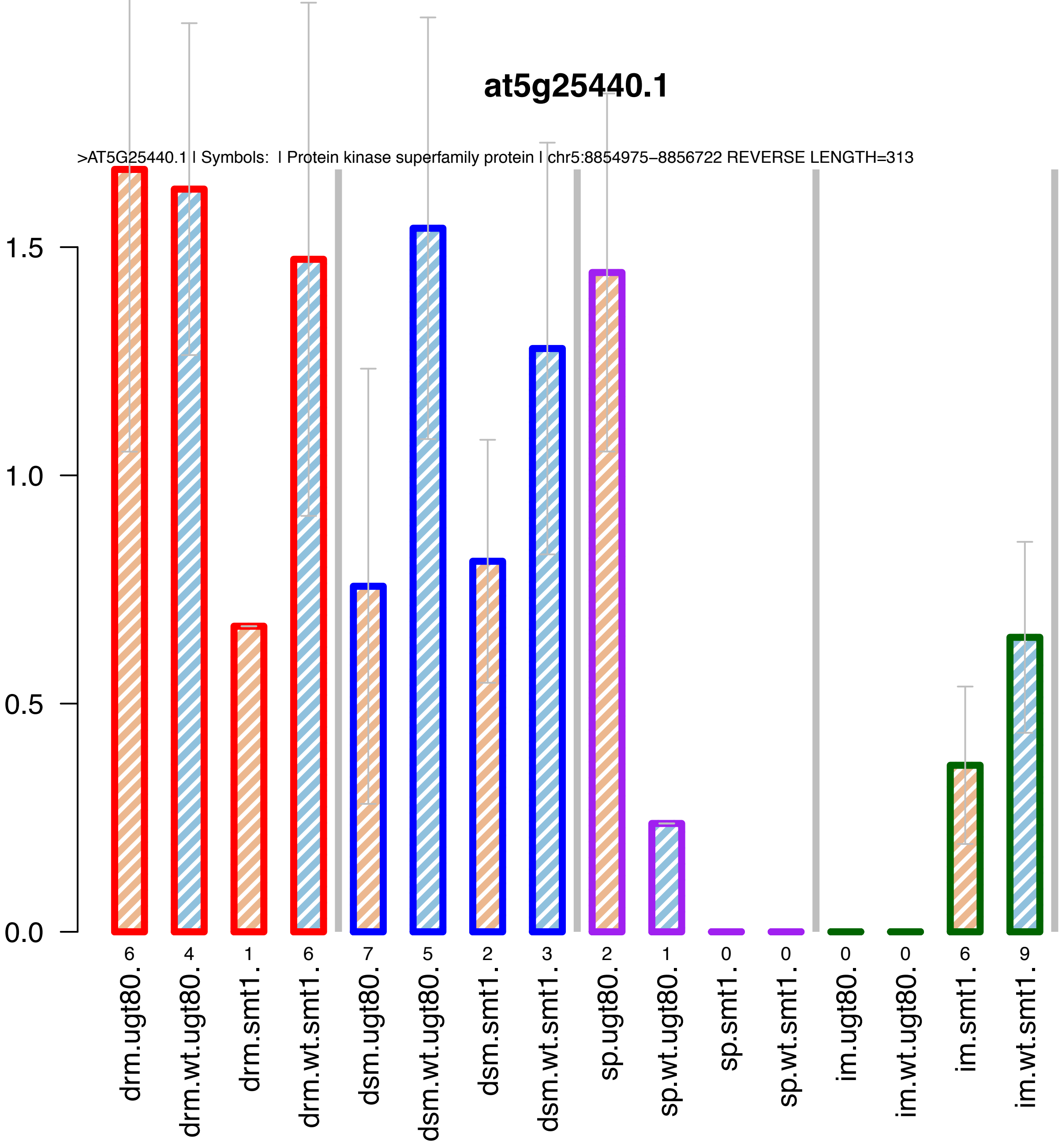

at5g25440.1
>AT5G25440.1 | Symbols: | Protein kinase superfamily protein | chr5:8854975-8856722 REVERSE LENGTH=313
1.5
1.0
0.5
0.0
6
4
1
6
7
5
2
3
2
1
0
0
0
0
6
9
drm.ugt80.
drm.wt.ugt80.
drm.smt1.
drm.wt.smt1.
dsm.ugt80.
dsm.wt.ugt80.
dsm.smt1.
dsm.wt.smt1.
sp.ugt80.
sp.wt.ugt80.
sp.smt1.
sp.wt.smt1.
im.ugt80.
im.wt.ugt80.
im.smt1.
im.wt.smt1.

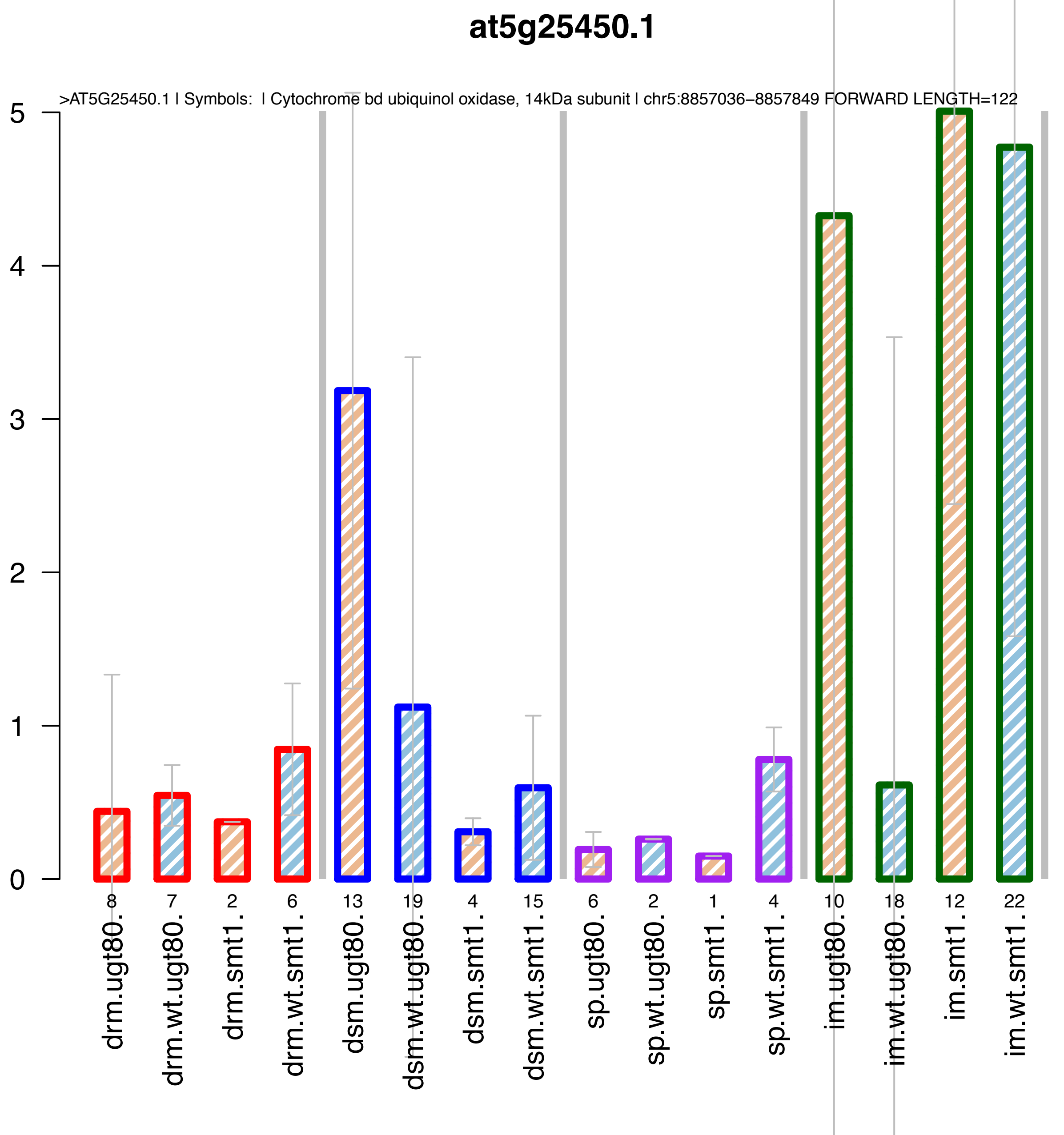
at5g25450.1
>AT5G25450.1 | Symbols: | Cytochrome bd ubiquinol oxidase, 14kDa subunit | chr5:8857036-8857849 FORWARD LENGTH=122
0
1
2
3
4
5
8
7
2
6
13
19
4
15
6
2
1
4
10
18
12
22
drm.ugt80.
drm.wt.ugt80.
drm.smt1.
drm.wt.smt1.
dsm.ugt80.
dsm.wt.ugt80.
dsm.smt1.
dsm.wt.smt1.
sp.ugt80.
sp.wt.ugt80.
sp.smt1.
sp.wt.smt1.
im.ugt80.
im.wt.ugt80.
im.smt1.
im.wt.smt1.

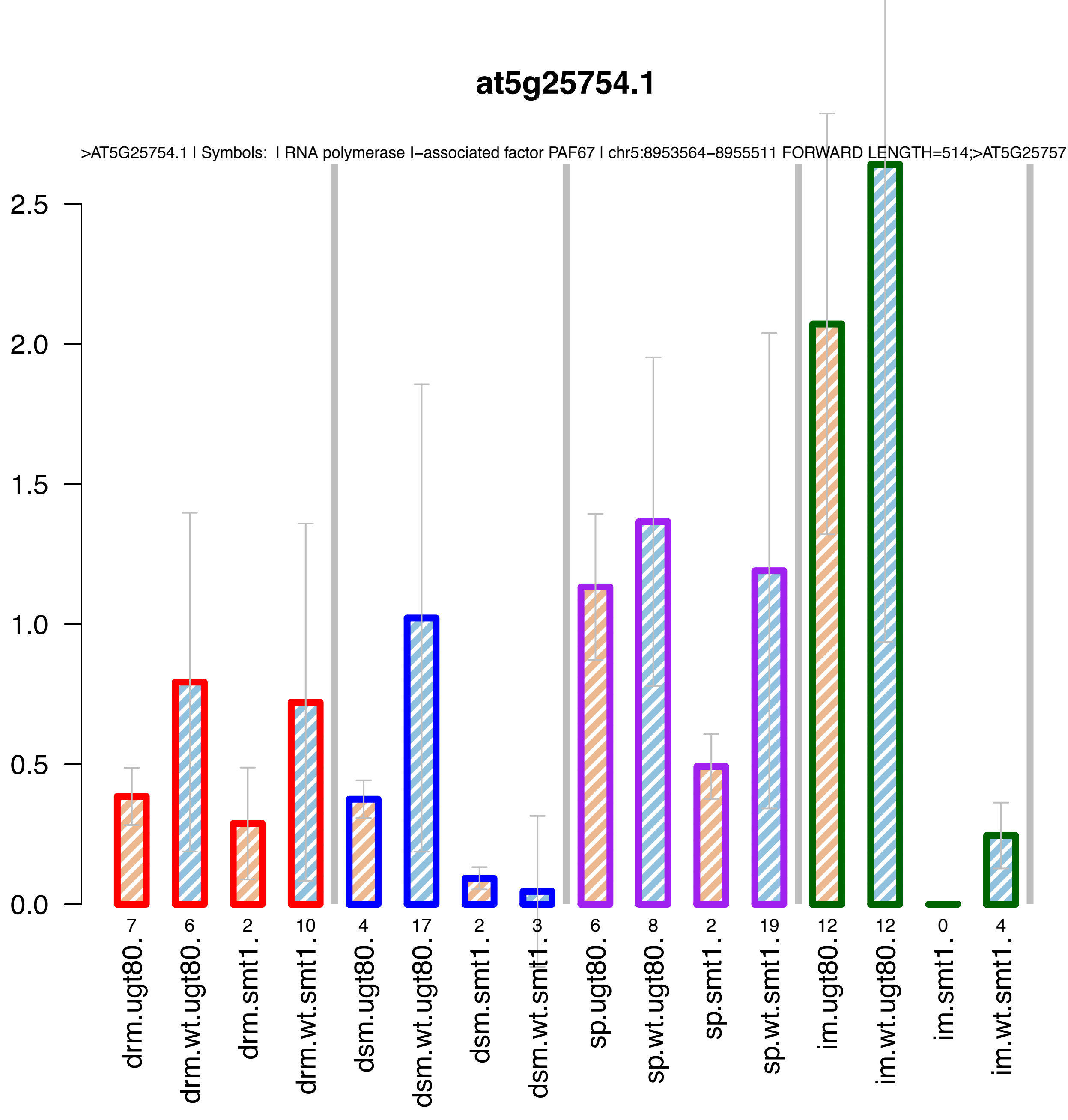

at5g25754.1
>AT5G25754.1 | Symbols: | RNA polymerase I−associated factor PAF67 | chr5:8953564−8955511 FORWARD LENGTH=514;>AT5G25757
2.5
2.0
1.5
1.0
0.5
0.0
7
6
2
10
4
17
2
3
6
8
2
19
12
12
0
4
drm.ugt80.
drm.wt.ugt80.
drm.smt1.
drm.wt.smt1.
dsm.ugt80.
dsm.wt.ugt80.
dsm.smt1.
dsm.wt.smt1.
sp.ugt80.
sp.wt.ugt80.
sp.smt1.
sp.wt.smt1.
im.ugt80.
im.wt.ugt80.
im.smt1.
im.wt.smt1.

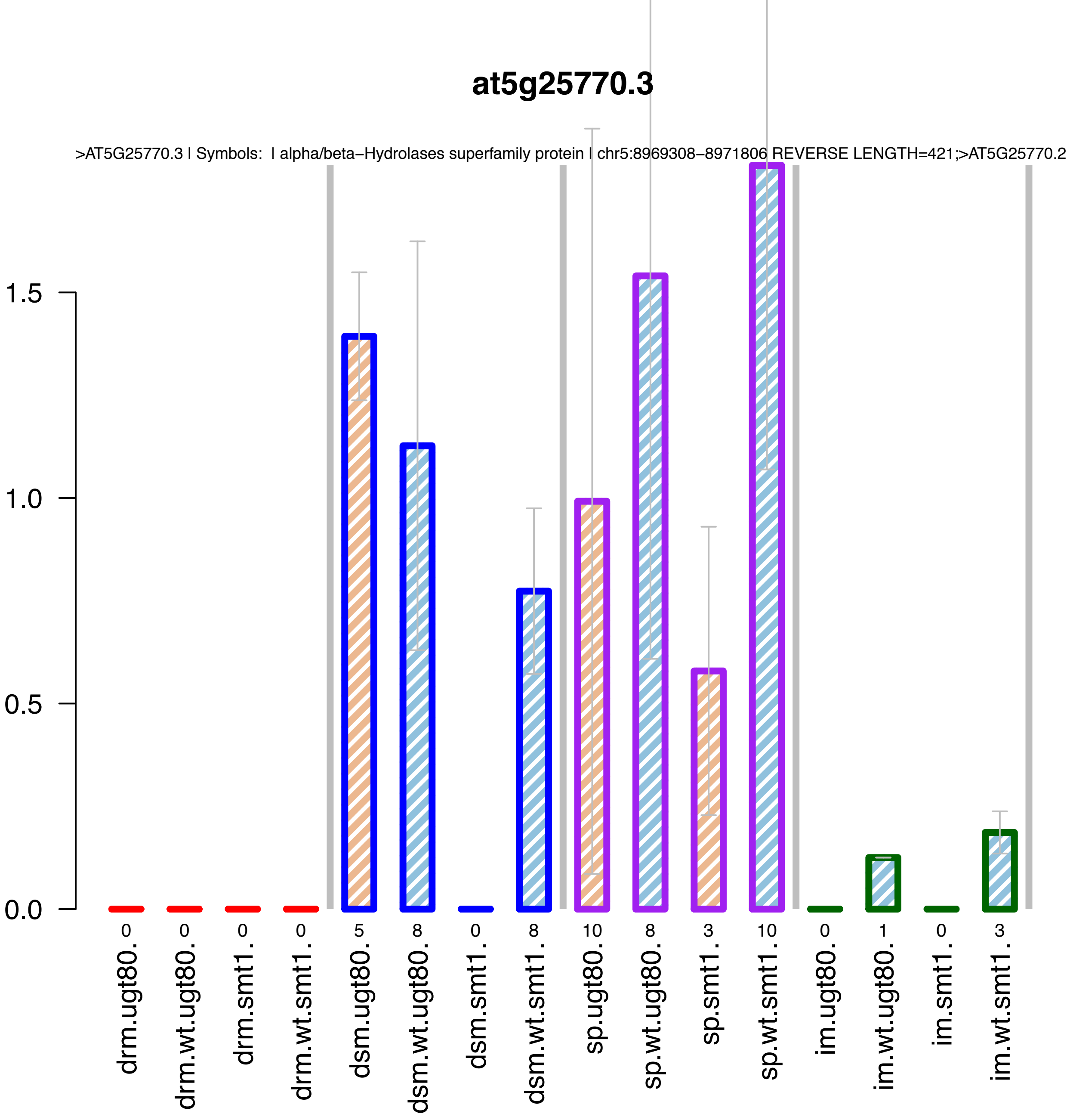

at5g25770.3
>AT5G25770.3 | Symbols: | alpha/beta–Hydrolases superfamily protein | chr5:8969308–8971806 REVERSE LENGTH=421;>AT5G25770.2
0.0
0.5
1.0
1.5
0
0
0
0
5
8
0
8
10
8
3
10
0
1
0
3
drm.ugt80.
drm.wt.ugt80.
drm.smt1.
drm.wt.smt1.
dsm.ugt80.
dsm.wt.ugt80.
dsm.smt1.
dsm.wt.smt1.
sp.ugt80.
sp.wt.ugt80.
sp.smt1.
sp.wt.smt1.
im.ugt80.
im.wt.ugt80.
im.smt1.
im.wt.smt1.

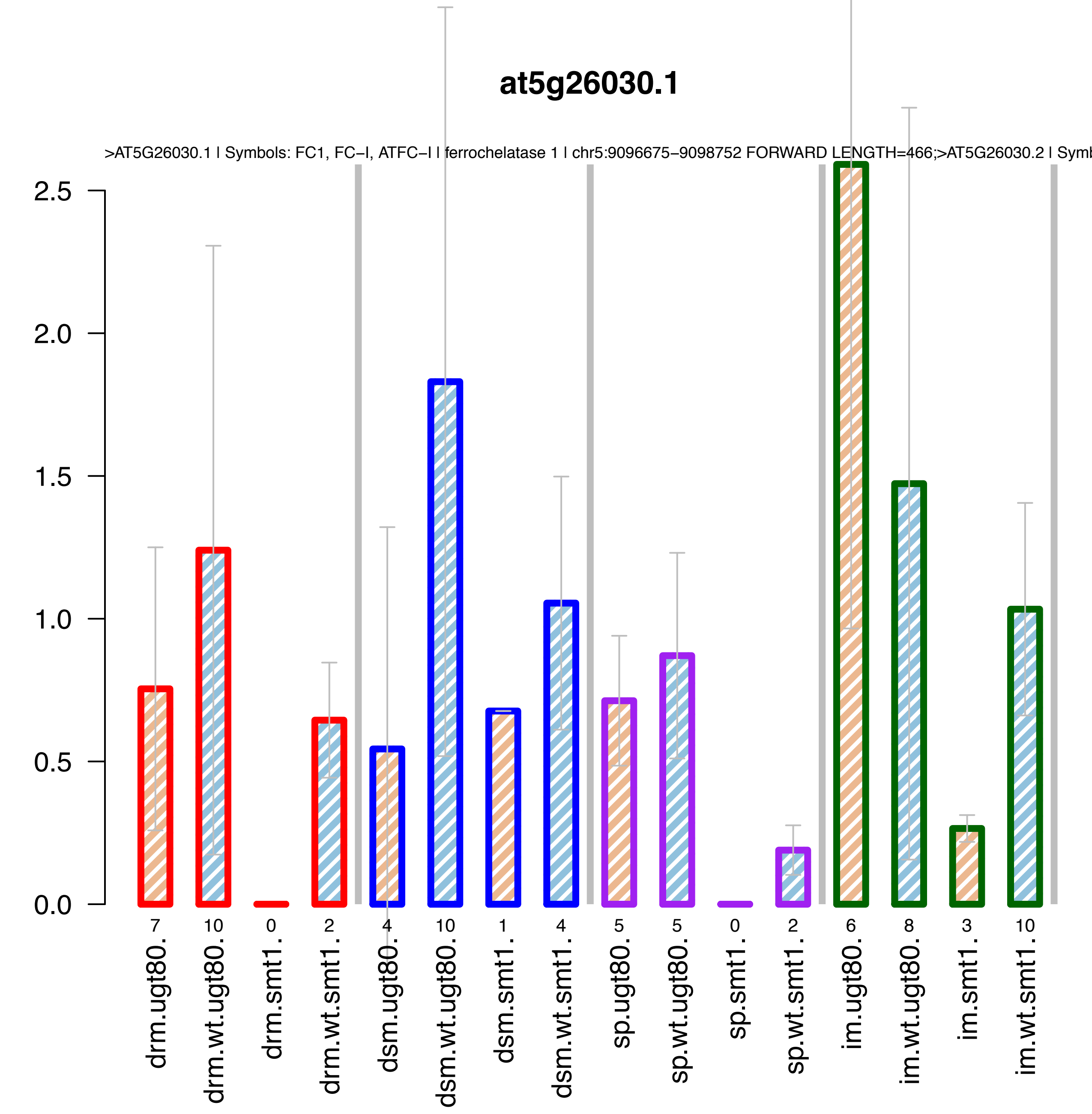

at5g26030.1
>AT5G26030.1 | Symbols: FC1, FC−I, ATFC−I | ferrochelatase 1 | chr5:9096675−9098752 FORWARD LENGTH=466;>AT5G26030.2 | Symb
0.0
0.5
1.0
1.5
2.0
2.5
7
10
0
2
4
10
1
4
5
5
0
2
6
8
3
10
drm.ugt80.
drm.wt.ugt80.
drm.smt1.
drm.wt.smt1.
dsm.ugt80.
dsm.wt.ugt80.
dsm.smt1.
dsm.wt.smt1.
sp.ugt80.
sp.wt.ugt80.
sp.smt1.
sp.wt.smt1.
im.ugt80.
im.wt.ugt80.
im.smt1.
im.wt.smt1.

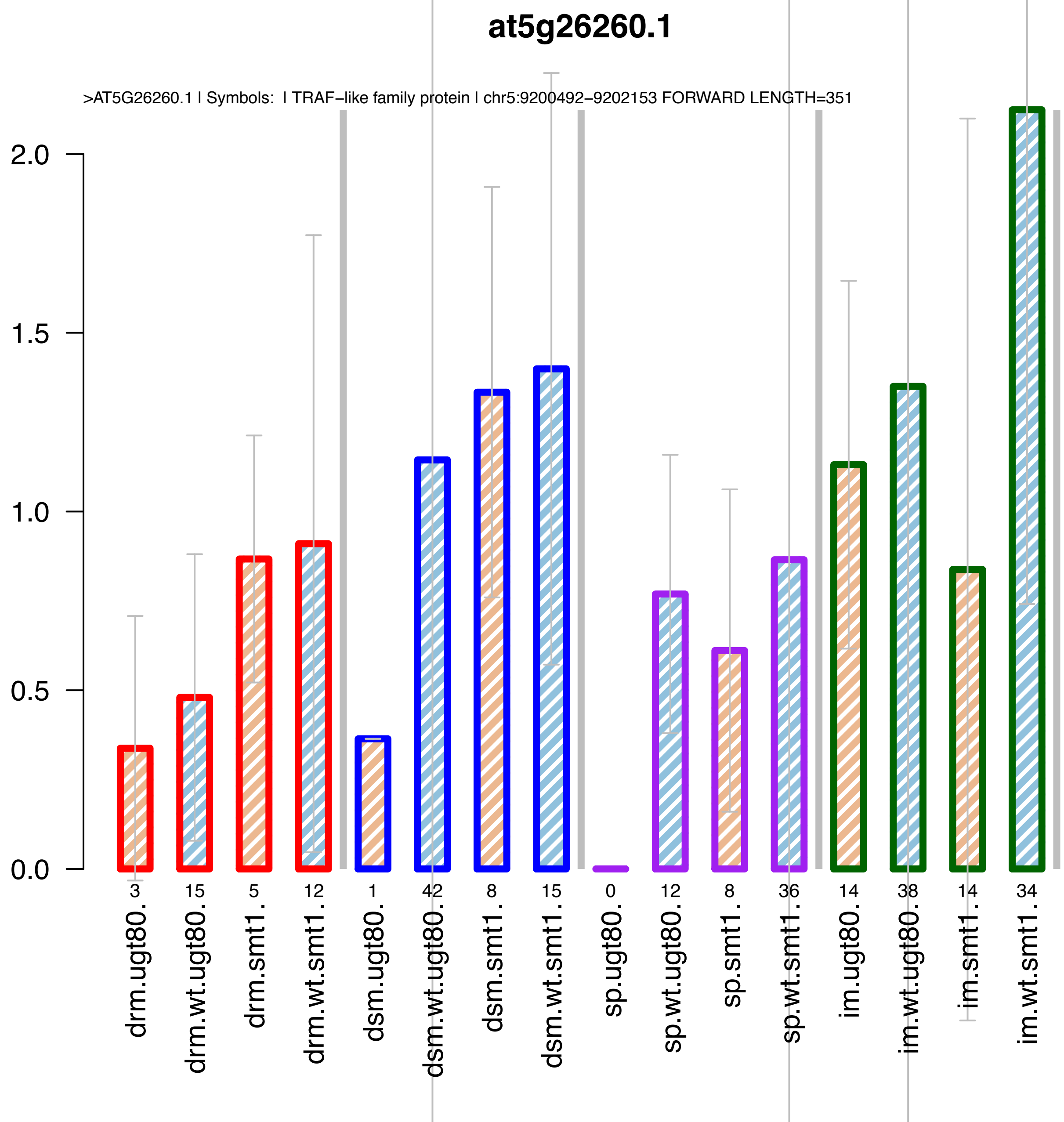

at5g26260.1
>AT5G26260.1 | Symbols: | TRAF−like family protein | chr5:9200492−9202153 FORWARD LENGTH=351
2.0
1.5
1.0
0.5
0.0
3
15
5
12
1
42
8
15
0
12
8
36
14
38
14
34
drm.ugt80..
drm.wt.ugt80..
drm.smt1..
drm.wt.smt1..
dsm.ugt80..
dsm.wt.ugt80..
dsm.smt1..
dsm.wt.smt1..
sp.ugt80..
sp.wt.ugt80..
sp.smt1..
sp.wt.smt1..
im.ugt80..
im.wt.ugt80..
im.smt1..
im.wt.smt1..

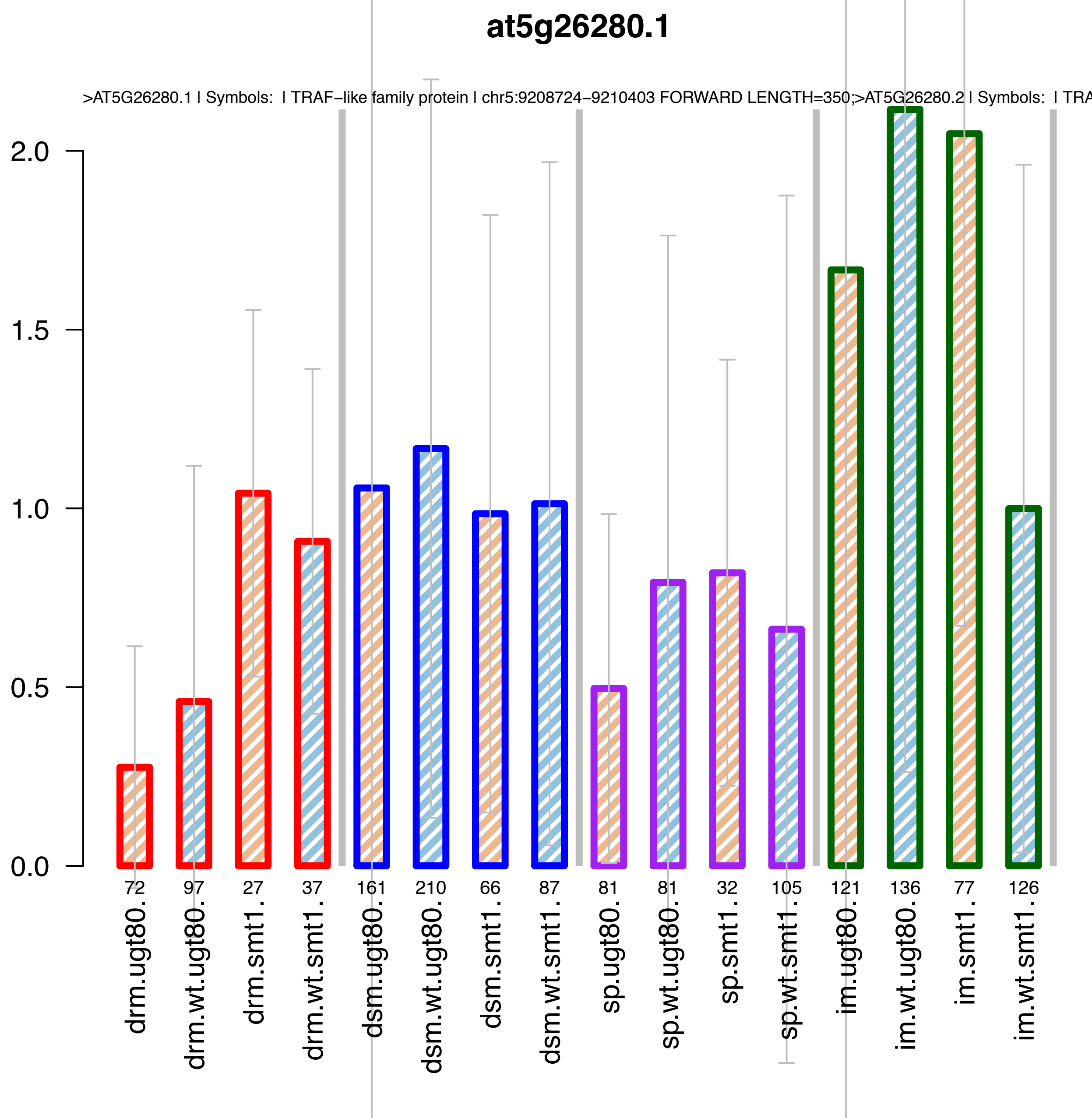

at5g26280.1
>AT5G26280.1 | Symbols: | TRAF−like family protein | chr5:9208724−9210403 FORWARD LENGTH=350;>AT5G26280.2 | Symbols: | TRA
2.0
1.5
1.0
0.5
0.0
72
97
27
37
161
210
66
87
81
81
32
105
121
136
77
126
drm.ugt80.
drm.wt.ugt80.
drm.smt1.
drm.wt.smt1.
dsm.ugt80.
dsm.wt.ugt80.
dsm.smt1.
dsm.wt.smt1.
sp.ugt80.
sp.wt.ugt80.
sp.smt1.
sp.wt.smt1.
im.ugt80.
im.wt.ugt80.
im.smt1.
im.wt.smt1.

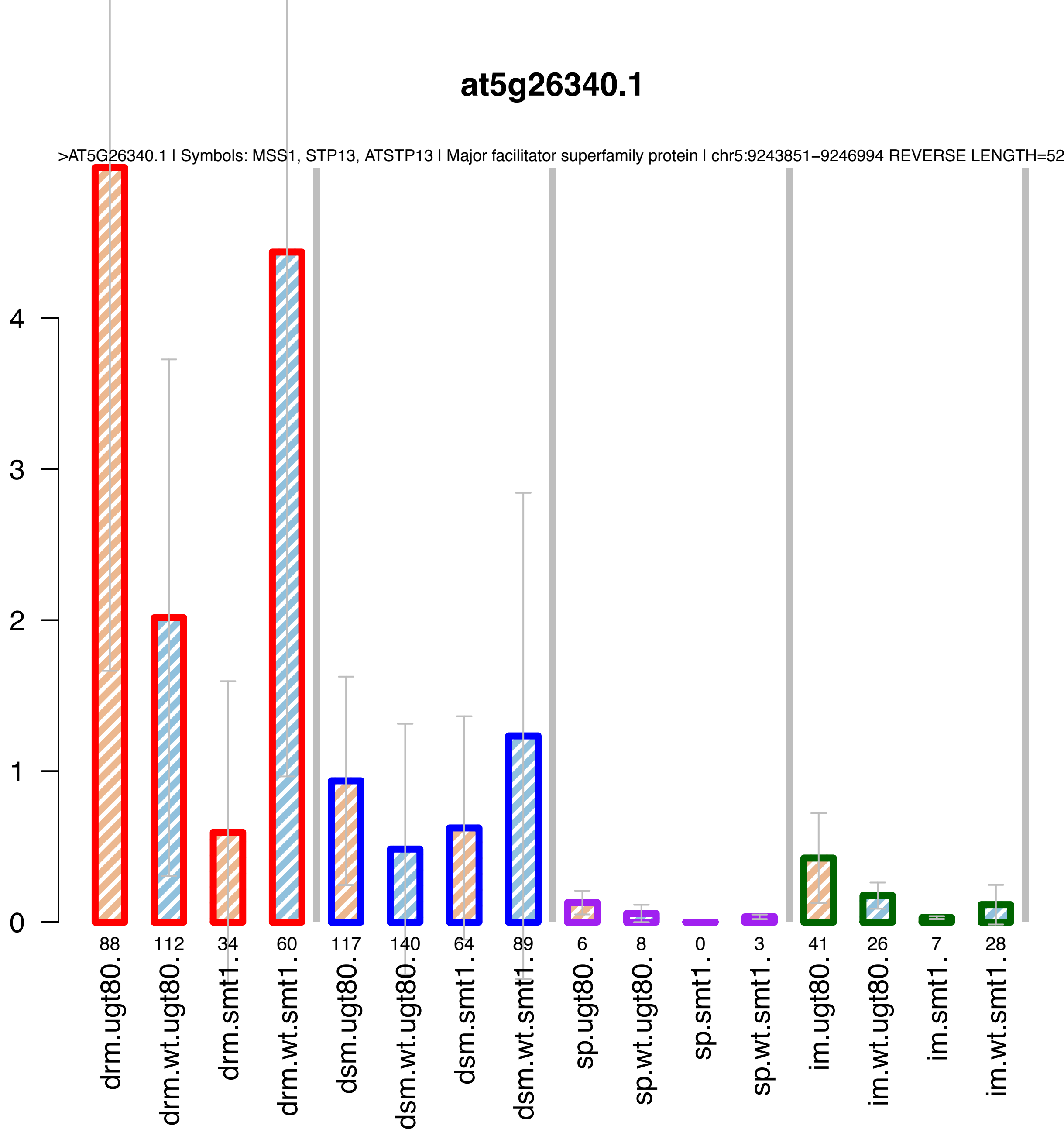

at5g26340.1
>AT5G26340.1 | Symbols: MSS1, STP13, ATSTP13 | Major facilitator superfamily protein | chr5:9243851-9246994 REVERSE LENGTH=52
0
1
2
3
4
88
112
34
60
117
140
64
89
6
8
0
3
41
26
7
28
drm.ugt80.
drm.wt.ugt80.
drm.smt1.
drm.wt.smt1.
dsm.ugt80.
dsm.wt.ugt80.
dsm.smt1.
dsm.wt.smt1.
sp.ugt80.
sp.wt.ugt80.
sp.smt1.
sp.wt.smt1.
im.ugt80.
im.wt.ugt80.
im.smt1.
im.wt.smt1.

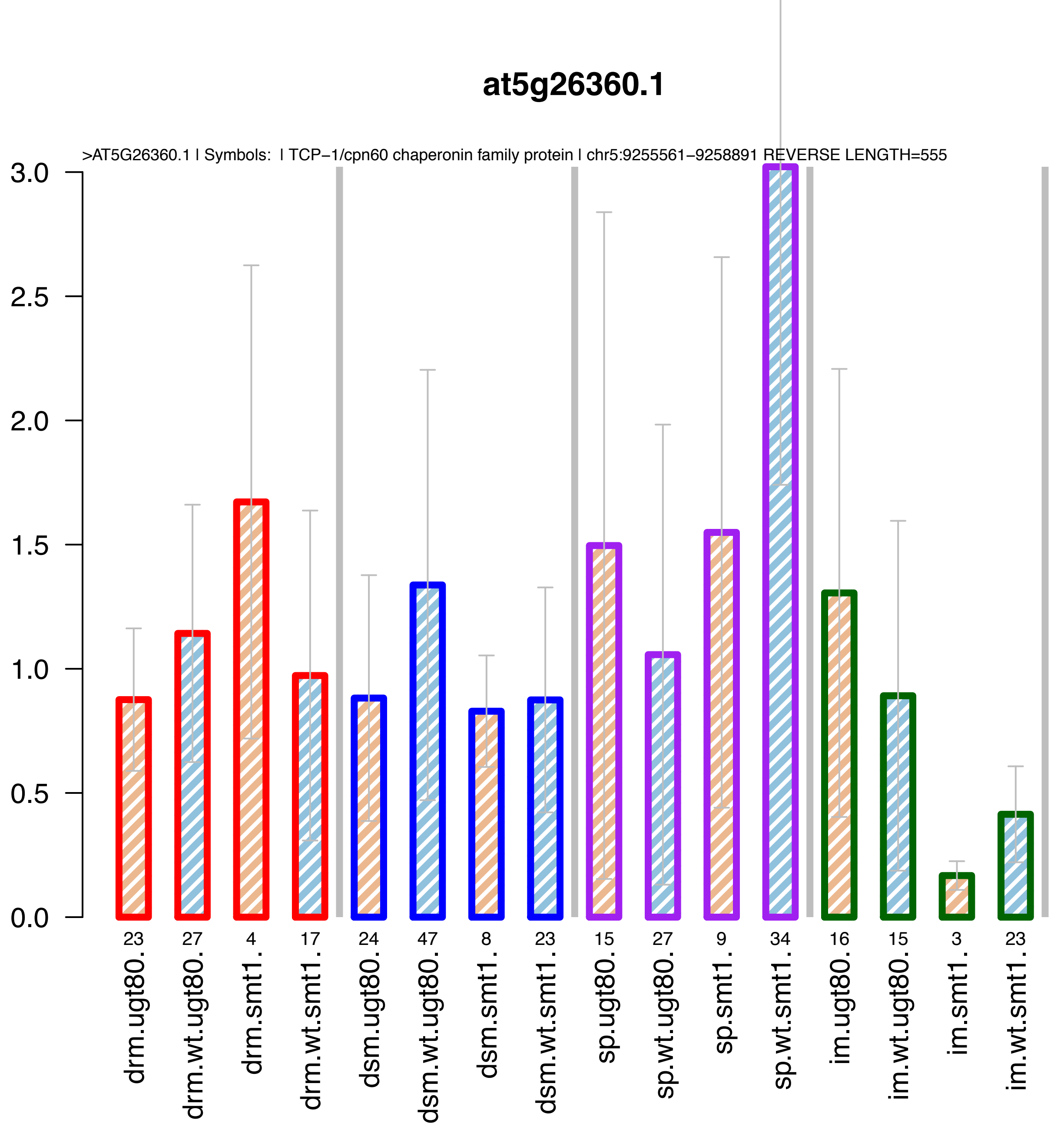

at5g26360.1
>AT5G26360.1 | Symbols: | TCP−1/cpn60 chaperonin family protein | chr5:9255561−9258891 REVERSE LENGTH=555
3.0
2.5
2.0
1.5
1.0
0.5
0.0
23
27
4
17
24
47
8
23
15
27
9
34
16
15
3
23
drm.ugt80..
drm.wt.ugt80..
drm.smt1..
drm.wt.smt1..
dsm.ugt80..
dsm.wt.ugt80..
dsm.smt1..
dsm.wt.smt1..
sp.ugt80..
sp.wt.ugt80..
sp.smt1..
sp.wt.smt1..
im.ugt80..
im.wt.ugt80..
im.smt1..
im.wt.smt1..

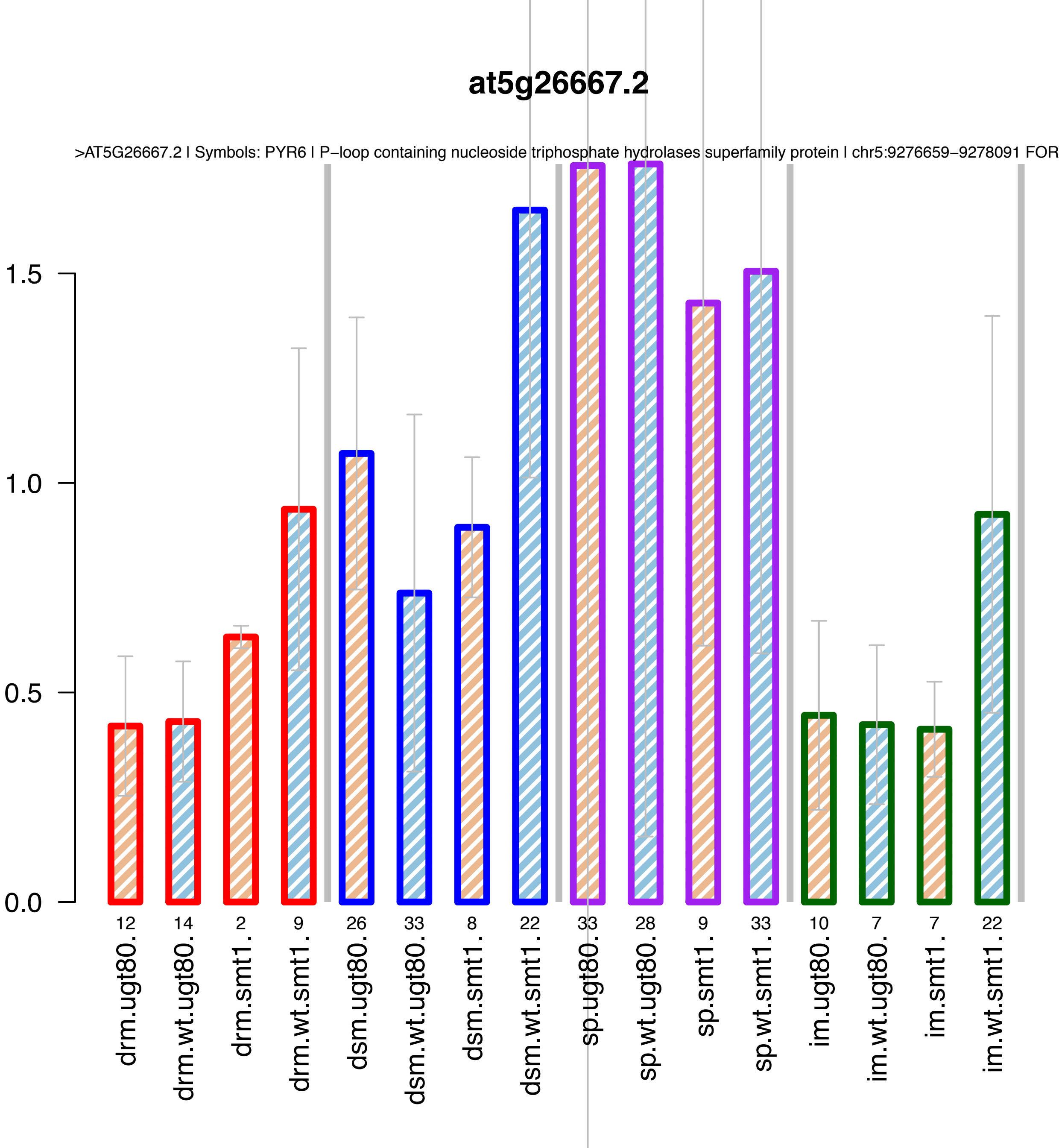

at5g26667.2
>AT5G26667.2 | Symbols: PYR6 | P−loop containing nucleoside triphosphate hydrolases superfamily protein | chr5:9276659−9278091 FOR
0.0
0.5
1.0
1.5
12
14
2
9
26
33
8
22
33
28
9
33
10
7
7
22
drm.ugt80..
drm.wt.ugt80..
drm.smt1..
drm.wt.smt1..
dsm.ugt80..
dsm.wt.ugt80..
dsm.smt1..
dsm.wt.smt1..
sp.ugt80..
sp.wt.ugt80..
sp.smt1..
sp.wt.smt1..
im.ugt80..
im.wt.ugt80..
im.smt1..
im.wt.smt1..

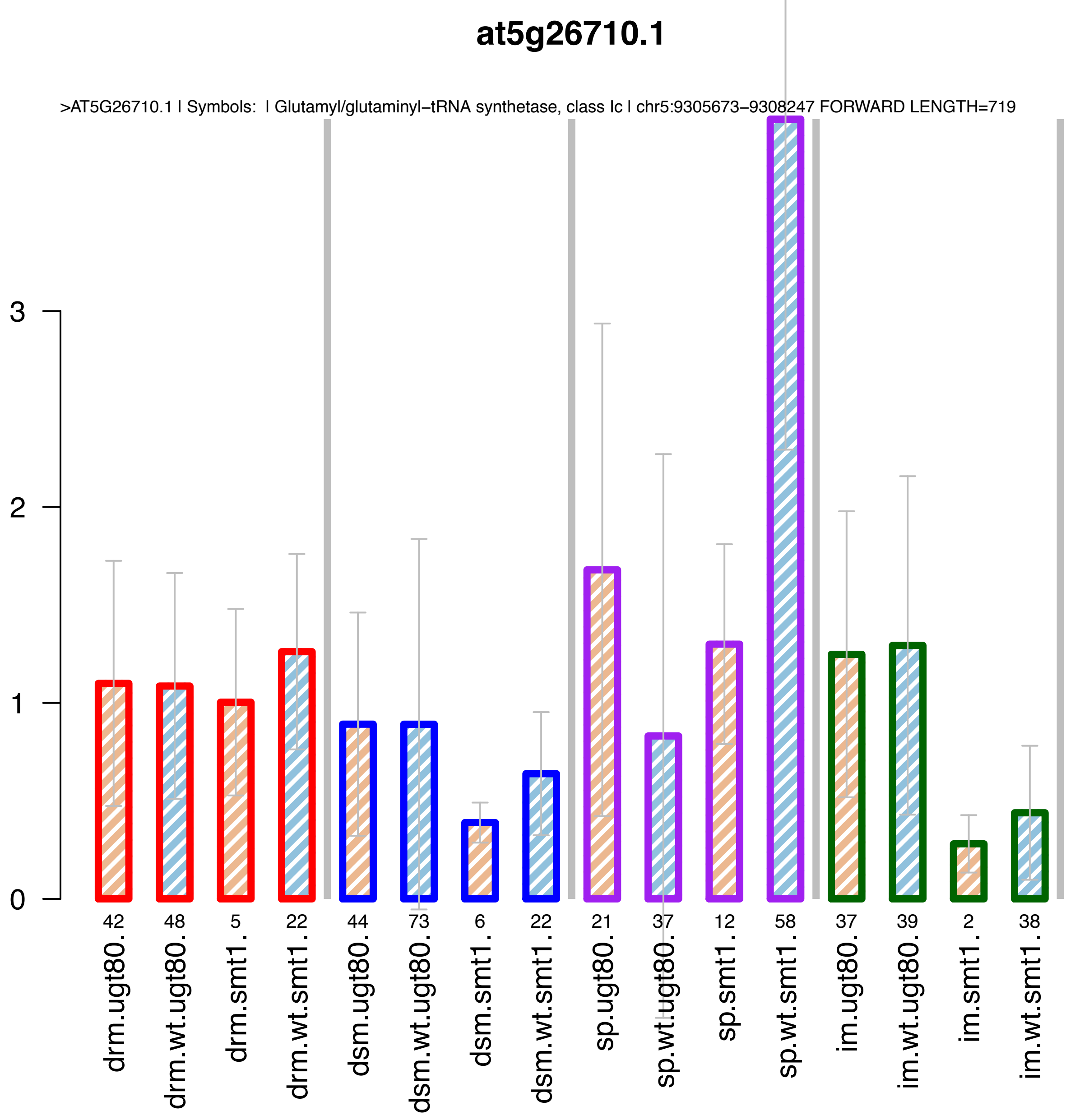

at5g26710.1
>AT5G26710.1 | Symbols: | Glutamyl/glutaminyl−tRNA synthetase, class Ic | chr5:9305673−9308247 FORWARD LENGTH=719
0
1
2
3
42
48
5
22
44
73
6
22
21
37
12
58
37
39
2
38
drm.ugt80..
drm.wt.ugt80..
drm.smt1..
drm.wt.smt1..
dsm.ugt80..
dsm.wt.ugt80..
dsm.smt1..
dsm.wt.smt1..
sp.ugt80..
sp.wt.ugt80..
sp.smt1..
sp.wt.smt1..
im.ugt80..
im.wt.ugt80..
im.smt1..
im.wt.smt1..

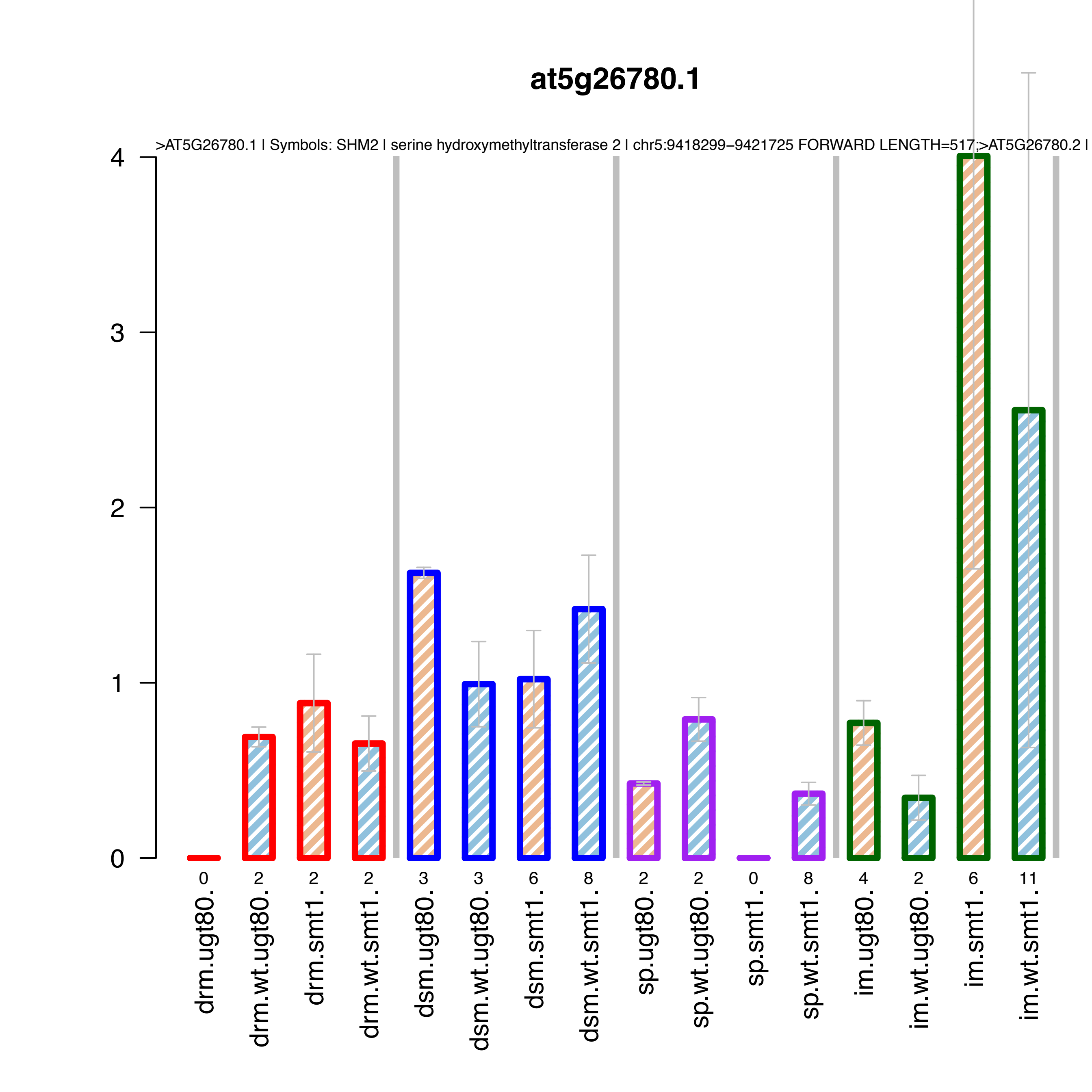
at5g26780.1
>AT5G26780.1 | Symbols: SHM2 | serine hydroxymethyltransferase 2 | chr5:9418299-9421725 FORWARD LENGTH=517;>AT5G26780.2 |
0
1
2
3
4
0
2
2
2
3
3
6
8
2
2
0
8
4
2
6
11
drm.ugt80.
drm.wt.ugt80.
drm.smt1.
drm.wt.smt1.
dsm.ugt80.
dsm.wt.ugt80.
dsm.smt1.
dsm.wt.smt1.
sp.ugt80.
sp.wt.ugt80.
sp.smt1.
sp.wt.smt1.
im.ugt80.
im.wt.ugt80.
im.smt1.
im.wt.smt1.

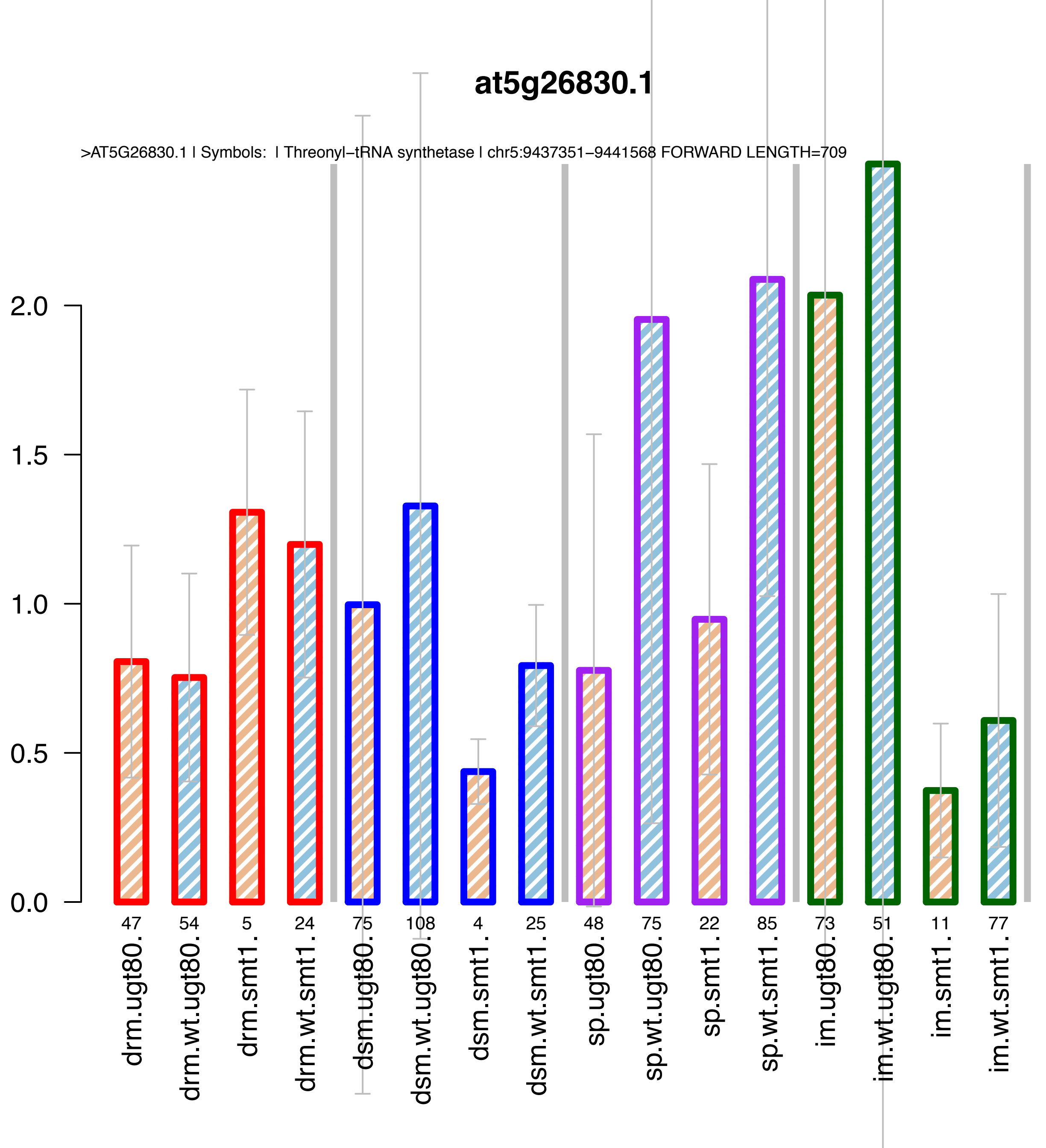

at5g26830.1
>AT5G26830.1 | Symbols: | Threonyl−tRNA synthetase | chr5:9437351−9441568 FORWARD LENGTH=709
2.0
1.5
1.0
0.5
0.0
47
54
5
24
75
108
4
25
48
75
22
85
73
51
11
77
drm.ugt80..
drm.wt.ugt80..
drm.smt1..
drm.wt.smt1..
dsm.ugt80..
dsm.wt.ugt80..
dsm.smt1..
dsm.wt.smt1..
sp.ugt80..
sp.wt.ugt80..
sp.smt1..
sp.wt.smt1..
im.ugt80..
im.wt.ugt80..
im.smt1..
im.wt.smt1..

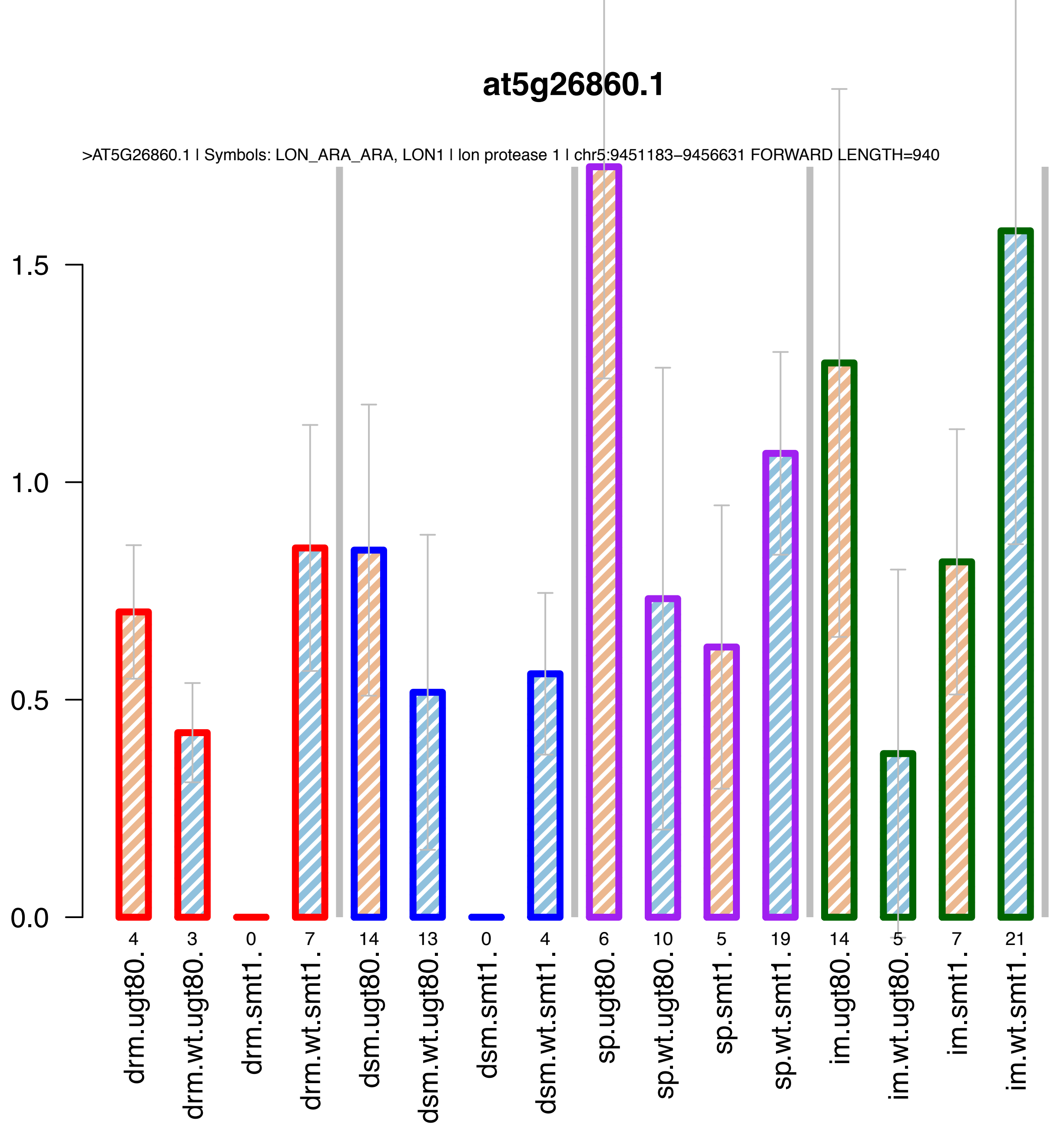

at5g26860.1
>AT5G26860.1 | Symbols: LON_ARA_ARA, LON1 | lon protease 1 | chr5:9451183-9456631 FORWARD LENGTH=940
0.0
0.5
1.0
1.5
4
3
0
7
14
13
0
4
6
10
5
19
14
5
7
21
drm.ugt80..
drm.wt.ugt80..
drm.smt1..
drm.wt.smt1..
dsm.ugt80..
dsm.wt.ugt80..
dsm.smt1..
dsm.wt.smt1..
sp.ugt80..
sp.wt.ugt80..
sp.smt1..
sp.wt.smt1..
im.ugt80..
im.wt.ugt80..
im.smt1..
im.wt.smt1..

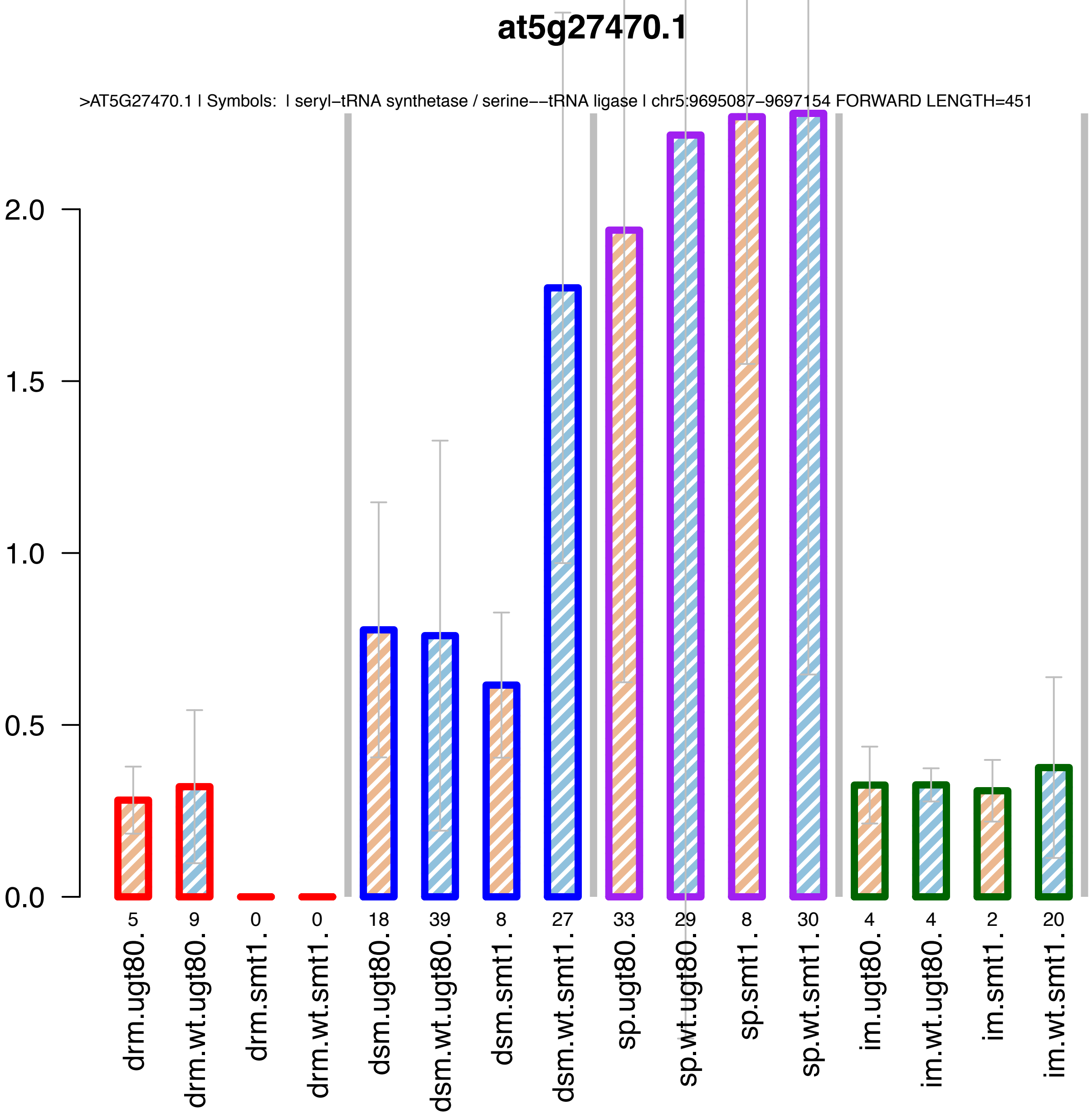

at5g27470.1
>AT5G27470.1 | Symbols: | seryl−tRNA synthetase / serine−−tRNA ligase | chr5:9695087−9697154 FORWARD LENGTH=451
0.0
0.5
1.0
1.5
2.0
5
9
0
0
18
39
8
27
33
29
8
30
4
4
2
20
drm.ugt80.
drm.wt.ugt80.
drm.smt1.
drm.wt.smt1.
dsm.ugt80.
dsm.wt.ugt80.
dsm.smt1.
dsm.wt.smt1.
sp.ugt80.
sp.wt.ugt80.
sp.smt1.
sp.wt.smt1.
im.ugt80.
im.wt.ugt80.
im.smt1.
im.wt.smt1.

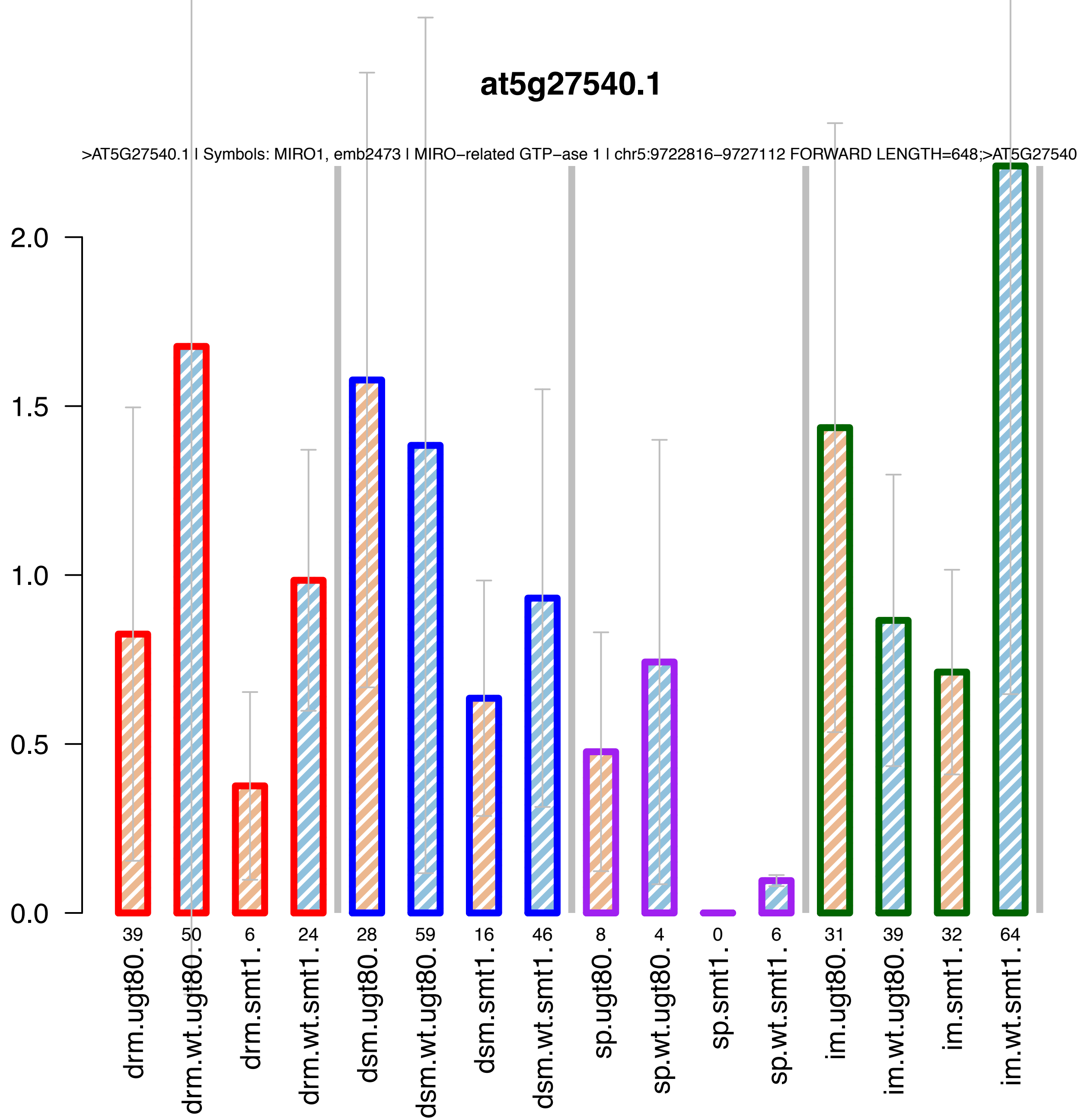
at5g27540.1
>AT5G27540.1 | Symbols: MIRO1, emb2473 | MIRO−related GTP−ase 1 | chr5:9722816−9727112 FORWARD LENGTH=648;>AT5G27540
2.0
1.5
1.0
0.5
0.0
39
50
6
24
28
59
16
46
8
4
0
6
31
39
32
64
drm.ugt80.
drm.wt.ugt80.
drm.smt1.
drm.wt.smt1.
dsm.ugt80.
dsm.wt.ugt80.
dsm.smt1.
dsm.wt.smt1.
sp.ugt80.
sp.wt.ugt80.
sp.smt1.
sp.wt.smt1.
im.ugt80.
im.wt.ugt80.
im.smt1.
im.wt.smt1.

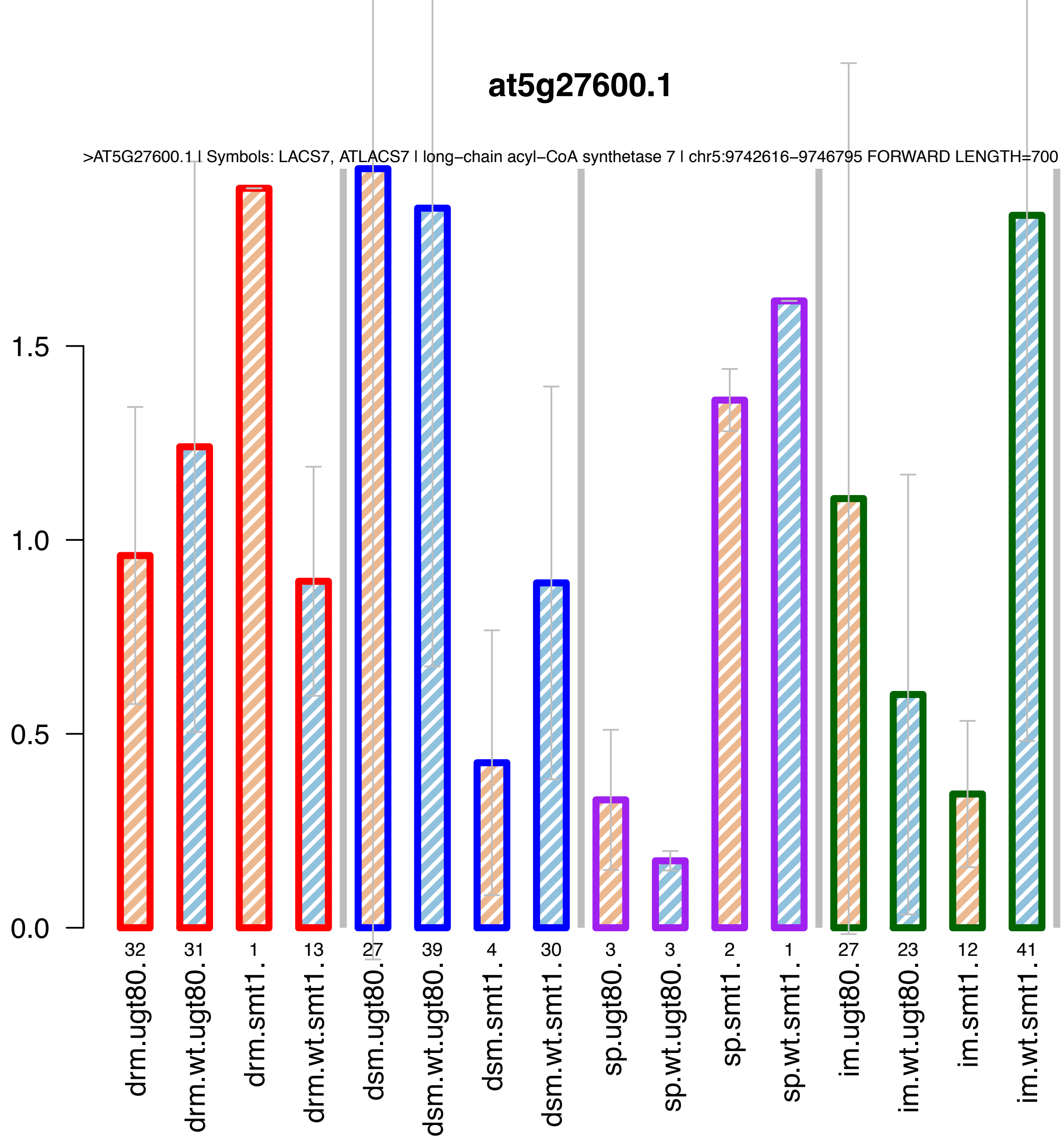

at5g27600.1
>AT5G27600.1 | Symbols: LACS7, ATLACS7 | long-chain acyl-CoA synthetase 7 | chr5:9742616-9746795 FORWARD LENGTH=700
0.0
0.5
1.0
1.5
32
31
1
13
27
39
4
30
3
3
2
1
27
23
12
41
drm.ugt80.
drm.wt.ugt80.
drm.smt1.
drm.wt.smt1.
dsm.ugt80.
dsm.wt.ugt80.
dsm.smt1.
dsm.wt.smt1.
sp.ugt80.
sp.wt.ugt80.
sp.smt1.
sp.wt.smt1.
im.ugt80.
im.wt.ugt80.
im.smt1.
im.wt.smt1.

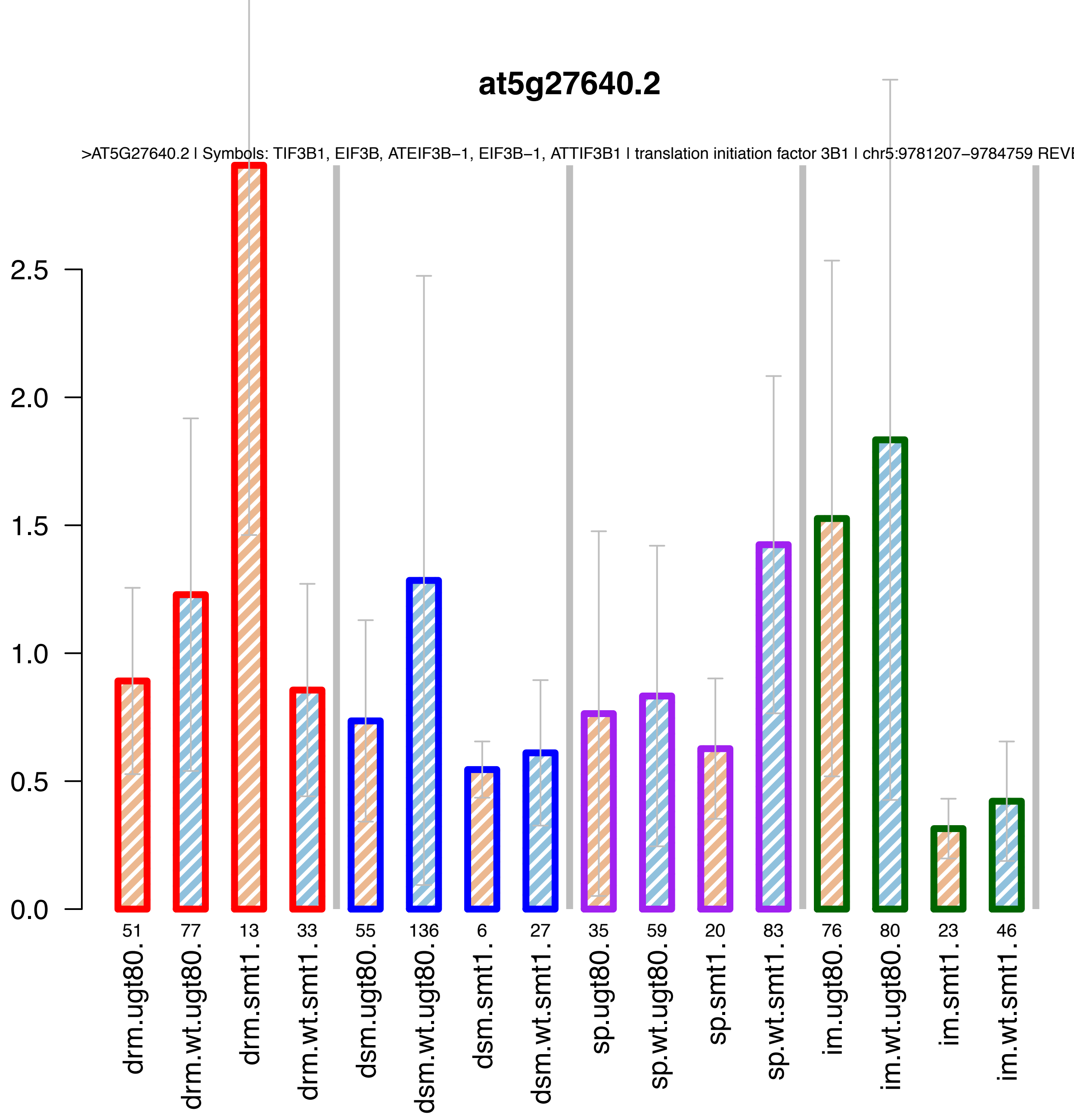

at5g27640.2
>AT5G27640.2 | Symbols: TIF3B1, EIF3B, ATEIF3B-1, EIF3B-1, ATTIF3B1 | translation initiation factor 3B1 | chr5:9781207-9784759 REVE
0.0
0.5
1.0
1.5
2.0
2.5
51
77
13
33
55
136
6
27
35
59
20
83
76
80
23
46
drm.ugt80..
drm.wt.ugt80..
drm.smt1..
drm.wt.smt1..
dsm.ugt80..
dsm.wt.ugt80..
dsm.smt1..
dsm.wt.smt1..
sp.ugt80..
sp.wt.ugt80..
sp.smt1..
sp.wt.smt1..
im.ugt80..
im.wt.ugt80..
im.smt1..
im.wt.smt1..

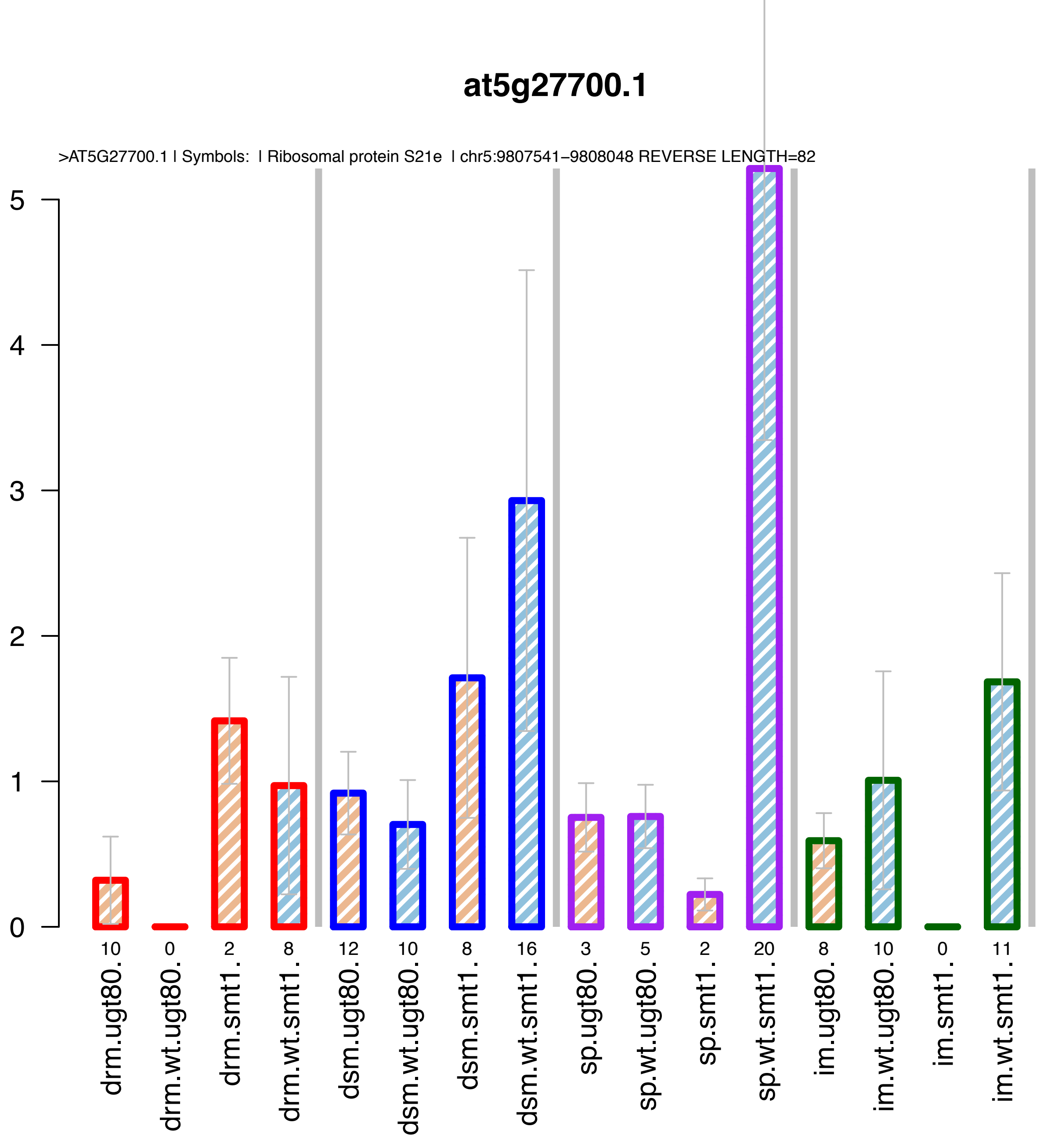

at5g27700.1
>AT5G27700.1 | Symbols: | Ribosomal protein S21e | chr5:9807541-9808048 REVERSE LENGTH=82
0
1
2
3
4
5
10
0
2
8
12
10
8
16
3
5
2
20
8
10
0
11
drm.ugt80.
drm.wt.ugt80.
drm.smt1.
drm.wt.smt1.
dsm.ugt80.
dsm.wt.ugt80.
dsm.smt1.
dsm.wt.smt1.
sp.ugt80.
sp.wt.ugt80.
sp.smt1.
sp.wt.smt1.
im.ugt80.
im.wt.ugt80.
im.smt1.
im.wt.smt1.

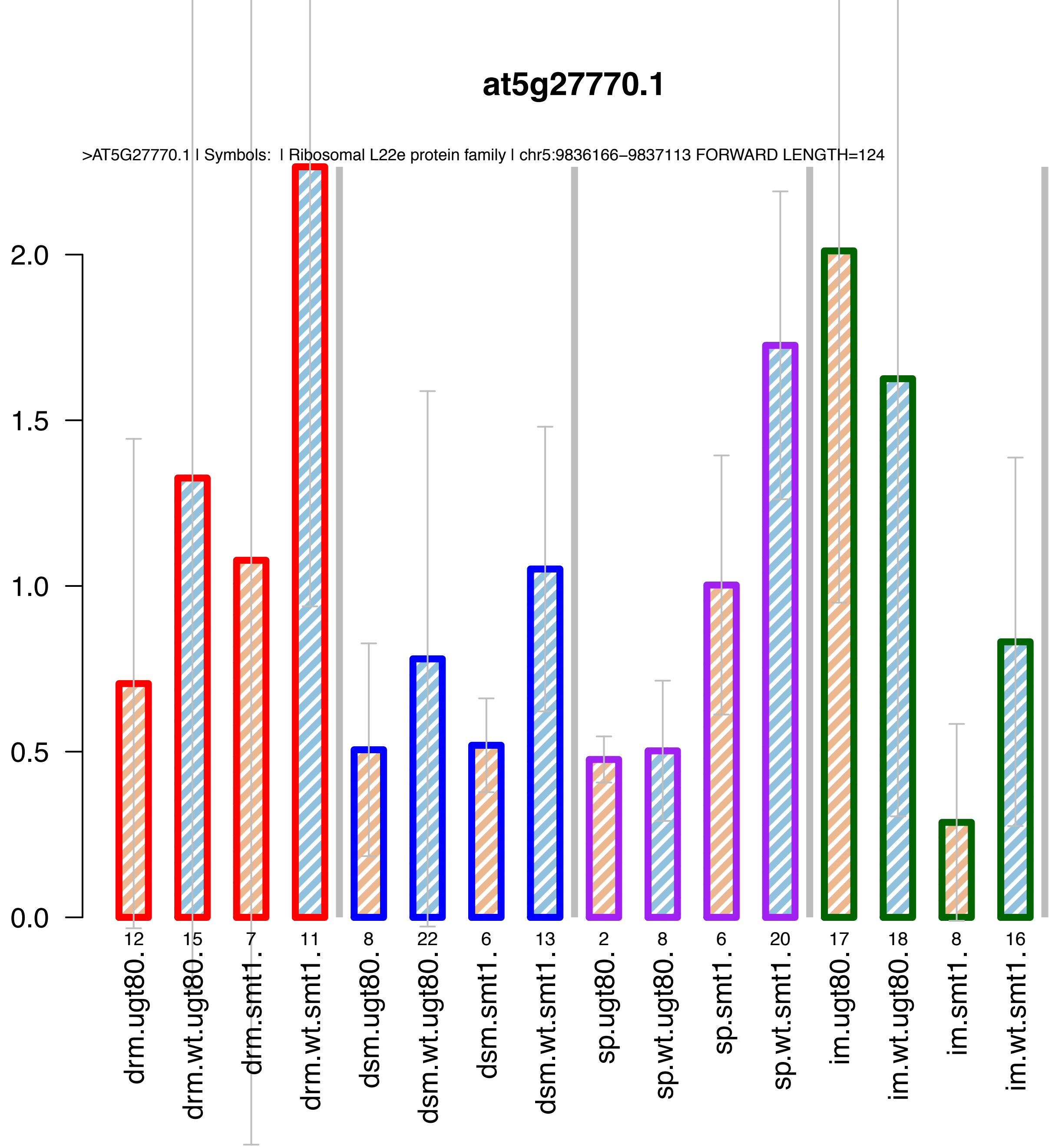

at5g27770.1
>AT5G27770.1 | Symbols: | Ribosomal L22e protein family | chr5:9836166-9837113 FORWARD LENGTH=124
2.0
1.5
1.0
0.5
0.0
12
15
7
11
8
22
6
13
2
8
6
20
17
18
8
16
drm.ugt80.
drm.wt.ugt80.
drm.smt1.
drm.wt.smt1.
dsm.ugt80.
dsm.wt.ugt80.
dsm.smt1.
dsm.wt.smt1.
sp.ugt80.
sp.wt.ugt80.
sp.smt1.
sp.wt.smt1.
im.ugt80.
im.wt.ugt80.
im.smt1.
im.wt.smt1.

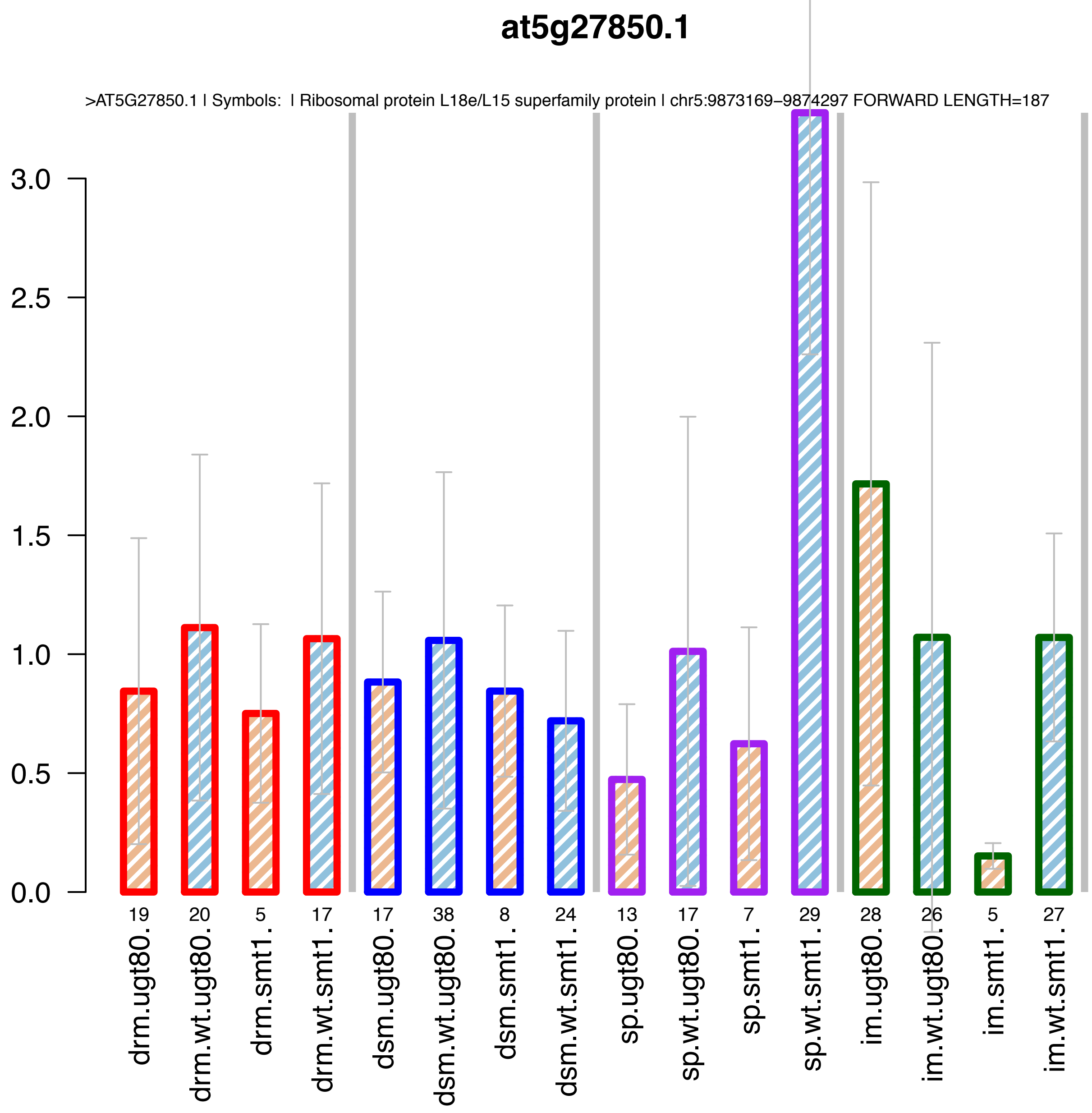

at5g27850.1
>AT5G27850.1 | Symbols: | Ribosomal protein L18e/L15 superfamily protein | chr5:9873169–9874297 FORWARD LENGTH=187
3.0
2.5
2.0
1.5
1.0
0.5
0.0
19
20
5
17
17
38
8
24
13
17
7
29
28
26
5
27
drm.ugt80..
drm.wt.ugt80..
drm.smt1..
drm.wt.smt1..
dsm.ugt80..
dsm.wt.ugt80..
dsm.smt1..
dsm.wt.smt1..
sp.ugt80..
sp.wt.ugt80..
sp.smt1..
sp.wt.smt1..
im.ugt80..
im.wt.ugt80..
im.smt1..
im.wt.smt1..

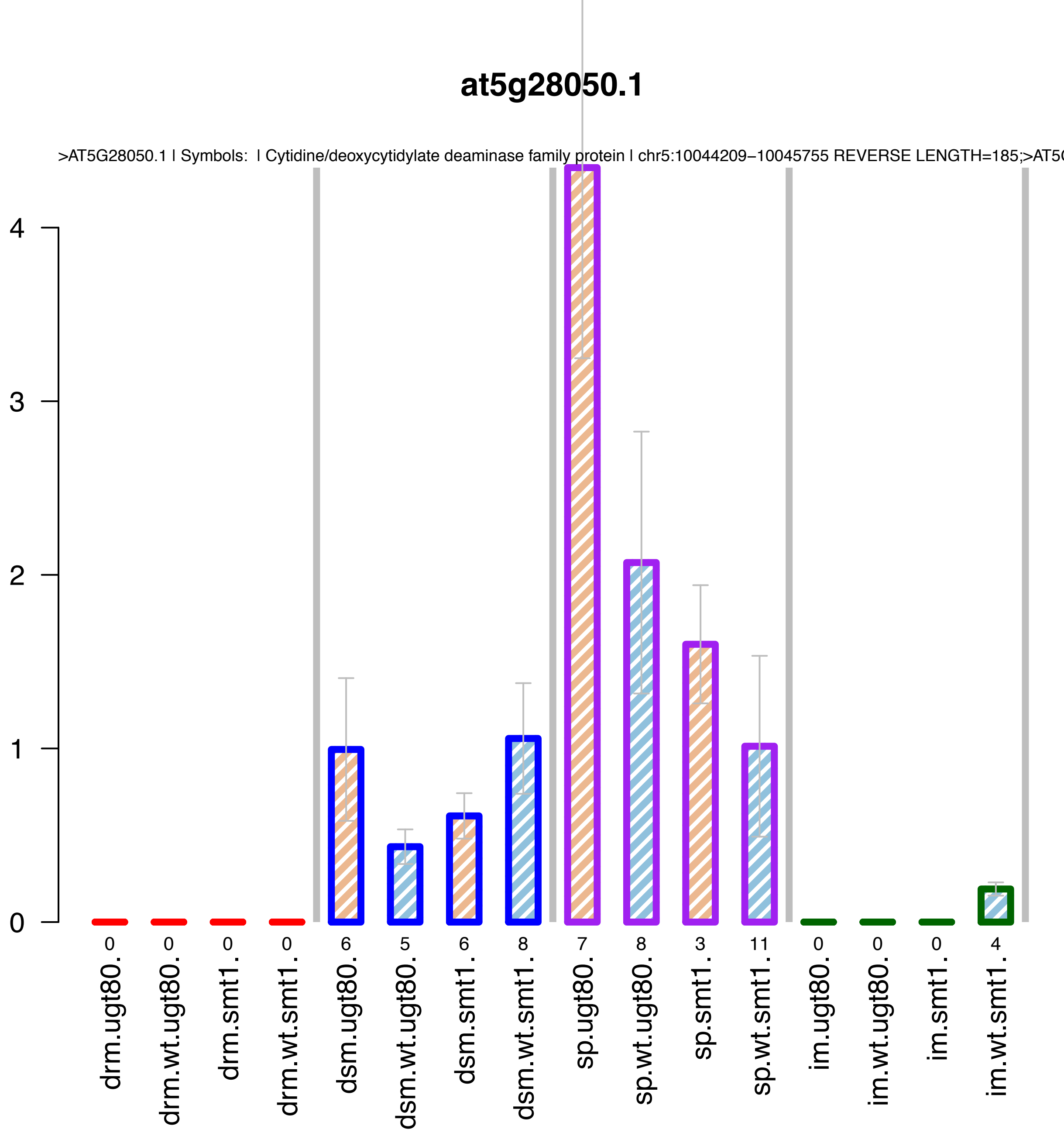

at5g28050.1
>AT5G28050.1 | Symbols: | Cytidine/deoxycytidylate deaminase family protein | chr5:10044209-10045755 REVERSE LENGTH=185;>AT5
0
1
2
3
4
0
0
0
0
6
5
6
8
7
8
3
11
0
0
0
4
drm.ugt80.
drm.wt.ugt80.
drm.smt1.
drm.wt.smt1.
dsm.ugt80.
dsm.wt.ugt80.
dsm.smt1.
dsm.wt.smt1.
sp.ugt80.
sp.wt.ugt80.
sp.smt1.
sp.wt.smt1.
im.ugt80.
im.wt.ugt80.
im.smt1.
im.wt.smt1.

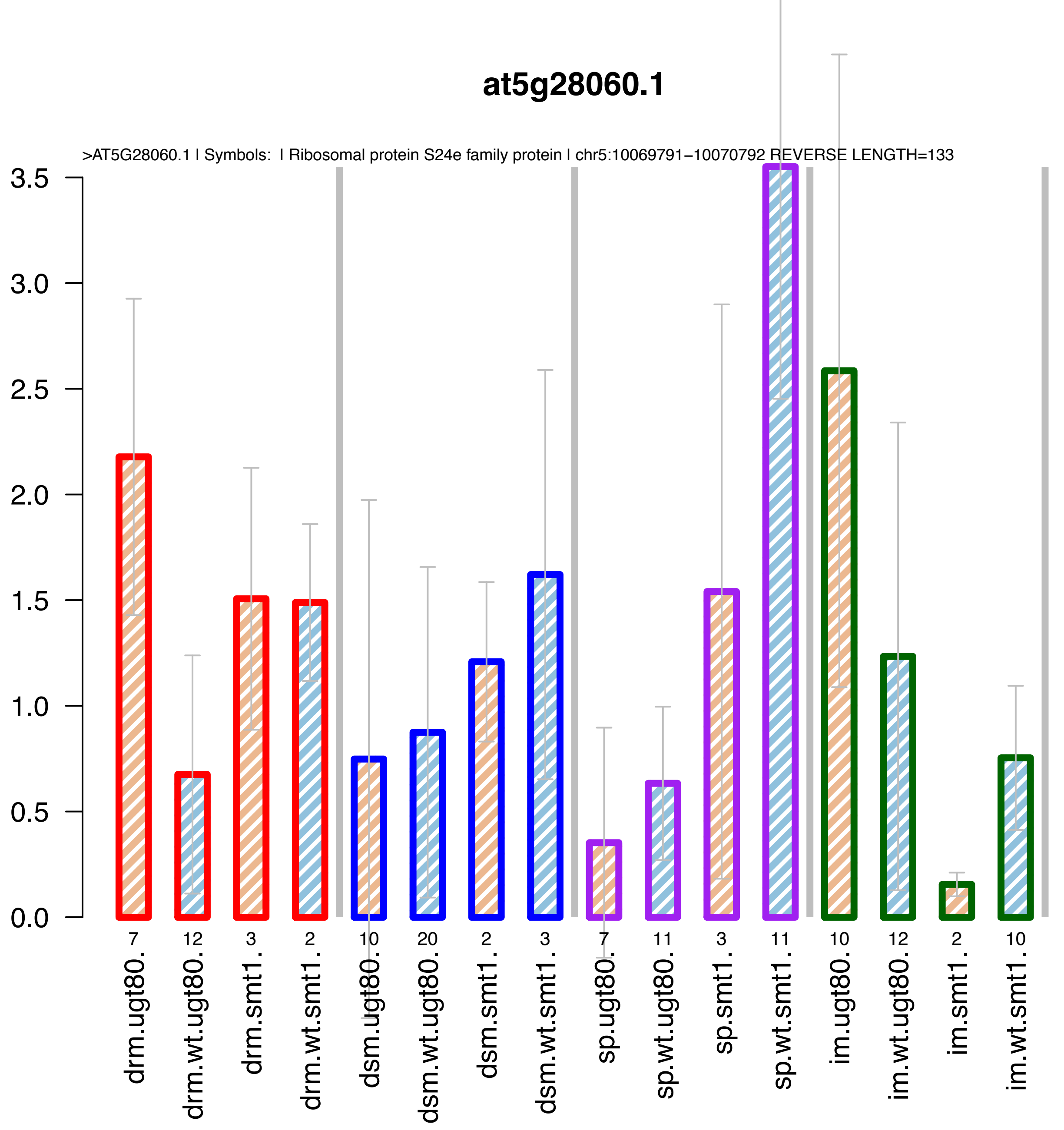
at5g28060.1
>AT5G28060.1 | Symbols: | Ribosomal protein S24e family protein | chr5:10069791-10070792 REVERSE LENGTH=133
0.0
0.5
1.0
1.5
2.0
2.5
3.0
3.5
7
12
3
2
10
20
2
3
7
11
3
11
10
12
2
10
drm.ugt80.
drm.wt.ugt80.
drm.smt1.
drm.wt.smt1.
dsm.ugt80.
dsm.wt.ugt80.
dsm.smt1.
dsm.wt.smt1.
sp.ugt80.
sp.wt.ugt80.
sp.smt1.
sp.wt.smt1.
im.ugt80.
im.wt.ugt80.
im.smt1.
im.wt.smt1.

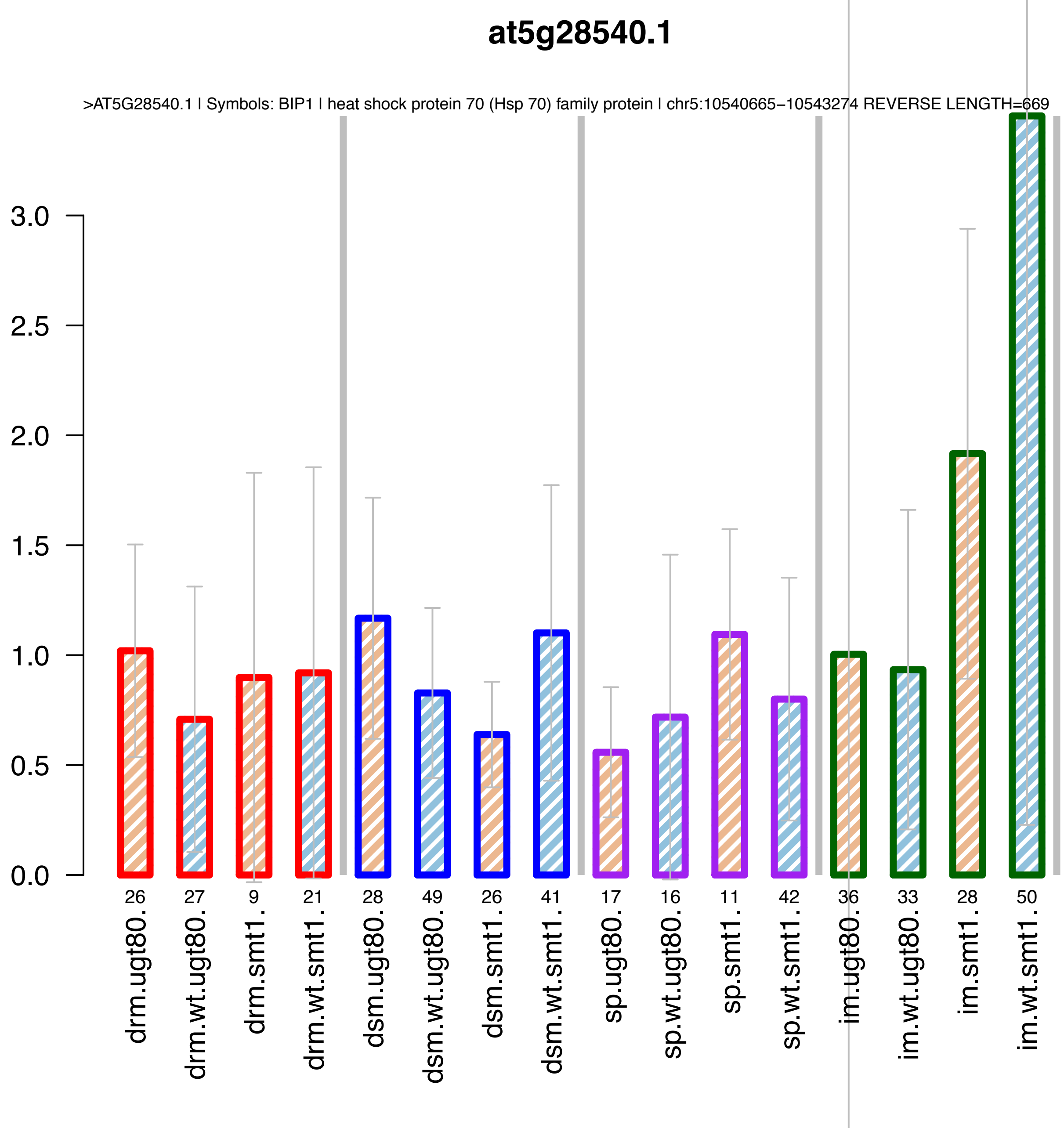
at5g28540.1
>AT5G28540.1 | Symbols: BIP1 | heat shock protein 70 (Hsp 70) family protein | chr5:10540665-10543274 REVERSE LENGTH=669
0.0
0.5
1.0
1.5
2.0
2.5
3.0
26
27
9
21
28
49
26
41
17
16
11
42
36
33
28
50
drm.ugt80..
drm.wt.ugt80..
drm.smt1..
drm.wt.smt1..
dsm.ugt80..
dsm.wt.ugt80..
dsm.smt1..
dsm.wt.smt1..
sp.ugt80..
sp.wt.ugt80..
sp.smt1..
sp.wt.smt1..
im.ugt80..
im.wt.ugt80..
im.smt1..
im.wt.smt1..

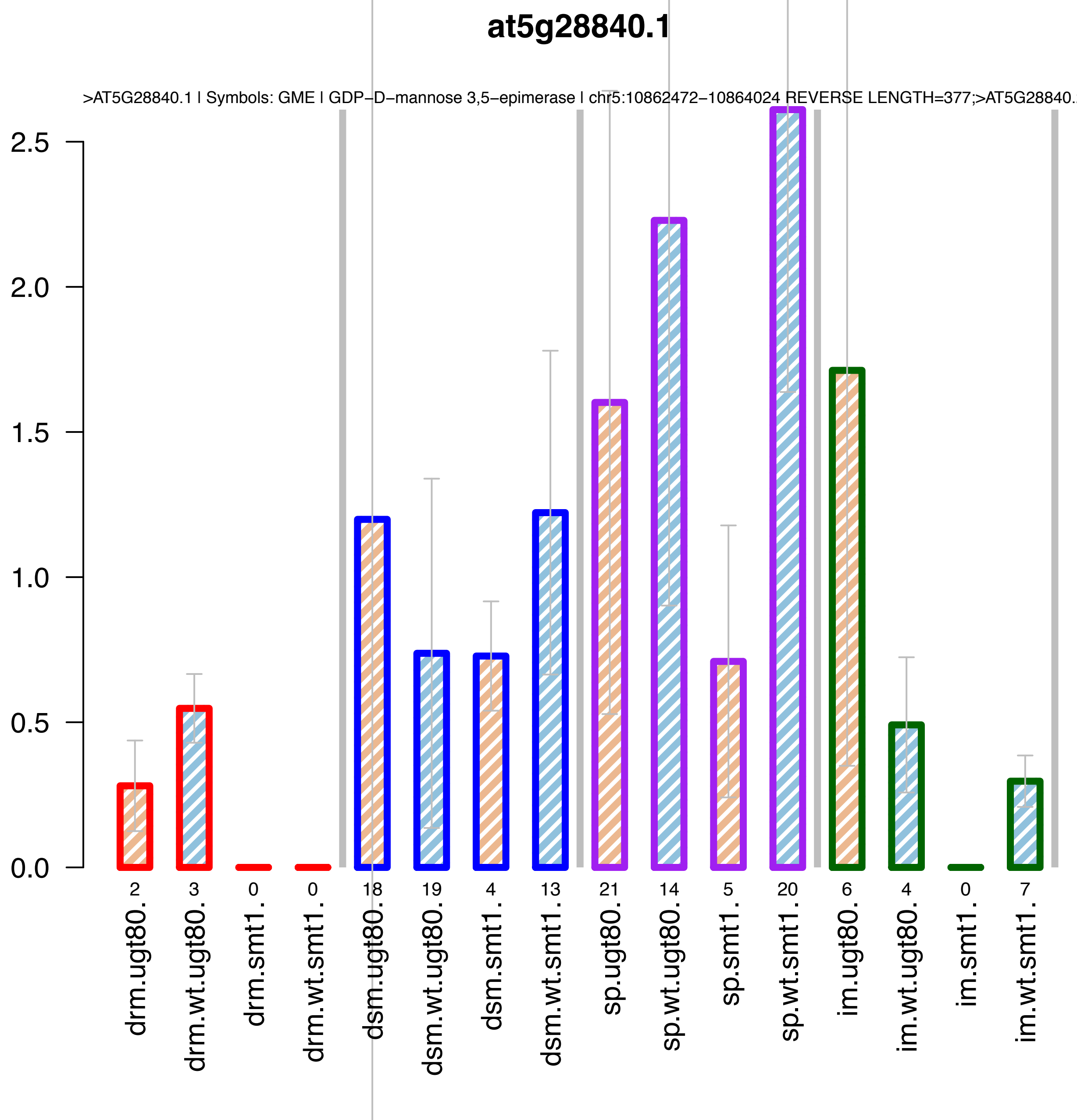

at5g28840.1
>AT5G28840.1 | Symbols: GME | GDP−D−mannose 3,5−epimerase | chr5:10862472−10864024 REVERSE LENGTH=377;>AT5G28840.2 |
0.0
0.5
1.0
1.5
2.0
2.5
2
3
0
0
18
19
4
13
21
14
5
20
6
4
0
7
drm.ugt80..
drm.wt.ugt80..
drm.smt1..
drm.wt.smt1..
dsm.ugt80..
dsm.wt.ugt80..
dsm.smt1..
dsm.wt.smt1..
sp.ugt80..
sp.wt.ugt80..
sp.smt1..
sp.wt.smt1..
im.ugt80..
im.wt.ugt80..
im.smt1..
im.wt.smt1..

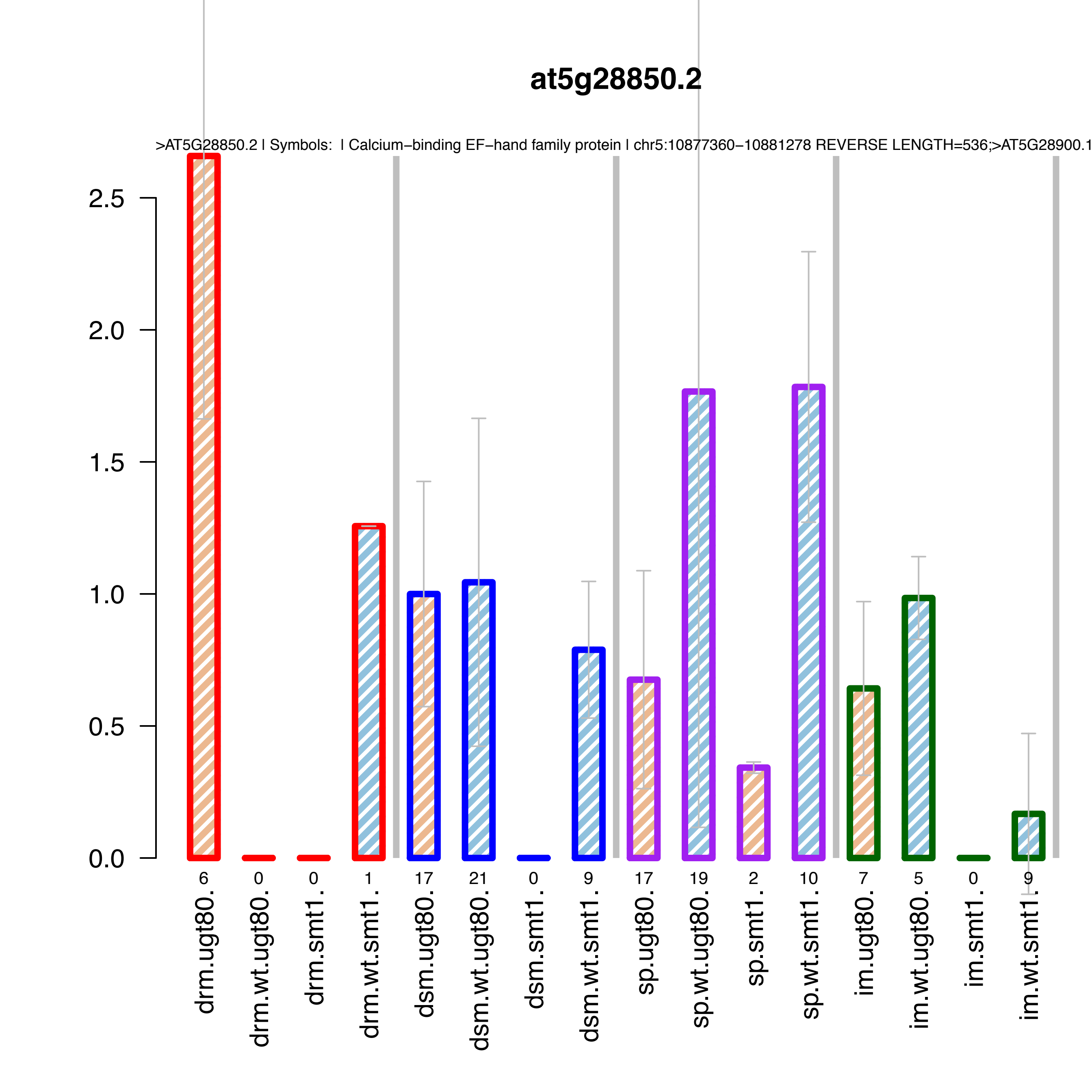
at5g28850.2
>AT5G28850.2 | Symbols: | Calcium−binding EF−hand family protein | chr5:10877360−10881278 REVERSE LENGTH=536;>AT5G28900.1
0.0
0.5
1.0
1.5
2.0
2.5
6
0
0
1
17
21
0
9
17
19
2
10
7
5
0
9
drm.ugt80.
drm.wt.ugt80.
drm.smt1.
drm.wt.smt1.
dsm.ugt80.
dsm.wt.ugt80.
dsm.smt1.
dsm.wt.smt1.
sp.ugt80.
sp.wt.ugt80.
sp.smt1.
sp.wt.smt1.
im.ugt80.
im.wt.ugt80.
im.smt1.
im.wt.smt1.

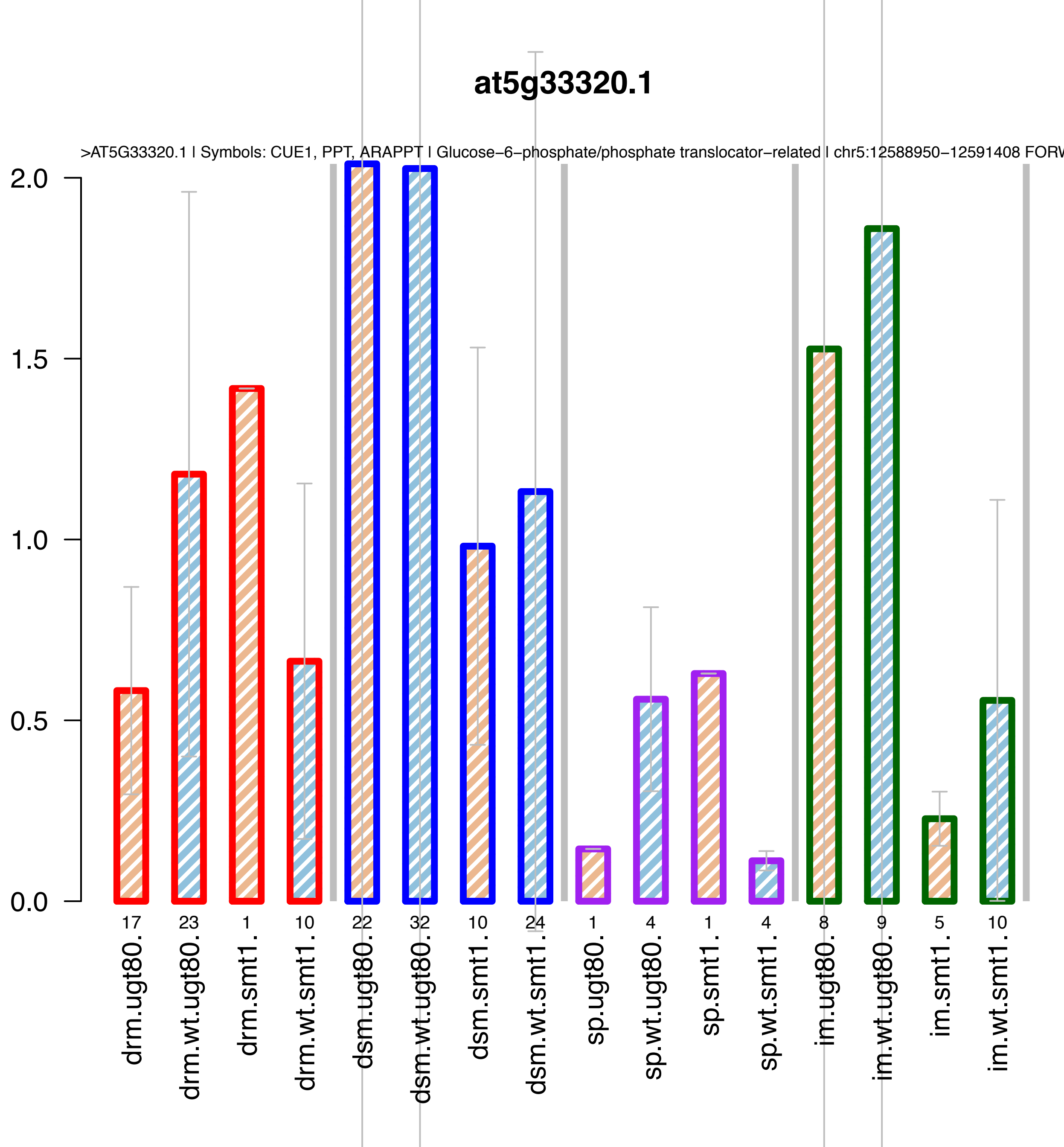

at5g33320.1
>AT5G33320.1 | Symbols: CUE1, PPT, ARAPPT | Glucose−6−phosphate/phosphate translocator−related | chr5:12588950−12591408 FORW
2.0
1.5
1.0
0.5
0.0
17
23
1
10
22
32
10
24
1
4
1
4
8
9
5
10
drm.ugt80.
drm.wt.ugt80.
drm.smt1.
drm.wt.smt1.
dsm.ugt80.
dsm.wt.ugt80.
dsm.smt1.
dsm.wt.smt1.
sp.ugt80.
sp.wt.ugt80.
sp.smt1.
sp.wt.smt1.
im.ugt80.
im.wt.ugt80.
im.smt1.
im.wt.smt1.

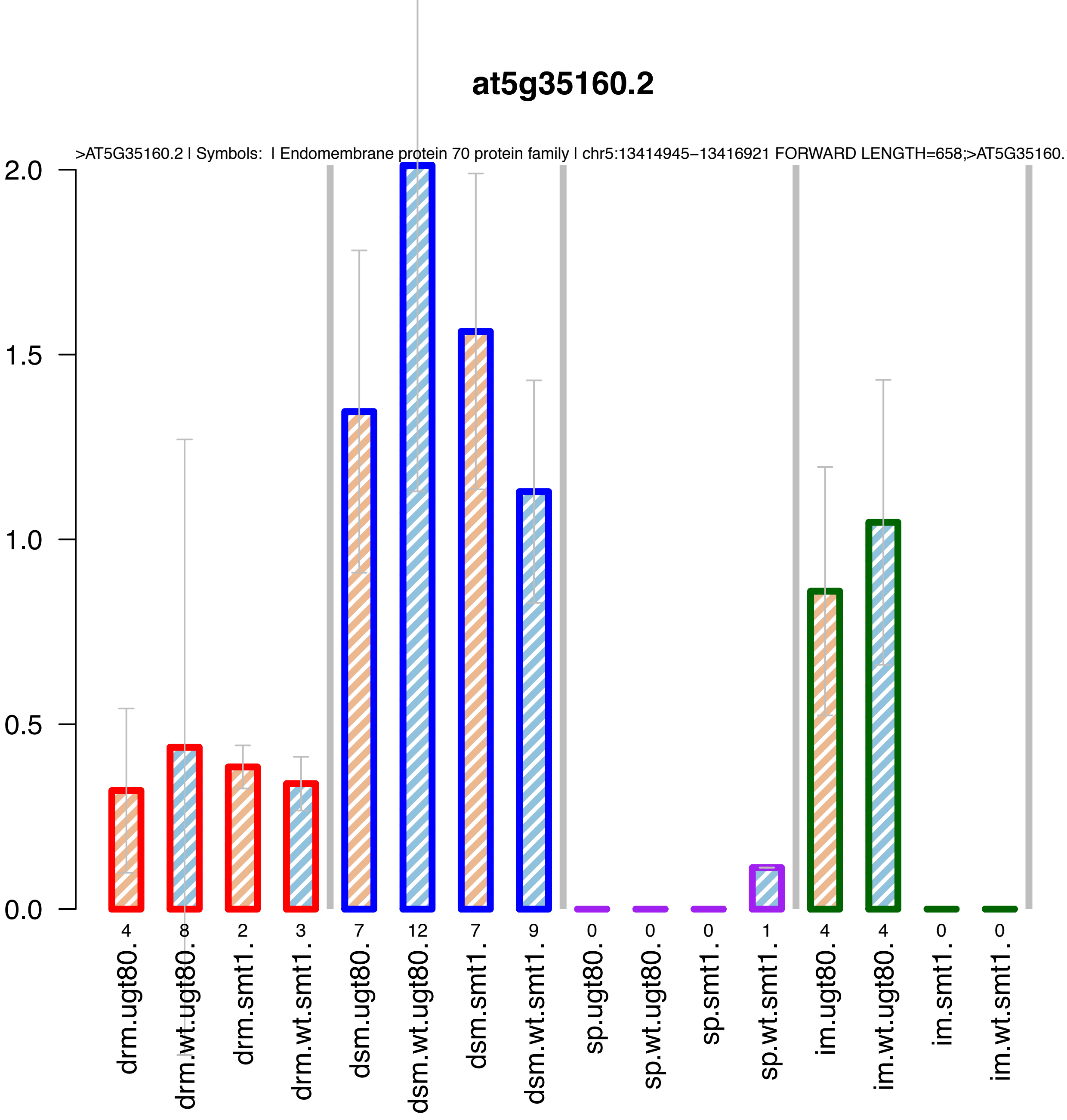

at5g35160.2
>AT5G35160.2 | Symbols: | Endomembrane protein 70 protein family | chr5:13414945-13416921 FORWARD LENGTH=658;>AT5G35160.
2.0
1.5
1.0
0.5
0.0
4
8
2
3
7
12
7
9
0
0
0
1
4
4
0
0
drm.ugt80.
drm.wt.ugt80.
drm.smt1.
drm.wt.smt1.
dsm.ugt80.
dsm.wt.ugt80.
dsm.smt1.
dsm.wt.smt1.
sp.ugt80.
sp.wt.ugt80.
sp.smt1.
sp.wt.smt1.
im.ugt80.
im.wt.ugt80.
im.smt1.
im.wt.smt1.

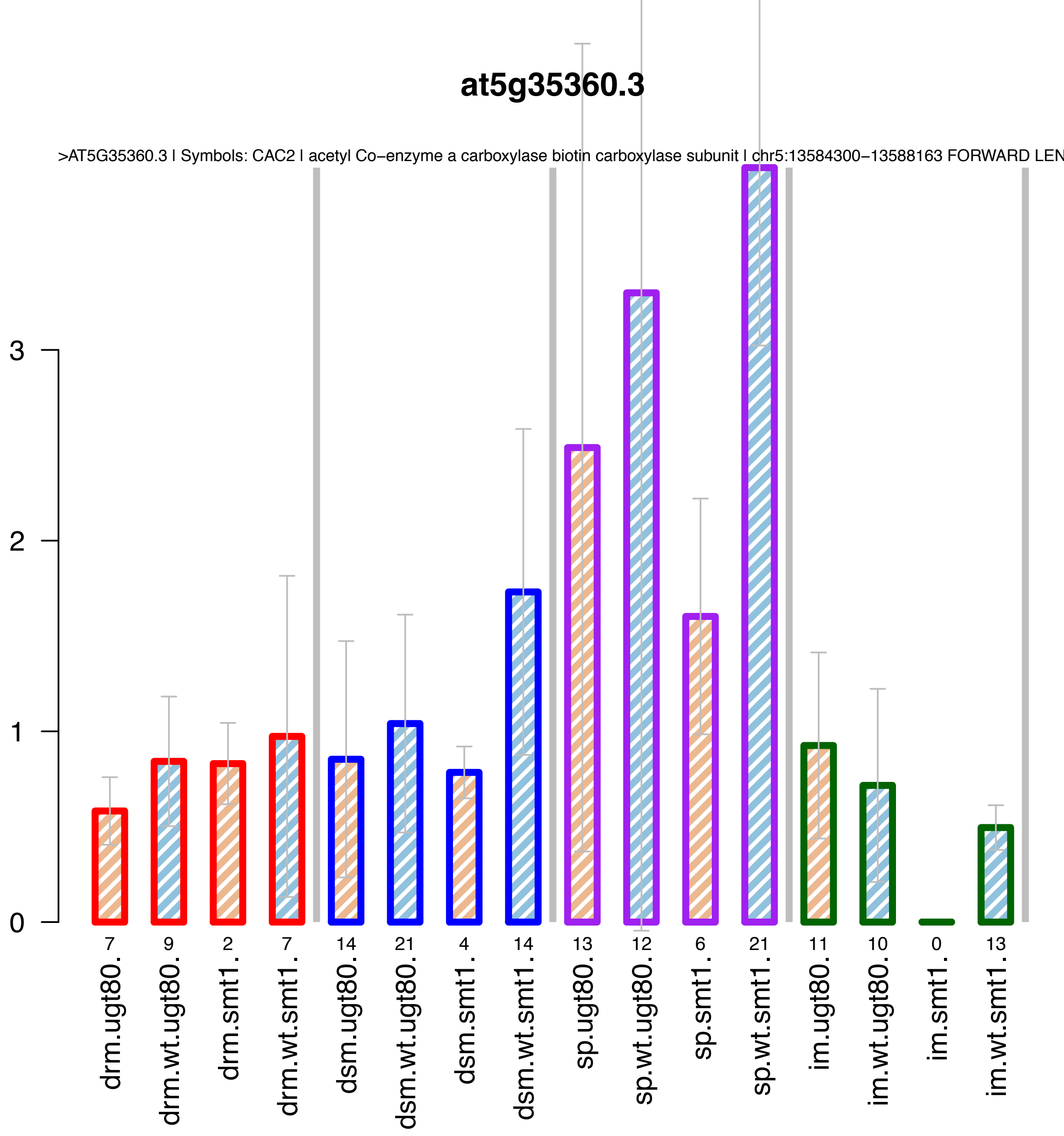

at5g35360.3
>AT5G35360.3 | Symbols: CAC2 | acetyl Co−enzyme a carboxylase biotin carboxylase subunit | chr5:13584300−13588163 FORWARD LEN
0
1
2
3
7
9
2
7
14
21
4
14
13
12
6
21
11
10
0
13
drm.ugt80..
drm.wt.ugt80..
drm.smt1..
drm.wt.smt1..
dsm.ugt80..
dsm.wt.ugt80..
dsm.smt1..
dsm.wt.smt1..
sp.ugt80..
sp.wt.ugt80..
sp.smt1..
sp.wt.smt1..
im.ugt80..
im.wt.ugt80..
im.smt1..
im.wt.smt1..

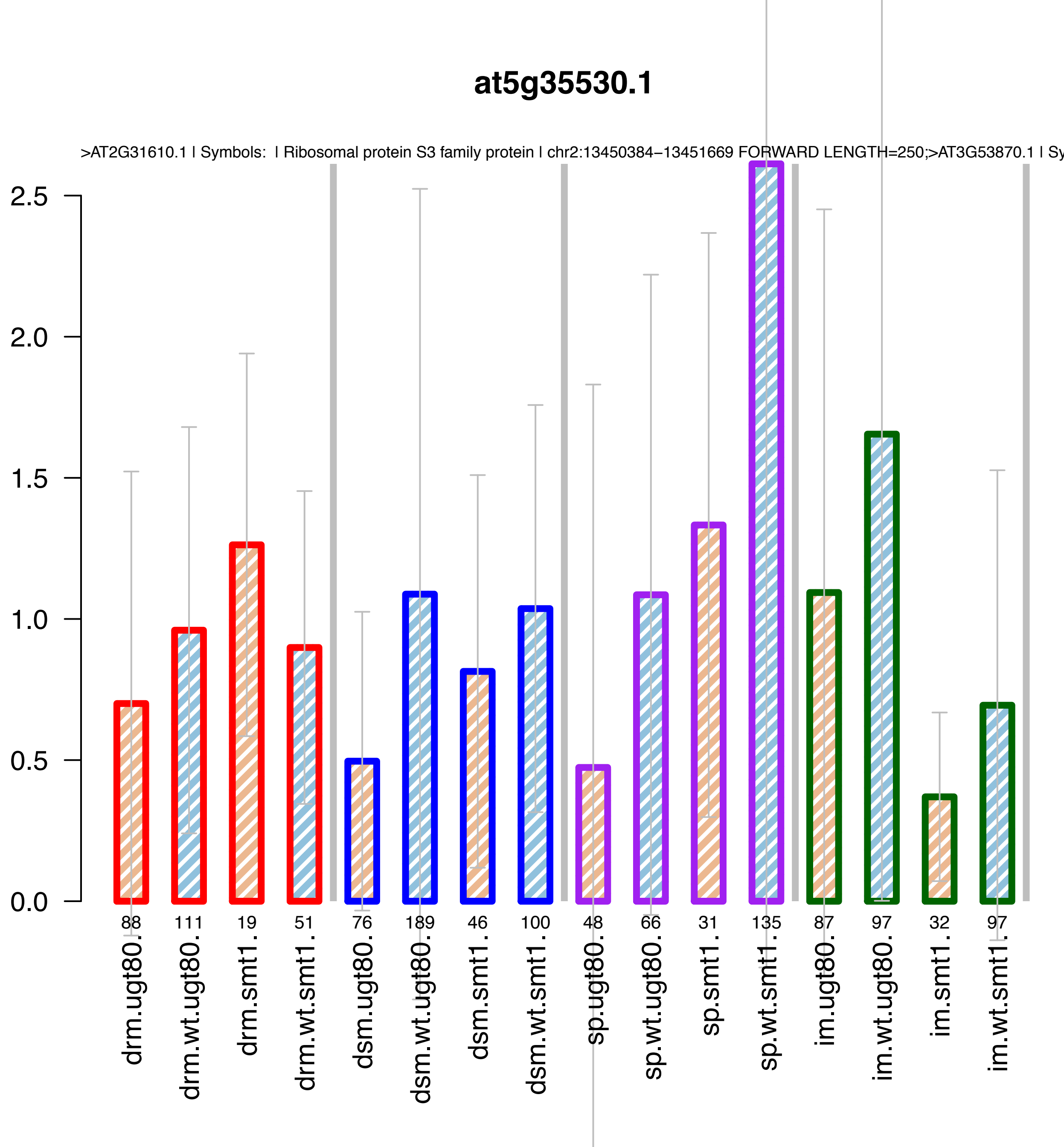

at5g35530.1
>AT2G31610.1 | Symbols: | Ribosomal protein S3 family protein | chr2:13450384-13451669 FORWARD LENGTH=250;>AT3G53870.1 | Sy
2.5
2.0
1.5
1.0
0.5
0.0
88
111
19
51
76
189
46
100
48
66
31
135
87
97
32
97
drm.ugt80..
drm.wt.ugt80..
drm.smt1..
drm.wt.smt1..
dsm.ugt80..
dsm.wt.ugt80..
dsm.smt1..
dsm.wt.smt1..
sp.ugt80..
sp.wt.ugt80..
sp.smt1..
sp.wt.smt1..
im.ugt80..
im.wt.ugt80..
im.smt1..
im.wt.smt1..

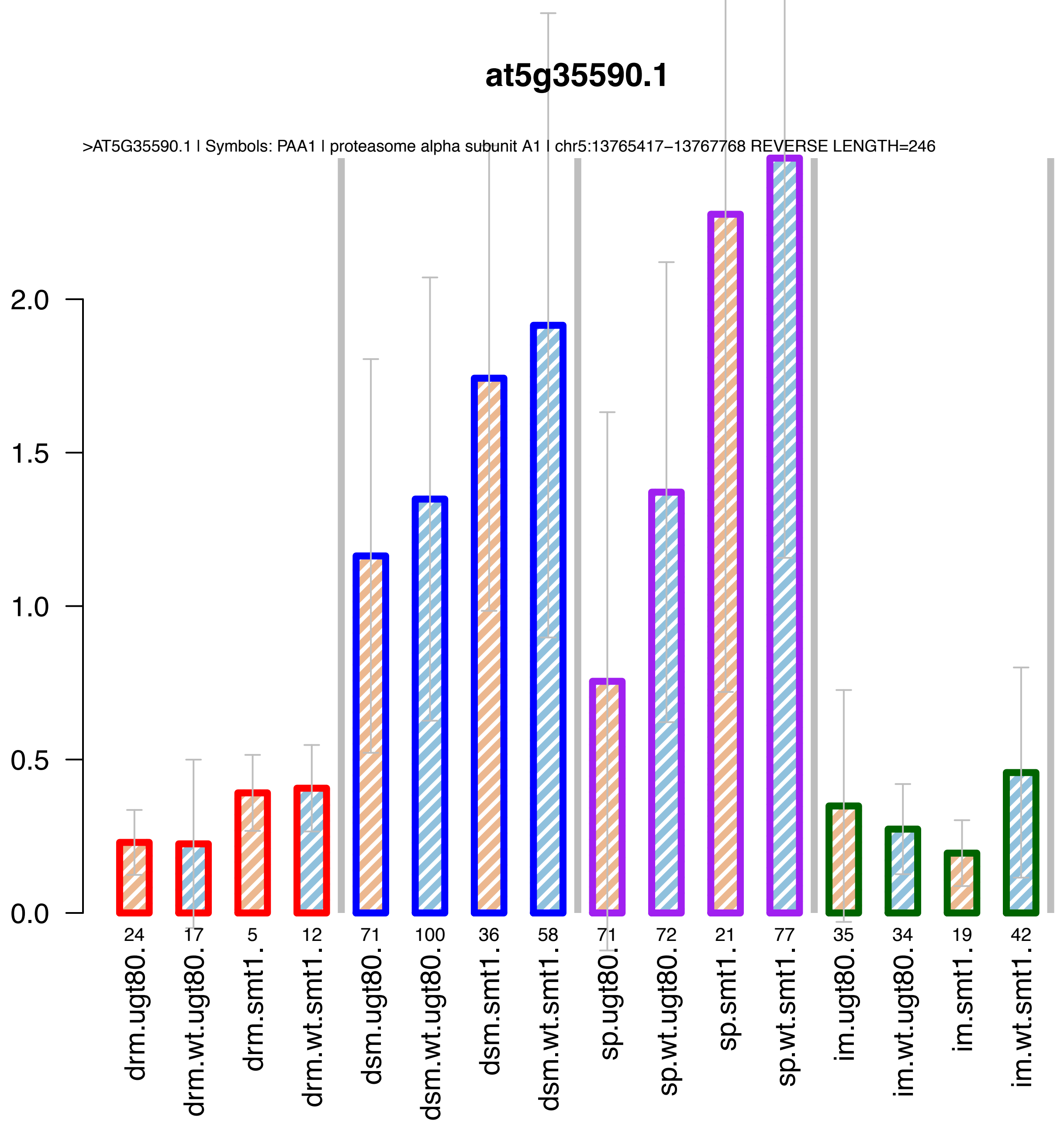

at5g35590.1
>AT5G35590.1 | Symbols: PAA1 | proteasome alpha subunit A1 | chr5:13765417−13767768 REVERSE LENGTH=246
2.0
1.5
1.0
0.5
0.0
24
17
5
12
71
100
36
58
71
72
21
77
35
34
19
42
drm.ugt80..
drm.wt.ugt80..
drm.smt1..
drm.wt.smt1..
dsm.ugt80..
dsm.wt.ugt80..
dsm.smt1..
dsm.wt.smt1..
sp.ugt80..
sp.wt.ugt80..
sp.smt1..
sp.wt.smt1..
im.ugt80..
im.wt.ugt80..
im.smt1..
im.wt.smt1..

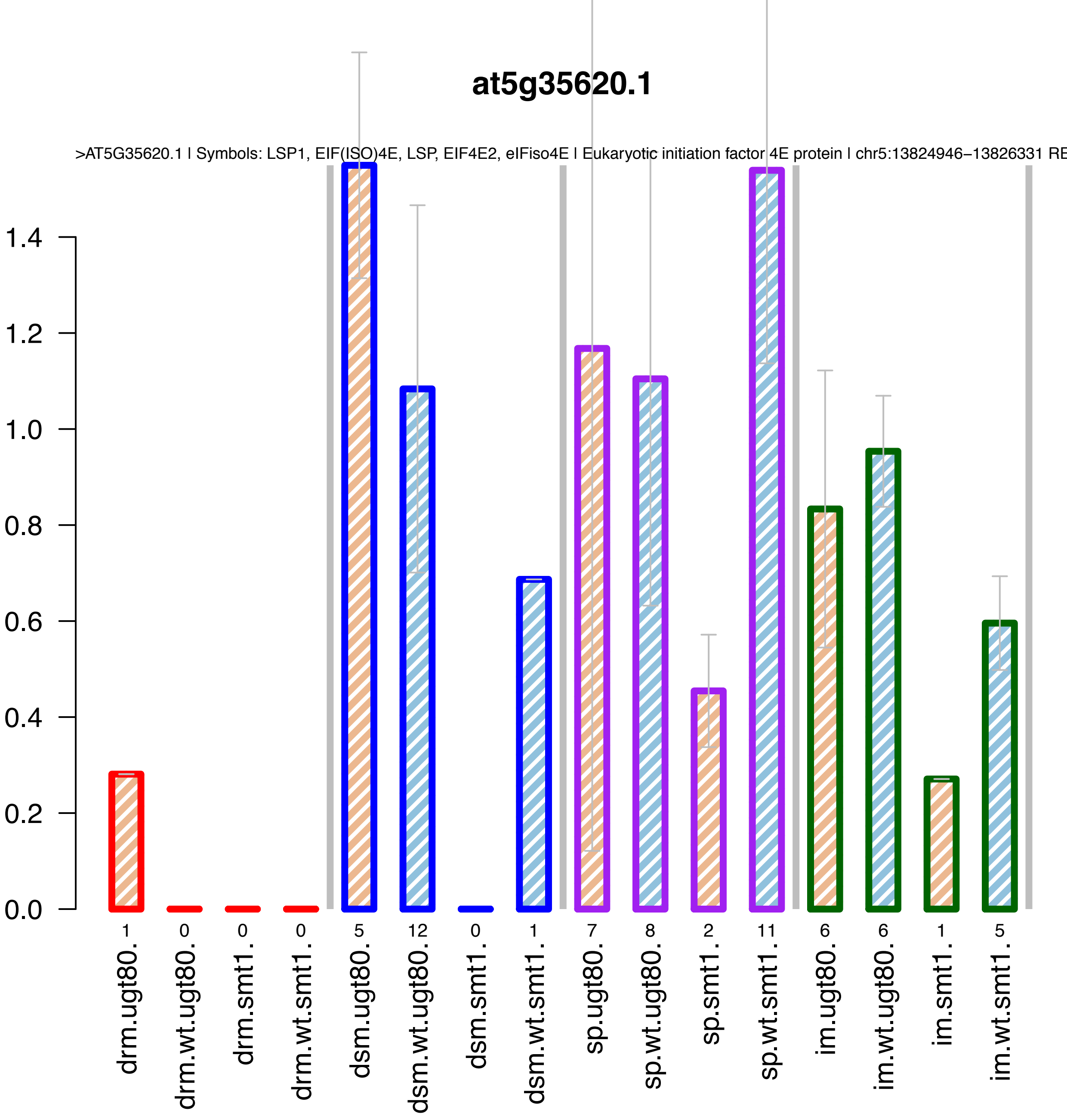

at5g35620.1
>AT5G35620.1 | Symbols: LSP1, EIF(ISO)4E, LSP, EIF4E2, eIFiso4E | Eukaryotic initiation factor 4E protein | chr5:13824946-13826331 RE
1.4
1.2
1.0
0.8
0.6
0.4
0.2
0.0
1
0
0
0
5
12
0
1
7
8
2
11
6
6
1
5
drm.ugt80.
drm.wt.ugt80.
drm.smt1.
drm.wt.smt1.
dsm.ugt80.
dsm.wt.ugt80.
dsm.smt1.
dsm.wt.smt1.
sp.ugt80.
sp.wt.ugt80.
sp.smt1.
sp.wt.smt1.
im.ugt80.
im.wt.ugt80.
im.smt1.
im.wt.smt1.

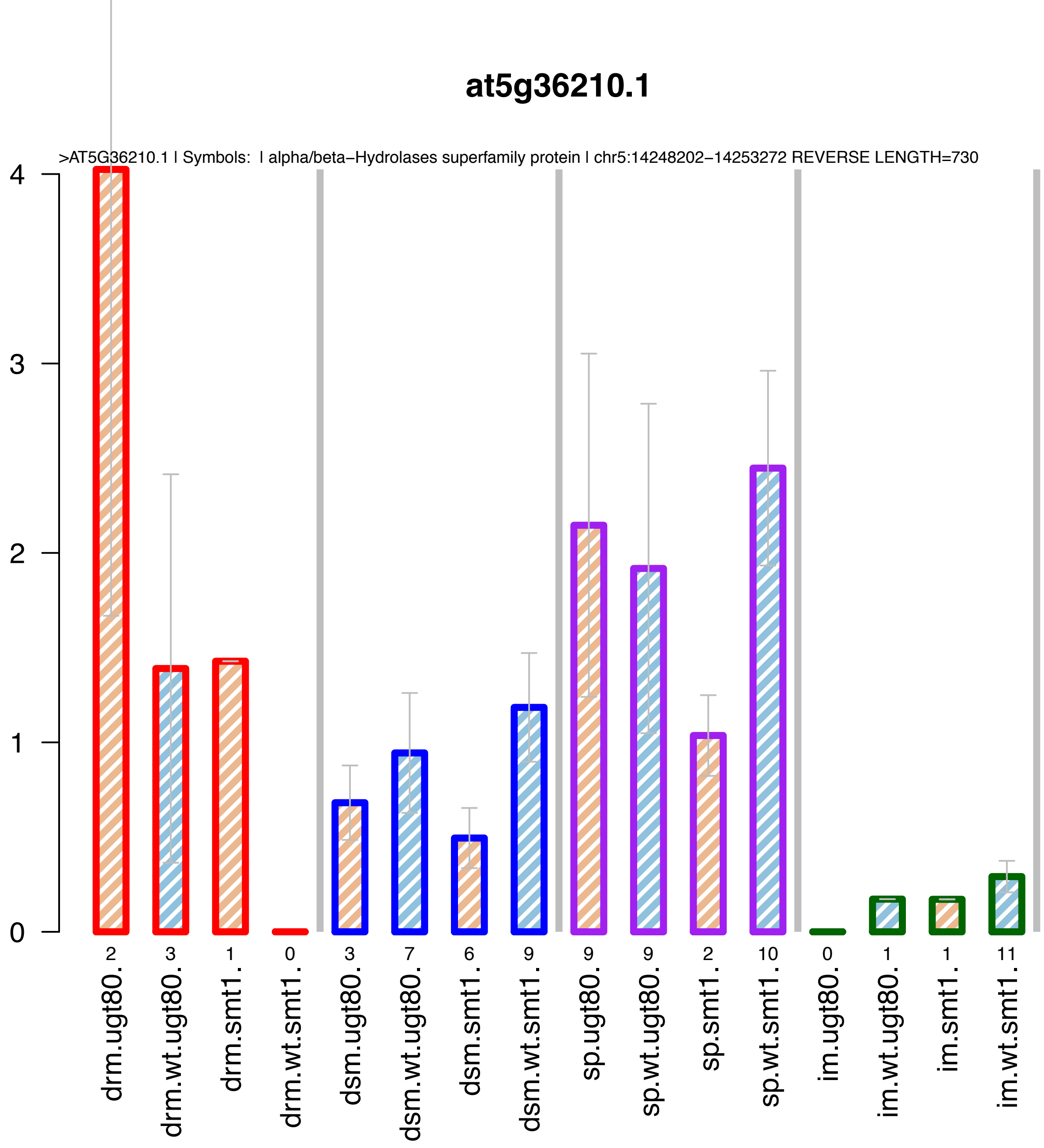

at5g36210.1
>AT5G36210.1 | Symbols: | alpha/beta−Hydrolases superfamily protein | chr5:14248202−14253272 REVERSE LENGTH=730
0
1
2
3
4
2
3
1
0
3
7
6
9
9
9
2
10
0
1
1
11
drm.ugt80..
drm.wt.ugt80..
drm.smt1..
drm.wt.smt1..
dsm.ugt80..
dsm.wt.ugt80..
dsm.smt1..
dsm.wt.smt1..
sp.ugt80..
sp.wt.ugt80..
sp.smt1..
sp.wt.smt1..
im.ugt80..
im.wt.ugt80..
im.smt1..
im.wt.smt1..

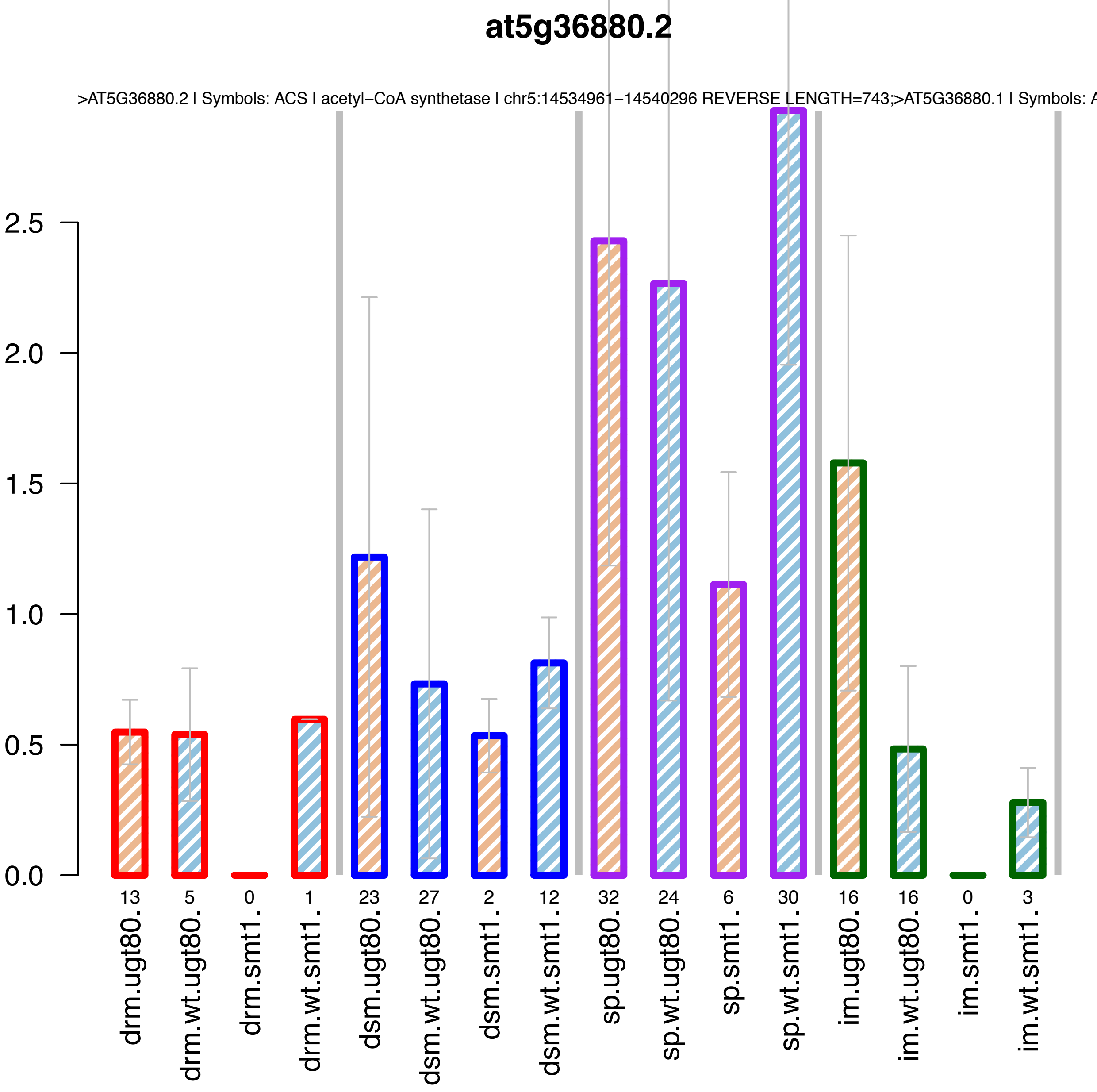

at5g36880.2
>AT5G36880.2 | Symbols: ACS | acetyl−CoA synthetase | chr5:14534961−14540296 REVERSE LENGTH=743;>AT5G36880.1 | Symbols: A
0.0
0.5
1.0
1.5
2.0
2.5
13
5
0
1
23
27
2
12
32
24
6
30
16
16
0
3
drm.ugt80..
drm.wt.ugt80..
drm.smt1..
drm.wt.smt1..
dsm.ugt80..
dsm.wt.ugt80..
dsm.smt1..
dsm.wt.smt1..
sp.ugt80..
sp.wt.ugt80..
sp.smt1..
sp.wt.smt1..
im.ugt80..
im.wt.ugt80..
im.smt1..
im.wt.smt1..

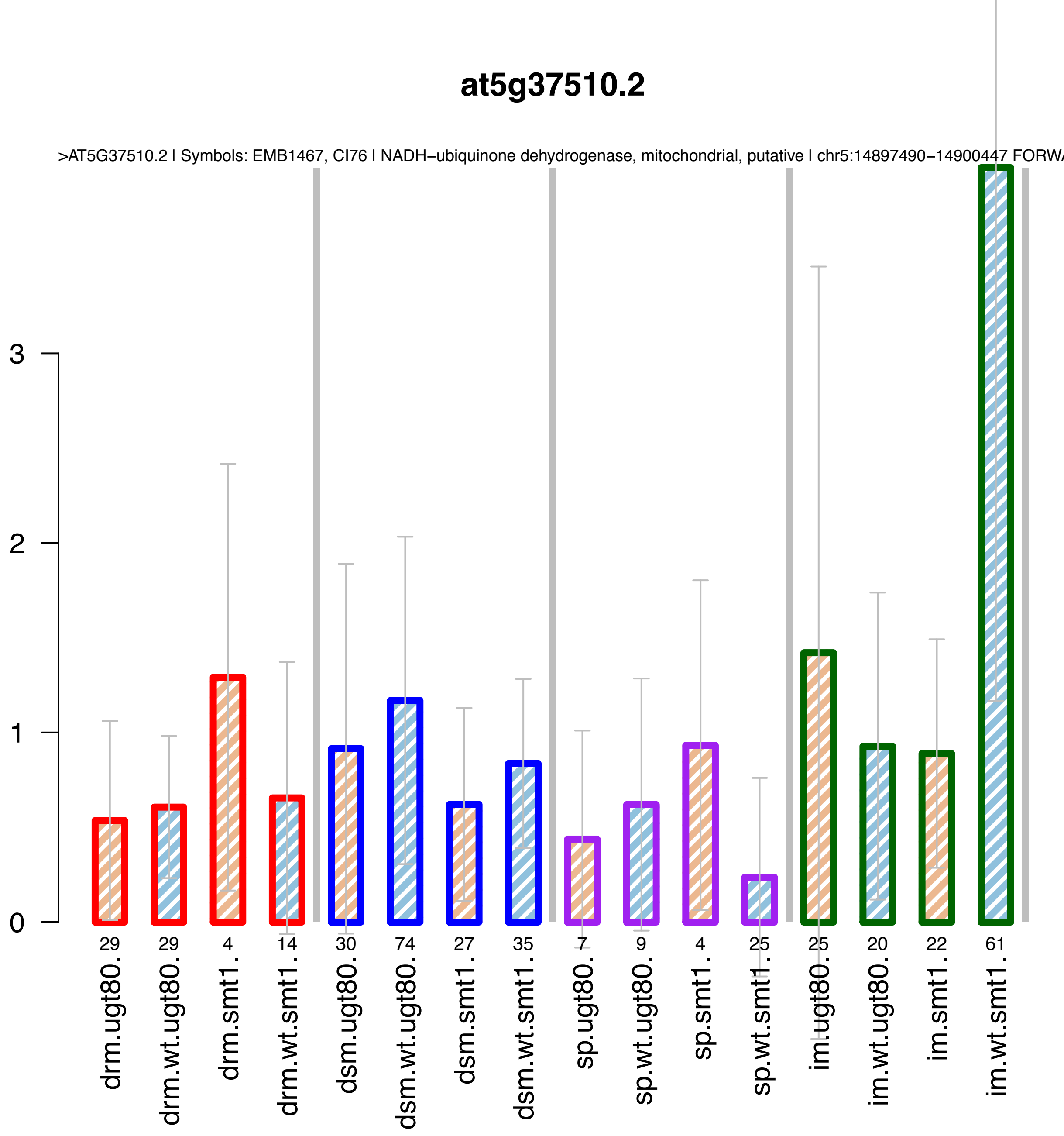

at5g37510.2
>AT5G37510.2 | Symbols: EMB1467, CI76 | NADH−ubiquinone dehydrogenase, mitochondrial, putative | chr5:14897490−14900447 FORWA
0
1
2
3
29
29
4
14
30
74
27
35
7
9
4
25
25
20
22
61
drm.ugt80.
drm.wt.ugt80.
drm.smt1.
drm.wt.smt1.
dsm.ugt80.
dsm.wt.ugt80.
dsm.smt1.
dsm.wt.smt1.
sp.ugt80.
sp.wt.ugt80.
sp.smt1.
sp.wt.smt1.
im.ugt80.
im.wt.ugt80.
im.smt1.
im.wt.smt1.

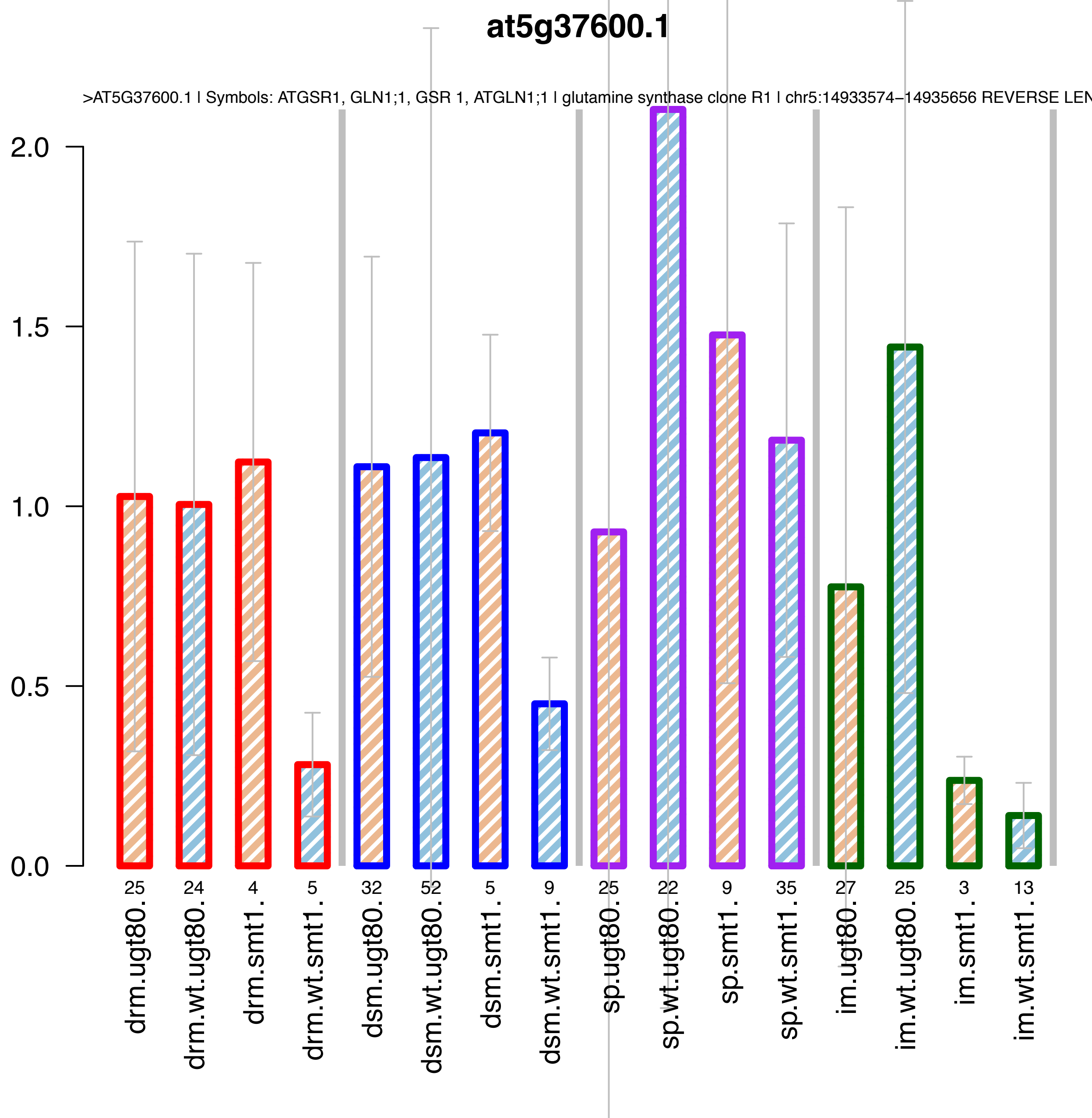

at5g37600.1
>AT5G37600.1 | Symbols: ATGSR1, GLN1;1, GSR 1, ATGLN1;1 | glutamine synthase clone R1 | chr5:14933574-14935656 REVERSE LEN
2.0
1.5
1.0
0.5
0.0
25
24
4
5
32
52
5
9
25
22
9
35
27
25
3
13
drm.ugt80.
drm.wt.ugt80.
drm.smt1.
drm.wt.smt1.
dsm.ugt80.
dsm.wt.ugt80.
dsm.smt1.
dsm.wt.smt1.
sp.ugt80.
sp.wt.ugt80.
sp.smt1.
sp.wt.smt1.
im.ugt80.
im.wt.ugt80.
im.smt1.
im.wt.smt1.

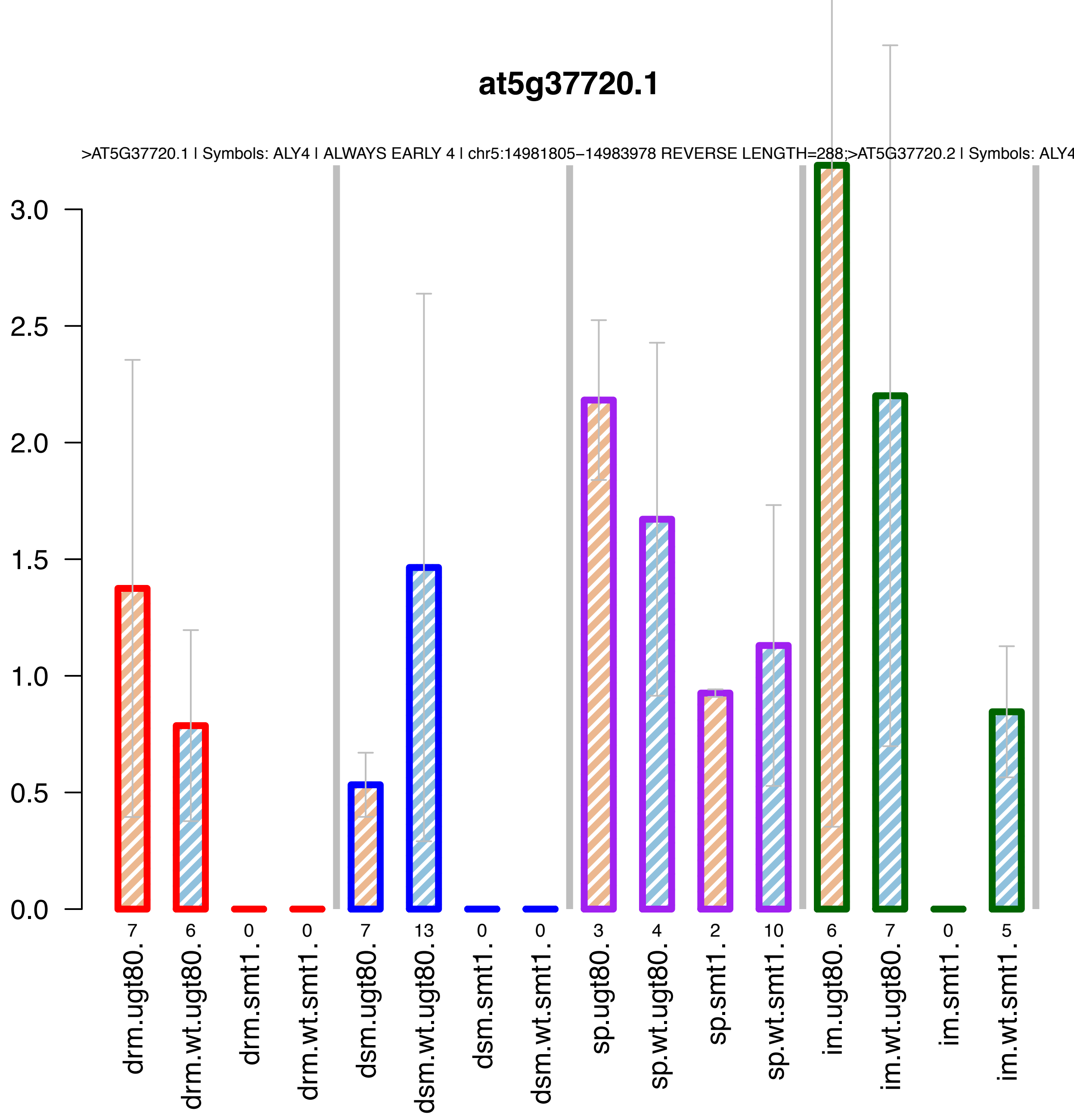

at5g37720.1
>AT5G37720.1 | Symbols: ALY4 | ALWAYS EARLY 4 | chr5:14981805–14983978 REVERSE LENGTH=288;>AT5G37720.2 | Symbols: ALY4
0.0
0.5
1.0
1.5
2.0
2.5
3.0
7
6
0
0
7
13
0
0
3
4
2
10
6
7
0
5
drm.ugt80.
drm.wt.ugt80.
drm.smt1.
drm.wt.smt1.
dsm.ugt80.
dsm.wt.ugt80.
dsm.smt1.
dsm.wt.smt1.
sp.ugt80.
sp.wt.ugt80.
sp.smt1.
sp.wt.smt1.
im.ugt80.
im.wt.ugt80.
im.smt1.
im.wt.smt1.

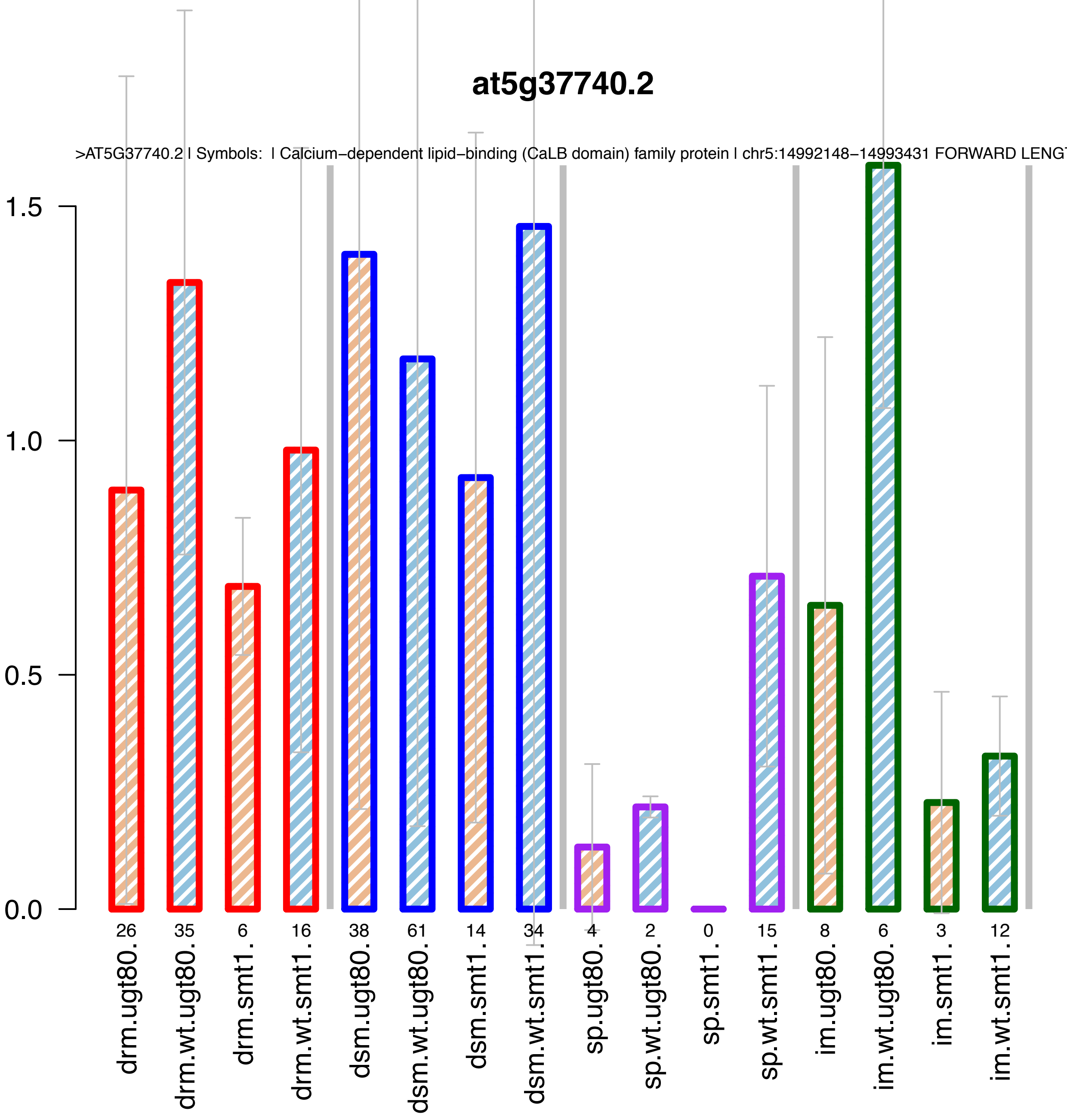

at5g37740.2
>AT5G37740.2 | Symbols: | Calcium−dependent lipid−binding (CaLB domain) family protein | chr5:14992148−14993431 FORWARD LENG
1.5
1.0
0.5
0.0
26
35
6
16
38
61
14
34
4
2
0
15
8
6
3
12
drm.ugt80..
drm.wt.ugt80..
drm.smt1..
drm.wt.smt1..
dsm.ugt80..
dsm.wt.ugt80..
dsm.smt1..
dsm.wt.smt1..
sp.ugt80..
sp.wt.ugt80..
sp.smt1..
sp.wt.smt1..
im.ugt80..
im.wt.ugt80..
im.smt1..
im.wt.smt1..

# at5g37770.1

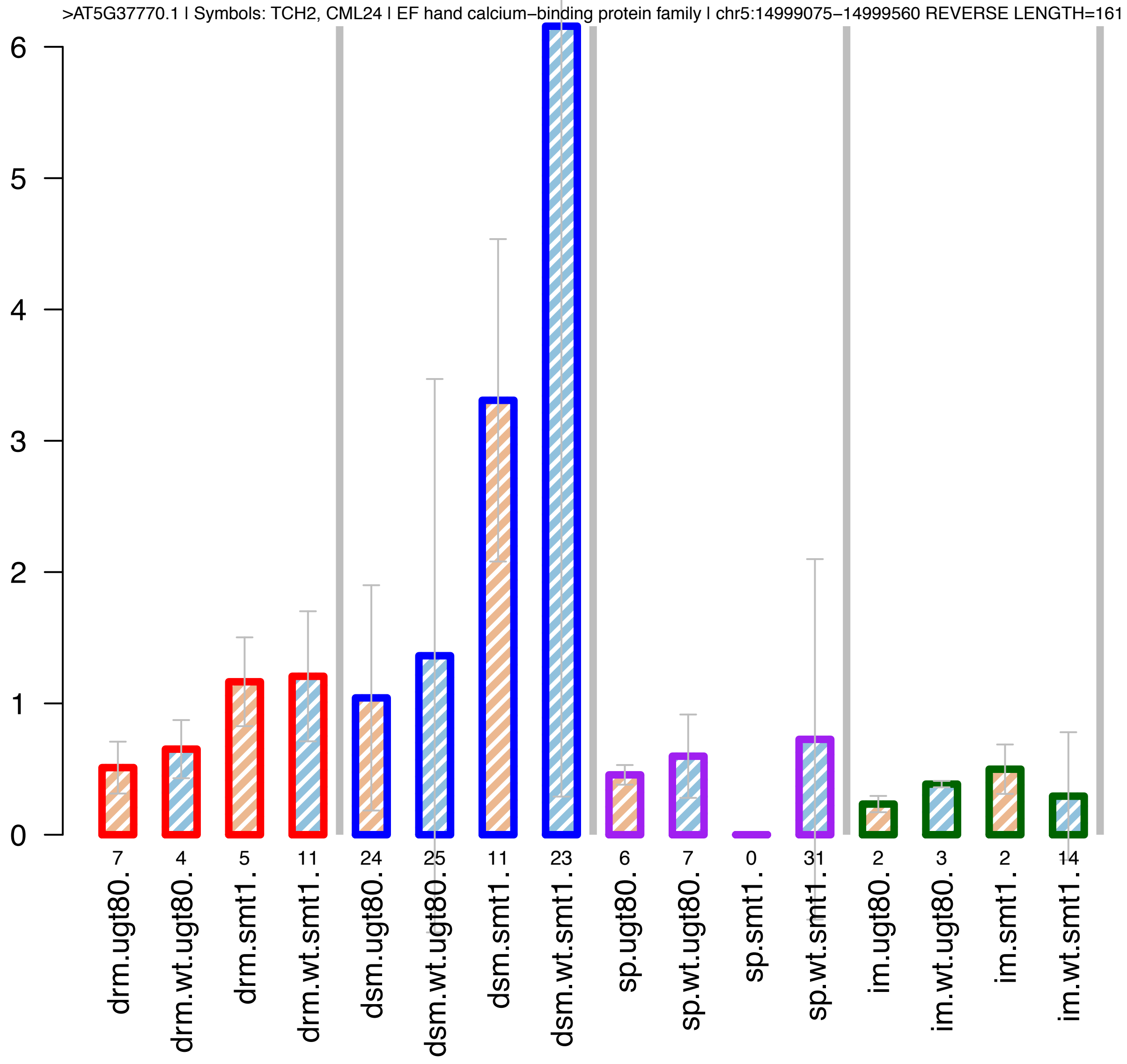

>AT5G37770.1 | Symbols: TCH2, CML24 | EF hand calcium−binding protein family | chr5:14999075−14999560 REVERSE LENGTH=161
0
1
2
3
4
5
6
7
4
5
11
24
25
11
23
6
7
0
31
2
3
2
14
drm.ugt80.
drm.wt.ugt80.
drm.smt1.
drm.wt.smt1.
dsm.ugt80.
dsm.wt.ugt80.
dsm.smt1.
dsm.wt.smt1.
sp.ugt80.
sp.wt.ugt80.
sp.smt1.
sp.wt.smt1.
im.ugt80.
im.wt.ugt80.
im.smt1.
im.wt.smt1.

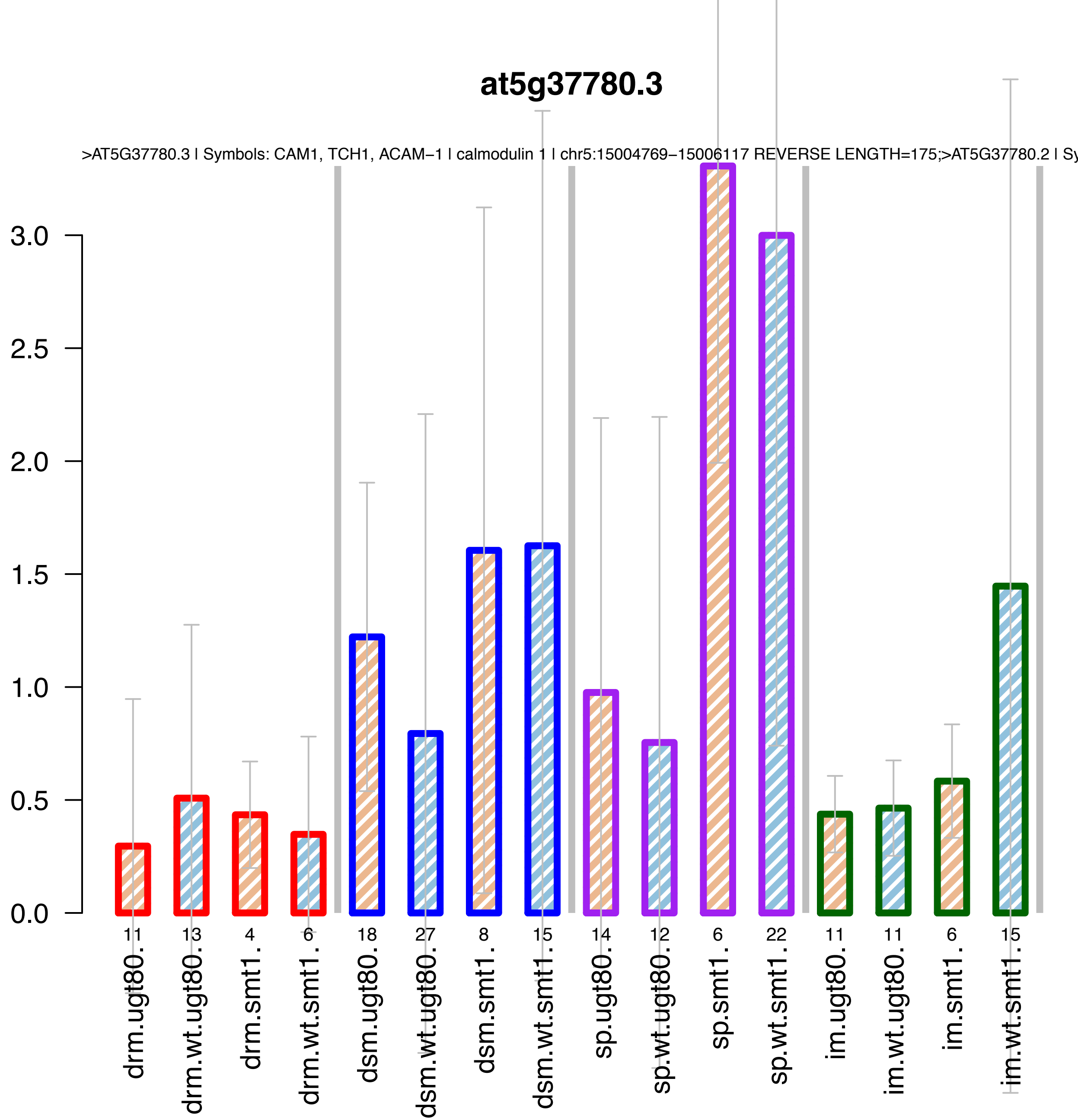
at5g37780.3
>AT5G37780.3 | Symbols: CAM1, TCH1, ACAM−1 | calmodulin 1 | chr5:15004769−15006117 REVERSE LENGTH=175;>AT5G37780.2 | Sy
0.0
0.5
1.0
1.5
2.0
2.5
3.0
11
13
4
6
18
27
8
15
14
12
6
22
11
11
6
15
drm.ugt80..
drm.wt.ugt80..
drm.smt1..
drm.wt.smt1..
dsm.ugt80..
dsm.wt.ugt80..
dsm.smt1..
dsm.wt.smt1..
sp.ugt80..
sp.wt.ugt80..
sp.smt1..
sp.wt.smt1..
im.ugt80..
im.wt.ugt80..
im.smt1..
im.wt.smt1..

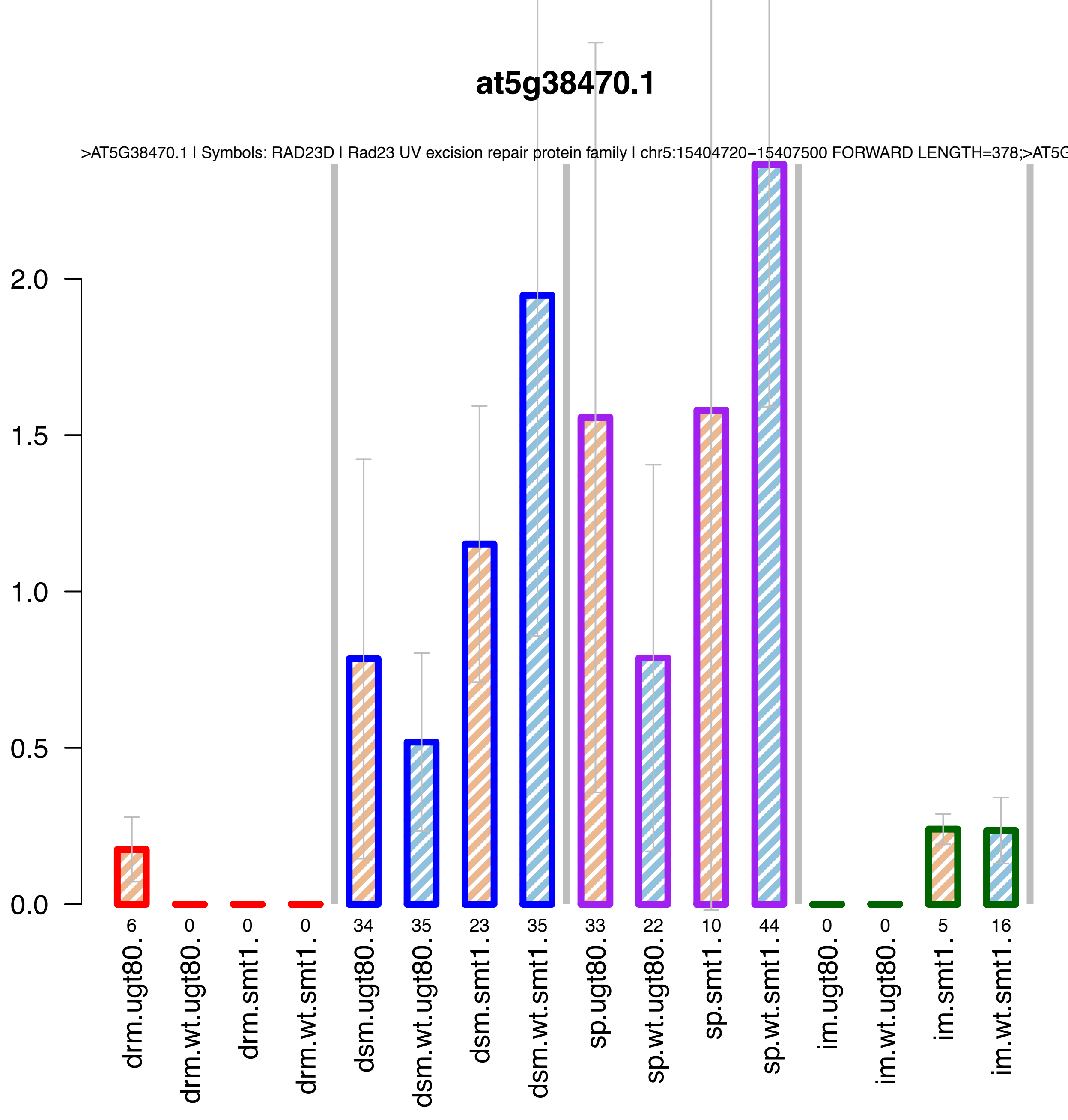

at5g38470.1
>AT5G38470.1 | Symbols: RAD23D | Rad23 UV excision repair protein family | chr5:15404720–15407500 FORWARD LENGTH=378;>AT5G
2.0
1.5
1.0
0.5
0.0
6
0
0
0
34
35
23
35
33
22
10
44
0
0
5
16
drm.ugt80.
drm.wt.ugt80.
drm.smt1.
drm.wt.smt1.
dsm.ugt80.
dsm.wt.ugt80.
dsm.smt1.
dsm.wt.smt1.
sp.ugt80.
sp.wt.ugt80.
sp.smt1.
sp.wt.smt1.
im.ugt80.
im.wt.ugt80.
im.smt1.
im.wt.smt1.

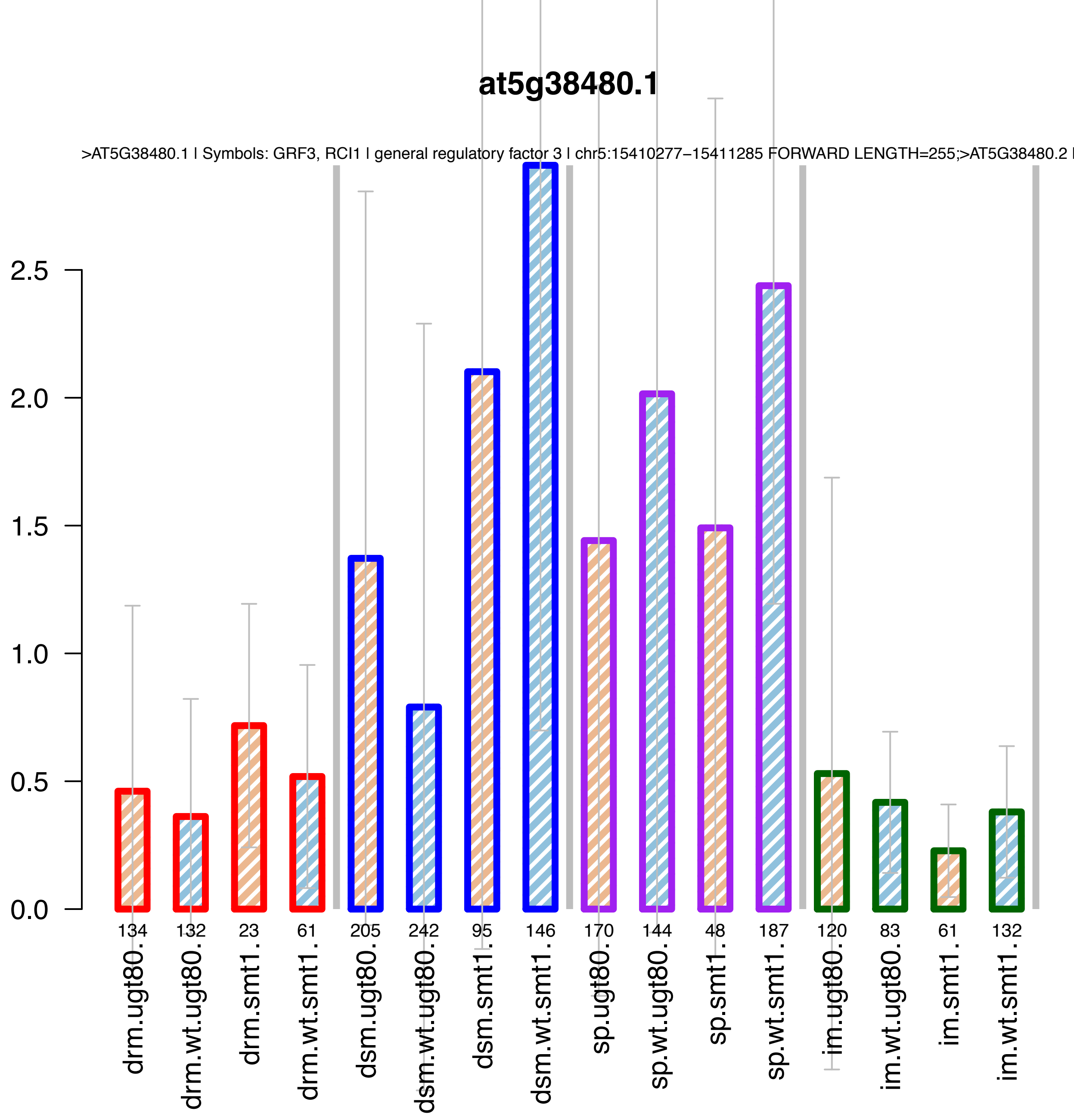

at5g38480.1
>AT5G38480.1 | Symbols: GRF3, RCI1 | general regulatory factor 3 | chr5:15410277-15411285 FORWARD LENGTH=255;>AT5G38480.2 |
0.0
0.5
1.0
1.5
2.0
2.5
134
132
23
61
205
242
95
146
170
144
48
187
120
83
61
132
drm.ugt80..
drm.wt.ugt80..
drm.smt1..
drm.wt.smt1..
dsm.ugt80..
dsm.wt.ugt80..
dsm.smt1..
dsm.wt.smt1..
sp.ugt80..
sp.wt.ugt80..
sp.smt1..
sp.wt.smt1..
im.ugt80..
im.wt.ugt80..
im.smt1..
im.wt.smt1..

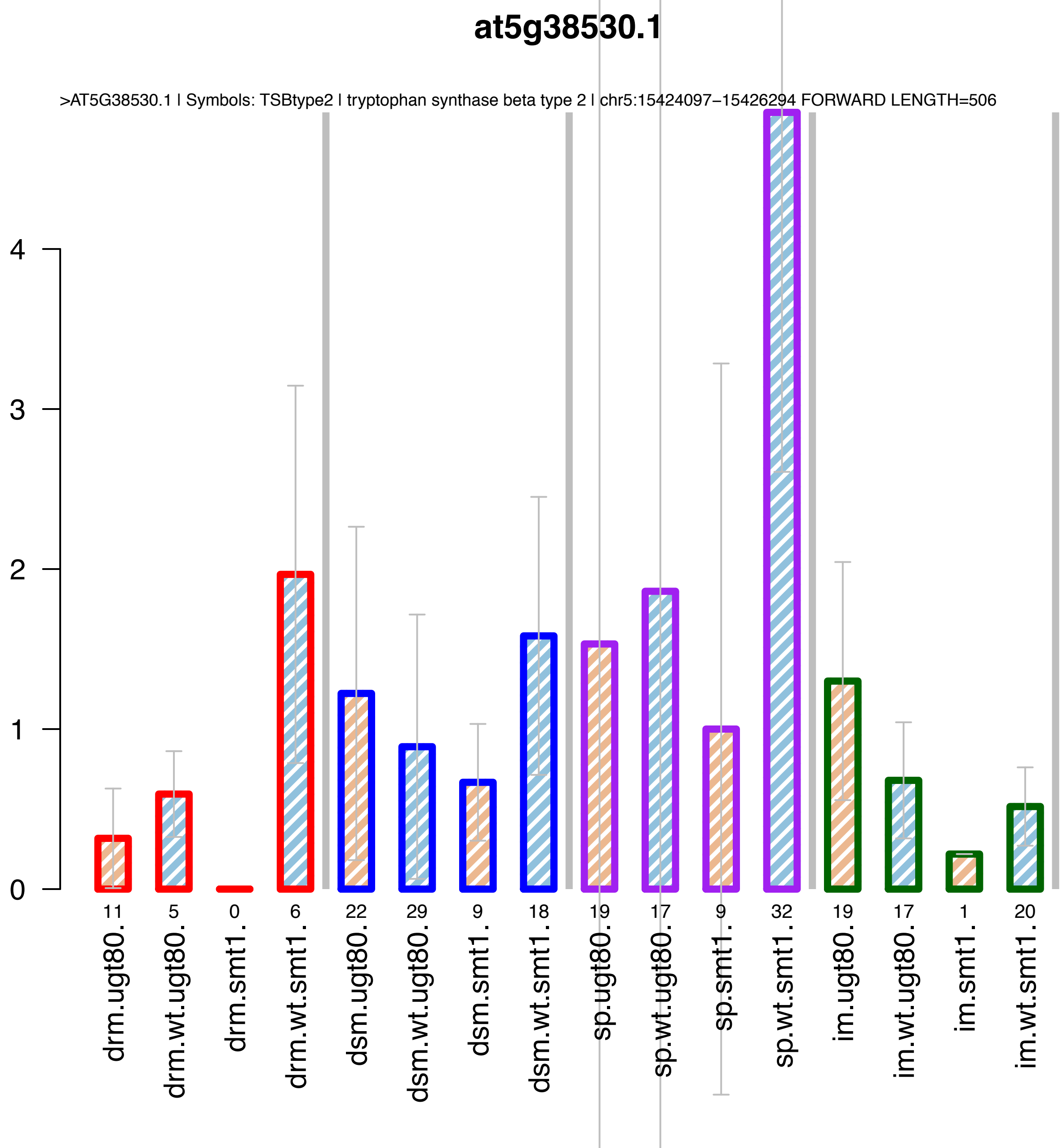

at5g38530.1
>AT5G38530.1 | Symbols: TSBtype2 | tryptophan synthase beta type 2 | chr5:15424097−15426294 FORWARD LENGTH=506
0
1
2
3
4
11
5
0
6
22
29
9
18
19
17
9
32
19
17
1
20
drm.ugt80.
drm.wt.ugt80.
drm.smt1.
drm.wt.smt1.
dsm.ugt80.
dsm.wt.ugt80.
dsm.smt1.
dsm.wt.smt1.
sp.ugt80.
sp.wt.ugt80.
sp.smt1.
sp.wt.smt1.
im.ugt80.
im.wt.ugt80.
im.smt1.
im.wt.smt1.

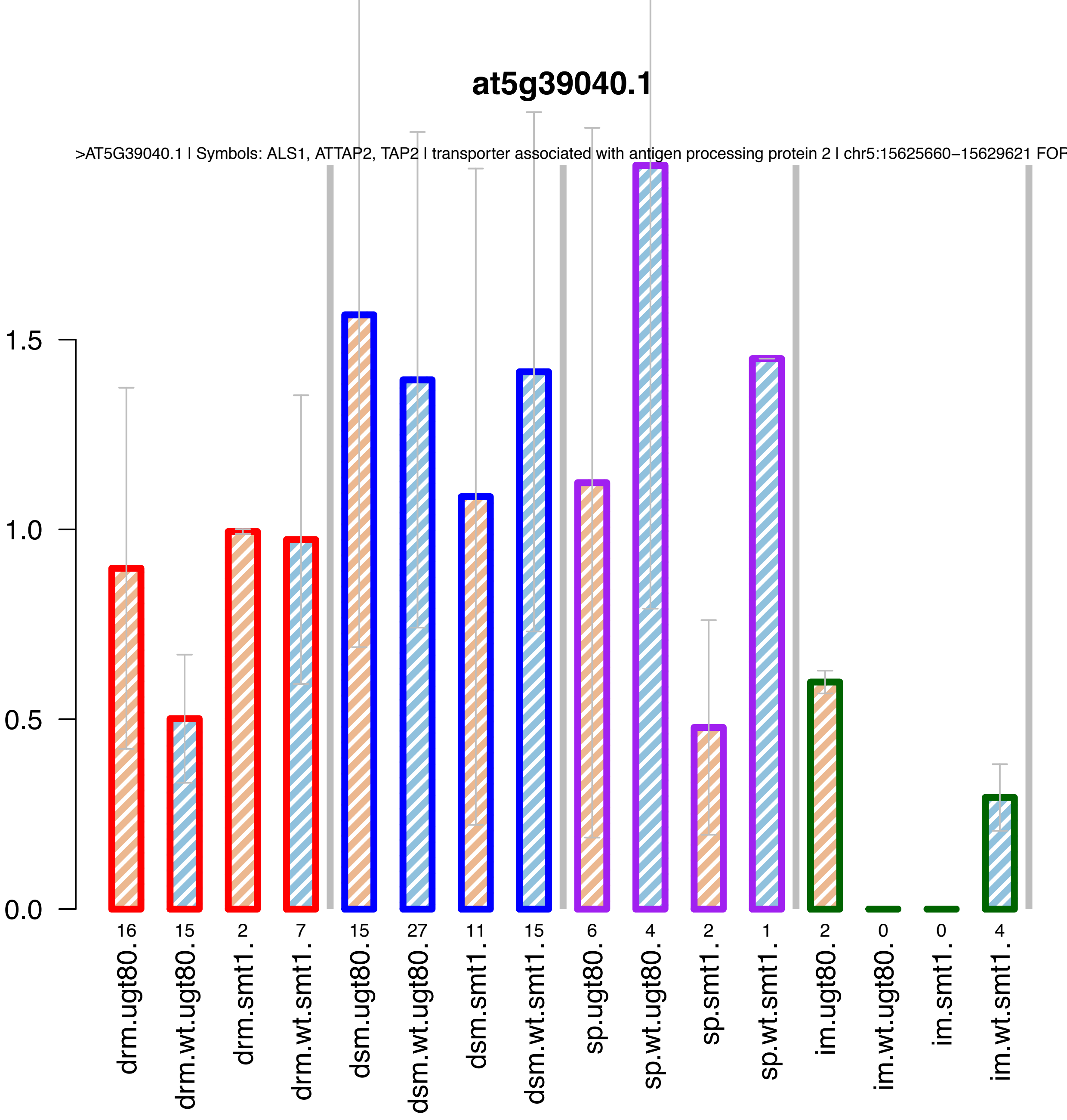

at5g39040.1
>AT5G39040.1 | Symbols: ALS1, ATTAP2, TAP2 | transporter associated with antigen processing protein 2 | chr5:15625660-15629621 FOR
1.5
1.0
0.5
0.0
16
15
2
7
15
27
11
15
6
4
2
1
2
0
0
4
drm.ugt80..
drm.wt.ugt80..
drm.smt1..
drm.wt.smt1..
dsm.ugt80..
dsm.wt.ugt80..
dsm.smt1..
dsm.wt.smt1..
sp.ugt80..
sp.wt.ugt80..
sp.smt1..
sp.wt.smt1..
im.ugt80..
im.wt.ugt80..
im.smt1..
im.wt.smt1..

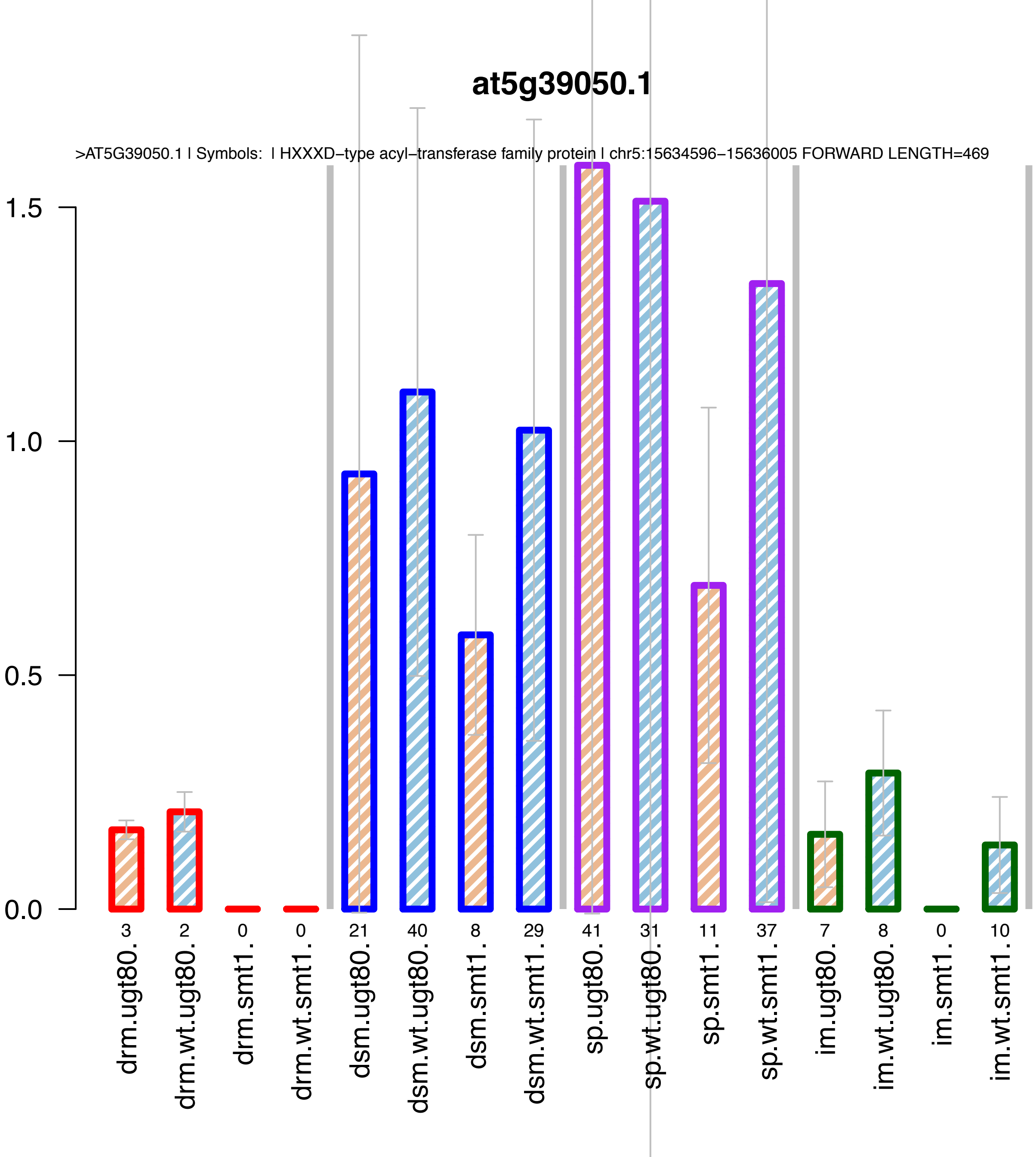

at5g39050.1
>AT5G39050.1 | Symbols: | HXXXD−type acyl−transferase family protein | chr5:15634596−15636005 FORWARD LENGTH=469
1.5
1.0
0.5
0.0
3
2
0
0
21
40
8
29
41
31
11
37
7
8
0
10
drm.ugt80.
drm.wt.ugt80.
drm.smt1.
drm.wt.smt1.
dsm.ugt80.
dsm.wt.ugt80.
dsm.smt1.
dsm.wt.smt1.
sp.ugt80.
sp.wt.ugt80.
sp.smt1.
sp.wt.smt1.
im.ugt80.
im.wt.ugt80.
im.smt1.
im.wt.smt1.

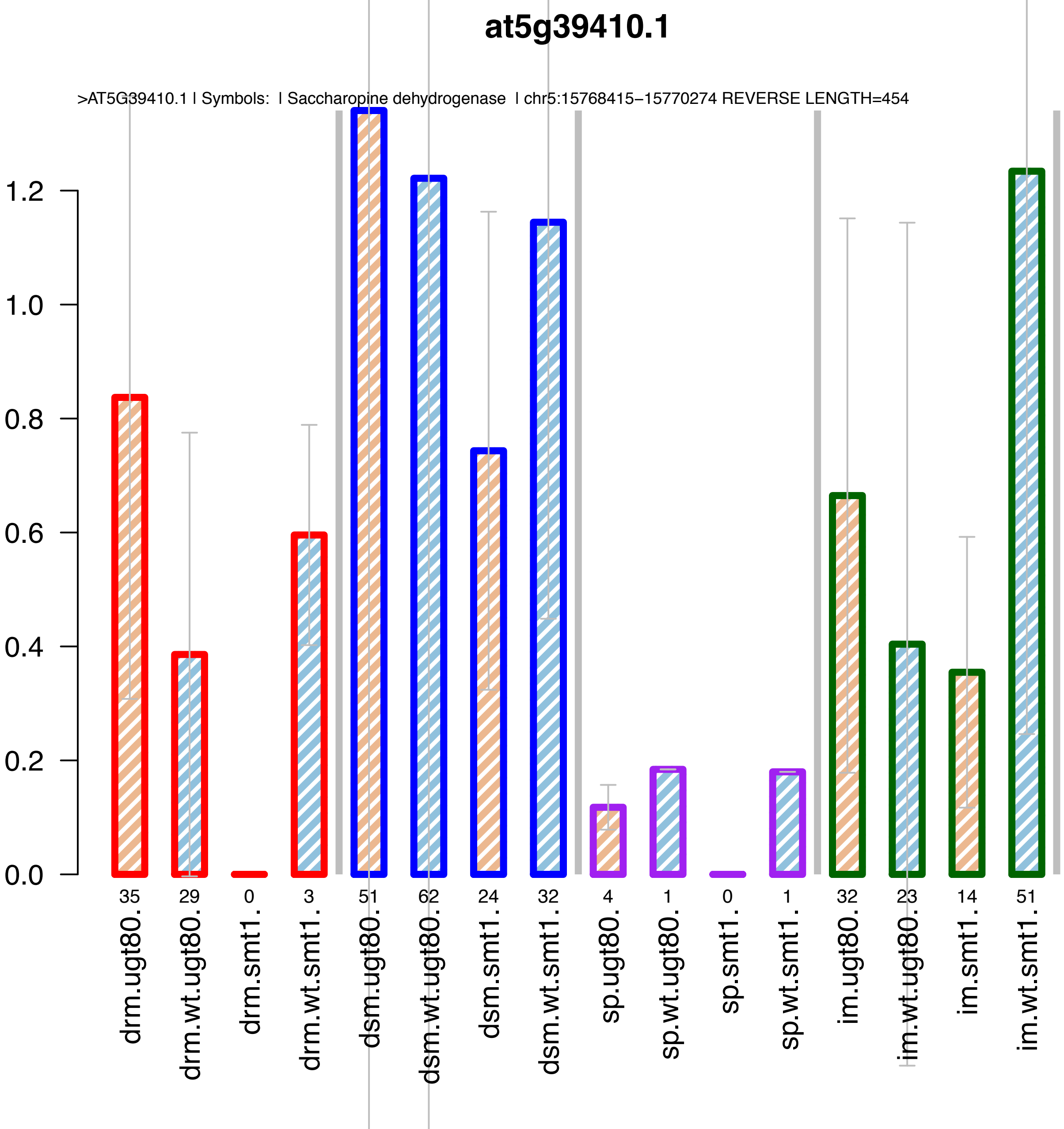

at5g39410.1
>AT5G39410.1 | Symbols: | Saccharopine dehydrogenase | chr5:15768415−15770274 REVERSE LENGTH=454
1.2
1.0
0.8
0.6
0.4
0.2
0.0
35
29
0
3
51
62
24
32
4
1
0
1
32
23
14
51
drm.ugt80.
drm.wt.ugt80.
drm.smt1.
drm.wt.smt1.
dsm.ugt80.
dsm.wt.ugt80.
dsm.smt1.
dsm.wt.smt1.
sp.ugt80.
sp.wt.ugt80.
sp.smt1.
sp.wt.smt1.
im.ugt80.
im.wt.ugt80.
im.smt1.
im.wt.smt1.

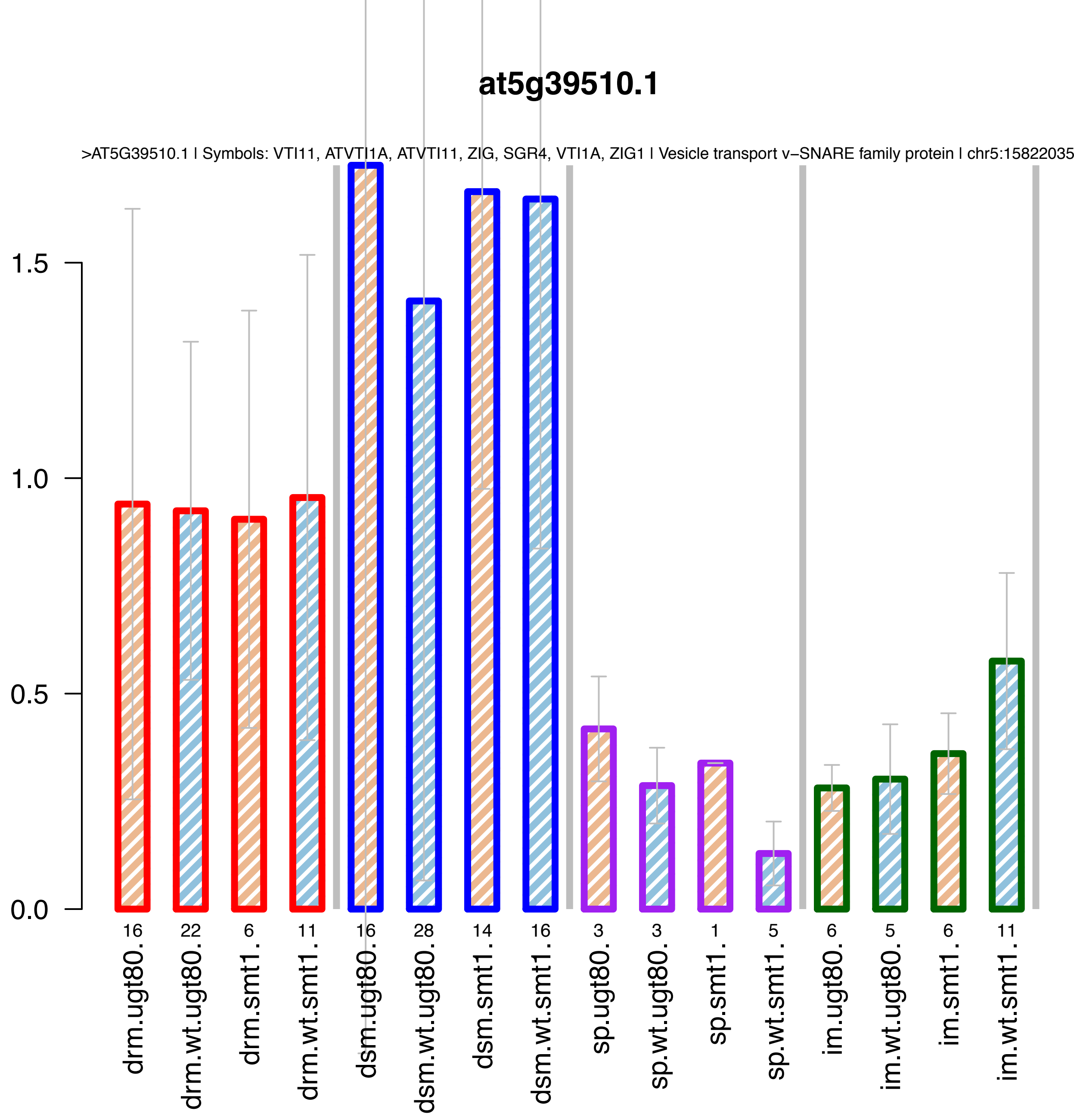

at5g39510.1
>AT5G39510.1 | Symbols: VTI11, ATVTI1A, ATVTI11, ZIG, SGR4, VTI1A, ZIG1 | Vesicle transport v−SNARE family protein | chr5:15822035
0.0
0.5
1.0
1.5
16
22
6
11
16
28
14
16
3
3
1
5
6
5
6
11
drm.ugt80.
drm.wt.ugt80.
drm.smt1.
drm.wt.smt1.
dsm.ugt80.
dsm.wt.ugt80.
dsm.smt1.
dsm.wt.smt1.
sp.ugt80.
sp.wt.ugt80.
sp.smt1.
sp.wt.smt1.
im.ugt80.
im.wt.ugt80.
im.smt1.
im.wt.smt1.

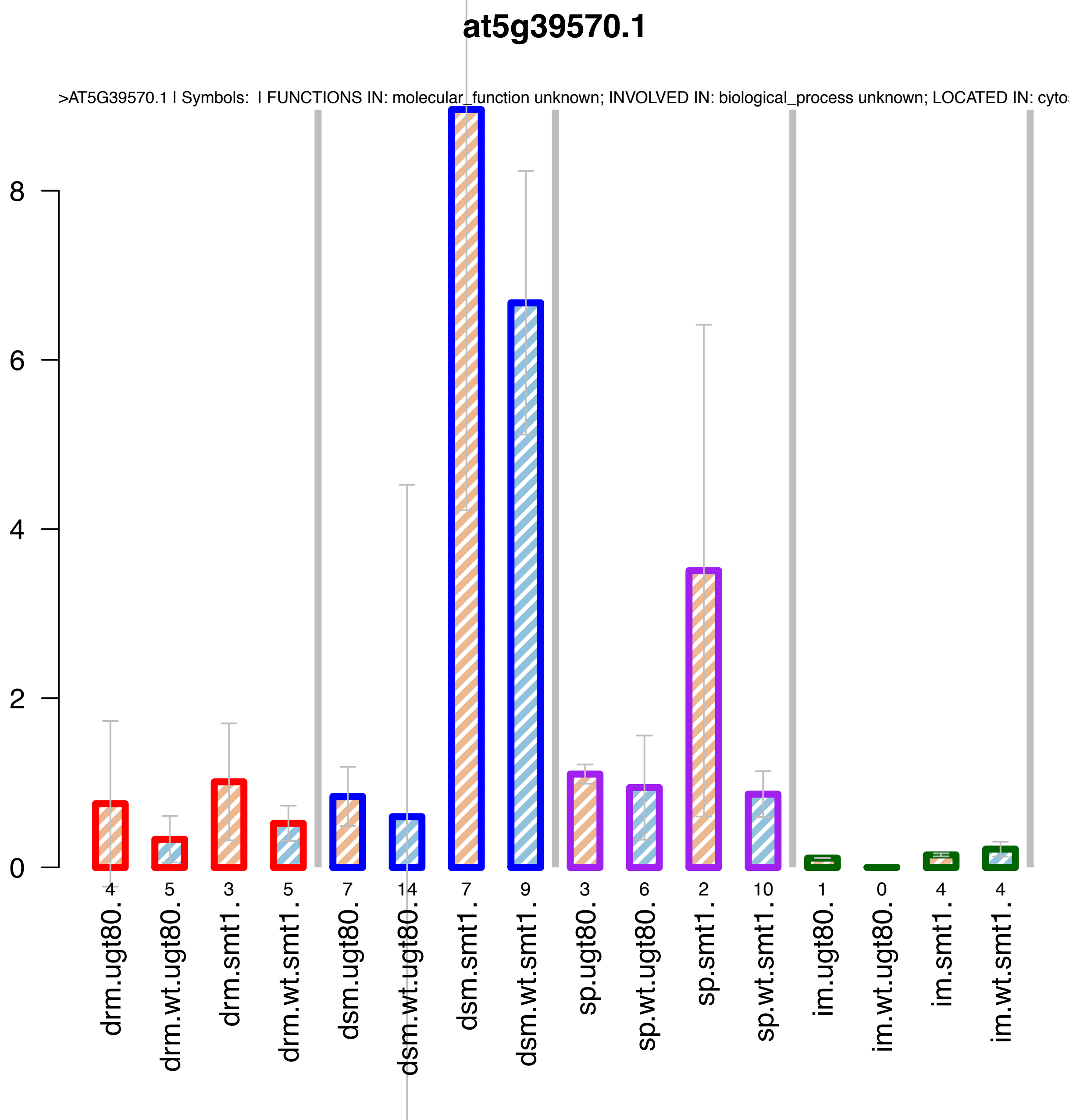

at5g39570.1
>AT5G39570.1 | Symbols: | FUNCTIONS IN: molecular_function unknown; INVOLVED IN: biological_process unknown; LOCATED IN: cyto
0
2
4
6
8
4
5
3
5
7
14
7
9
3
6
2
10
1
0
4
4
drm.ugt80.
drm.wt.ugt80.
drm.smt1.
drm.wt.smt1.
dsm.ugt80.
dsm.wt.ugt80.
dsm.smt1.
dsm.wt.smt1.
sp.ugt80.
sp.wt.ugt80.
sp.smt1.
sp.wt.smt1.
im.ugt80.
im.wt.ugt80.
im.smt1.
im.wt.smt1.

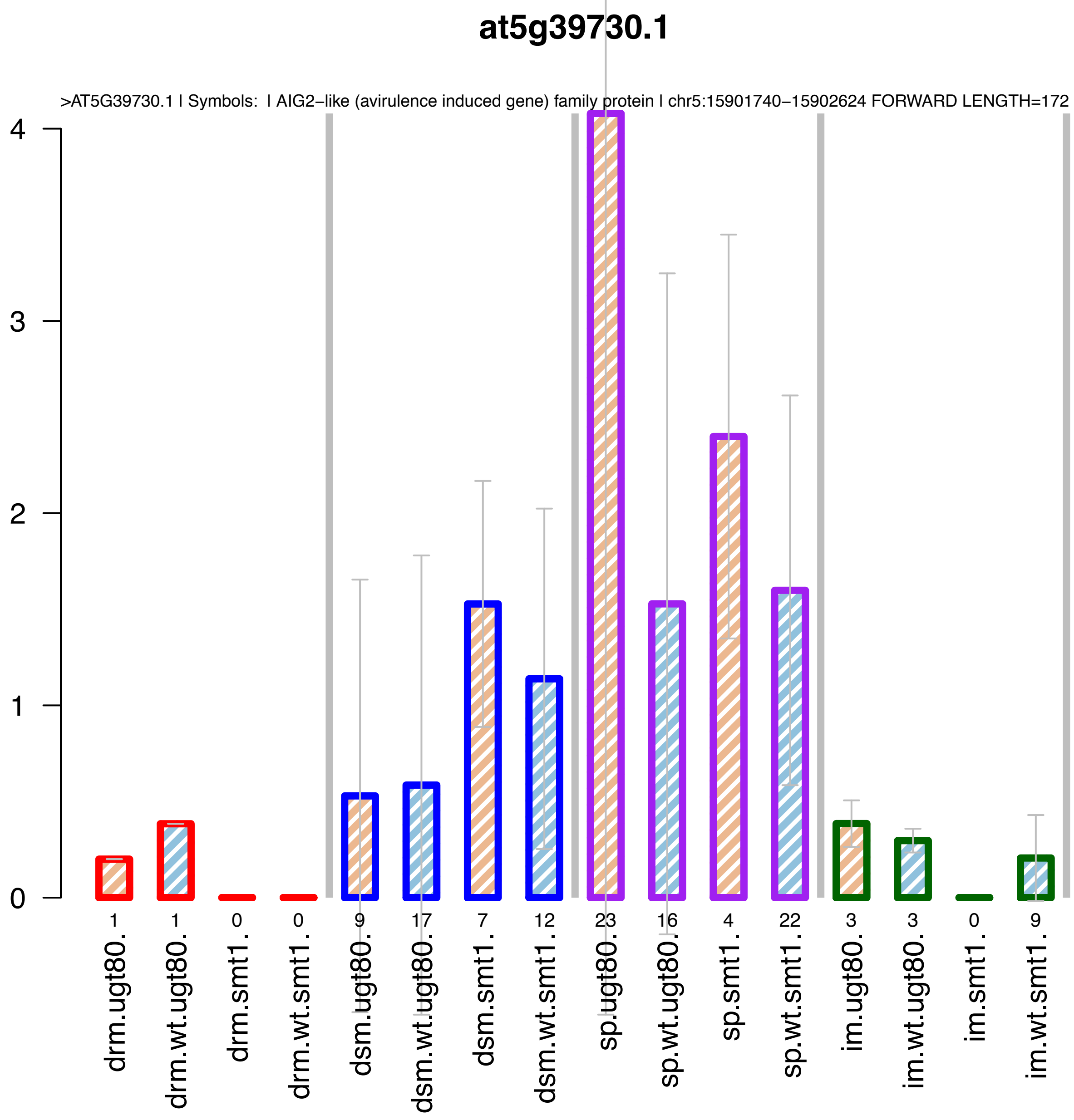

at5g39730.1
>AT5G39730.1 | Symbols: | AIG2-like (avirulence induced gene) family protein | chr5:15901740-15902624 FORWARD LENGTH=172
0
1
2
3
4
1
1
0
0
9
17
7
12
23
16
4
22
3
3
0
9
drm.ugt80.
drm.wt.ugt80.
drm.smt1.
drm.wt.smt1.
dsm.ugt80.
dsm.wt.ugt80.
dsm.smt1.
dsm.wt.smt1.
sp.ugt80.
sp.wt.ugt80.
sp.smt1.
sp.wt.smt1.
im.ugt80.
im.wt.ugt80.
im.smt1.
im.wt.smt1.

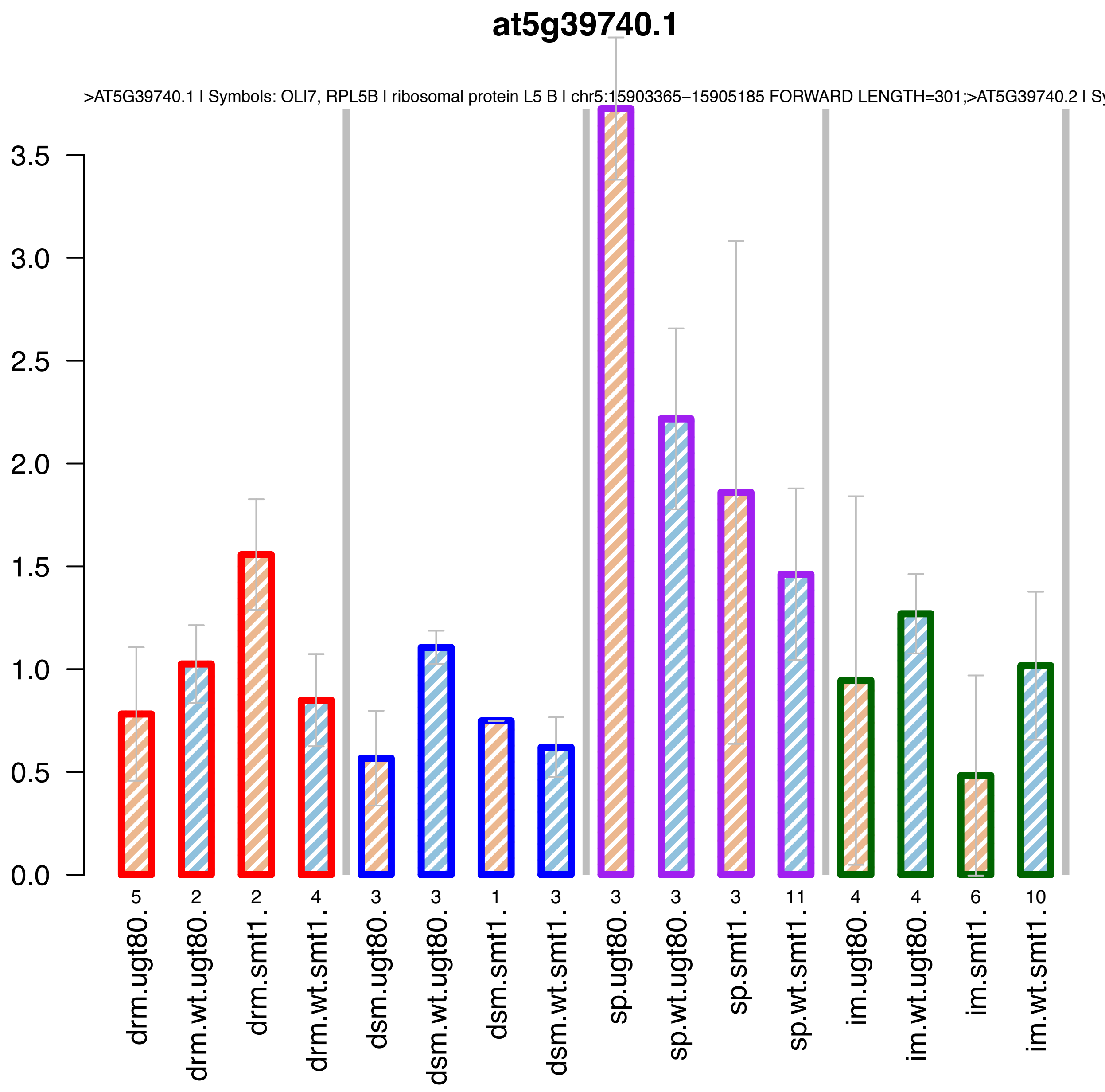
at5g39740.1
>AT5G39740.1 | Symbols: OLI7, RPL5B | ribosomal protein L5 B | chr5:15903365−15905185 FORWARD LENGTH=301;>AT5G39740.2 | Sy
0.0
0.5
1.0
1.5
2.0
2.5
3.0
3.5
5
2
2
4
3
3
1
3
3
3
3
11
4
4
6
10
drm.ugt80..
drm.wt.ugt80..
drm.smt1..
drm.wt.smt1..
dsm.ugt80..
dsm.wt.ugt80..
dsm.smt1..
dsm.wt.smt1..
sp.ugt80..
sp.wt.ugt80..
sp.smt1..
sp.wt.smt1..
im.ugt80..
im.wt.ugt80..
im.smt1..
im.wt.smt1..

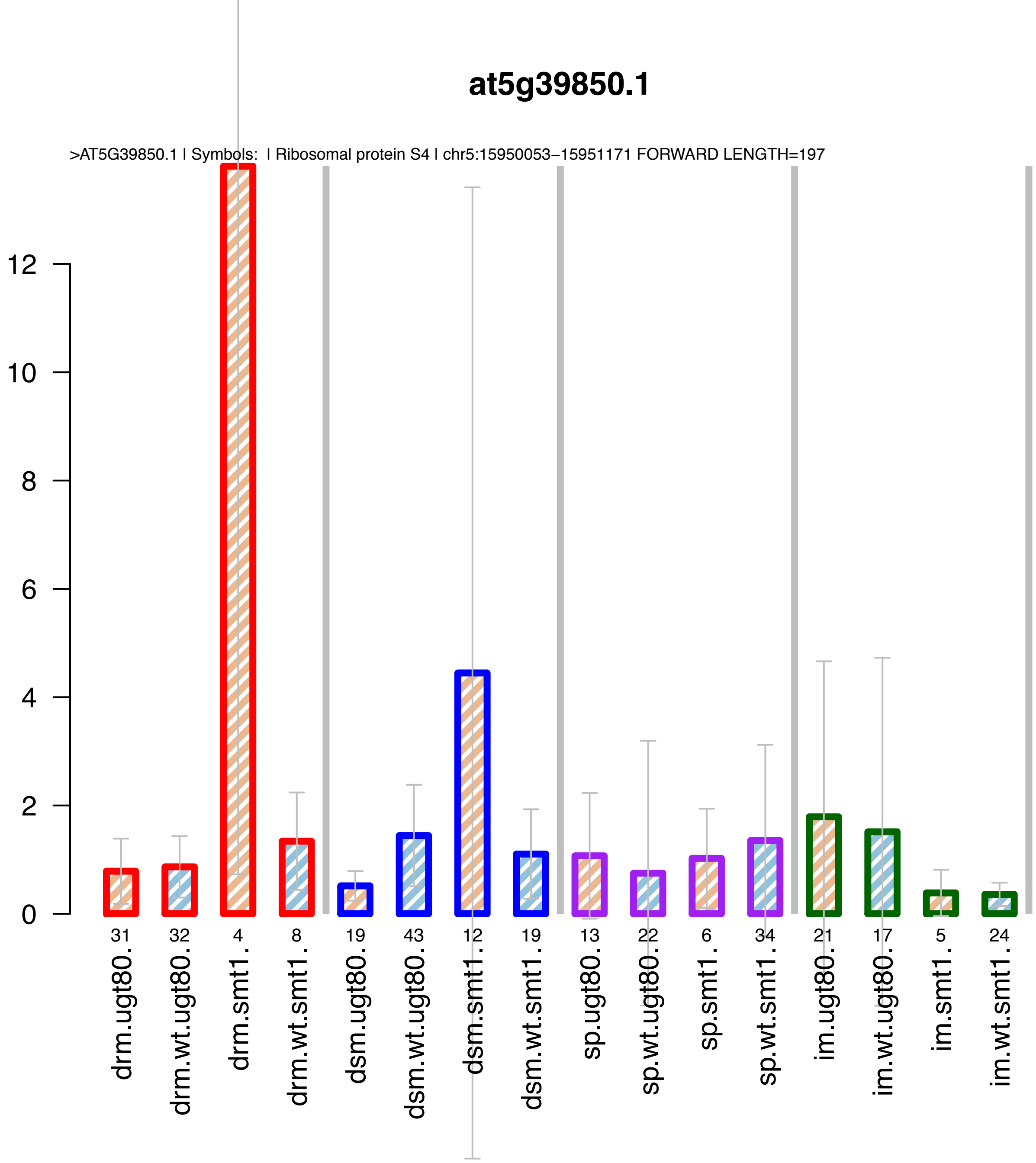

at5g39850.1
>AT5G39850.1 | Symbols: | Ribosomal protein S4 | chr5:15950053-15951171 FORWARD LENGTH=197
0
2
4
6
8
10
12
31
32
4
8
19
43
12
19
13
22
6
34
21
17
5
24
drm.ugt80.
drm.wt.ugt80.
drm.smt1.
drm.wt.smt1.
dsm.ugt80.
dsm.wt.ugt80.
dsm.smt1.
dsm.wt.smt1.
sp.ugt80.
sp.wt.ugt80.
sp.smt1.
sp.wt.smt1.
im.ugt80.
im.wt.ugt80.
im.smt1.
im.wt.smt1.

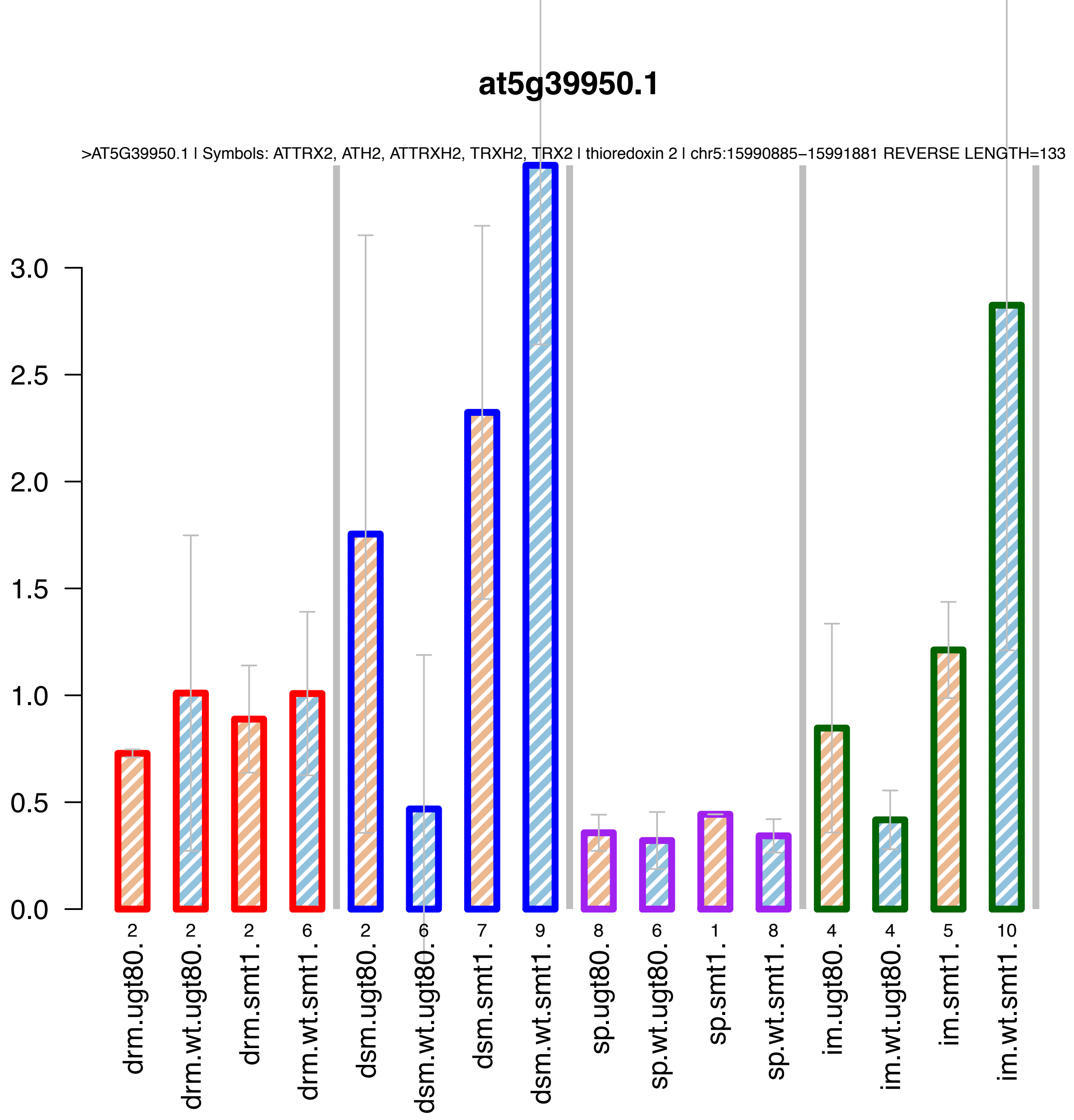

at5g39950.1
>AT5G39950.1 | Symbols: ATTRX2, ATH2, ATTRXH2, TRXH2, TRX2 | thioredoxin 2 | chr5:15990885−15991881 REVERSE LENGTH=133
0.0
0.5
1.0
1.5
2.0
2.5
3.0
2
2
2
6
2
6
7
9
8
6
1
8
4
4
5
10
drm.ugt80..
drm.wt.ugt80..
drm.smt1..
drm.wt.smt1..
dsm.ugt80..
dsm.wt.ugt80..
dsm.smt1..
dsm.wt.smt1..
sp.ugt80..
sp.wt.ugt80..
sp.smt1..
sp.wt.smt1..
im.ugt80..
im.wt.ugt80..
im.smt1..
im.wt.smt1..

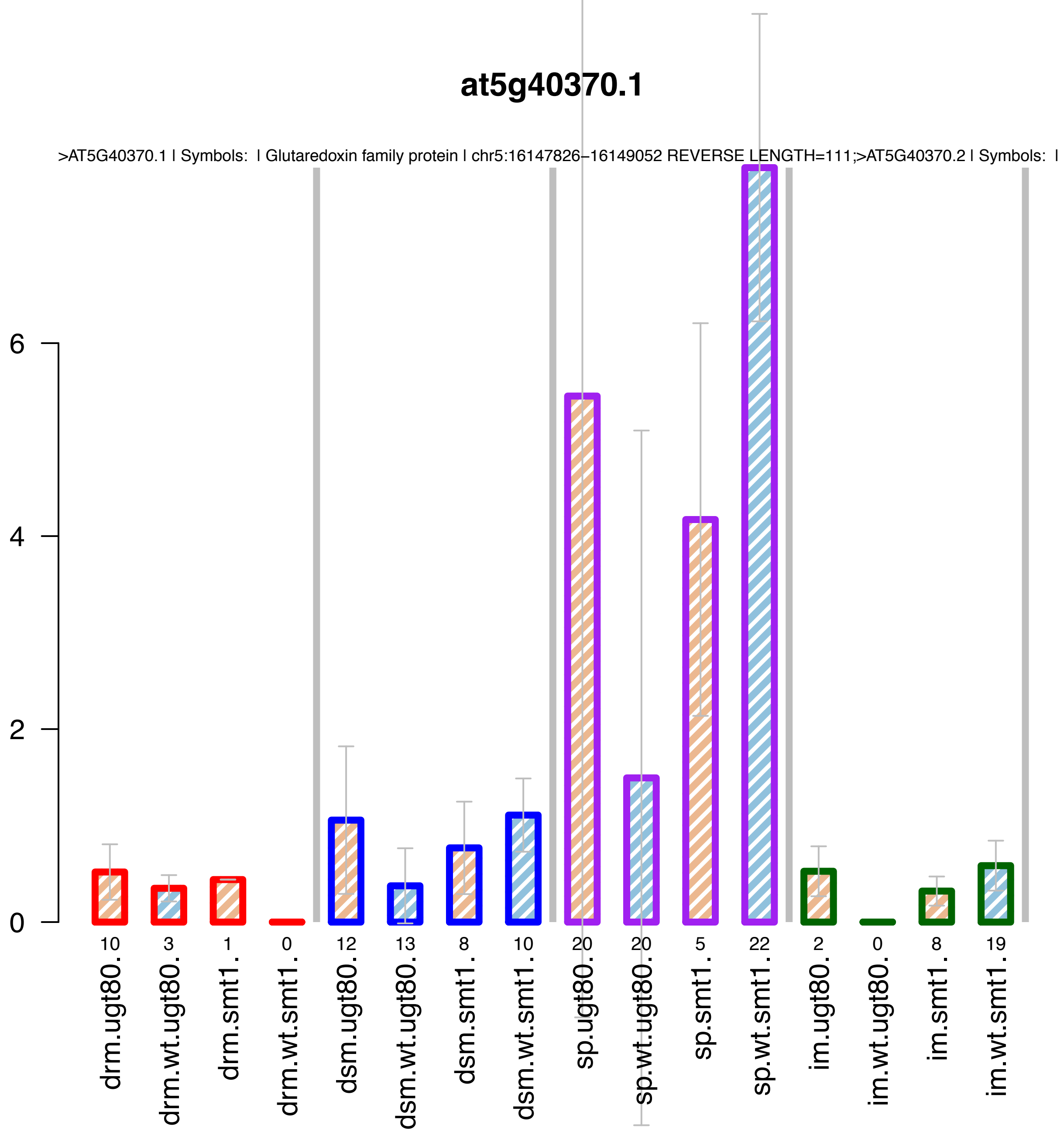

at5g40370.1
>AT5G40370.1 | Symbols: | Glutaredoxin family protein | chr5:16147826-16149052 REVERSE LENGTH=111;>AT5G40370.2 | Symbols: |
0
2
4
6
10
3
1
0
12
13
8
10
20
20
5
22
2
0
8
19
drm.ugt80..
drm.wt.ugt80..
drm.smt1..
drm.wt.smt1..
dsm.ugt80..
dsm.wt.ugt80..
dsm.smt1..
dsm.wt.smt1..
sp.ugt80..
sp.wt.ugt80..
sp.smt1..
sp.wt.smt1..
im.ugt80..
im.wt.ugt80..
im.smt1..
im.wt.smt1..

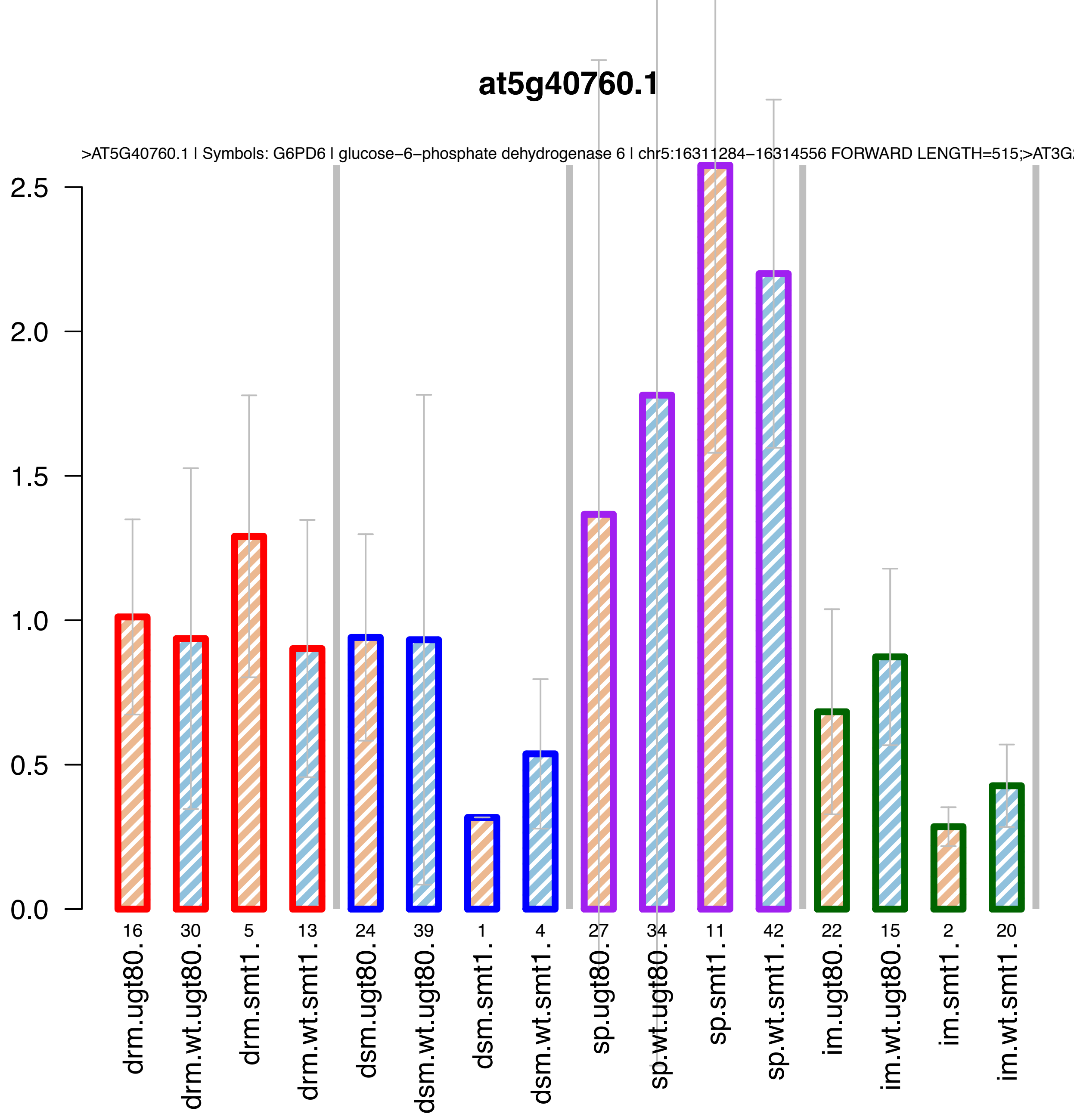

at5g40760.1
>AT5G40760.1 | Symbols: G6PD6 | glucose−6−phosphate dehydrogenase 6 | chr5:16311284−16314556 FORWARD LENGTH=515;>AT3G
0.0
0.5
1.0
1.5
2.0
2.5
16
30
5
13
24
39
1
4
27
34
11
42
22
15
2
20
drm.ugt80..
drm.wt.ugt80..
drm.smt1..
drm.wt.smt1..
dsm.ugt80..
dsm.wt.ugt80..
dsm.smt1..
dsm.wt.smt1..
sp.ugt80..
sp.wt.ugt80..
sp.smt1..
sp.wt.smt1..
im.ugt80..
im.wt.ugt80..
im.smt1..
im.wt.smt1..

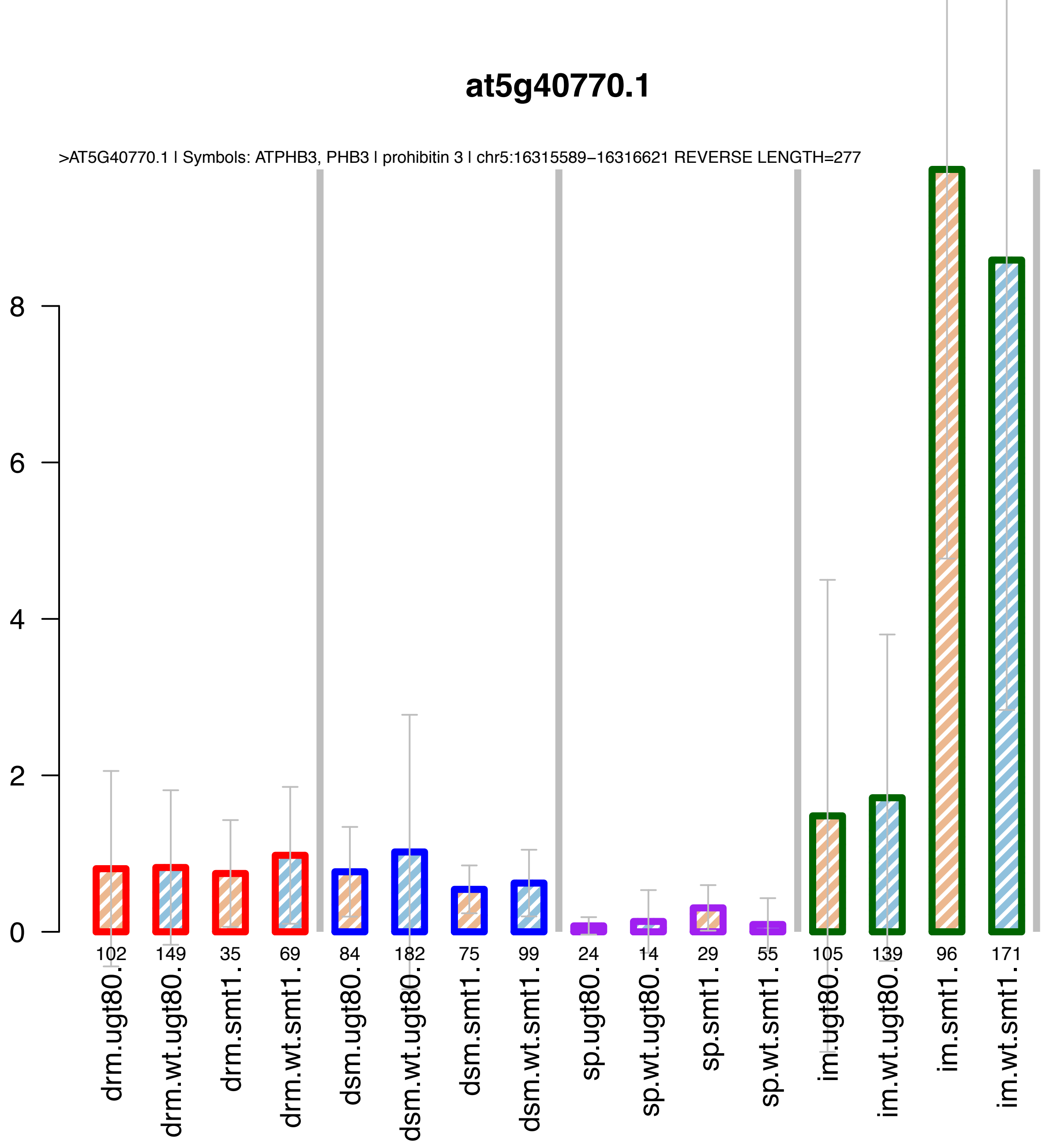
at5g40770.1
>AT5G40770.1 | Symbols: ATPHB3, PHB3 | prohibitin 3 | chr5:16315589−16316621 REVERSE LENGTH=277
0
2
4
6
8
102
149
35
69
84
182
75
99
24
14
29
55
105
139
96
171
drm.ugt80..
drm.wt.ugt80..
drm.smt1..
drm.wt.smt1..
dsm.ugt80..
dsm.wt.ugt80..
dsm.smt1..
dsm.wt.smt1..
sp.ugt80..
sp.wt.ugt80..
sp.smt1..
sp.wt.smt1..
im.ugt80..
im.wt.ugt80..
im.smt1..
im.wt.smt1..

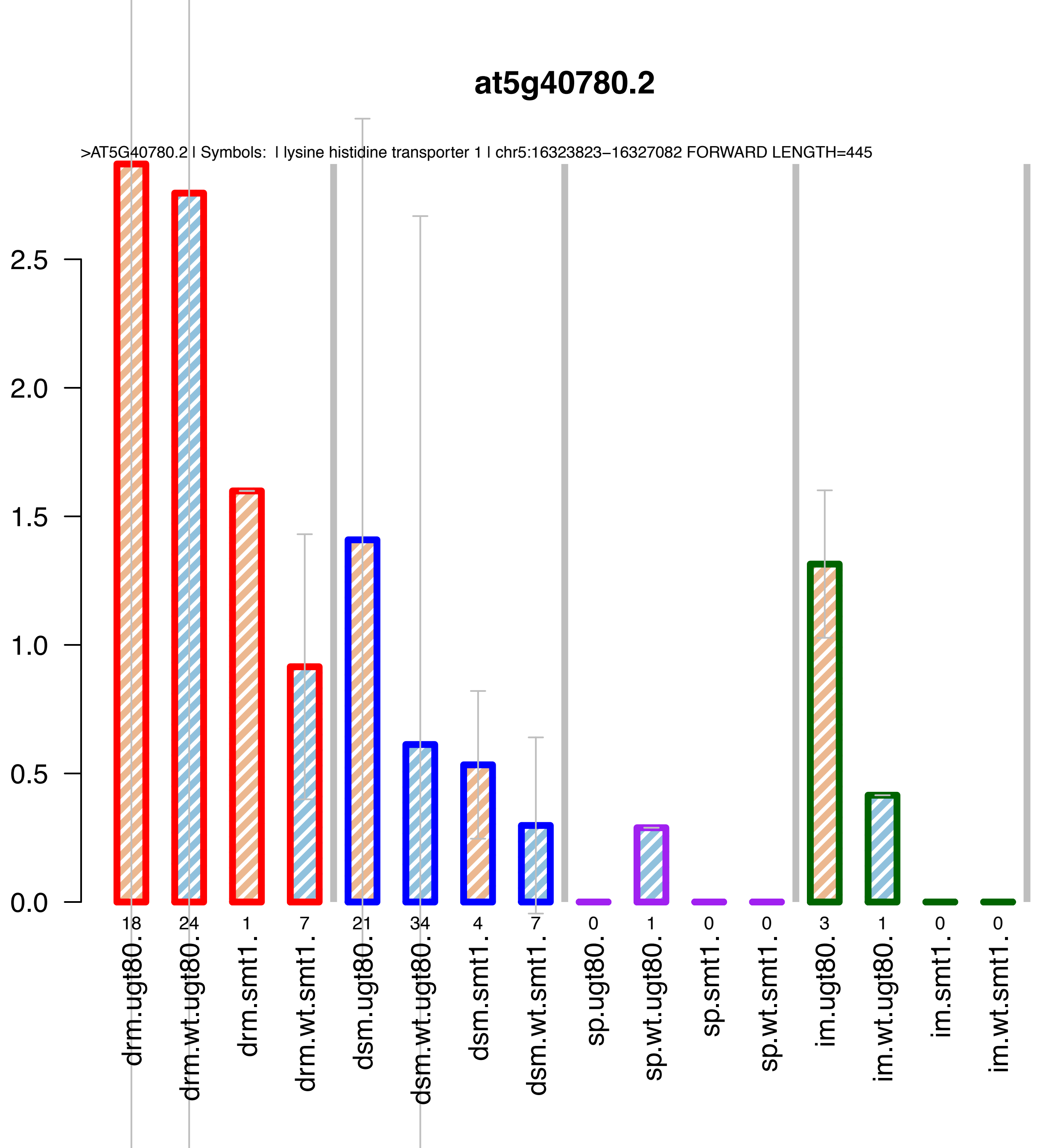

at5g40780.2
>AT5G40780.2 | Symbols: | lysine histidine transporter 1 | chr5:16323823-16327082 FORWARD LENGTH=445
0.0
0.5
1.0
1.5
2.0
2.5
18
24
1
7
21
34
4
7
0
1
0
0
3
1
0
0
drm.ugt80.
drm.wt.ugt80.
drm.smt1.
drm.wt.smt1.
dsm.ugt80.
dsm.wt.ugt80.
dsm.smt1.
dsm.wt.smt1.
sp.ugt80.
sp.wt.ugt80.
sp.smt1.
sp.wt.smt1.
im.ugt80.
im.wt.ugt80.
im.smt1.
im.wt.smt1.

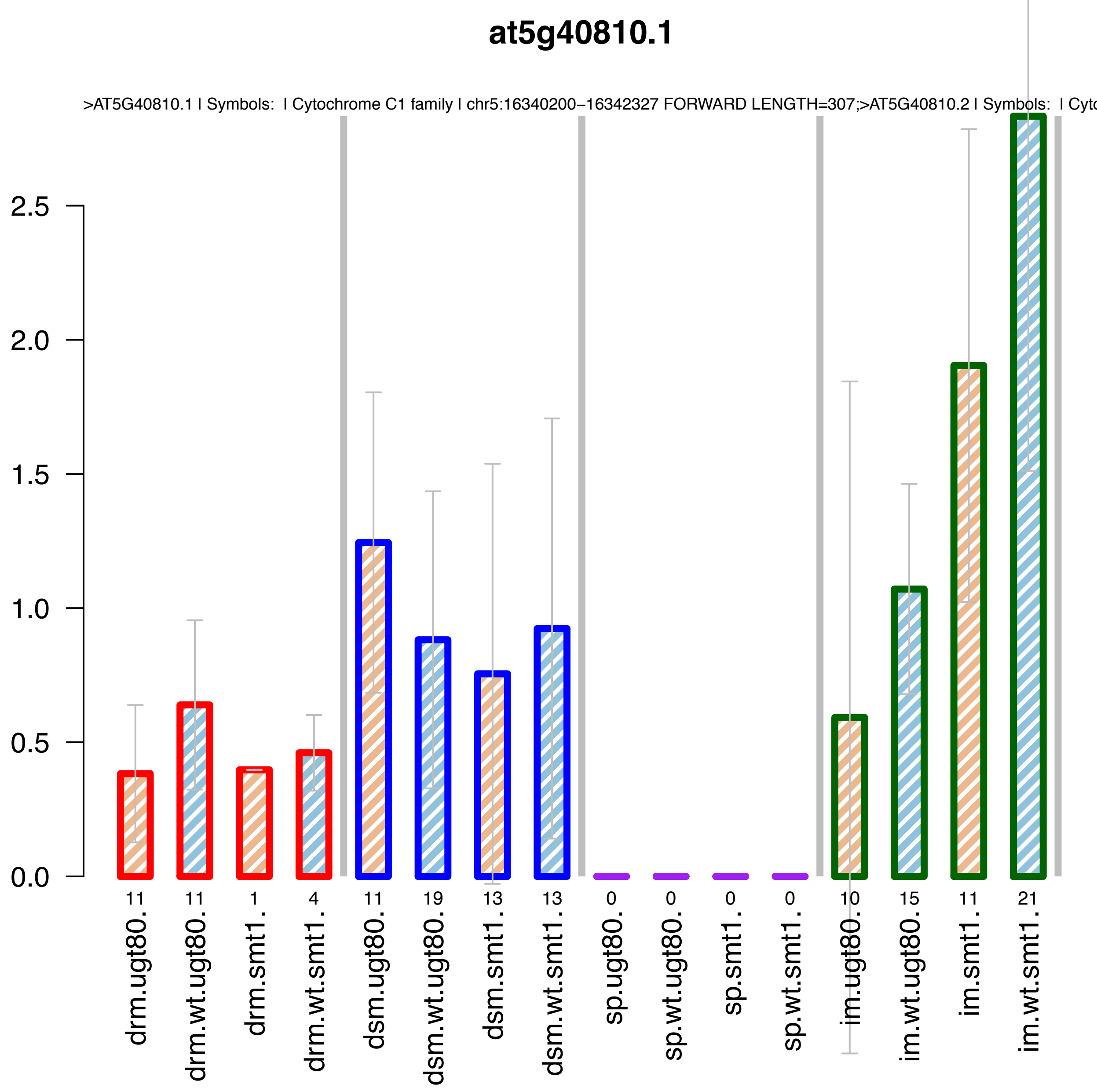

at5g40810.1
>AT5G40810.1 | Symbols: | Cytochrome C1 family | chr5:16340200-16342327 FORWARD LENGTH=307;>AT5G40810.2 | Symbols: | Cyto
2.5
2.0
1.5
1.0
0.5
0.0
11
11
1
4
11
19
13
13
0
0
0
0
10
15
11
21
drm.ugt80.
drm.wt.ugt80.
drm.smt1.
drm.wt.smt1.
dsm.ugt80.
dsm.wt.ugt80.
dsm.smt1.
dsm.wt.smt1.
sp.ugt80.
sp.wt.ugt80.
sp.smt1.
sp.wt.smt1.
im.ugt80.
im.wt.ugt80.
im.smt1.
im.wt.smt1.

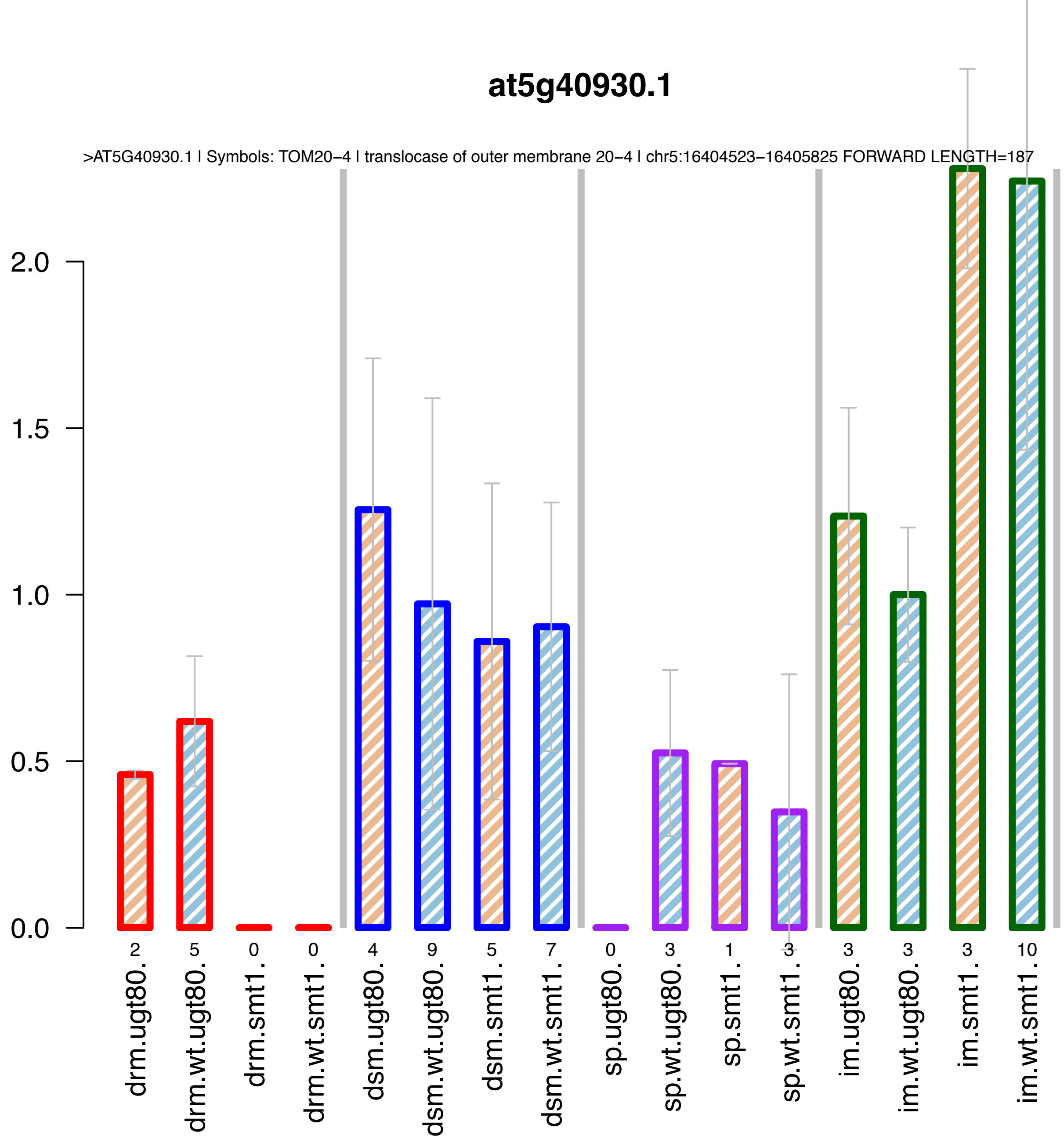

at5g40930.1
>AT5G40930.1 | Symbols: TOM20-4 | translocase of outer membrane 20-4 | chr5:16404523-16405825 FORWARD LENGTH=187
2.0
1.5
1.0
0.5
0.0
2
5
0
0
4
9
5
7
0
3
1
3
3
3
3
10
drm.ugt80..
drm.wt.ugt80..
drm.smt1..
drm.wt.smt1..
dsm.ugt80..
dsm.wt.ugt80..
dsm.smt1..
dsm.wt.smt1..
sp.ugt80..
sp.wt.ugt80..
sp.smt1..
sp.wt.smt1..
im.ugt80..
im.wt.ugt80..
im.smt1..
im.wt.smt1..

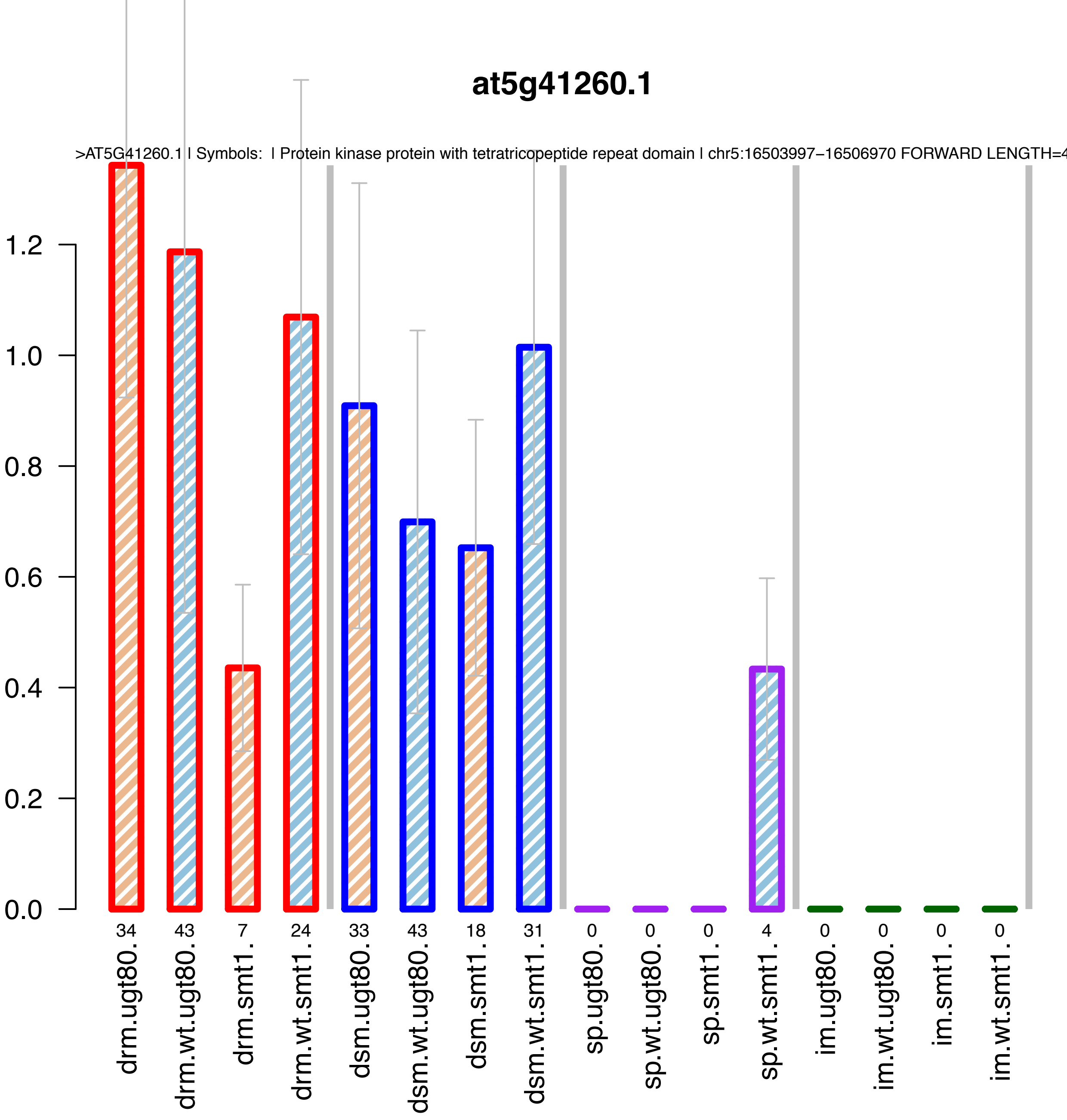

at5g41260.1
>AT5G41260.1 | Symbols: | Protein kinase protein with tetratricopeptide repeat domain | chr5:16503997-16506970 FORWARD LENGTH=4
0.0
0.2
0.4
0.6
0.8
1.0
1.2
34
43
7
24
33
43
18
31
0
0
0
4
0
0
0
0
drm.ugt80.
drm.wt.ugt80.
drm.smt1.
drm.wt.smt1.
dsm.ugt80.
dsm.wt.ugt80.
dsm.smt1.
dsm.wt.smt1.
sp.ugt80.
sp.wt.ugt80.
sp.smt1.
sp.wt.smt1.
im.ugt80.
im.wt.ugt80.
im.smt1.
im.wt.smt1.

# at5g41520.1

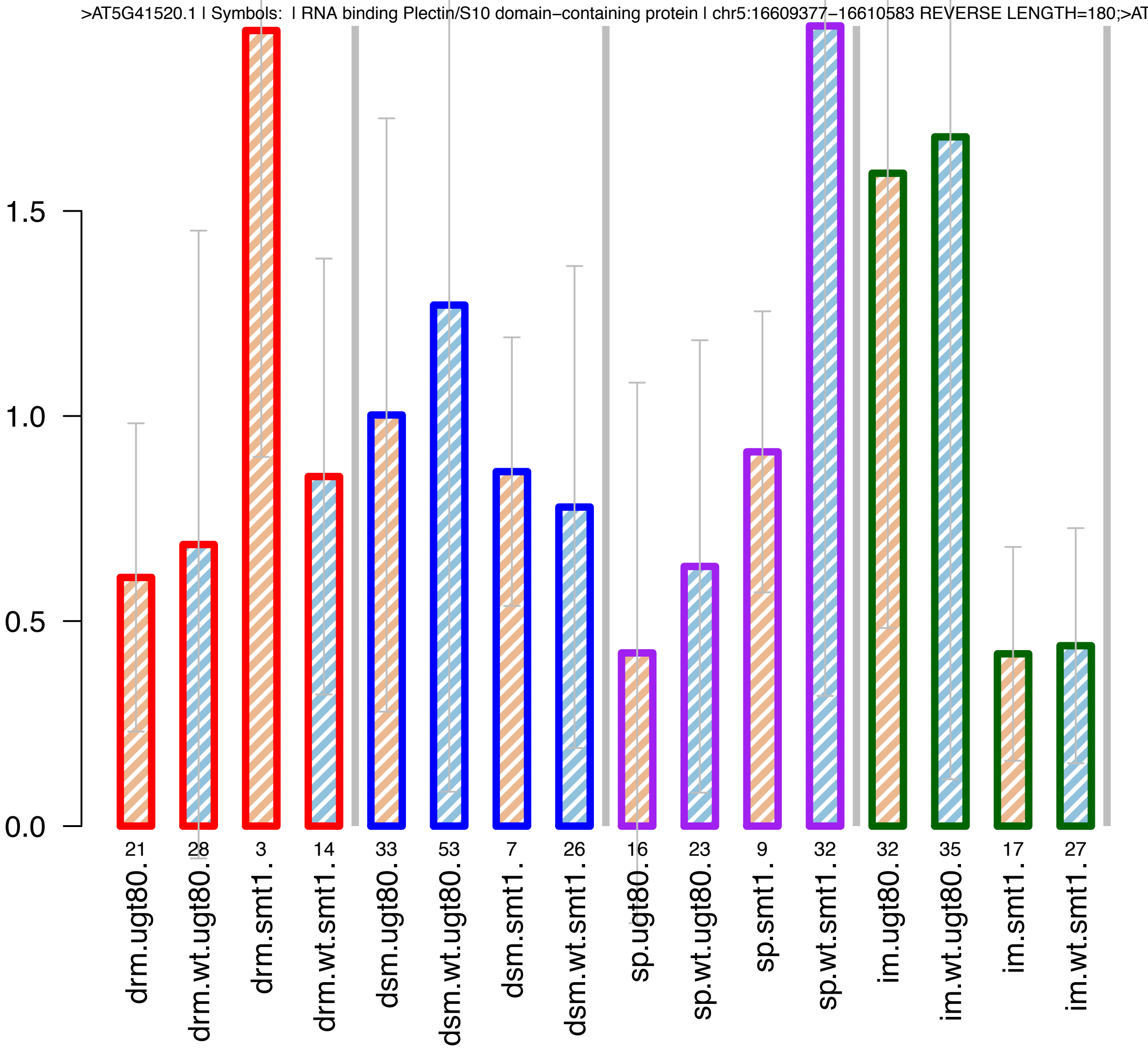

>AT5G41520.1 | Symbols: | RNA binding Plectin/S10 domain-containing protein | chr5:16609377-16610583 REVERSE LENGTH=180;>AT
1.5
1.0
0.5
0.0
21
28
3
14
33
53
7
26
16
23
9
32
32
35
17
27
drm.ugt80..
drm.wt.ugt80..
drm.smt1..
drm.wt.smt1..
dsm.ugt80..
dsm.wt.ugt80..
dsm.smt1..
dsm.wt.smt1..
sp.ugt80..
sp.wt.ugt80..
sp.smt1..
sp.wt.smt1..
im.ugt80..
im.wt.ugt80..
im.smt1..
im.wt.smt1..

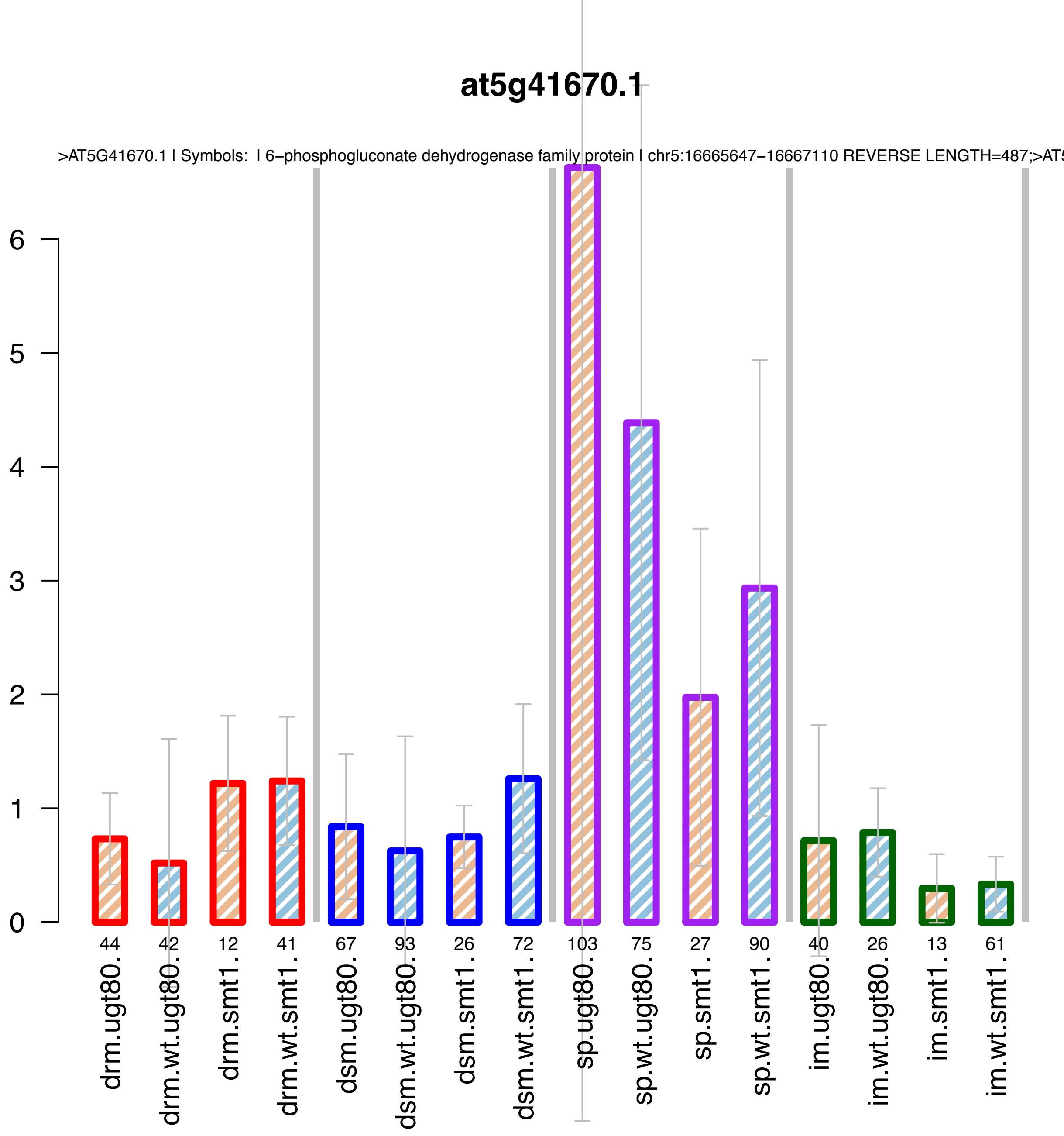

at5g41670.1
>AT5G41670.1 | Symbols: | 6-phosphogluconate dehydrogenase family protein | chr5:16665647-16667110 REVERSE LENGTH=487;>AT5
0
1
2
3
4
5
6
44
42
12
41
67
93
26
72
103
75
27
90
40
26
13
61
drm.ugt80..
drm.wt.ugt80..
drm.smt1..
drm.wt.smt1..
dsm.ugt80..
dsm.wt.ugt80..
dsm.smt1..
dsm.wt.smt1..
sp.ugt80..
sp.wt.ugt80..
sp.smt1..
sp.wt.smt1..
im.ugt80..
im.wt.ugt80..
im.smt1..
im.wt.smt1..

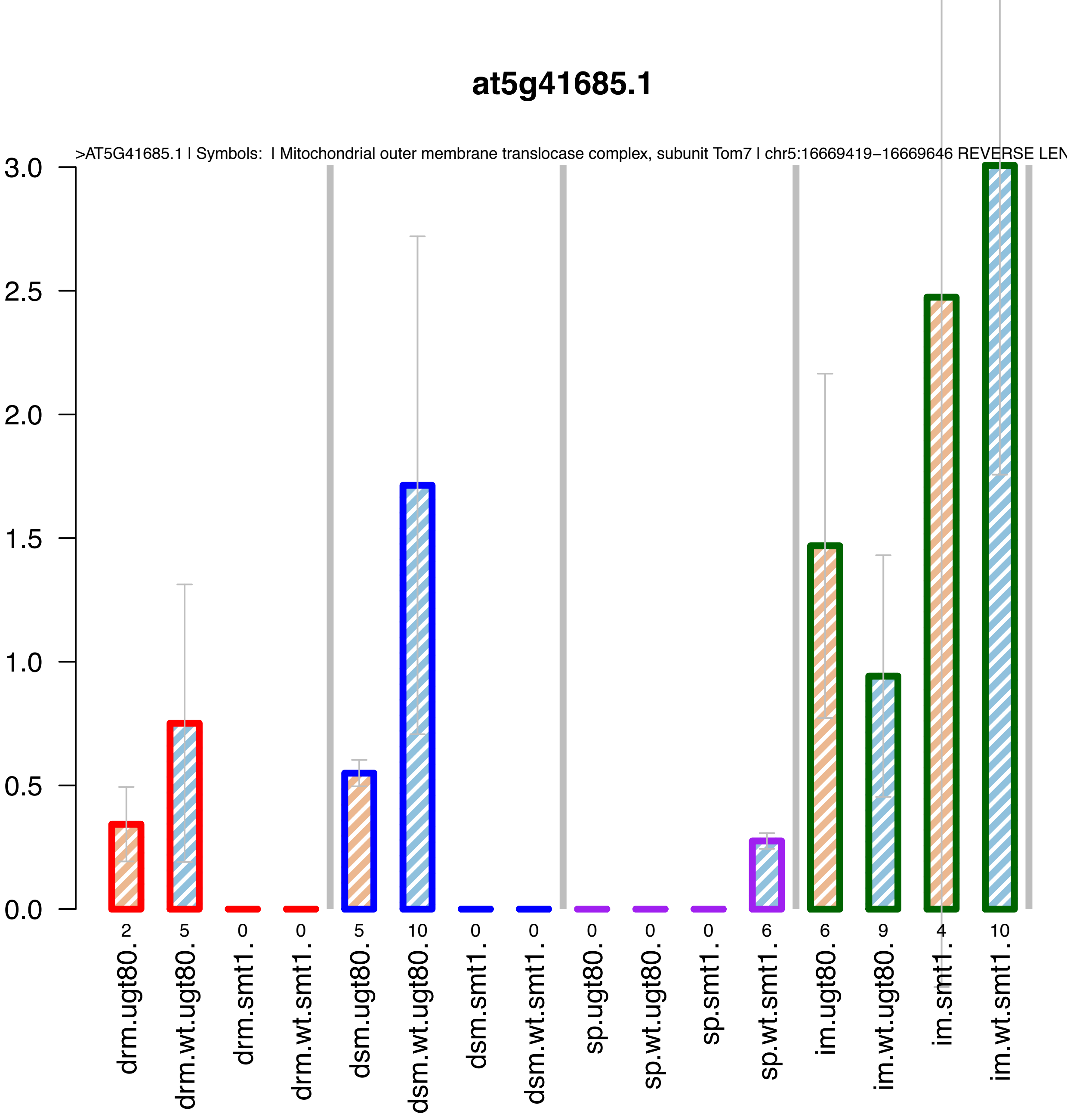

at5g41685.1
>AT5G41685.1 | Symbols: | Mitochondrial outer membrane translocase complex, subunit Tom7 | chr5:16669419-16669646 REVERSE LEN
3.0
2.5
2.0
1.5
1.0
0.5
0.0
2
5
0
0
5
10
0
0
0
0
0
6
6
9
4
10
drm.ugt80.
drm.wt.ugt80.
drm.smt1.
drm.wt.smt1.
dsm.ugt80.
dsm.wt.ugt80.
dsm.smt1.
dsm.wt.smt1.
sp.ugt80.
sp.wt.ugt80.
sp.smt1.
sp.wt.smt1.
im.ugt80.
im.wt.ugt80.
im.smt1.
im.wt.smt1.

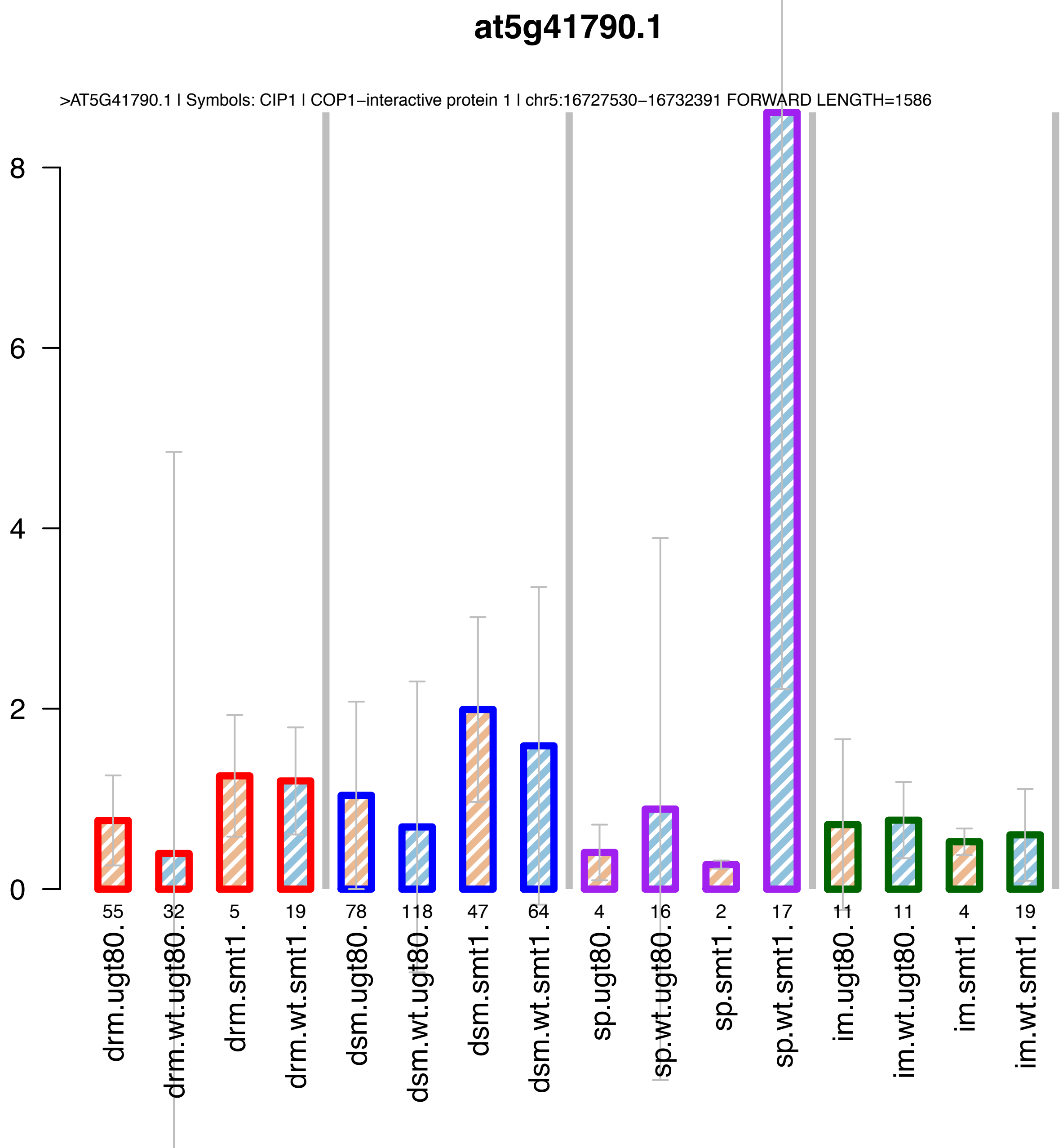

at5g41790.1
>AT5G41790.1 | Symbols: CIP1 | COP1−interactive protein 1 | chr5:16727530−16732391 FORWARD LENGTH=1586
0
2
4
6
8
55
32
5
19
78
118
47
64
4
16
2
17
11
11
4
19
drm.ugt80..
drm.wt.ugt80..
drm.smt1..
drm.wt.smt1..
dsm.ugt80..
dsm.wt.ugt80..
dsm.smt1..
dsm.wt.smt1..
sp.ugt80..
sp.wt.ugt80..
sp.smt1..
sp.wt.smt1..
im.ugt80..
im.wt.ugt80..
im.smt1..
im.wt.smt1..

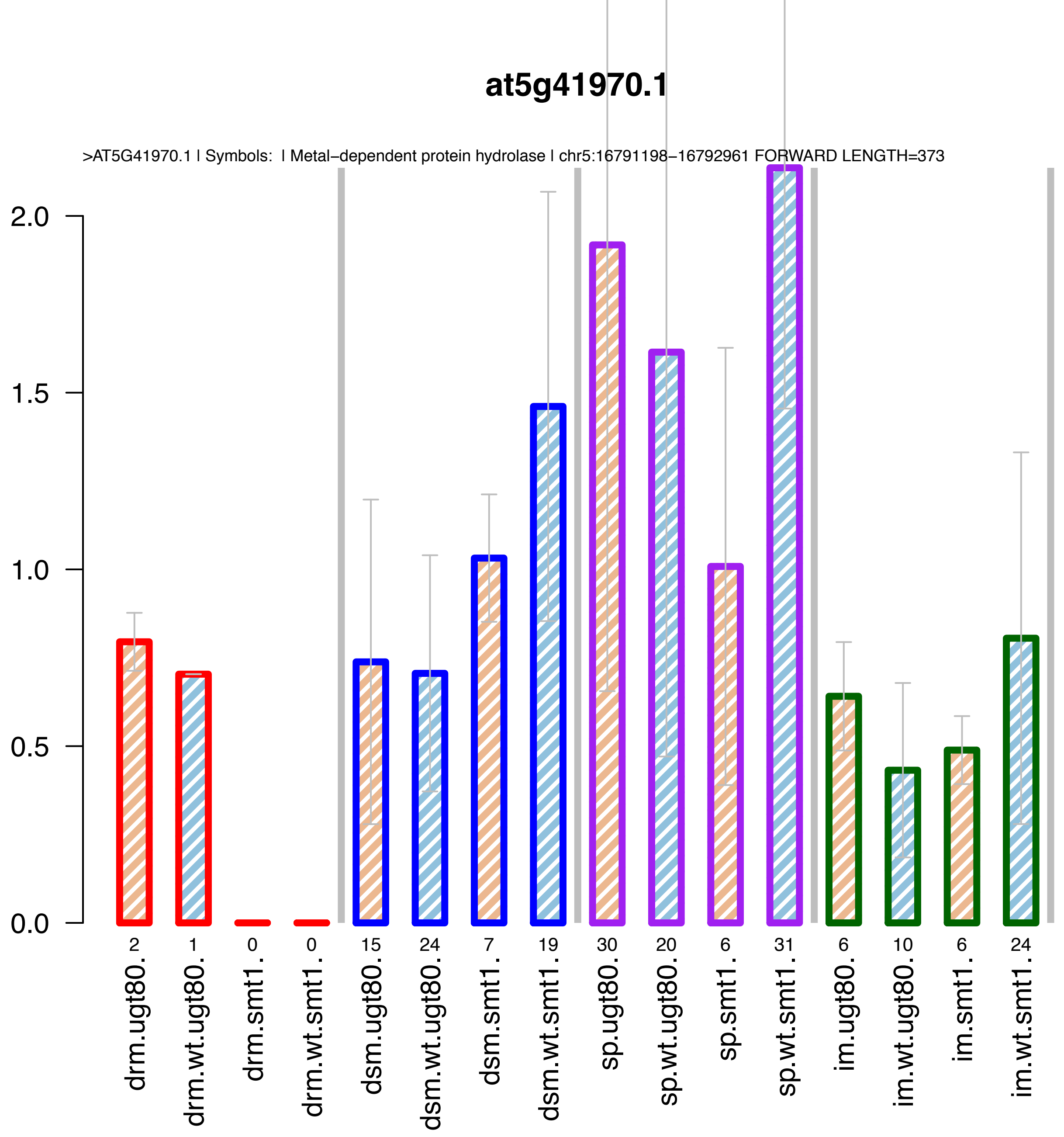

at5g41970.1
>AT5G41970.1 | Symbols: | Metal−dependent protein hydrolase | chr5:16791198−16792961 FORWARD LENGTH=373
0.0
0.5
1.0
1.5
2.0
2
1
0
0
15
24
7
19
30
20
6
31
6
10
6
24
drm.ugt80..
drm.wt.ugt80..
drm.smt1..
drm.wt.smt1..
dsm.ugt80..
dsm.wt.ugt80..
dsm.smt1..
dsm.wt.smt1..
sp.ugt80..
sp.wt.ugt80..
sp.smt1..
sp.wt.smt1..
im.ugt80..
im.wt.ugt80..
im.smt1..
im.wt.smt1..

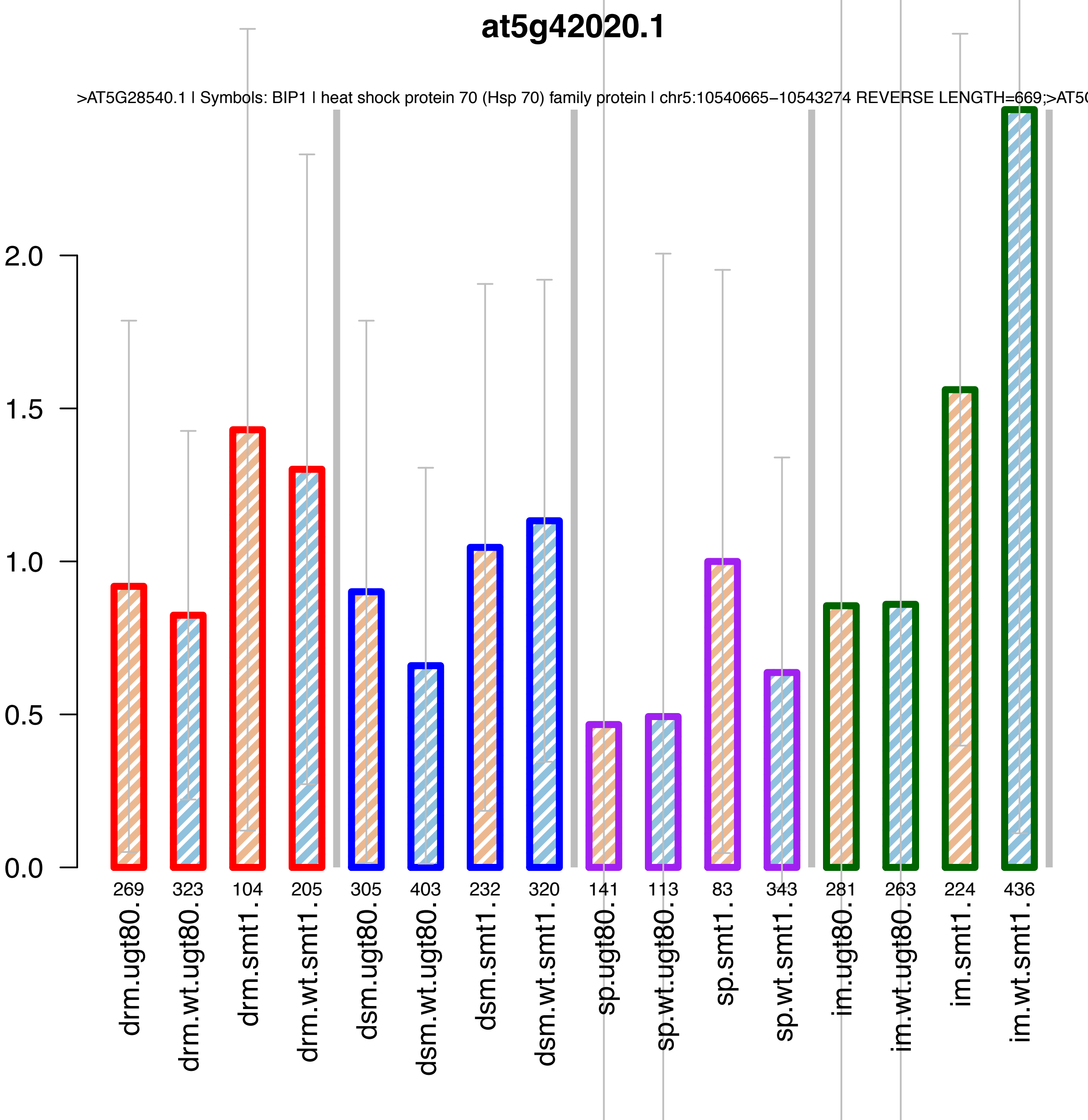

at5g42020.1
>AT5G28540.1 | Symbols: BIP1 | heat shock protein 70 (Hsp 70) family protein | chr5:10540665-10543274 REVERSE LENGTH=669;>AT5
2.0
1.5
1.0
0.5
0.0
269
323
104
205
305
403
232
320
141
113
83
343
281
263
224
436
drm.ugt80..
drm.wt.ugt80..
drm.smt1..
drm.wt.smt1..
dsm.ugt80..
dsm.wt.ugt80..
dsm.smt1..
dsm.wt.smt1..
sp.ugt80..
sp.wt.ugt80..
sp.smt1..
sp.wt.smt1..
im.ugt80..
im.wt.ugt80..
im.smt1..
im.wt.smt1..

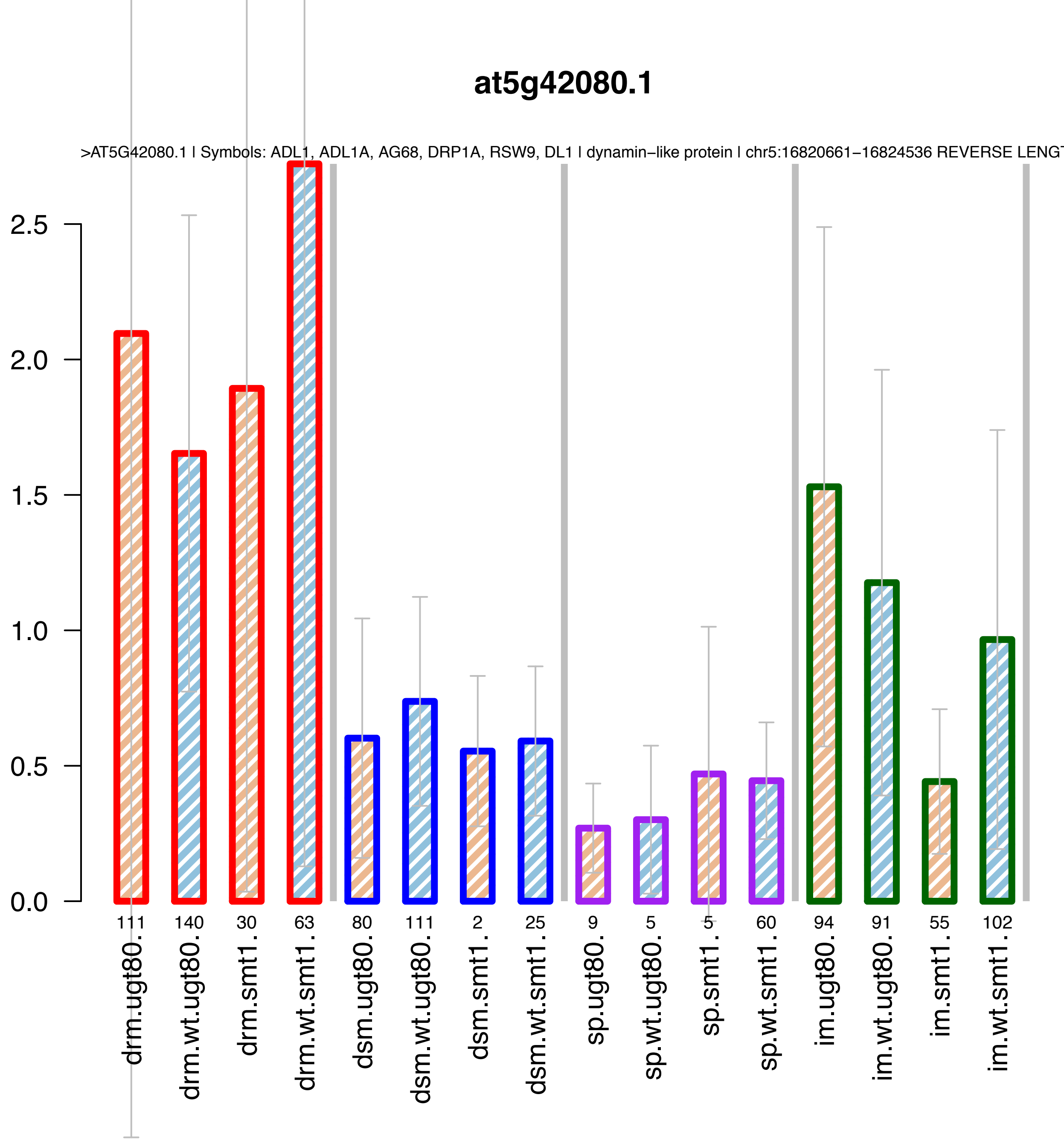

at5g42080.1
>AT5G42080.1 | Symbols: ADL1, ADL1A, AG68, DRP1A, RSW9, DL1 | dynamin−like protein | chr5:16820661−16824536 REVERSE LENGT
2.5
2.0
1.5
1.0
0.5
0.0
111
140
30
63
80
111
2
25
9
5
5
60
94
91
55
102
drm.ugt80..
drm.wt.ugt80..
drm.smt1..
drm.wt.smt1..
dsm.ugt80..
dsm.wt.ugt80..
dsm.smt1..
dsm.wt.smt1..
sp.ugt80..
sp.wt.ugt80..
sp.smt1..
sp.wt.smt1..
im.ugt80..
im.wt.ugt80..
im.smt1..
im.wt.smt1..

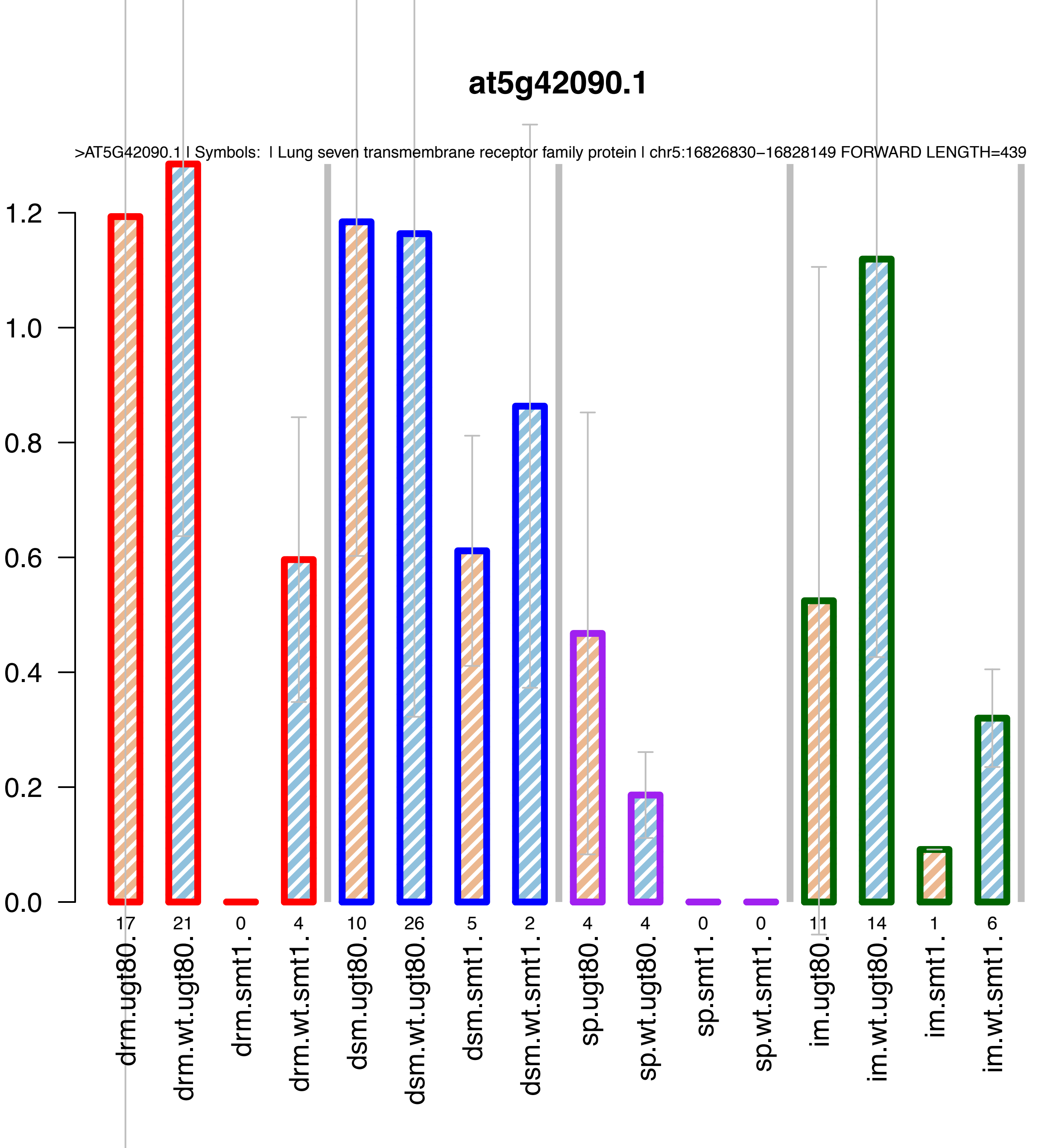

at5g42090.1
>AT5G42090.1 | Symbols: | Lung seven transmembrane receptor family protein | chr5:16826830-16828149 FORWARD LENGTH=439
1.2
1.0
0.8
0.6
0.4
0.2
0.0
17
21
0
4
10
26
5
2
4
4
0
0
11
14
1
6
drm.ugt80.
drm.wt.ugt80.
drm.smt1.
drm.wt.smt1.
dsm.ugt80.
dsm.wt.ugt80.
dsm.smt1.
dsm.wt.smt1.
sp.ugt80.
sp.wt.ugt80.
sp.smt1.
sp.wt.smt1.
im.ugt80.
im.wt.ugt80.
im.smt1.
im.wt.smt1.

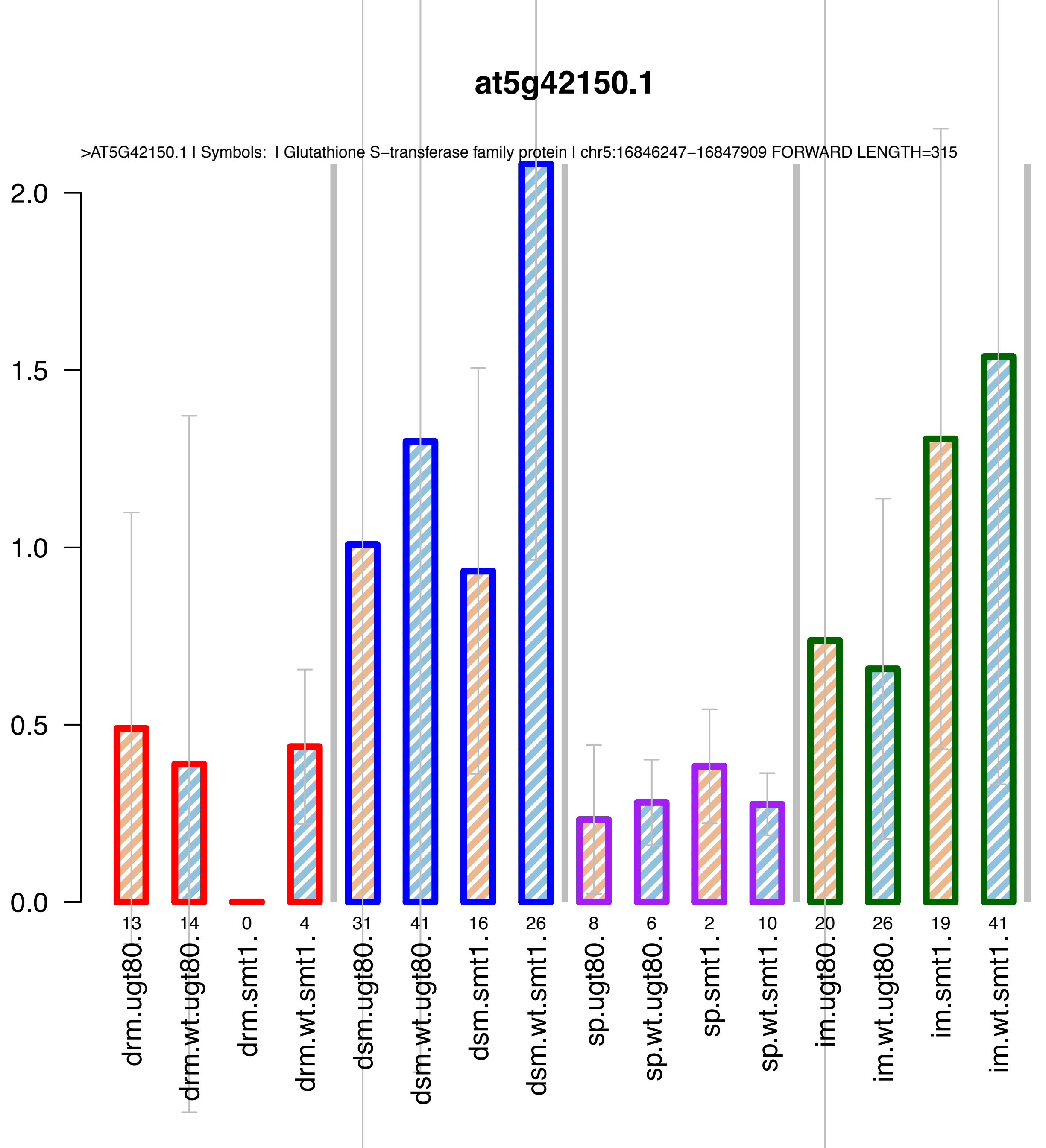

at5g42150.1
>AT5G42150.1 | Symbols: | Glutathione S−transferase family protein | chr5:16846247−16847909 FORWARD LENGTH=315
2.0
1.5
1.0
0.5
0.0
13
14
0
4
31
41
16
26
8
6
2
10
20
26
19
41
drm.ugt80.
drm.wt.ugt80.
drm.smt1.
drm.wt.smt1.
dsm.ugt80.
dsm.wt.ugt80.
dsm.smt1.
dsm.wt.smt1.
sp.ugt80.
sp.wt.ugt80.
sp.smt1.
sp.wt.smt1.
im.ugt80.
im.wt.ugt80.
im.smt1.
im.wt.smt1.

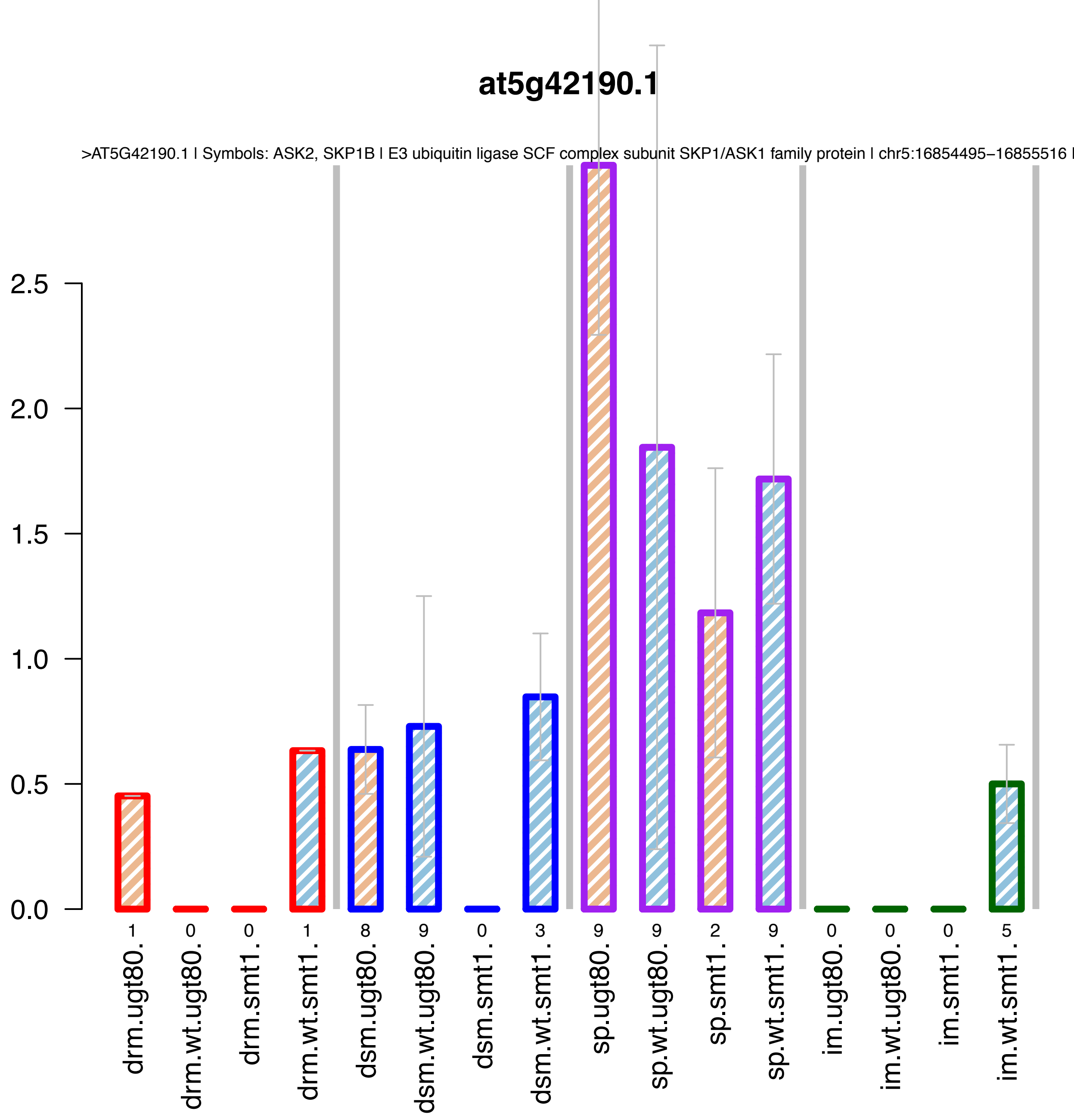

at5g42190.1
>AT5G42190.1 | Symbols: ASK2, SKP1B | E3 ubiquitin ligase SCF complex subunit SKP1/ASK1 family protein | chr5:16854495-16855516 |
2.5
2.0
1.5
1.0
0.5
0.0
1
0
0
1
8
9
0
3
9
9
2
9
0
0
0
5
drm.ugt80.
drm.wt.ugt80.
drm.smt1.
drm.wt.smt1.
dsm.ugt80.
dsm.wt.ugt80.
dsm.smt1.
dsm.wt.smt1.
sp.ugt80.
sp.wt.ugt80.
sp.smt1.
sp.wt.smt1.
im.ugt80.
im.wt.ugt80.
im.smt1.
im.wt.smt1.

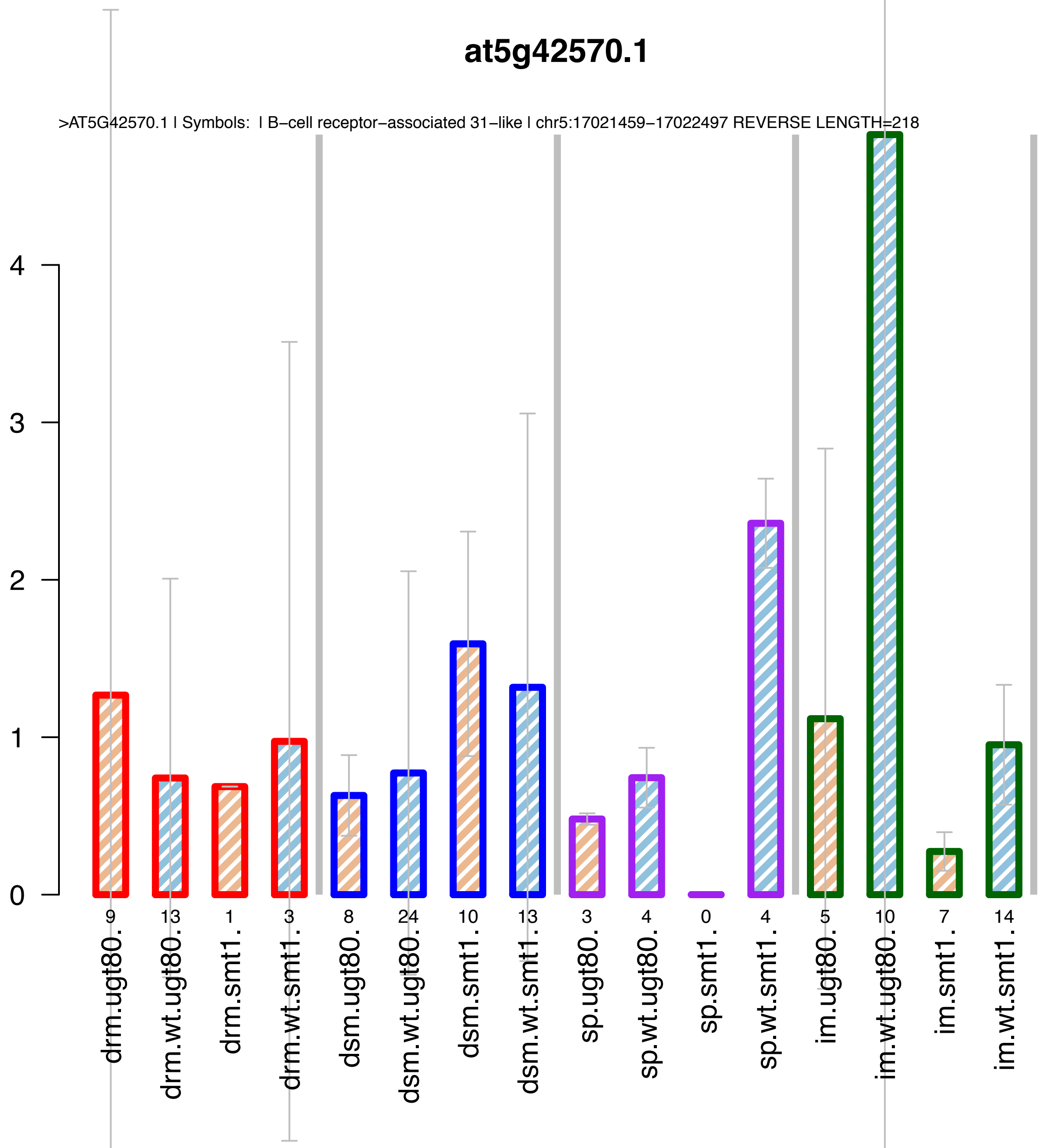

at5g42570.1
>AT5G42570.1 | Symbols: | B−cell receptor−associated 31−like | chr5:17021459−17022497 REVERSE LENGTH=218
0
1
2
3
4
9
13
1
3
8
24
10
13
3
4
0
4
5
10
7
14
drm.ugt80..
drm.wt.ugt80..
drm.smt1..
drm.wt.smt1..
dsm.ugt80..
dsm.wt.ugt80..
dsm.smt1..
dsm.wt.smt1..
sp.ugt80..
sp.wt.ugt80..
sp.smt1..
sp.wt.smt1..
im.ugt80..
im.wt.ugt80..
im.smt1..
im.wt.smt1..

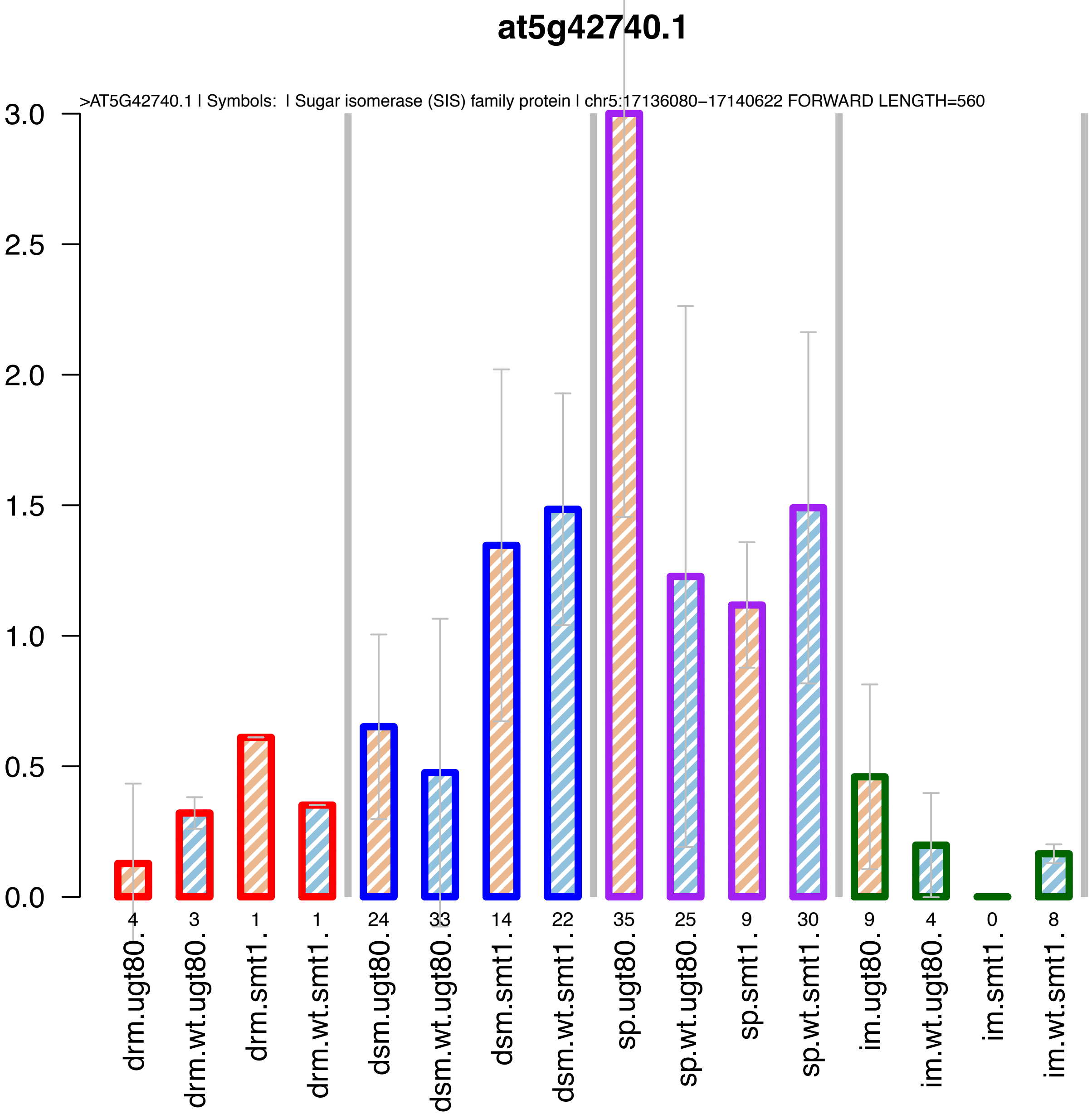

at5g42740.1
>AT5G42740.1 | Symbols: | Sugar isomerase (SIS) family protein | chr5:17136080-17140622 FORWARD LENGTH=560
3.0
2.5
2.0
1.5
1.0
0.5
0.0
4
3
1
1
24
33
14
22
35
25
9
30
9
4
0
8
drm.ugt80.
drm.wt.ugt80.
drm.smt1.
drm.wt.smt1.
dsm.ugt80.
dsm.wt.ugt80.
dsm.smt1.
dsm.wt.smt1.
sp.ugt80.
sp.wt.ugt80.
sp.smt1.
sp.wt.smt1.
im.ugt80.
im.wt.ugt80.
im.smt1.
im.wt.smt1.

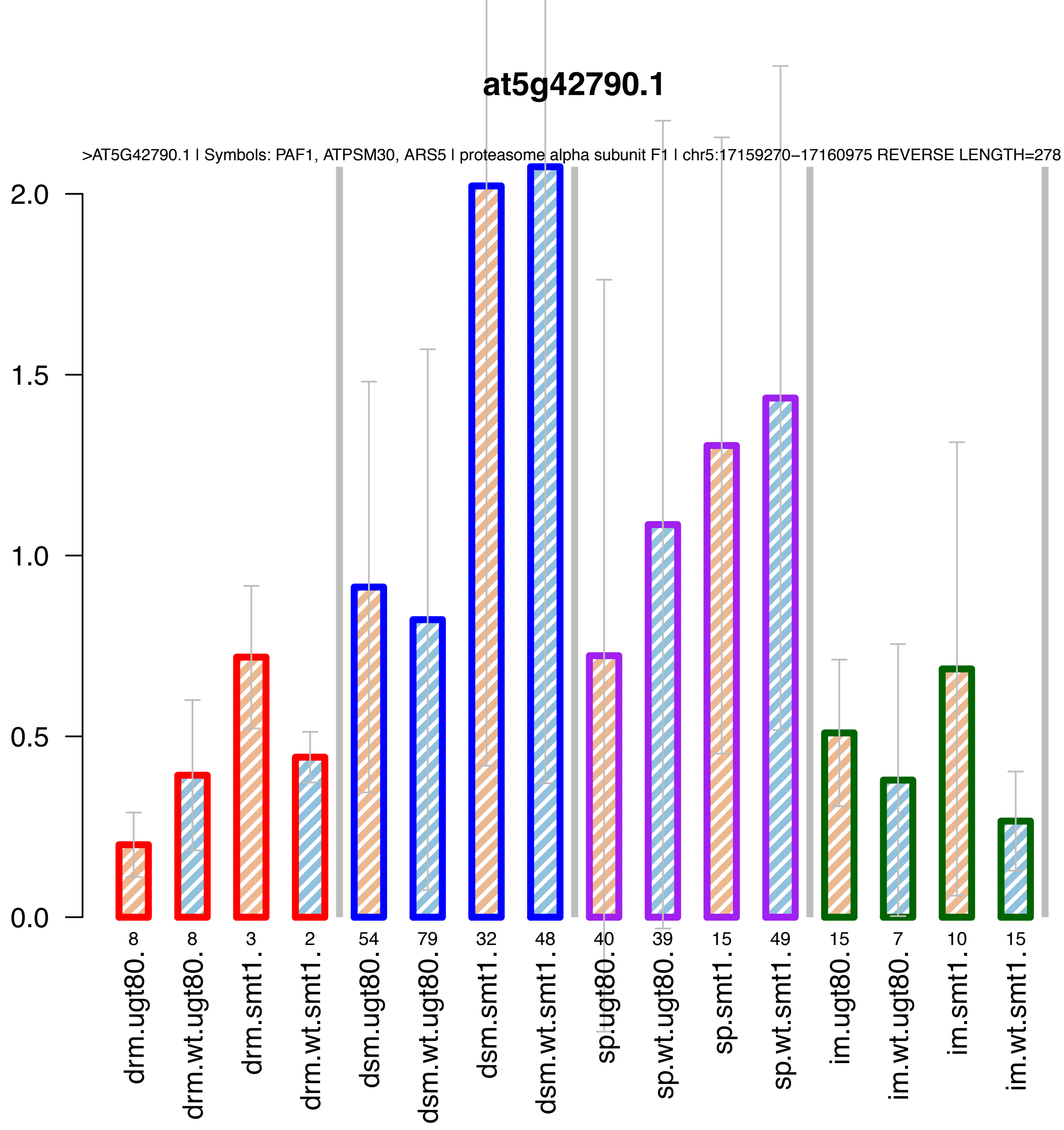

at5g42790.1
>AT5G42790.1 | Symbols: PAF1, ATPSM30, ARS5 | proteasome alpha subunit F1 | chr5:17159270–17160975 REVERSE LENGTH=278
2.0
1.5
1.0
0.5
0.0
8
8
3
2
54
79
32
48
40
39
15
49
15
7
10
15
drm.ugt80..
drm.wt.ugt80..
drm.smt1..
drm.wt.smt1..
dsm.ugt80..
dsm.wt.ugt80..
dsm.smt1..
dsm.wt.smt1..
sp.ugt80..
sp.wt.ugt80..
sp.smt1..
sp.wt.smt1..
im.ugt80..
im.wt.ugt80..
im.smt1..
im.wt.smt1..

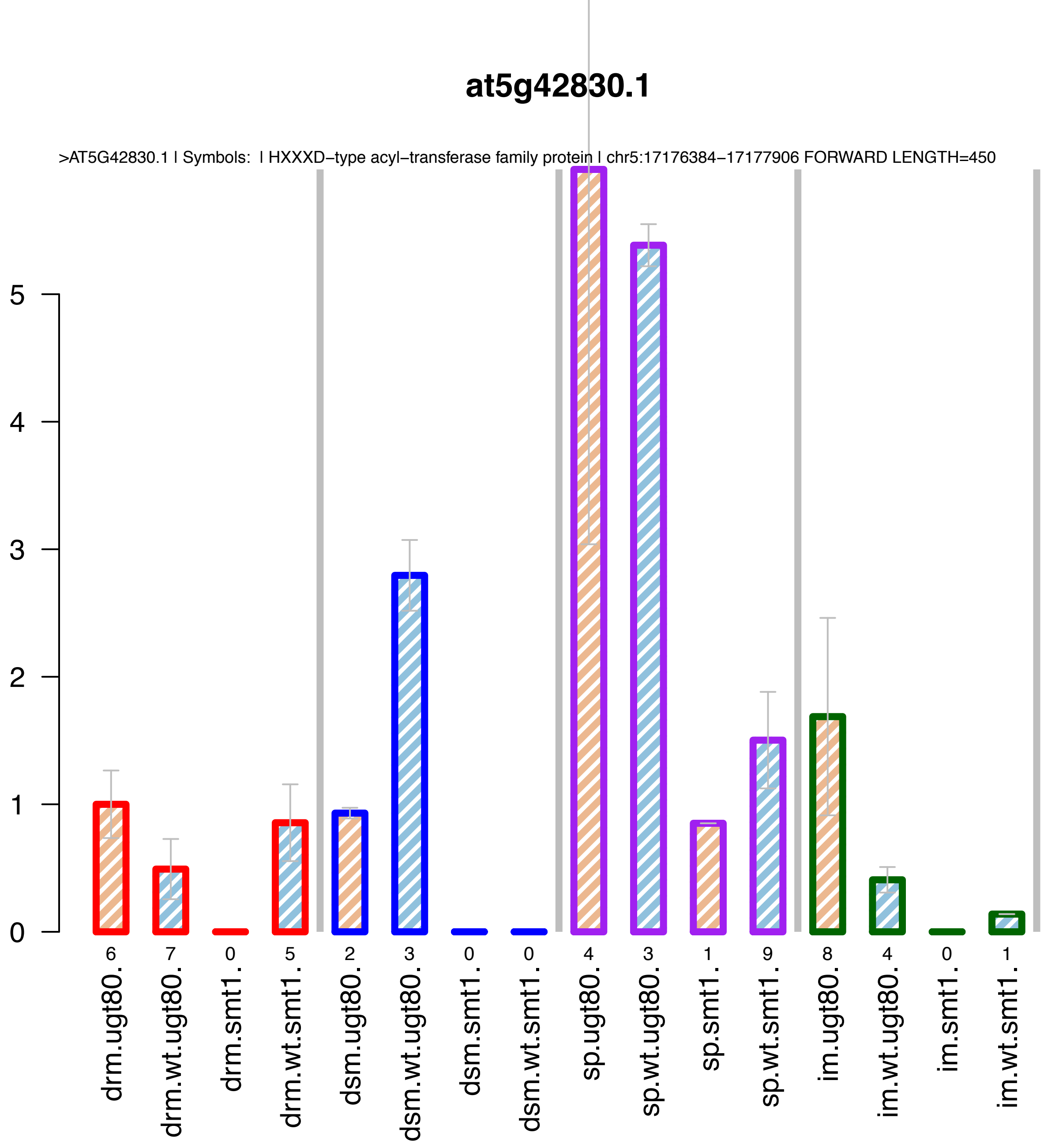

at5g42830.1
>AT5G42830.1 | Symbols: | HXXXD−type acyl−transferase family protein | chr5:17176384−17177906 FORWARD LENGTH=450
0
1
2
3
4
5
6
7
0
5
2
3
0
0
4
3
1
9
8
4
0
1
drm.ugt80.
drm.wt.ugt80.
drm.smt1.
drm.wt.smt1.
dsm.ugt80.
dsm.wt.ugt80.
dsm.smt1.
dsm.wt.smt1.
sp.ugt80.
sp.wt.ugt80.
sp.smt1.
sp.wt.smt1.
im.ugt80.
im.wt.ugt80.
im.smt1.
im.wt.smt1.

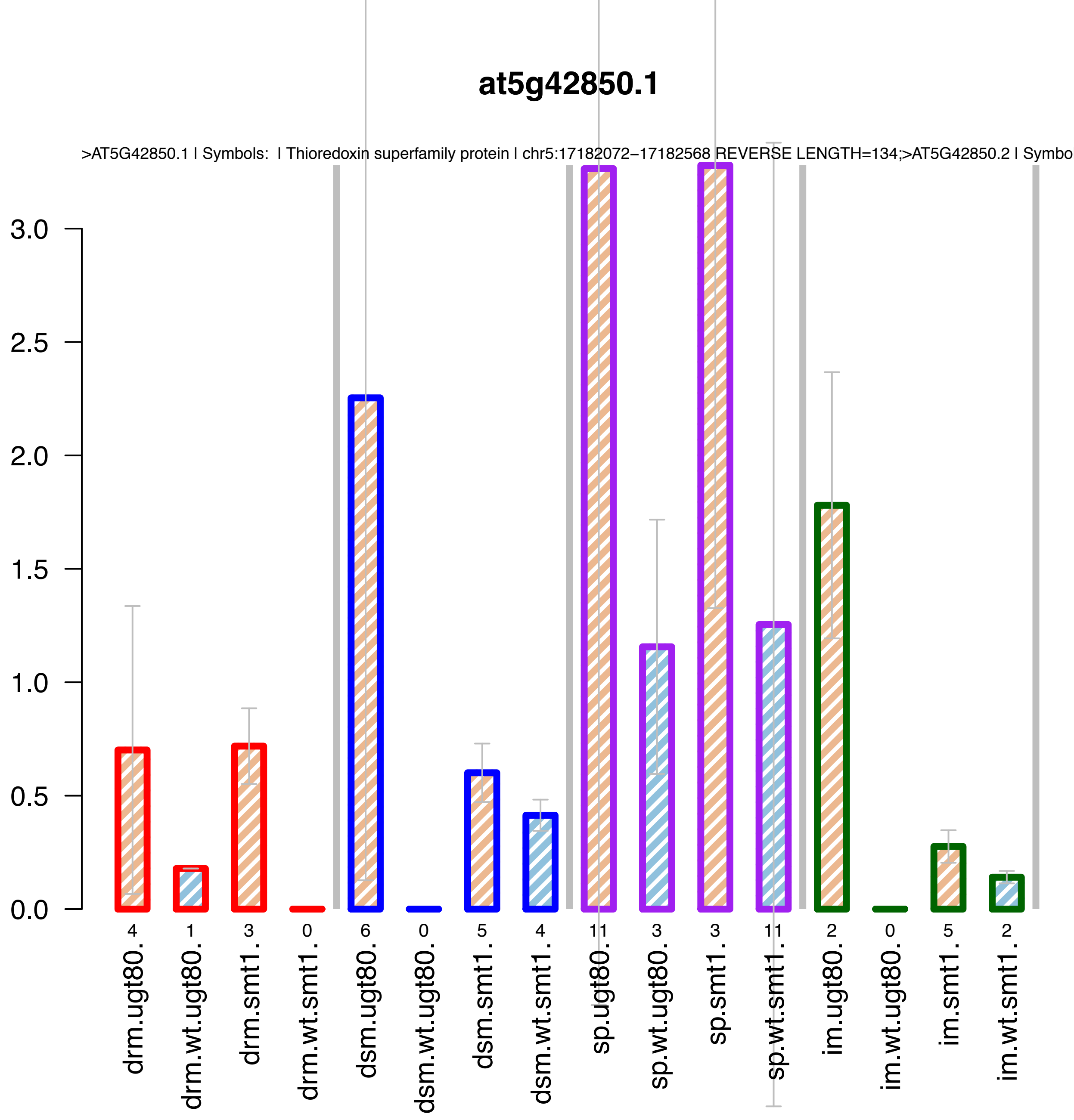

at5g42850.1
>AT5G42850.1 | Symbols: | Thioredoxin superfamily protein | chr5:17182072-17182568 REVERSE LENGTH=134;>AT5G42850.2 | Symbo
3.0
2.5
2.0
1.5
1.0
0.5
0.0
4
1
3
0
6
0
5
4
11
3
3
11
2
0
5
2
drm.ugt80.
drm.wt.ugt80.
drm.smt1.
drm.wt.smt1.
dsm.ugt80.
dsm.wt.ugt80.
dsm.smt1.
dsm.wt.smt1.
sp.ugt80.
sp.wt.ugt80.
sp.smt1.
sp.wt.smt1.
im.ugt80.
im.wt.ugt80.
im.smt1.
im.wt.smt1.

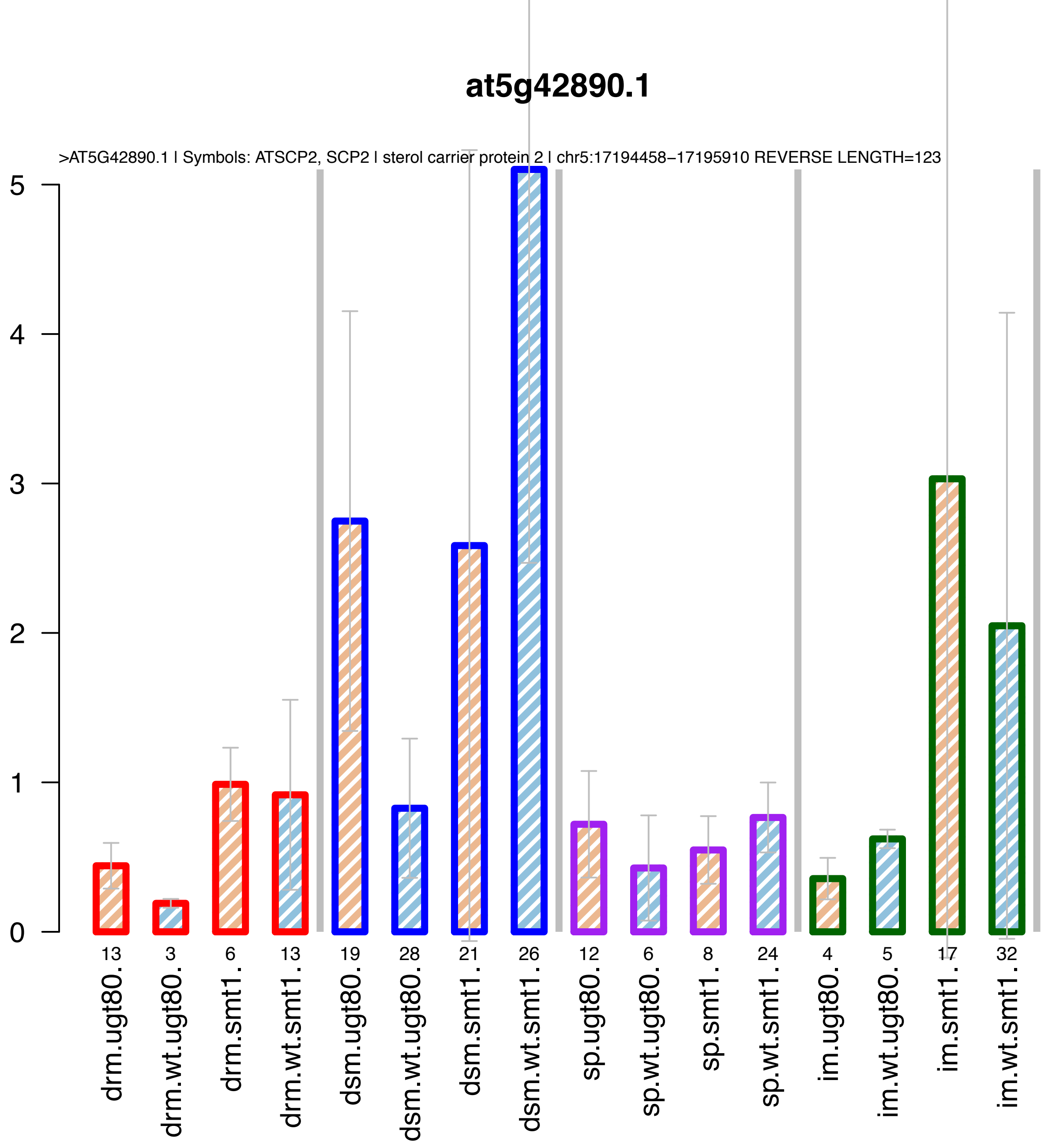

at5g42890.1
>AT5G42890.1 | Symbols: ATSCP2, SCP2 | sterol carrier protein 2 | chr5:17194458−17195910 REVERSE LENGTH=123
0
1
2
3
4
5
13
3
6
13
19
28
21
26
12
6
8
24
4
5
17
32
drm.ugt80..
drm.wt.ugt80..
drm.smt1..
drm.wt.smt1..
dsm.ugt80..
dsm.wt.ugt80..
dsm.smt1..
dsm.wt.smt1..
sp.ugt80..
sp.wt.ugt80..
sp.smt1..
sp.wt.smt1..
im.ugt80..
im.wt.ugt80..
im.smt1..
im.wt.smt1..

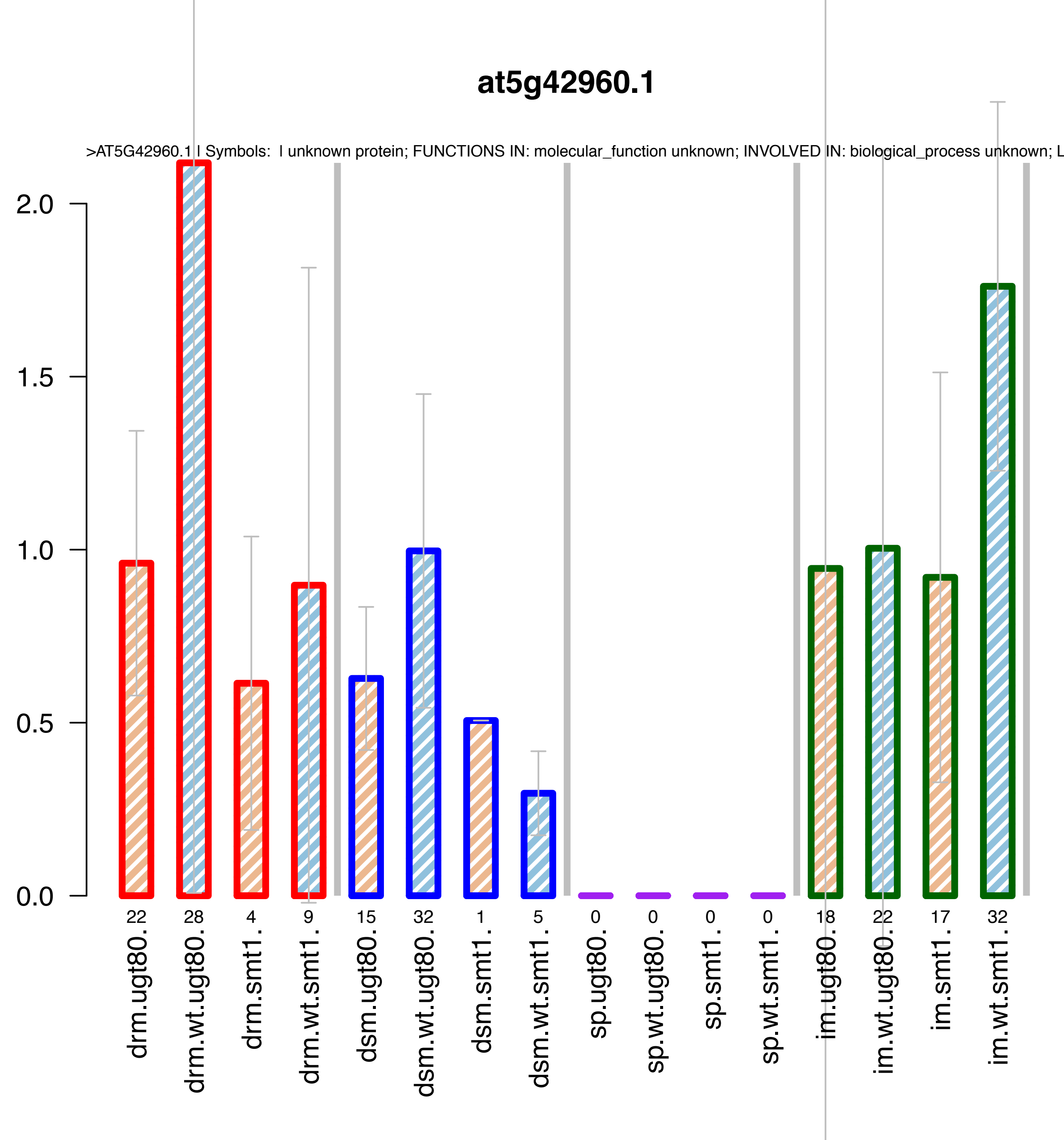
at5g42960.1
>AT5G42960.1 | Symbols: | unknown protein; FUNCTIONS IN: molecular_function unknown; INVOLVED IN: biological_process unknown; L
2.0
1.5
1.0
0.5
0.0
22
28
4
9
15
32
1
5
0
0
0
0
18
22
17
32
drm.ugt80..
drm.wt.ugt80..
drm.smt1..
drm.wt.smt1..
dsm.ugt80..
dsm.wt.ugt80..
dsm.smt1..
dsm.wt.smt1..
sp.ugt80..
sp.wt.ugt80..
sp.smt1..
sp.wt.smt1..
im.ugt80..
im.wt.ugt80..
im.smt1..
im.wt.smt1..

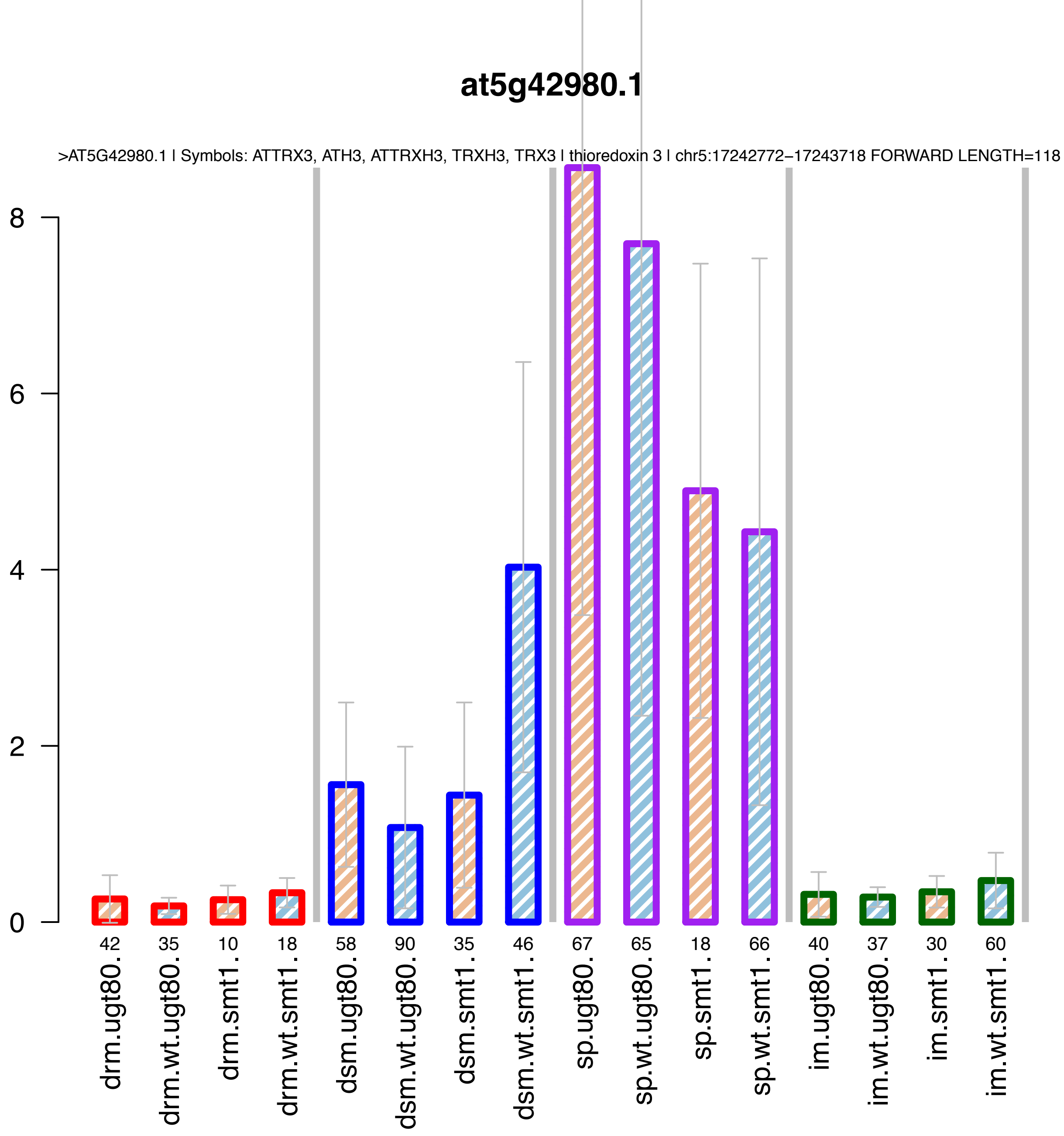

at5g42980.1
>AT5G42980.1 | Symbols: ATTRX3, ATH3, ATTRXH3, TRXH3, TRX3 | thioredoxin 3 | chr5:17242772-17243718 FORWARD LENGTH=118
0
2
4
6
8
42
35
10
18
58
90
35
46
67
65
18
66
40
37
30
60
drm.ugt80.
drm.wt.ugt80.
drm.smt1.
drm.wt.smt1.
dsm.ugt80.
dsm.wt.ugt80.
dsm.smt1.
dsm.wt.smt1.
sp.ugt80.
sp.wt.ugt80.
sp.smt1.
sp.wt.smt1.
im.ugt80.
im.wt.ugt80.
im.smt1.
im.wt.smt1.

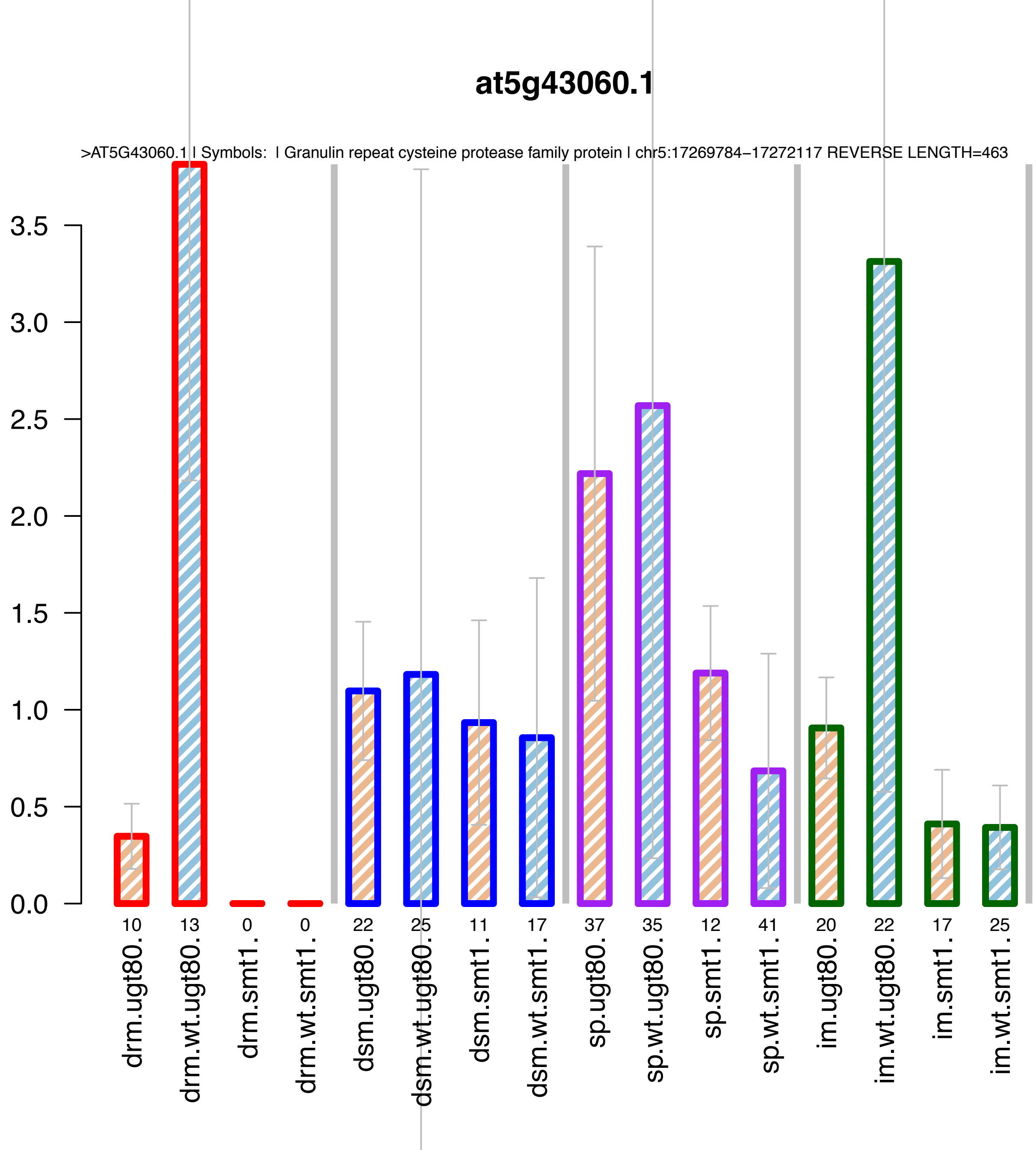

at5g43060.1
>AT5G43060.1 | Symbols: | Granulin repeat cysteine protease family protein | chr5:17269784-17272117 REVERSE LENGTH=463
0.0
0.5
1.0
1.5
2.0
2.5
3.0
3.5
10
13
0
0
22
25
11
17
37
35
12
41
20
22
17
25
drm.ugt80..
drm.wt.ugt80..
drm.smt1..
drm.wt.smt1..
dsm.ugt80..
dsm.wt.ugt80..
dsm.smt1..
dsm.wt.smt1..
sp.ugt80..
sp.wt.ugt80..
sp.smt1..
sp.wt.smt1..
im.ugt80..
im.wt.ugt80..
im.smt1..
im.wt.smt1..

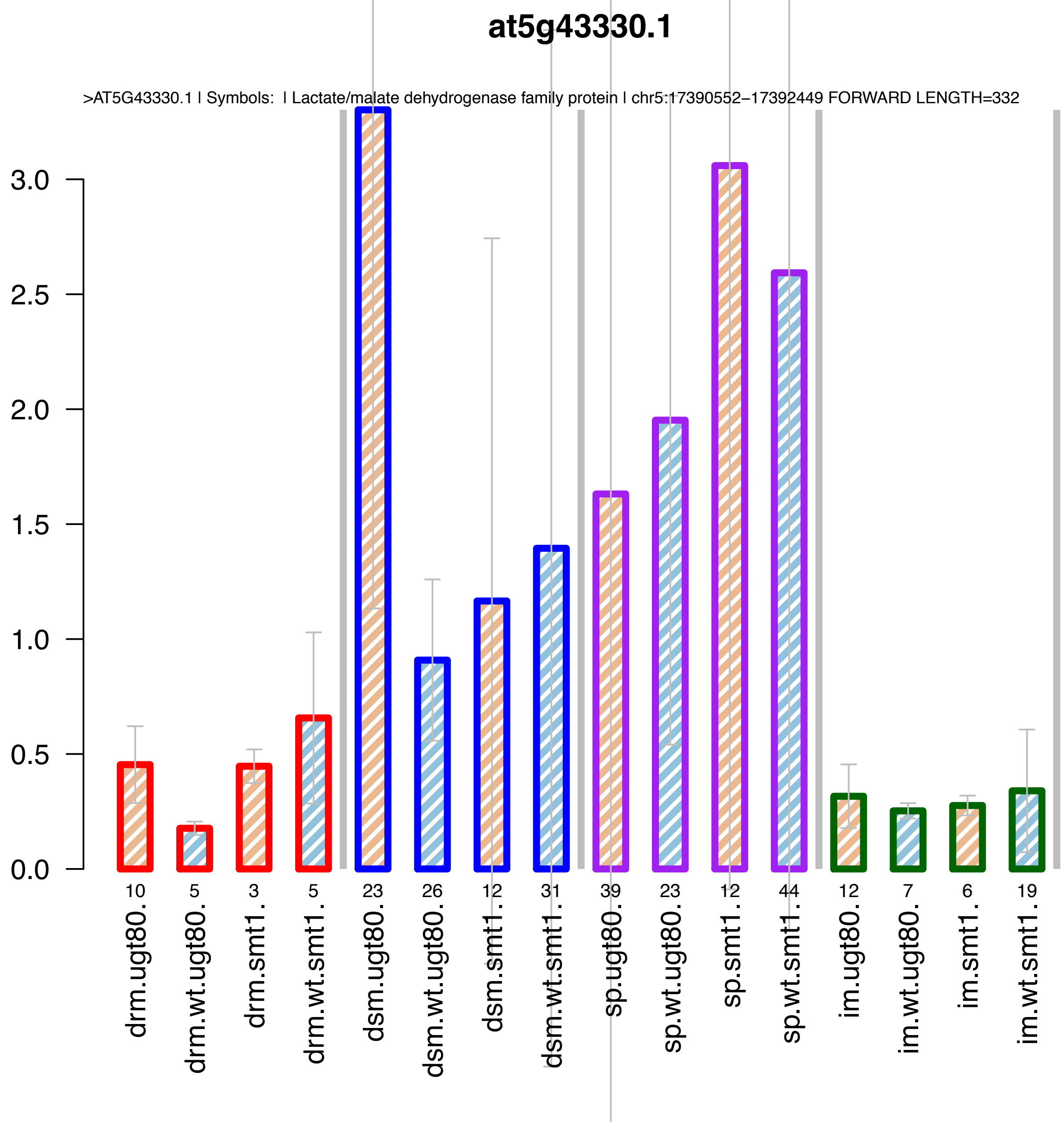
at5g43330.1
>AT5G43330.1 | Symbols: | Lactate/malate dehydrogenase family protein | chr5:17390552-17392449 FORWARD LENGTH=332
3.0
2.5
2.0
1.5
1.0
0.5
0.0
10
5
3
5
23
26
12
31
39
23
12
44
12
7
6
19
drm.ugt80.
drm.wt.ugt80.
drm.smt1.
drm.wt.smt1.
dsm.ugt80.
dsm.wt.ugt80.
dsm.smt1.
dsm.wt.smt1.
sp.ugt80.
sp.wt.ugt80.
sp.smt1.
sp.wt.smt1.
im.ugt80.
im.wt.ugt80.
im.smt1.
im.wt.smt1.

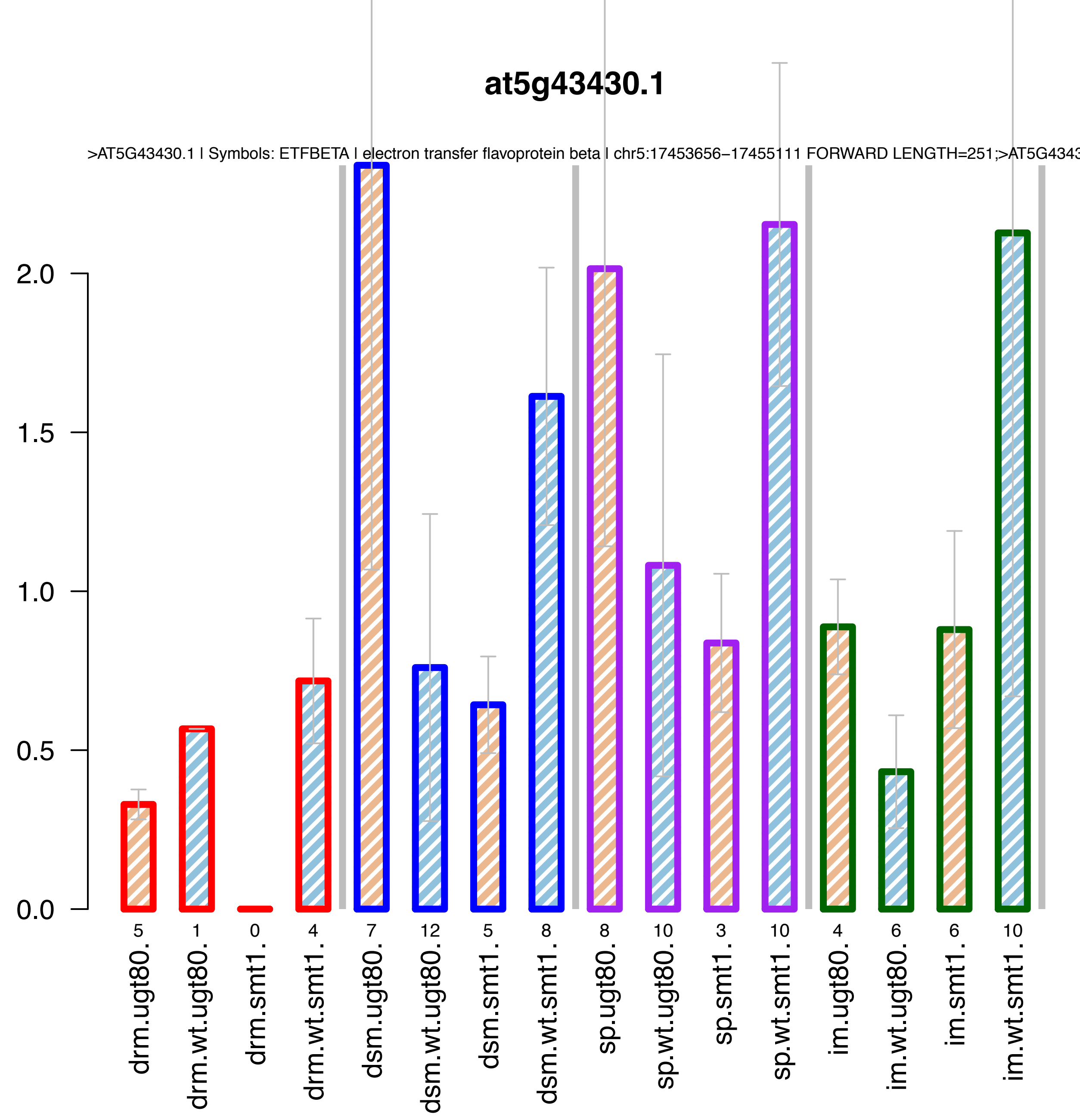

at5g43430.1
>AT5G43430.1 | Symbols: ETFBETA | electron transfer flavoprotein beta | chr5:17453656-17455111 FORWARD LENGTH=251;>AT5G4343
0.0
0.5
1.0
1.5
2.0
5
1
0
4
7
12
5
8
8
10
3
10
4
6
6
10
drm.ugt80.
drm.wt.ugt80.
drm.smt1.
drm.wt.smt1.
dsm.ugt80.
dsm.wt.ugt80.
dsm.smt1.
dsm.wt.smt1.
sp.ugt80.
sp.wt.ugt80.
sp.smt1.
sp.wt.smt1.
im.ugt80.
im.wt.ugt80.
im.smt1.
im.wt.smt1.

# at5g43780.1

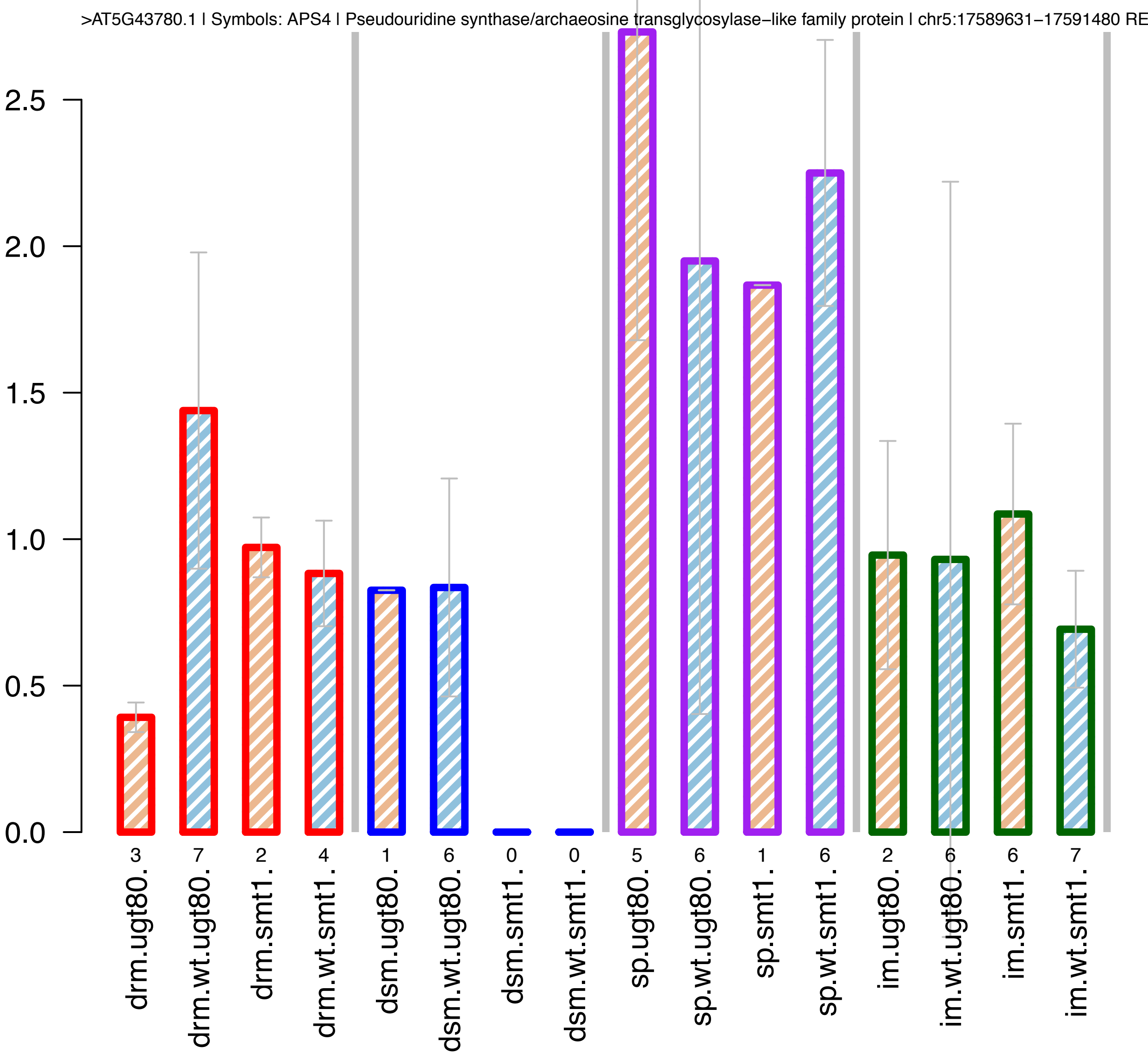

>AT5G43780.1 | Symbols: APS4 | Pseudouridine synthase/archaeosine transglycosylase−like family protein | chr5:17589631−17591480 RE
2.5
2.0
1.5
1.0
0.5
0.0
3
7
2
4
1
6
0
0
5
6
1
6
2
6
6
7
drm.ugt80.
drm.wt.ugt80.
drm.smt1.
drm.wt.smt1.
dsm.ugt80.
dsm.wt.ugt80.
dsm.smt1.
dsm.wt.smt1.
sp.ugt80.
sp.wt.ugt80.
sp.smt1.
sp.wt.smt1.
im.ugt80.
im.wt.ugt80.
im.smt1.
im.wt.smt1.

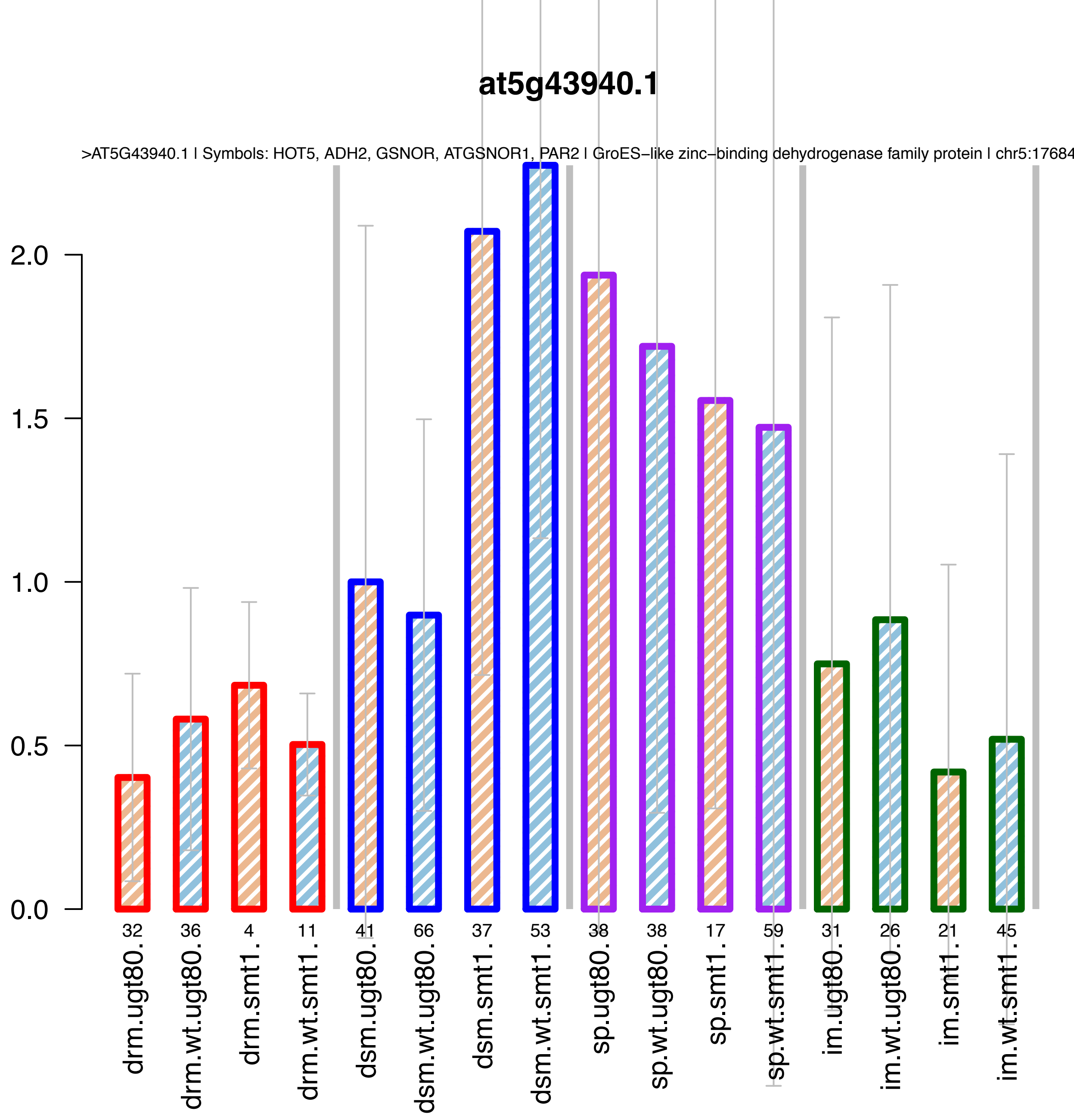
at5g43940.1
>AT5G43940.1 | Symbols: HOT5, ADH2, GSNOR, ATGSNOR1, PAR2 | GroES−like zinc−binding dehydrogenase family protein | chr5:17684
2.0
1.5
1.0
0.5
0.0
32
36
4
11
41
66
37
53
38
38
17
59
31
26
21
45
drm.ugt80..
drm.wt.ugt80..
drm.smt1..
drm.wt.smt1..
dsm.ugt80..
dsm.wt.ugt80..
dsm.smt1..
dsm.wt.smt1..
sp.ugt80..
sp.wt.ugt80..
sp.smt1..
sp.wt.smt1..
im.ugt80..
im.wt.ugt80..
im.smt1..
im.wt.smt1..

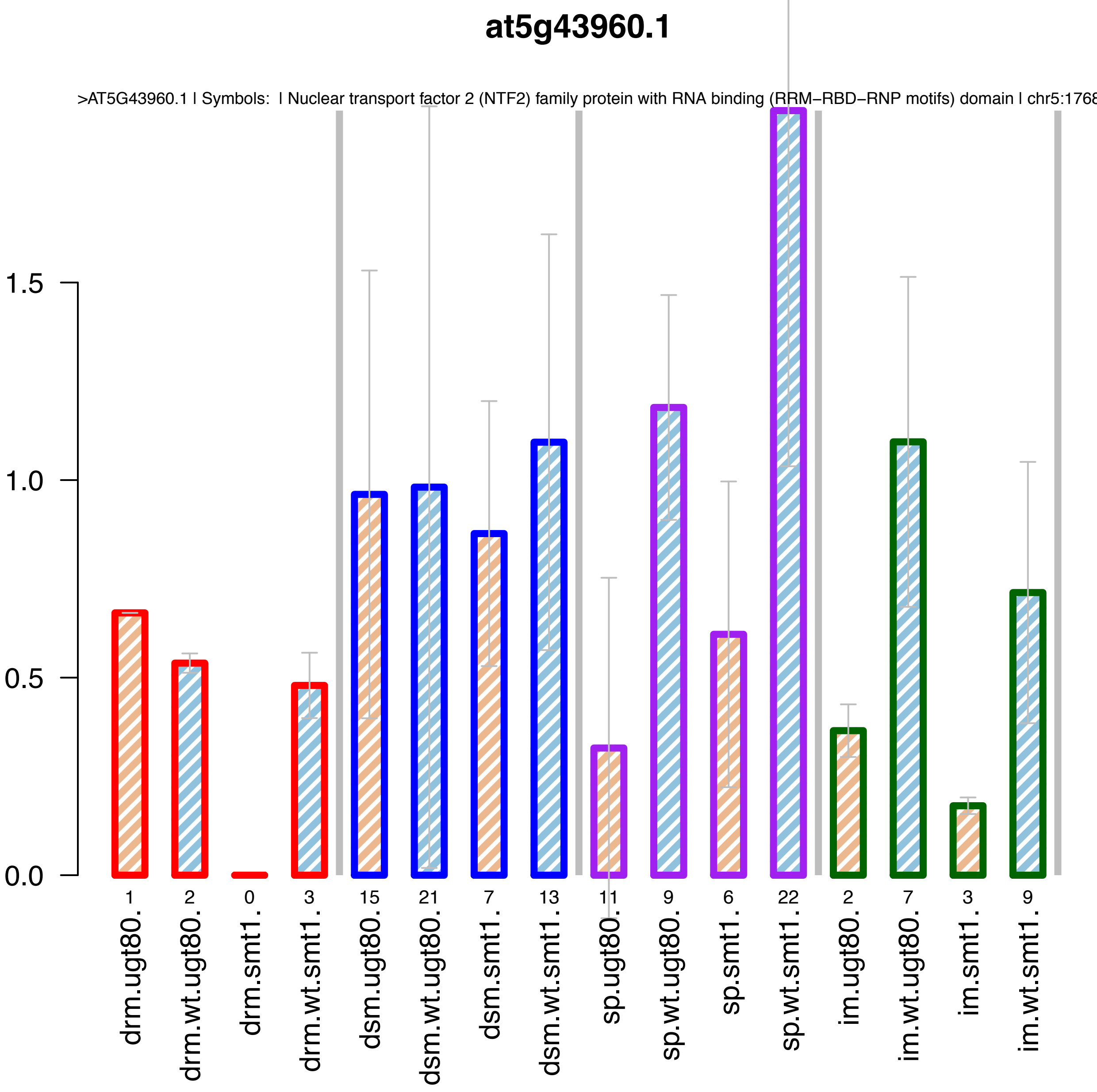
at5g43960.1
>AT5G43960.1 | Symbols: | Nuclear transport factor 2 (NTF2) family protein with RNA binding (RRM-RBD-RNP motifs) domain | chr5:1768
1.5
1.0
0.5
0.0
1
2
0
3
15
21
7
13
11
9
6
22
2
7
3
9
drm.ugt80.
drm.wt.ugt80.
drm.smt1.
drm.wt.smt1.
dsm.ugt80.
dsm.wt.ugt80.
dsm.smt1.
dsm.wt.smt1.
sp.ugt80.
sp.wt.ugt80.
sp.smt1.
sp.wt.smt1.
im.ugt80.
im.wt.ugt80.
im.smt1.
im.wt.smt1.

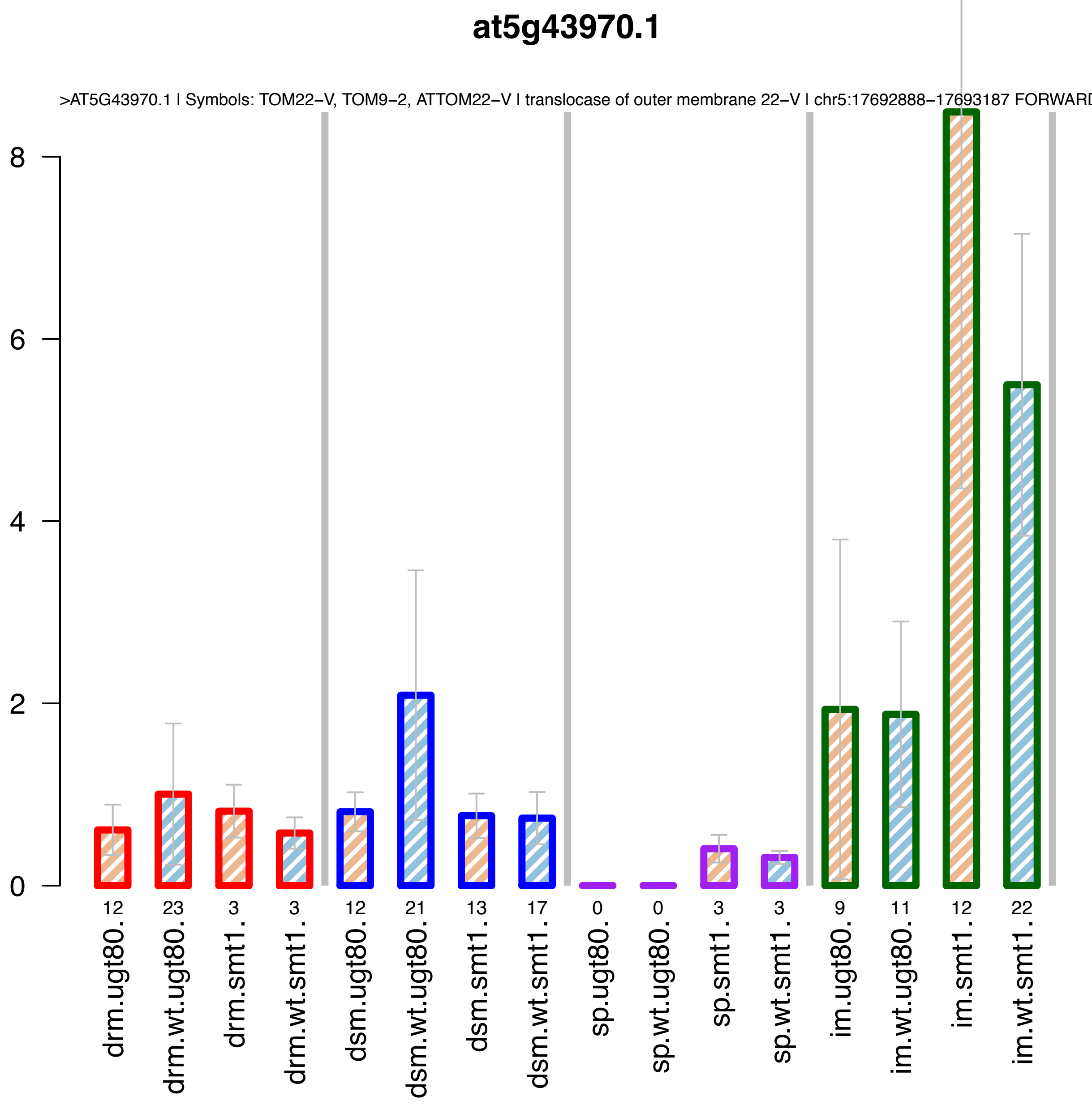

at5g43970.1
>AT5G43970.1 | Symbols: TOM22−V, TOM9−2, ATTOM22−V | translocase of outer membrane 22−V | chr5:17692888−17693187 FORWARD
8
6
4
2
0
12
23
3
3
12
21
13
17
0
0
3
3
9
11
12
22
drm.ugt80.
drm.wt.ugt80.
drm.smt1.
drm.wt.smt1.
dsm.ugt80.
dsm.wt.ugt80.
dsm.smt1.
dsm.wt.smt1.
sp.ugt80.
sp.wt.ugt80.
sp.smt1.
sp.wt.smt1.
im.ugt80.
im.wt.ugt80.
im.smt1.
im.wt.smt1.

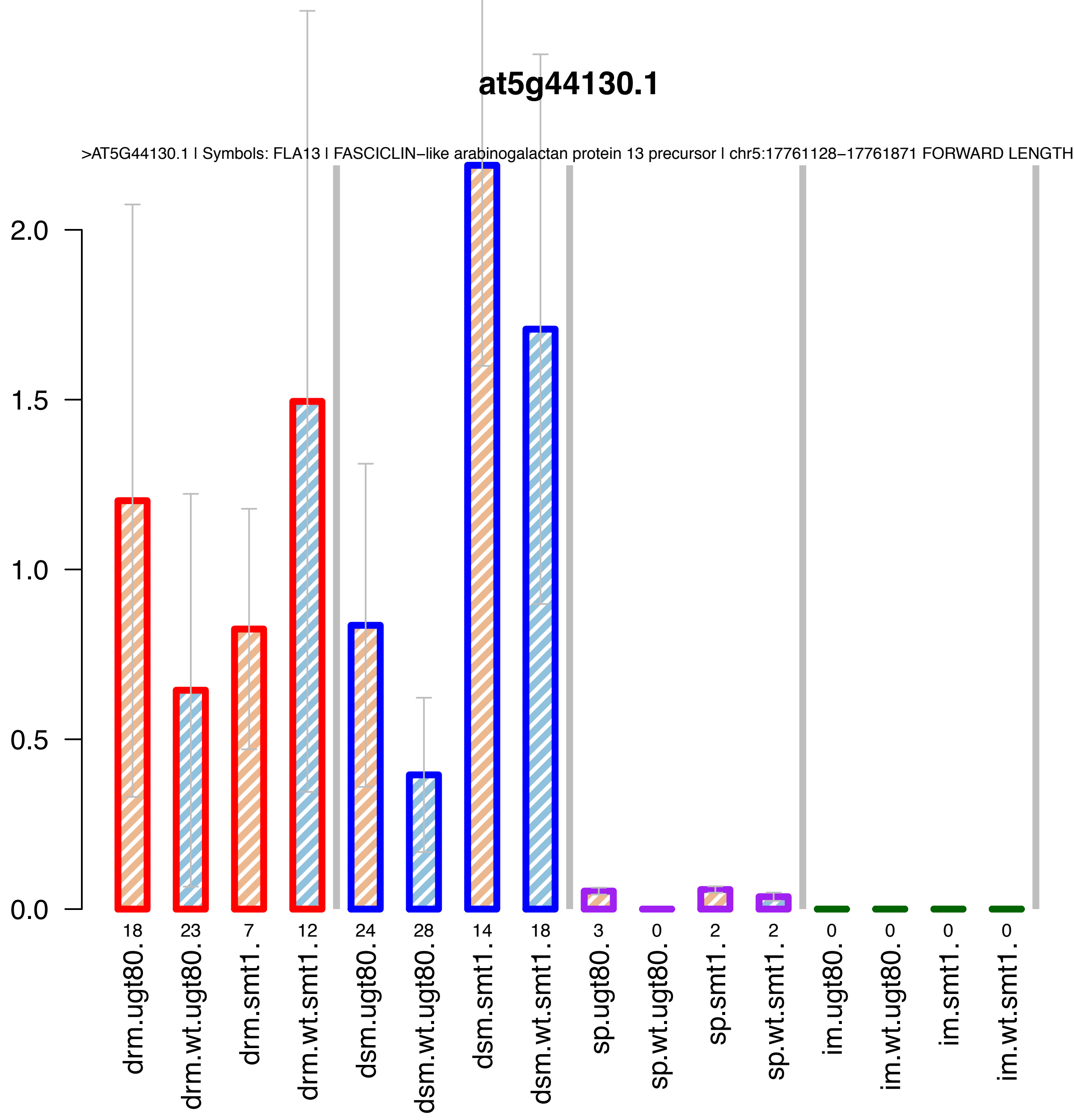

at5g44130.1
>AT5G44130.1 | Symbols: FLA13 | FASCICLIN−like arabinogalactan protein 13 precursor | chr5:17761128−17761871 FORWARD LENGTH
2.0
1.5
1.0
0.5
0.0
18
23
7
12
24
28
14
18
3
0
2
2
0
0
0
0
drm.ugt80.
drm.wt.ugt80.
drm.smt1.
drm.wt.smt1.
dsm.ugt80.
dsm.wt.ugt80.
dsm.smt1.
dsm.wt.smt1.
sp.ugt80.
sp.wt.ugt80.
sp.smt1.
sp.wt.smt1.
im.ugt80.
im.wt.ugt80.
im.smt1.
im.wt.smt1.

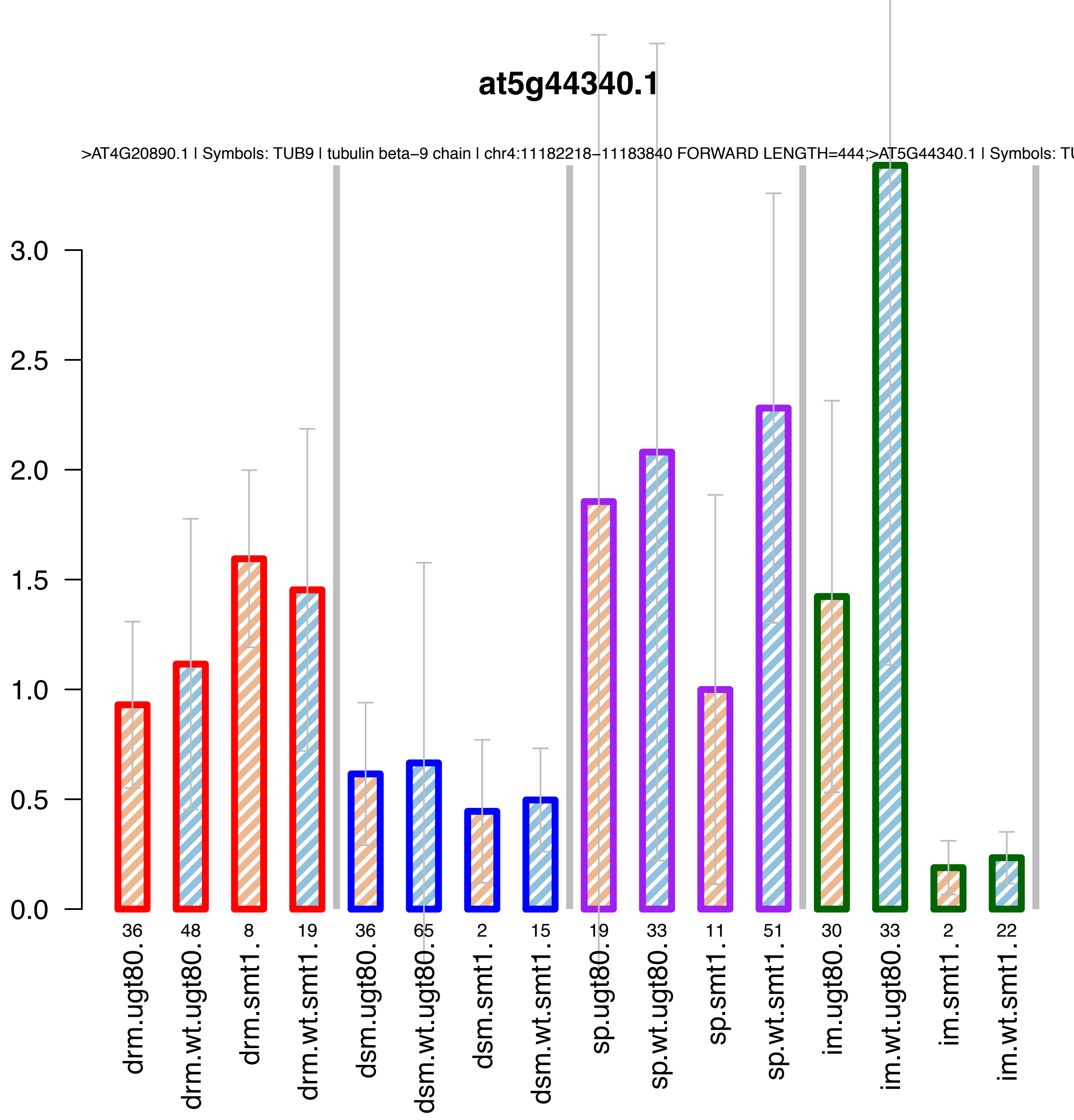

at5g44340.1
>AT4G20890.1 | Symbols: TUB9 | tubulin beta-9 chain | chr4:11182218-11183840 FORWARD LENGTH=444;>AT5G44340.1 | Symbols: TU
0.0
0.5
1.0
1.5
2.0
2.5
3.0
36
48
8
19
36
65
2
15
19
33
11
51
30
33
2
22
drm.ugt80.
drm.wt.ugt80.
drm.smt1.
drm.wt.smt1.
dsm.ugt80.
dsm.wt.ugt80.
dsm.smt1.
dsm.wt.smt1.
sp.ugt80.
sp.wt.ugt80.
sp.smt1.
sp.wt.smt1.
im.ugt80.
im.wt.ugt80.
im.smt1.
im.wt.smt1.

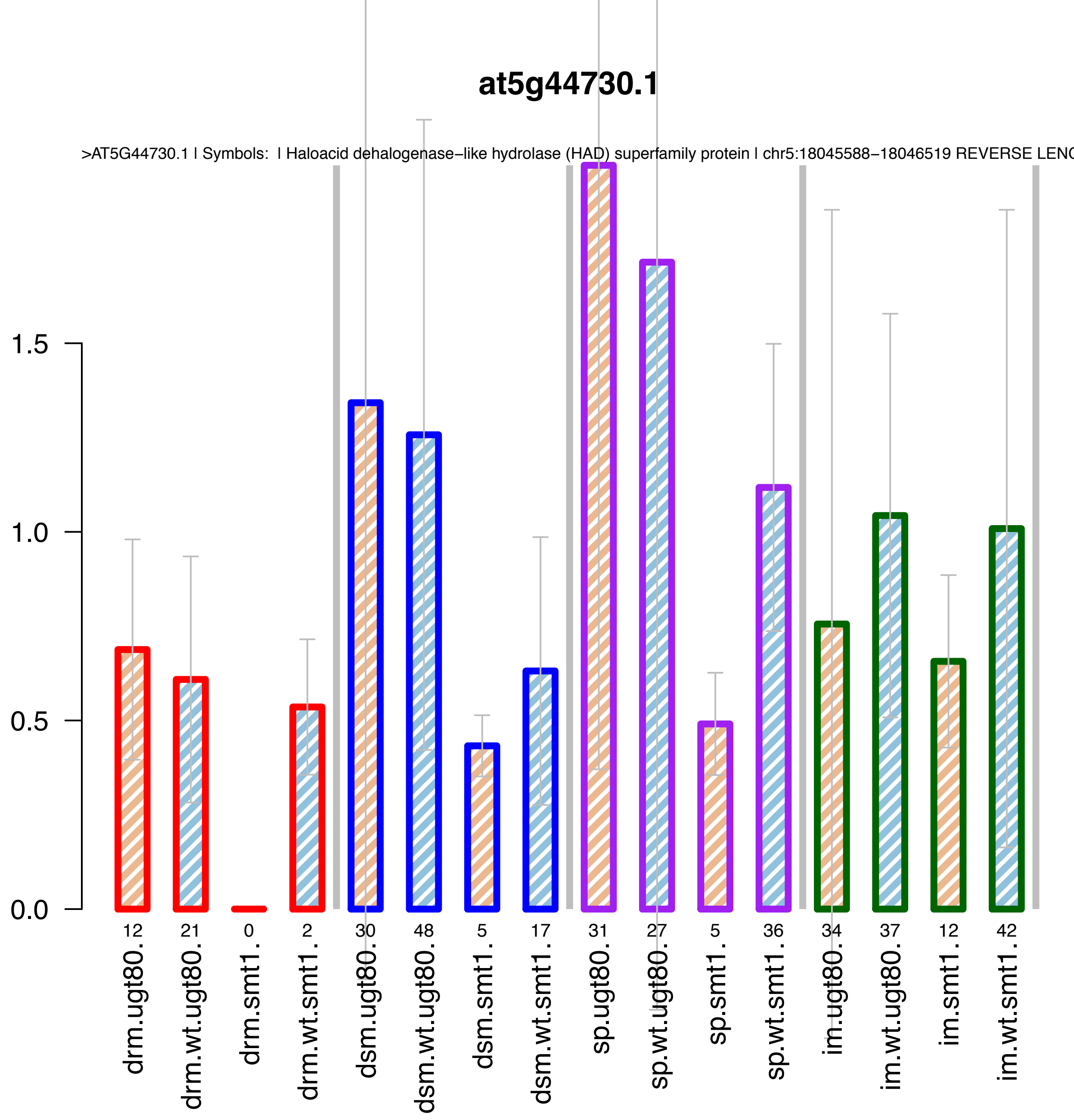

at5g44730.1
>AT5G44730.1 | Symbols: | Haloacid dehalogenase−like hydrolase (HAD) superfamily protein | chr5:18045588−18046519 REVERSE LENG
1.5
1.0
0.5
0.0
12
21
0
2
30
48
5
17
31
27
5
36
34
37
12
42
drm.ugt80..
drm.wt.ugt80..
drm.smt1..
drm.wt.smt1..
dsm.ugt80..
dsm.wt.ugt80..
dsm.smt1..
dsm.wt.smt1..
sp.ugt80..
sp.wt.ugt80..
sp.smt1..
sp.wt.smt1..
im.ugt80..
im.wt.ugt80..
im.smt1..
im.wt.smt1..

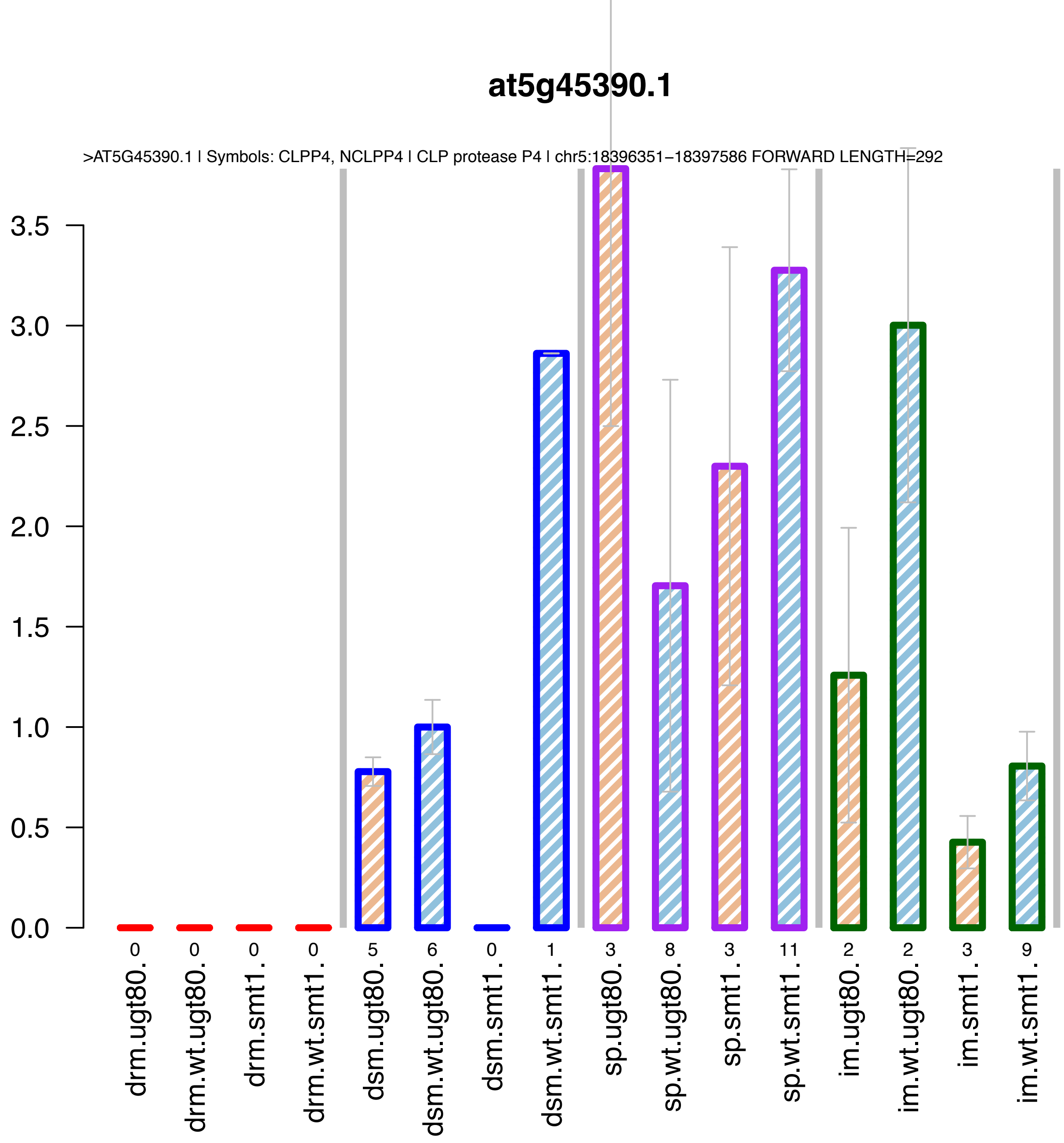

at5g45390.1
>AT5G45390.1 | Symbols: CLPP4, NCLPP4 | CLP protease P4 | chr5:18396351–18397586 FORWARD LENGTH=292
0.0
0.5
1.0
1.5
2.0
2.5
3.0
3.5
0 0 0 0 5 6 0 1 3 8 3 11 2 2 3 9
drm.ugt80.
drm.wt.ugt80.
drm.smt1.
drm.wt.smt1.
dsm.ugt80.
dsm.wt.ugt80.
dsm.smt1.
dsm.wt.smt1.
sp.ugt80.
sp.wt.ugt80.
sp.smt1.
sp.wt.smt1.
im.ugt80.
im.wt.ugt80.
im.smt1.
im.wt.smt1.

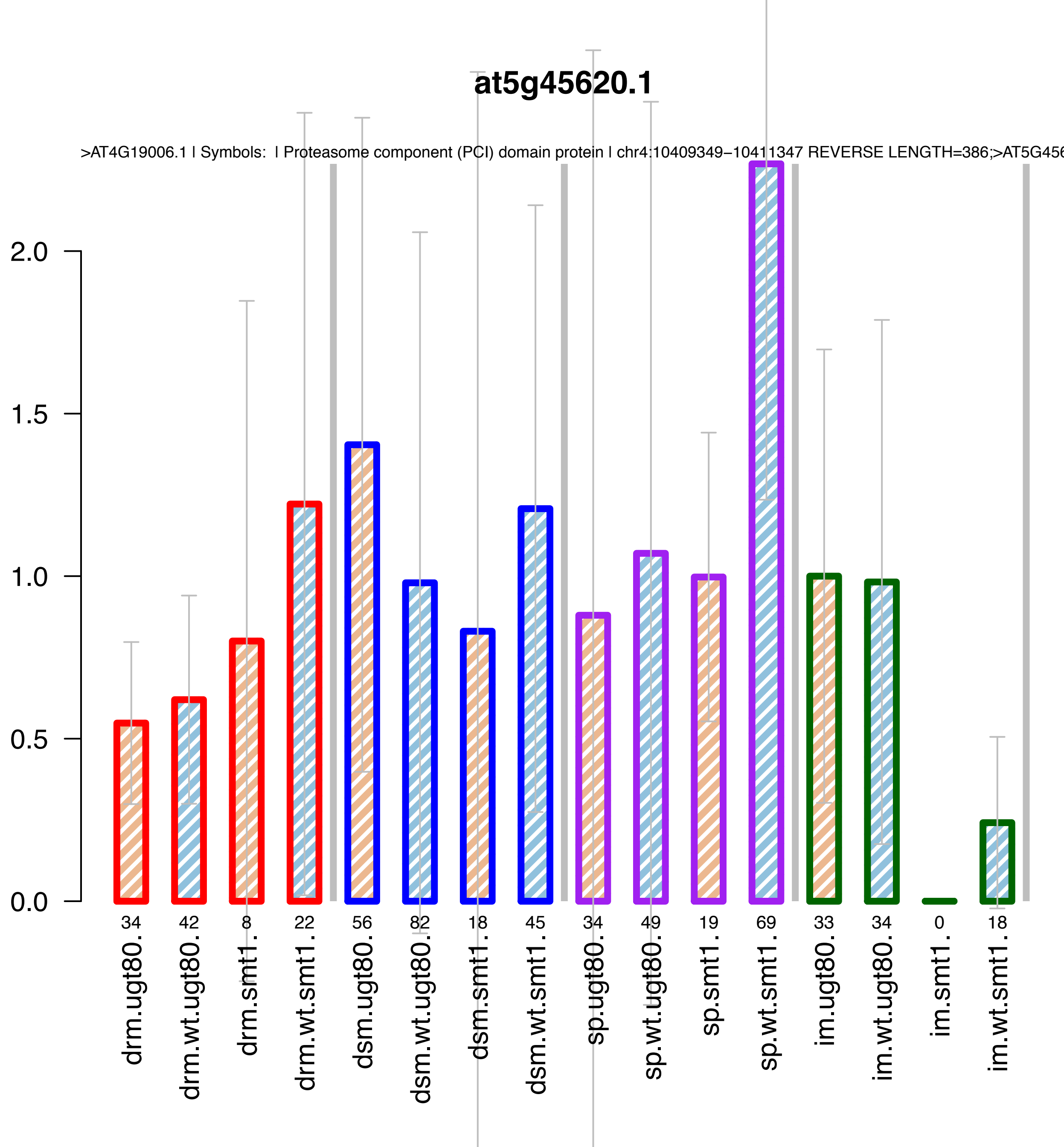

at5g45620.1
>AT4G19006.1 | Symbols: | Proteasome component (PCI) domain protein | chr4:10409349-10411347 REVERSE LENGTH=386;>AT5G456
2.0
1.5
1.0
0.5
0.0
34
42
8
22
56
82
18
45
34
49
19
69
33
34
0
18
drm.ugt80.
drm.wt.ugt80.
drm.smt1.
drm.wt.smt1.
dsm.ugt80.
dsm.wt.ugt80.
dsm.smt1.
dsm.wt.smt1.
sp.ugt80.
sp.wt.ugt80.
sp.smt1.
sp.wt.smt1.
im.ugt80.
im.wt.ugt80.
im.smt1.
im.wt.smt1.

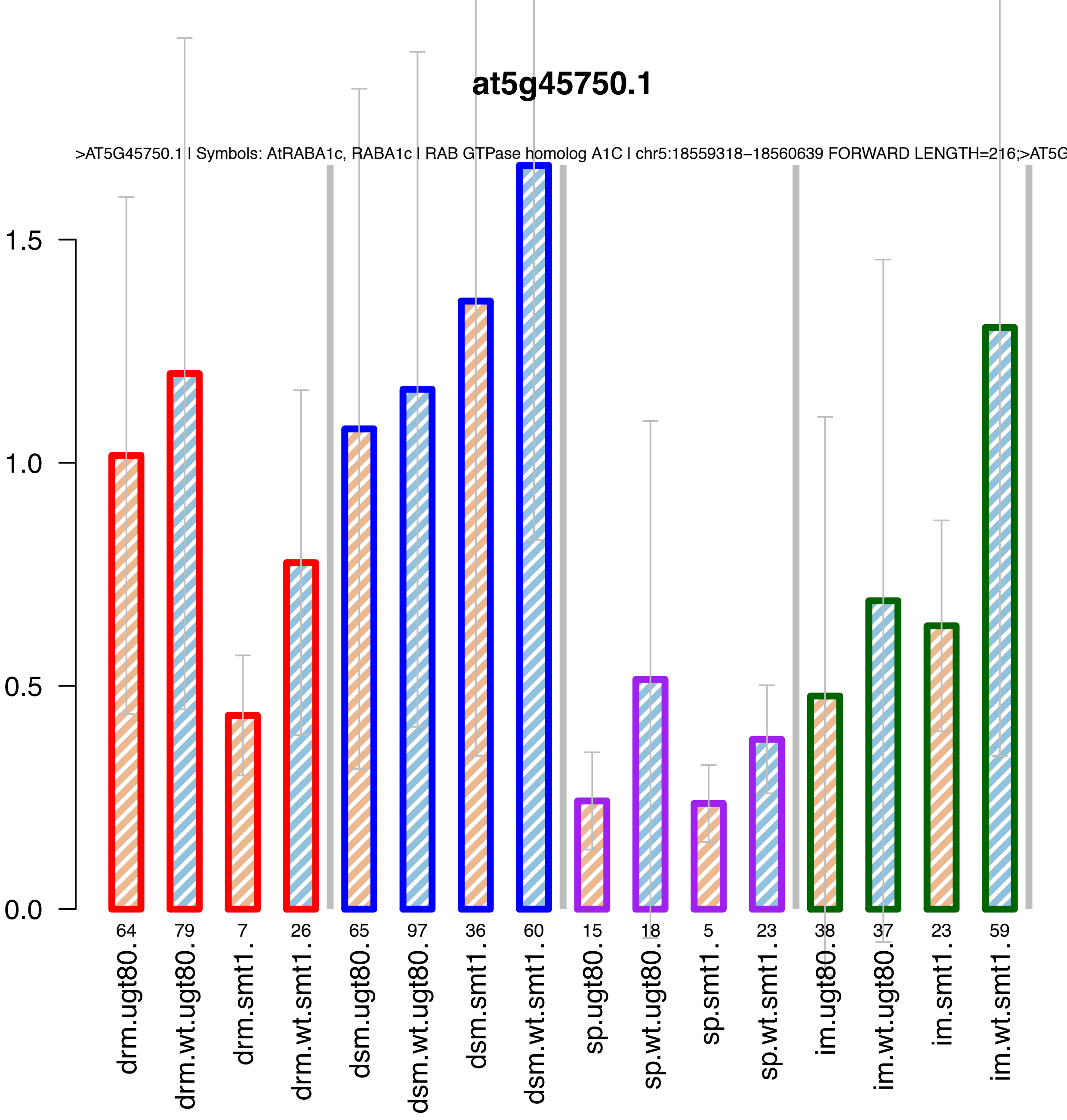

at5g45750.1
>AT5G45750.1 | Symbols: AtRABA1c, RABA1c | RAB GTPase homolog A1C | chr5:18559318−18560639 FORWARD LENGTH=216;>AT5G
1.5
1.0
0.5
0.0
64
79
7
26
65
97
36
60
15
18
5
23
38
37
23
59
drm.ugt80..
drm.wt.ugt80..
drm.smt1..
drm.wt.smt1..
dsm.ugt80..
dsm.wt.ugt80..
dsm.smt1..
dsm.wt.smt1..
sp.ugt80..
sp.wt.ugt80..
sp.smt1..
sp.wt.smt1..
im.ugt80..
im.wt.ugt80..
im.smt1..
im.wt.smt1..

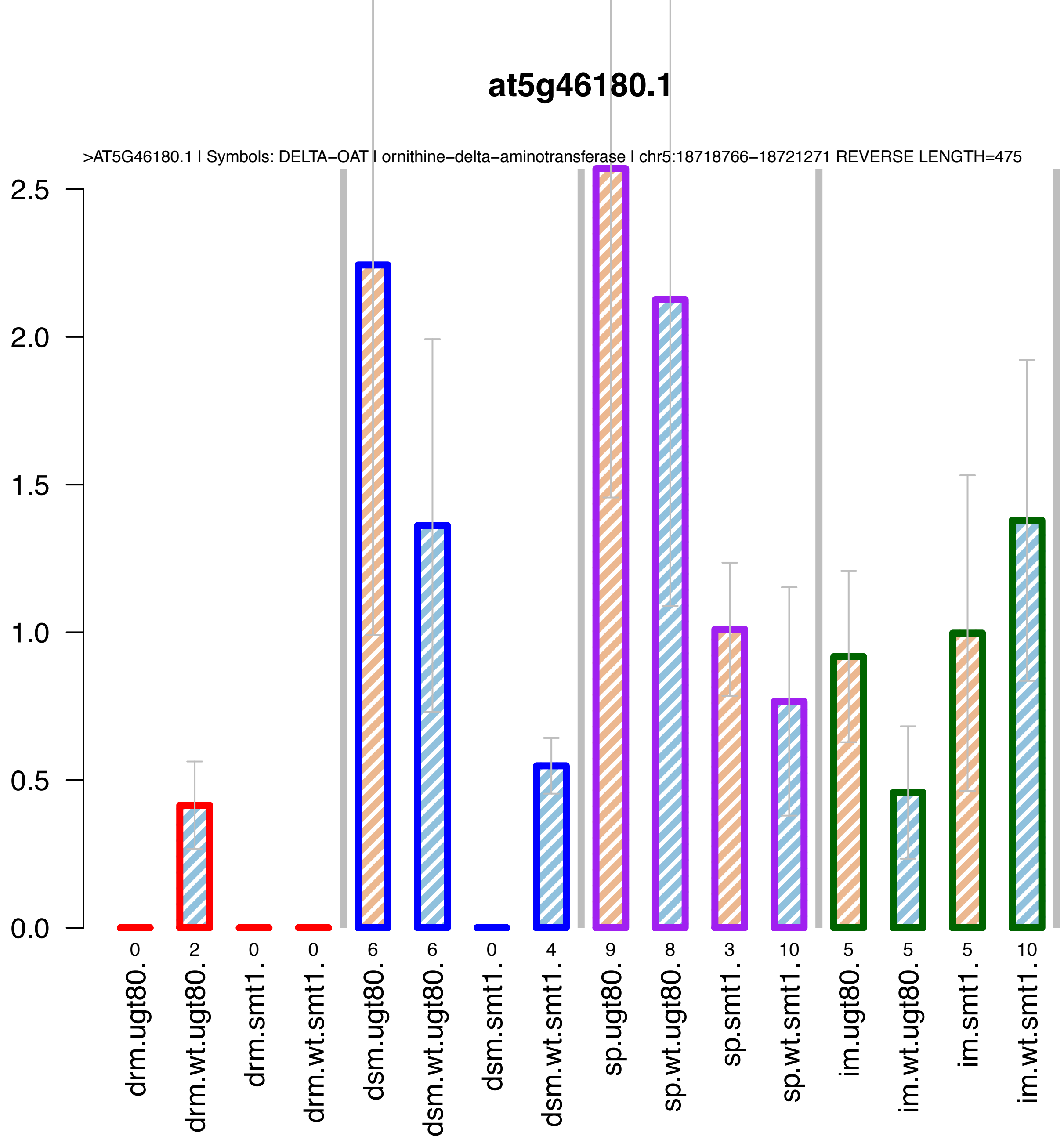
at5g46180.1
>AT5G46180.1 | Symbols: DELTA−OAT | ornithine−delta−aminotransferase | chr5:18718766−18721271 REVERSE LENGTH=475
0.0
0.5
1.0
1.5
2.0
2.5
0
2
0
0
6
6
0
4
9
8
3
10
5
5
5
10
drm.ugt80.
drm.wt.ugt80.
drm.smt1.
drm.wt.smt1.
dsm.ugt80.
dsm.wt.ugt80.
dsm.smt1.
dsm.wt.smt1.
sp.ugt80.
sp.wt.ugt80.
sp.smt1.
sp.wt.smt1.
im.ugt80.
im.wt.ugt80.
im.smt1.
im.wt.smt1.

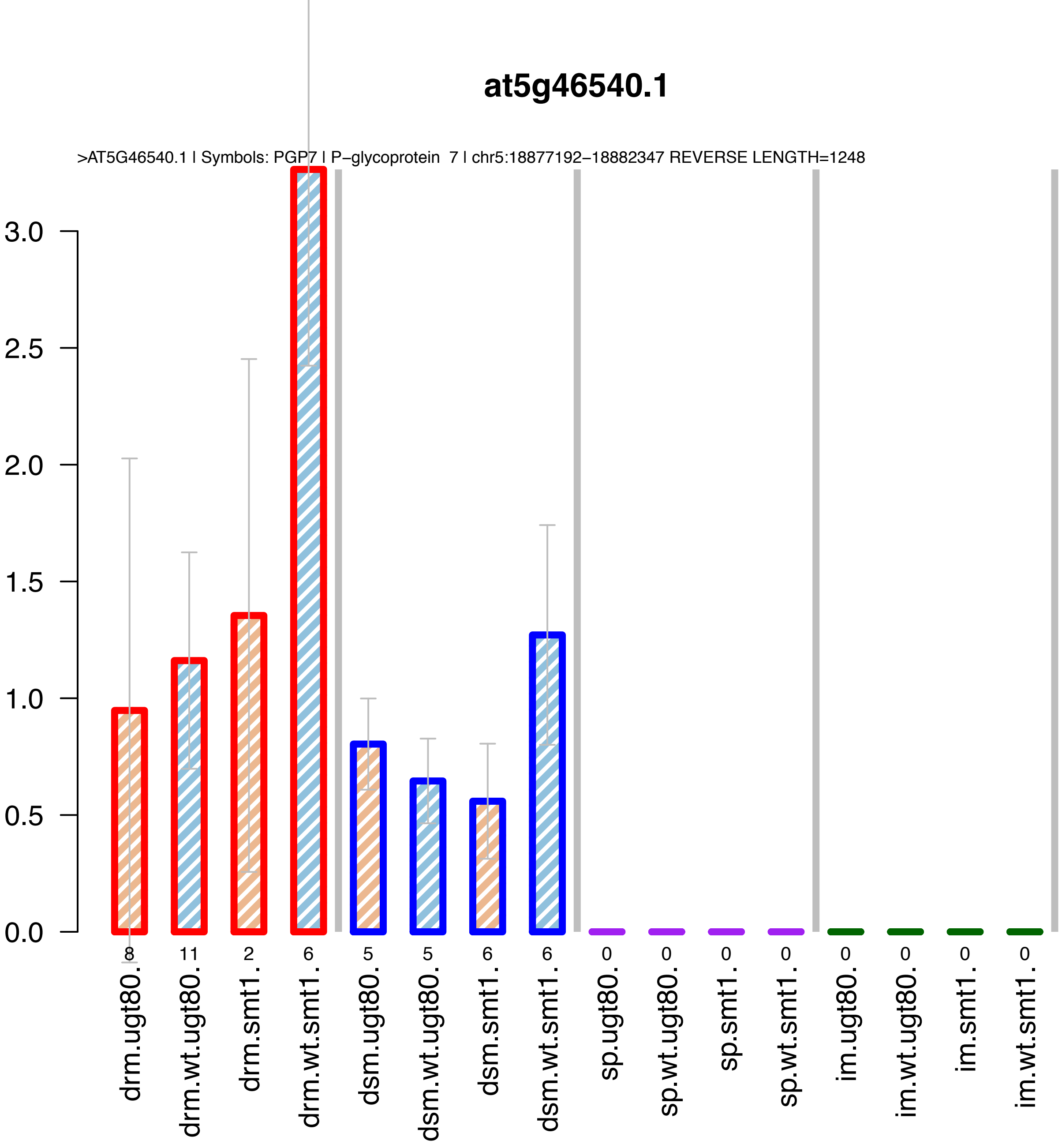

at5g46540.1
>AT5G46540.1 | Symbols: PGP7 | P−glycoprotein 7 | chr5:18877192−18882347 REVERSE LENGTH=1248
3.0
2.5
2.0
1.5
1.0
0.5
0.0
8
11
2
6
5
5
6
6
0
0
0
0
0
0
0
0
drm.ugt80.
drm.wt.ugt80.
drm.smt1.
drm.wt.smt1.
dsm.ugt80.
dsm.wt.ugt80.
dsm.smt1.
dsm.wt.smt1.
sp.ugt80.
sp.wt.ugt80.
sp.smt1.
sp.wt.smt1.
im.ugt80.
im.wt.ugt80.
im.smt1.
im.wt.smt1.

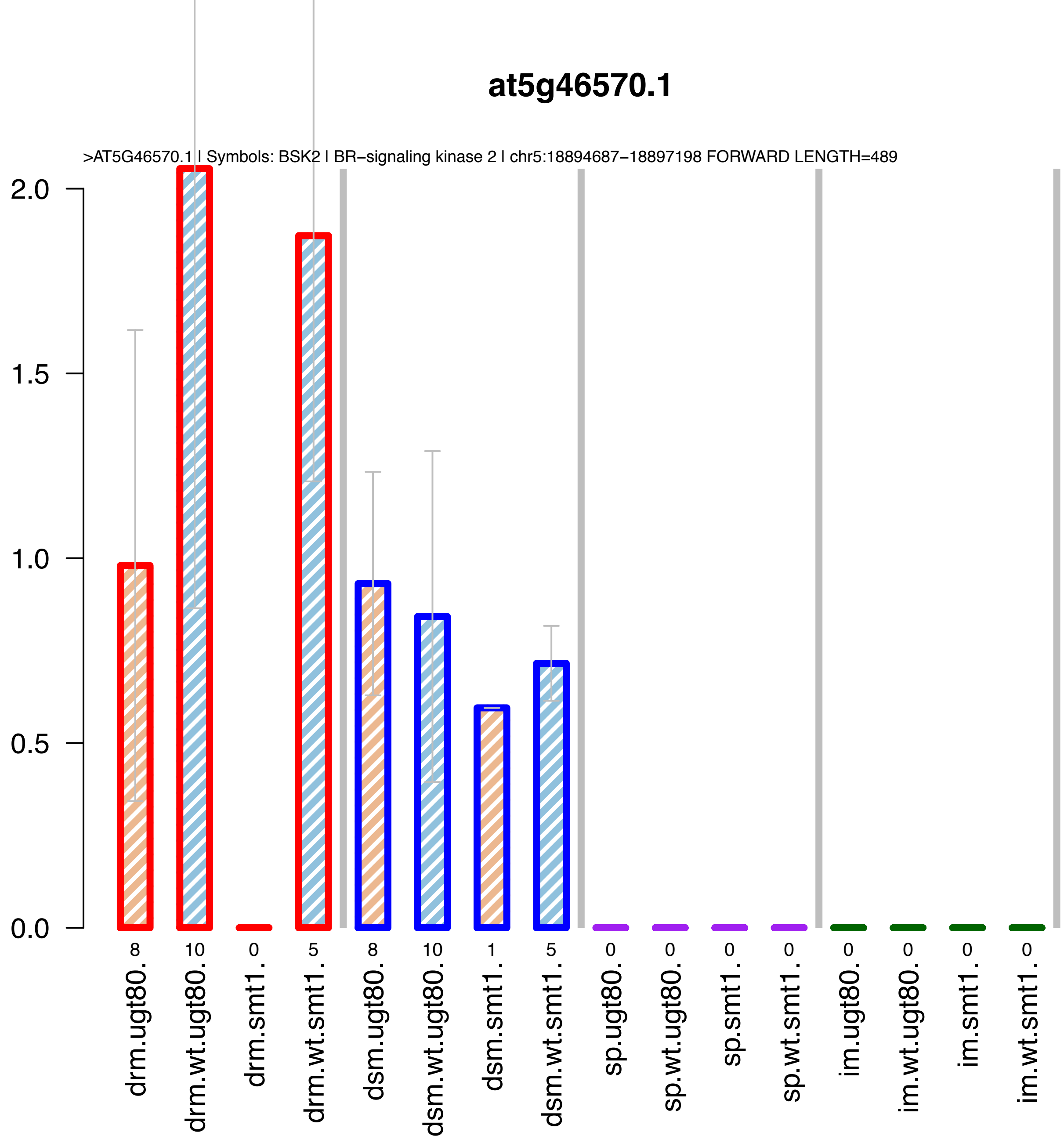

at5g46570.1
>AT5G46570.1 | Symbols: BSK2 | BR−signaling kinase 2 | chr5:18894687−18897198 FORWARD LENGTH=489
2.0
1.5
1.0
0.5
0.0
8
10
0
5
8
10
1
5
0
0
0
0
0
0
0
0
drm.ugt80.
drm.wt.ugt80.
drm.smt1.
drm.wt.smt1.
dsm.ugt80.
dsm.wt.ugt80.
dsm.smt1.
dsm.wt.smt1.
sp.ugt80.
sp.wt.ugt80.
sp.smt1.
sp.wt.smt1.
im.ugt80.
im.wt.ugt80.
im.smt1.
im.wt.smt1.

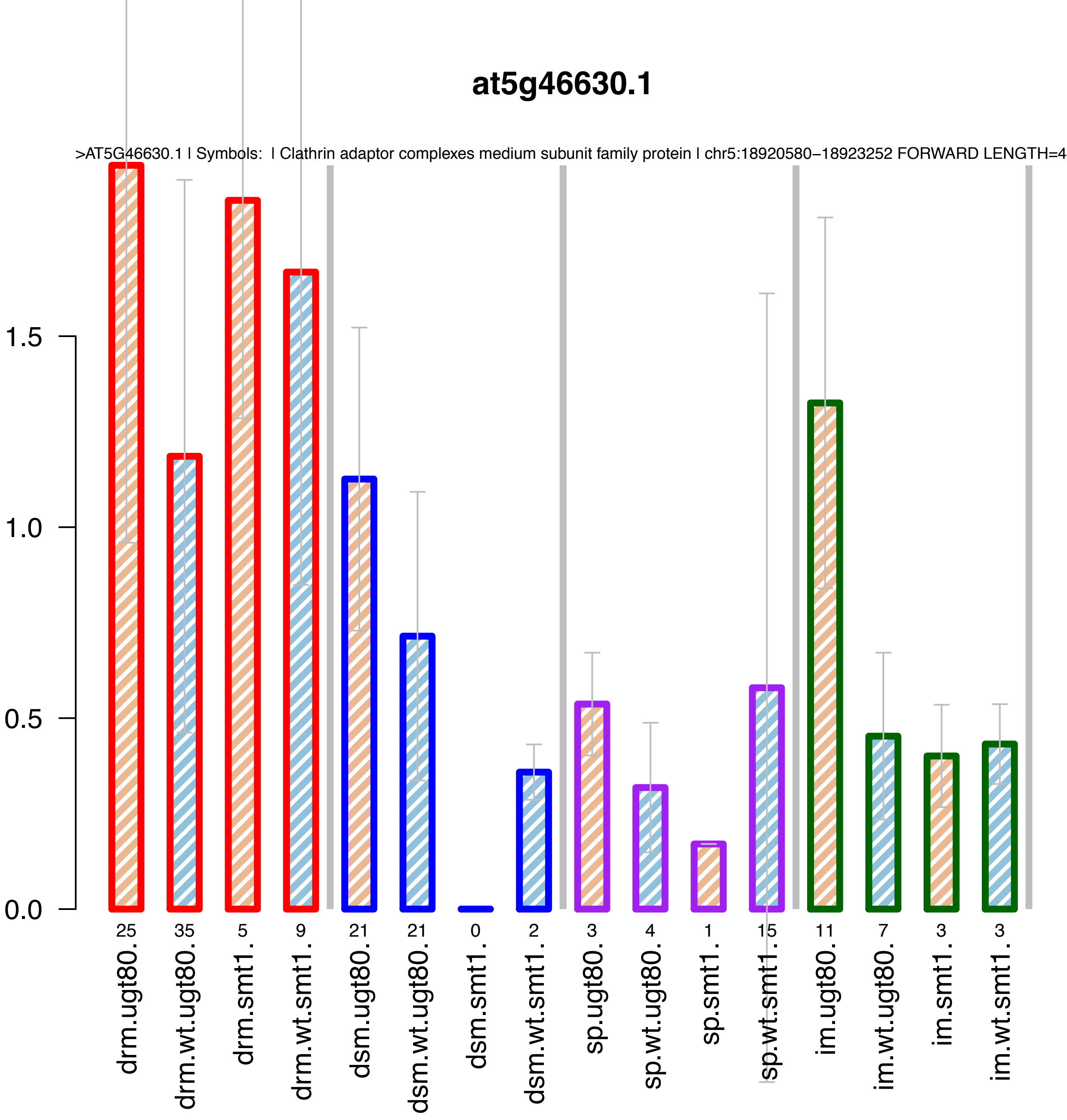

at5g46630.1
>AT5G46630.1 | Symbols: | Clathrin adaptor complexes medium subunit family protein | chr5:18920580-18923252 FORWARD LENGTH=4
1.5
1.0
0.5
0.0
25
35
5
9
21
21
0
2
3
4
1
15
11
7
3
3
drm.ugt80.
drm.wt.ugt80.
drm.smt1.
drm.wt.smt1.
dsm.ugt80.
dsm.wt.ugt80.
dsm.smt1.
dsm.wt.smt1.
sp.ugt80.
sp.wt.ugt80.
sp.smt1.
sp.wt.smt1.
im.ugt80.
im.wt.ugt80.
im.smt1.
im.wt.smt1.

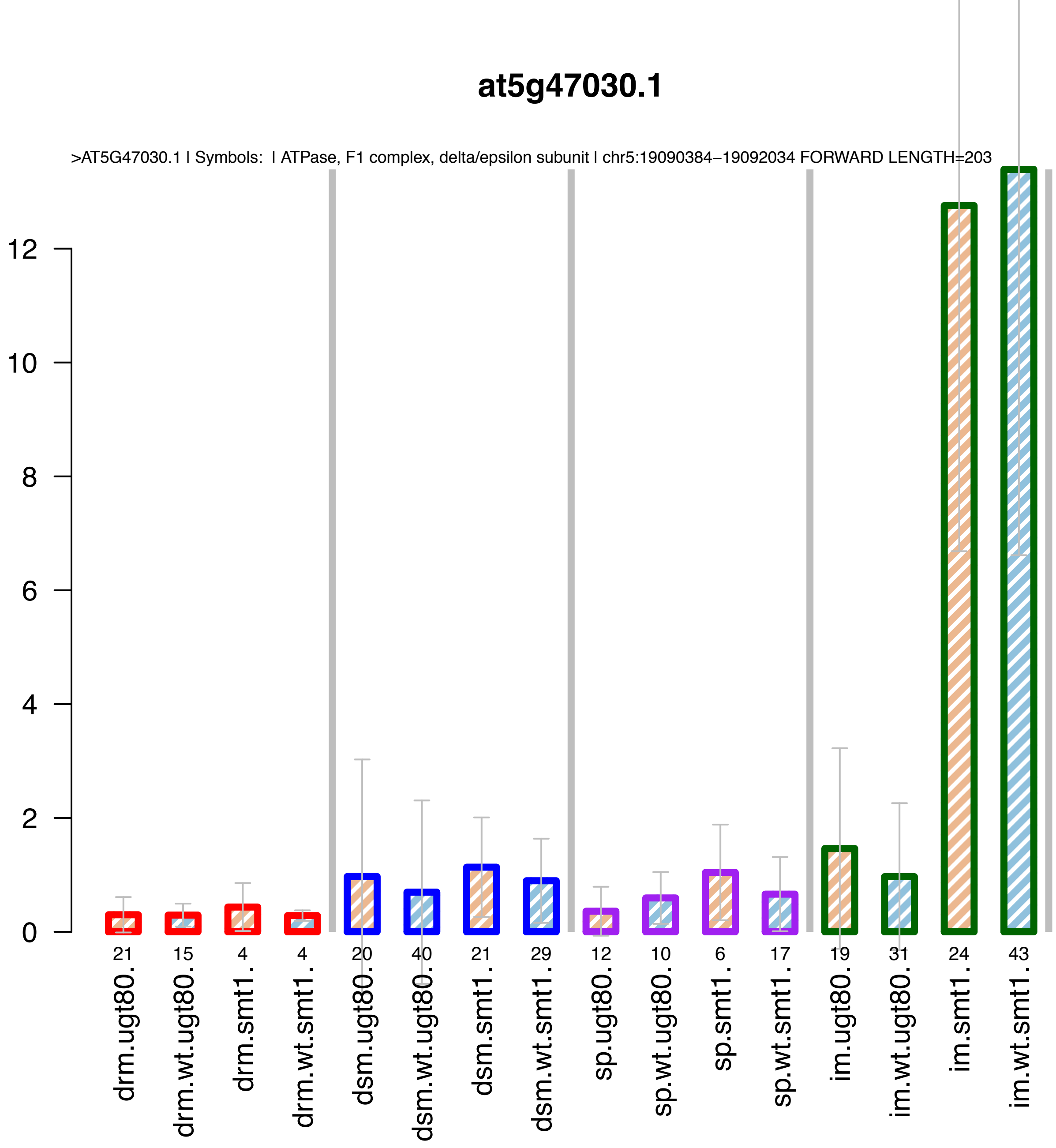

at5g47030.1
>AT5G47030.1 | Symbols: | ATPase, F1 complex, delta/epsilon subunit | chr5:19090384−19092034 FORWARD LENGTH=203
0
2
4
6
8
10
12
21
15
4
4
20
40
21
29
12
10
6
17
19
31
24
43
drm.ugt80..
drm.wt.ugt80..
drm.smt1..
drm.wt.smt1..
dsm.ugt80..
dsm.wt.ugt80..
dsm.smt1..
dsm.wt.smt1..
sp.ugt80..
sp.wt.ugt80..
sp.smt1..
sp.wt.smt1..
im.ugt80..
im.wt.ugt80..
im.smt1..
im.wt.smt1..

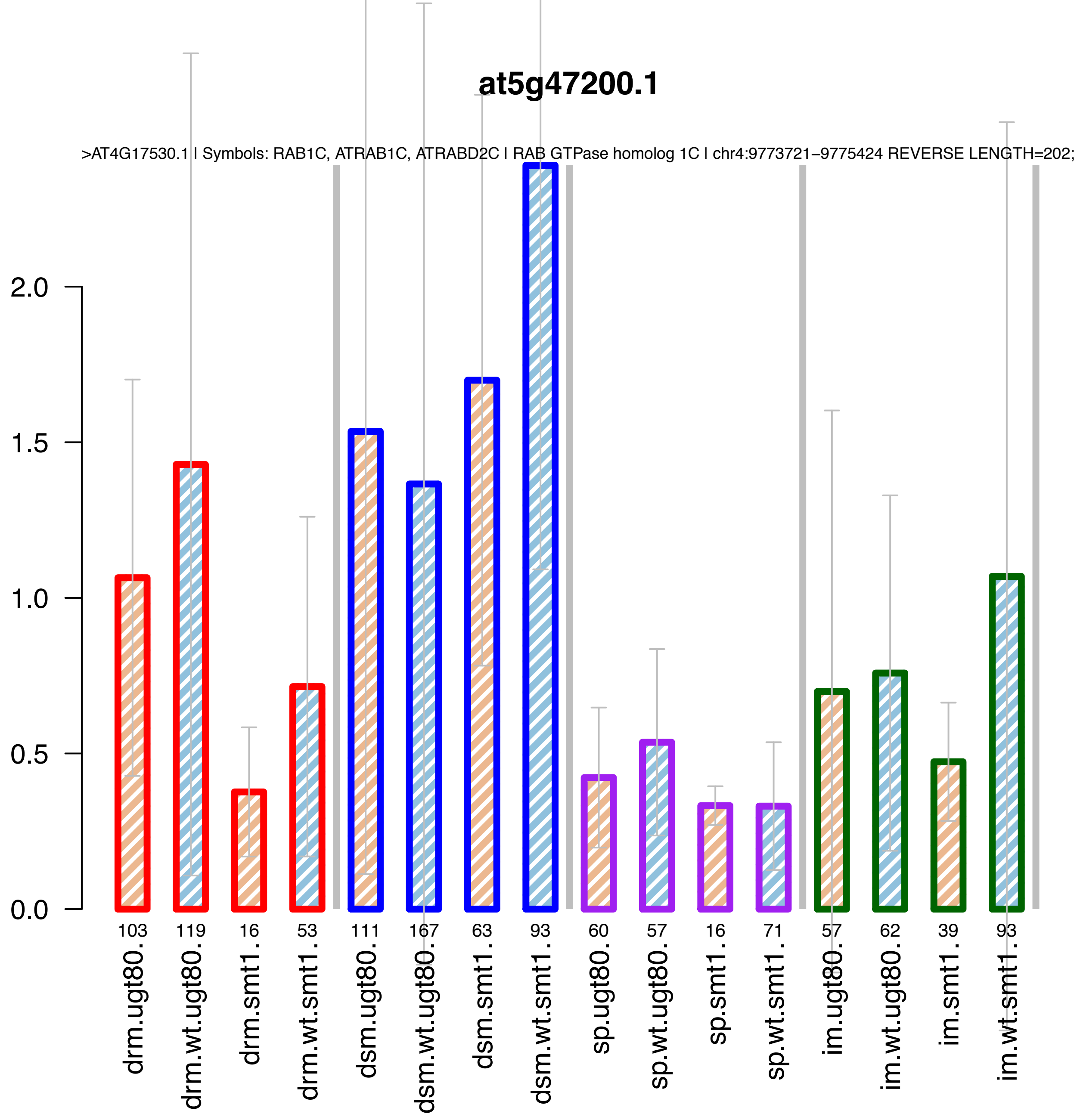

at5g47200.1
>AT4G17530.1 | Symbols: RAB1C, ATRAB1C, ATRABD2C | RAB GTPase homolog 1C | chr4:9773721−9775424 REVERSE LENGTH=202;
0.0
0.5
1.0
1.5
2.0
103
119
16
53
111
167
63
93
60
57
16
71
57
62
39
93
drm.ugt80..
drm.wt.ugt80..
drm.smt1..
drm.wt.smt1..
dsm.ugt80..
dsm.wt.ugt80..
dsm.smt1..
dsm.wt.smt1..
sp.ugt80..
sp.wt.ugt80..
sp.smt1..
sp.wt.smt1..
im.ugt80..
im.wt.ugt80..
im.smt1..
im.wt.smt1..

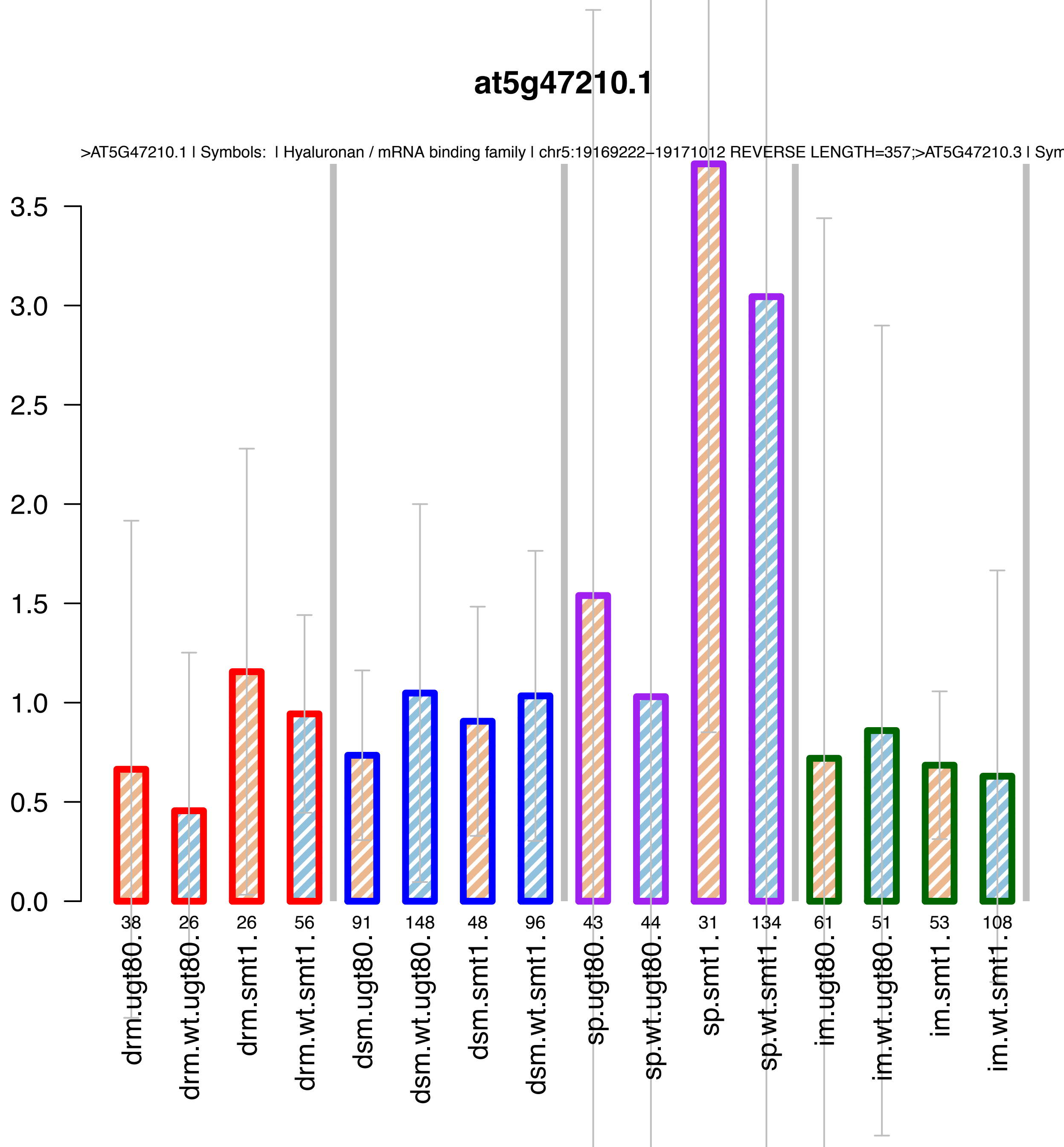

at5g47210.1
>AT5G47210.1 | Symbols: | Hyaluronan / mRNA binding family | chr5:19169222-19171012 REVERSE LENGTH=357;>AT5G47210.3 | Sym
0.0
0.5
1.0
1.5
2.0
2.5
3.0
3.5
38
26
26
56
91
148
48
96
43
44
31
134
61
51
53
108
drm.ugt80..
drm.wt.ugt80..
drm.smt1..
drm.wt.smt1..
dsm.ugt80..
dsm.wt.ugt80..
dsm.smt1..
dsm.wt.smt1..
sp.ugt80..
sp.wt.ugt80..
sp.smt1..
sp.wt.smt1..
im.ugt80..
im.wt.ugt80..
im.smt1..
im.wt.smt1..

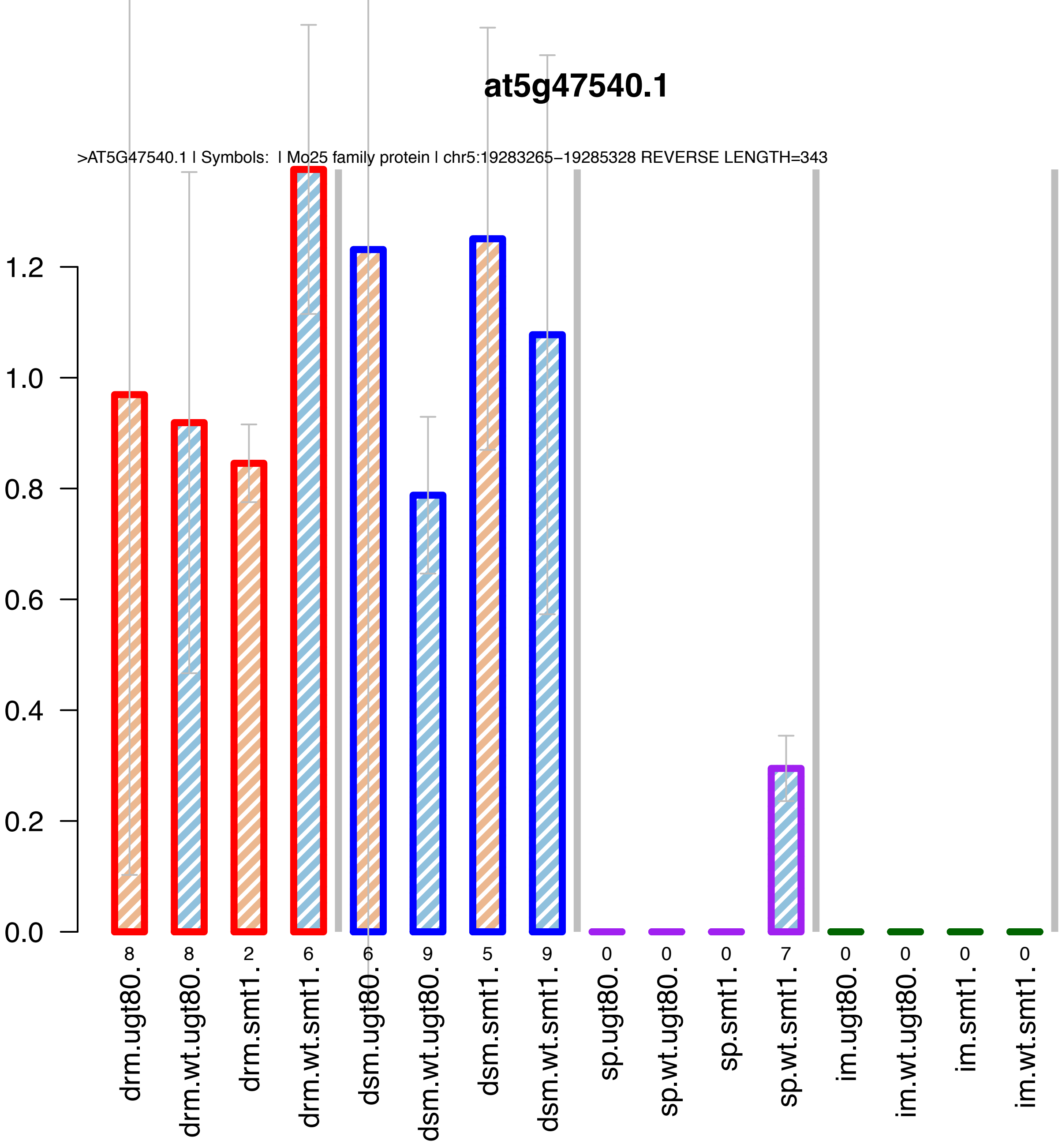

at5g47540.1
>AT5G47540.1 | Symbols: | Mo25 family protein | chr5:19283265-19285328 REVERSE LENGTH=343
1.2
1.0
0.8
0.6
0.4
0.2
0.0
8
8
2
6
6
9
5
9
0
0
0
7
0
0
0
0
drm.ugt80.
drm.wt.ugt80.
drm.smt1.
drm.wt.smt1.
dsm.ugt80.
dsm.wt.ugt80.
dsm.smt1.
dsm.wt.smt1.
sp.ugt80.
sp.wt.ugt80.
sp.smt1.
sp.wt.smt1.
im.ugt80.
im.wt.ugt80.
im.smt1.
im.wt.smt1.

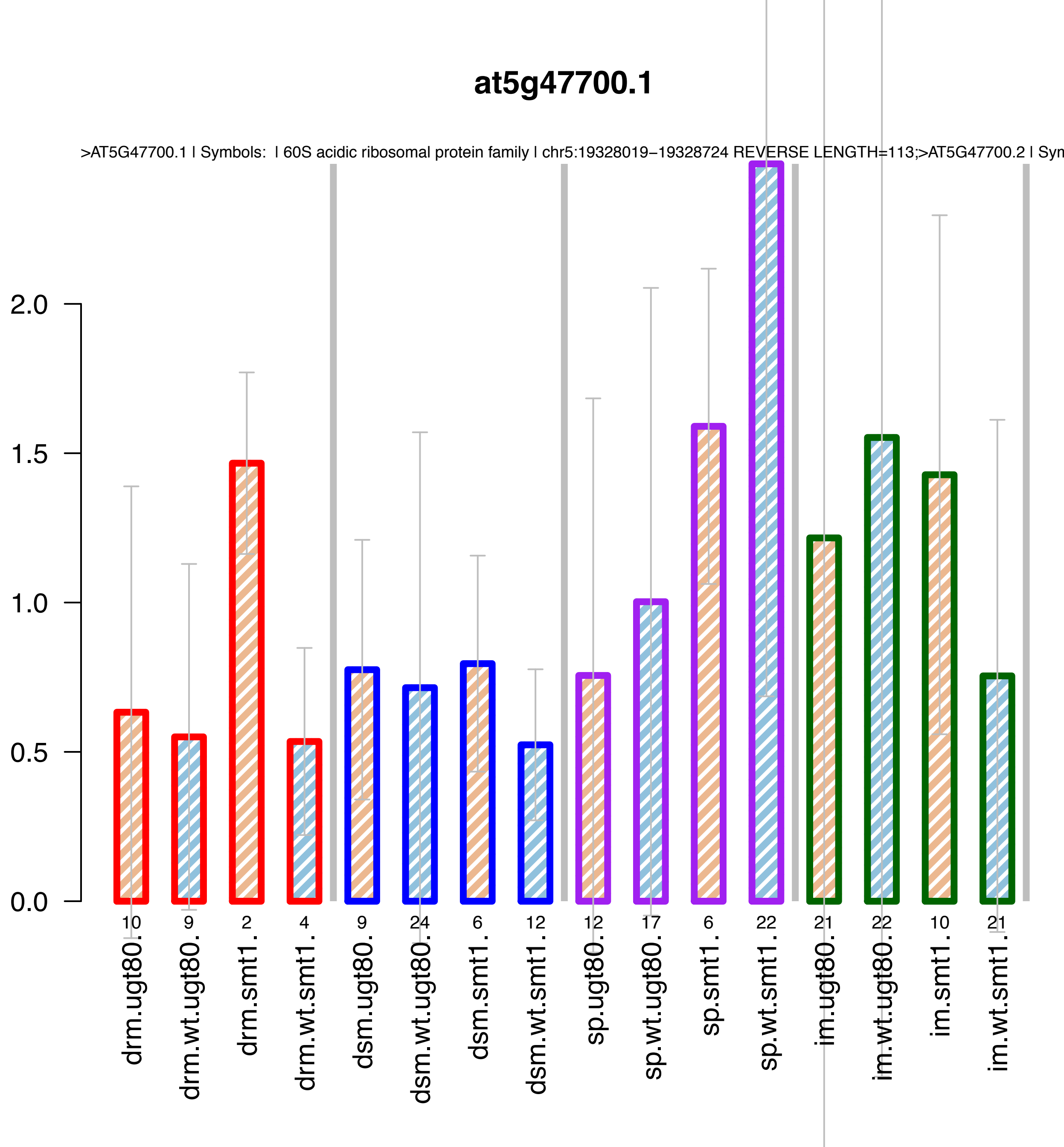

at5g47700.1
>AT5G47700.1 | Symbols: | 60S acidic ribosomal protein family | chr5:19328019-19328724 REVERSE LENGTH=113;>AT5G47700.2 | Sym
2.0
1.5
1.0
0.5
0.0
10
9
2
4
9
24
6
12
12
17
6
22
21
22
10
21
drm.ugt80.
drm.wt.ugt80.
drm.smt1.
drm.wt.smt1.
dsm.ugt80.
dsm.wt.ugt80.
dsm.smt1.
dsm.wt.smt1.
sp.ugt80.
sp.wt.ugt80.
sp.smt1.
sp.wt.smt1.
im.ugt80.
im.wt.ugt80.
im.smt1.
im.wt.smt1.

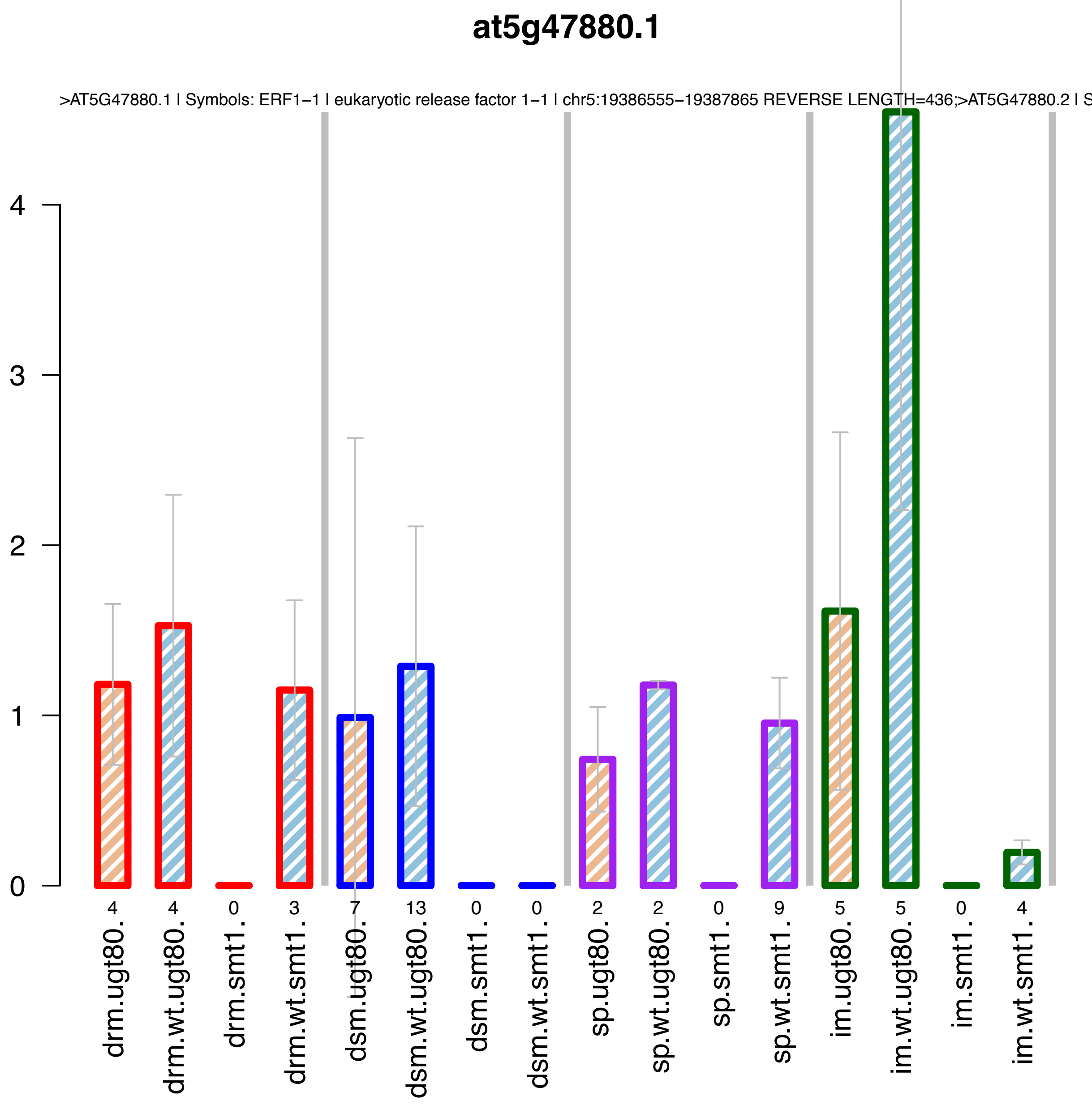

at5g47880.1
>AT5G47880.1 | Symbols: ERF1−1 | eukaryotic release factor 1−1 | chr5:19386555−19387865 REVERSE LENGTH=436;>AT5G47880.2 | S
0
1
2
3
4
4 4 0 3 7 13 0 0 2 2 0 9 5 5 0 4
drm.ugt80.
drm.wt.ugt80.
drm.smt1.
drm.wt.smt1.
dsm.ugt80.
dsm.wt.ugt80.
dsm.smt1.
dsm.wt.smt1.
sp.ugt80.
sp.wt.ugt80.
sp.smt1.
sp.wt.smt1.
im.ugt80.
im.wt.ugt80.
im.smt1.
im.wt.smt1.

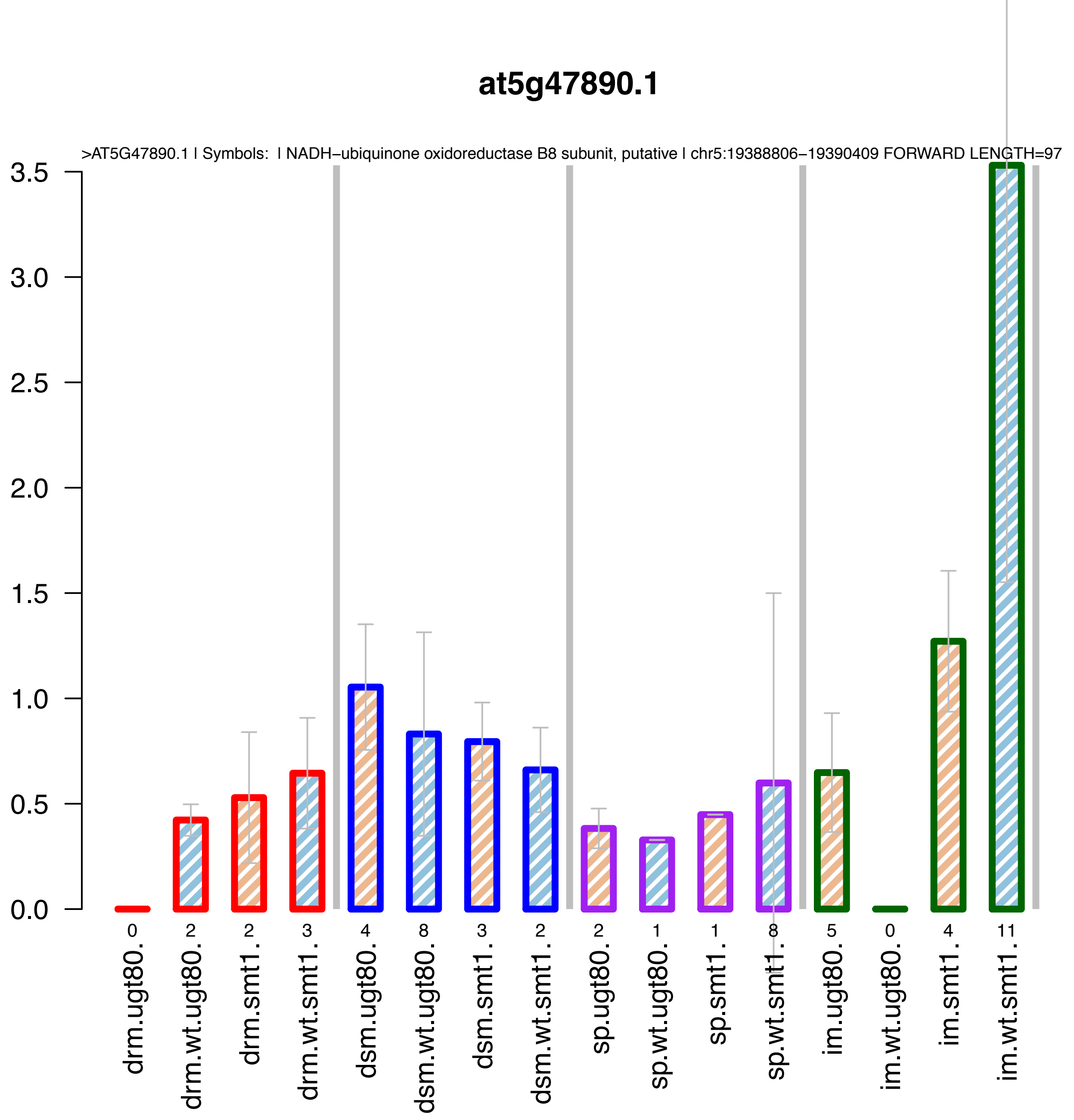

at5g47890.1
>AT5G47890.1 | Symbols: | NADH–ubiquinone oxidoreductase B8 subunit, putative | chr5:19388806–19390409 FORWARD LENGTH=97
3.5
3.0
2.5
2.0
1.5
1.0
0.5
0.0
0 2 2 3 4 8 3 2 2 1 1 8 5 0 4 11
drm.ugt80.
drm.wt.ugt80.
drm.smt1.
drm.wt.smt1.
dsm.ugt80.
dsm.wt.ugt80.
dsm.smt1.
dsm.wt.smt1.
sp.ugt80.
sp.wt.ugt80.
sp.smt1.
sp.wt.smt1.
im.ugt80.
im.wt.ugt80.
im.smt1.
im.wt.smt1.

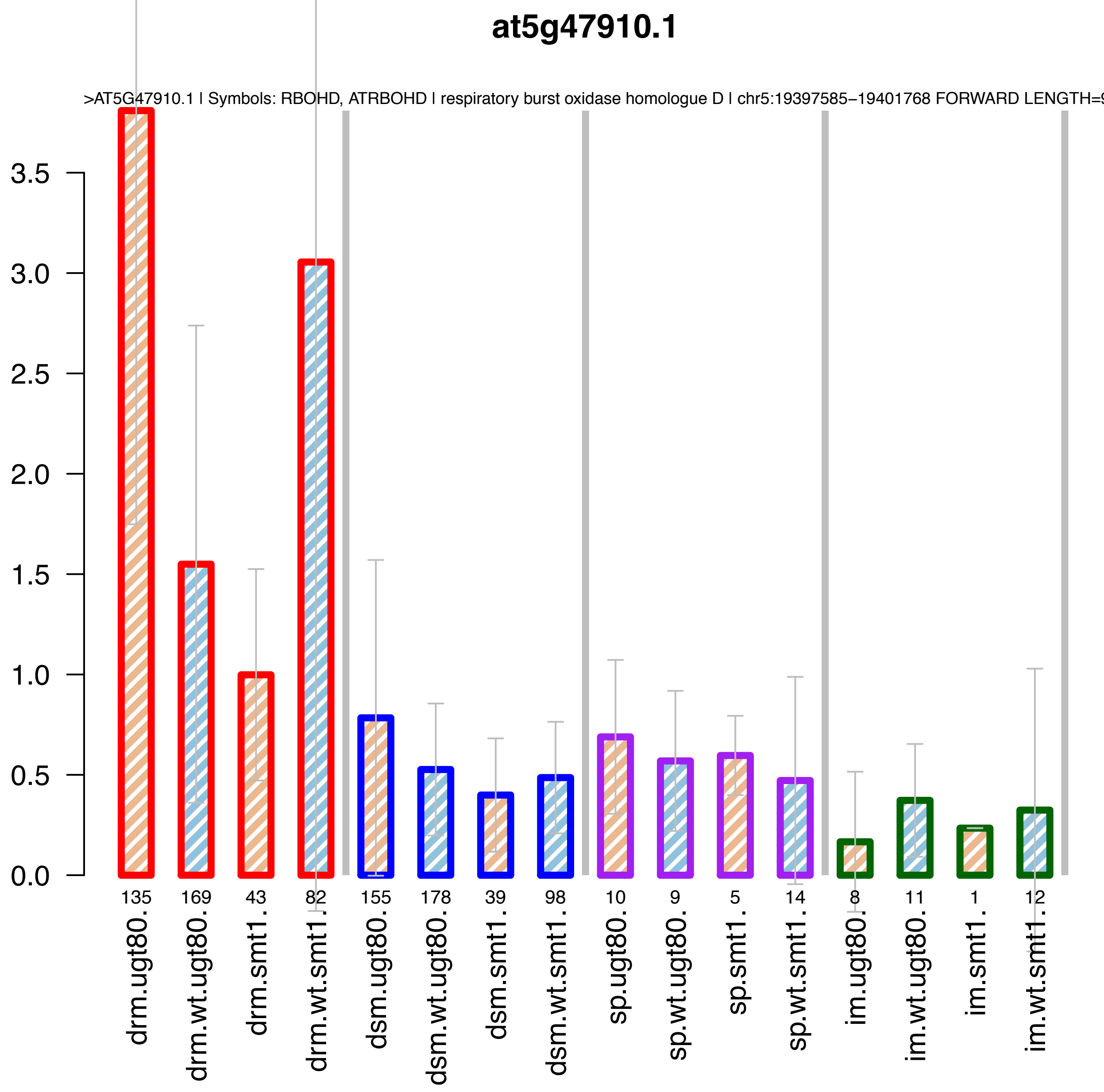

at5g47910.1
>AT5G47910.1 | Symbols: RBOHD, ATRBOHD | respiratory burst oxidase homologue D | chr5:19397585-19401768 FORWARD LENGTH=9
3.5
3.0
2.5
2.0
1.5
1.0
0.5
0.0
135
169
43
82
155
178
39
98
10
9
5
14
8
11
1
12
drm.ugt80..
drm.wt.ugt80..
drm.smt1..
drm.wt.smt1..
dsm.ugt80..
dsm.wt.ugt80..
dsm.smt1..
dsm.wt.smt1..
sp.ugt80..
sp.wt.ugt80..
sp.smt1..
sp.wt.smt1..
im.ugt80..
im.wt.ugt80..
im.smt1..
im.wt.smt1..

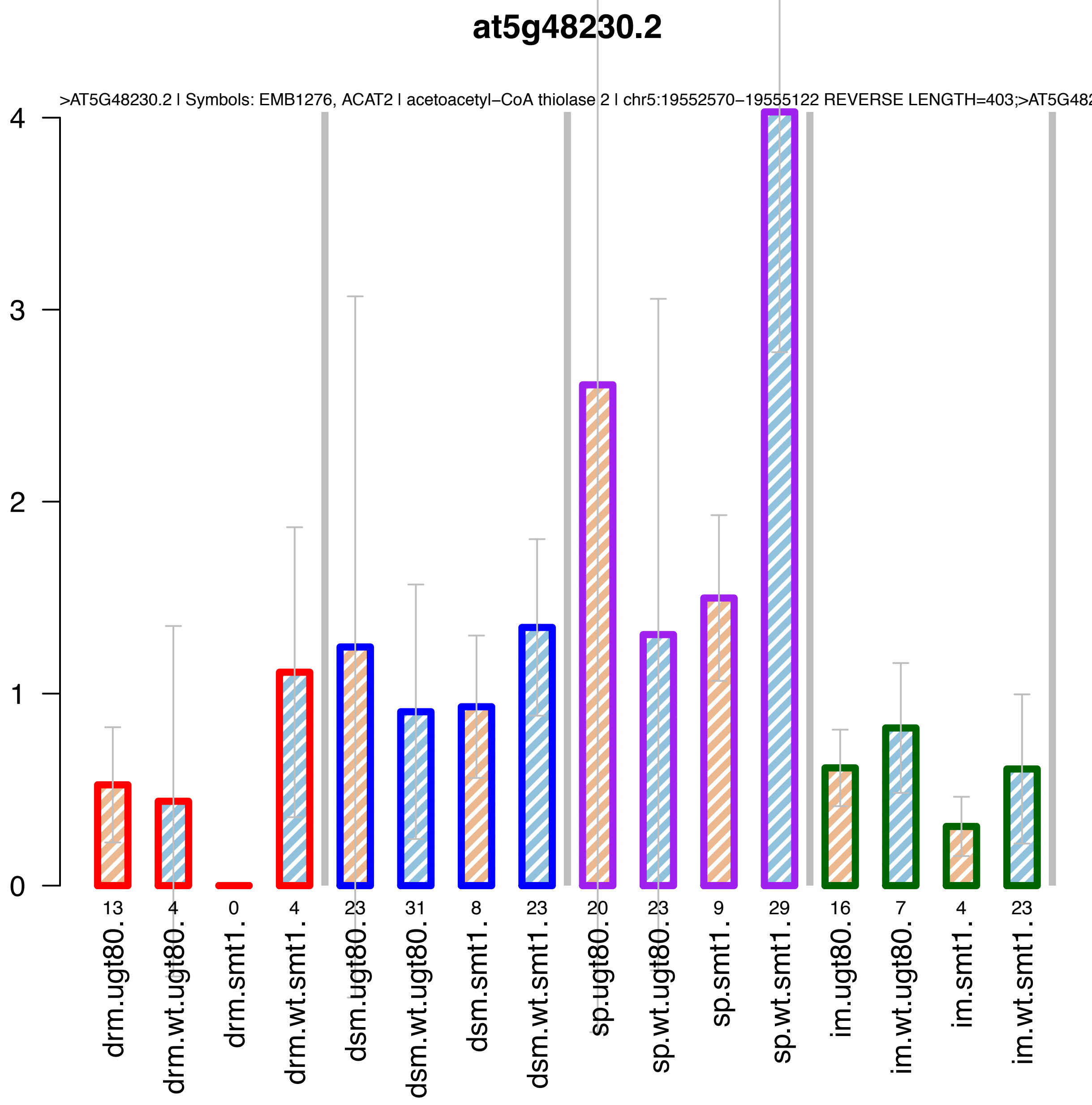
at5g48230.2
>AT5G48230.2 | Symbols: EMB1276, ACAT2 | acetoacetyl−CoA thiolase 2 | chr5:19552570−19555122 REVERSE LENGTH=403;>AT5G482
0
1
2
3
4
13
4
0
4
23
31
8
23
20
23
9
29
16
7
4
23
drm.ugt80.
drm.wt.ugt80.
drm.smt1.
drm.wt.smt1.
dsm.ugt80.
dsm.wt.ugt80.
dsm.smt1.
dsm.wt.smt1.
sp.ugt80.
sp.wt.ugt80.
sp.smt1.
sp.wt.smt1.
im.ugt80.
im.wt.ugt80.
im.smt1.
im.wt.smt1.

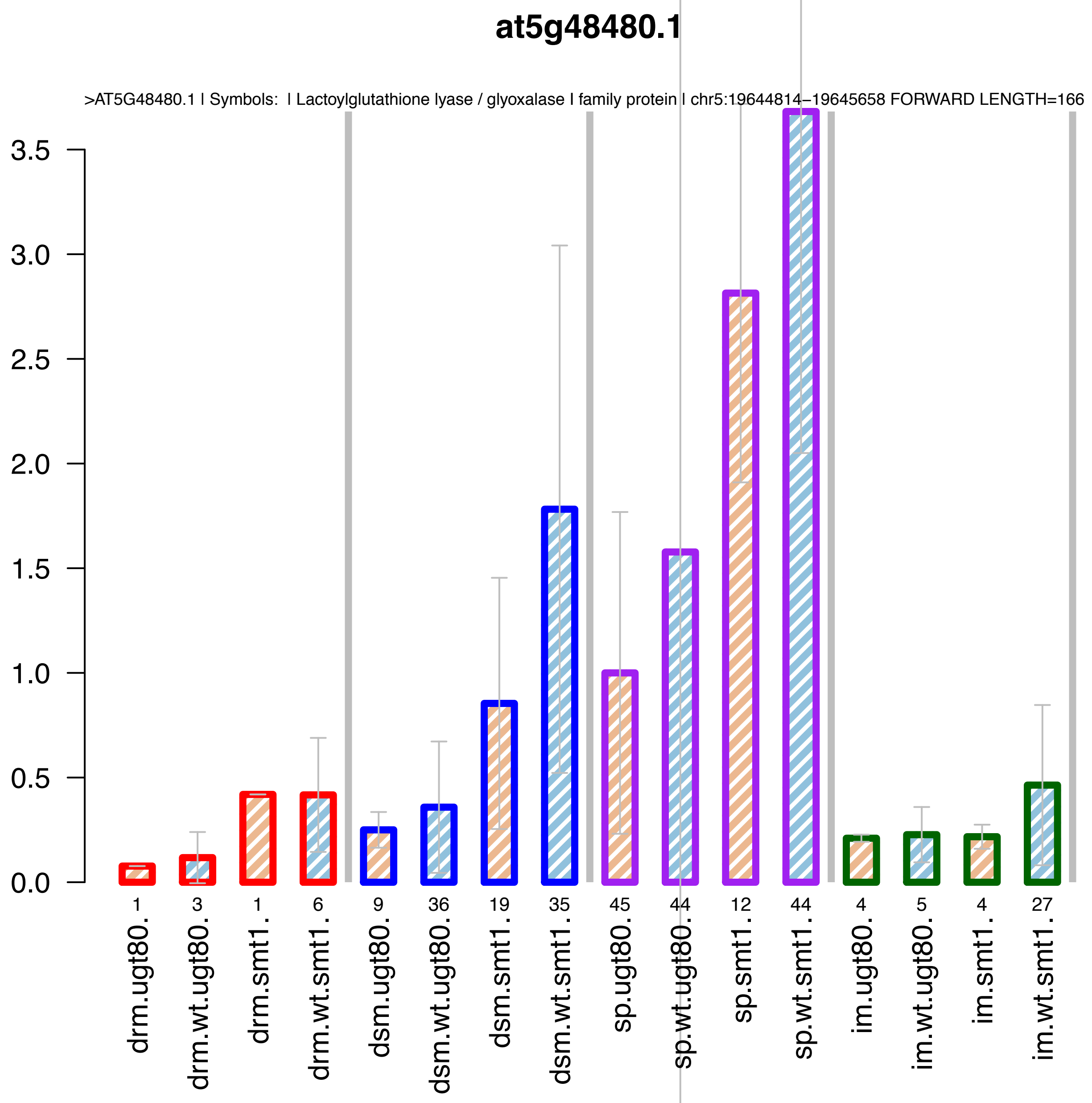

at5g48480.1
>AT5G48480.1 | Symbols: | Lactoylglutathione lyase / glyoxalase I family protein | chr5:19644814-19645658 FORWARD LENGTH=166
0.0
0.5
1.0
1.5
2.0
2.5
3.0
3.5
1 3 1 6 9 36 19 35 45 44 12 44 4 5 4 27
drm.ugt80.
drm.wt.ugt80.
drm.smt1.
drm.wt.smt1.
dsm.ugt80.
dsm.wt.ugt80.
dsm.smt1.
dsm.wt.smt1.
sp.ugt80.
sp.wt.ugt80.
sp.smt1.
sp.wt.smt1.
im.ugt80.
im.wt.ugt80.
im.smt1.
im.wt.smt1.

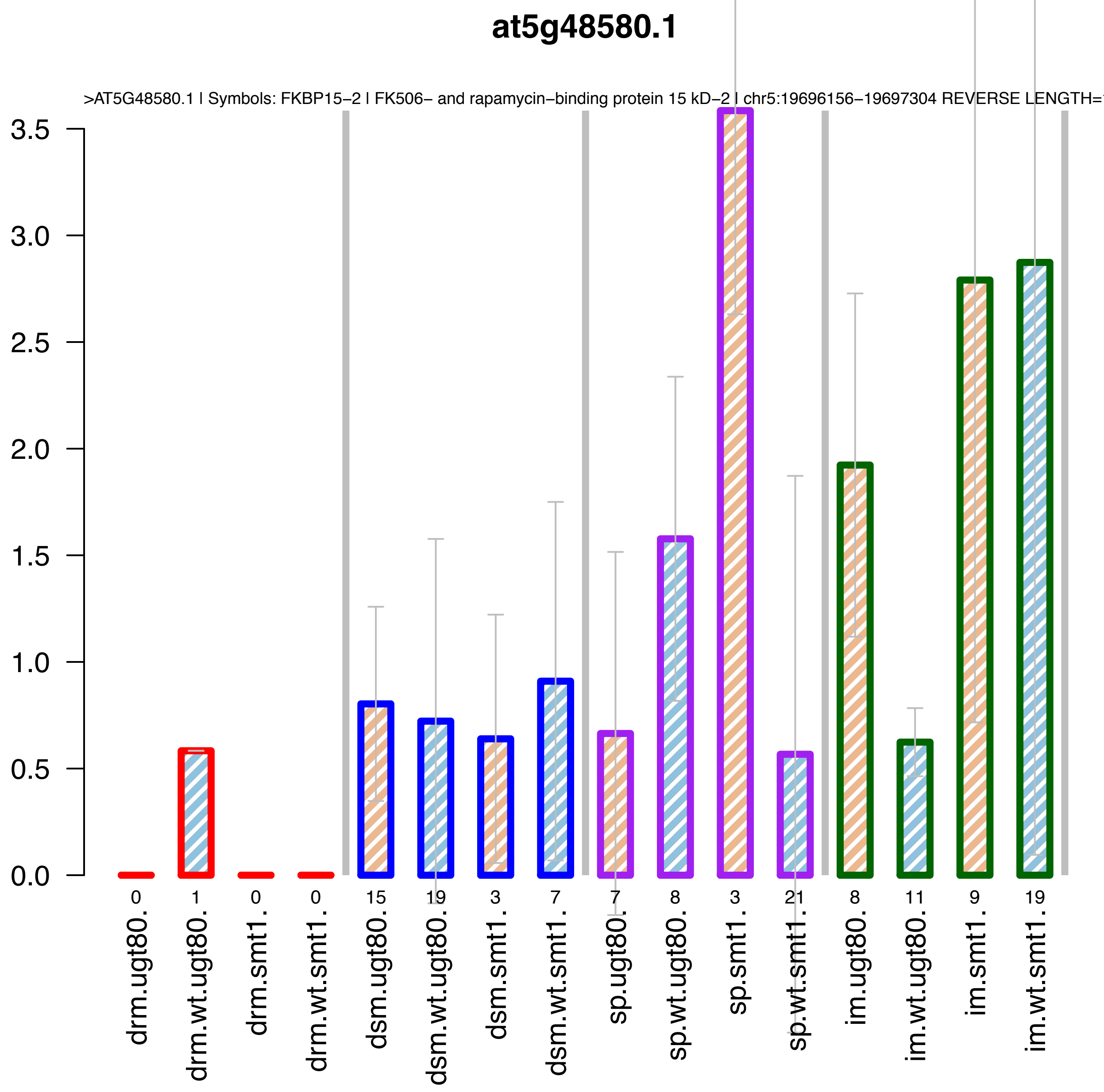

at5g48580.1
>AT5G48580.1 | Symbols: FKBP15−2 | FK506− and rapamycin−binding protein 15 kD−2 | chr5:19696156−19697304 REVERSE LENGTH=
3.5
3.0
2.5
2.0
1.5
1.0
0.5
0.0
0
1
0
0
15
19
3
7
7
8
3
21
8
11
9
19
drm.ugt80.
drm.wt.ugt80.
drm.smt1.
drm.wt.smt1.
dsm.ugt80.
dsm.wt.ugt80.
dsm.smt1.
dsm.wt.smt1.
sp.ugt80.
sp.wt.ugt80.
sp.smt1.
sp.wt.smt1.
im.ugt80.
im.wt.ugt80.
im.smt1.
im.wt.smt1.

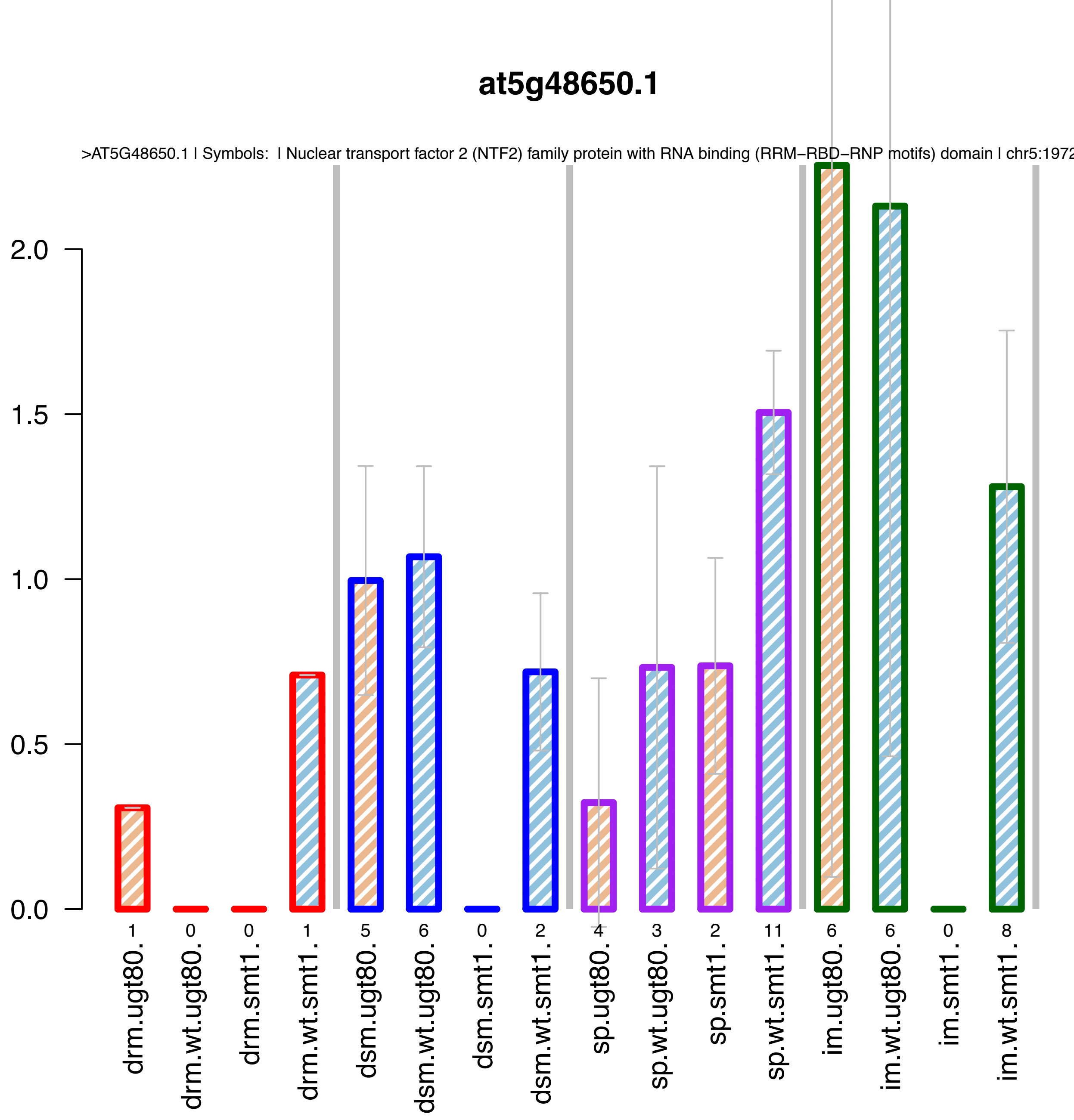

at5g48650.1
>AT5G48650.1 | Symbols: | Nuclear transport factor 2 (NTF2) family protein with RNA binding (RRM−RBD−RNP motifs) domain | chr5:1972
2.0
1.5
1.0
0.5
0.0
1
0
0
1
5
6
0
2
4
3
2
11
6
6
0
8
drm.ugt80.
drm.wt.ugt80.
drm.smt1.
drm.wt.smt1.
dsm.ugt80.
dsm.wt.ugt80.
dsm.smt1.
dsm.wt.smt1.
sp.ugt80.
sp.wt.ugt80.
sp.smt1.
sp.wt.smt1.
im.ugt80.
im.wt.ugt80.
im.smt1.
im.wt.smt1.

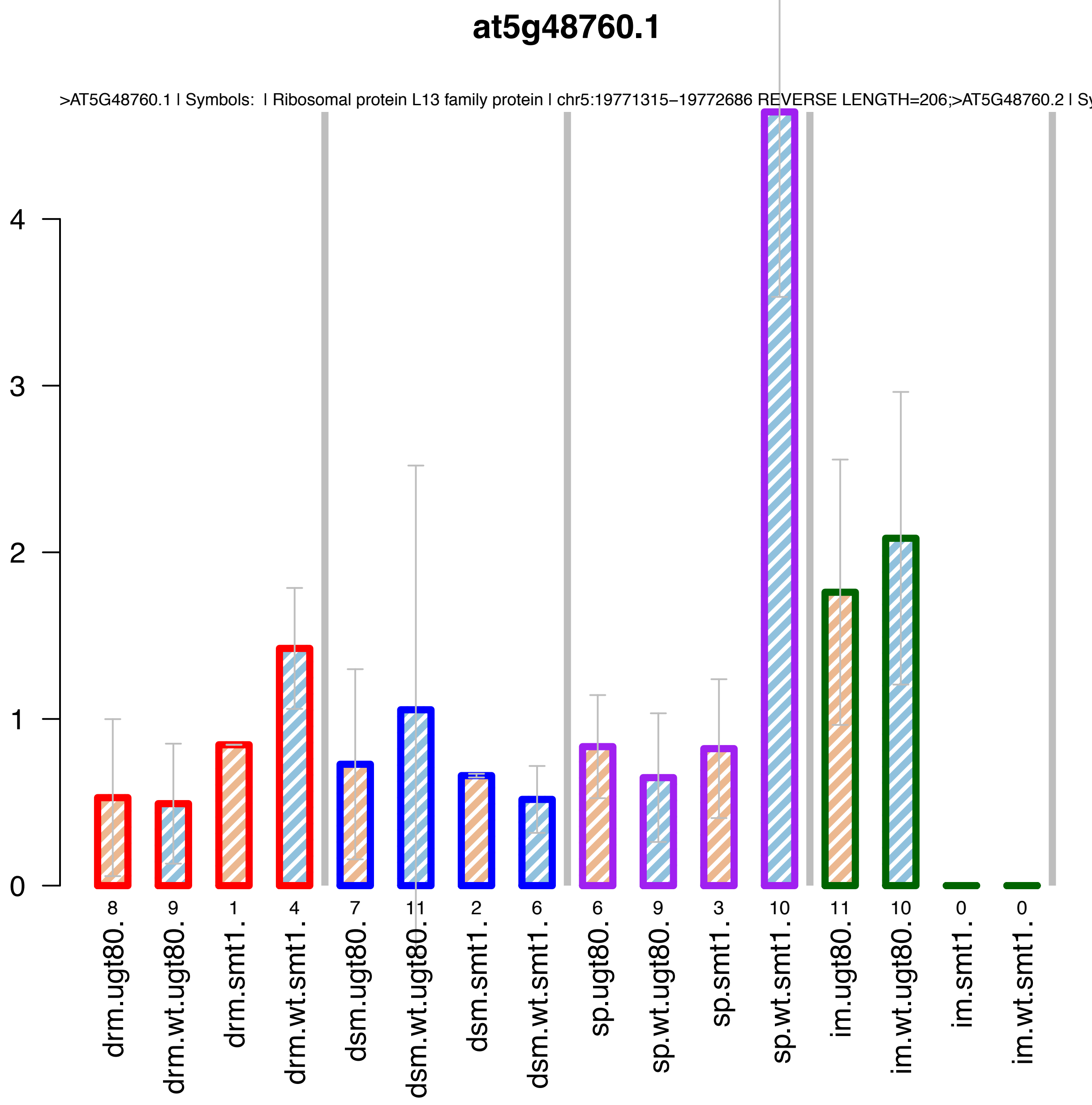

at5g48760.1
>AT5G48760.1 | Symbols: | Ribosomal protein L13 family protein | chr5:19771315-19772686 REVERSE LENGTH=206;>AT5G48760.2 | Sy
0
1
2
3
4
8
9
1
4
7
11
2
6
6
9
3
10
11
10
0
0
drm.ugt80.
drm.wt.ugt80.
drm.smt1.
drm.wt.smt1.
dsm.ugt80.
dsm.wt.ugt80.
dsm.smt1.
dsm.wt.smt1.
sp.ugt80.
sp.wt.ugt80.
sp.smt1.
sp.wt.smt1.
im.ugt80.
im.wt.ugt80.
im.smt1.
im.wt.smt1.

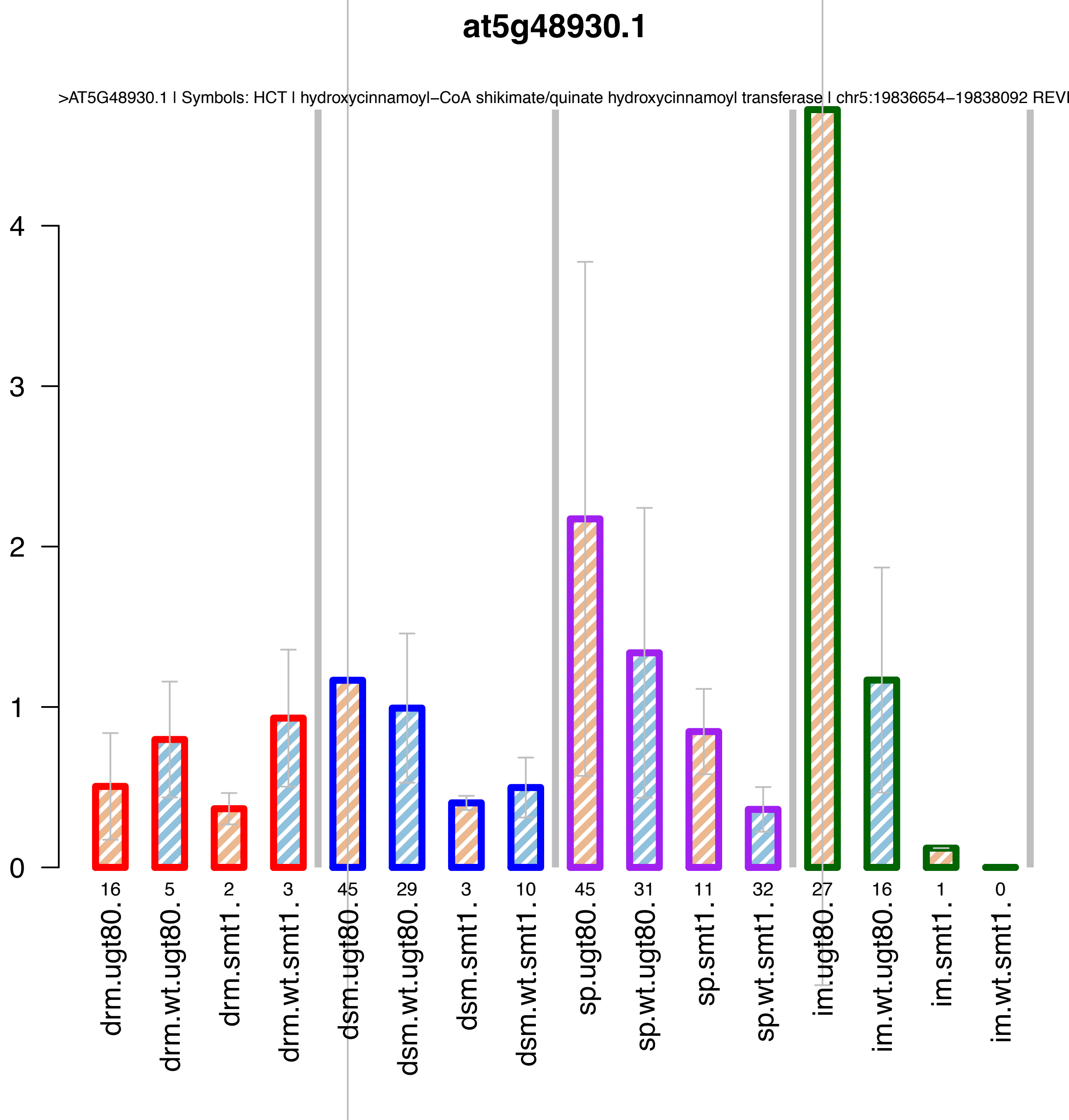

at5g48930.1
>AT5G48930.1 | Symbols: HCT | hydroxycinnamoyl−CoA shikimate/quinate hydroxycinnamoyl transferase | chr5:19836654−19838092 REVE
0
1
2
3
4
16
5
2
3
45
29
3
10
45
31
11
32
27
16
1
0
drm.ugt80.
drm.wt.ugt80.
drm.smt1.
drm.wt.smt1.
dsm.ugt80.
dsm.wt.ugt80.
dsm.smt1.
dsm.wt.smt1.
sp.ugt80.
sp.wt.ugt80.
sp.smt1.
sp.wt.smt1.
im.ugt80.
im.wt.ugt80.
im.smt1.
im.wt.smt1.

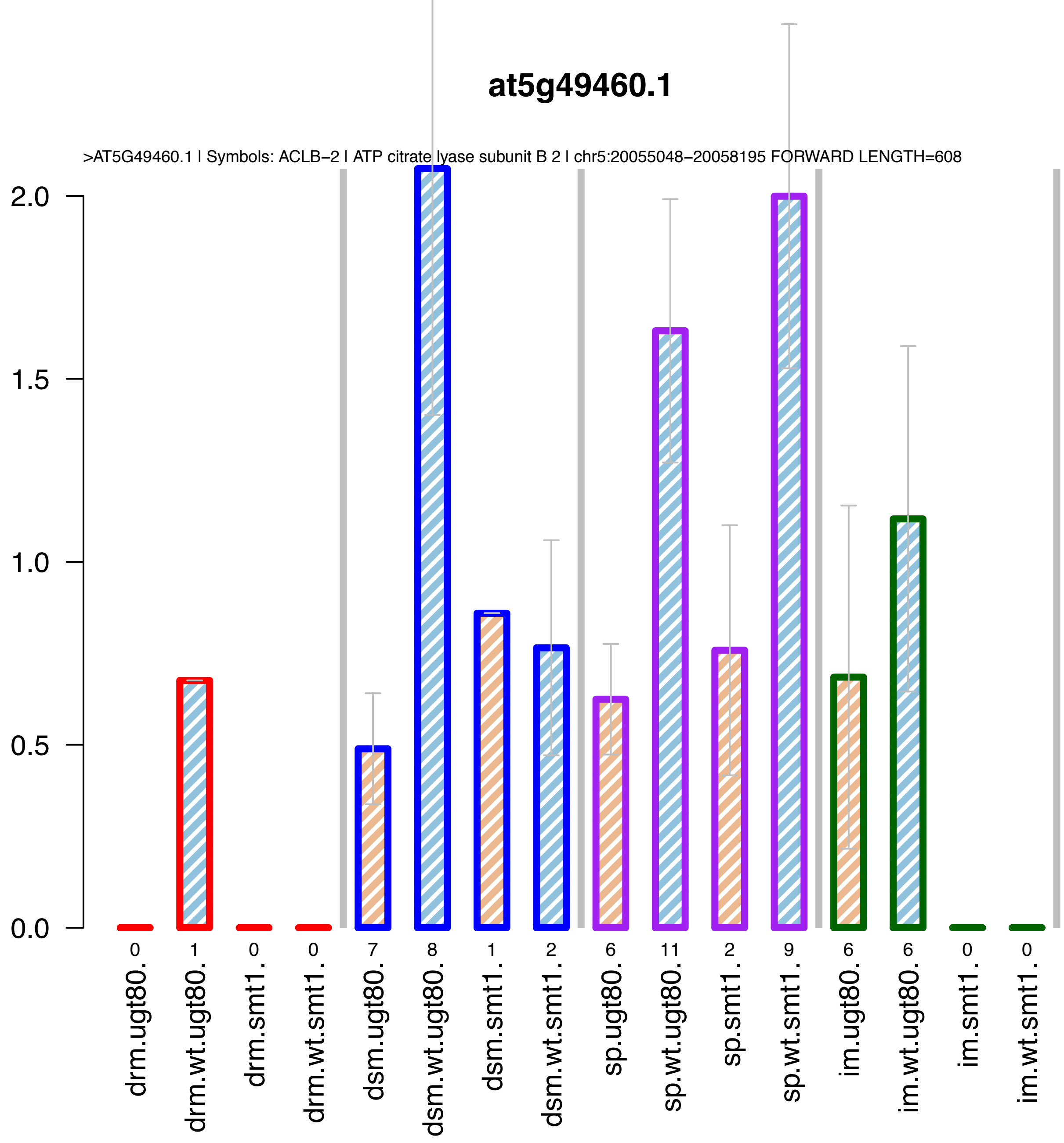

at5g49460.1
>AT5G49460.1 | Symbols: ACLB−2 | ATP citrate lyase subunit B 2 | chr5:20055048−20058195 FORWARD LENGTH=608
2.0
1.5
1.0
0.5
0.0
0
1
0
0
7
8
1
2
6
11
2
9
6
6
0
0
drm.ugt80.
drm.wt.ugt80.
drm.smt1.
drm.wt.smt1.
dsm.ugt80.
dsm.wt.ugt80.
dsm.smt1.
dsm.wt.smt1.
sp.ugt80.
sp.wt.ugt80.
sp.smt1.
sp.wt.smt1.
im.ugt80.
im.wt.ugt80.
im.smt1.
im.wt.smt1.

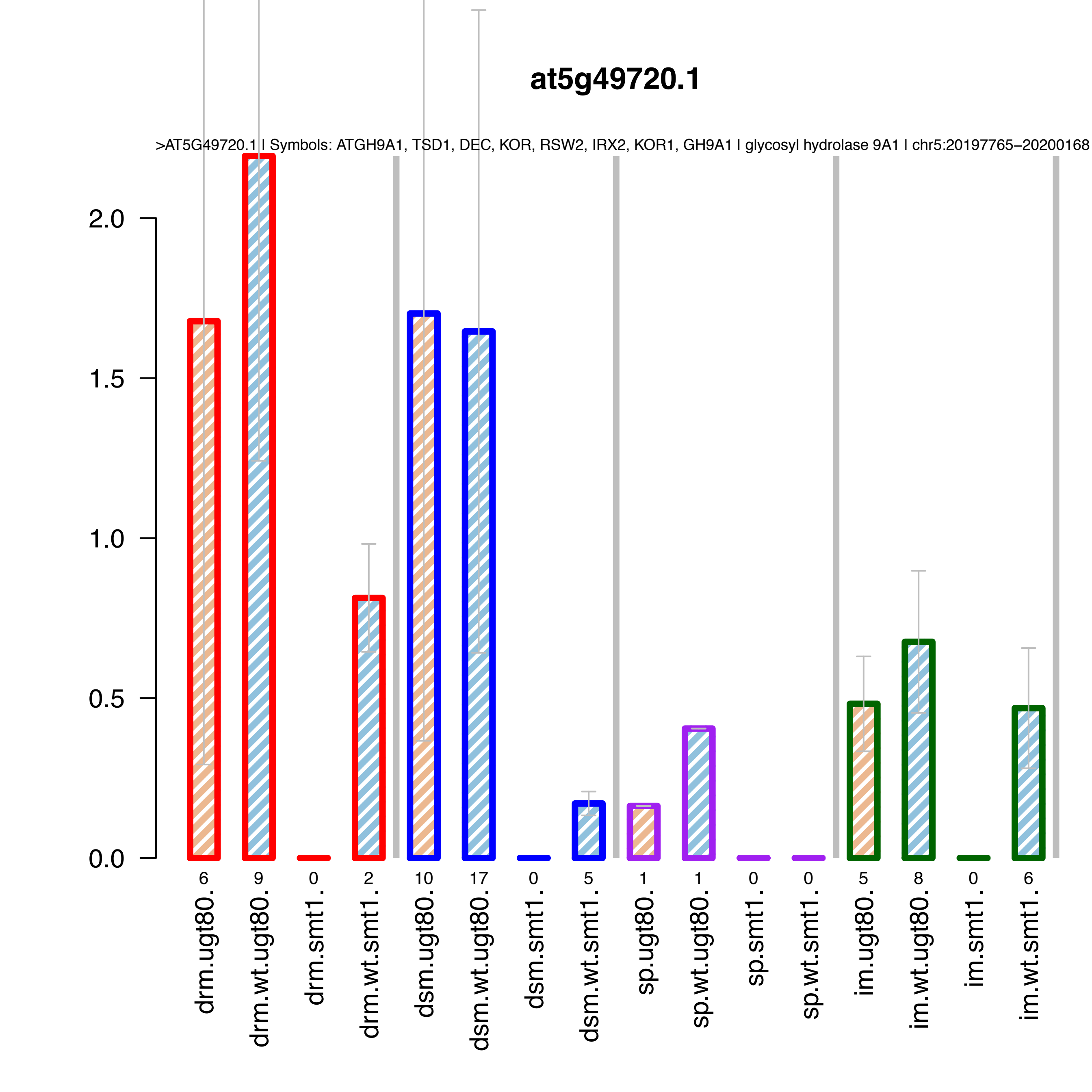

at5g49720.1
>AT5G49720.1 | Symbols: ATGH9A1, TSD1, DEC, KOR, RSW2, IRX2, KOR1, GH9A1 | glycosyl hydrolase 9A1 | chr5:20197765-20200168
2.0
1.5
1.0
0.5
0.0
6
9
0
2
10
17
0
5
1
1
0
0
5
8
0
6
drm.ugt80.
drm.wt.ugt80.
drm.smt1.
drm.wt.smt1.
dsm.ugt80.
dsm.wt.ugt80.
dsm.smt1.
dsm.wt.smt1.
sp.ugt80.
sp.wt.ugt80.
sp.smt1.
sp.wt.smt1.
im.ugt80.
im.wt.ugt80.
im.smt1.
im.wt.smt1.

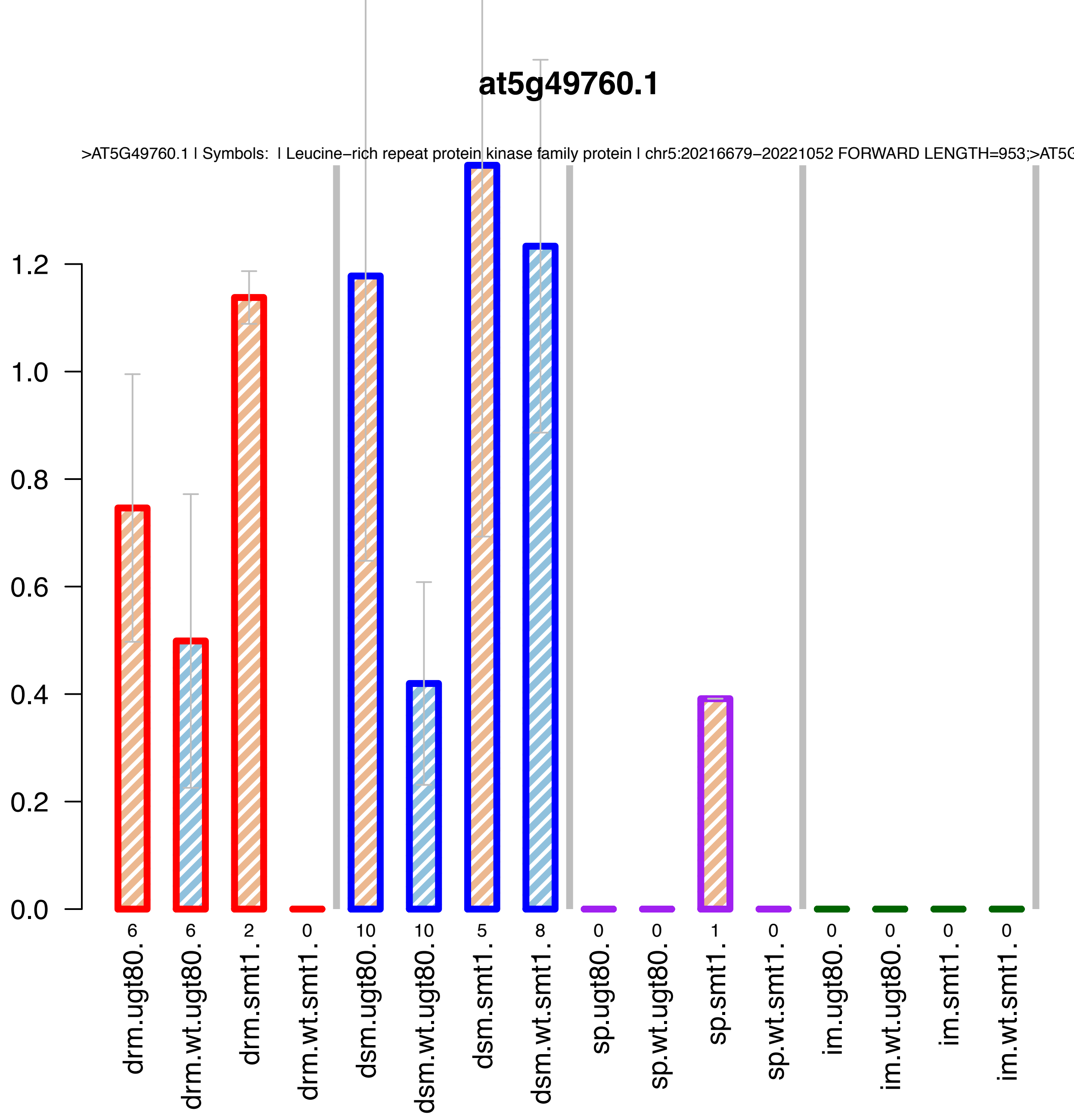

at5g49760.1
>AT5G49760.1 | Symbols: | Leucine−rich repeat protein kinase family protein | chr5:20216679−20221052 FORWARD LENGTH=953;>AT5G
0.0
0.2
0.4
0.6
0.8
1.0
1.2
6
6
2
0
10
10
5
8
0
0
1
0
0
0
0
0
drm.ugt80.
drm.wt.ugt80.
drm.smt1.
drm.wt.smt1.
dsm.ugt80.
dsm.wt.ugt80.
dsm.smt1.
dsm.wt.smt1.
sp.ugt80.
sp.wt.ugt80.
sp.smt1.
sp.wt.smt1.
im.ugt80.
im.wt.ugt80.
im.smt1.
im.wt.smt1.

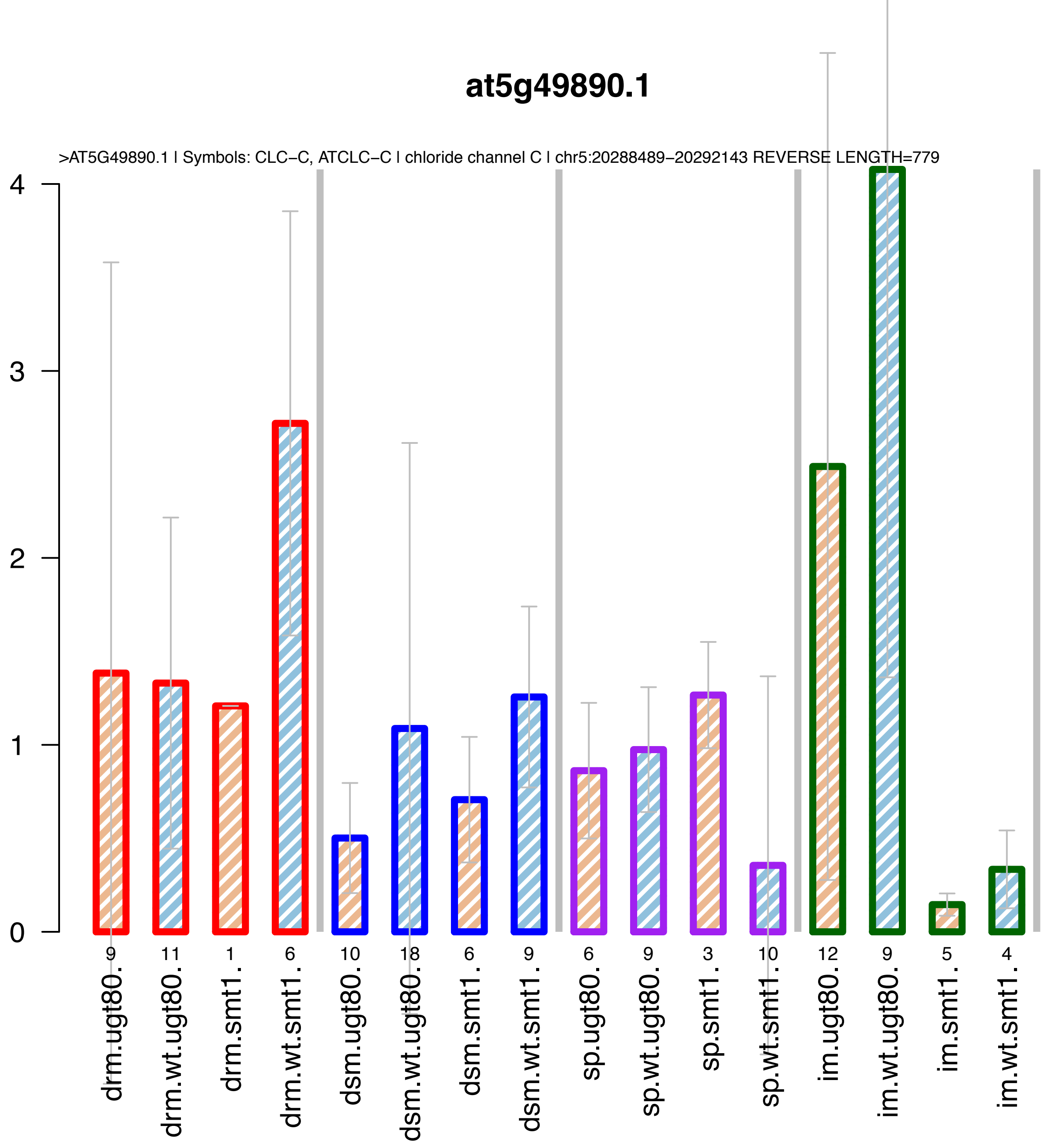

at5g49890.1
>AT5G49890.1 | Symbols: CLC−C, ATCLC−C | chloride channel C | chr5:20288489−20292143 REVERSE LENGTH=779
0
1
2
3
4
9
11
1
6
10
18
6
9
6
9
3
10
12
9
5
4
drm.ugt80.
drm.wt.ugt80.
drm.smt1.
drm.wt.smt1.
dsm.ugt80.
dsm.wt.ugt80.
dsm.smt1.
dsm.wt.smt1.
sp.ugt80.
sp.wt.ugt80.
sp.smt1.
sp.wt.smt1.
im.ugt80.
im.wt.ugt80.
im.smt1.
im.wt.smt1.

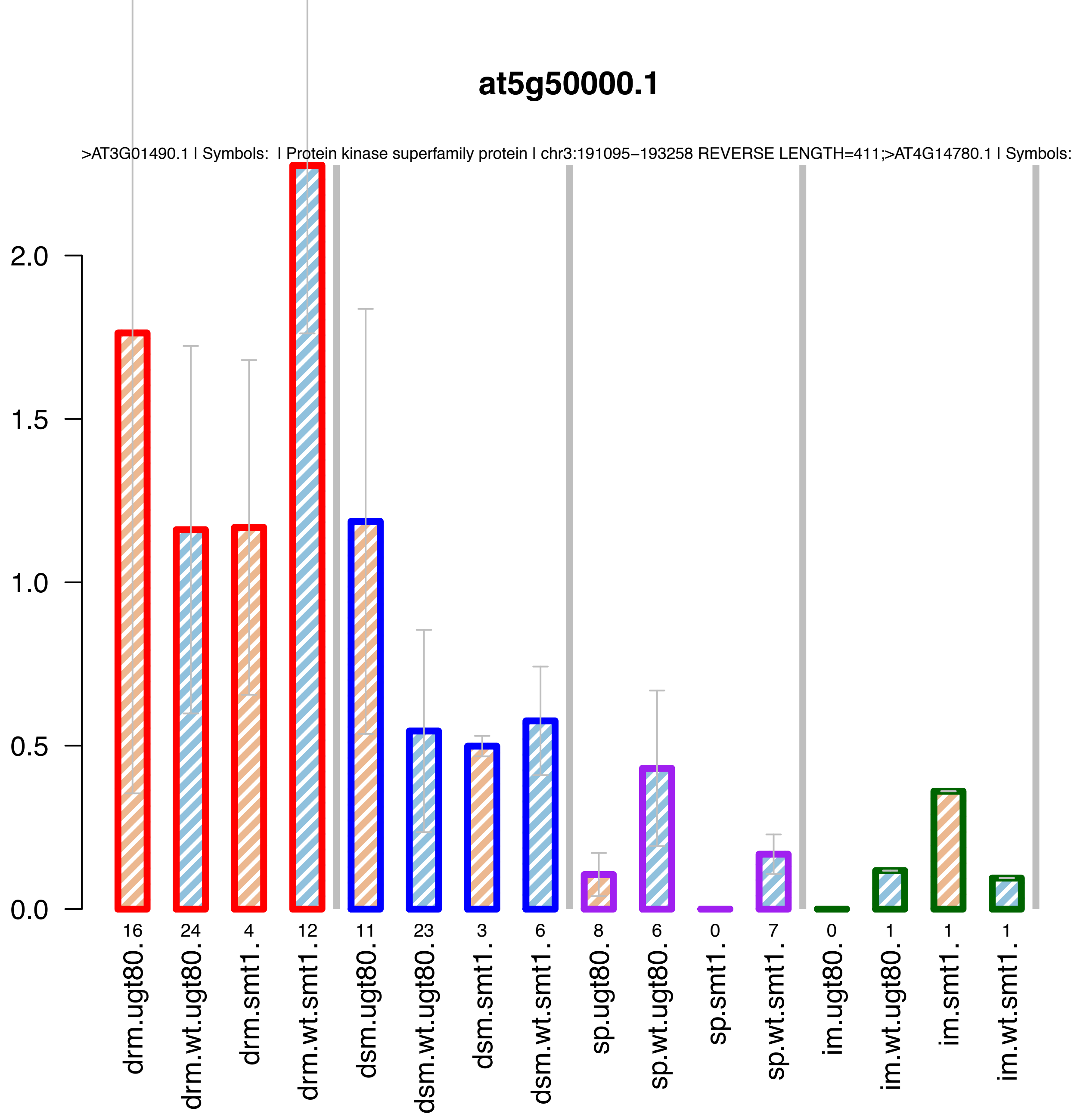

at5g50000.1
>AT3G01490.1 | Symbols: | Protein kinase superfamily protein | chr3:191095-193258 REVERSE LENGTH=411;>AT4G14780.1 | Symbols:
2.0
1.5
1.0
0.5
0.0
16
24
4
12
11
23
3
6
8
6
0
7
0
1
1
1
drm.ugt80.
drm.wt.ugt80.
drm.smt1.
drm.wt.smt1.
dsm.ugt80.
dsm.wt.ugt80.
dsm.smt1.
dsm.wt.smt1.
sp.ugt80.
sp.wt.ugt80.
sp.smt1.
sp.wt.smt1.
im.ugt80.
im.wt.ugt80.
im.smt1.
im.wt.smt1.

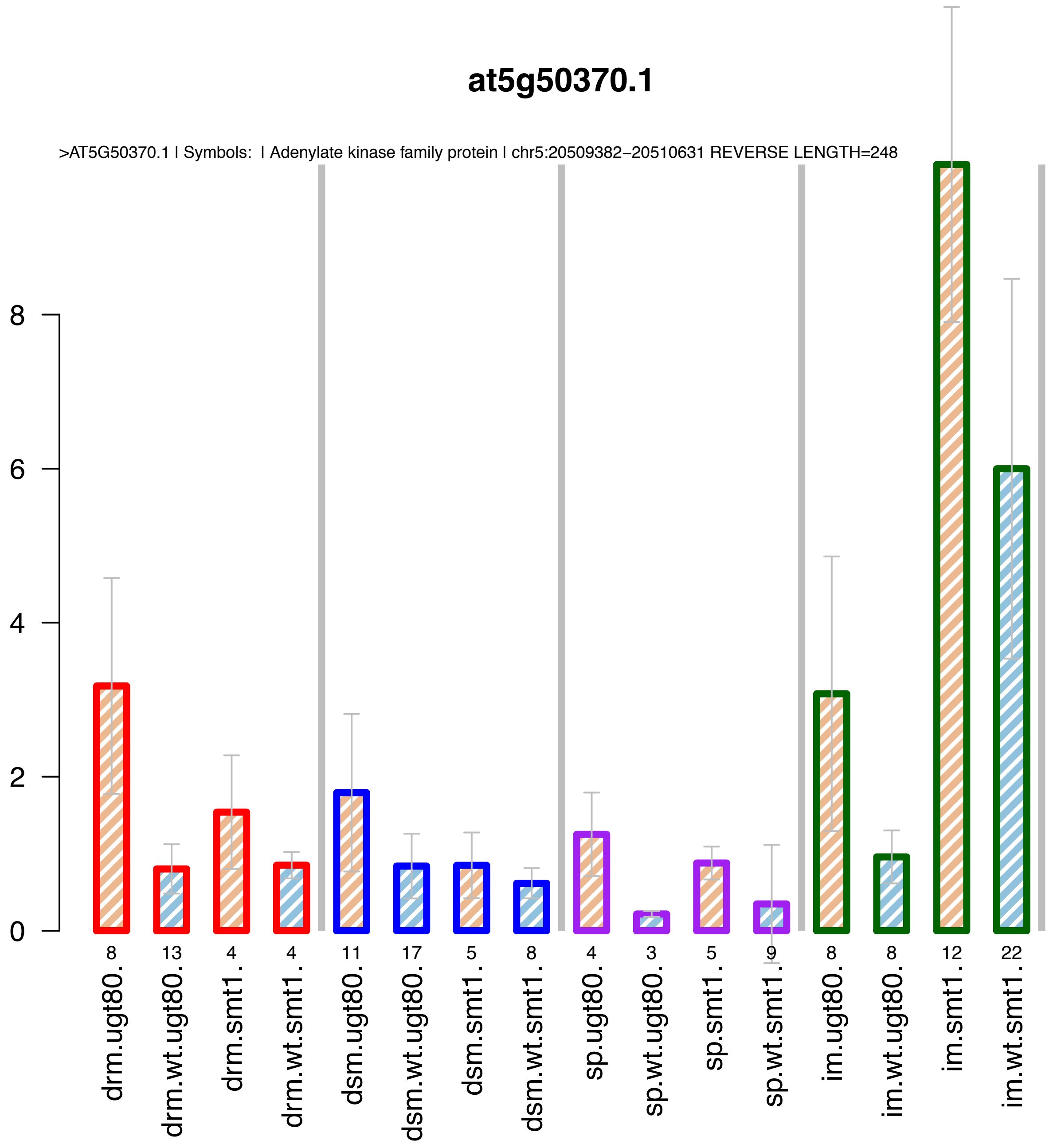

at5g50370.1
>AT5G50370.1 | Symbols: | Adenylate kinase family protein | chr5:20509382-20510631 REVERSE LENGTH=248
0
2
4
6
8
8
13
4
4
11
17
5
8
4
3
5
9
8
8
12
22
drm.ugt80.
drm.wt.ugt80.
drm.smt1.
drm.wt.smt1.
dsm.ugt80.
dsm.wt.ugt80.
dsm.smt1.
dsm.wt.smt1.
sp.ugt80.
sp.wt.ugt80.
sp.smt1.
sp.wt.smt1.
im.ugt80.
im.wt.ugt80.
im.smt1.
im.wt.smt1.

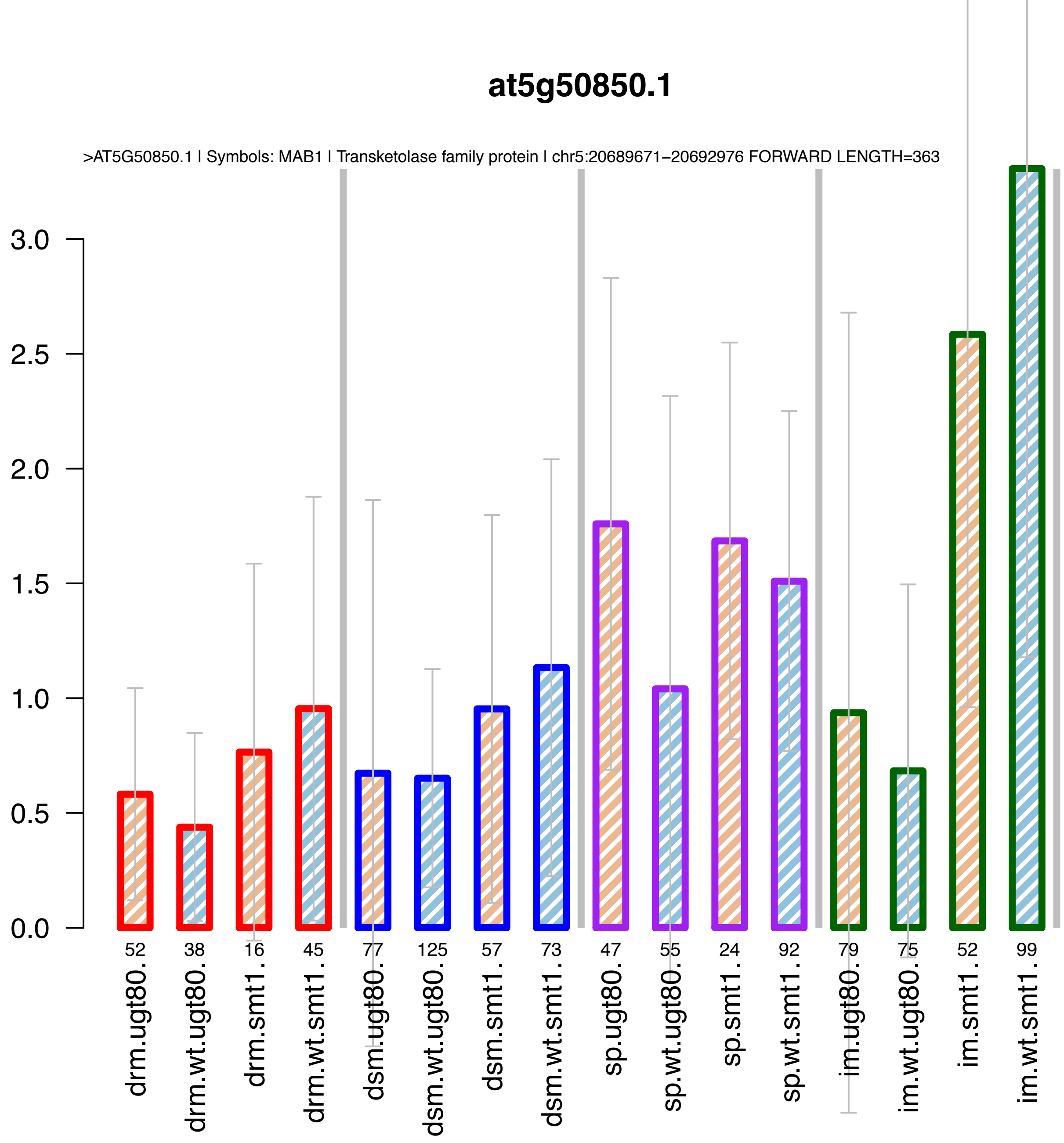

at5g50850.1
>AT5G50850.1 | Symbols: MAB1 | Transketolase family protein | chr5:20689671–20692976 FORWARD LENGTH=363
3.0
2.5
2.0
1.5
1.0
0.5
0.0
52
38
16
45
77
125
57
73
47
55
24
92
79
75
52
99
drm.ugt80..
drm.wt.ugt80..
drm.smt1..
drm.wt.smt1..
dsm.ugt80..
dsm.wt.ugt80..
dsm.smt1..
dsm.wt.smt1..
sp.ugt80..
sp.wt.ugt80..
sp.smt1..
sp.wt.smt1..
im.ugt80..
im.wt.ugt80..
im.smt1..
im.wt.smt1..

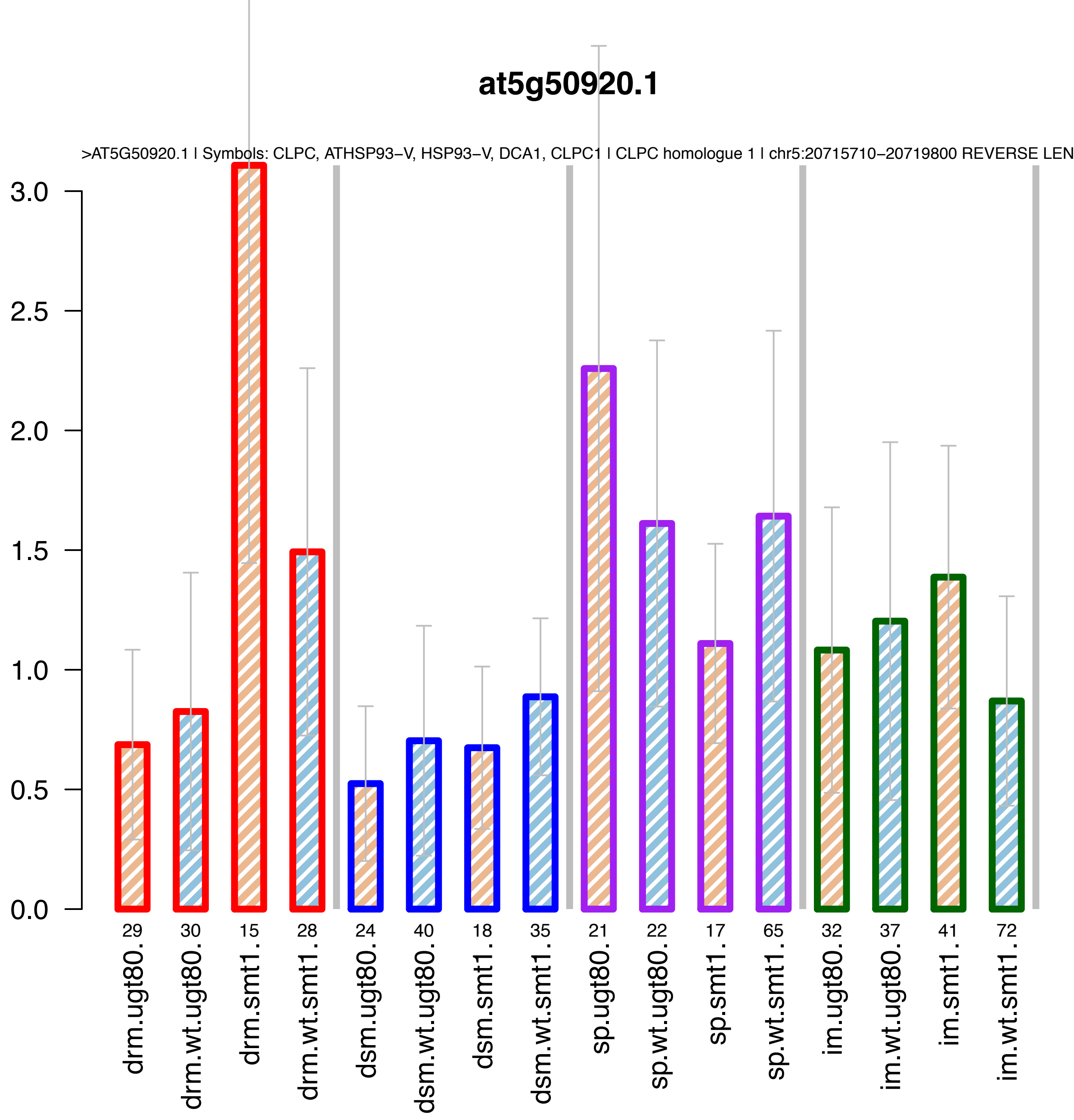

at5g50920.1
>AT5G50920.1 | Symbols: CLPC, ATHSP93−V, HSP93−V, DCA1, CLPC1 | CLPC homologue 1 | chr5:20715710−20719800 REVERSE LEN
3.0
2.5
2.0
1.5
1.0
0.5
0.0
29
30
15
28
24
40
18
35
21
22
17
65
32
37
41
72
drm.ugt80..
drm.wt.ugt80..
drm.smt1..
drm.wt.smt1..
dsm.ugt80..
dsm.wt.ugt80..
dsm.smt1..
dsm.wt.smt1..
sp.ugt80..
sp.wt.ugt80..
sp.smt1..
sp.wt.smt1..
im.ugt80..
im.wt.ugt80..
im.smt1..
im.wt.smt1..

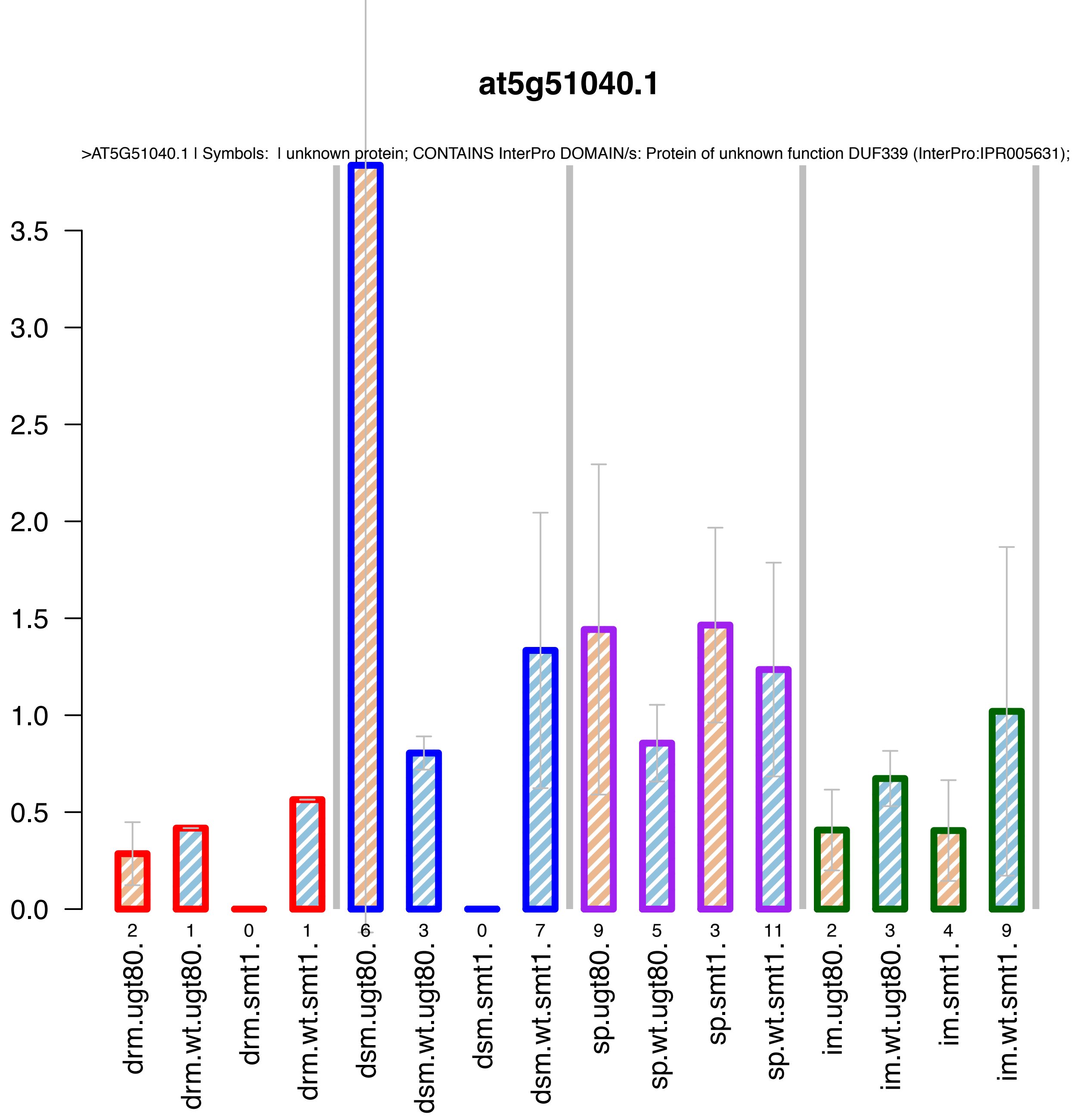

at5g51040.1
>AT5G51040.1 | Symbols: | unknown protein; CONTAINS InterPro DOMAIN/s: Protein of unknown function DUF339 (InterPro:IPR005631);
0.0
0.5
1.0
1.5
2.0
2.5
3.0
3.5
2
1
0
1
6
3
0
7
9
5
3
11
2
3
4
9
drm.ugt80.
drm.wt.ugt80.
drm.smt1.
drm.wt.smt1.
dsm.ugt80.
dsm.wt.ugt80.
dsm.smt1.
dsm.wt.smt1.
sp.ugt80.
sp.wt.ugt80.
sp.smt1.
sp.wt.smt1.
im.ugt80.
im.wt.ugt80.
im.smt1.
im.wt.smt1.

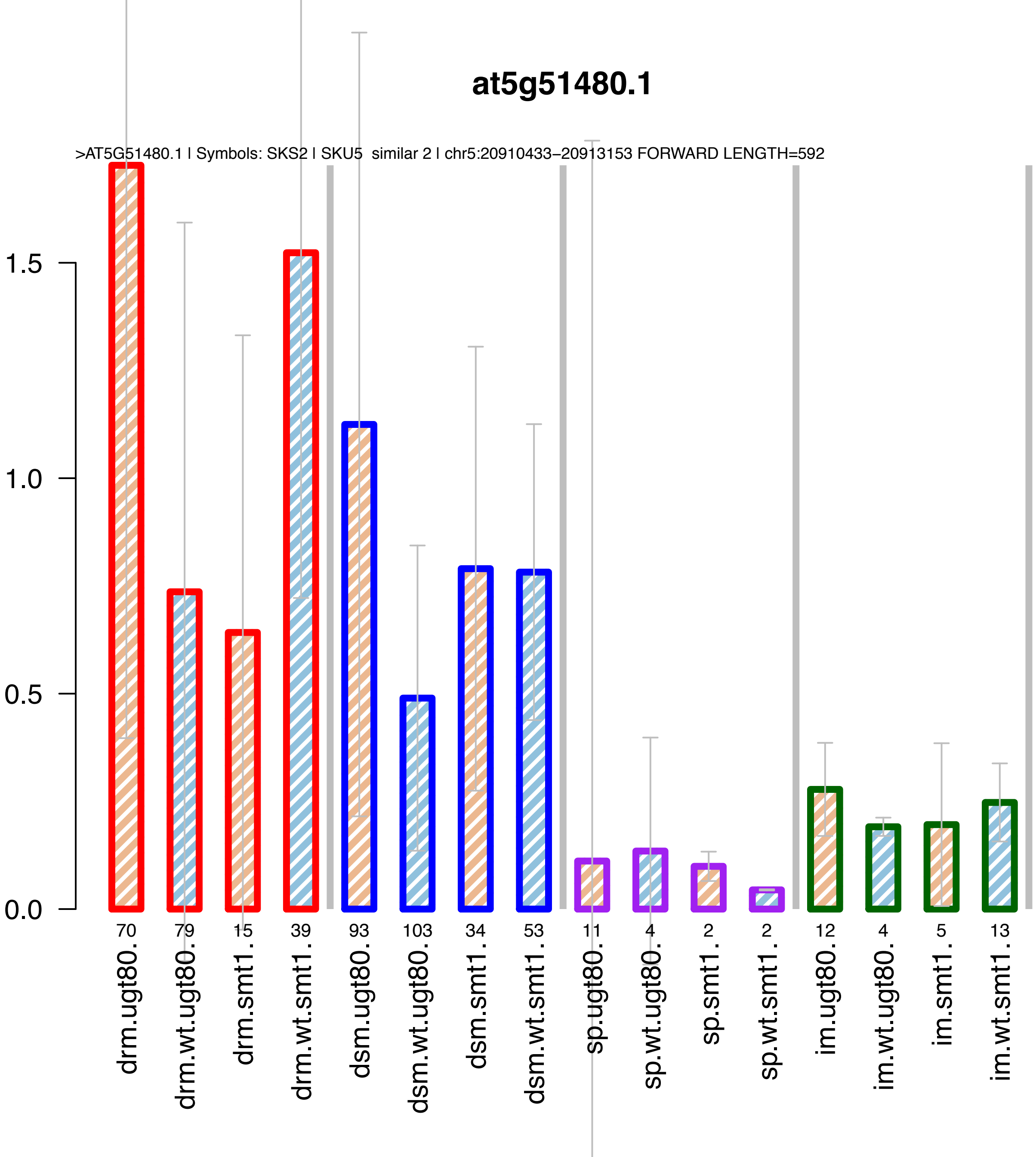
at5g51480.1
>AT5G51480.1 | Symbols: SKS2 | SKU5 similar 2 | chr5:20910433-20913153 FORWARD LENGTH=592
1.5
1.0
0.5
0.0
70
79
15
39
93
103
34
53
11
4
2
2
12
4
5
13
drm.ugt80..
drm.wt.ugt80..
drm.smt1..
drm.wt.smt1..
dsm.ugt80..
dsm.wt.ugt80..
dsm.smt1..
dsm.wt.smt1..
sp.ugt80..
sp.wt.ugt80..
sp.smt1..
sp.wt.smt1..
im.ugt80..
im.wt.ugt80..
im.smt1..
im.wt.smt1..

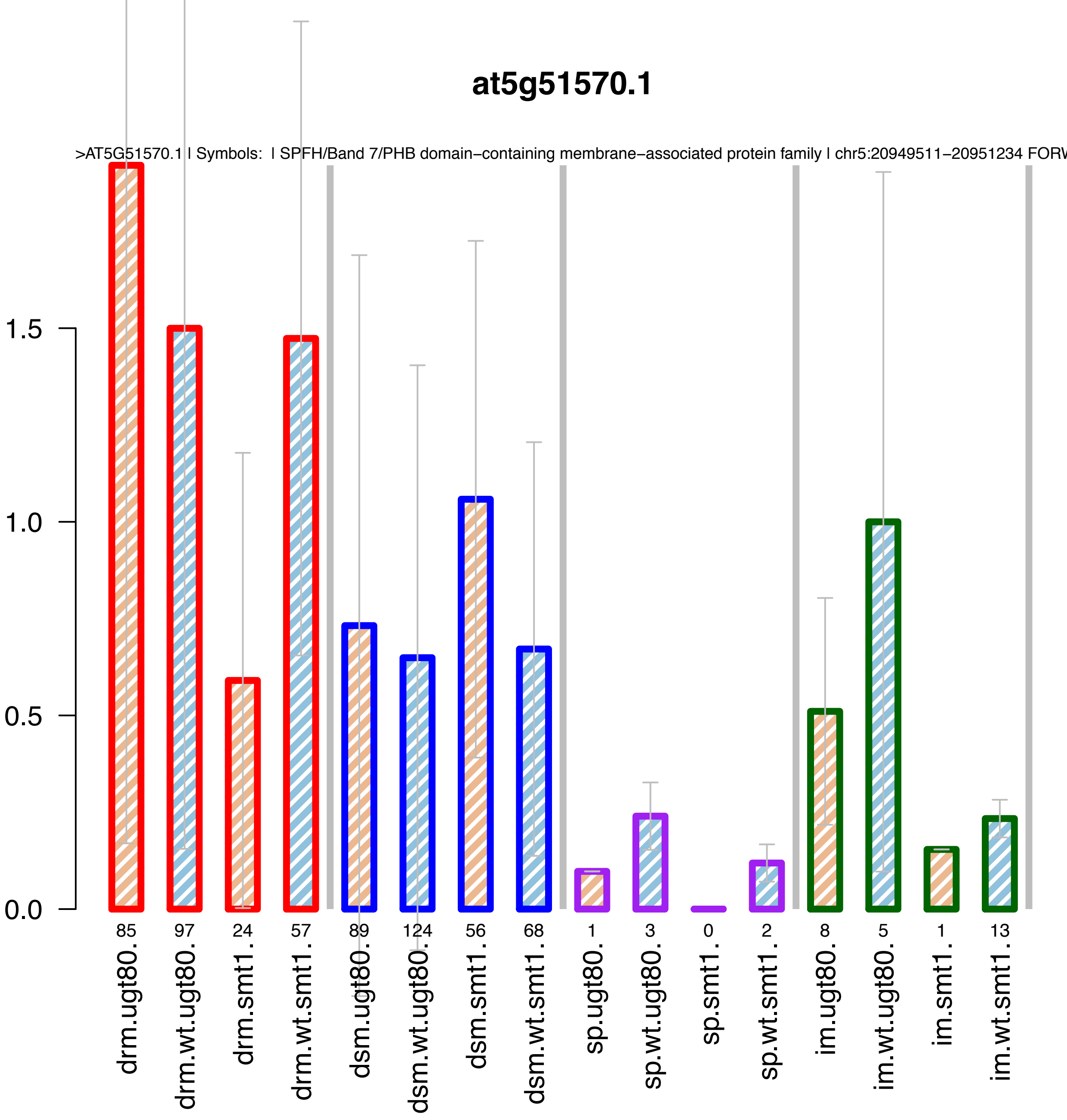

at5g51570.1
>AT5G51570.1 | Symbols: | SPFH/Band 7/PHB domain−containing membrane−associated protein family | chr5:20949511−20951234 FORW
1.5
1.0
0.5
0.0
85
97
24
57
89
124
56
68
1
3
0
2
8
5
1
13
drm.ugt80..
drm.wt.ugt80..
drm.smt1..
drm.wt.smt1..
dsm.ugt80..
dsm.wt.ugt80..
dsm.smt1..
dsm.wt.smt1..
sp.ugt80..
sp.wt.ugt80..
sp.smt1..
sp.wt.smt1..
im.ugt80..
im.wt.ugt80..
im.smt1..
im.wt.smt1..

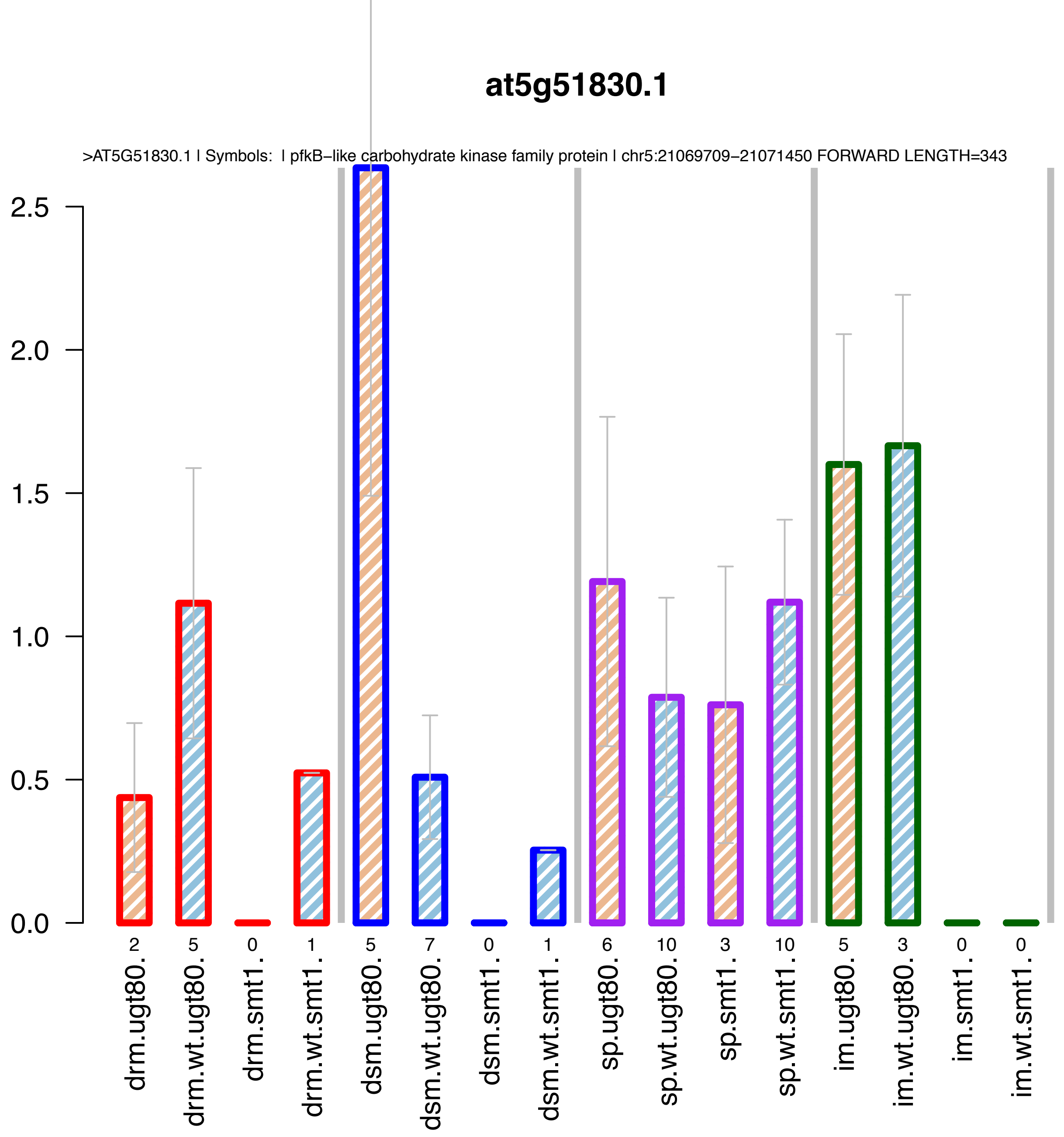

at5g51830.1
>AT5G51830.1 | Symbols: | pfkB–like carbohydrate kinase family protein | chr5:21069709–21071450 FORWARD LENGTH=343
0.0
0.5
1.0
1.5
2.0
2.5
2
5
0
1
5
7
0
1
6
10
3
10
5
3
0
0
drm.ugt80.
drm.wt.ugt80.
drm.smt1.
drm.wt.smt1.
dsm.ugt80.
dsm.wt.ugt80.
dsm.smt1.
dsm.wt.smt1.
sp.ugt80.
sp.wt.ugt80.
sp.smt1.
sp.wt.smt1.
im.ugt80.
im.wt.ugt80.
im.smt1.
im.wt.smt1.

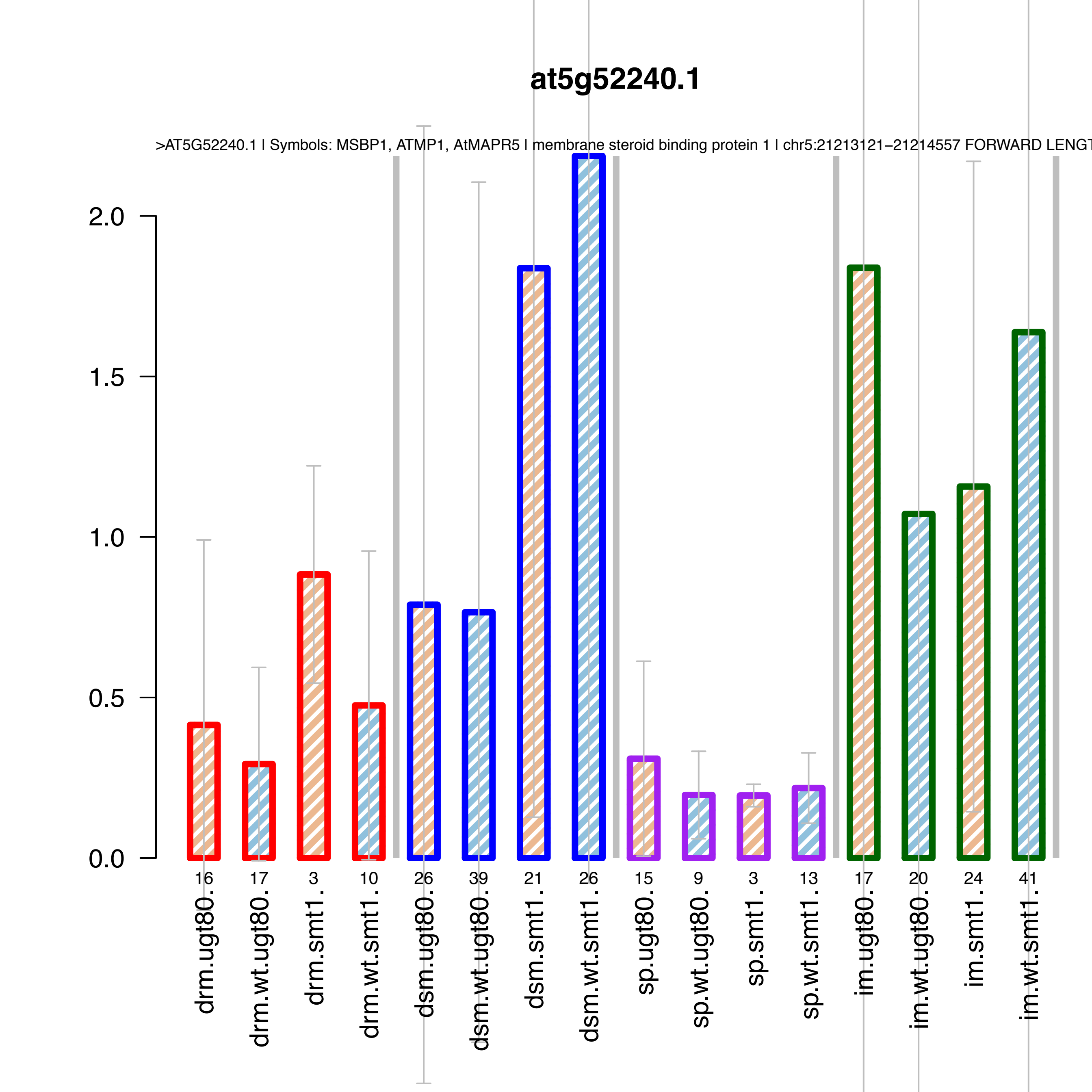

at5g52240.1
>AT5G52240.1 | Symbols: MSBP1, ATMP1, AtMAPR5 | membrane steroid binding protein 1 | chr5:21213121-21214557 FORWARD LENGT
2.0
1.5
1.0
0.5
0.0
16
17
3
10
26
39
21
26
15
9
3
13
17
20
24
41
drm.ugt80..
drm.wt.ugt80..
drm.smt1..
drm.wt.smt1..
dsm.ugt80..
dsm.wt.ugt80..
dsm.smt1..
dsm.wt.smt1..
sp.ugt80..
sp.wt.ugt80..
sp.smt1..
sp.wt.smt1..
im.ugt80..
im.wt.ugt80..
im.smt1..
im.wt.smt1..

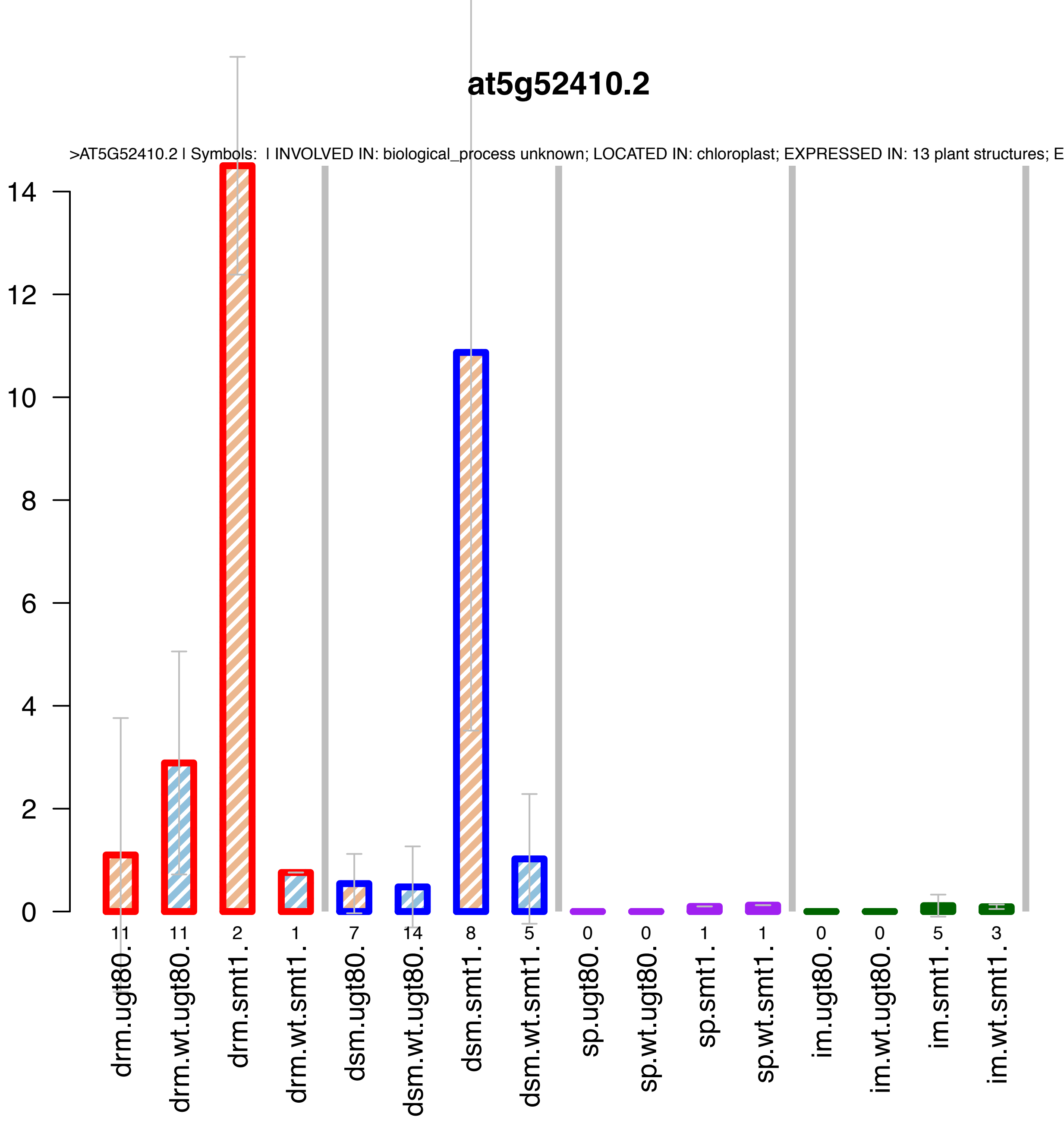

at5g52410.2
>AT5G52410.2 | Symbols: | INVOLVED IN: biological_process unknown; LOCATED IN: chloroplast; EXPRESSED IN: 13 plant structures; E
14
12
10
8
6
4
2
0
11
11
2
1
7
14
8
5
0
0
1
1
0
0
5
3
drm.ugt80.
drm.wt.ugt80.
drm.smt1.
drm.wt.smt1.
dsm.ugt80.
dsm.wt.ugt80.
dsm.smt1.
dsm.wt.smt1.
sp.ugt80.
sp.wt.ugt80.
sp.smt1.
sp.wt.smt1.
im.ugt80.
im.wt.ugt80.
im.smt1.
im.wt.smt1.

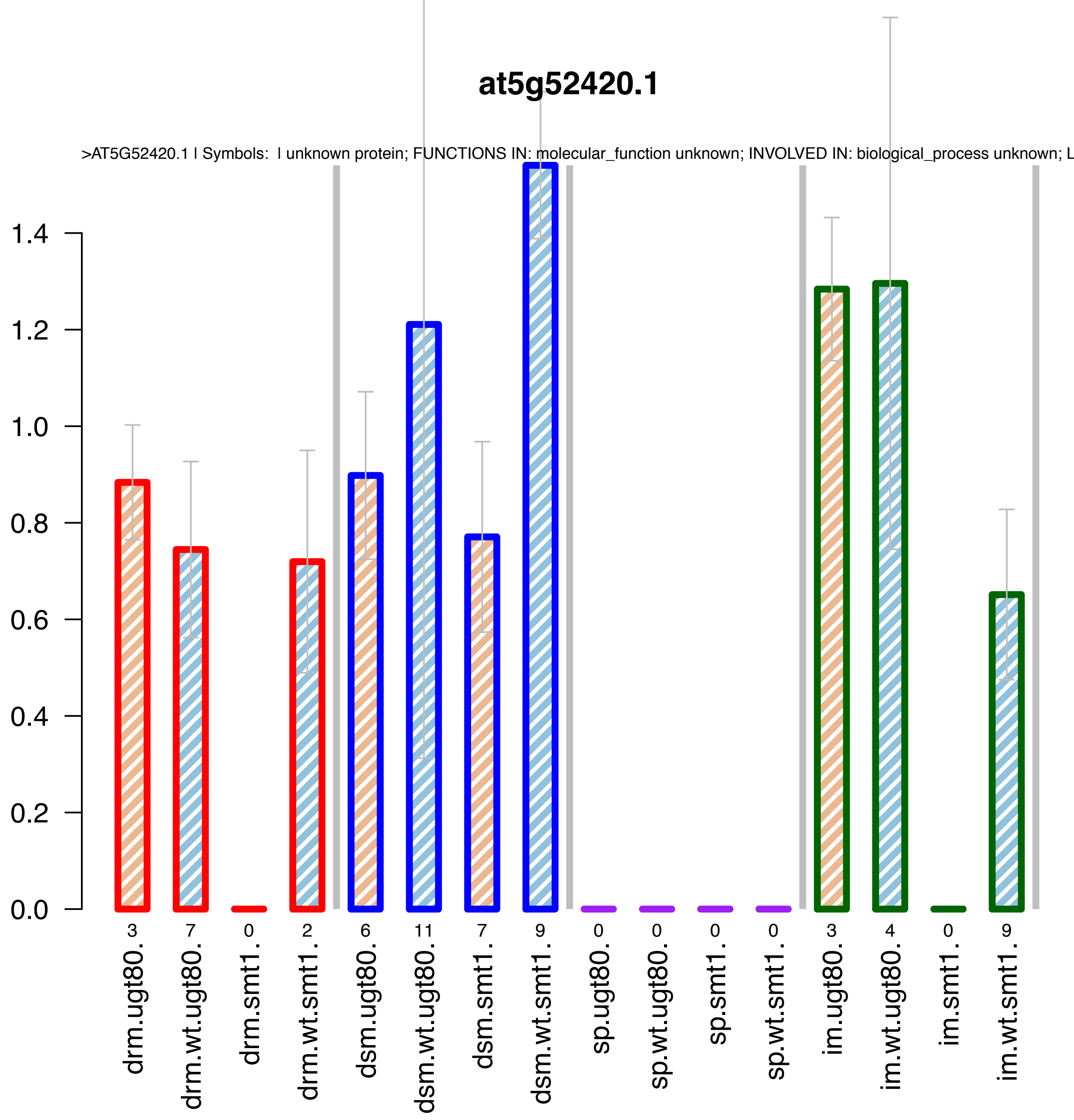

at5g52420.1
>AT5G52420.1 | Symbols: | unknown protein; FUNCTIONS IN: molecular_function unknown; INVOLVED IN: biological_process unknown; L
1.4
1.2
1.0
0.8
0.6
0.4
0.2
0.0
3
7
0
2
6
11
7
9
0
0
0
0
3
4
0
9
drm.ugt80.
drm.wt.ugt80.
drm.smt1.
drm.wt.smt1.
dsm.ugt80.
dsm.wt.ugt80.
dsm.smt1.
dsm.wt.smt1.
sp.ugt80.
sp.wt.ugt80.
sp.smt1.
sp.wt.smt1.
im.ugt80.
im.wt.ugt80.
im.smt1.
im.wt.smt1.

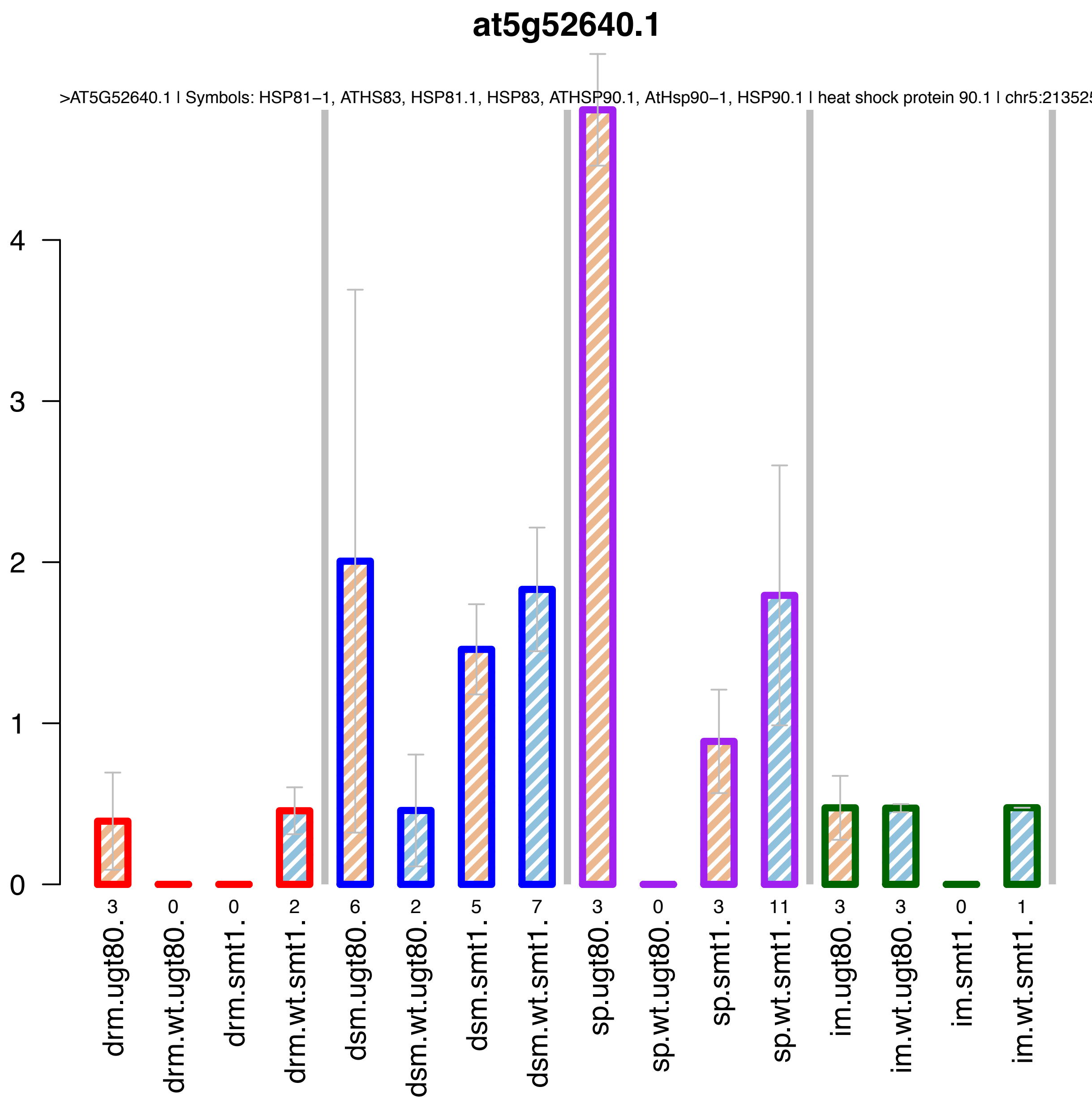

at5g52640.1
>AT5G52640.1 | Symbols: HSP81−1, ATHS83, HSP81.1, HSP83, ATHSP90.1, AtHsp90−1, HSP90.1 | heat shock protein 90.1 | chr5:21352
0
1
2
3
4
3
0
0
2
6
2
5
7
3
0
3
11
3
3
0
1
drm.ugt80.
drm.wt.ugt80.
drm.smt1.
drm.wt.smt1.
dsm.ugt80.
dsm.wt.ugt80.
dsm.smt1.
dsm.wt.smt1.
sp.ugt80.
sp.wt.ugt80.
sp.smt1.
sp.wt.smt1.
im.ugt80.
im.wt.ugt80.
im.smt1.
im.wt.smt1.

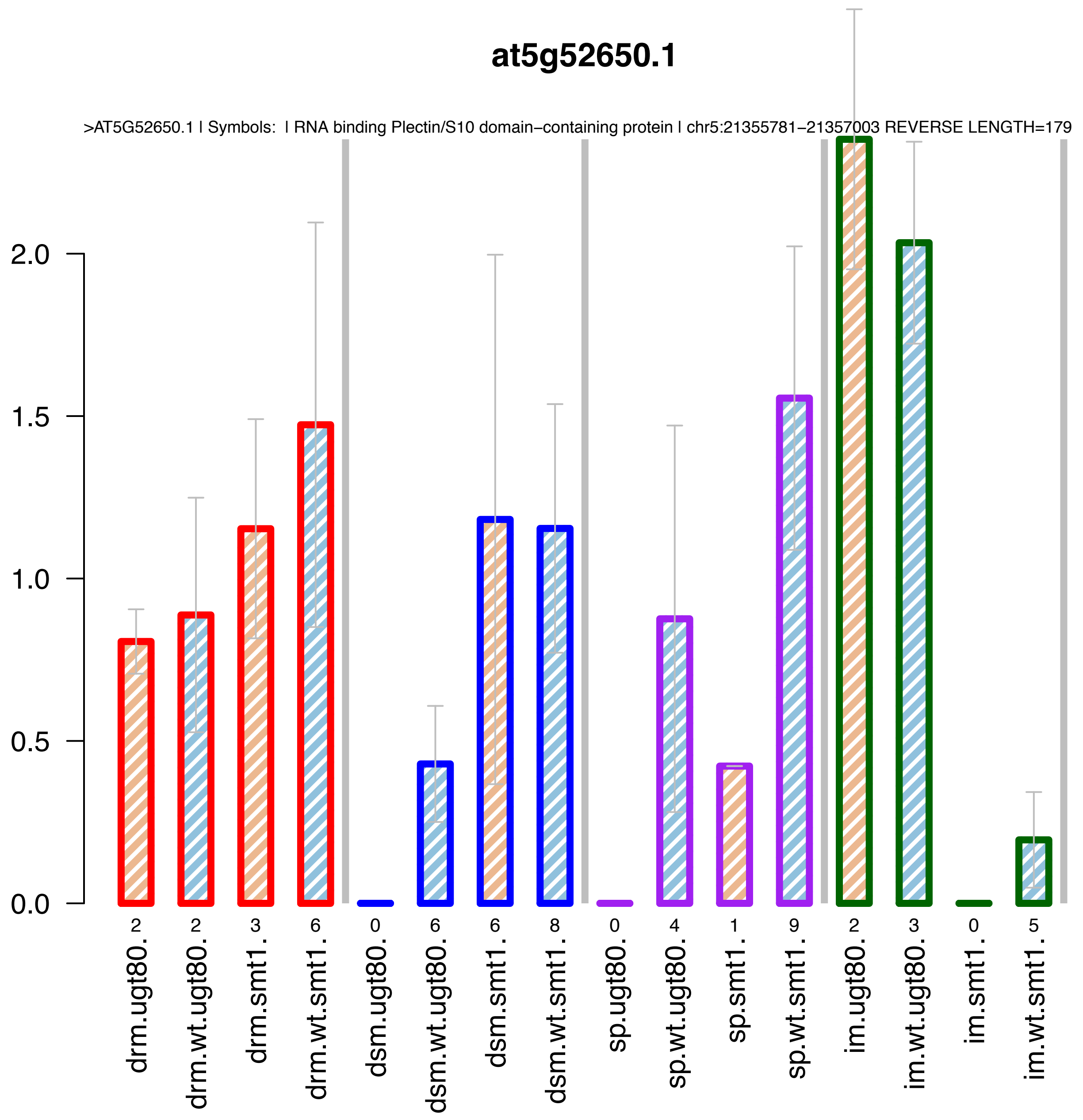

at5g52650.1
>AT5G52650.1 | Symbols: | RNA binding Plectin/S10 domain−containing protein | chr5:21355781−21357003 REVERSE LENGTH=179
0.0
0.5
1.0
1.5
2.0
2
2
3
6
0
6
6
8
0
4
1
9
2
3
0
5
drm.ugt80..
drm.wt.ugt80..
drm.smt1..
drm.wt.smt1..
dsm.ugt80..
dsm.wt.ugt80..
dsm.smt1..
dsm.wt.smt1..
sp.ugt80..
sp.wt.ugt80..
sp.smt1..
sp.wt.smt1..
im.ugt80..
im.wt.ugt80..
im.smt1..
im.wt.smt1..

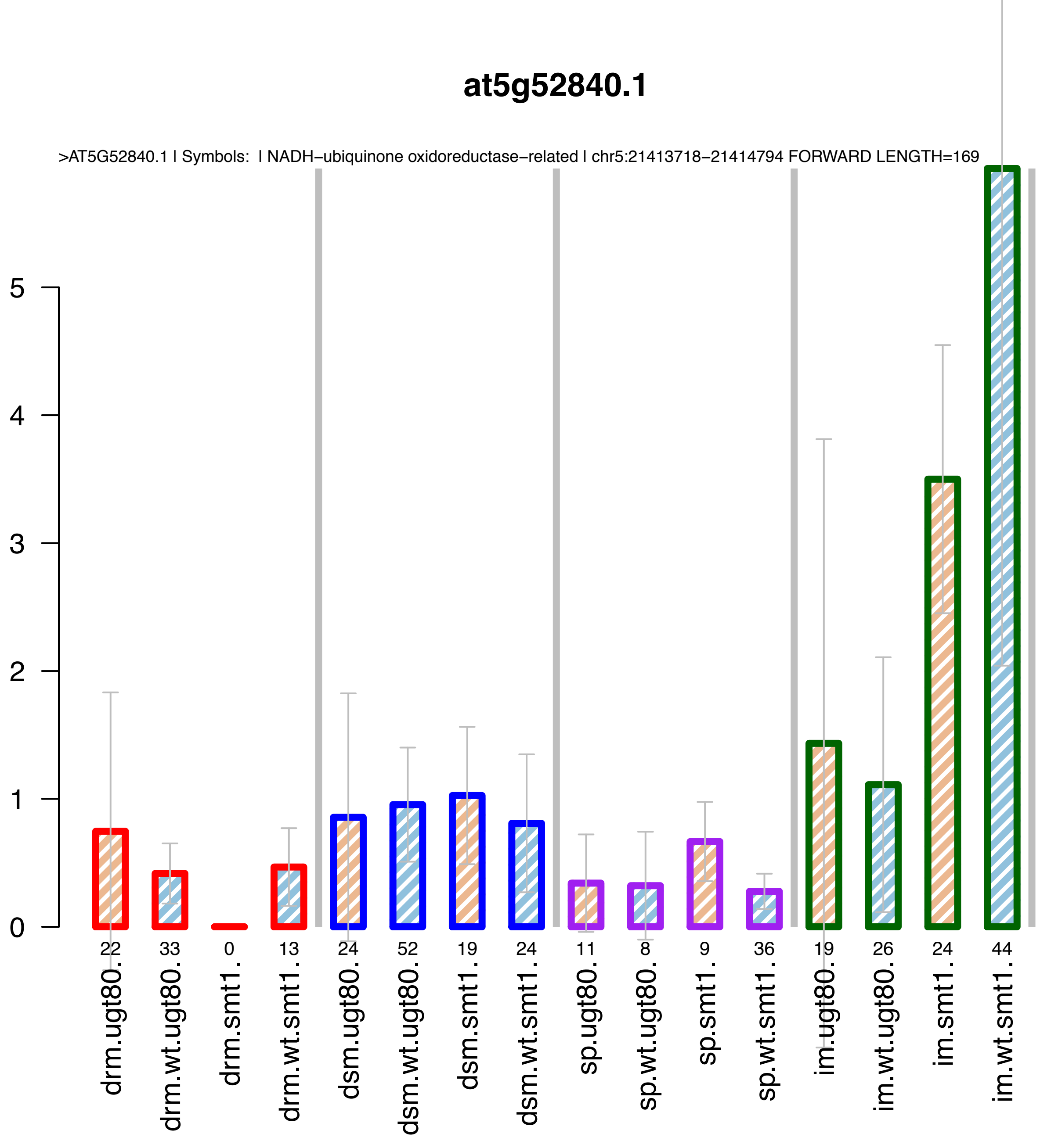

at5g52840.1
>AT5G52840.1 | Symbols: | NADH−ubiquinone oxidoreductase−related | chr5:21413718−21414794 FORWARD LENGTH=169
0
1
2
3
4
5
22
33
0
13
24
52
19
24
11
8
9
36
19
26
24
44
drm.ugt80..
drm.wt.ugt80..
drm.smt1..
drm.wt.smt1..
dsm.ugt80..
dsm.wt.ugt80..
dsm.smt1..
dsm.wt.smt1..
sp.ugt80..
sp.wt.ugt80..
sp.smt1..
sp.wt.smt1..
im.ugt80..
im.wt.ugt80..
im.smt1..
im.wt.smt1..

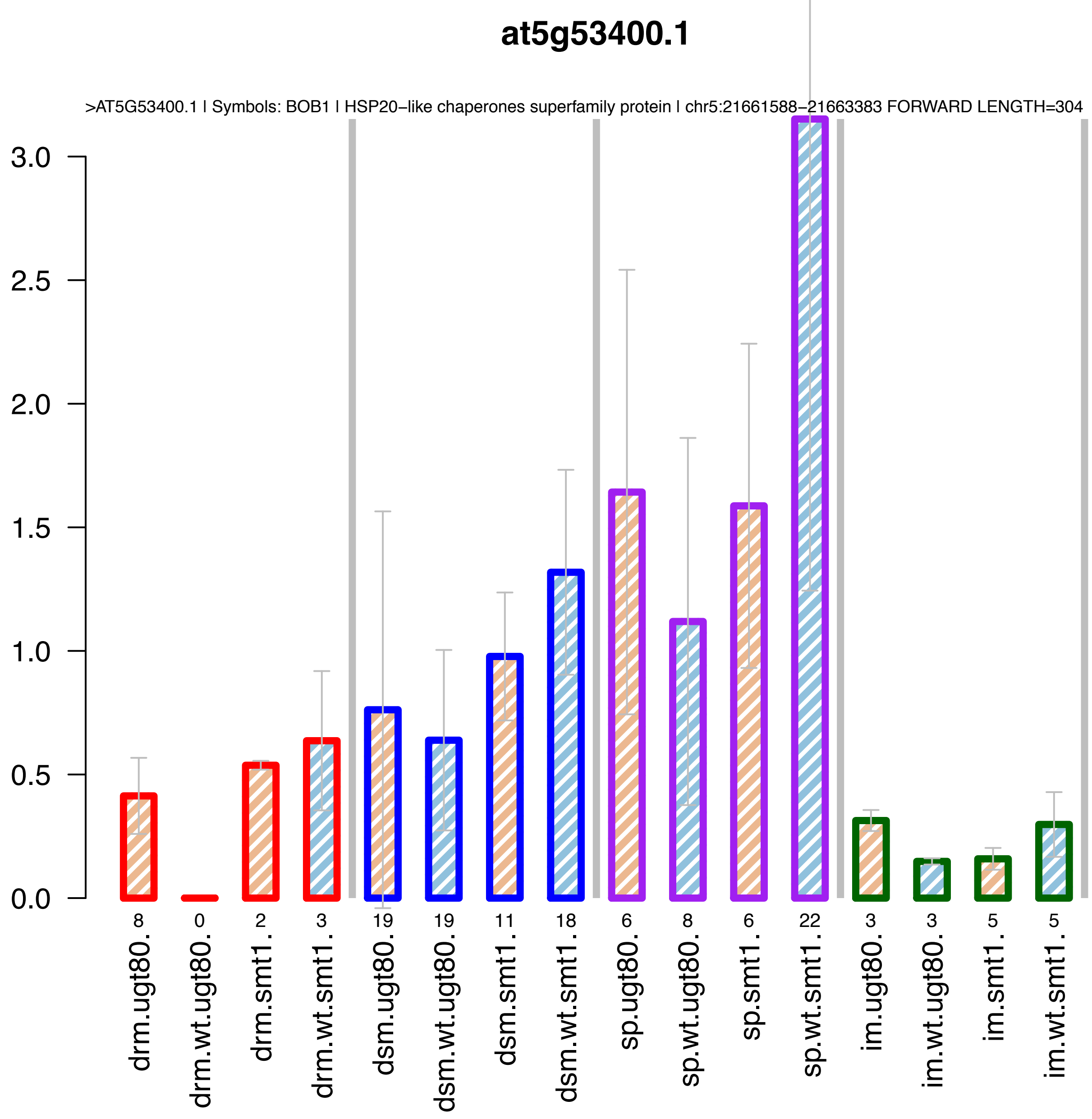

at5g53400.1
>AT5G53400.1 | Symbols: BOB1 | HSP20−like chaperones superfamily protein | chr5:21661588−21663383 FORWARD LENGTH=304
3.0
2.5
2.0
1.5
1.0
0.5
0.0
8
0
2
3
19
19
11
18
6
8
6
22
3
3
5
5
drm.ugt80.
drm.wt.ugt80.
drm.smt1.
drm.wt.smt1.
dsm.ugt80.
dsm.wt.ugt80.
dsm.smt1.
dsm.wt.smt1.
sp.ugt80.
sp.wt.ugt80.
sp.smt1.
sp.wt.smt1.
im.ugt80.
im.wt.ugt80.
im.smt1.
im.wt.smt1.

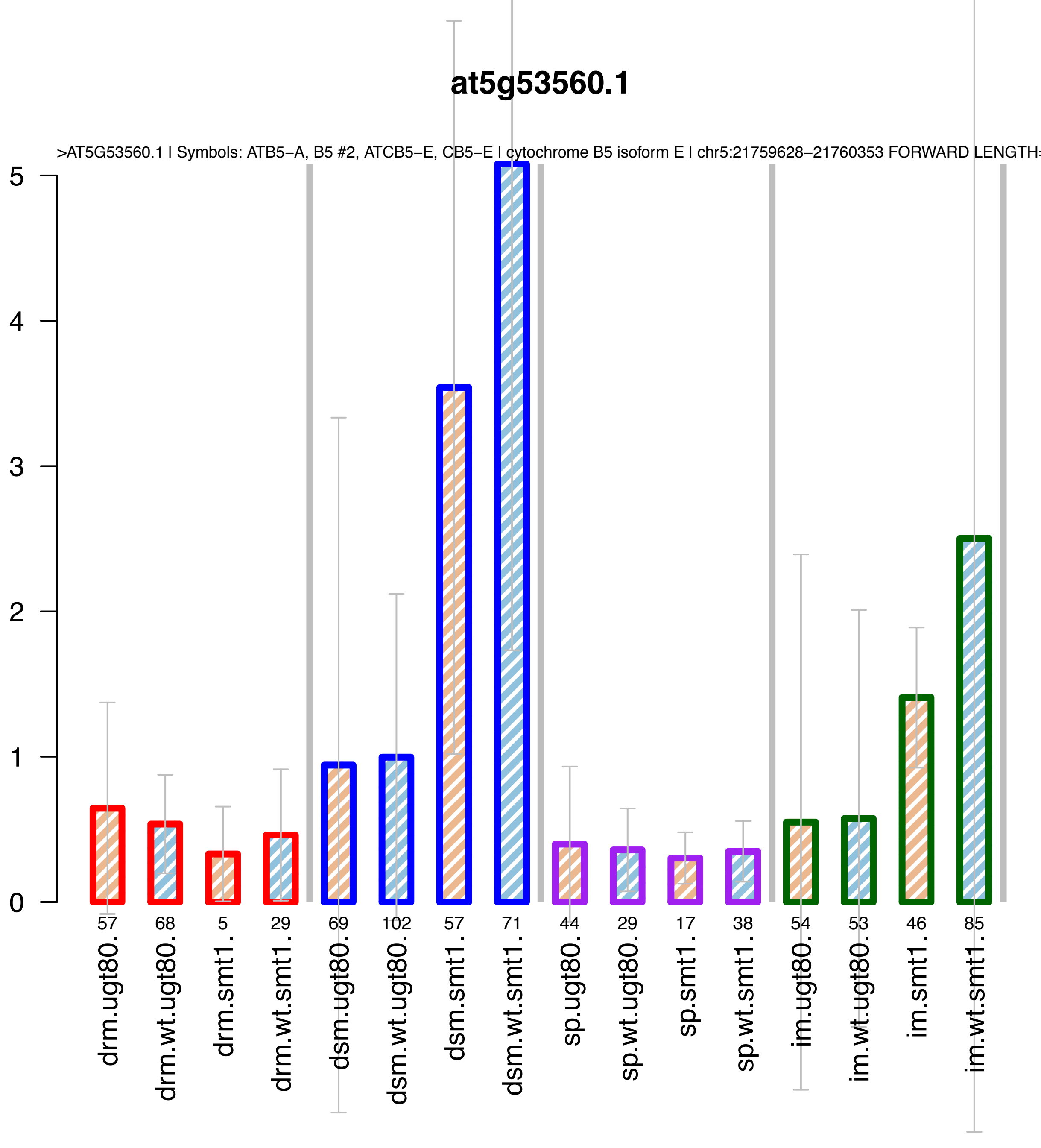

at5g53560.1
>AT5G53560.1 | Symbols: ATB5−A, B5 #2, ATCB5−E, CB5−E | cytochrome B5 isoform E | chr5:21759628−21760353 FORWARD LENGTH=
0
1
2
3
4
5
57
68
5
29
69
102
57
71
44
29
17
38
54
53
46
85
drm.ugt80.
drm.wt.ugt80.
drm.smt1.
drm.wt.smt1.
dsm.ugt80.
dsm.wt.ugt80.
dsm.smt1.
dsm.wt.smt1.
sp.ugt80.
sp.wt.ugt80.
sp.smt1.
sp.wt.smt1.
im.ugt80.
im.wt.ugt80.
im.smt1.
im.wt.smt1.

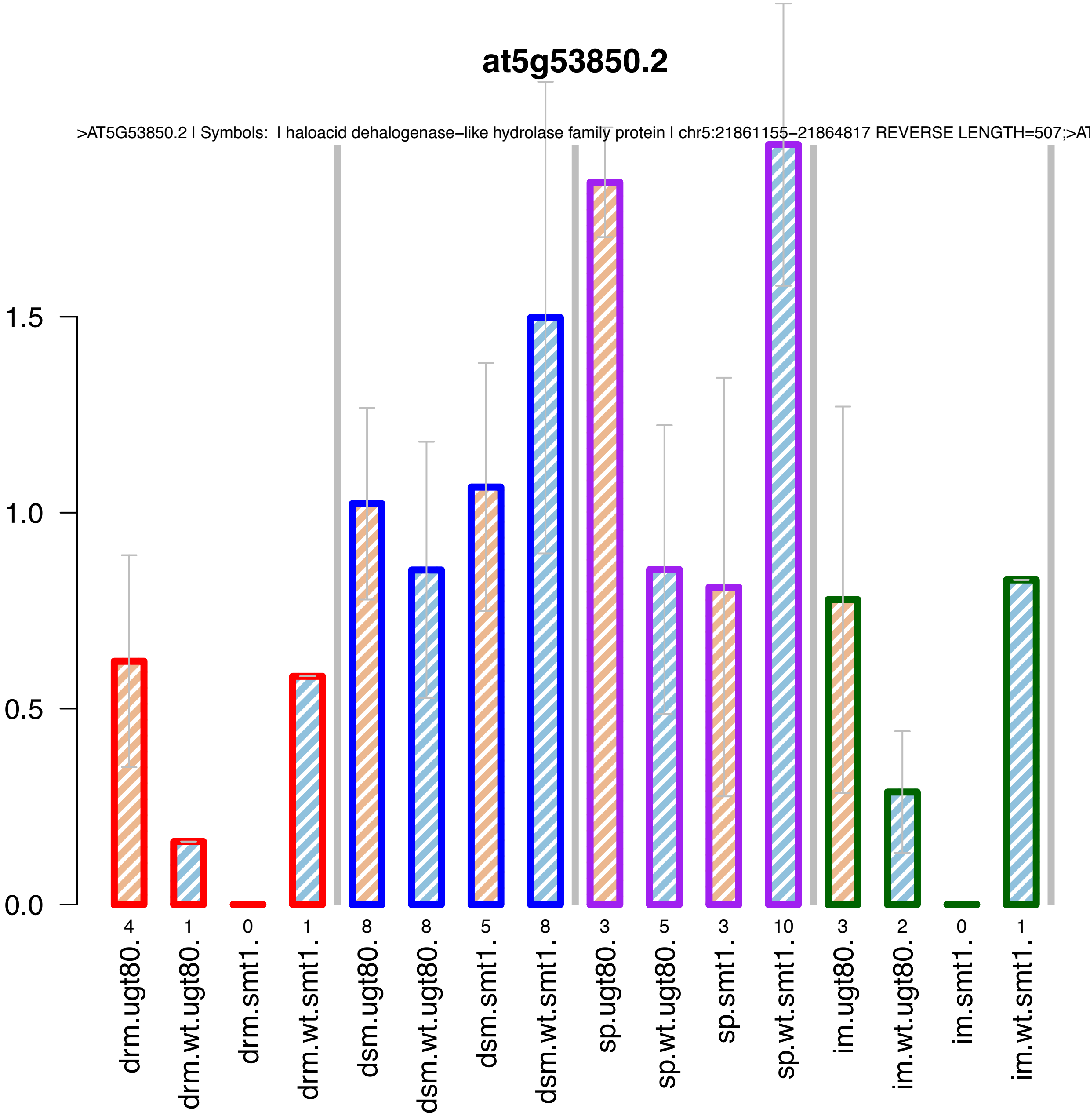
at5g53850.2
>AT5G53850.2 | Symbols: | haloacid dehalogenase-like hydrolase family protein | chr5:21861155-21864817 REVERSE LENGTH=507;>AT
0.0
0.5
1.0
1.5
4
1
0
1
8
8
5
8
3
5
3
10
3
2
0
1
drm.ugt80..
drm.wt.ugt80..
drm.smt1..
drm.wt.smt1..
dsm.ugt80..
dsm.wt.ugt80..
dsm.smt1..
dsm.wt.smt1..
sp.ugt80..
sp.wt.ugt80..
sp.smt1..
sp.wt.smt1..
im.ugt80..
im.wt.ugt80..
im.smt1..
im.wt.smt1..

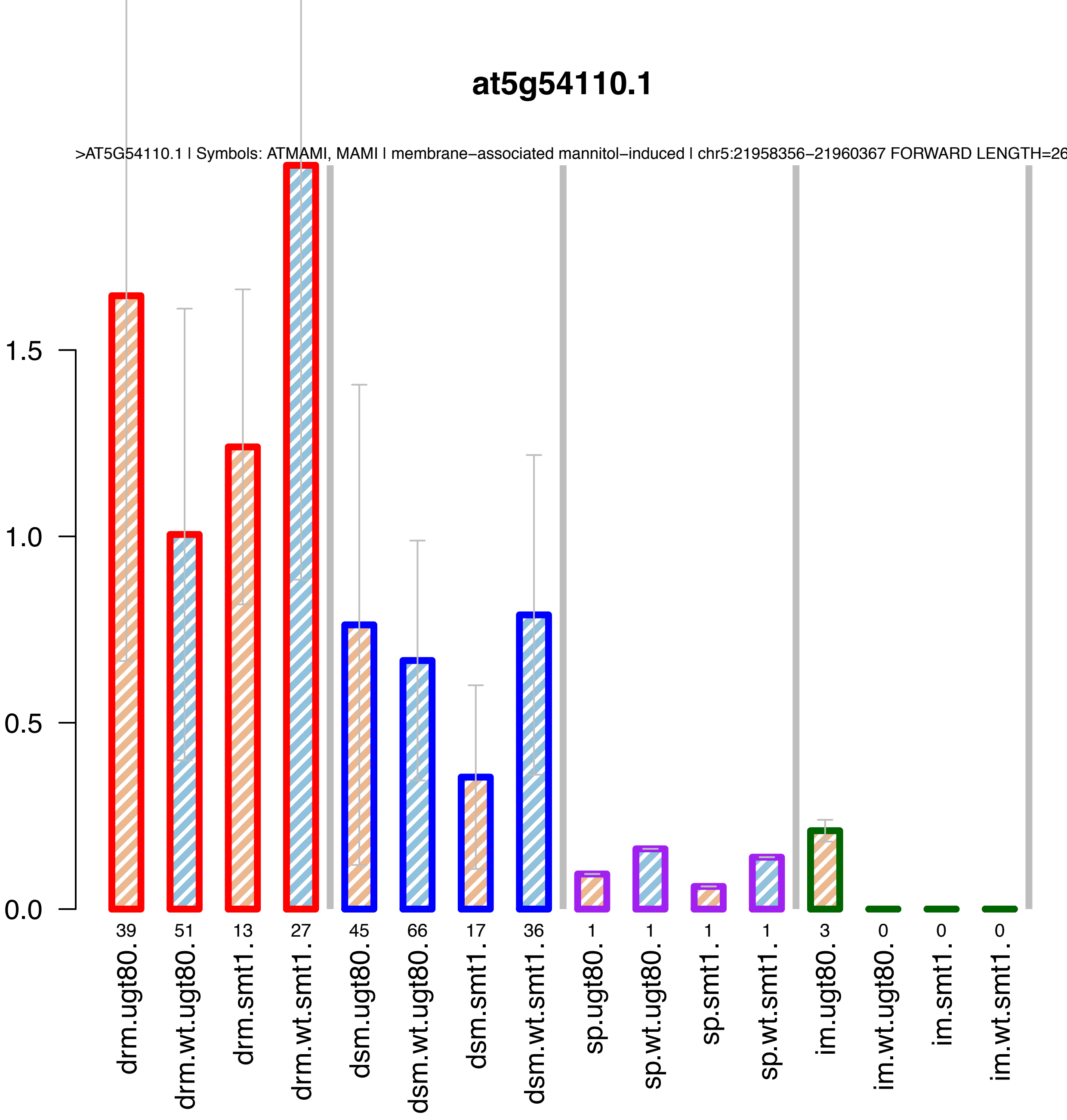

at5g54110.1
>AT5G54110.1 | Symbols: ATMAMI, MAMI | membrane−associated mannitol−induced | chr5:21958356−21960367 FORWARD LENGTH=26
1.5
1.0
0.5
0.0
39
51
13
27
45
66
17
36
1
1
1
1
3
0
0
0
drm.ugt80.
drm.wt.ugt80.
drm.smt1.
drm.wt.smt1.
dsm.ugt80.
dsm.wt.ugt80.
dsm.smt1.
dsm.wt.smt1.
sp.ugt80.
sp.wt.ugt80.
sp.smt1.
sp.wt.smt1.
im.ugt80.
im.wt.ugt80.
im.smt1.
im.wt.smt1.

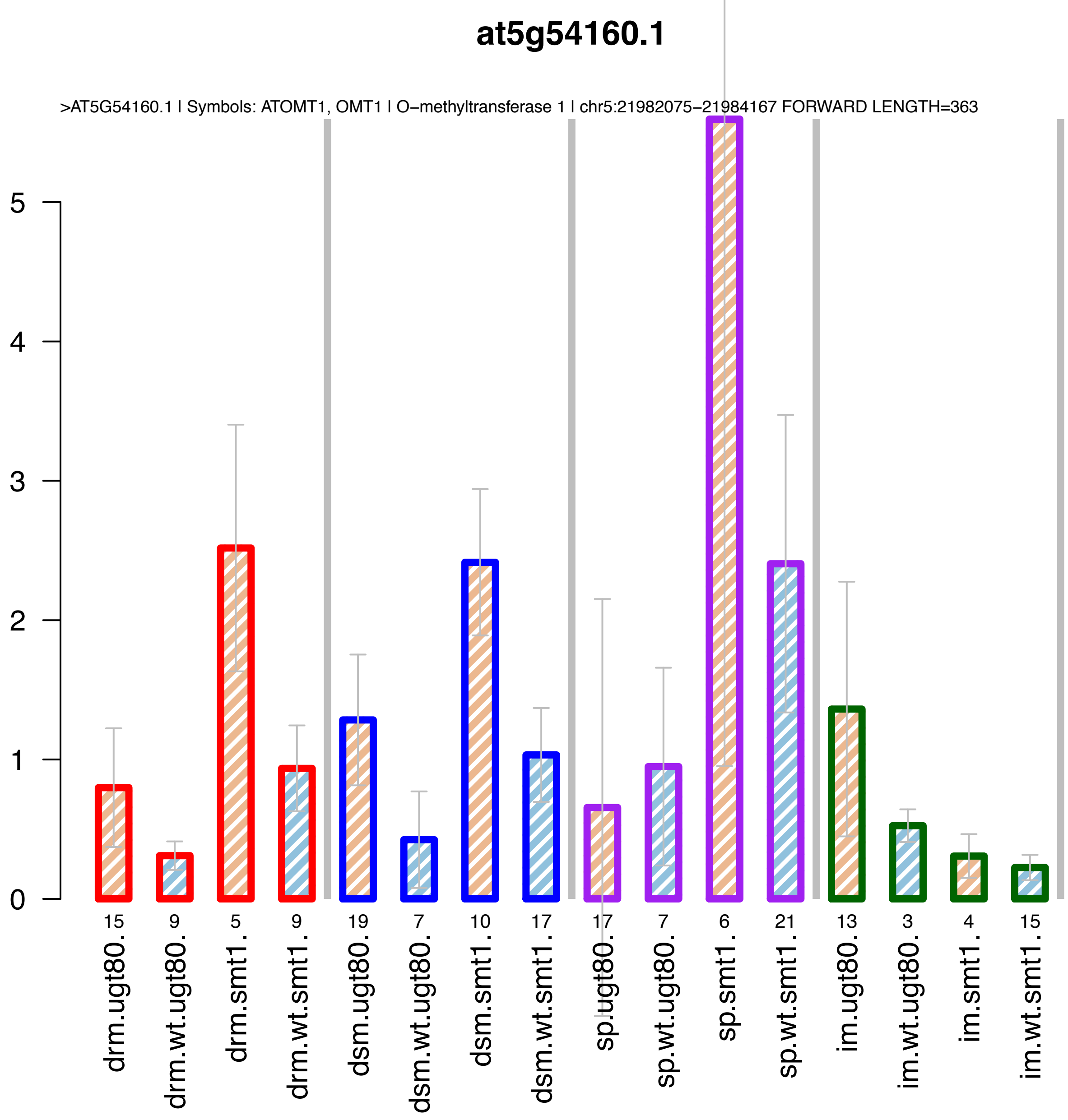

at5g54160.1
>AT5G54160.1 | Symbols: ATOMT1, OMT1 | O−methyltransferase 1 | chr5:21982075−21984167 FORWARD LENGTH=363
0
1
2
3
4
5
15
9
5
9
19
7
10
17
17
7
6
21
13
3
4
15
drm.ugt80.
drm.wt.ugt80.
drm.smt1.
drm.wt.smt1.
dsm.ugt80.
dsm.wt.ugt80.
dsm.smt1.
dsm.wt.smt1.
sp.ugt80.
sp.wt.ugt80.
sp.smt1.
sp.wt.smt1.
im.ugt80.
im.wt.ugt80.
im.smt1.
im.wt.smt1.

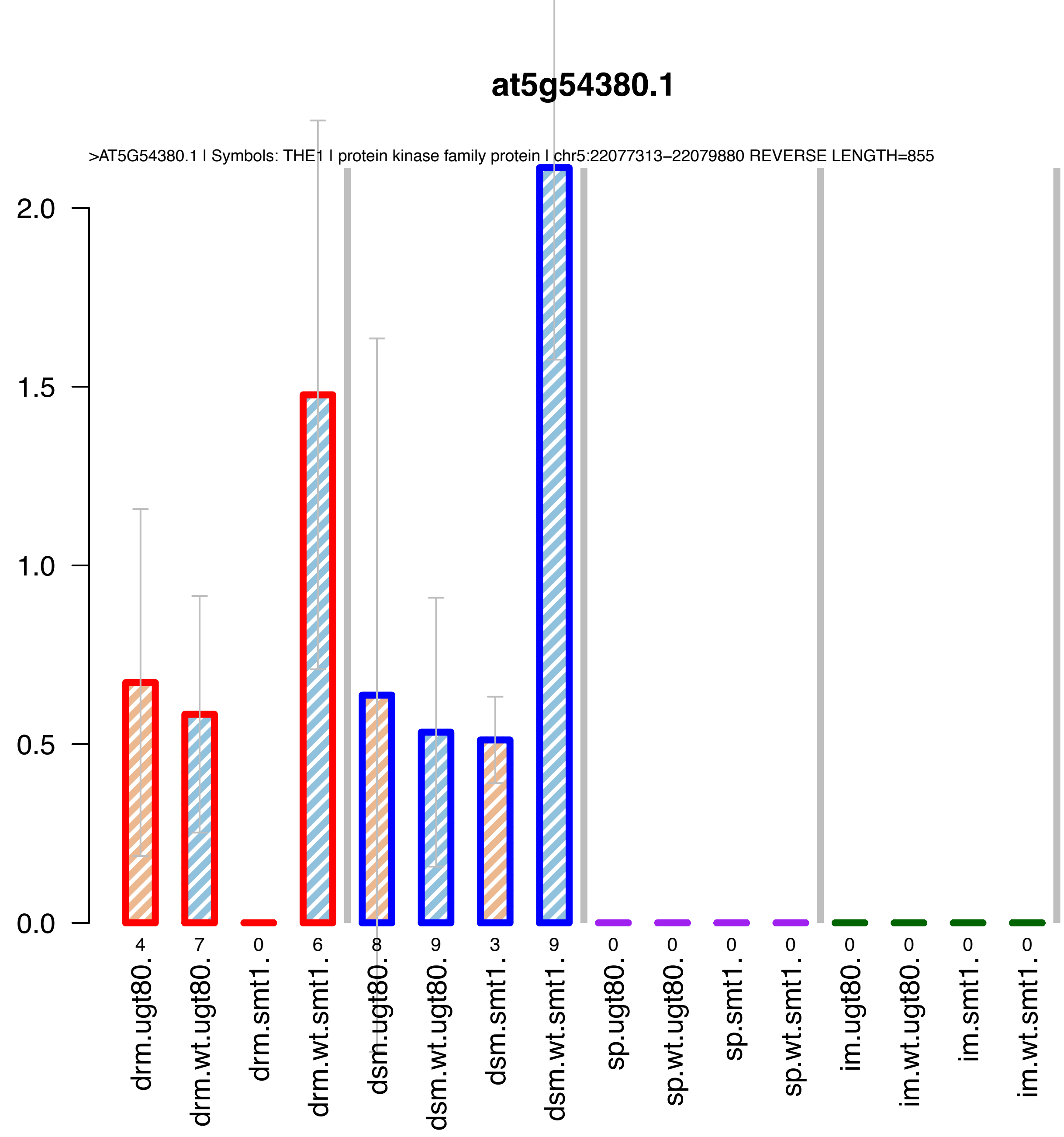

at5g54380.1
>AT5G54380.1 | Symbols: THE1 | protein kinase family protein | chr5:22077313–22079880 REVERSE LENGTH=855
2.0
1.5
1.0
0.5
0.0
4
7
0
6
8
9
3
9
0
0
0
0
0
0
0
0
drm.ugt80.
drm.wt.ugt80.
drm.smt1.
drm.wt.smt1.
dsm.ugt80.
dsm.wt.ugt80.
dsm.smt1.
dsm.wt.smt1.
sp.ugt80.
sp.wt.ugt80.
sp.smt1.
sp.wt.smt1.
im.ugt80.
im.wt.ugt80.
im.smt1.
im.wt.smt1.

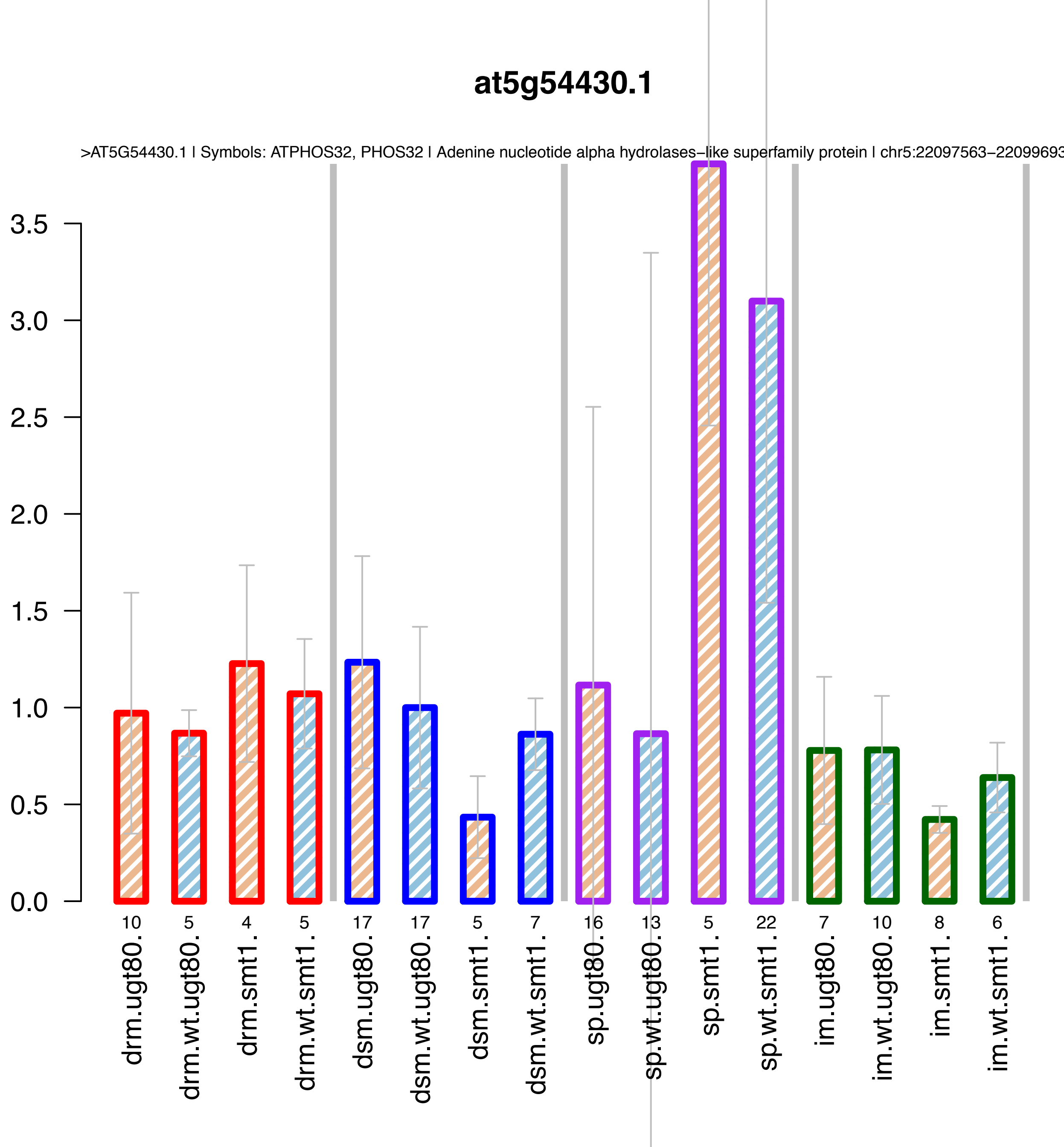

at5g54430.1
>AT5G54430.1 | Symbols: ATPHOS32, PHOS32 | Adenine nucleotide alpha hydrolases−like superfamily protein | chr5:22097563−22099693
3.5
3.0
2.5
2.0
1.5
1.0
0.5
0.0
10
5
4
5
17
17
5
7
16
13
5
22
7
10
8
6
drm.ugt80.
drm.wt.ugt80.
drm.smt1.
drm.wt.smt1.
dsm.ugt80.
dsm.wt.ugt80.
dsm.smt1.
dsm.wt.smt1.
sp.ugt80.
sp.wt.ugt80.
sp.smt1.
sp.wt.smt1.
im.ugt80.
im.wt.ugt80.
im.smt1.
im.wt.smt1.

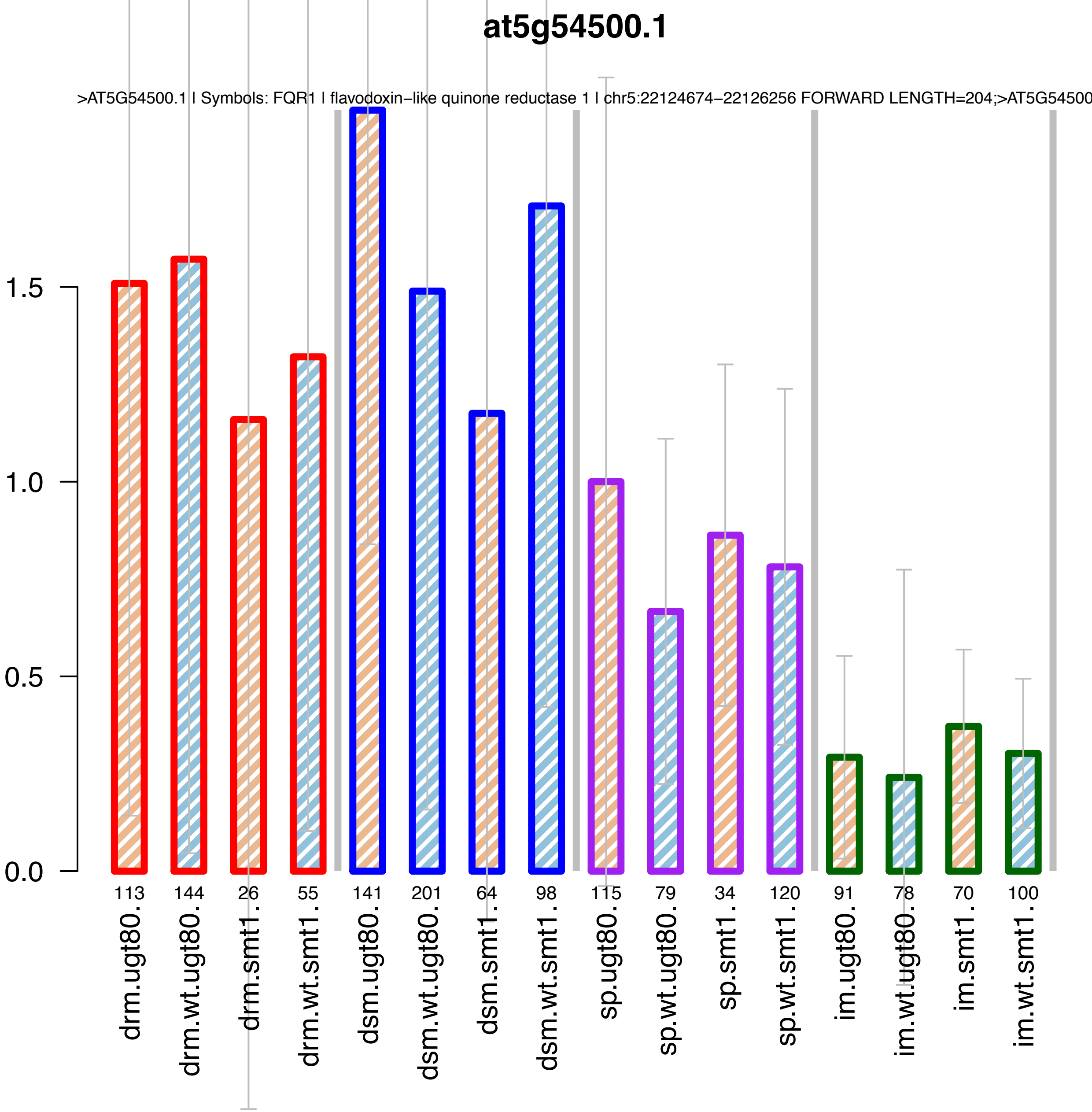
at5g54500.1
>AT5G54500.1 | Symbols: FQR1 | flavodoxin−like quinone reductase 1 | chr5:22124674−22126256 FORWARD LENGTH=204;>AT5G54500
0.0
0.5
1.0
1.5
113
144
26
55
141
201
64
98
115
79
34
120
91
78
70
100
drm.ugt80..
drm.wt.ugt80..
drm.smt1..
drm.wt.smt1..
dsm.ugt80..
dsm.wt.ugt80..
dsm.smt1..
dsm.wt.smt1..
sp.ugt80..
sp.wt.ugt80..
sp.smt1..
sp.wt.smt1..
im.ugt80..
im.wt.ugt80..
im.smt1..
im.wt.smt1..

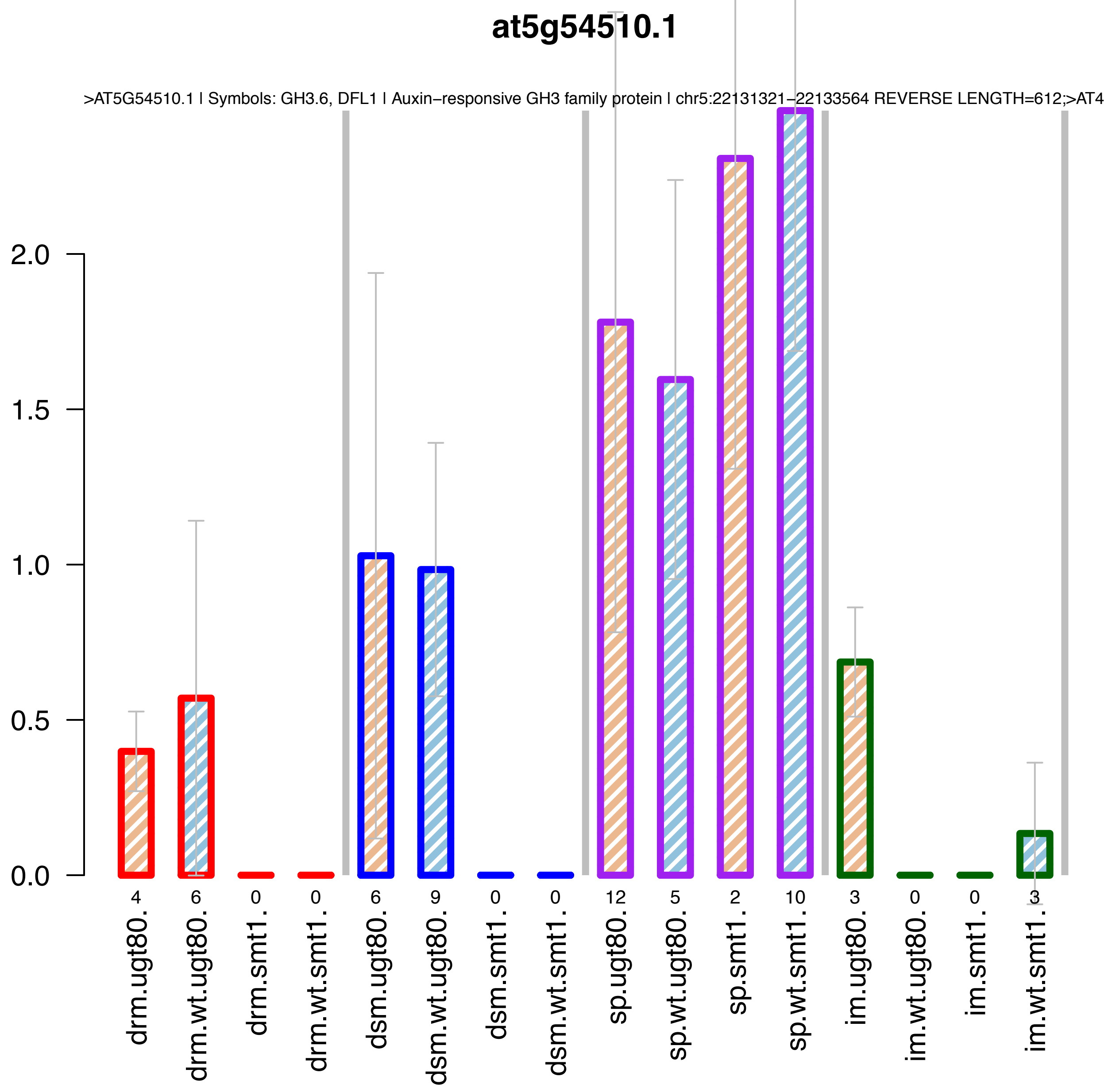
at5g54510.1
>AT5G54510.1 | Symbols: GH3.6, DFL1 | Auxin−responsive GH3 family protein | chr5:22131321−22133564 REVERSE LENGTH=612;>AT4
0.0
0.5
1.0
1.5
2.0
4
6
0
0
6
9
0
0
12
5
2
10
3
0
0
3
drm.ugt80.
drm.wt.ugt80.
drm.smt1.
drm.wt.smt1.
dsm.ugt80.
dsm.wt.ugt80.
dsm.smt1.
dsm.wt.smt1.
sp.ugt80.
sp.wt.ugt80.
sp.smt1.
sp.wt.smt1.
im.ugt80.
im.wt.ugt80.
im.smt1.
im.wt.smt1.

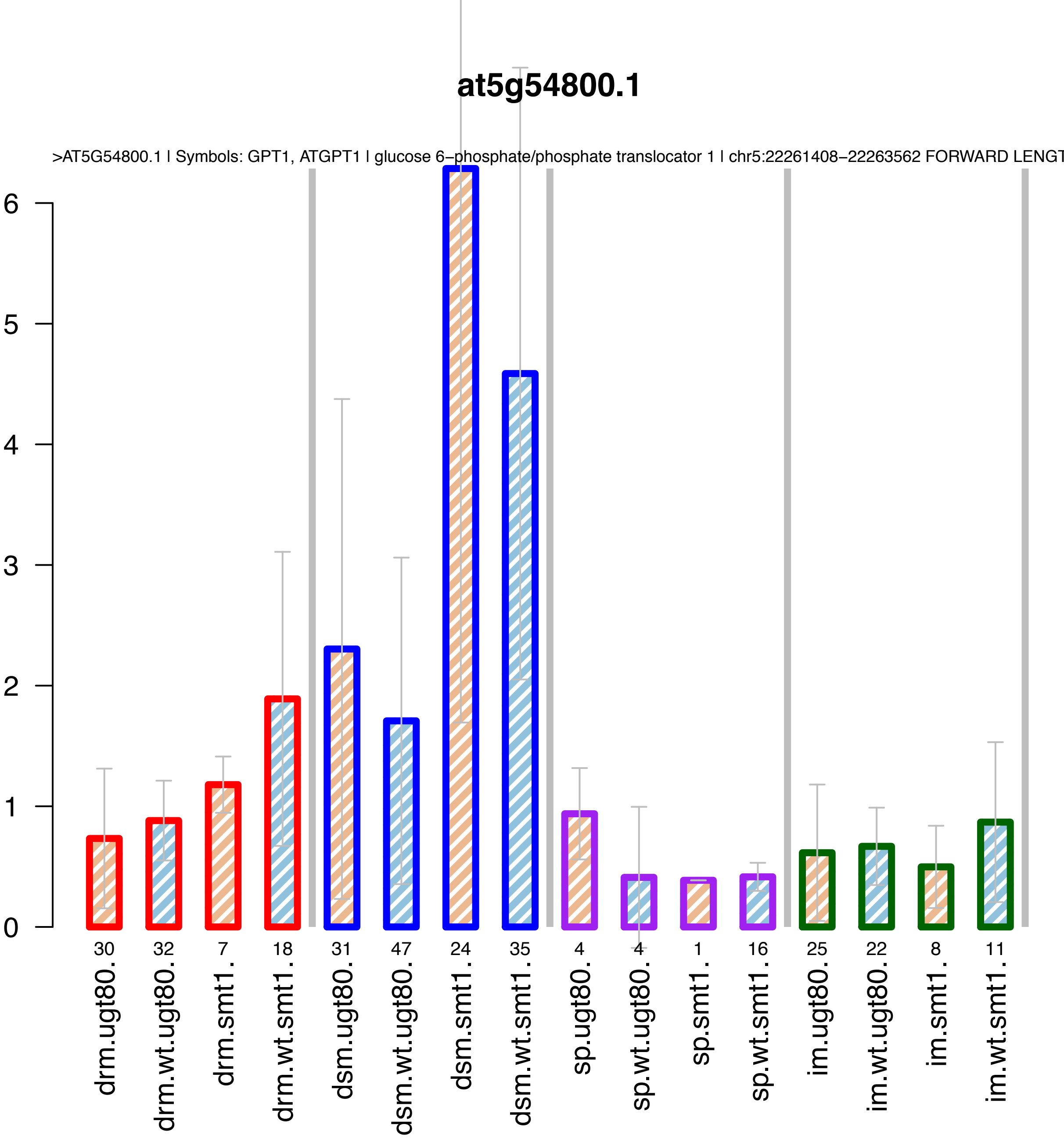

at5g54800.1
>AT5G54800.1 | Symbols: GPT1, ATGPT1 | glucose 6−phosphate/phosphate translocator 1 | chr5:22261408−22263562 FORWARD LENGT
0
1
2
3
4
5
6
30
32
7
18
31
47
24
35
4
4
1
16
25
22
8
11
drm.ugt80..
drm.wt.ugt80..
drm.smt1..
drm.wt.smt1..
dsm.ugt80..
dsm.wt.ugt80..
dsm.smt1..
dsm.wt.smt1..
sp.ugt80..
sp.wt.ugt80..
sp.smt1..
sp.wt.smt1..
im.ugt80..
im.wt.ugt80..
im.smt1..
im.wt.smt1..

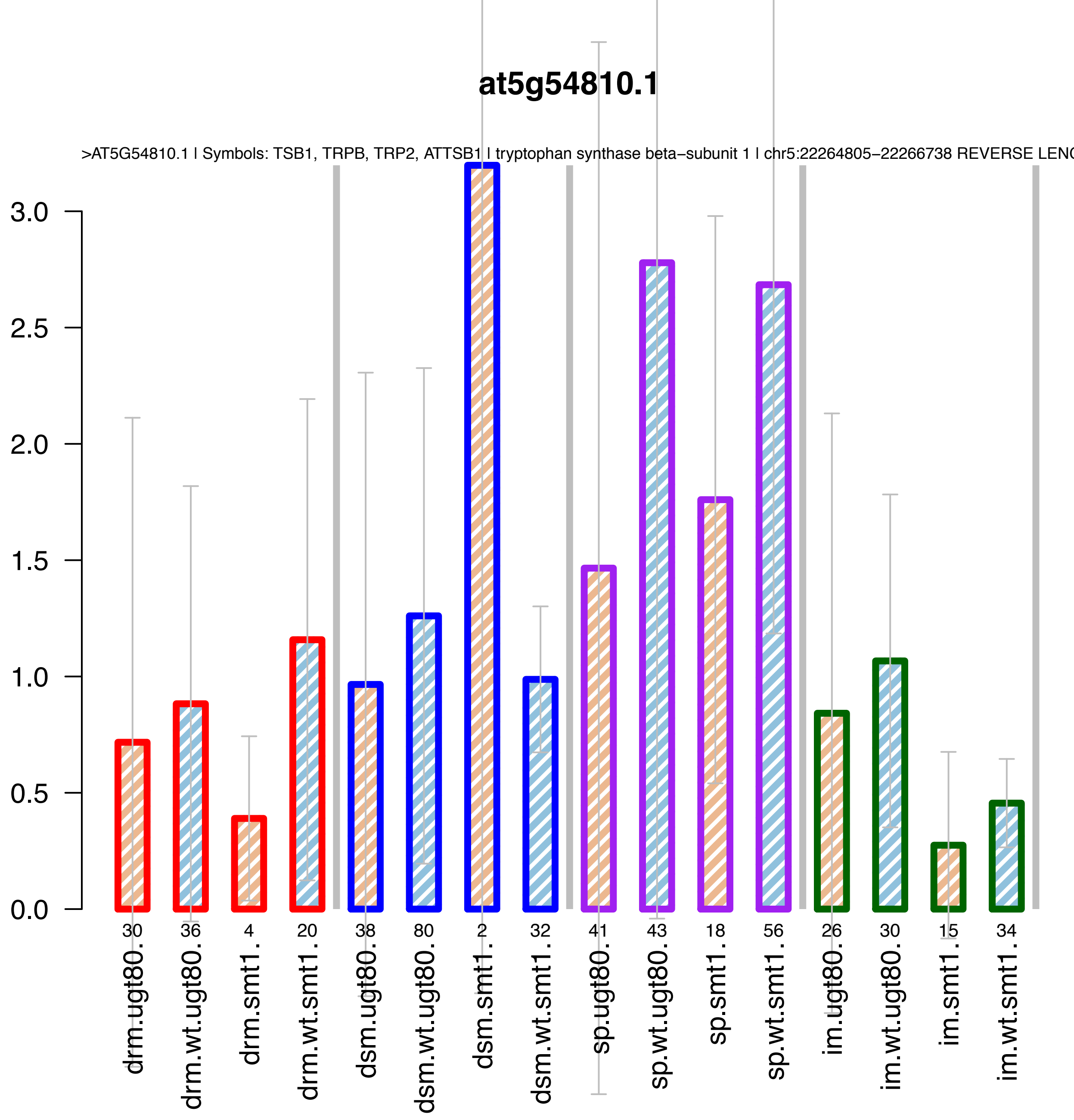

at5g54810.1
>AT5G54810.1 | Symbols: TSB1, TRPB, TRP2, ATTSB1 | tryptophan synthase beta−subunit 1 | chr5:22264805−22266738 REVERSE LENG
3.0
2.5
2.0
1.5
1.0
0.5
0.0
30
36
4
20
38
80
2
32
41
43
18
56
26
30
15
34
drm.ugt80.
drm.wt.ugt80.
drm.smt1.
drm.wt.smt1.
dsm.ugt80.
dsm.wt.ugt80.
dsm.smt1.
dsm.wt.smt1.
sp.ugt80.
sp.wt.ugt80.
sp.smt1.
sp.wt.smt1.
im.ugt80.
im.wt.ugt80.
im.smt1.
im.wt.smt1.

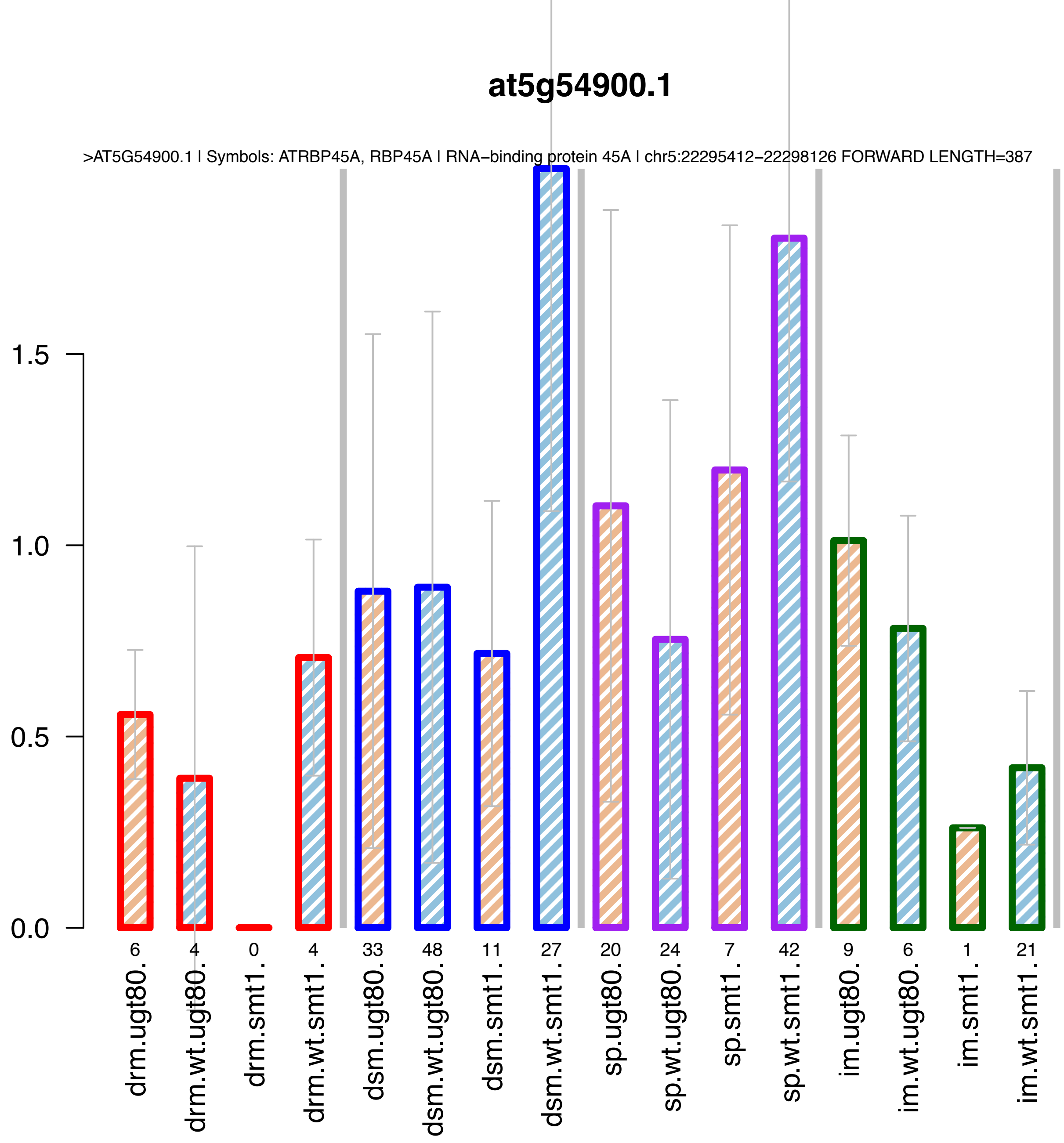

at5g54900.1
>AT5G54900.1 | Symbols: ATRBP45A, RBP45A | RNA−binding protein 45A | chr5:22295412−22298126 FORWARD LENGTH=387
0.0
0.5
1.0
1.5
6
4
0
4
33
48
11
27
20
24
7
42
9
6
1
21
drm.ugt80..
drm.wt.ugt80..
drm.smt1..
drm.wt.smt1..
dsm.ugt80..
dsm.wt.ugt80..
dsm.smt1..
dsm.wt.smt1..
sp.ugt80..
sp.wt.ugt80..
sp.smt1..
sp.wt.smt1..
im.ugt80..
im.wt.ugt80..
im.smt1..
im.wt.smt1..

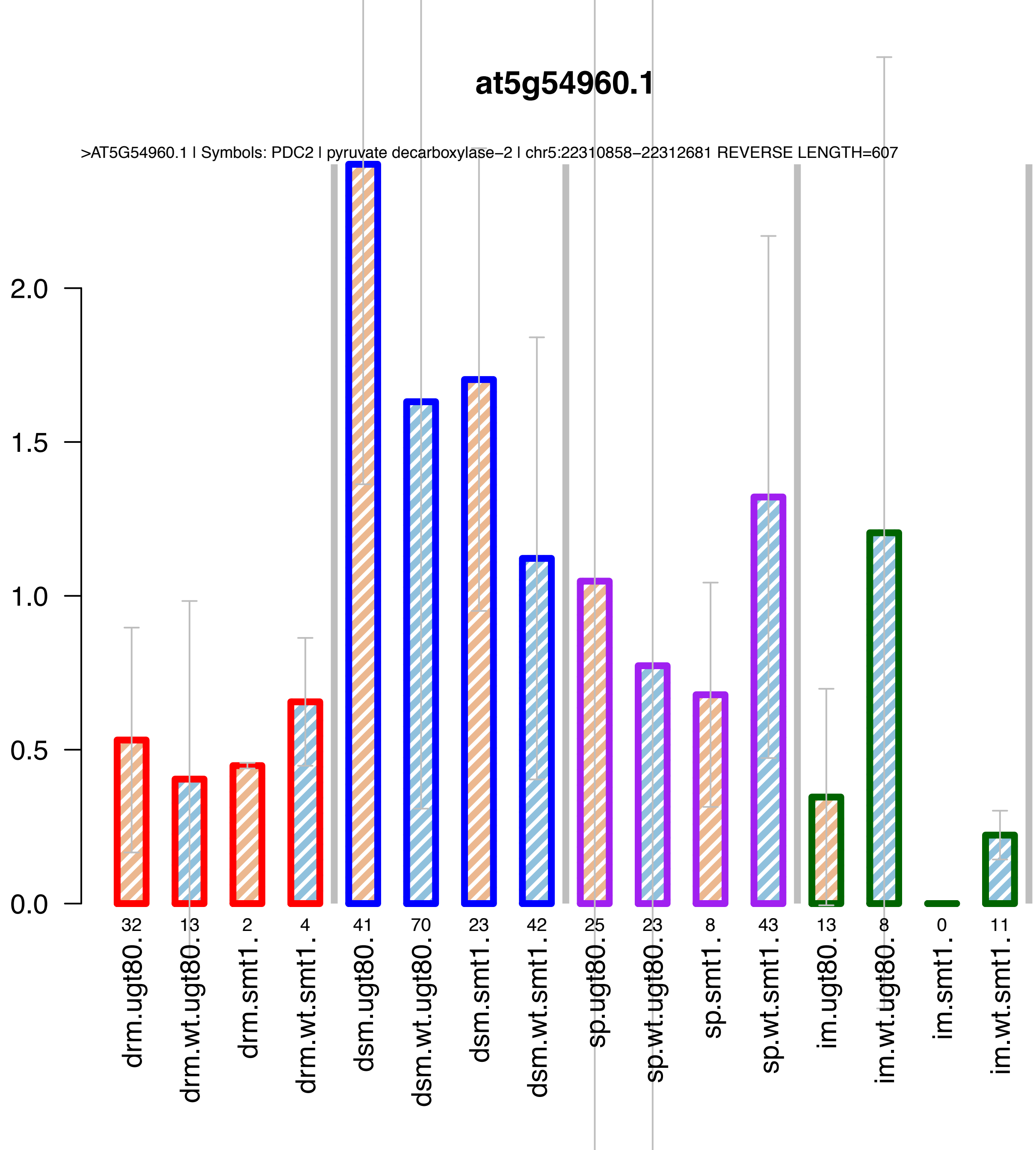
at5g54960.1
>AT5G54960.1 | Symbols: PDC2 | pyruvate decarboxylase−2 | chr5:22310858−22312681 REVERSE LENGTH=607
2.0
1.5
1.0
0.5
0.0
32
13
2
4
41
70
23
42
25
23
8
43
13
8
0
11
drm.ugt80..
drm.wt.ugt80..
drm.smt1..
drm.wt.smt1..
dsm.ugt80..
dsm.wt.ugt80..
dsm.smt1..
dsm.wt.smt1..
sp.ugt80..
sp.wt.ugt80..
sp.smt1..
sp.wt.smt1..
im.ugt80..
im.wt.ugt80..
im.smt1..
im.wt.smt1..

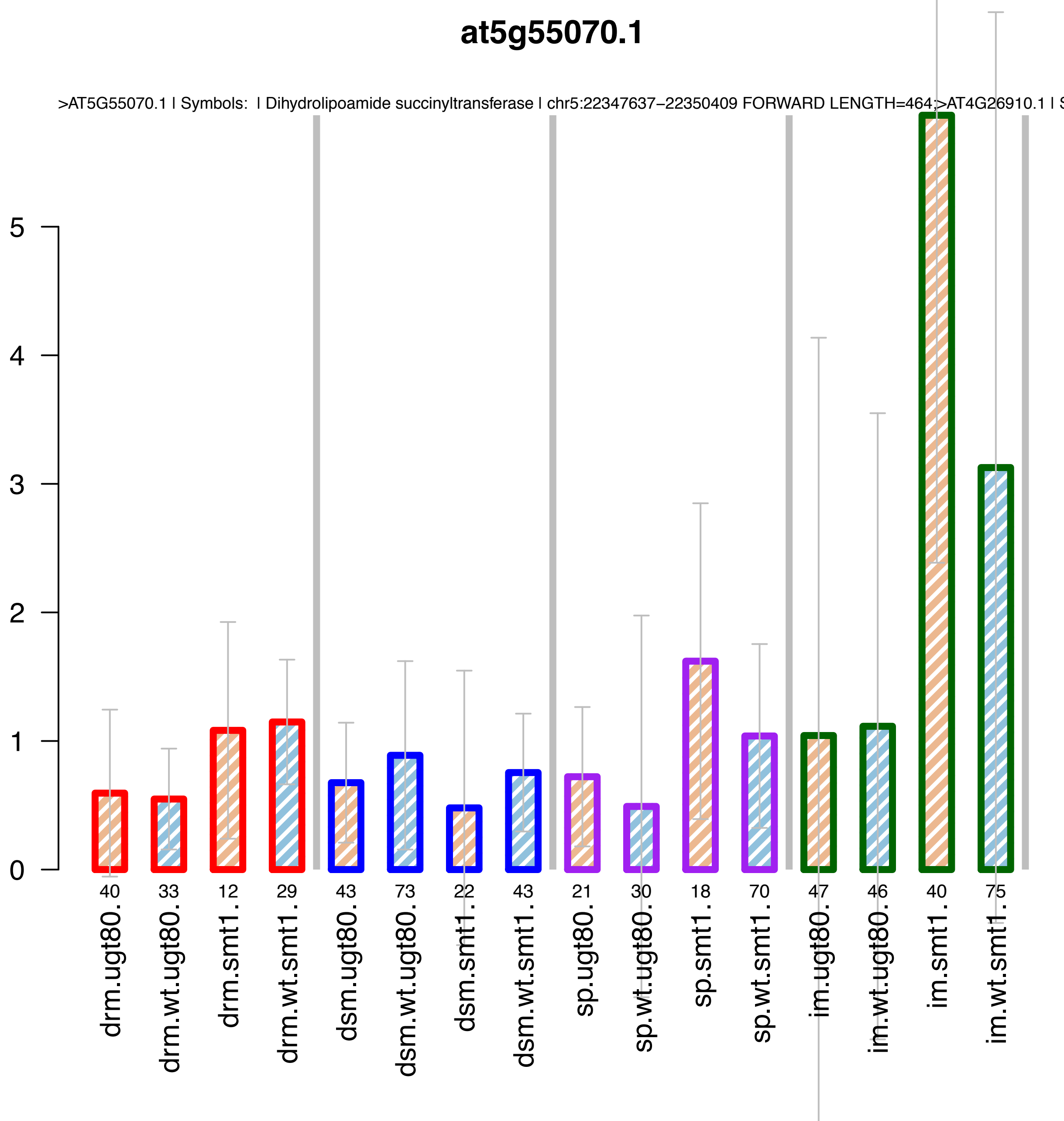

at5g55070.1
>AT5G55070.1 | Symbols: | Dihydrolipoamide succinyltransferase | chr5:22347637-22350409 FORWARD LENGTH=464;>AT4G26910.1 | S
0
1
2
3
4
5
40
33
12
29
43
73
22
43
21
30
18
70
47
46
40
75
drm.ugt80..
drm.wt.ugt80..
drm.smt1..
drm.wt.smt1..
dsm.ugt80..
dsm.wt.ugt80..
dsm.smt1..
dsm.wt.smt1..
sp.ugt80..
sp.wt.ugt80..
sp.smt1..
sp.wt.smt1..
im.ugt80..
im.wt.ugt80..
im.smt1..
im.wt.smt1..

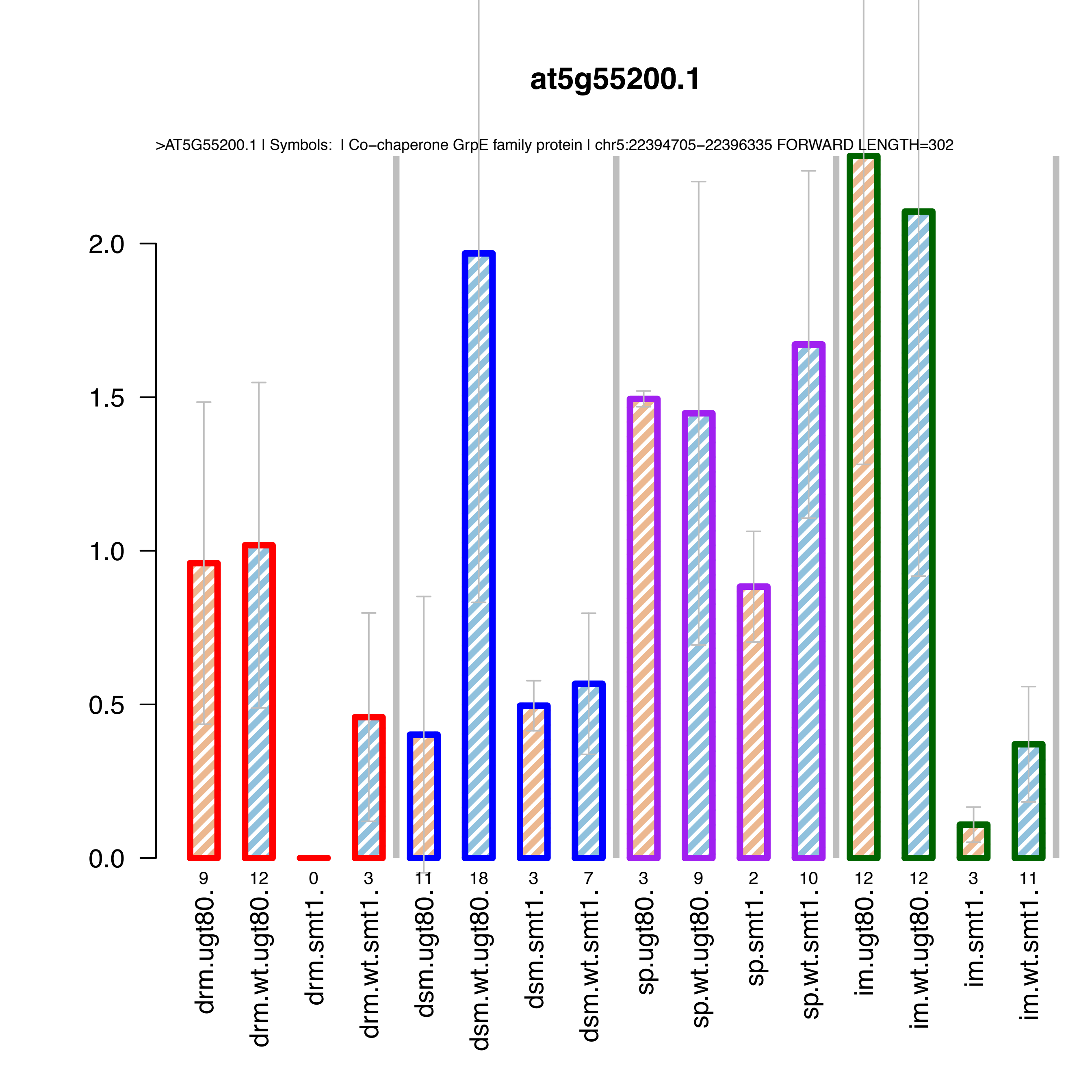

at5g55200.1
>AT5G55200.1 | Symbols: | Co−chaperone GrpE family protein | chr5:22394705−22396335 FORWARD LENGTH=302
2.0
1.5
1.0
0.5
0.0
9
12
0
3
11
18
3
7
3
9
2
10
12
12
3
11
drm.ugt80..
drm.wt.ugt80..
drm.smt1..
drm.wt.smt1..
dsm.ugt80..
dsm.wt.ugt80..
dsm.smt1..
dsm.wt.smt1..
sp.ugt80..
sp.wt.ugt80..
sp.smt1..
sp.wt.smt1..
im.ugt80..
im.wt.ugt80..
im.smt1..
im.wt.smt1..

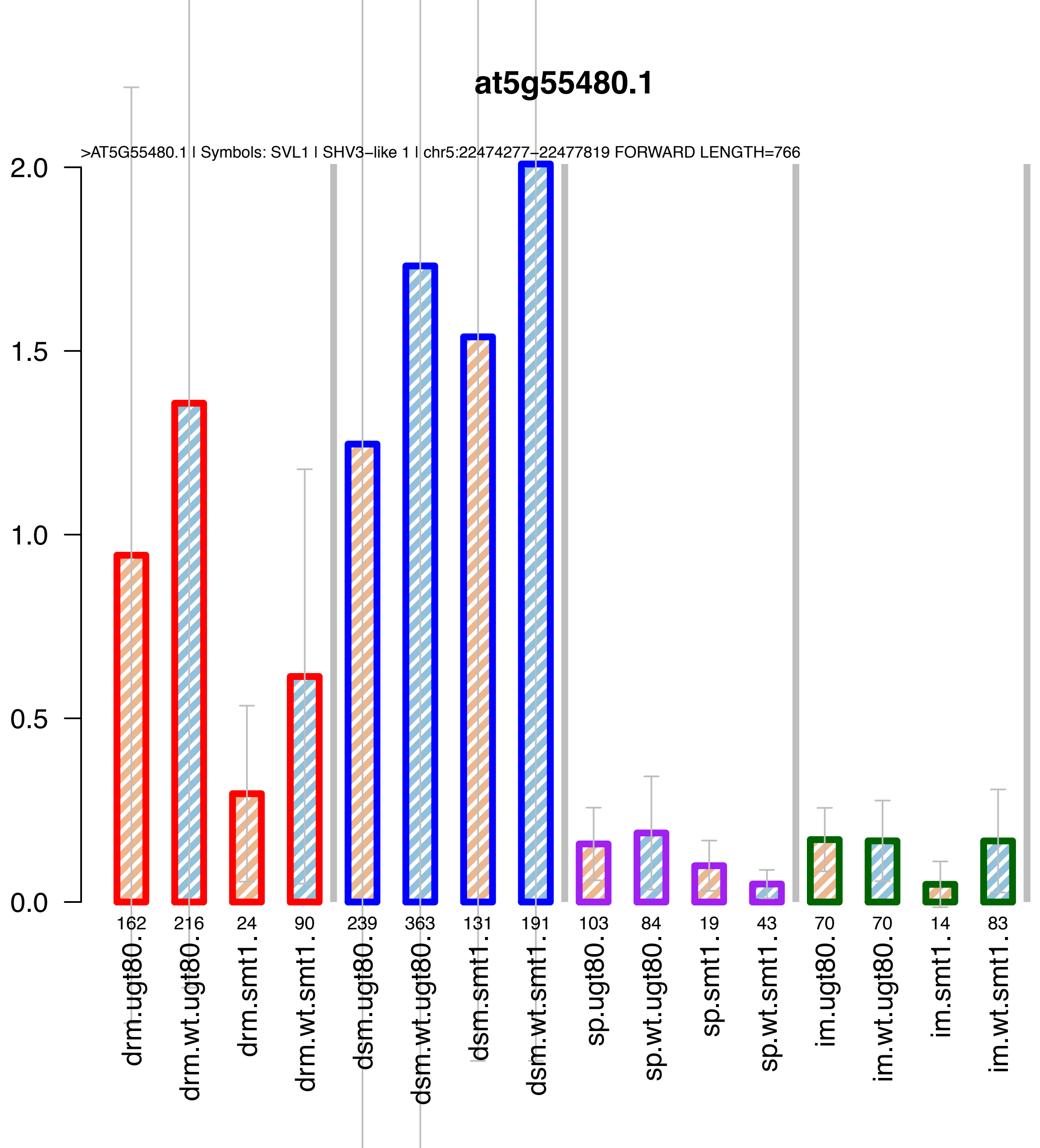

at5g55480.1
>AT5G55480.1 | Symbols: SVL1 | SHV3-like 1 | chr5:22474277-22477819 FORWARD LENGTH=766
2.0
1.5
1.0
0.5
0.0
162
216
24
90
239
363
131
191
103
84
19
43
70
70
14
83
drm.ugt80.
drm.wt.ugt80.
drm.smt1.
drm.wt.smt1.
dsm.ugt80.
dsm.wt.ugt80.
dsm.smt1.
dsm.wt.smt1.
sp.ugt80.
sp.wt.ugt80.
sp.smt1.
sp.wt.smt1.
im.ugt80.
im.wt.ugt80.
im.smt1.
im.wt.smt1.

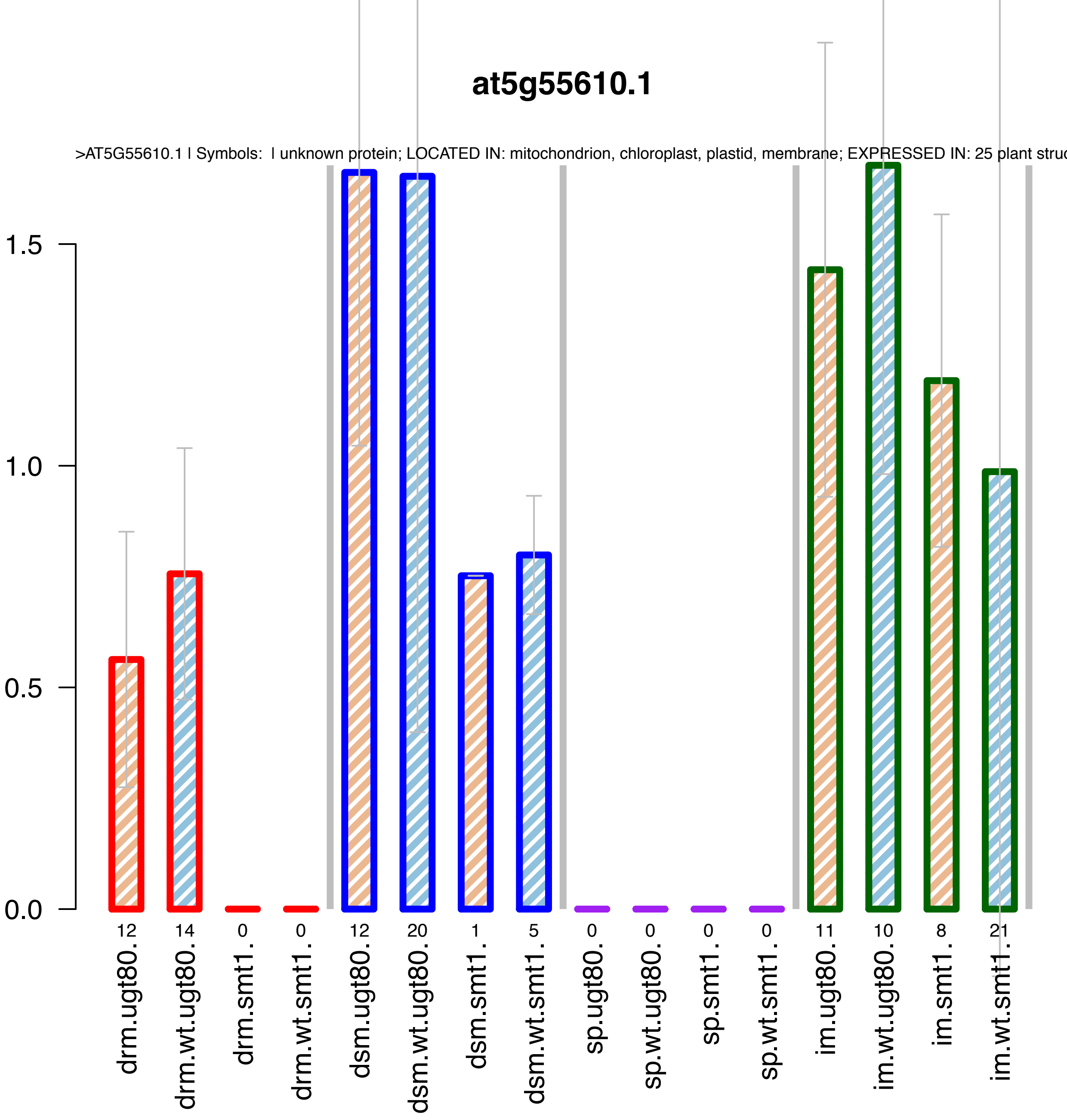

at5g55610.1
>AT5G55610.1 | Symbols: | unknown protein; LOCATED IN: mitochondrion, chloroplast, plastid, membrane; EXPRESSED IN: 25 plant struc
1.5
1.0
0.5
0.0
12
14
0
0
12
20
1
5
0
0
0
0
11
10
8
21
drm.ugt80.
drm.wt.ugt80.
drm.smt1.
drm.wt.smt1.
dsm.ugt80.
dsm.wt.ugt80.
dsm.smt1.
dsm.wt.smt1.
sp.ugt80.
sp.wt.ugt80.
sp.smt1.
sp.wt.smt1.
im.ugt80.
im.wt.ugt80.
im.smt1.
im.wt.smt1.

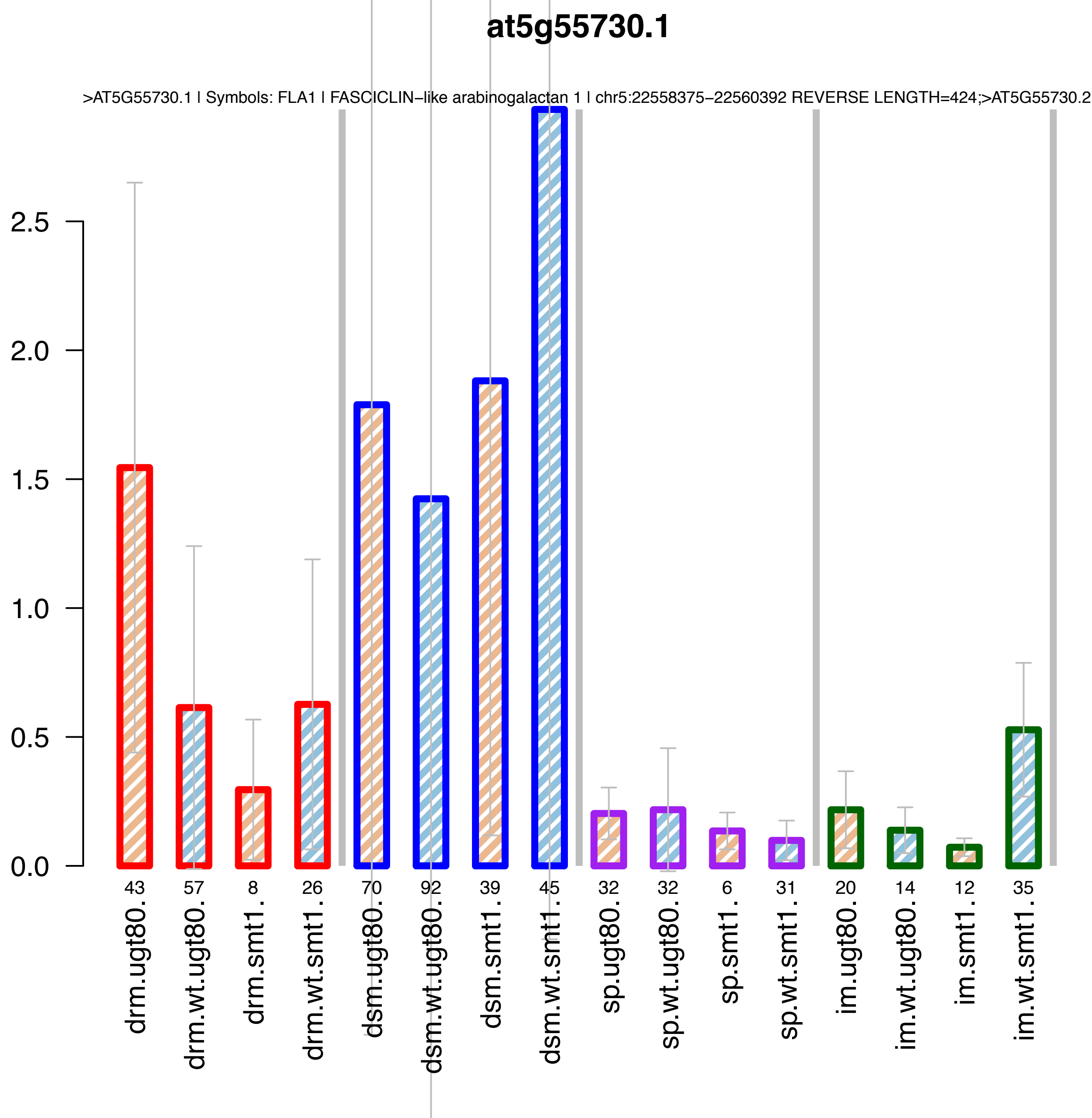

at5g55730.1
>AT5G55730.1 | Symbols: FLA1 | FASCICLIN−like arabinogalactan 1 | chr5:22558375−22560392 REVERSE LENGTH=424;>AT5G55730.2
2.5
2.0
1.5
1.0
0.5
0.0
43
57
8
26
70
92
39
45
32
32
6
31
20
14
12
35
drm.ugt80..
drm.wt.ugt80..
drm.smt1..
drm.wt.smt1..
dsm.ugt80..
dsm.wt.ugt80..
dsm.smt1..
dsm.wt.smt1..
sp.ugt80..
sp.wt.ugt80..
sp.smt1..
sp.wt.smt1..
im.ugt80..
im.wt.ugt80..
im.smt1..
im.wt.smt1..

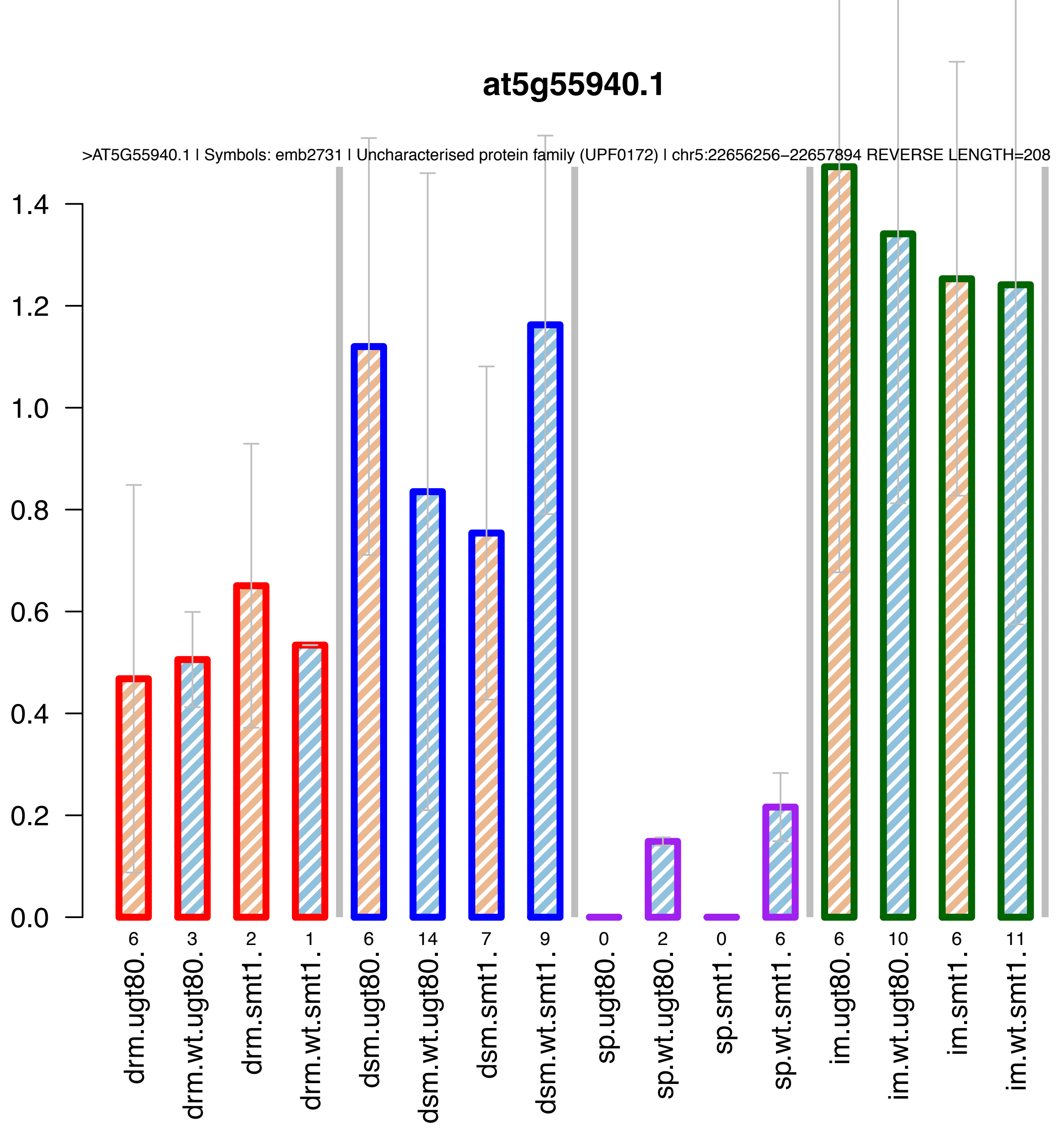

at5g55940.1
>AT5G55940.1 | Symbols: emb2731 | Uncharacterised protein family (UPF0172) | chr5:22656256−22657894 REVERSE LENGTH=208
0.0
0.2
0.4
0.6
0.8
1.0
1.2
1.4
6
3
2
1
6
14
7
9
0
2
0
6
6
10
6
11
drm.ugt80..
drm.wt.ugt80..
drm.smt1..
drm.wt.smt1..
dsm.ugt80..
dsm.wt.ugt80..
dsm.smt1..
dsm.wt.smt1..
sp.ugt80..
sp.wt.ugt80..
sp.smt1..
sp.wt.smt1..
im.ugt80..
im.wt.ugt80..
im.smt1..
im.wt.smt1..

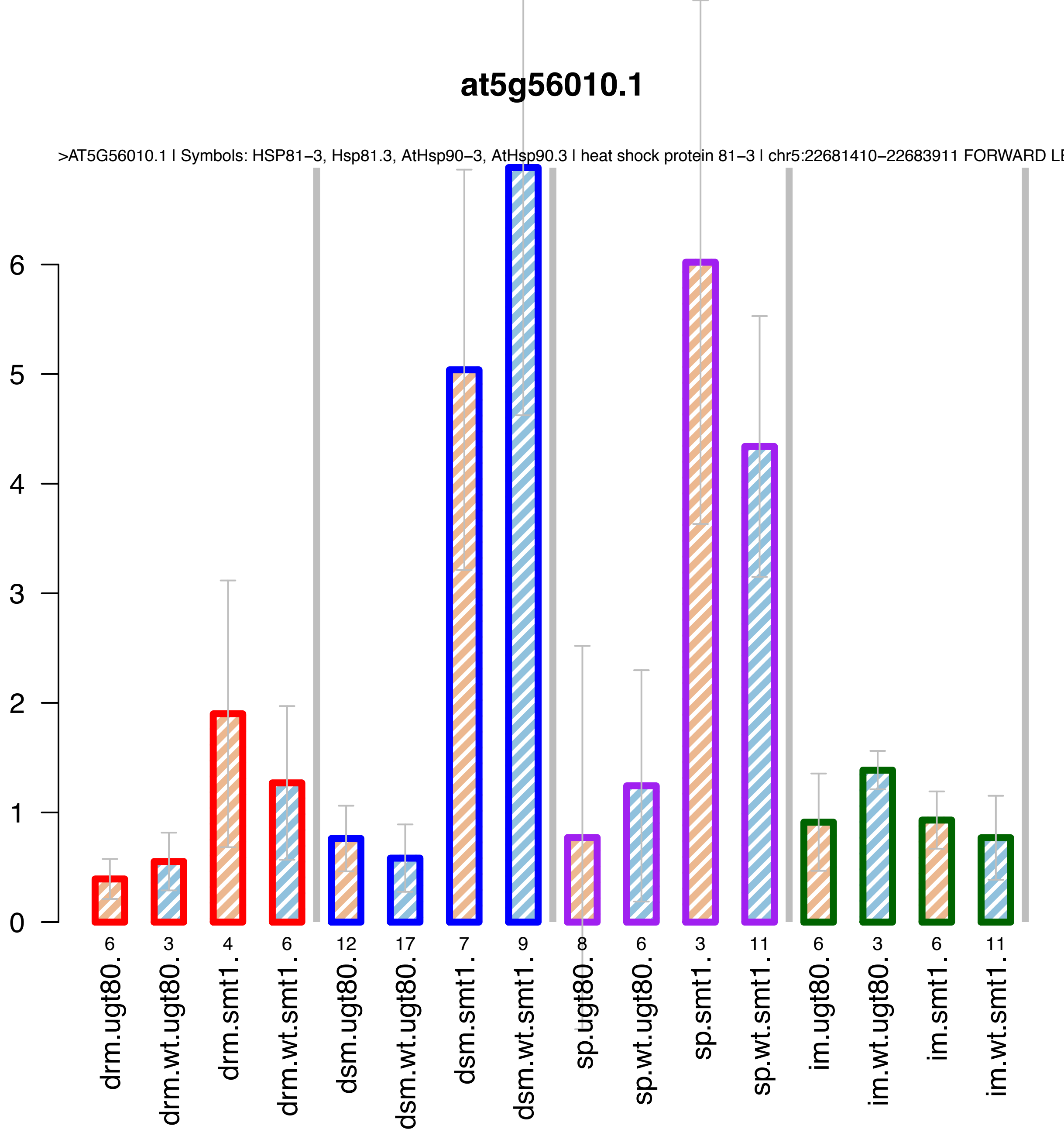
at5g56010.1
>AT5G56010.1 | Symbols: HSP81–3, Hsp81.3, AtHsp90–3, AtHsp90.3 | heat shock protein 81–3 | chr5:22681410–22683911 FORWARD LE
0
1
2
3
4
5
6
6
3
4
6
12
17
7
9
8
6
3
11
6
3
6
11
drm.ugt80.
drm.wt.ugt80.
drm.smt1.
drm.wt.smt1.
dsm.ugt80.
dsm.wt.ugt80.
dsm.smt1.
dsm.wt.smt1.
sp.ugt80.
sp.wt.ugt80.
sp.smt1.
sp.wt.smt1.
im.ugt80.
im.wt.ugt80.
im.smt1.
im.wt.smt1.

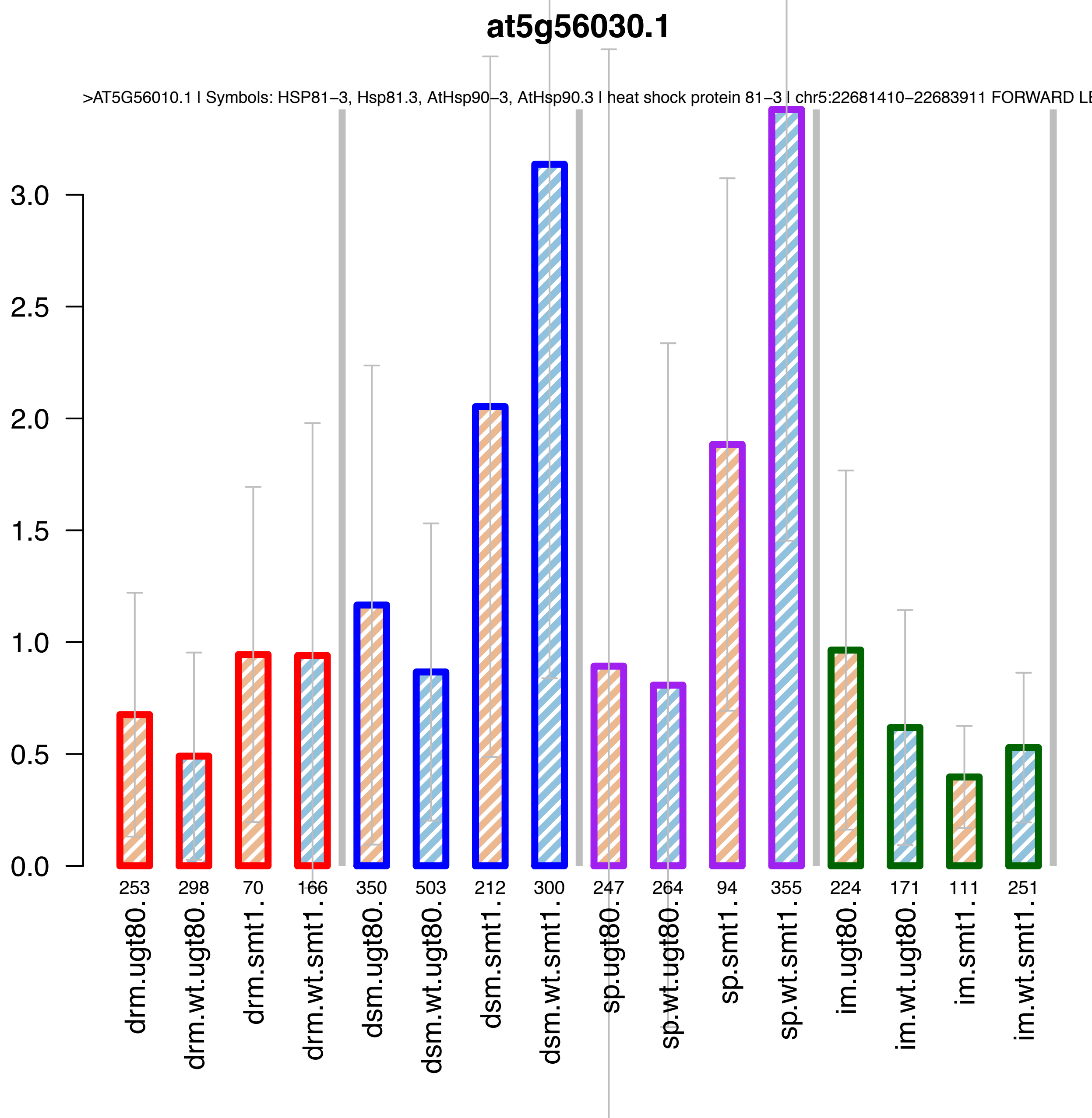

at5g56030.1
>AT5G56010.1 | Symbols: HSP81−3, Hsp81.3, AtHsp90−3, AtHsp90.3 | heat shock protein 81−3 | chr5:22681410−22683911 FORWARD LE
0.0
0.5
1.0
1.5
2.0
2.5
3.0
253
298
70
166
350
503
212
300
247
264
94
355
224
171
111
251
drm.ugt80..
drm.wt.ugt80..
drm.smt1..
drm.wt.smt1..
dsm.ugt80..
dsm.wt.ugt80..
dsm.smt1..
dsm.wt.smt1..
sp.ugt80..
sp.wt.ugt80..
sp.smt1..
sp.wt.smt1..
im.ugt80..
im.wt.ugt80..
im.smt1..
im.wt.smt1..

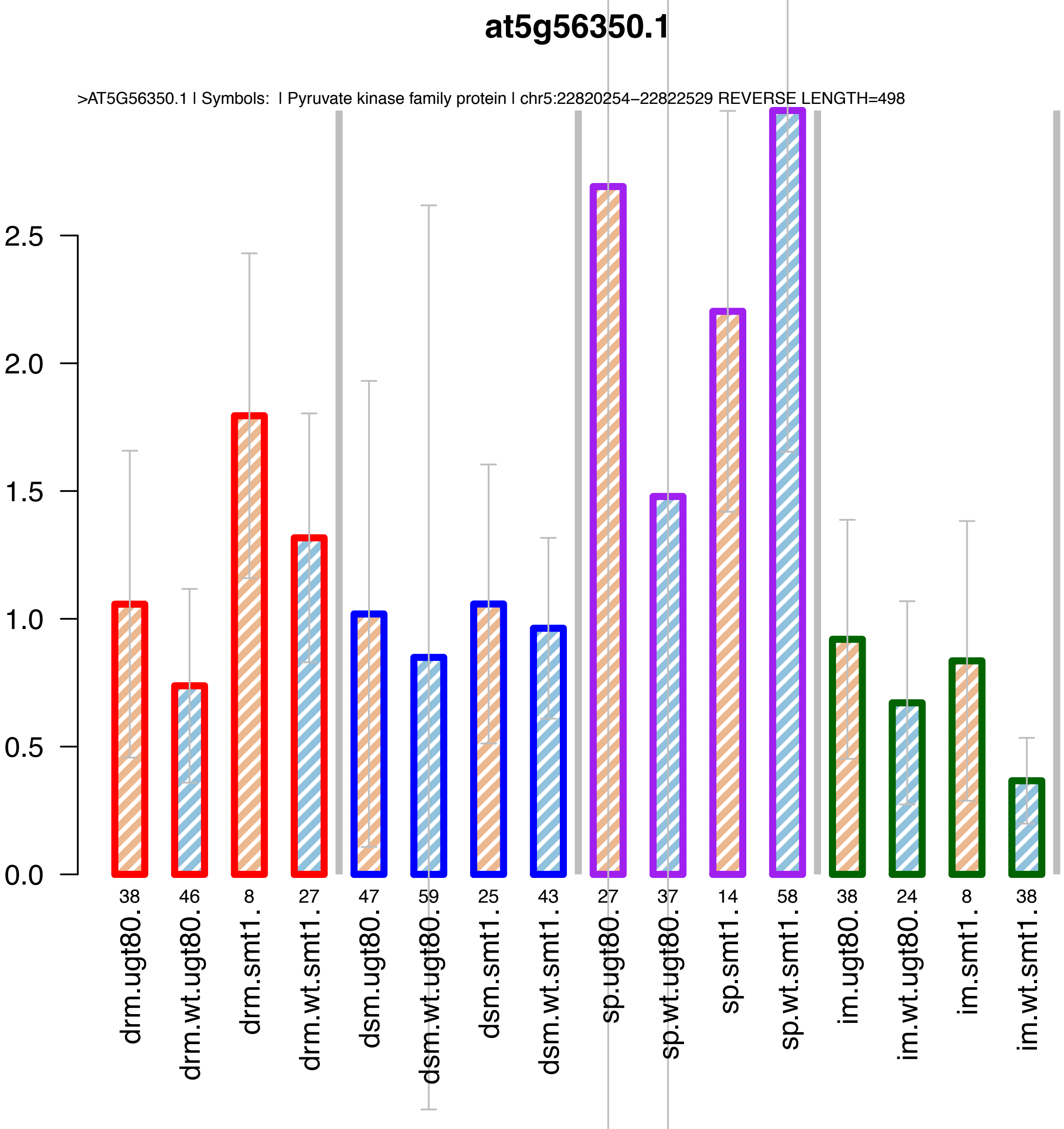

at5g56350.1
>AT5G56350.1 | Symbols: | Pyruvate kinase family protein | chr5:22820254−22822529 REVERSE LENGTH=498
0.0
0.5
1.0
1.5
2.0
2.5
38
46
8
27
47
59
25
43
27
37
14
58
38
24
8
38
drm.ugt80.
drm.wt.ugt80.
drm.smt1.
drm.wt.smt1.
dsm.ugt80.
dsm.wt.ugt80.
dsm.smt1.
dsm.wt.smt1.
sp.ugt80.
sp.wt.ugt80.
sp.smt1.
sp.wt.smt1.
im.ugt80.
im.wt.ugt80.
im.smt1.
im.wt.smt1.

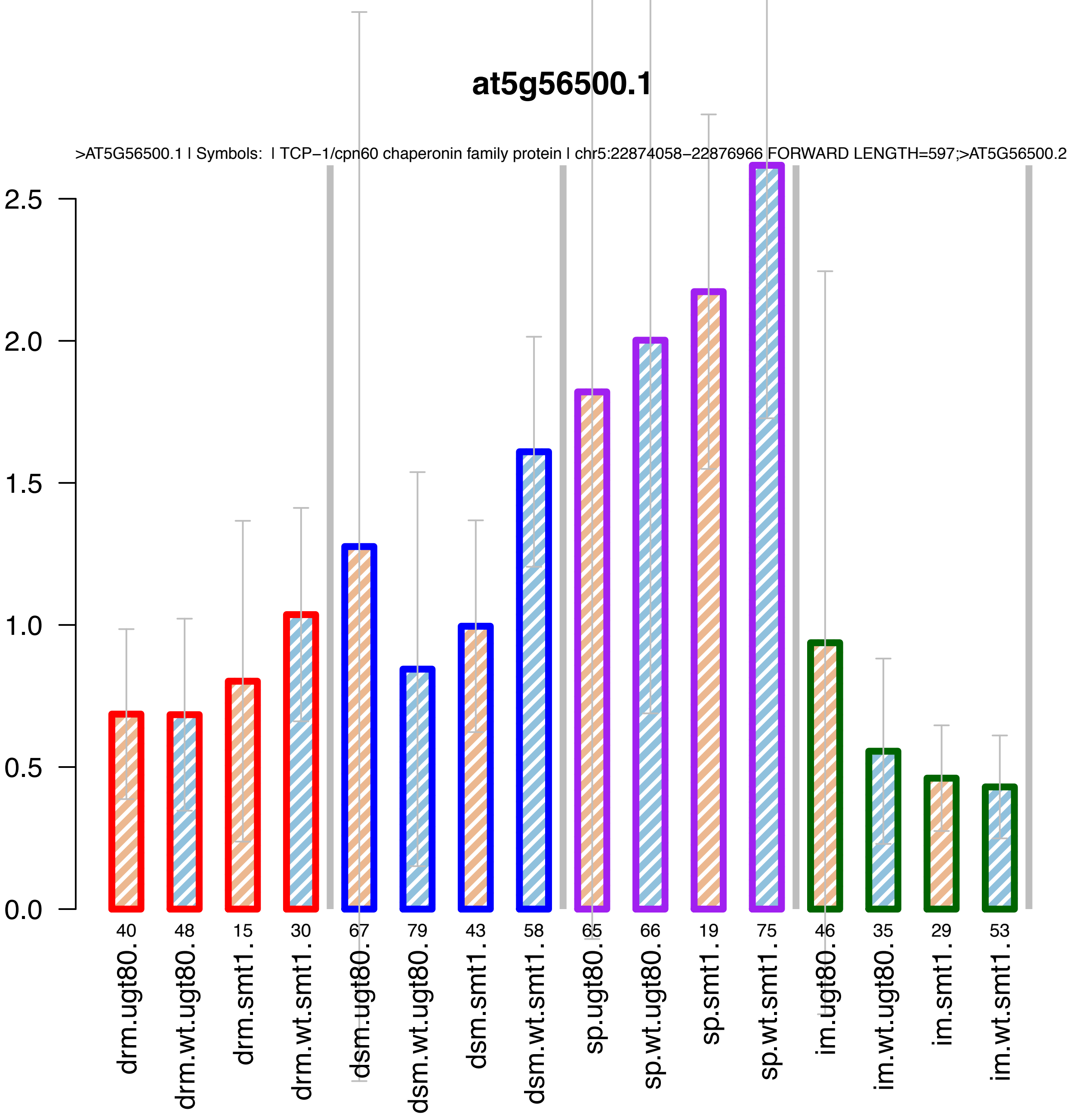

at5g56500.1
>AT5G56500.1 | Symbols: | TCP−1/cpn60 chaperonin family protein | chr5:22874058−22876966 FORWARD LENGTH=597;>AT5G56500.2
2.5
2.0
1.5
1.0
0.5
0.0
40
48
15
30
67
79
43
58
65
66
19
75
46
35
29
53
drm.ugt80..
drm.wt.ugt80..
drm.smt1..
drm.wt.smt1..
dsm.ugt80..
dsm.wt.ugt80..
dsm.smt1..
dsm.wt.smt1..
sp.ugt80..
sp.wt.ugt80..
sp.smt1..
sp.wt.smt1..
im.ugt80..
im.wt.ugt80..
im.smt1..
im.wt.smt1..

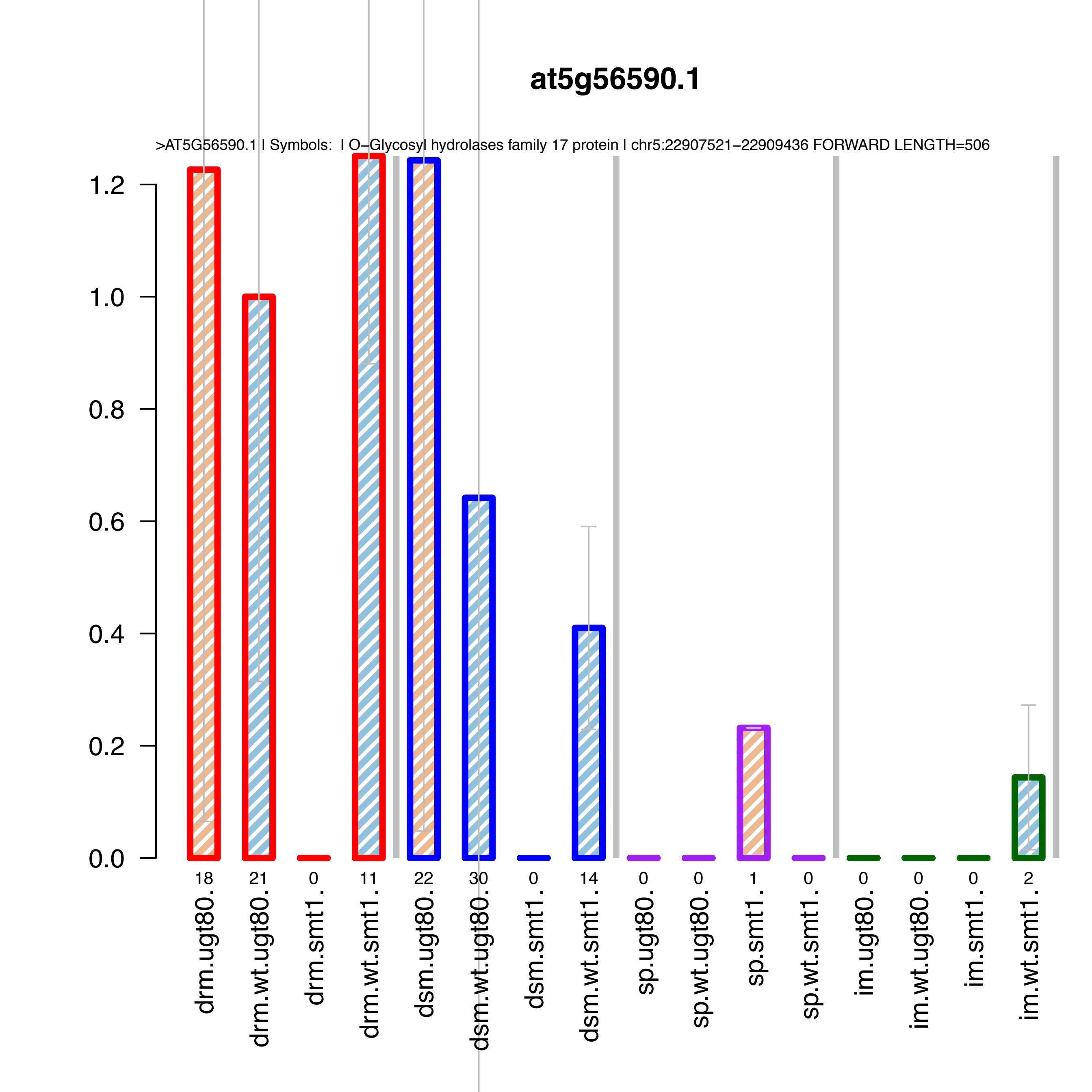

at5g56590.1
>AT5G56590.1 | Symbols: | O−Glycosyl hydrolases family 17 protein | chr5:22907521−22909436 FORWARD LENGTH=506
0.0
0.2
0.4
0.6
0.8
1.0
1.2
18
21
0
11
22
30
0
14
0
0
1
0
0
0
0
2
drm.ugt80..
drm.wt.ugt80..
drm.smt1..
drm.wt.smt1..
dsm.ugt80..
dsm.wt.ugt80..
dsm.smt1..
dsm.wt.smt1..
sp.ugt80..
sp.wt.ugt80..
sp.smt1..
sp.wt.smt1..
im.ugt80..
im.wt.ugt80..
im.smt1..
im.wt.smt1..

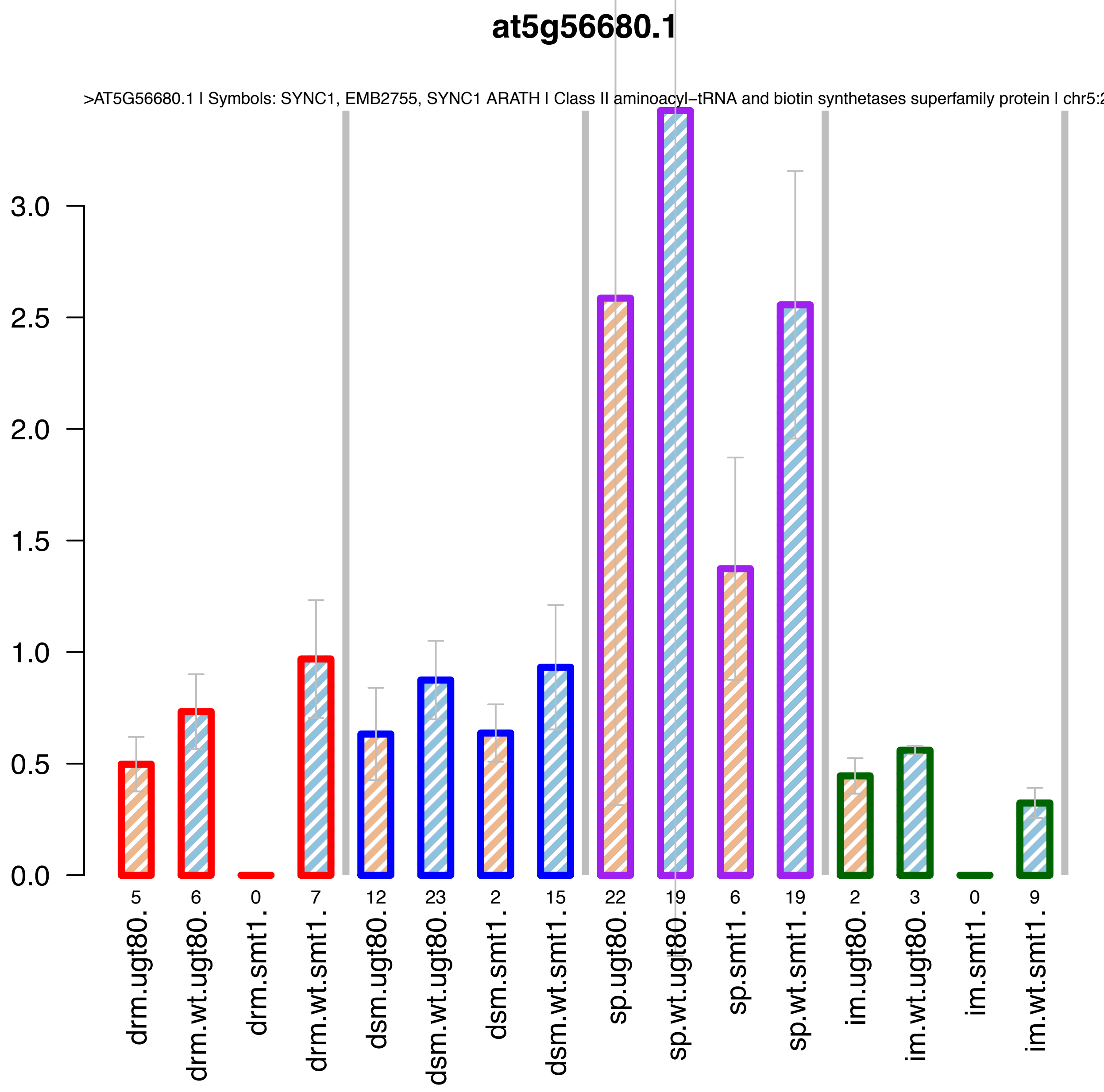

at5g56680.1
>AT5G56680.1 | Symbols: SYNC1, EMB2755, SYNC1 ARATH | Class II aminoacyl–tRNA and biotin synthetases superfamily protein | chr5:2
0.0
0.5
1.0
1.5
2.0
2.5
3.0
5
6
0
7
12
23
2
15
22
19
6
19
2
3
0
9
drm.ugt80.
drm.wt.ugt80.
drm.smt1.
drm.wt.smt1.
dsm.ugt80.
dsm.wt.ugt80.
dsm.smt1.
dsm.wt.smt1.
sp.ugt80.
sp.wt.ugt80.
sp.smt1.
sp.wt.smt1.
im.ugt80.
im.wt.ugt80.
im.smt1.
im.wt.smt1.

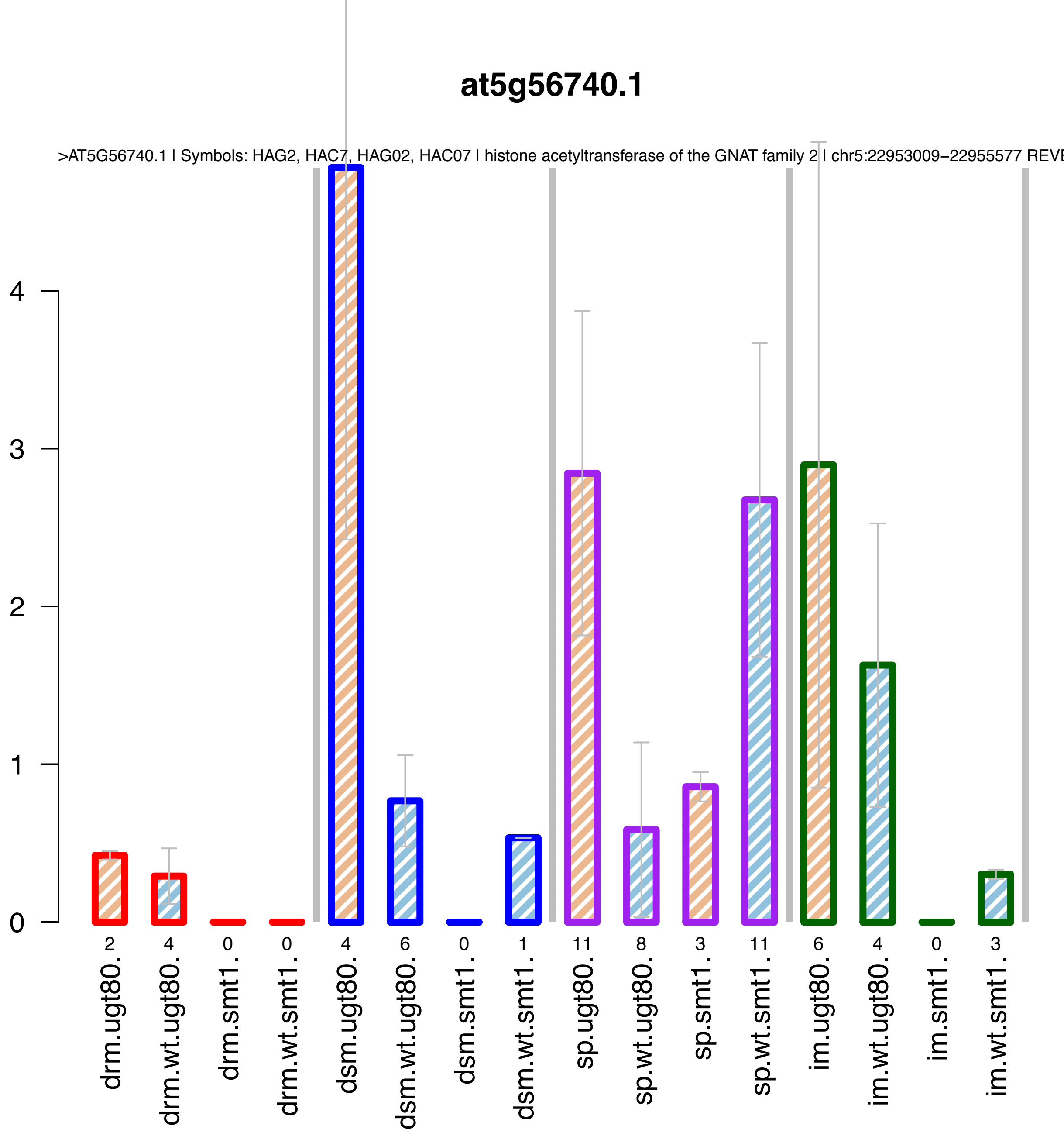
at5g56740.1
>AT5G56740.1 | Symbols: HAG2, HAC7, HAG02, HAC07 | histone acetyltransferase of the GNAT family 2 | chr5:22953009-22955577 REVE
4
3
2
1
0
2
4
0
0
4
6
0
1
11
8
3
11
6
4
0
3
drm.ugt80.
drm.wt.ugt80.
drm.smt1.
drm.wt.smt1.
dsm.ugt80.
dsm.wt.ugt80.
dsm.smt1.
dsm.wt.smt1.
sp.ugt80.
sp.wt.ugt80.
sp.smt1.
sp.wt.smt1.
im.ugt80.
im.wt.ugt80.
im.smt1.
im.wt.smt1.

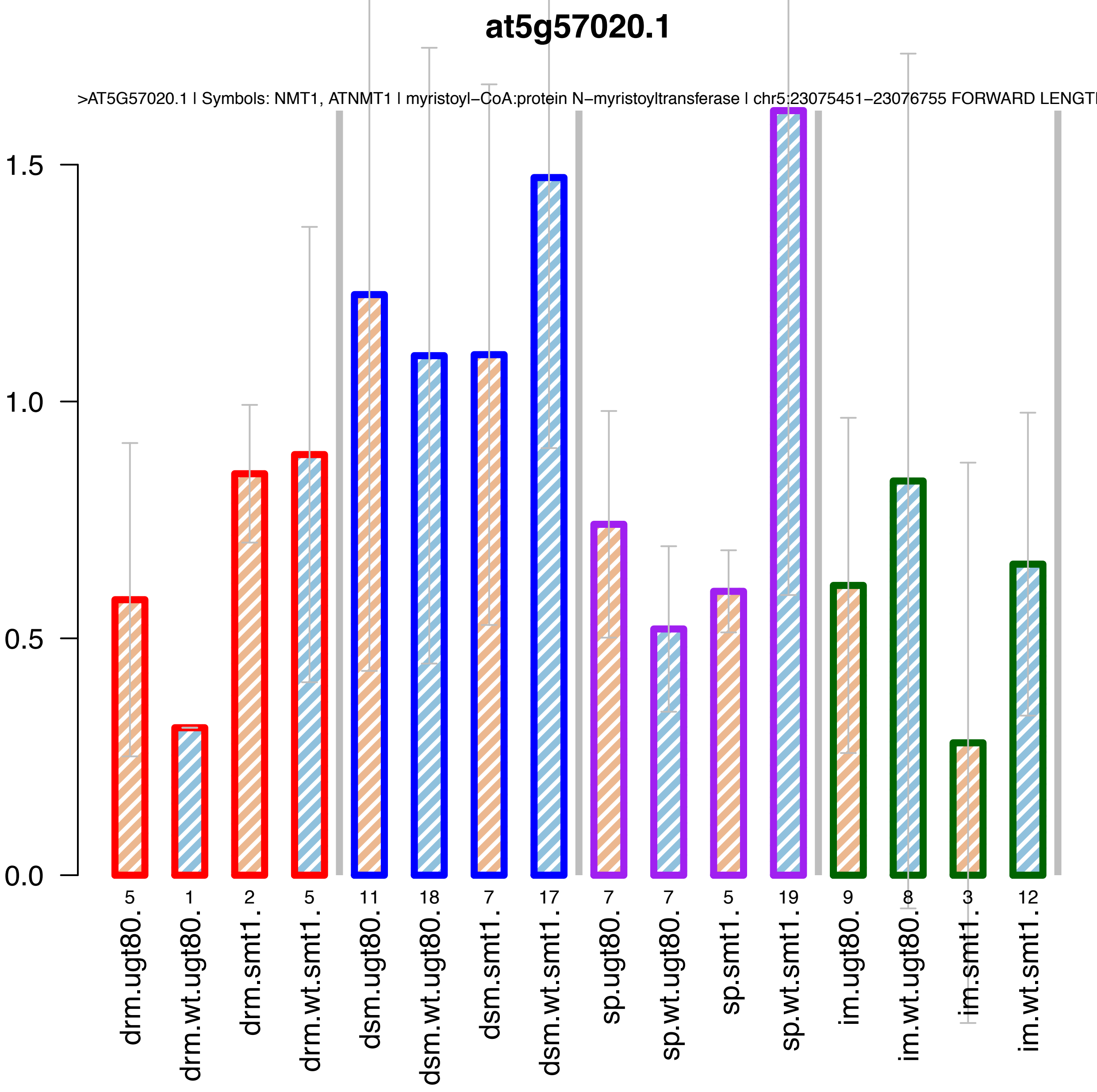

at5g57020.1
>AT5G57020.1 | Symbols: NMT1, ATNMT1 | myristoyl−CoA:protein N−myristoyltransferase | chr5:23075451−23076755 FORWARD LENGTH
1.5
1.0
0.5
0.0
5
1
2
5
11
18
7
17
7
7
5
19
9
8
3
12
drm.ugt80..
drm.wt.ugt80..
drm.smt1..
drm.wt.smt1..
dsm.ugt80..
dsm.wt.ugt80..
dsm.smt1..
dsm.wt.smt1..
sp.ugt80..
sp.wt.ugt80..
sp.smt1..
sp.wt.smt1..
im.ugt80..
im.wt.ugt80..
im.smt1..
im.wt.smt1..

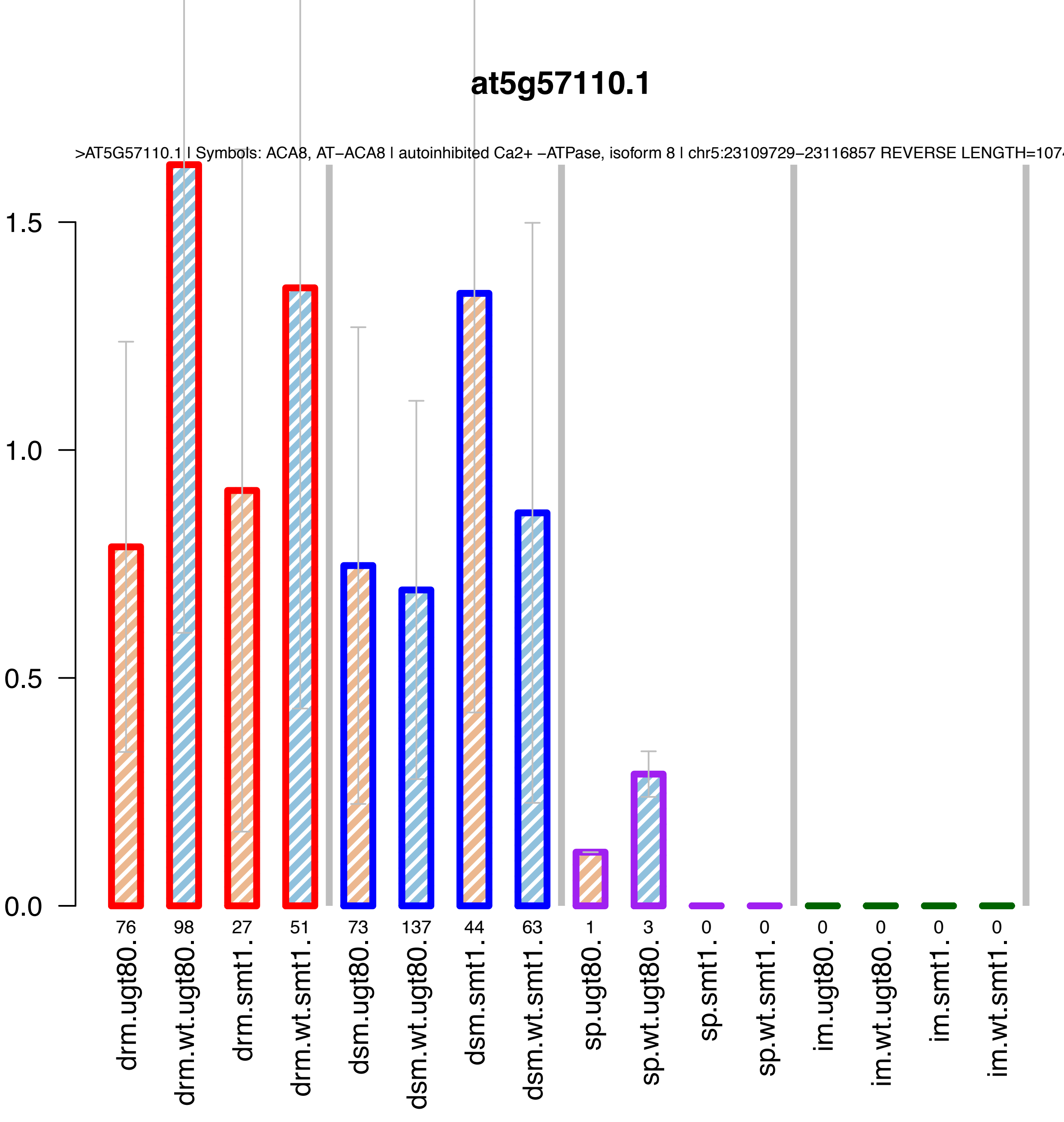

at5g57110.1
>AT5G57110.1 | Symbols: ACA8, AT-ACA8 | autoinhibited Ca2+ -ATPase, isoform 8 | chr5:23109729-23116857 REVERSE LENGTH=107
1.5
1.0
0.5
0.0
76
98
27
51
73
137
44
63
1
3
0
0
0
0
0
0
drm.ugt80.
drm.wt.ugt80.
drm.smt1.
drm.wt.smt1.
dsm.ugt80.
dsm.wt.ugt80.
dsm.smt1.
dsm.wt.smt1.
sp.ugt80.
sp.wt.ugt80.
sp.smt1.
sp.wt.smt1.
im.ugt80.
im.wt.ugt80.
im.smt1.
im.wt.smt1.

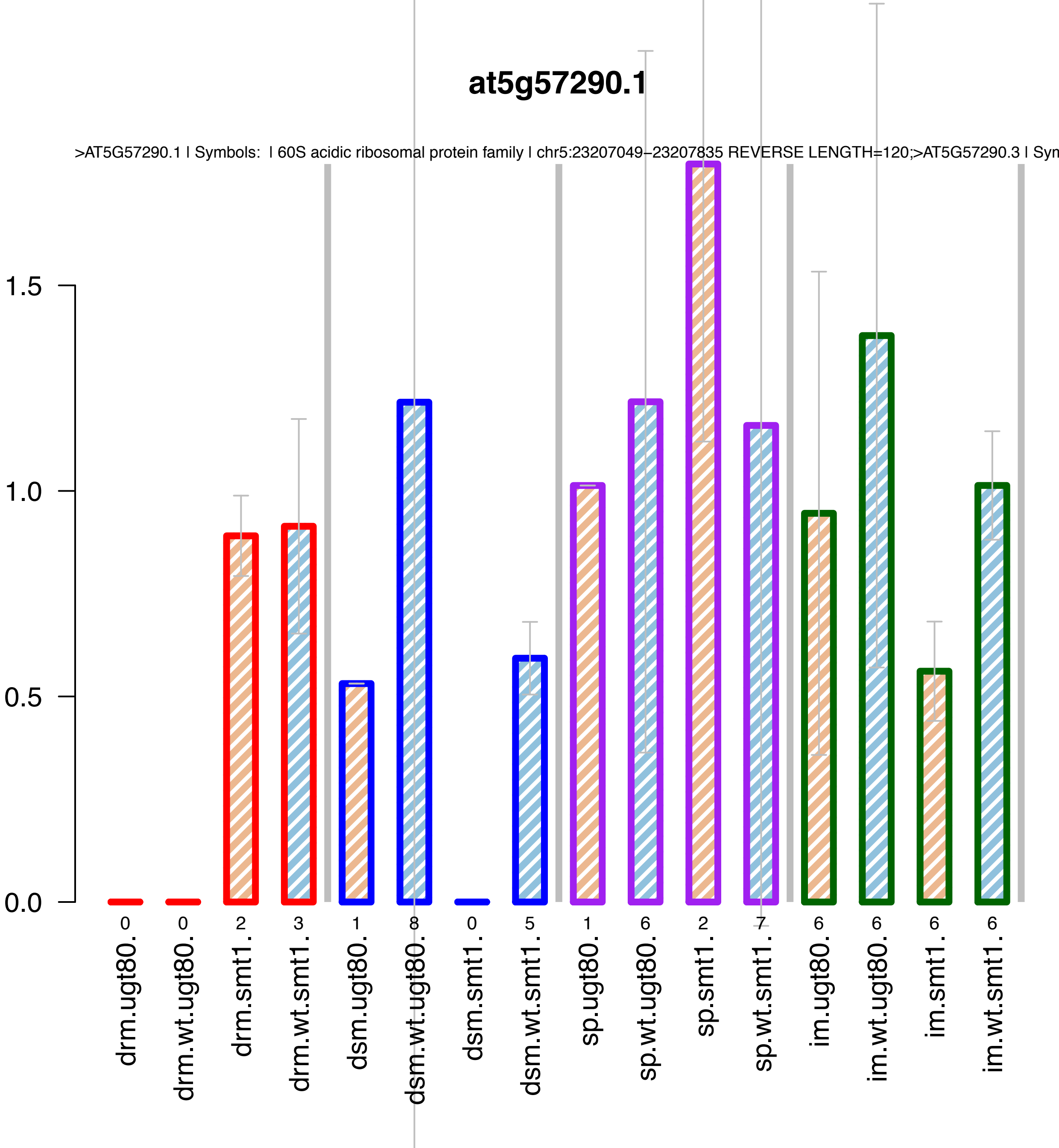

at5g57290.1
>AT5G57290.1 | Symbols: | 60S acidic ribosomal protein family | chr5:23207049-23207835 REVERSE LENGTH=120;>AT5G57290.3 | Sym
1.5
1.0
0.5
0.0
0
0
2
3
1
8
0
5
1
6
2
7
6
6
6
6
drm.ugt80..
drm.wt.ugt80..
drm.smt1..
drm.wt.smt1..
dsm.ugt80..
dsm.wt.ugt80..
dsm.smt1..
dsm.wt.smt1..
sp.ugt80..
sp.wt.ugt80..
sp.smt1..
sp.wt.smt1..
im.ugt80..
im.wt.ugt80..
im.smt1..
im.wt.smt1..

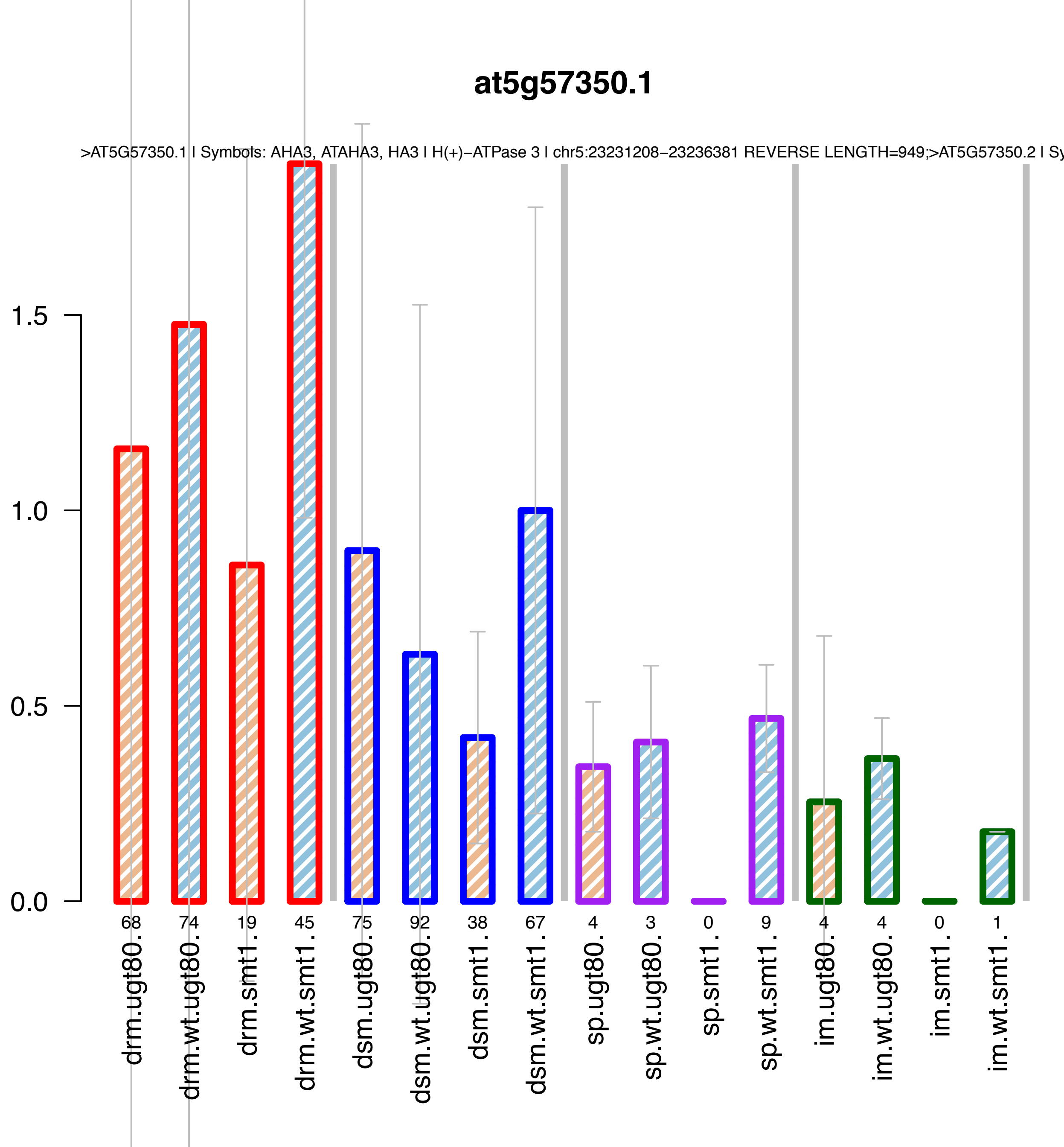
at5g57350.1
>AT5G57350.1 | Symbols: AHA3, ATAHA3, HA3 | H(+)−ATPase 3 | chr5:23231208−23236381 REVERSE LENGTH=949;>AT5G57350.2 | Sy
1.5
1.0
0.5
0.0
68
74
19
45
75
92
38
67
4
3
0
9
4
4
0
1
drm.ugt80..
drm.wt.ugt80..
drm.smt1..
drm.wt.smt1..
dsm.ugt80..
dsm.wt.ugt80..
dsm.smt1..
dsm.wt.smt1..
sp.ugt80..
sp.wt.ugt80..
sp.smt1..
sp.wt.smt1..
im.ugt80..
im.wt.ugt80..
im.smt1..
im.wt.smt1..

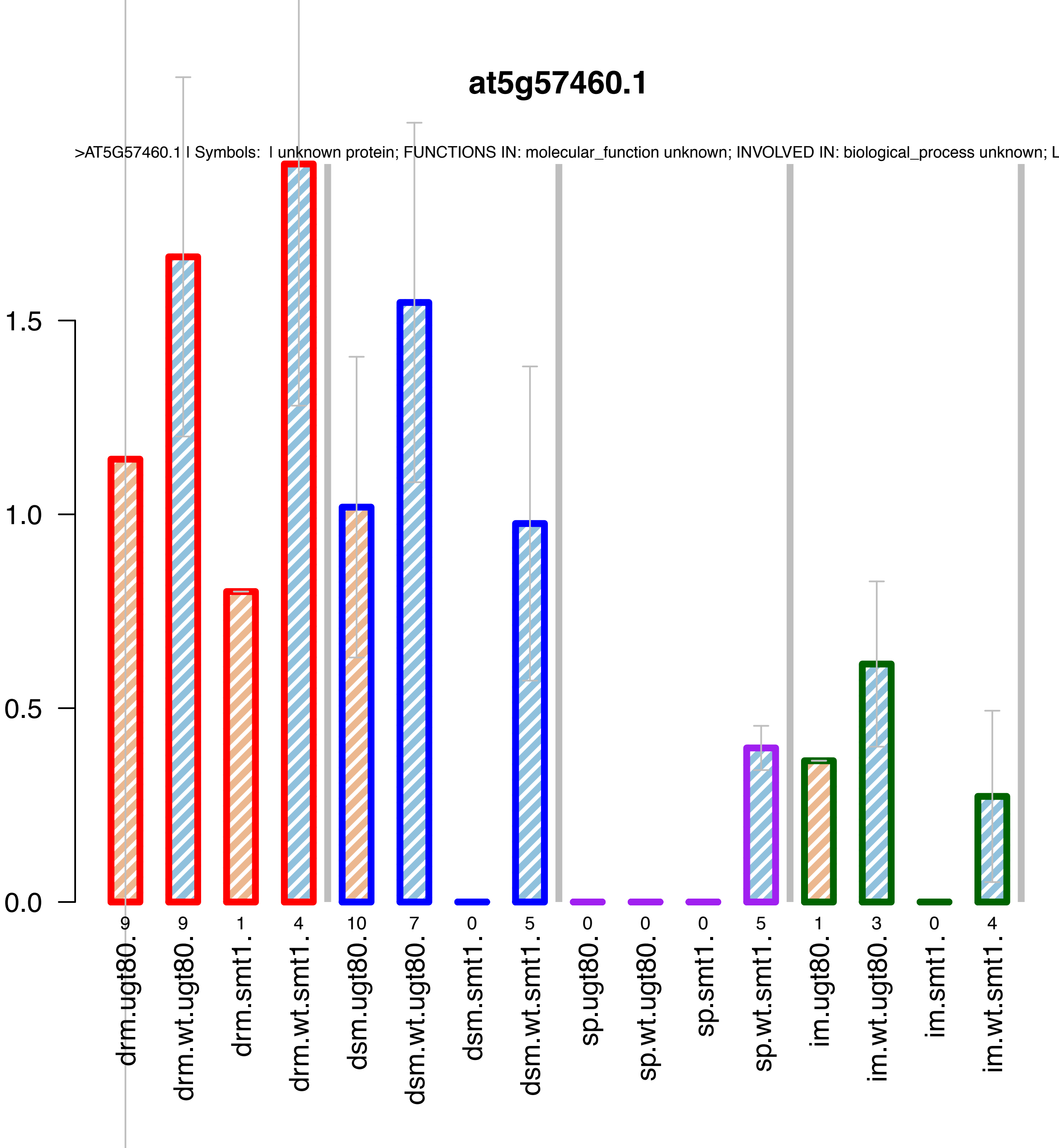

at5g57460.1
>AT5G57460.1 | Symbols: | unknown protein; FUNCTIONS IN: molecular_function unknown; INVOLVED IN: biological_process unknown; L
1.5
1.0
0.5
0.0
9
9
1
4
10
7
0
5
0
0
0
5
1
3
0
4
drm.ugt80.
drm.wt.ugt80.
drm.smt1.
drm.wt.smt1.
dsm.ugt80.
dsm.wt.ugt80.
dsm.smt1.
dsm.wt.smt1.
sp.ugt80.
sp.wt.ugt80.
sp.smt1.
sp.wt.smt1.
im.ugt80.
im.wt.ugt80.
im.smt1.
im.wt.smt1.

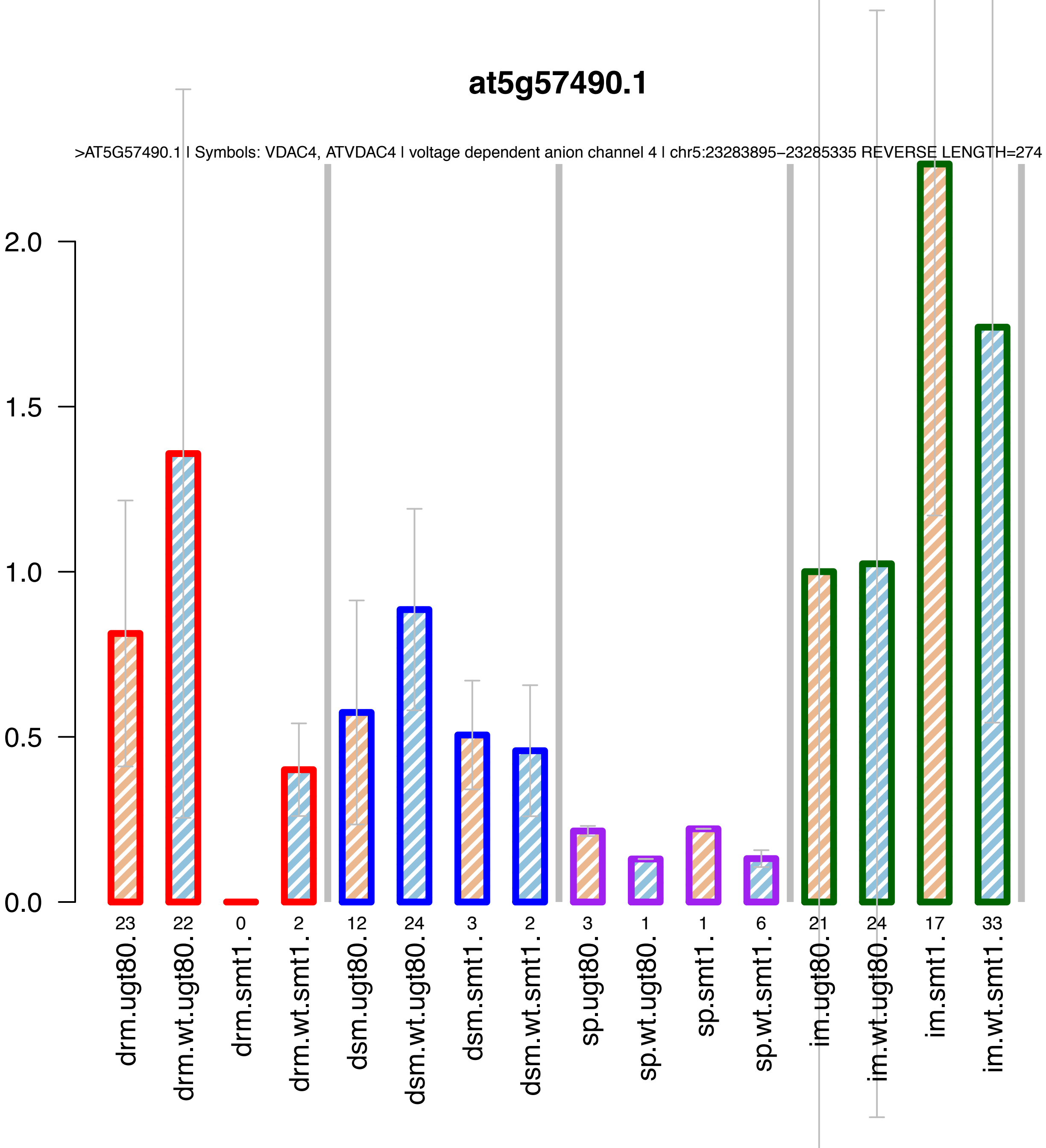

at5g57490.1
>AT5G57490.1 | Symbols: VDAC4, ATVDAC4 | voltage dependent anion channel 4 | chr5:23283895-23285335 REVERSE LENGTH=274
0.0
0.5
1.0
1.5
2.0
23
22
0
2
12
24
3
2
3
1
1
6
21
24
17
33
drm.ugt80.
drm.wt.ugt80.
drm.smt1.
drm.wt.smt1.
dsm.ugt80.
dsm.wt.ugt80.
dsm.smt1.
dsm.wt.smt1.
sp.ugt80.
sp.wt.ugt80.
sp.smt1.
sp.wt.smt1.
im.ugt80.
im.wt.ugt80.
im.smt1.
im.wt.smt1.

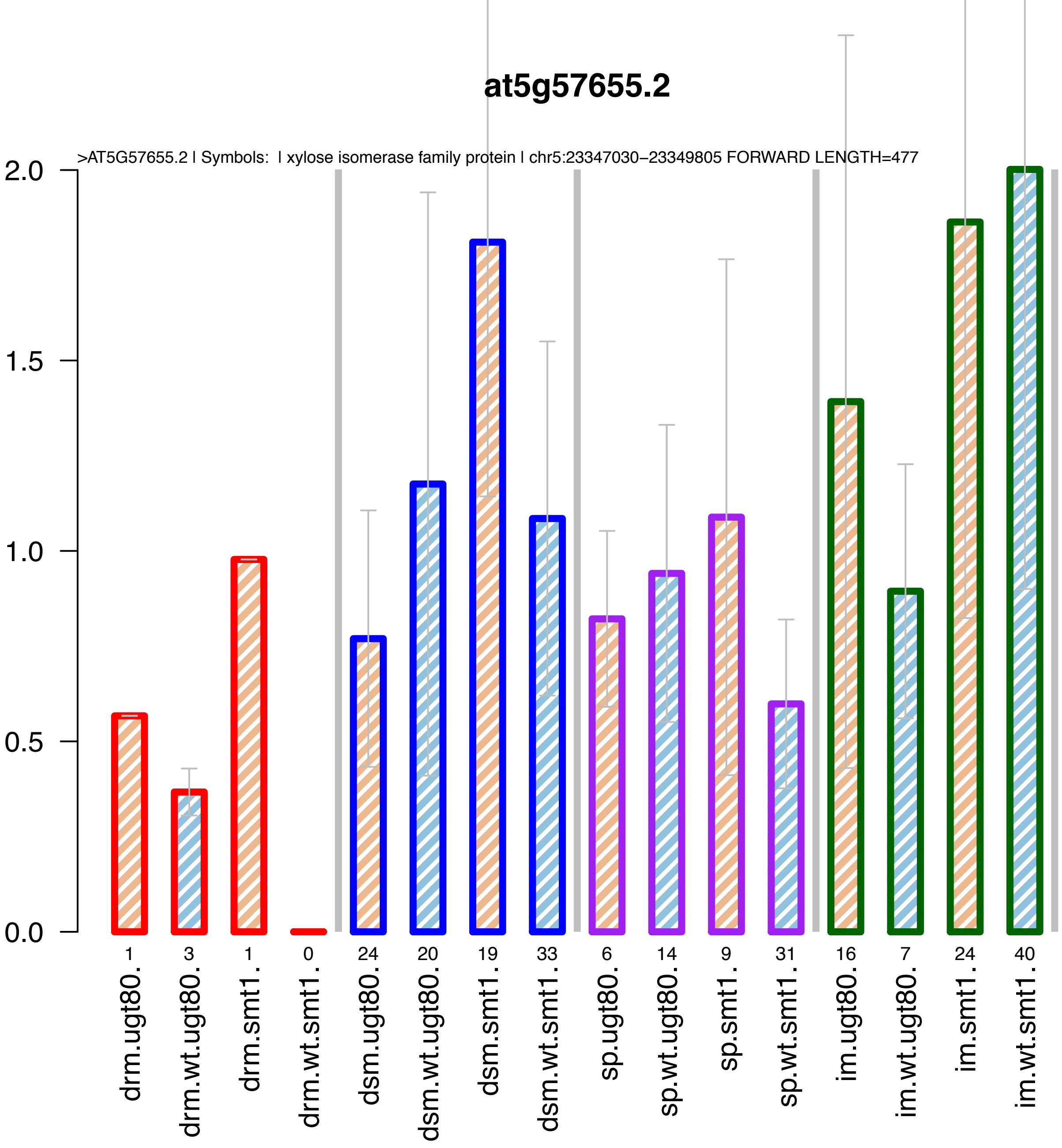

at5g57655.2
>AT5G57655.2 | Symbols: | xylose isomerase family protein | chr5:23347030–23349805 FORWARD LENGTH=477
2.0
1.5
1.0
0.5
0.0
1
3
1
0
24
20
19
33
6
14
9
31
16
7
24
40
drm.ugt80..
drm.wt.ugt80..
drm.smt1..
drm.wt.smt1..
dsm.ugt80..
dsm.wt.ugt80..
dsm.smt1..
dsm.wt.smt1..
sp.ugt80..
sp.wt.ugt80..
sp.smt1..
sp.wt.smt1..
im.ugt80..
im.wt.ugt80..
im.smt1..
im.wt.smt1..

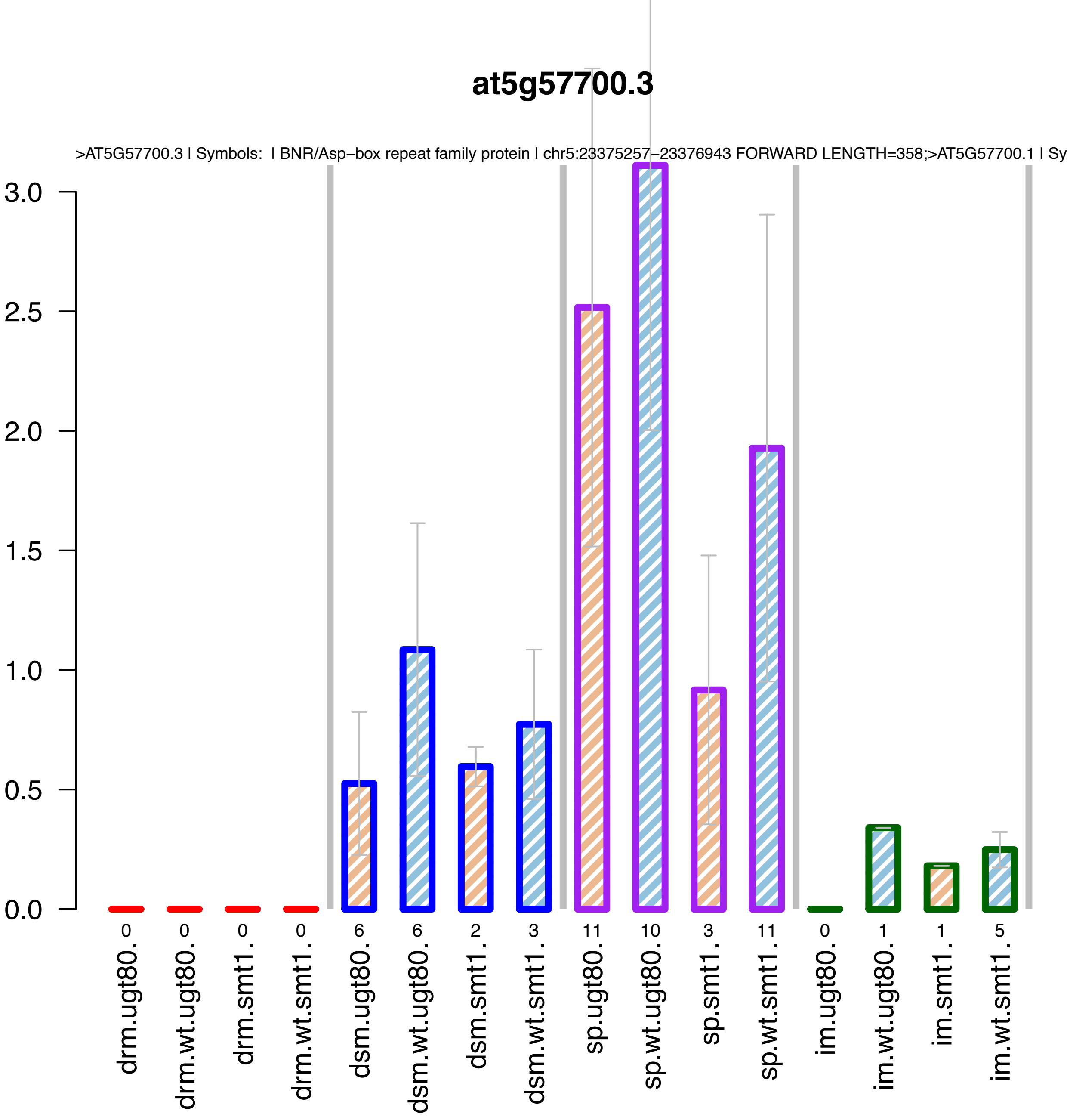

at5g57700.3
>AT5G57700.3 | Symbols: | BNR/Asp−box repeat family protein | chr5:23375257−23376943 FORWARD LENGTH=358;>AT5G57700.1 | Sy
3.0
2.5
2.0
1.5
1.0
0.5
0.0
0 0 0 0 6 6 2 3 11 10 3 11 0 1 1 5
drm.ugt80.
drm.wt.ugt80.
drm.smt1.
drm.wt.smt1.
dsm.ugt80.
dsm.wt.ugt80.
dsm.smt1.
dsm.wt.smt1.
sp.ugt80.
sp.wt.ugt80.
sp.smt1.
sp.wt.smt1.
im.ugt80.
im.wt.ugt80.
im.smt1.
im.wt.smt1.

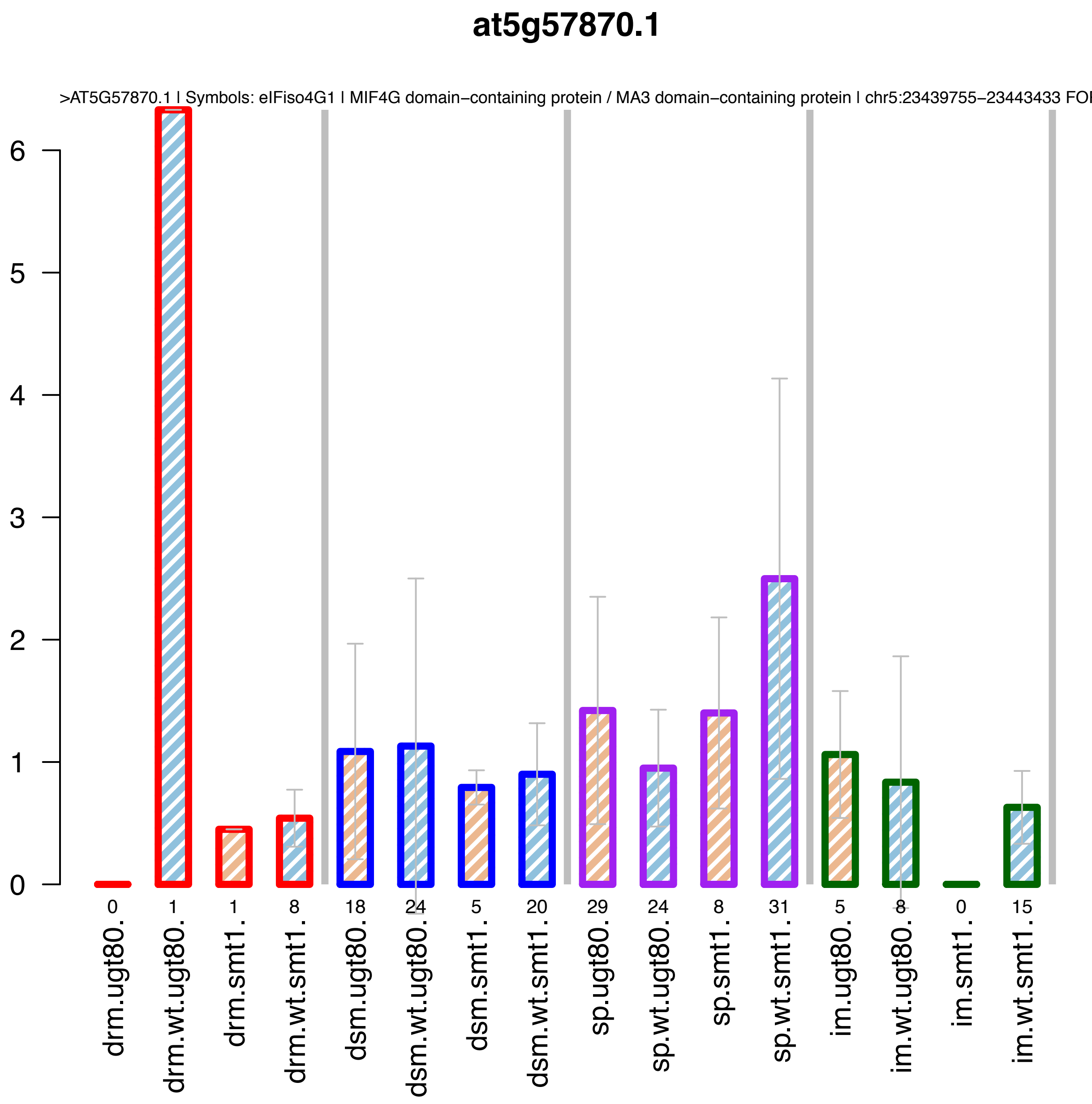
at5g57870.1
>AT5G57870.1 | Symbols: eIFiso4G1 | MIF4G domain−containing protein / MA3 domain−containing protein | chr5:23439755−23443433 FO
0
1
2
3
4
5
6
0 1 1 8 18 24 5 20 29 24 8 31 5 8 0 15
drm.ugt80.
drm.wt.ugt80.
drm.smt1.
drm.wt.smt1.
dsm.ugt80.
dsm.wt.ugt80.
dsm.smt1.
dsm.wt.smt1.
sp.ugt80.
sp.wt.ugt80.
sp.smt1.
sp.wt.smt1.
im.ugt80.
im.wt.ugt80.
im.smt1.
im.wt.smt1.

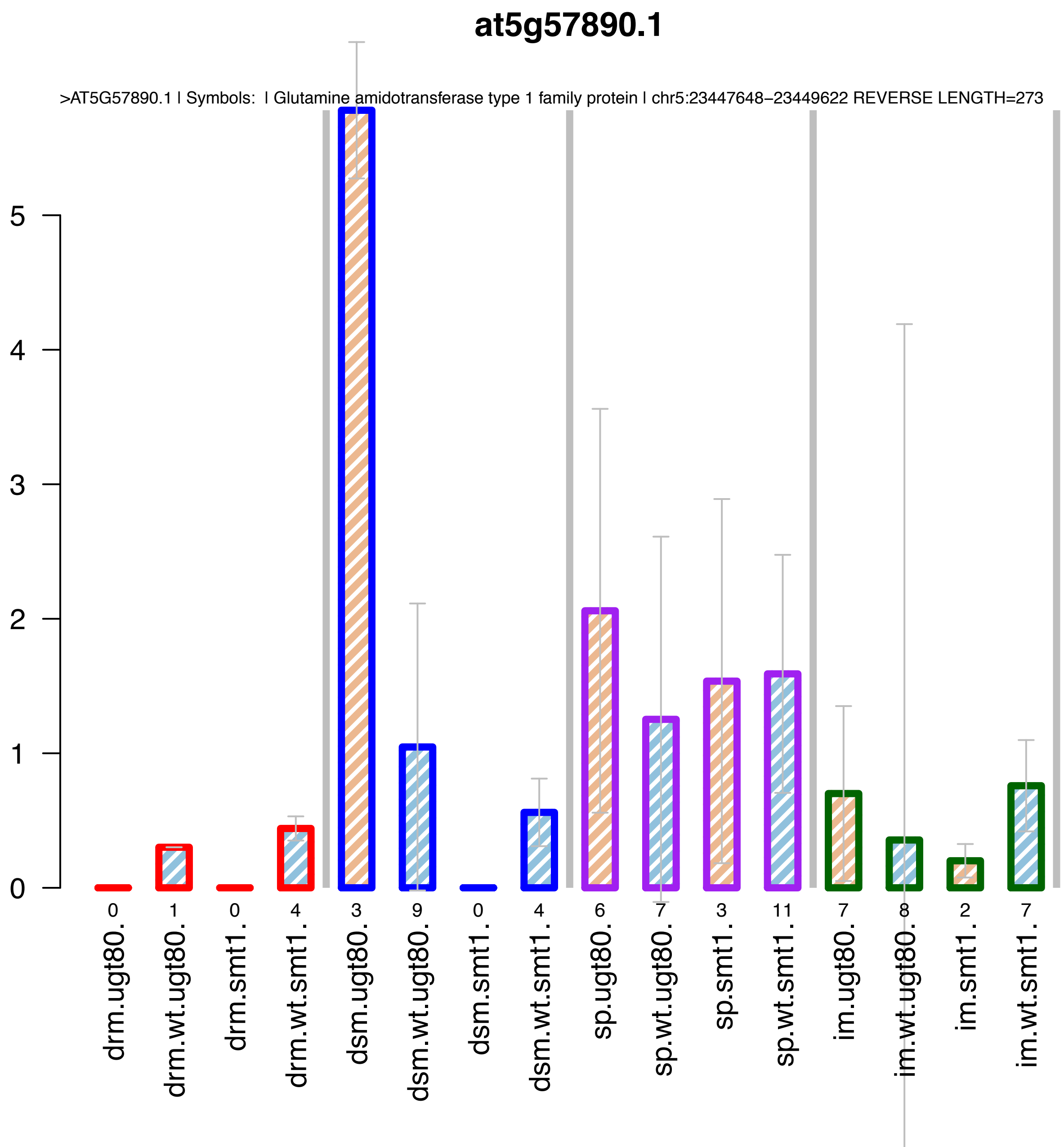

at5g57890.1
>AT5G57890.1 | Symbols: | Glutamine amidotransferase type 1 family protein | chr5:23447648-23449622 REVERSE LENGTH=273
0
1
2
3
4
5
0 1 0 4 3 9 0 4 6 7 3 11 7 8 2 7
drm.ugt80.
drm.wt.ugt80.
drm.smt1.
drm.wt.smt1.
dsm.ugt80.
dsm.wt.ugt80.
dsm.smt1.
dsm.wt.smt1.
sp.ugt80.
sp.wt.ugt80.
sp.smt1.
sp.wt.smt1.
im.ugt80.
im.wt.ugt80.
im.smt1.
im.wt.smt1.

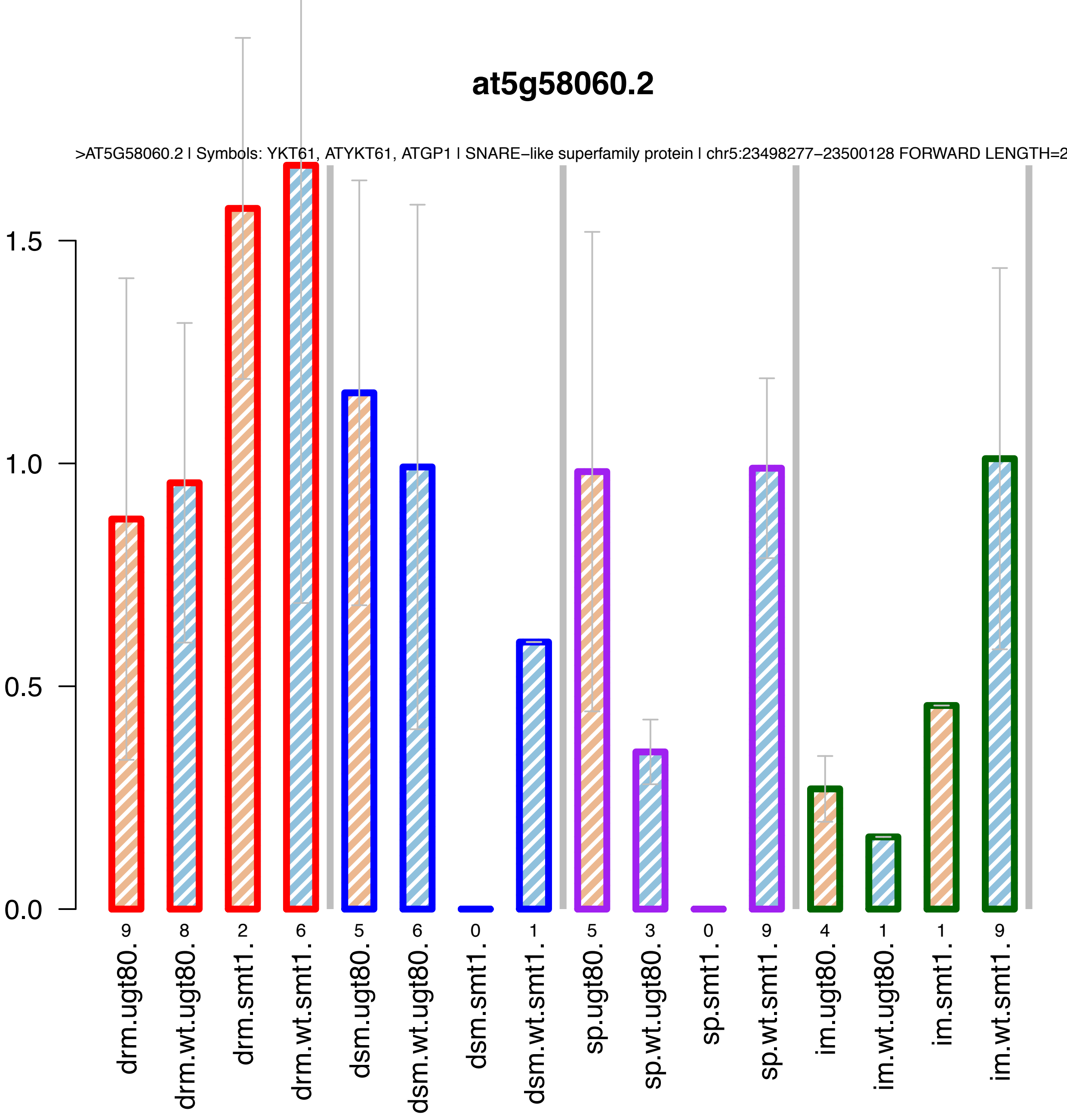

at5g58060.2
>AT5G58060.2 | Symbols: YKT61, ATYKT61, ATGP1 | SNARE−like superfamily protein | chr5:23498277−23500128 FORWARD LENGTH=2
0.0
0.5
1.0
1.5
9 8 2 6 5 6 0 1 5 3 0 9 4 1 1 9
drm.ugt80..
drm.wt.ugt80..
drm.smt1..
drm.wt.smt1..
dsm.ugt80..
dsm.wt.ugt80..
dsm.smt1..
dsm.wt.smt1..
sp.ugt80..
sp.wt.ugt80..
sp.smt1..
sp.wt.smt1..
im.ugt80..
im.wt.ugt80..
im.smt1..
im.wt.smt1..

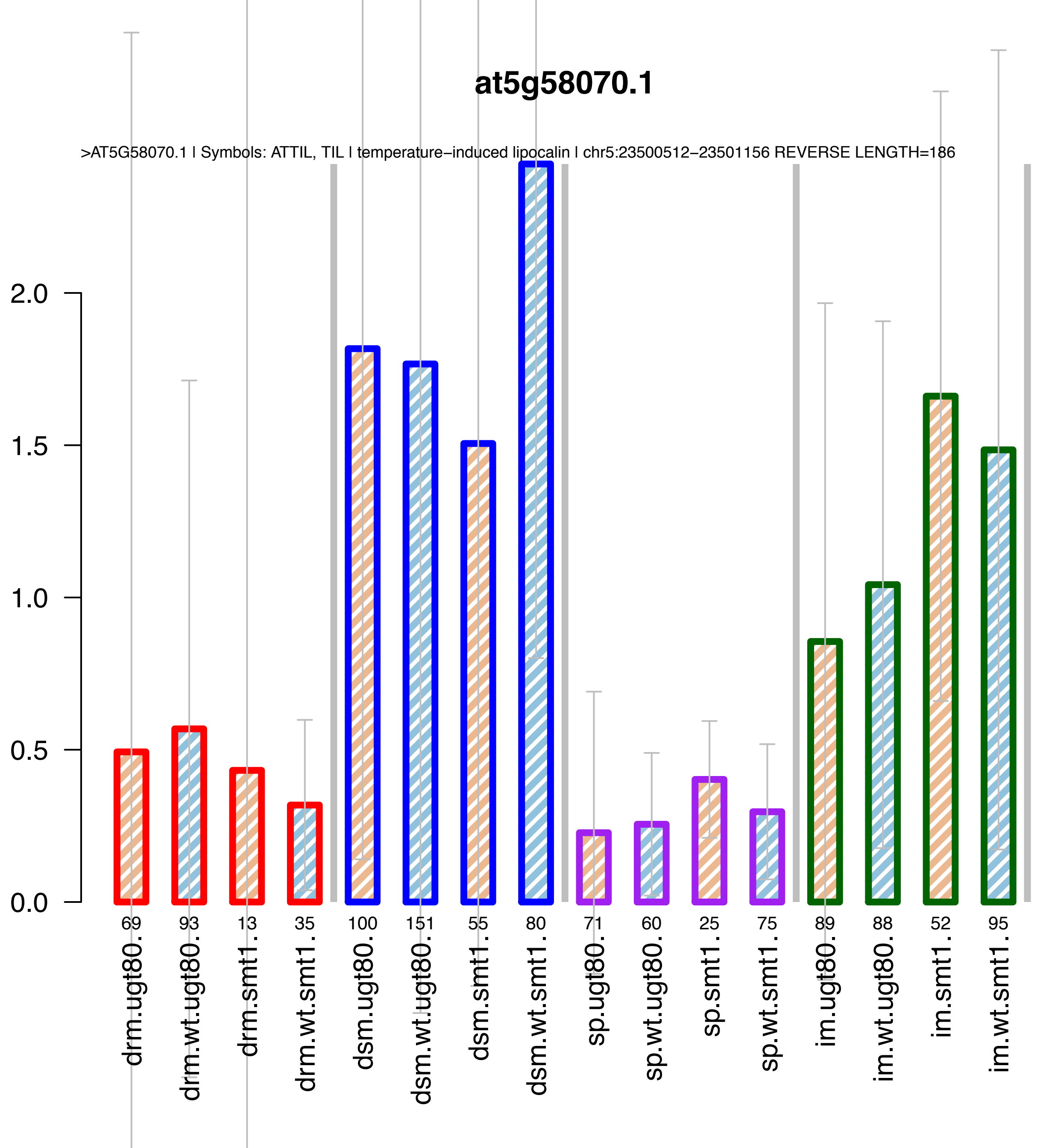

at5g58070.1
>AT5G58070.1 | Symbols: ATTIL, TIL | temperature−induced lipocalin | chr5:23500512−23501156 REVERSE LENGTH=186
0.0
0.5
1.0
1.5
2.0
69
93
13
35
100
151
55
80
71
60
25
75
89
88
52
95
drm.ugt80..
drm.wt.ugt80..
drm.smt1..
drm.wt.smt1..
dsm.ugt80..
dsm.wt.ugt80..
dsm.smt1..
dsm.wt.smt1..
sp.ugt80..
sp.wt.ugt80..
sp.smt1..
sp.wt.smt1..
im.ugt80..
im.wt.ugt80..
im.smt1..
im.wt.smt1..

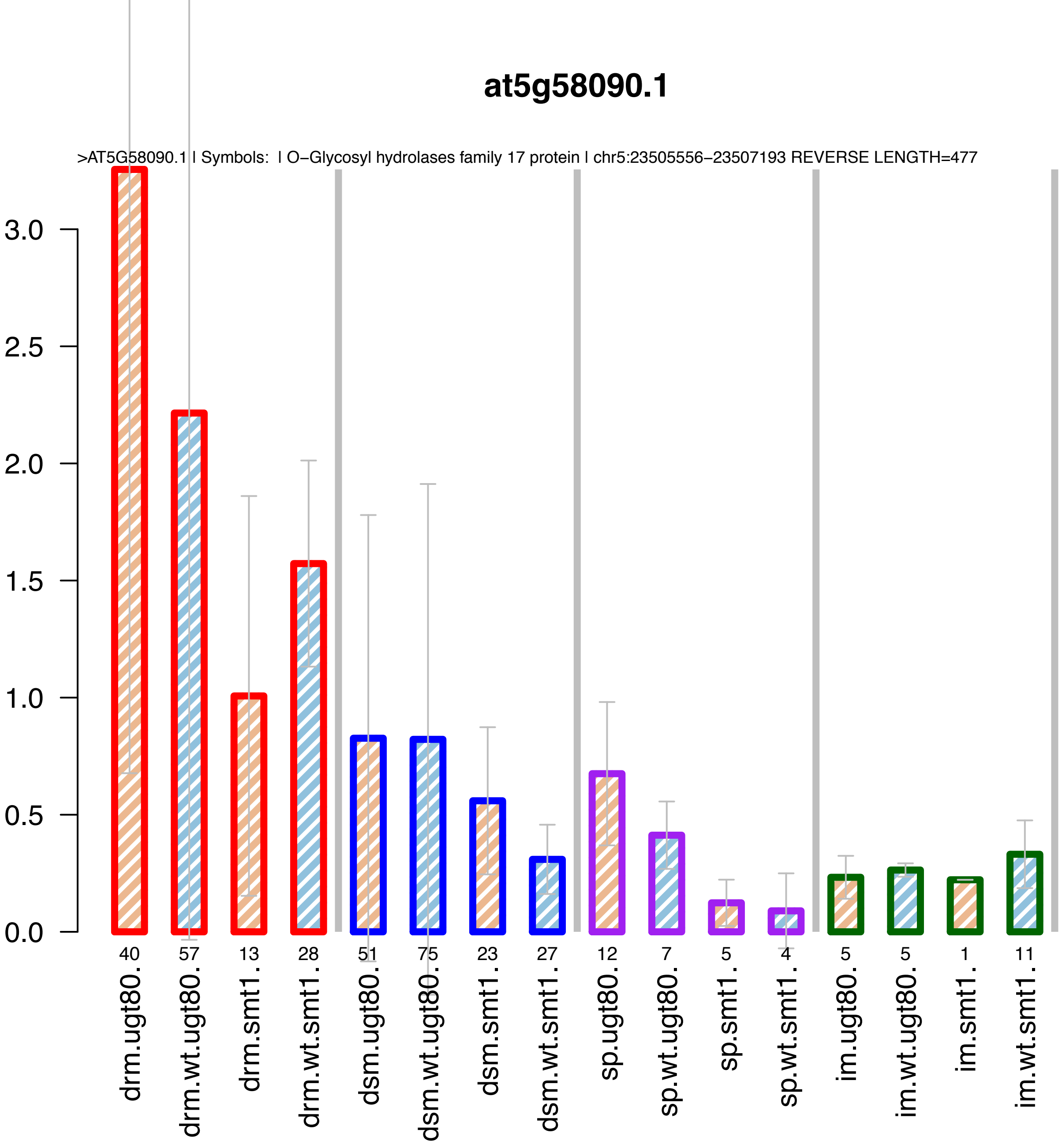

at5g58090.1
>AT5G58090.1 | Symbols: | O−Glycosyl hydrolases family 17 protein | chr5:23505556−23507193 REVERSE LENGTH=477
3.0
2.5
2.0
1.5
1.0
0.5
0.0
40
57
13
28
51
75
23
27
12
7
5
4
5
5
1
11
drm.ugt80..
drm.wt.ugt80..
drm.smt1..
drm.wt.smt1..
dsm.ugt80..
dsm.wt.ugt80..
dsm.smt1..
dsm.wt.smt1..
sp.ugt80..
sp.wt.ugt80..
sp.smt1..
sp.wt.smt1..
im.ugt80..
im.wt.ugt80..
im.smt1..
im.wt.smt1..

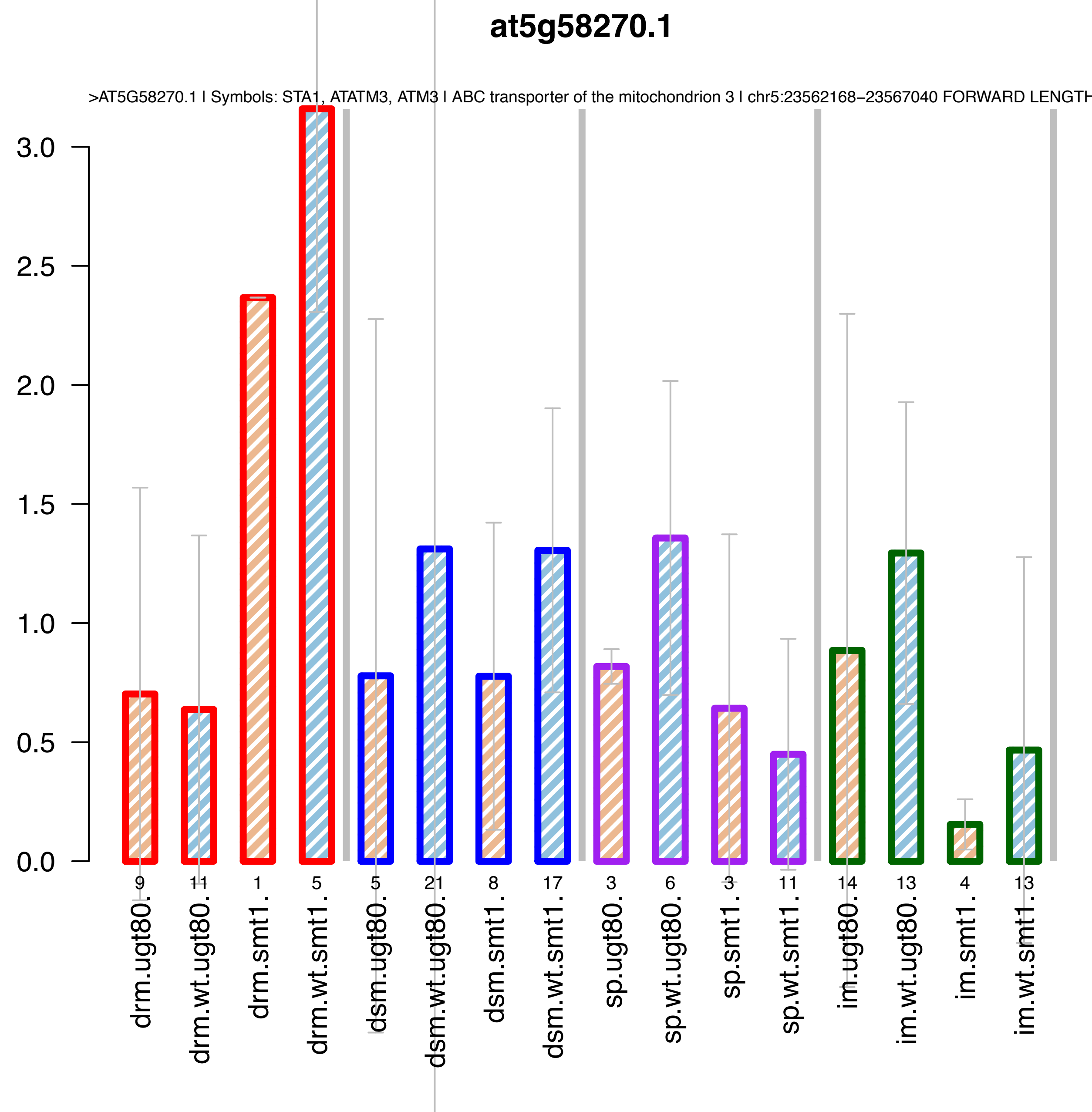

at5g58270.1
>AT5G58270.1 | Symbols: STA1, ATATM3, ATM3 | ABC transporter of the mitochondrion 3 | chr5:23562168-23567040 FORWARD LENGTH
3.0
2.5
2.0
1.5
1.0
0.5
0.0
9
11
1
5
5
21
8
17
3
6
3
11
14
13
4
13
drm.ugt80.
drm.wt.ugt80.
drm.smt1.
drm.wt.smt1.
dsm.ugt80.
dsm.wt.ugt80.
dsm.smt1.
dsm.wt.smt1.
sp.ugt80.
sp.wt.ugt80.
sp.smt1.
sp.wt.smt1.
im.ugt80.
im.wt.ugt80.
im.smt1.
im.wt.smt1.

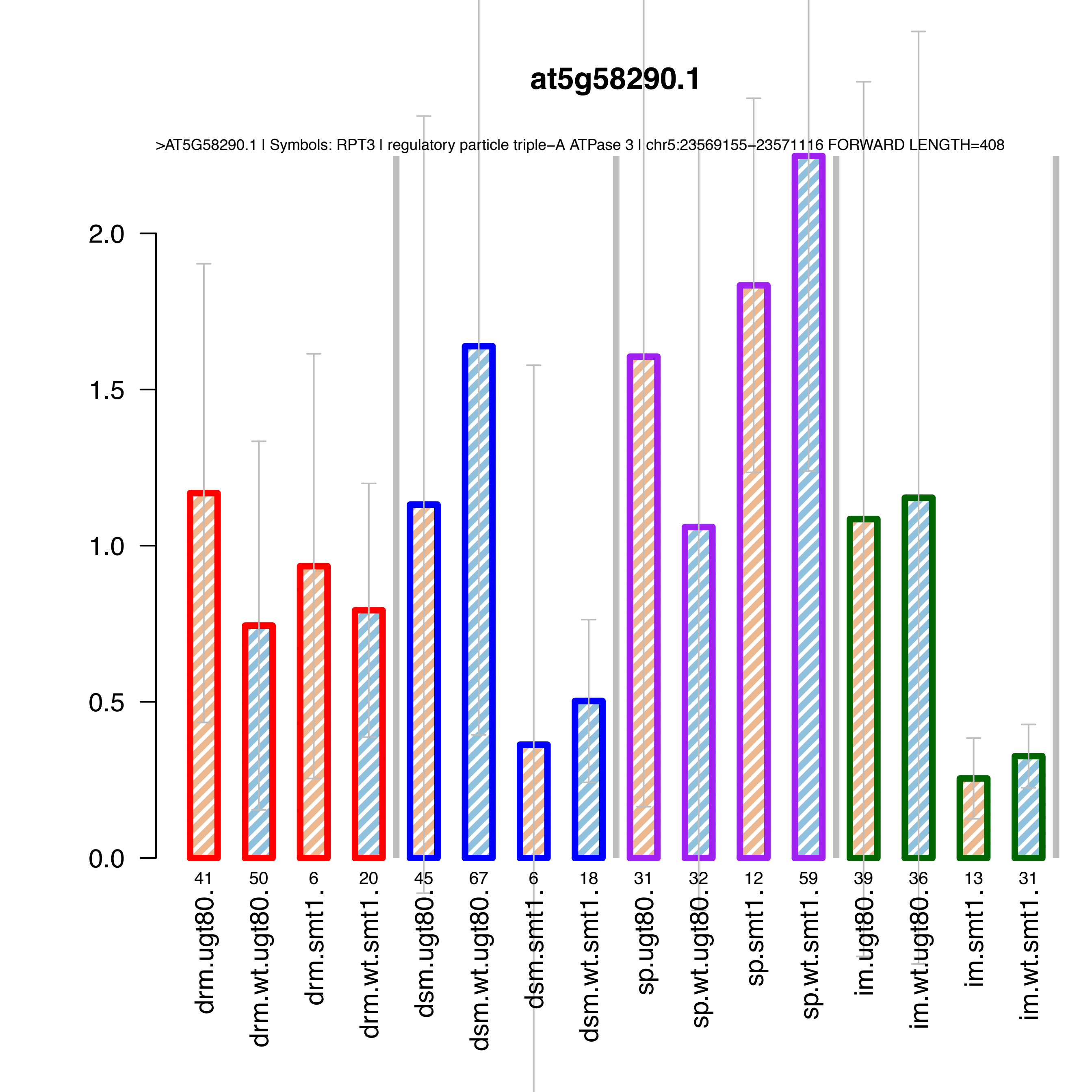

at5g58290.1
>AT5G58290.1 | Symbols: RPT3 | regulatory particle triple-A ATPase 3 | chr5:23569155-23571116 FORWARD LENGTH=408
2.0
1.5
1.0
0.5
0.0
41
50
6
20
45
67
6
18
31
32
12
59
39
36
13
31
drm.ugt80..
drm.wt.ugt80..
drm.smt1..
drm.wt.smt1..
dsm.ugt80..
dsm.wt.ugt80..
dsm.smt1..
dsm.wt.smt1..
sp.ugt80..
sp.wt.ugt80..
sp.smt1..
sp.wt.smt1..
im.ugt80..
im.wt.ugt80..
im.smt1..
im.wt.smt1..

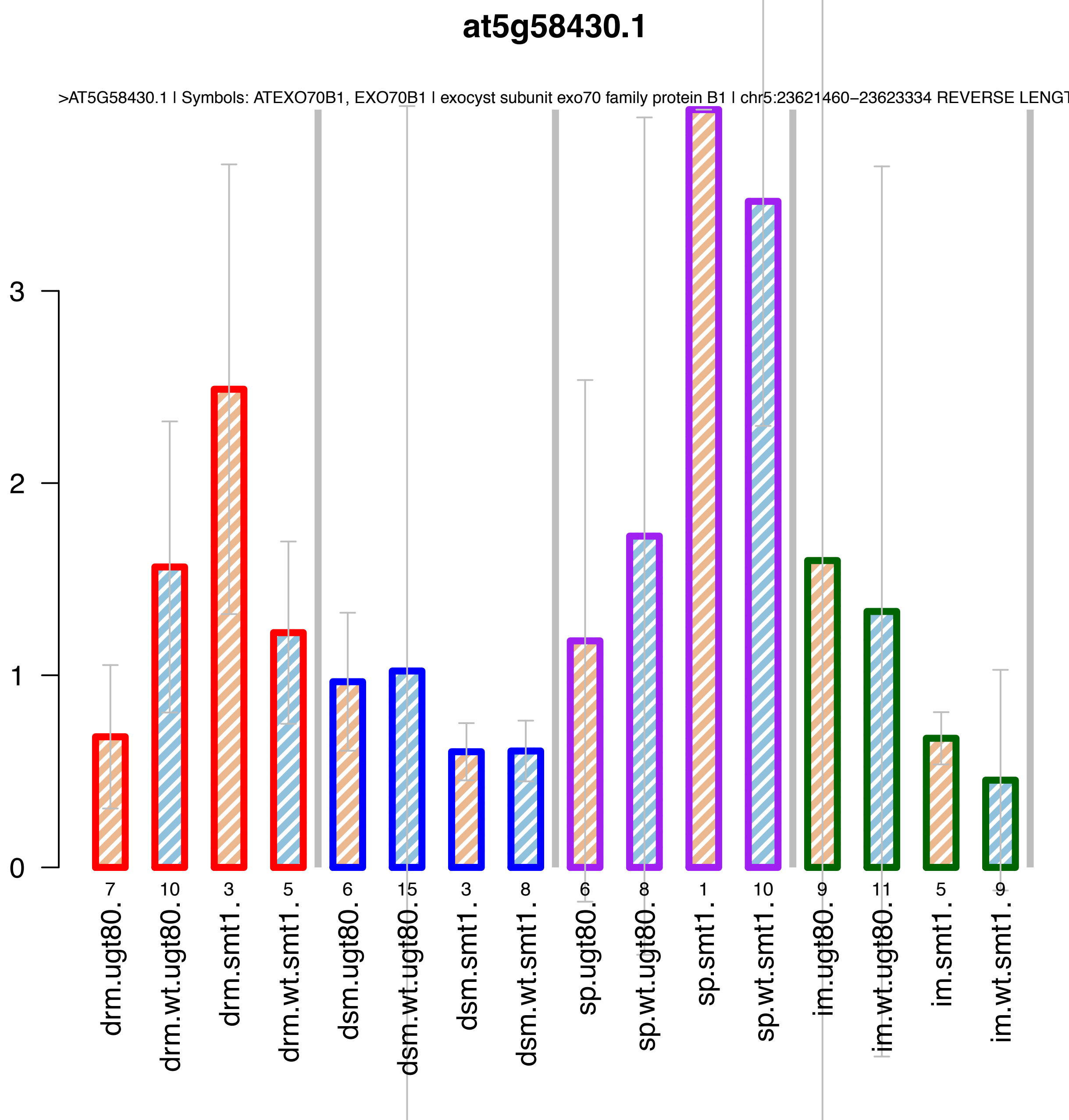

at5g58430.1
>AT5G58430.1 | Symbols: ATEXO70B1, EXO70B1 | exocyst subunit exo70 family protein B1 | chr5:23621460−23623334 REVERSE LENGT
0
1
2
3
7
10
3
5
6
15
3
8
6
8
1
10
9
11
5
9
drm.ugt80.
drm.wt.ugt80.
drm.smt1.
drm.wt.smt1.
dsm.ugt80.
dsm.wt.ugt80.
dsm.smt1.
dsm.wt.smt1.
sp.ugt80.
sp.wt.ugt80.
sp.smt1.
sp.wt.smt1.
im.ugt80.
im.wt.ugt80.
im.smt1.
im.wt.smt1.

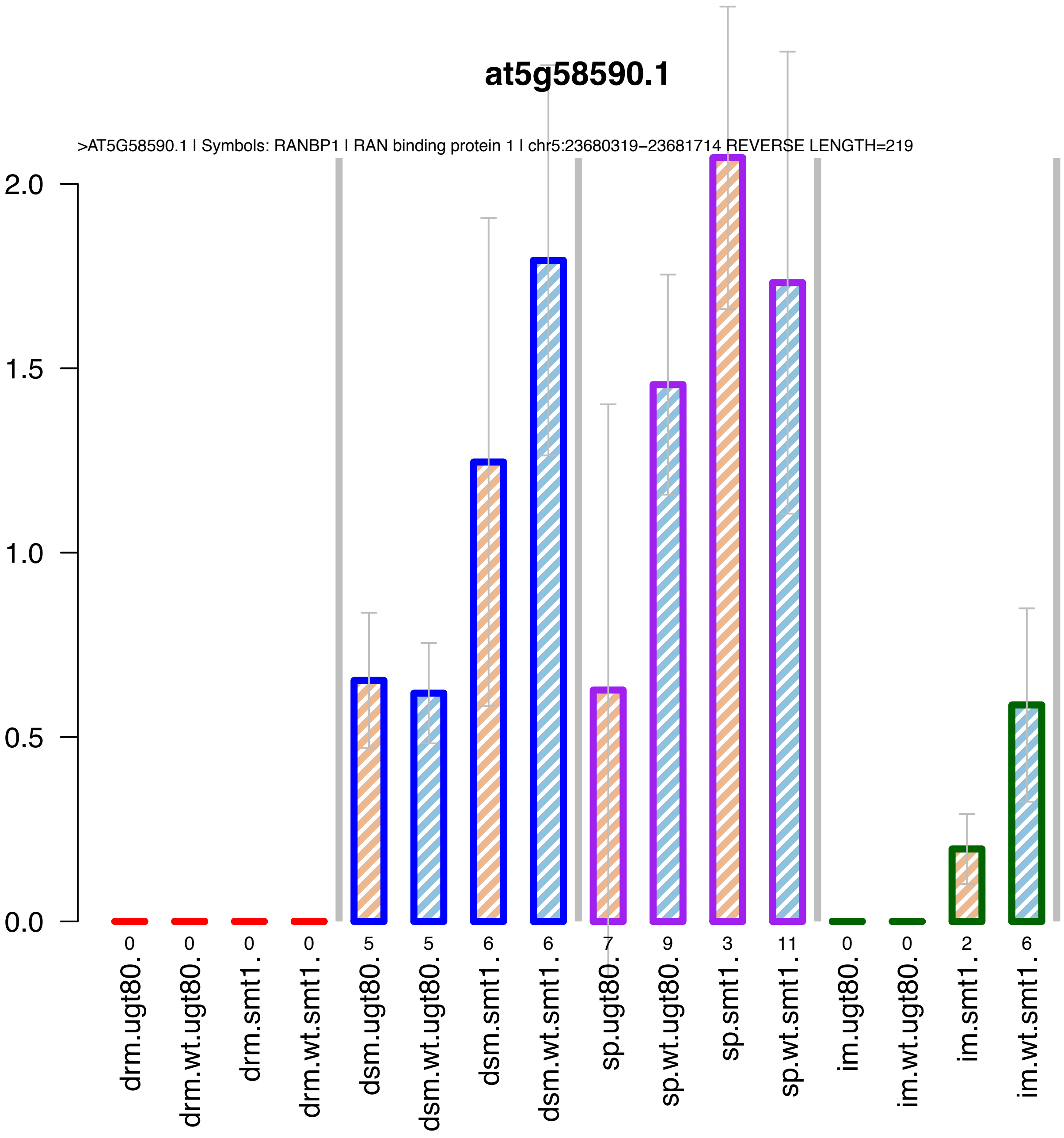

at5g58590.1
>AT5G58590.1 | Symbols: RANBP1 | RAN binding protein 1 | chr5:23680319-23681714 REVERSE LENGTH=219
2.0
1.5
1.0
0.5
0.0
0
0
0
0
5
5
6
6
7
9
3
11
0
0
2
6
drm.ugt80.
drm.wt.ugt80.
drm.smt1.
drm.wt.smt1.
dsm.ugt80.
dsm.wt.ugt80.
dsm.smt1.
dsm.wt.smt1.
sp.ugt80.
sp.wt.ugt80.
sp.smt1.
sp.wt.smt1.
im.ugt80.
im.wt.ugt80.
im.smt1.
im.wt.smt1.

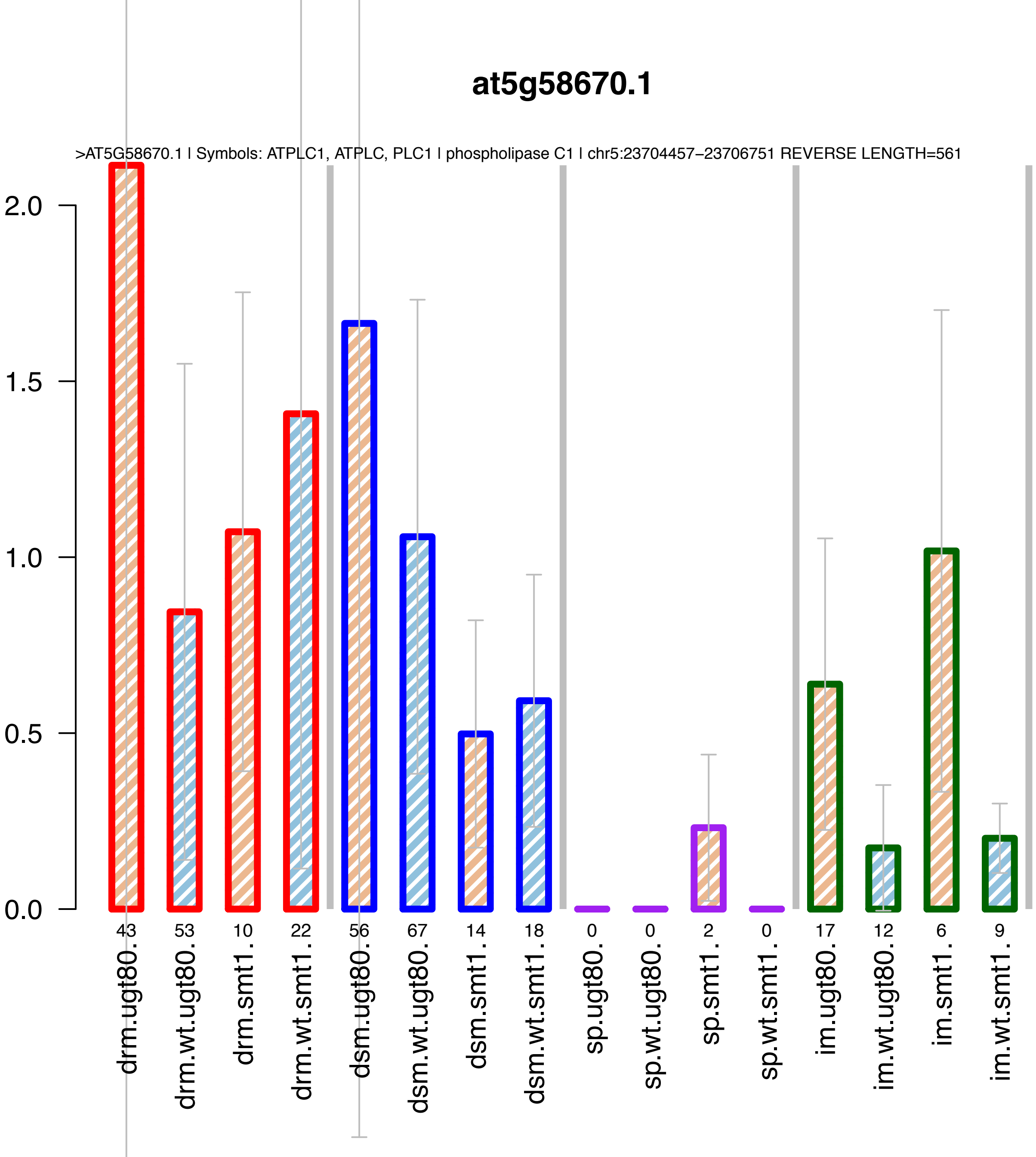
at5g58670.1
>AT5G58670.1 | Symbols: ATPLC1, ATPLC, PLC1 | phospholipase C1 | chr5:23704457−23706751 REVERSE LENGTH=561
2.0
1.5
1.0
0.5
0.0
43
53
10
22
56
67
14
18
0
0
2
0
17
12
6
9
drm.ugt80.
drm.wt.ugt80.
drm.smt1.
drm.wt.smt1.
dsm.ugt80.
dsm.wt.ugt80.
dsm.smt1.
dsm.wt.smt1.
sp.ugt80.
sp.wt.ugt80.
sp.smt1.
sp.wt.smt1.
im.ugt80.
im.wt.ugt80.
im.smt1.
im.wt.smt1.

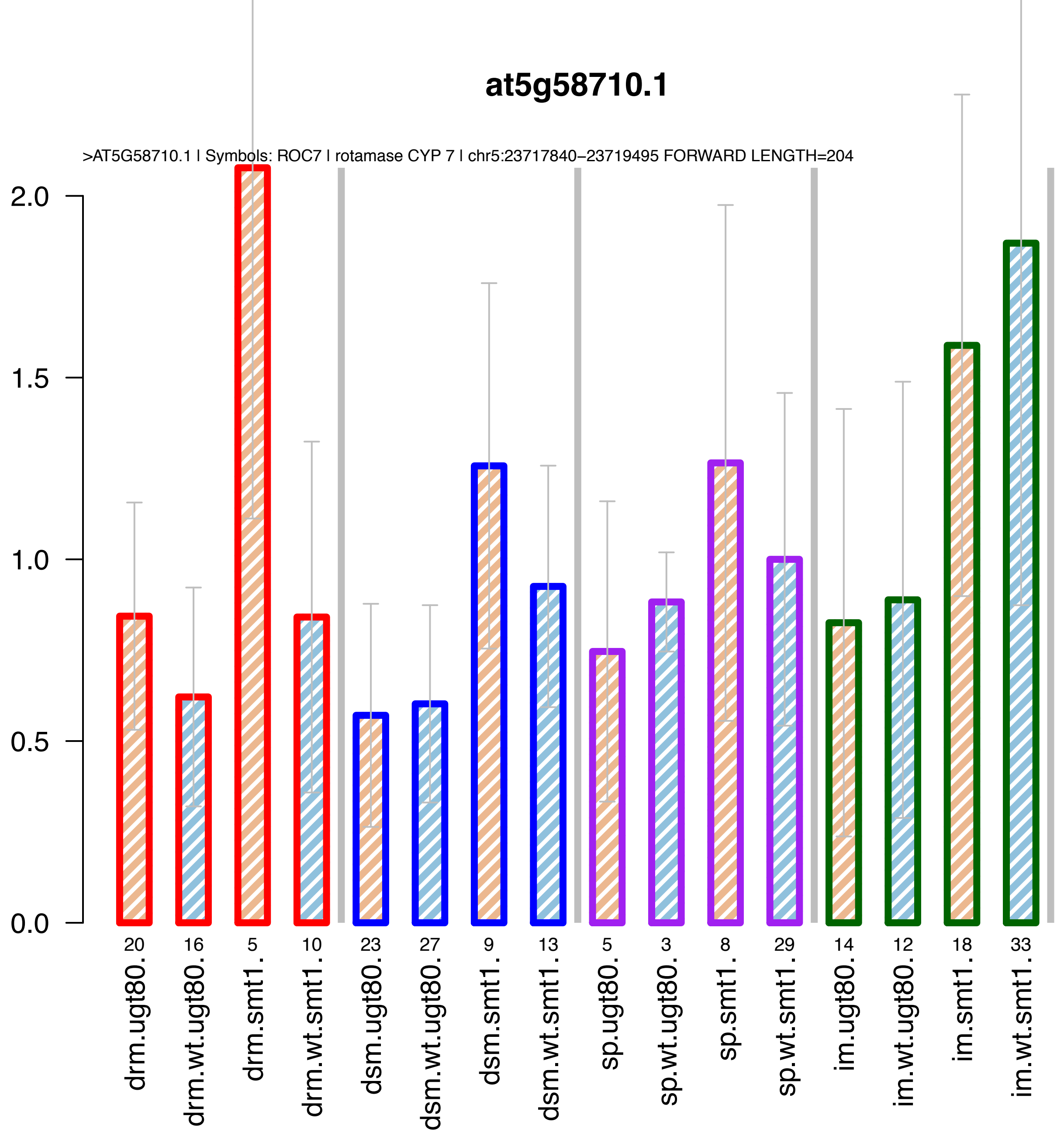

at5g58710.1
>AT5G58710.1 | Symbols: ROC7 | rotamase CYP 7 | chr5:23717840-23719495 FORWARD LENGTH=204
2.0
1.5
1.0
0.5
0.0
20
16
5
10
23
27
9
13
5
3
8
29
14
12
18
33
drm.ugt80..
drm.wt.ugt80..
drm.smt1..
drm.wt.smt1..
dsm.ugt80..
dsm.wt.ugt80..
dsm.smt1..
dsm.wt.smt1..
sp.ugt80..
sp.wt.ugt80..
sp.smt1..
sp.wt.smt1..
im.ugt80..
im.wt.ugt80..
im.smt1..
im.wt.smt1..

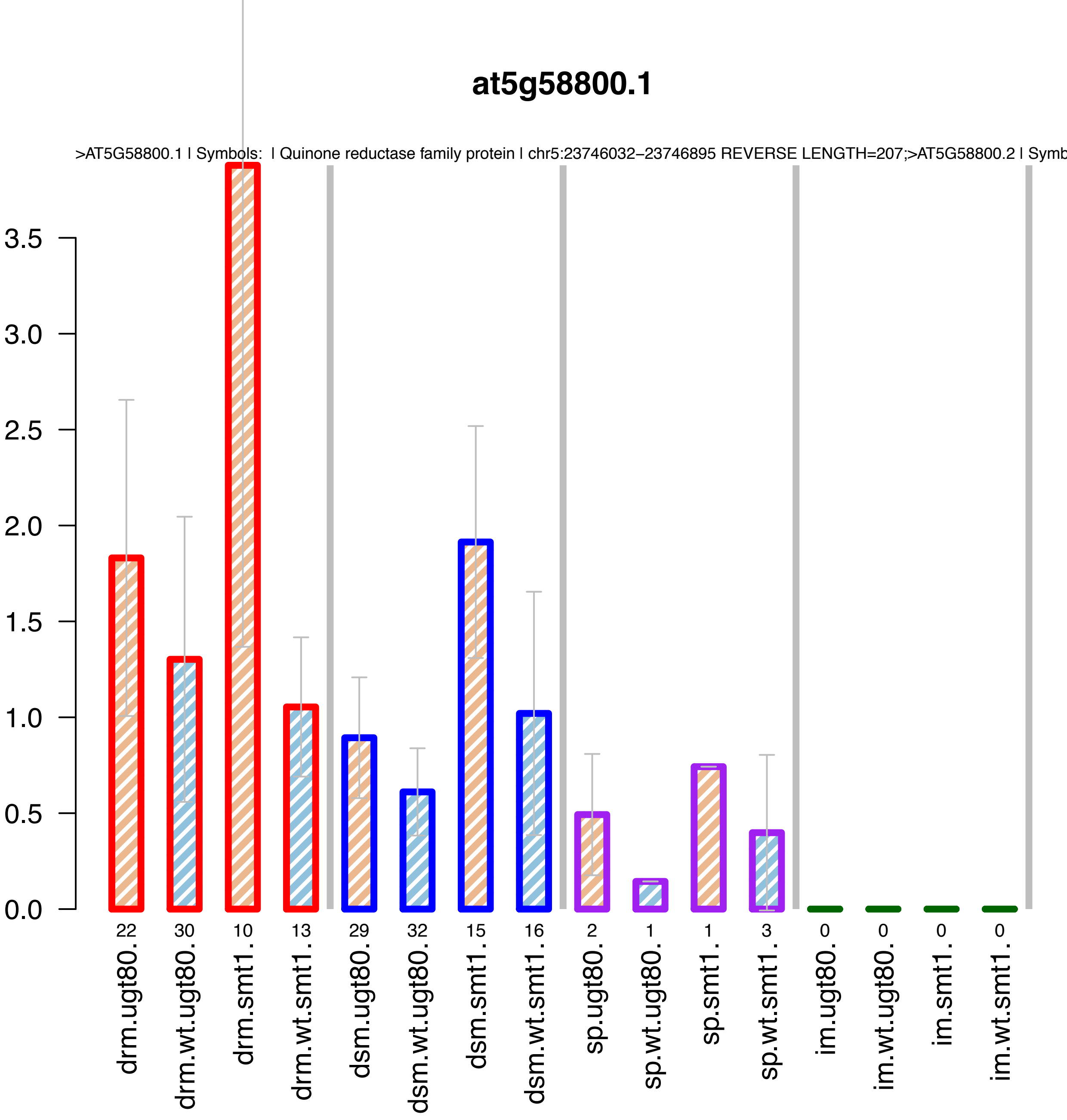
at5g58800.1
>AT5G58800.1 | Symbols: | Quinone reductase family protein | chr5:23746032–23746895 REVERSE LENGTH=207;>AT5G58800.2 | Symb
0.0
0.5
1.0
1.5
2.0
2.5
3.0
3.5
22
30
10
13
29
32
15
16
2
1
1
3
0
0
0
0
drm.ugt80.
drm.wt.ugt80.
drm.smt1.
drm.wt.smt1.
dsm.ugt80.
dsm.wt.ugt80.
dsm.smt1.
dsm.wt.smt1.
sp.ugt80.
sp.wt.ugt80.
sp.smt1.
sp.wt.smt1.
im.ugt80.
im.wt.ugt80.
im.smt1.
im.wt.smt1.

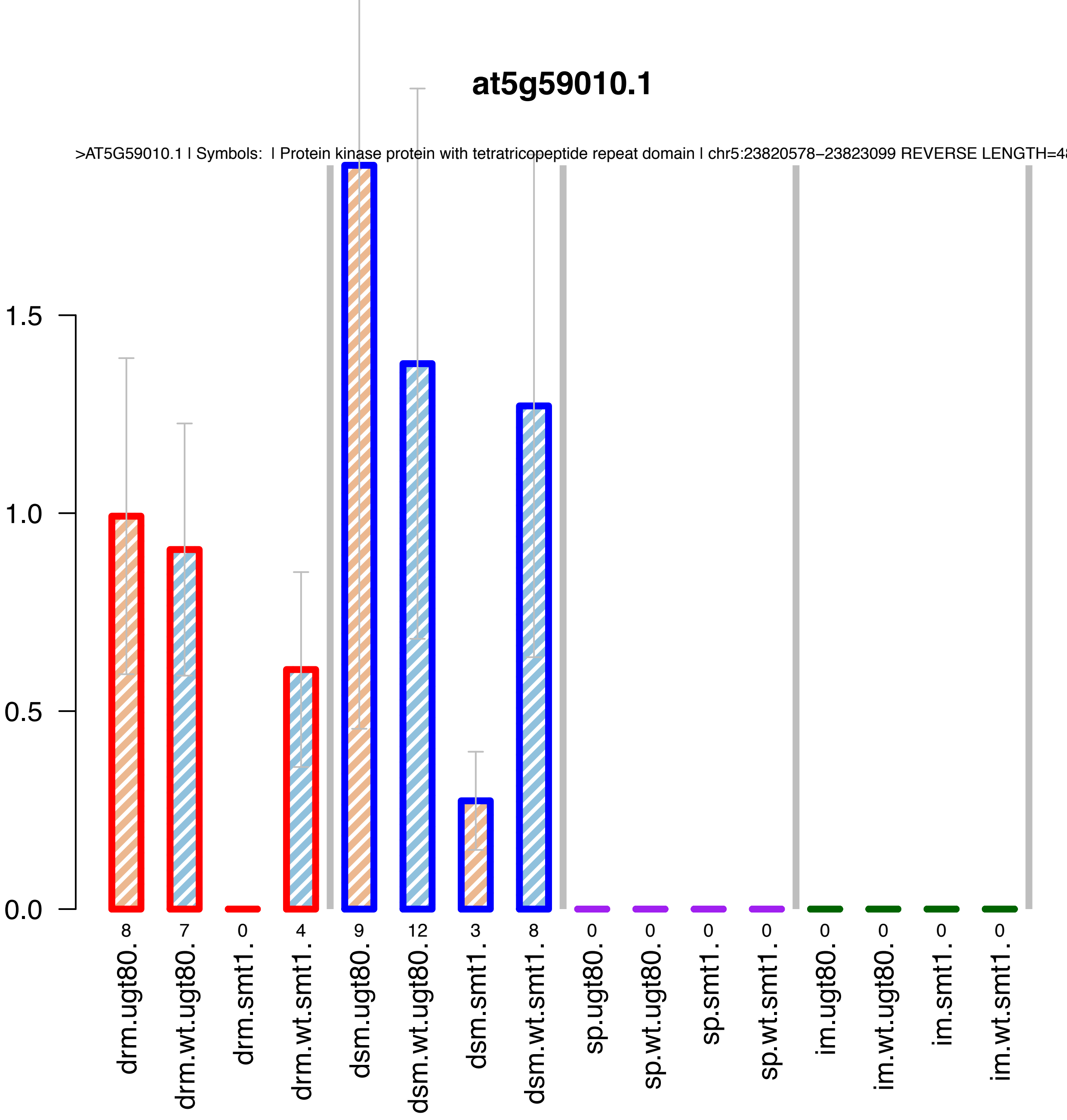
at5g59010.1
>AT5G59010.1 | Symbols: | Protein kinase protein with tetratricopeptide repeat domain | chr5:23820578-23823099 REVERSE LENGTH=4
1.5
1.0
0.5
0.0
8
7
0
4
9
12
3
8
0
0
0
0
0
0
0
0
drm.ugt80.
drm.wt.ugt80.
drm.smt1.
drm.wt.smt1.
dsm.ugt80.
dsm.wt.ugt80.
dsm.smt1.
dsm.wt.smt1.
sp.ugt80.
sp.wt.ugt80.
sp.smt1.
sp.wt.smt1.
im.ugt80.
im.wt.ugt80.
im.smt1.
im.wt.smt1.

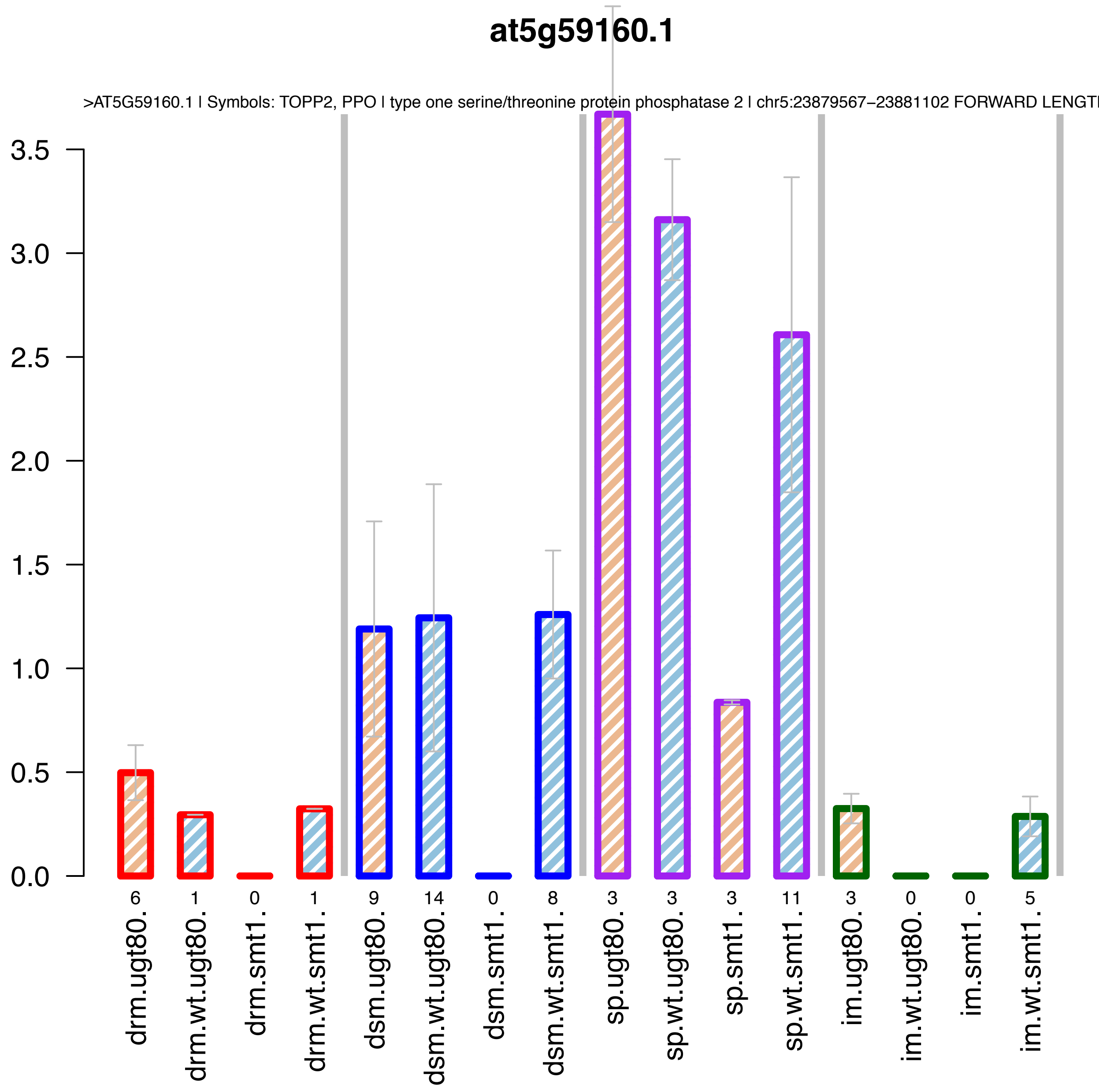

at5g59160.1
>AT5G59160.1 | Symbols: TOPP2, PPO | type one serine/threonine protein phosphatase 2 | chr5:23879567-23881102 FORWARD LENGT
3.5
3.0
2.5
2.0
1.5
1.0
0.5
0.0
6
1
0
1
9
14
0
8
3
3
3
11
3
0
0
5
drm.ugt80.
drm.wt.ugt80.
drm.smt1.
drm.wt.smt1.
dsm.ugt80.
dsm.wt.ugt80.
dsm.smt1.
dsm.wt.smt1.
sp.ugt80.
sp.wt.ugt80.
sp.smt1.
sp.wt.smt1.
im.ugt80.
im.wt.ugt80.
im.smt1.
im.wt.smt1.

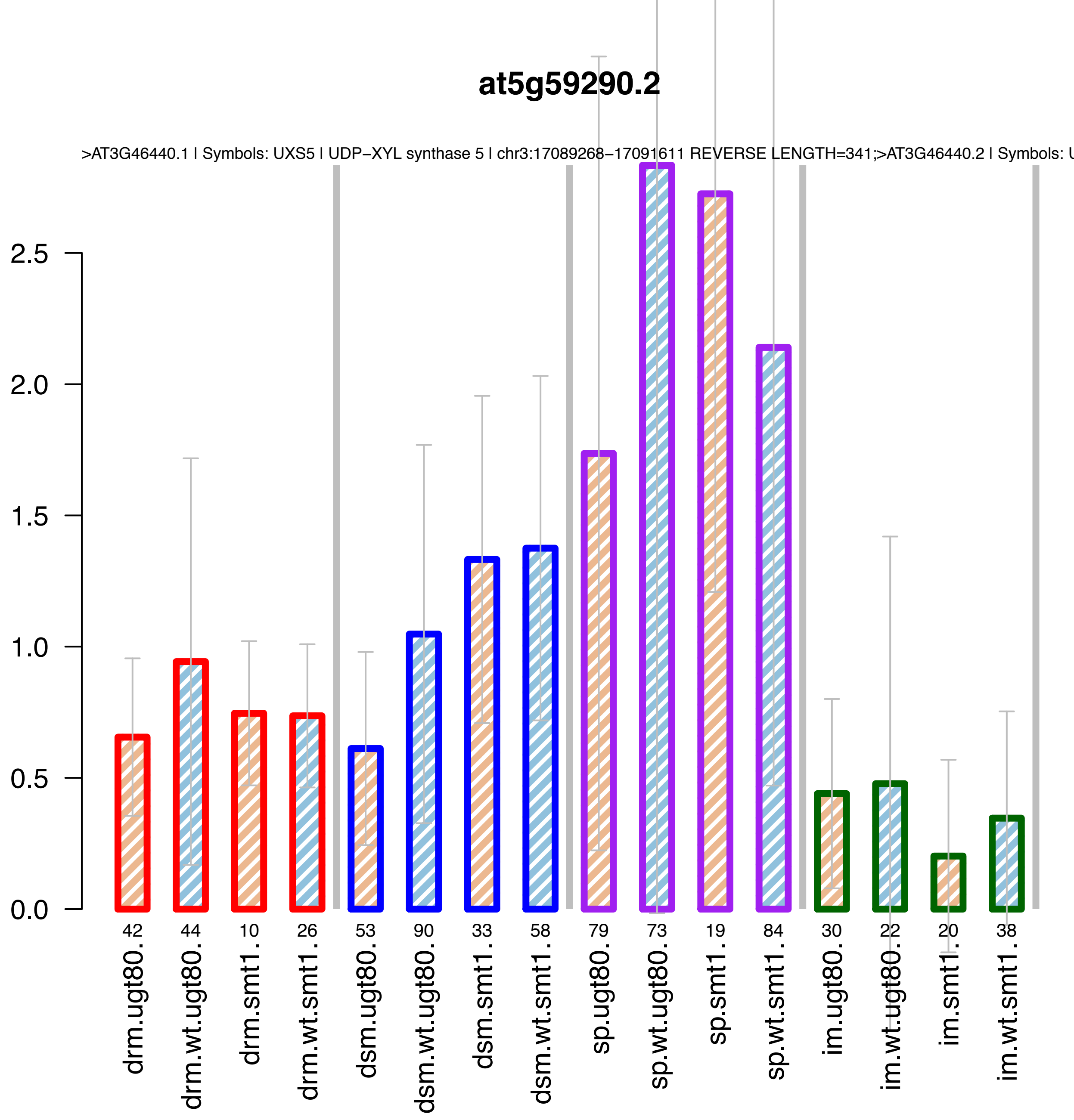

at5g59290.2
>AT3G46440.1 | Symbols: UXS5 | UDP−XYL synthase 5 | chr3:17089268−17091611 REVERSE LENGTH=341;>AT3G46440.2 | Symbols: U
0.0
0.5
1.0
1.5
2.0
2.5
42
44
10
26
53
90
33
58
79
73
19
84
30
22
20
38
drm.ugt80..
drm.wt.ugt80..
drm.smt1..
drm.wt.smt1..
dsm.ugt80..
dsm.wt.ugt80..
dsm.smt1..
dsm.wt.smt1..
sp.ugt80..
sp.wt.ugt80..
sp.smt1..
sp.wt.smt1..
im.ugt80..
im.wt.ugt80..
im.smt1..
im.wt.smt1..

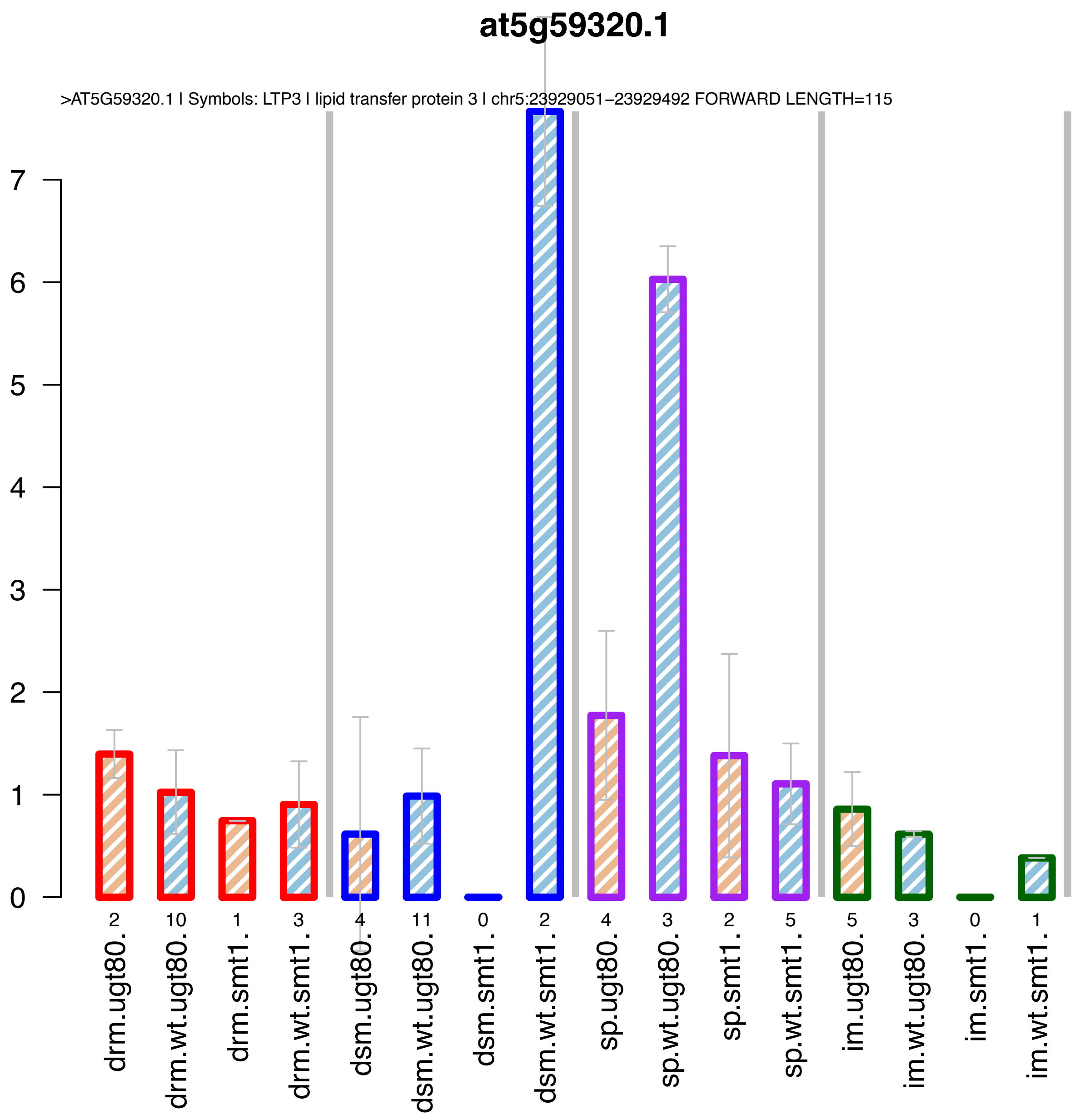
at5g59320.1
>AT5G59320.1 | Symbols: LTP3 | lipid transfer protein 3 | chr5:23929051-23929492 FORWARD LENGTH=115
0
1
2
3
4
5
6
7
2
10
1
3
4
11
0
2
4
3
2
5
5
3
0
1
drm.ugt80..
drm.wt.ugt80..
drm.smt1..
drm.wt.smt1..
dsm.ugt80..
dsm.wt.ugt80..
dsm.smt1..
dsm.wt.smt1..
sp.ugt80..
sp.wt.ugt80..
sp.smt1..
sp.wt.smt1..
im.ugt80..
im.wt.ugt80..
im.smt1..
im.wt.smt1..

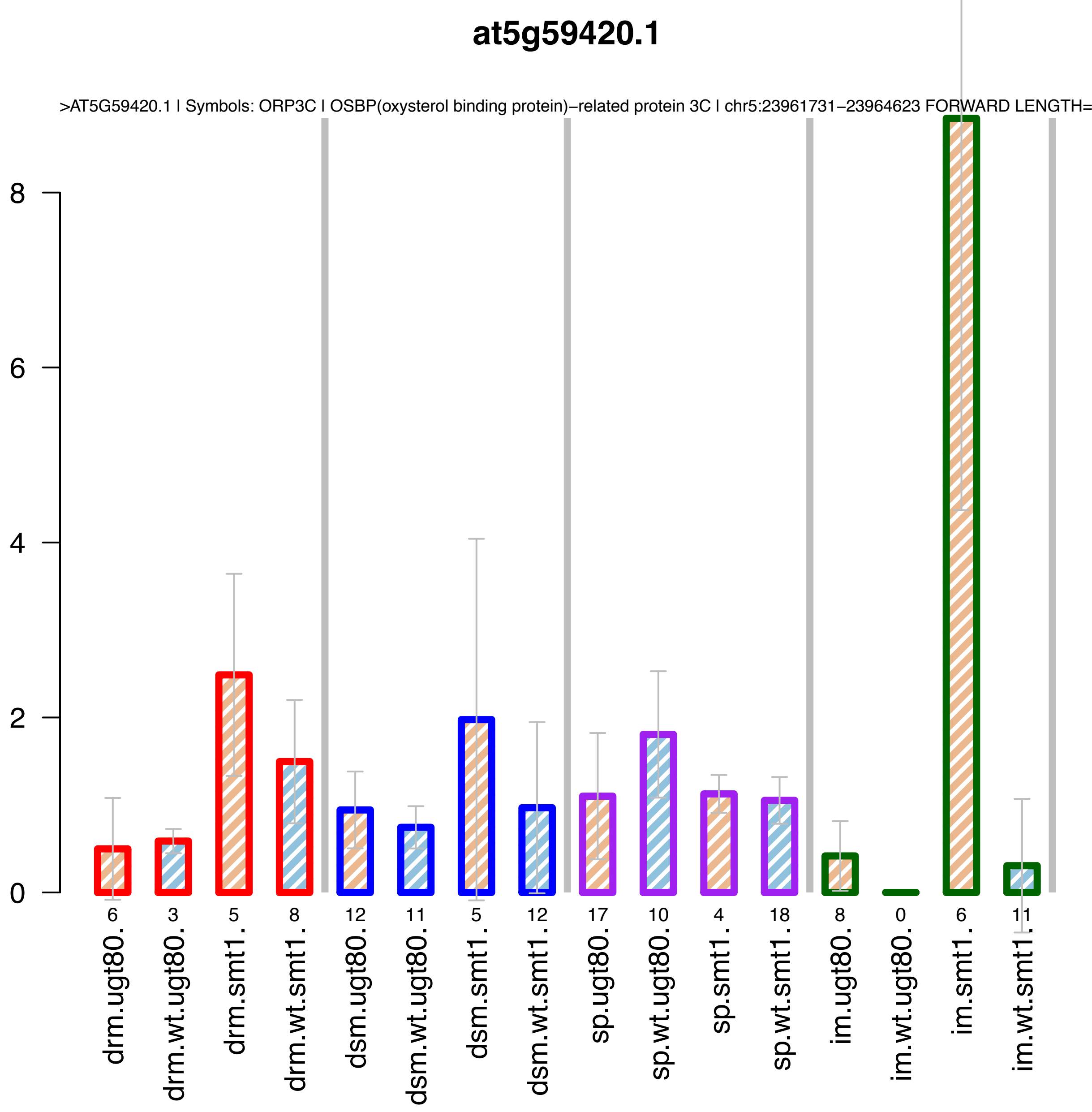

at5g59420.1
>AT5G59420.1 | Symbols: ORP3C | OSBP(oxysterol binding protein)−related protein 3C | chr5:23961731−23964623 FORWARD LENGTH=
0
2
4
6
8
6
3
5
8
12
11
5
12
17
10
4
18
8
0
6
11
drm.ugt80.
drm.wt.ugt80.
drm.smt1.
drm.wt.smt1.
dsm.ugt80.
dsm.wt.ugt80.
dsm.smt1.
dsm.wt.smt1.
sp.ugt80.
sp.wt.ugt80.
sp.smt1.
sp.wt.smt1.
im.ugt80.
im.wt.ugt80.
im.smt1.
im.wt.smt1.

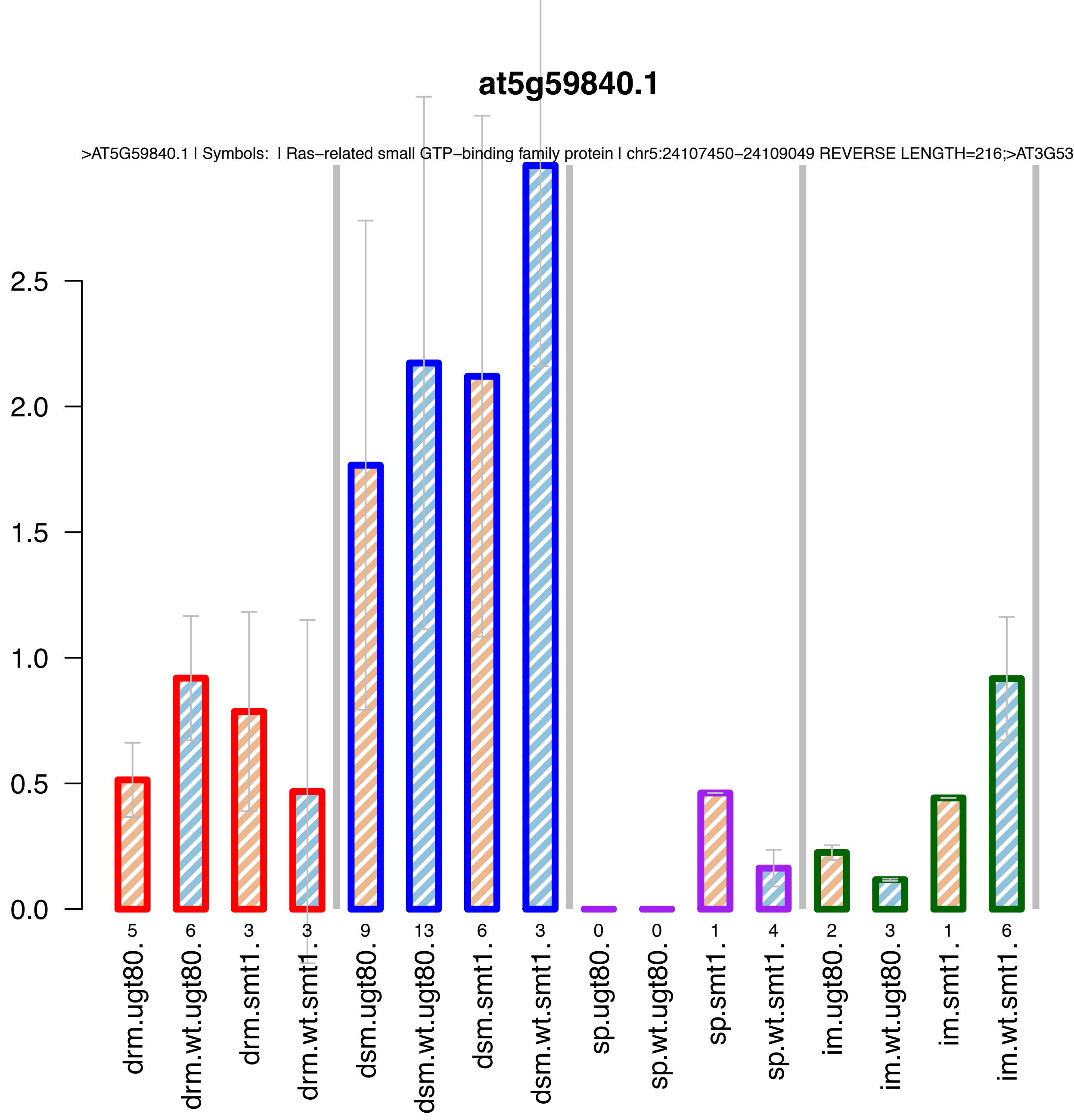

at5g59840.1
>AT5G59840.1 | Symbols: | Ras−related small GTP−binding family protein | chr5:24107450−24109049 REVERSE LENGTH=216;>AT3G53
0.0
0.5
1.0
1.5
2.0
2.5
5
6
3
3
9
13
6
3
0
0
1
4
2
3
1
6
drm.ugt80..
drm.wt.ugt80..
drm.smt1..
drm.wt.smt1..
dsm.ugt80..
dsm.wt.ugt80..
dsm.smt1..
dsm.wt.smt1..
sp.ugt80..
sp.wt.ugt80..
sp.smt1..
sp.wt.smt1..
im.ugt80..
im.wt.ugt80..
im.smt1..
im.wt.smt1..

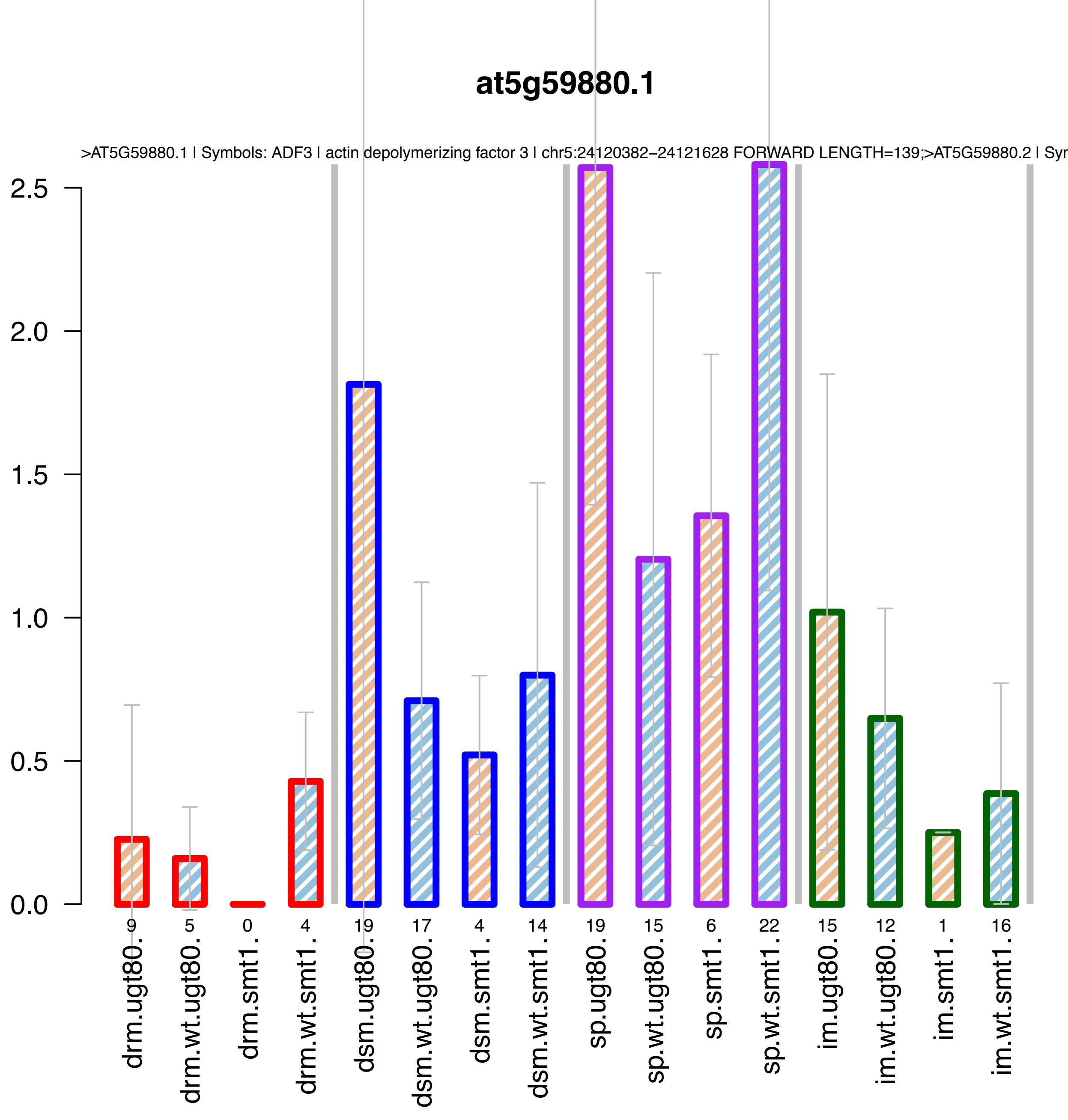

at5g59880.1
>AT5G59880.1 | Symbols: ADF3 | actin depolymerizing factor 3 | chr5:24120382-24121628 FORWARD LENGTH=139;>AT5G59880.2 | Syr
2.5
2.0
1.5
1.0
0.5
0.0
9
5
0
4
19
17
4
14
19
15
6
22
15
12
1
16
drm.ugt80..
drm.wt.ugt80..
drm.smt1..
drm.wt.smt1..
dsm.ugt80..
dsm.wt.ugt80..
dsm.smt1..
dsm.wt.smt1..
sp.ugt80..
sp.wt.ugt80..
sp.smt1..
sp.wt.smt1..
im.ugt80..
im.wt.ugt80..
im.smt1..
im.wt.smt1..

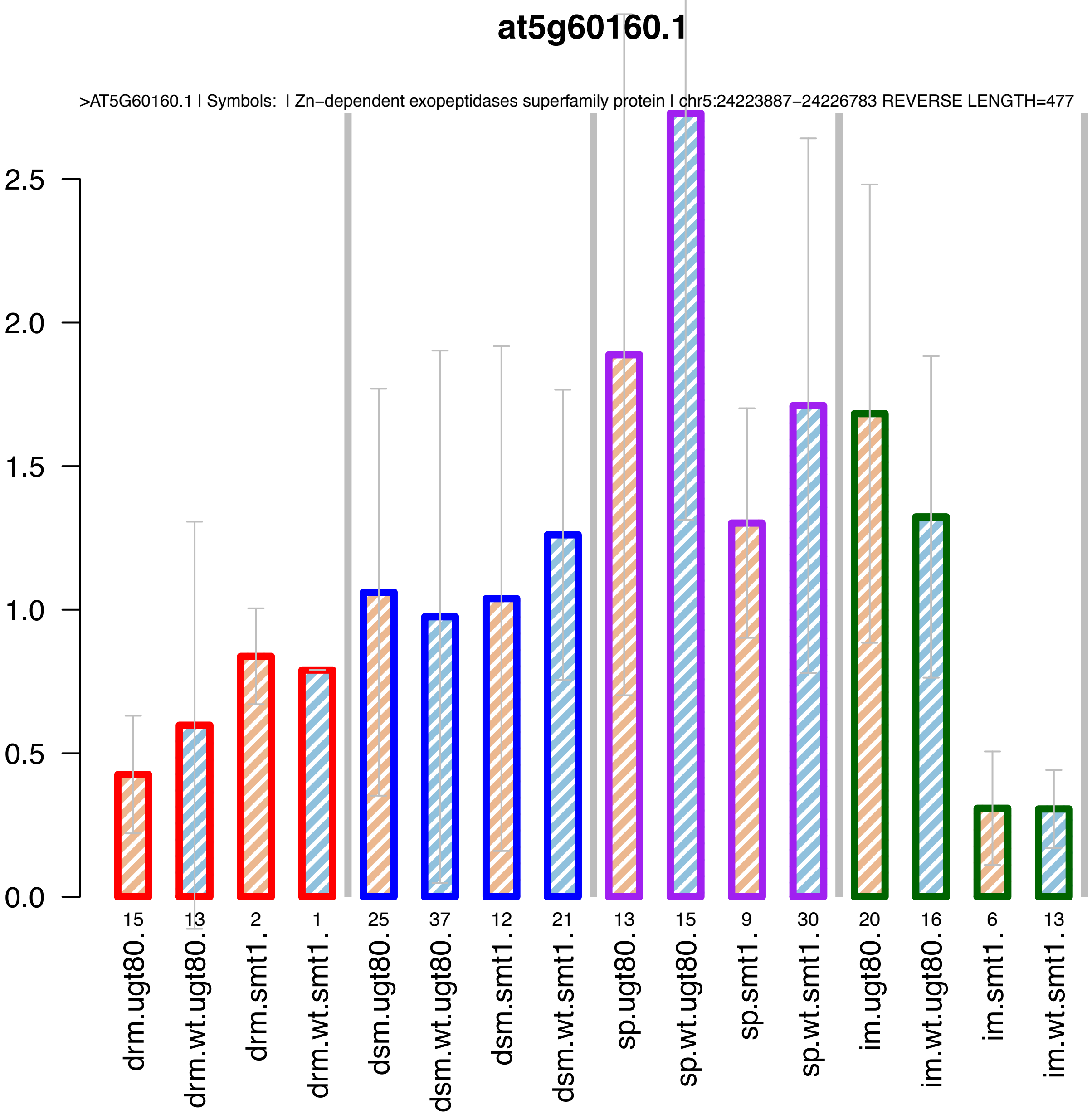

at5g60160.1
>AT5G60160.1 | Symbols: | Zn−dependent exopeptidases superfamily protein | chr5:24223887−24226783 REVERSE LENGTH=477
0.0
0.5
1.0
1.5
2.0
2.5
15
13
2
1
25
37
12
21
13
15
9
30
20
16
6
13
drm.ugt80.
drm.wt.ugt80.
drm.smt1.
drm.wt.smt1.
dsm.ugt80.
dsm.wt.ugt80.
dsm.smt1.
dsm.wt.smt1.
sp.ugt80.
sp.wt.ugt80.
sp.smt1.
sp.wt.smt1.
im.ugt80.
im.wt.ugt80.
im.smt1.
im.wt.smt1.

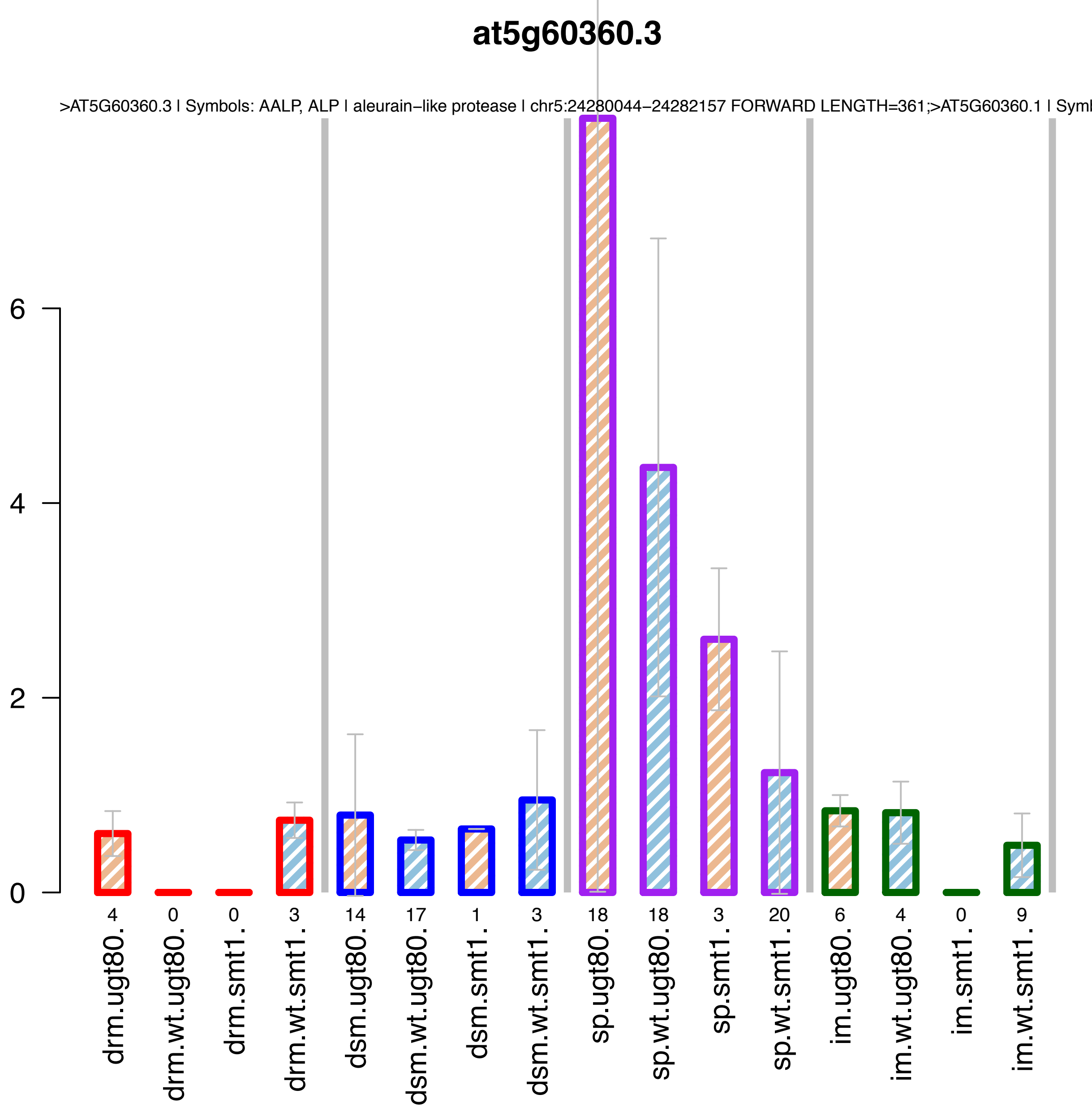

at5g60360.3
>AT5G60360.3 | Symbols: AALP, ALP | aleurain−like protease | chr5:24280044−24282157 FORWARD LENGTH=361;>AT5G60360.1 | Symb
0
2
4
6
4
0
0
3
14
17
1
3
18
18
3
20
6
4
0
9
drm.ugt80.
drm.wt.ugt80.
drm.smt1.
drm.wt.smt1.
dsm.ugt80.
dsm.wt.ugt80.
dsm.smt1.
dsm.wt.smt1.
sp.ugt80.
sp.wt.ugt80.
sp.smt1.
sp.wt.smt1.
im.ugt80.
im.wt.ugt80.
im.smt1.
im.wt.smt1.

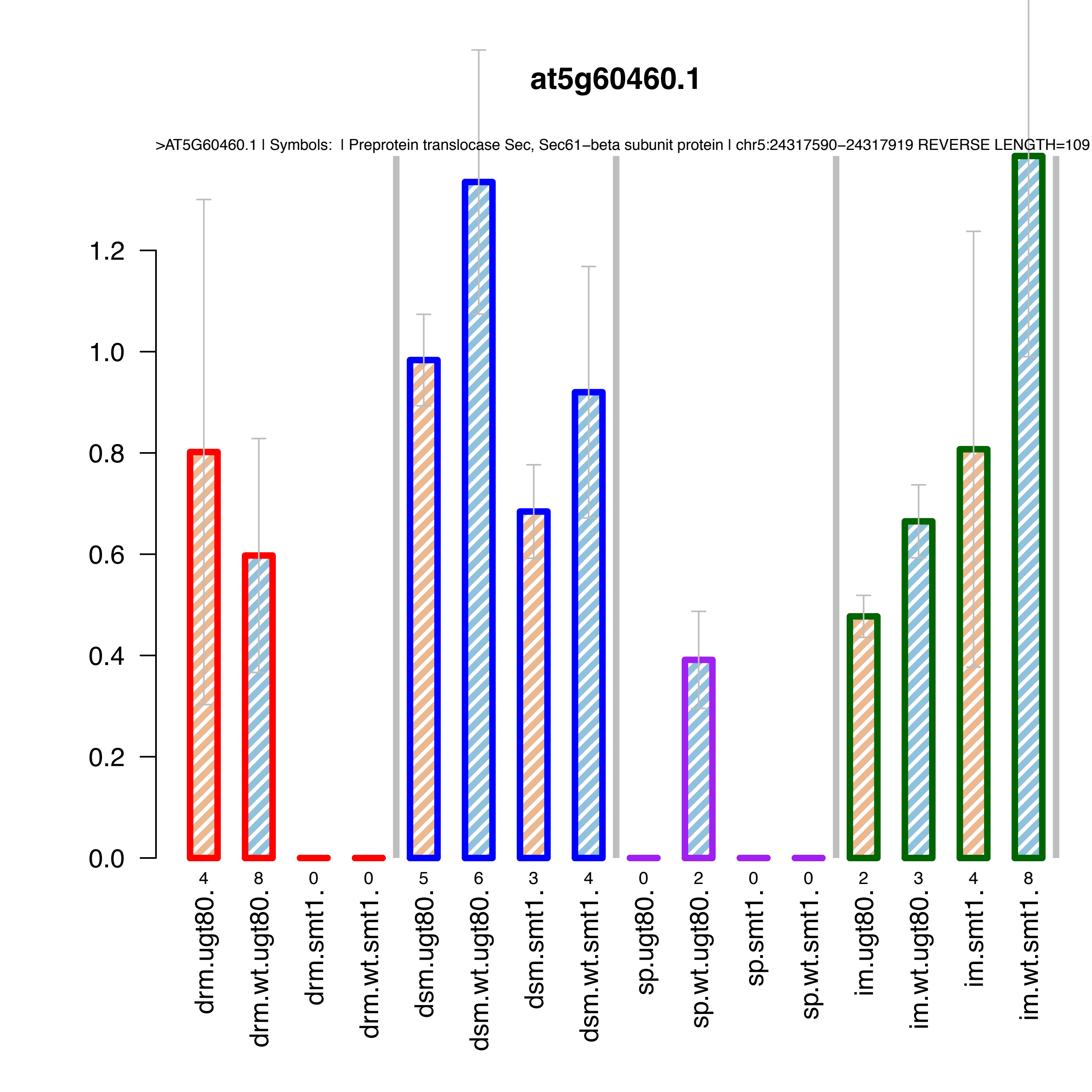

at5g60460.1
>AT5G60460.1 | Symbols: | Preprotein translocase Sec, Sec61−beta subunit protein | chr5:24317590−24317919 REVERSE LENGTH=109
0.0
0.2
0.4
0.6
0.8
1.0
1.2
4
8
0
0
5
6
3
4
0
2
0
0
2
3
4
8
drm.ugt80..
drm.wt.ugt80..
drm.smt1..
drm.wt.smt1..
dsm.ugt80..
dsm.wt.ugt80..
dsm.smt1..
dsm.wt.smt1..
sp.ugt80..
sp.wt.ugt80..
sp.smt1..
sp.wt.smt1..
im.ugt80..
im.wt.ugt80..
im.smt1..
im.wt.smt1..

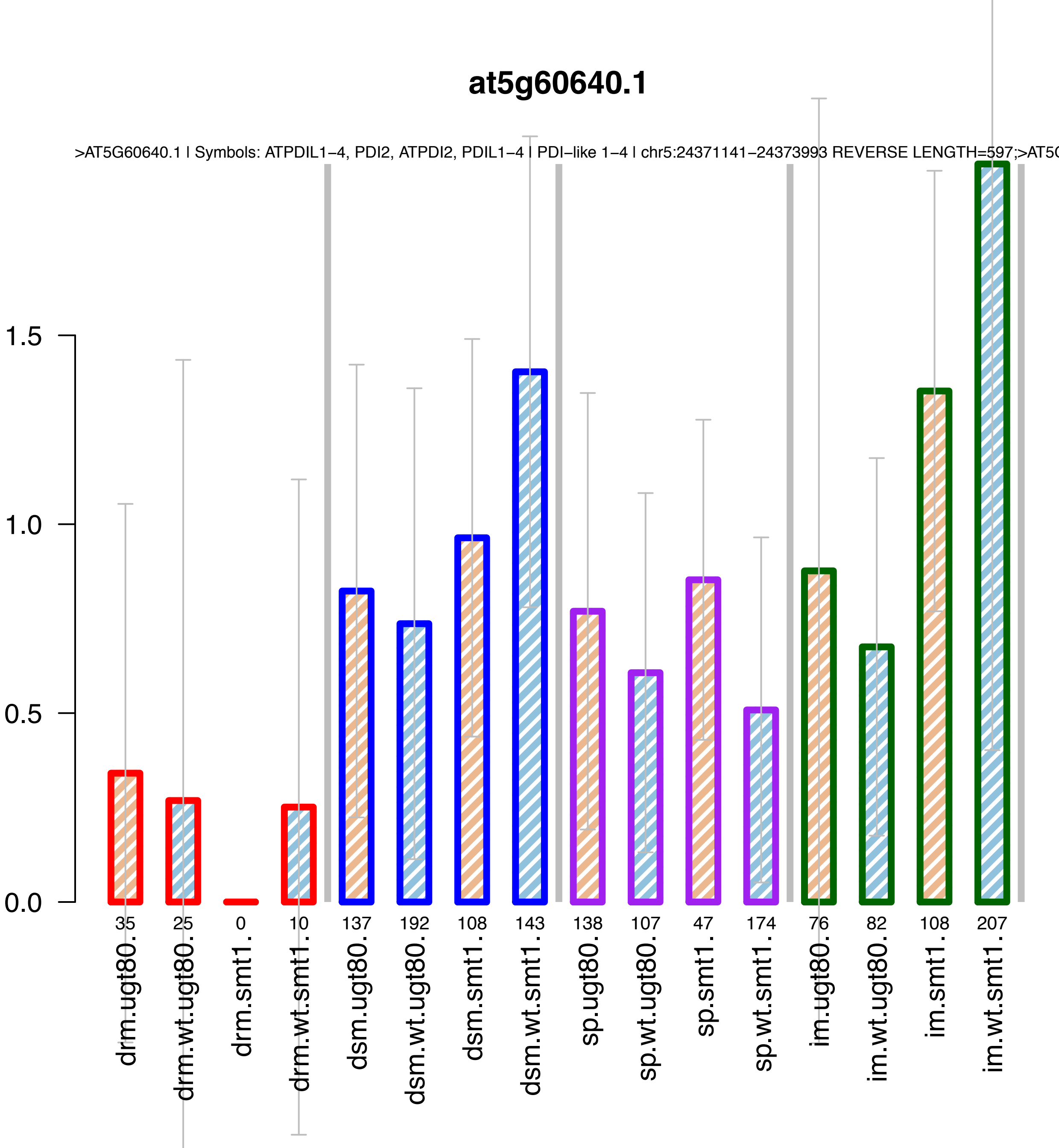

at5g60640.1
>AT5G60640.1 | Symbols: ATPDIL1-4, PDI2, ATPDI2, PDIL1-4 | PDI-like 1-4 | chr5:24371141-24373993 REVERSE LENGTH=597;>AT5
1.5
1.0
0.5
0.0
35
25
0
10
137
192
108
143
138
107
47
174
76
82
108
207
drm.ugt80..
drm.wt.ugt80..
drm.smt1..
drm.wt.smt1..
dsm.ugt80..
dsm.wt.ugt80..
dsm.smt1..
dsm.wt.smt1..
sp.ugt80..
sp.wt.ugt80..
sp.smt1..
sp.wt.smt1..
im.ugt80..
im.wt.ugt80..
im.smt1..
im.wt.smt1..

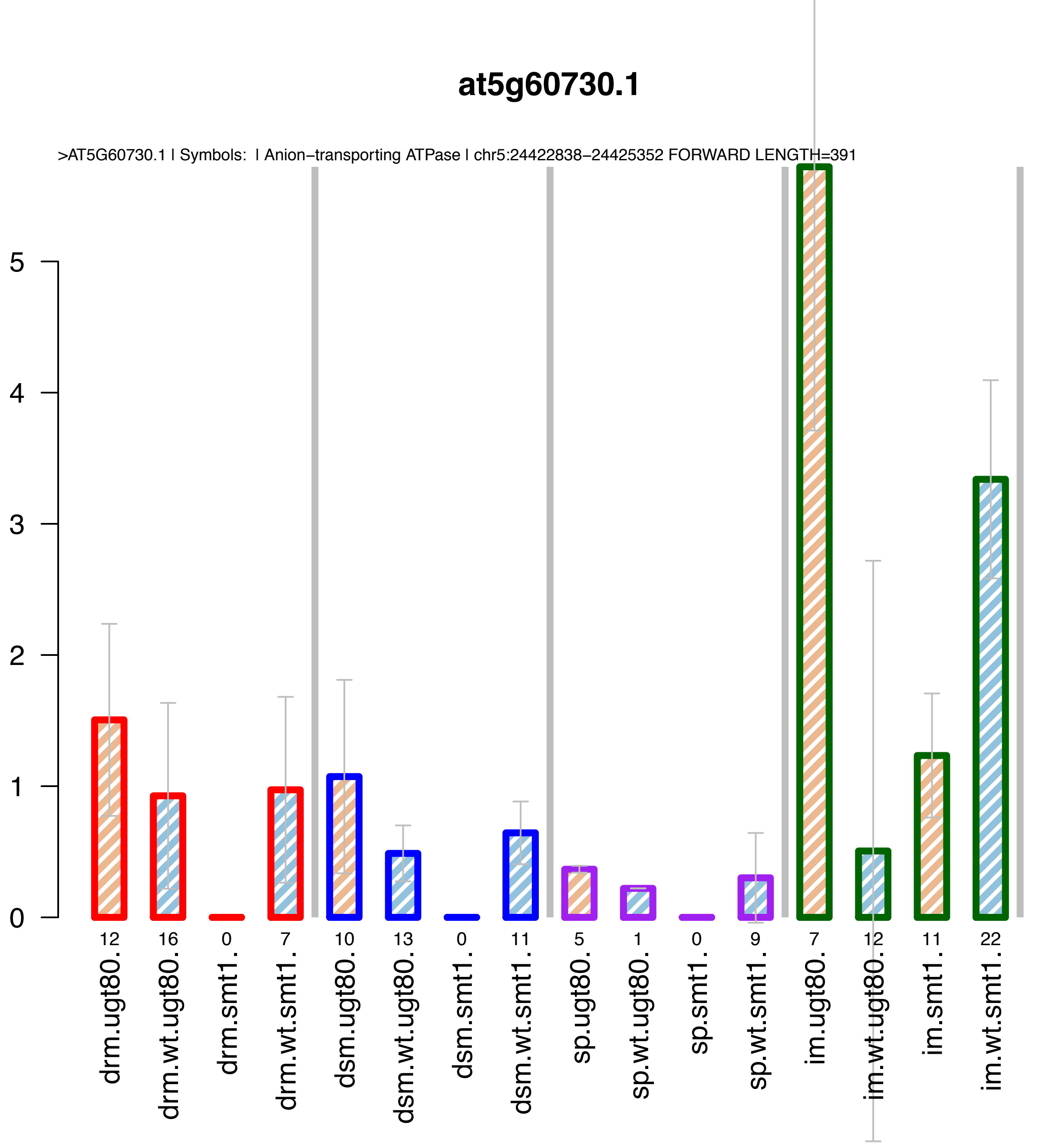

at5g60730.1
>AT5G60730.1 | Symbols: | Anion−transporting ATPase | chr5:24422838−24425352 FORWARD LENGTH=391
0
1
2
3
4
5
12
16
0
7
10
13
0
11
5
1
0
9
7
12
11
22
drm.ugt80..
drm.wt.ugt80..
drm.smt1..
drm.wt.smt1..
dsm.ugt80..
dsm.wt.ugt80..
dsm.smt1..
dsm.wt.smt1..
sp.ugt80..
sp.wt.ugt80..
sp.smt1..
sp.wt.smt1..
im.ugt80..
im.wt.ugt80..
im.smt1..
im.wt.smt1..

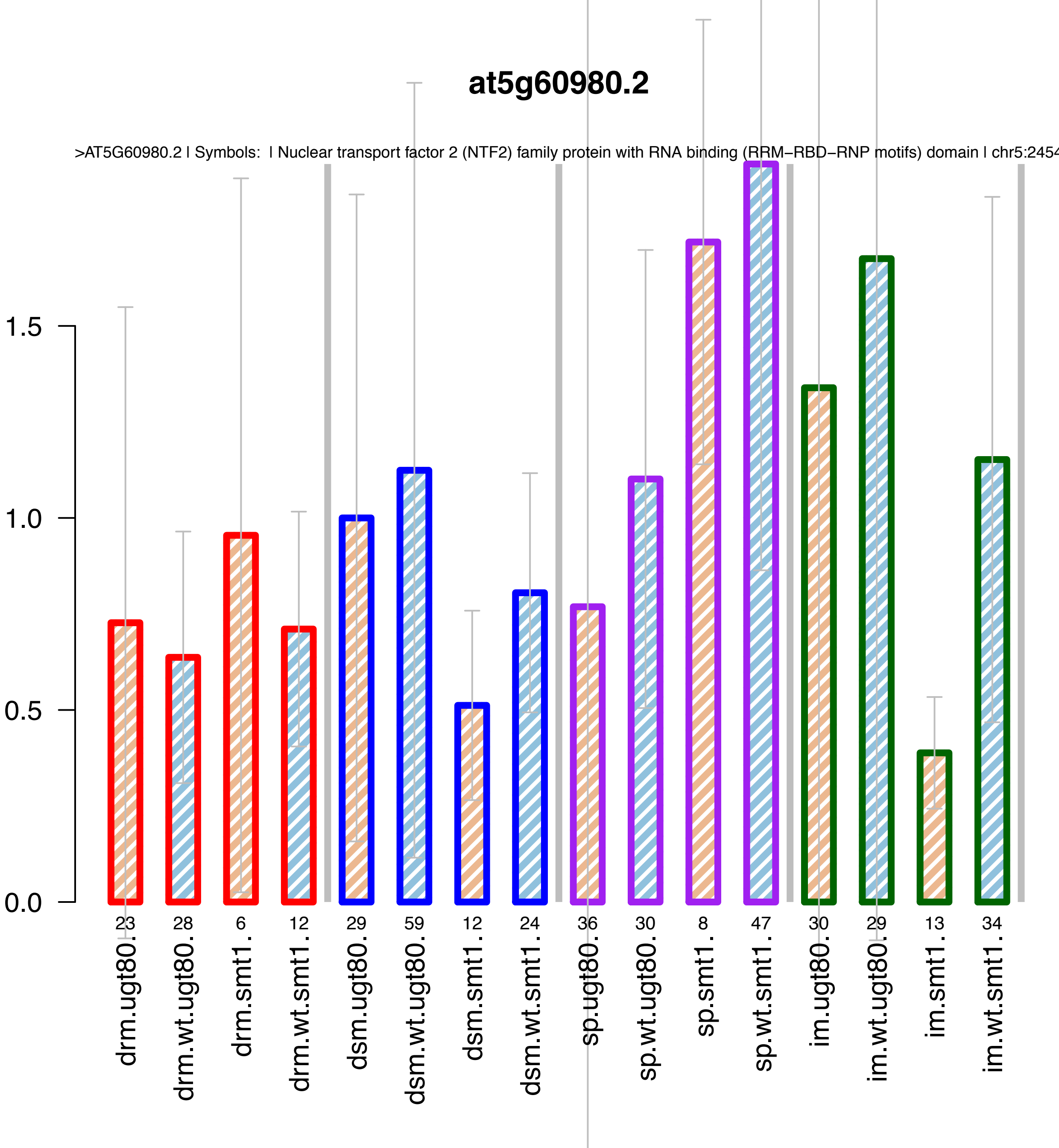
at5g60980.2
>AT5G60980.2 | Symbols: | Nuclear transport factor 2 (NTF2) family protein with RNA binding (RRM-RBD-RNP motifs) domain | chr5:2454
0.0
0.5
1.0
1.5
23
28
6
12
29
59
12
24
36
30
8
47
30
29
13
34
drm.ugt80.
drm.wt.ugt80.
drm.smt1.
drm.wt.smt1.
dsm.ugt80.
dsm.wt.ugt80.
dsm.smt1.
dsm.wt.smt1.
sp.ugt80.
sp.wt.ugt80.
sp.smt1.
sp.wt.smt1.
im.ugt80.
im.wt.ugt80.
im.smt1.
im.wt.smt1.

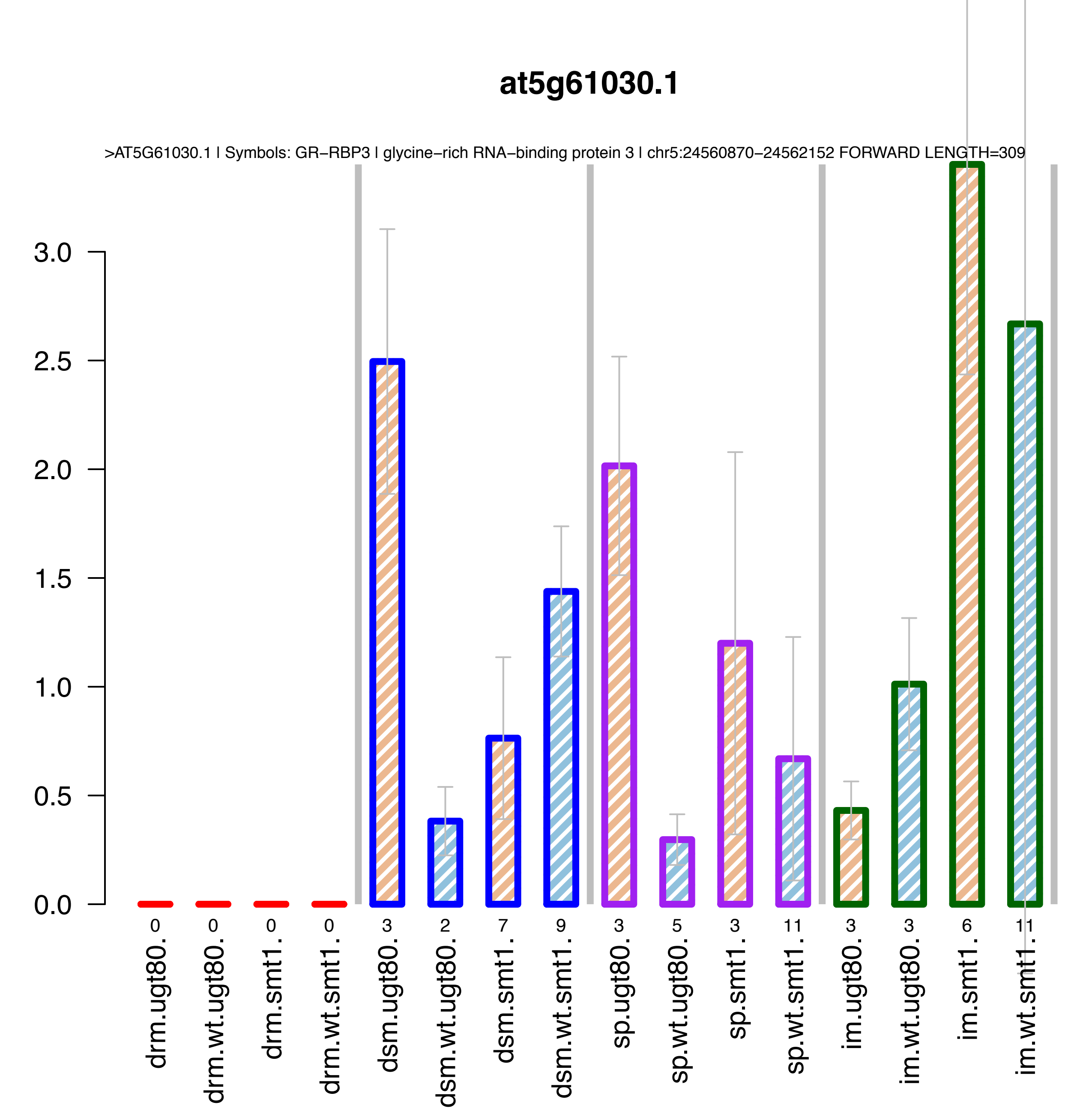

at5g61030.1
>AT5G61030.1 | Symbols: GR-RBP3 | glycine-rich RNA-binding protein 3 | chr5:24560870-24562152 FORWARD LENGTH=309
0.0
0.5
1.0
1.5
2.0
2.5
3.0
0
0
0
0
3
2
7
9
3
5
3
11
3
3
6
11
drm.ugt80.
drm.wt.ugt80.
drm.smt1.
drm.wt.smt1.
dsm.ugt80.
dsm.wt.ugt80.
dsm.smt1.
dsm.wt.smt1.
sp.ugt80.
sp.wt.ugt80.
sp.smt1.
sp.wt.smt1.
im.ugt80.
im.wt.ugt80.
im.smt1.
im.wt.smt1.

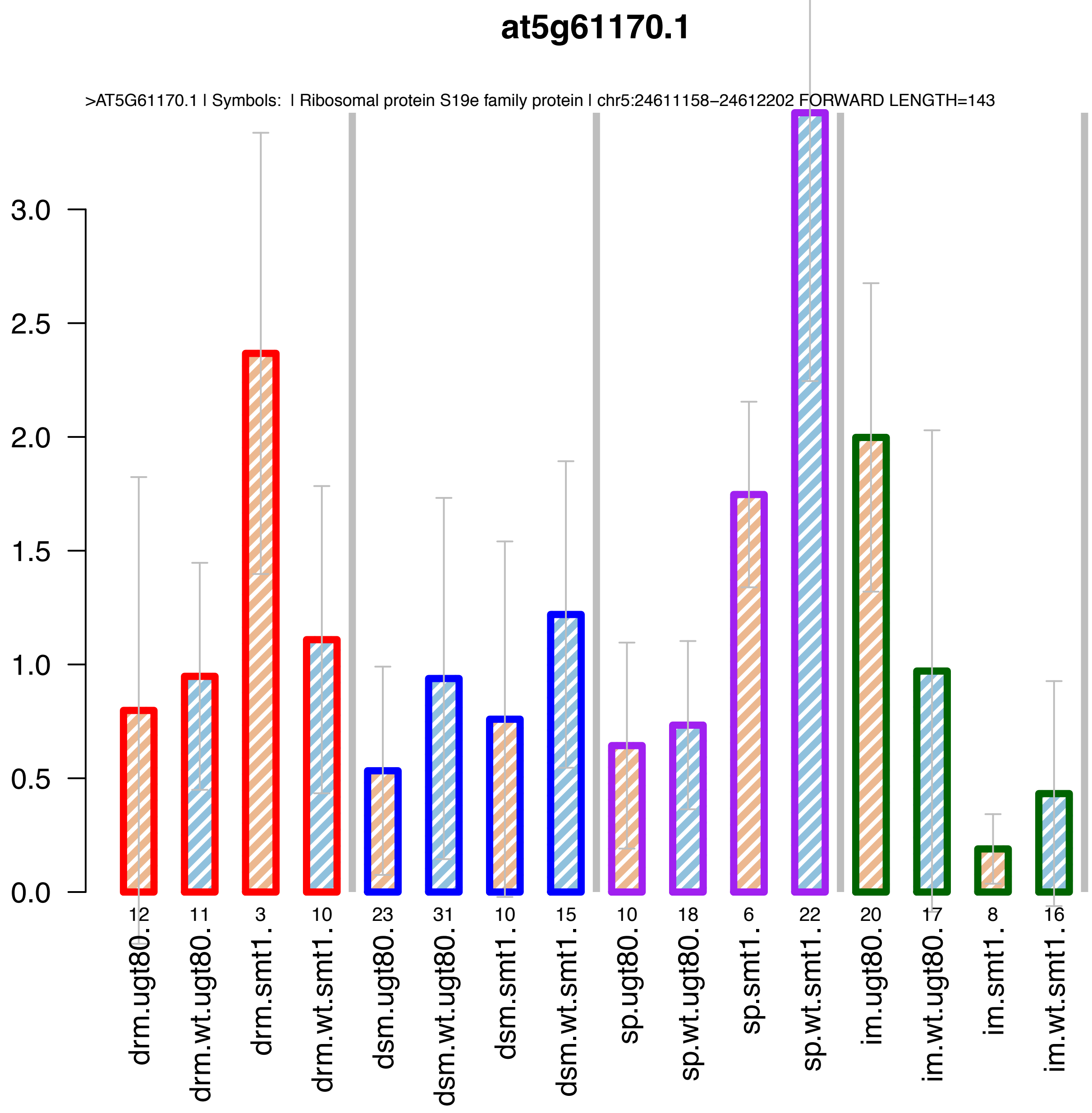

at5g61170.1
>AT5G61170.1 | Symbols: | Ribosomal protein S19e family protein | chr5:24611158-24612202 FORWARD LENGTH=143
0.0
0.5
1.0
1.5
2.0
2.5
3.0
12
11
3
10
23
31
10
15
10
18
6
22
20
17
8
16
drm.ugt80..
drm.wt.ugt80..
drm.smt1..
drm.wt.smt1..
dsm.ugt80..
dsm.wt.ugt80..
dsm.smt1..
dsm.wt.smt1..
sp.ugt80..
sp.wt.ugt80..
sp.smt1..
sp.wt.smt1..
im.ugt80..
im.wt.ugt80..
im.smt1..
im.wt.smt1..

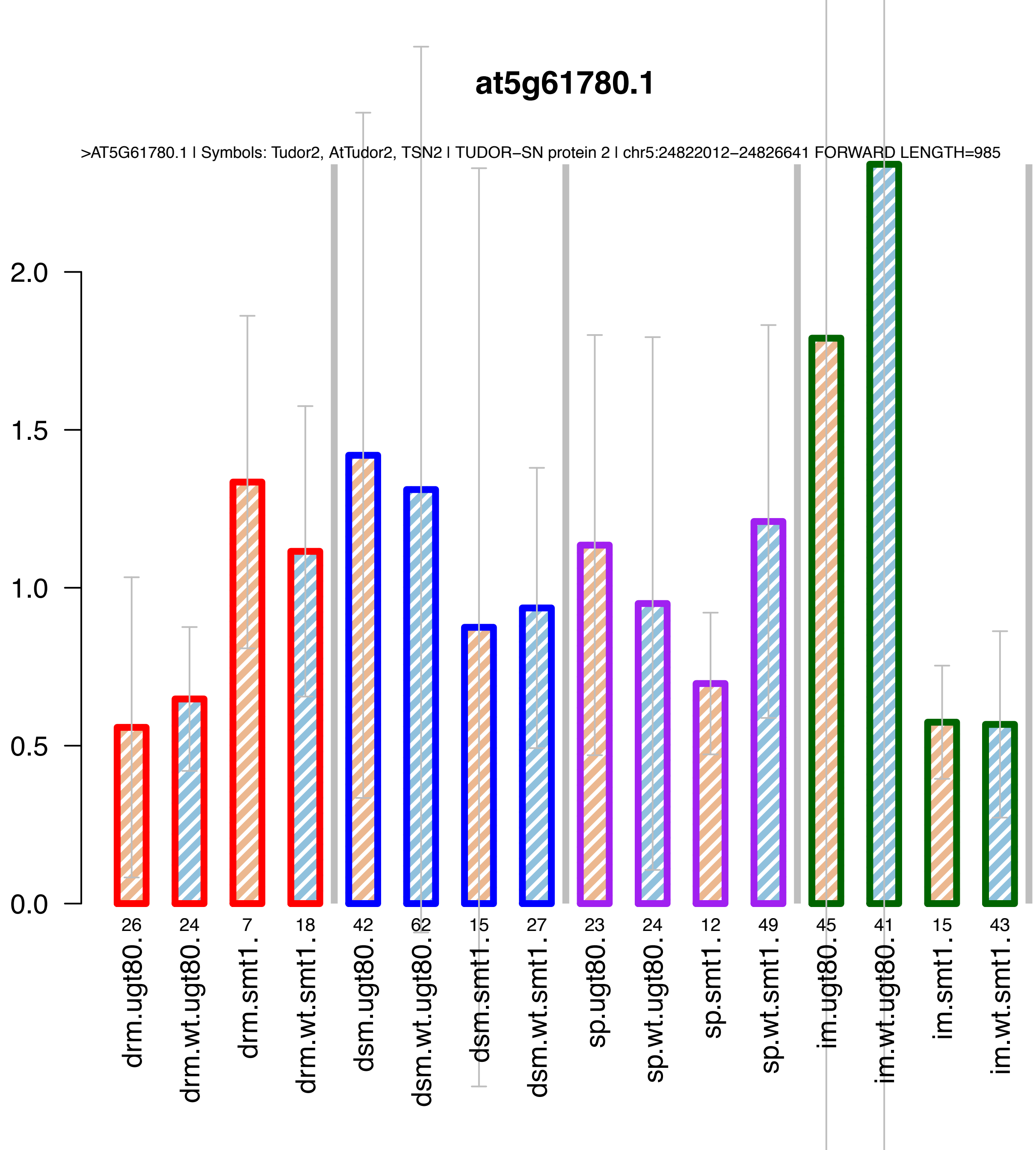

at5g61780.1
>AT5G61780.1 | Symbols: Tudor2, AtTudor2, TSN2 | TUDOR−SN protein 2 | chr5:24822012−24826641 FORWARD LENGTH=985
0.0
0.5
1.0
1.5
2.0
26
24
7
18
42
62
15
27
23
24
12
49
45
41
15
43
drm.ugt80.
drm.wt.ugt80.
drm.smt1.
drm.wt.smt1.
dsm.ugt80.
dsm.wt.ugt80.
dsm.smt1.
dsm.wt.smt1.
sp.ugt80.
sp.wt.ugt80.
sp.smt1.
sp.wt.smt1.
im.ugt80.
im.wt.ugt80.
im.smt1.
im.wt.smt1.

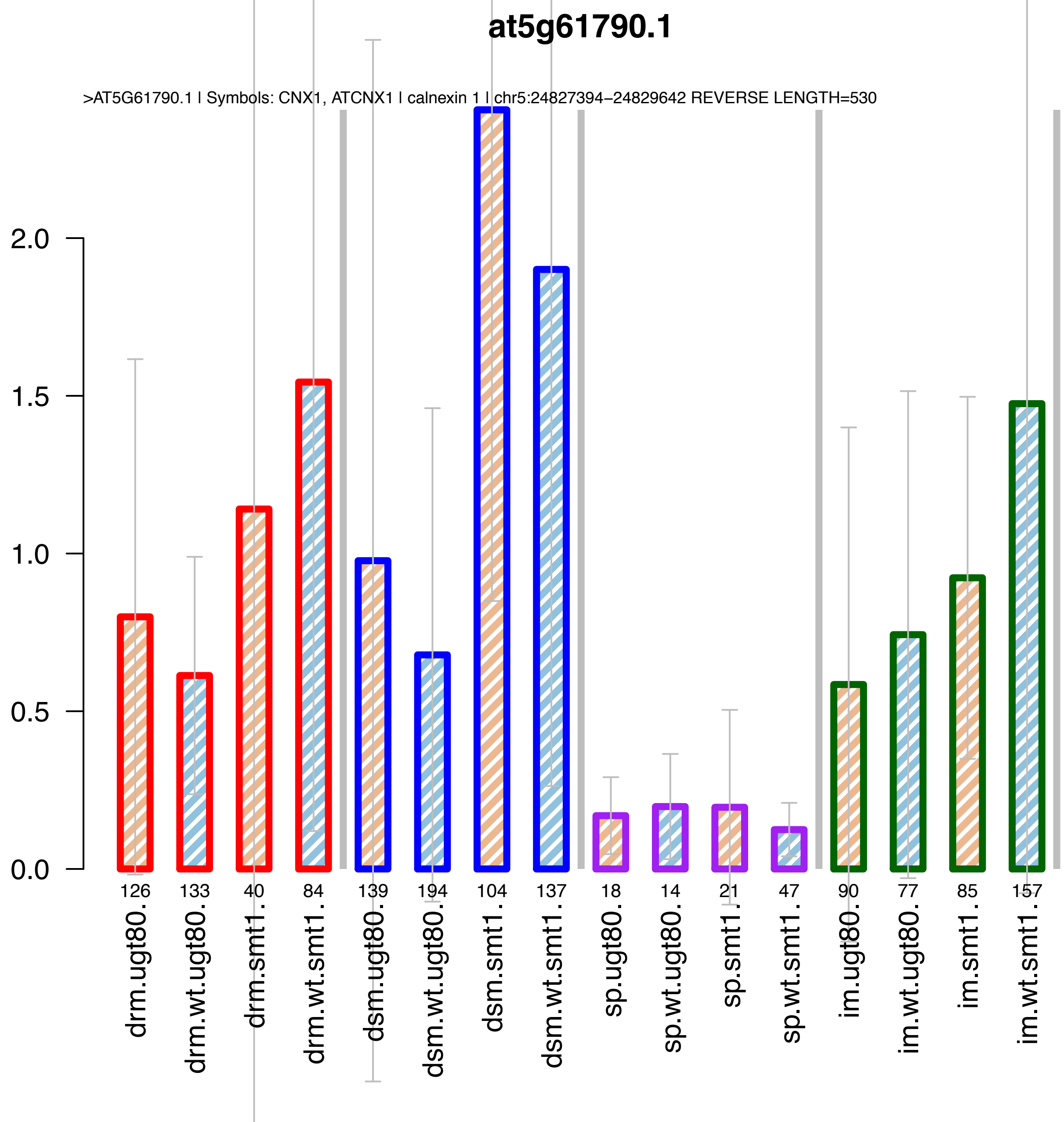

at5g61790.1
>AT5G61790.1 | Symbols: CNX1, ATCNX1 | calnexin 1 | chr5:24827394-24829642 REVERSE LENGTH=530
2.0
1.5
1.0
0.5
0.0
126
133
40
84
139
194
104
137
18
14
21
47
90
77
85
157
drm.ugt80..
drm.wt.ugt80..
drm.smt1..
drm.wt.smt1..
dsm.ugt80..
dsm.wt.ugt80..
dsm.smt1..
dsm.wt.smt1..
sp.ugt80..
sp.wt.ugt80..
sp.smt1..
sp.wt.smt1..
im.ugt80..
im.wt.ugt80..
im.smt1..
im.wt.smt1..

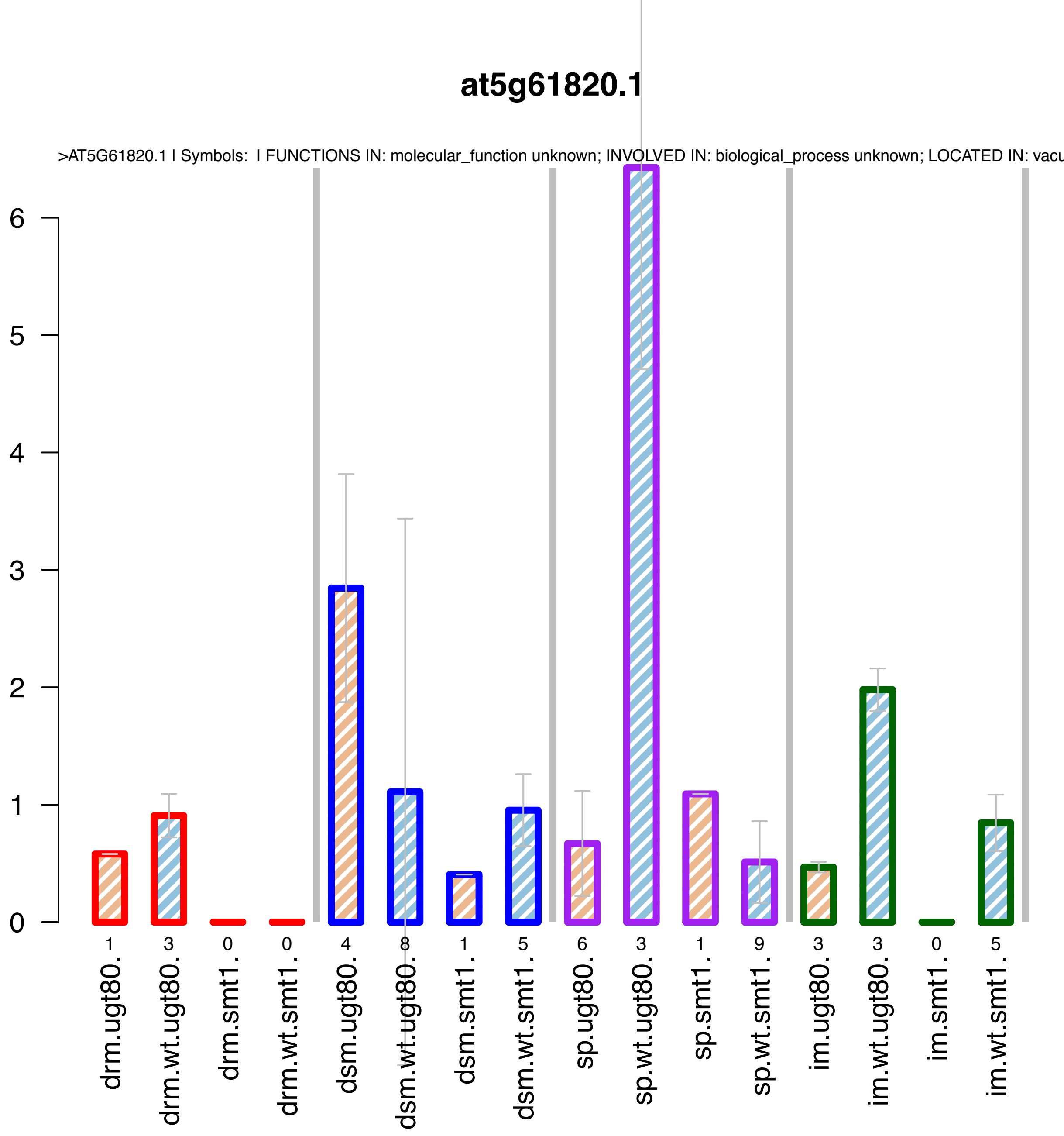

at5g61820.1
>AT5G61820.1 | Symbols: | FUNCTIONS IN: molecular_function unknown; INVOLVED IN: biological_process unknown; LOCATED IN: vacu
0
1
2
3
4
5
6
1
3
0
0
4
8
1
5
6
3
1
9
3
3
0
5
drm.ugt80.
drm.wt.ugt80.
drm.smt1.
drm.wt.smt1.
dsm.ugt80.
dsm.wt.ugt80.
dsm.smt1.
dsm.wt.smt1.
sp.ugt80.
sp.wt.ugt80.
sp.smt1.
sp.wt.smt1.
im.ugt80.
im.wt.ugt80.
im.smt1.
im.wt.smt1.

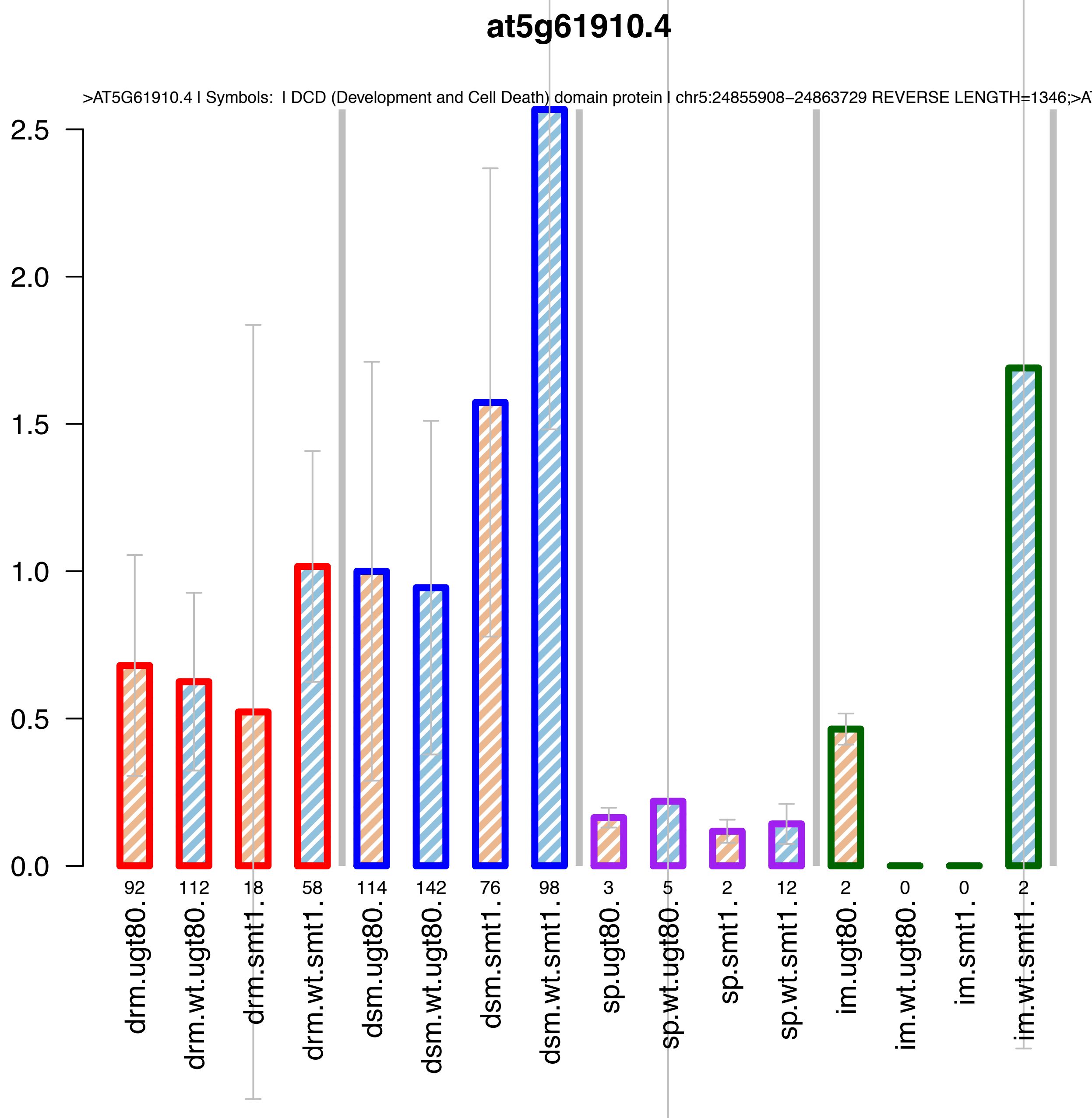
at5g61910.4
>AT5G61910.4 | Symbols: | DCD (Development and Cell Death) domain protein | chr5:24855908-24863729 REVERSE LENGTH=1346;>A
2.5
2.0
1.5
1.0
0.5
0.0
92
112
18
58
114
142
76
98
3
5
2
12
2
0
0
2
drm.ugt80.
drm.wt.ugt80.
drm.smt1.
drm.wt.smt1.
dsm.ugt80.
dsm.wt.ugt80.
dsm.smt1.
dsm.wt.smt1.
sp.ugt80.
sp.wt.ugt80.
sp.smt1.
sp.wt.smt1.
im.ugt80.
im.wt.ugt80.
im.smt1.
im.wt.smt1.

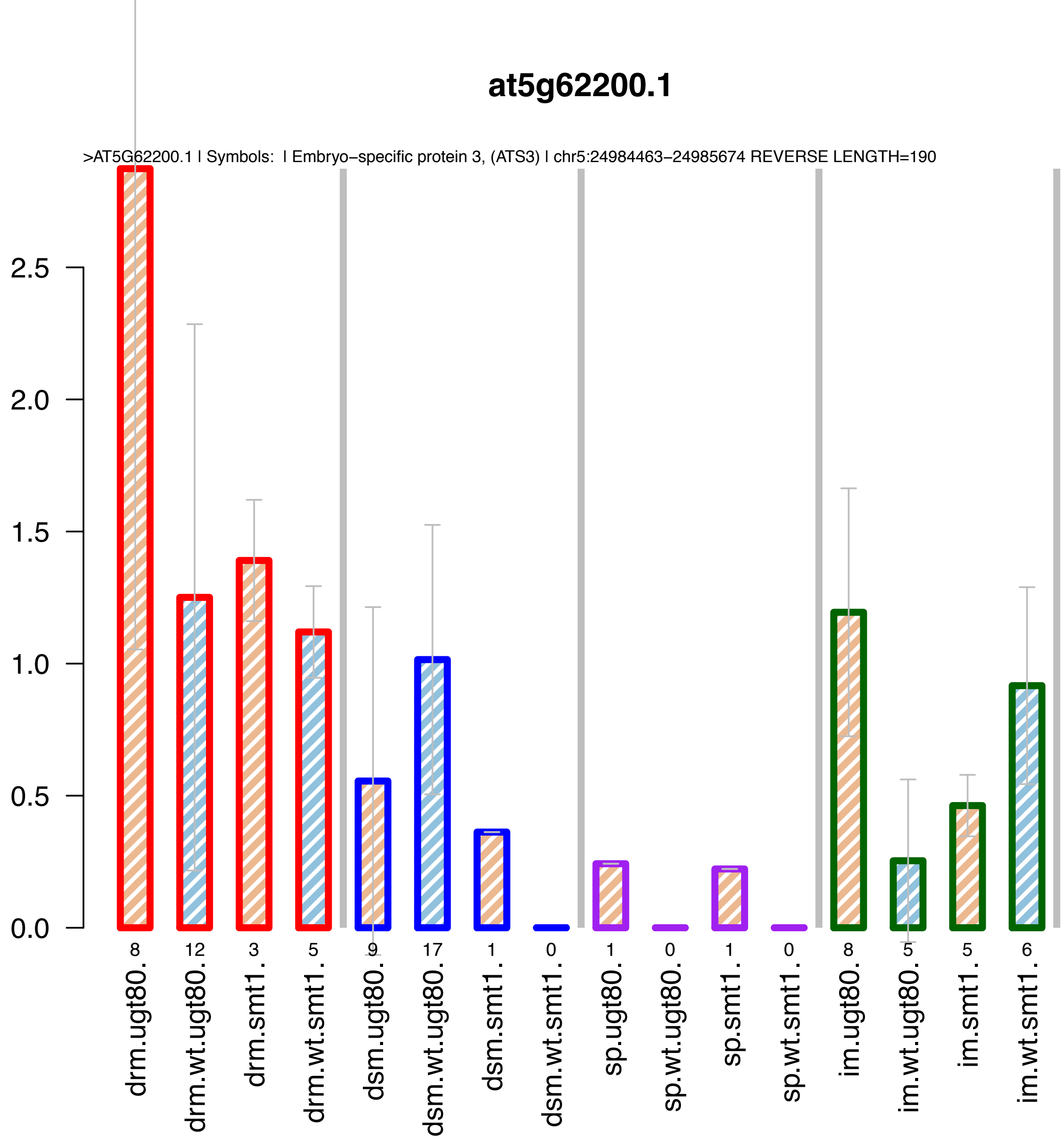

at5g62200.1
>AT5G62200.1 | Symbols: | Embryo−specific protein 3, (ATS3) | chr5:24984463−24985674 REVERSE LENGTH=190
0.0
0.5
1.0
1.5
2.0
2.5
8
12
3
5
9
17
1
0
1
0
1
0
8
5
5
6
drm.ugt80.
drm.wt.ugt80.
drm.smt1.
drm.wt.smt1.
dsm.ugt80.
dsm.wt.ugt80.
dsm.smt1.
dsm.wt.smt1.
sp.ugt80.
sp.wt.ugt80.
sp.smt1.
sp.wt.smt1.
im.ugt80.
im.wt.ugt80.
im.smt1.
im.wt.smt1.

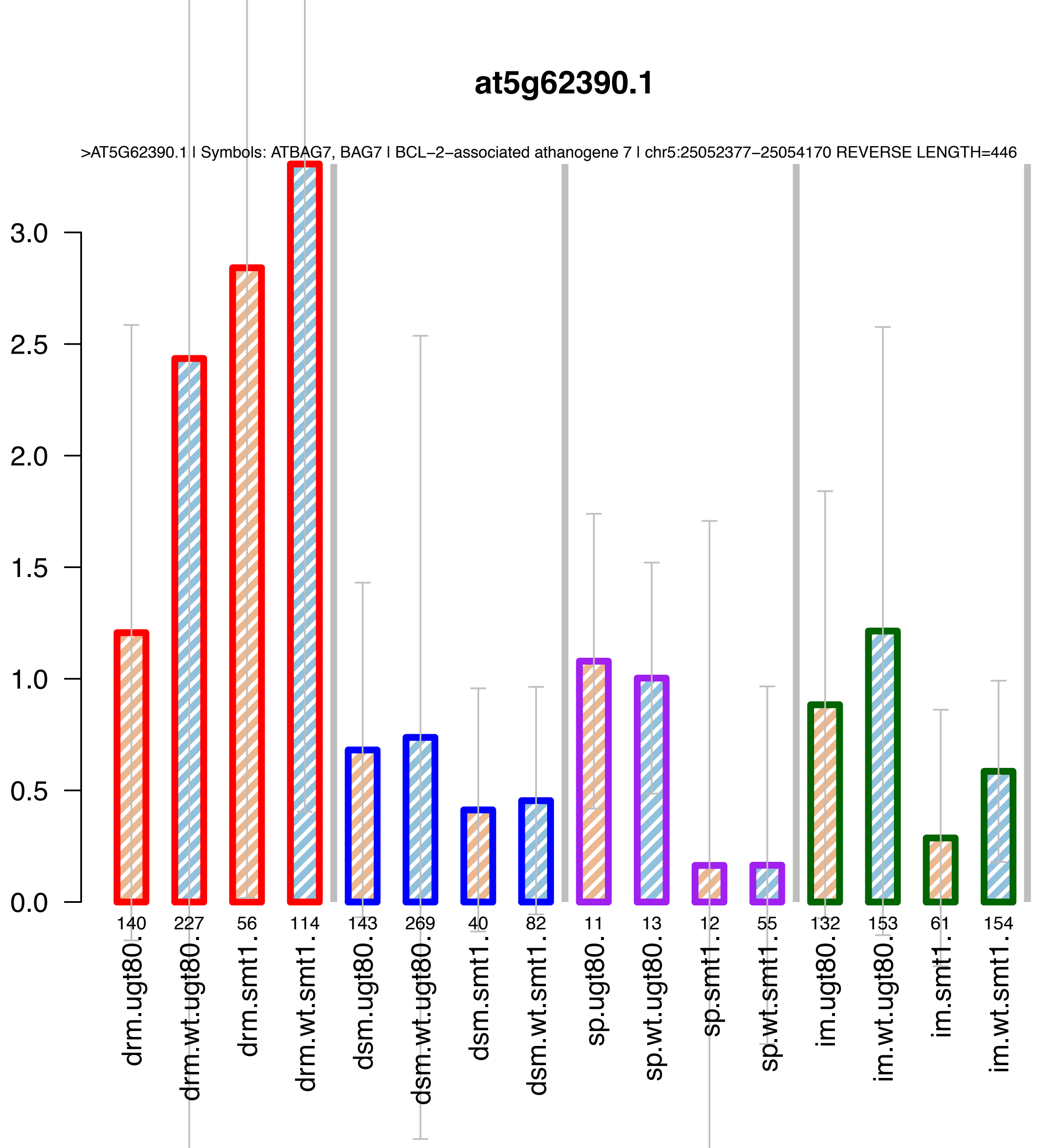

at5g62390.1
>AT5G62390.1 | Symbols: ATBAG7, BAG7 | BCL−2−associated athanogene 7 | chr5:25052377−25054170 REVERSE LENGTH=446
3.0
2.5
2.0
1.5
1.0
0.5
0.0
140
227
56
114
143
269
40
82
11
13
12
55
132
153
61
154
drm.ugt80..
drm.wt.ugt80..
drm.smt1..
drm.wt.smt1..
dsm.ugt80..
dsm.wt.ugt80..
dsm.smt1..
dsm.wt.smt1..
sp.ugt80..
sp.wt.ugt80..
sp.smt1..
sp.wt.smt1..
im.ugt80..
im.wt.ugt80..
im.smt1..
im.wt.smt1..

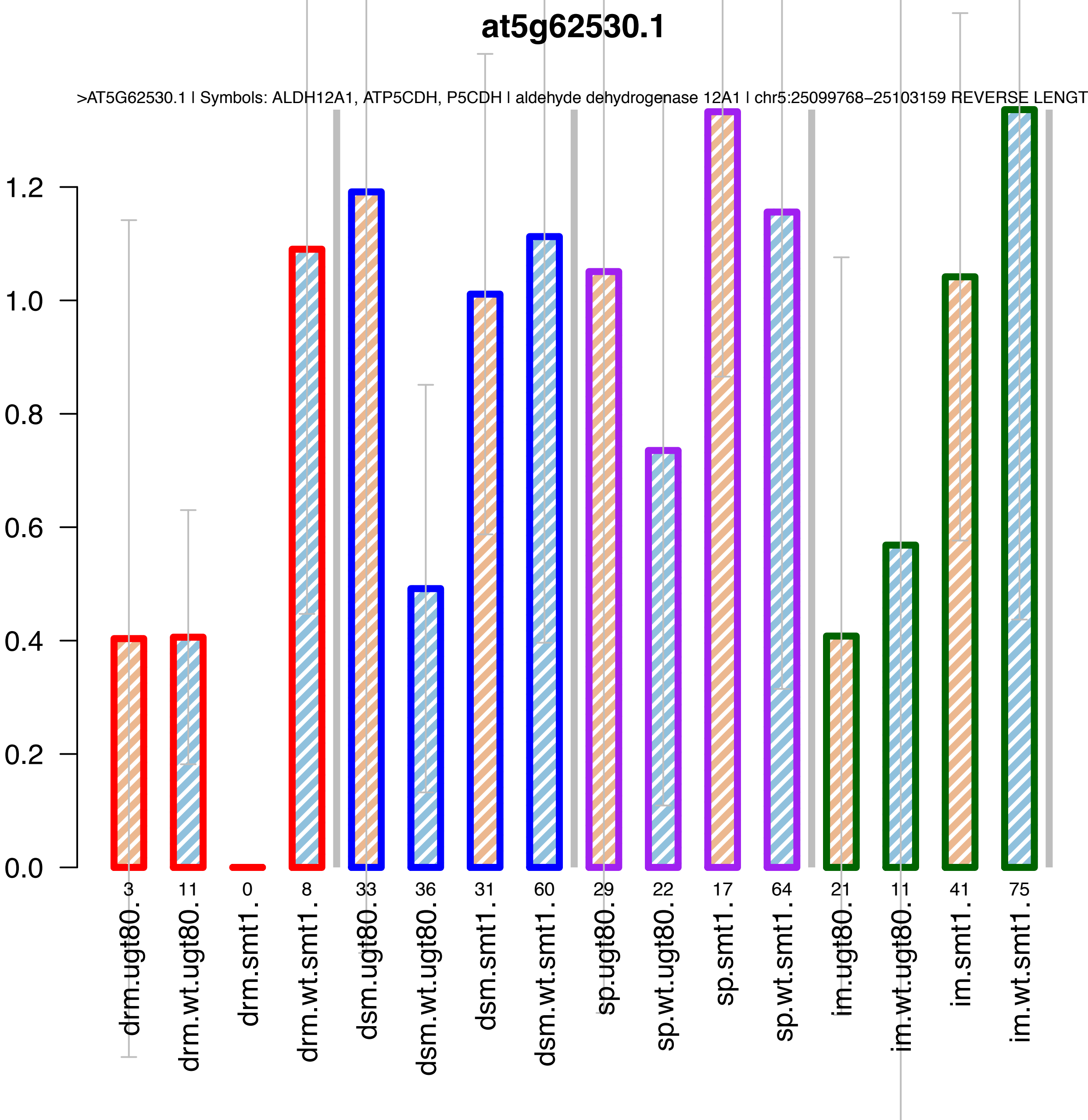

at5g62530.1
>AT5G62530.1 | Symbols: ALDH12A1, ATP5CDH, P5CDH | aldehyde dehydrogenase 12A1 | chr5:25099768-25103159 REVERSE LENGT
0.0
0.2
0.4
0.6
0.8
1.0
1.2
3
11
0
8
33
36
31
60
29
22
17
64
21
11
41
75
drm.ugt80..
drm.wt.ugt80..
drm.smt1..
drm.wt.smt1..
dsm.ugt80..
dsm.wt.ugt80..
dsm.smt1..
dsm.wt.smt1..
sp.ugt80..
sp.wt.ugt80..
sp.smt1..
sp.wt.smt1..
im.ugt80..
im.wt.ugt80..
im.smt1..
im.wt.smt1..

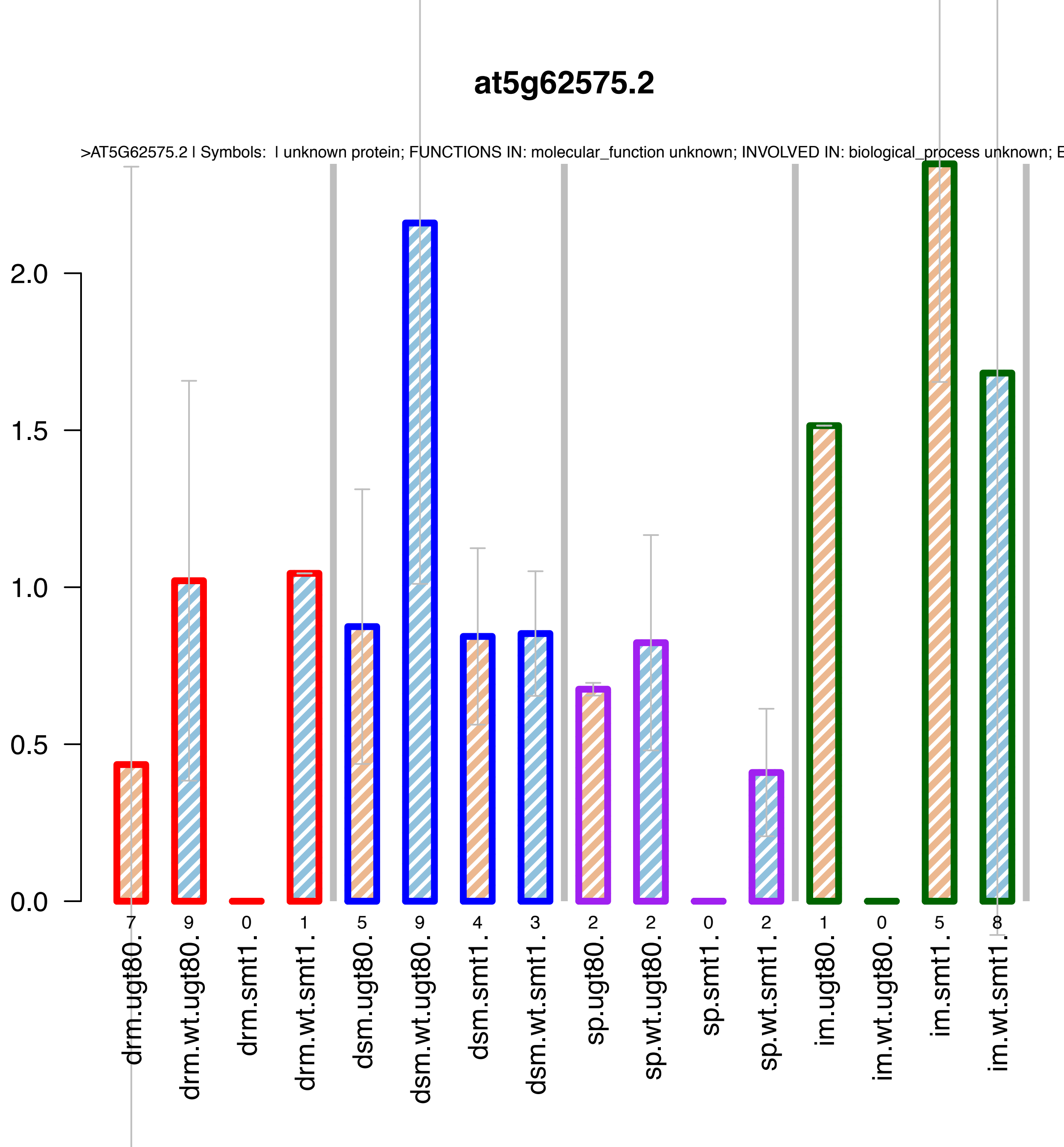

at5g62575.2
>AT5G62575.2 | Symbols: | unknown protein; FUNCTIONS IN: molecular_function unknown; INVOLVED IN: biological_process unknown; E
2.0
1.5
1.0
0.5
0.0
7 9 0 1 5 9 4 3 2 2 0 2 1 0 5 8
drm.ugt80.
drm.wt.ugt80.
drm.smt1.
drm.wt.smt1.
dsm.ugt80.
dsm.wt.ugt80.
dsm.smt1.
dsm.wt.smt1.
sp.ugt80.
sp.wt.ugt80.
sp.smt1.
sp.wt.smt1.
im.ugt80.
im.wt.ugt80.
im.smt1.
im.wt.smt1.

# at5g62630.1

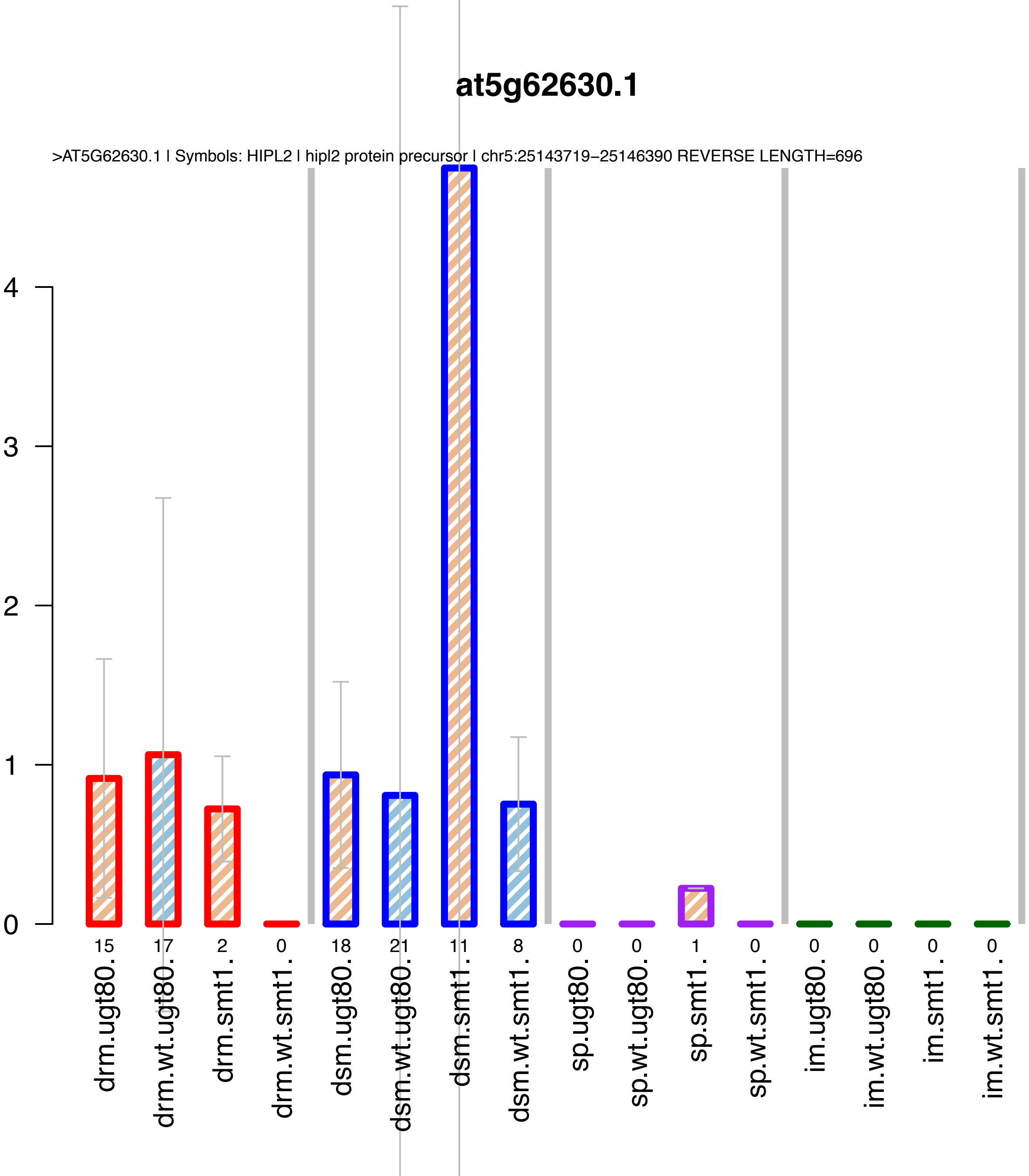

>AT5G62630.1 | Symbols: HIPL2 | hipl2 protein precursor | chr5:25143719-25146390 REVERSE LENGTH=696
0
1
2
3
4
15
17
2
0
18
21
11
8
0
0
1
0
0
0
0
0
drm.ugt80.
drm.wt.ugt80.
drm.smt1.
drm.wt.smt1.
dsm.ugt80.
dsm.wt.ugt80.
dsm.smt1.
dsm.wt.smt1.
sp.ugt80.
sp.wt.ugt80.
sp.smt1.
sp.wt.smt1.
im.ugt80.
im.wt.ugt80.
im.smt1.
im.wt.smt1.

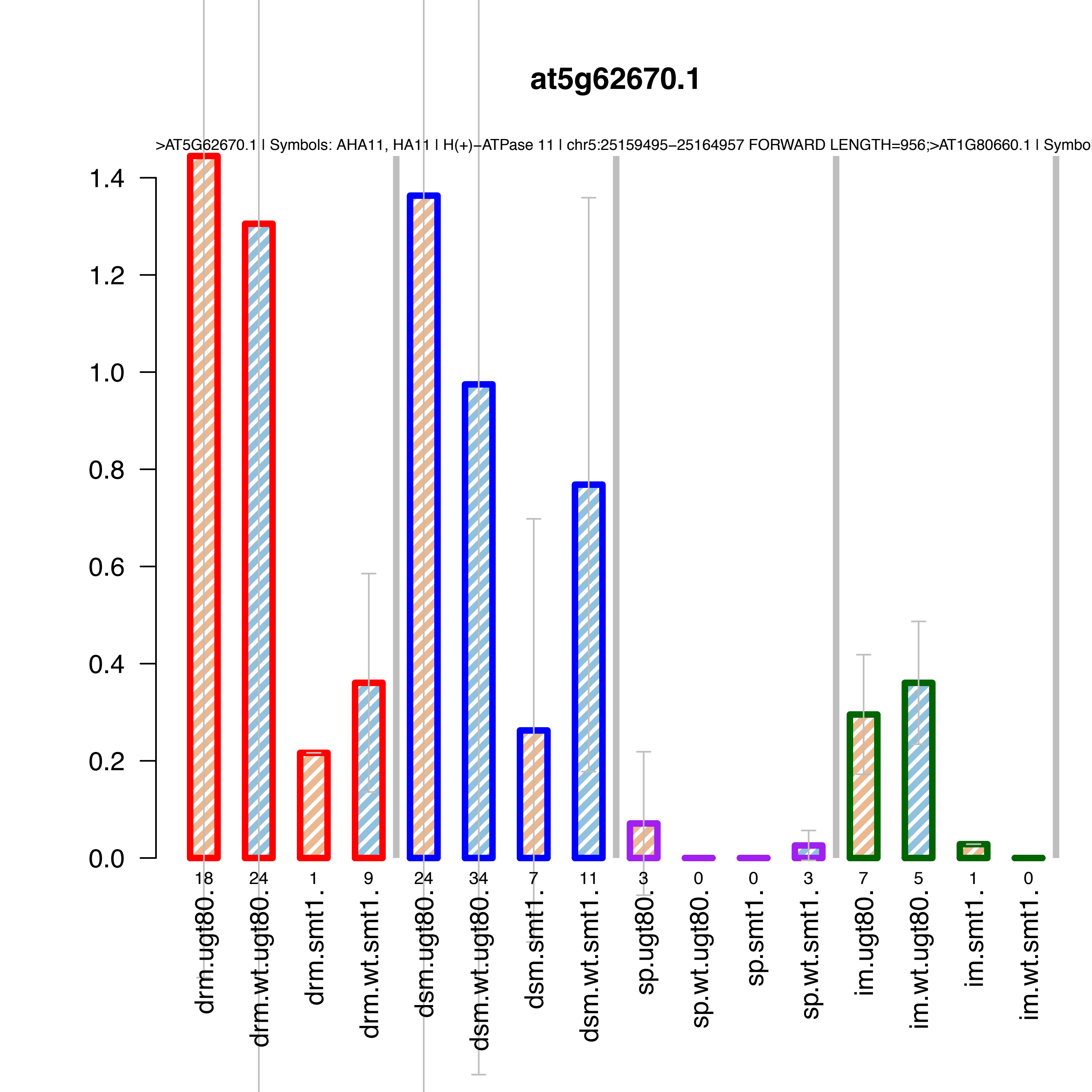

at5g62670.1
>AT5G62670.1 | Symbols: AHA11, HA11 | H(+)-ATPase 11 | chr5:25159495-25164957 FORWARD LENGTH=956;>AT1G80660.1 | Symbo
1.4
1.2
1.0
0.8
0.6
0.4
0.2
0.0
18
24
1
9
24
34
7
11
3
0
0
3
7
5
1
0
drm.ugt80..
drm.wt.ugt80..
drm.smt1..
drm.wt.smt1..
dsm.ugt80..
dsm.wt.ugt80..
dsm.smt1..
dsm.wt.smt1..
sp.ugt80..
sp.wt.ugt80..
sp.smt1..
sp.wt.smt1..
im.ugt80..
im.wt.ugt80..
im.smt1..
im.wt.smt1..

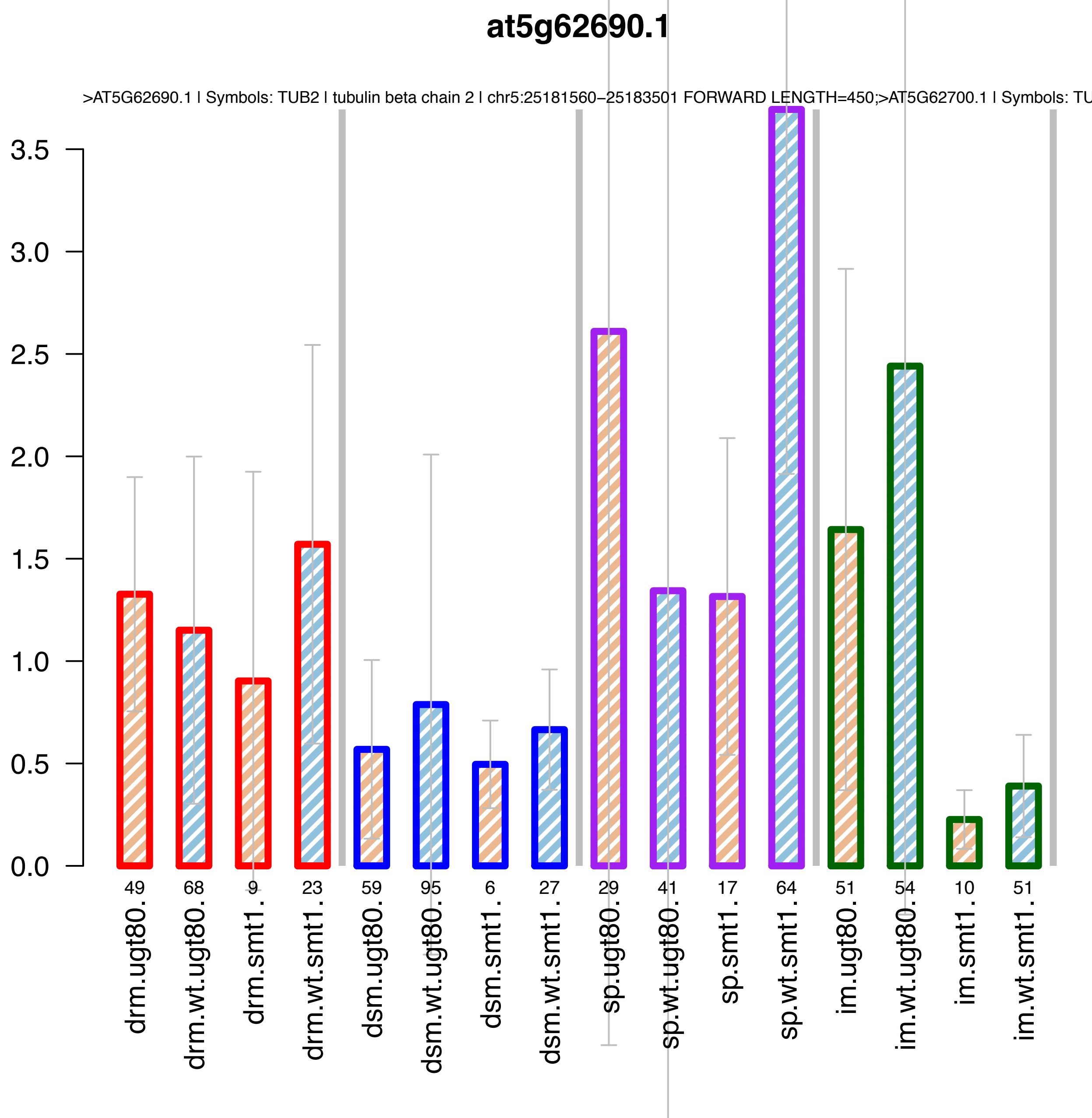

at5g62690.1
>AT5G62690.1 | Symbols: TUB2 | tubulin beta chain 2 | chr5:25181560-25183501 FORWARD LENGTH=450;>AT5G62700.1 | Symbols: TU
3.5
3.0
2.5
2.0
1.5
1.0
0.5
0.0
49
68
9
23
59
95
6
27
29
41
17
64
51
54
10
51
drm.ugt80.
drm.wt.ugt80.
drm.smt1.
drm.wt.smt1.
dsm.ugt80.
dsm.wt.ugt80.
dsm.smt1.
dsm.wt.smt1.
sp.ugt80.
sp.wt.ugt80.
sp.smt1.
sp.wt.smt1.
im.ugt80.
im.wt.ugt80.
im.smt1.
im.wt.smt1.

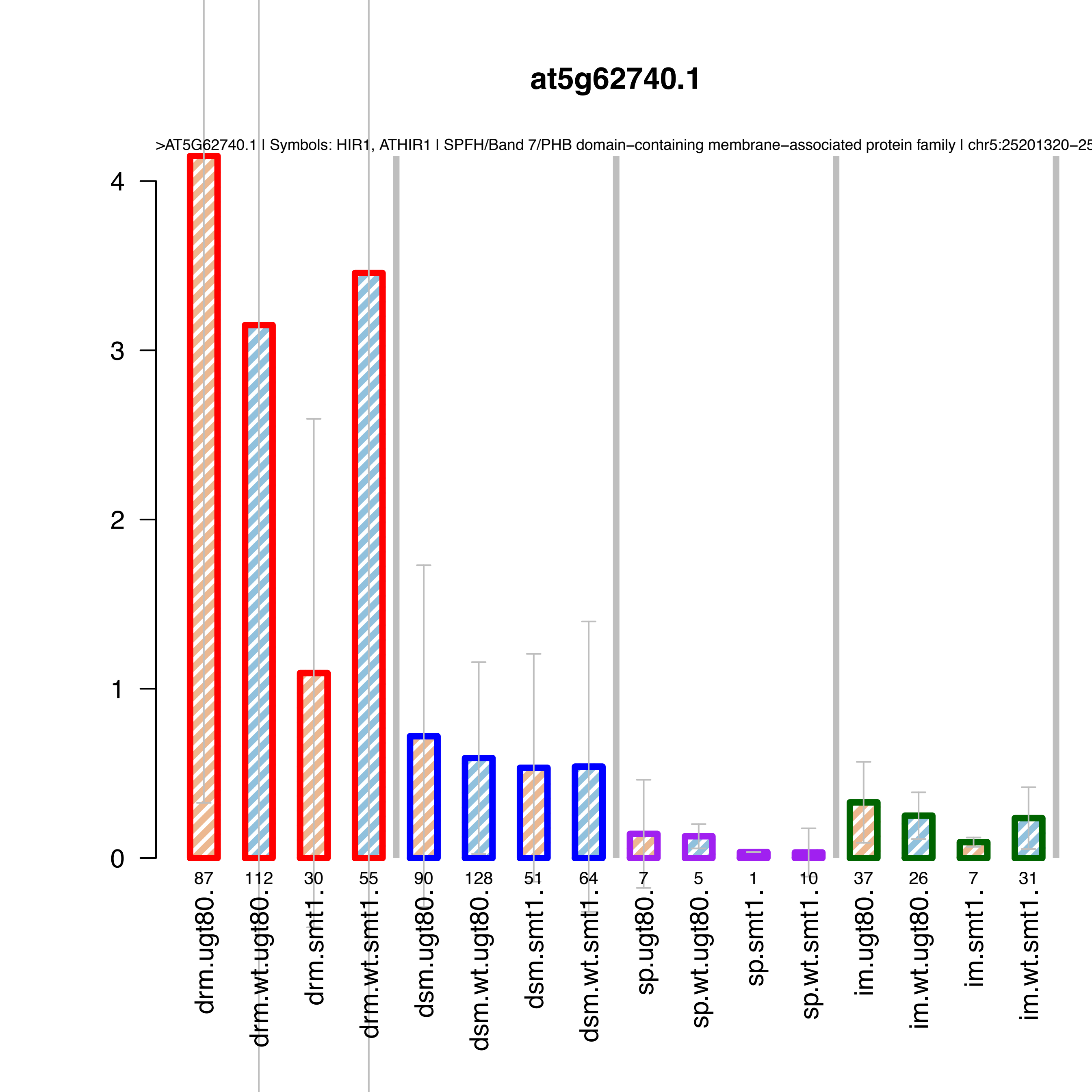

at5g62740.1
>AT5G62740.1 | Symbols: HIR1, ATHIR1 | SPFH/Band 7/PHB domain-containing membrane-associated protein family | chr5:25201320-25
4
3
2
1
0
87
112
30
55
90
128
51
64
7
5
1
10
37
26
7
31
drm.ugt80.
drm.wt.ugt80.
drm.smt1.
drm.wt.smt1.
dsm.ugt80.
dsm.wt.ugt80.
dsm.smt1.
dsm.wt.smt1.
sp.ugt80.
sp.wt.ugt80.
sp.smt1.
sp.wt.smt1.
im.ugt80.
im.wt.ugt80.
im.smt1.
im.wt.smt1.

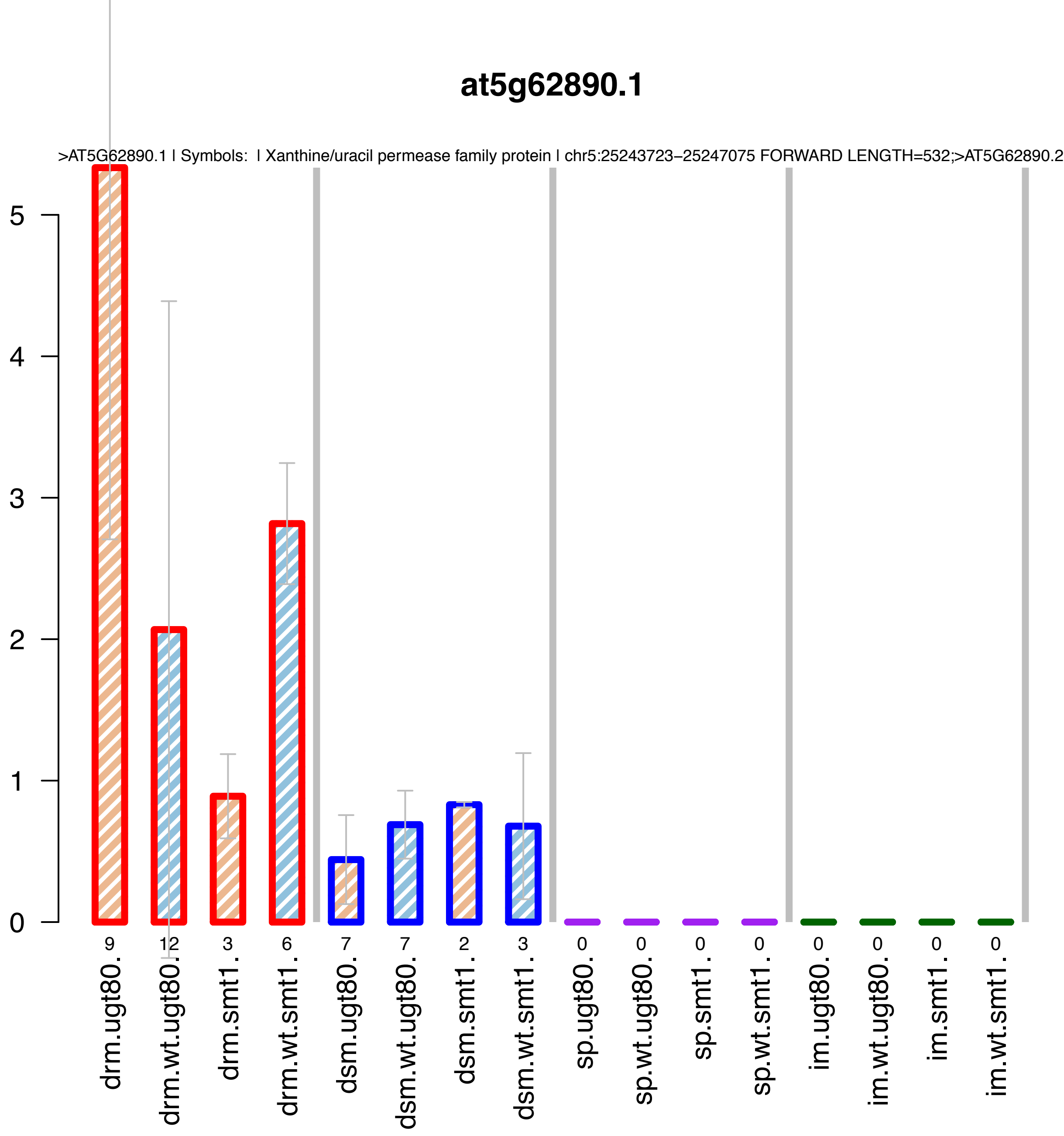
at5g62890.1
>AT5G62890.1 | Symbols: | Xanthine/uracil permease family protein | chr5:25243723-25247075 FORWARD LENGTH=532;>AT5G62890.2
0
1
2
3
4
5
9
12
3
6
7
7
2
3
0
0
0
0
0
0
0
0
drm.ugt80.
drm.wt.ugt80.
drm.smt1.
drm.wt.smt1.
dsm.ugt80.
dsm.wt.ugt80.
dsm.smt1.
dsm.wt.smt1.
sp.ugt80.
sp.wt.ugt80.
sp.smt1.
sp.wt.smt1.
im.ugt80.
im.wt.ugt80.
im.smt1.
im.wt.smt1.

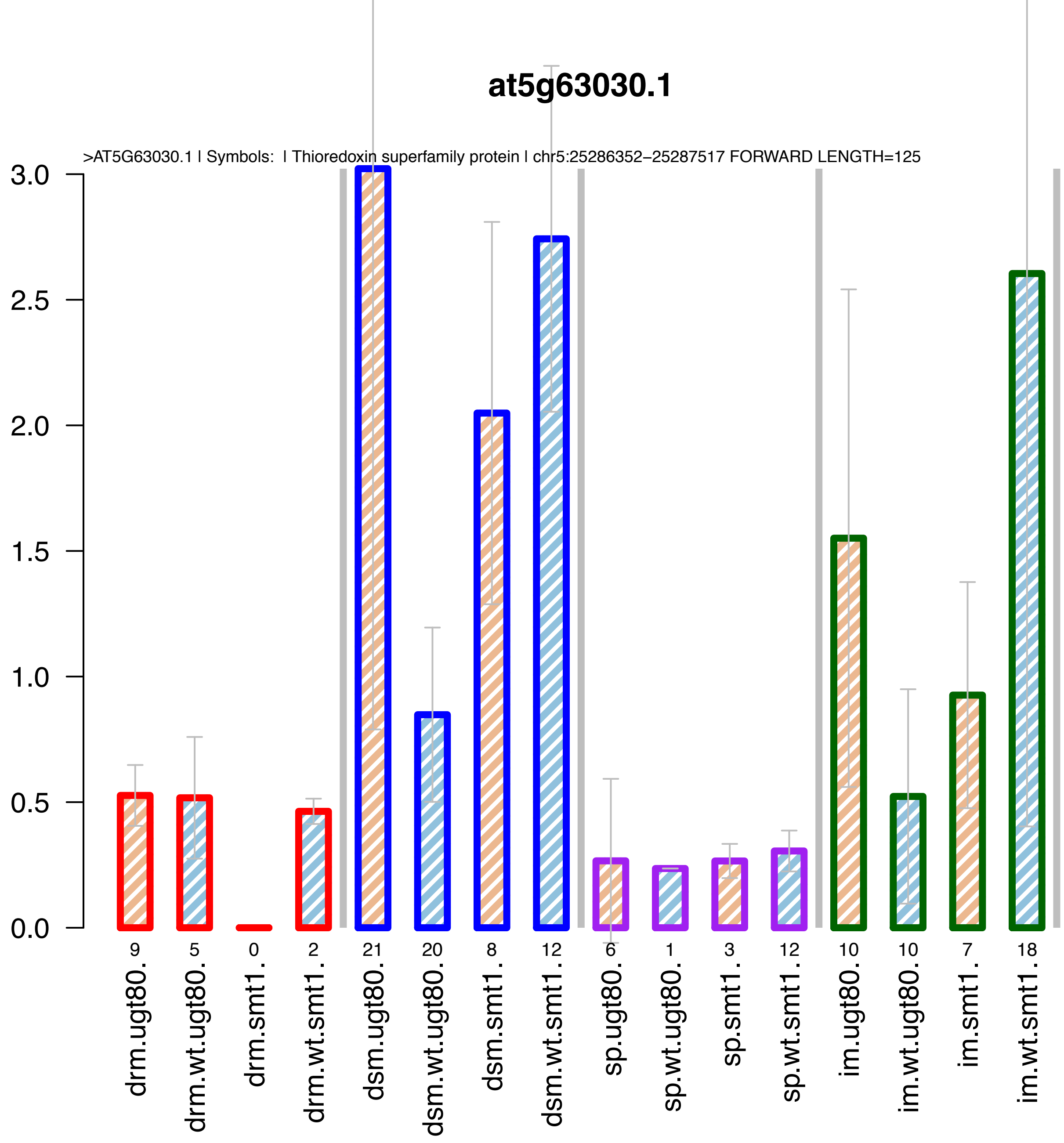

at5g63030.1
>AT5G63030.1 | Symbols: | Thioredoxin superfamily protein | chr5:25286352-25287517 FORWARD LENGTH=125
3.0
2.5
2.0
1.5
1.0
0.5
0.0
9
5
0
2
21
20
8
12
6
1
3
12
10
10
7
18
drm.ugt80..
drm.wt.ugt80..
drm.smt1..
drm.wt.smt1..
dsm.ugt80..
dsm.wt.ugt80..
dsm.smt1..
dsm.wt.smt1..
sp.ugt80..
sp.wt.ugt80..
sp.smt1..
sp.wt.smt1..
im.ugt80..
im.wt.ugt80..
im.smt1..
im.wt.smt1..

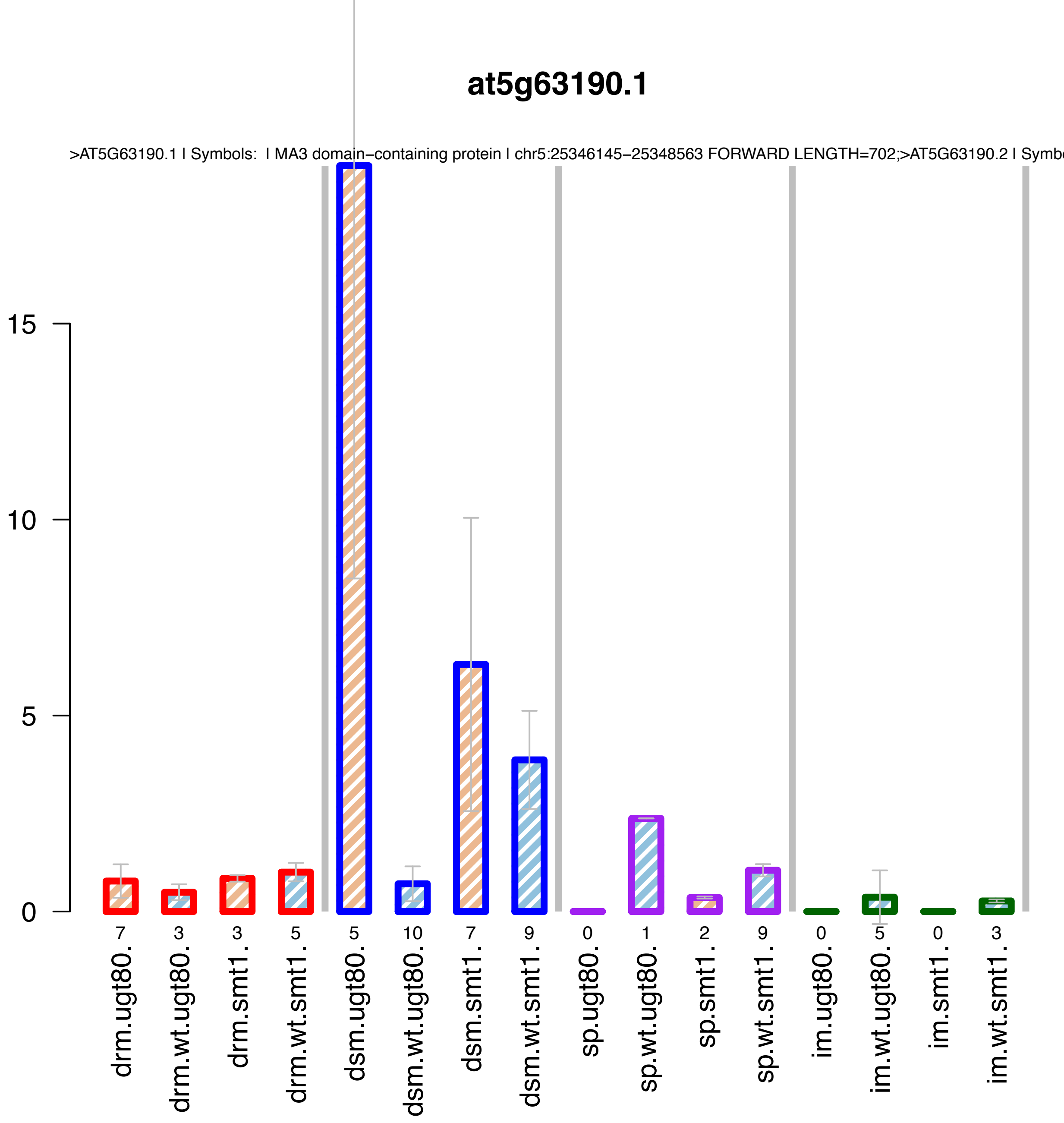
at5g63190.1
>AT5G63190.1 | Symbols: | MA3 domain−containing protein | chr5:25346145−25348563 FORWARD LENGTH=702;>AT5G63190.2 | Symbo
15
10
5
0
7
3
3
5
5
10
7
9
0
1
2
9
0
5
0
3
drm.ugt80.
drm.wt.ugt80.
drm.smt1.
drm.wt.smt1.
dsm.ugt80.
dsm.wt.ugt80.
dsm.smt1.
dsm.wt.smt1.
sp.ugt80.
sp.wt.ugt80.
sp.smt1.
sp.wt.smt1.
im.ugt80.
im.wt.ugt80.
im.smt1.
im.wt.smt1.

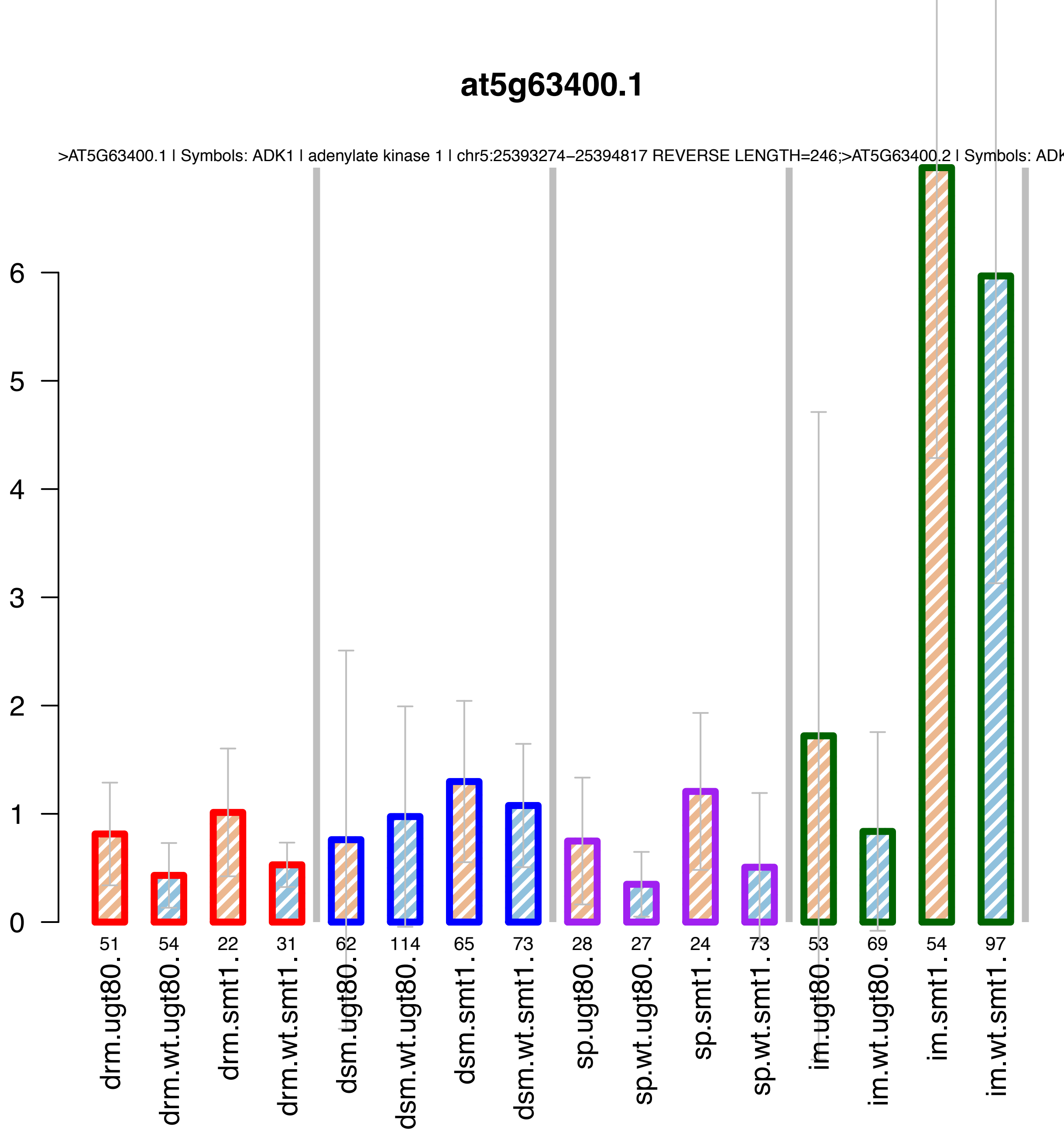

at5g63400.1
>AT5G63400.1 | Symbols: ADK1 | adenylate kinase 1 | chr5:25393274-25394817 REVERSE LENGTH=246;>AT5G63400.2 | Symbols: ADK
0
1
2
3
4
5
6
51
54
22
31
62
114
65
73
28
27
24
73
53
69
54
97
drm.ugt80..
drm.wt.ugt80..
drm.smt1..
drm.wt.smt1..
dsm.ugt80..
dsm.wt.ugt80..
dsm.smt1..
dsm.wt.smt1..
sp.ugt80..
sp.wt.ugt80..
sp.smt1..
sp.wt.smt1..
im.ugt80..
im.wt.ugt80..
im.smt1..
im.wt.smt1..

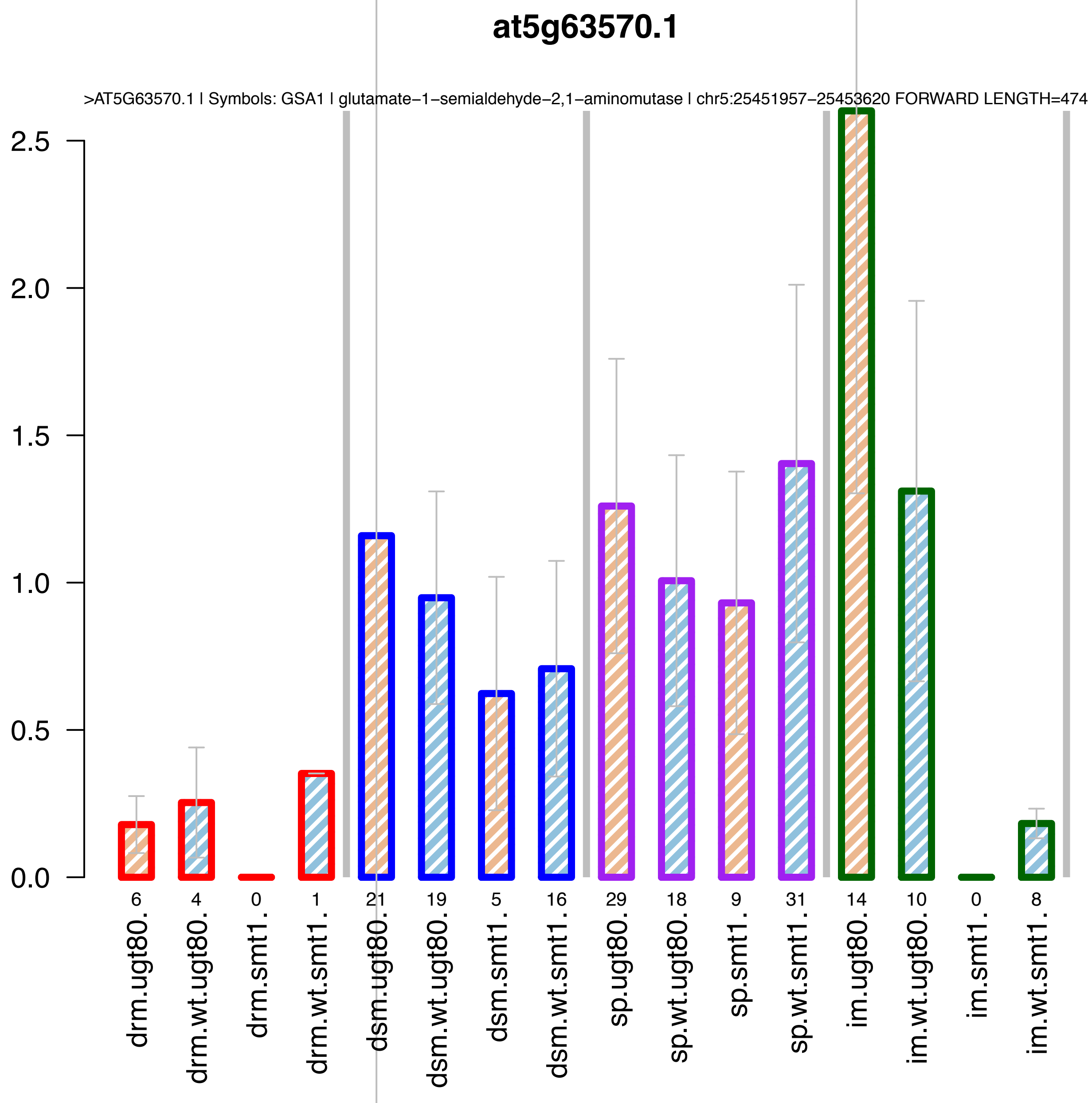

at5g63570.1
>AT5G63570.1 | Symbols: GSA1 | glutamate−1−semialdehyde−2,1−aminomutase | chr5:25451957−25453620 FORWARD LENGTH=474
2.5
2.0
1.5
1.0
0.5
0.0
6
4
0
1
21
19
5
16
29
18
9
31
14
10
0
8
drm.ugt80.
drm.wt.ugt80.
drm.smt1.
drm.wt.smt1.
dsm.ugt80.
dsm.wt.ugt80.
dsm.smt1.
dsm.wt.smt1.
sp.ugt80.
sp.wt.ugt80.
sp.smt1.
sp.wt.smt1.
im.ugt80.
im.wt.ugt80.
im.smt1.
im.wt.smt1.

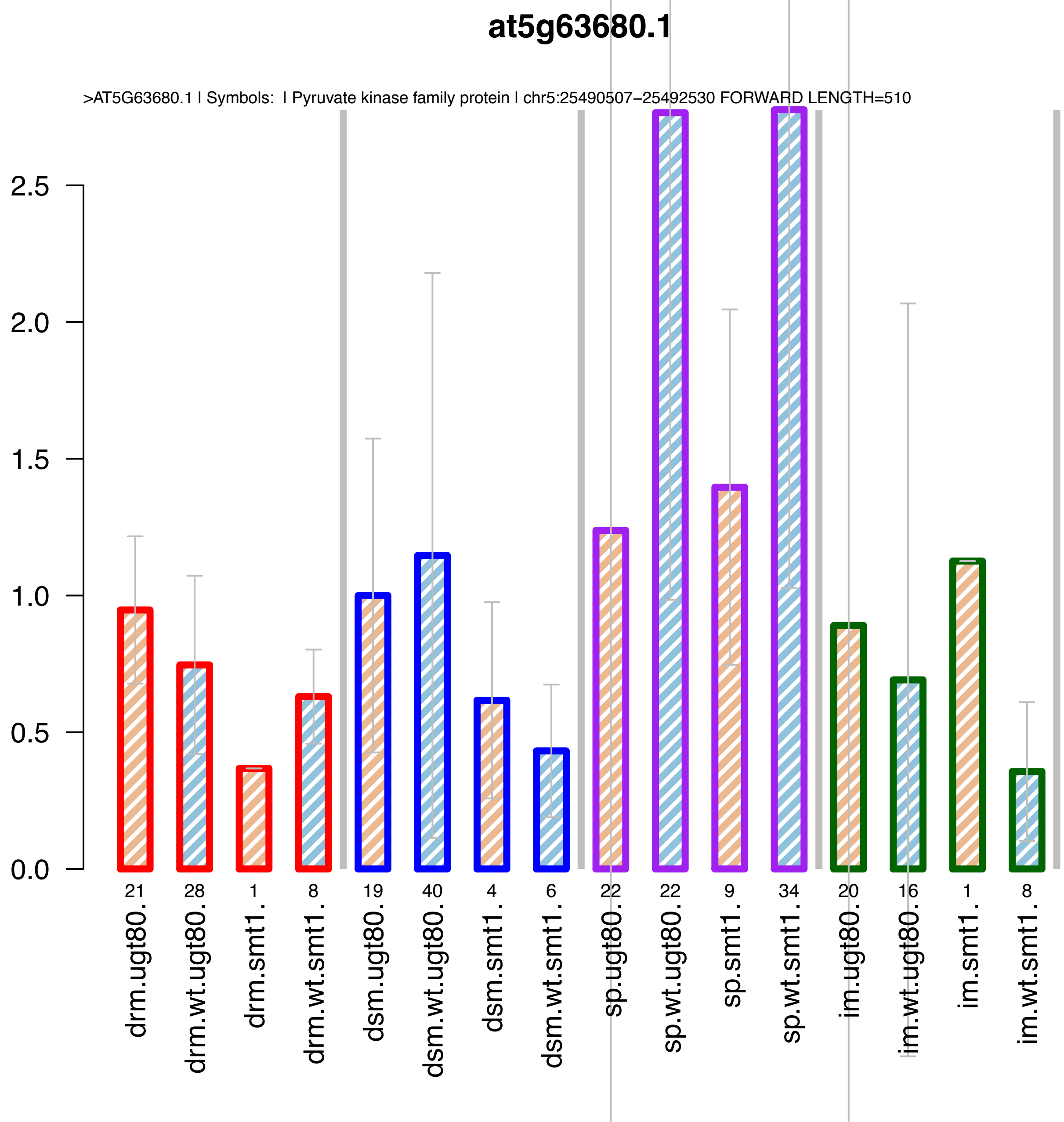

at5g63680.1
>AT5G63680.1 | Symbols: | Pyruvate kinase family protein | chr5:25490507-25492530 FORWARD LENGTH=510
0.0
0.5
1.0
1.5
2.0
2.5
21
28
1
8
19
40
4
6
22
22
9
34
20
16
1
8
drm.ugt80..
drm.wt.ugt80..
drm.smt1..
drm.wt.smt1..
dsm.ugt80..
dsm.wt.ugt80..
dsm.smt1..
dsm.wt.smt1..
sp.ugt80..
sp.wt.ugt80..
sp.smt1..
sp.wt.smt1..
im.ugt80..
im.wt.ugt80..
im.smt1..
im.wt.smt1..

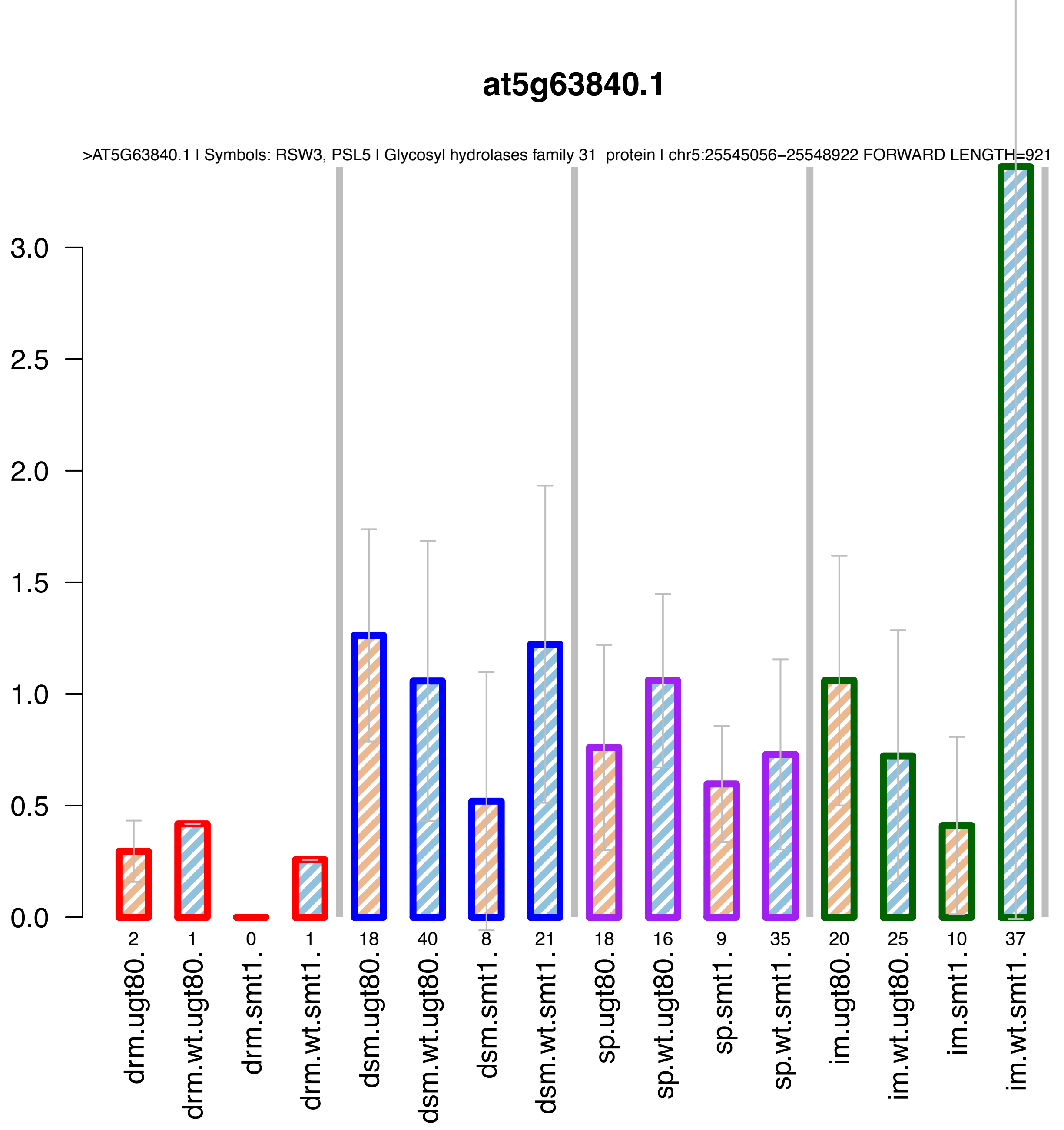

at5g63840.1
>AT5G63840.1 | Symbols: RSW3, PSL5 | Glycosyl hydrolases family 31 protein | chr5:25545056-25548922 FORWARD LENGTH=921
3.0
2.5
2.0
1.5
1.0
0.5
0.0
2
1
0
1
18
40
8
21
18
16
9
35
20
25
10
37
drm.ugt80.
drm.wt.ugt80.
drm.smt1.
drm.wt.smt1.
dsm.ugt80.
dsm.wt.ugt80.
dsm.smt1.
dsm.wt.smt1.
sp.ugt80.
sp.wt.ugt80.
sp.smt1.
sp.wt.smt1.
im.ugt80.
im.wt.ugt80.
im.smt1.
im.wt.smt1.

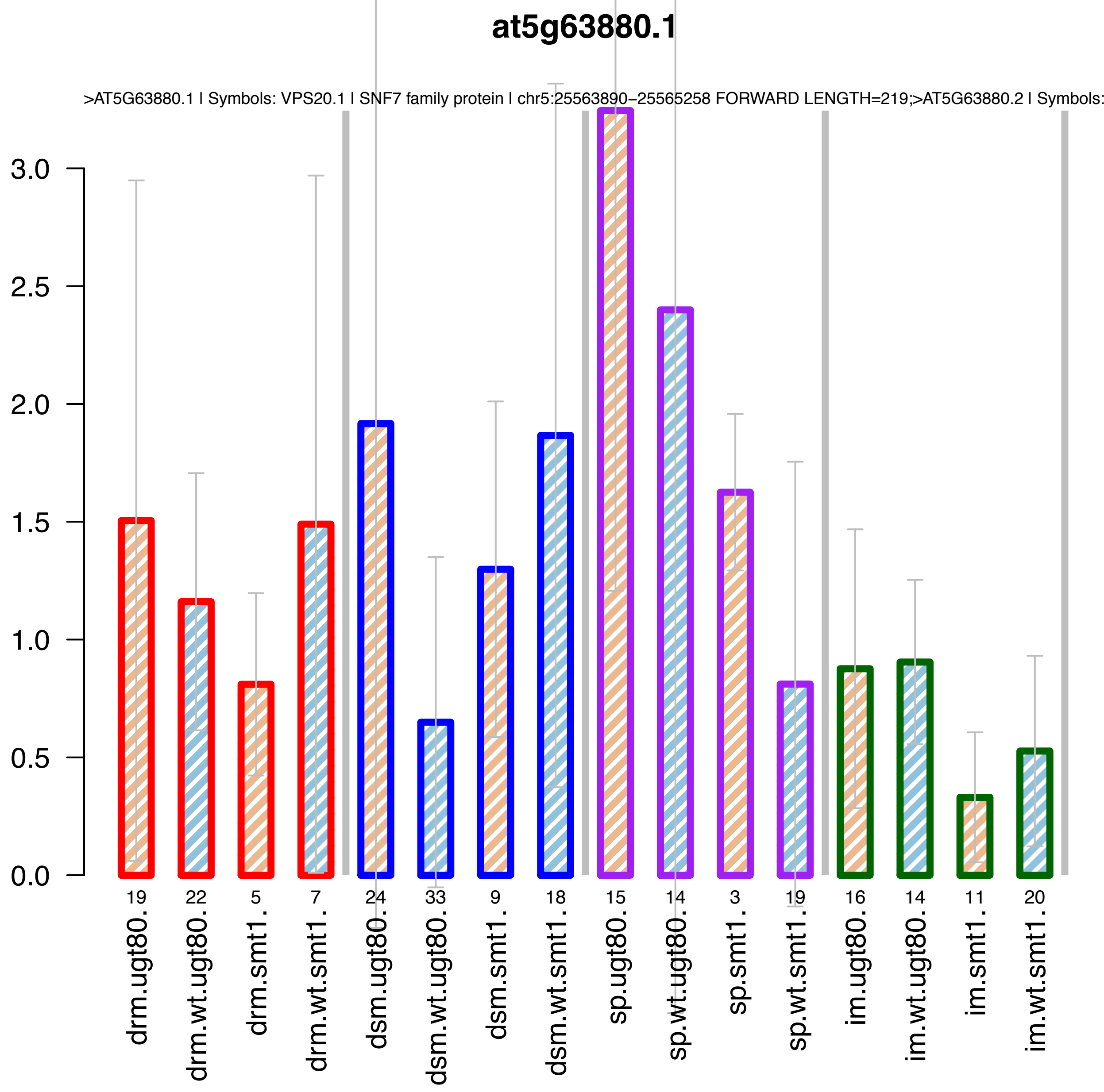

at5g63880.1
>AT5G63880.1 | Symbols: VPS20.1 | SNF7 family protein | chr5:25563890-25565258 FORWARD LENGTH=219;>AT5G63880.2 | Symbols:
3.0
2.5
2.0
1.5
1.0
0.5
0.0
19
22
5
7
24
33
9
18
15
14
3
19
16
14
11
20
drm.ugt80..
drm.wt.ugt80..
drm.smt1..
drm.wt.smt1..
dsm.ugt80..
dsm.wt.ugt80..
dsm.smt1..
dsm.wt.smt1..
sp.ugt80..
sp.wt.ugt80..
sp.smt1..
sp.wt.smt1..
im.ugt80..
im.wt.ugt80..
im.smt1..
im.wt.smt1..

# at5g63890.2

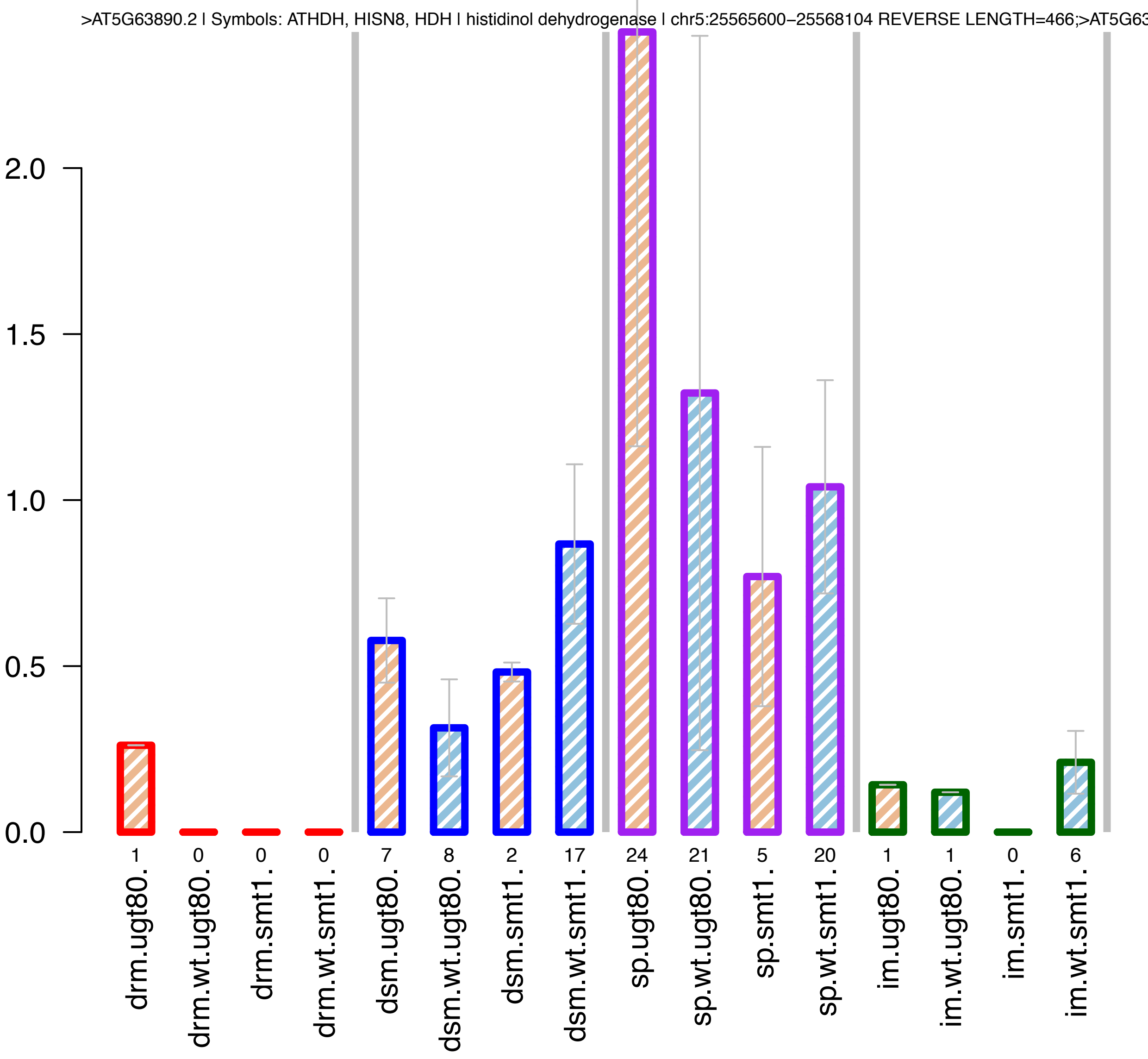

>AT5G63890.2 | Symbols: ATHDH, HISN8, HDH | histidinol dehydrogenase | chr5:25565600−25568104 REVERSE LENGTH=466;>AT5G63
2.0
1.5
1.0
0.5
0.0
1
0
0
0
7
8
2
17
24
21
5
20
1
1
0
6
drm.ugt80.
drm.wt.ugt80.
drm.smt1.
drm.wt.smt1.
dsm.ugt80.
dsm.wt.ugt80.
dsm.smt1.
dsm.wt.smt1.
sp.ugt80.
sp.wt.ugt80.
sp.smt1.
sp.wt.smt1.
im.ugt80.
im.wt.ugt80.
im.smt1.
im.wt.smt1.

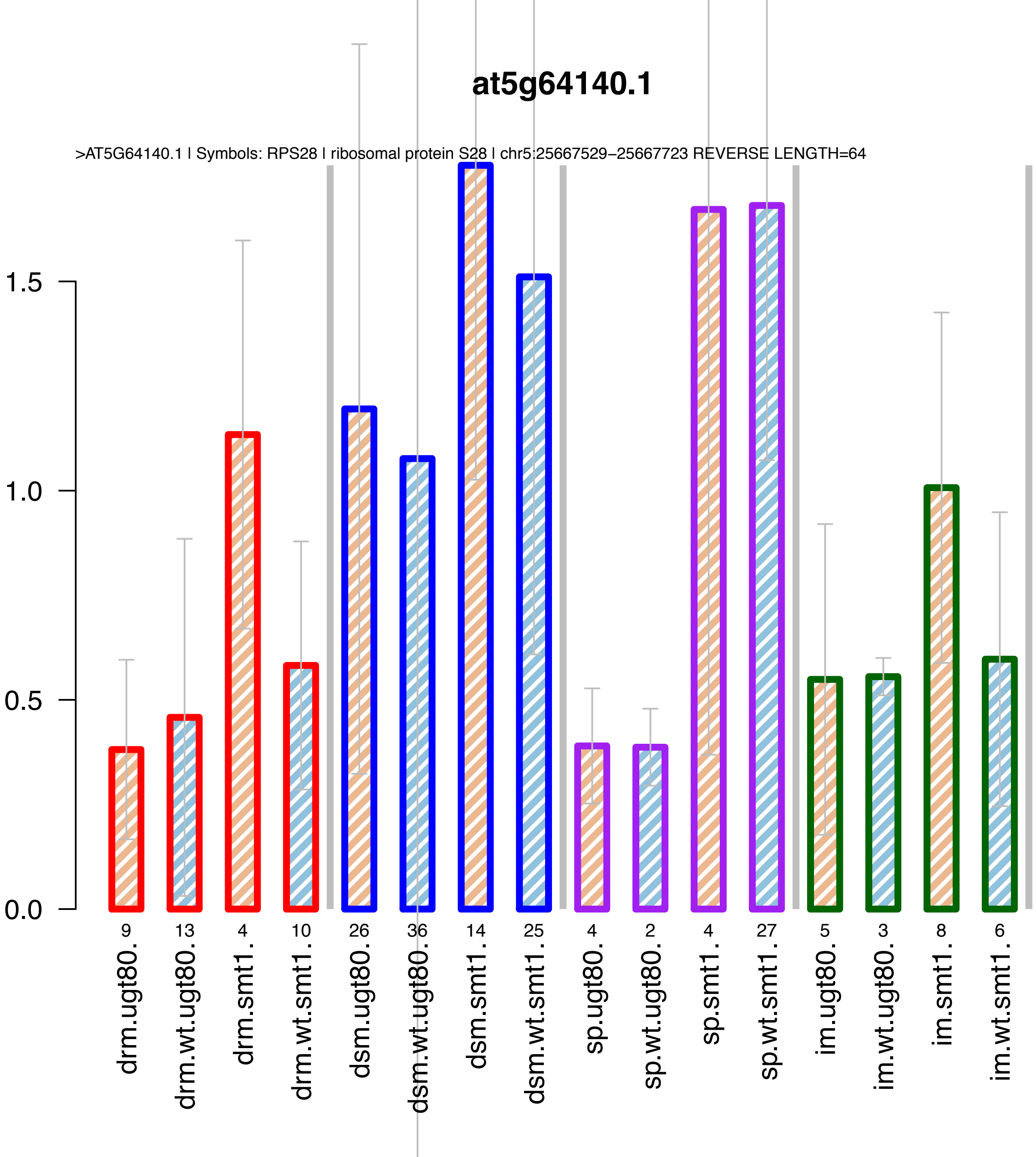

at5g64140.1
>AT5G64140.1 | Symbols: RPS28 | ribosomal protein S28 | chr5:25667529-25667723 REVERSE LENGTH=64
0.0
0.5
1.0
1.5
9
13
4
10
26
36
14
25
4
2
4
27
5
3
8
6
drm.ugt80..
drm.wt.ugt80..
drm.smt1..
drm.wt.smt1..
dsm.ugt80..
dsm.wt.ugt80..
dsm.smt1..
dsm.wt.smt1..
sp.ugt80..
sp.wt.ugt80..
sp.smt1..
sp.wt.smt1..
im.ugt80..
im.wt.ugt80..
im.smt1..
im.wt.smt1..

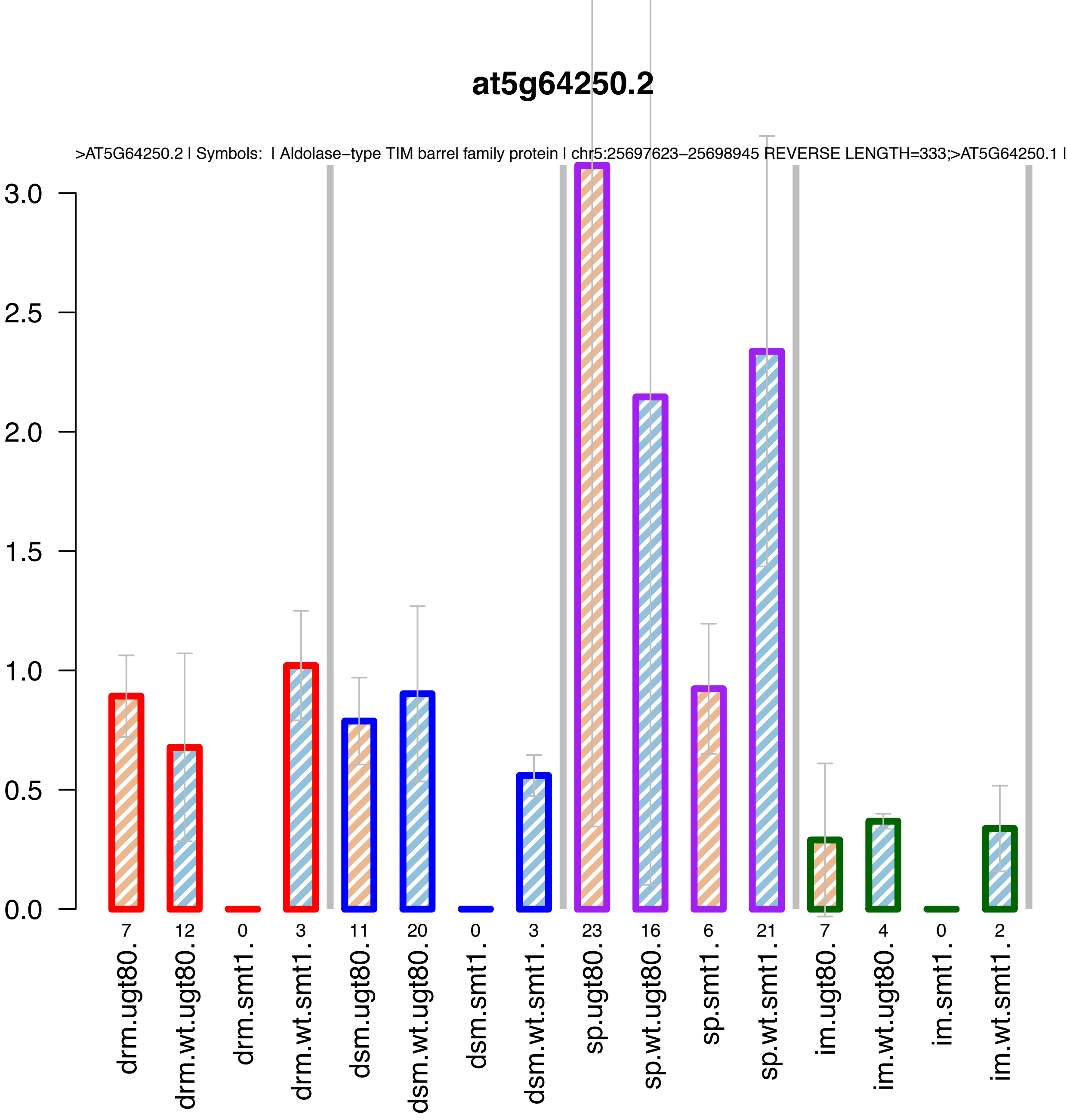

at5g64250.2
>AT5G64250.2 | Symbols: | Aldolase−type TIM barrel family protein | chr5:25697623−25698945 REVERSE LENGTH=333;>AT5G64250.1 |
3.0
2.5
2.0
1.5
1.0
0.5
0.0
7
12
0
3
11
20
0
3
23
16
6
21
7
4
0
2
drm.ugt80.
drm.wt.ugt80.
drm.smt1.
drm.wt.smt1.
dsm.ugt80.
dsm.wt.ugt80.
dsm.smt1.
dsm.wt.smt1.
sp.ugt80.
sp.wt.ugt80.
sp.smt1.
sp.wt.smt1.
im.ugt80.
im.wt.ugt80.
im.smt1.
im.wt.smt1.

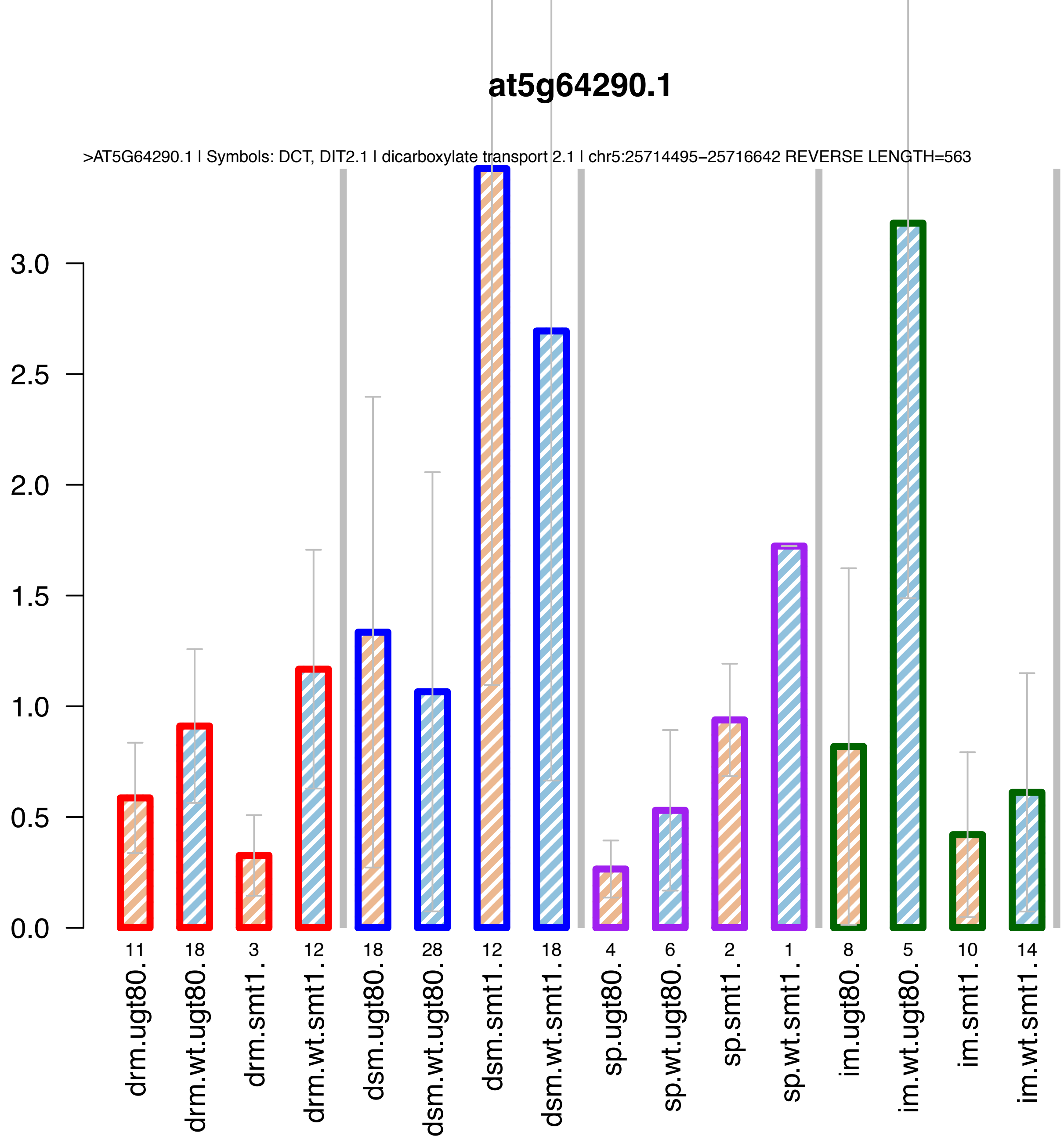

at5g64290.1
>AT5G64290.1 | Symbols: DCT, DIT2.1 | dicarboxylate transport 2.1 | chr5:25714495–25716642 REVERSE LENGTH=563
3.0
2.5
2.0
1.5
1.0
0.5
0.0
11
18
3
12
18
28
12
18
4
6
2
1
8
5
10
14
drm.ugt80..
drm.wt.ugt80..
drm.smt1..
drm.wt.smt1..
dsm.ugt80..
dsm.wt.ugt80..
dsm.smt1..
dsm.wt.smt1..
sp.ugt80..
sp.wt.ugt80..
sp.smt1..
sp.wt.smt1..
im.ugt80..
im.wt.ugt80..
im.smt1..
im.wt.smt1..

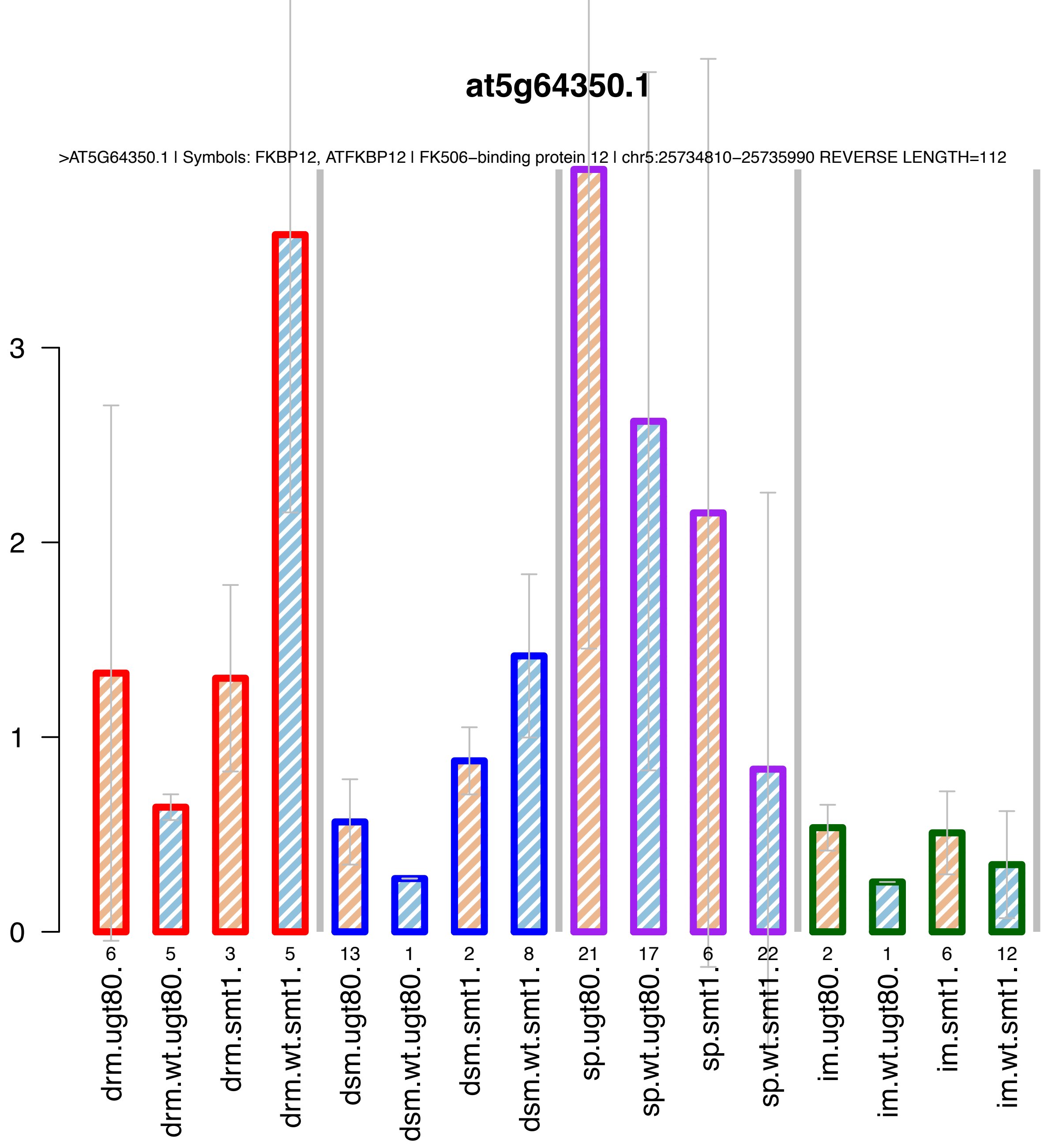

at5g64350.1
>AT5G64350.1 | Symbols: FKBP12, ATFKBP12 | FK506−binding protein 12 | chr5:25734810−25735990 REVERSE LENGTH=112
0
1
2
3
6
5
3
5
13
1
2
8
21
17
6
22
2
1
6
12
drm.ugt80.
drm.wt.ugt80.
drm.smt1.
drm.wt.smt1.
dsm.ugt80.
dsm.wt.ugt80.
dsm.smt1.
dsm.wt.smt1.
sp.ugt80.
sp.wt.ugt80.
sp.smt1.
sp.wt.smt1.
im.ugt80.
im.wt.ugt80.
im.smt1.
im.wt.smt1.

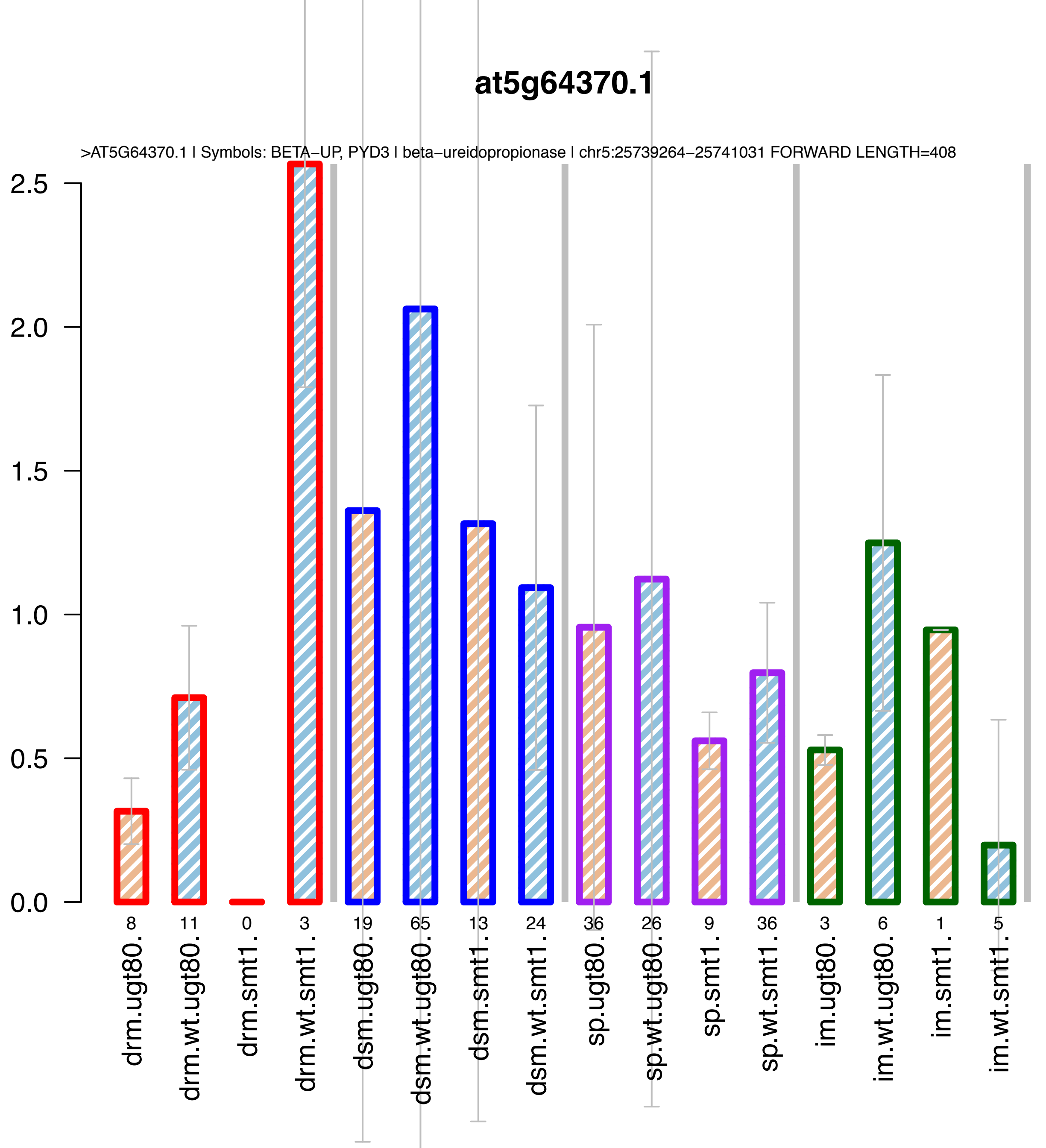

at5g64370.1
>AT5G64370.1 | Symbols: BETA−UP, PYD3 | beta−ureidopropionase | chr5:25739264−25741031 FORWARD LENGTH=408
2.5
2.0
1.5
1.0
0.5
0.0
8
11
0
3
19
65
13
24
36
26
9
36
3
6
1
5
drm.ugt80.
drm.wt.ugt80.
drm.smt1.
drm.wt.smt1.
dsm.ugt80.
dsm.wt.ugt80.
dsm.smt1.
dsm.wt.smt1.
sp.ugt80.
sp.wt.ugt80.
sp.smt1.
sp.wt.smt1.
im.ugt80.
im.wt.ugt80.
im.smt1.
im.wt.smt1.

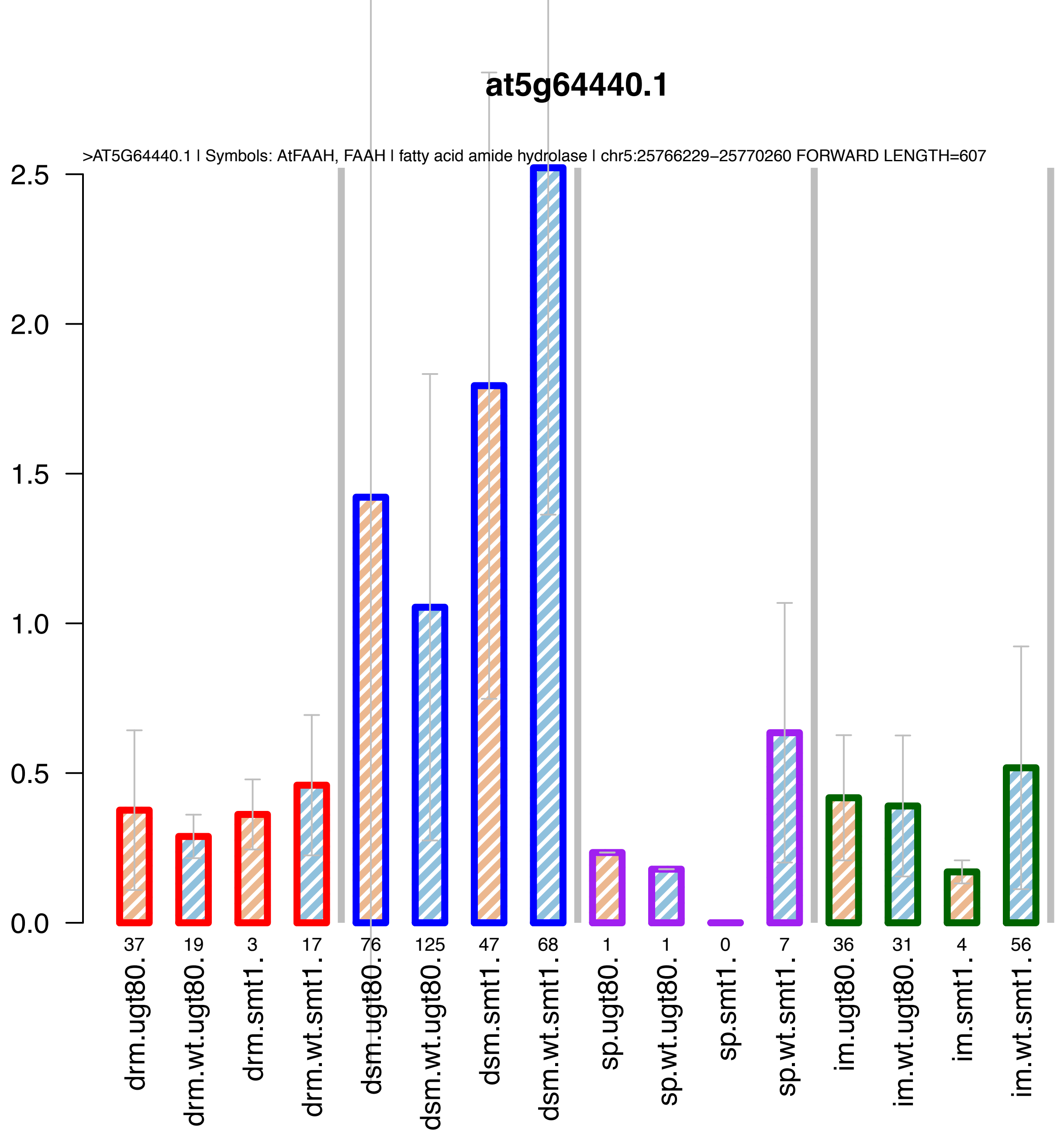

at5g64440.1
>AT5G64440.1 | Symbols: AtFAAH, FAAH | fatty acid amide hydrolase | chr5:25766229-25770260 FORWARD LENGTH=607
2.5
2.0
1.5
1.0
0.5
0.0
37
19
3
17
76
125
47
68
1
1
0
7
36
31
4
56
drm.ugt80.
drm.wt.ugt80.
drm.smt1.
drm.wt.smt1.
dsm.ugt80.
dsm.wt.ugt80.
dsm.smt1.
dsm.wt.smt1.
sp.ugt80.
sp.wt.ugt80.
sp.smt1.
sp.wt.smt1.
im.ugt80.
im.wt.ugt80.
im.smt1.
im.wt.smt1.

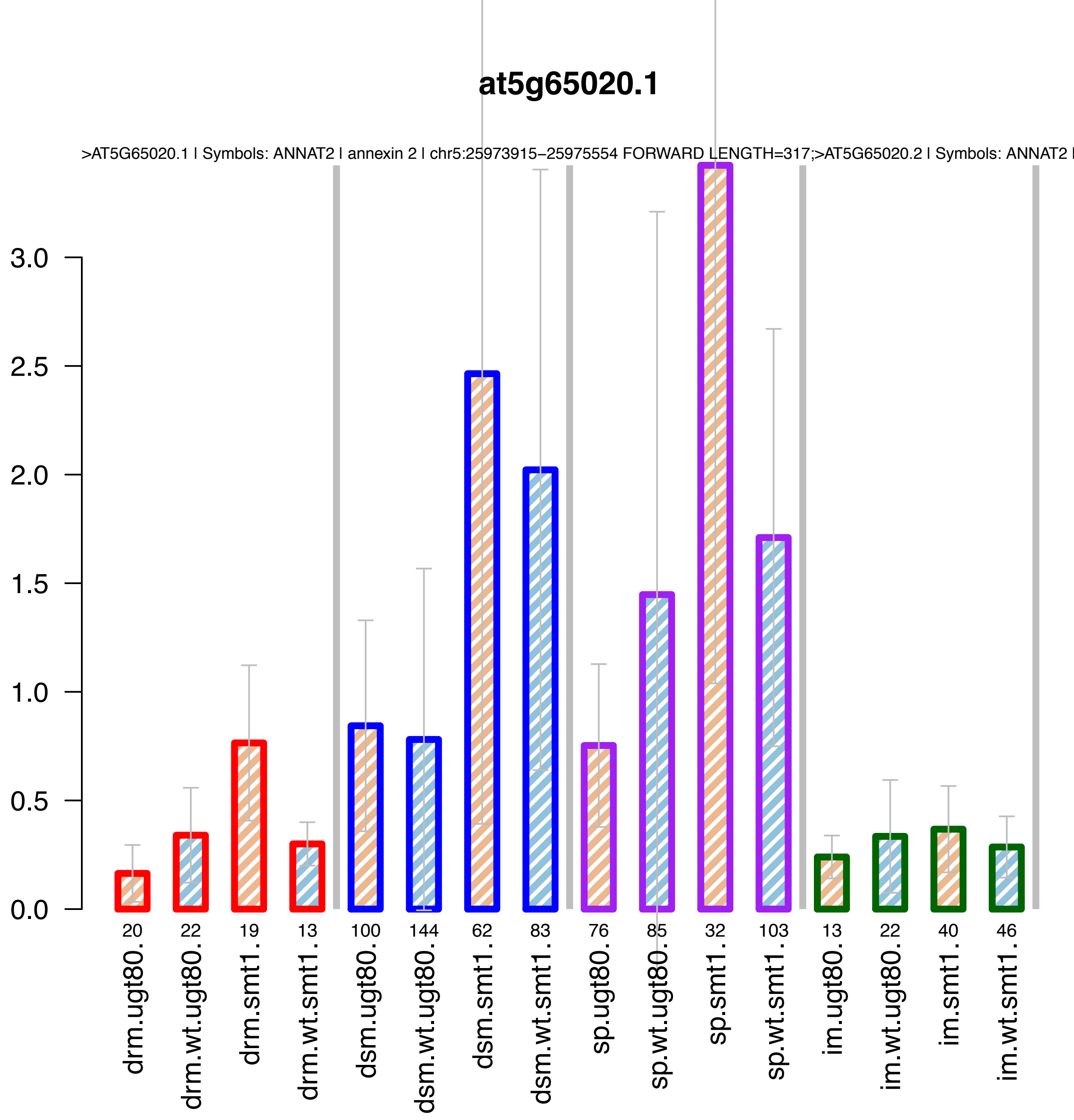
at5g65020.1
>AT5G65020.1 | Symbols: ANNAT2 | annexin 2 | chr5:25973915-25975554 FORWARD LENGTH=317;>AT5G65020.2 | Symbols: ANNAT2 |
3.0
2.5
2.0
1.5
1.0
0.5
0.0
20
22
19
13
100
144
62
83
76
85
32
103
13
22
40
46
drm.ugt80..
drm.wt.ugt80..
drm.smt1..
drm.wt.smt1..
dsm.ugt80..
dsm.wt.ugt80..
dsm.smt1..
dsm.wt.smt1..
sp.ugt80..
sp.wt.ugt80..
sp.smt1..
sp.wt.smt1..
im.ugt80..
im.wt.ugt80..
im.smt1..
im.wt.smt1..

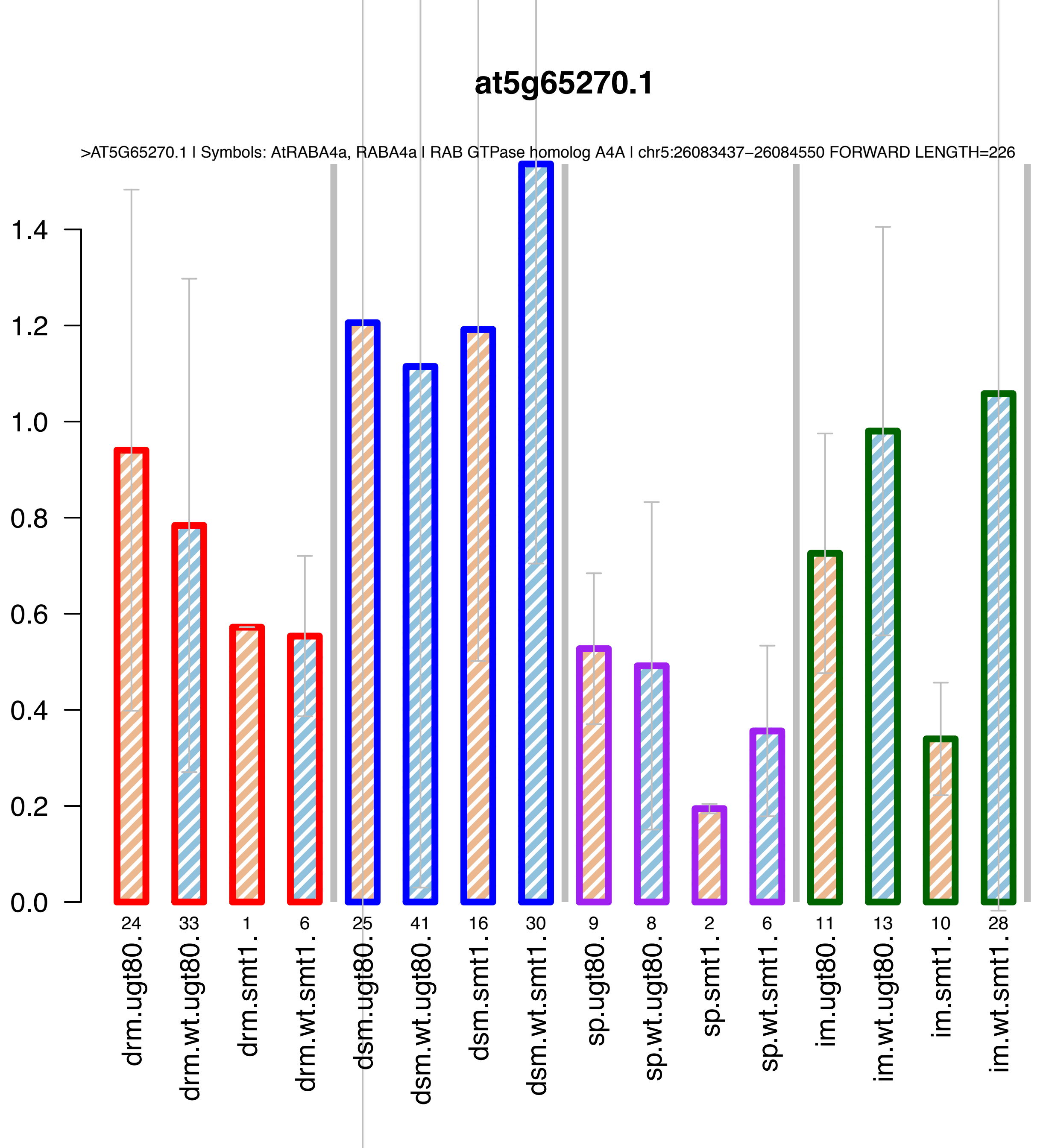

at5g65270.1
>AT5G65270.1 | Symbols: AtRABA4a, RABA4a | RAB GTPase homolog A4A | chr5:26083437-26084550 FORWARD LENGTH=226
0.0
0.2
0.4
0.6
0.8
1.0
1.2
1.4
24
33
1
6
25
41
16
30
9
8
2
6
11
13
10
28
drm.ugt80..
drm.wt.ugt80..
drm.smt1..
drm.wt.smt1..
dsm.ugt80..
dsm.wt.ugt80..
dsm.smt1..
dsm.wt.smt1..
sp.ugt80..
sp.wt.ugt80..
sp.smt1..
sp.wt.smt1..
im.ugt80..
im.wt.ugt80..
im.smt1..
im.wt.smt1..

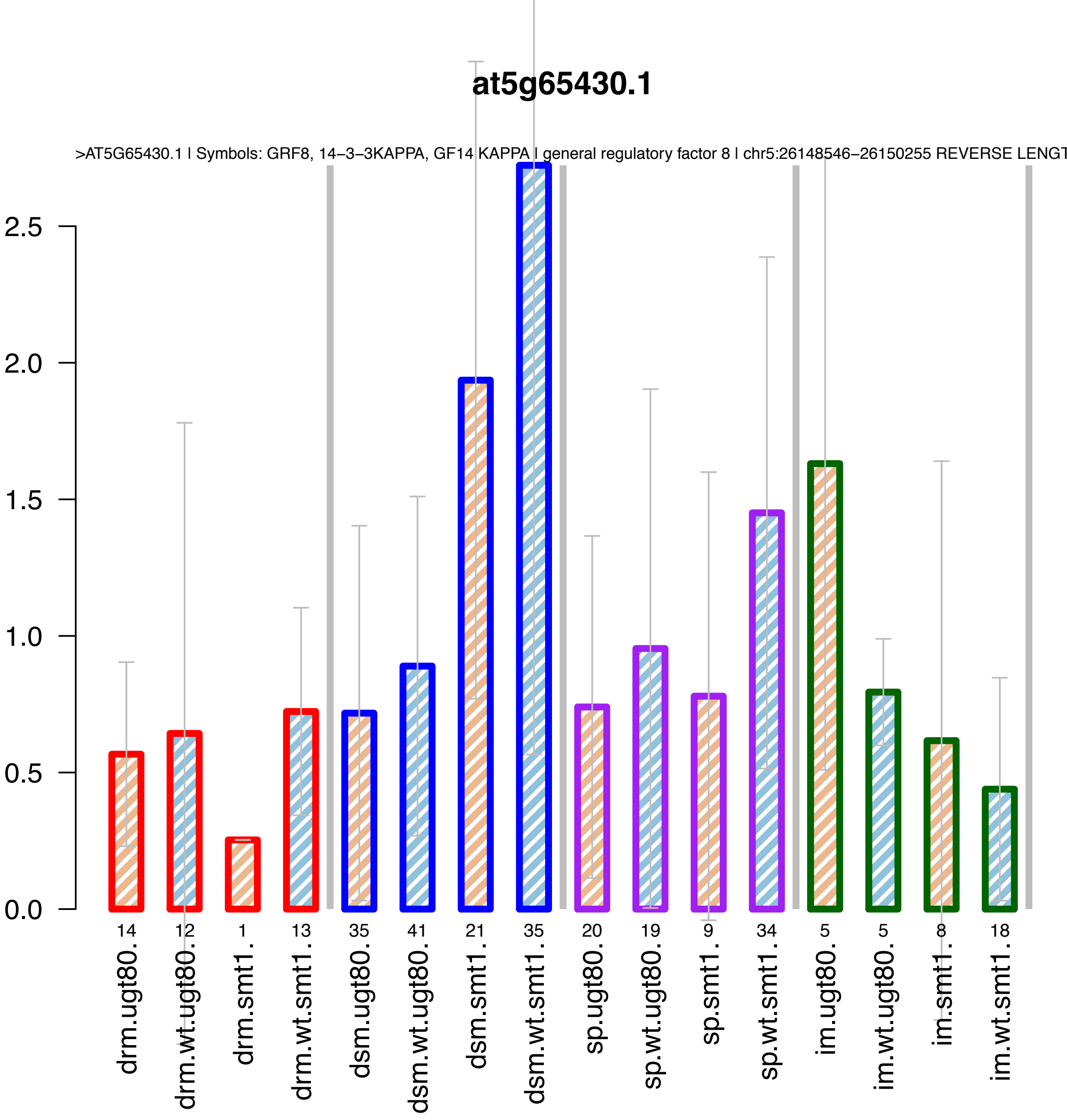
at5g65430.1
>AT5G65430.1 | Symbols: GRF8, 14-3-3KAPPA, GF14 KAPPA | general regulatory factor 8 | chr5:26148546-26150255 REVERSE LENGT
2.5
2.0
1.5
1.0
0.5
0.0
14
12
1
13
35
41
21
35
20
19
9
34
5
5
8
18
drm.ugt80..
drm.wt.ugt80..
drm.smt1..
drm.wt.smt1..
dsm.ugt80..
dsm.wt.ugt80..
dsm.smt1..
dsm.wt.smt1..
sp.ugt80..
sp.wt.ugt80..
sp.smt1..
sp.wt.smt1..
im.ugt80..
im.wt.ugt80..
im.smt1..
im.wt.smt1..

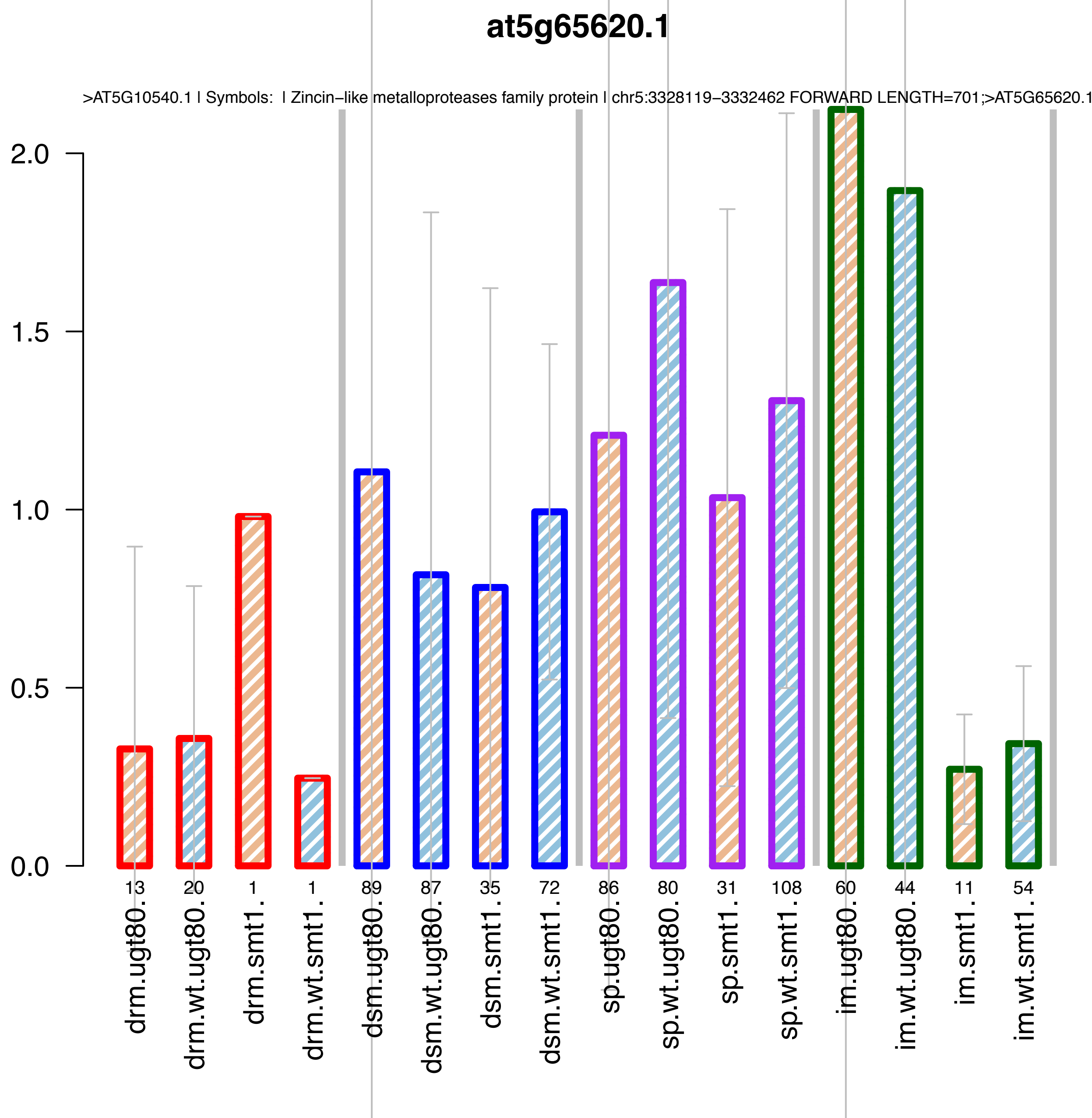

at5g65620.1
>AT5G10540.1 | Symbols: | Zincin–like metalloproteases family protein | chr5:3328119–3332462 FORWARD LENGTH=701;>AT5G65620.1
2.0
1.5
1.0
0.5
0.0
13
20
1
1
89
87
35
72
86
80
31
108
60
44
11
54
drm.ugt80..
drm.wt.ugt80..
drm.smt1..
drm.wt.smt1..
dsm.ugt80..
dsm.wt.ugt80..
dsm.smt1..
dsm.wt.smt1..
sp.ugt80..
sp.wt.ugt80..
sp.smt1..
sp.wt.smt1..
im.ugt80..
im.wt.ugt80..
im.smt1..
im.wt.smt1..

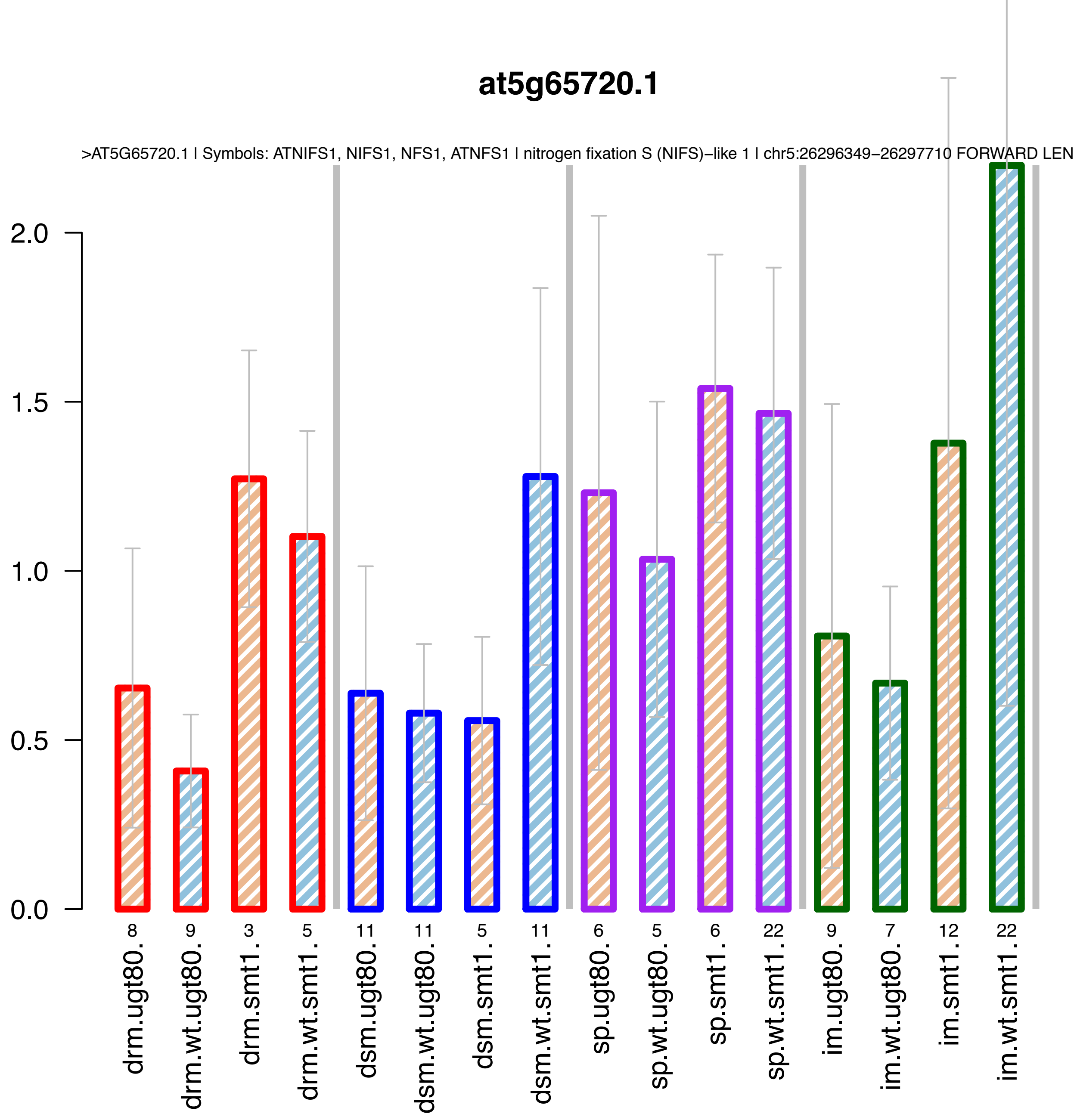

at5g65720.1
>AT5G65720.1 | Symbols: ATNIFS1, NIFS1, NFS1, ATNFS1 | nitrogen fixation S (NIFS)−like 1 | chr5:26296349−26297710 FORWARD LEN
0.0
0.5
1.0
1.5
2.0
8
9
3
5
11
11
5
11
6
5
6
22
9
7
12
22
drm.ugt80.
drm.wt.ugt80.
drm.smt1.
drm.wt.smt1.
dsm.ugt80.
dsm.wt.ugt80.
dsm.smt1.
dsm.wt.smt1.
sp.ugt80.
sp.wt.ugt80.
sp.smt1.
sp.wt.smt1.
im.ugt80.
im.wt.ugt80.
im.smt1.
im.wt.smt1.

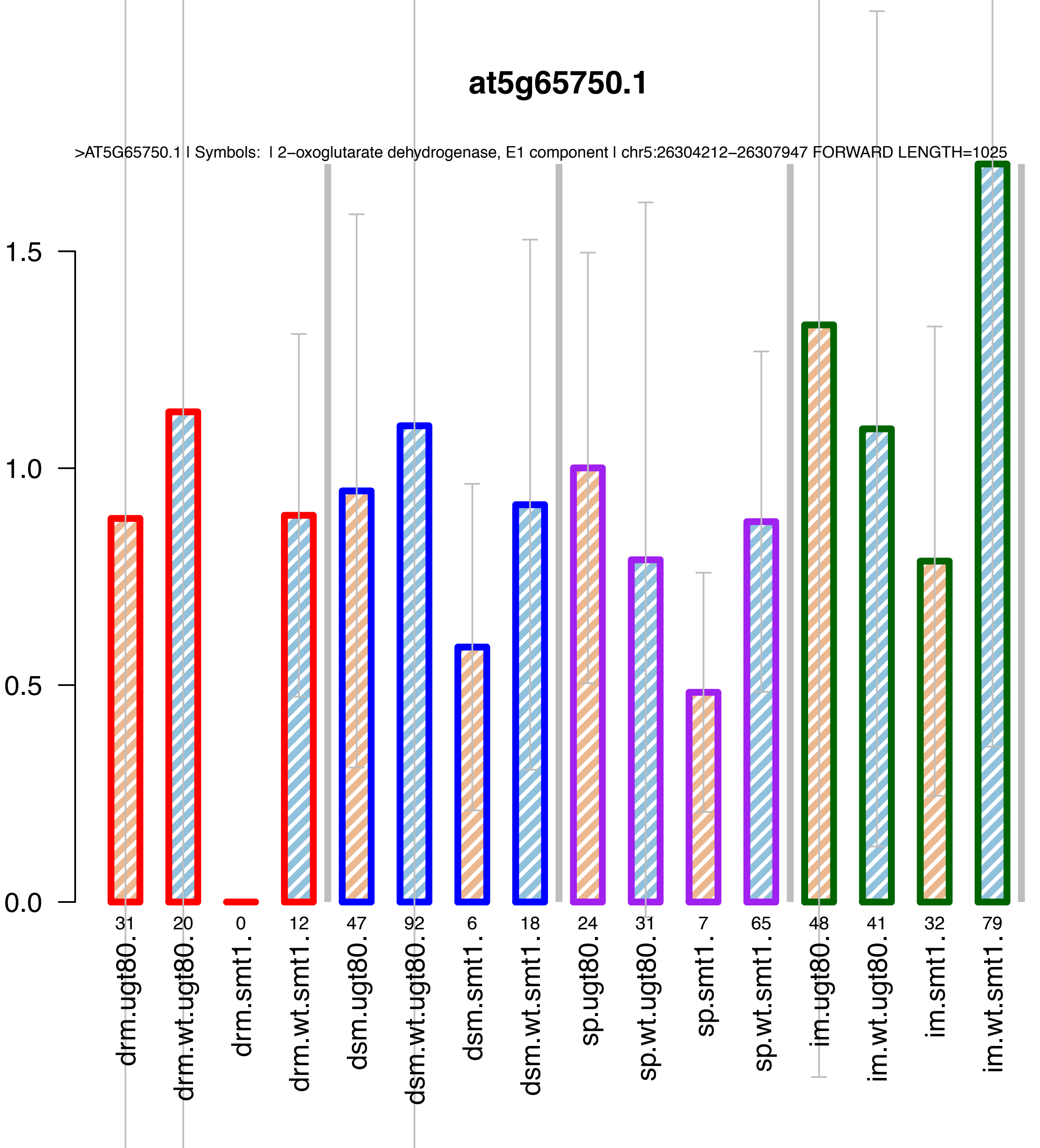

at5g65750.1
>AT5G65750.1 | Symbols: | 2−oxoglutarate dehydrogenase, E1 component | chr5:26304212−26307947 FORWARD LENGTH=1025
0.0
0.5
1.0
1.5
31
20
0
12
47
92
6
18
24
31
7
65
48
41
32
79
drm.ugt80..
drm.wt.ugt80..
drm.smt1..
drm.wt.smt1..
dsm.ugt80..
dsm.wt.ugt80..
dsm.smt1..
dsm.wt.smt1..
sp.ugt80..
sp.wt.ugt80..
sp.smt1..
sp.wt.smt1..
im.ugt80..
im.wt.ugt80..
im.smt1..
im.wt.smt1..

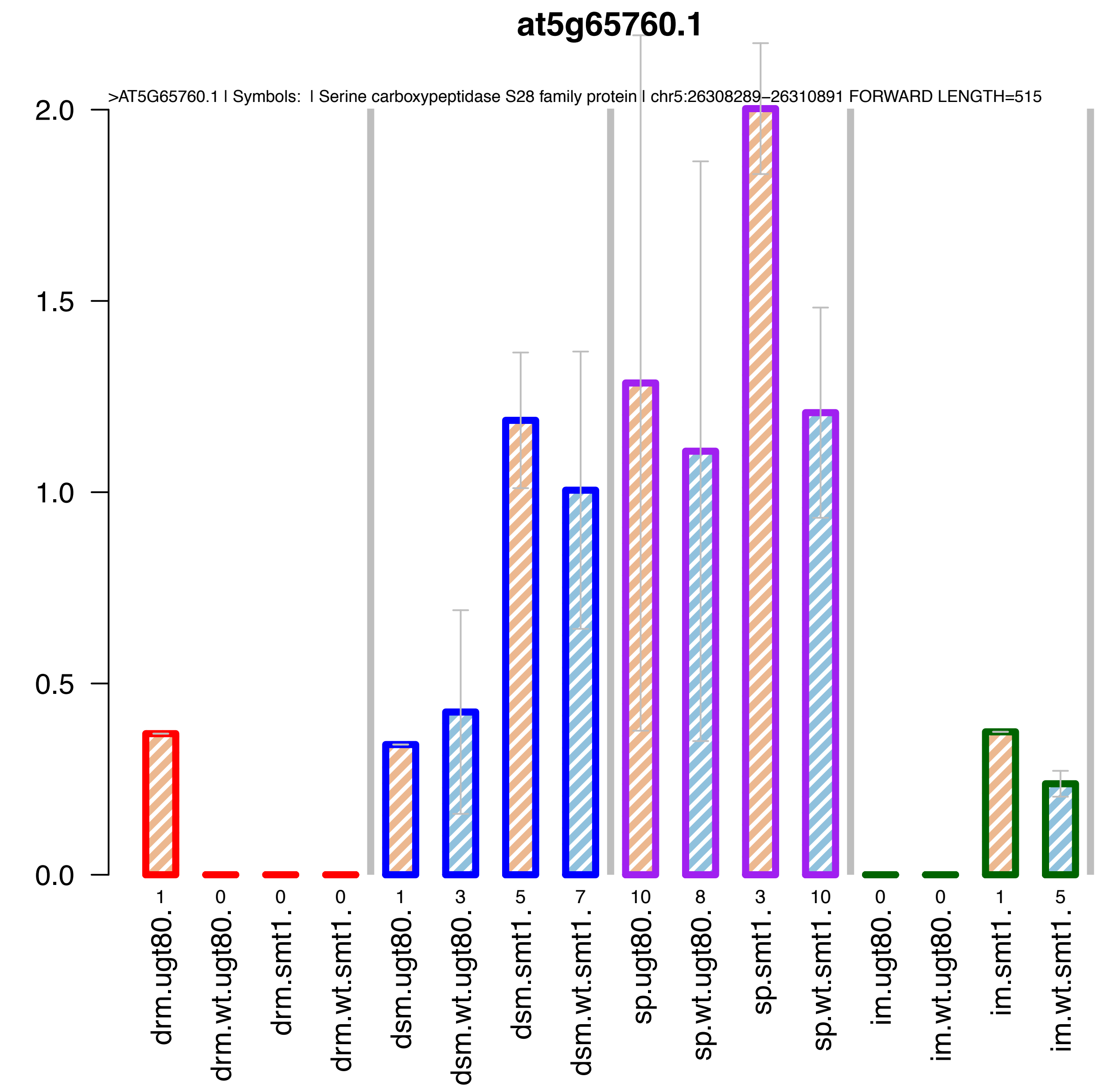

at5g65760.1
>AT5G65760.1 | Symbols: | Serine carboxypeptidase S28 family protein | chr5:26308289–26310891 FORWARD LENGTH=515
2.0
1.5
1.0
0.5
0.0
1
0
0
0
1
3
5
7
10
8
3
10
0
0
1
5
drm.ugt80.
drm.wt.ugt80.
drm.smt1.
drm.wt.smt1.
dsm.ugt80.
dsm.wt.ugt80.
dsm.smt1.
dsm.wt.smt1.
sp.ugt80.
sp.wt.ugt80.
sp.smt1.
sp.wt.smt1.
im.ugt80.
im.wt.ugt80.
im.smt1.
im.wt.smt1.

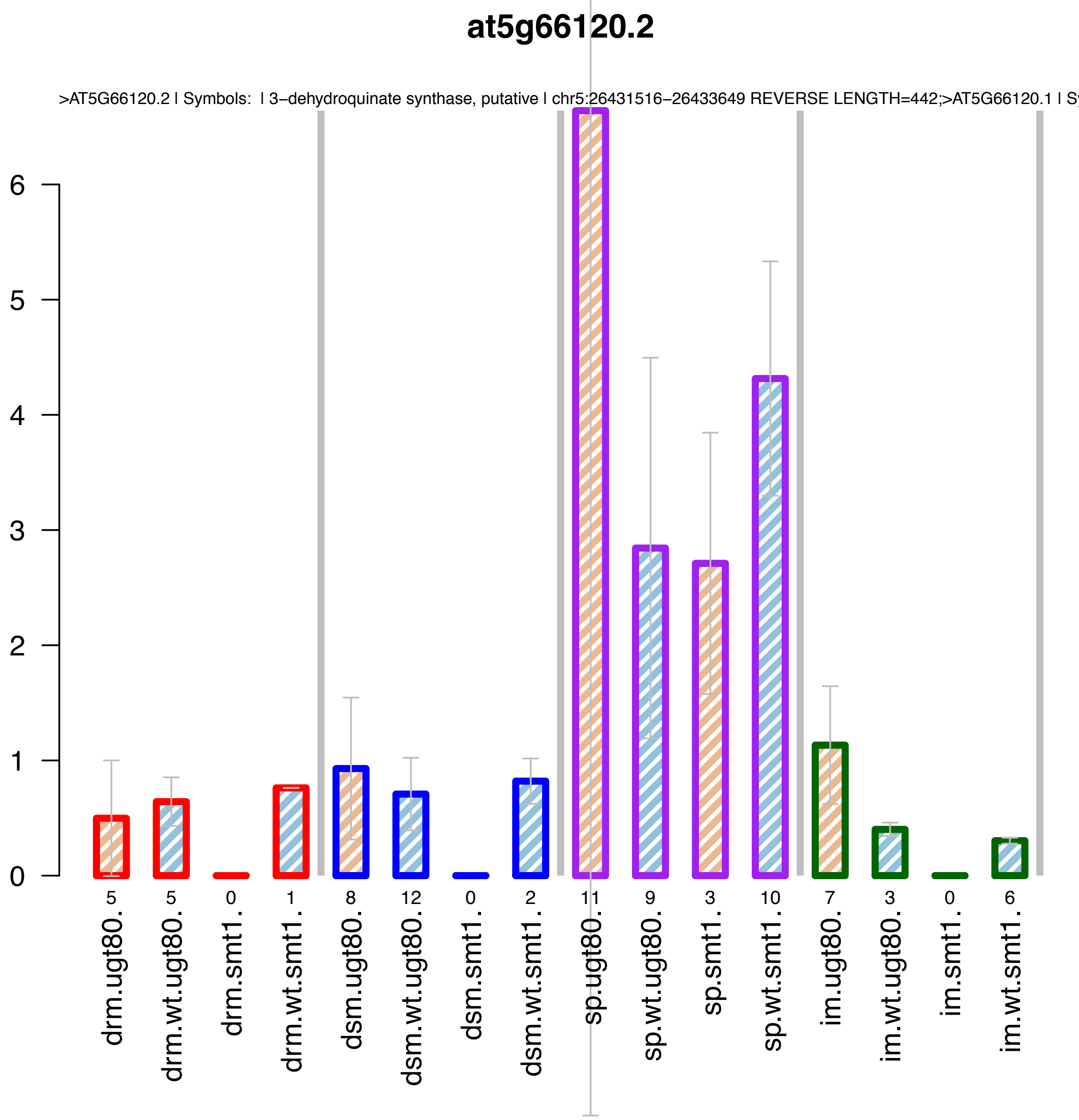
at5g66120.2
>AT5G66120.2 | Symbols: | 3−dehydroquinate synthase, putative | chr5:26431516−26433649 REVERSE LENGTH=442;>AT5G66120.1 | S
0
1
2
3
4
5
6
5
5
0
1
8
12
0
2
11
9
3
10
7
3
0
6
drm.ugt80.
drm.wt.ugt80.
drm.smt1.
drm.wt.smt1.
dsm.ugt80.
dsm.wt.ugt80.
dsm.smt1.
dsm.wt.smt1.
sp.ugt80.
sp.wt.ugt80.
sp.smt1.
sp.wt.smt1.
im.ugt80.
im.wt.ugt80.
im.smt1.
im.wt.smt1.

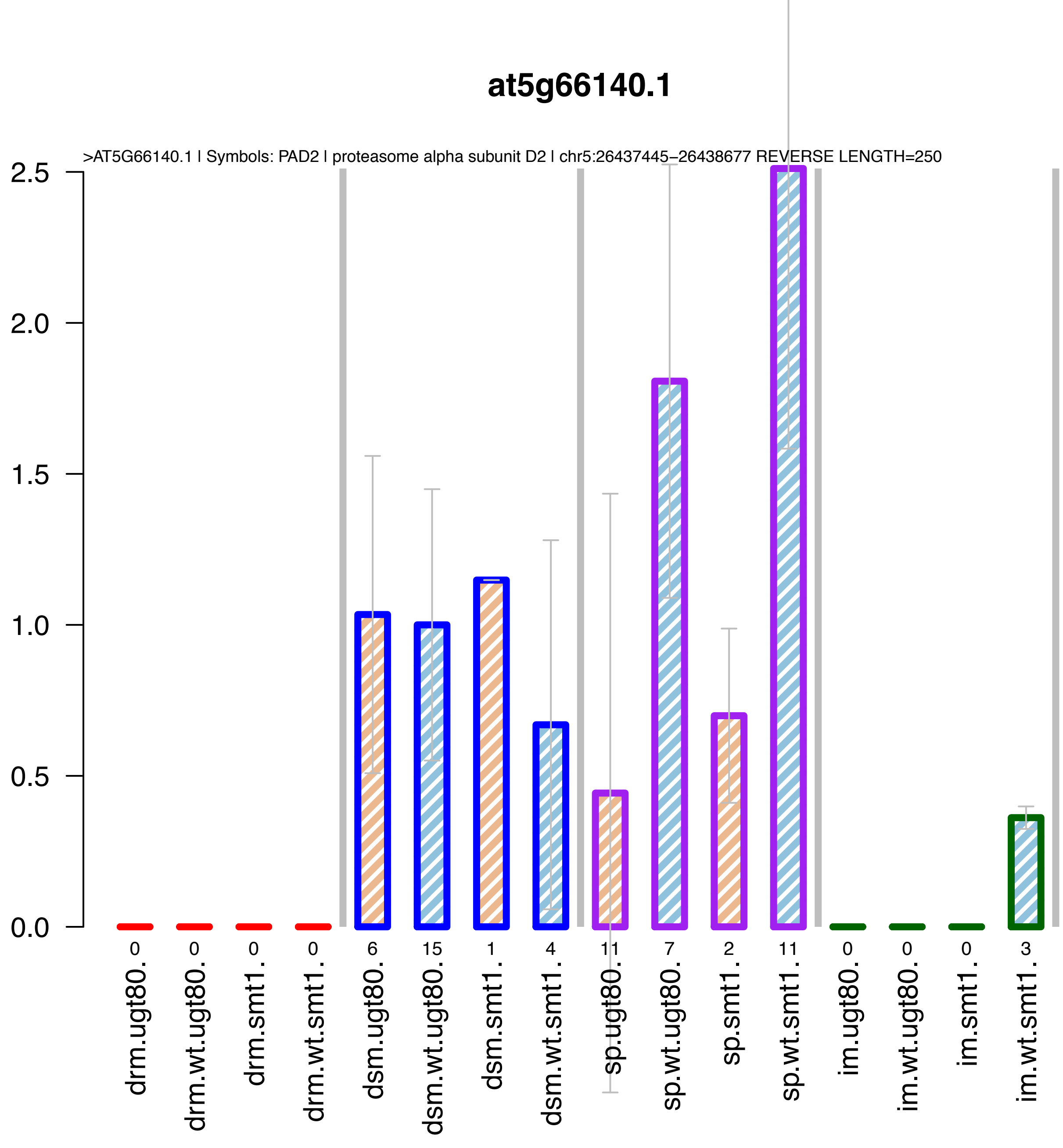

at5g66140.1
>AT5G66140.1 | Symbols: PAD2 | proteasome alpha subunit D2 | chr5:26437445-26438677 REVERSE LENGTH=250
2.5
2.0
1.5
1.0
0.5
0.0
0
0
0
0
6
15
1
4
11
7
2
11
0
0
0
3
drm.ugt80.
drm.wt.ugt80.
drm.smt1.
drm.wt.smt1.
dsm.ugt80.
dsm.wt.ugt80.
dsm.smt1.
dsm.wt.smt1.
sp.ugt80.
sp.wt.ugt80.
sp.smt1.
sp.wt.smt1.
im.ugt80.
im.wt.ugt80.
im.smt1.
im.wt.smt1.

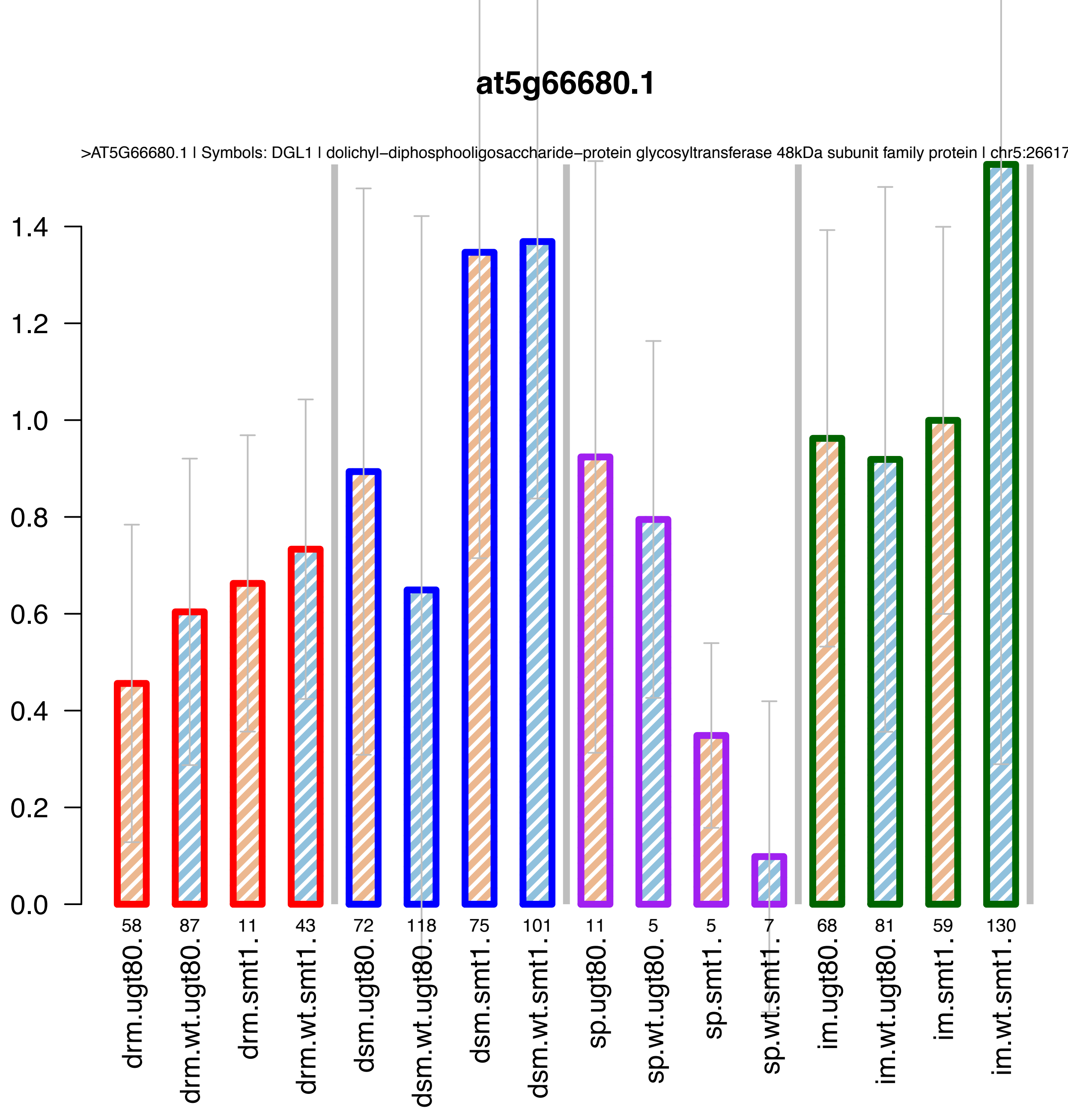

at5g66680.1
>AT5G66680.1 | Symbols: DGL1 | dolichyl−diphosphooligosaccharide−protein glycosyltransferase 48kDa subunit family protein | chr5:26617
0.0
0.2
0.4
0.6
0.8
1.0
1.2
1.4
58
87
11
43
72
118
75
101
11
5
5
7
68
81
59
130
drm.ugt80..
drm.wt.ugt80..
drm.smt1..
drm.wt.smt1..
dsm.ugt80..
dsm.wt.ugt80..
dsm.smt1..
dsm.wt.smt1..
sp.ugt80..
sp.wt.ugt80..
sp.smt1..
sp.wt.smt1..
im.ugt80..
im.wt.ugt80..
im.smt1..
im.wt.smt1..

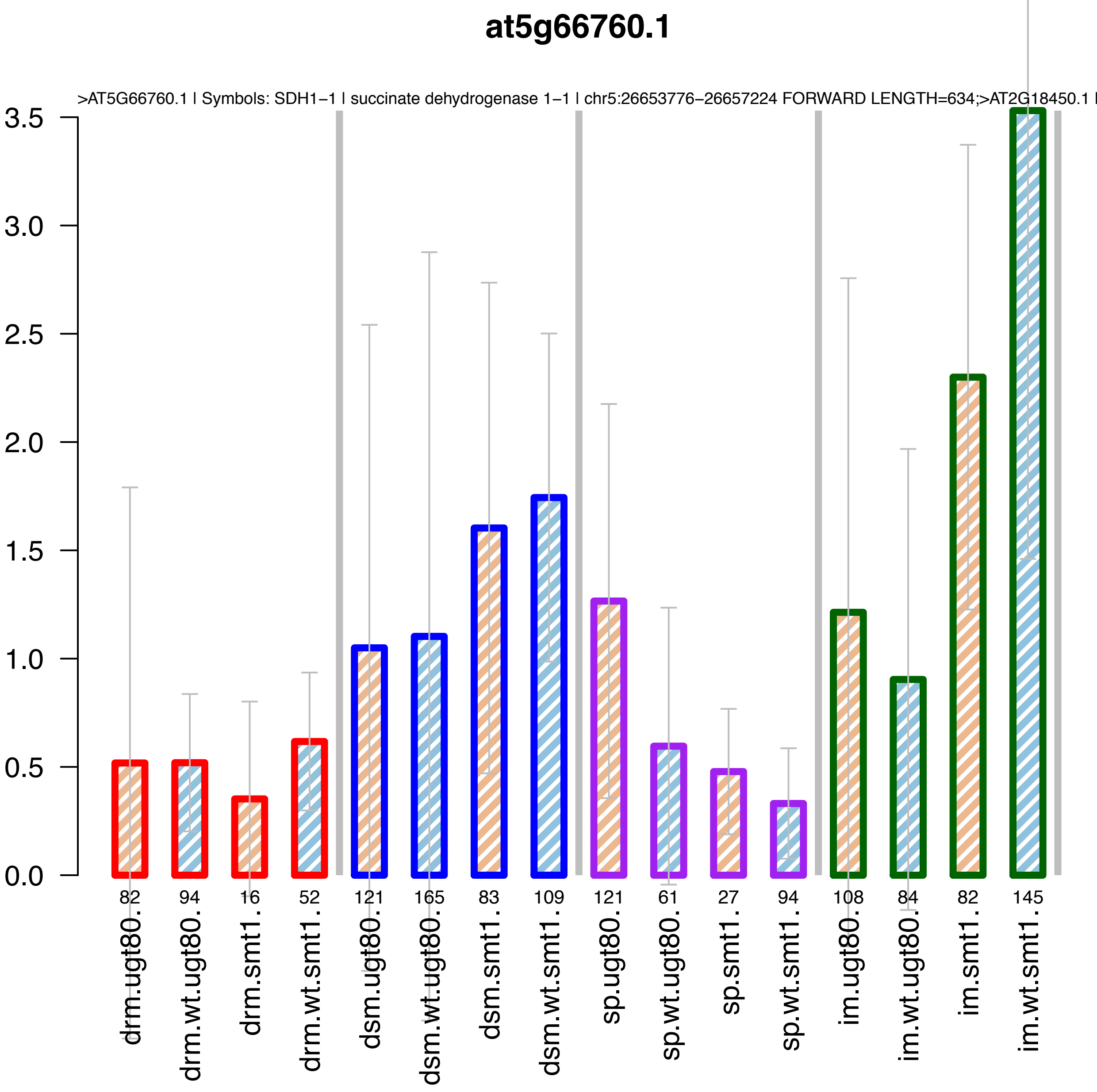

at5g66760.1
>AT5G66760.1 | Symbols: SDH1−1 | succinate dehydrogenase 1−1 | chr5:26653776−26657224 FORWARD LENGTH=634;>AT2G18450.1 |
3.5
3.0
2.5
2.0
1.5
1.0
0.5
0.0
82
94
16
52
121
165
83
109
121
61
27
94
108
84
82
145
drm.ugt80..
drm.wt.ugt80..
drm.smt1..
drm.wt.smt1..
dsm.ugt80..
dsm.wt.ugt80..
dsm.smt1..
dsm.wt.smt1..
sp.ugt80..
sp.wt.ugt80..
sp.smt1..
sp.wt.smt1..
im.ugt80..
im.wt.ugt80..
im.smt1..
im.wt.smt1..

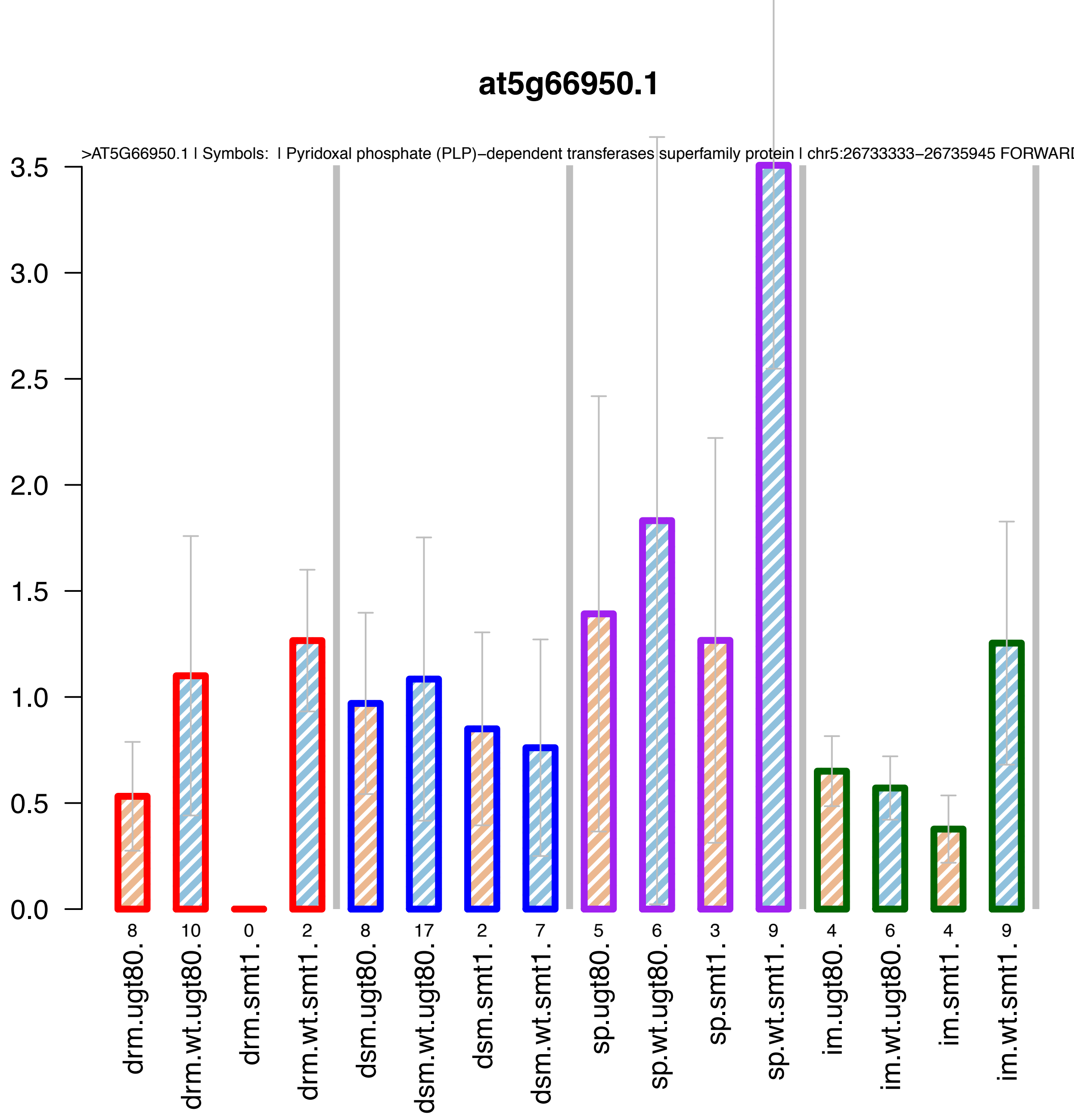

at5g66950.1
>AT5G66950.1 | Symbols: | Pyridoxal phosphate (PLP)-dependent transferases superfamily protein | chr5:26733333-26735945 FORWARD
3.5
3.0
2.5
2.0
1.5
1.0
0.5
0.0
8
10
0
2
8
17
2
7
5
6
3
9
4
6
4
9
drm.ugt80.
drm.wt.ugt80.
drm.smt1.
drm.wt.smt1.
dsm.ugt80.
dsm.wt.ugt80.
dsm.smt1.
dsm.wt.smt1.
sp.ugt80.
sp.wt.ugt80.
sp.smt1.
sp.wt.smt1.
im.ugt80.
im.wt.ugt80.
im.smt1.
im.wt.smt1.

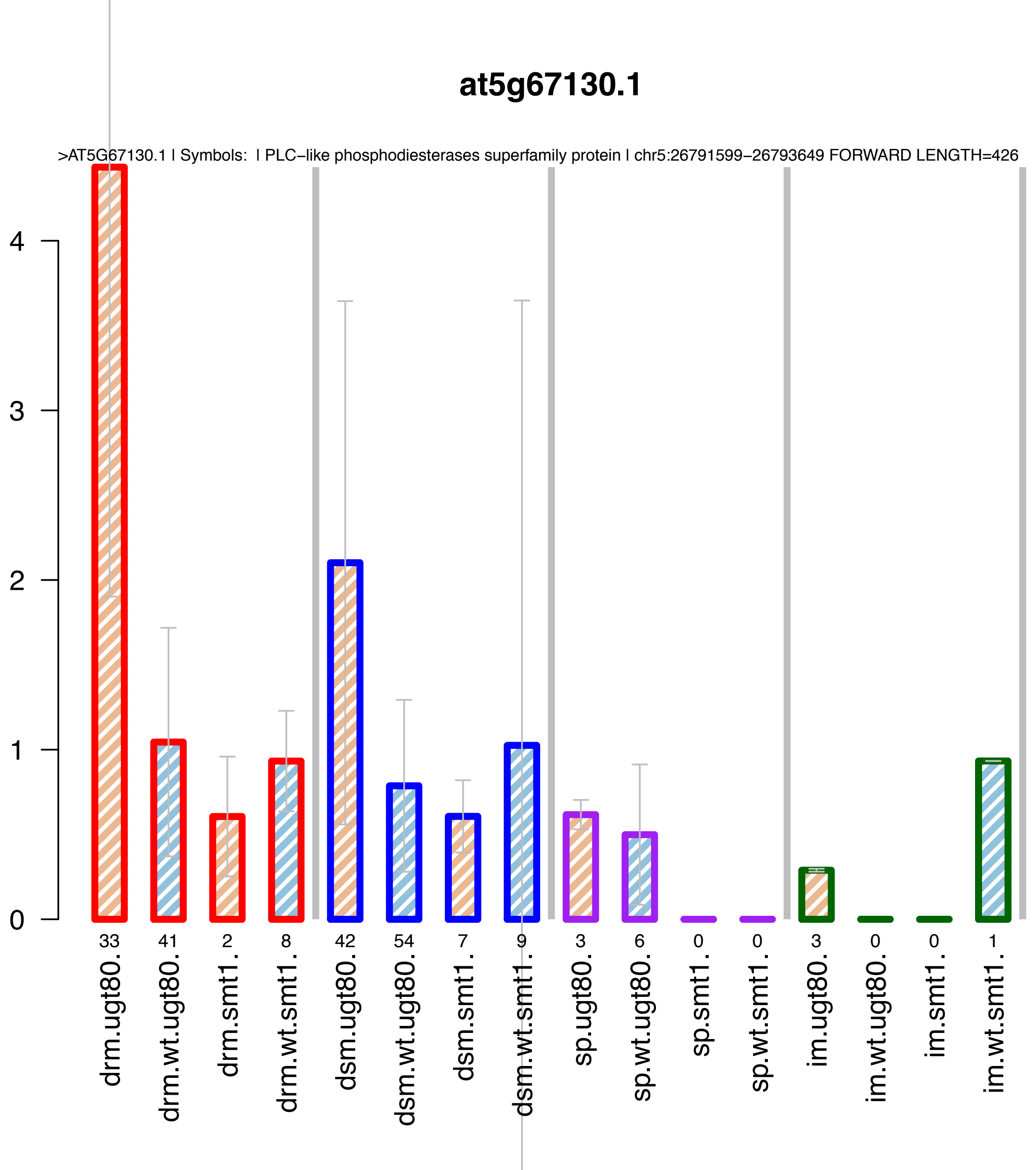

at5g67130.1
>AT5G67130.1 | Symbols: | PLC−like phosphodiesterases superfamily protein | chr5:26791599−26793649 FORWARD LENGTH=426
0
1
2
3
4
33
41
2
8
42
54
7
9
3
6
0
0
3
0
0
1
drm.ugt80..
drm.wt.ugt80..
drm.smt1..
drm.wt.smt1..
dsm.ugt80..
dsm.wt.ugt80..
dsm.smt1..
dsm.wt.smt1..
sp.ugt80..
sp.wt.ugt80..
sp.smt1..
sp.wt.smt1..
im.ugt80..
im.wt.ugt80..
im.smt1..
im.wt.smt1..

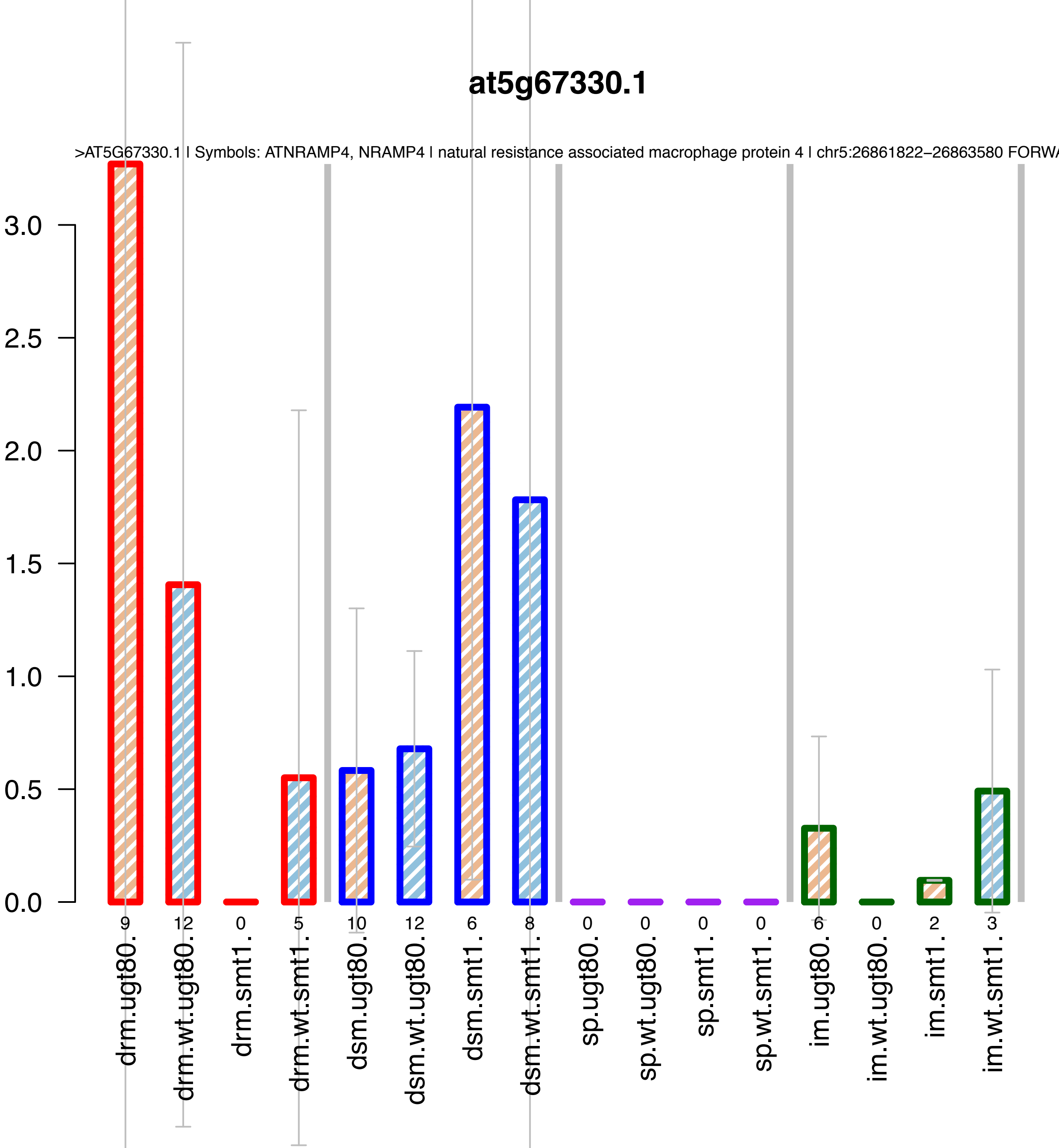

at5g67330.1
>AT5G67330.1 | Symbols: ATNRAMP4, NRAMP4 | natural resistance associated macrophage protein 4 | chr5:26861822-26863580 FORW
3.0
2.5
2.0
1.5
1.0
0.5
0.0
9
12
0
5
10
12
6
8
0
0
0
0
6
0
2
3
drm.ugt80.
drm.wt.ugt80.
drm.smt1.
drm.wt.smt1.
dsm.ugt80.
dsm.wt.ugt80.
dsm.smt1.
dsm.wt.smt1.
sp.ugt80.
sp.wt.ugt80.
sp.smt1.
sp.wt.smt1.
im.ugt80.
im.wt.ugt80.
im.smt1.
im.wt.smt1.

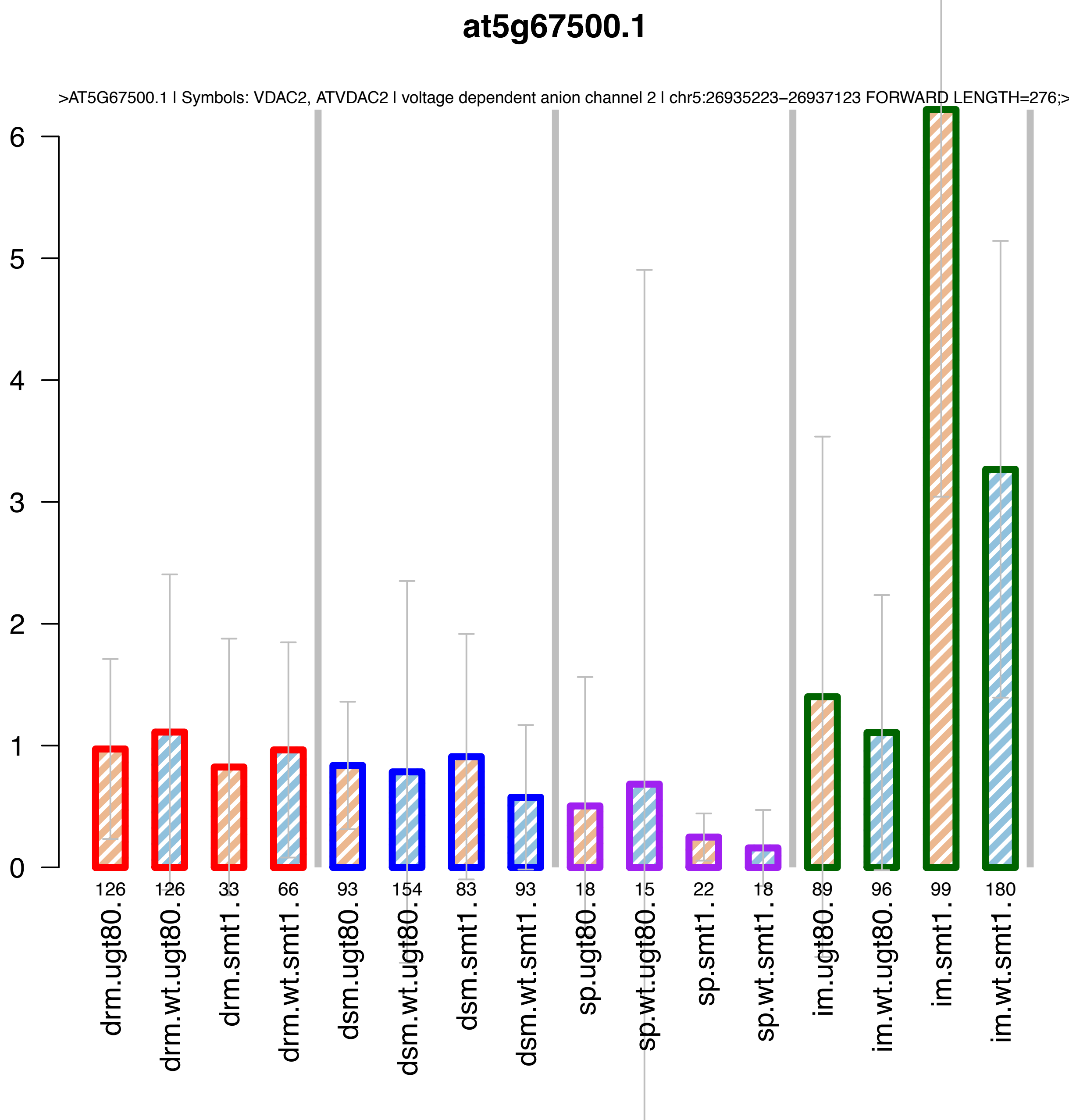
at5g67500.1
>AT5G67500.1 | Symbols: VDAC2, ATVDAC2 | voltage dependent anion channel 2 | chr5:26935223-26937123 FORWARD LENGTH=276;>
0
1
2
3
4
5
6
126
126
33
66
93
154
83
93
18
15
22
18
89
96
99
180
drm.ugt80..
drm.wt.ugt80..
drm.smt1..
drm.wt.smt1..
dsm.ugt80..
dsm.wt.ugt80..
dsm.smt1..
dsm.wt.smt1..
sp.ugt80..
sp.wt.ugt80..
sp.smt1..
sp.wt.smt1..
im.ugt80..
im.wt.ugt80..
im.smt1..
im.wt.smt1..

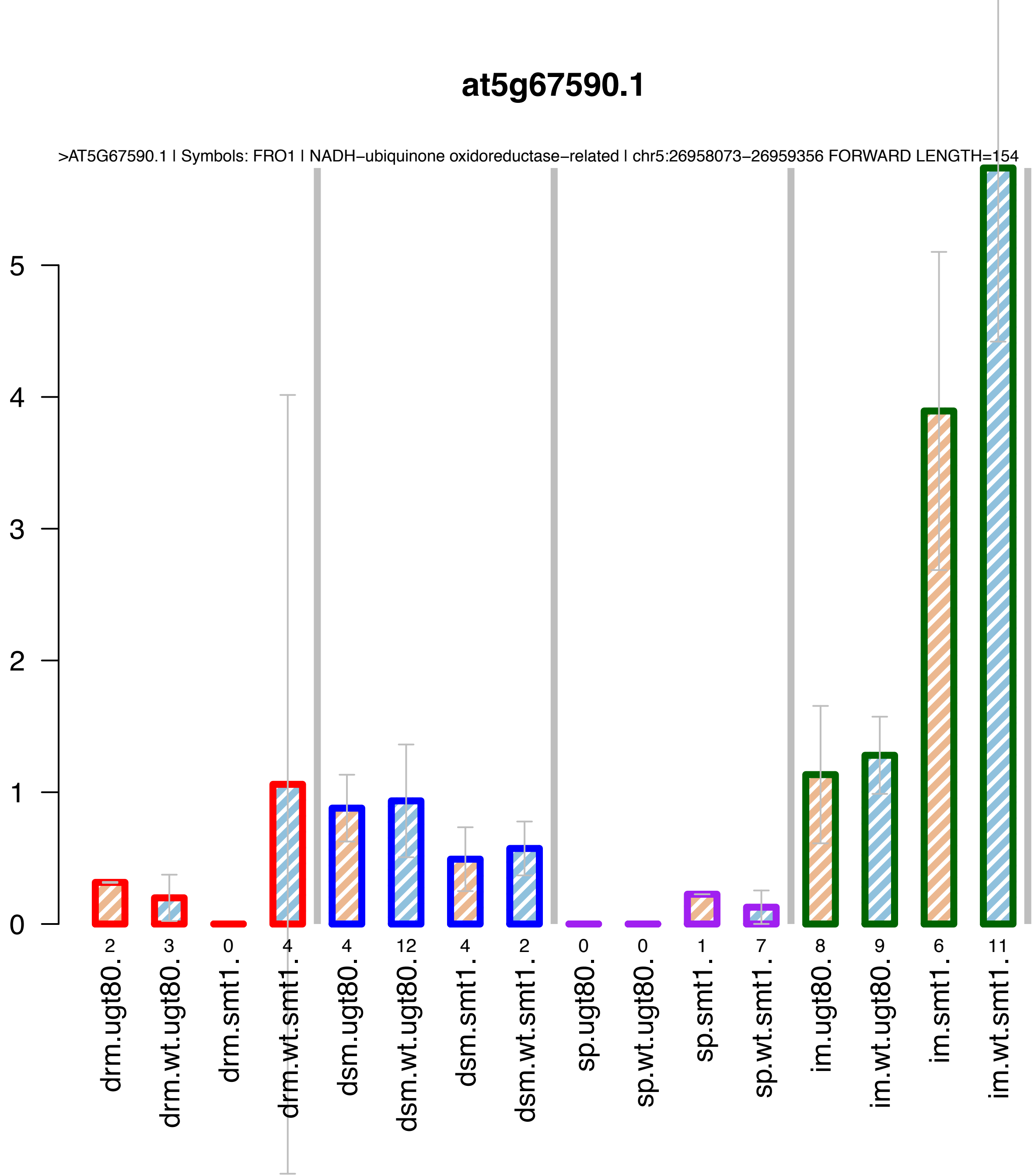

at5g67590.1
>AT5G67590.1 | Symbols: FRO1 | NADH−ubiquinone oxidoreductase−related | chr5:26958073−26959356 FORWARD LENGTH=154
0
1
2
3
4
5
2
3
0
4
4
12
4
2
0
0
1
7
8
9
6
11
drm.ugt80.
drm.wt.ugt80.
drm.smt1.
drm.wt.smt1.
dsm.ugt80.
dsm.wt.ugt80.
dsm.smt1.
dsm.wt.smt1.
sp.ugt80.
sp.wt.ugt80.
sp.smt1.
sp.wt.smt1.
im.ugt80.
im.wt.ugt80.
im.smt1.
im.wt.smt1.

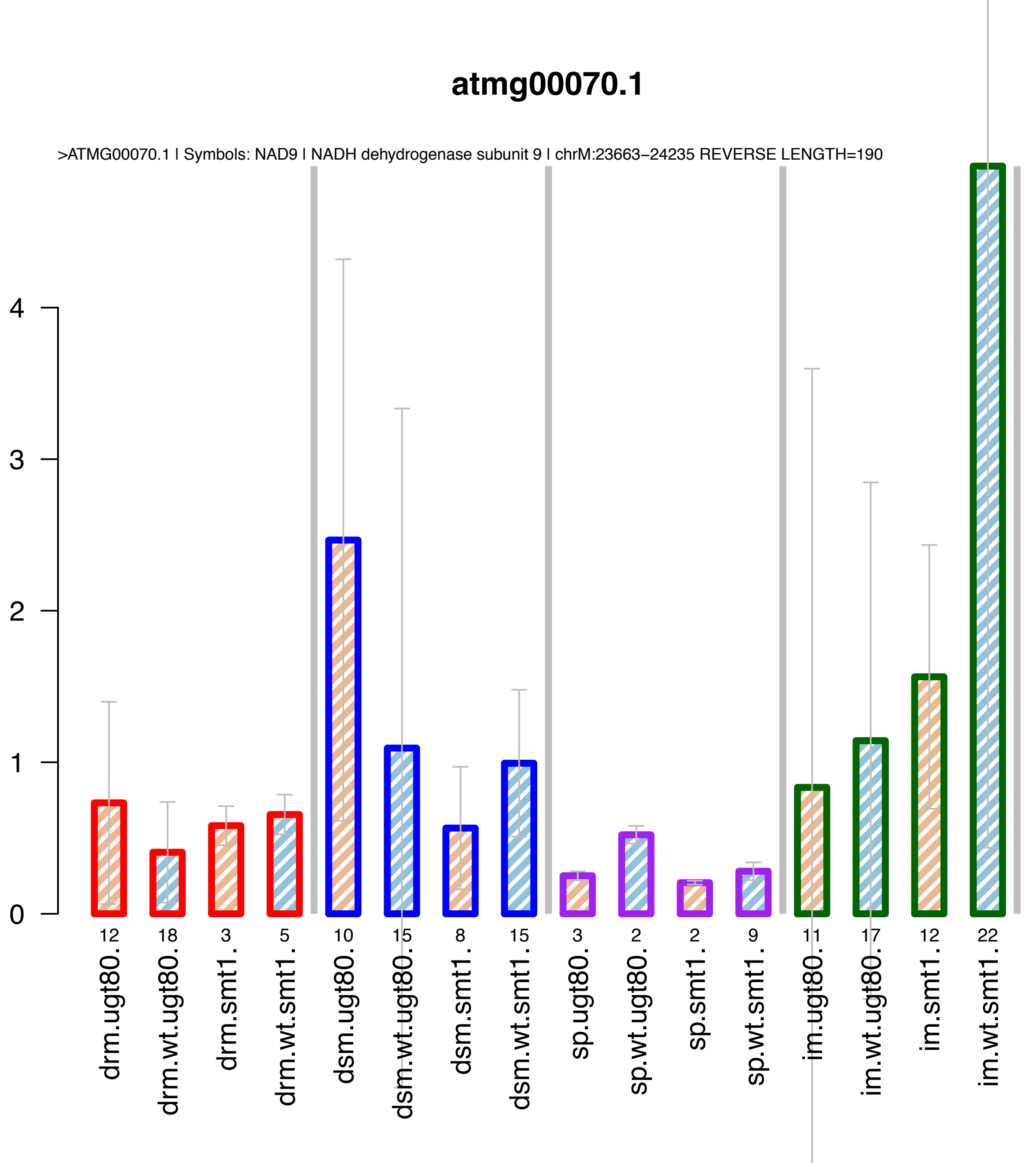

atmg00070.1
>ATMG00070.1 | Symbols: NAD9 | NADH dehydrogenase subunit 9 | chrM:23663−24235 REVERSE LENGTH=190
0
1
2
3
4
12
18
3
5
10
15
8
15
3
2
2
9
11
17
12
22
drm.ugt80..
drm.wt.ugt80..
drm.smt1..
drm.wt.smt1..
dsm.ugt80..
dsm.wt.ugt80..
dsm.smt1..
dsm.wt.smt1..
sp.ugt80..
sp.wt.ugt80..
sp.smt1..
sp.wt.smt1..
im.ugt80..
im.wt.ugt80..
im.smt1..
im.wt.smt1..

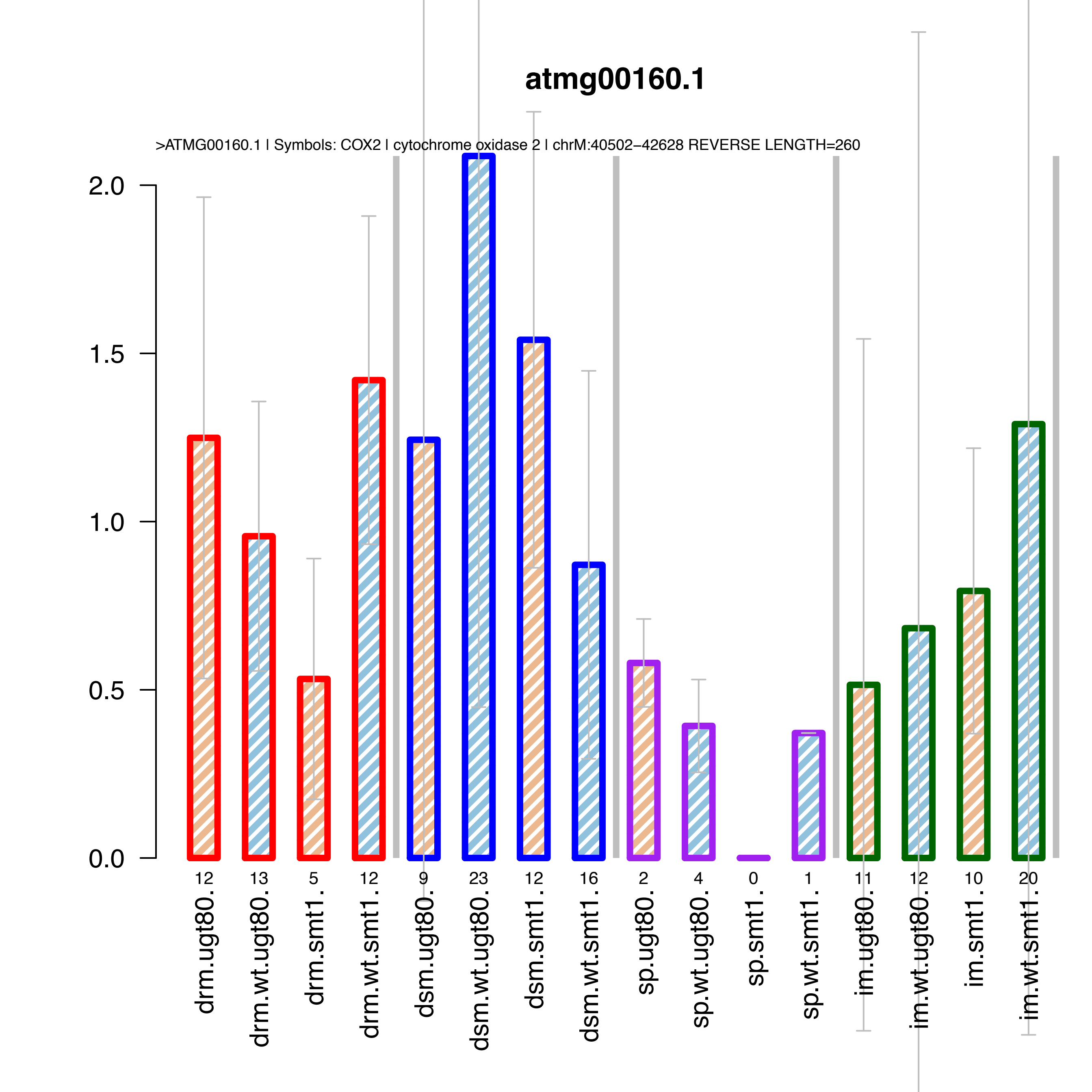

atmg00160.1
>ATMG00160.1 | Symbols: COX2 | cytochrome oxidase 2 | chrM:40502-42628 REVERSE LENGTH=260
2.0
1.5
1.0
0.5
0.0
12
13
5
12
9
23
12
16
2
4
0
1
11
12
10
20
drm.ugt80..
drm.wt.ugt80..
drm.smt1..
drm.wt.smt1..
dsm.ugt80..
dsm.wt.ugt80..
dsm.smt1..
dsm.wt.smt1..
sp.ugt80..
sp.wt.ugt80..
sp.smt1..
sp.wt.smt1..
im.ugt80..
im.wt.ugt80..
im.smt1..
im.wt.smt1..

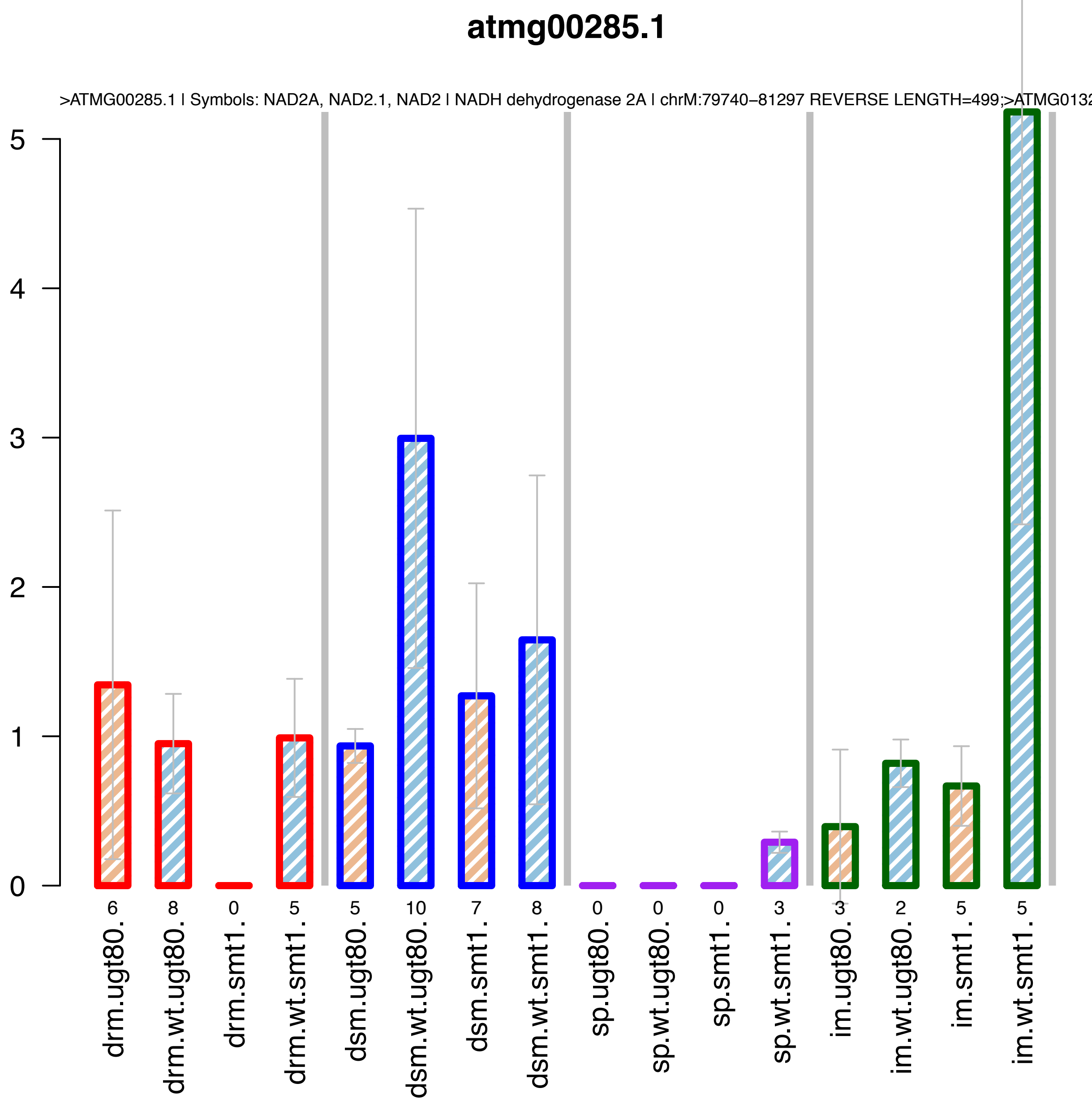

atmg00285.1
>ATMG00285.1 | Symbols: NAD2A, NAD2.1, NAD2 | NADH dehydrogenase 2A | chrM:79740-81297 REVERSE LENGTH=499;>ATMG0132
0
1
2
3
4
5
6
8
0
5
5
10
7
8
0
0
0
3
3
2
5
5
drm.ugt80.
drm.wt.ugt80.
drm.smt1.
drm.wt.smt1.
dsm.ugt80.
dsm.wt.ugt80.
dsm.smt1.
dsm.wt.smt1.
sp.ugt80.
sp.wt.ugt80.
sp.smt1.
sp.wt.smt1.
im.ugt80.
im.wt.ugt80.
im.smt1.
im.wt.smt1.

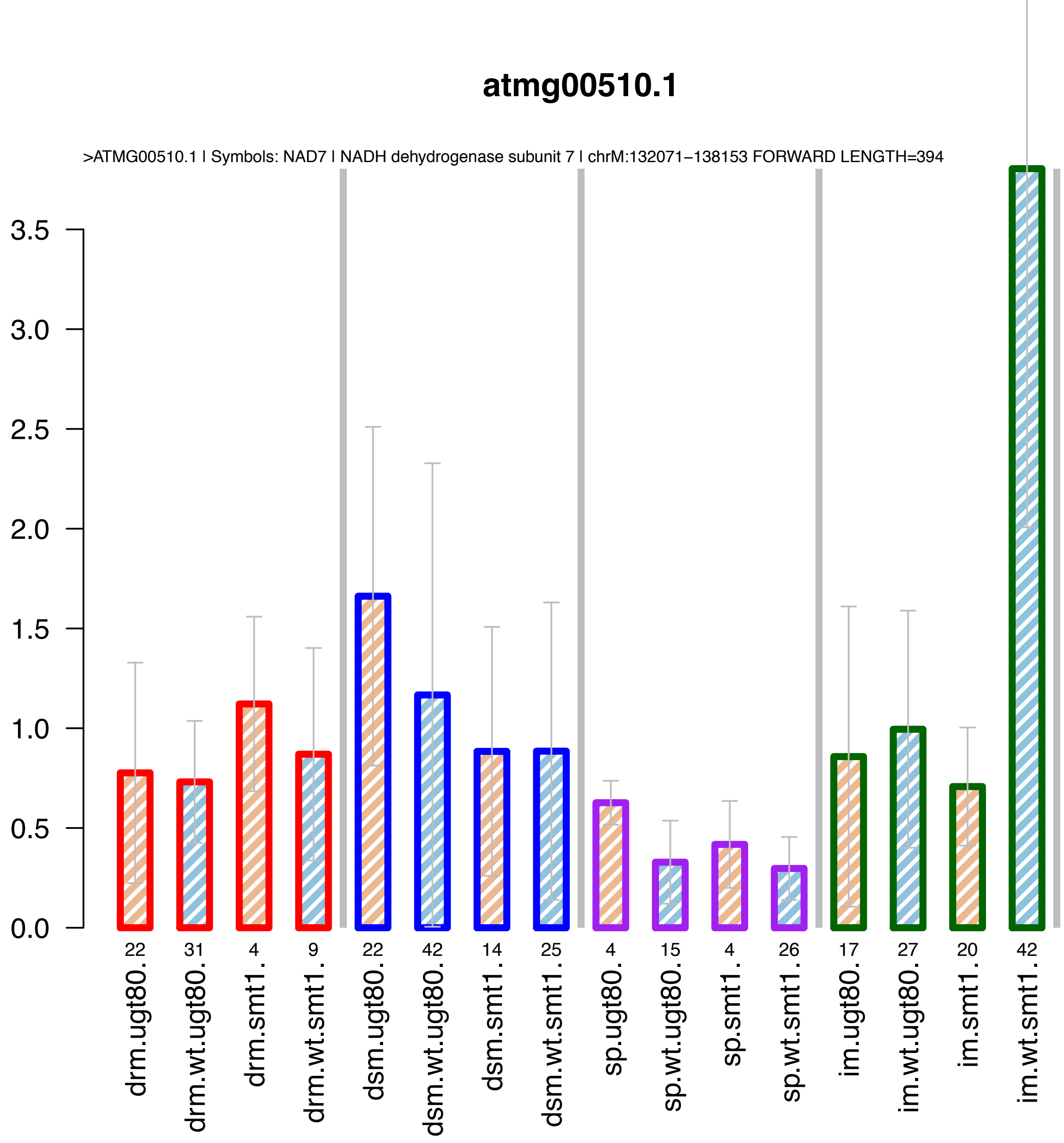

atmg00510.1
>ATMG00510.1 | Symbols: NAD7 | NADH dehydrogenase subunit 7 | chrM:132071−138153 FORWARD LENGTH=394
0.0
0.5
1.0
1.5
2.0
2.5
3.0
3.5
22
31
4
9
22
42
14
25
4
15
4
26
17
27
20
42
drm.ugt80..
drm.wt.ugt80..
drm.smt1..
drm.wt.smt1..
dsm.ugt80..
dsm.wt.ugt80..
dsm.smt1..
dsm.wt.smt1..
sp.ugt80..
sp.wt.ugt80..
sp.smt1..
sp.wt.smt1..
im.ugt80..
im.wt.ugt80..
im.smt1..
im.wt.smt1..

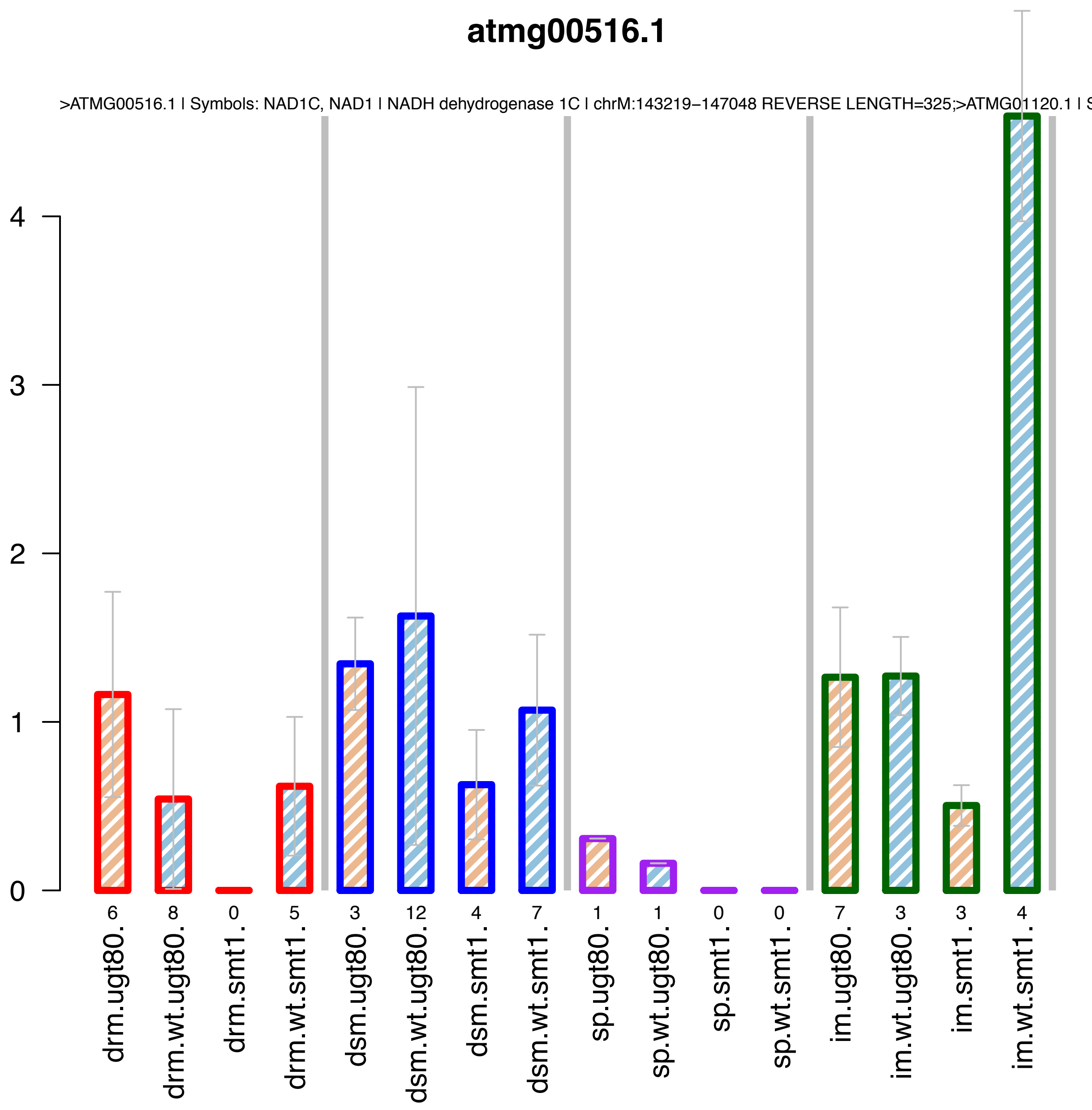
atmg00516.1
>ATMG00516.1 | Symbols: NAD1C, NAD1 | NADH dehydrogenase 1C | chrM:143219-147048 REVERSE LENGTH=325;>ATMG01120.1 | S
0
1
2
3
4
6
8
0
5
3
12
4
7
1
1
0
0
7
3
3
4
drm.ugt80.
drm.wt.ugt80.
drm.smt1.
drm.wt.smt1.
dsm.ugt80.
dsm.wt.ugt80.
dsm.smt1.
dsm.wt.smt1.
sp.ugt80.
sp.wt.ugt80.
sp.smt1.
sp.wt.smt1.
im.ugt80.
im.wt.ugt80.
im.smt1.
im.wt.smt1.

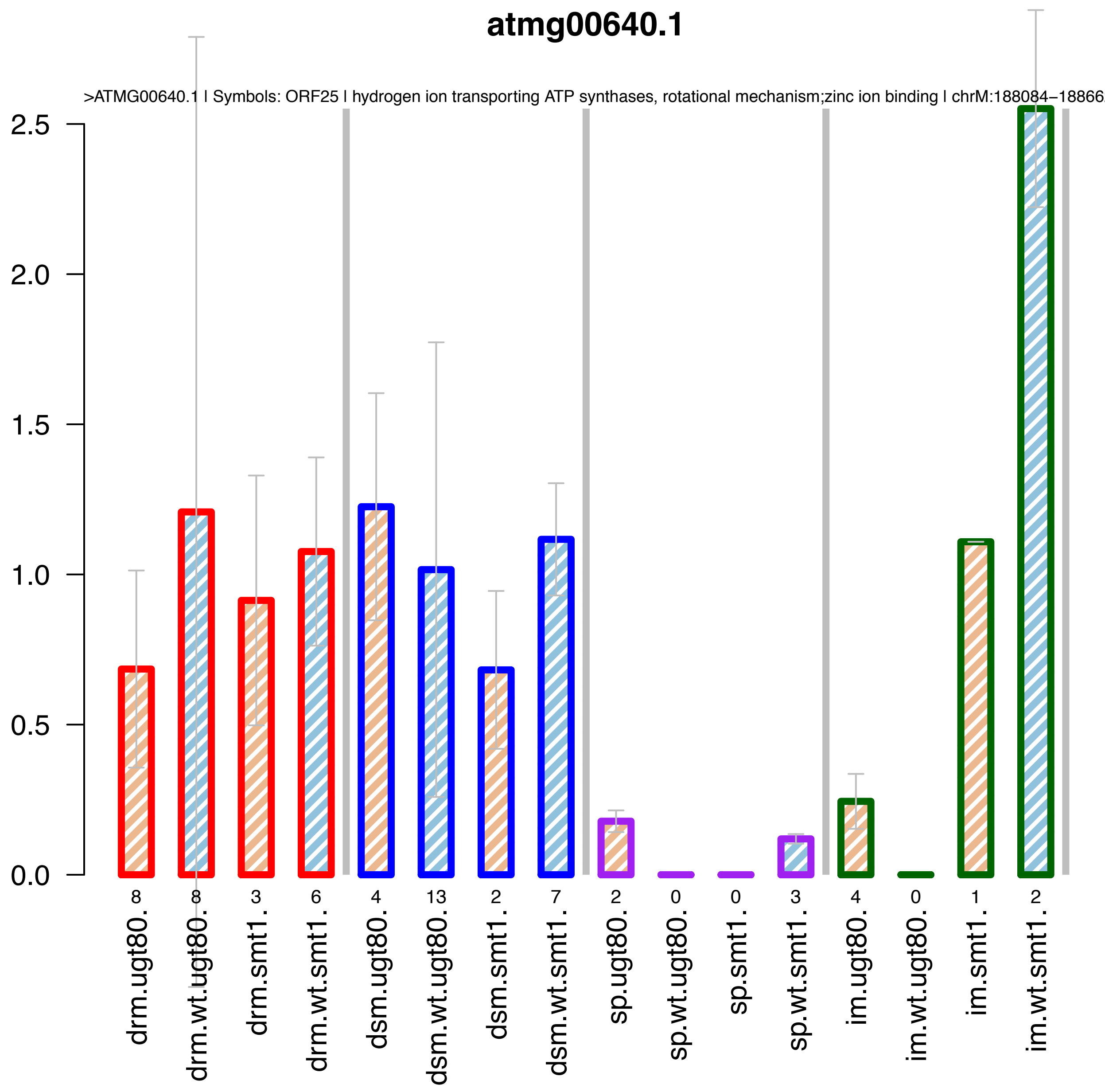

atmg00640.1
>ATMG00640.1 | Symbols: ORF25 | hydrogen ion transporting ATP synthases, rotational mechanism;zinc ion binding | chrM:188084–18866
0.0
0.5
1.0
1.5
2.0
2.5
8
8
3
6
4
13
2
7
2
0
0
3
4
0
1
2
drm.ugt80..
drm.wt.ugt80..
drm.smt1..
drm.wt.smt1..
dsm.ugt80..
dsm.wt.ugt80..
dsm.smt1..
dsm.wt.smt1..
sp.ugt80..
sp.wt.ugt80..
sp.smt1..
sp.wt.smt1..
im.ugt80..
im.wt.ugt80..
im.smt1..
im.wt.smt1..
